# Supplementary material for: Characterization of missing values in untargeted MS-based metabolomics data and evaluation of missing data handling strategies
Source: Metabolomics. 2018 Sep 20;14(10):128. doi: 10.1007/s11306-018-1420-2 (PMC6153696; doi:10.1007/s11306-018-1420-2)
Supplement: Supplementary file 1 — Supplementary material 1 (PDF 24335 KB) [file 11306_2018_1420_MOESM1_ESM.pdf]

Characterization of missingness in untargeted MS-based metabolomics data sets and evaluation of missing data handling strategies

Kieu Trinh Do[¶], Simone Wahl[¶], Johannes Raffler, Sophie Molnos, Michael Laimighofer, Jerzy Adamski, Karsten Suhre, Konstantin Strauch, Annette Peters, Christian Gieger, Claudia Langenberg, Isobel D. Stewart, Fabian J. Theis, Harald Grallert, Gabi Kastenmüller[#], Jan Krumsiek[#]

Supporting Information File S1: LOD tendency

As an explorative method to visualize the missingness mechanism of a metabolite m, a second (auxiliary) metabolite m_{aux} showing the strongest Pearson correlation to m was determined. Comparing the distribution of concentrations of metabolite m_{aux} for observations with and without observed m can provide information about whether or not missingness in m is systematic.

In the following, for all metabolites (x-axis) with more than 10% and less than 70% missing values a scatterplot with its auxiliary metabolite (y-axis) is shown. Blue dots correspond to observed concentrations in both metabolites. Red dots represent concentrations of the auxiliary metabolite in samples with missing values of the investigated metabolite. $\mathbf{corr}$ and $\mathbf{p}$ are Pearson correlation coefficient and corresponding p-value.

The boxplots compare the concentrations of the auxiliary metabolite between samples with missing and samples with measured values of the given metabolite. $\mathbf{p_{Wst}}$ is the p-value of a performed Wilcoxon-Mann-Whitney test. An LOD tendency is assumed is $\mathbf{p_{Wst}}$ is below $\boldsymbol{\alpha = 0.05}$ after Bonferroni correction for multiple testing.

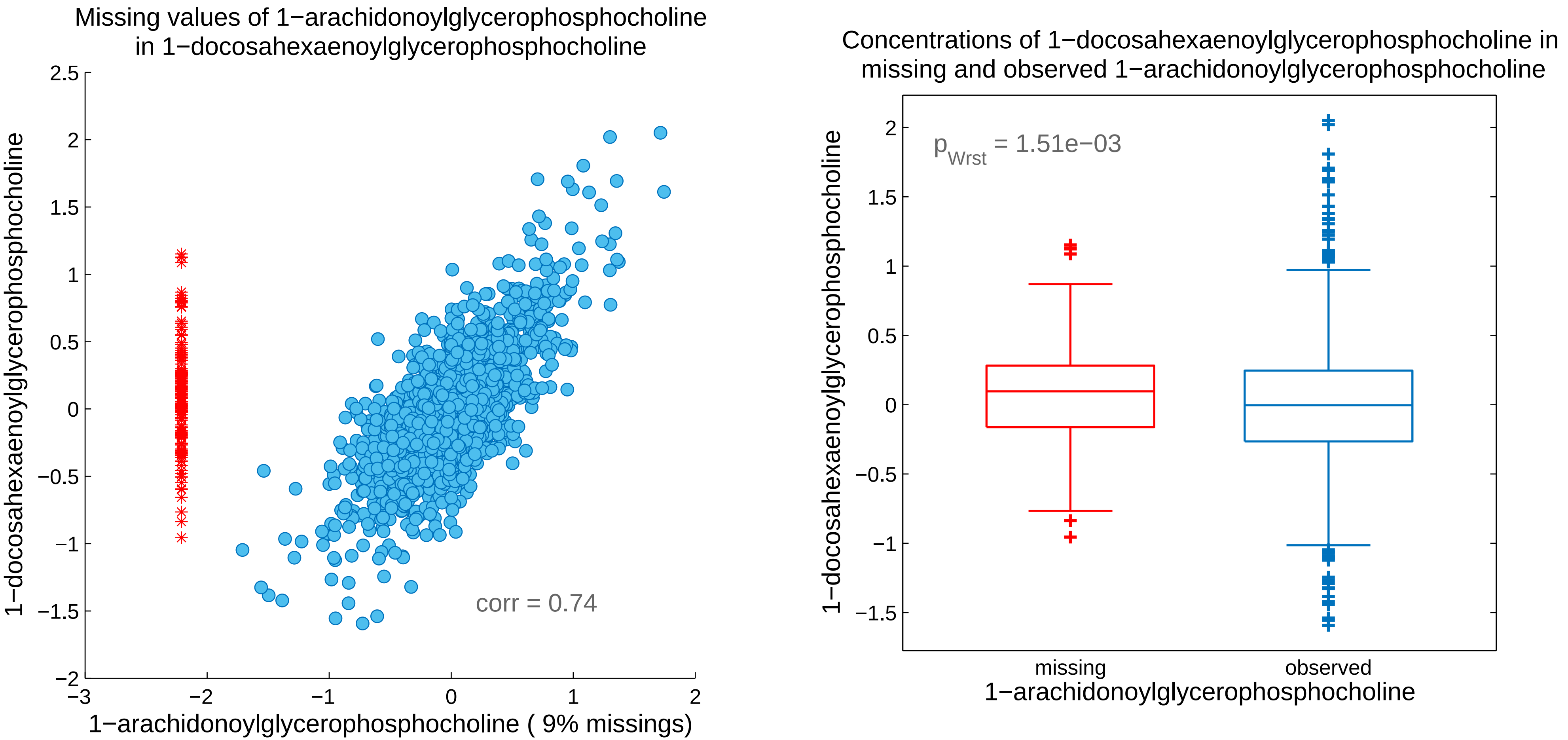

Missing values of 1−arachidonoylglycerophosphocholine in 1−docosahexaenoylglycerophosphocholine
1−docosahexaenoylglycerophosphocholine
1−arachidonoylglycerophosphocholine (9% missings)
corr = 0.74
Concentrations of 1−docosahexaenoylglycerophosphocholine in missing and observed 1−arachidonoylglycerophosphocholine
p_{Wrst} = 1.51e−03
missing
observed
1−arachidonoylglycerophosphocholine

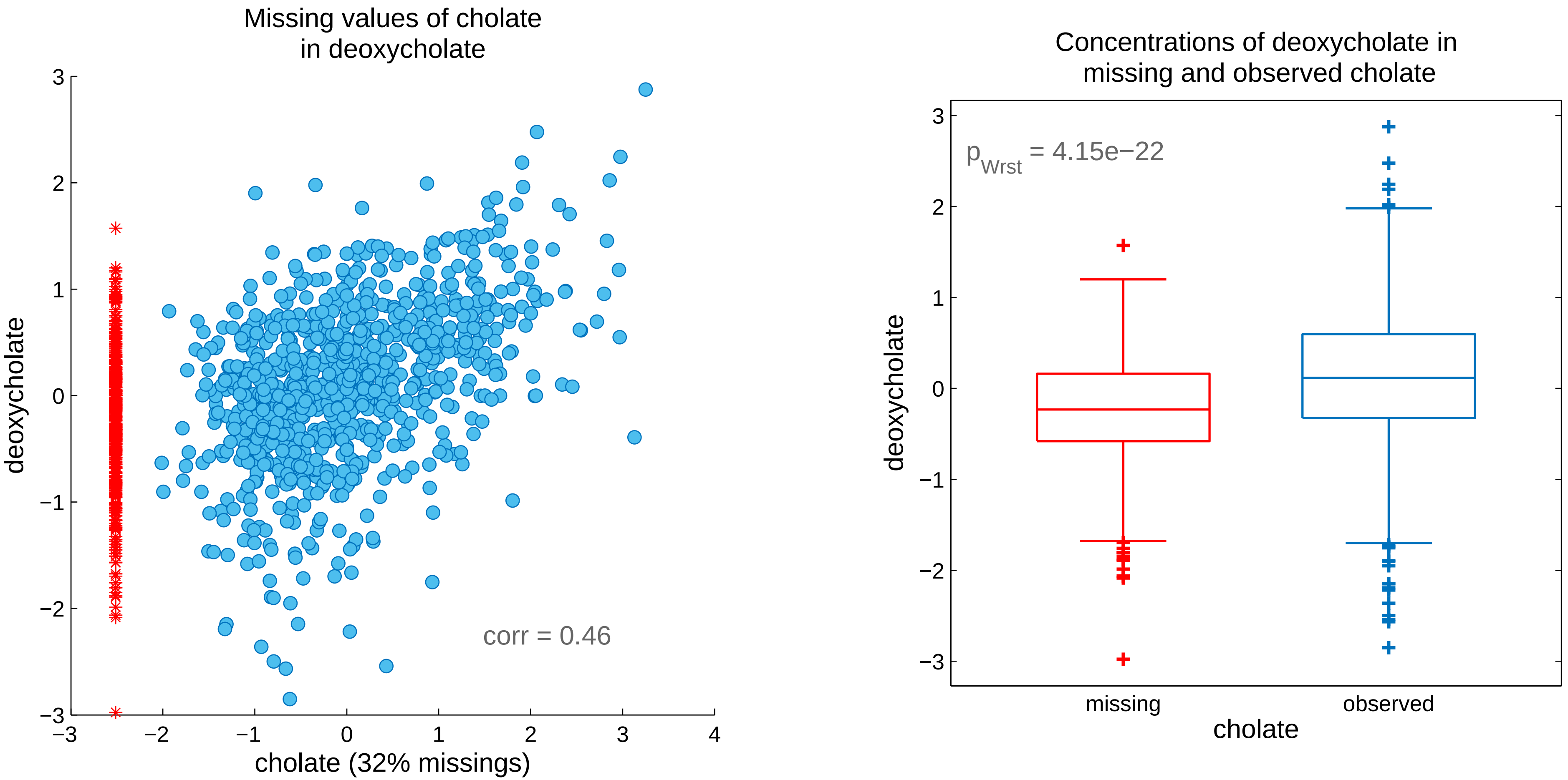

Missing values of cholate in deoxycholate
deoxycholate
cholate (32% missings)
corr = 0.46
3
2
1
0
−1
−2
−3
−3
−2
−1
0
1
2
3
4
Concentrations of deoxycholate in missing and observed cholate
p_{Wrst} = 4.15e−22
deoxycholate
3
2
1
0
−1
−2
−3
missing
observed
cholate

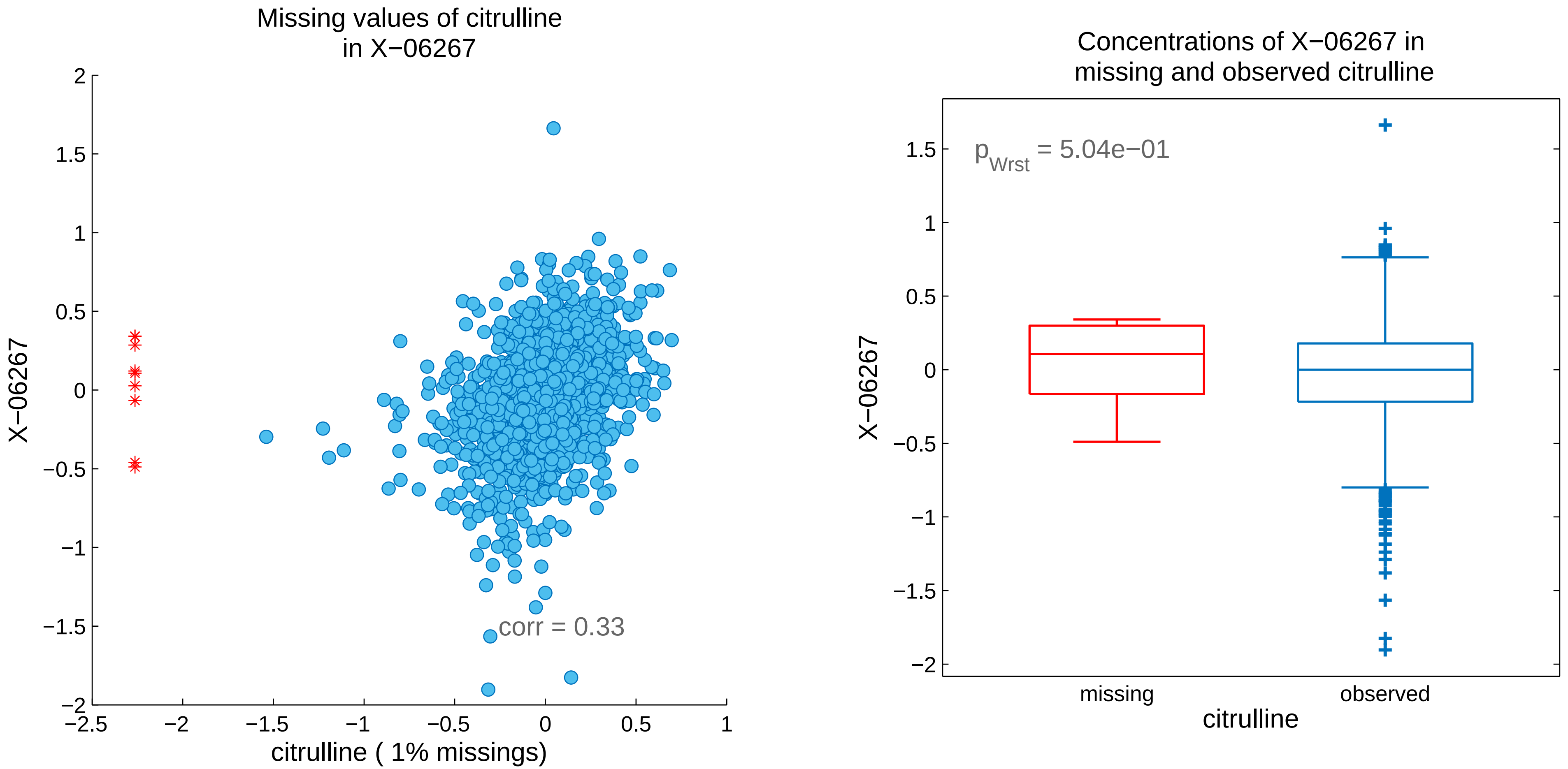
Missing values of citrulline in X−06267
X−06267
citrulline (1% missings)
corr = 0.33
Concentrations of X−06267 in missing and observed citrulline
p_{Wrst} = 5.04e−01
X−06267
missing
observed
citrulline

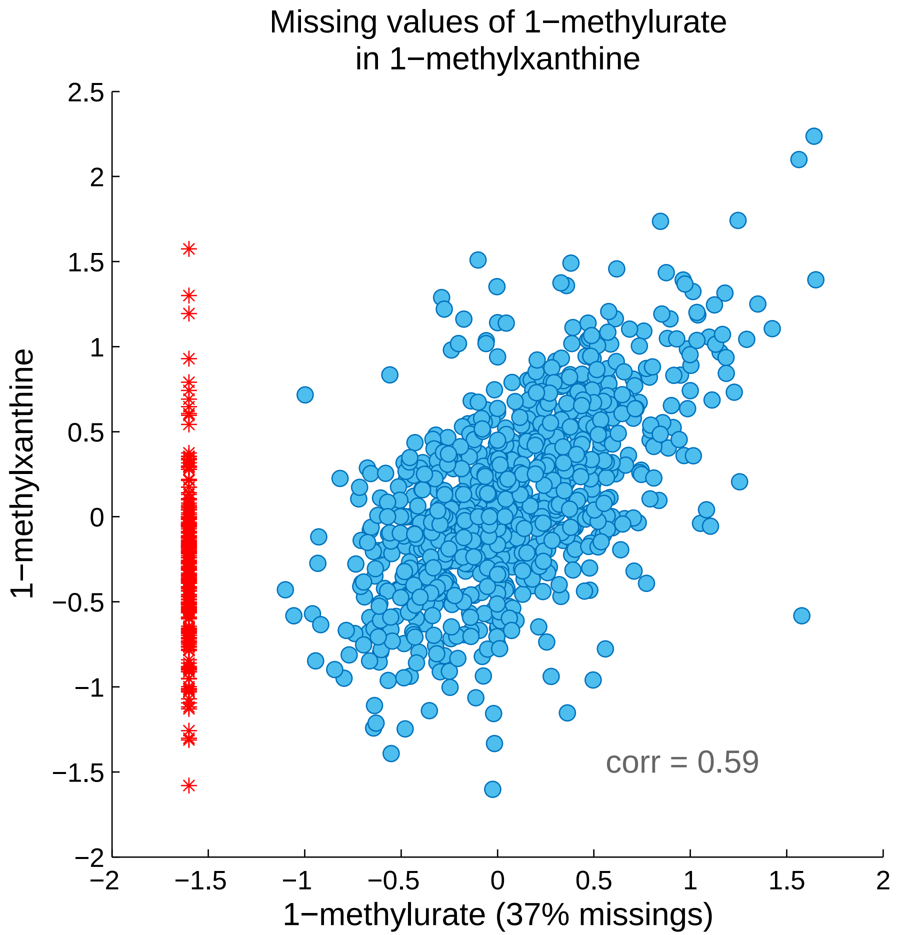

Missing values of 1−methylurate in 1−methylxanthine
1−methylxanthine
1−methylurate (37% missings)
corr = 0.59
2.5
2
1.5
1
0.5
0
−0.5
−1
−1.5
−2

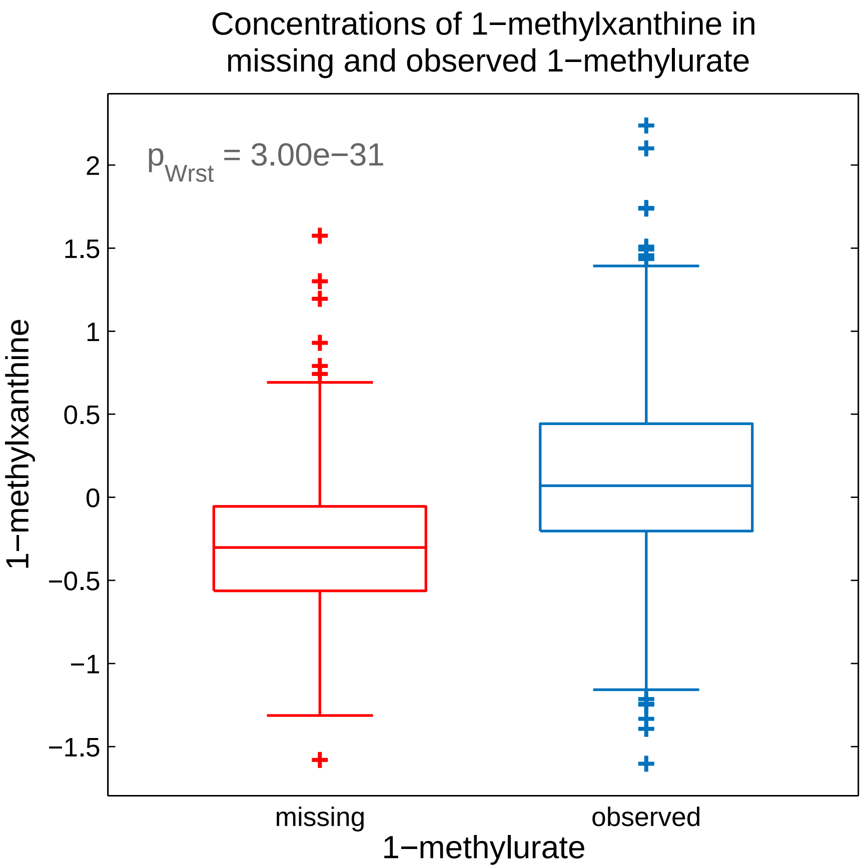

Concentrations of 1−methylxanthine in missing and observed 1−methylurate
p_Wrst = 3.00e−31
1−methylxanthine
missing
observed
1−methylurate

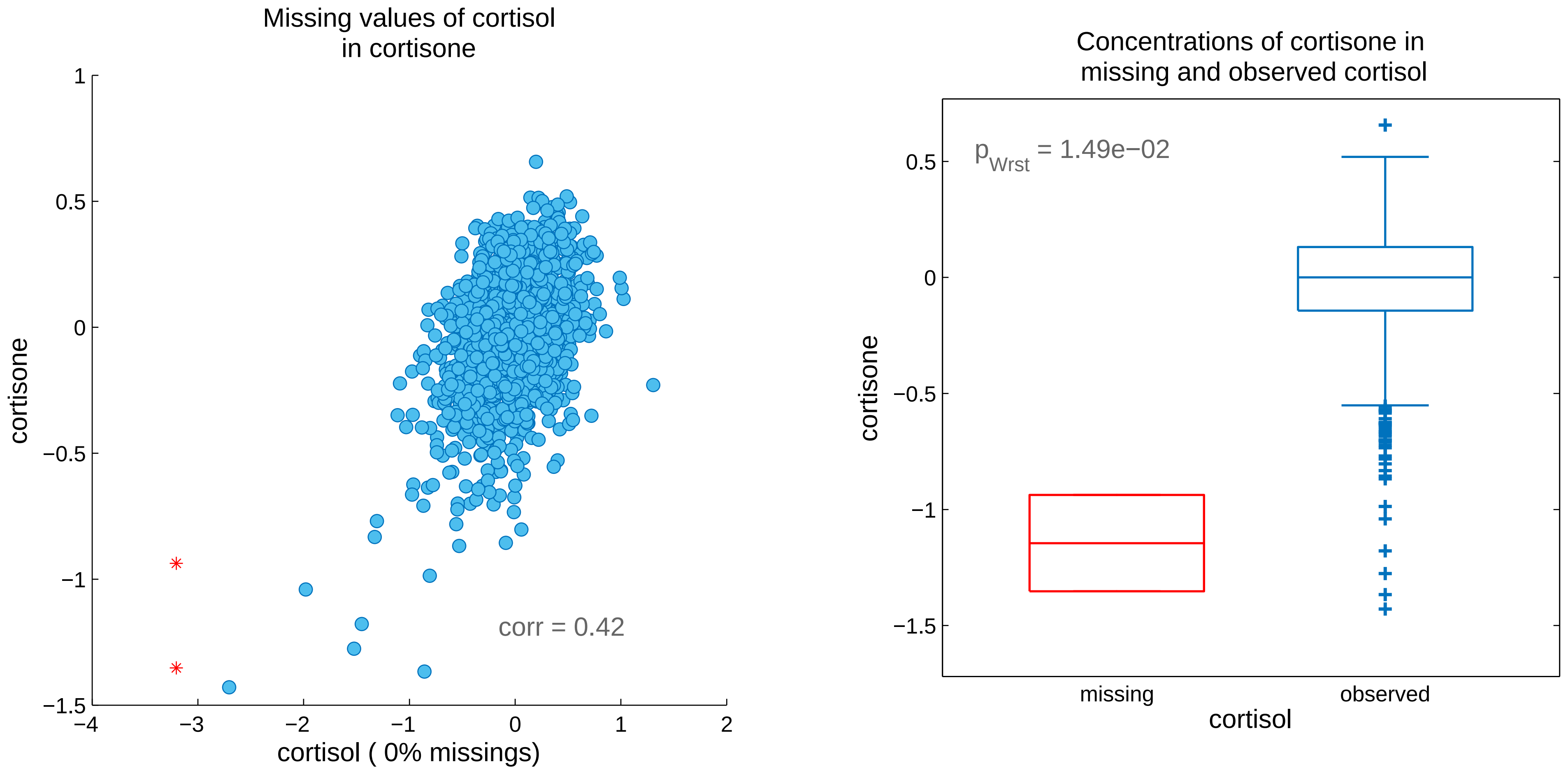

Missing values of cortisol
in cortisone
cortisone
cortisol (0% missings)
corr = 0.42
Concentrations of cortisone in
missing and observed cortisol
p_{Wrst} = 1.49e−02
cortisone
missing
observed
cortisol

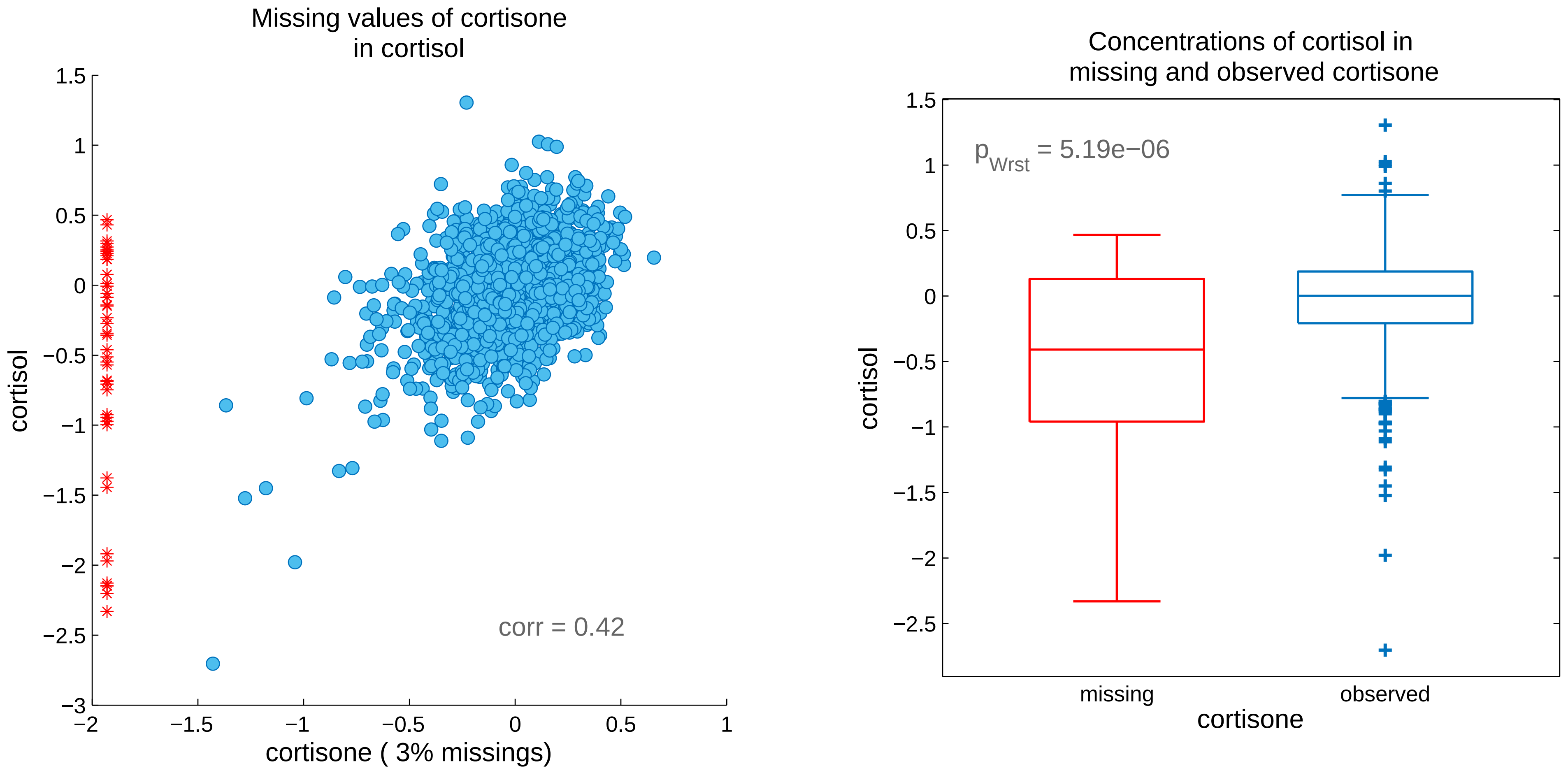
Missing values of cortisone in cortisol
cortisol
cortisone (3% missings)
corr = 0.42
Concentrations of cortisol in missing and observed cortisone
cortisol
p_{Wrst} = 5.19e−06
missing
observed
cortisone

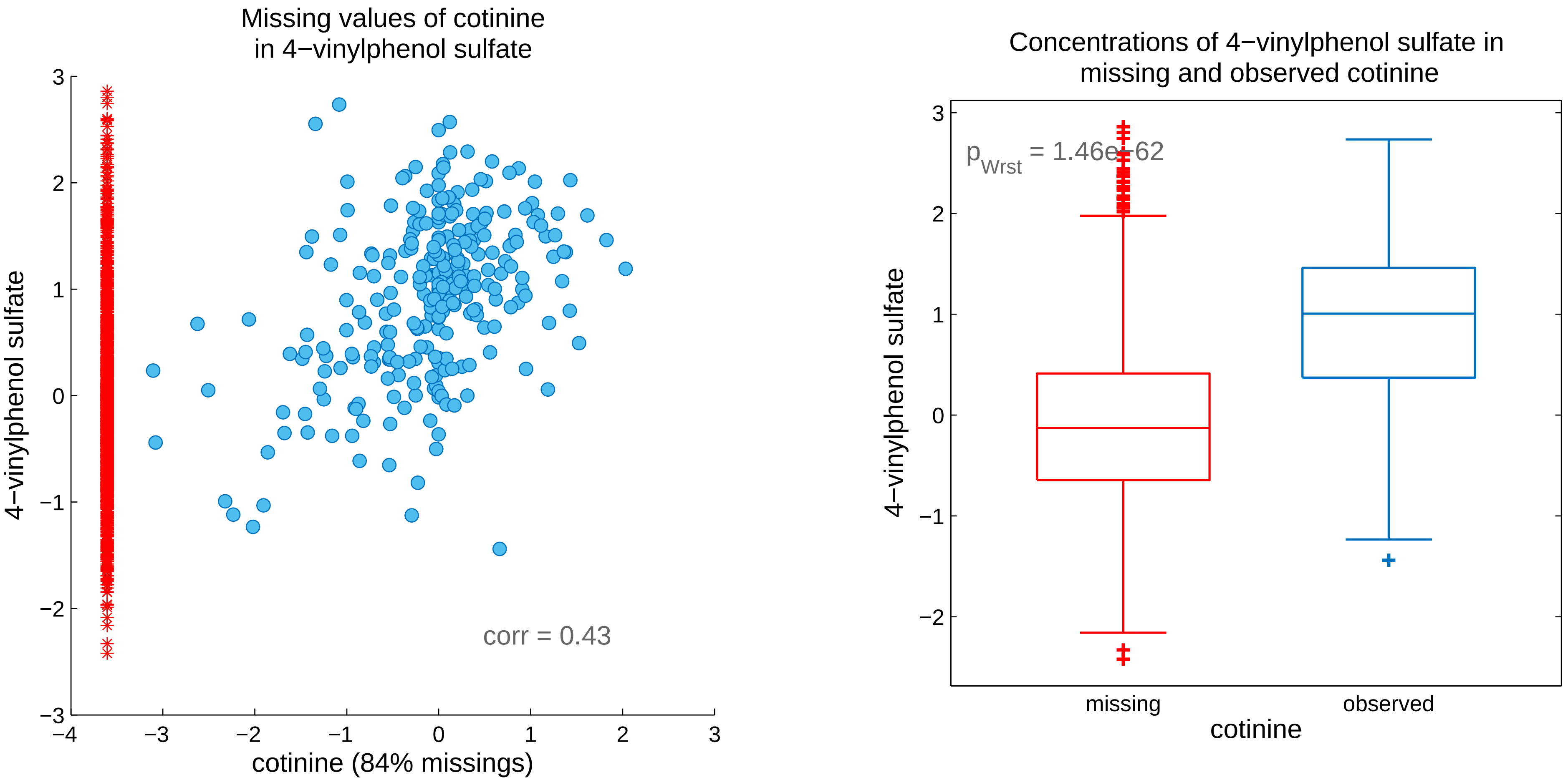
Missing values of cotinine
in 4−vinylphenol sulfate
4−vinylphenol sulfate
cotinine (84% missings)
corr = 0.43
3
2
1
0
−1
−2
−3
−4
−3
−2
−1
0
1
2
3
Concentrations of 4−vinylphenol sulfate in
missing and observed cotinine
p_{Wrst} = 1.46e−62
4−vinylphenol sulfate
3
2
1
0
−1
−2
missing
observed
cotinine

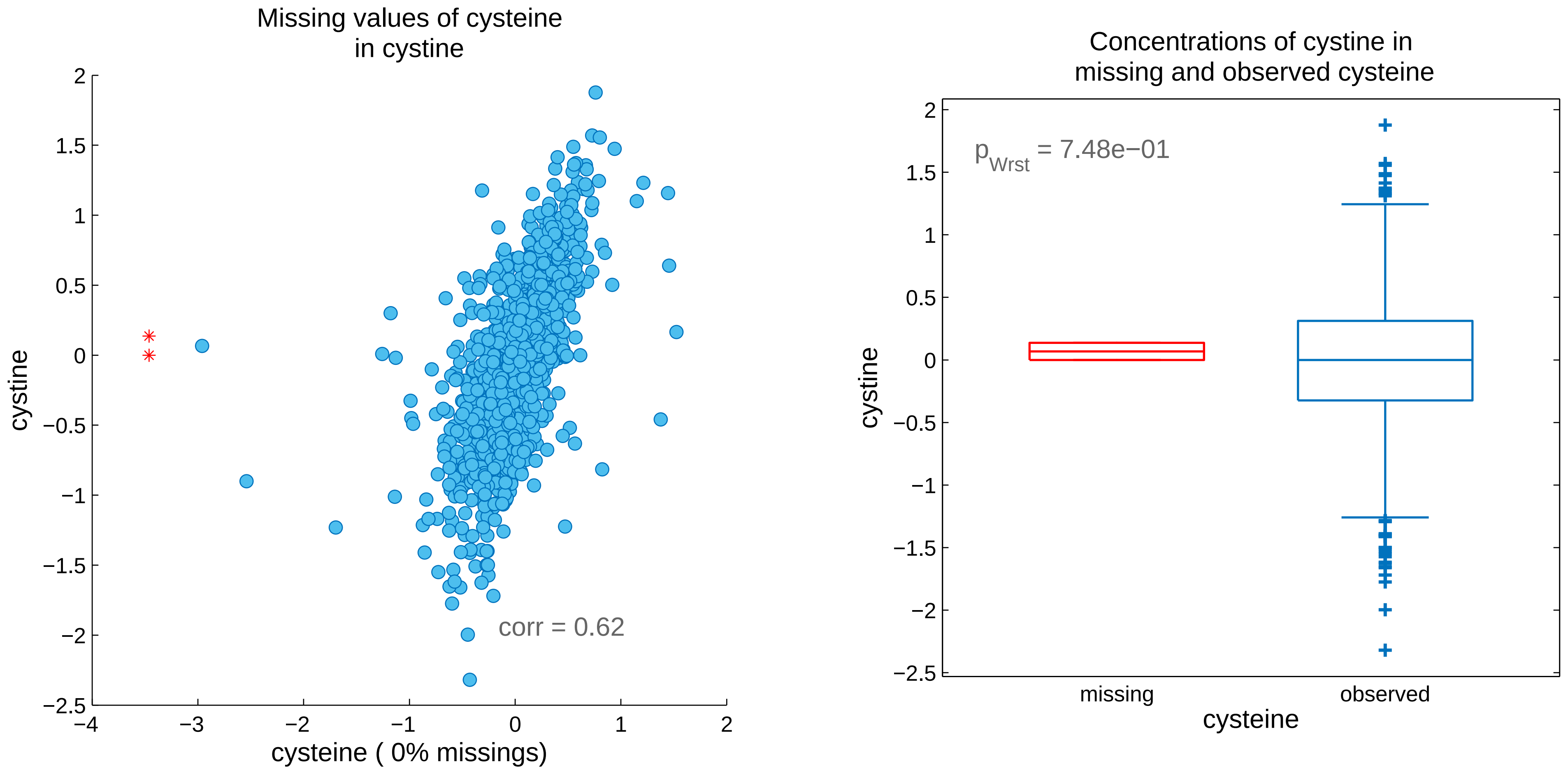
Missing values of cysteine
in cystine
corr = 0.62
cystine
cysteine (0% missings)
Concentrations of cystine in
missing and observed cysteine
p_{Wrst} = 7.48e−01
cystine
missing
observed
cysteine

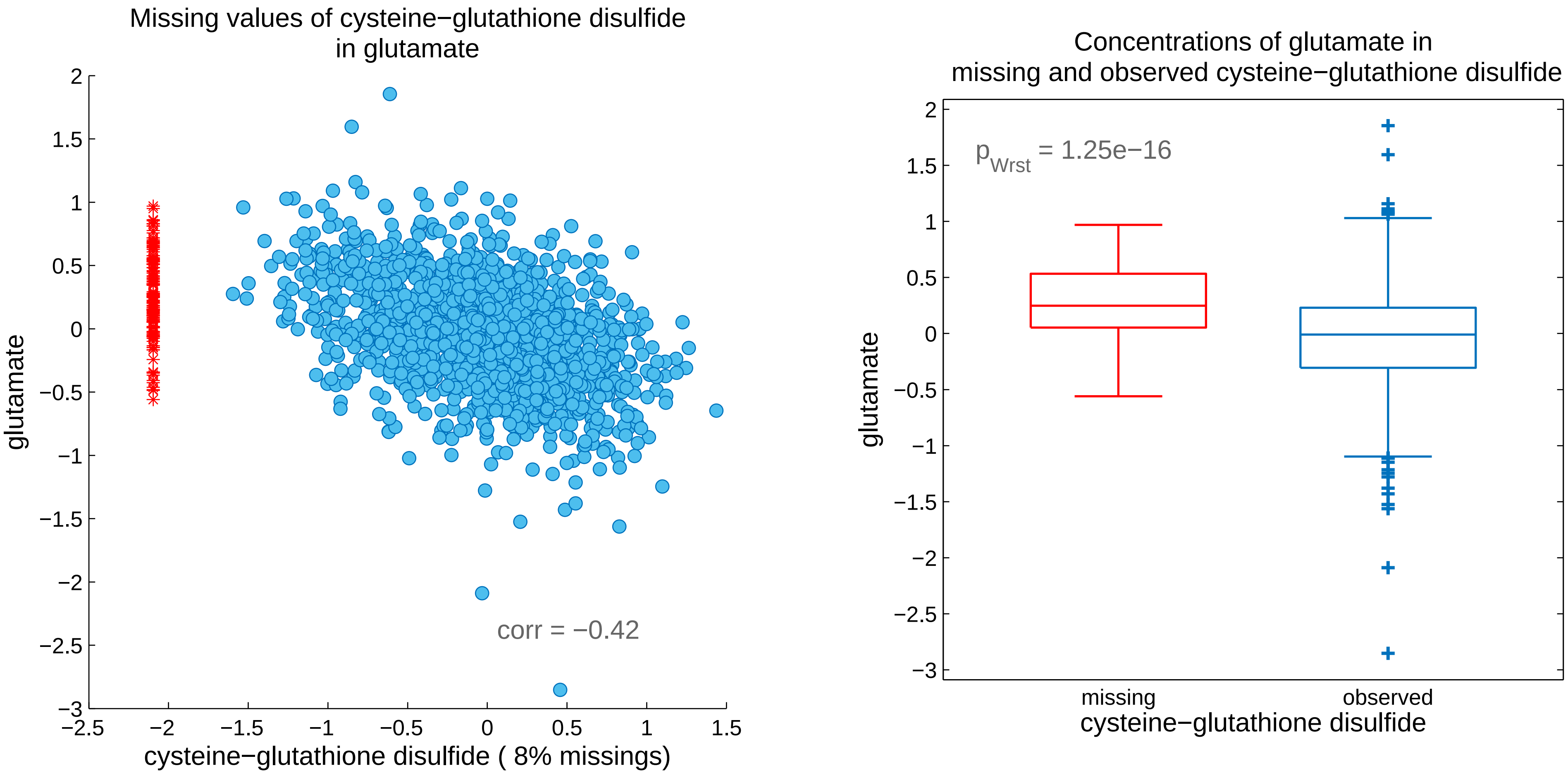

Missing values of cysteine−glutathione disulfide in glutamate
glutamate
cysteine−glutathione disulfide (8% missings)
corr = −0.42
Concentrations of glutamate in missing and observed cysteine−glutathione disulfide
p_{Wrst} = 1.25e−16
glutamate
missing
observed
cysteine−glutathione disulfide

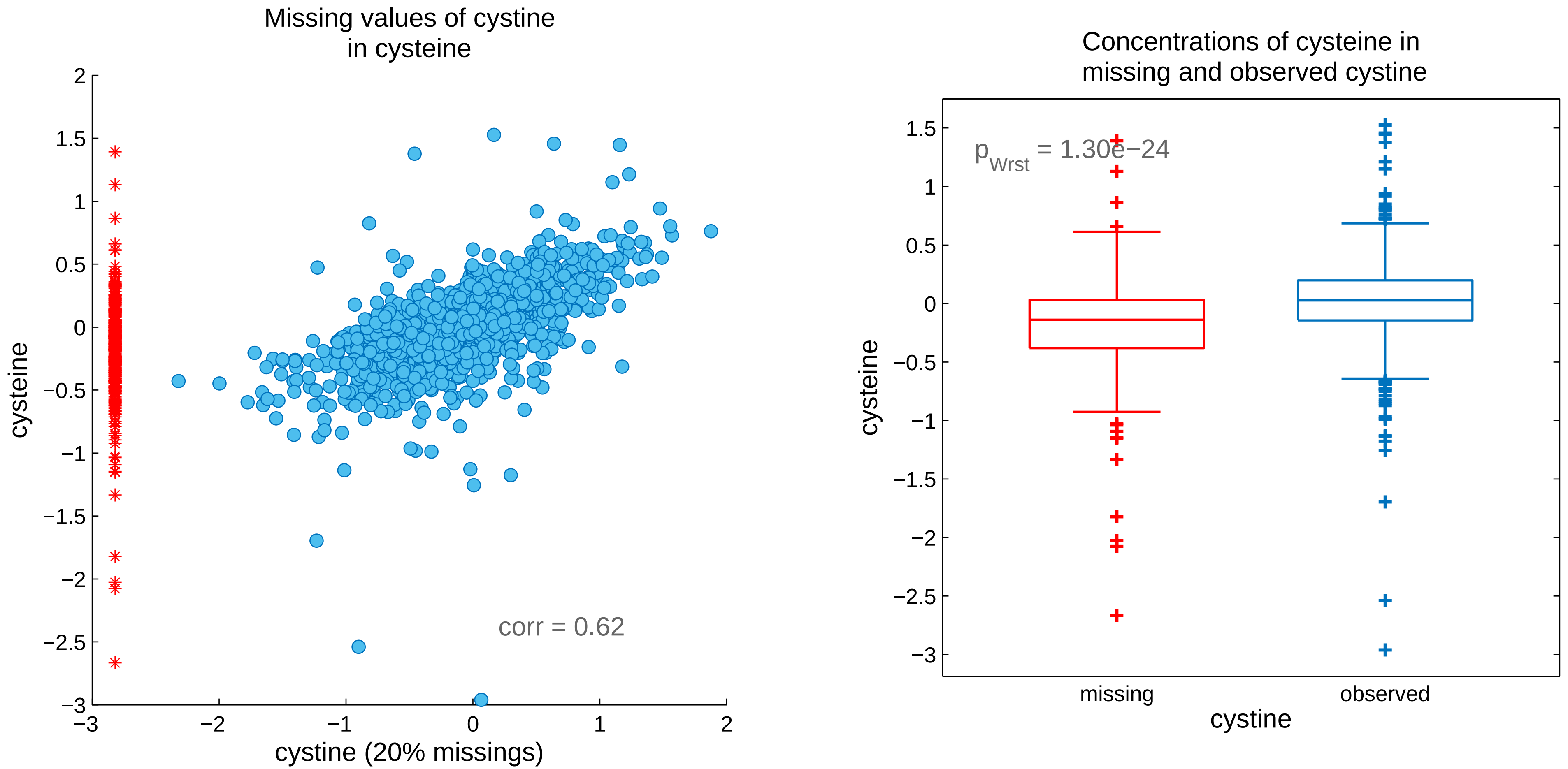
Missing values of cystine
in cysteine
cysteine
cystine (20% missings)
corr = 0.62
Concentrations of cysteine in
missing and observed cystine
p_Wrst = 1.30e−24
cysteine
missing
observed
cystine

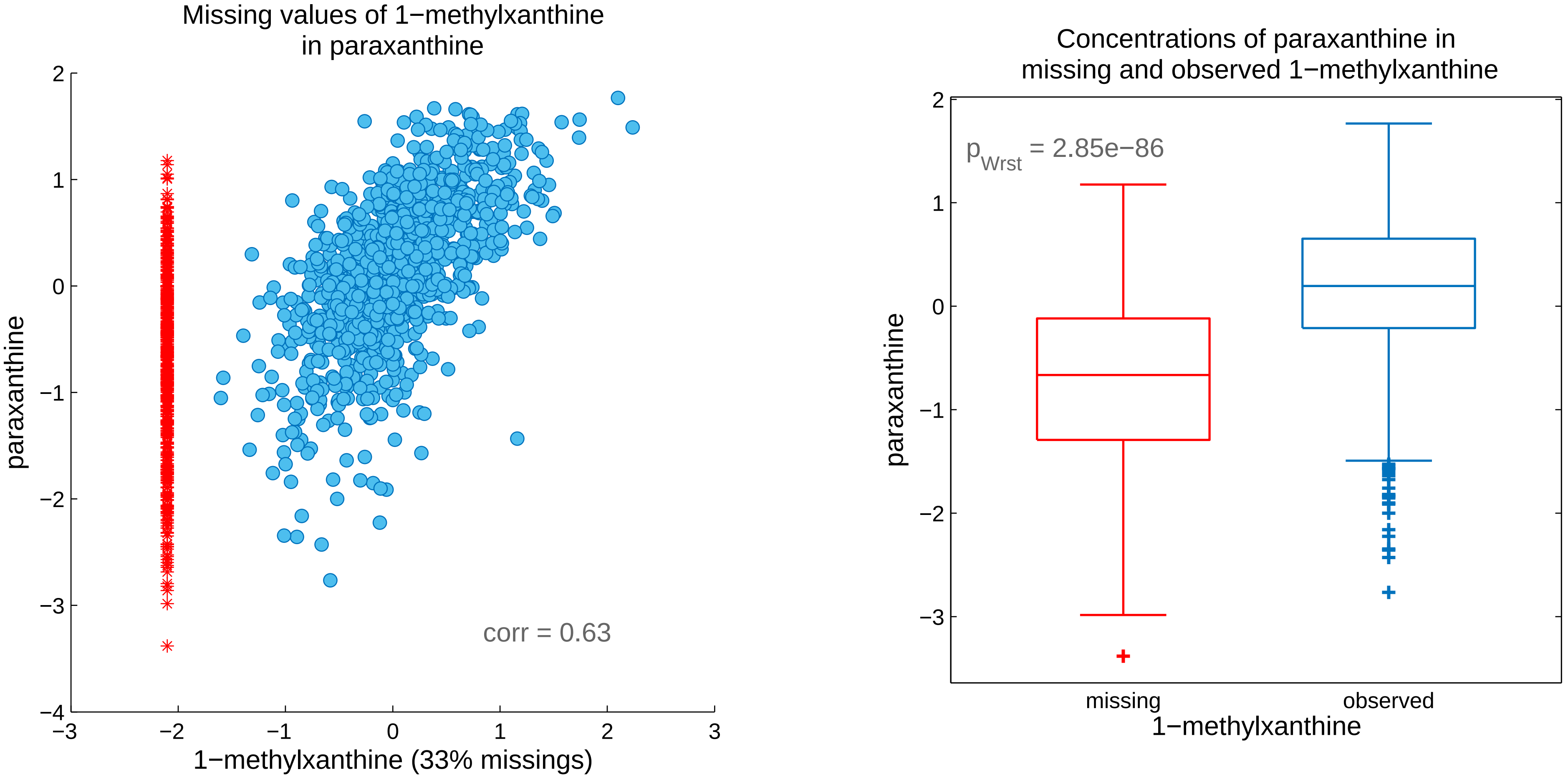
Missing values of 1−methylxanthine in paraxanthine
paraxanthine
1−methylxanthine (33% missings)
corr = 0.63
2
1
0
−1
−2
−3
−4
−3
−2
−1
0
1
2
3
Concentrations of paraxanthine in missing and observed 1−methylxanthine
p_{Wrst} = 2.85e−86
paraxanthine
missing
observed
1−methylxanthine

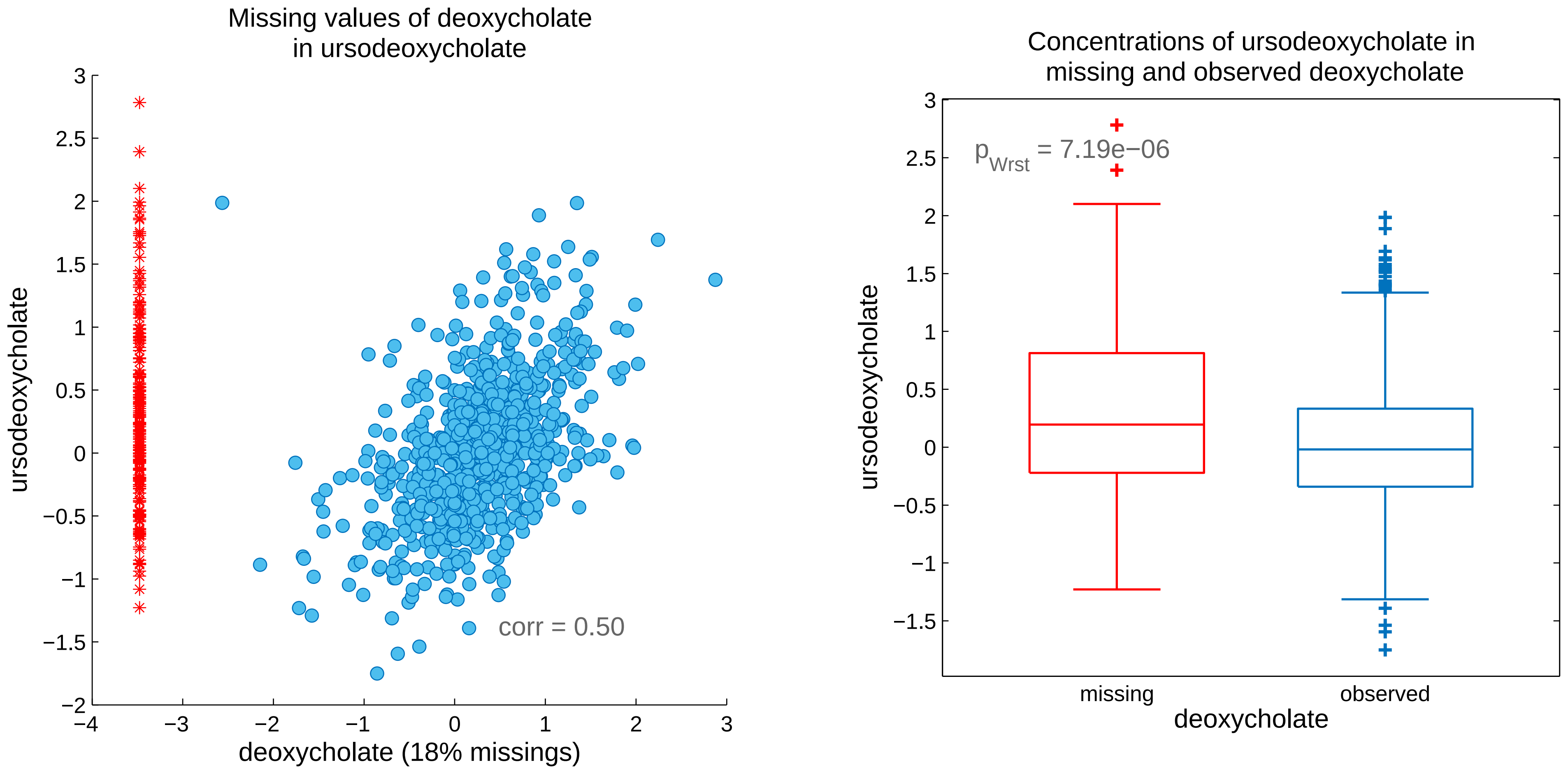
Missing values of deoxycholate in ursodeoxycholate
ursodeoxycholate
deoxycholate (18% missings)
corr = 0.50
3
2.5
2
1.5
1
0.5
0
−0.5
−1
−1.5
−2
−4
−3
−2
−1
0
1
2
3
Concentrations of ursodeoxycholate in missing and observed deoxycholate
p_{Wrst} = 7.19e−06
ursodeoxycholate
deoxycholate
missing
observed
3
2.5
2
1.5
1
0.5
0
−0.5
−1
−1.5

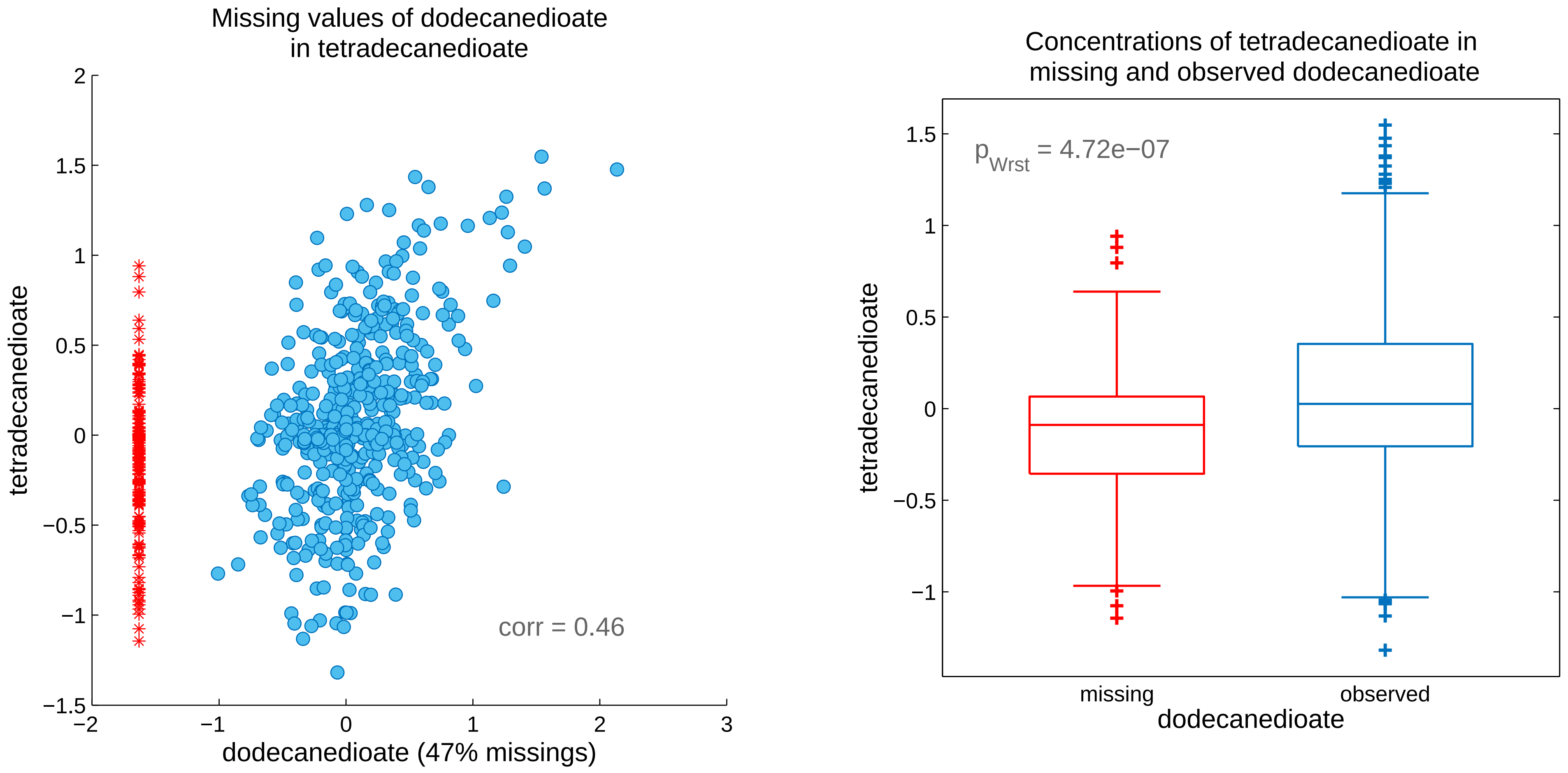
Missing values of dodecanedioate
in tetradecanedioate
tetradecanedioate
dodecanedioate (47% missings)
corr = 0.46
2
1.5
1
0.5
0
−0.5
−1
−1.5
−2
−1
0
1
2
3
Concentrations of tetradecanedioate in
missing and observed dodecanedioate
p_{Wrst} = 4.72e−07
tetradecanedioate
1.5
1
0.5
0
−0.5
−1
missing
observed
dodecanedioate

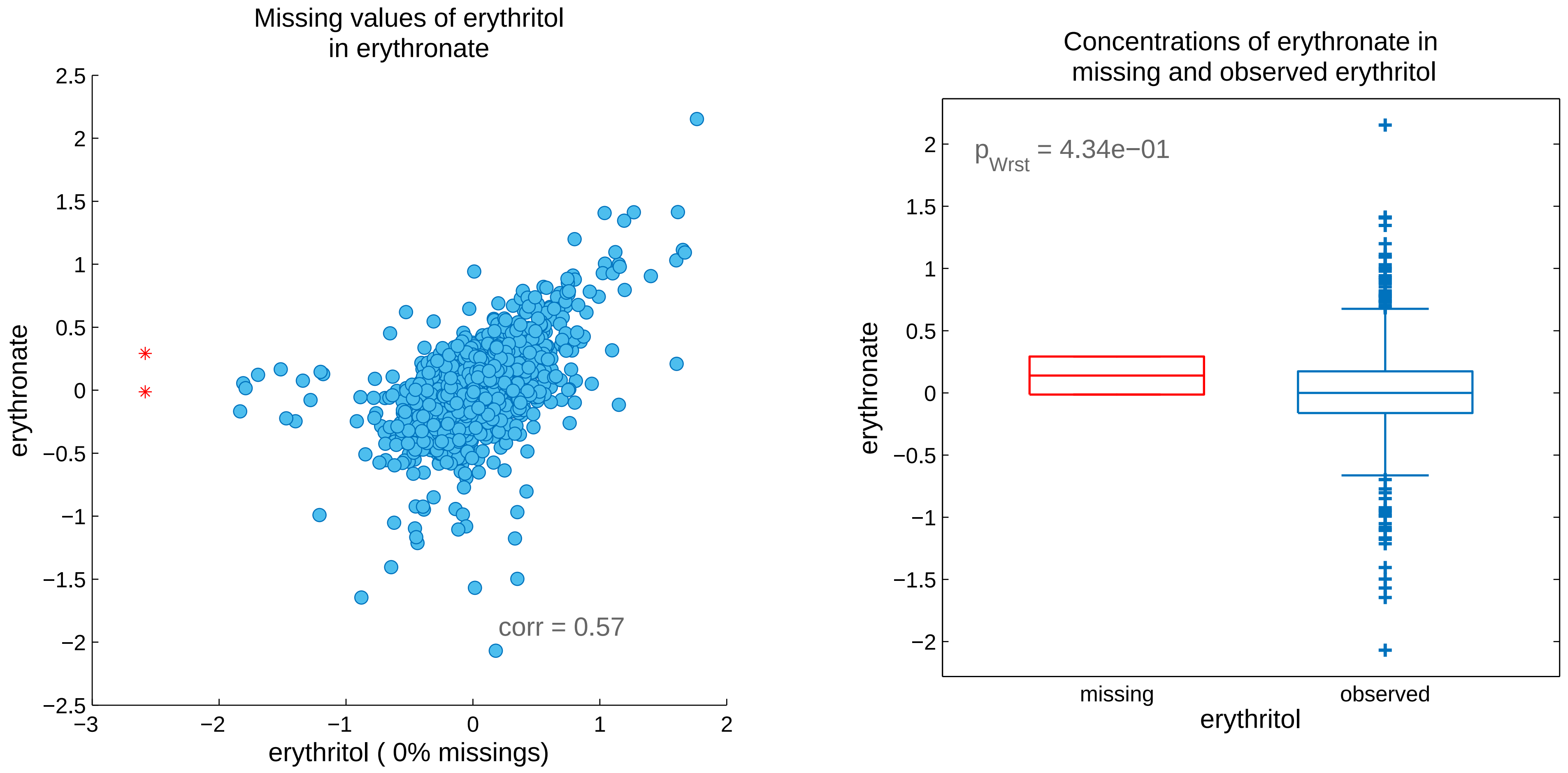

Missing values of erythritol in erythronate
erythronate
erythritol (0% missings)
corr = 0.57
Concentrations of erythronate in missing and observed erythritol
p_{Wrst} = 4.34e−01
missing
observed
erythritol

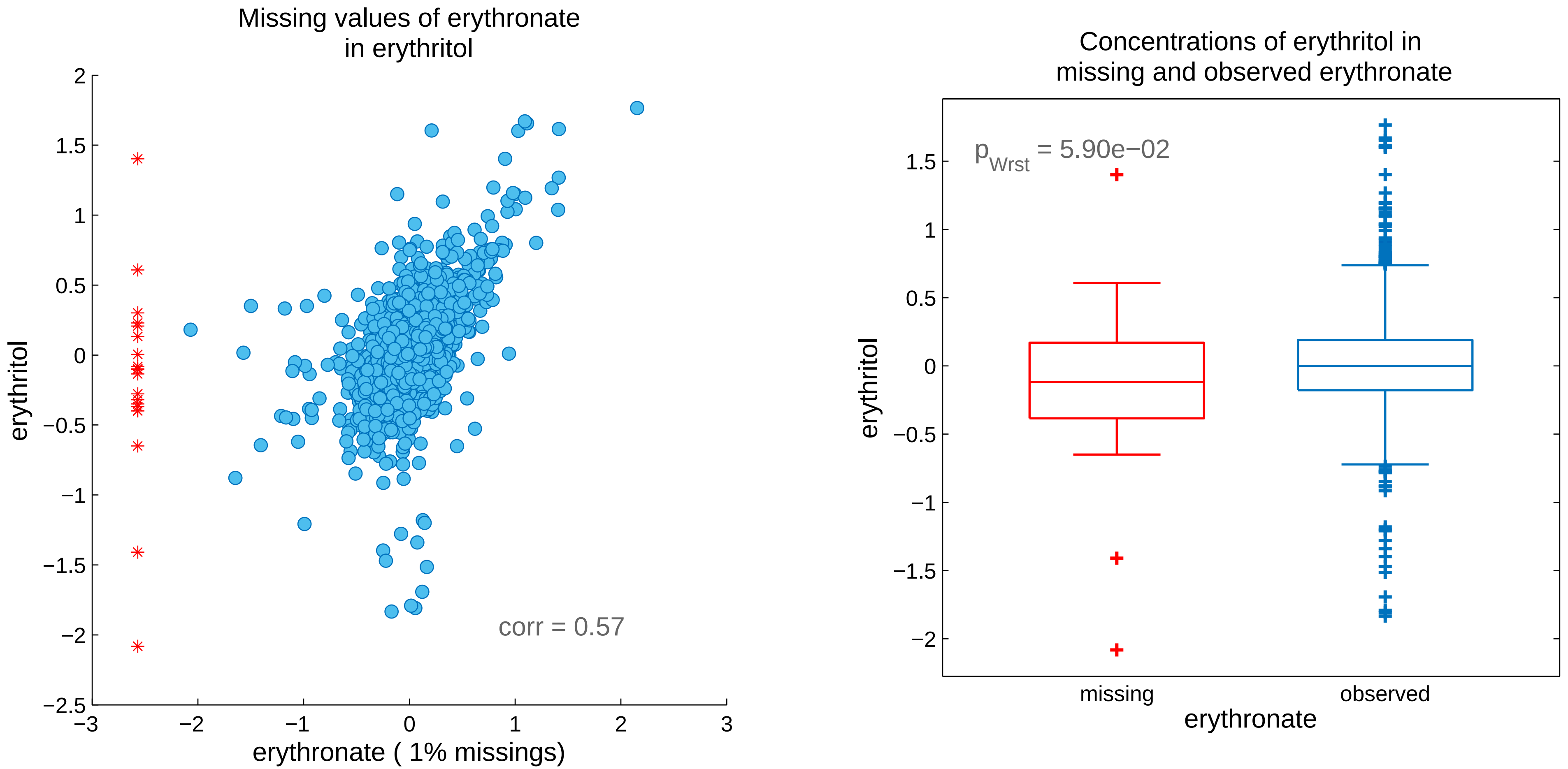
Missing values of erythronate in erythritol
erythritol
erythronate (1% missings)
corr = 0.57
2
1.5
1
0.5
0
−0.5
−1
−1.5
−2
−2.5
−3
−2
−1
0
1
2
3
Concentrations of erythritol in missing and observed erythronate
p_{Wrst} = 5.90e−02
erythritol
erythronate
missing
observed

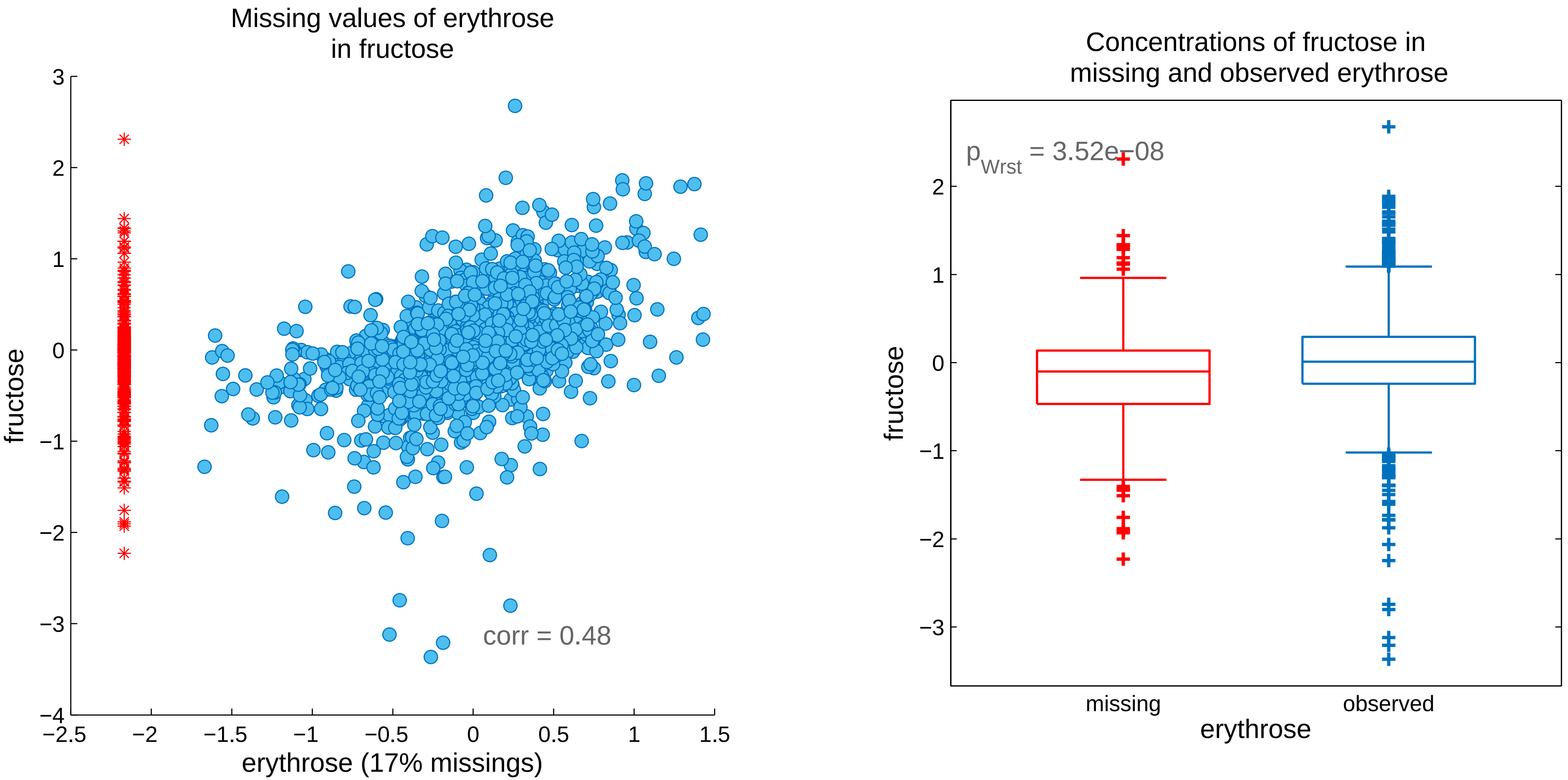

Missing values of erythrose
in fructose
fructose
erythrose (17% missings)
corr = 0.48
3
2
1
0
−1
−2
−3
−4
−2.5
−2
−1.5
−1
−0.5
0
0.5
1
1.5
Concentrations of fructose in
missing and observed erythrose
p_Wrst = 3.52e−08
fructose
2
1
0
−1
−2
−3
missing
observed
erythrose

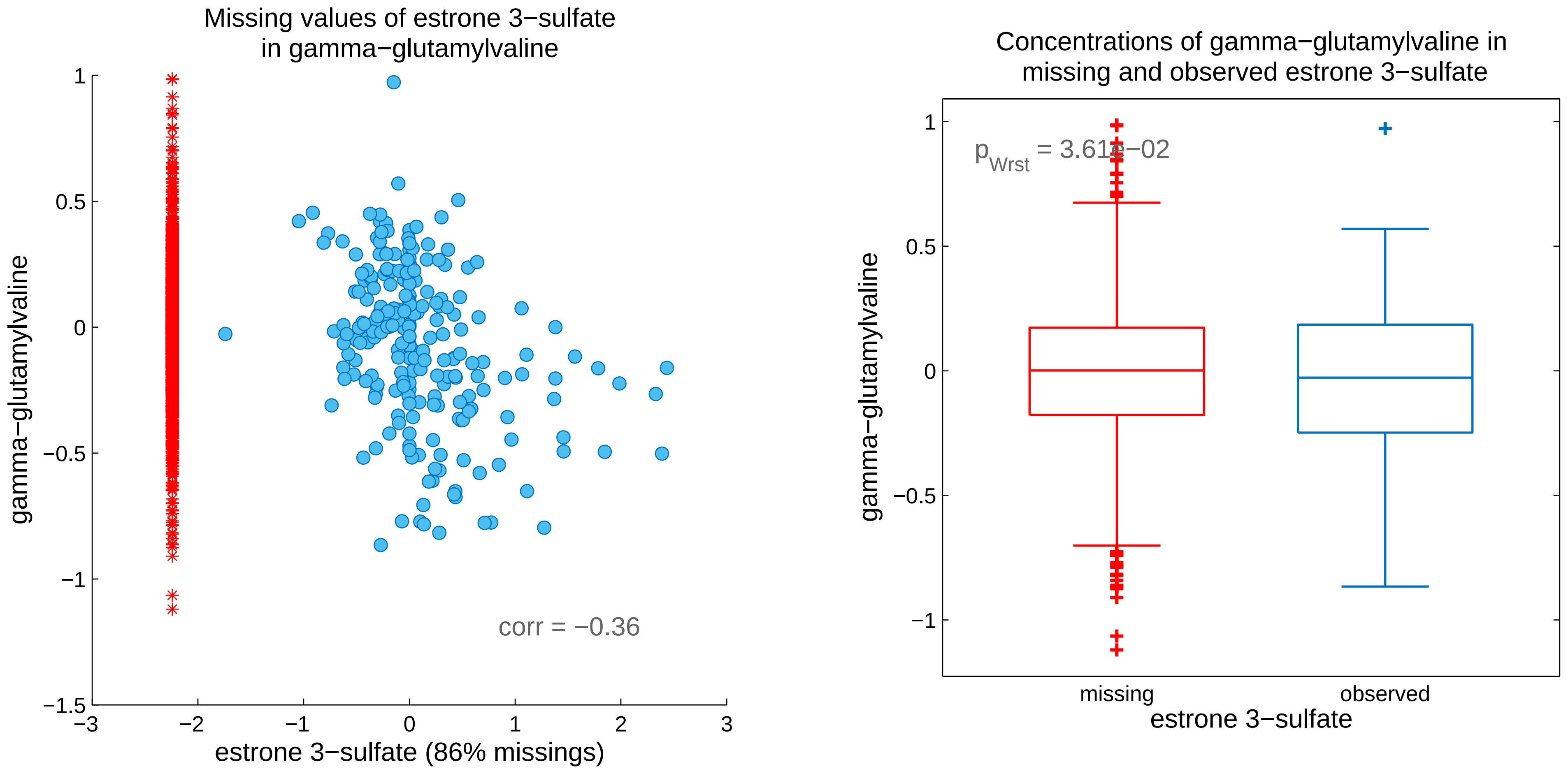

Missing values of estrone 3−sulfate
in gamma−glutamylvaline
gamma−glutamylvaline
estrone 3−sulfate (86% missings)
corr = −0.36
Concentrations of gamma−glutamylvaline in
missing and observed estrone 3−sulfate
p_Wrst = 3.61e−02
gamma−glutamylvaline
missing
observed
estrone 3−sulfate

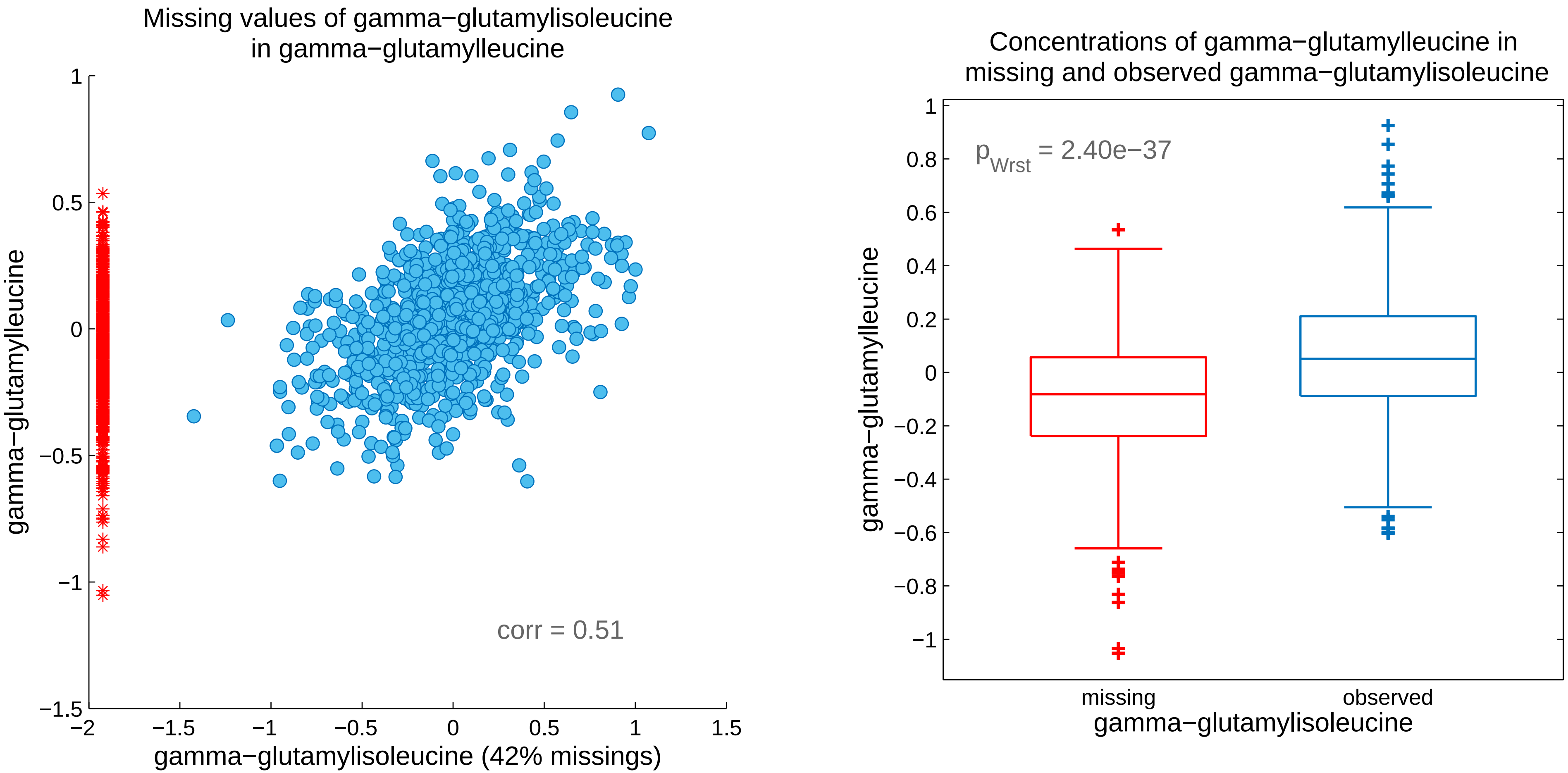
Missing values of gamma−glutamylisoleucine in gamma−glutamylleucine
gamma−glutamylleucine
gamma−glutamylisoleucine (42% missings)
corr = 0.51
1
0.5
0
−0.5
−1
−1.5
−2
−1.5
−1
−0.5
0
0.5
1
1.5
Concentrations of gamma−glutamylleucine in missing and observed gamma−glutamylisoleucine
p_{Wrst} = 2.40e−37
gamma−glutamylleucine
1
0.8
0.6
0.4
0.2
0
−0.2
−0.4
−0.6
−0.8
−1
missing
observed
gamma−glutamylisoleucine

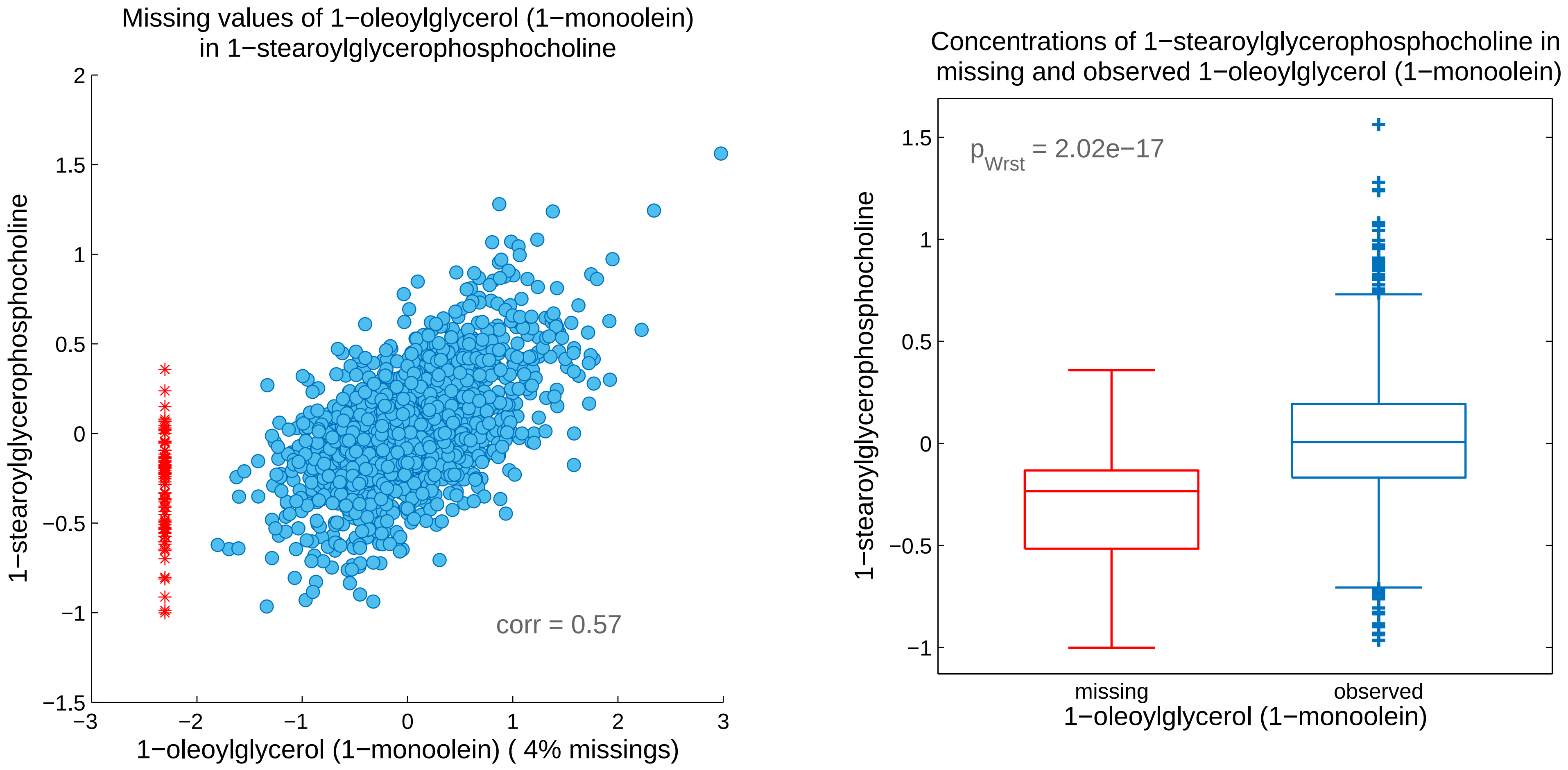
Missing values of 1−oleoylglycerol (1−monoolein) in 1−stearoylglycerophosphocholine
1−stearoylglycerophosphocholine
1−oleoylglycerol (1−monoolein) (4% missings)
corr = 0.57
2
1.5
1
0.5
0
−0.5
−1
−1.5
−3
−2
−1
0
1
2
3
Concentrations of 1−stearoylglycerophosphocholine in missing and observed 1−oleoylglycerol (1−monoolein)
p_Wrst = 2.02e−17
1.5
1
0.5
0
−0.5
−1
missing
observed
1−oleoylglycerol (1−monoolein)

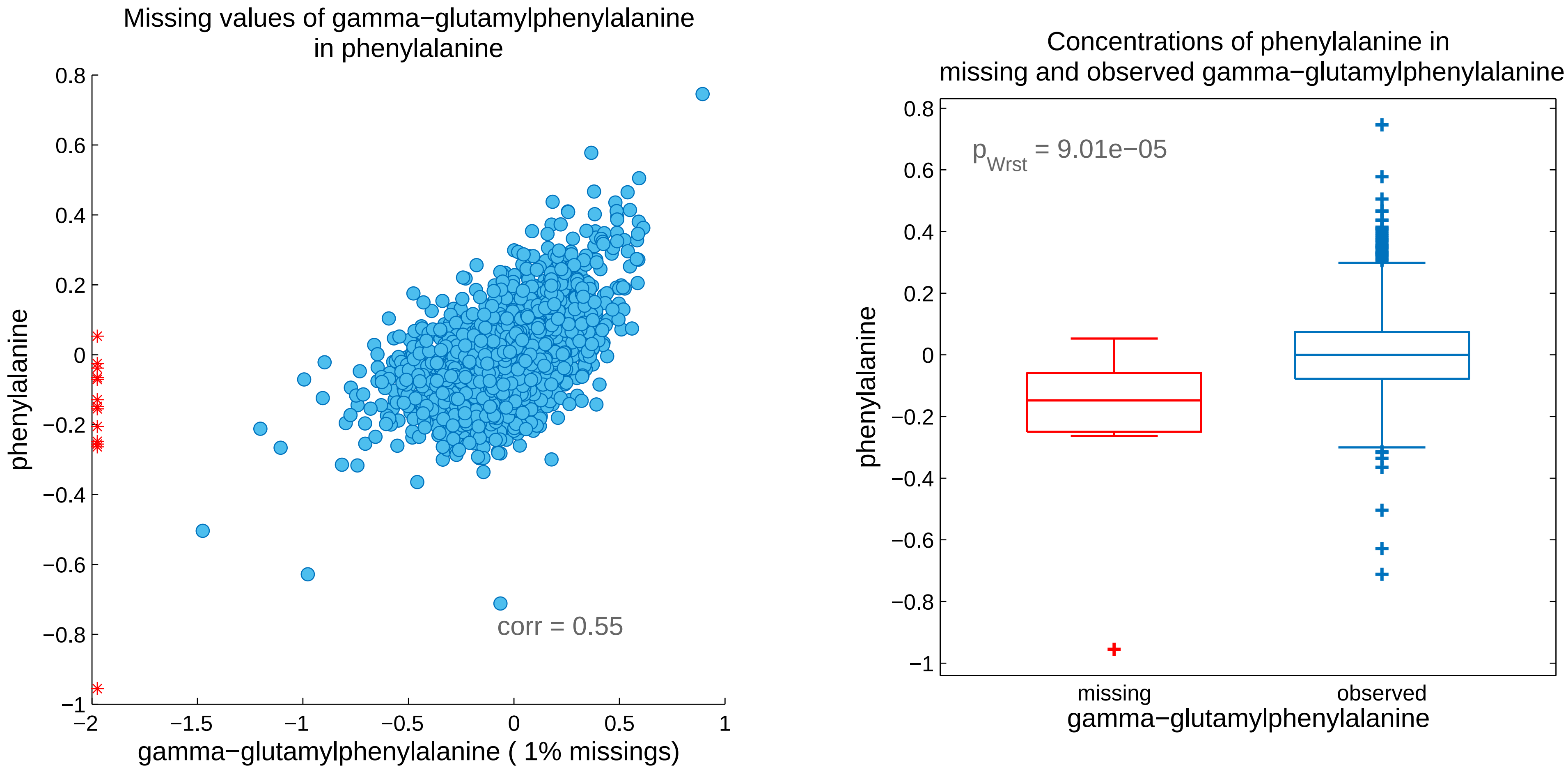

Missing values of gamma−glutamylphenylalanine in phenylalanine
phenylalanine
gamma−glutamylphenylalanine (1% missings)
corr = 0.55
0.8
0.6
0.4
0.2
0
−0.2
−0.4
−0.6
−0.8
−1
−2
−1.5
−1
−0.5
0
0.5
1
Concentrations of phenylalanine in missing and observed gamma−glutamylphenylalanine
phenylalanine
p_{Wrst} = 9.01e−05
missing
observed
gamma−glutamylphenylalanine

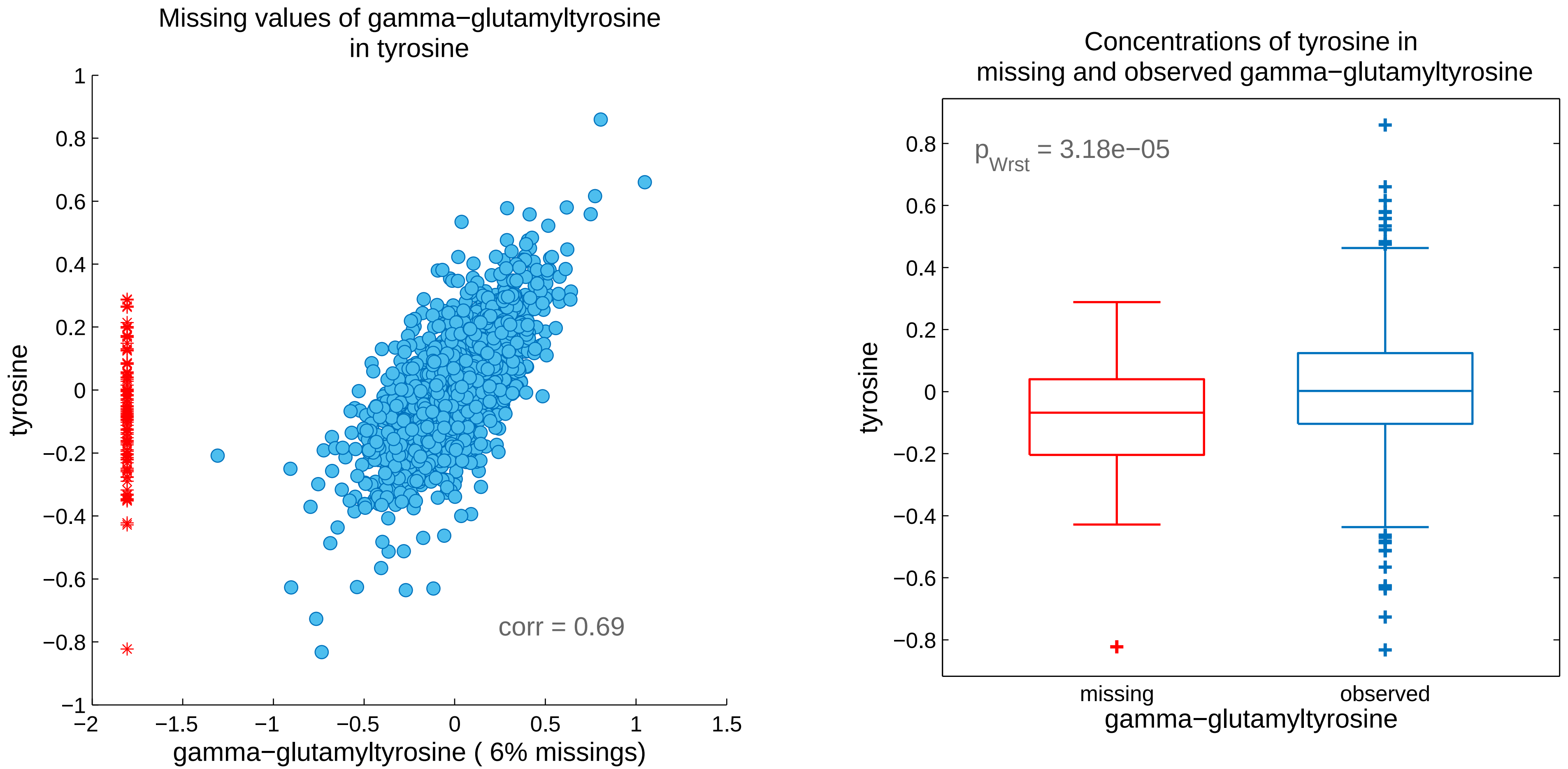
Missing values of gamma−glutamyltyrosine
in tyrosine
tyrosine
gamma−glutamyltyrosine (6% missings)
corr = 0.69
1
0.8
0.6
0.4
0.2
0
−0.2
−0.4
−0.6
−0.8
−1
−2
−1.5
−1
−0.5
0
0.5
1
1.5
Concentrations of tyrosine in
missing and observed gamma−glutamyltyrosine
p Wrst = 3.18e−05
tyrosine
missing
observed
gamma−glutamyltyrosine

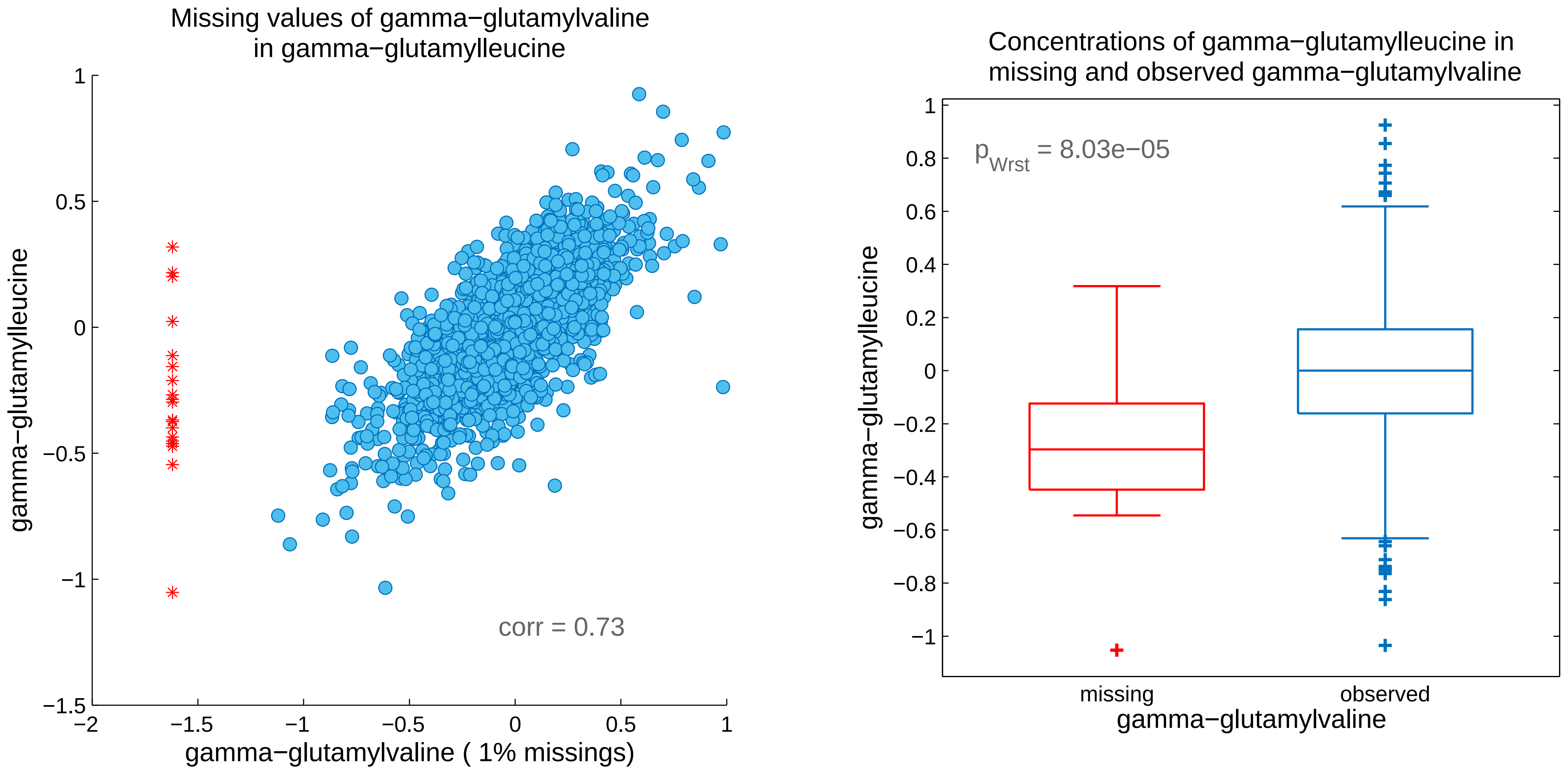
Missing values of gamma−glutamylvaline
in gamma−glutamylleucine
gamma−glutamylleucine
gamma−glutamylvaline (1% missings)
corr = 0.73
1
0.5
0
−0.5
−1
−1.5
−2
−1.5
−1
−0.5
0
0.5
1
Concentrations of gamma−glutamylleucine in
missing and observed gamma−glutamylvaline
p_{Wrst} = 8.03e−05
gamma−glutamylleucine
gamma−glutamylvaline
missing
observed
1
0.8
0.6
0.4
0.2
0
−0.2
−0.4
−0.6
−0.8
−1

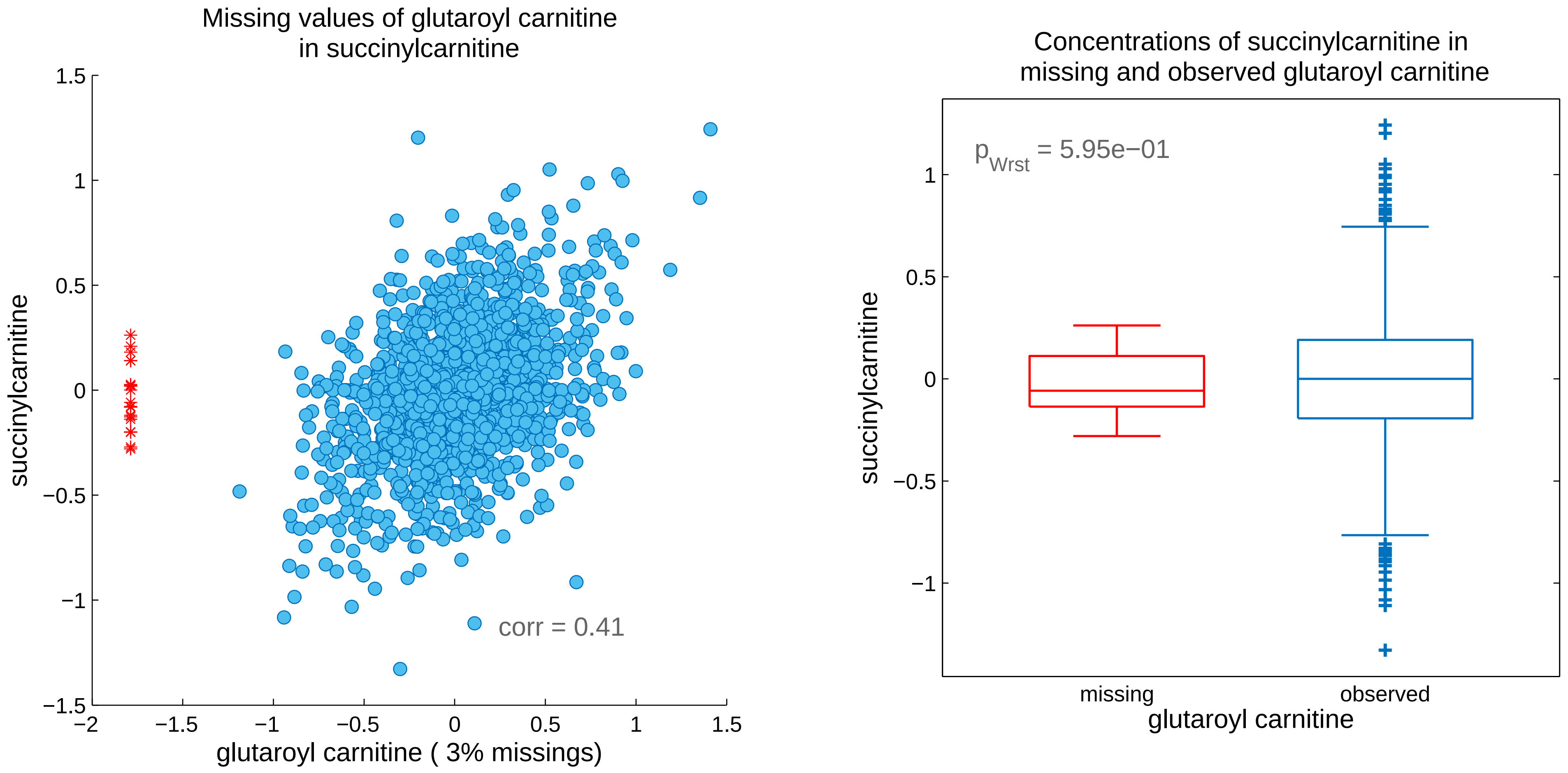
Missing values of glutaroyl carnitine
in succinylcarnitine
succinylcarnitine
glutaroyl carnitine (3% missings)
corr = 0.41
1.5
1
0.5
0
−0.5
−1
−1.5
−2
−1.5
−1
−0.5
0
0.5
1
1.5
Concentrations of succinylcarnitine in
missing and observed glutaroyl carnitine
p_{Wrst} = 5.95e−01
succinylcarnitine
1
0.5
0
−0.5
−1
missing
observed
glutaroyl carnitine

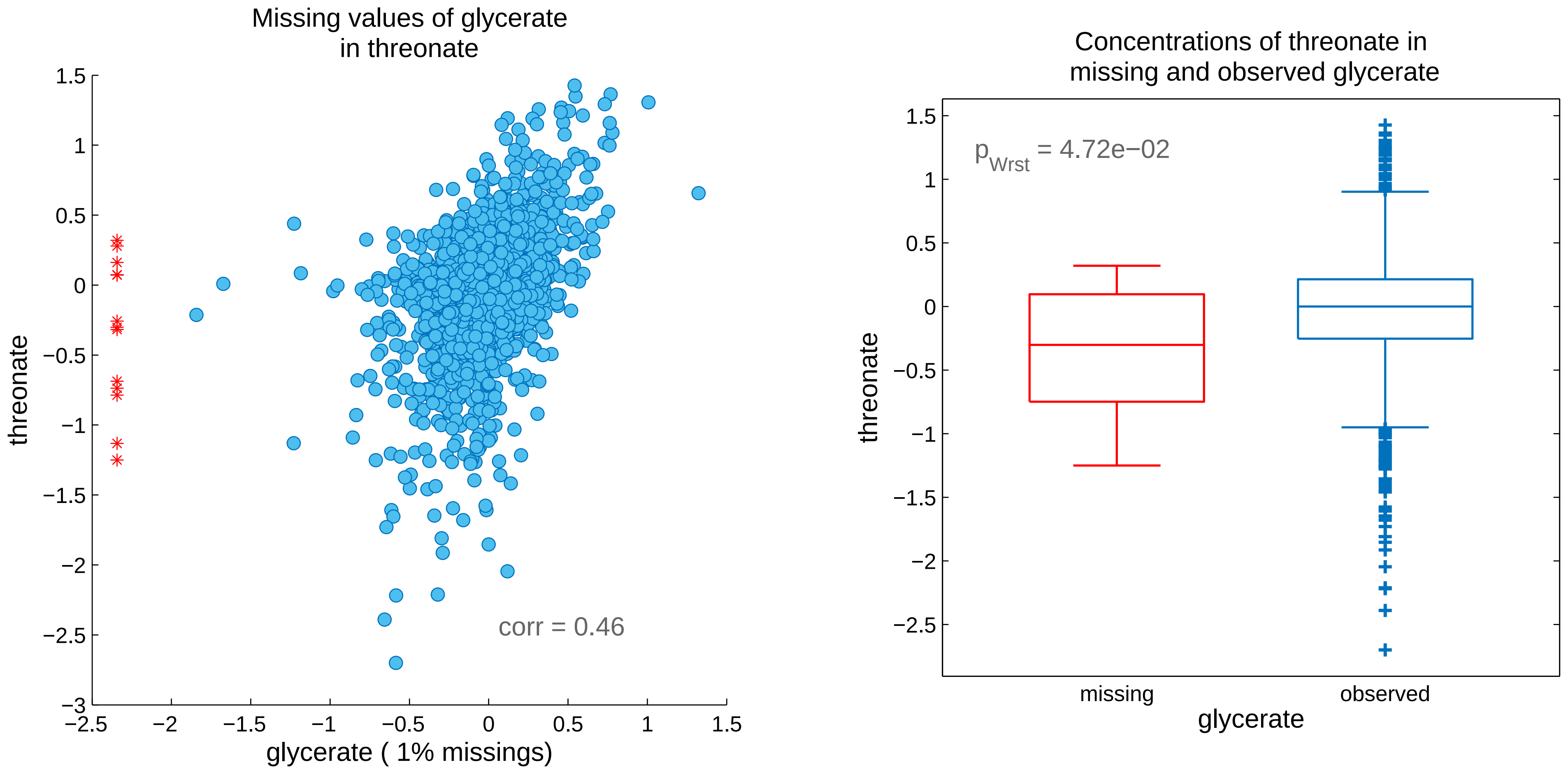

Missing values of glycerate in threonate
threonate
glycerate (1% missings)
corr = 0.46
1.5
1
0.5
0
−0.5
−1
−1.5
−2
−2.5
−3
−2.5
−2
−1.5
−1
−0.5
0
0.5
1
1.5
Concentrations of threonate in missing and observed glycerate
p_{Wrst} = 4.72e−02
threonate
missing
observed
glycerate

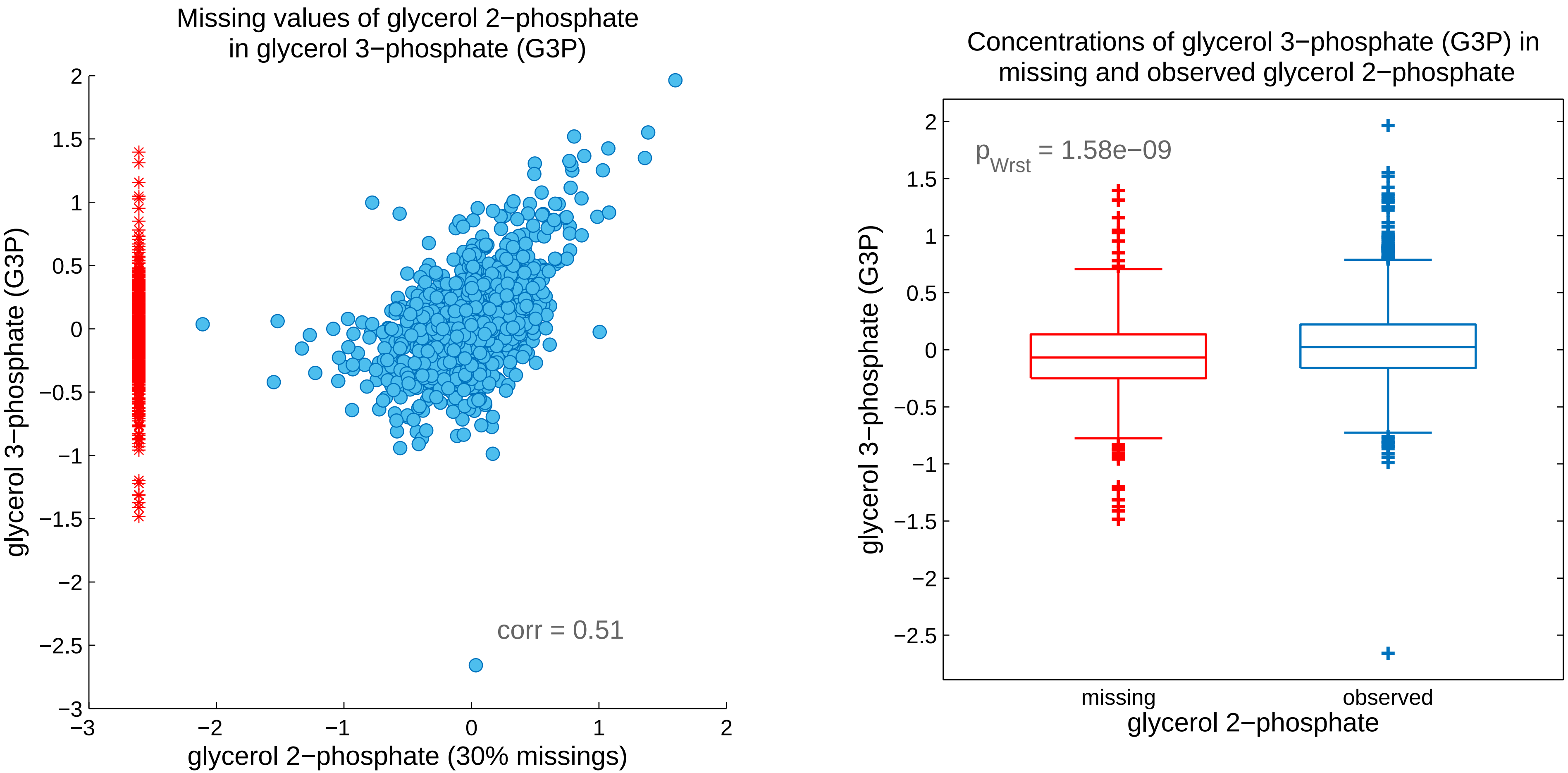
Missing values of glycerol 2−phosphate in glycerol 3−phosphate (G3P)
glycerol 3−phosphate (G3P)
glycerol 2−phosphate (30% missings)
corr = 0.51
Concentrations of glycerol 3−phosphate (G3P) in missing and observed glycerol 2−phosphate
p_Wrst = 1.58e−09
glycerol 3−phosphate (G3P)
missing
observed
glycerol 2−phosphate

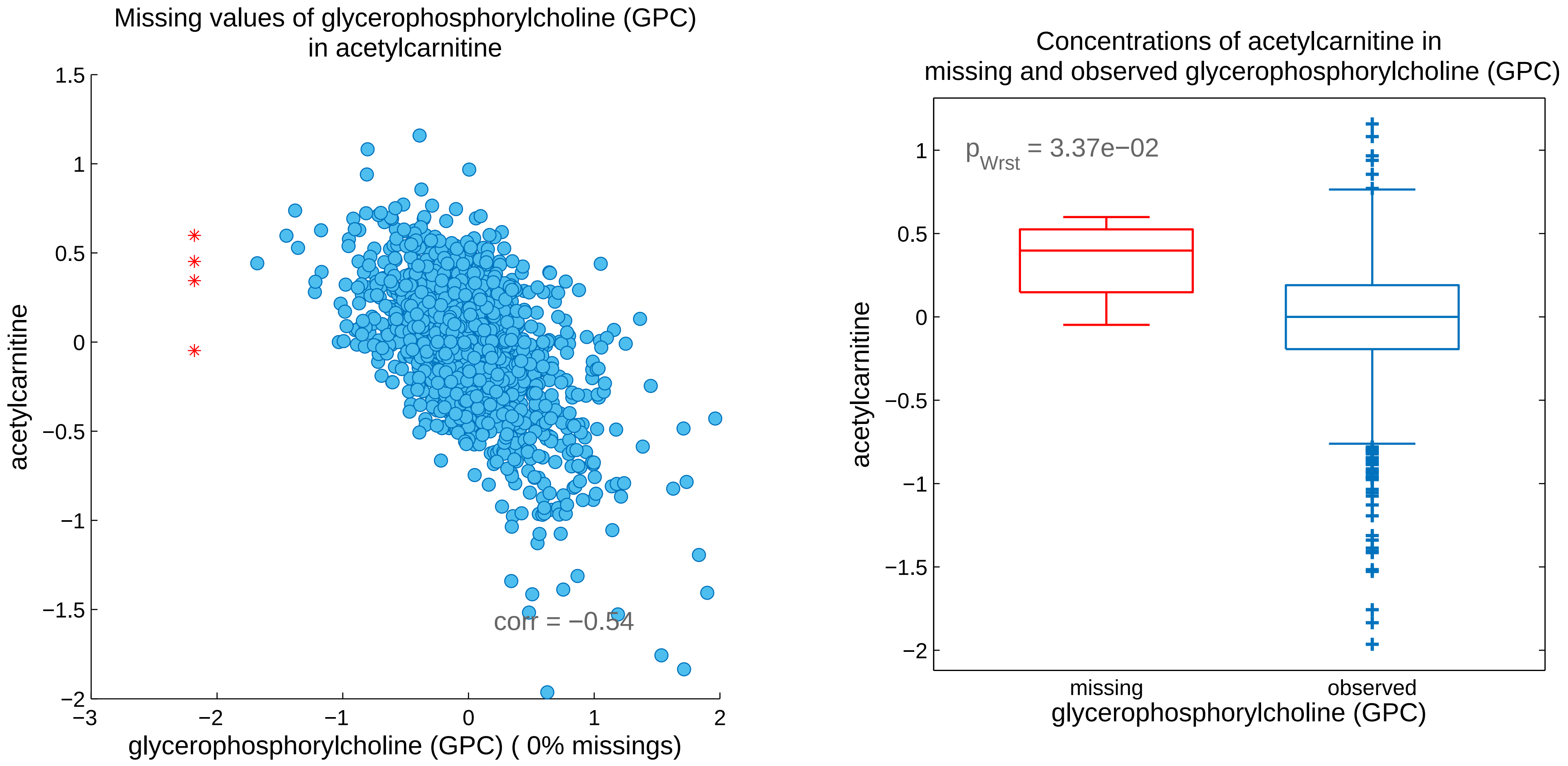

Missing values of glycerophosphorylcholine (GPC) in acetylcarnitine
acetylcarnitine
glycerophosphorylcholine (GPC) (0% missings)
corr = −0.54
Concentrations of acetylcarnitine in missing and observed glycerophosphorylcholine (GPC)
p_{Wrst} = 3.37e−02
acetylcarnitine
missing
observed
glycerophosphorylcholine (GPC)

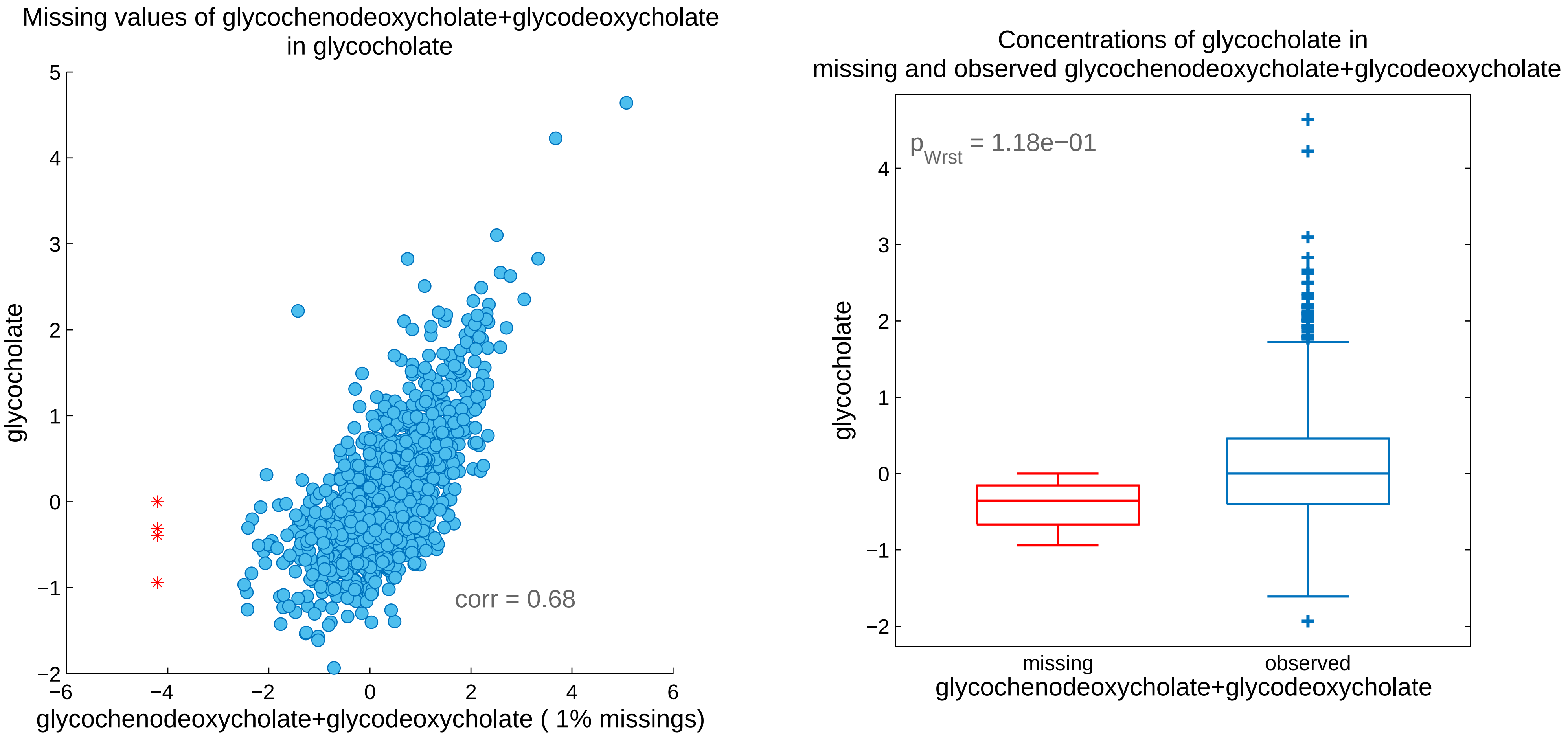
Missing values of glycochenodeoxycholate+glycodeoxycholate
in glycocholate
glycocholate
glycochenodeoxycholate+glycodeoxycholate (1% missings)
corr = 0.68
Concentrations of glycocholate in
missing and observed glycochenodeoxycholate+glycodeoxycholate
p Wrst = 1.18e−01
glycocholate
missing
observed
glycochenodeoxycholate+glycodeoxycholate

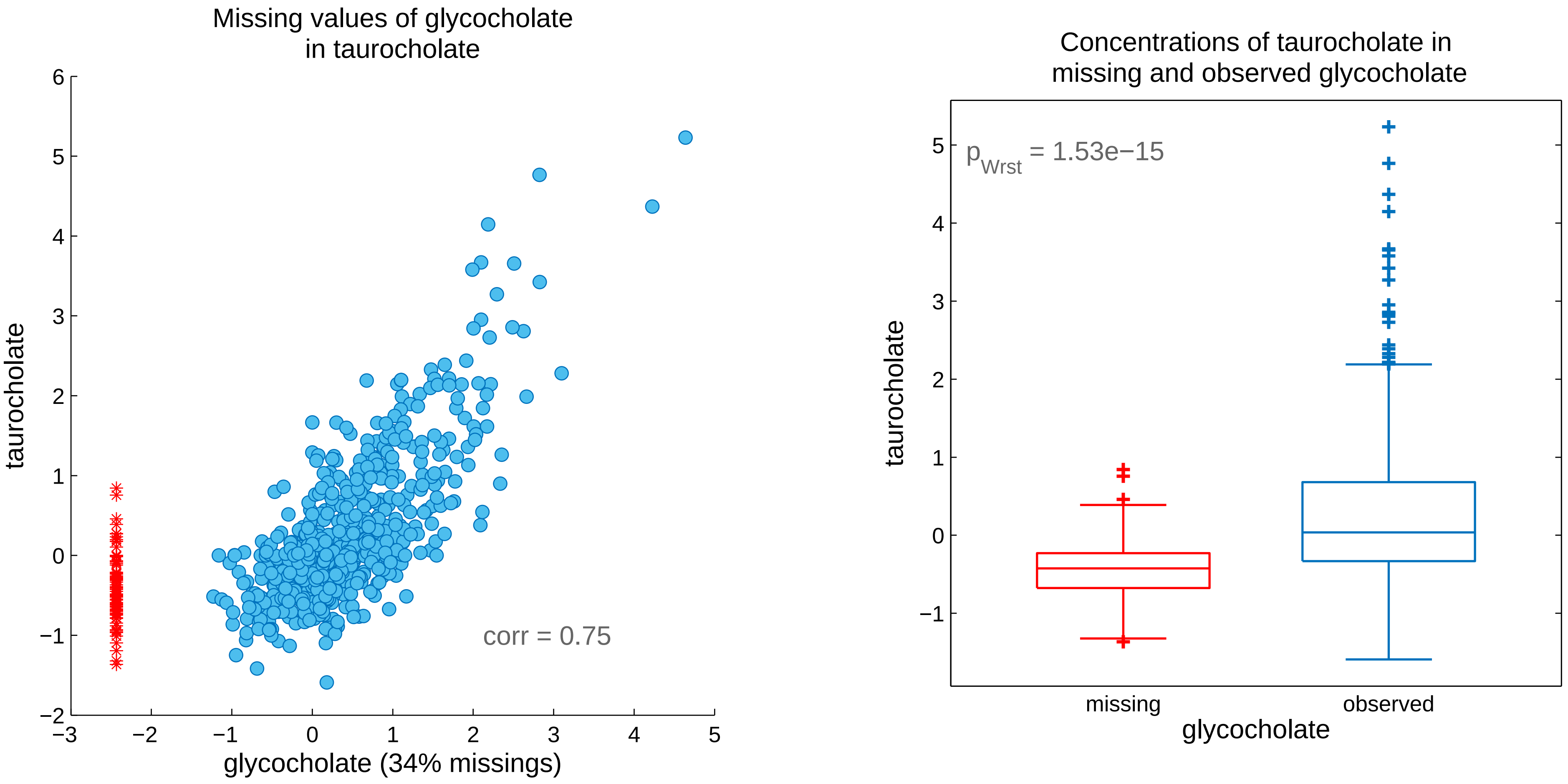

Missing values of glycocholate
in taurocholate
taurocholate
glycocholate (34% missings)
corr = 0.75
Concentrations of taurocholate in
missing and observed glycocholate
p_{Wrst} = 1.53e−15
taurocholate
missing
observed
glycocholate

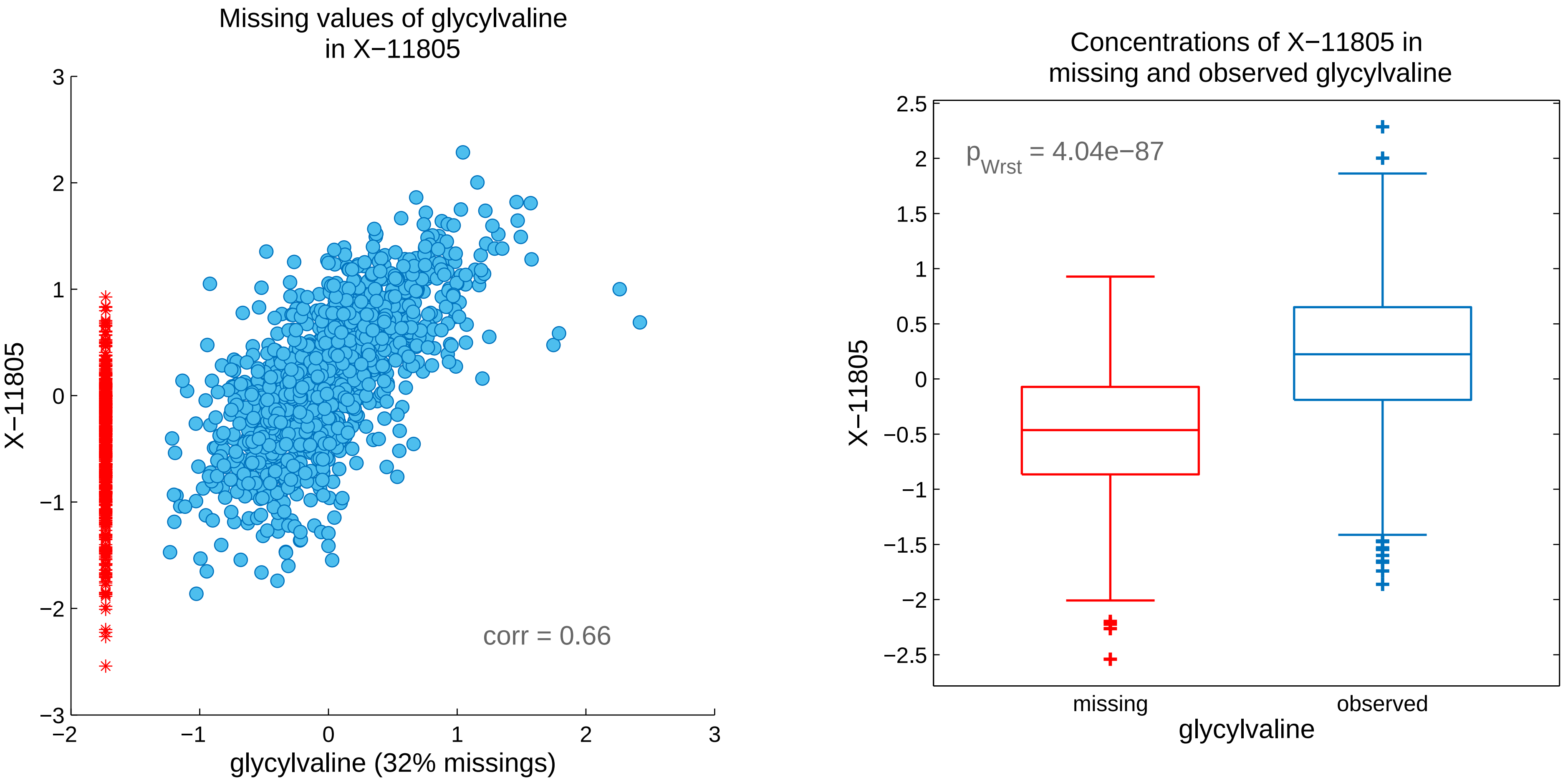

Missing values of glycylvaline in X−11805
X−11805
glycylvaline (32% missings)
corr = 0.66
Concentrations of X−11805 in missing and observed glycylvaline
X−11805
p_{Wrst} = 4.04e−87
missing
observed
glycylvaline

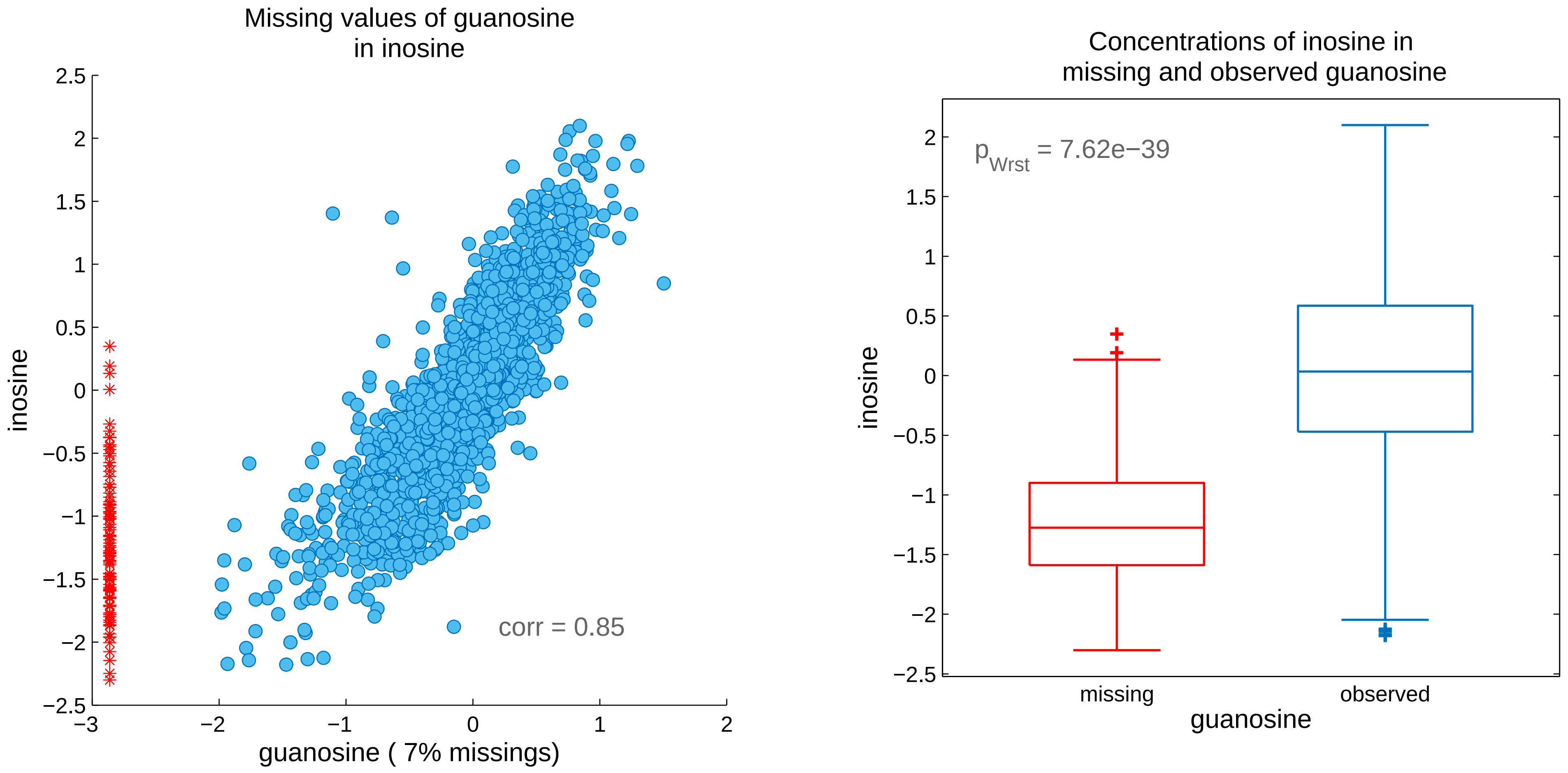
Missing values of guanosine
in inosine
inosine
guanosine (7% missings)
corr = 0.85
2.5
2
1.5
1
0.5
0
−0.5
−1
−1.5
−2
−2.5
−3
−2
−1
0
1
2
Concentrations of inosine in
missing and observed guanosine
p_{Wrst} = 7.62e−39
inosine
guanosine
missing
observed

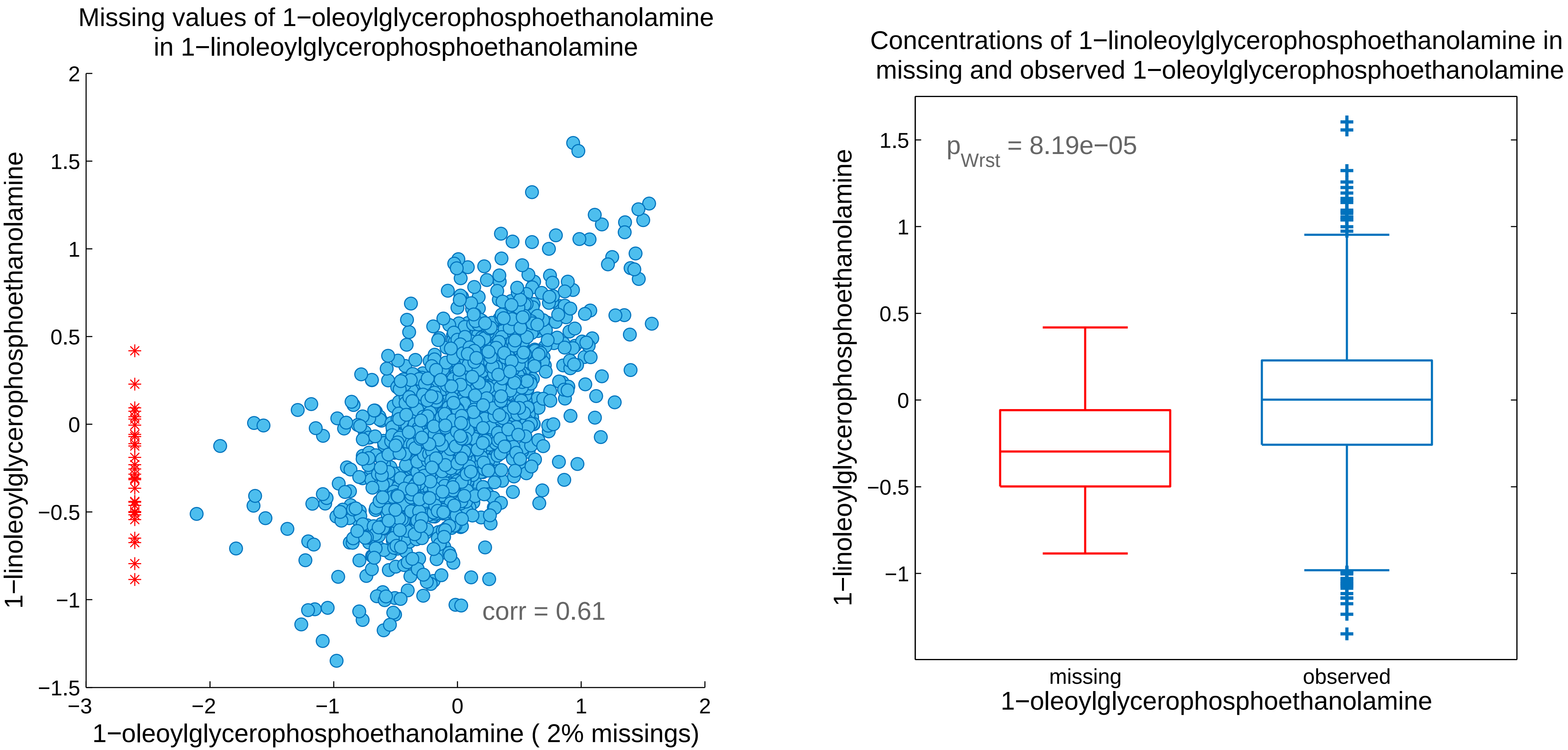
Missing values of 1−oleoylglycerophosphoethanolamine in 1−linoleoylglycerophosphoethanolamine
1−linoleoylglycerophosphoethanolamine
1−oleoylglycerophosphoethanolamine (2% missings)
corr = 0.61
2
1.5
1
0.5
0
−0.5
−1
−1.5
−3
−2
−1
0
1
2
Concentrations of 1−linoleoylglycerophosphoethanolamine in missing and observed 1−oleoylglycerophosphoethanolamine
p_{Wrst} = 8.19e−05
1−linoleoylglycerophosphoethanolamine
1.5
1
0.5
0
−0.5
−1
missing
observed
1−oleoylglycerophosphoethanolamine

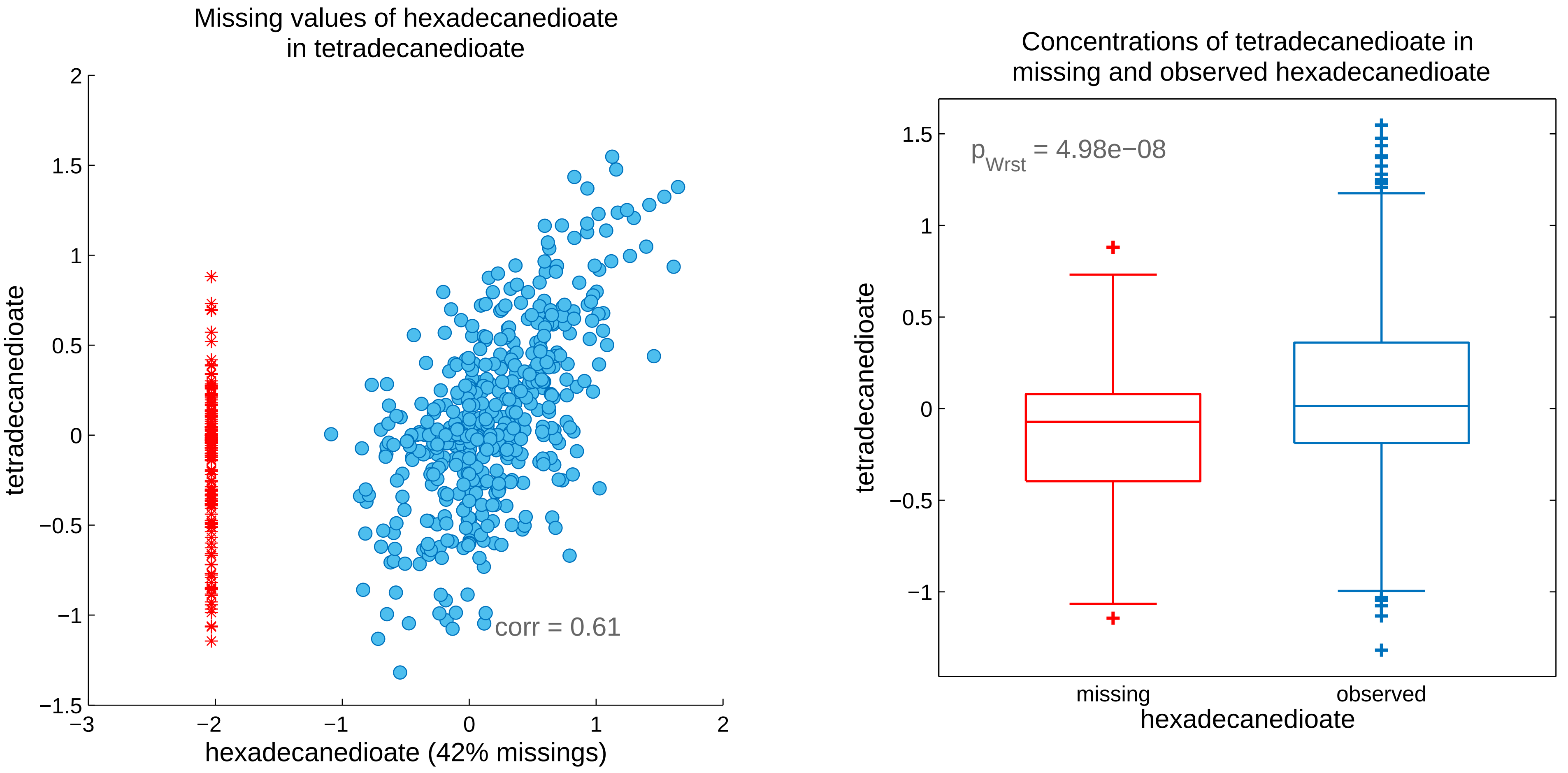
Missing values of hexadecanedioate in tetradecanedioate
tetradecanedioate
hexadecanedioate (42% missings)
corr = 0.61
2
1.5
1
0.5
0
−0.5
−1
−1.5
−3
−2
−1
0
1
2
Concentrations of tetradecanedioate in missing and observed hexadecanedioate
p_{Wrst} = 4.98e−08
tetradecanedioate
1.5
1
0.5
0
−0.5
−1
missing
observed
hexadecanedioate

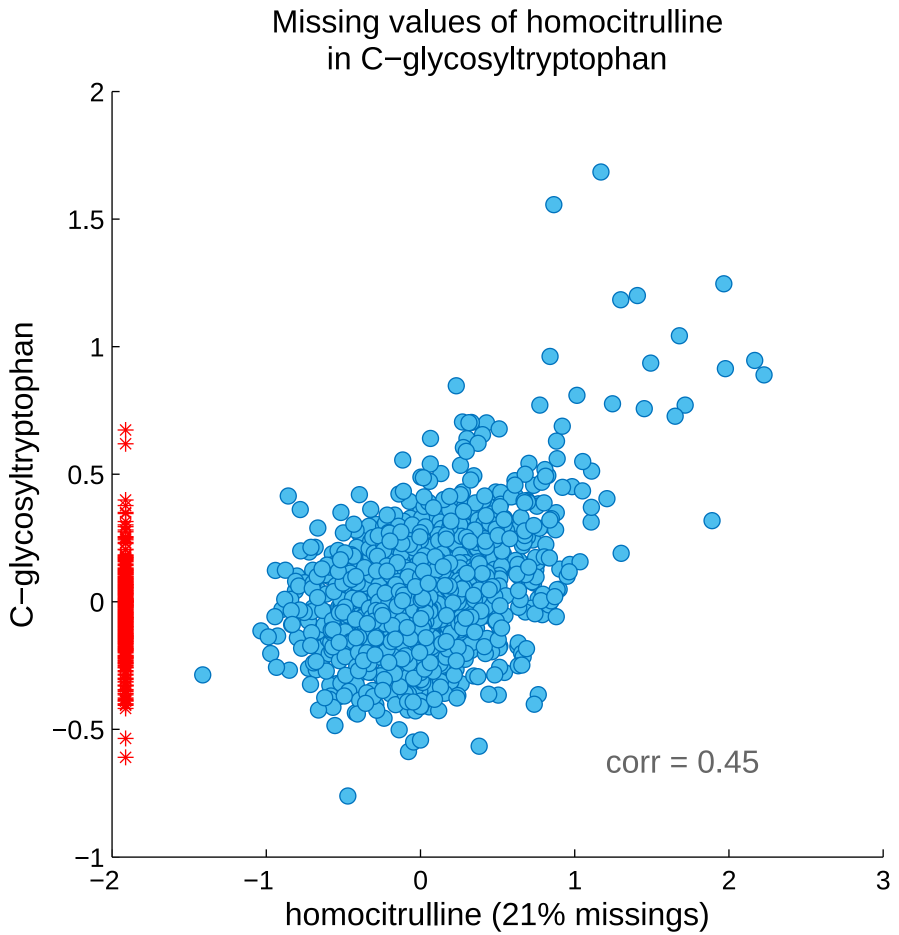

Missing values of homocitrulline
in C−glycosyltryptophan
C−glycosyltryptophan
homocitrulline (21% missings)
corr = 0.45
2
1.5
1
0.5
0
−0.5
−1
−2
−1
0
1
2
3

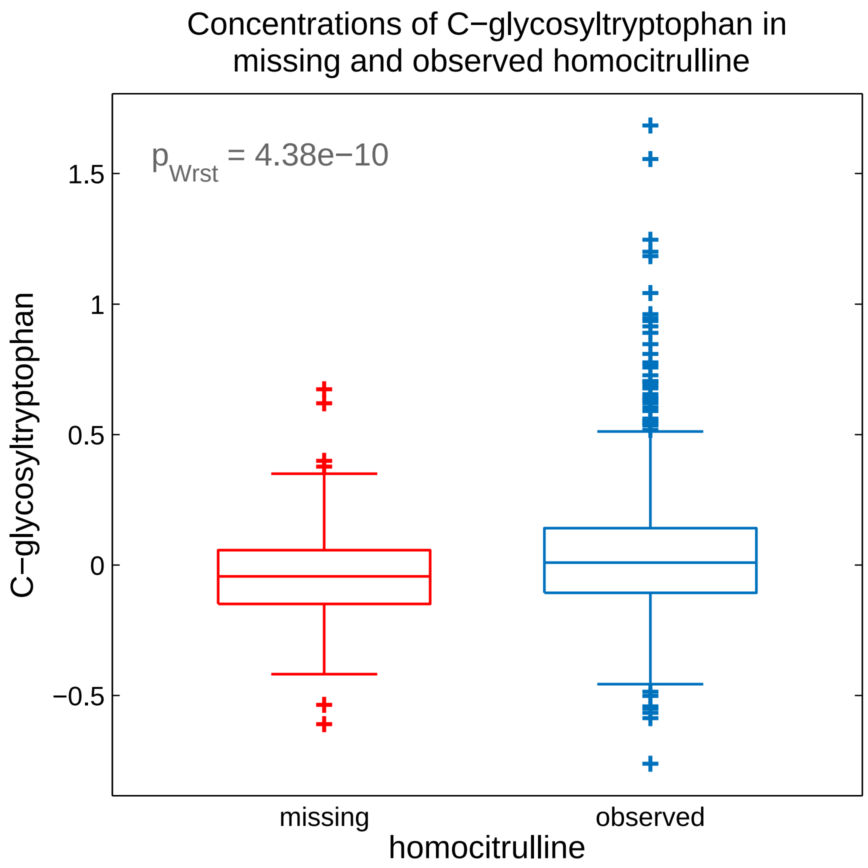

Concentrations of C−glycosyltryptophan in
missing and observed homocitrulline
p_{Wrst} = 4.38e−10
C−glycosyltryptophan
1.5
1
0.5
0
−0.5
missing
observed
homocitrulline

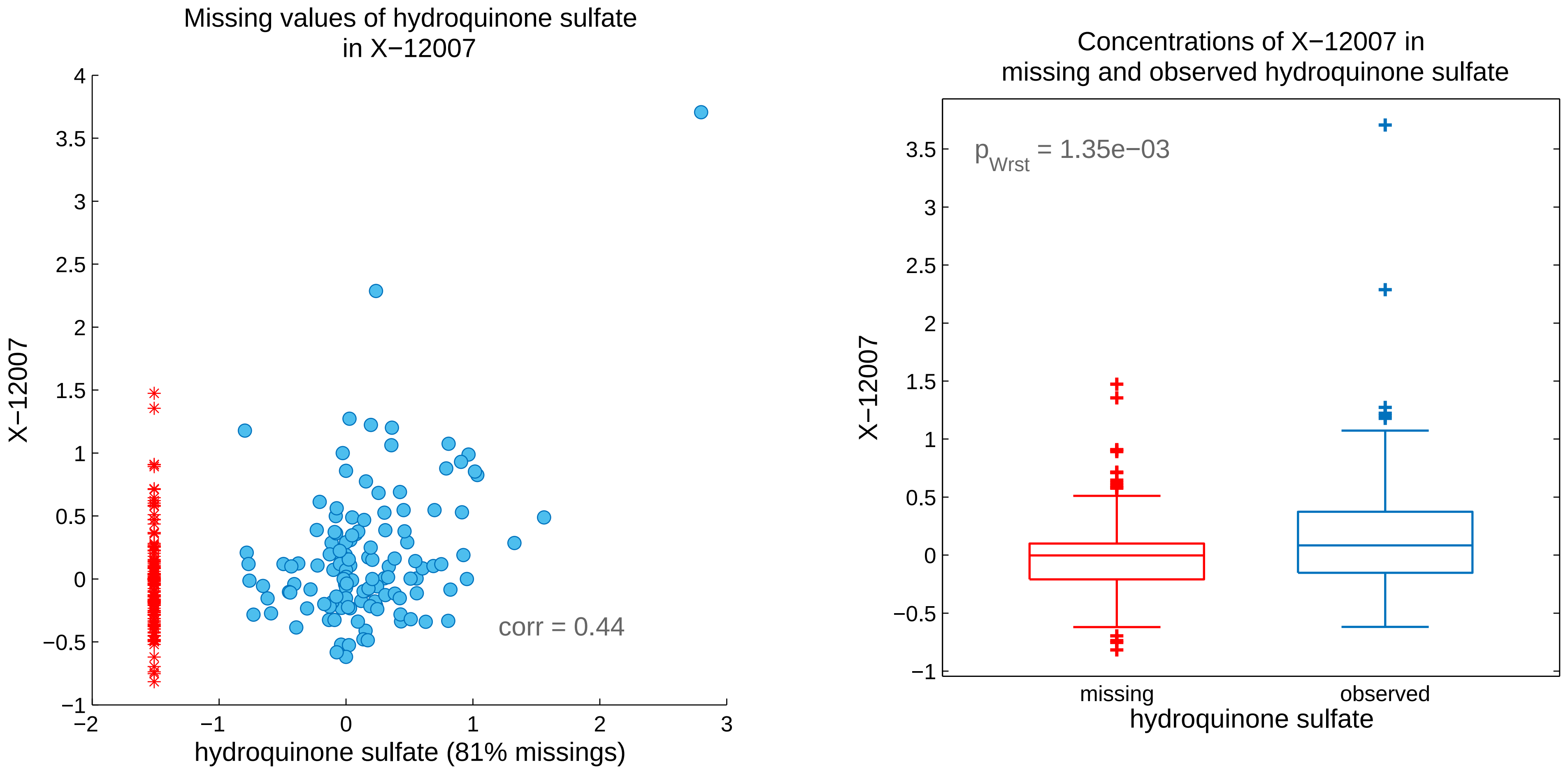

Missing values of hydroquinone sulfate in X−12007
X−12007
hydroquinone sulfate (81% missings)
corr = 0.44
Concentrations of X−12007 in missing and observed hydroquinone sulfate
p_{Wrst} = 1.35e−03
X−12007
missing
observed
hydroquinone sulfate

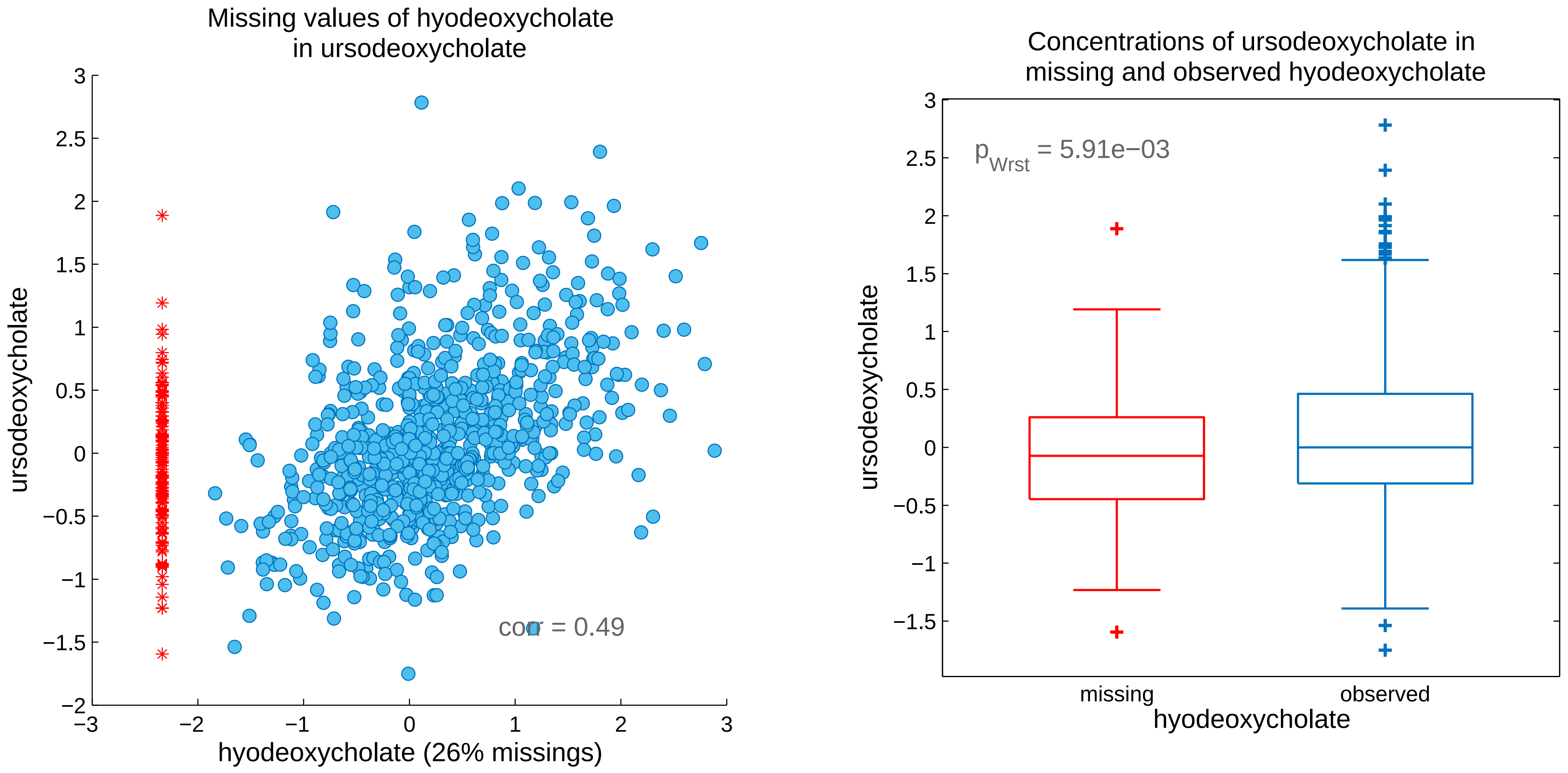

Missing values of hyodeoxycholate in ursodeoxycholate
ursodeoxycholate
hyodeoxycholate (26% missings)
corr = 0.49
Concentrations of ursodeoxycholate in missing and observed hyodeoxycholate
p_{Wrst} = 5.91e−03
ursodeoxycholate
missing
observed
hyodeoxycholate

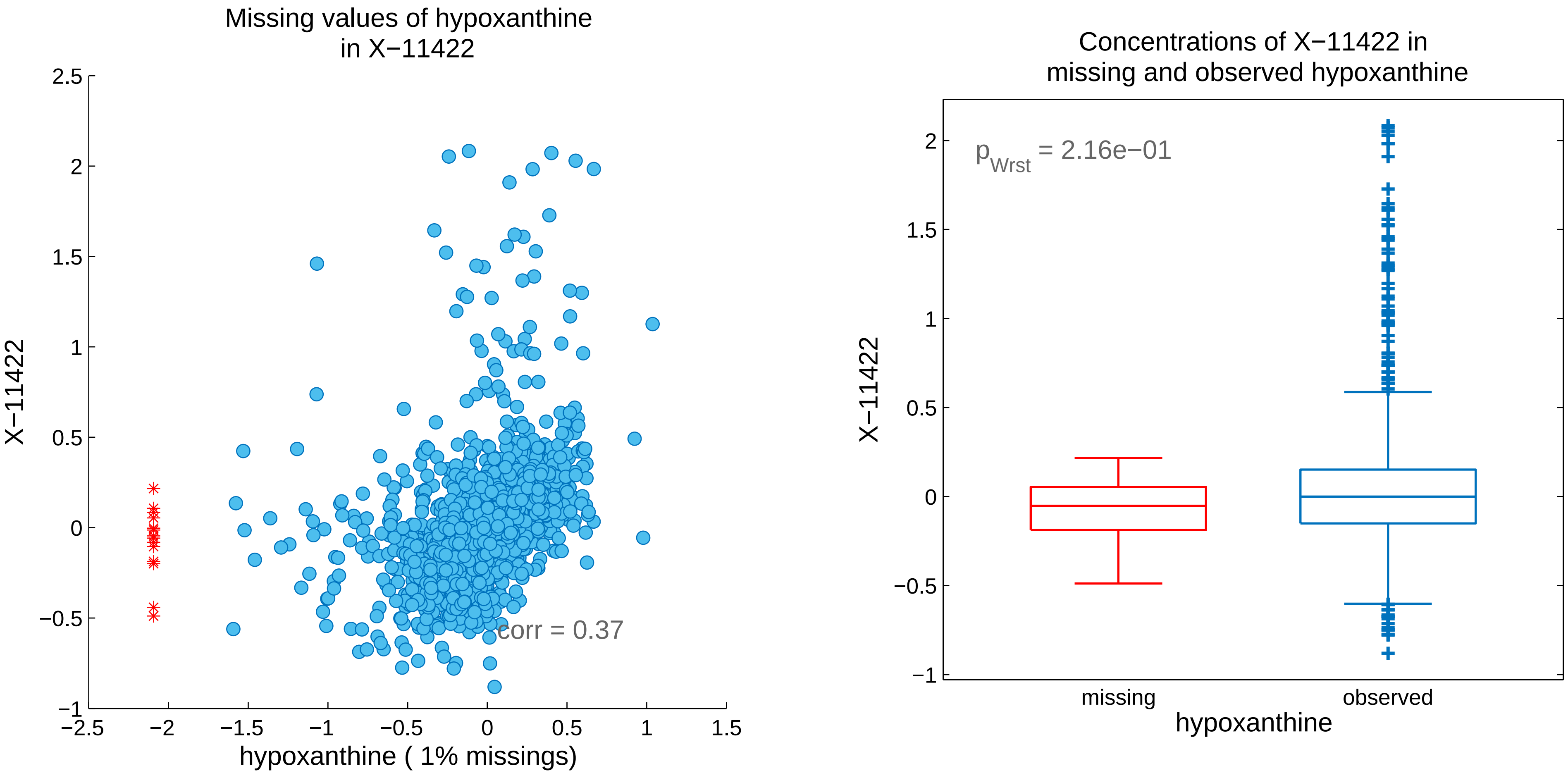

Missing values of hypoxanthine in X−11422
X−11422
hypoxanthine (1% missings)
corr = 0.37
Concentrations of X−11422 in missing and observed hypoxanthine
p_Wrst = 2.16e−01
X−11422
missing
observed
hypoxanthine

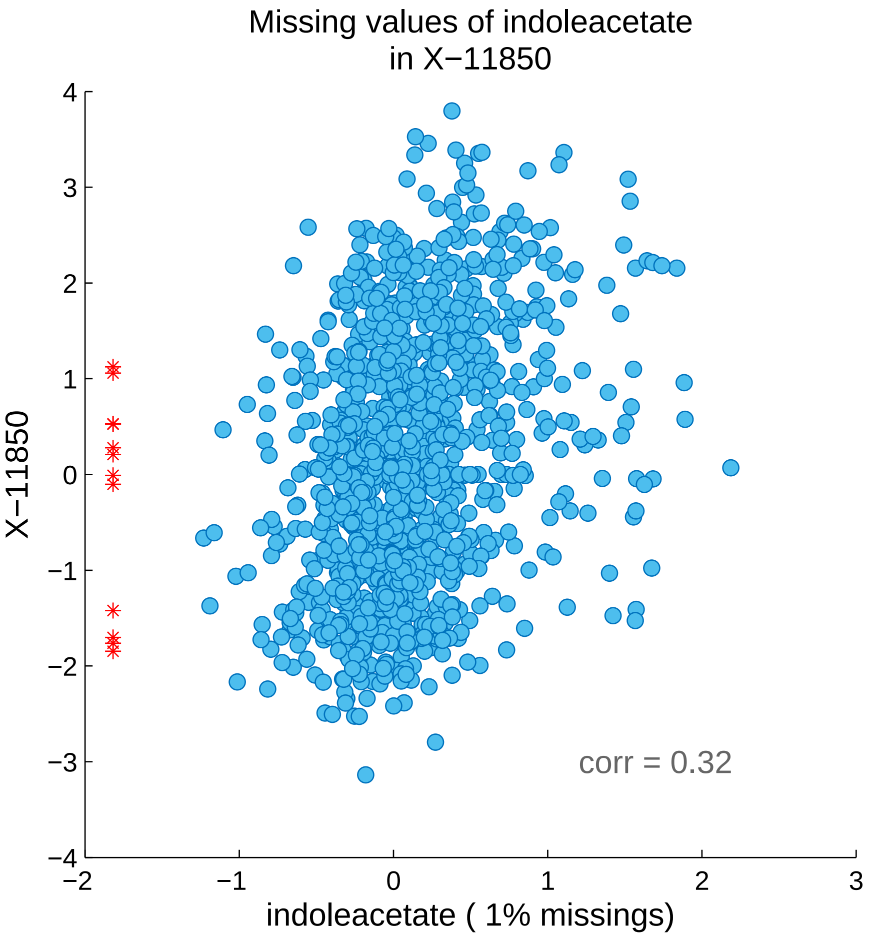

Missing values of indoleacetate
in X−11850
X−11850
indoleacetate (1% missings)
corr = 0.32

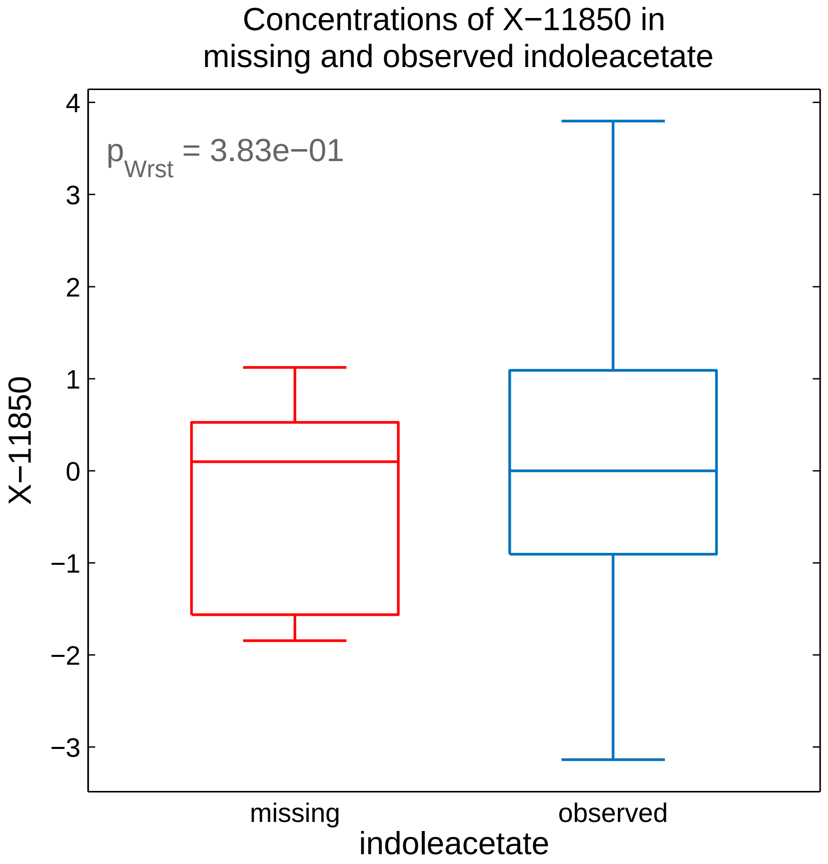

Concentrations of X−11850 in
missing and observed indoleacetate
p_{Wrst} = 3.83e−01
X−11850
missing
observed
indoleacetate

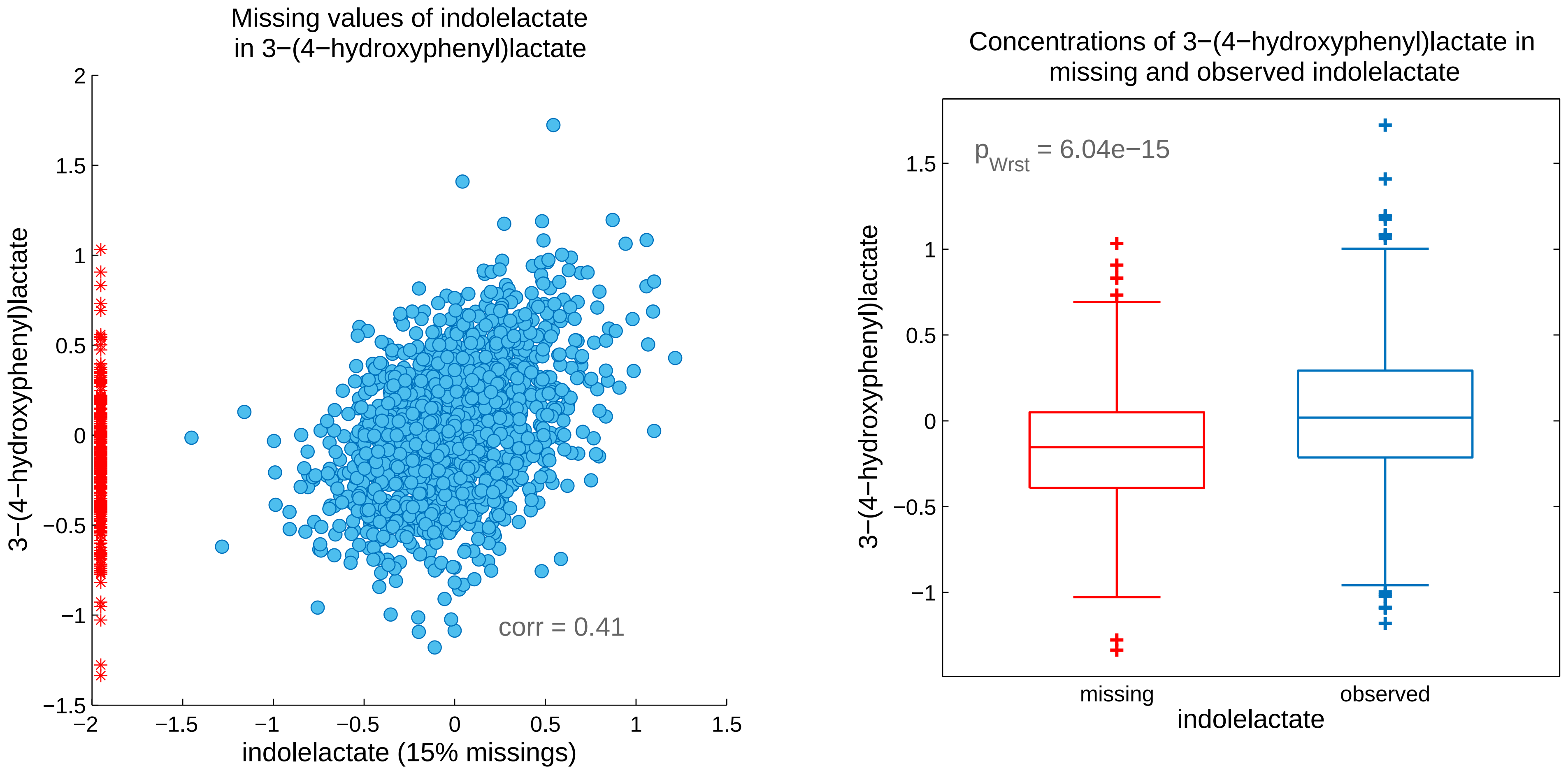
Missing values of indolelactate
in 3−(4−hydroxyphenyl)lactate
3−(4−hydroxyphenyl)lactate
indolelactate (15% missings)
corr = 0.41
2
1.5
1
0.5
0
−0.5
−1
−1.5
−2
−1.5
−1
−0.5
0
0.5
1
1.5
Concentrations of 3−(4−hydroxyphenyl)lactate in
missing and observed indolelactate
p_Wrst = 6.04e−15
3−(4−hydroxyphenyl)lactate
1.5
1
0.5
0
−0.5
−1
missing
observed
indolelactate

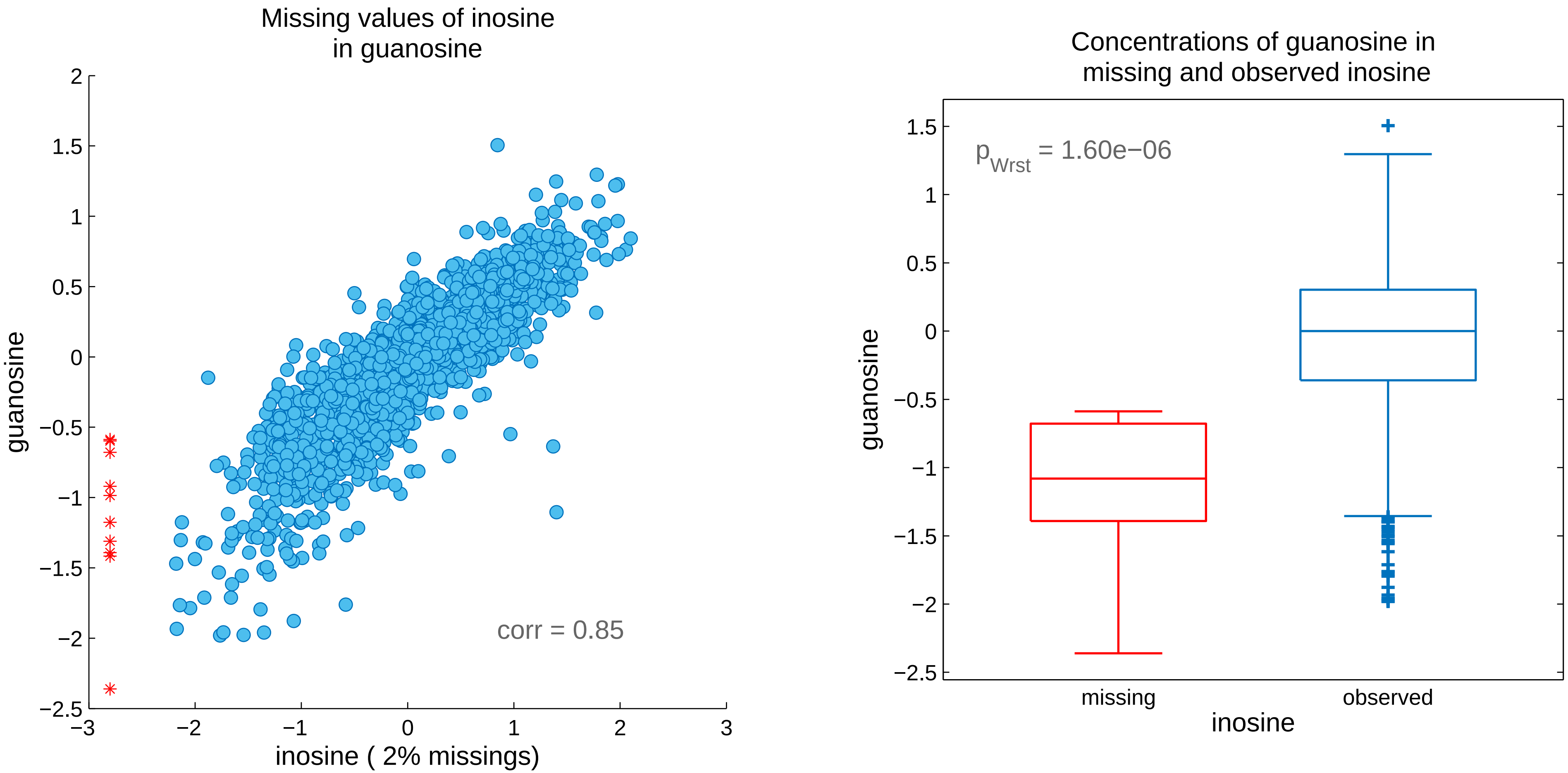
Missing values of inosine
in guanosine
guanosine
inosine (2% missings)
corr = 0.85
Concentrations of guanosine in
missing and observed inosine
p_Wrst = 1.60e−06
guanosine
missing
observed
inosine

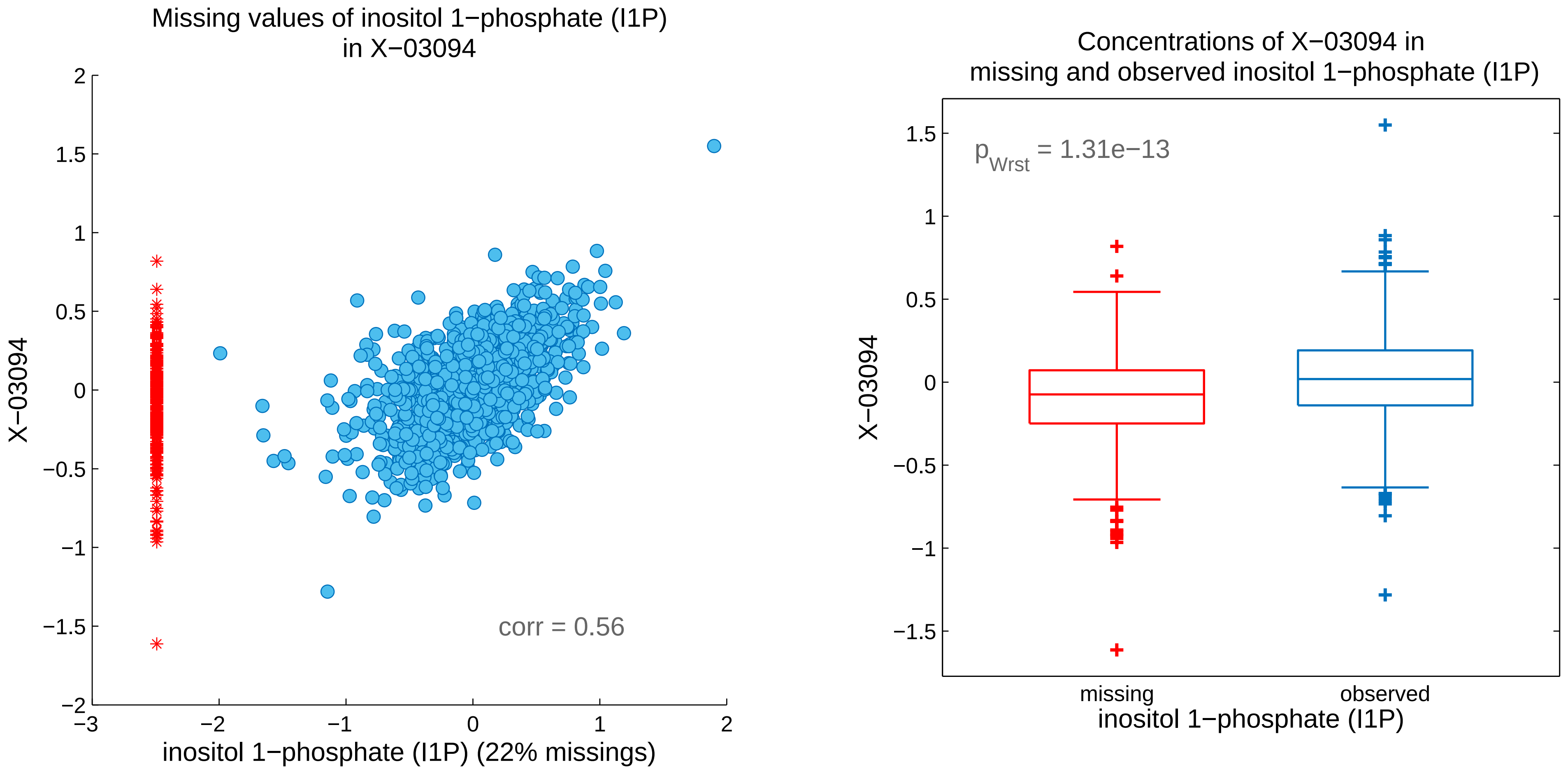

Missing values of inositol 1−phosphate (I1P) in X−03094
X−03094
inositol 1−phosphate (I1P) (22% missings)
corr = 0.56
Concentrations of X−03094 in missing and observed inositol 1−phosphate (I1P)
p_{Wrst} = 1.31e−13
X−03094
missing
observed
inositol 1−phosphate (I1P)

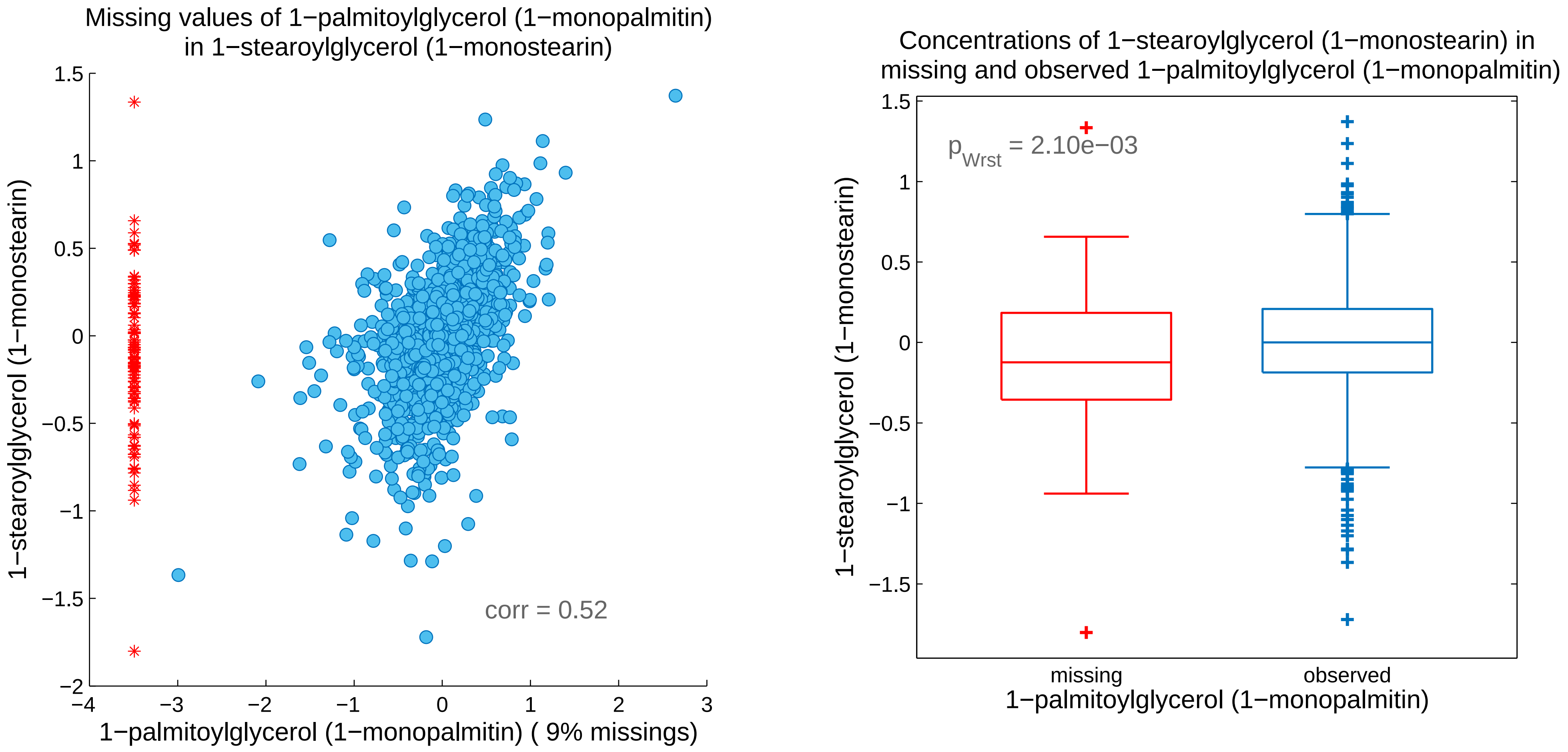
Missing values of 1−palmitoylglycerol (1−monopalmitin) in 1−stearoylglycerol (1−monostearin)
1−stearoylglycerol (1−monostearin)
1−palmitoylglycerol (1−monopalmitin) (9% missings)
corr = 0.52
Concentrations of 1−stearoylglycerol (1−monostearin) in missing and observed 1−palmitoylglycerol (1−monopalmitin)
p_{Wrst} = 2.10e−03
missing
observed
1−palmitoylglycerol (1−monopalmitin)

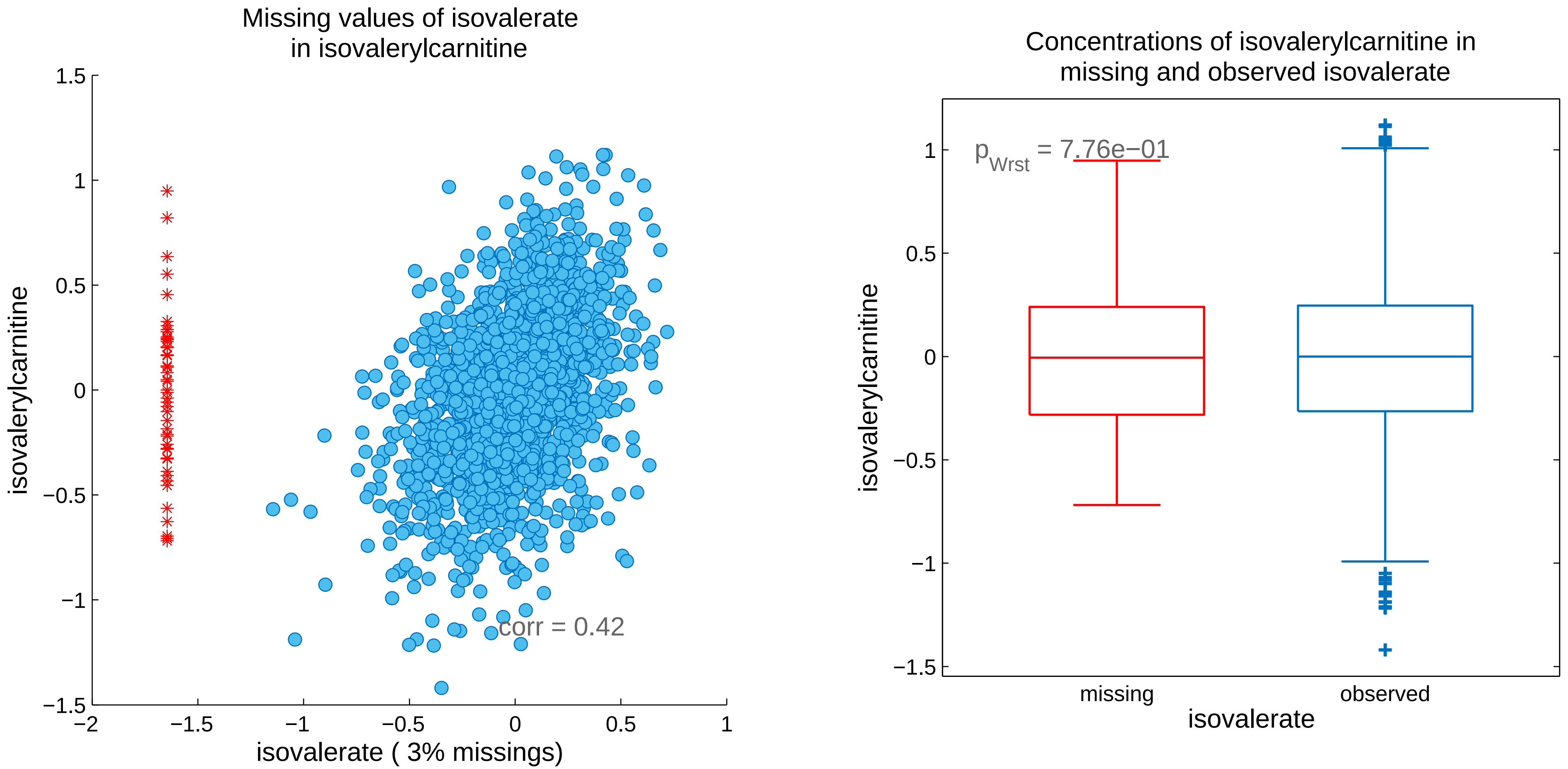

Missing values of isovalerate in isovalerylcarnitine
isovalerylcarnitine
isovalerate (3% missings)
corr = 0.42
1.5
1
0.5
0
−0.5
−1
−1.5
−2
−1.5
−1
−0.5
0
0.5
1
Concentrations of isovalerylcarnitine in missing and observed isovalerate
p_Wrst = 7.76e−01
isovalerylcarnitine
missing
observed
isovalerate

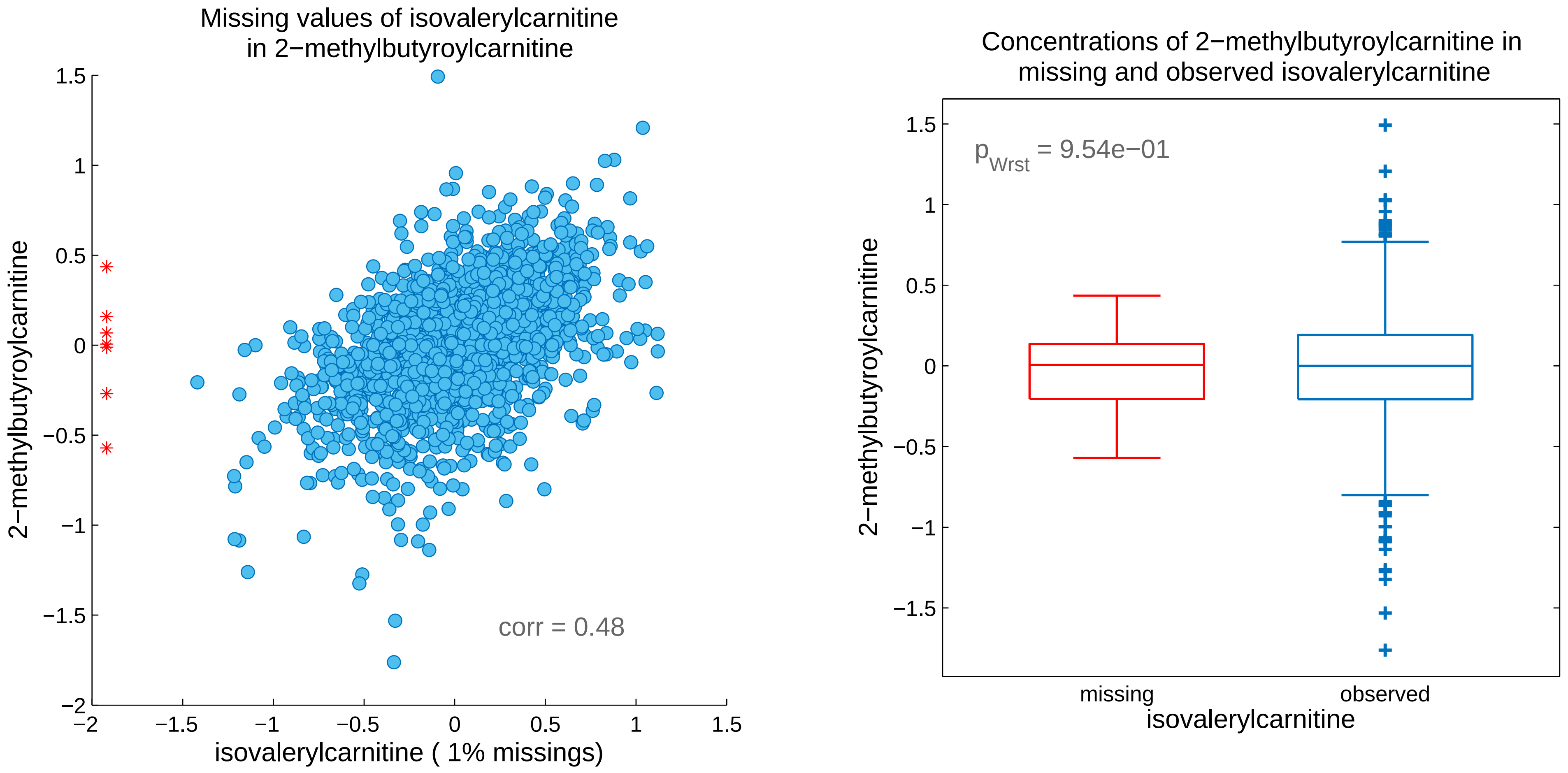
Missing values of isovalerylcarnitine in 2−methylbutyroylcarnitine
2−methylbutyroylcarnitine
isovalerylcarnitine (1% missings)
corr = 0.48
1.5
1
0.5
0
−0.5
−1
−1.5
−2
−1.5
−1
−0.5
0
0.5
1
1.5
Concentrations of 2−methylbutyroylcarnitine in missing and observed isovalerylcarnitine
p_Wrst = 9.54e−01
2−methylbutyroylcarnitine
missing
observed
isovalerylcarnitine

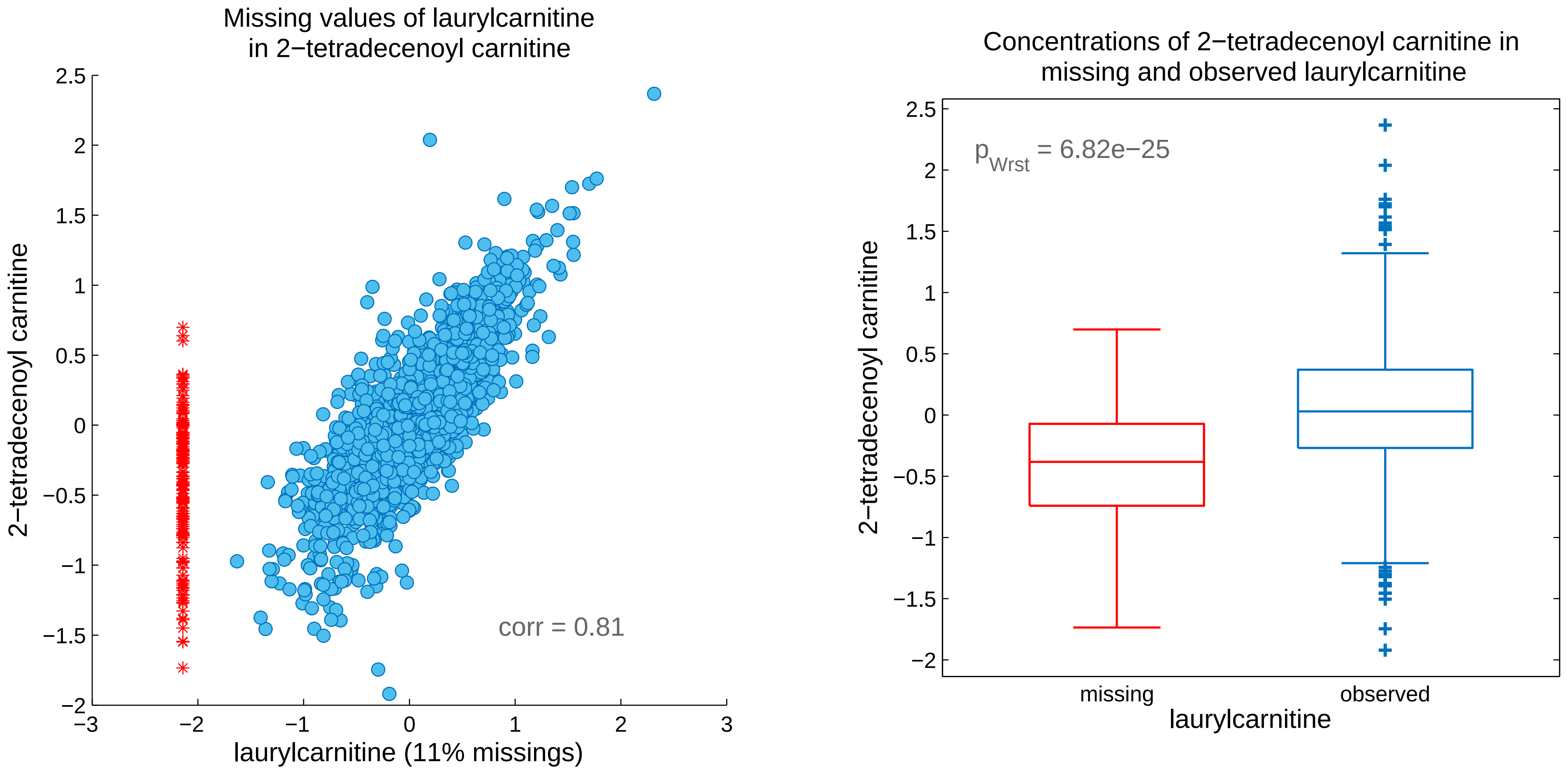

Missing values of laurylcarnitine in 2−tetradecenoyl carnitine
2−tetradecenoyl carnitine
laurylcarnitine (11% missings)
corr = 0.81
Concentrations of 2−tetradecenoyl carnitine in missing and observed laurylcarnitine
p_{Wrst} = 6.82e−25
2−tetradecenoyl carnitine
missing
observed
laurylcarnitine

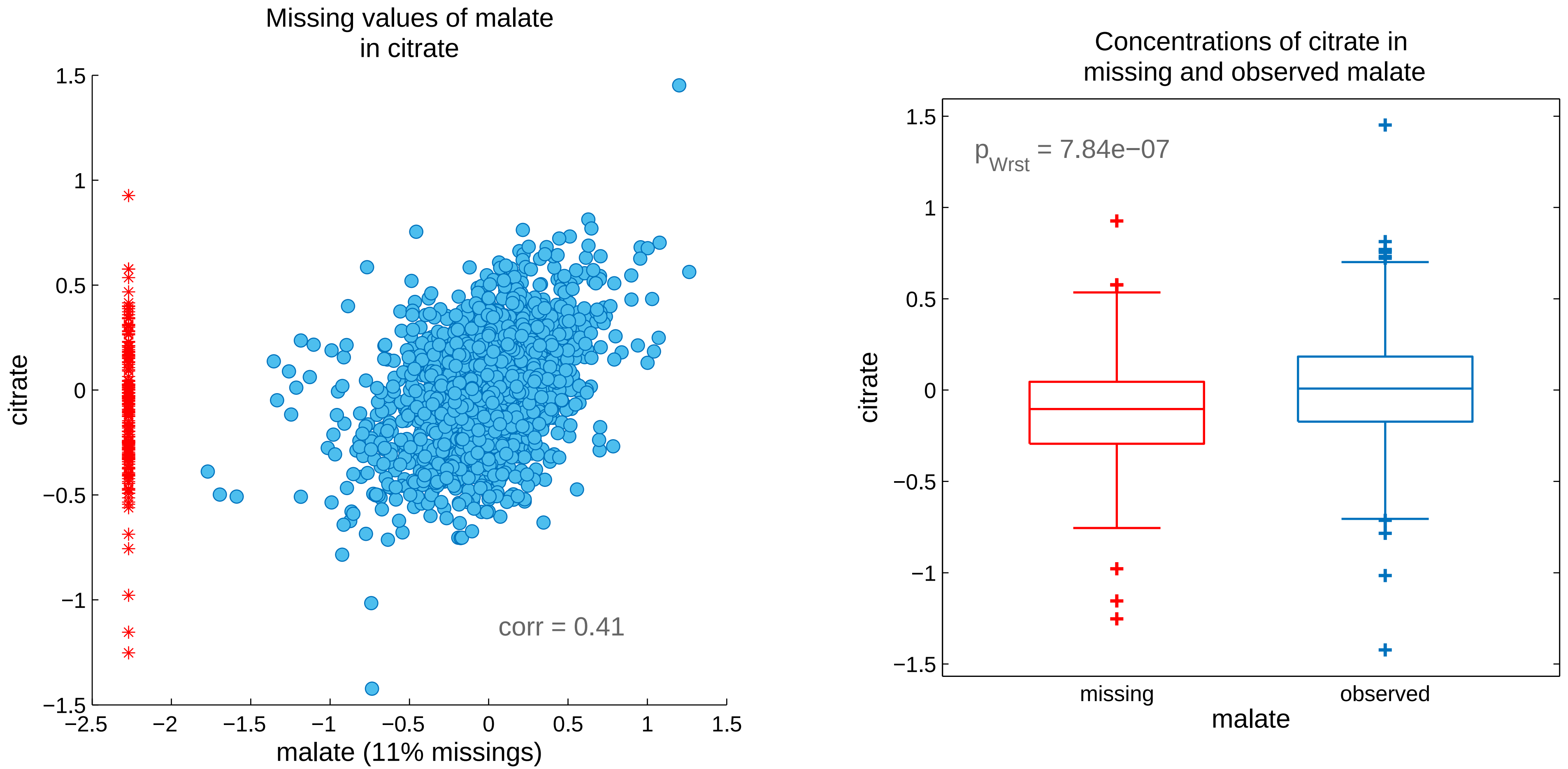
Missing values of malate
in citrate
citrate
malate (11% missings)
corr = 0.41
Concentrations of citrate in
missing and observed malate
p_{Wrst} = 7.84e−07
citrate
missing
observed
malate

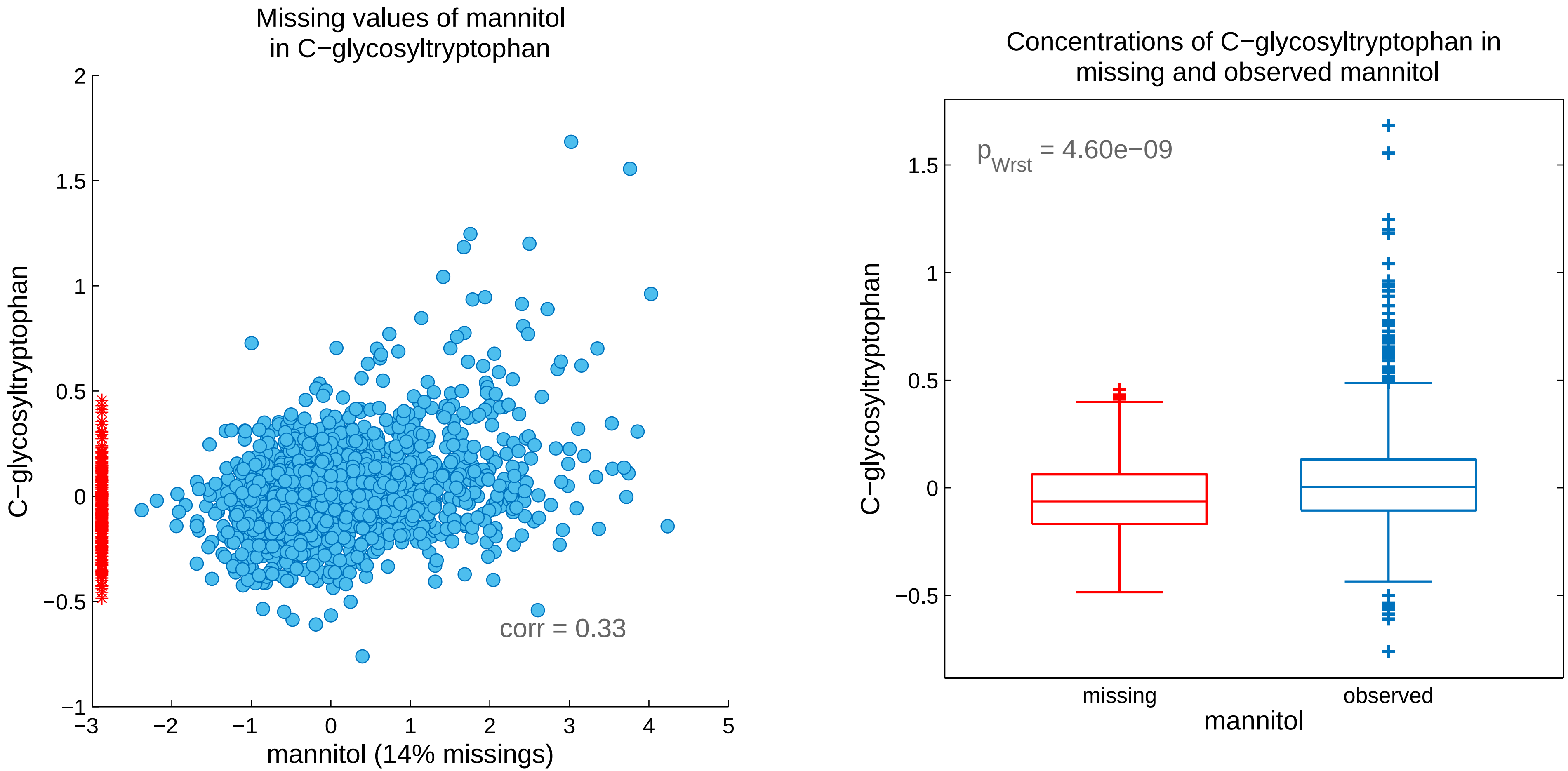
Missing values of mannitol
in C−glycosyltryptophan
C−glycosyltryptophan
mannitol (14% missings)
corr = 0.33
Concentrations of C−glycosyltryptophan in
missing and observed mannitol
p_{Wrst} = 4.60e−09
C−glycosyltryptophan
missing
observed
mannitol

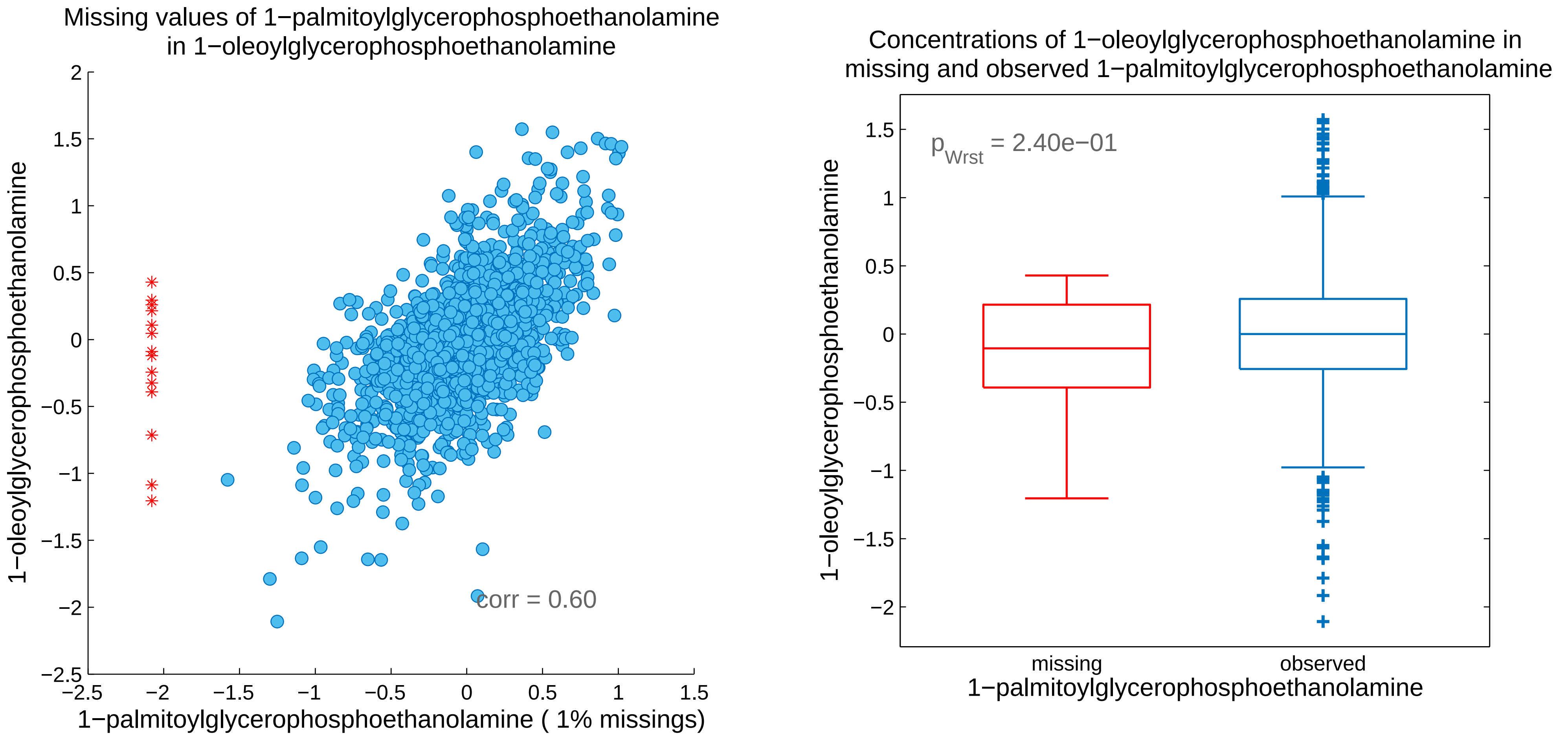
Missing values of 1−palmitoylglycerophosphoethanolamine in 1−oleoylglycerophosphoethanolamine
1−oleoylglycerophosphoethanolamine
1−palmitoylglycerophosphoethanolamine (1% missings)
corr = 0.60
Concentrations of 1−oleoylglycerophosphoethanolamine in missing and observed 1−palmitoylglycerophosphoethanolamine
p_{Wrst} = 2.40e−01
missing
observed
1−palmitoylglycerophosphoethanolamine

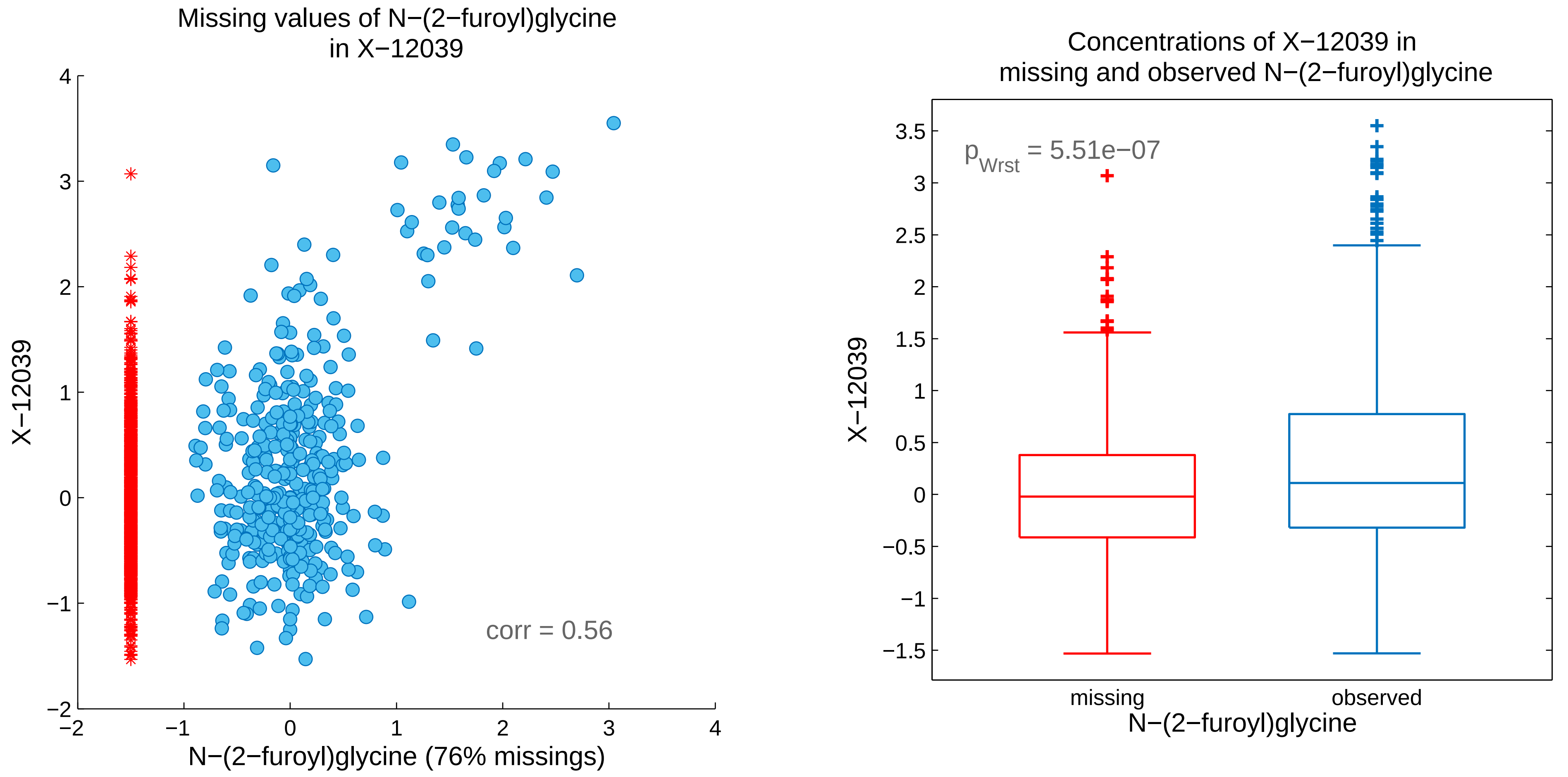

Missing values of N−(2−furoyl)glycine in X−12039
X−12039
N−(2−furoyl)glycine (76% missings)
corr = 0.56
Concentrations of X−12039 in missing and observed N−(2−furoyl)glycine
p_{Wrst} = 5.51e−07
X−12039
missing
observed
N−(2−furoyl)glycine

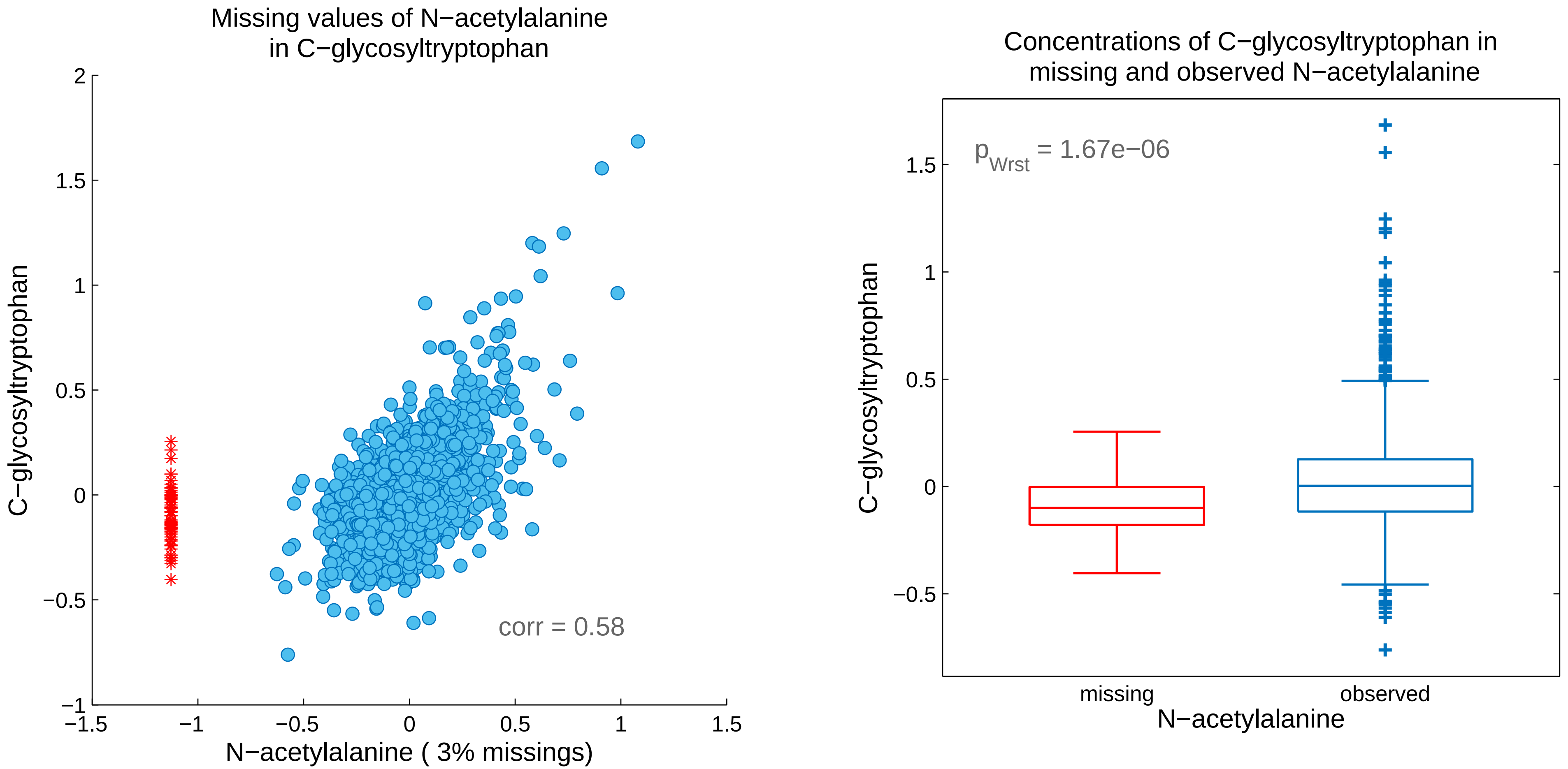

Missing values of N−acetylalanine
in C−glycosyltryptophan
C−glycosyltryptophan
N−acetylalanine (3% missings)
corr = 0.58
Concentrations of C−glycosyltryptophan in
missing and observed N−acetylalanine
p_Wrst = 1.67e−06
C−glycosyltryptophan
missing
observed
N−acetylalanine

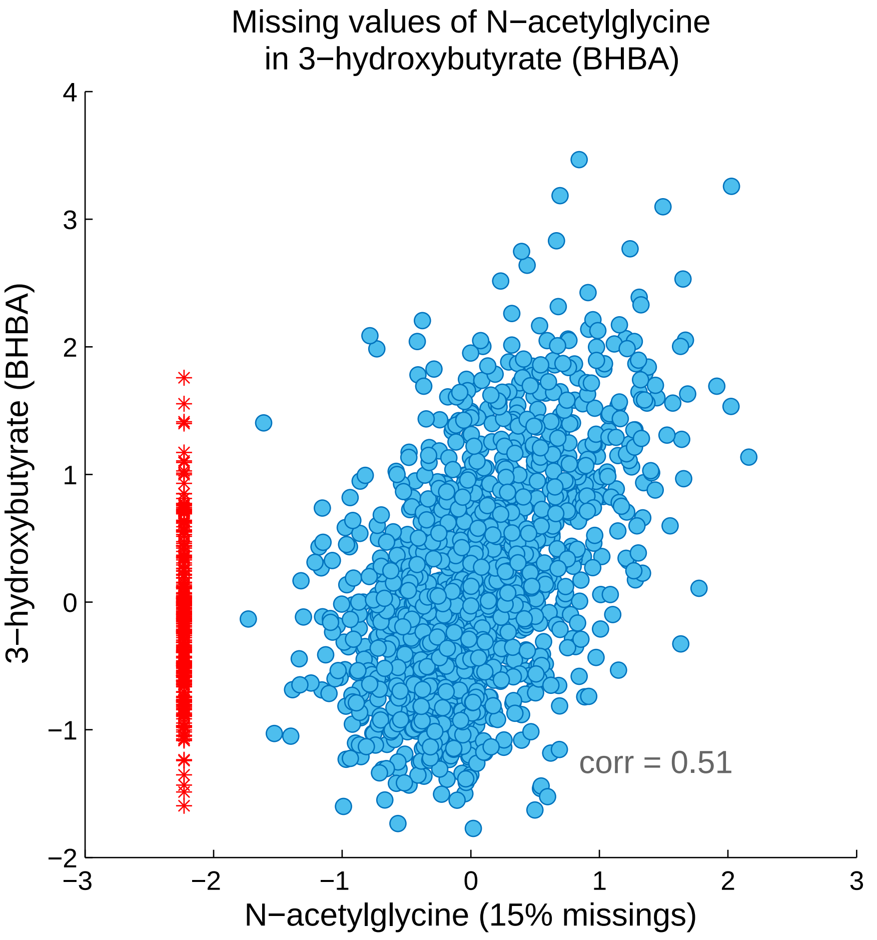

Missing values of N−acetylglycine
in 3−hydroxybutyrate (BHBA)
3−hydroxybutyrate (BHBA)
N−acetylglycine (15% missings)
corr = 0.51
−3
−2
−1
0
1
2
3
4

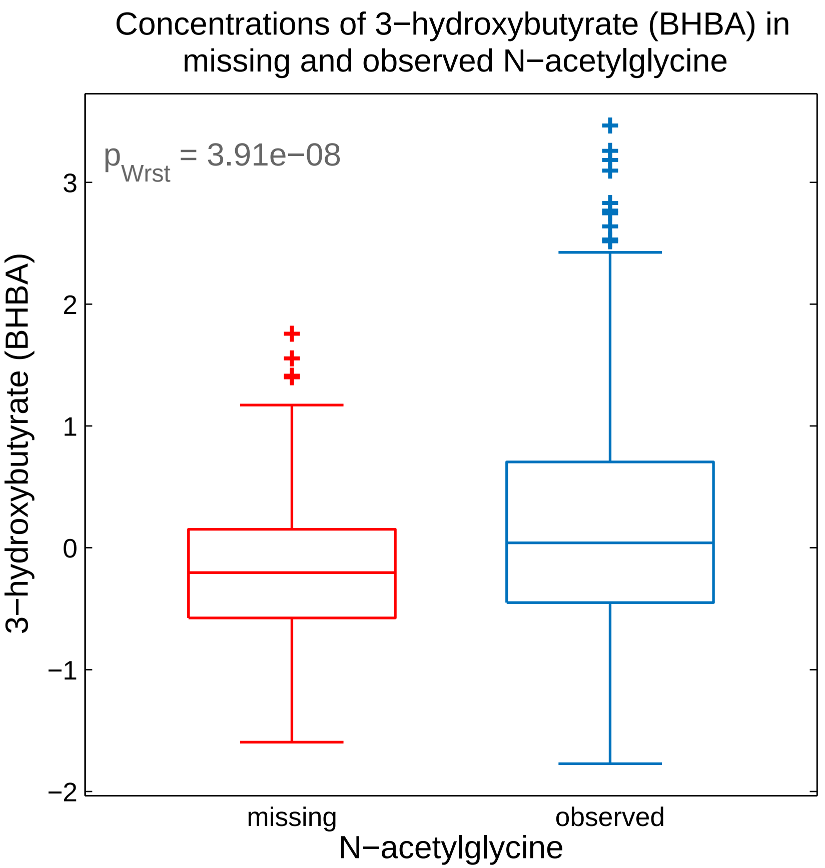

Concentrations of 3−hydroxybutyrate (BHBA) in
missing and observed N−acetylglycine
p_Wrst = 3.91e−08
3−hydroxybutyrate (BHBA)
missing
observed
N−acetylglycine
−2
−1
0
1
2
3

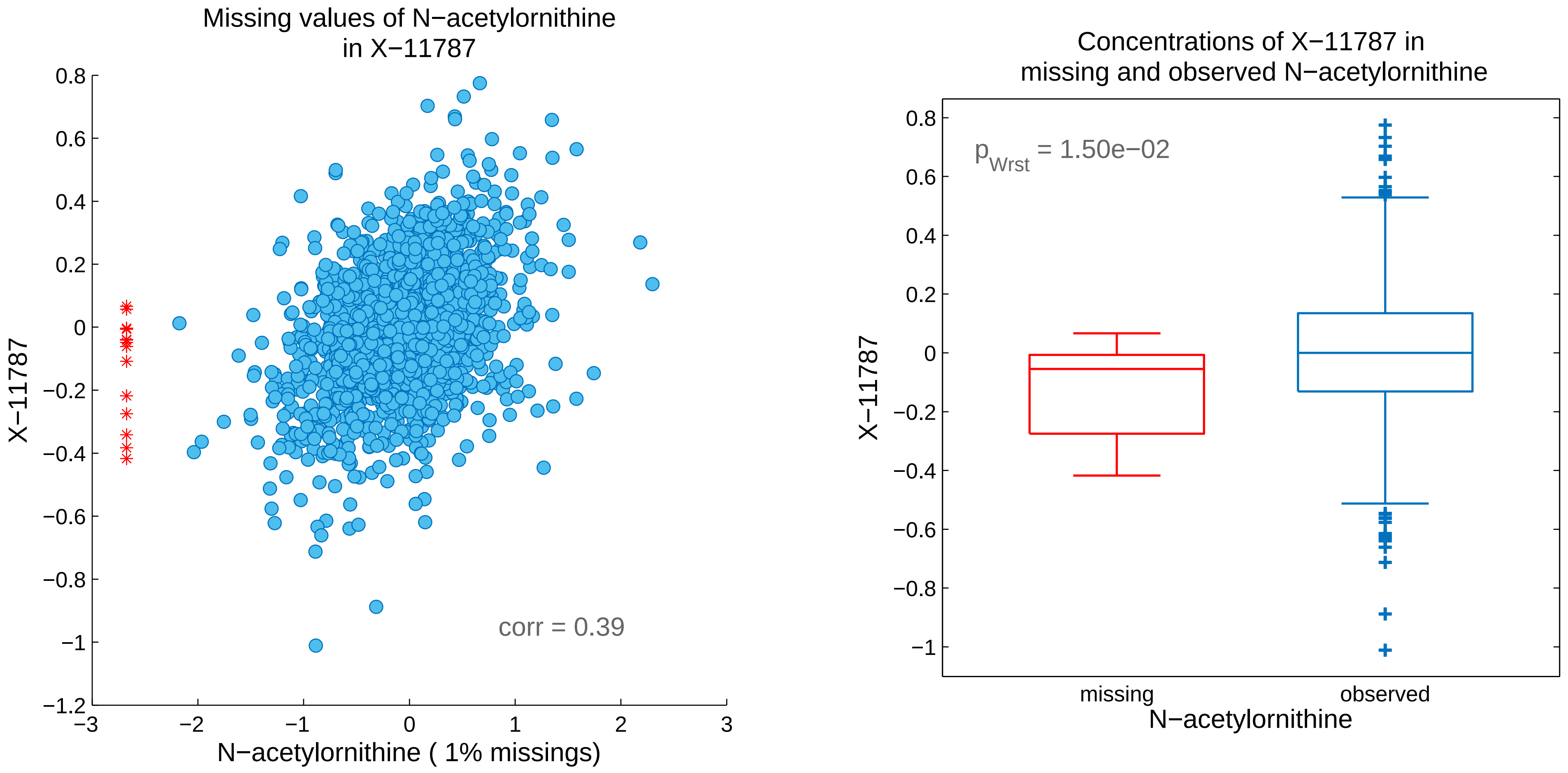
Missing values of N−acetylornithine
in X−11787
X−11787
N−acetylornithine (1% missings)
corr = 0.39
Concentrations of X−11787 in
missing and observed N−acetylornithine
p_{Wrst} = 1.50e−02
X−11787
missing
observed
N−acetylornithine

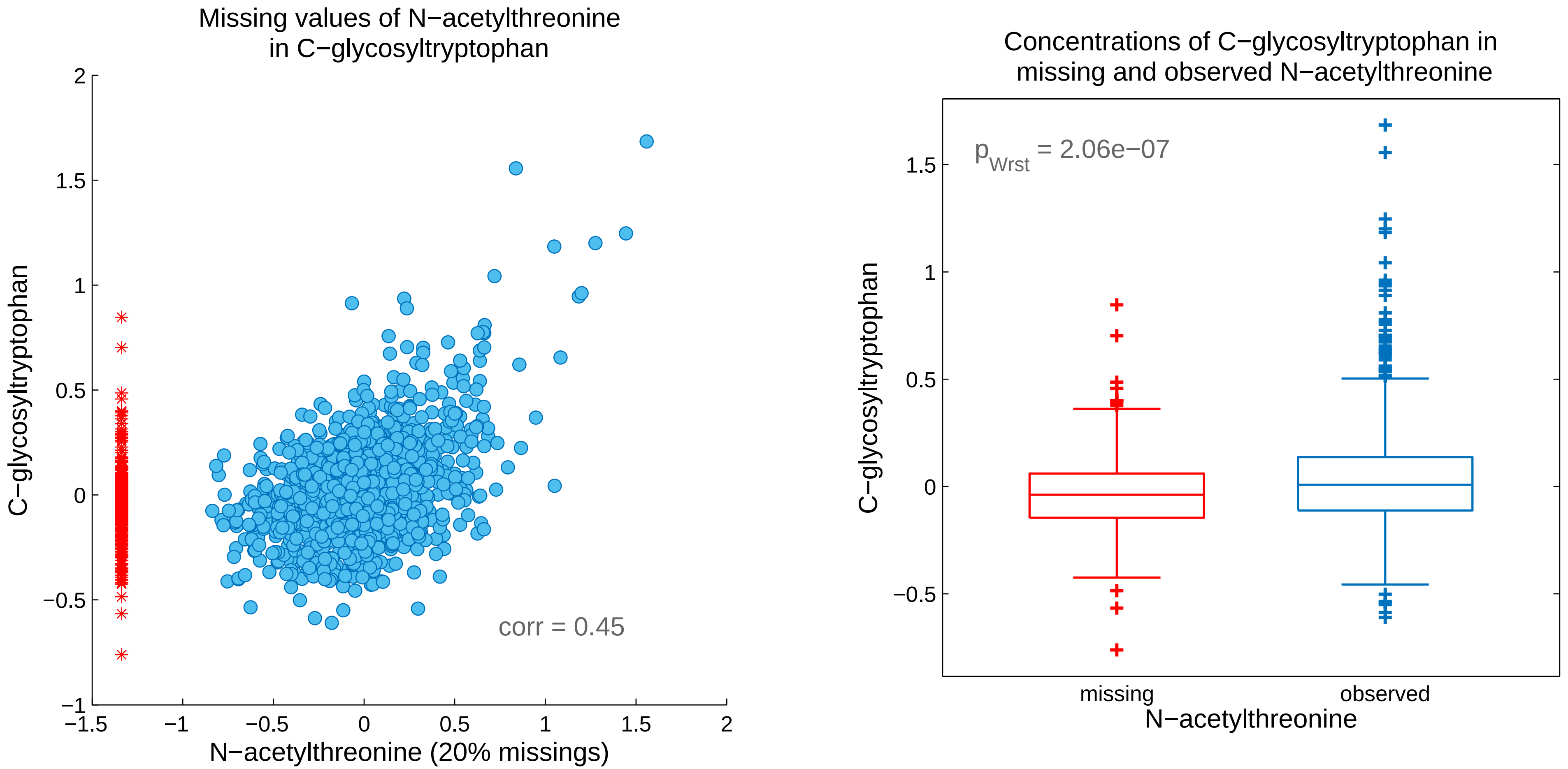
Missing values of N−acetylthreonine in C−glycosyltryptophan
C−glycosyltryptophan
N−acetylthreonine (20% missings)
corr = 0.45
2
1.5
1
0.5
0
−0.5
−1
−1.5
−1
−0.5
0
0.5
1
1.5
2
Concentrations of C−glycosyltryptophan in missing and observed N−acetylthreonine
p_{Wrst} = 2.06e−07
C−glycosyltryptophan
1.5
1
0.5
0
−0.5
missing
observed
N−acetylthreonine

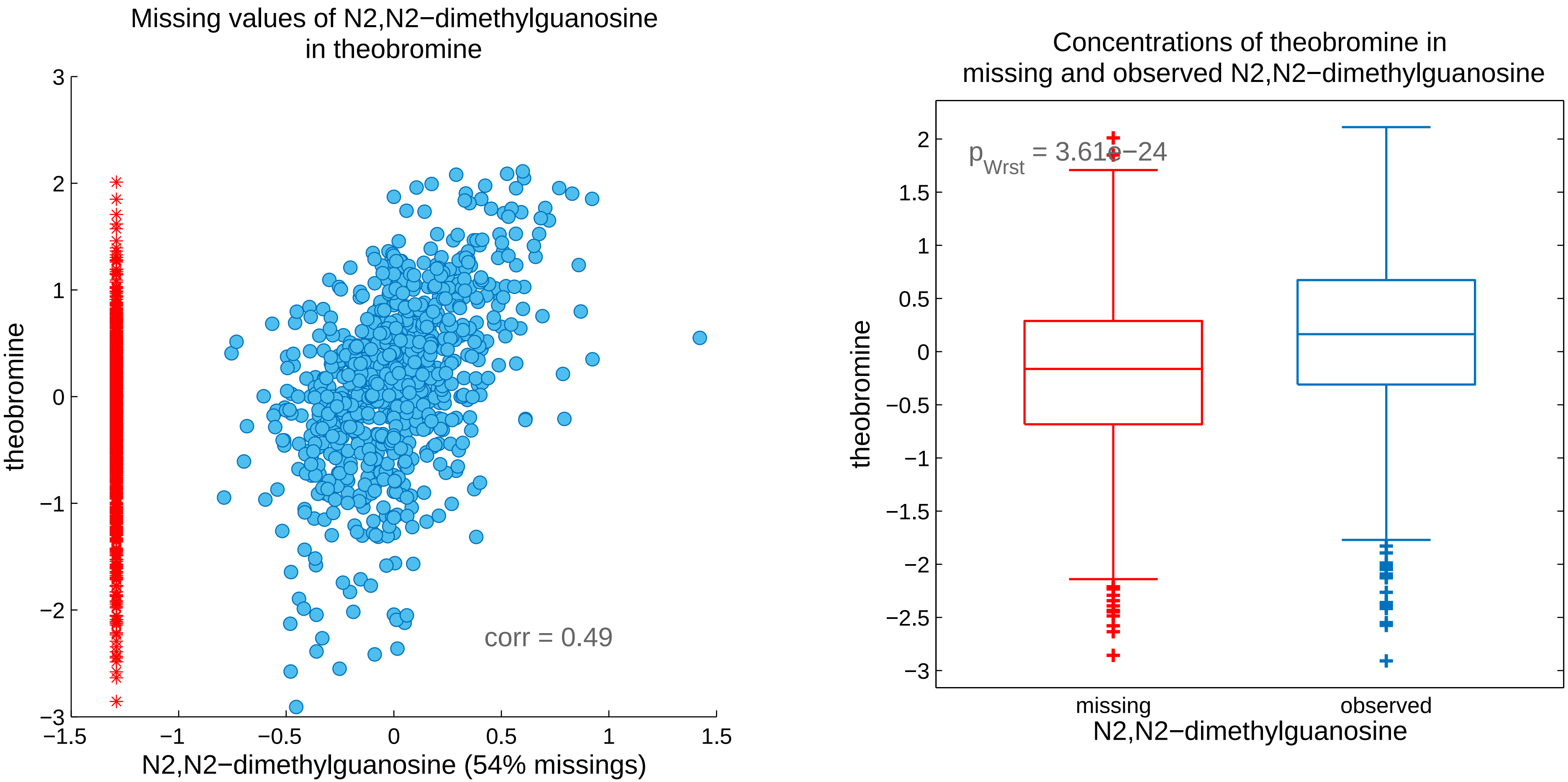

Missing values of N2,N2−dimethylguanosine in theobromine
theobromine
N2,N2−dimethylguanosine (54% missings)
corr = 0.49
3
2
1
0
−1
−2
−3
−1.5
−1
−0.5
0
0.5
1
1.5
Concentrations of theobromine in missing and observed N2,N2−dimethylguanosine
p_{Wrst} = 3.61e−24
theobromine
2
1.5
1
0.5
0
−0.5
−1
−1.5
−2
−2.5
−3
missing
observed
N2,N2−dimethylguanosine

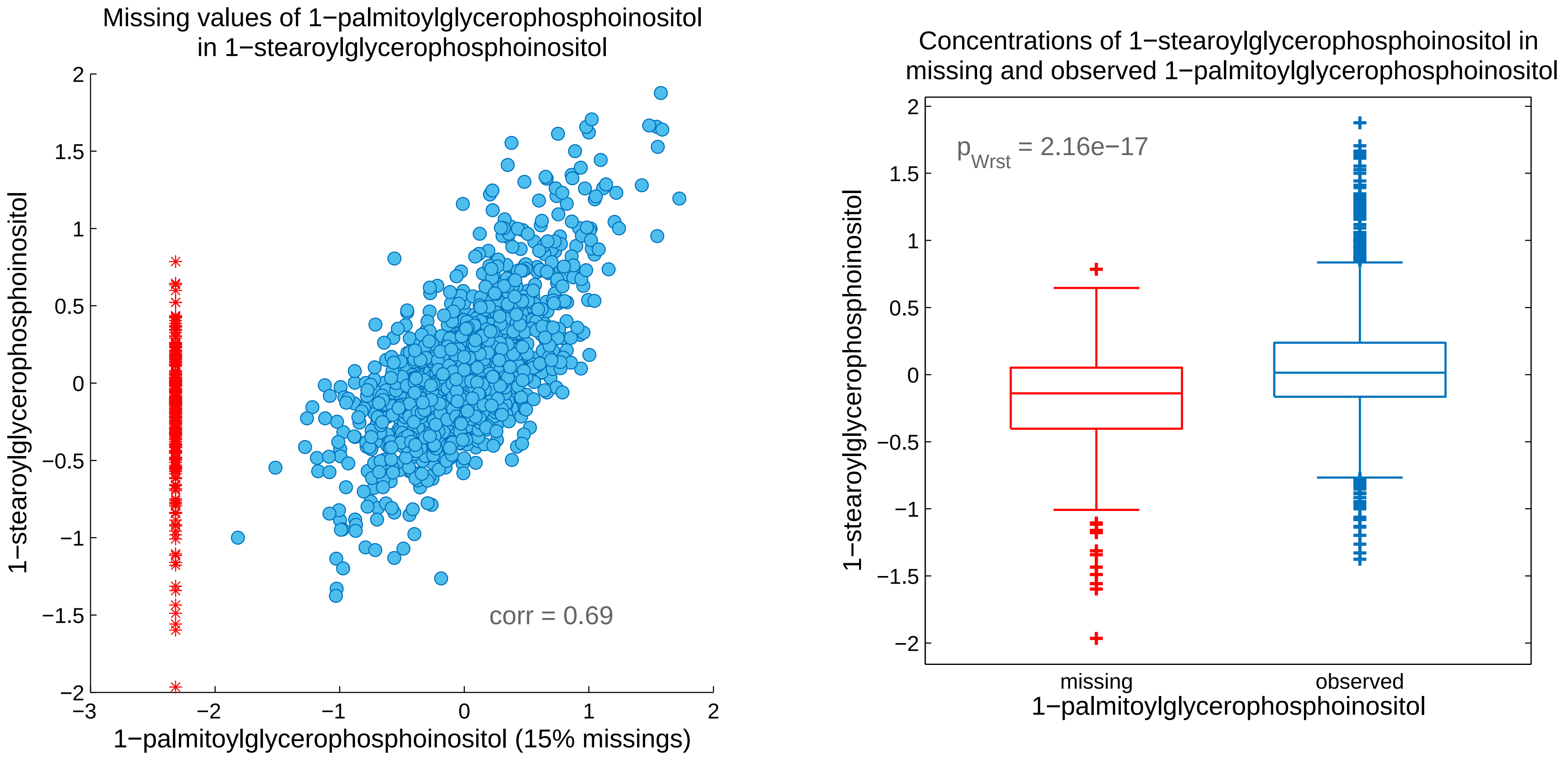
Missing values of 1−palmitoylglycerophosphoinositol in 1−stearoylglycerophosphoinositol
1−stearoylglycerophosphoinositol
1−palmitoylglycerophosphoinositol (15% missings)
corr = 0.69
Concentrations of 1−stearoylglycerophosphoinositol in missing and observed 1−palmitoylglycerophosphoinositol
p_Wrst = 2.16e−17
missing
observed
1−palmitoylglycerophosphoinositol

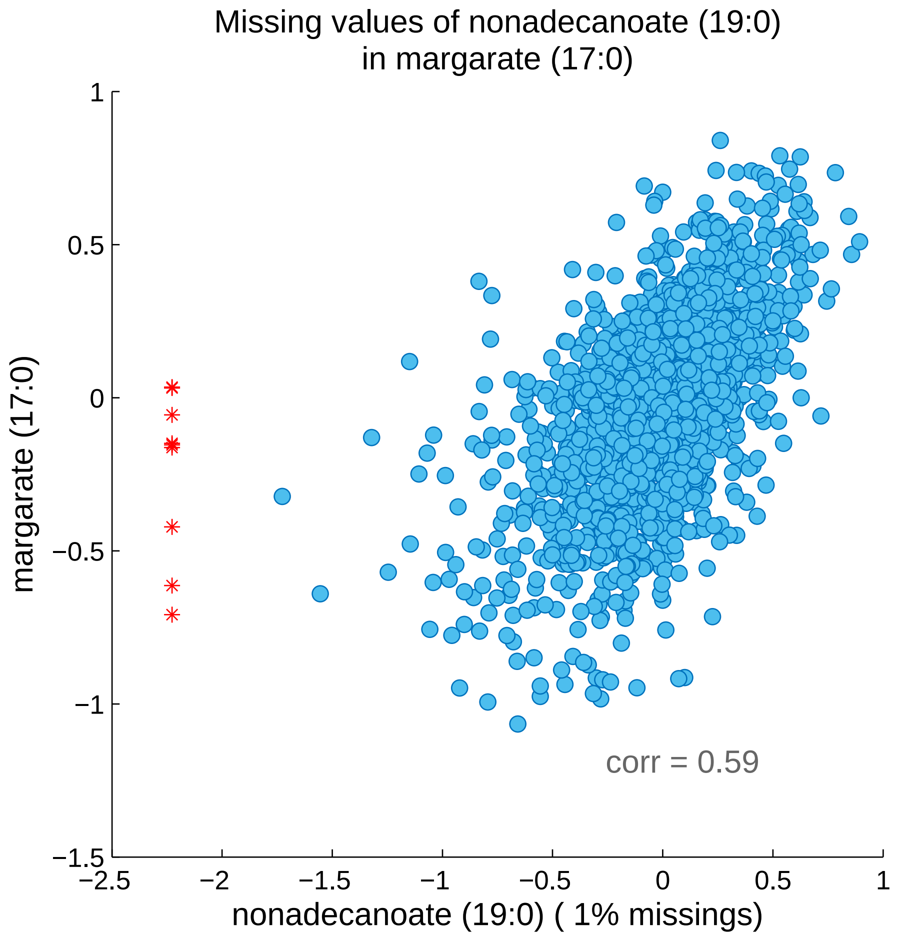

Missing values of nonadecanoate (19:0)
in margarate (17:0)
margarate (17:0)
nonadecanoate (19:0) (1% missings)
corr = 0.59
1
0.5
0
−0.5
−1
−1.5
−2.5
−2
−1.5
−1
−0.5
0
0.5
1

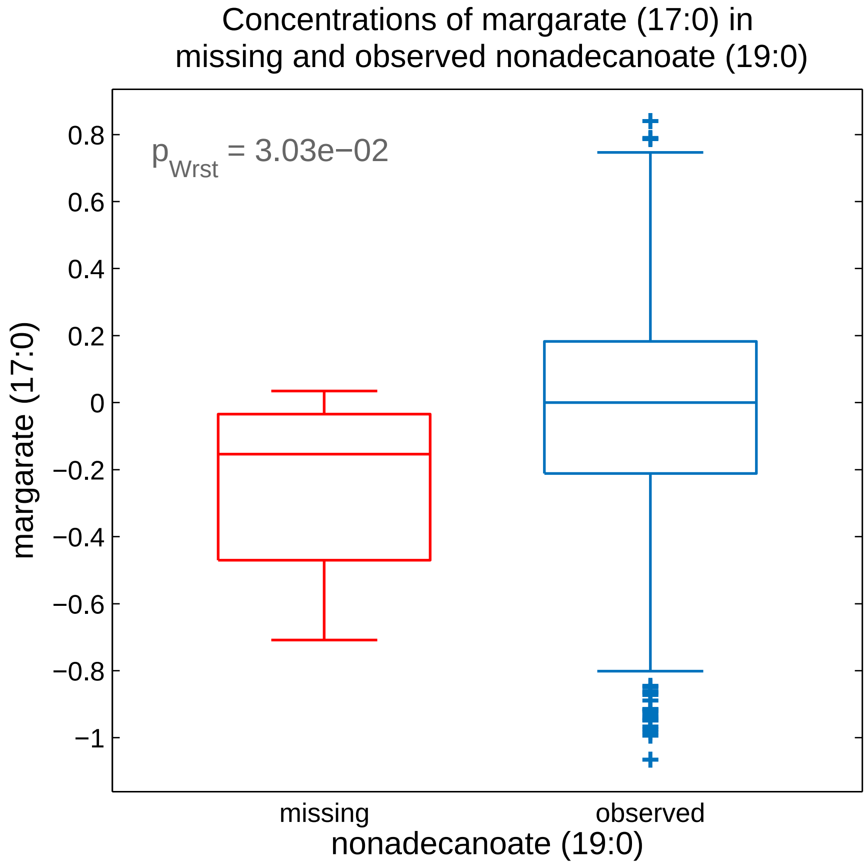

Concentrations of margarate (17:0) in
missing and observed nonadecanoate (19:0)
p_Wrst = 3.03e−02
margarate (17:0)
0.8
0.6
0.4
0.2
0
−0.2
−0.4
−0.6
−0.8
−1
missing
observed
nonadecanoate (19:0)

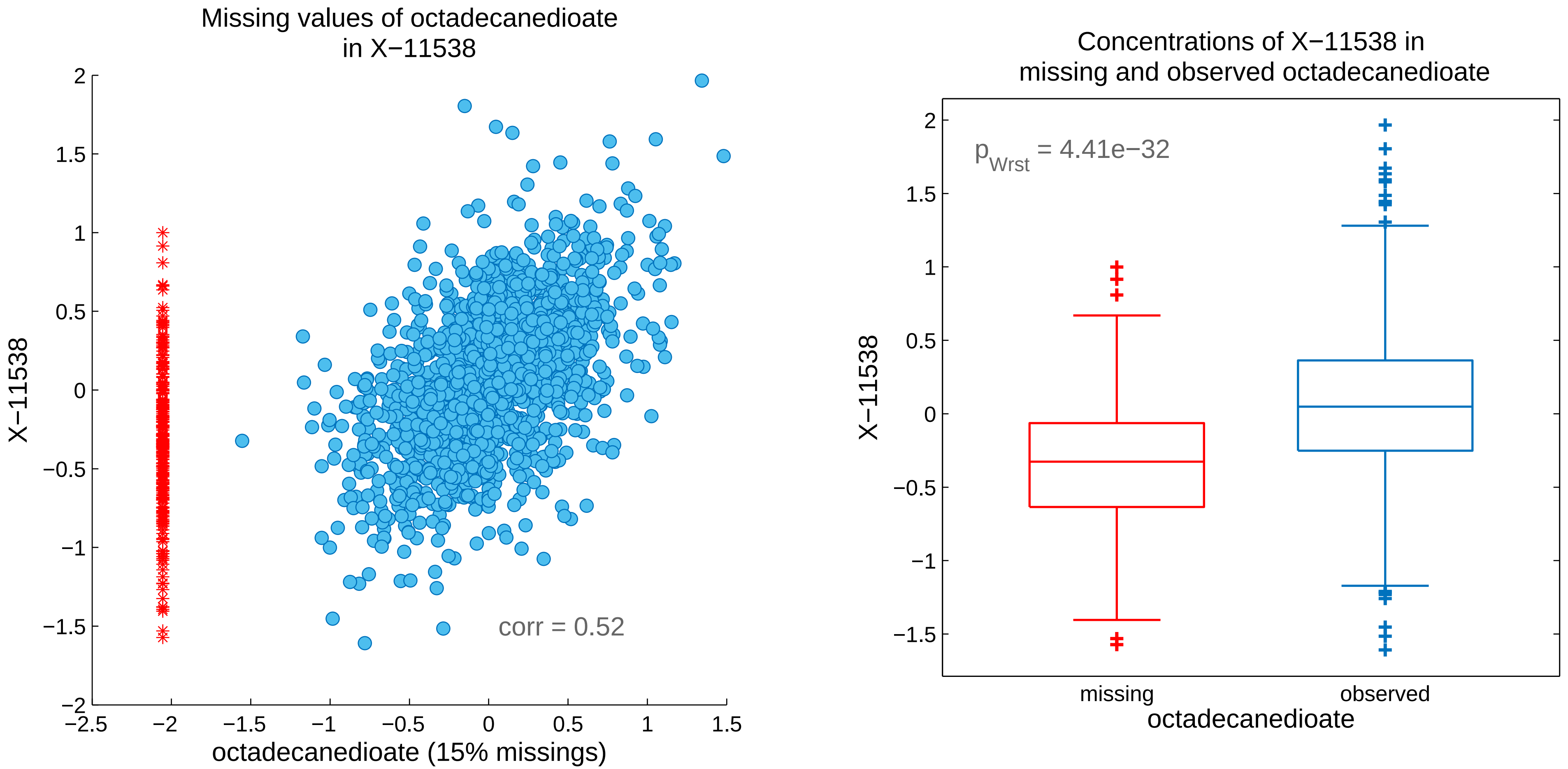
Missing values of octadecanedioate in X−11538
X−11538
octadecanedioate (15% missings)
corr = 0.52
Concentrations of X−11538 in missing and observed octadecanedioate
p_Wrst = 4.41e−32
missing
observed
octadecanedioate

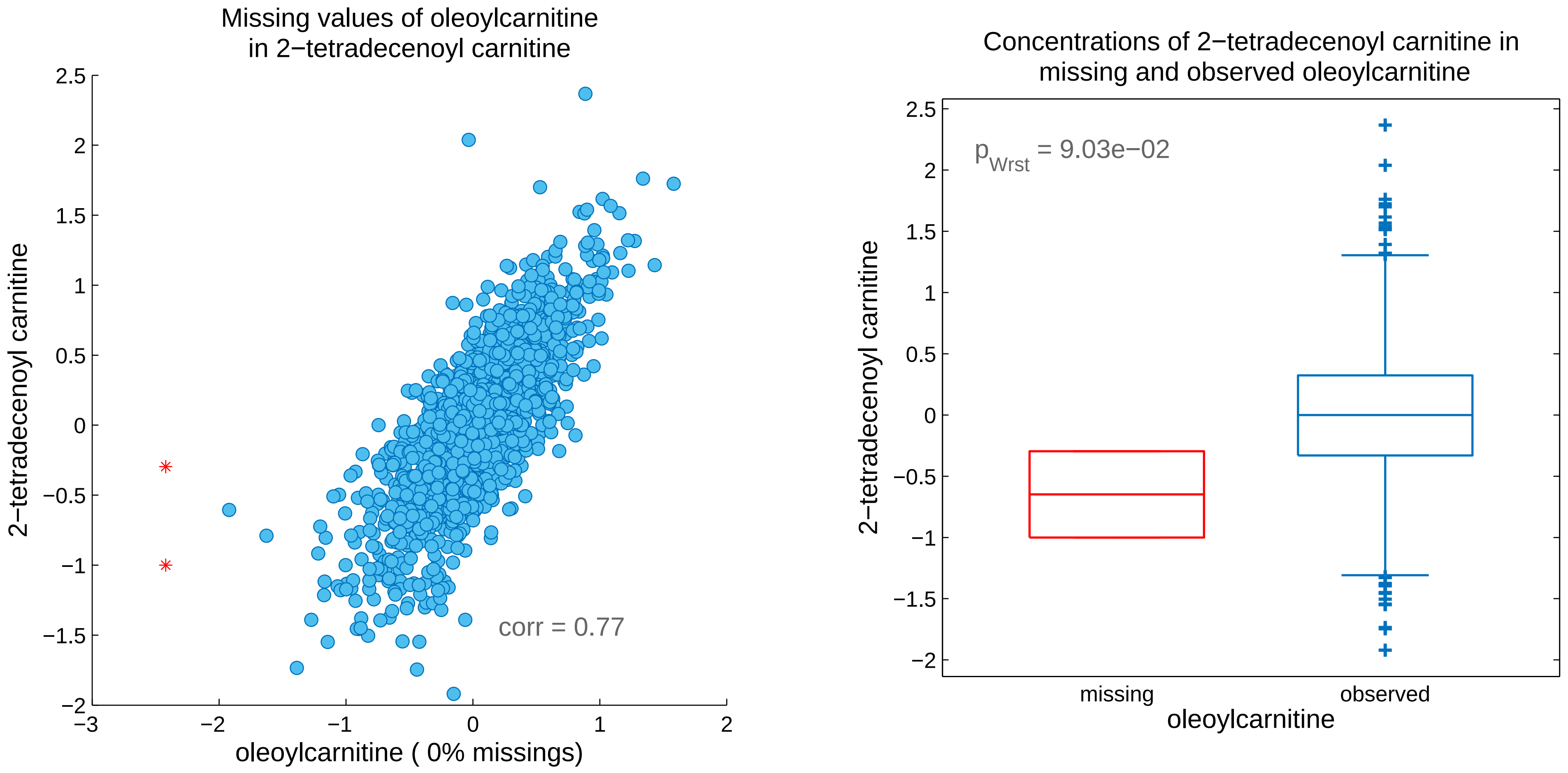
Missing values of oleoylcarnitine
in 2−tetradecenoyl carnitine
2−tetradecenoyl carnitine
oleoylcarnitine (0% missings)
corr = 0.77
Concentrations of 2−tetradecenoyl carnitine in
missing and observed oleoylcarnitine
p_{Wrst} = 9.03e−02
2−tetradecenoyl carnitine
missing
observed
oleoylcarnitine

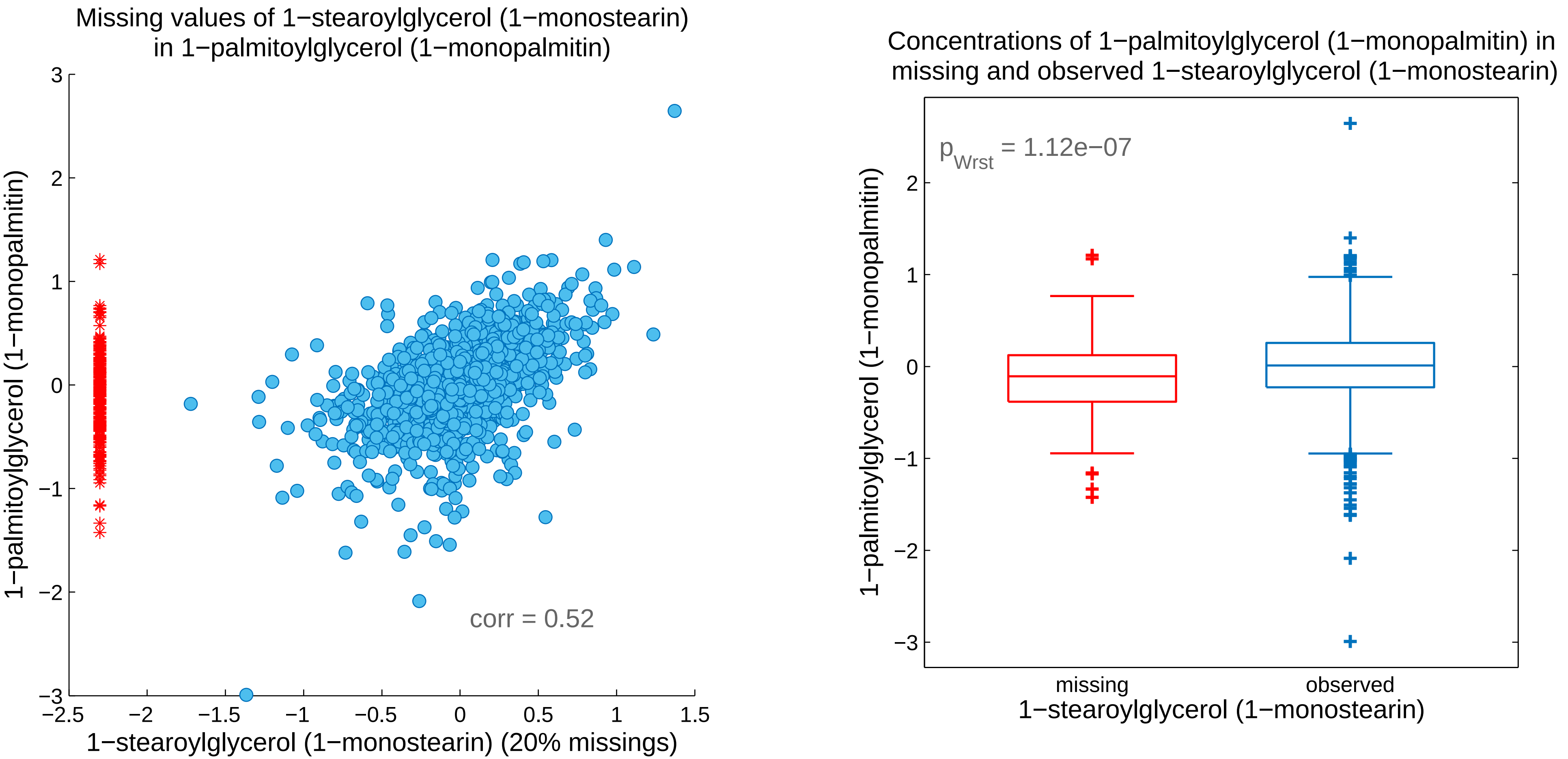
Missing values of 1−stearoylglycerol (1−monostearin) in 1−palmitoylglycerol (1−monopalmitin)
1−palmitoylglycerol (1−monopalmitin)
1−stearoylglycerol (1−monostearin) (20% missings)
corr = 0.52
3
2
1
0
−1
−2
−3
−2.5
−2
−1.5
−1
−0.5
0
0.5
1
1.5
Concentrations of 1−palmitoylglycerol (1−monopalmitin) in missing and observed 1−stearoylglycerol (1−monostearin)
p_{Wrst} = 1.12e−07
1−palmitoylglycerol (1−monopalmitin)
2
1
0
−1
−2
−3
missing
observed
1−stearoylglycerol (1−monostearin)

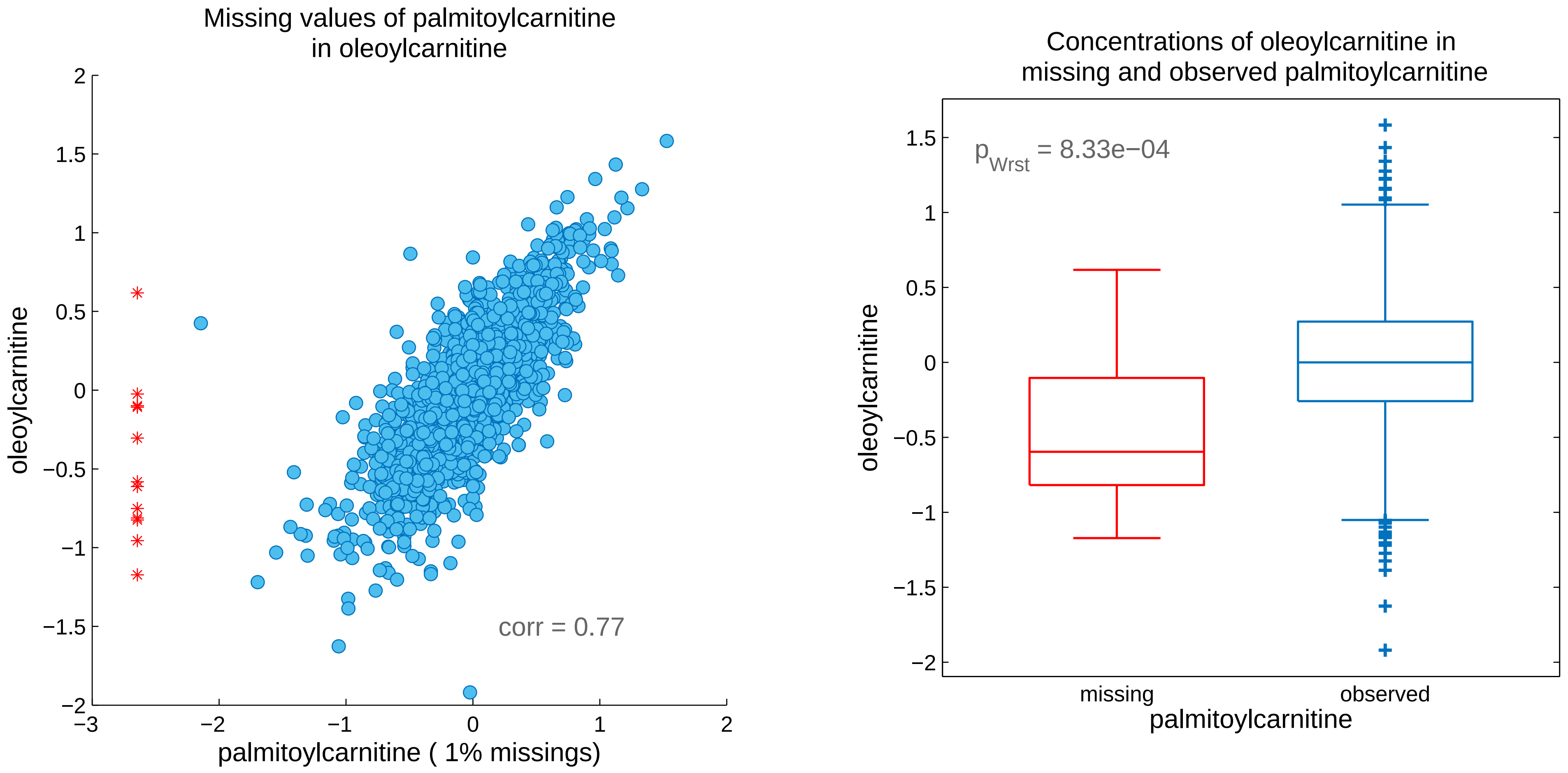
Missing values of palmitoylcarnitine in oleoylcarnitine
oleoylcarnitine
palmitoylcarnitine (1% missings)
corr = 0.77
2
1.5
1
0.5
0
−0.5
−1
−1.5
−2
−3
−2
−1
0
1
2
Concentrations of oleoylcarnitine in missing and observed palmitoylcarnitine
oleoylcarnitine
palmitoylcarnitine
p_Wrst = 8.33e−04
missing
observed
1.5
1
0.5
0
−0.5
−1
−1.5
−2

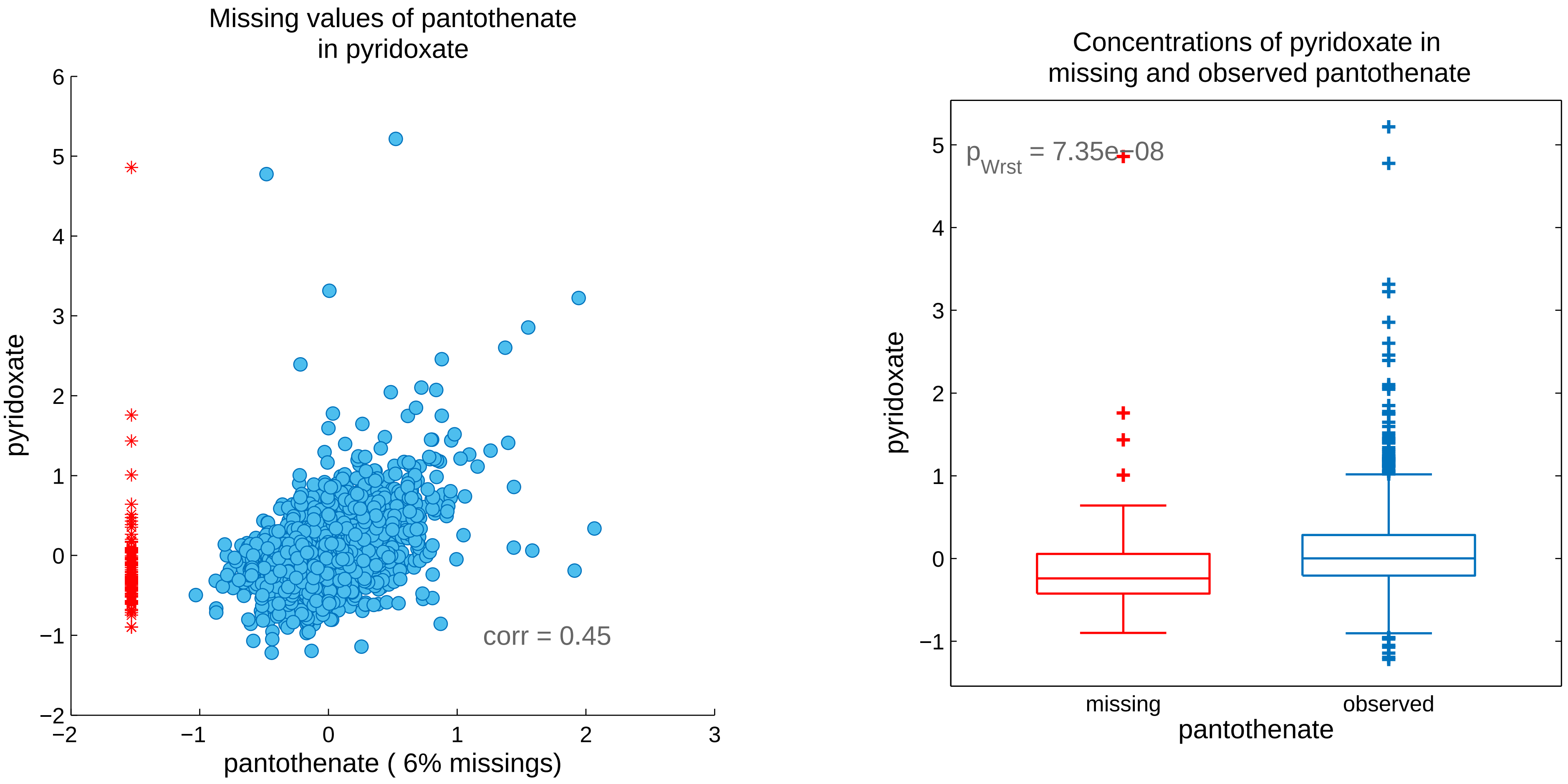

Missing values of pantothenate in pyridoxate
pyridoxate
pantothenate (6% missings)
corr = 0.45
6
5
4
3
2
1
0
−1
−2
−2
−1
0
1
2
3
Concentrations of pyridoxate in missing and observed pantothenate
p_{Wrst} = 7.35e−08
pyridoxate
5
4
3
2
1
0
−1
missing
observed
pantothenate

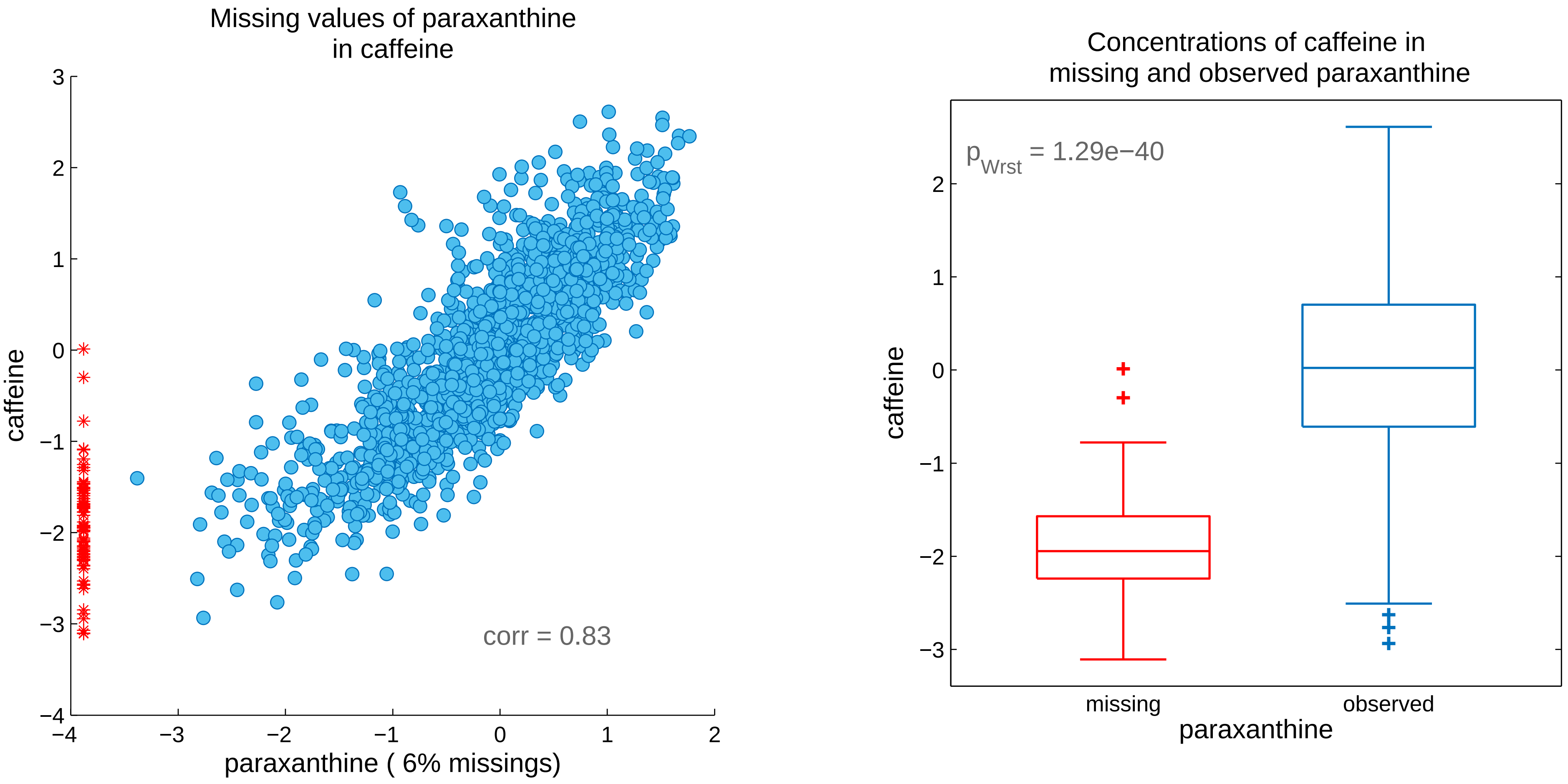

Missing values of paraxanthine in caffeine
caffeine
paraxanthine (6% missings)
corr = 0.83
3
2
1
0
−1
−2
−3
−4
−3
−2
−1
0
1
2
Concentrations of caffeine in missing and observed paraxanthine
p_Wrst = 1.29e−40
caffeine
missing
observed
paraxanthine

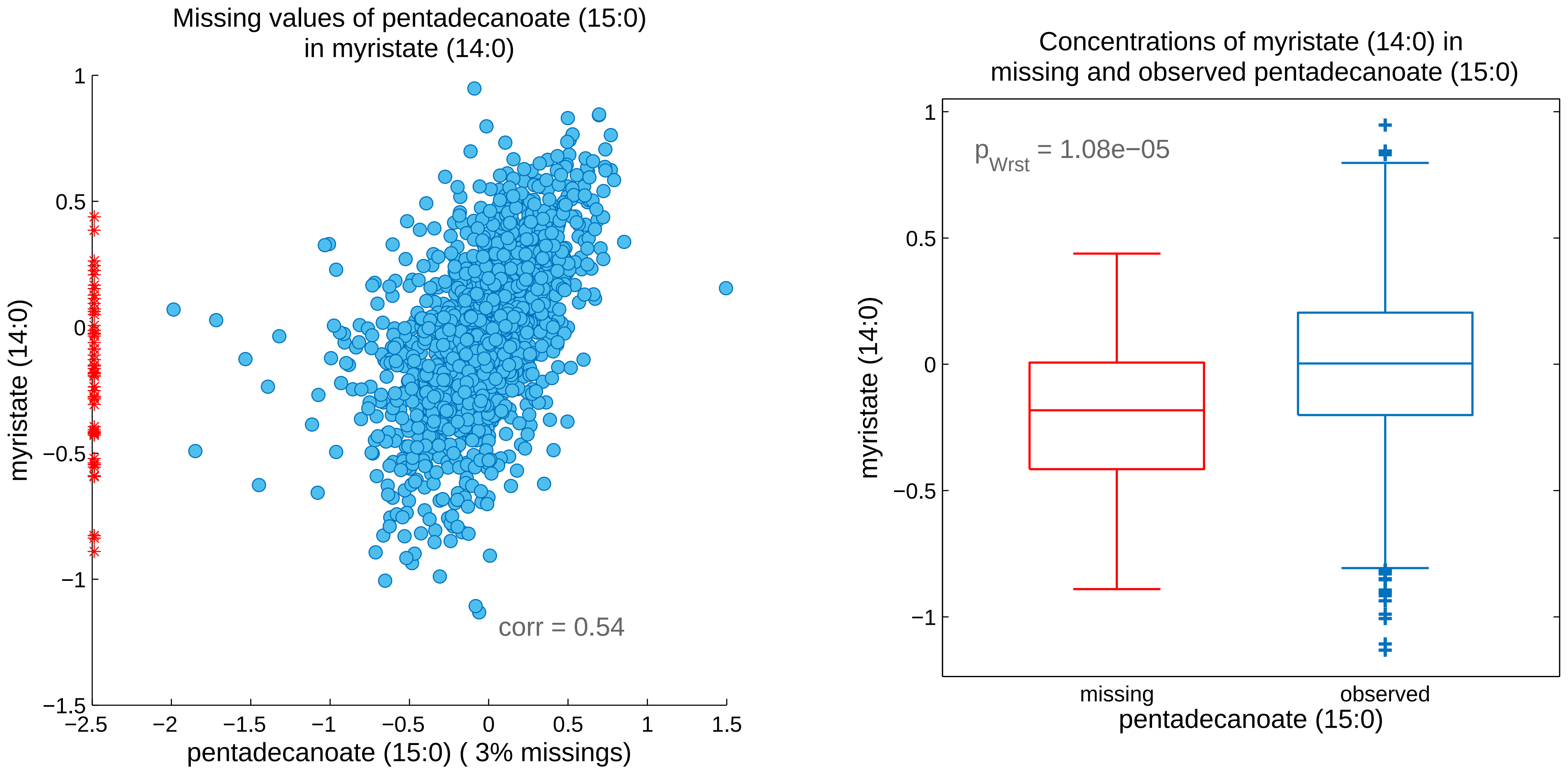
Missing values of pentadecanoate (15:0)
in myristate (14:0)
myristate (14:0)
pentadecanoate (15:0) (3% missings)
corr = 0.54
1
0.5
0
−0.5
−1
−1.5
−2.5
−2
−1.5
−1
−0.5
0
0.5
1
1.5
Concentrations of myristate (14:0) in
missing and observed pentadecanoate (15:0)
p_{Wrst} = 1.08e−05
myristate (14:0)
missing
observed
pentadecanoate (15:0)

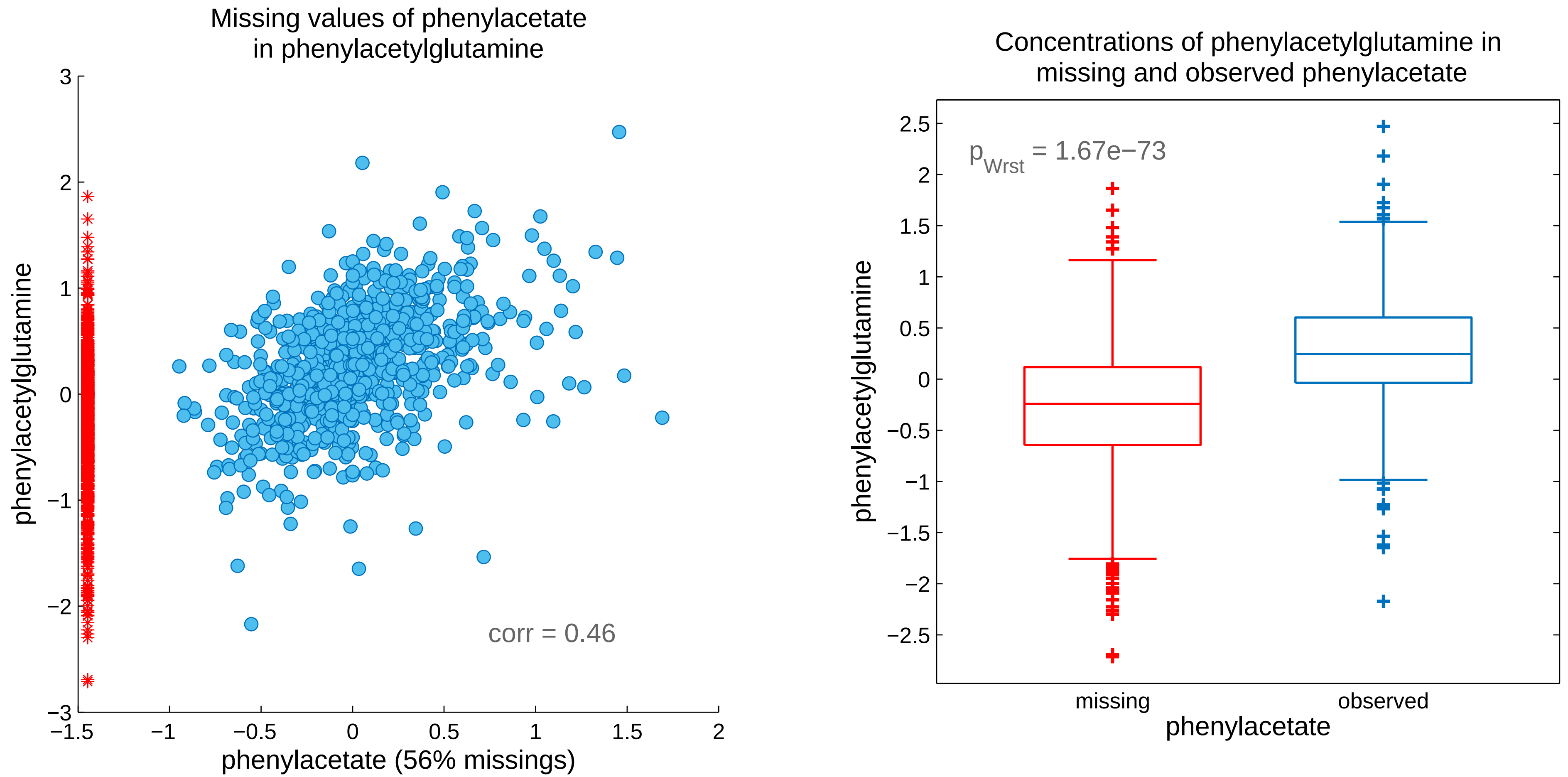
Missing values of phenylacetate in phenylacetylglutamine
phenylacetylglutamine
phenylacetate (56% missings)
corr = 0.46
Concentrations of phenylacetylglutamine in missing and observed phenylacetate
p_{Wrst} = 1.67e−73
phenylacetylglutamine
missing
observed
phenylacetate

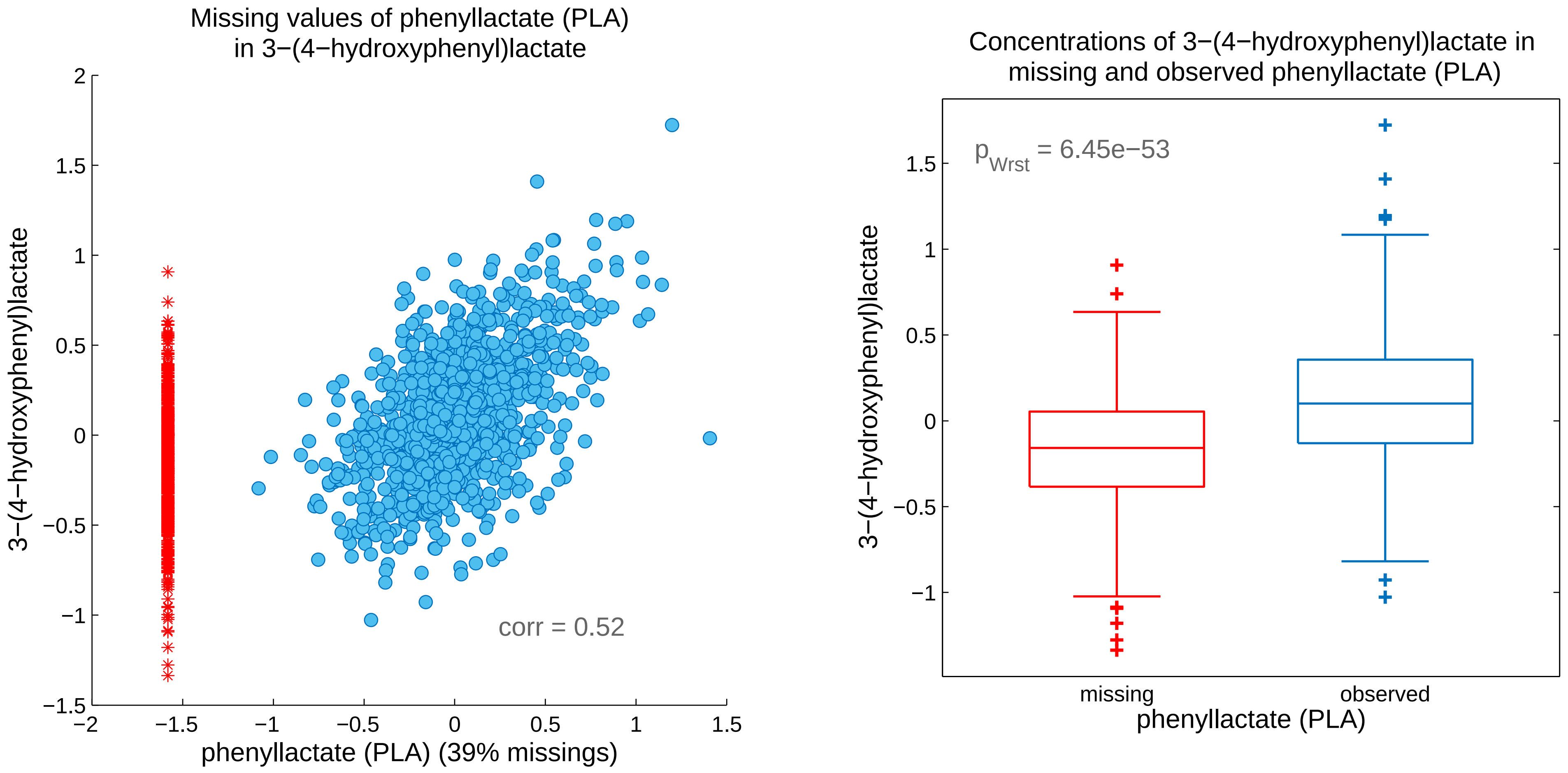

Missing values of phenyllactate (PLA)
in 3−(4−hydroxyphenyl)lactate
3−(4−hydroxyphenyl)lactate
phenyllactate (PLA) (39% missings)
corr = 0.52
2
1.5
1
0.5
0
−0.5
−1
−1.5
−2
−1.5
−1
−0.5
0
0.5
1
1.5
Concentrations of 3−(4−hydroxyphenyl)lactate in
missing and observed phenyllactate (PLA)
p_{Wrst} = 6.45e−53
3−(4−hydroxyphenyl)lactate
1.5
1
0.5
0
−0.5
−1
missing
observed
phenyllactate (PLA)

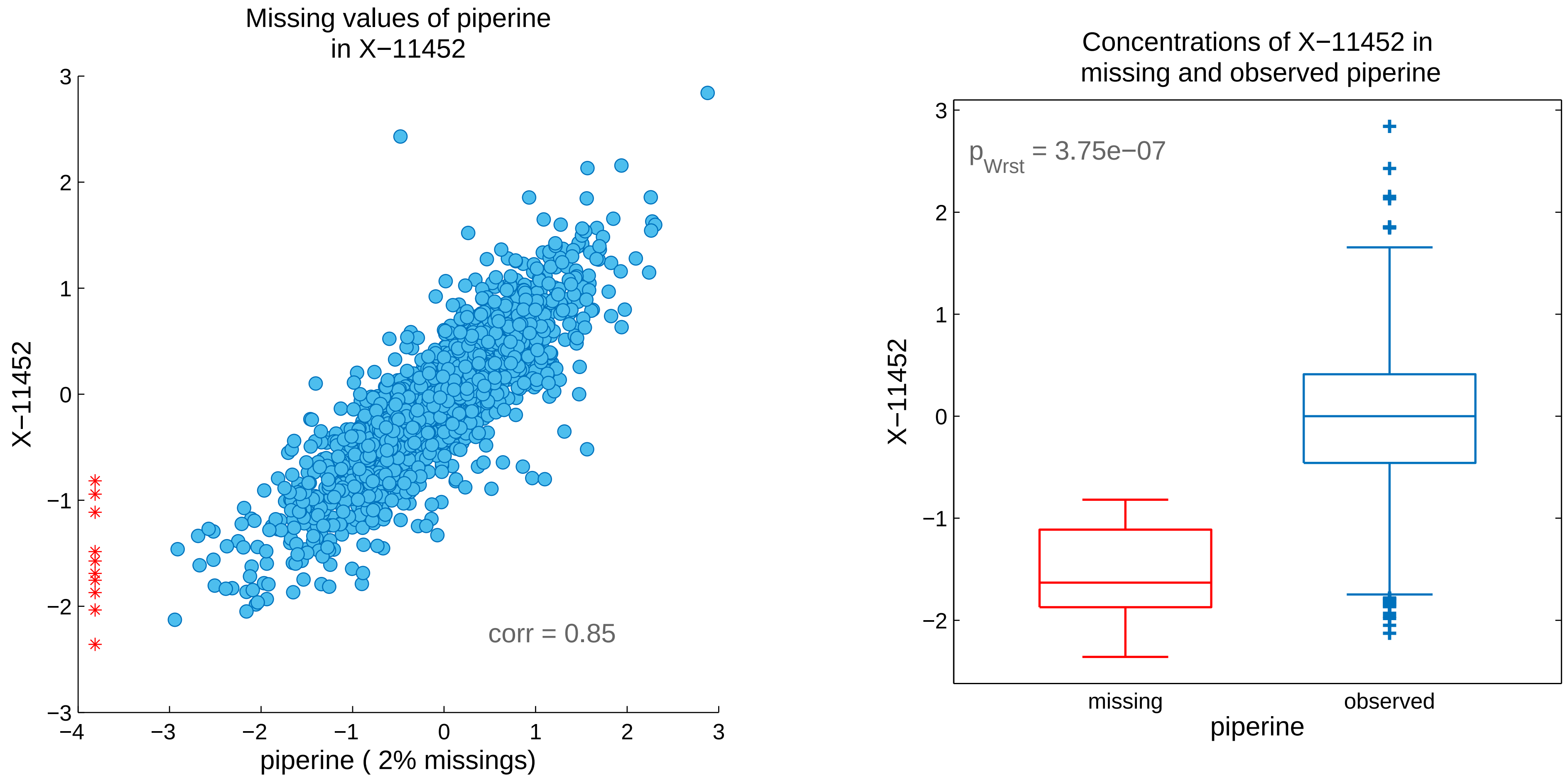
Missing values of piperine
in X−11452
X−11452
piperine (2% missings)
corr = 0.85
Concentrations of X−11452 in
missing and observed piperine
p_Wrst = 3.75e−07
X−11452
missing
observed
piperine

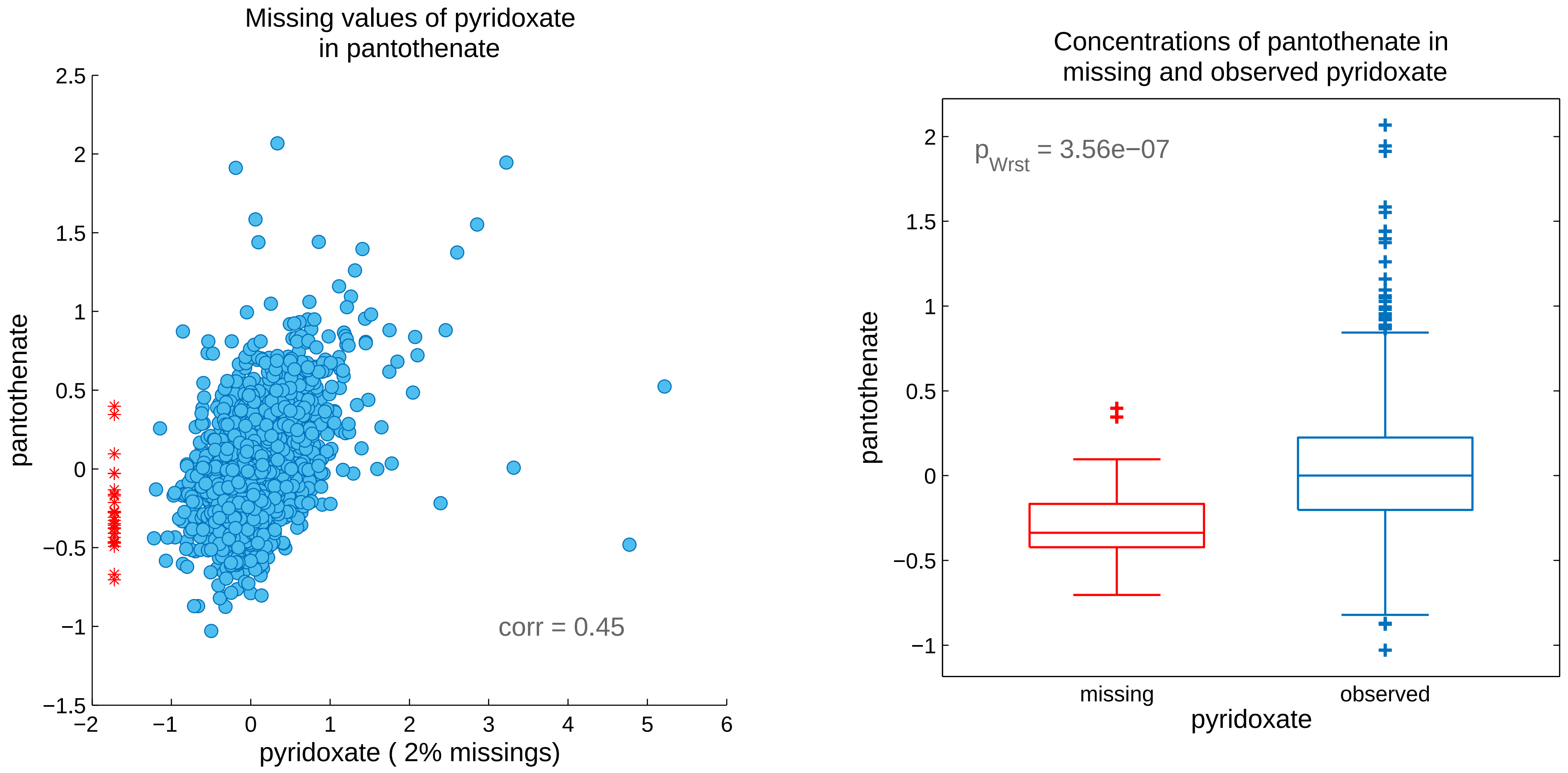

Missing values of pyridoxate
in pantothenate
pantothenate
pyridoxate (2% missings)
corr = 0.45
2.5
2
1.5
1
0.5
0
−0.5
−1
−1.5
−2
−1
0
1
2
3
4
5
6
Concentrations of pantothenate in
missing and observed pyridoxate
pWrst = 3.56e−07
pantothenate
pyridoxate
missing
observed
2
1.5
1
0.5
0
−0.5
−1

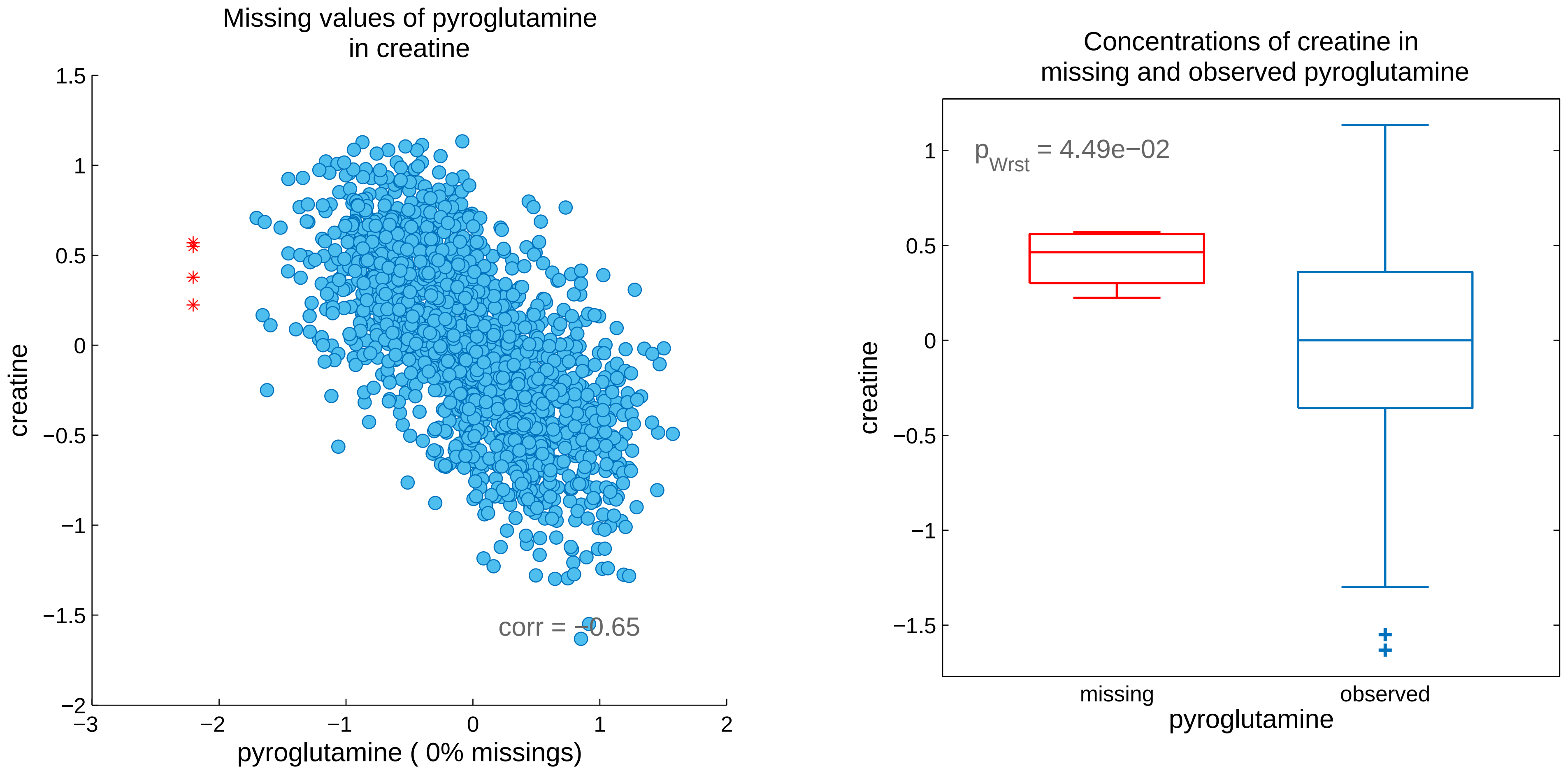

Missing values of pyroglutamine in creatine
1.5
1
0.5
0
−0.5
−1
−1.5
−2
creatine
corr = −0.65
−3
−2
−1
0
1
2
pyroglutamine (0% missings)
Concentrations of creatine in missing and observed pyroglutamine
p_{Wrst} = 4.49e−02
1
0.5
0
−0.5
−1
−1.5
creatine
missing
observed
pyroglutamine

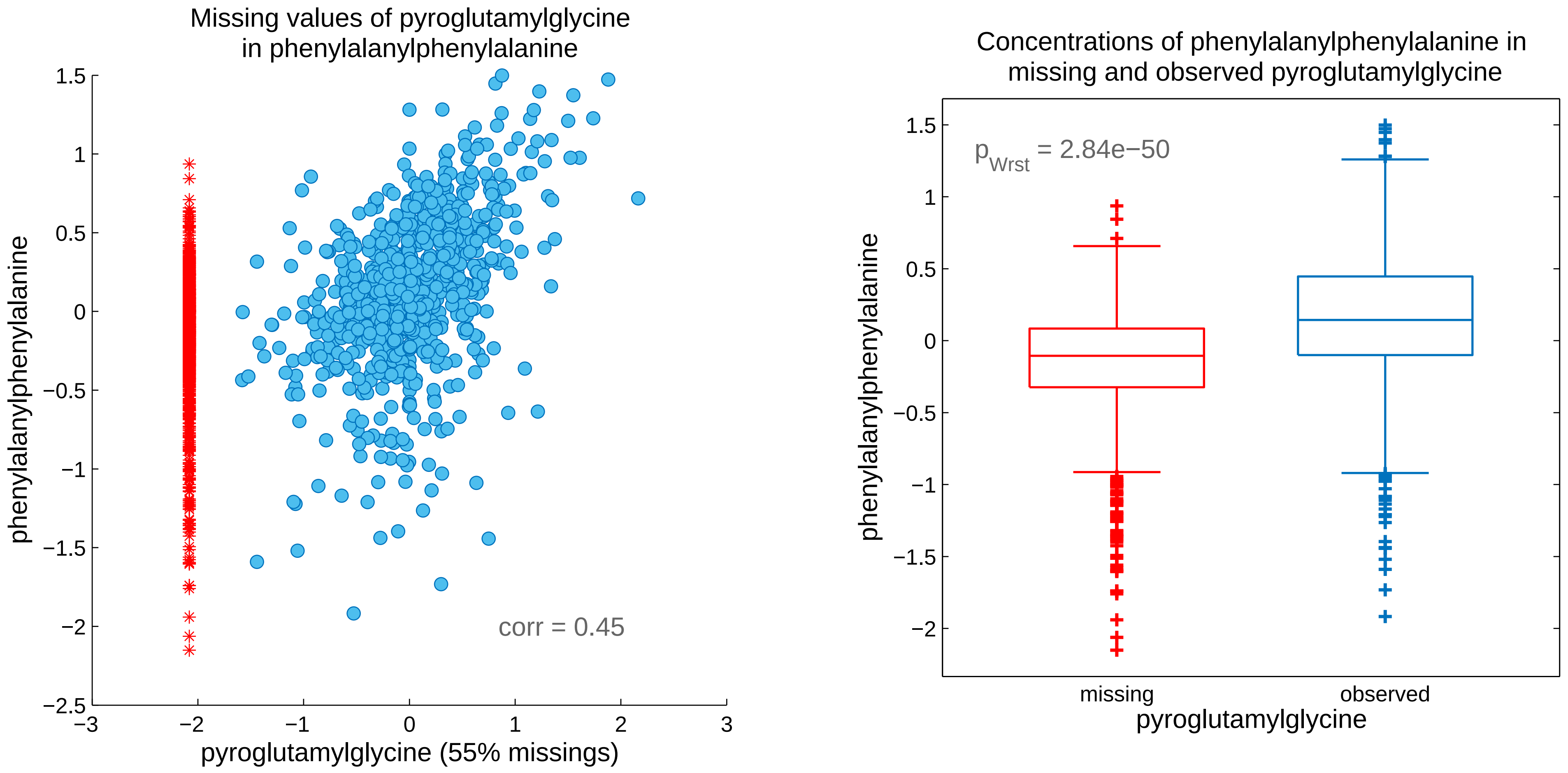

Missing values of pyroglutamylglycine in phenylalanylphenylalanine
phenylalanylphenylalanine
pyroglutamylglycine (55% missings)
corr = 0.45
Concentrations of phenylalanylphenylalanine in missing and observed pyroglutamylglycine
p_{Wrst} = 2.84e−50
phenylalanylphenylalanine
missing
observed
pyroglutamylglycine

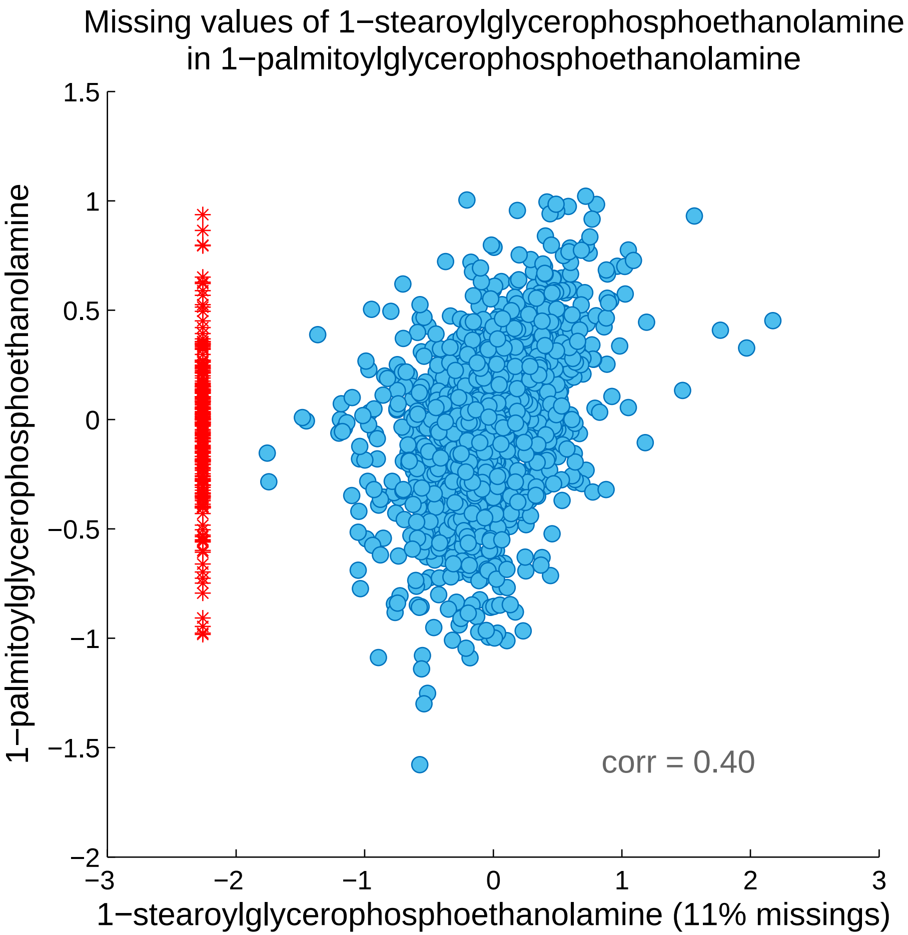
Missing values of 1−stearoylglycerophosphoethanolamine
in 1−palmitoylglycerophosphoethanolamine
1−palmitoylglycerophosphoethanolamine
1−stearoylglycerophosphoethanolamine (11% missings)
corr = 0.40
1.5
1
0.5
0
−0.5
−1
−1.5
−2
−3
−2
−1
0
1
2
3

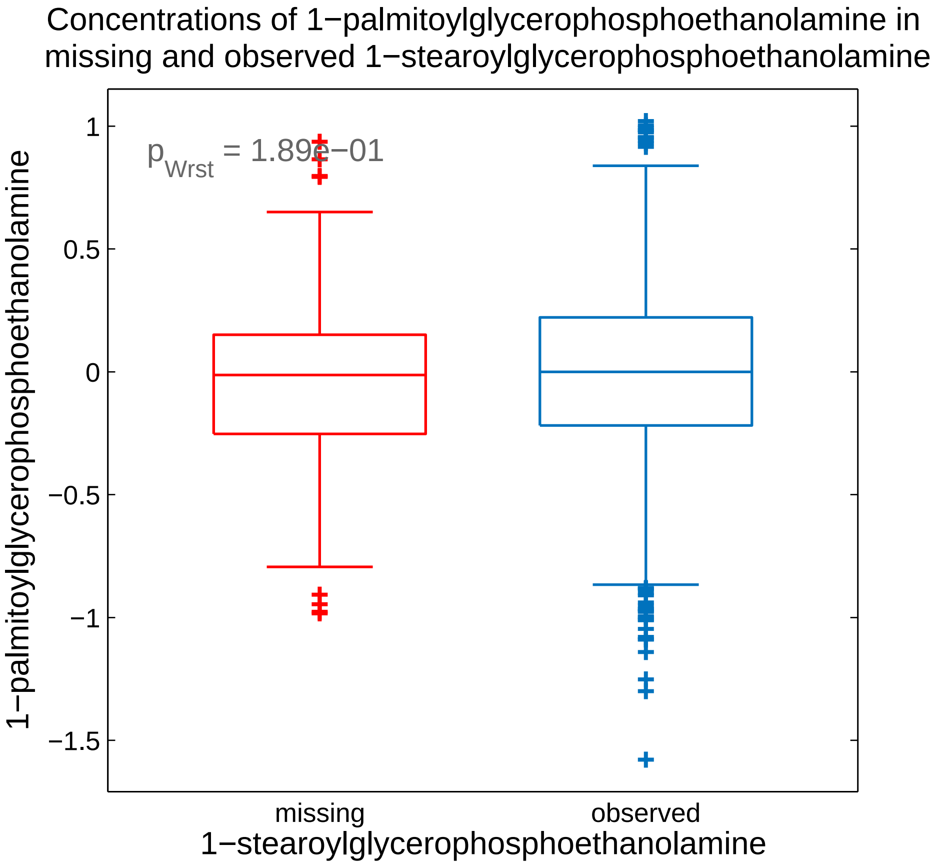
Concentrations of 1−palmitoylglycerophosphoethanolamine in
missing and observed 1−stearoylglycerophosphoethanolamine
p_{Wrst} = 1.89e−01
1−palmitoylglycerophosphoethanolamine
1
0.5
0
−0.5
−1
−1.5
missing
observed
1−stearoylglycerophosphoethanolamine

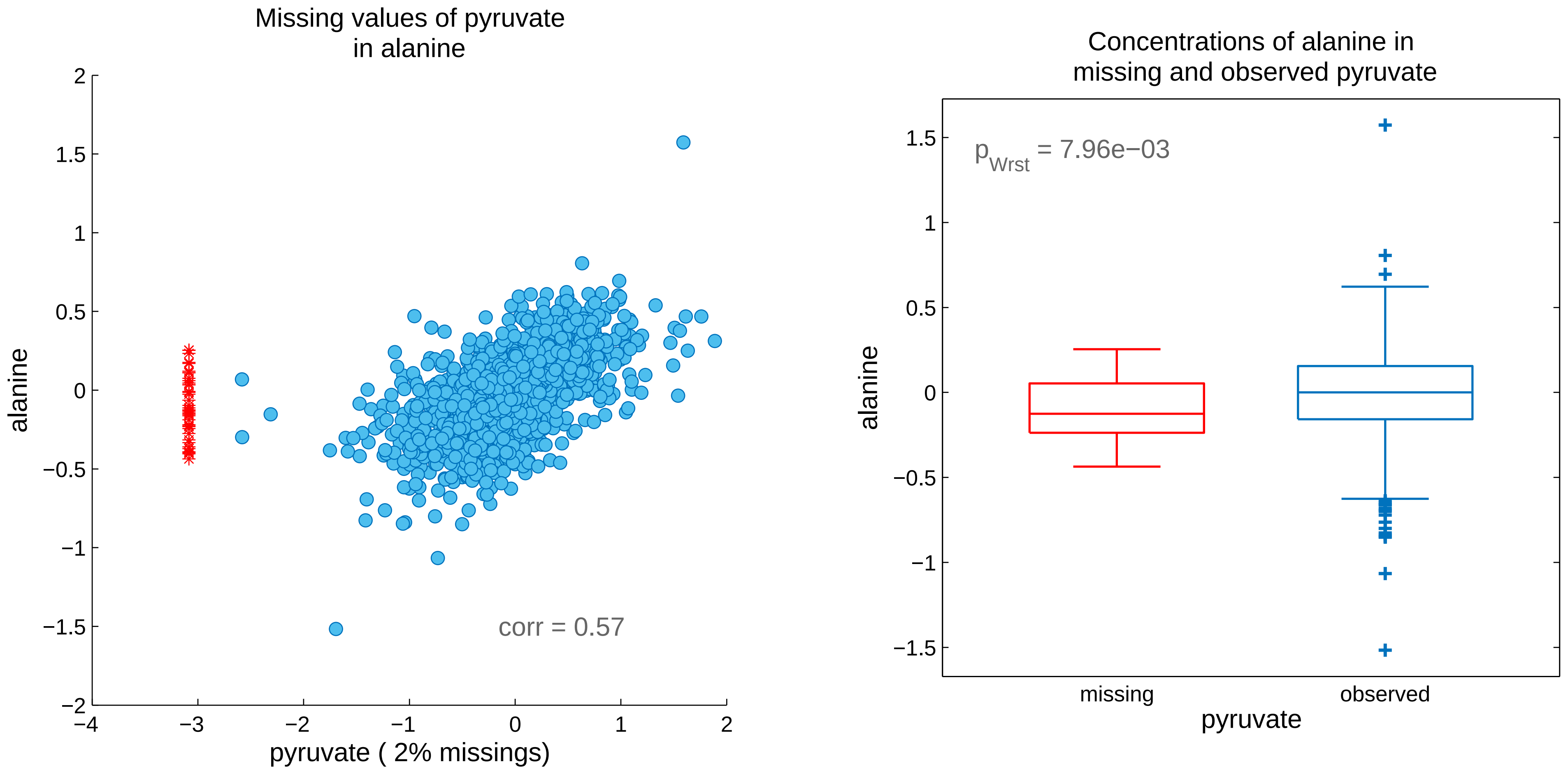

Missing values of pyruvate
in alanine
alanine
pyruvate (2% missings)
corr = 0.57
2
1.5
1
0.5
0
−0.5
−1
−1.5
−2
−4
−3
−2
−1
0
1
2
Concentrations of alanine in
missing and observed pyruvate
p_Wrst = 7.96e−03
alanine
1.5
1
0.5
0
−0.5
−1
−1.5
missing
observed
pyruvate

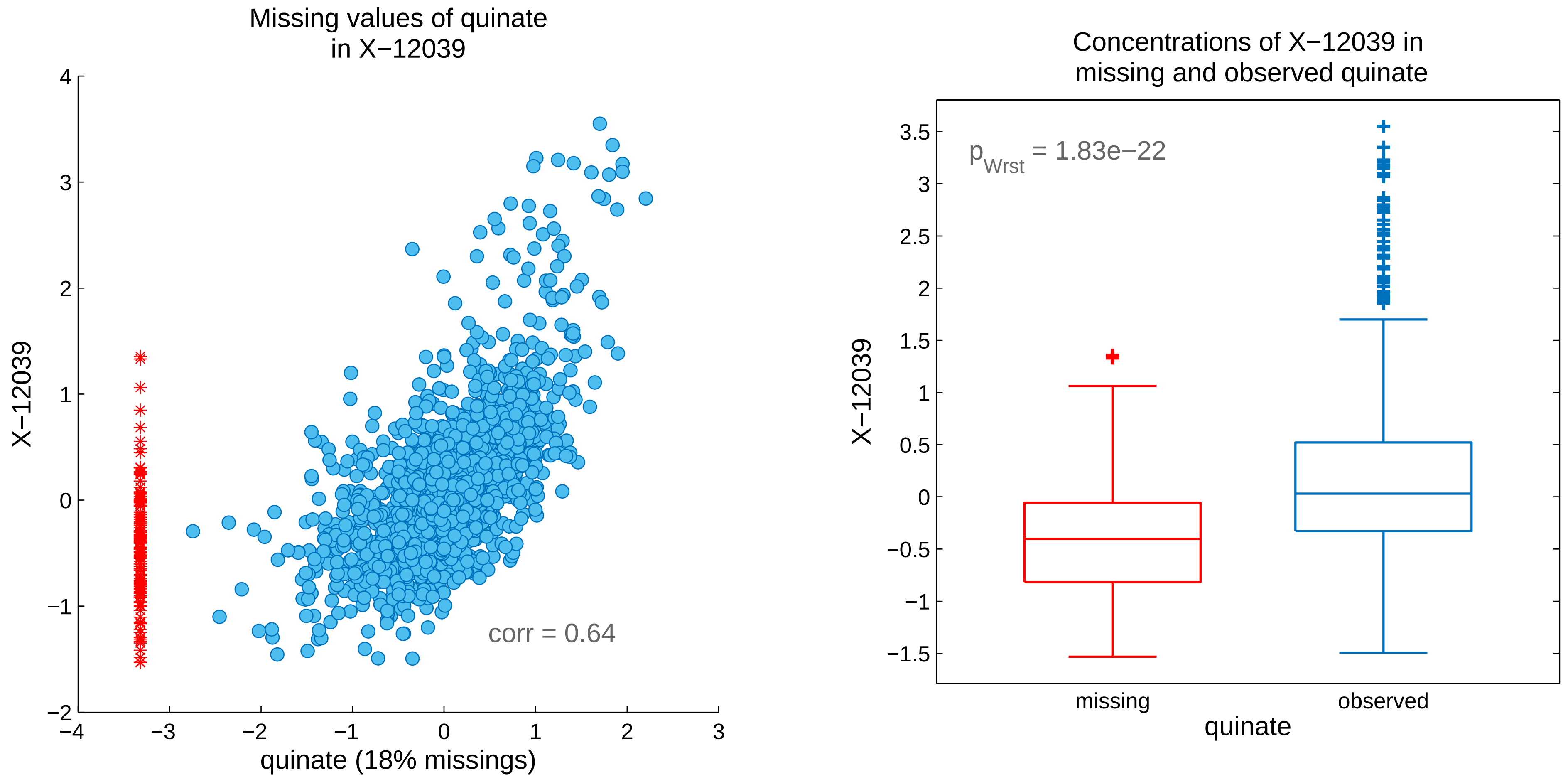

Missing values of quinate in X−12039
X−12039
quinate (18% missings)
corr = 0.64
Concentrations of X−12039 in missing and observed quinate
p_{Wrst} = 1.83e−22
X−12039
missing
observed
quinate

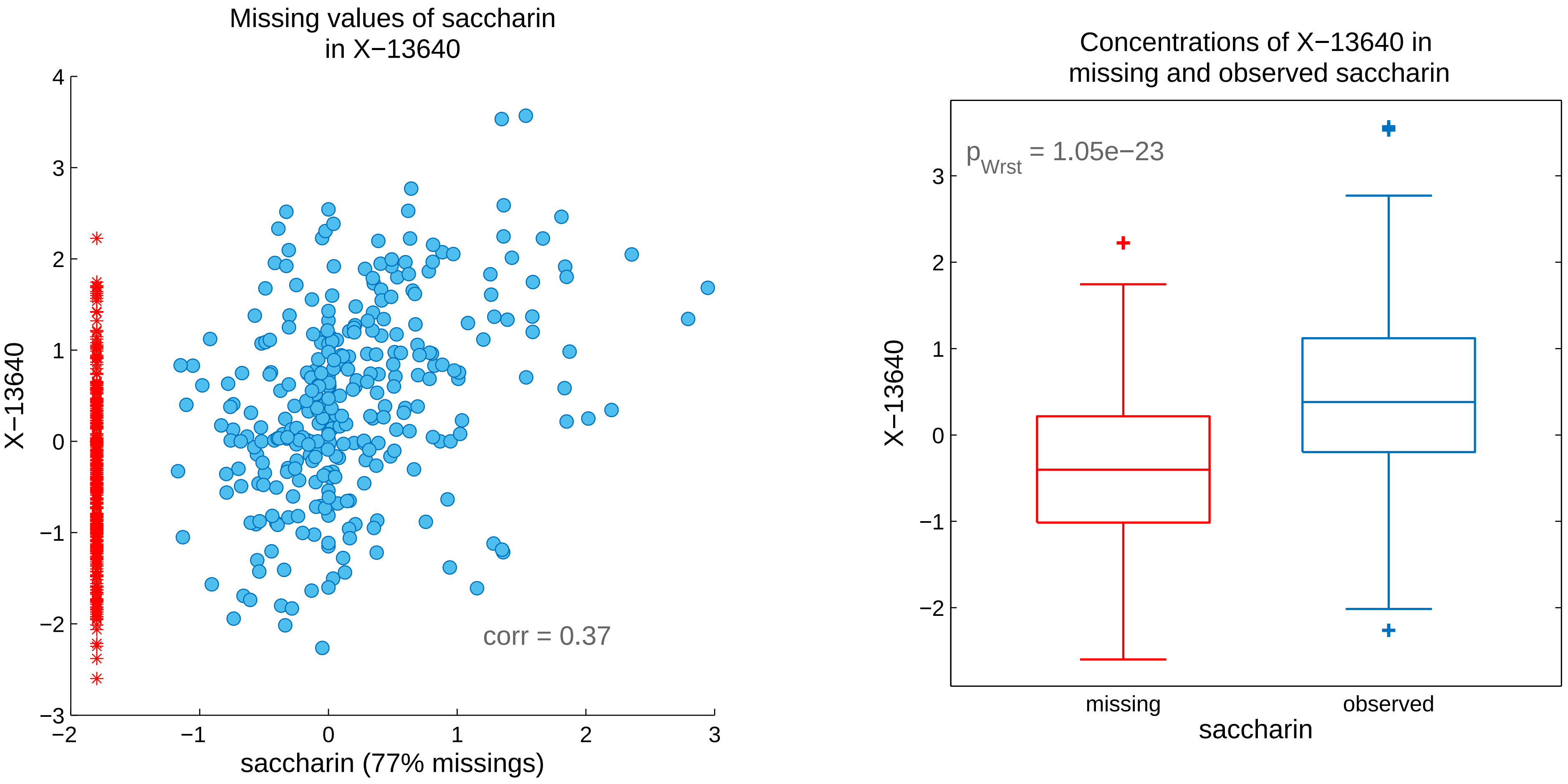
Missing values of saccharin
in X−13640
X−13640
saccharin (77% missings)
corr = 0.37
Concentrations of X−13640 in
missing and observed saccharin
p_Wrst = 1.05e−23
X−13640
missing
observed
saccharin

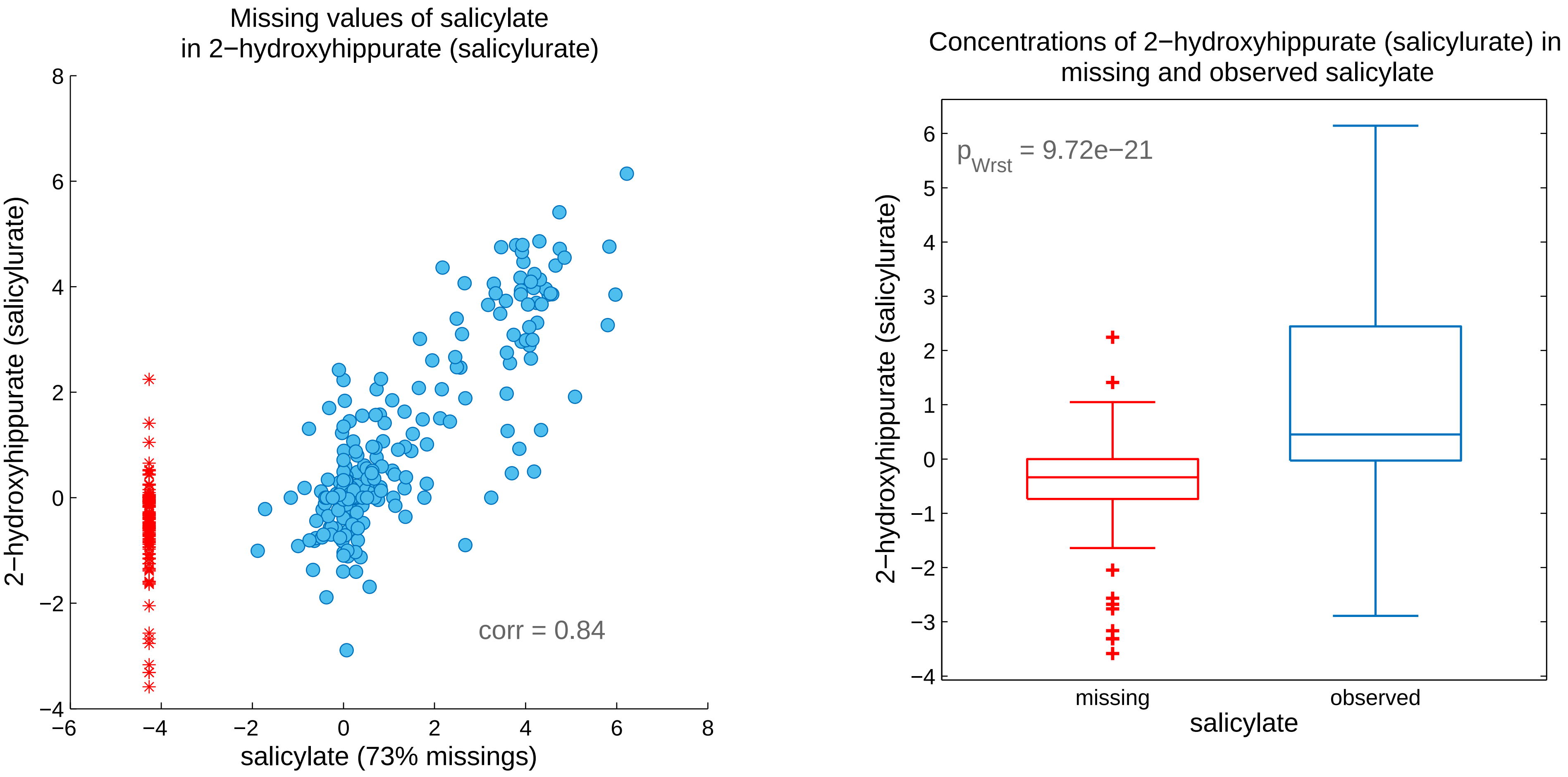
Missing values of salicylate
in 2−hydroxyhippurate (salicylurate)
2−hydroxyhippurate (salicylurate)
corr = 0.84
salicylate (73% missings)
Concentrations of 2−hydroxyhippurate (salicylurate) in
missing and observed salicylate
p_{Wrst} = 9.72e−21
2−hydroxyhippurate (salicylurate)
missing
observed
salicylate

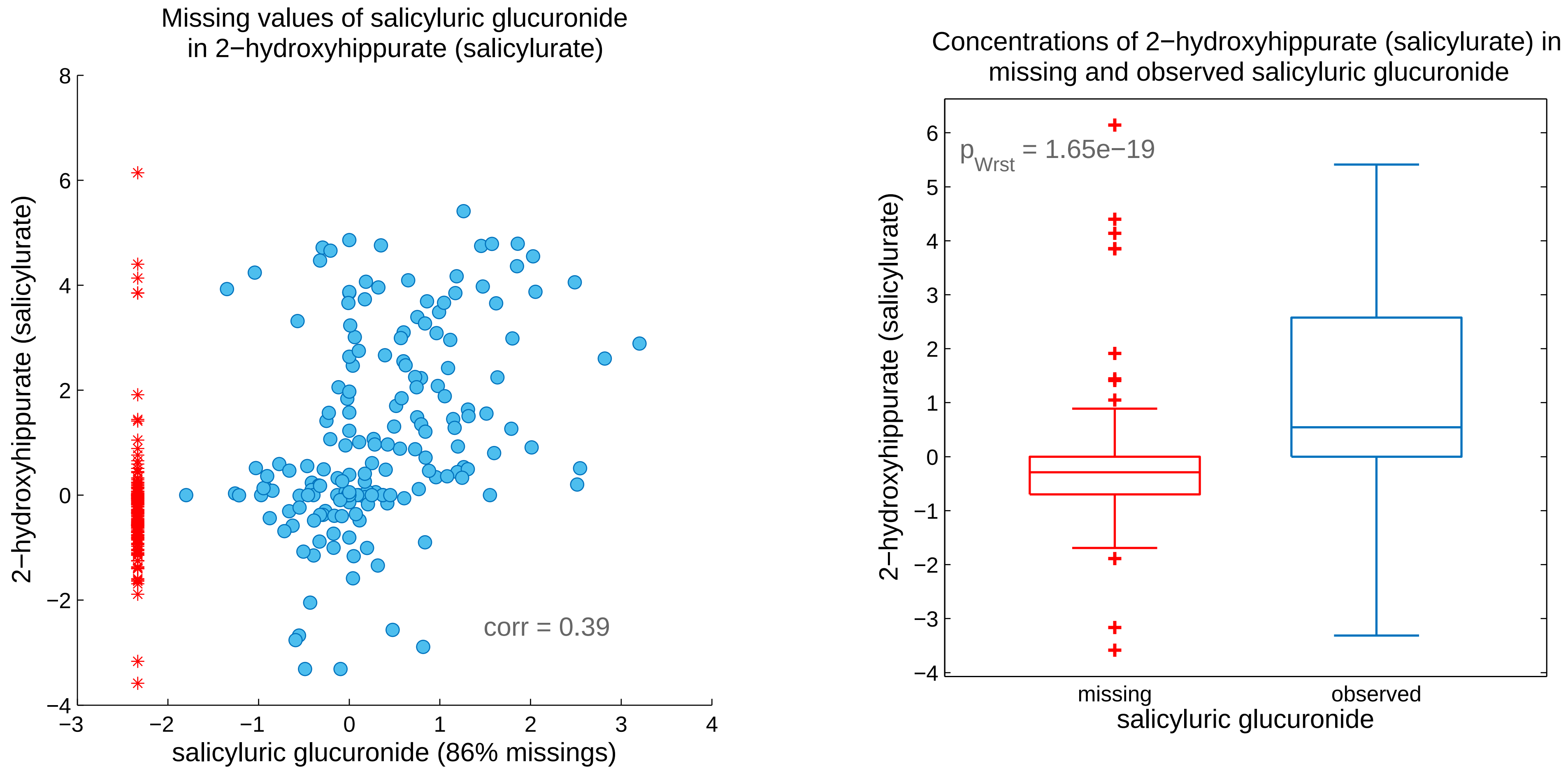

Missing values of salicyluric glucuronide
in 2−hydroxyhippurate (salicylurate)
2−hydroxyhippurate (salicylurate)
salicyluric glucuronide (86% missings)
corr = 0.39
Concentrations of 2−hydroxyhippurate (salicylurate) in
missing and observed salicyluric glucuronide
p_Wrst = 1.65e−19
2−hydroxyhippurate (salicylurate)
missing
observed
salicyluric glucuronide

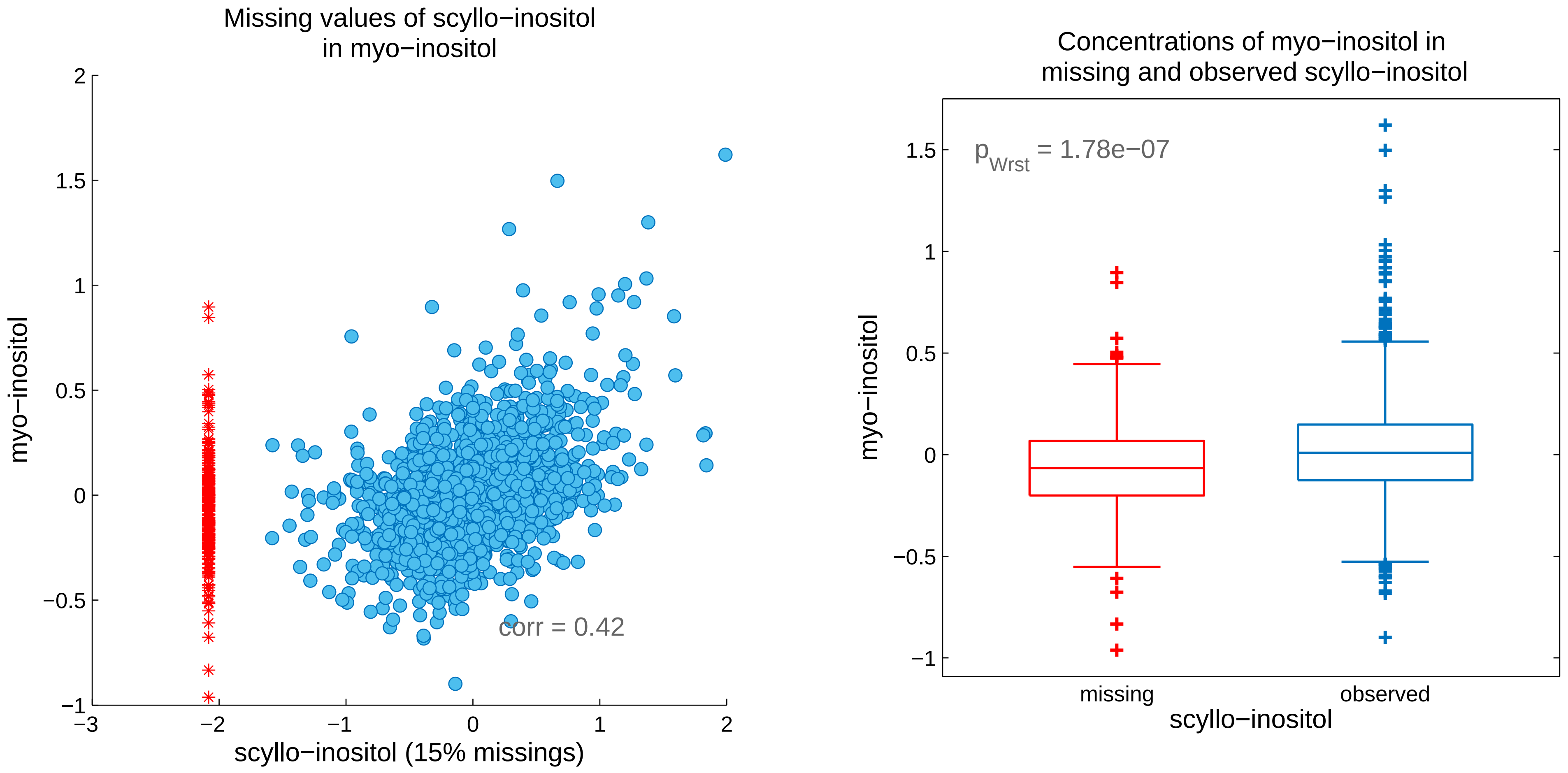

Missing values of scyllo−inositol in myo−inositol
myo−inositol
scyllo−inositol (15% missings)
corr = 0.42
2
1.5
1
0.5
0
−0.5
−1
−3
−2
−1
0
1
2
Concentrations of myo−inositol in missing and observed scyllo−inositol
p_{Wrst} = 1.78e−07
myo−inositol
1.5
1
0.5
0
−0.5
−1
missing
observed
scyllo−inositol

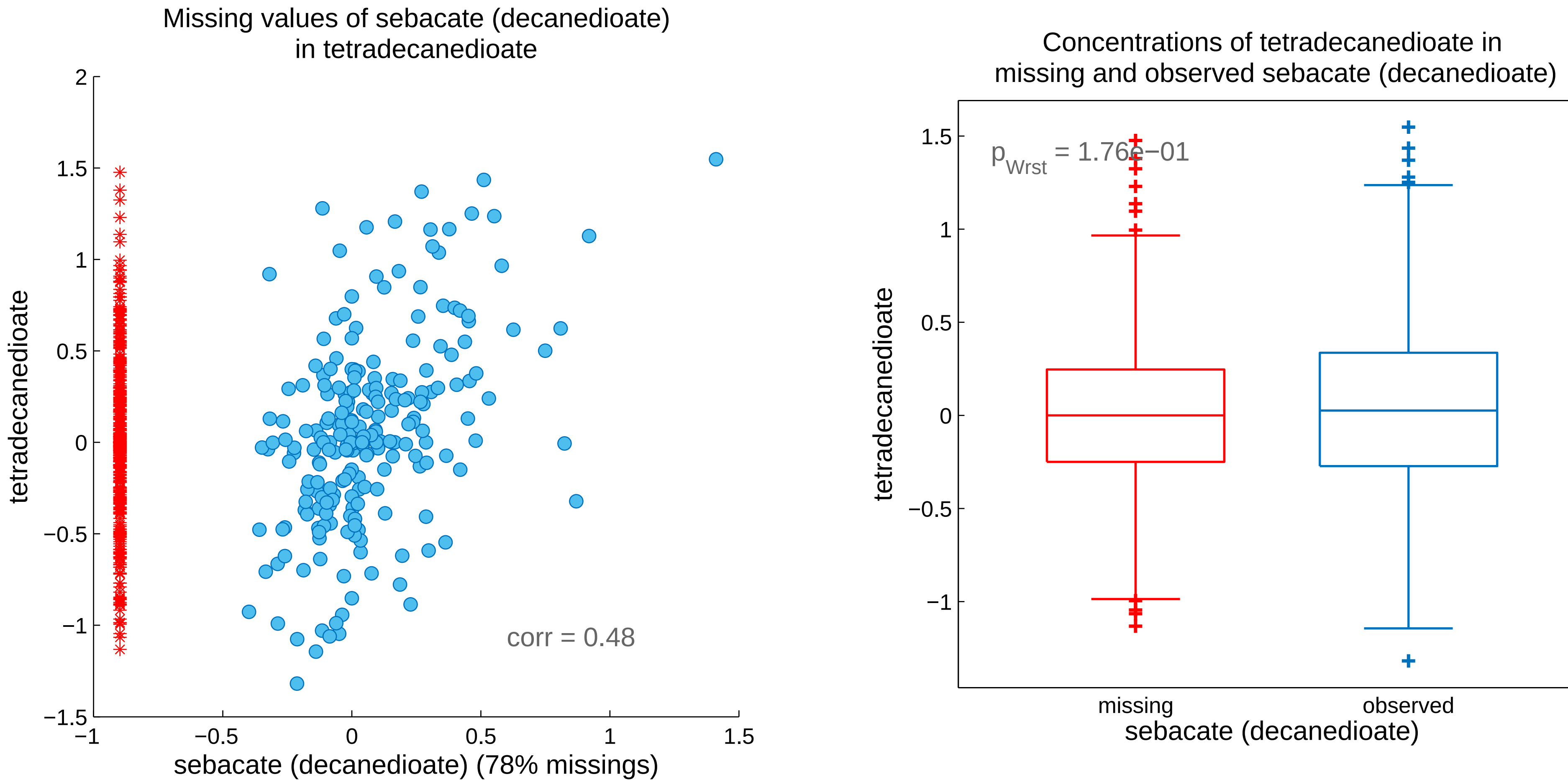

Missing values of sebacate (decanedioate) in tetradecanedioate
tetradecanedioate
sebacate (decanedioate) (78% missings)
corr = 0.48
2
1.5
1
0.5
0
−0.5
−1
−1.5
−1
−0.5
0
0.5
1
1.5
Concentrations of tetradecanedioate in missing and observed sebacate (decanedioate)
p_{Wrst} = 1.76e−01
tetradecanedioate
missing
observed
sebacate (decanedioate)

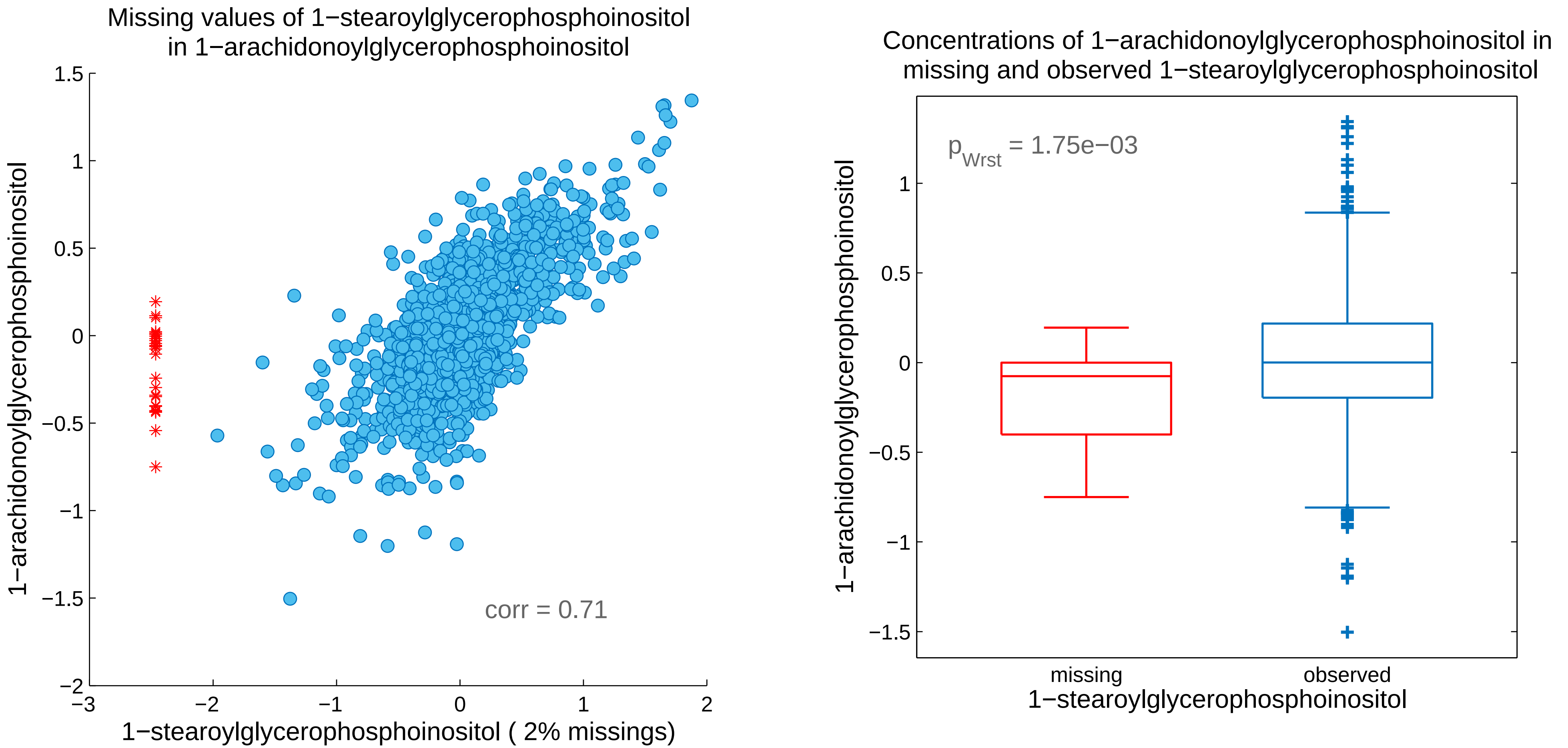
Missing values of 1−stearoylglycerophosphoinositol in 1−arachidonoylglycerophosphoinositol
1−arachidonoylglycerophosphoinositol
1−stearoylglycerophosphoinositol (2% missings)
corr = 0.71
1.5
1
0.5
0
−0.5
−1
−1.5
−2
−3
−2
−1
0
1
2
Concentrations of 1−arachidonoylglycerophosphoinositol in missing and observed 1−stearoylglycerophosphoinositol
p_{Wrst} = 1.75e−03
1−arachidonoylglycerophosphoinositol
missing
observed
1−stearoylglycerophosphoinositol

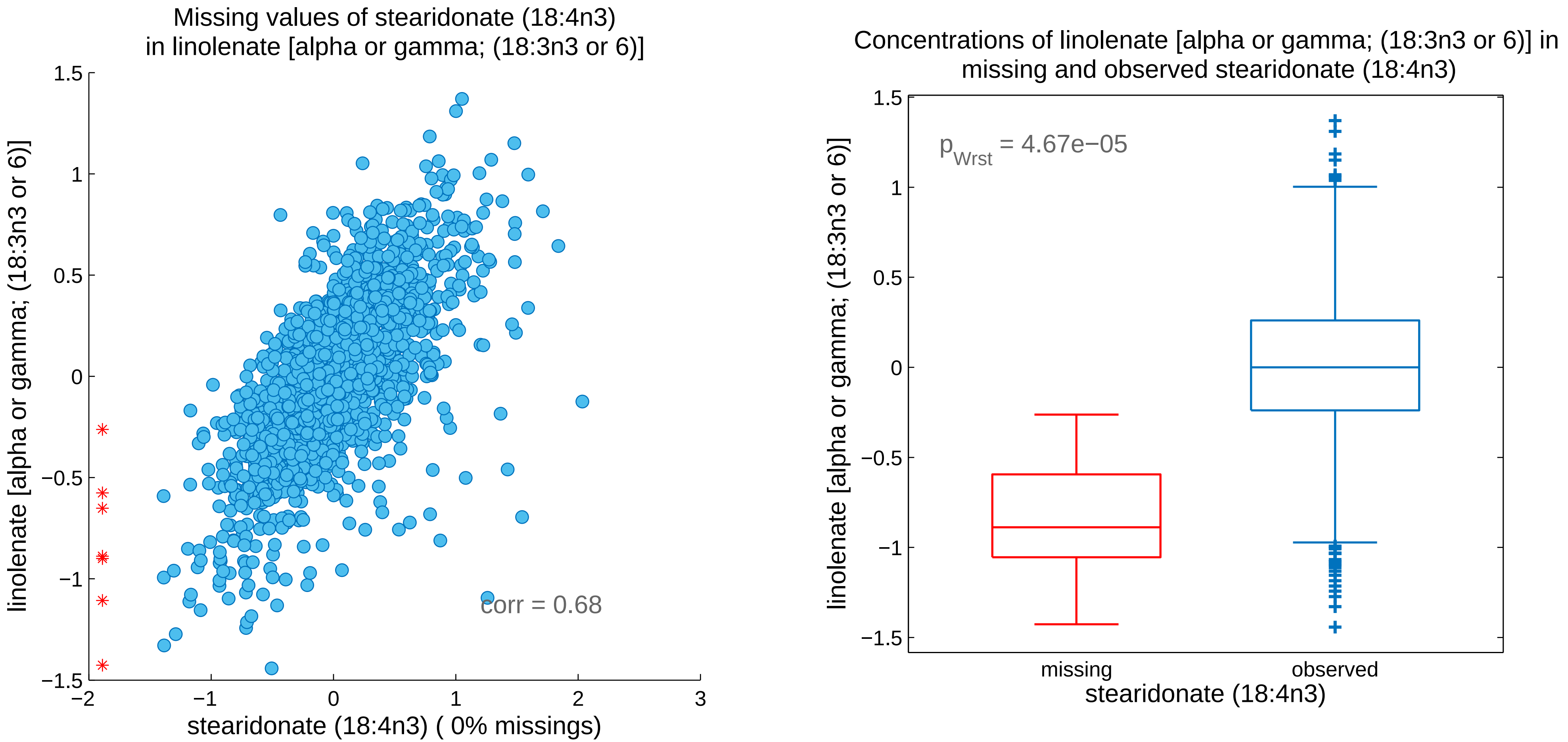

Missing values of stearidonate (18:4n3)
in linolenate [alpha or gamma; (18:3n3 or 6)]
linolenate [alpha or gamma; (18:3n3 or 6)]
stearidonate (18:4n3) (0% missings)
corr = 0.68
Concentrations of linolenate [alpha or gamma; (18:3n3 or 6)] in
missing and observed stearidonate (18:4n3)
p_{Wrst} = 4.67e−05
missing
observed
stearidonate (18:4n3)

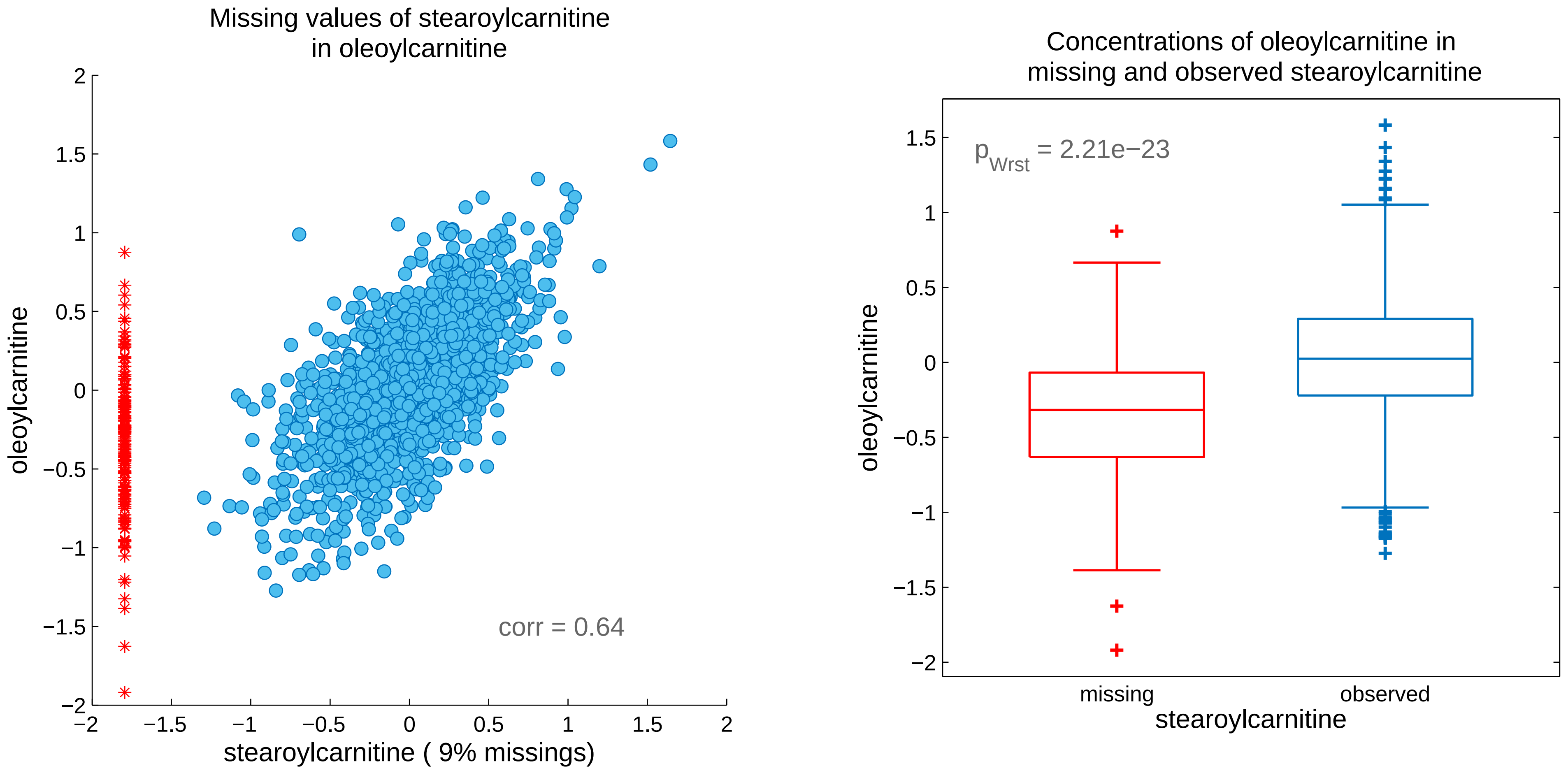

Missing values of stearoylcarnitine in oleoylcarnitine
oleoylcarnitine
stearoylcarnitine (9% missings)
corr = 0.64
Concentrations of oleoylcarnitine in missing and observed stearoylcarnitine
p_{Wrst} = 2.21e−23
oleoylcarnitine
missing
observed
stearoylcarnitine

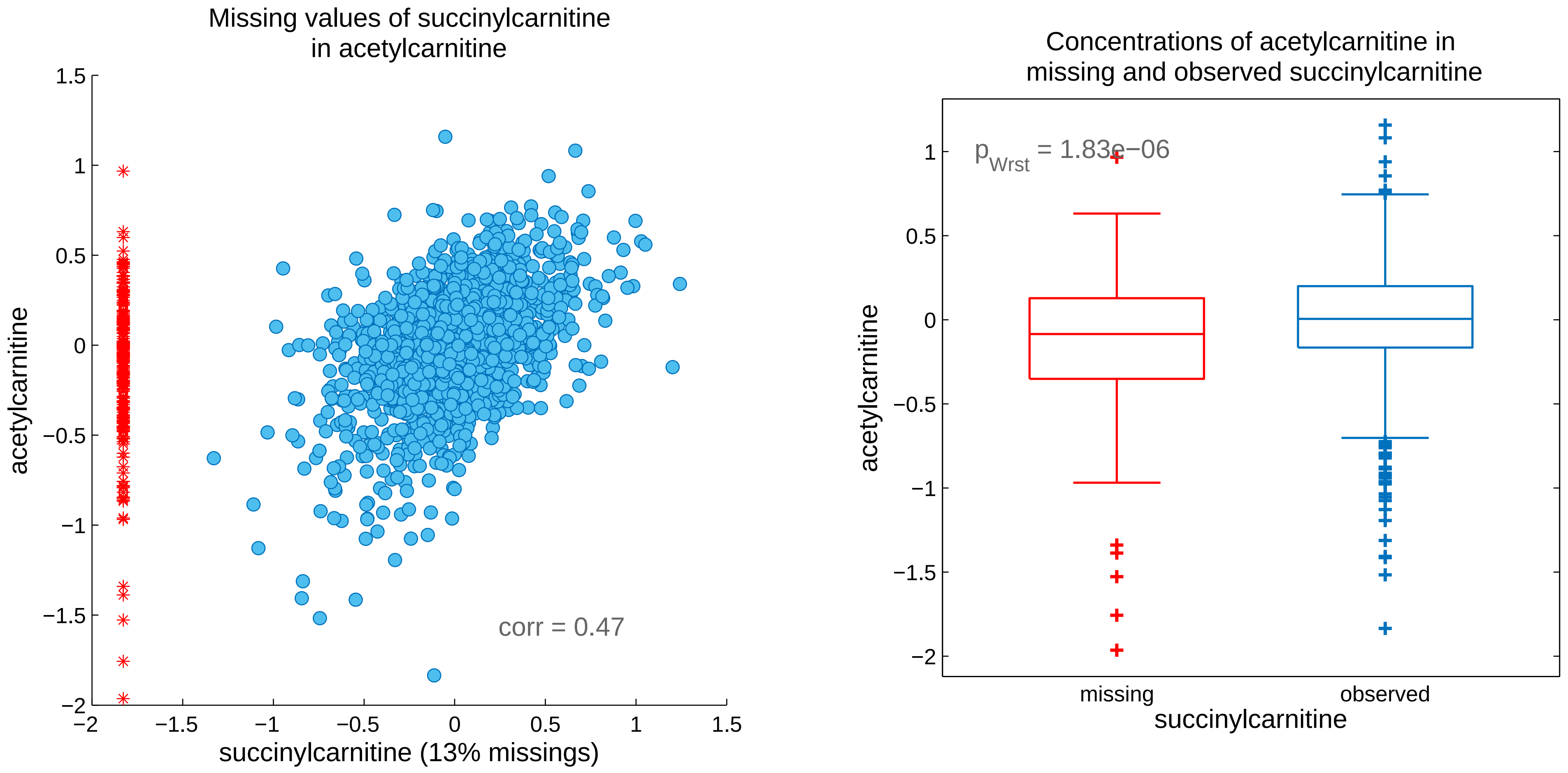

Missing values of succinylcarnitine in acetylcarnitine
acetylcarnitine
succinylcarnitine (13% missings)
corr = 0.47
Concentrations of acetylcarnitine in missing and observed succinylcarnitine
p_{Wrst} = 1.83e−06
acetylcarnitine
missing
observed
succinylcarnitine

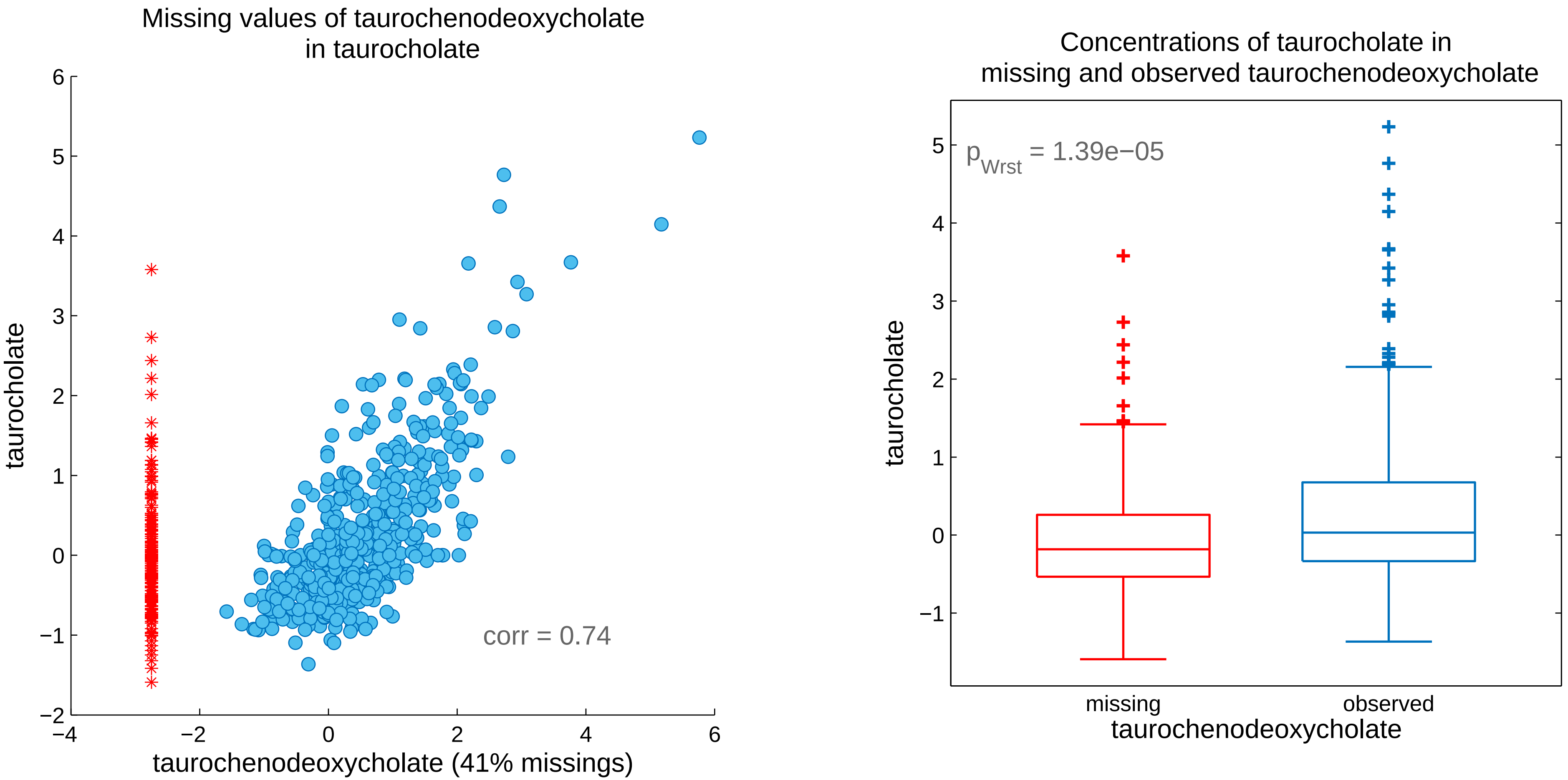
Missing values of taurochenodeoxycholate in taurocholate
taurocholate
taurochenodeoxycholate (41% missings)
corr = 0.74
Concentrations of taurocholate in missing and observed taurochenodeoxycholate
p_{Wrst} = 1.39e−05
taurocholate
missing
observed
taurochenodeoxycholate

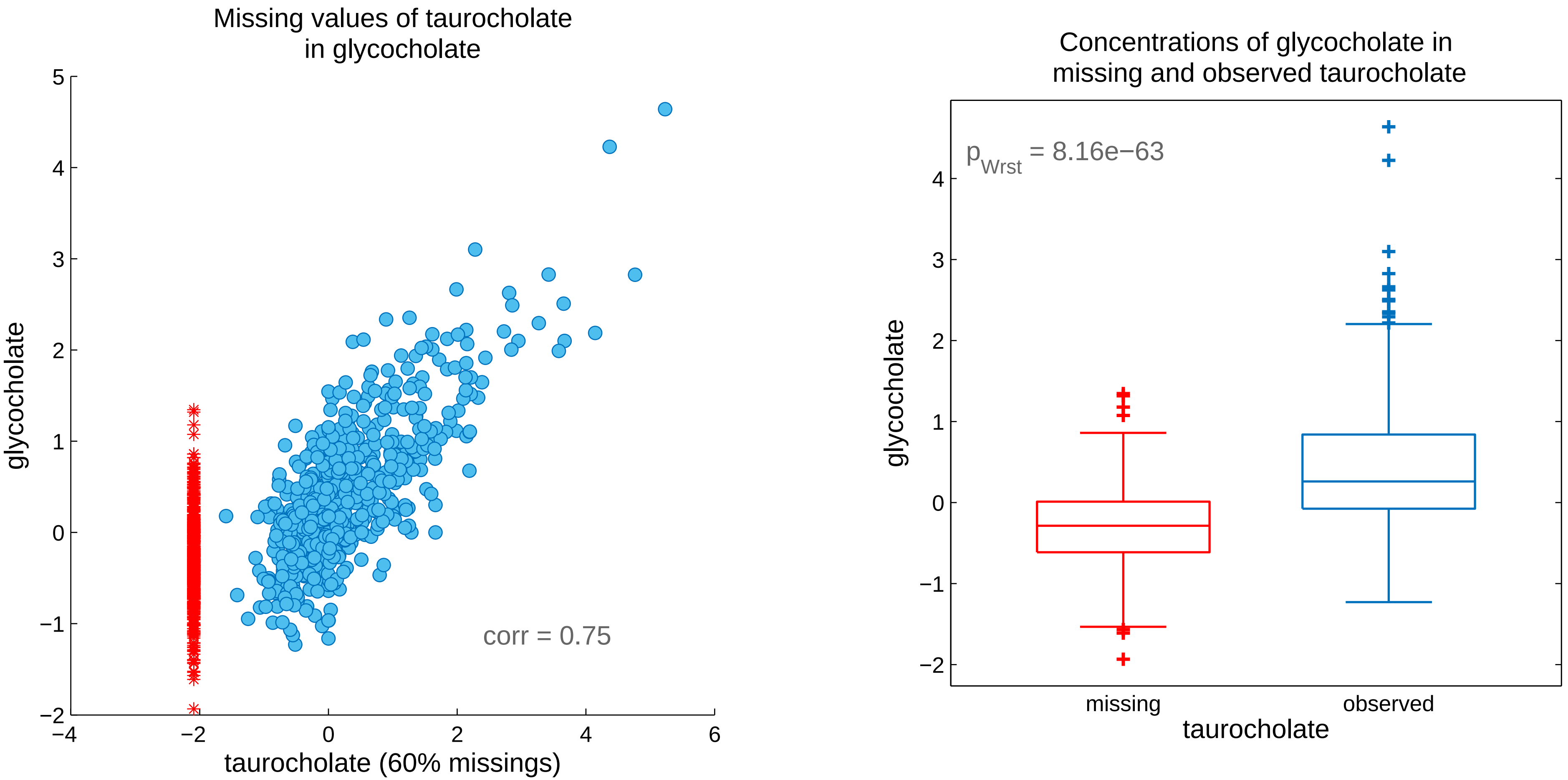
Missing values of taurocholate in glycocholate
glycocholate
taurocholate (60% missings)
corr = 0.75
5
4
3
2
1
0
−1
−2
−4
−2
0
2
4
6
Concentrations of glycocholate in missing and observed taurocholate
p_{Wrst} = 8.16e−63
glycocholate
taurocholate
missing
observed
4
3
2
1
0
−1
−2

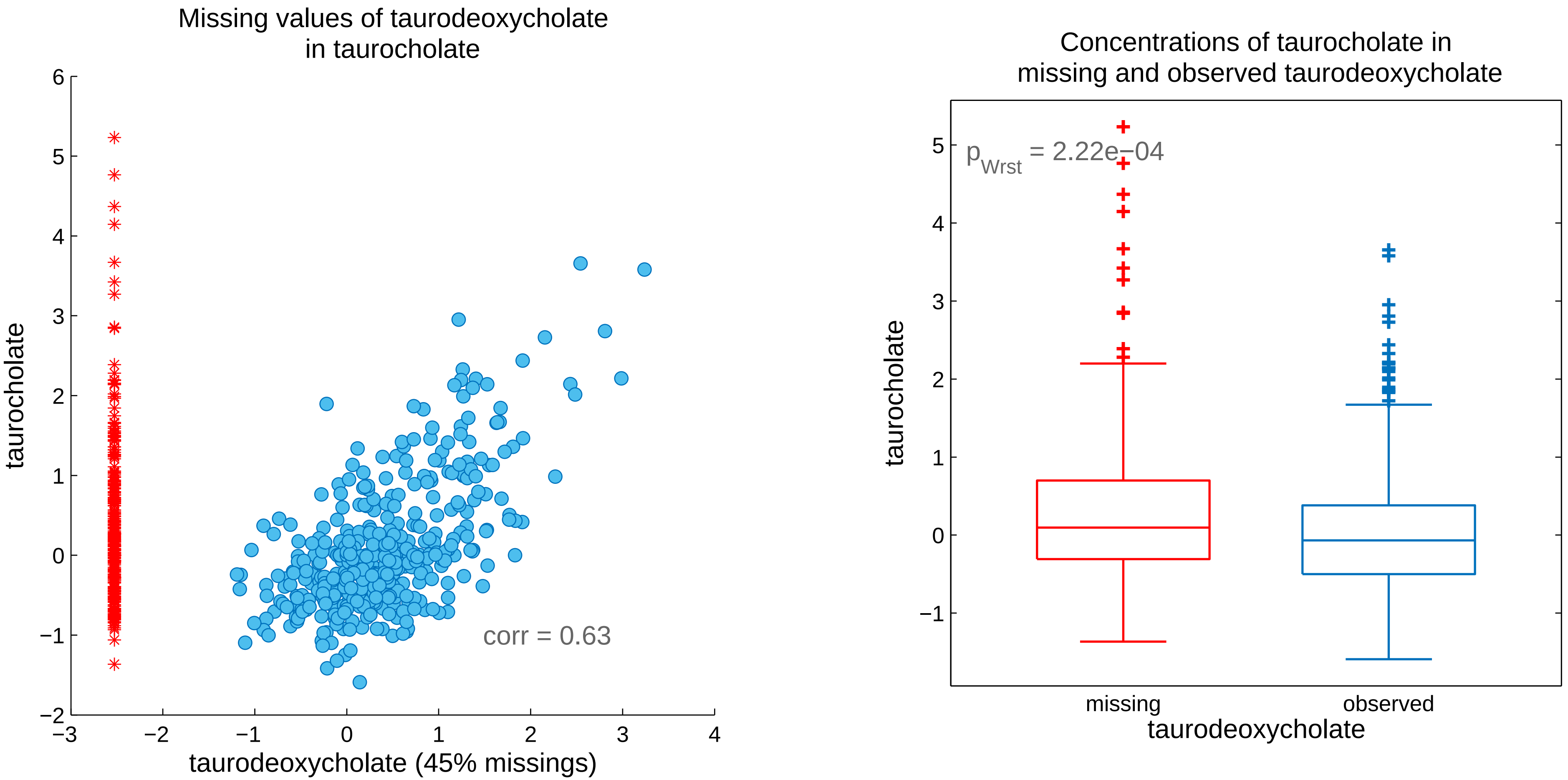

Missing values of taurodeoxycholate in taurocholate
taurocholate
taurodeoxycholate (45% missings)
corr = 0.63
Concentrations of taurocholate in missing and observed taurodeoxycholate
p_{Wrst} = 2.22e−04
taurocholate
missing
observed
taurodeoxycholate

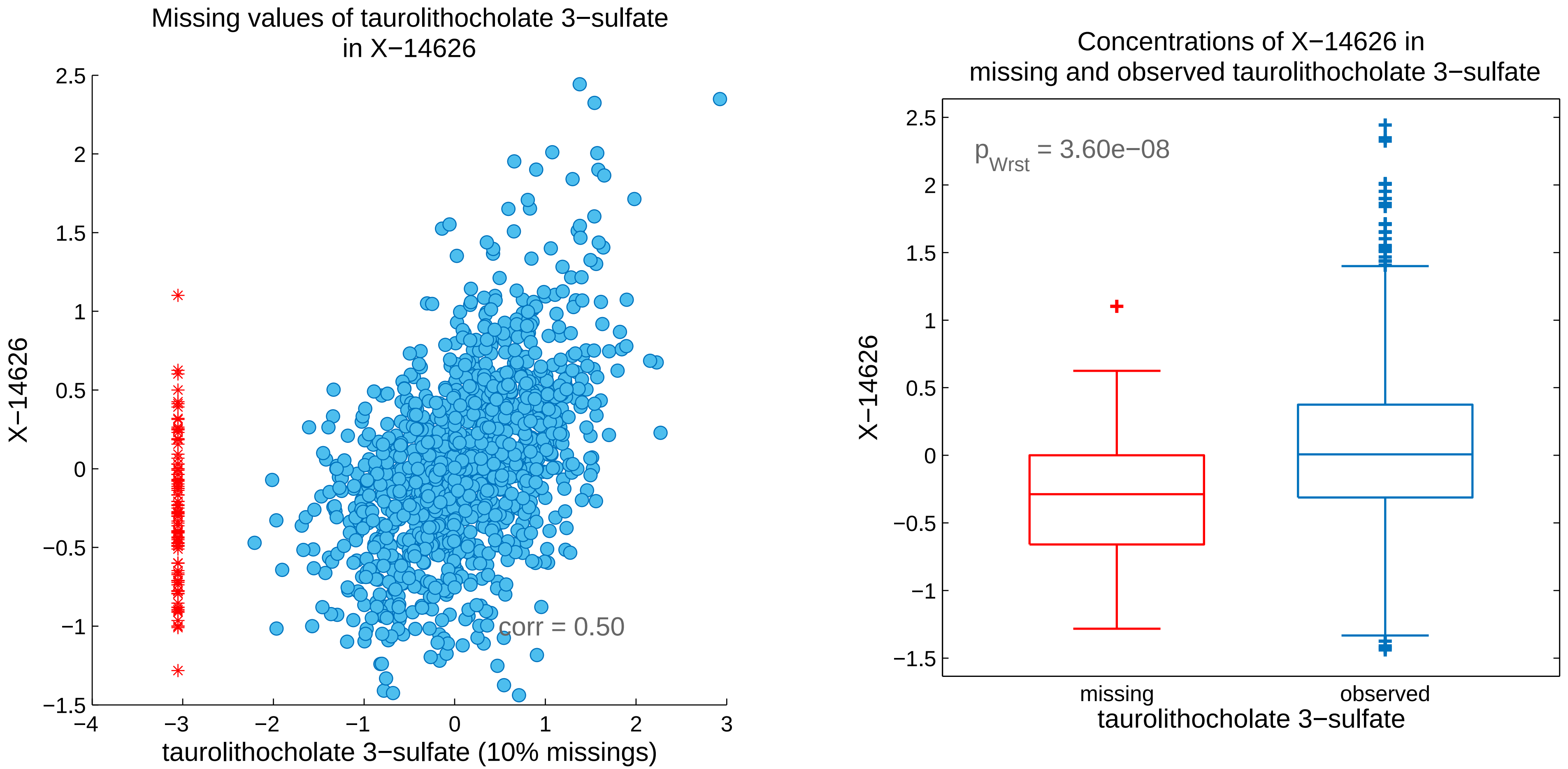

Missing values of taurolithocholate 3−sulfate in X−14626
X−14626
taurolithocholate 3−sulfate (10% missings)
corr = 0.50
Concentrations of X−14626 in missing and observed taurolithocholate 3−sulfate
p_{Wrst} = 3.60e−08
X−14626
missing
observed
taurolithocholate 3−sulfate

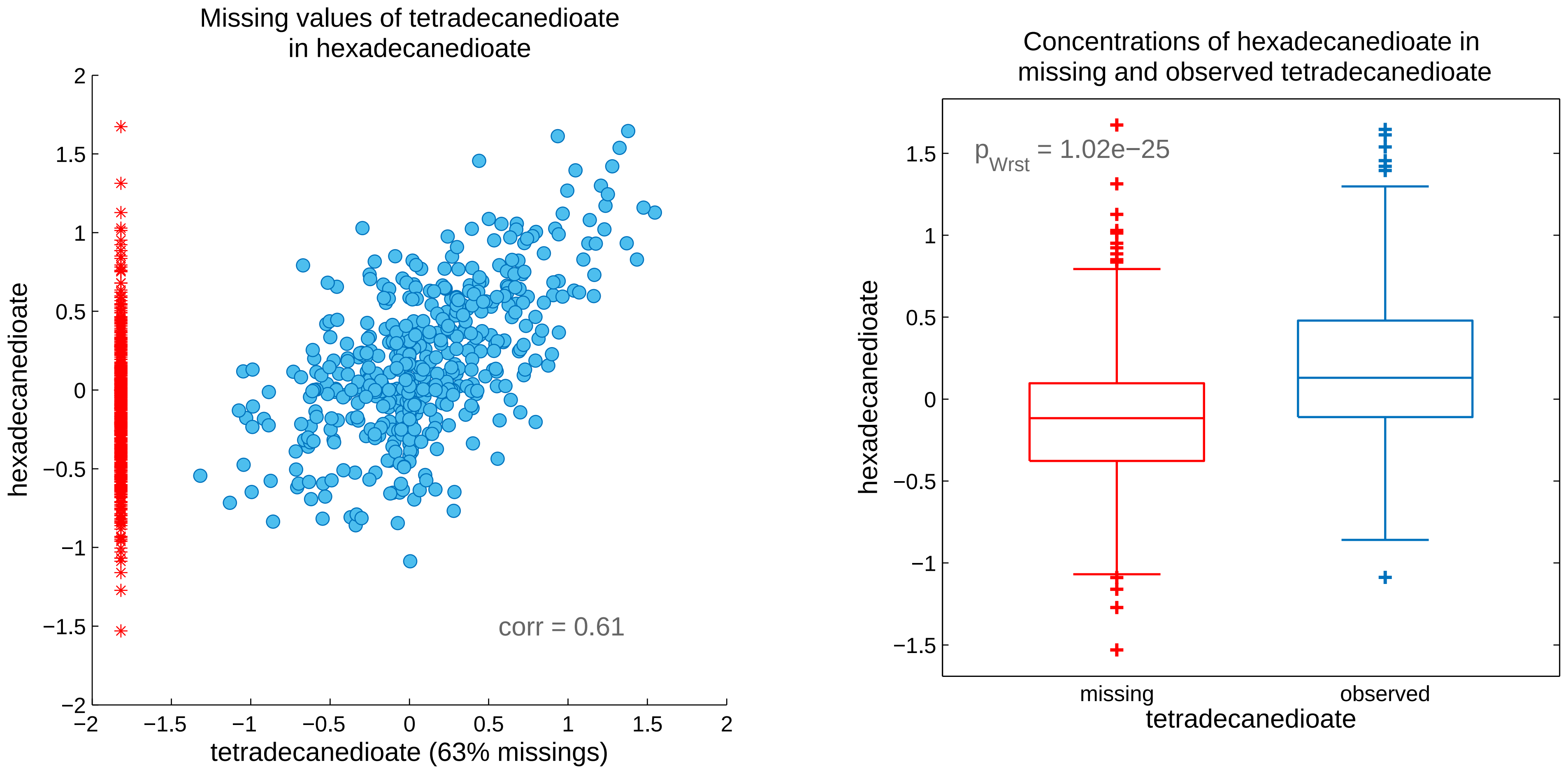
Missing values of tetradecanedioate
in hexadecanedioate
hexadecanedioate
tetradecanedioate (63% missings)
corr = 0.61
2
1.5
1
0.5
0
−0.5
−1
−1.5
−2
−2
−1.5
−1
−0.5
0
0.5
1
1.5
2
Concentrations of hexadecanedioate in
missing and observed tetradecanedioate
p_Wrst = 1.02e−25
hexadecanedioate
1.5
1
0.5
0
−0.5
−1
−1.5
missing
observed
tetradecanedioate

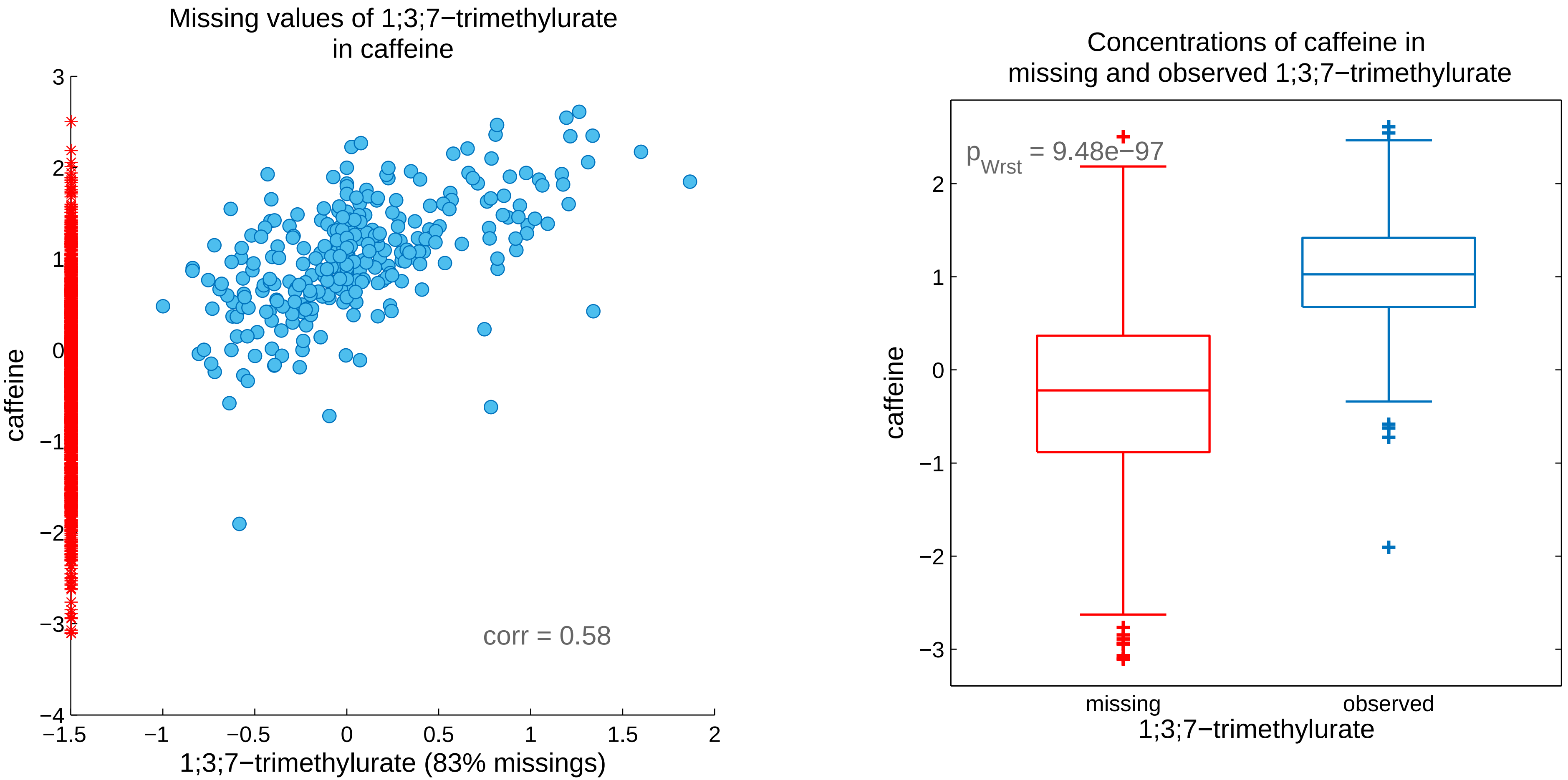
Missing values of 1;3;7−trimethylurate
in caffeine
caffeine
1;3;7−trimethylurate (83% missings)
corr = 0.58
3
2
1
0
−1
−2
−3
−4
−1.5
−1
−0.5
0
0.5
1
1.5
2
Concentrations of caffeine in
missing and observed 1;3;7−trimethylurate
p_{Wrst} = 9.48e−97
caffeine
missing
observed
1;3;7−trimethylurate

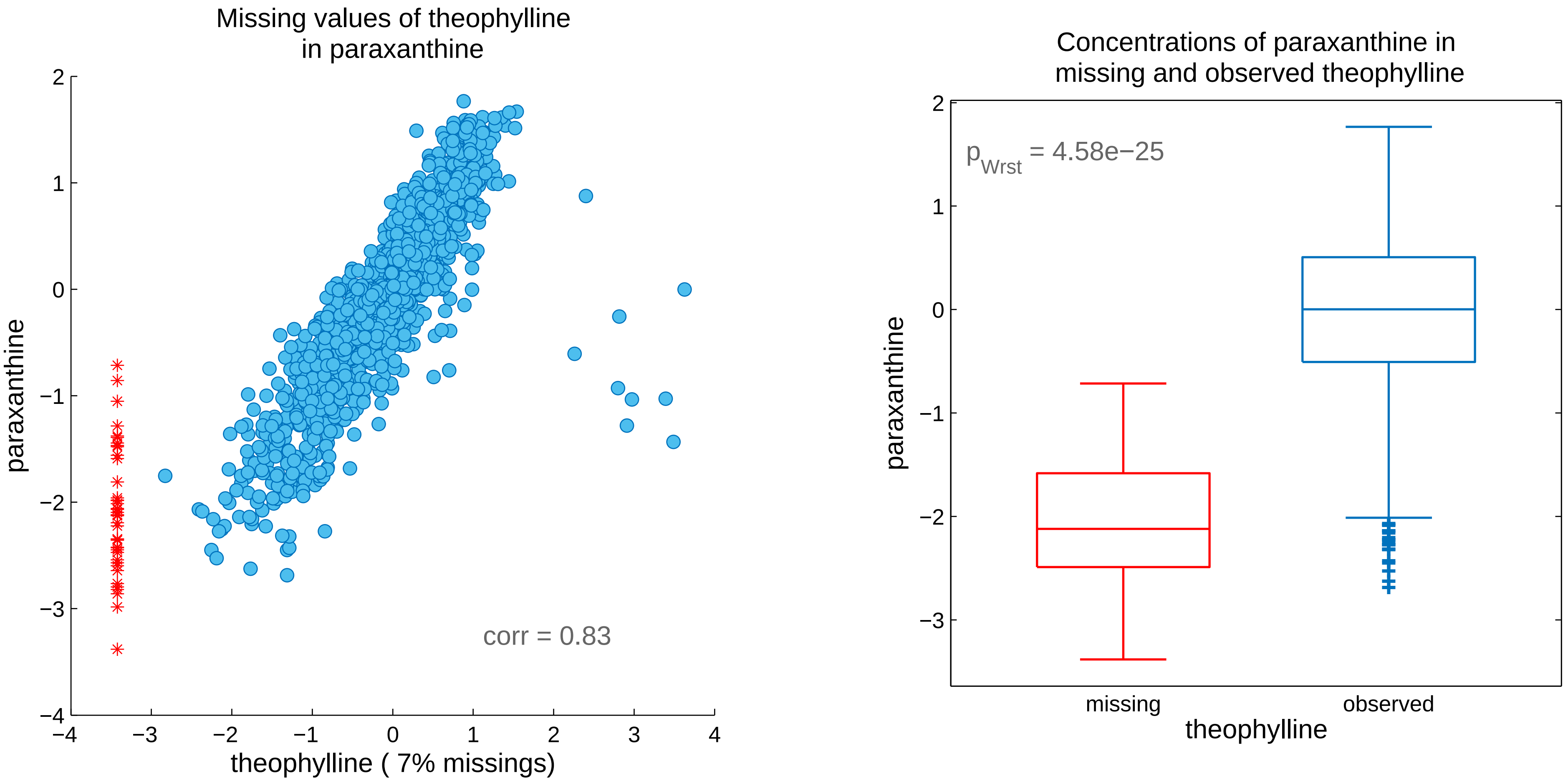
Missing values of theophylline
in paraxanthine
paraxanthine
theophylline (7% missings)
corr = 0.83
2
1
0
−1
−2
−3
−4
−4
−3
−2
−1
0
1
2
3
4
Concentrations of paraxanthine in
missing and observed theophylline
p_{Wrst} = 4.58e−25
paraxanthine
theophylline
missing
observed
2
1
0
−1
−2
−3

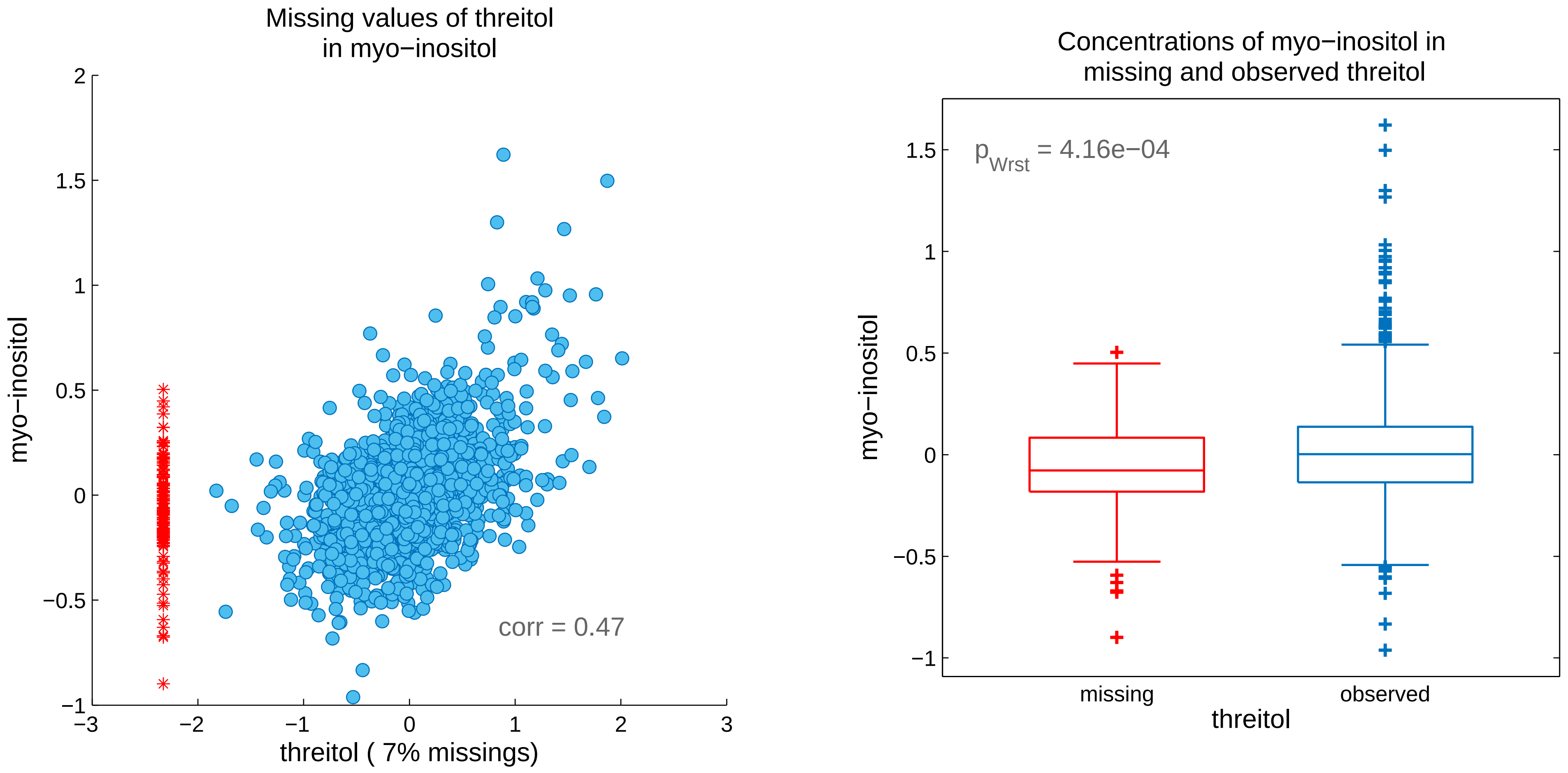

Missing values of threitol
in myo−inositol
myo−inositol
threitol (7% missings)
corr = 0.47
Concentrations of myo−inositol in
missing and observed threitol
p_{Wrst} = 4.16e−04
myo−inositol
missing
observed
threitol

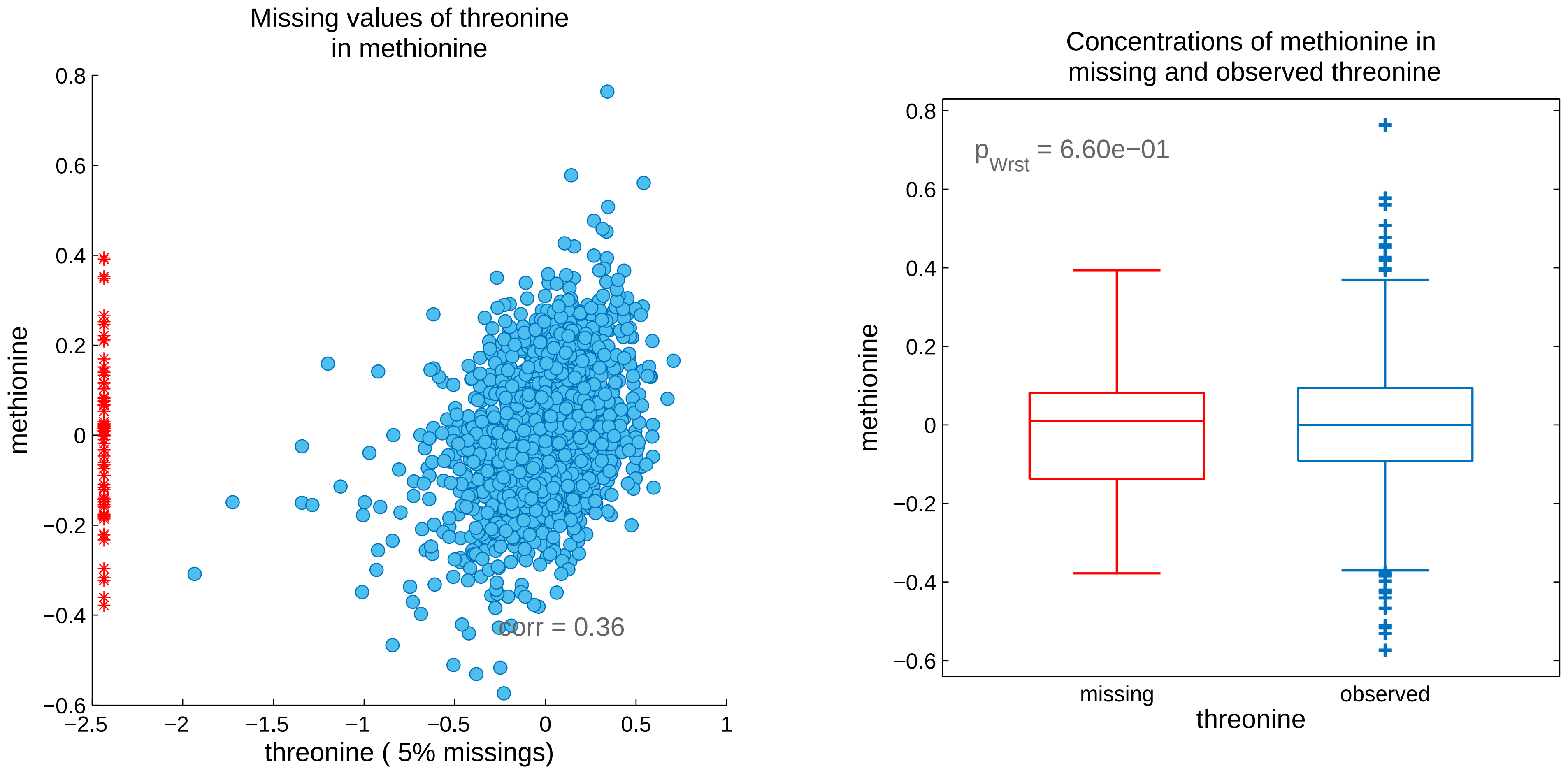

Missing values of threonine in methionine
methionine
threonine (5% missings)
corr = 0.36
0.8
0.6
0.4
0.2
0
−0.2
−0.4
−0.6
−2.5
−2
−1.5
−1
−0.5
0
0.5
1
Concentrations of methionine in missing and observed threonine
p_{Wrst} = 6.60e−01
methionine
threonine
missing
observed

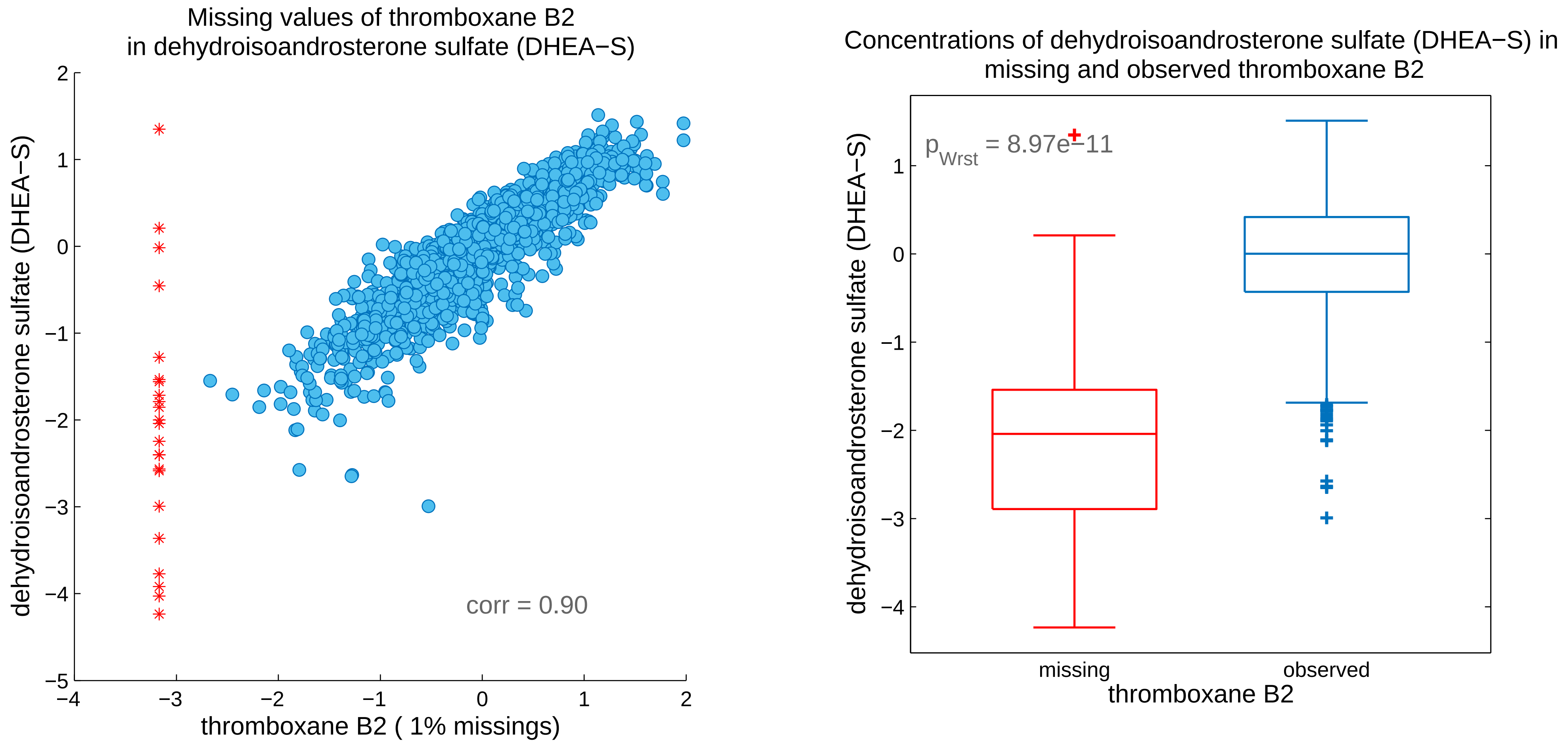
Missing values of thromboxane B2
in dehydroisoandrosterone sulfate (DHEA−S)
dehydroisoandrosterone sulfate (DHEA−S)
thromboxane B2 (1% missings)
corr = 0.90
Concentrations of dehydroisoandrosterone sulfate (DHEA−S) in
missing and observed thromboxane B2
p_Wrst = 8.97e−11
dehydroisoandrosterone sulfate (DHEA−S)
missing
observed
thromboxane B2

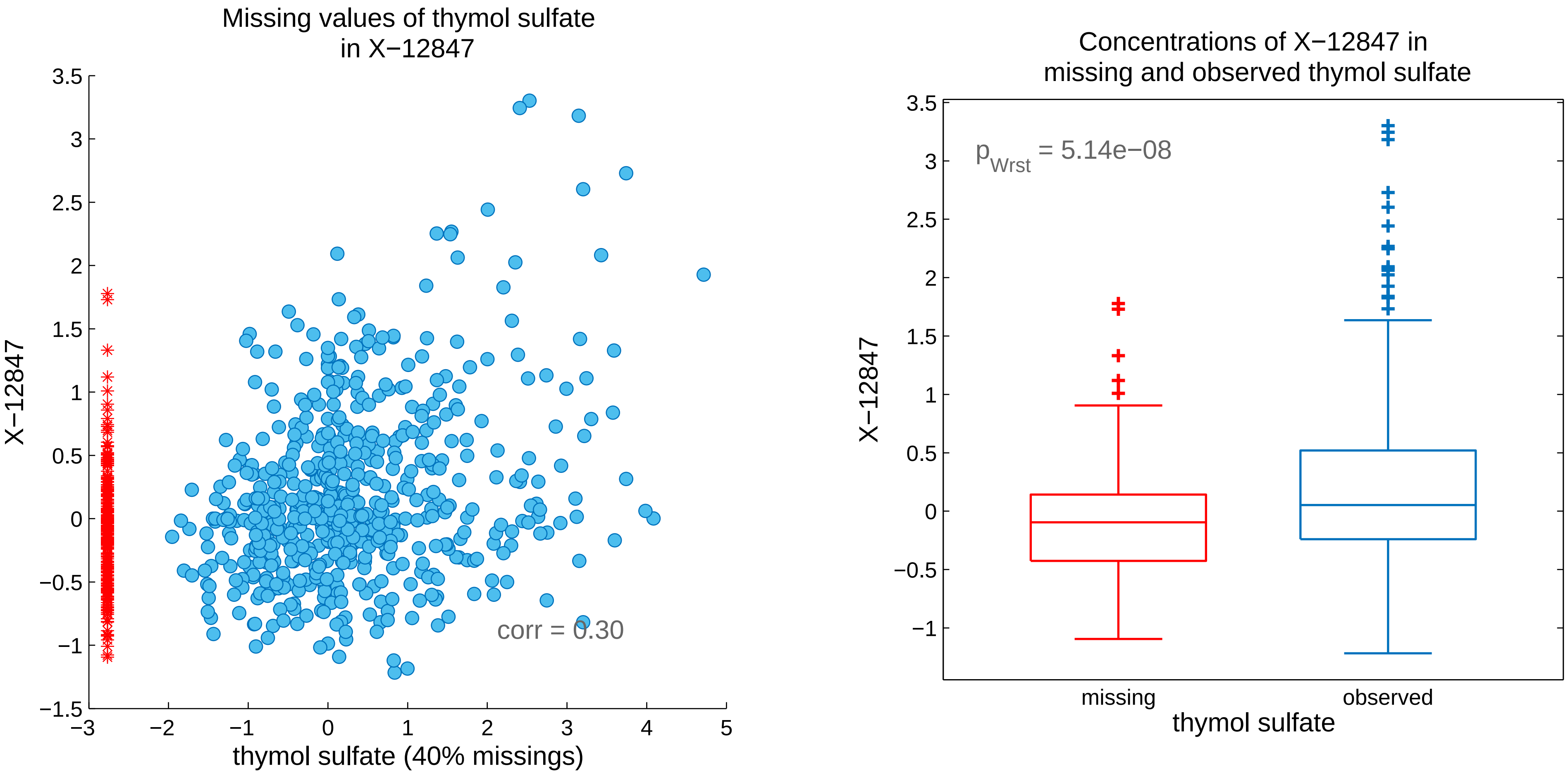

Missing values of thymol sulfate in X−12847
X−12847
thymol sulfate (40% missings)
corr = 0.30
Concentrations of X−12847 in missing and observed thymol sulfate
p_{Wrst} = 5.14e−08
X−12847
missing
observed
thymol sulfate

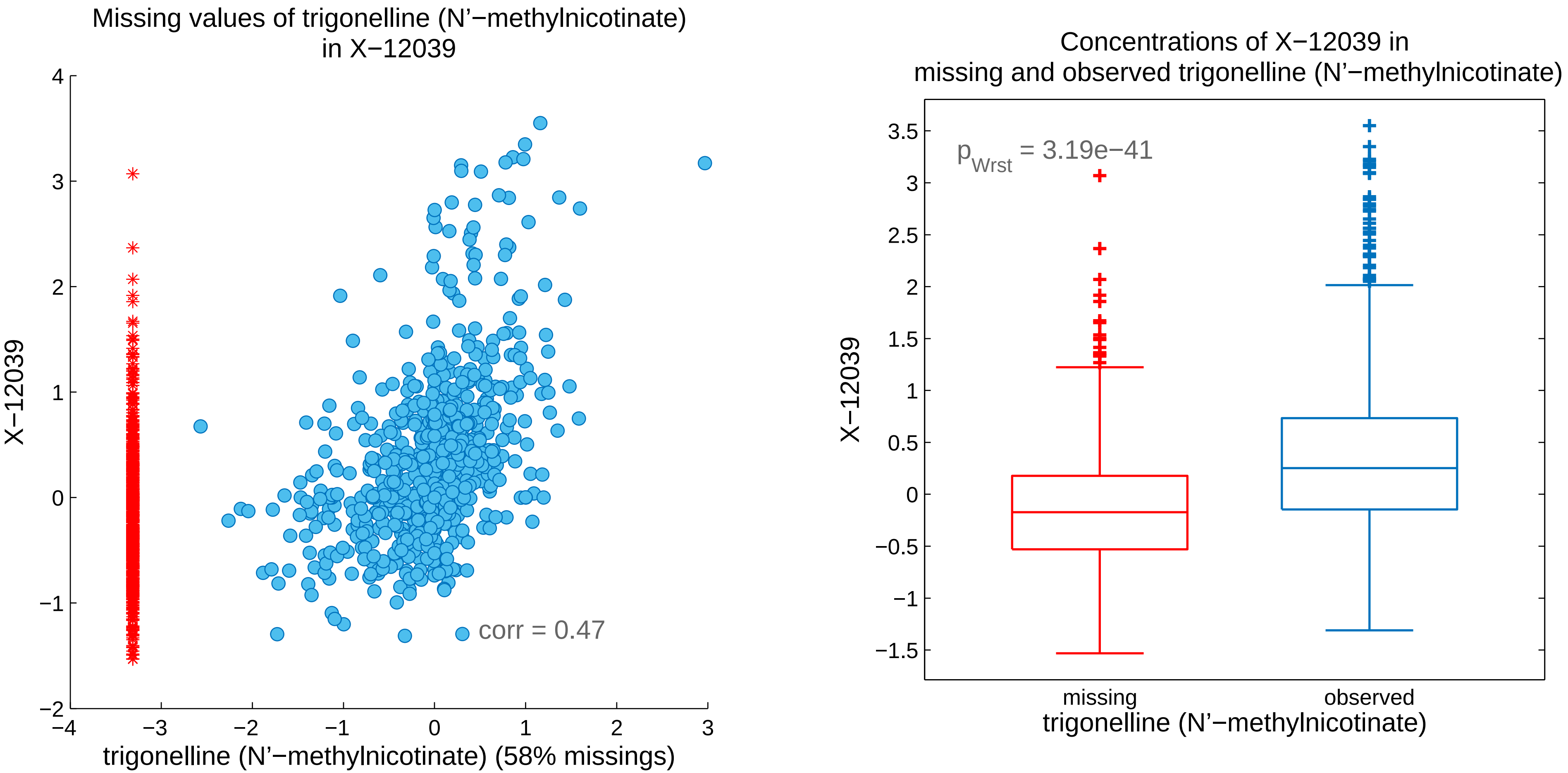
Missing values of trigonelline (N'−methylnicotinate) in X−12039
X−12039
trigonelline (N'−methylnicotinate) (58% missings)
corr = 0.47
4
3
2
1
0
−1
−2
−4
−3
−2
−1
0
1
2
3
Concentrations of X−12039 in missing and observed trigonelline (N'−methylnicotinate)
p_{Wrst} = 3.19e−41
X−12039
3.5
3
2.5
2
1.5
1
0.5
0
−0.5
−1
−1.5
missing
observed
trigonelline (N'−methylnicotinate)

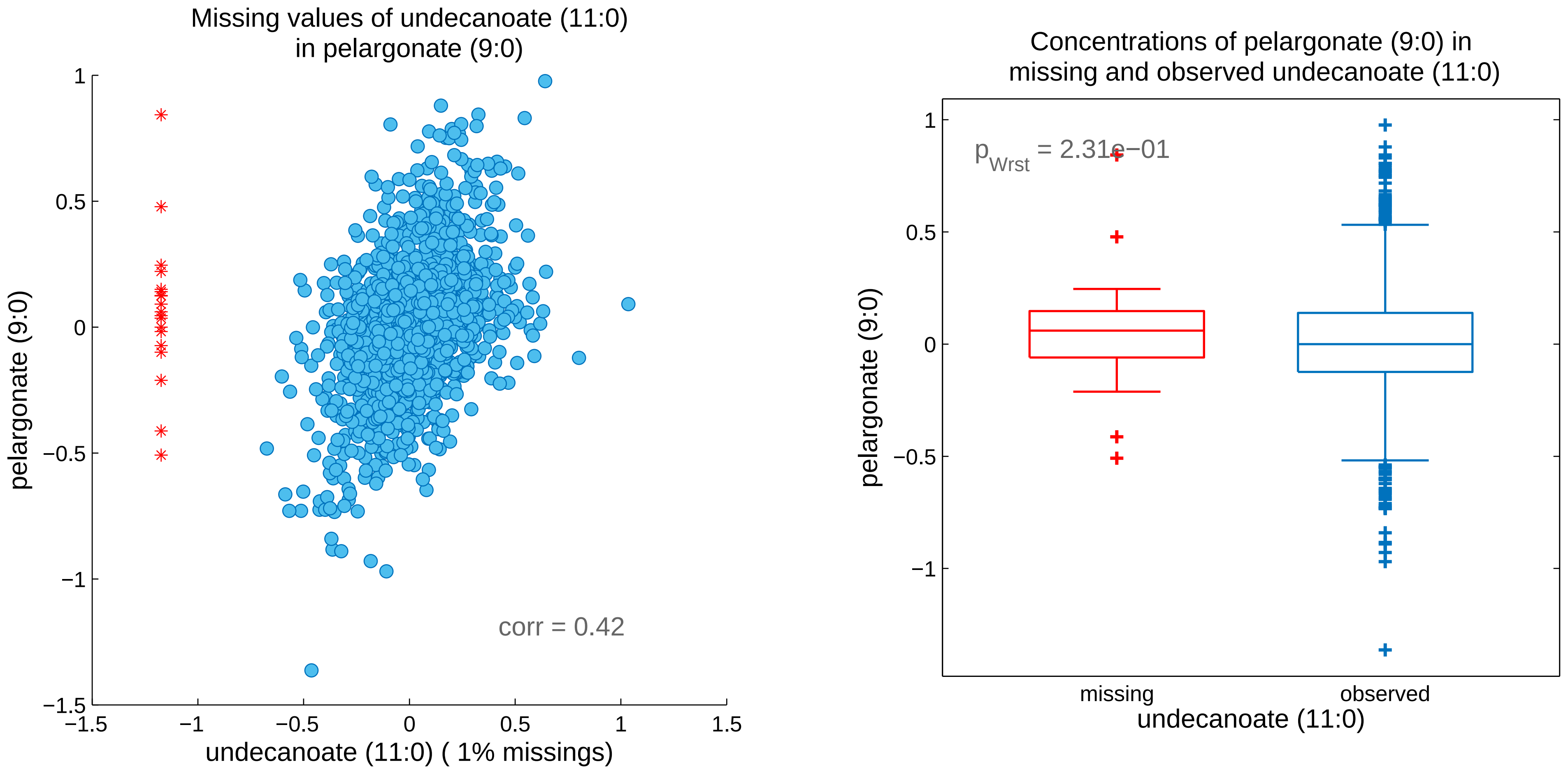

Missing values of undecanoate (11:0) in pelargonate (9:0)
corr = 0.42
pelargonate (9:0)
undecanoate (11:0) (1% missings)
Concentrations of pelargonate (9:0) in missing and observed undecanoate (11:0)
p_{Wrst} = 2.31e−01
pelargonate (9:0)
missing
observed
undecanoate (11:0)

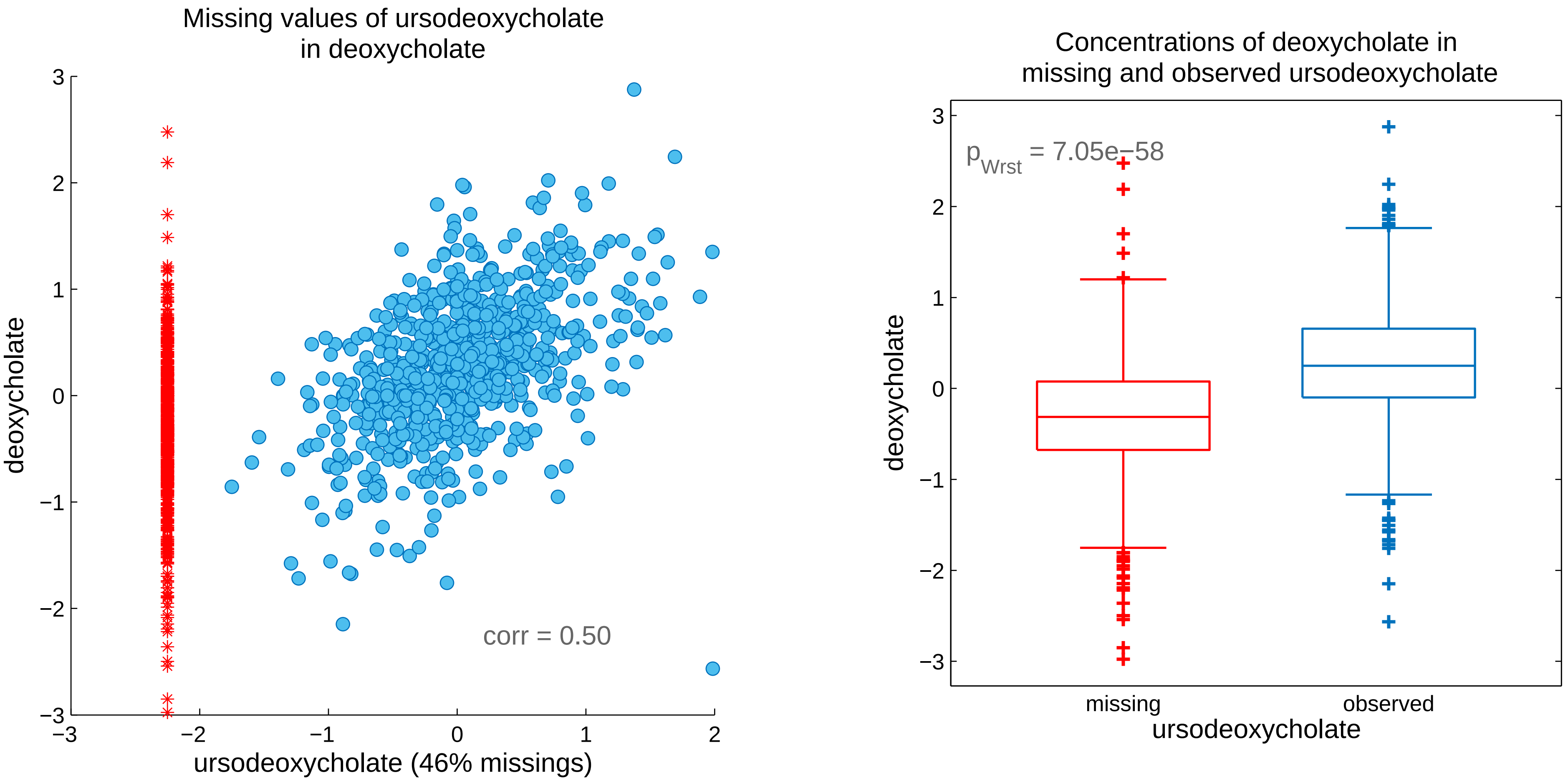
Missing values of ursodeoxycholate in deoxycholate
deoxycholate
ursodeoxycholate (46% missings)
corr = 0.50
Concentrations of deoxycholate in missing and observed ursodeoxycholate
p_{Wrst} = 7.05e−58
deoxycholate
missing
observed
ursodeoxycholate

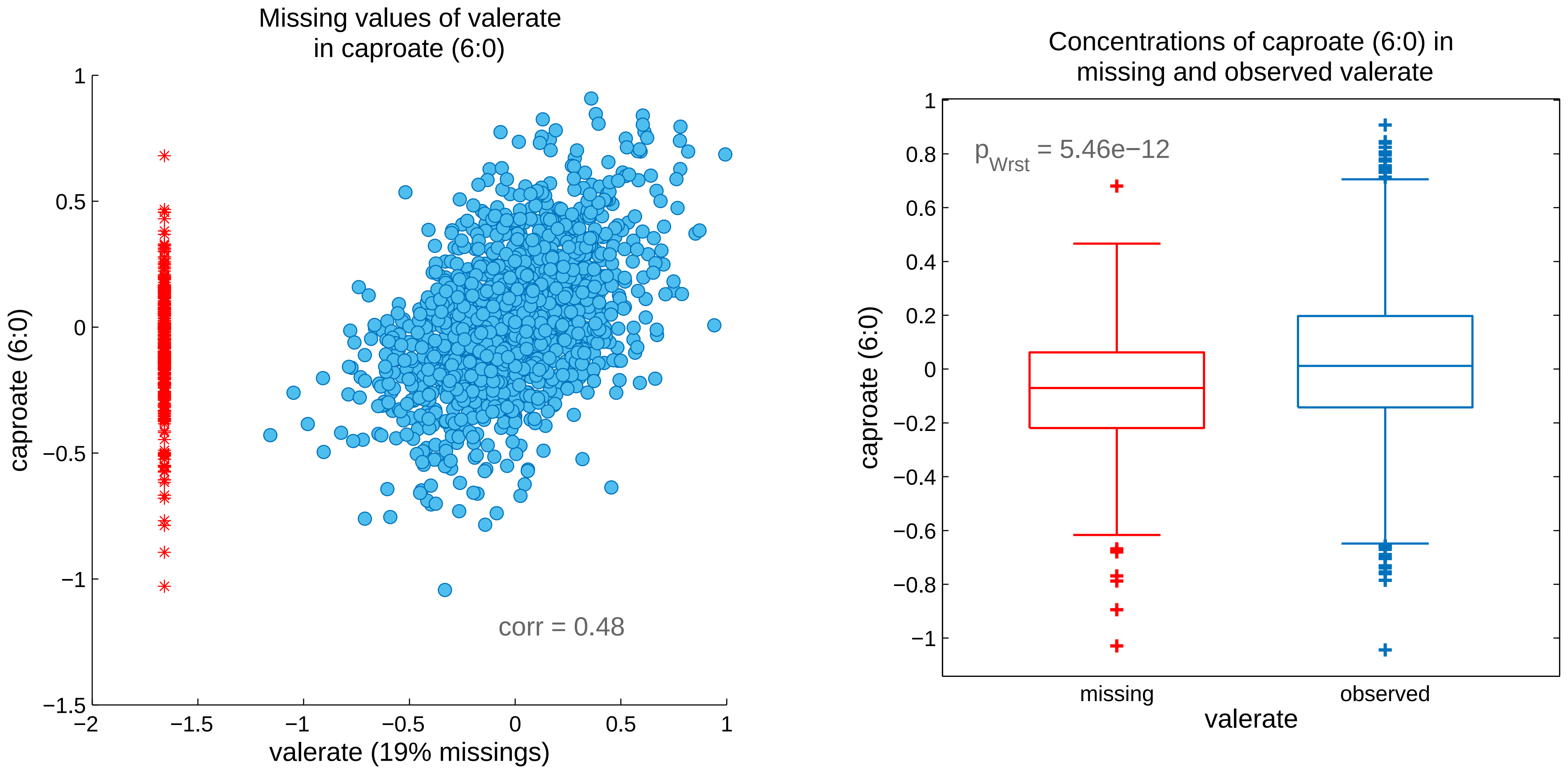

Missing values of valerate in caproate (6:0)
caproate (6:0)
valerate (19% missings)
corr = 0.48
1
0.5
0
−0.5
−1
−1.5
−2
−1.5
−1
−0.5
0
0.5
1
Concentrations of caproate (6:0) in missing and observed valerate
p_Wrst = 5.46e−12
caproate (6:0)
valerate
missing
observed
1
0.8
0.6
0.4
0.2
0
−0.2
−0.4
−0.6
−0.8
−1

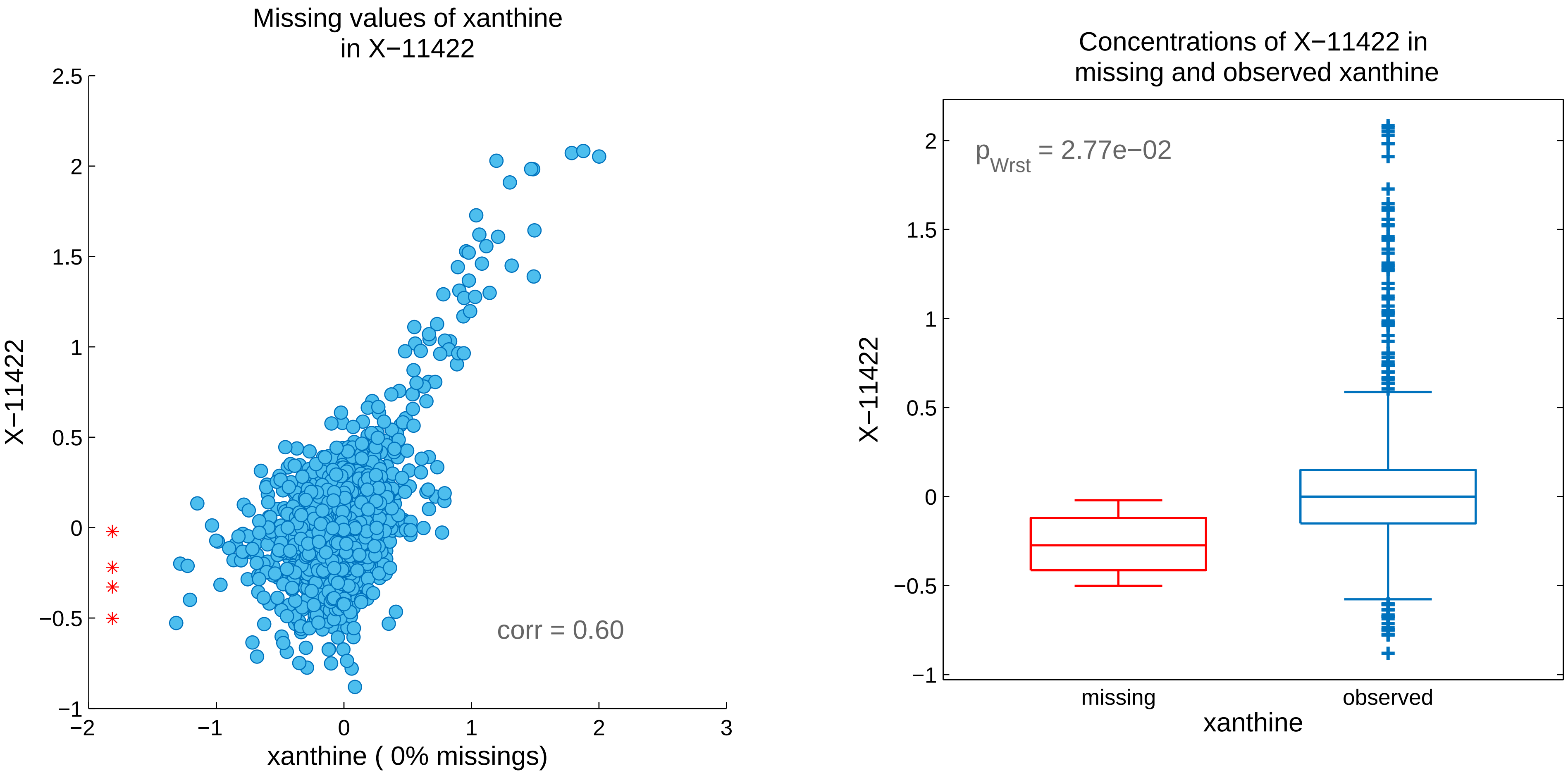

Missing values of xanthine in X−11422
X−11422
xanthine (0% missings)
corr = 0.60
Concentrations of X−11422 in missing and observed xanthine
p_{Wrst} = 2.77e−02
X−11422
missing
observed
xanthine

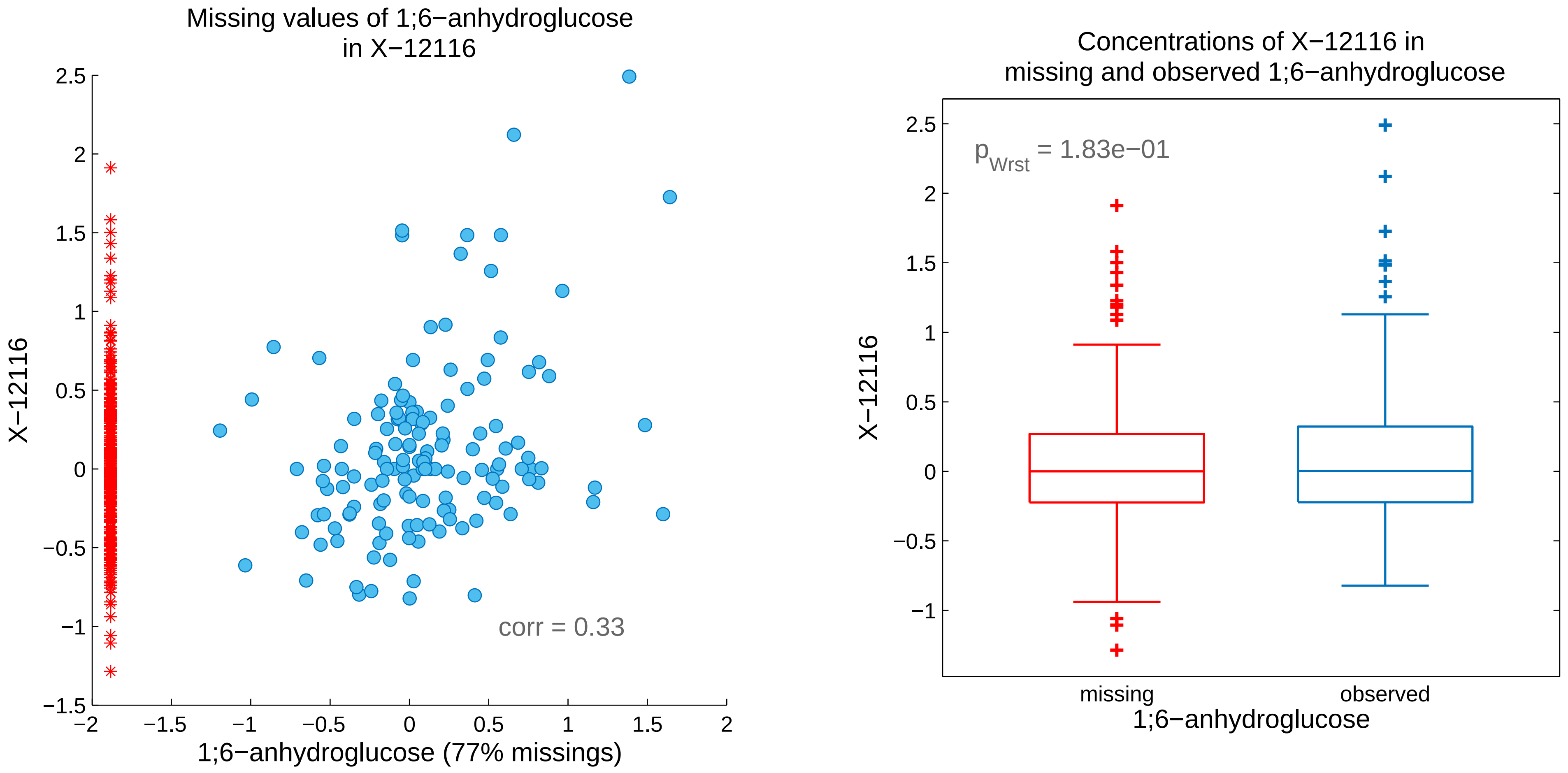

Missing values of 1;6−anhydroglucose in X−12116
X−12116
1;6−anhydroglucose (77% missings)
corr = 0.33
Concentrations of X−12116 in missing and observed 1;6−anhydroglucose
p_{Wrst} = 1.83e−01
missing
observed
1;6−anhydroglucose

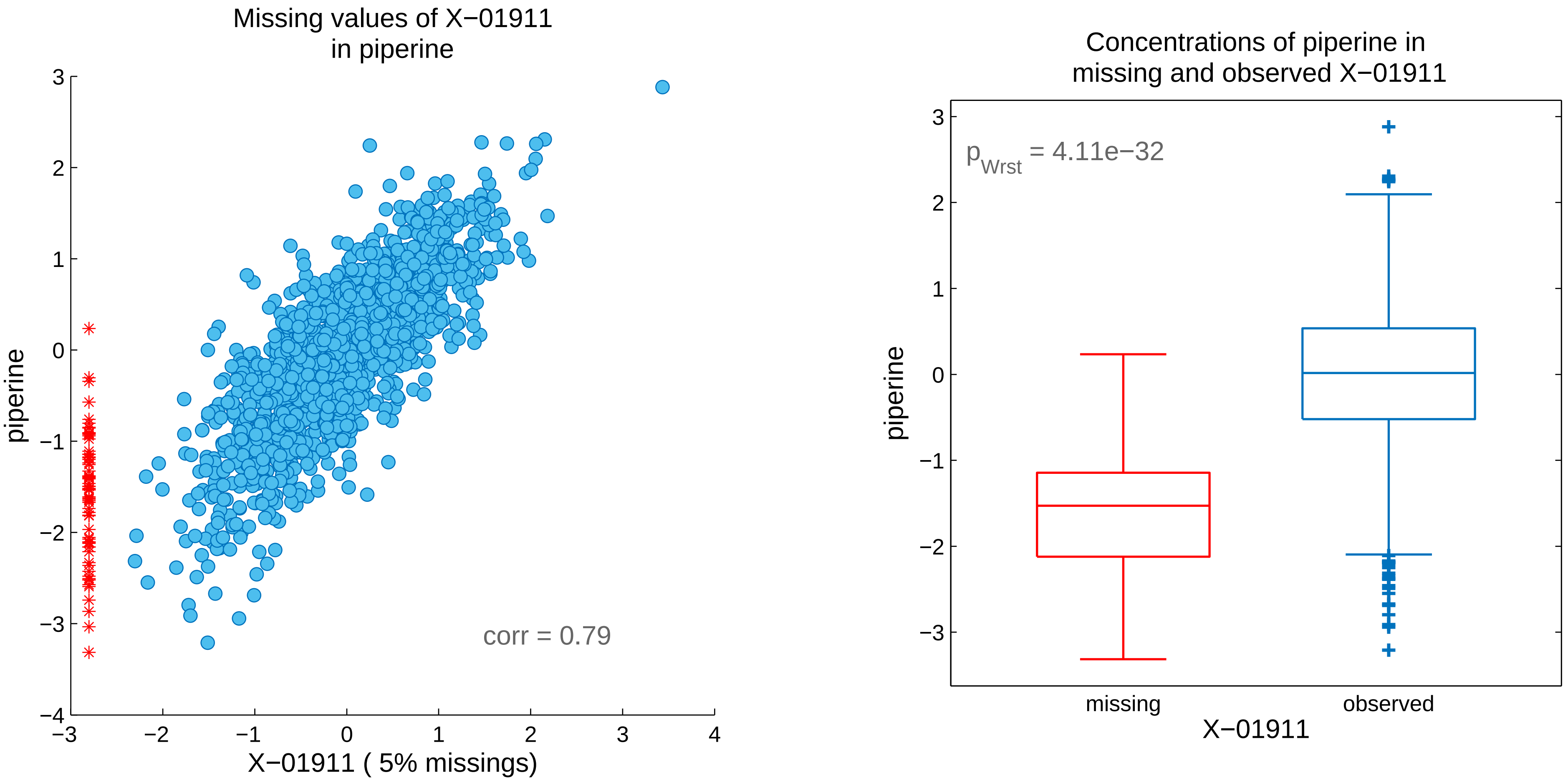

Missing values of X−01911 in piperine
piperine
X−01911 (5% missings)
corr = 0.79
Concentrations of piperine in missing and observed X−01911
p_{Wrst} = 4.11e−32
piperine
missing
observed
X−01911

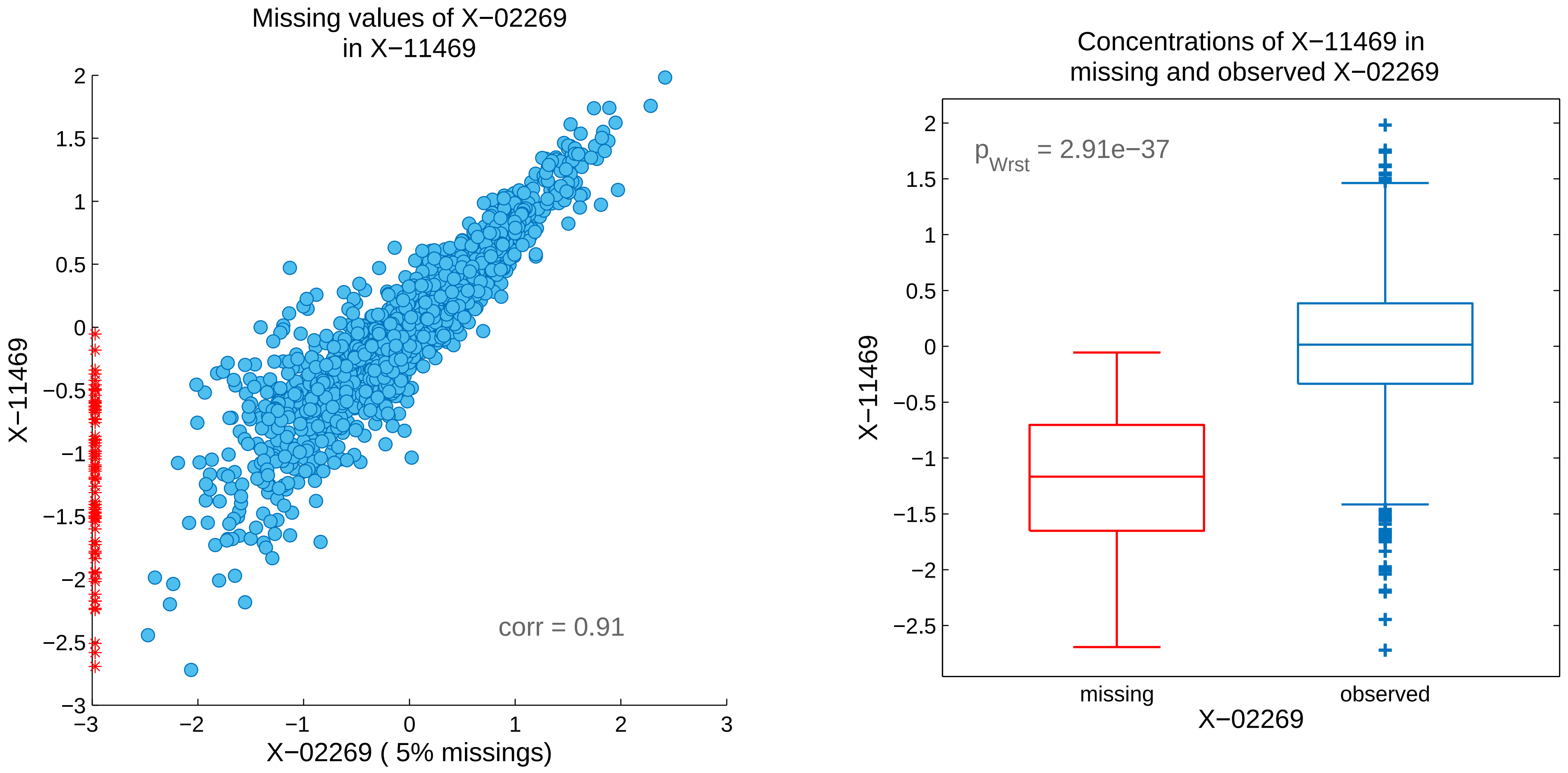
Missing values of X−02269
in X−11469
X−11469
X−02269 (5% missings)
corr = 0.91
2
1.5
1
0.5
0
−0.5
−1
−1.5
−2
−2.5
−3
−3
−2
−1
0
1
2
3
Concentrations of X−11469 in
missing and observed X−02269
p_{Wrst} = 2.91e−37
X−11469
X−02269
missing
observed

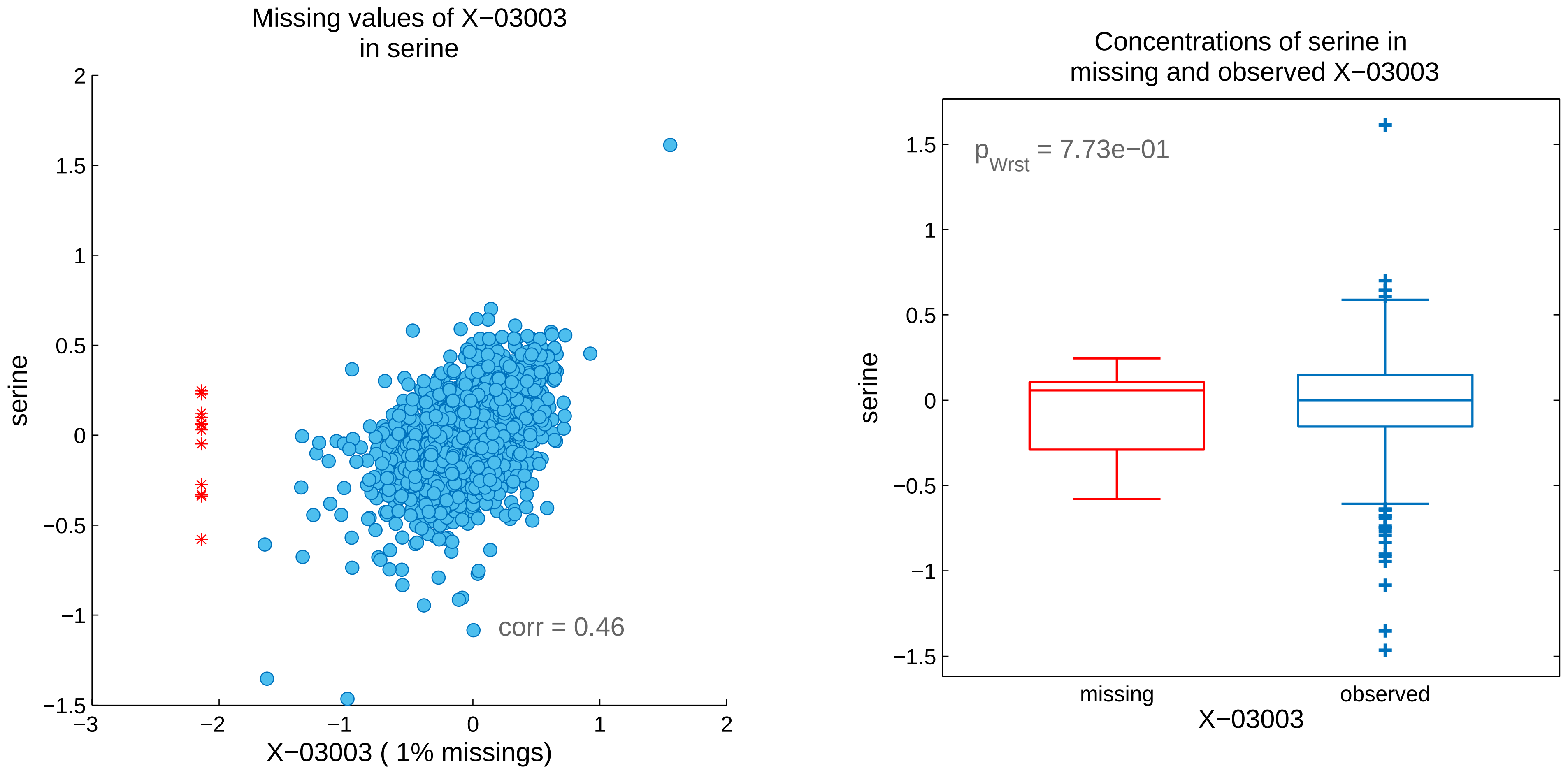
Missing values of X−03003
in serine
serine
X−03003 (1% missings)
corr = 0.46
Concentrations of serine in
missing and observed X−03003
p_Wrst = 7.73e−01
serine
missing
observed
X−03003

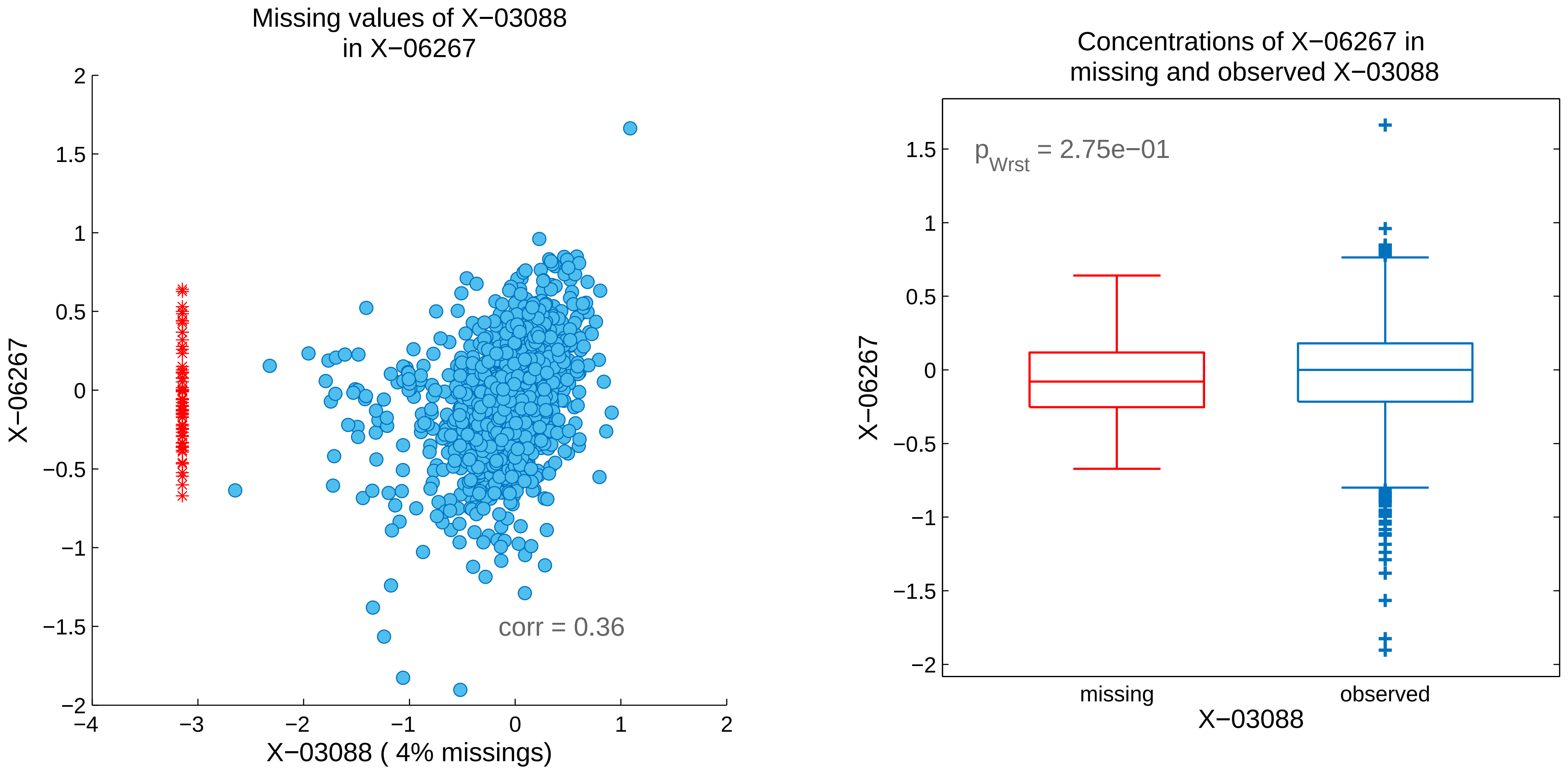

Missing values of X−03088
in X−06267
corr = 0.36
X−06267
X−03088 (4% missings)
Concentrations of X−06267 in
missing and observed X−03088
p_{Wrst} = 2.75e−01
X−06267
missing
observed
X−03088

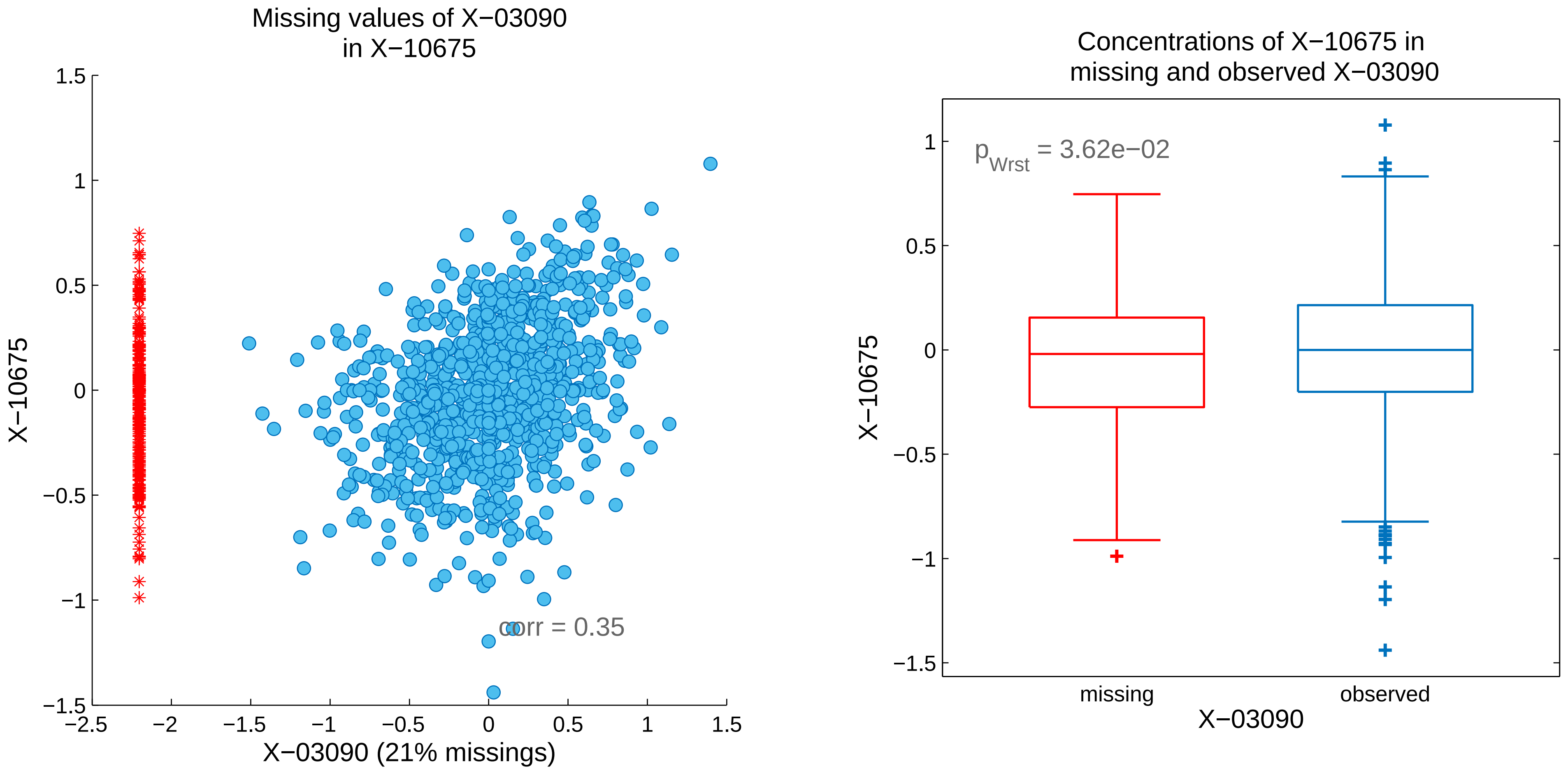
Missing values of X−03090
in X−10675
X−10675
X−03090 (21% missings)
corr = 0.35
Concentrations of X−10675 in
missing and observed X−03090
p_{Wrst} = 3.62e−02
X−10675
missing
observed
X−03090

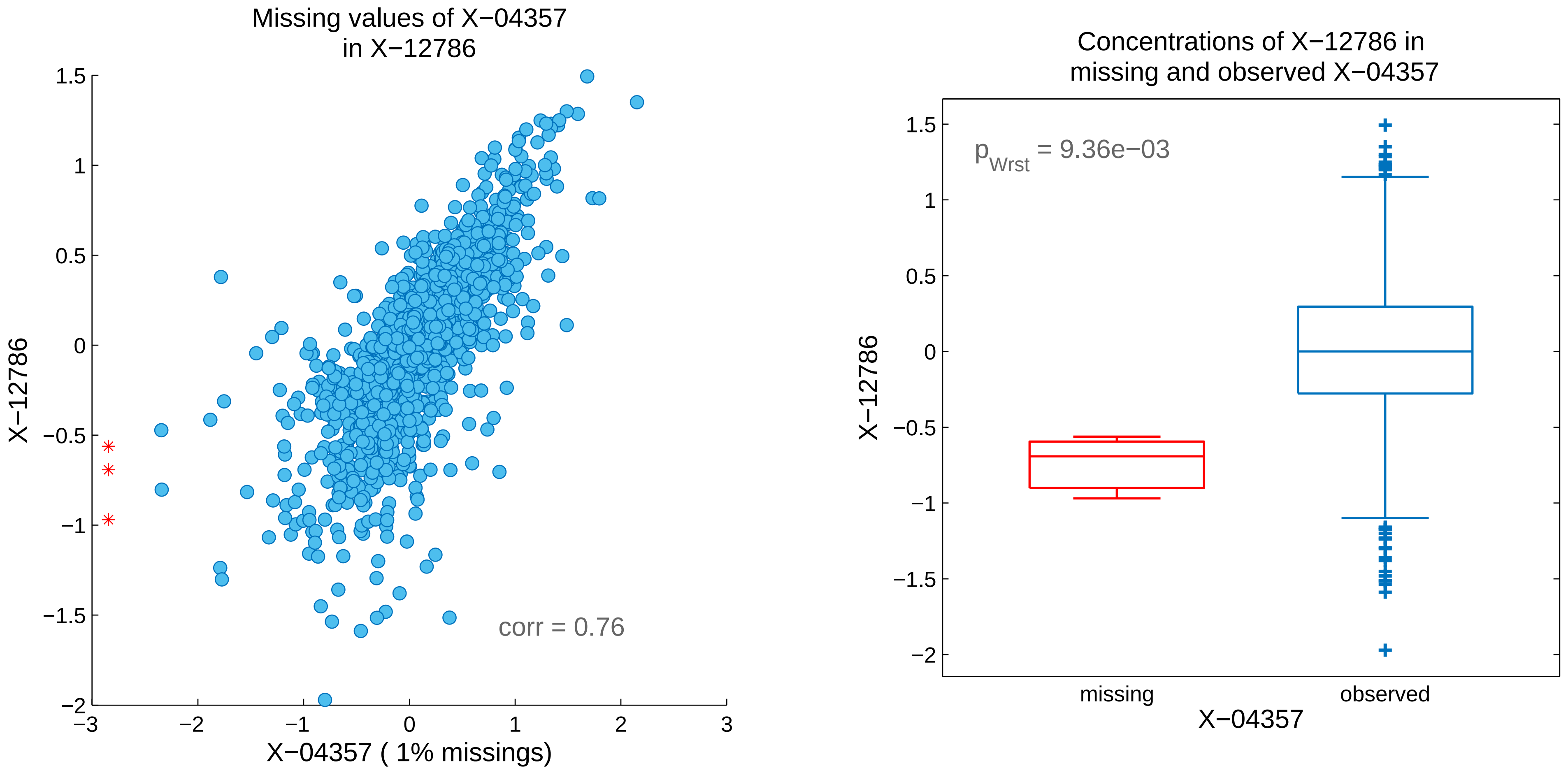

Missing values of X−04357
in X−12786
X−12786
X−04357 (1% missings)
corr = 0.76
Concentrations of X−12786 in
missing and observed X−04357
p_{Wrst} = 9.36e−03
X−12786
missing
observed
X−04357

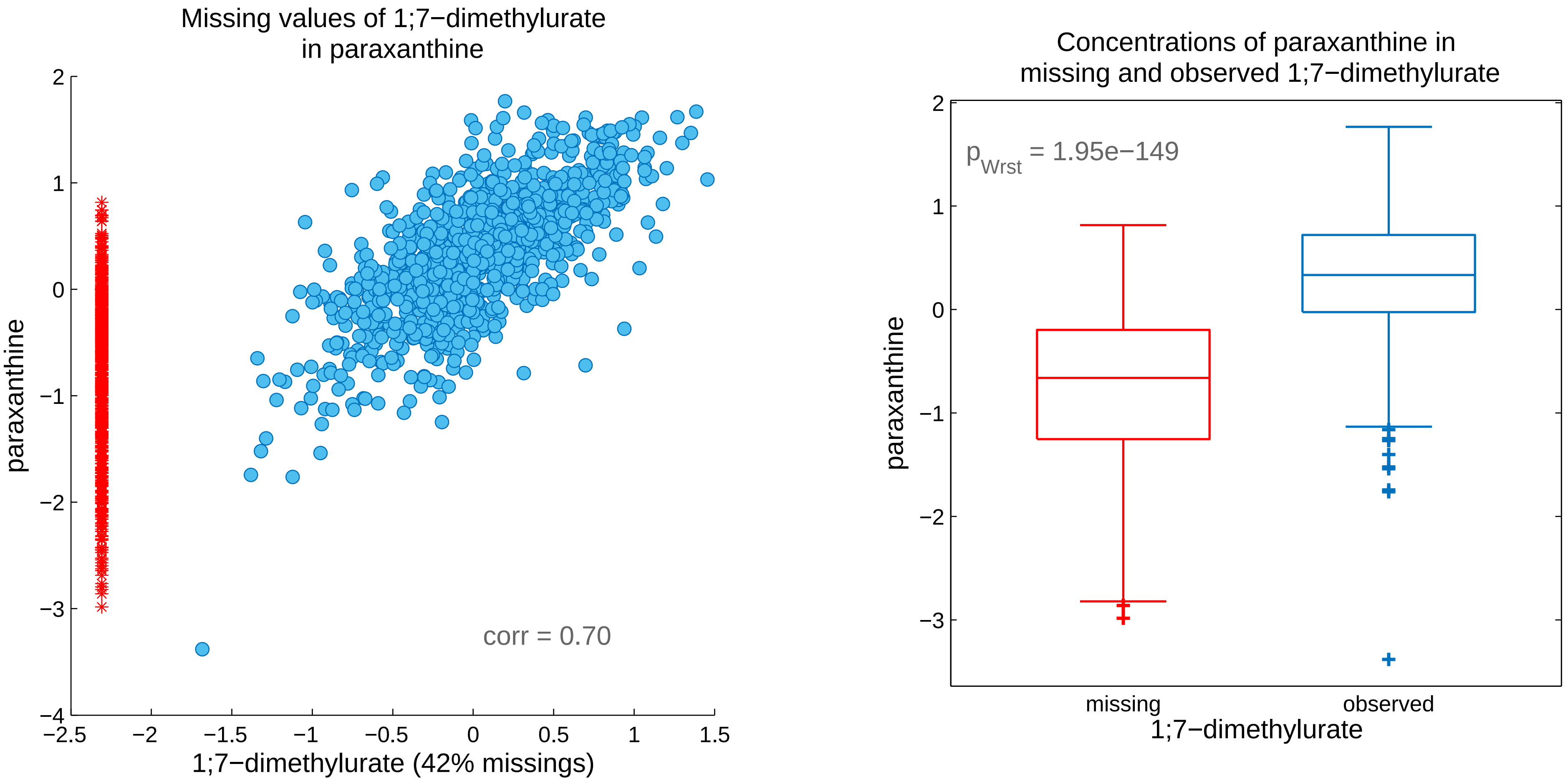
Missing values of 1;7−dimethylurate in paraxanthine
paraxanthine
1;7−dimethylurate (42% missings)
corr = 0.70
2
1
0
−1
−2
−3
−4
−2.5
−2
−1.5
−1
−0.5
0
0.5
1
1.5
Concentrations of paraxanthine in missing and observed 1;7−dimethylurate
p_{Wrst} = 1.95e−149
paraxanthine
missing
observed
1;7−dimethylurate

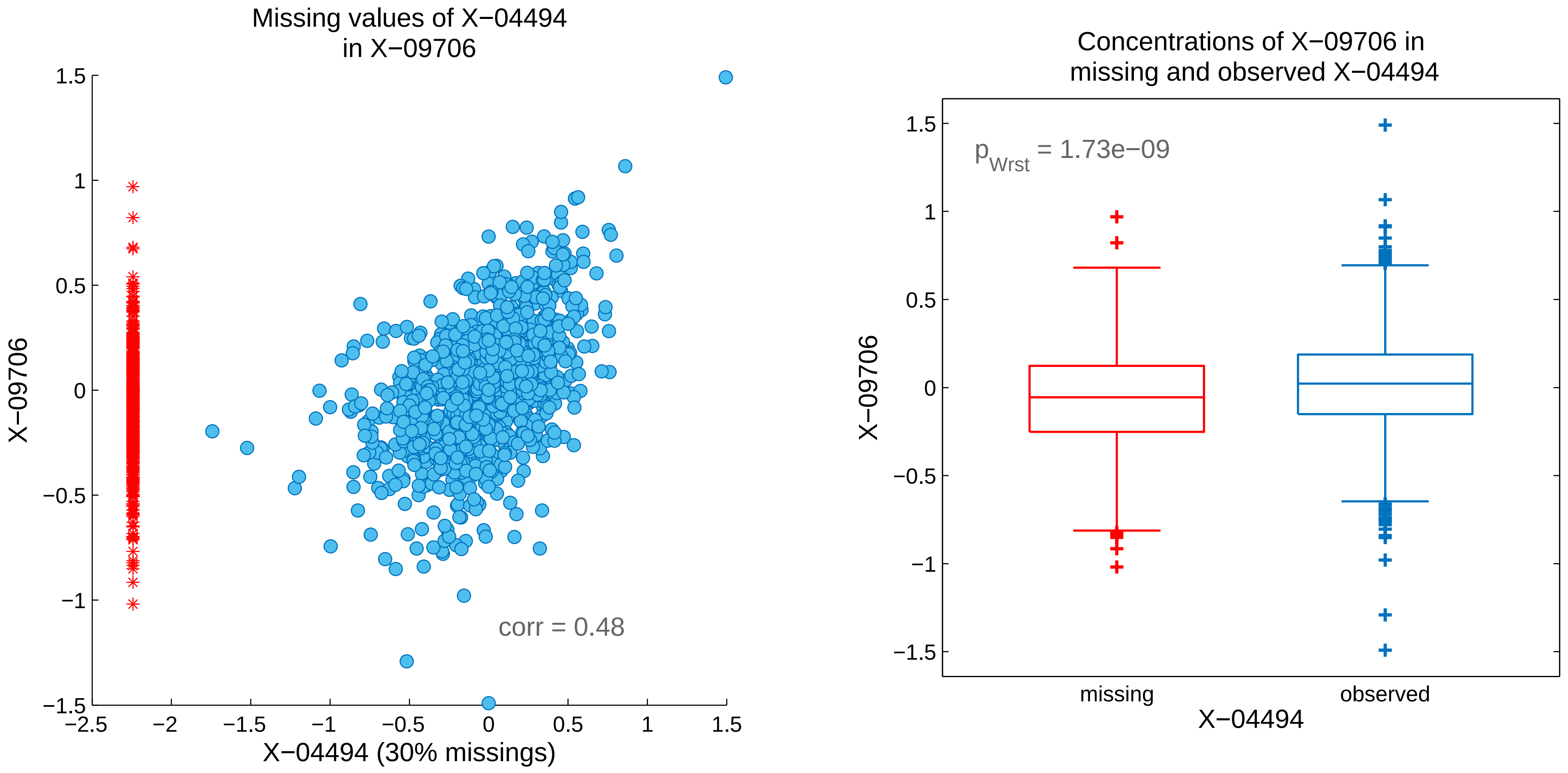

Missing values of X−04494
in X−09706
X−09706
X−04494 (30% missings)
corr = 0.48
Concentrations of X−09706 in
missing and observed X−04494
p_{Wrst} = 1.73e−09
X−09706
missing
observed
X−04494

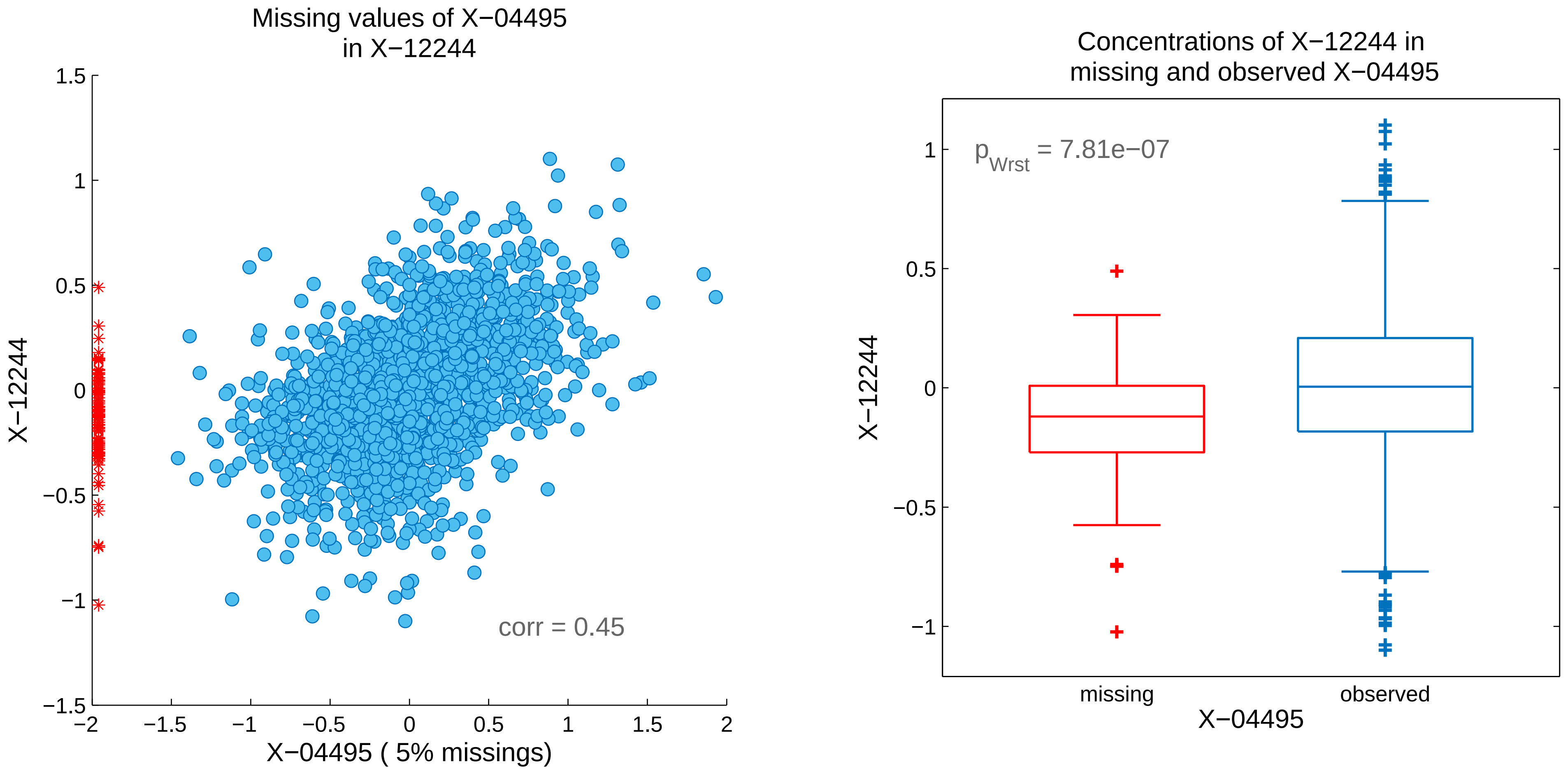
Missing values of X−04495
in X−12244
X−12244
X−04495 (5% missings)
corr = 0.45
Concentrations of X−12244 in
missing and observed X−04495
p_{Wrst} = 7.81e−07
X−12244
missing
observed
X−04495

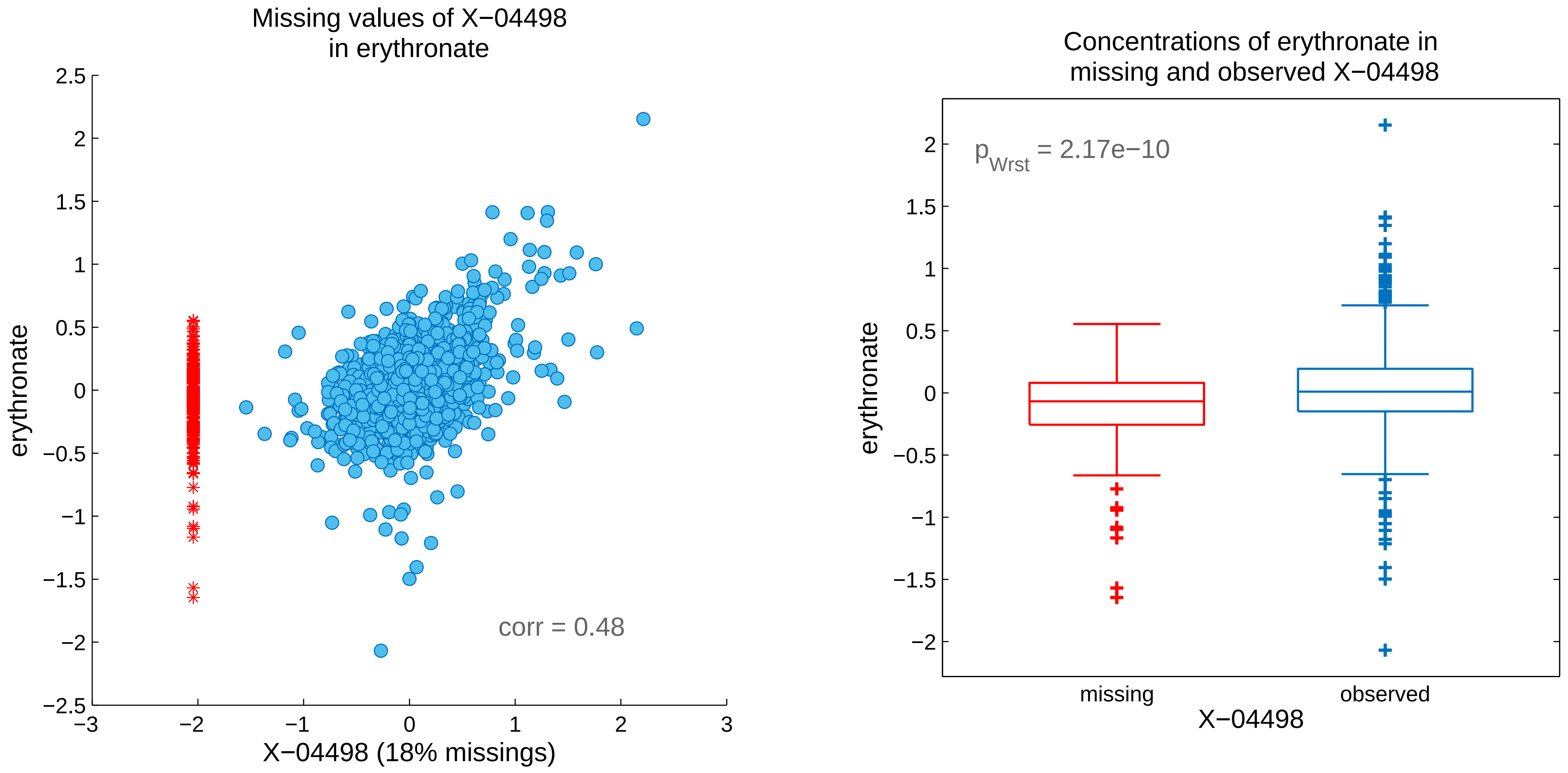

Missing values of X−04498
in erythronate
erythronate
X−04498 (18% missings)
corr = 0.48
Concentrations of erythronate in
missing and observed X−04498
erythronate
p_{Wrst} = 2.17e−10
missing
observed
X−04498

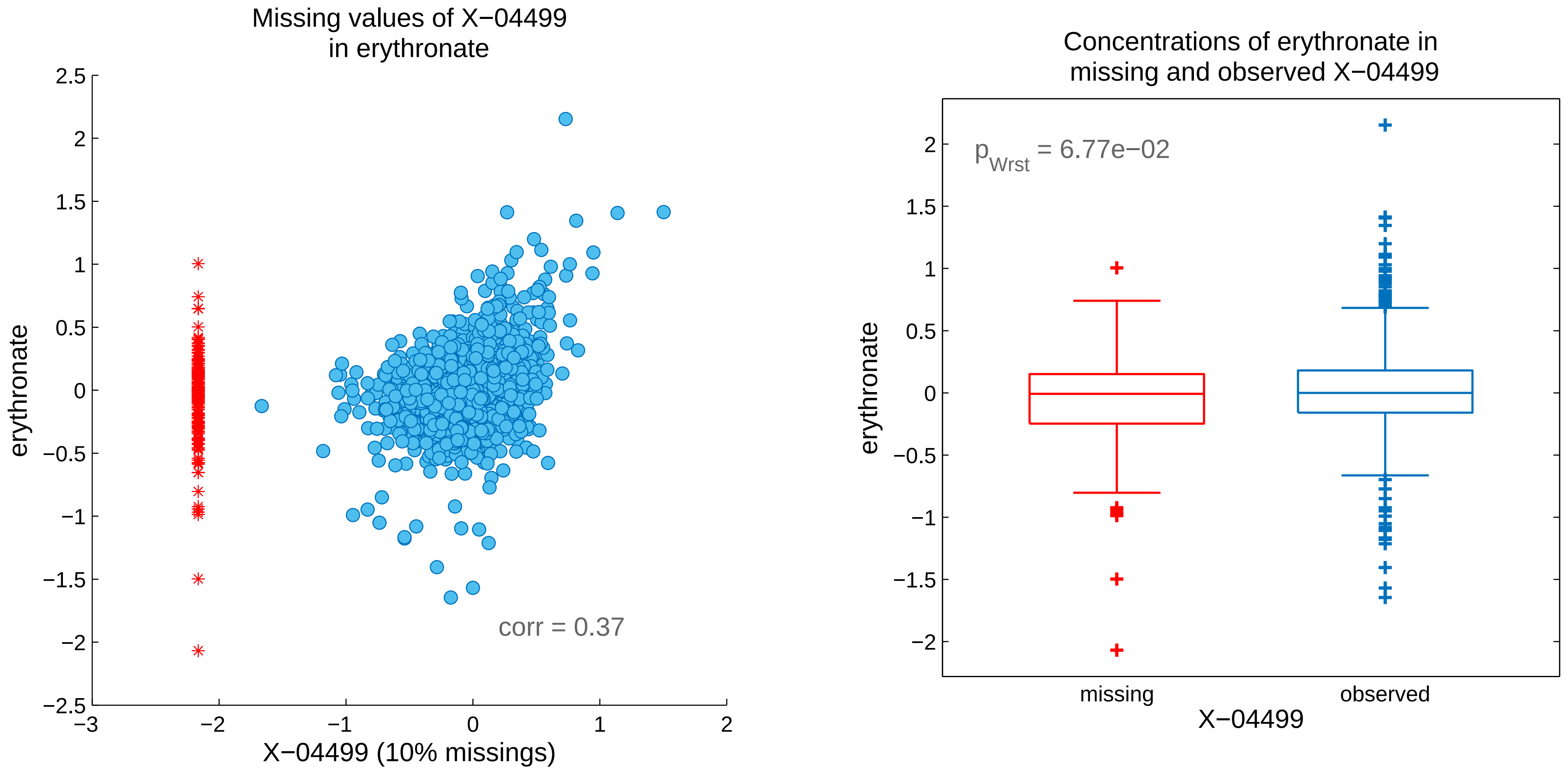
Missing values of X−04499
in erythronate
erythronate
X−04499 (10% missings)
corr = 0.37
2.5
2
1.5
1
0.5
0
−0.5
−1
−1.5
−2
−2.5
−3
−2
−1
0
1
2
Concentrations of erythronate in
missing and observed X−04499
p_{Wrst} = 6.77e−02
erythronate
missing
observed
X−04499

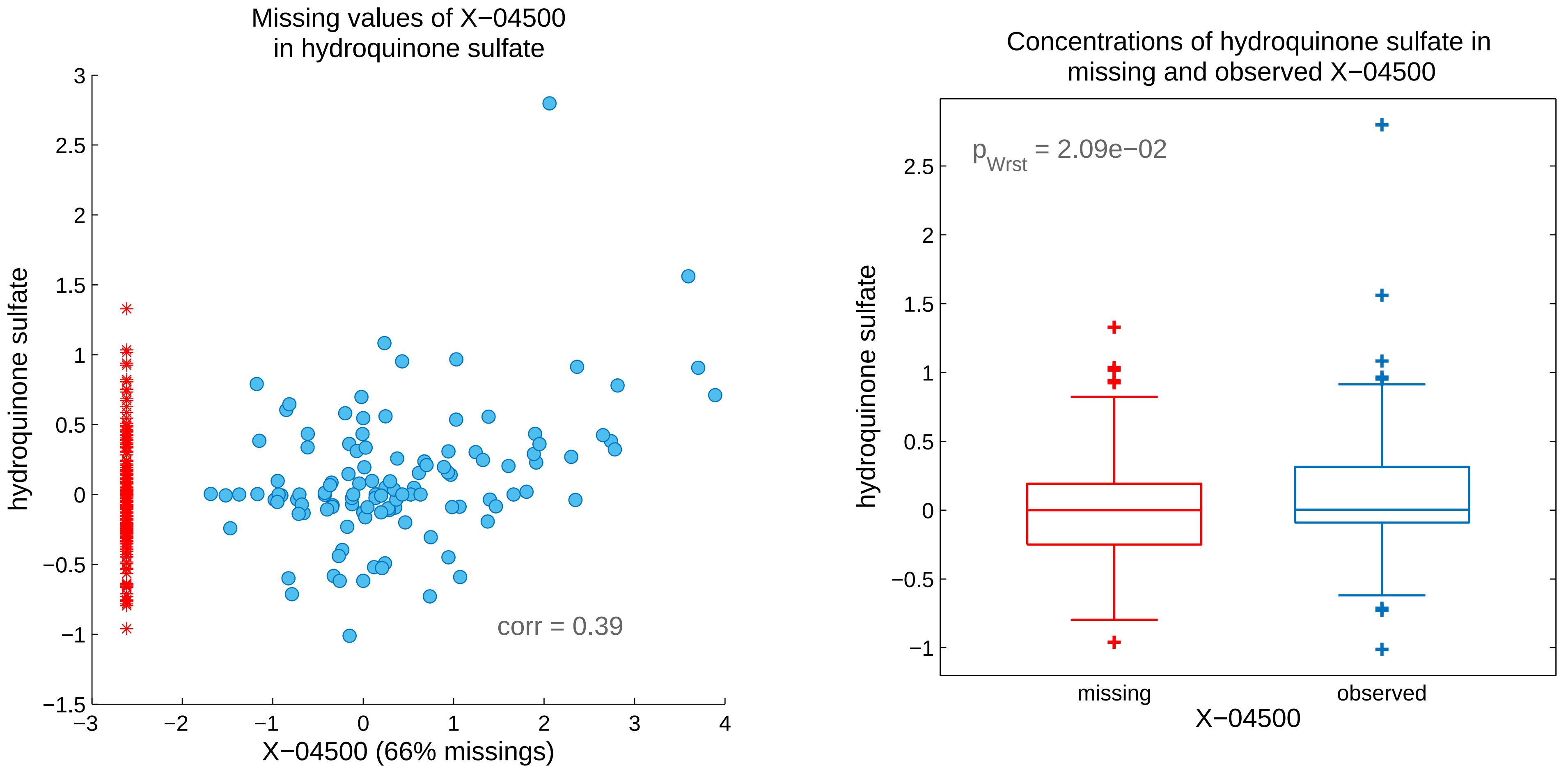
Missing values of X−04500
in hydroquinone sulfate
hydroquinone sulfate
X−04500 (66% missings)
corr = 0.39
Concentrations of hydroquinone sulfate in
missing and observed X−04500
p_{Wrst} = 2.09e−02
hydroquinone sulfate
missing
observed
X−04500

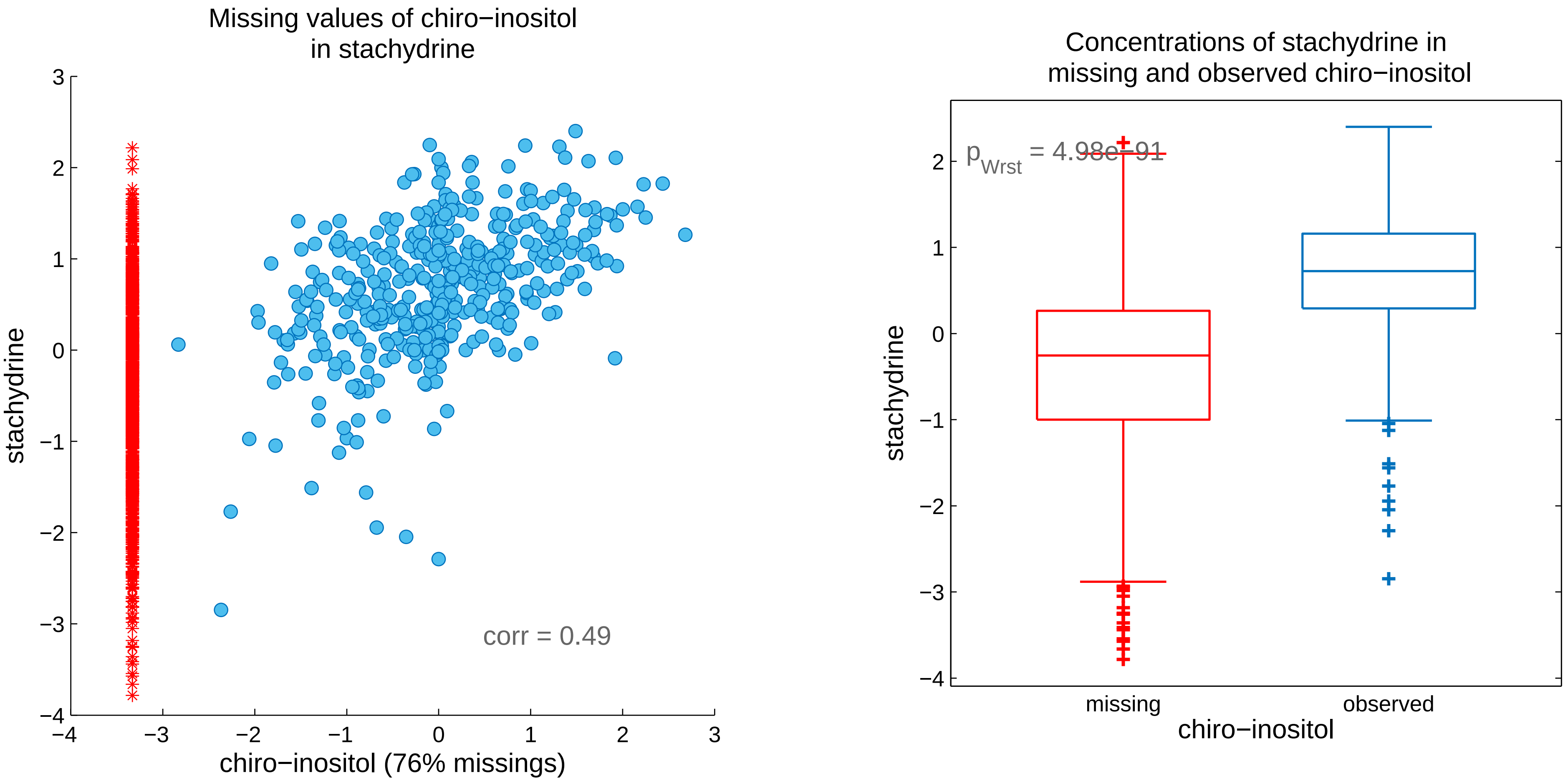

Missing values of chiro−inositol in stachydrine
stachydrine
chiro−inositol (76% missings)
corr = 0.49
Concentrations of stachydrine in missing and observed chiro−inositol
p_{Wrst} = 4.98e−91
stachydrine
missing
observed
chiro−inositol

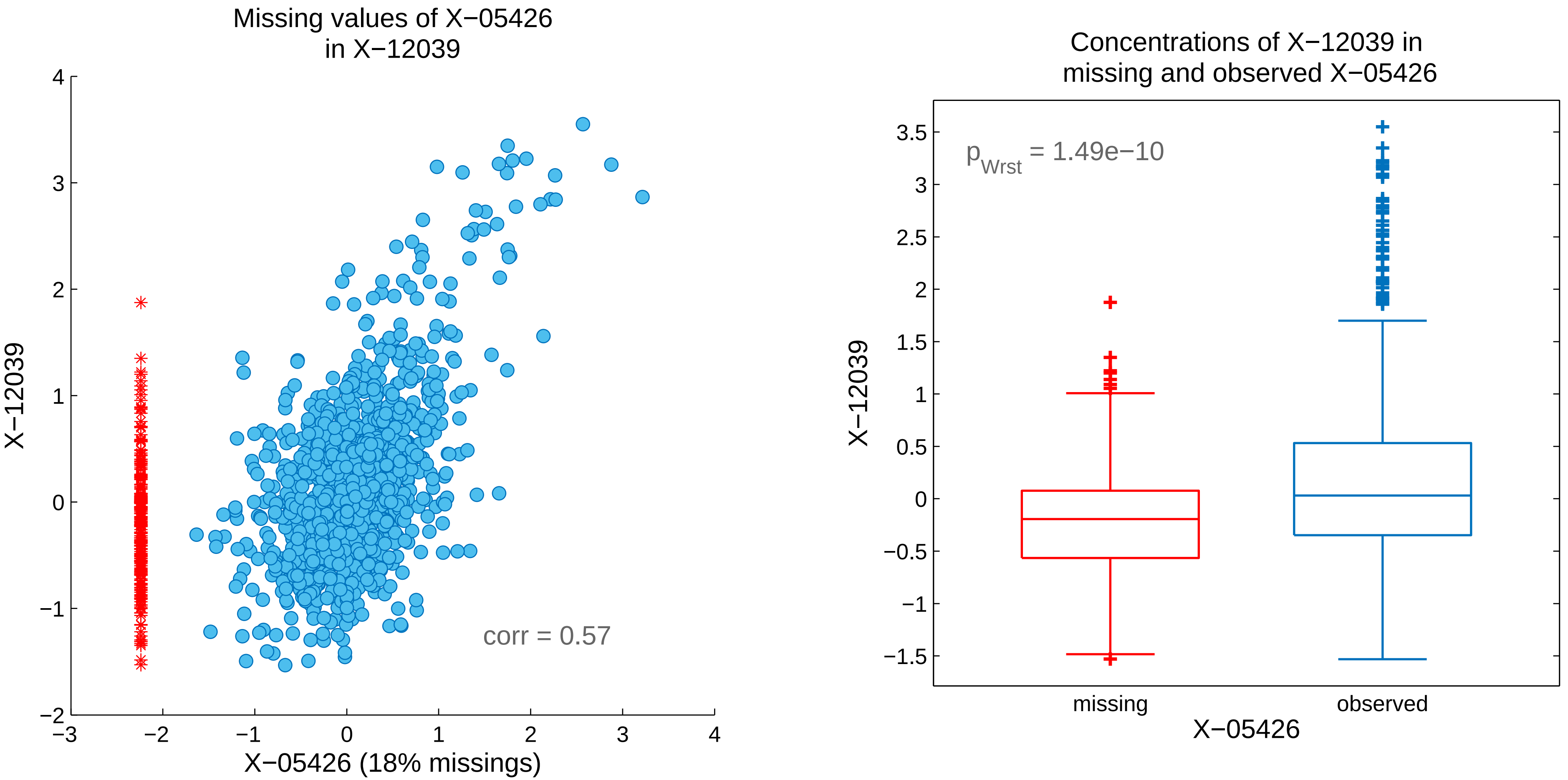
Missing values of X−05426
in X−12039
X−12039
X−05426 (18% missings)
corr = 0.57
4
3
2
1
0
−1
−2
−3
−2
−1
0
1
2
3
4
Concentrations of X−12039 in
missing and observed X−05426
p_{Wrst} = 1.49e−10
X−12039
3.5
3
2.5
2
1.5
1
0.5
0
−0.5
−1
−1.5
missing
observed
X−05426

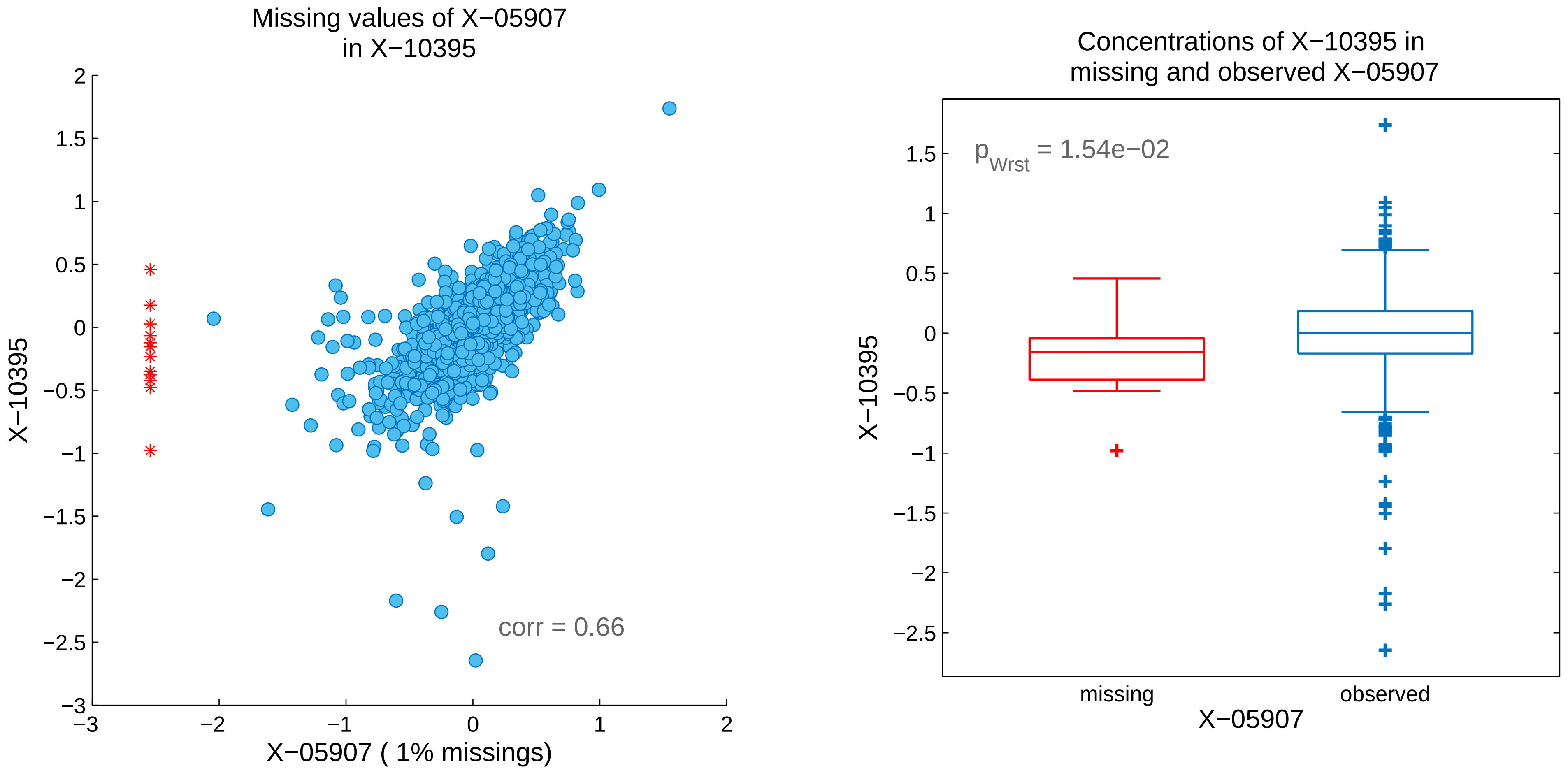

Missing values of X−05907
in X−10395
X−10395
X−05907 (1% missings)
corr = 0.66
Concentrations of X−10395 in
missing and observed X−05907
p_{Wrst} = 1.54e−02
X−10395
missing
observed
X−05907

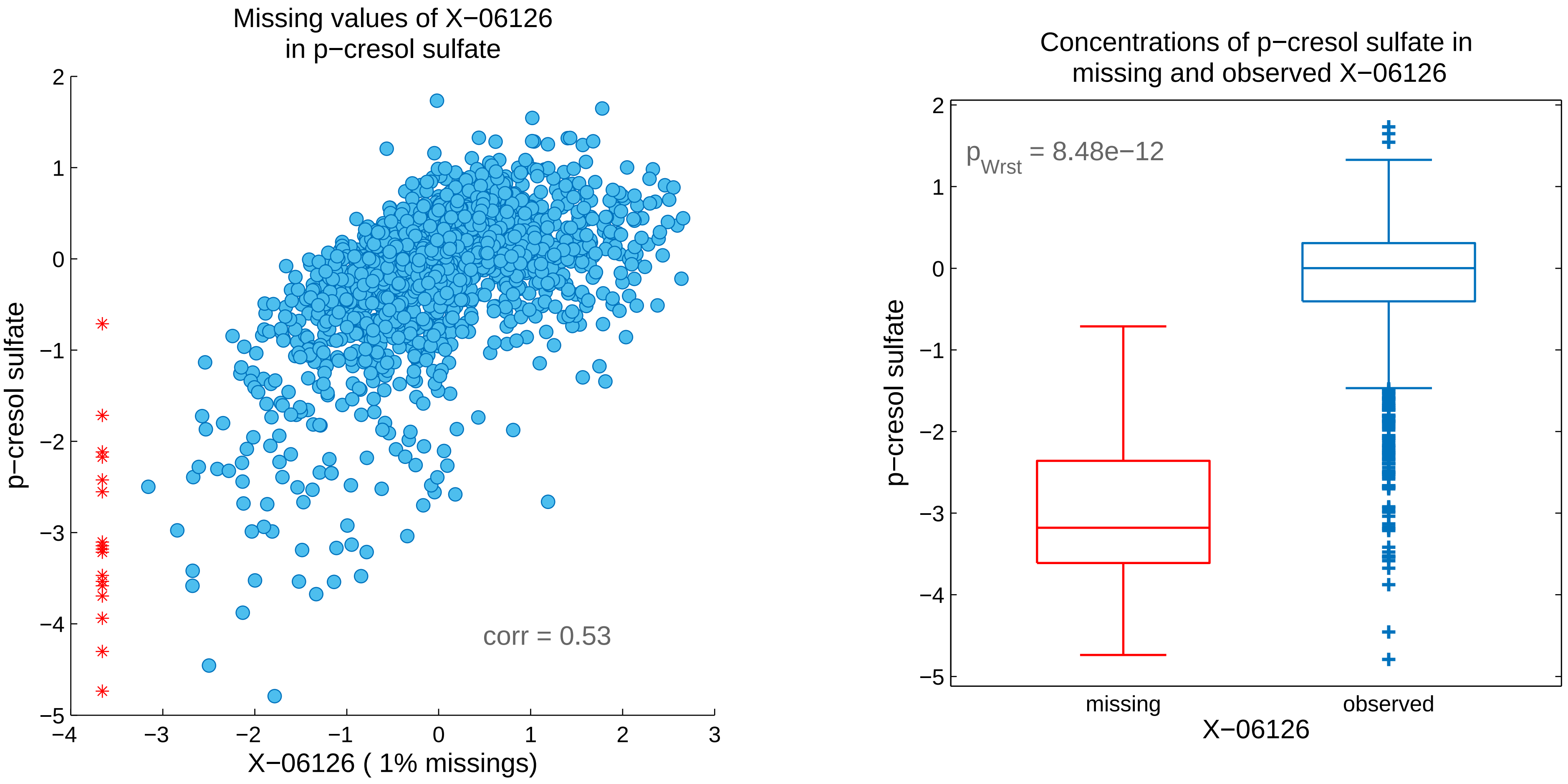

Missing values of X−06126 in p−cresol sulfate
p−cresol sulfate
X−06126 (1% missings)
corr = 0.53
Concentrations of p−cresol sulfate in missing and observed X−06126
p_{Wrst} = 8.48e−12
missing
observed
X−06126

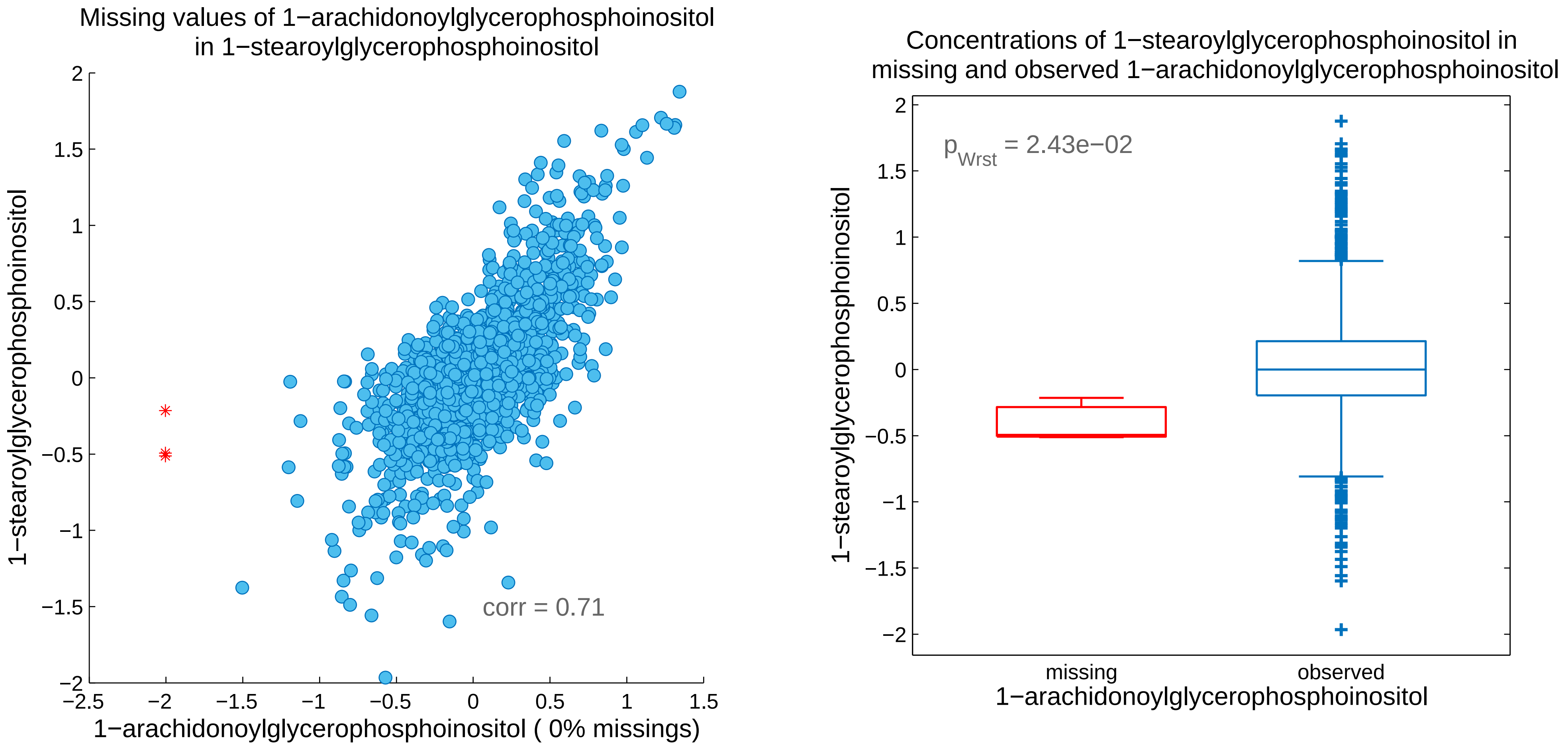
Missing values of 1−arachidonoylglycerophosphoinositol in 1−stearoylglycerophosphoinositol
1−stearoylglycerophosphoinositol
1−arachidonoylglycerophosphoinositol (0% missings)
corr = 0.71
Concentrations of 1−stearoylglycerophosphoinositol in missing and observed 1−arachidonoylglycerophosphoinositol
p_{Wrst} = 2.43e−02
1−stearoylglycerophosphoinositol
missing
observed
1−arachidonoylglycerophosphoinositol

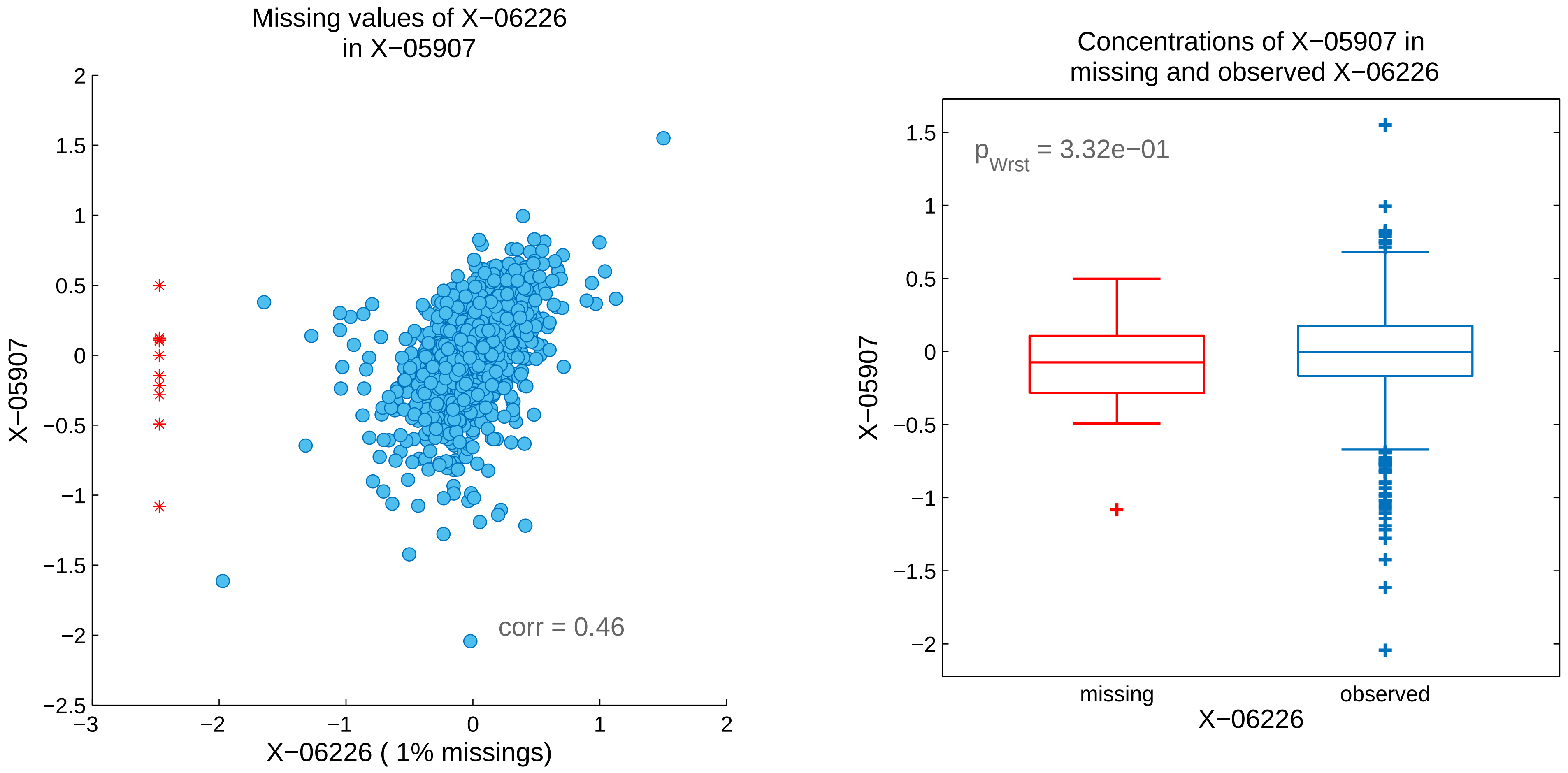
Missing values of X−06226 in X−05907
X−05907
X−06226 (1% missings)
corr = 0.46
Concentrations of X−05907 in missing and observed X−06226
p_{Wrst} = 3.32e−01
X−05907
missing
observed
X−06226

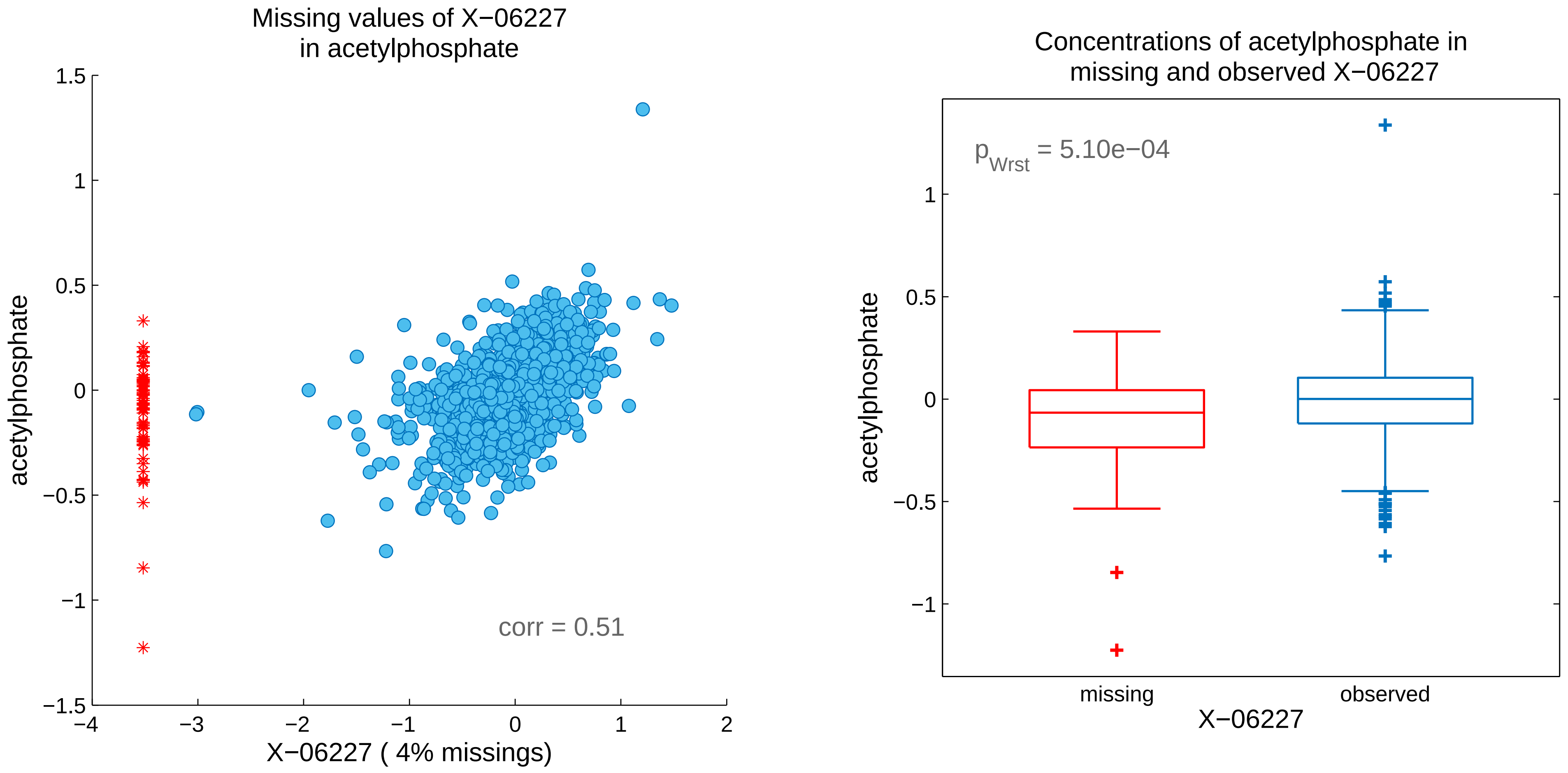
Missing values of X−06227
in acetylphosphate
acetylphosphate
X−06227 (4% missings)
corr = 0.51
Concentrations of acetylphosphate in
missing and observed X−06227
p_Wrst = 5.10e−04
acetylphosphate
missing
observed
X−06227

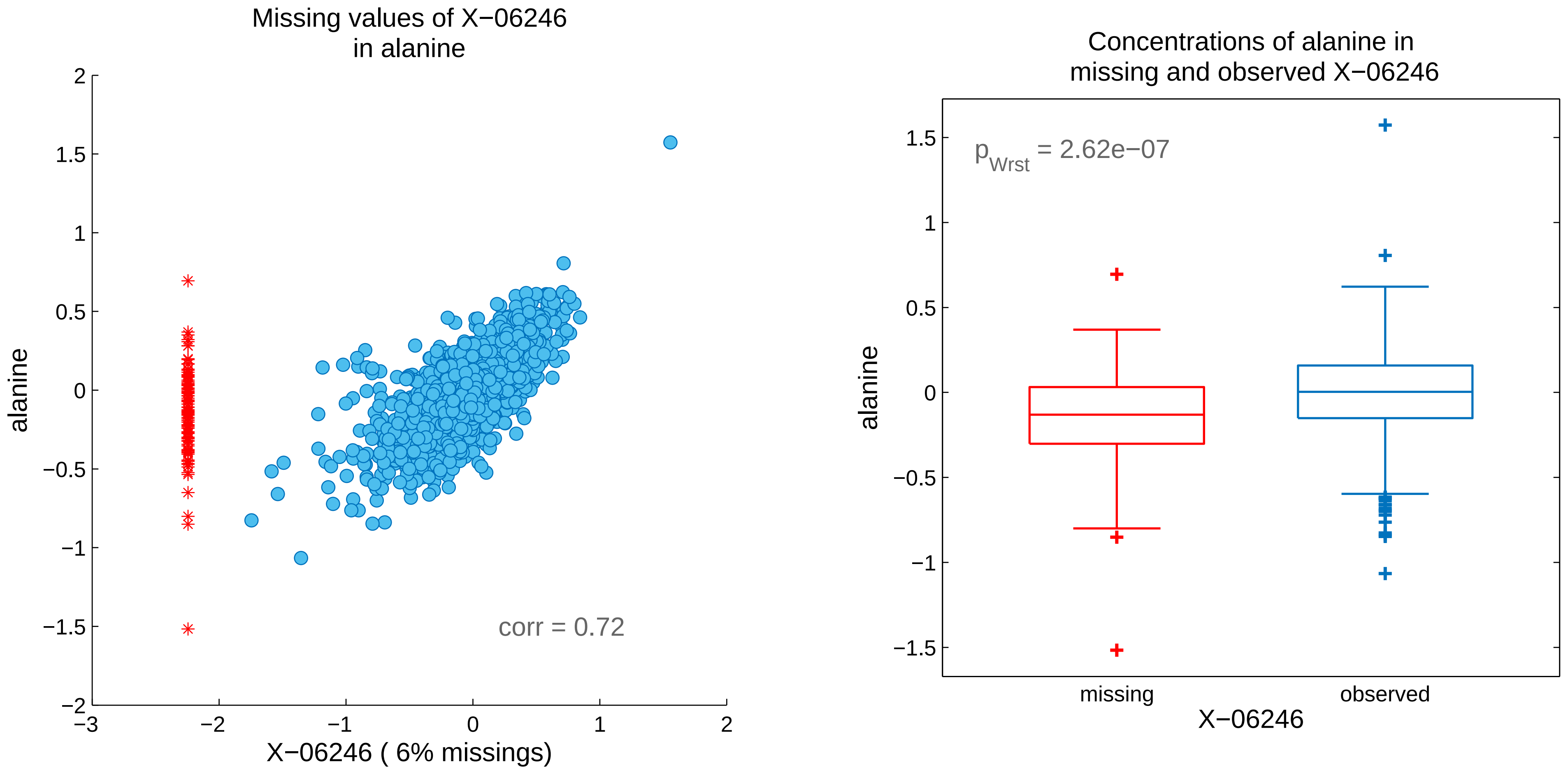

Missing values of X−06246
in alanine
alanine
X−06246 (6% missings)
corr = 0.72
Concentrations of alanine in
missing and observed X−06246
p_{Wrst} = 2.62e−07
alanine
missing
observed
X−06246

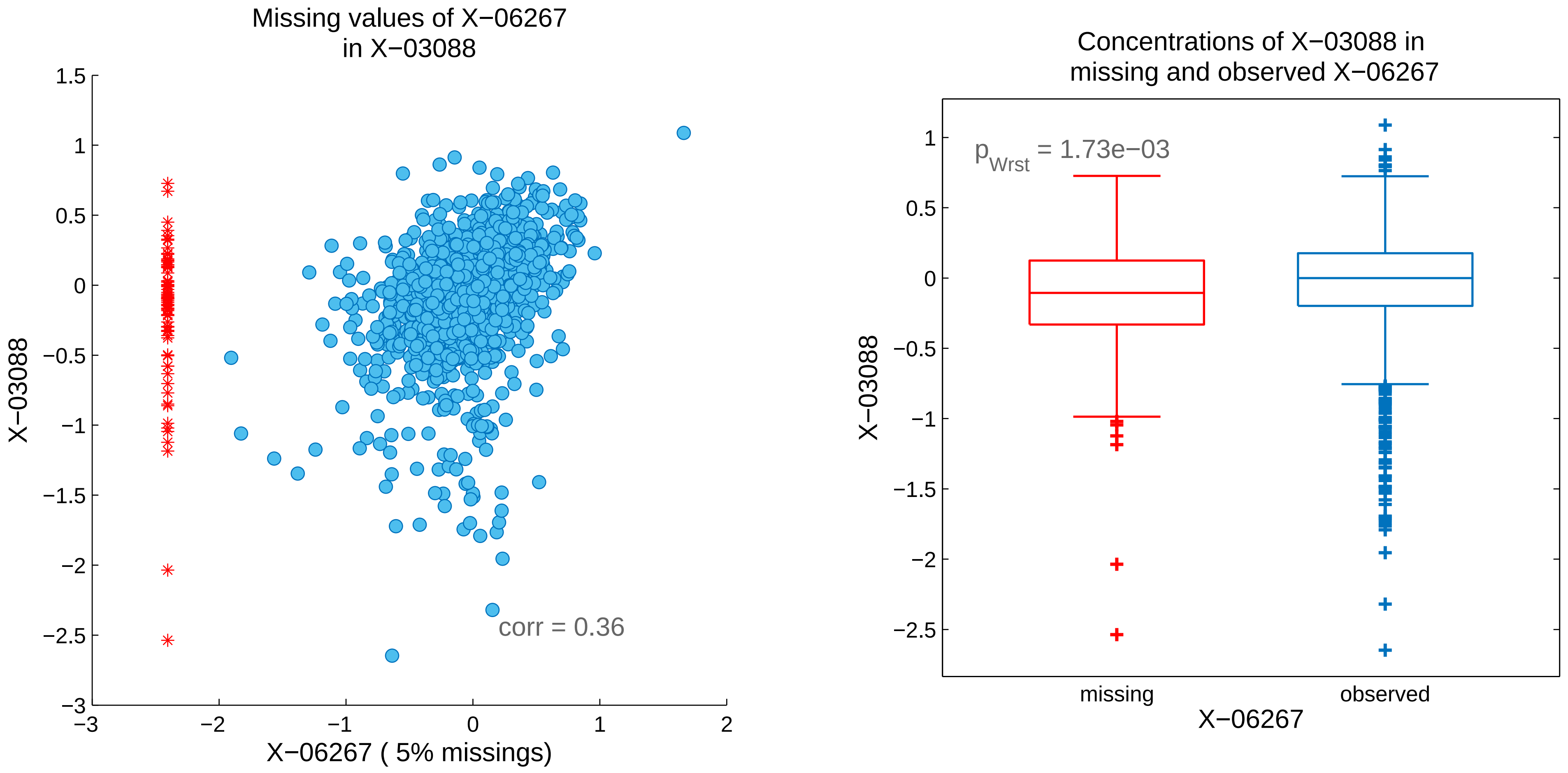

Missing values of X−06267
in X−03088
X−03088
X−06267 (5% missings)
corr = 0.36
Concentrations of X−03088 in
missing and observed X−06267
p_{Wrst} = 1.73e−03
X−03088
missing
observed
X−06267

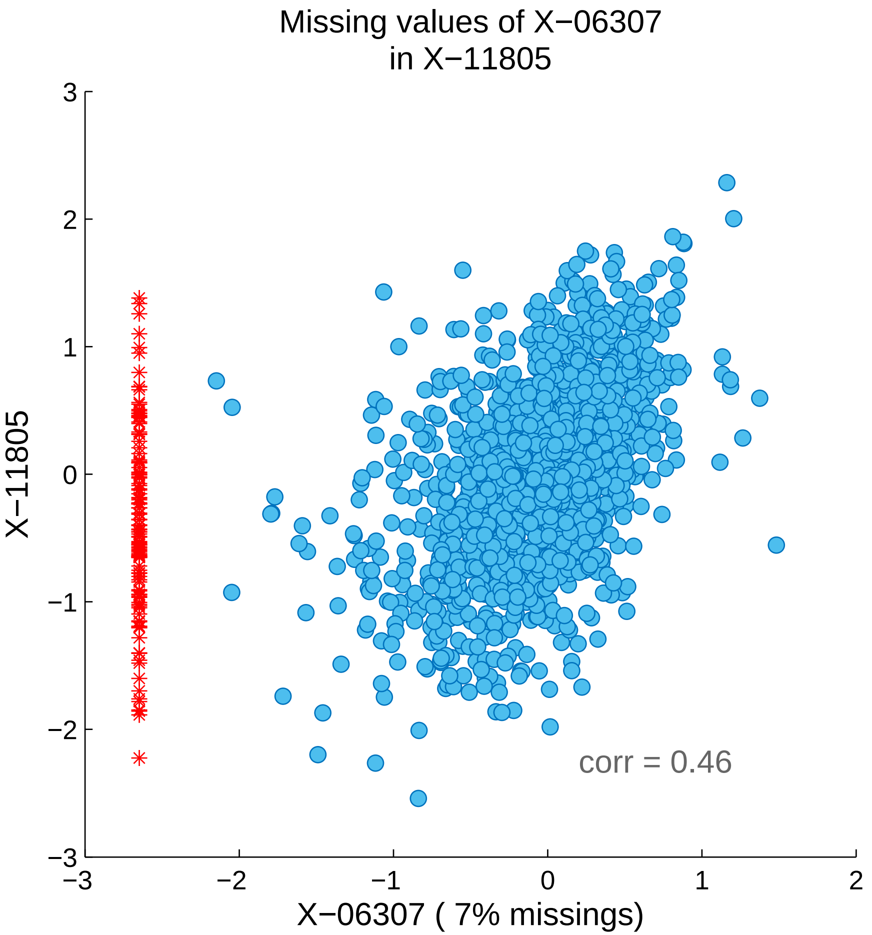
Missing values of X−06307
in X−11805
X−11805
X−06307 (7% missings)
corr = 0.46
3
2
1
0
−1
−2
−3
−3
−2
−1
0
1
2

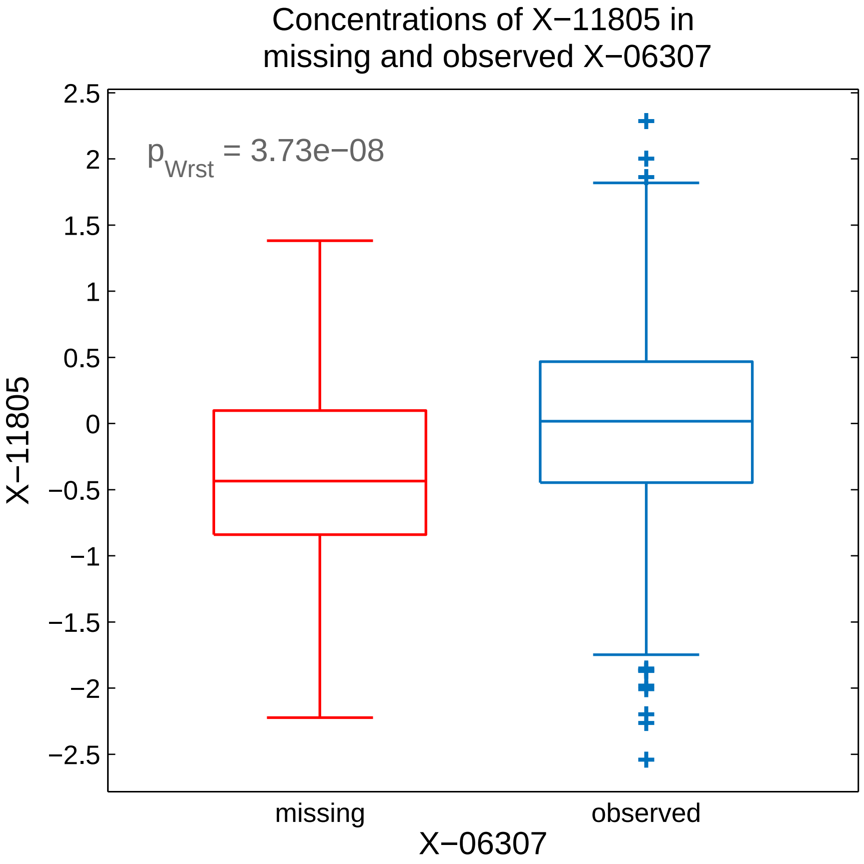
Concentrations of X−11805 in
missing and observed X−06307
p_{Wrst} = 3.73e−08
X−11805
X−06307
missing
observed
2.5
2
1.5
1
0.5
0
−0.5
−1
−1.5
−2
−2.5

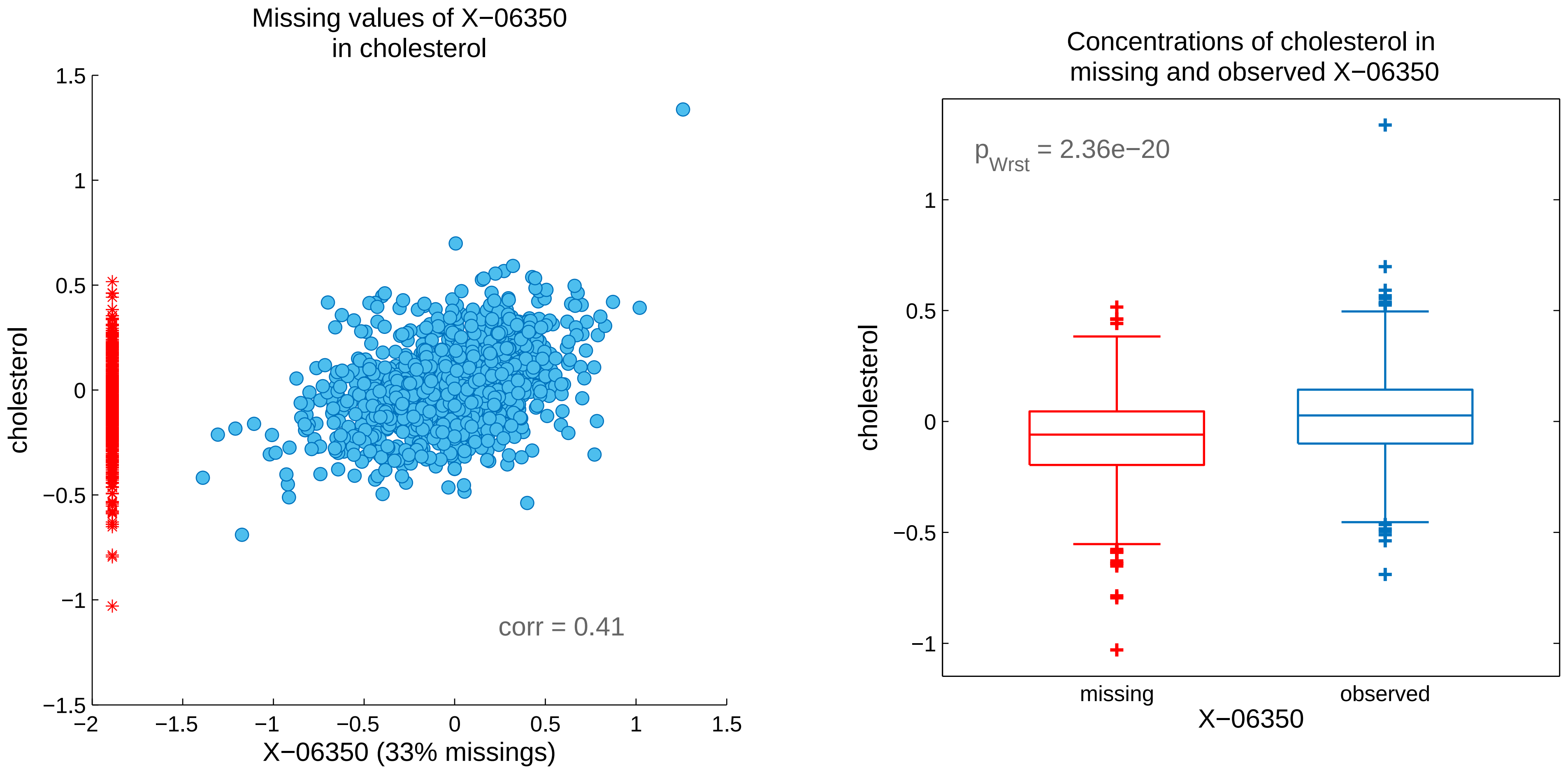

Missing values of X−06350
in cholesterol
1.5
1
0.5
0
−0.5
−1
−1.5
cholesterol
−2
−1.5
−1
−0.5
0
0.5
1
1.5
X−06350 (33% missings)
corr = 0.41
Concentrations of cholesterol in
missing and observed X−06350
p_Wrst = 2.36e−20
1
0.5
0
−0.5
−1
cholesterol
missing
observed
X−06350

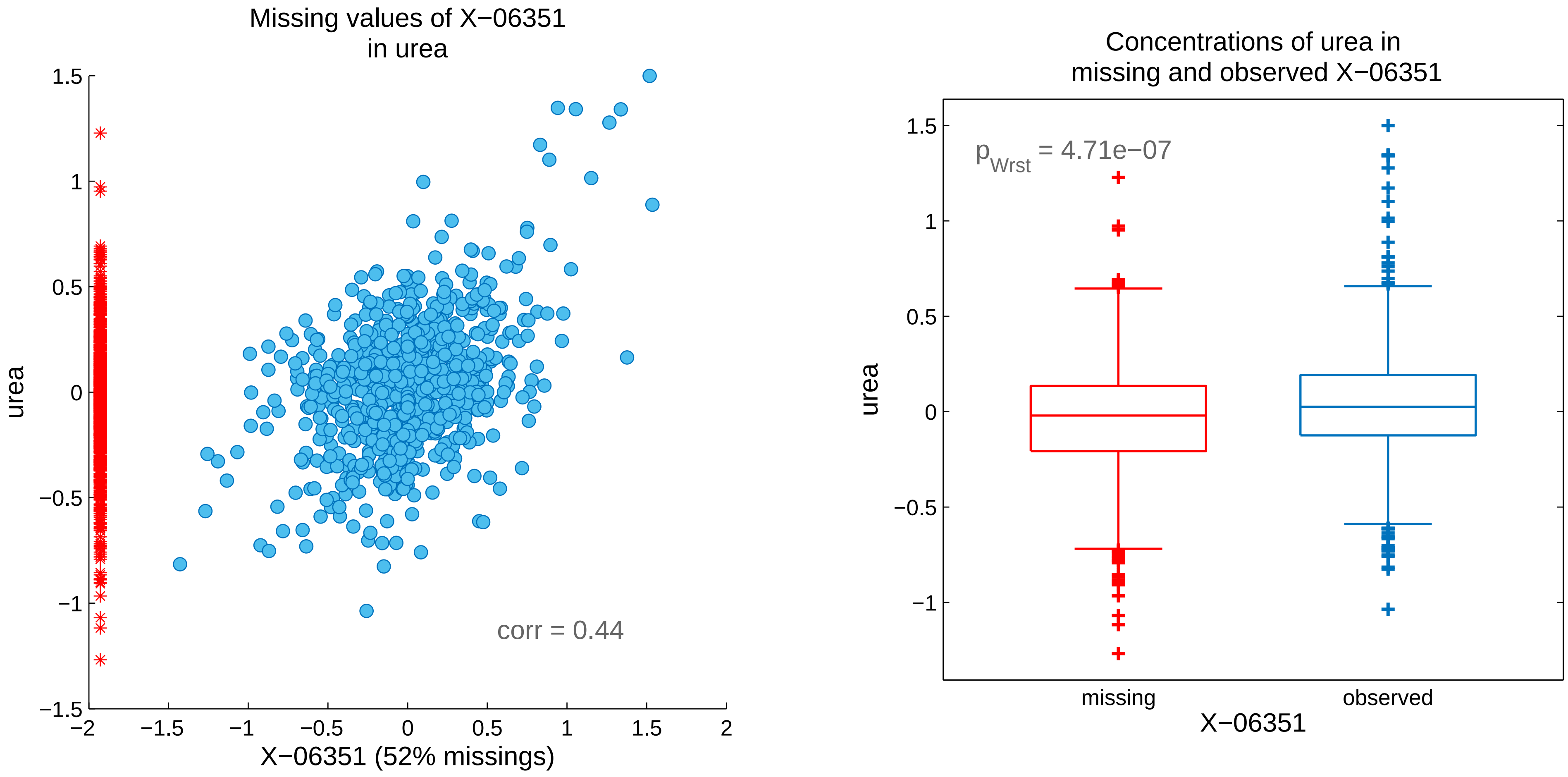

Missing values of X−06351 in urea
corr = 0.44
urea
X−06351 (52% missings)
Concentrations of urea in missing and observed X−06351
p_{Wrst} = 4.71e−07
urea
missing
observed
X−06351

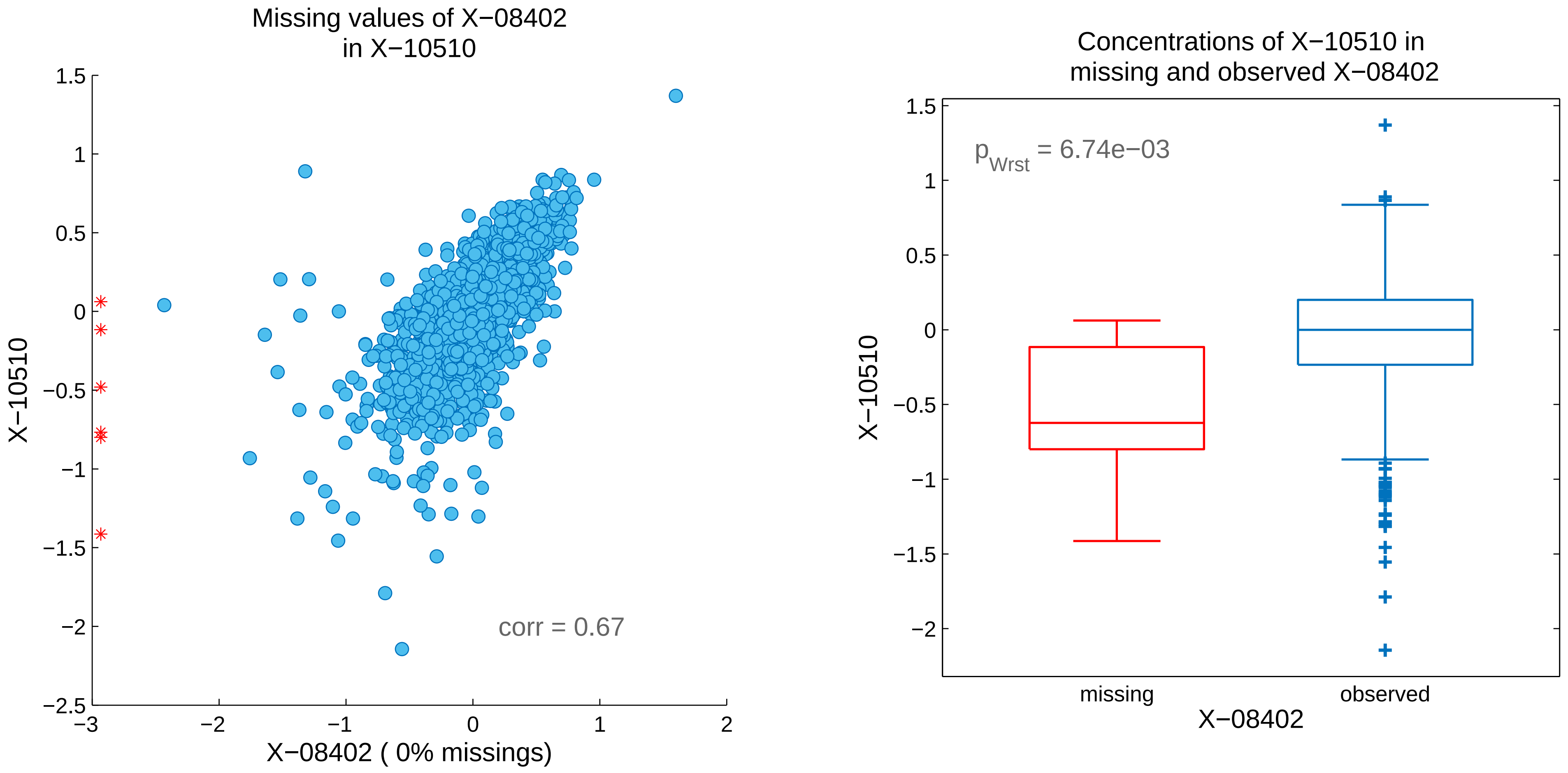

Missing values of X−08402
in X−10510
X−10510
X−08402 (0% missings)
corr = 0.67
1.5
1
0.5
0
−0.5
−1
−1.5
−2
−2.5
−3
−2
−1
0
1
2
Concentrations of X−10510 in
missing and observed X−08402
p_{Wrst} = 6.74e−03
X−10510
missing
observed
X−08402

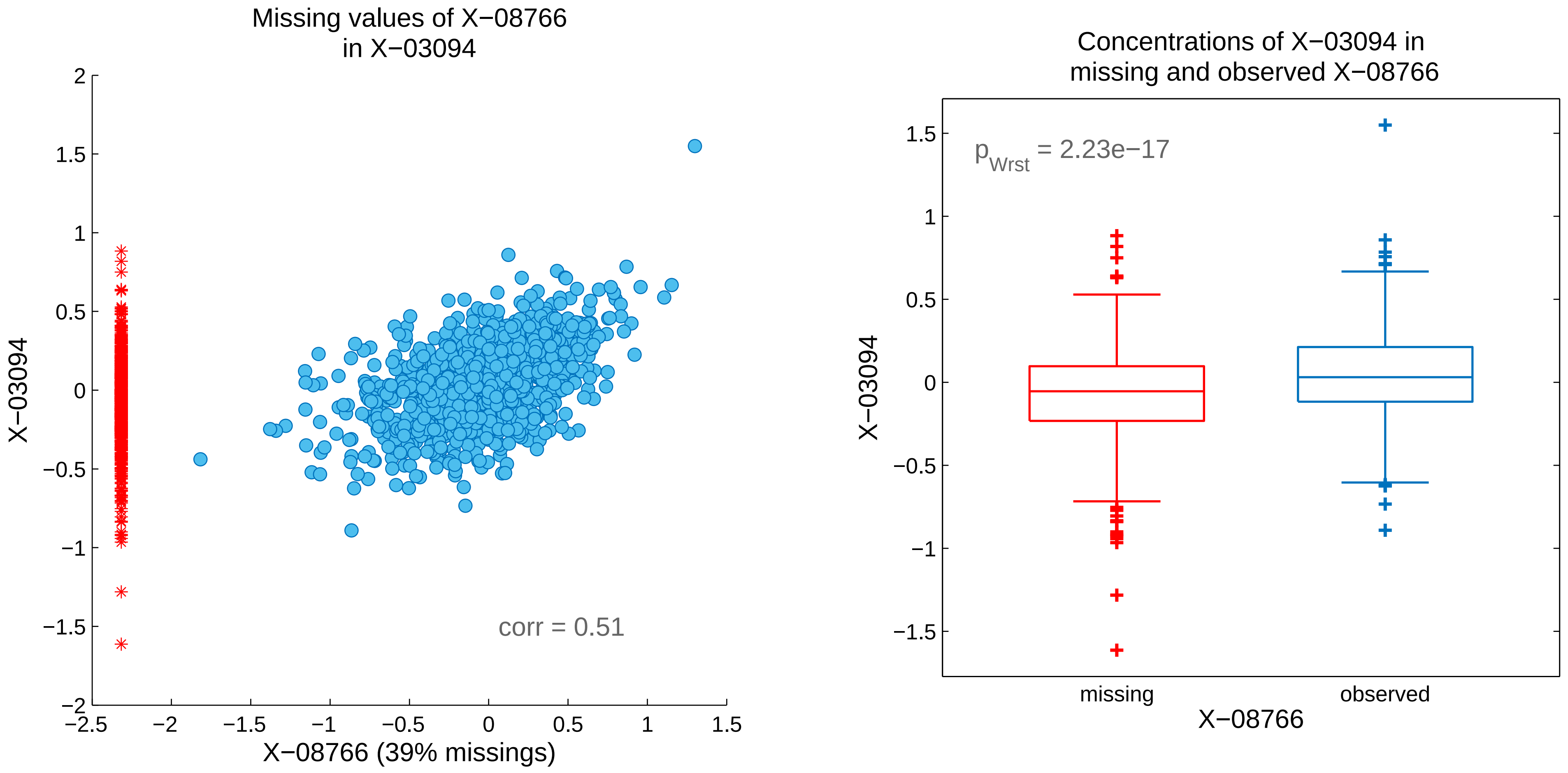

Missing values of X−08766
in X−03094
X−03094
X−08766 (39% missings)
corr = 0.51
Concentrations of X−03094 in
missing and observed X−08766
p$_{Wrst}$ = 2.23e−17
X−03094
missing
observed
X−08766

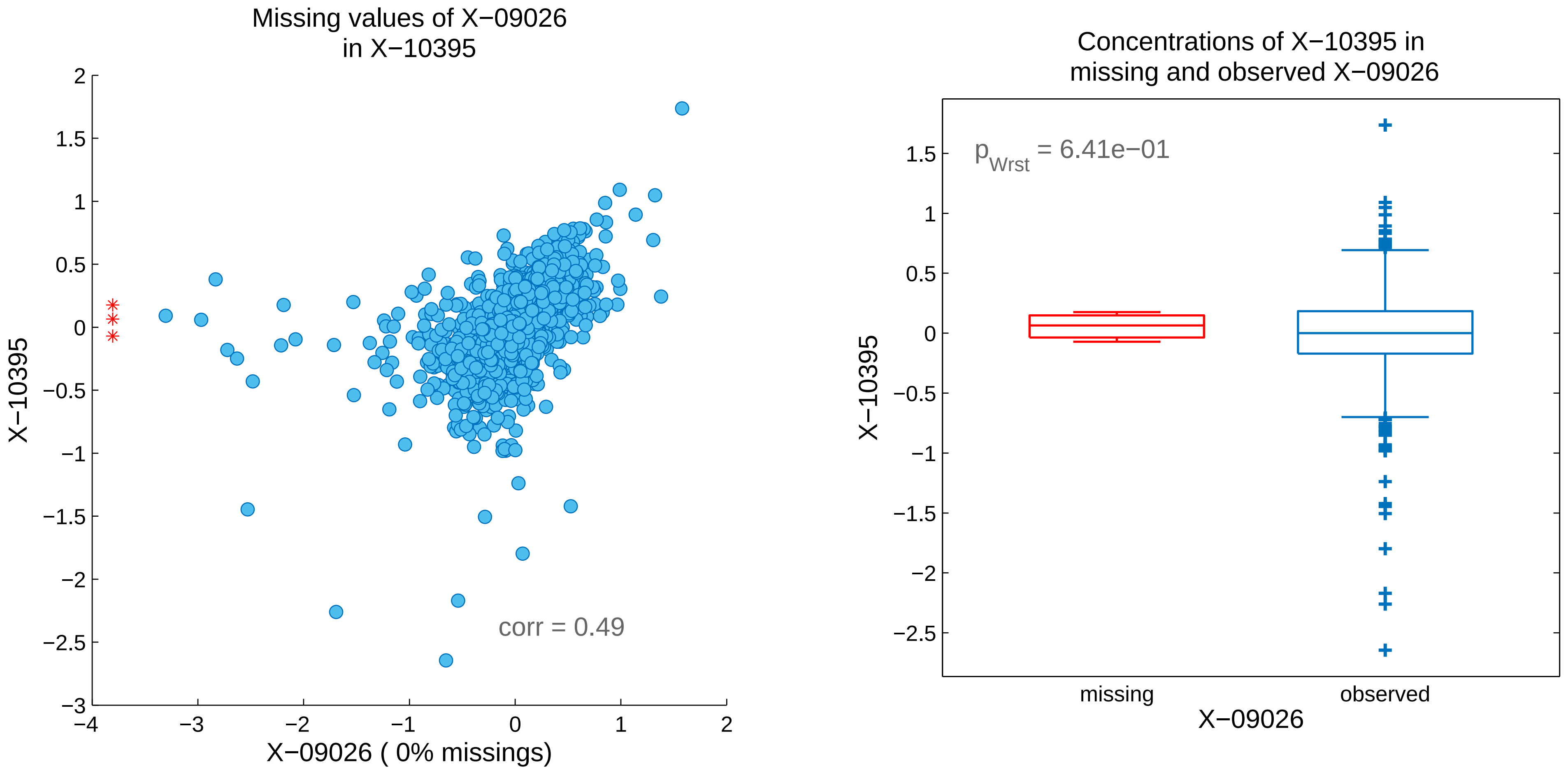

Missing values of X−09026
in X−10395
X−10395
X−09026 (0% missings)
corr = 0.49
Concentrations of X−10395 in
missing and observed X−09026
p_{Wrst} = 6.41e−01
X−10395
missing
observed
X−09026

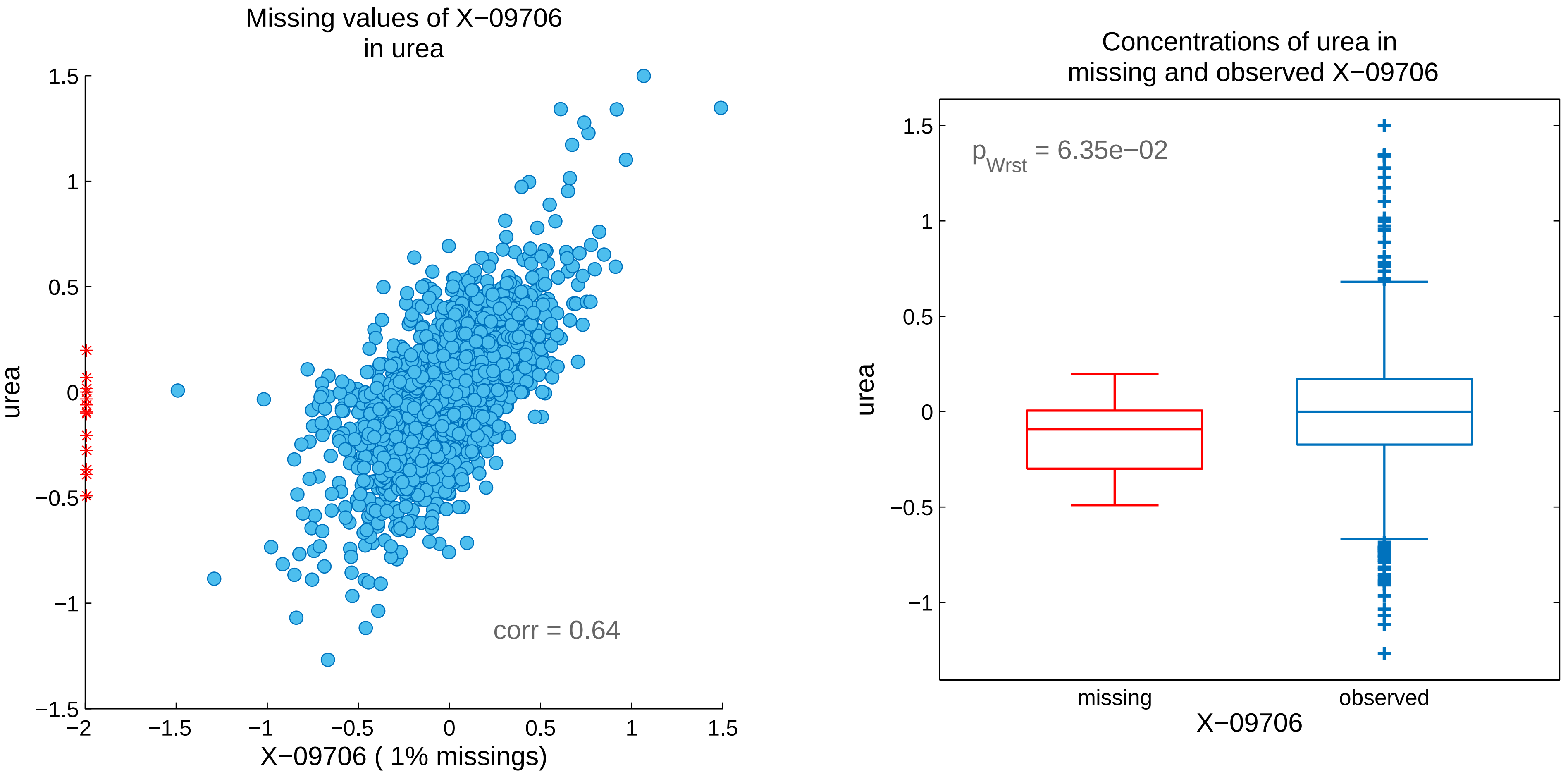

Missing values of X−09706
in urea
corr = 0.64
urea
X−09706 (1% missings)
Concentrations of urea in
missing and observed X−09706
p_Wrst = 6.35e−02
urea
missing
observed
X−09706

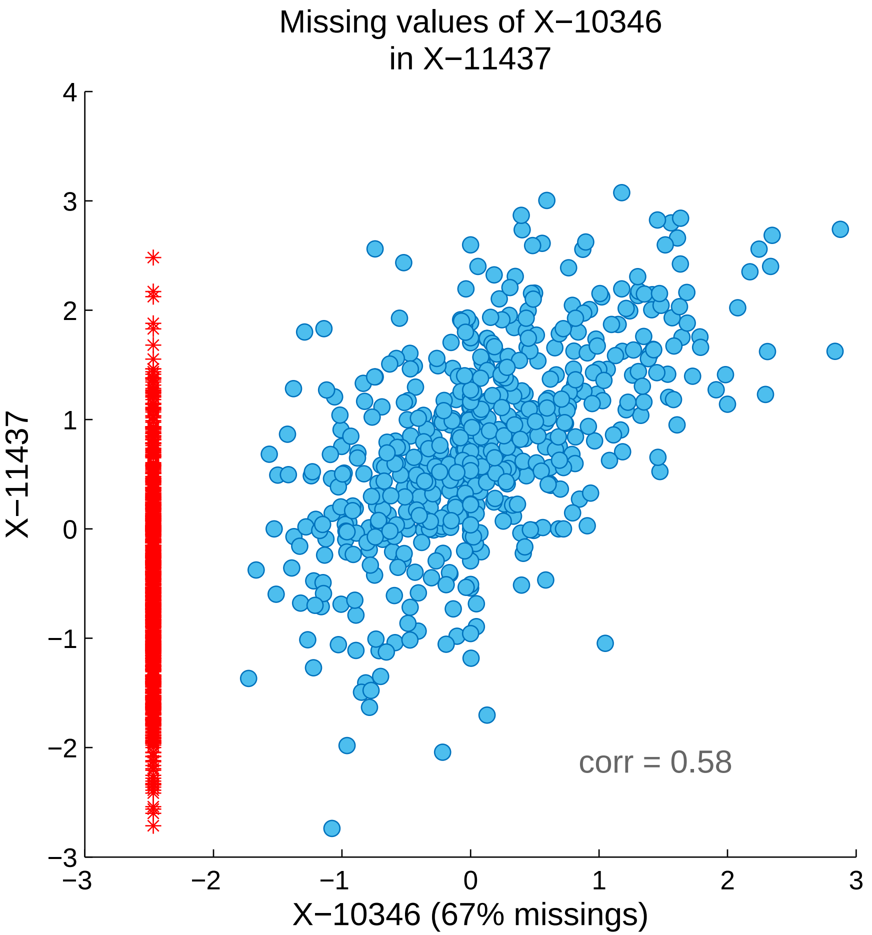
Missing values of X−10346
in X−11437
X−11437
X−10346 (67% missings)
corr = 0.58
4
3
2
1
0
−1
−2
−3
−3
−2
−1
0
1
2
3

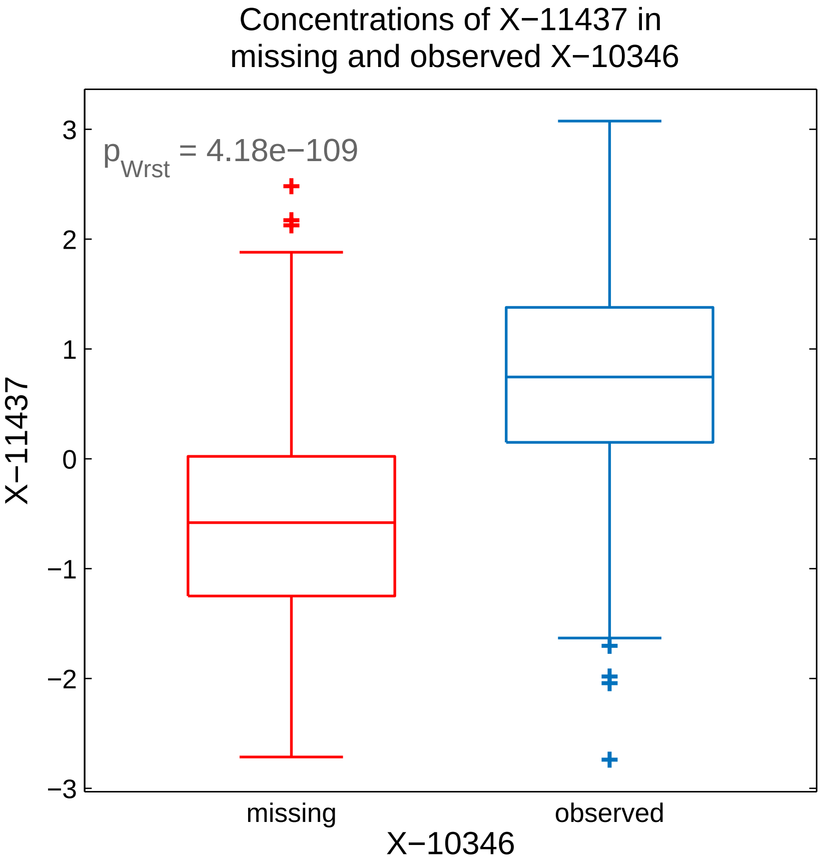
Concentrations of X−11437 in
missing and observed X−10346
p_Wrst = 4.18e−109
X−11437
X−10346
missing
observed
3
2
1
0
−1
−2
−3

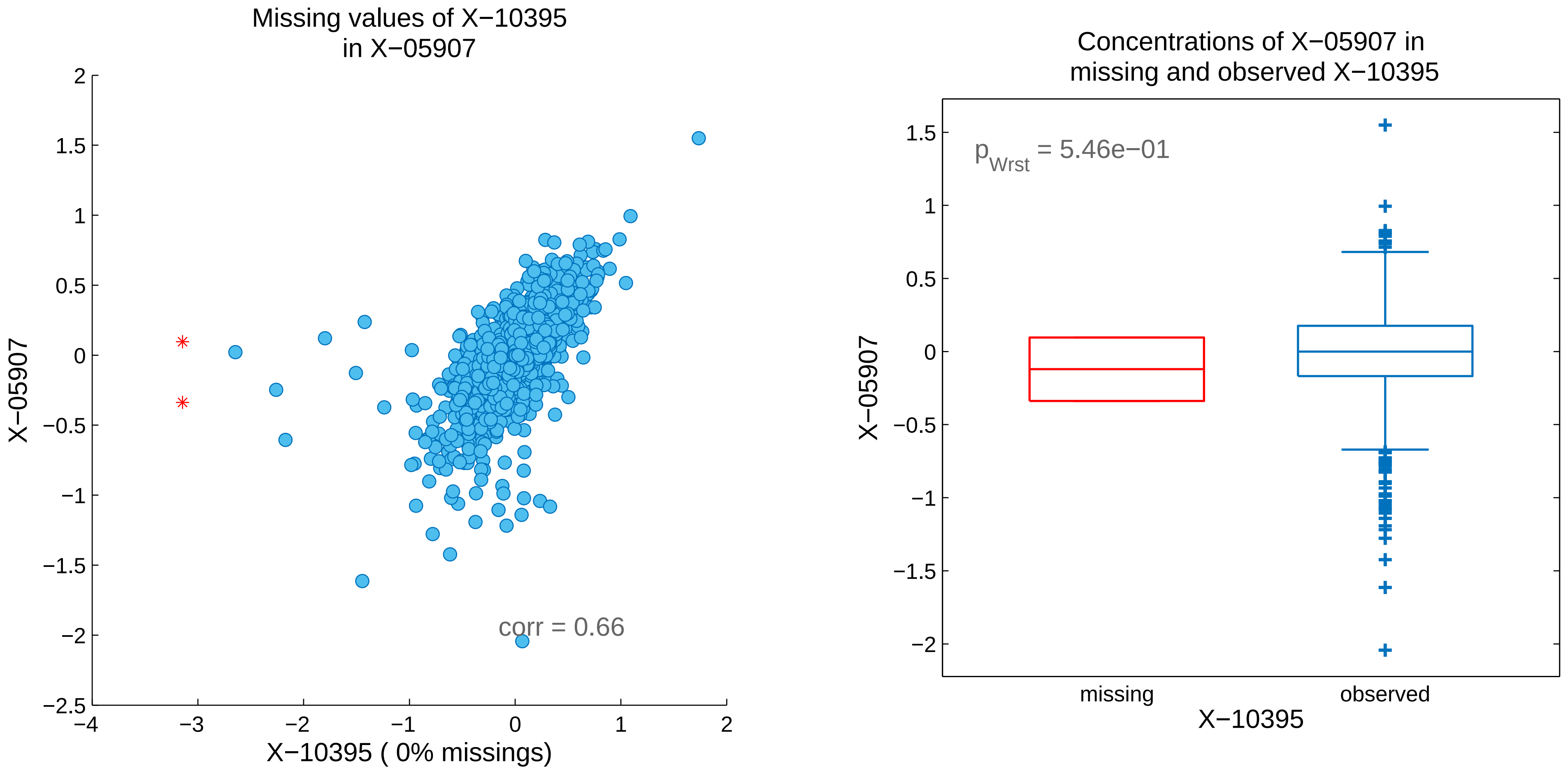

Missing values of X−10395
in X−05907
X−05907
X−10395 (0% missings)
corr = 0.66
Concentrations of X−05907 in
missing and observed X−10395
p_{Wrst} = 5.46e−01
X−05907
missing
observed
X−10395

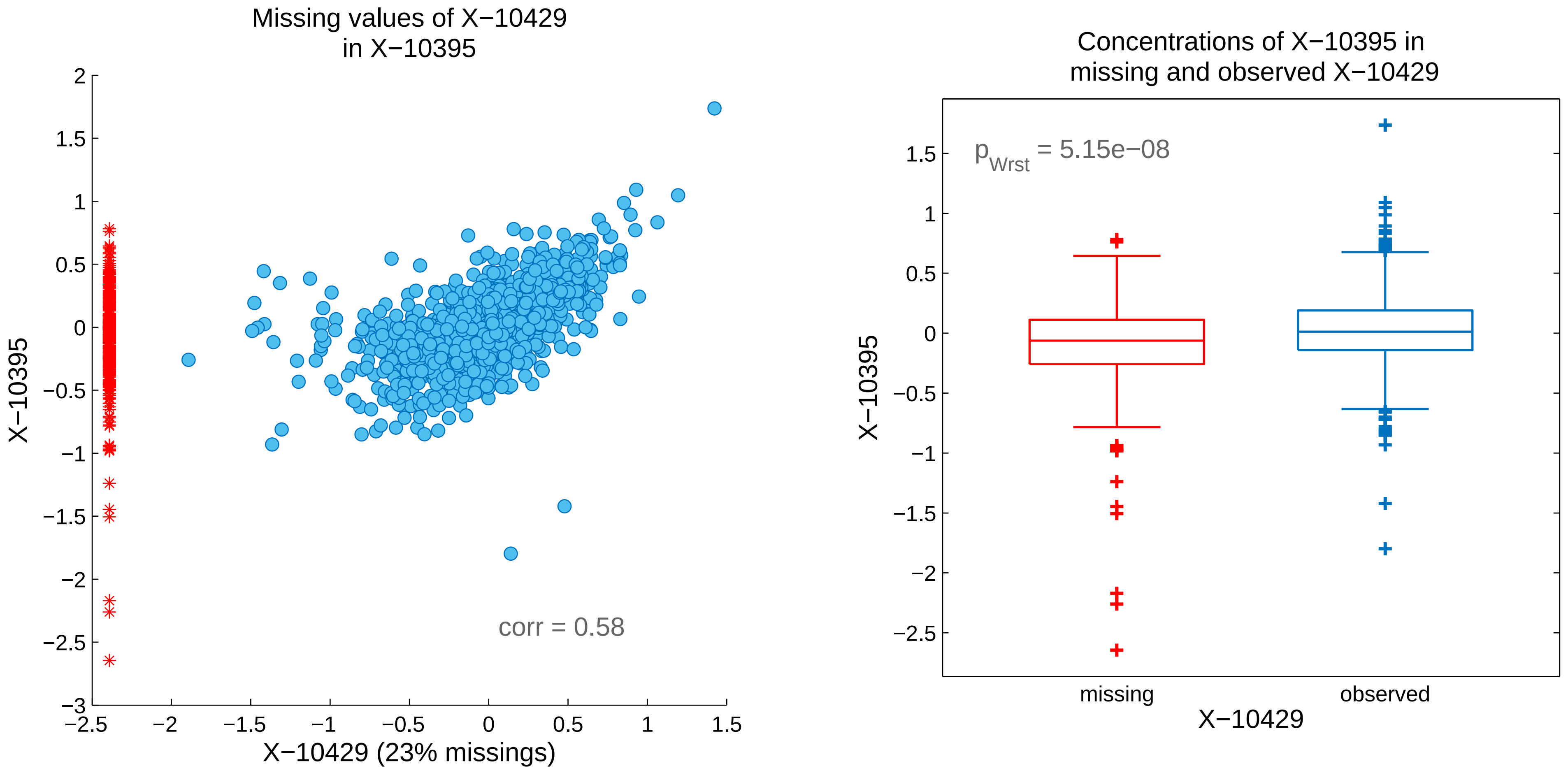

Missing values of X−10429
in X−10395
X−10395
X−10429 (23% missings)
corr = 0.58
2
1.5
1
0.5
0
−0.5
−1
−1.5
−2
−2.5
−3
−2.5
−2
−1.5
−1
−0.5
0
0.5
1
1.5
Concentrations of X−10395 in
missing and observed X−10429
p_{Wrst} = 5.15e−08
X−10395
missing
observed
X−10429

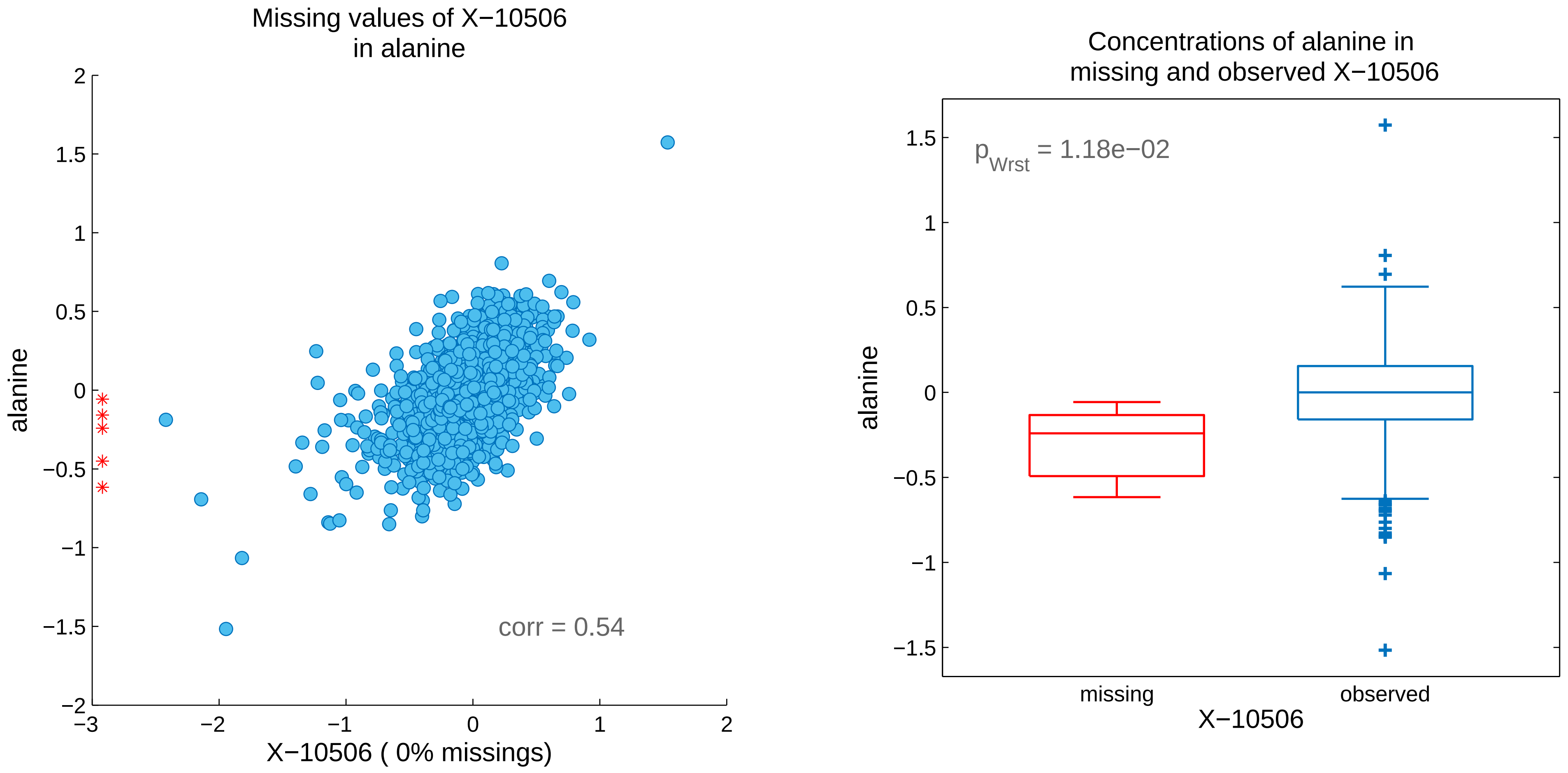

Missing values of X−10506
in alanine
alanine
X−10506 (0% missings)
corr = 0.54
Concentrations of alanine in
missing and observed X−10506
p_Wrst = 1.18e−02
alanine
missing
observed
X−10506

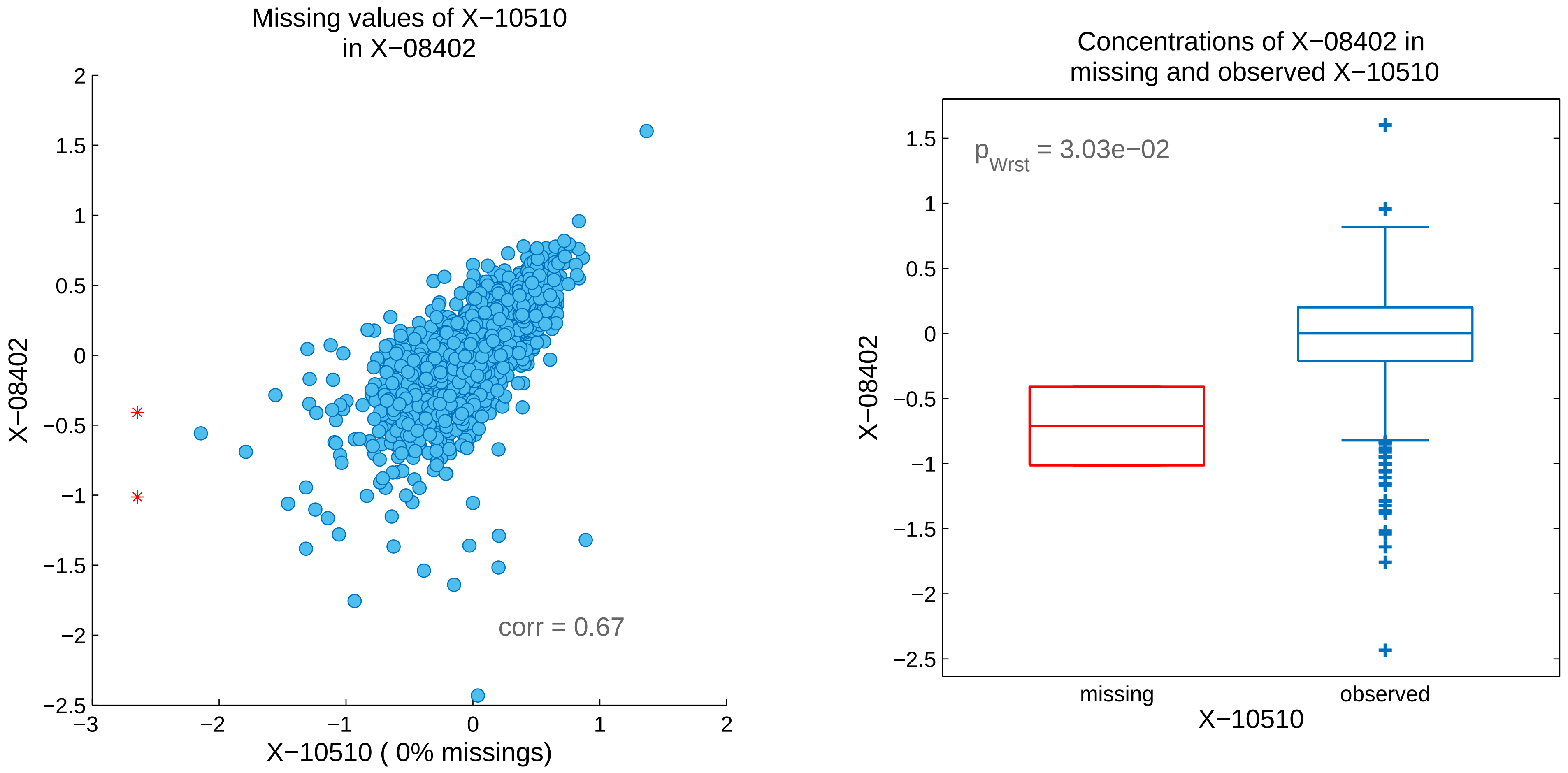

Missing values of X−10510 in X−08402
X−08402
X−10510 (0% missings)
corr = 0.67
Concentrations of X−08402 in missing and observed X−10510
p_{Wrst} = 3.03e−02
X−08402
missing
observed
X−10510

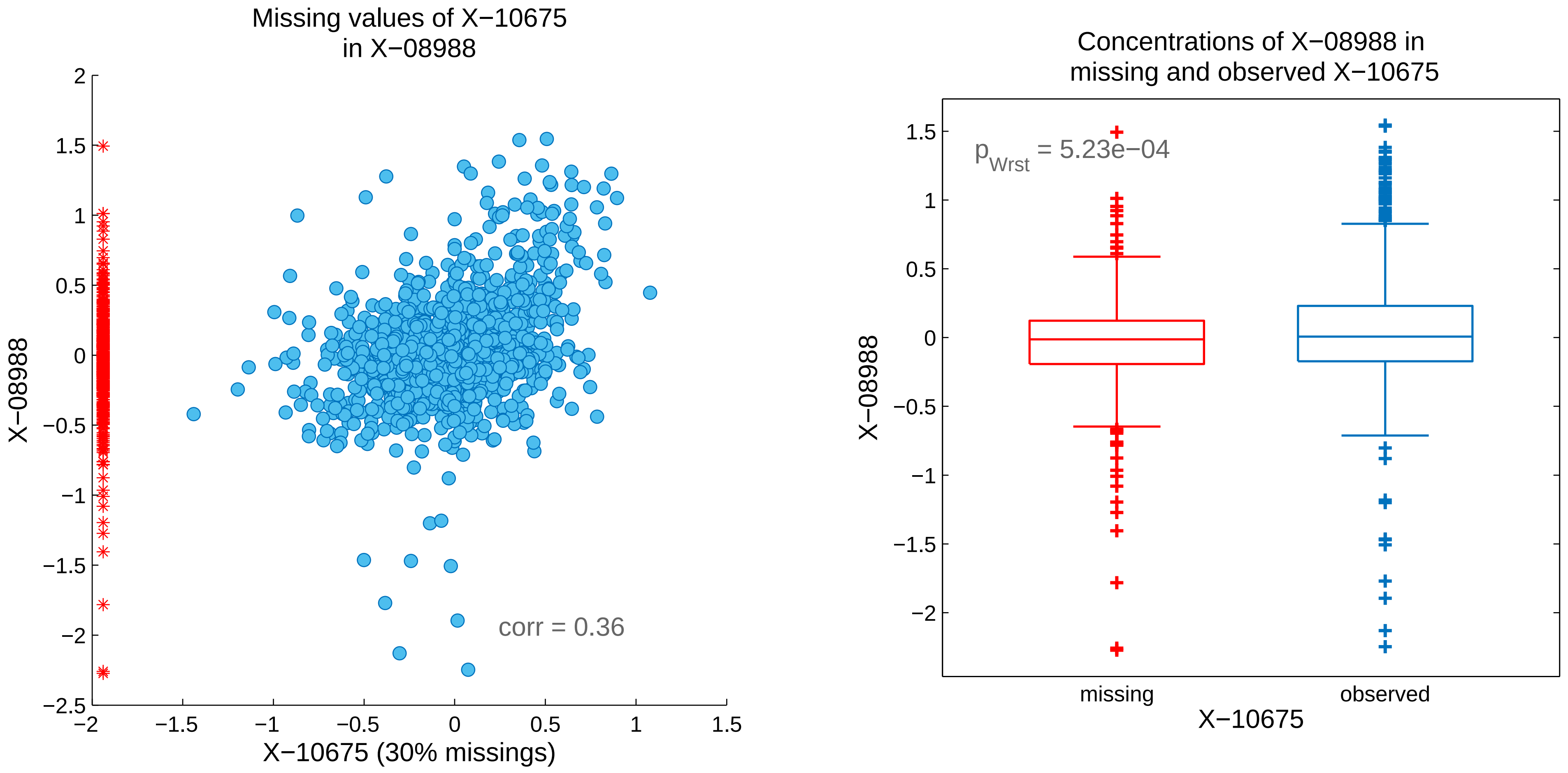
Missing values of X−10675
in X−08988
X−08988
X−10675 (30% missings)
corr = 0.36
Concentrations of X−08988 in
missing and observed X−10675
p_Wrst = 5.23e−04
X−08988
missing
observed
X−10675

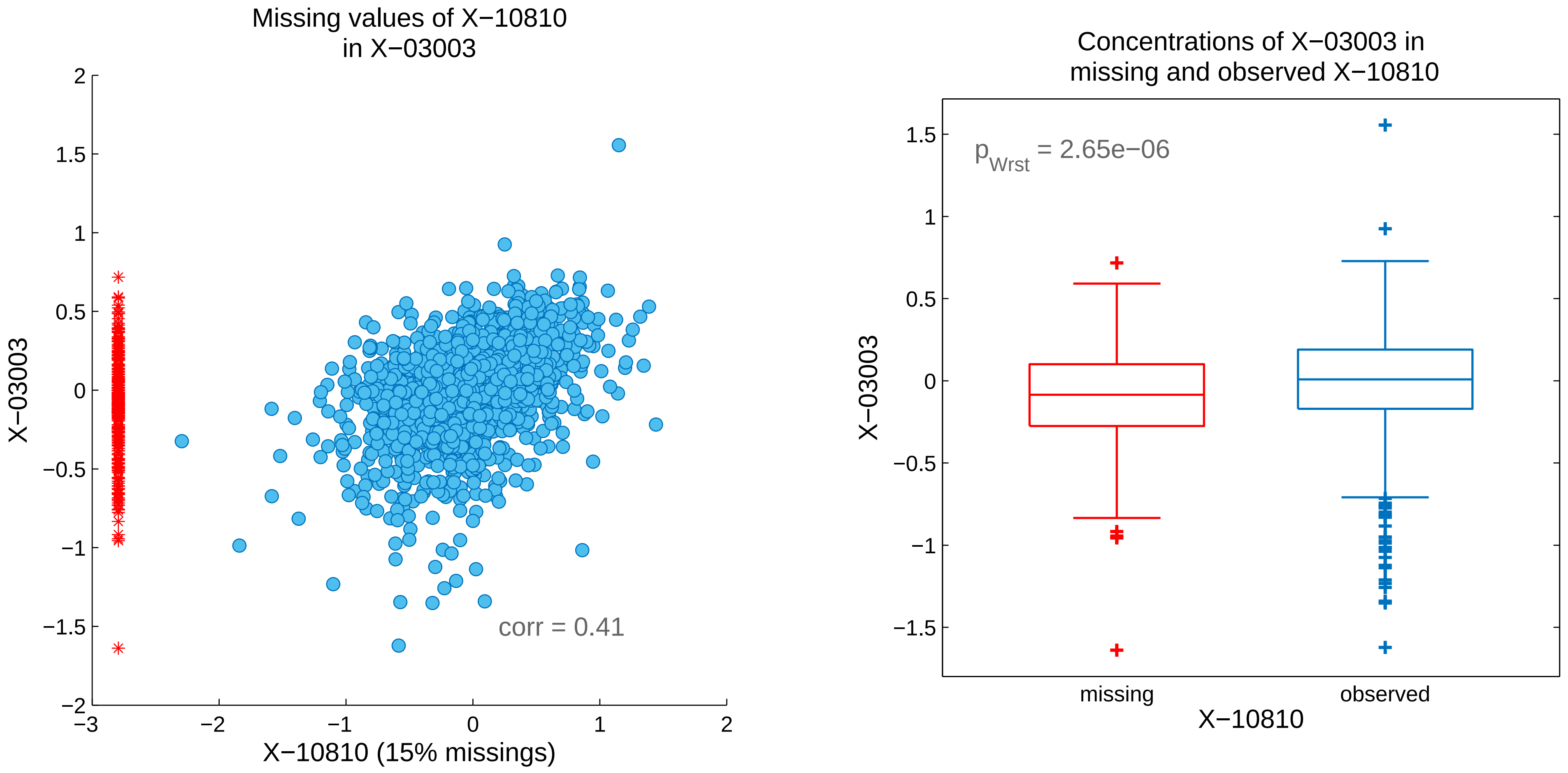

Missing values of X−10810
in X−03003
corr = 0.41
X−03003
X−10810 (15% missings)
Concentrations of X−03003 in
missing and observed X−10810
p_Wrst = 2.65e−06
X−03003
missing
observed
X−10810

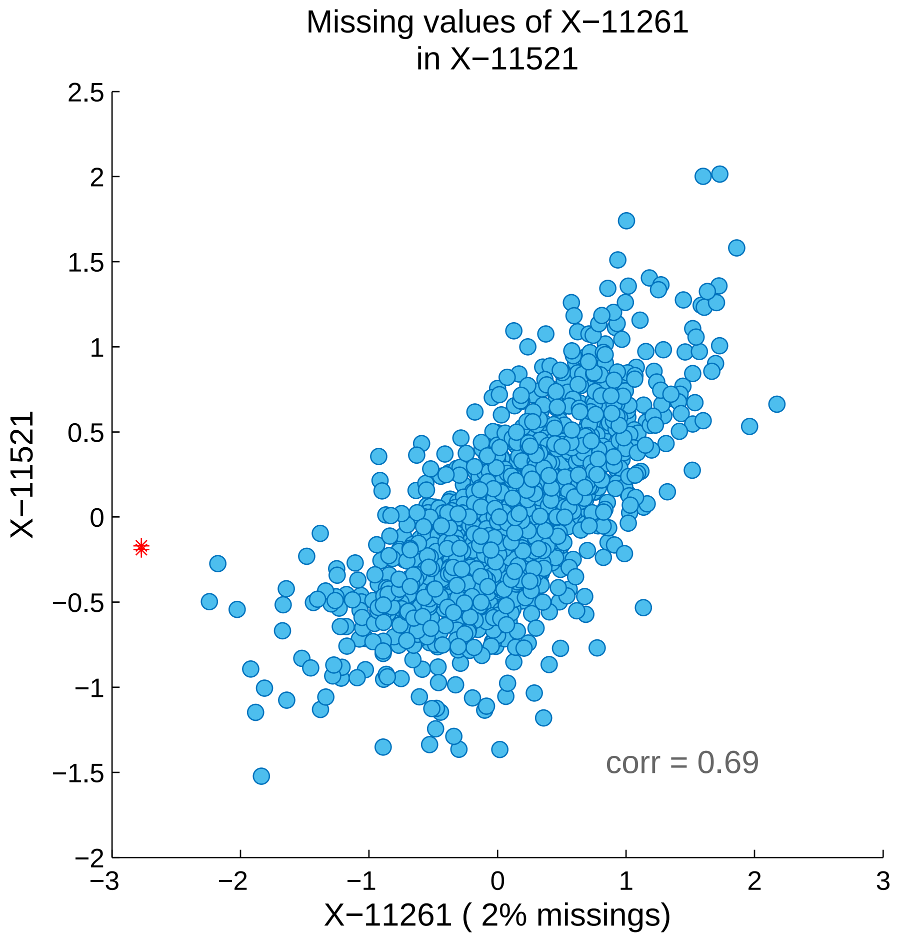
Missing values of X−11261
in X−11521
2.5
2
1.5
1
0.5
0
−0.5
−1
−1.5
−2
X−11521
−3
−2
−1
0
1
2
3
X−11261 (2% missings)
corr = 0.69

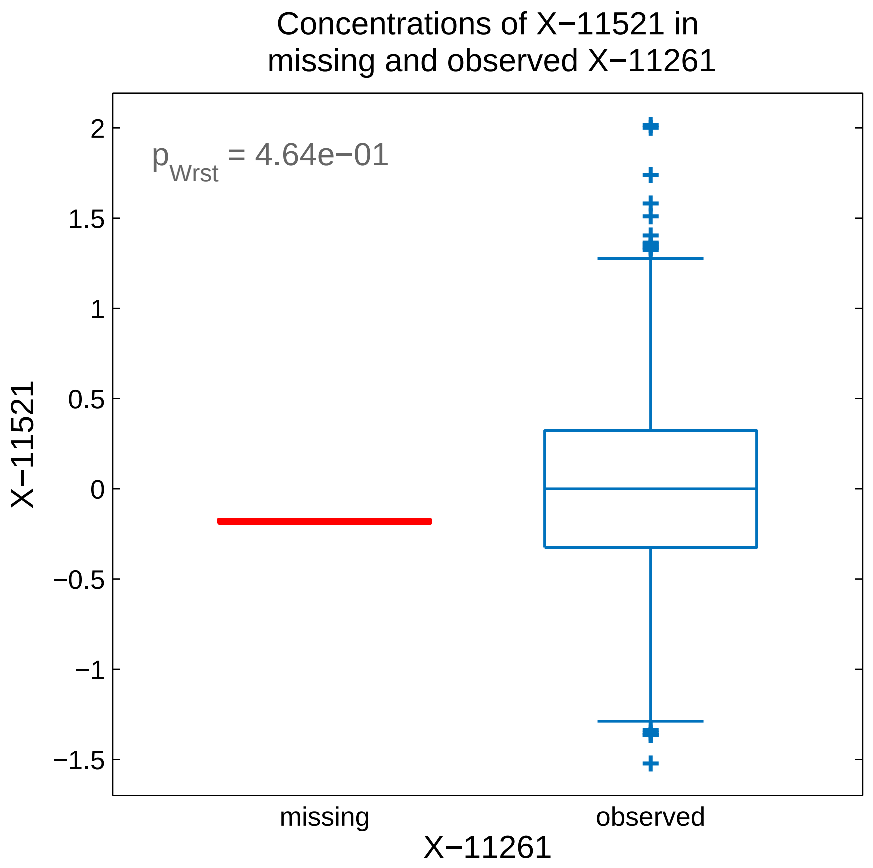
Concentrations of X−11521 in
missing and observed X−11261
2
1.5
1
0.5
0
−0.5
−1
−1.5
X−11521
p_Wrst = 4.64e−01
missing
observed
X−11261

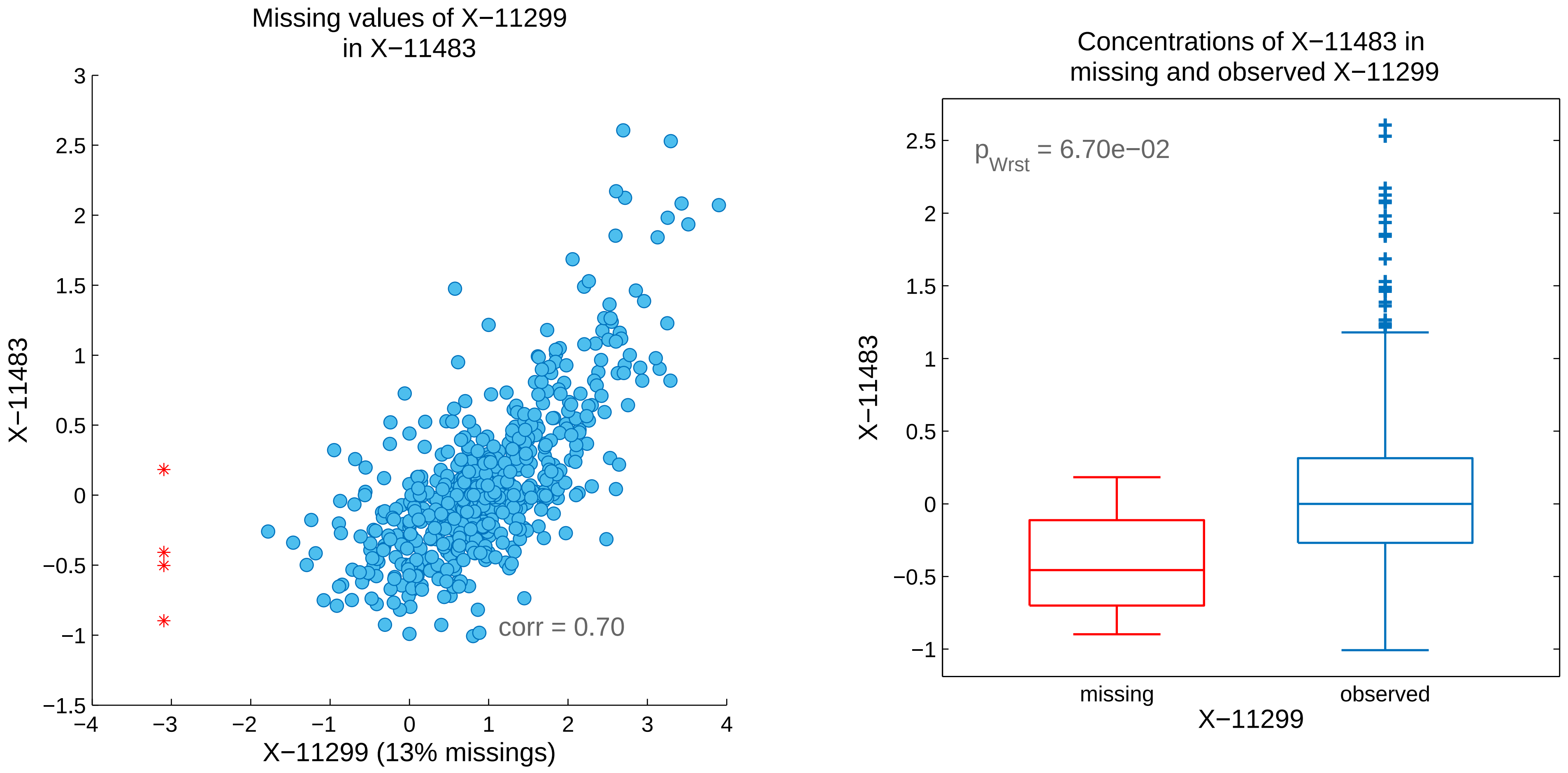

Missing values of X−11299 in X−11483
X−11483
X−11299 (13% missings)
corr = 0.70
Concentrations of X−11483 in missing and observed X−11299
p_{Wrst} = 6.70e−02
X−11483
missing
observed
X−11299

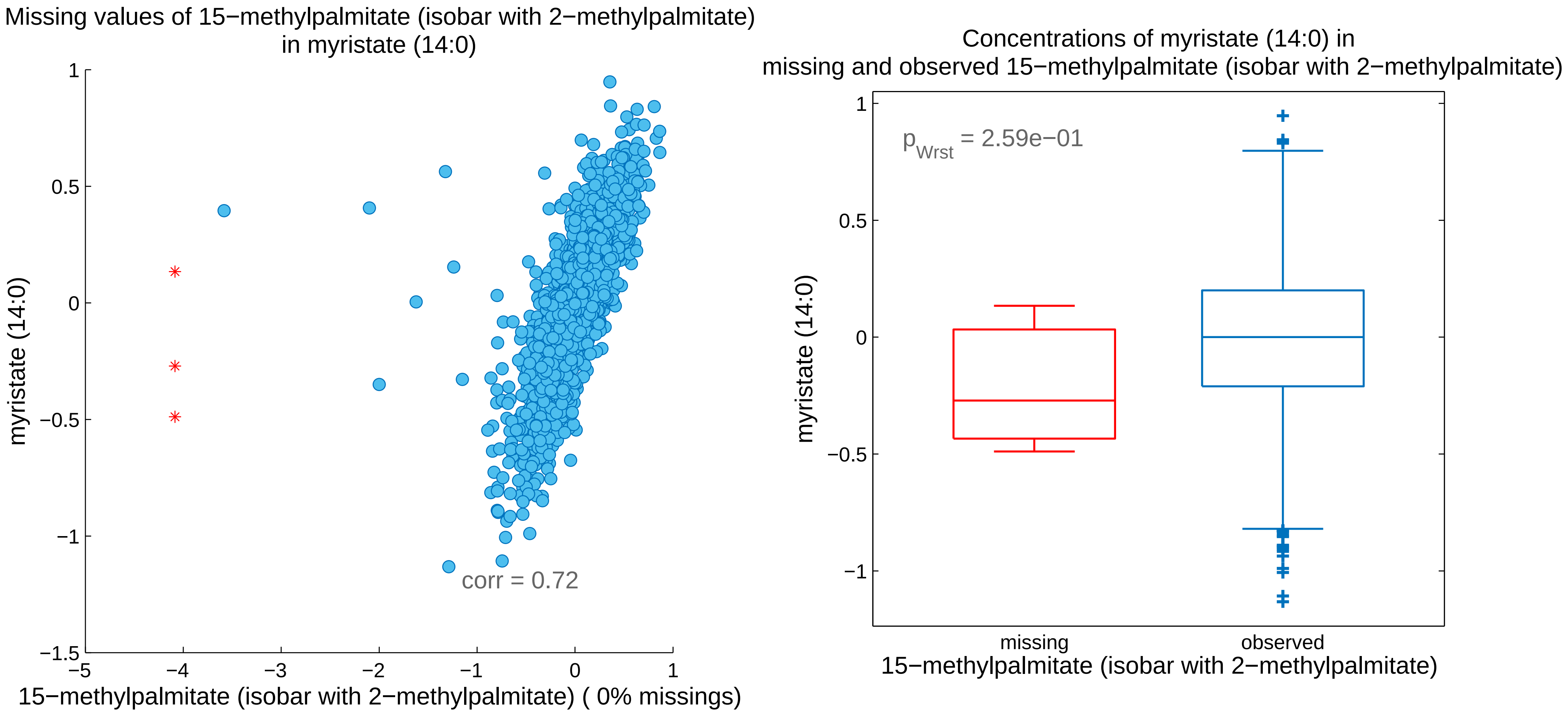

Missing values of 15−methylpalmitate (isobar with 2−methylpalmitate) in myristate (14:0)
myristate (14:0)
15−methylpalmitate (isobar with 2−methylpalmitate) (0% missings)
corr = 0.72
1
0.5
0
−0.5
−1
−1.5
−5
−4
−3
−2
−1
0
1
Concentrations of myristate (14:0) in missing and observed 15−methylpalmitate (isobar with 2−methylpalmitate)
p_{Wrst} = 2.59e−01
myristate (14:0)
missing
observed
15−methylpalmitate (isobar with 2−methylpalmitate)

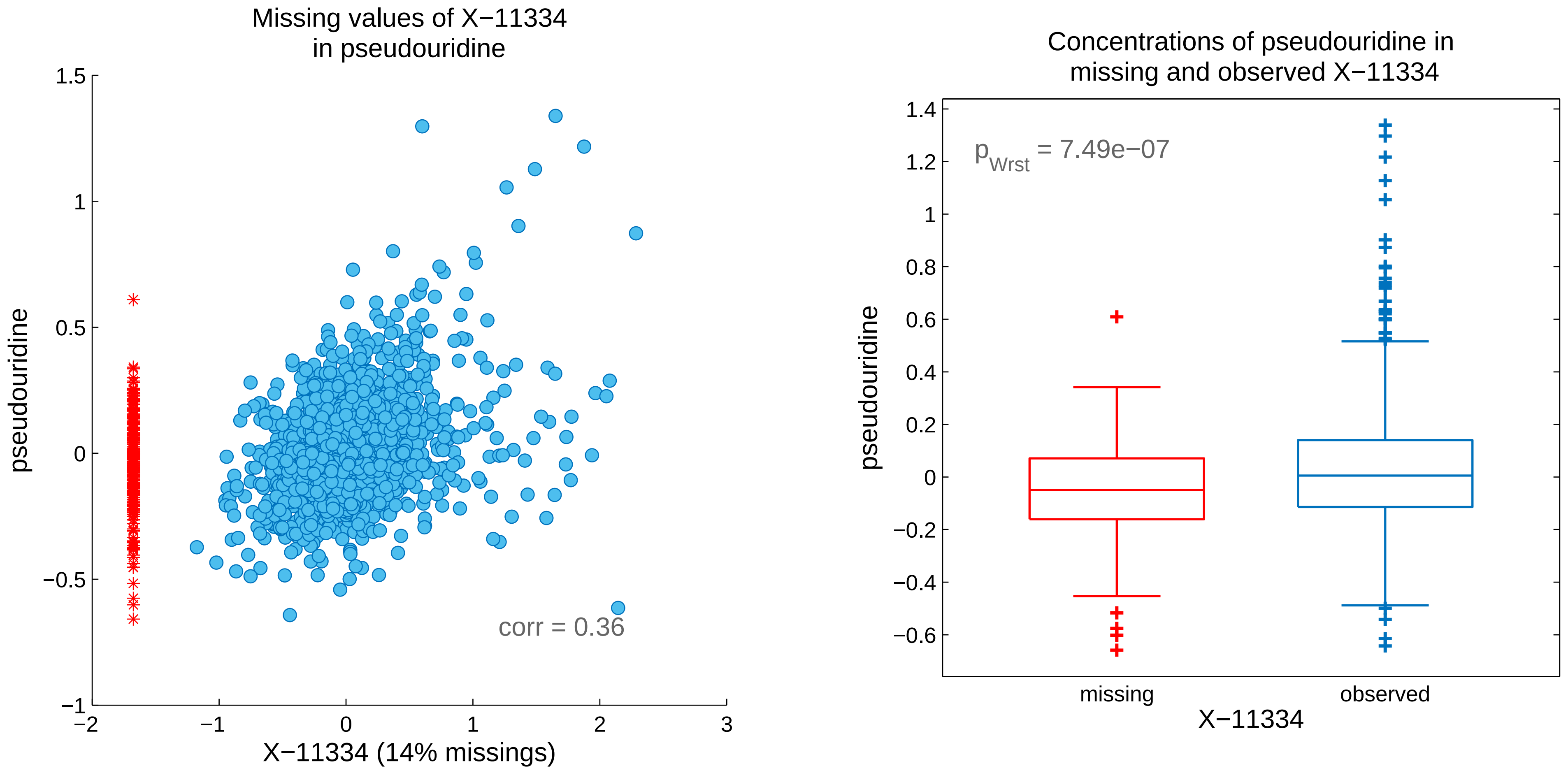

Missing values of X−11334 in pseudouridine
pseudouridine
X−11334 (14% missings)
corr = 0.36
Concentrations of pseudouridine in missing and observed X−11334
p_{Wrst} = 7.49e−07
pseudouridine
missing
observed
X−11334

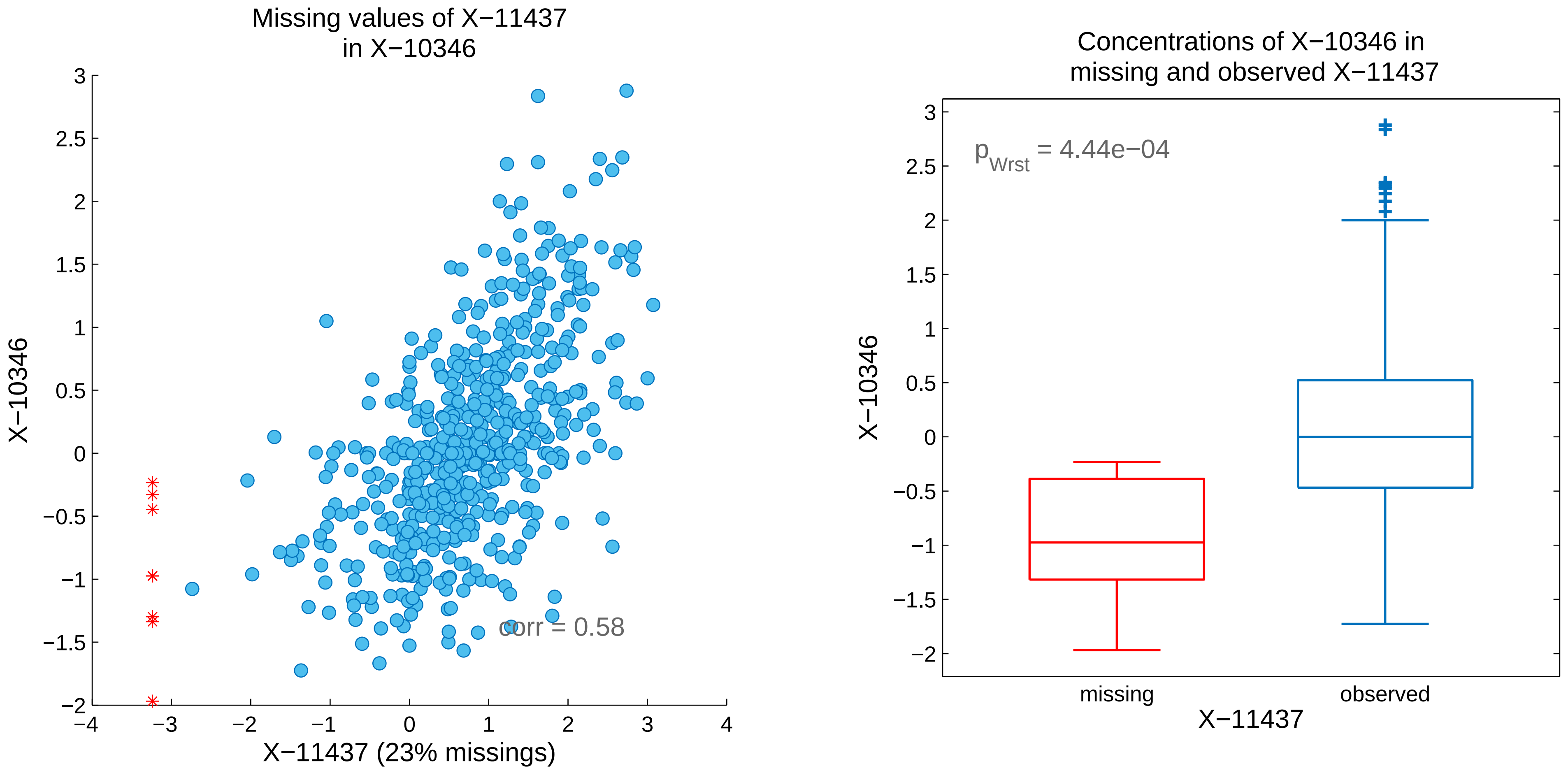

Missing values of X−11437
in X−10346
X−10346
X−11437 (23% missings)
corr = 0.58
3
2.5
2
1.5
1
0.5
0
−0.5
−1
−1.5
−2
−4
−3
−2
−1
0
1
2
3
4
Concentrations of X−10346 in
missing and observed X−11437
p_Wrst = 4.44e−04
X−10346
X−11437
missing
observed
3
2.5
2
1.5
1
0.5
0
−0.5
−1
−1.5
−2

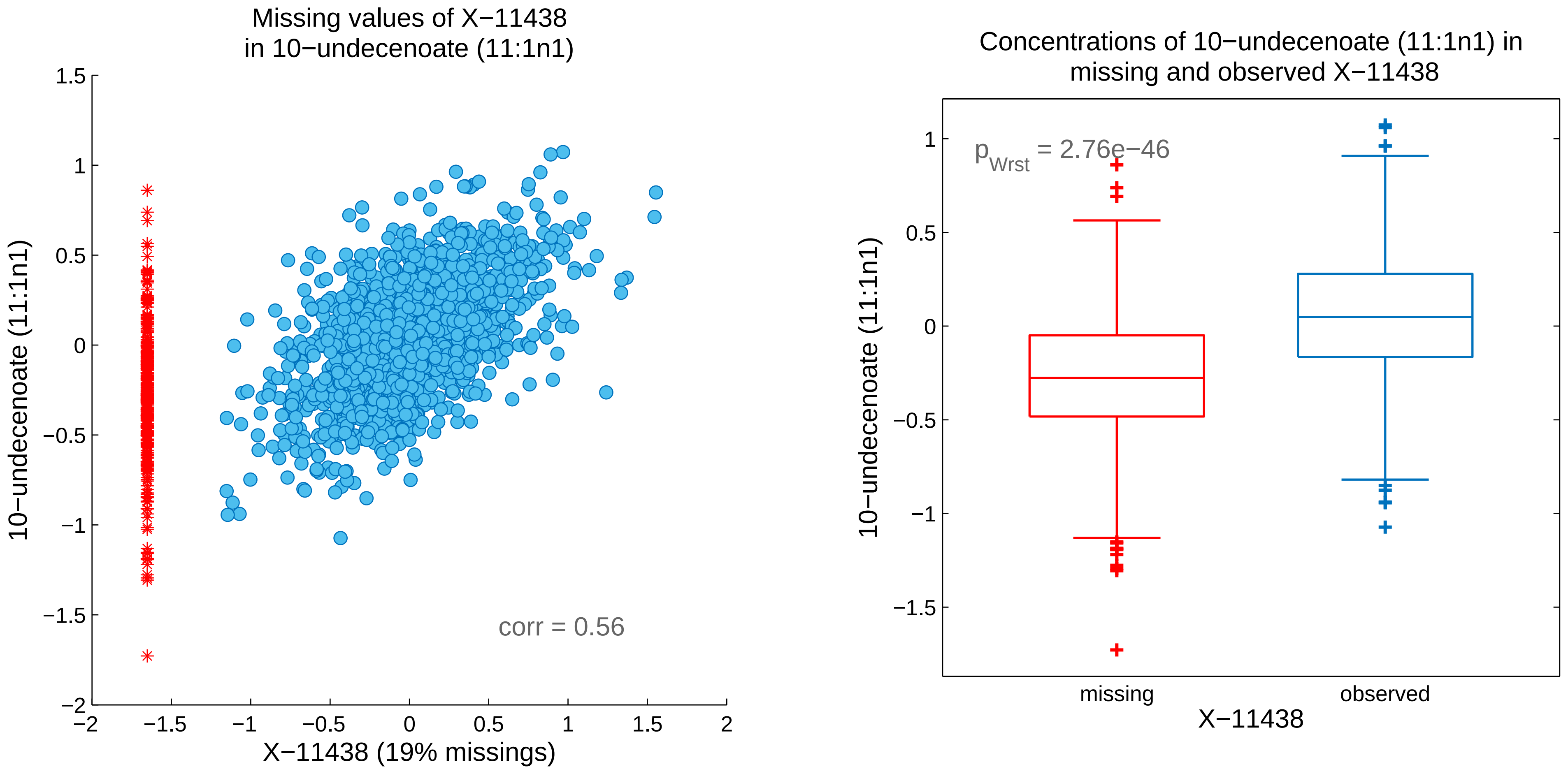

Missing values of X−11438
in 10−undecenoate (11:1n1)
10−undecenoate (11:1n1)
X−11438 (19% missings)
corr = 0.56
Concentrations of 10−undecenoate (11:1n1) in
missing and observed X−11438
p_{Wrst} = 2.76e−46
10−undecenoate (11:1n1)
missing
observed
X−11438

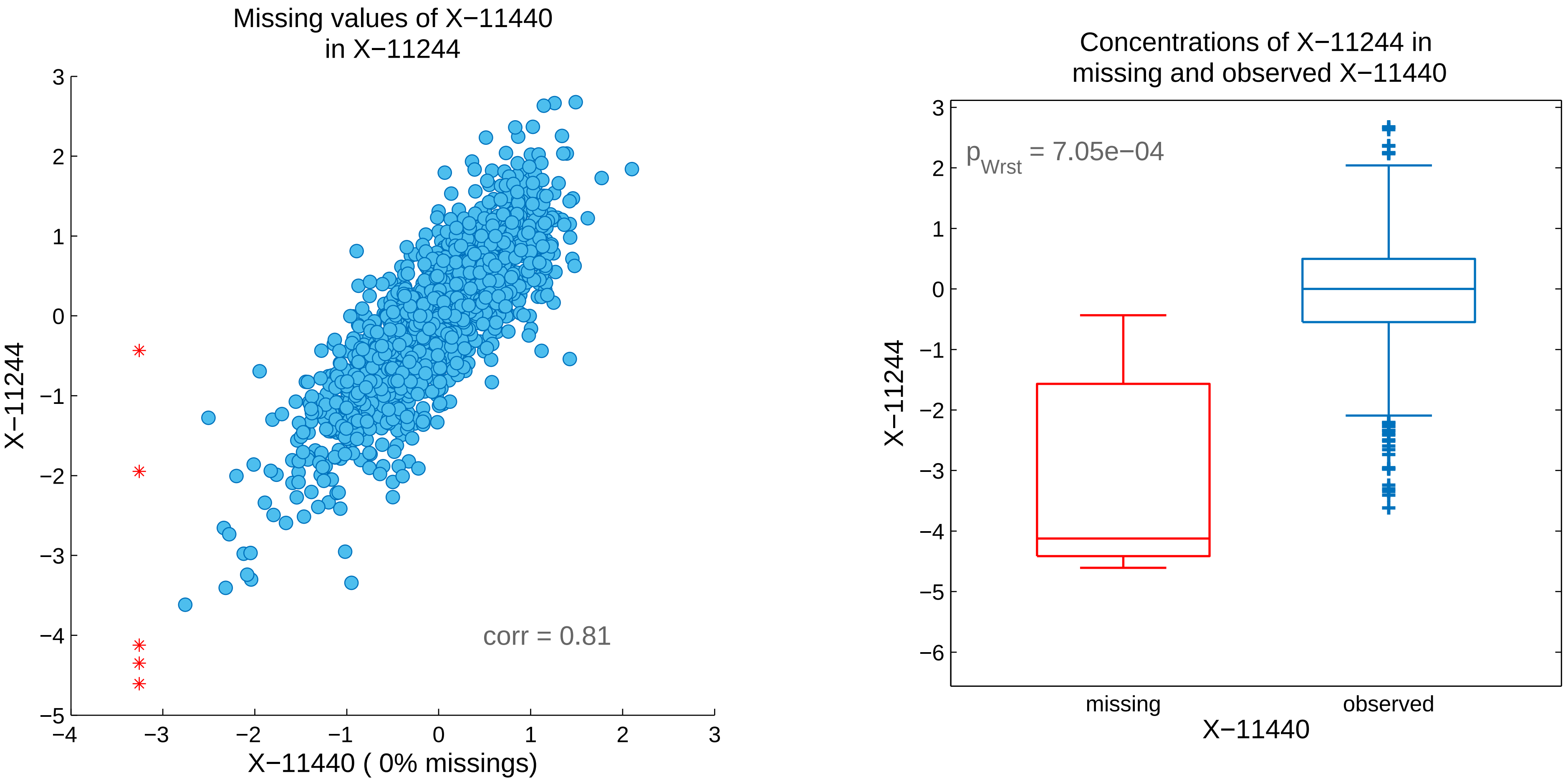

Missing values of X−11440
in X−11244
X−11244
X−11440 (0% missings)
corr = 0.81
Concentrations of X−11244 in
missing and observed X−11440
p_{Wrst} = 7.05e−04
X−11244
missing
observed
X−11440

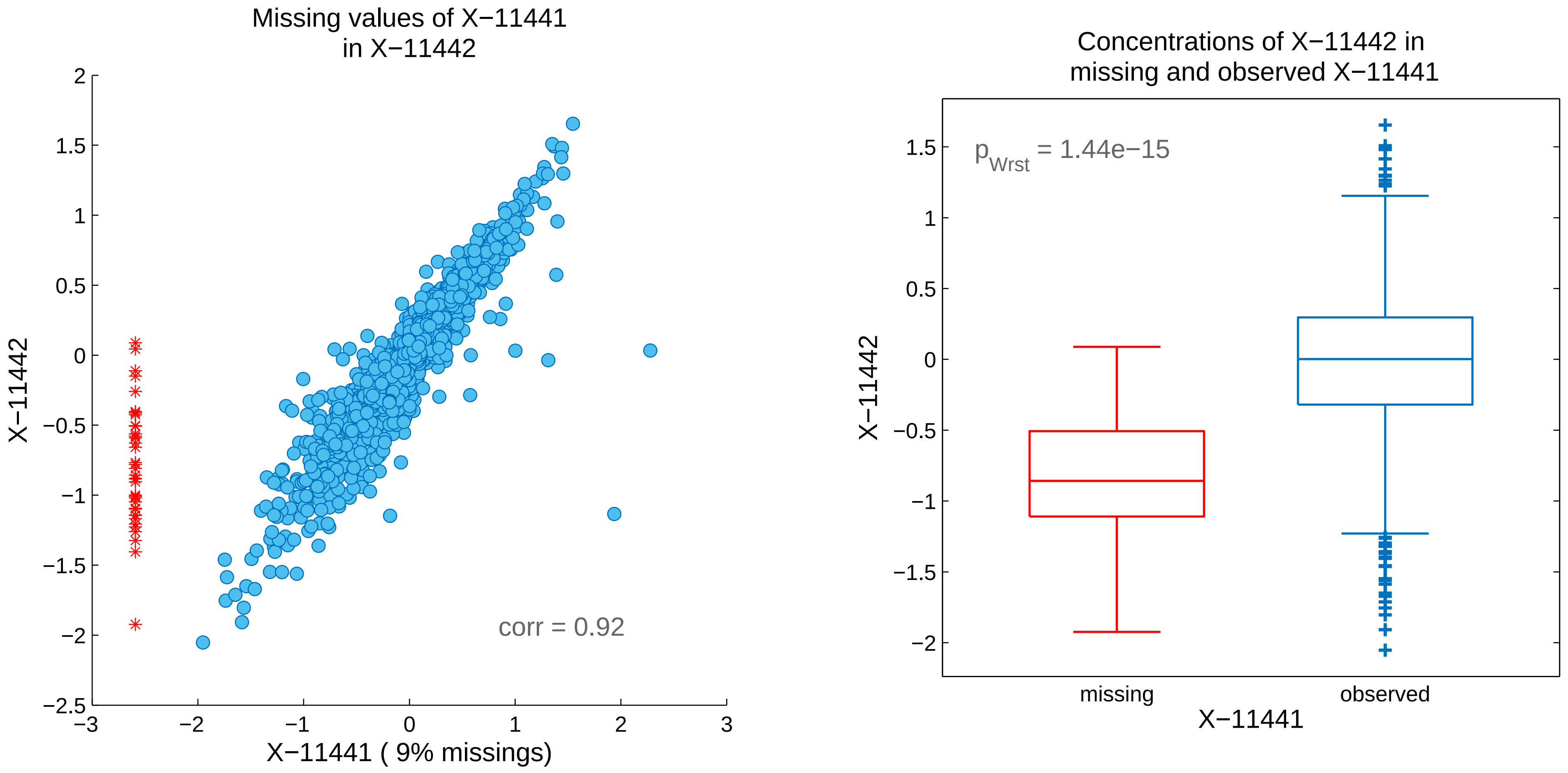

Missing values of X−11441
in X−11442
X−11442
X−11441 (9% missings)
corr = 0.92
Concentrations of X−11442 in
missing and observed X−11441
p_{Wrst} = 1.44e−15
X−11442
missing
observed
X−11441

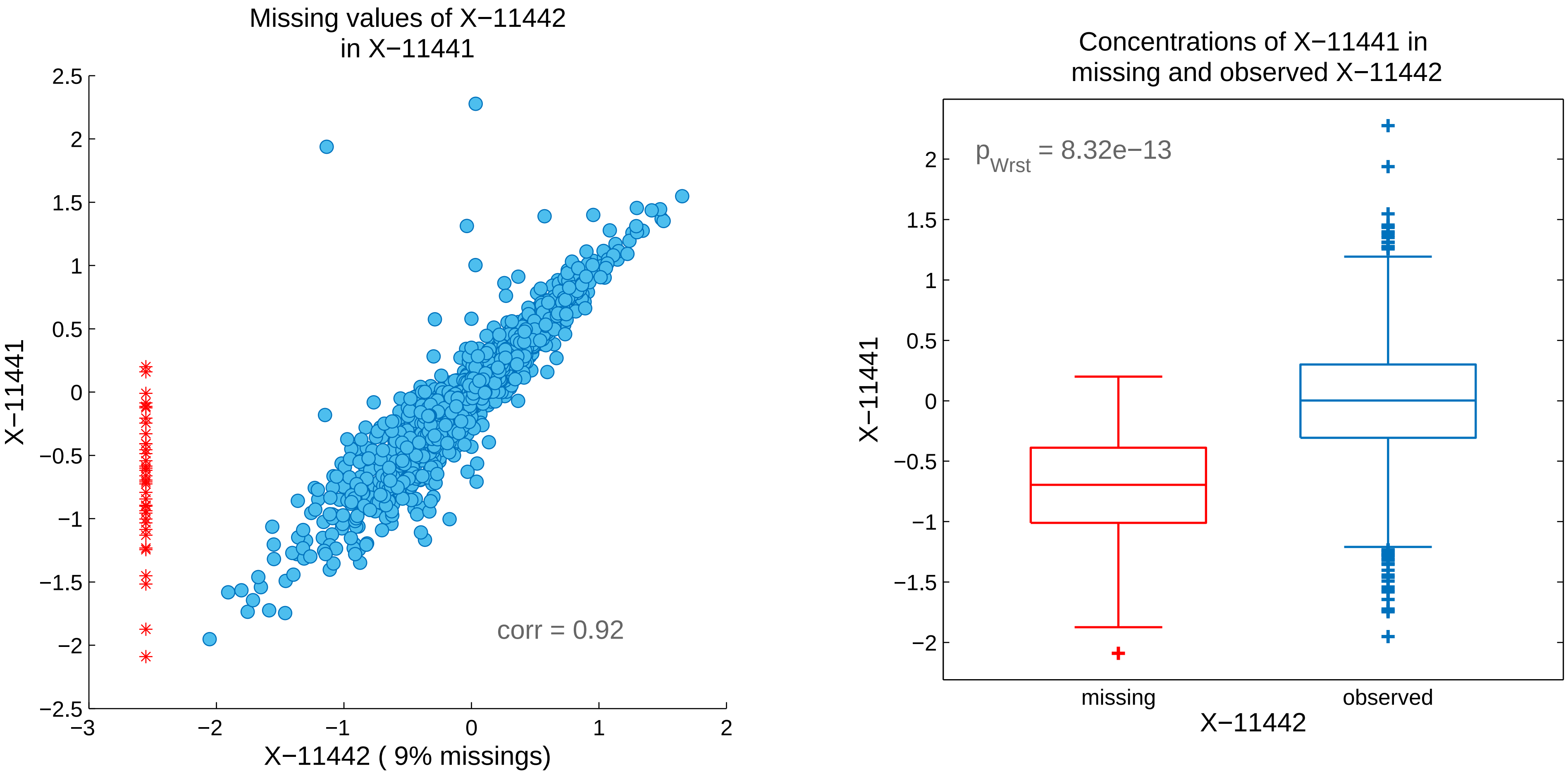
Missing values of X−11442
in X−11441
corr = 0.92
X−11441
X−11442 (9% missings)
Concentrations of X−11441 in
missing and observed X−11442
p_{Wrst} = 8.32e−13
X−11441
missing
observed
X−11442

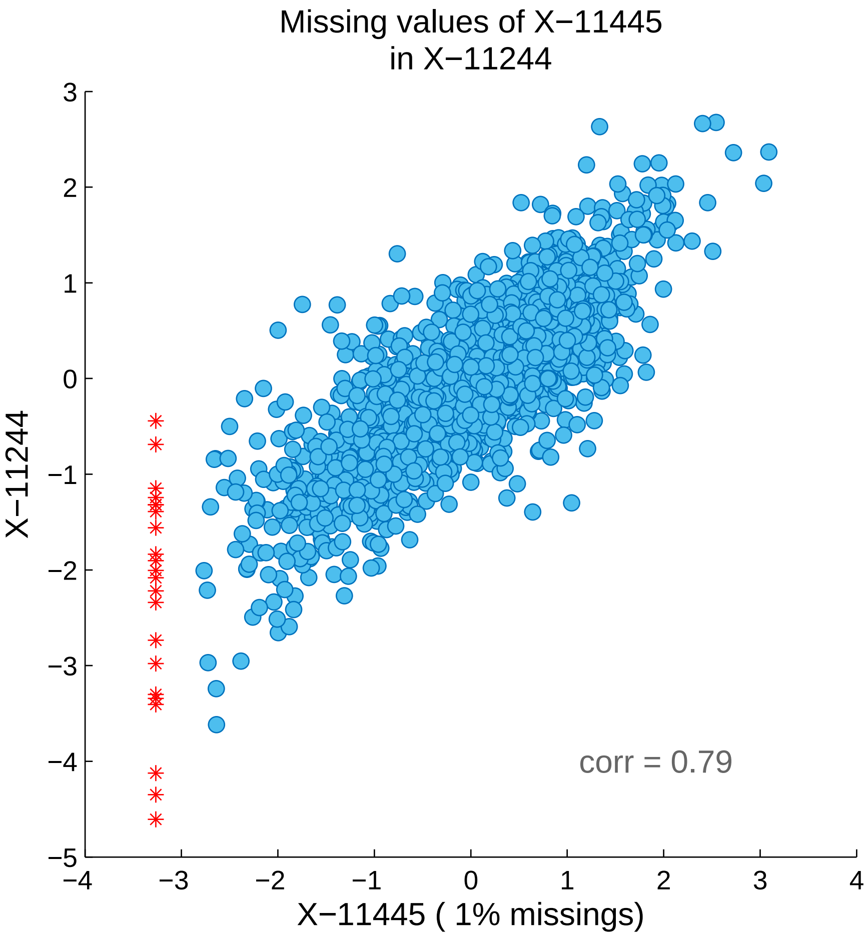

Missing values of X−11445
in X−11244
X−11244
X−11445 (1% missings)
corr = 0.79
3
2
1
0
−1
−2
−3
−4
−5
−4
−3
−2
−1
0
1
2
3
4

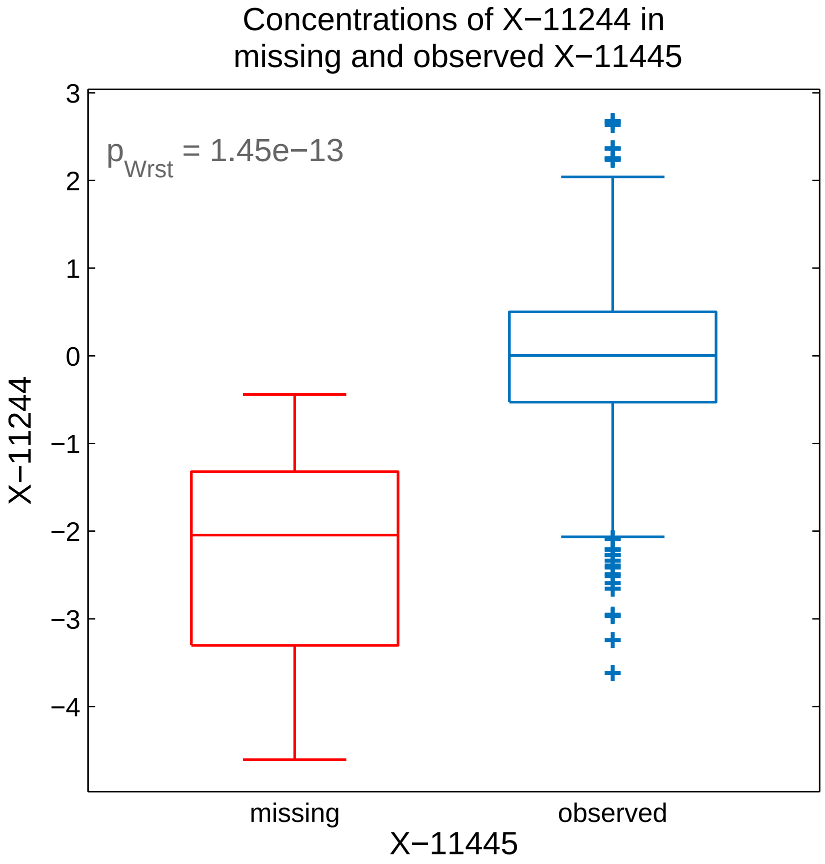

Concentrations of X−11244 in
missing and observed X−11445
p_{Wrst} = 1.45e−13
X−11244
X−11445
missing
observed
3
2
1
0
−1
−2
−3
−4

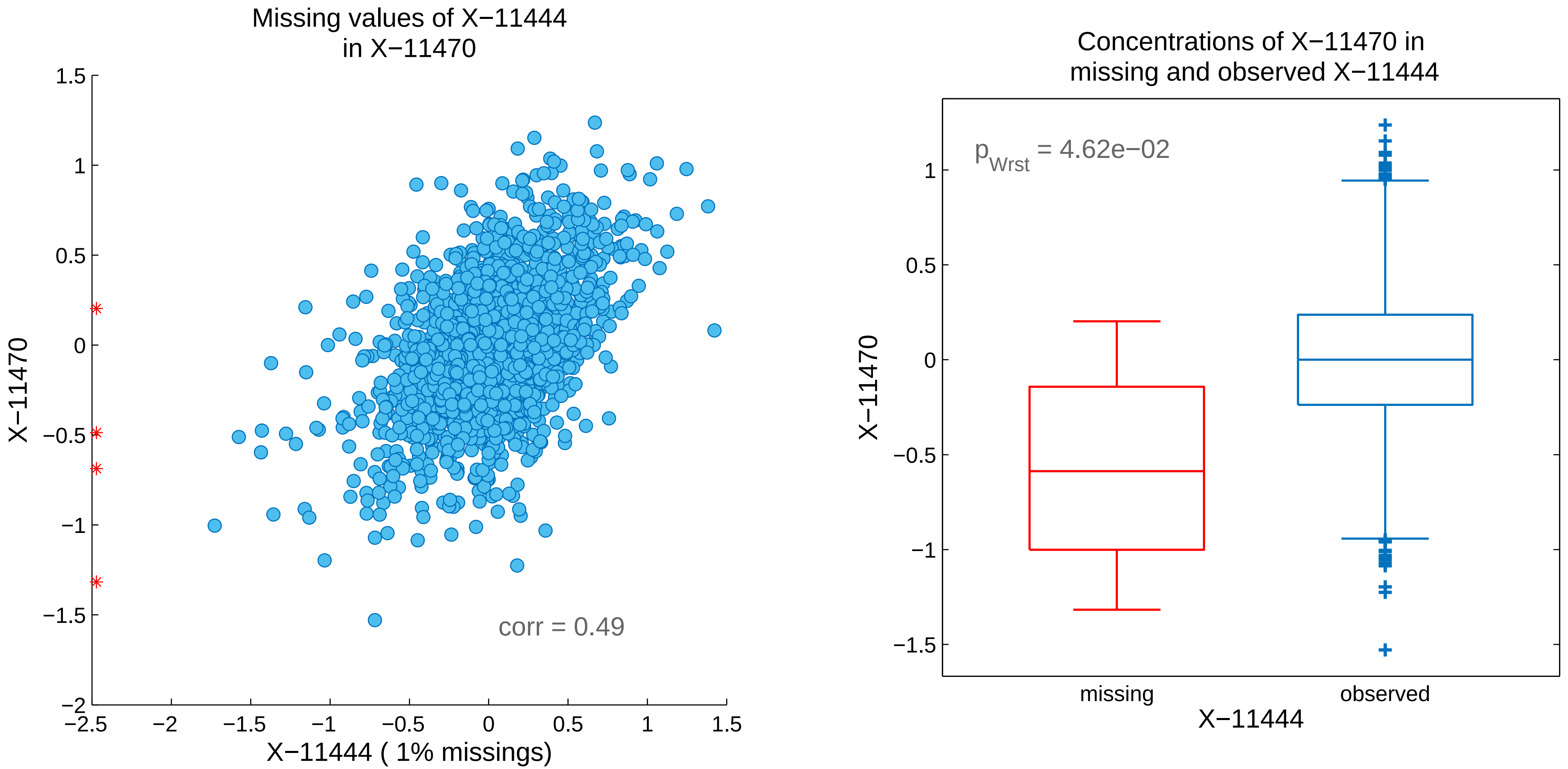

Missing values of X−11444 in X−11470
X−11470
X−11444 (1% missings)
corr = 0.49
Concentrations of X−11470 in missing and observed X−11444
p_{Wrst} = 4.62e−02
X−11470
missing
observed
X−11444

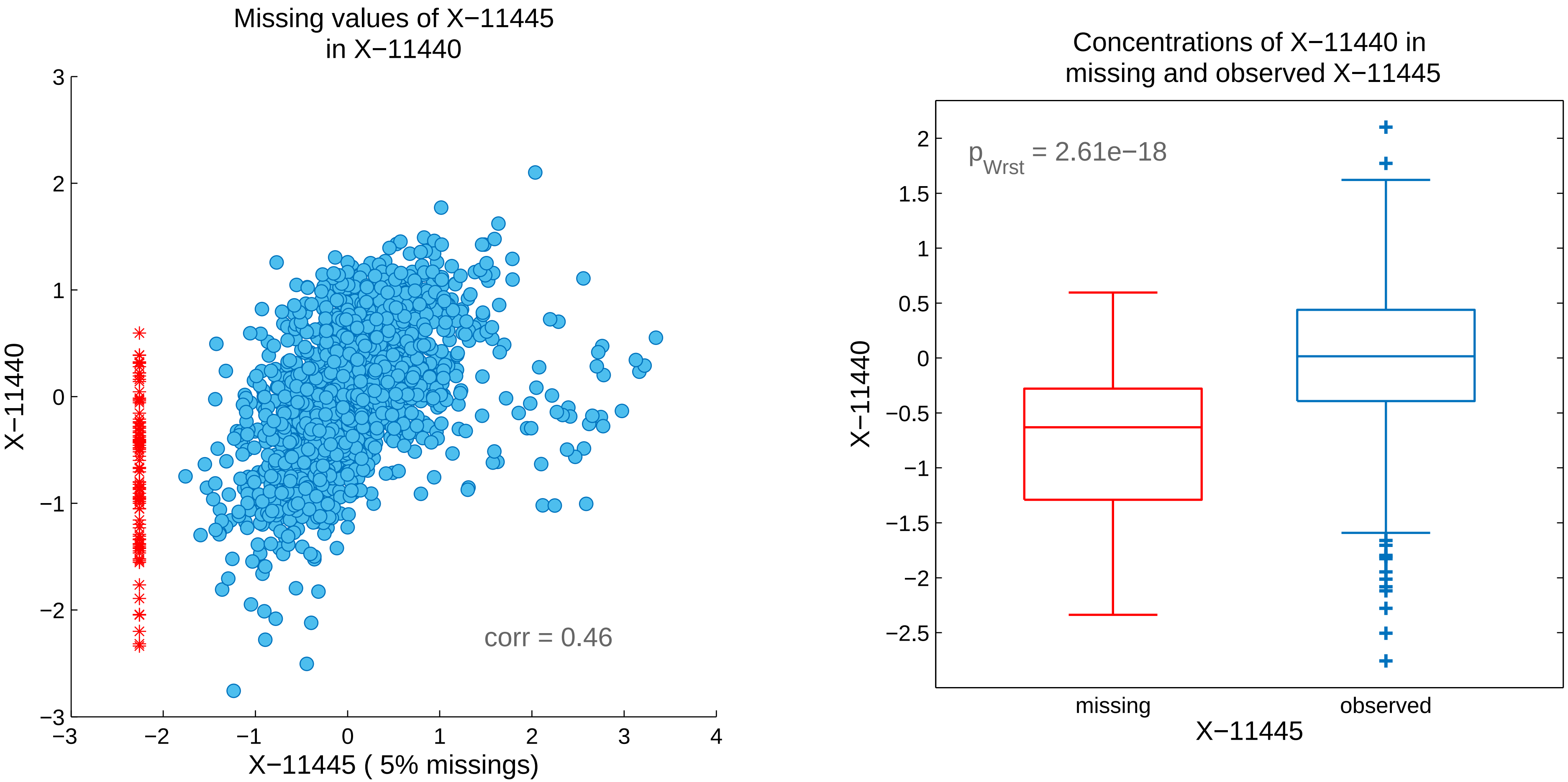

Missing values of X−11445
in X−11440
corr = 0.46
X−11440
X−11445 (5% missings)
Concentrations of X−11440 in
missing and observed X−11445
p Wrst = 2.61e−18
X−11440
missing
observed
X−11445

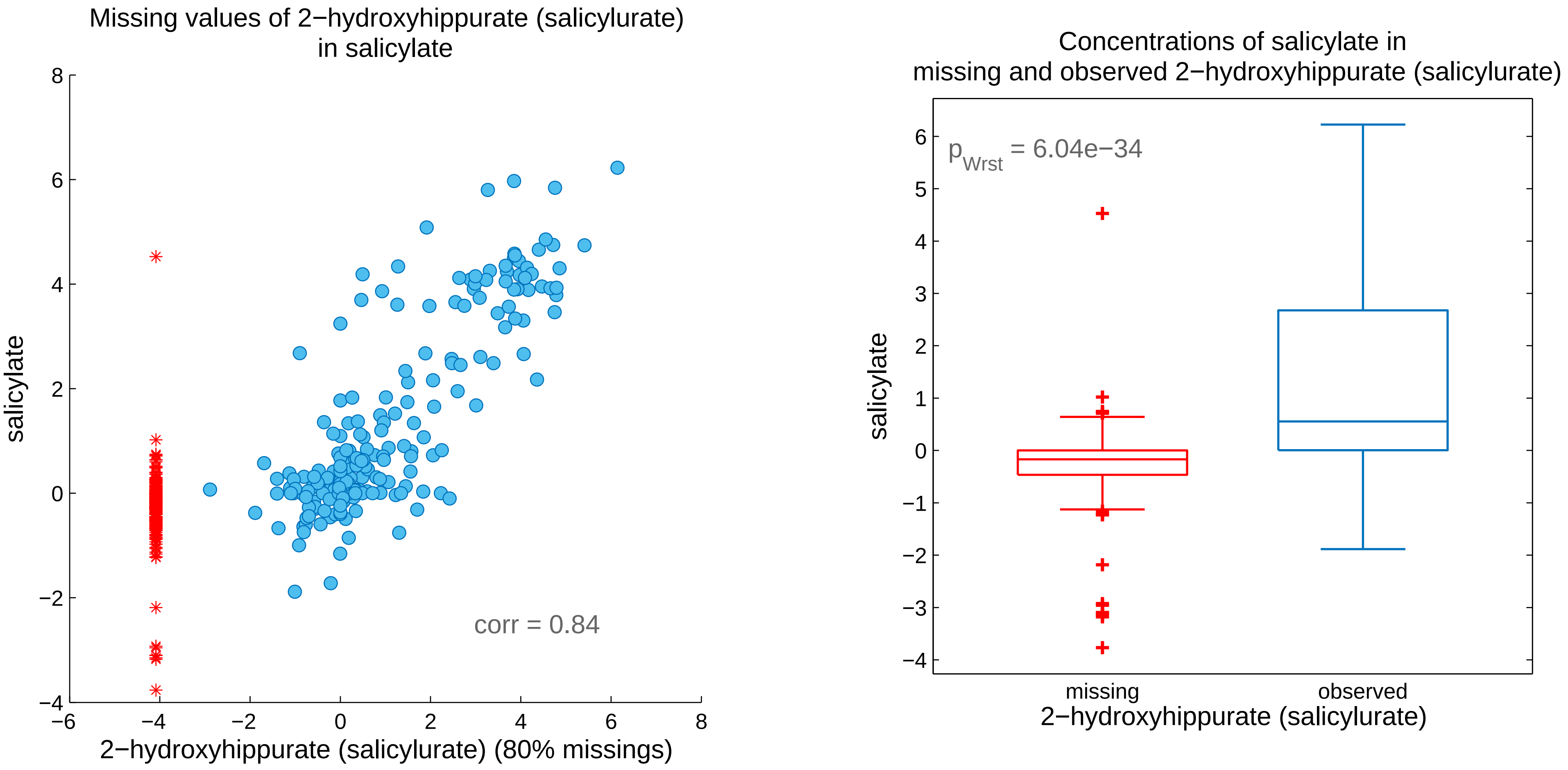

Missing values of 2−hydroxyhippurate (salicylurate) in salicylate
salicylate
2−hydroxyhippurate (salicylurate) (80% missings)
corr = 0.84
8
6
4
2
0
−2
−4
−6
−4
−2
0
2
4
6
8
Concentrations of salicylate in missing and observed 2−hydroxyhippurate (salicylurate)
p_{Wrst} = 6.04e−34
salicylate
missing
observed
2−hydroxyhippurate (salicylurate)
6
5
4
3
2
1
0
−1
−2
−3
−4

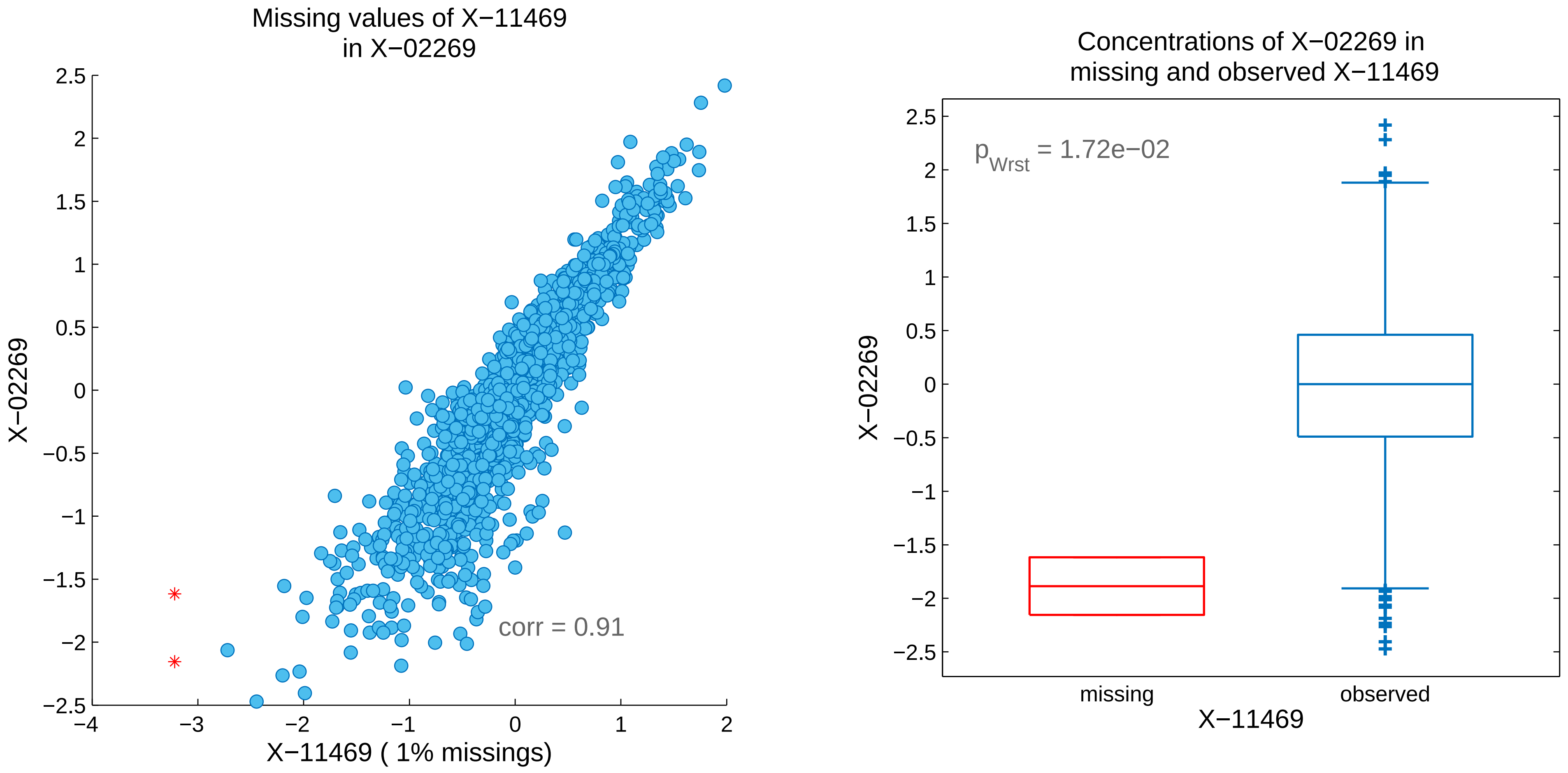
Missing values of X−11469
in X−02269
X−02269
X−11469 (1% missings)
corr = 0.91
Concentrations of X−02269 in
missing and observed X−11469
p_{Wrst} = 1.72e−02
X−02269
missing
observed
X−11469

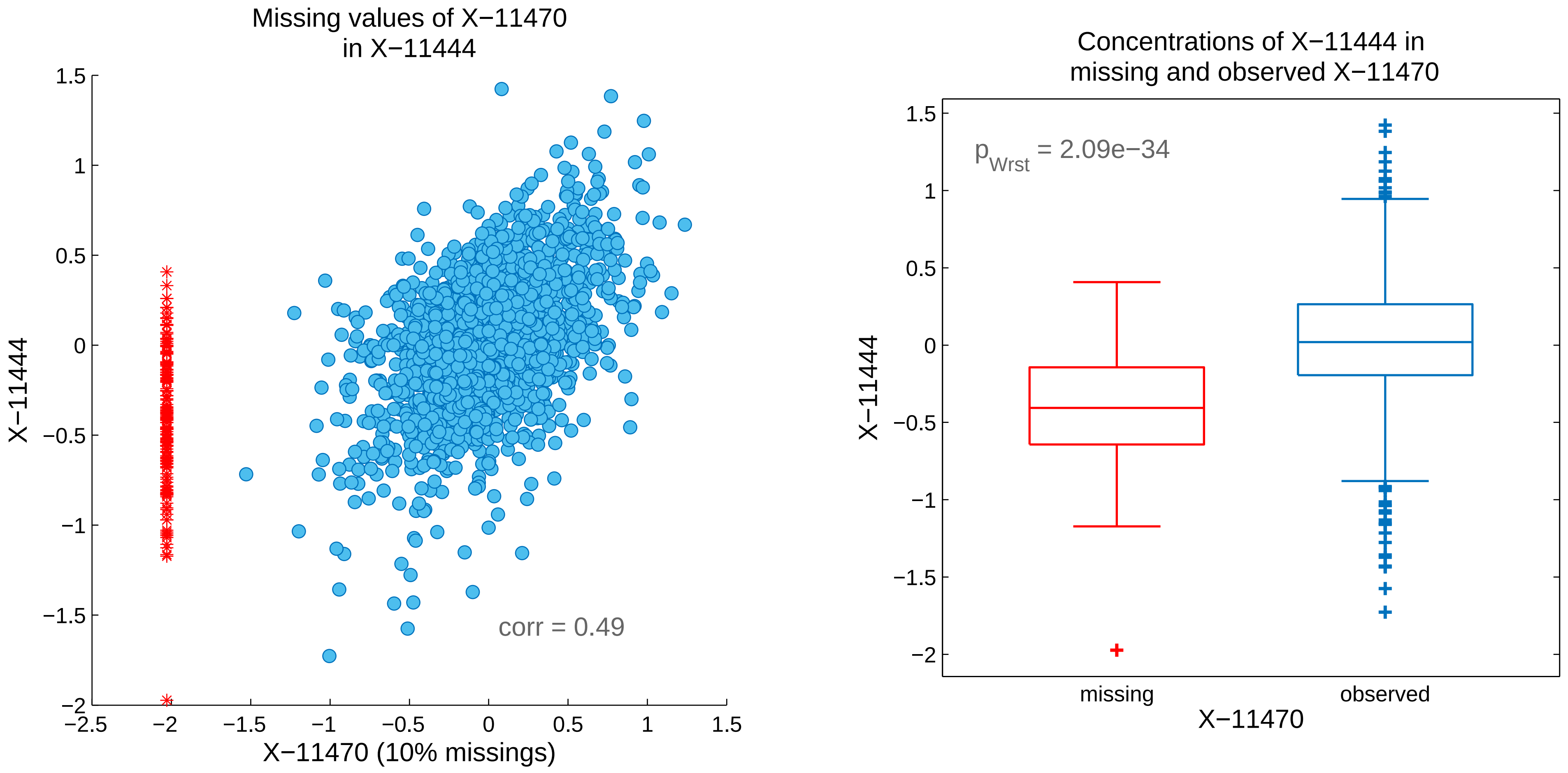

Missing values of X−11470
in X−11444
1.5
1
0.5
0
−0.5
−1
−1.5
−2
X−11444
−2.5
−2
−1.5
−1
−0.5
0
0.5
1
1.5
X−11470 (10% missings)
corr = 0.49
Concentrations of X−11444 in
missing and observed X−11470
p_Wrst = 2.09e−34
X−11444
missing
observed
X−11470

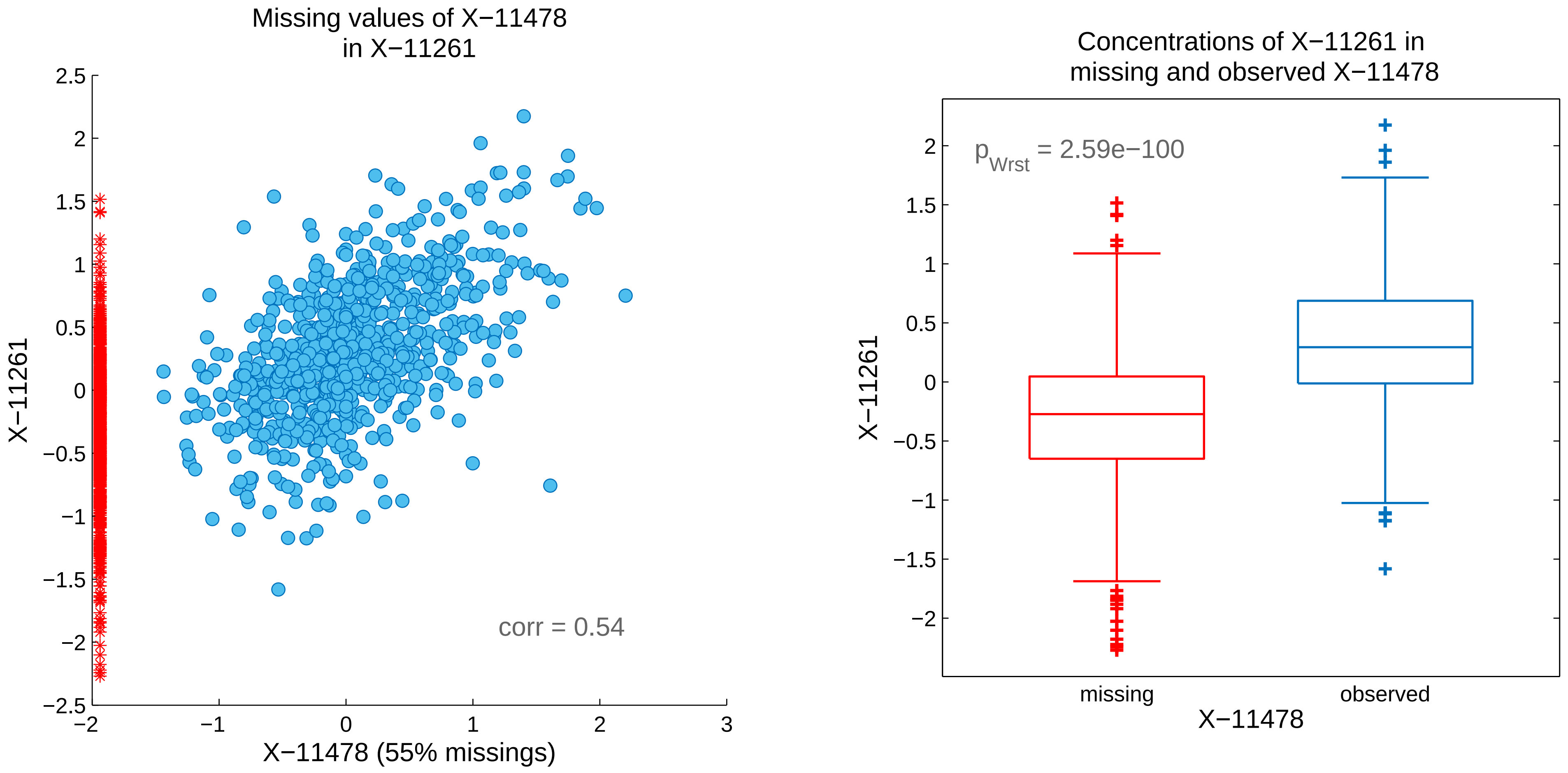

Missing values of X−11478 in X−11261
X−11261
X−11478 (55% missings)
corr = 0.54
Concentrations of X−11261 in missing and observed X−11478
p_{Wrst} = 2.59e−100
X−11261
missing
observed
X−11478

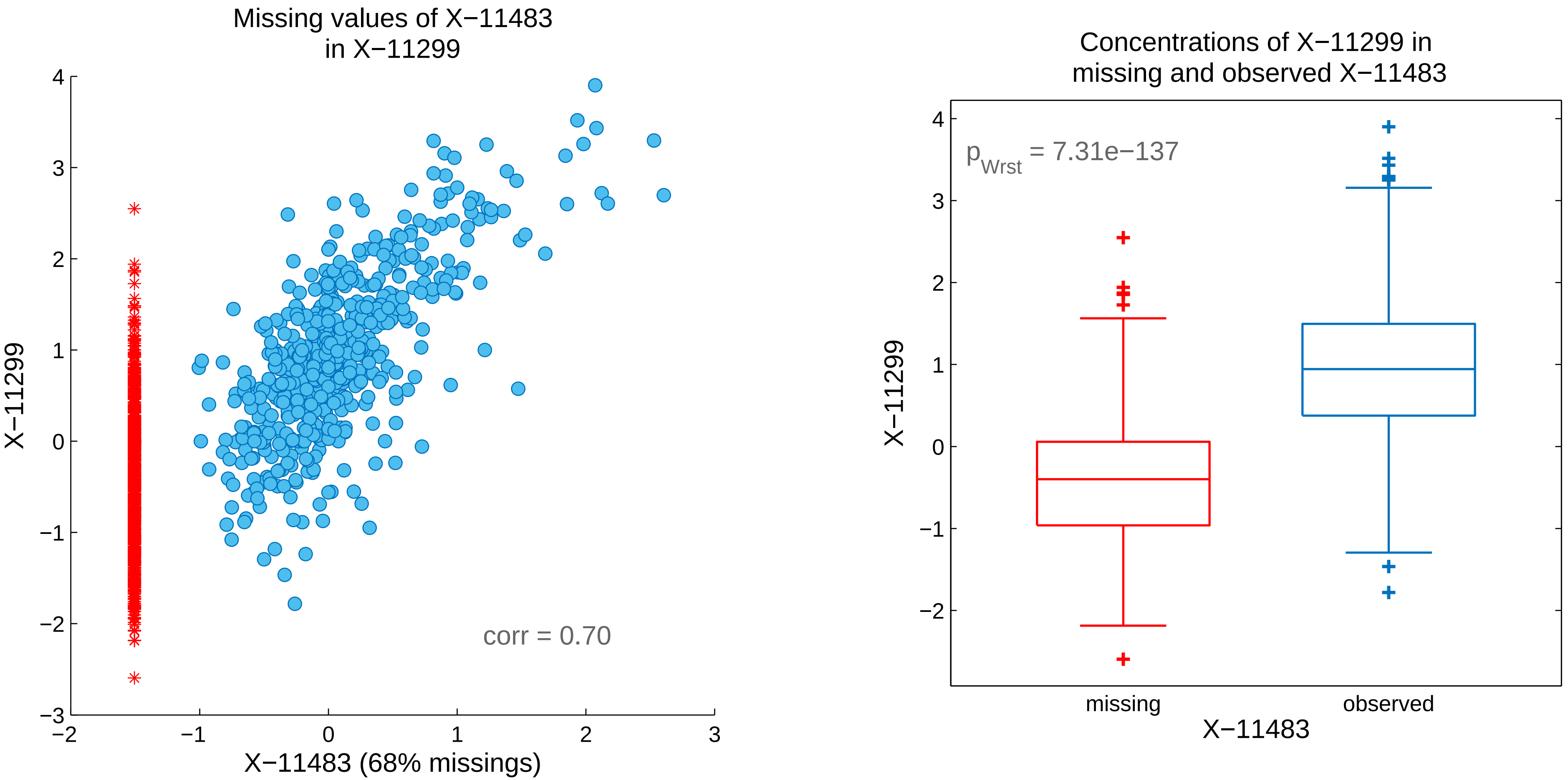

Missing values of X−11483 in X−11299
X−11299
X−11483 (68% missings)
corr = 0.70
Concentrations of X−11299 in missing and observed X−11483
p_{Wrst} = 7.31e−137
missing
observed
X−11483

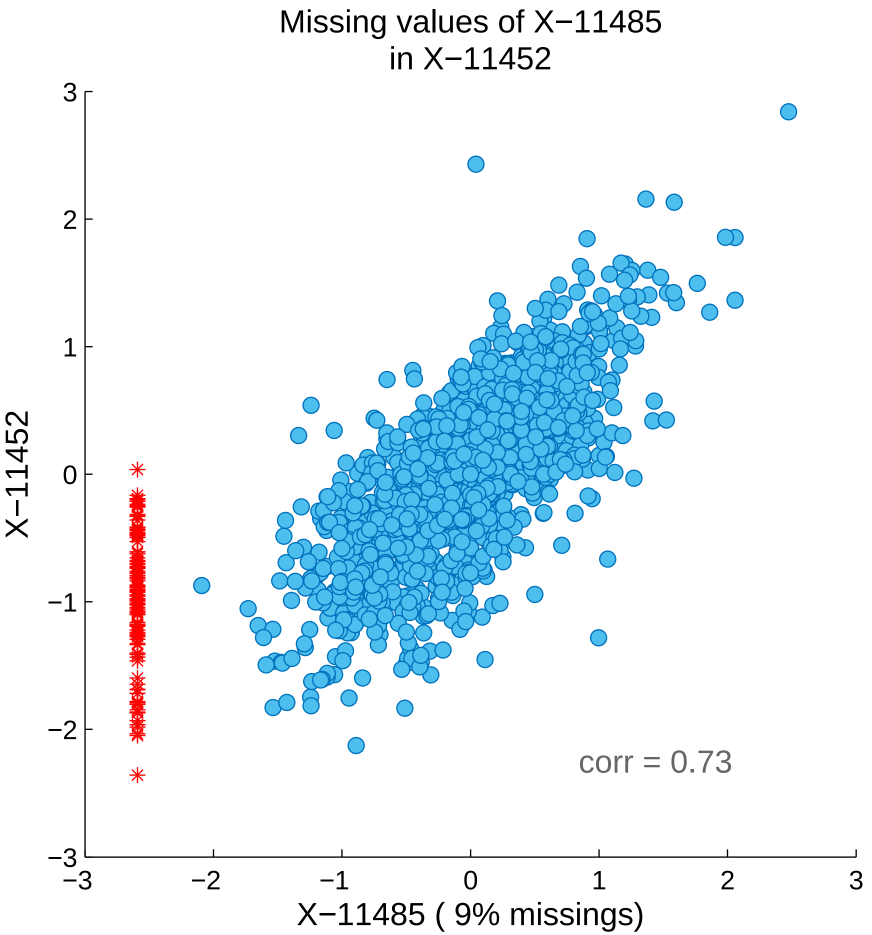

Missing values of X−11485
in X−11452
X−11452
X−11485 (9% missings)
corr = 0.73
3
2
1
0
−1
−2
−3
−3
−2
−1
0
1
2
3

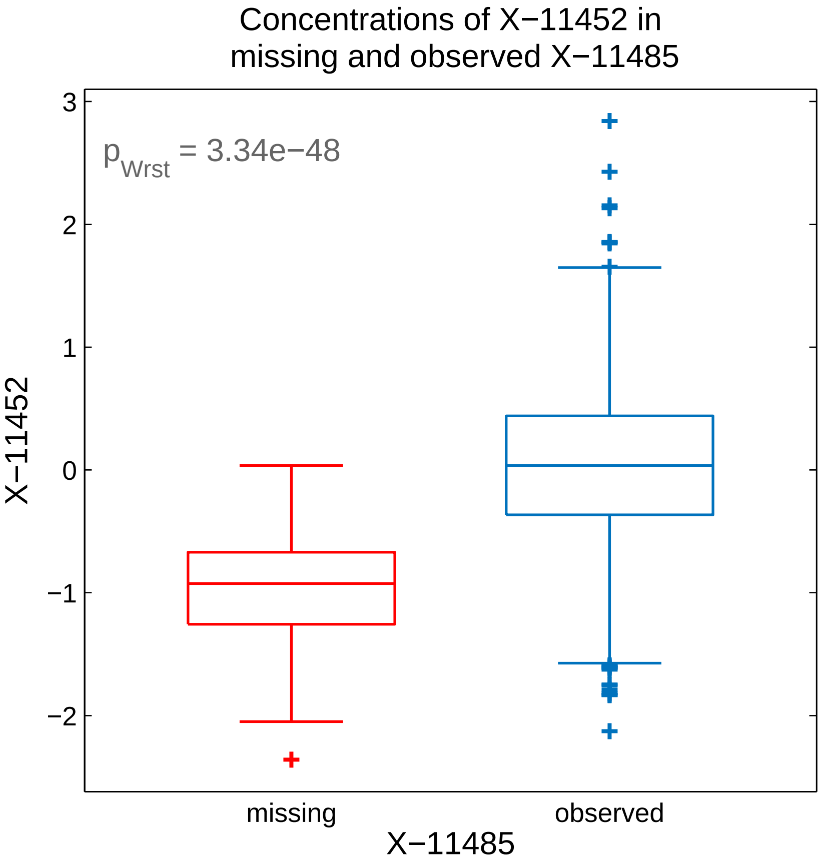

Concentrations of X−11452 in
missing and observed X−11485
p_Wrst = 3.34e−48
X−11452
3
2
1
0
−1
−2
missing
observed
X−11485

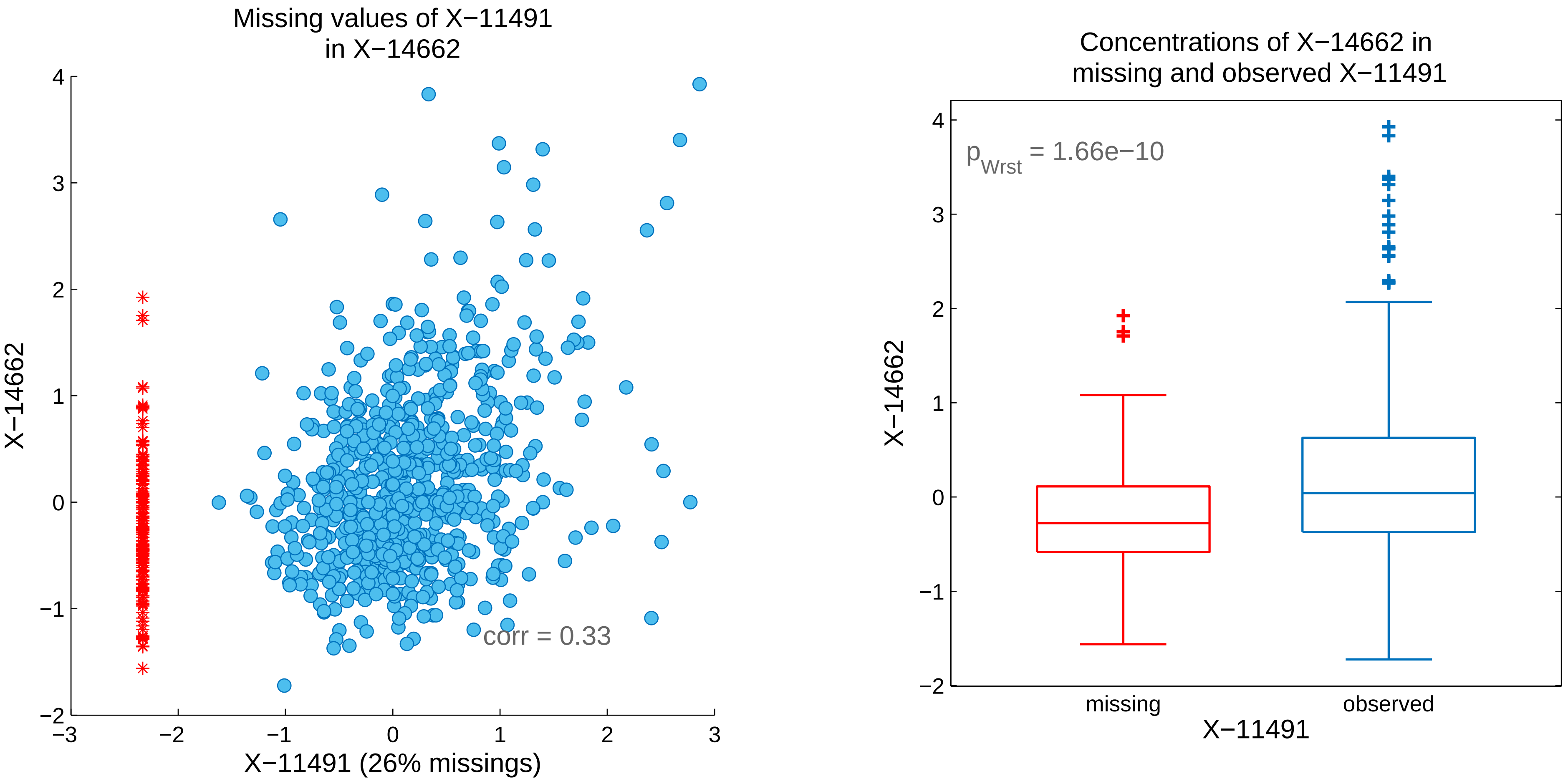

Missing values of X−11491
in X−14662
X−14662
X−11491 (26% missings)
corr = 0.33
Concentrations of X−14662 in
missing and observed X−11491
p_{Wrst} = 1.66e−10
X−14662
missing
observed
X−11491

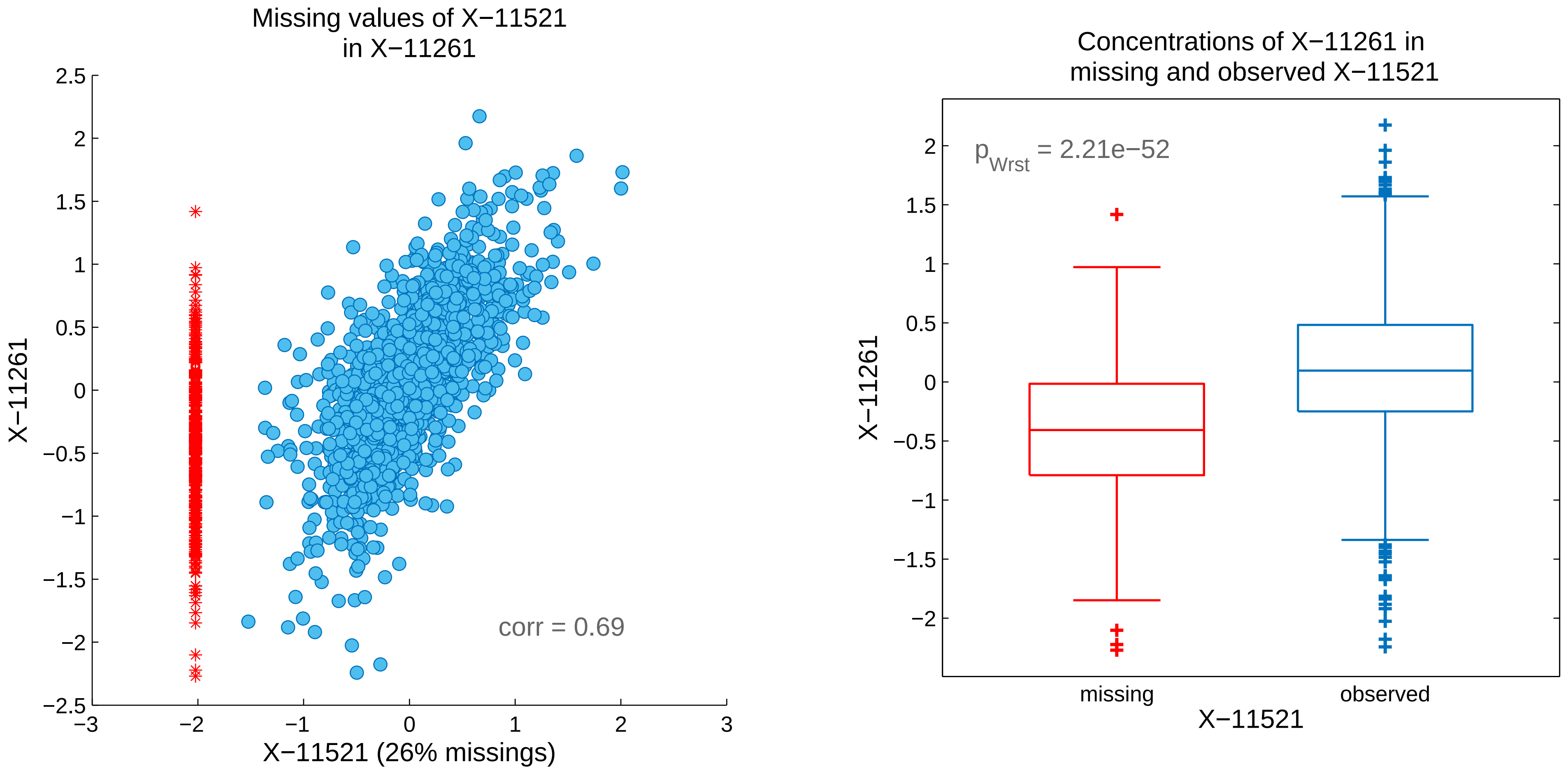
Missing values of X−11521
in X−11261
X−11261
X−11521 (26% missings)
corr = 0.69
Concentrations of X−11261 in
missing and observed X−11521
p_Wrst = 2.21e−52
X−11261
missing
observed
X−11521

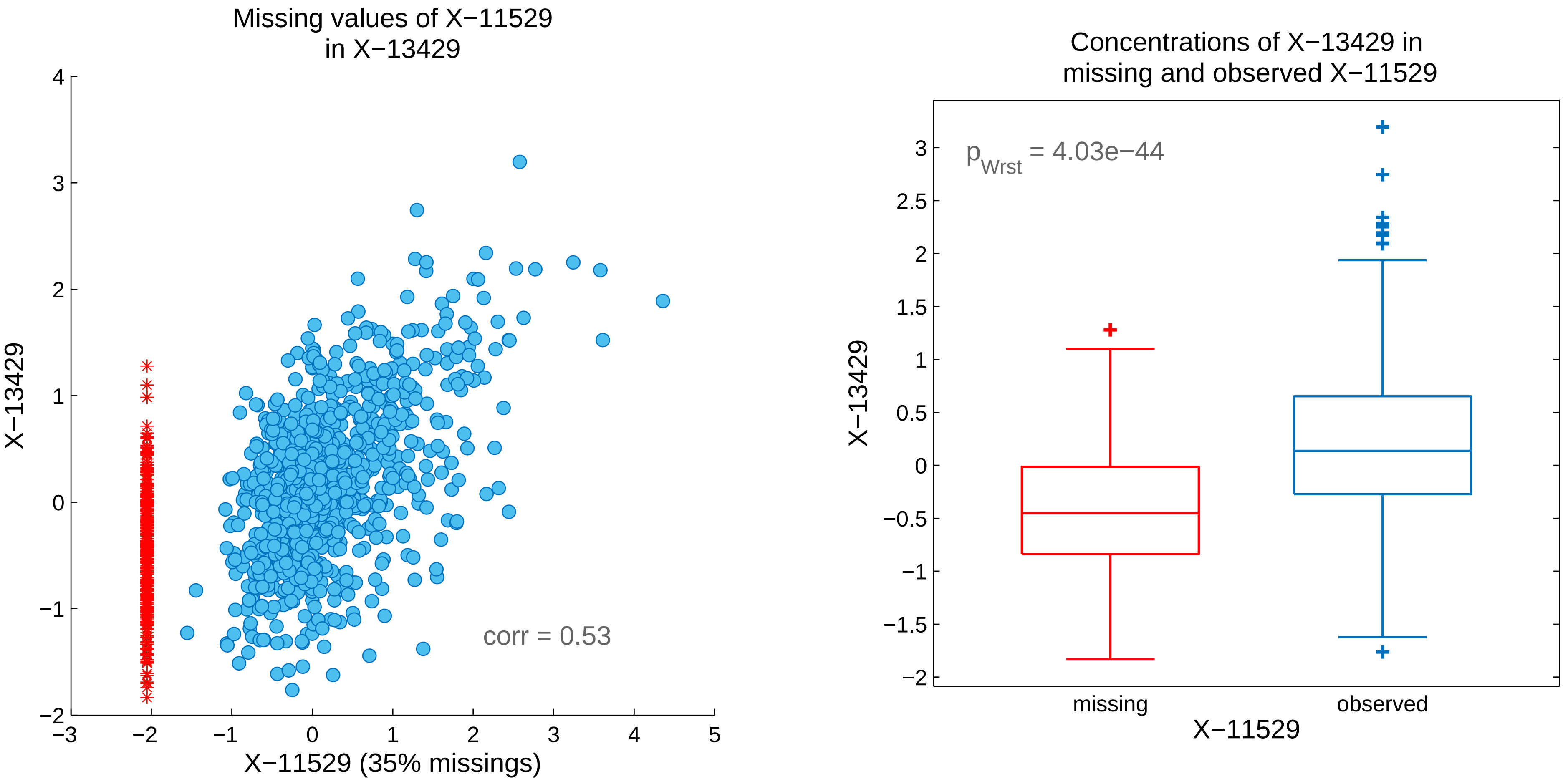

Missing values of X−11529
in X−13429
X−13429
X−11529 (35% missings)
corr = 0.53
Concentrations of X−13429 in
missing and observed X−11529
p_{Wrst} = 4.03e−44
X−13429
missing
observed
X−11529

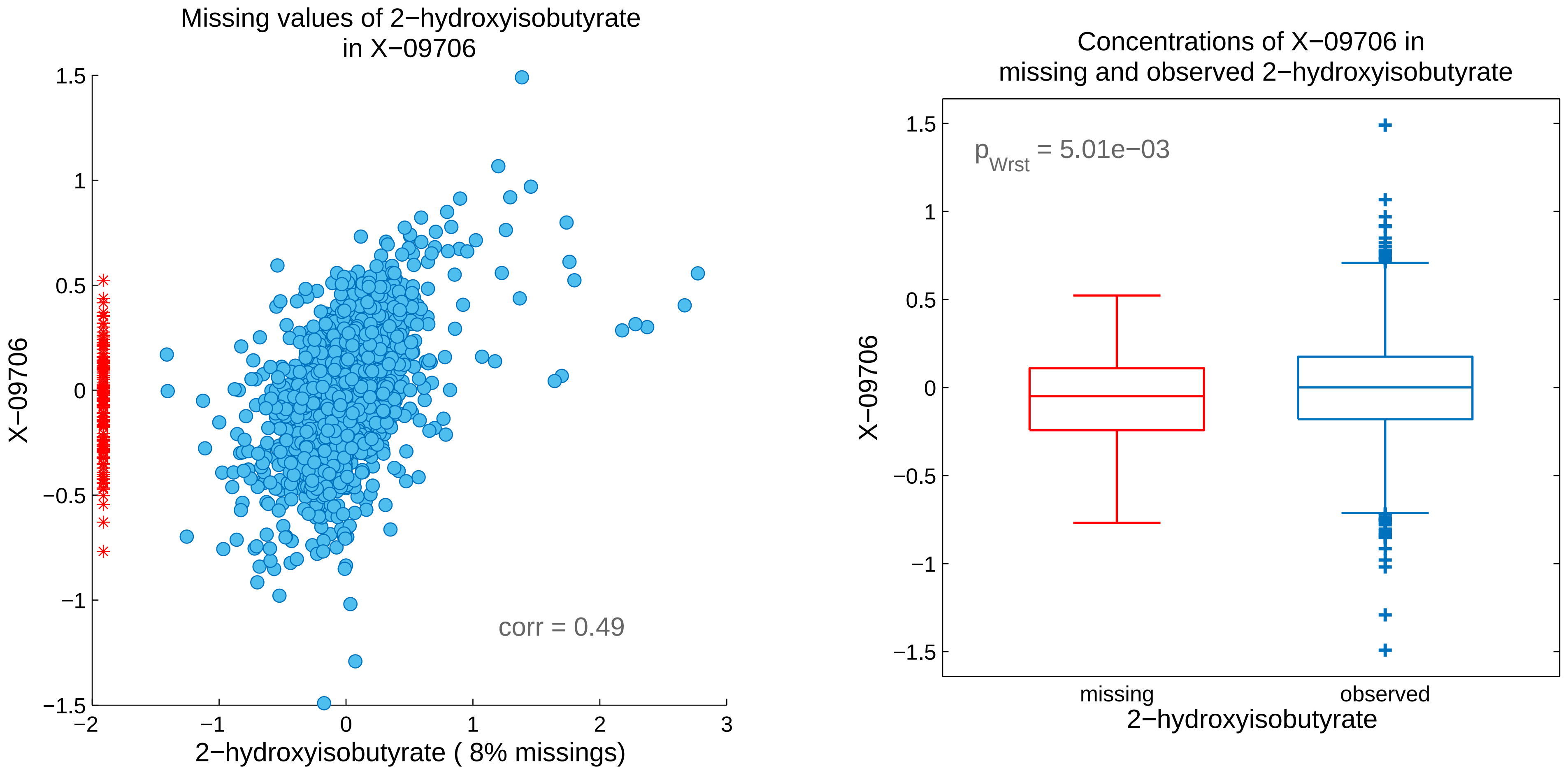

Missing values of 2−hydroxyisobutyrate in X−09706
X−09706
2−hydroxyisobutyrate (8% missings)
corr = 0.49
Concentrations of X−09706 in missing and observed 2−hydroxyisobutyrate
p_{Wrst} = 5.01e−03
X−09706
missing
observed
2−hydroxyisobutyrate

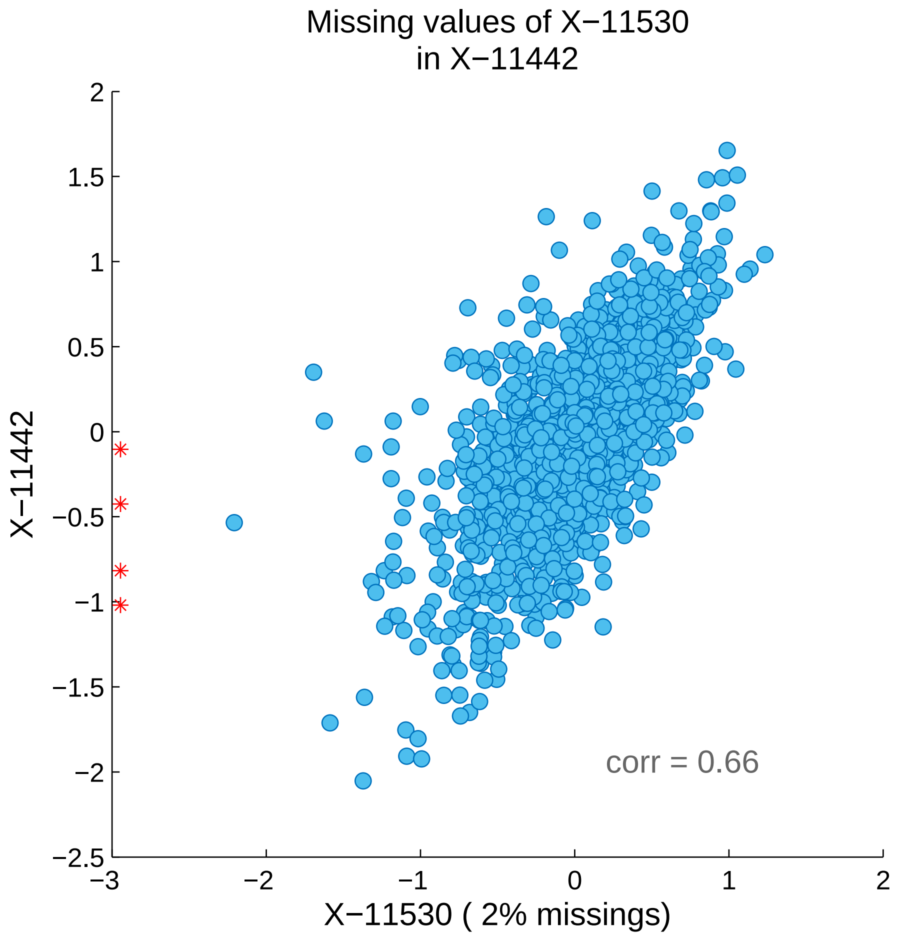

Missing values of X−11530
in X−11442
corr = 0.66
X−11442
X−11530 (2% missings)

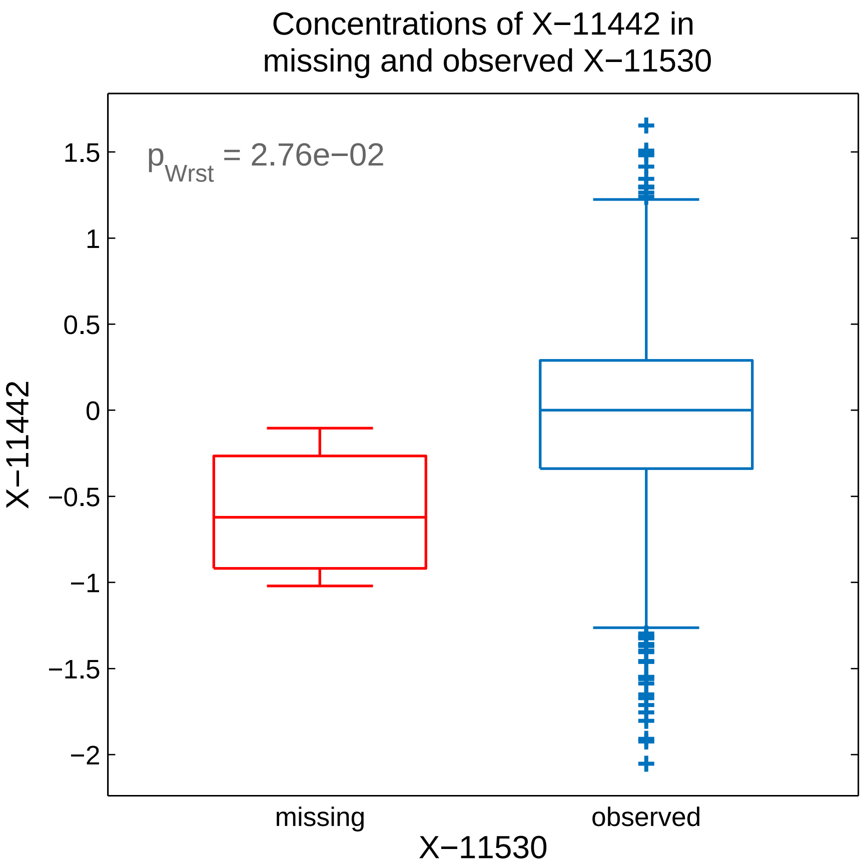

Concentrations of X−11442 in
missing and observed X−11530
p_Wrst = 2.76e−02
X−11442
missing
observed
X−11530

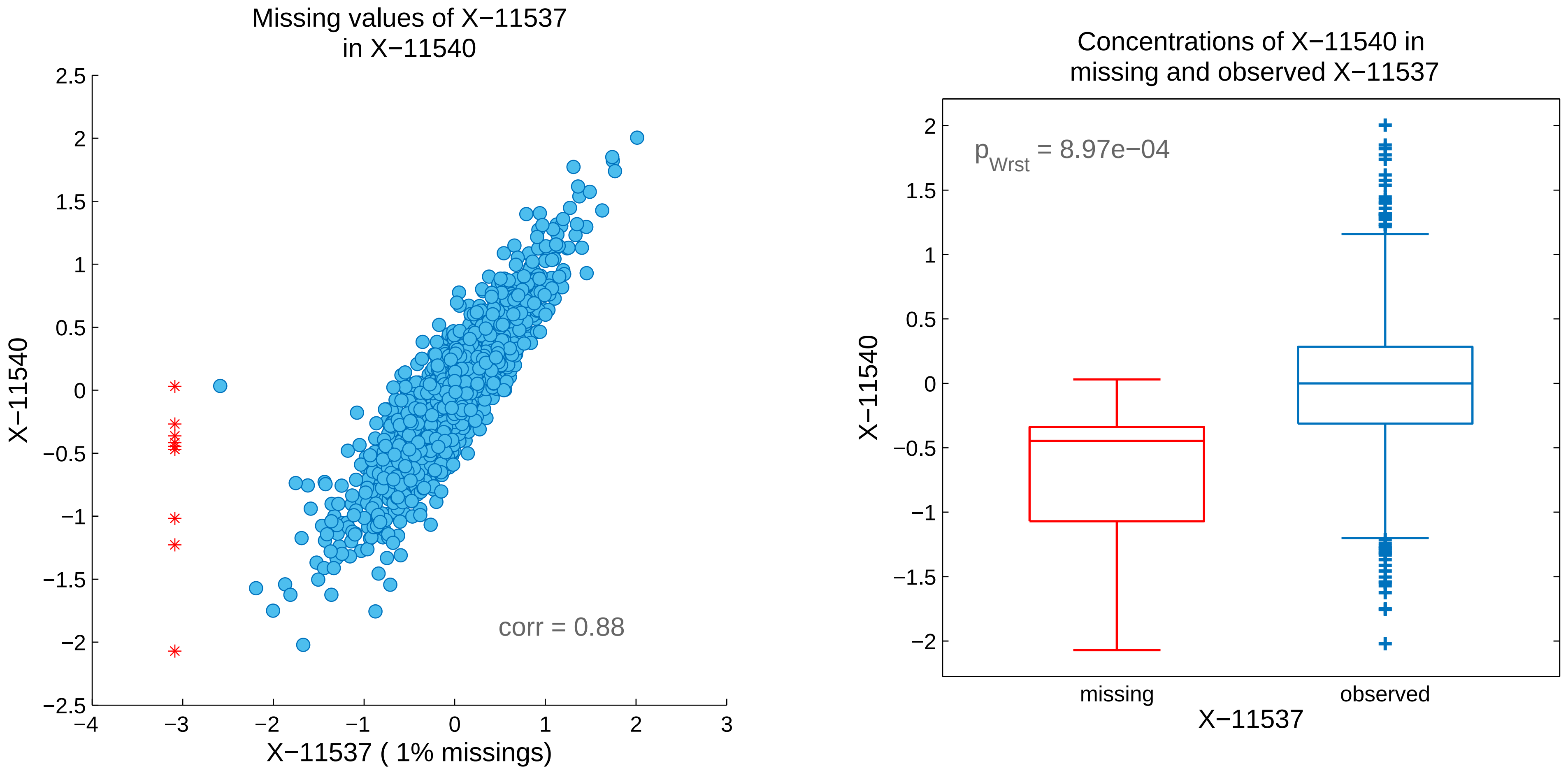
Missing values of X−11537
in X−11540
X−11540
X−11537 (1% missings)
corr = 0.88
Concentrations of X−11540 in
missing and observed X−11537
X−11540
X−11537
p_{Wrst} = 8.97e−04
missing
observed

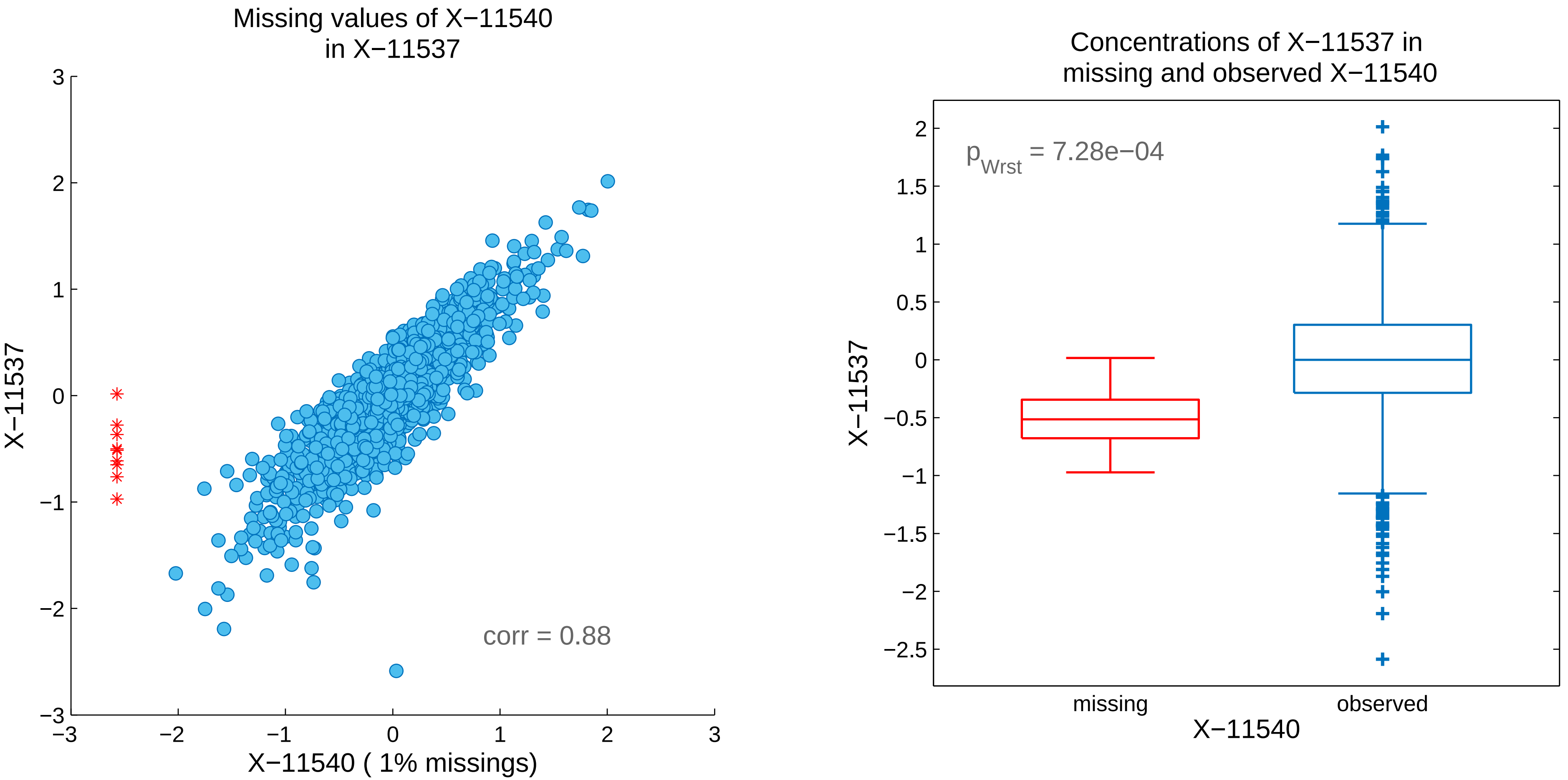

Missing values of X−11540
in X−11537
X−11537
X−11540 (1% missings)
corr = 0.88
Concentrations of X−11537 in
missing and observed X−11540
p_{Wrst} = 7.28e−04
X−11537
missing
observed
X−11540

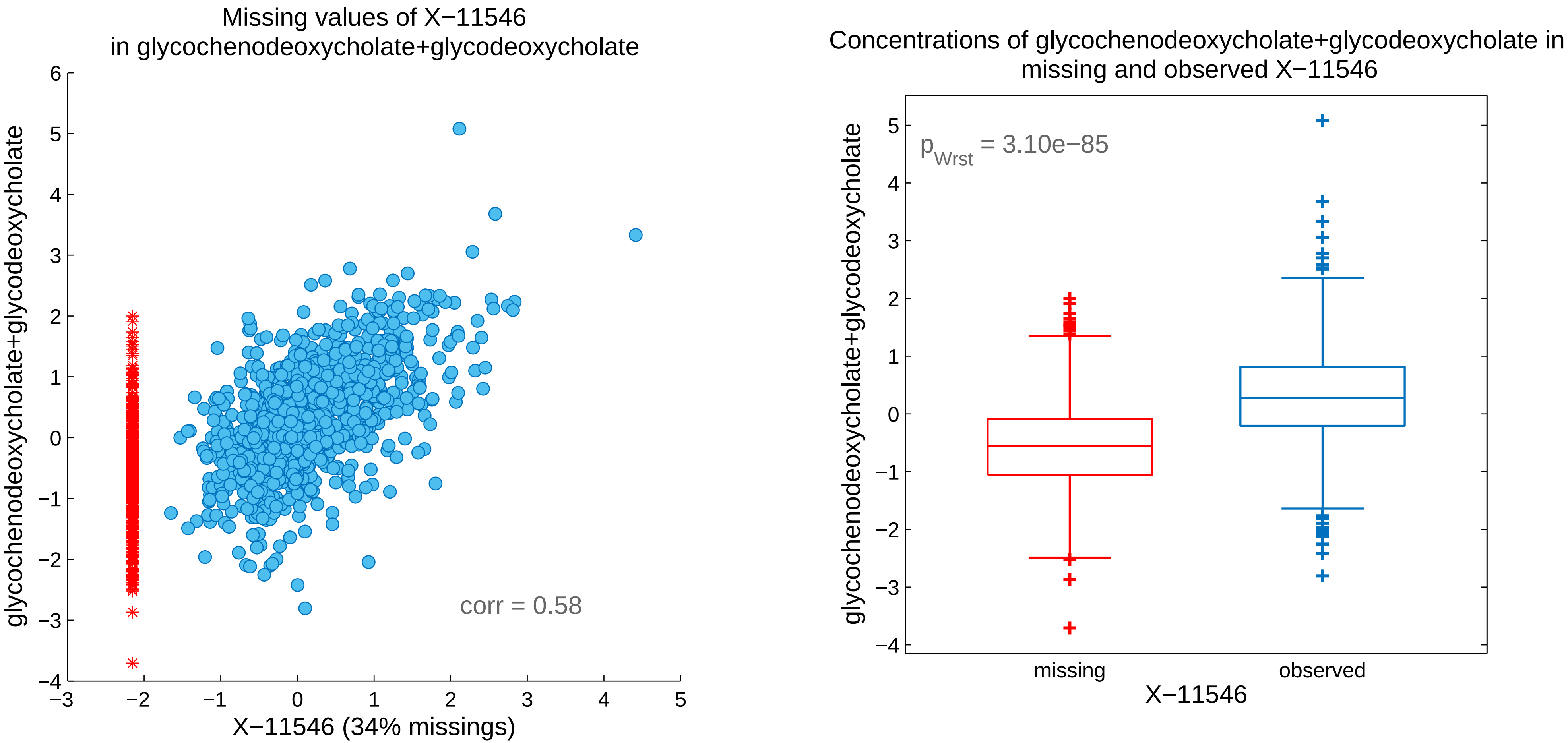

Missing values of X−11546
in glycochenodeoxycholate+glycodeoxycholate
glycochenodeoxycholate+glycodeoxycholate
X−11546 (34% missings)
corr = 0.58
Concentrations of glycochenodeoxycholate+glycodeoxycholate in
missing and observed X−11546
glycochenodeoxycholate+glycodeoxycholate
p_Wrst = 3.10e−85
missing
observed
X−11546

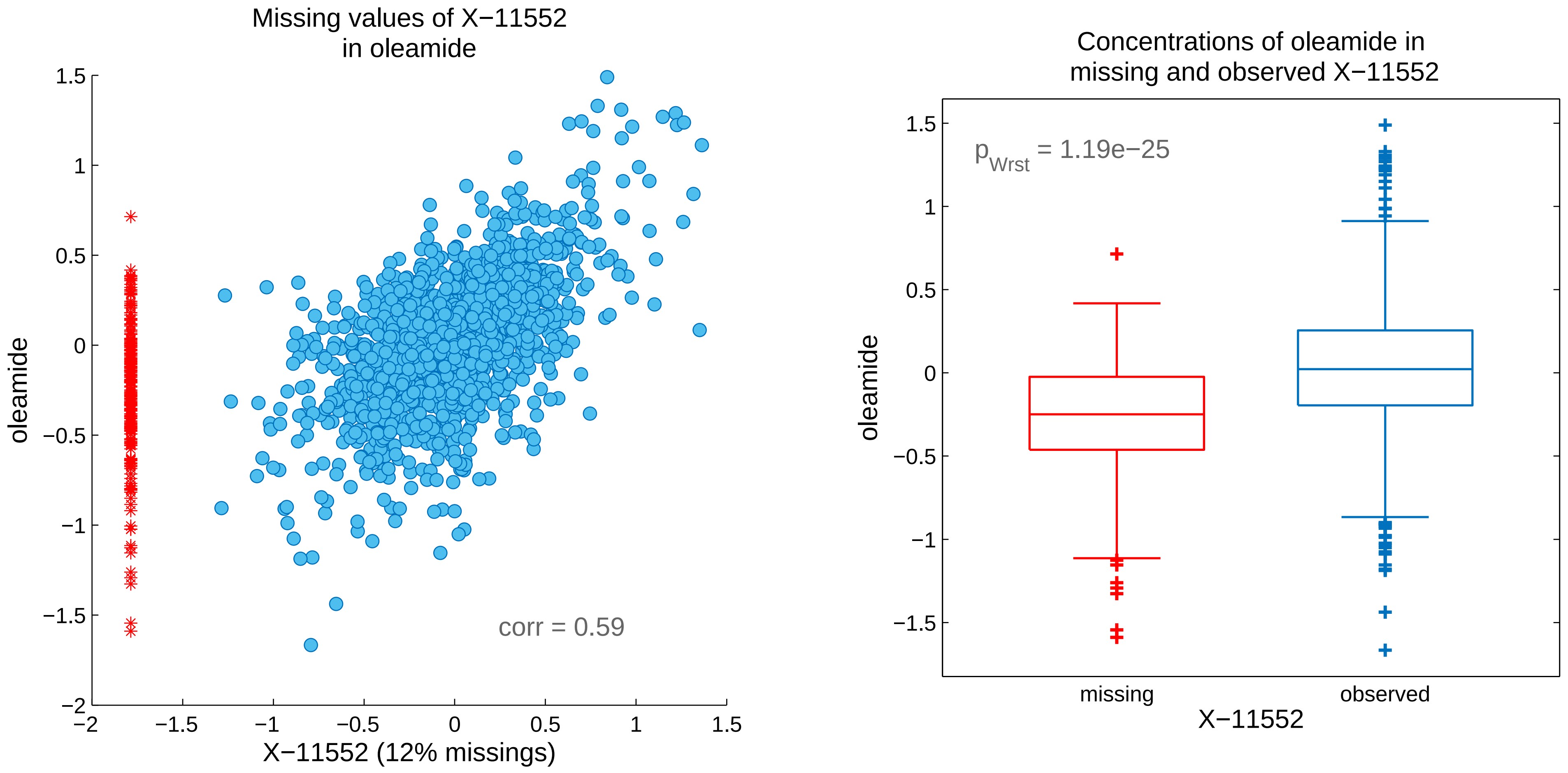

Missing values of X−11552
in oleamide
oleamide
X−11552 (12% missings)
corr = 0.59
1.5
1
0.5
0
−0.5
−1
−1.5
−2
−2
−1.5
−1
−0.5
0
0.5
1
1.5
Concentrations of oleamide in
missing and observed X−11552
p_{Wrst} = 1.19e−25
oleamide
1.5
1
0.5
0
−0.5
−1
−1.5
missing
observed
X−11552

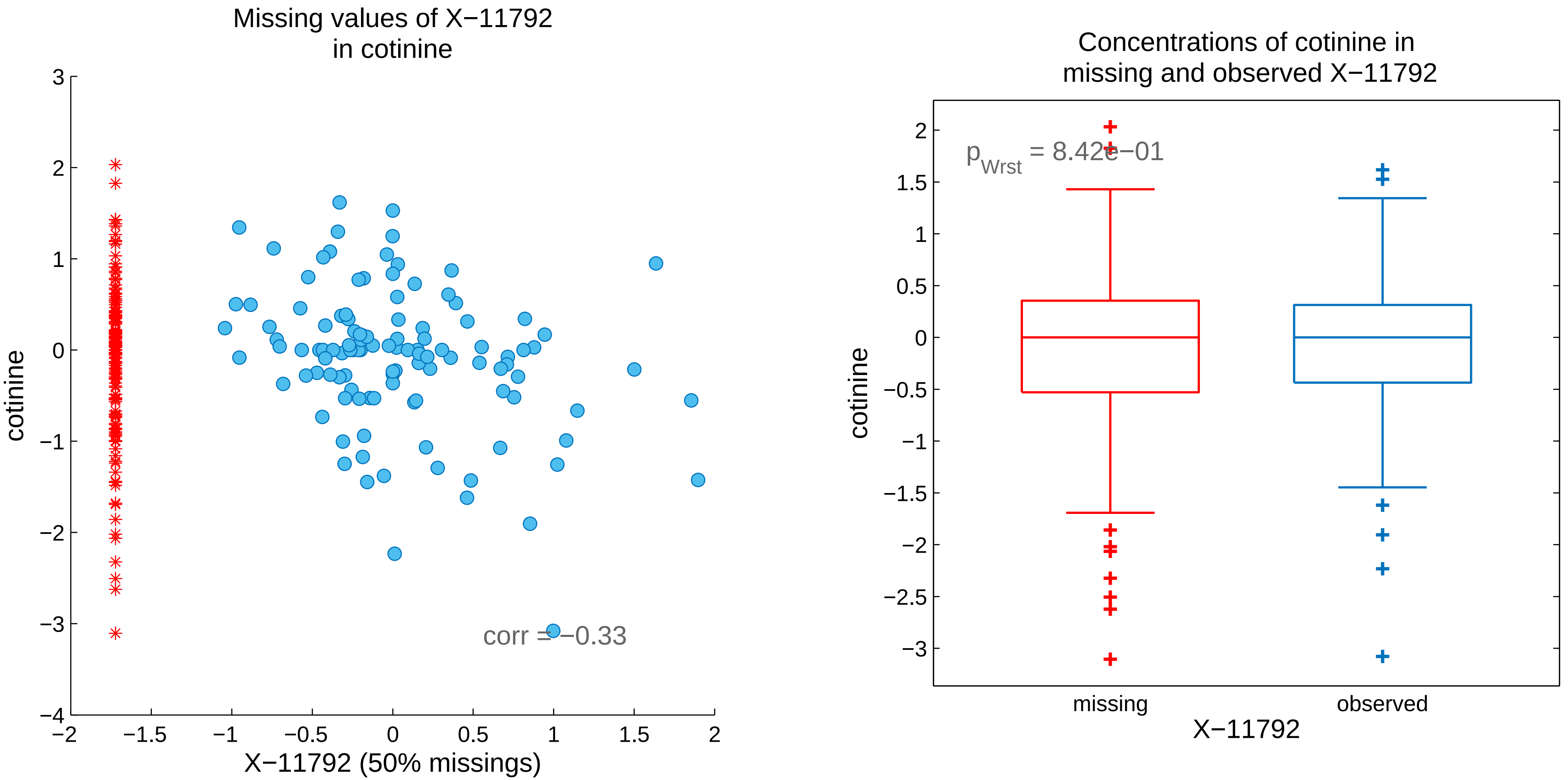
Missing values of X−11792
in cotinine
cotinine
X−11792 (50% missings)
corr = −0.33
Concentrations of cotinine in
missing and observed X−11792
p_{Wrst} = 8.42e−01
cotinine
missing
observed
X−11792

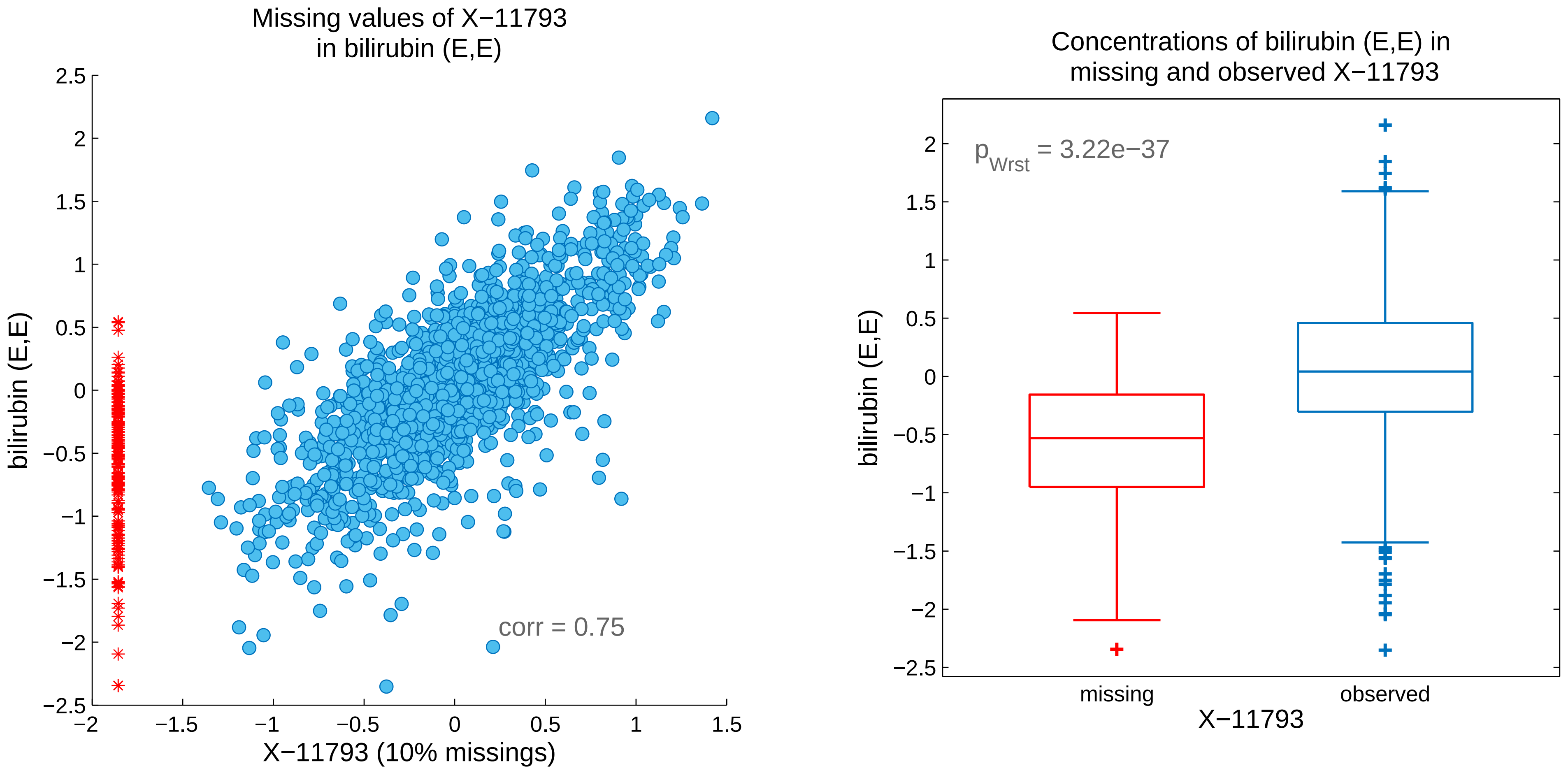

Missing values of X−11793 in bilirubin (E,E)
bilirubin (E,E)
X−11793 (10% missings)
corr = 0.75
Concentrations of bilirubin (E,E) in missing and observed X−11793
p_Wrst = 3.22e−37
bilirubin (E,E)
missing
observed
X−11793

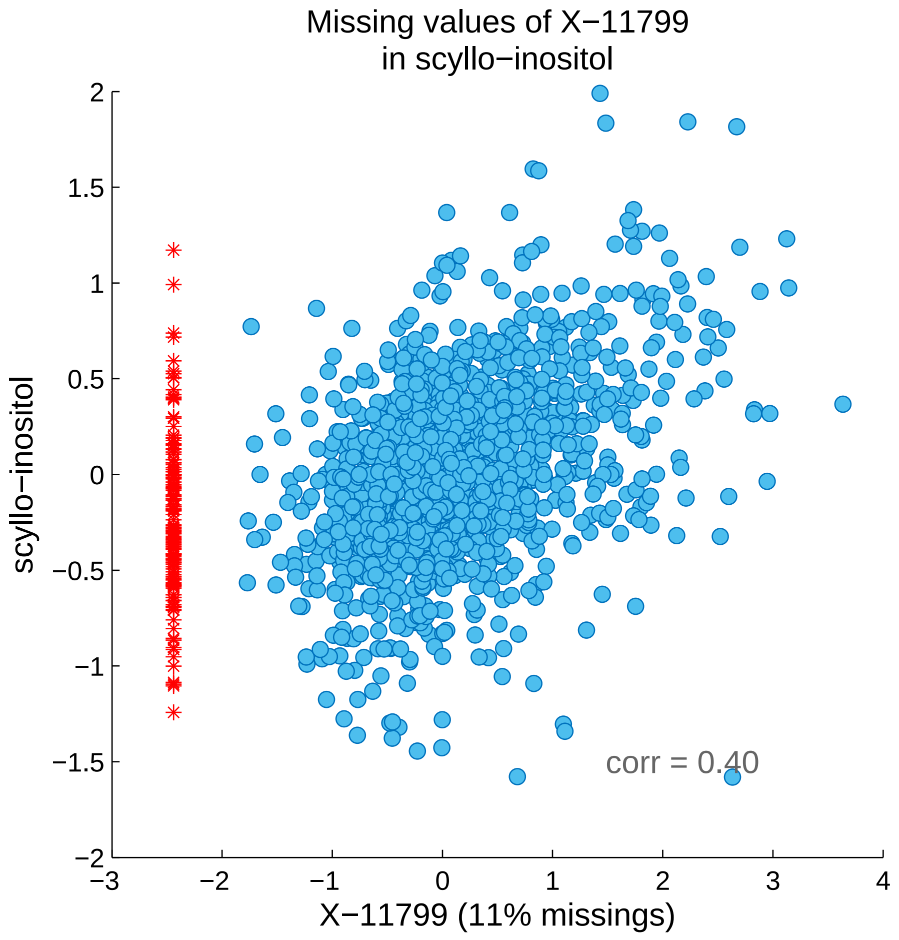
Missing values of X−11799
in scyllo−inositol
scyllo−inositol
X−11799 (11% missings)
corr = 0.40

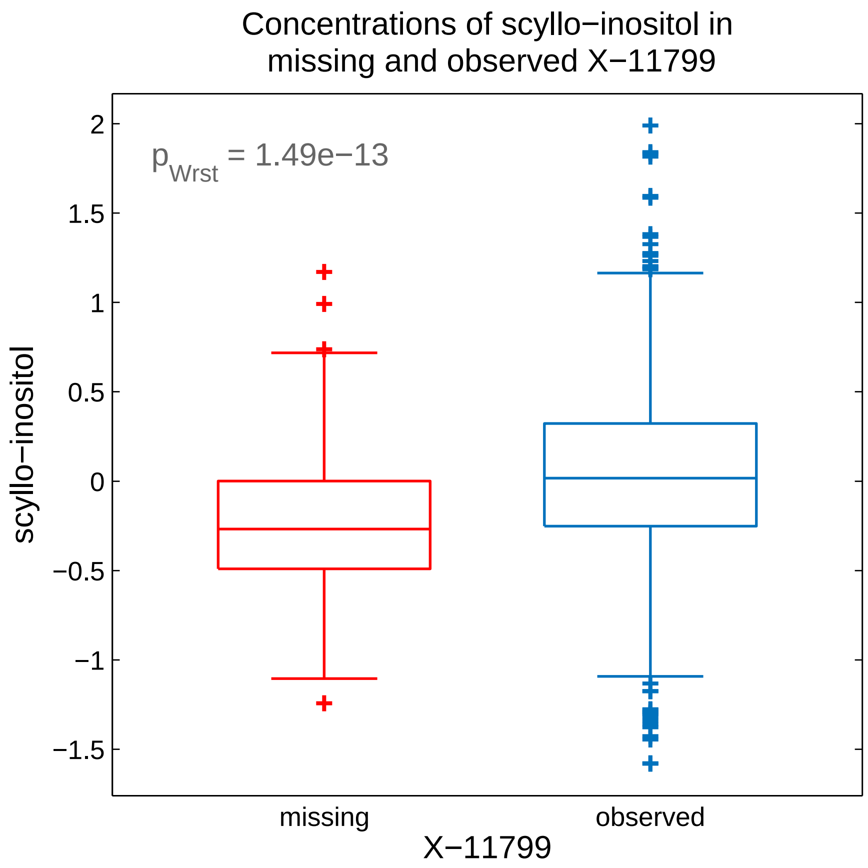
Concentrations of scyllo−inositol in
missing and observed X−11799
p_{Wrst} = 1.49e−13
scyllo−inositol
missing
observed
X−11799

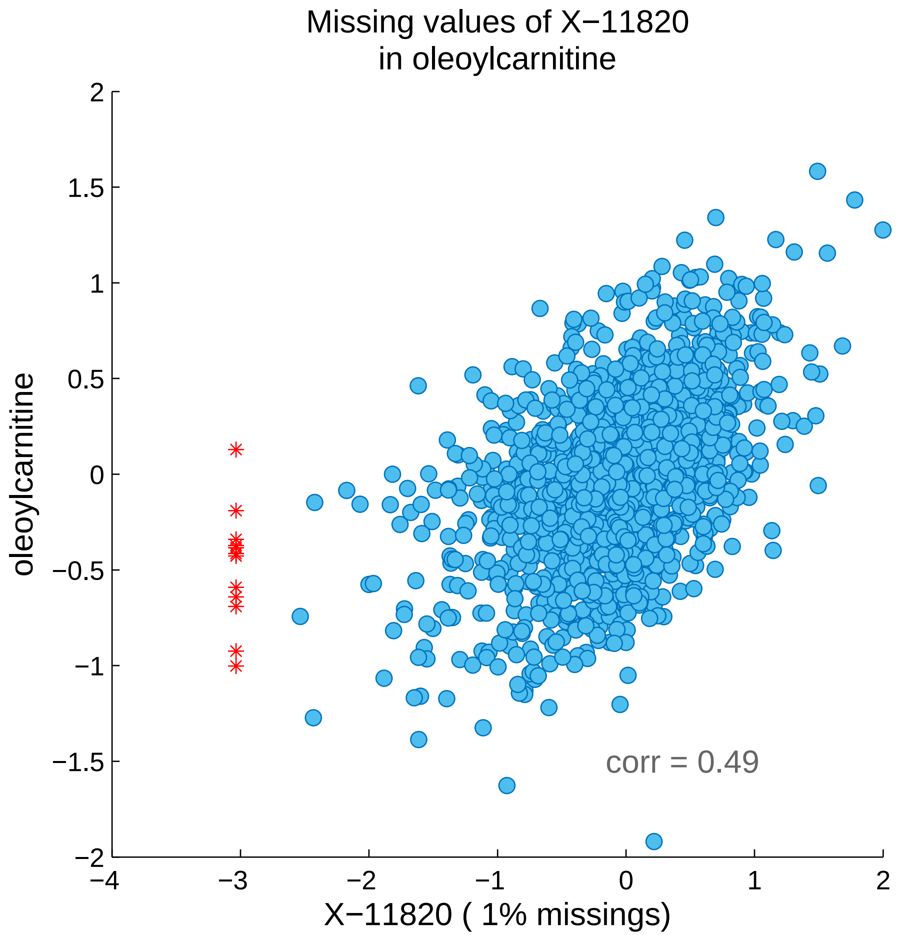
Missing values of X−11820
in oleoylcarnitine
oleoylcarnitine
X−11820 (1% missings)
corr = 0.49
2
1.5
1
0.5
0
−0.5
−1
−1.5
−2
−4
−3
−2
−1
0
1
2

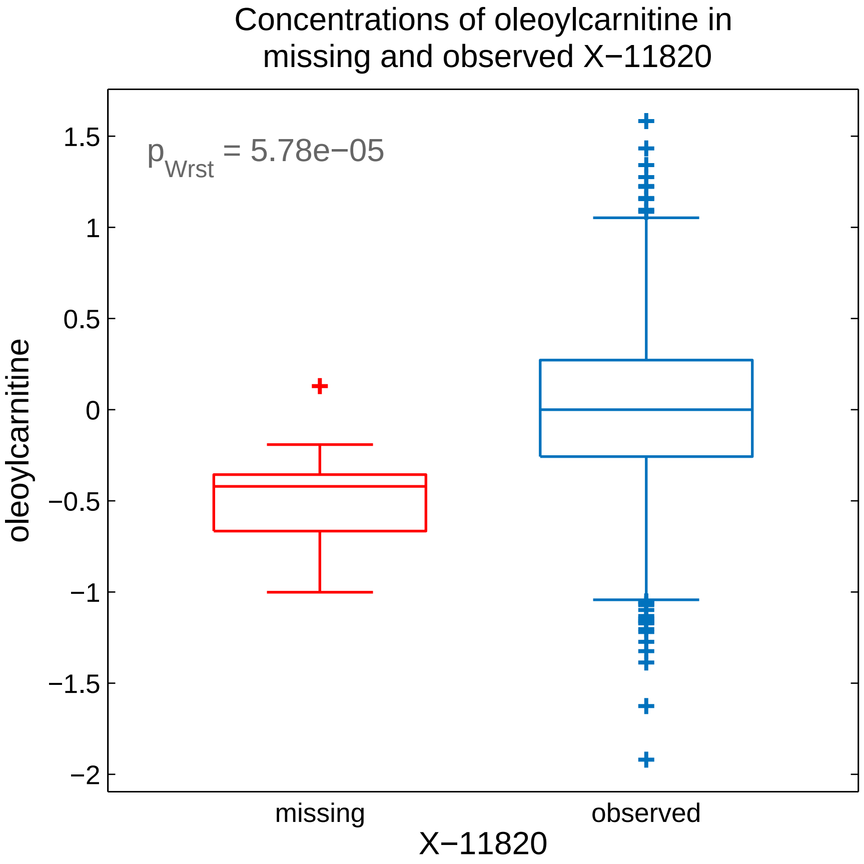
Concentrations of oleoylcarnitine in
missing and observed X−11820
p_{Wrst} = 5.78e−05
oleoylcarnitine
X−11820
missing
observed
1.5
1
0.5
0
−0.5
−1
−1.5
−2

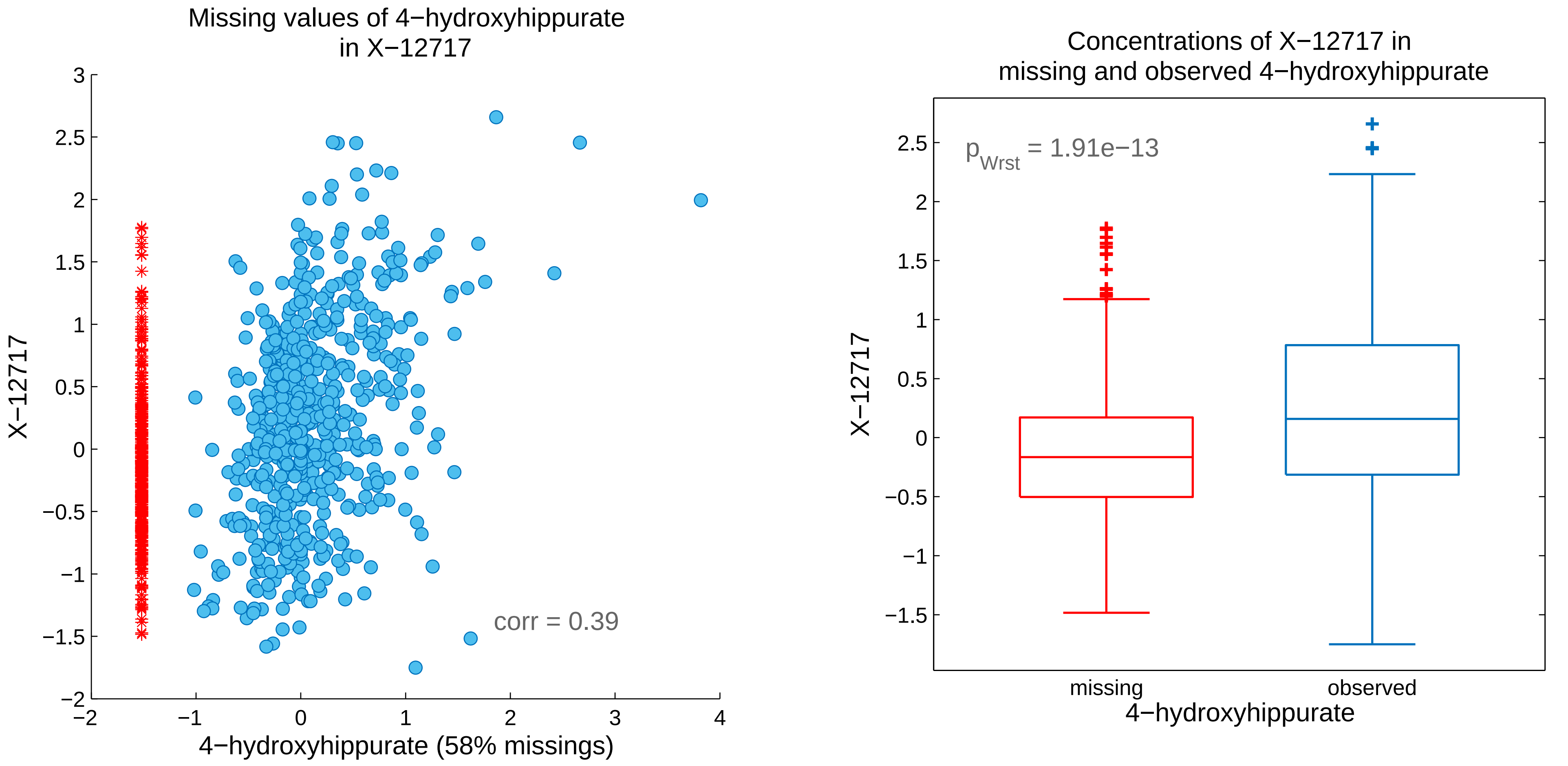

Missing values of 4−hydroxyhippurate in X−12717
X−12717
4−hydroxyhippurate (58% missings)
corr = 0.39
Concentrations of X−12717 in missing and observed 4−hydroxyhippurate
p_Wrst = 1.91e−13
missing
observed
4−hydroxyhippurate

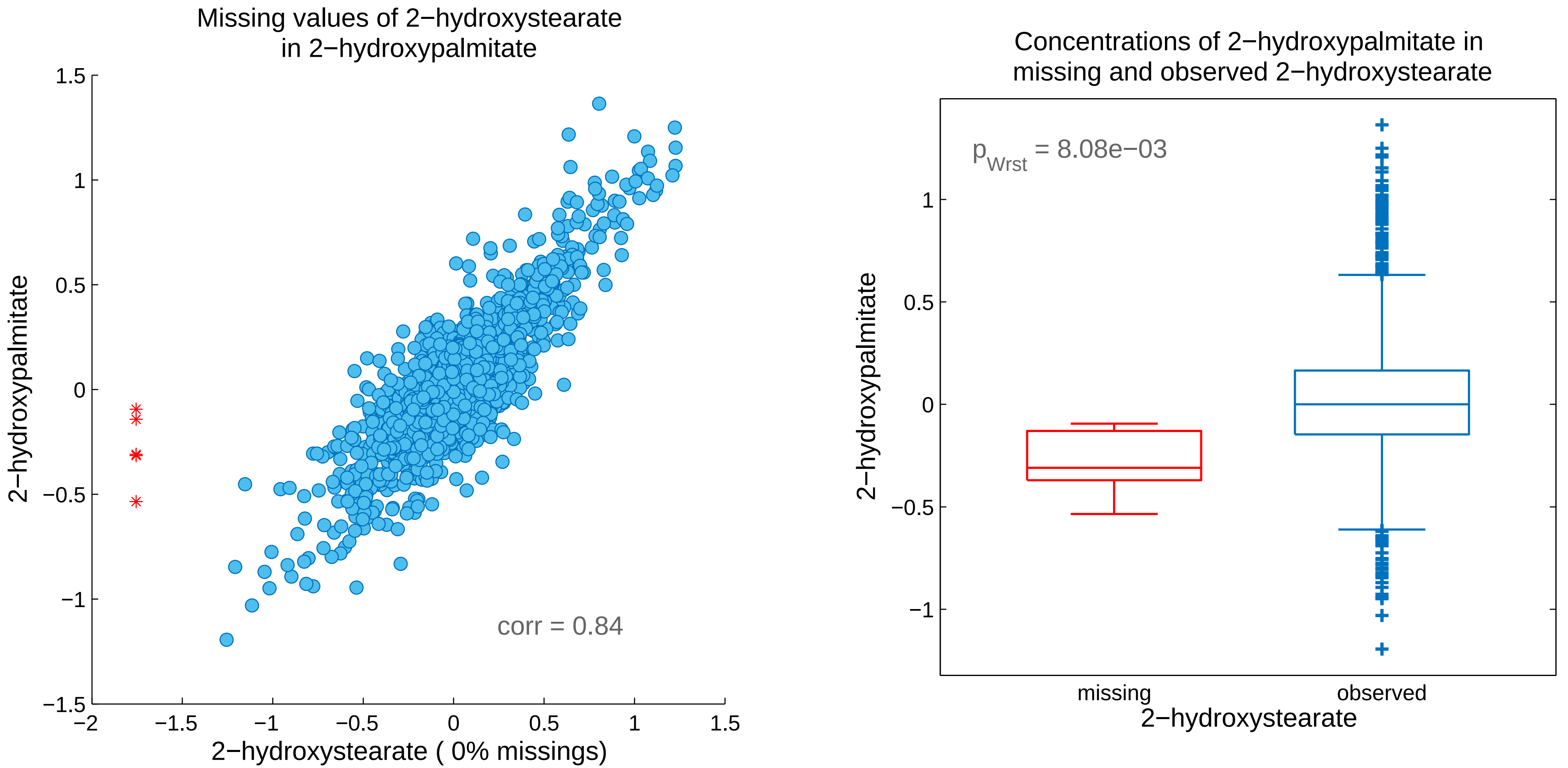

Missing values of 2−hydroxystearate in 2−hydroxypalmitate
2−hydroxypalmitate
2−hydroxystearate (0% missings)
corr = 0.84
1.5
1
0.5
0
−0.5
−1
−1.5
−2
Concentrations of 2−hydroxypalmitate in missing and observed 2−hydroxystearate
p_Wrst = 8.08e−03
2−hydroxypalmitate
missing
observed
2−hydroxystearate

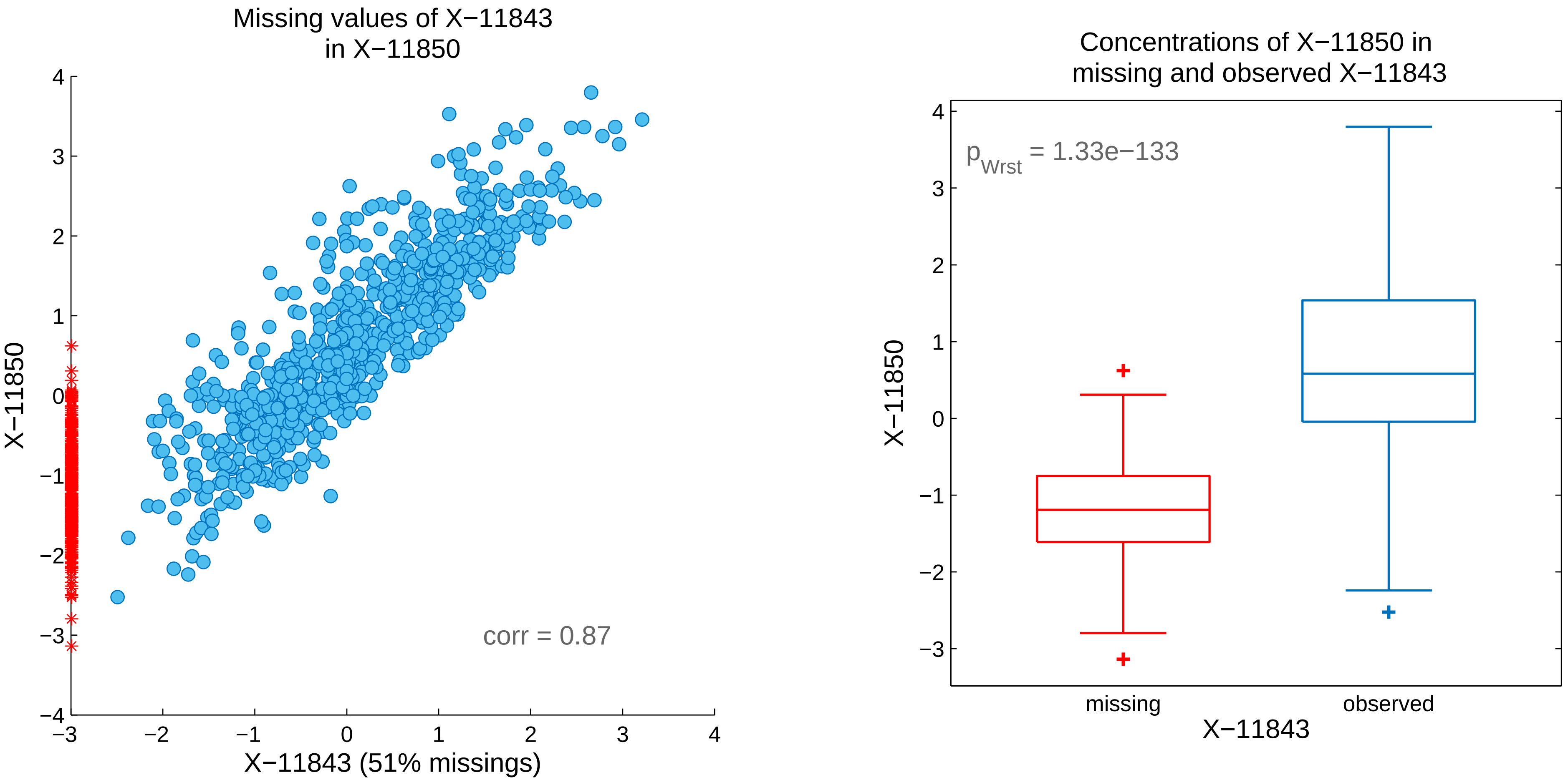
Missing values of X−11843
in X−11850
X−11850
X−11843 (51% missings)
corr = 0.87
Concentrations of X−11850 in
missing and observed X−11843
p_{Wrst} = 1.33e−133
X−11850
missing
observed
X−11843

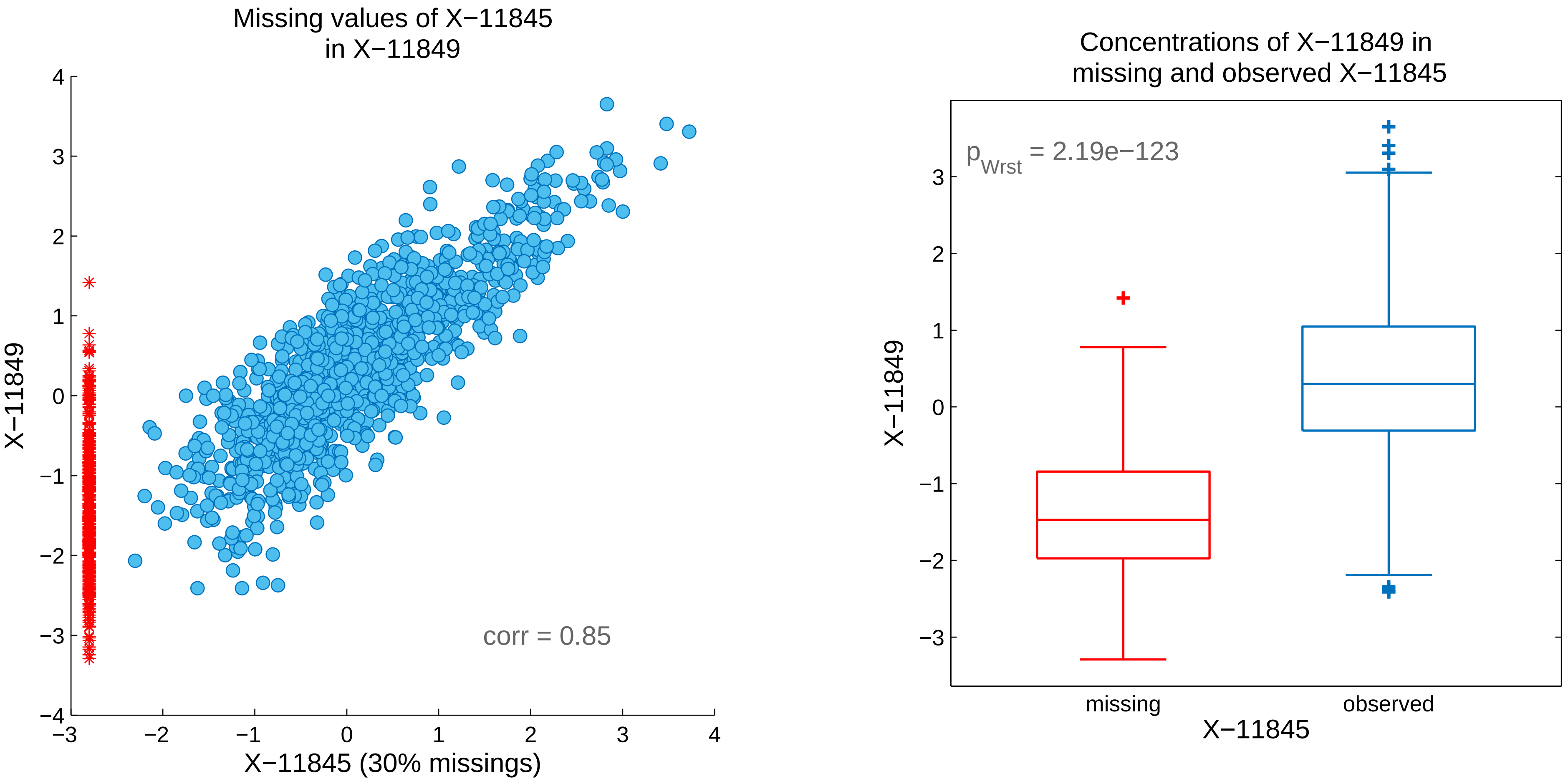

Missing values of X−11845
in X−11849
X−11849
X−11845 (30% missings)
corr = 0.85
Concentrations of X−11849 in
missing and observed X−11845
p_{Wrst} = 2.19e−123
X−11849
missing
observed
X−11845

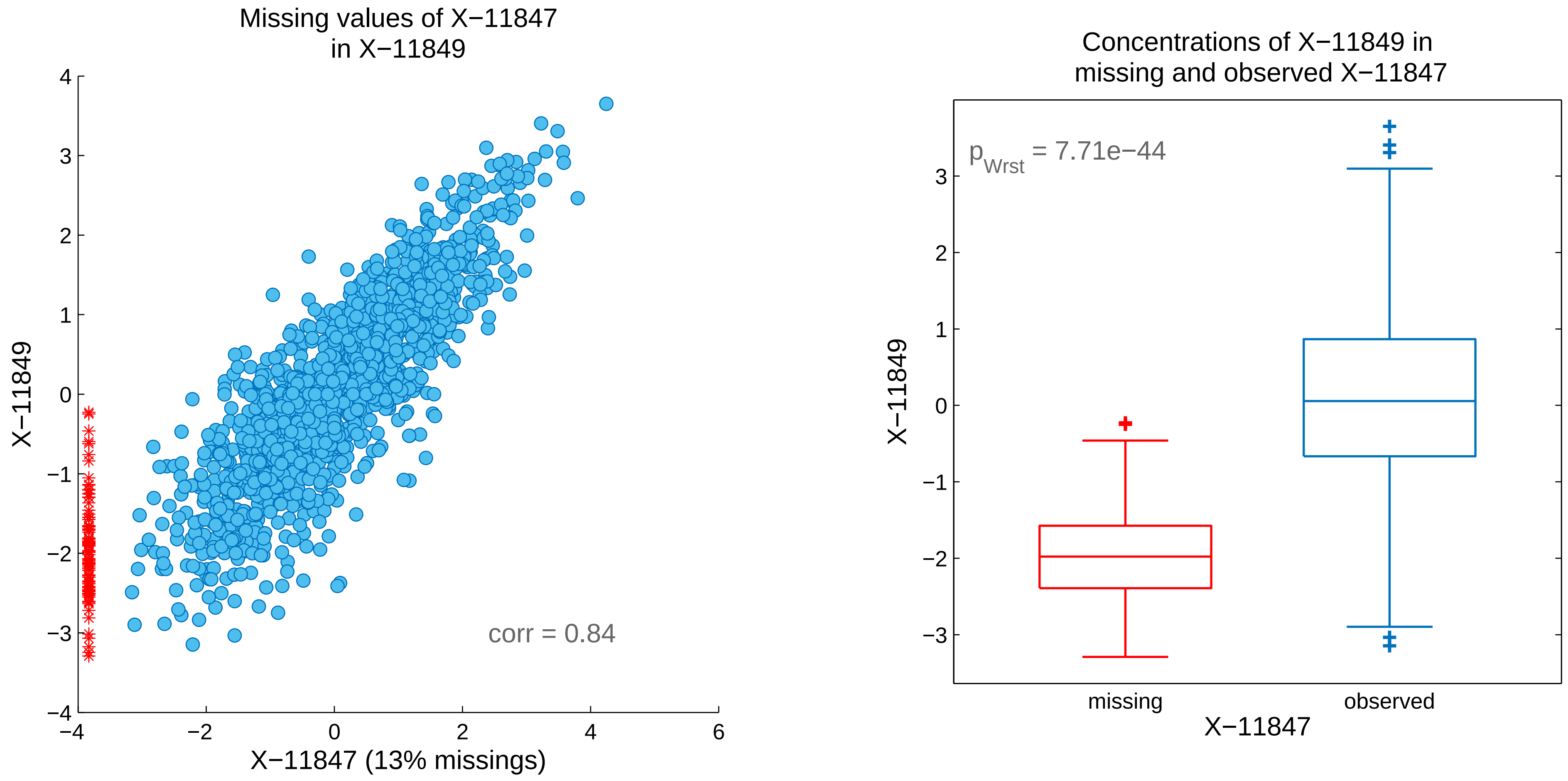
Missing values of X−11847
in X−11849
X−11849
X−11847 (13% missings)
corr = 0.84
Concentrations of X−11849 in
missing and observed X−11847
p_{Wrst} = 7.71e−44
X−11849
missing
observed
X−11847

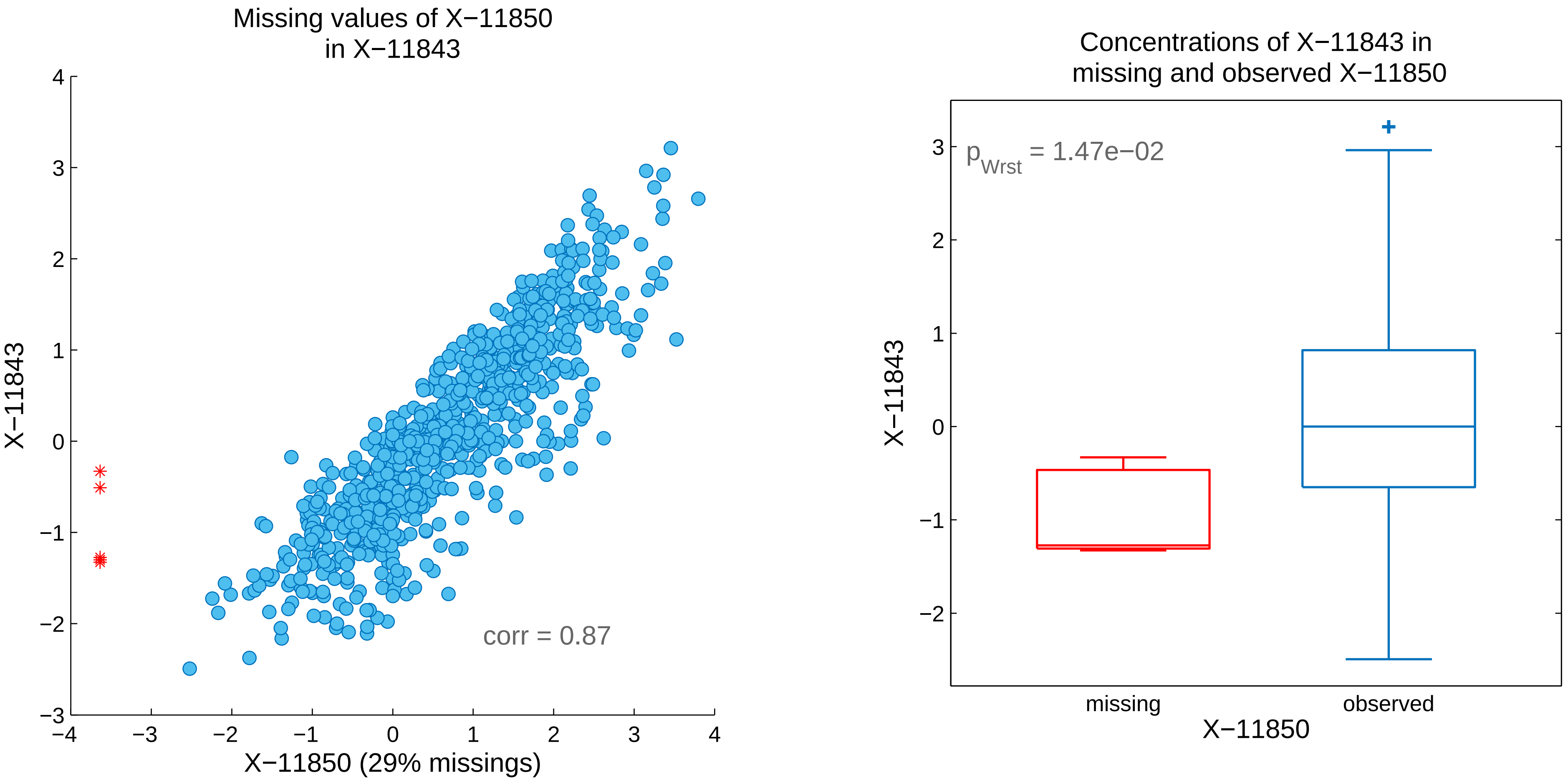
Missing values of X−11850
in X−11843
X−11843
X−11850 (29% missings)
corr = 0.87
4
3
2
1
0
−1
−2
−3
−4
−3
−2
−1
0
1
2
3
4
Concentrations of X−11843 in
missing and observed X−11850
p_{Wrst} = 1.47e−02
X−11843
3
2
1
0
−1
−2
missing
observed
X−11850

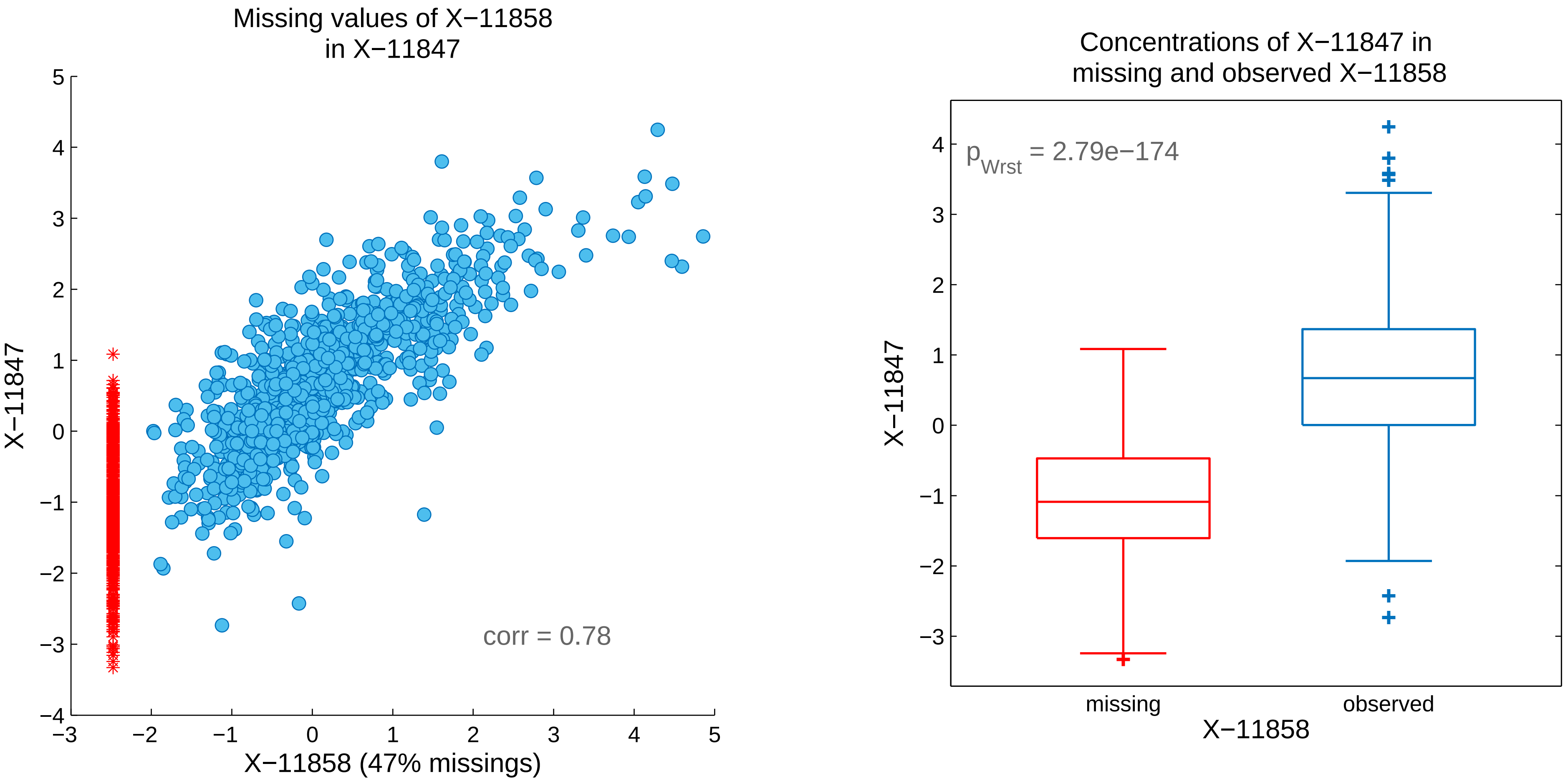

Missing values of X−11858
in X−11847
X−11847
X−11858 (47% missings)
corr = 0.78
Concentrations of X−11847 in
missing and observed X−11858
p_Wrst = 2.79e−174
X−11847
missing
observed
X−11858

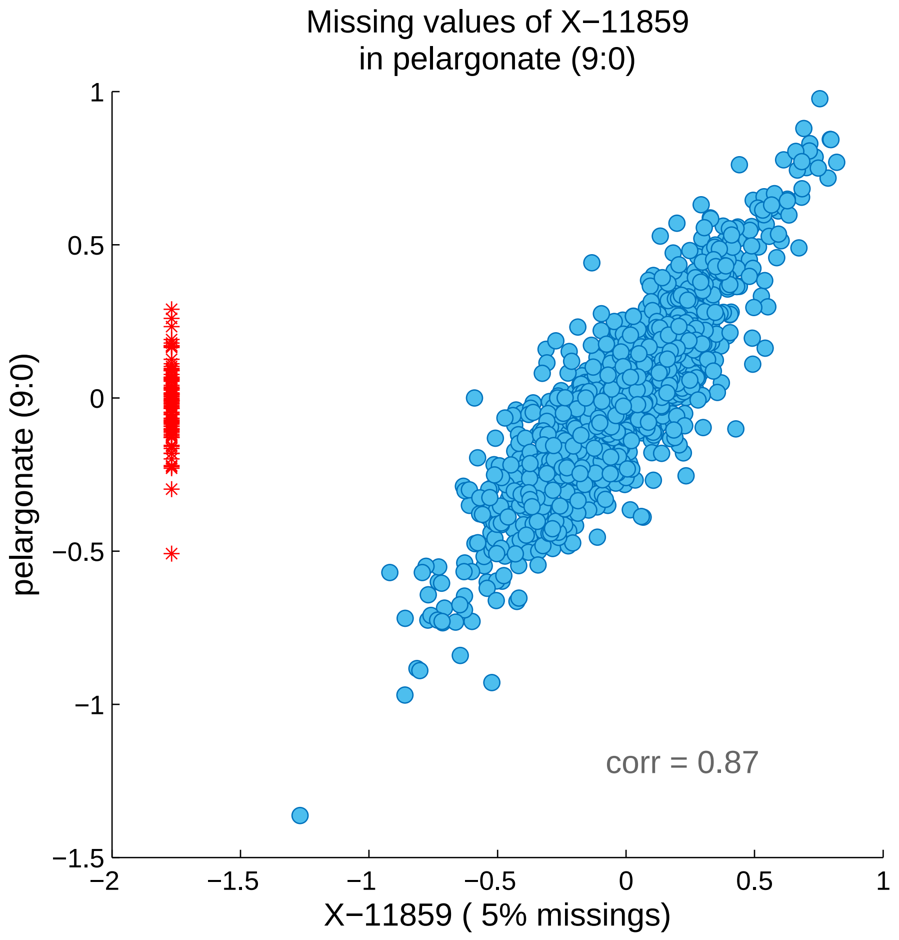
Missing values of X−11859
in pelargonate (9:0)
corr = 0.87
pelargonate (9:0)
X−11859 (5% missings)
1
0.5
0
−0.5
−1
−1.5
−2
−1.5
−1
−0.5
0
0.5
1

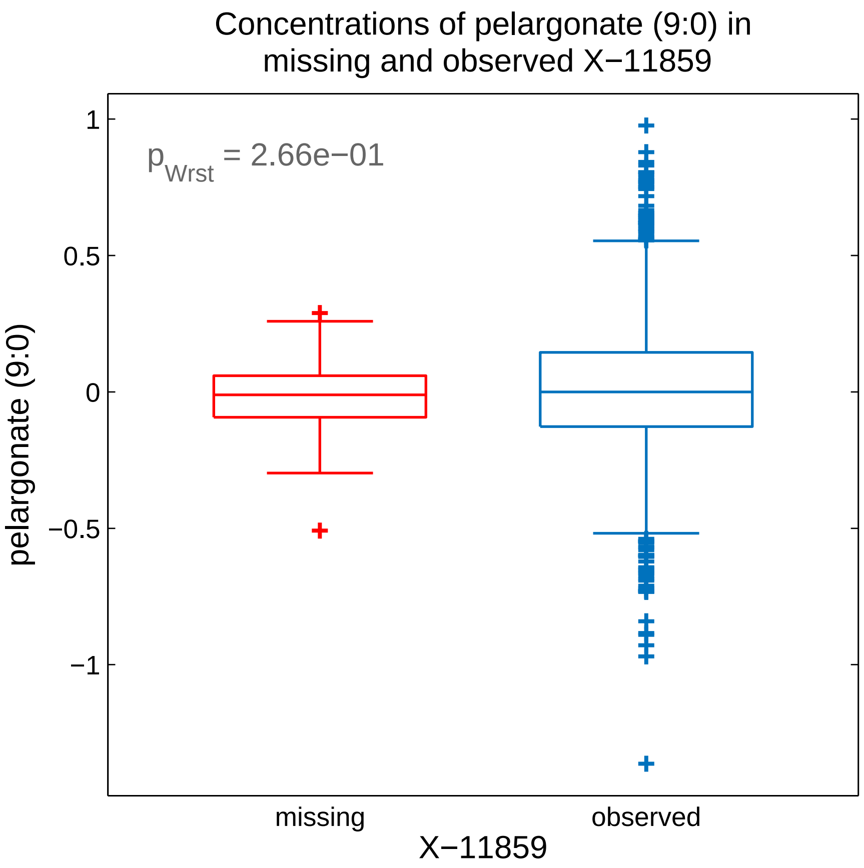
Concentrations of pelargonate (9:0) in
missing and observed X−11859
p_Wrst = 2.66e−01
pelargonate (9:0)
1
0.5
0
−0.5
−1
missing
observed
X−11859

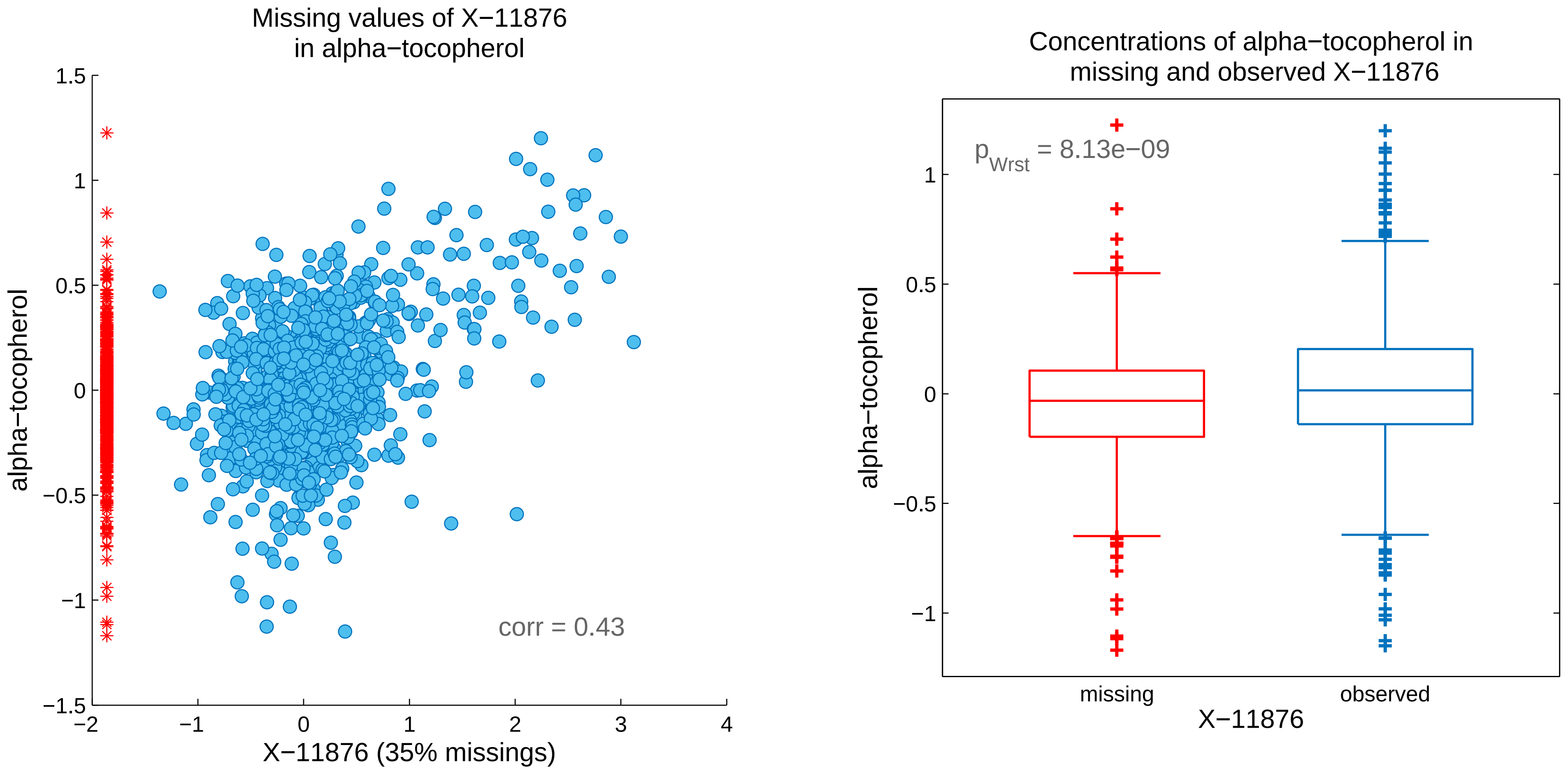

Missing values of X−11876
in alpha−tocopherol
alpha−tocopherol
X−11876 (35% missings)
corr = 0.43
1.5
1
0.5
0
−0.5
−1
−1.5
−2
−1
0
1
2
3
4
Concentrations of alpha−tocopherol in
missing and observed X−11876
p_{Wrst} = 8.13e−09
alpha−tocopherol
1
0.5
0
−0.5
−1
missing
observed
X−11876

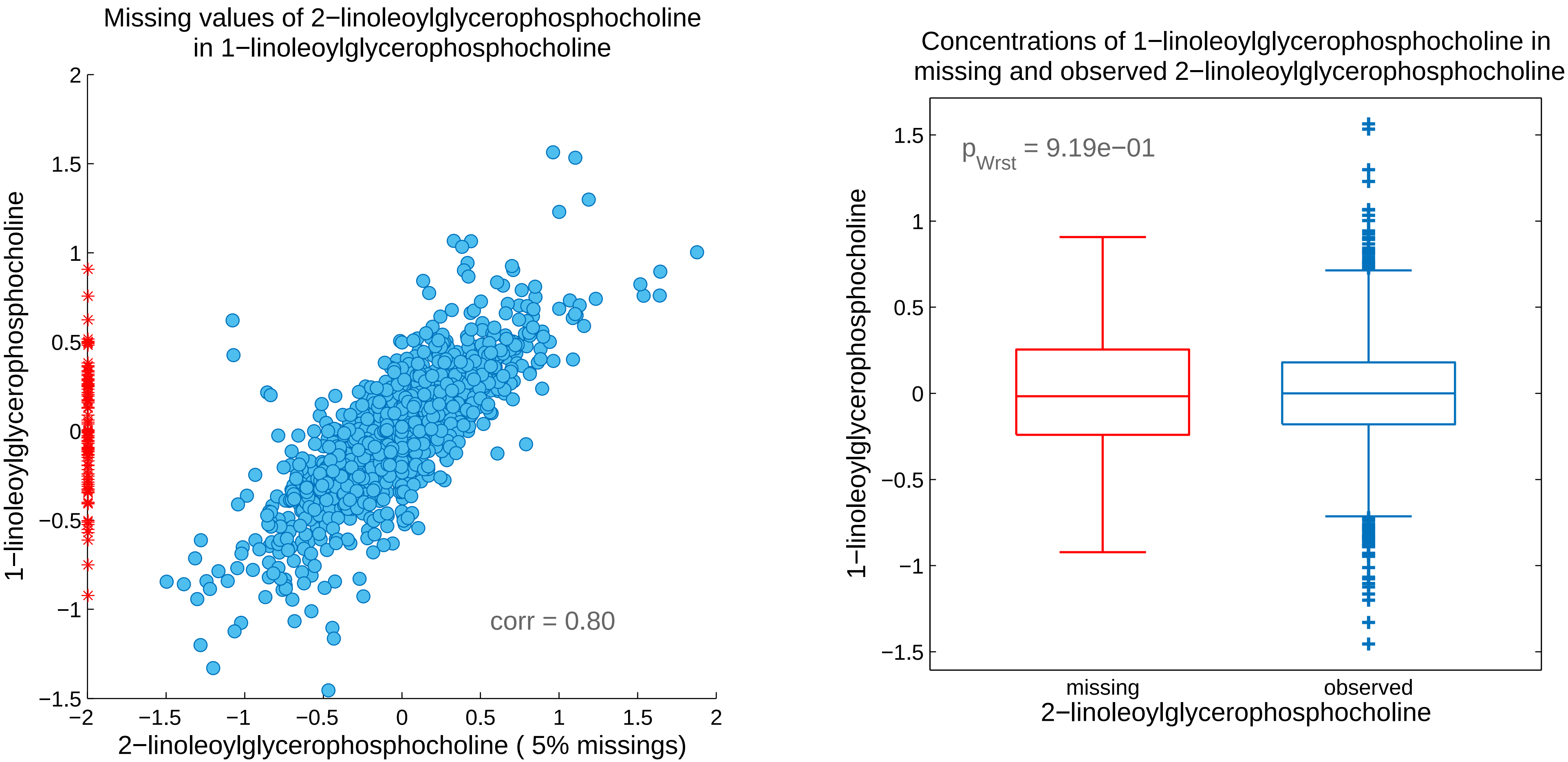

Missing values of 2−linoleoylglycerophosphocholine
in 1−linoleoylglycerophosphocholine
1−linoleoylglycerophosphocholine
2−linoleoylglycerophosphocholine (5% missings)
corr = 0.80
2
1.5
1
0.5
0
−0.5
−1
−1.5
−2
−1.5
−1
−0.5
0
0.5
1
1.5
2
Concentrations of 1−linoleoylglycerophosphocholine in
missing and observed 2−linoleoylglycerophosphocholine
p_Wrst = 9.19e−01
1−linoleoylglycerophosphocholine
1.5
1
0.5
0
−0.5
−1
−1.5
missing
observed
2−linoleoylglycerophosphocholine

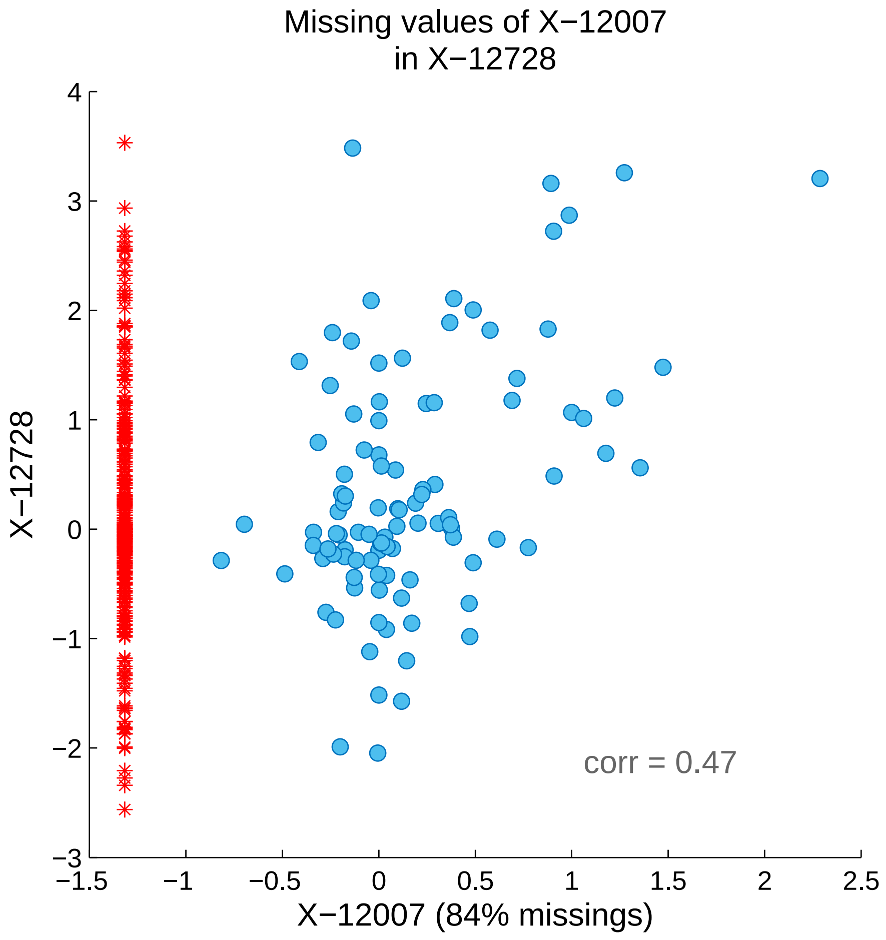

Missing values of X−12007
in X−12728
X−12728
X−12007 (84% missings)
corr = 0.47

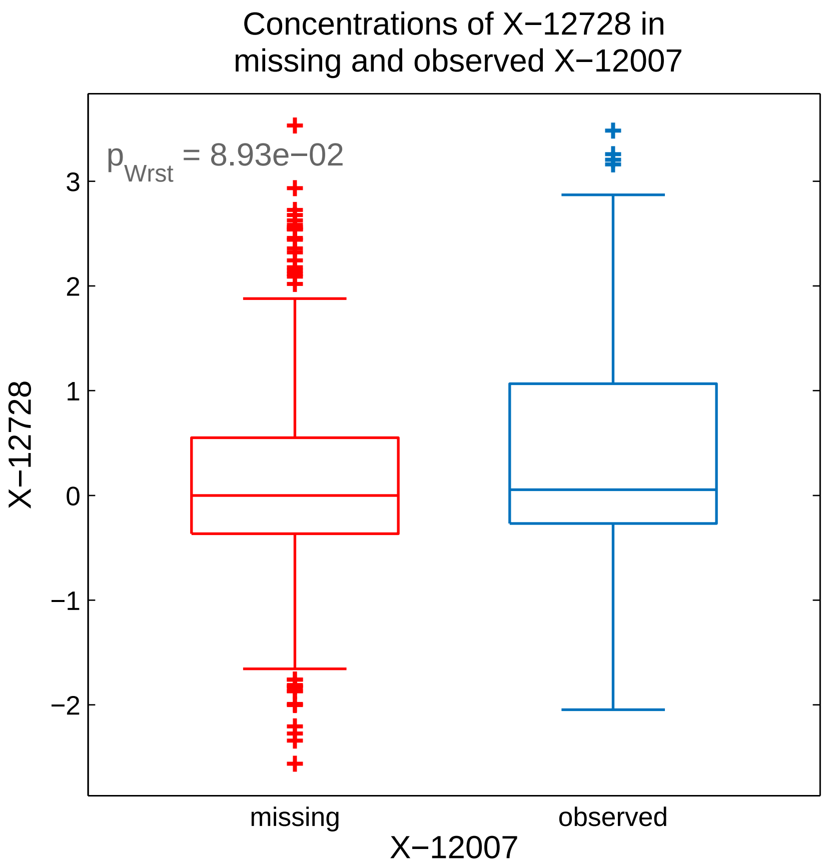

Concentrations of X−12728 in
missing and observed X−12007
p_{Wrst} = 8.93e−02
X−12728
missing
observed
X−12007

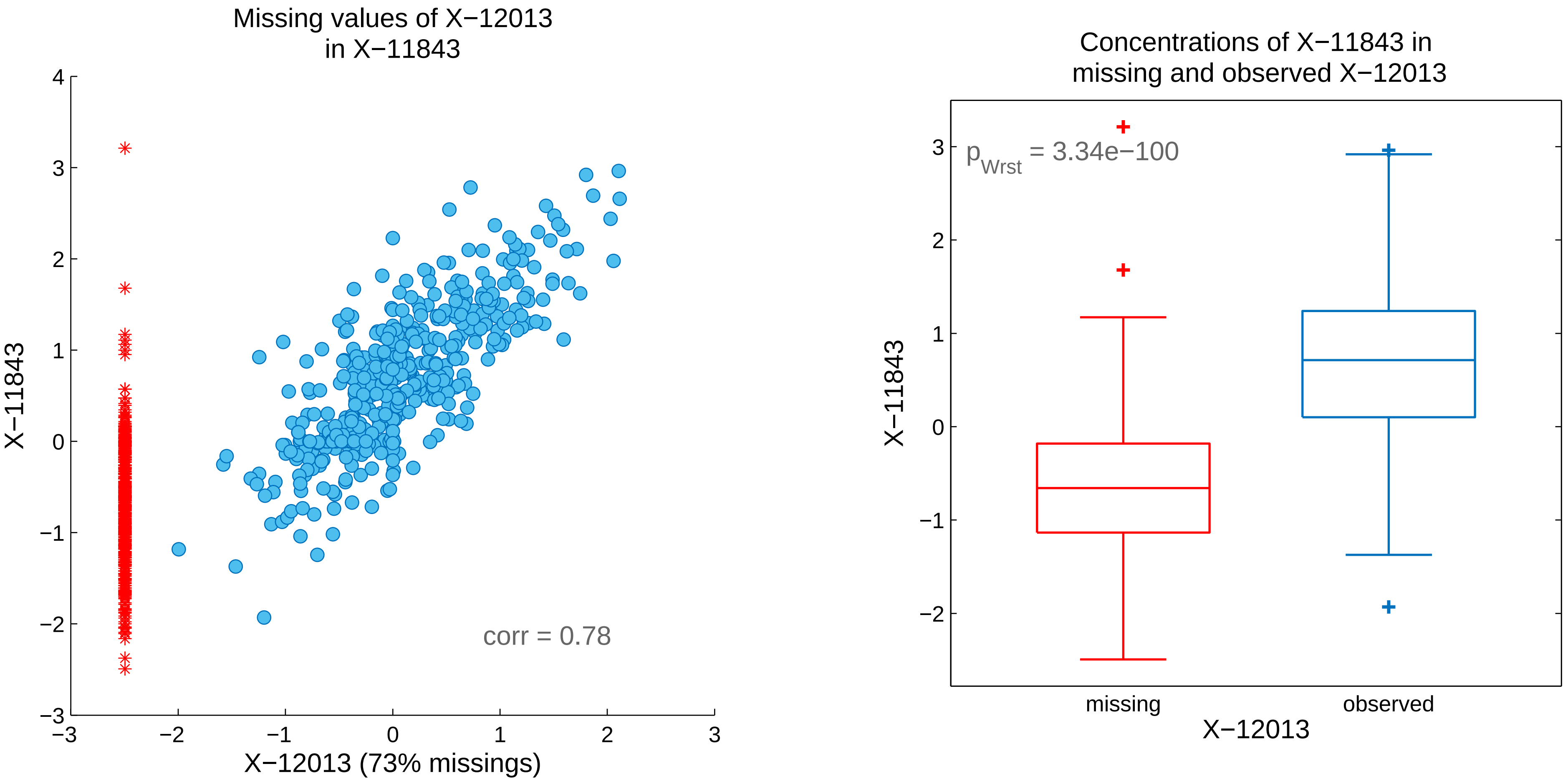
Missing values of X−12013
in X−11843
X−11843
X−12013 (73% missings)
corr = 0.78
Concentrations of X−11843 in
missing and observed X−12013
p_Wrst = 3.34e−100
missing
observed
X−12013

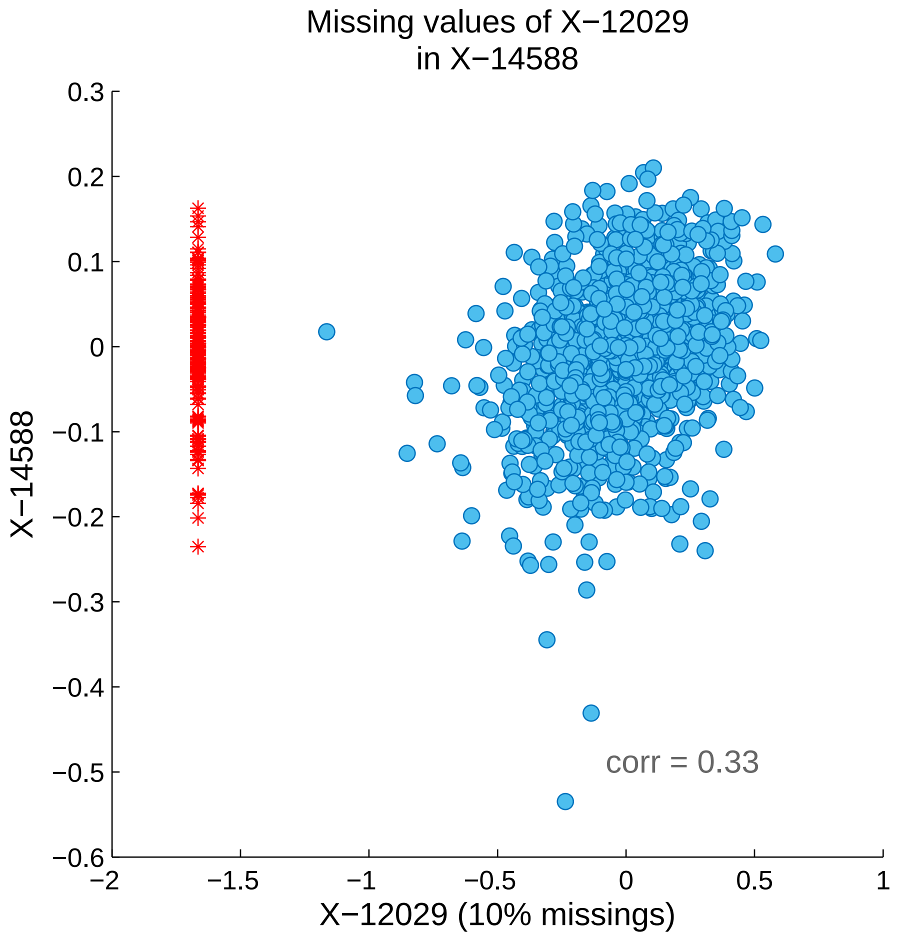
Missing values of X−12029
in X−14588
X−14588
X−12029 (10% missings)
corr = 0.33

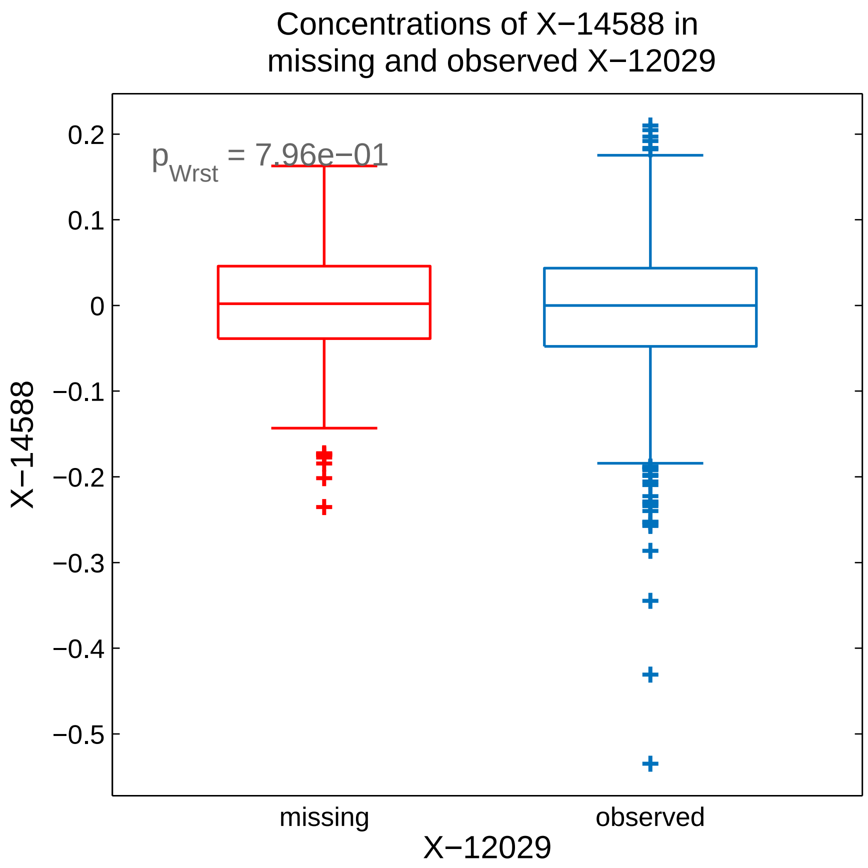
Concentrations of X−14588 in
missing and observed X−12029
p_{Wrst} = 7.96e−01
X−14588
missing
observed
X−12029

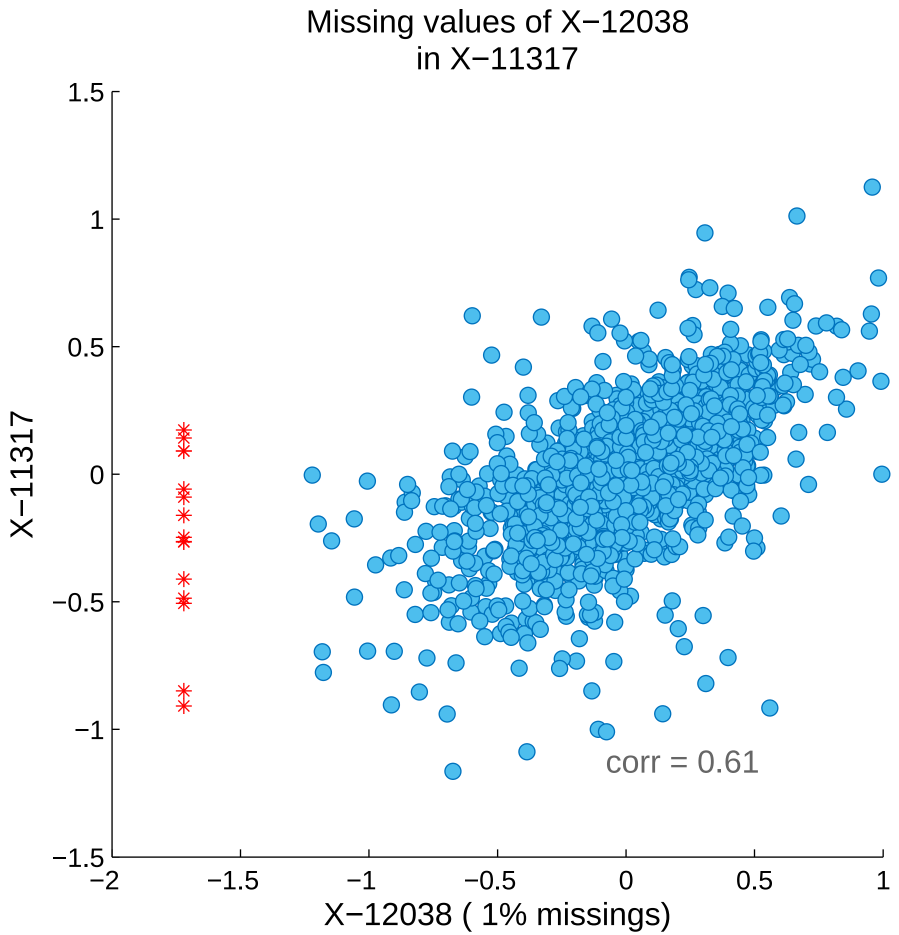

Missing values of X−12038
in X−11317
1.5
1
0.5
0
−0.5
−1
−1.5
X−11317
−2
−1.5
−1
−0.5
0
0.5
1
corr = 0.61
X−12038 (1% missings)

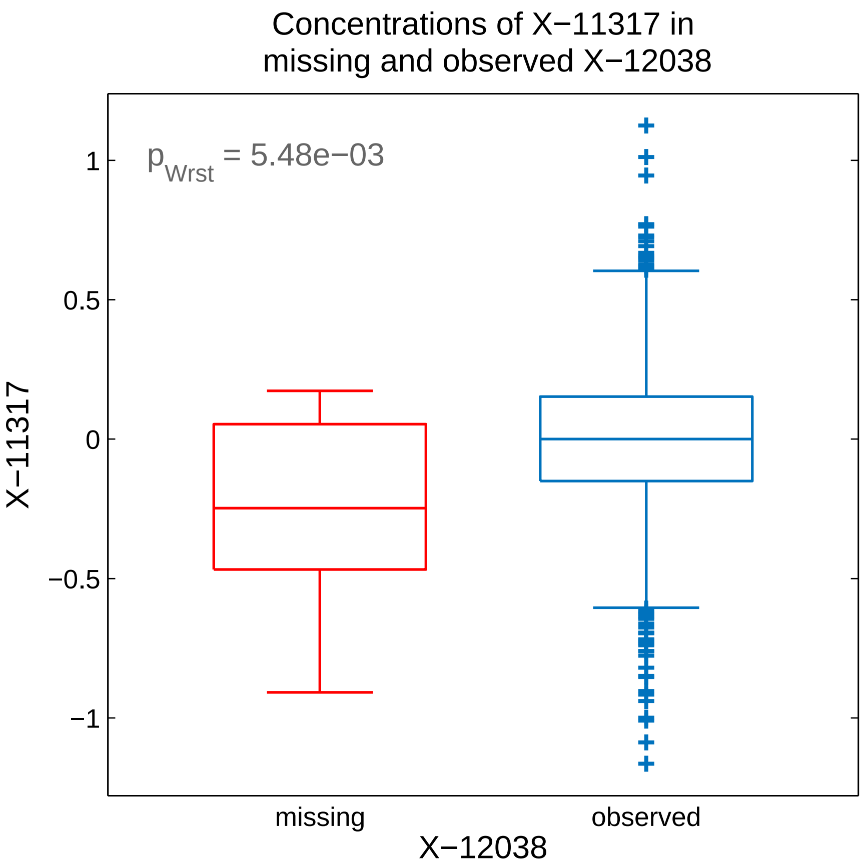

Concentrations of X−11317 in
missing and observed X−12038
p_{Wrst} = 5.48e−03
1
0.5
0
−0.5
−1
X−11317
missing
observed
X−12038

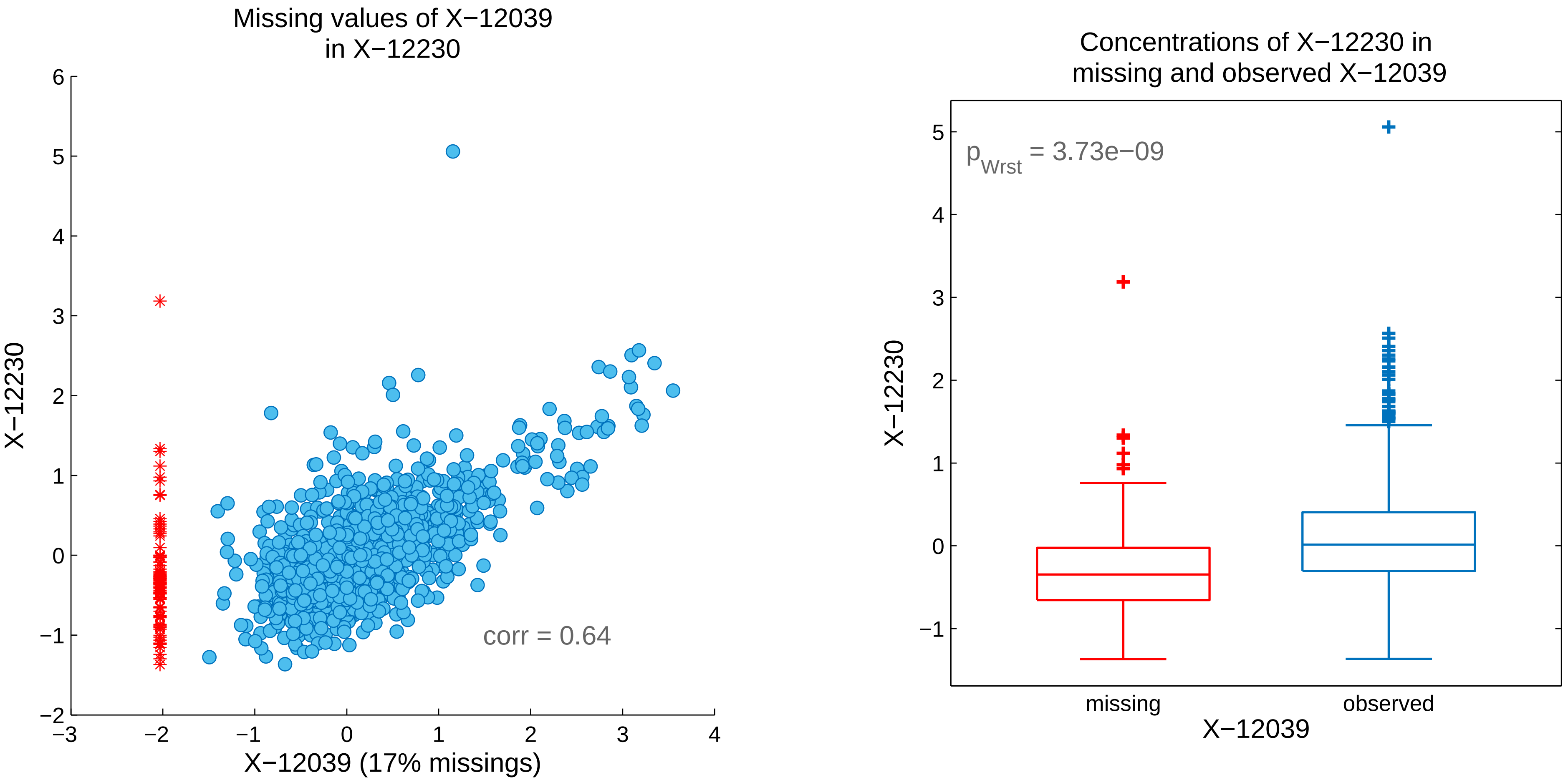
Missing values of X−12039
in X−12230
corr = 0.64
X−12230
X−12039 (17% missings)
−3
−2
−1
0
1
2
3
4
−2
−1
0
1
2
3
4
5
6
Concentrations of X−12230 in
missing and observed X−12039
p_{Wrst} = 3.73e−09
X−12230
−1
0
1
2
3
4
5
missing
observed
X−12039

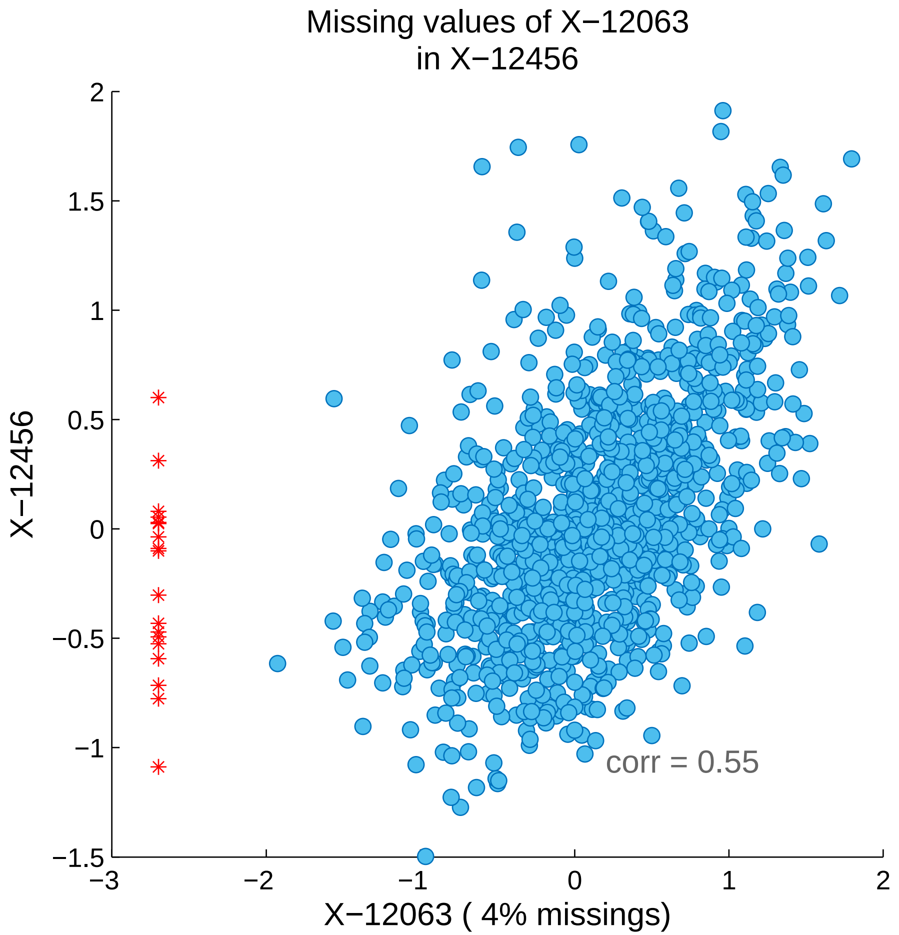

Missing values of X−12063
in X−12456
X−12456
X−12063 (4% missings)
corr = 0.55
2
1.5
1
0.5
0
−0.5
−1
−1.5
−3
−2
−1
0
1
2

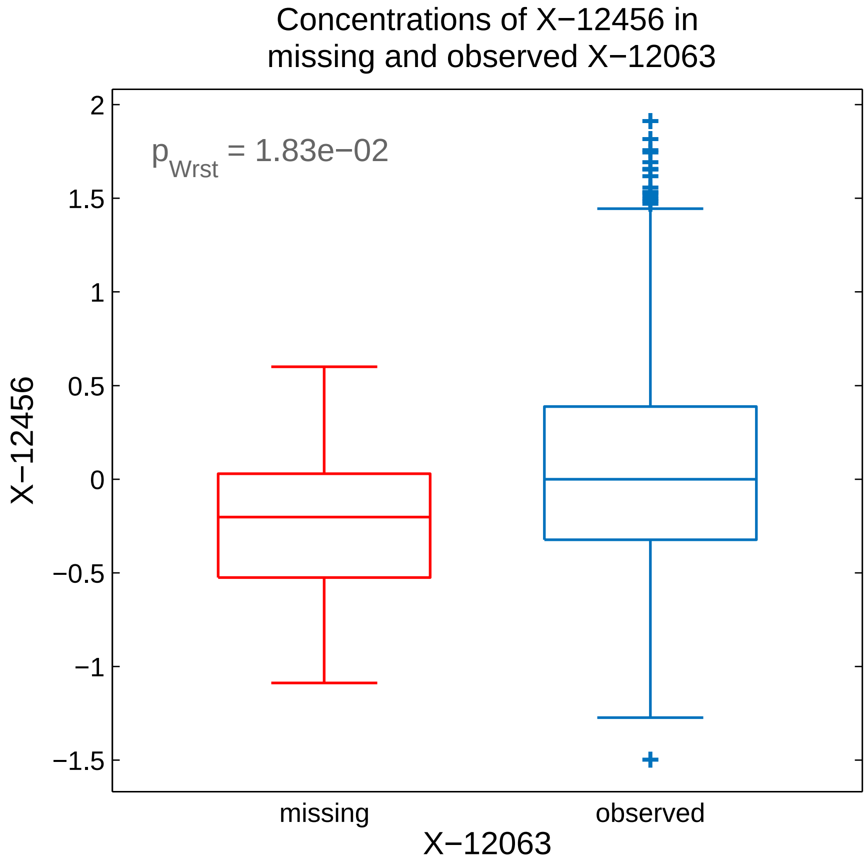

Concentrations of X−12456 in
missing and observed X−12063
p_{Wrst} = 1.83e−02
X−12456
missing
observed
X−12063
2
1.5
1
0.5
0
−0.5
−1
−1.5

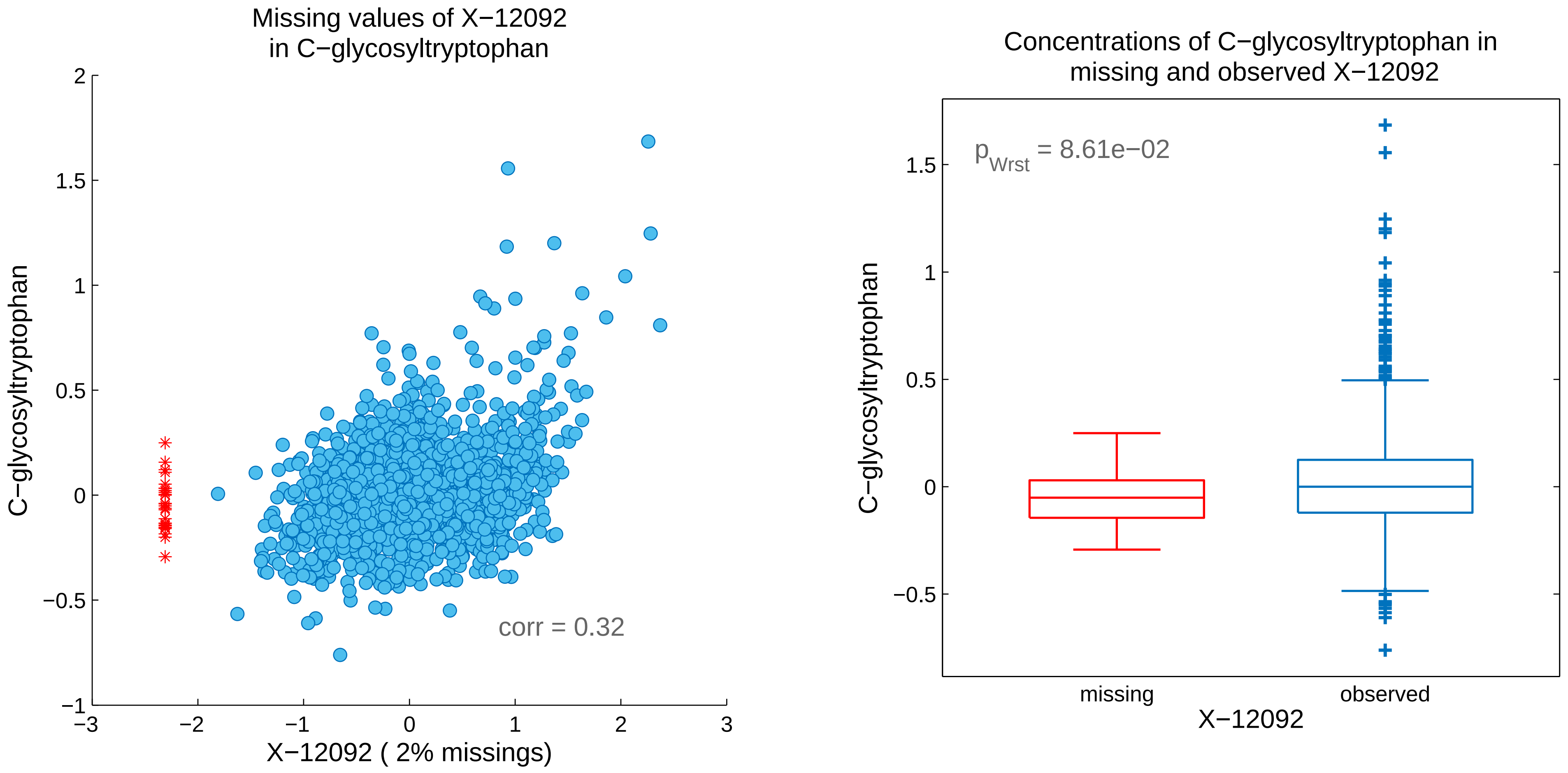
Missing values of X−12092
in C−glycosylttryptophan
C−glycosyltryptophan
X−12092 (2% missings)
corr = 0.32
Concentrations of C−glycosyltryptophan in
missing and observed X−12092
p_{Wrst} = 8.61e−02
missing
observed
X−12092

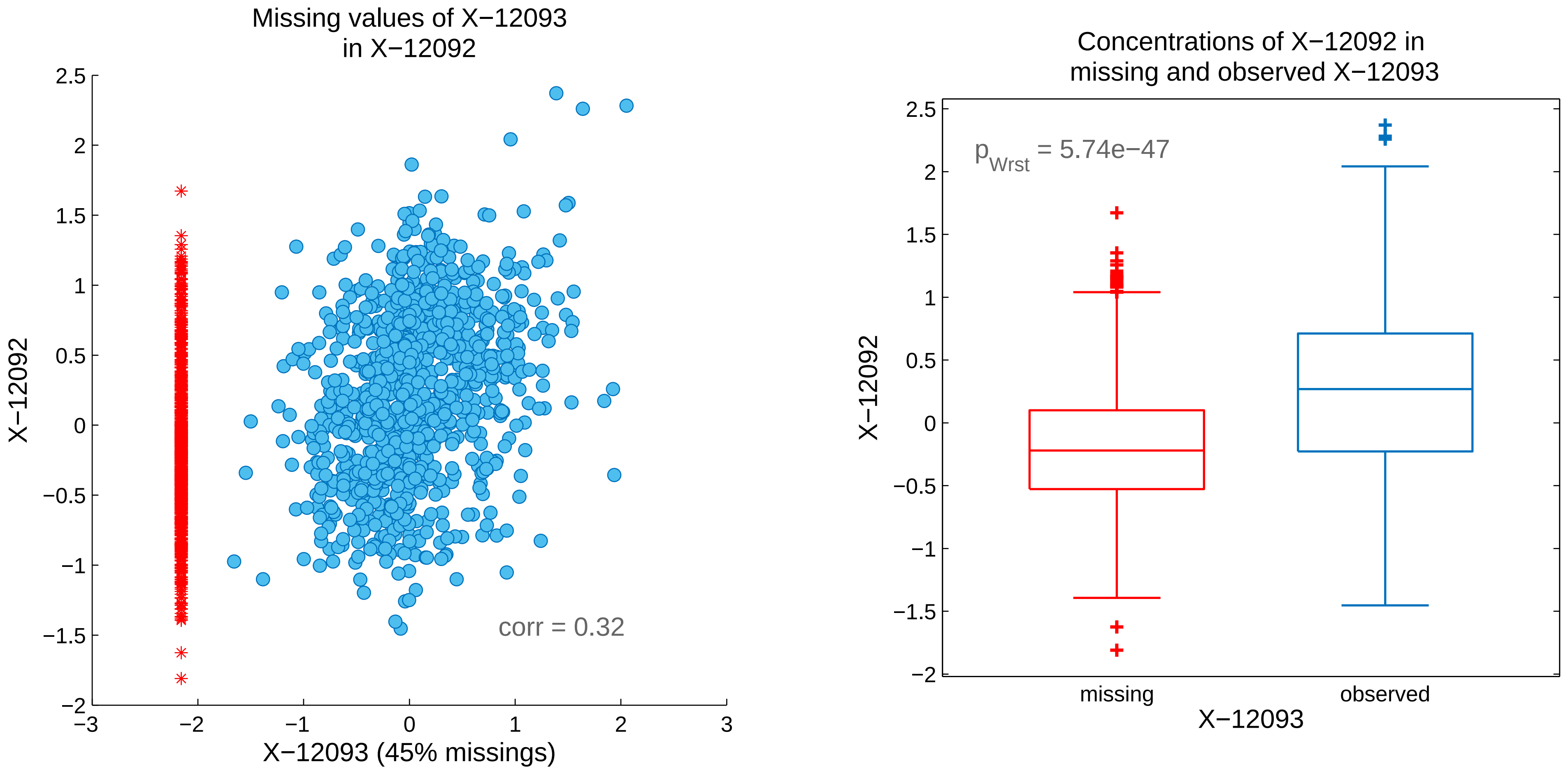
Missing values of X−12093
in X−12092
X−12092
X−12093 (45% missings)
corr = 0.32
Concentrations of X−12092 in
missing and observed X−12093
p_{Wrst} = 5.74e−47
X−12092
missing
observed
X−12093

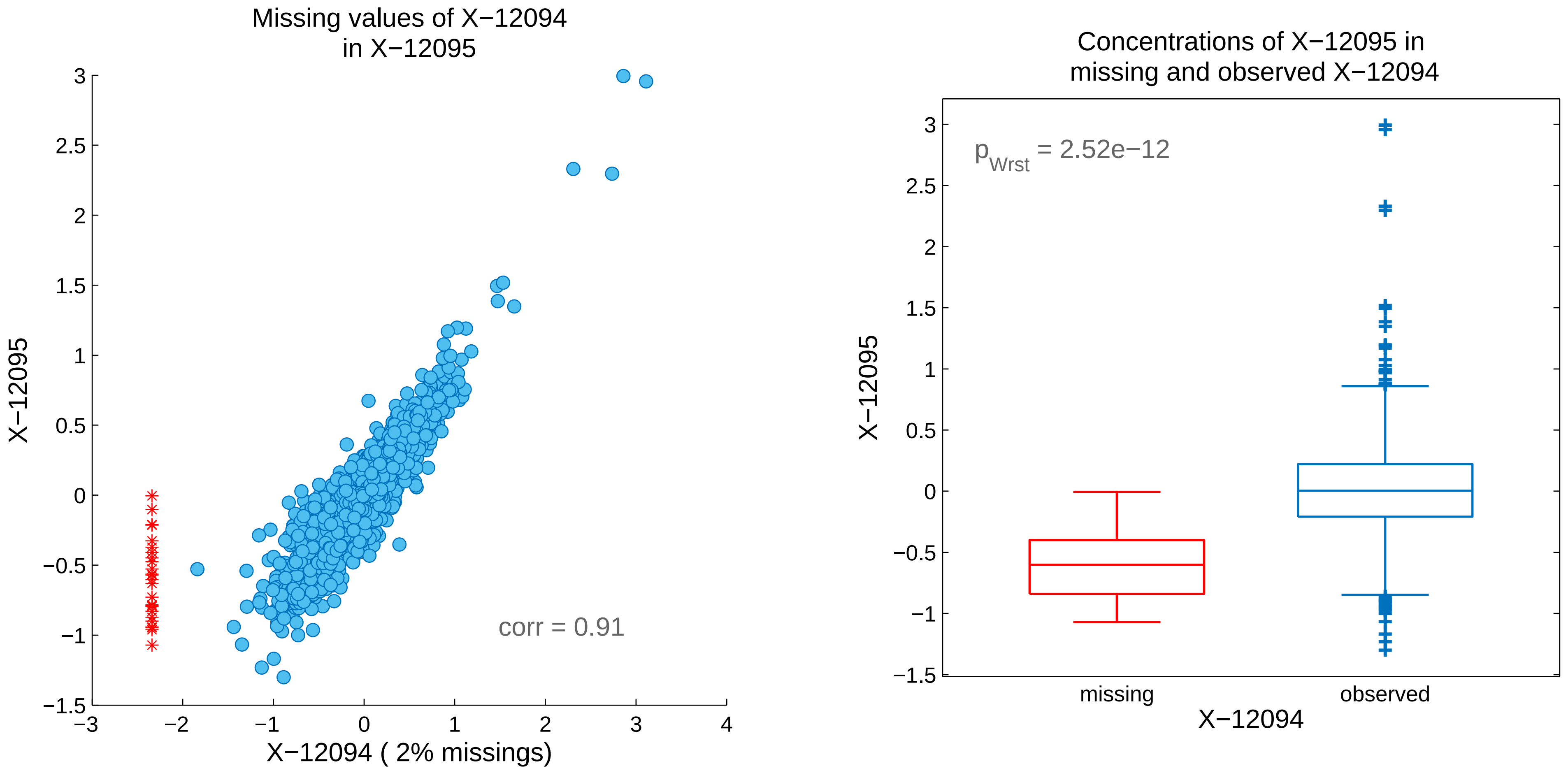
Missing values of X−12094
in X−12095
X−12095
X−12094 (2% missings)
corr = 0.91
Concentrations of X−12095 in
missing and observed X−12094
p_{Wrst} = 2.52e−12
X−12095
missing
observed
X−12094

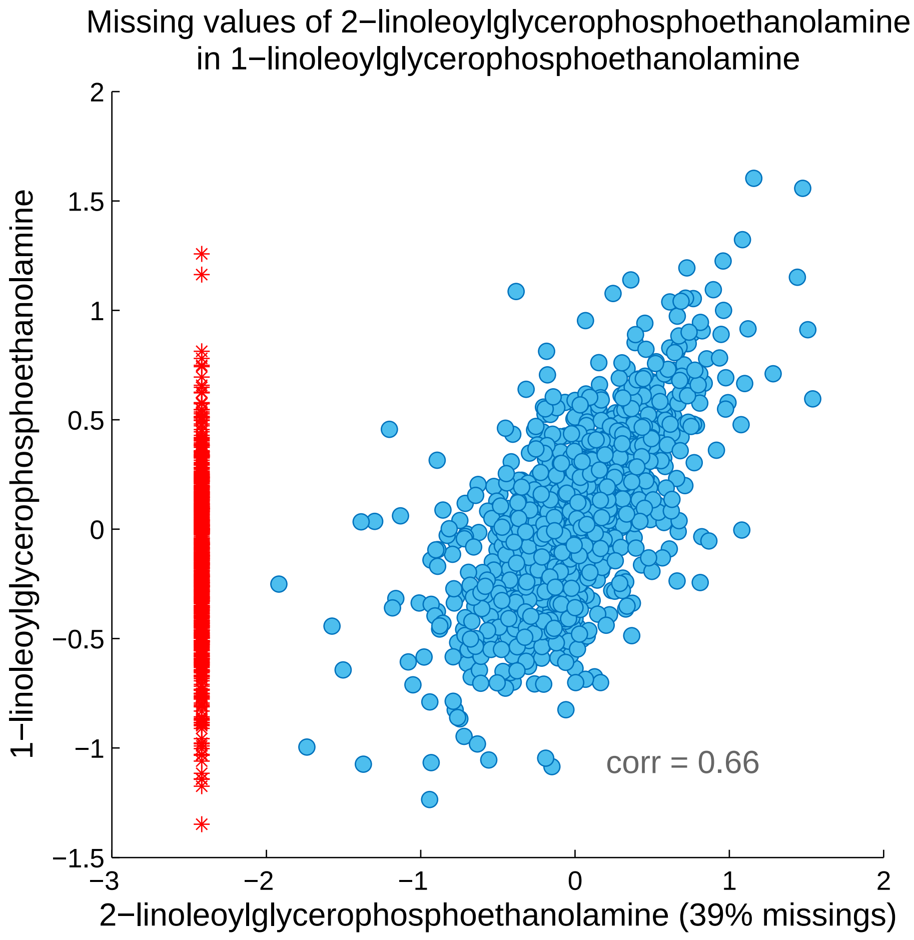
Missing values of 2−linoleoylglycerophosphoethanolamine in 1−linoleoylglycerophosphoethanolamine
1−linoleoylglycerophosphoethanolamine
2−linoleoylglycerophosphoethanolamine (39% missings)
corr = 0.66
2
1.5
1
0.5
0
−0.5
−1
−1.5
−3
−2
−1
0
1
2

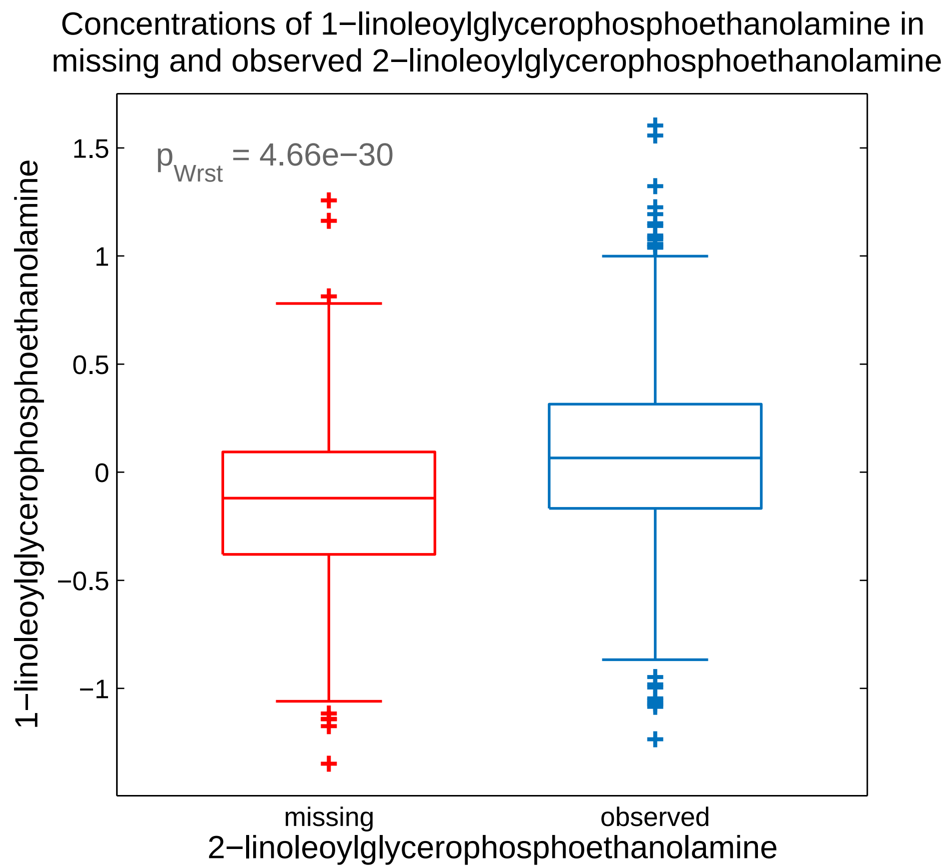
Concentrations of 1−linoleoylglycerophosphoethanolamine in missing and observed 2−linoleoylglycerophosphoethanolamine
p_Wrst = 4.66e−30
1−linoleoylglycerophosphoethanolamine
missing
observed
2−linoleoylglycerophosphoethanolamine
1.5
1
0.5
0
−0.5
−1

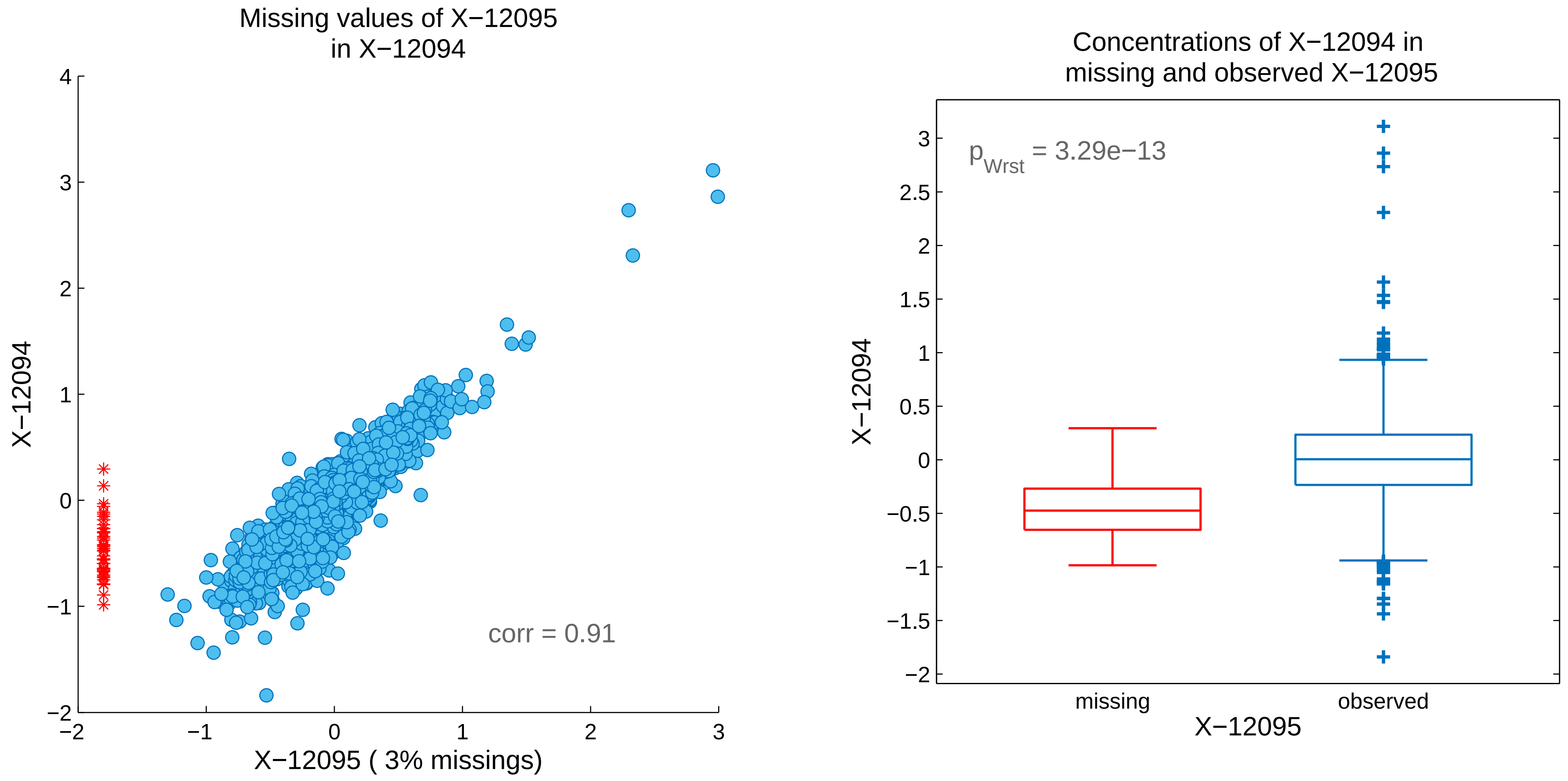

Missing values of X−12095
in X−12094
X−12094
X−12095 (3% missings)
corr = 0.91
Concentrations of X−12094 in
missing and observed X−12095
p_{Wrst} = 3.29e−13
X−12094
missing
observed
X−12095

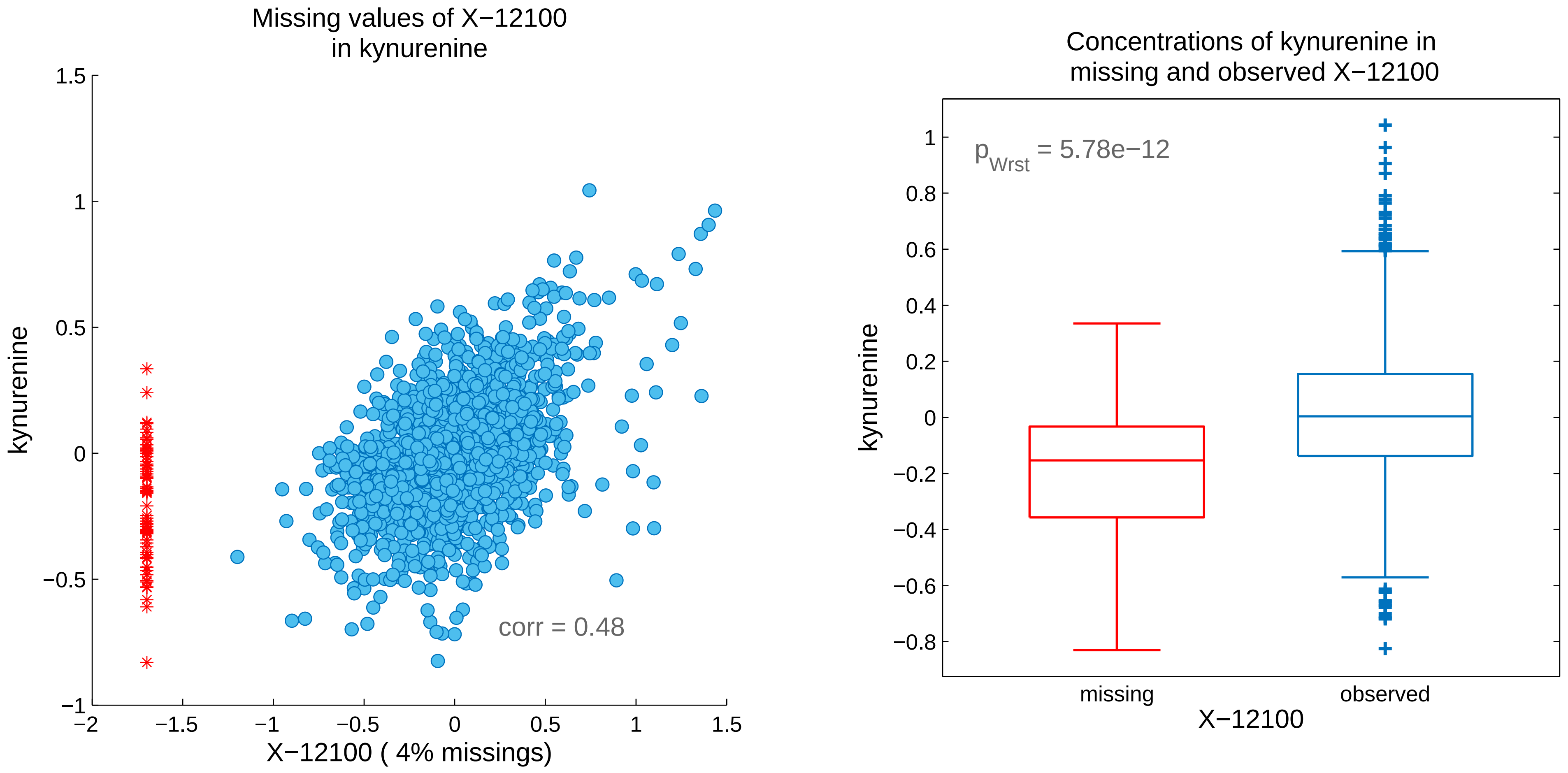

Missing values of X−12100
in kynurenine
1.5
1
0.5
0
−0.5
−1
kynurenine
−2
−1.5
−1
−0.5
0
0.5
1
1.5
X−12100 (4% missings)
corr = 0.48
Concentrations of kynurenine in
missing and observed X−12100
p_{Wrst} = 5.78e−12
1
0.8
0.6
0.4
0.2
0
−0.2
−0.4
−0.6
−0.8
kynurenine
missing
observed
X−12100

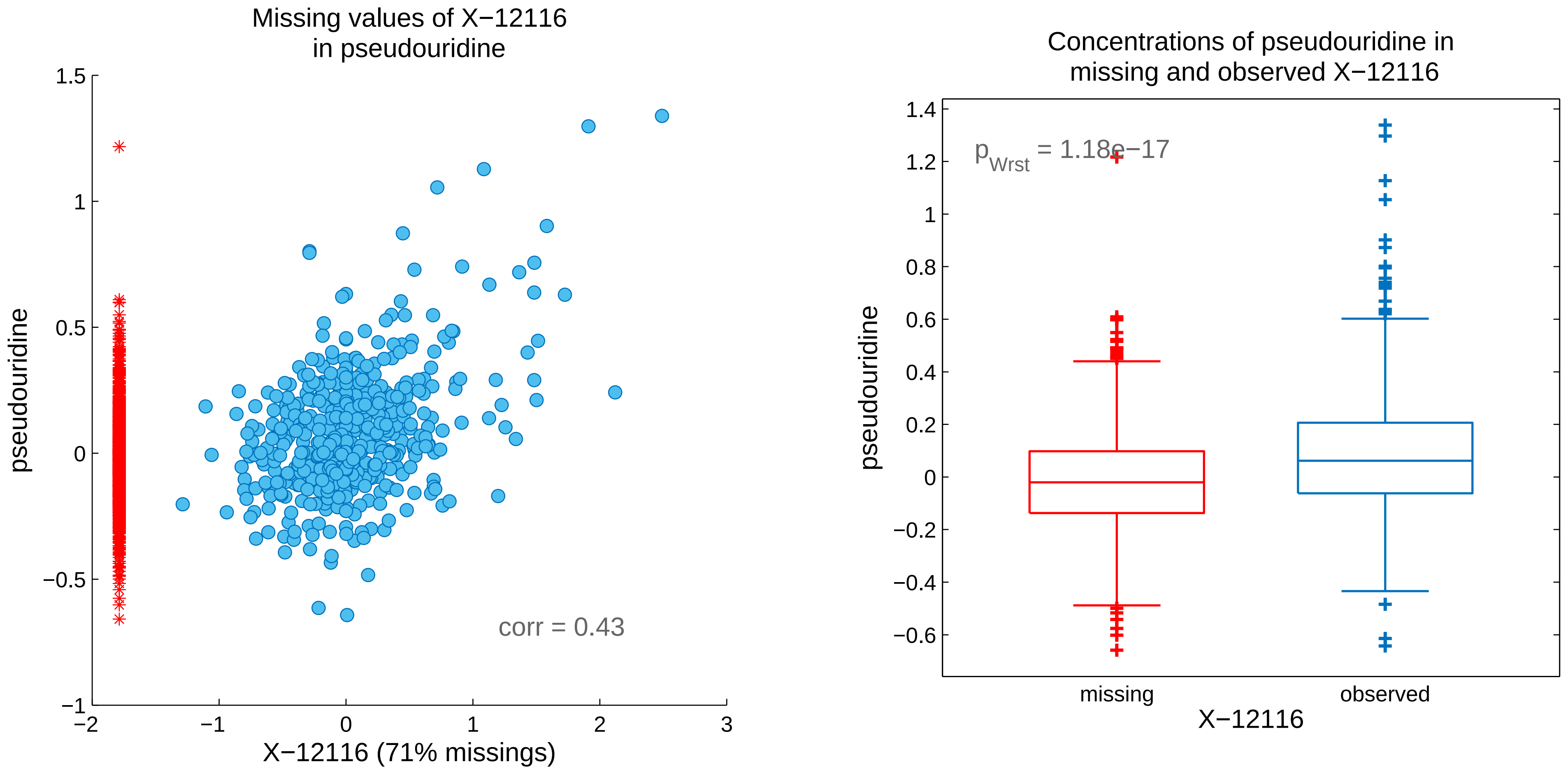

Missing values of X−12116
in pseudouridine
pseudouridine
X−12116 (71% missings)
corr = 0.43
Concentrations of pseudouridine in
missing and observed X−12116
pseudouridine
p_{Wrst} = 1.18e−17
missing
observed
X−12116

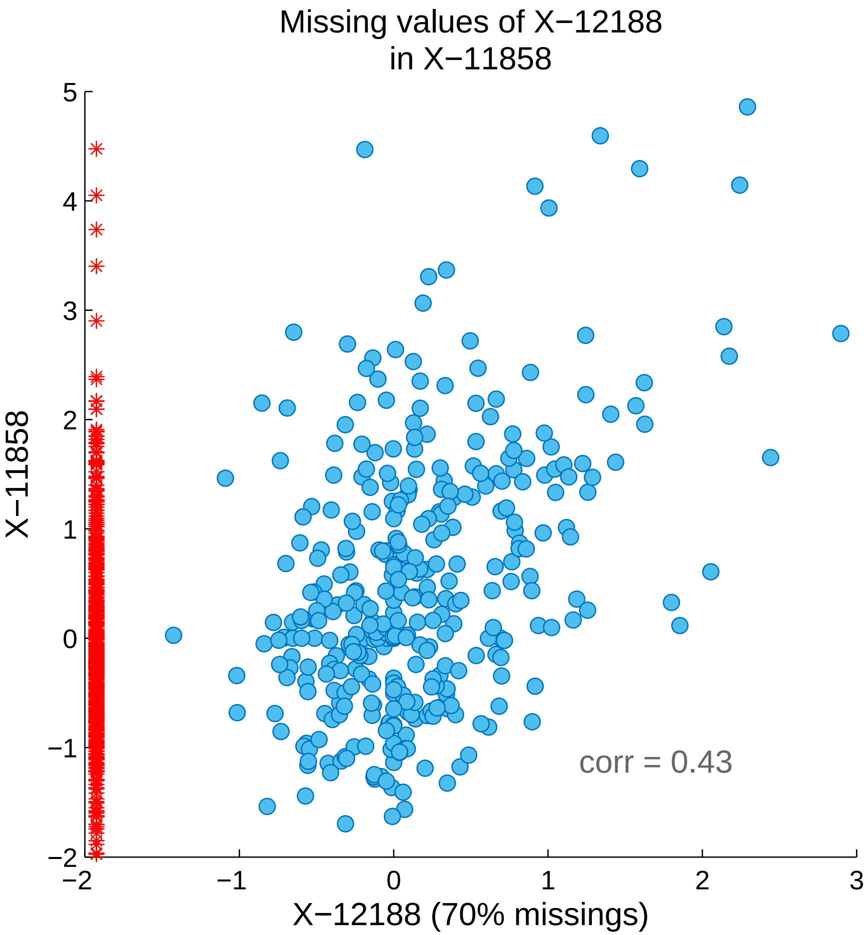
Missing values of X−12188
in X−11858
X−11858
X−12188 (70% missings)
corr = 0.43

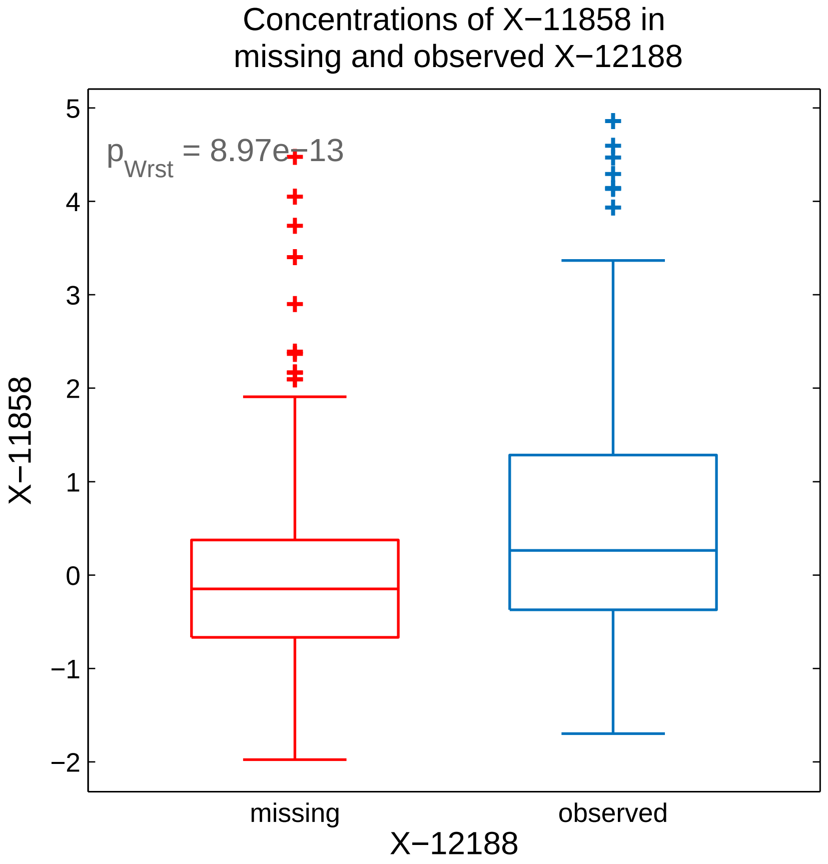
Concentrations of X−11858 in
missing and observed X−12188
p_{Wrst} = 8.97e−13
X−11858
missing
observed
X−12188

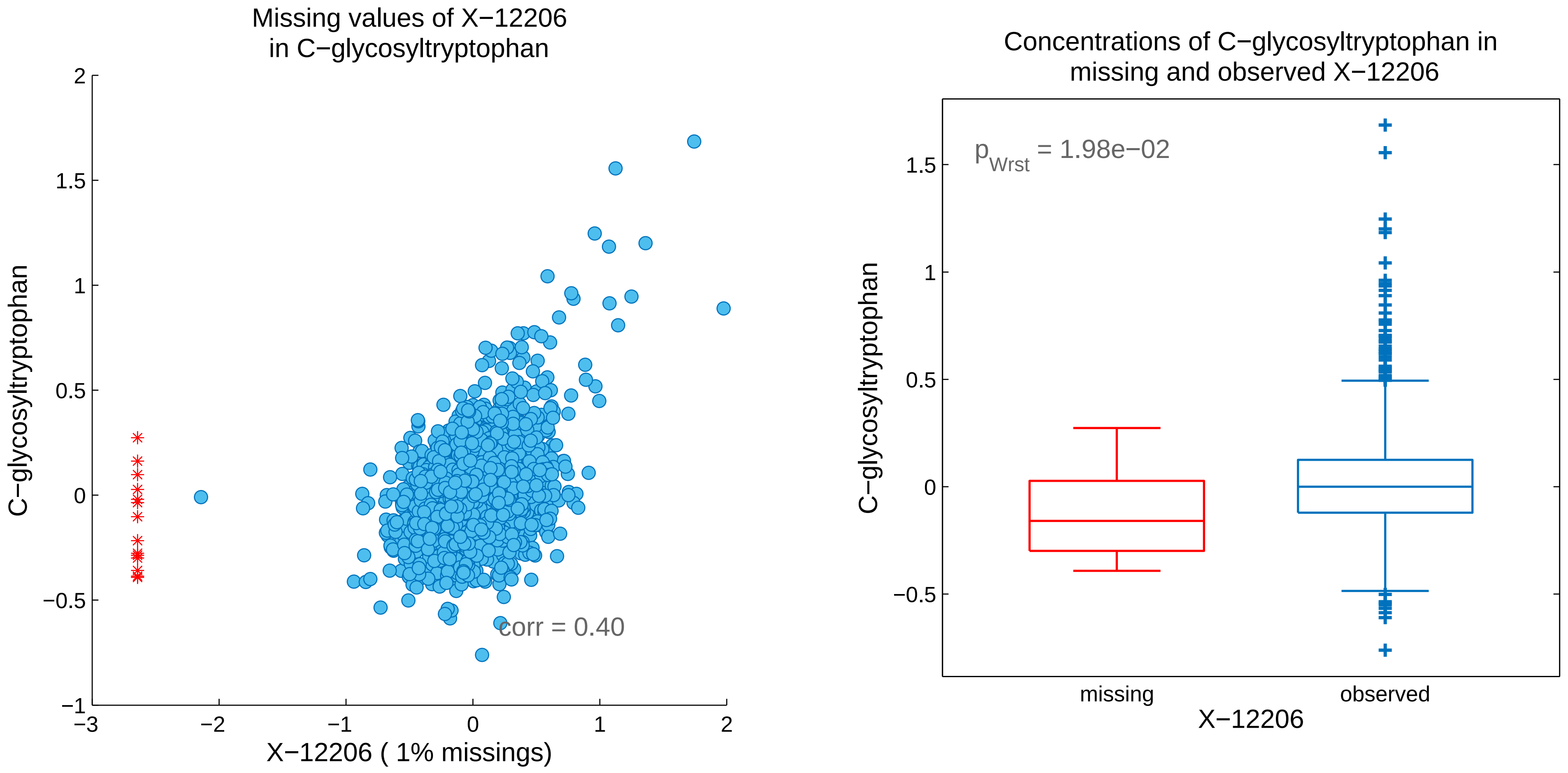
Missing values of X−12206
in C−glycosyltryptophan
C−glycosyltryptophan
X−12206 (1% missings)
corr = 0.40
Concentrations of C−glycosyltryptophan in
missing and observed X−12206
p_Wrst = 1.98e−02
C−glycosyltryptophan
missing
observed
X−12206

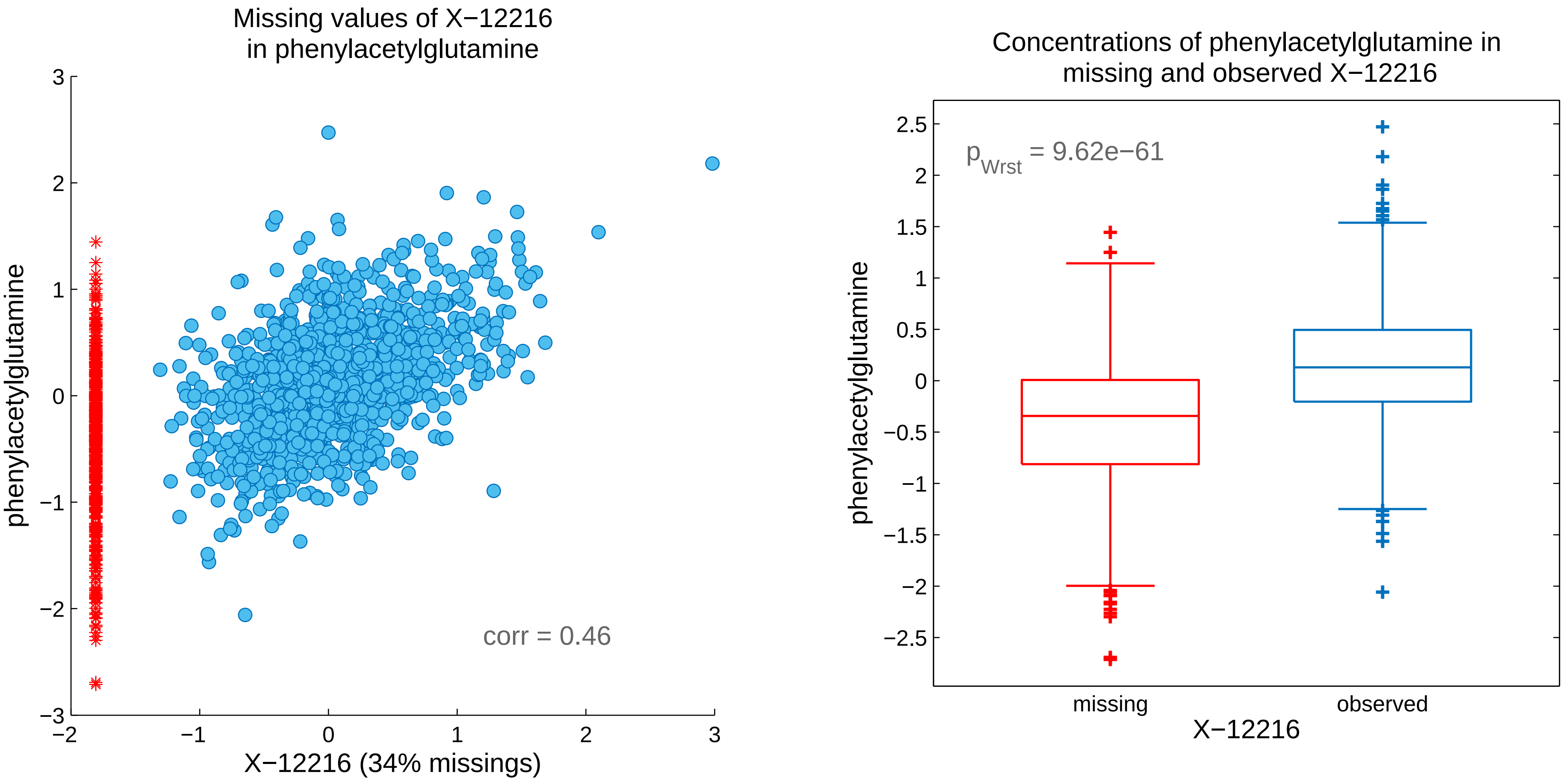
Missing values of X−12216
in phenylacetylglutamine
phenylacetylglutamine
X−12216 (34% missings)
corr = 0.46
Concentrations of phenylacetylglutamine in
missing and observed X−12216
phenylacetylglutamine
p_Wrst = 9.62e−61
missing
observed
X−12216

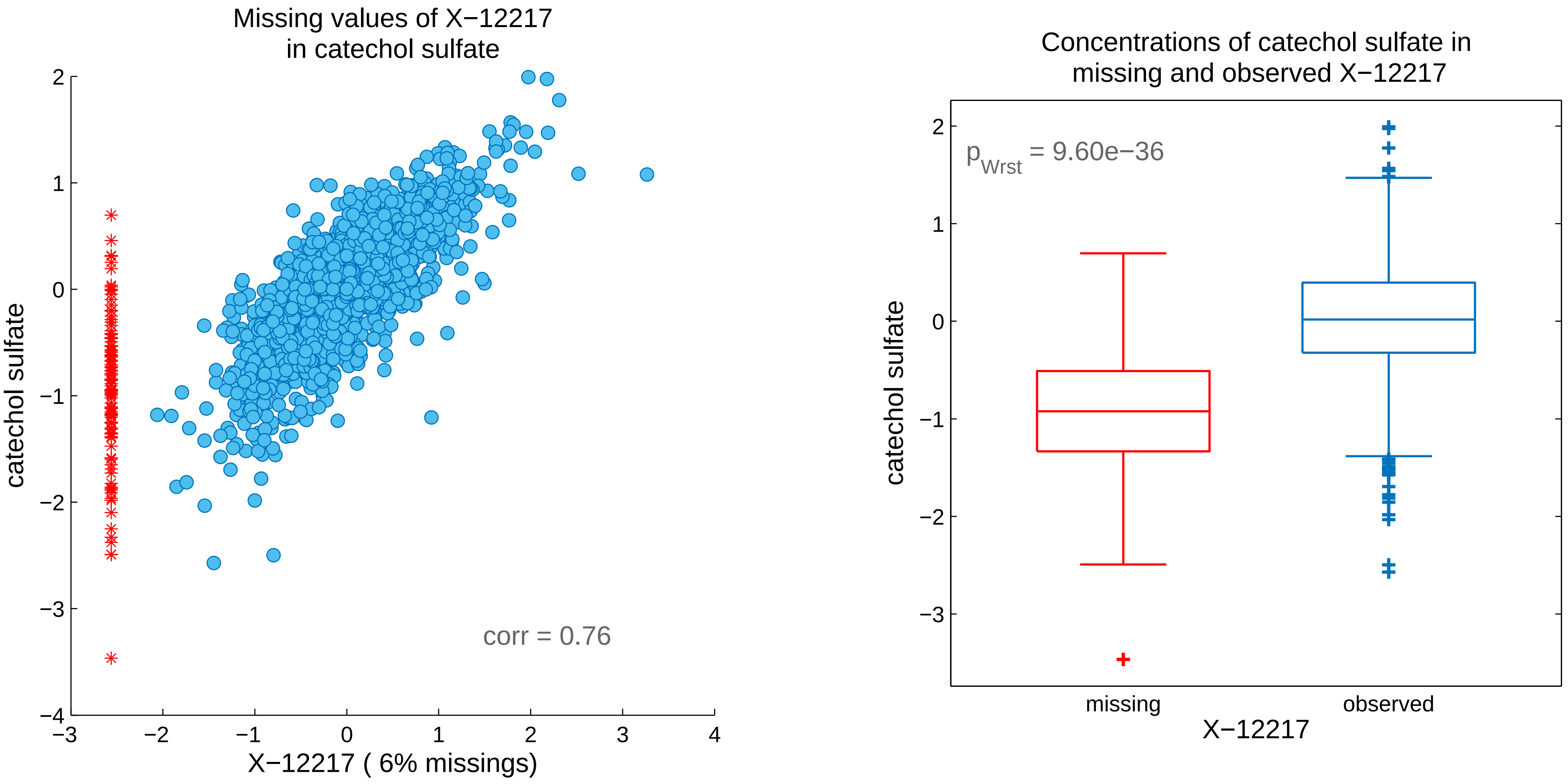
Missing values of X−12217
in catechol sulfate
catechol sulfate
corr = 0.76
X−12217 (6% missings)
Concentrations of catechol sulfate in
missing and observed X−12217
p_{Wrst} = 9.60e−36
catechol sulfate
missing
observed
X−12217

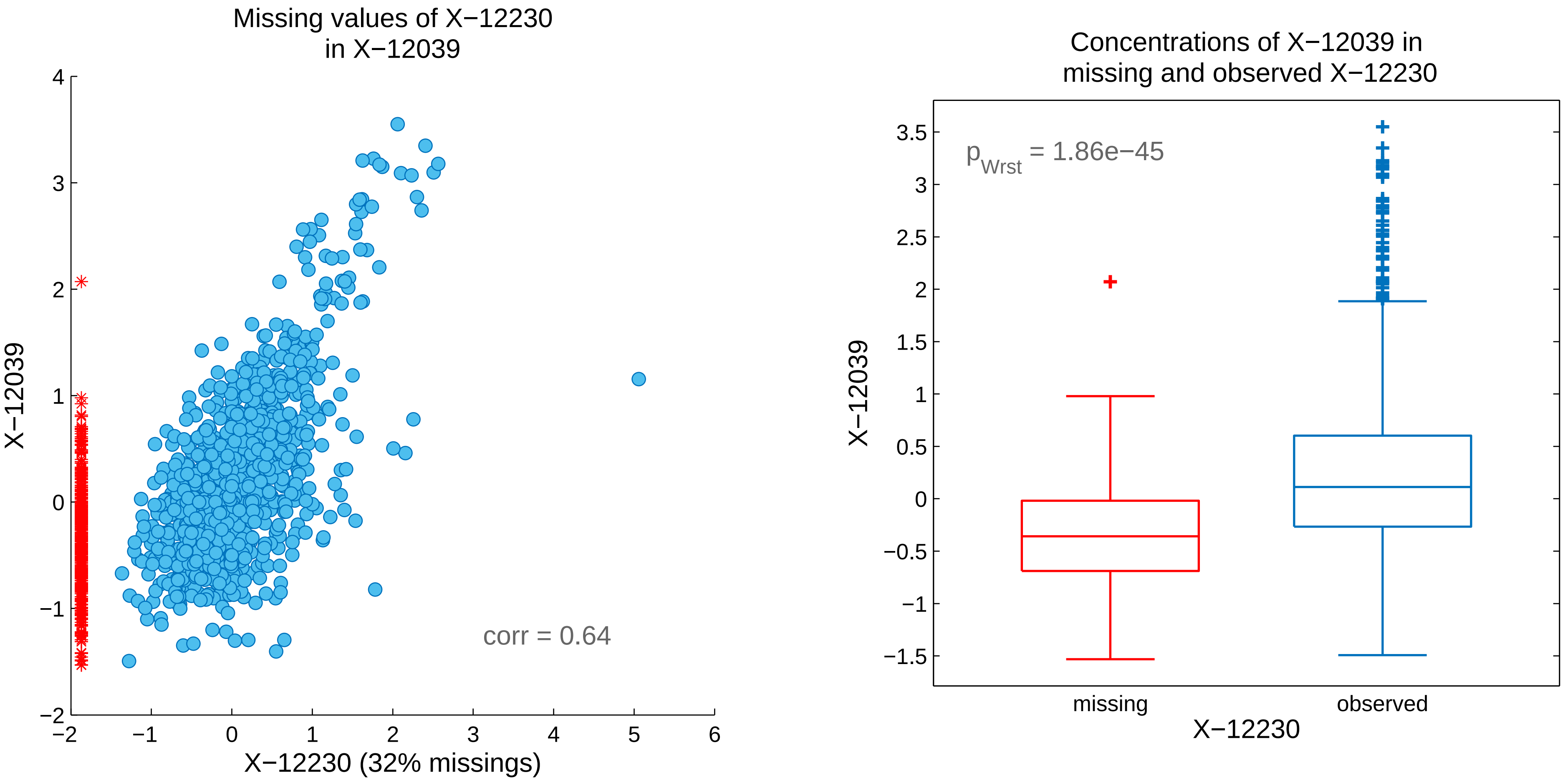

Missing values of X−12230 in X−12039
X−12039
X−12230 (32% missings)
corr = 0.64
Concentrations of X−12039 in missing and observed X−12230
p_{Wrst} = 1.86e−45
X−12039
missing
observed
X−12230

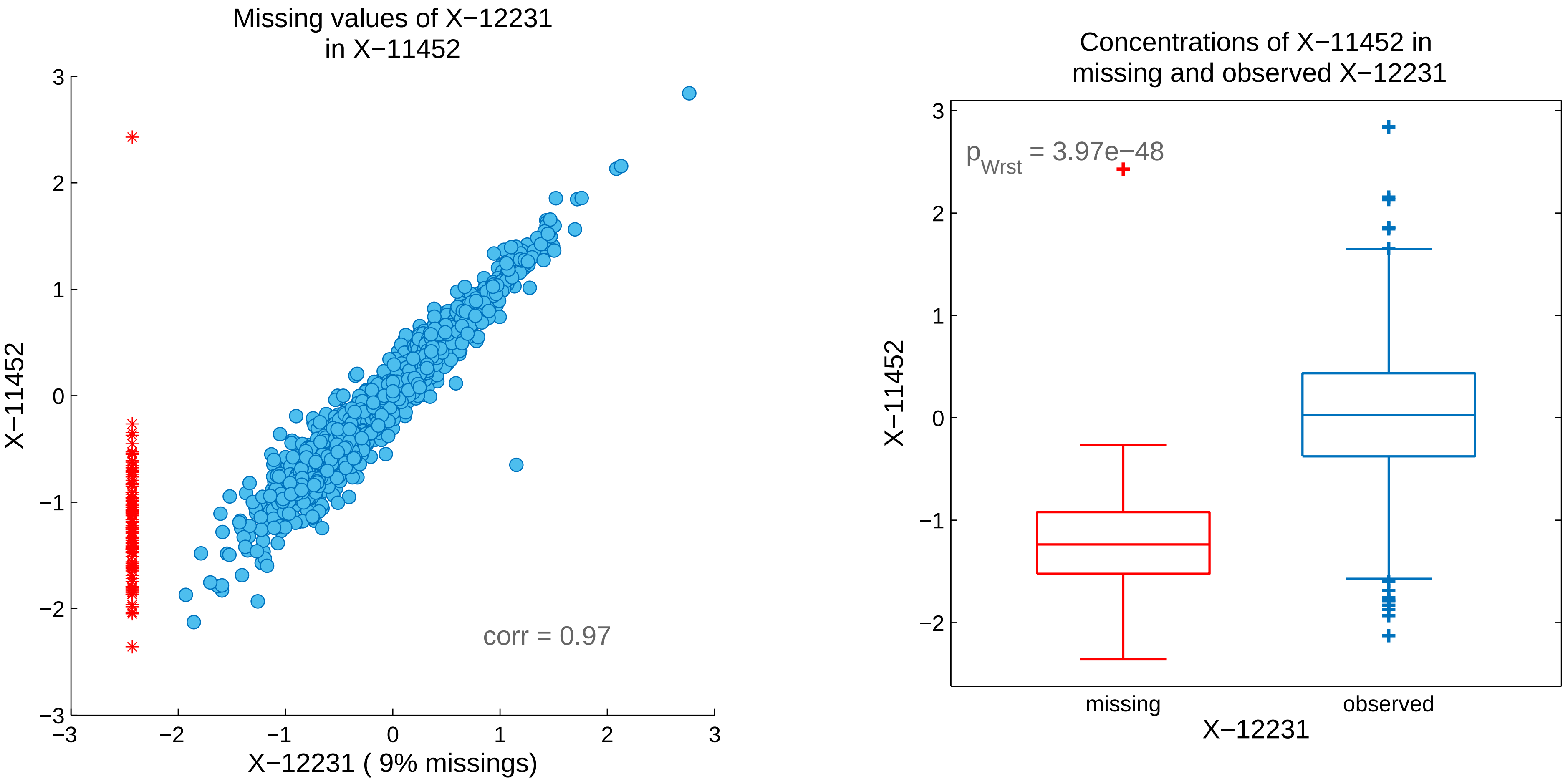
Missing values of X−12231 in X−11452
X−11452
X−12231 (9% missings)
corr = 0.97
Concentrations of X−11452 in missing and observed X−12231
p_{Wrst} = 3.97e−48
X−11452
missing
observed
X−12231

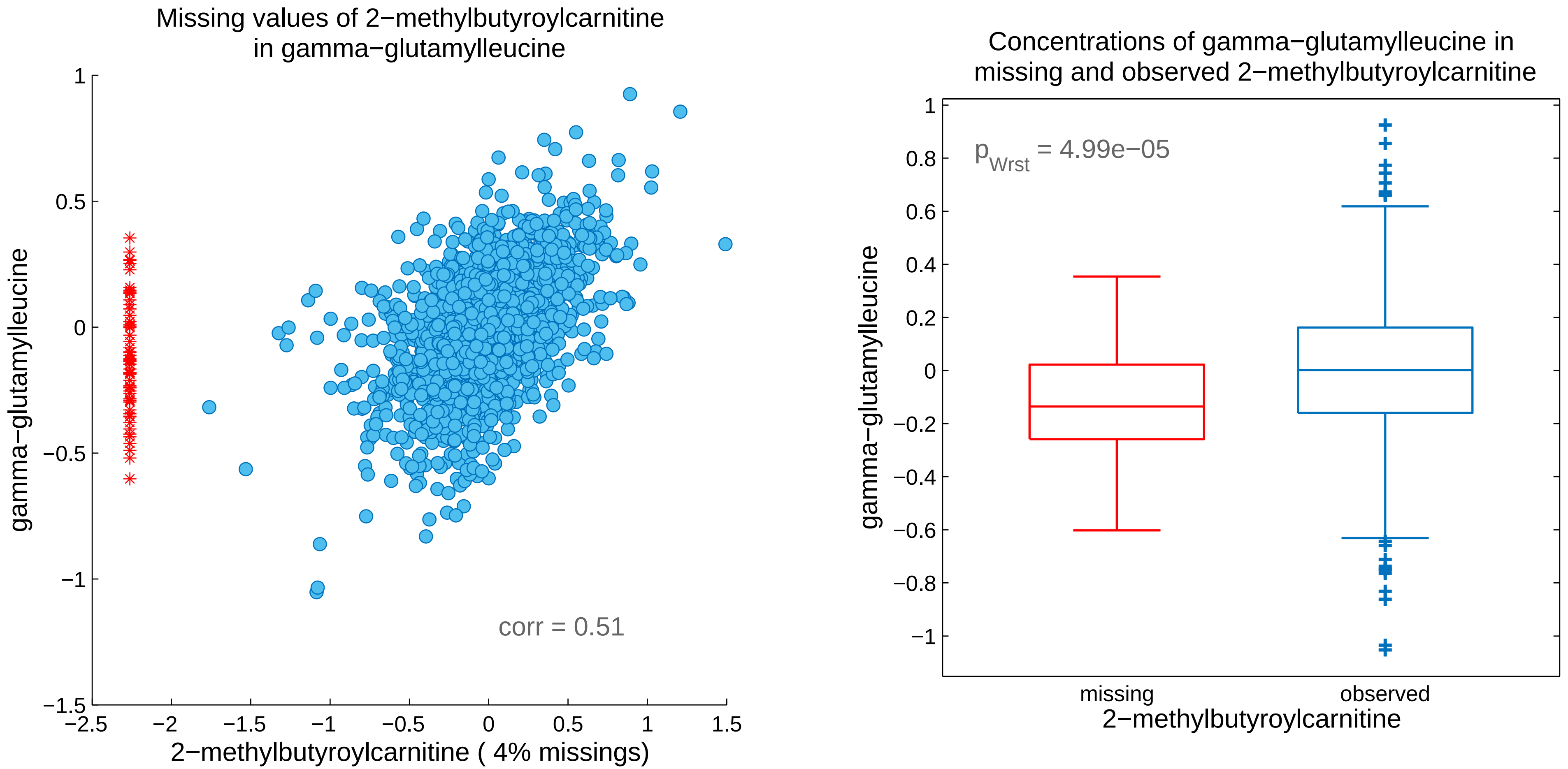

Missing values of 2−methylbutyroylcarnitine in gamma−glutamylleucine
gamma−glutamylleucine
2−methylbutyroylcarnitine (4% missings)
corr = 0.51
1
0.5
0
−0.5
−1
−1.5
−2.5
−2
−1.5
−1
−0.5
0
0.5
1
1.5
Concentrations of gamma−glutamylleucine in missing and observed 2−methylbutyroylcarnitine
p_{Wrst} = 4.99e−05
gamma−glutamylleucine
1
0.8
0.6
0.4
0.2
0
−0.2
−0.4
−0.6
−0.8
−1
missing
observed
2−methylbutyroylcarnitine

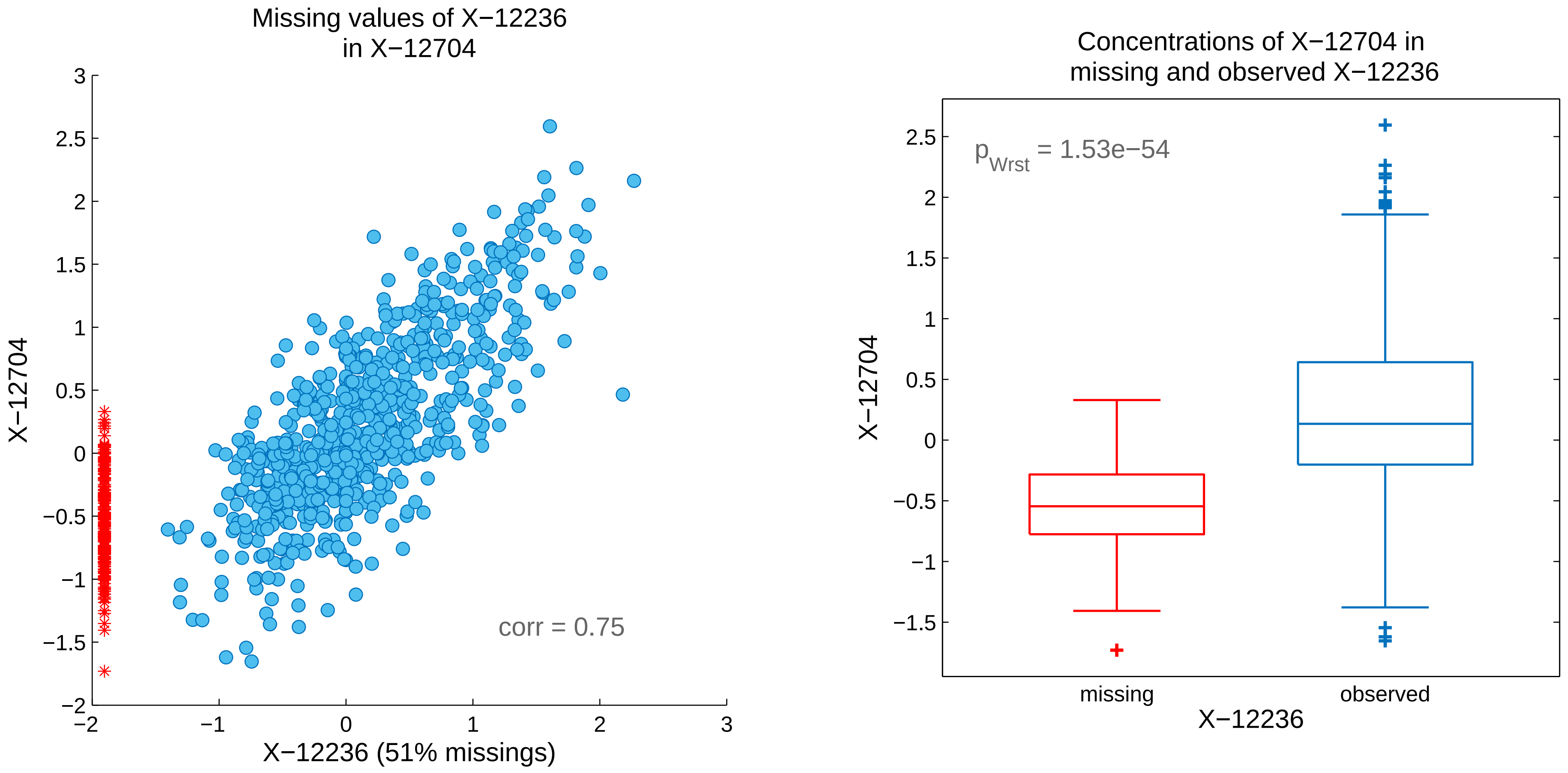

Missing values of X−12236
in X−12704
X−12704
X−12236 (51% missings)
corr = 0.75
Concentrations of X−12704 in
missing and observed X−12236
p_{Wrst} = 1.53e−54
X−12704
missing
observed
X−12236

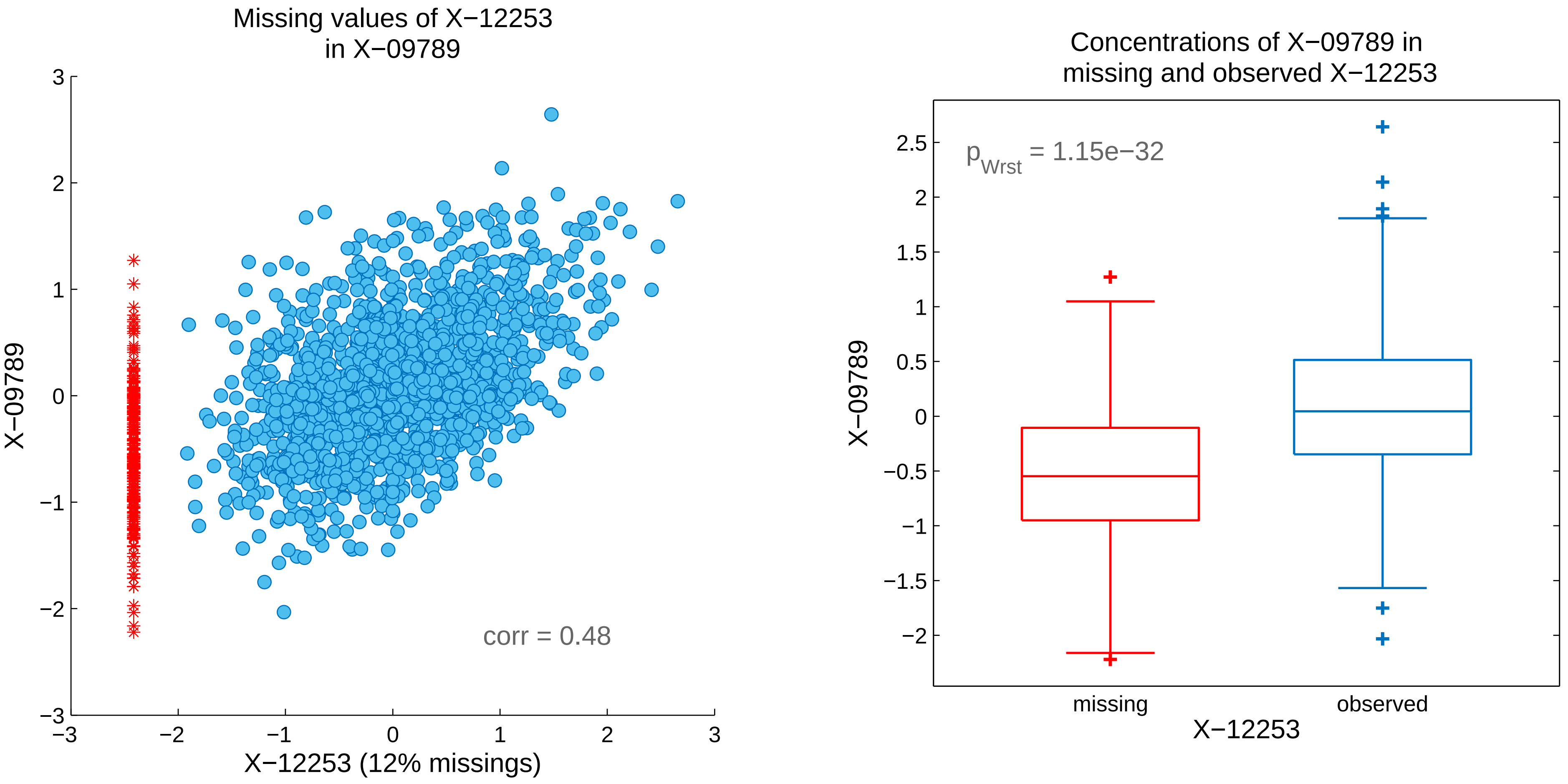

Missing values of X−12253
in X−09789
X−09789
X−12253 (12% missings)
corr = 0.48
Concentrations of X−09789 in
missing and observed X−12253
p_{Wrst} = 1.15e−32
X−09789
missing
observed
X−12253

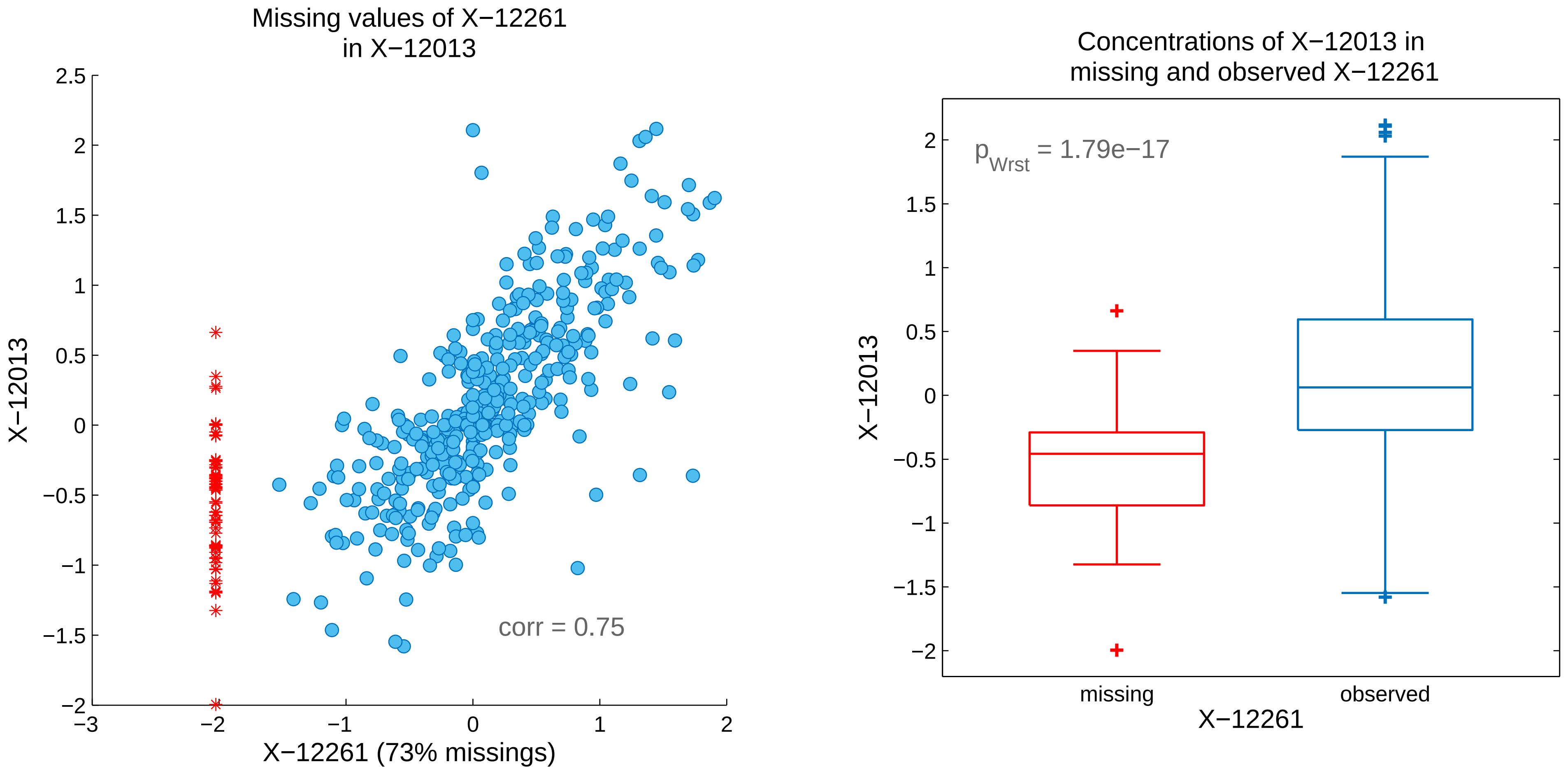

Missing values of X−12261 in X−12013
X−12013
X−12261 (73% missings)
corr = 0.75
Concentrations of X−12013 in missing and observed X−12261
p_{Wrst} = 1.79e−17
X−12013
missing
observed
X−12261

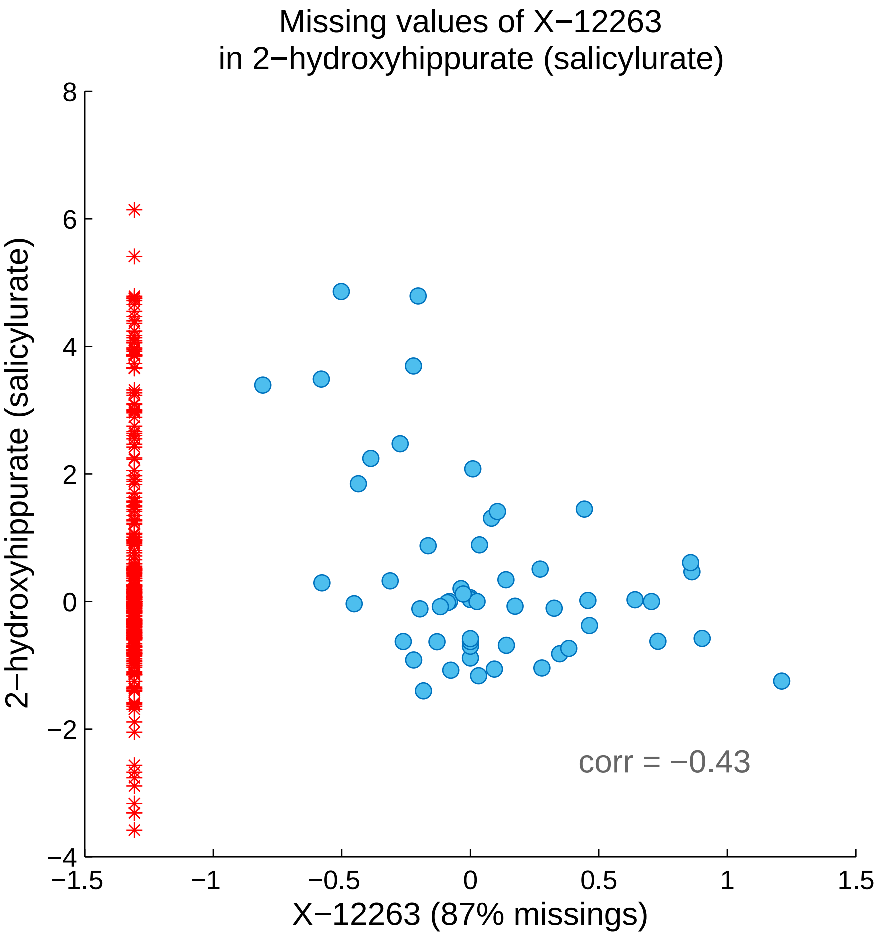
Missing values of X−12263
in 2−hydroxyhippurate (salicylurate)
2−hydroxyhippurate (salicylurate)
X−12263 (87% missings)
corr = −0.43
8
6
4
2
0
−2
−4
−1.5
−1
−0.5
0
0.5
1
1.5

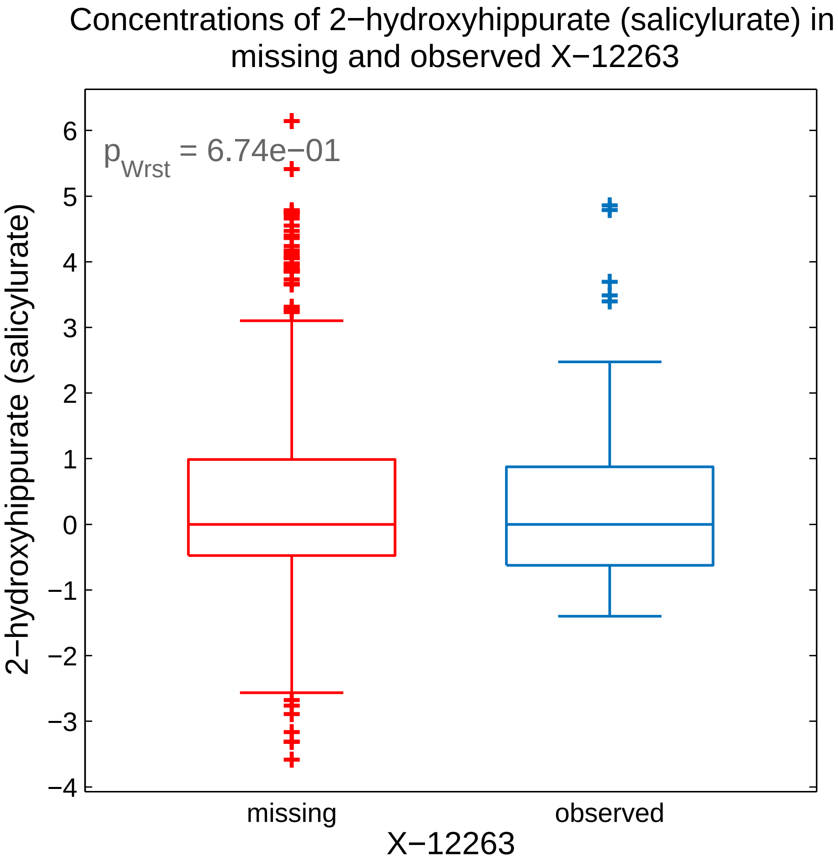
Concentrations of 2−hydroxyhippurate (salicylurate) in
missing and observed X−12263
p_{Wrst} = 6.74e−01
2−hydroxyhippurate (salicylurate)
X−12263
missing
observed
6
5
4
3
2
1
0
−1
−2
−3
−4

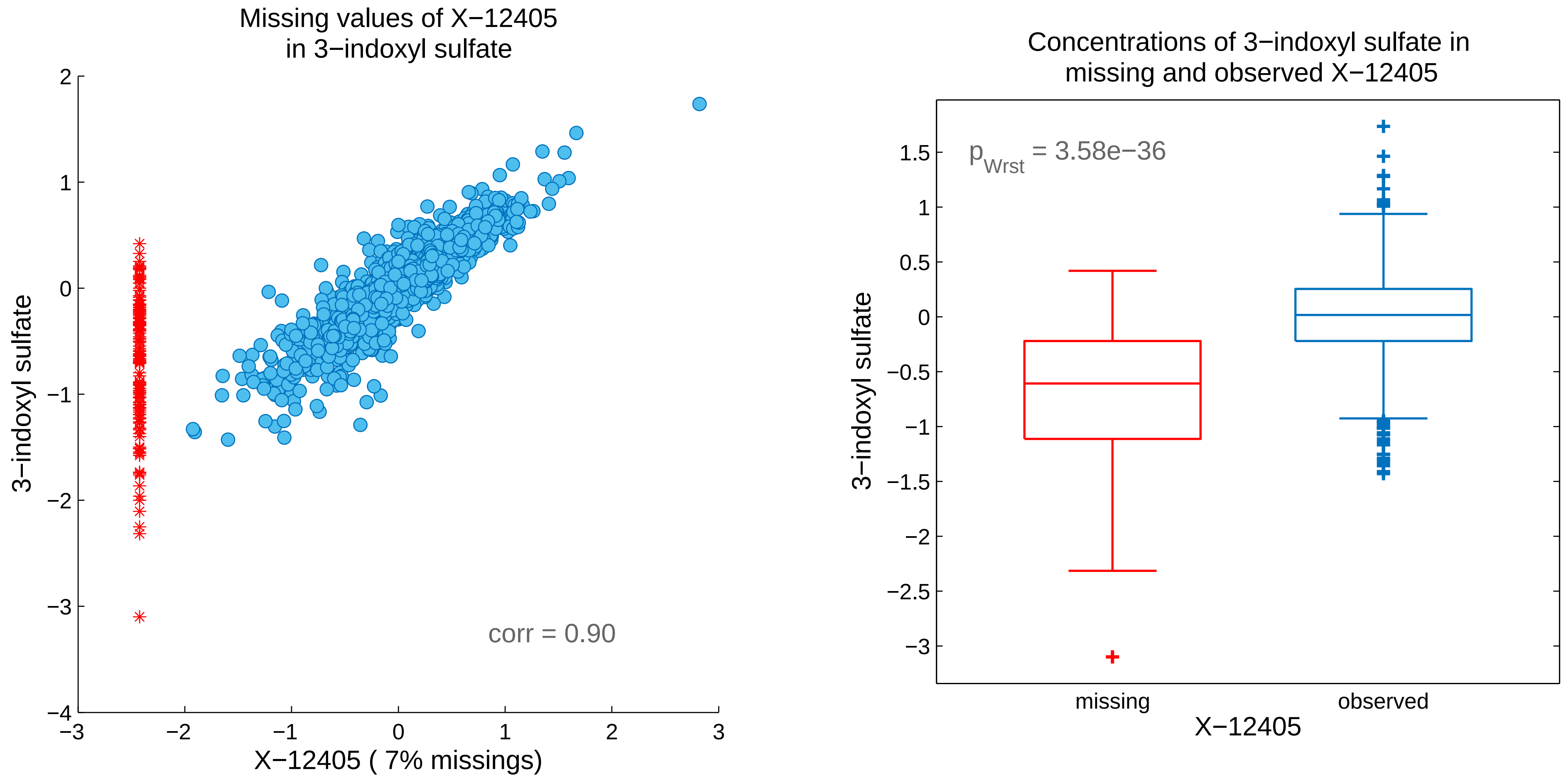
Missing values of X−12405
in 3−indoxyl sulfate
3−indoxyl sulfate
X−12405 (7% missings)
corr = 0.90
Concentrations of 3−indoxyl sulfate in
missing and observed X−12405
p_{Wrst} = 3.58e−36
3−indoxyl sulfate
missing
observed
X−12405

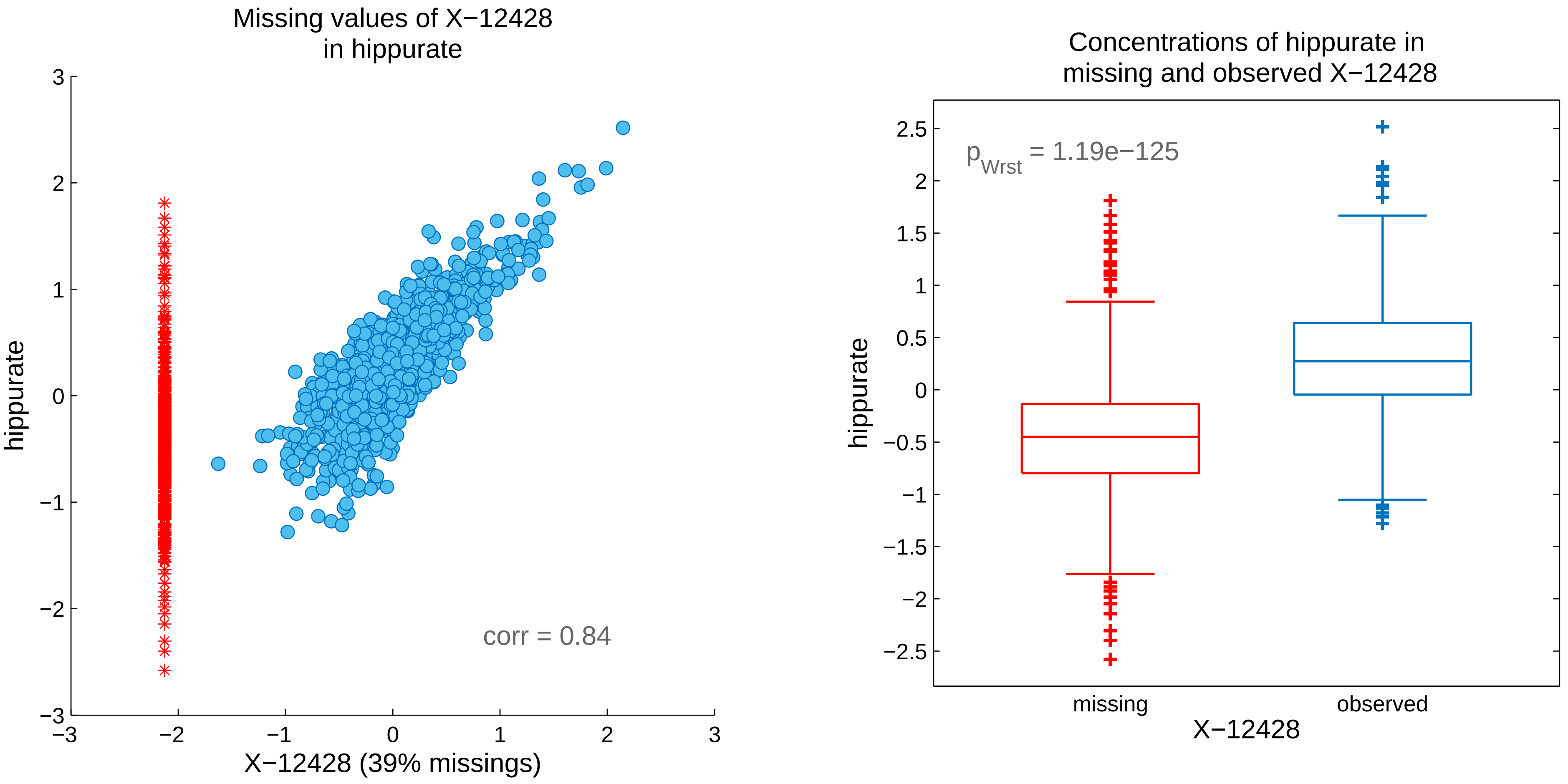

Missing values of X−12428
in hippurate
hippurate
X−12428 (39% missings)
corr = 0.84
Concentrations of hippurate in
missing and observed X−12428
p_Wrst = 1.19e−125
hippurate
missing
observed
X−12428

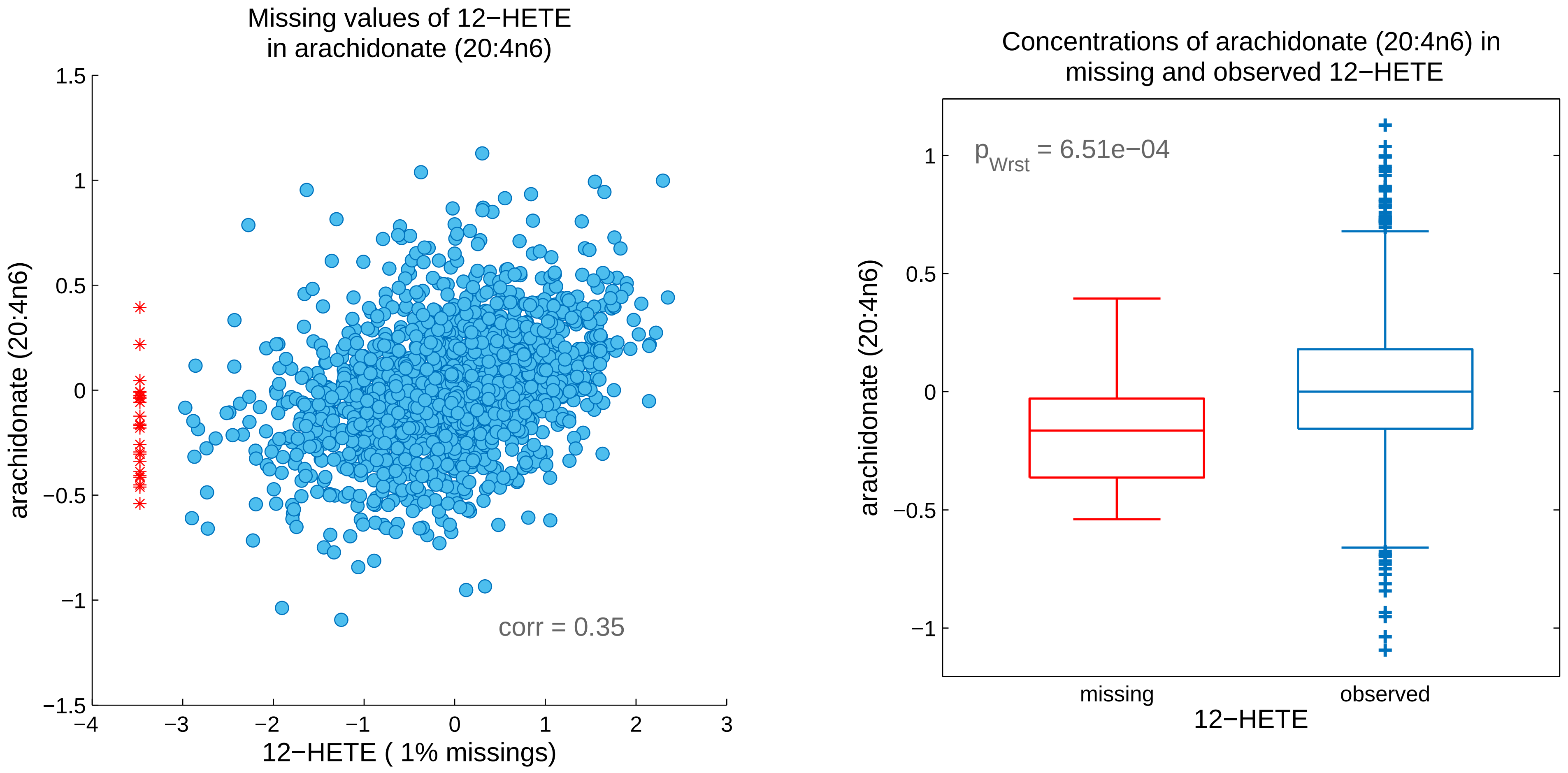

Missing values of 12−HETE in arachidonate (20:4n6)
arachidonate (20:4n6)
12−HETE (1% missings)
corr = 0.35
Concentrations of arachidonate (20:4n6) in missing and observed 12−HETE
p_Wrst = 6.51e−04
arachidonate (20:4n6)
missing
observed
12−HETE

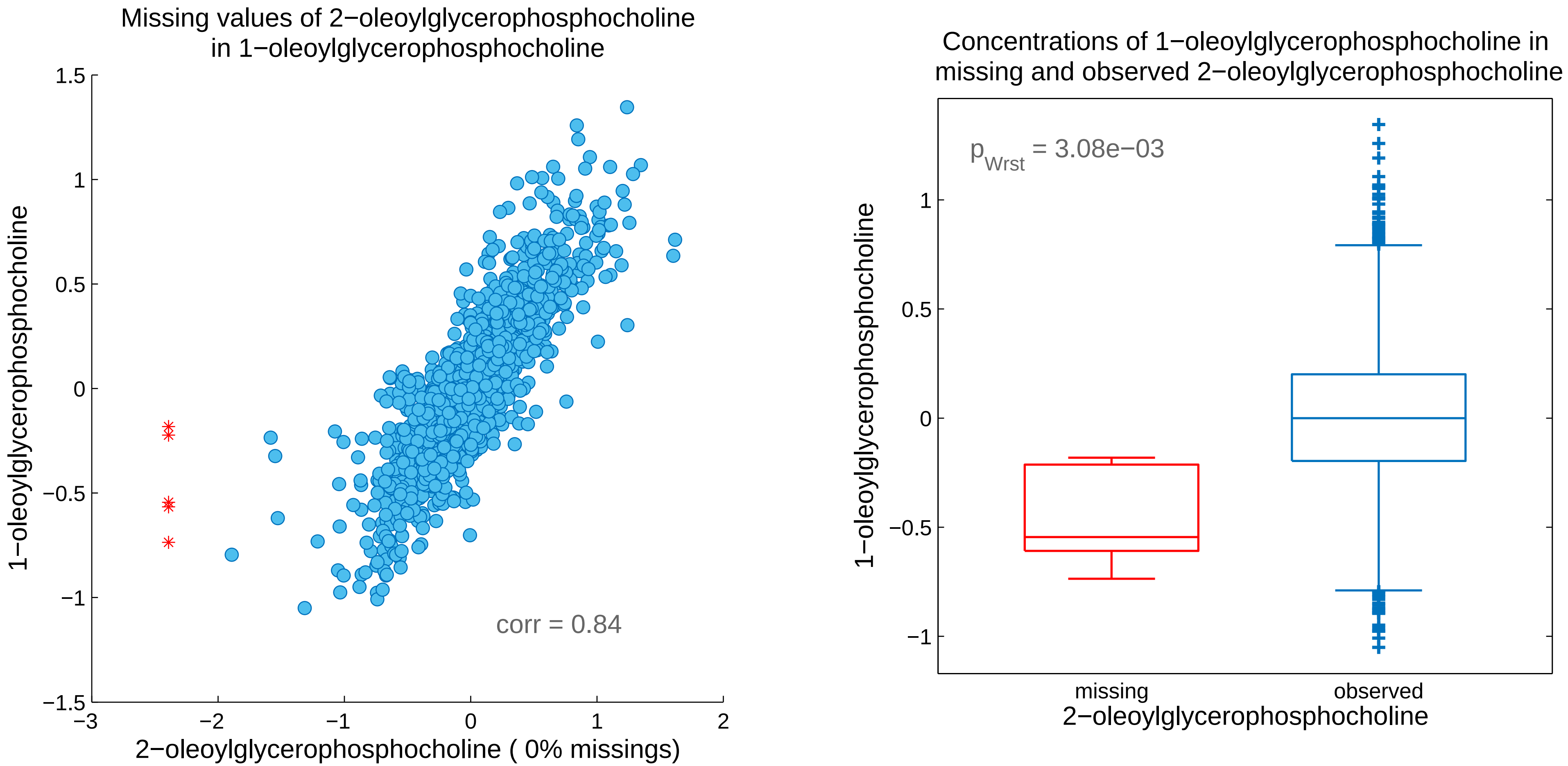

Missing values of 2−oleoylglycerophosphocholine
in 1−oleoylglycerophosphocholine
1−oleoylglycerophosphocholine
2−oleoylglycerophosphocholine (0% missings)
corr = 0.84
1.5
1
0.5
0
−0.5
−1
−1.5
−3
−2
−1
0
1
2
Concentrations of 1−oleoylglycerophosphocholine in
missing and observed 2−oleoylglycerophosphocholine
p_{Wrst} = 3.08e−03
1−oleoylglycerophosphocholine
1
0.5
0
−0.5
−1
missing
observed
2−oleoylglycerophosphocholine

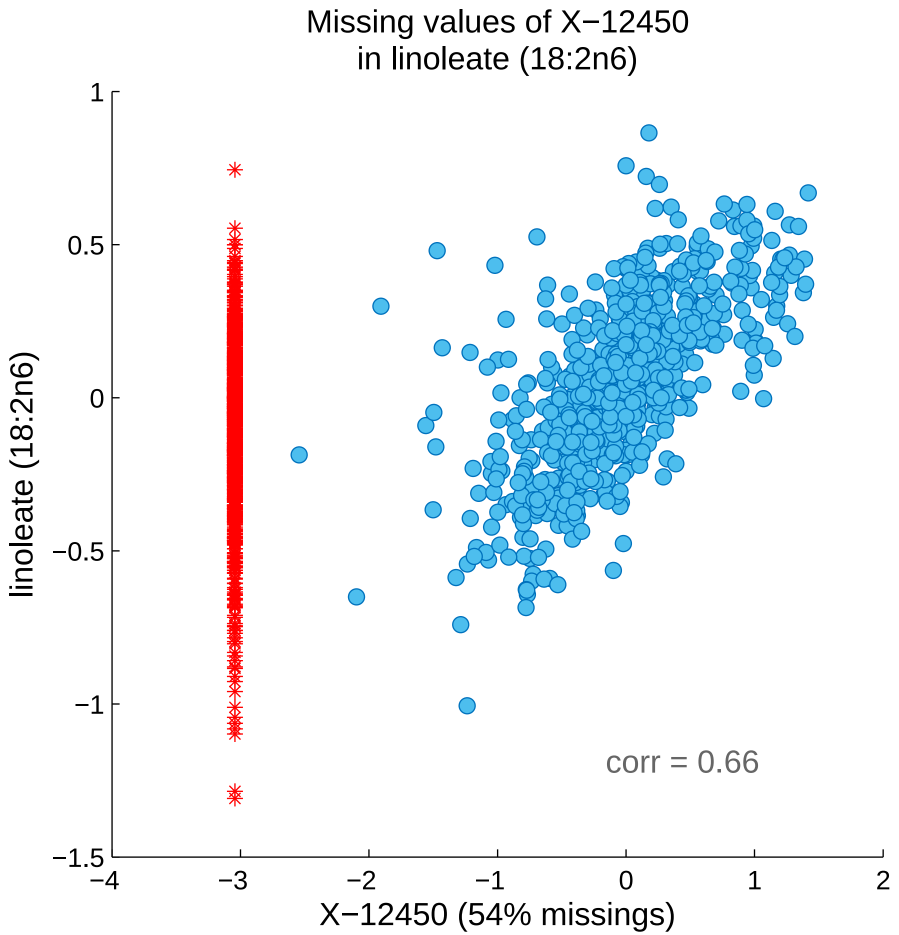

Missing values of X−12450
in linoleate (18:2n6)
linoleate (18:2n6)
X−12450 (54% missings)
corr = 0.66
1
0.5
0
−0.5
−1
−1.5
−4
−3
−2
−1
0
1
2

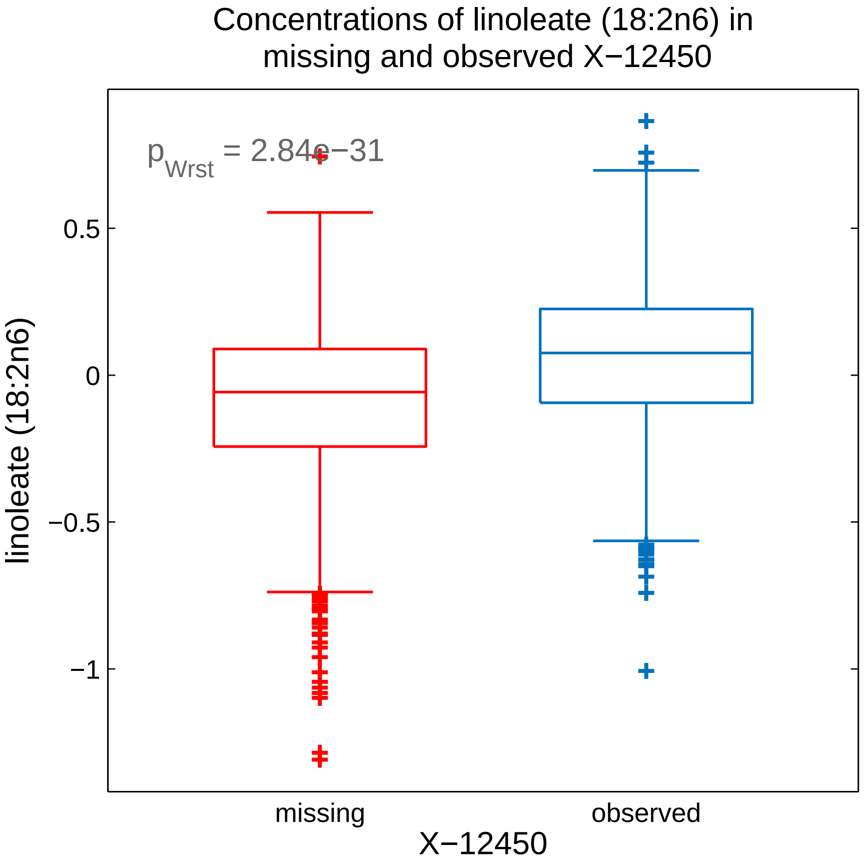

Concentrations of linoleate (18:2n6) in
missing and observed X−12450
p_{Wrst} = 2.84e−31
linoleate (18:2n6)
0.5
0
−0.5
−1
missing
observed
X−12450

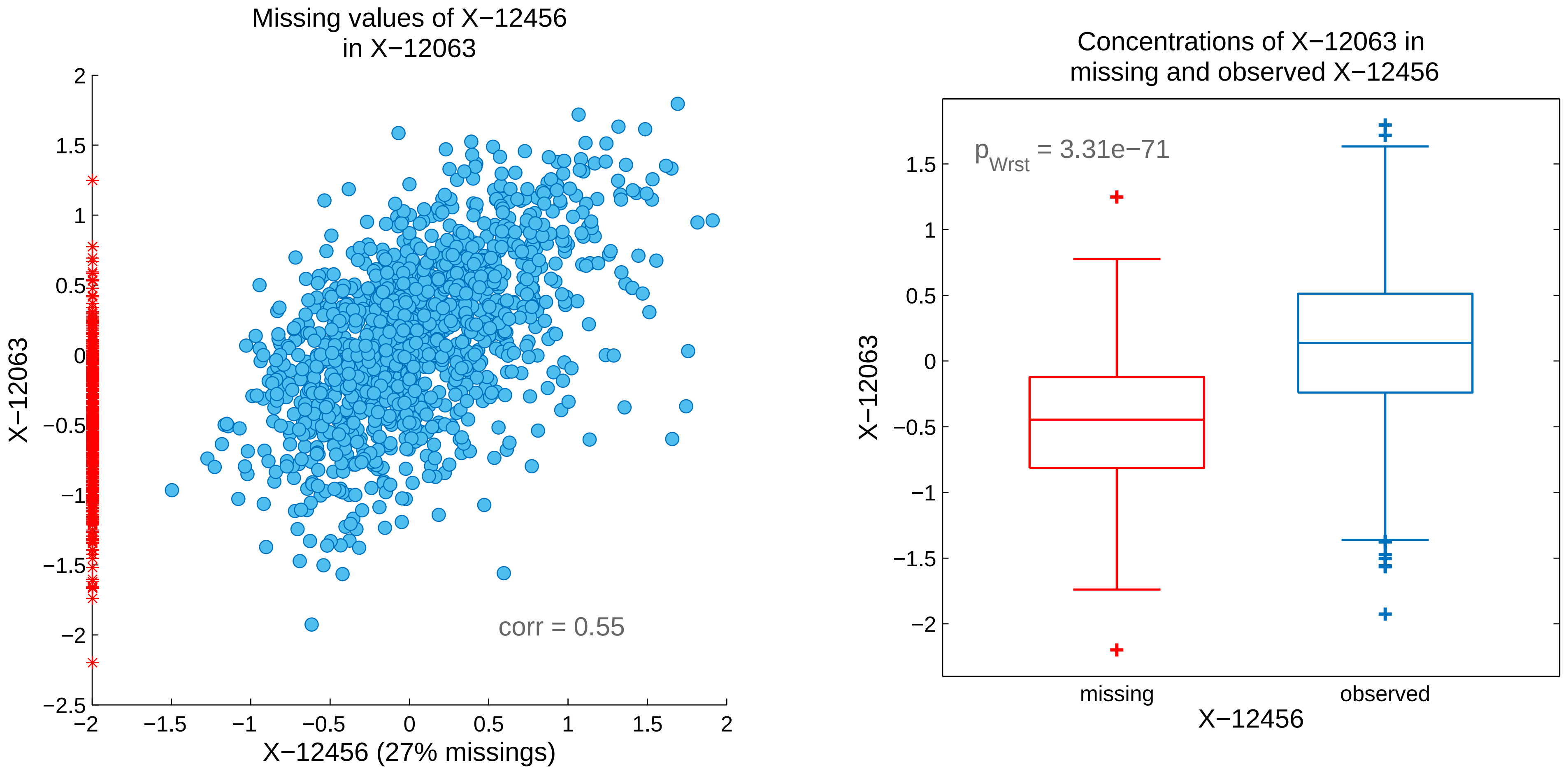

Missing values of X−12456 in X−12063
X−12063
X−12456 (27% missings)
corr = 0.55
Concentrations of X−12063 in missing and observed X−12456
p_Wrst = 3.31e−71
X−12063
missing
observed
X−12456

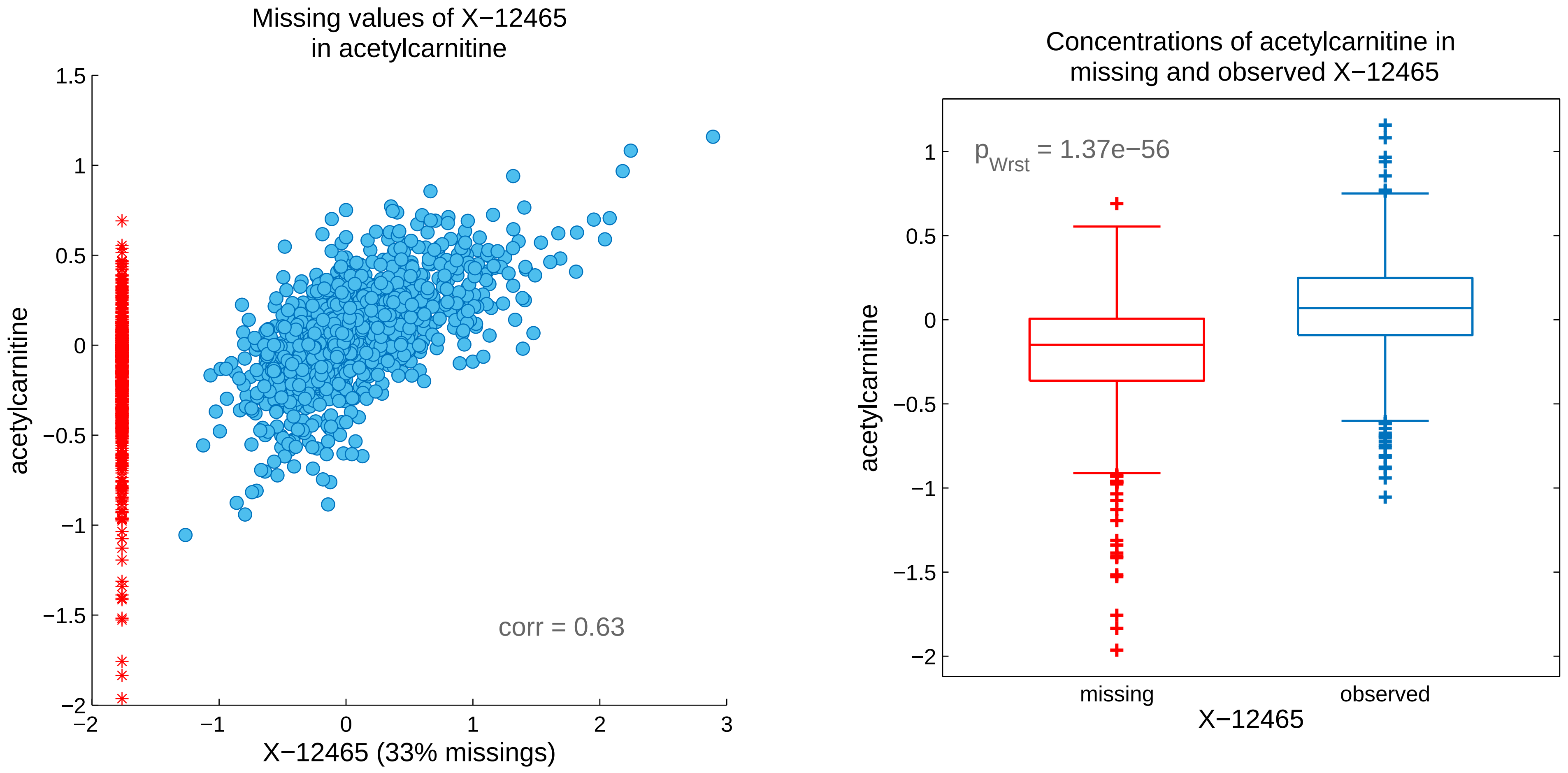

Missing values of X−12465
in acetylcarnitine
acetylcarnitine
X−12465 (33% missings)
corr = 0.63
1.5
1
0.5
0
−0.5
−1
−1.5
−2
−2
−1
0
1
2
3
Concentrations of acetylcarnitine in
missing and observed X−12465
p_{Wrst} = 1.37e−56
acetylcarnitine
1
0.5
0
−0.5
−1
−1.5
−2
missing
observed
X−12465

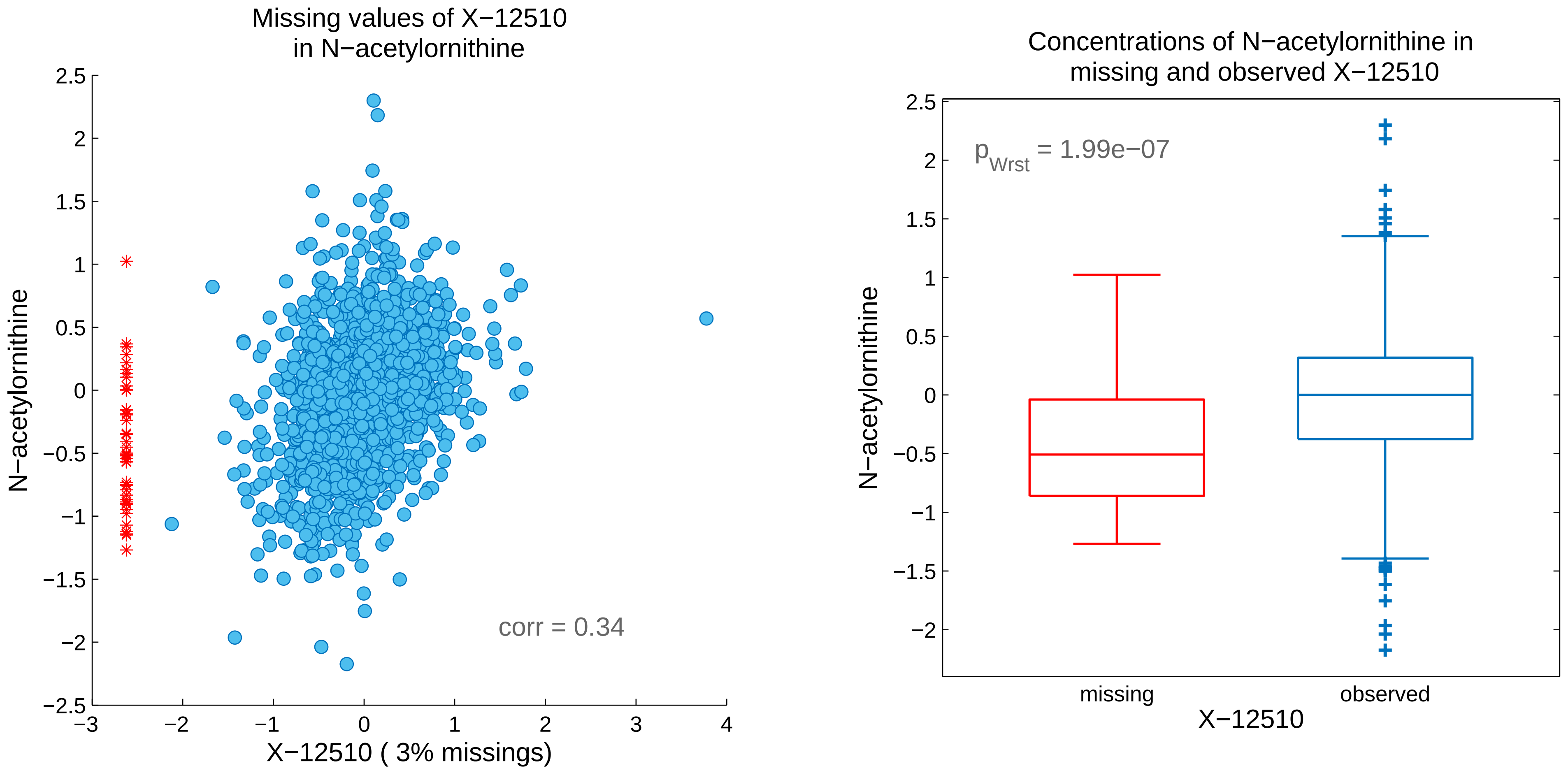
Missing values of X−12510
in N−acetylornithine
N−acetylornithine
corr = 0.34
X−12510 (3% missings)
Concentrations of N−acetylornithine in
missing and observed X−12510
p_Wrst = 1.99e−07
N−acetylornithine
missing
observed
X−12510

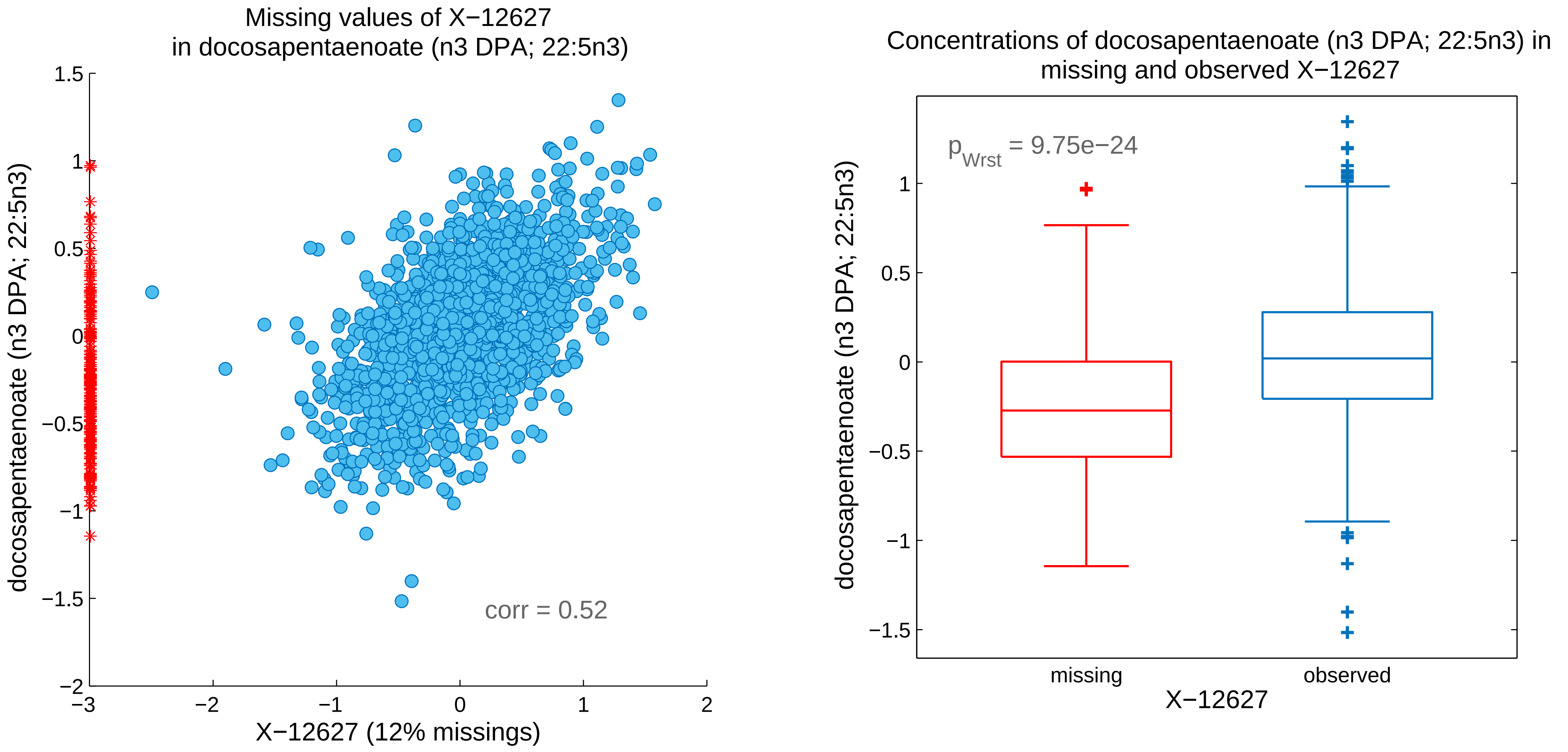

Missing values of X−12627
in docosapentaenoate (n3 DPA; 22:5n3)
docosapentaenoate (n3 DPA; 22:5n3)
X−12627 (12% missings)
corr = 0.52
1.5
1
0.5
0
−0.5
−1
−1.5
−2
−3
−2
−1
0
1
2
Concentrations of docosapentaenoate (n3 DPA; 22:5n3) in
missing and observed X−12627
p_Wrst = 9.75e−24
docosapentaenoate (n3 DPA; 22:5n3)
missing
observed
X−12627

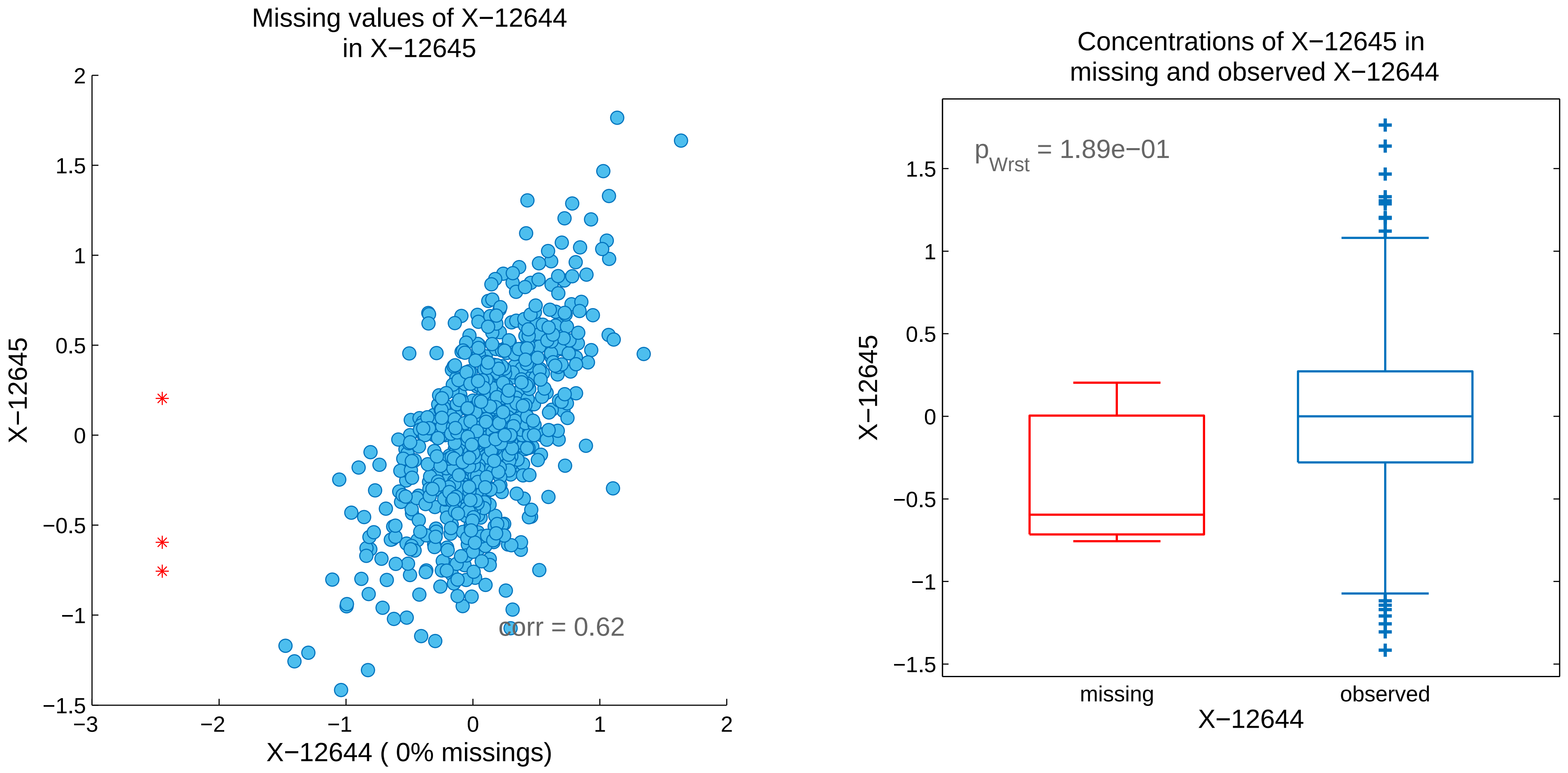

Missing values of X−12644
in X−12645
X−12645
X−12644 (0% missings)
corr = 0.62
Concentrations of X−12645 in
missing and observed X−12644
p_{Wrst} = 1.89e−01
X−12645
missing
observed
X−12644

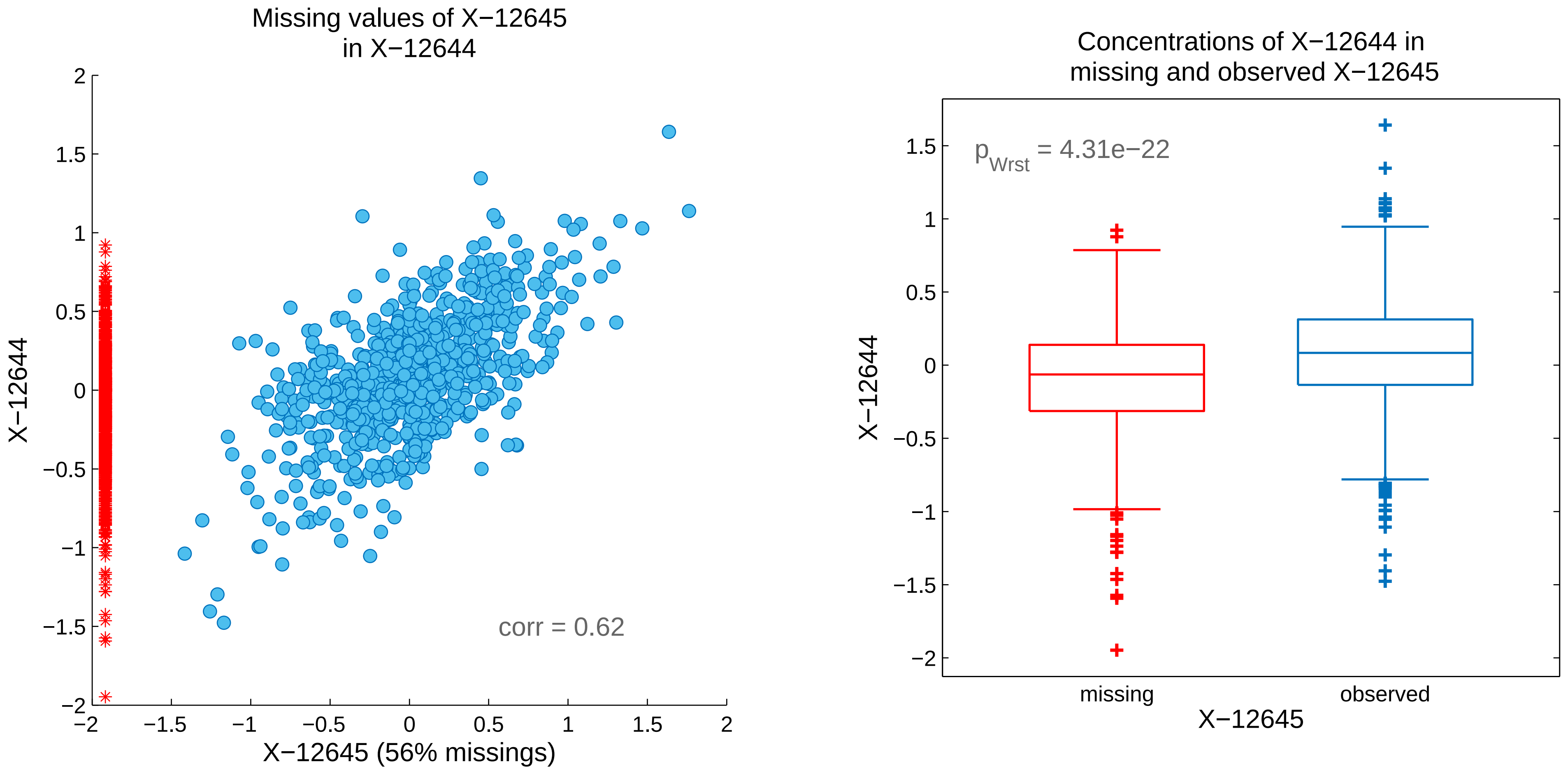
Missing values of X−12645
in X−12644
X−12644
X−12645 (56% missings)
corr = 0.62
Concentrations of X−12644 in
missing and observed X−12645
p_{Wrst} = 4.31e−22
X−12644
missing
observed
X−12645

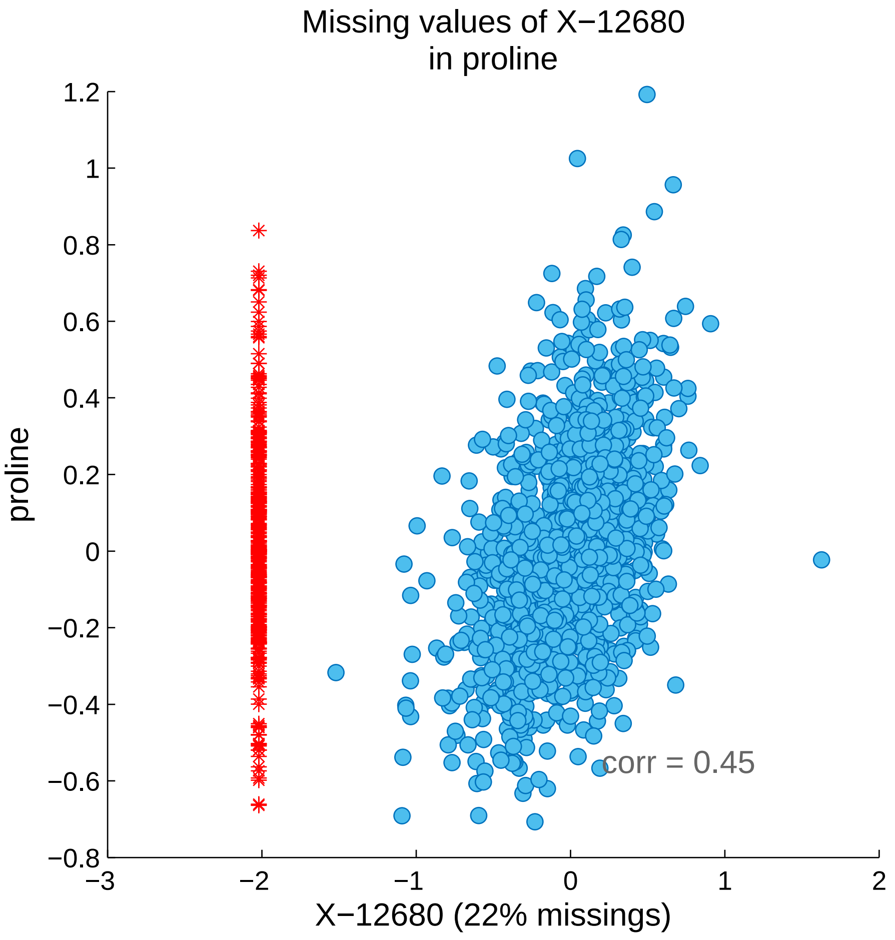

Missing values of X−12680
in proline
proline
X−12680 (22% missings)
corr = 0.45

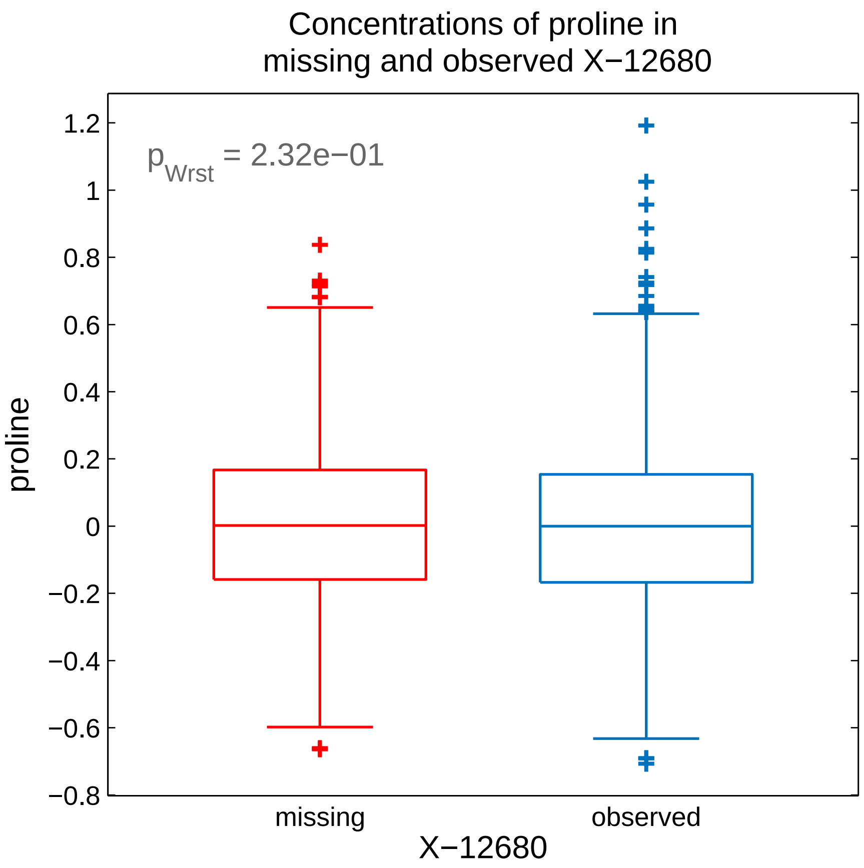

Concentrations of proline in
missing and observed X−12680
p_Wrst = 2.32e−01
proline
missing
observed
X−12680

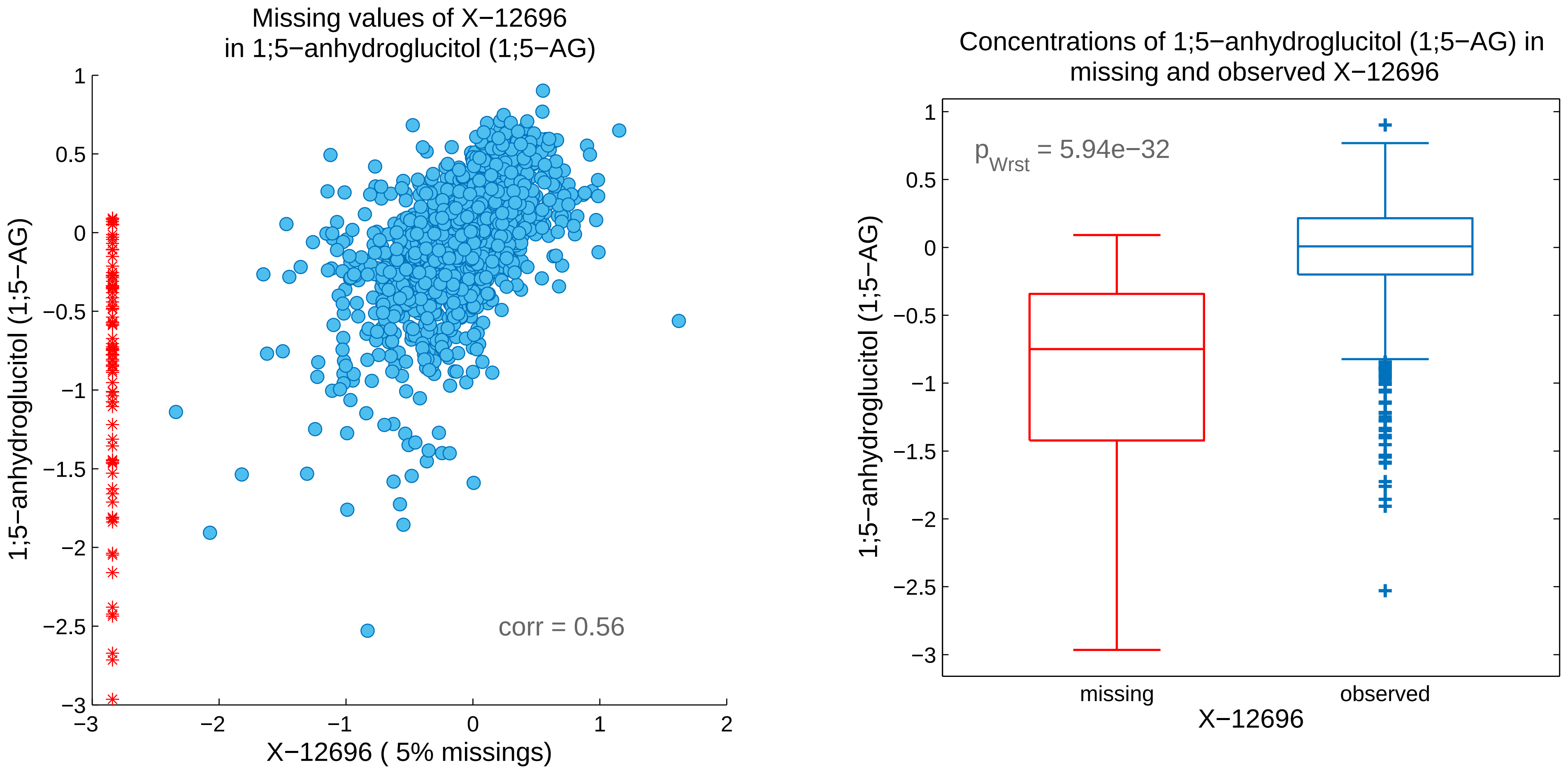
Missing values of X−12696
in 1;5−anhydroglucitol (1;5−AG)
1;5−anhydroglucitol (1;5−AG)
X−12696 (5% missings)
corr = 0.56
1
0.5
0
−0.5
−1
−1.5
−2
−2.5
−3
−3
−2
−1
0
1
2
Concentrations of 1;5−anhydroglucitol (1;5−AG) in
missing and observed X−12696
p_Wrst = 5.94e−32
1
0.5
0
−0.5
−1
−1.5
−2
−2.5
−3
missing
observed
X−12696

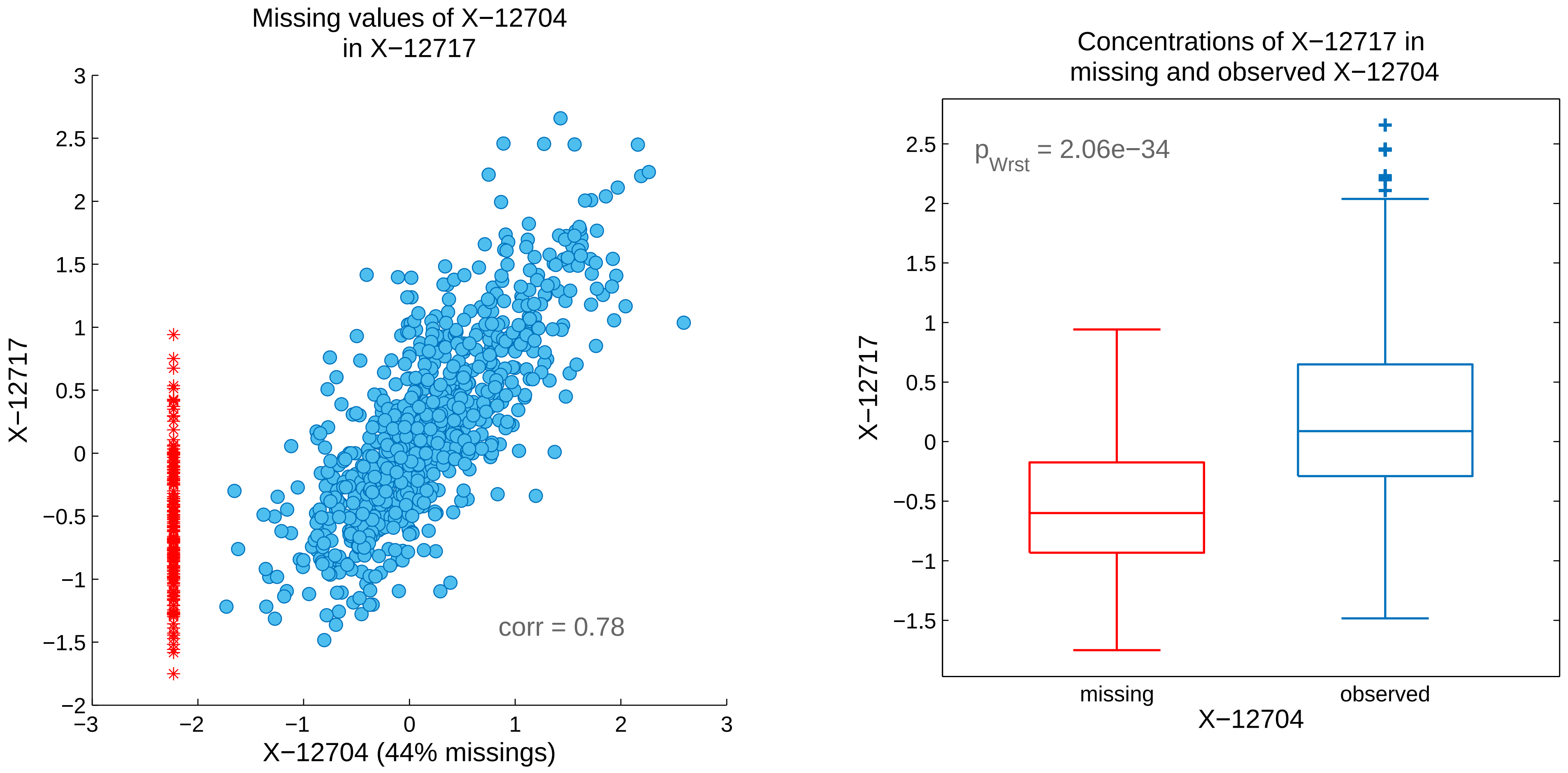
Missing values of X−12704
in X−12717
X−12717
X−12704 (44% missings)
corr = 0.78
Concentrations of X−12717 in
missing and observed X−12704
p_{Wrst} = 2.06e−34
X−12717
missing
observed
X−12704

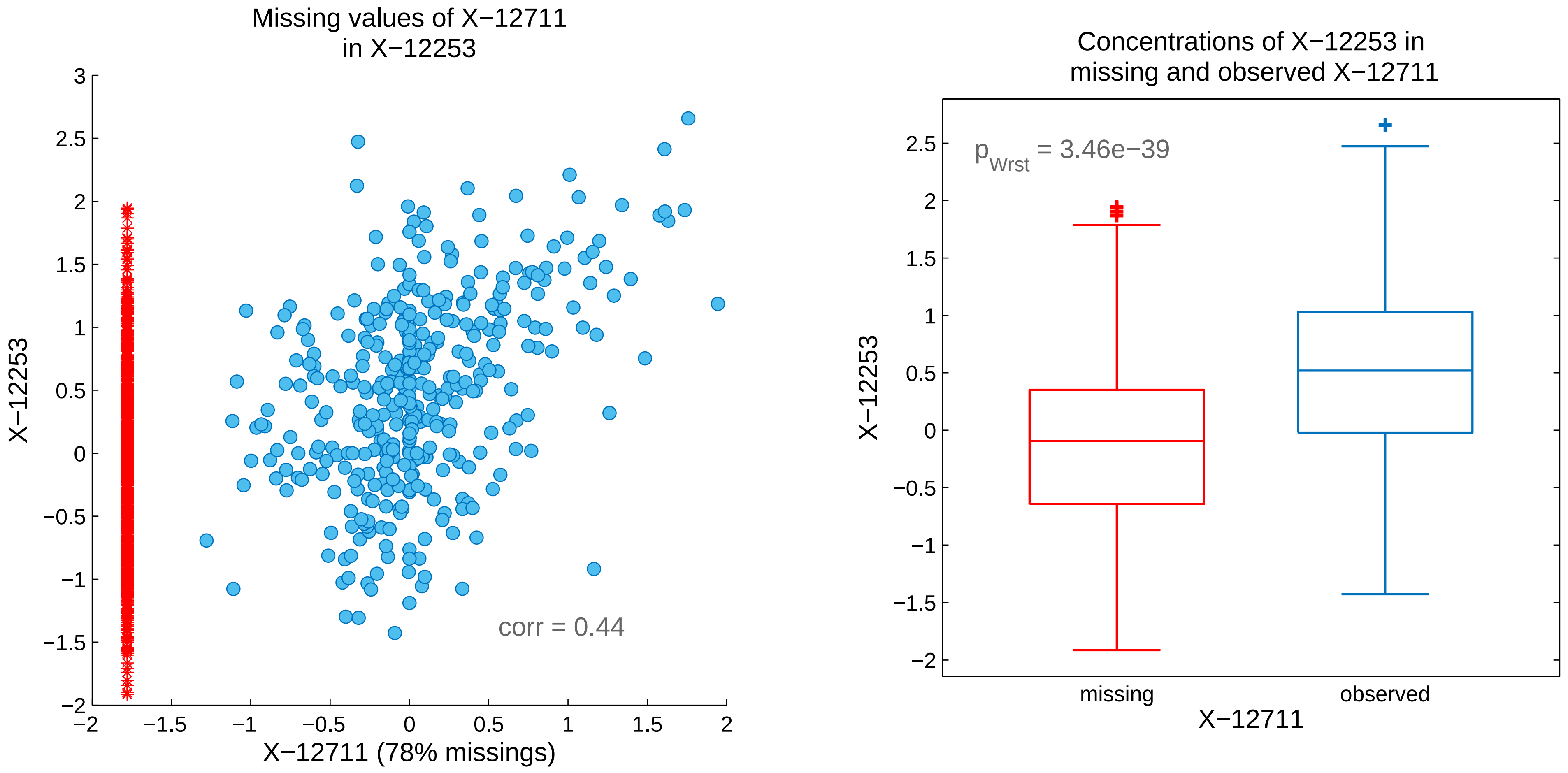
Missing values of X−12711
in X−12253
X−12253
X−12711 (78% missings)
corr = 0.44
Concentrations of X−12253 in
missing and observed X−12711
p_{Wrst} = 3.46e−39
X−12253
missing
observed
X−12711

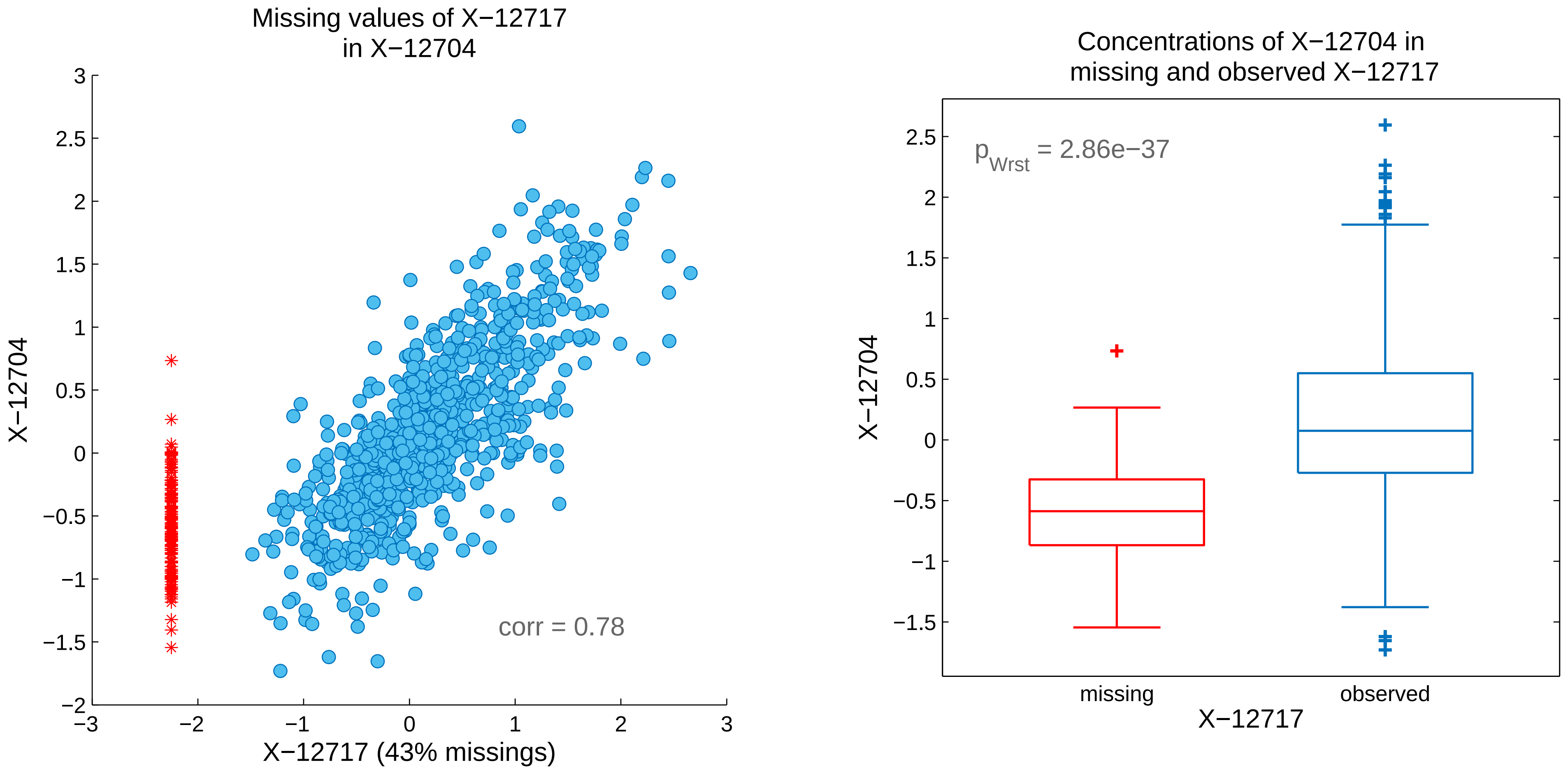

Missing values of X−12717
in X−12704
X−12704
X−12717 (43% missings)
corr = 0.78
3
2.5
2
1.5
1
0.5
0
−0.5
−1
−1.5
−2
−3
−2
−1
0
1
2
3
Concentrations of X−12704 in
missing and observed X−12717
p_Wrst = 2.86e−37
X−12704
X−12717
missing
observed
2.5
2
1.5
1
0.5
0
−0.5
−1
−1.5

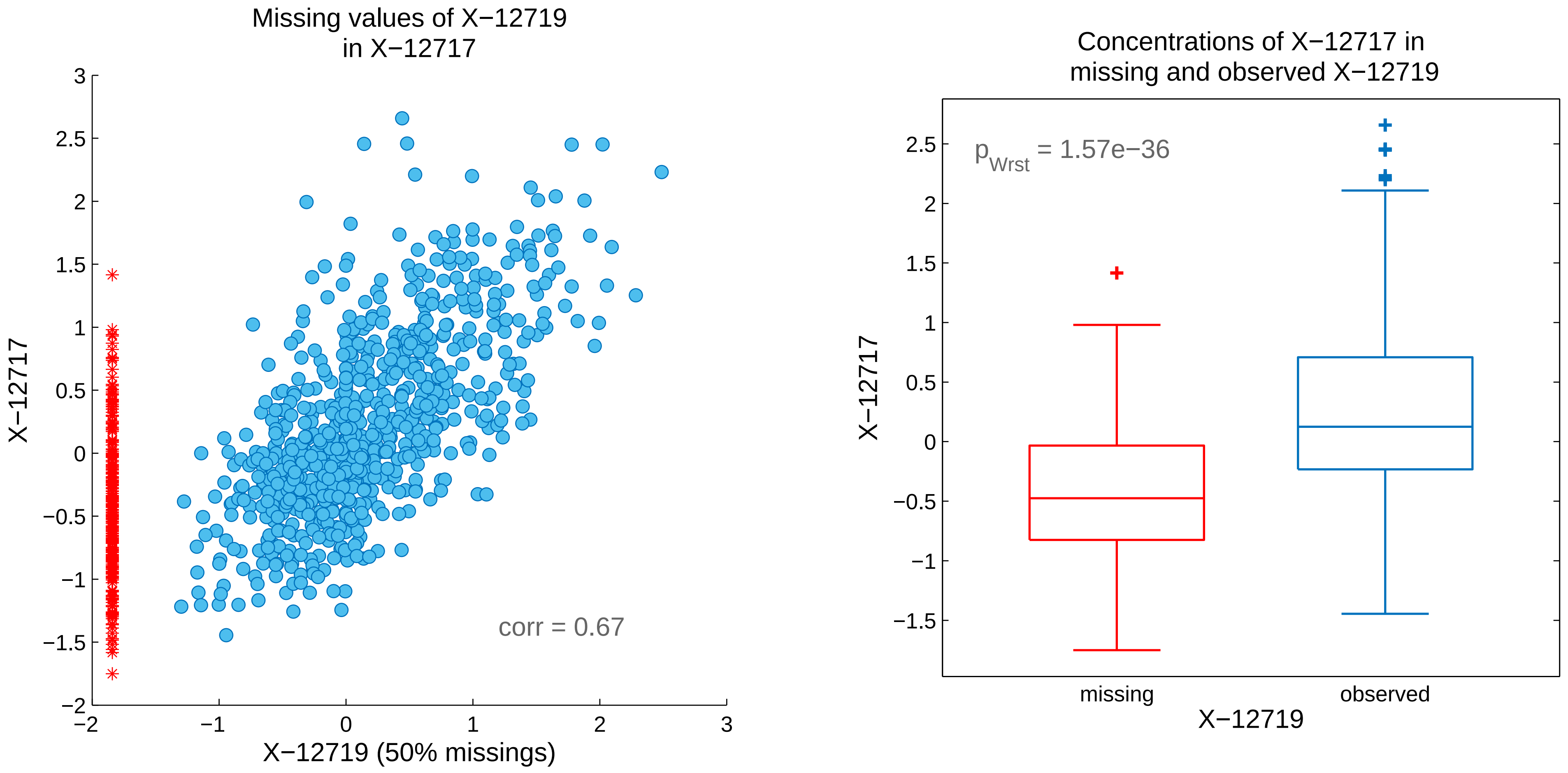

Missing values of X−12719
in X−12717
X−12717
X−12719 (50% missings)
corr = 0.67
Concentrations of X−12717 in
missing and observed X−12719
p_{Wrst} = 1.57e−36
X−12717
missing
observed
X−12719

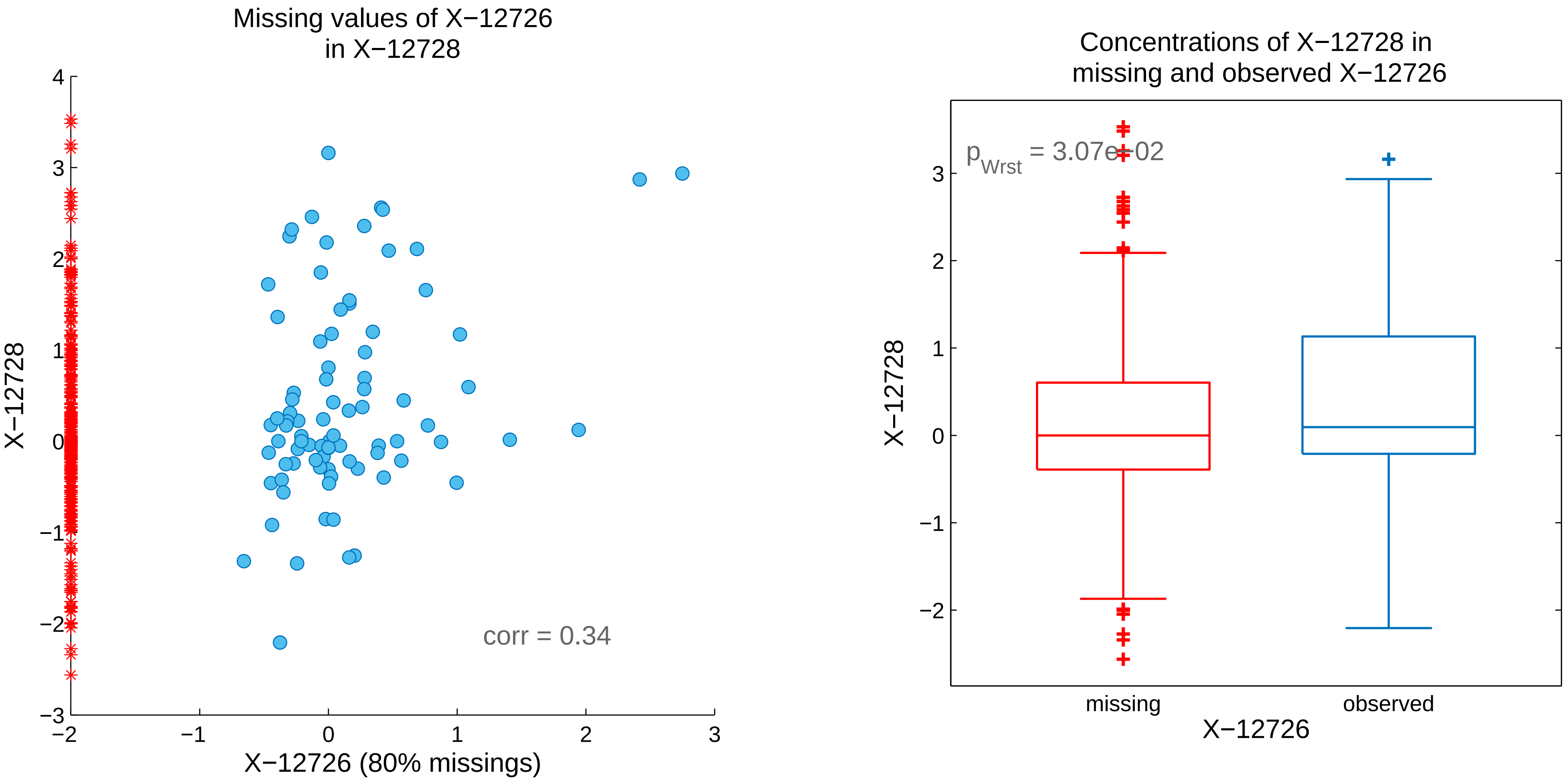

Missing values of X−12726
in X−12728
X−12728
X−12726 (80% missings)
corr = 0.34
Concentrations of X−12728 in
missing and observed X−12726
p_Wrst = 3.07e−02
missing
observed
X−12726

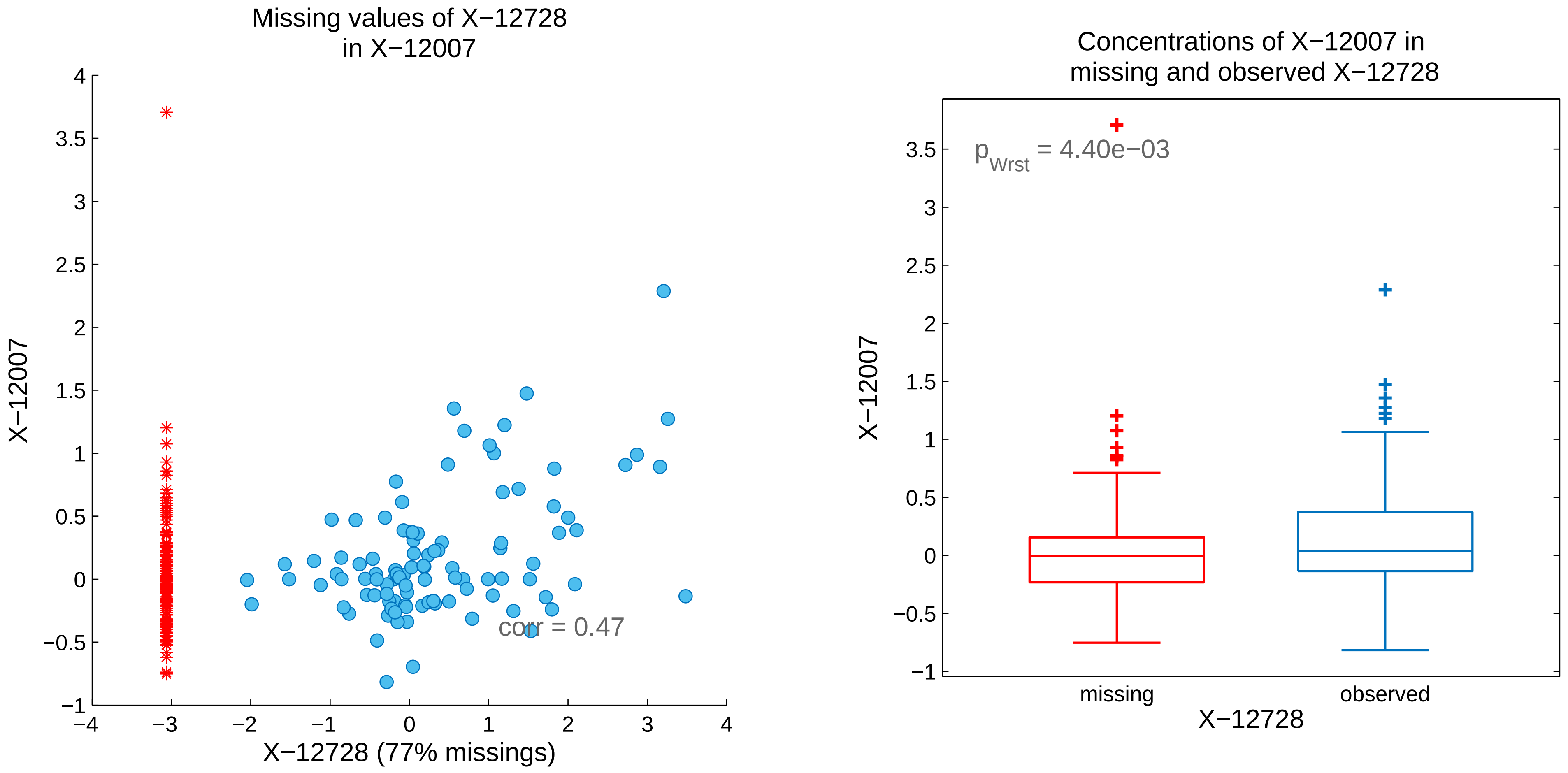

Missing values of X−12728
in X−12007
X−12007
X−12728 (77% missings)
corr = 0.47
Concentrations of X−12007 in
missing and observed X−12728
p_{Wrst} = 4.40e−03
X−12007
missing
observed
X−12728

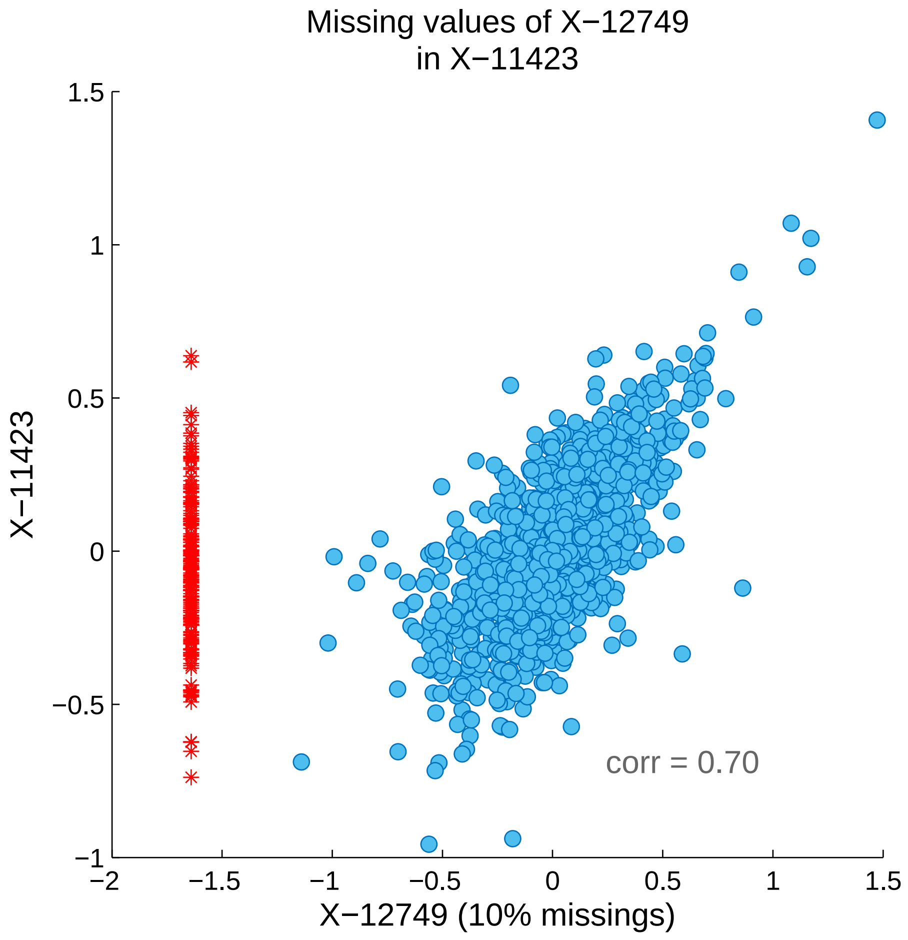
Missing values of X−12749
in X−11423
X−11423
X−12749 (10% missings)
corr = 0.70
1.5
1
0.5
0
−0.5
−1
−2
−1.5
−0.5

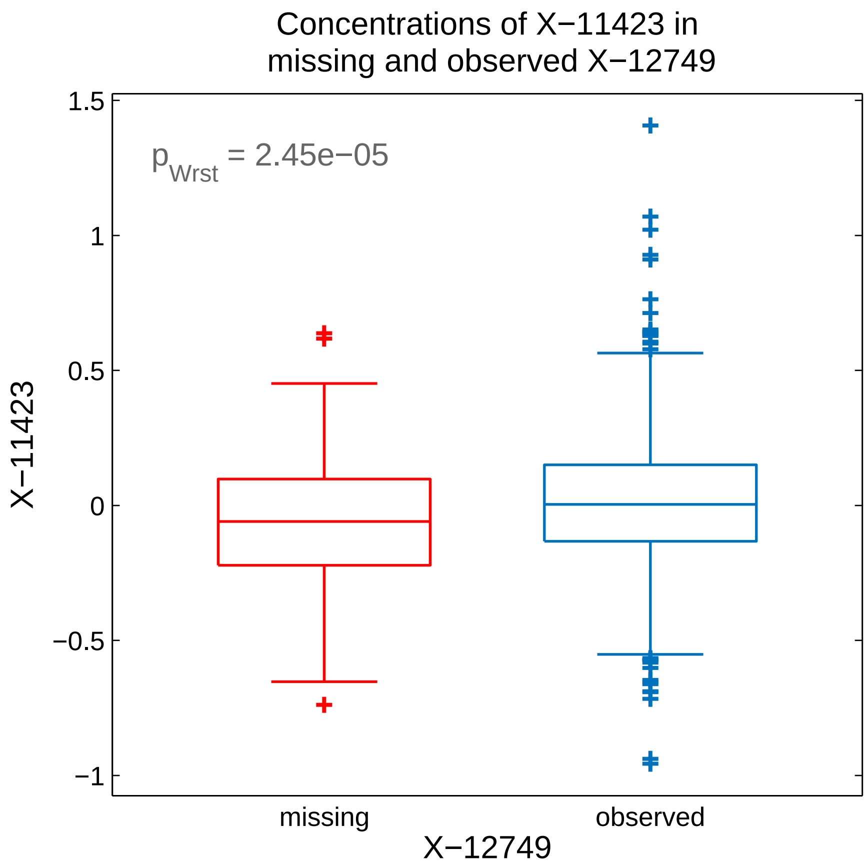
Concentrations of X−11423 in
missing and observed X−12749
p_{Wrst} = 2.45e−05
X−11423
missing
observed
X−12749
1.5
1
0.5
0
−0.5
−1

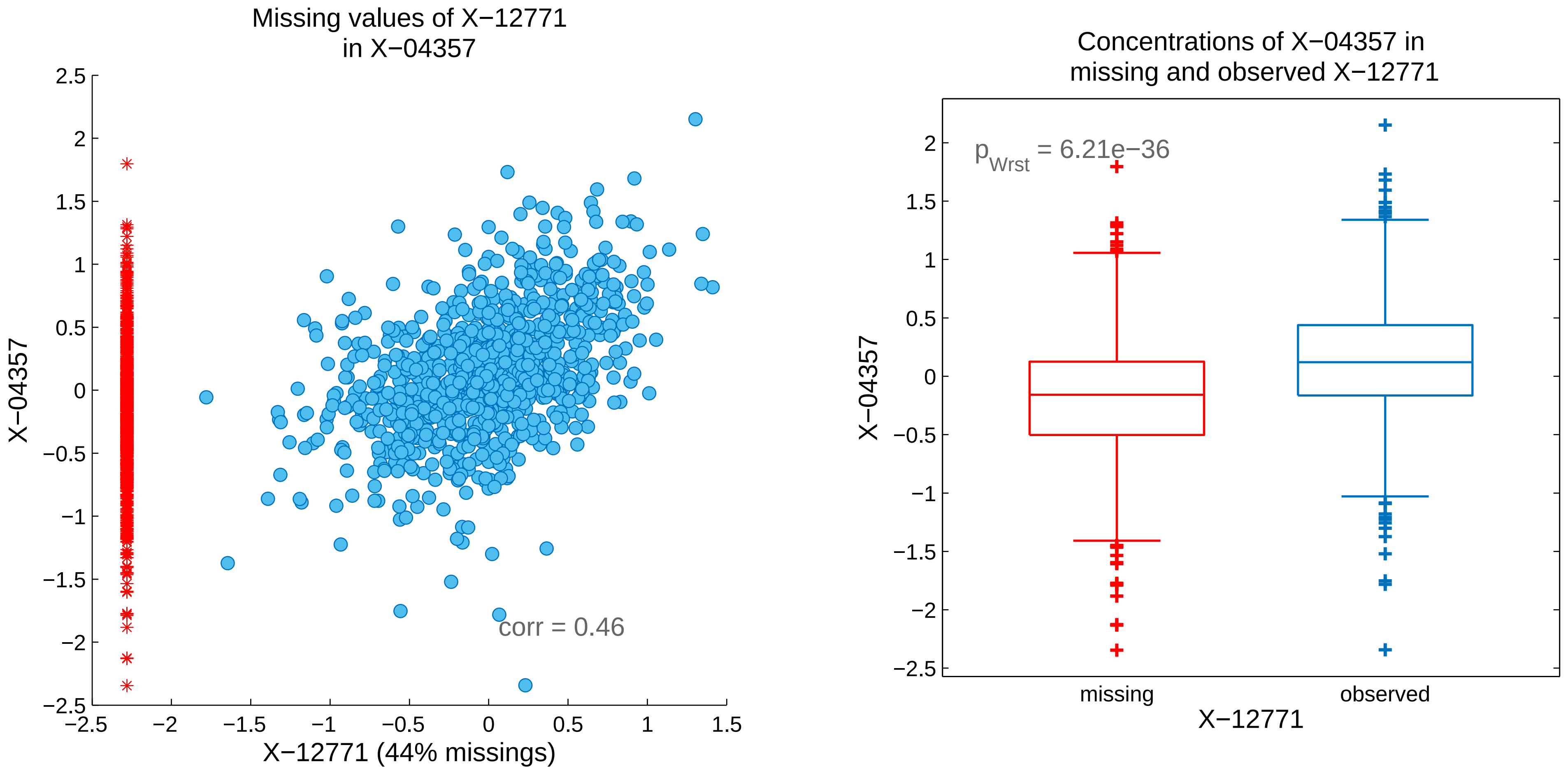

Missing values of X−12771
in X−04357
X−04357
X−12771 (44% missings)
corr = 0.46
Concentrations of X−04357 in
missing and observed X−12771
p_Wrst = 6.21e−36
X−04357
missing
observed
X−12771

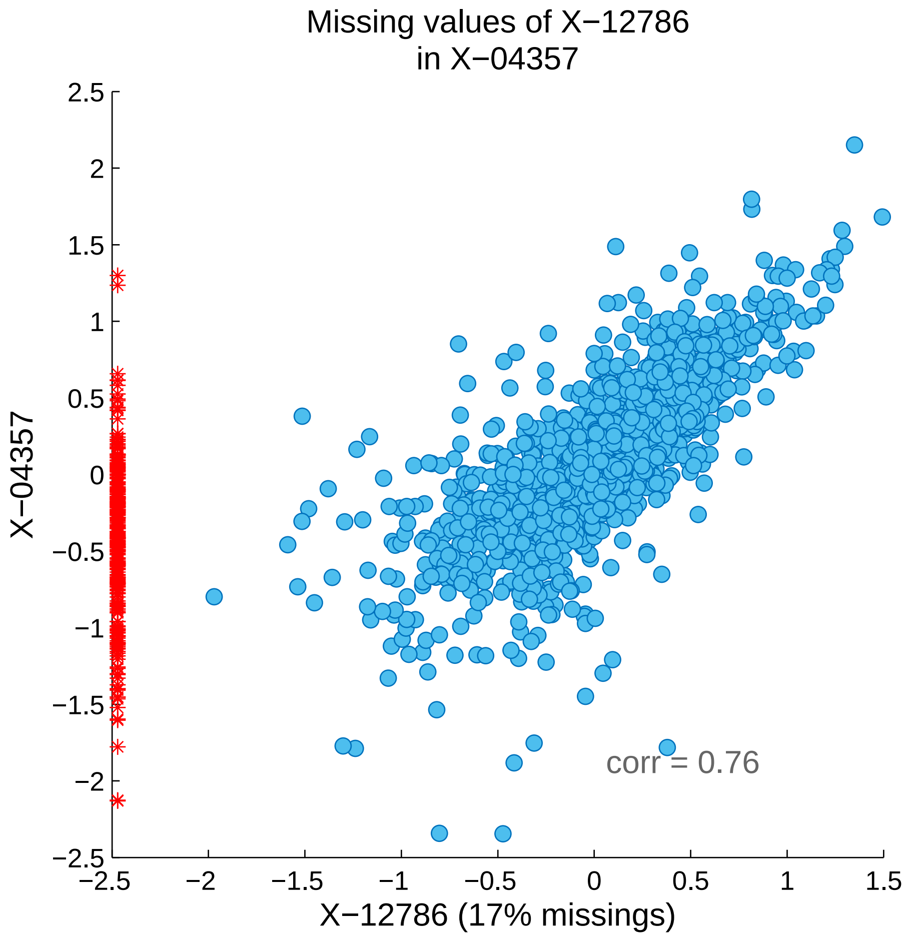

Missing values of X−12786
in X−04357
X−04357
X−12786 (17% missings)
corr = 0.76
2.5
2
1.5
1
0.5
0
−0.5
−1
−1.5
−2
−2.5
−2.5
−2
−1.5
−1
−0.5
0
0.5
1
1.5

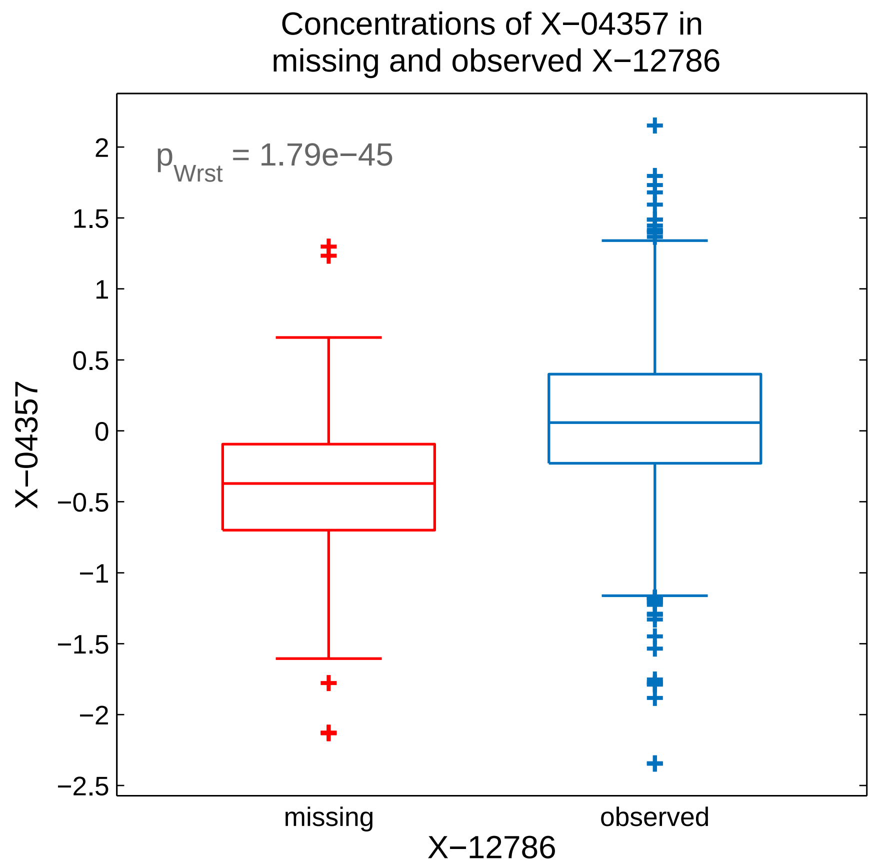

Concentrations of X−04357 in
missing and observed X−12786
p_{Wrst} = 1.79e−45
X−04357
X−12786
missing
observed
2
1.5
1
0.5
0
−0.5
−1
−1.5
−2
−2.5

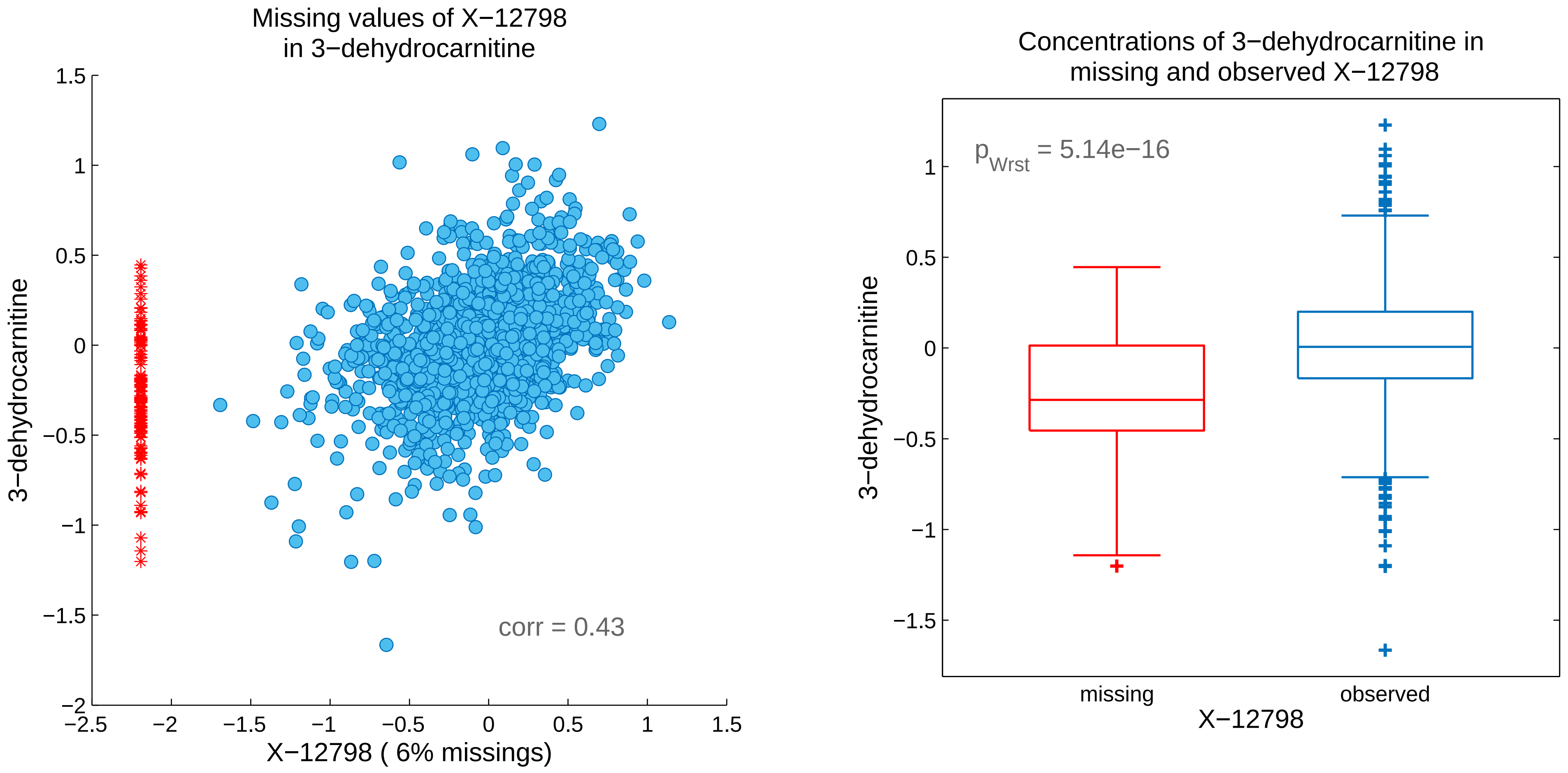

Missing values of X−12798
in 3−dehydrocarnitine
3−dehydrocarnitine
X−12798 (6% missings)
corr = 0.43
1.5
1
0.5
0
−0.5
−1
−1.5
−2
−2.5
−2
−1.5
−1
−0.5
0
0.5
1
1.5
Concentrations of 3−dehydrocarnitine in
missing and observed X−12798
p_{Wrst} = 5.14e−16
3−dehydrocarnitine
1
0.5
0
−0.5
−1
−1.5
missing
observed
X−12798

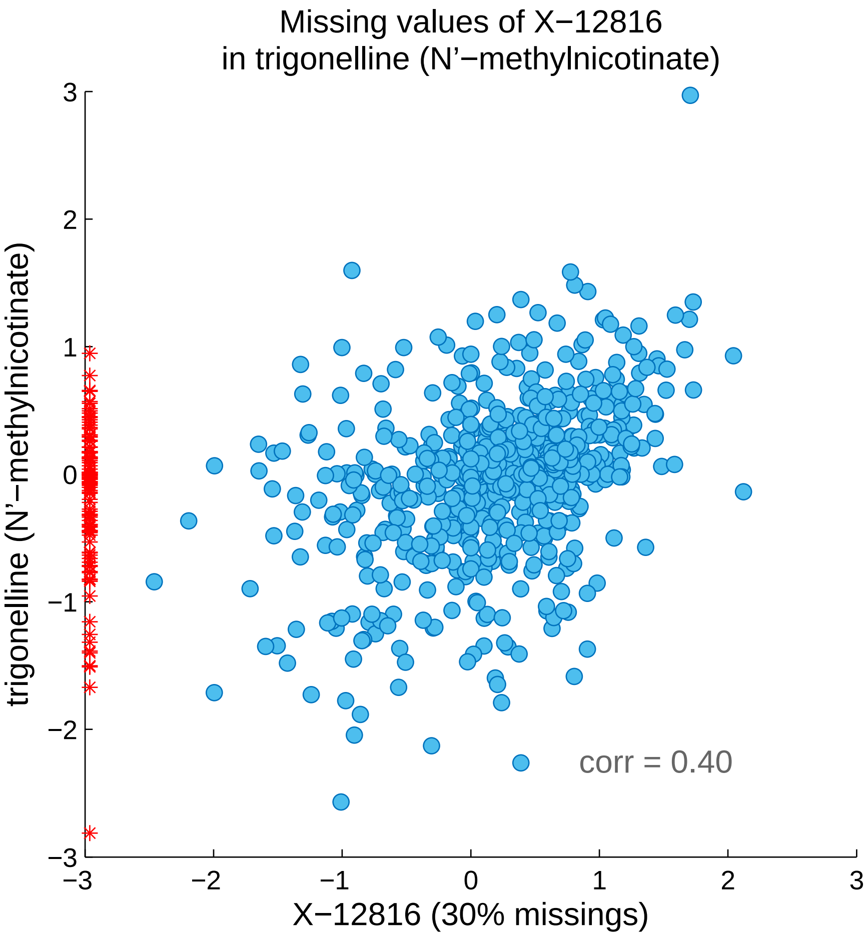
Missing values of X−12816
in trigonelline (N'−methylnicotinate)
trigonelline (N'−methylnicotinate)
X−12816 (30% missings)
corr = 0.40
3
2
1
0
−1
−2
−3
−3
−2
−1
0
1
2
3

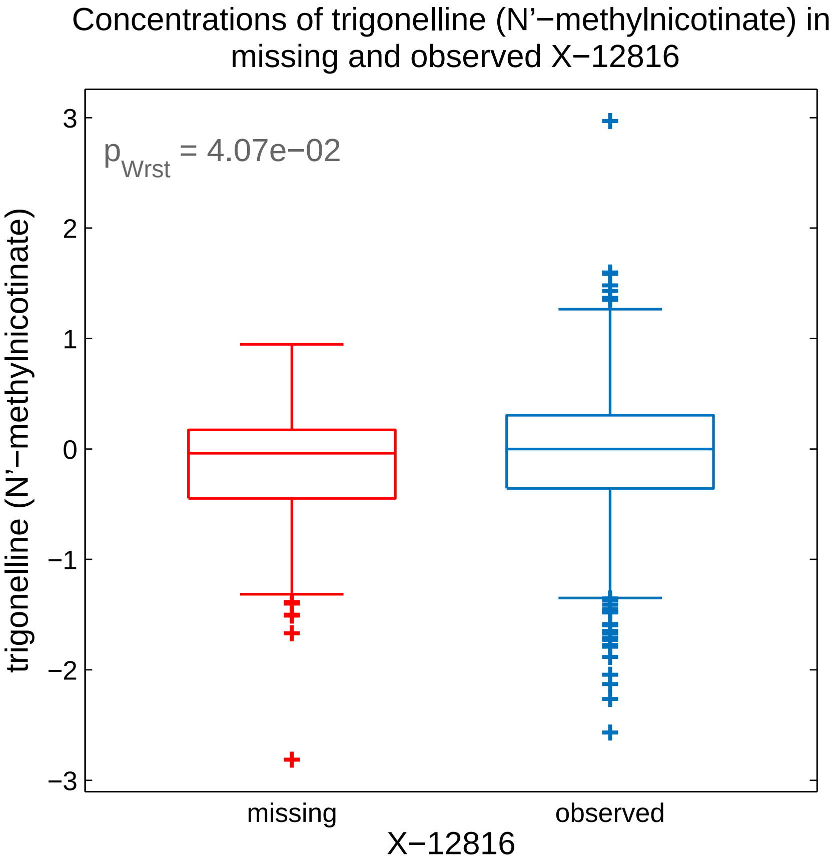
Concentrations of trigonelline (N'−methylnicotinate) in
missing and observed X−12816
p Wrst = 4.07e−02
trigonelline (N'−methylnicotinate)
3
2
1
0
−1
−2
−3
missing
observed
X−12816

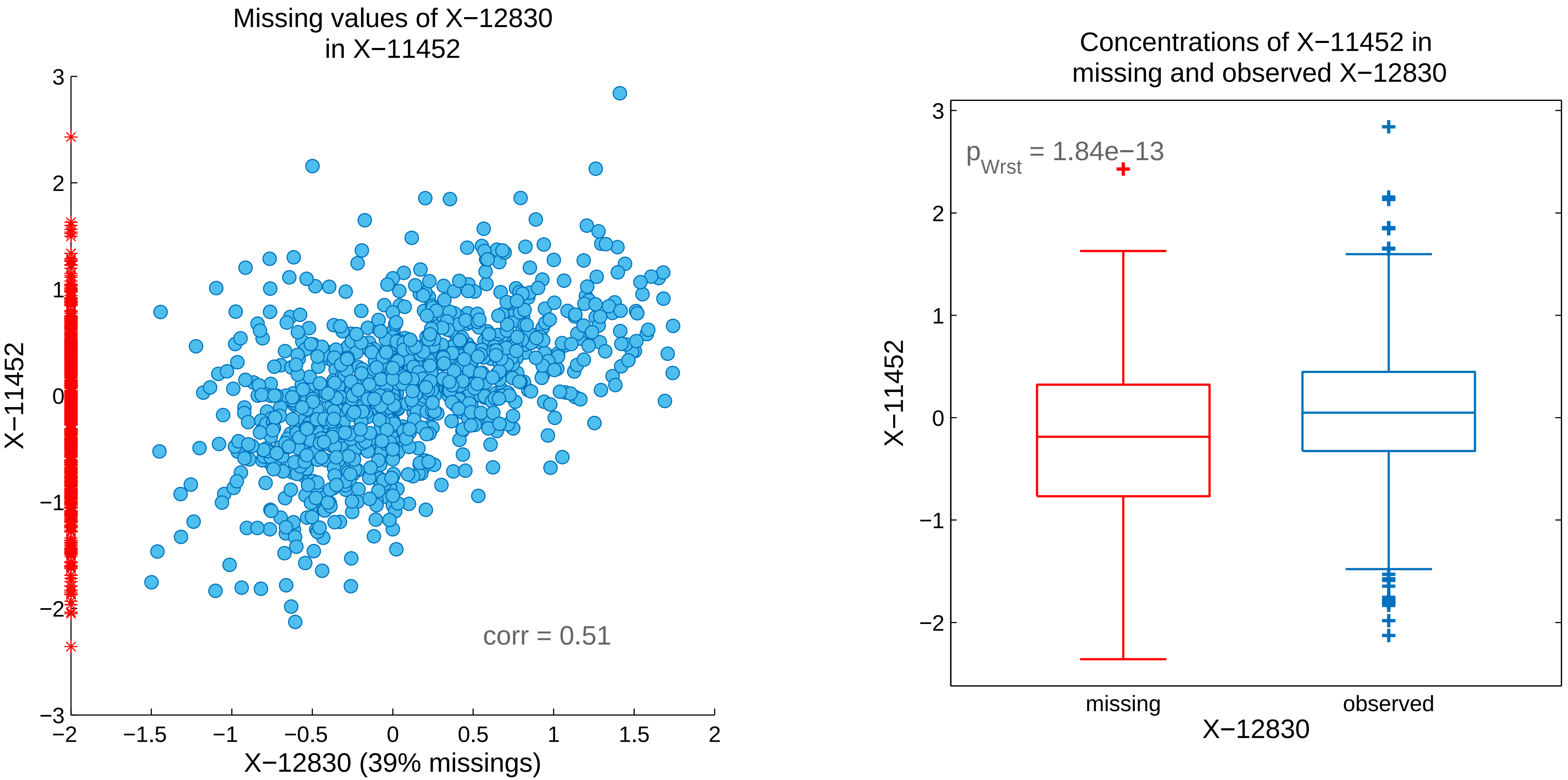

Missing values of X−12830
in X−11452
X−11452
X−12830 (39% missings)
corr = 0.51
Concentrations of X−11452 in
missing and observed X−12830
p_Wrst = 1.84e−13
X−11452
missing
observed
X−12830

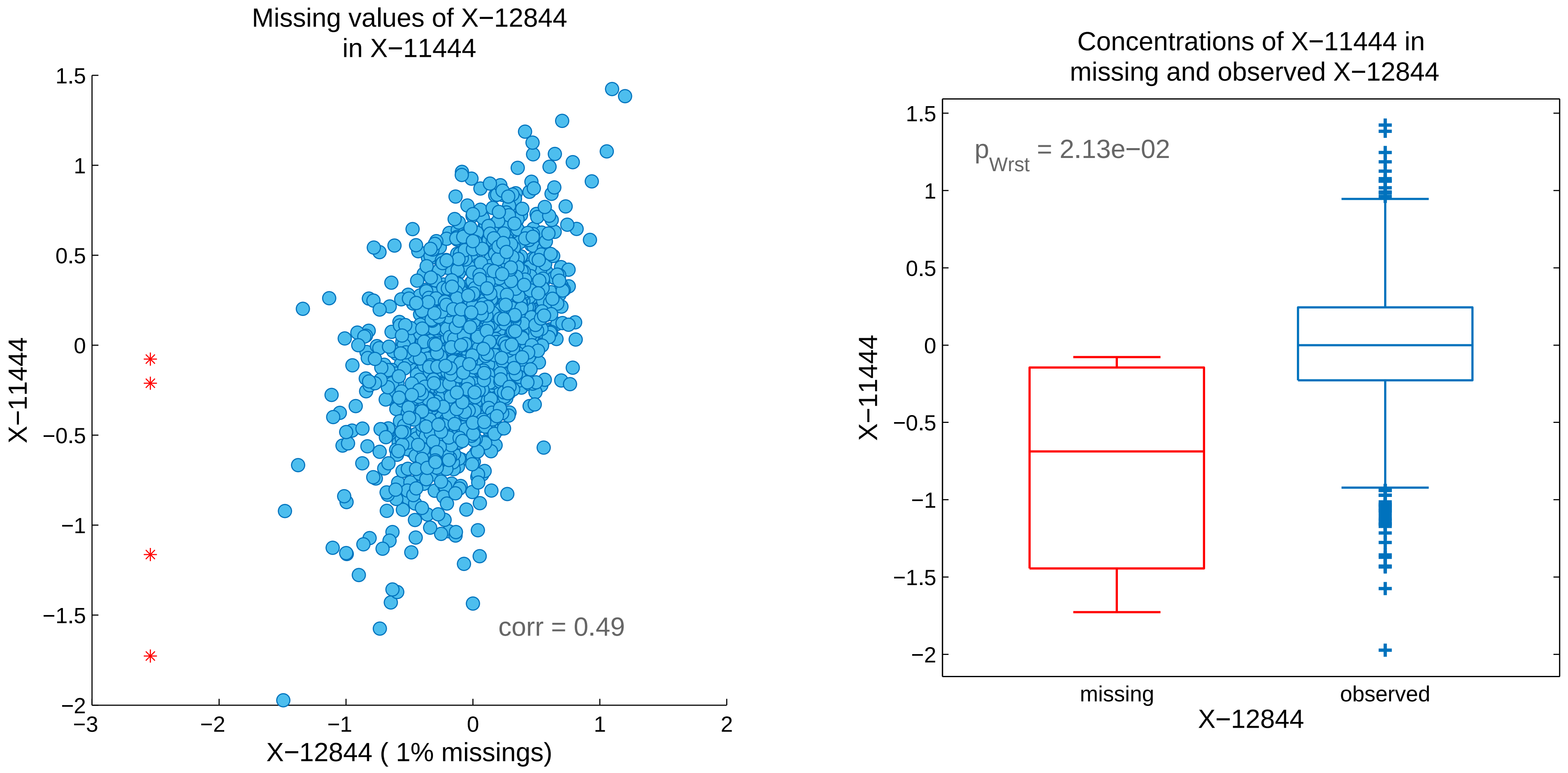

Missing values of X−12844
in X−11444
X−11444
X−12844 (1% missings)
corr = 0.49
1.5
1
0.5
0
−0.5
−1
−1.5
−2
−3
−2
−1
0
1
2
Concentrations of X−11444 in
missing and observed X−12844
p_Wrst = 2.13e−02
X−11444
missing
observed
X−12844

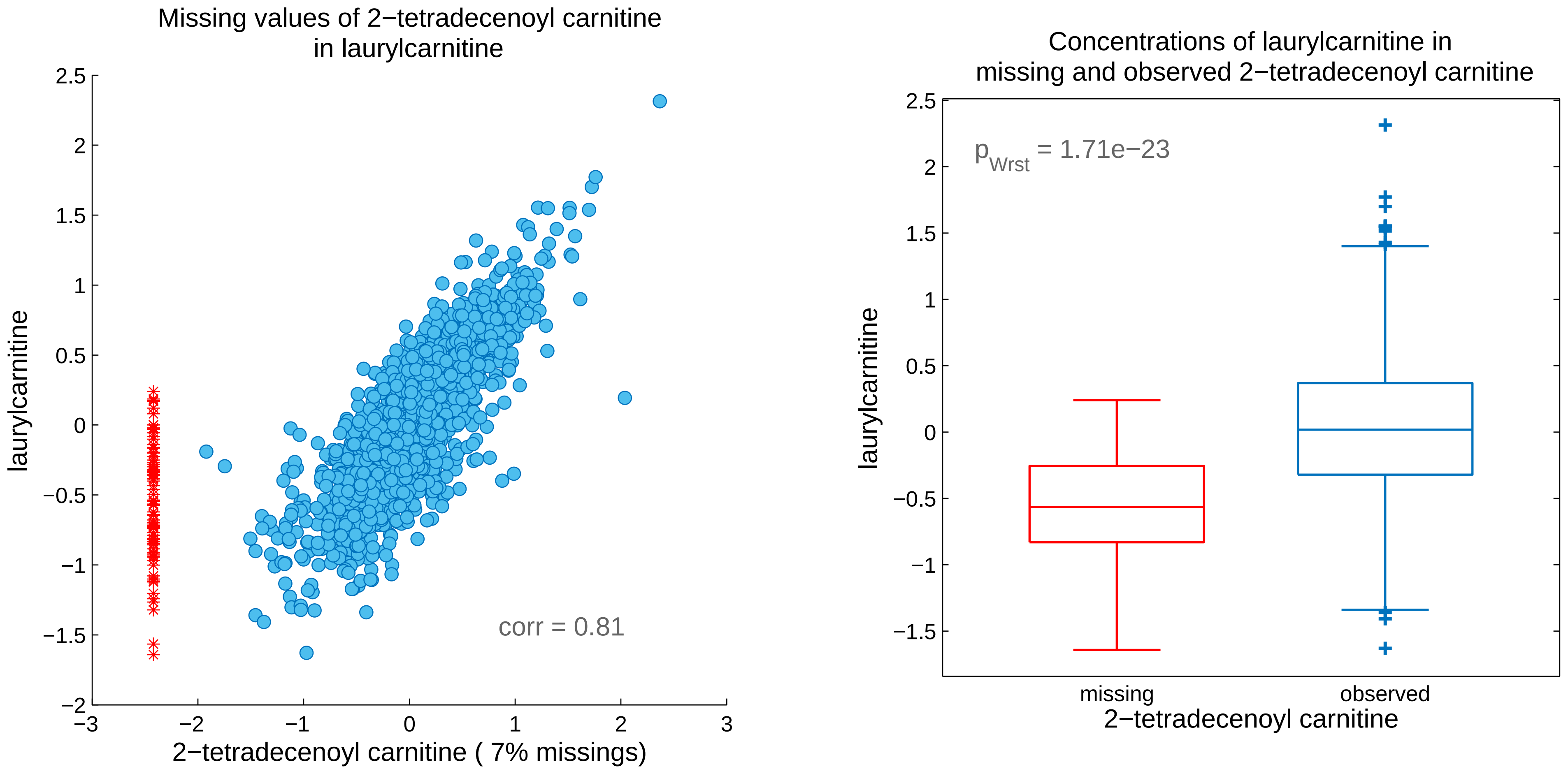
Missing values of 2−tetradecenoyl carnitine in laurylcarnitine
laurylcarnitine
2−tetradecenoyl carnitine (7% missings)
corr = 0.81
Concentrations of laurylcarnitine in missing and observed 2−tetradecenoyl carnitine
laurylcarnitine
p_{Wrst} = 1.71e−23
missing
observed
2−tetradecenoyl carnitine

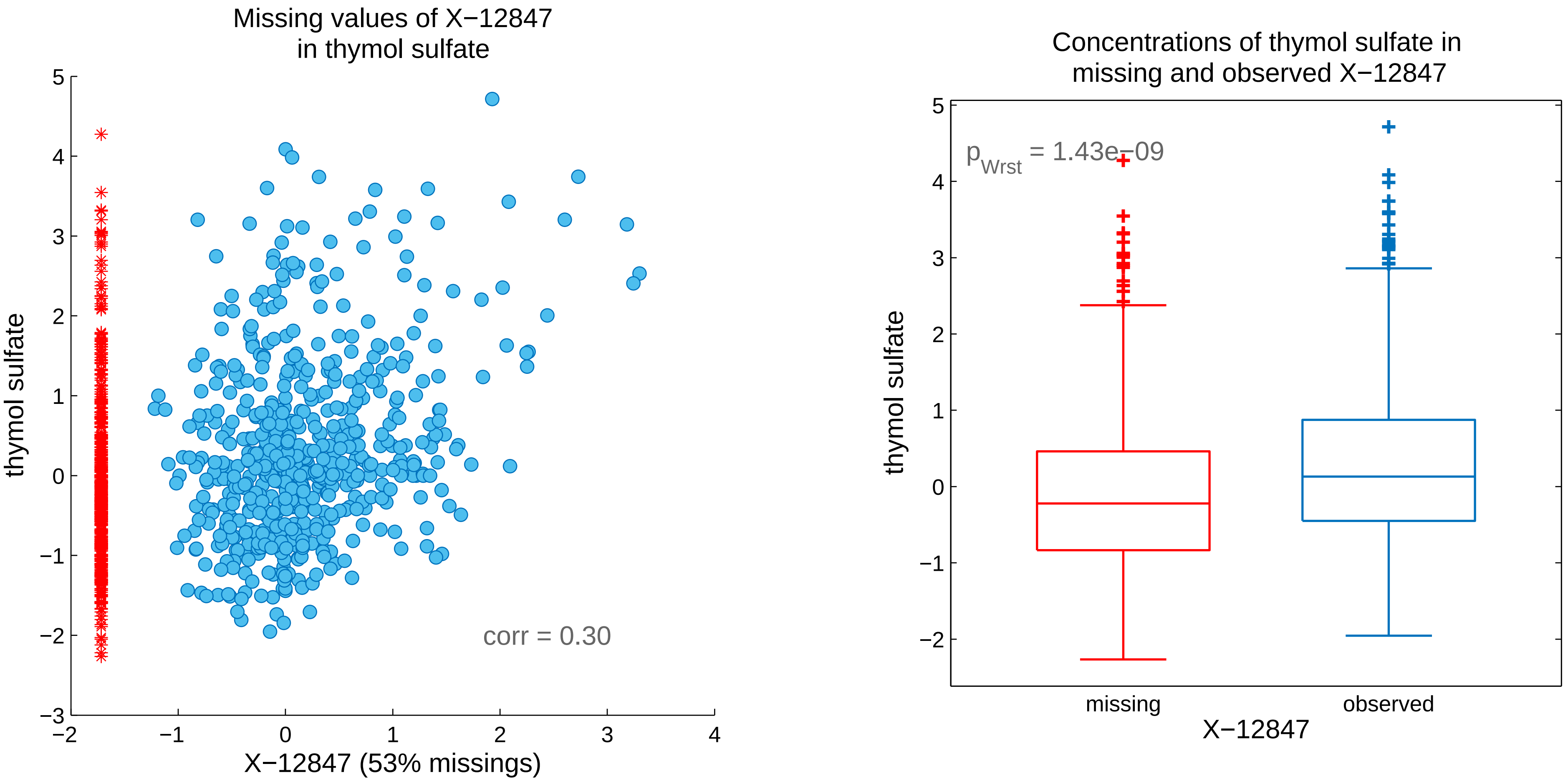

Missing values of X−12847
in thymol sulfate
thymol sulfate
X−12847 (53% missings)
corr = 0.30
Concentrations of thymol sulfate in
missing and observed X−12847
p_{Wrst} = 1.43e−09
thymol sulfate
missing
observed
X−12847

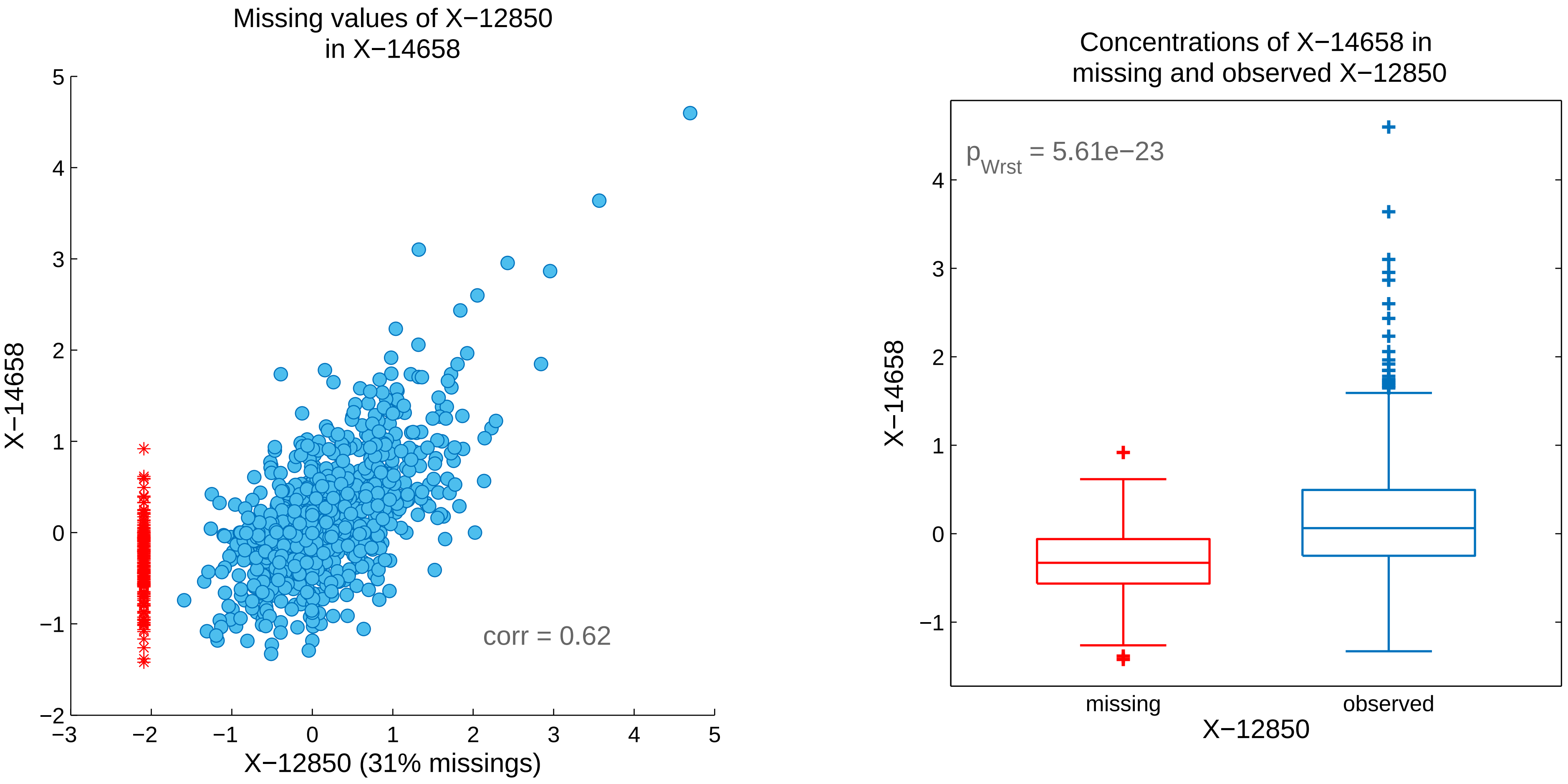

Missing values of X−12850
in X−14658
X−14658
X−12850 (31% missings)
corr = 0.62
Concentrations of X−14658 in
missing and observed X−12850
p$_{Wrst}$ = 5.61e−23
X−14658
missing
observed
X−12850

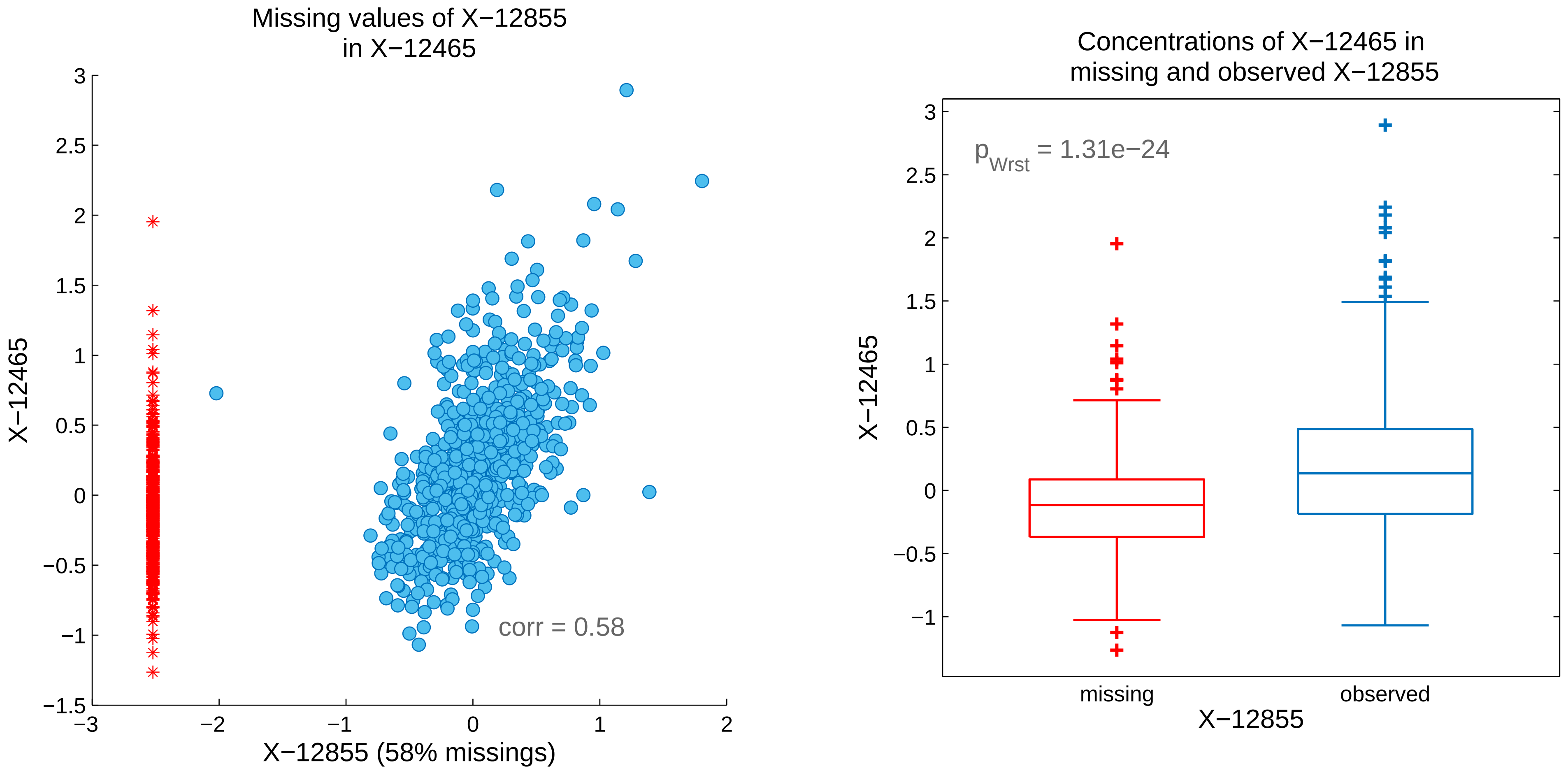

Missing values of X−12855 in X−12465
X−12465
X−12855 (58% missings)
corr = 0.58
Concentrations of X−12465 in missing and observed X−12855
p_{Wrst} = 1.31e−24
X−12465
missing
observed
X−12855

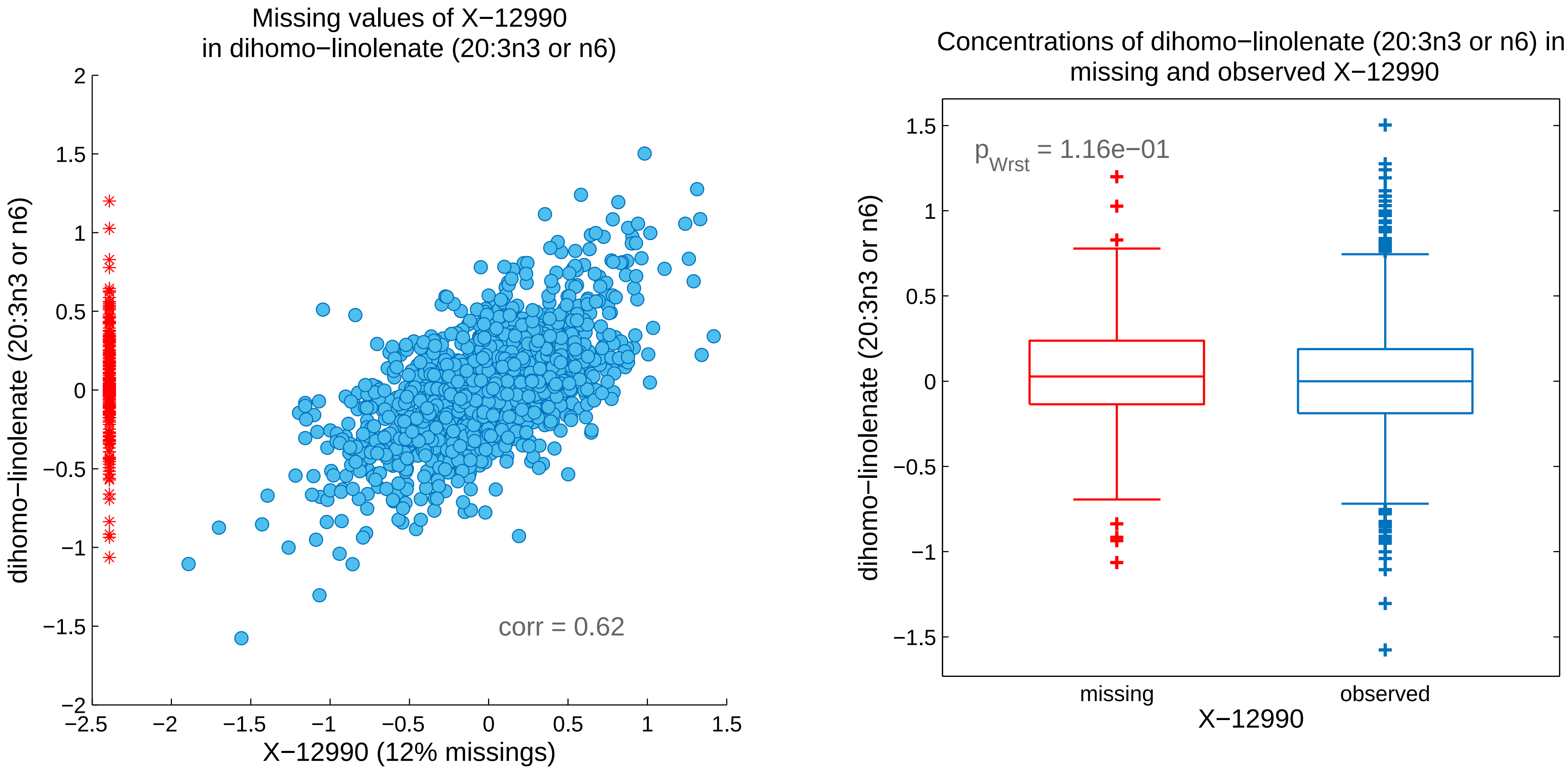

Missing values of X−12990
in dihomo−linolenate (20:3n3 or n6)
dihomo−linolenate (20:3n3 or n6)
X−12990 (12% missings)
corr = 0.62
2
1.5
1
0.5
0
−0.5
−1
−1.5
−2
−2.5
−2
−1.5
−1
−0.5
0
0.5
1
1.5
Concentrations of dihomo−linolenate (20:3n3 or n6) in
missing and observed X−12990
p_{Wrst} = 1.16e−01
dihomo−linolenate (20:3n3 or n6)
missing
observed
X−12990

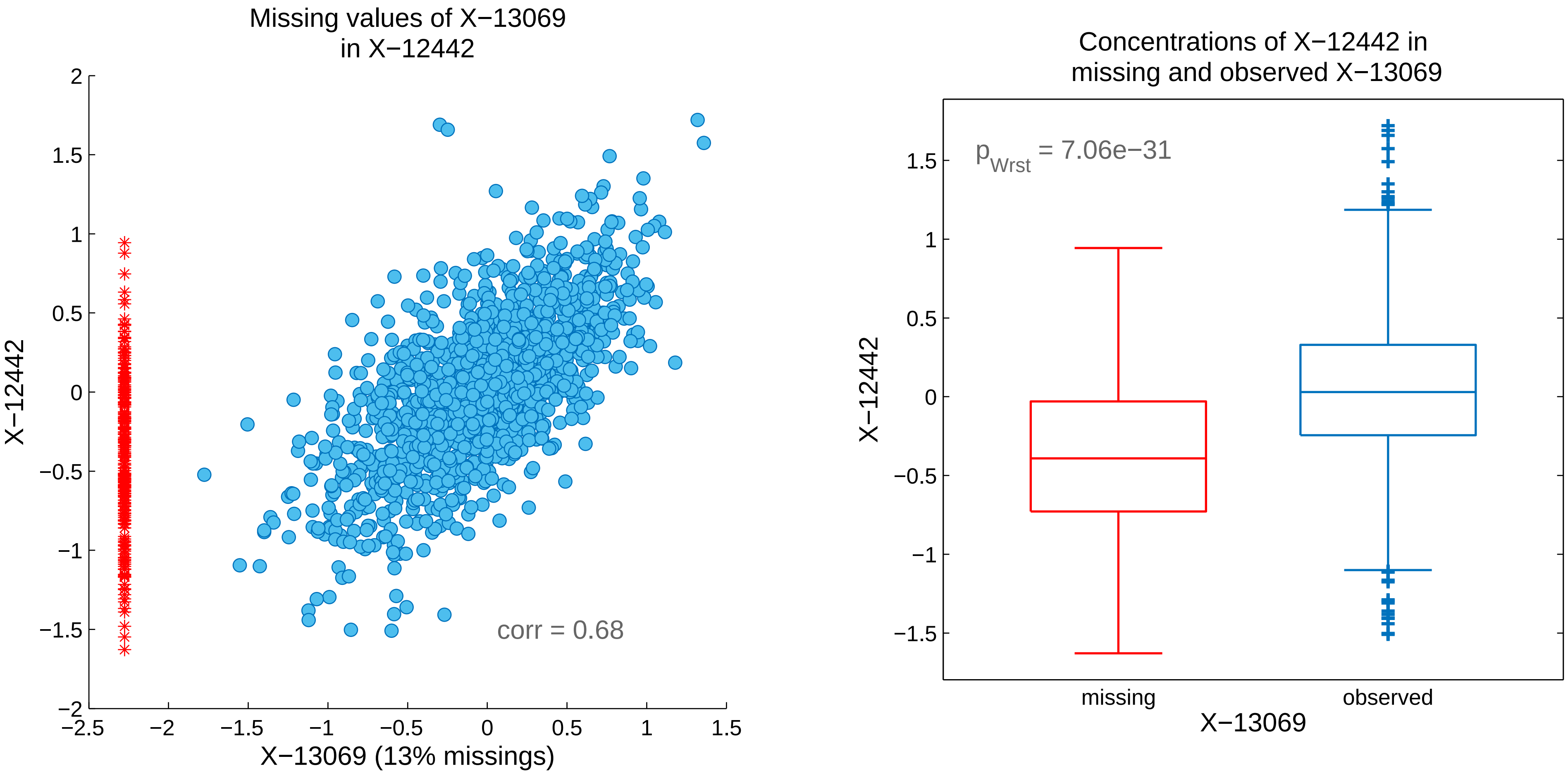

Missing values of X−13069 in X−12442
X−12442
X−13069 (13% missings)
corr = 0.68
Concentrations of X−12442 in missing and observed X−13069
p_{Wrst} = 7.06e−31
X−12442
missing
observed
X−13069

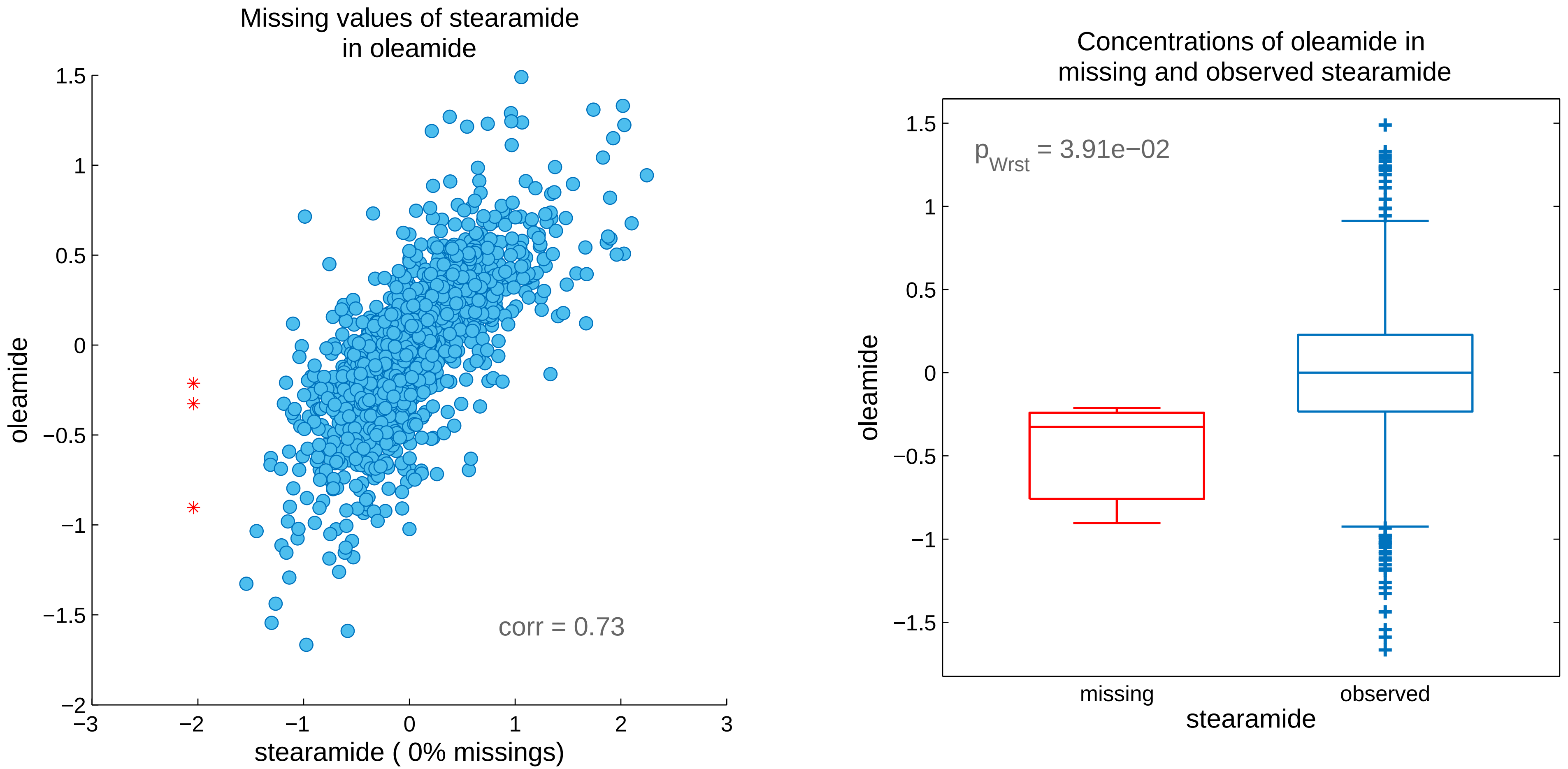
Missing values of stearamide
in oleamide
oleamide
stearamide (0% missings)
corr = 0.73
1.5
1
0.5
0
−0.5
−1
−1.5
−2
−3
−2
−1
0
1
2
3
Concentrations of oleamide in
missing and observed stearamide
p_Wrst = 3.91e−02
oleamide
missing
observed
stearamide

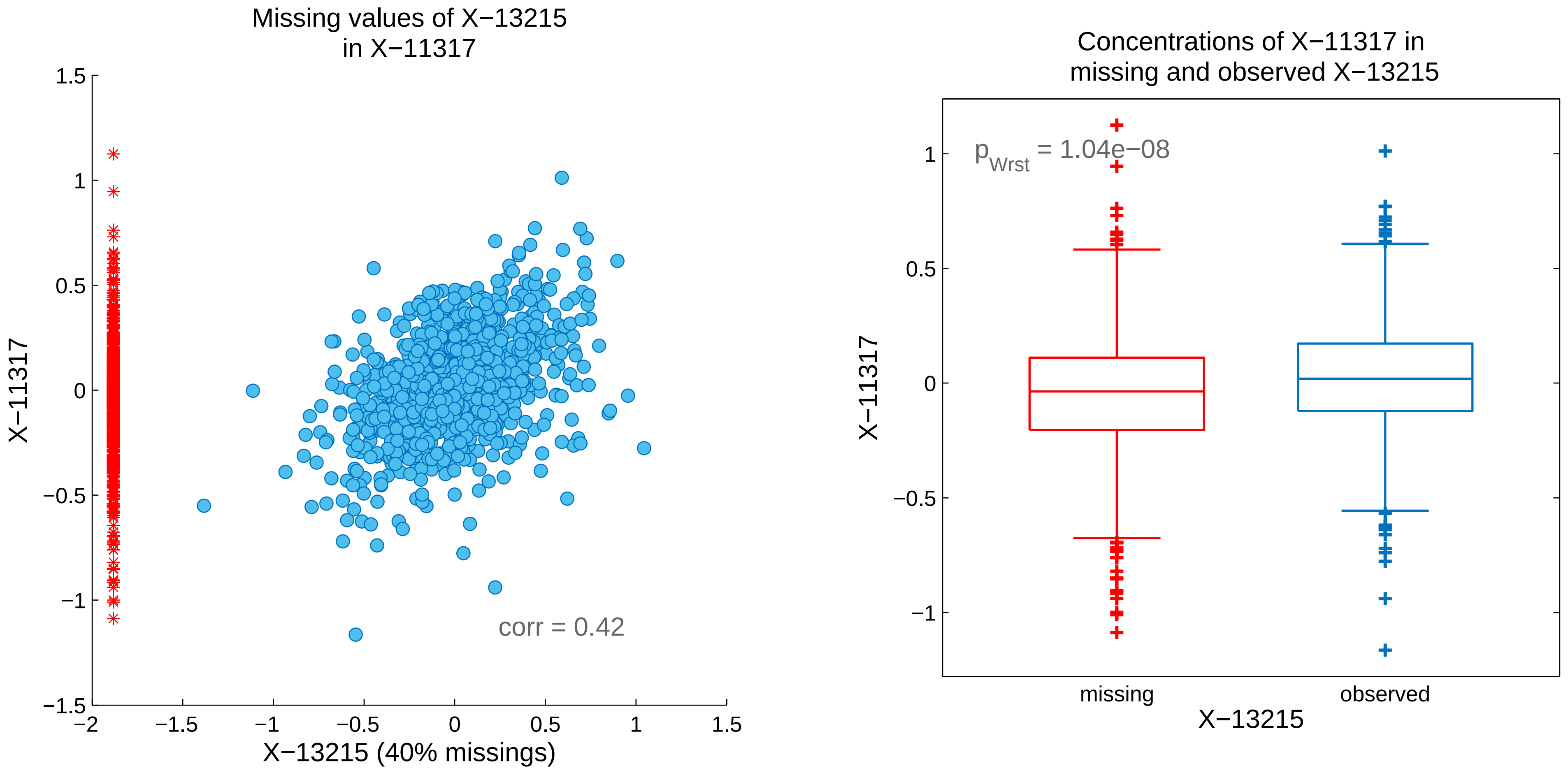
Missing values of X−13215
in X−11317
1.5
1
0.5
0
−0.5
−1
−1.5
X−11317
−2
−1.5
−1
−0.5
0
0.5
1
1.5
X−13215 (40% missings)
corr = 0.42
Concentrations of X−11317 in
missing and observed X−13215
p_{Wrst} = 1.04e−08
missing
observed
X−13215

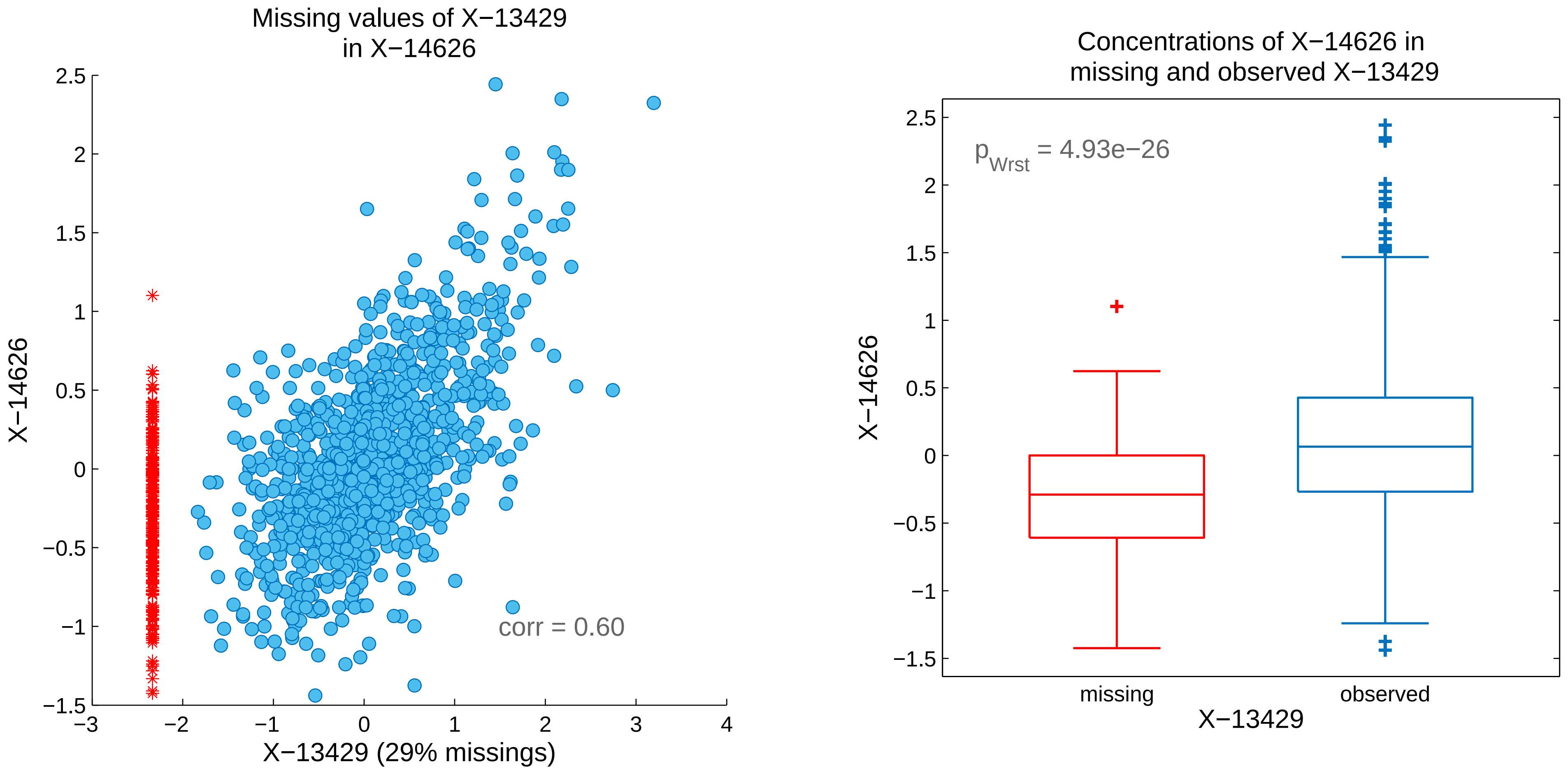

Missing values of X−13429 in X−14626
X−14626
X−13429 (29% missings)
corr = 0.60
2.5
2
1.5
1
0.5
0
−0.5
−1
−1.5
−3
−2
−1
0
1
2
3
4
Concentrations of X−14626 in missing and observed X−13429
p_{Wrst} = 4.93e−26
X−14626
X−13429
missing
observed

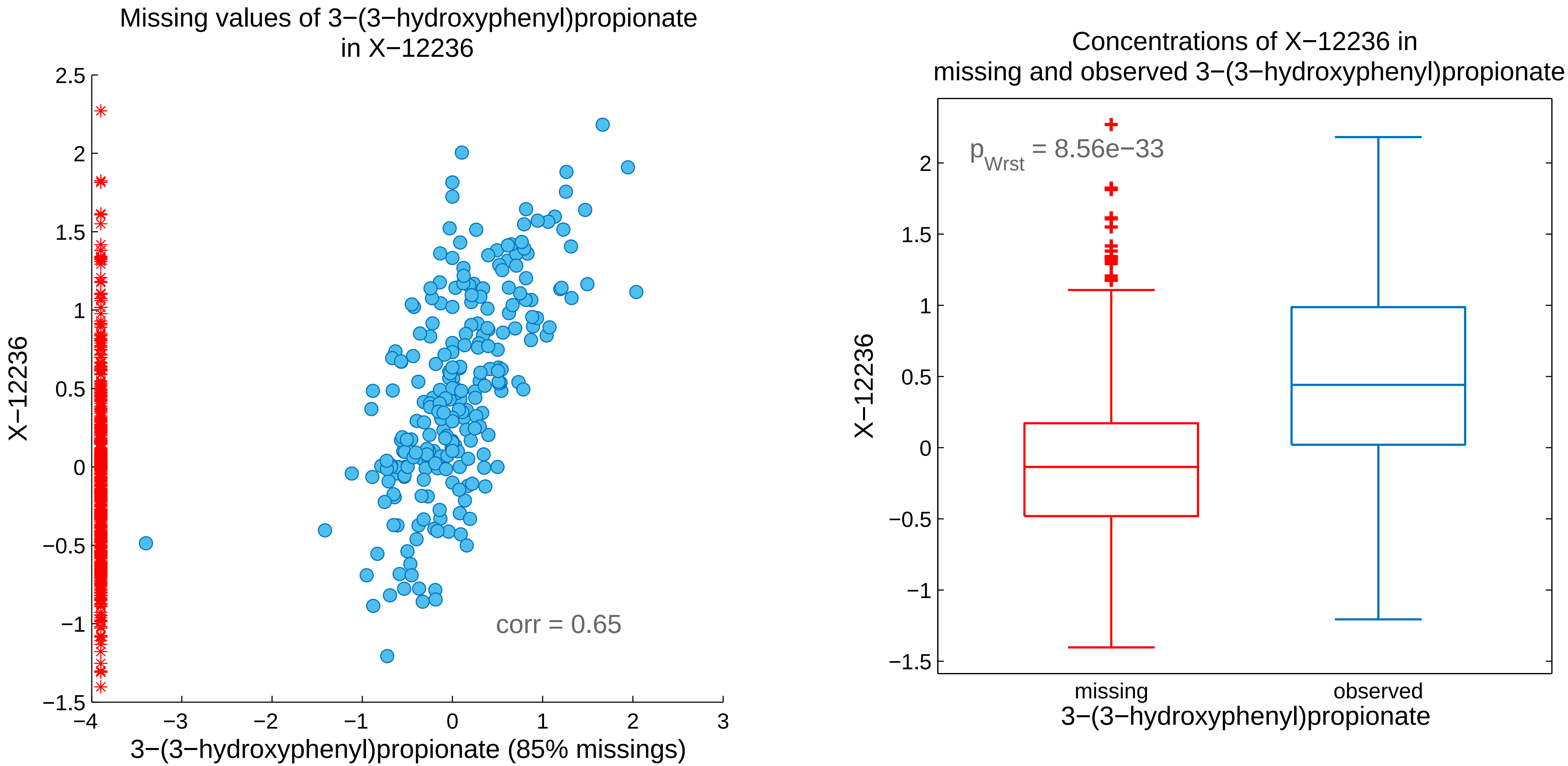

Missing values of 3−(3−hydroxyphenyl)propionate in X−12236
X−12236
3−(3−hydroxyphenyl)propionate (85% missings)
corr = 0.65
Concentrations of X−12236 in missing and observed 3−(3−hydroxyphenyl)propionate
p_{Wrst} = 8.56e−33
missing
observed
3−(3−hydroxyphenyl)propionate

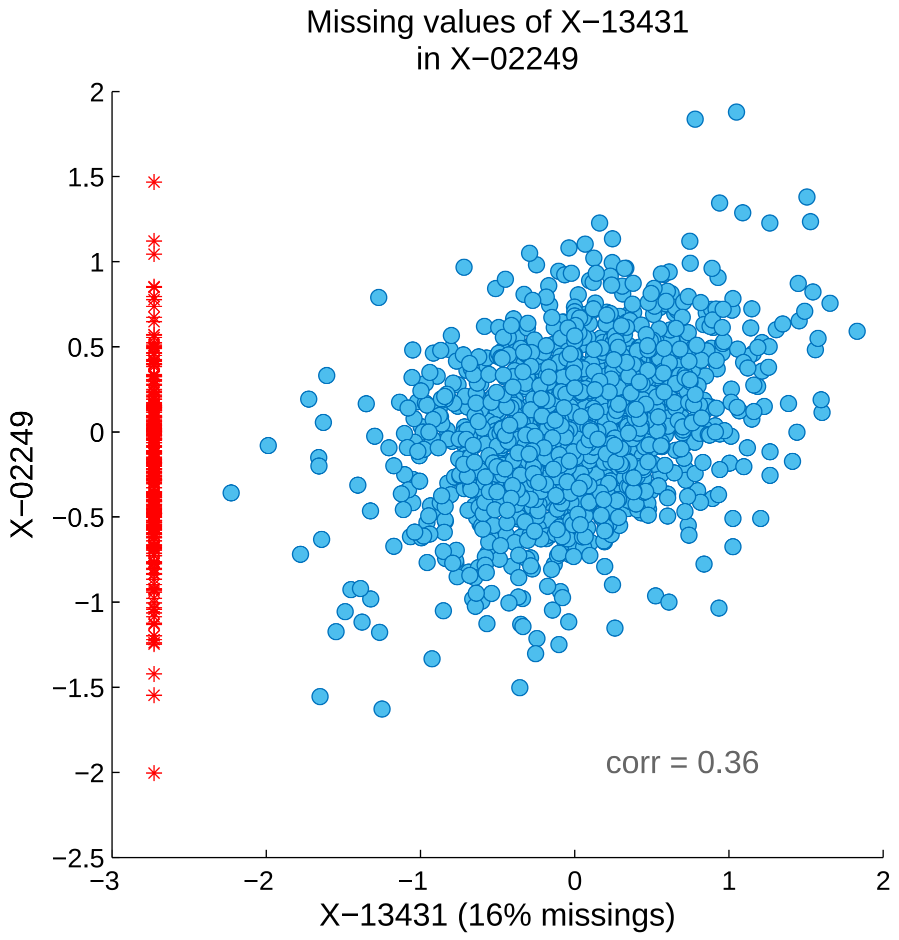
Missing values of X−13431
in X−02249
corr = 0.36
X−02249
X−13431 (16% missings)
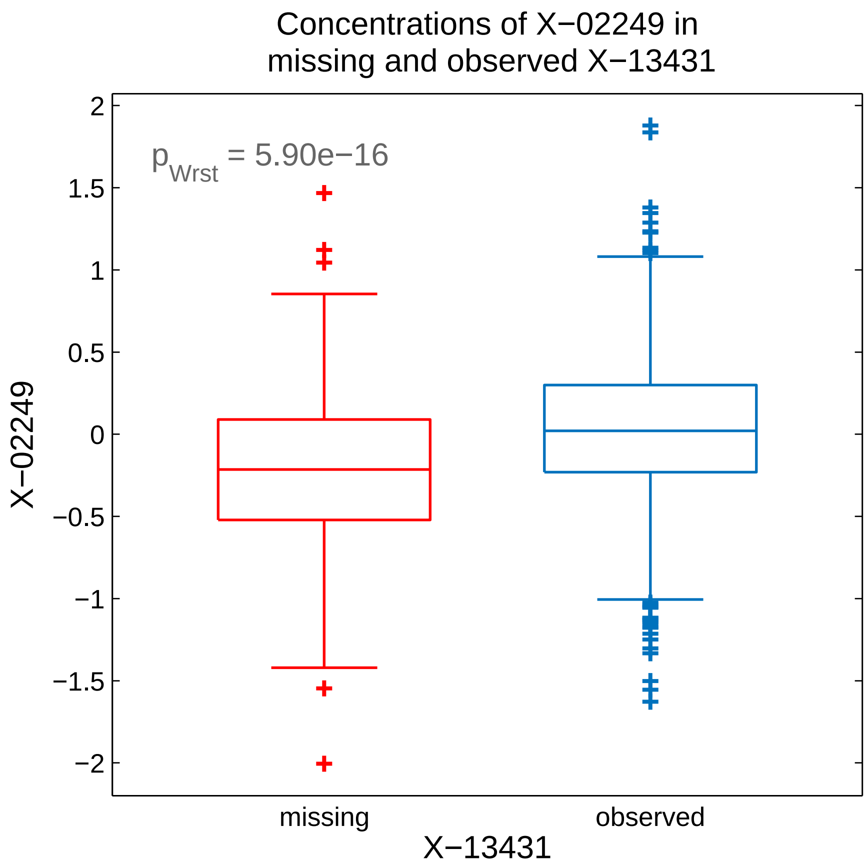
Concentrations of X−02249 in
missing and observed X−13431
p_Wrst = 5.90e−16
X−02249
missing
observed
X−13431

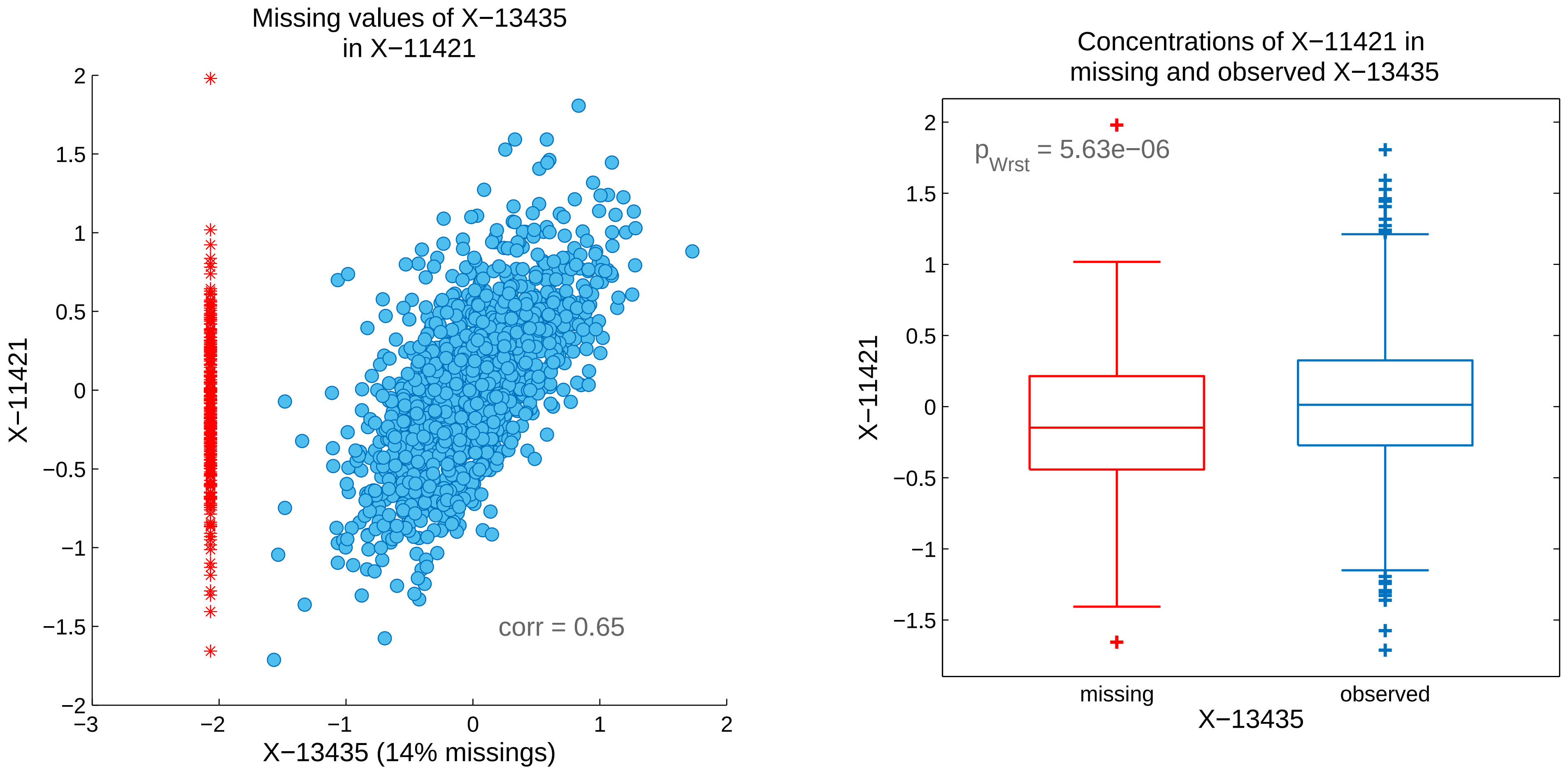

Missing values of X−13435
in X−11421
X−11421
X−13435 (14% missings)
corr = 0.65
2
1.5
1
0.5
0
−0.5
−1
−1.5
−2
−3
−2
−1
0
1
2
Concentrations of X−11421 in
missing and observed X−13435
p_{Wrst} = 5.63e−06
X−11421
missing
observed
X−13435

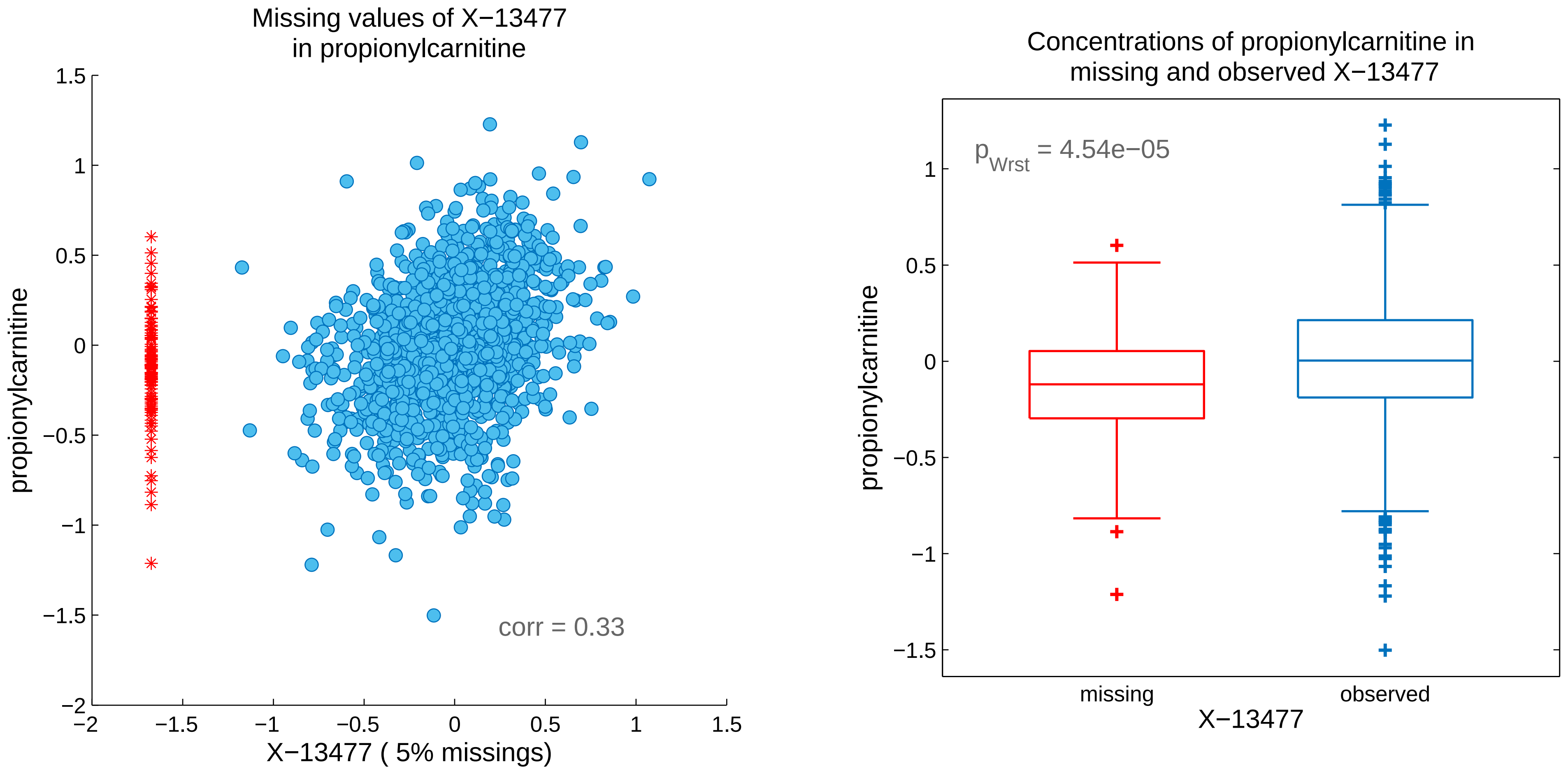

Missing values of X−13477 in propionylcarnitine
propionylcarnitine
X−13477 (5% missings)
corr = 0.33
Concentrations of propionylcarnitine in missing and observed X−13477
propionylcarnitine
p_{Wrst} = 4.54e−05
missing
observed
X−13477

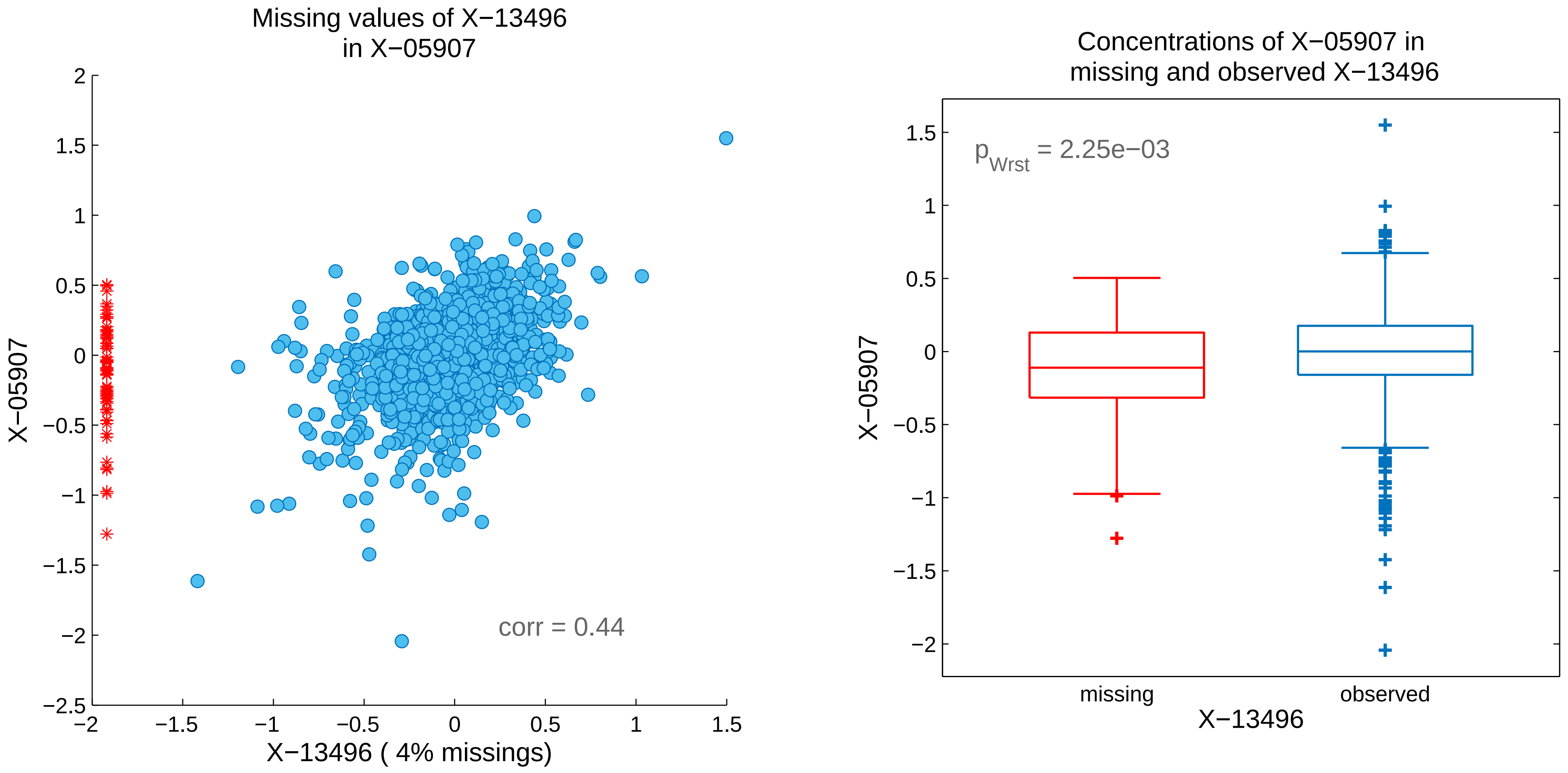

Missing values of X−13496
in X−05907
X−05907
X−13496 (4% missings)
corr = 0.44
Concentrations of X−05907 in
missing and observed X−13496
p_{Wrst} = 2.25e−03
X−05907
missing
observed
X−13496

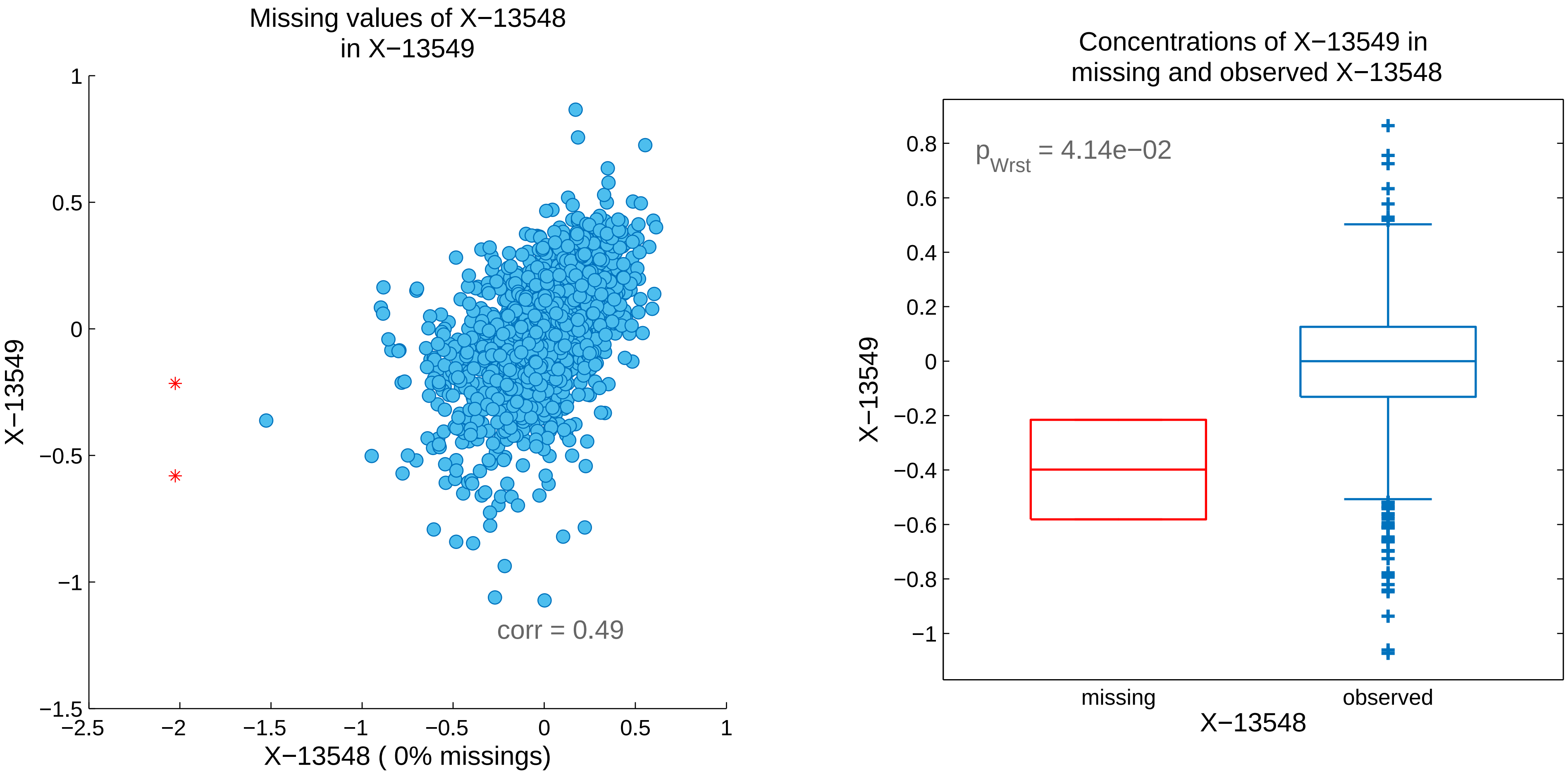
Missing values of X−13548
in X−13549
corr = 0.49
X−13549
X−13548 (0% missings)
1
0.5
0
−0.5
−1
−1.5
−2.5
−2
−1.5
−1
−0.5
0
0.5
1
Concentrations of X−13549 in
missing and observed X−13548
p_Wrst = 4.14e−02
X−13549
0.8
0.6
0.4
0.2
0
−0.2
−0.4
−0.6
−0.8
−1
missing
observed
X−13548

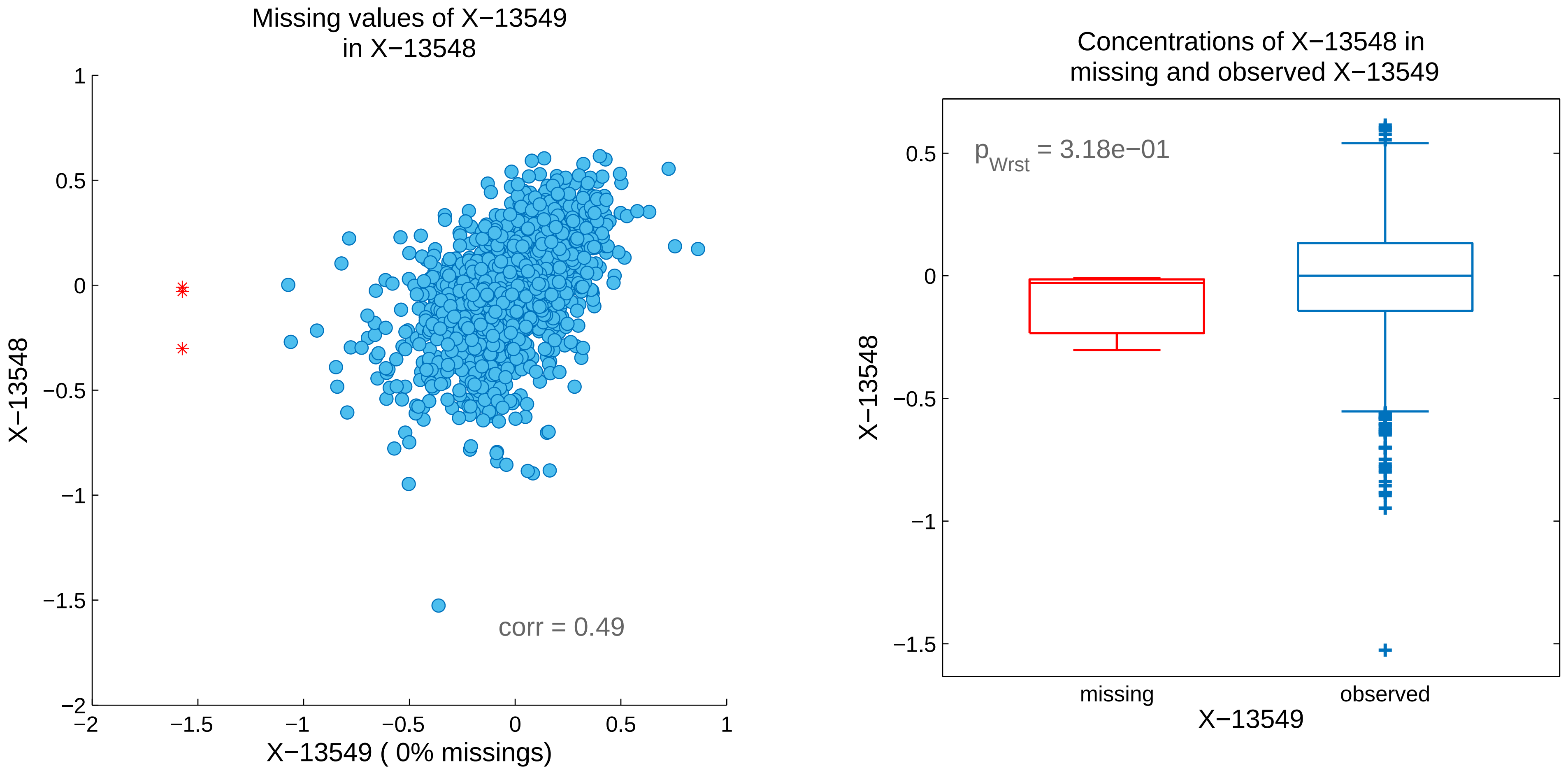
Missing values of X−13549
in X−13548
X−13548
X−13549 (0% missings)
corr = 0.49
1
0.5
0
−0.5
−1
−1.5
−2
−2
−1.5
−1
−0.5
0
0.5
1
Concentrations of X−13548 in
missing and observed X−13549
p_{Wrst} = 3.18e−01
X−13548
0.5
0
−0.5
−1
−1.5
missing
observed
X−13549

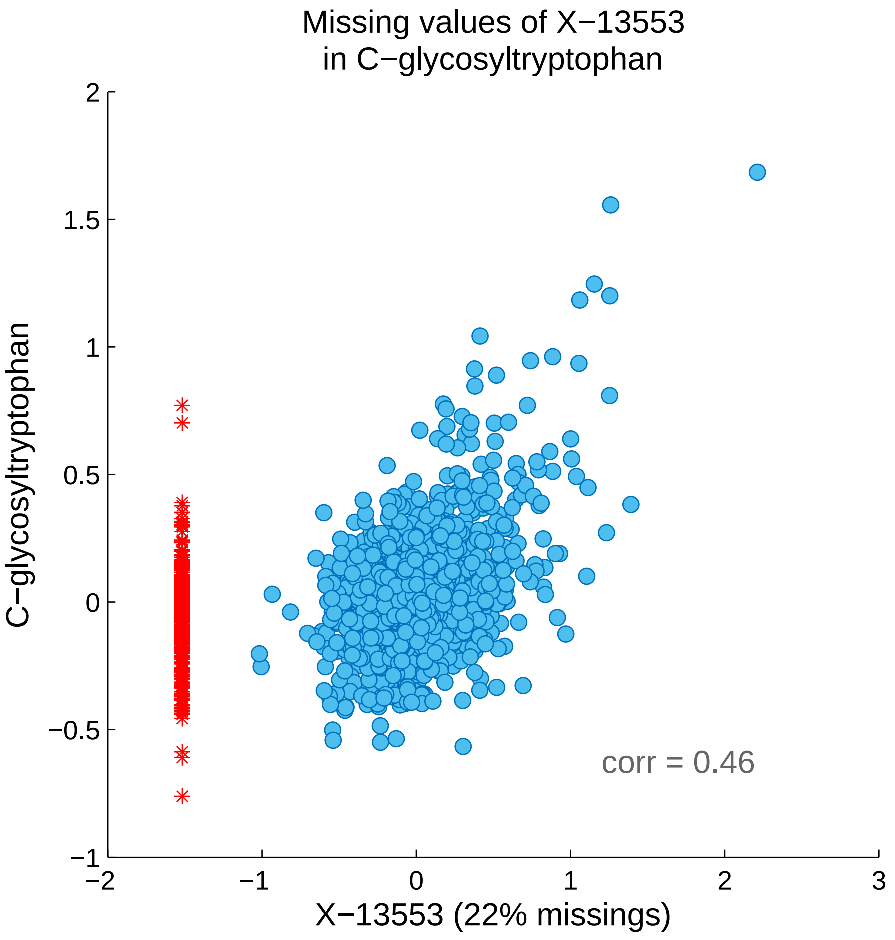
Missing values of X−13553
in C−glycosyltryptophan
C−glycosyltryptophan
X−13553 (22% missings)
corr = 0.46
2
1.5
1
0.5
0
−0.5
−1
−2
−1
0
1
2
3

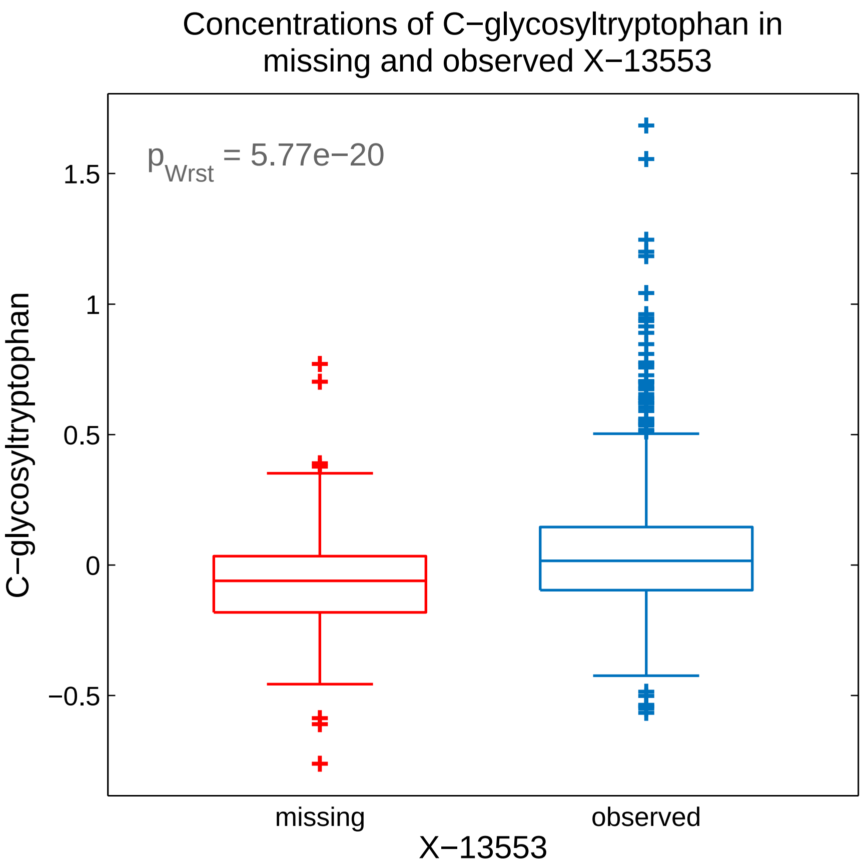
Concentrations of C−glycosyltryptophan in
missing and observed X−13553
p_{Wrst} = 5.77e−20
C−glycosyltryptophan
1.5
1
0.5
0
−0.5
missing
observed
X−13553

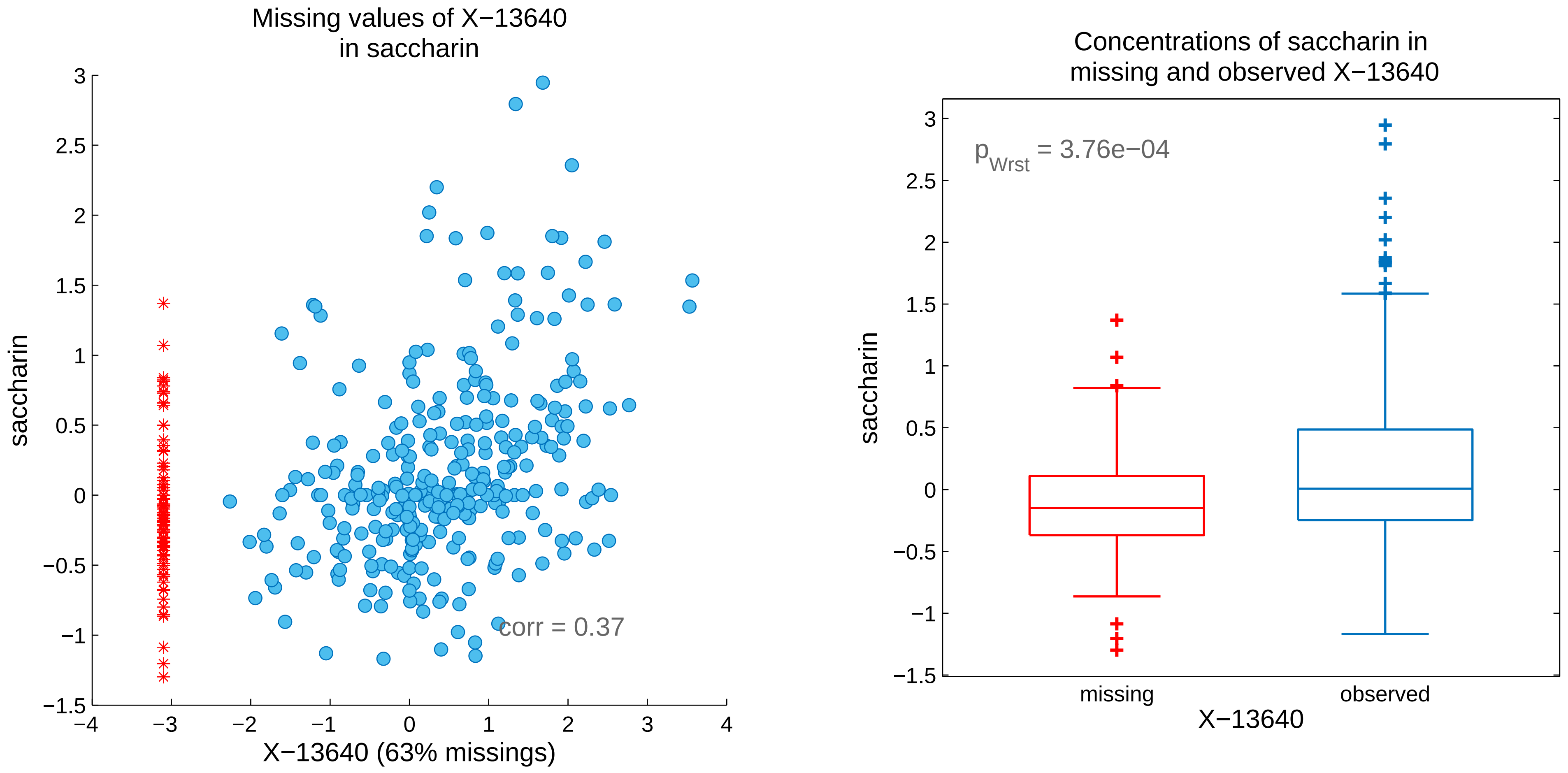

Missing values of X−13640
in saccharin
saccharin
X−13640 (63% missings)
corr = 0.37
Concentrations of saccharin in
missing and observed X−13640
p_{Wrst} = 3.76e−04
saccharin
missing
observed
X−13640

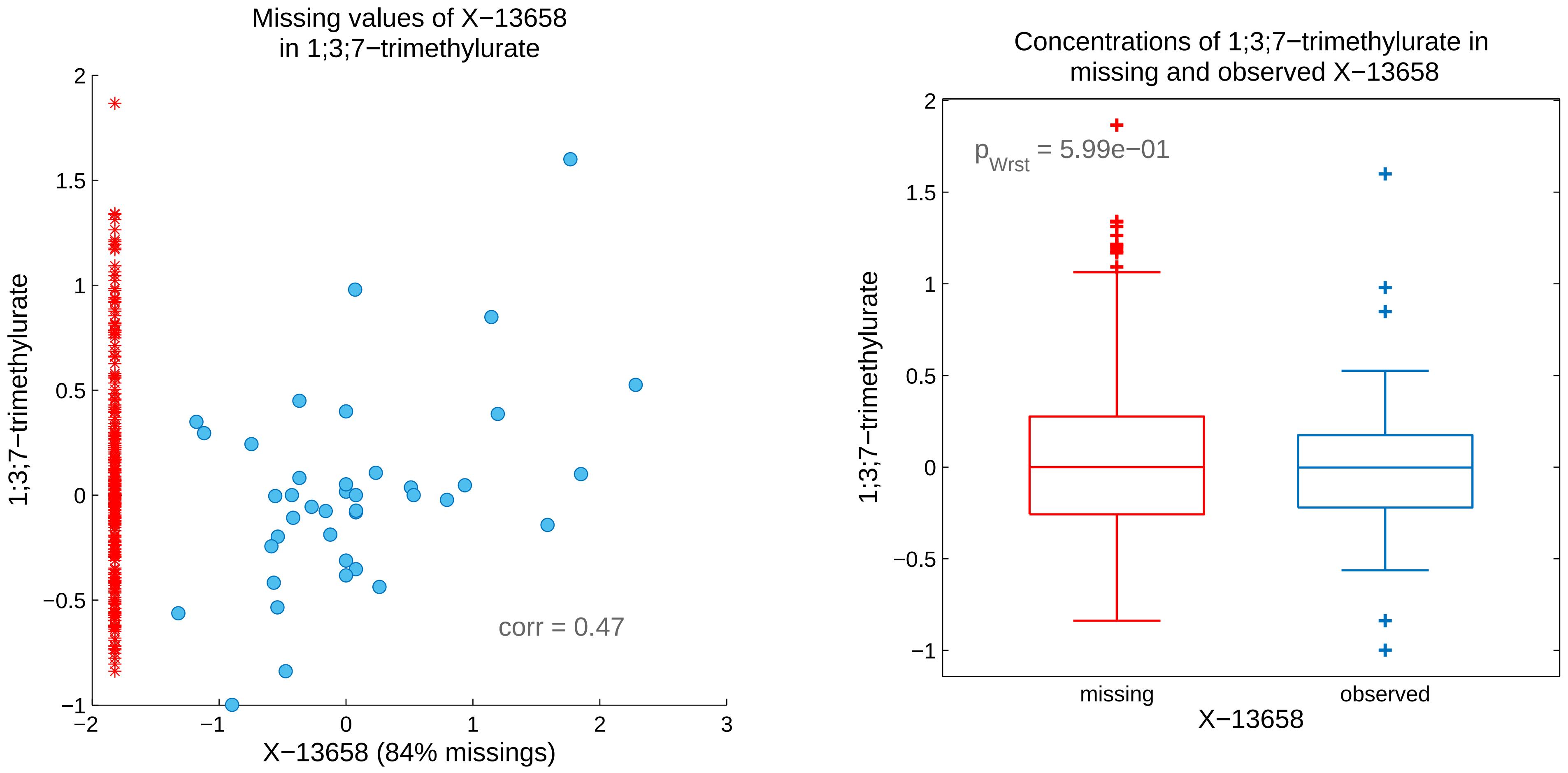
Missing values of X−13658
in 1;3;7−trimethylurate
1;3;7−trimethylurate
X−13658 (84% missings)
corr = 0.47
Concentrations of 1;3;7−trimethylurate in
missing and observed X−13658
p_Wrst = 5.99e−01
1;3;7−trimethylurate
missing
observed
X−13658

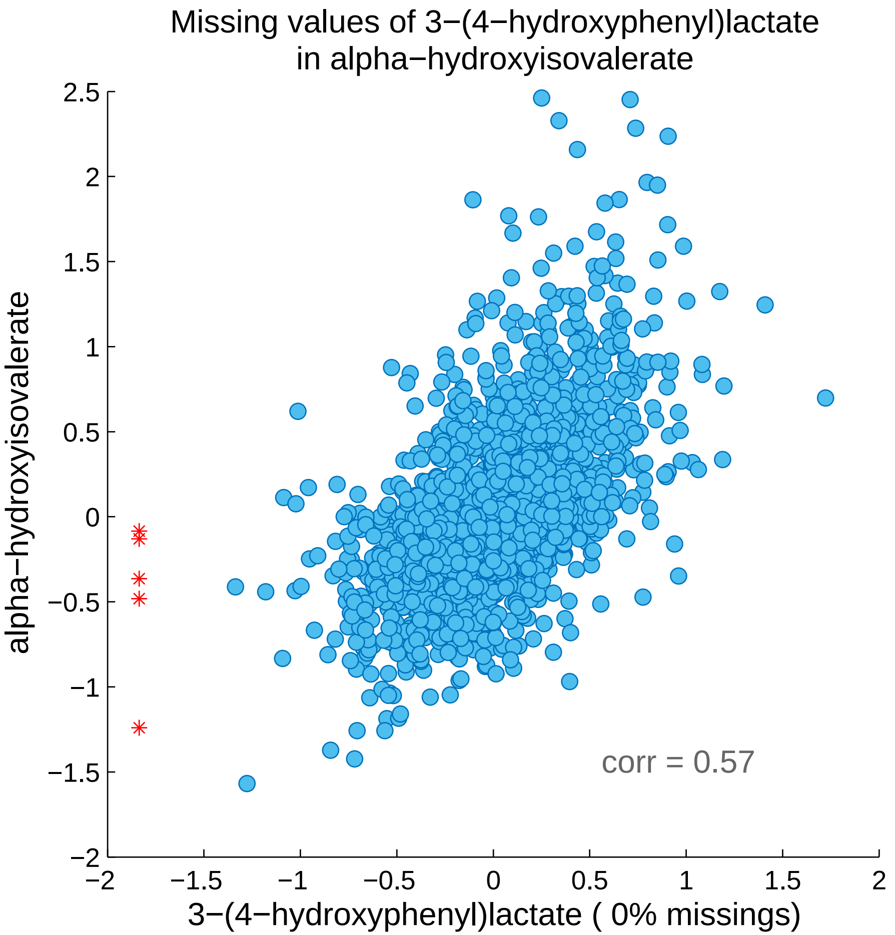

Missing values of 3−(4−hydroxyphenyl)lactate in alpha−hydroxyisovalerate
alpha−hydroxyisovalerate
3−(4−hydroxyphenyl)lactate (0% missings)
corr = 0.57

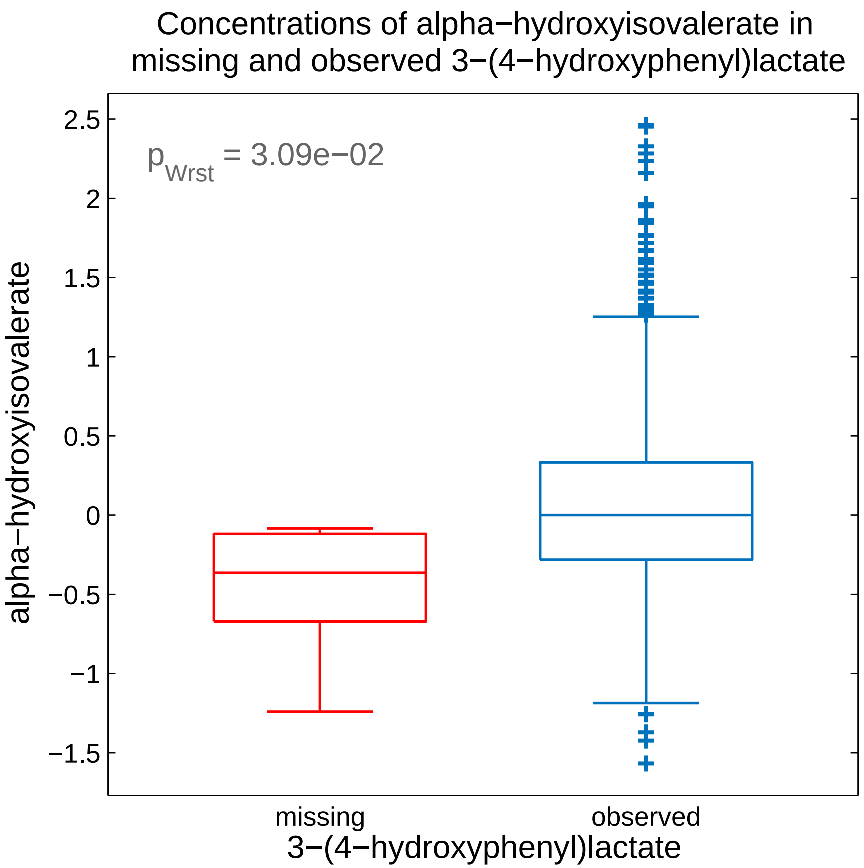

Concentrations of alpha−hydroxyisovalerate in missing and observed 3−(4−hydroxyphenyl)lactate
alpha−hydroxyisovalerate
p_Wrst = 3.09e−02
missing
observed
3−(4−hydroxyphenyl)lactate

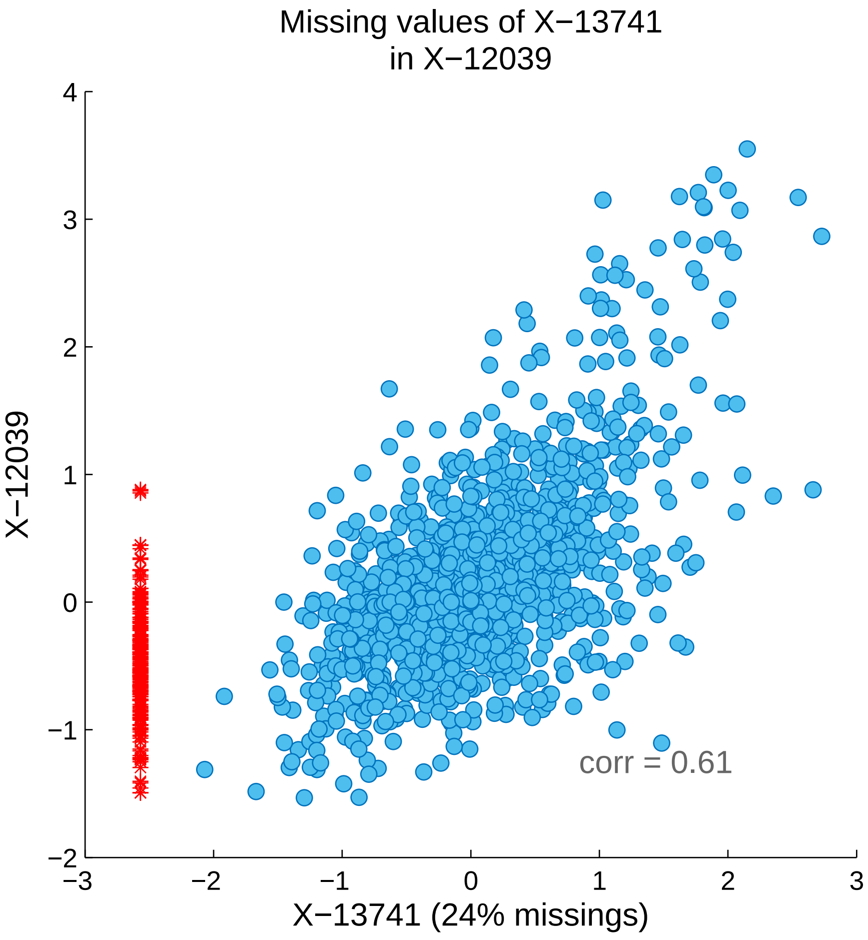
Missing values of X−13741
in X−12039
X−12039
X−13741 (24% missings)
corr = 0.61

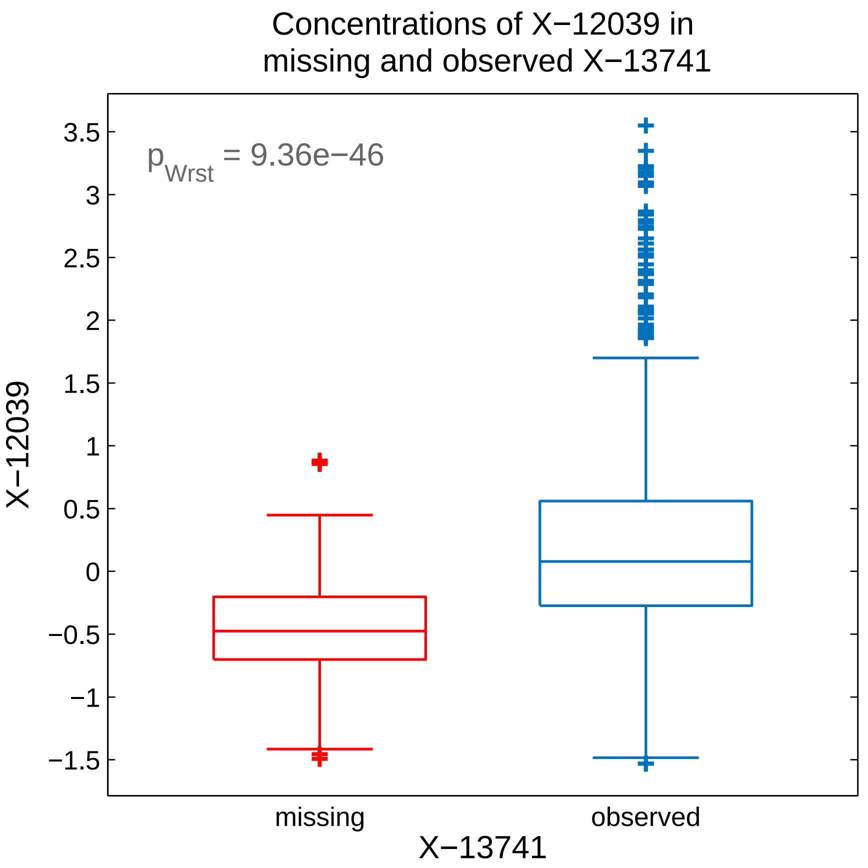
Concentrations of X−12039 in
missing and observed X−13741
p_{Wrst} = 9.36e−46
X−12039
missing
observed
X−13741

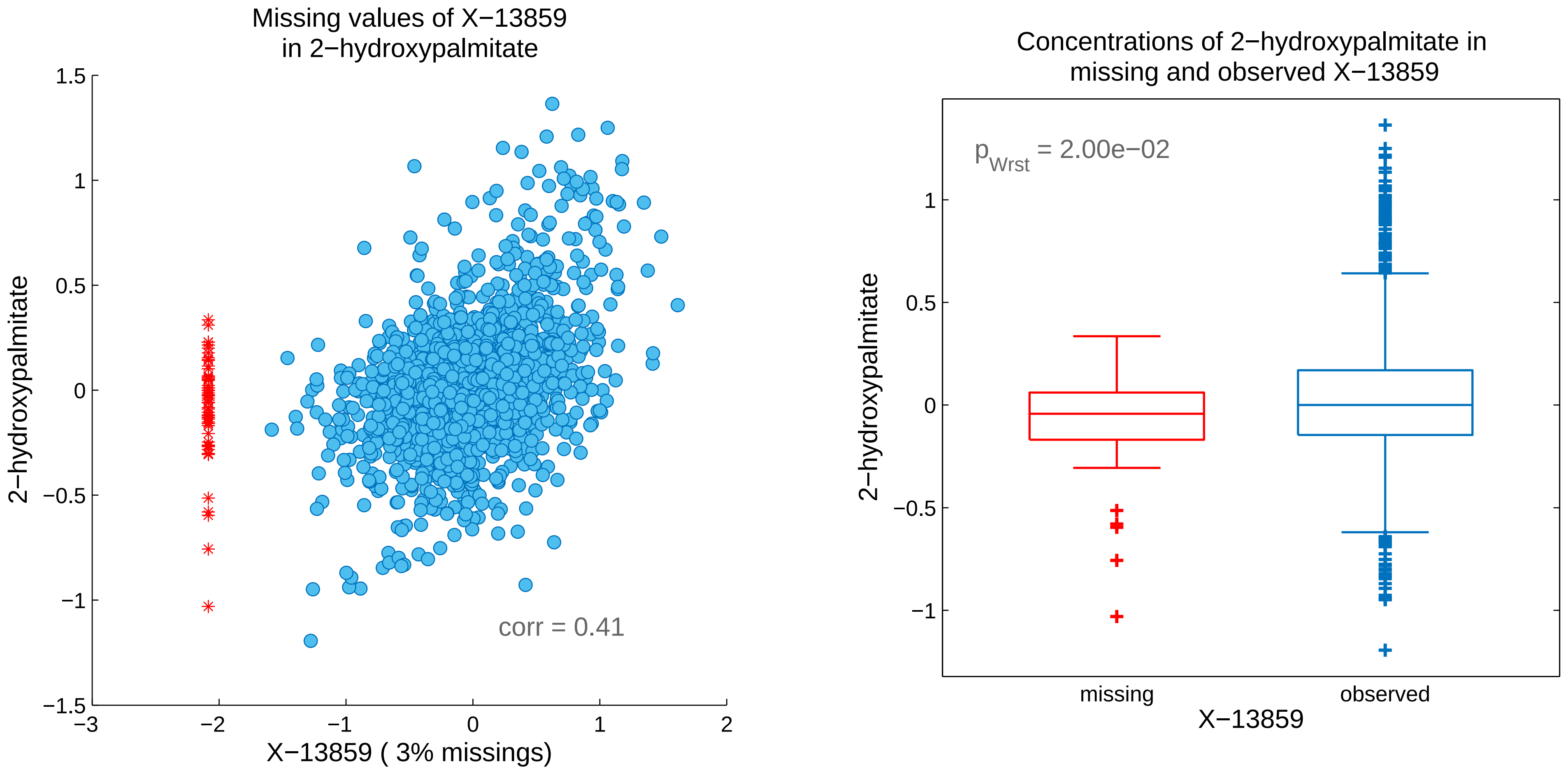

Missing values of X−13859
in 2−hydroxypalmitate
2−hydroxypalmitate
X−13859 (3% missings)
corr = 0.41
Concentrations of 2−hydroxypalmitate in
missing and observed X−13859
p_{Wrst} = 2.00e−02
2−hydroxypalmitate
missing
observed
X−13859

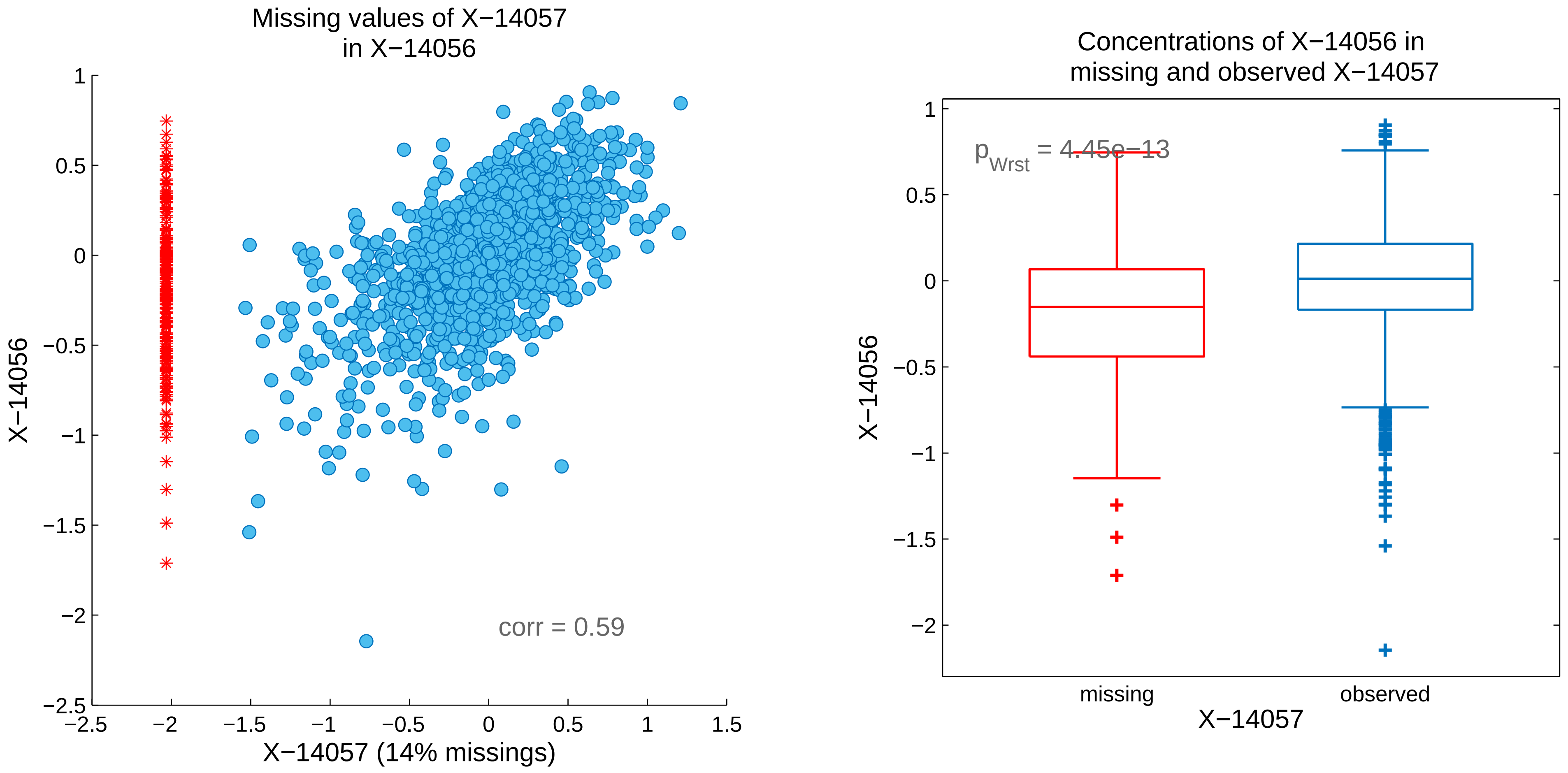

Missing values of X−14057
in X−14056
X−14056
X−14057 (14% missings)
corr = 0.59
Concentrations of X−14056 in
missing and observed X−14057
p_{Wrst} = 4.45e−13
missing
observed
X−14057

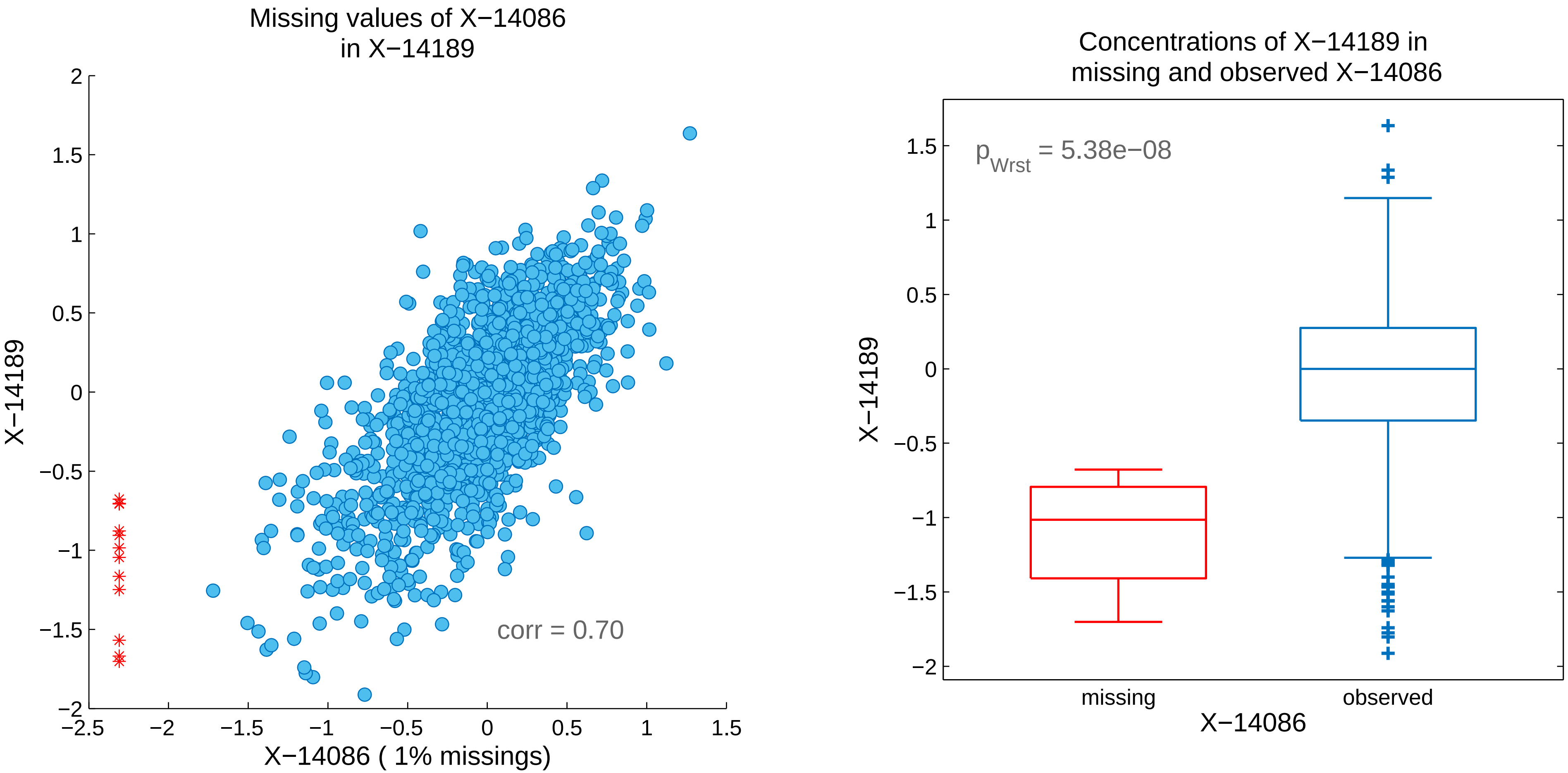
Missing values of X−14086
in X−14189
X−14189
X−14086 (1% missings)
corr = 0.70
Concentrations of X−14189 in
missing and observed X−14086
p_Wrst = 5.38e−08
X−14189
missing
observed
X−14086

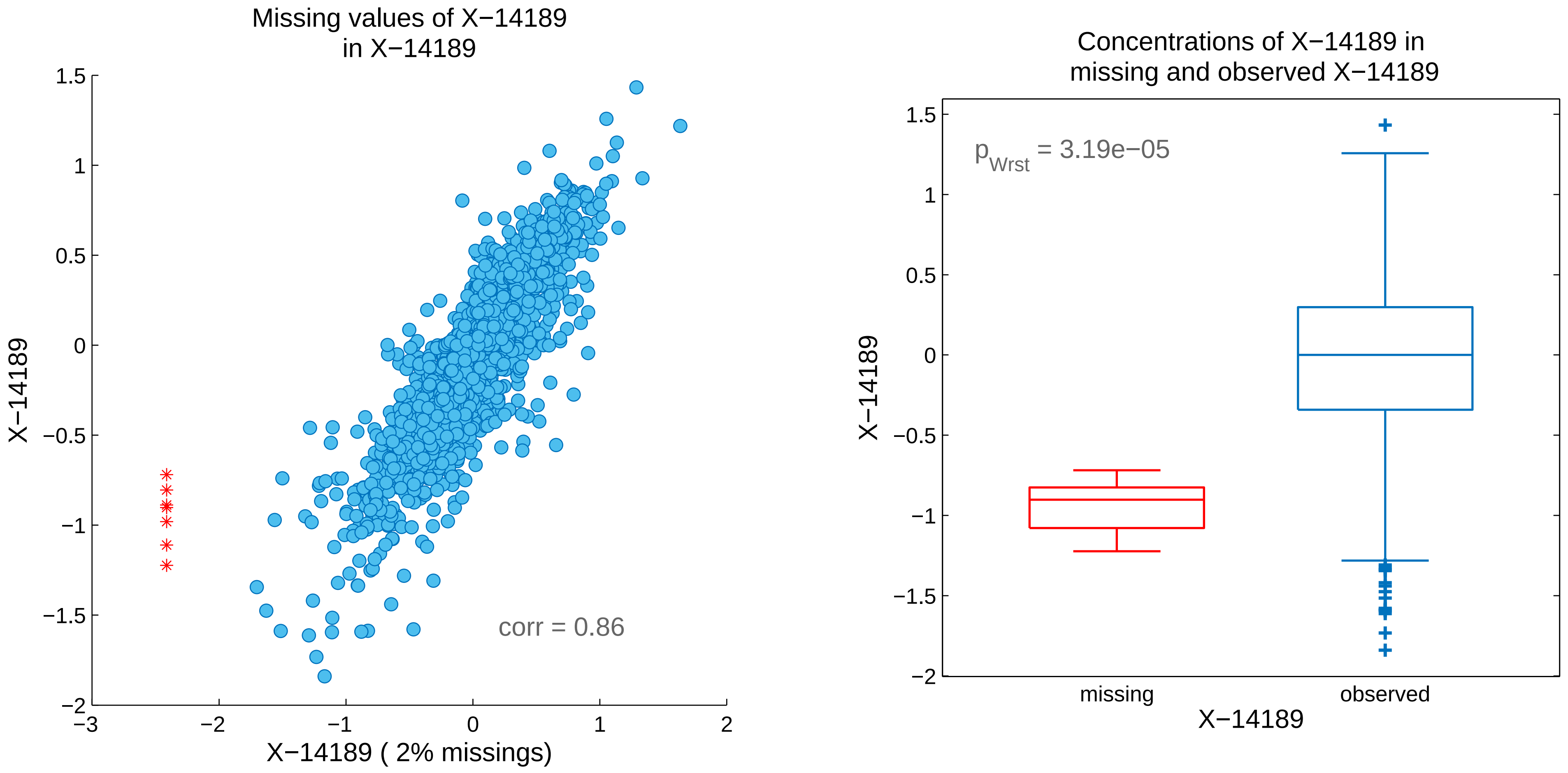

Missing values of X−14189
in X−14189
1.5
1
0.5
0
−0.5
−1
−1.5
−2
−3
−2
−1
0
1
2
X−14189
X−14189 (2% missings)
corr = 0.86
Concentrations of X−14189 in
missing and observed X−14189
1.5
1
0.5
0
−0.5
−1
−1.5
−2
p_Wrst = 3.19e−05
X−14189
missing
observed
X−14189

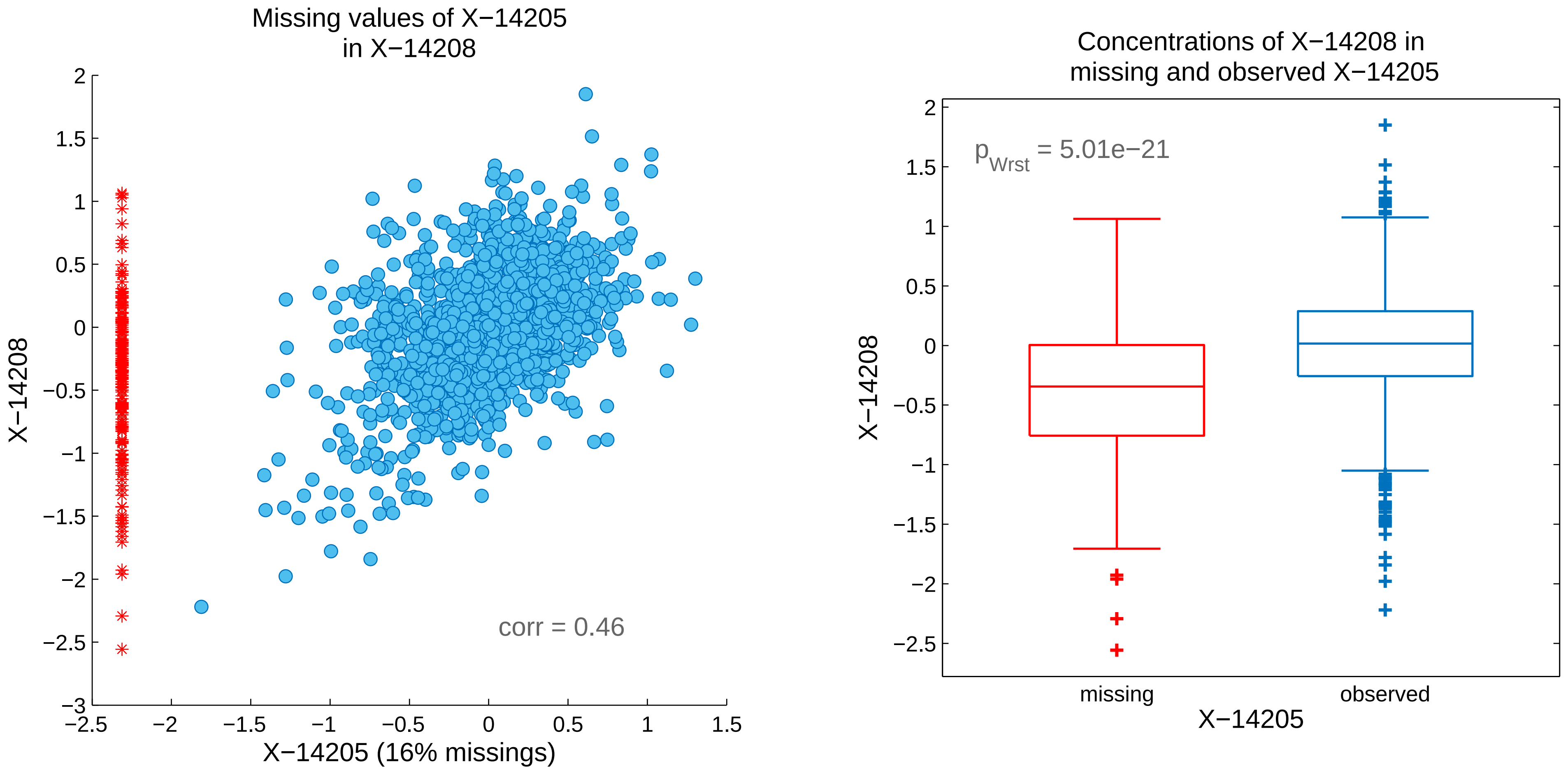

Missing values of X−14205
in X−14208
X−14208
X−14205 (16% missings)
corr = 0.46
Concentrations of X−14208 in
missing and observed X−14205
p_{Wrst} = 5.01e−21
X−14208
missing
observed
X−14205

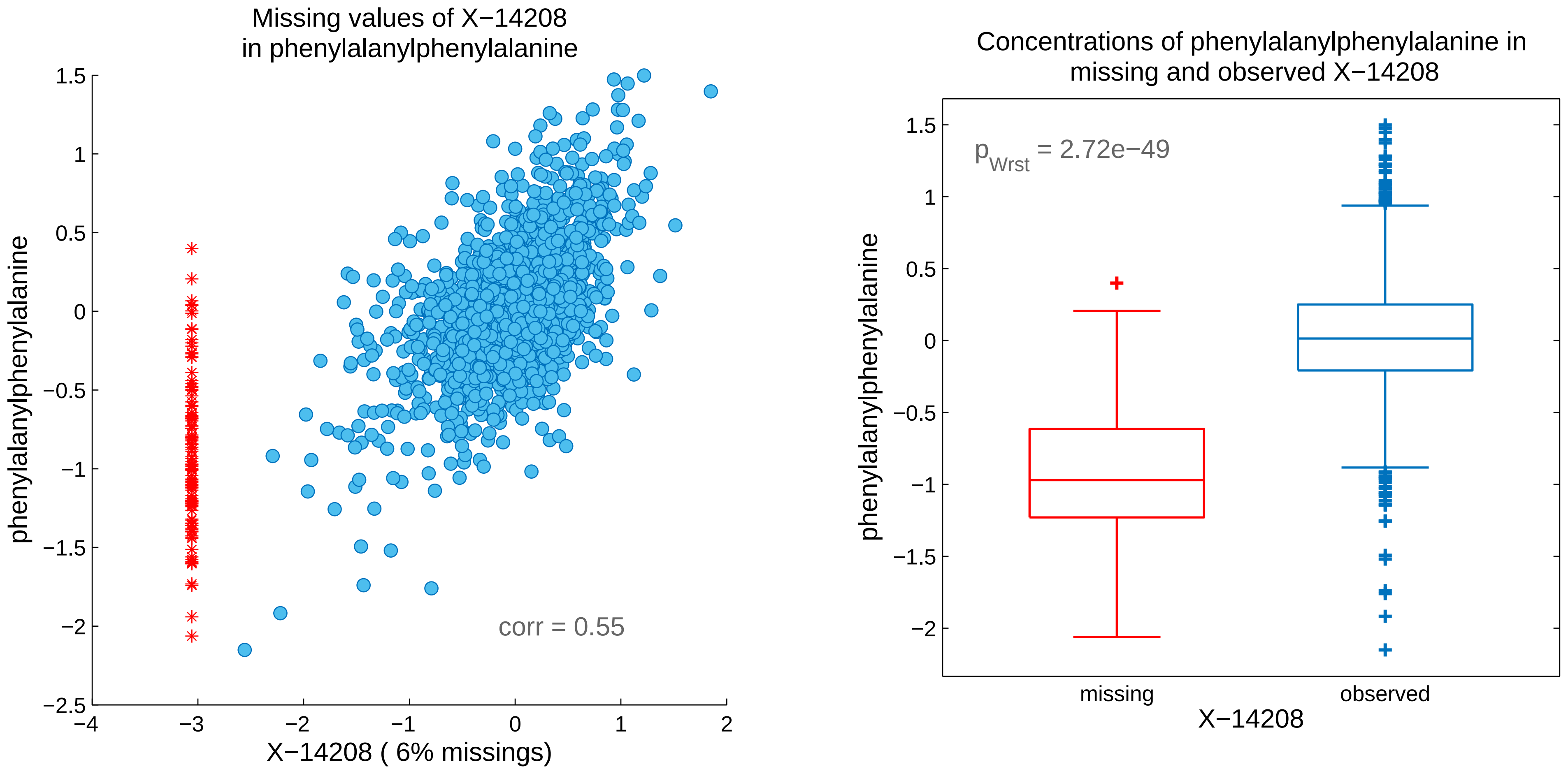
Missing values of X−14208
in phenylalanylphenylalanine
phenylalanylphenylalanine
X−14208 (6% missings)
corr = 0.55
Concentrations of phenylalanylphenylalanine in
missing and observed X−14208
p_Wrst = 2.72e−49
phenylalanylphenylalanine
missing
observed
X−14208

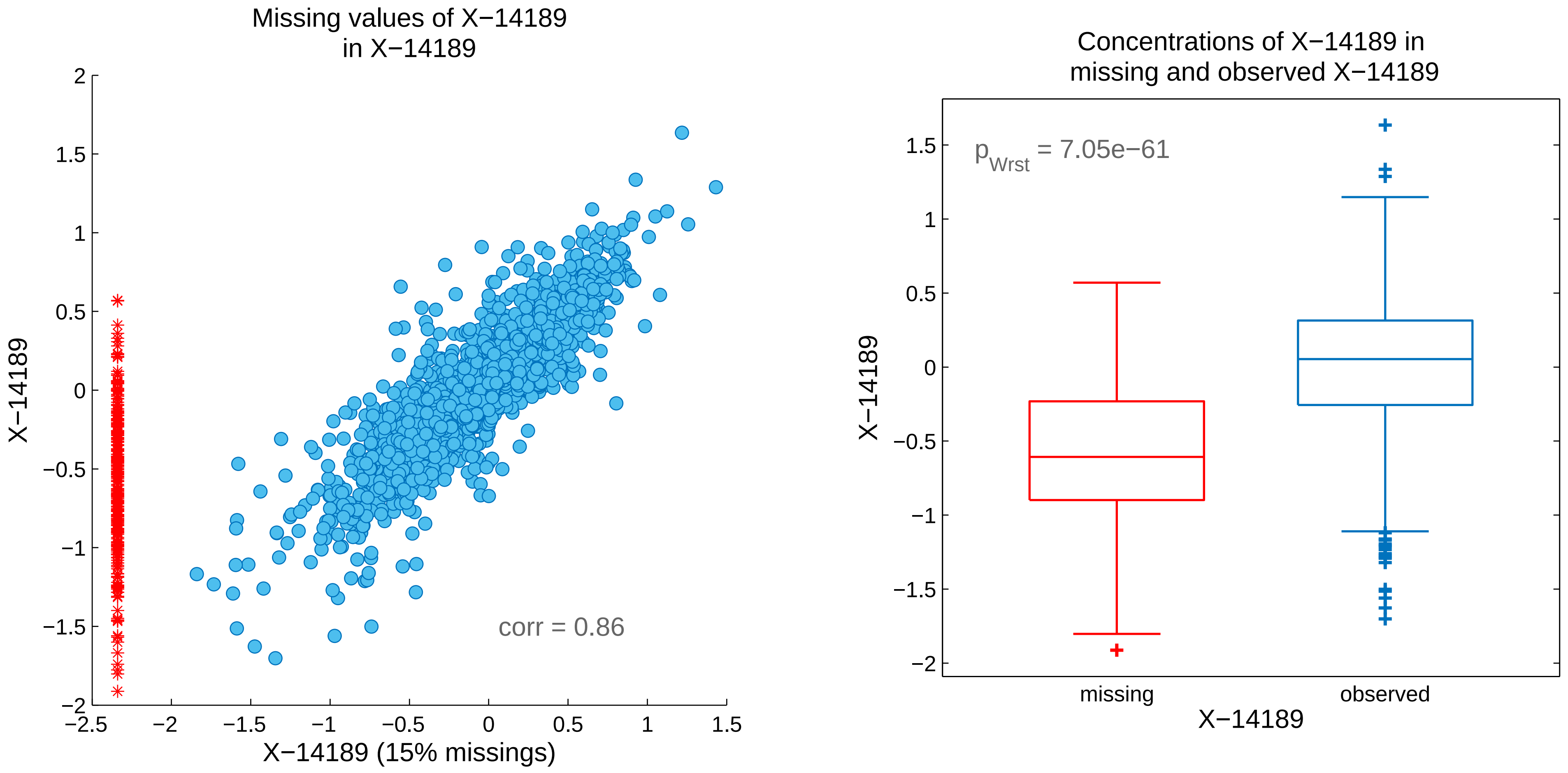

Missing values of X−14189
in X−14189
X−14189
corr = 0.86
X−14189 (15% missings)
Concentrations of X−14189 in
missing and observed X−14189
p_{Wrst} = 7.05e−61
X−14189
missing
observed
X−14189

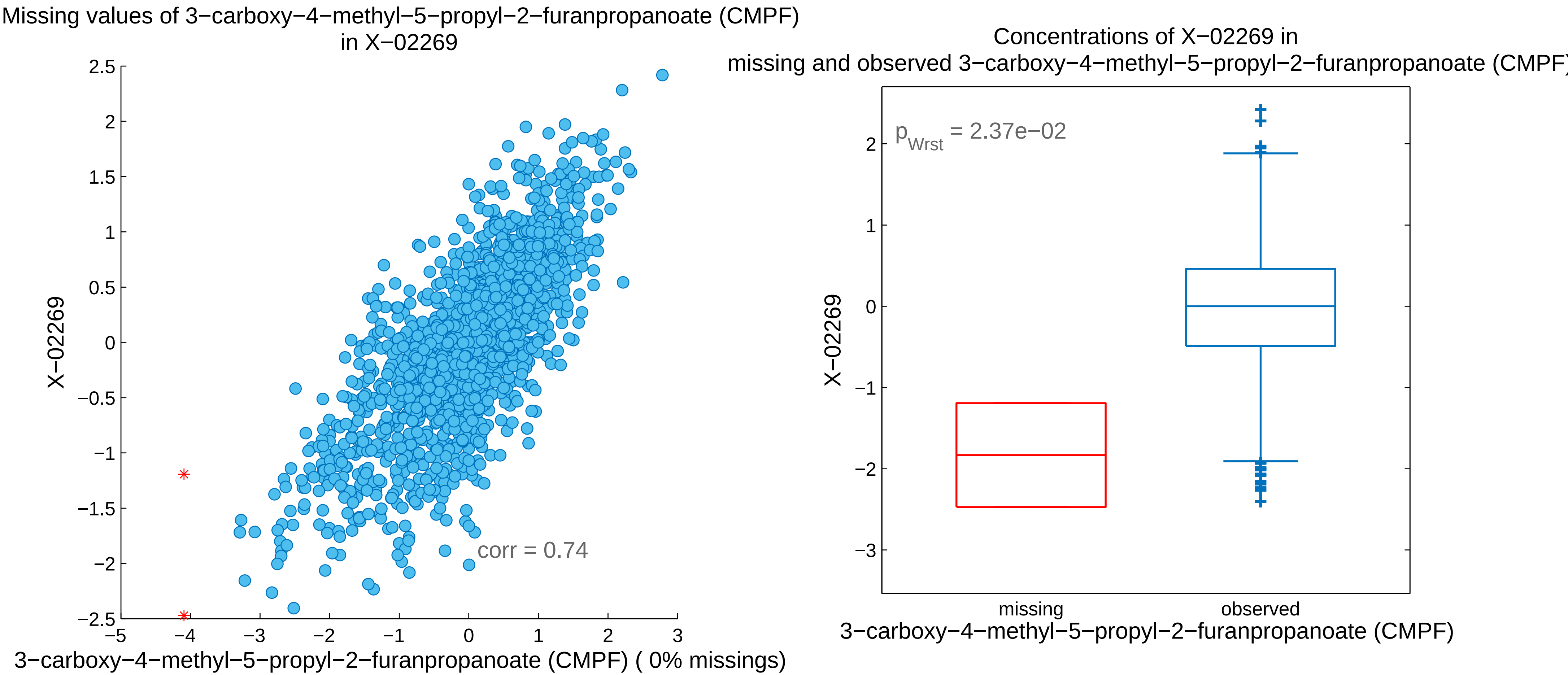

Missing values of 3−carboxy−4−methyl−5−propyl−2−furanpropanoate (CMPF) in X−02269
X−02269
corr = 0.74
3−carboxy−4−methyl−5−propyl−2−furanpropanoate (CMPF) (0% missings)
Concentrations of X−02269 in missing and observed 3−carboxy−4−methyl−5−propyl−2−furanpropanoate (CMPF)
p_{Wrst} = 2.37e−02
X−02269
missing
observed
3−carboxy−4−methyl−5−propyl−2−furanpropanoate (CMPF)

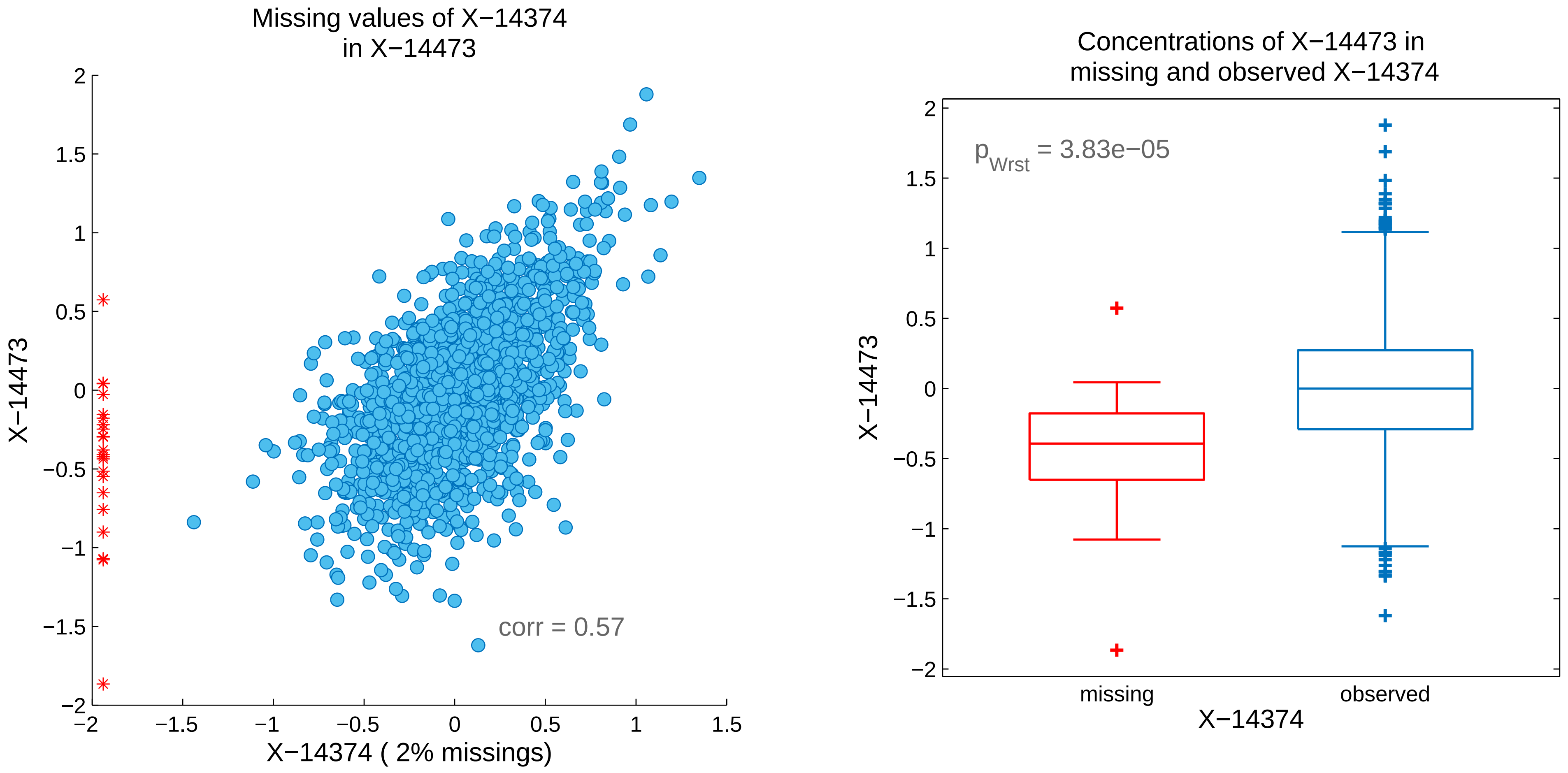

Missing values of X−14374 in X−14473
X−14473
X−14374 (2% missings)
corr = 0.57
Concentrations of X−14473 in missing and observed X−14374
p_{Wrst} = 3.83e−05
X−14473
missing
observed
X−14374

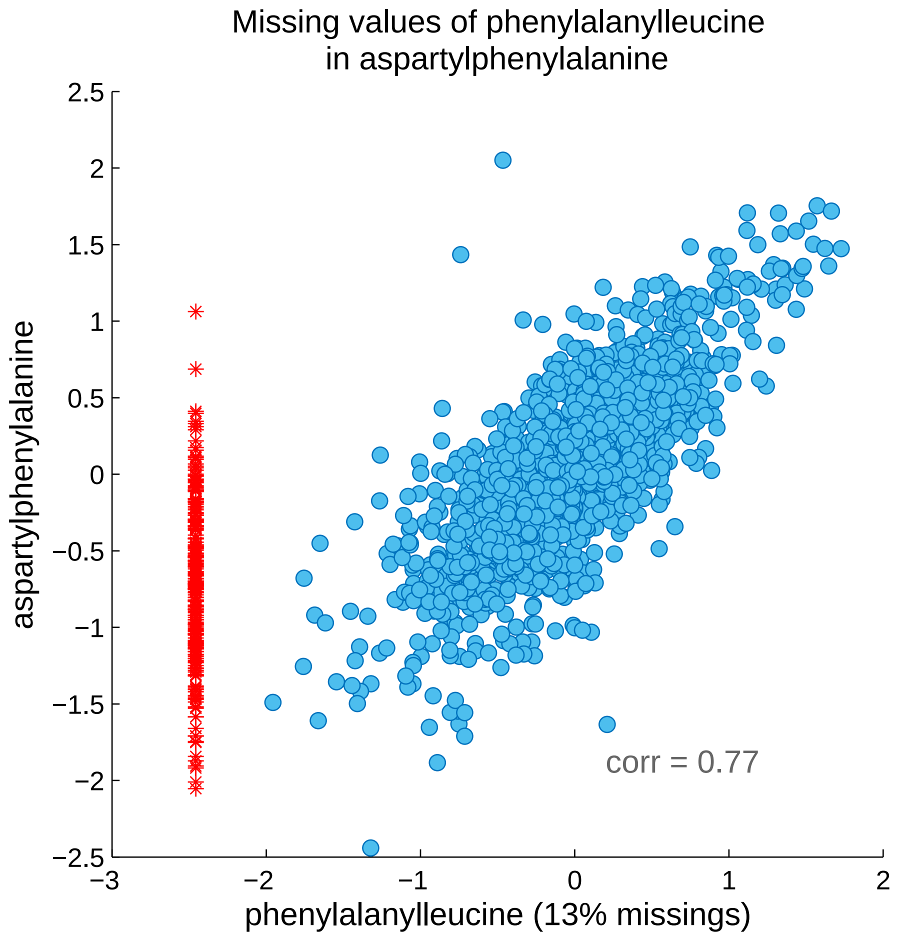

Missing values of phenylalanylleucine
in aspartylphenylalanine
aspartylphenylalanine
phenylalanylleucine (13% missings)
corr = 0.77

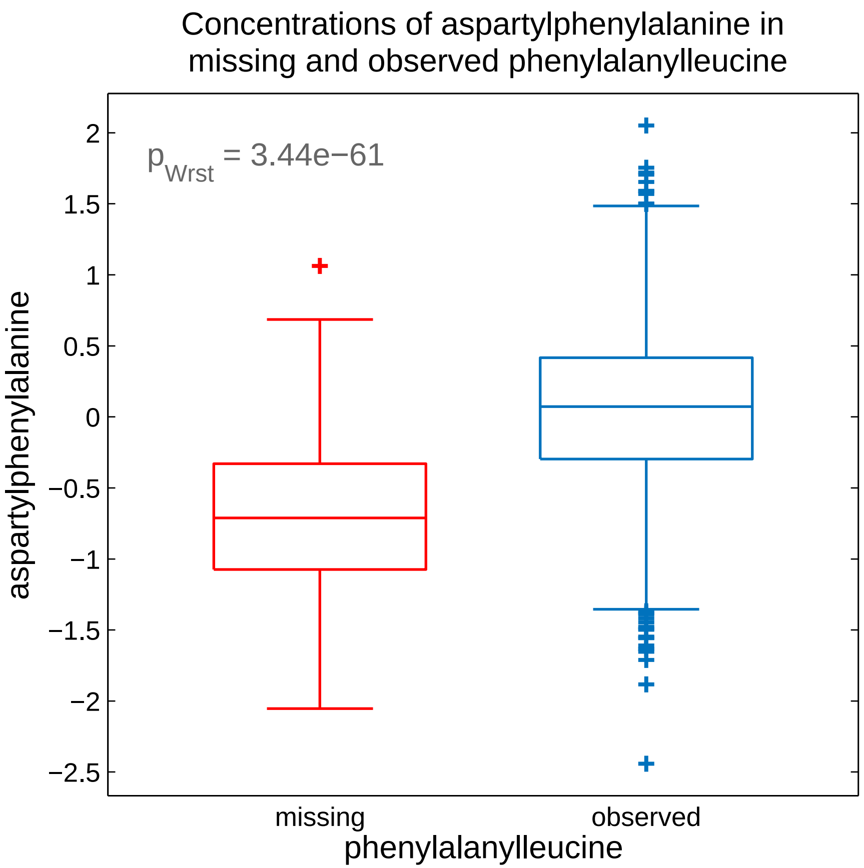

Concentrations of aspartylphenylalanine in
missing and observed phenylalanylleucine
p_{Wrst} = 3.44e−61
aspartylphenylalanine
missing
observed
phenylalanylleucine

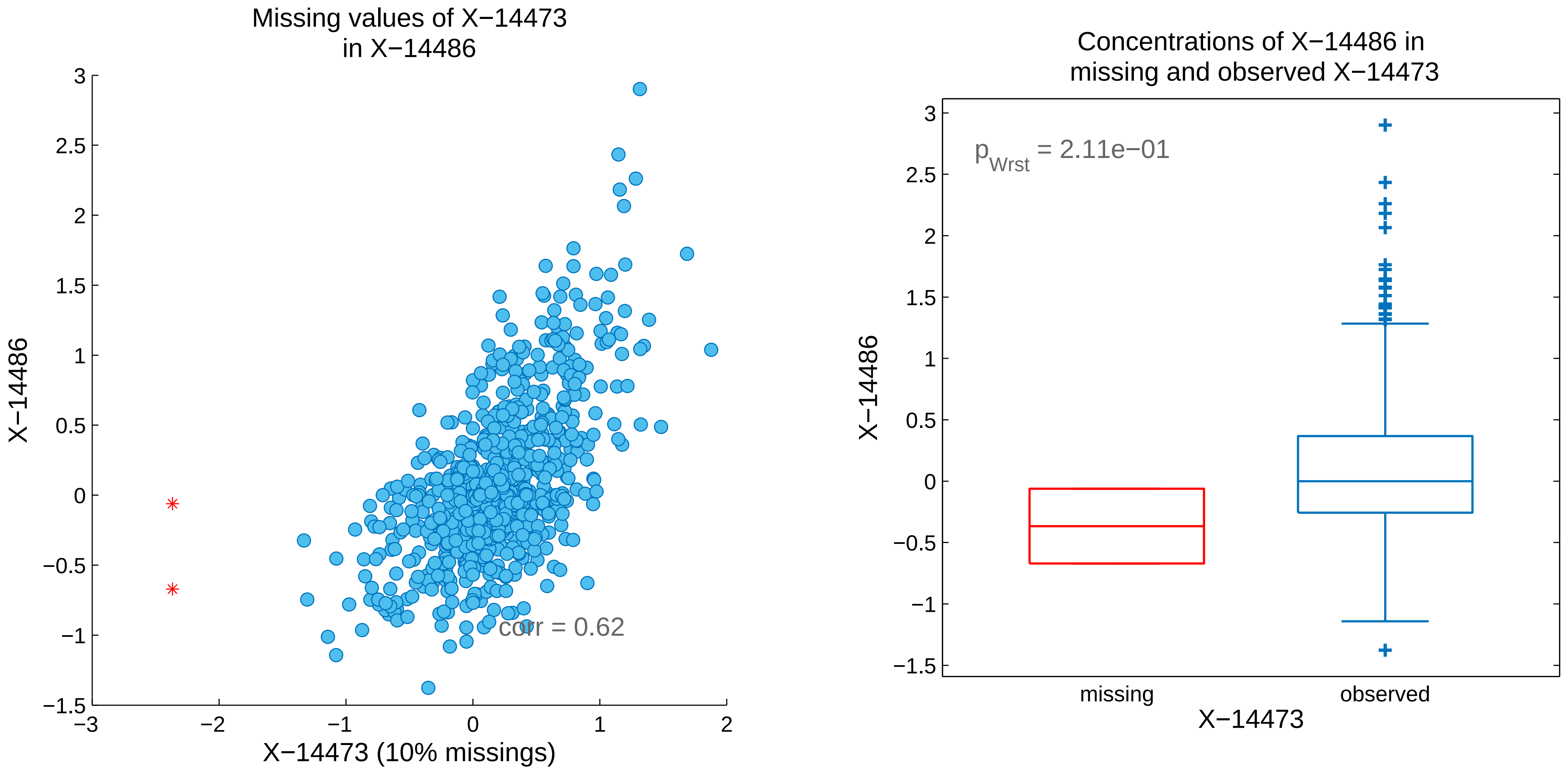

Missing values of X−14473
in X−14486
3
2.5
2
1.5
1
0.5
0
−0.5
−1
−1.5
X−14486
−3
−2
−1
0
1
2
X−14473 (10% missings)
corr = 0.62
Concentrations of X−14486 in
missing and observed X−14473
p_Wrst = 2.11e−01
3
2.5
2
1.5
1
0.5
0
−0.5
−1
−1.5
X−14486
missing
observed
X−14473

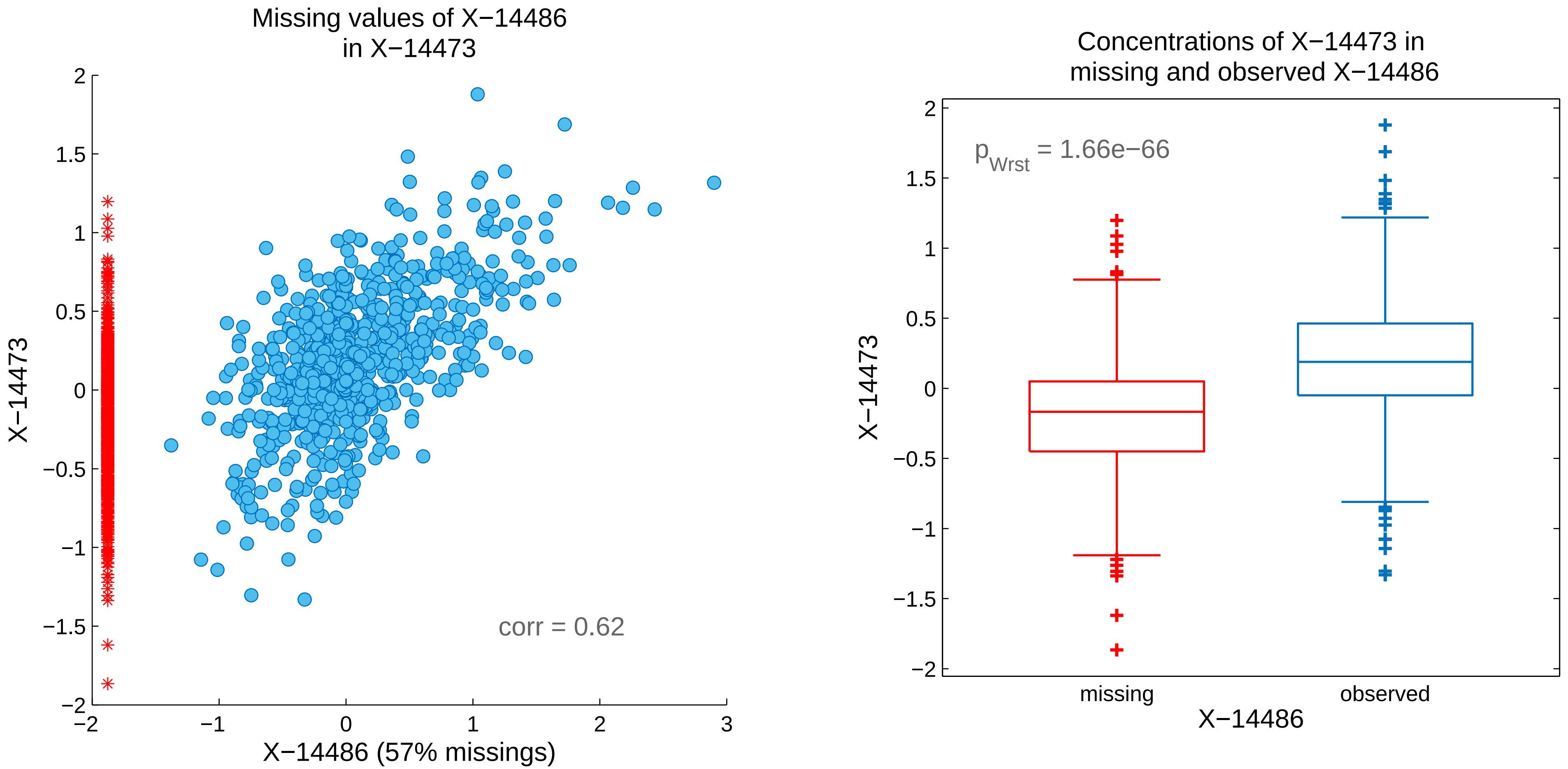
Missing values of X−14486
in X−14473
X−14473
X−14486 (57% missings)
corr = 0.62
2
1.5
1
0.5
0
−0.5
−1
−1.5
−2
−2
−1
0
1
2
3
Concentrations of X−14473 in
missing and observed X−14486
p_{Wrst} = 1.66e−66
X−14473
X−14486
missing
observed

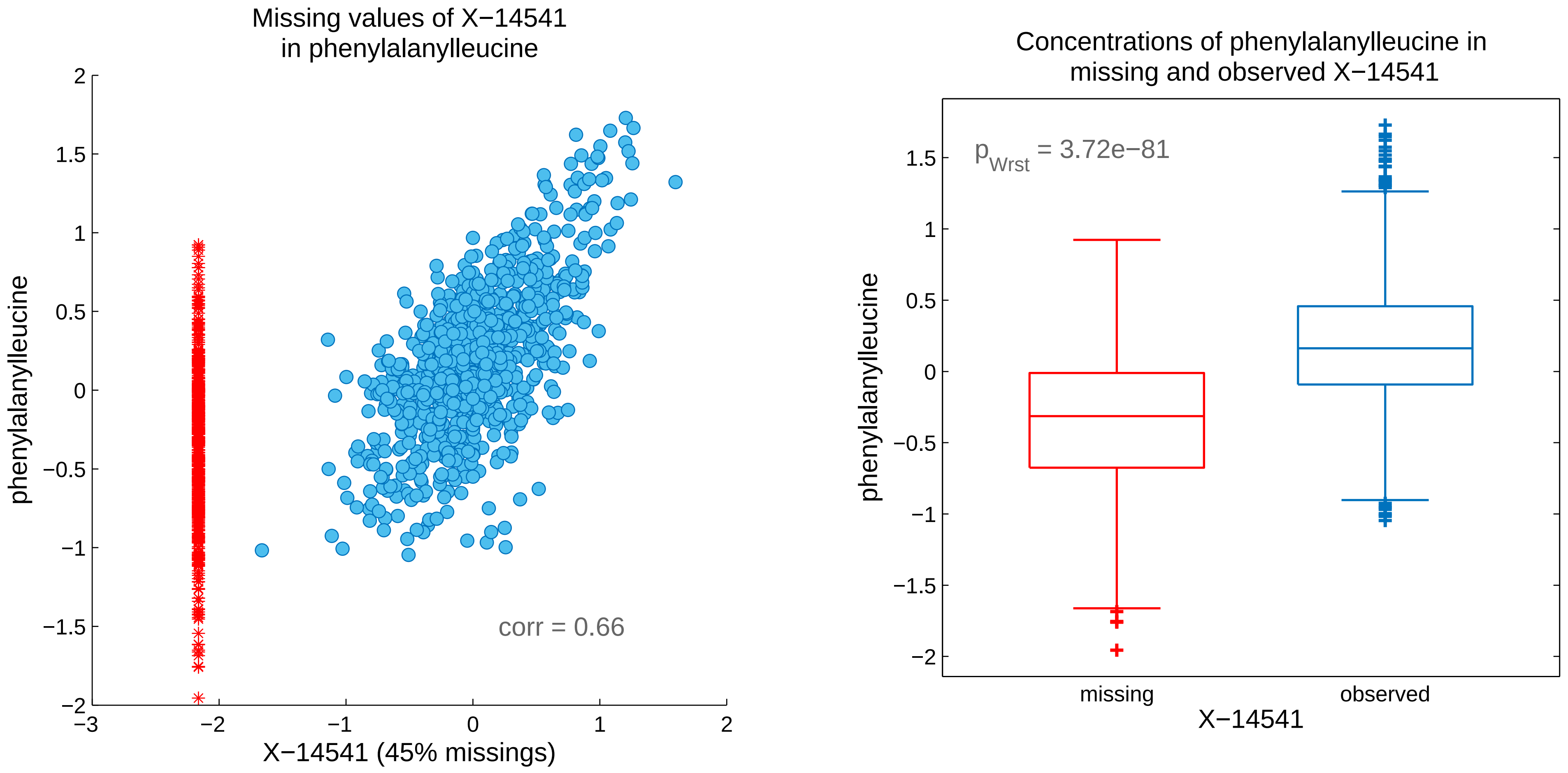

Missing values of X−14541
in phenylalanylleucine
phenylalanylleucine
X−14541 (45% missings)
corr = 0.66
2
1.5
1
0.5
0
−0.5
−1
−1.5
−2
−3
−2
−1
0
1
2
Concentrations of phenylalanylleucine in
missing and observed X−14541
p_{Wrst} = 3.72e−81
phenylalanylleucine
1.5
1
0.5
0
−0.5
−1
−1.5
−2
missing
observed
X−14541

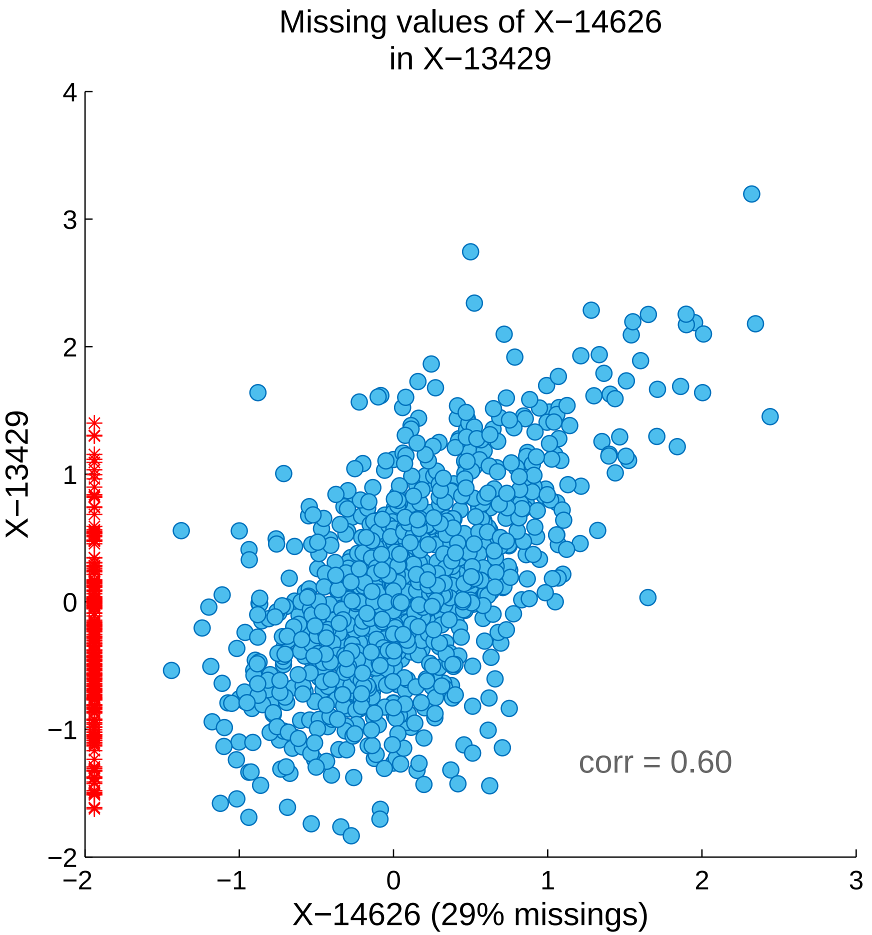
Missing values of X−14626
in X−13429
X−13429
X−14626 (29% missings)
corr = 0.60
4
3
2
1
0
−1
−2
−2
−1
0
1
2
3

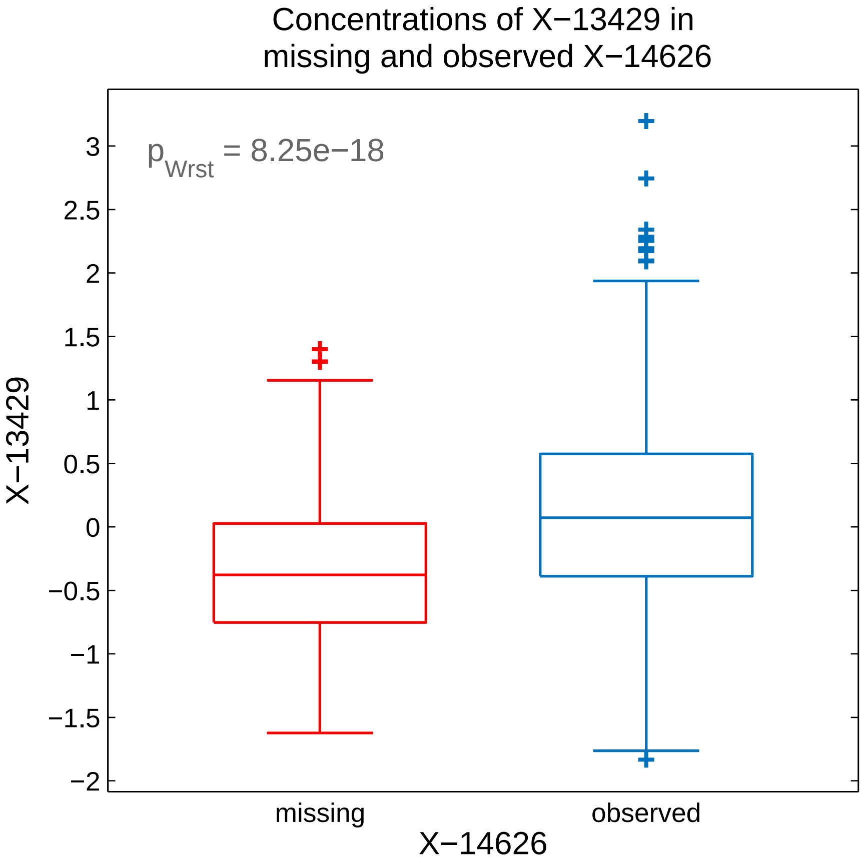
Concentrations of X−13429 in
missing and observed X−14626
p_Wrst = 8.25e−18
X−13429
X−14626
missing
observed
3
2.5
2
1.5
1
0.5
0
−0.5
−1
−1.5
−2

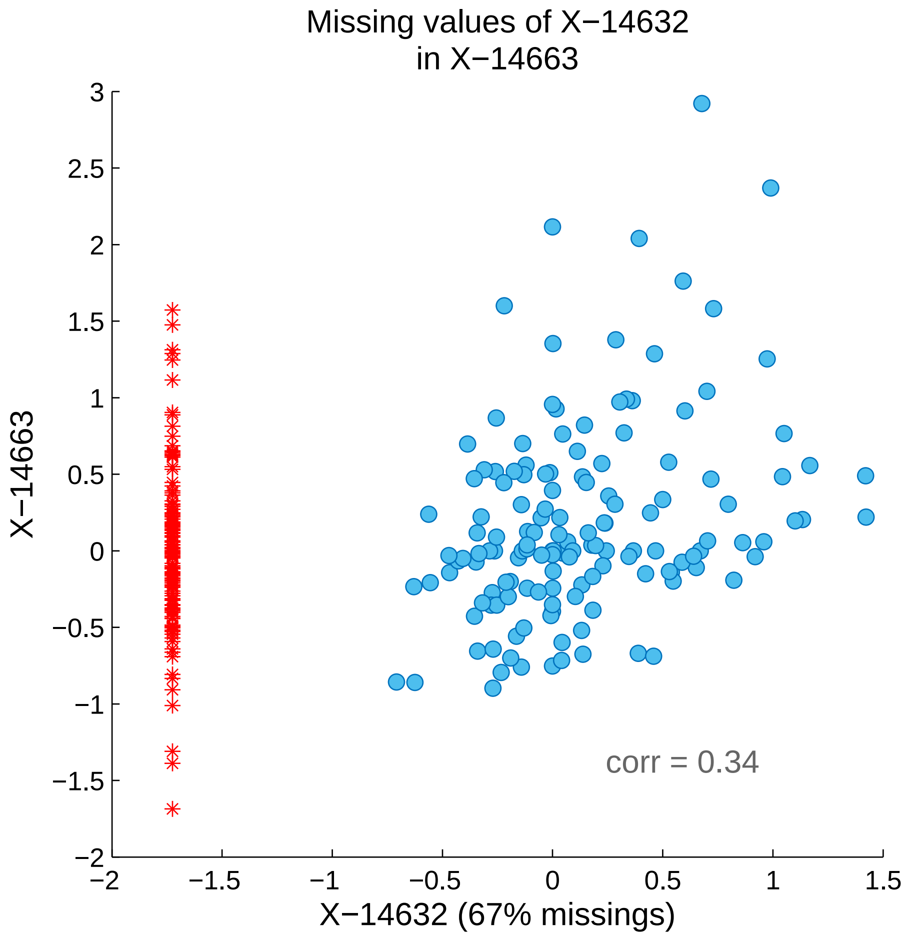

Missing values of X−14632
in X−14663
corr = 0.34
X−14663
X−14632 (67% missings)

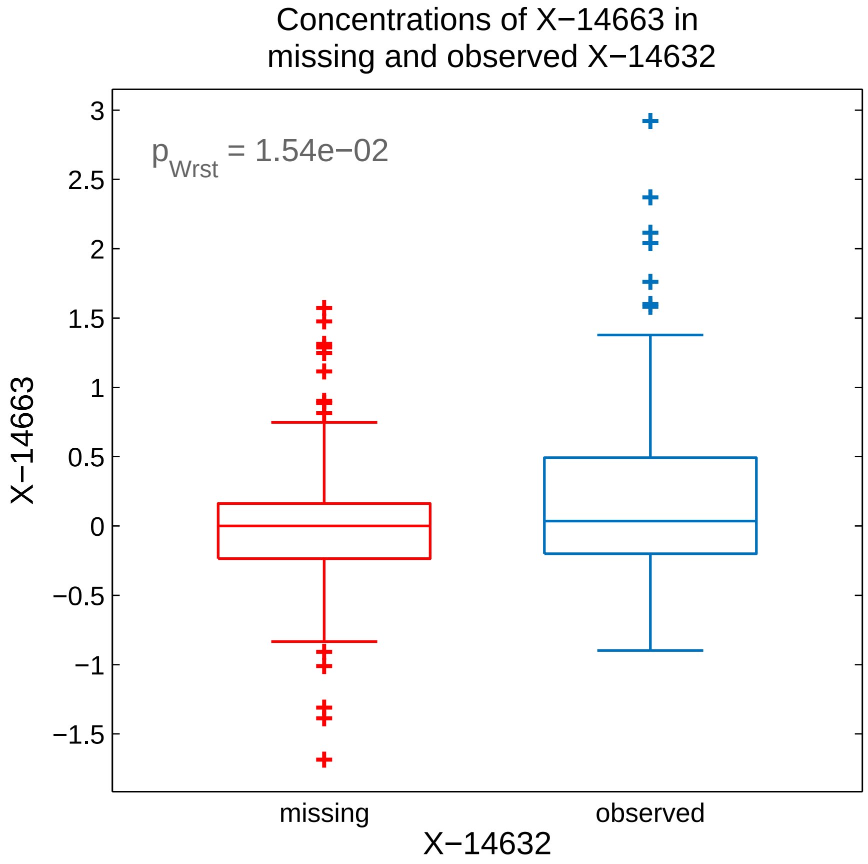

Concentrations of X−14663 in
missing and observed X−14632
p_{Wrst} = 1.54e−02
X−14663
missing
observed
X−14632

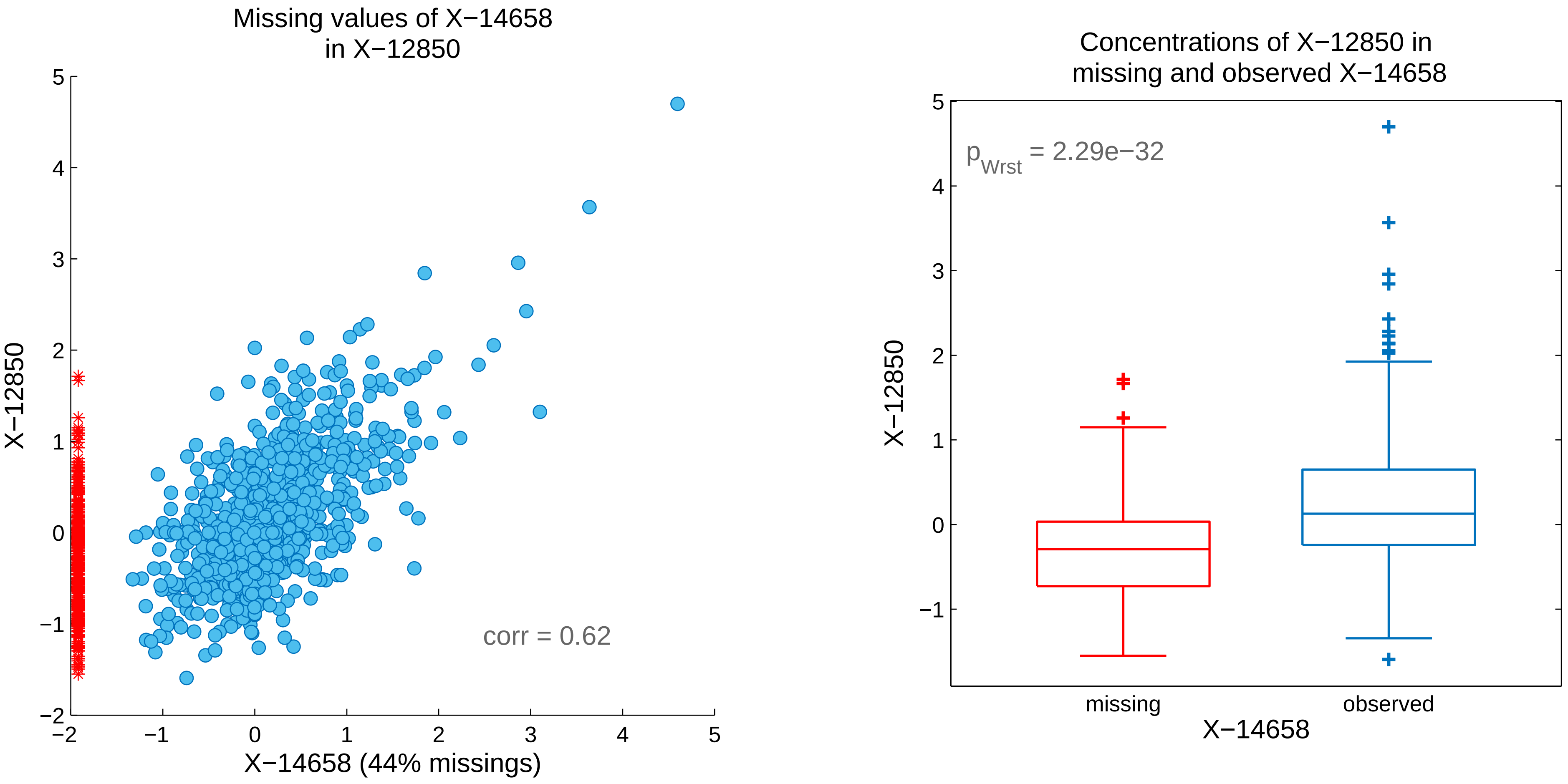
Missing values of X−14658
in X−12850
X−12850
X−14658 (44% missings)
corr = 0.62
−2
−1
0
1
2
3
4
5
Concentrations of X−12850 in
missing and observed X−14658
p_{Wrst} = 2.29e−32
X−12850
missing
observed
X−14658

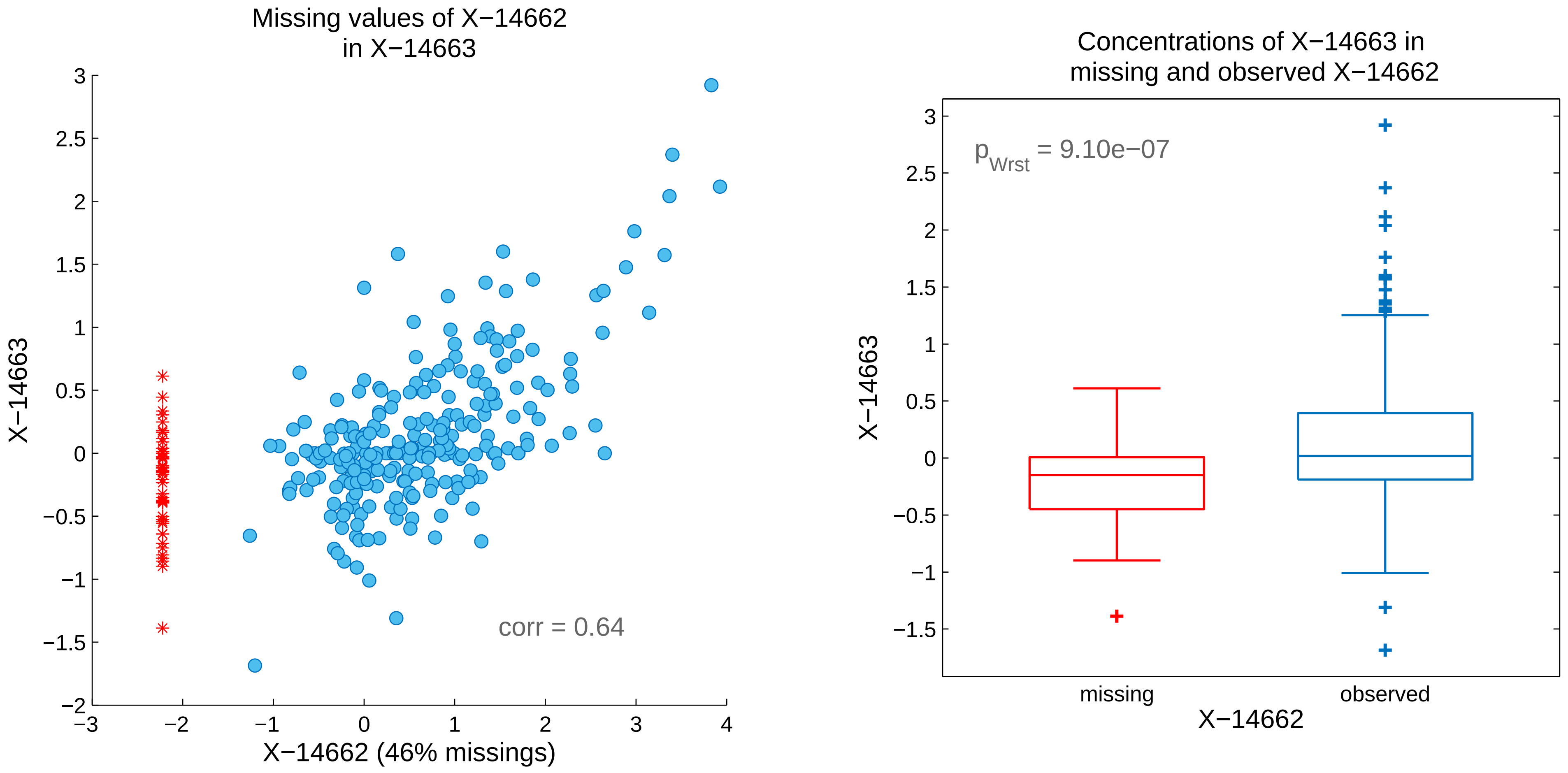

Missing values of X−14662 in X−14663
corr = 0.64
X−14663
X−14662 (46% missings)
Concentrations of X−14663 in missing and observed X−14662
p_{Wrst} = 9.10e−07
X−14663
missing
observed
X−14662

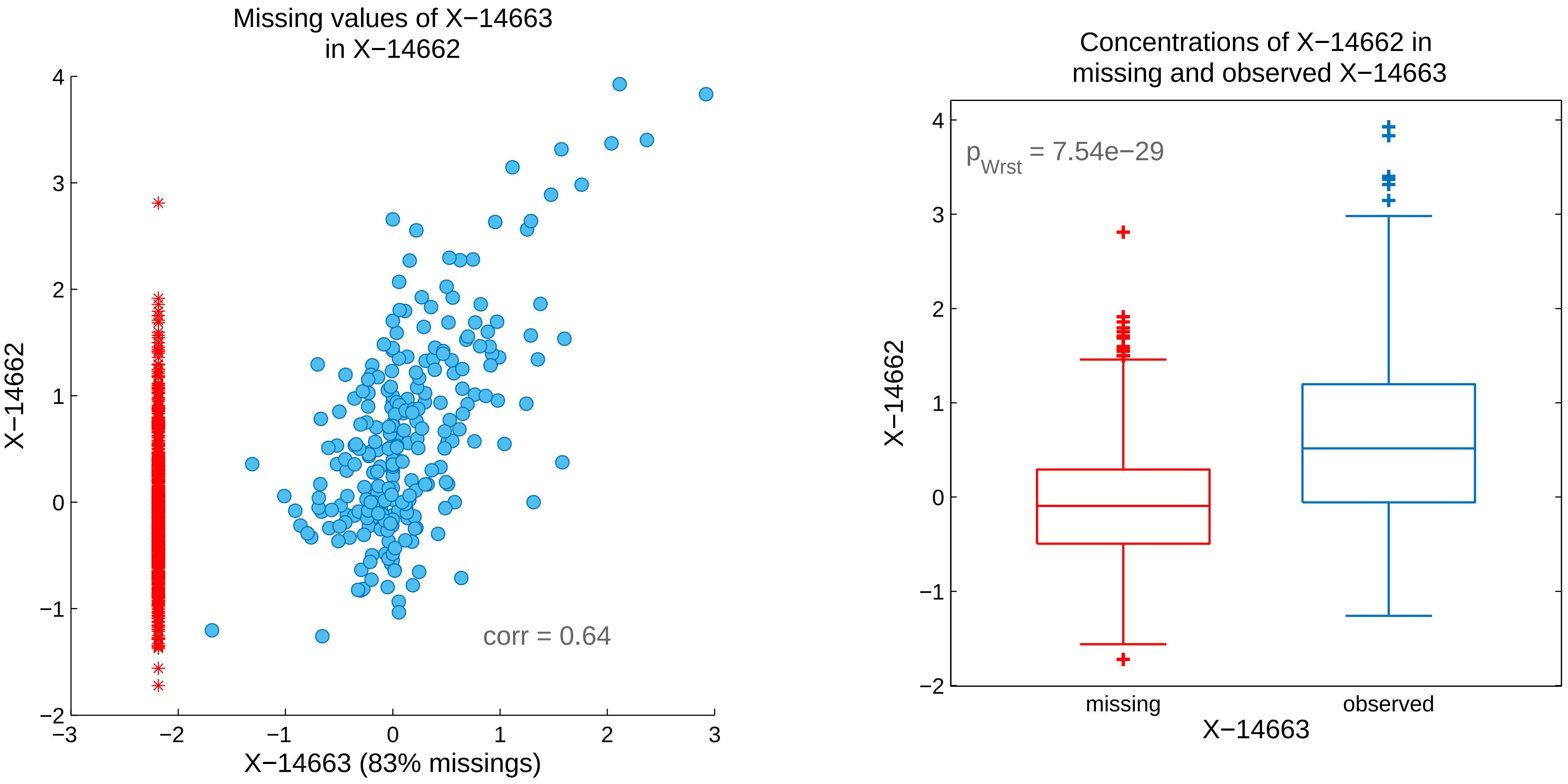
Missing values of X−14663
in X−14662
corr = 0.64
X−14662
X−14663 (83% missings)
Concentrations of X−14662 in
missing and observed X−14663
p_Wrst = 7.54e−29
missing
observed
X−14663
X−14662

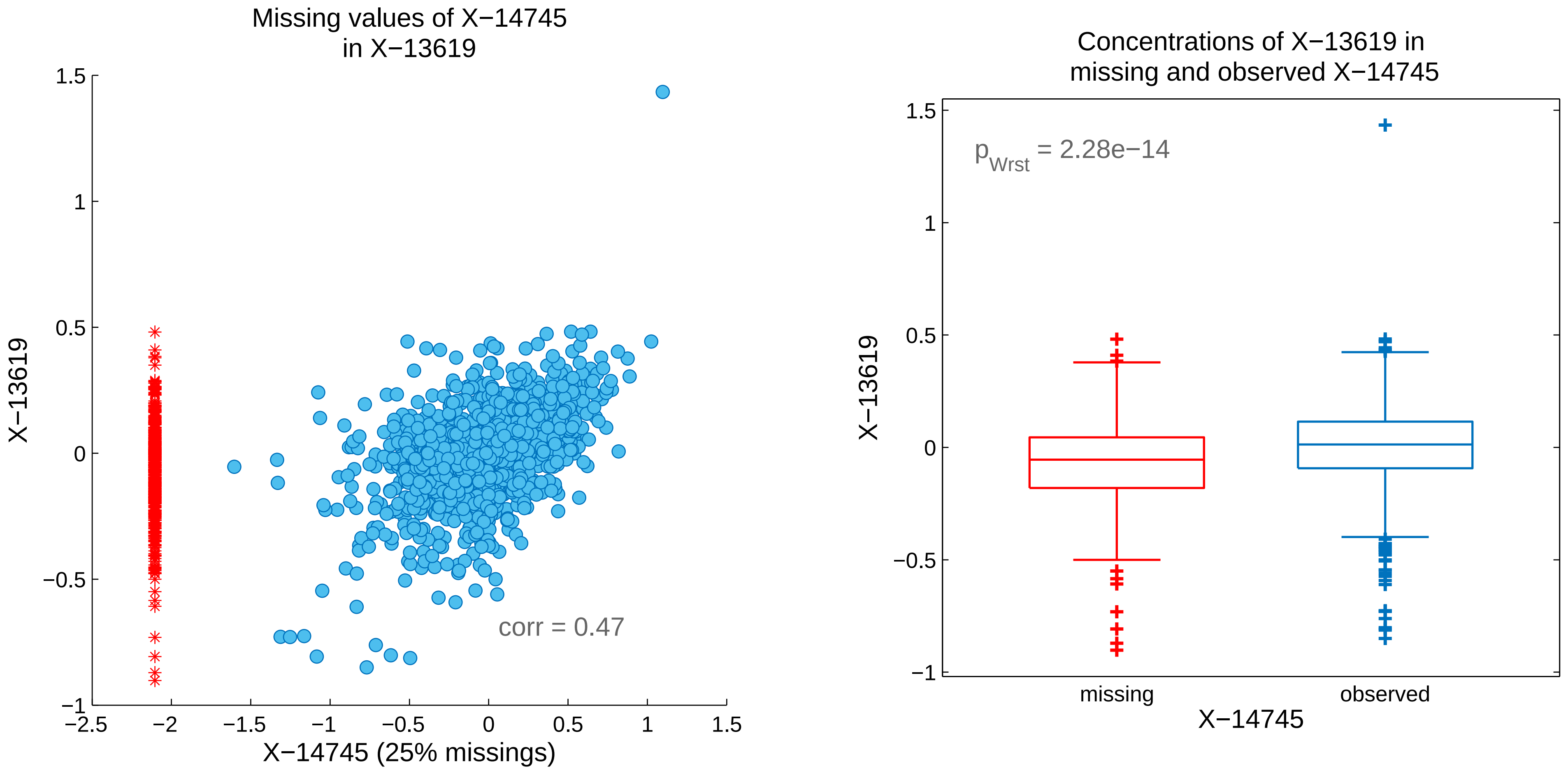

Missing values of X−14745
in X−13619
1.5
1
0.5
0
−0.5
−1
X−13619
−2.5
−2
−1.5
−1
−0.5
0
0.5
1
1.5
X−14745 (25% missings)
corr = 0.47
Concentrations of X−13619 in
missing and observed X−14745
p$_{Wrst}$ = 2.28e−14
1.5
1
0.5
0
−0.5
−1
X−13619
missing
observed
X−14745

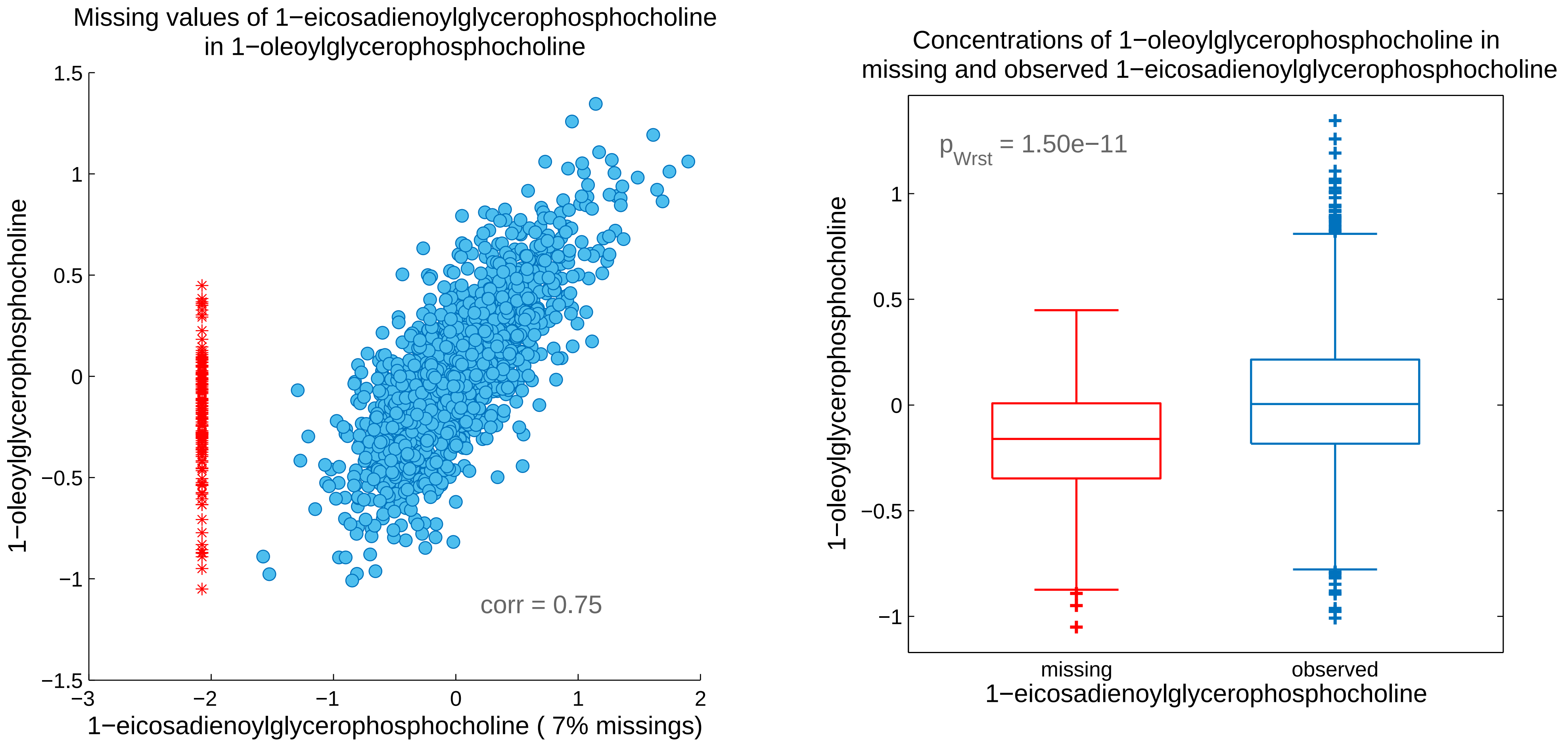
Missing values of 1−eicosadienoylglycerophosphocholine in 1−oleoylglycerophosphocholine
1−oleoylglycerophosphocholine
1−eicosadienoylglycerophosphocholine (7% missings)
corr = 0.75
Concentrations of 1−oleoylglycerophosphocholine in missing and observed 1−eicosadienoylglycerophosphocholine
p_Wrst = 1.50e−11
missing
observed
1−eicosadienoylglycerophosphocholine
1−oleoylglycerophosphocholine

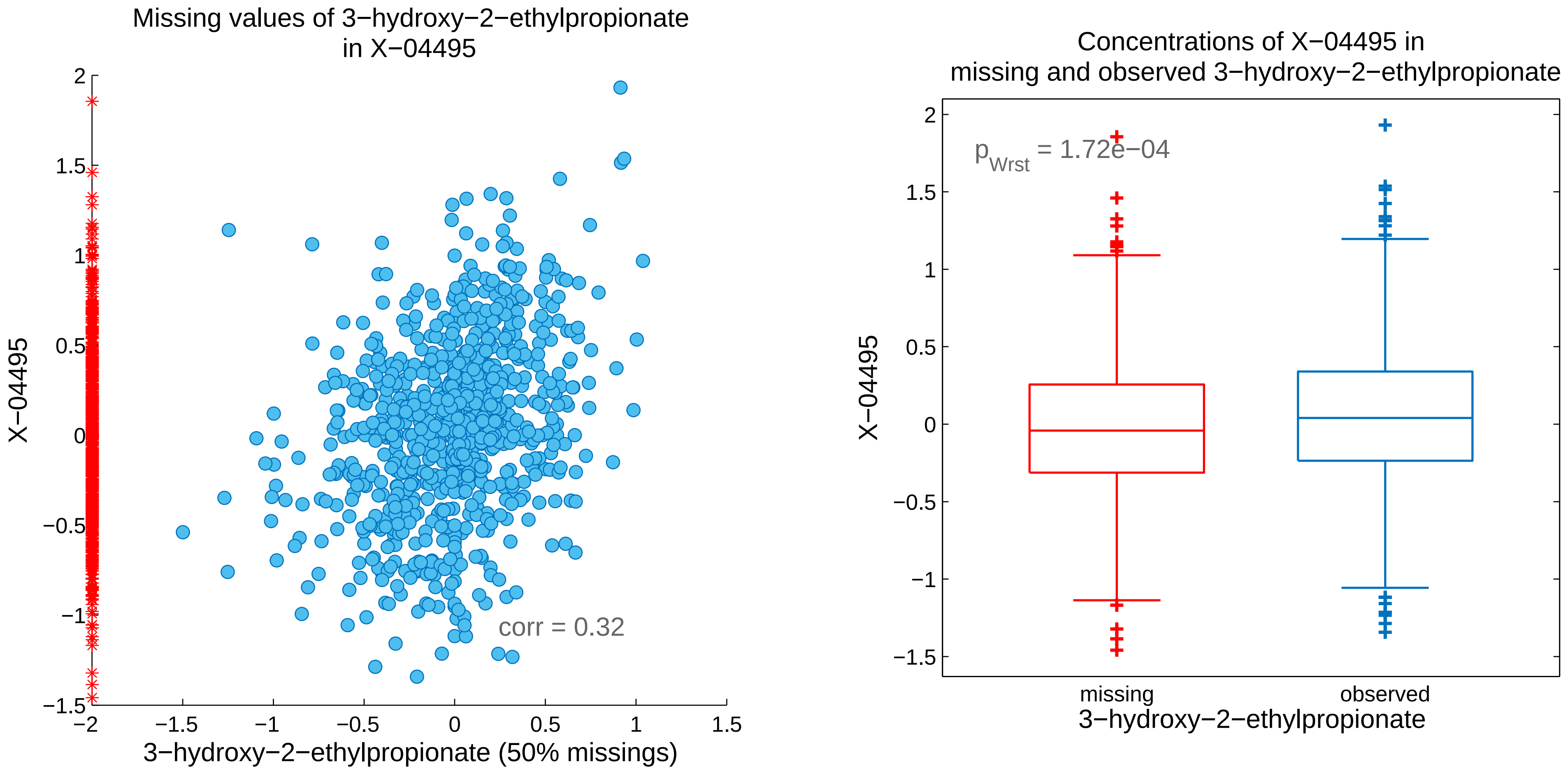

Missing values of 3−hydroxy−2−ethylpropionate in X−04495
X−04495
corr = 0.32
3−hydroxy−2−ethylpropionate (50% missings)
Concentrations of X−04495 in missing and observed 3−hydroxy−2−ethylpropionate
p_Wrst = 1.72e−04
missing
observed
3−hydroxy−2−ethylpropionate

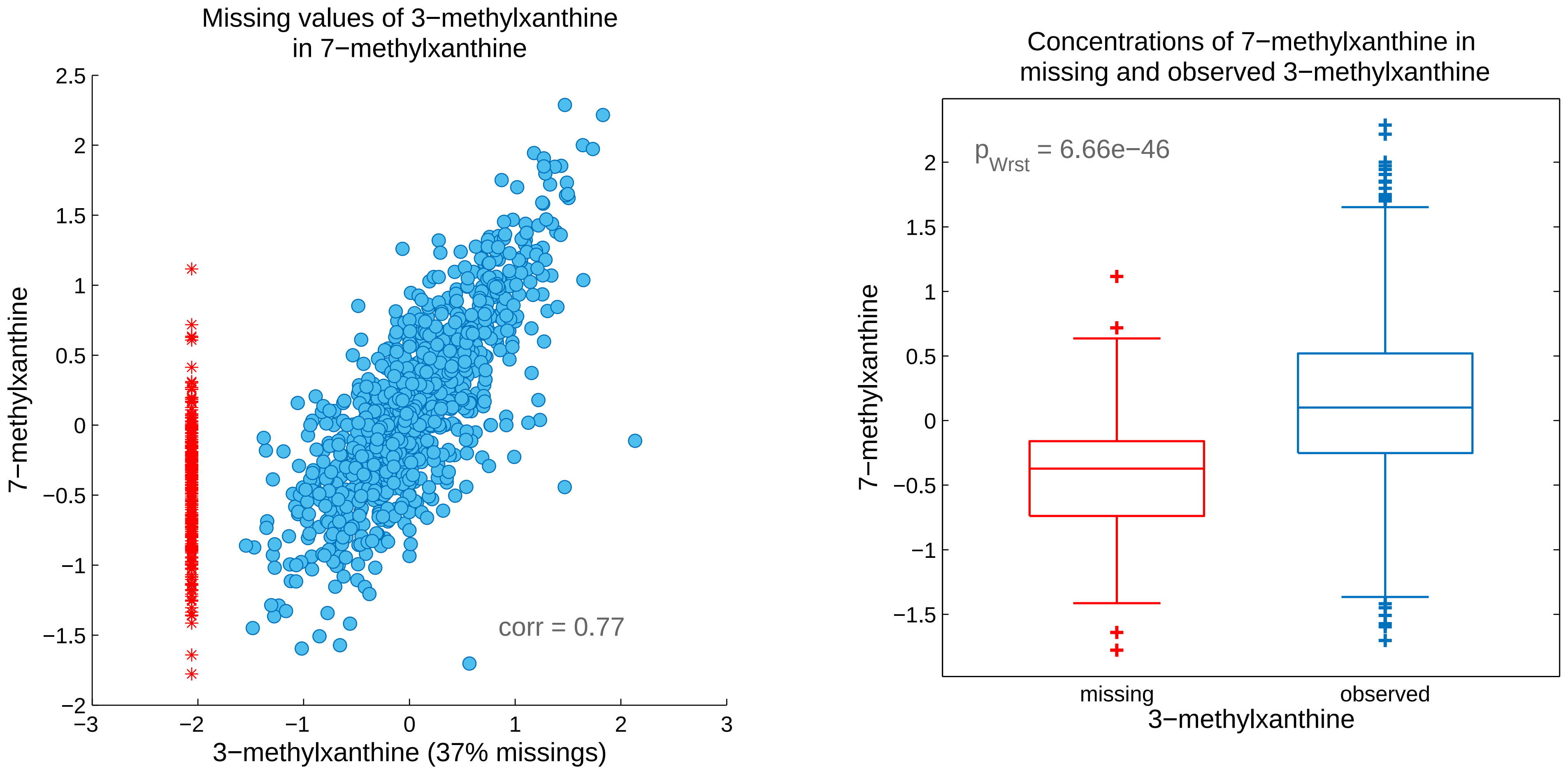
Missing values of 3−methylxanthine in 7−methylxanthine
7−methylxanthine
3−methylxanthine (37% missings)
corr = 0.77
Concentrations of 7−methylxanthine in missing and observed 3−methylxanthine
p_{Wrst} = 6.66e−46
7−methylxanthine
missing
observed
3−methylxanthine

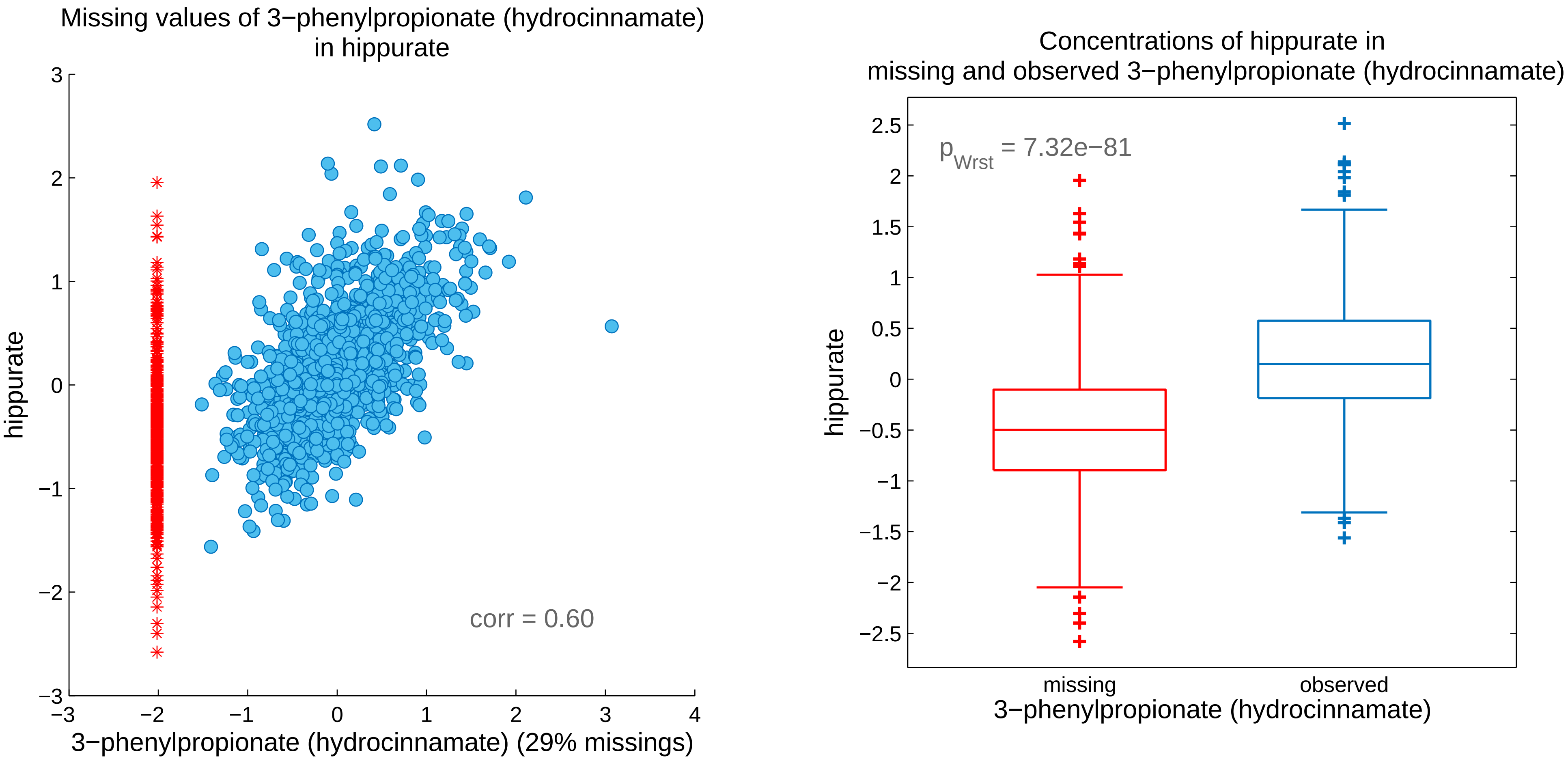
Missing values of 3−phenylpropionate (hydrocinnamate) in hippurate
hippurate
3−phenylpropionate (hydrocinnamate) (29% missings)
corr = 0.60
Concentrations of hippurate in missing and observed 3−phenylpropionate (hydrocinnamate)
p_{Wrst} = 7.32e−81
hippurate
missing
observed
3−phenylpropionate (hydrocinnamate)

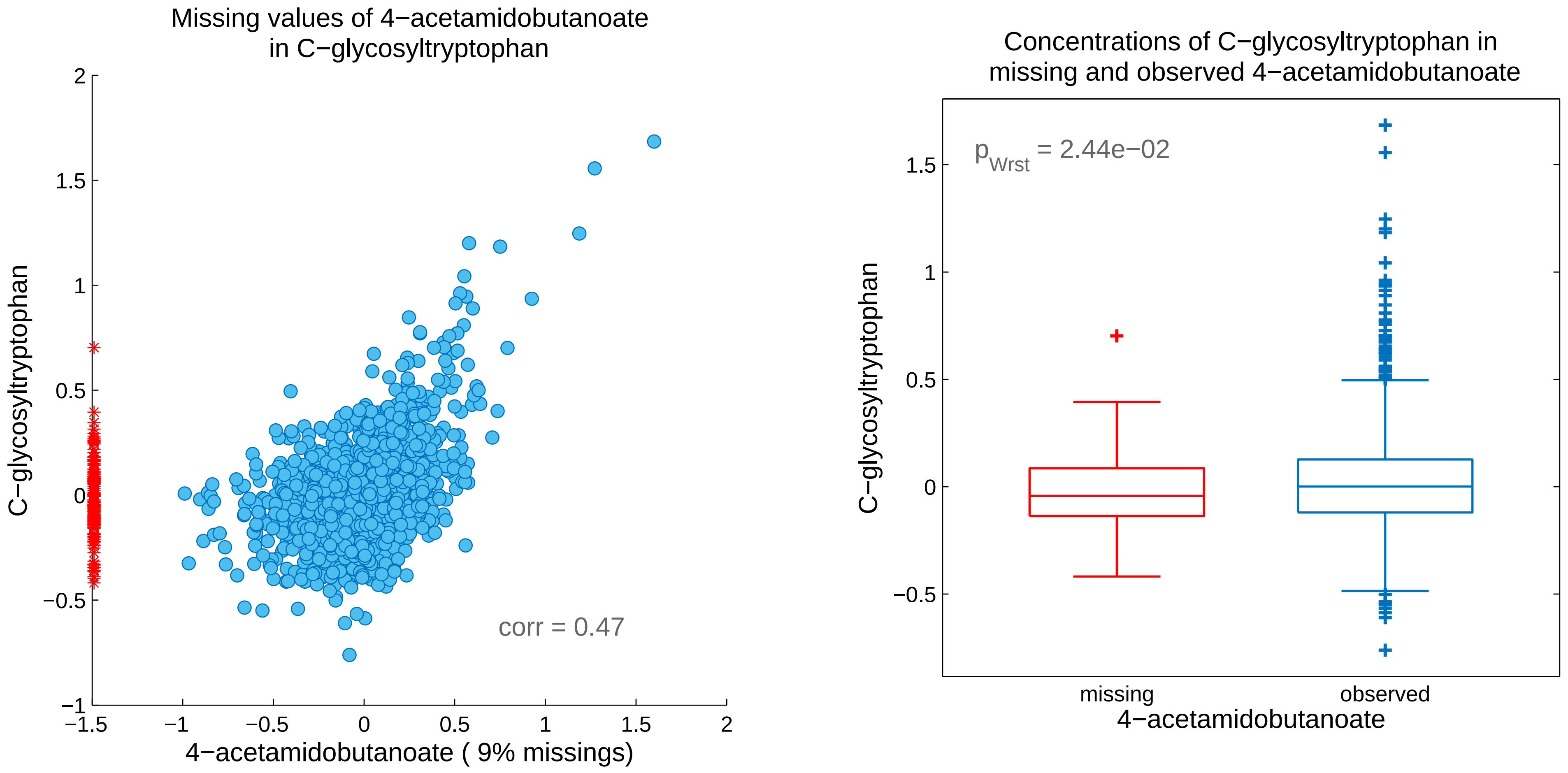

Missing values of 4−acetamidobutanoate in C−glycosyltryptophan
C−glycosyltryptophan
4−acetamidobutanoate (9% missings)
corr = 0.47
Concentrations of C−glycosyltryptophan in missing and observed 4−acetamidobutanoate
C−glycosyltryptophan
p_Wrst = 2.44e−02
missing
observed
4−acetamidobutanoate

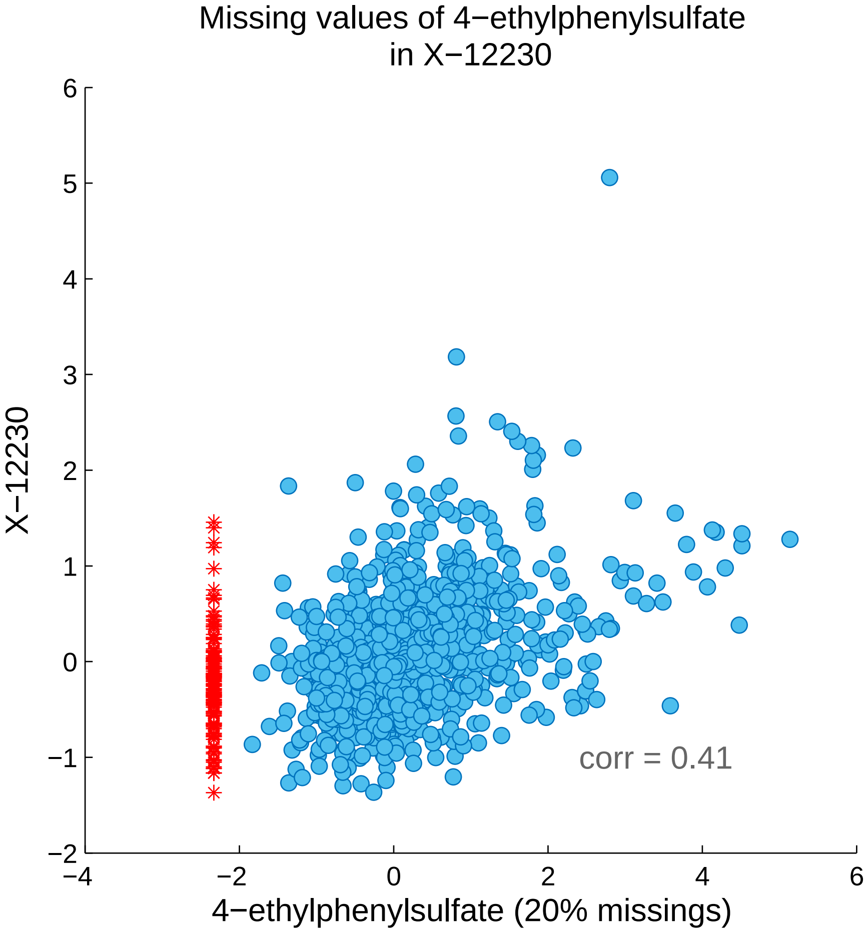

Missing values of 4−ethylphenylsulfate in X−12230
X−12230
4−ethylphenylsulfate (20% missings)
corr = 0.41
−4
−2
0
2
4
6
−2
−1
0
1
2
3
4
5
6

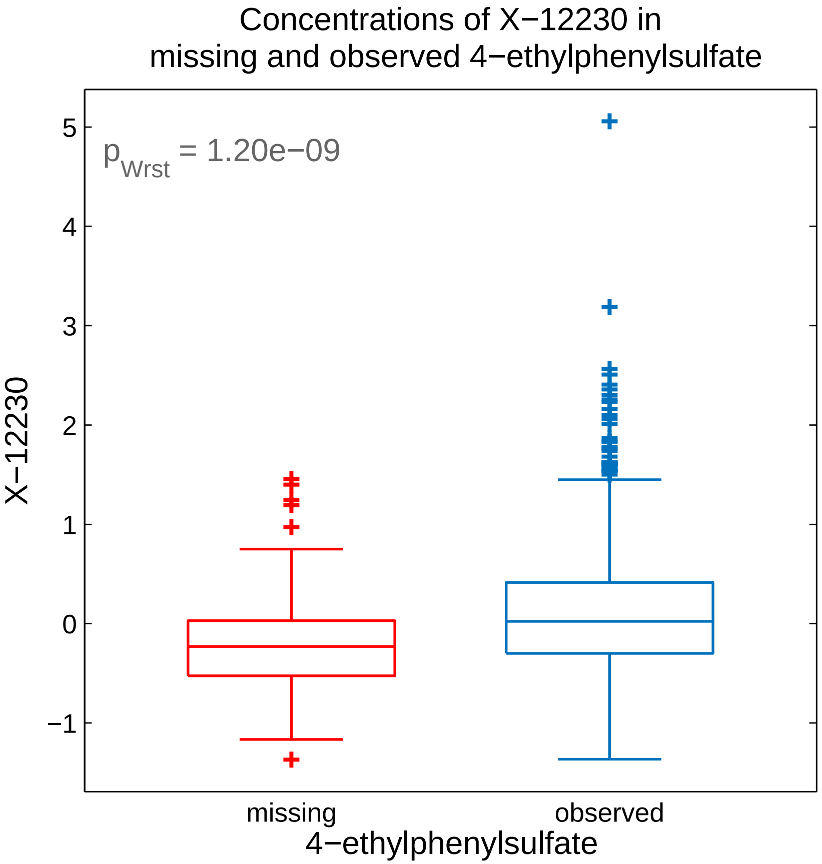

Concentrations of X−12230 in missing and observed 4−ethylphenylsulfate
p_{Wrst} = 1.20e−09
X−12230
missing
observed
4−ethylphenylsulfate
−1
0
1
2
3
4
5

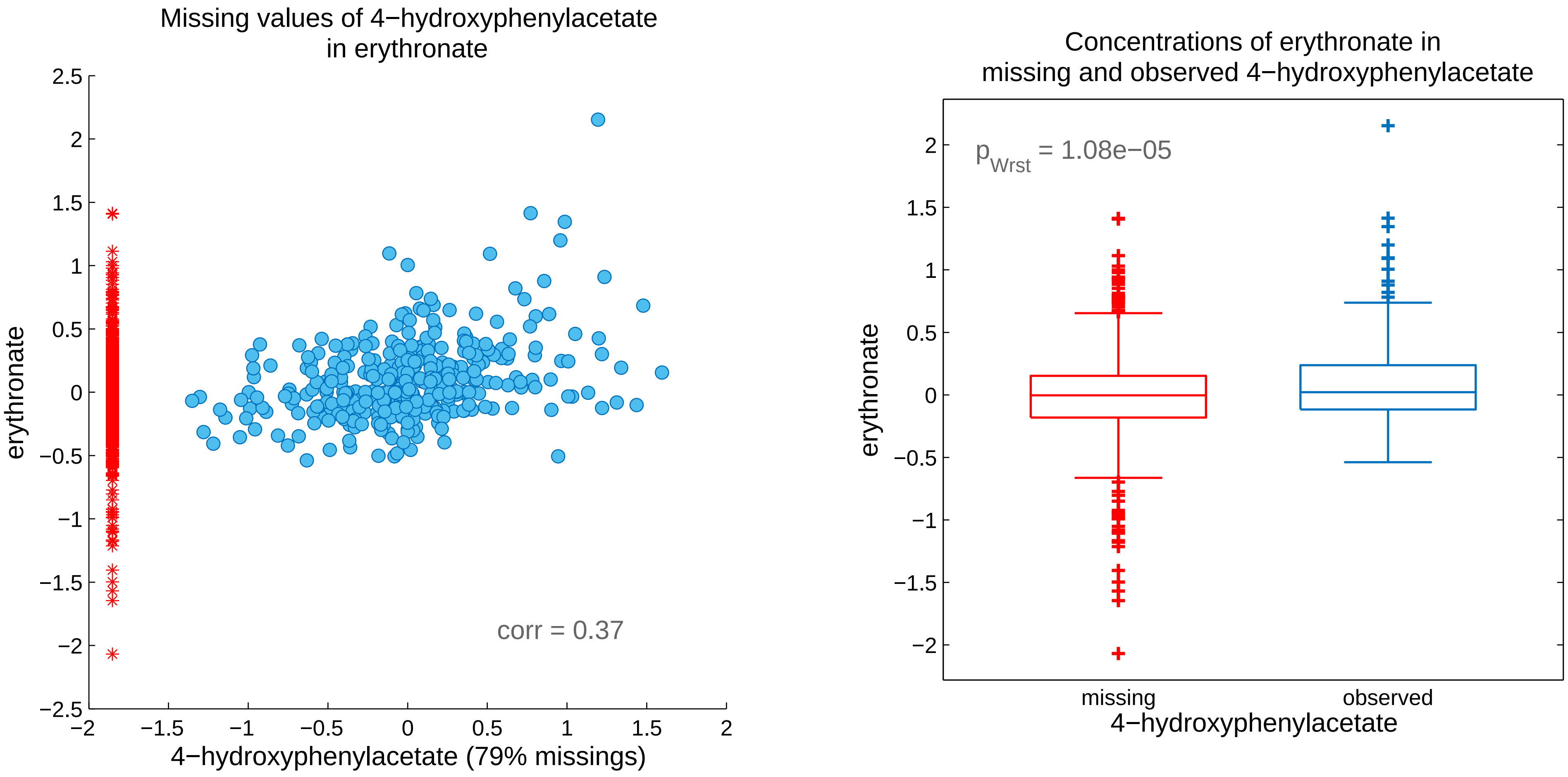
Missing values of 4−hydroxyphenylacetate in erythronate
erythronate
4−hydroxyphenylacetate (79% missings)
corr = 0.37
Concentrations of erythronate in missing and observed 4−hydroxyphenylacetate
p_{Wrst} = 1.08e−05
erythronate
missing
observed
4−hydroxyphenylacetate

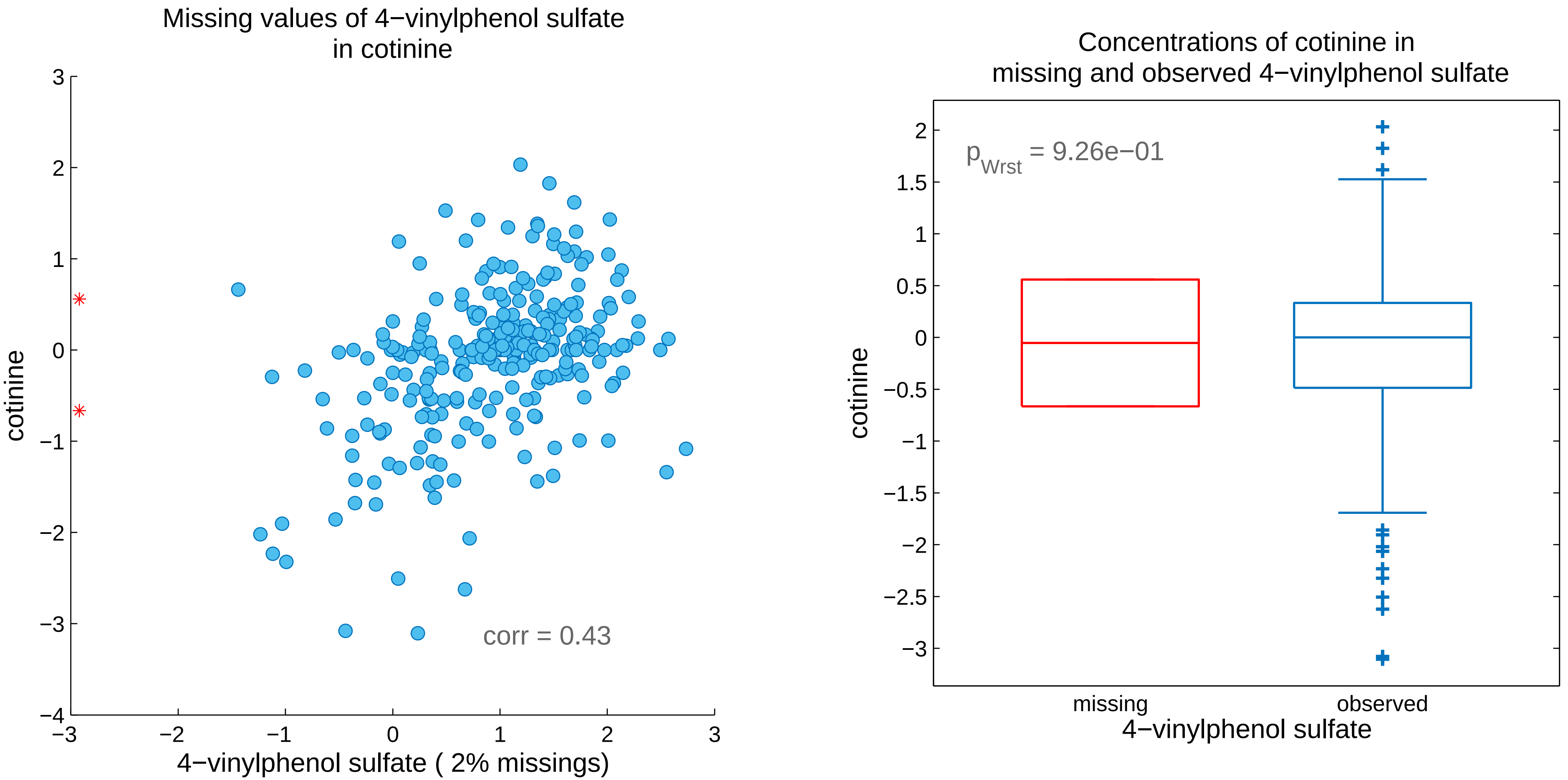

Missing values of 4−vinylphenol sulfate
in cotinine
cotinine
4−vinylphenol sulfate (2% missings)
corr = 0.43
Concentrations of cotinine in
missing and observed 4−vinylphenol sulfate
p Wrst = 9.26e−01
cotinine
missing
observed
4−vinylphenol sulfate

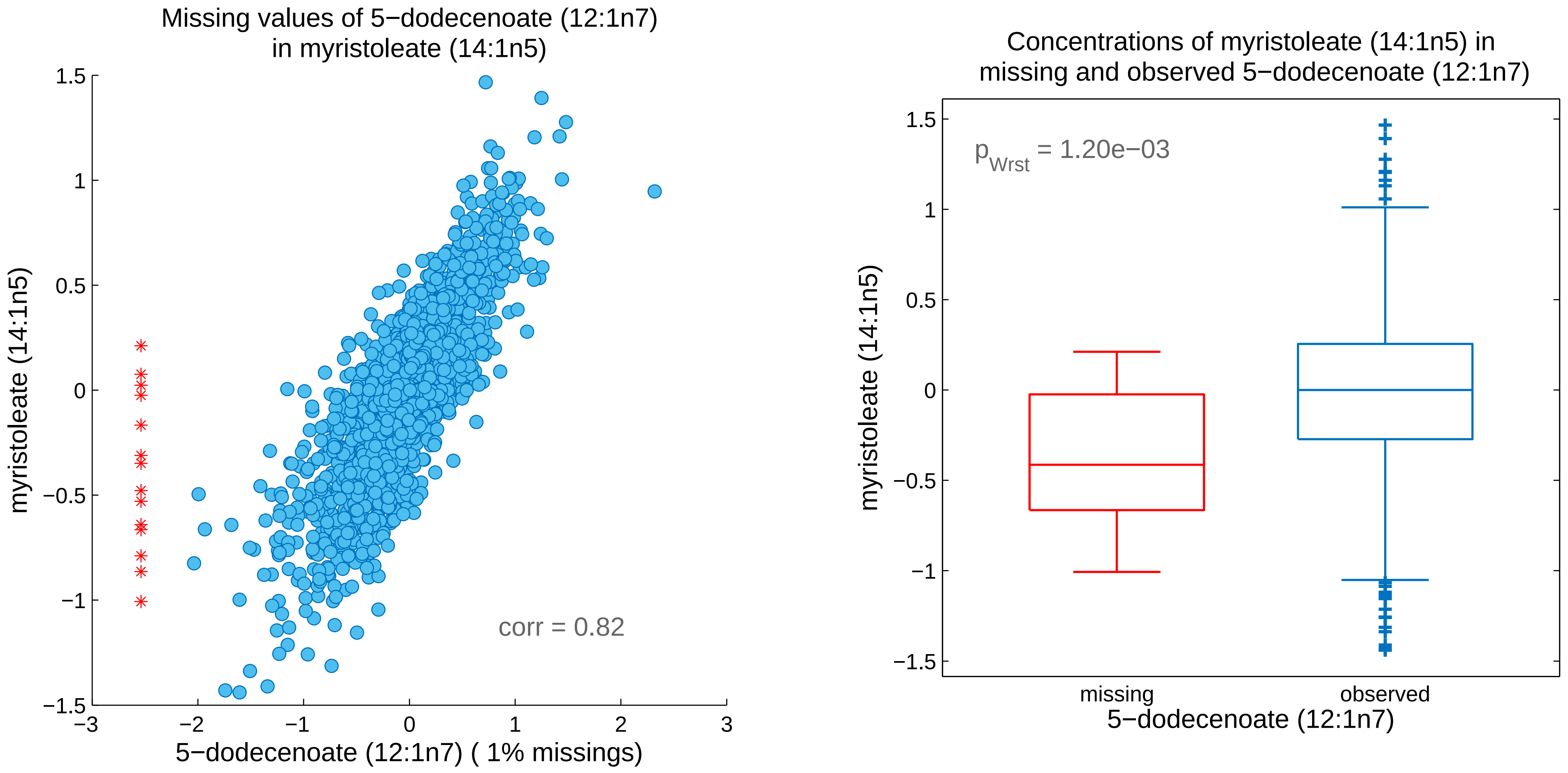
Missing values of 5−dodecenoate (12:1n7) in myristoleate (14:1n5)
myristoleate (14:1n5)
5−dodecenoate (12:1n7) (1% missings)
corr = 0.82
Concentrations of myristoleate (14:1n5) in missing and observed 5−dodecenoate (12:1n7)
p_Wrst = 1.20e−03
missing
observed
5−dodecenoate (12:1n7)

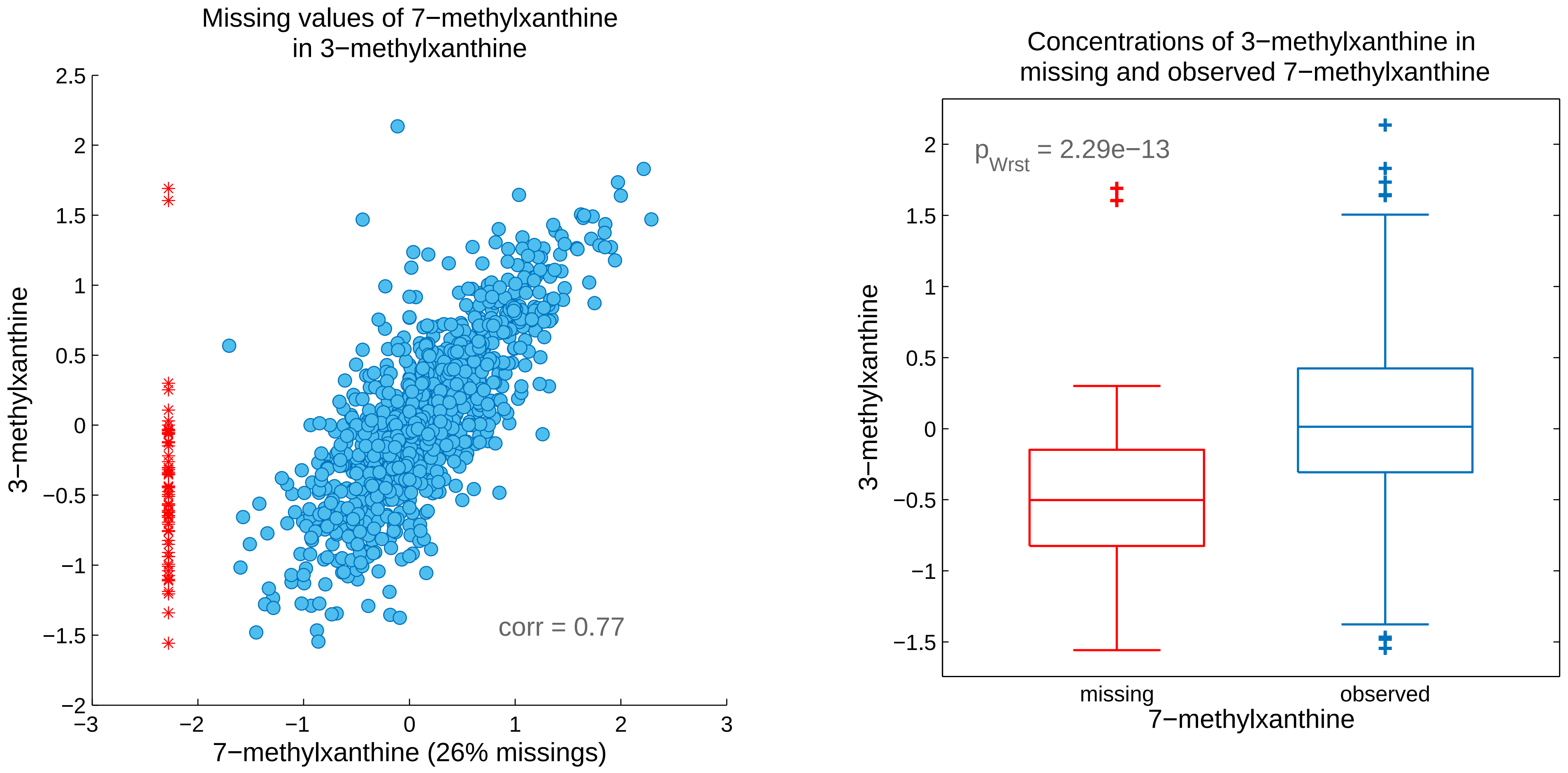

Missing values of 7−methylxanthine in 3−methylxanthine
3−methylxanthine
7−methylxanthine (26% missings)
corr = 0.77
Concentrations of 3−methylxanthine in missing and observed 7−methylxanthine
p_{Wrst} = 2.29e−13
3−methylxanthine
missing
observed
7−methylxanthine

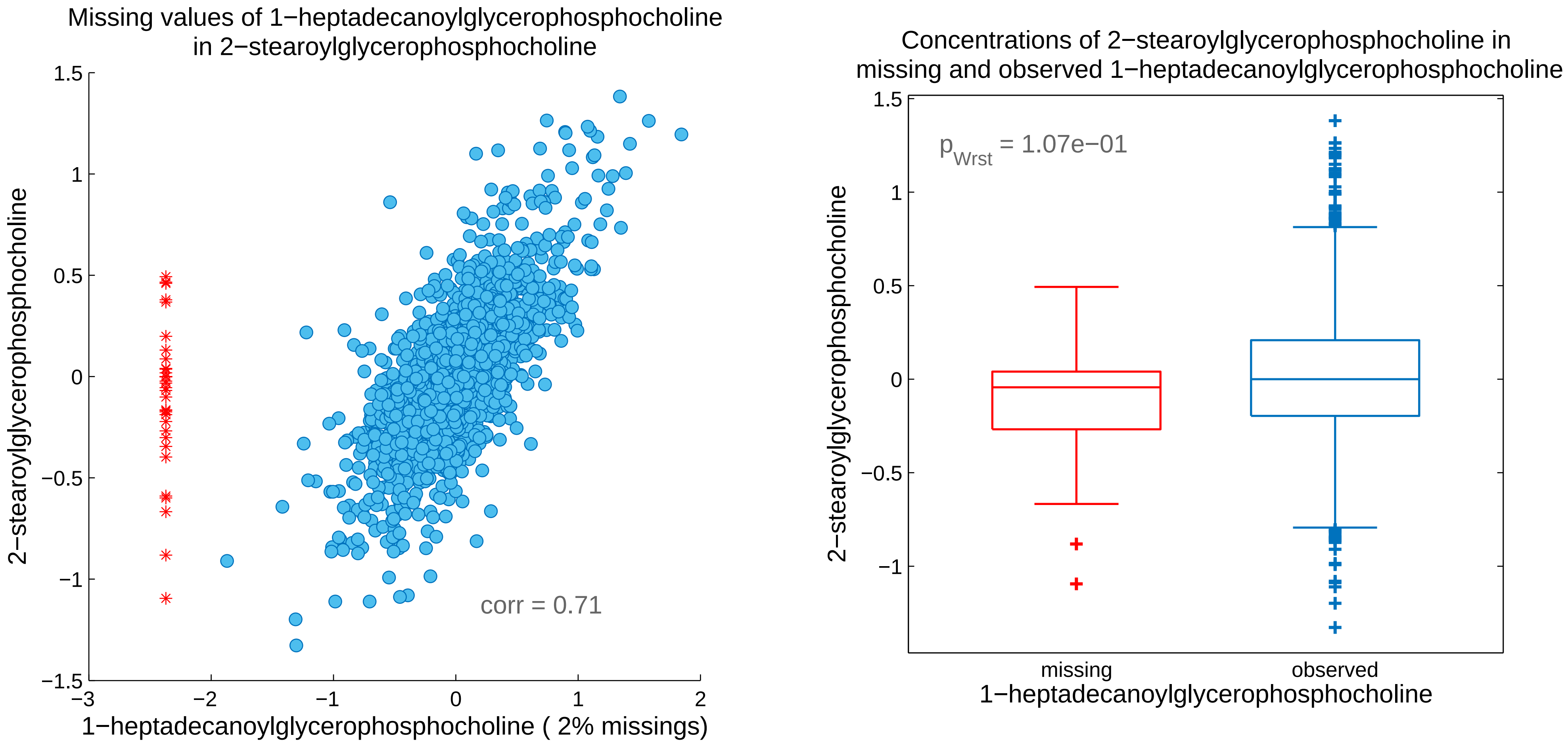
Missing values of 1−heptadecanoylglycerophosphocholine in 2−stearoylglycerophosphocholine
2−stearoylglycerophosphocholine
1−heptadecanoylglycerophosphocholine (2% missings)
corr = 0.71
1.5
1
0.5
0
−0.5
−1
−1.5
−3
−2
−1
0
1
2
Concentrations of 2−stearoylglycerophosphocholine in missing and observed 1−heptadecanoylglycerophosphocholine
p_Wrst = 1.07e−01
2−stearoylglycerophosphocholine
missing
observed
1−heptadecanoylglycerophosphocholine

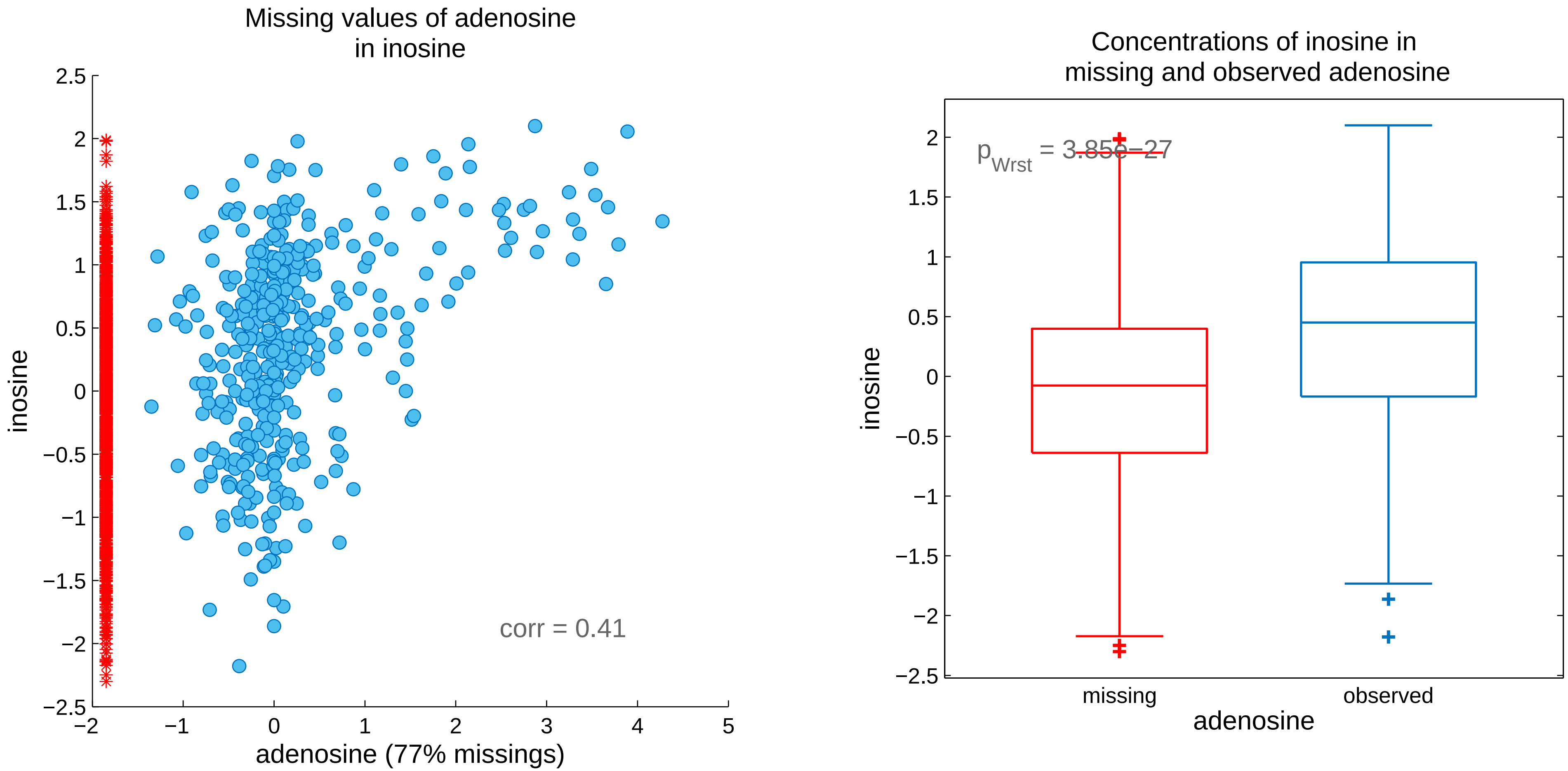

Missing values of adenosine in inosine
inosine
adenosine (77% missings)
corr = 0.41
Concentrations of inosine in missing and observed adenosine
pWrst = 3.85e−27
inosine
missing
observed
adenosine

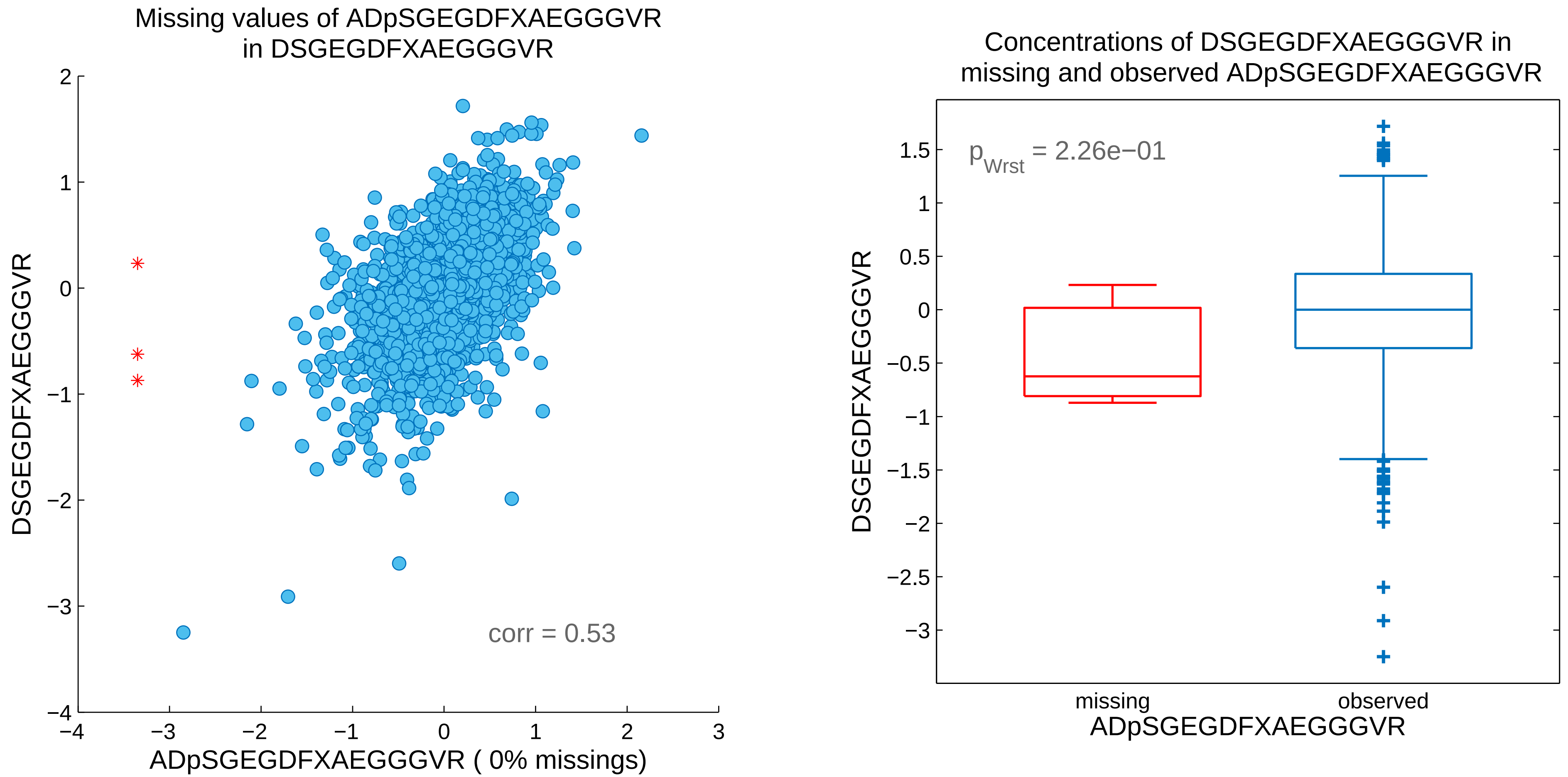
Missing values of ADpSGEGDFXAEGGGVR
in DSGEGDFXAEGGGVR
DSGEGDFXAEGGGVR
ADpSGEGDFXAEGGGVR (0% missings)
corr = 0.53
Concentrations of DSGEGDFXAEGGGVR in
missing and observed ADpSGEGDFXAEGGGVR
p_{Wrst} = 2.26e−01
missing
observed
ADpSGEGDFXAEGGGVR

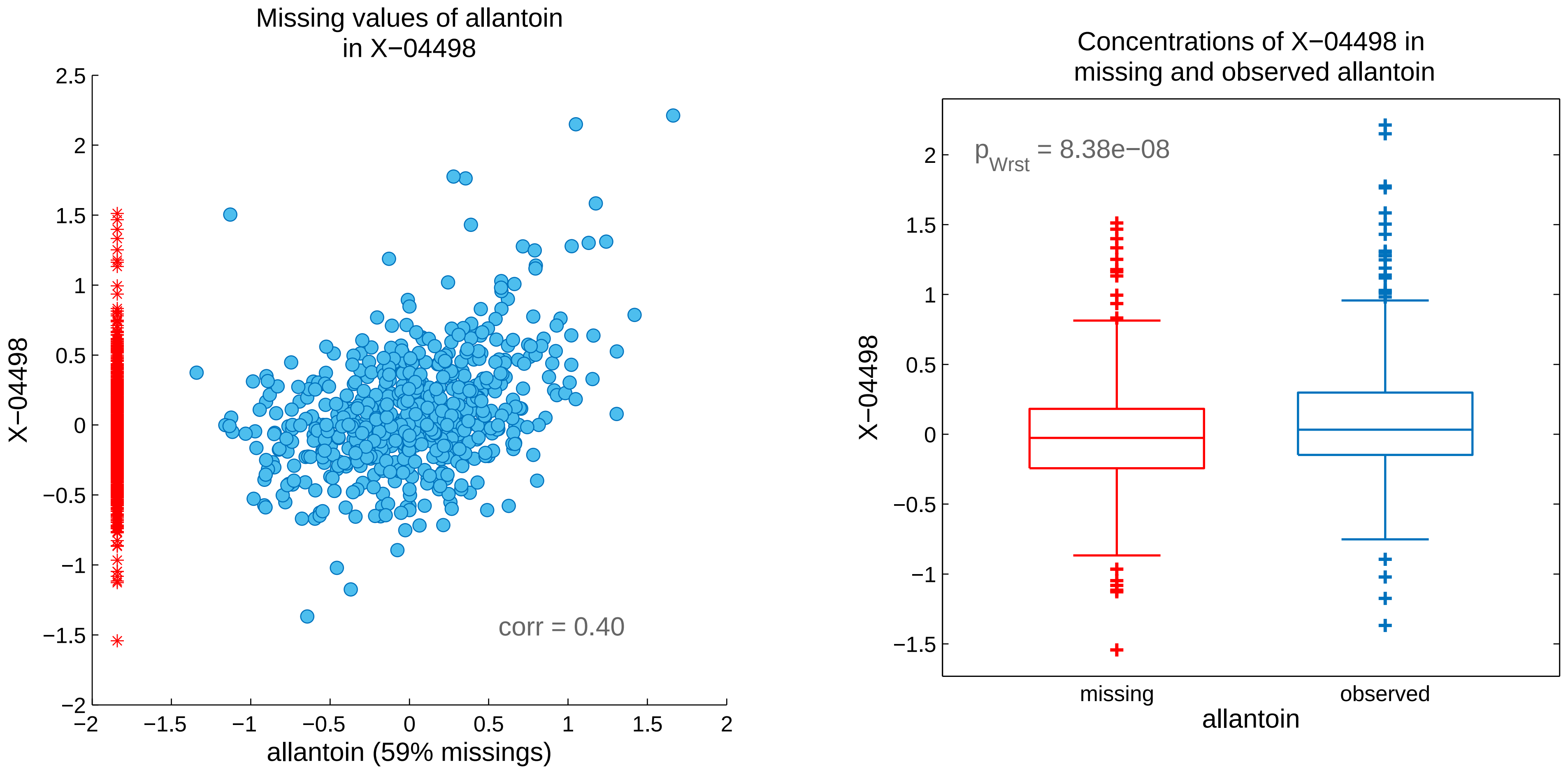
Missing values of allantoin
in X−04498
X−04498
allantoin (59% missings)
corr = 0.40
2.5
2
1.5
1
0.5
0
−0.5
−1
−1.5
−2
−2
−1.5
−1
−0.5
0
0.5
1
1.5
2
Concentrations of X−04498 in
missing and observed allantoin
p_{Wrst} = 8.38e−08
X−04498
2
1.5
1
0.5
0
−0.5
−1
−1.5
missing
observed
allantoin

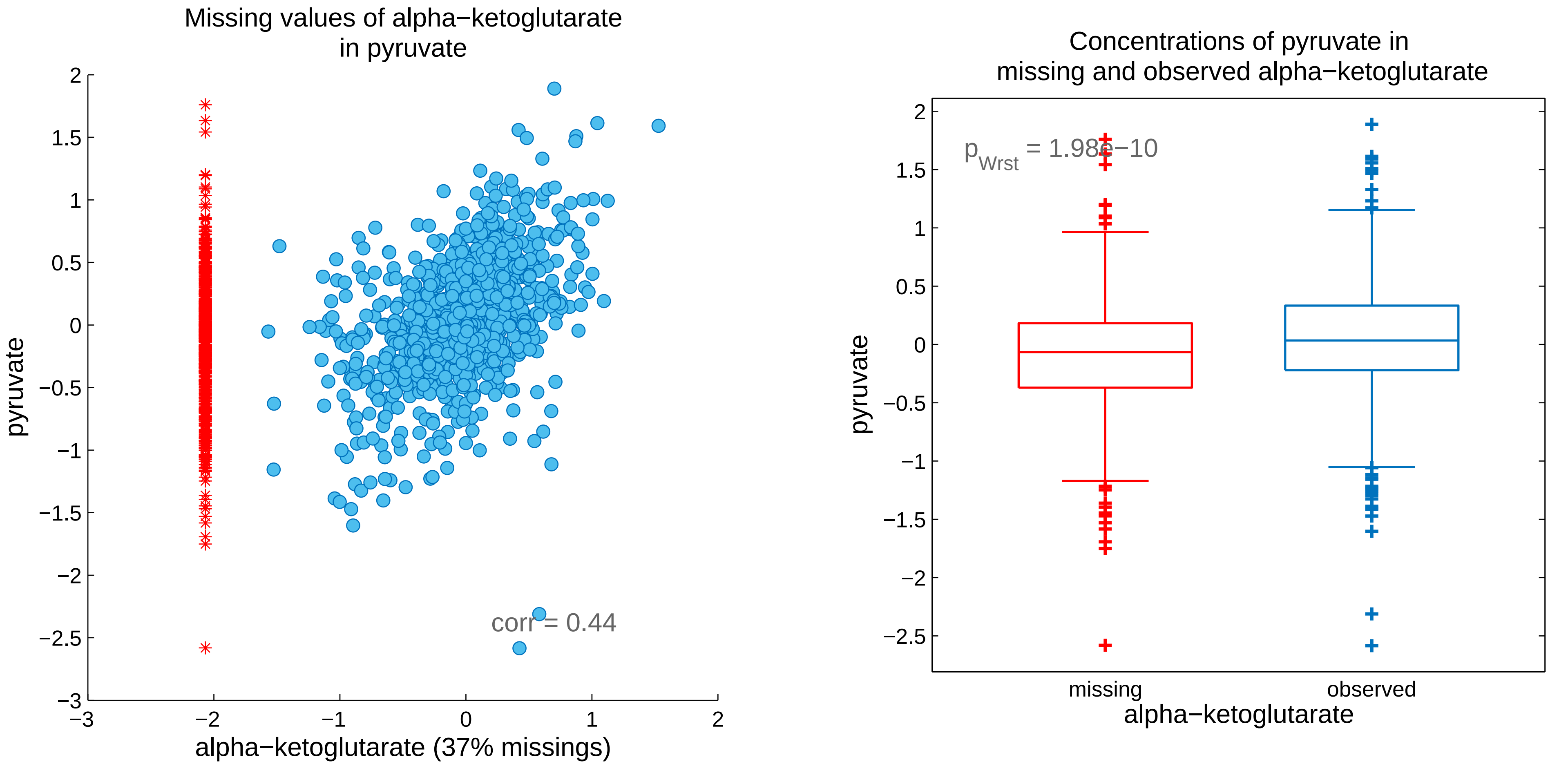
Missing values of alpha−ketoglutarate
in pyruvate
pyruvate
alpha−ketoglutarate (37% missings)
corr = 0.44
Concentrations of pyruvate in
missing and observed alpha−ketoglutarate
pyruvate
p_{Wrst} = 1.98e−10
missing
observed
alpha−ketoglutarate

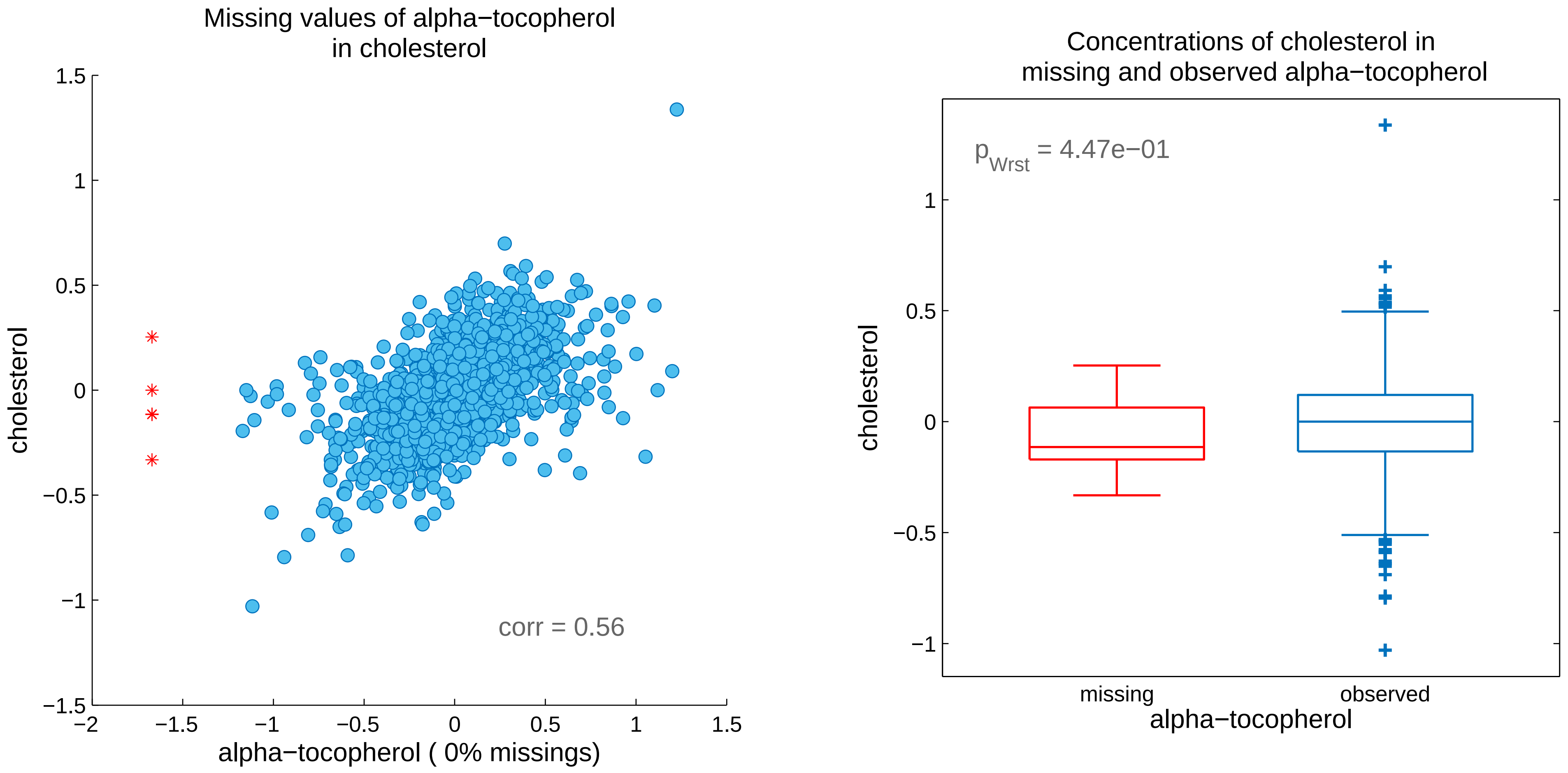

Missing values of alpha−tocopherol in cholesterol
cholesterol
alpha−tocopherol (0% missings)
corr = 0.56
1.5
1
0.5
0
−0.5
−1
−1.5
−2
−1.5
−1
−0.5
0
0.5
1
1.5
Concentrations of cholesterol in missing and observed alpha−tocopherol
p_Wrst = 4.47e−01
cholesterol
missing
observed
alpha−tocopherol

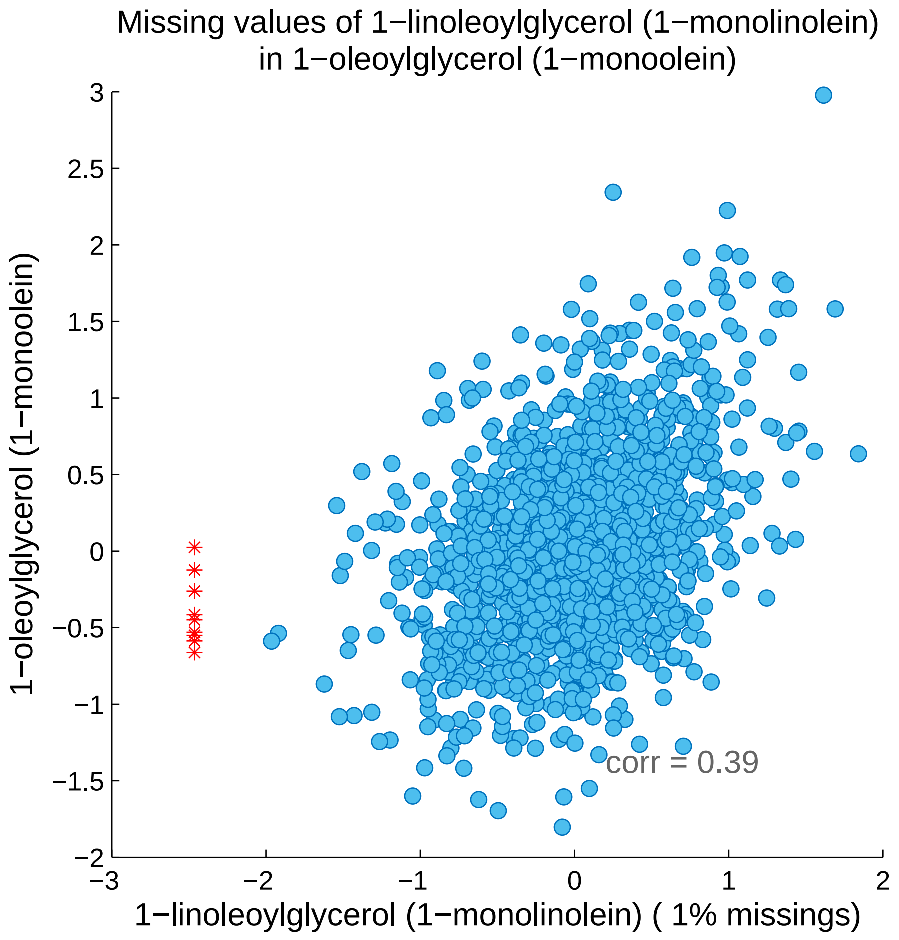
Missing values of 1−linoleoylglycerol (1−monolinolein) in 1−oleoylglycerol (1−monoolein)
corr = 0.39
1−oleoylglycerol (1−monoolein)
1−linoleoylglycerol (1−monolinolein) (1% missings)

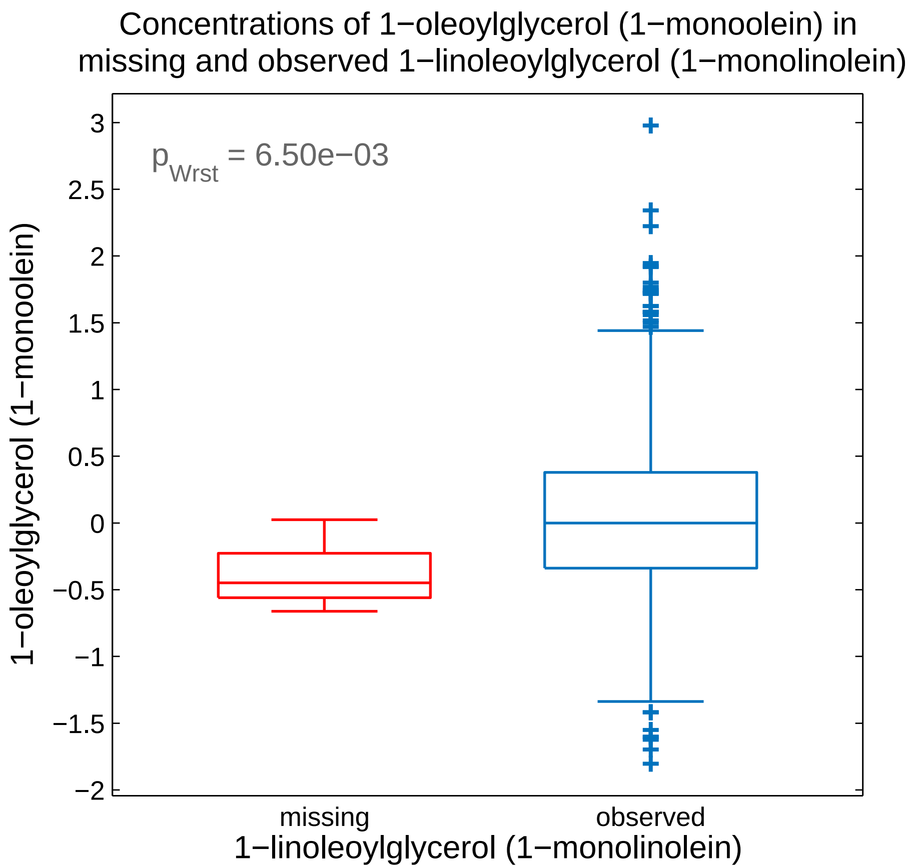
Concentrations of 1−oleoylglycerol (1−monoolein) in missing and observed 1−linoleoylglycerol (1−monolinolein)
p_Wrst = 6.50e−03
1−oleoylglycerol (1−monoolein)
missing
observed
1−linoleoylglycerol (1−monolinolein)

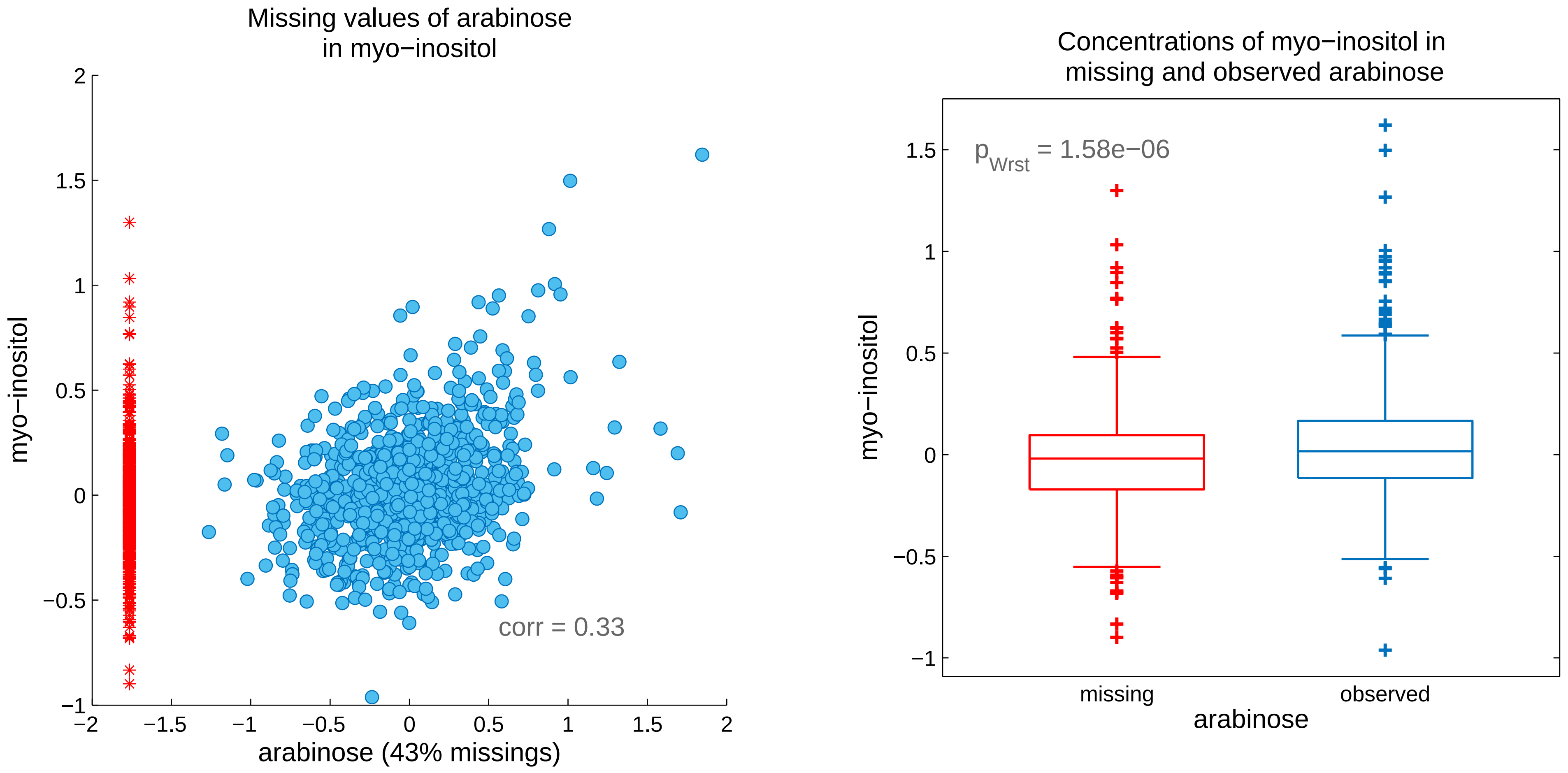
Missing values of arabinose in myo−inositol
myo−inositol
arabinose (43% missings)
corr = 0.33
Concentrations of myo−inositol in missing and observed arabinose
p_{Wrst} = 1.58e−06
myo−inositol
missing
observed
arabinose

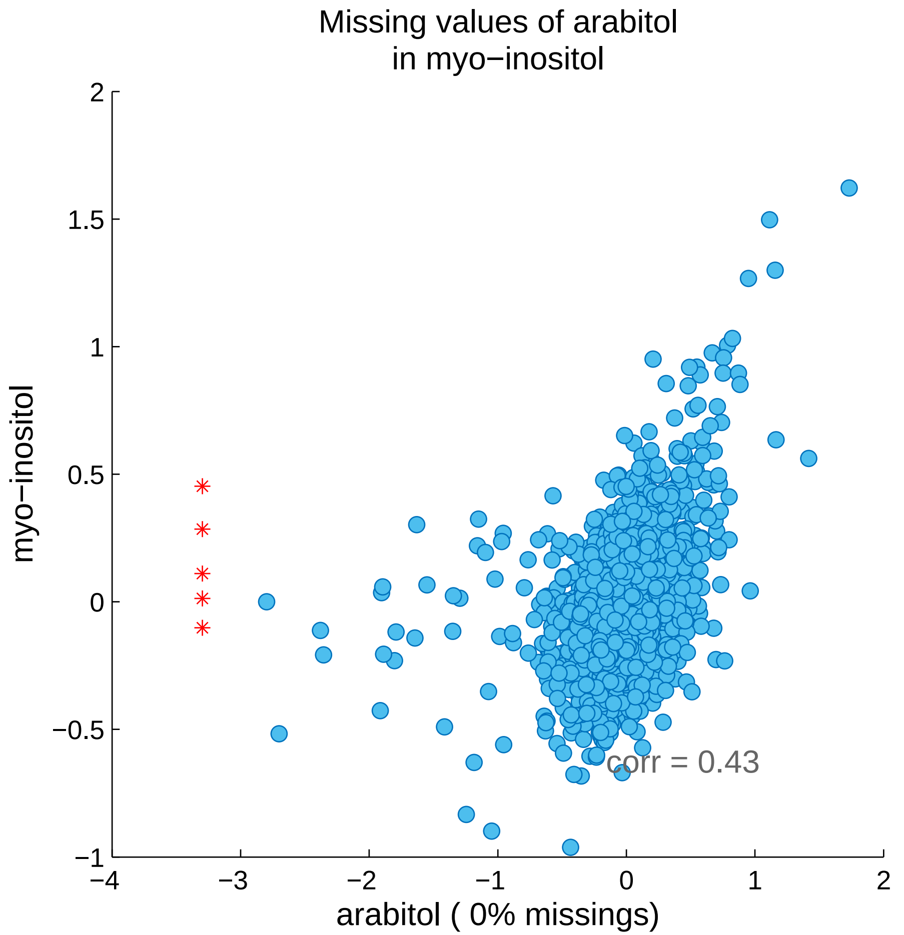

Missing values of arabitol
in myo−inositol
myo−inositol
arabitol (0% missings)
corr = 0.43
2
1.5
1
0.5
0
−0.5
−1
−4
−3
−2
−1
0
1
2

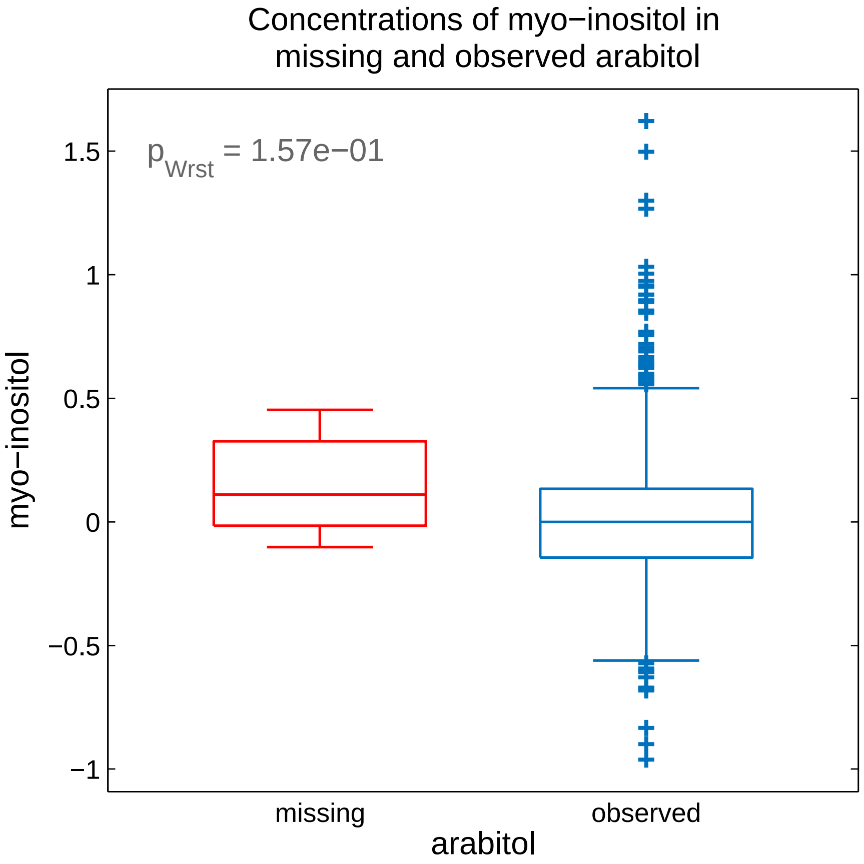

Concentrations of myo−inositol in
missing and observed arabitol
p_{Wrst} = 1.57e−01
myo−inositol
1.5
1
0.5
0
−0.5
−1
missing
observed
arabitol

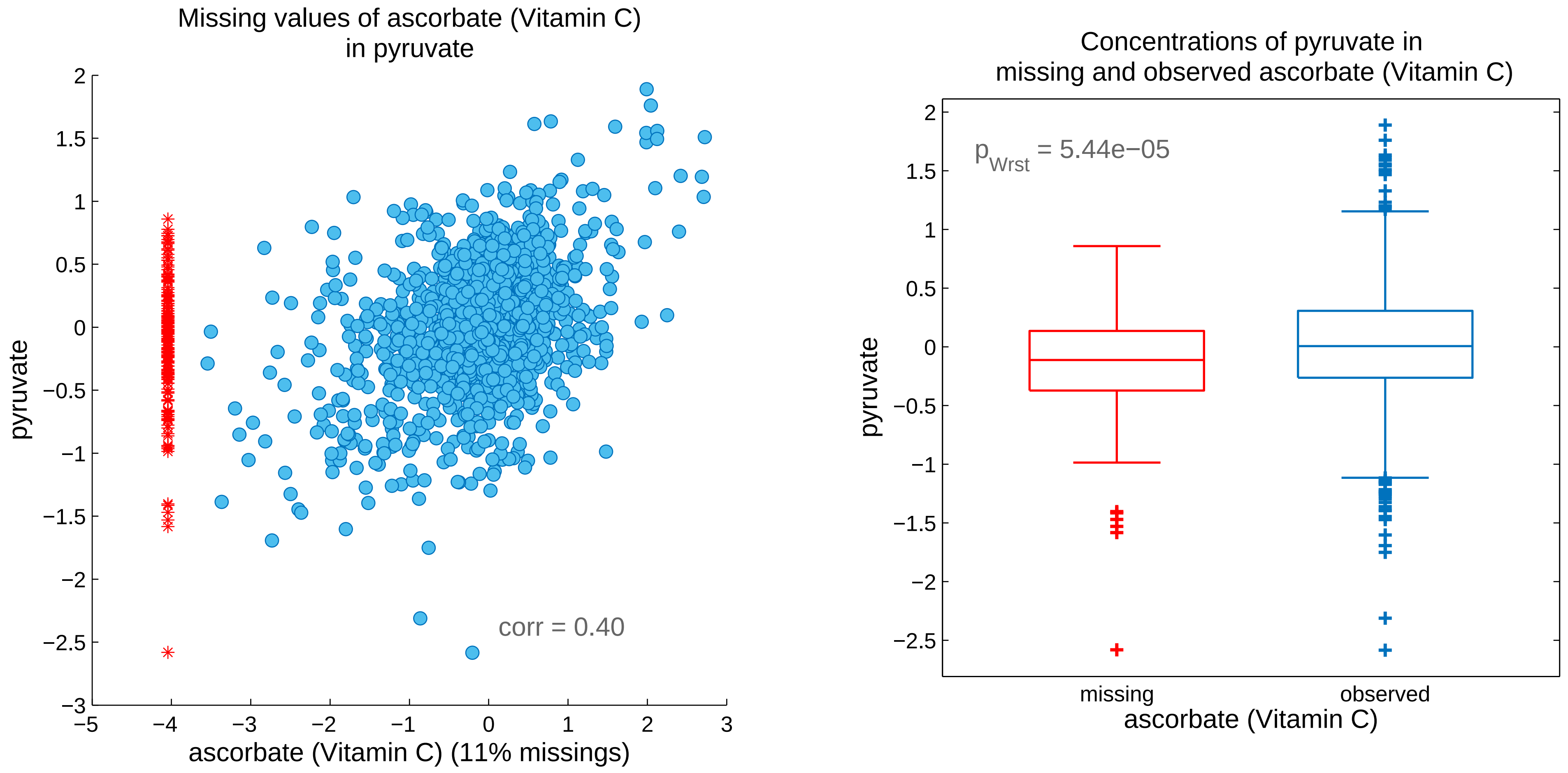

Missing values of ascorbate (Vitamin C)
in pyruvate
pyruvate
ascorbate (Vitamin C) (11% missings)
corr = 0.40
2
1.5
1
0.5
0
−0.5
−1
−1.5
−2
−2.5
−3
−5
−4
−3
−2
−1
0
1
2
3
Concentrations of pyruvate in
missing and observed ascorbate (Vitamin C)
p_Wrst = 5.44e−05
pyruvate
missing
observed
ascorbate (Vitamin C)

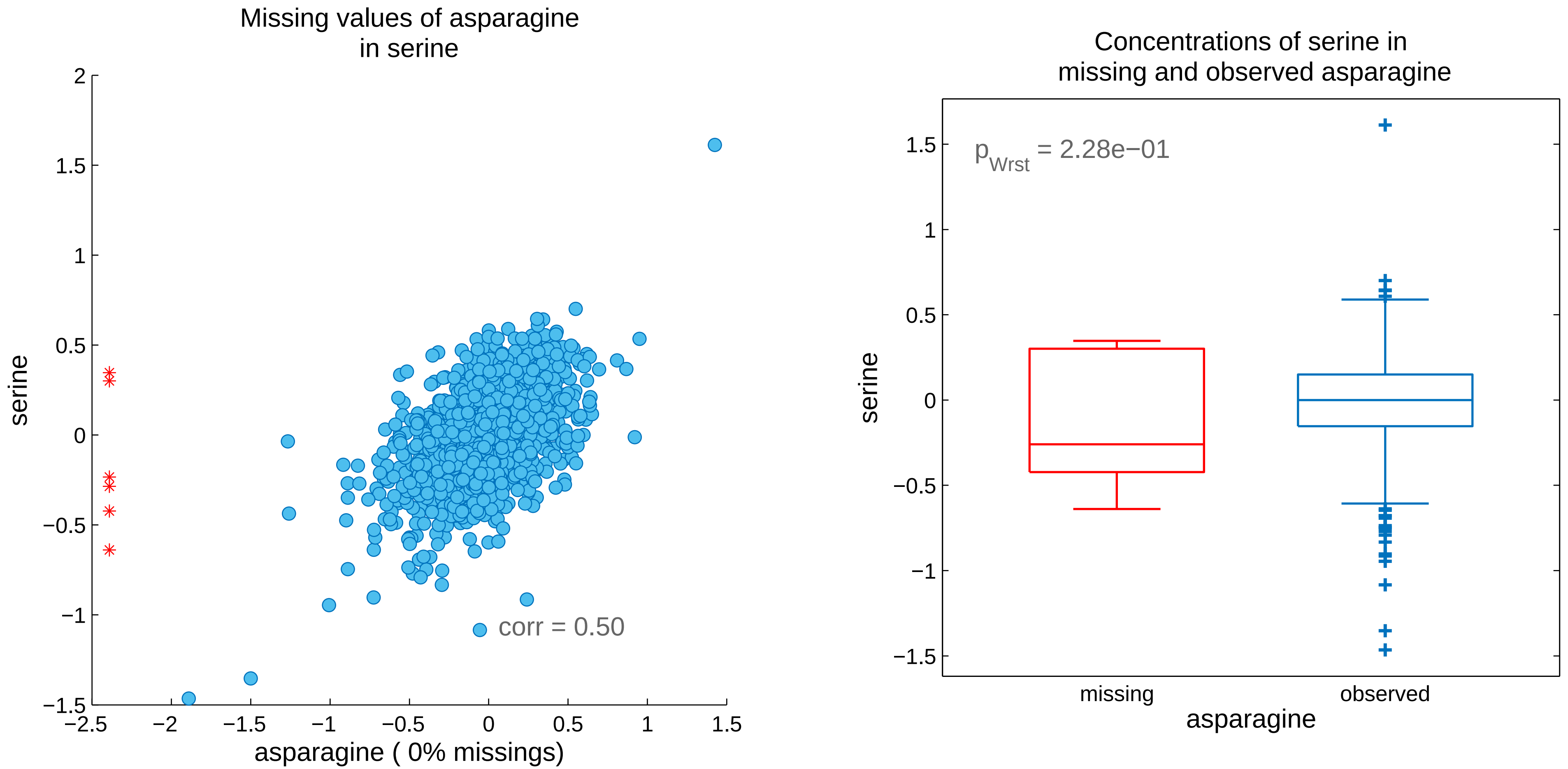

Missing values of asparagine in serine
serine
asparagine (0% missings)
corr = 0.50
Concentrations of serine in missing and observed asparagine
p_{Wrst} = 2.28e−01
serine
missing
observed
asparagine

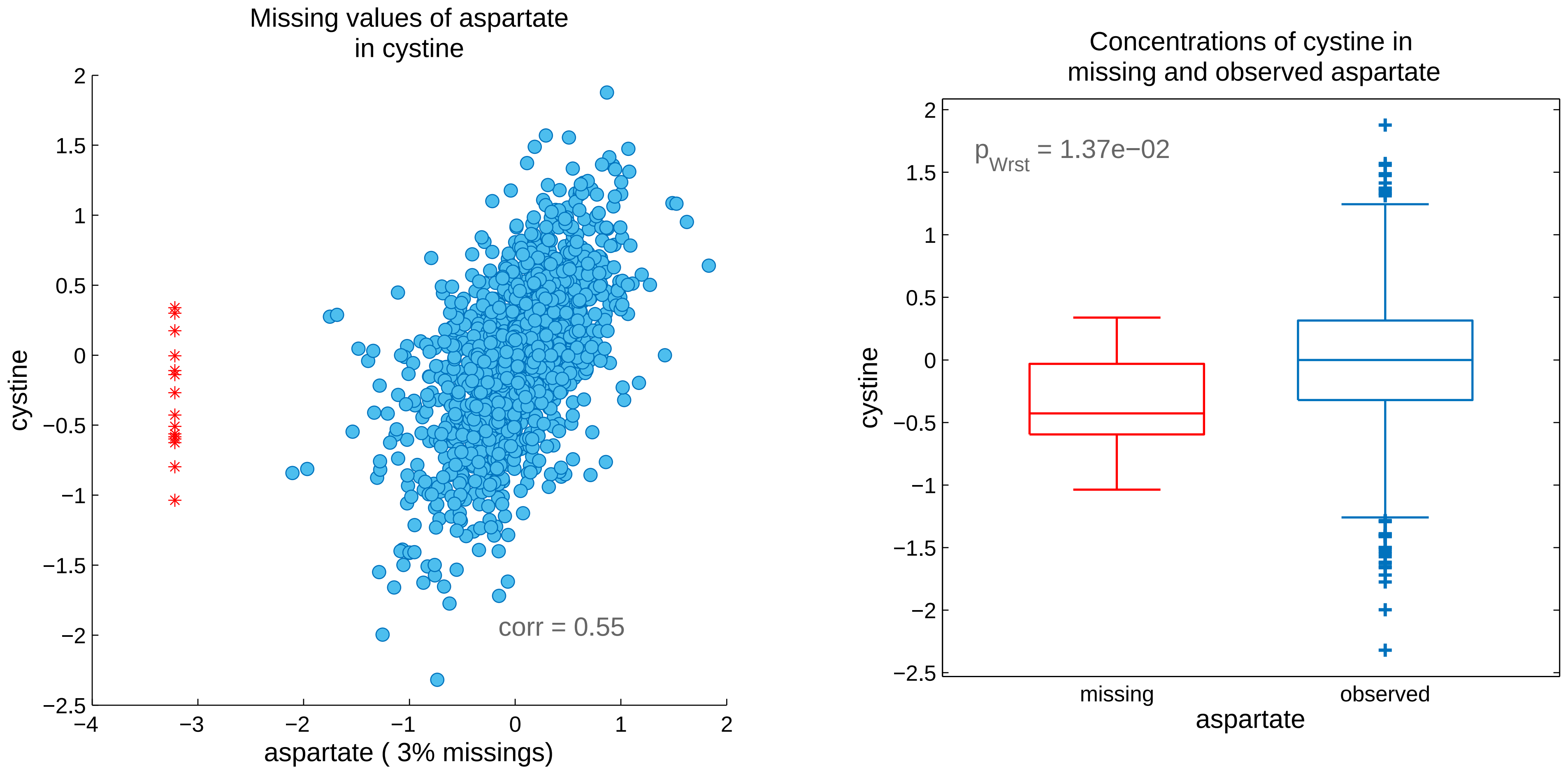

Missing values of aspartate in cystine
cystine
aspartate (3% missings)
corr = 0.55
Concentrations of cystine in missing and observed aspartate
p_{Wrst} = 1.37e−02
cystine
missing
observed
aspartate

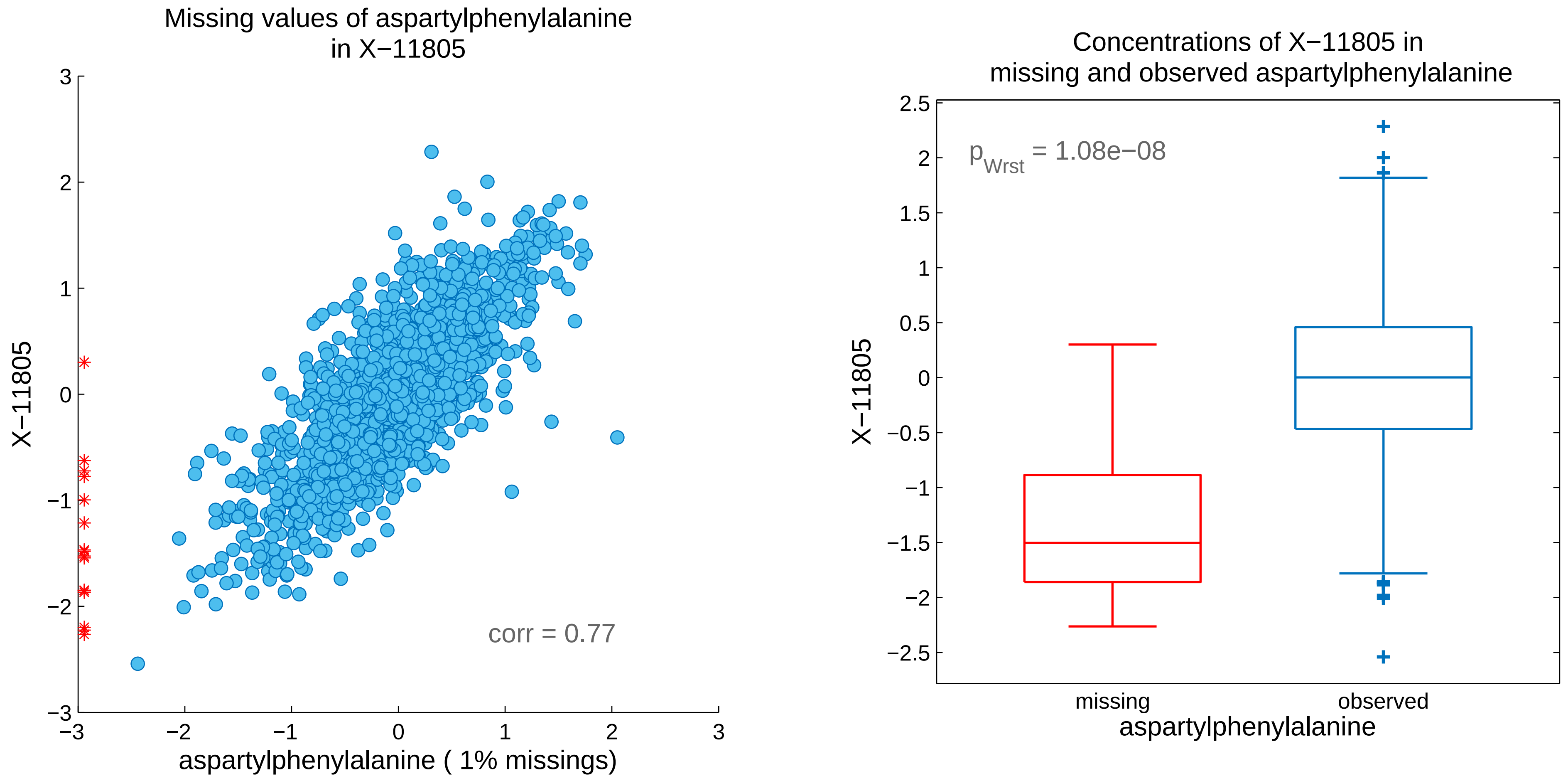

Missing values of aspartylphenylalanine in X−11805
X−11805
aspartylphenylalanine (1% missings)
corr = 0.77
Concentrations of X−11805 in missing and observed aspartylphenylalanine
X−11805
p_{Wrst} = 1.08e−08
missing
observed
aspartylphenylalanine

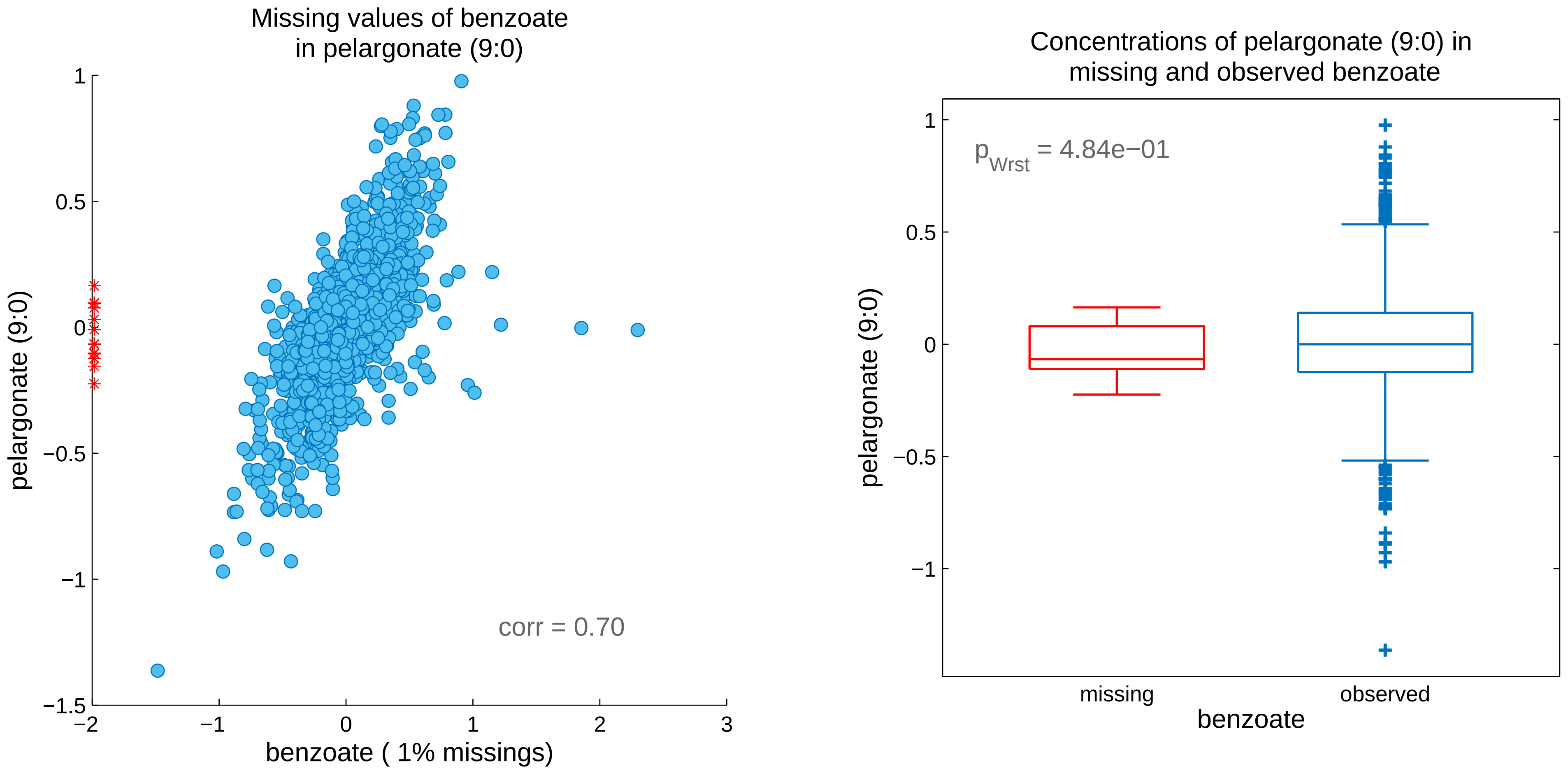

Missing values of benzoate in pelargonate (9:0)
pelargonate (9:0)
benzoate (1% missings)
corr = 0.70
Concentrations of pelargonate (9:0) in missing and observed benzoate
p_{Wrst} = 4.84e−01
pelargonate (9:0)
missing
observed
benzoate

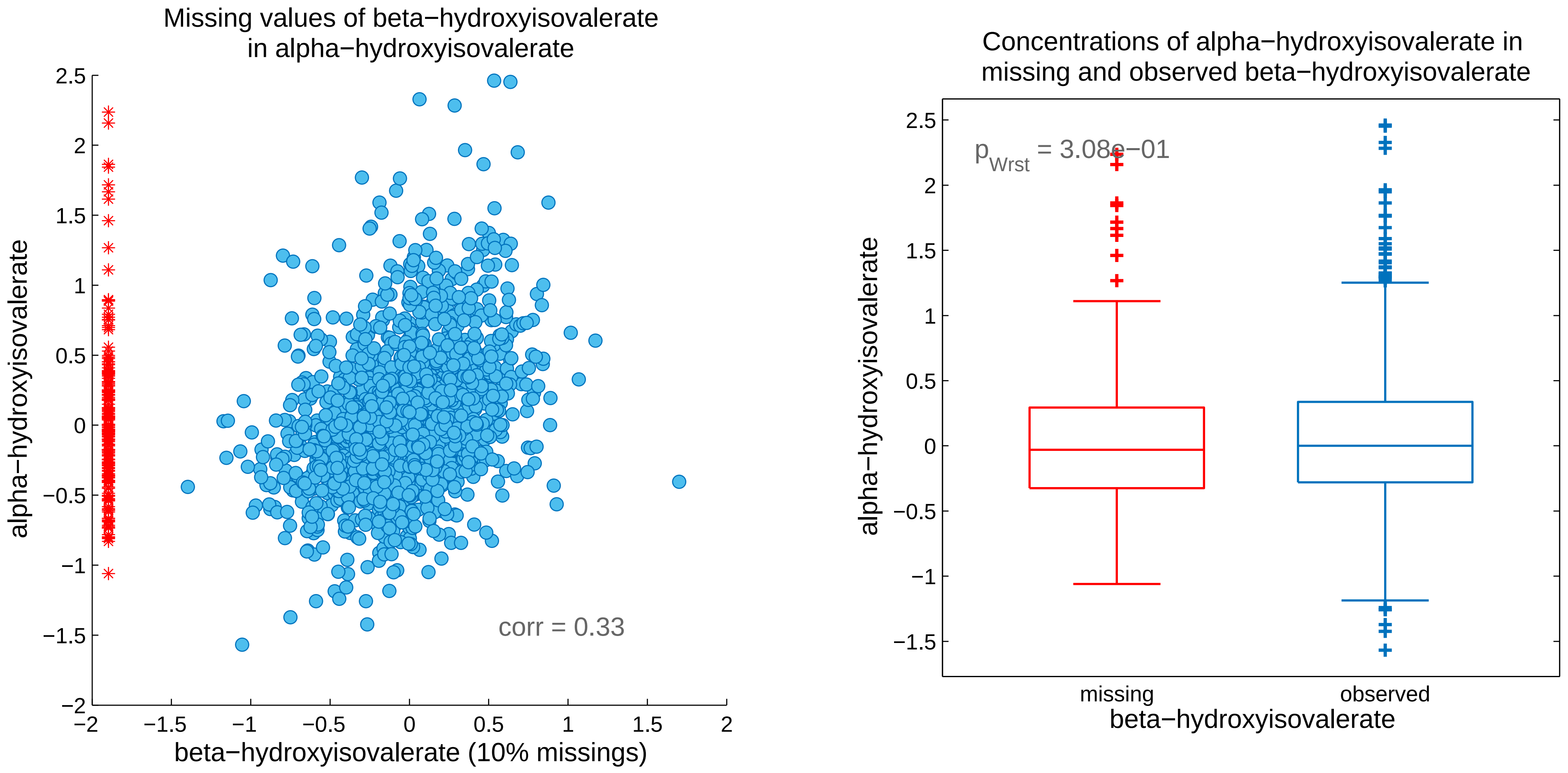
Missing values of beta−hydroxyisovalerate in alpha−hydroxyisovalerate
alpha−hydroxyisovalerate
beta−hydroxyisovalerate (10% missings)
corr = 0.33
Concentrations of alpha−hydroxyisovalerate in missing and observed beta−hydroxyisovalerate
p_{Wrst} = 3.08e−01
alpha−hydroxyisovalerate
missing
observed
beta−hydroxyisovalerate

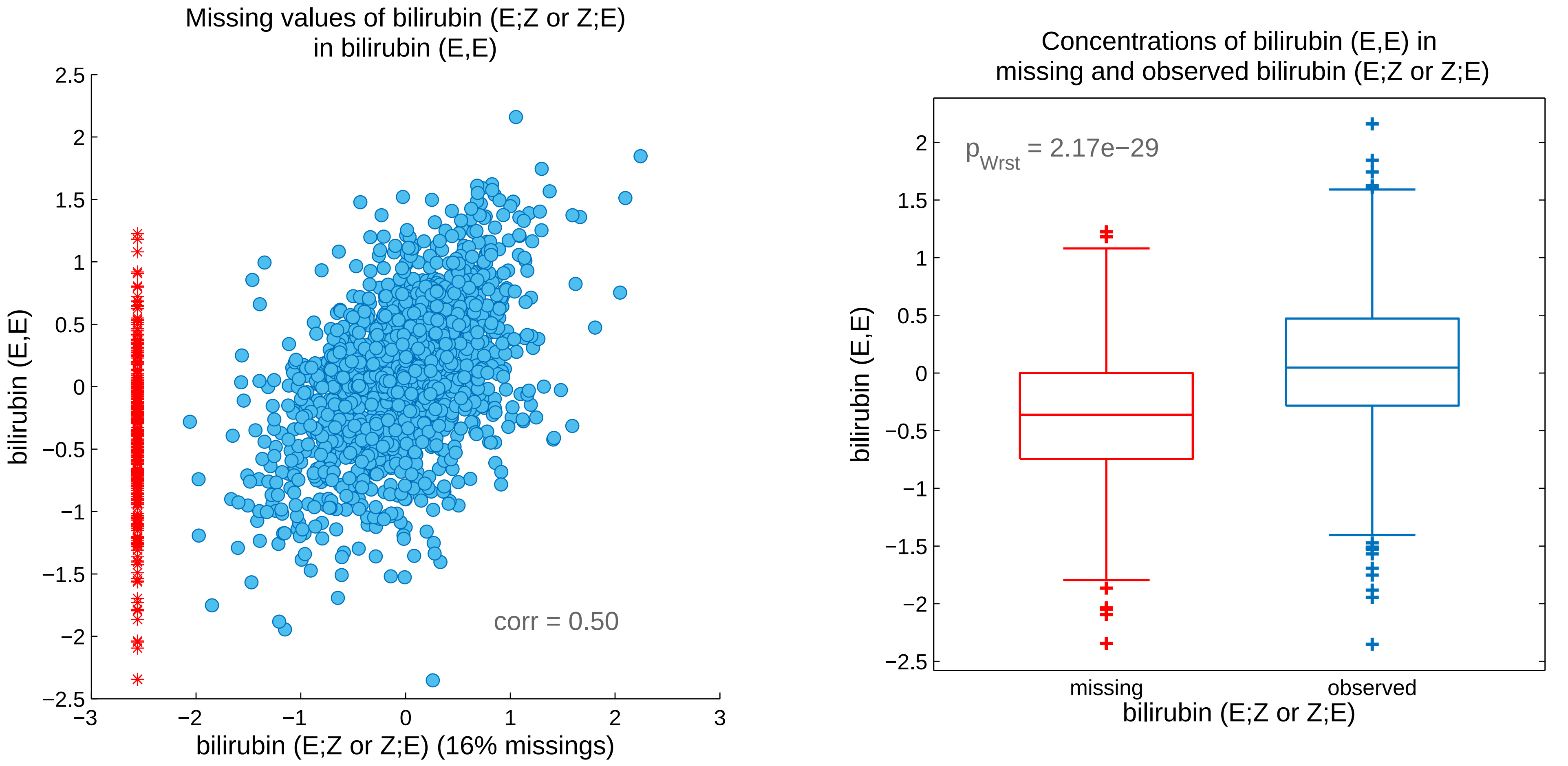

Missing values of bilirubin (E;Z or Z;E) in bilirubin (E,E)
bilirubin (E,E)
bilirubin (E;Z or Z;E) (16% missings)
corr = 0.50
Concentrations of bilirubin (E,E) in missing and observed bilirubin (E;Z or Z;E)
p_Wrst = 2.17e−29
bilirubin (E,E)
missing
observed
bilirubin (E;Z or Z;E)

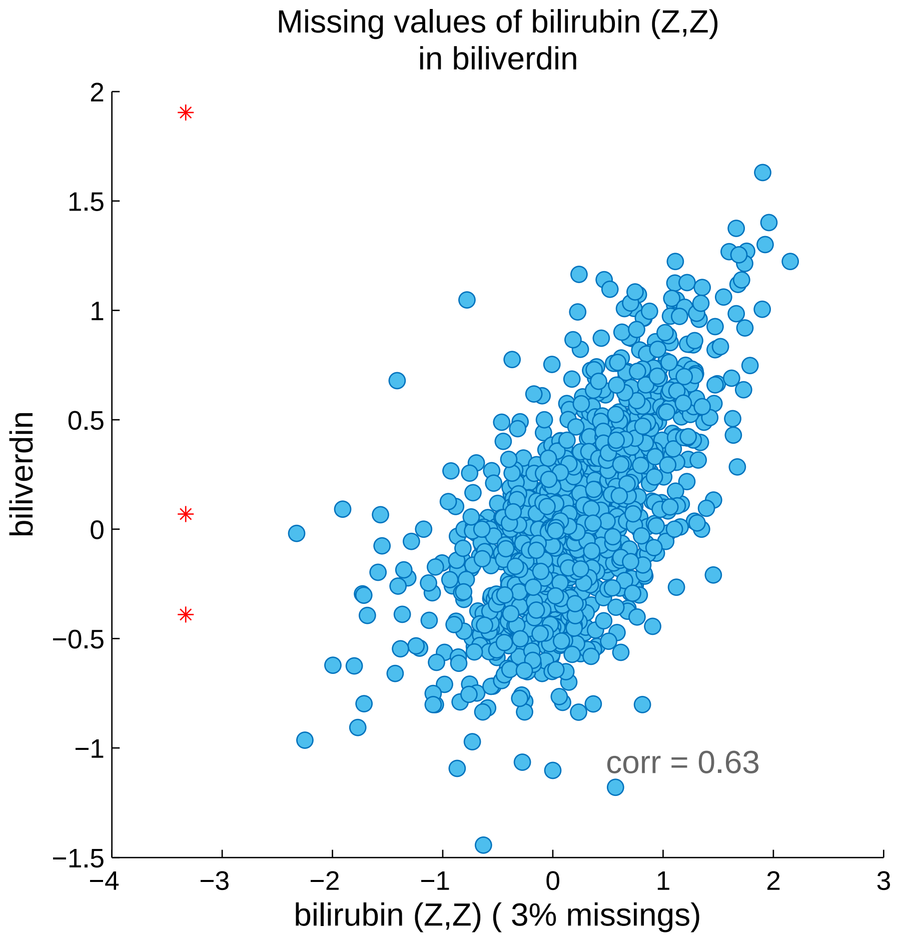

Missing values of bilirubin (Z,Z)
in biliverdin
biliverdin
bilirubin (Z,Z) (3% missings)
corr = 0.63
2
1.5
1
0.5
0
−0.5
−1
−1.5
−4
−3
−2
−1
0
1
2
3

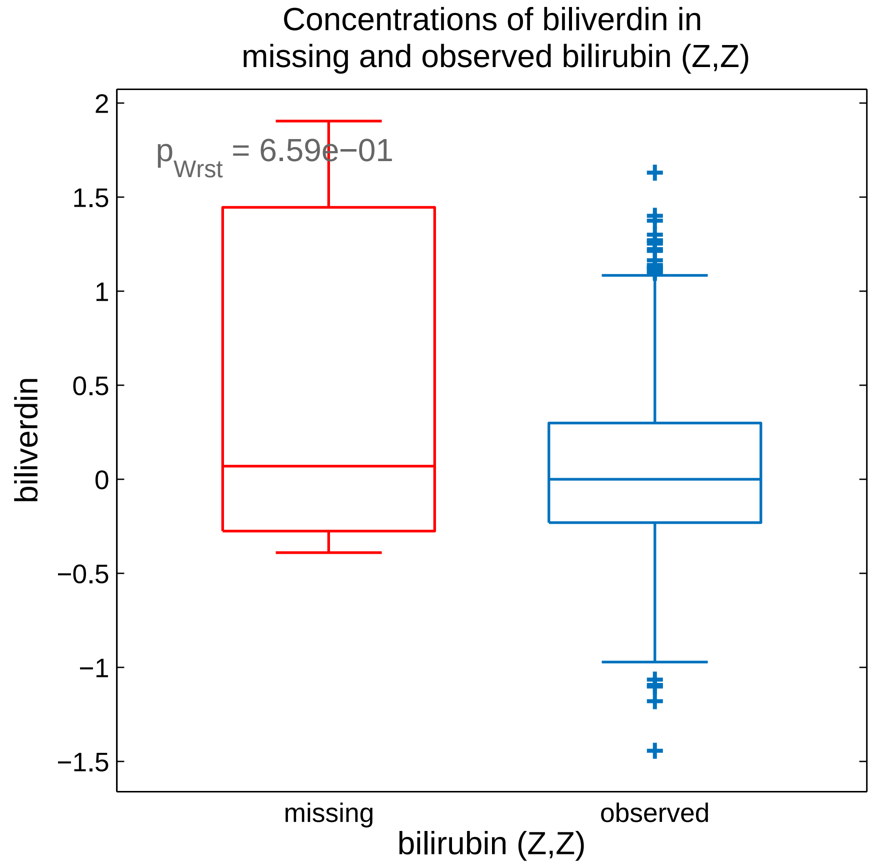

Concentrations of biliverdin in
missing and observed bilirubin (Z,Z)
p_Wrst = 6.59e−01
biliverdin
missing
observed
bilirubin (Z,Z)
2
1.5
1
0.5
0
−0.5
−1
−1.5

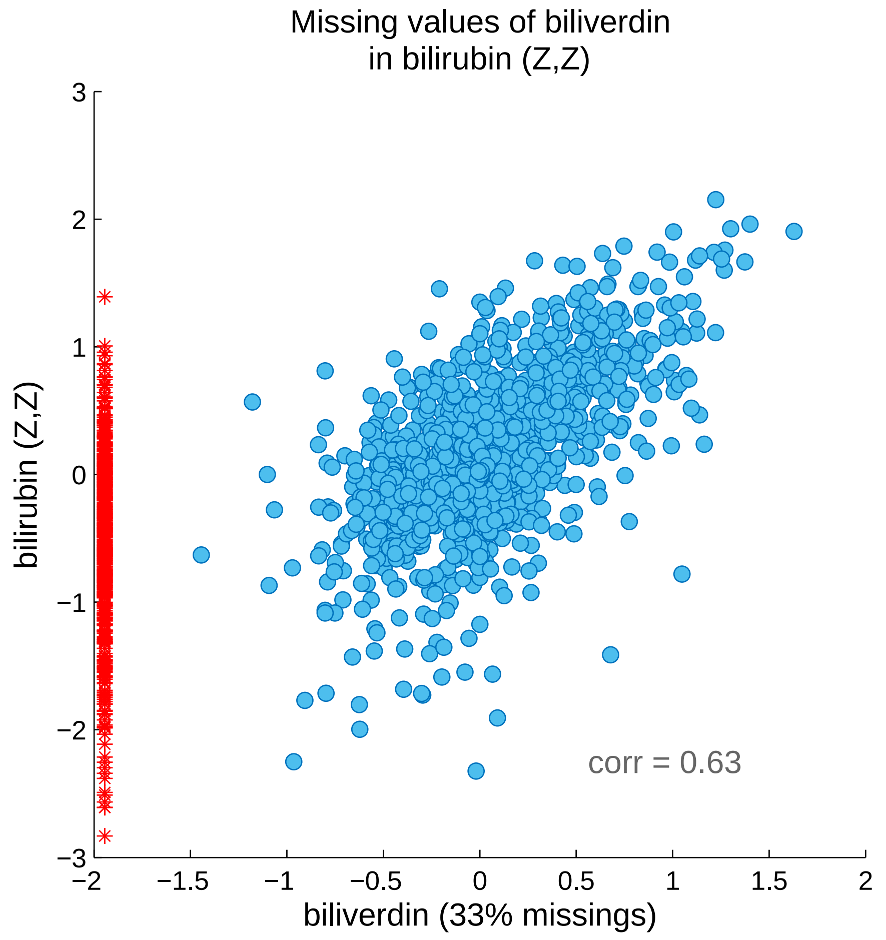

Missing values of biliverdin
in bilirubin (Z,Z)
bilirubin (Z,Z)
biliverdin (33% missings)
corr = 0.63
3
2
1
0
−1
−2
−3
−2
−1.5
−1
−0.5
0
0.5
1
1.5
2

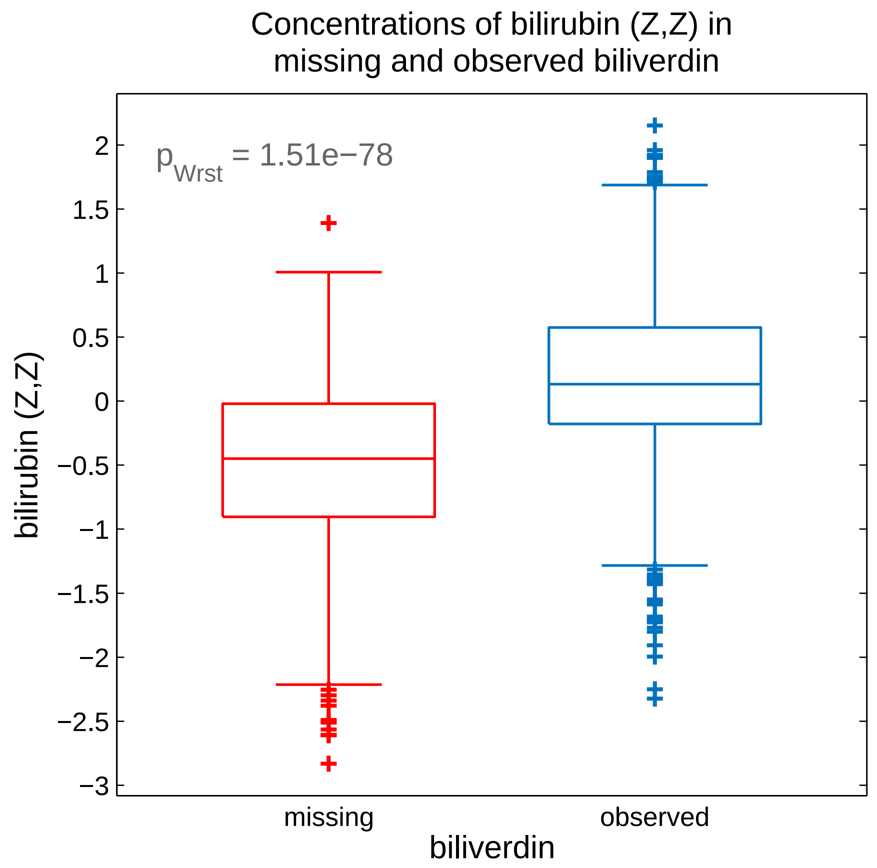

Concentrations of bilirubin (Z,Z) in
missing and observed biliverdin
p_{Wrst} = 1.51e−78
bilirubin (Z,Z)
biliverdin
missing
observed
2
1.5
1
0.5
0
−0.5
−1
−1.5
−2
−2.5
−3

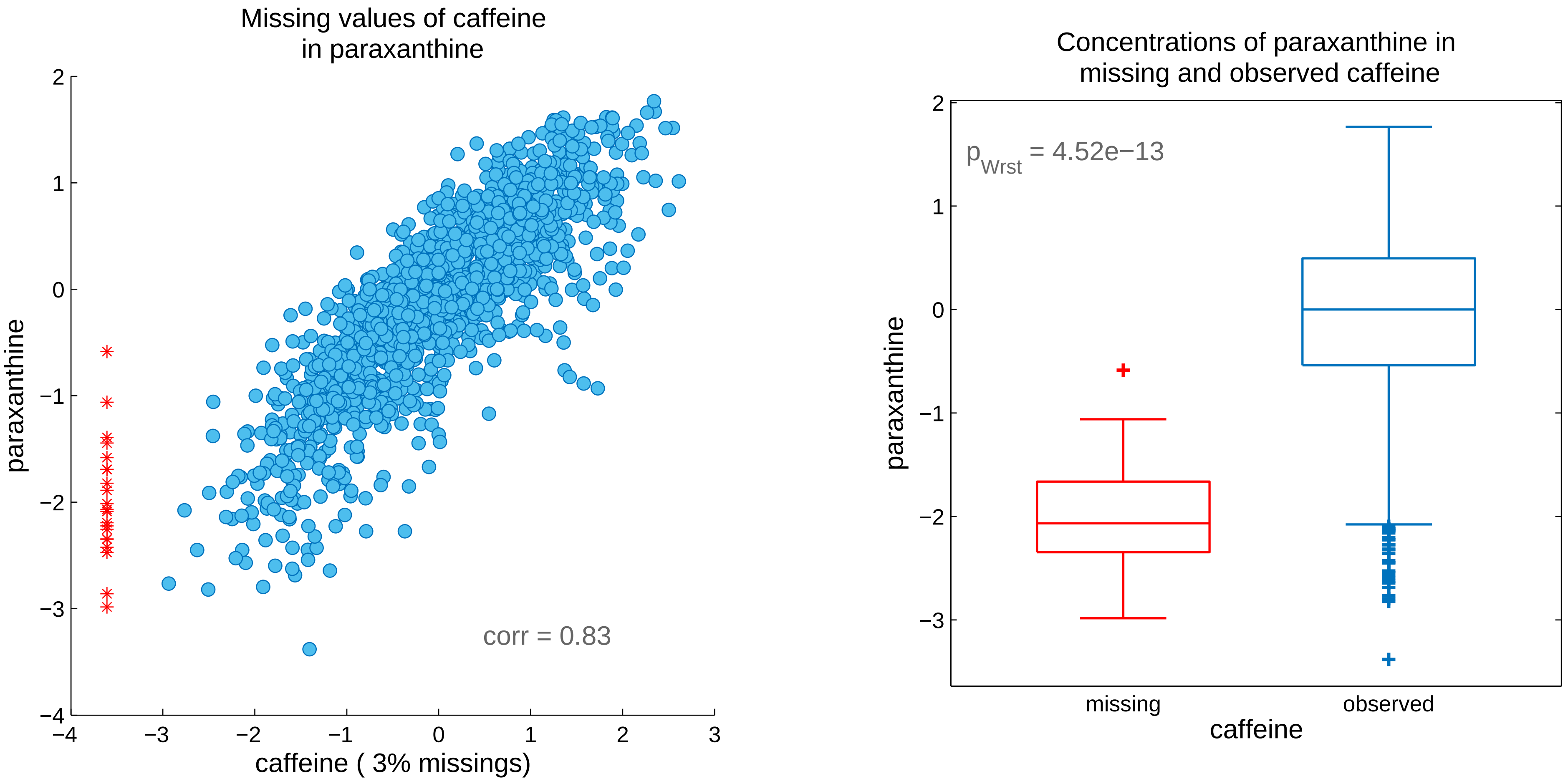

Missing values of caffeine in paraxanthine
paraxanthine
caffeine (3% missings)
corr = 0.83
2
1
0
−1
−2
−3
−4
−4
−3
−2
−1
0
1
2
3
Concentrations of paraxanthine in missing and observed caffeine
p_{Wrst} = 4.52e−13
paraxanthine
caffeine
missing
observed
2
1
0
−1
−2
−3
