# Supplementary material for: Characterization of missing values in untargeted MS-based metabolomics data and evaluation of missing data handling strategies
Source: Metabolomics. 2018 Sep 20;14(10):128. doi: 10.1007/s11306-018-1420-2 (PMC6153696; doi:10.1007/s11306-018-1420-2)
Supplement: Supplementary file 2 — Supplementary material 2 (PDF 26281 KB) [file 11306_2018_1420_MOESM2_ESM.pdf]

Characterization of missingness in untargeted MS-based metabolomics data sets and evaluation of missing data handling strategies

Kieu Trinh Do¶, Simone Wahl¶, Johannes Raffler, Sophie Molnos, Michael Laimighofer, Jerzy Adamski, Karsten Suhre, Konstantin Strauch, Annette Peters, Christian Gieger, Claudia Langenberg, Isobel D. Stewart, Fabian J. Theis, Harald Grallert, Gabi Kastenmüller#, Jan Krumsiek#

Supporting Information File S2: Runday-dependent densities in relation with missingness

The distribution of each metabolite is shown before (top panel) and after runday normalization (bottom panel). Runday normalization was performed with runday median. The colors of the distributions correspond to the amount of missing values in the respective runday.

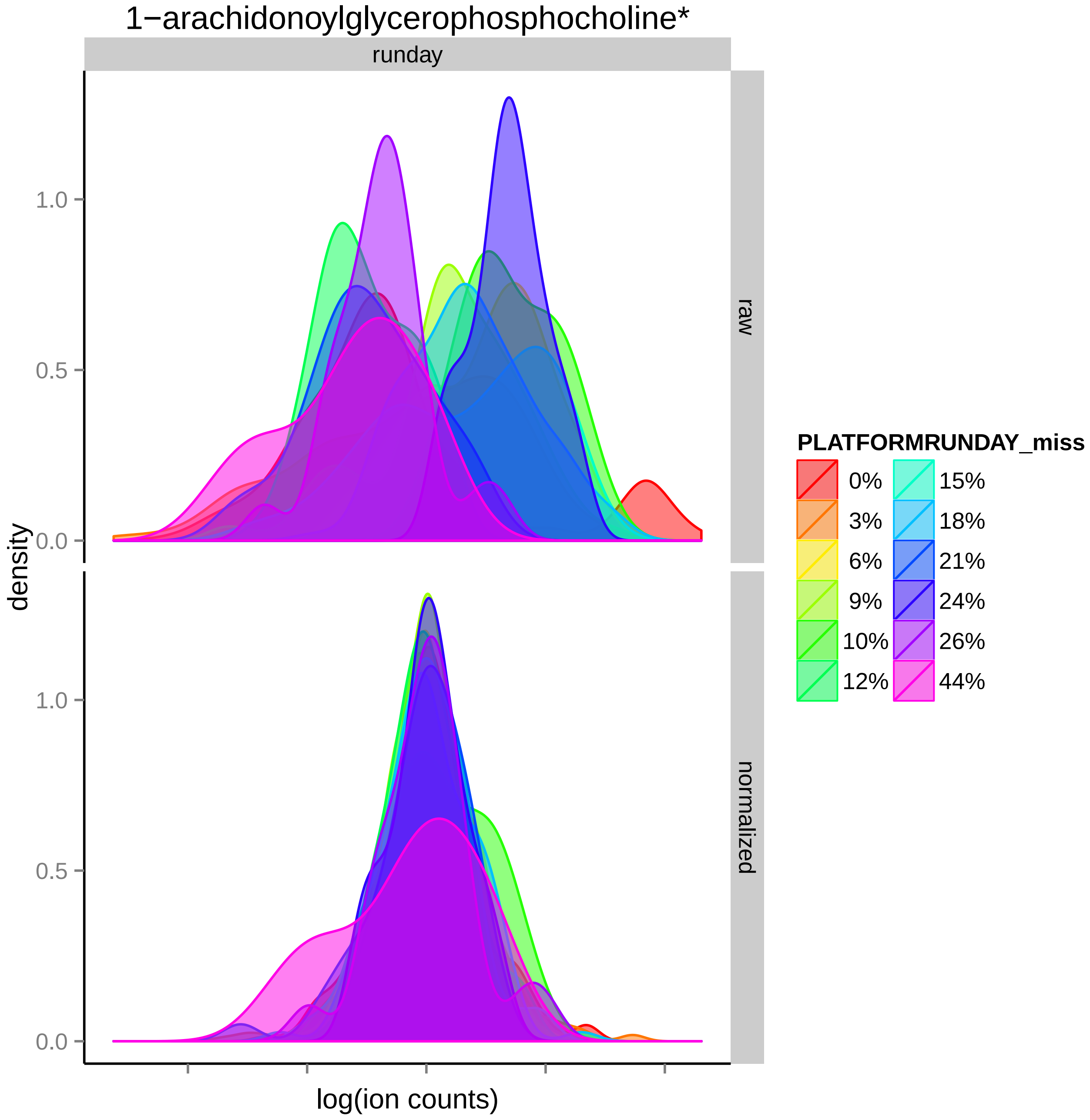

1−arachidonoylglycerophosphocholine*
runday
raw
normalized
density
log(ion counts)
1.0
0.5
0.0
PLATFORMRUNDAY_miss
0%
3%
6%
9%
10%
12%
15%
18%
21%
24%
26%
44%

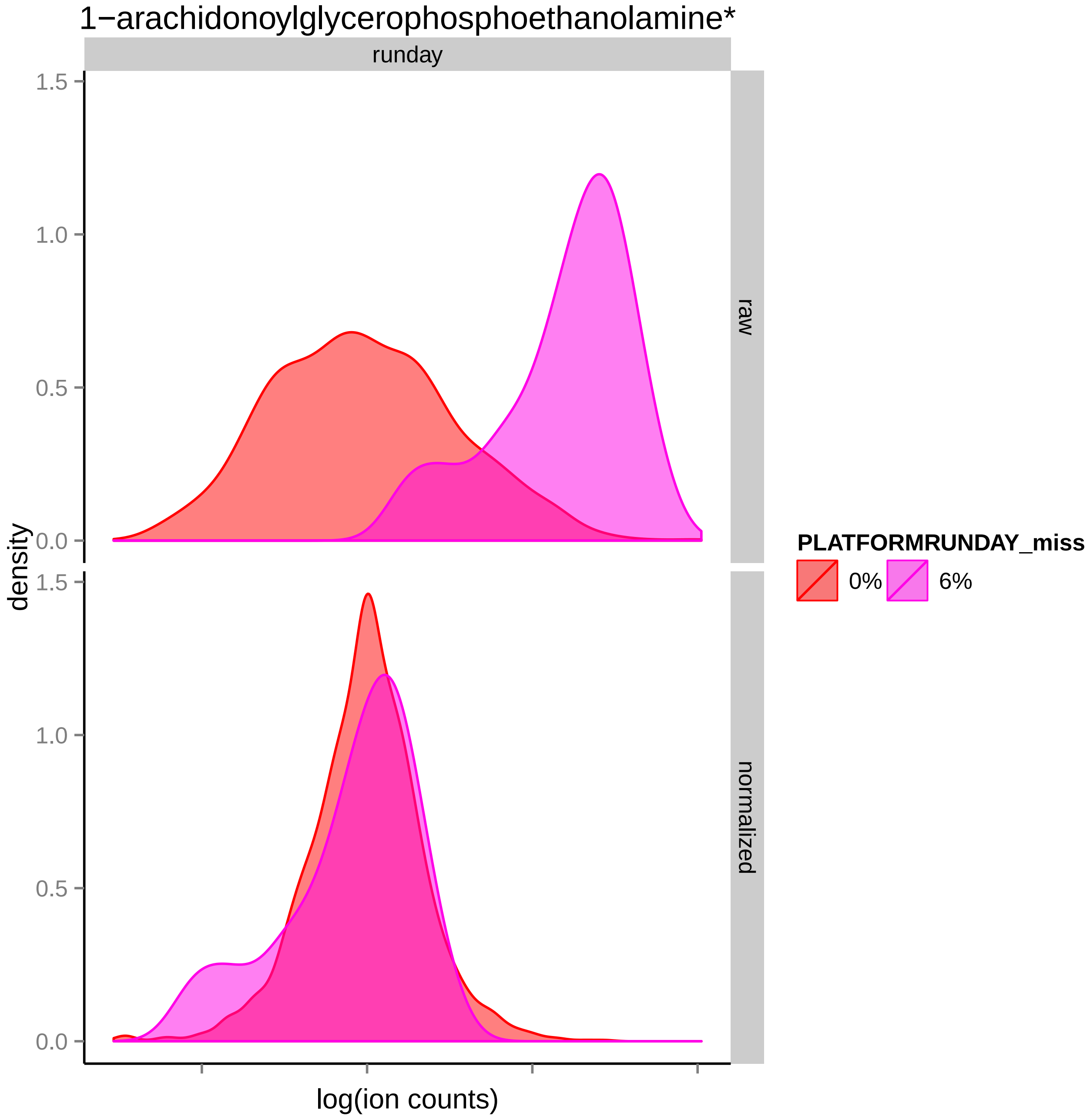
1−arachidonoylglycerophosphoethanolamine*
runday
raw
normalized
1.5
1.0
0.5
0.0
density
log(ion counts)
PLATFORMRUNDAY_miss
0%
6%

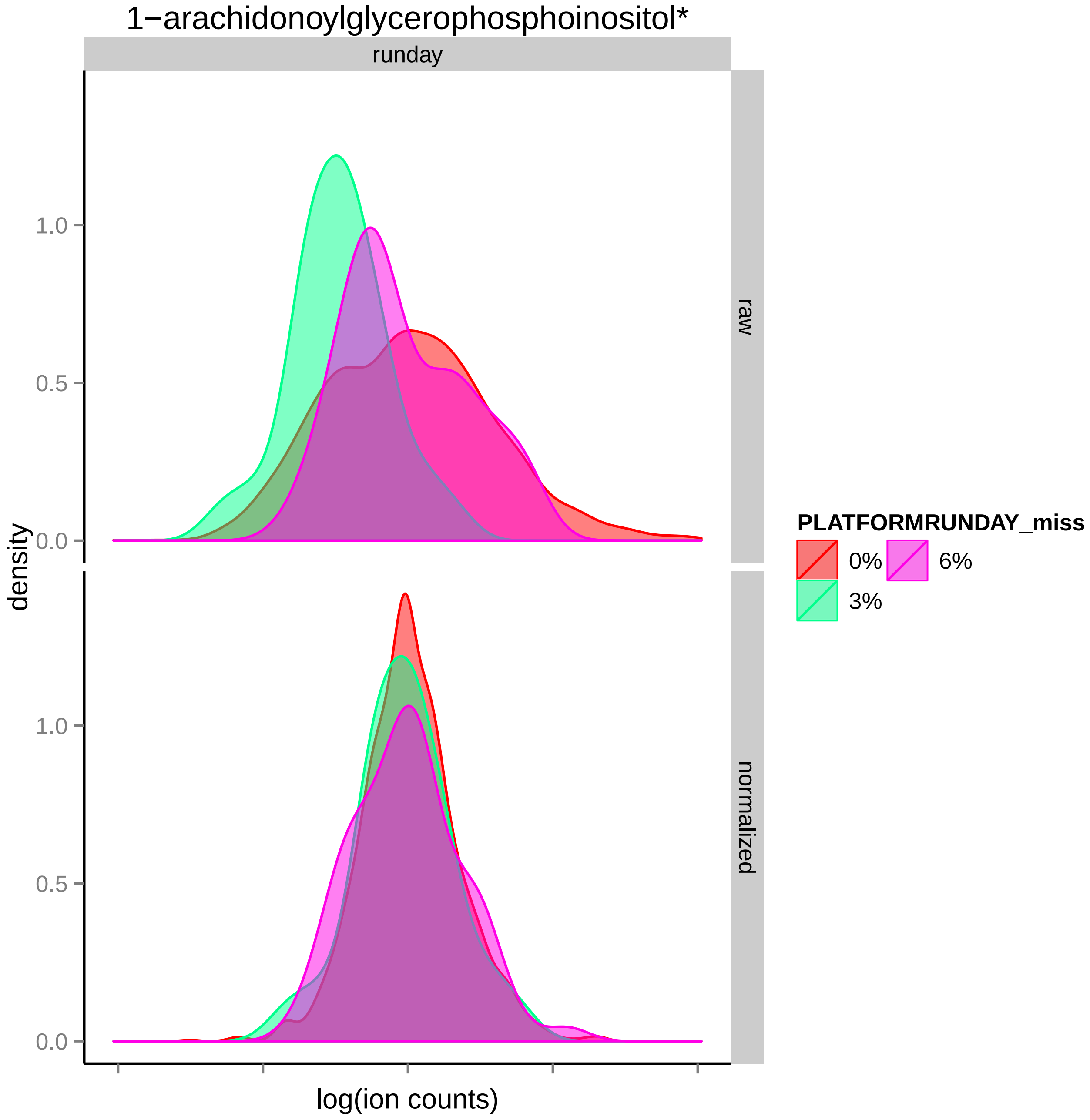
1−arachidonoylglycerophosphoinositol*
runday
raw
normalized
1.0
0.5
0.0
1.0
0.5
0.0
density
log(ion counts)
PLATFORMRUNDAY_miss
0%
6%
3%

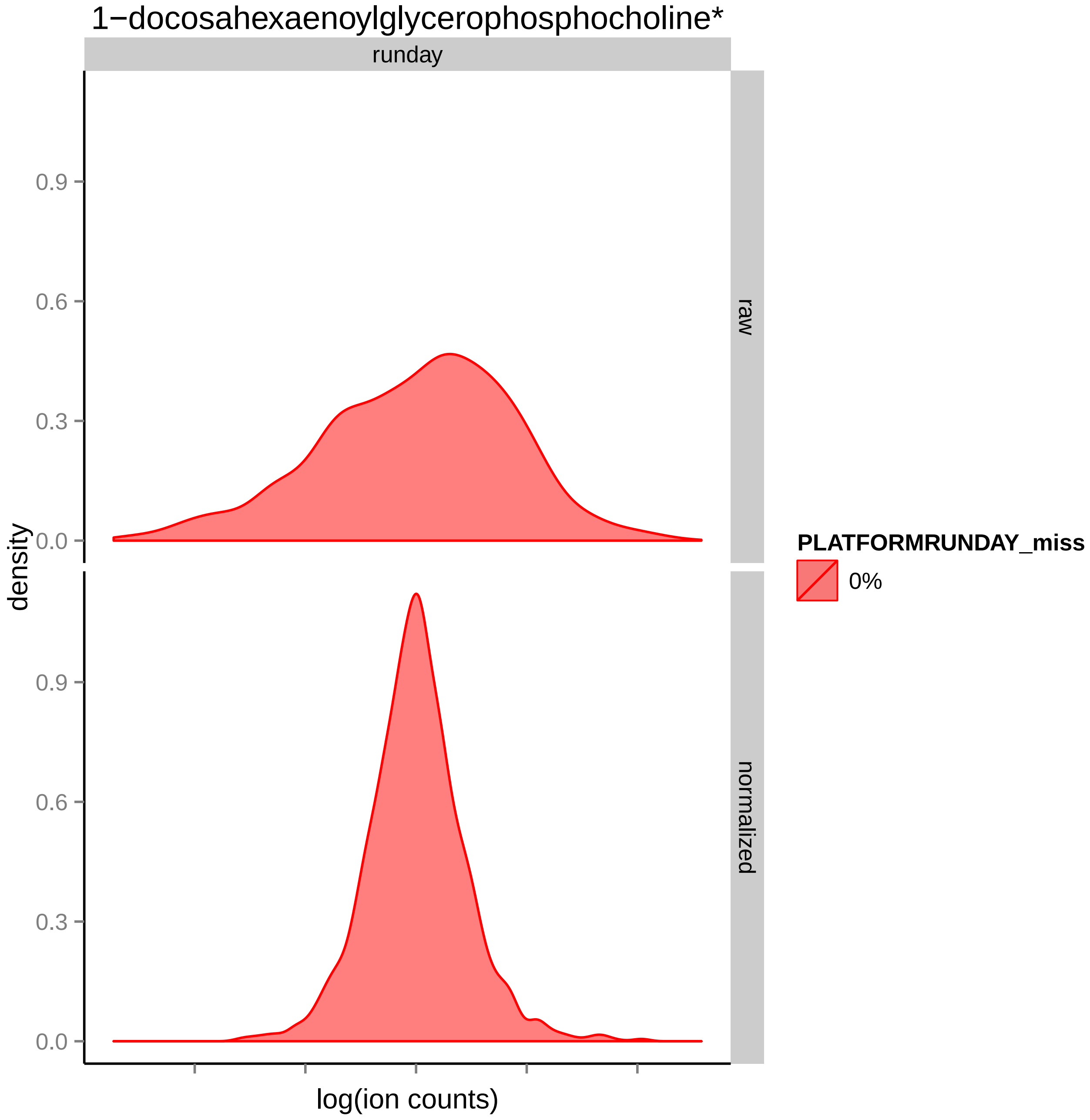
1−docosahexaenoylglycerophosphocholine*
runday
raw
normalized
density
0.0
0.3
0.6
0.9
log(ion counts)
PLATFORMRUNDAY_miss
0%

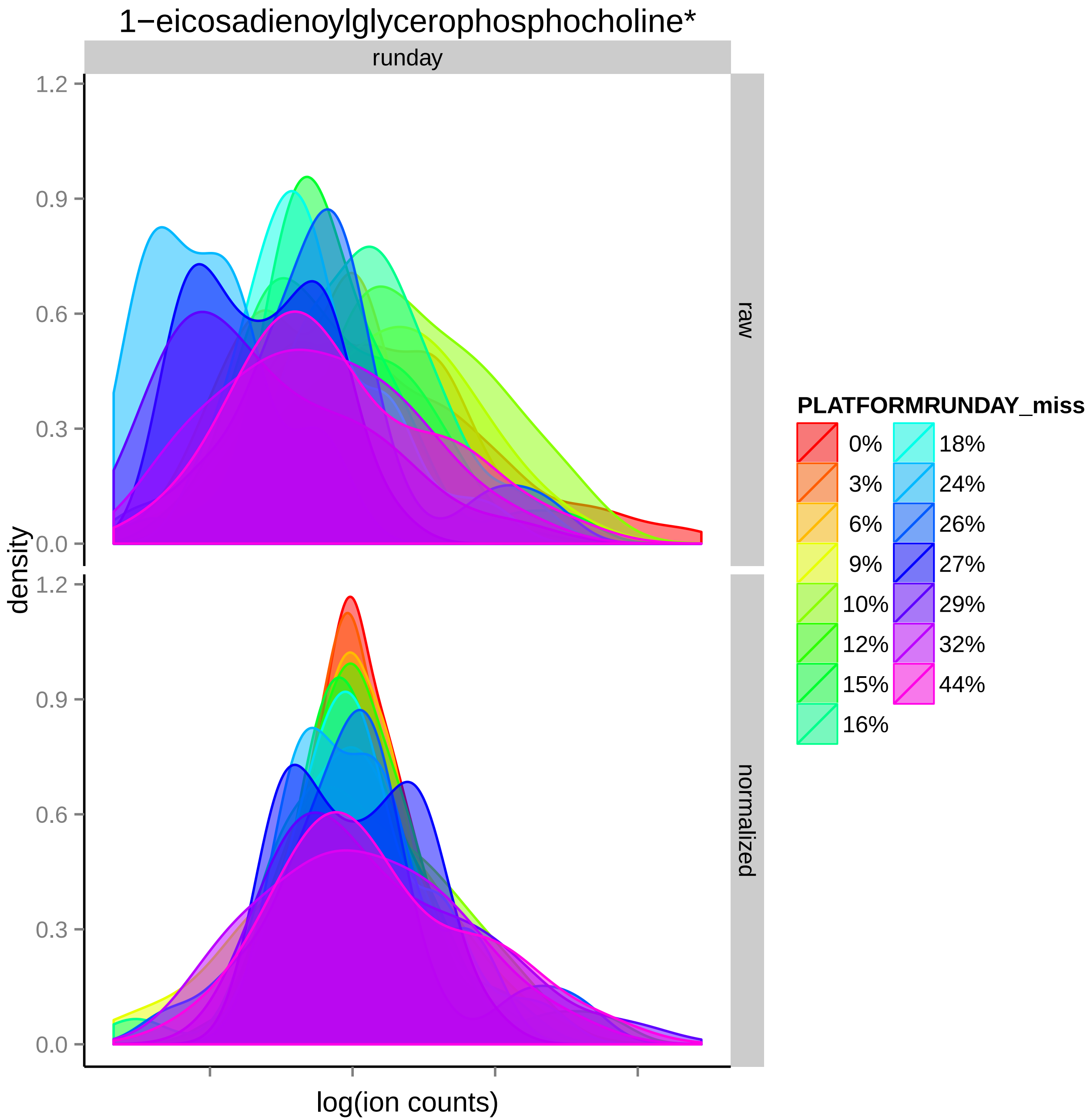

1−eicosadienoylglycerophosphocholine*
runday
raw
normalized
density
log(ion counts)
1.2
0.9
0.6
0.3
0.0
PLATFORMRUNDAY_miss
0%
3%
6%
9%
10%
12%
15%
16%
18%
24%
26%
27%
29%
32%
44%

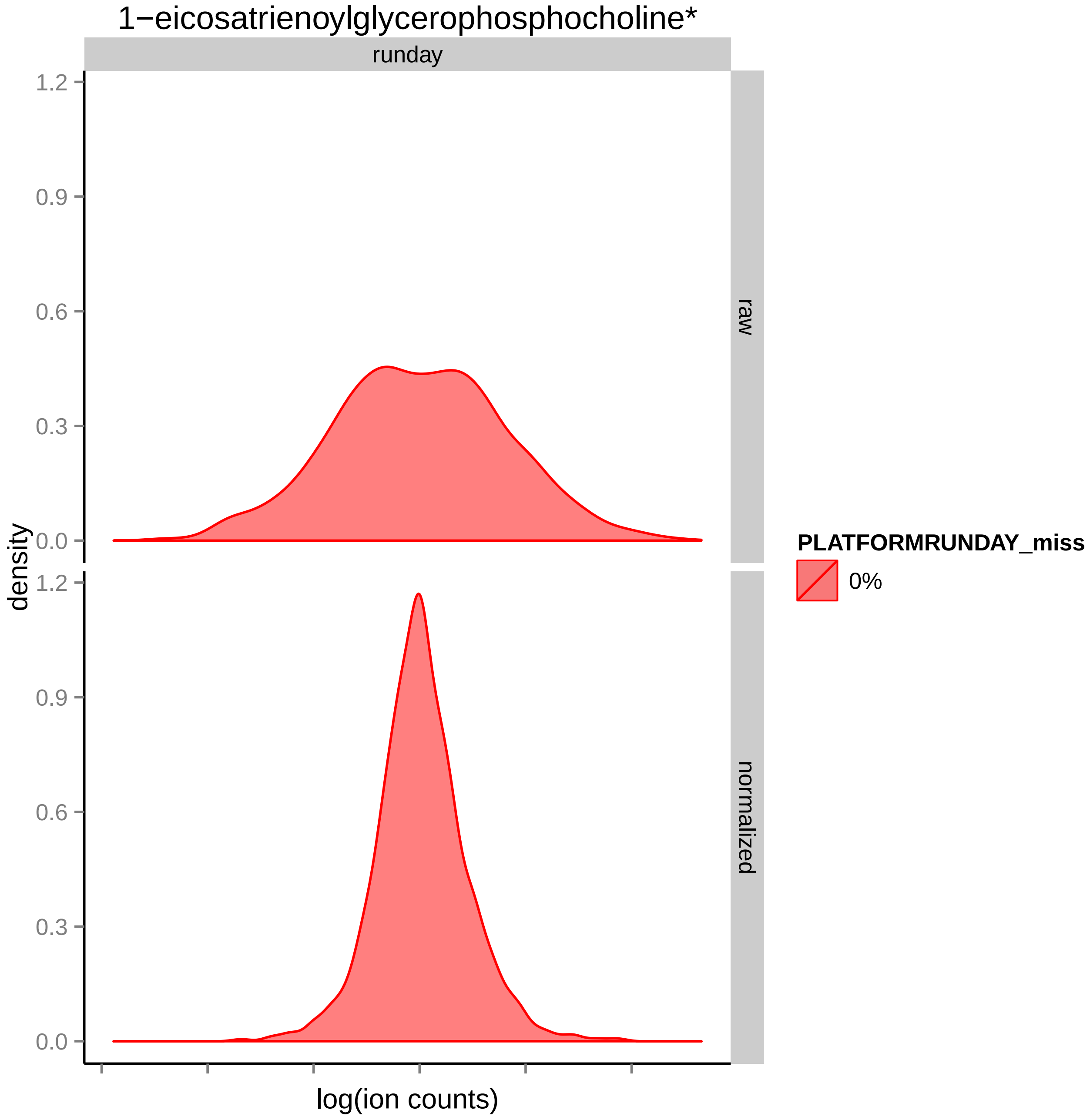
1−eicosatrienoylglycerophosphocholine*
runday
raw
normalized
density
1.2
0.9
0.6
0.3
0.0
log(ion counts)
PLATFORMRUNDAY_miss
0%

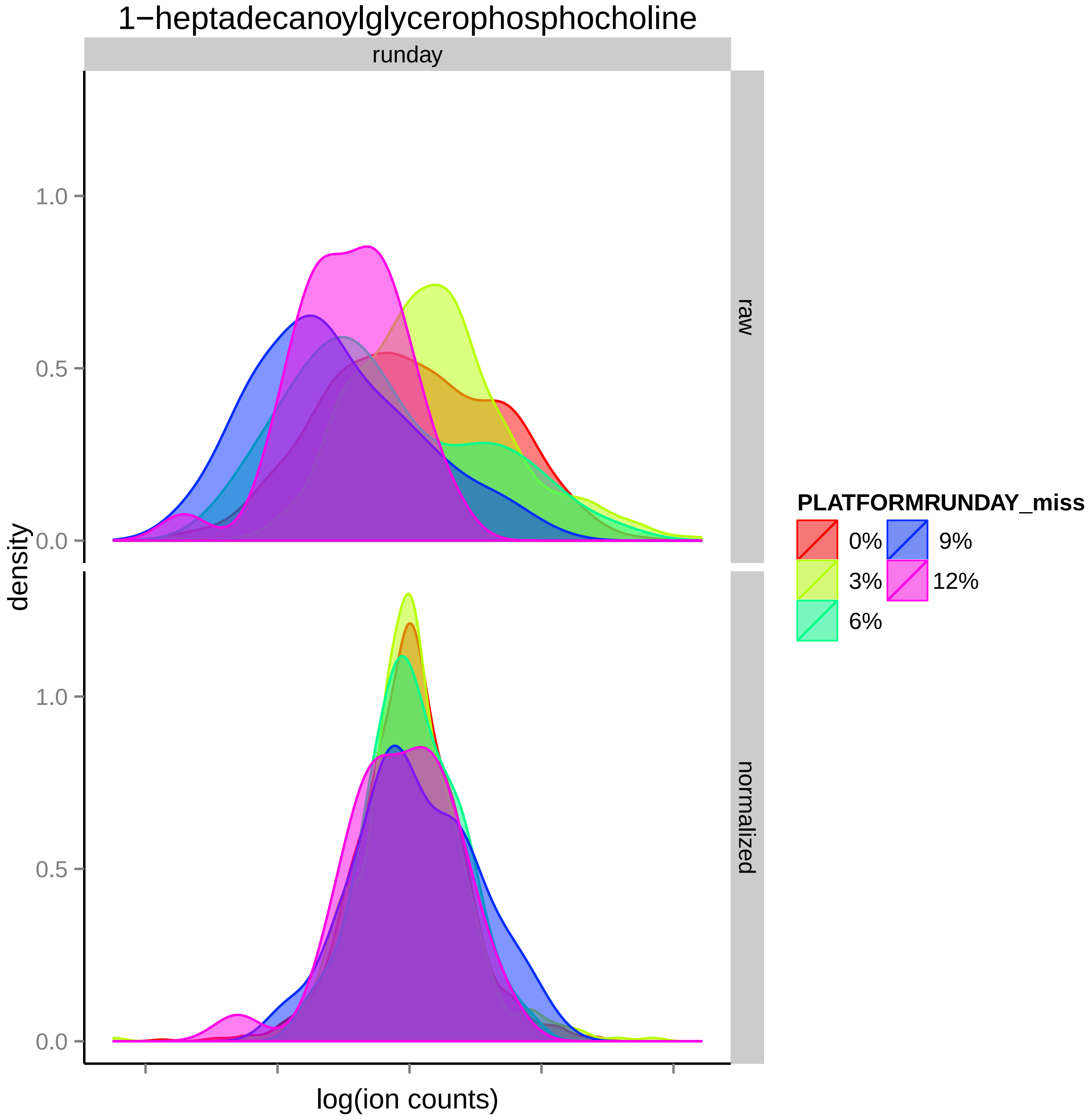
1−heptadecanoylglycerophosphocholine
runday
raw
normalized
density
log(ion counts)
1.0
0.5
0.0
1.0
0.5
0.0
PLATFORMRUNDAY_miss
0%
3%
6%
9%
12%

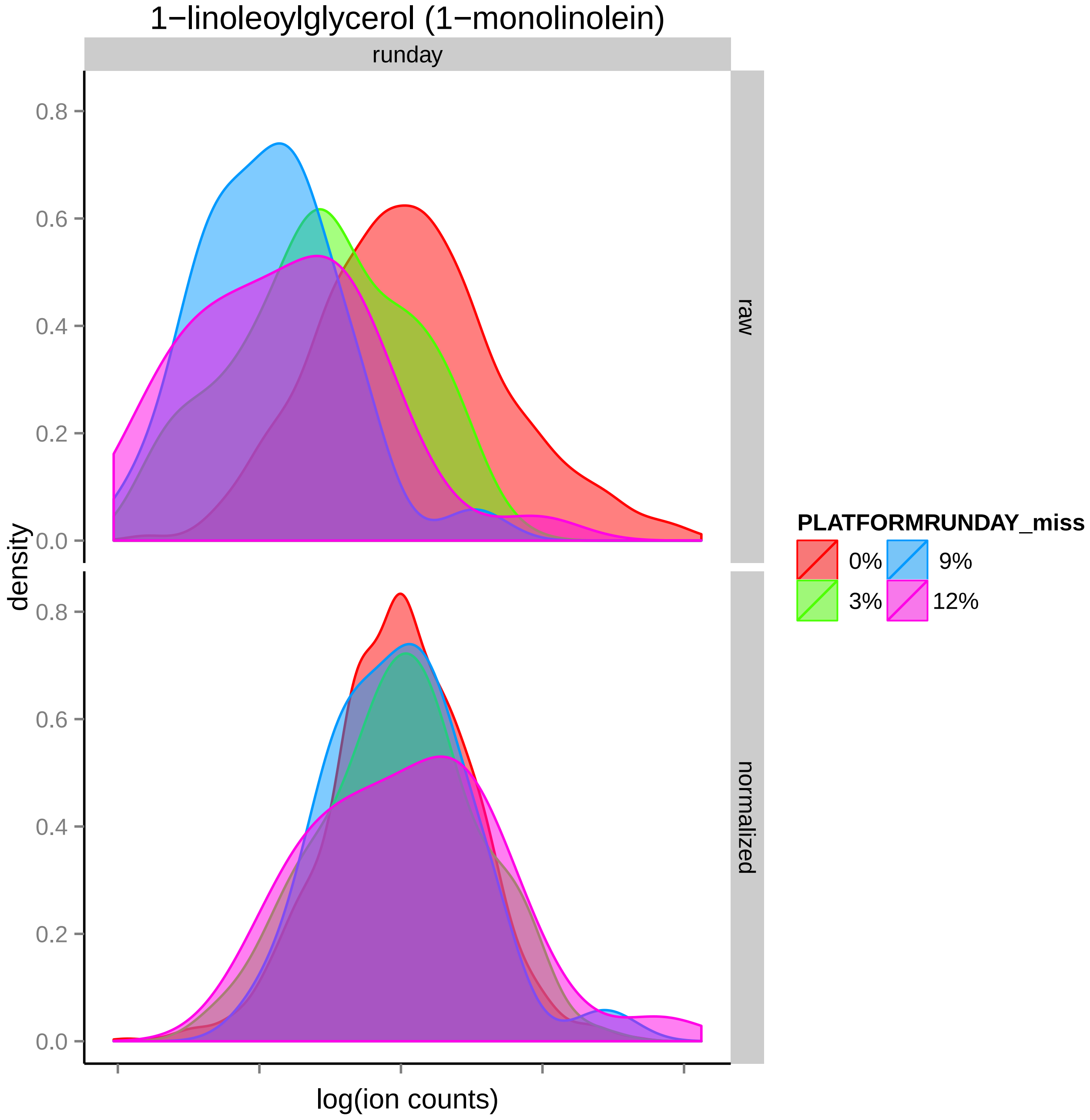
1−linoleoylglycerol (1−monolinolein)
runday
raw
normalized
density
log(ion counts)
0.0
0.2
0.4
0.6
0.8
PLATFORMRUNDAY_miss
0%
9%
3%
12%

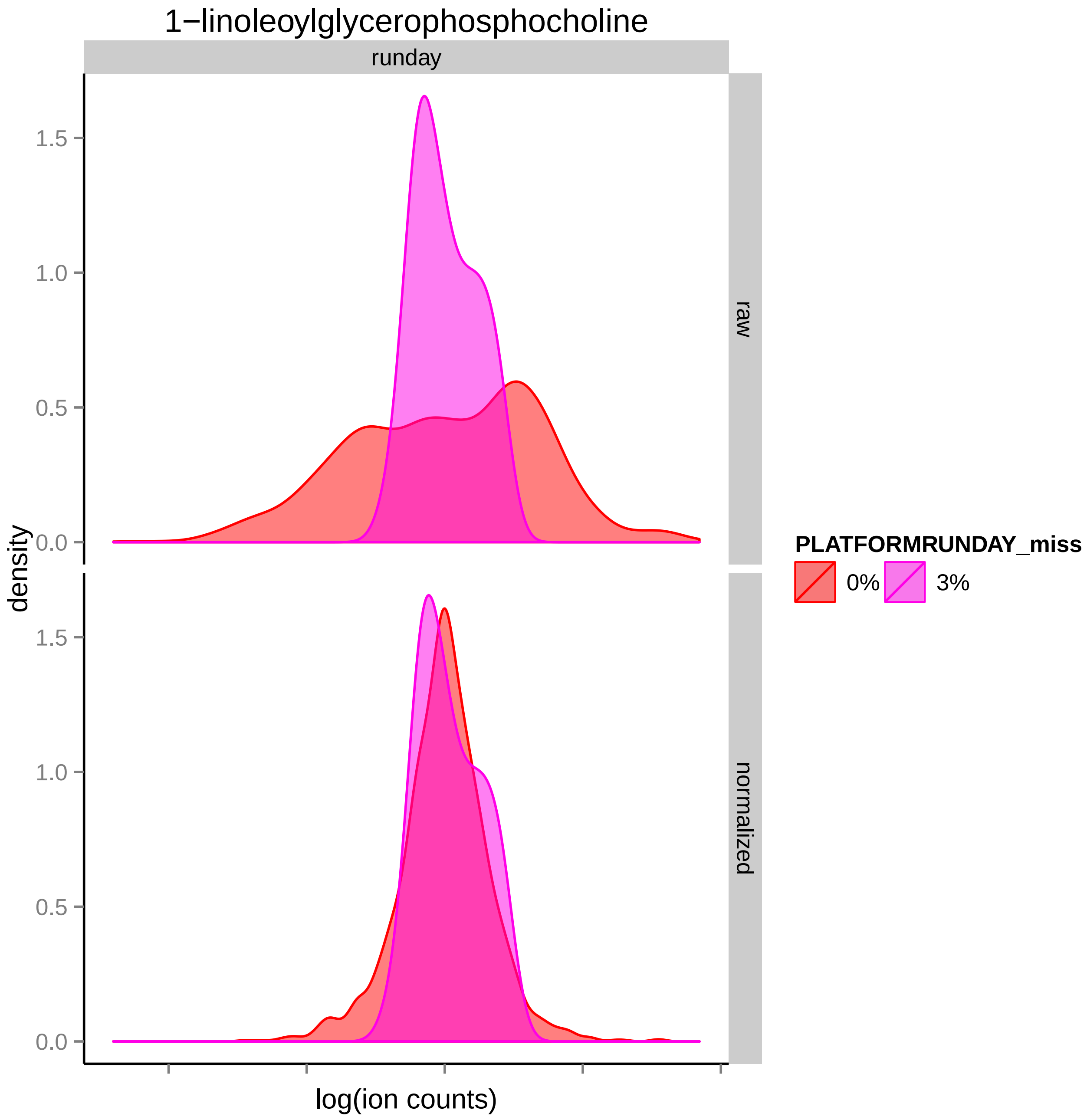

1−linoleoylglycerophosphocholine
runday
raw
normalized
density
log(ion counts)
0.0
0.5
1.0
1.5
PLATFORMRUNDAY_miss
0%
3%

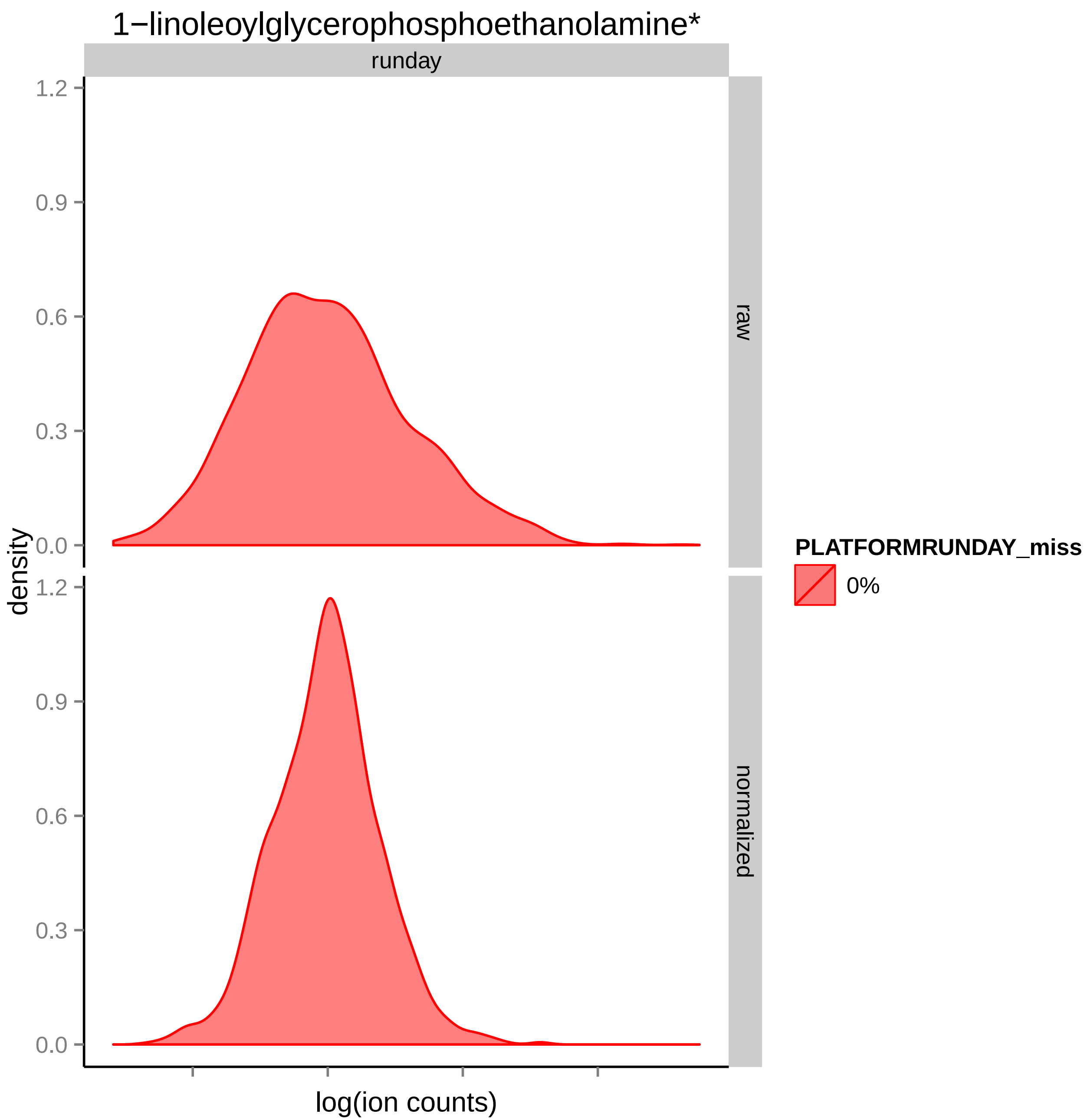
1−linoleoylglycerophosphoethanolamine*
runday
raw
normalized
1.2
0.9
0.6
0.3
0.0
density
log(ion counts)
PLATFORMRUNDAY_miss
0%

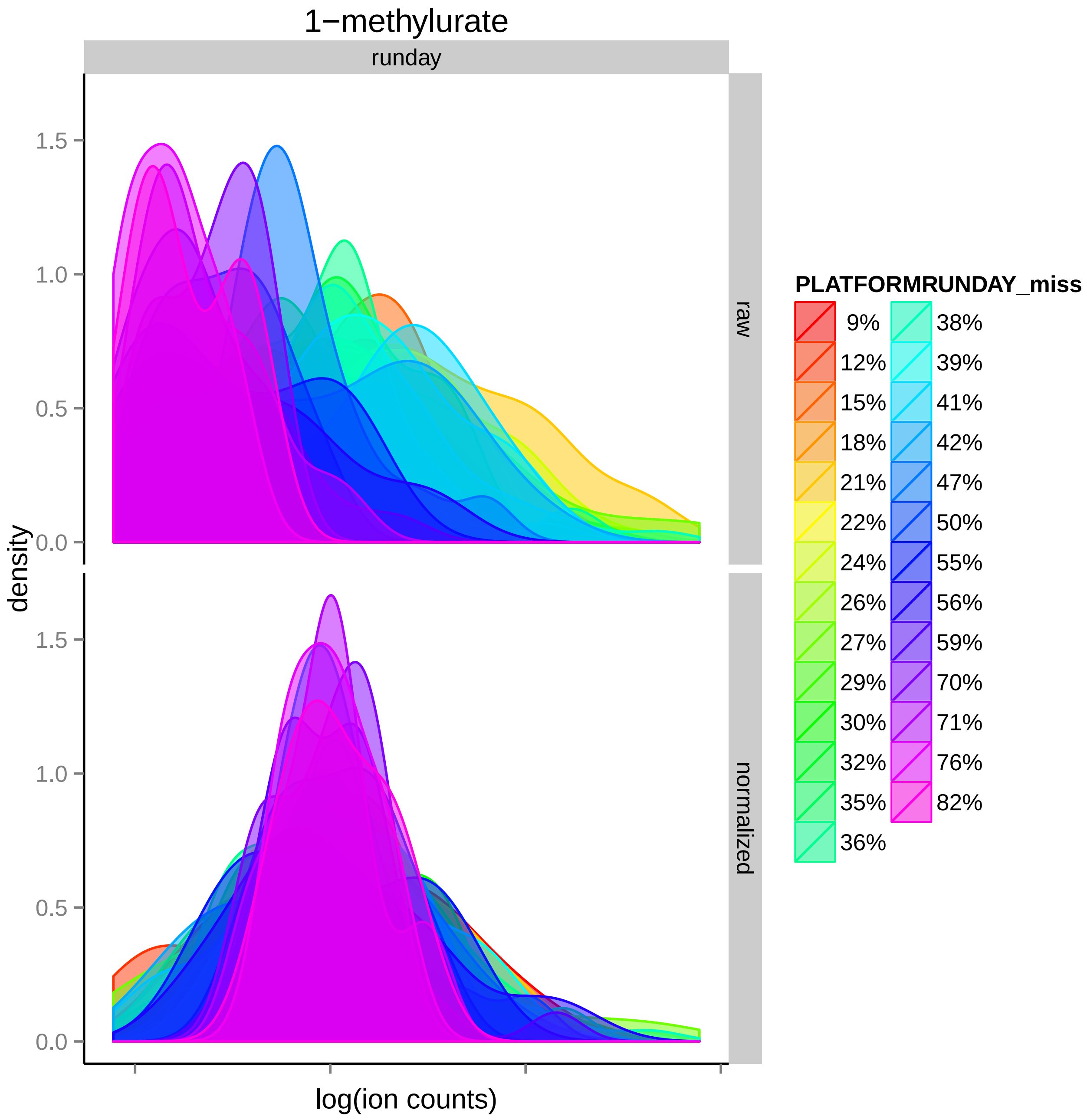

1−methylurate
runday
raw
normalized
density
log(ion counts)
1.5
1.0
0.5
0.0
PLATFORMRUNDAY_miss
9%
12%
15%
18%
21%
22%
24%
26%
27%
29%
30%
32%
35%
36%
38%
39%
41%
42%
47%
50%
55%
56%
59%
70%
71%
76%
82%

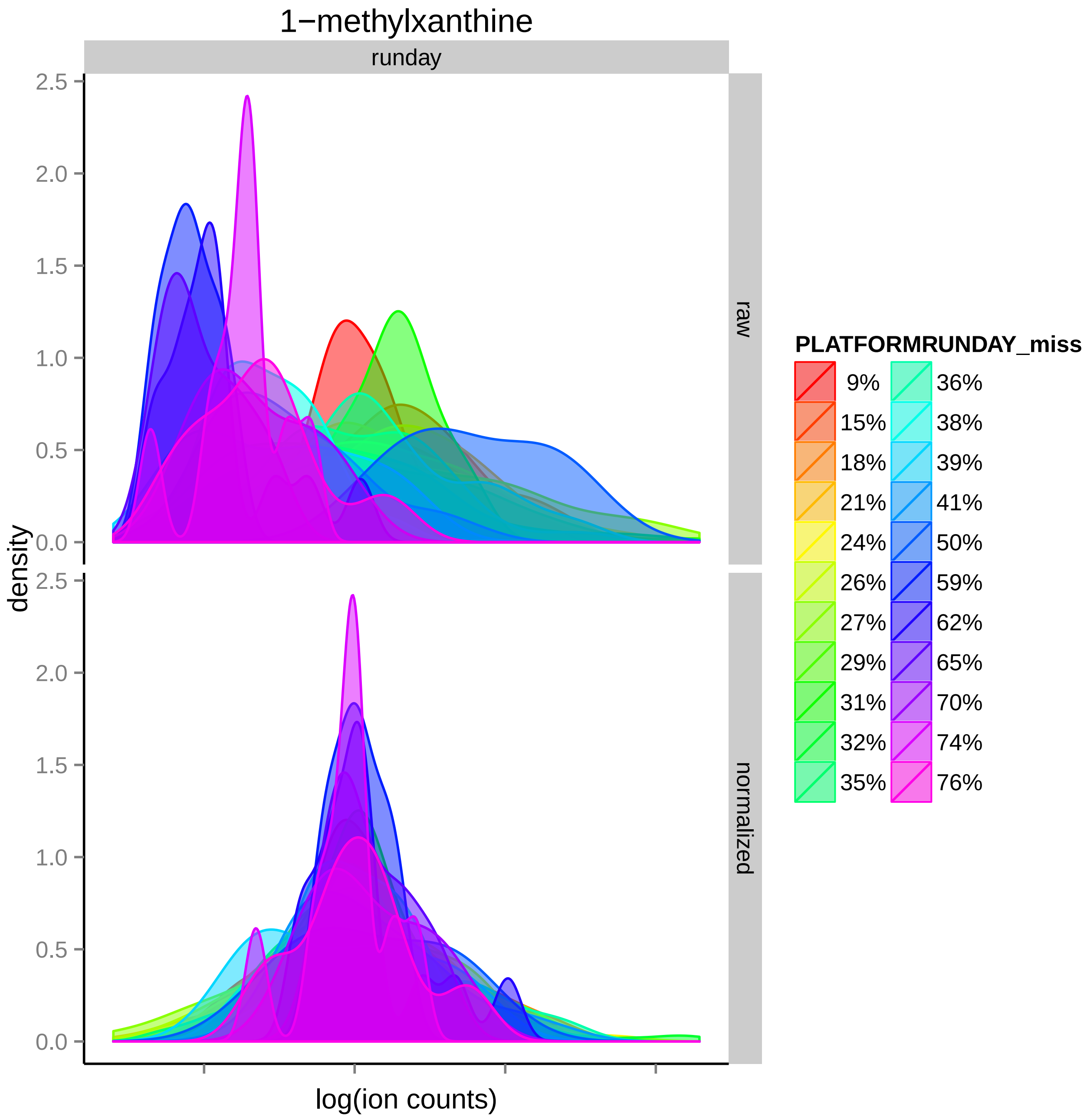
1−methylxanthine
runday
raw
normalized
density
log(ion counts)
0.0
0.5
1.0
1.5
2.0
2.5
PLATFORMRUNDAY_miss
9%
15%
18%
21%
24%
26%
27%
29%
31%
32%
35%
36%
38%
39%
41%
50%
59%
62%
65%
70%
74%
76%

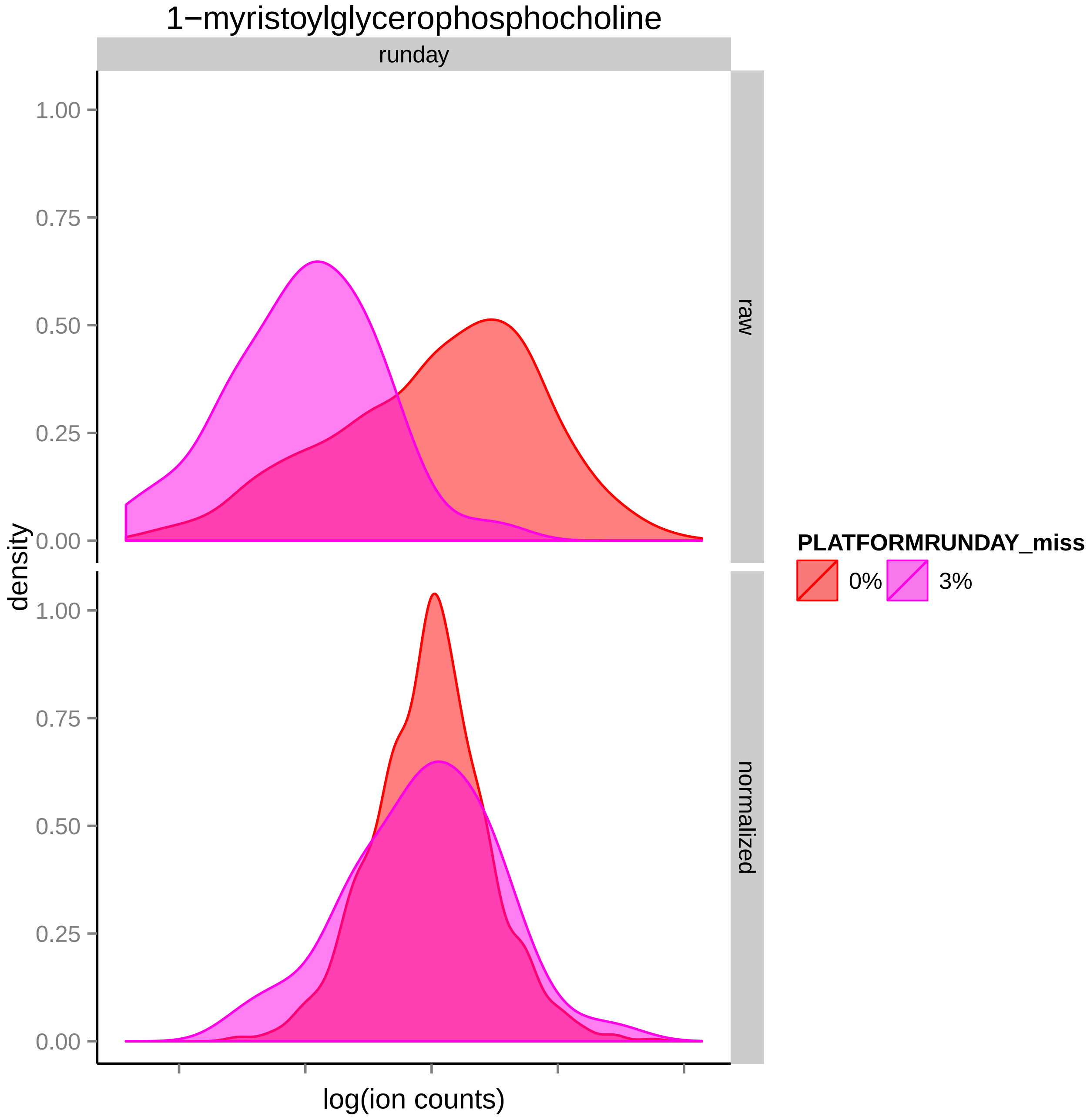

1−myristoylglycerophosphocholine
runday
raw
normalized
density
log(ion counts)
1.00
0.75
0.50
0.25
0.00
PLATFORMRUNDAY_miss
0%
3%

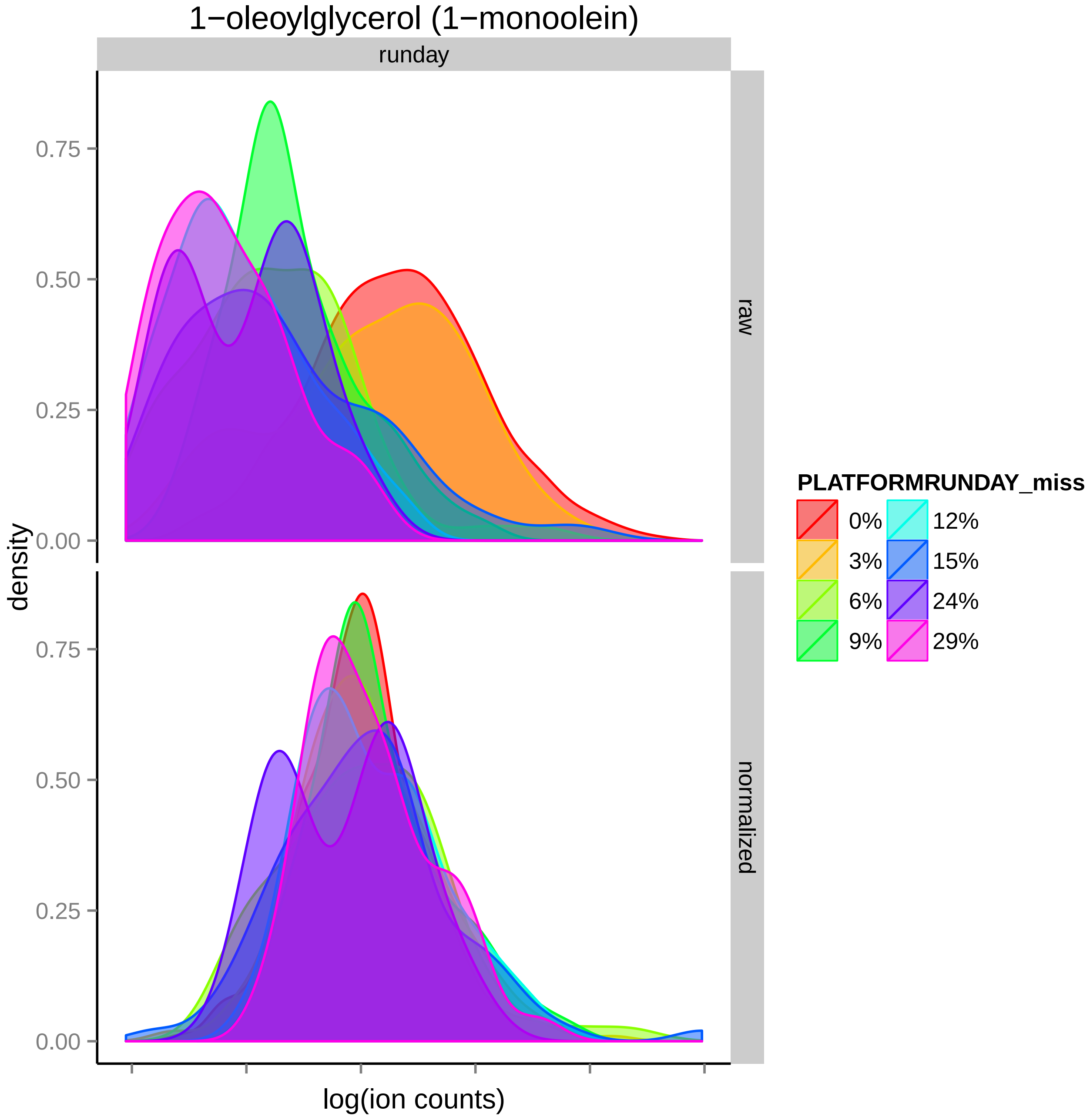

1−oleoylglycerol (1−monoolein)
runday
raw
normalized
density
log(ion counts)
0.00
0.25
0.50
0.75
PLATFORMRUNDAY_miss
0%
3%
6%
9%
12%
15%
24%
29%

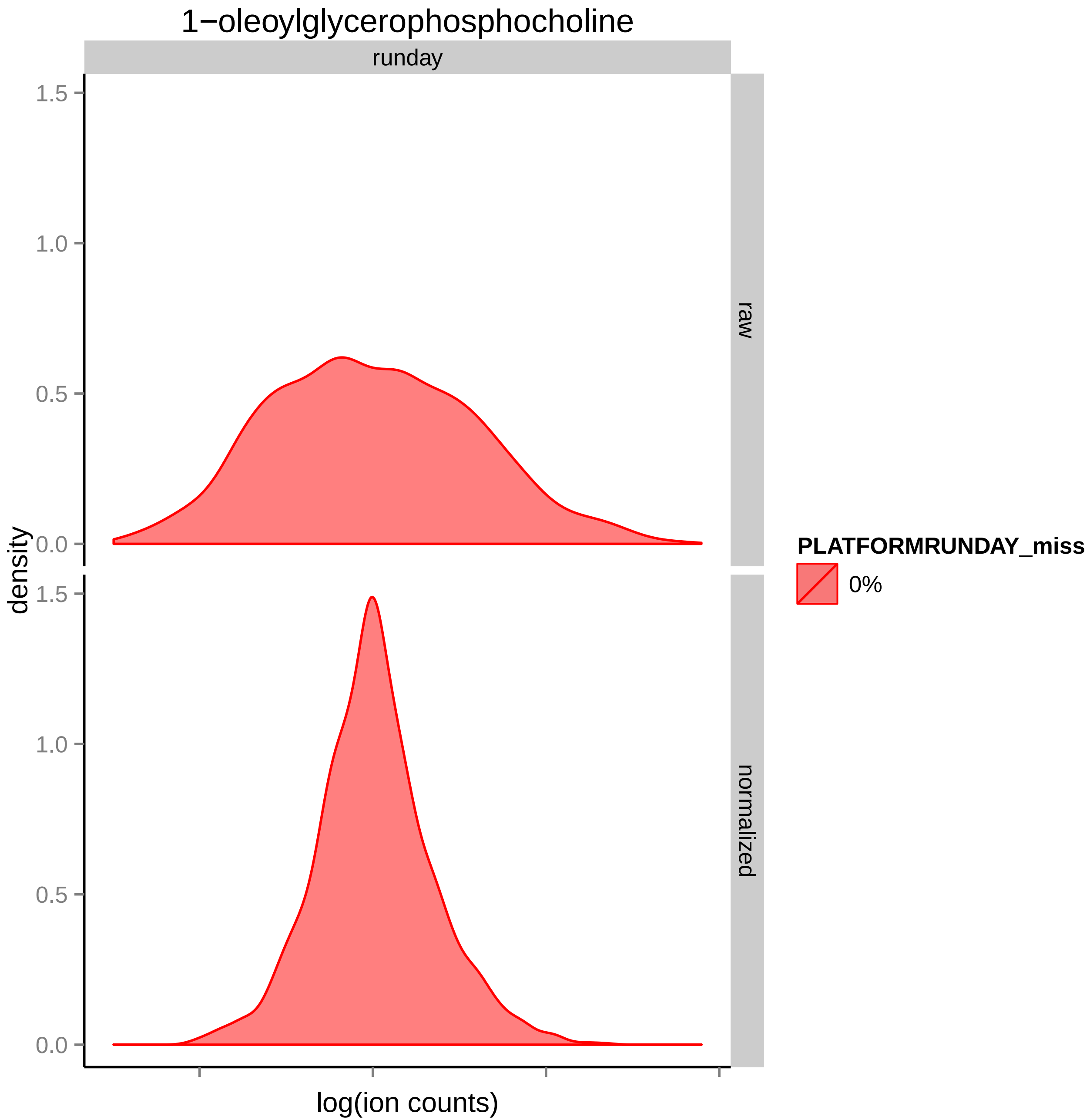
1−oleoylglycerophosphocholine
runday
raw
normalized
density
1.5
1.0
0.5
0.0
log(ion counts)
PLATFORMRUNDAY_miss
0%

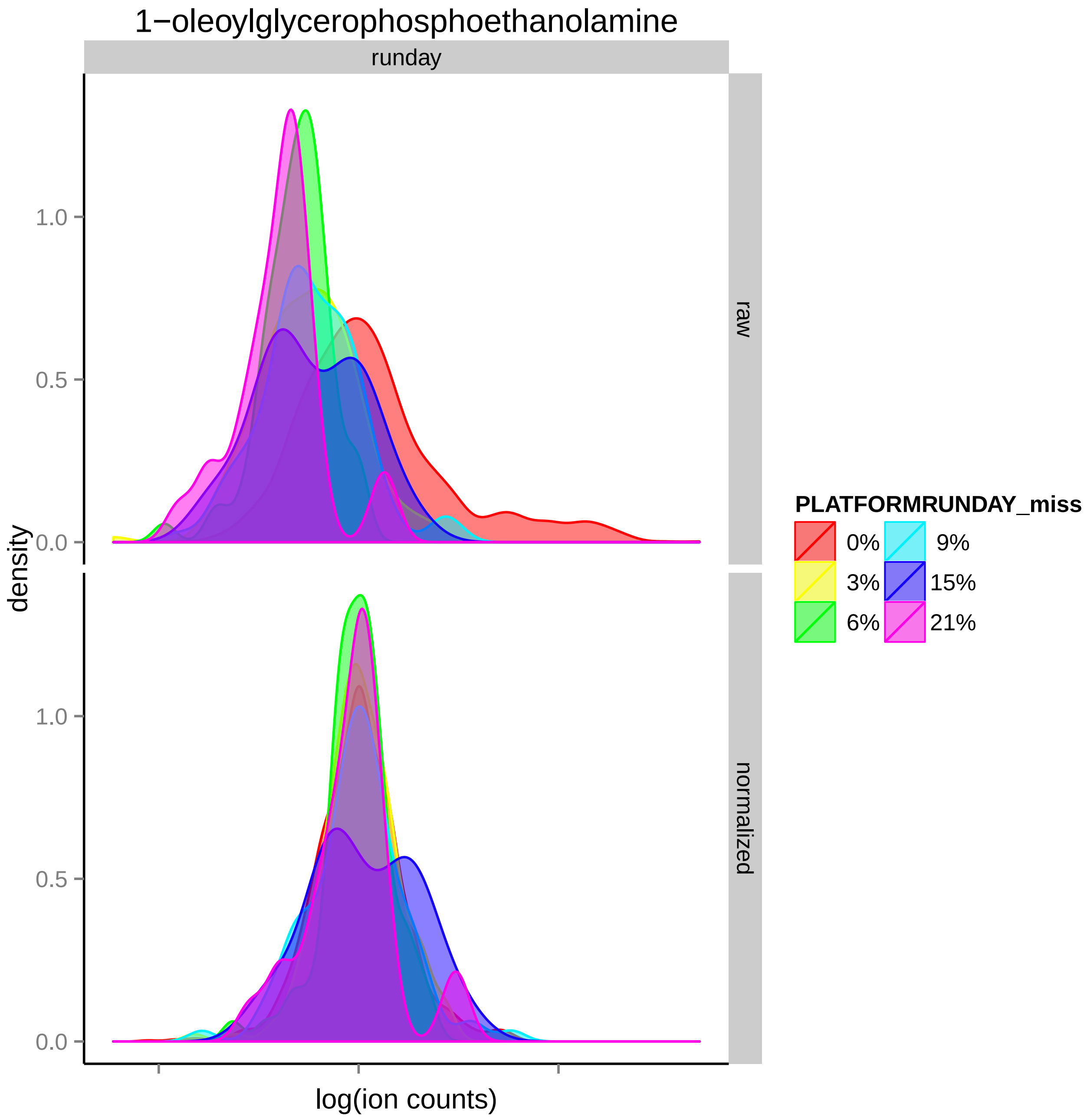
1−oleoylglycerophosphoethanolamine
runday
raw
normalized
density
log(ion counts)
1.0
0.5
0.0
PLATFORMRUNDAY_miss
0%
3%
6%
9%
15%
21%

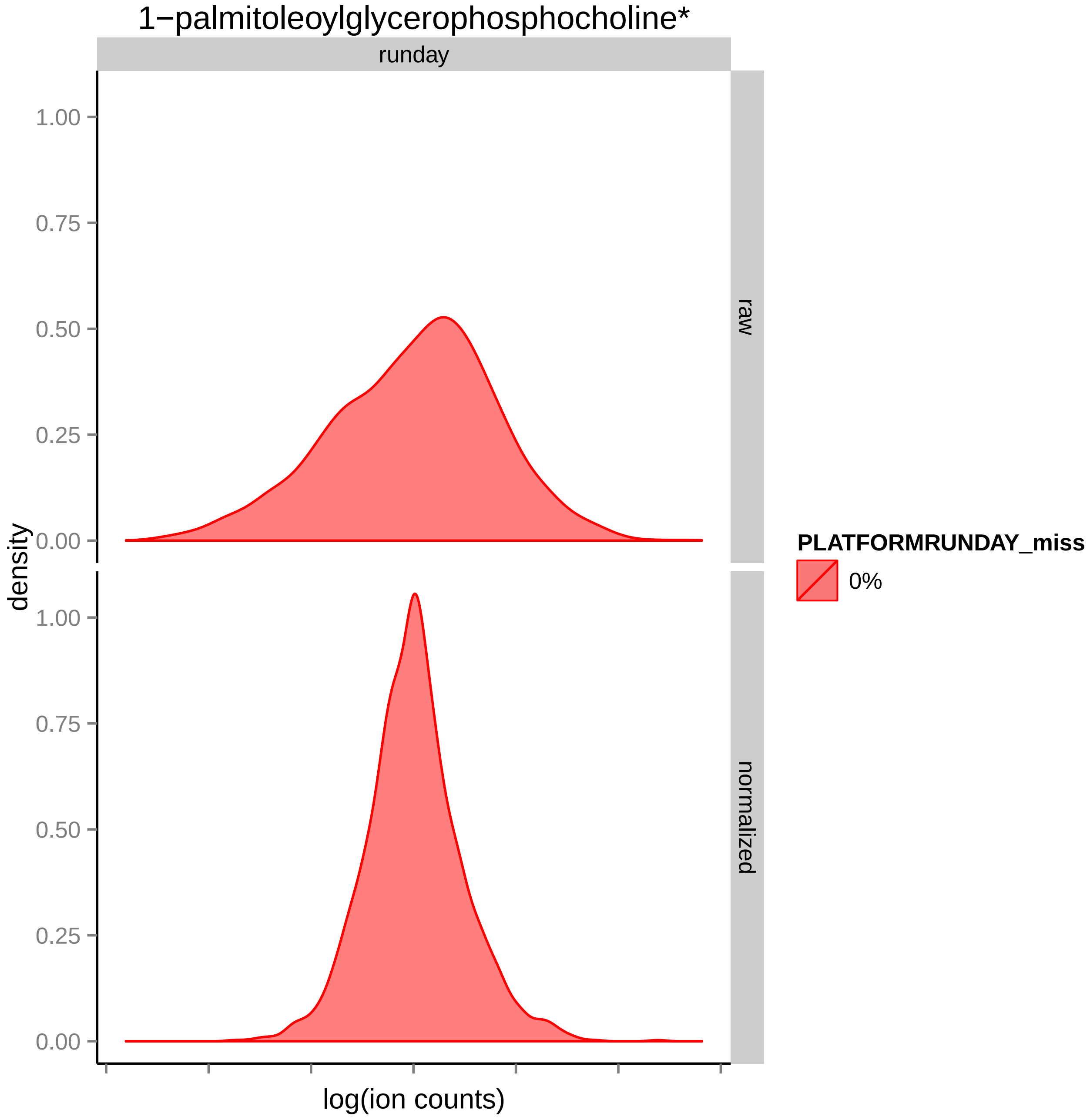
1−palmitoleoylglycerophosphocholine*
runday
raw
normalized
density
1.00
0.75
0.50
0.25
0.00
log(ion counts)
PLATFORMRUNDAY_miss
0%

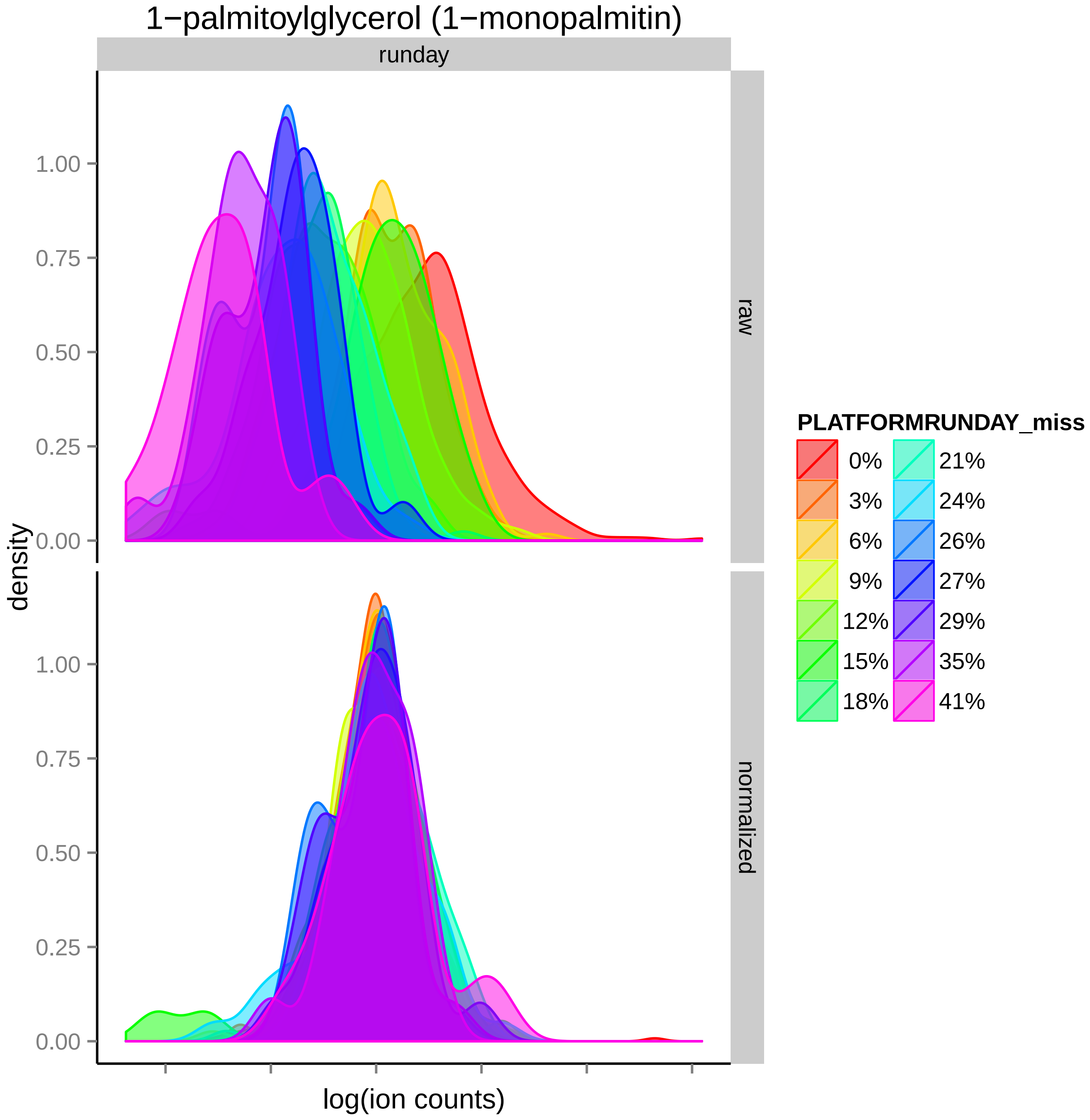

1−palmitoylglycerol (1−monopalmitin)
runday
raw
normalized
density
1.00
0.75
0.50
0.25
0.00
log(ion counts)
PLATFORMRUNDAY_miss
0%
3%
6%
9%
12%
15%
18%
21%
24%
26%
27%
29%
35%
41%

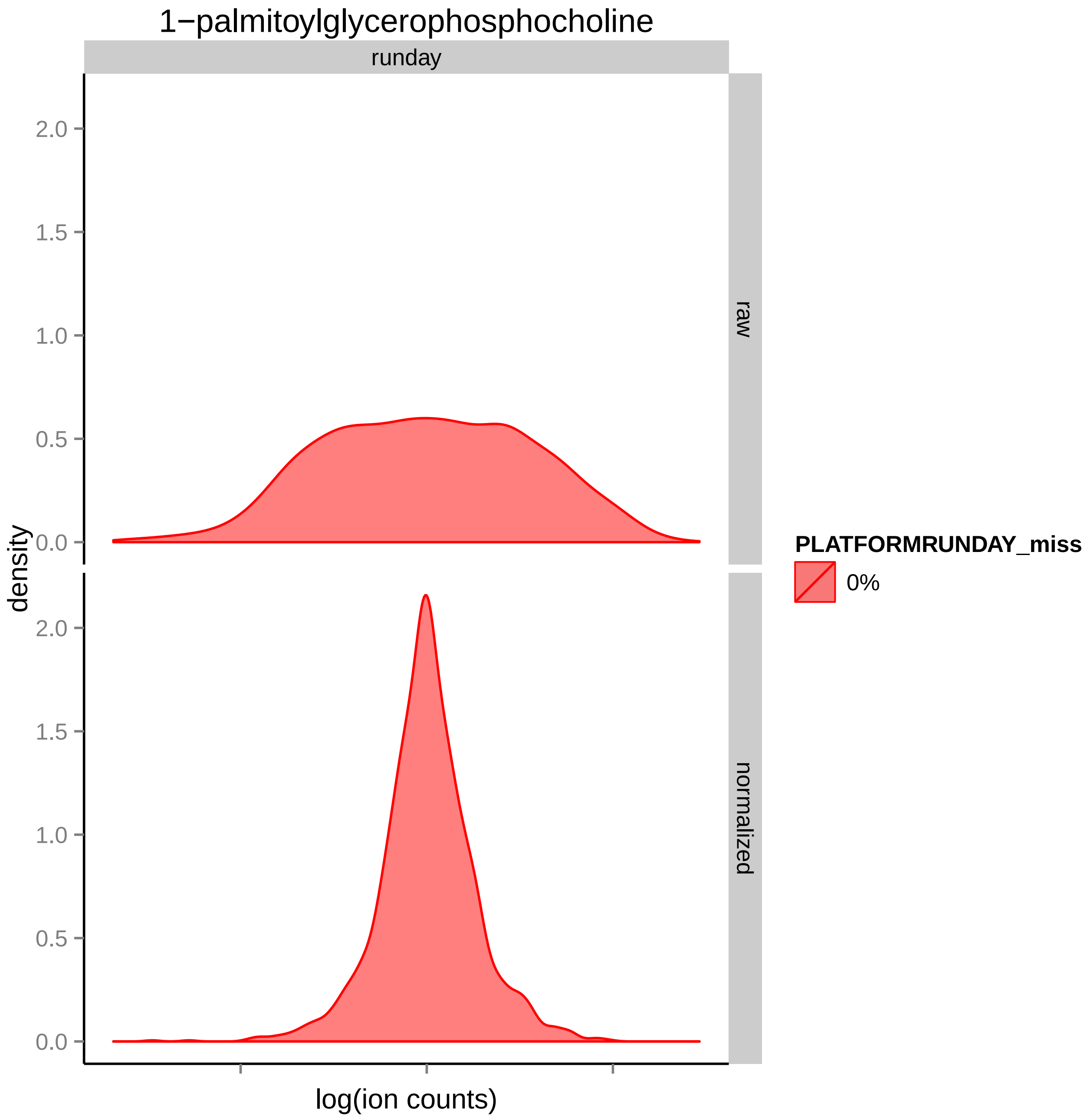

1−palmitoylglycerophosphocholine
runday
raw
normalized
density
log(ion counts)
2.0
1.5
1.0
0.5
0.0
PLATFORMRUNDAY_miss
0%

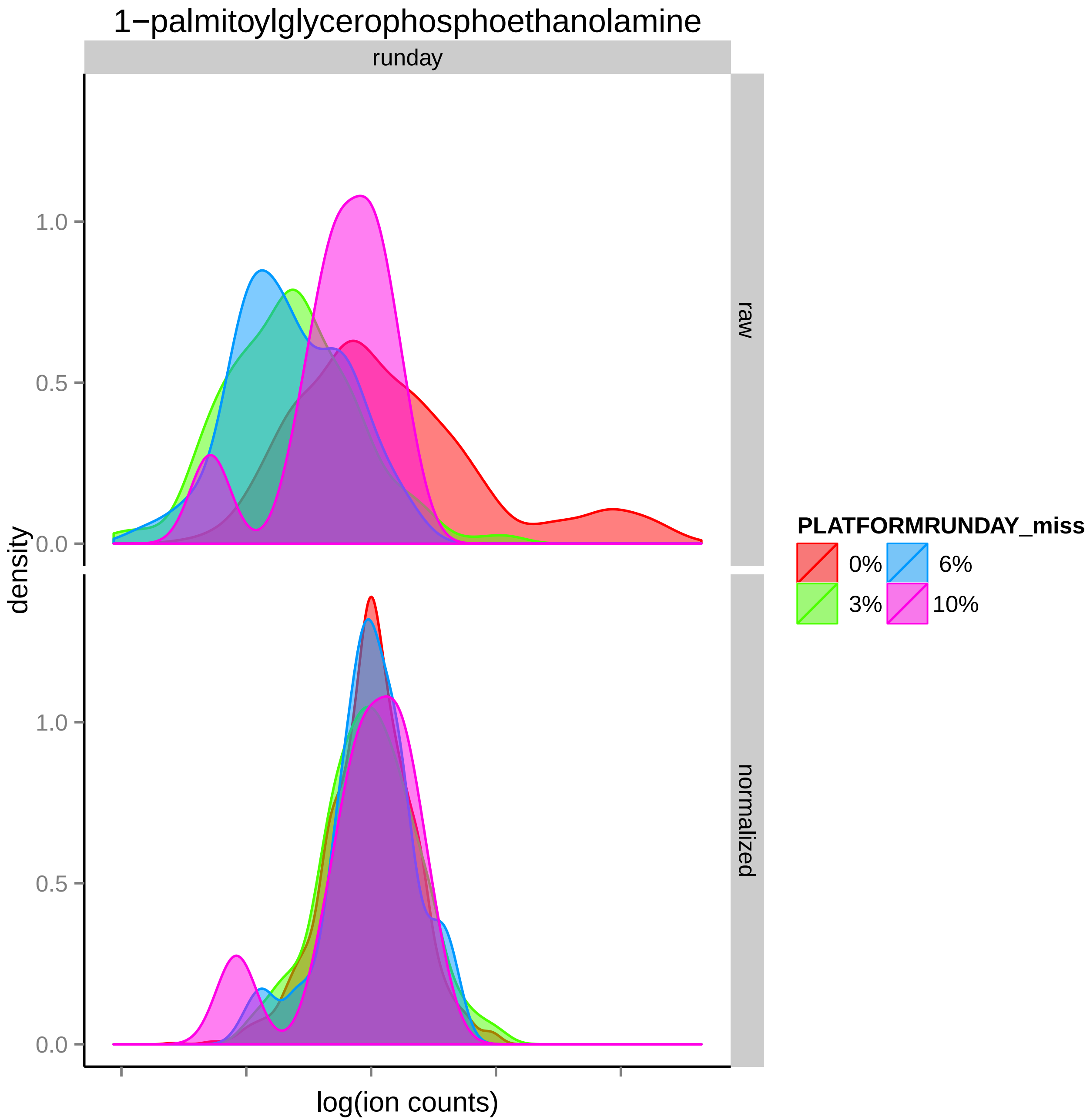

1−palmitoylglycerophosphoethanolamine
runday
raw
normalized
density
1.0
0.5
0.0
log(ion counts)
PLATFORMRUNDAY_miss
0%
6%
3%
10%

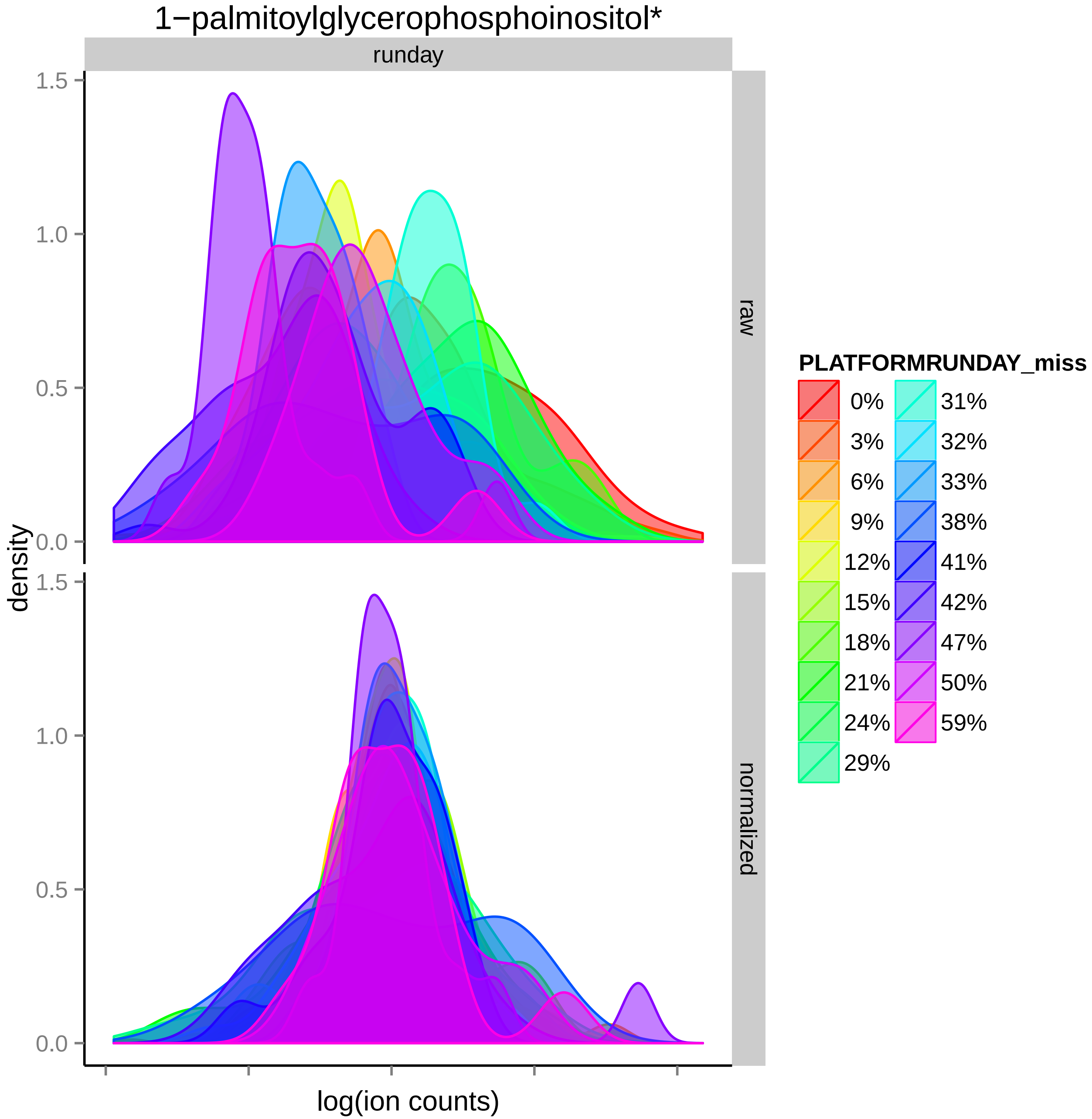
1−palmitoylglycerophosphoinositol*
runday
raw
normalized
density
log(ion counts)
1.5
1.0
0.5
0.0
PLATFORMRUNDAY_miss
0%
3%
6%
9%
12%
15%
18%
21%
24%
29%
31%
32%
33%
38%
41%
42%
47%
50%
59%

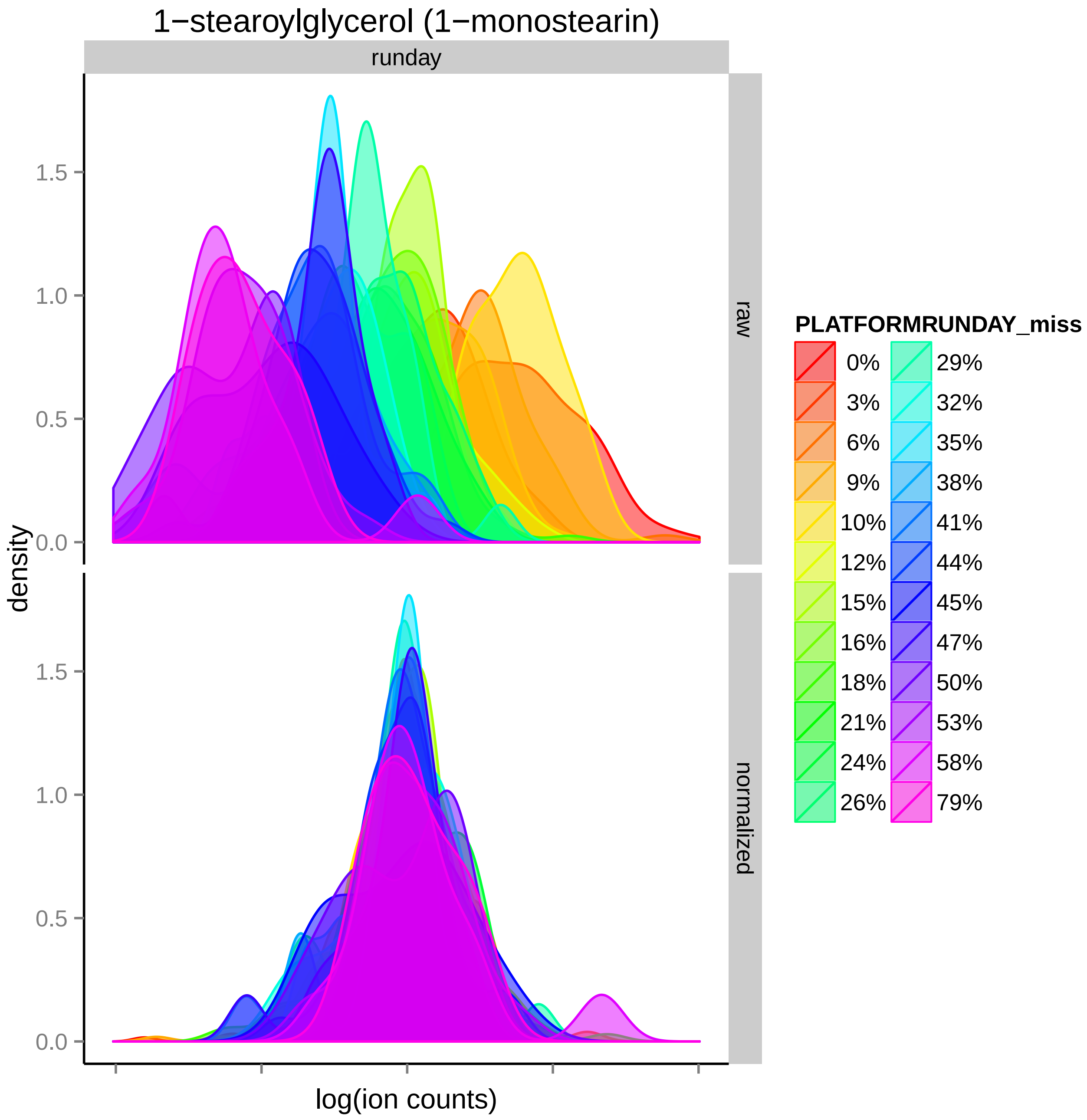

1−stearoylglycerol (1−monostearin)
runday
raw
normalized
density
log(ion counts)
0.0
0.5
1.0
1.5
PLATFORMRUNDAY_miss
0%
3%
6%
9%
10%
12%
15%
16%
18%
21%
24%
26%
29%
32%
35%
38%
41%
44%
45%
47%
50%
53%
58%
79%

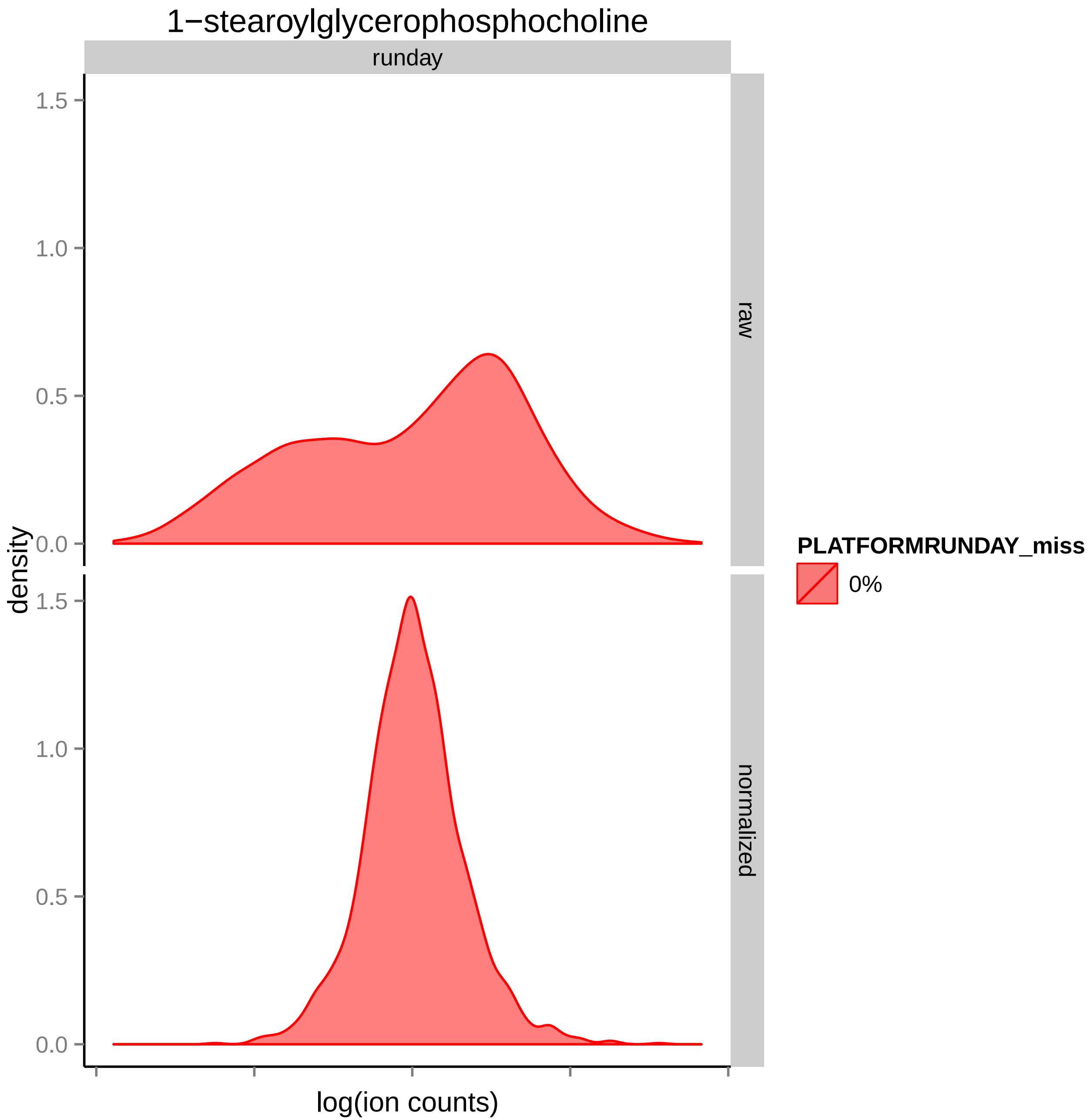
1−stearoylglycerophosphocholine
runday
raw
normalized
density
1.5
1.0
0.5
0.0
1.5
1.0
0.5
0.0
log(ion counts)
PLATFORMRUNDAY_miss
0%

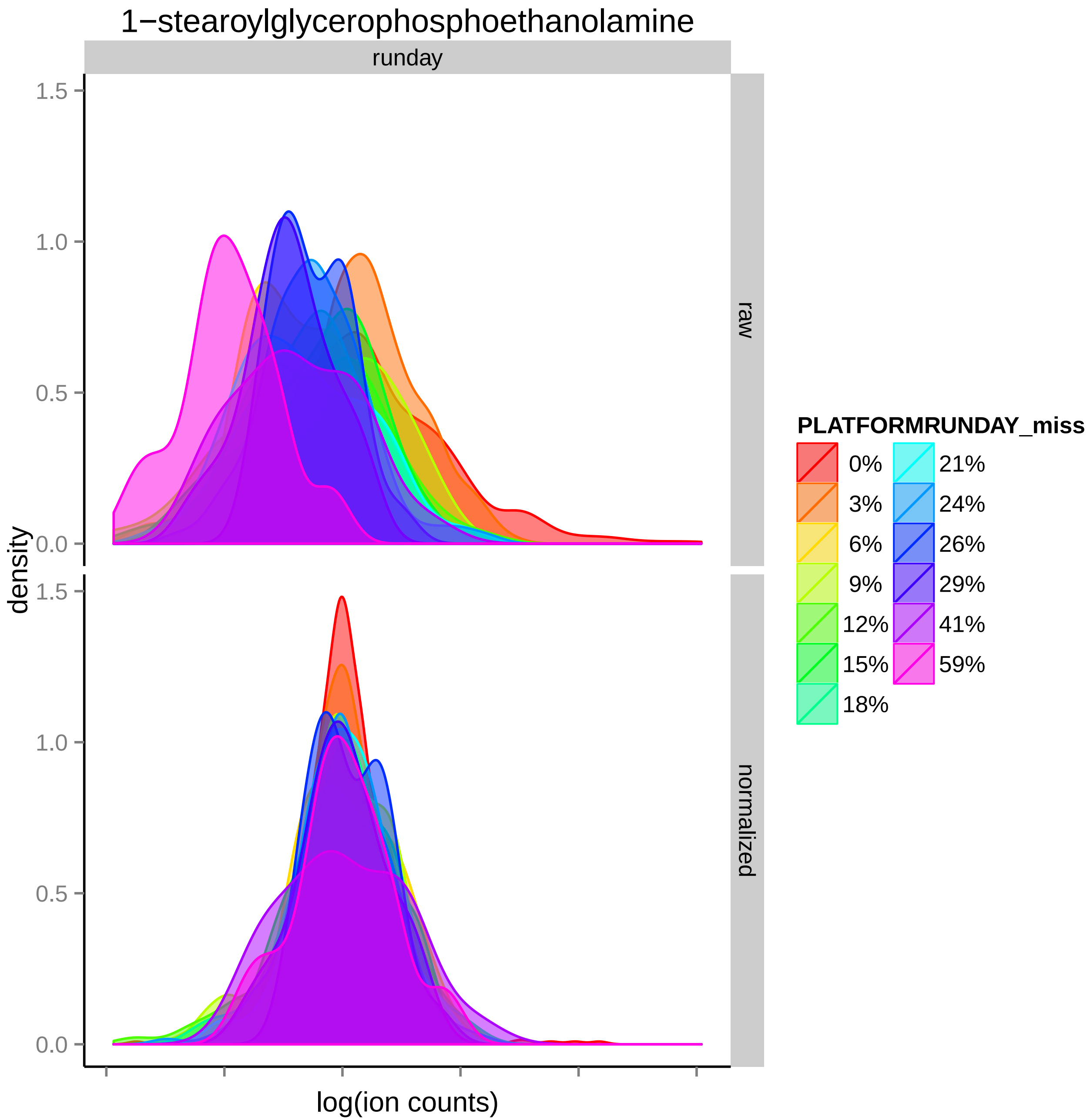
1−stearoylglycerophosphoethanolamine
runday
raw
normalized
density
log(ion counts)
1.5
1.0
0.5
0.0
PLATFORMRUNDAY_miss
0%
3%
6%
9%
12%
15%
18%
21%
24%
26%
29%
41%
59%

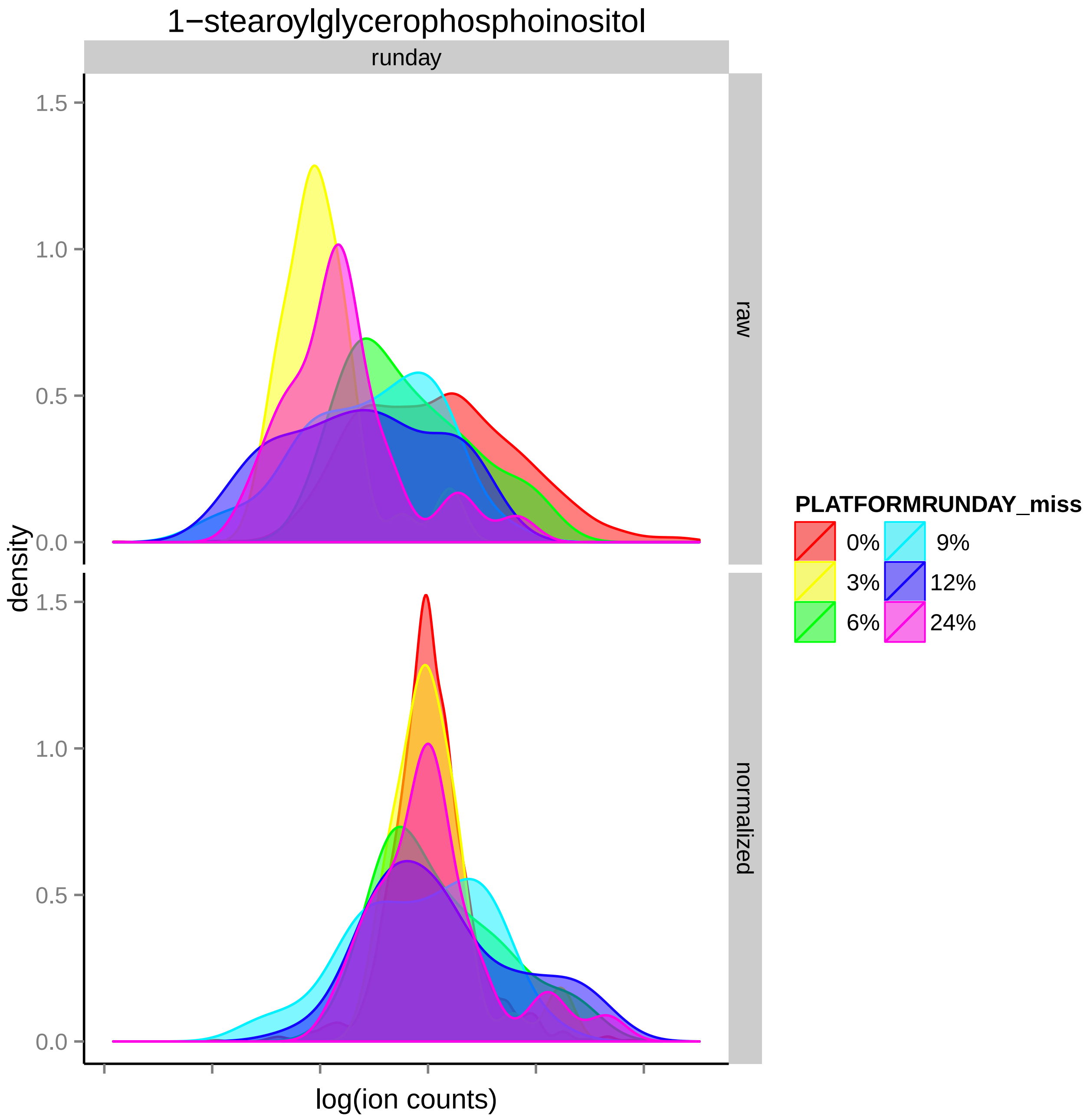
1−stearoylglycerophosphoinositol
runday
raw
normalized
density
log(ion counts)
1.5
1.0
0.5
0.0
PLATFORMRUNDAY_miss
0%
3%
6%
9%
12%
24%

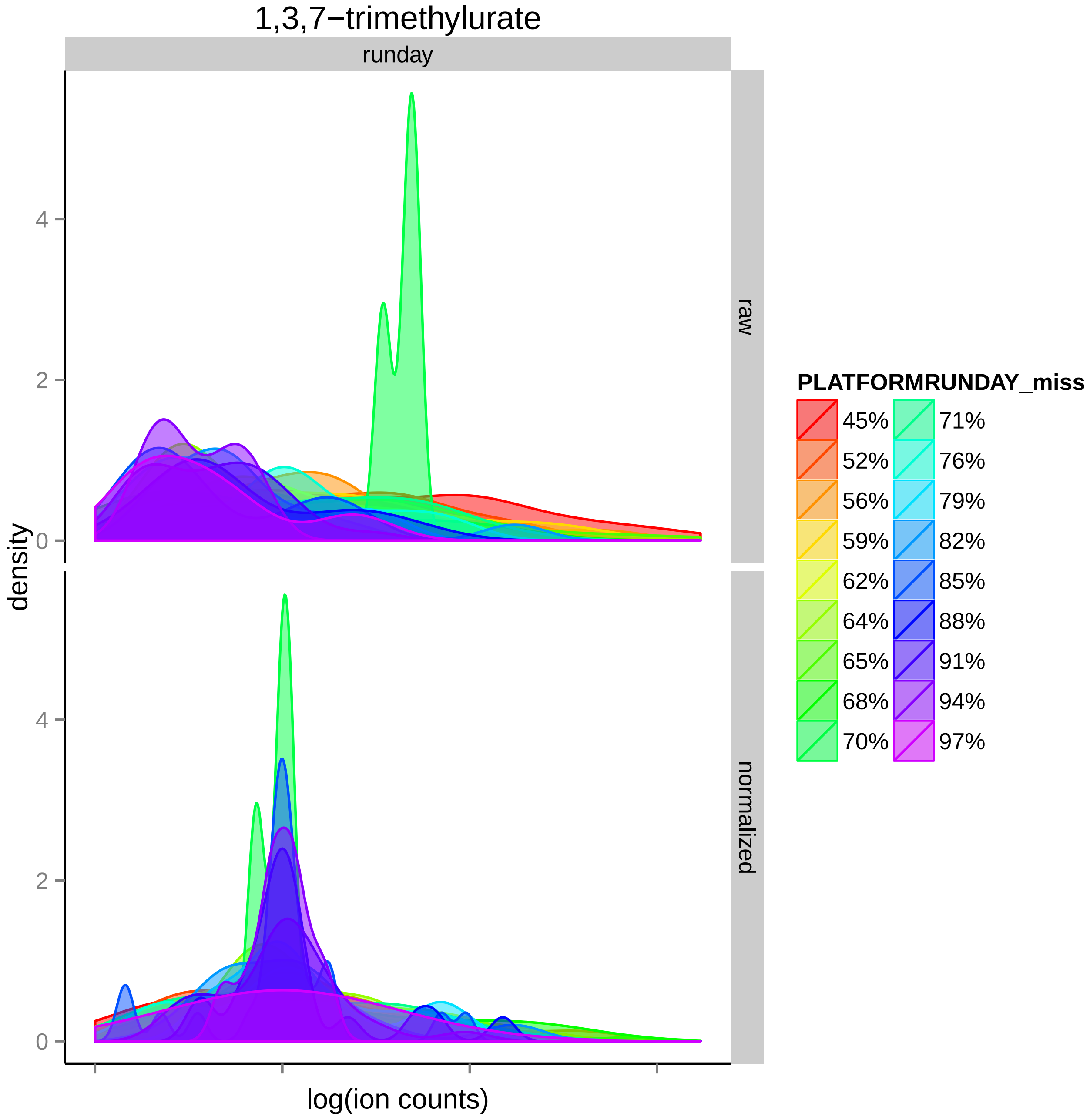
1,3,7−trimethylurate
runday
raw
normalized
density
log(ion counts)
0
2
4
0
2
4
PLATFORMRUNDAY_miss
45%
52%
56%
59%
62%
64%
65%
68%
70%
71%
76%
79%
82%
85%
88%
91%
94%
97%

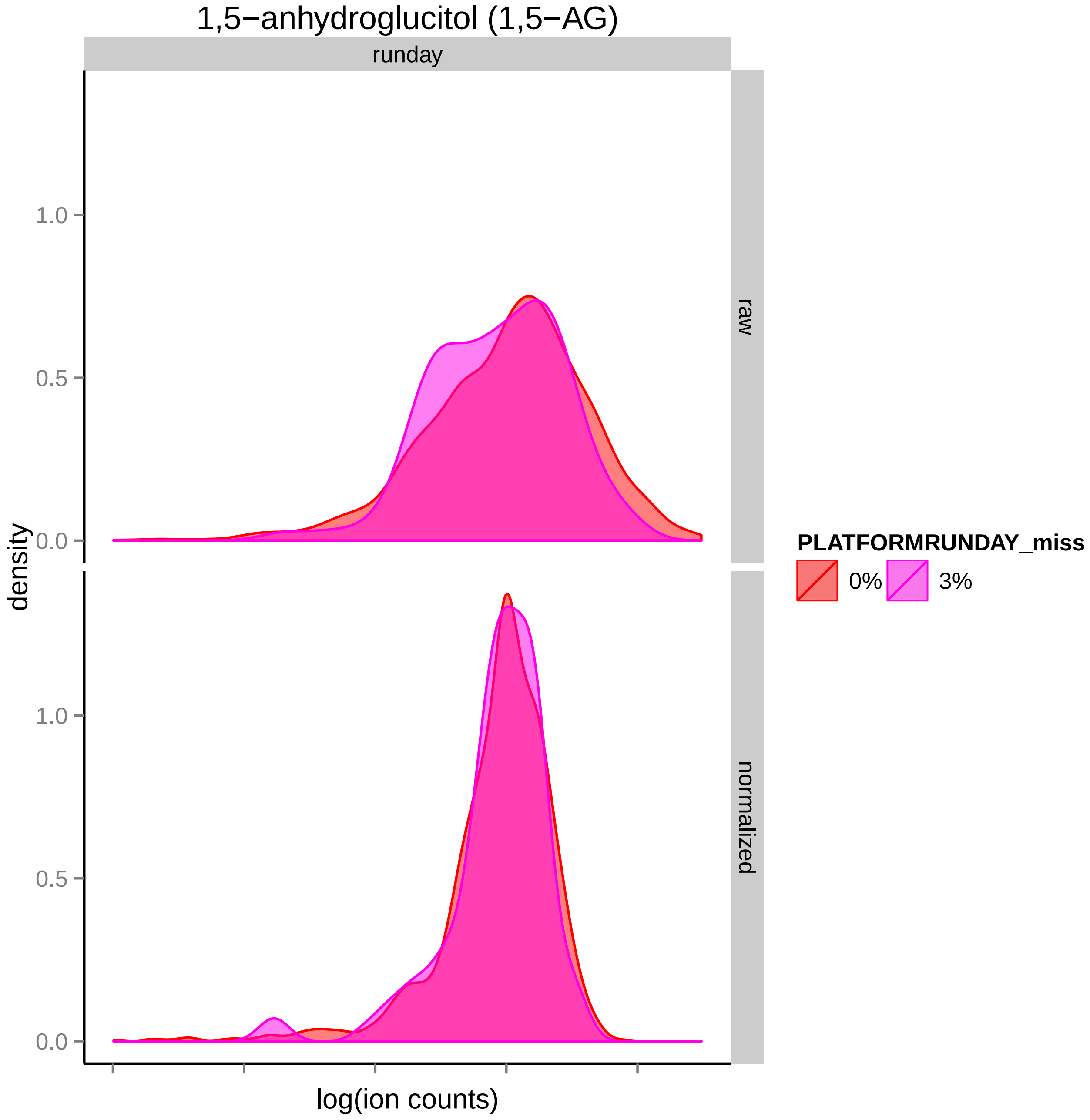
1,5−anhydroglucitol (1,5−AG)
runday
raw
normalized
density
log(ion counts)
0.0
0.5
1.0
0.0
0.5
1.0
PLATFORMRUNDAY_miss
0%
3%

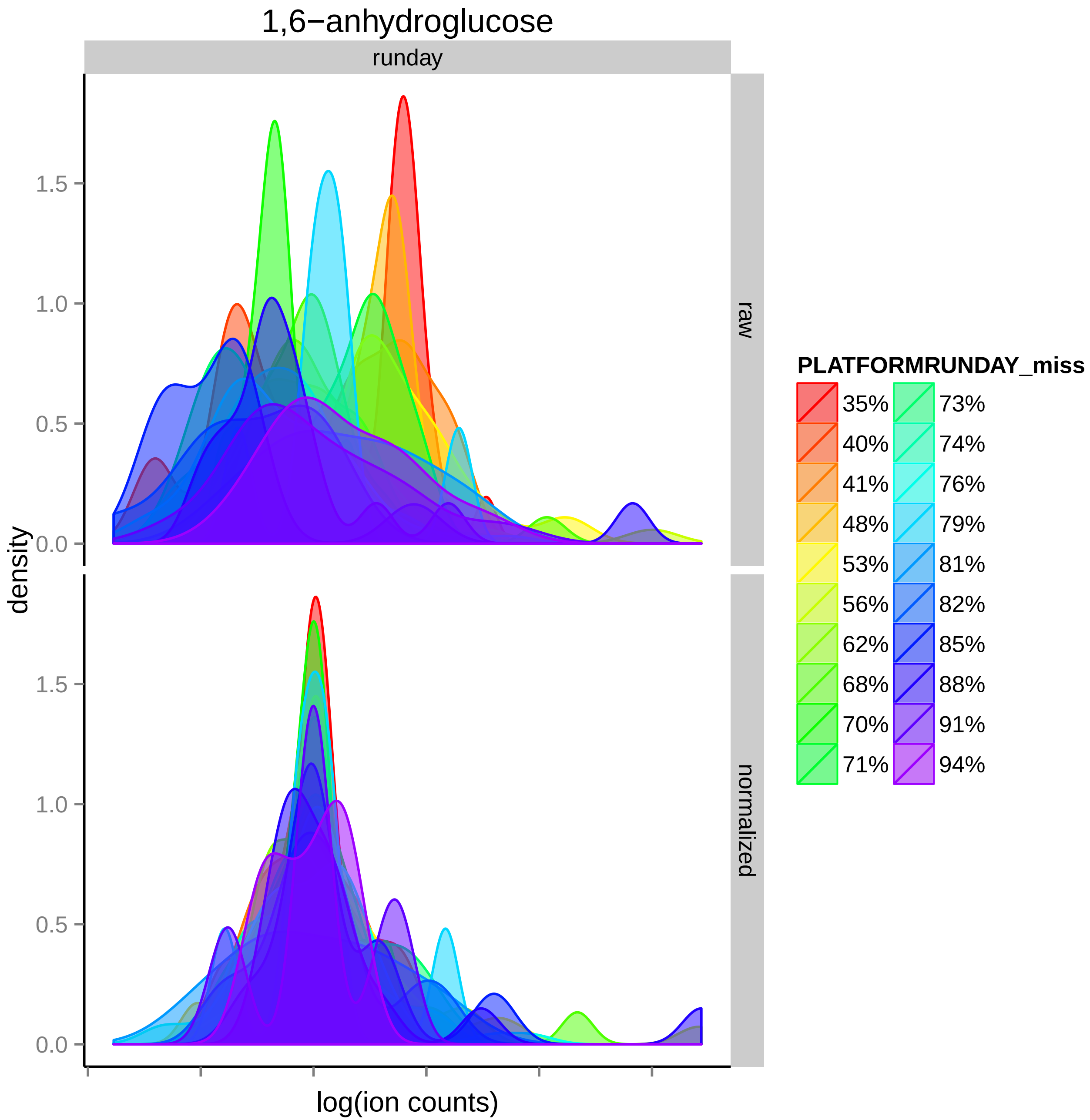
1,6−anhydroglucose
runday
raw
normalized
density
log(ion counts)
0.0
0.5
1.0
1.5
PLATFORMRUNDAY_miss
35%
40%
41%
48%
53%
56%
62%
68%
70%
71%
73%
74%
76%
79%
81%
82%
85%
88%
91%
94%

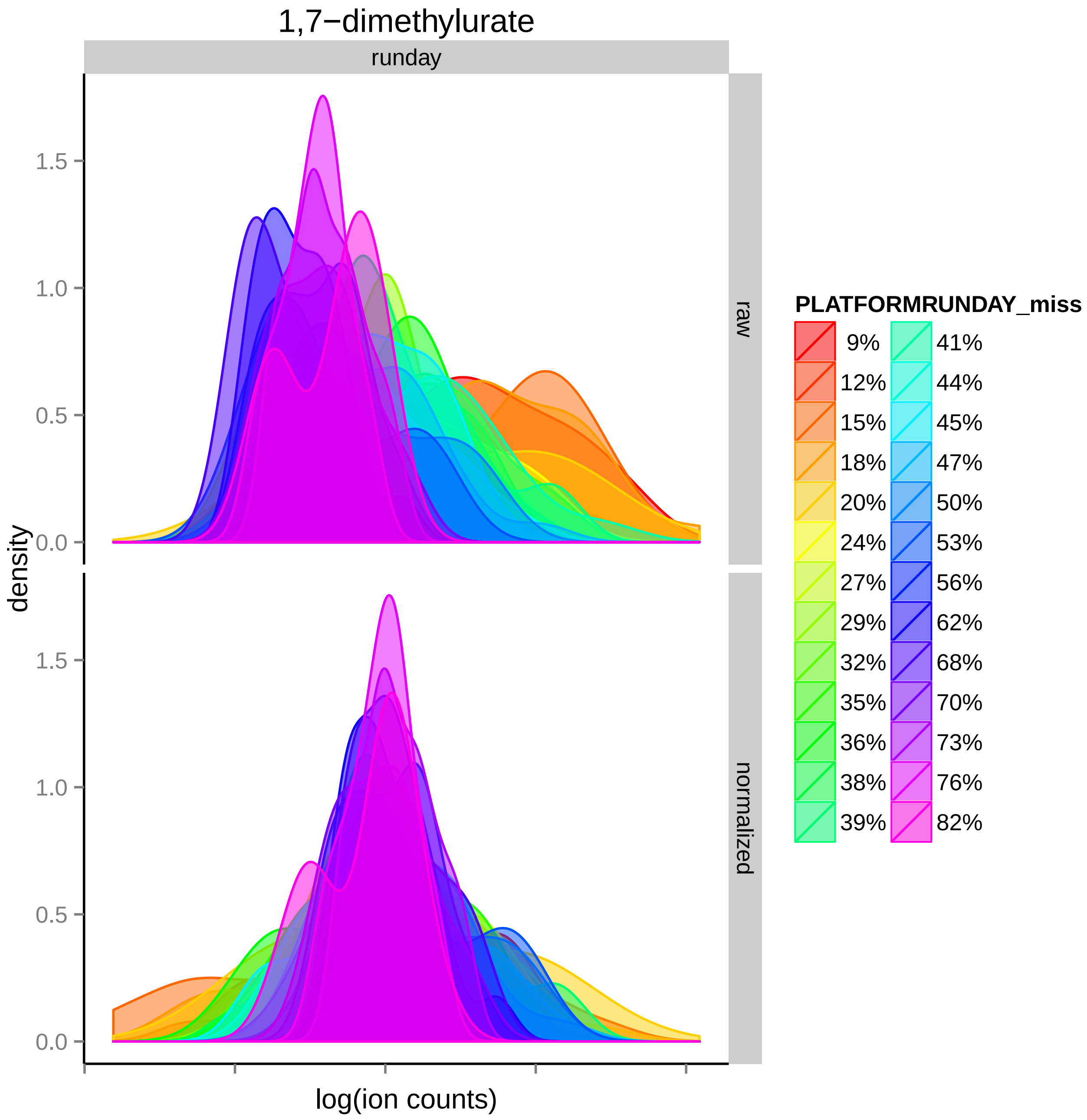
1,7−dimethylurate
runday
raw
normalized
density
log(ion counts)
0.0
0.5
1.0
1.5
PLATFORMRUNDAY_miss
9%
12%
15%
18%
20%
24%
27%
29%
32%
35%
36%
38%
39%
41%
44%
45%
47%
50%
53%
56%
62%
68%
70%
73%
76%
82%

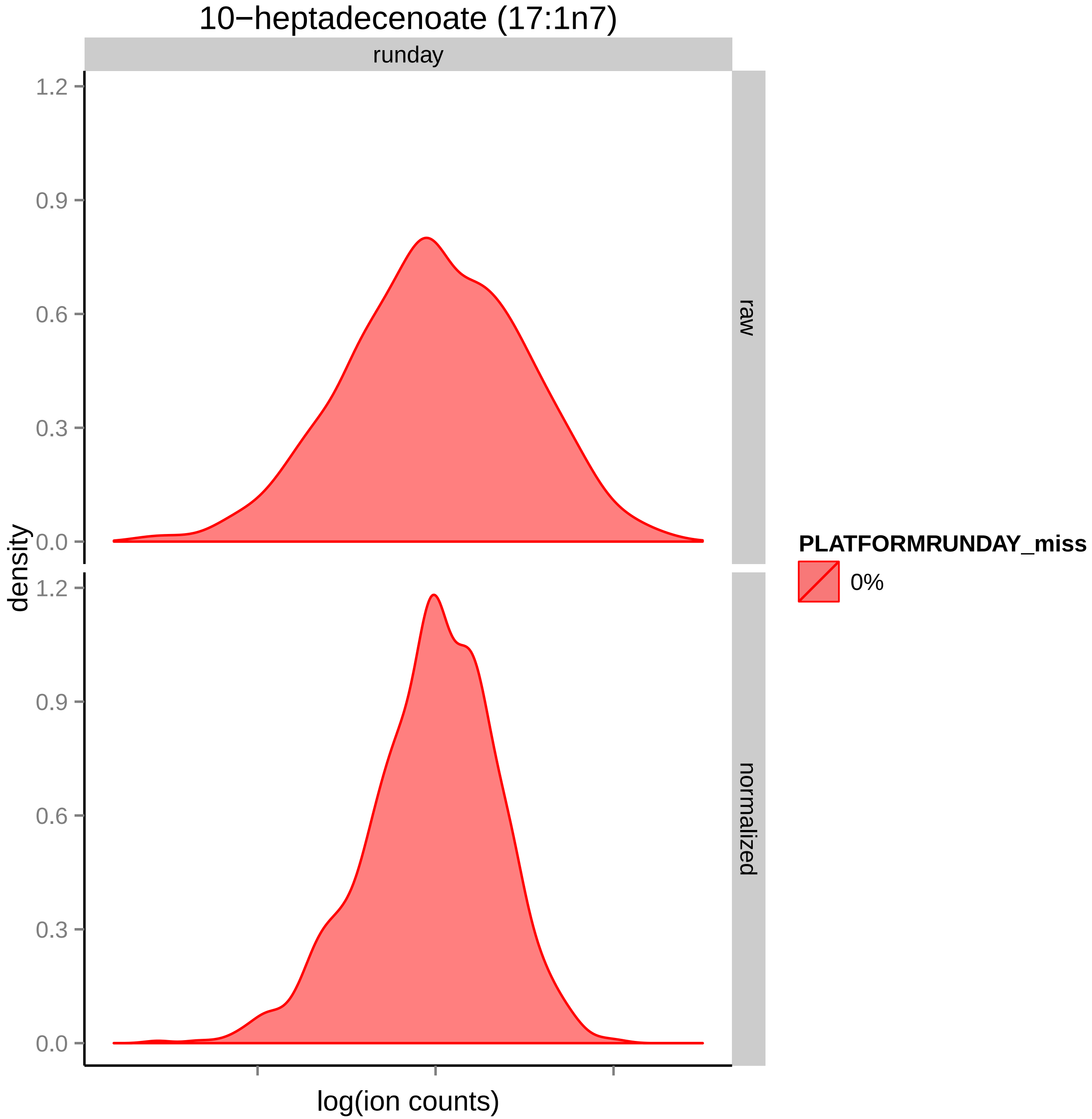

10−heptadecenoate (17:1n7)
runday
raw
normalized
density
log(ion counts)
1.2
0.9
0.6
0.3
0.0
PLATFORMRUNDAY_miss
0%

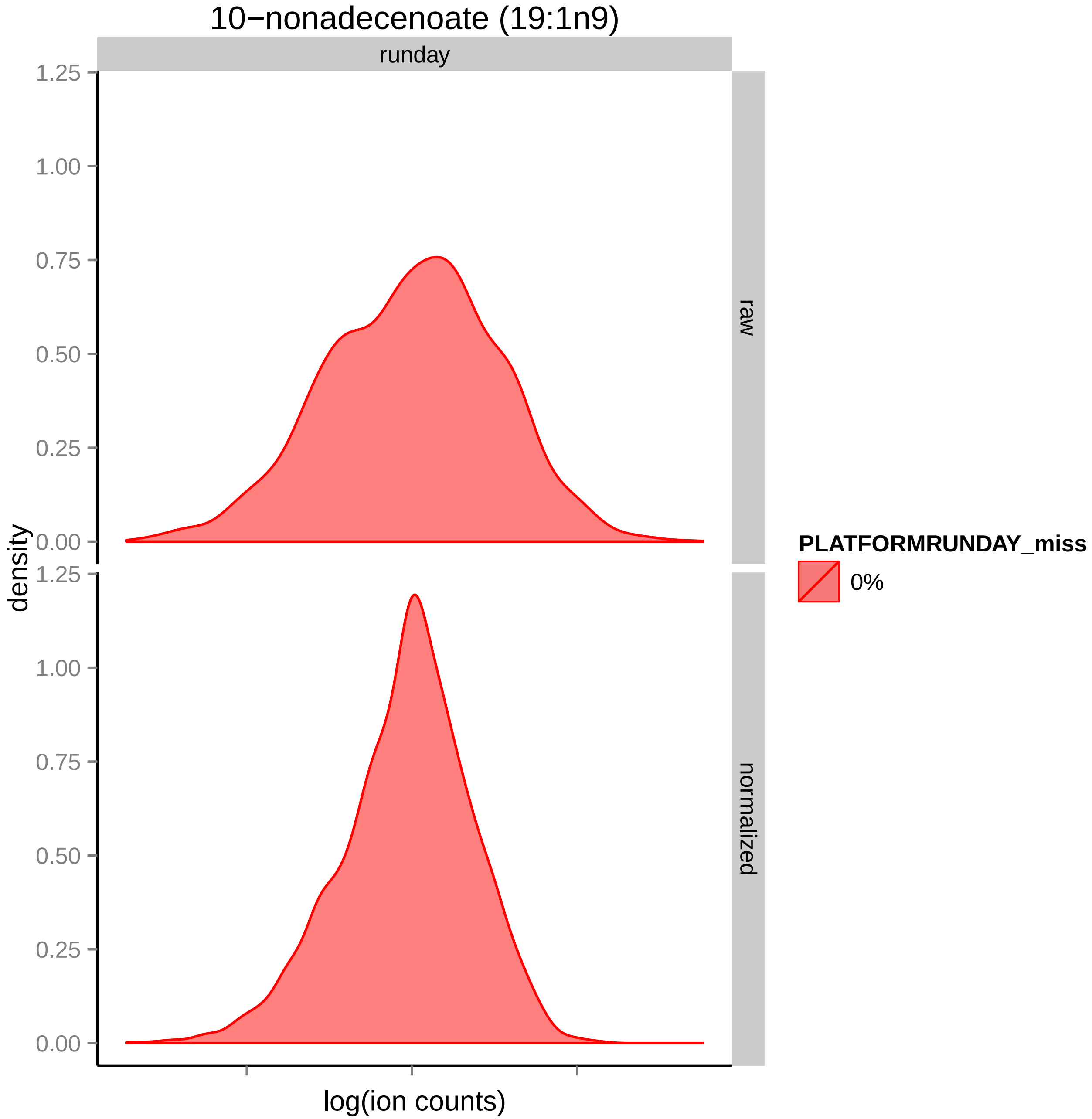
10−nonadecenoate (19:1n9)
runday
raw
normalized
density
log(ion counts)
1.25
1.00
0.75
0.50
0.25
0.00
PLATFORMRUNDAY_miss
0%

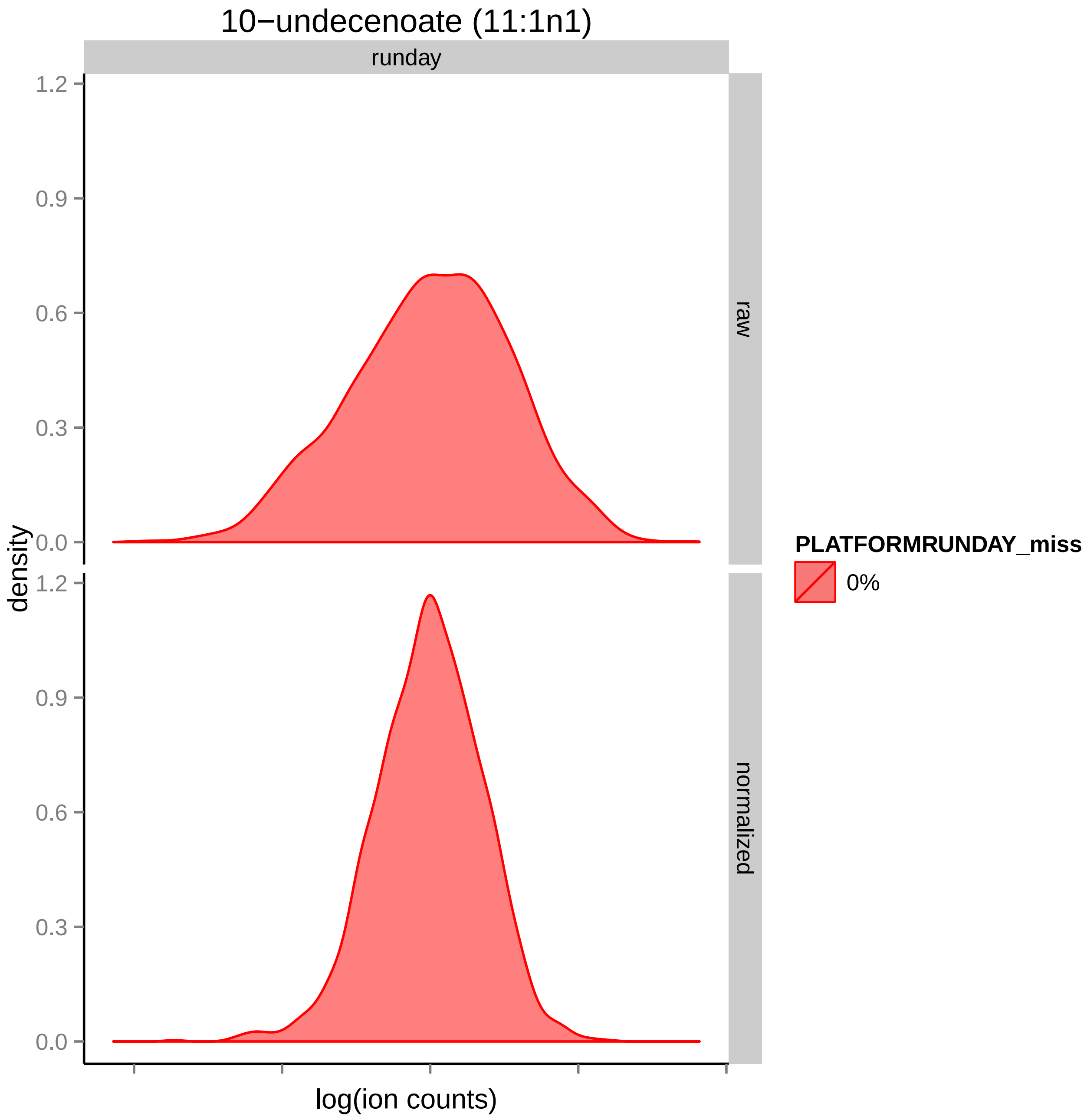

10−undecenoate (11:1n1)
runday
raw
normalized
density
log(ion counts)
1.2
0.9
0.6
0.3
0.0
PLATFORMRUNDAY_miss
0%

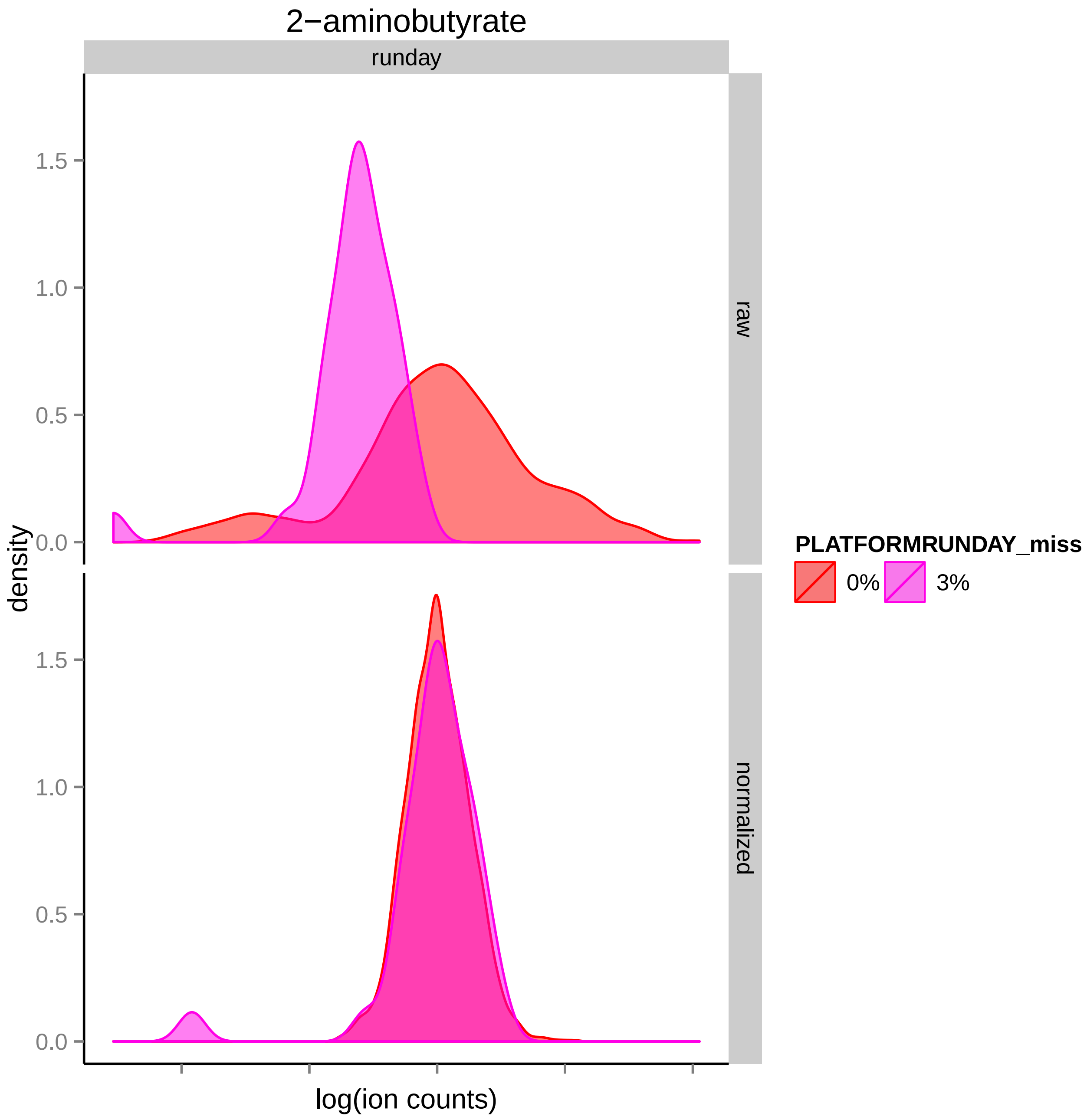

2−aminobutyrate
runday
raw
normalized
density
log(ion counts)
1.5
1.0
0.5
0.0
PLATFORMRUNDAY_miss
0%
3%

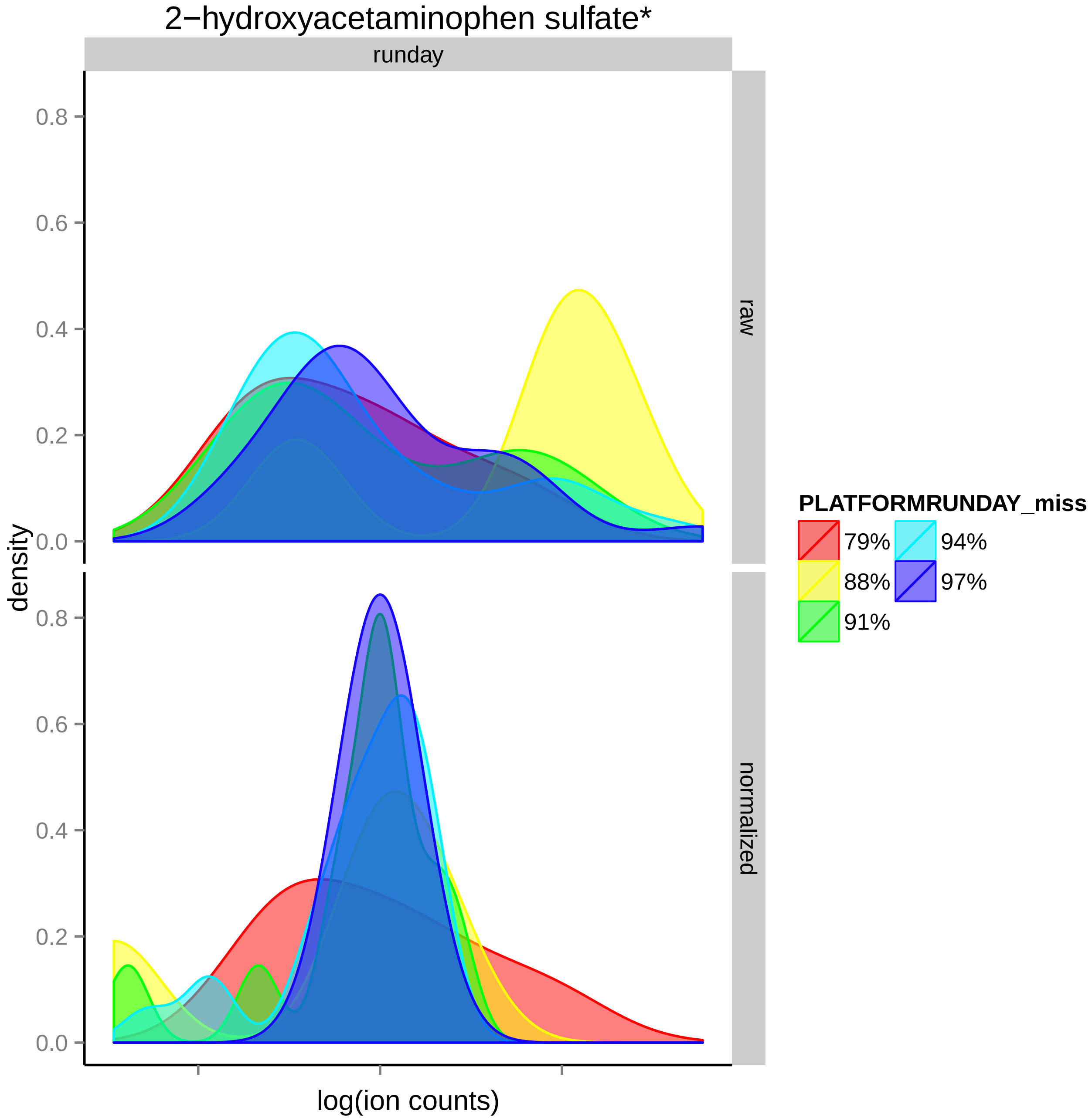

2−hydroxyacetaminophen sulfate*
runday
raw
normalized
density
0.0
0.2
0.4
0.6
0.8
log(ion counts)
PLATFORMRUNDAY_miss
79%
88%
91%
94%
97%

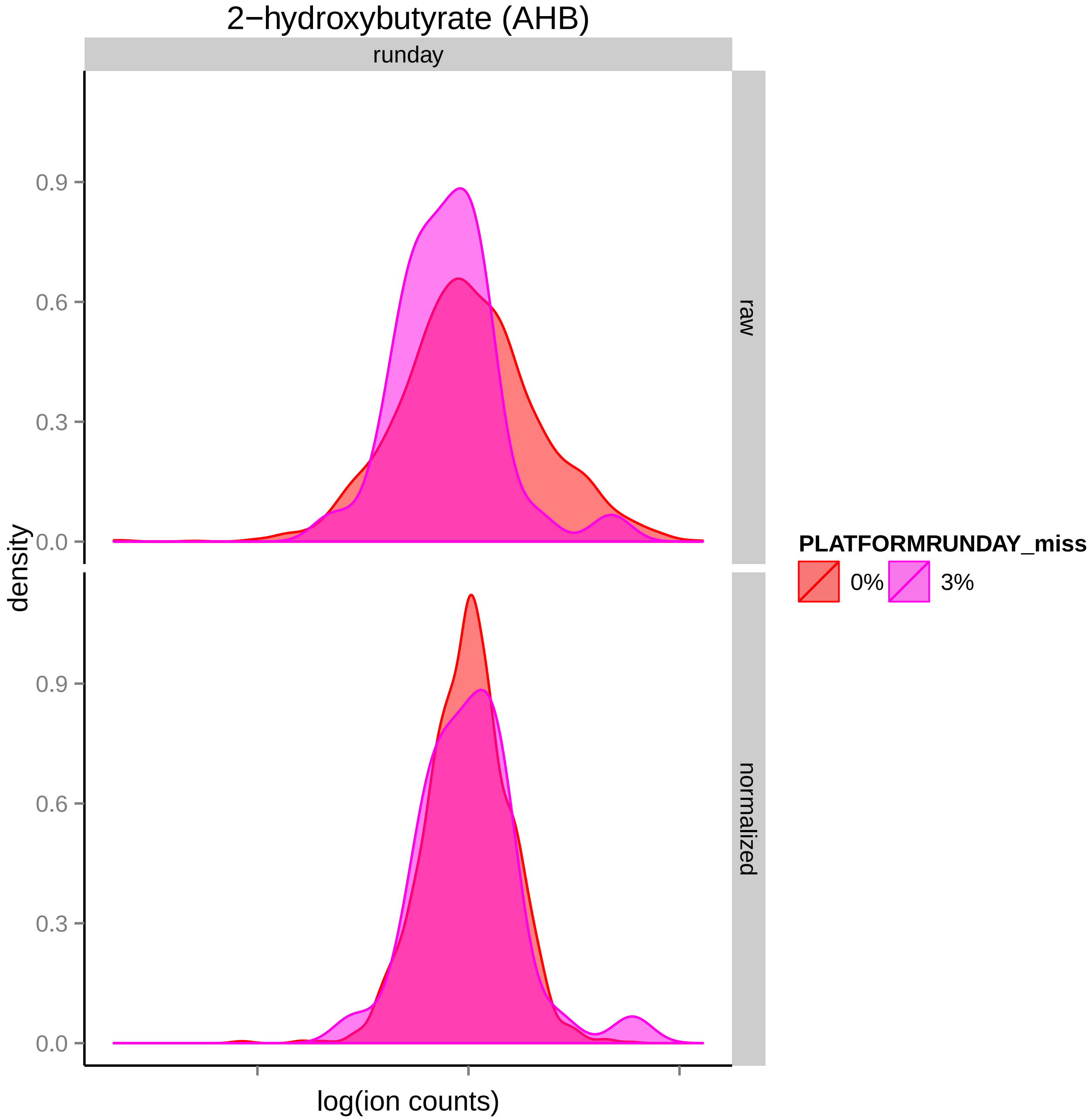
2−hydroxybutyrate (AHB)
runday
raw
normalized
density
0.0
0.3
0.6
0.9
log(ion counts)
PLATFORMRUNDAY_miss
0%
3%

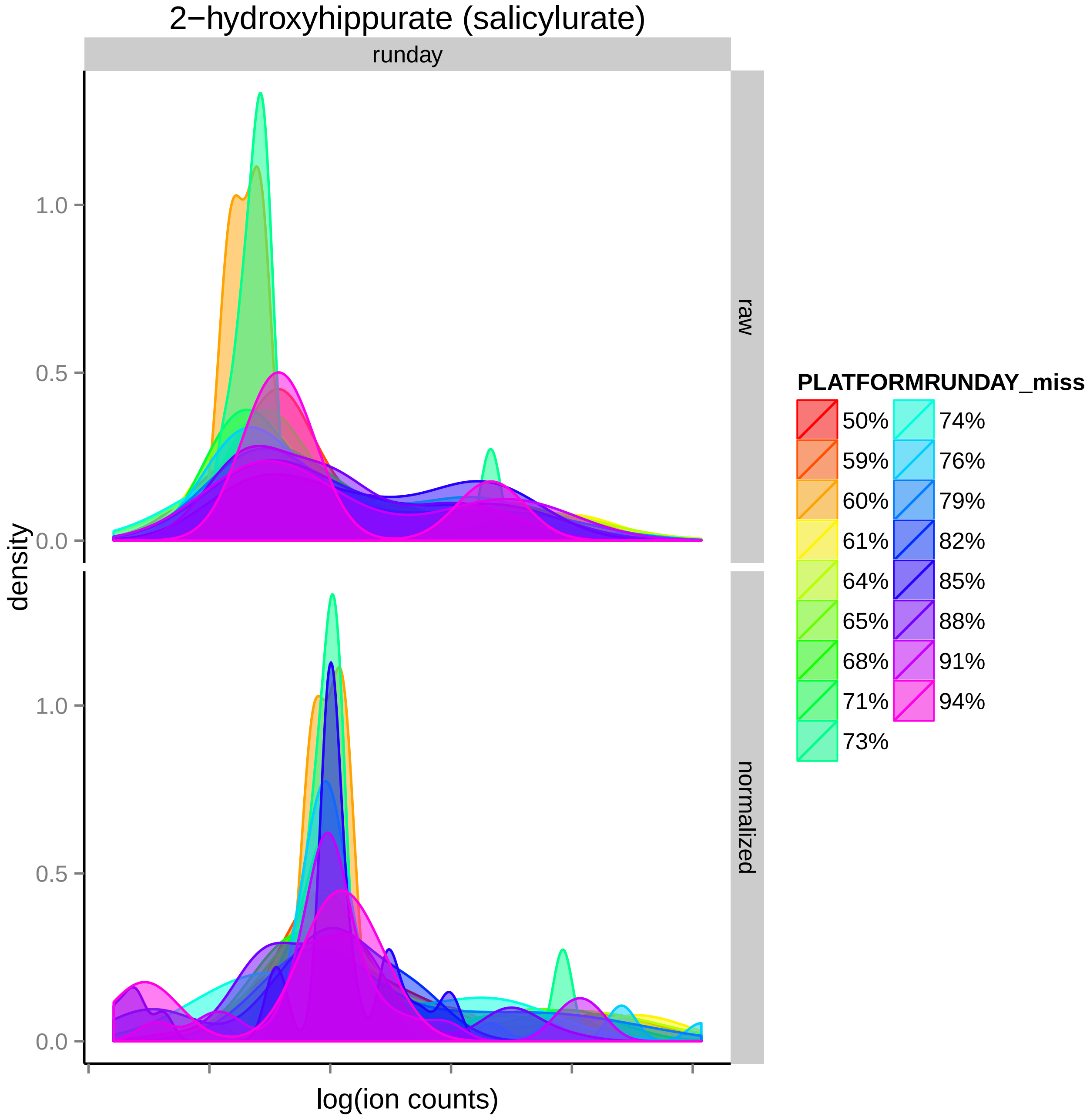
2−hydroxyhippurate (salicylurate)
runday
raw
normalized
density
log(ion counts)
1.0
0.5
0.0
1.0
0.5
0.0
PLATFORMRUNDAY_miss
50%
59%
60%
61%
64%
65%
68%
71%
73%
74%
76%
79%
82%
85%
88%
91%
94%

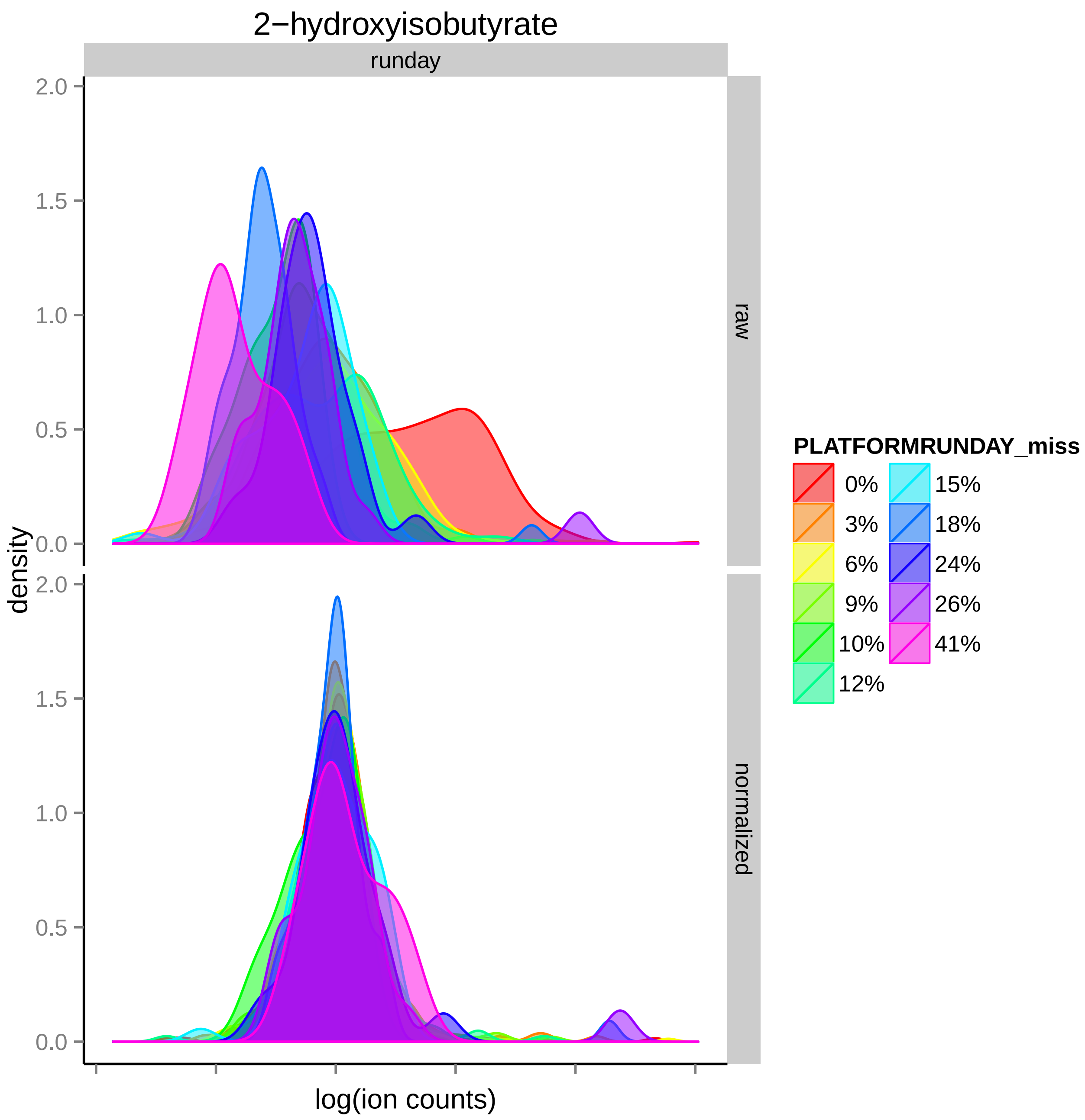
2−hydroxyisobutyrate
runday
raw
normalized
density
log(ion counts)
2.0
1.5
1.0
0.5
0.0
PLATFORMRUNDAY_miss
0%
3%
6%
9%
10%
12%
15%
18%
24%
26%
41%

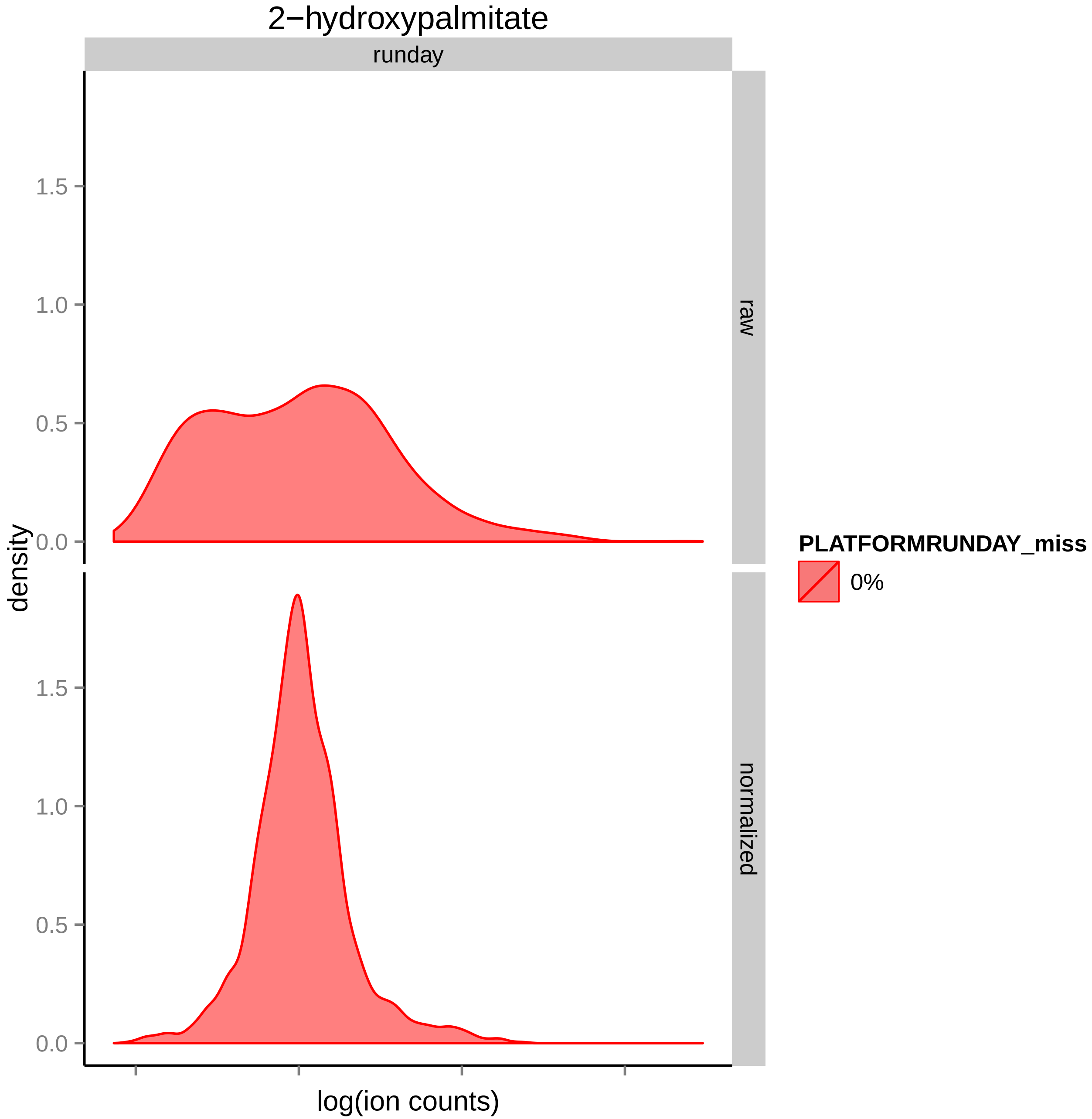

2−hydroxypalmitate
runday
raw
normalized
density
1.5
1.0
0.5
0.0
log(ion counts)
PLATFORMRUNDAY_miss
0%

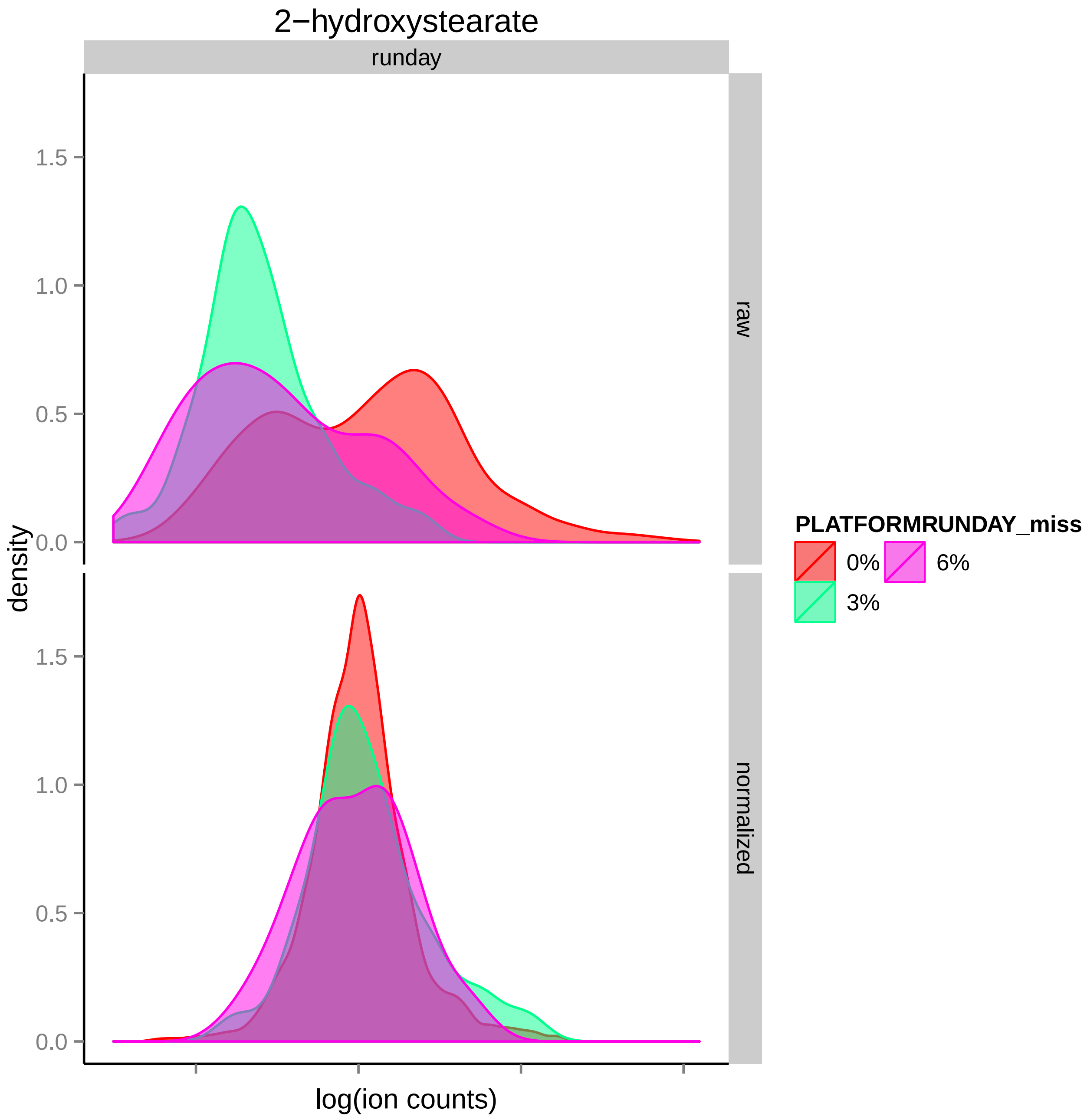
2−hydroxystearate
runday
raw
normalized
density
1.5
1.0
0.5
0.0
log(ion counts)
PLATFORMRUNDAY_miss
0%
6%
3%

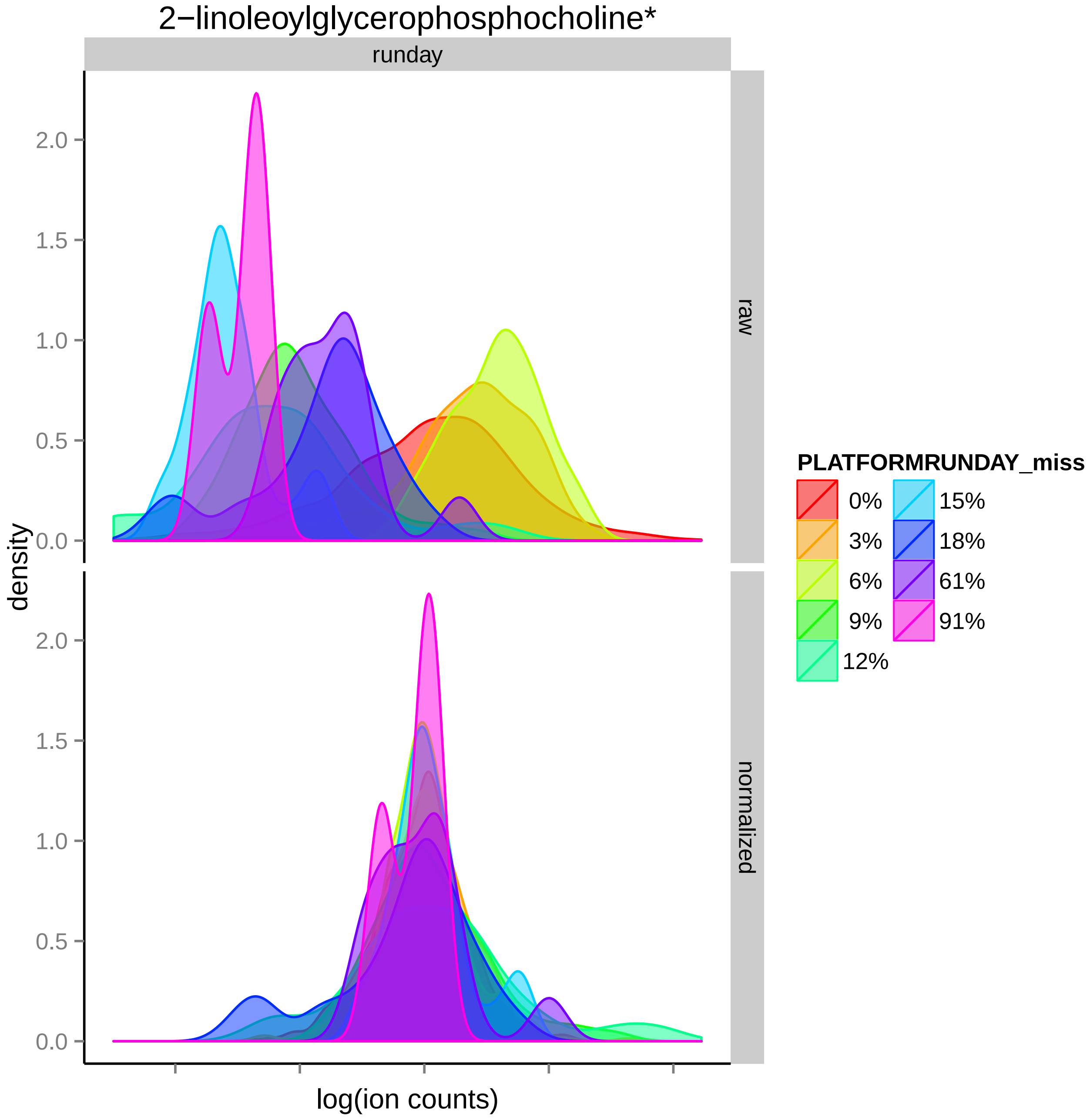

2−linoleoylglycerophosphocholine*
runday
raw
normalized
density
log(ion counts)
0.0
0.5
1.0
1.5
2.0
PLATFORMRUNDAY_miss
0%
3%
6%
9%
12%
15%
18%
61%
91%

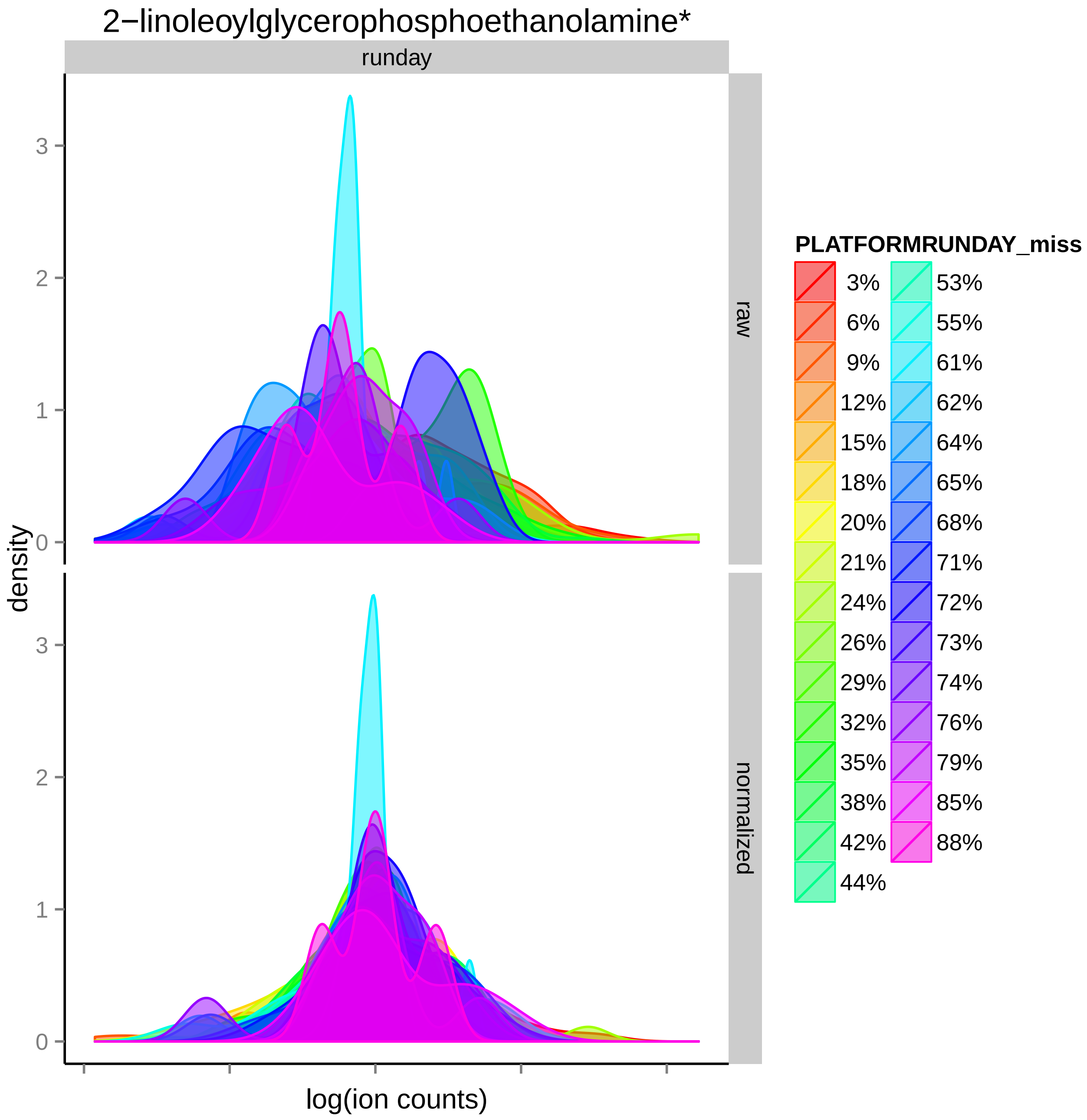

2−linoleoylglycerophosphoethanolamine*
runday
raw
normalized
density
log(ion counts)
0
1
2
3
0
1
2
3
PLATFORMRUNDAY_miss
3%
6%
9%
12%
15%
18%
20%
21%
24%
26%
29%
32%
35%
38%
42%
44%
53%
55%
61%
62%
64%
65%
68%
71%
72%
73%
74%
76%
79%
85%
88%

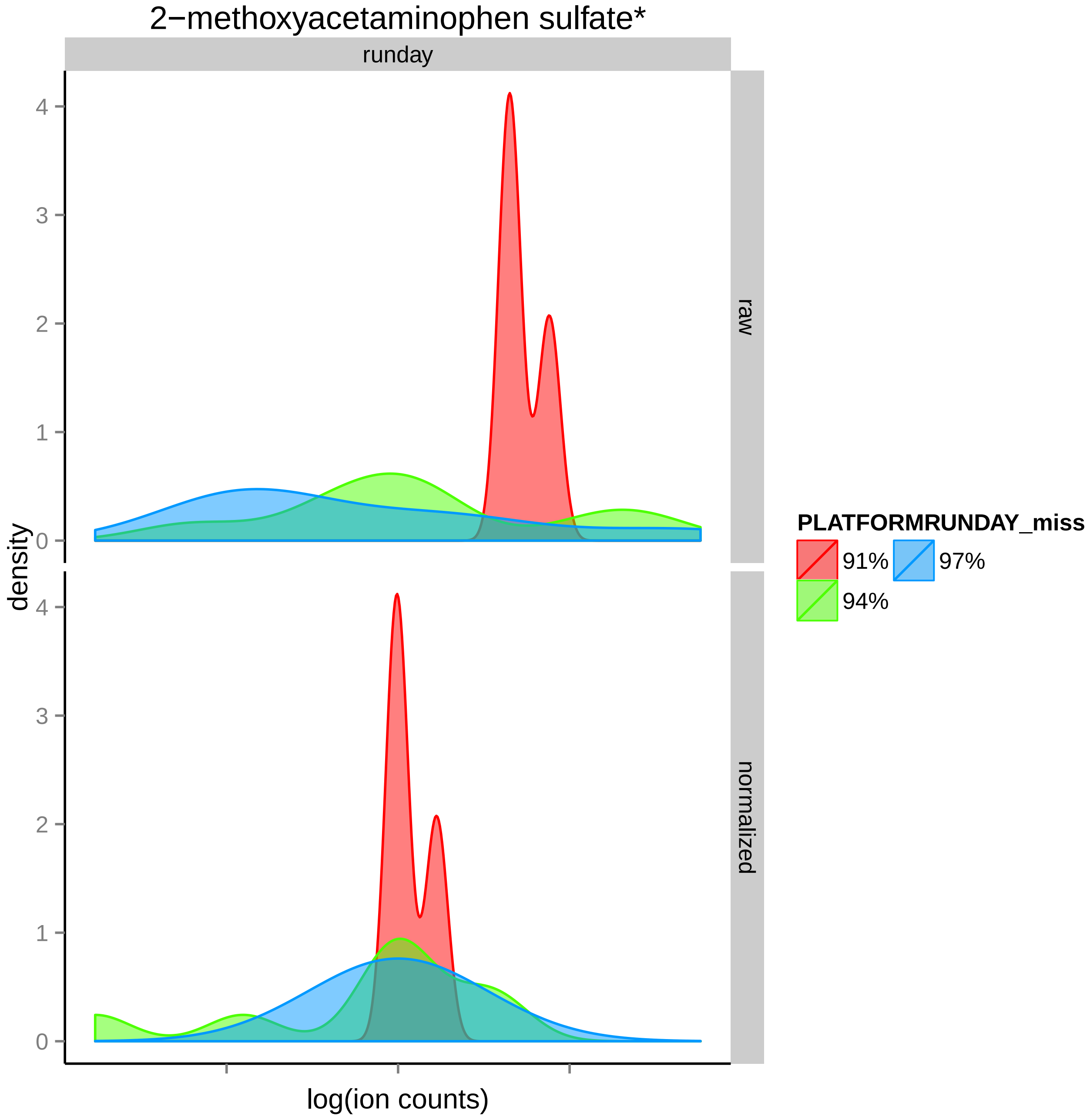

2−methoxyacetaminophen sulfate*
runday
raw
normalized
density
log(ion counts)
0
1
2
3
4
PLATFORMRUNDAY_miss
91%
94%
97%

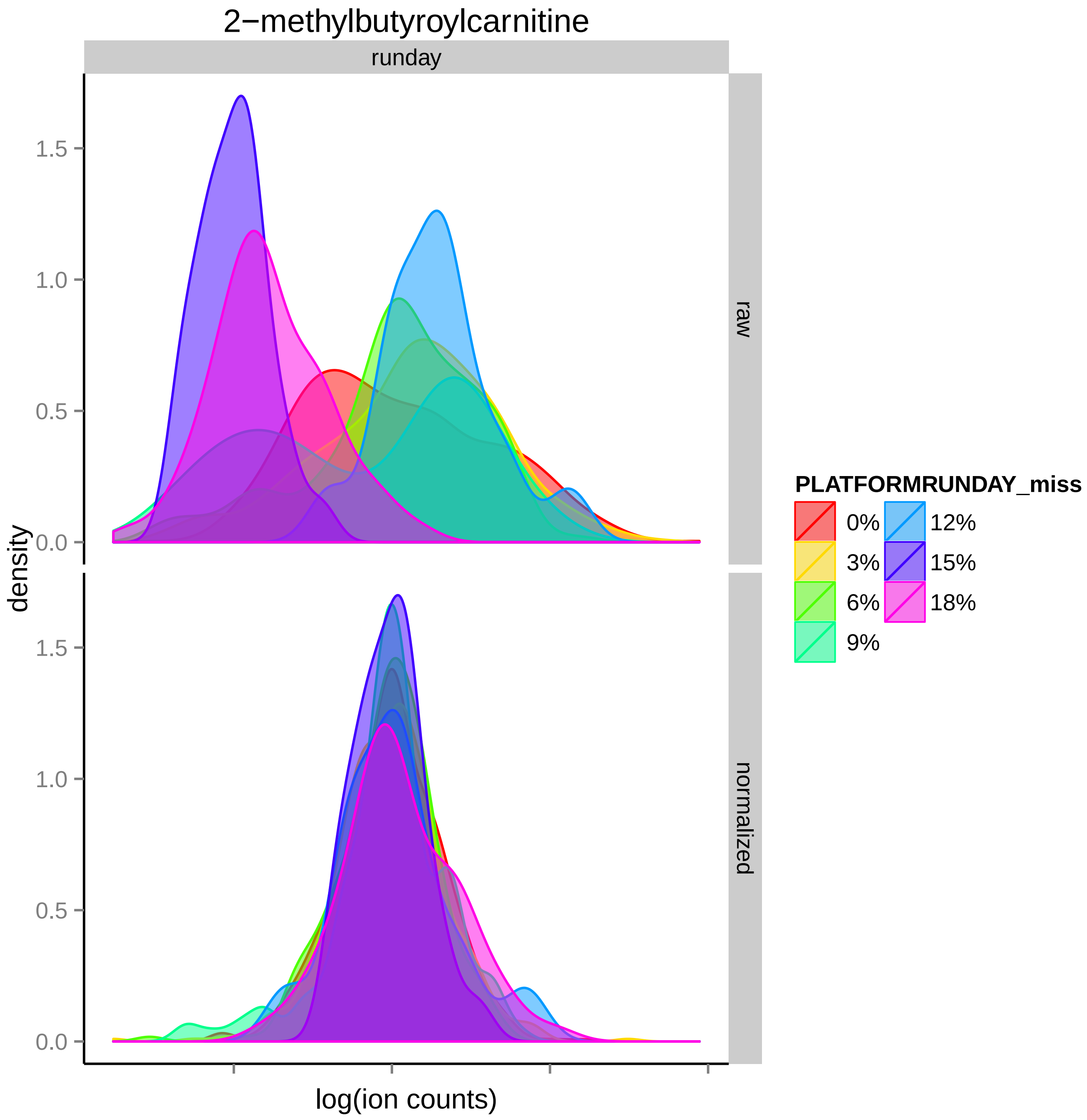

2−methylbutyroylcarnitine
runday
raw
normalized
density
log(ion counts)
0.0
0.5
1.0
1.5
PLATFORMRUNDAY_miss
0%
3%
6%
9%
12%
15%
18%

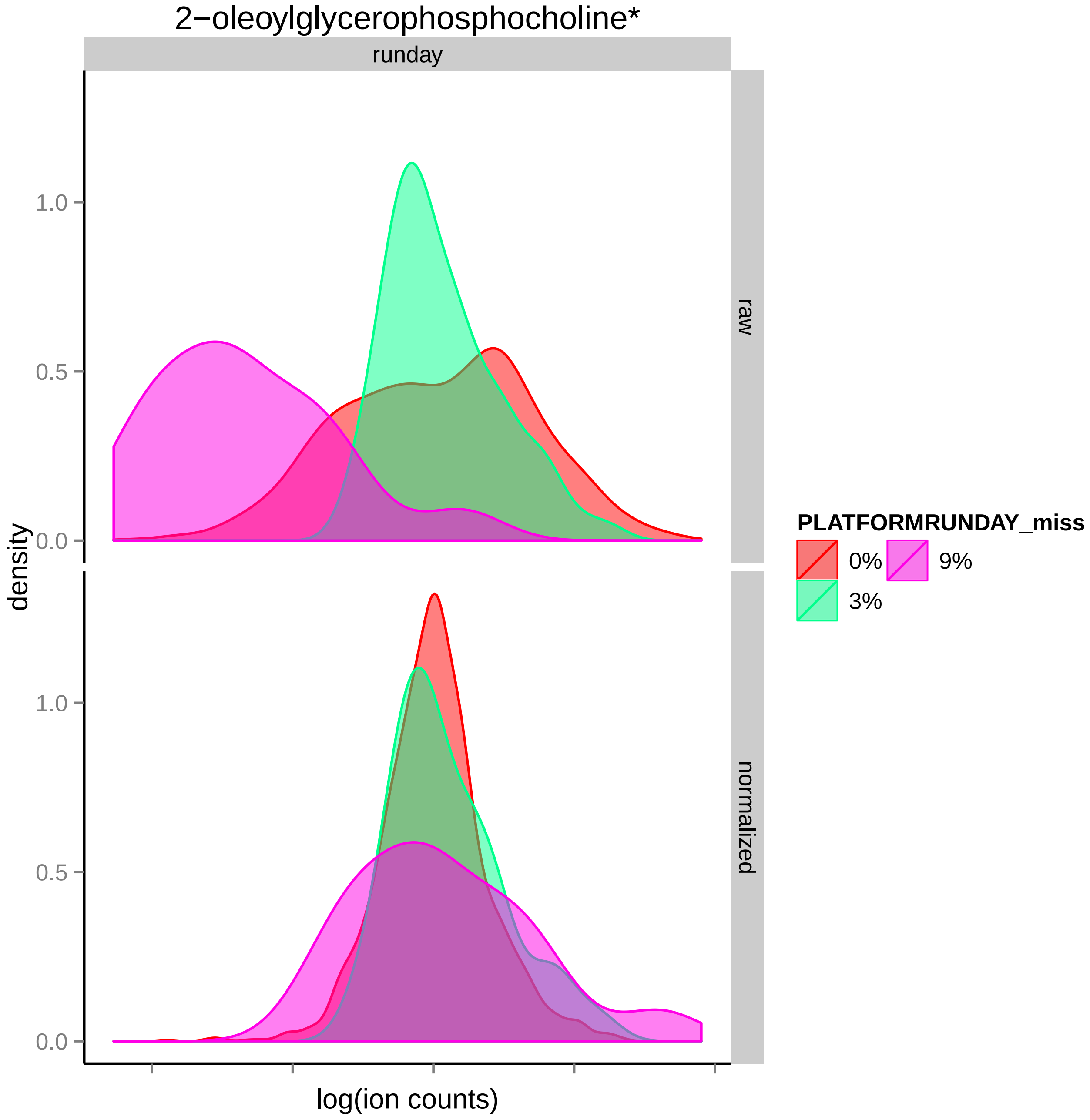
2−oleoylglycerophosphocholine*
runday
raw
normalized
density
log(ion counts)
1.0
0.5
0.0
PLATFORMRUNDAY_miss
0%
3%
9%

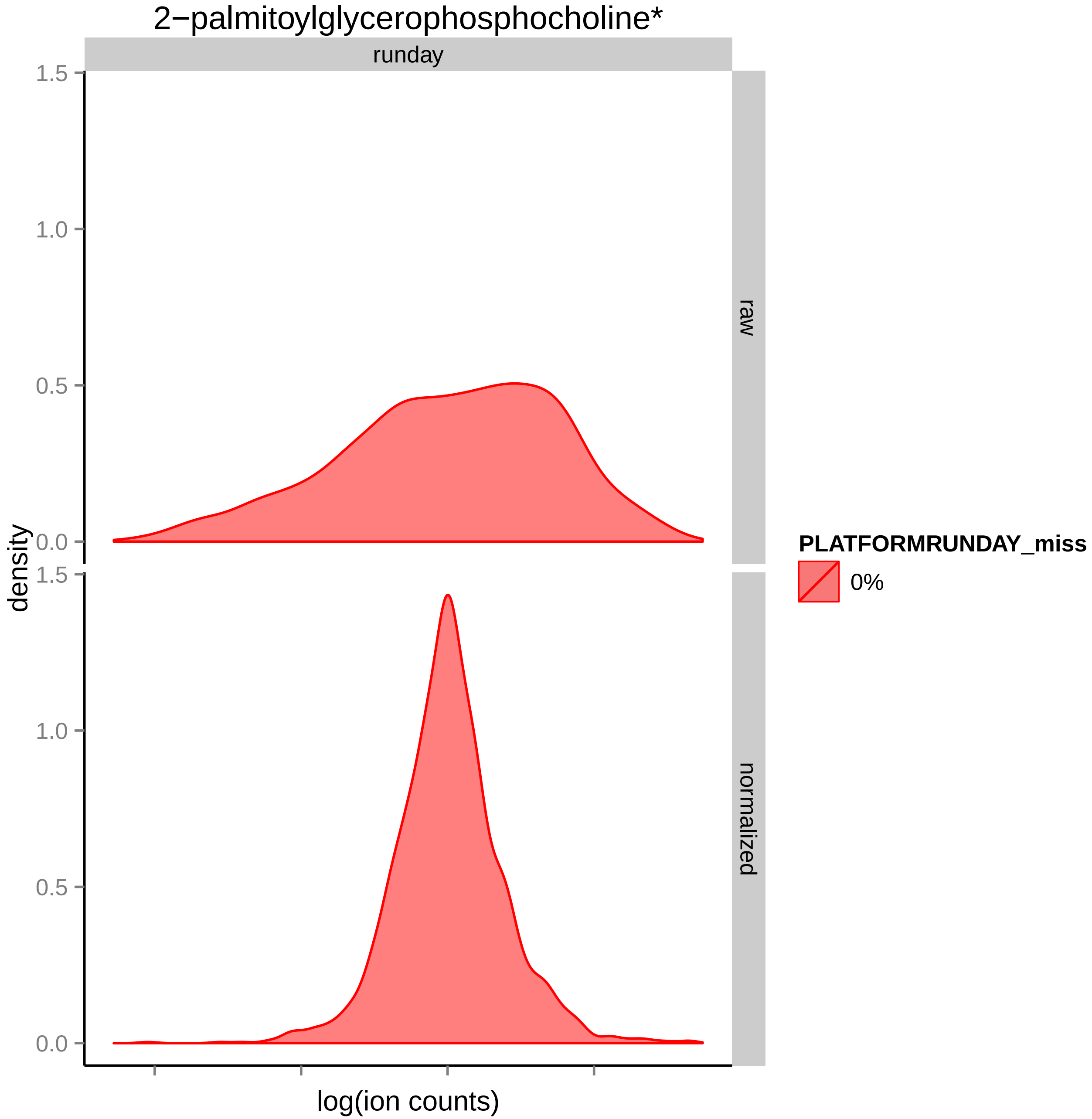
2−palmitoylglycerophosphocholine*
runday
raw
normalized
density
1.5
1.0
0.5
0.0
log(ion counts)
PLATFORMRUNDAY_miss
0%

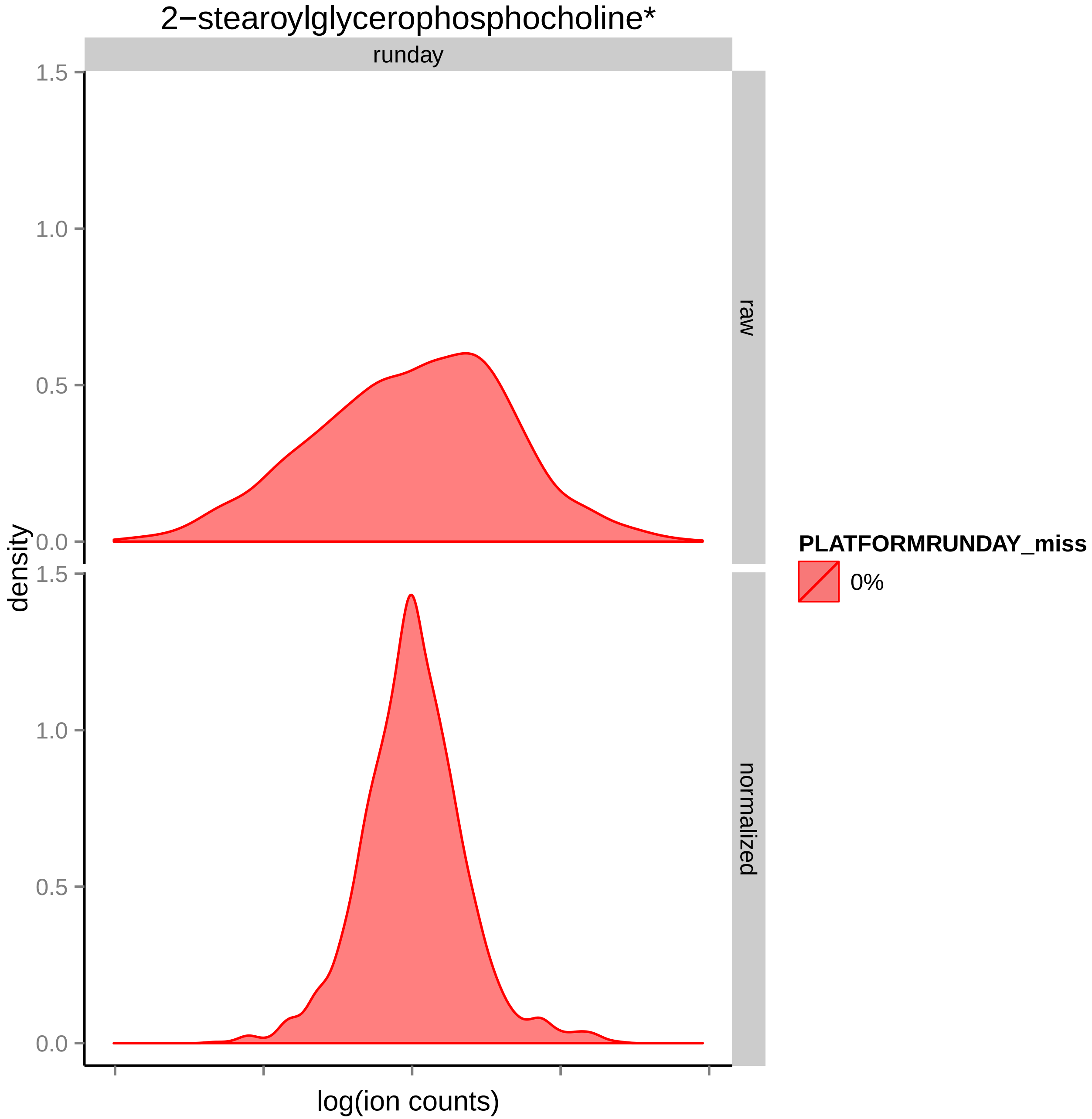

2−stearoylglycerophosphocholine*
runday
raw
normalized
density
log(ion counts)
1.5
1.0
0.5
0.0
PLATFORMRUNDAY_miss
0%

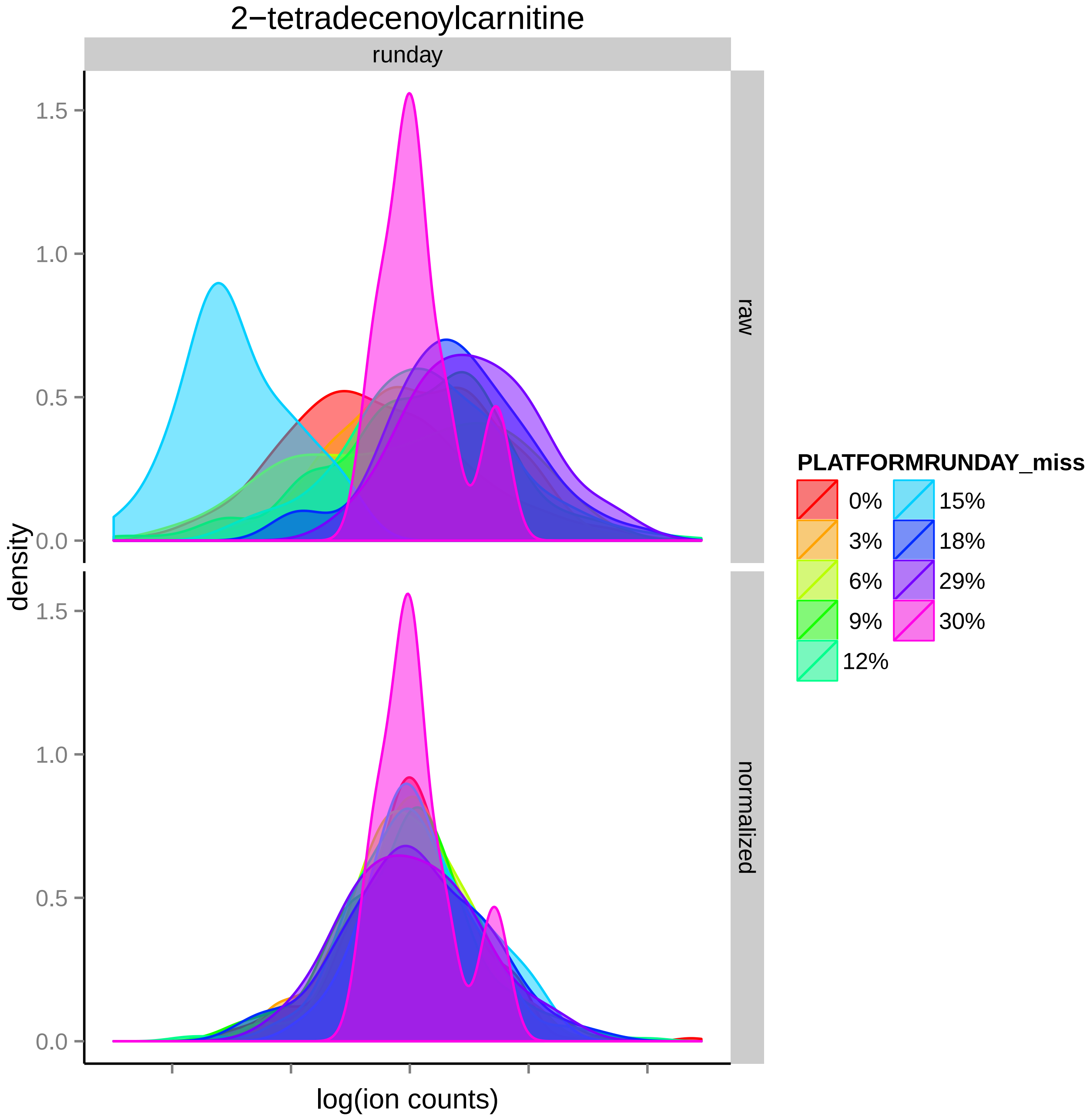
2−tetradecenoylcarnitine
runday
raw
normalized
density
log(ion counts)
1.5
1.0
0.5
0.0
PLATFORMRUNDAY_miss
0%
3%
6%
9%
12%
15%
18%
29%
30%

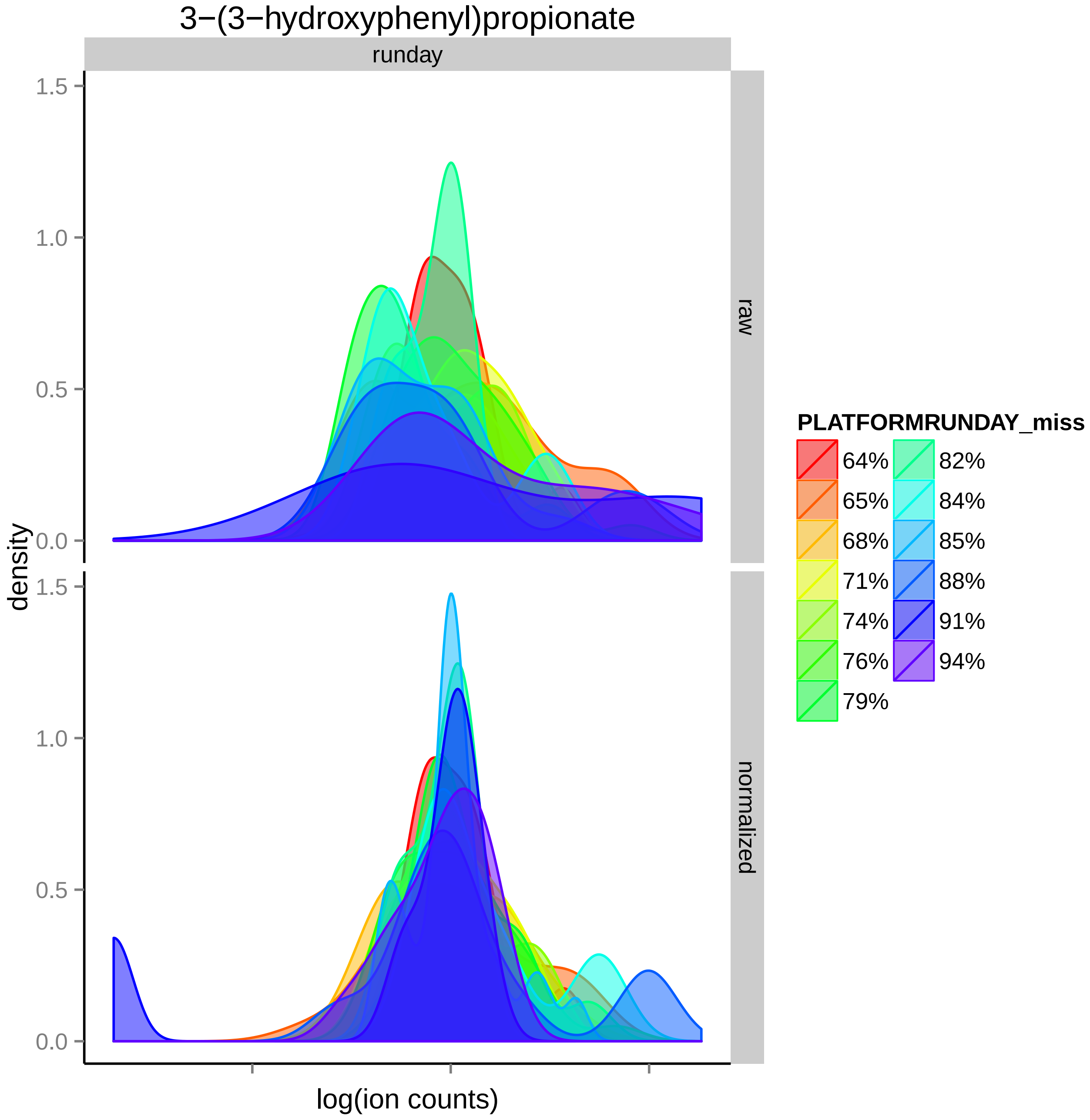
3−(3−hydroxyphenyl)propionate
runday
raw
normalized
density
log(ion counts)
0.0
0.5
1.0
1.5
PLATFORMRUNDAY_miss
64%
65%
68%
71%
74%
76%
79%
82%
84%
85%
88%
91%
94%

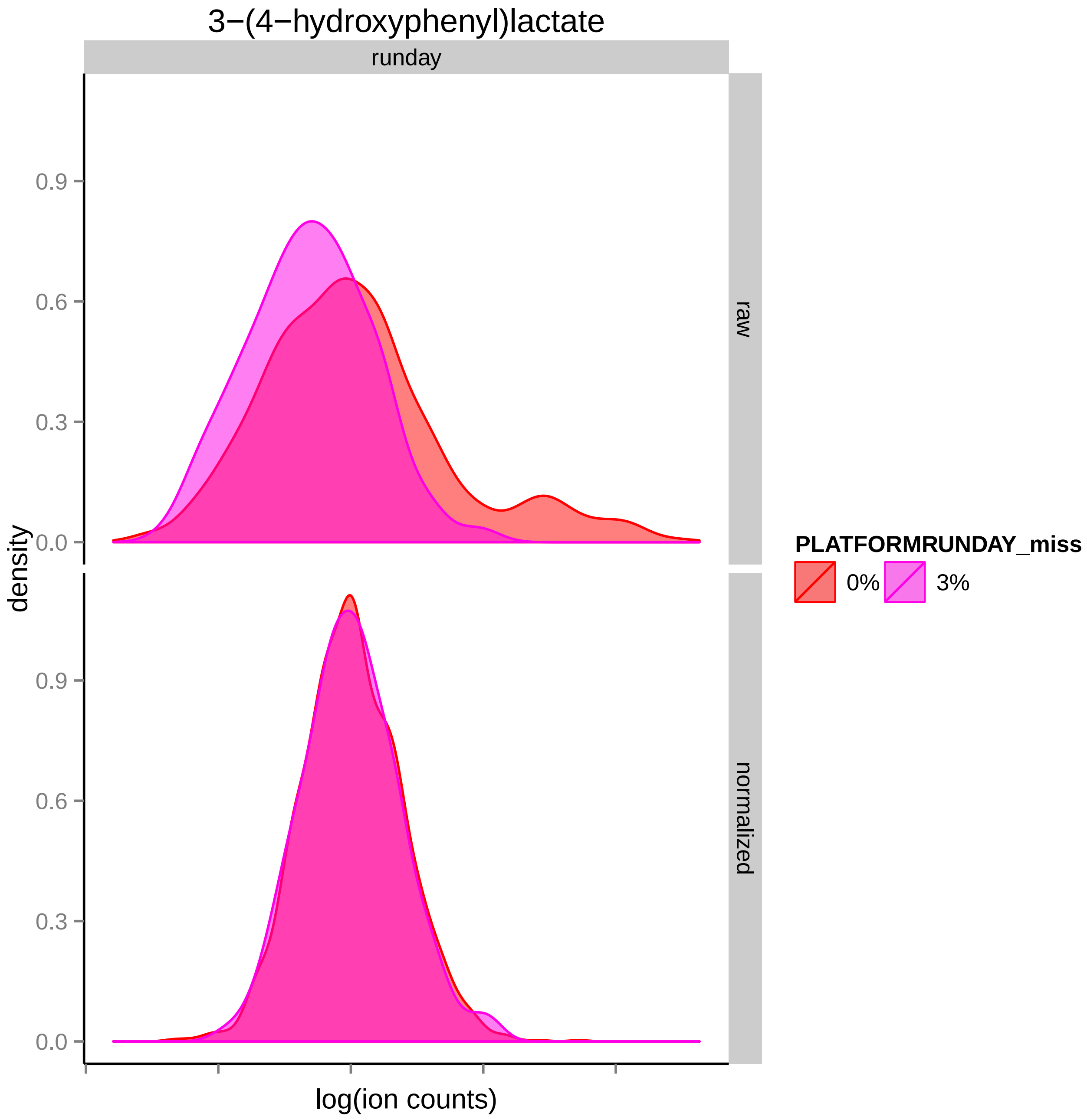

3−(4−hydroxyphenyl)lactate
runday
raw
normalized
density
0.9
0.6
0.3
0.0
0.9
0.6
0.3
0.0
log(ion counts)
PLATFORMRUNDAY_miss
0%
3%

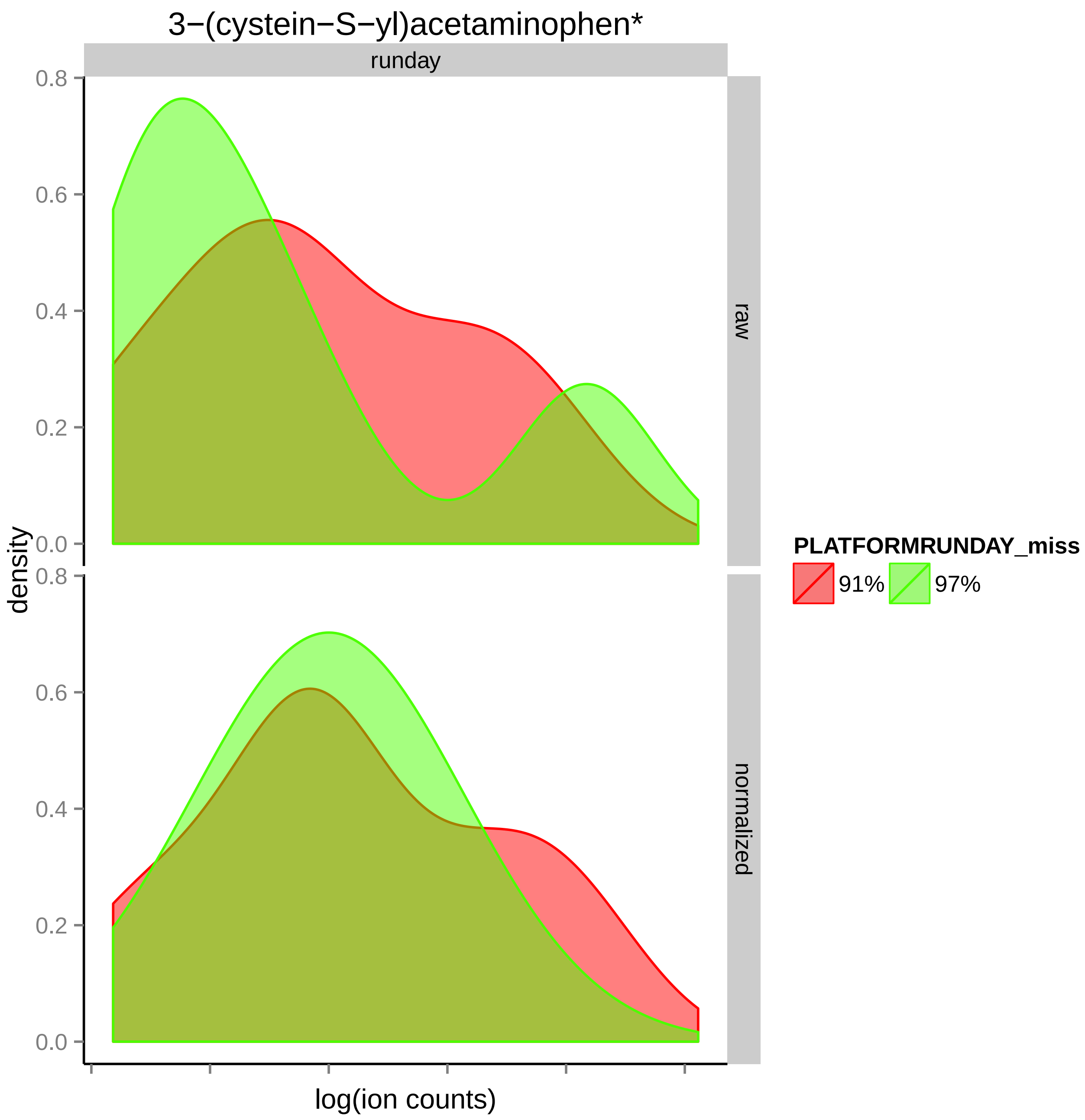

3−(cystein−S−yl)acetaminophen*
runday
raw
normalized
density
0.8
0.6
0.4
0.2
0.0
log(ion counts)
PLATFORMRUNDAY_miss
91%
97%

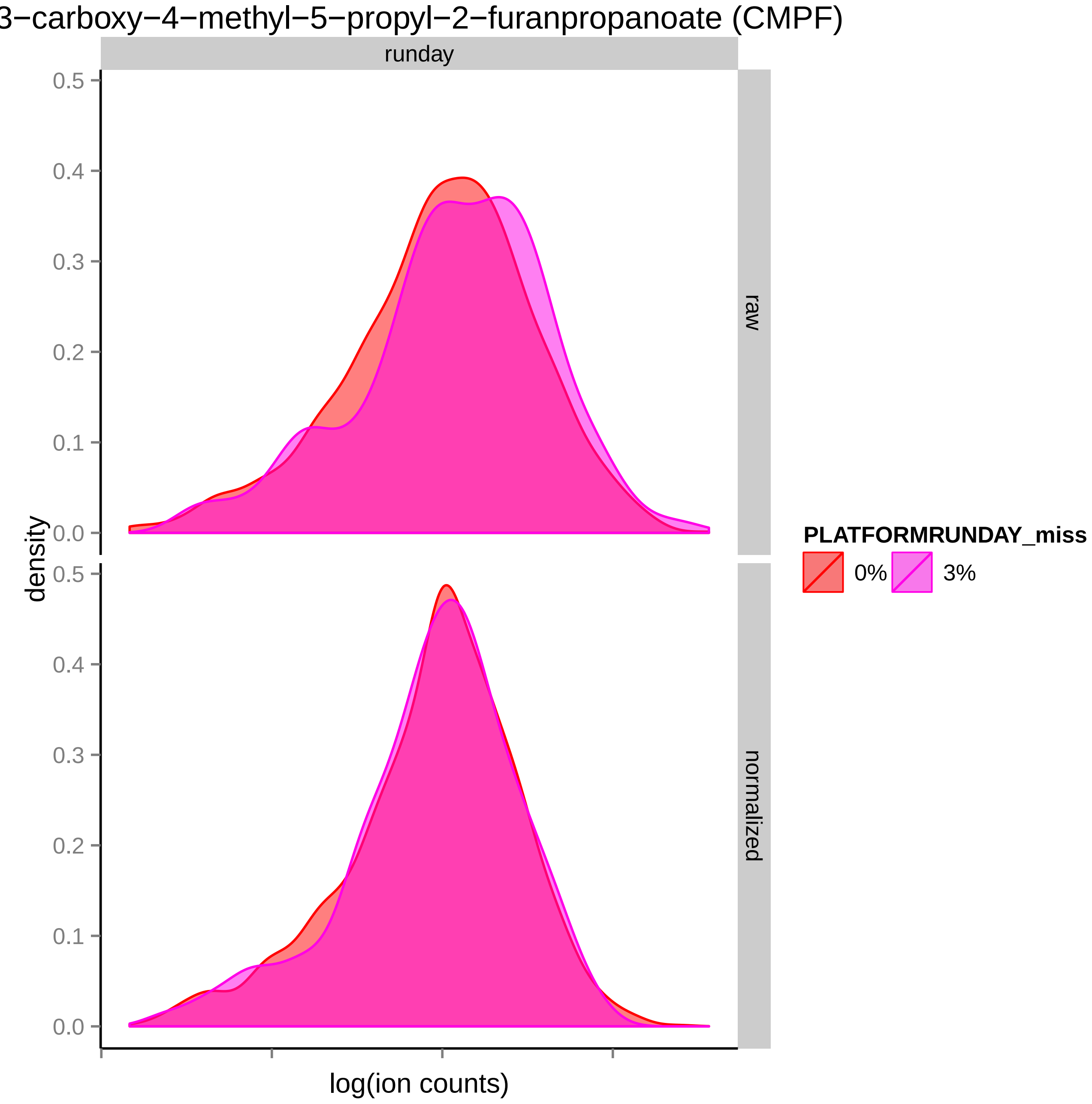

3−carboxy−4−methyl−5−propyl−2−furanpropanoate (CMPF)
runday
raw
normalized
density
0.0
0.1
0.2
0.3
0.4
0.5
log(ion counts)
PLATFORMRUNDAY_miss
0%
3%

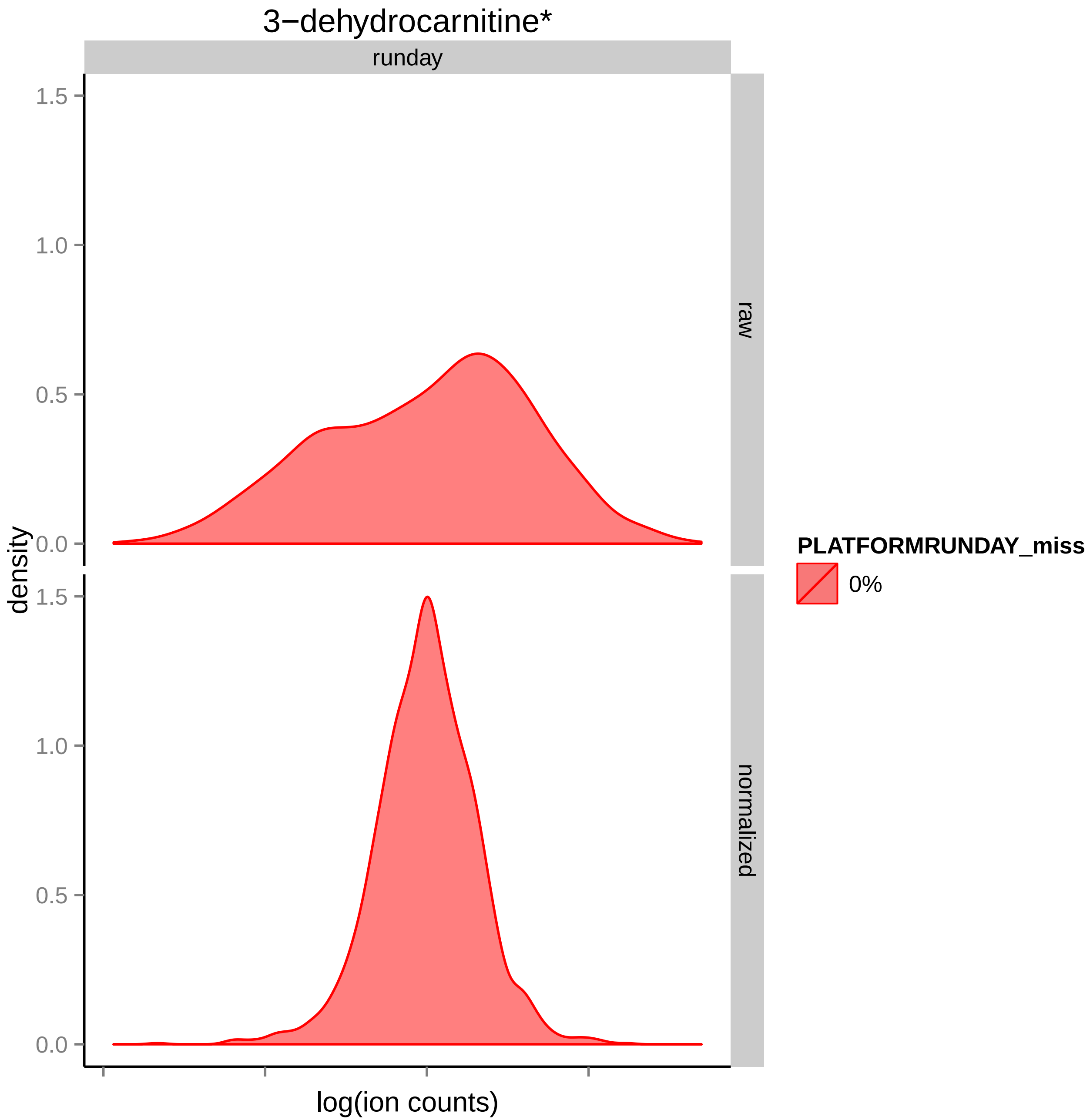

3−dehydrocarnitine*
runday
raw
normalized
density
1.5
1.0
0.5
0.0
log(ion counts)
PLATFORMRUNDAY_miss
0%

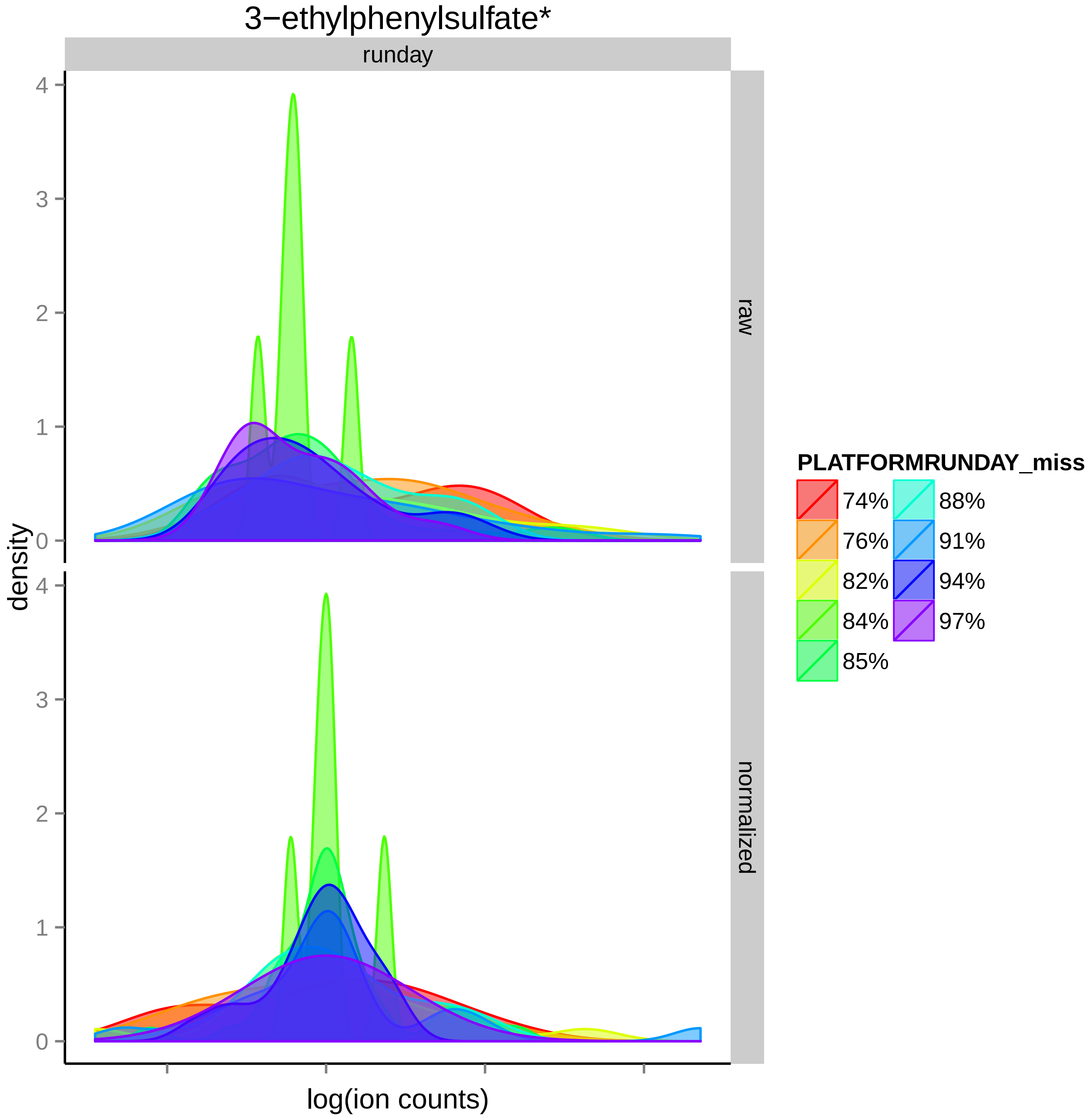
3−ethylphenylsulfate*
runday
raw
normalized
density
log(ion counts)
0
1
2
3
4
PLATFORMRUNDAY_miss
74%
76%
82%
84%
85%
88%
91%
94%
97%

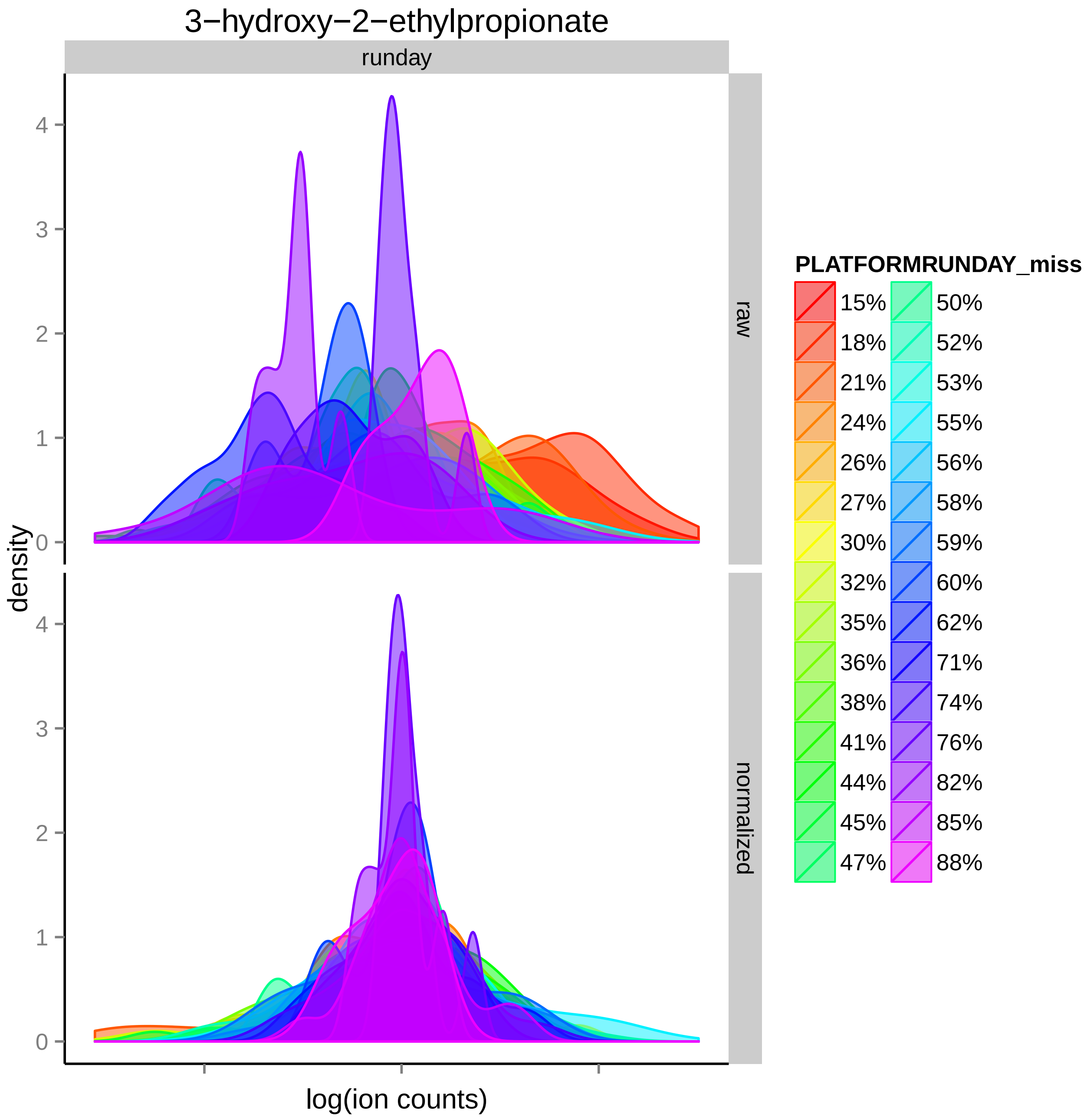

3−hydroxy−2−ethylpropionate
runday
raw
normalized
density
log(ion counts)
0
1
2
3
4
PLATFORMRUNDAY_miss
15%
18%
21%
24%
26%
27%
30%
32%
35%
36%
38%
41%
44%
45%
47%
50%
52%
53%
55%
56%
58%
59%
60%
62%
71%
74%
76%
82%
85%
88%

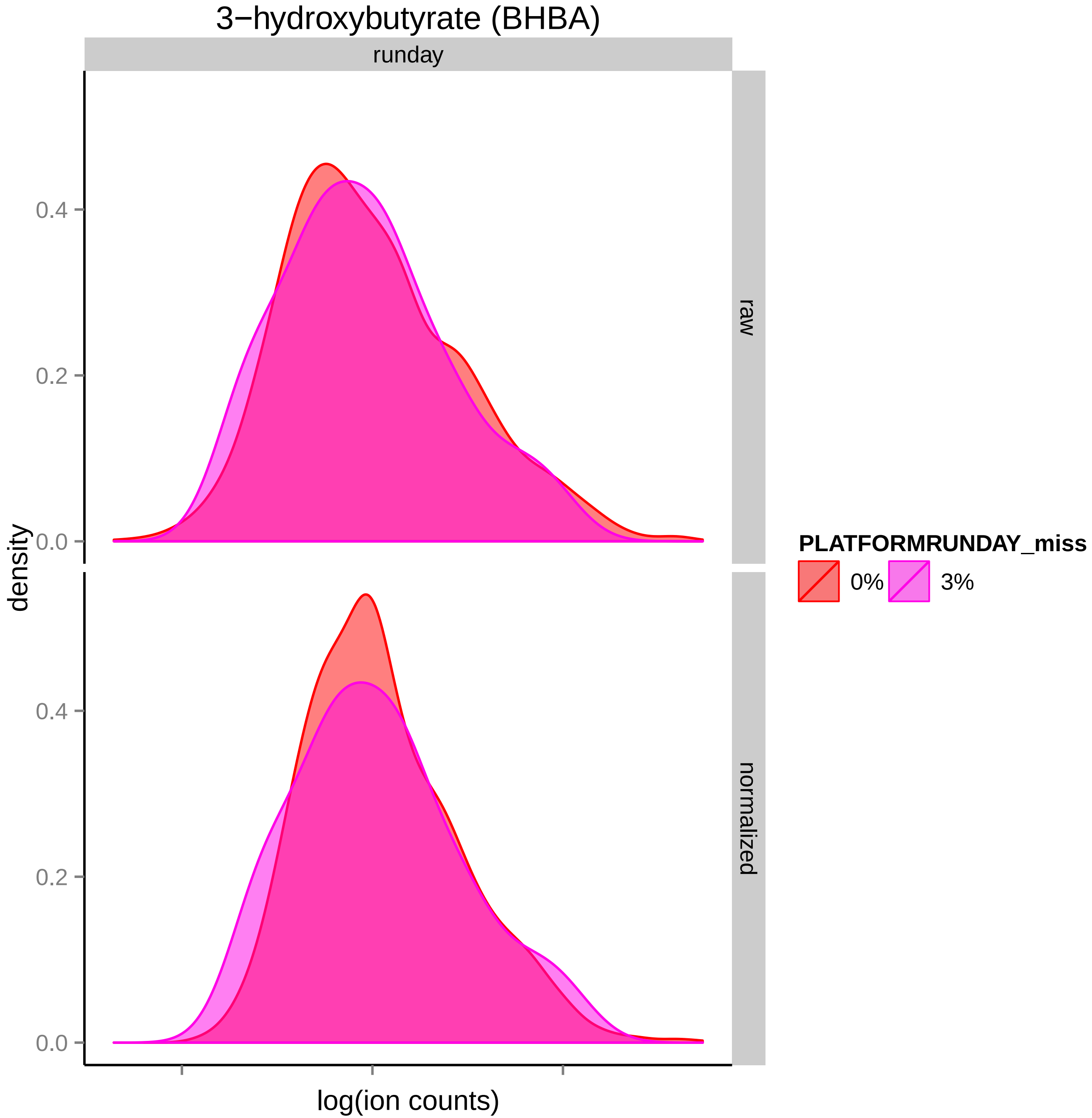
3−hydroxybutyrate (BHBA)
runday
raw
normalized
density
0.0
0.2
0.4
log(ion counts)
PLATFORMRUNDAY_miss
0%
3%

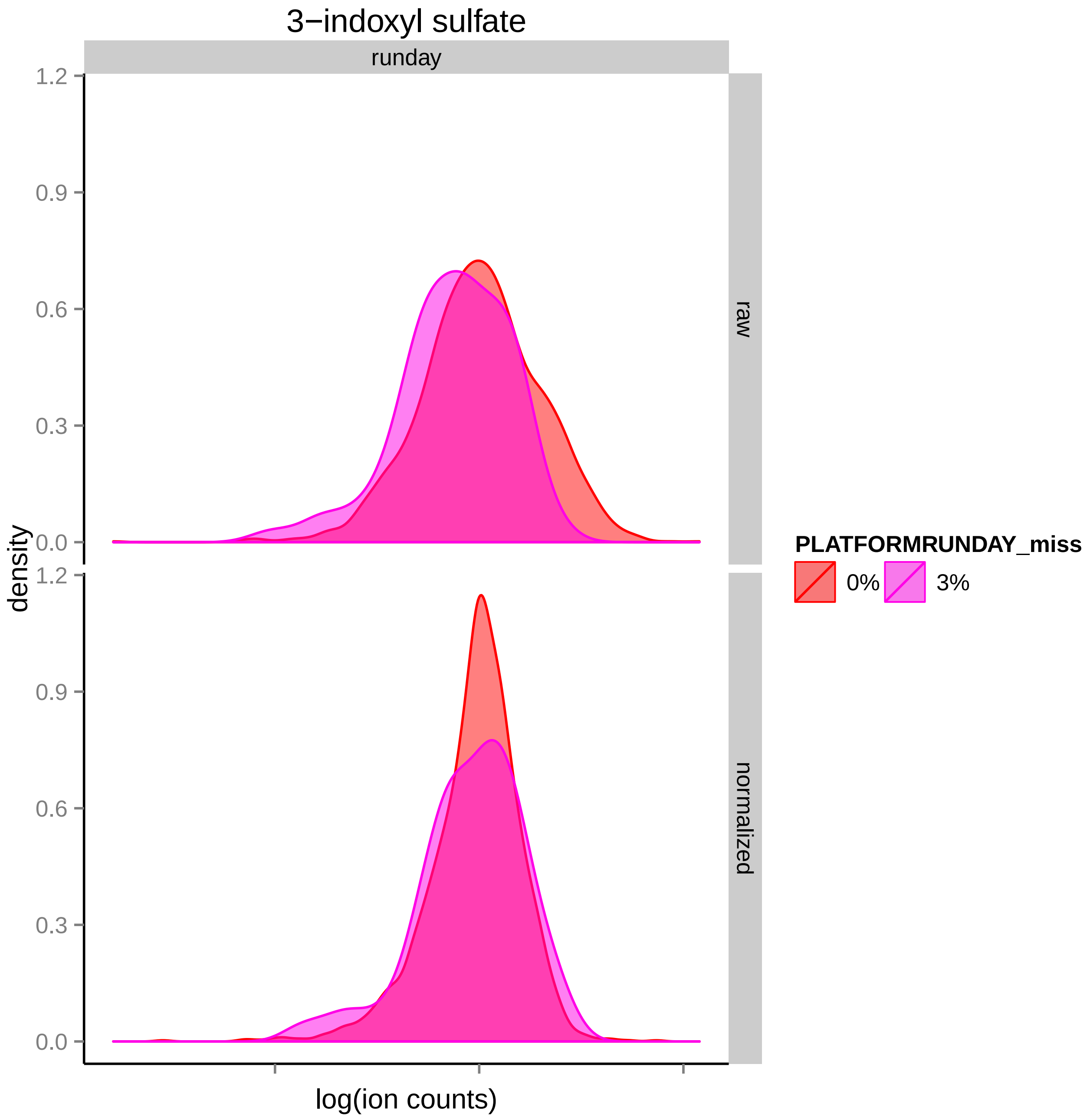
3−indoxyl sulfate
runday
raw
normalized
density
1.2
0.9
0.6
0.3
0.0
log(ion counts)
PLATFORMRUNDAY_miss
0%
3%

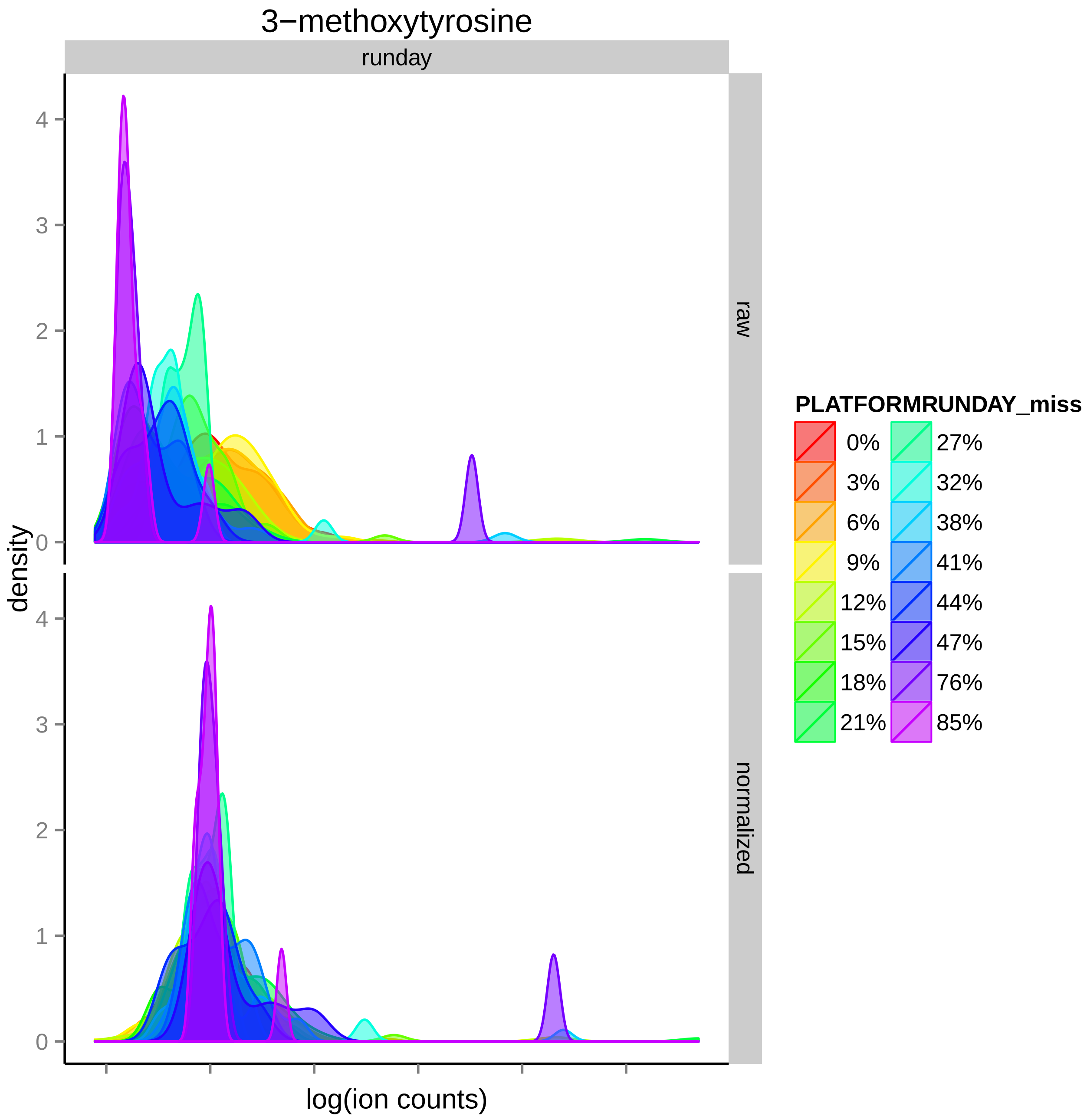

3−methoxytyrosine
runday
raw
normalized
density
log(ion counts)
0
1
2
3
4
PLATFORMRUNDAY_miss
0%
3%
6%
9%
12%
15%
18%
21%
27%
32%
38%
41%
44%
47%
76%
85%

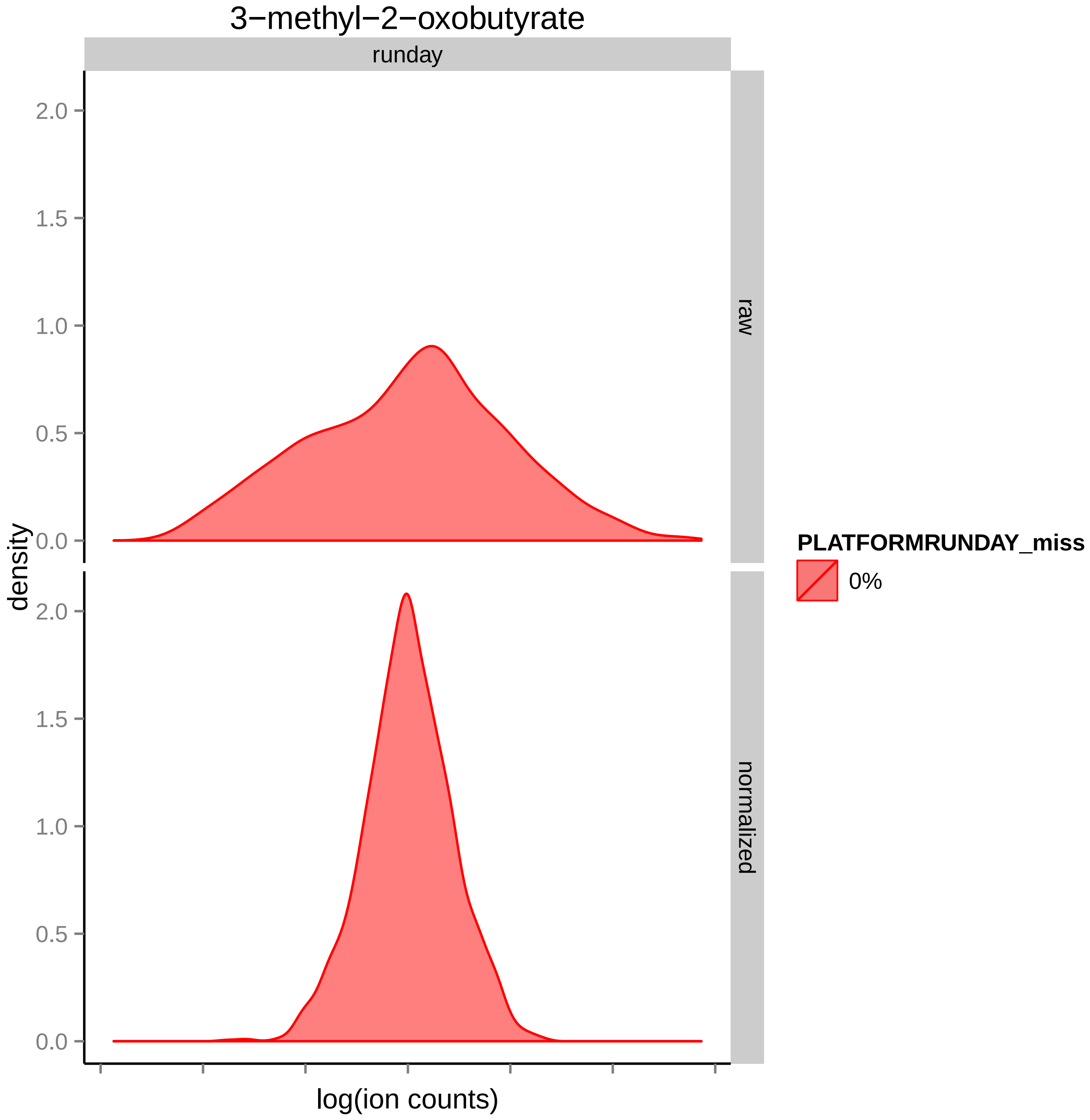
3−methyl−2−oxobutyrate
runday
raw
normalized
density
log(ion counts)
2.0
1.5
1.0
0.5
0.0
PLATFORMRUNDAY_miss
0%

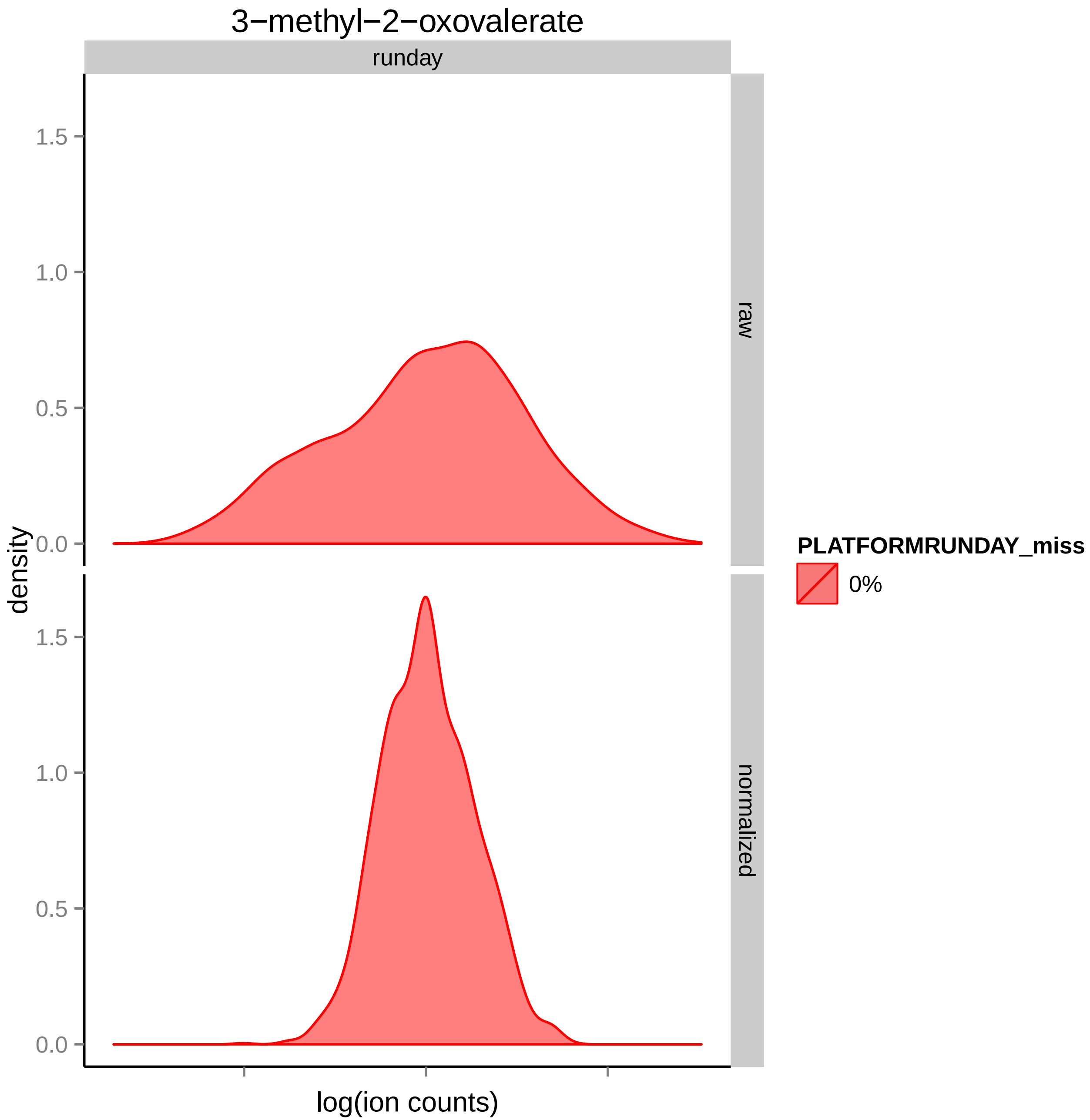
3−methyl−2−oxovalerate
runday
raw
normalized
density
1.5
1.0
0.5
0.0
1.5
1.0
0.5
0.0
log(ion counts)
PLATFORMRUNDAY_miss
0%

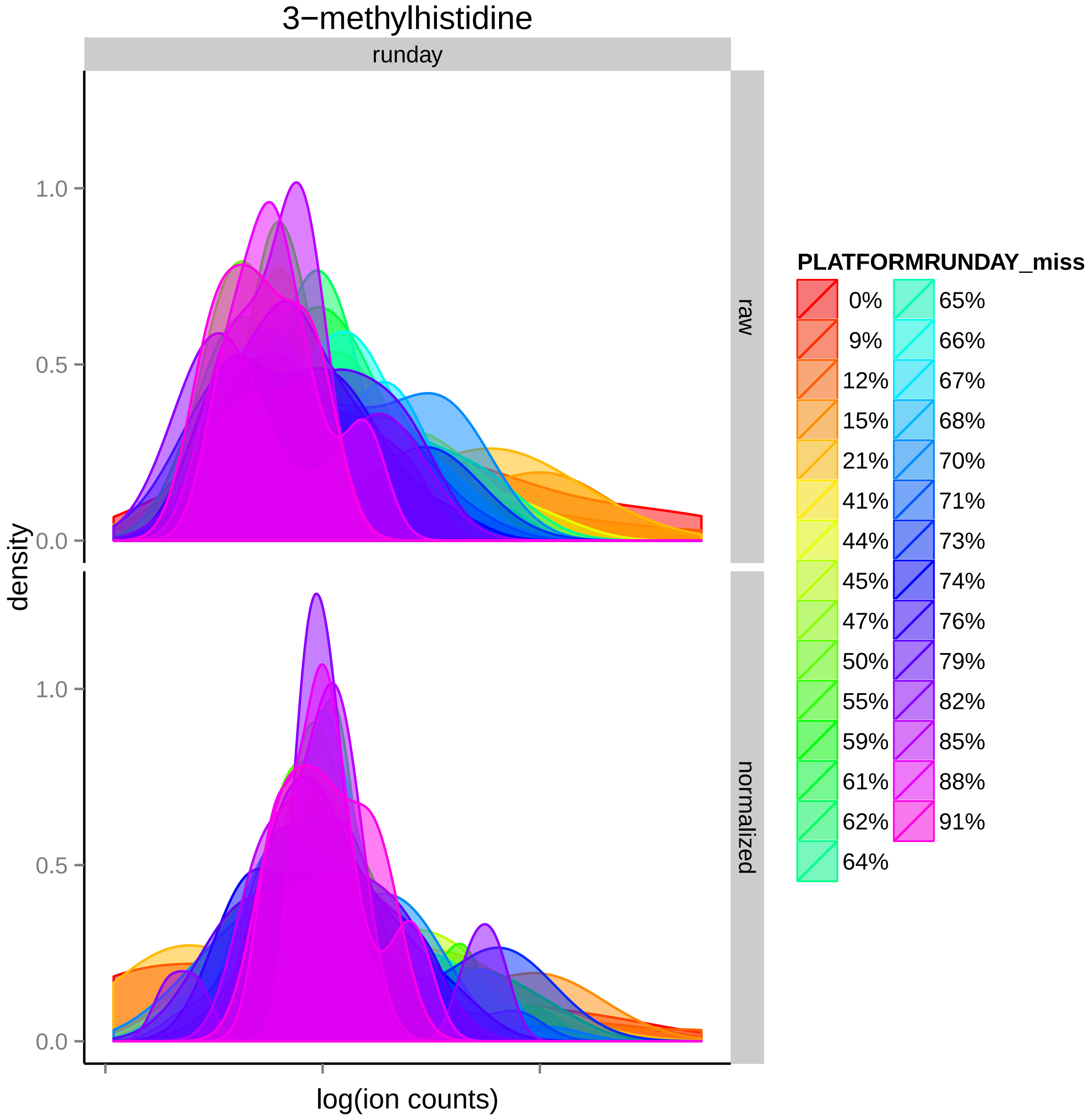
3−methylhistidine
runday
raw
normalized
density
log(ion counts)
1.0
0.5
0.0
1.0
0.5
0.0
PLATFORMRUNDAY_miss
0%
9%
12%
15%
21%
41%
44%
45%
47%
50%
55%
59%
61%
62%
64%
65%
66%
67%
68%
70%
71%
73%
74%
76%
79%
82%
85%
88%
91%

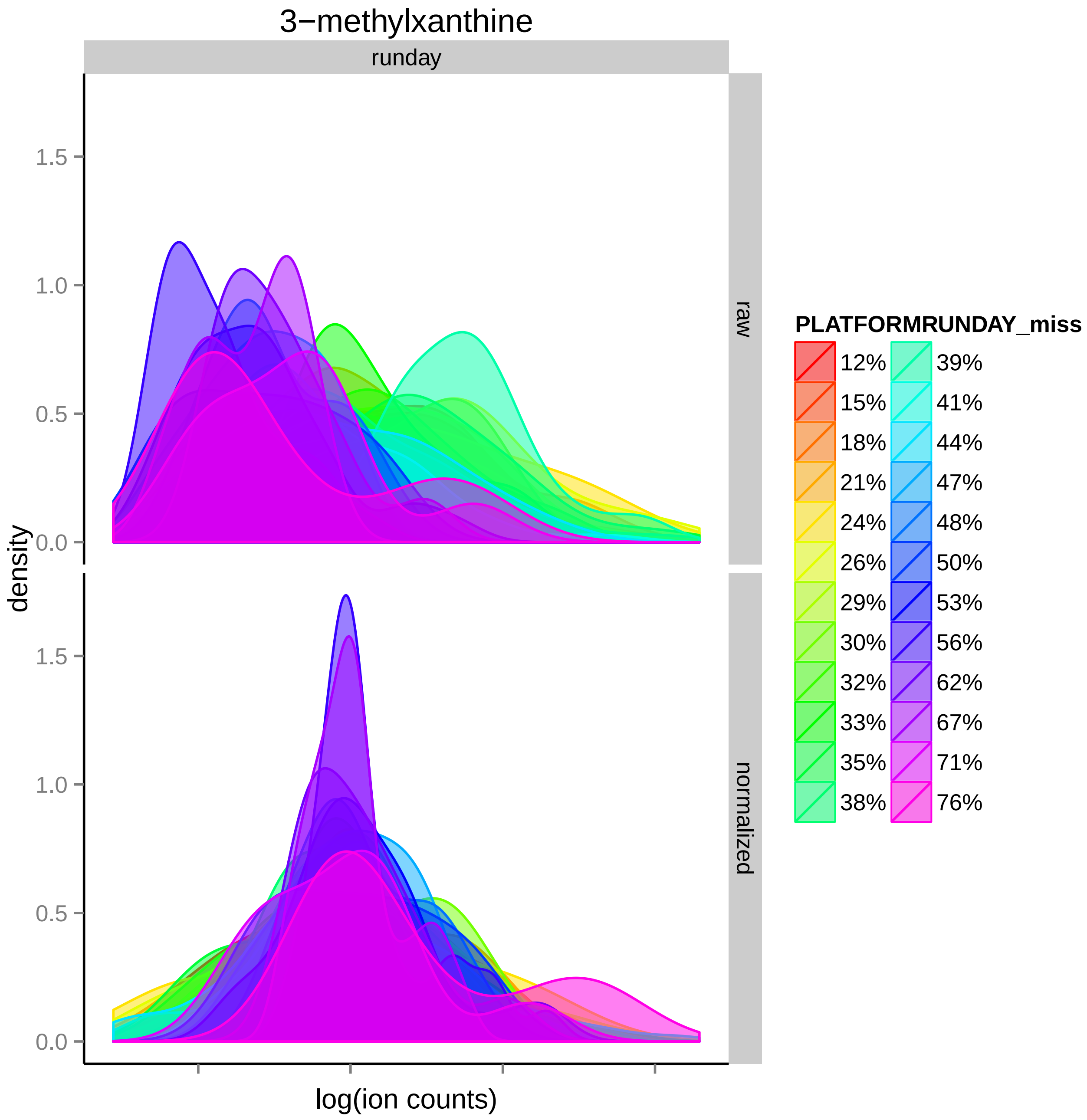

3−methylxanthine
runday
raw
normalized
density
log(ion counts)
0.0
0.5
1.0
1.5
PLATFORMRUNDAY_miss
12%
15%
18%
21%
24%
26%
29%
30%
32%
33%
35%
38%
39%
41%
44%
47%
48%
50%
53%
56%
62%
67%
71%
76%

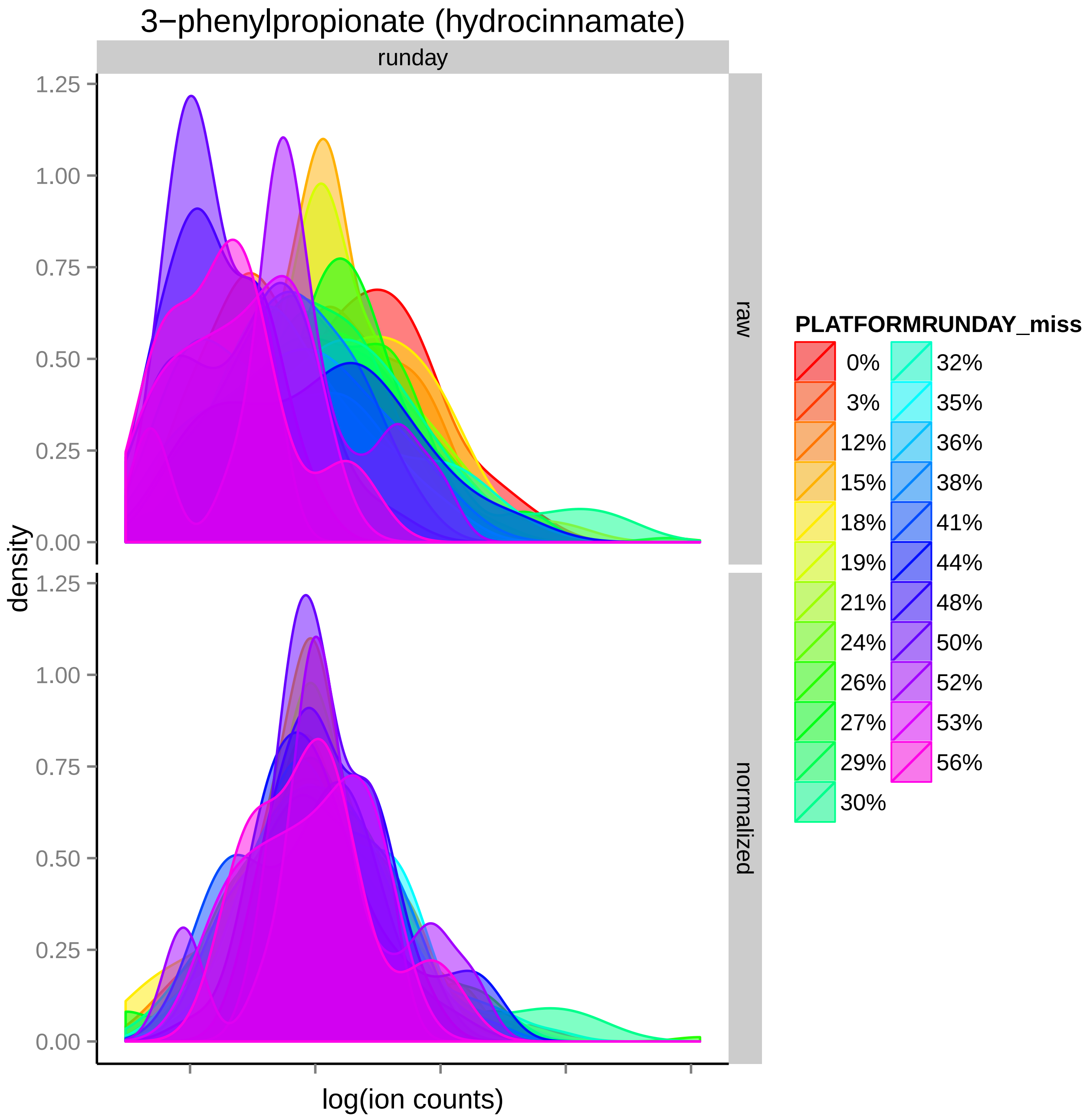

3−phenylpropionate (hydrocinnamate)
runday
raw
normalized
density
log(ion counts)
1.25
1.00
0.75
0.50
0.25
0.00
PLATFORMRUNDAY_miss
0%
3%
12%
15%
18%
19%
21%
24%
26%
27%
29%
30%
32%
35%
36%
38%
41%
44%
48%
50%
52%
53%
56%

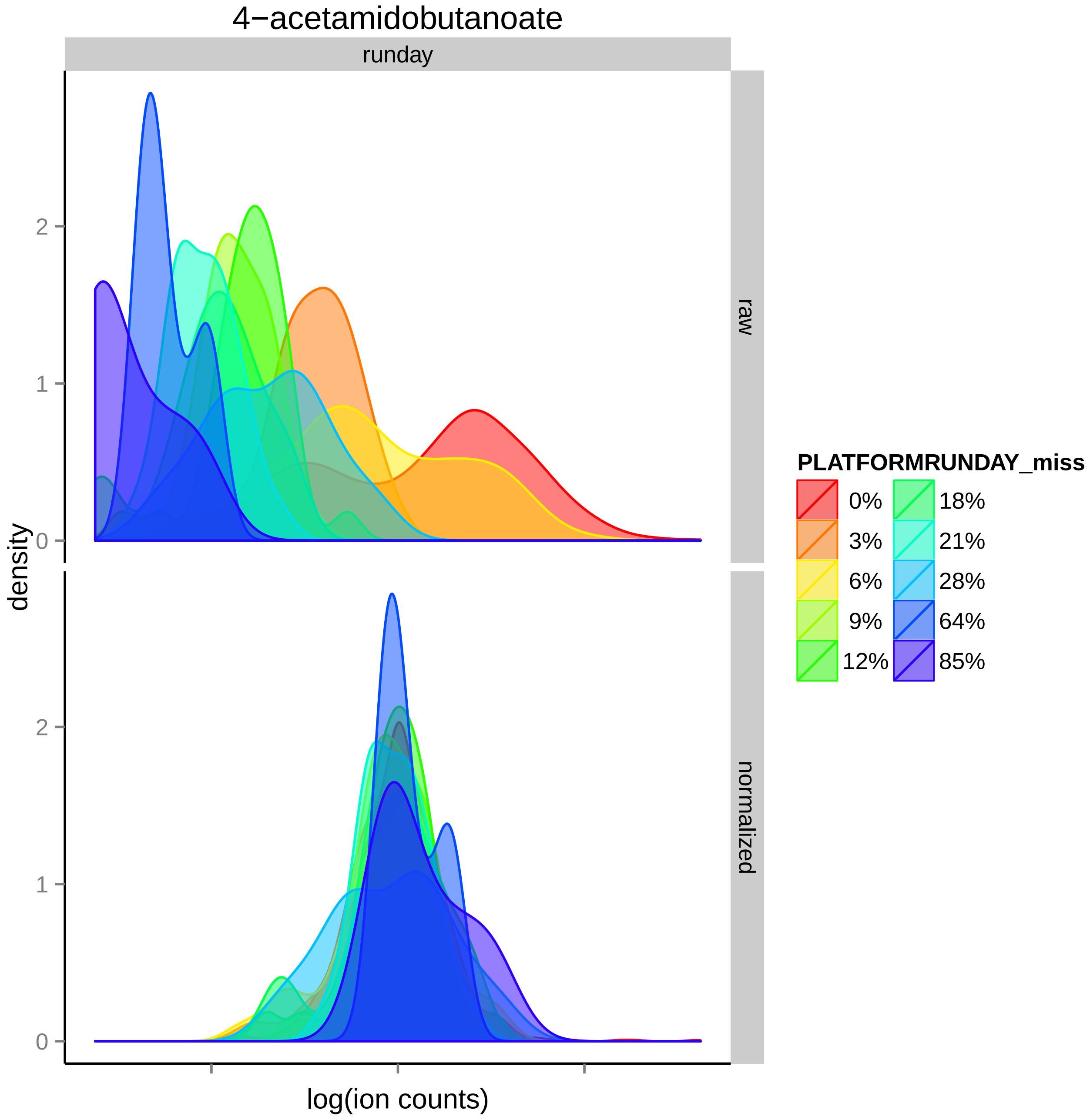
4−acetamidobutanoate
runday
raw
normalized
density
log(ion counts)
0
1
2
PLATFORMRUNDAY_miss
0%
3%
6%
9%
12%
18%
21%
28%
64%
85%

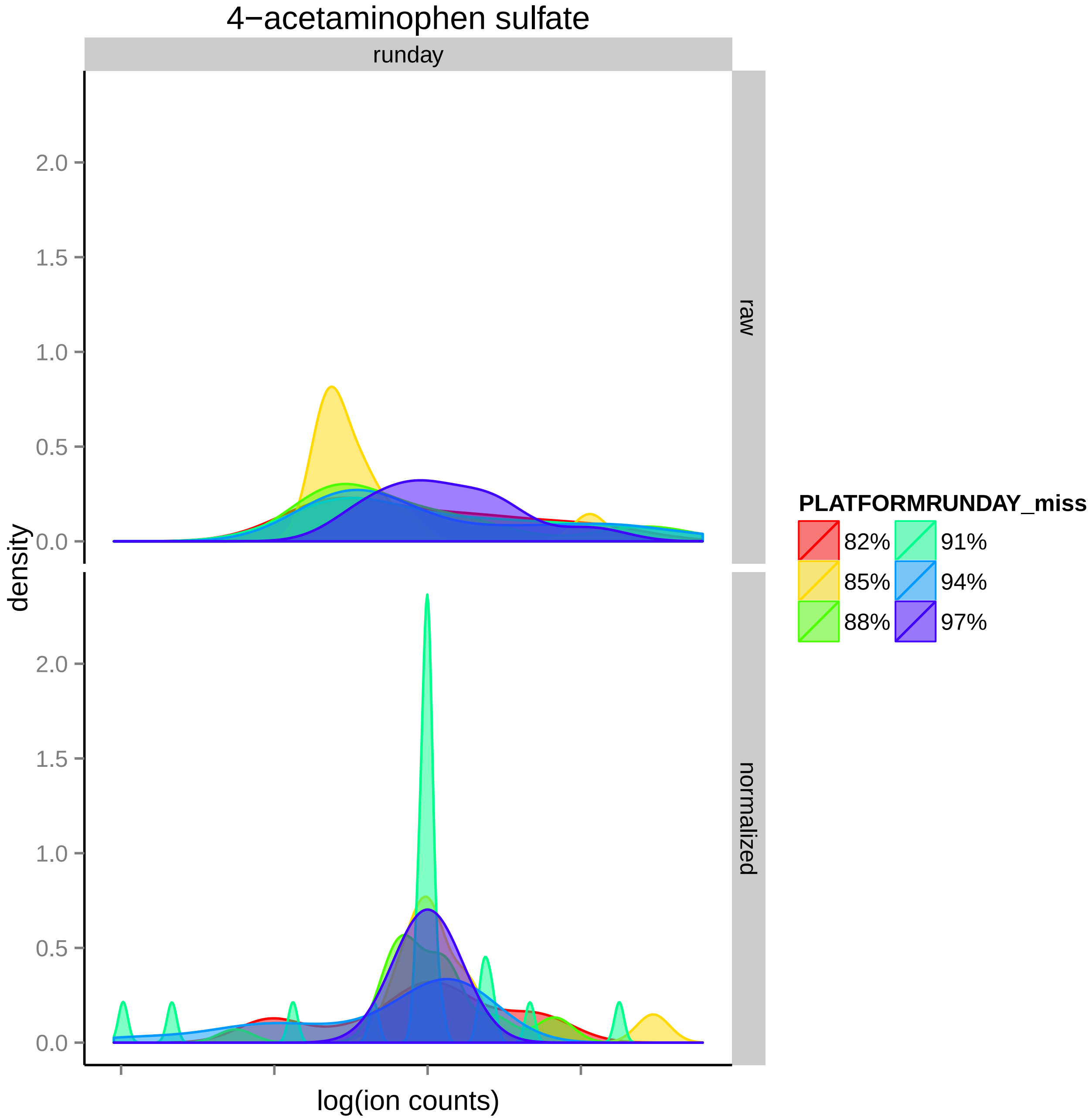

4−acetaminophen sulfate
runday
raw
normalized
density
2.0
1.5
1.0
0.5
0.0
log(ion counts)
PLATFORMRUNDAY_miss
82%
85%
88%
91%
94%
97%

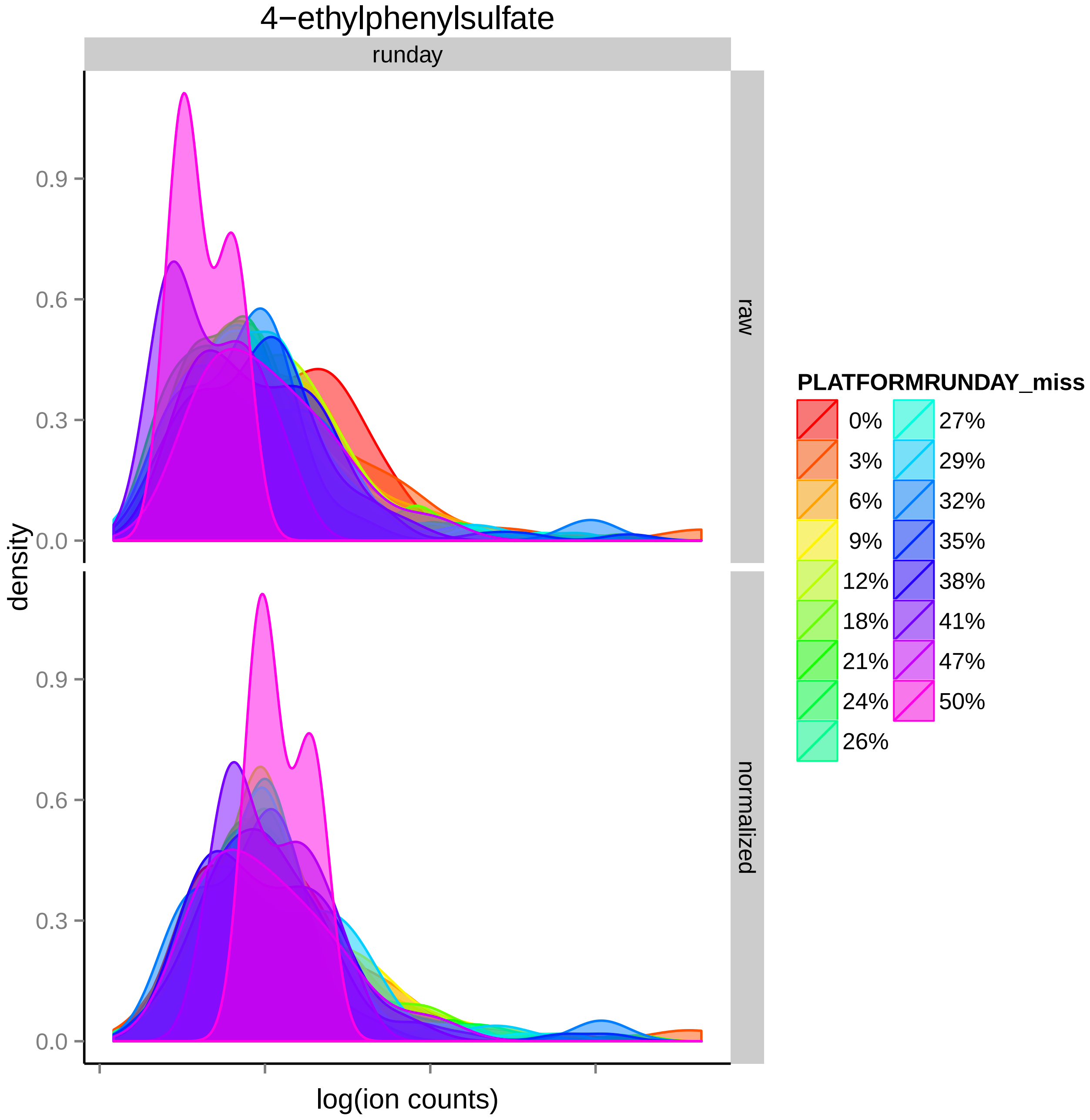
4−ethylphenylsulfate
runday
raw
normalized
density
log(ion counts)
0.0
0.3
0.6
0.9
PLATFORMRUNDAY_miss
0%
3%
6%
9%
12%
18%
21%
24%
26%
27%
29%
32%
35%
38%
41%
47%
50%

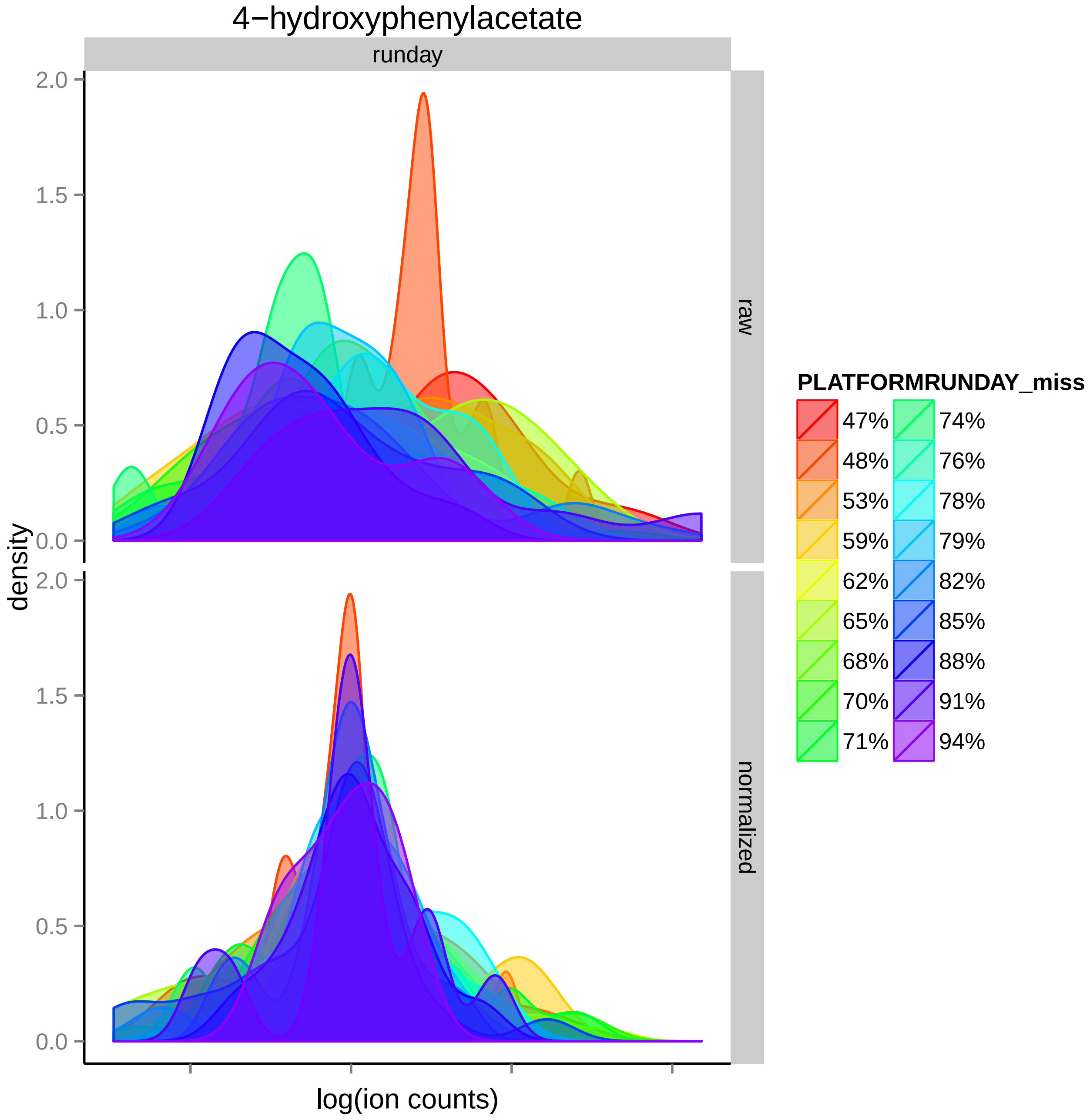
4−hydroxyphenylacetate
runday
raw
normalized
density
log(ion counts)
2.0
1.5
1.0
0.5
0.0
PLATFORMRUNDAY_miss
47%
48%
53%
59%
62%
65%
68%
70%
71%
74%
76%
78%
79%
82%
85%
88%
91%
94%

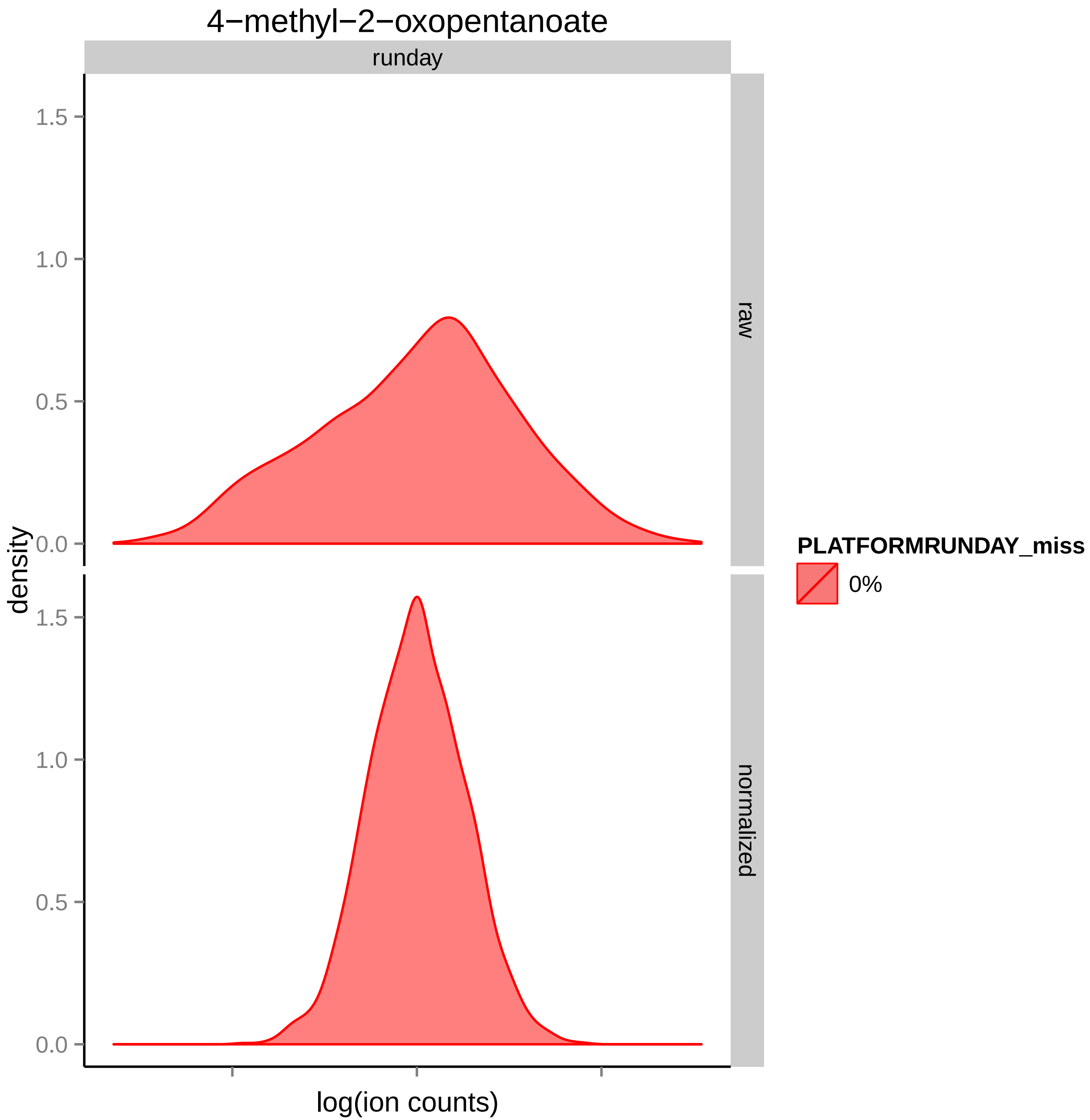

4−methyl−2−oxopentanoate
runday
raw
normalized
density
1.5
1.0
0.5
0.0
log(ion counts)
PLATFORMRUNDAY_miss
0%

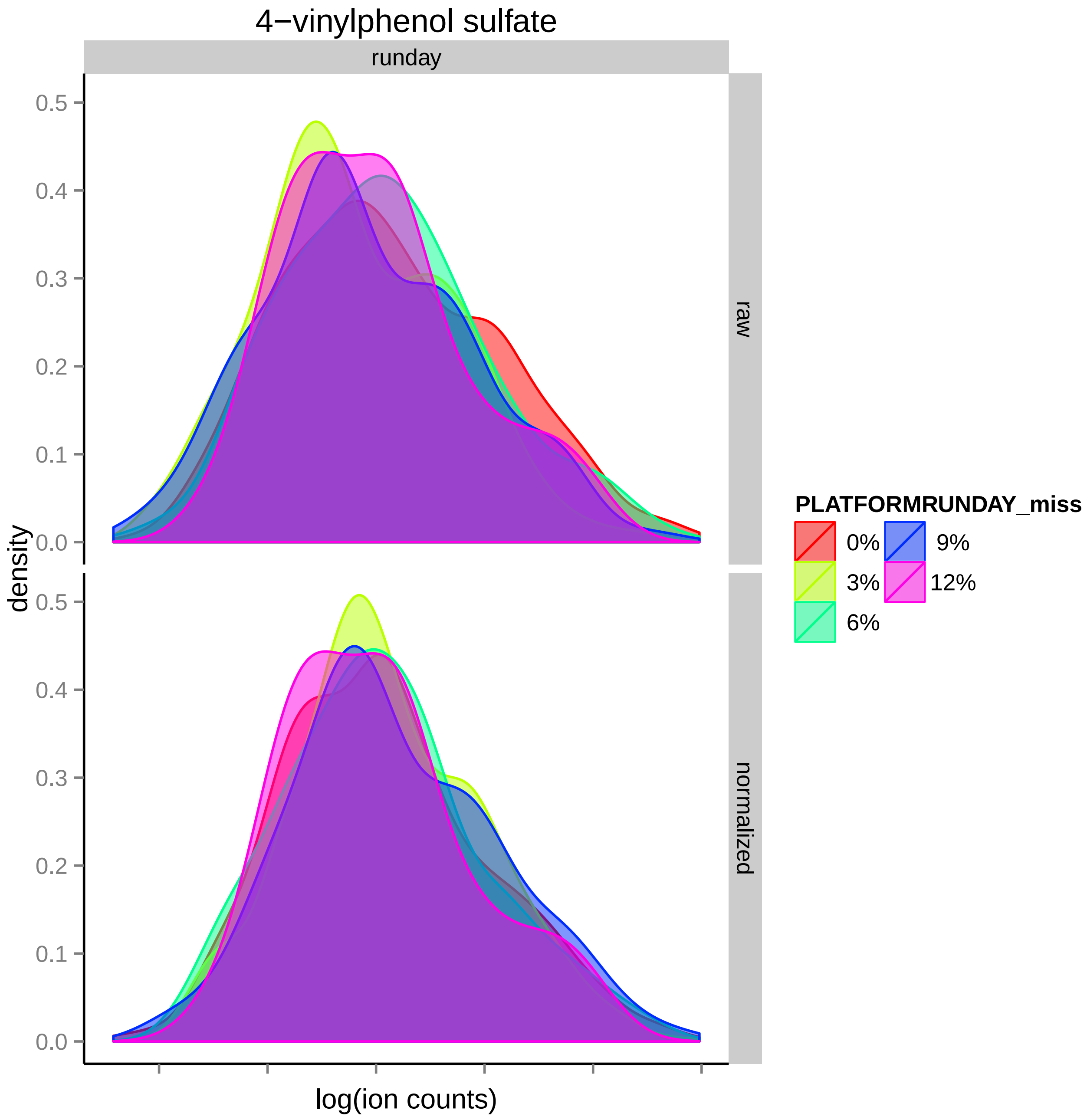

4−vinylphenol sulfate
runday
raw
normalized
density
0.5
0.4
0.3
0.2
0.1
0.0
log(ion counts)
PLATFORMRUNDAY_miss
0%
3%
6%
9%
12%

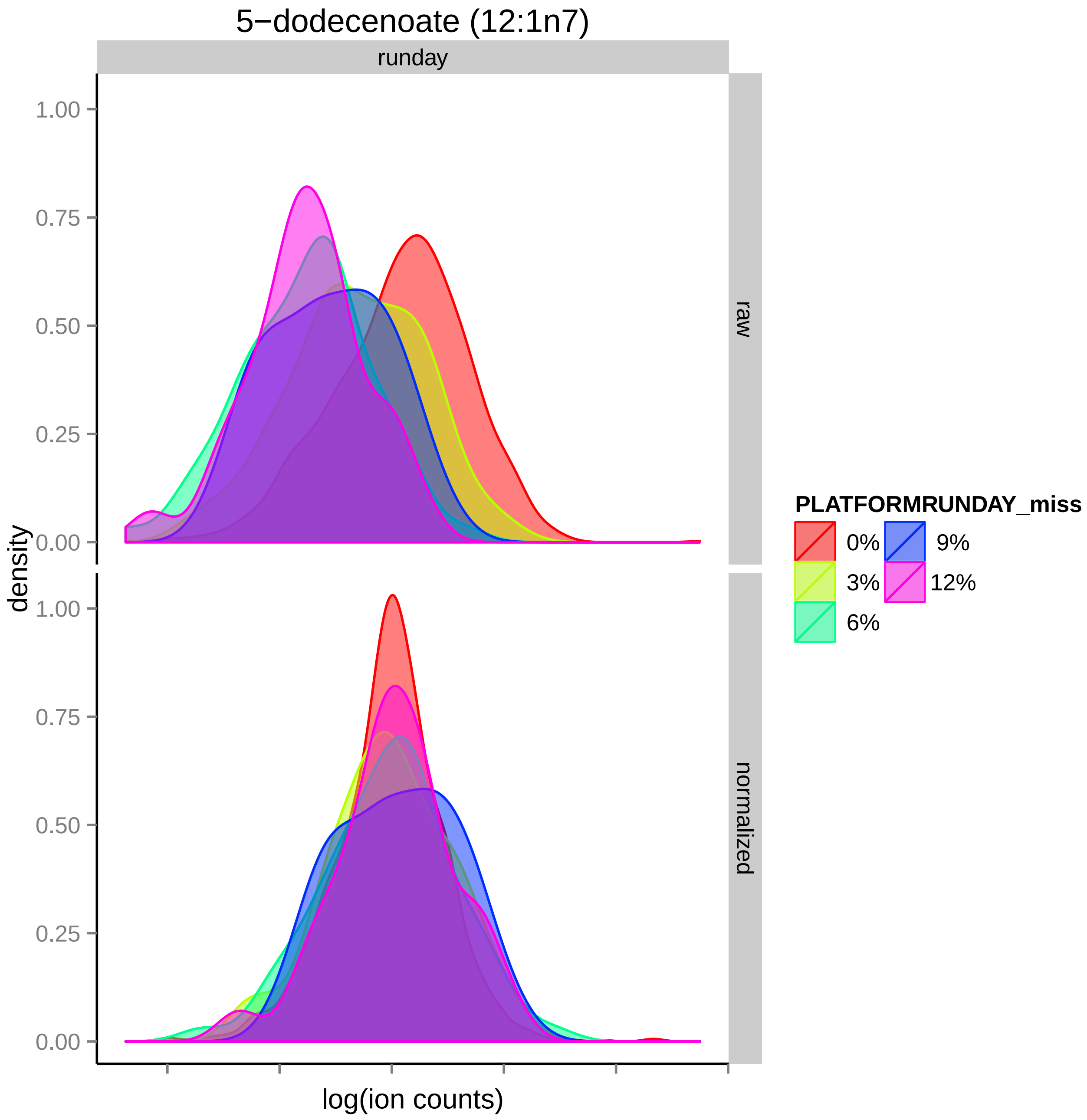
5−dodecenoate (12:1n7)
runday
raw
normalized
density
log(ion counts)
1.00
0.75
0.50
0.25
0.00
PLATFORMRUNDAY_miss
0%
3%
6%
9%
12%

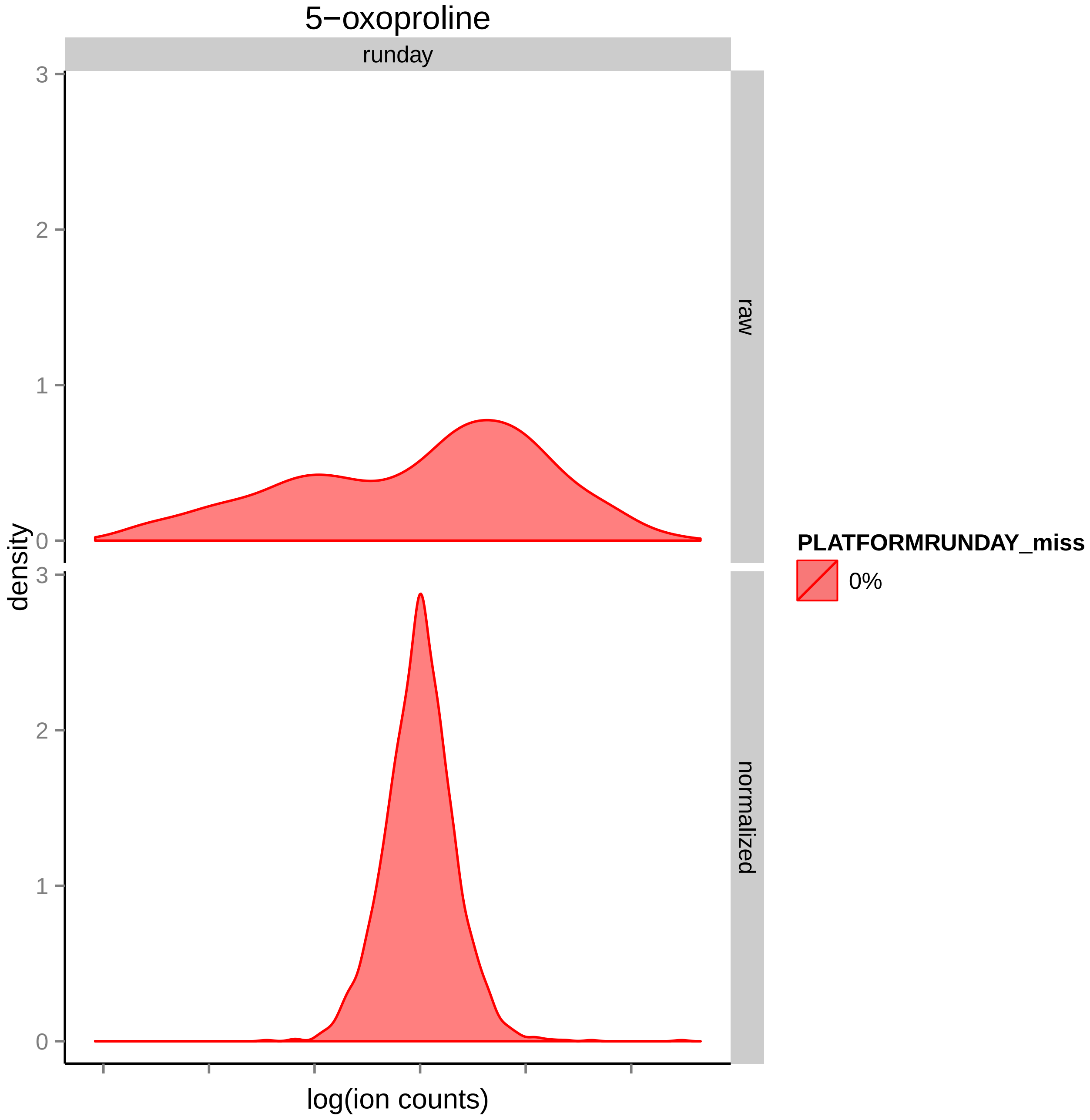

5−oxoproline
runday
raw
normalized
density
log(ion counts)
0
1
2
3
PLATFORMRUNDAY_miss
0%

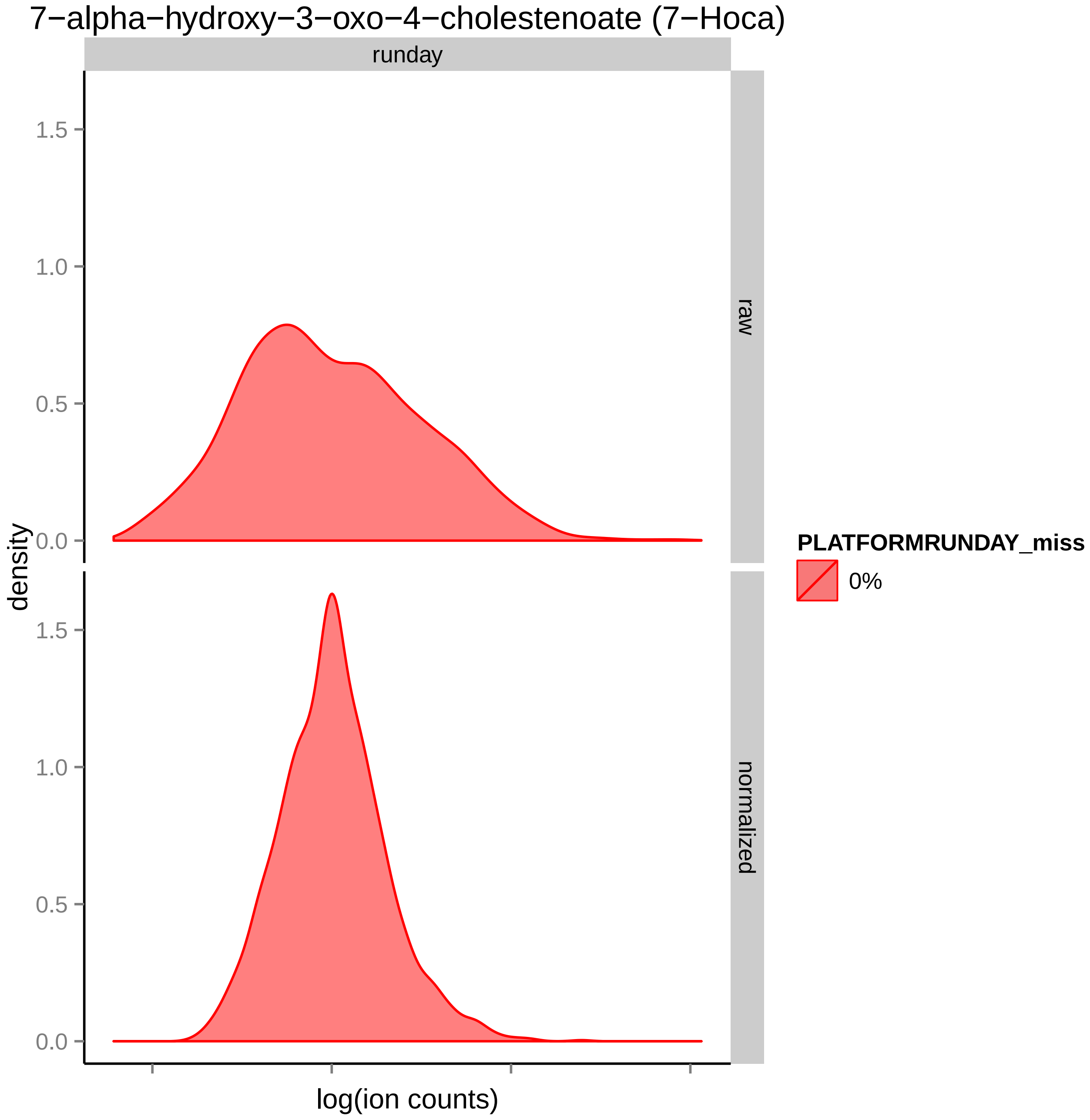

7−alpha−hydroxy−3−oxo−4−cholestenoate (7−Hoca)
runday
raw
normalized
density
1.5
1.0
0.5
0.0
log(ion counts)
PLATFORMRUNDAY_miss
0%

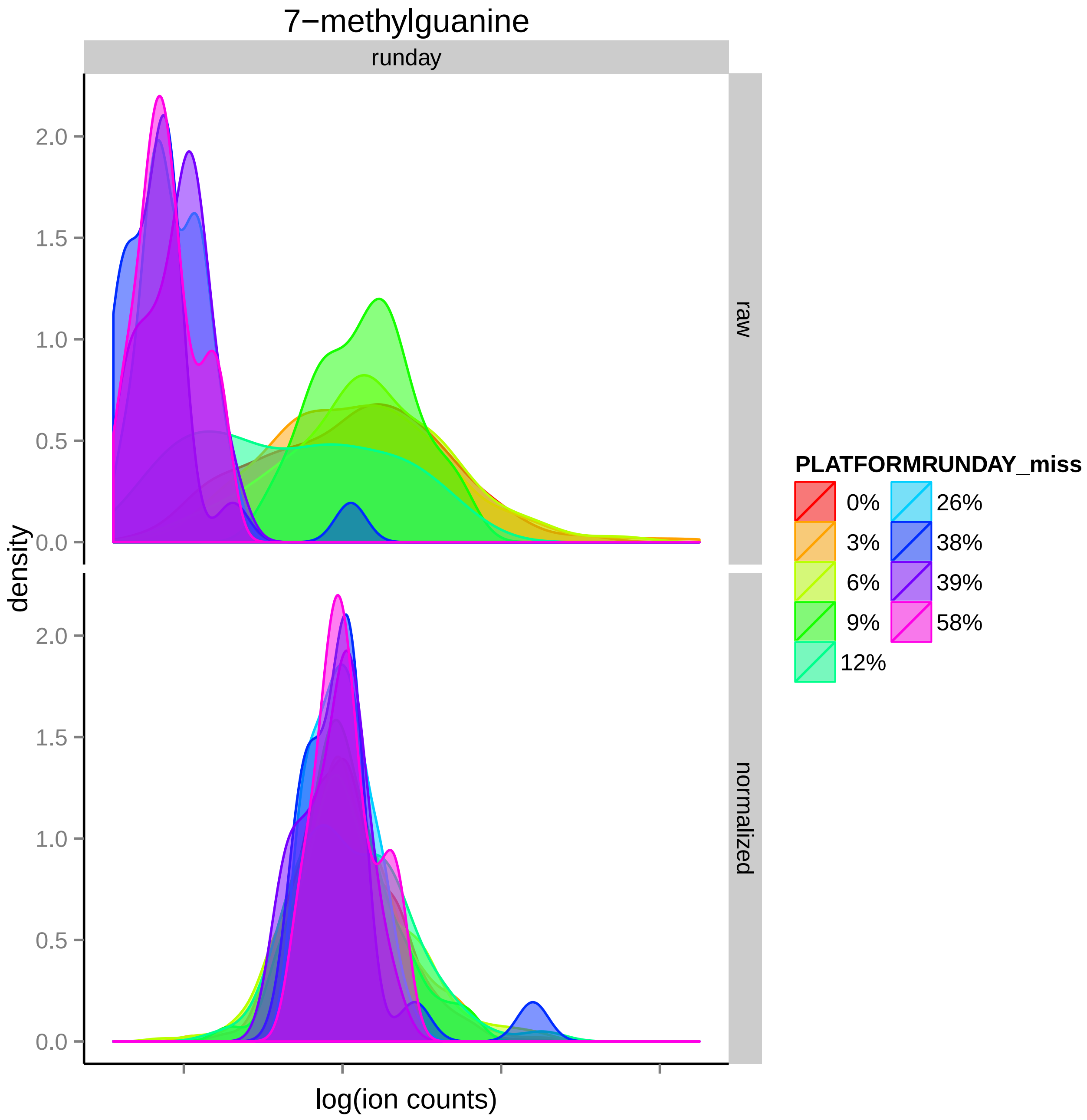

7−methylguanine
runday
raw
normalized
density
log(ion counts)
2.0
1.5
1.0
0.5
0.0
PLATFORMRUNDAY_miss
0%
3%
6%
9%
12%
26%
38%
39%
58%

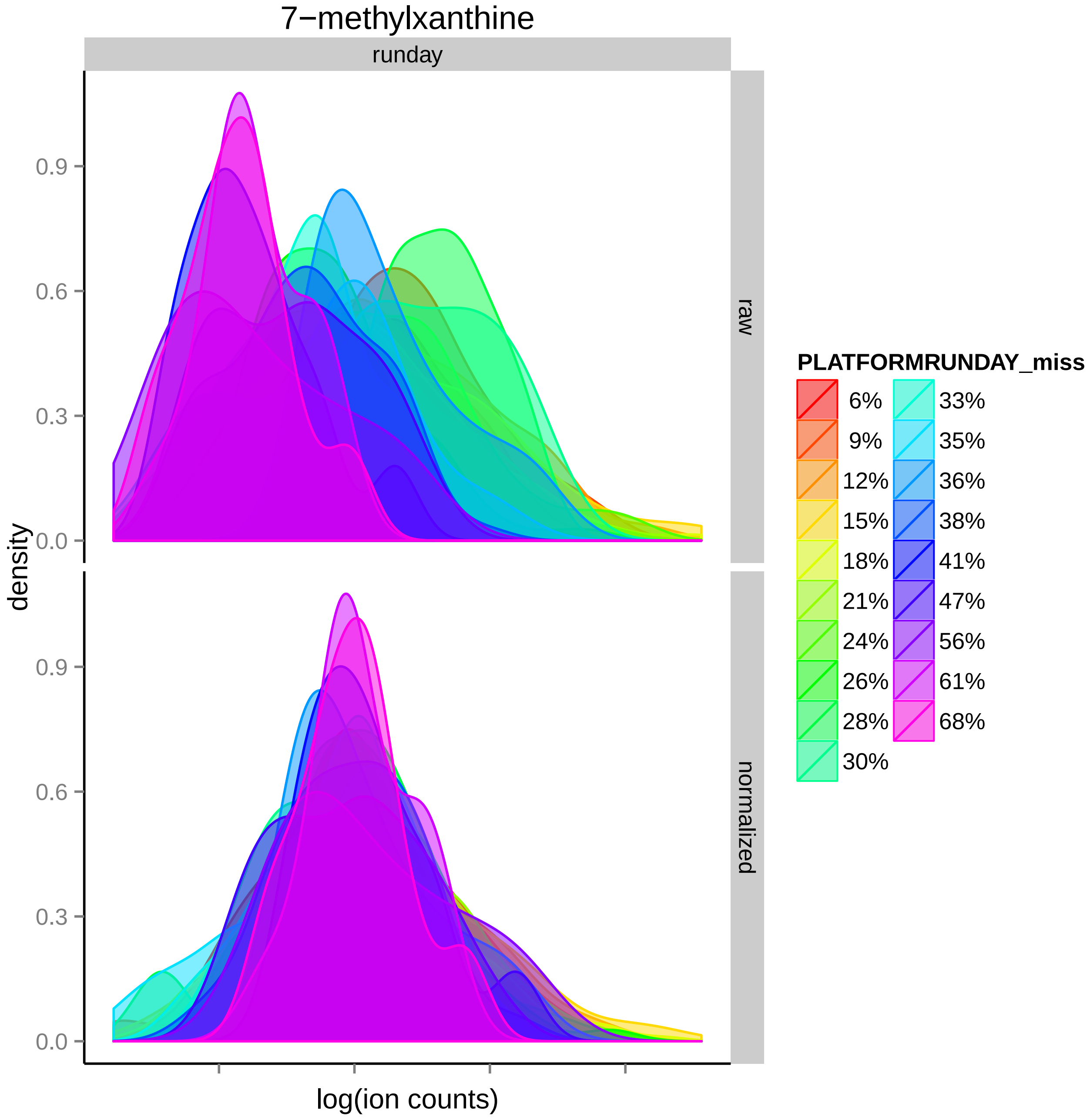
7−methylxanthine
runday
raw
normalized
density
0.0
0.3
0.6
0.9
log(ion counts)
PLATFORMRUNDAY_miss
6%
9%
12%
15%
18%
21%
24%
26%
28%
30%
33%
35%
36%
38%
41%
47%
56%
61%
68%

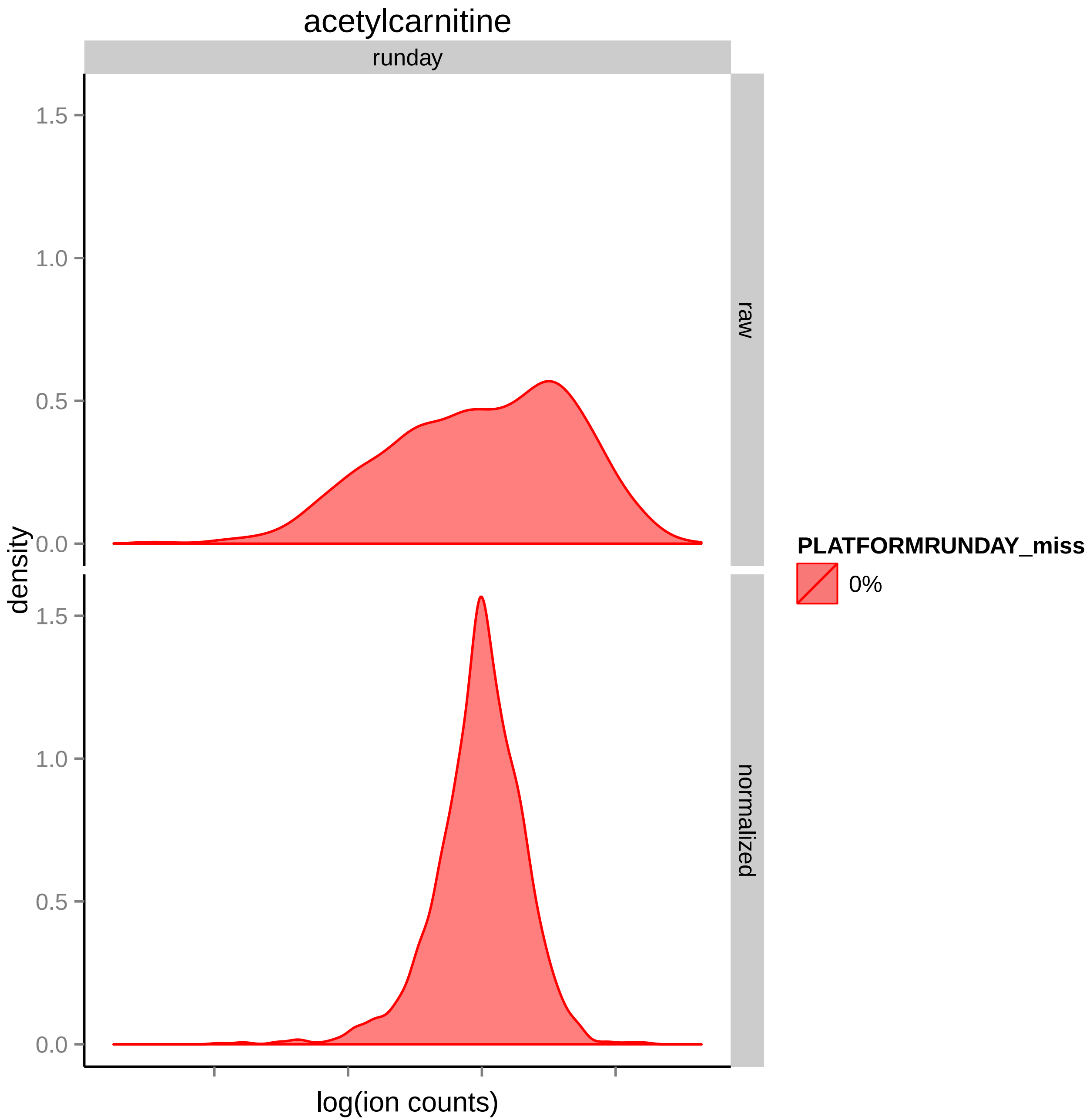
acetylcarnitine
runday
raw
normalized
density
log(ion counts)
0.0
0.5
1.0
1.5
PLATFORMRUNDAY_miss
0%

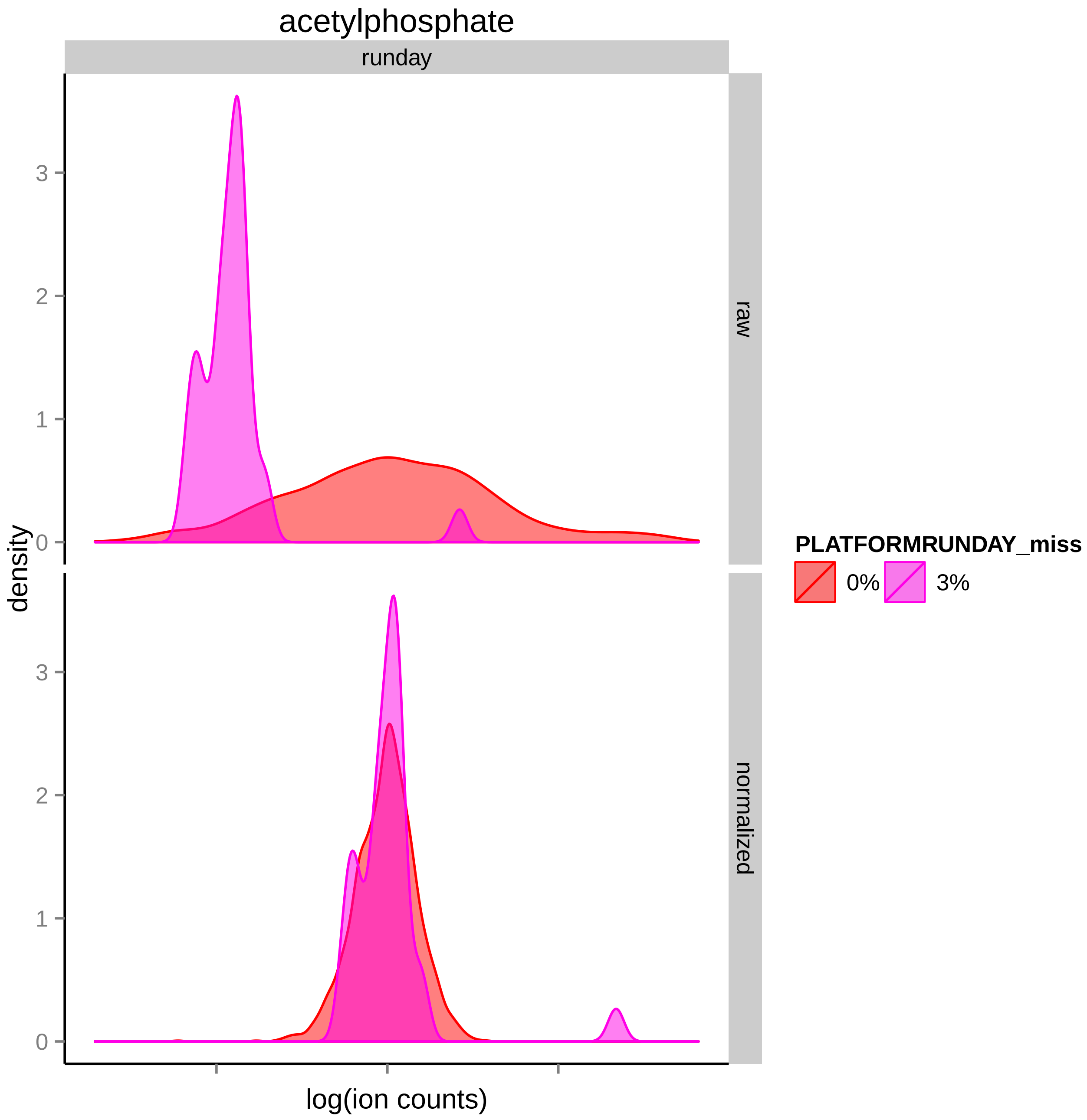

acetylphosphate
runday
raw
normalized
density
log(ion counts)
0
1
2
3
PLATFORMRUNDAY_miss
0%
3%

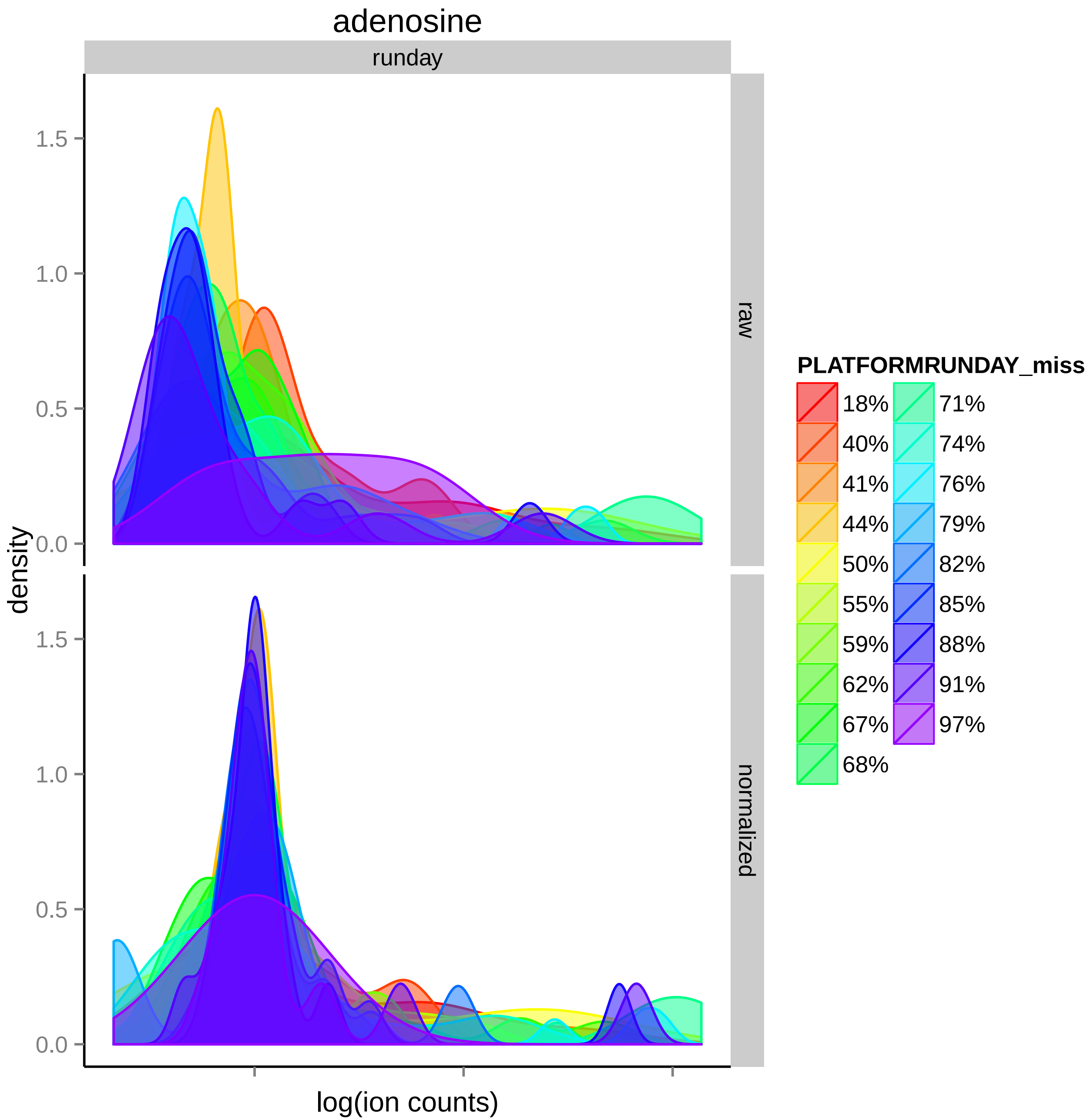

adenosine
runday
raw
normalized
density
log(ion counts)
1.5
1.0
0.5
0.0
PLATFORMRUNDAY_miss
18%
40%
41%
44%
50%
55%
59%
62%
67%
68%
71%
74%
76%
79%
82%
85%
88%
91%
97%

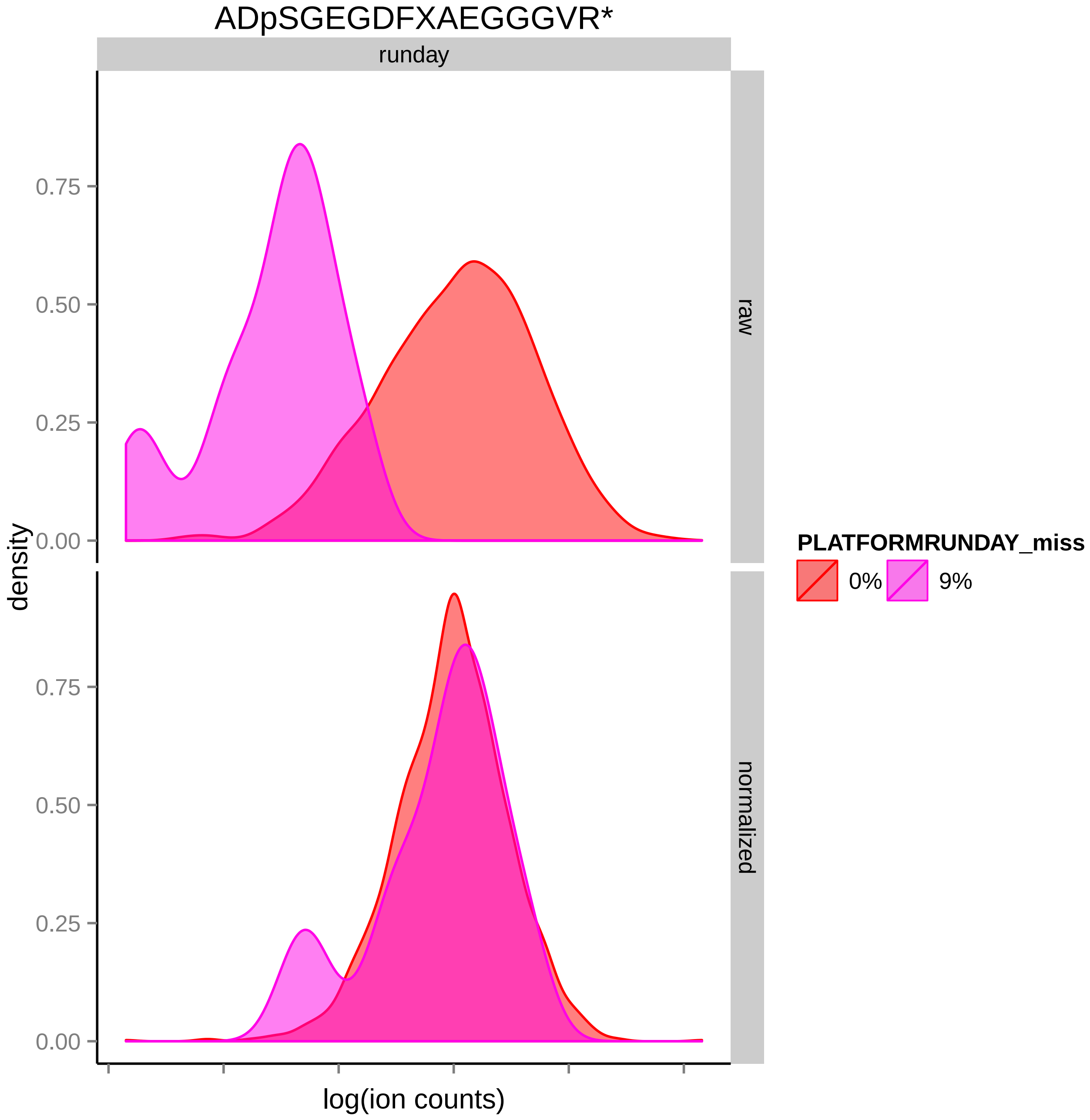

ADpSGEGDFXAEGGGVR*
runday
raw
normalized
density
0.00
0.25
0.50
0.75
0.00
0.25
0.50
0.75
log(ion counts)
PLATFORMRUNDAY_miss
0%
9%

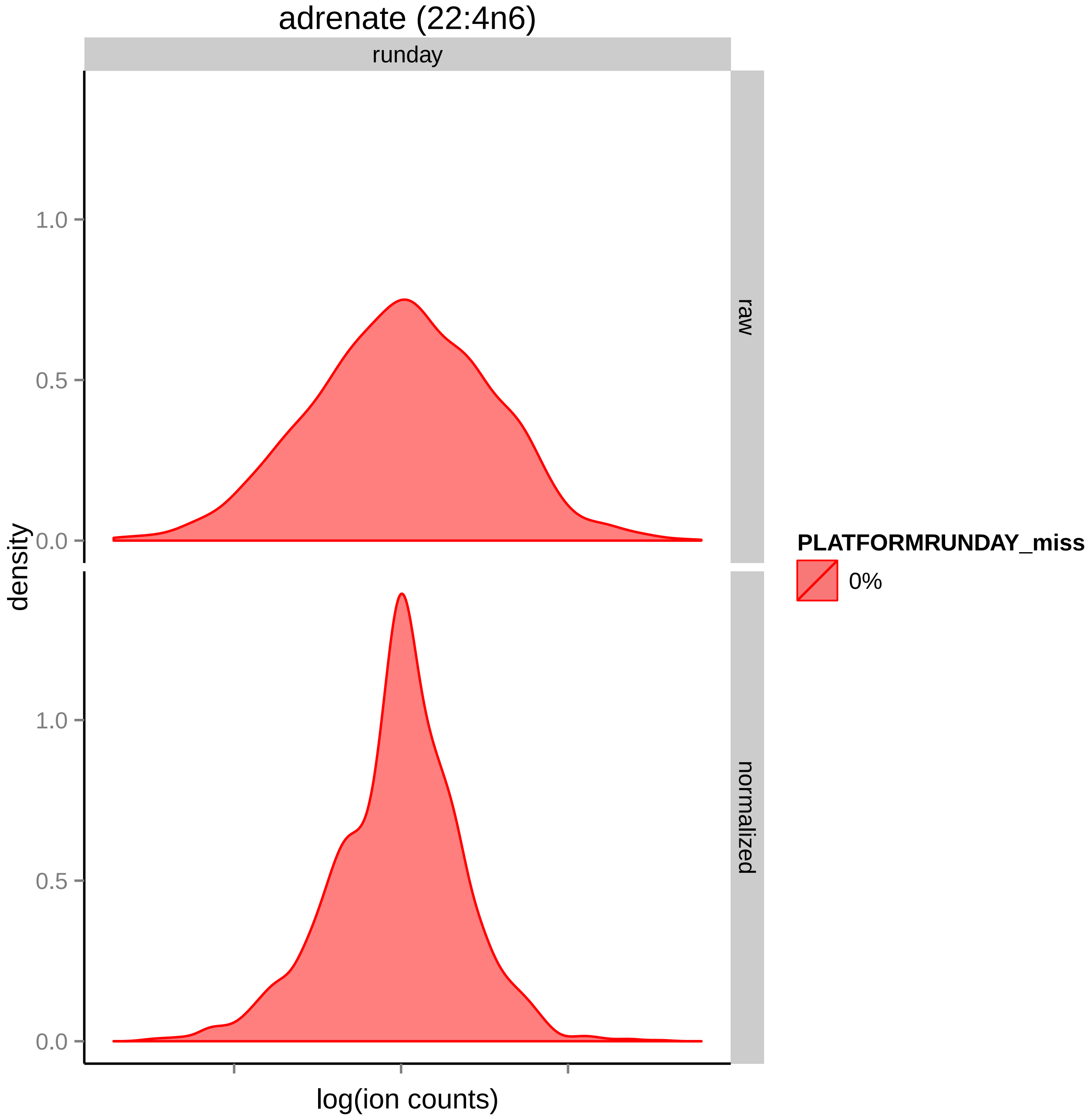

adrenate (22:4n6)
runday
raw
normalized
density
1.0
0.5
0.0
1.0
0.5
0.0
log(ion counts)
PLATFORMRUNDAY_miss
0%

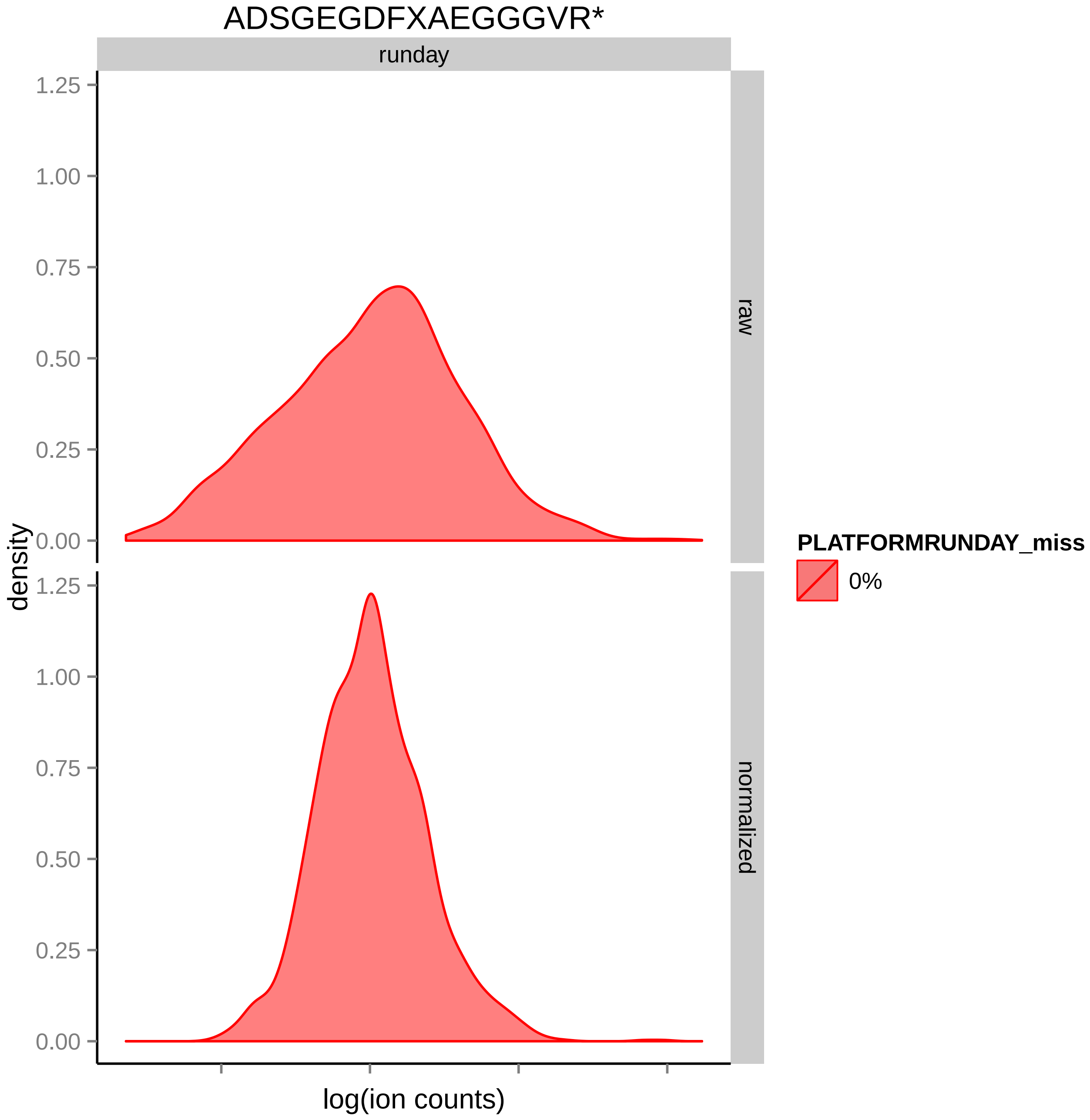

ADSGEGDFXAEGGGVR*
runday
raw
normalized
density
1.25
1.00
0.75
0.50
0.25
0.00
log(ion counts)
PLATFORMRUNDAY_miss
0%

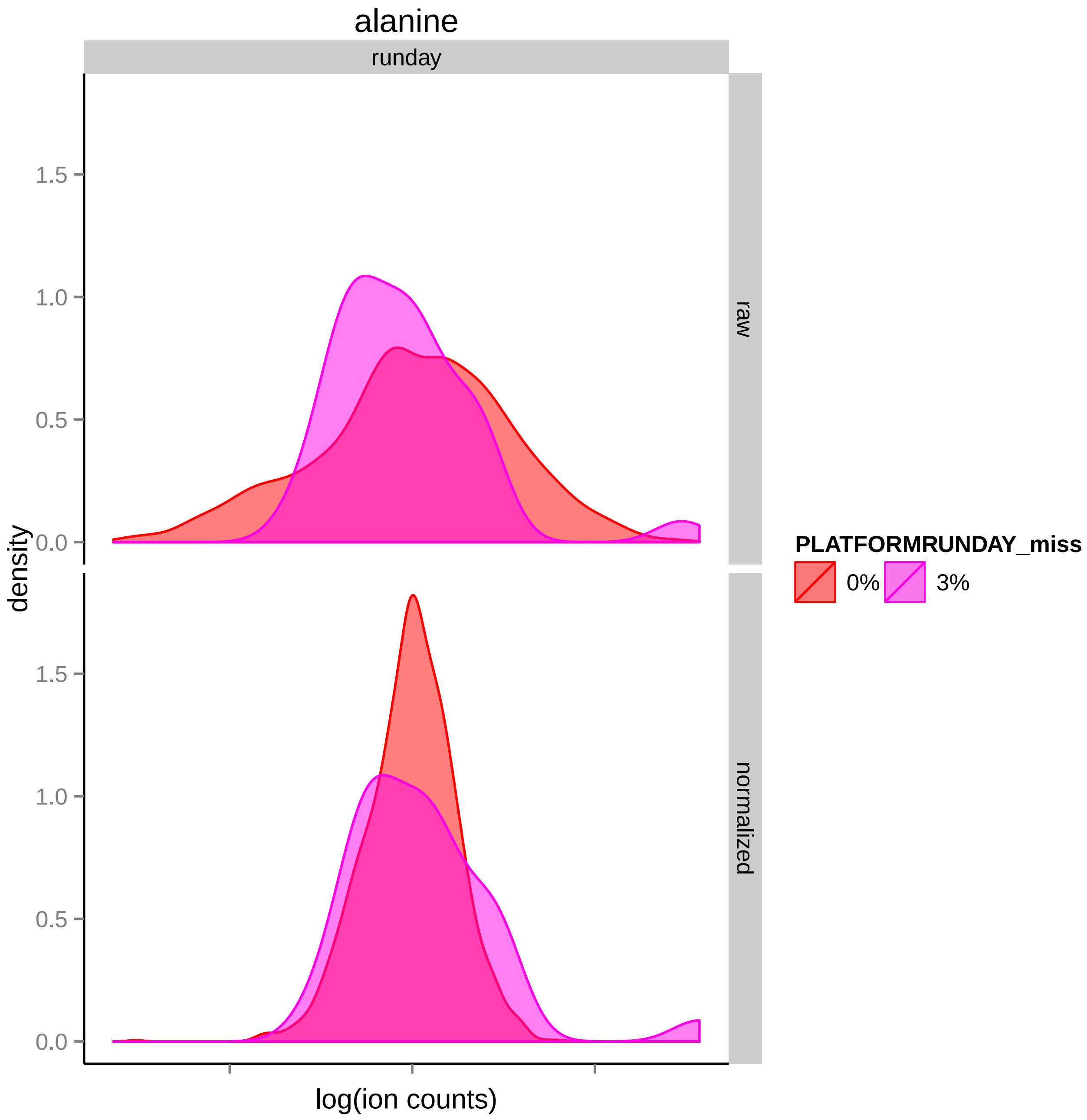

alanine
runday
raw
normalized
density
log(ion counts)
1.5
1.0
0.5
0.0
1.5
1.0
0.5
0.0
PLATFORMRUNDAY_miss
0%
3%

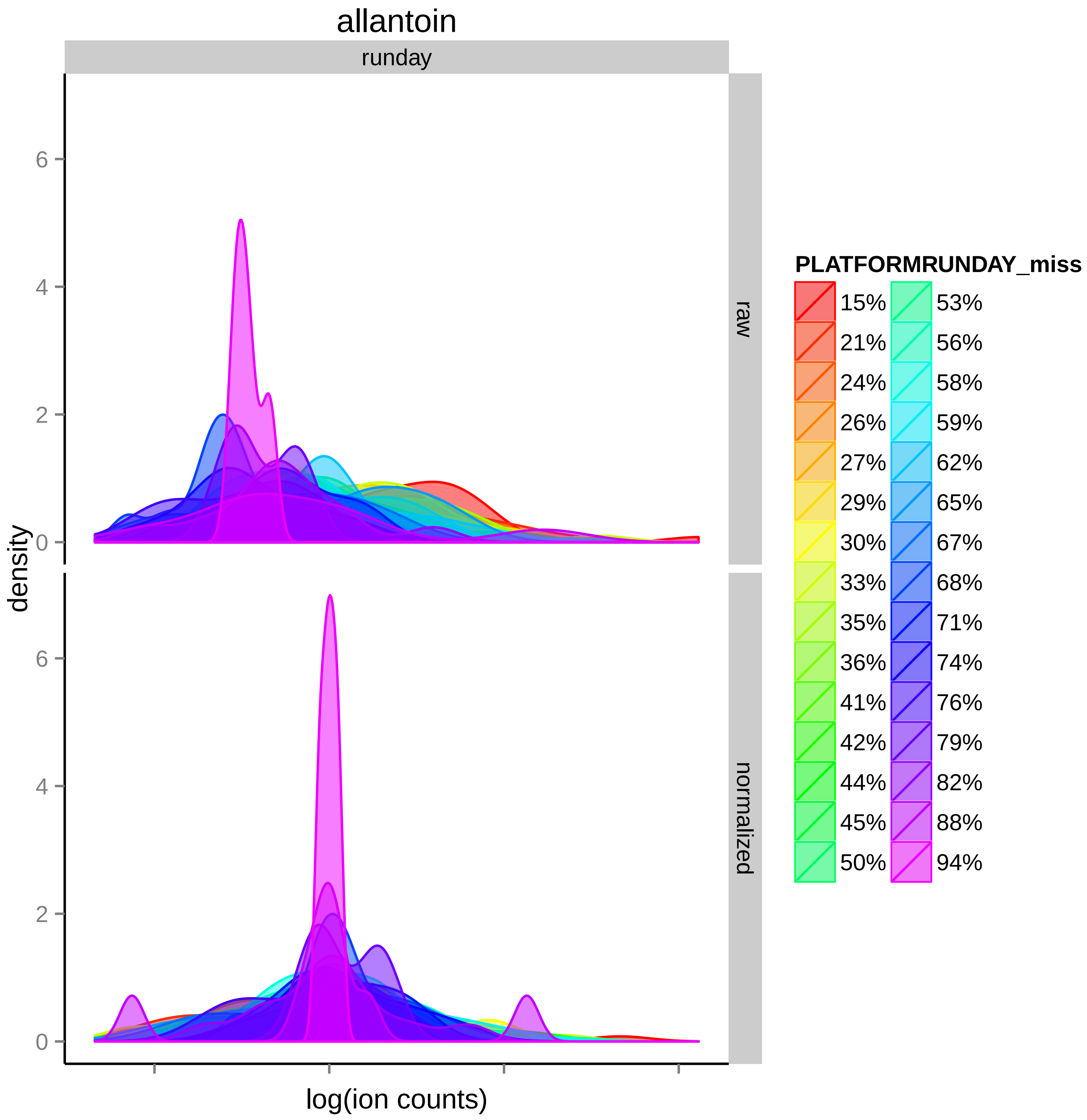
allantoin
runday
raw
normalized
density
log(ion counts)
6
4
2
0
PLATFORMRUNDAY_miss
15%
21%
24%
26%
27%
29%
30%
33%
35%
36%
41%
42%
44%
45%
50%
53%
56%
58%
59%
62%
65%
67%
68%
71%
74%
76%
79%
82%
88%
94%

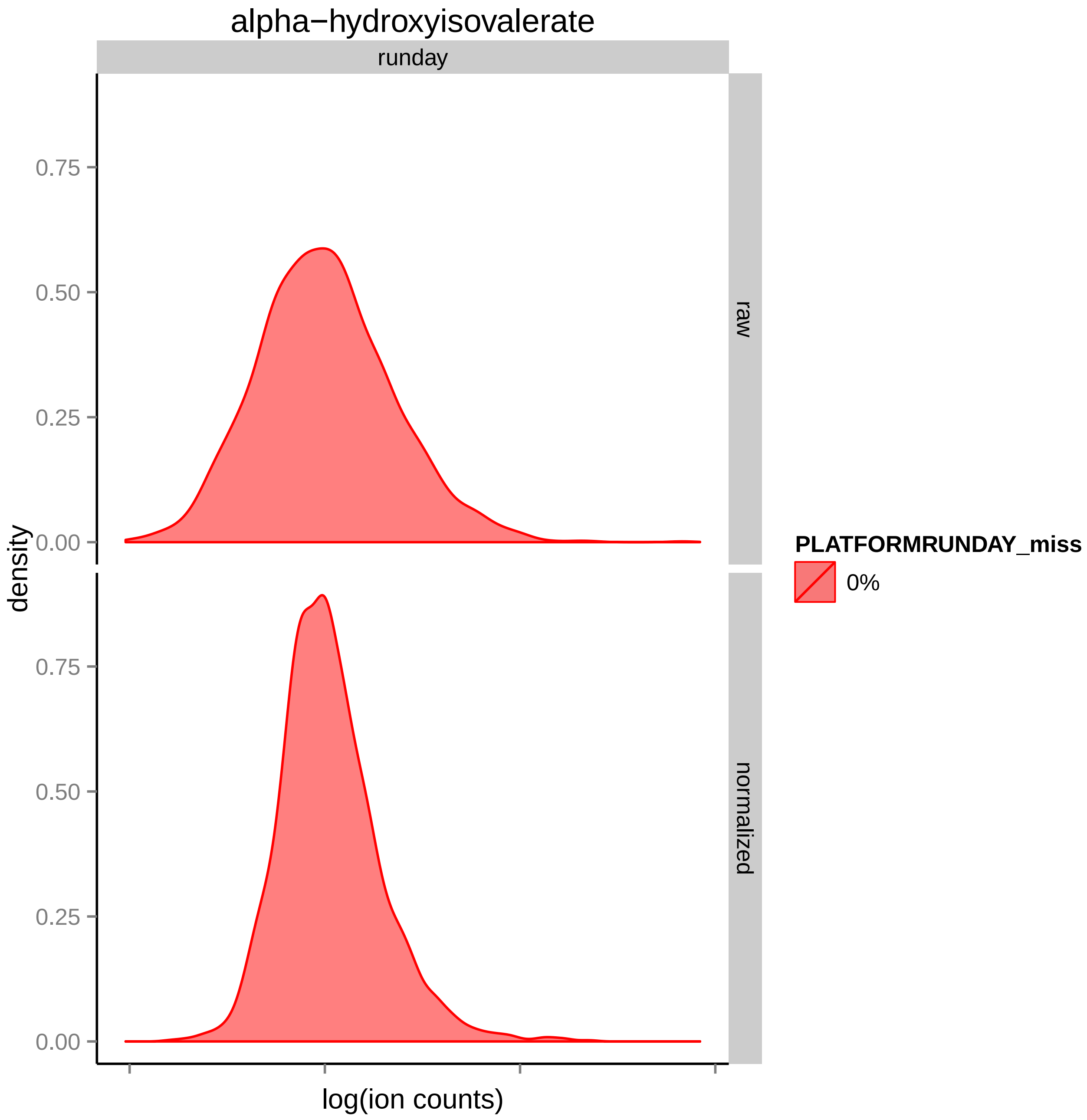
alpha−hydroxyisovalerate
runday
raw
normalized
density
0.00
0.25
0.50
0.75
log(ion counts)
PLATFORMRUNDAY_miss
0%

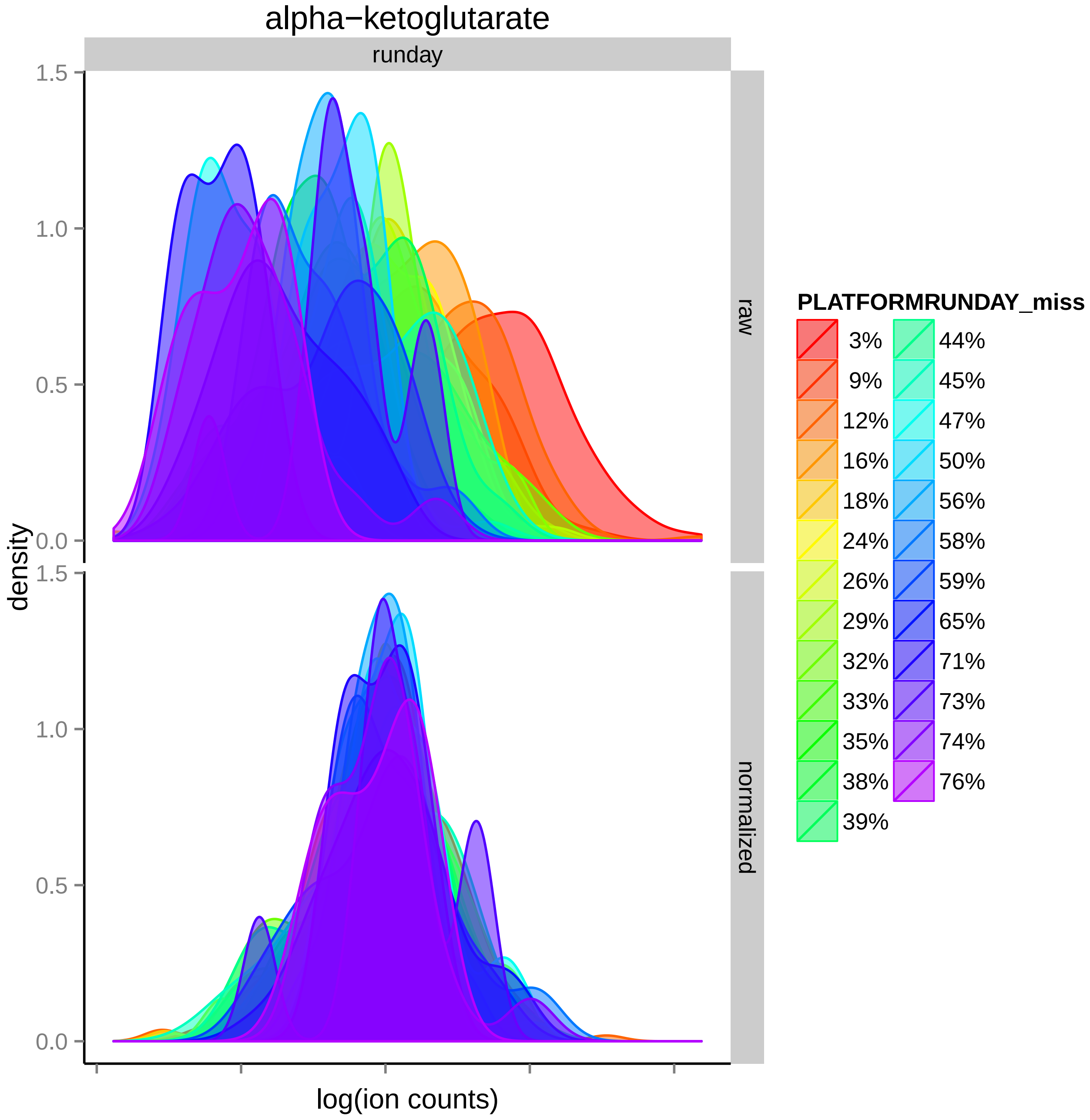

alpha−ketoglutarate
runday
raw
normalized
density
log(ion counts)
1.5
1.0
0.5
0.0
PLATFORMRUNDAY_miss
3%
9%
12%
16%
18%
24%
26%
29%
32%
33%
35%
38%
39%
44%
45%
47%
50%
56%
58%
59%
65%
71%
73%
74%
76%

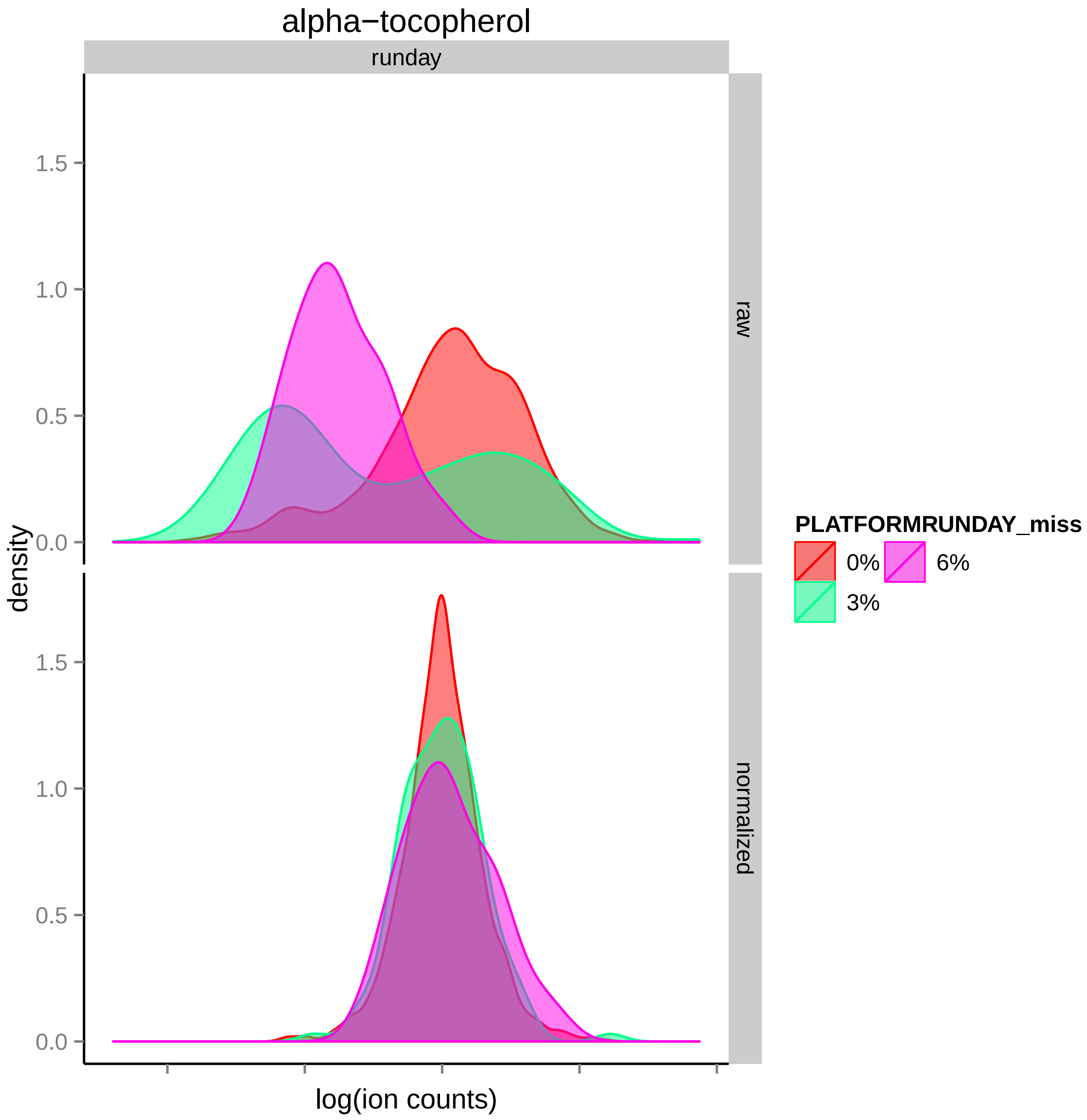

alpha−tocopherol
runday
raw
normalized
density
1.5
1.0
0.5
0.0
log(ion counts)
PLATFORMRUNDAY_miss
0%
6%
3%

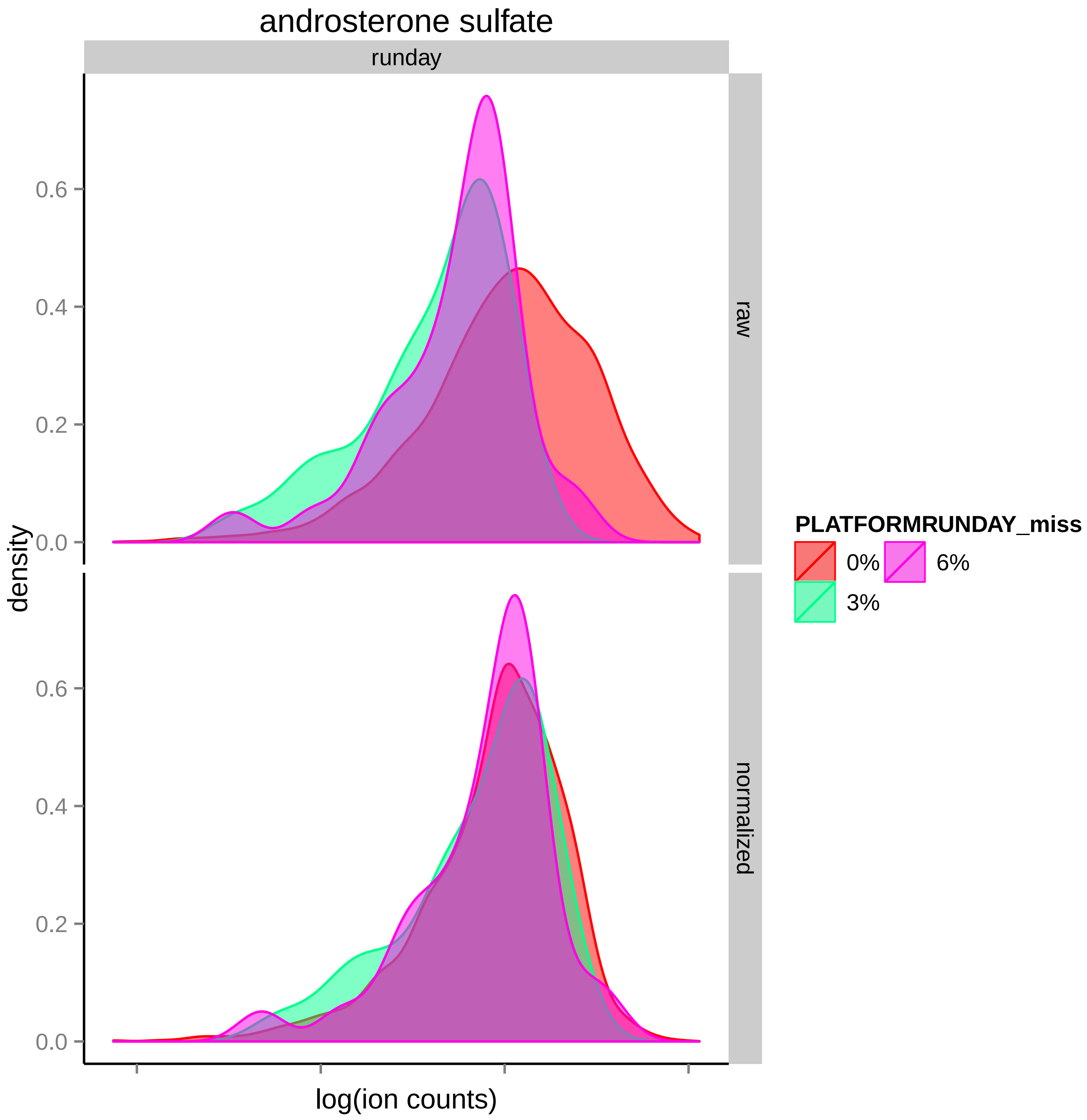

androsterone sulfate
runday
raw
normalized
density
0.0
0.2
0.4
0.6
log(ion counts)
PLATFORMRUNDAY_miss
0%
3%
6%

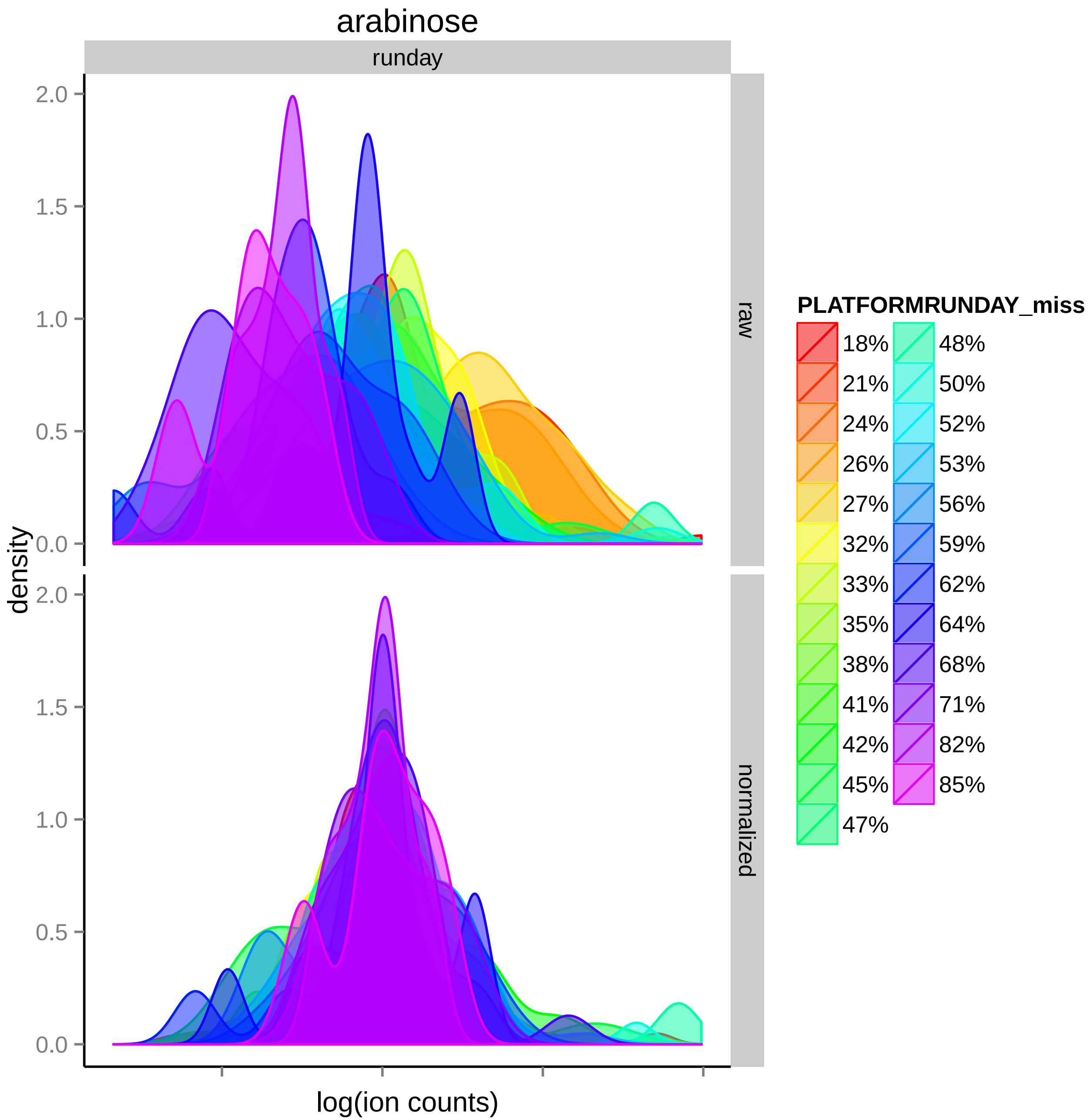

arabinose
runday
raw
normalized
density
2.0
1.5
1.0
0.5
0.0
log(ion counts)
PLATFORMRUNDAY_miss
18%
21%
24%
26%
27%
32%
33%
35%
38%
41%
42%
45%
47%
48%
50%
52%
53%
56%
59%
62%
64%
68%
71%
82%
85%

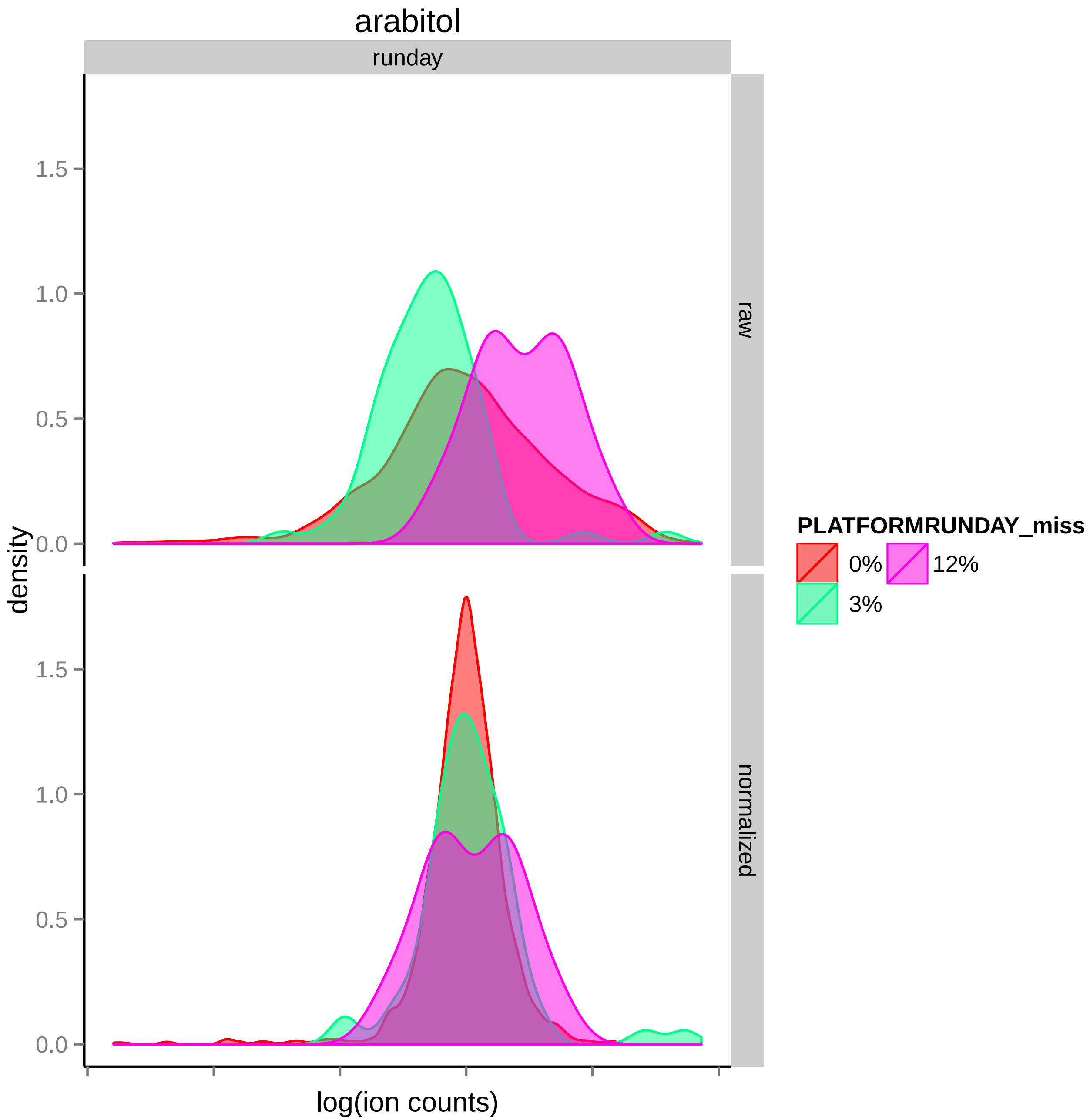

arabitol
runday
raw
normalized
density
log(ion counts)
0.0
0.5
1.0
1.5
PLATFORMRUNDAY_miss
0%
12%
3%

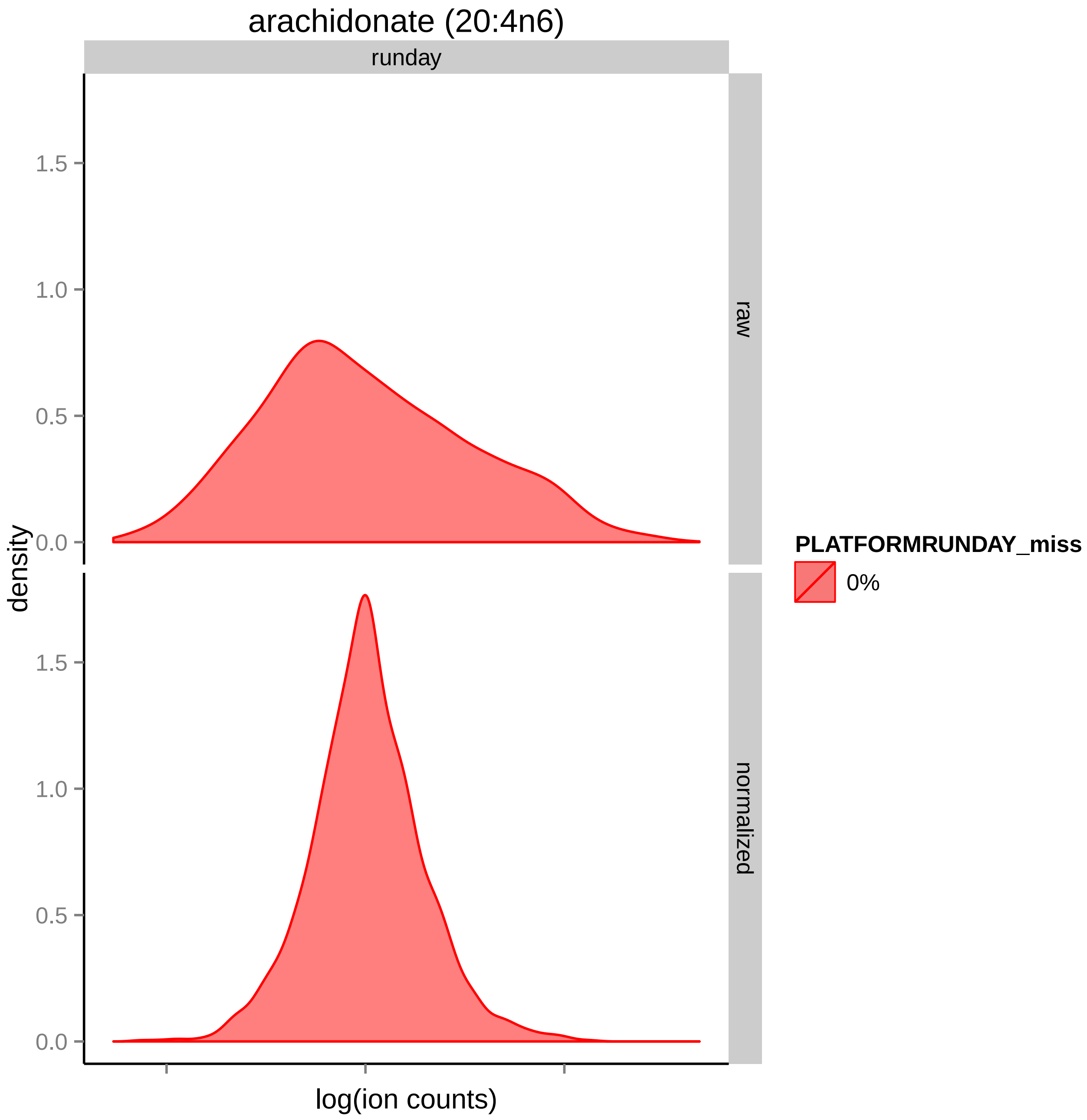

arachidonate (20:4n6)
runday
raw
normalized
density
0.0
0.5
1.0
1.5
log(ion counts)
PLATFORMRUNDAY_miss
0%

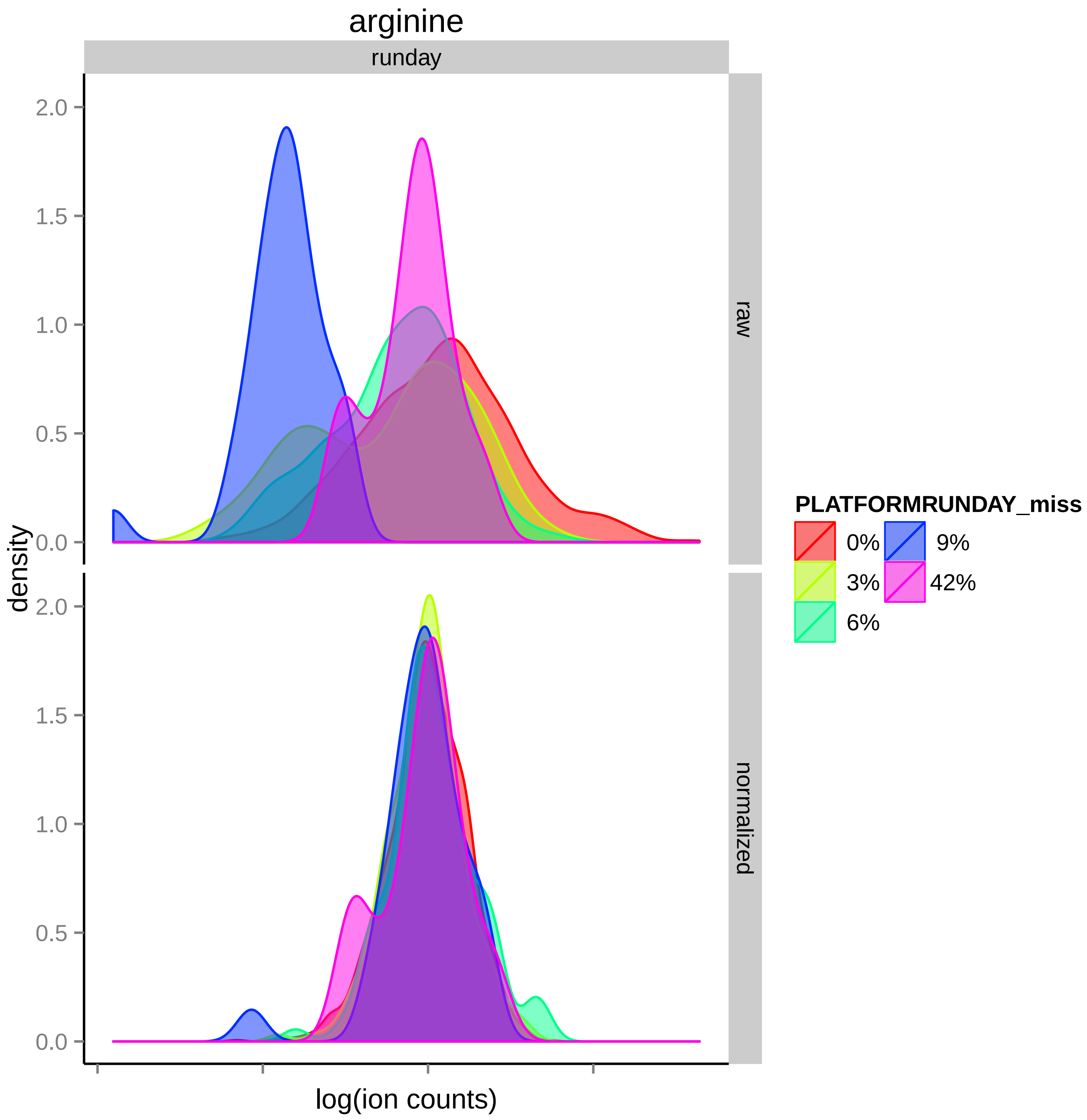

arginine
runday
raw
normalized
density
2.0
1.5
1.0
0.5
0.0
log(ion counts)
PLATFORMRUNDAY_miss
0%
9%
3%
42%
6%

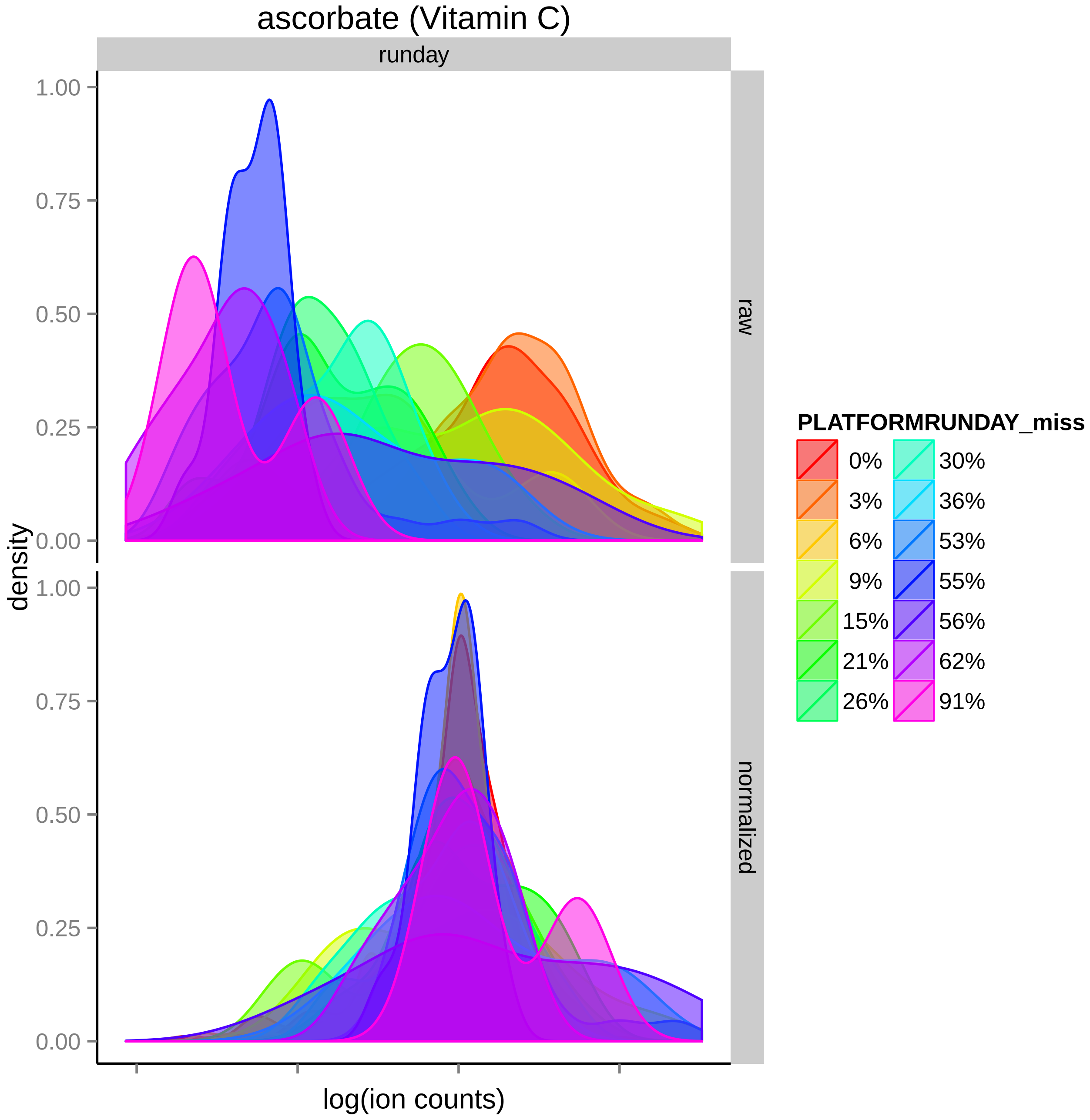

ascorbate (Vitamin C)
runday
raw
normalized
density
log(ion counts)
1.00
0.75
0.50
0.25
0.00
PLATFORMRUNDAY_miss
0%
3%
6%
9%
15%
21%
26%
30%
36%
53%
55%
56%
62%
91%

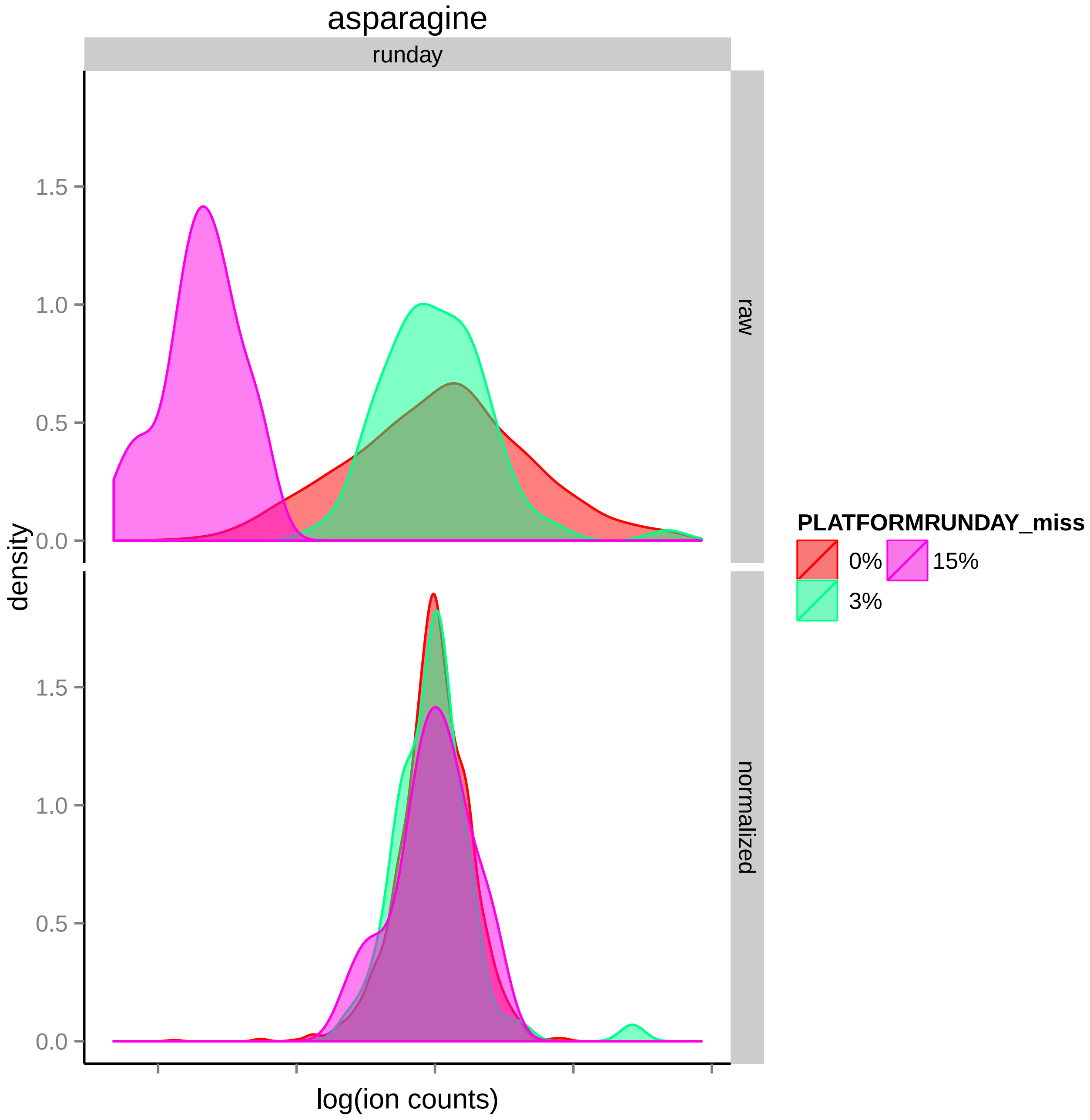
asparagine
runday
raw
normalized
density
1.5
1.0
0.5
0.0
log(ion counts)
PLATFORMRUNDAY_miss
0%
15%
3%

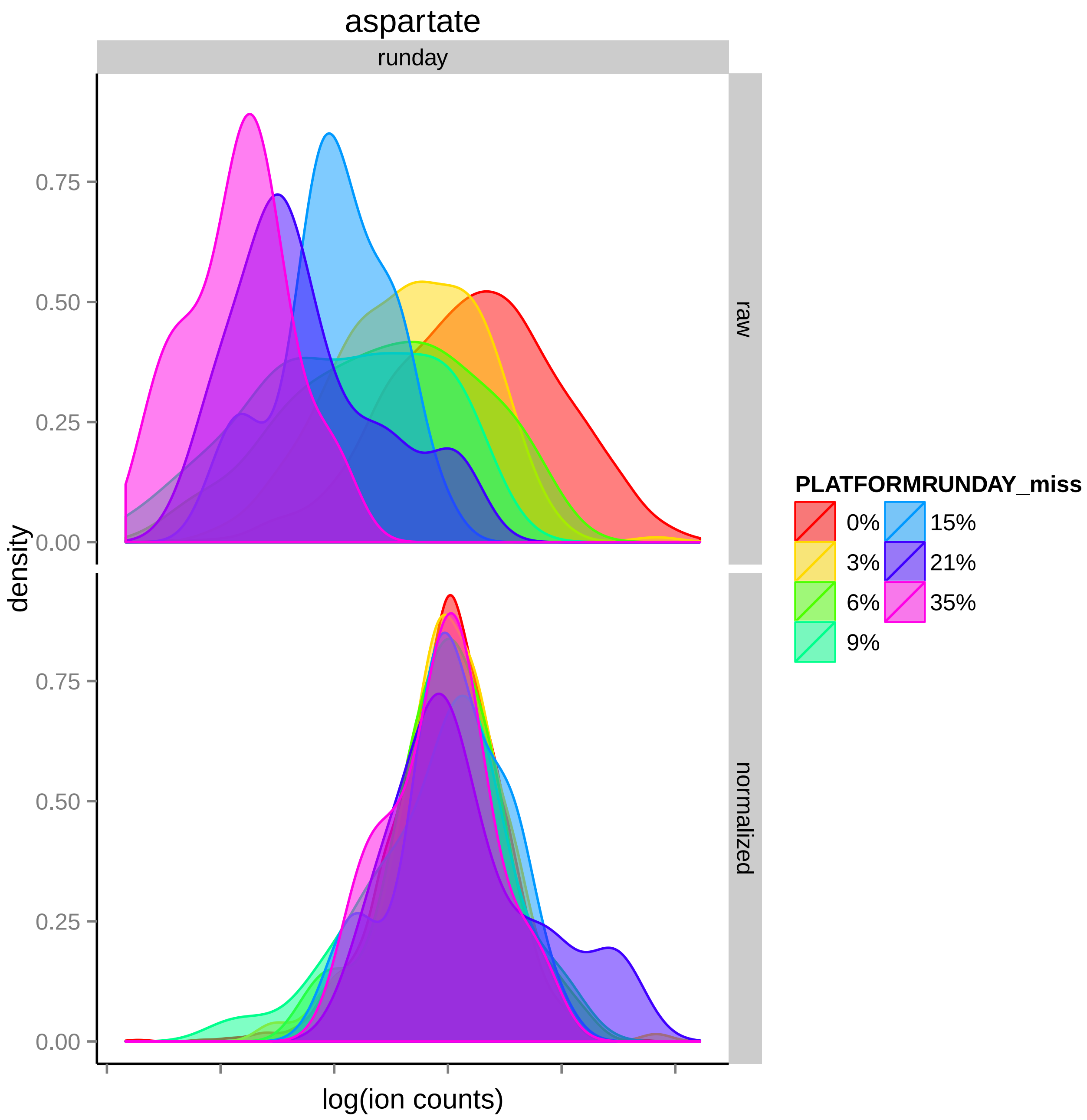

aspartate
runday
raw
normalized
density
0.00
0.25
0.50
0.75
log(ion counts)
PLATFORMRUNDAY_miss
0%
3%
6%
9%
15%
21%
35%

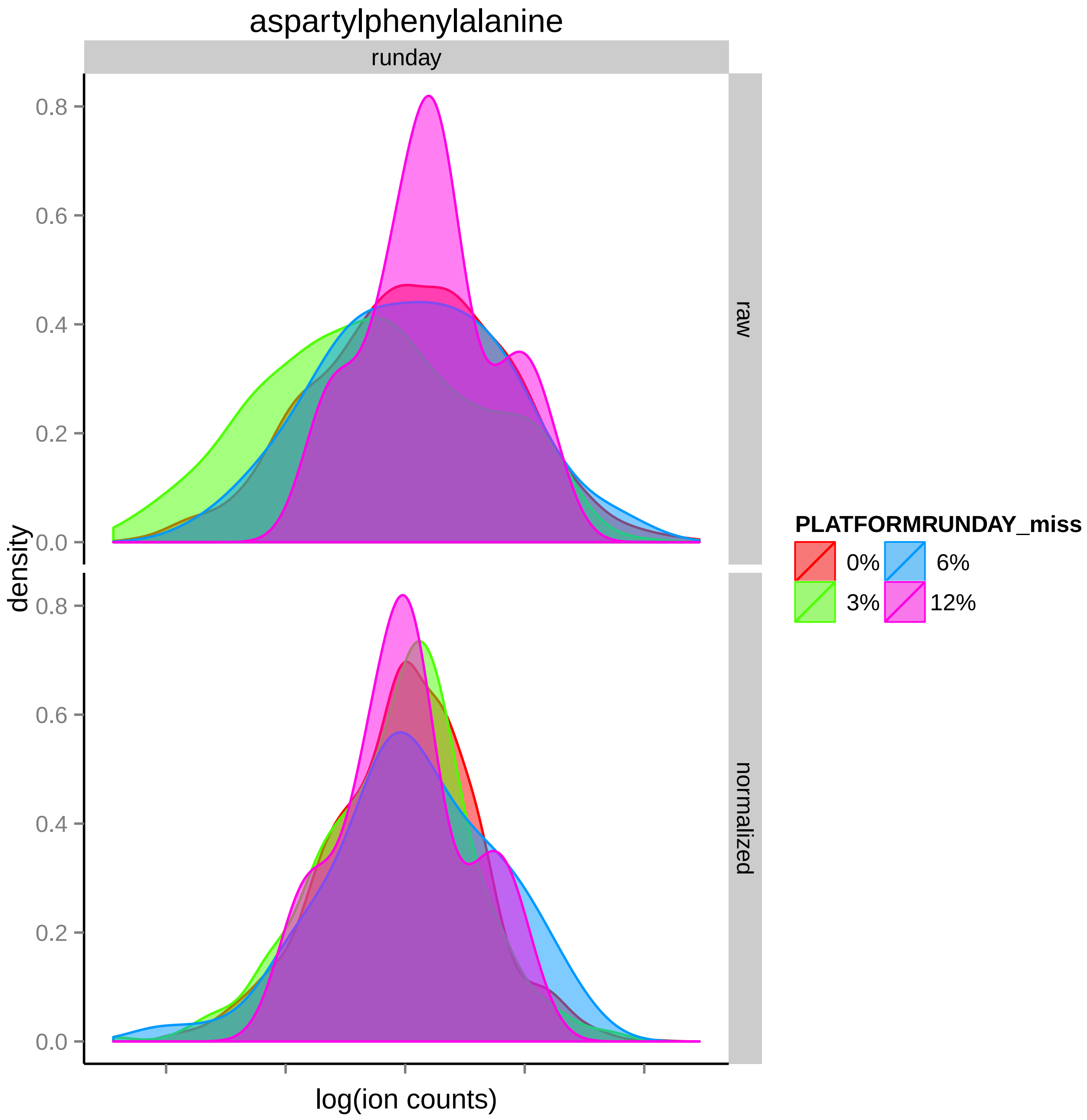
aspartylphenylalanine
runday
raw
normalized
density
0.0
0.2
0.4
0.6
0.8
log(ion counts)
PLATFORMRUNDAY_miss
0%
3%
6%
12%

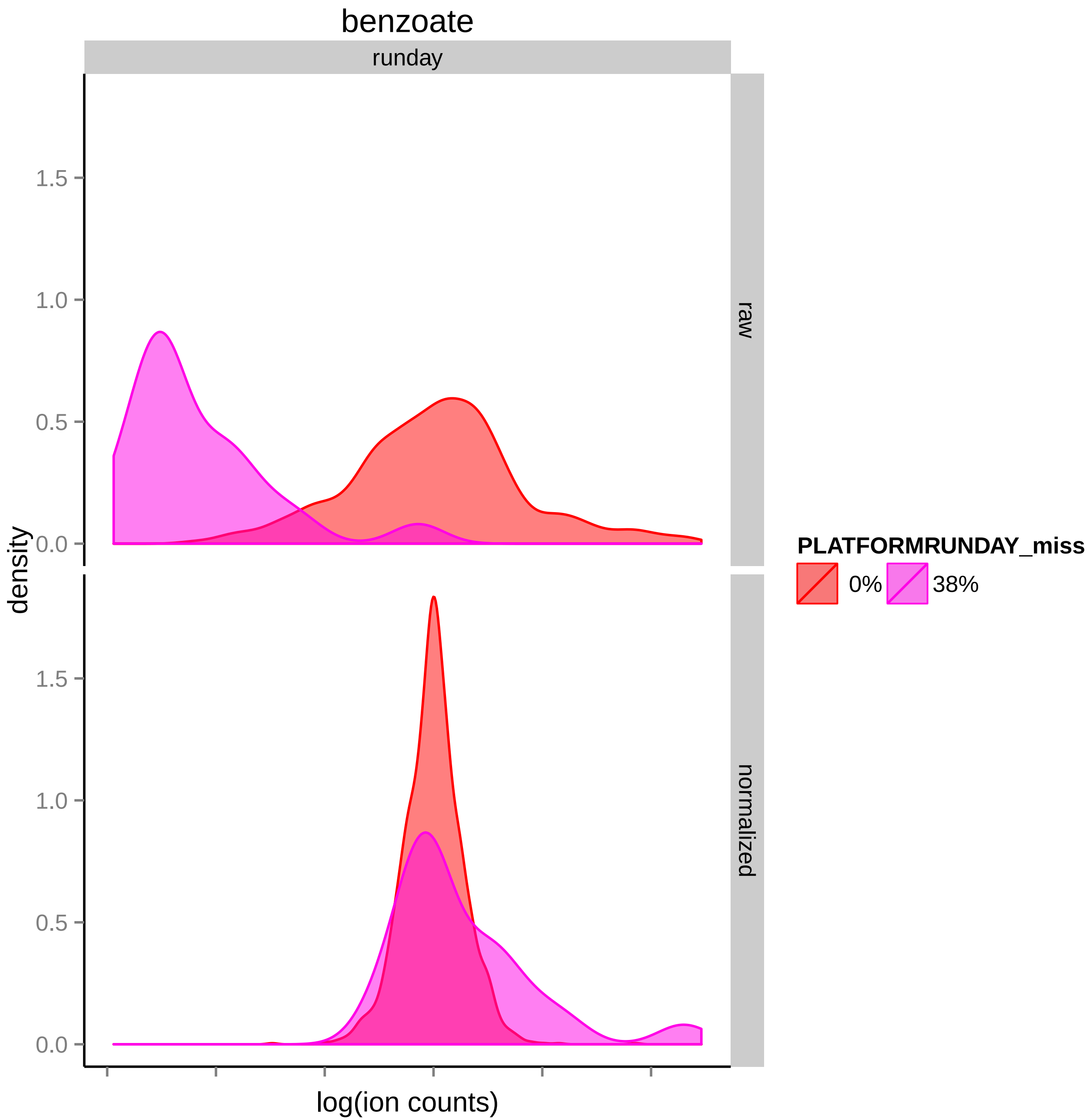
benzoate
runday
raw
normalized
density
1.5
1.0
0.5
0.0
1.5
1.0
0.5
0.0
log(ion counts)
PLATFORMRUNDAY_miss
0%
38%

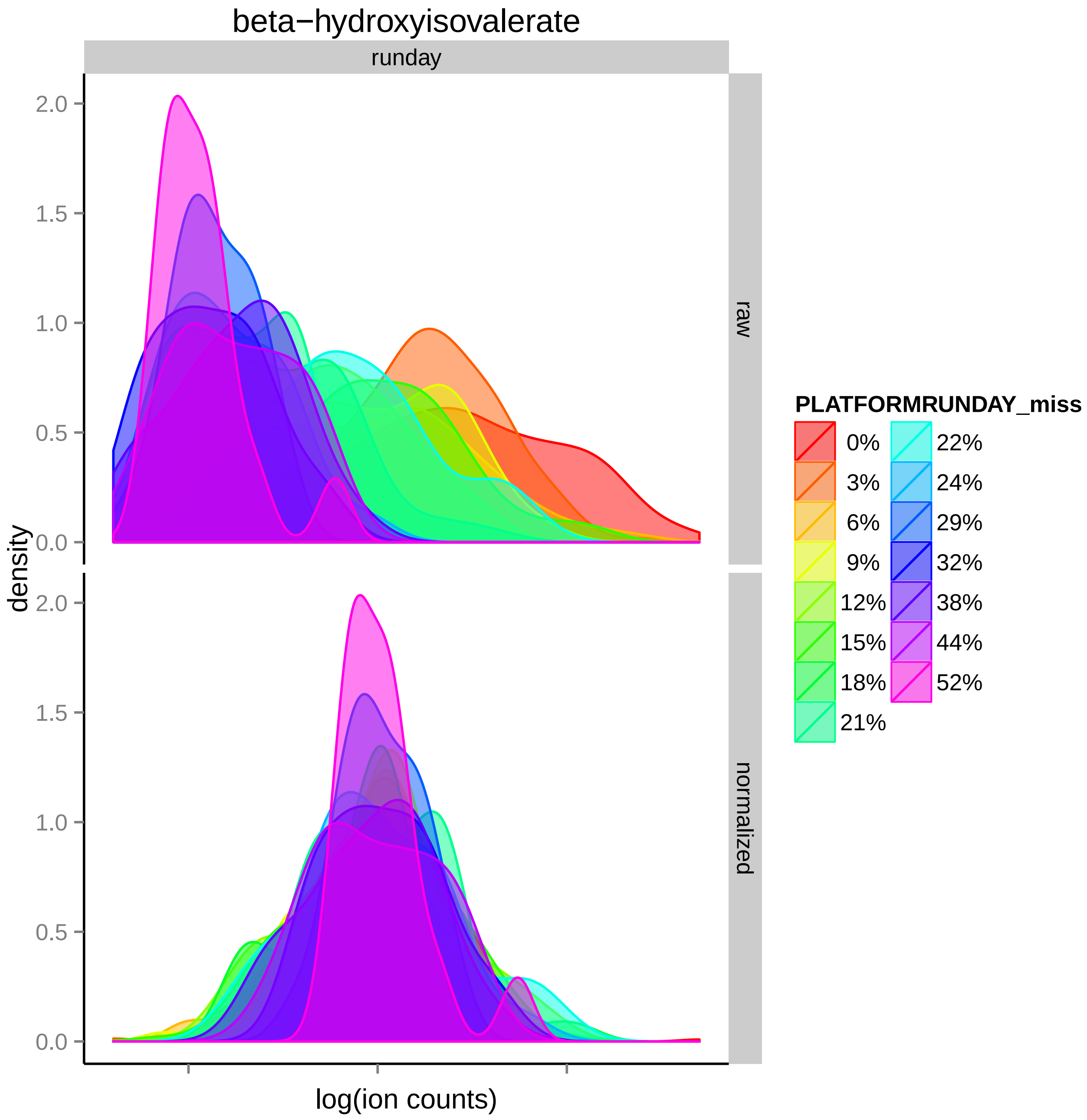
beta−hydroxyisovalerate
runday
raw
normalized
density
log(ion counts)
0.0
0.5
1.0
1.5
2.0
PLATFORMRUNDAY_miss
0%
3%
6%
9%
12%
15%
18%
21%
22%
24%
29%
32%
38%
44%
52%

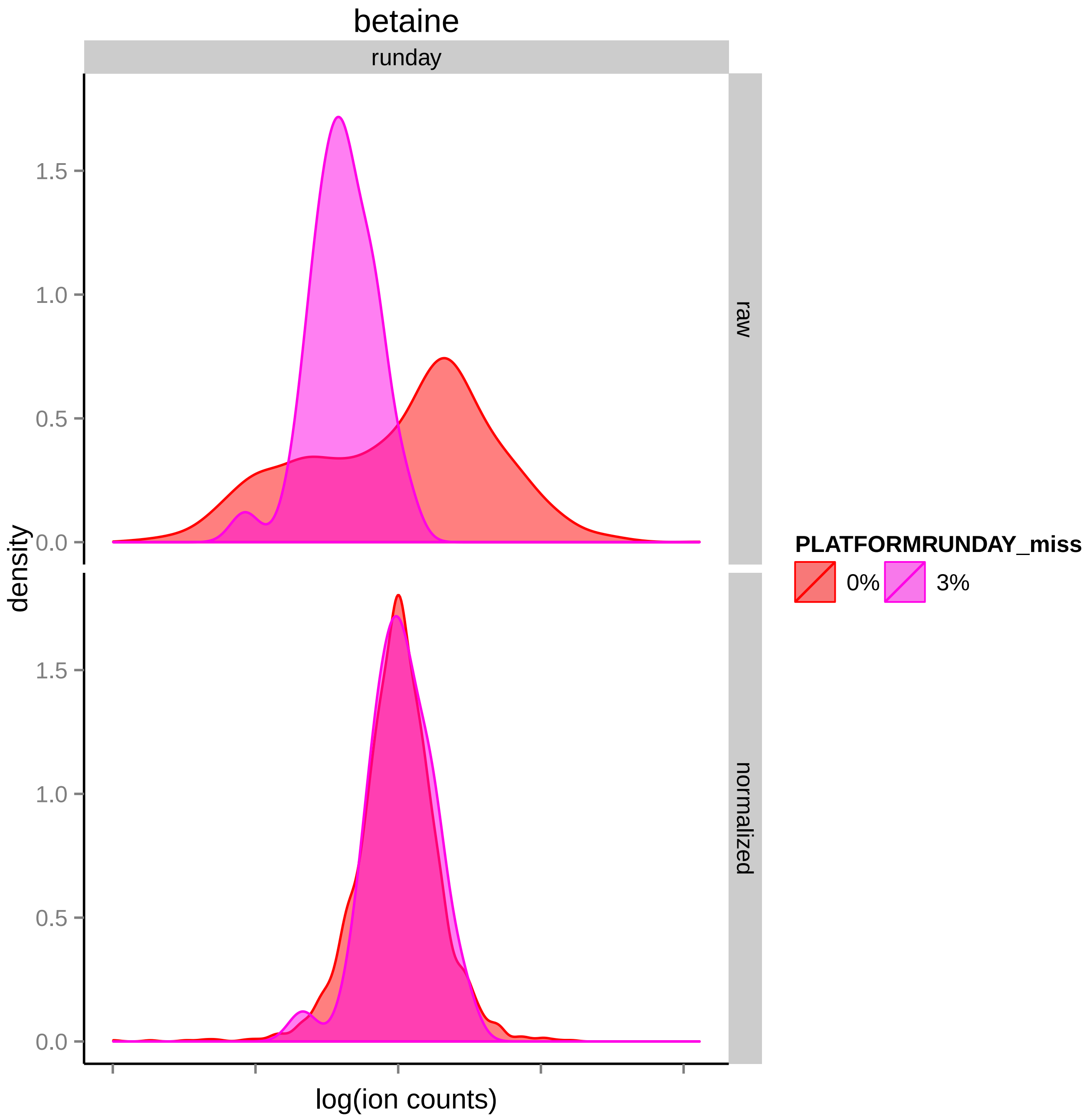
betaine
runday
raw
normalized
density
1.5
1.0
0.5
0.0
log(ion counts)
PLATFORMRUNDAY_miss
0%
3%

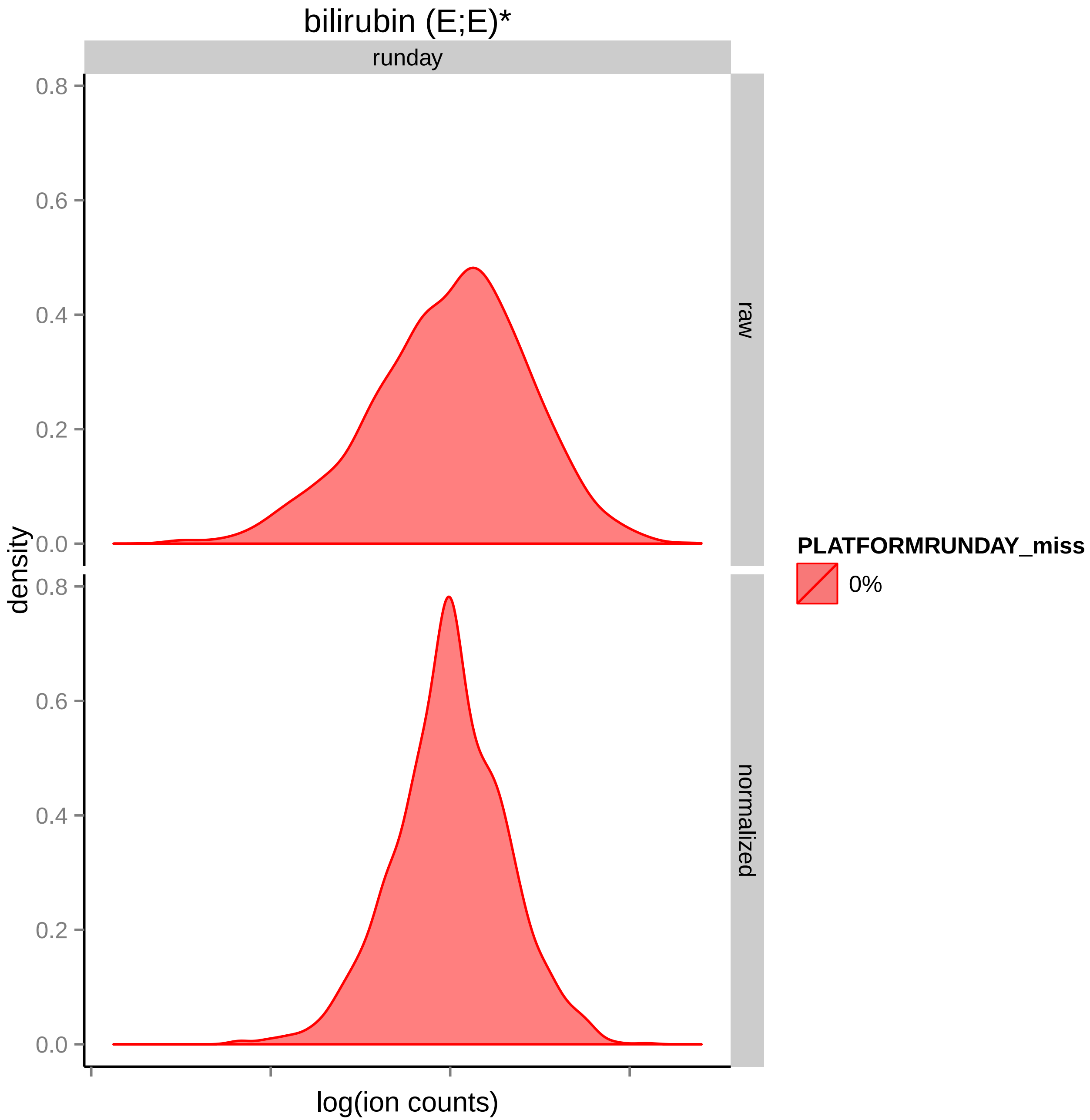
bilirubin (E;E)*
runday
raw
normalized
0.8
0.6
0.4
0.2
0.0
density
log(ion counts)
PLATFORMRUNDAY_miss
0%

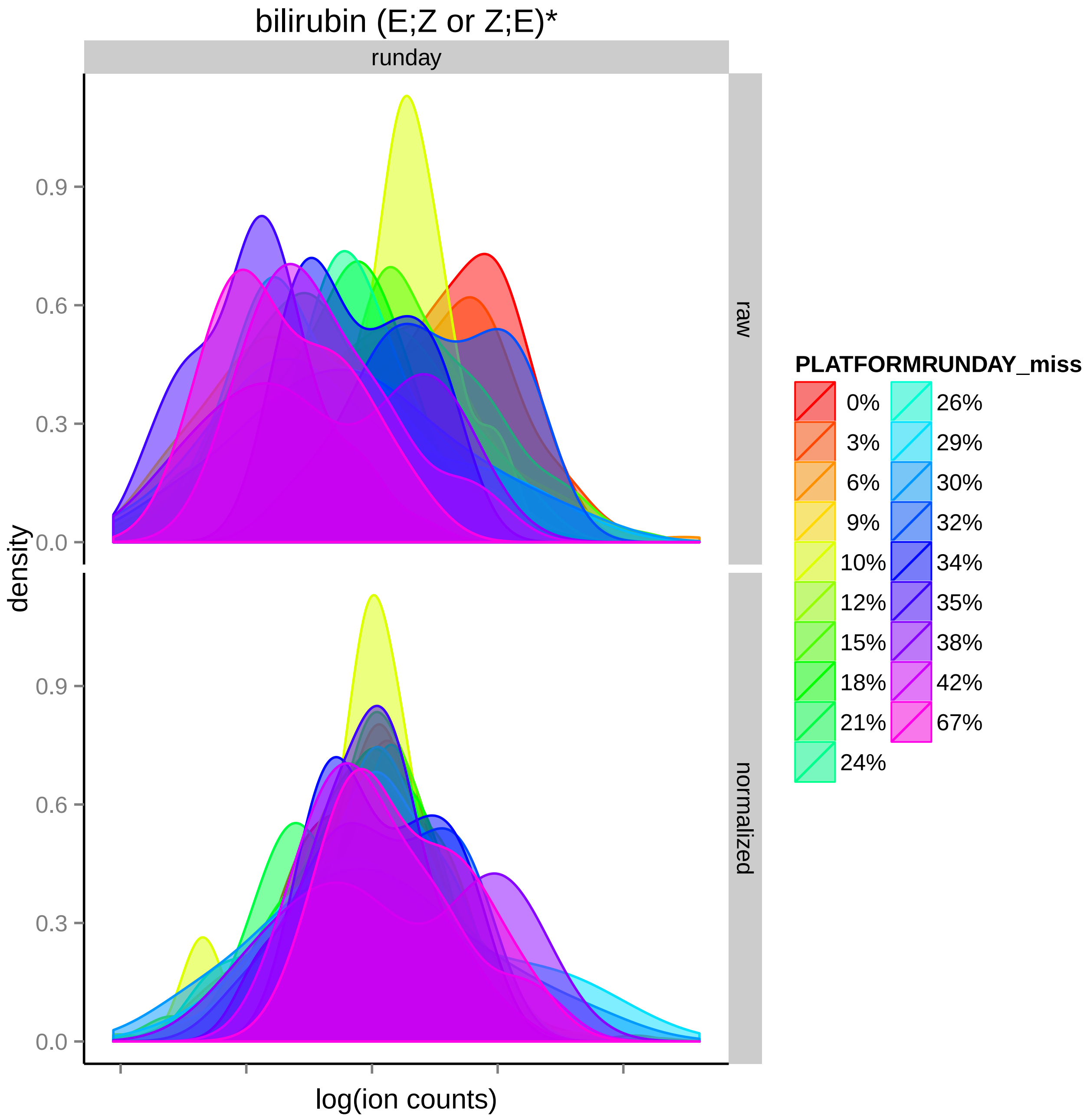
bilirubin (E;Z or Z;E)*
runday
raw
normalized
density
log(ion counts)
0.0
0.3
0.6
0.9
PLATFORMRUNDAY_miss
0%
3%
6%
9%
10%
12%
15%
18%
21%
24%
26%
29%
30%
32%
34%
35%
38%
42%
67%

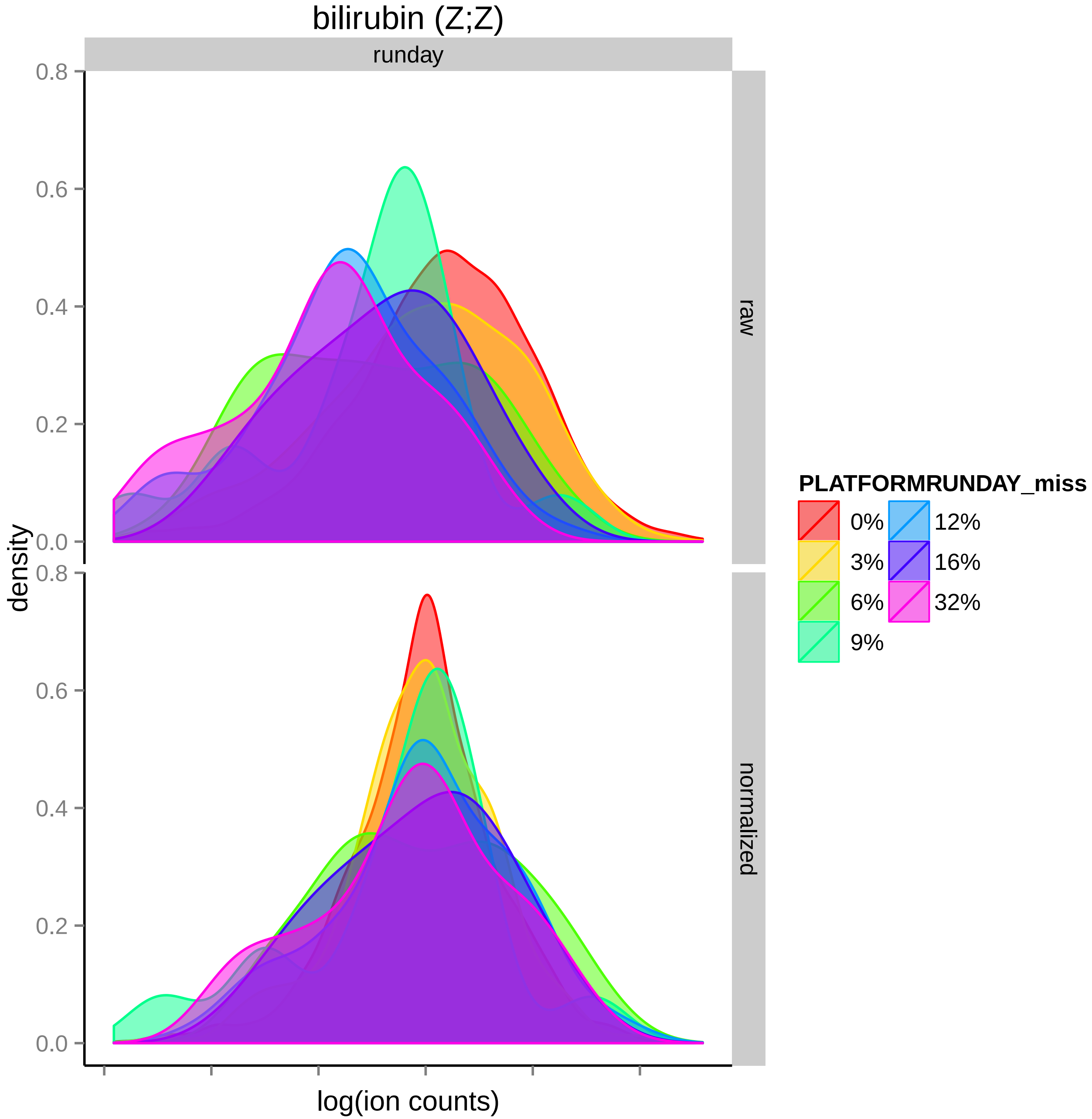
bilirubin (Z;Z)
runday
raw
normalized
density
log(ion counts)
0.0
0.2
0.4
0.6
0.8
PLATFORMRUNDAY_miss
0%
3%
6%
9%
12%
16%
32%

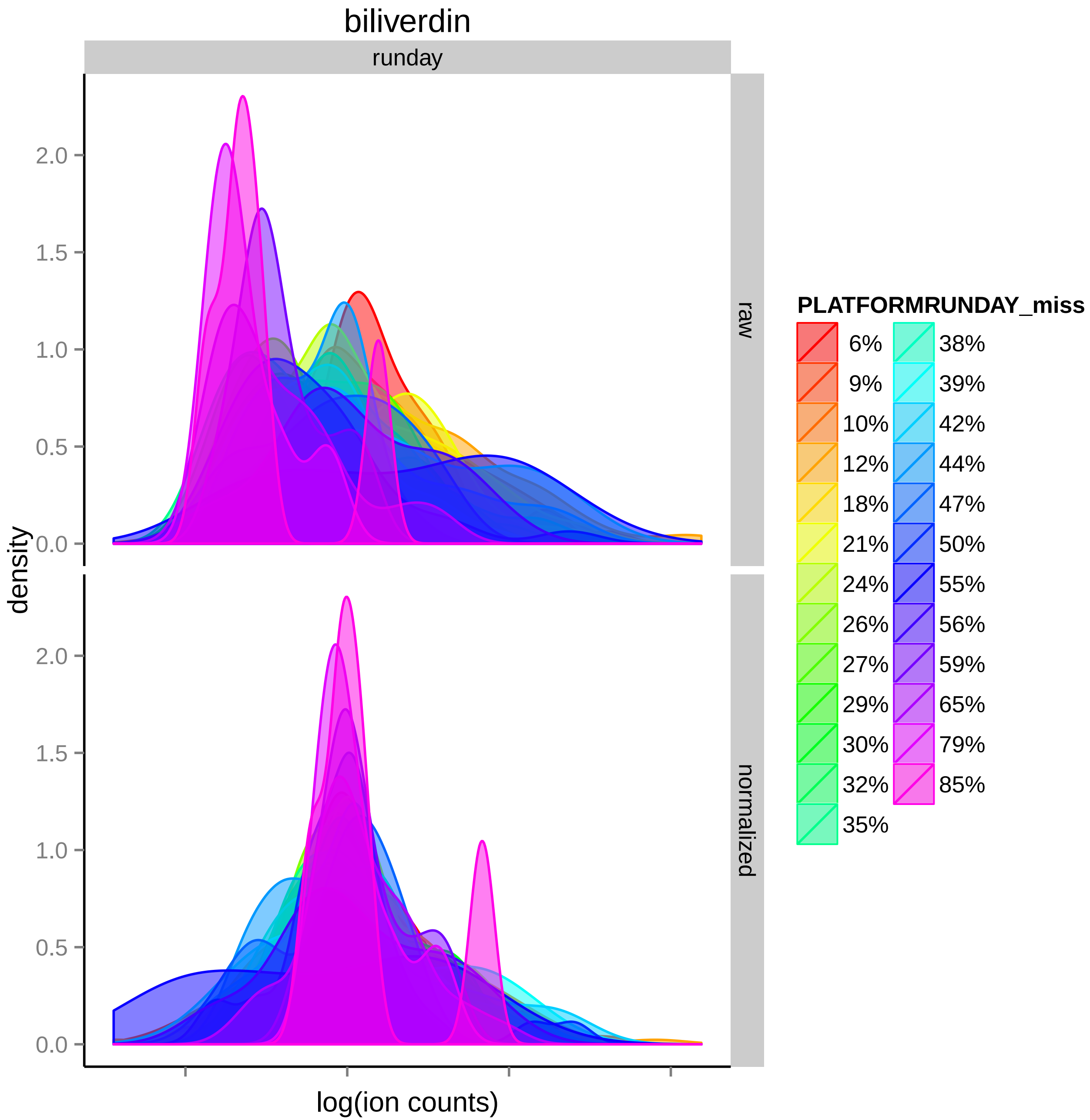
biliverdin
runday
raw
normalized
density
log(ion counts)
2.0
1.5
1.0
0.5
0.0
PLATFORMRUNDAY_miss
6%
9%
10%
12%
18%
21%
24%
26%
27%
29%
30%
32%
35%
38%
39%
42%
44%
47%
50%
55%
56%
59%
65%
79%
85%

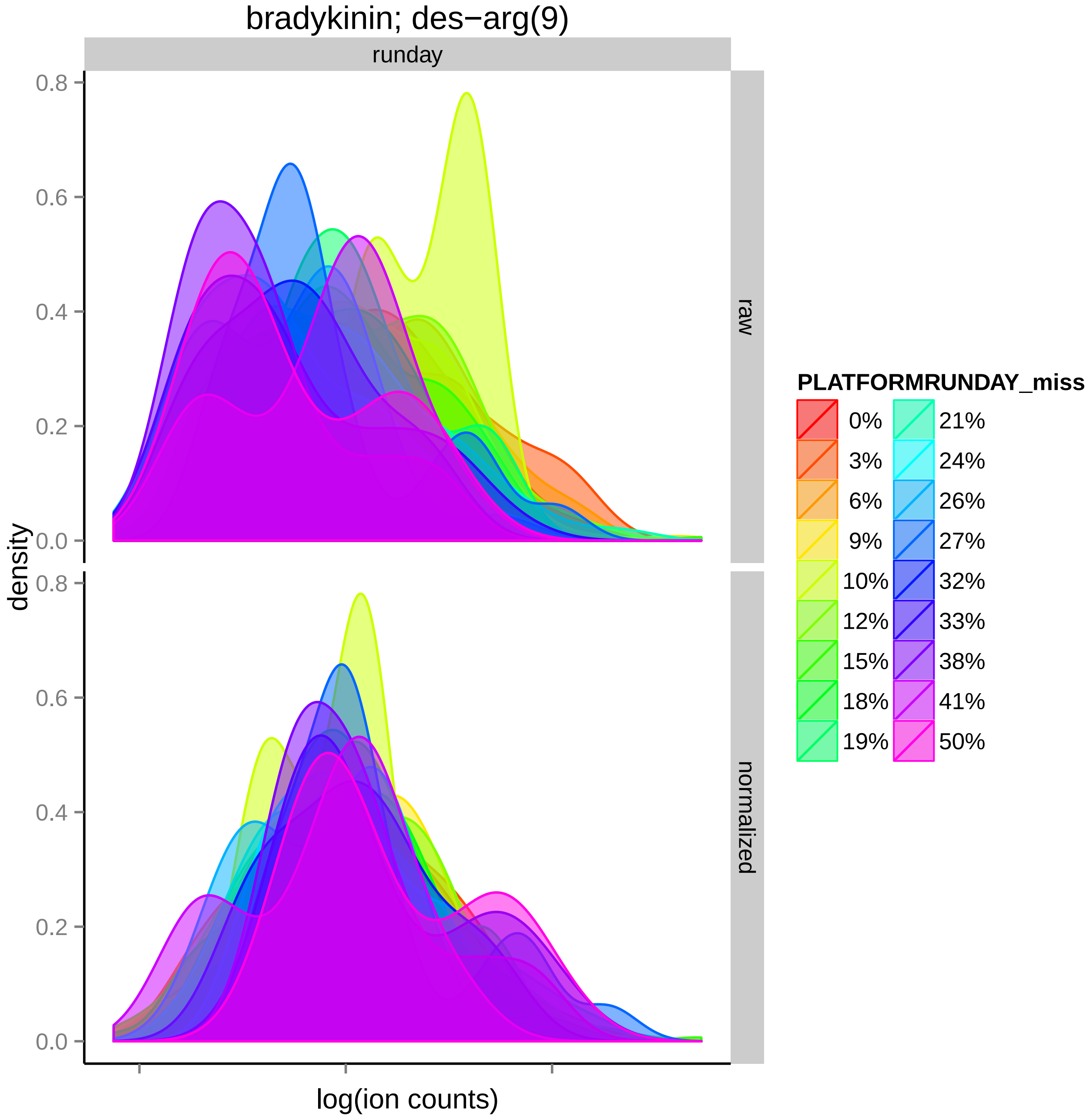

bradykinin; des−arg(9)
runday
raw
normalized
density
log(ion counts)
0.0
0.2
0.4
0.6
0.8
PLATFORMRUNDAY_miss
0%
3%
6%
9%
10%
12%
15%
18%
19%
21%
24%
26%
27%
32%
33%
38%
41%
50%

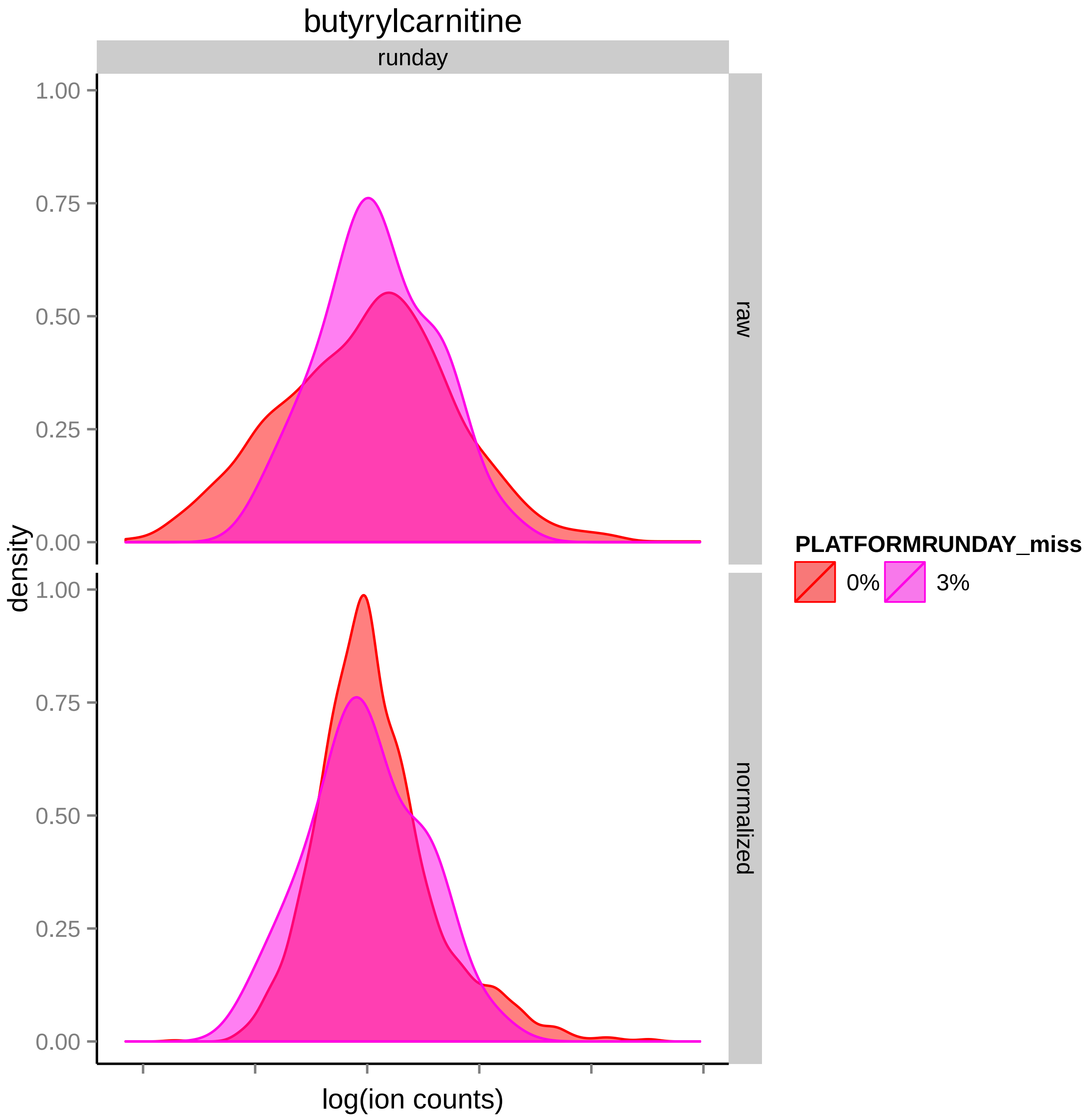
butyrylcarnitine
runday
raw
normalized
density
log(ion counts)
1.00
0.75
0.50
0.25
0.00
PLATFORMRUNDAY_miss
0%
3%

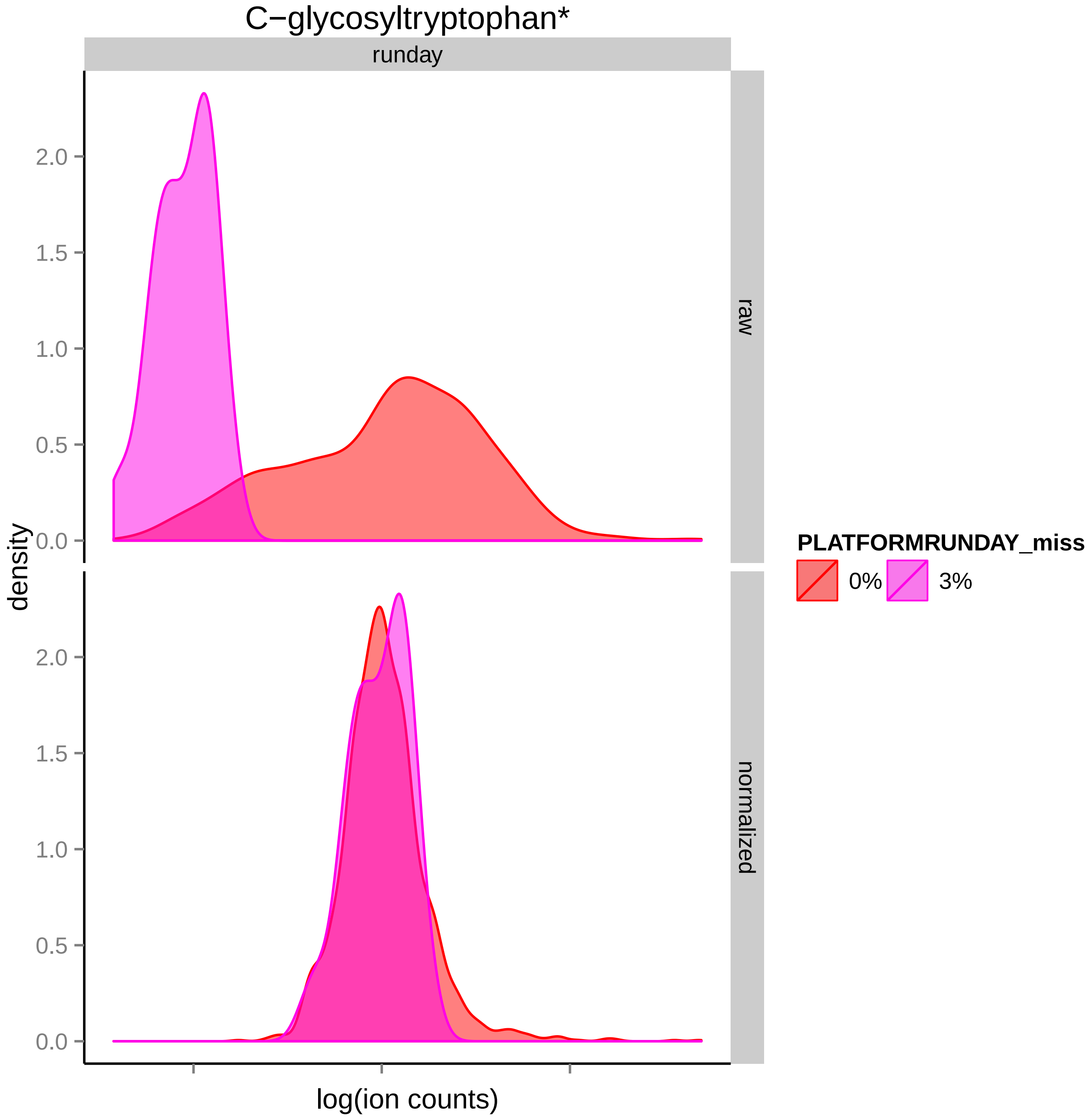
C−glycosyltryptophan*
runday
raw
normalized
density
log(ion counts)
2.0
1.5
1.0
0.5
0.0
PLATFORMRUNDAY_miss
0%
3%

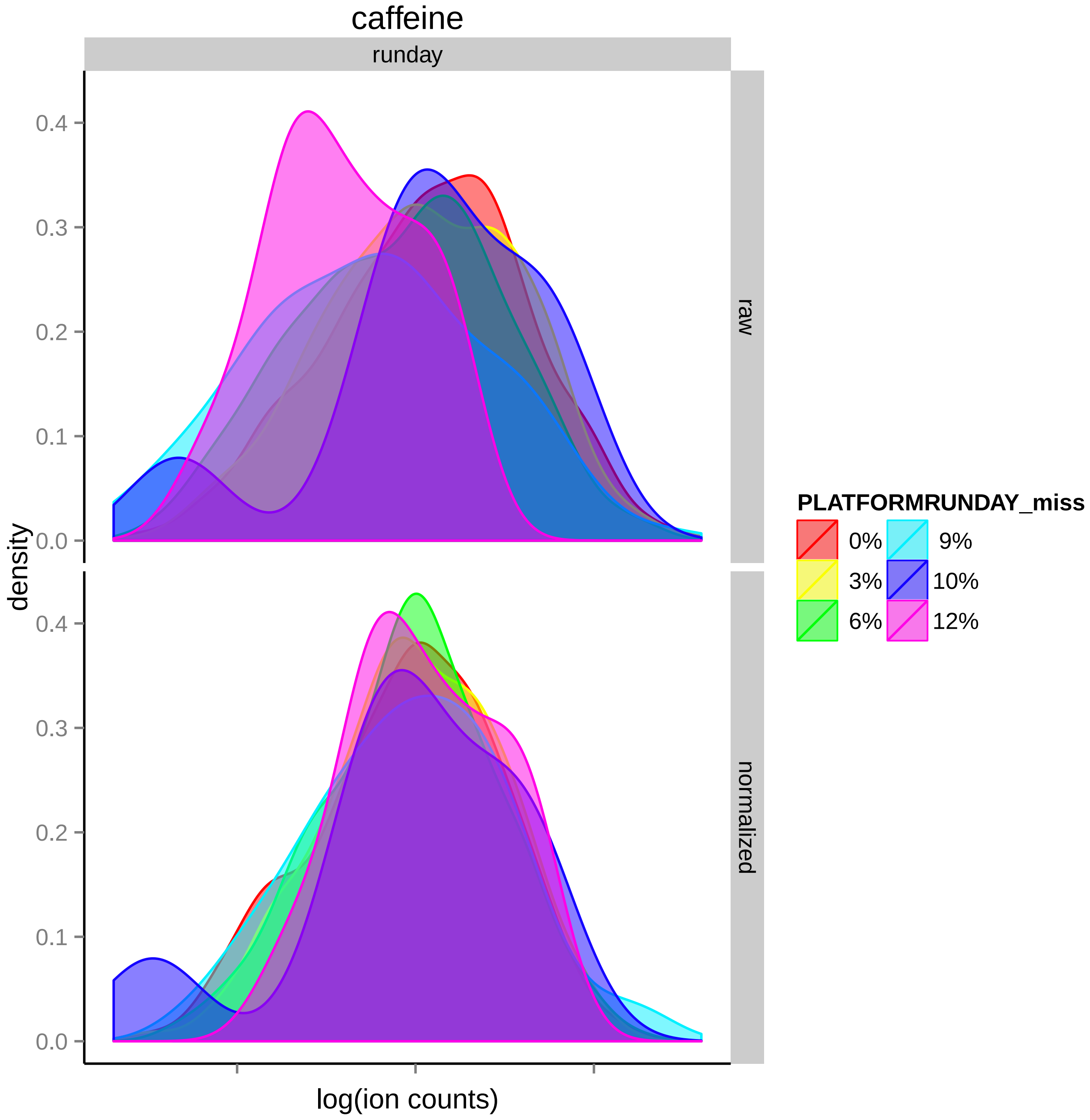

caffeine
runday
raw
normalized
density
0.4
0.3
0.2
0.1
0.0
log(ion counts)
PLATFORMRUNDAY_miss
0%
3%
6%
9%
10%
12%

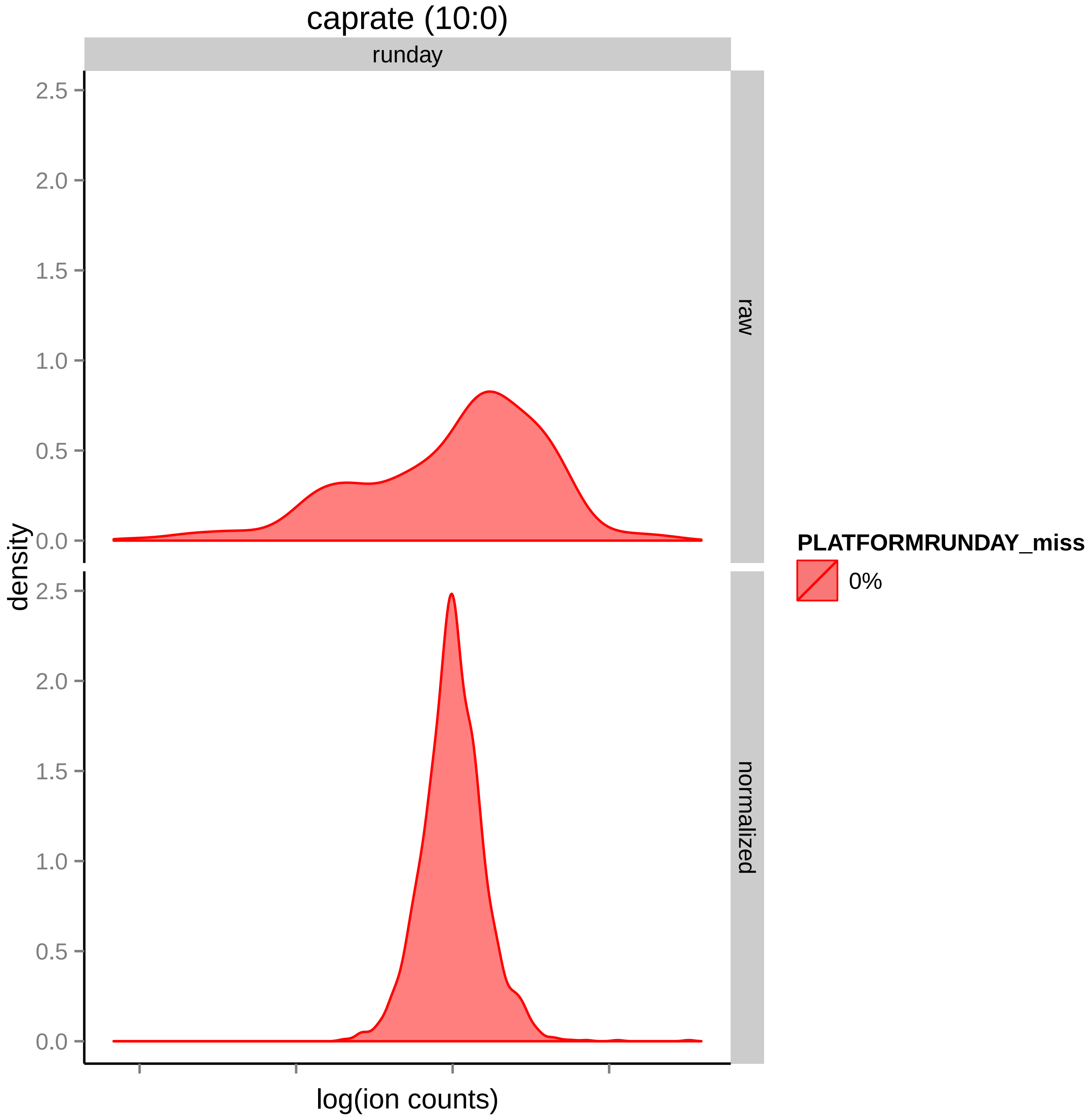
caprate (10:0)
runday
raw
normalized
density
log(ion counts)
PLATFORMRUNDAY_miss
0%
2.5
2.0
1.5
1.0
0.5
0.0

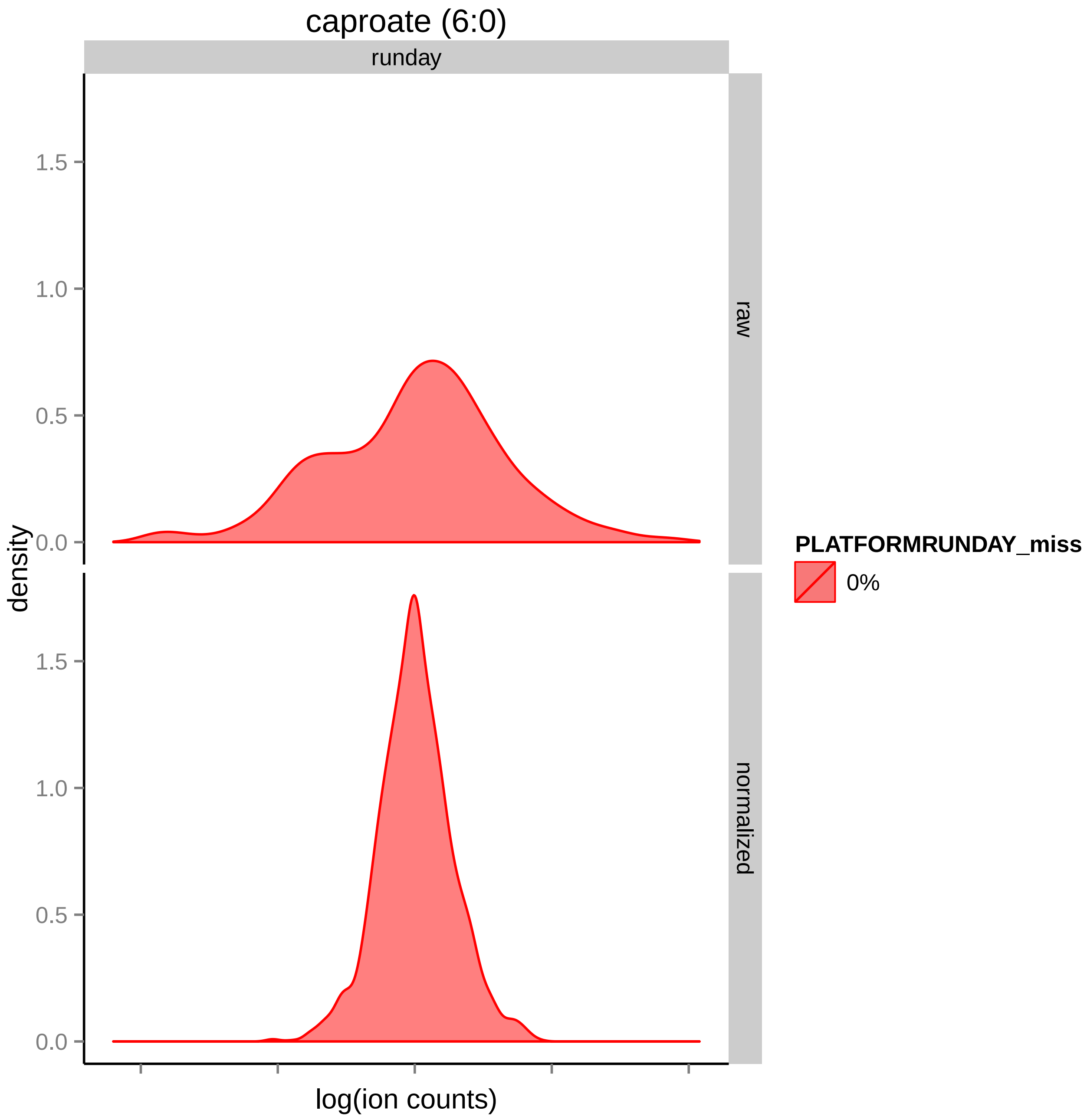
caproate (6:0)
runday
raw
normalized
density
1.5
1.0
0.5
0.0
1.5
1.0
0.5
0.0
log(ion counts)
PLATFORMRUNDAY_miss
0%

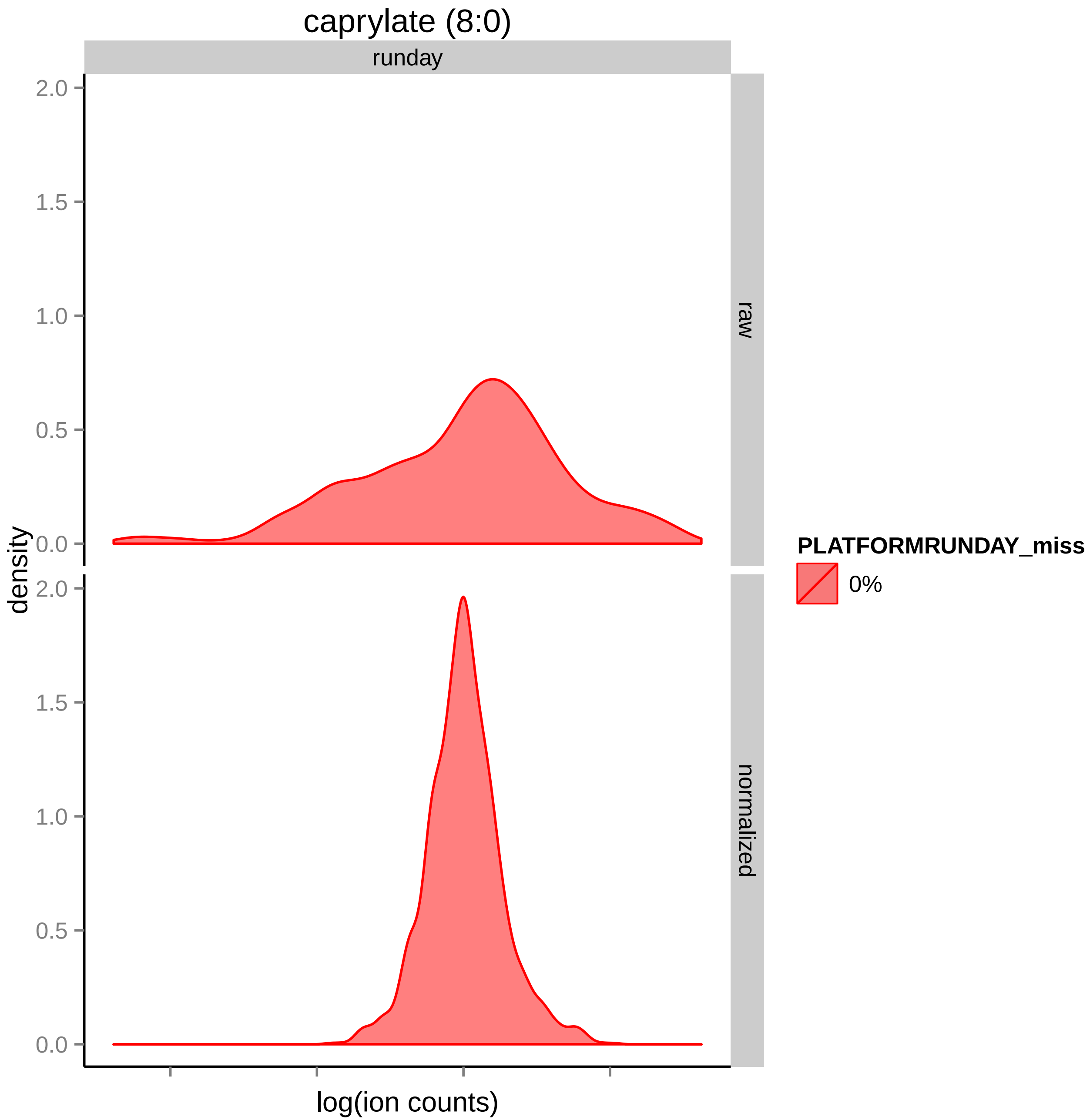

caprylate (8:0)
runday
raw
normalized
density
log(ion counts)
2.0
1.5
1.0
0.5
0.0
PLATFORMRUNDAY_miss
0%

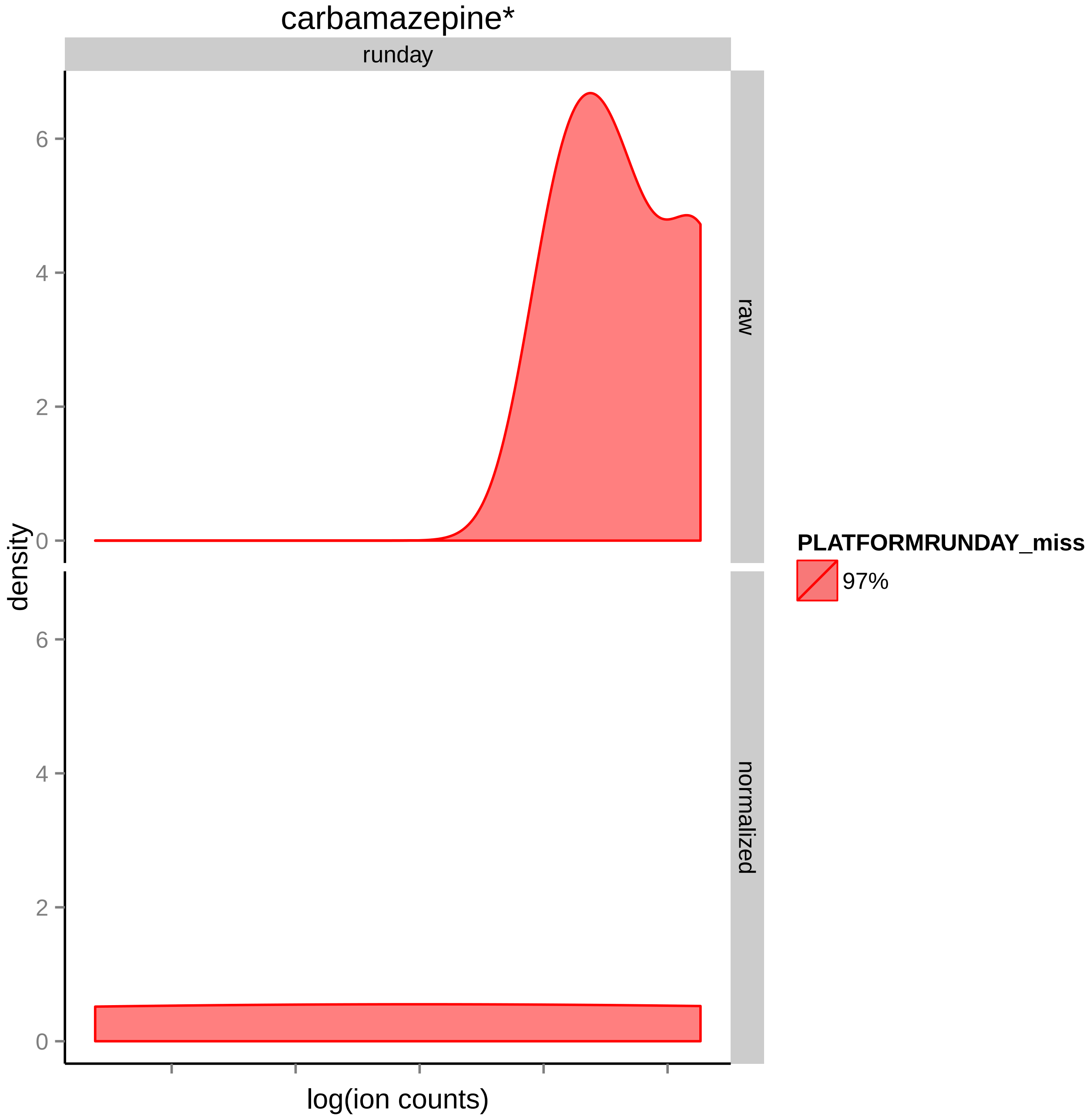
carbamazepine*
runday
raw
normalized
density
log(ion counts)
0
2
4
6
PLATFORMRUNDAY_miss
97%

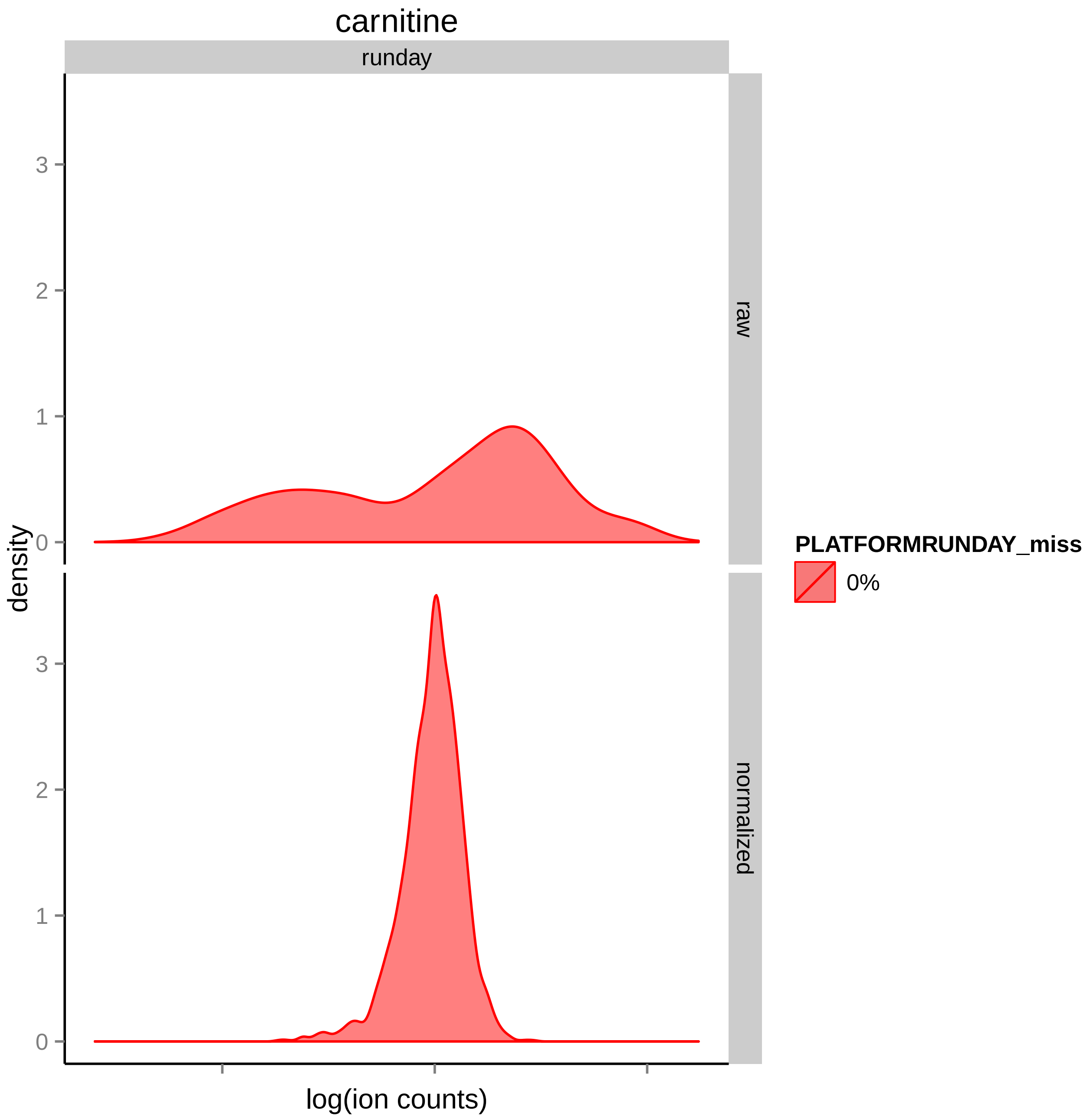
carnitine
runday
raw
normalized
density
log(ion counts)
0
1
2
3
0
1
2
3
PLATFORMRUNDAY_miss
0%

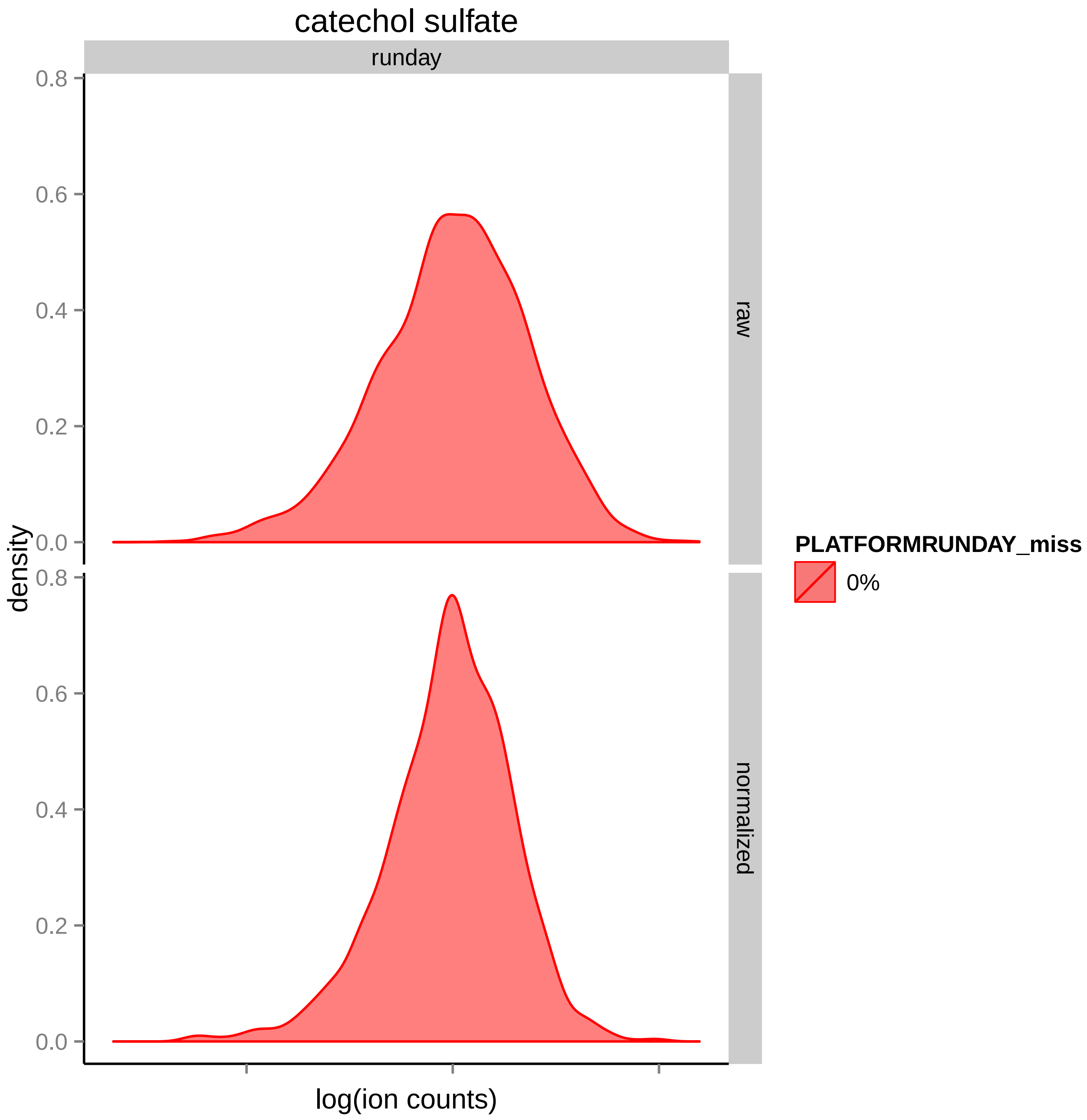
catechol sulfate
runday
raw
normalized
density
0.8
0.6
0.4
0.2
0.0
log(ion counts)
PLATFORMRUNDAY_miss
0%

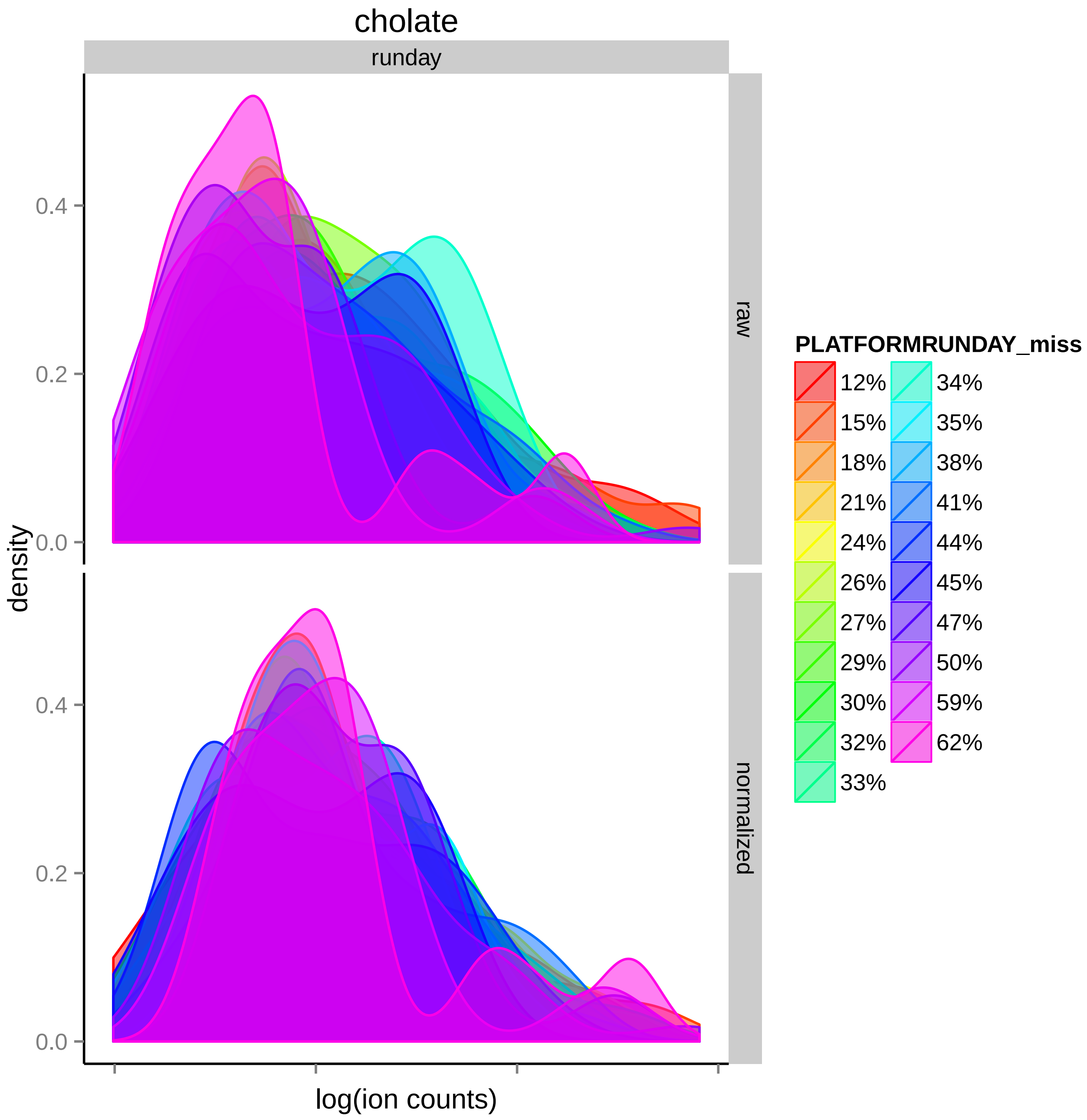
cholate
runday
raw
normalized
density
0.0
0.2
0.4
log(ion counts)
PLATFORMRUNDAY_miss
12%
15%
18%
21%
24%
26%
27%
29%
30%
32%
33%
34%
35%
38%
41%
44%
45%
47%
50%
59%
62%

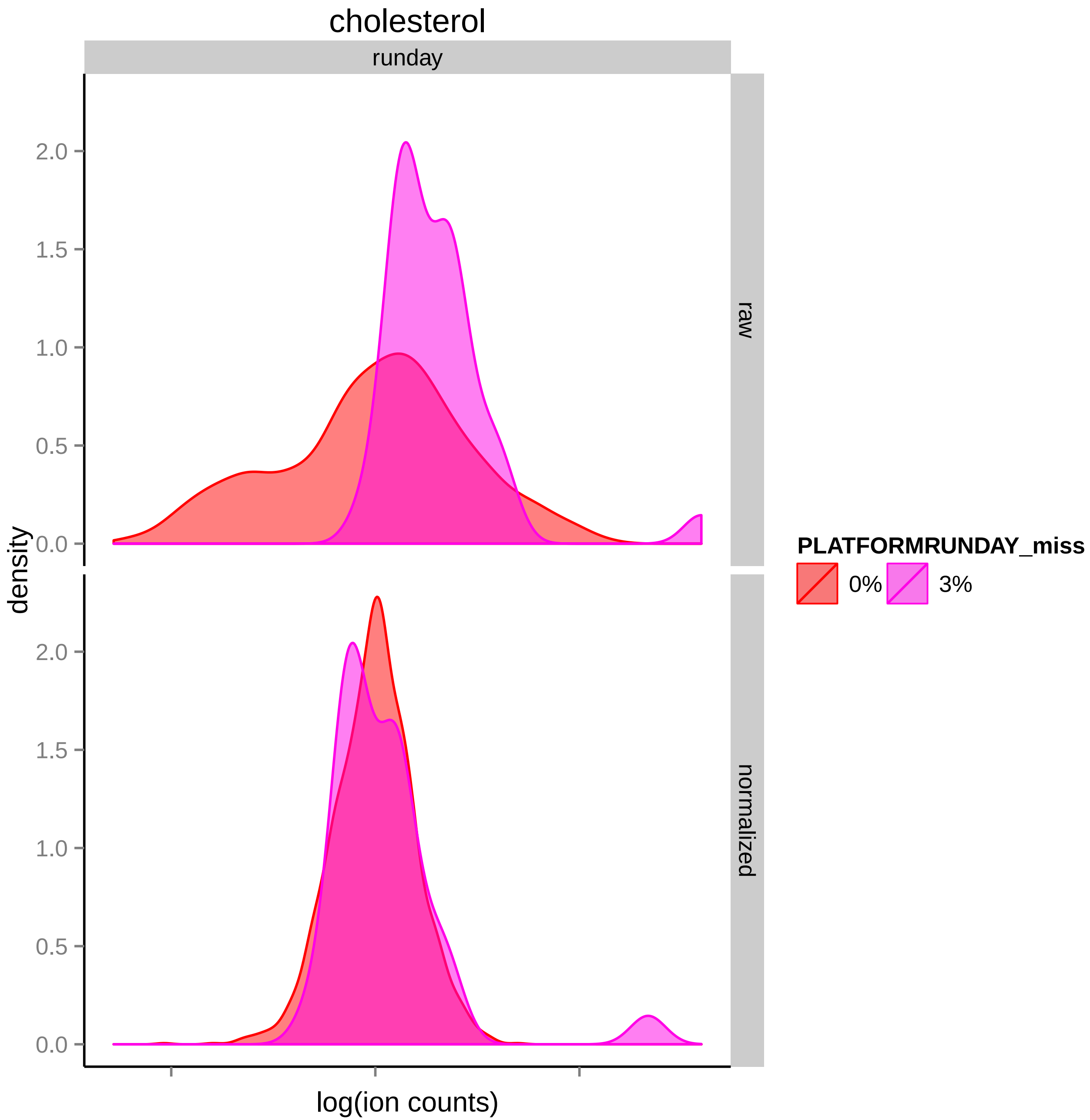
cholesterol
runday
raw
normalized
density
2.0
1.5
1.0
0.5
0.0
log(ion counts)
PLATFORMRUNDAY_miss
0%
3%

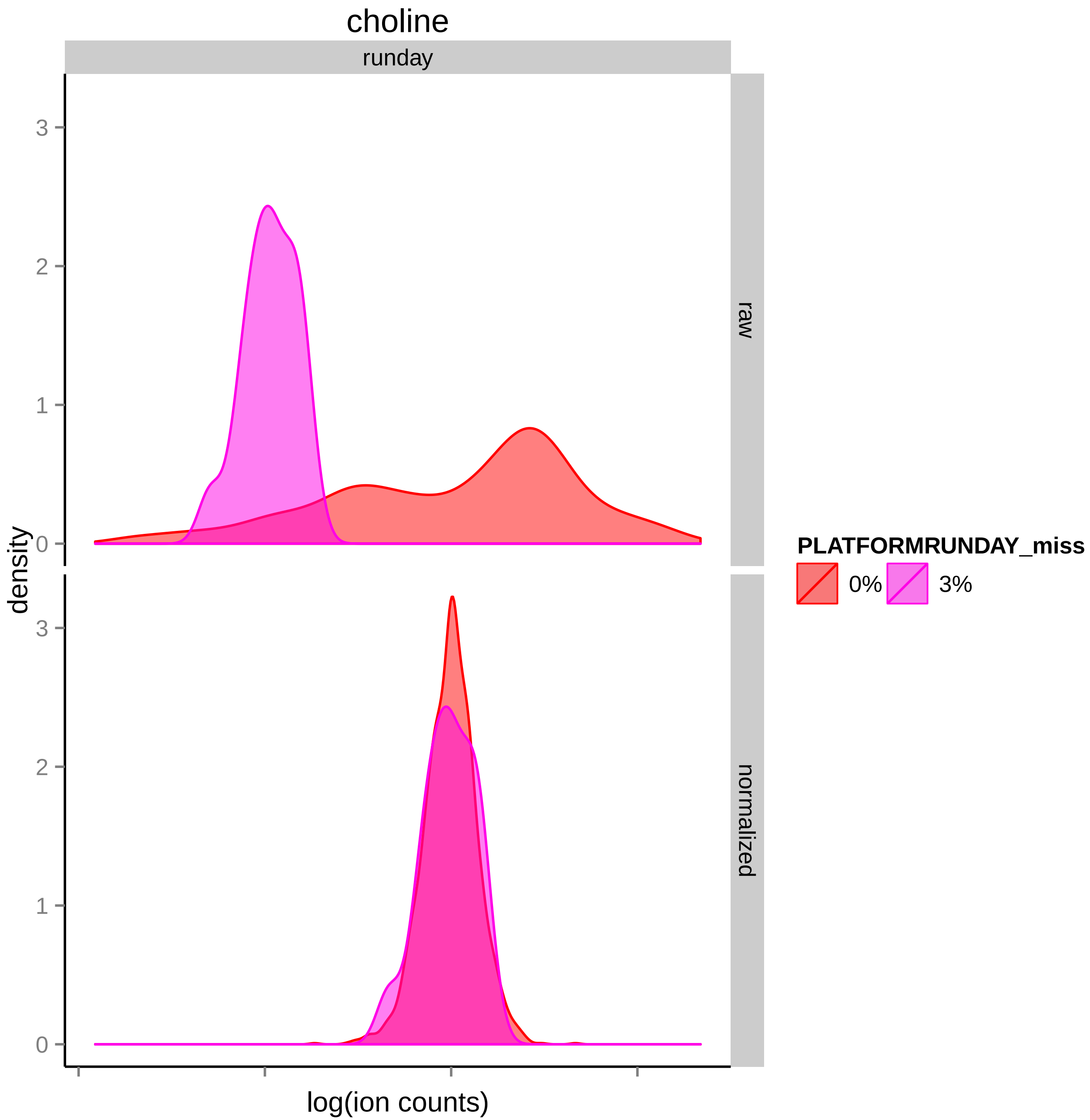

choline
runday
raw
normalized
density
log(ion counts)
0
1
2
3
PLATFORMRUNDAY_miss
0%
3%

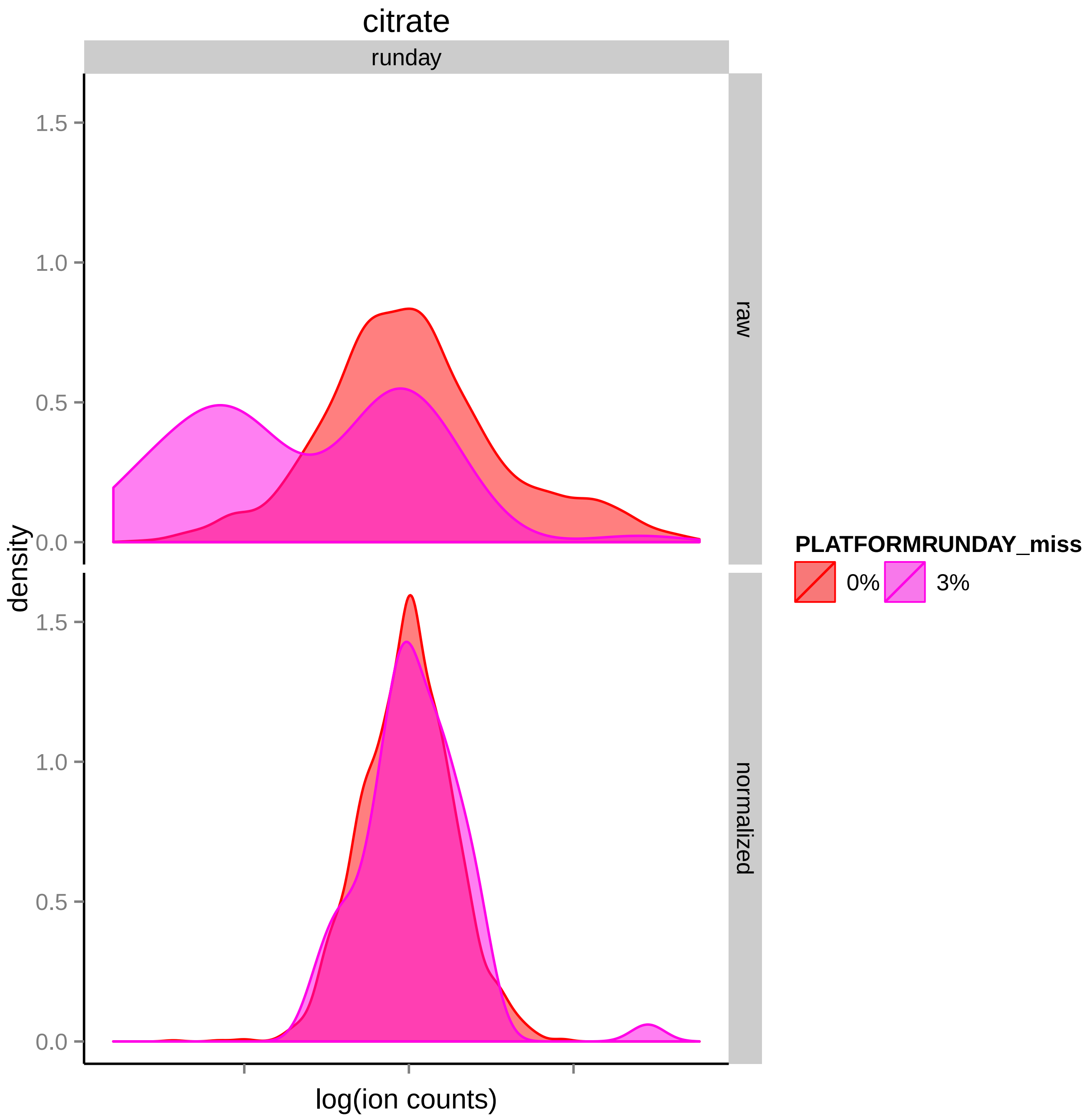
citrate
runday
raw
normalized
density
log(ion counts)
1.5
1.0
0.5
0.0
PLATFORMRUNDAY_miss
0%
3%

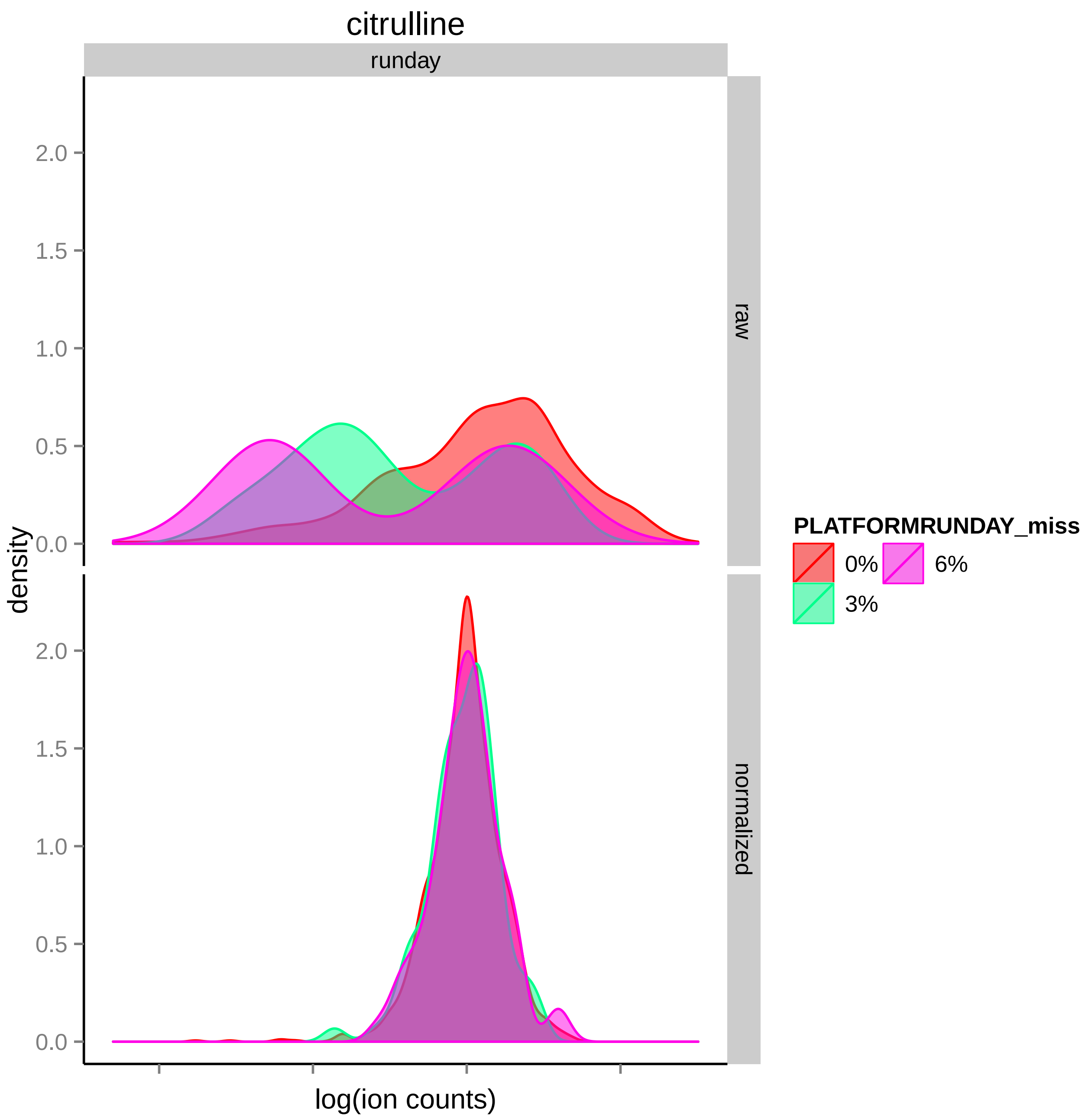

citrulline
runday
raw
normalized
density
log(ion counts)
0.0
0.5
1.0
1.5
2.0
PLATFORMRUNDAY_miss
0%
3%
6%

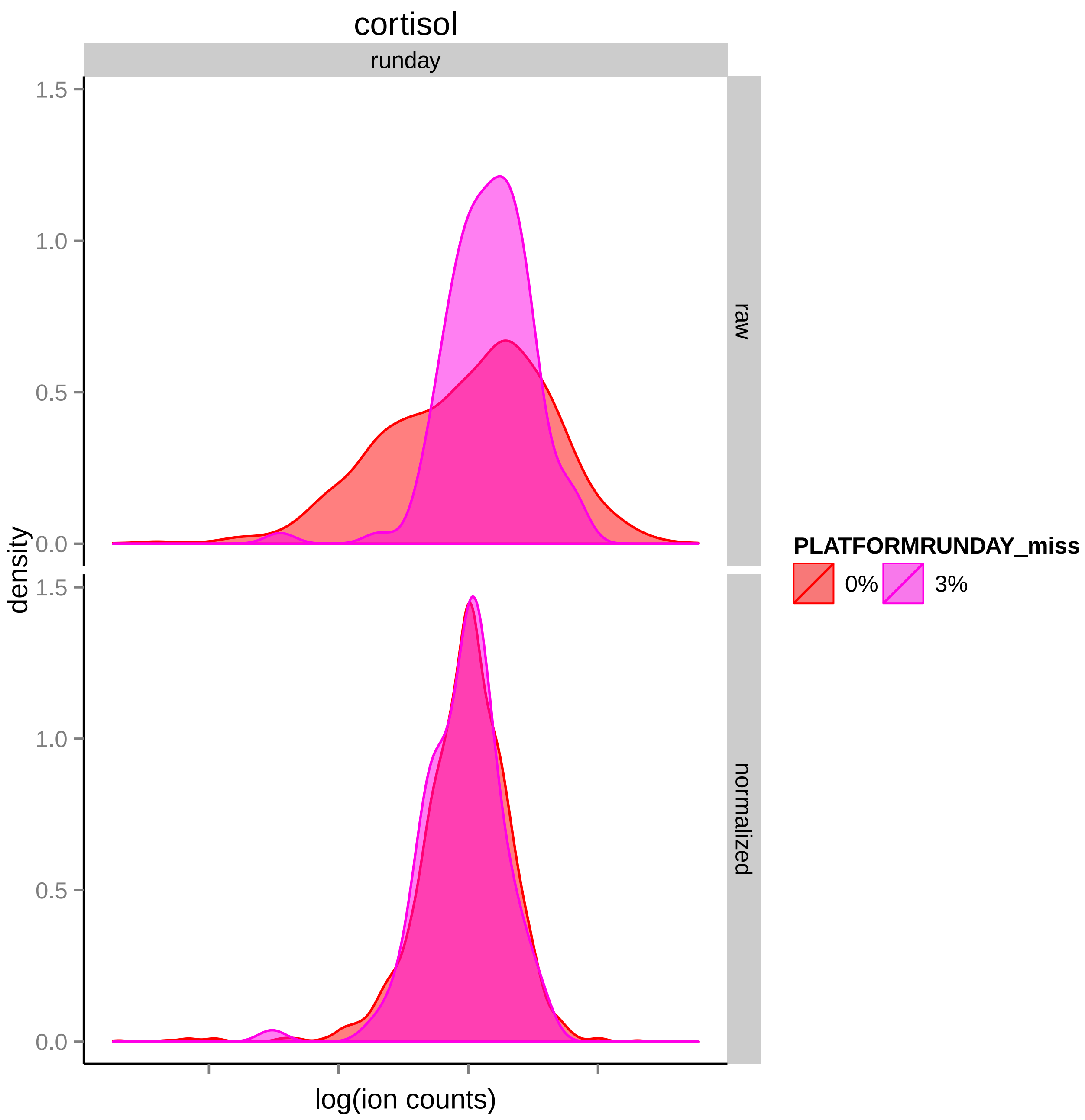

cortisol
runday
raw
normalized
density
1.5
1.0
0.5
0.0
log(ion counts)
PLATFORMRUNDAY_miss
0%
3%

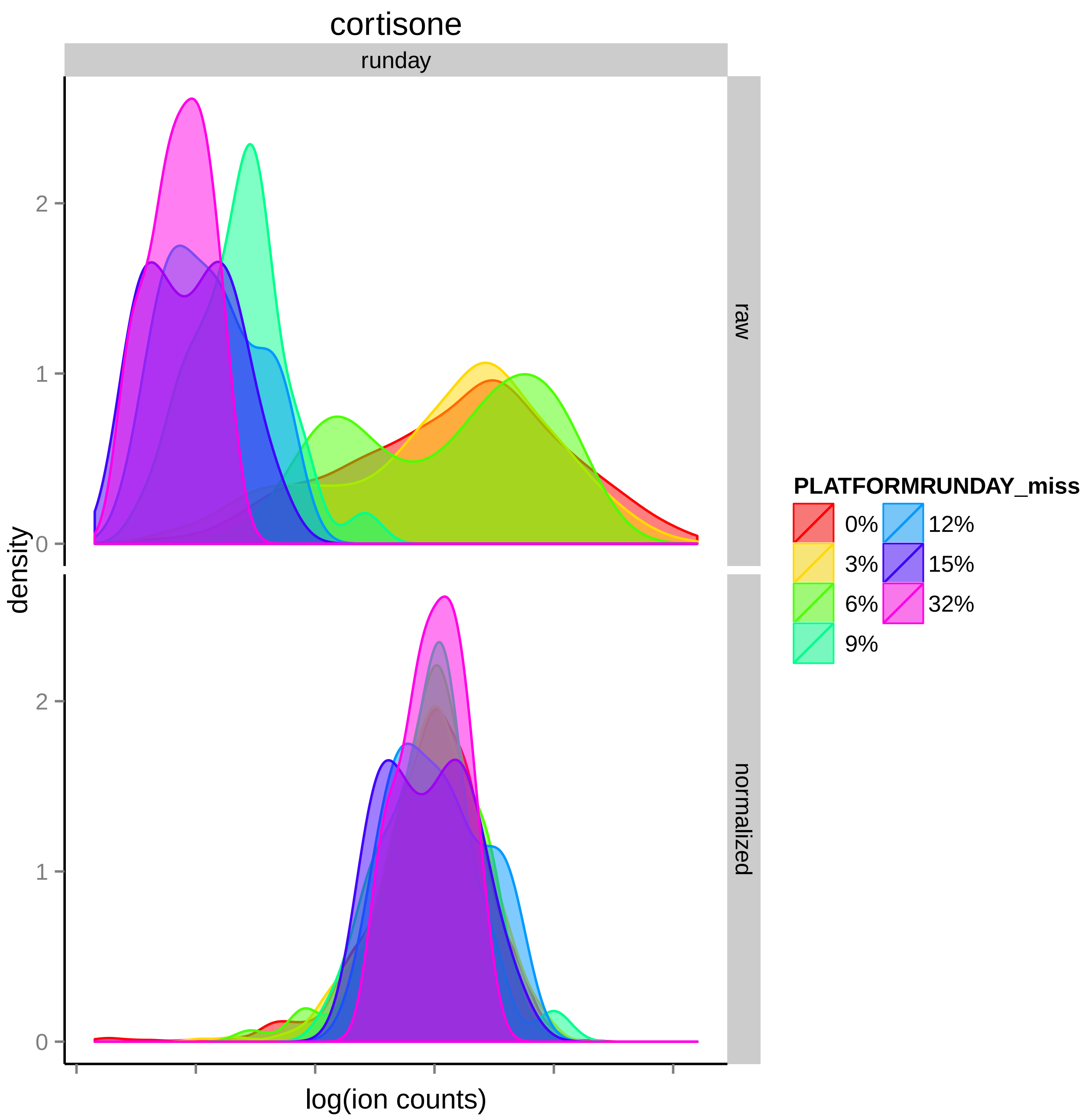
cortisone
runday
raw
normalized
density
log(ion counts)
0
1
2
0
1
2
PLATFORMRUNDAY_miss
0%
3%
6%
9%
12%
15%
32%

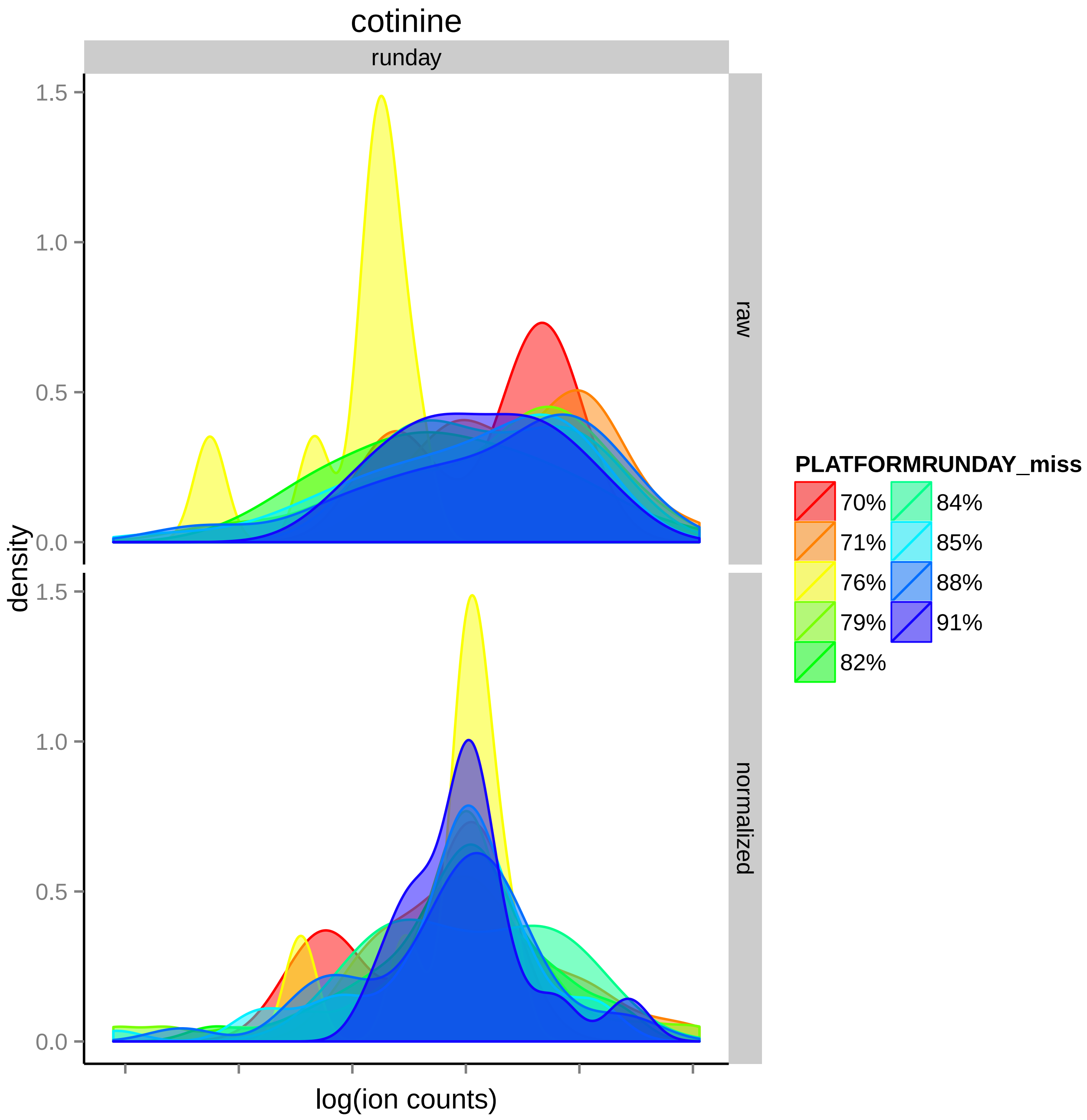
cotinine
runday
raw
normalized
density
log(ion counts)
1.5
1.0
0.5
0.0
PLATFORMRUNDAY_miss
70%
71%
76%
79%
82%
84%
85%
88%
91%

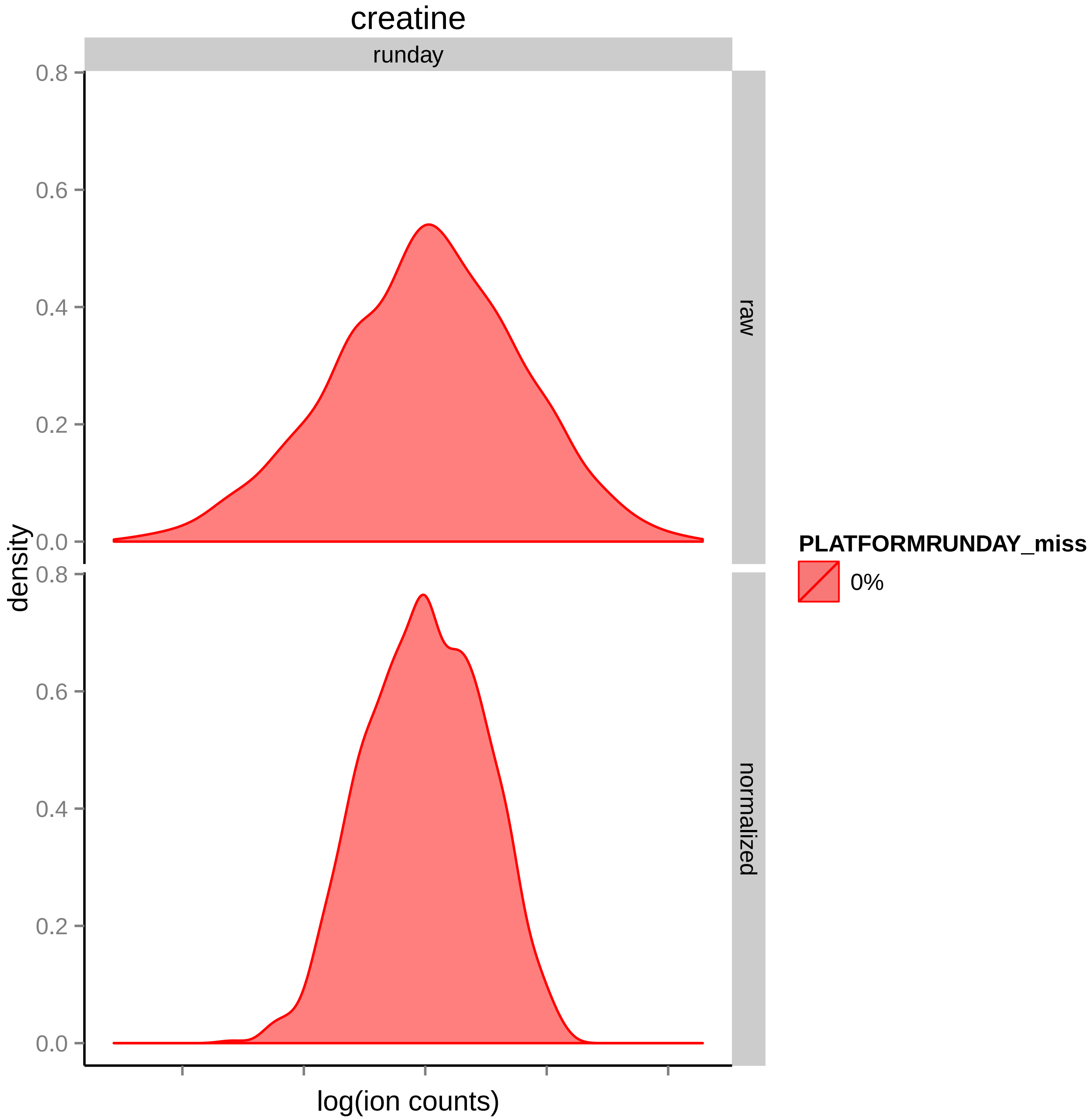

creatine
runday
raw
normalized
0.8
0.6
0.4
0.2
0.0
density
log(ion counts)
PLATFORMRUNDAY_miss
0%

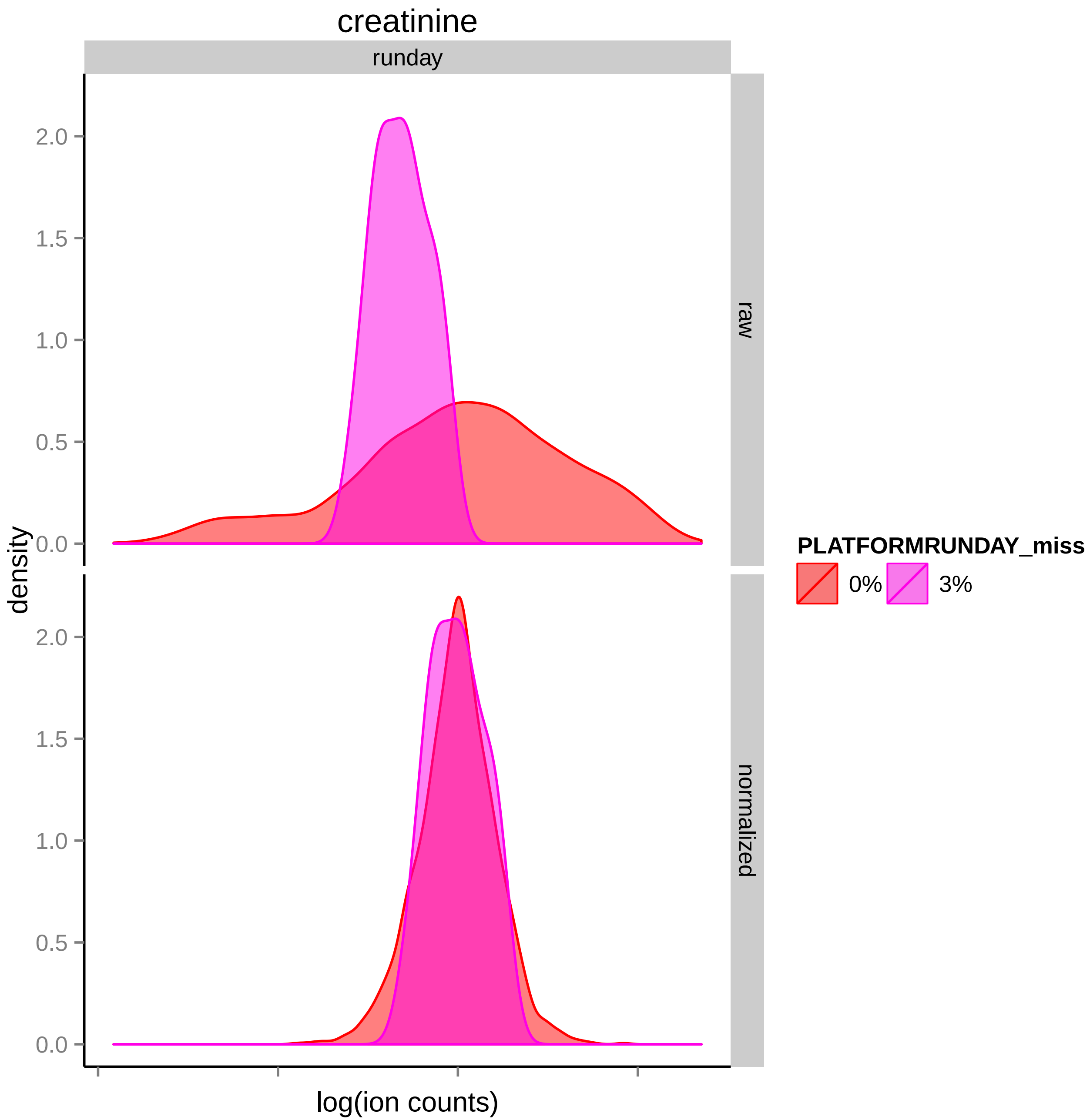

creatinine
runday
raw
normalized
density
log(ion counts)
2.0
1.5
1.0
0.5
0.0
PLATFORMRUNDAY_miss
0%
3%

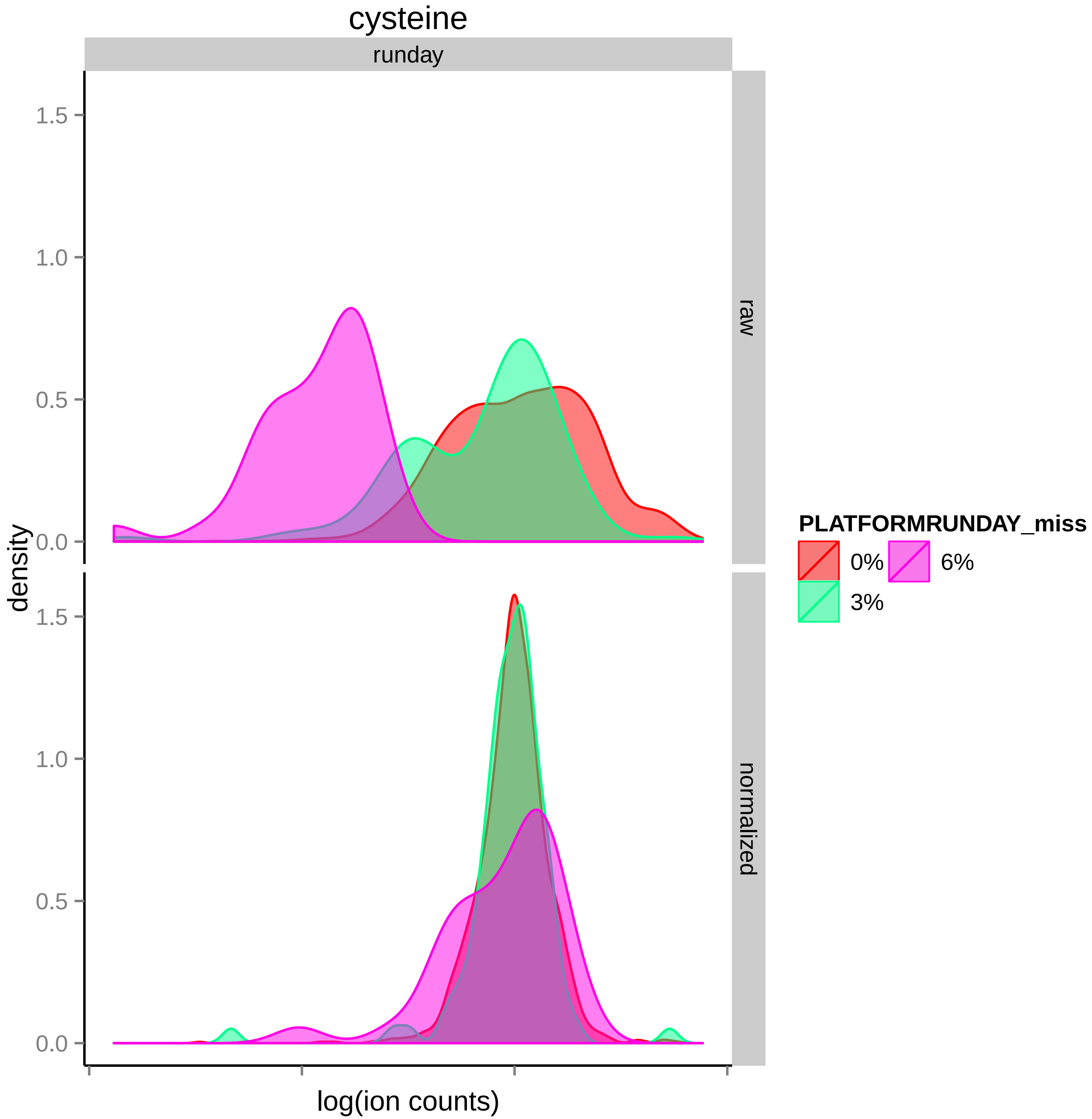

cysteine
runday
raw
normalized
density
log(ion counts)
1.5
1.0
0.5
0.0
PLATFORMRUNDAY_miss
0%
3%
6%

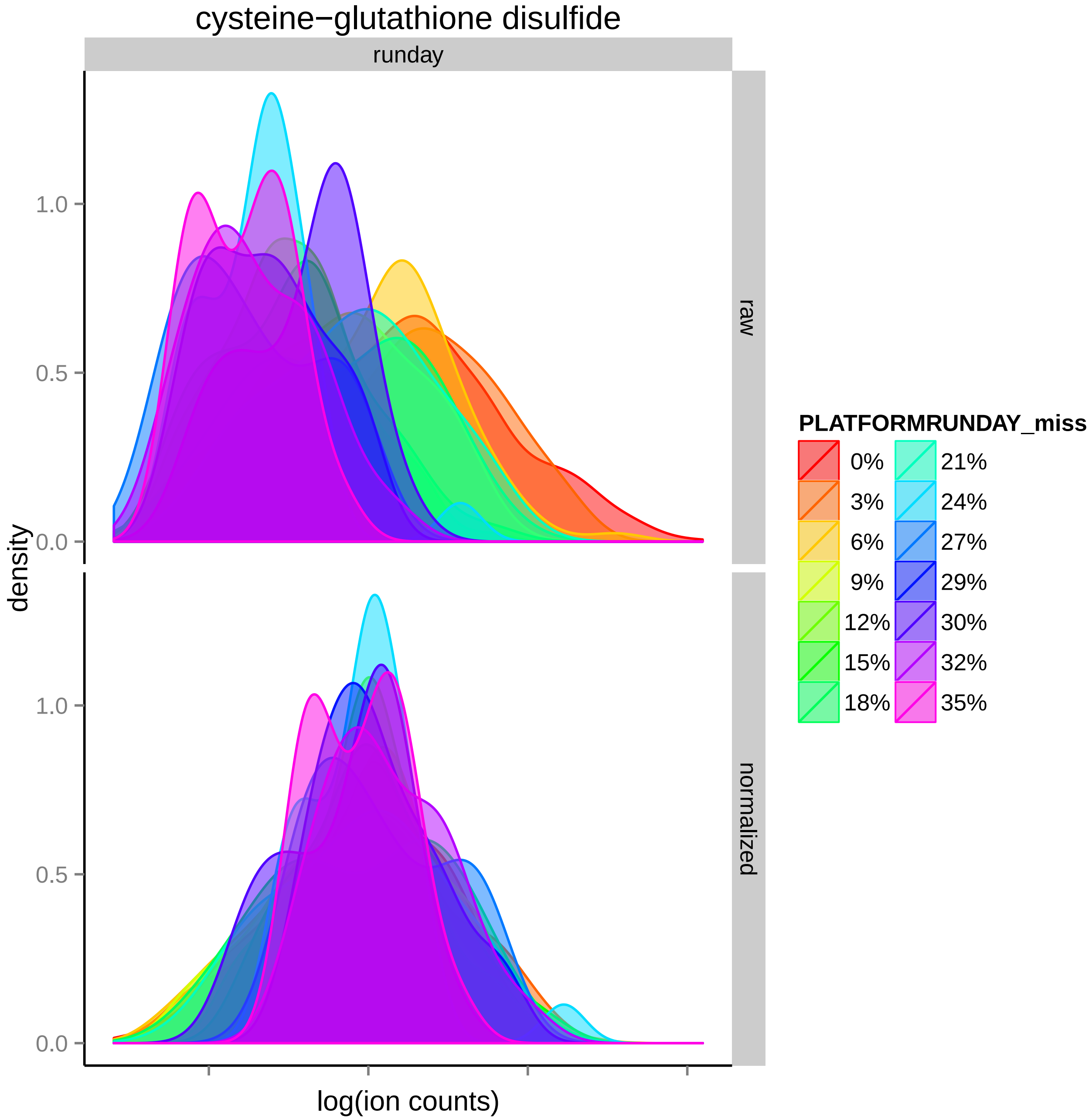
cysteine−glutathione disulfide
runday
raw
normalized
density
log(ion counts)
0.0
0.5
1.0
PLATFORMRUNDAY_miss
0%
3%
6%
9%
12%
15%
18%
21%
24%
27%
29%
30%
32%
35%

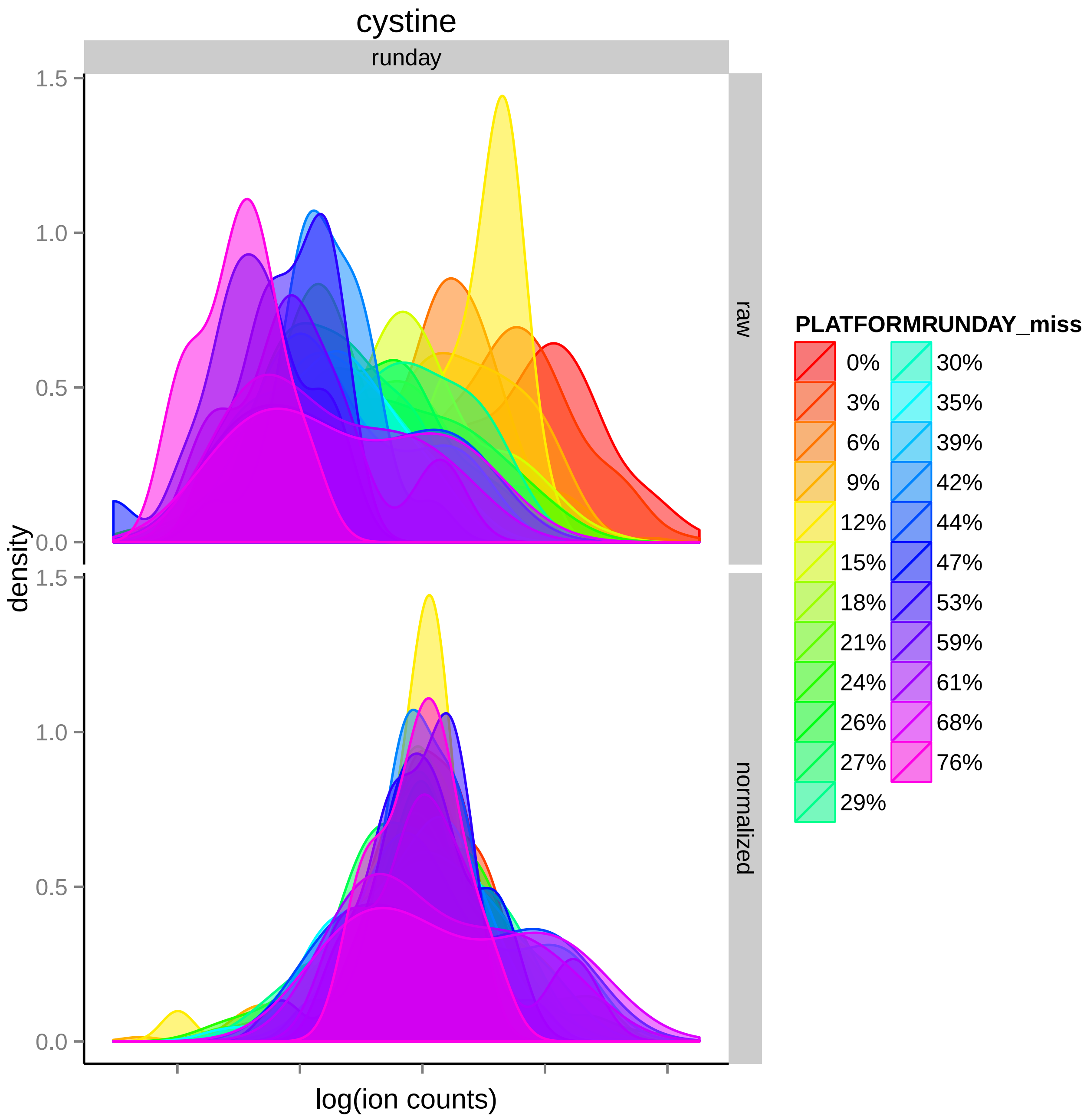
cystine
runday
raw
normalized
density
log(ion counts)
1.5
1.0
0.5
0.0
PLATFORMRUNDAY_miss
0%
3%
6%
9%
12%
15%
18%
21%
24%
26%
27%
29%
30%
35%
39%
42%
44%
47%
53%
59%
61%
68%
76%

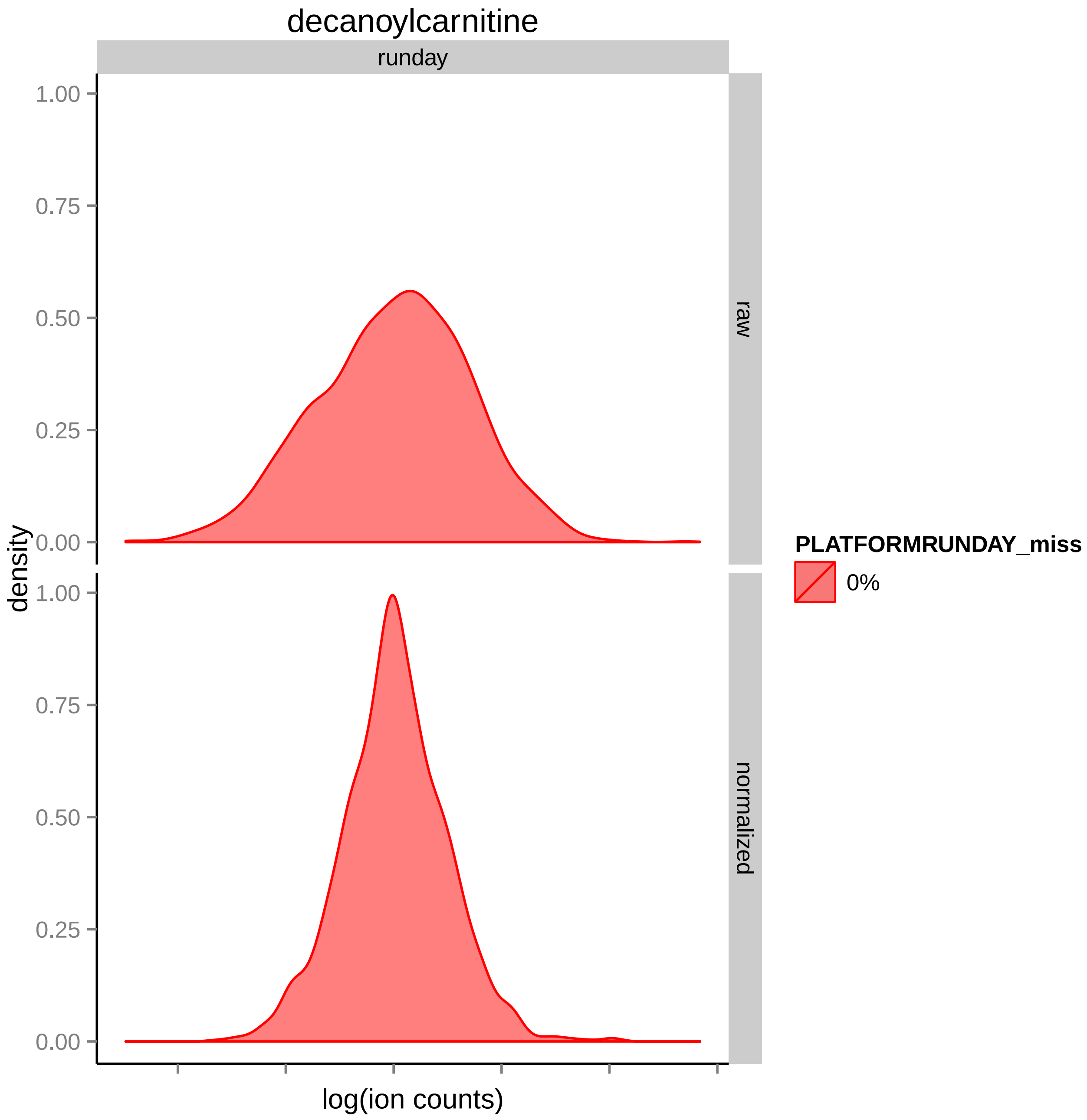

decanoylcarnitine
runday
raw
normalized
density
1.00
0.75
0.50
0.25
0.00
log(ion counts)
PLATFORMRUNDAY_miss
0%

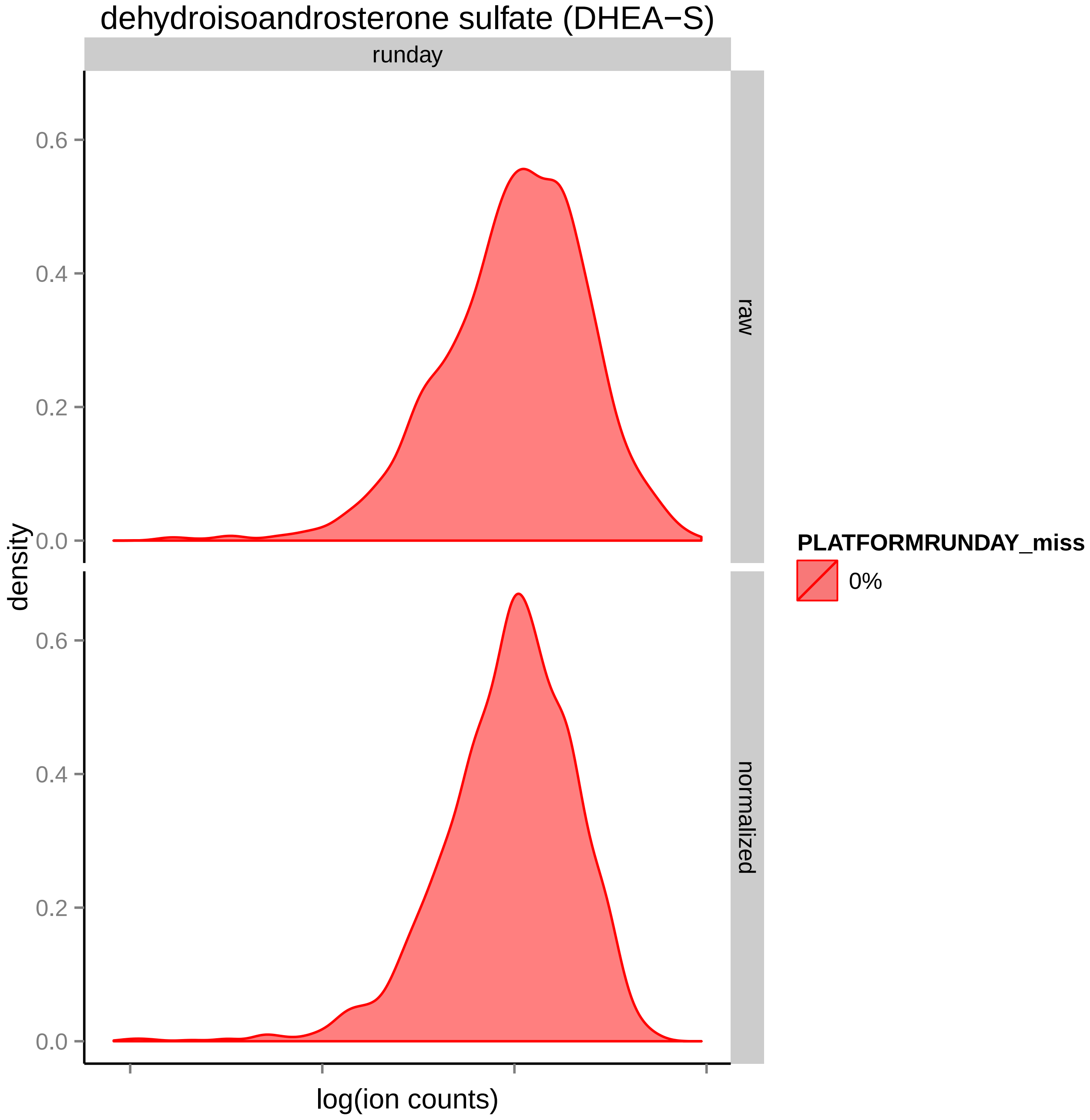
dehydroisoandrosterone sulfate (DHEA−S)
runday
raw
normalized
density
0.0
0.2
0.4
0.6
log(ion counts)
PLATFORMRUNDAY_miss
0%

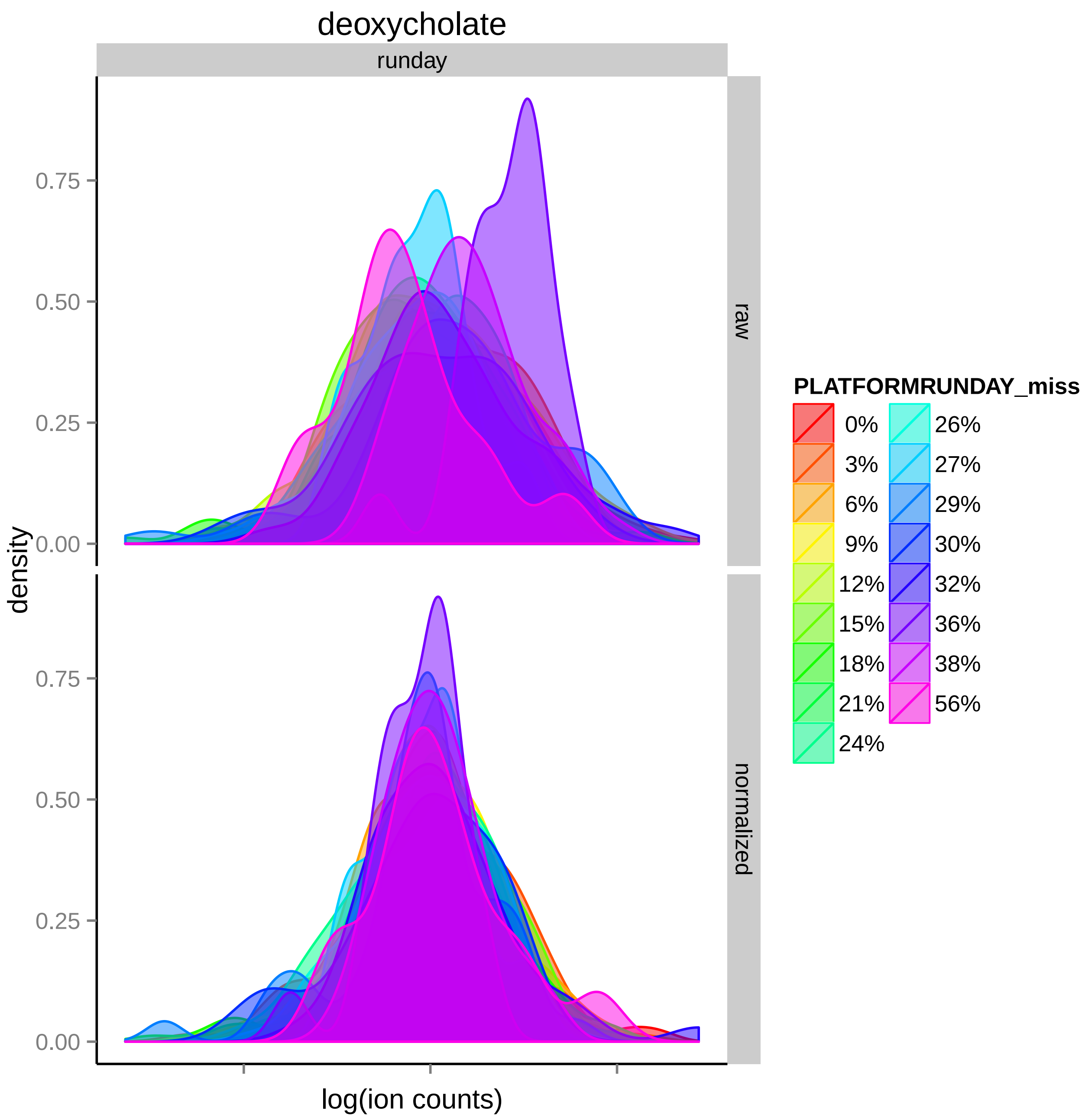
deoxycholate
runday
raw
normalized
density
0.00
0.25
0.50
0.75
log(ion counts)
PLATFORMRUNDAY_miss
0%
3%
6%
9%
12%
15%
18%
21%
24%
26%
27%
29%
30%
32%
36%
38%
56%

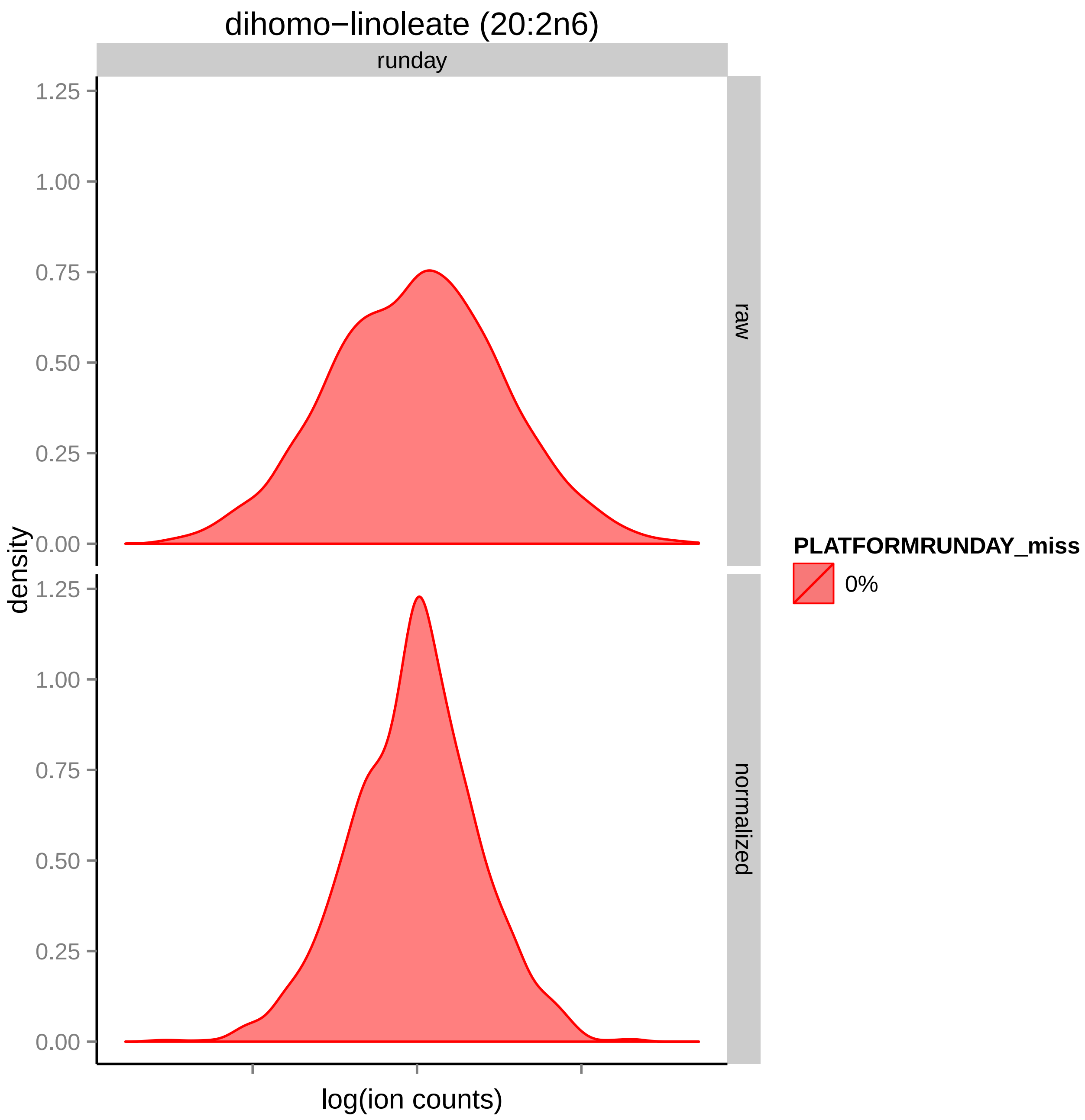
dihomo−linoleate (20:2n6)
runday
raw
normalized
density
log(ion counts)
1.25
1.00
0.75
0.50
0.25
0.00
PLATFORMRUNDAY_miss
0%

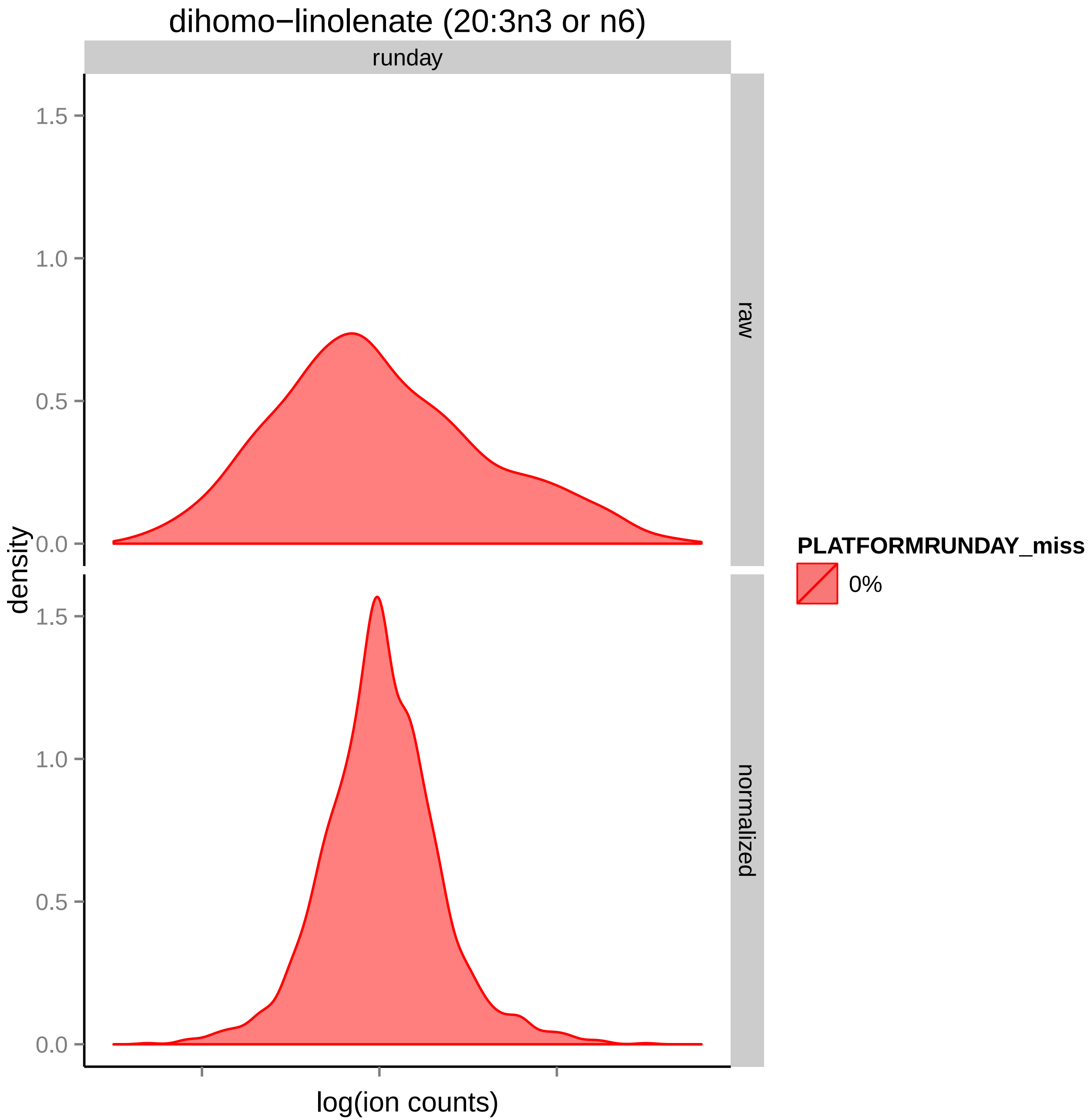

dihomo−linolenate (20:3n3 or n6)
runday
raw
normalized
density
1.5
1.0
0.5
0.0
log(ion counts)
PLATFORMRUNDAY_miss
0%

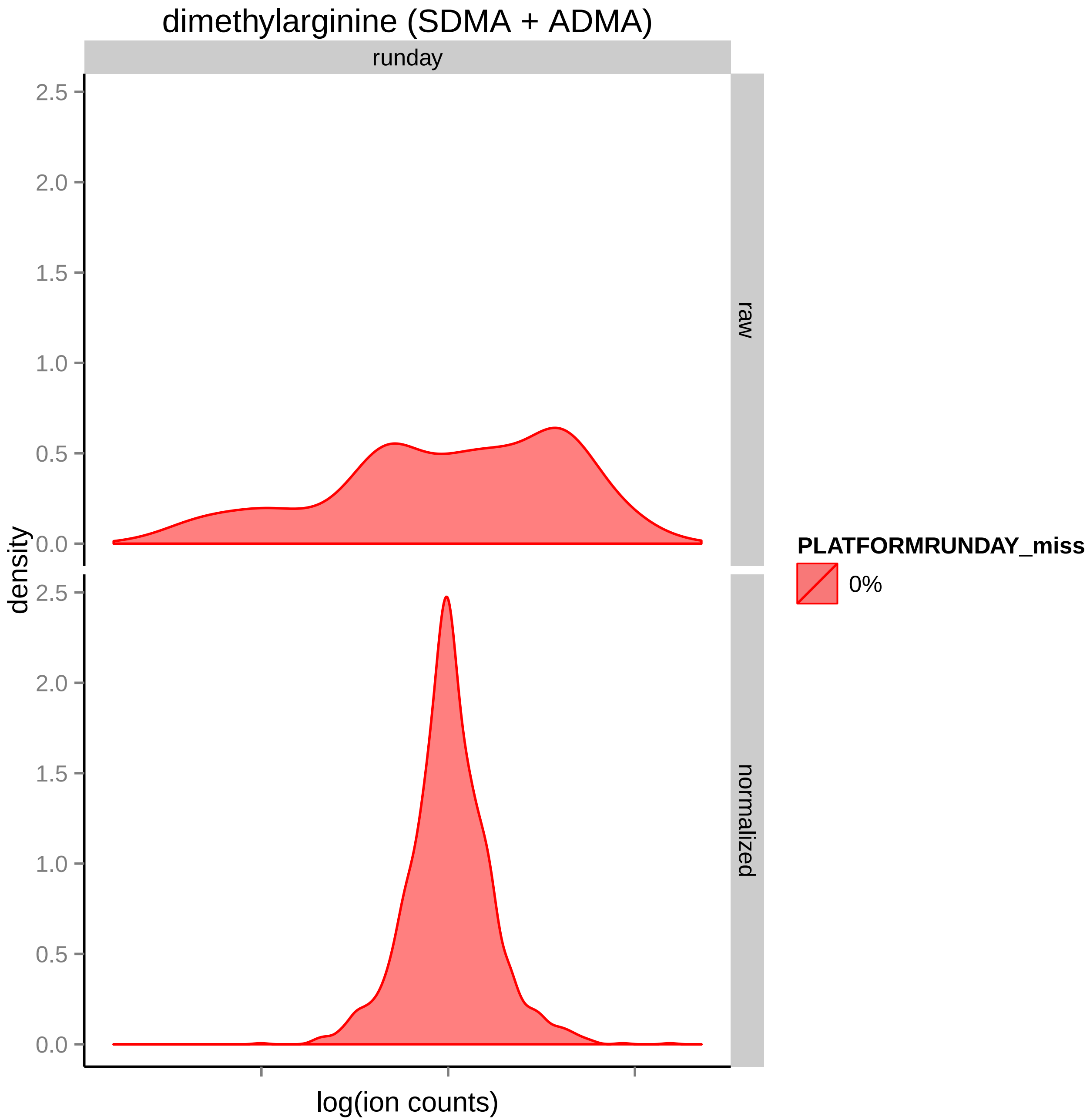

dimethylarginine (SDMA + ADMA)
runday
raw
normalized
density
2.5
2.0
1.5
1.0
0.5
0.0
log(ion counts)
PLATFORMRUNDAY_miss
0%

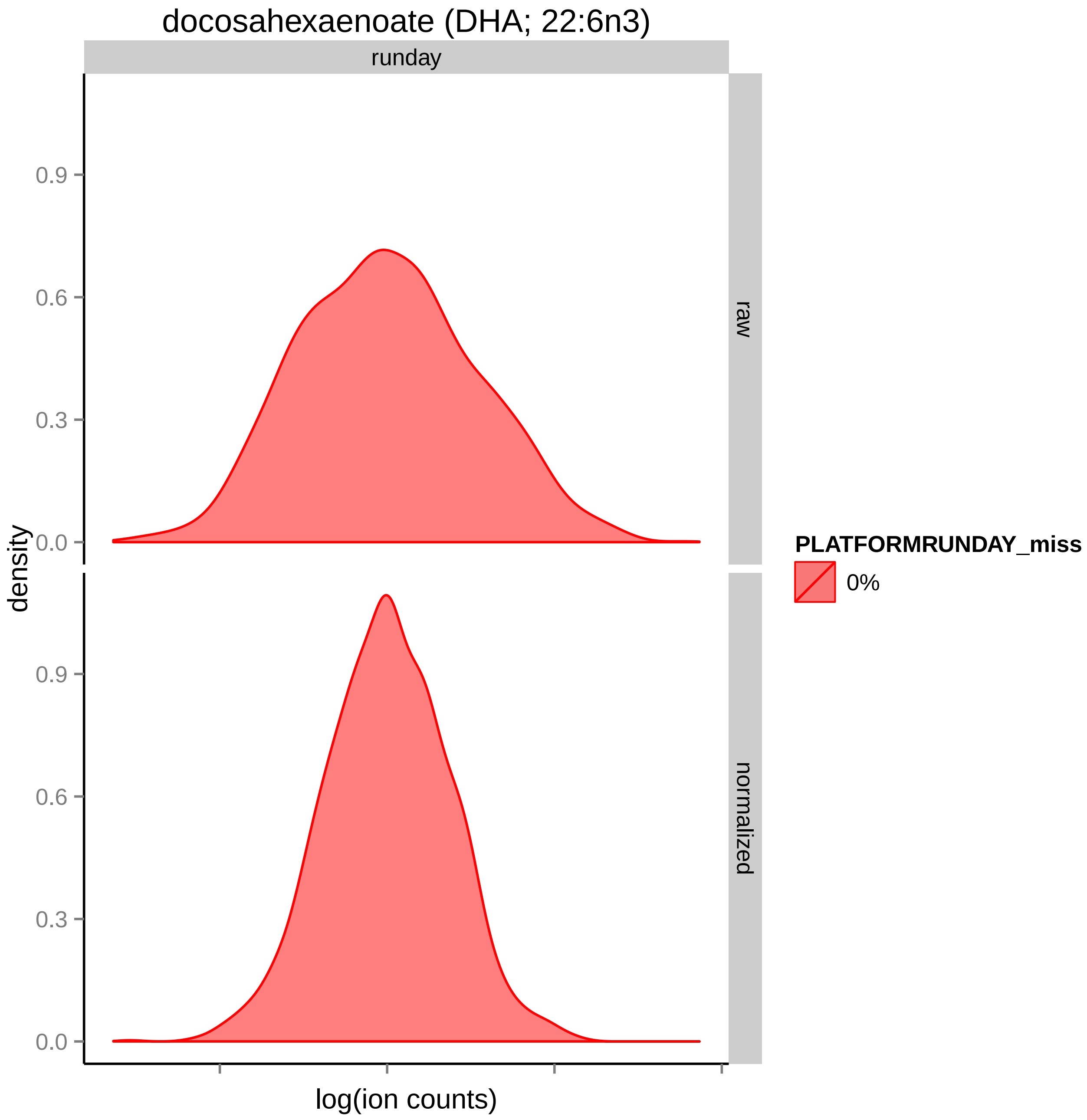
docosahexaenoate (DHA; 22:6n3)
runday
raw
normalized
density
log(ion counts)
0.0
0.3
0.6
0.9
PLATFORMRUNDAY_miss
0%

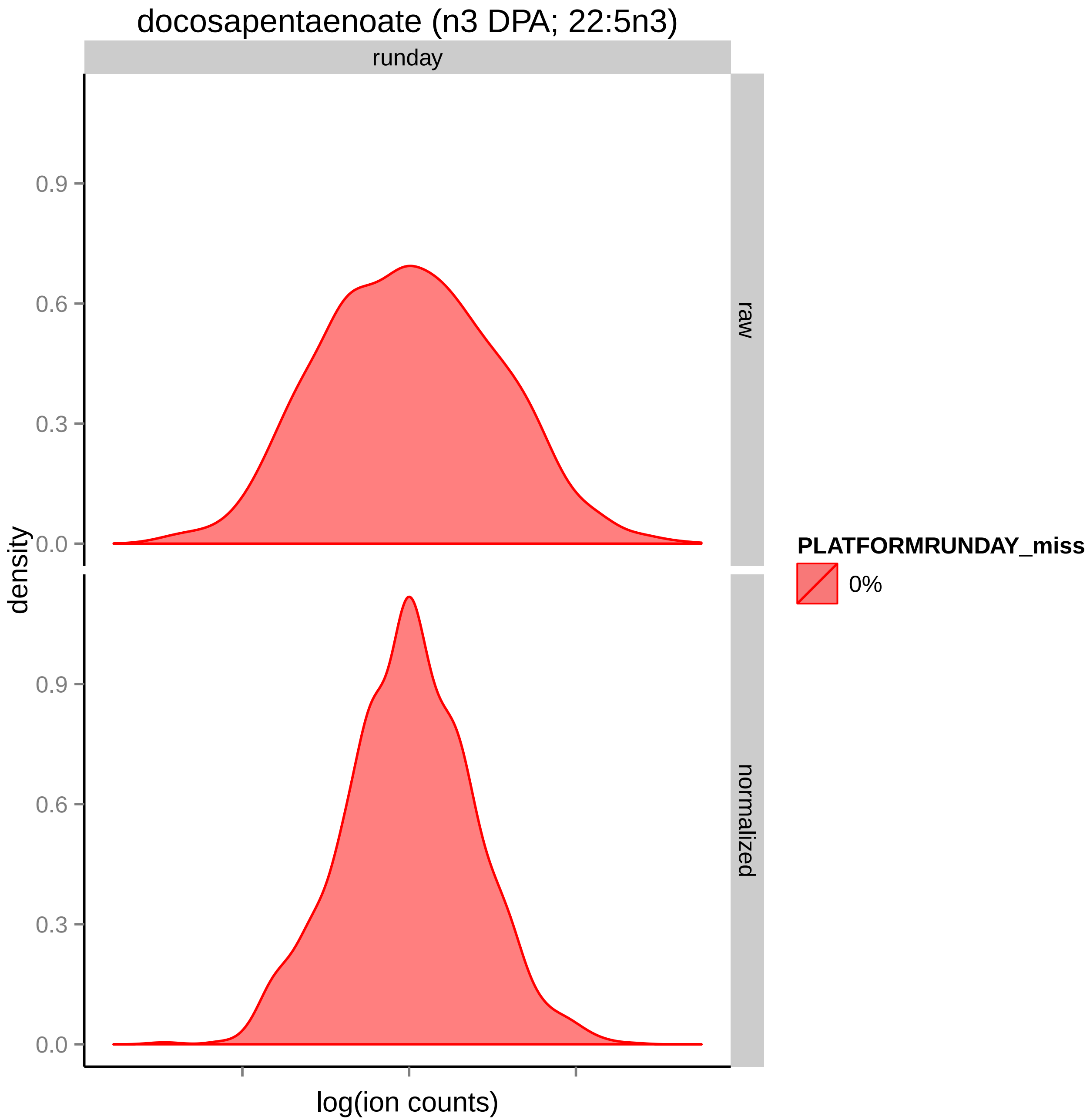
docosapentaenoate (n3 DPA; 22:5n3)
runday
raw
normalized
0.9
0.6
0.3
0.0
density
log(ion counts)
PLATFORMRUNDAY_miss
0%

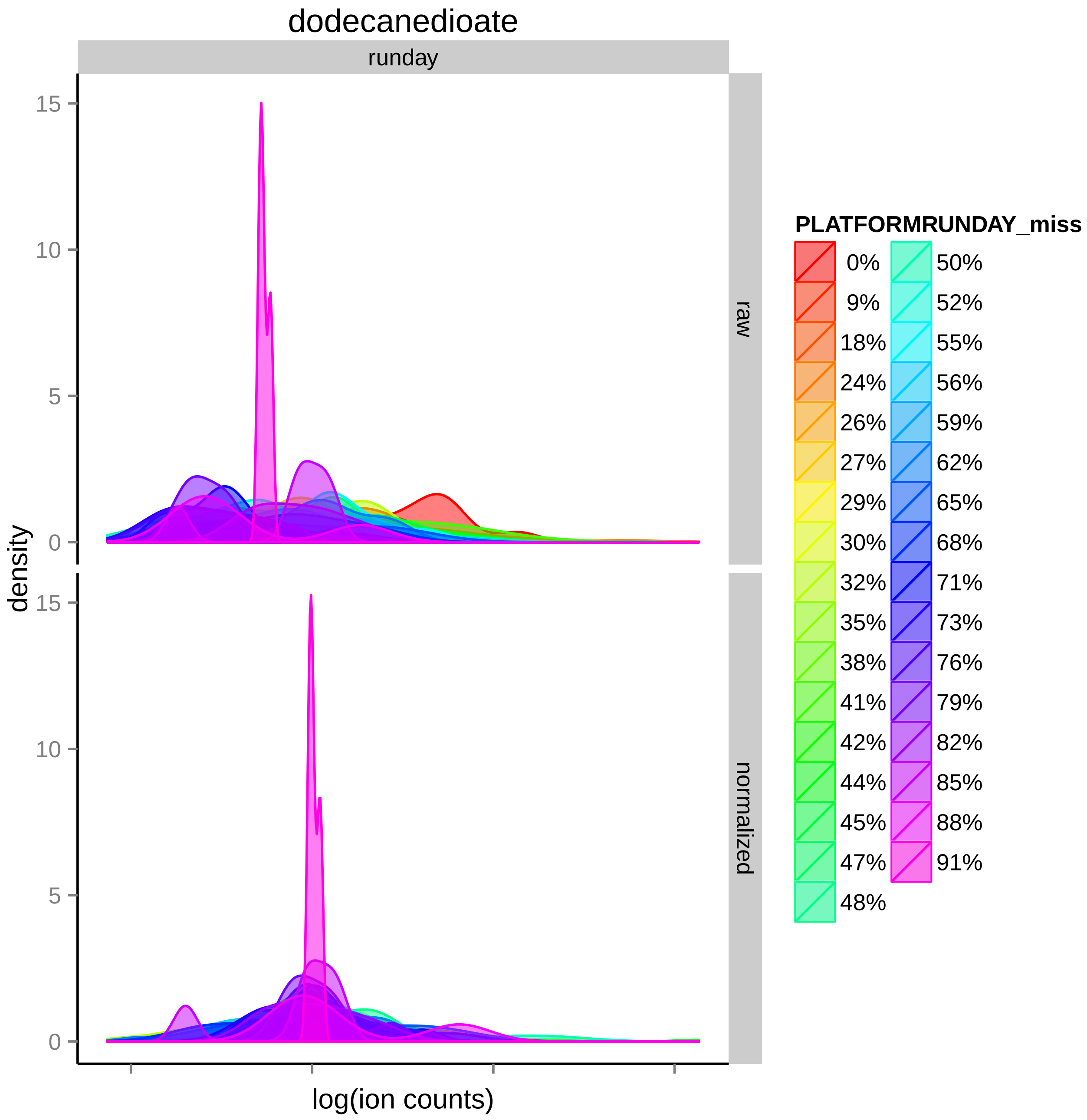
dodecanedioate
runday
raw
normalized
density
log(ion counts)
0
5
10
15
PLATFORMRUNDAY_miss
0%
9%
18%
24%
26%
27%
29%
30%
32%
35%
38%
41%
42%
44%
45%
47%
48%
50%
52%
55%
56%
59%
62%
65%
68%
71%
73%
76%
79%
82%
85%
88%
91%

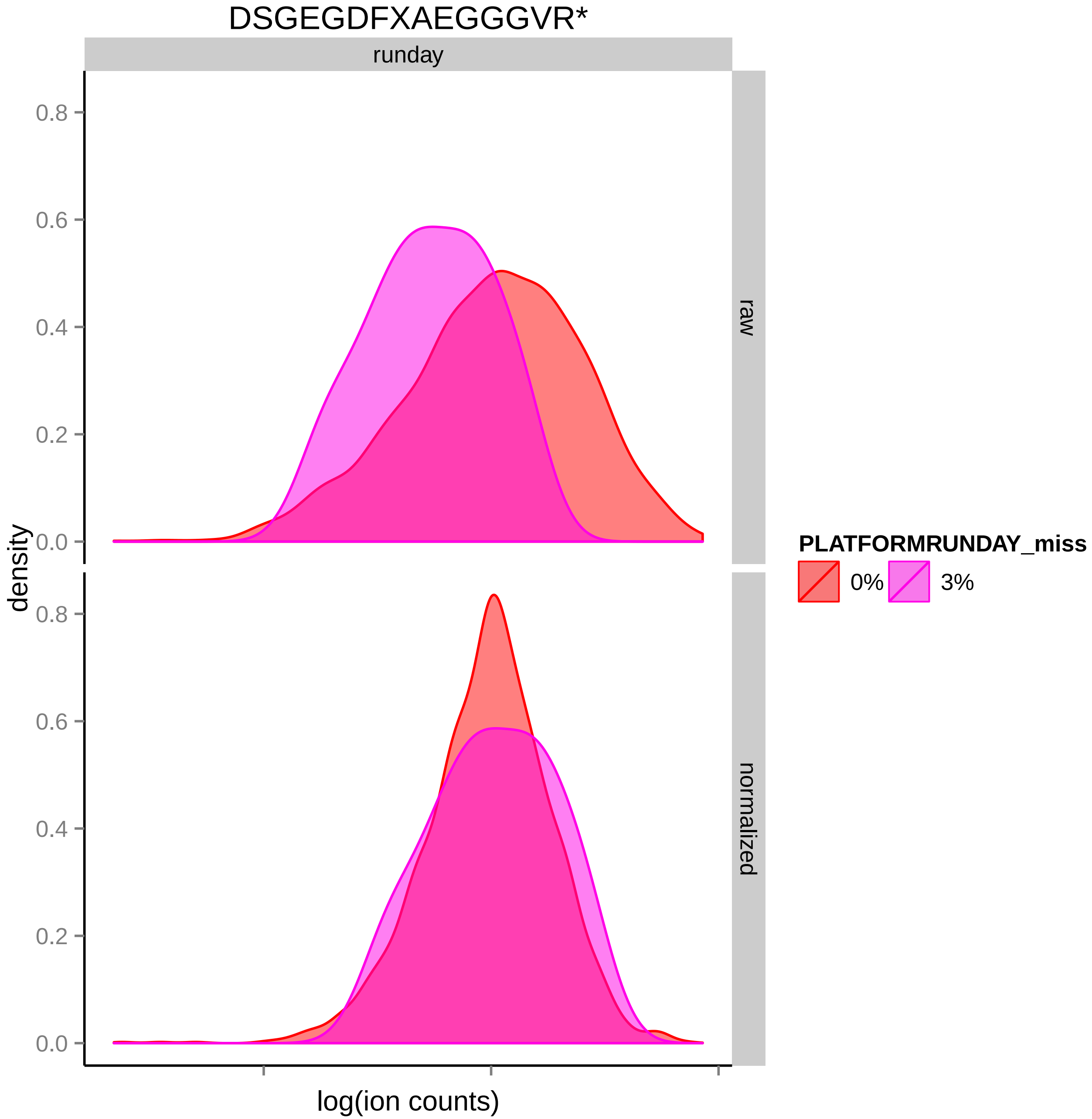

DSGEGDFXAEGGGVR*
runday
raw
normalized
density
0.0
0.2
0.4
0.6
0.8
log(ion counts)
PLATFORMRUNDAY_miss
0%
3%

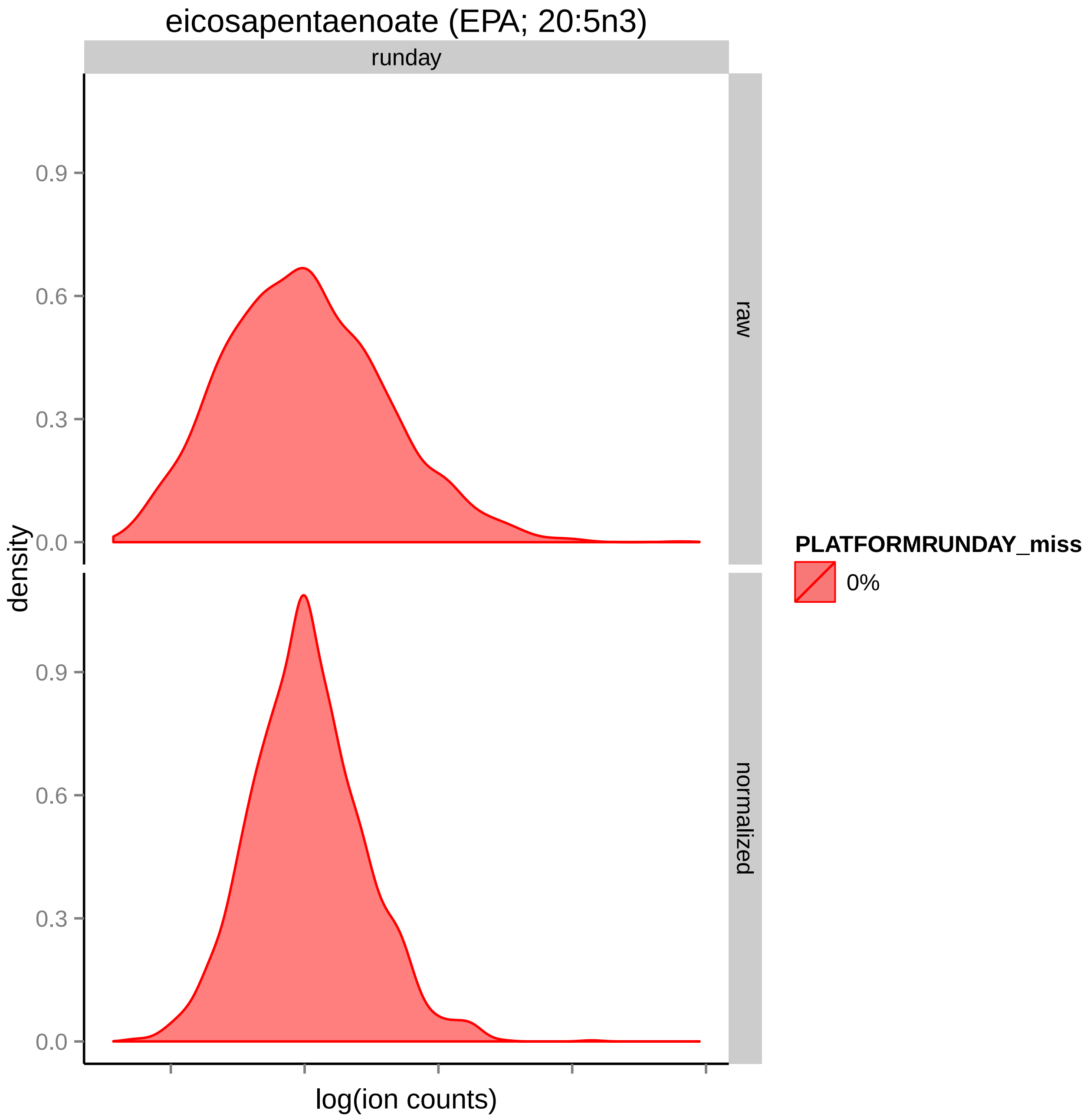

eicosapentaenoate (EPA; 20:5n3)
runday
raw
normalized
density
0.0
0.3
0.6
0.9
log(ion counts)
PLATFORMRUNDAY_miss
0%

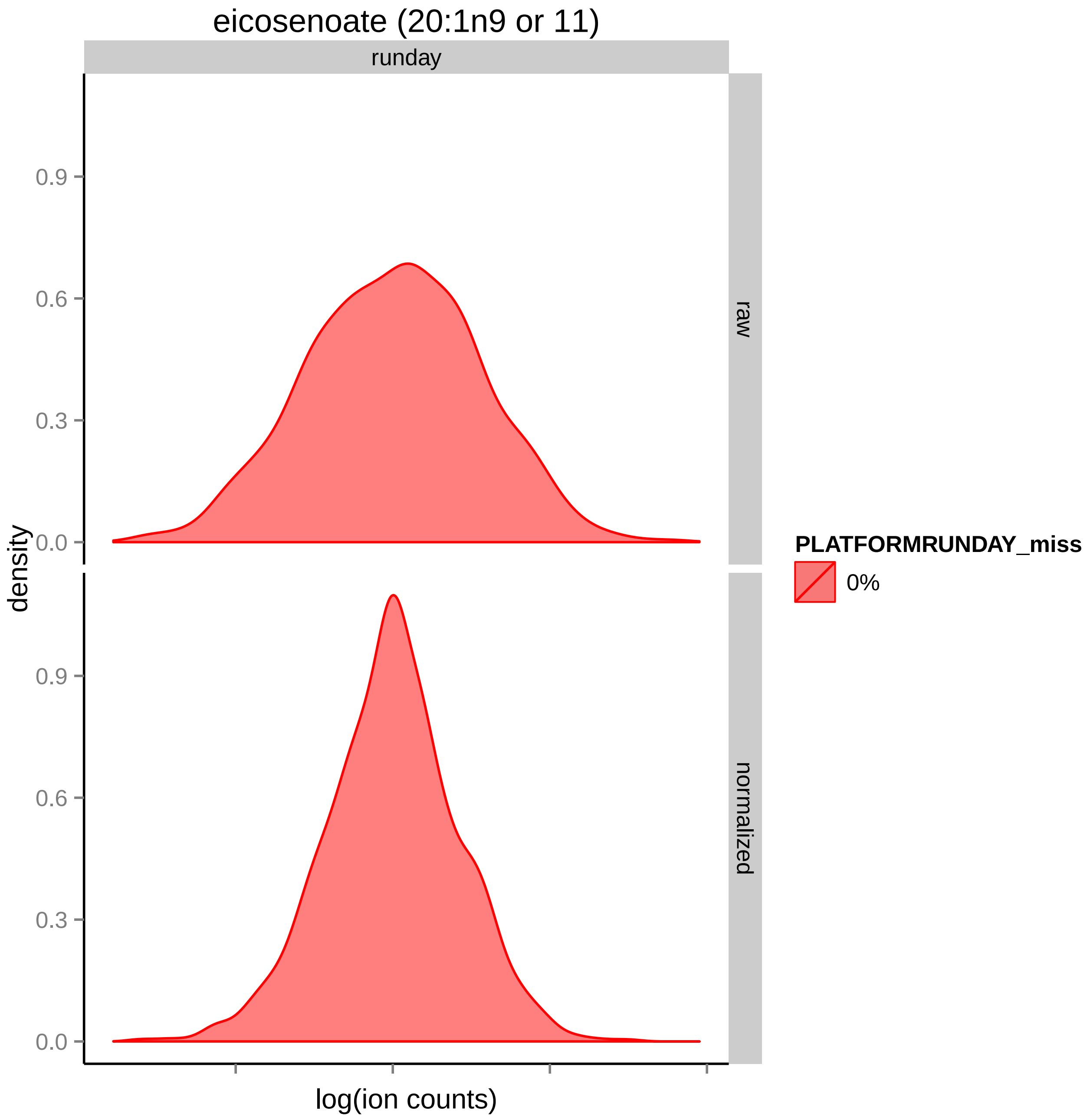
eicosenoate (20:1n9 or 11)
runday
raw
normalized
density
0.0
0.3
0.6
0.9
log(ion counts)
PLATFORMRUNDAY_miss
0%

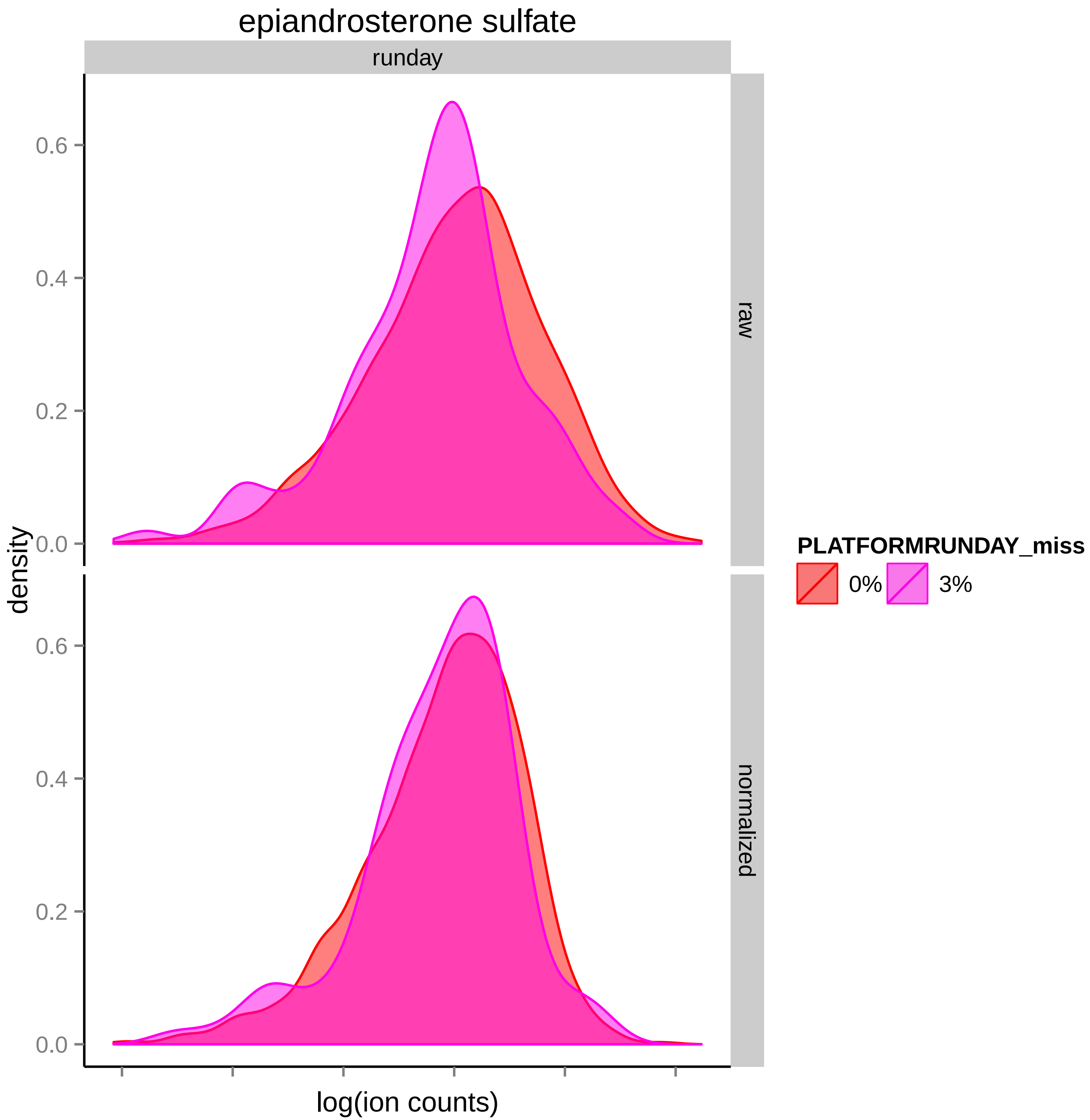

epiandrosterone sulfate
runday
raw
normalized
density
0.0
0.2
0.4
0.6
log(ion counts)
PLATFORMRUNDAY_miss
0%
3%

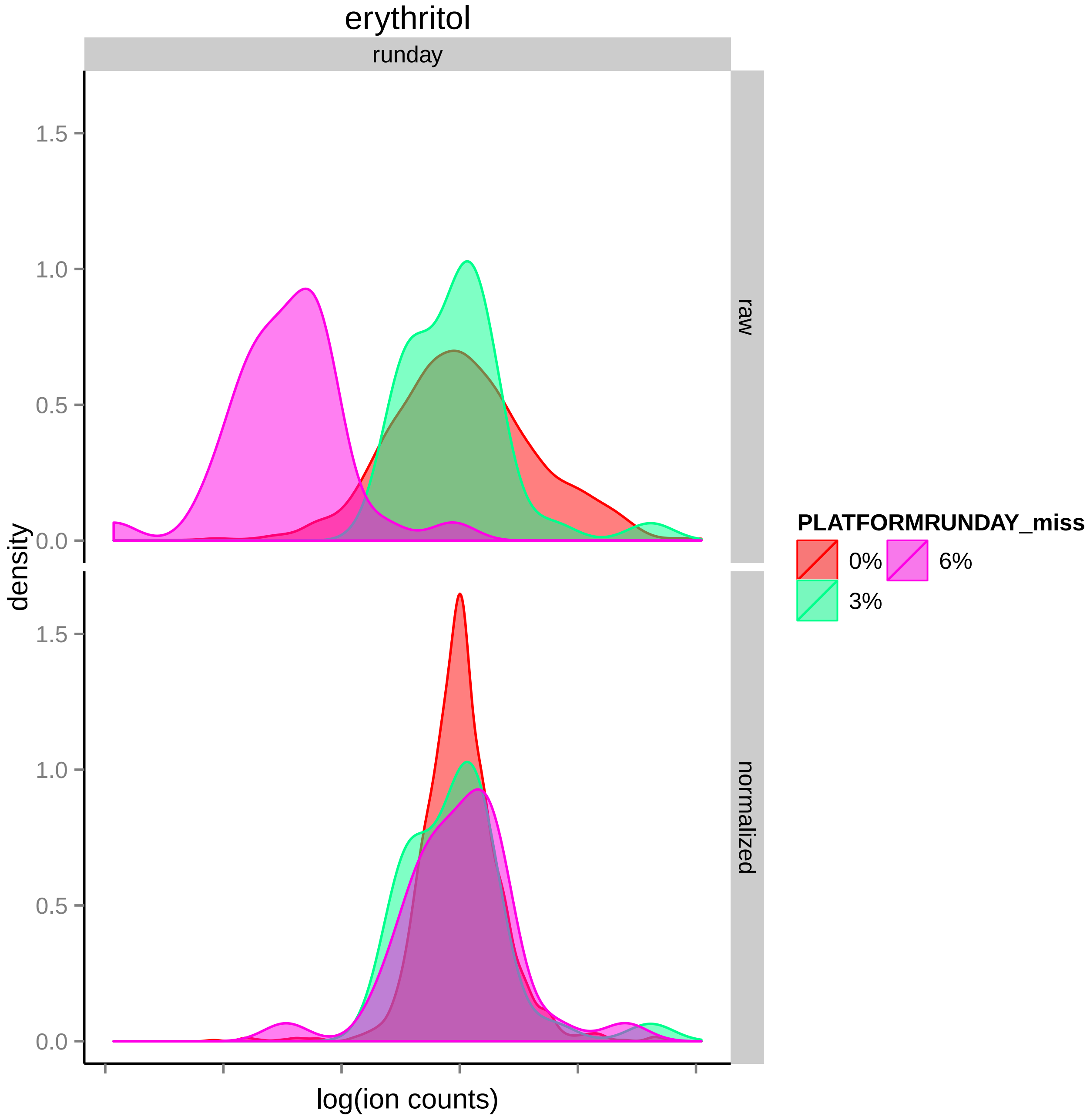
erythritol
runday
raw
normalized
density
1.5
1.0
0.5
0.0
log(ion counts)
PLATFORMRUNDAY_miss
0%
3%
6%

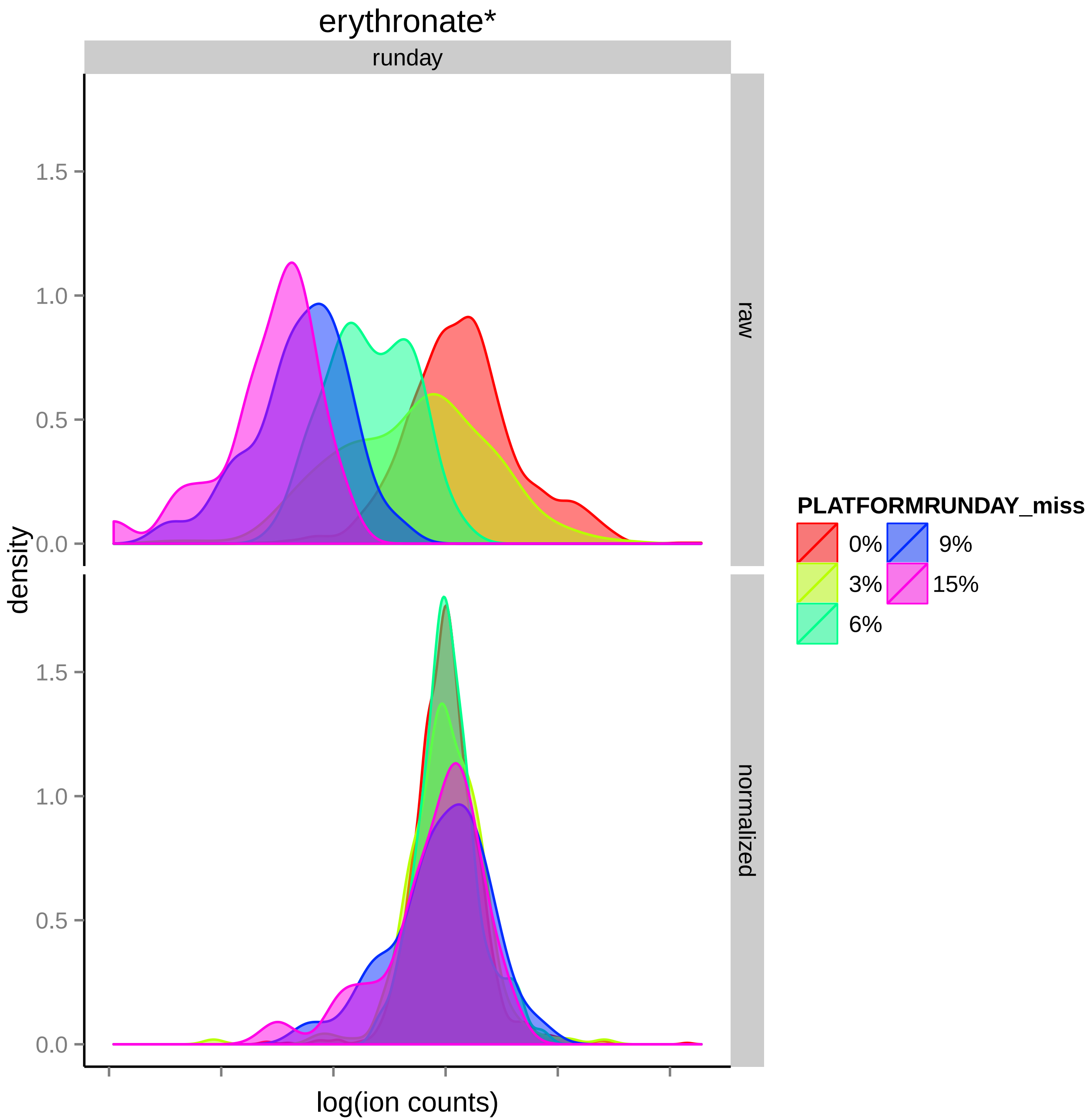

erythronate*
runday
raw
normalized
density
log(ion counts)
0.0
0.5
1.0
1.5
PLATFORMRUNDAY_miss
0%
3%
6%
9%
15%

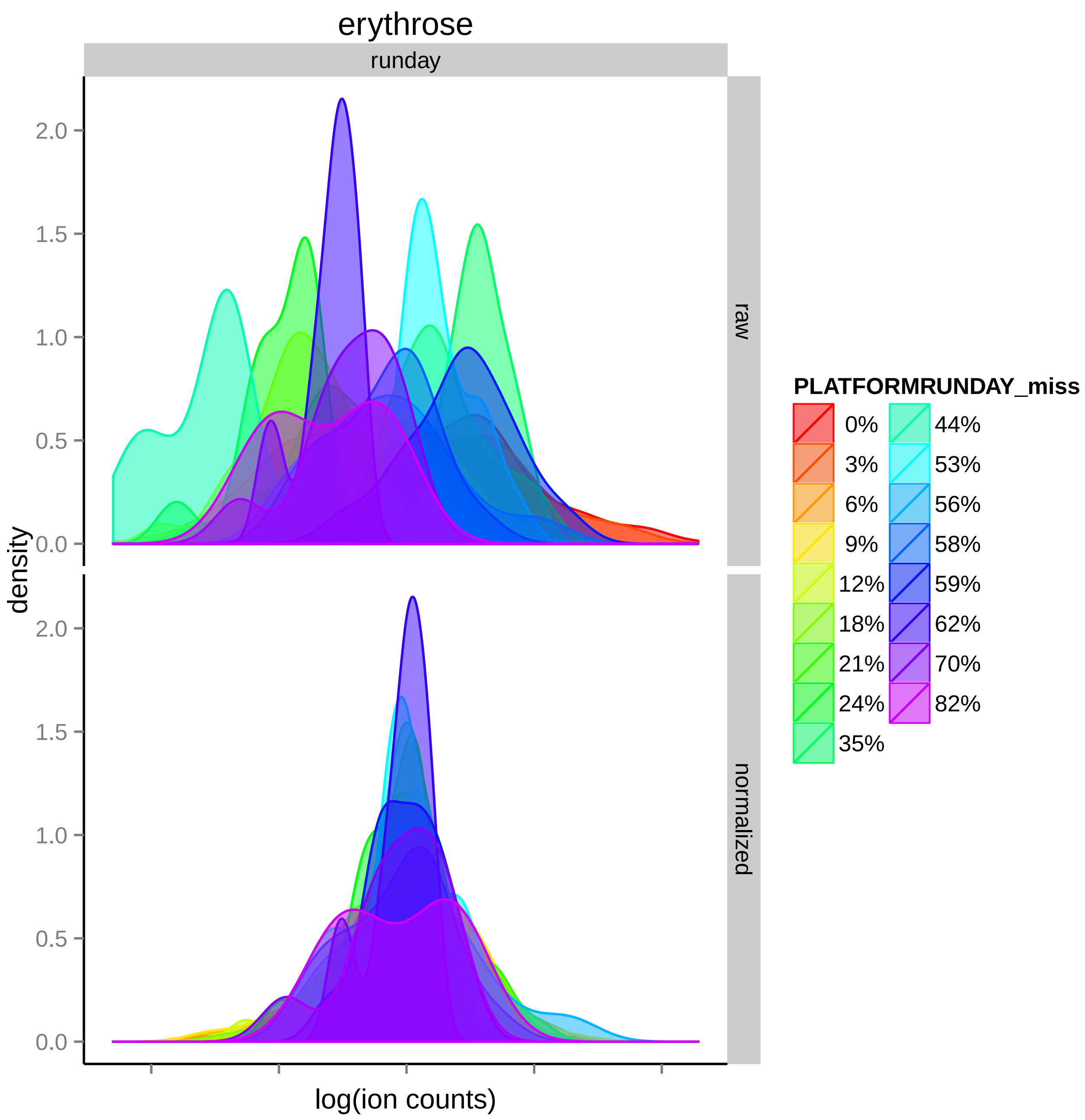

erythrose
runday
raw
normalized
density
2.0
1.5
1.0
0.5
0.0
log(ion counts)
PLATFORMRUNDAY_miss
0%
3%
6%
9%
12%
18%
21%
24%
35%
44%
53%
56%
58%
59%
62%
70%
82%

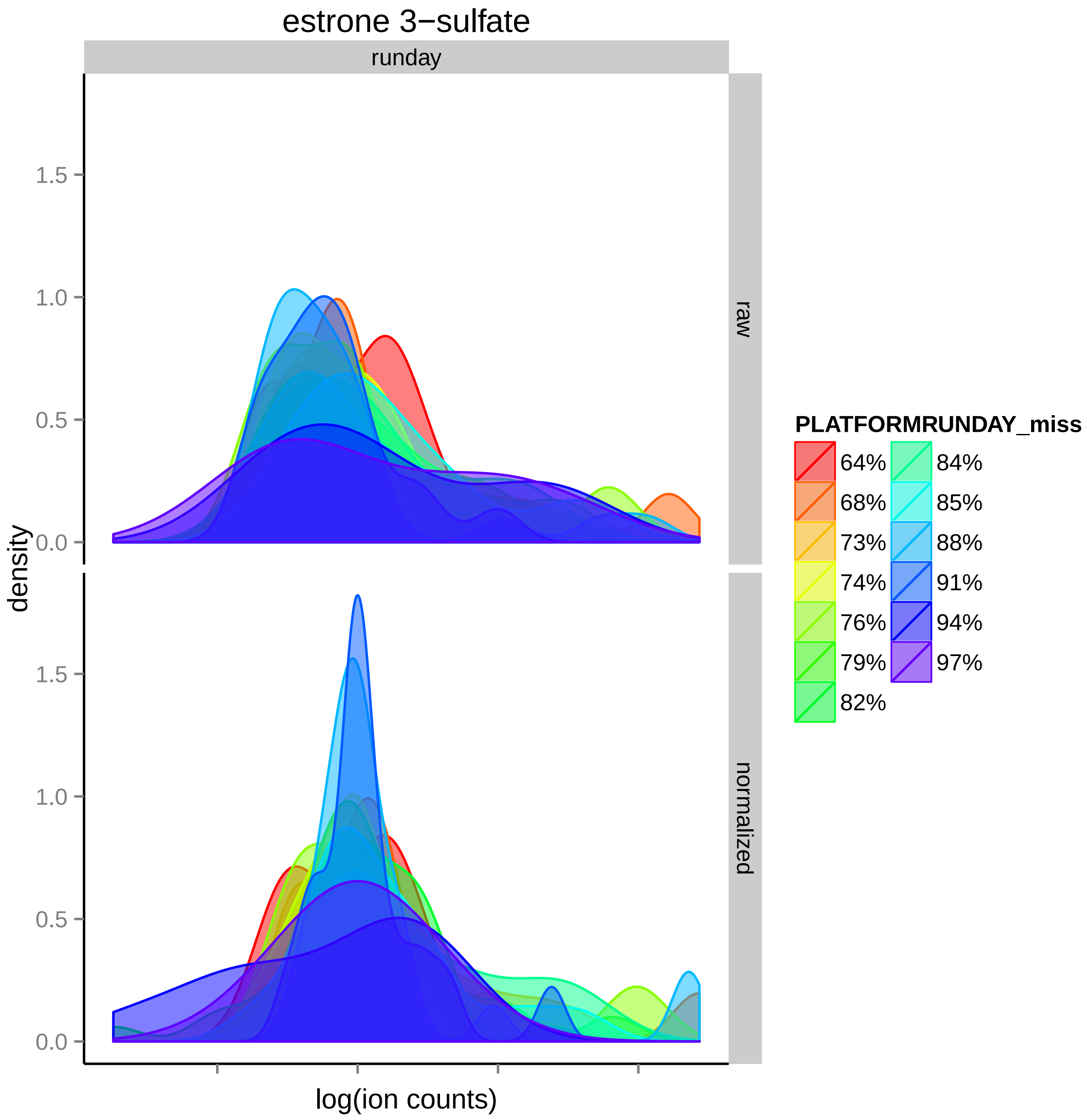
estrone 3−sulfate
runday
raw
normalized
density
log(ion counts)
0.0
0.5
1.0
1.5
PLATFORMRUNDAY_miss
64%
68%
73%
74%
76%
79%
82%
84%
85%
88%
91%
94%
97%

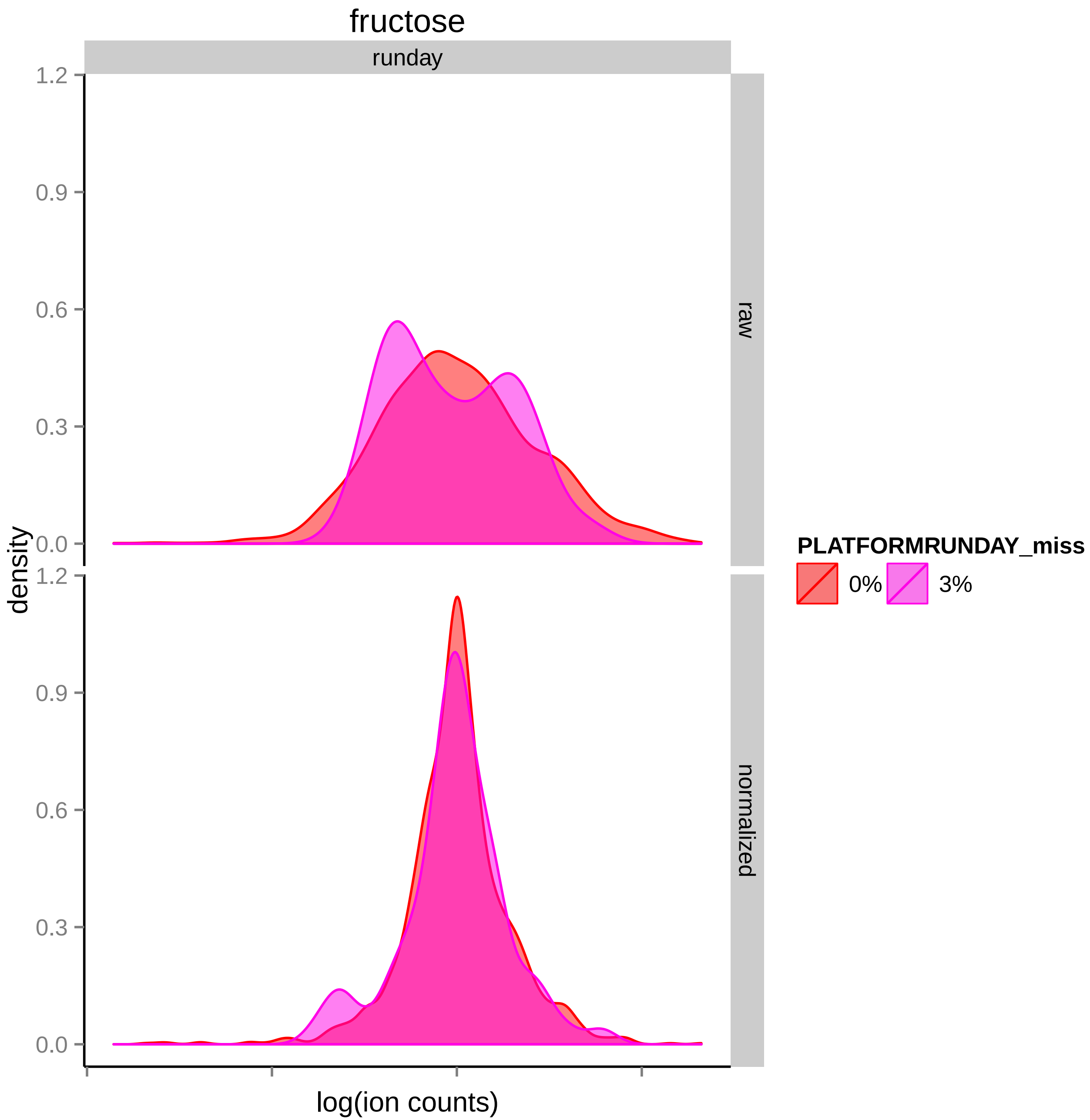
fructose
runday
raw
normalized
density
1.2
0.9
0.6
0.3
0.0
log(ion counts)
PLATFORMRUNDAY_miss
0%
3%

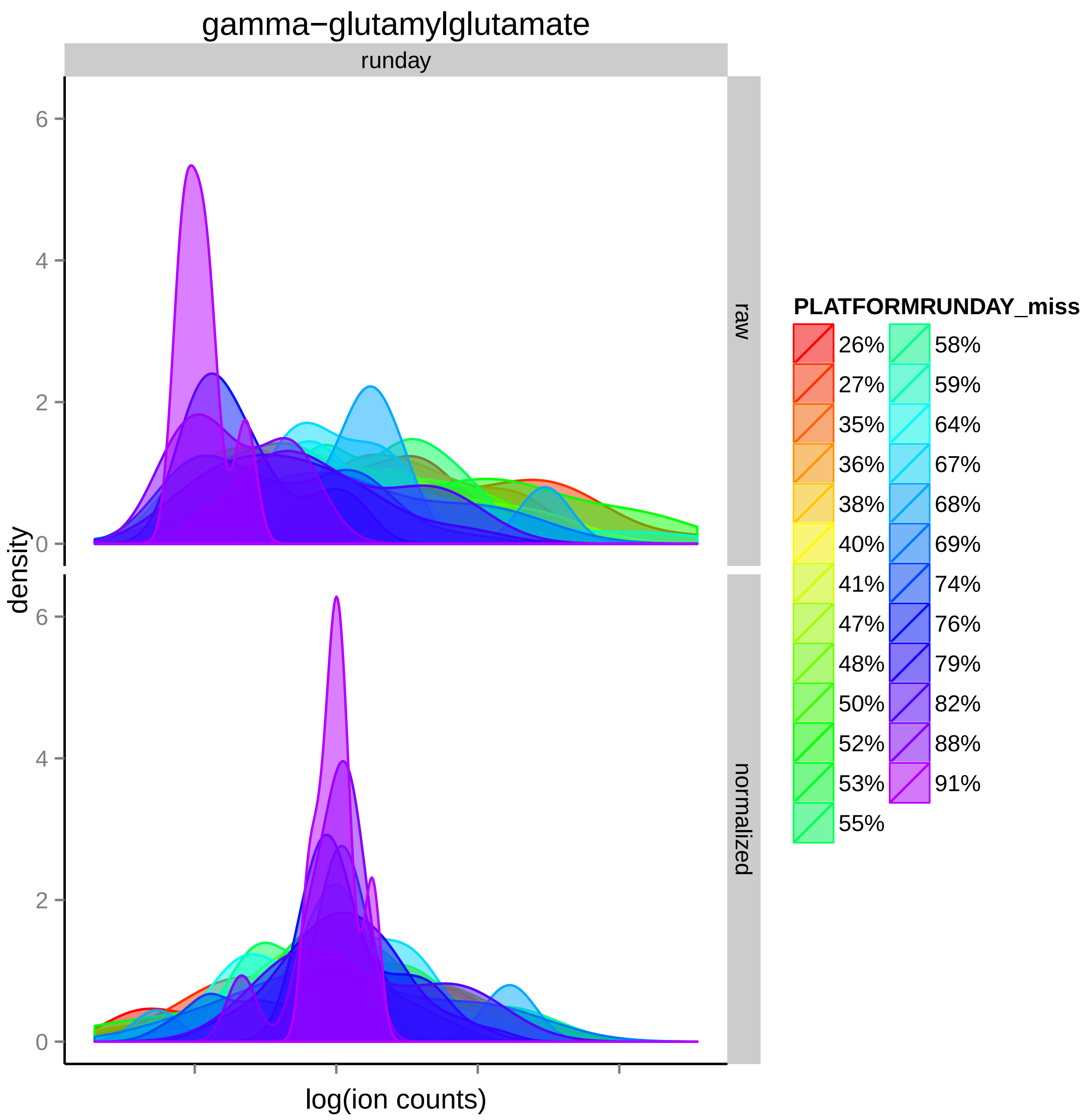
gamma−glutamylglutamate
runday
raw
normalized
density
log(ion counts)
0
2
4
6
PLATFORMRUNDAY_miss
26%
27%
35%
36%
38%
40%
41%
47%
48%
50%
52%
53%
55%
58%
59%
64%
67%
68%
69%
74%
76%
79%
82%
88%
91%

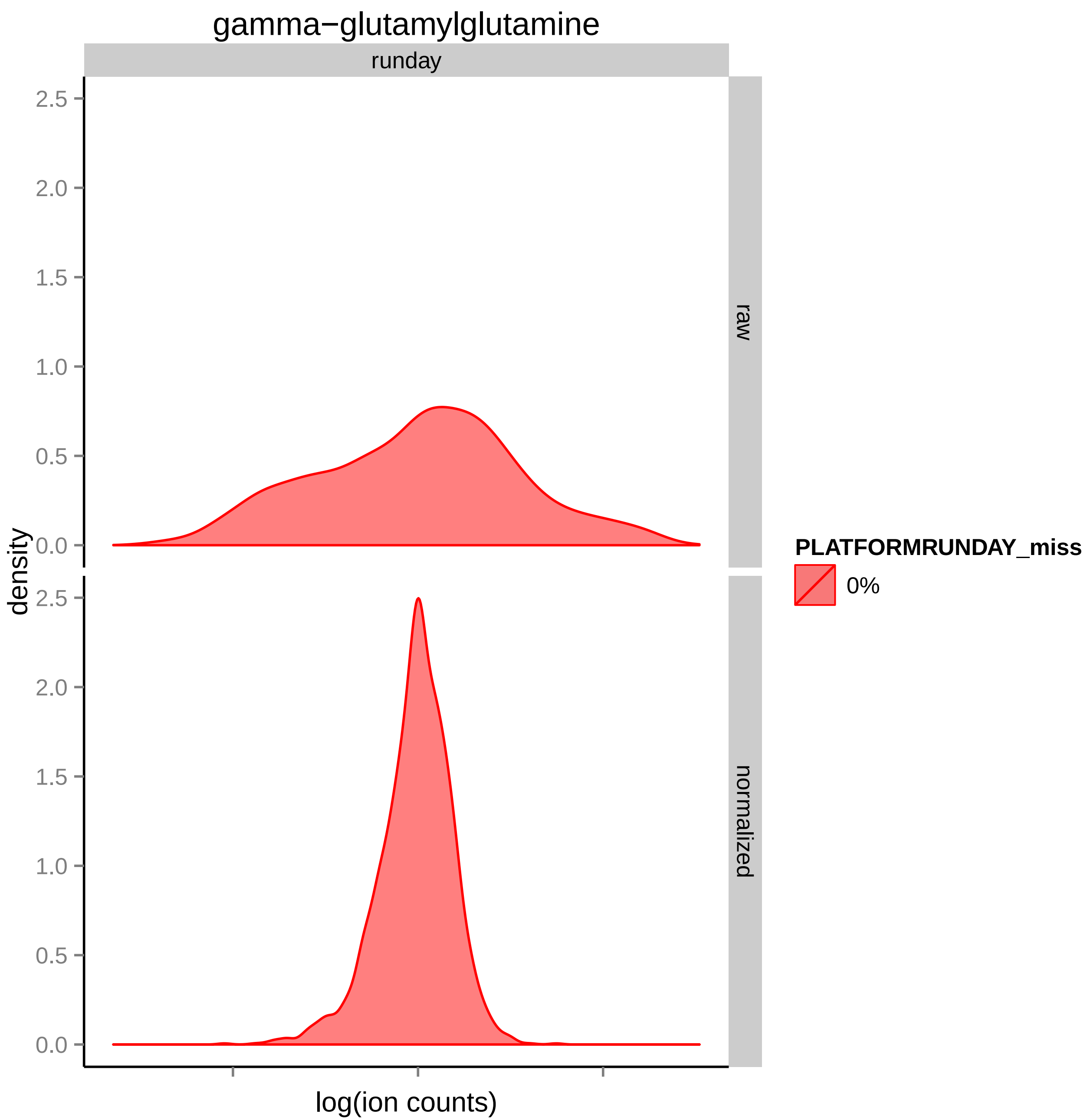
gamma−glutamylglutamine
runday
raw
normalized
density
2.5
2.0
1.5
1.0
0.5
0.0
log(ion counts)
PLATFORMRUNDAY_miss
0%

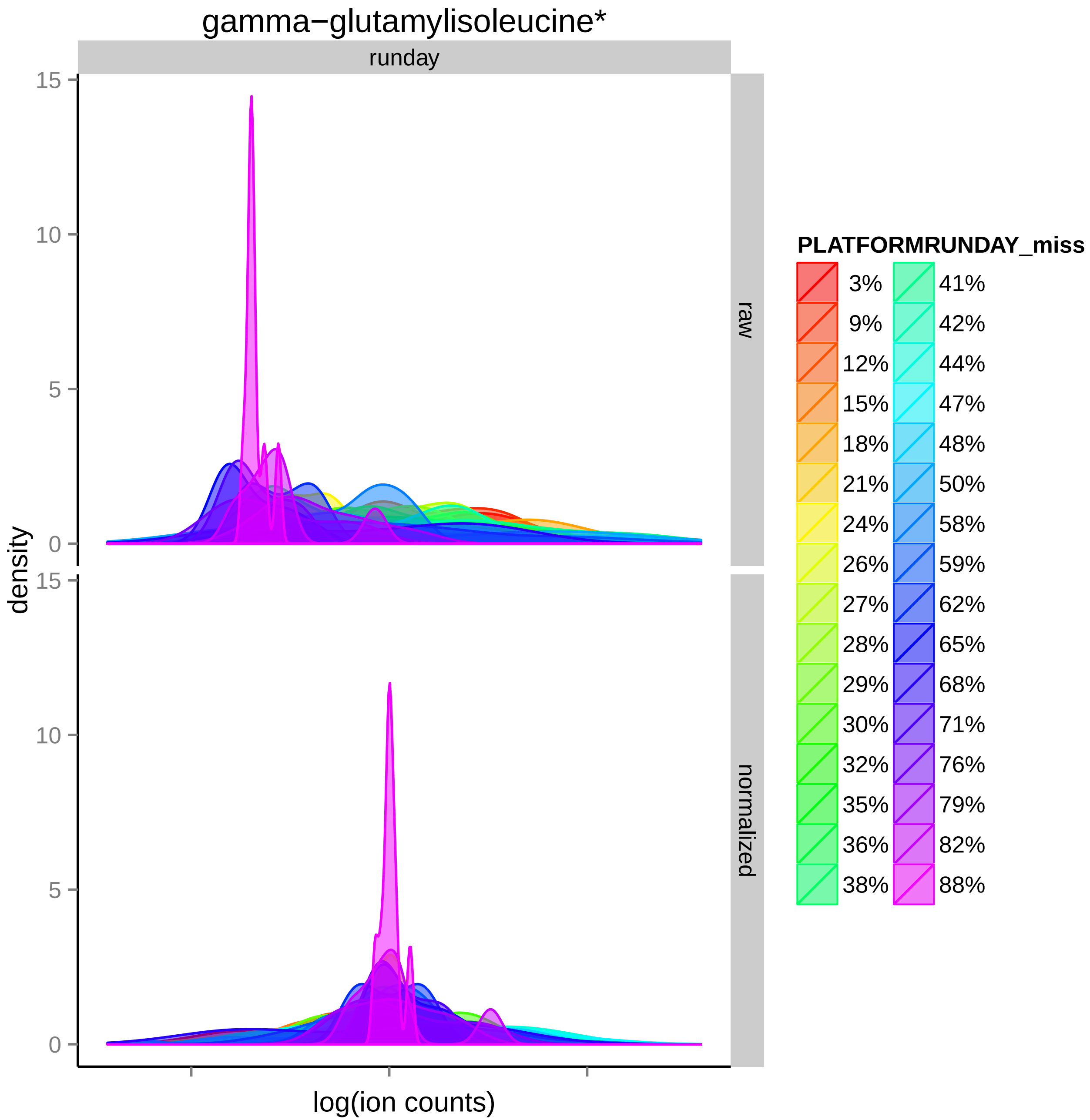

gamma−glutamylisoleucine*
runday
raw
normalized
density
log(ion counts)
15
10
5
0
PLATFORMRUNDAY_miss
3%
9%
12%
15%
18%
21%
24%
26%
27%
28%
29%
30%
32%
35%
36%
38%
41%
42%
44%
47%
48%
50%
58%
59%
62%
65%
68%
71%
76%
79%
82%
88%

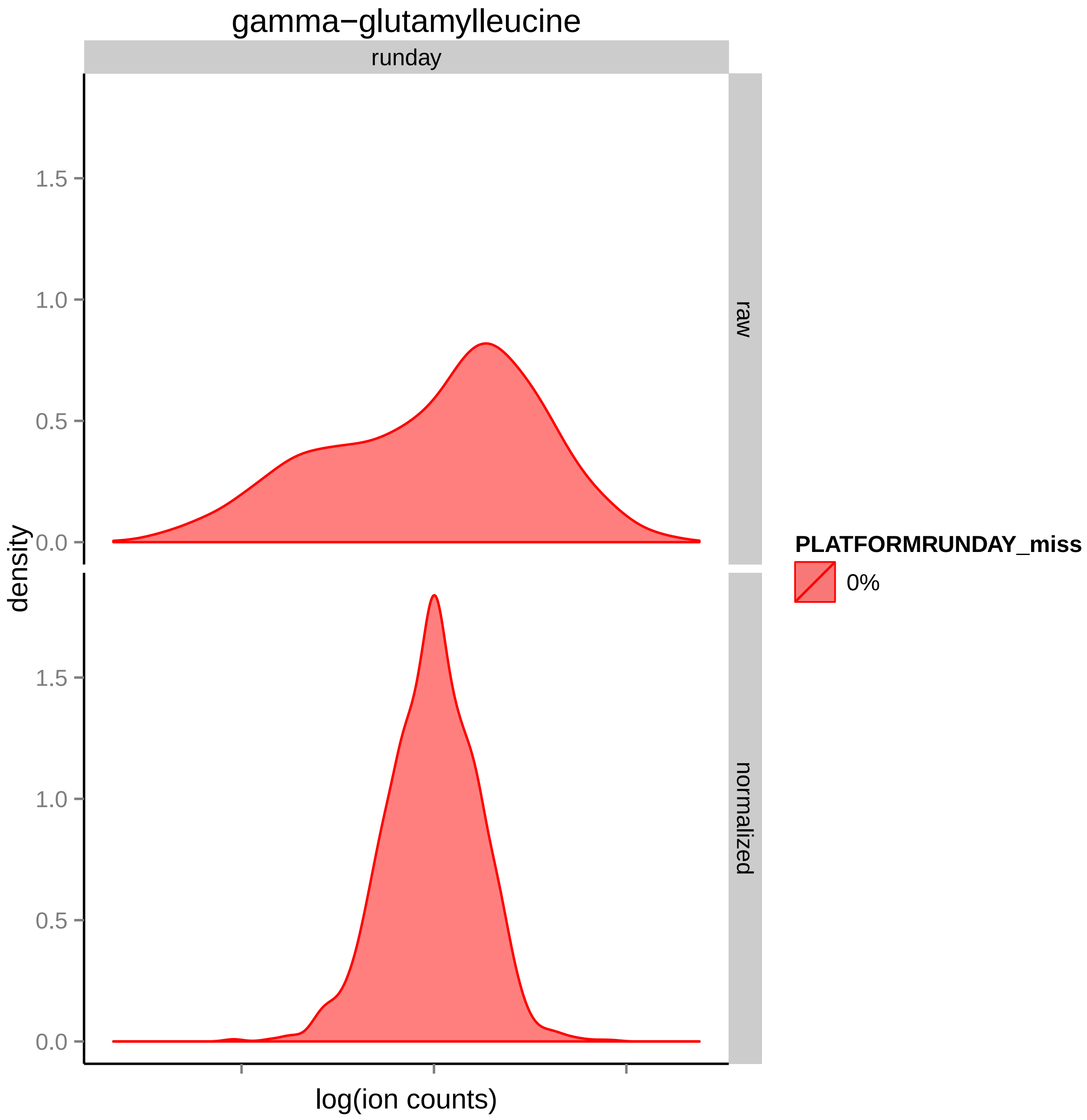
gamma−glutamylleucine
runday
raw
normalized
density
log(ion counts)
1.5
1.0
0.5
0.0
PLATFORMRUNDAY_miss
0%

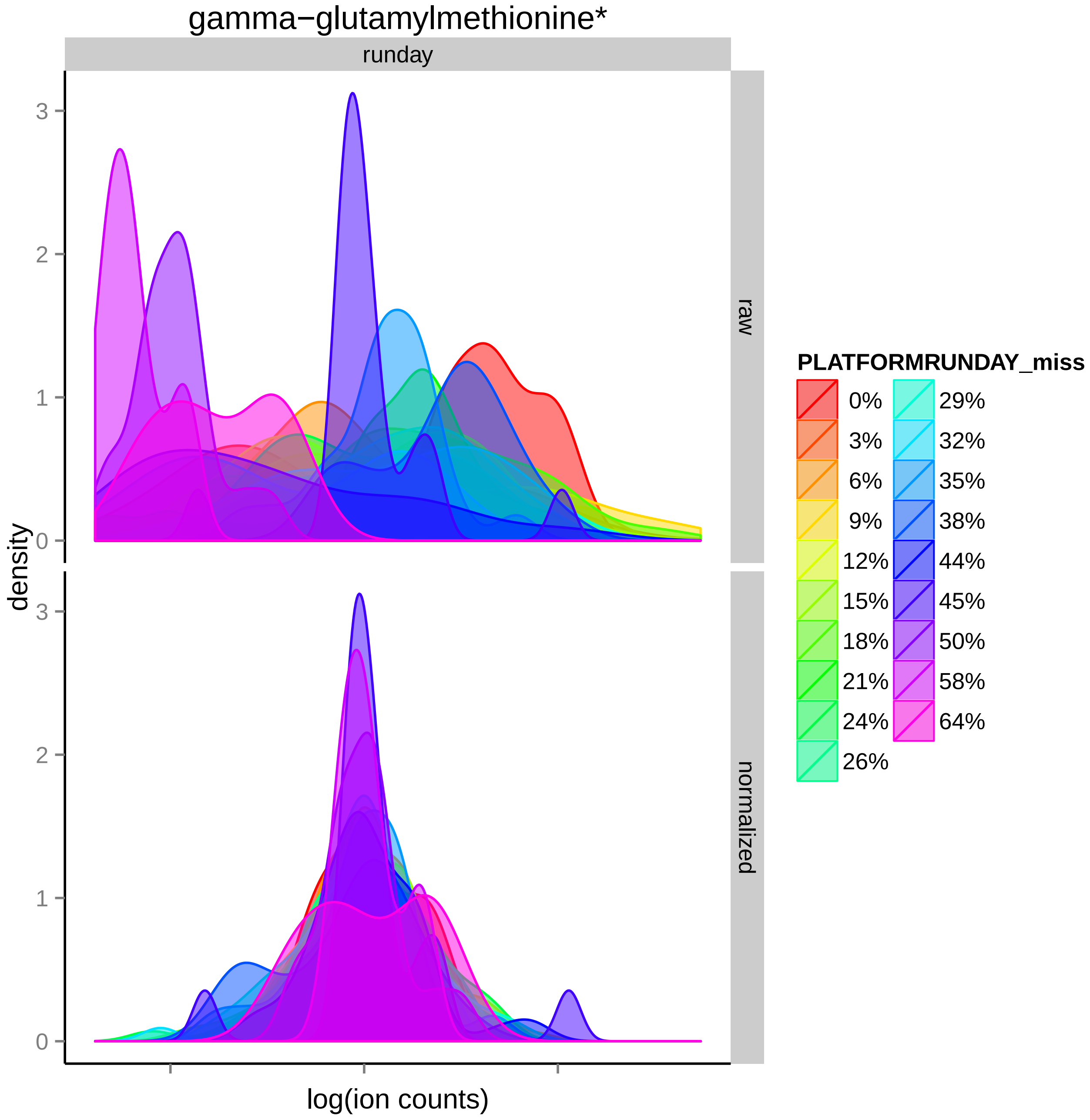

gamma−glutamylmethionine*
runday
raw
normalized
density
log(ion counts)
0
1
2
3
PLATFORMRUNDAY_miss
0%
3%
6%
9%
12%
15%
18%
21%
24%
26%
29%
32%
35%
38%
44%
45%
50%
58%
64%

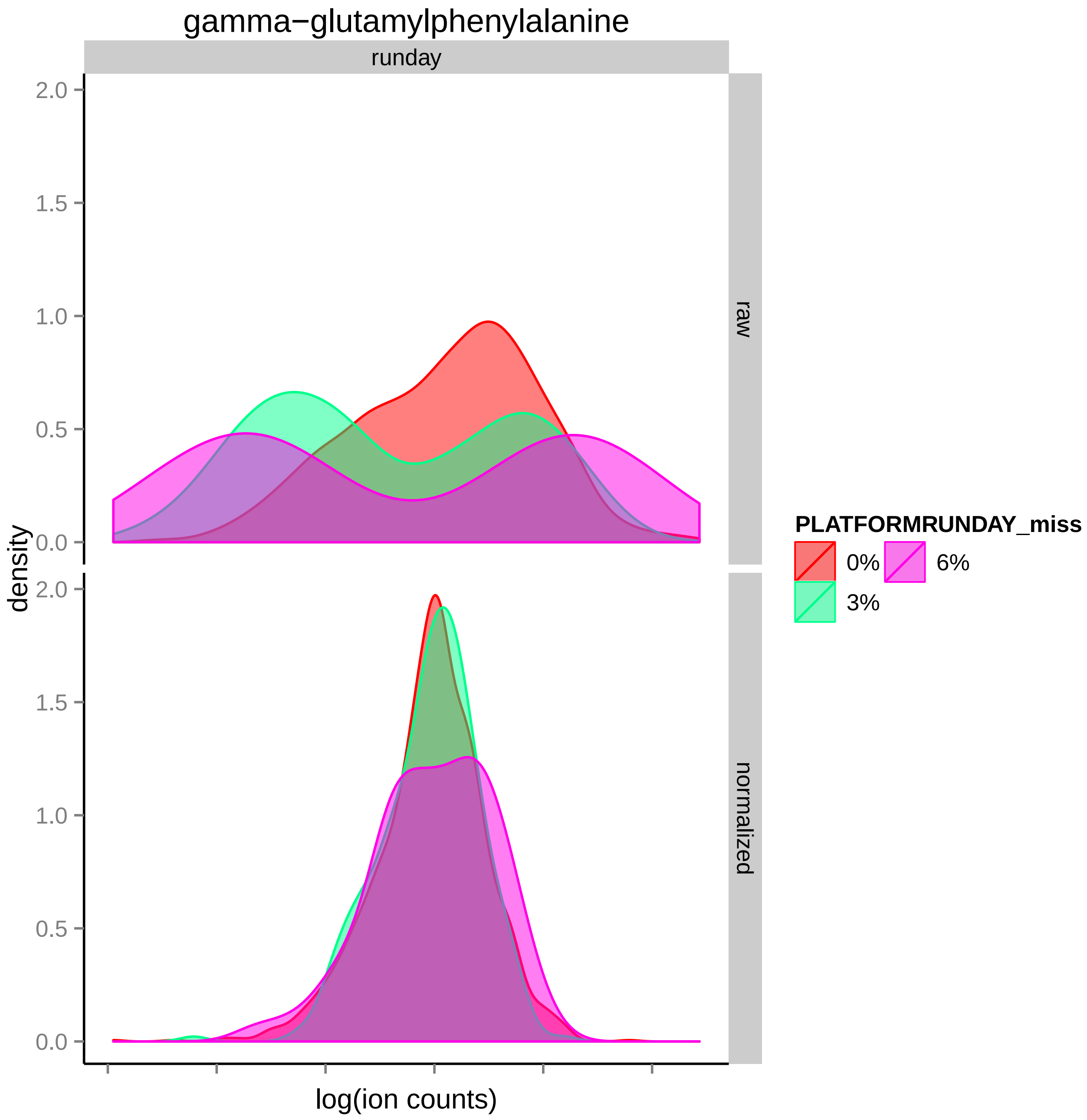

gamma−glutamylphenylalanine
runday
raw
normalized
density
log(ion counts)
2.0
1.5
1.0
0.5
0.0
PLATFORMRUNDAY_miss
0%
3%
6%

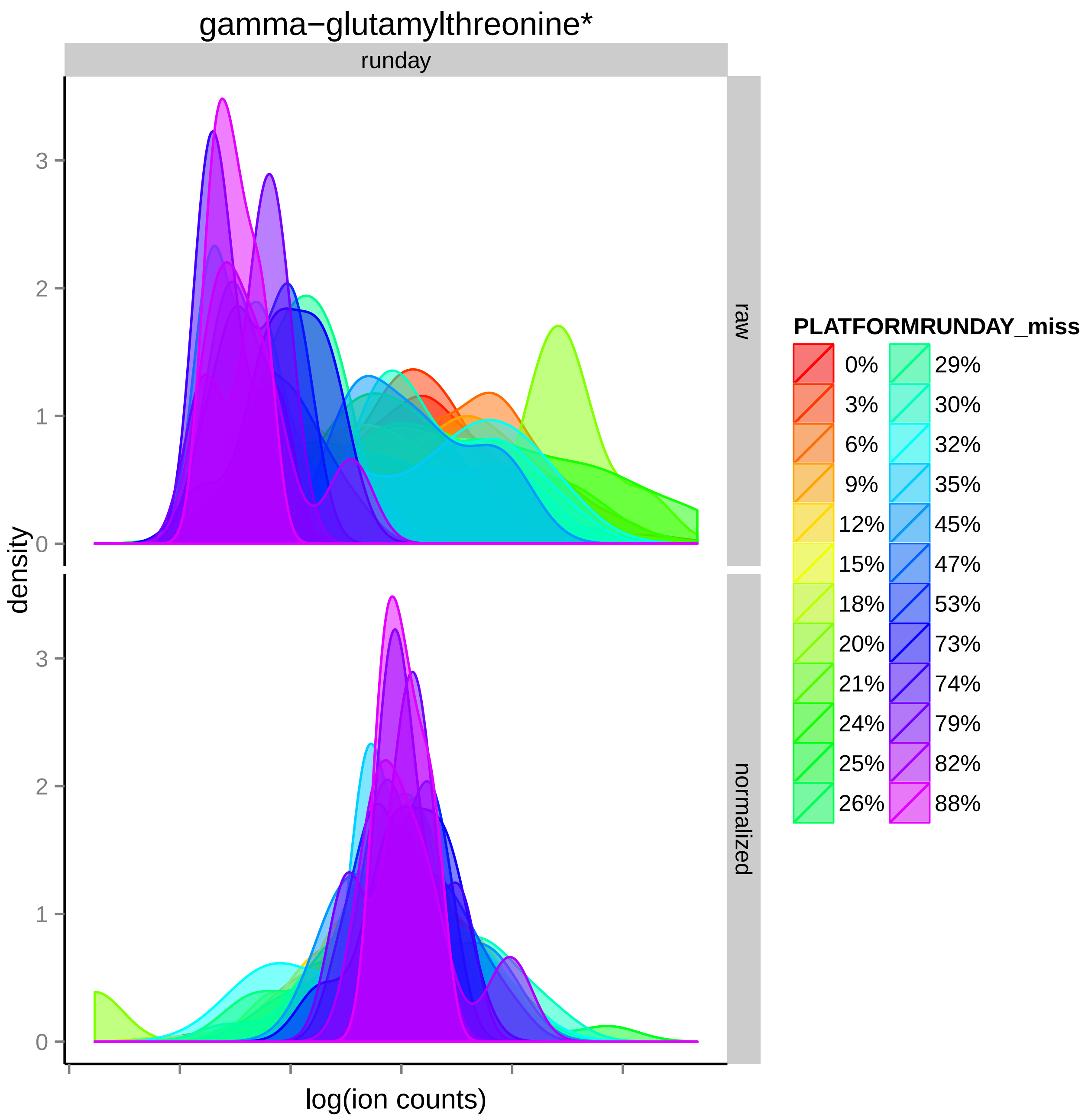
gamma−glutamylthreonine*
runday
raw
normalized
density
log(ion counts)
3
2
1
0
PLATFORMRUNDAY_miss
0%
3%
6%
9%
12%
15%
18%
20%
21%
24%
25%
26%
29%
30%
32%
35%
45%
47%
53%
73%
74%
79%
82%
88%

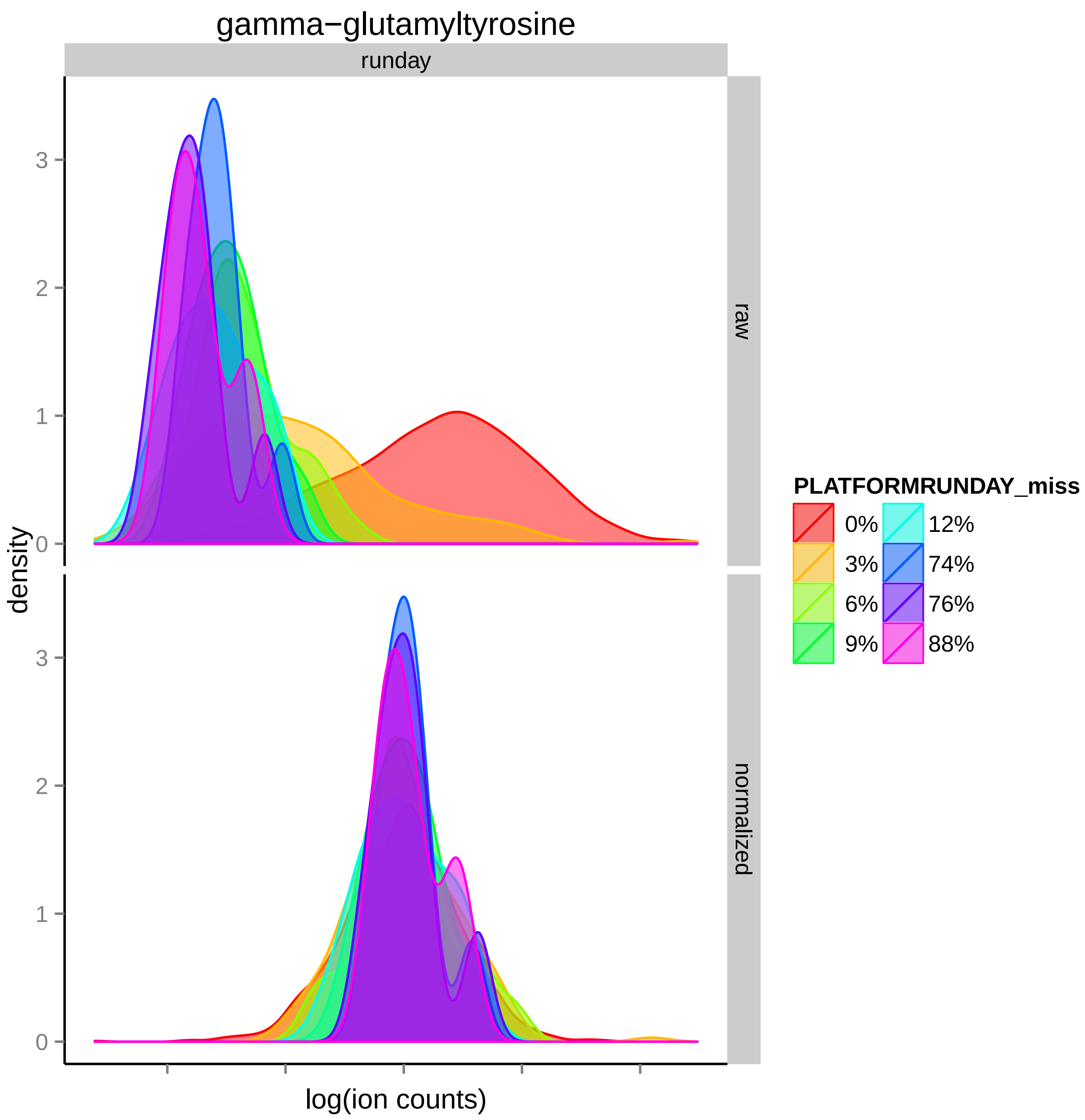

gamma−glutamyltyrosine
runday
raw
normalized
density
log(ion counts)
0
1
2
3
PLATFORMRUNDAY_miss
0%
3%
6%
9%
12%
74%
76%
88%

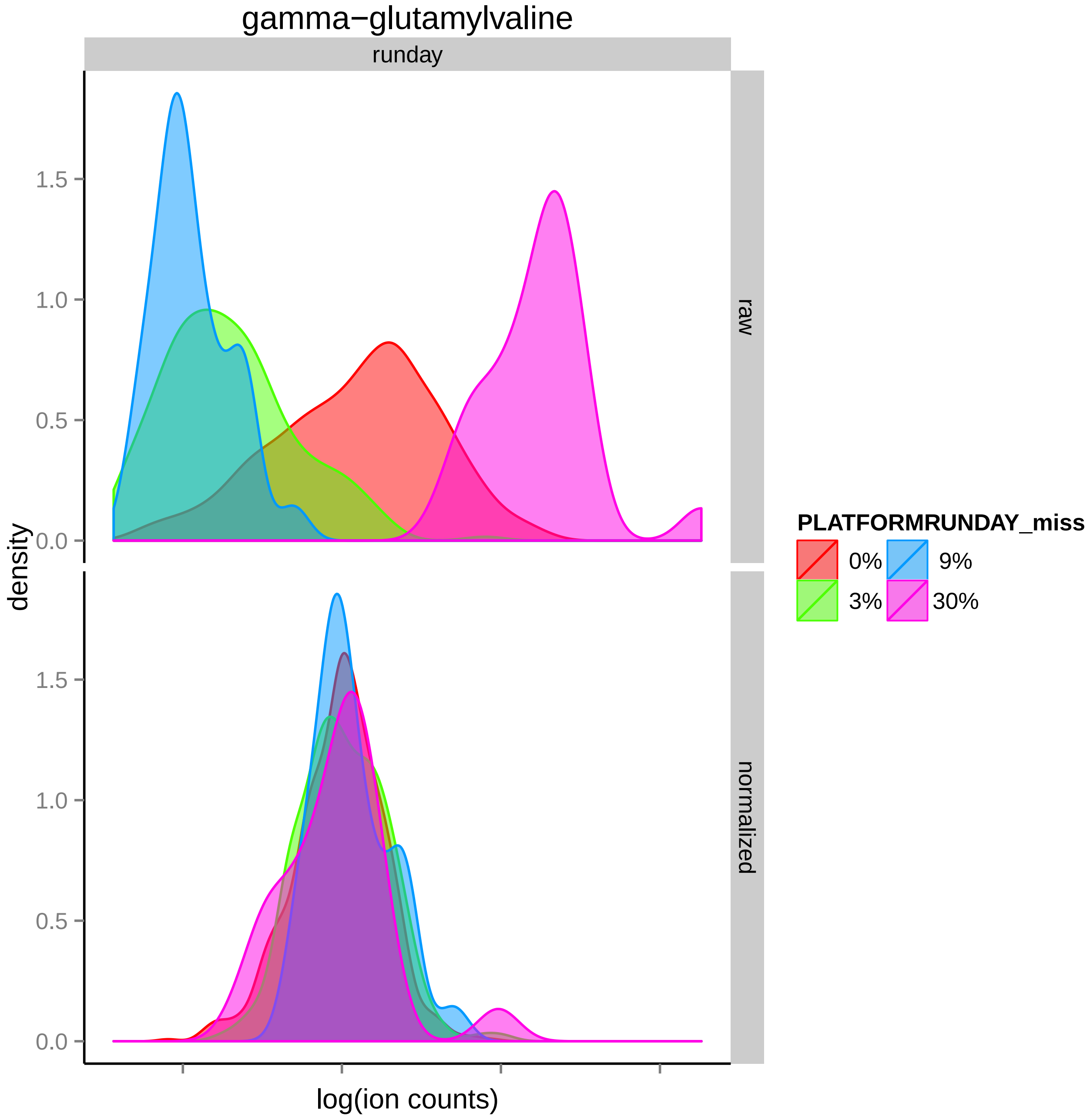
gamma−glutamylvaline
runday
raw
normalized
density
1.5
1.0
0.5
0.0
log(ion counts)
PLATFORMRUNDAY_miss
0%
9%
3%
30%

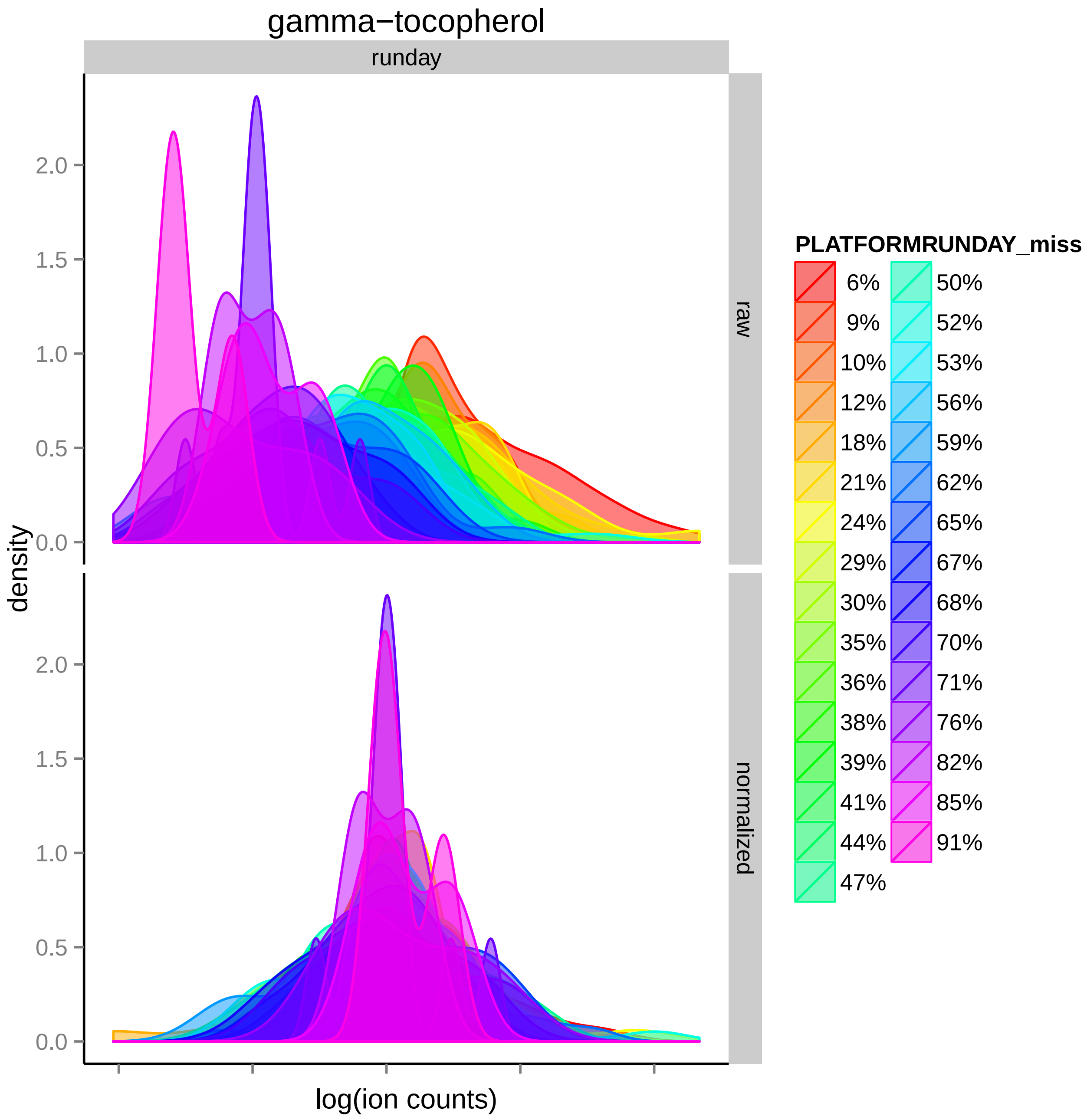
gamma−tocopherol
runday
raw
normalized
density
log(ion counts)
0.0
0.5
1.0
1.5
2.0
PLATFORMRUNDAY_miss
6%
9%
10%
12%
18%
21%
24%
29%
30%
35%
36%
38%
39%
41%
44%
47%
50%
52%
53%
56%
59%
62%
65%
67%
68%
70%
71%
76%
82%
85%
91%

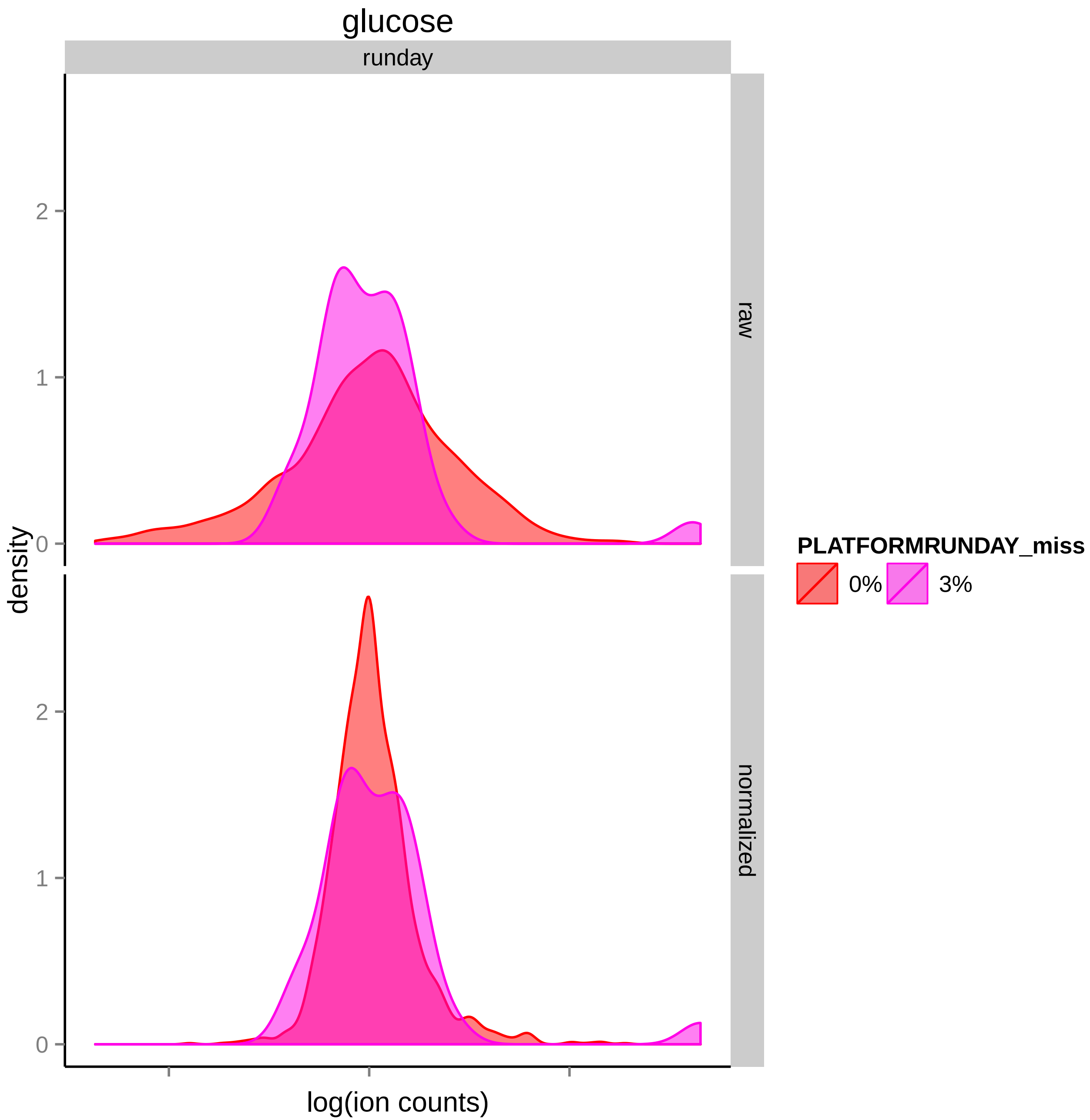
glucose
runday
raw
normalized
density
log(ion counts)
0
1
2
0
1
2
PLATFORMRUNDAY_miss
0%
3%

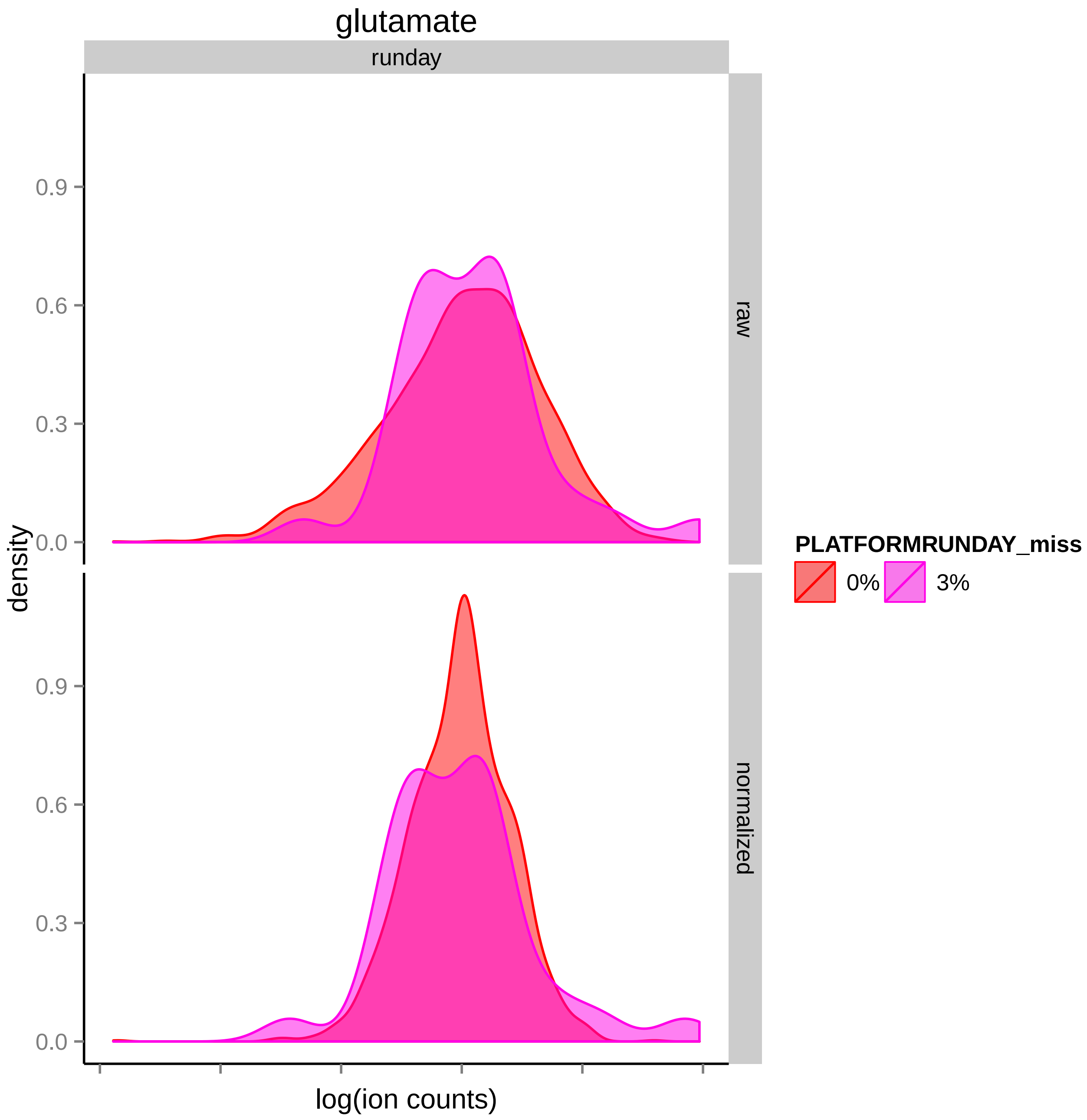
glutamate
runday
raw
normalized
density
0.9
0.6
0.3
0.0
0.9
0.6
0.3
0.0
log(ion counts)
PLATFORMRUNDAY_miss
0%
3%

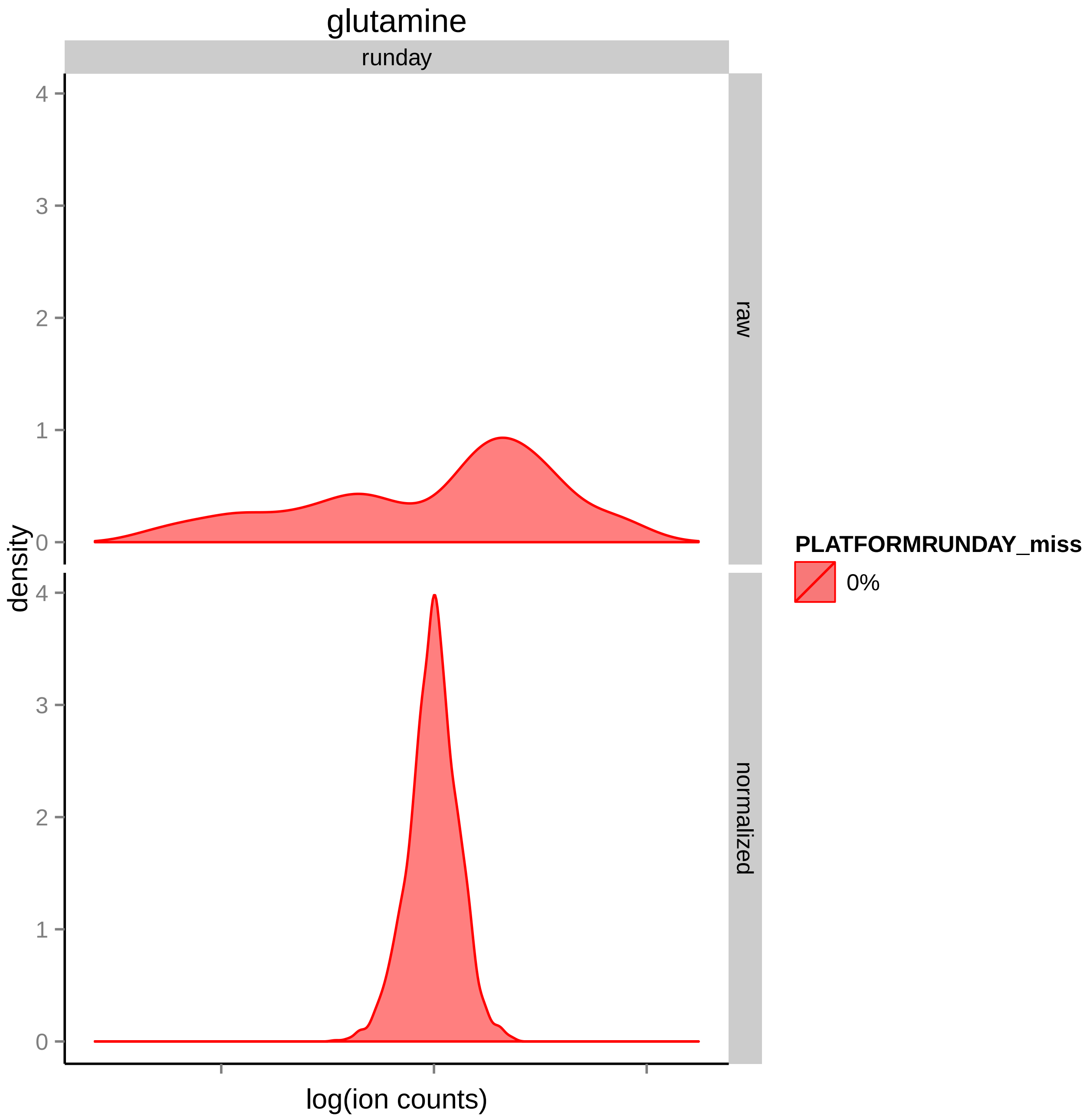

glutamine
runday
raw
normalized
density
log(ion counts)
4
3
2
1
0
PLATFORMRUNDAY_miss
0%

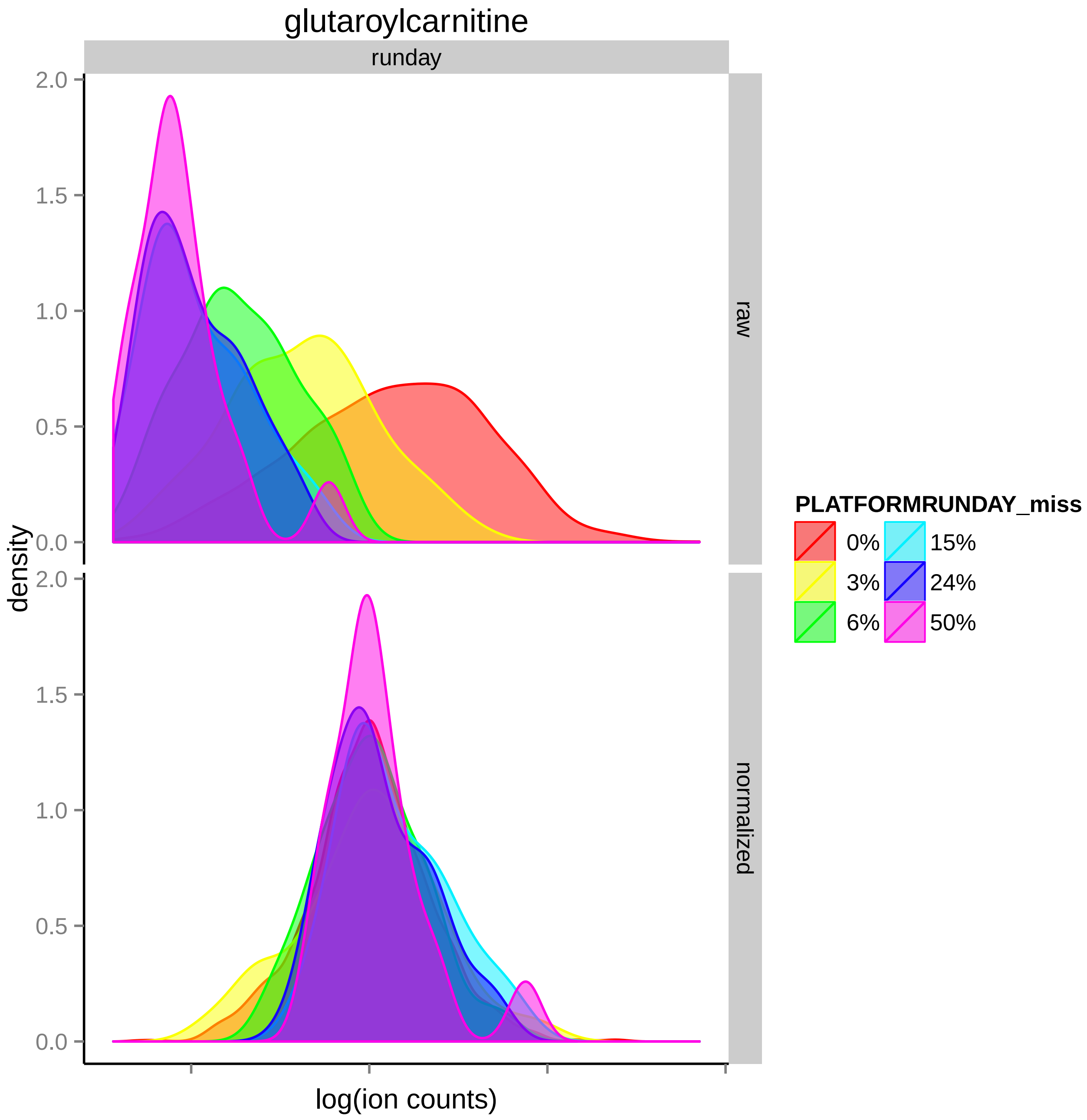
glutaroylcarnitine
runday
raw
normalized
density
2.0
1.5
1.0
0.5
0.0
2.0
1.5
1.0
0.5
0.0
log(ion counts)
PLATFORMRUNDAY_miss
0%
3%
6%
15%
24%
50%

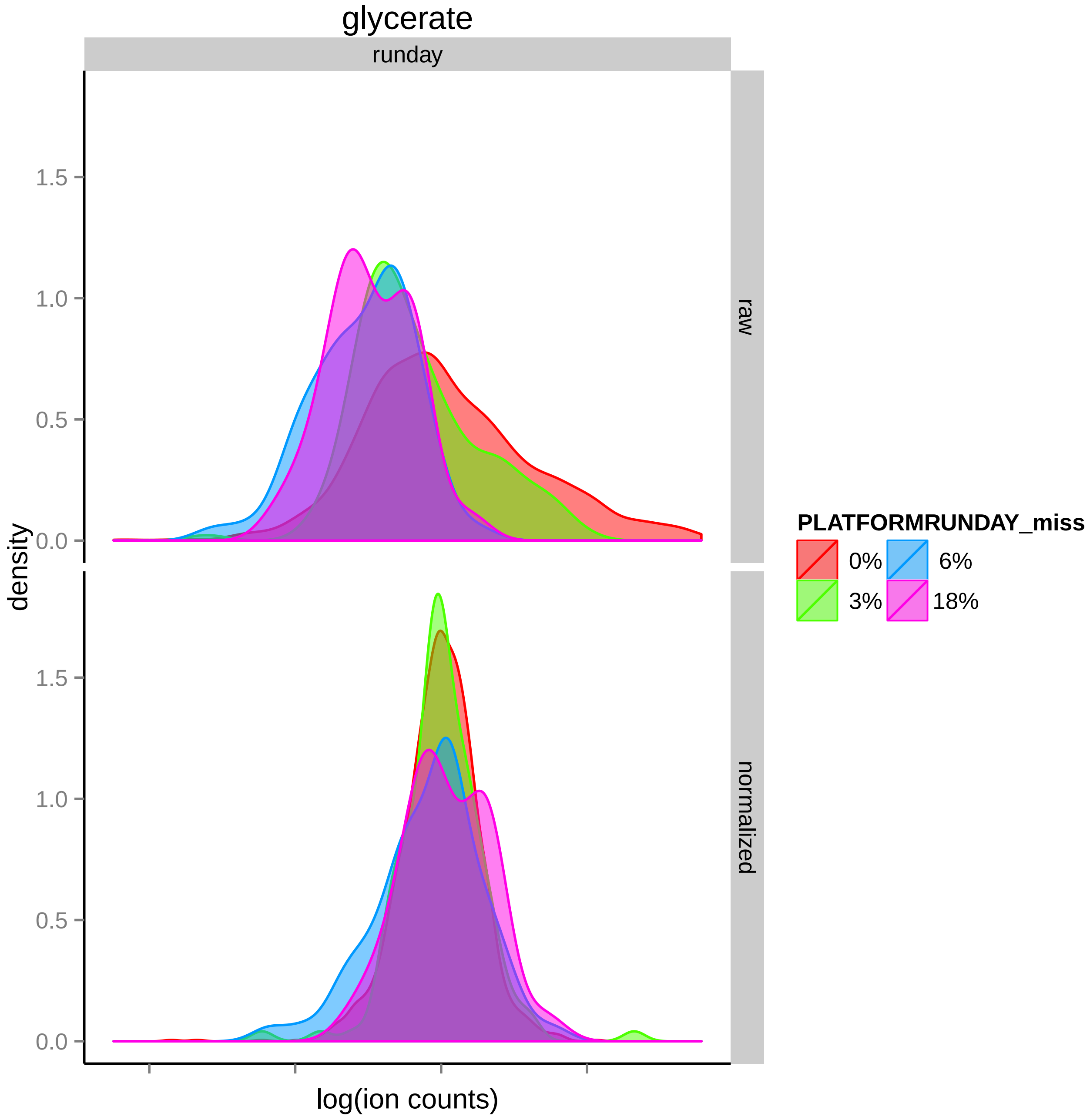

glycerate
runday
raw
normalized
density
1.5
1.0
0.5
0.0
log(ion counts)
PLATFORMRUNDAY_miss
0%
6%
3%
18%

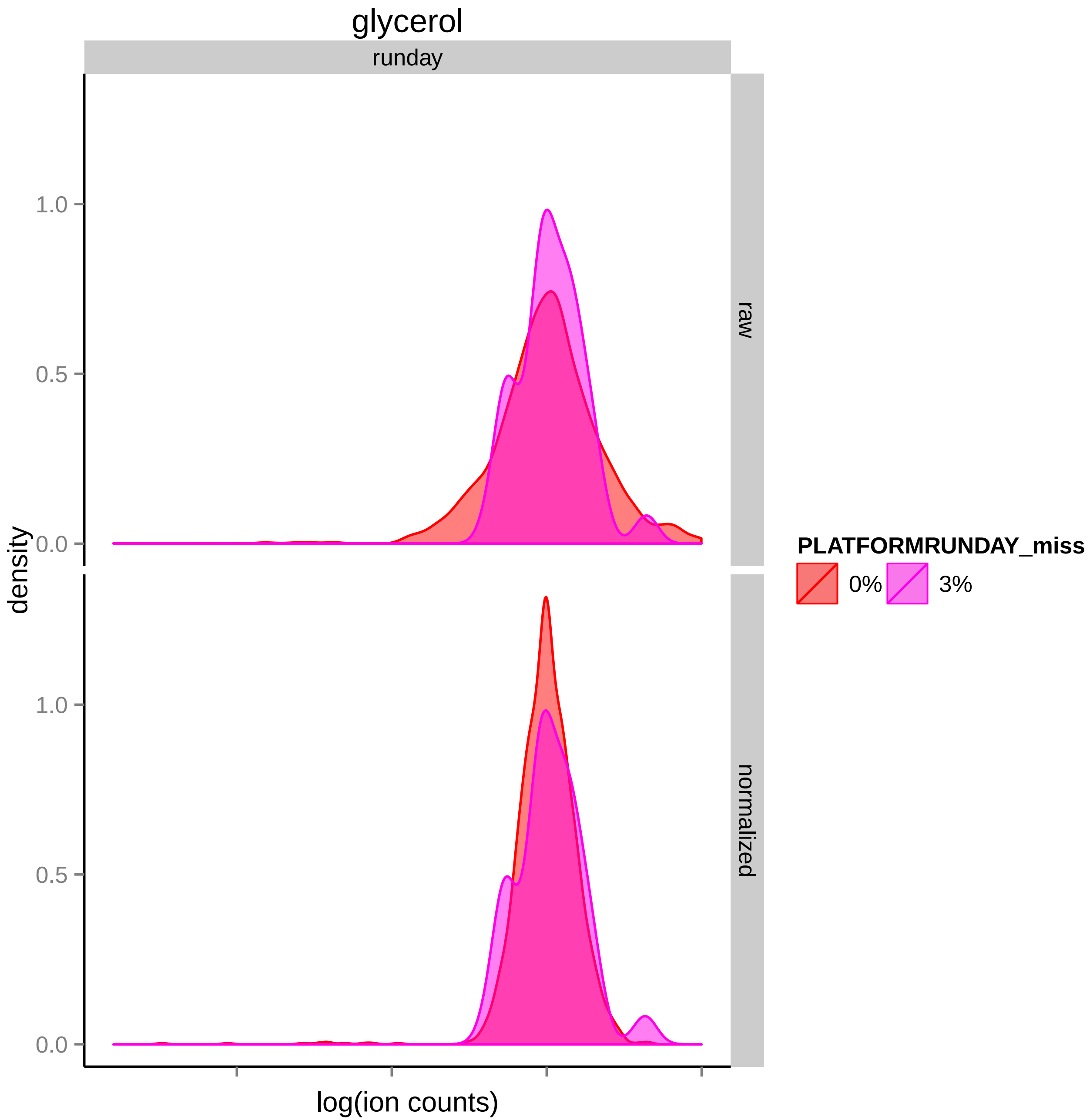

glycerol
runday
raw
normalized
density
1.0
0.5
0.0
1.0
0.5
0.0
log(ion counts)
PLATFORMRUNDAY_miss
0%
3%

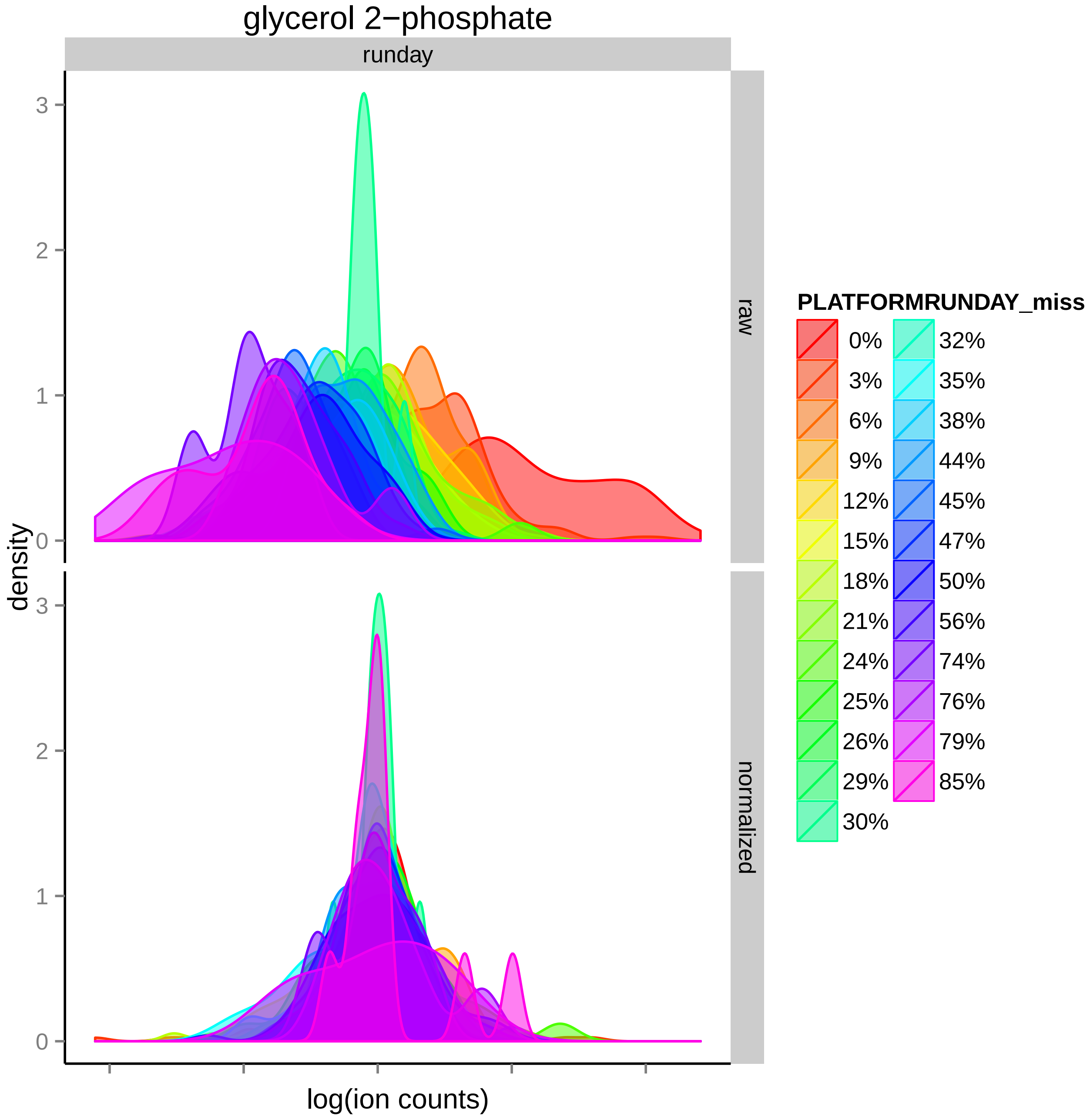

glycerol 2−phosphate
runday
raw
normalized
density
log(ion counts)
3
2
1
0
PLATFORMRUNDAY_miss
0%
3%
6%
9%
12%
15%
18%
21%
24%
25%
26%
29%
30%
32%
35%
38%
44%
45%
47%
50%
56%
74%
76%
79%
85%

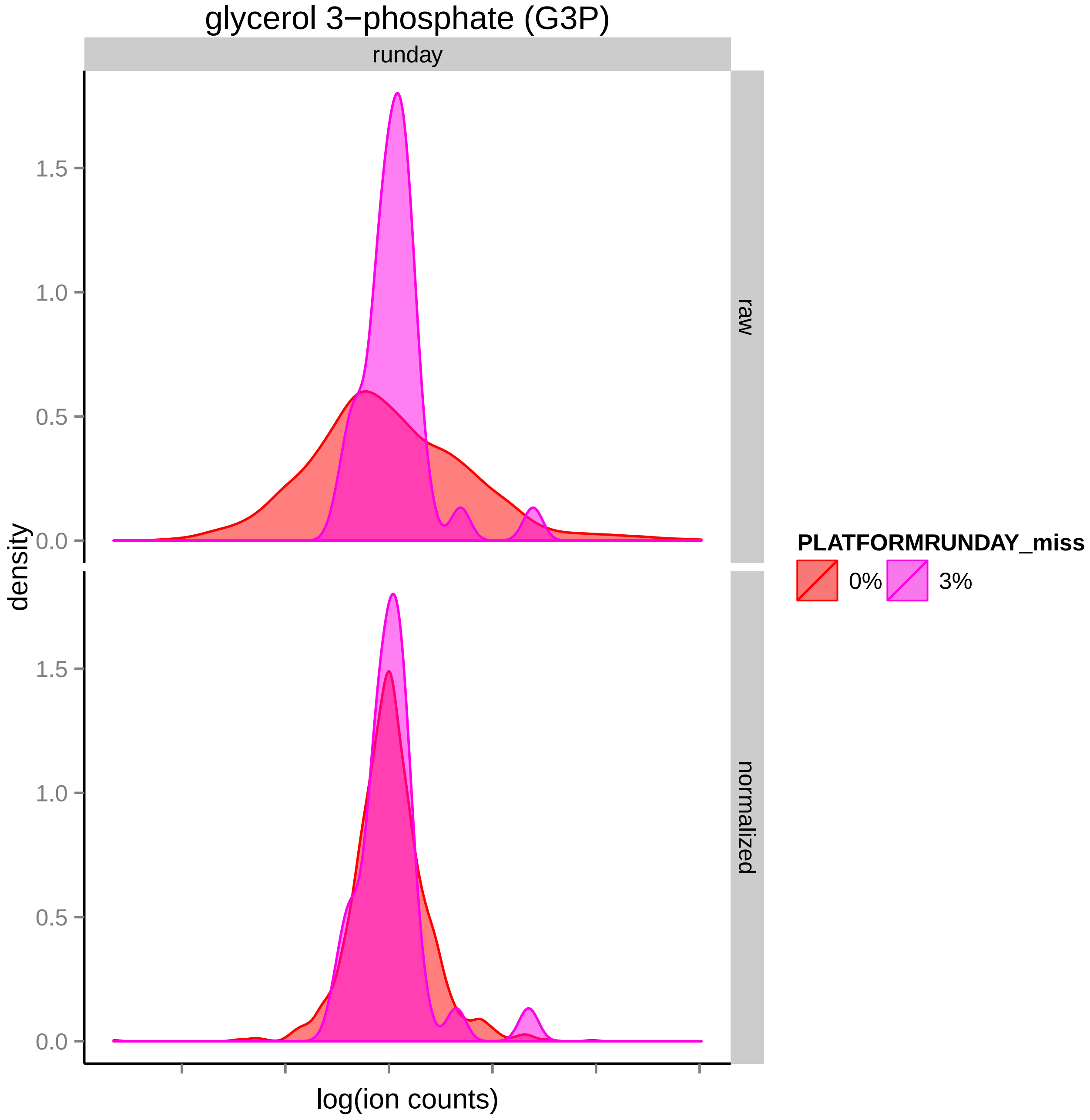

glycerol 3−phosphate (G3P)
runday
raw
normalized
density
0.0
0.5
1.0
1.5
log(ion counts)
PLATFORMRUNDAY_miss
0%
3%

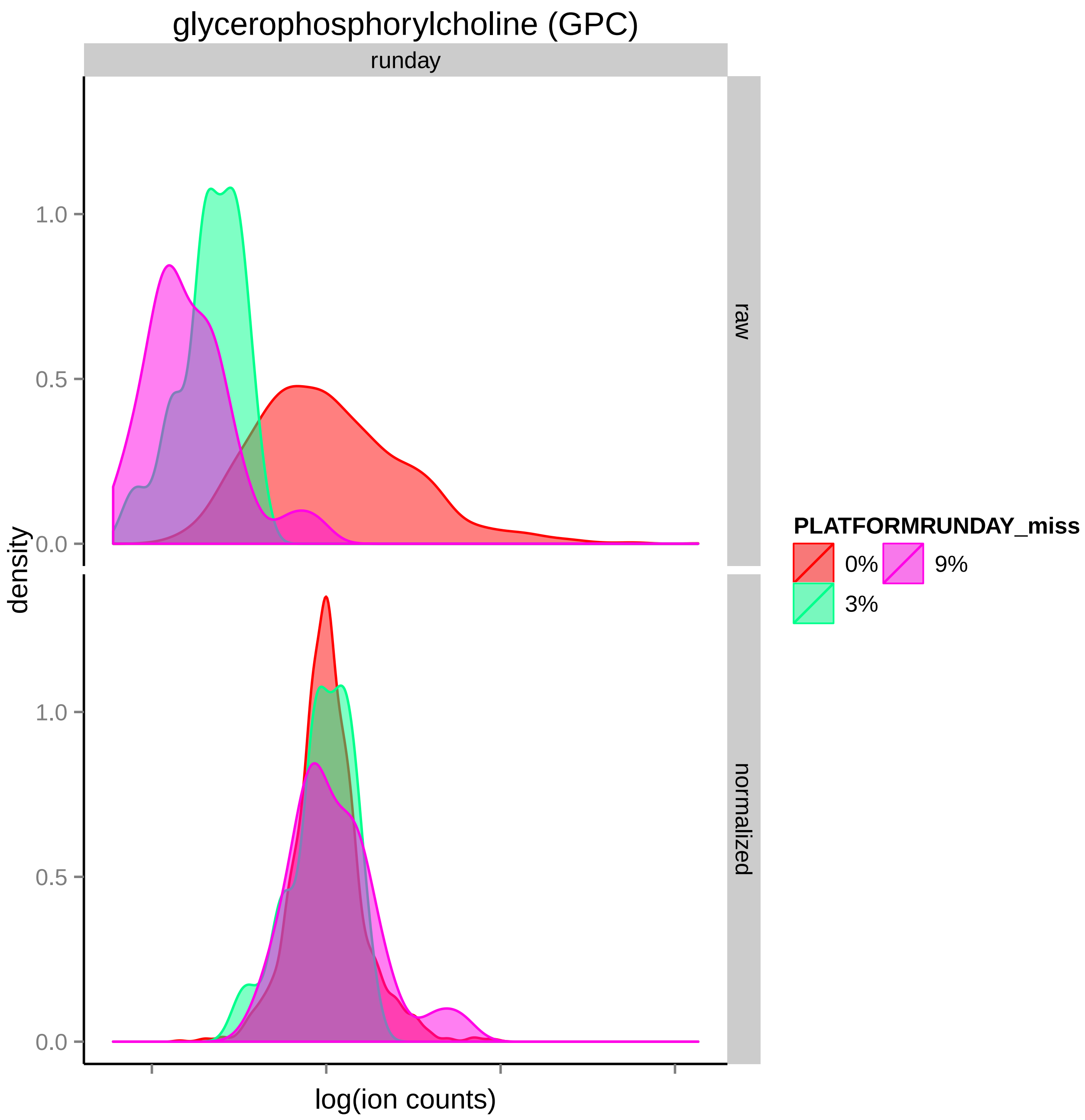
glycerophosphorylcholine (GPC)
runday
raw
normalized
density
1.0
0.5
0.0
log(ion counts)
PLATFORMRUNDAY_miss
0%
3%
9%

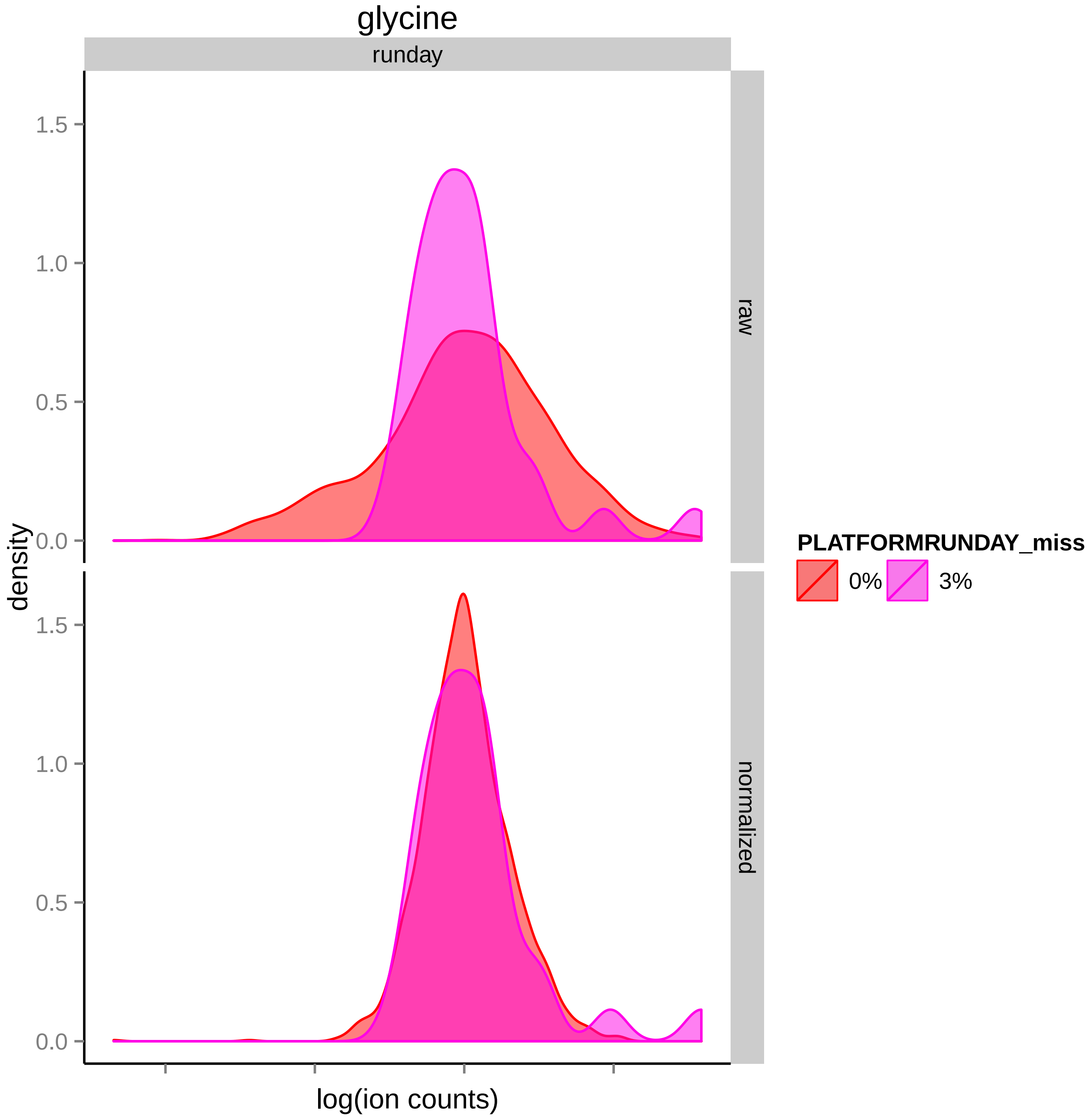

glycine
runday
raw
normalized
density
log(ion counts)
1.5
1.0
0.5
0.0
PLATFORMRUNDAY_miss
0%
3%

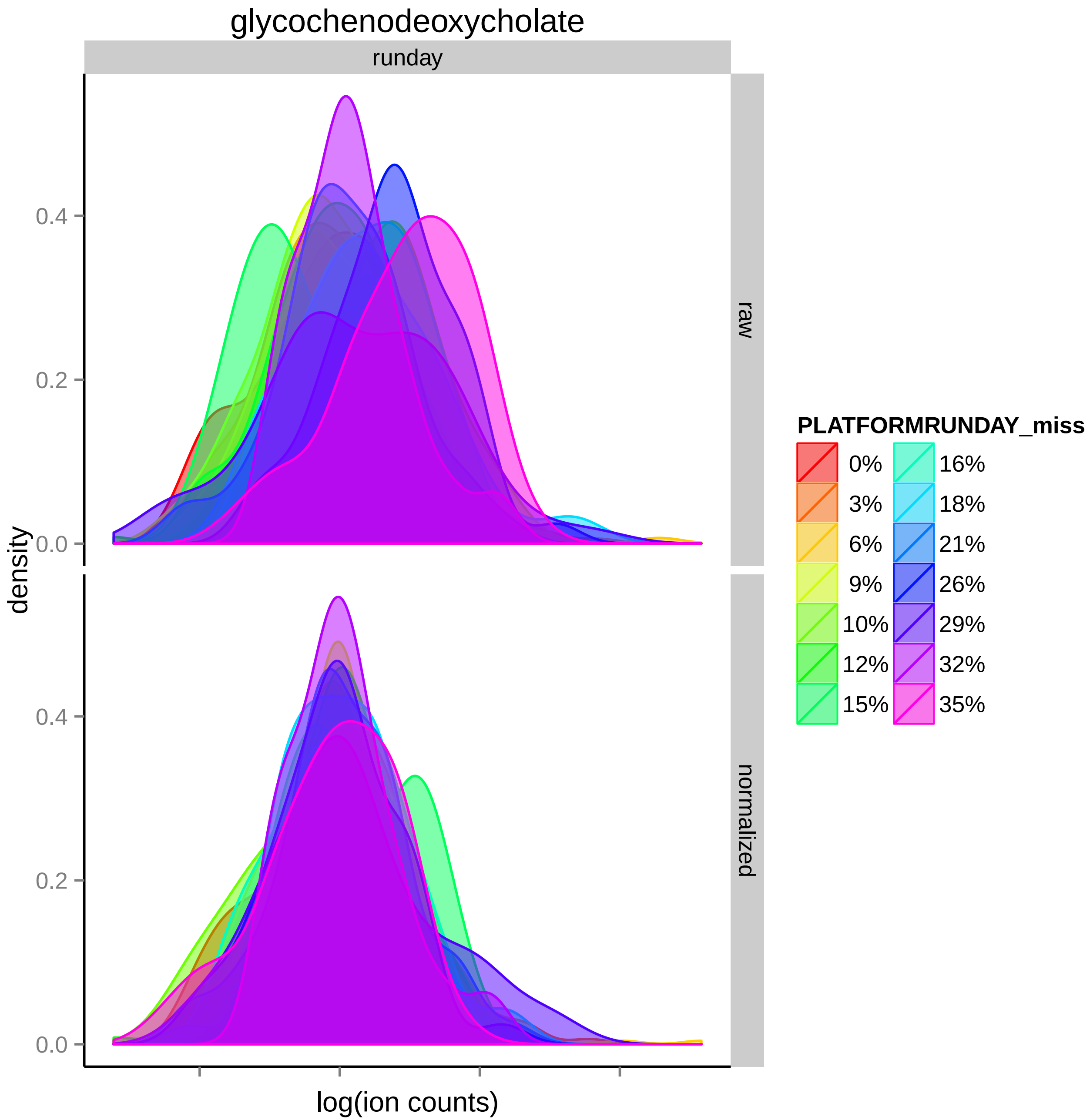

glycochenodeoxycholate
runday
raw
normalized
density
0.0
0.2
0.4
0.0
0.2
0.4
log(ion counts)
PLATFORMRUNDAY_miss
0%
3%
6%
9%
10%
12%
15%
16%
18%
21%
26%
29%
32%
35%

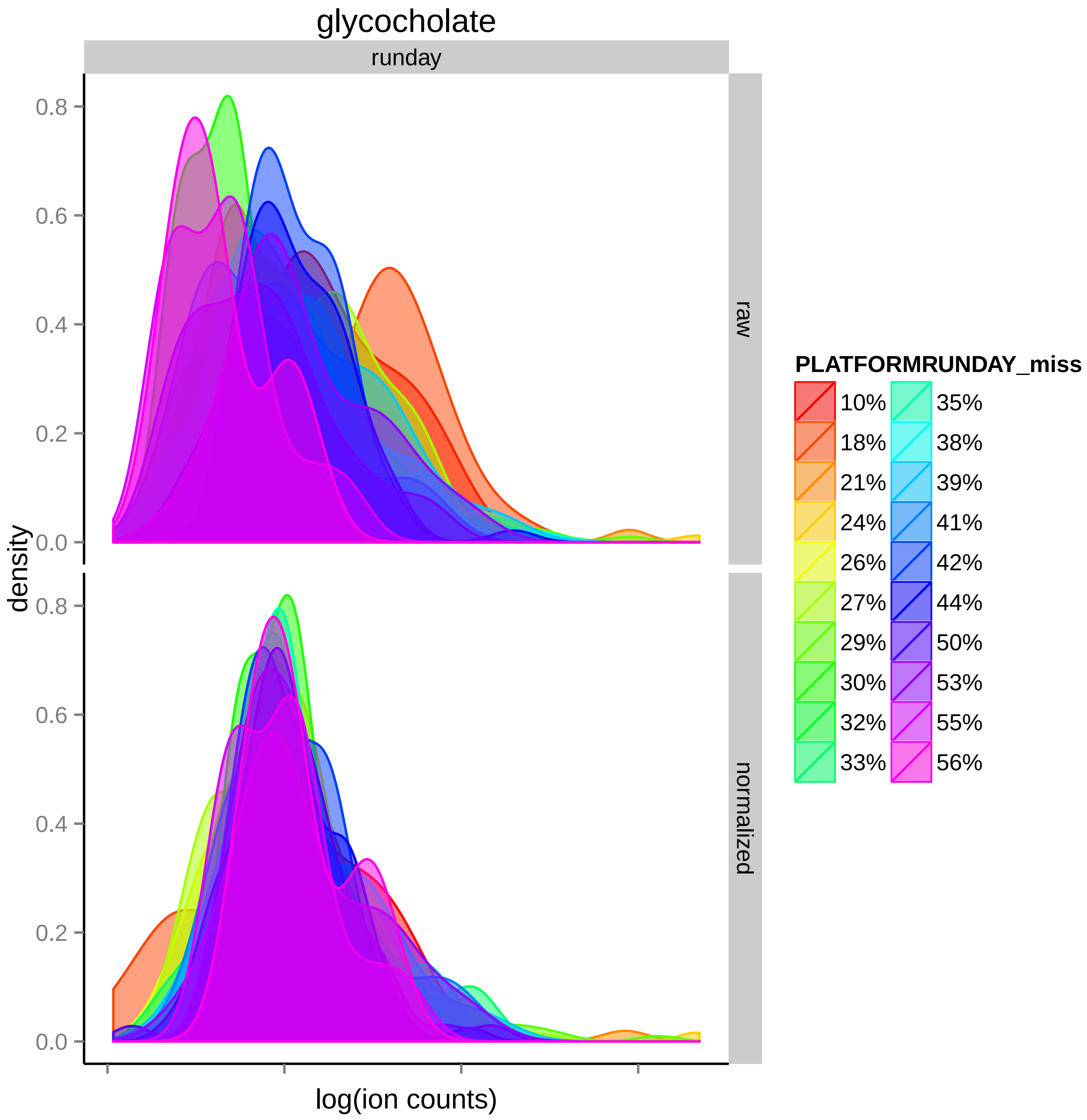
glycocholate
runday
raw
normalized
density
0.0
0.2
0.4
0.6
0.8
log(ion counts)
PLATFORMRUNDAY_miss
10%
18%
21%
24%
26%
27%
29%
30%
32%
33%
35%
38%
39%
41%
42%
44%
50%
53%
55%
56%

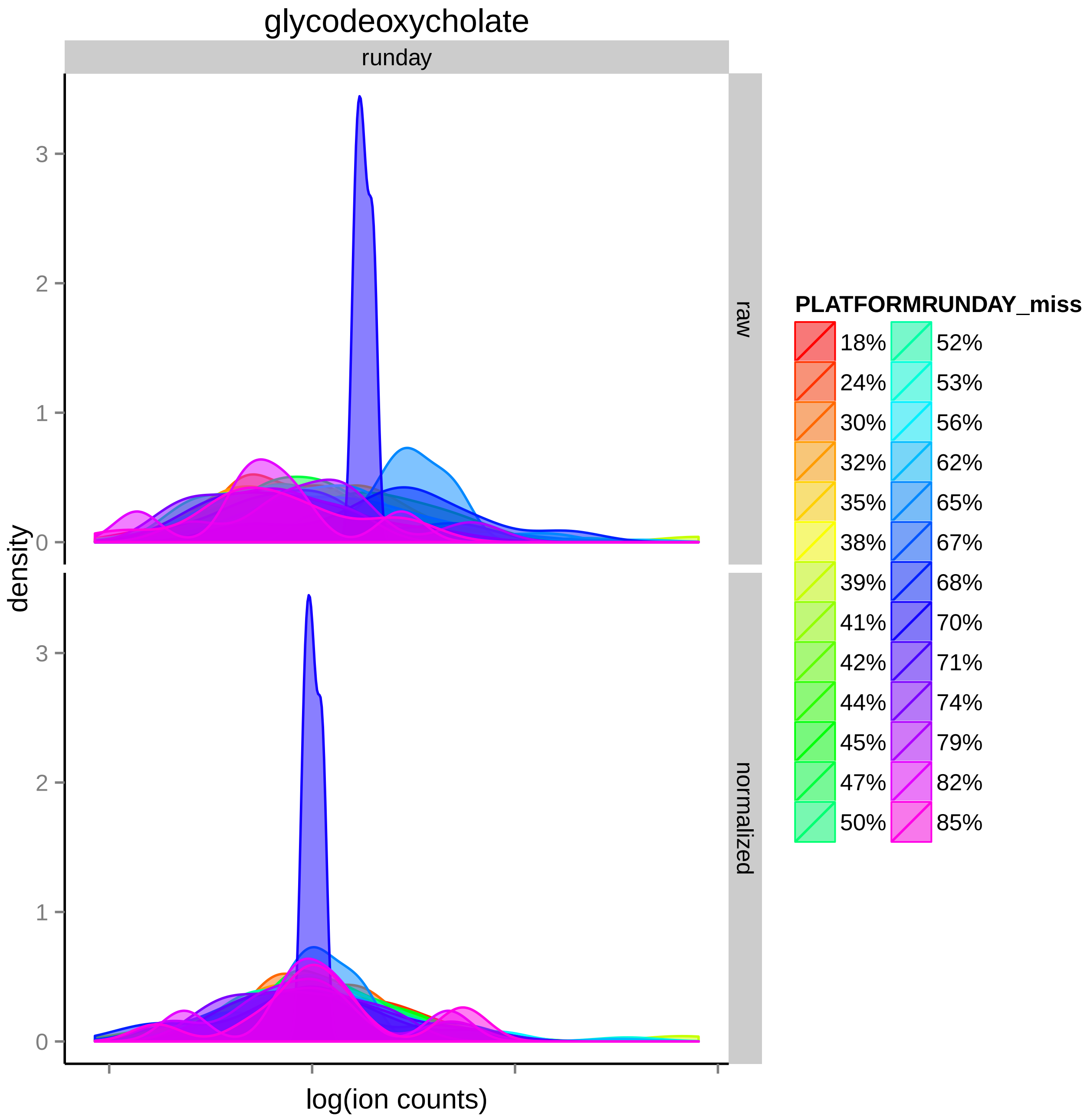

glycodeoxycholate
runday
raw
normalized
density
log(ion counts)
0
1
2
3
PLATFORMRUNDAY_miss
18%
24%
30%
32%
35%
38%
39%
41%
42%
44%
45%
47%
50%
52%
53%
56%
62%
65%
67%
68%
70%
71%
74%
79%
82%
85%

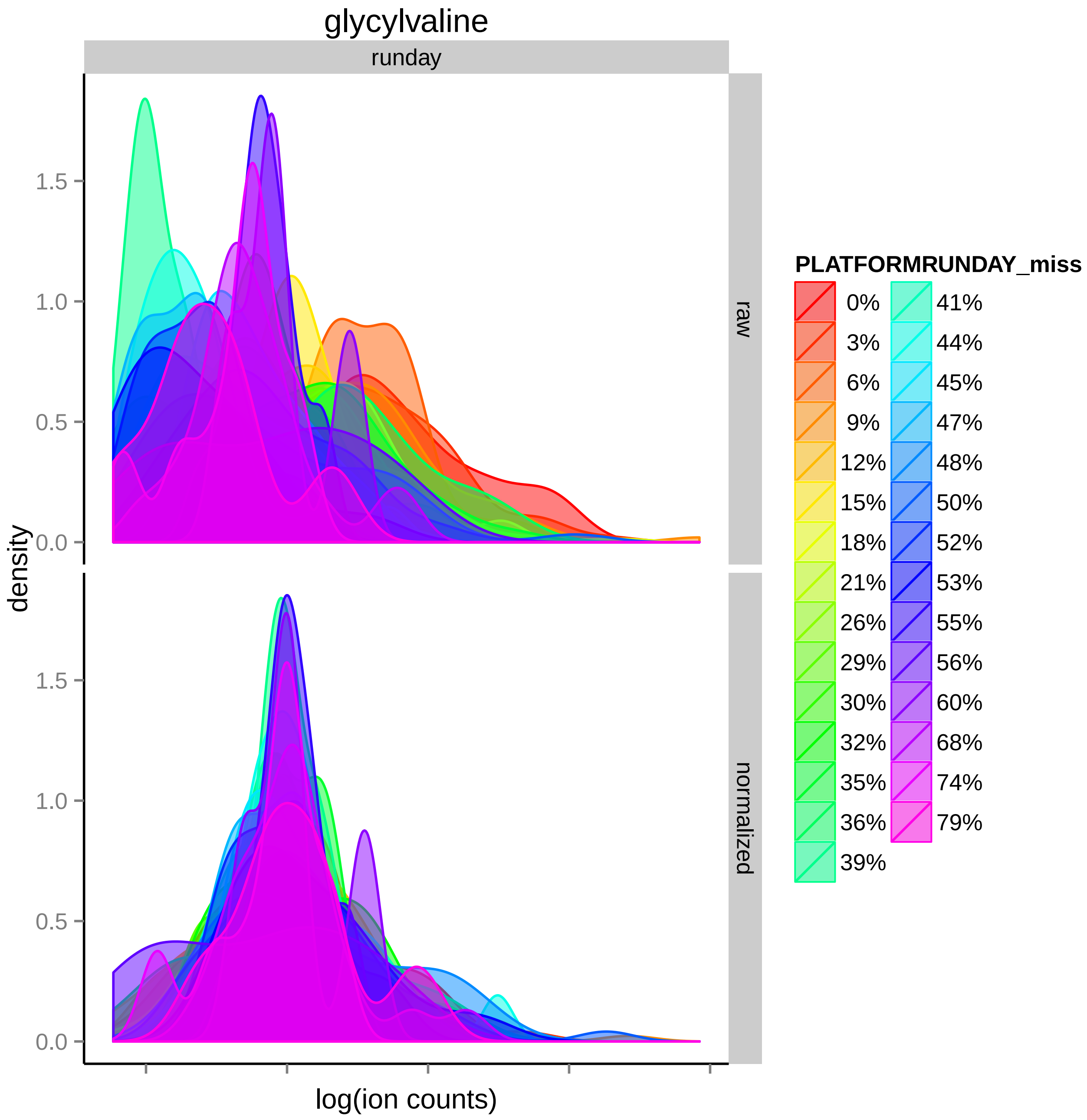
glycylvaline
runday
raw
normalized
density
log(ion counts)
0.0
0.5
1.0
1.5
PLATFORMRUNDAY_miss
0%
3%
6%
9%
12%
15%
18%
21%
26%
29%
30%
32%
35%
36%
39%
41%
44%
45%
47%
48%
50%
52%
53%
55%
56%
60%
68%
74%
79%

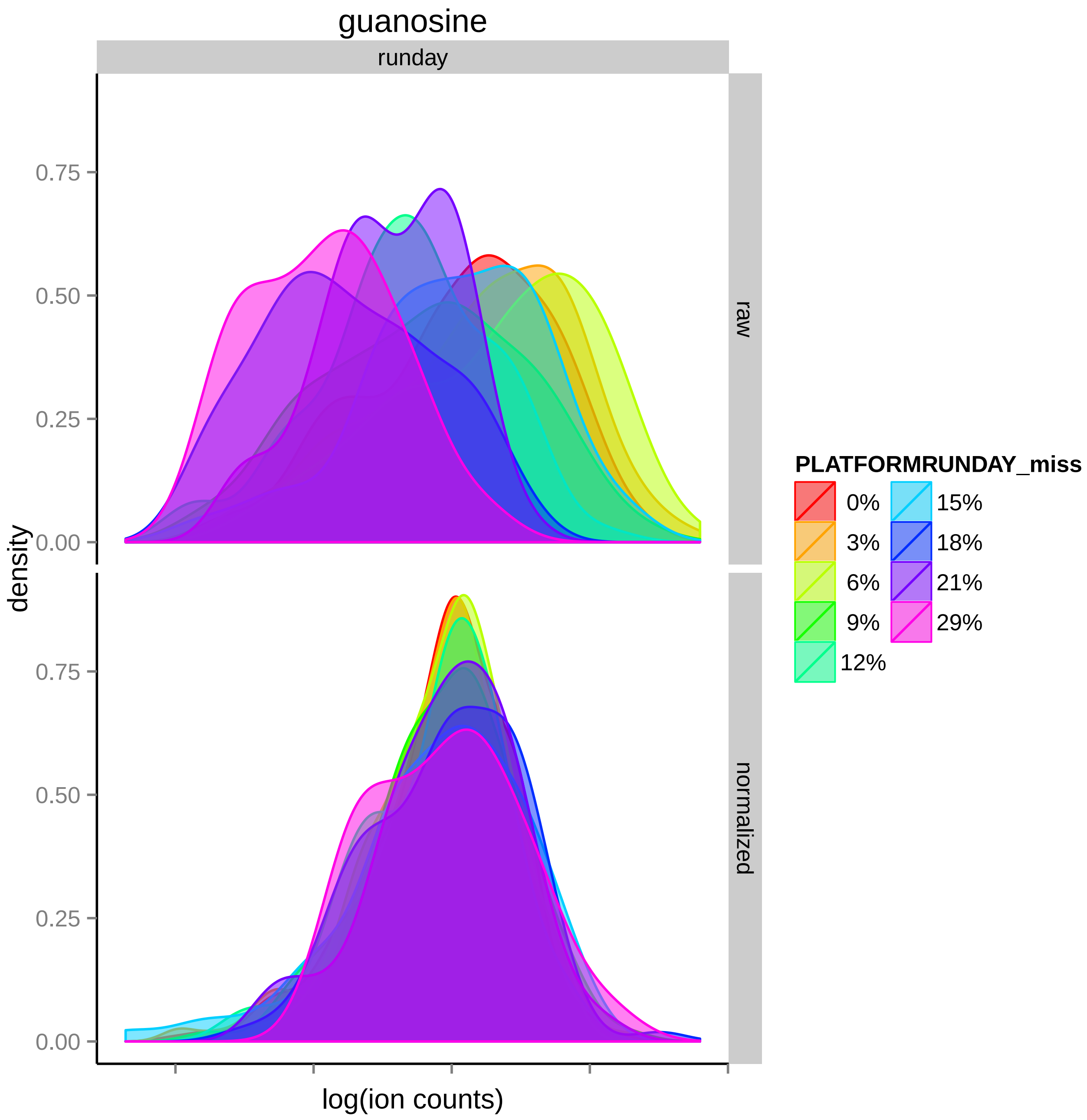

guanosine
runday
raw
normalized
density
log(ion counts)
0.00
0.25
0.50
0.75
PLATFORMRUNDAY_miss
0%
3%
6%
9%
12%
15%
18%
21%
29%

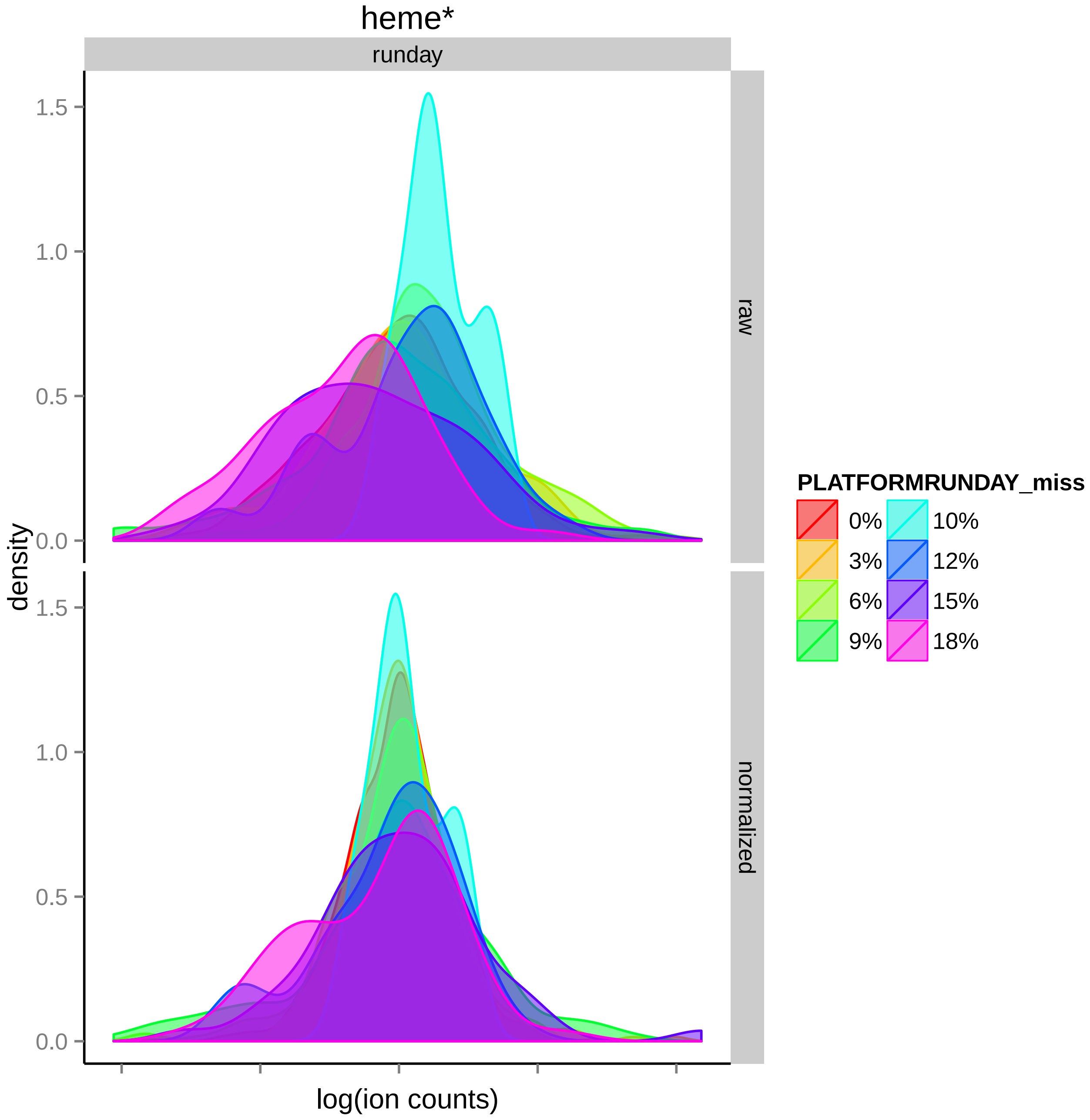

heme*
runday
raw
normalized
1.5
1.0
0.5
0.0
density
log(ion counts)
PLATFORMRUNDAY_miss
0%
3%
6%
9%
10%
12%
15%
18%

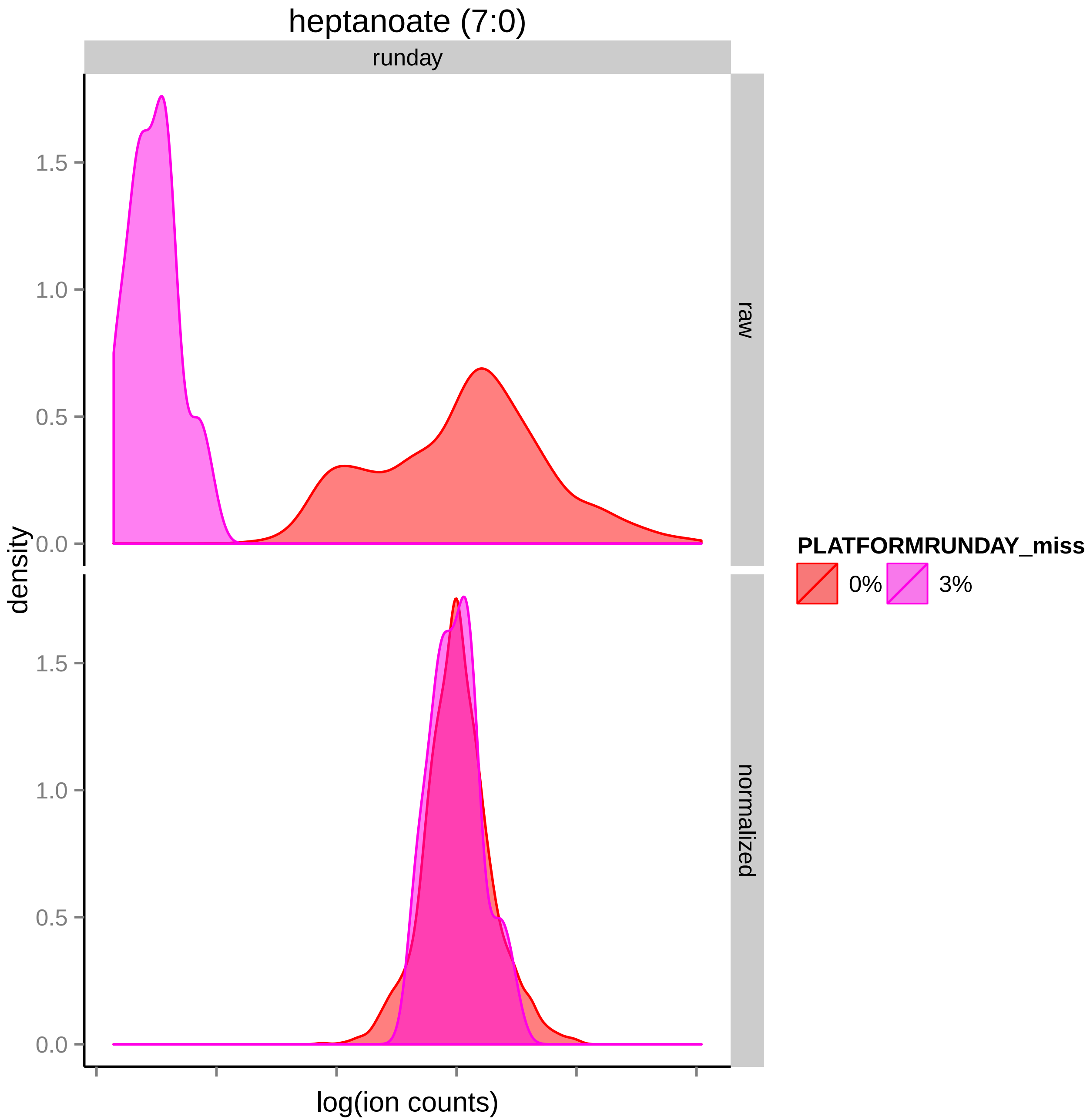

heptanoate (7:0)
runday
raw
normalized
density
1.5
1.0
0.5
0.0
log(ion counts)
PLATFORMRUNDAY_miss
0%
3%

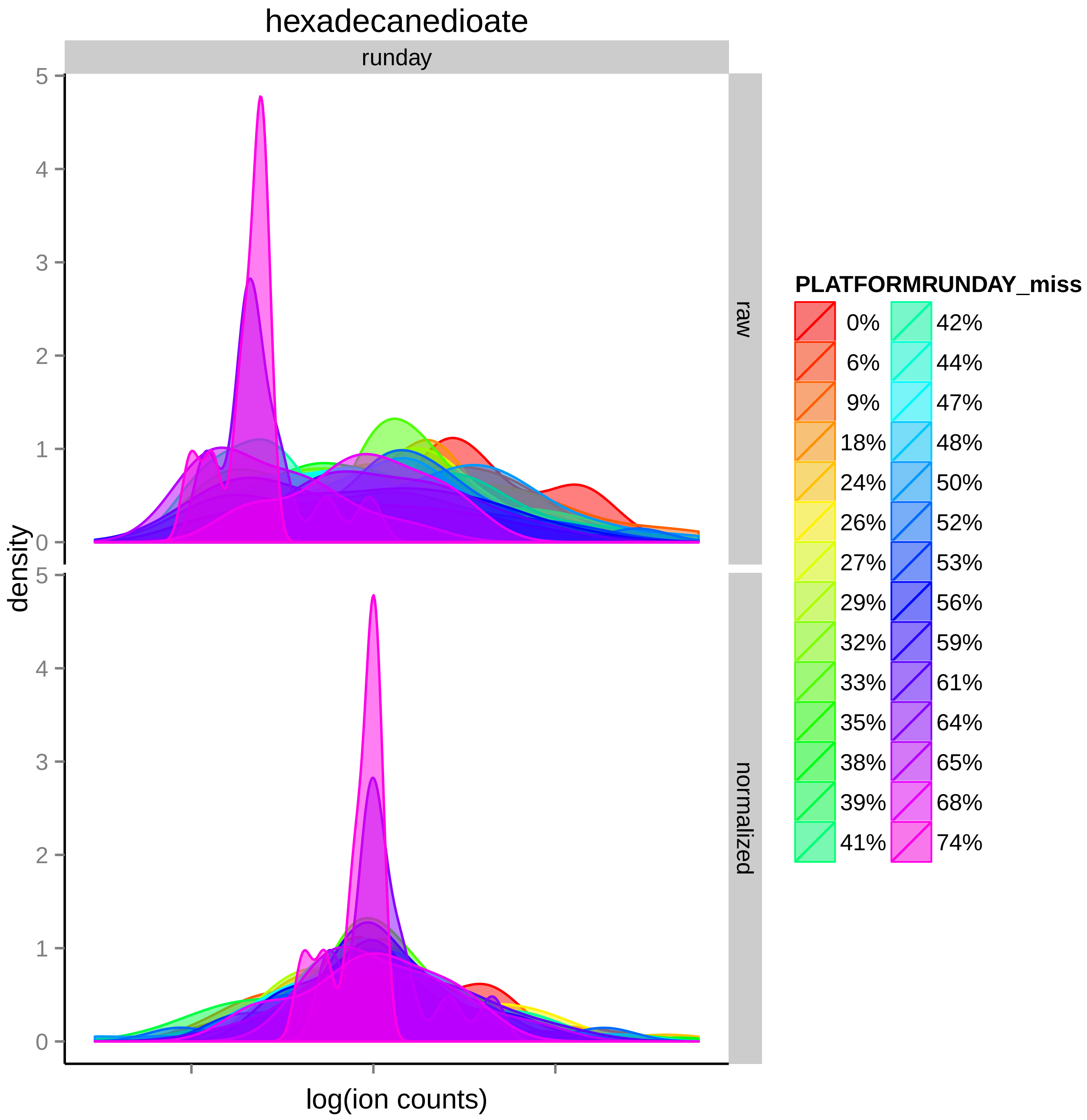
hexadecanedioate
runday
raw
normalized
density
log(ion counts)
0
1
2
3
4
5
PLATFORMRUNDAY_miss
0%
6%
9%
18%
24%
26%
27%
29%
32%
33%
35%
38%
39%
41%
42%
44%
47%
48%
50%
52%
53%
56%
59%
61%
64%
65%
68%
74%

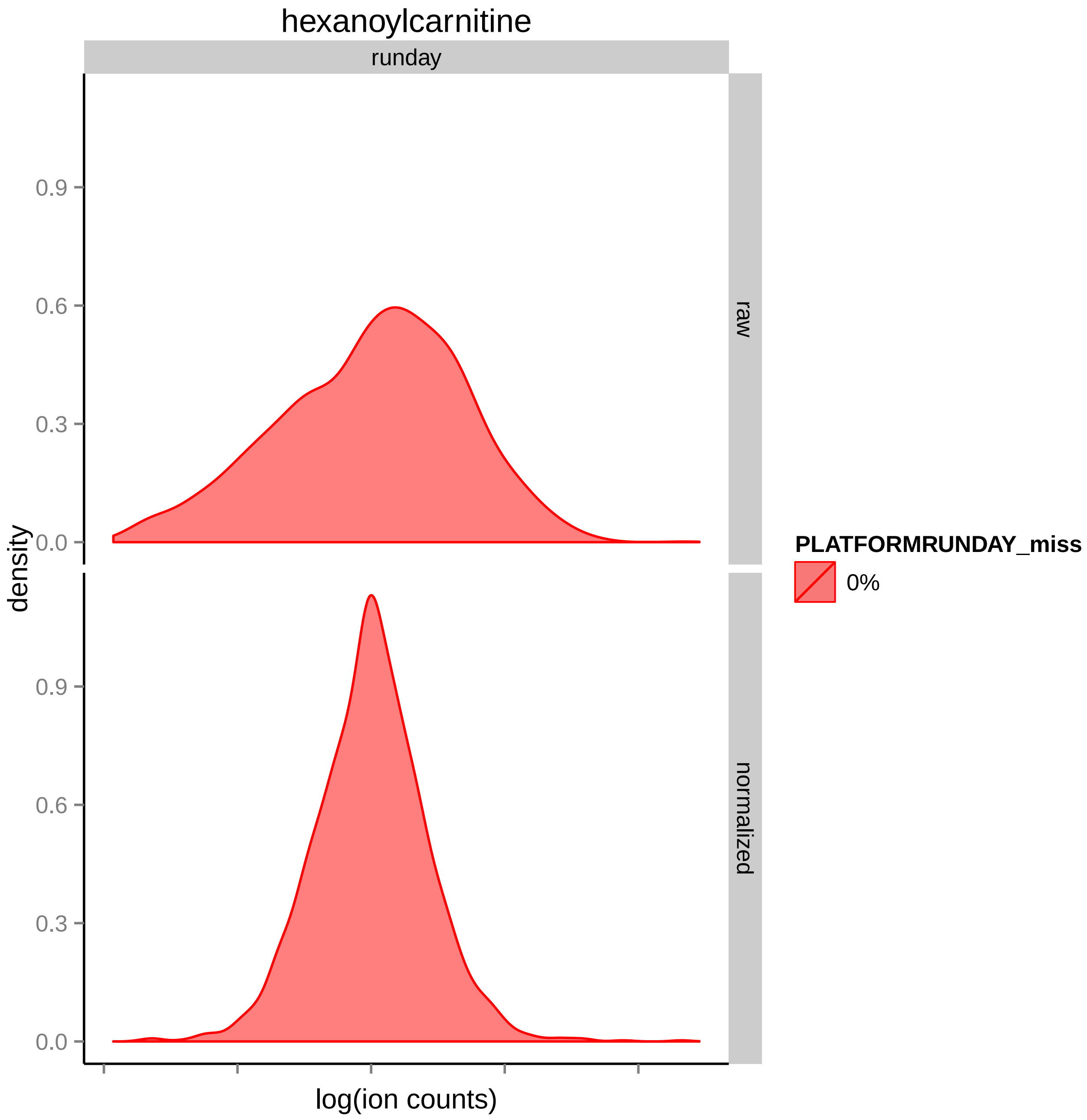

hexanoylcarnitine
runday
raw
normalized
density
0.0
0.3
0.6
0.9
log(ion counts)
PLATFORMRUNDAY_miss
0%

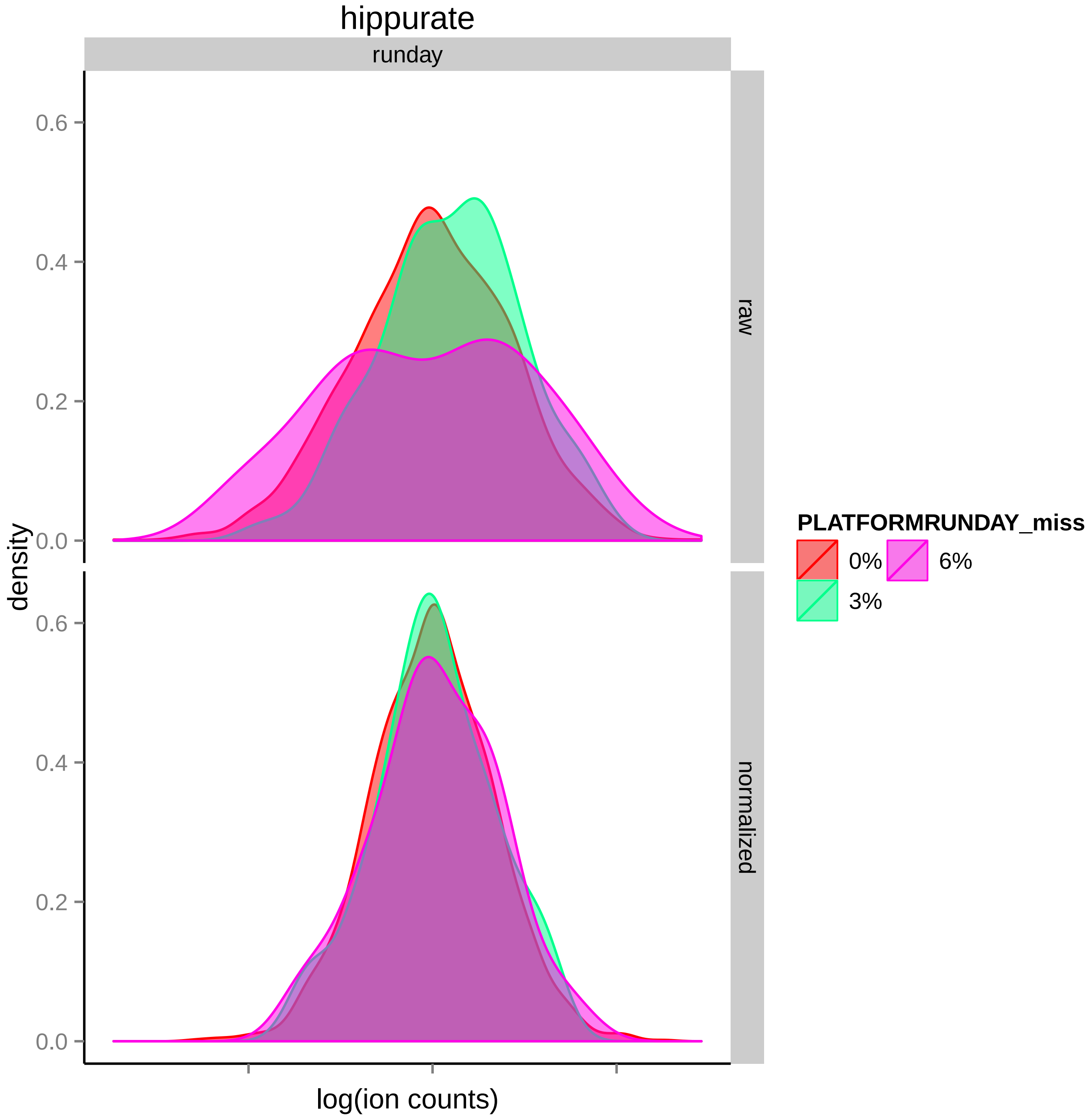

hippurate
runday
raw
normalized
density
0.0
0.2
0.4
0.6
log(ion counts)
PLATFORMRUNDAY_miss
0%
3%
6%

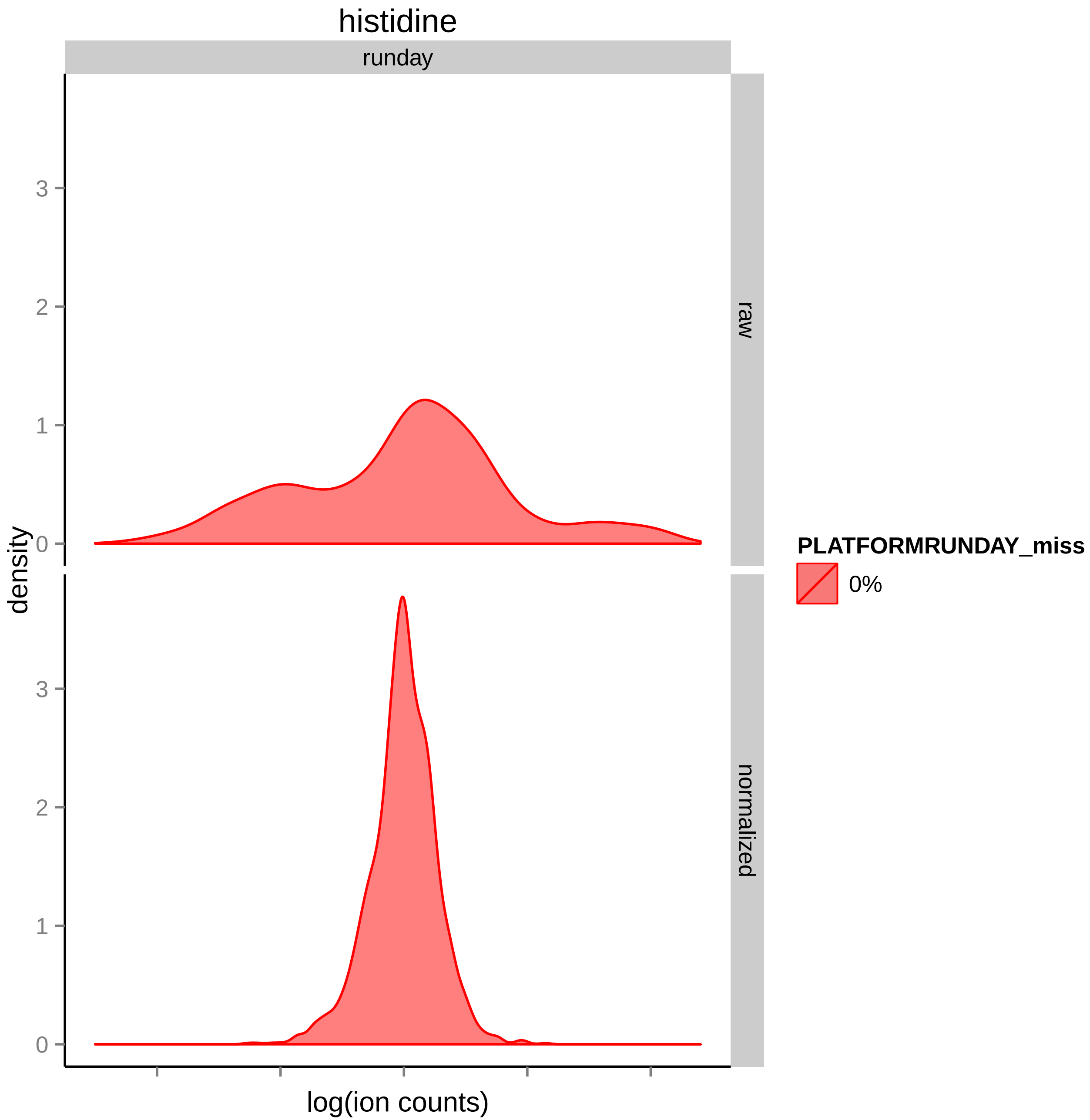

histidine
runday
raw
normalized
density
log(ion counts)
0
1
2
3
PLATFORMRUNDAY_miss
0%

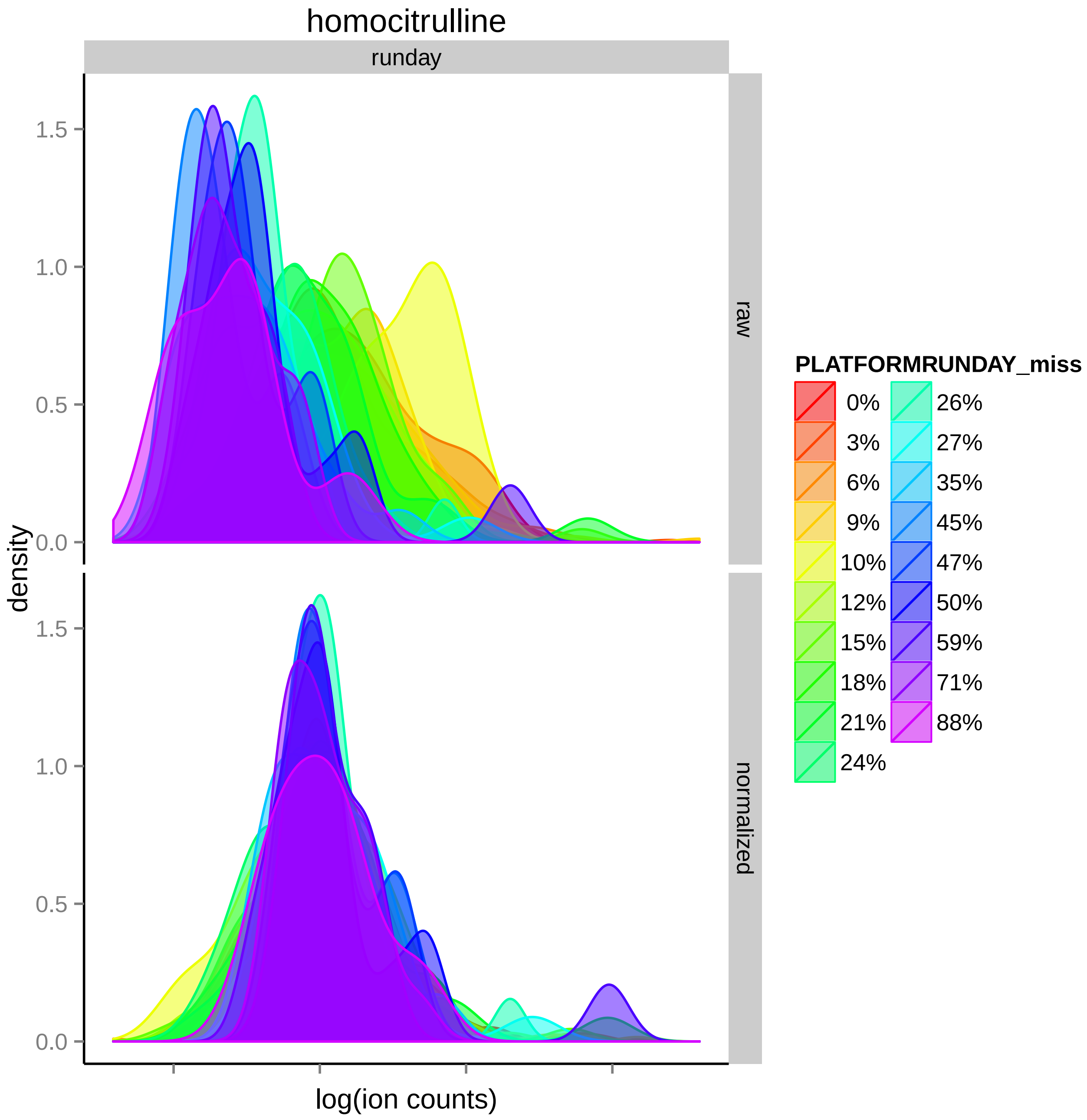
homocitrulline
runday
raw
normalized
density
log(ion counts)
1.5
1.0
0.5
0.0
1.5
1.0
0.5
0.0
PLATFORMRUNDAY_miss
0%
3%
6%
9%
10%
12%
15%
18%
21%
24%
26%
27%
35%
45%
47%
50%
59%
71%
88%

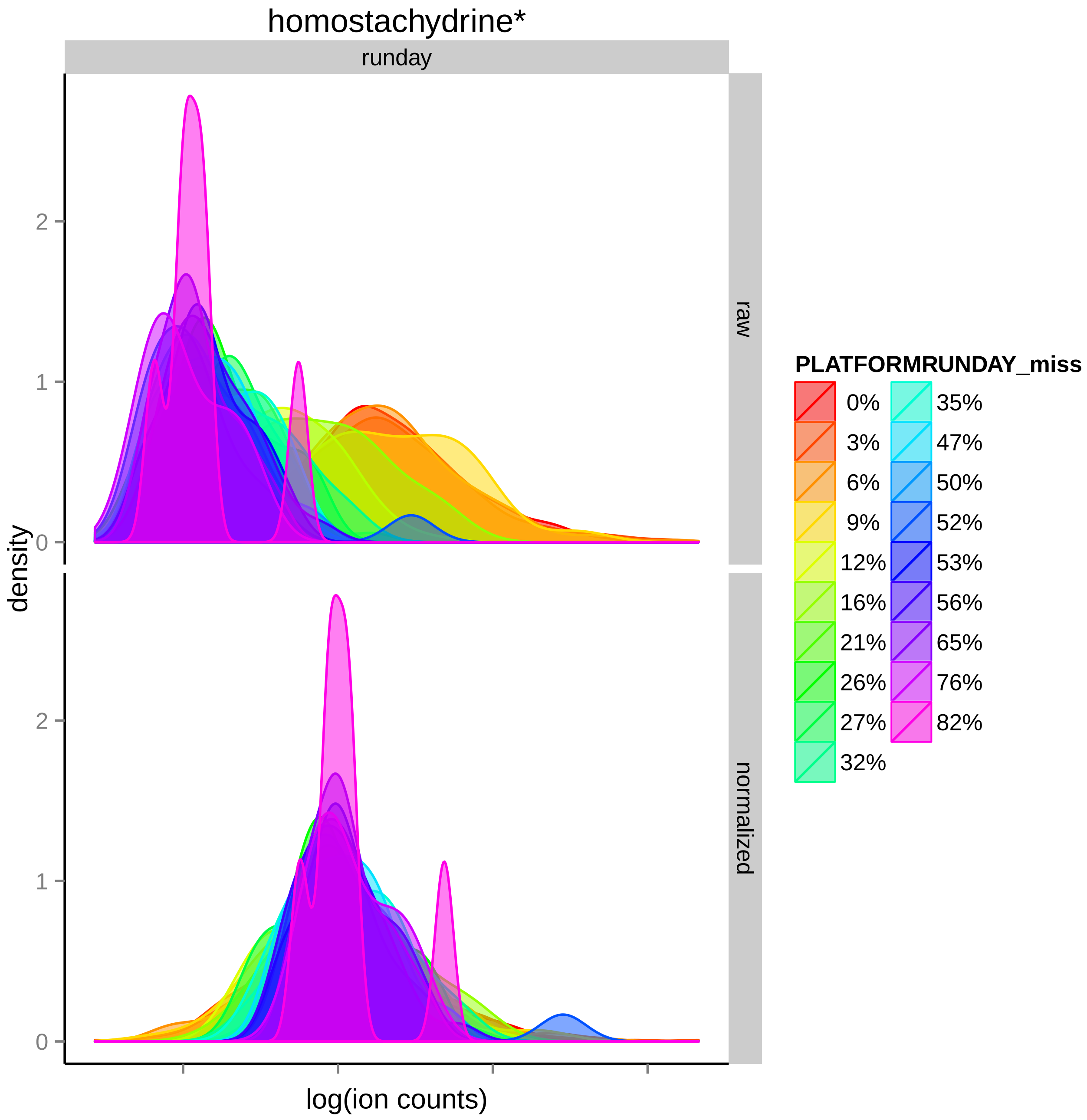
homostachydrine*
runday
raw
normalized
density
log(ion counts)
0
1
2
0
1
2
PLATFORMRUNDAY_miss
0%
3%
6%
9%
12%
16%
21%
26%
27%
32%
35%
47%
50%
52%
53%
56%
65%
76%
82%

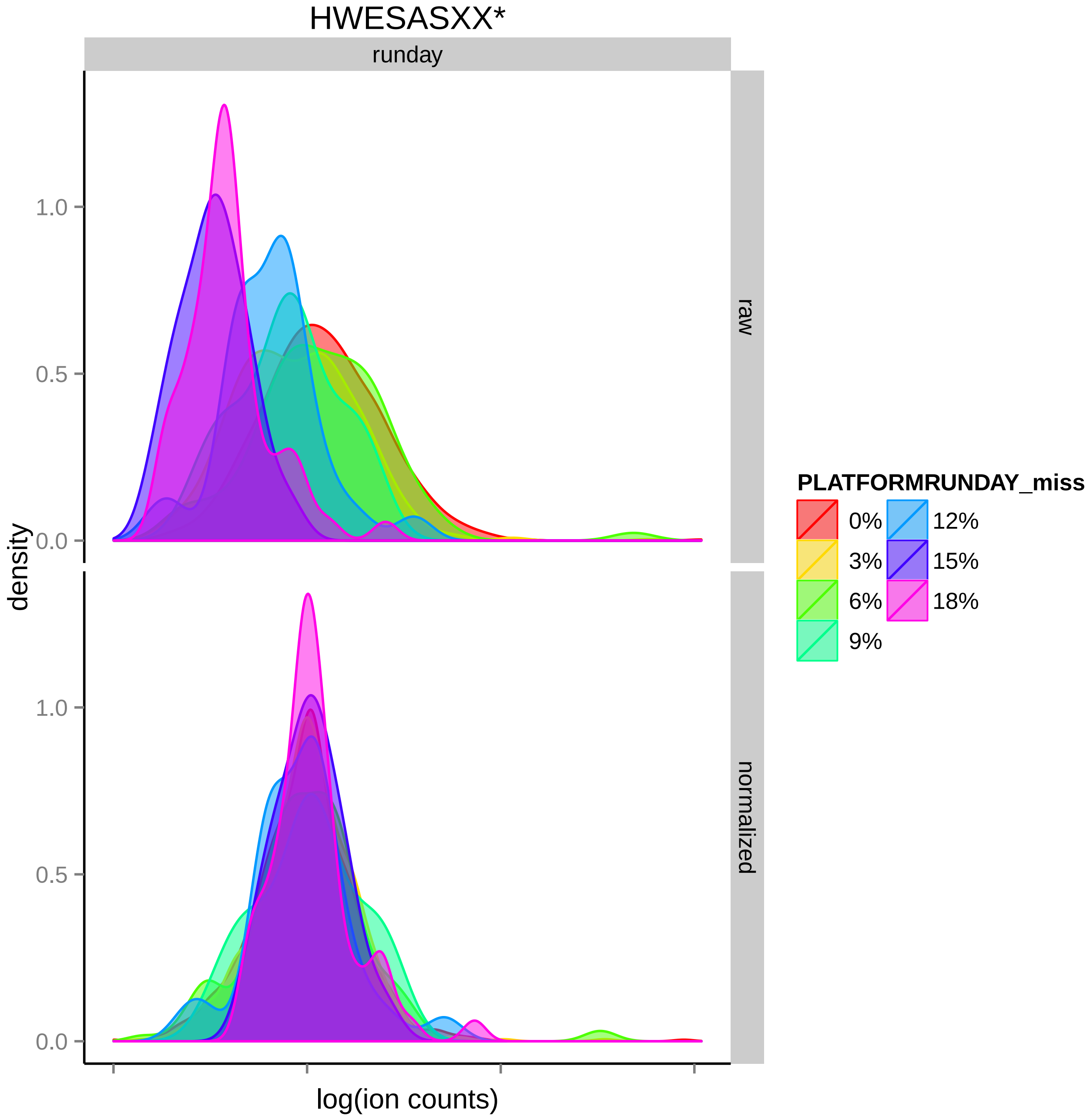

HWESASXX*
runday
raw
normalized
density
log(ion counts)
1.0
0.5
0.0
1.0
0.5
0.0
PLATFORMRUNDAY_miss
0%
3%
6%
9%
12%
15%
18%

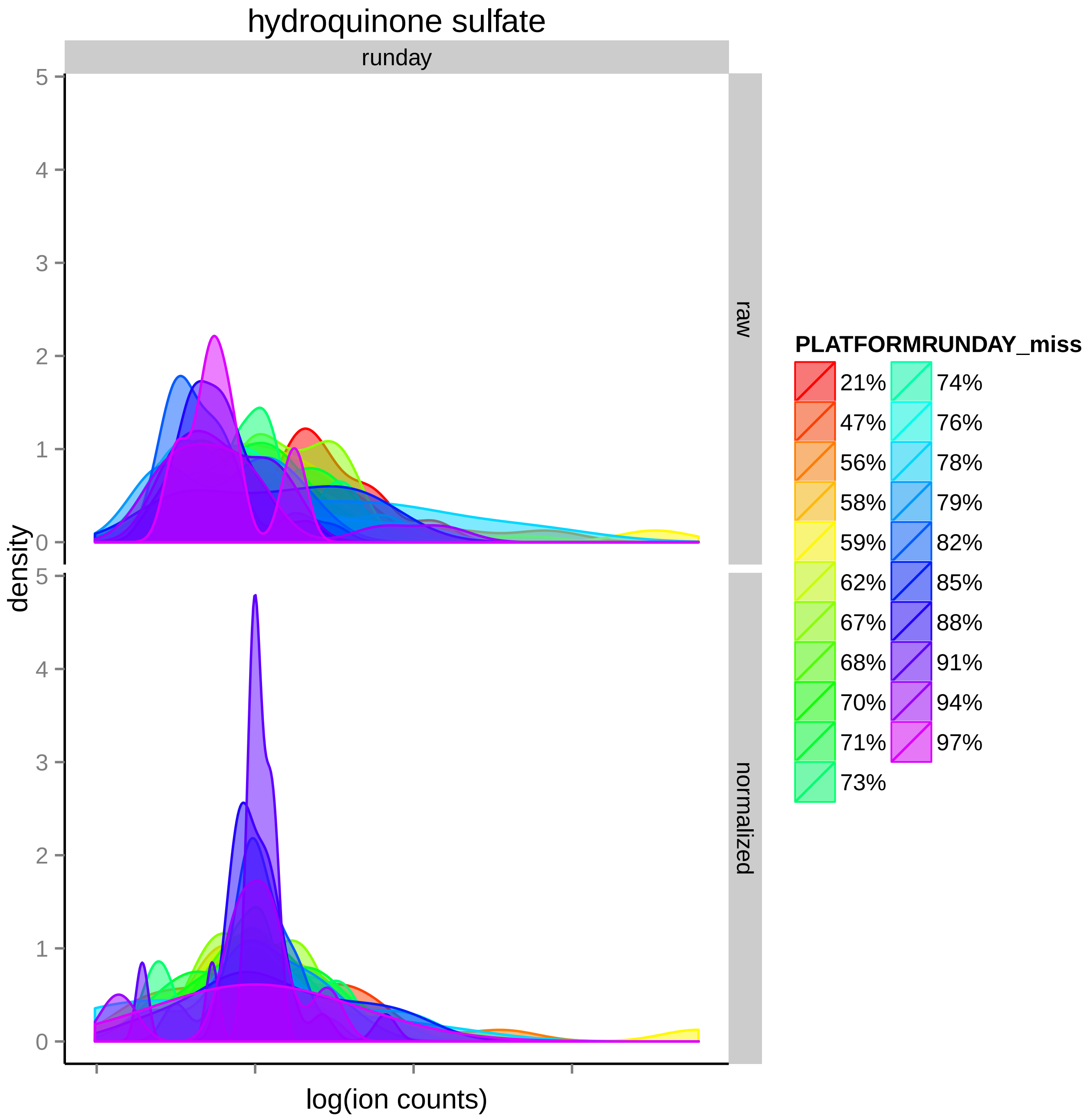

hydroquinone sulfate
runday
raw
normalized
density
log(ion counts)
0
1
2
3
4
5
PLATFORMRUNDAY_miss
21%
47%
56%
58%
59%
62%
67%
68%
70%
71%
73%
74%
76%
78%
79%
82%
85%
88%
91%
94%
97%

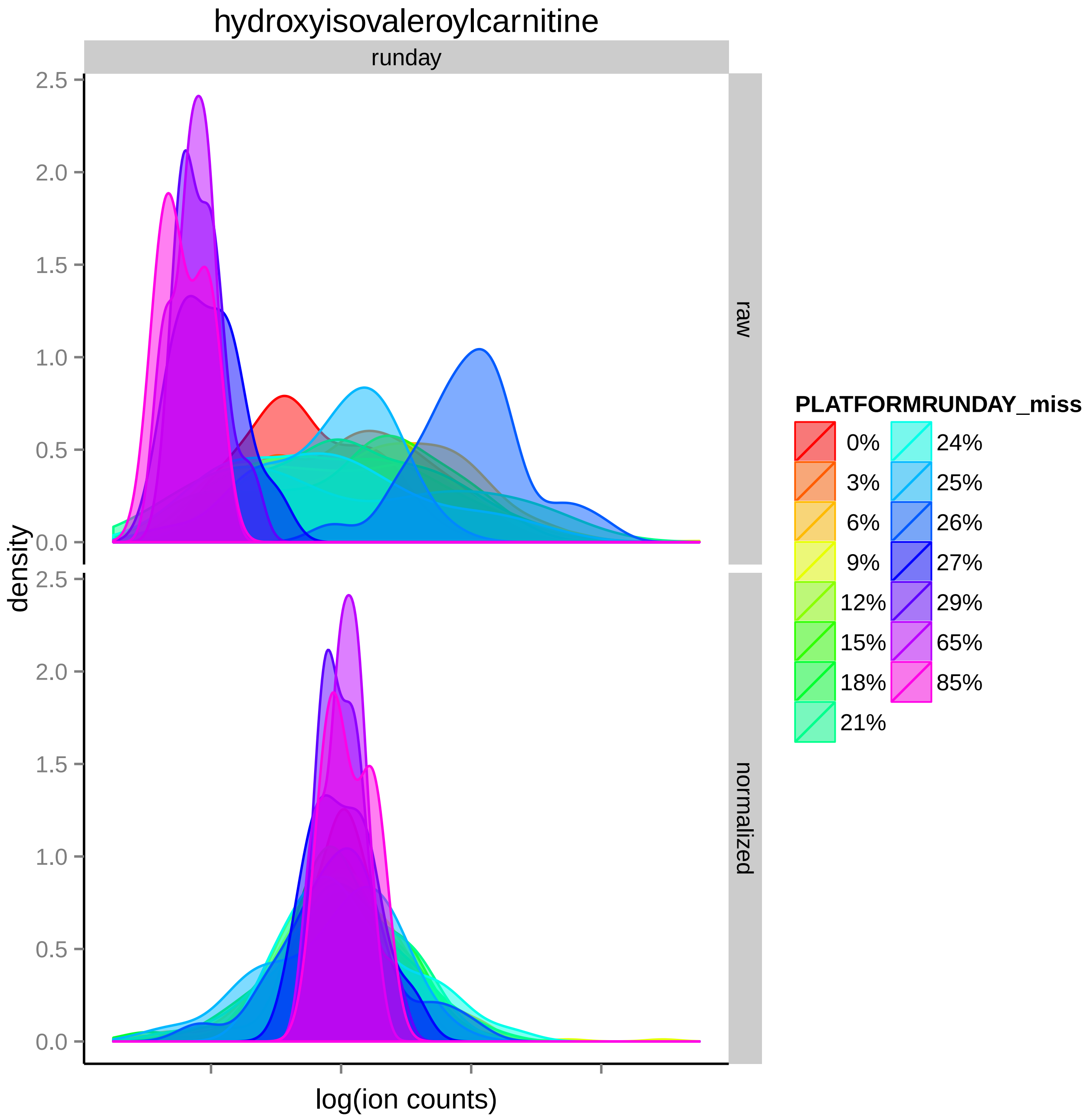

hydroxyisovaleroylcarnitine
runday
raw
normalized
density
log(ion counts)
0.0
0.5
1.0
1.5
2.0
2.5
PLATFORMRUNDAY_miss
0%
3%
6%
9%
12%
15%
18%
21%
24%
25%
26%
27%
29%
65%
85%

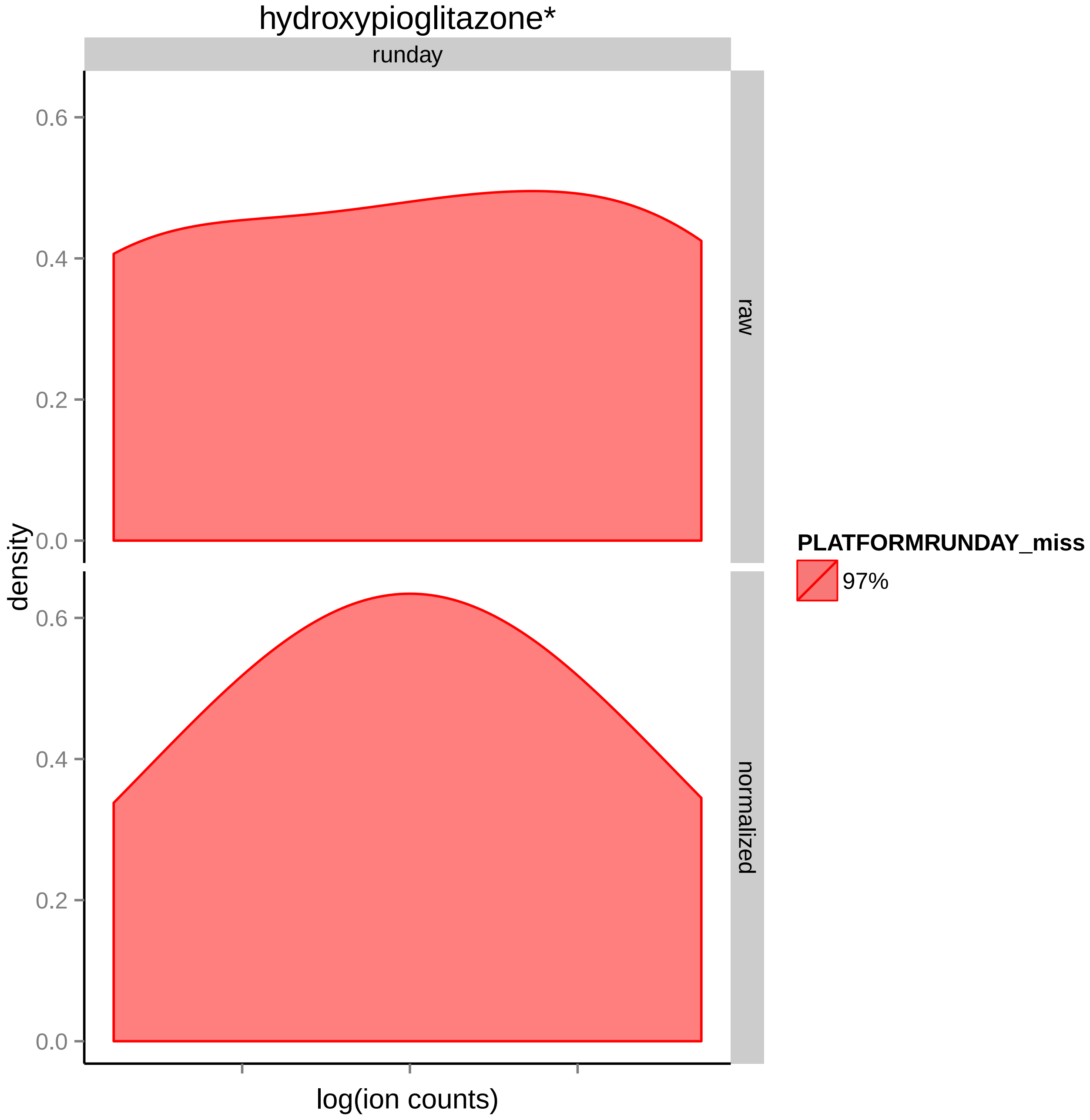
hydroxypioglitazone*
runday
raw
normalized
density
0.6
0.4
0.2
0.0
0.6
0.4
0.2
0.0
log(ion counts)
PLATFORMRUNDAY_miss
97%

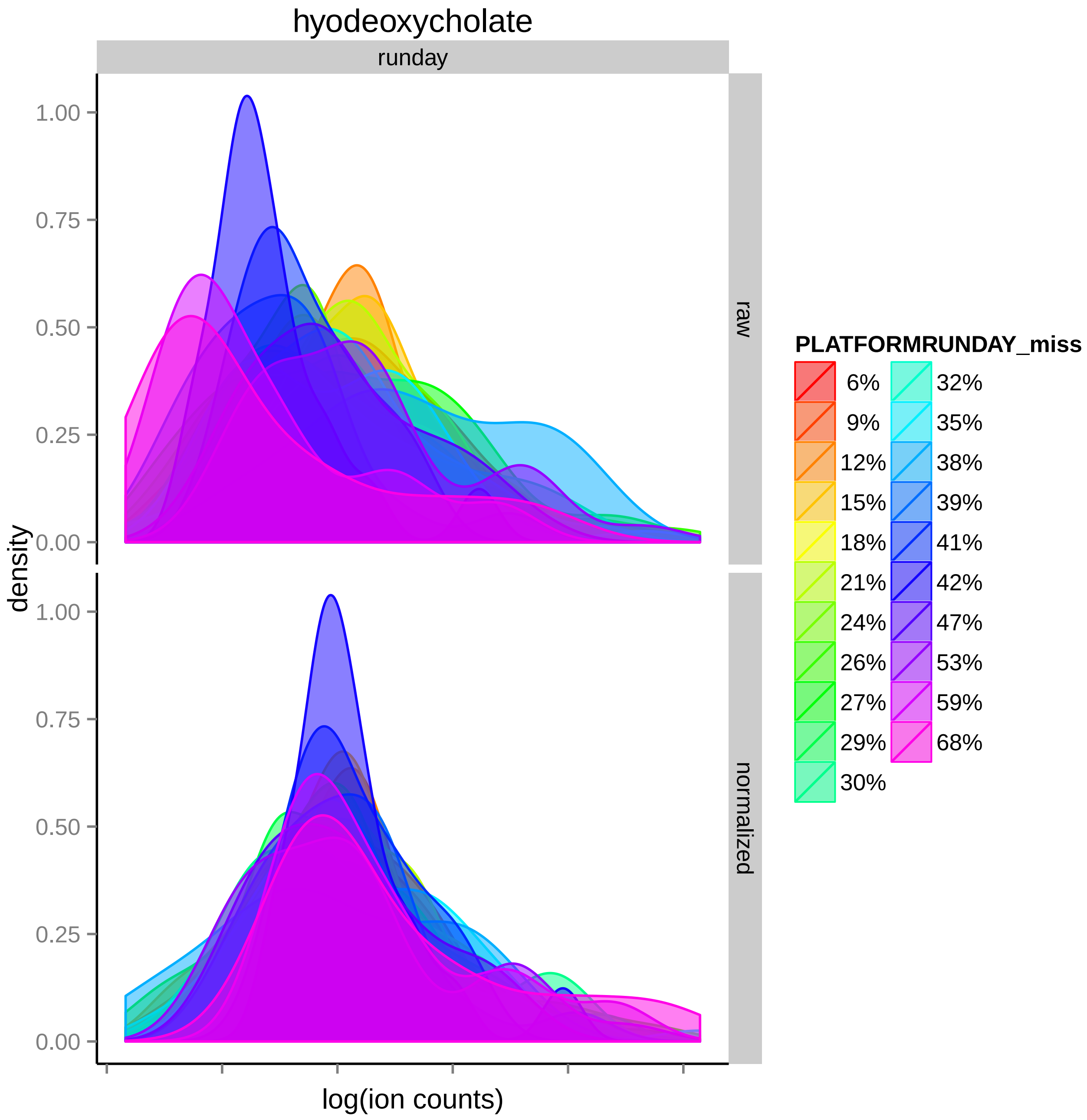

hyodeoxycholate
runday
raw
normalized
density
1.00
0.75
0.50
0.25
0.00
log(ion counts)
PLATFORMRUNDAY_miss
6%
9%
12%
15%
18%
21%
24%
26%
27%
29%
30%
32%
35%
38%
39%
41%
42%
47%
53%
59%
68%

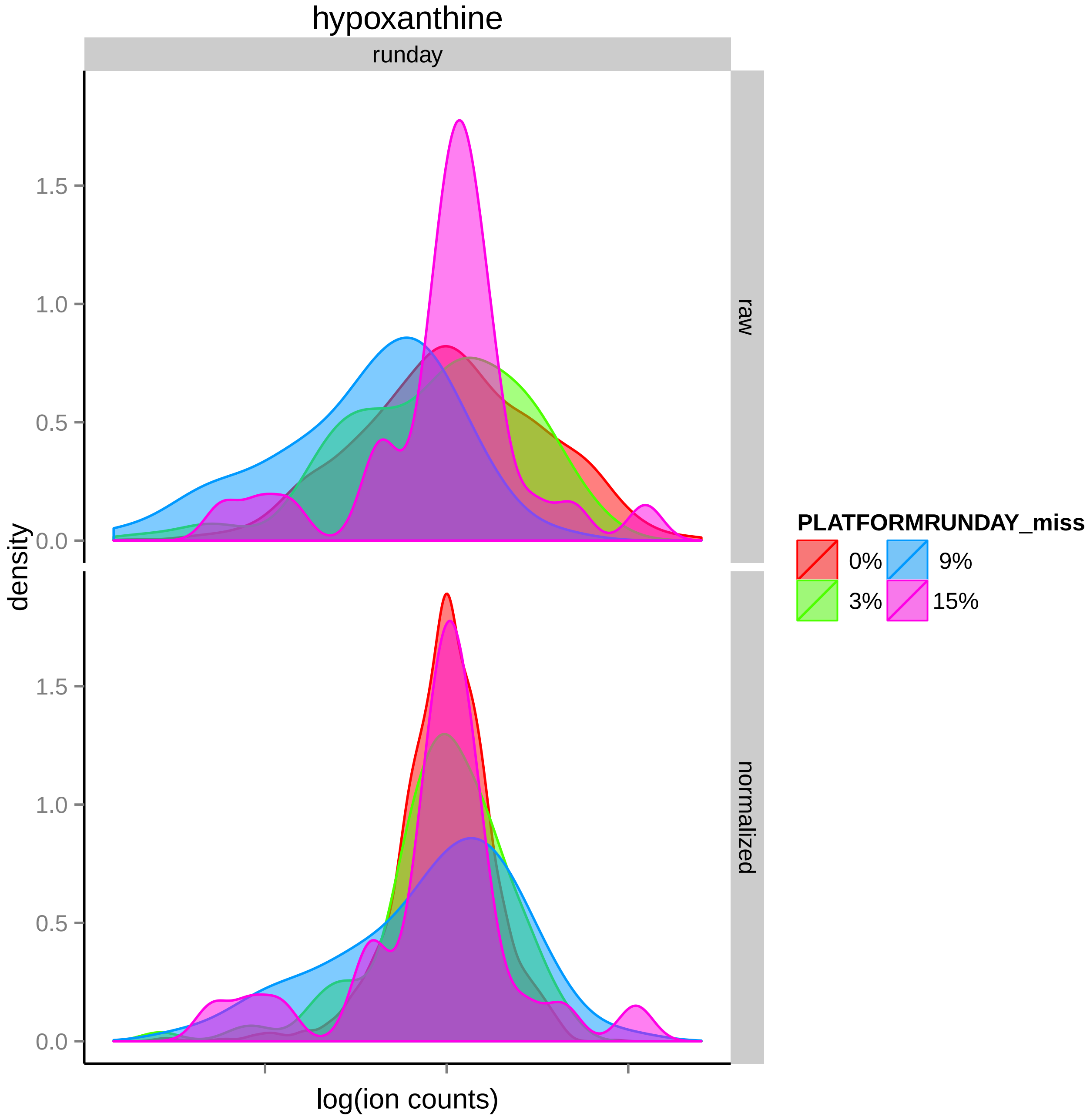
hypoxanthine
runday
raw
normalized
density
1.5
1.0
0.5
0.0
log(ion counts)
PLATFORMRUNDAY_miss
0%
9%
3%
15%

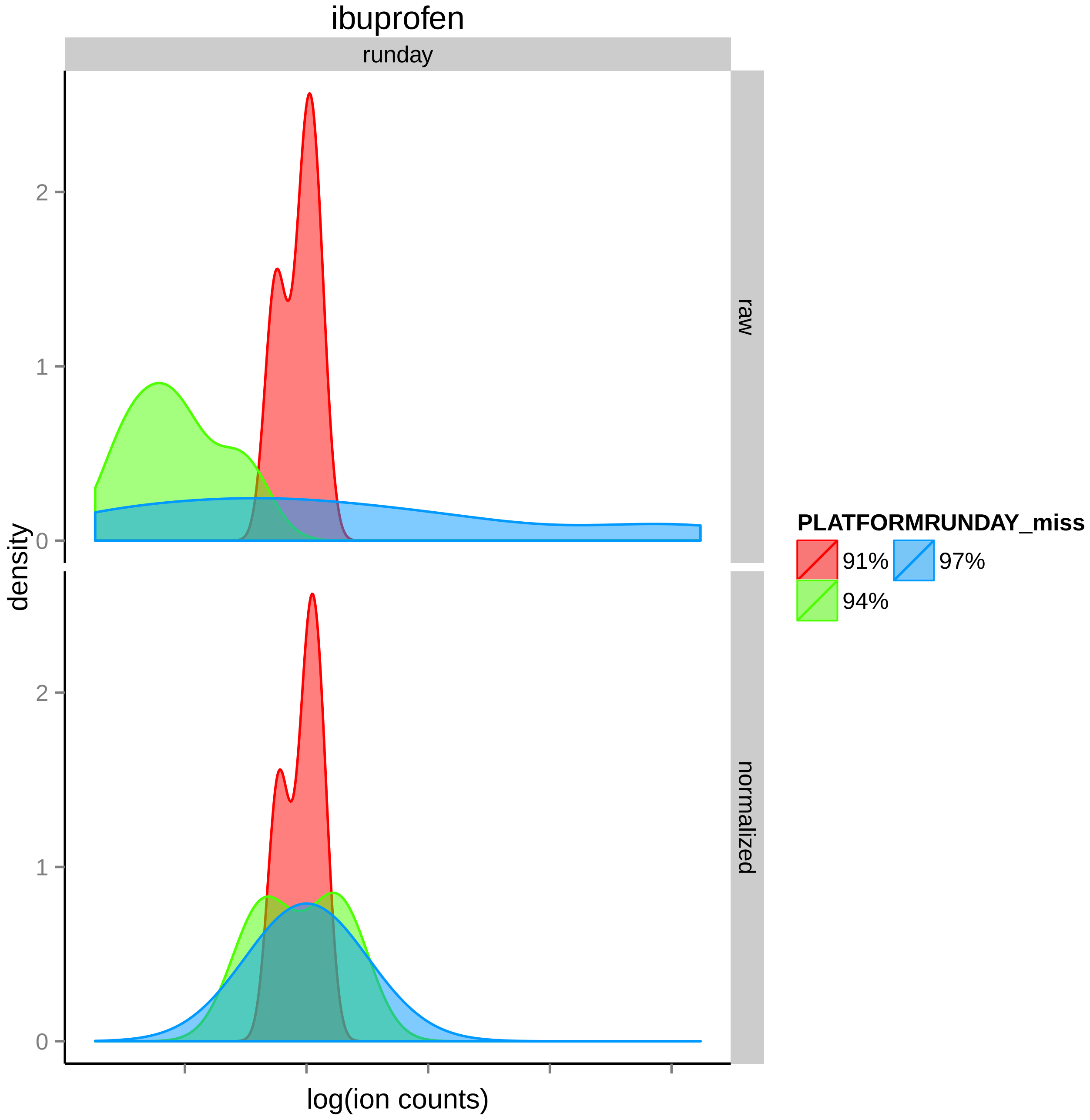

ibuprofen
runday
raw
normalized
density
log(ion counts)
2
1
0
2
1
0
PLATFORMRUNDAY_miss
91%
97%
94%

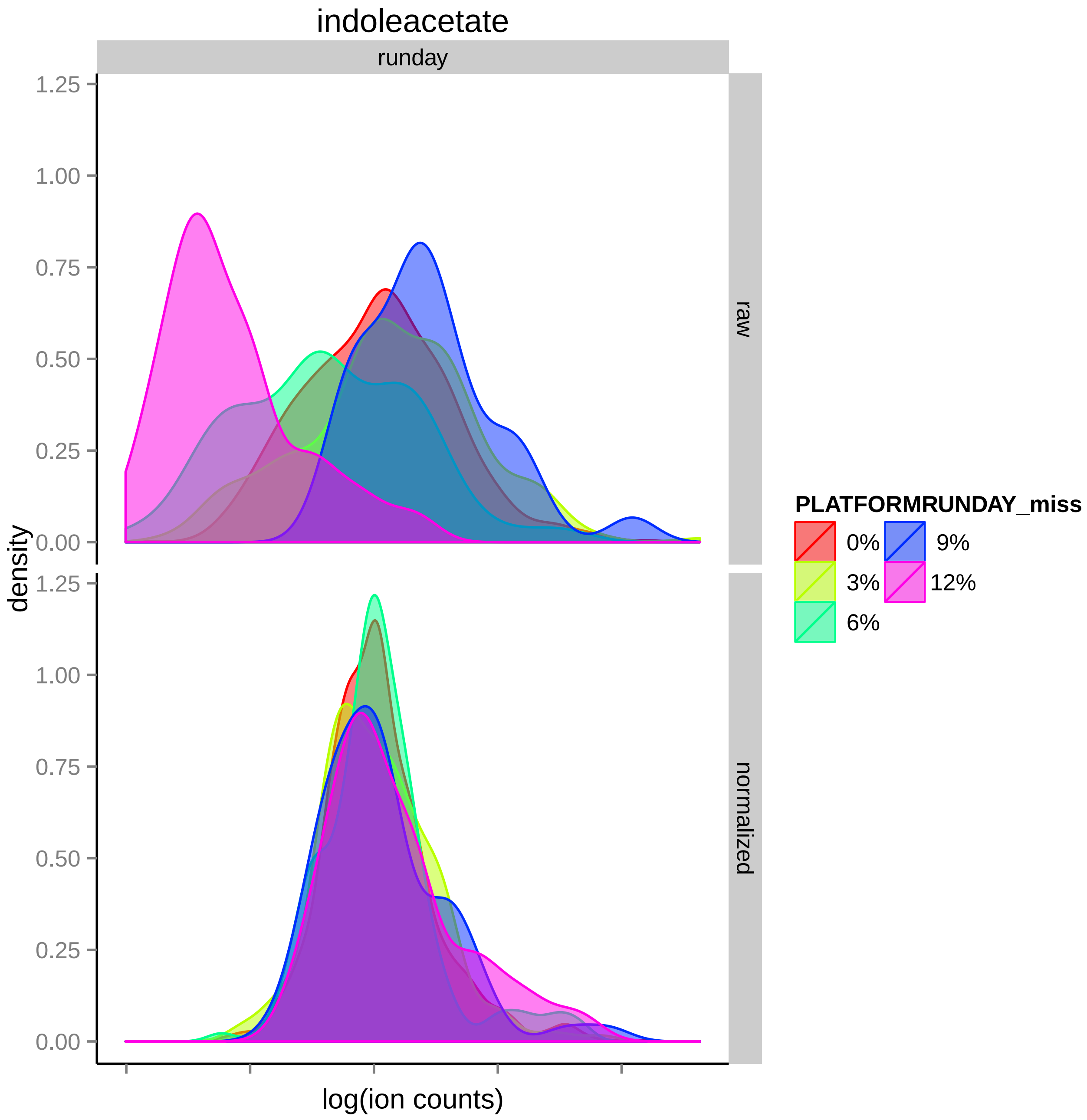
indoleacetate
runday
raw
normalized
density
log(ion counts)
1.25
1.00
0.75
0.50
0.25
0.00
PLATFORMRUNDAY_miss
0%
3%
6%
9%
12%

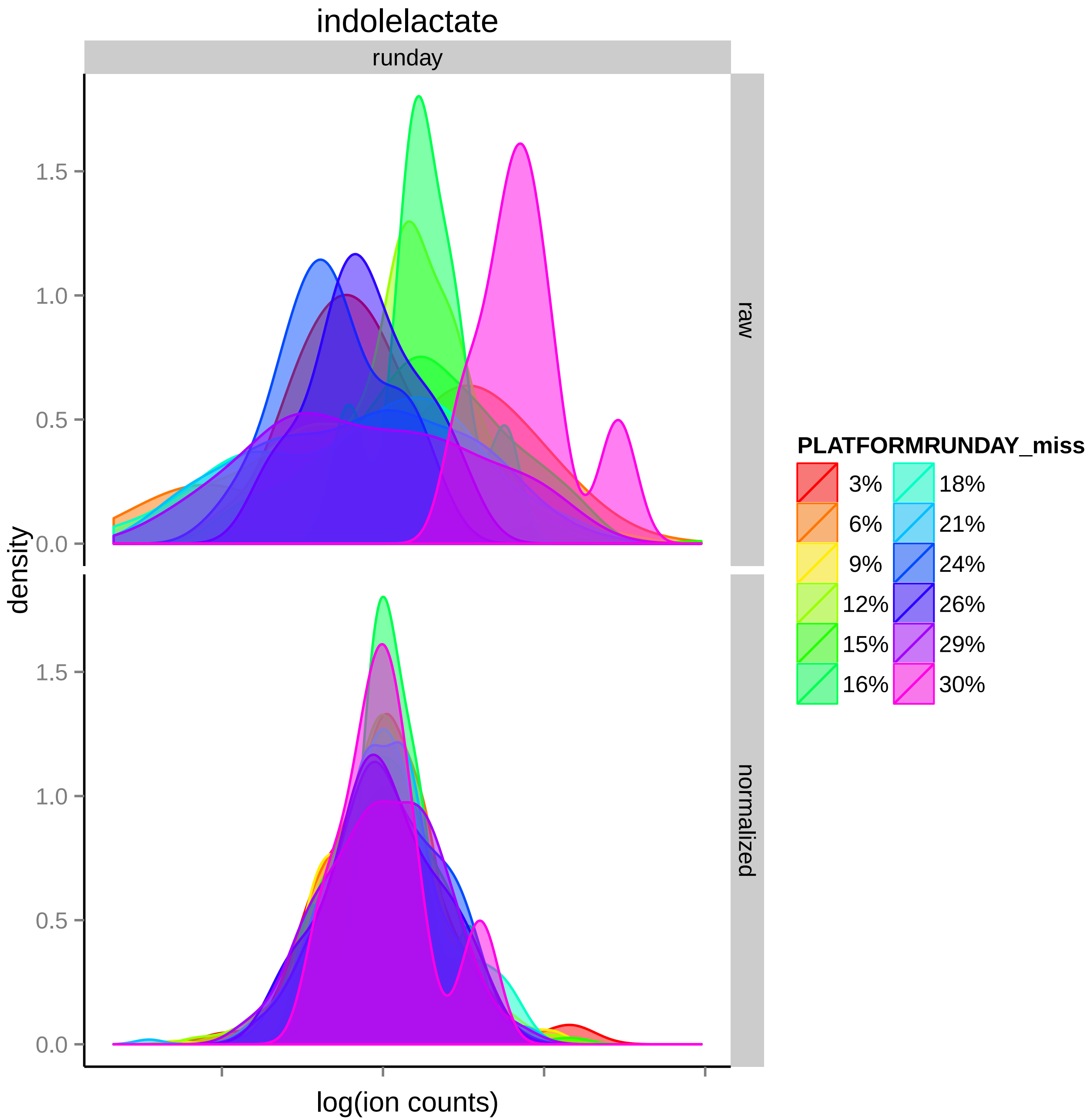
indolelactate
runday
raw
normalized
density
log(ion counts)
1.5
1.0
0.5
0.0
PLATFORMRUNDAY_miss
3%
6%
9%
12%
15%
16%
18%
21%
24%
26%
29%
30%

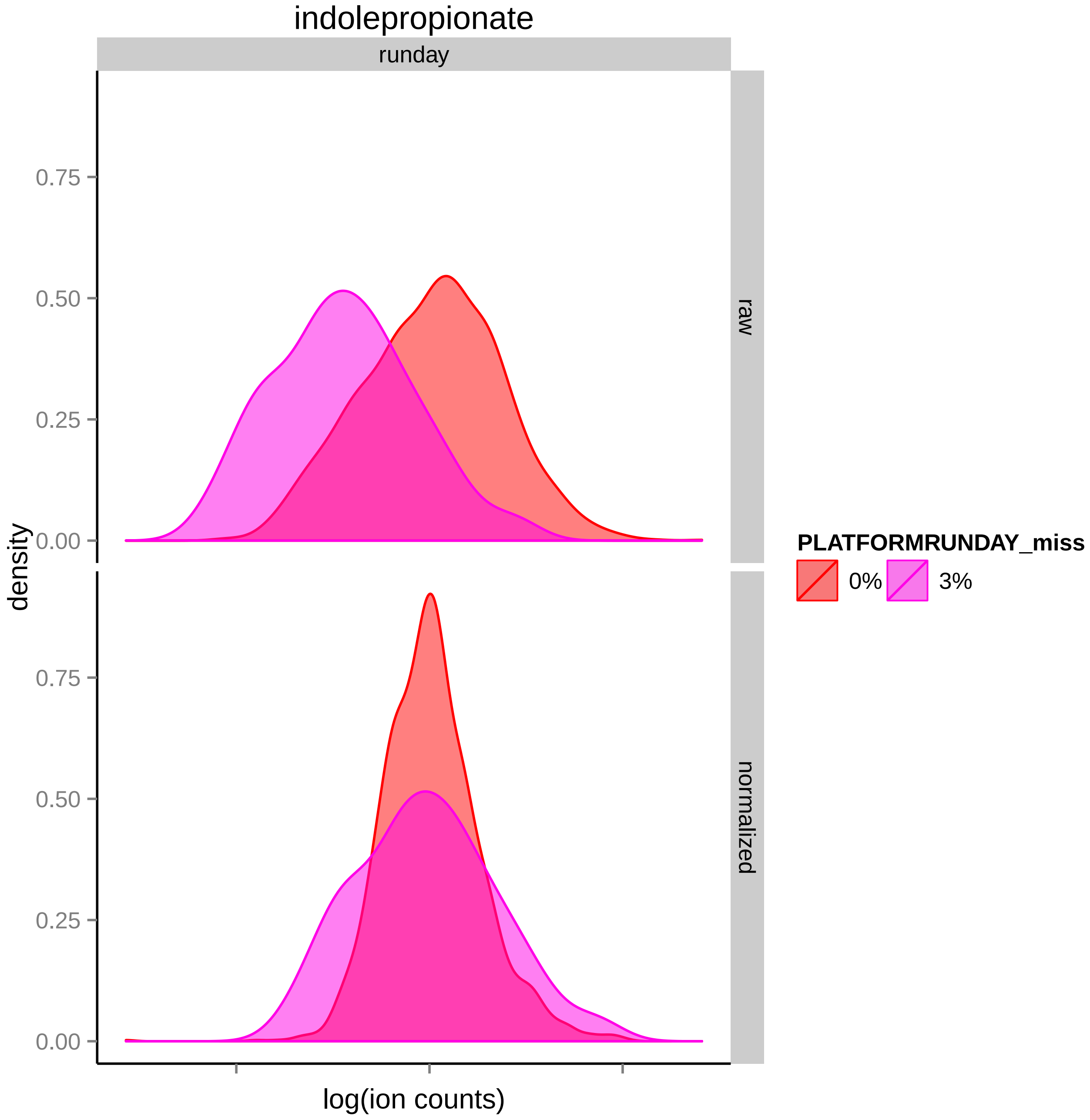

indolepropionate
runday
raw
normalized
0.75
0.50
0.25
0.00
density
log(ion counts)
PLATFORMRUNDAY_miss
0%
3%

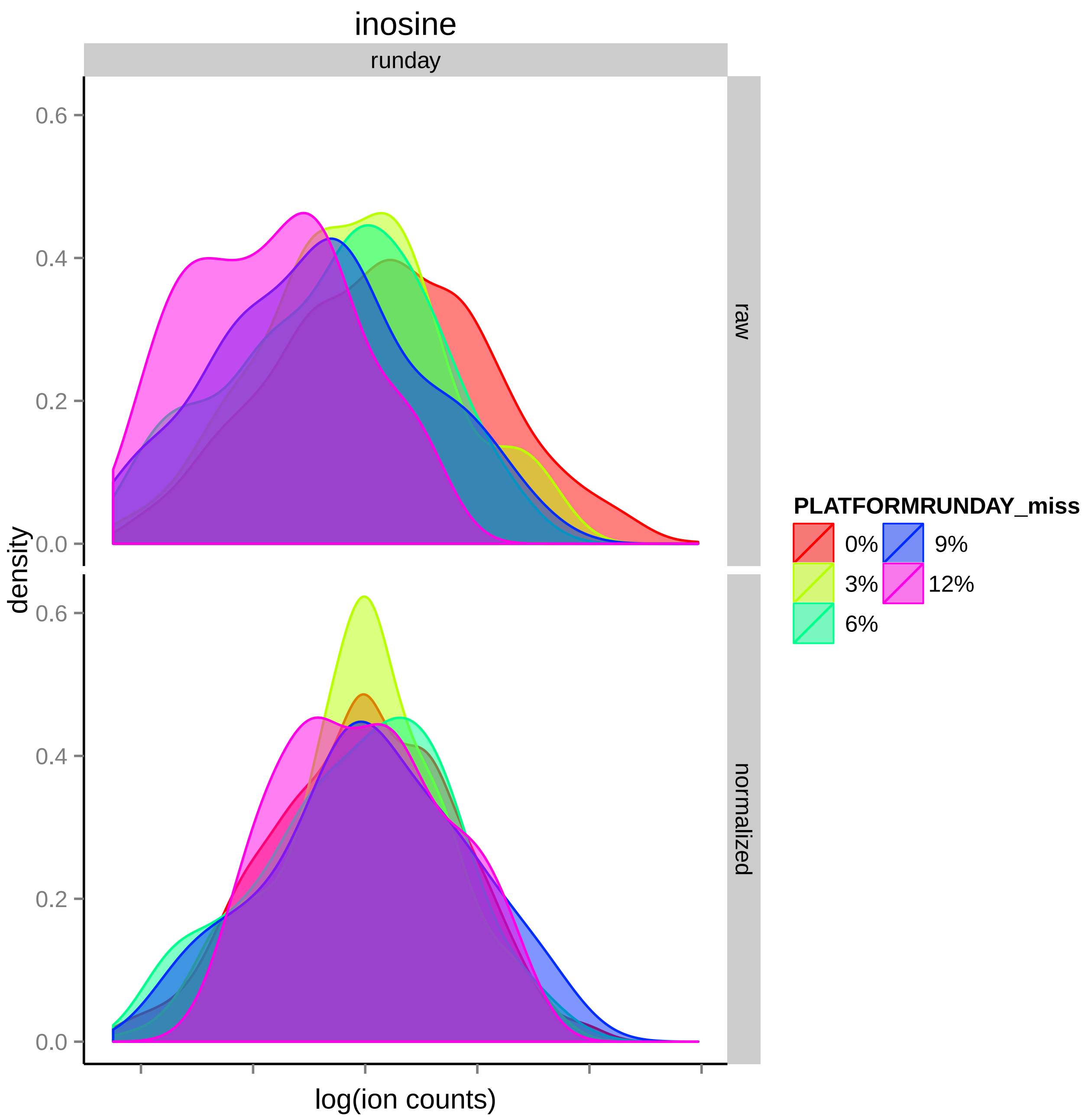

inosine
runday
raw
normalized
density
0.0
0.2
0.4
0.6
log(ion counts)
PLATFORMRUNDAY_miss
0%
3%
6%
9%
12%

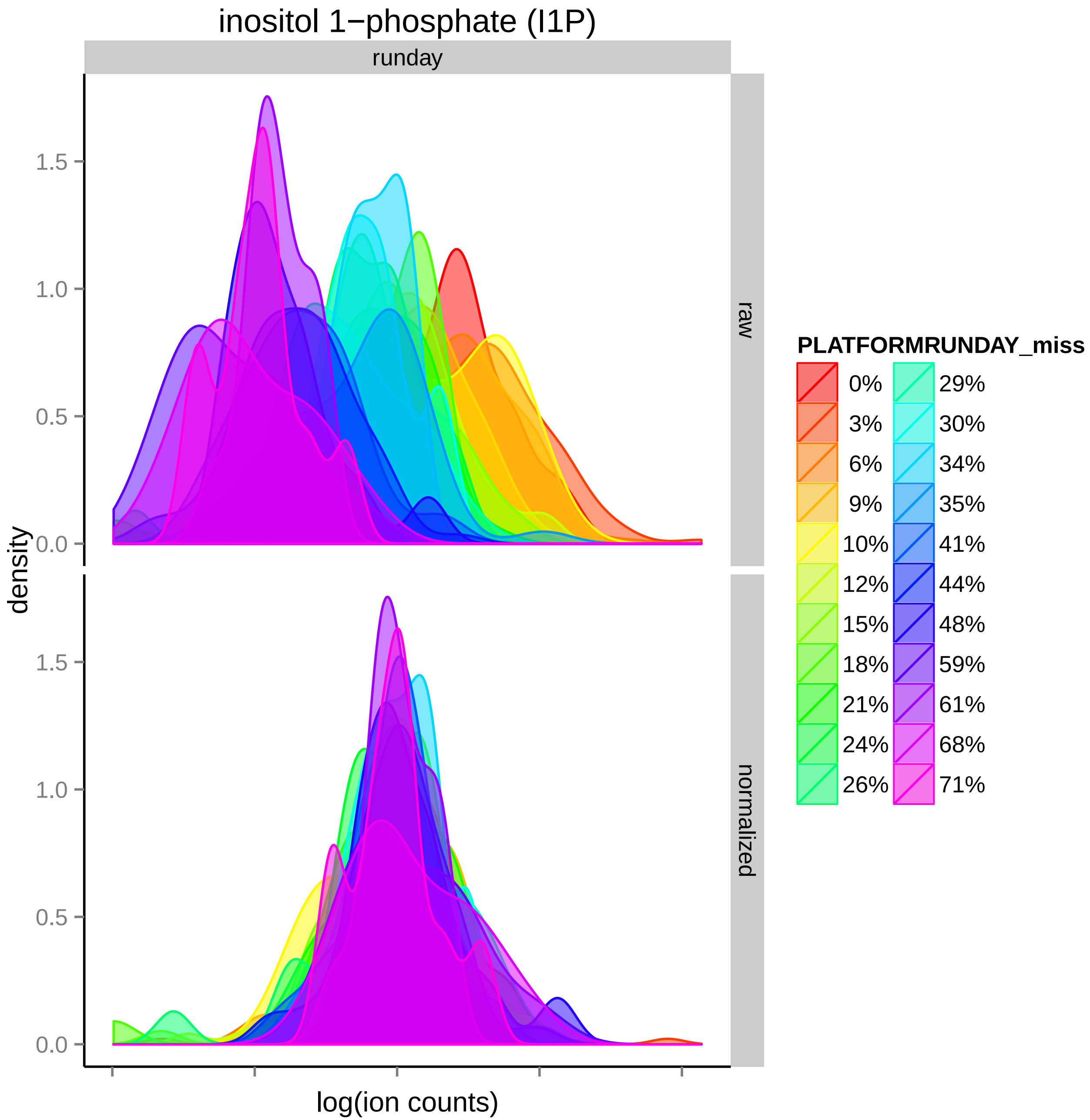

inositol 1−phosphate (I1P)
runday
raw
normalized
density
log(ion counts)
1.5
1.0
0.5
0.0
PLATFORMRUNDAY_miss
0%
3%
6%
9%
10%
12%
15%
18%
21%
24%
26%
29%
30%
34%
35%
41%
44%
48%
59%
61%
68%
71%

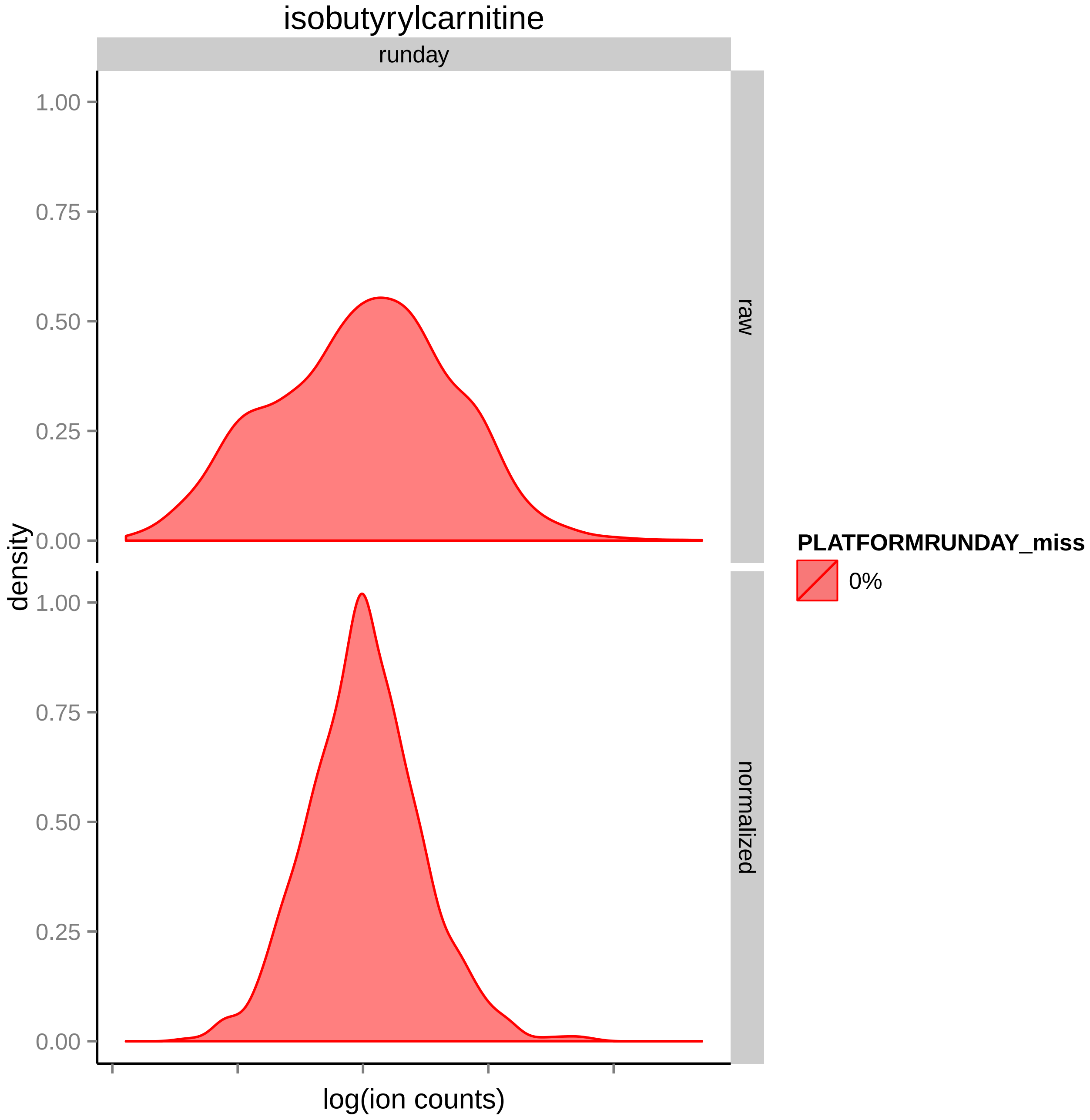

isobutyrylcarnitine
runday
raw
normalized
density
1.00
0.75
0.50
0.25
0.00
log(ion counts)
PLATFORMRUNDAY_miss
0%

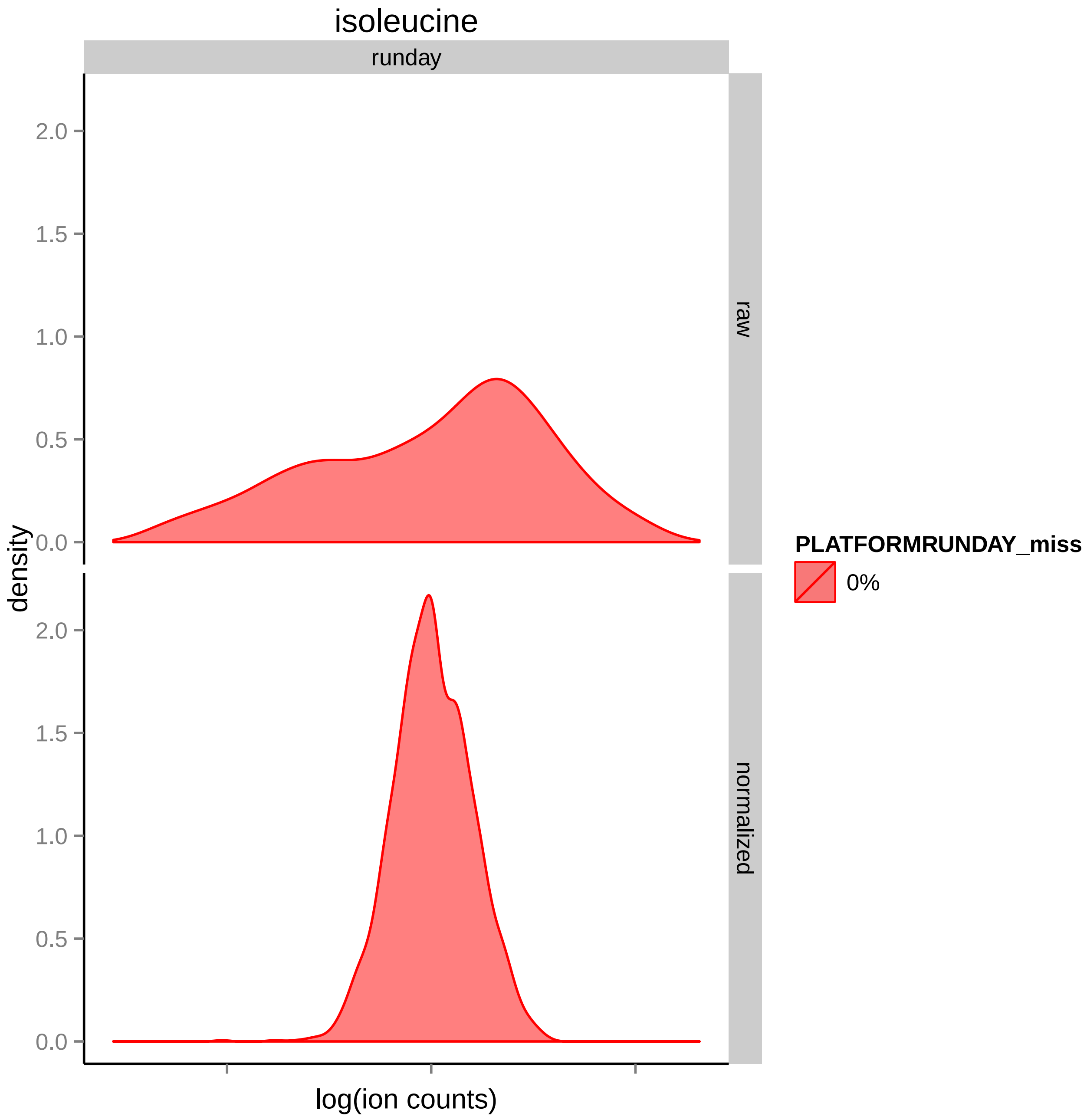

isoleucine
runday
raw
normalized
density
2.0
1.5
1.0
0.5
0.0
log(ion counts)
PLATFORMRUNDAY_miss
0%

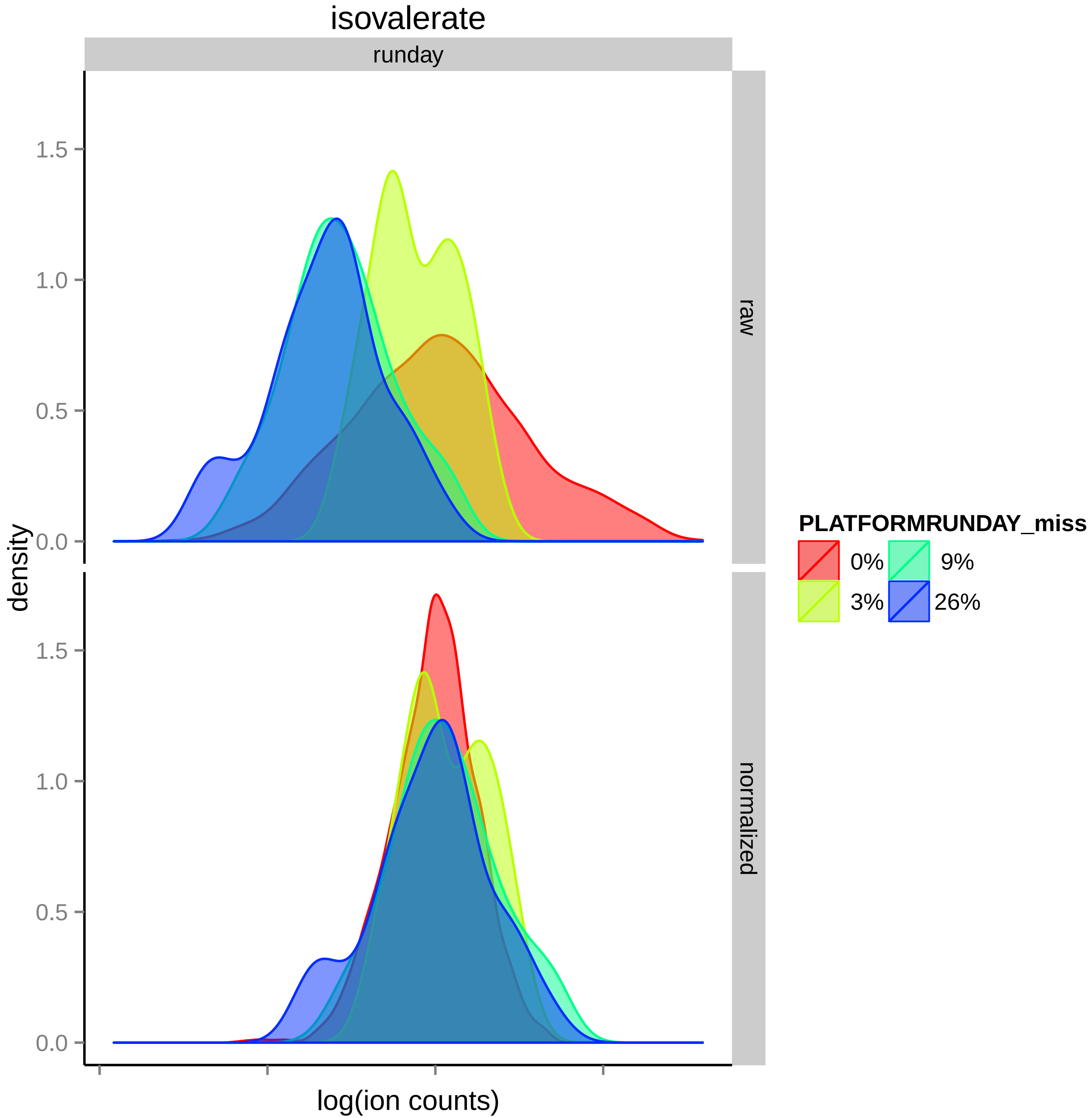
isovalerate
runday
raw
normalized
density
log(ion counts)
1.5
1.0
0.5
0.0
1.5
1.0
0.5
0.0
PLATFORMRUNDAY_miss
0%
9%
3%
26%

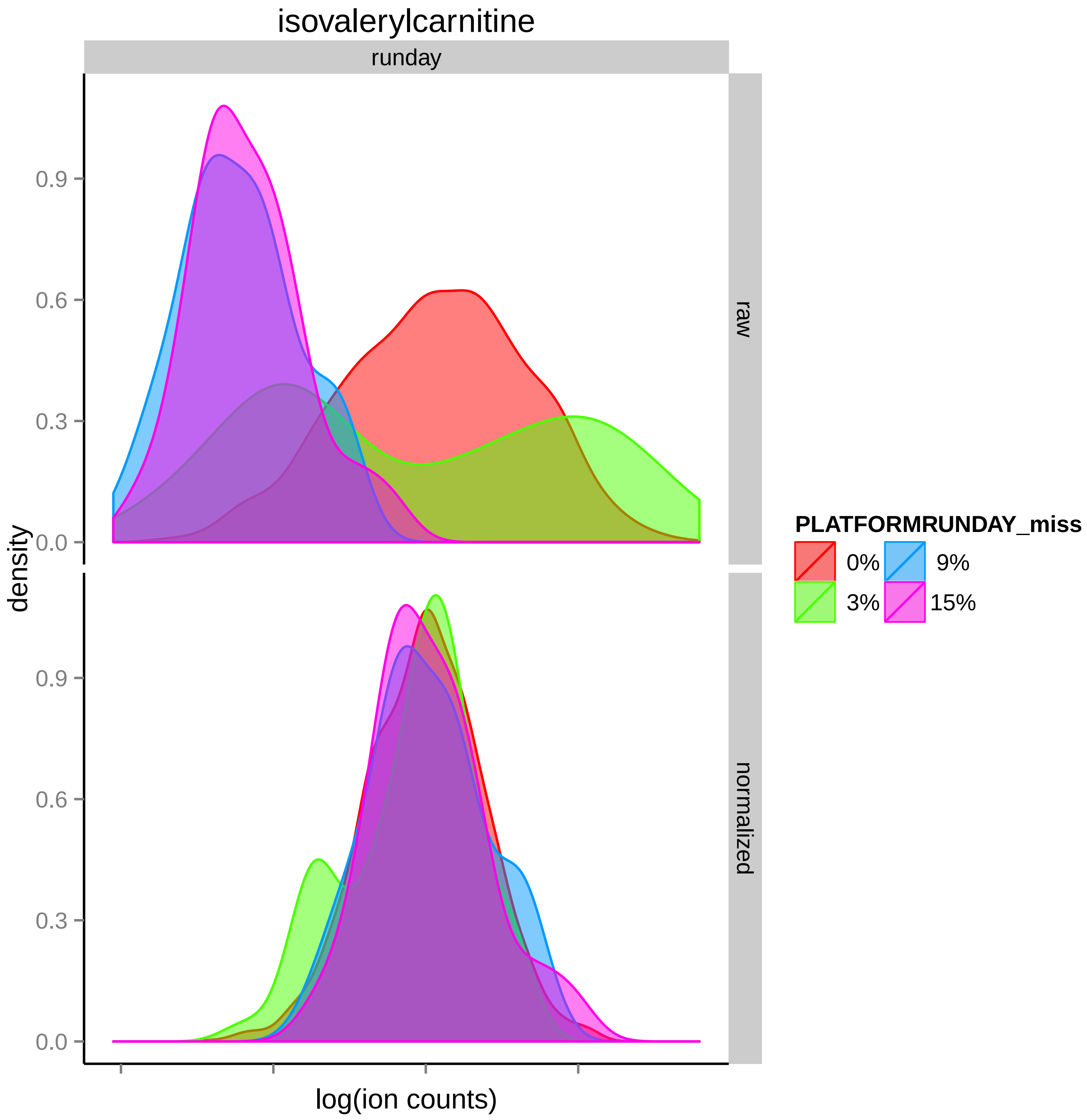
isovalerylcarnitine
runday
raw
normalized
density
0.0
0.3
0.6
0.9
log(ion counts)
PLATFORMRUNDAY_miss
0%
9%
3%
15%

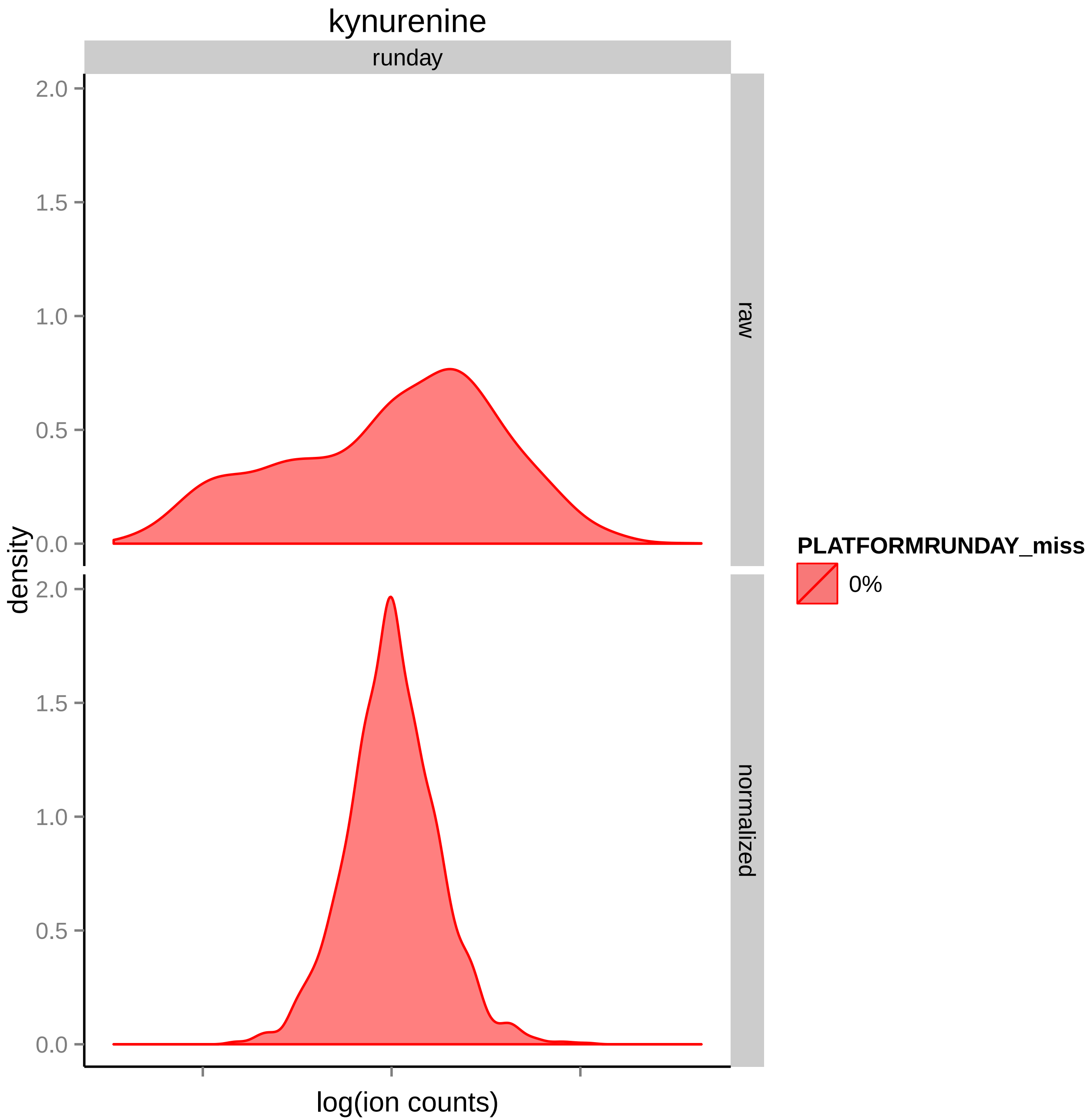
kynurenine
runday
raw
normalized
density
log(ion counts)
2.0
1.5
1.0
0.5
0.0
PLATFORMRUNDAY_miss
0%

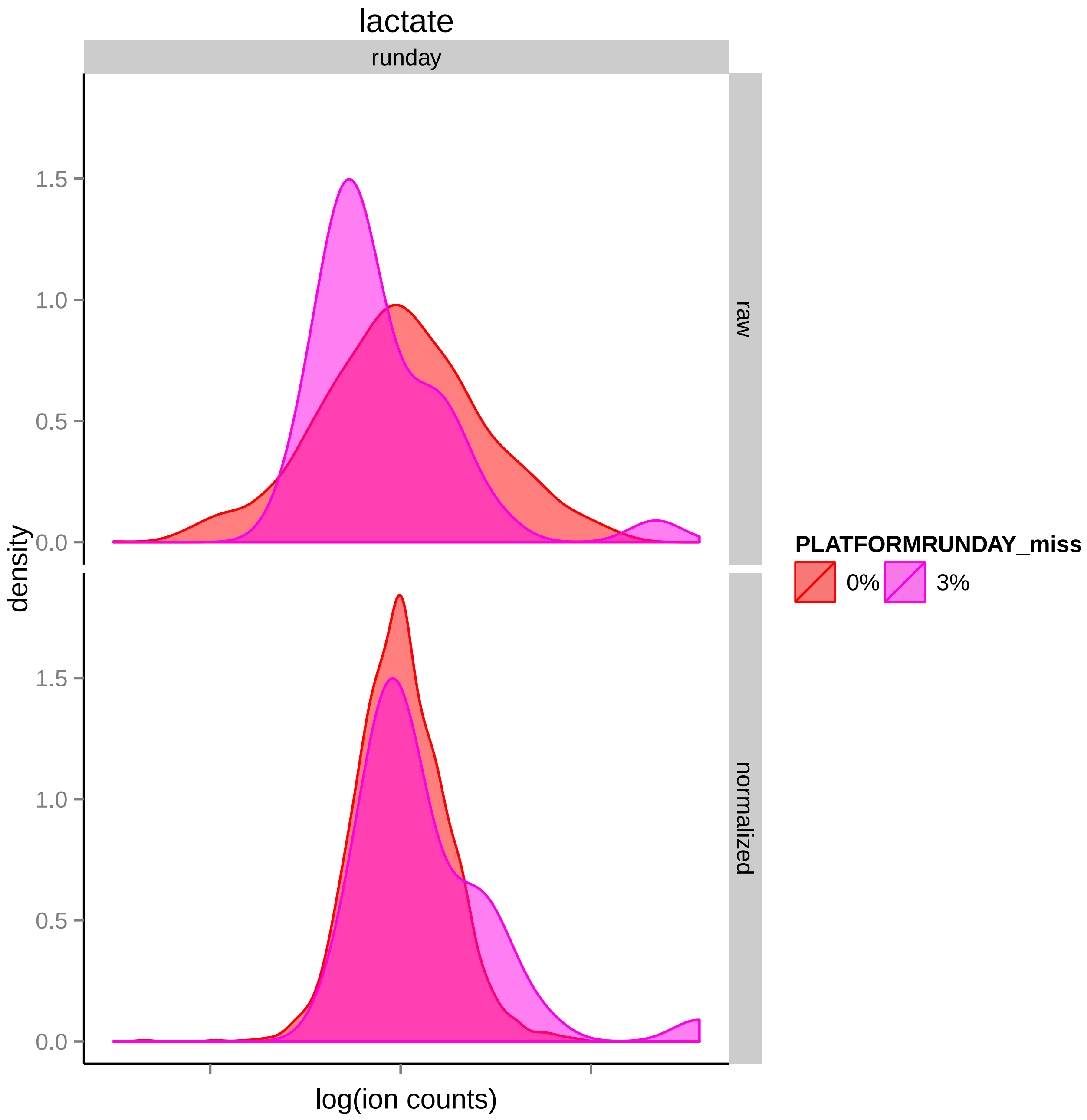
lactate
runday
raw
normalized
density
1.5
1.0
0.5
0.0
log(ion counts)
PLATFORMRUNDAY_miss
0%
3%

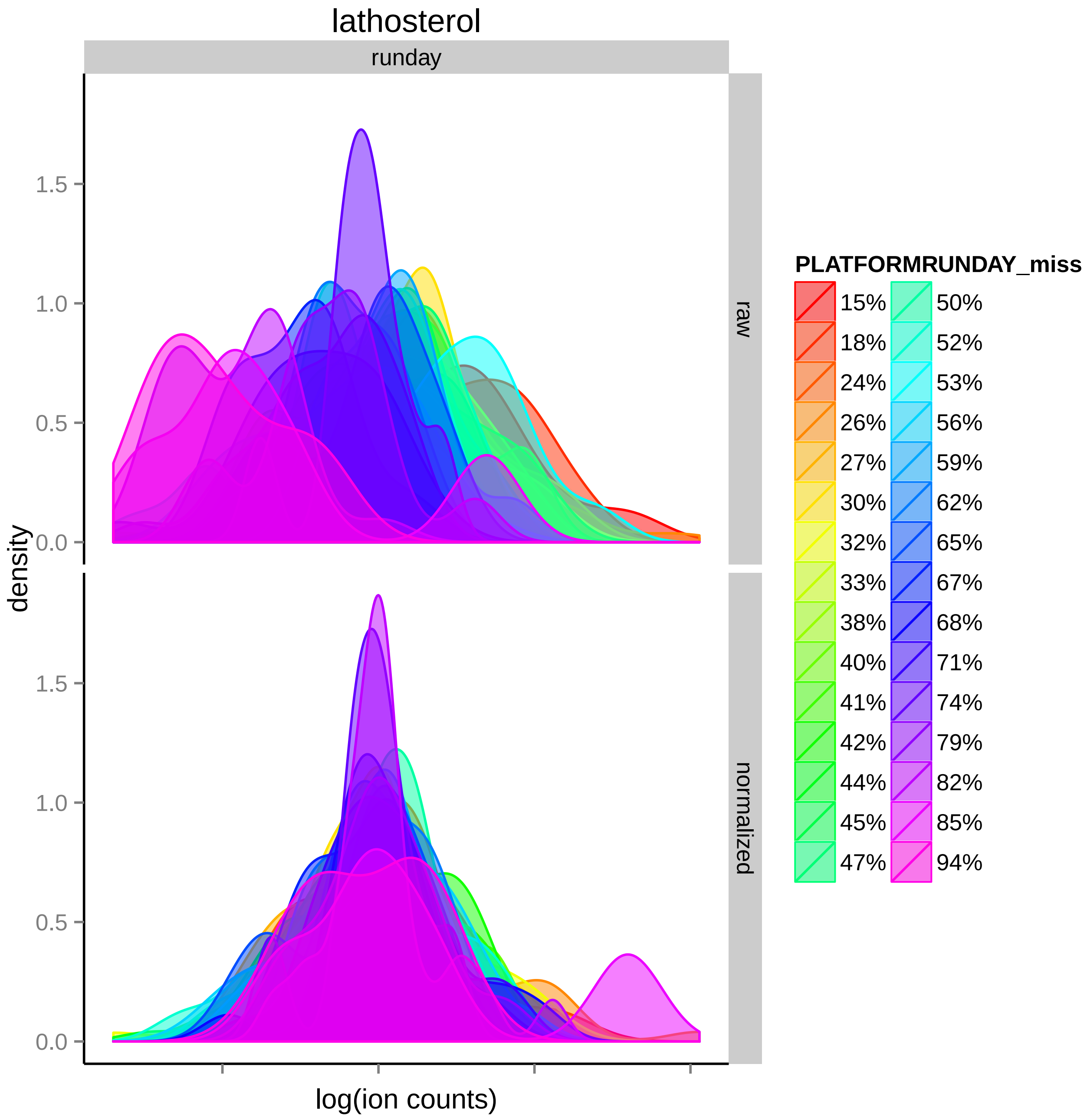
lathosterol
runday
raw
normalized
density
log(ion counts)
1.5
1.0
0.5
0.0
PLATFORMRUNDAY_miss
15%
18%
24%
26%
27%
30%
32%
33%
38%
40%
41%
42%
44%
45%
47%
50%
52%
53%
56%
59%
62%
65%
67%
68%
71%
74%
79%
82%
85%
94%

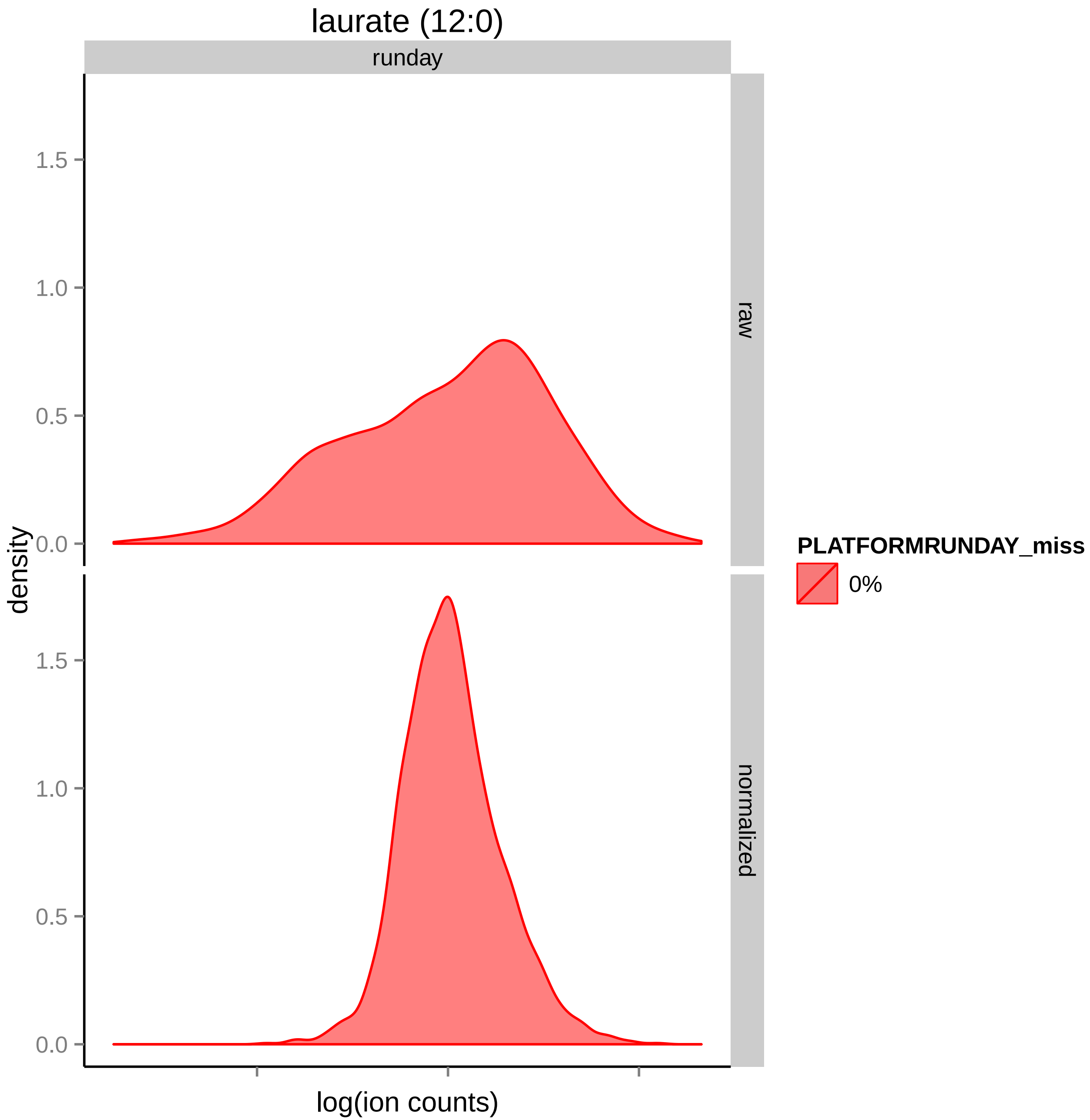

laurate (12:0)
runday
raw
normalized
density
1.5
1.0
0.5
0.0
1.5
1.0
0.5
0.0
log(ion counts)
PLATFORMRUNDAY_miss
0%

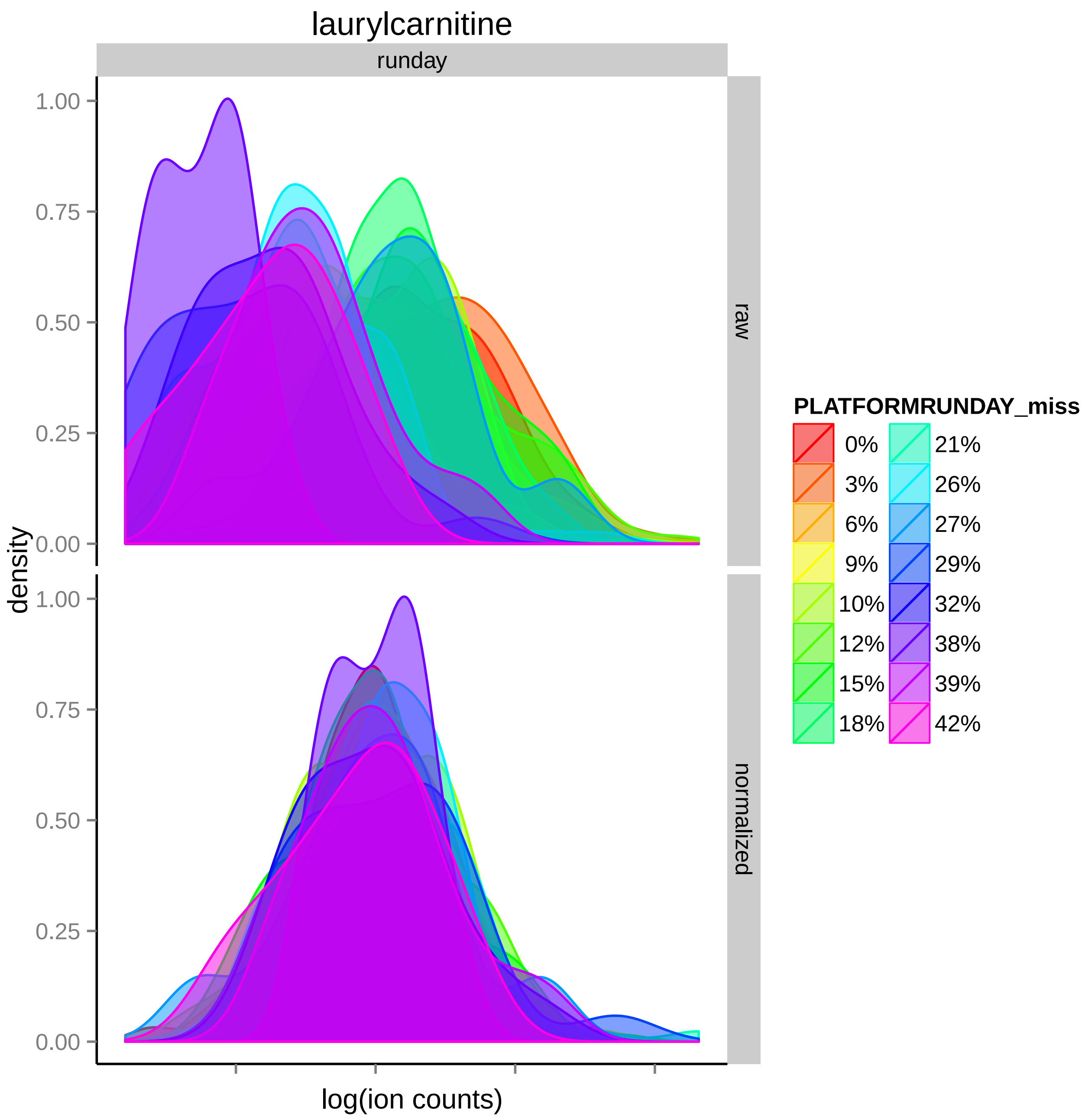
laurylcarnitine
runday
raw
normalized
density
1.00
0.75
0.50
0.25
0.00
log(ion counts)
PLATFORMRUNDAY_miss
0%
3%
6%
9%
10%
12%
15%
18%
21%
26%
27%
29%
32%
38%
39%
42%

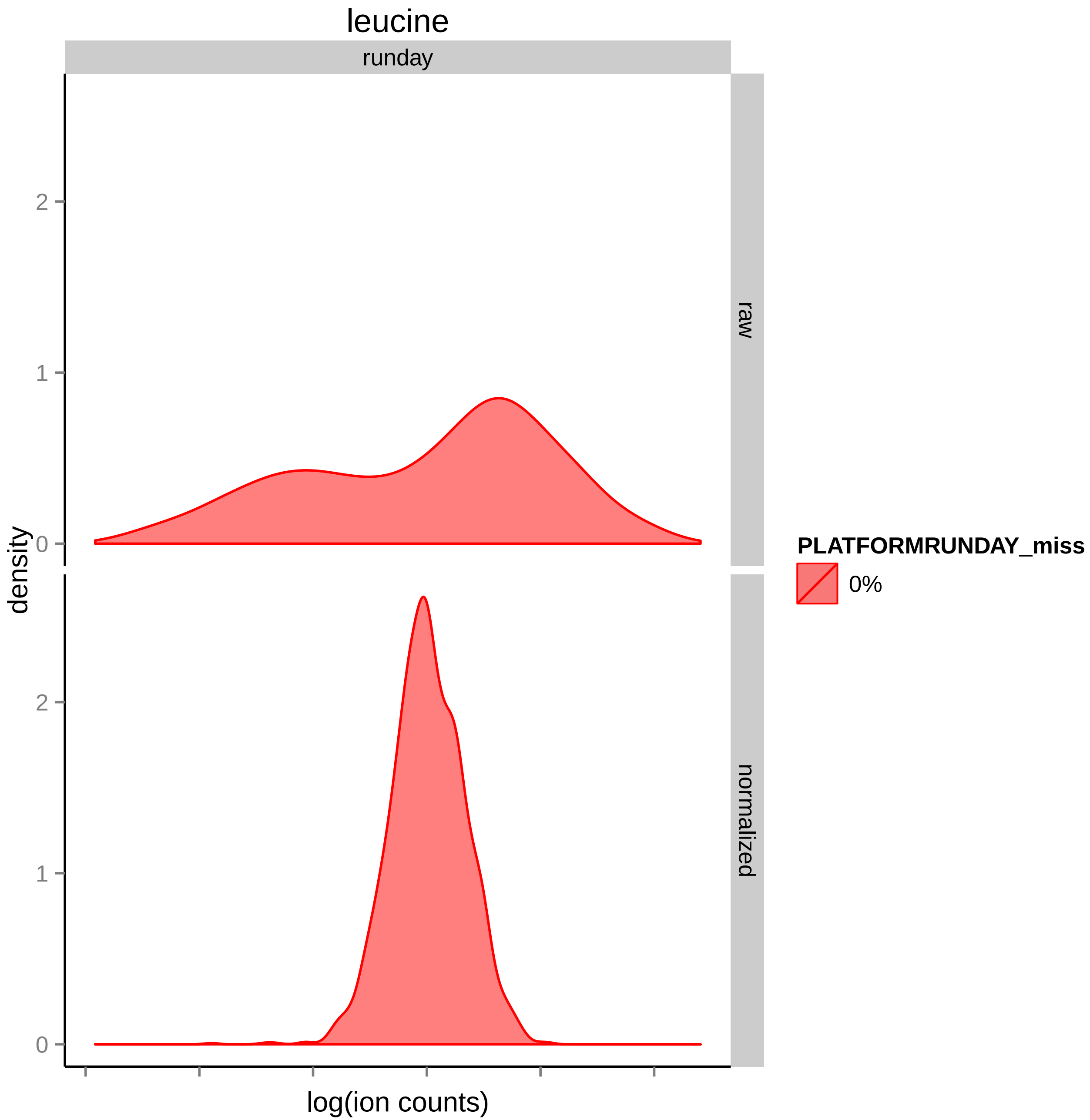
leucine
runday
raw
normalized
density
log(ion counts)
2
1
0
2
1
0
PLATFORMRUNDAY_miss
0%

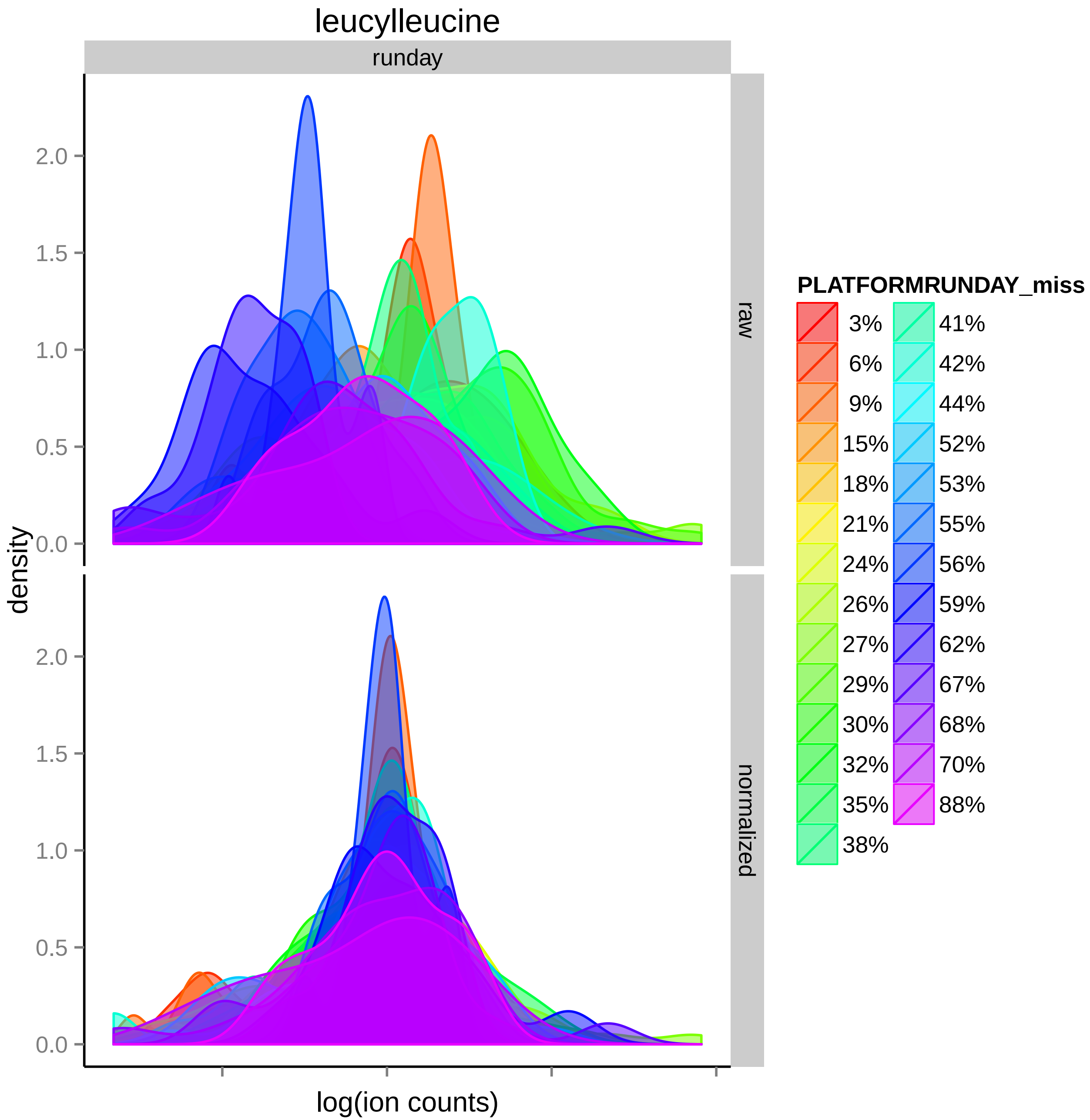

leucylleucine
runday
raw
normalized
density
log(ion counts)
2.0
1.5
1.0
0.5
0.0
PLATFORMRUNDAY_miss
3%
6%
9%
15%
18%
21%
24%
26%
27%
29%
30%
32%
35%
38%
41%
42%
44%
52%
53%
55%
56%
59%
62%
67%
68%
70%
88%

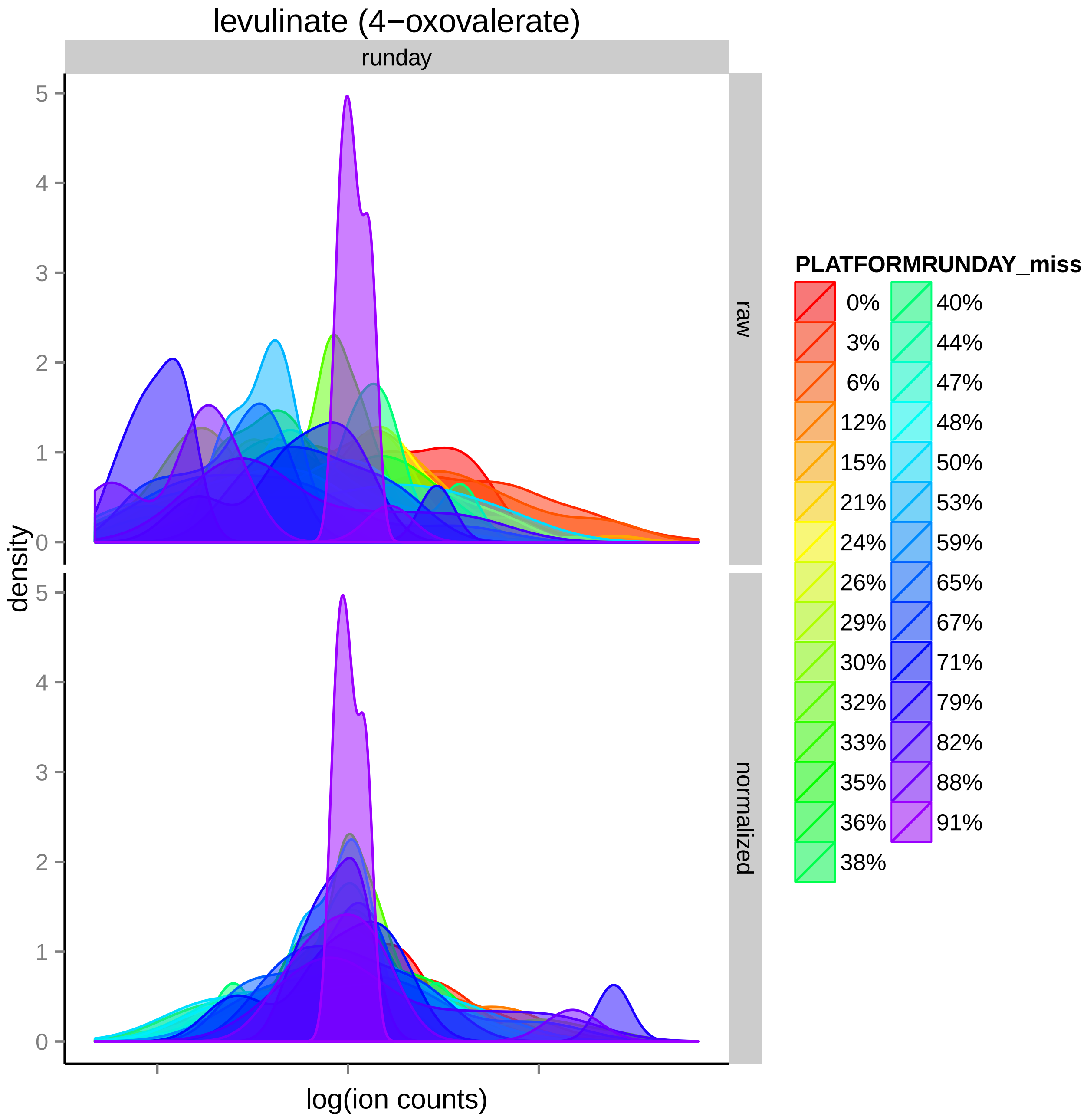

levulinate (4−oxovalerate)
runday
density
log(ion counts)
raw
normalized
0
1
2
3
4
5
PLATFORMRUNDAY_miss
0%
3%
6%
12%
15%
21%
24%
26%
29%
30%
32%
33%
35%
36%
38%
40%
44%
47%
48%
50%
53%
59%
65%
67%
71%
79%
82%
88%
91%

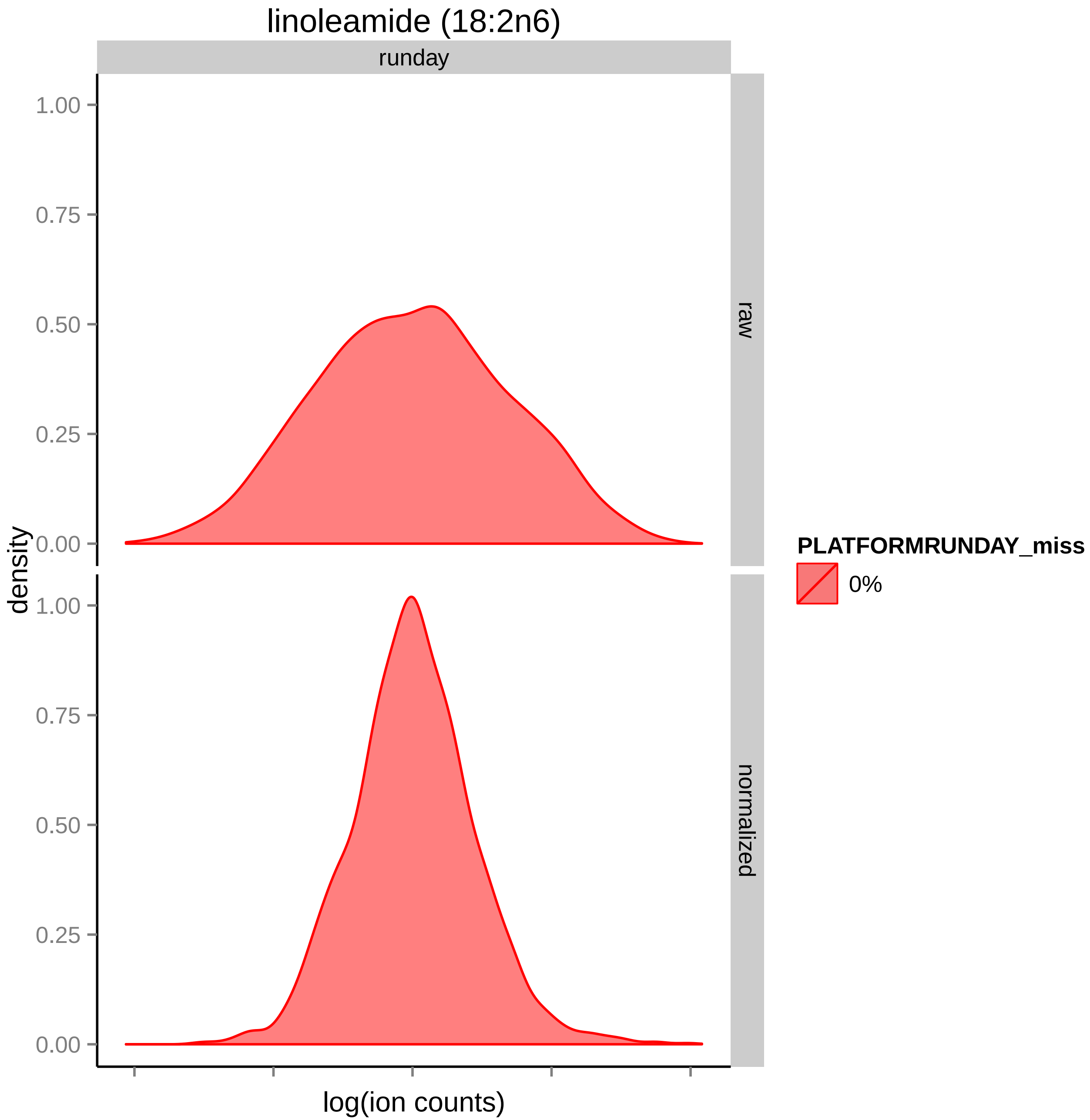

linoleamide (18:2n6)
runday
raw
normalized
density
log(ion counts)
1.00
0.75
0.50
0.25
0.00
PLATFORMRUNDAY_miss
0%

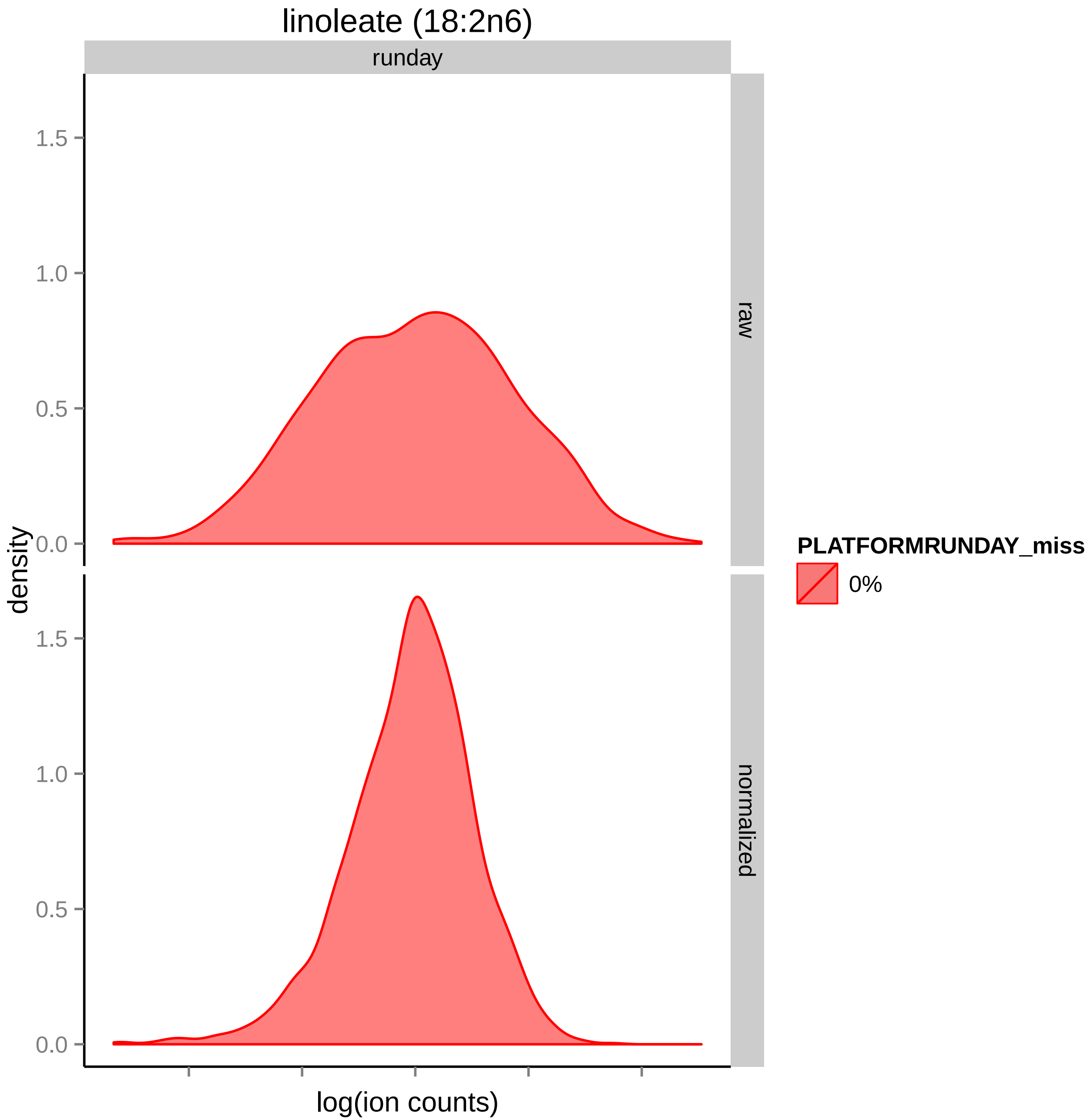

linoleate (18:2n6)
runday
raw
normalized
density
log(ion counts)
1.5
1.0
0.5
0.0
1.5
1.0
0.5
0.0
PLATFORMRUNDAY_miss
0%

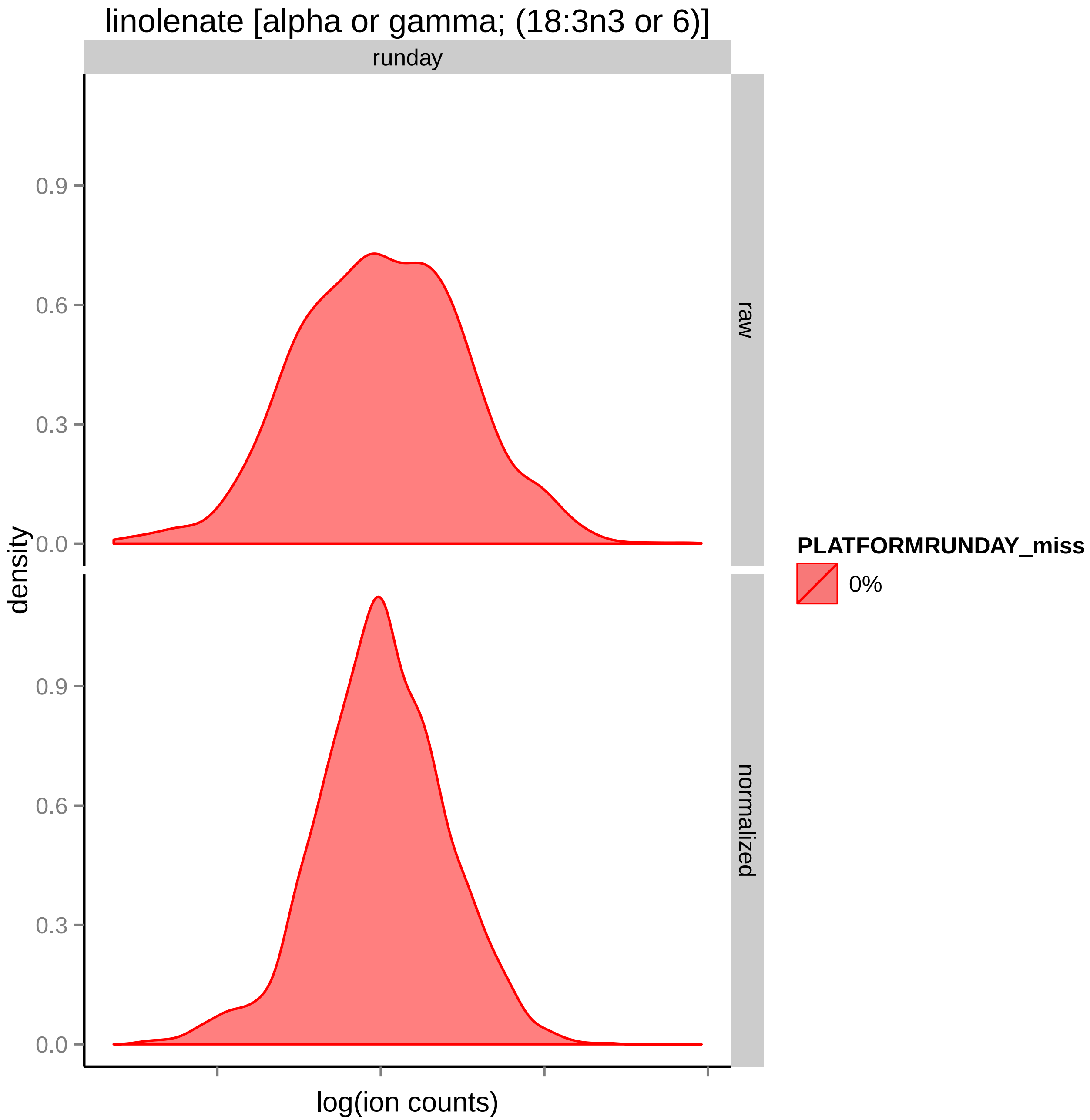

linolenate [alpha or gamma; (18:3n3 or 6)]
runday
raw
normalized
density
0.0
0.3
0.6
0.9
log(ion counts)
PLATFORMRUNDAY_miss
0%

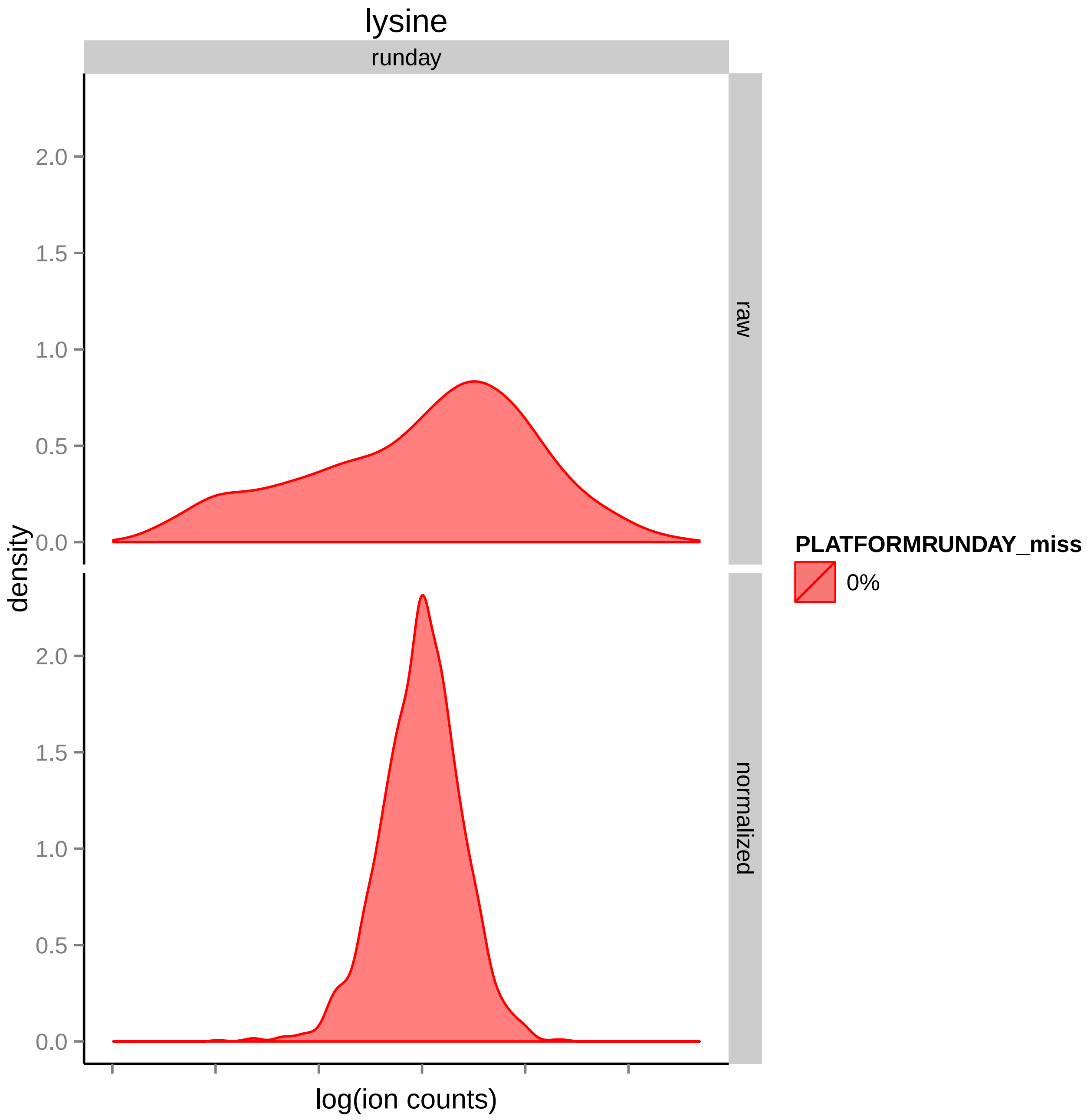
lysine
runday
raw
normalized
density
log(ion counts)
2.0
1.5
1.0
0.5
0.0
PLATFORMRUNDAY_miss
0%

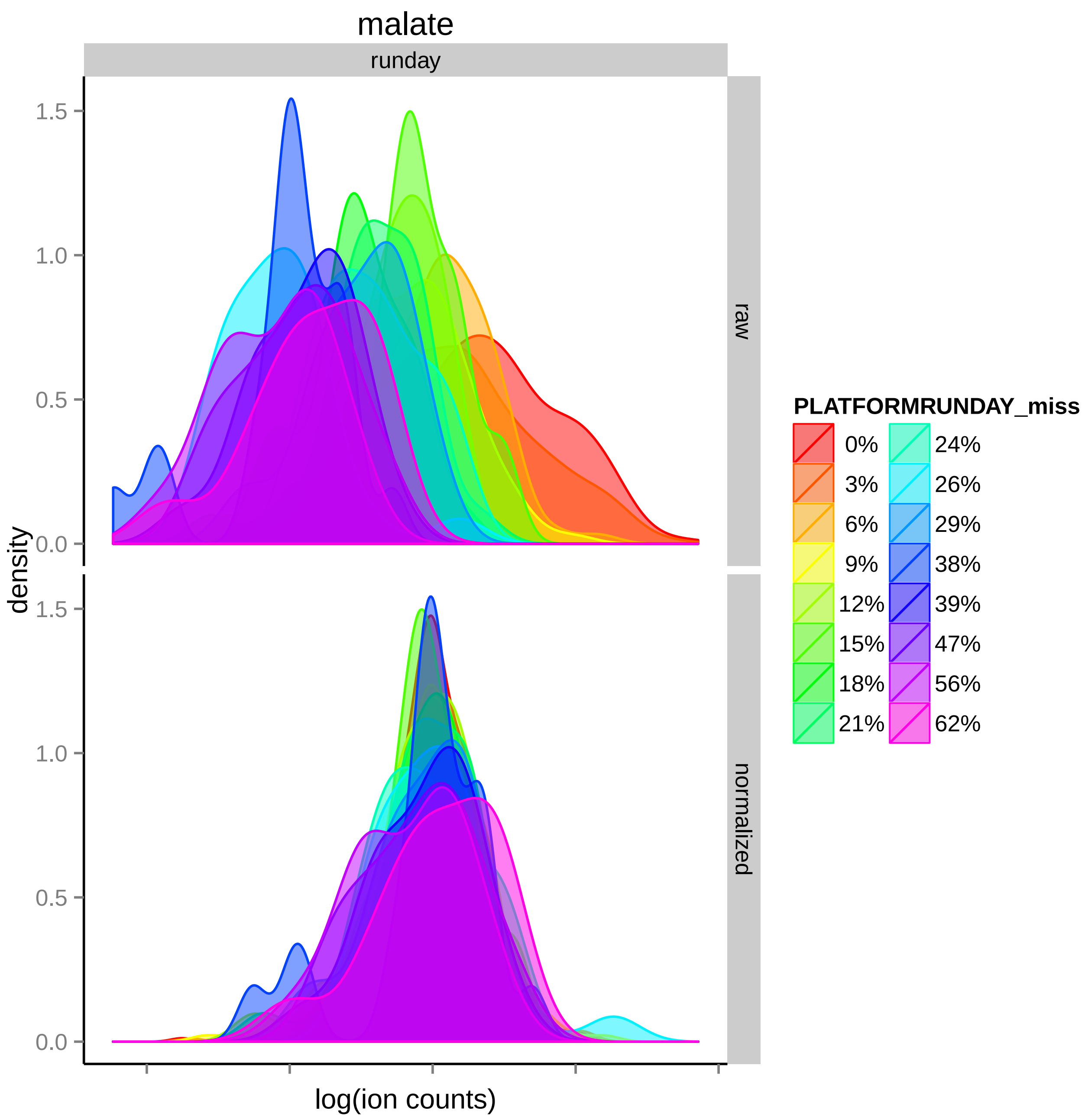
malate
runday
raw
normalized
density
log(ion counts)
1.5
1.0
0.5
0.0
PLATFORMRUNDAY_miss
0%
3%
6%
9%
12%
15%
18%
21%
24%
26%
29%
38%
39%
47%
56%
62%

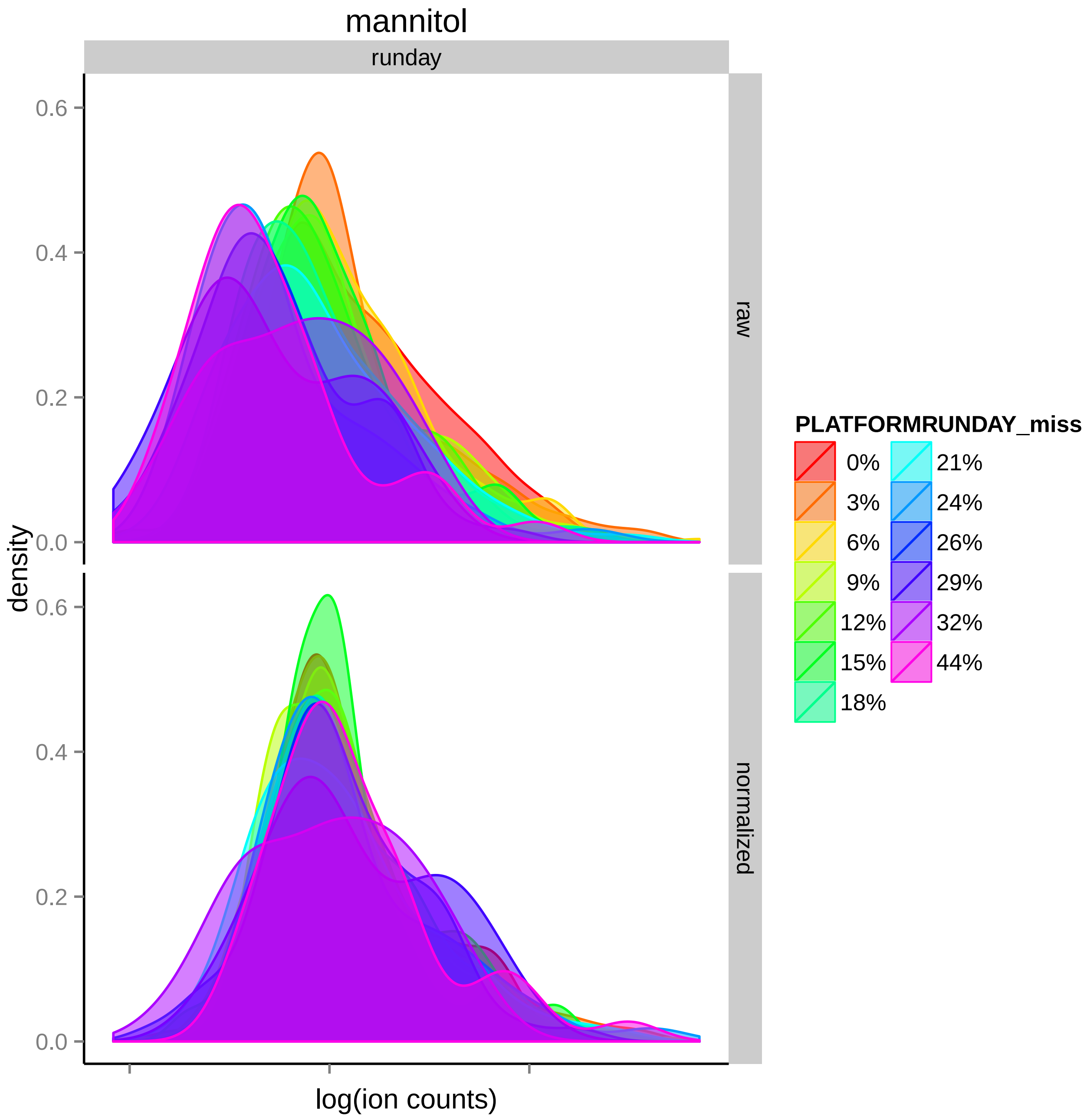

mannitol
runday
raw
normalized
density
log(ion counts)
0.0
0.2
0.4
0.6
PLATFORMRUNDAY_miss
0%
3%
6%
9%
12%
15%
18%
21%
24%
26%
29%
32%
44%

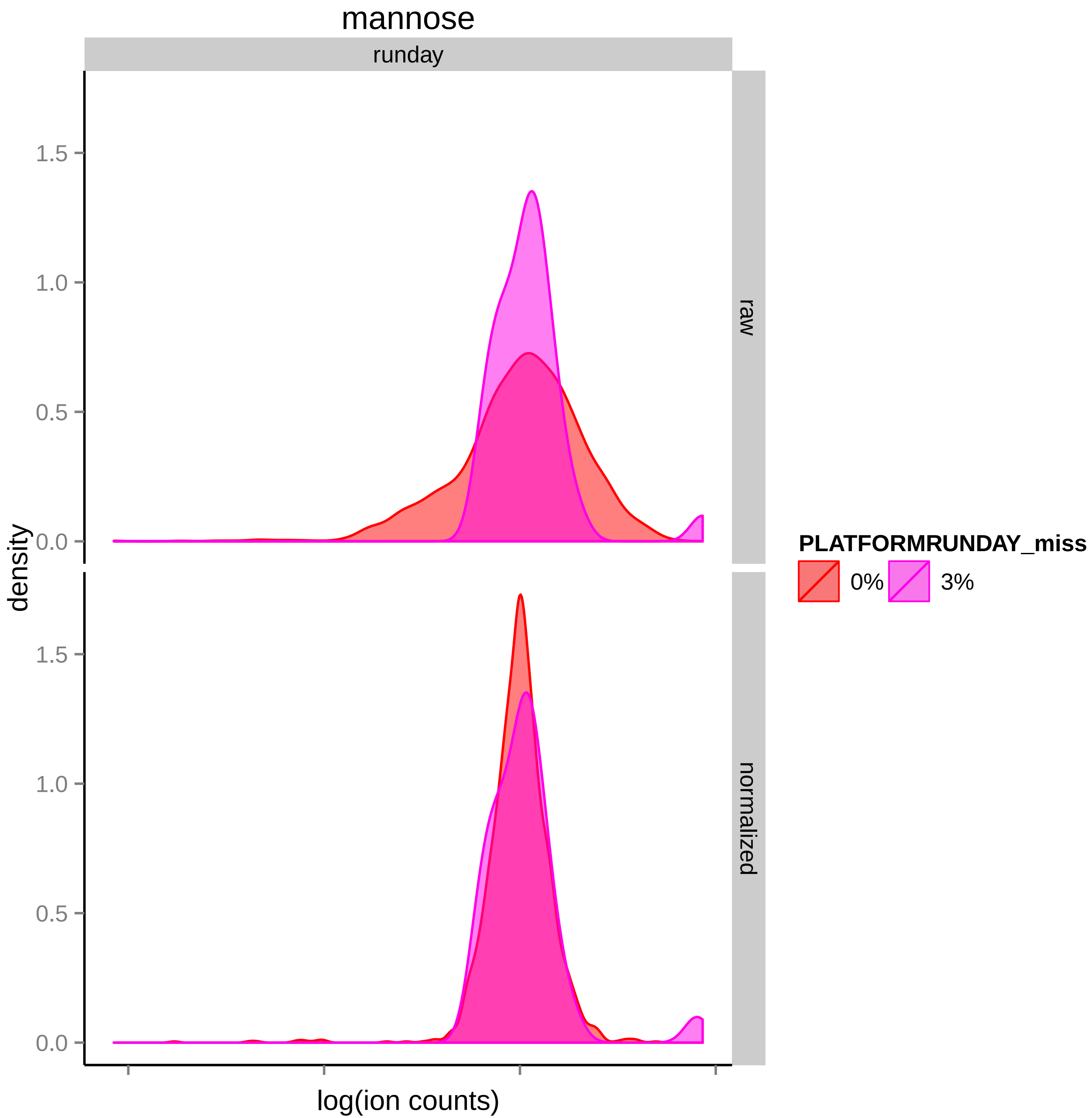
mannose
runday
raw
normalized
density
log(ion counts)
1.5
1.0
0.5
0.0
PLATFORMRUNDAY_miss
0%
3%

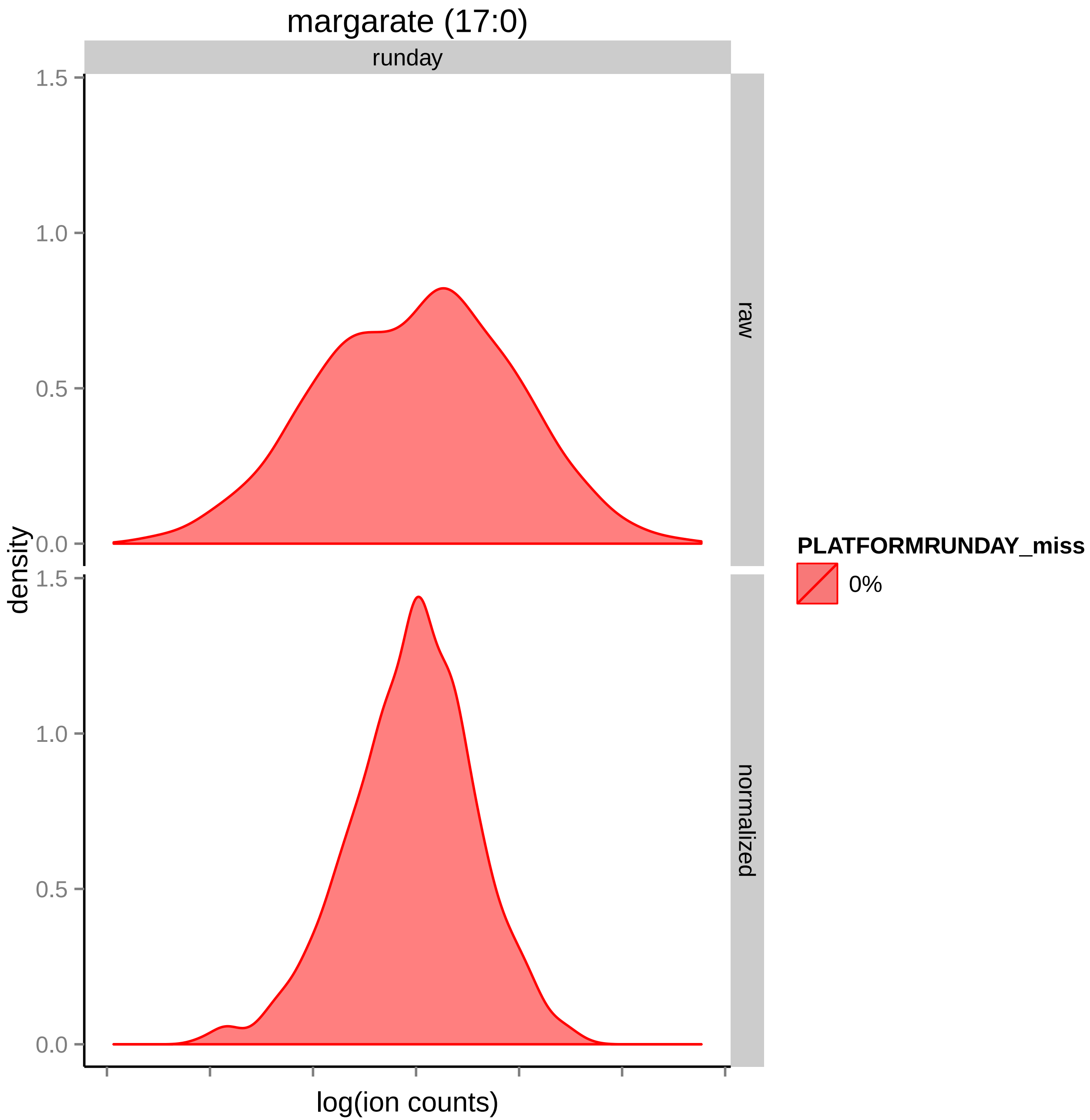

margarate (17:0)
runday
raw
normalized
density
log(ion counts)
1.5
1.0
0.5
0.0
PLATFORMRUNDAY_miss
0%

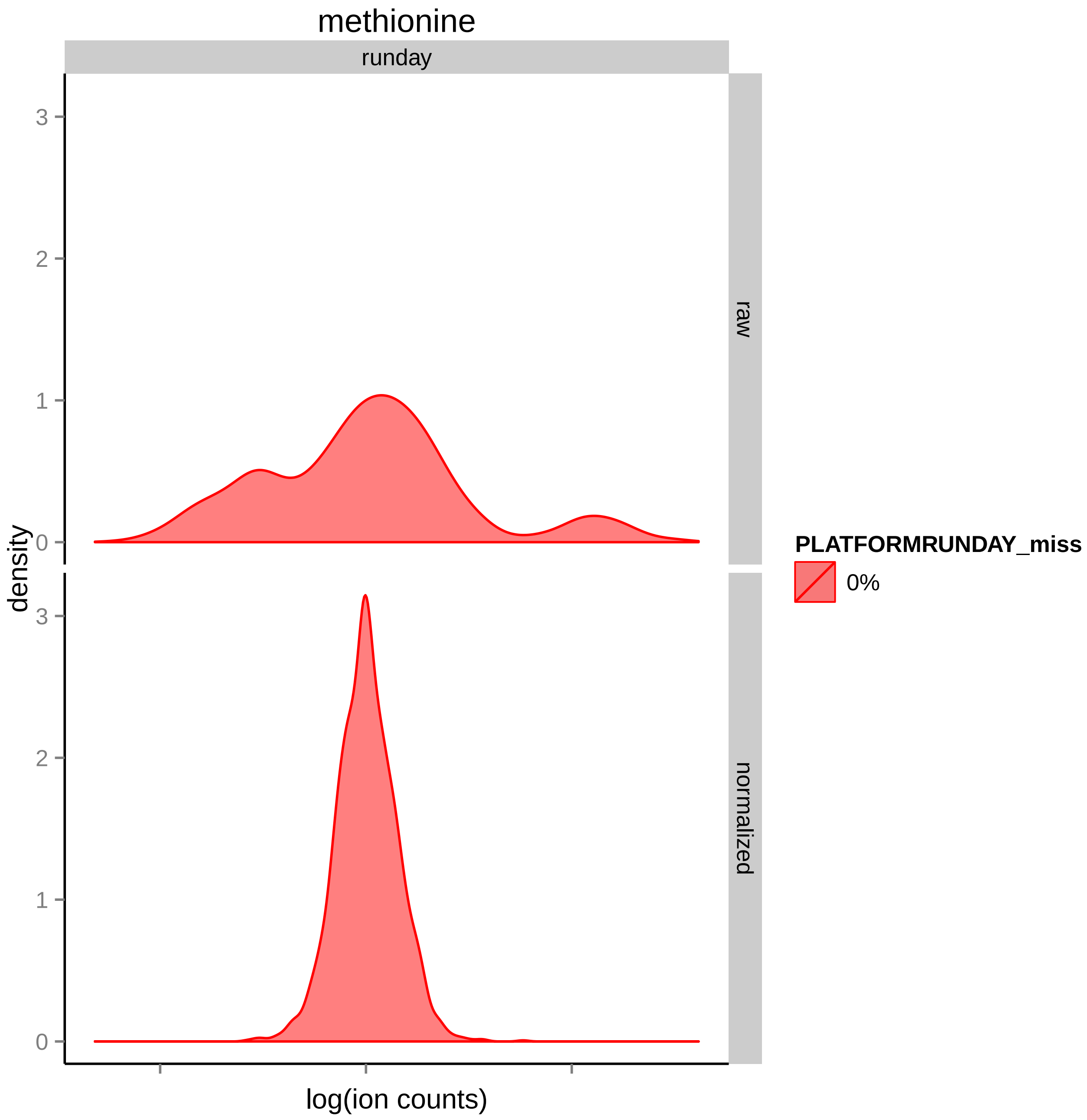
methionine
runday
raw
normalized
density
log(ion counts)
0
1
2
3
PLATFORMRUNDAY_miss
0%

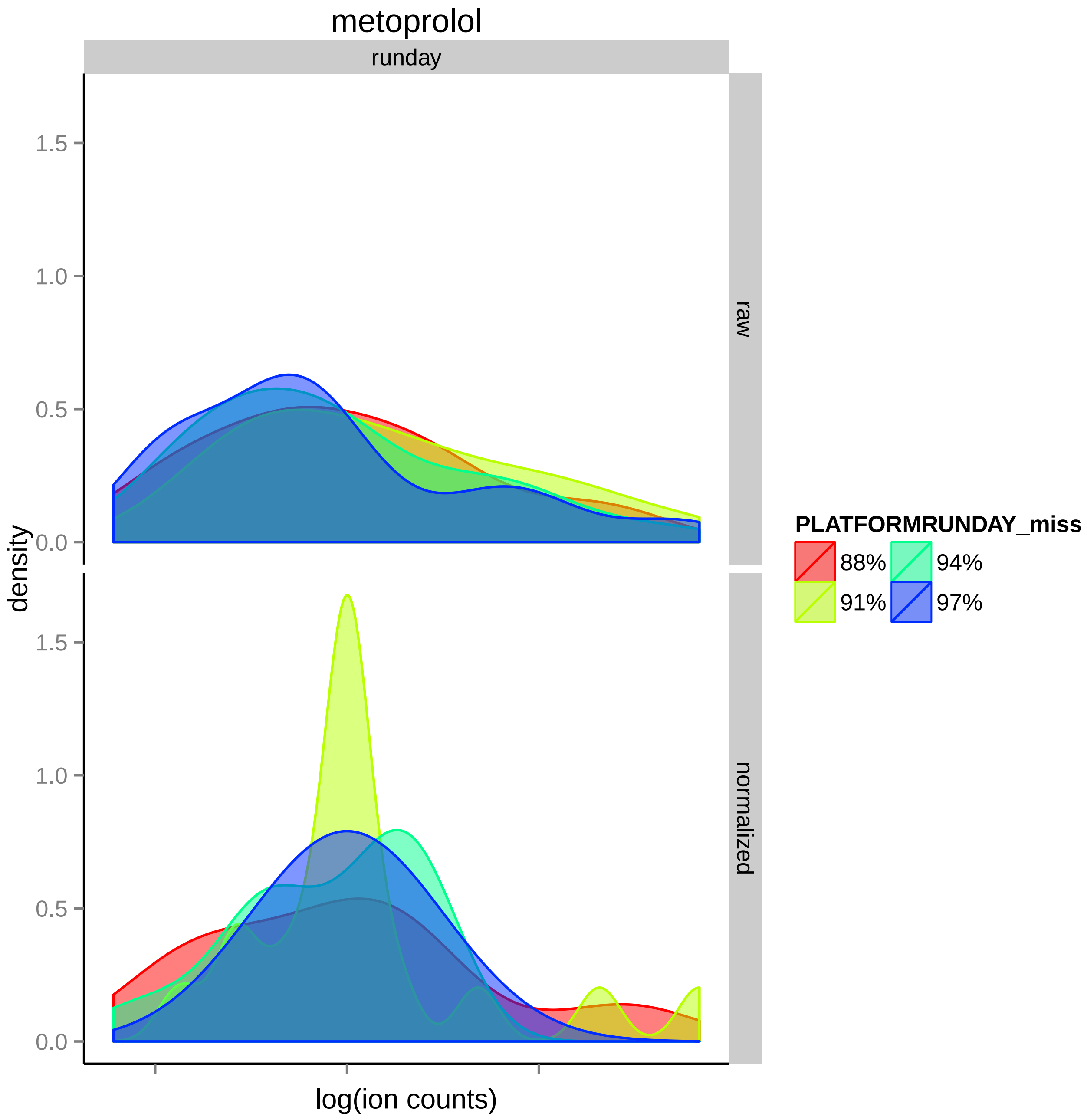

metoprolol
runday
raw
normalized
density
log(ion counts)
1.5
1.0
0.5
0.0
PLATFORMRUNDAY_miss
88%
91%
94%
97%

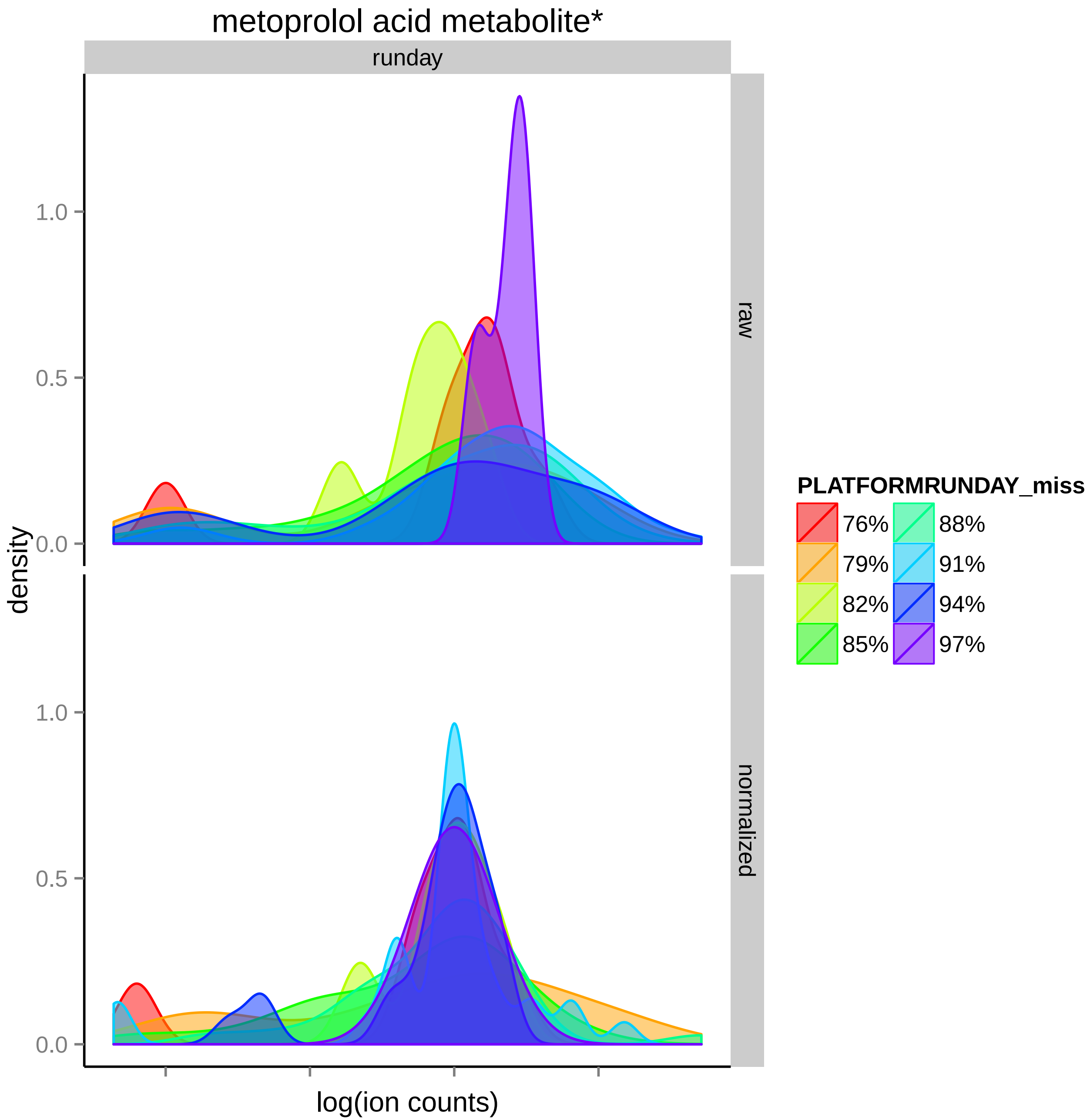

metoprolol acid metabolite*
runday
raw
normalized
density
log(ion counts)
1.0
0.5
0.0
1.0
0.5
0.0
PLATFORMRUNDAY_miss
76%
79%
82%
85%
88%
91%
94%
97%

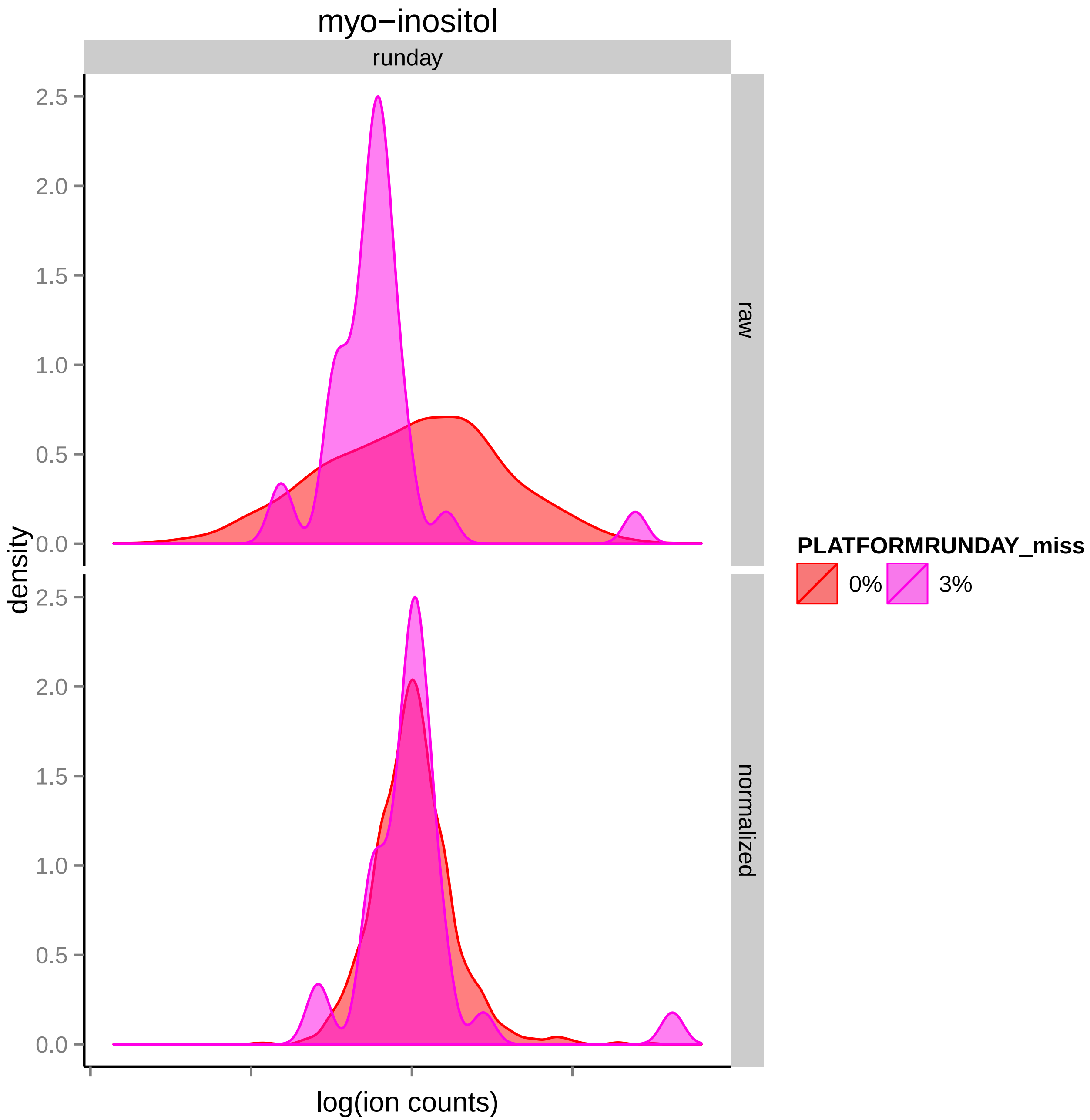
myo−inositol
runday
raw
normalized
density
2.5
2.0
1.5
1.0
0.5
0.0
log(ion counts)
PLATFORMRUNDAY_miss
0%
3%

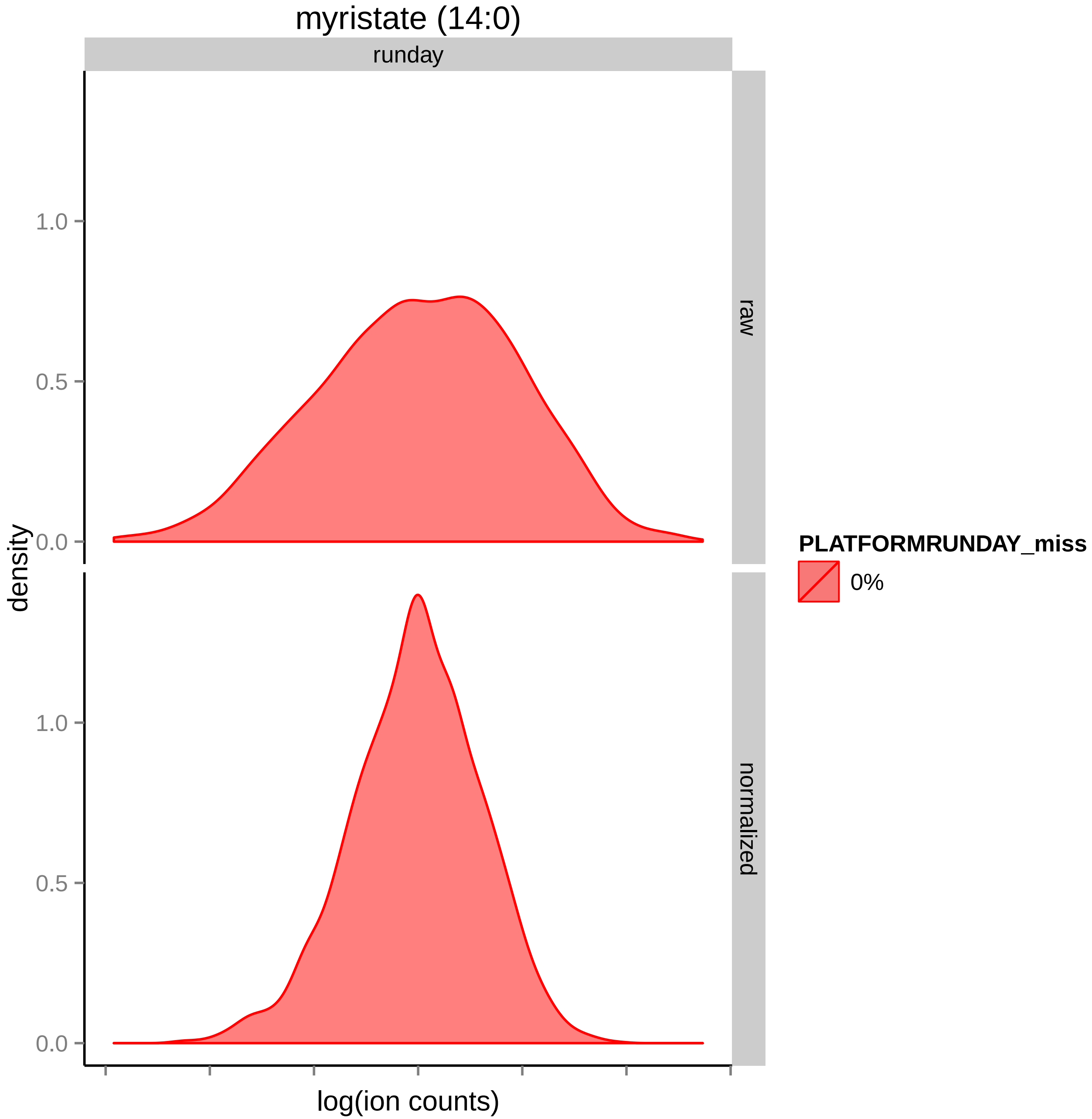
myristate (14:0)
runday
raw
normalized
density
1.0
0.5
0.0
1.0
0.5
0.0
log(ion counts)
PLATFORMRUNDAY_miss
0%

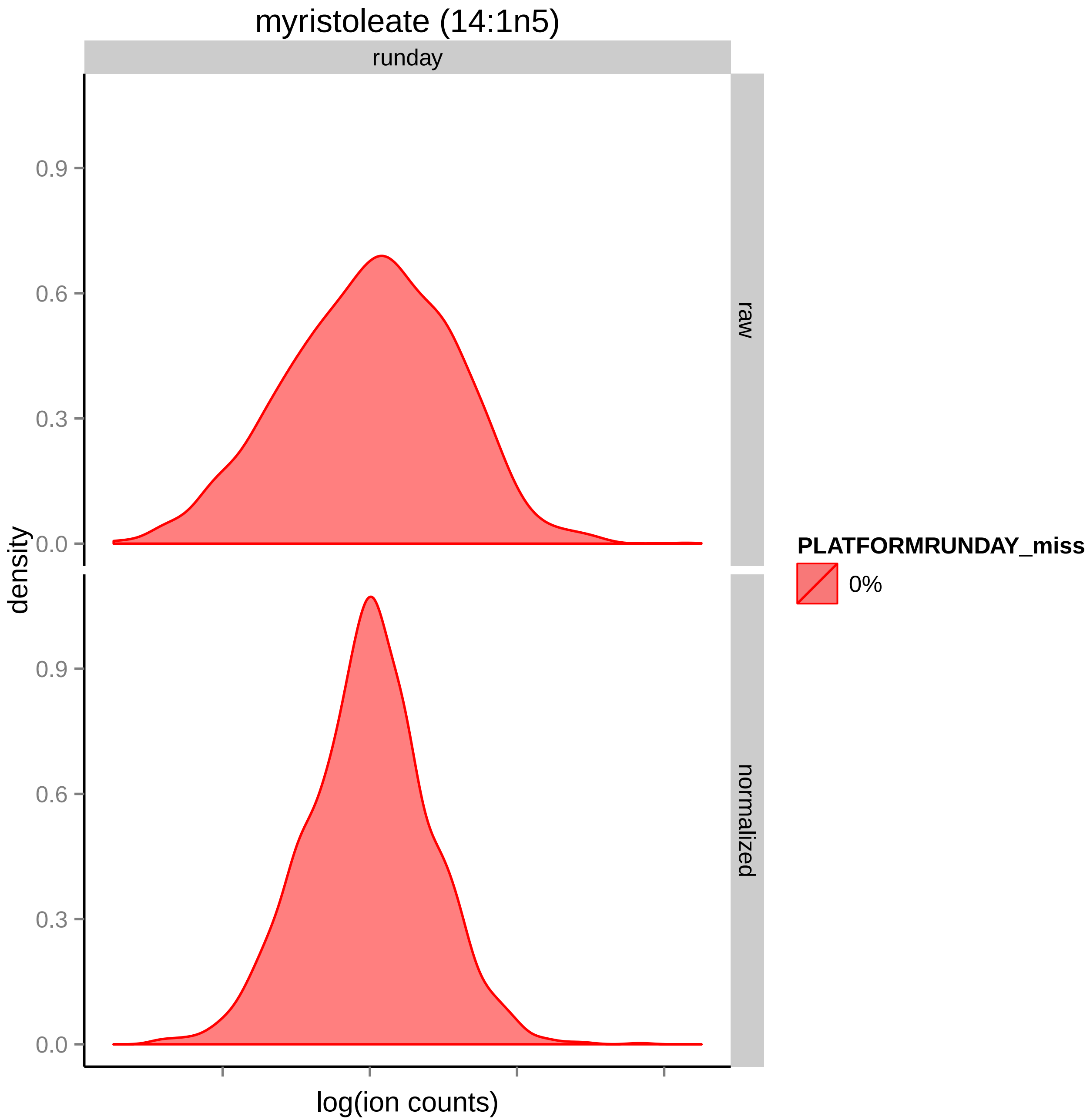
myristoleate (14:1n5)
runday
raw
normalized
density
0.9
0.6
0.3
0.0
log(ion counts)
PLATFORMRUNDAY_miss
0%

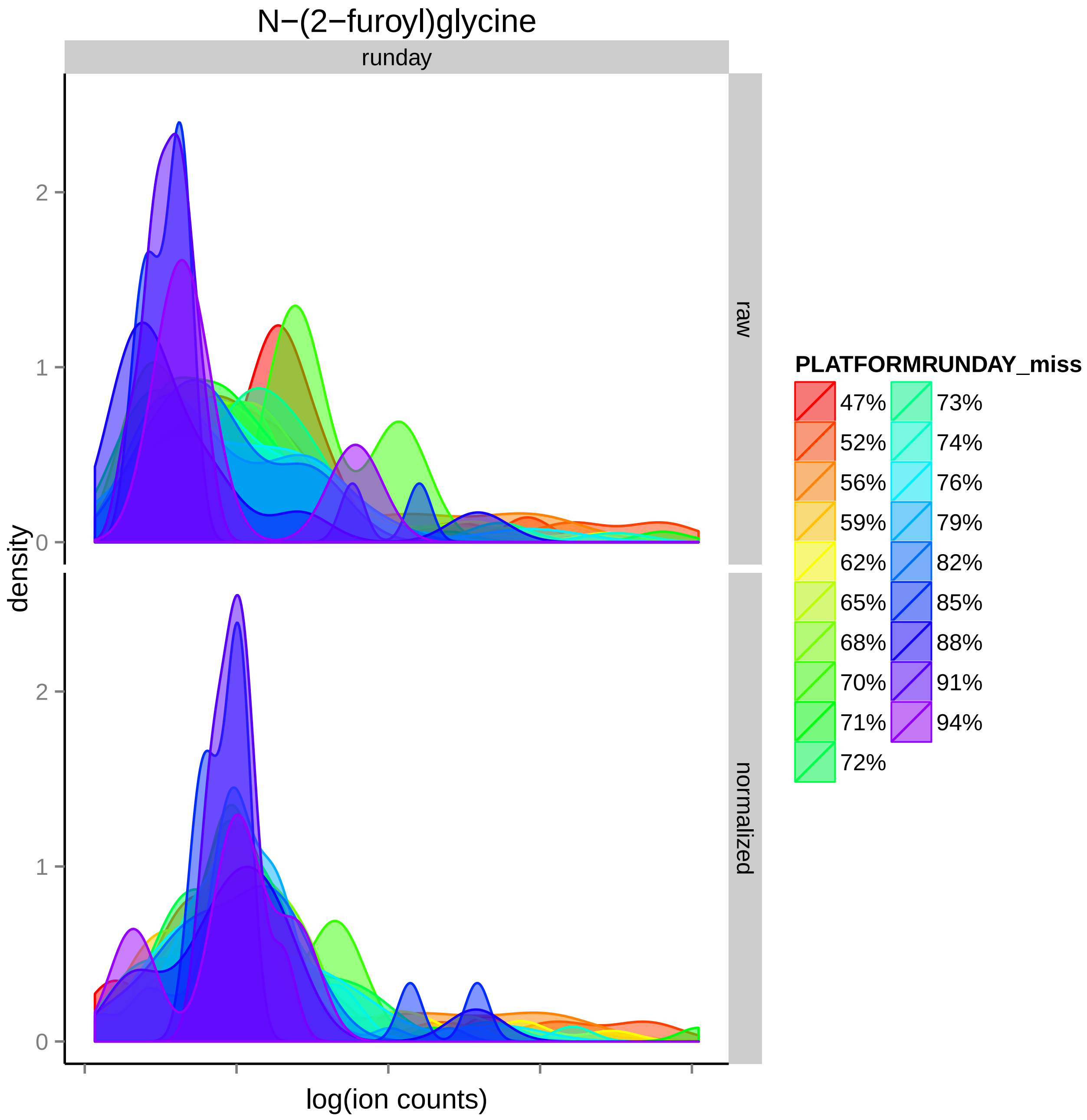

N−(2−furoyl)glycine
runday
raw
normalized
density
log(ion counts)
0
1
2
0
1
2
PLATFORMRUNDAY_miss
47%
52%
56%
59%
62%
65%
68%
70%
71%
72%
73%
74%
76%
79%
82%
85%
88%
91%
94%

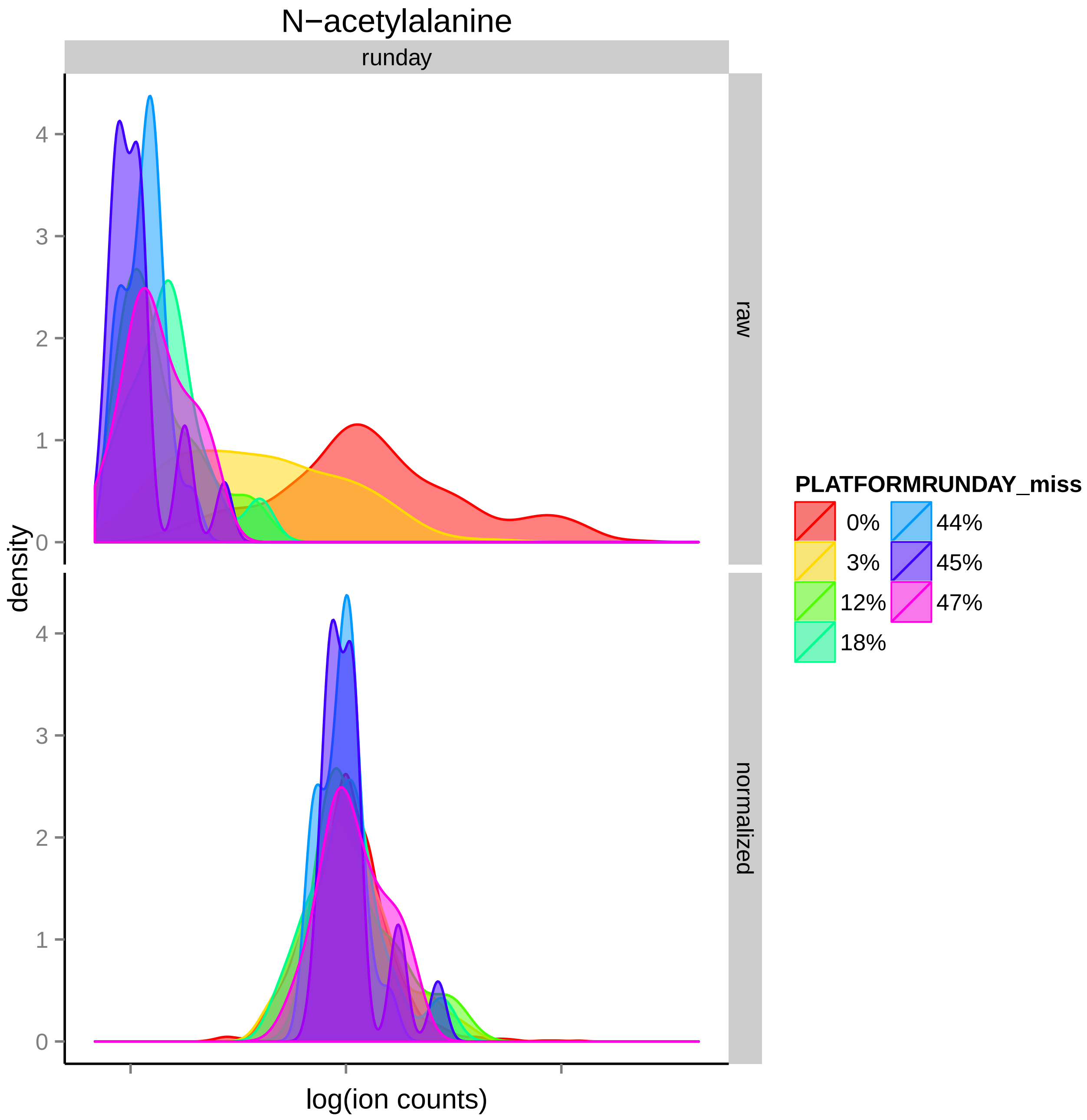
N−acetylalanine
runday
raw
normalized
density
log(ion counts)
0
1
2
3
4
PLATFORMRUNDAY_miss
0%
3%
12%
18%
44%
45%
47%

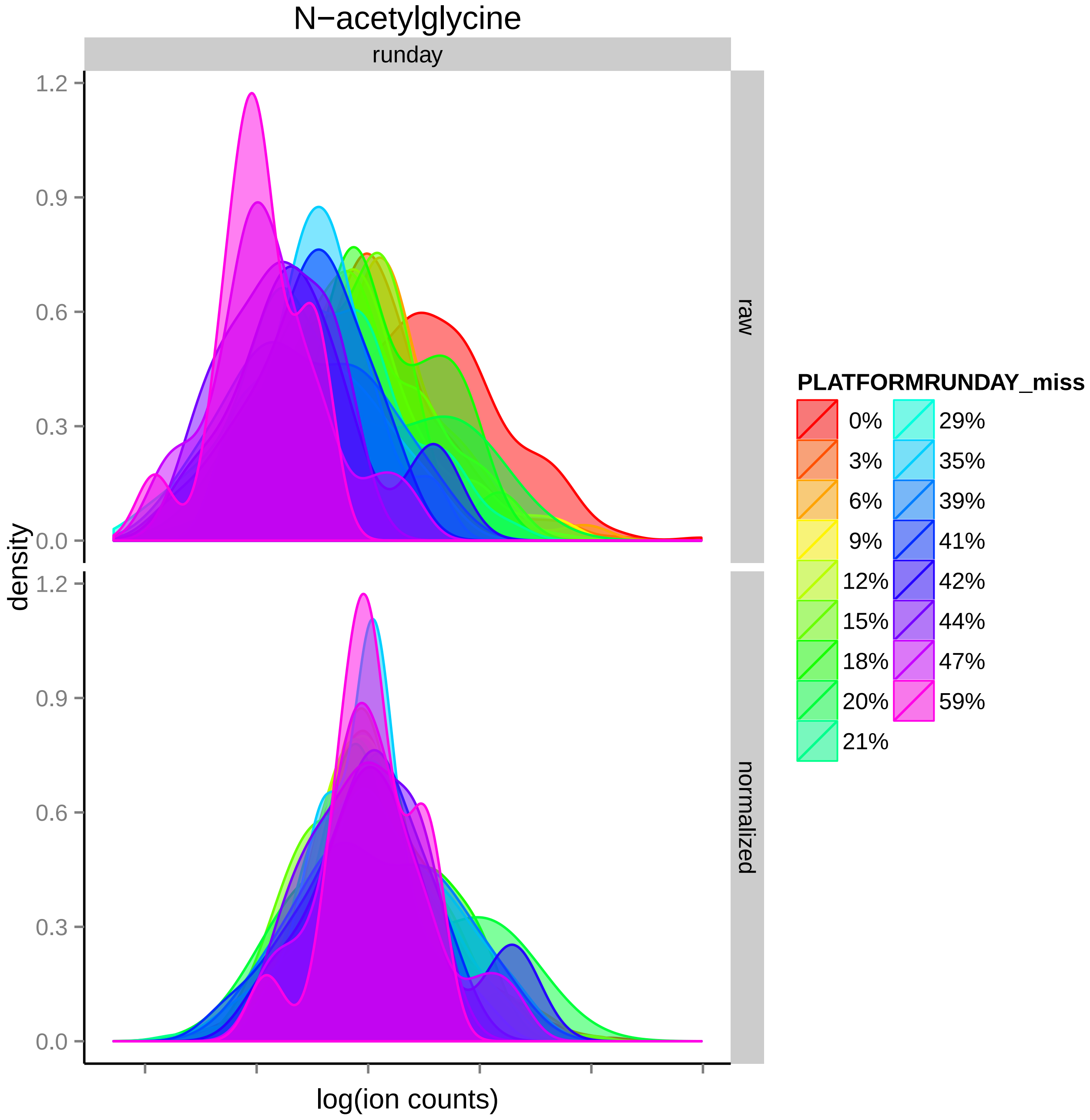

N−acetylglycine
runday
raw
normalized
density
log(ion counts)
1.2
0.9
0.6
0.3
0.0
PLATFORMRUNDAY_miss
0%
3%
6%
9%
12%
15%
18%
20%
21%
29%
35%
39%
41%
42%
44%
47%
59%

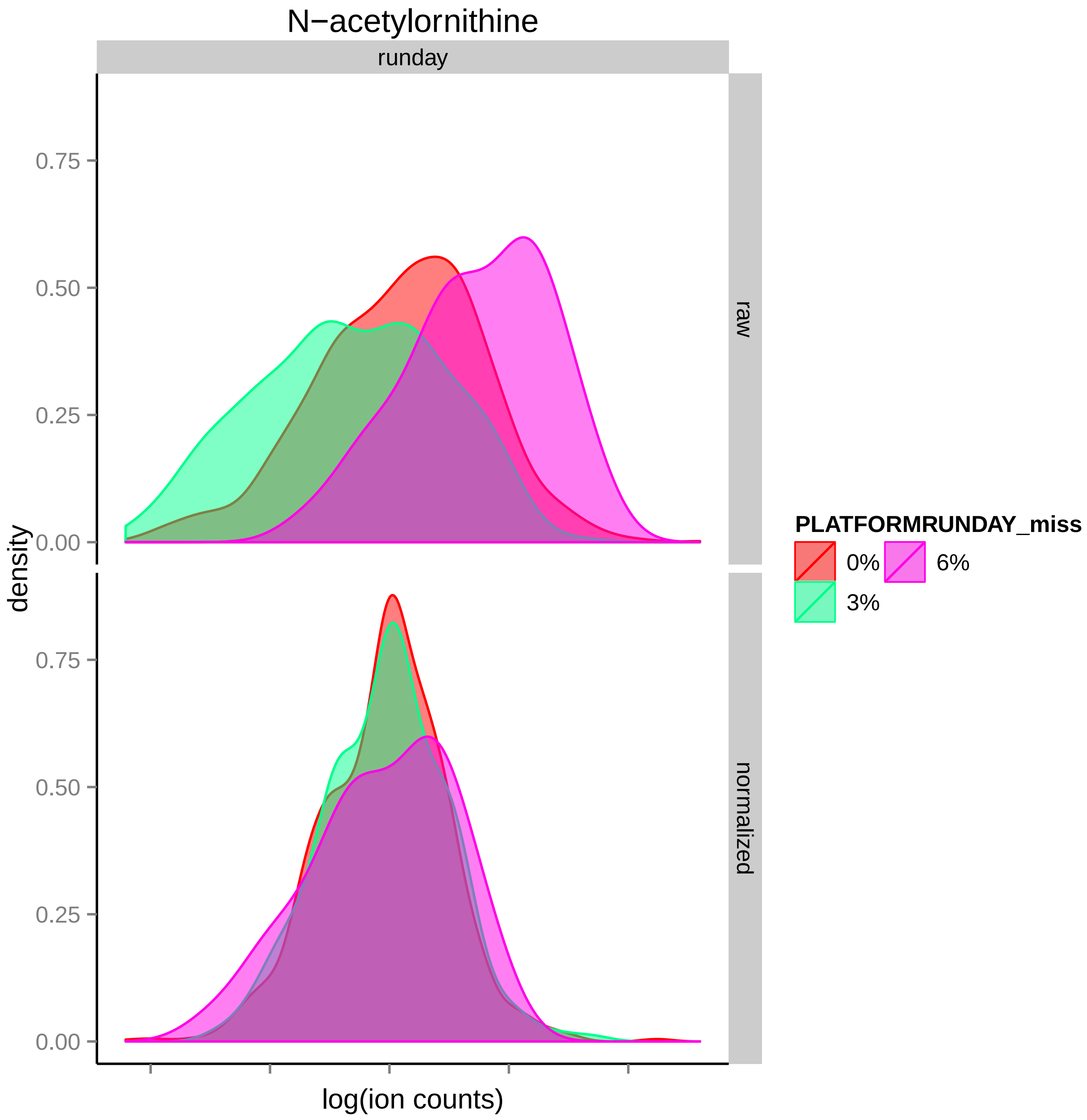
N−acetylornithine
runday
raw
normalized
density
0.00
0.25
0.50
0.75
log(ion counts)
PLATFORMRUNDAY_miss
0%
3%
6%

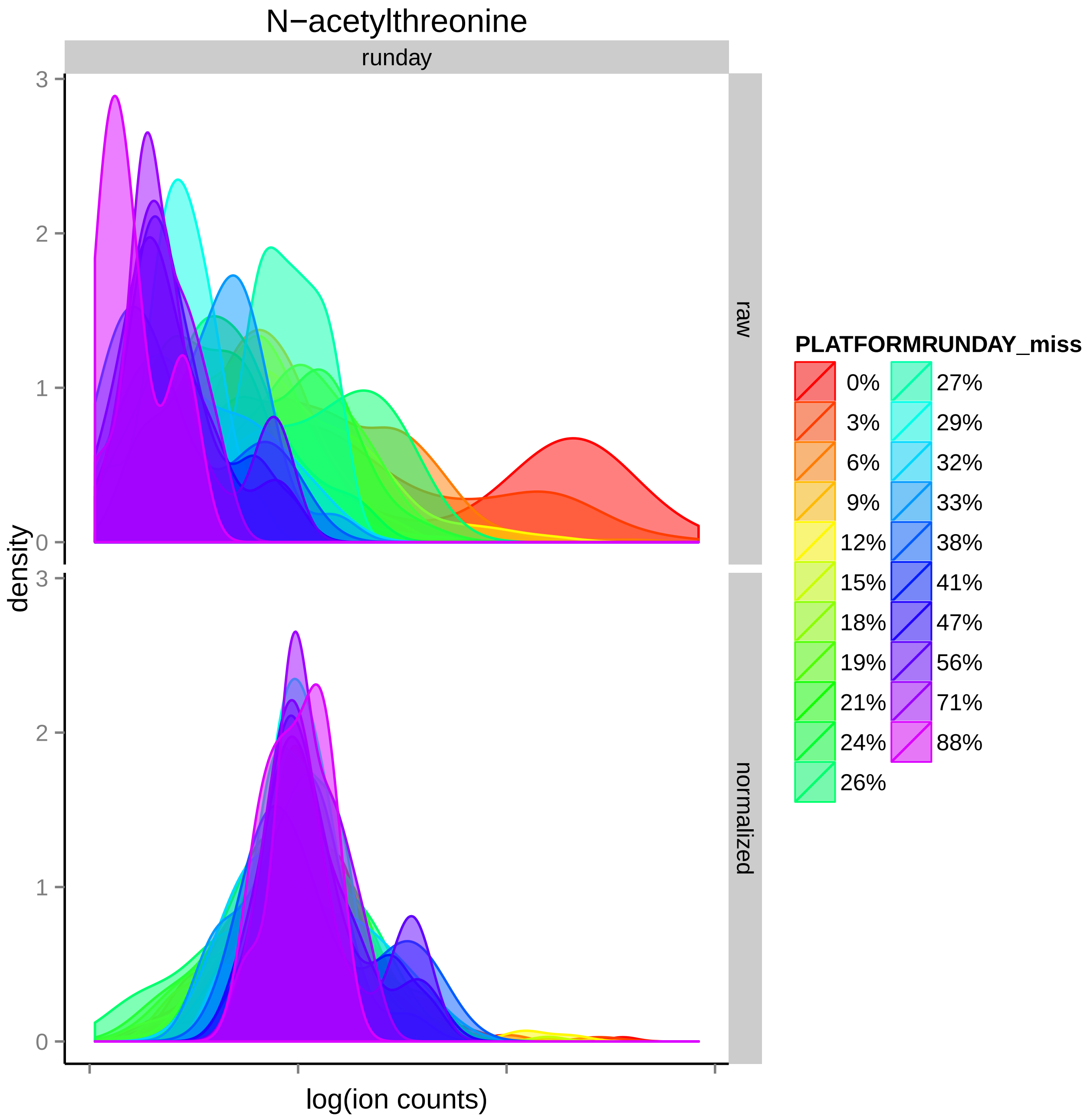
N−acetylthreonine
runday
raw
normalized
density
log(ion counts)
0
1
2
3
PLATFORMRUNDAY_miss
0%
3%
6%
9%
12%
15%
18%
19%
21%
24%
26%
27%
29%
32%
33%
38%
41%
47%
56%
71%
88%

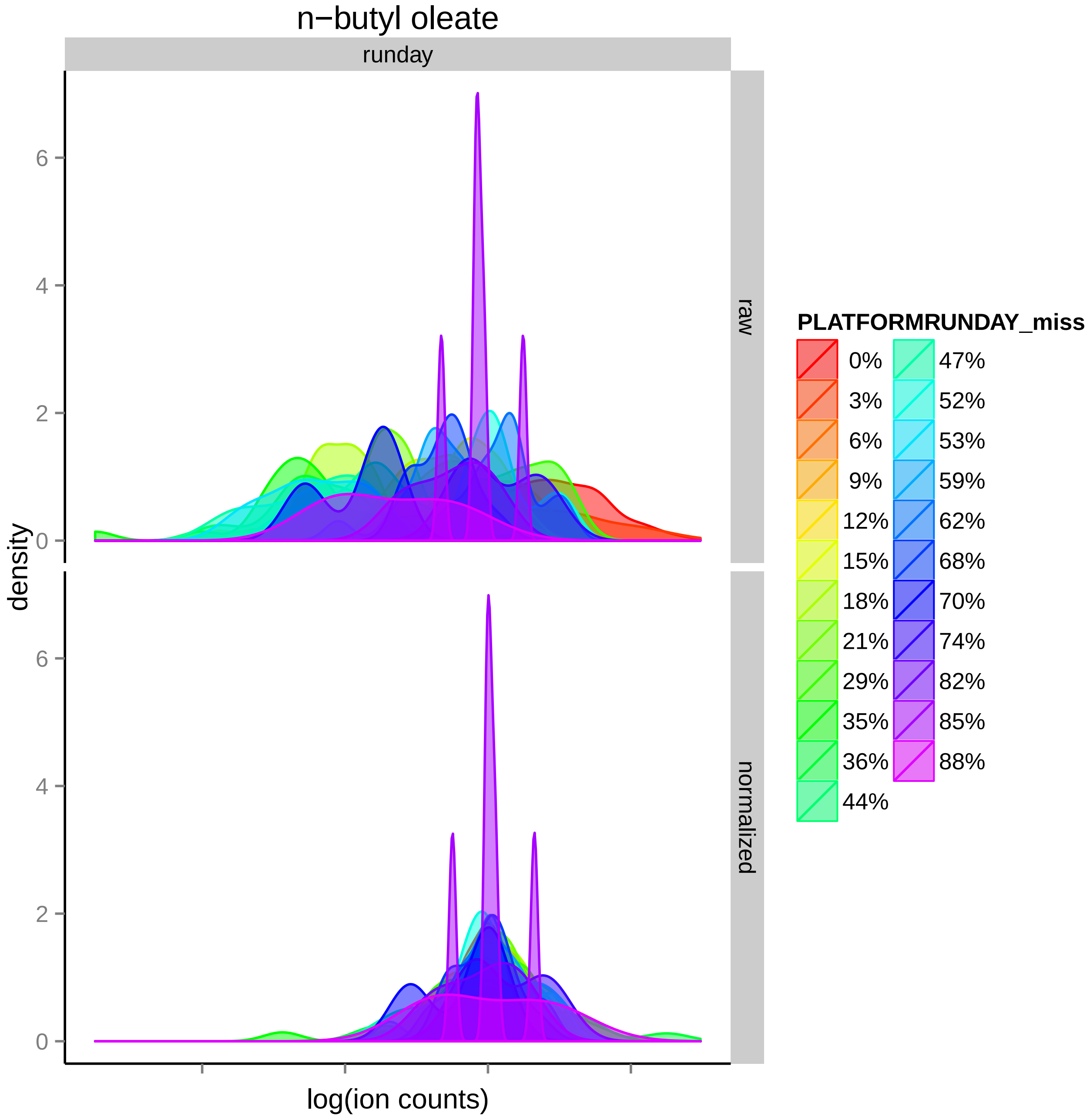
n−butyl oleate
runday
raw
normalized
density
log(ion counts)
0
2
4
6
0
2
4
6
PLATFORMRUNDAY_miss
0%
3%
6%
9%
12%
15%
18%
21%
29%
35%
36%
44%
47%
52%
53%
59%
62%
68%
70%
74%
82%
85%
88%

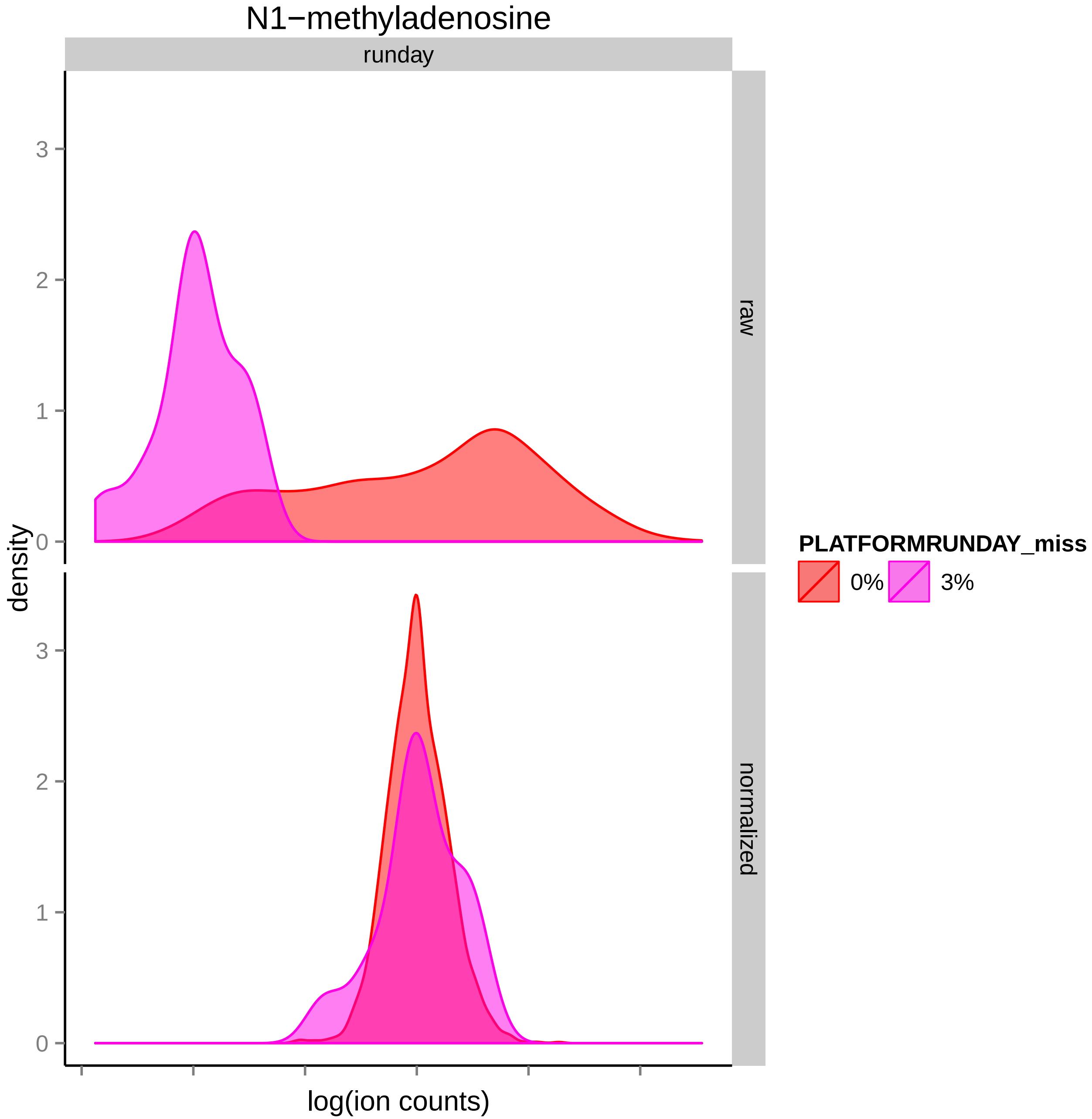

N1−methyladenosine
runday
raw
normalized
density
log(ion counts)
0
1
2
3
PLATFORMRUNDAY_miss
0%
3%

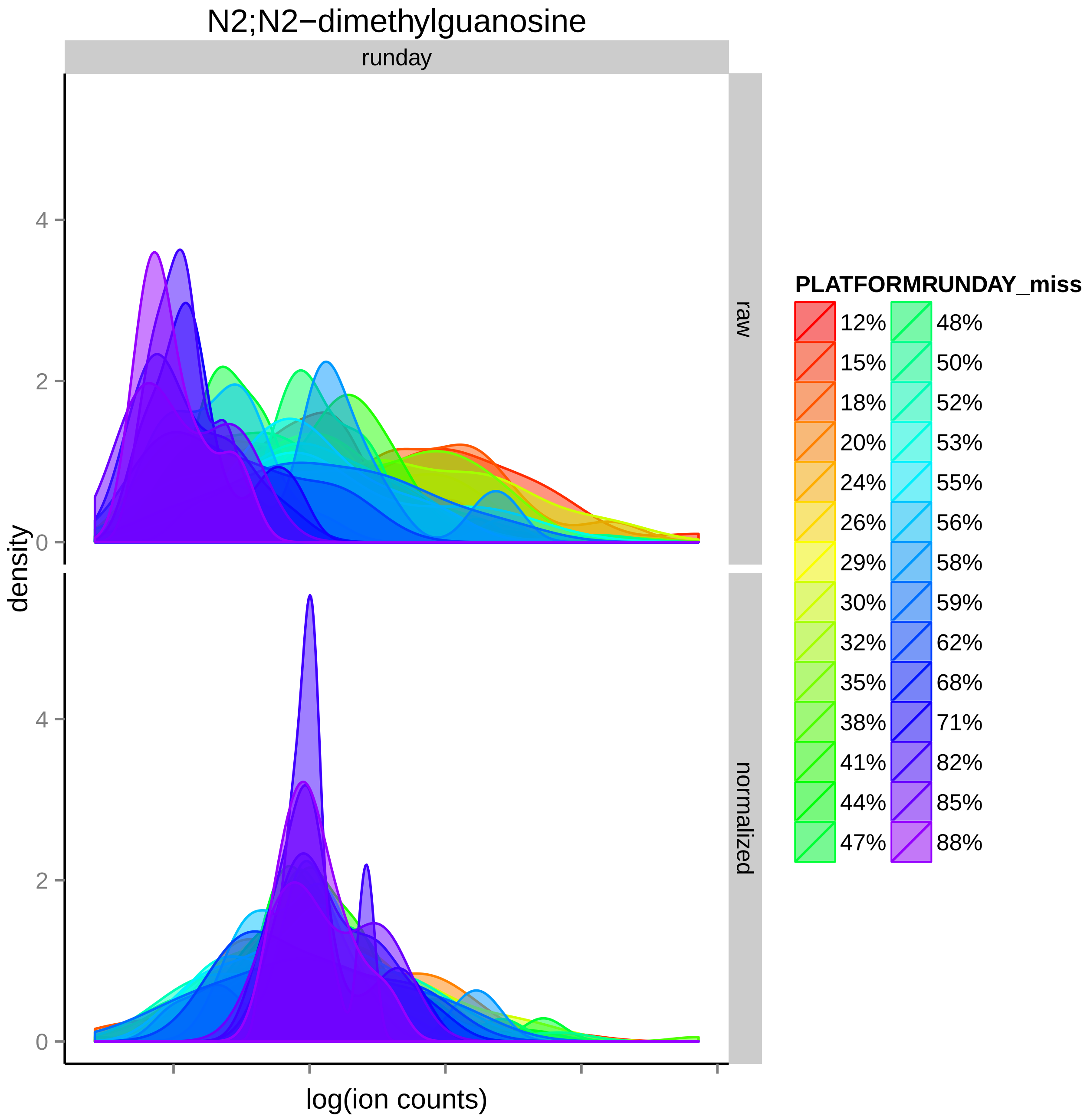
N2;N2−dimethylguanosine
runday
raw
normalized
density
log(ion counts)
0
2
4
0
2
4
PLATFORMRUNDAY_miss
12%
15%
18%
20%
24%
26%
29%
30%
32%
35%
38%
41%
44%
47%
48%
50%
52%
53%
55%
56%
58%
59%
62%
68%
71%
82%
85%
88%

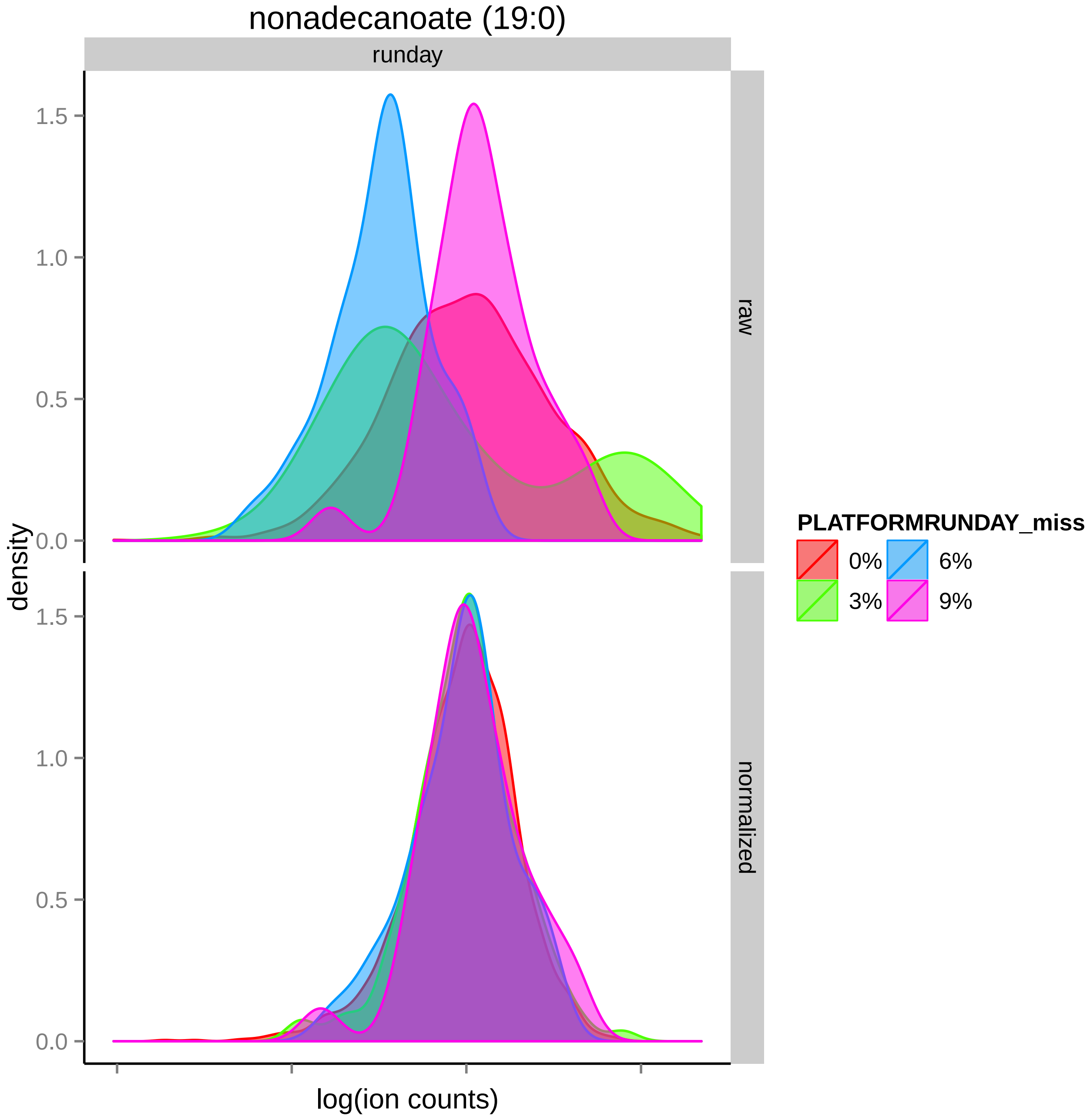

nonadecanoate (19:0)
runday
raw
normalized
density
log(ion counts)
1.5
1.0
0.5
0.0
PLATFORMRUNDAY_miss
0%
3%
6%
9%

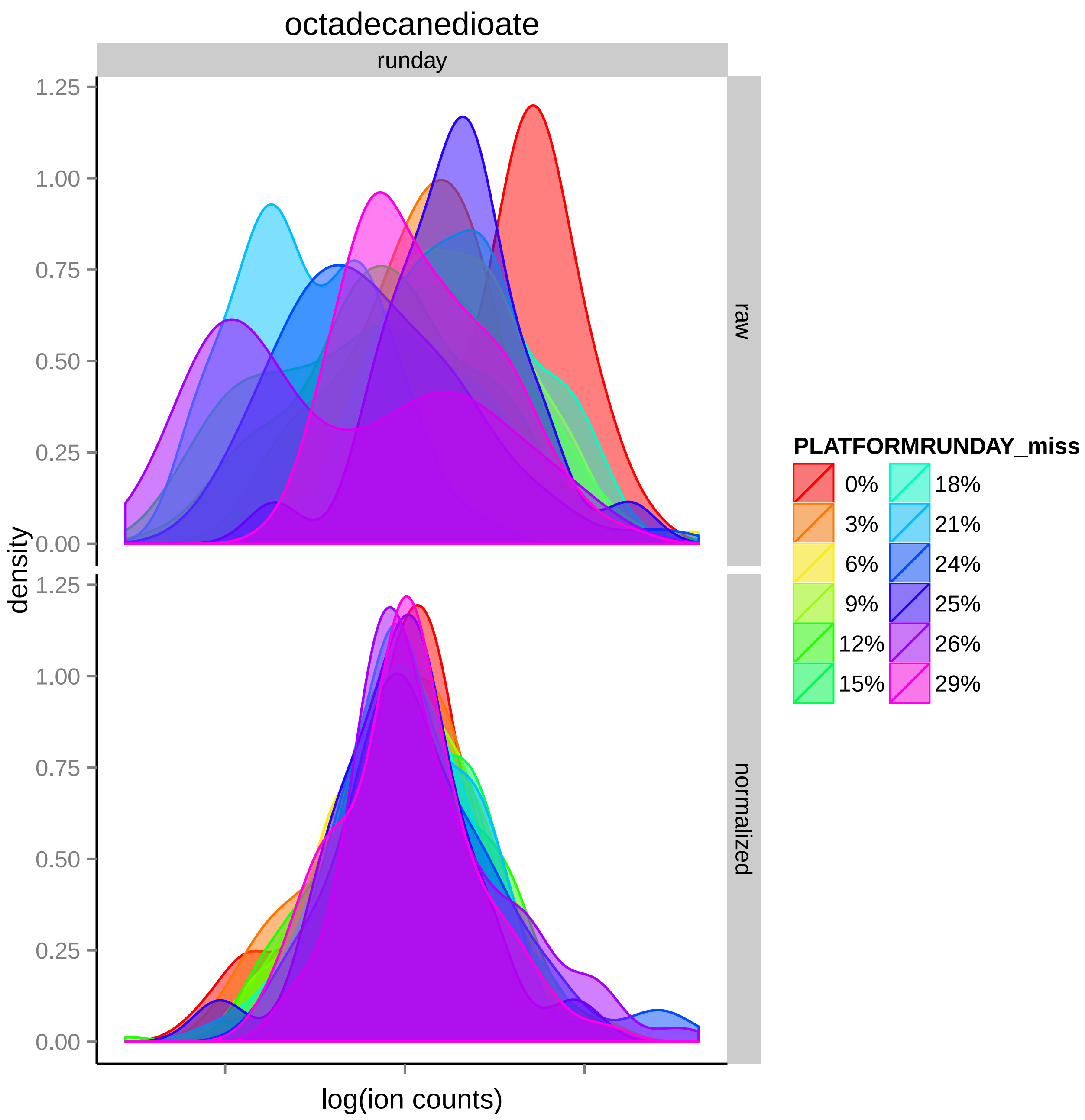

octadecanedioate
runday
raw
normalized
density
log(ion counts)
1.25
1.00
0.75
0.50
0.25
0.00
PLATFORMRUNDAY_miss
0%
3%
6%
9%
12%
15%
18%
21%
24%
25%
26%
29%

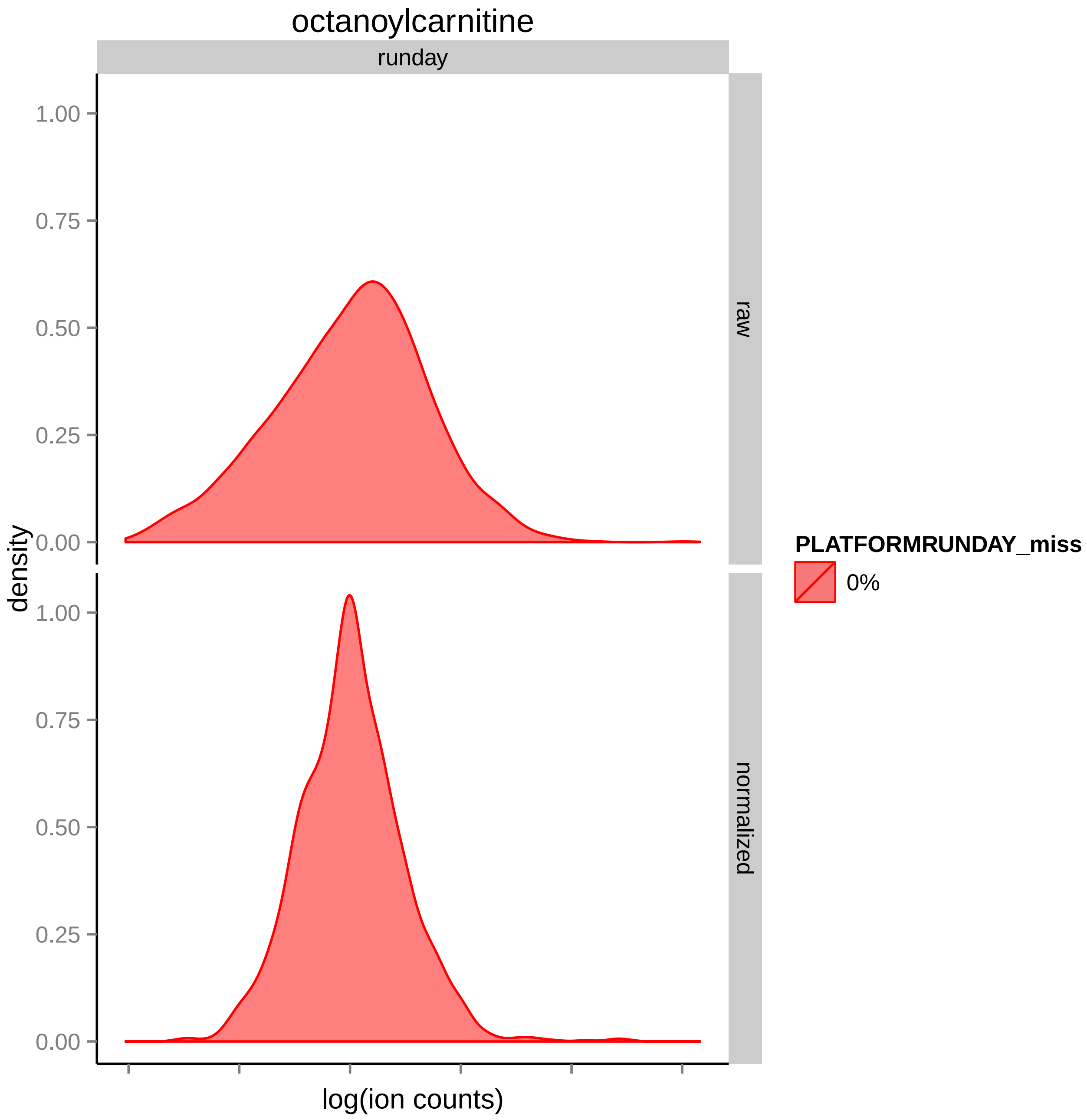

octanoylcarnitine
runday
raw
normalized
density
1.00
0.75
0.50
0.25
0.00
log(ion counts)
PLATFORMRUNDAY_miss
0%

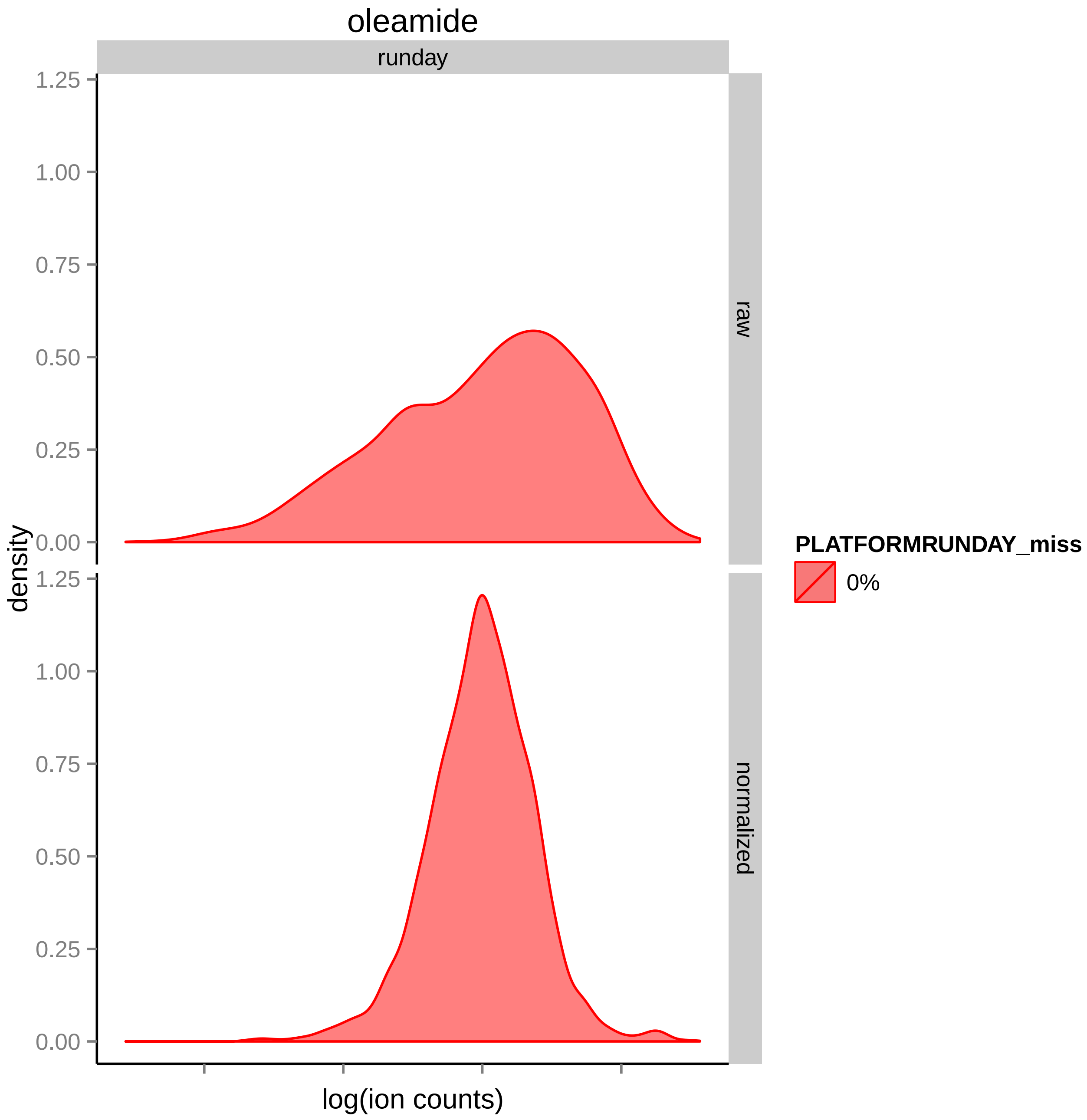

oleamide
runday
raw
normalized
density
log(ion counts)
1.25
1.00
0.75
0.50
0.25
0.00
PLATFORMRUNDAY_miss
0%

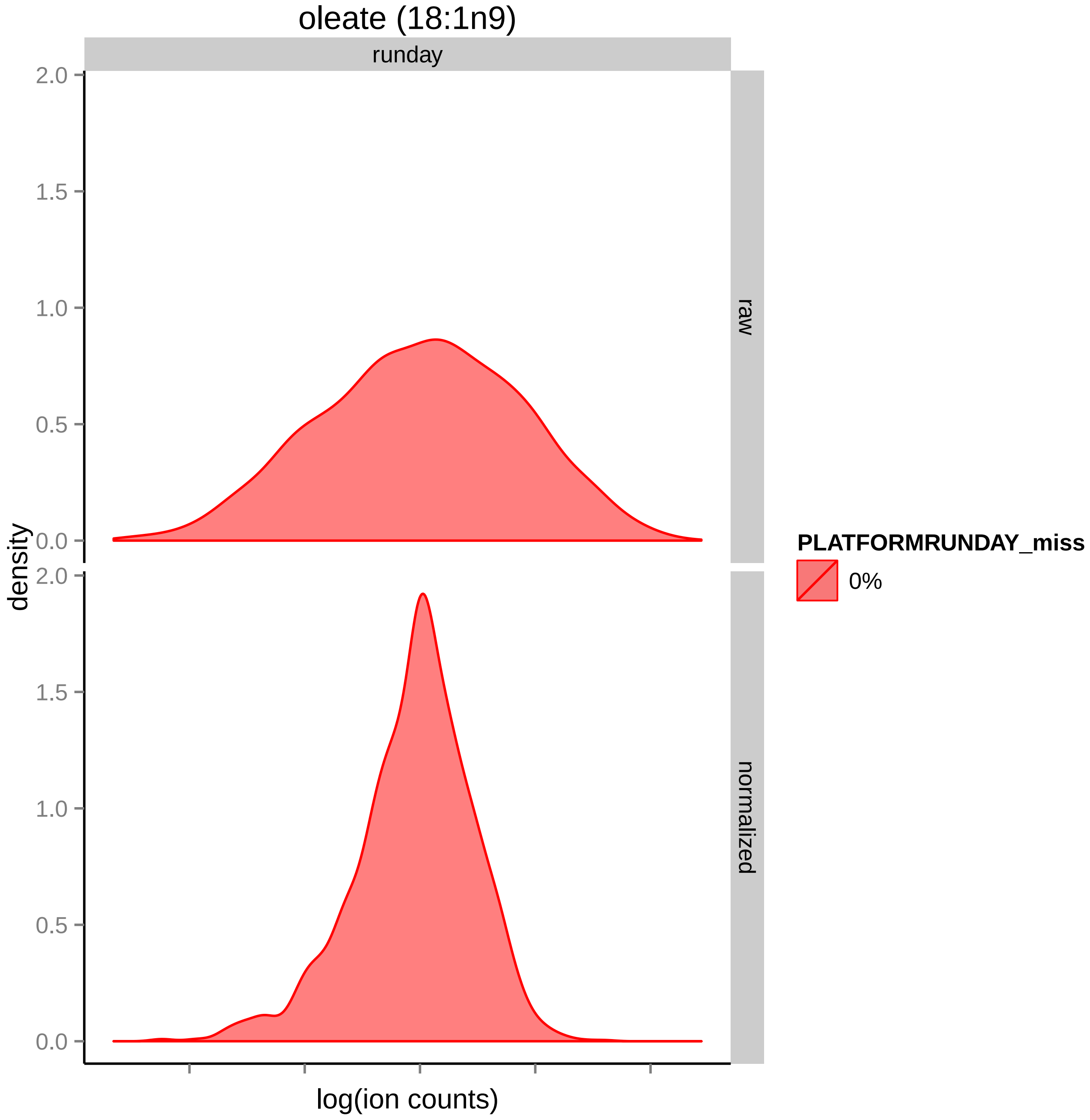

oleate (18:1n9)
runday
raw
normalized
density
log(ion counts)
2.0
1.5
1.0
0.5
0.0
PLATFORMRUNDAY_miss
0%

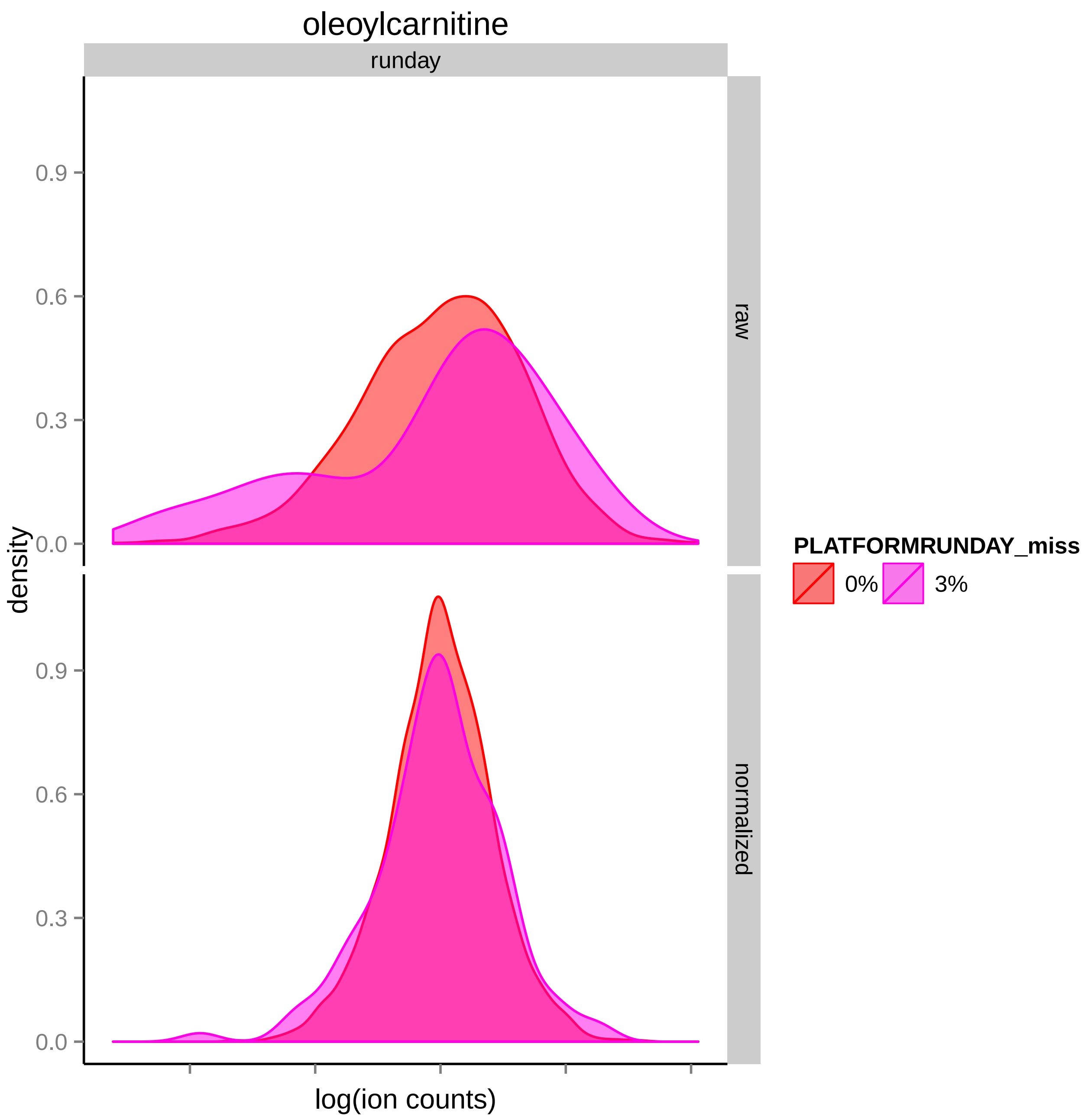
oleoylcarnitine
runday
raw
normalized
density
0.9
0.6
0.3
0.0
0.9
0.6
0.3
0.0
log(ion counts)
PLATFORMRUNDAY_miss
0%
3%

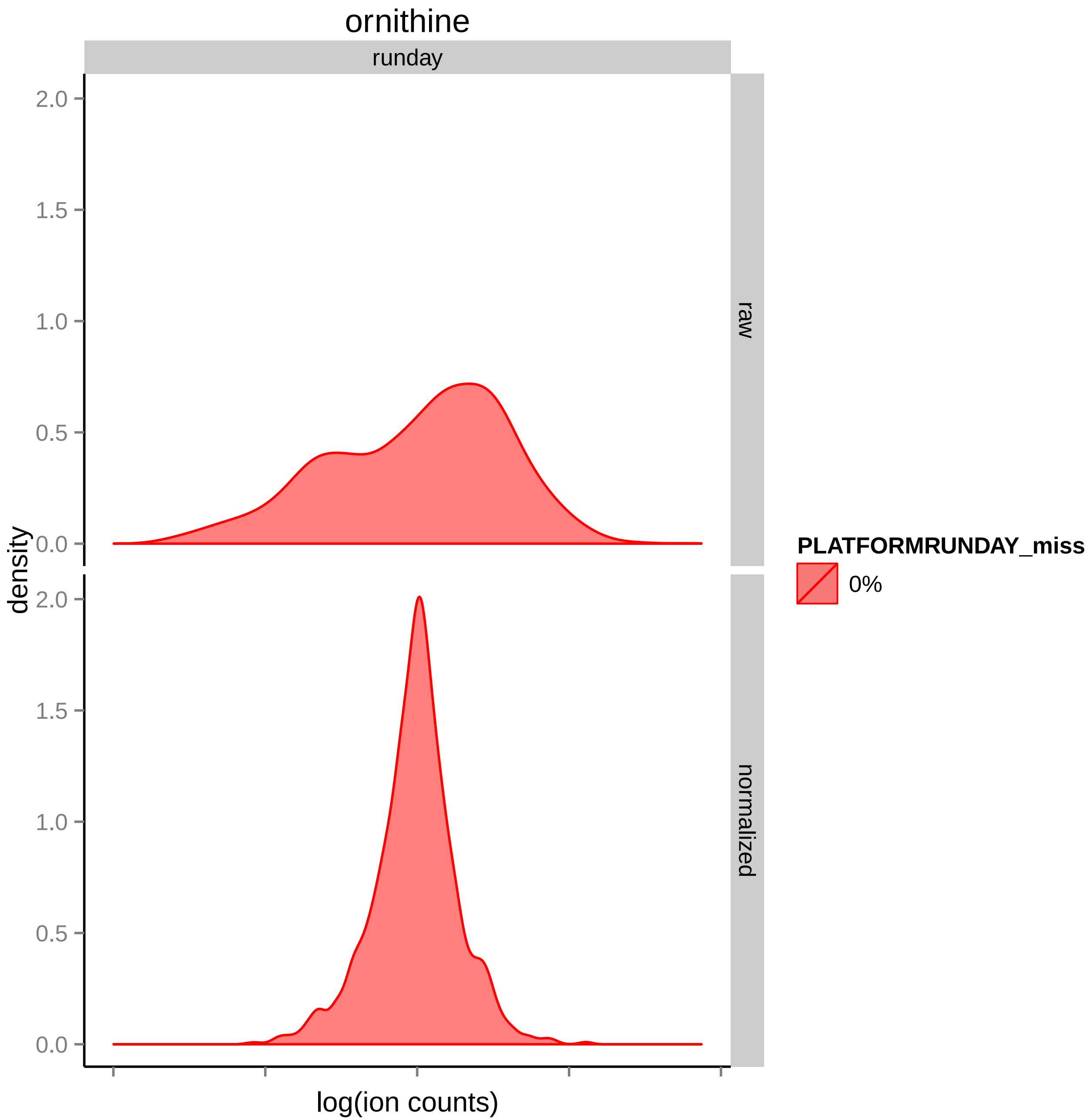
ornithine
runday
raw
normalized
density
2.0
1.5
1.0
0.5
0.0
log(ion counts)
PLATFORMRUNDAY_miss
0%

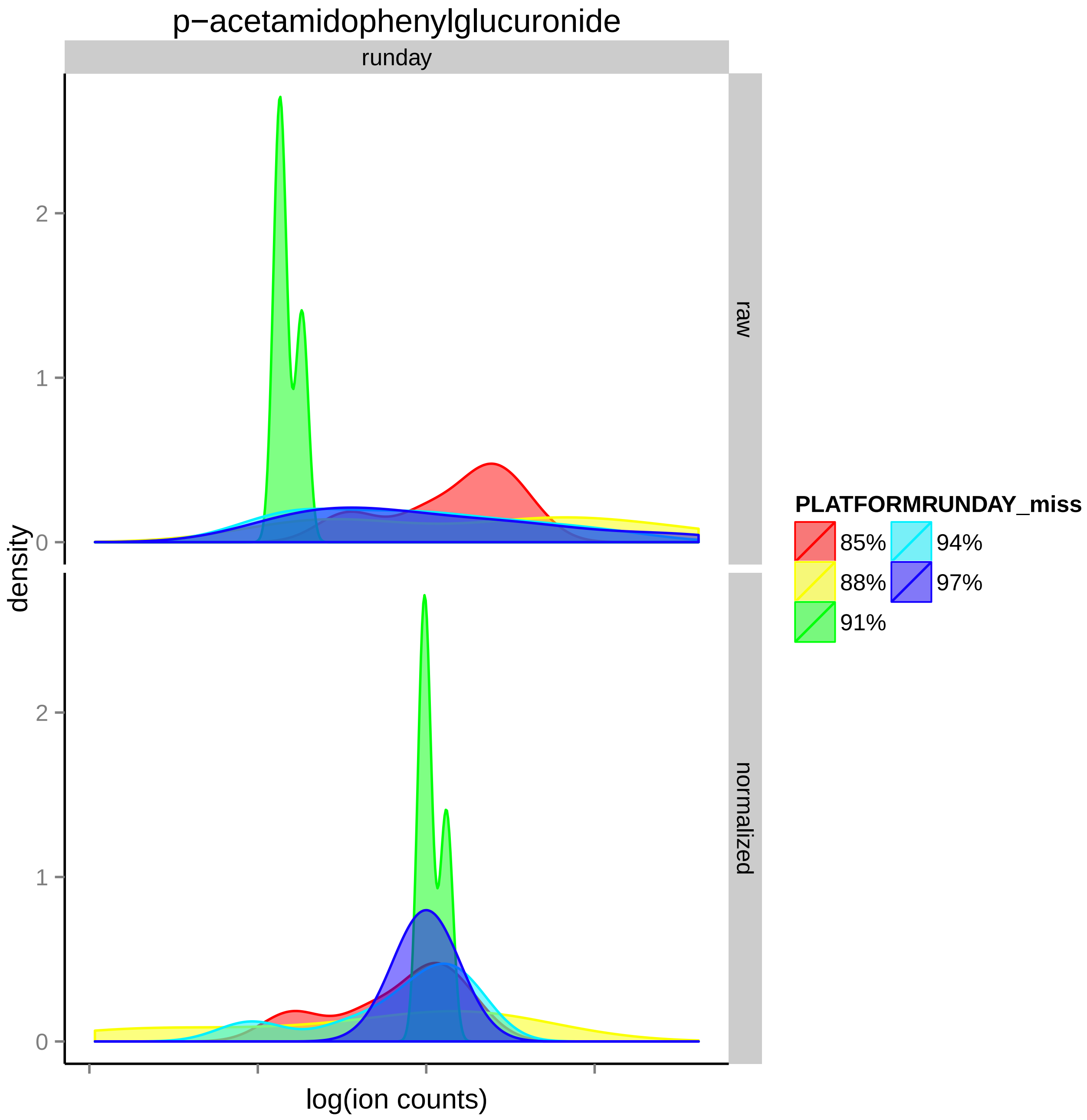
p−acetamidophenylglucuronide
runday
raw
normalized
density
log(ion counts)
0
1
2
0
1
2
PLATFORMRUNDAY_miss
85%
88%
91%
94%
97%

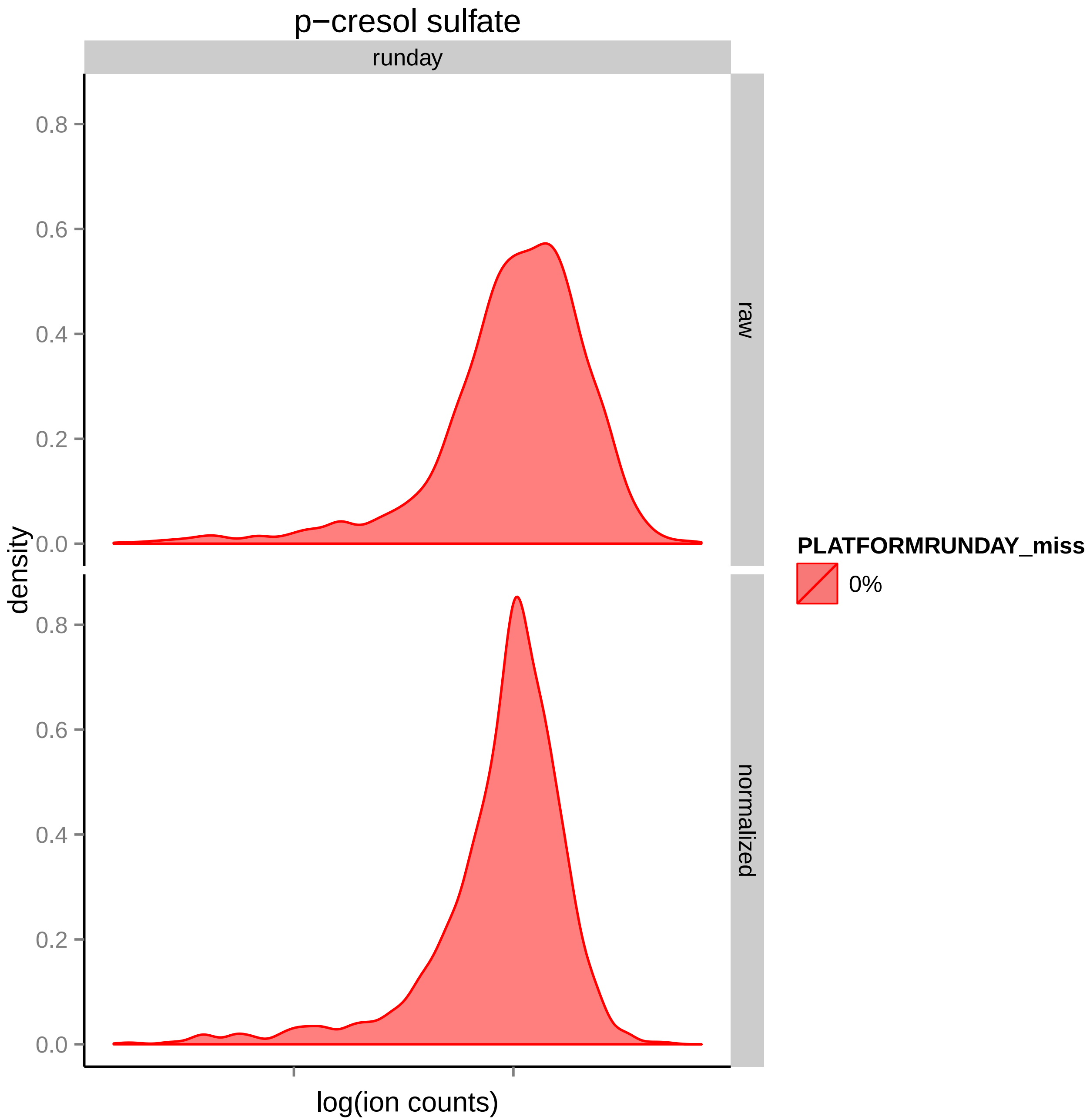

p−cresol sulfate
runday
raw
normalized
density
0.8
0.6
0.4
0.2
0.0
log(ion counts)
PLATFORMRUNDAY_miss
0%

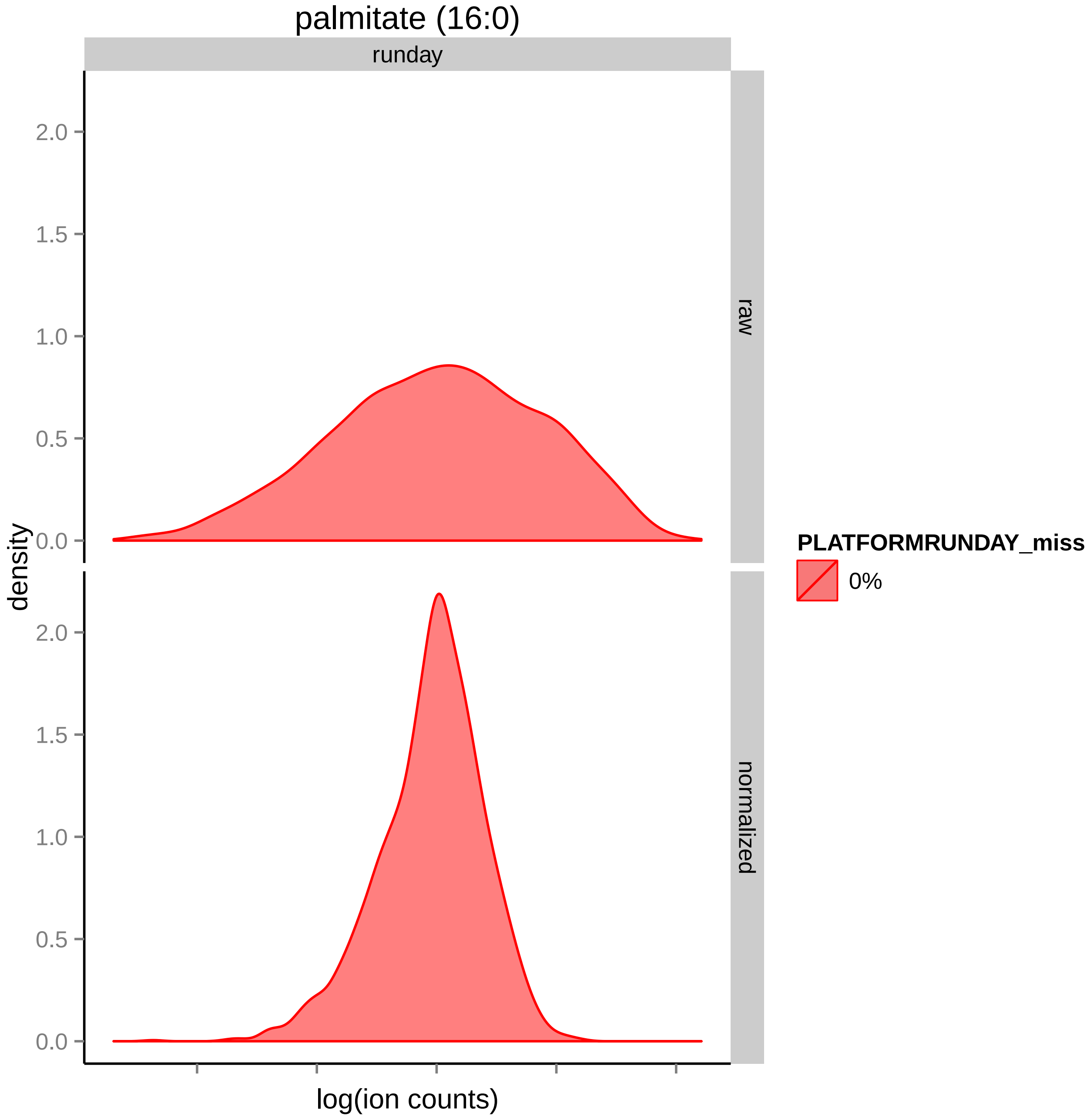

palmitate (16:0)
runday
raw
normalized
density
2.0
1.5
1.0
0.5
0.0
log(ion counts)
PLATFORMRUNDAY_miss
0%

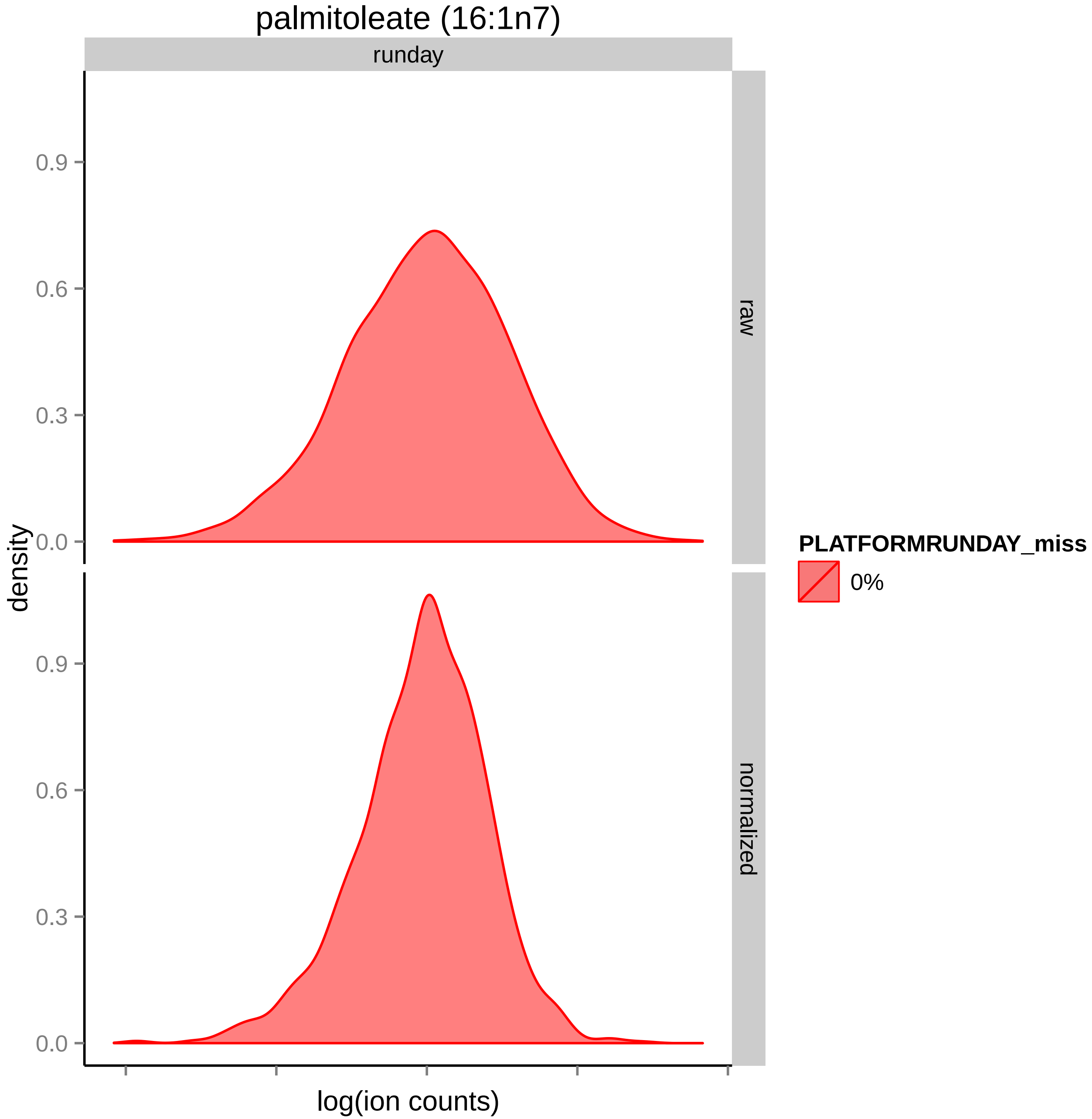
palmitoleate (16:1n7)
runday
raw
normalized
density
log(ion counts)
0.0
0.3
0.6
0.9
PLATFORMRUNDAY_miss
0%

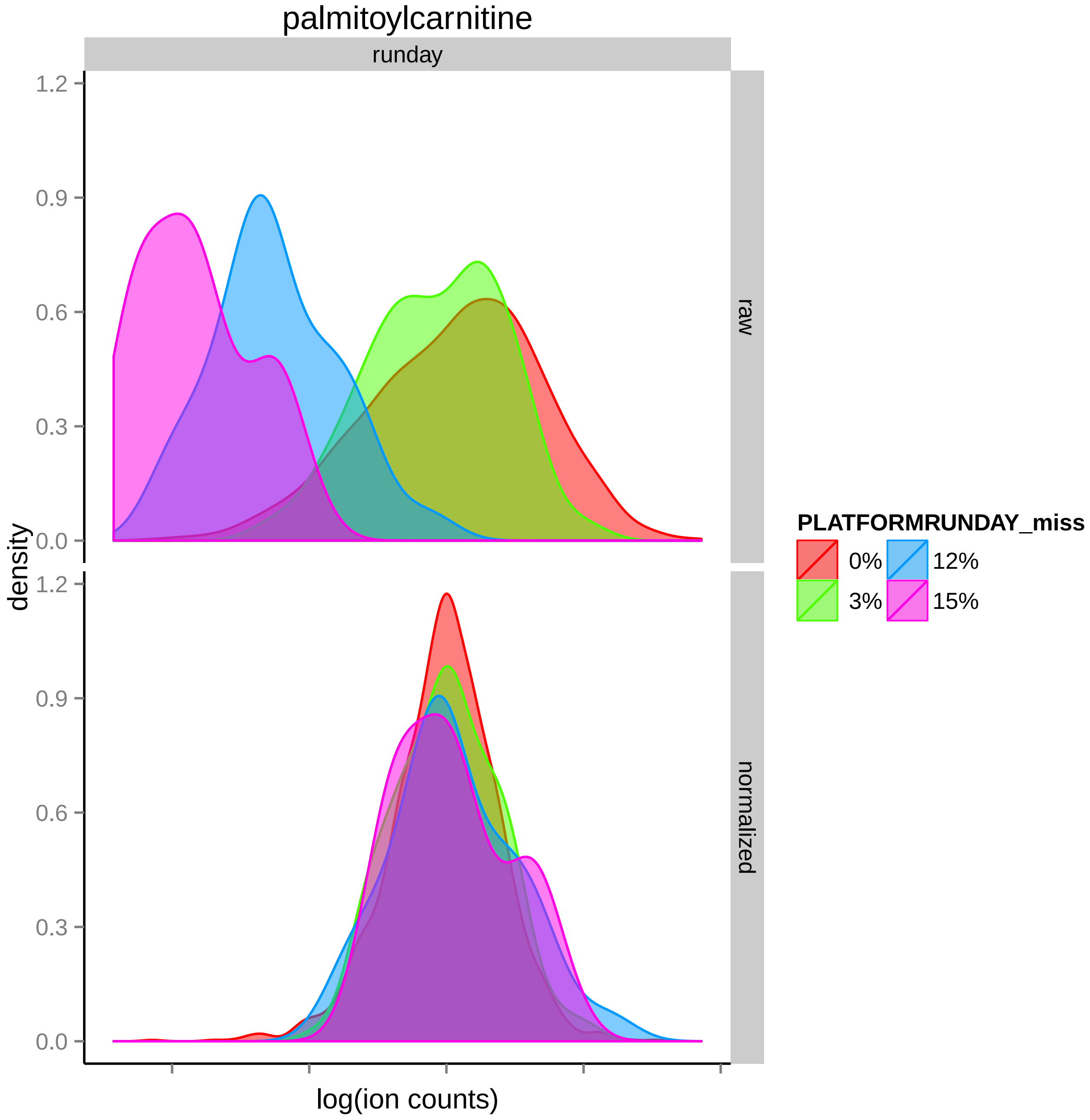
palmitoylcarnitine
runday
raw
normalized
density
log(ion counts)
1.2
0.9
0.6
0.3
0.0
PLATFORMRUNDAY_miss
0%
3%
12%
15%

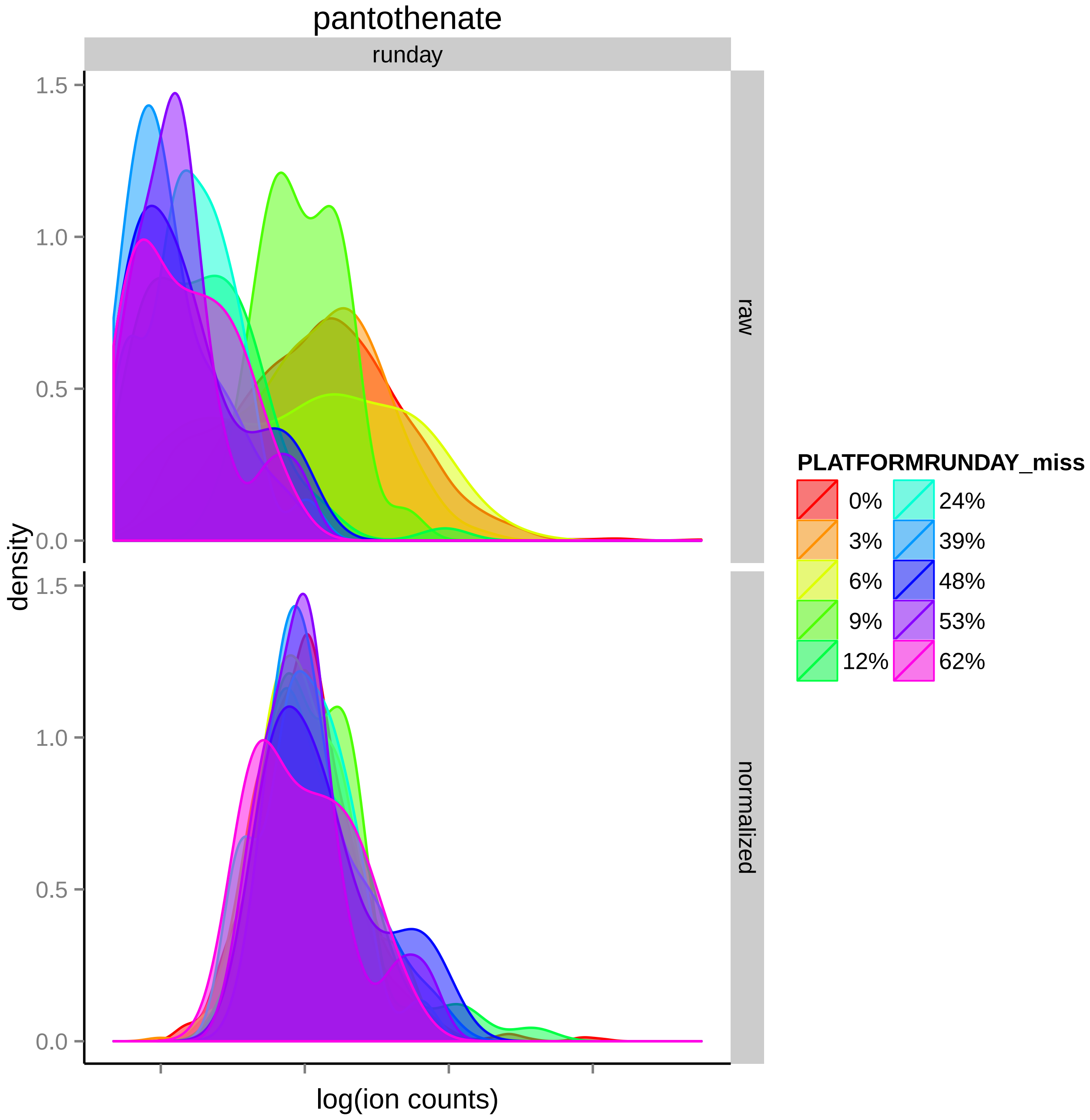

pantothenate
runday
raw
normalized
density
log(ion counts)
1.5
1.0
0.5
0.0
PLATFORMRUNDAY_miss
0%
3%
6%
9%
12%
24%
39%
48%
53%
62%

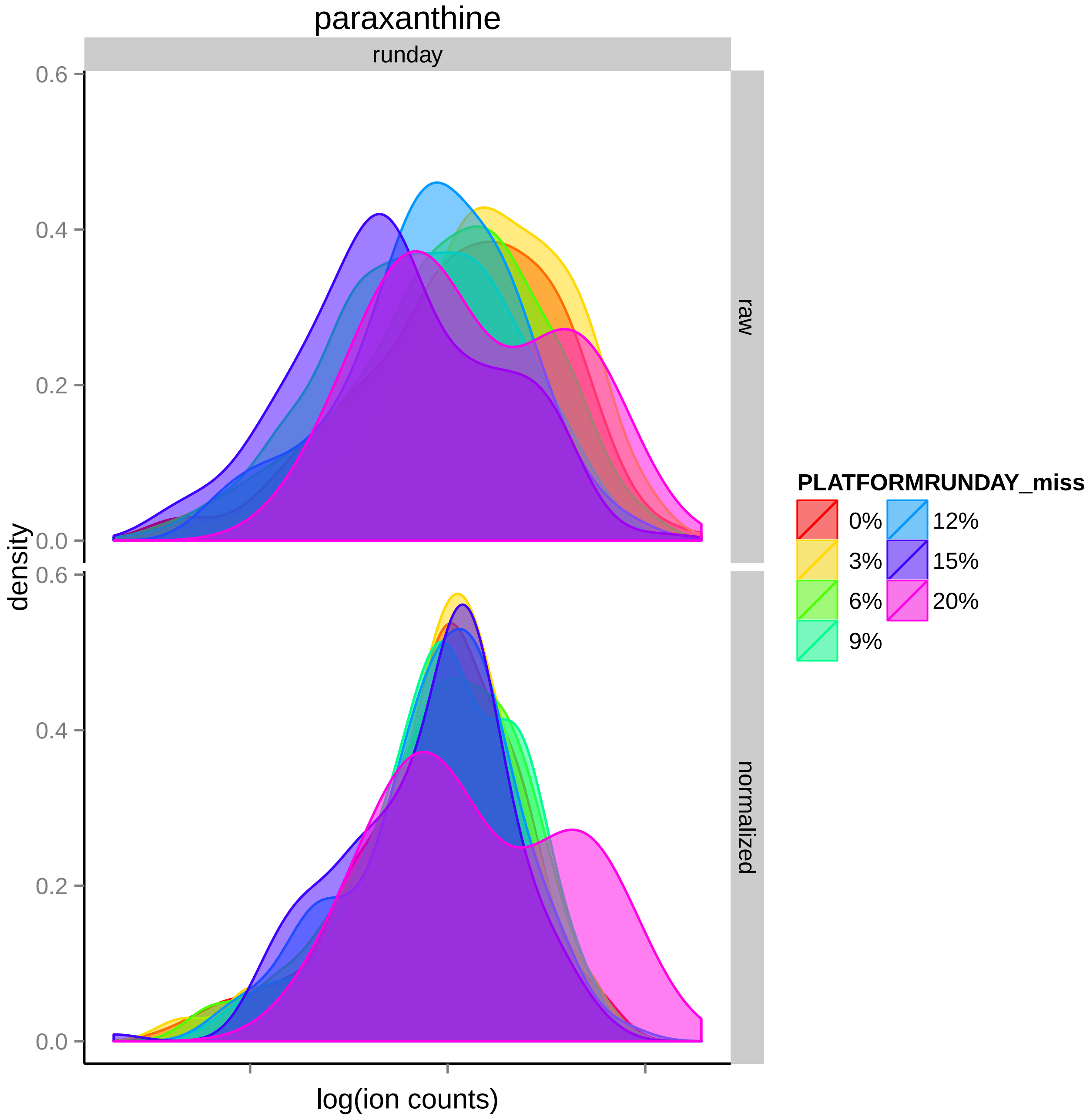

paraxanthine
runday
raw
normalized
density
0.0
0.2
0.4
0.6
log(ion counts)
PLATFORMRUNDAY_miss
0%
3%
6%
9%
12%
15%
20%

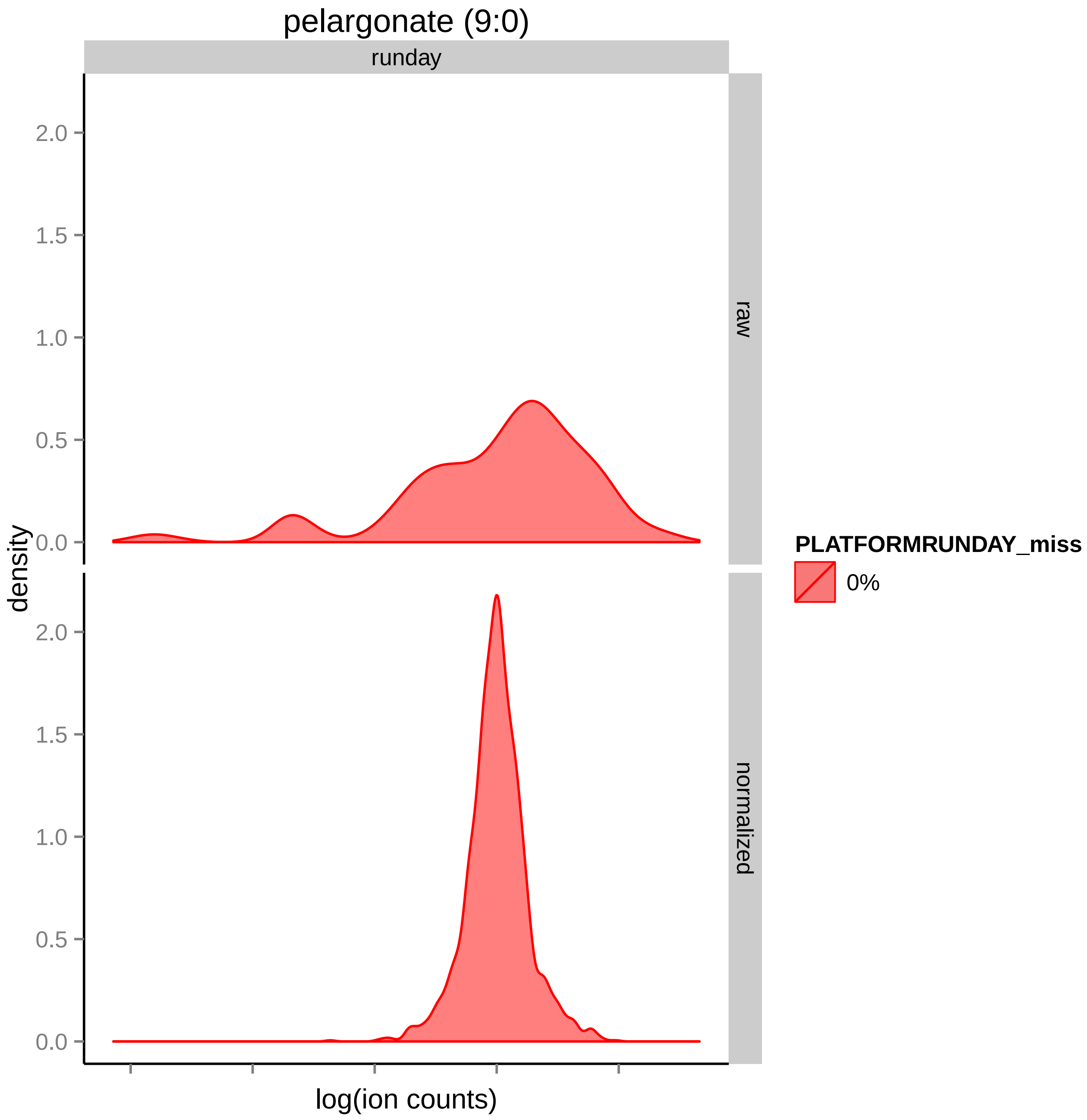

pelargonate (9:0)
runday
raw
normalized
density
2.0
1.5
1.0
0.5
0.0
log(ion counts)
PLATFORMRUNDAY_miss
0%

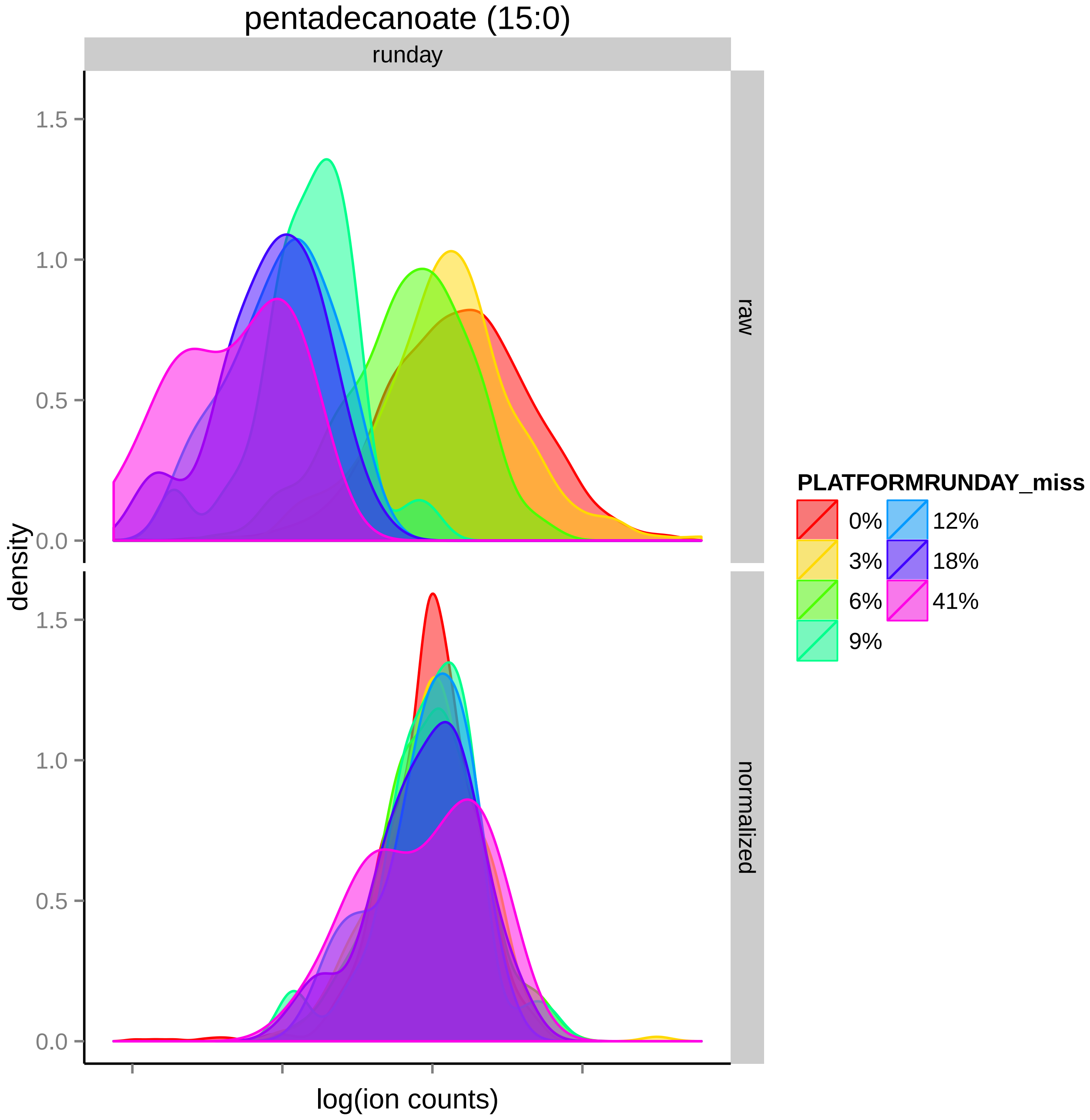
pentadecanoate (15:0)
runday
raw
normalized
density
log(ion counts)
1.5
1.0
0.5
0.0
1.5
1.0
0.5
0.0
PLATFORMRUNDAY_miss
0%
3%
6%
9%
12%
18%
41%

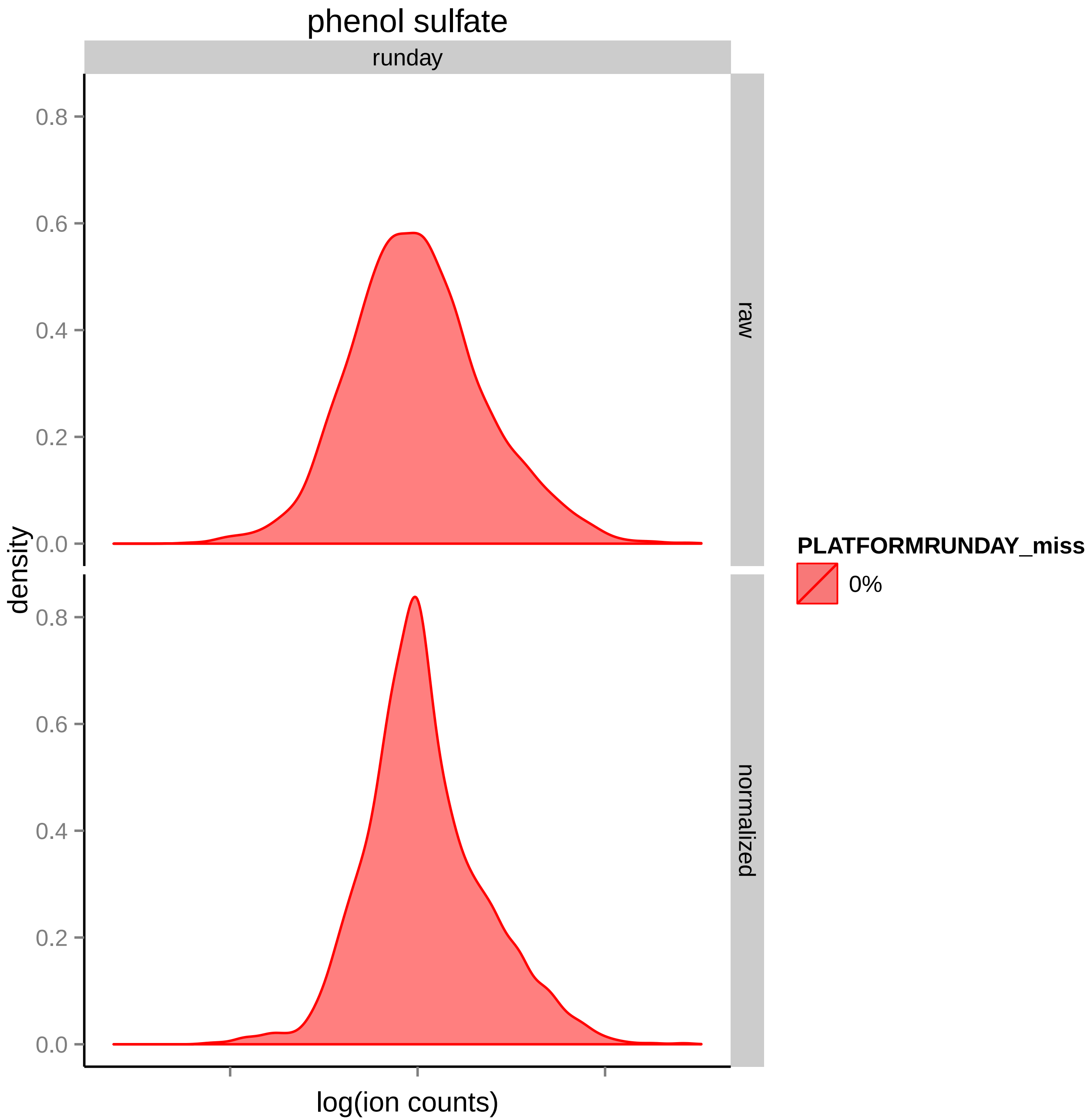

phenol sulfate
runday
raw
normalized
density
0.0
0.2
0.4
0.6
0.8
log(ion counts)
PLATFORMRUNDAY_miss
0%

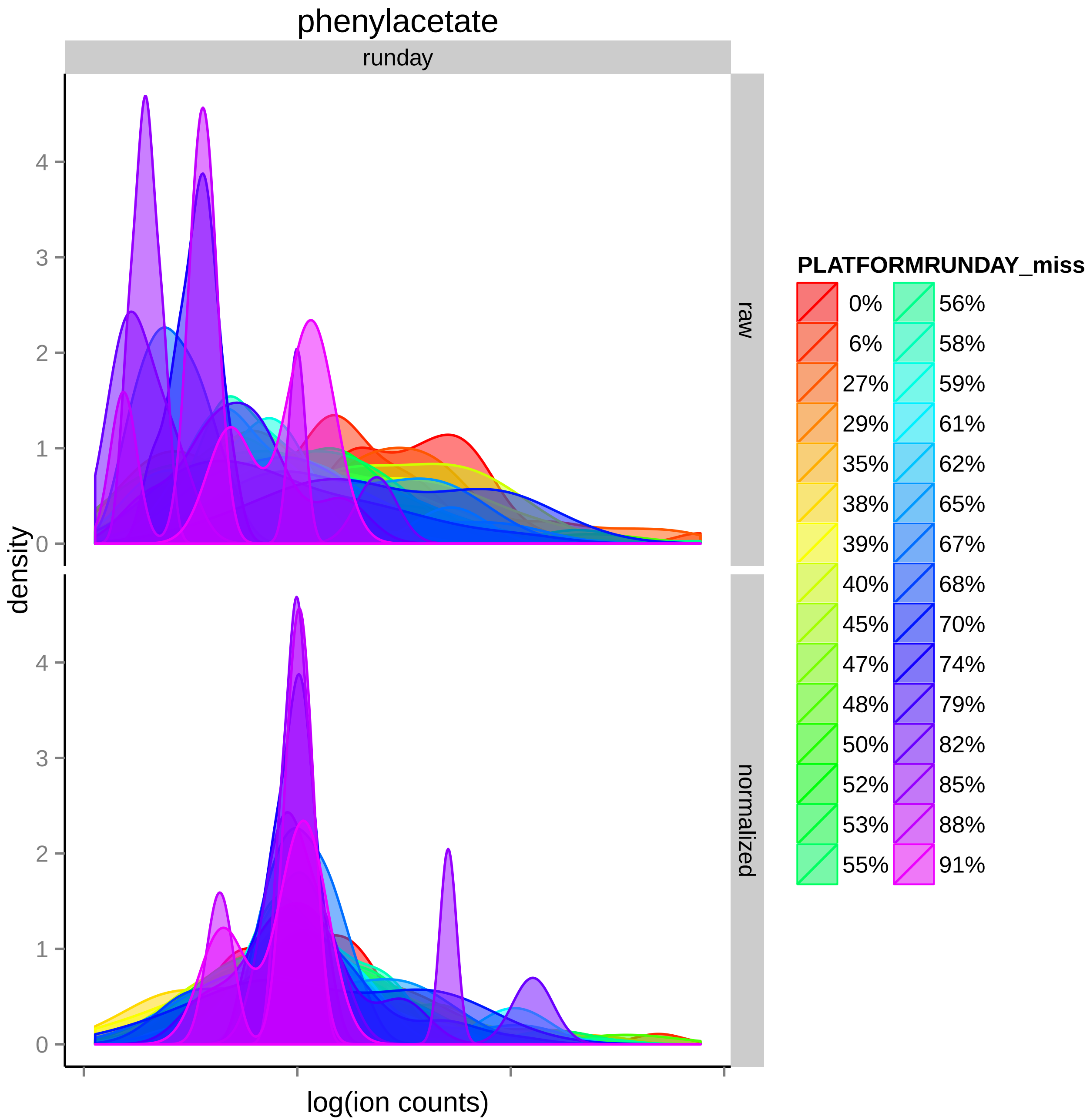

phenylacetate
runday
raw
normalized
density
log(ion counts)
0
1
2
3
4
PLATFORMRUNDAY_miss
0%
6%
27%
29%
35%
38%
39%
40%
45%
47%
48%
50%
52%
53%
55%
56%
58%
59%
61%
62%
65%
67%
68%
70%
74%
79%
82%
85%
88%
91%

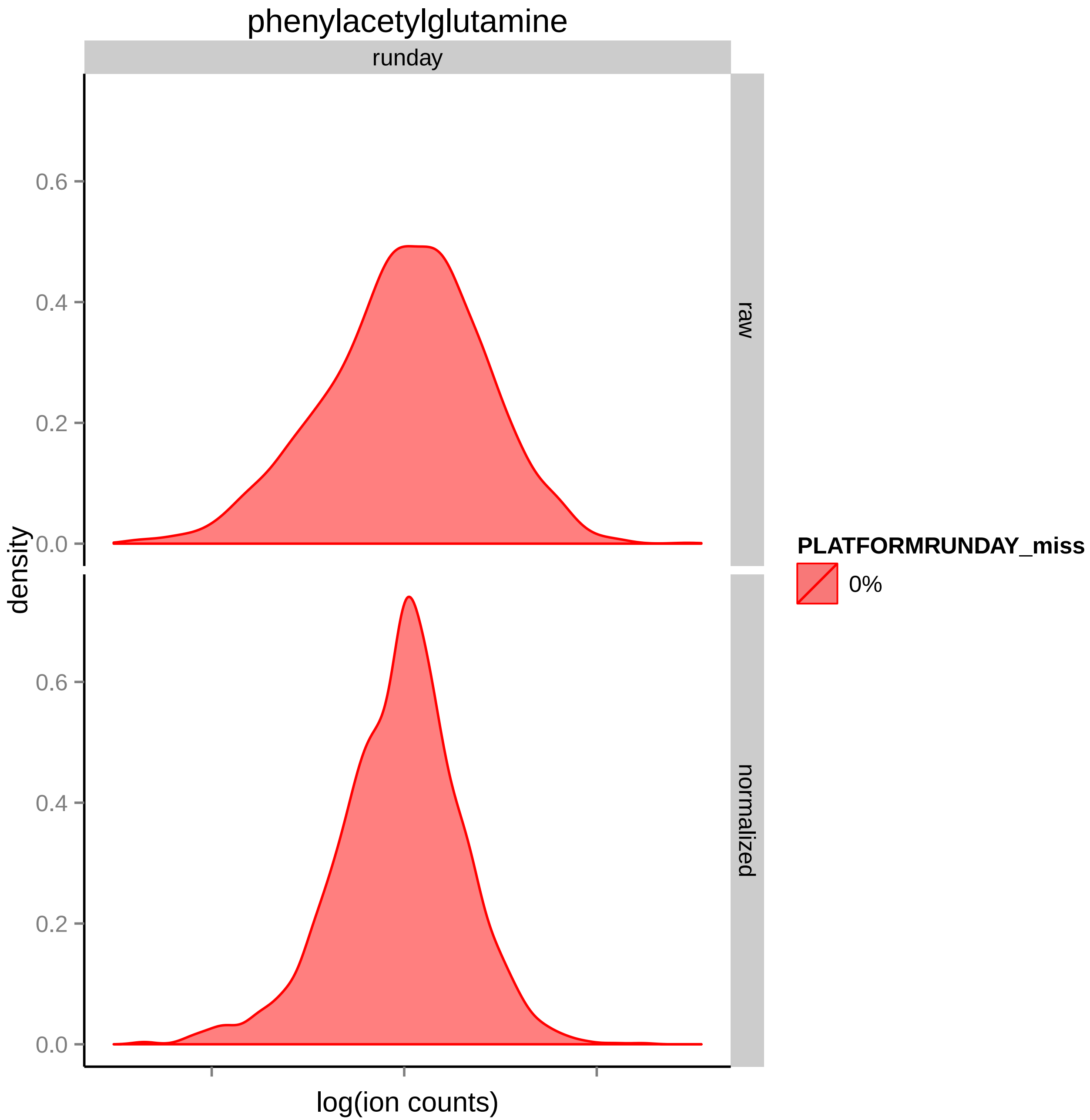

phenylacetylglutamine
runday
raw
normalized
density
0.0
0.2
0.4
0.6
log(ion counts)
PLATFORMRUNDAY_miss
0%

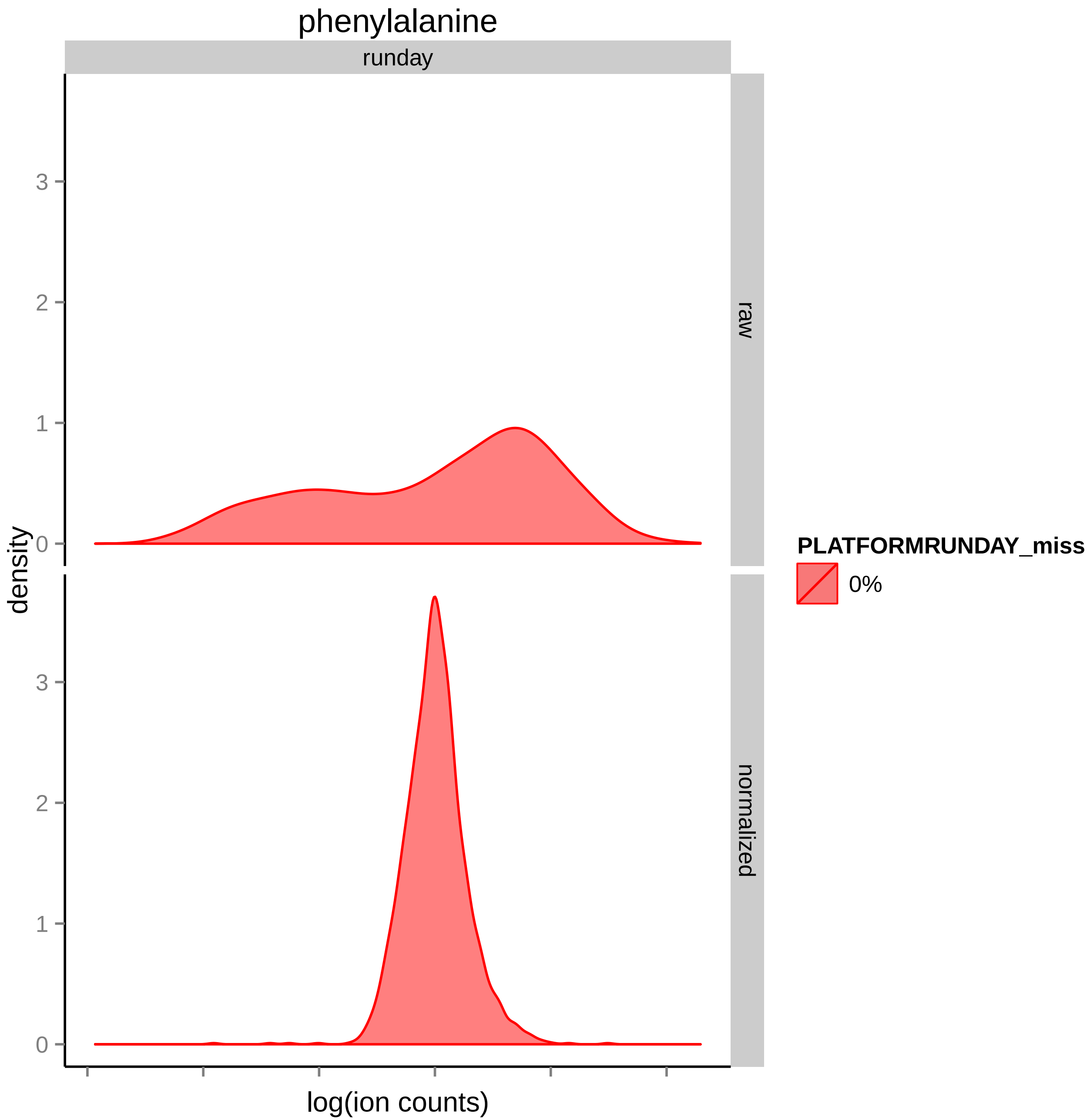
phenylalanine
runday
raw
normalized
density
log(ion counts)
0
1
2
3
0
1
2
3
PLATFORMRUNDAY_miss
0%

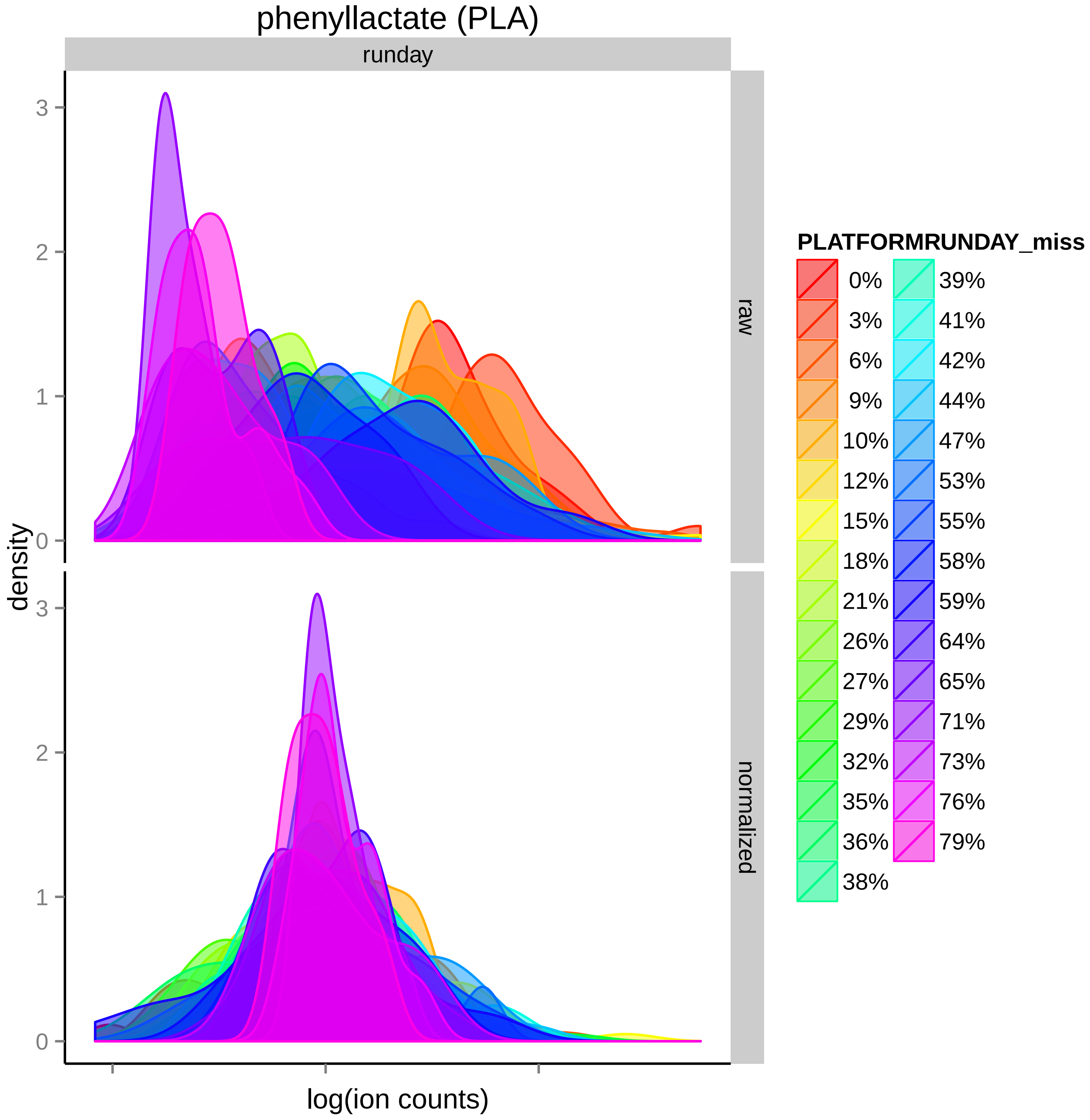
phenyllactate (PLA)
runday
raw
normalized
density
log(ion counts)
3
2
1
0
3
2
1
0
PLATFORMRUNDAY_miss
0%
3%
6%
9%
10%
12%
15%
18%
21%
26%
27%
29%
32%
35%
36%
38%
39%
41%
42%
44%
47%
53%
55%
58%
59%
64%
65%
71%
73%
76%
79%

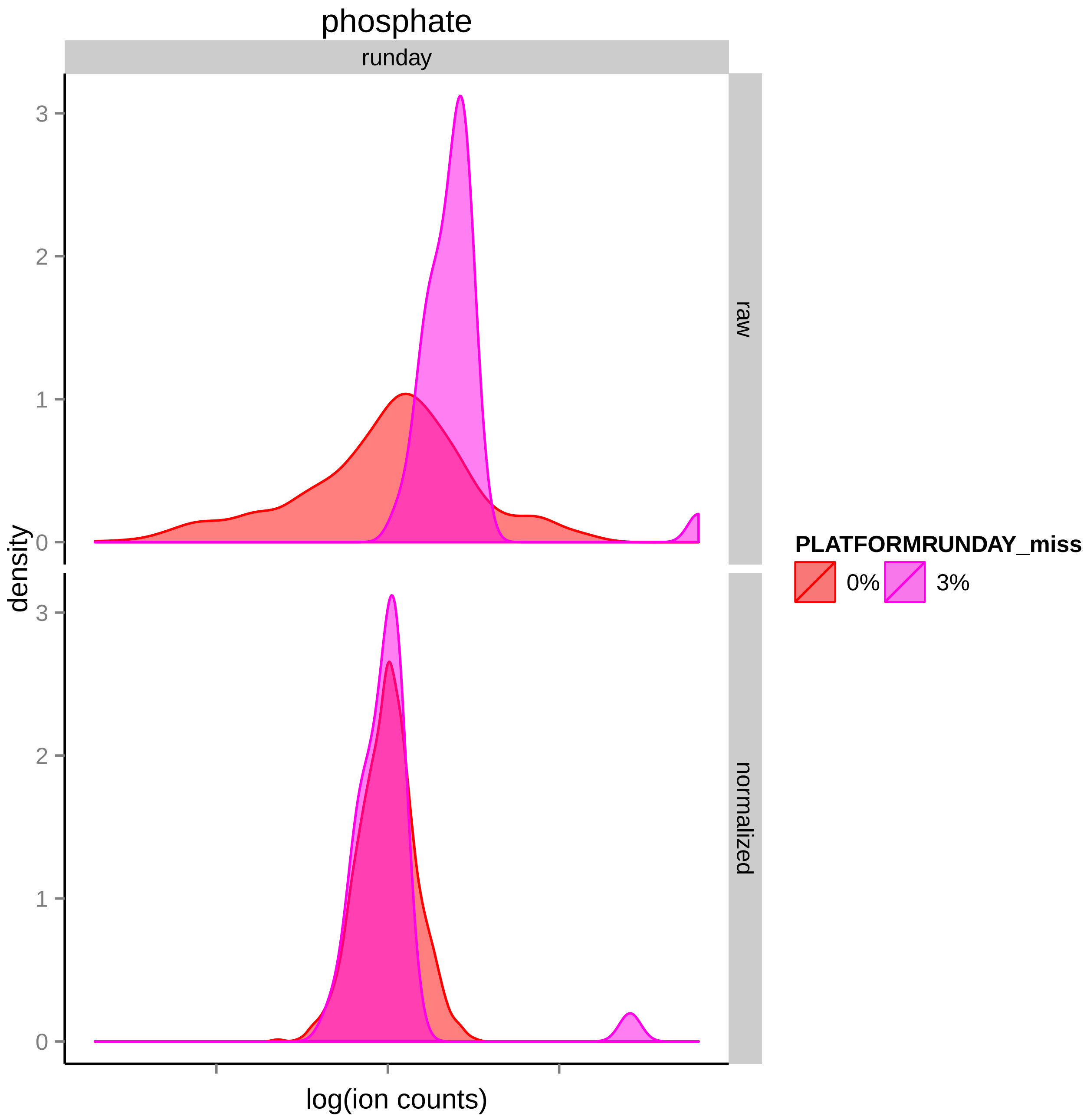
phosphate
runday
raw
normalized
density
log(ion counts)
0
1
2
3
0
1
2
3
PLATFORMRUNDAY_miss
0%
3%

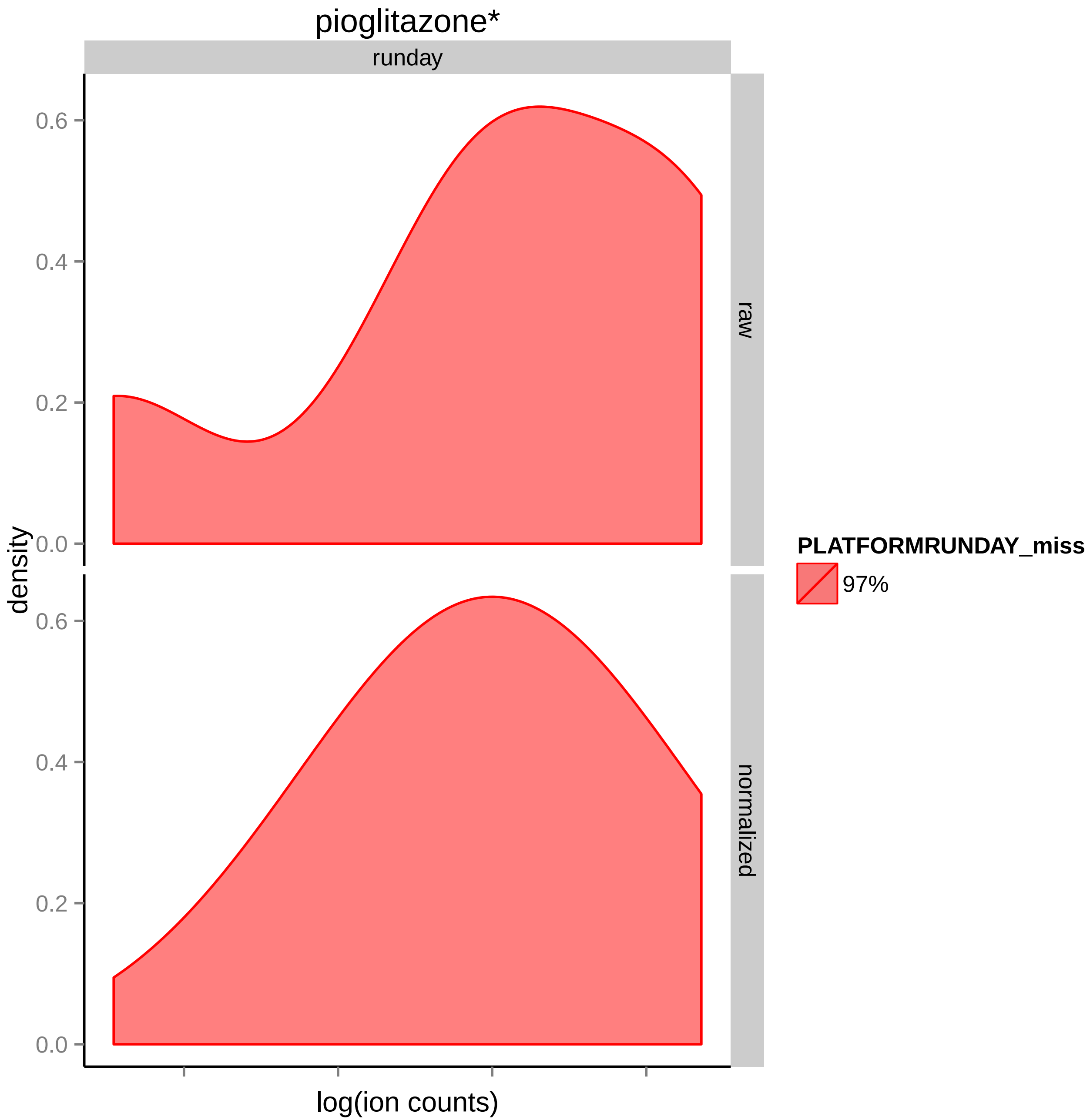
pioglitazone*
runday
raw
normalized
density
log(ion counts)
0.0
0.2
0.4
0.6
PLATFORMRUNDAY_miss
97%

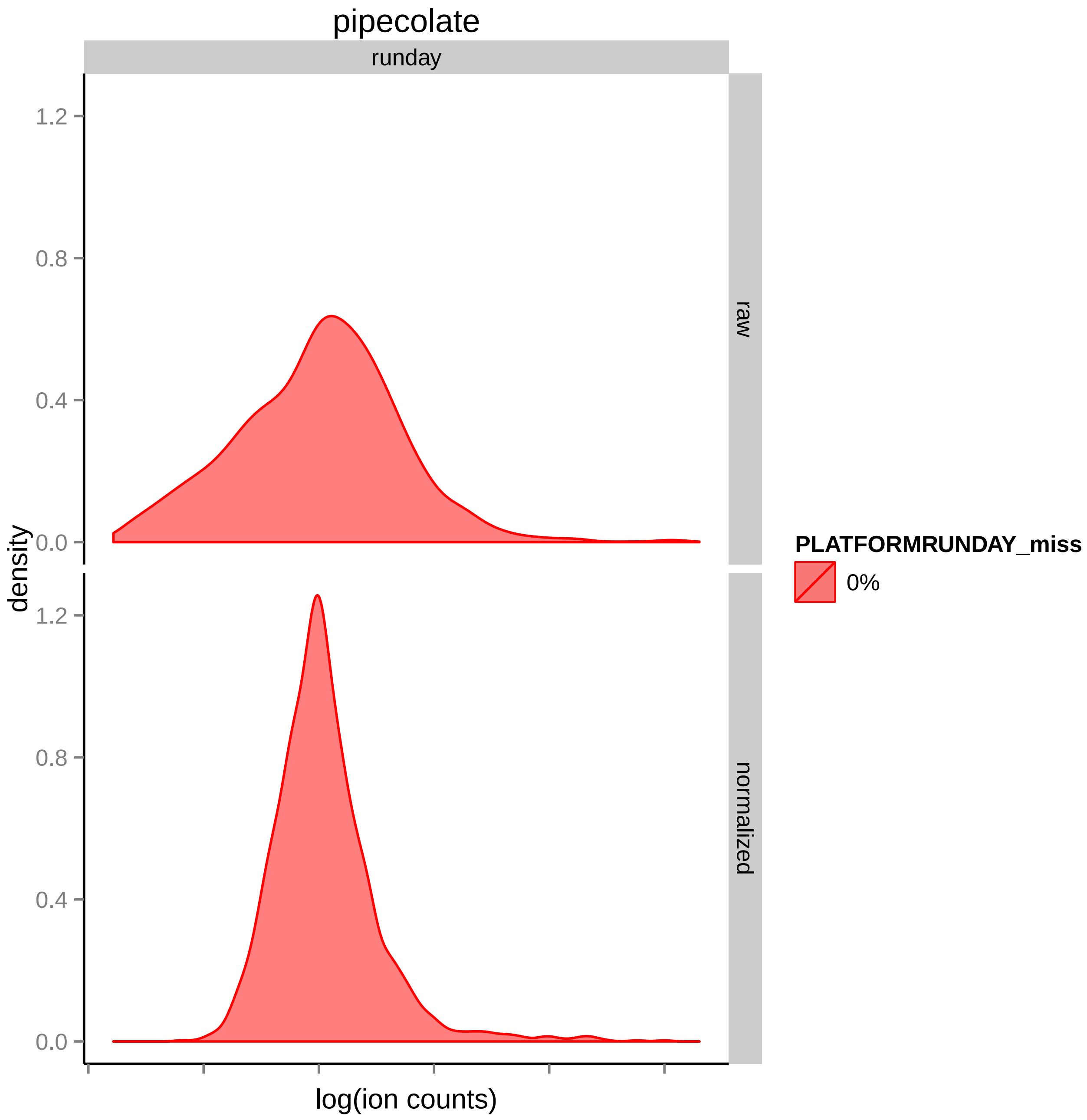

pipecolate
runday
raw
normalized
density
1.2
0.8
0.4
0.0
log(ion counts)
PLATFORMRUNDAY_miss
0%

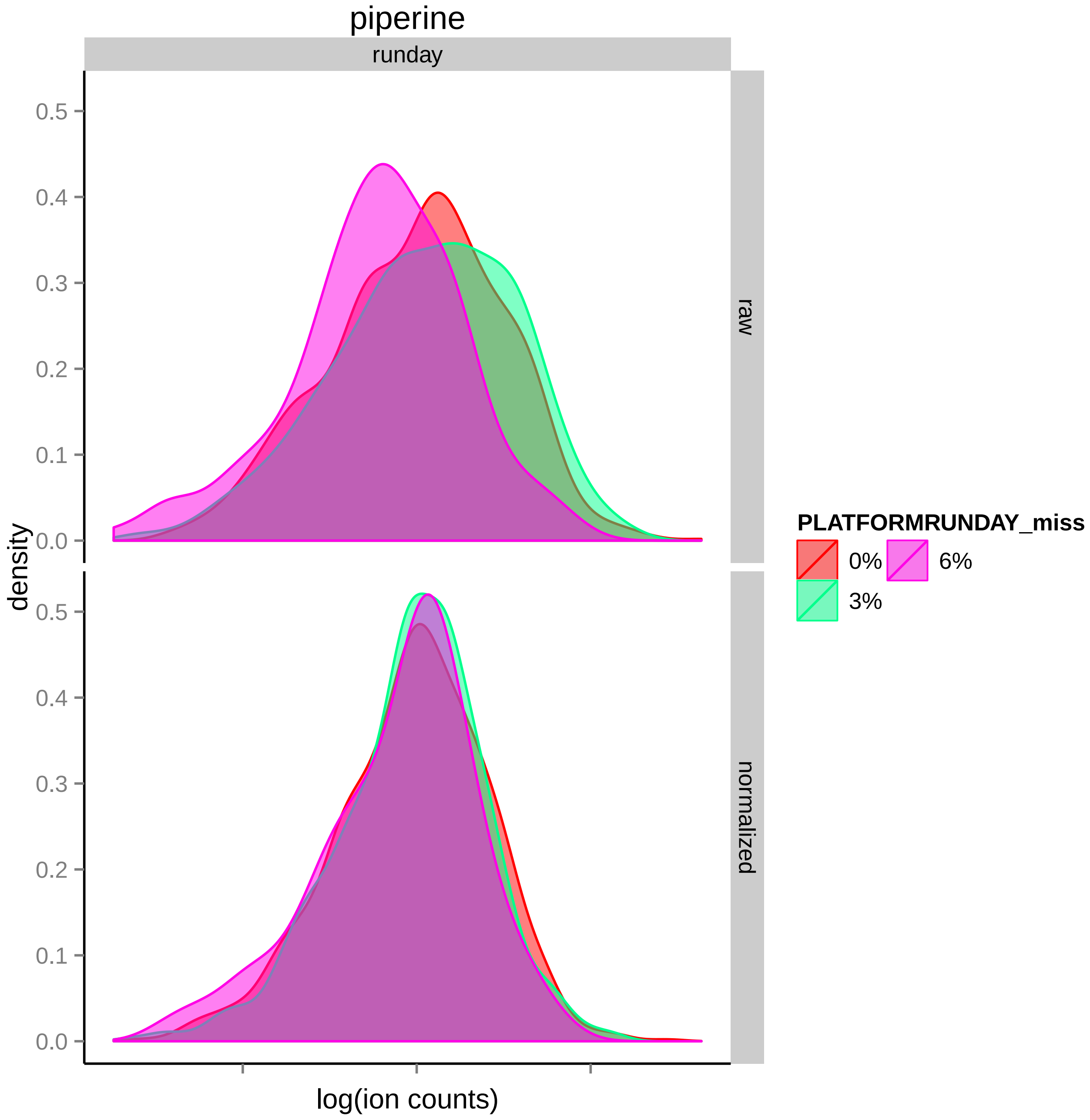

piperine
runday
raw
normalized
density
0.0
0.1
0.2
0.3
0.4
0.5
log(ion counts)
PLATFORMRUNDAY_miss
0%
3%
6%

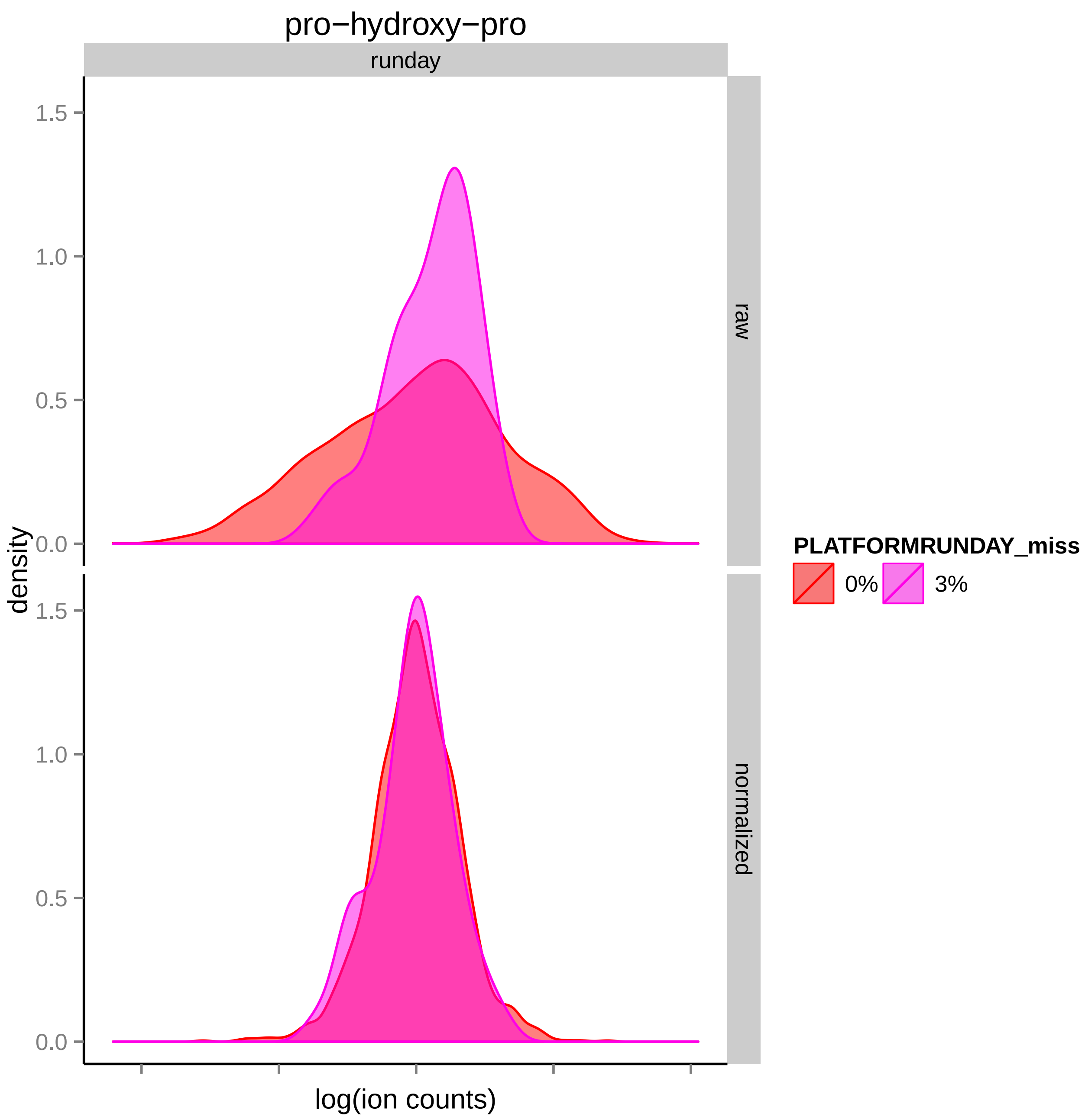

pro−hydroxy−pro
runday
raw
normalized
density
1.5
1.0
0.5
0.0
log(ion counts)
PLATFORMRUNDAY_miss
0%
3%

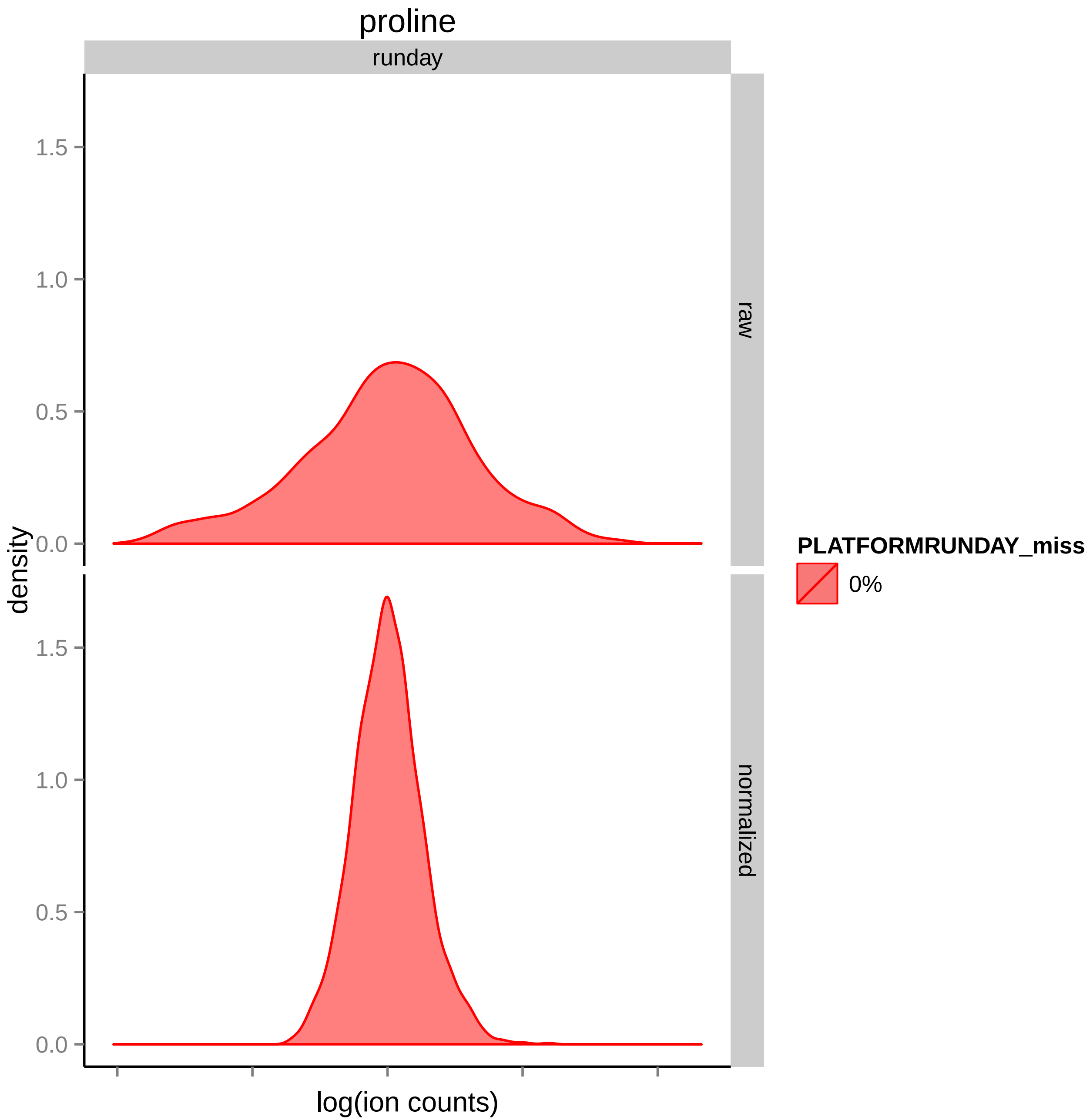

proline
runday
raw
normalized
density
1.5
1.0
0.5
0.0
log(ion counts)
PLATFORMRUNDAY_miss
0%

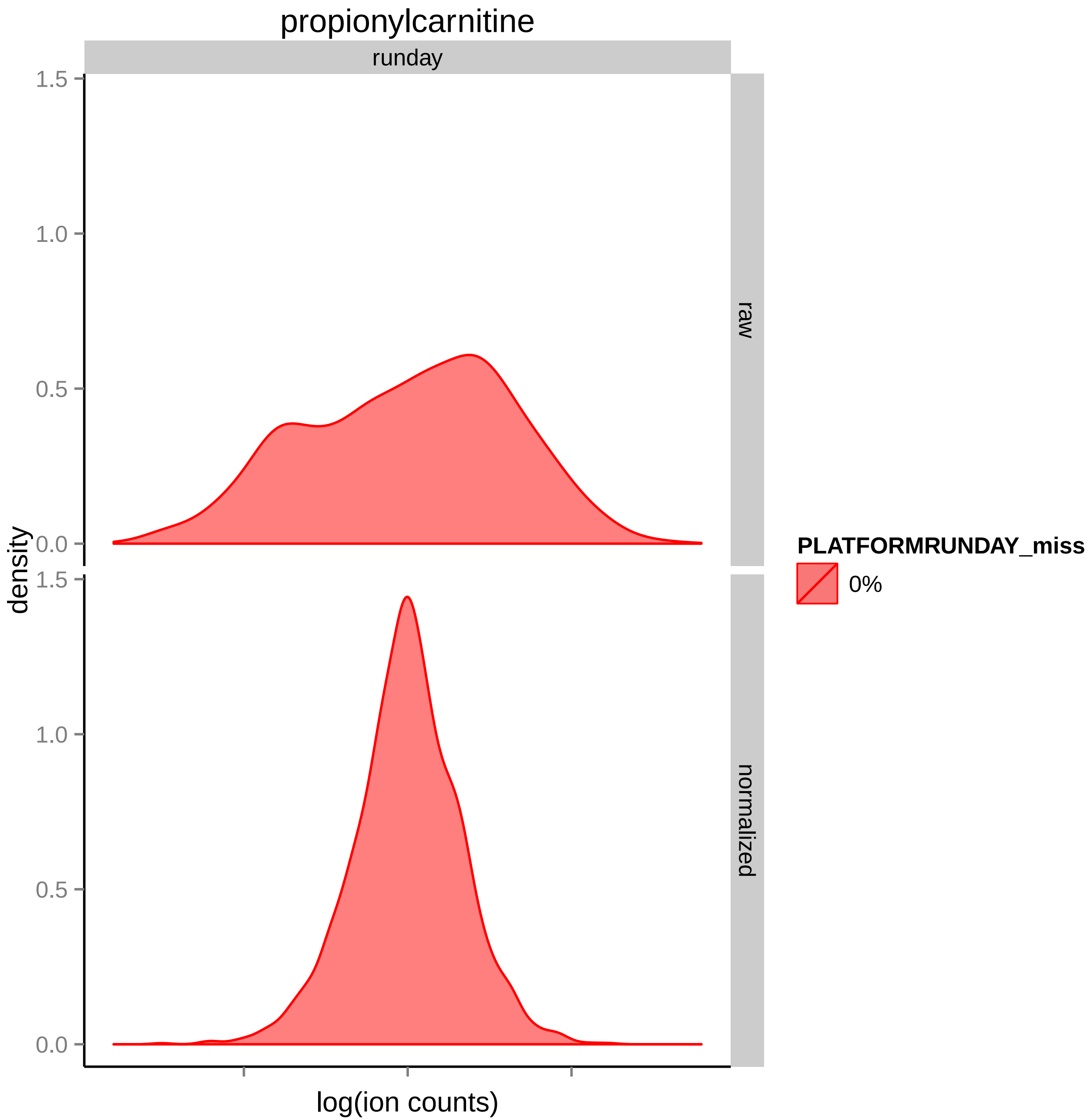
propionylcarnitine
runday
raw
normalized
density
log(ion counts)
0.0
0.5
1.0
1.5
PLATFORMRUNDAY_miss
0%

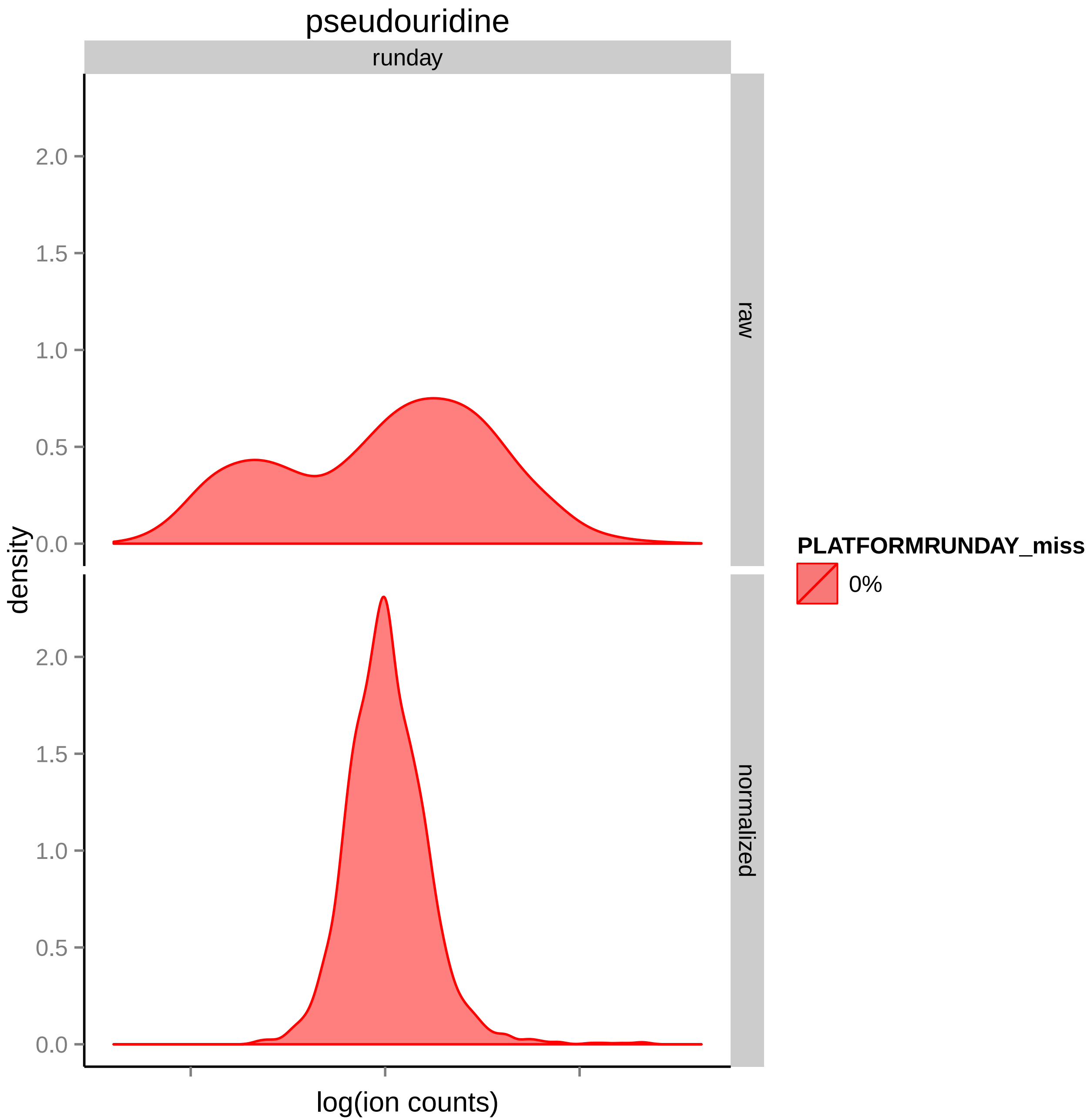
pseudouridine
runday
raw
normalized
density
log(ion counts)
2.0
1.5
1.0
0.5
0.0
2.0
1.5
1.0
0.5
0.0
PLATFORMRUNDAY_miss
0%

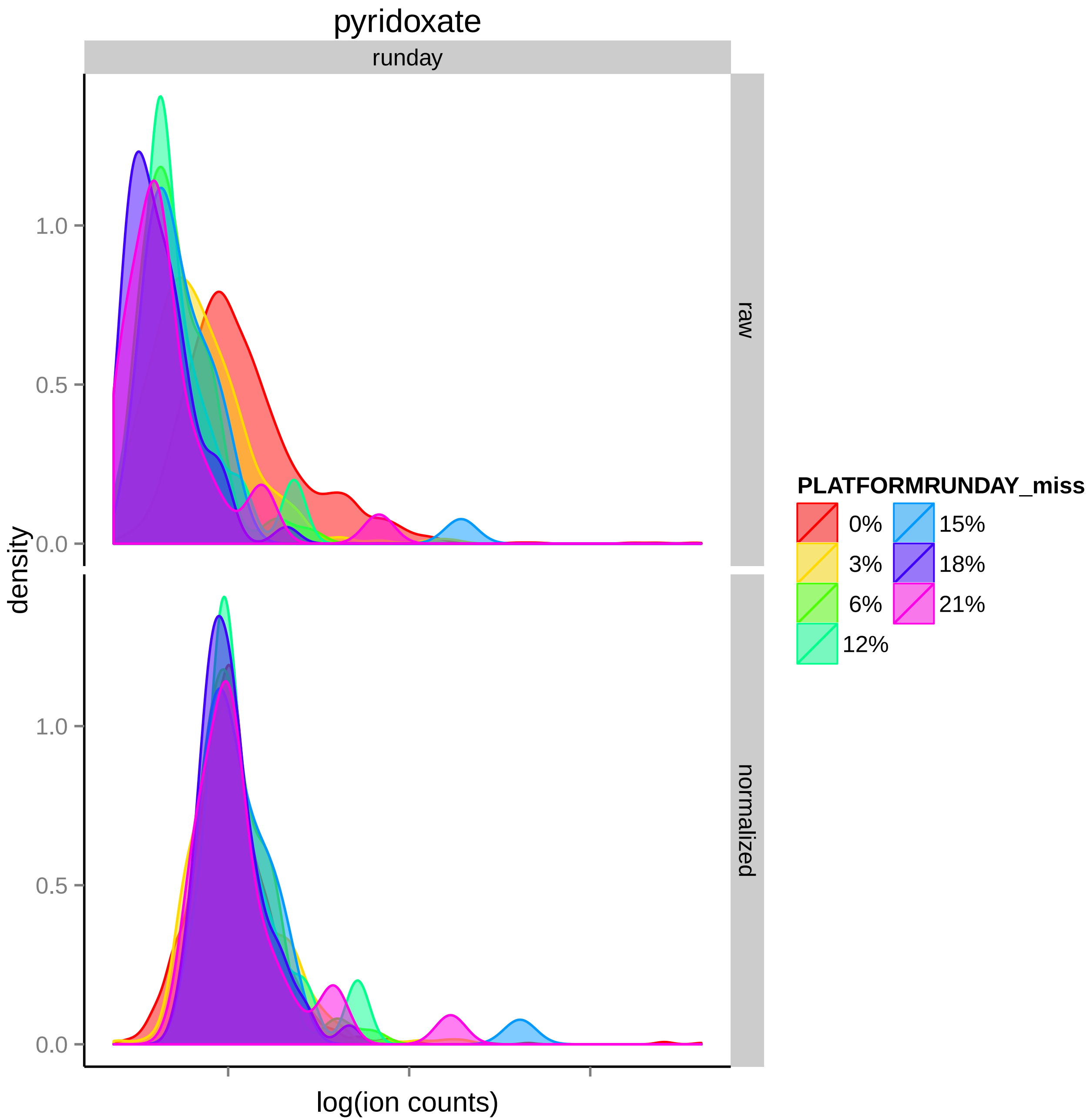

pyridoxate
runday
raw
normalized
density
log(ion counts)
1.0
0.5
0.0
1.0
0.5
0.0
PLATFORMRUNDAY_miss
0%
3%
6%
12%
15%
18%
21%

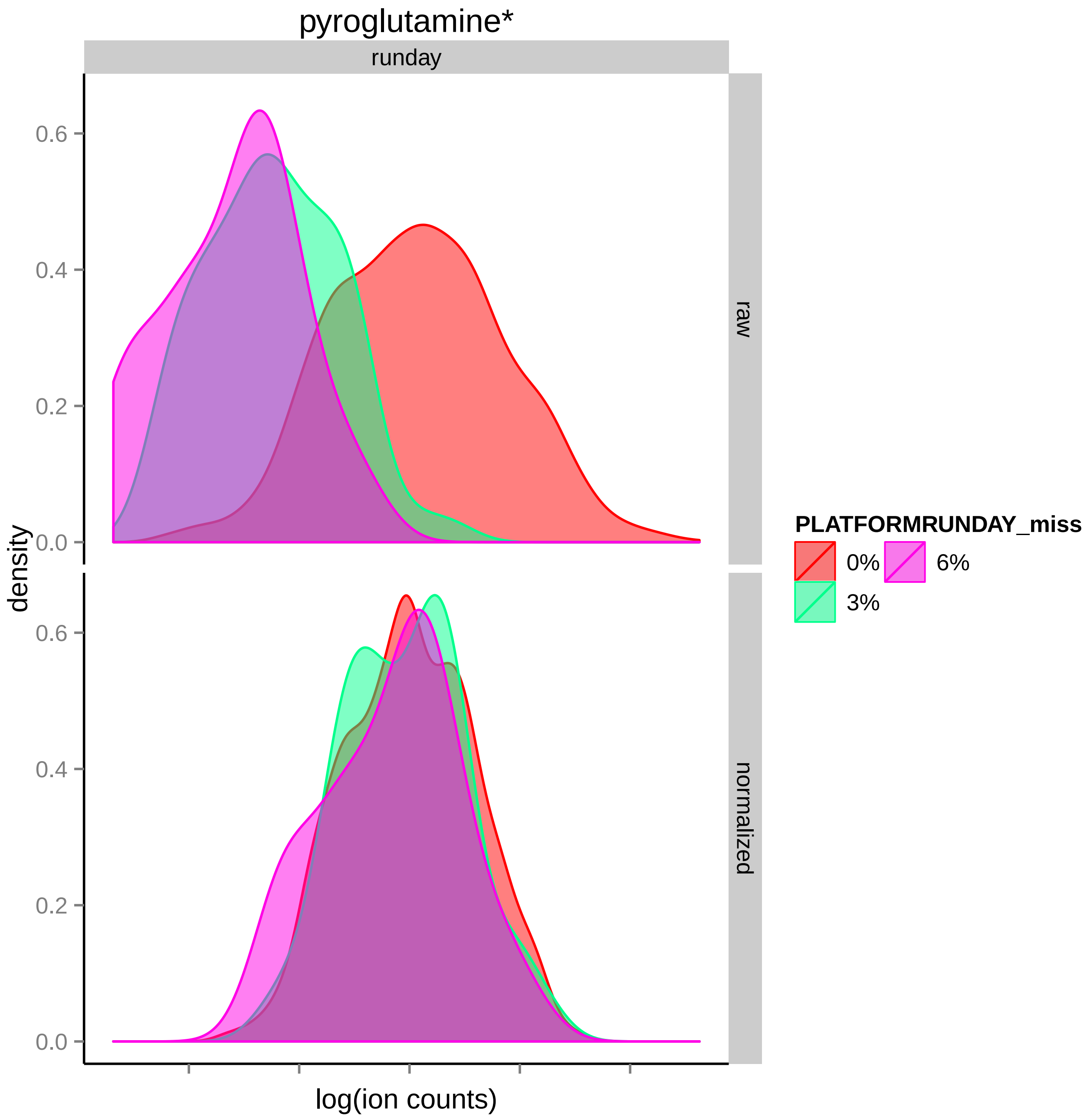

pyroglutamine*
runday
raw
normalized
density
0.0
0.2
0.4
0.6
log(ion counts)
PLATFORMRUNDAY_miss
0%
3%
6%

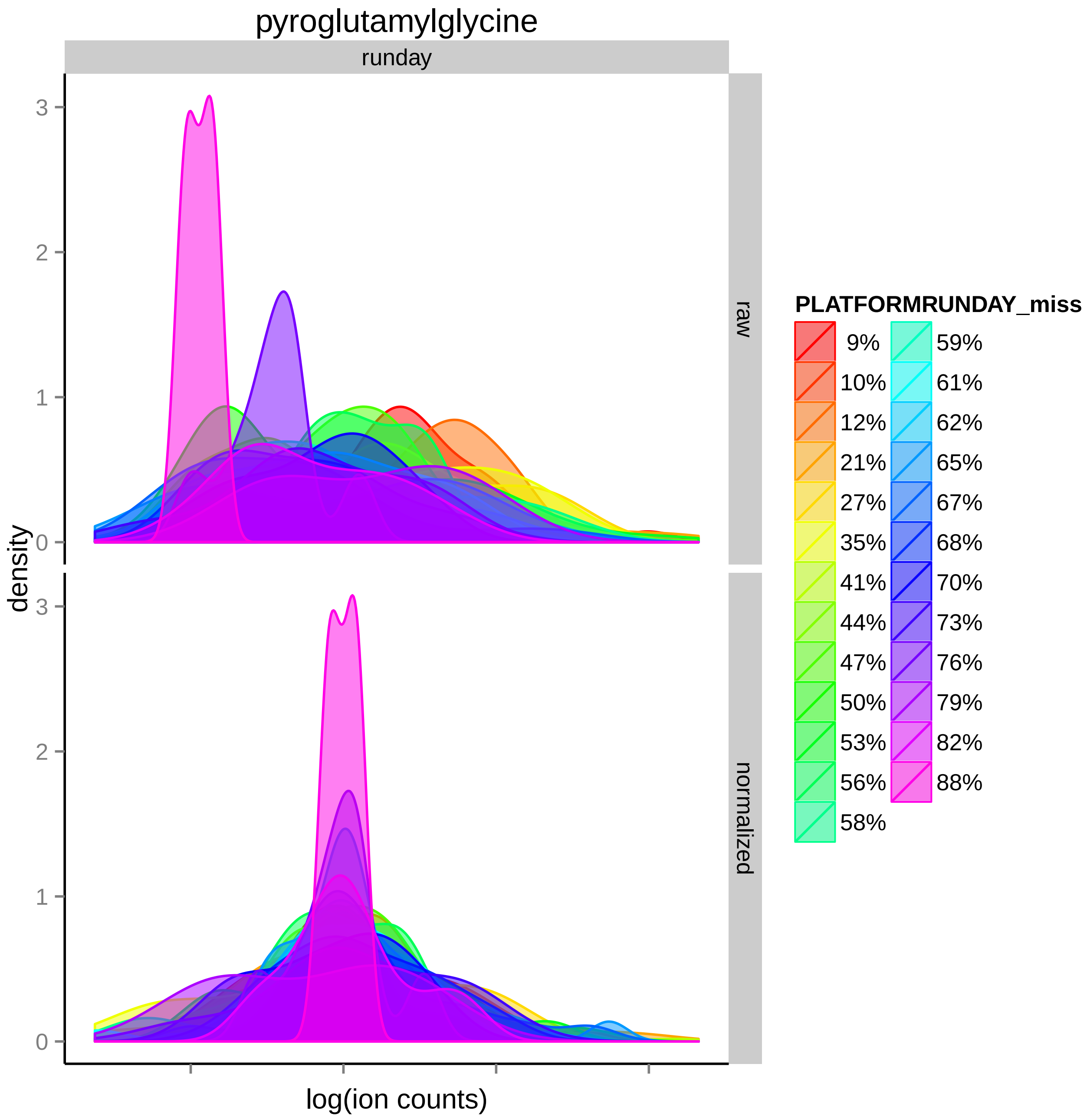

pyroglutamylglycine
runday
raw
normalized
density
log(ion counts)
0
1
2
3
PLATFORMRUNDAY_miss
9%
10%
12%
21%
27%
35%
41%
44%
47%
50%
53%
56%
58%
59%
61%
62%
65%
67%
68%
70%
73%
76%
79%
82%
88%

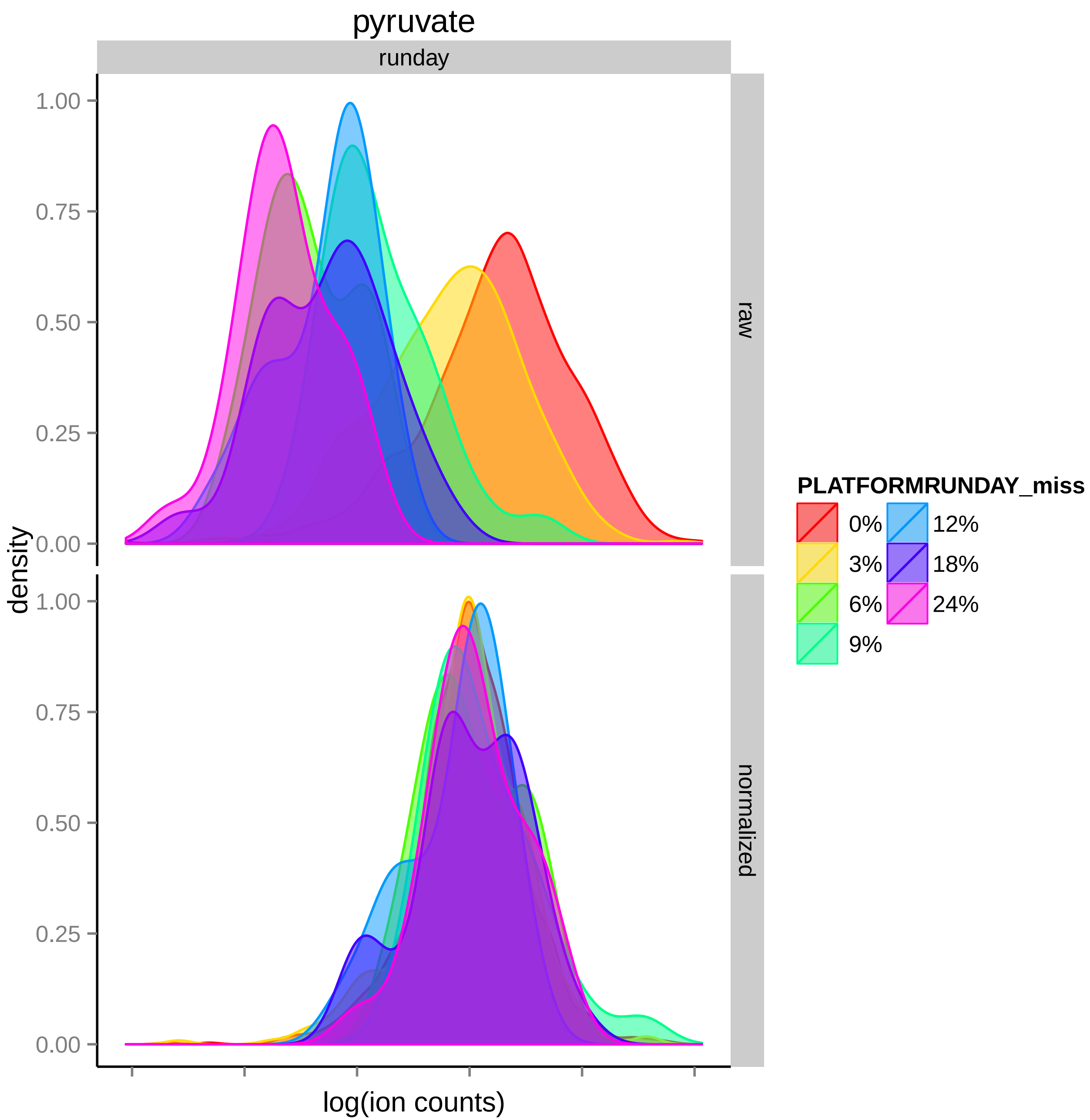
pyruvate
runday
raw
normalized
density
log(ion counts)
1.00
0.75
0.50
0.25
0.00
PLATFORMRUNDAY_miss
0%
3%
6%
9%
12%
18%
24%

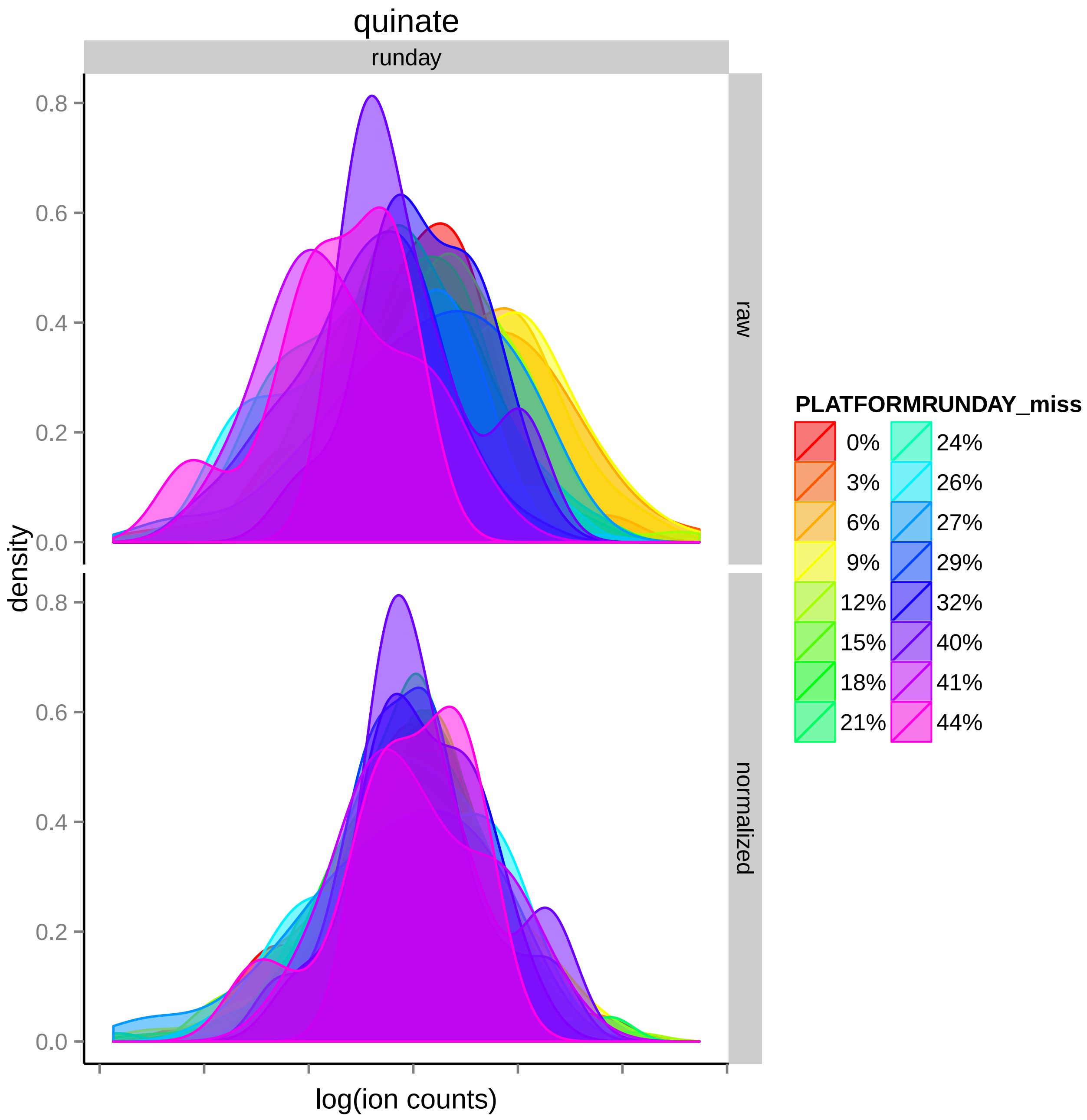
quinate
runday
raw
normalized
density
log(ion counts)
0.0
0.2
0.4
0.6
0.8
PLATFORMRUNDAY_miss
0%
3%
6%
9%
12%
15%
18%
21%
24%
26%
27%
29%
32%
40%
41%
44%

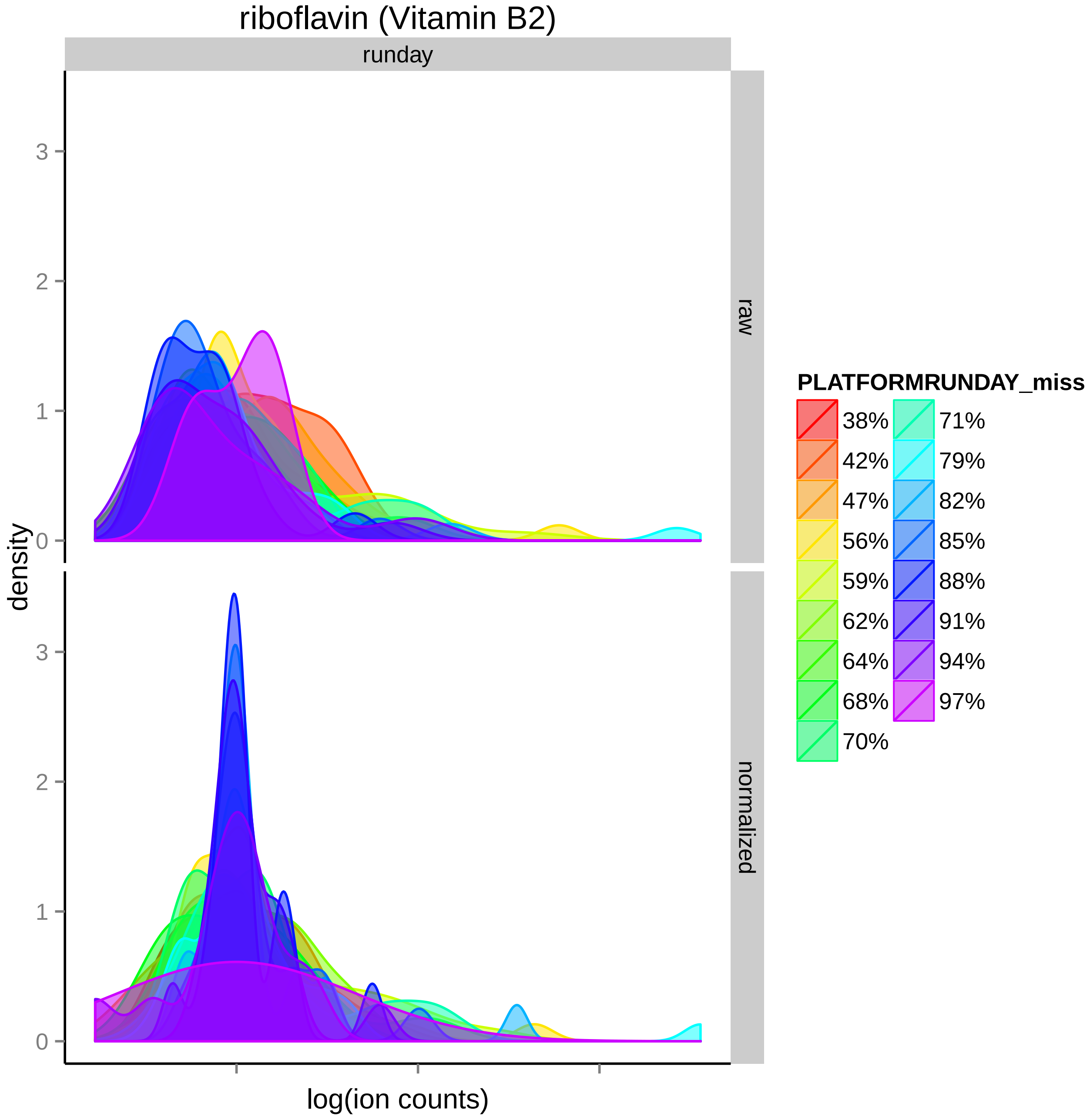

riboflavin (Vitamin B2)
runday
raw
normalized
density
log(ion counts)
0
1
2
3
PLATFORMRUNDAY_miss
38%
42%
47%
56%
59%
62%
64%
68%
70%
71%
79%
82%
85%
88%
91%
94%
97%

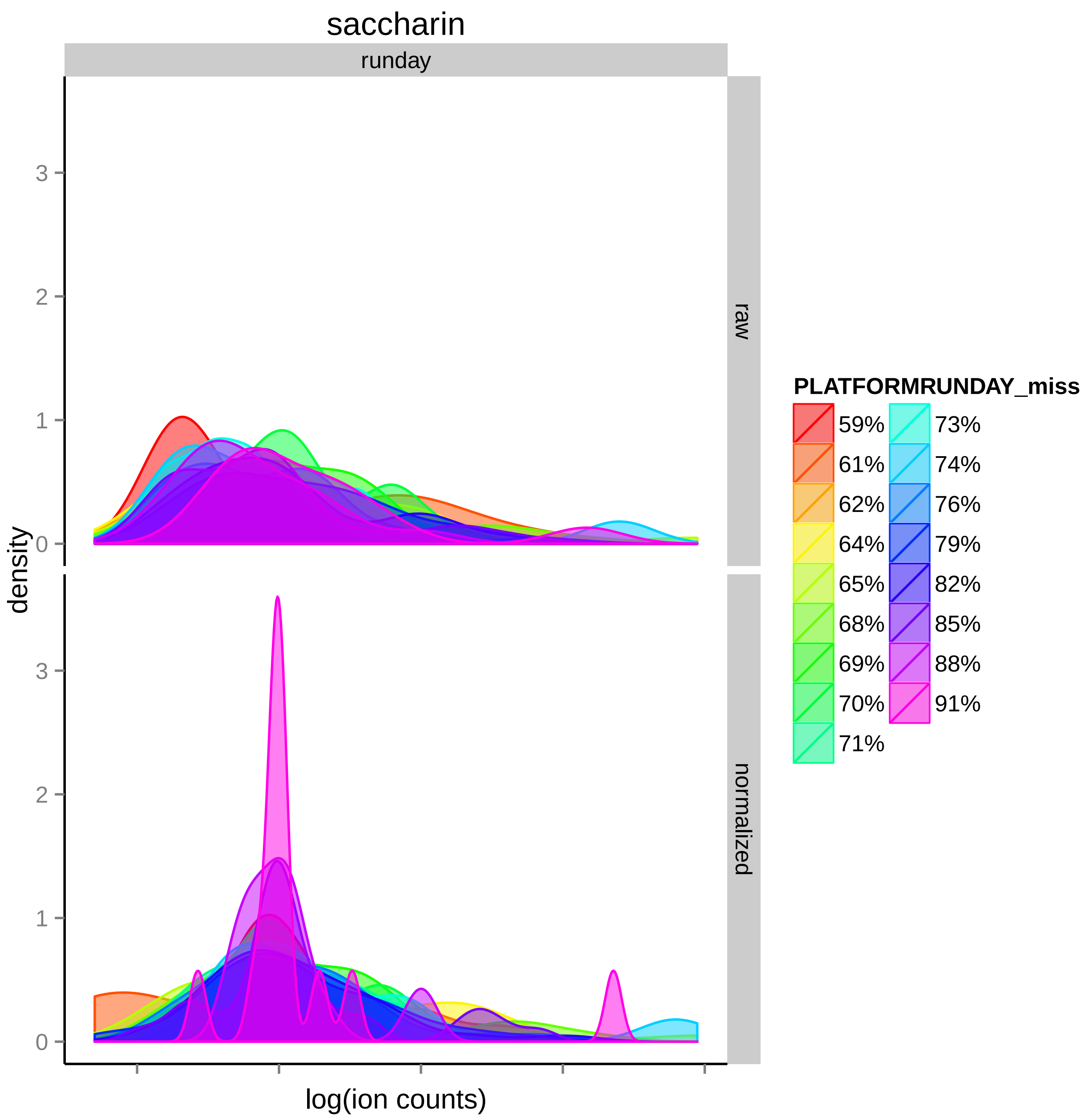
saccharin
runday
raw
normalized
density
log(ion counts)
0
1
2
3
PLATFORMRUNDAY_miss
59%
61%
62%
64%
65%
68%
69%
70%
71%
73%
74%
76%
79%
82%
85%
88%
91%

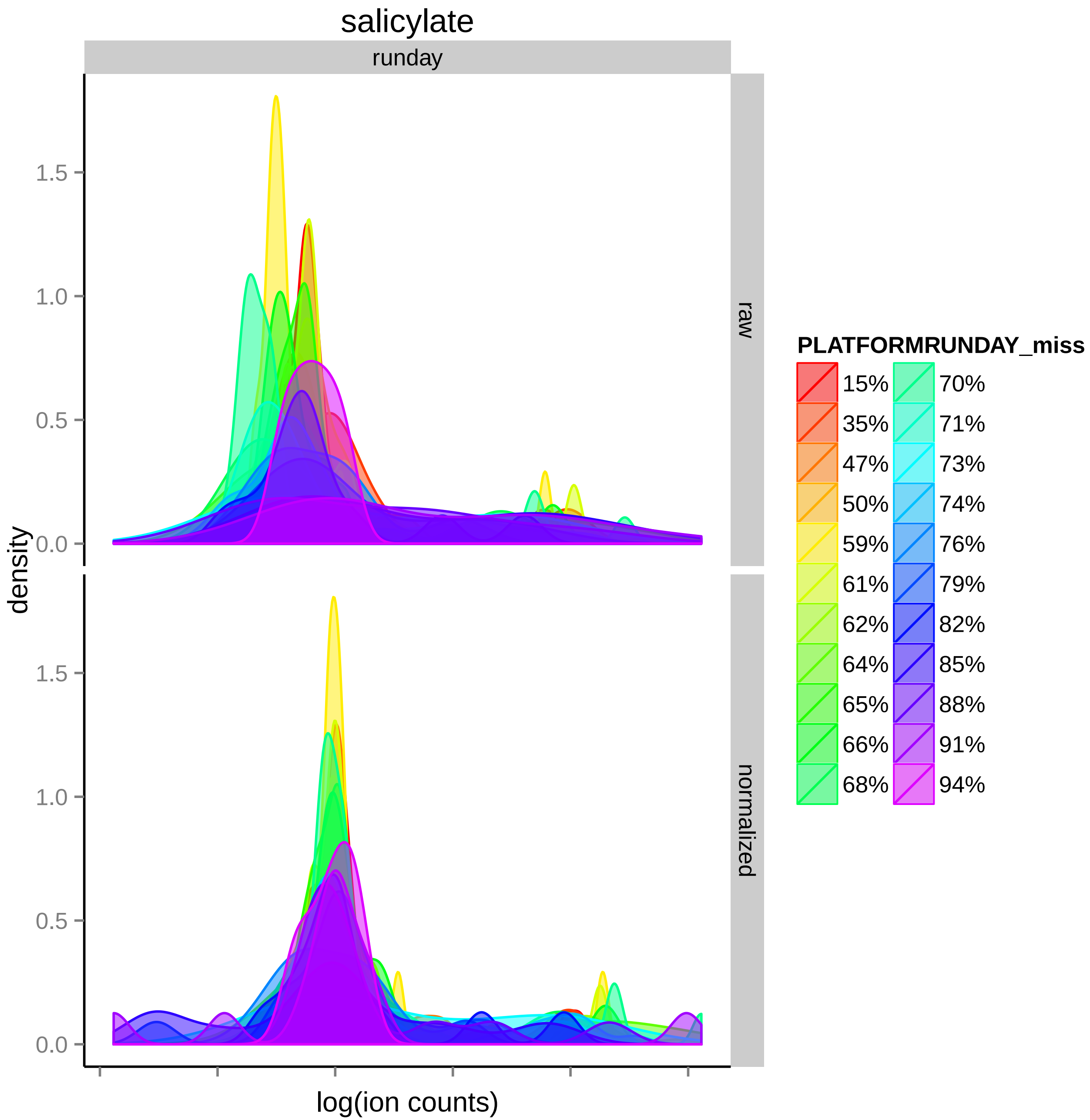

salicylate
runday
raw
normalized
density
log(ion counts)
0.0
0.5
1.0
1.5
PLATFORMRUNDAY_miss
15%
35%
47%
50%
59%
61%
62%
64%
65%
66%
68%
70%
71%
73%
74%
76%
79%
82%
85%
88%
91%
94%

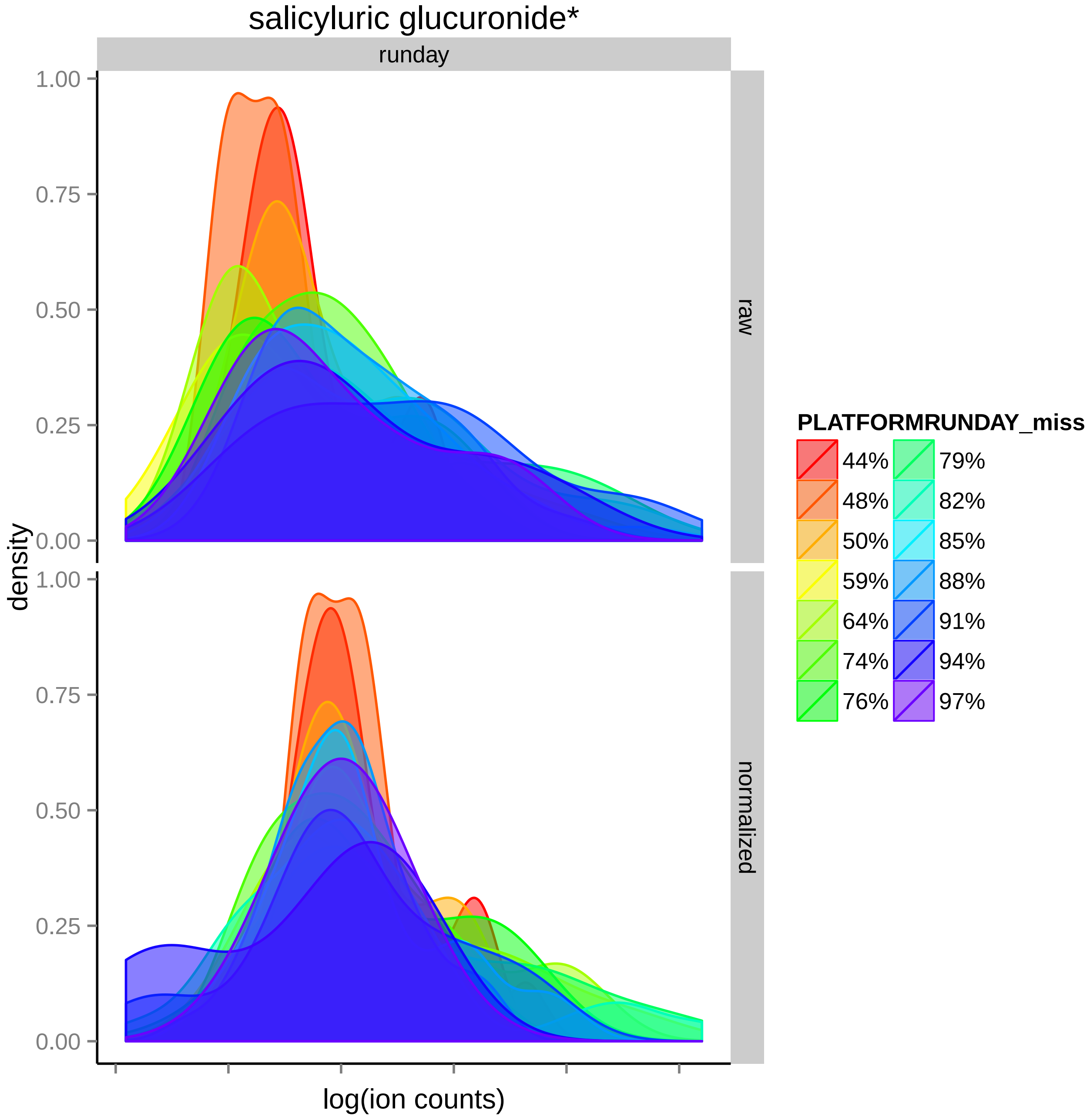
salicyluric glucuronide*
runday
raw
normalized
density
log(ion counts)
1.00
0.75
0.50
0.25
0.00
PLATFORMRUNDAY_miss
44%
48%
50%
59%
64%
74%
76%
79%
82%
85%
88%
91%
94%
97%

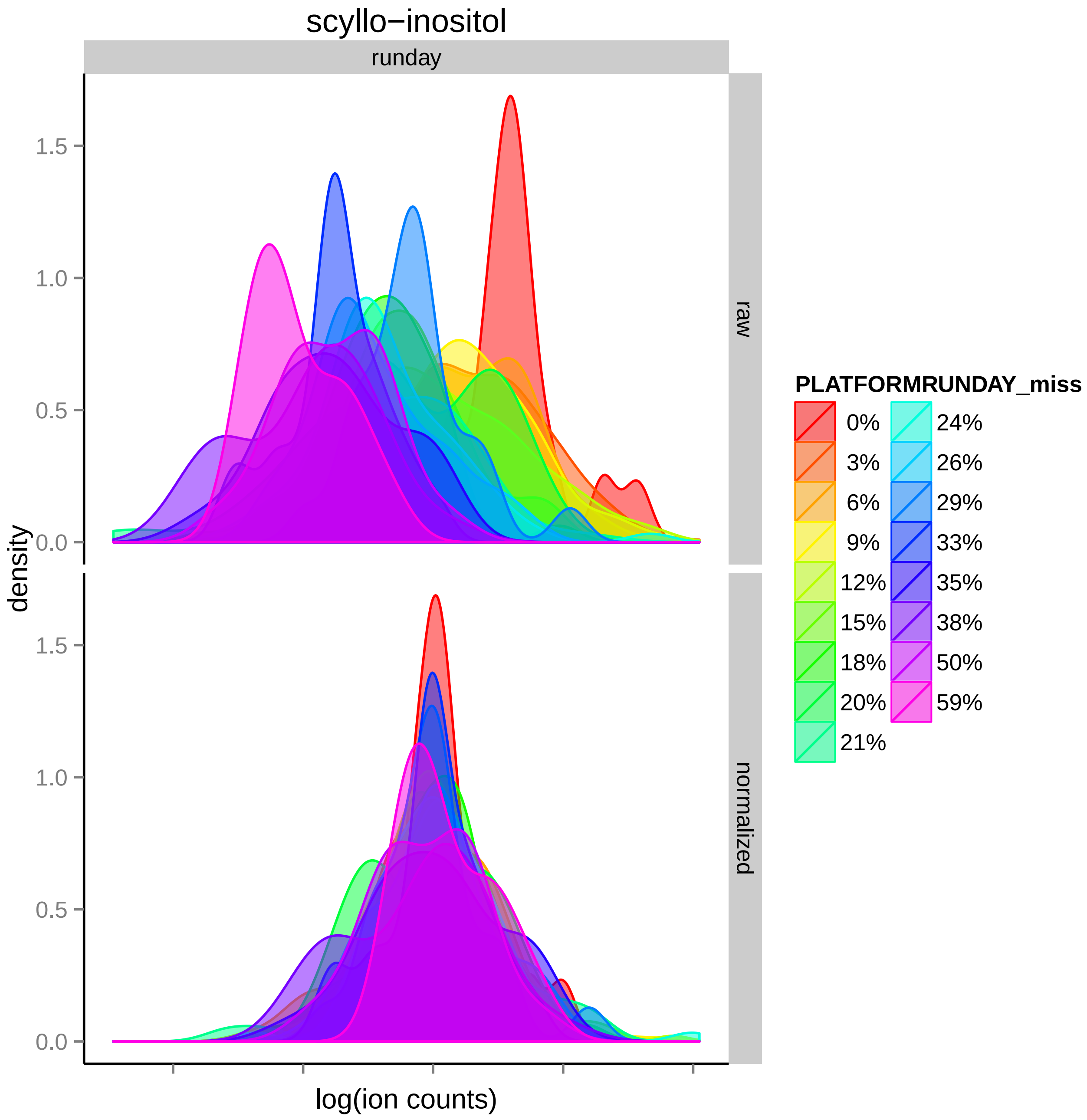
scyllo−inositol
runday
raw
normalized
density
log(ion counts)
0.0
0.5
1.0
1.5
PLATFORMRUNDAY_miss
0%
3%
6%
9%
12%
15%
18%
20%
21%
24%
26%
29%
33%
35%
38%
50%
59%

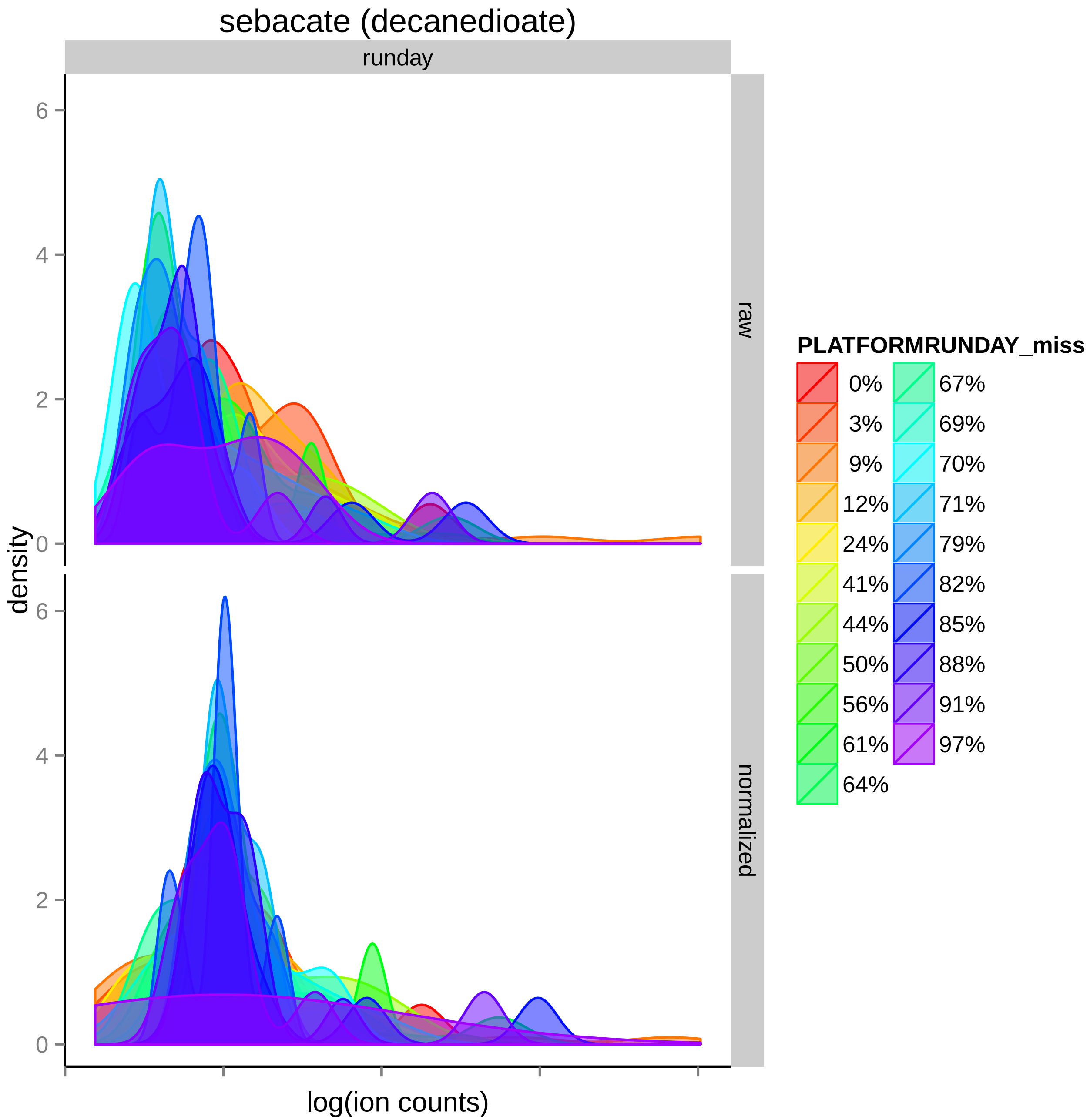

sebacate (decanedioate)
runday
raw
normalized
density
log(ion counts)
0
2
4
6
PLATFORMRUNDAY_miss
0%
3%
9%
12%
24%
41%
44%
50%
56%
61%
64%
67%
69%
70%
71%
79%
82%
85%
88%
91%
97%

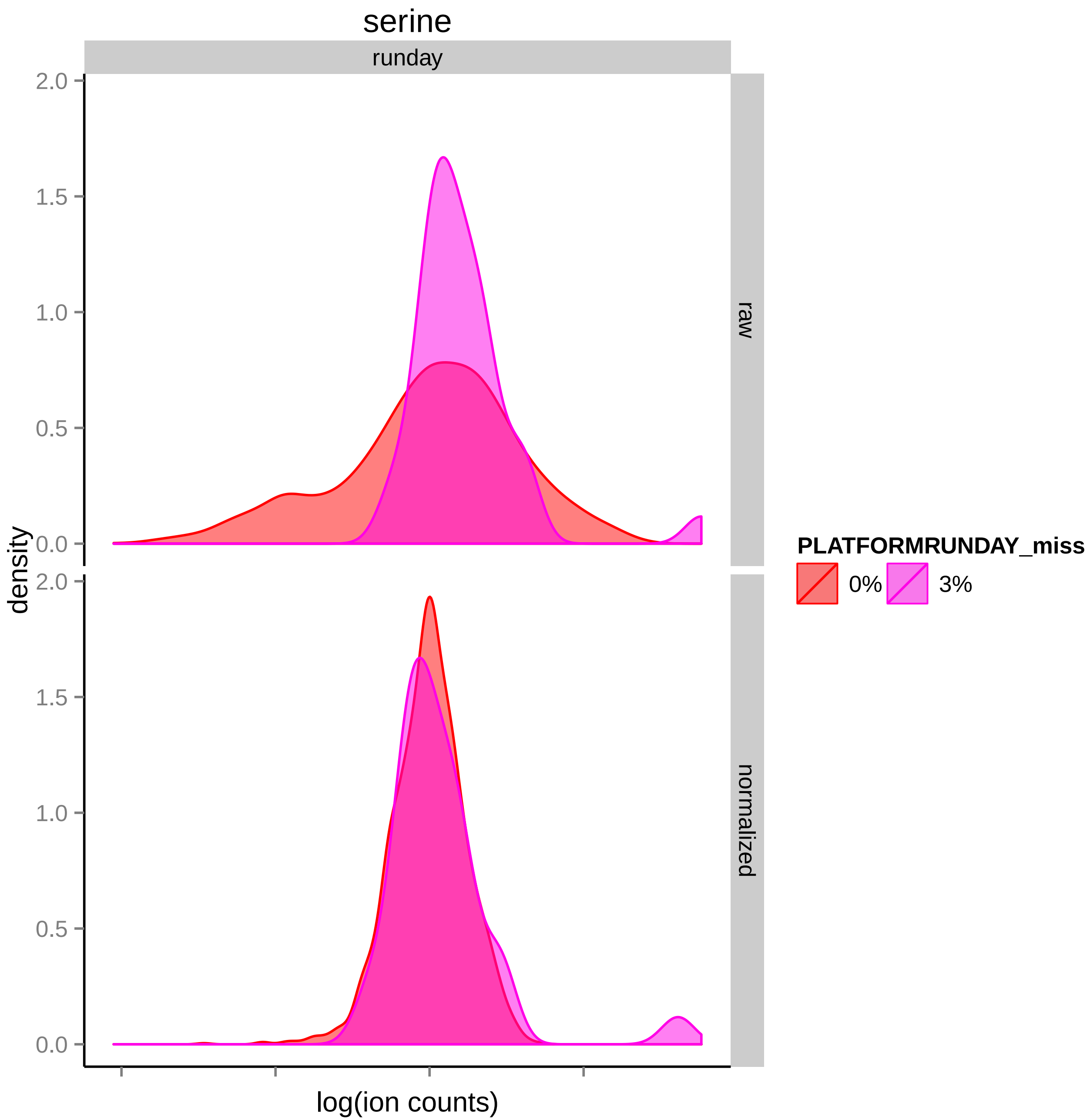
serine
runday
raw
normalized
density
log(ion counts)
2.0
1.5
1.0
0.5
0.0
PLATFORMRUNDAY_miss
0%
3%

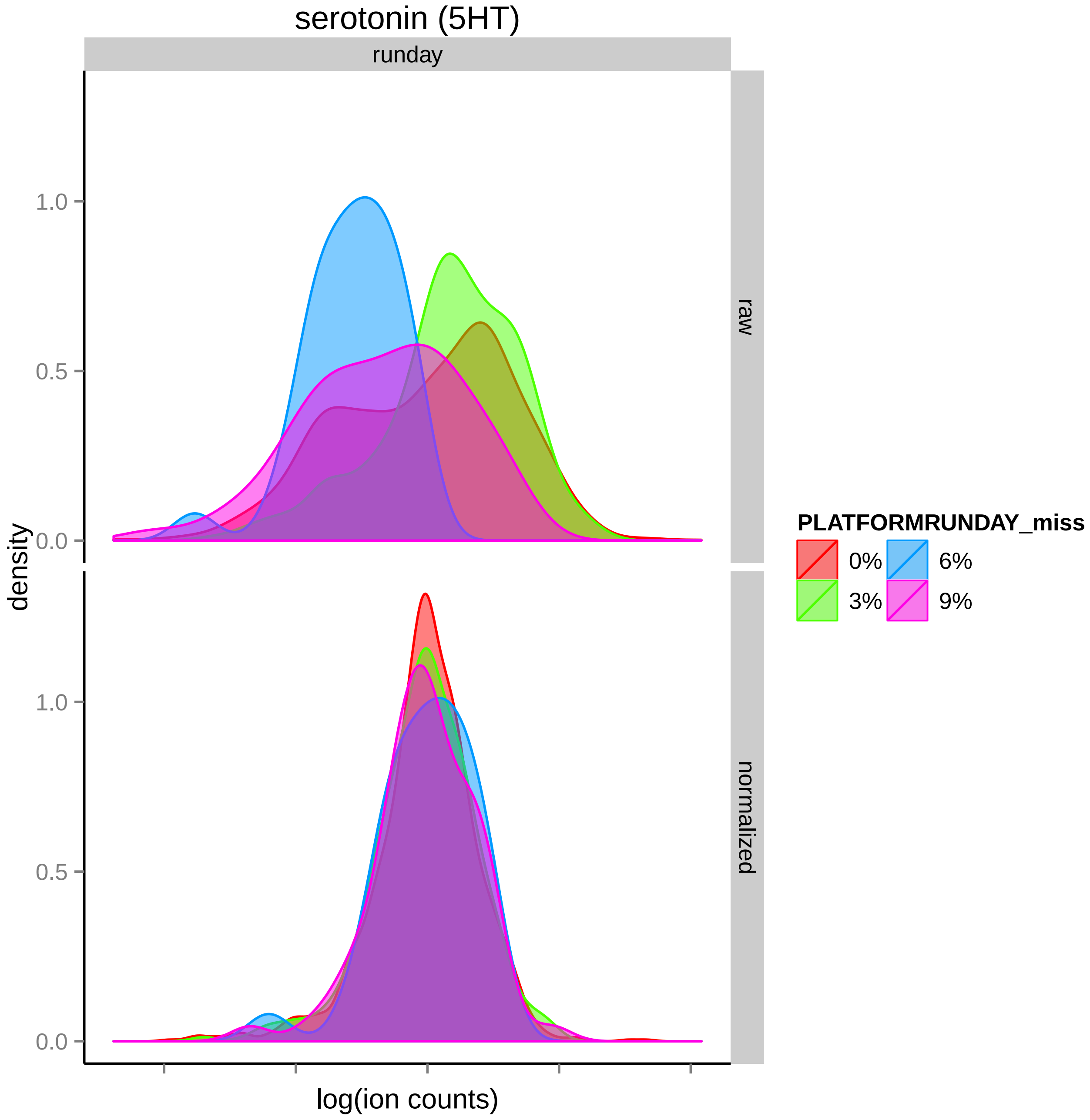
serotonin (5HT)
runday
raw
normalized
density
1.0
0.5
0.0
1.0
0.5
0.0
log(ion counts)
PLATFORMRUNDAY_miss
0%
6%
3%
9%

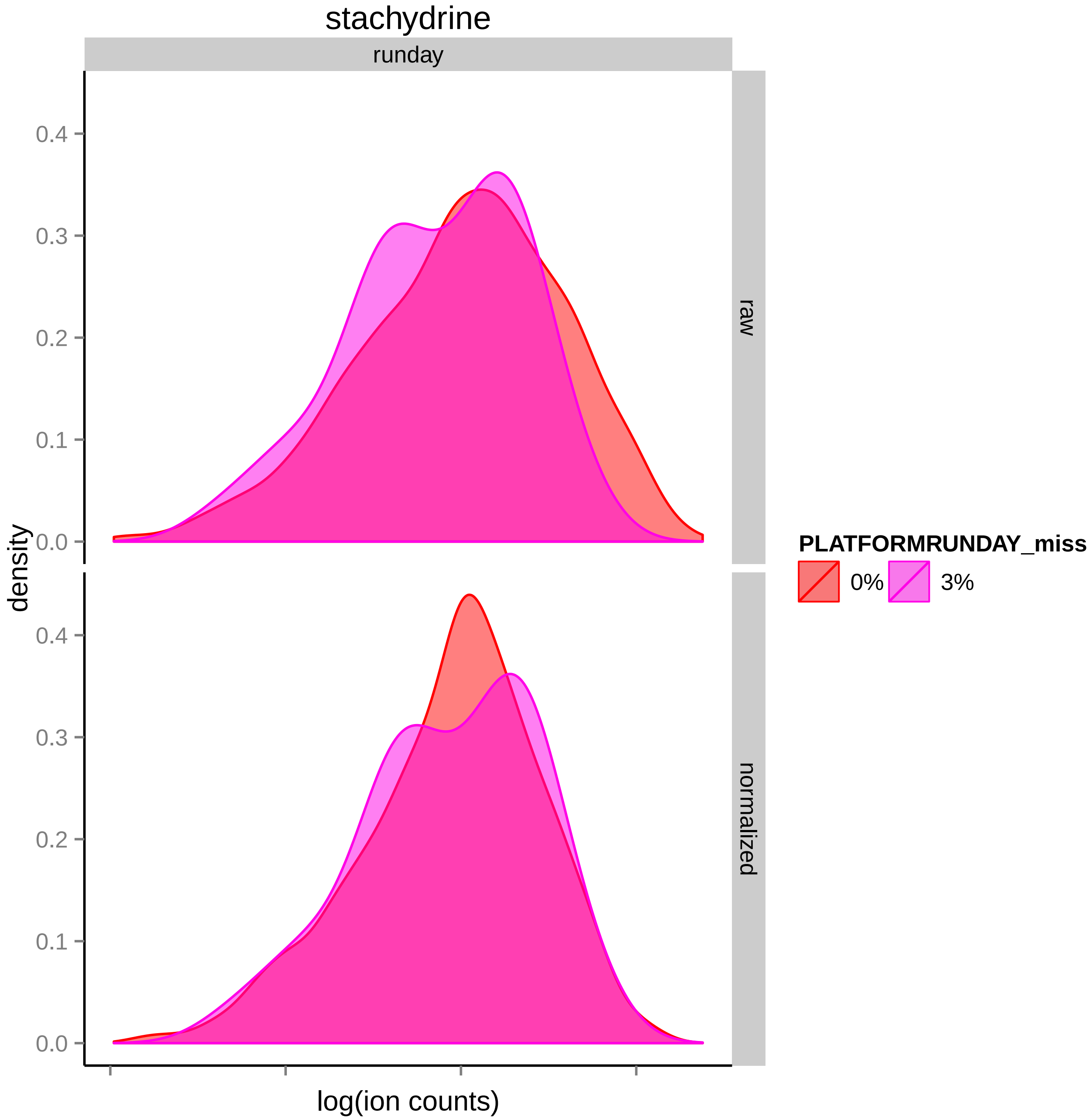
stachydrine
runday
raw
normalized
density
log(ion counts)
0.0
0.1
0.2
0.3
0.4
PLATFORMRUNDAY_miss
0%
3%

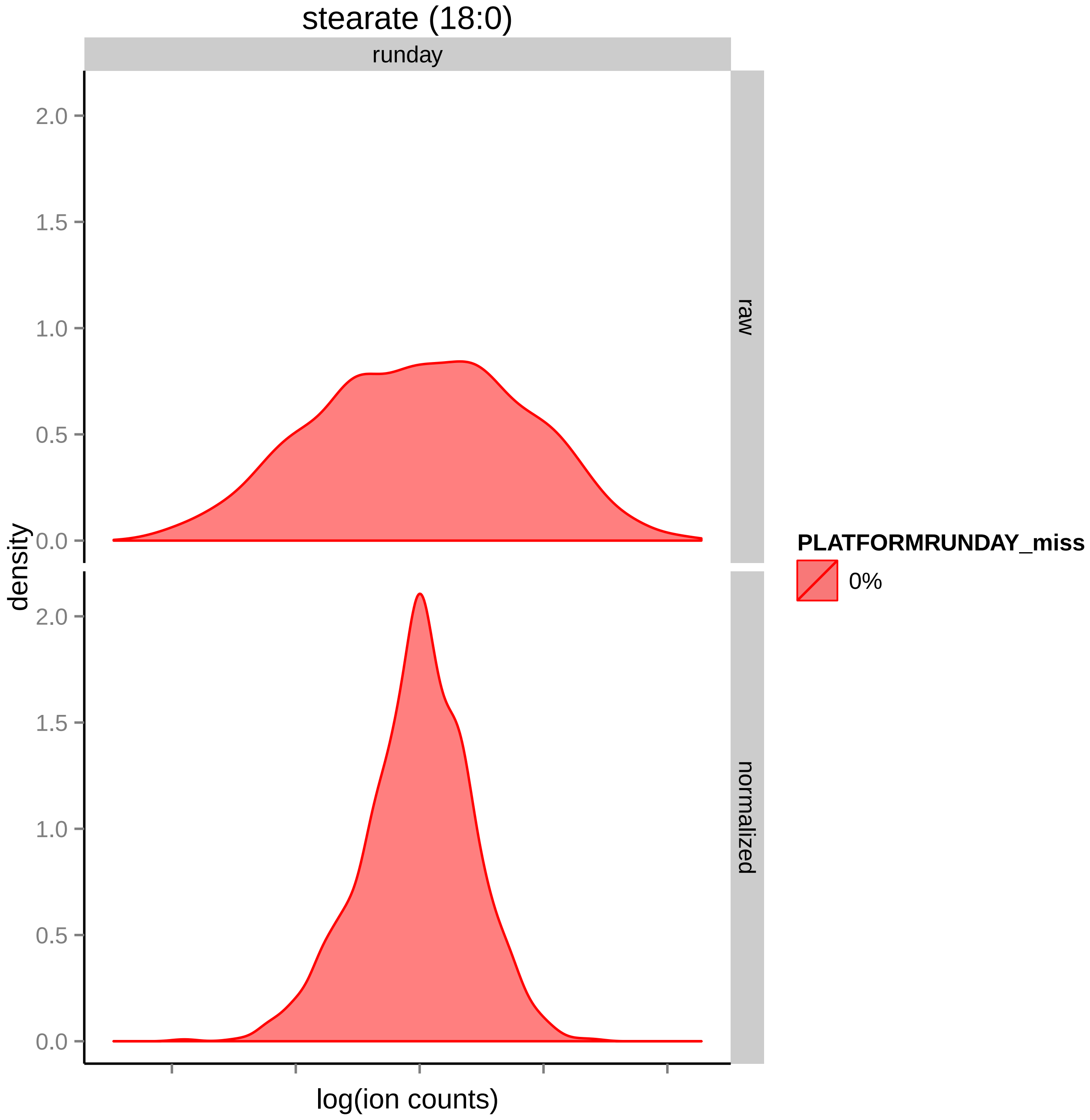

stearate (18:0)
runday
raw
normalized
density
2.0
1.5
1.0
0.5
0.0
log(ion counts)
PLATFORMRUNDAY_miss
0%

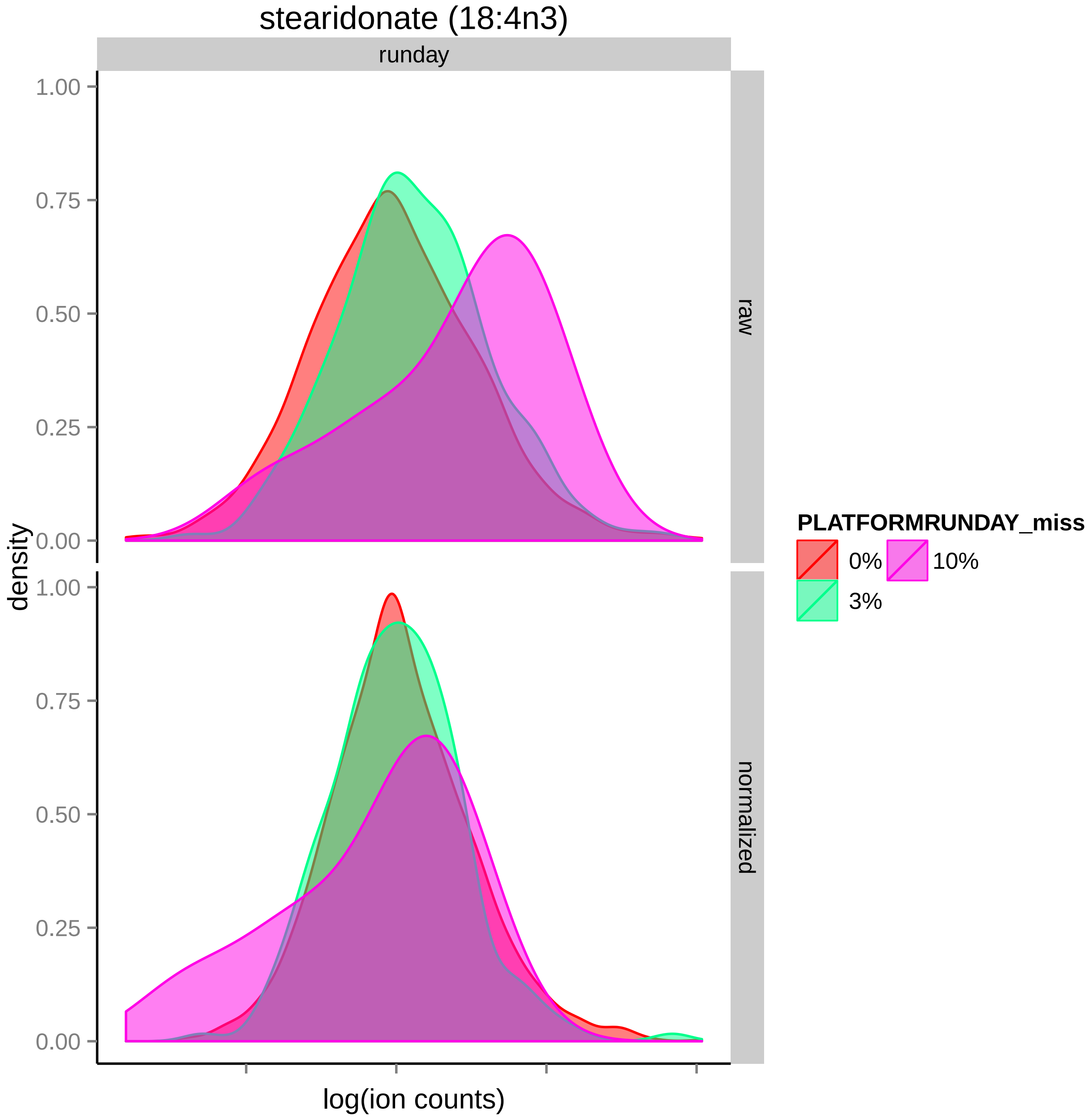
stearidonate (18:4n3)
runday
raw
normalized
density
log(ion counts)
1.00
0.75
0.50
0.25
0.00
PLATFORMRUNDAY_miss
0%
3%
10%

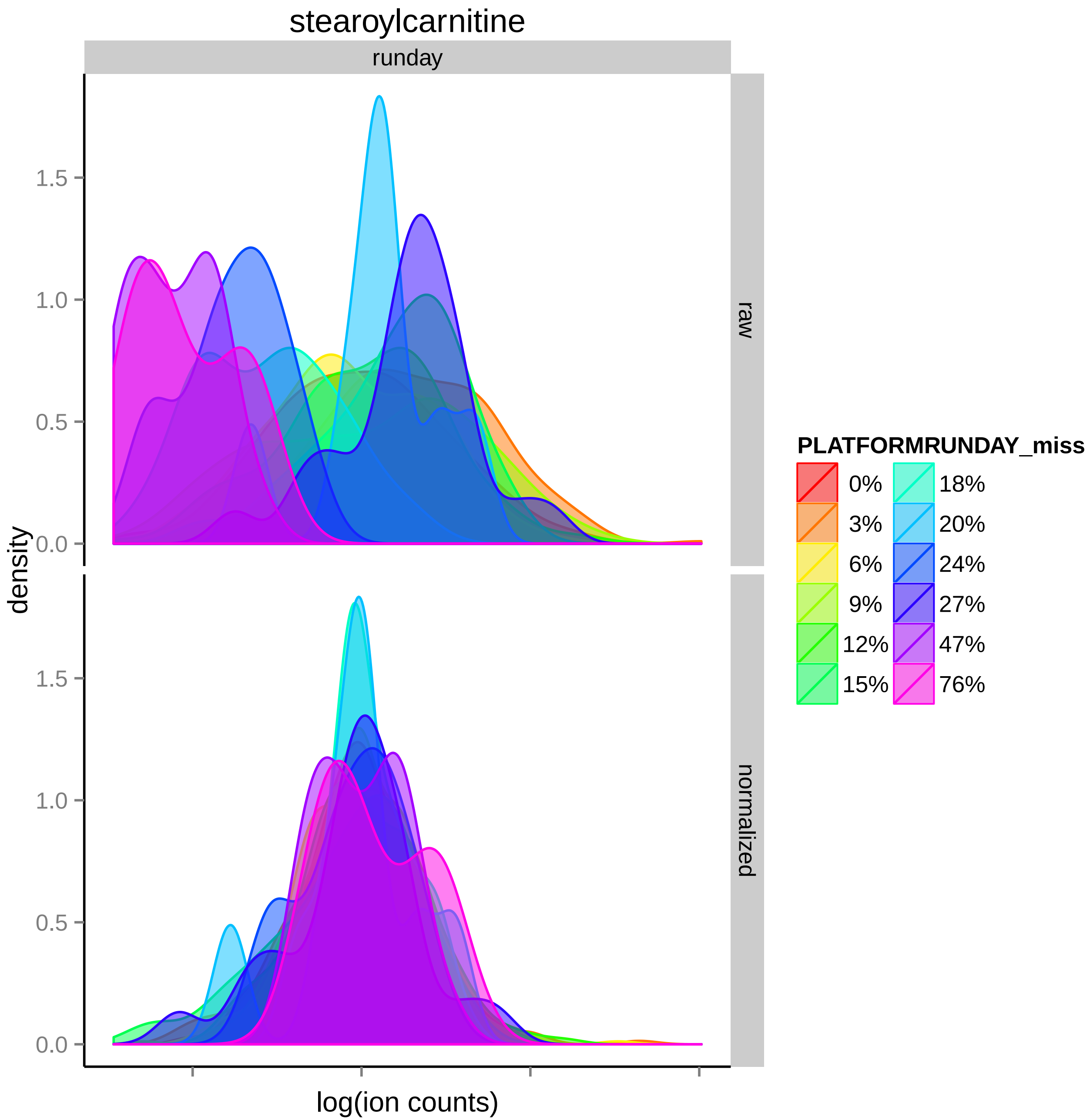
stearoylcarnitine
runday
raw
normalized
density
log(ion counts)
0.0
0.5
1.0
1.5
PLATFORMRUNDAY_miss
0%
3%
6%
9%
12%
15%
18%
20%
24%
27%
47%
76%

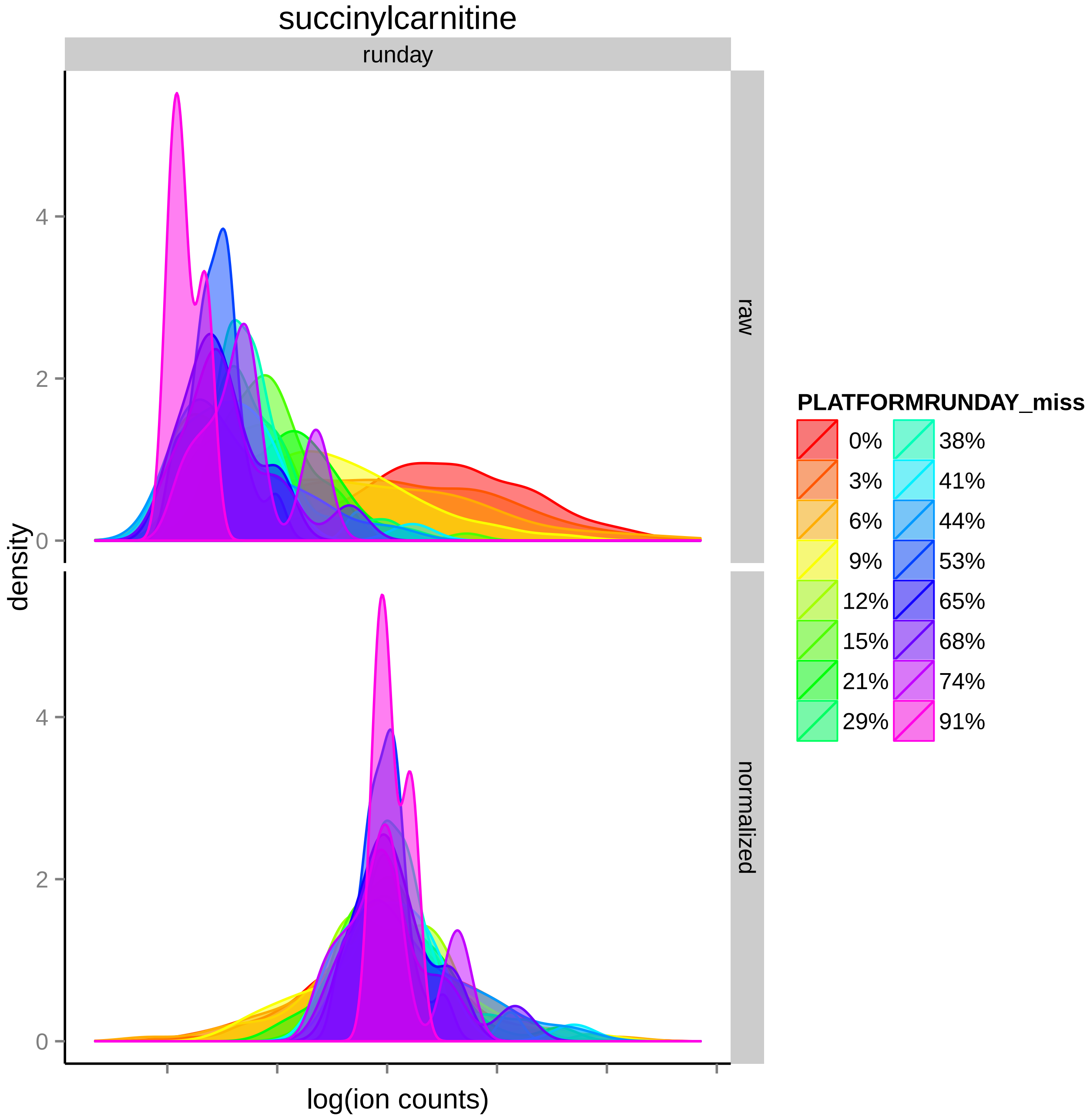

succinylcarnitine
runday
raw
normalized
density
log(ion counts)
0
2
4
PLATFORMRUNDAY_miss
0%
3%
6%
9%
12%
15%
21%
29%
38%
41%
44%
53%
65%
68%
74%
91%

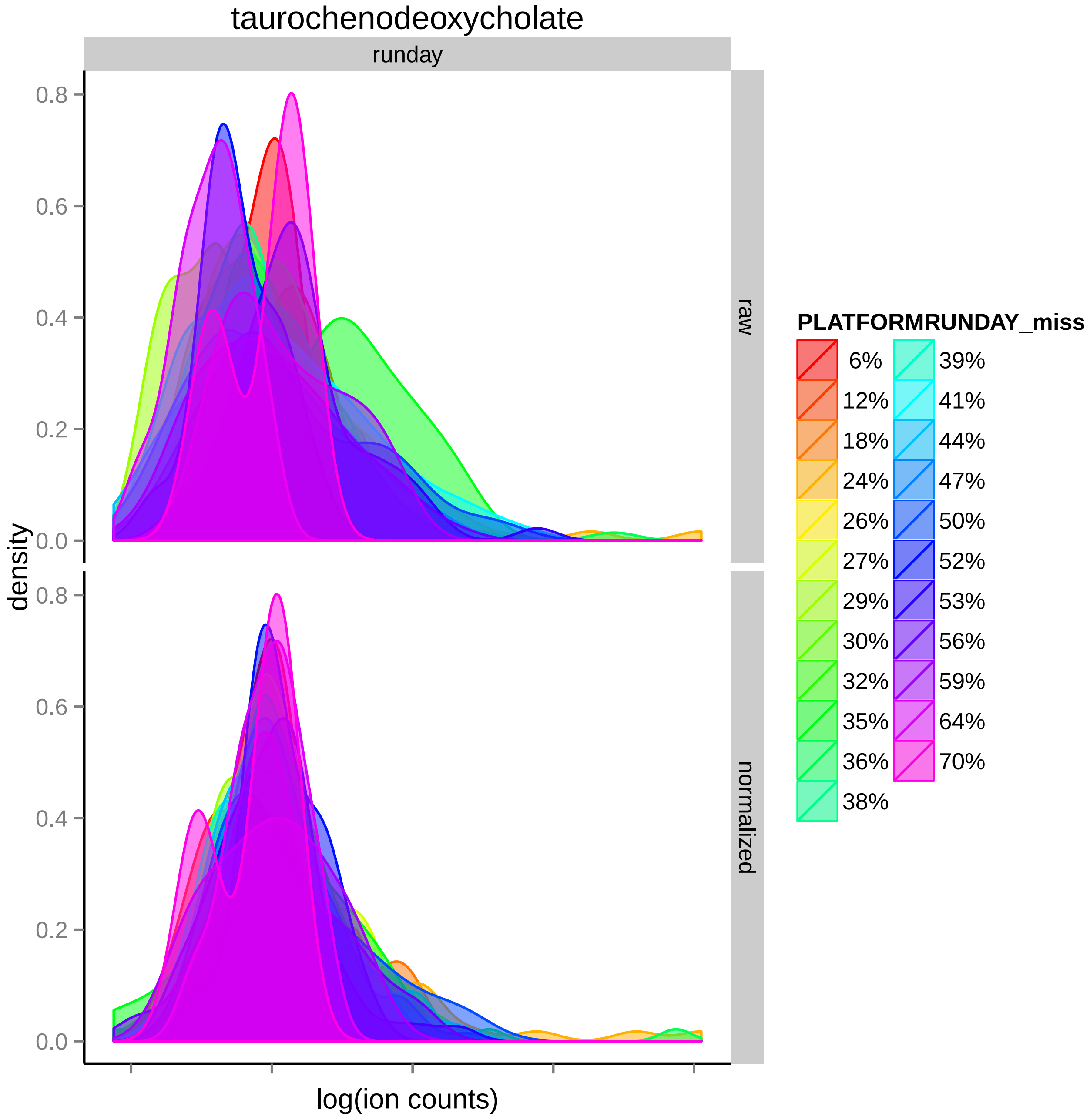

taurochenodeoxycholate
runday
raw
normalized
density
0.0
0.2
0.4
0.6
0.8
log(ion counts)
PLATFORMRUNDAY_miss
6%
12%
18%
24%
26%
27%
29%
30%
32%
35%
36%
38%
39%
41%
44%
47%
50%
52%
53%
56%
59%
64%
70%

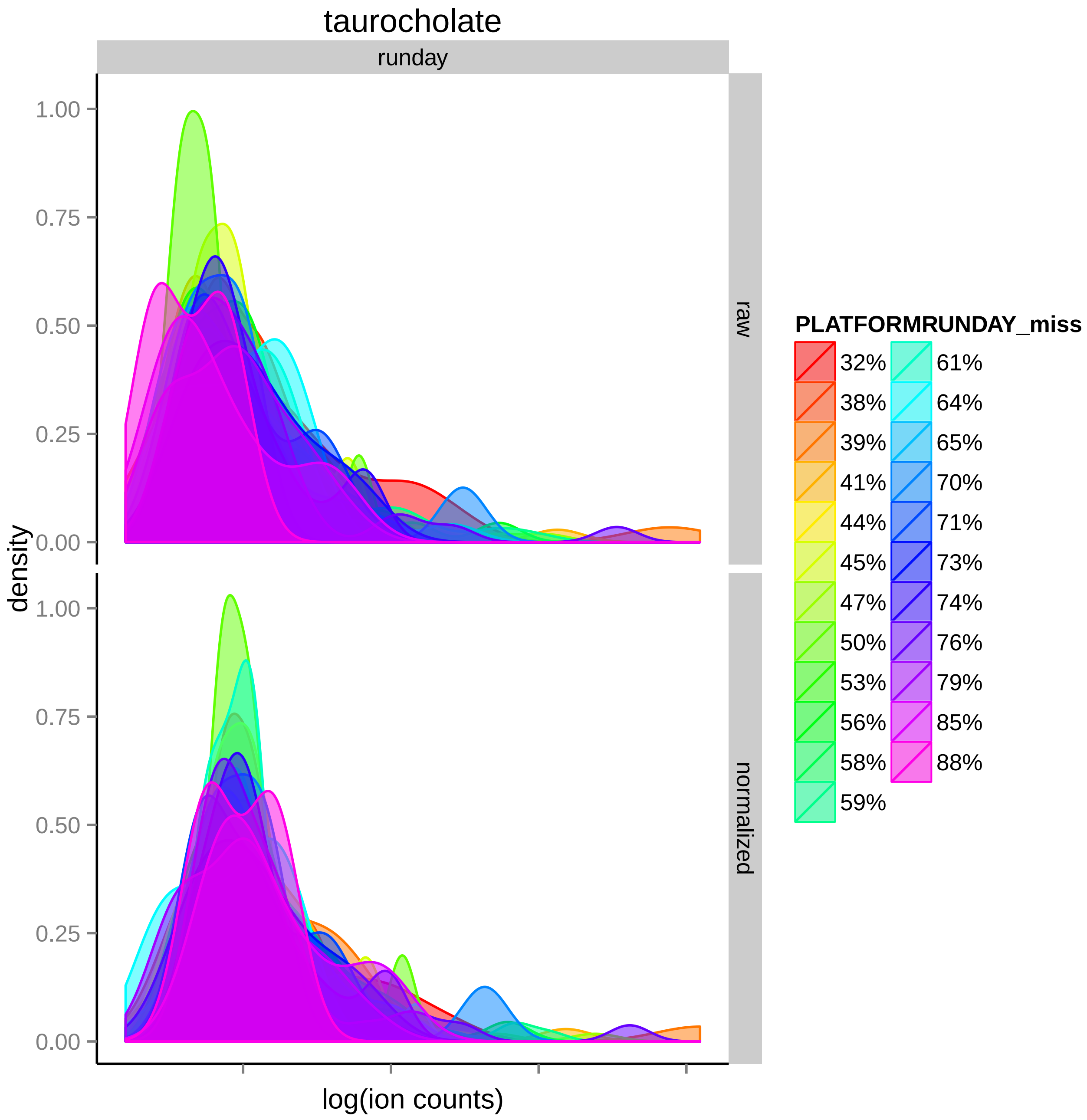

taurocholate
runday
raw
normalized
density
log(ion counts)
1.00
0.75
0.50
0.25
0.00
PLATFORMRUNDAY_miss
32%
38%
39%
41%
44%
45%
47%
50%
53%
56%
58%
59%
61%
64%
65%
70%
71%
73%
74%
76%
79%
85%
88%

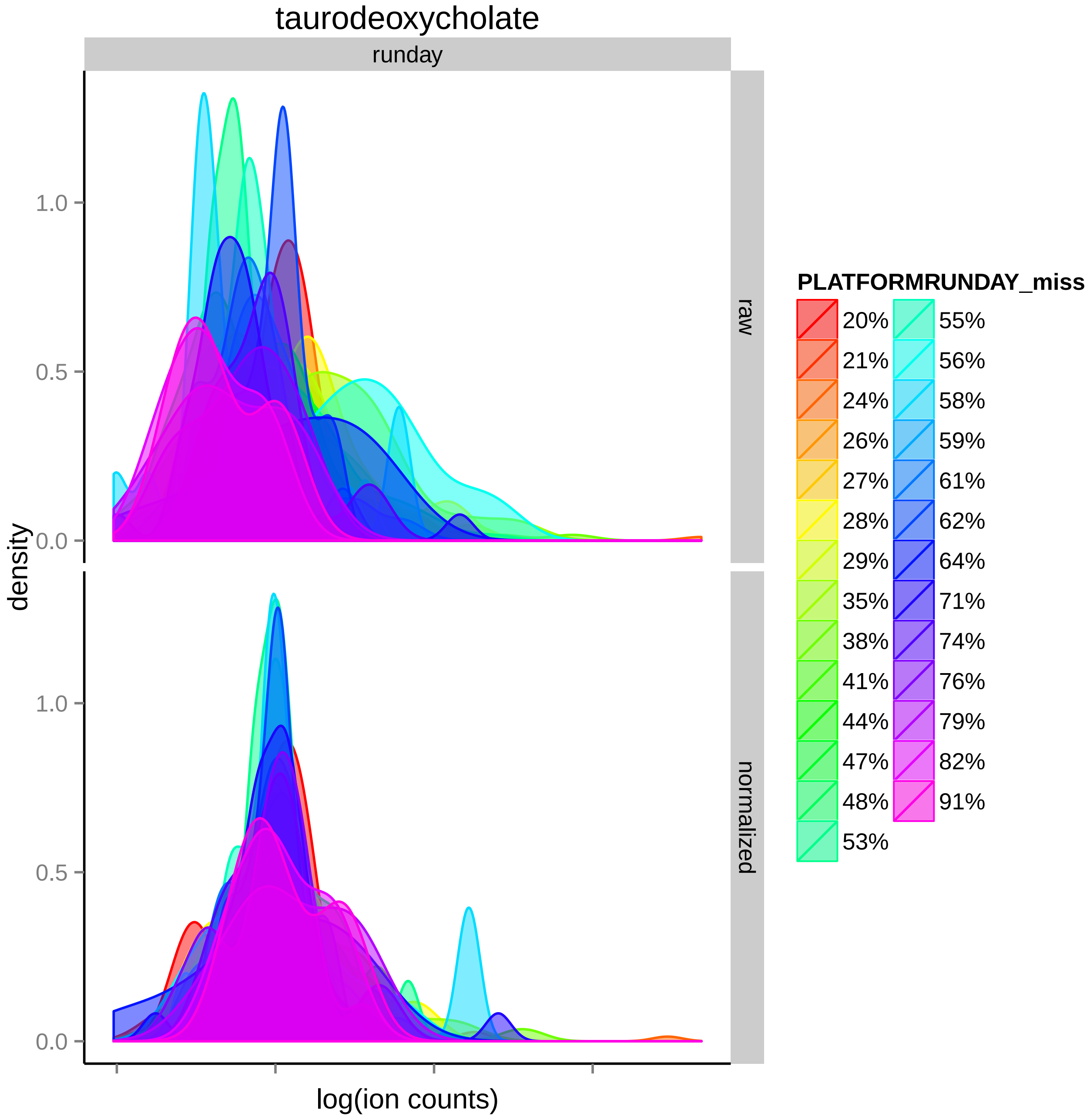

taurodeoxycholate
runday
raw
normalized
density
1.0
0.5
0.0
log(ion counts)
PLATFORMRUNDAY_miss
20%
21%
24%
26%
27%
28%
29%
35%
38%
41%
44%
47%
48%
53%
55%
56%
58%
59%
61%
62%
64%
71%
74%
76%
79%
82%
91%

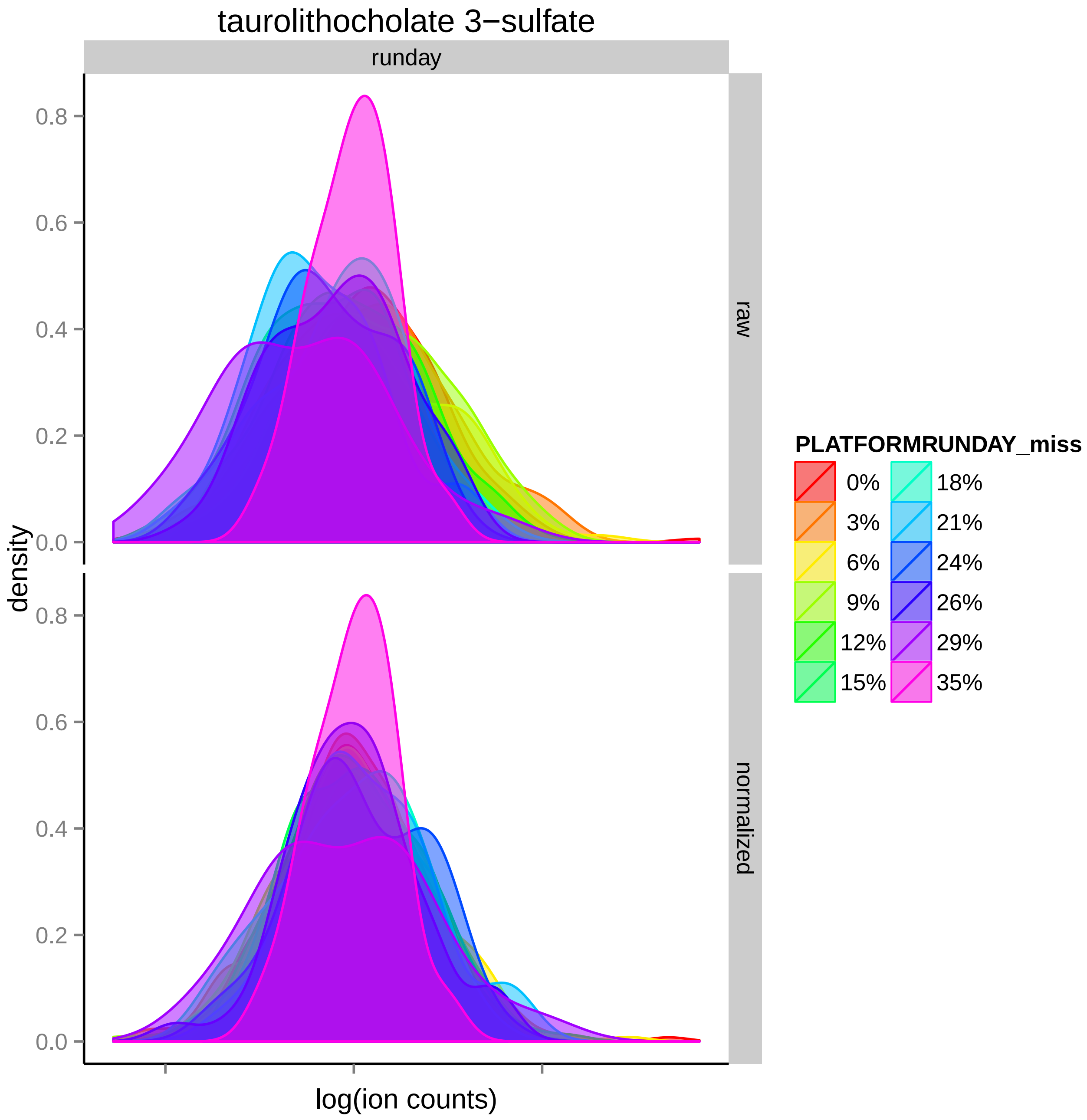
taurolithocholate 3−sulfate
runday
raw
normalized
density
log(ion counts)
0.0
0.2
0.4
0.6
0.8
PLATFORMRUNDAY_miss
0%
3%
6%
9%
12%
15%
18%
21%
24%
26%
29%
35%

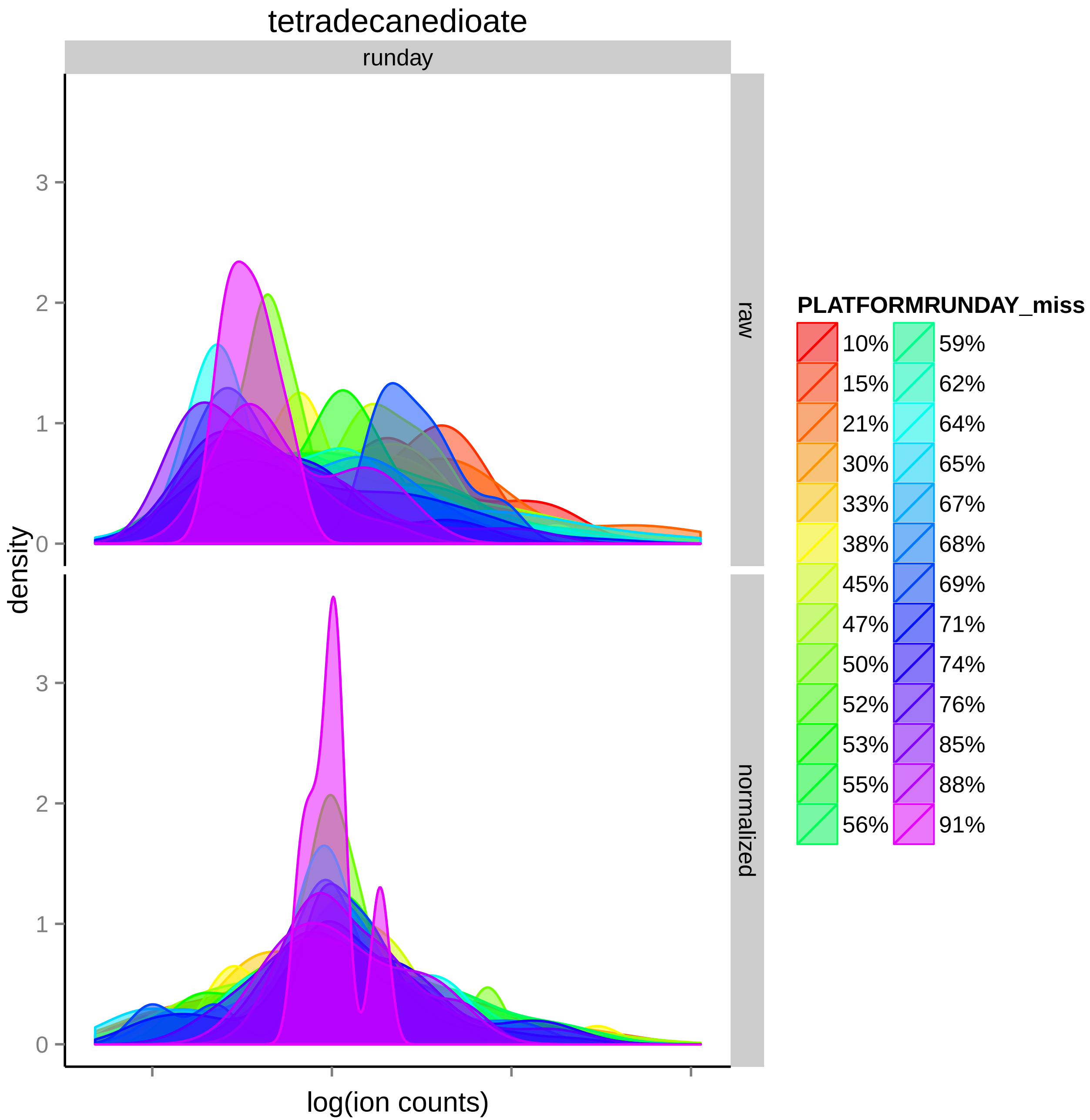
tetradecanedioate
runday
raw
normalized
density
log(ion counts)
0
1
2
3
PLATFORMRUNDAY_miss
10%
15%
21%
30%
33%
38%
45%
47%
50%
52%
53%
55%
56%
59%
62%
64%
65%
67%
68%
69%
71%
74%
76%
85%
88%
91%

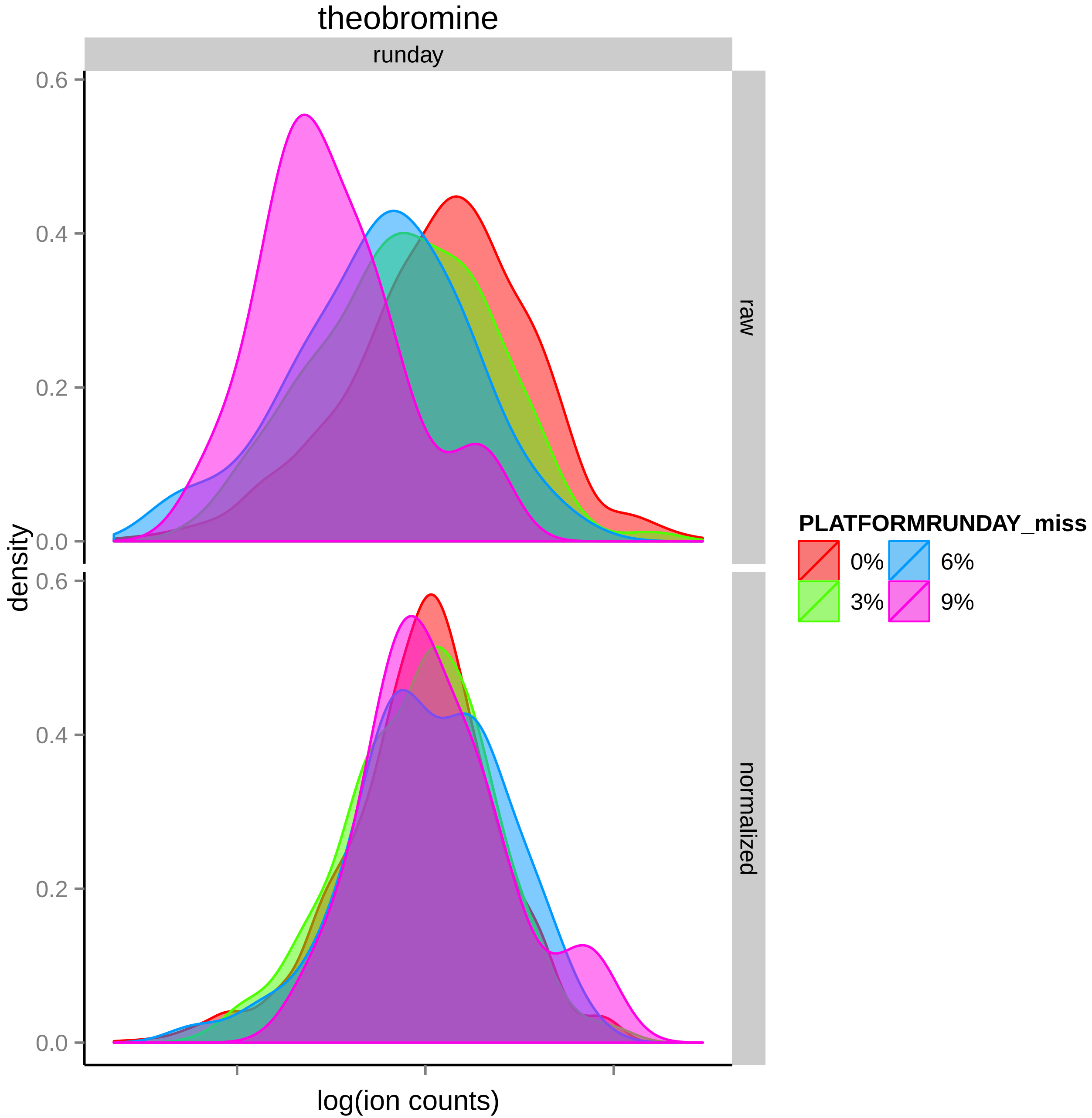
theobromine
runday
raw
normalized
density
0.0
0.2
0.4
0.6
log(ion counts)
PLATFORMRUNDAY_miss
0%
3%
6%
9%

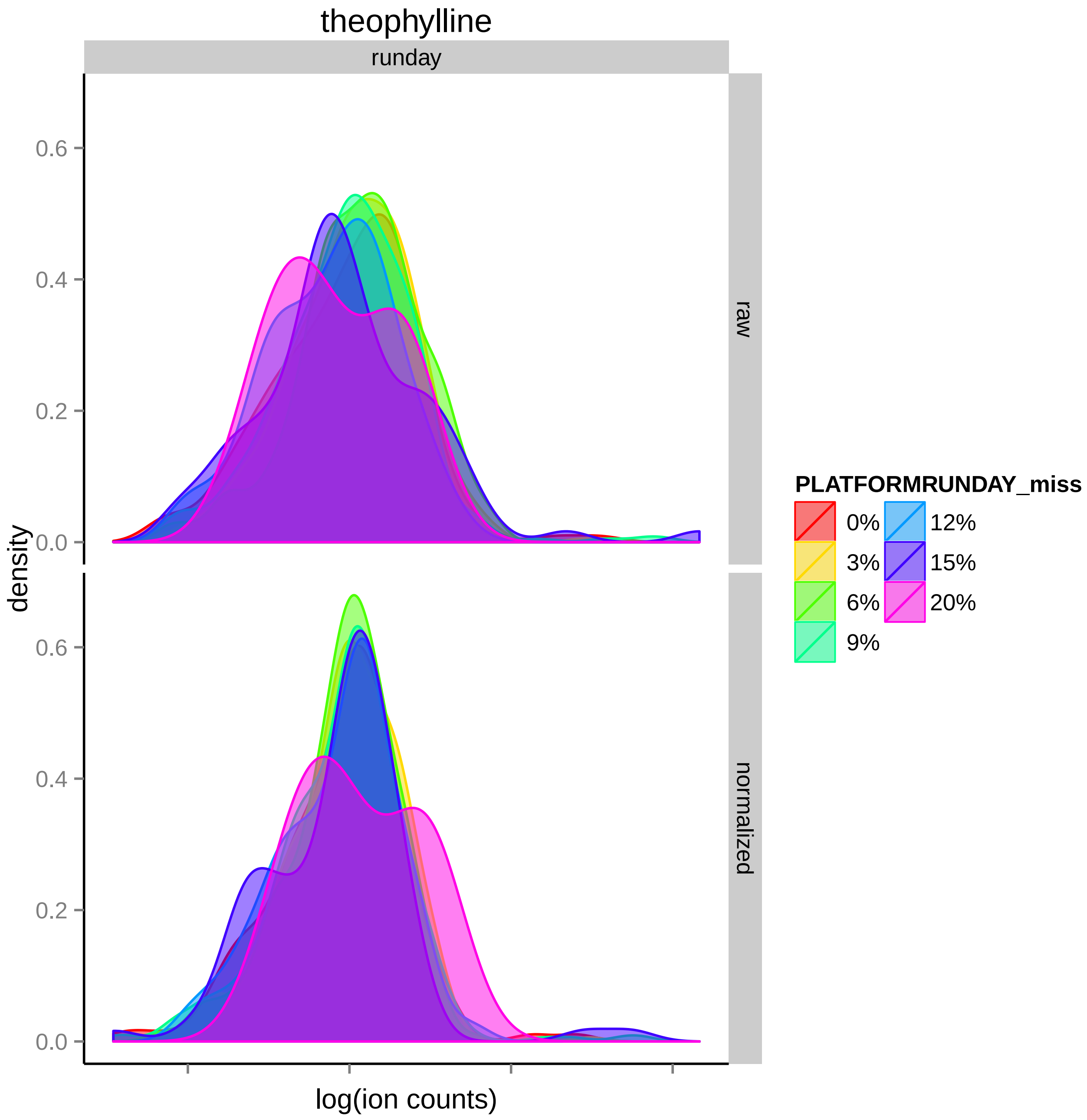
theophylline
runday
raw
normalized
density
0.0
0.2
0.4
0.6
log(ion counts)
PLATFORMRUNDAY_miss
0%
3%
6%
9%
12%
15%
20%

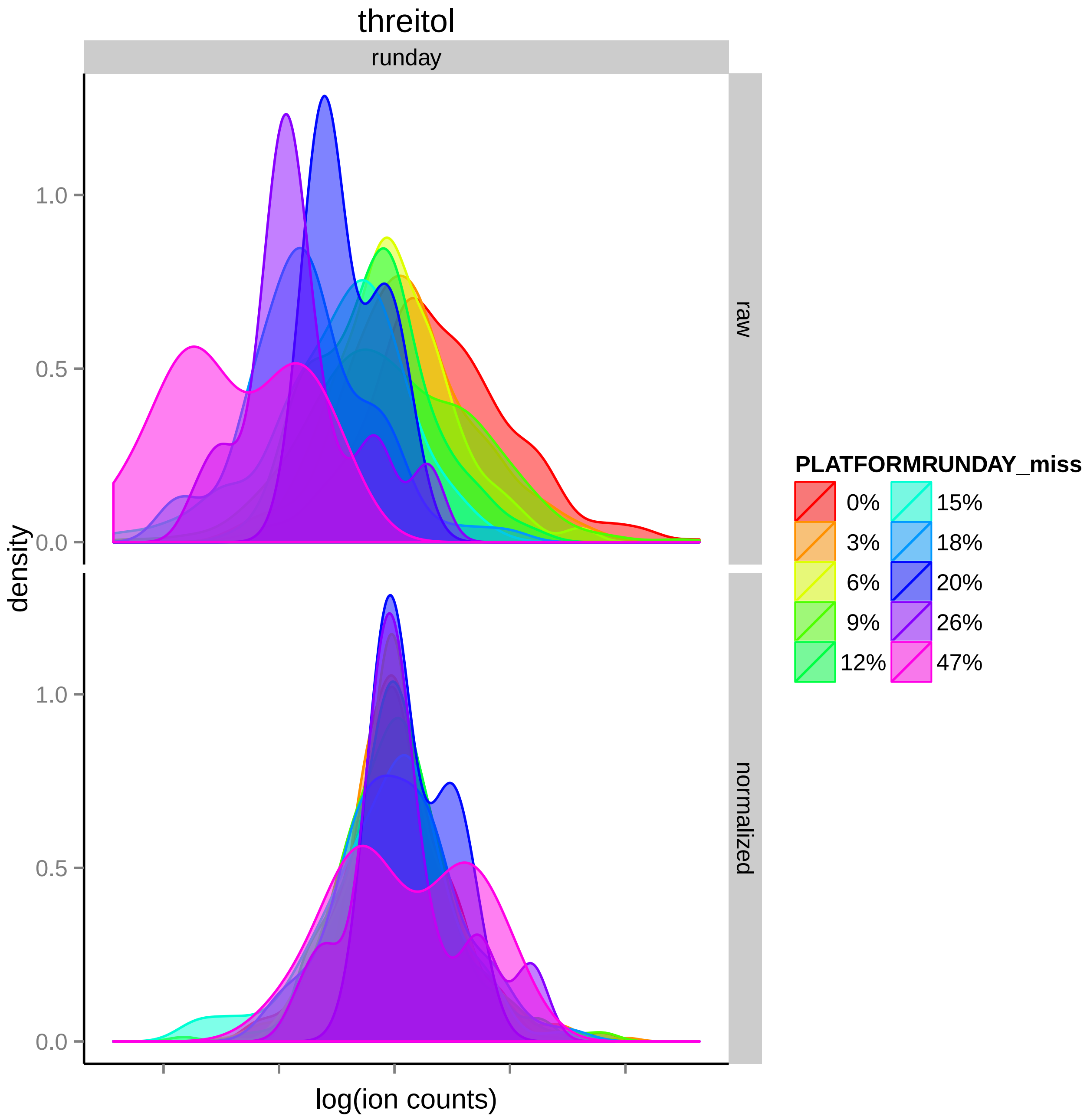

threitol
runday
raw
normalized
density
log(ion counts)
1.0
0.5
0.0
PLATFORMRUNDAY_miss
0%
3%
6%
9%
12%
15%
18%
20%
26%
47%

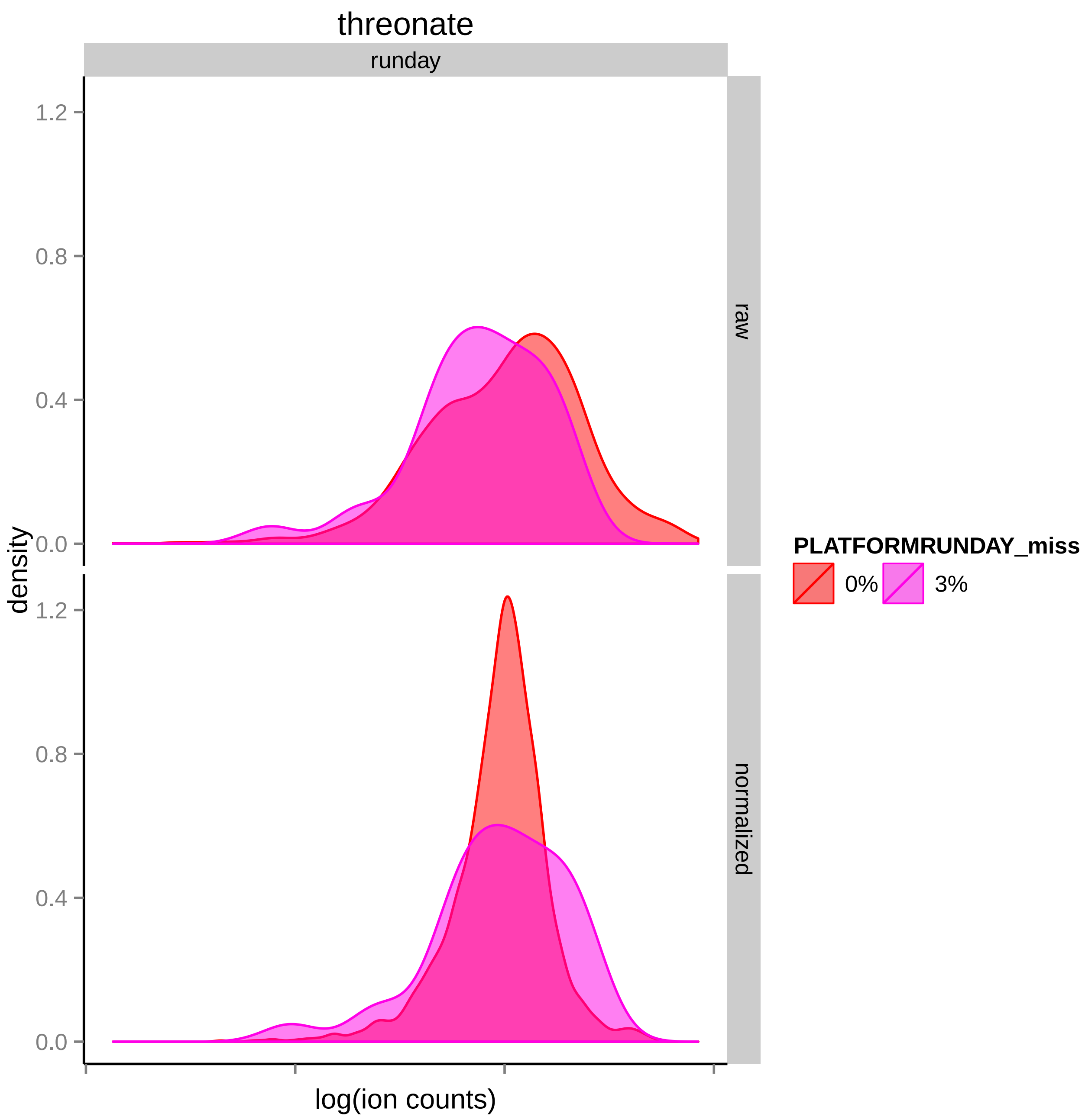

threonate
runday
raw
normalized
density
1.2
0.8
0.4
0.0
log(ion counts)
PLATFORMRUNDAY_miss
0%
3%

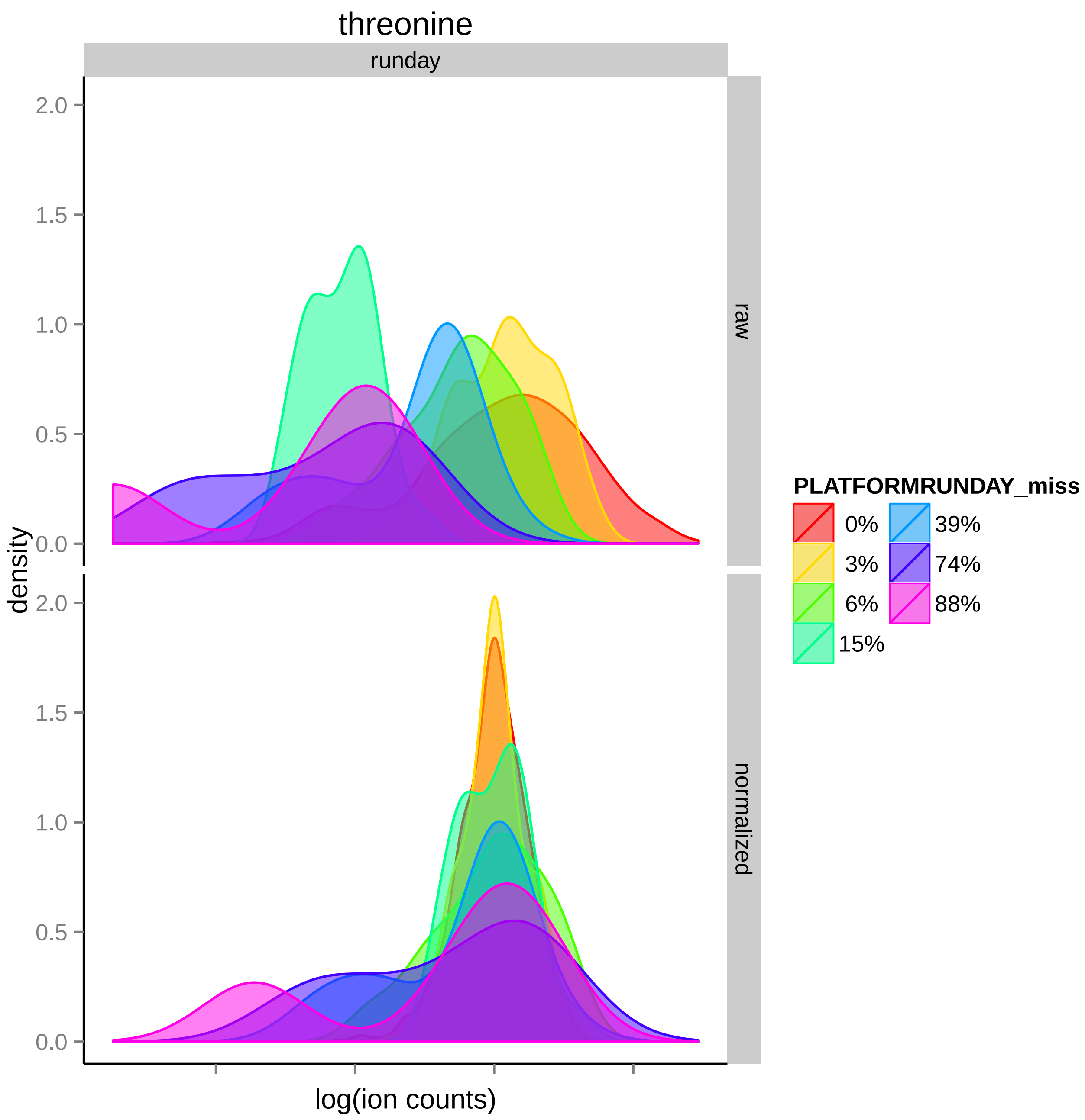
threonine
runday
raw
normalized
density
log(ion counts)
0.0
0.5
1.0
1.5
2.0
PLATFORMRUNDAY_miss
0%
3%
6%
15%
39%
74%
88%

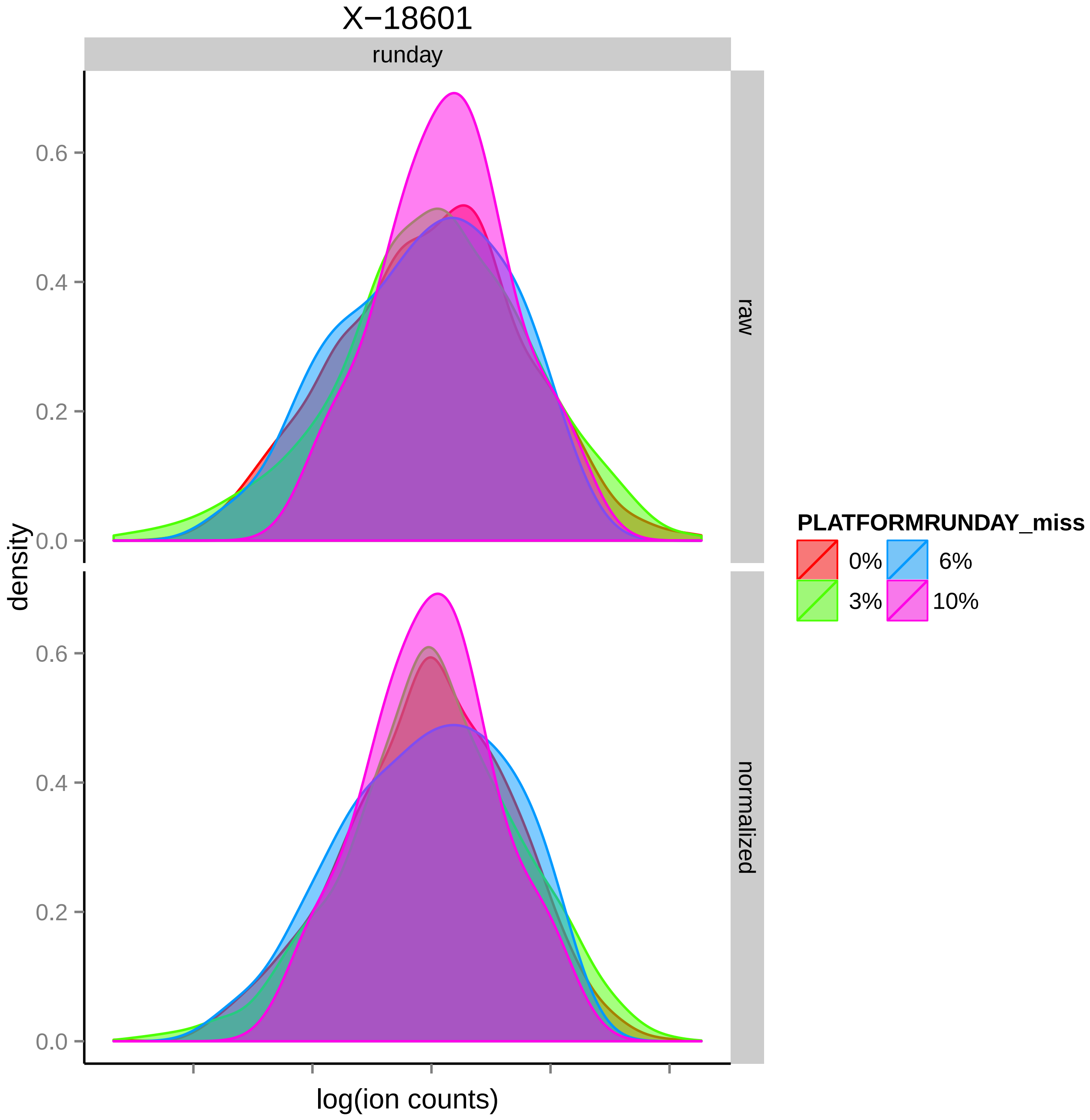
X−18601
runday
raw
normalized
density
0.0
0.2
0.4
0.6
log(ion counts)
PLATFORMRUNDAY_miss
0%
6%
3%
10%

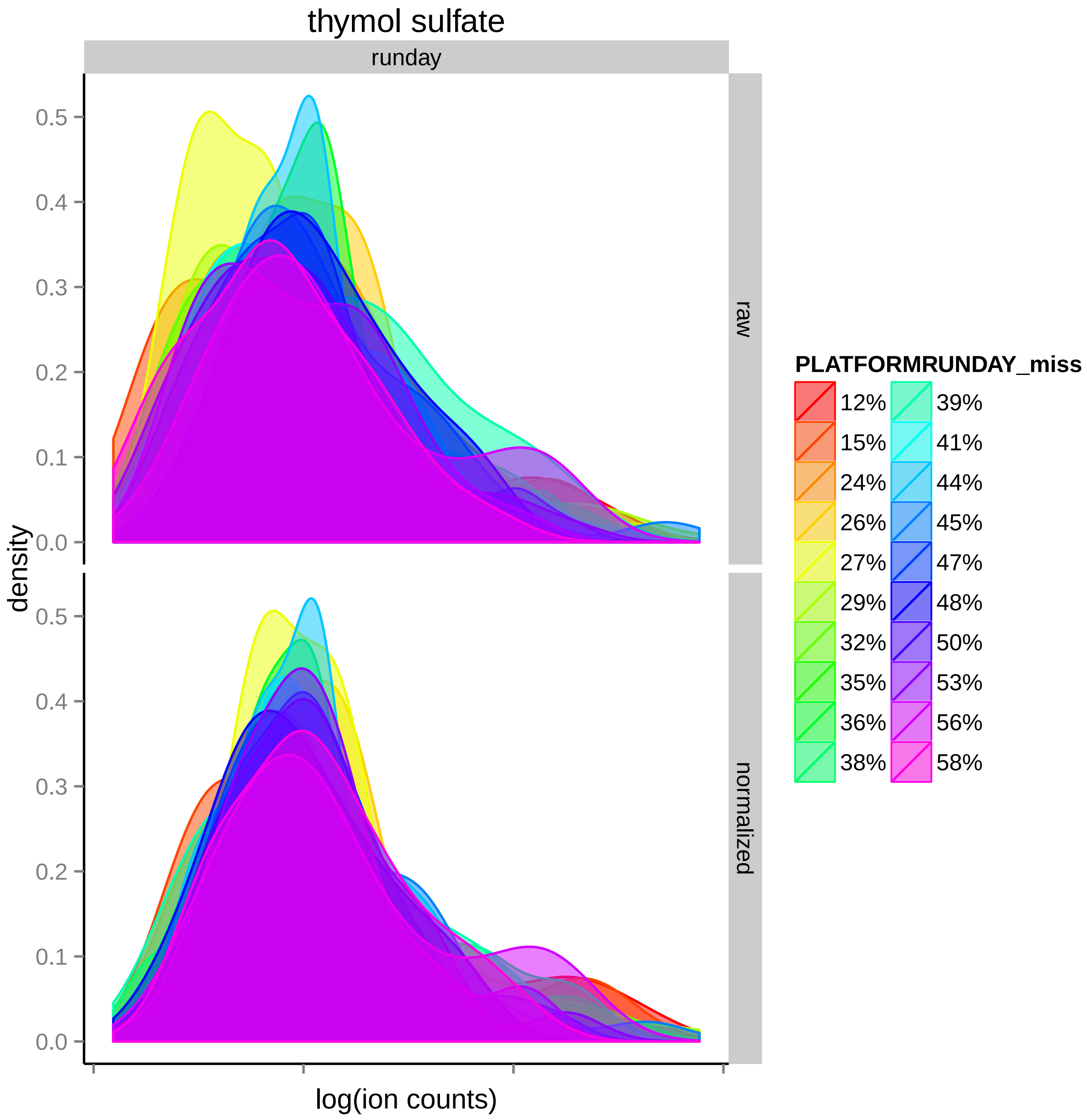

thymol sulfate
runday
raw
normalized
density
0.0
0.1
0.2
0.3
0.4
0.5
log(ion counts)
PLATFORMRUNDAY_miss
12%
15%
24%
26%
27%
29%
32%
35%
36%
38%
39%
41%
44%
45%
47%
48%
50%
53%
56%
58%

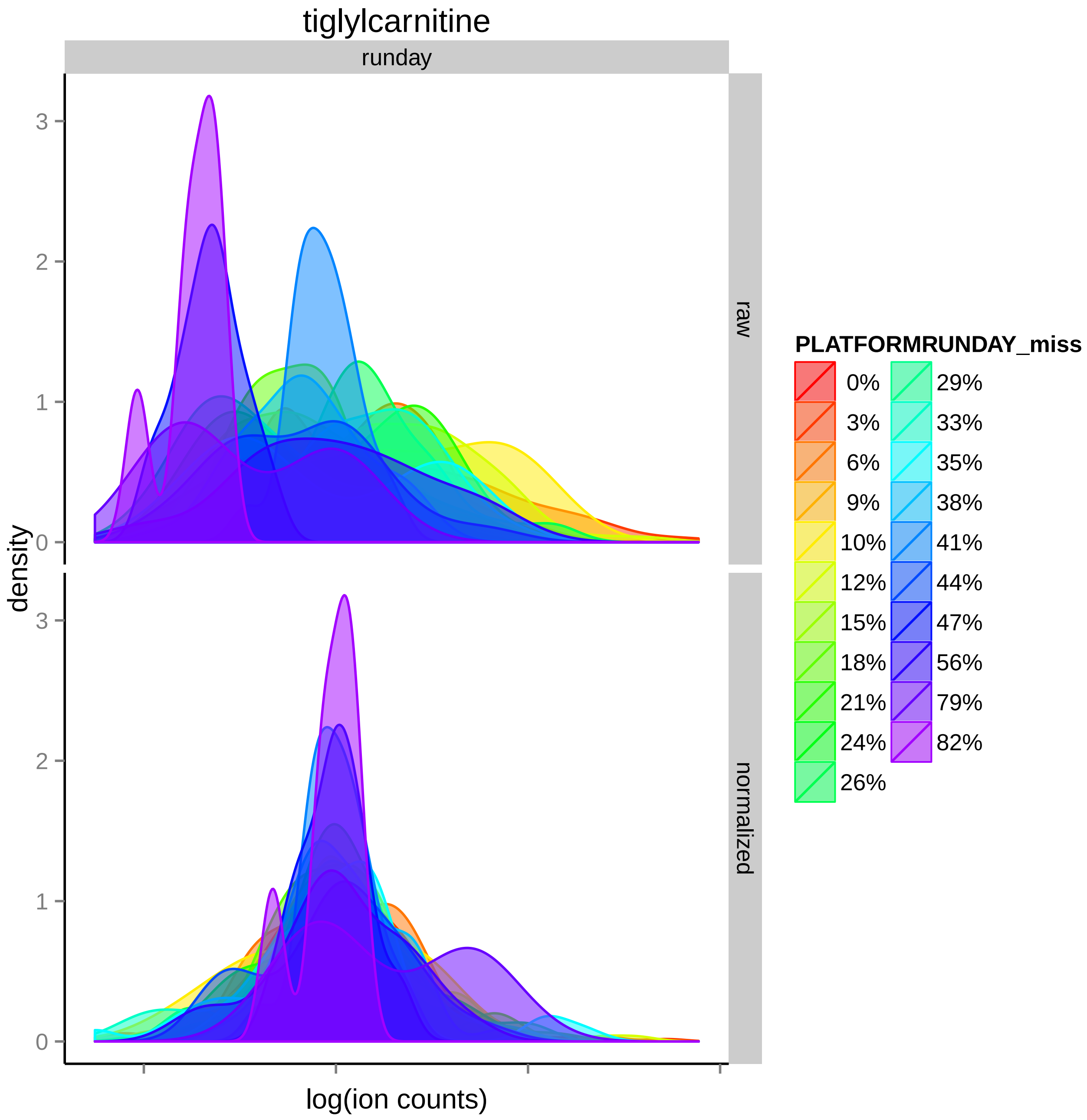

tiglylcarnitine
runday
raw
normalized
density
log(ion counts)
3
2
1
0
PLATFORMRUNDAY_miss
0%
3%
6%
9%
10%
12%
15%
18%
21%
24%
26%
29%
33%
35%
38%
41%
44%
47%
56%
79%
82%

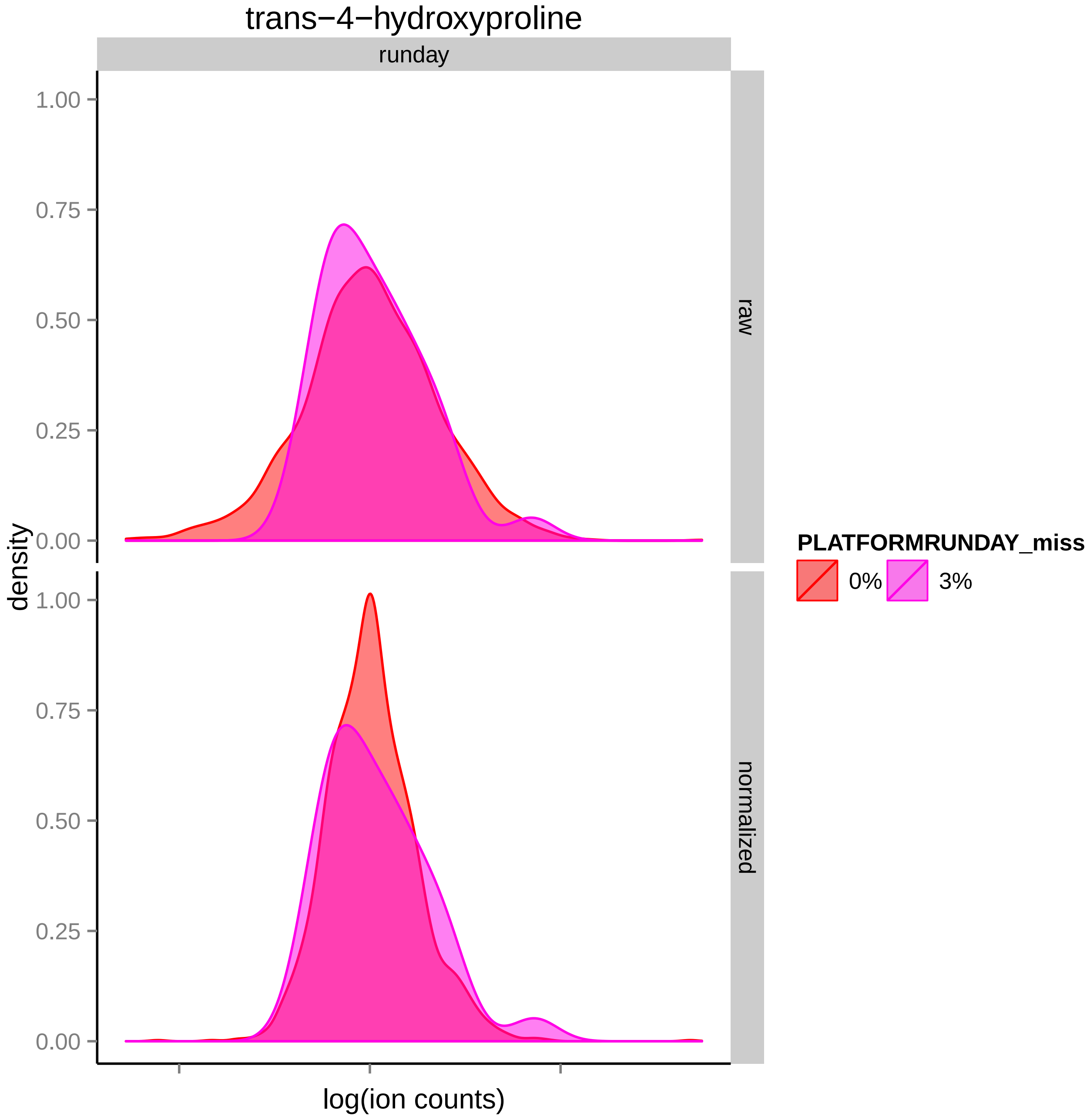

trans−4−hydroxyproline
runday
raw
normalized
density
1.00
0.75
0.50
0.25
0.00
log(ion counts)
PLATFORMRUNDAY_miss
0%
3%

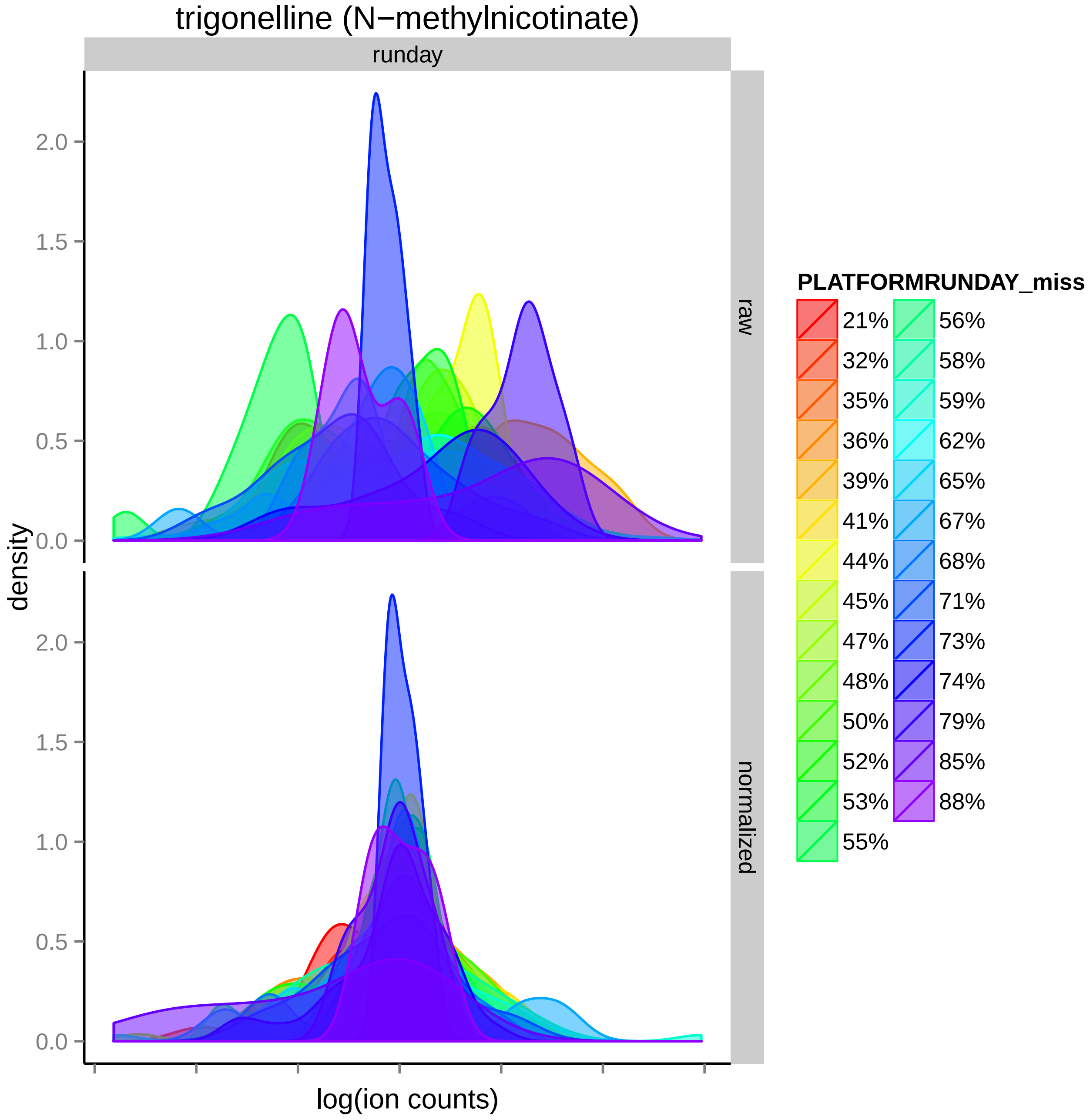

trigonelline (N−methylnicotinate)
runday
raw
normalized
density
log(ion counts)
0.0
0.5
1.0
1.5
2.0
PLATFORMRUNDAY_miss
21%
32%
35%
36%
39%
41%
44%
45%
47%
48%
50%
52%
53%
55%
56%
58%
59%
62%
65%
67%
68%
71%
73%
74%
79%
85%
88%

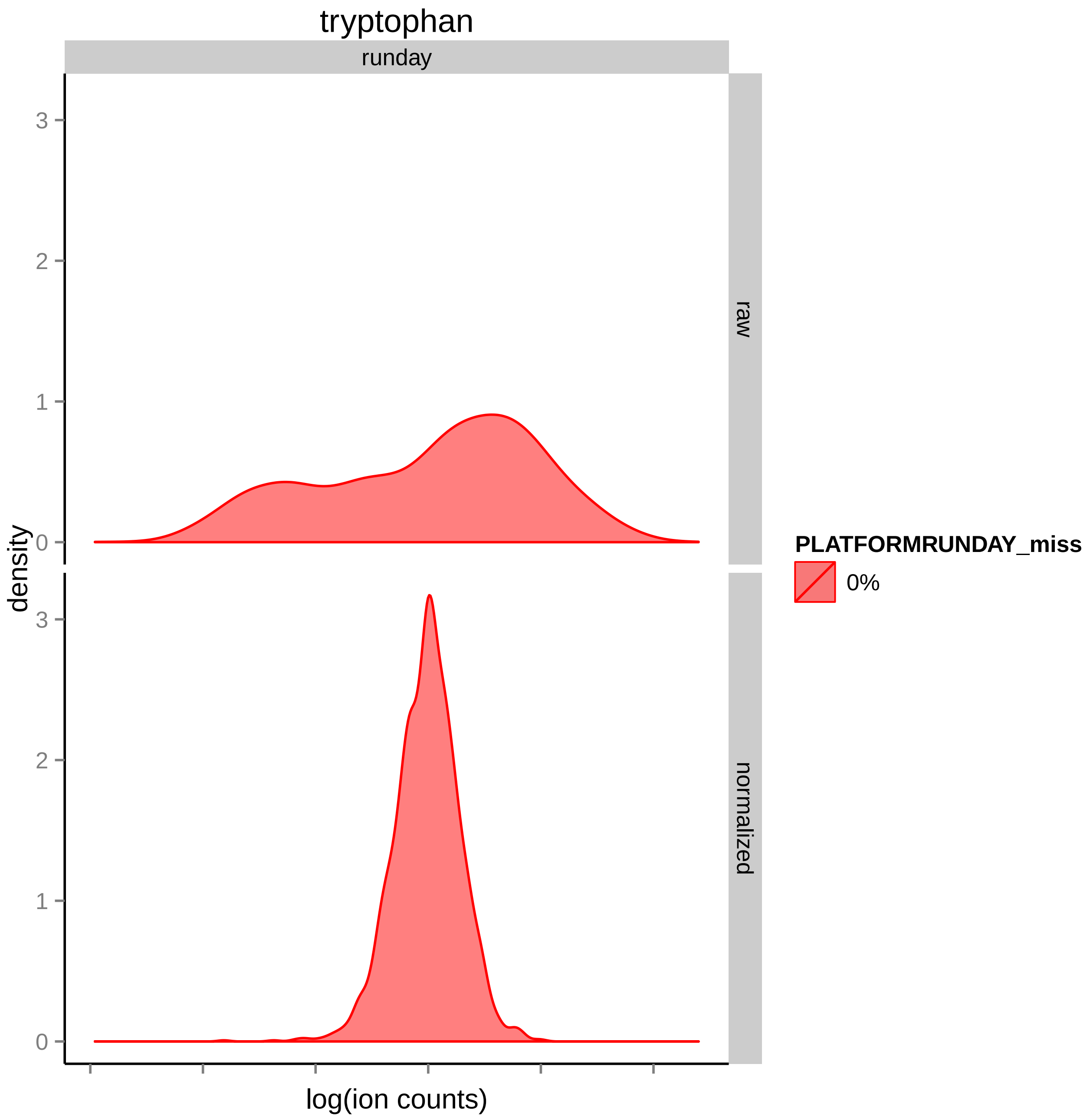

tryptophan
runday
raw
normalized
density
log(ion counts)
0
1
2
3
PLATFORMRUNDAY_miss
0%

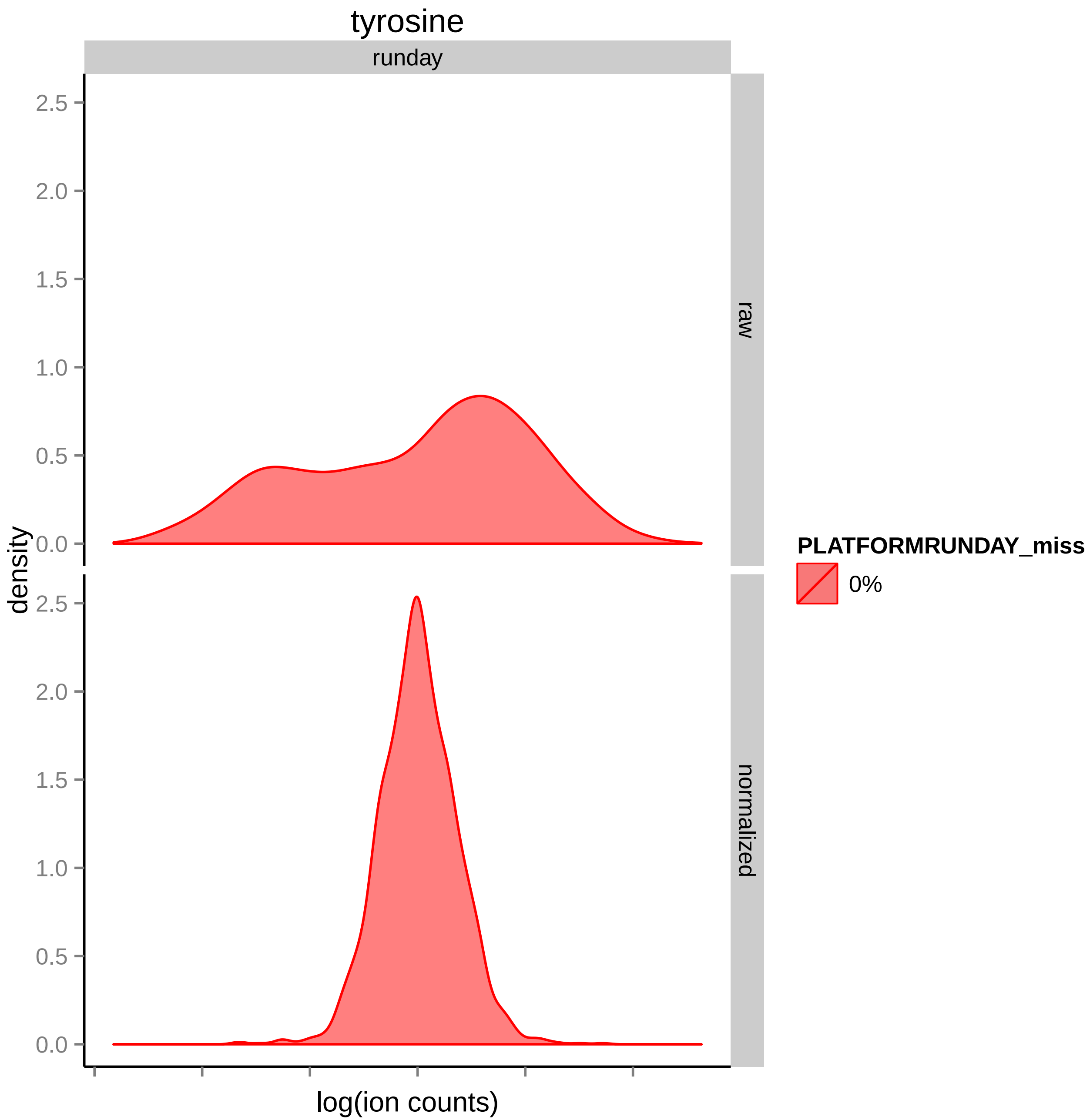

tyrosine
runday
raw
normalized
density
2.5
2.0
1.5
1.0
0.5
0.0
log(ion counts)
PLATFORMRUNDAY_miss
0%

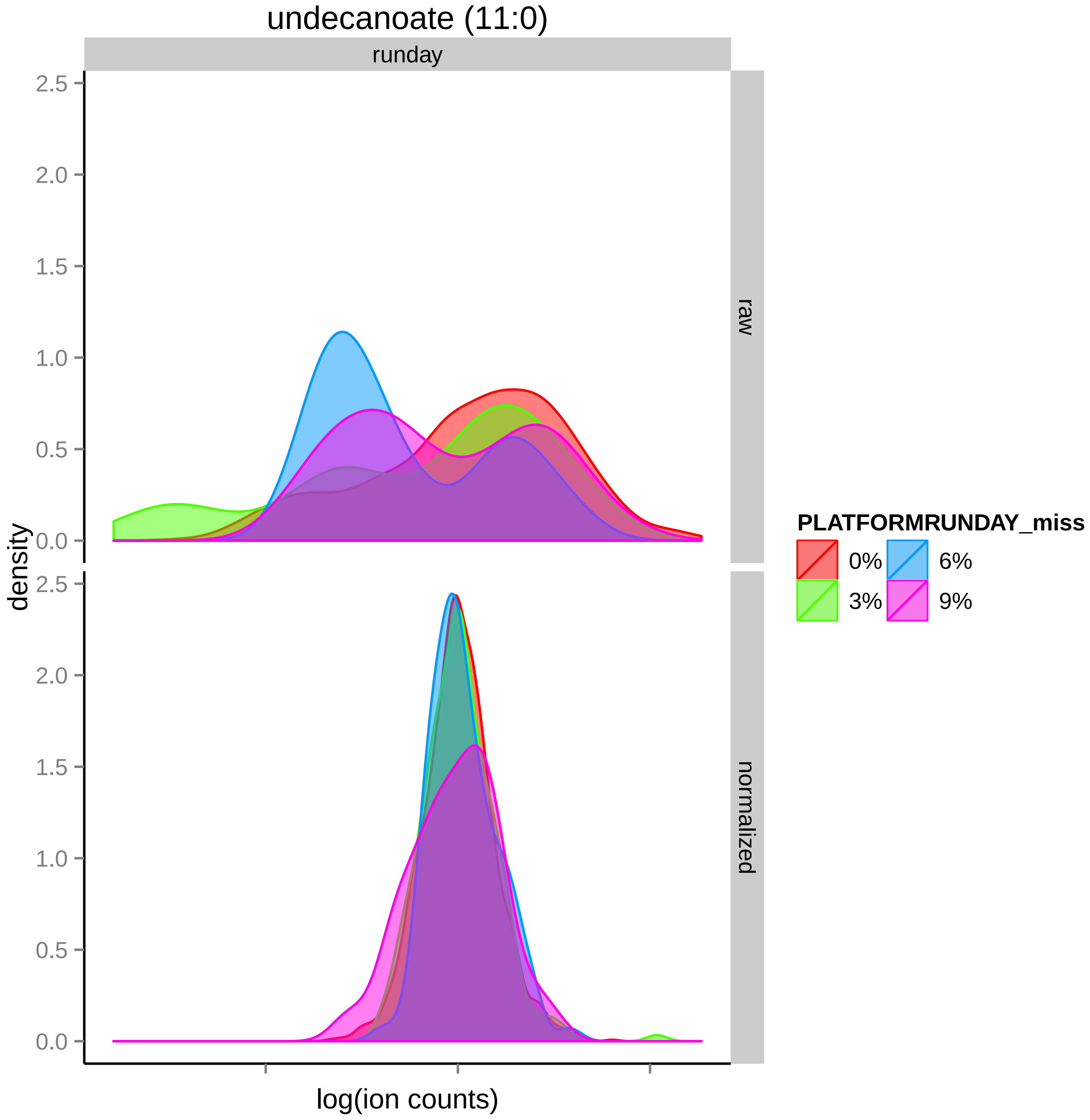

undecanoate (11:0)
runday
raw
normalized
density
log(ion counts)
0.0
0.5
1.0
1.5
2.0
2.5
PLATFORMRUNDAY_miss
0%
3%
6%
9%

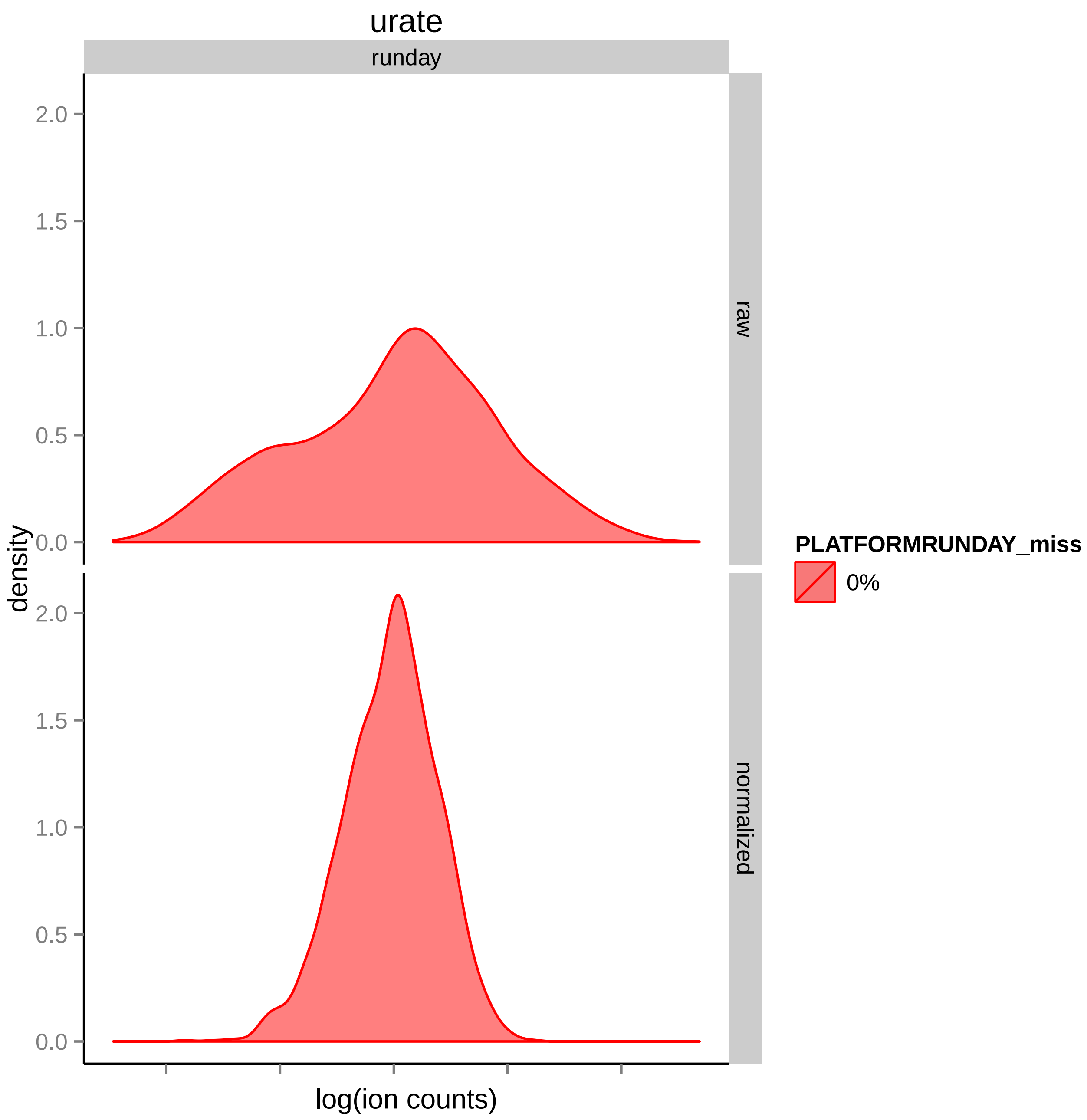

urate
runday
raw
normalized
density
2.0
1.5
1.0
0.5
0.0
2.0
1.5
1.0
0.5
0.0
log(ion counts)
PLATFORMRUNDAY_miss
0%

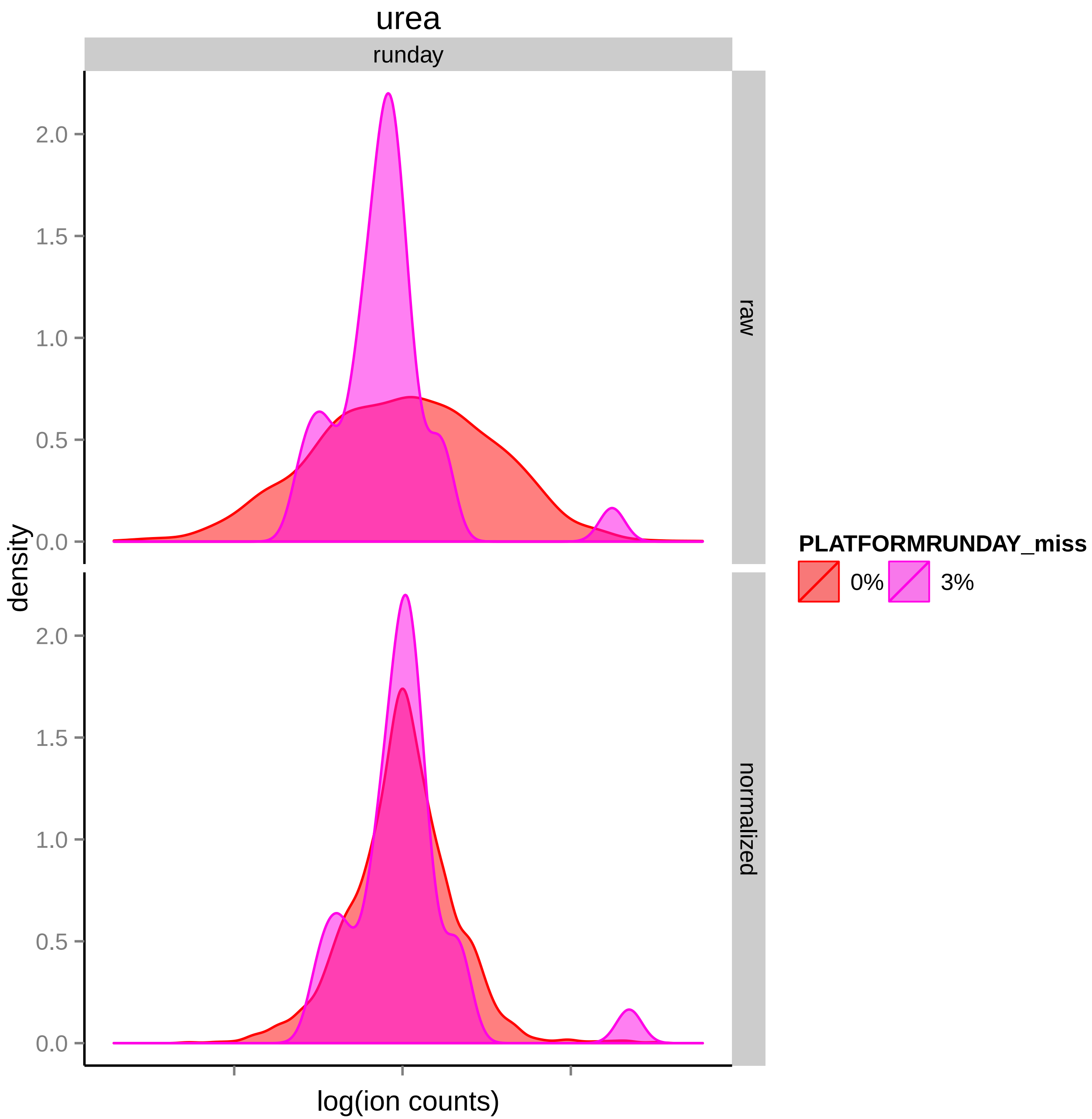

urea
runday
raw
normalized
density
2.0
1.5
1.0
0.5
0.0
log(ion counts)
PLATFORMRUNDAY_miss
0%
3%

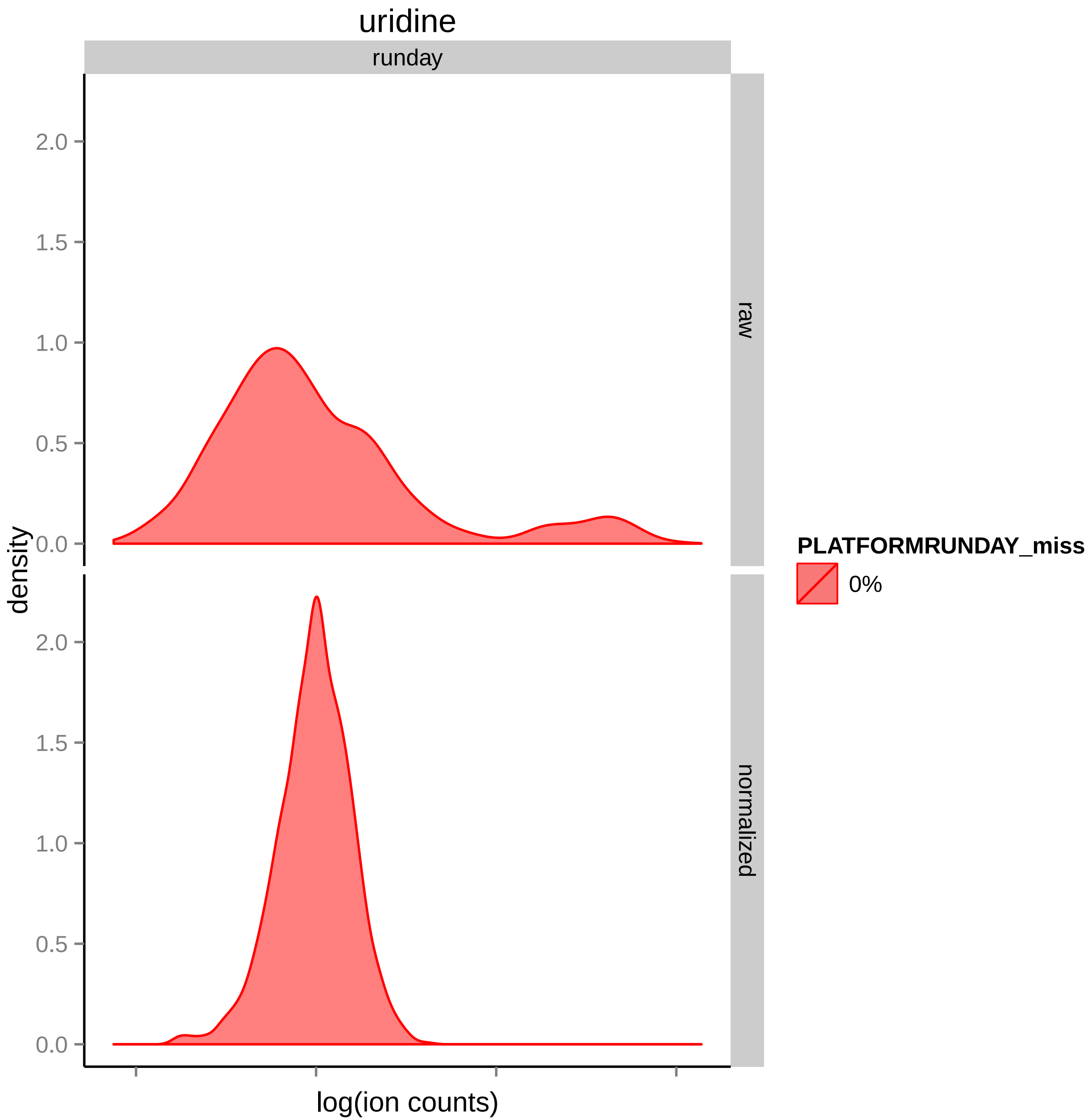

uridine
runday
raw
normalized
2.0
1.5
1.0
0.5
0.0
density
log(ion counts)
PLATFORMRUNDAY_miss
0%

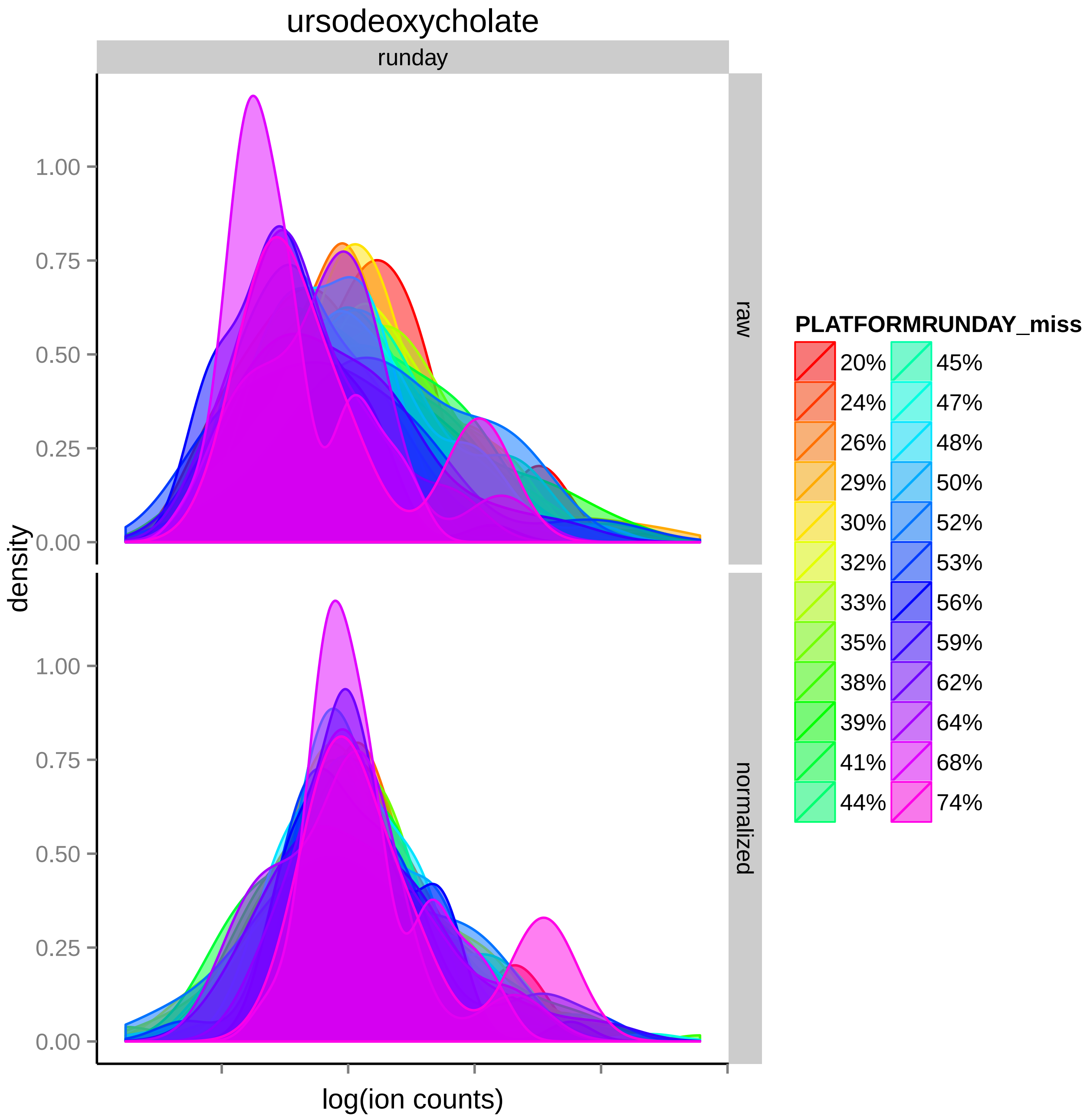
ursodeoxycholate
runday
raw
normalized
density
log(ion counts)
1.00
0.75
0.50
0.25
0.00
PLATFORMRUNDAY_miss
20%
24%
26%
29%
30%
32%
33%
35%
38%
39%
41%
44%
45%
47%
48%
50%
52%
53%
56%
59%
62%
64%
68%
74%

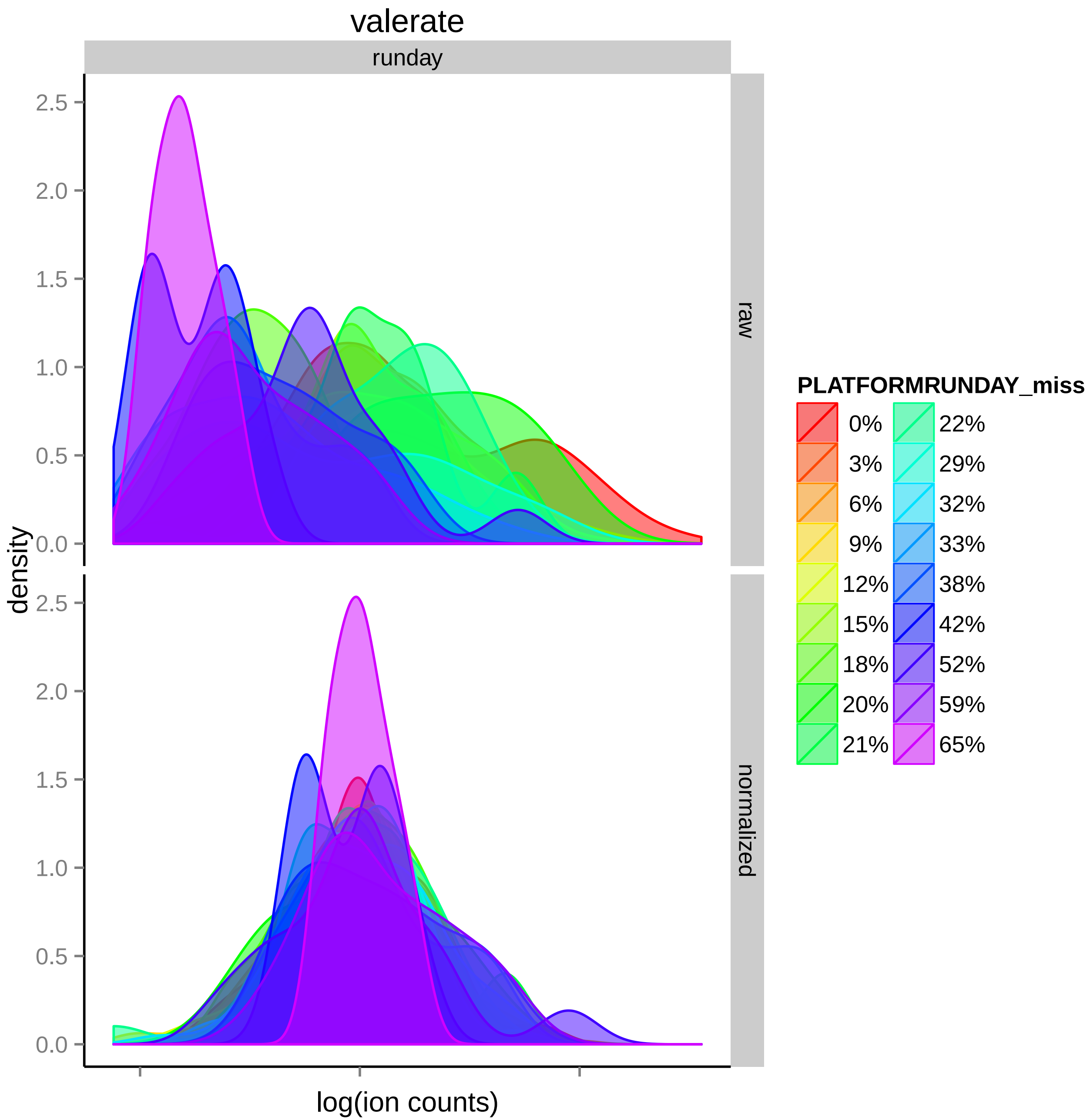

valerate
runday
raw
normalized
density
2.5
2.0
1.5
1.0
0.5
0.0
log(ion counts)
PLATFORMRUNDAY_miss
0%
3%
6%
9%
12%
15%
18%
20%
21%
22%
29%
32%
33%
38%
42%
52%
59%
65%

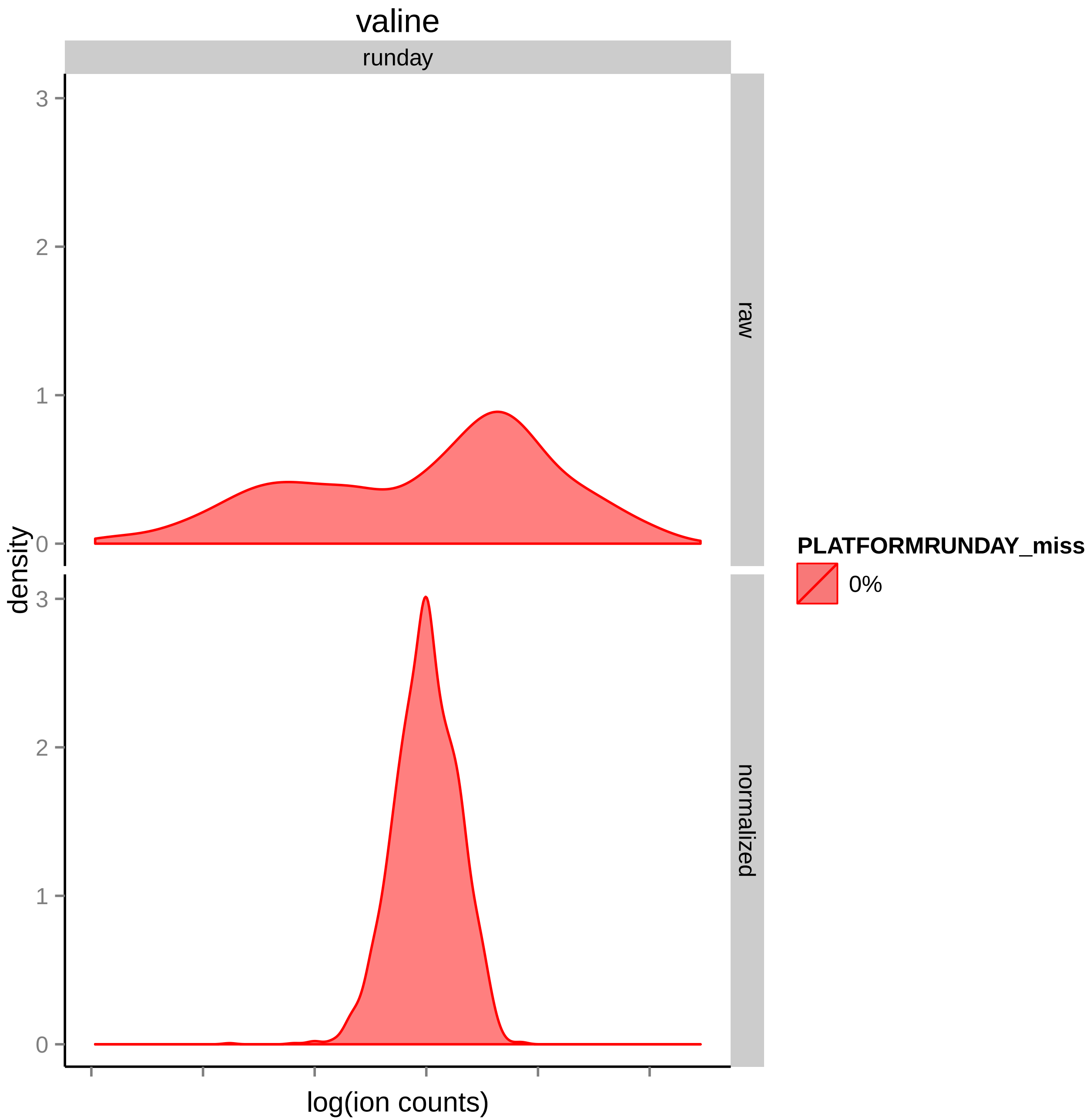

valine
runday
raw
normalized
density
3
2
1
0
log(ion counts)
PLATFORMRUNDAY_miss
0%

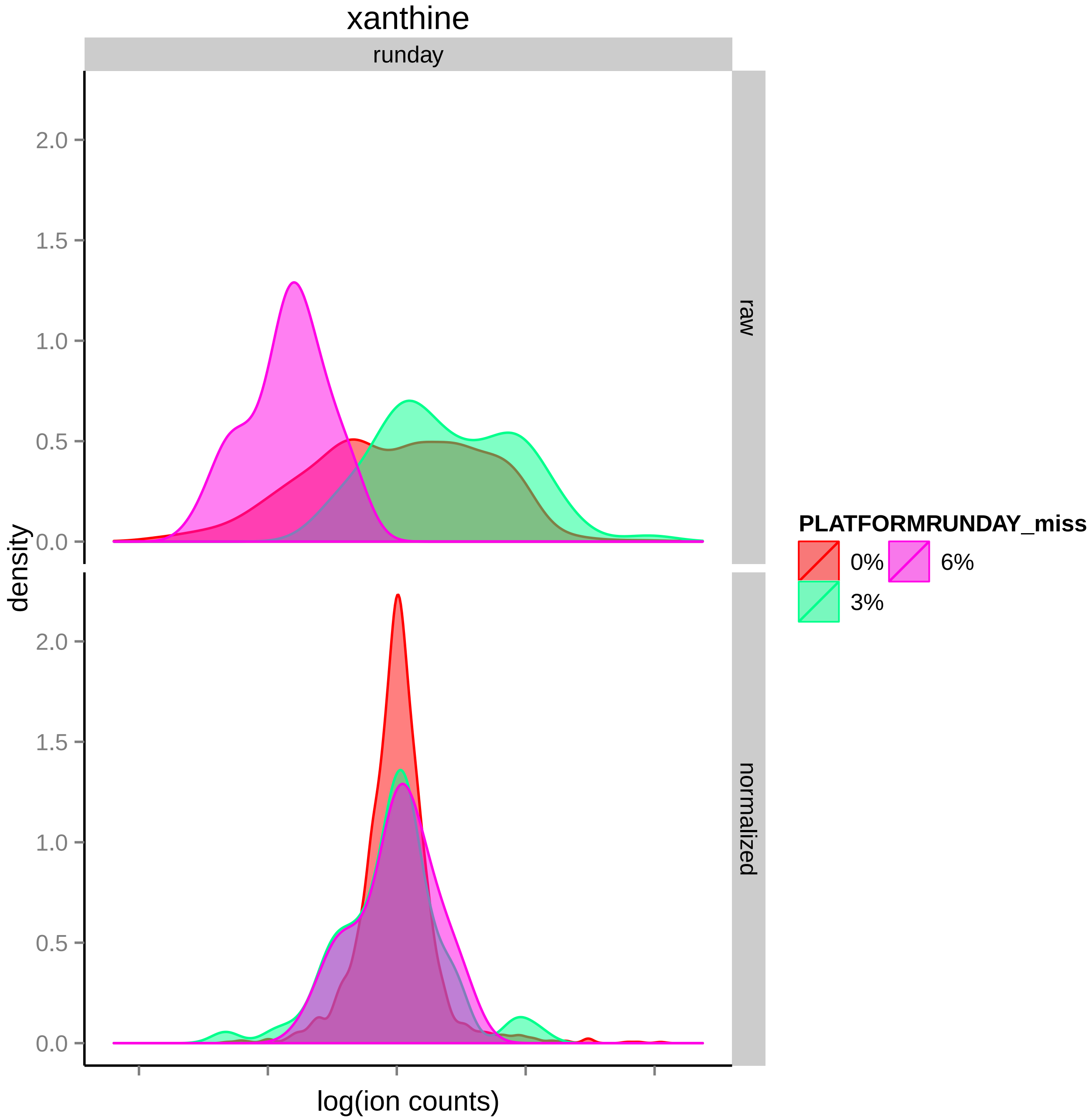

xanthine
runday
raw
normalized
density
2.0
1.5
1.0
0.5
0.0
log(ion counts)
PLATFORMRUNDAY_miss
0%
3%
6%

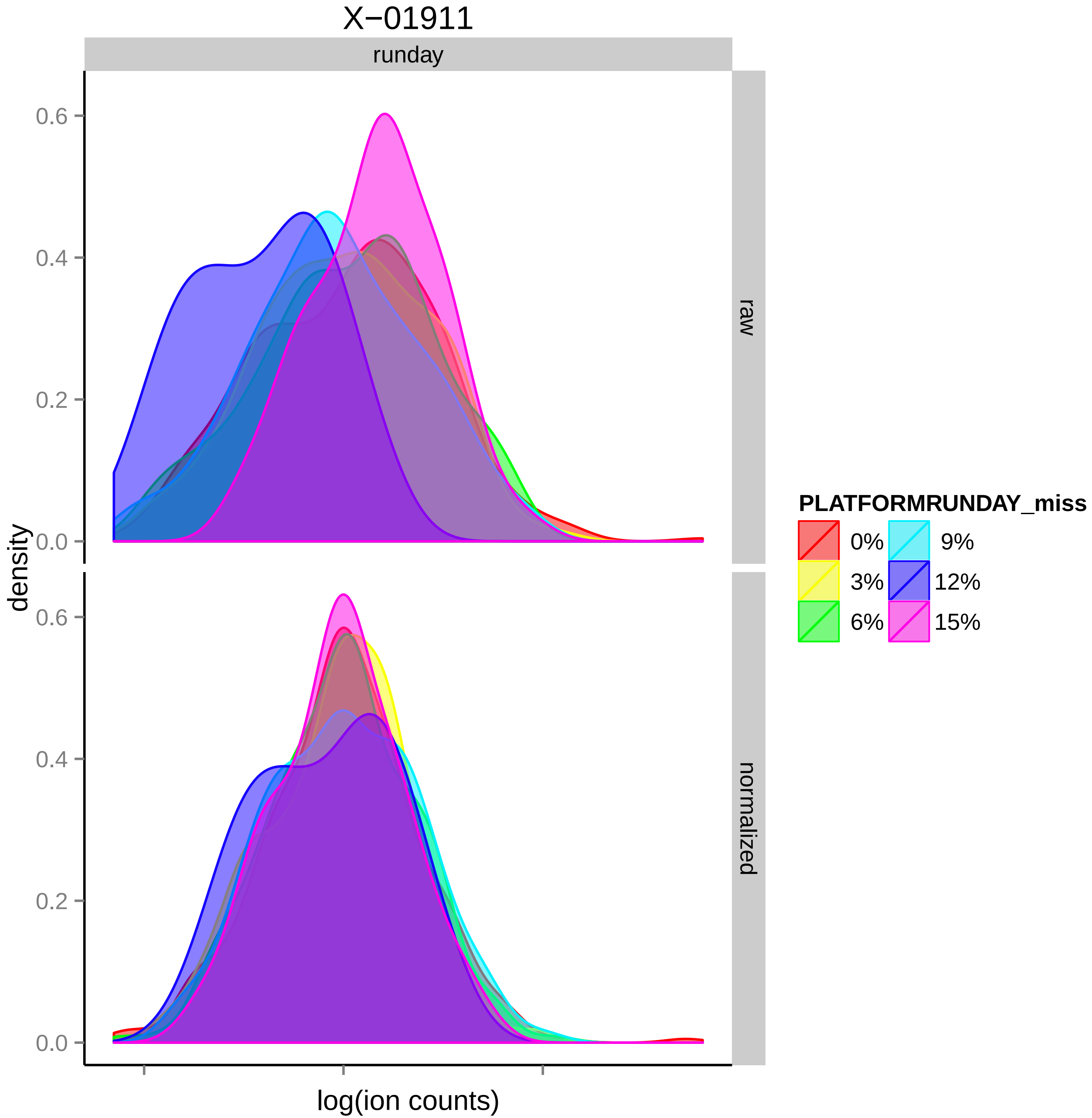

X−01911
runday
raw
normalized
density
0.6
0.4
0.2
0.0
log(ion counts)
PLATFORMRUNDAY_miss
0%
3%
6%
9%
12%
15%

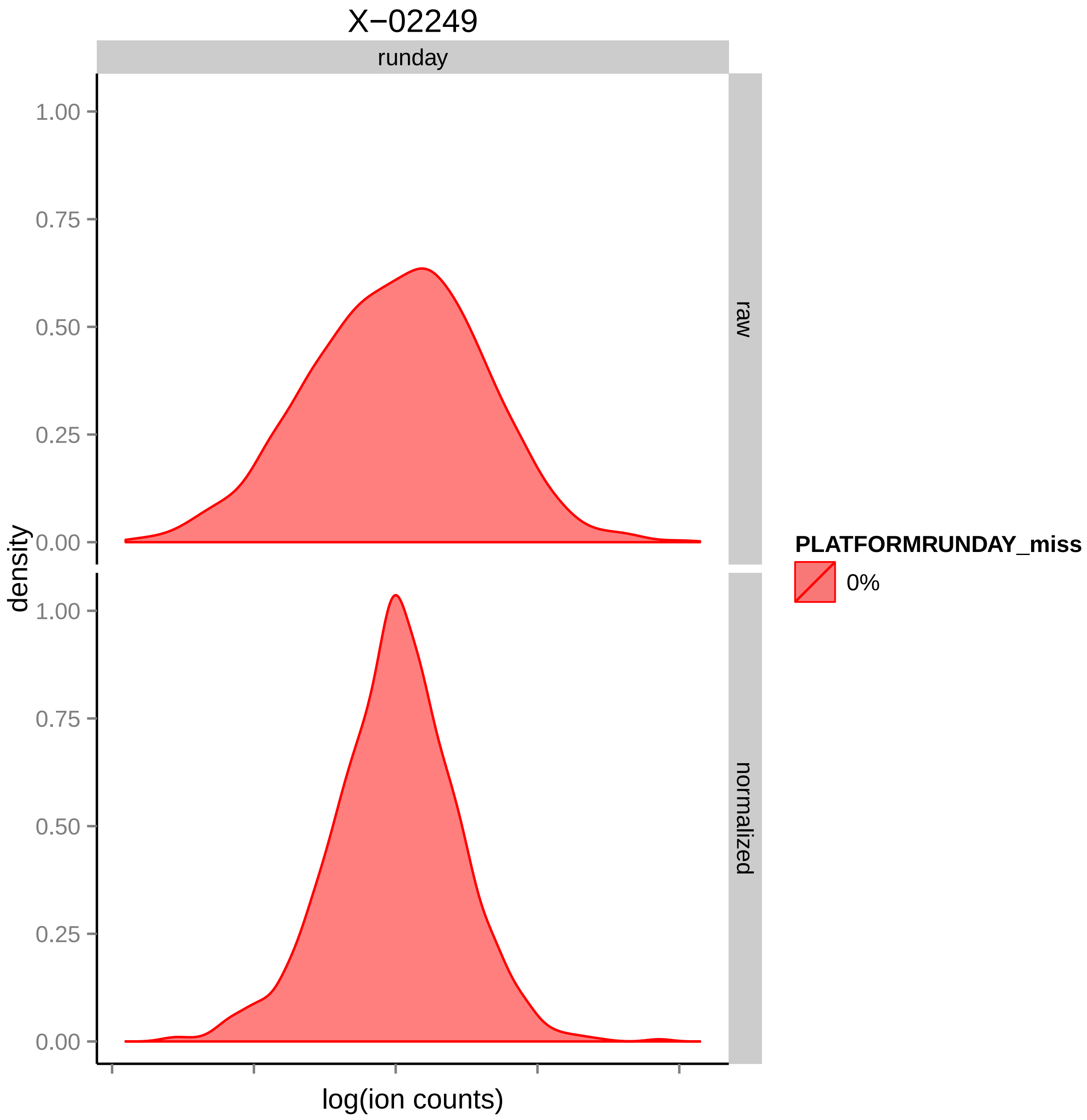
X−02249
runday
raw
normalized
density
1.00
0.75
0.50
0.25
0.00
log(ion counts)
PLATFORMRUNDAY_miss
0%

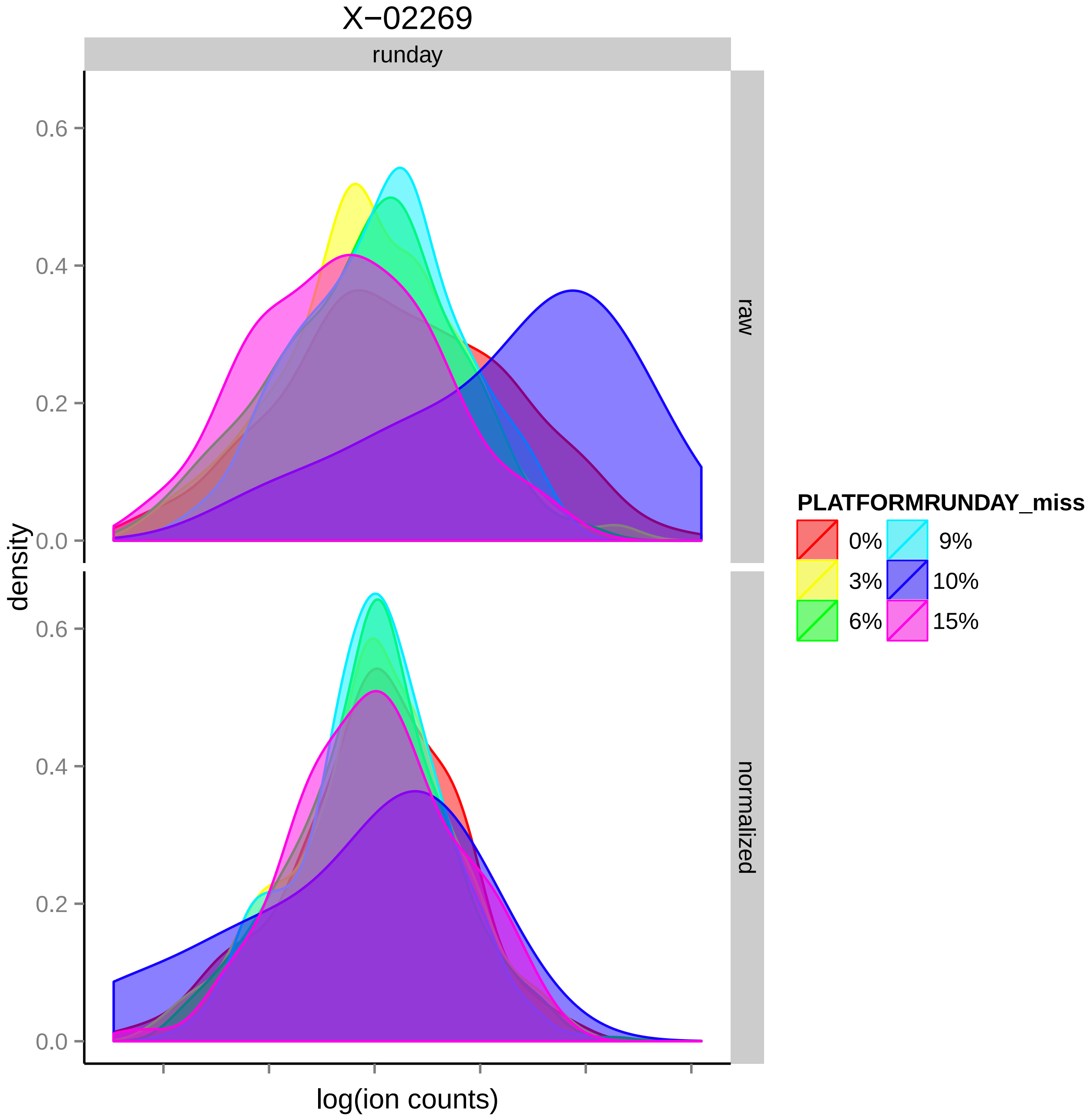

X−02269
runday
raw
normalized
density
0.0
0.2
0.4
0.6
log(ion counts)
PLATFORMRUNDAY_miss
0%
3%
6%
9%
10%
15%

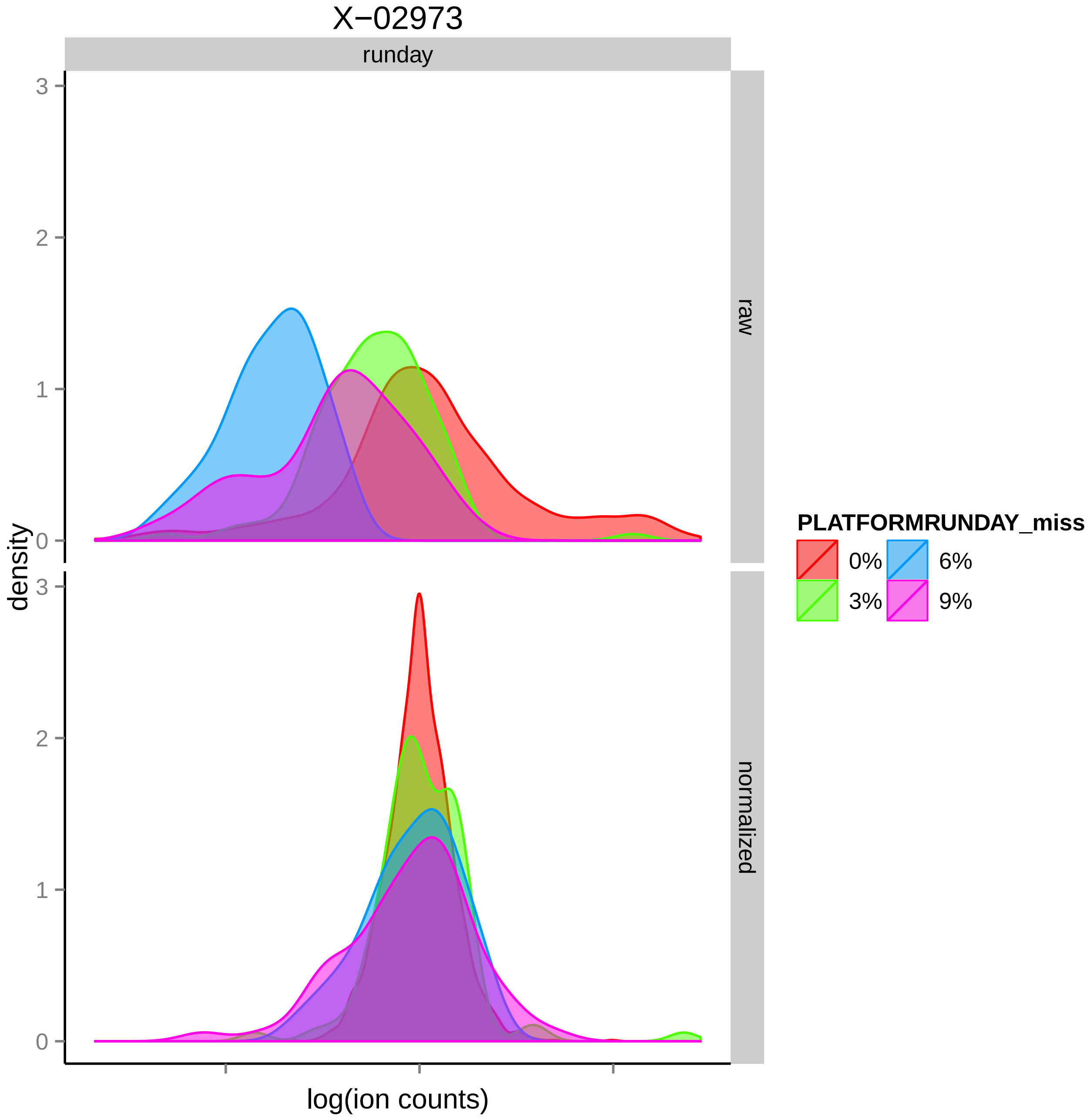

X−02973
runday
raw
normalized
density
log(ion counts)
3
2
1
0
3
2
1
0
PLATFORMRUNDAY_miss
0%
6%
3%
9%

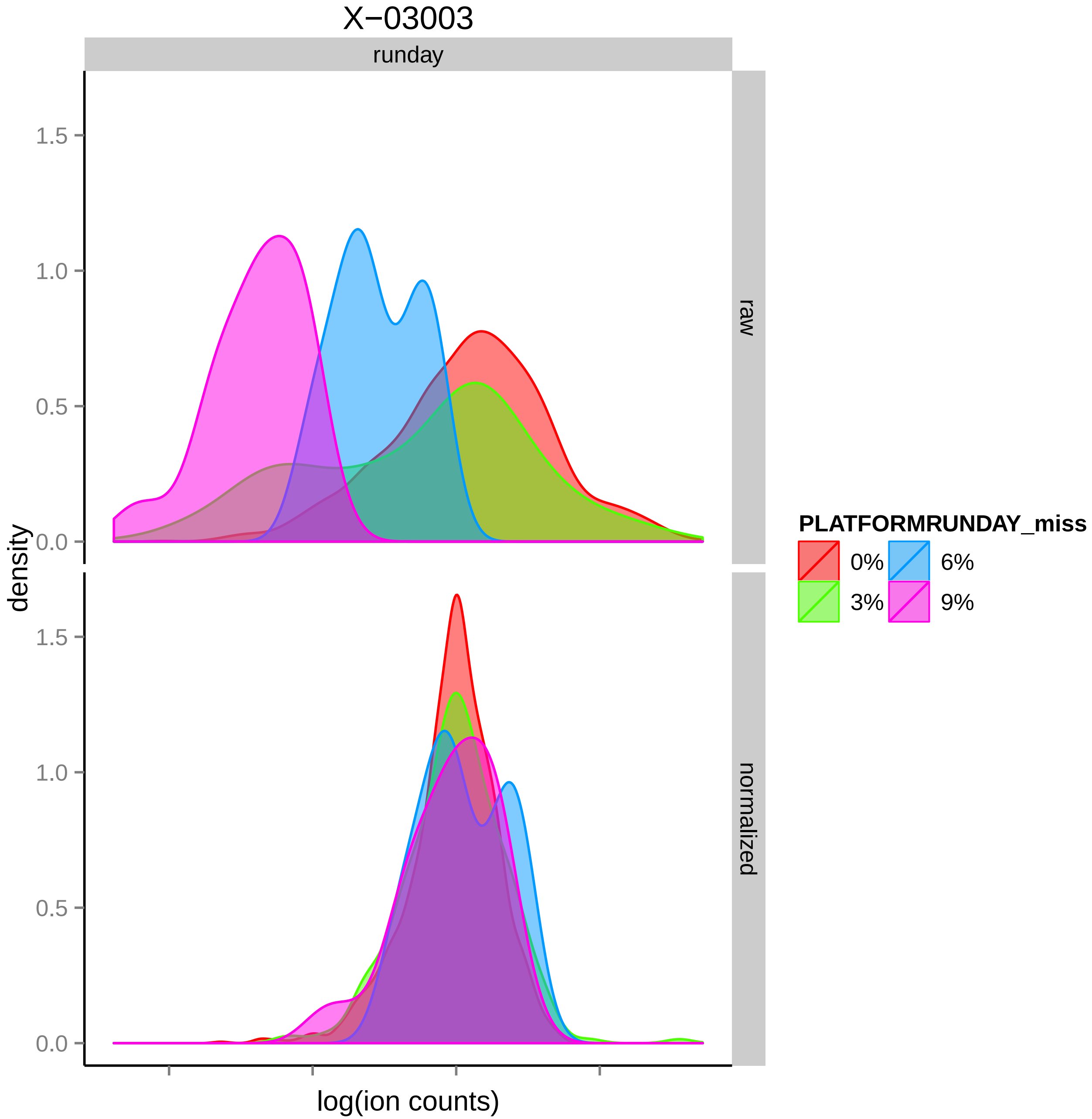
X−03003
runday
raw
normalized
density
log(ion counts)
1.5
1.0
0.5
0.0
PLATFORMRUNDAY_miss
0%
6%
3%
9%

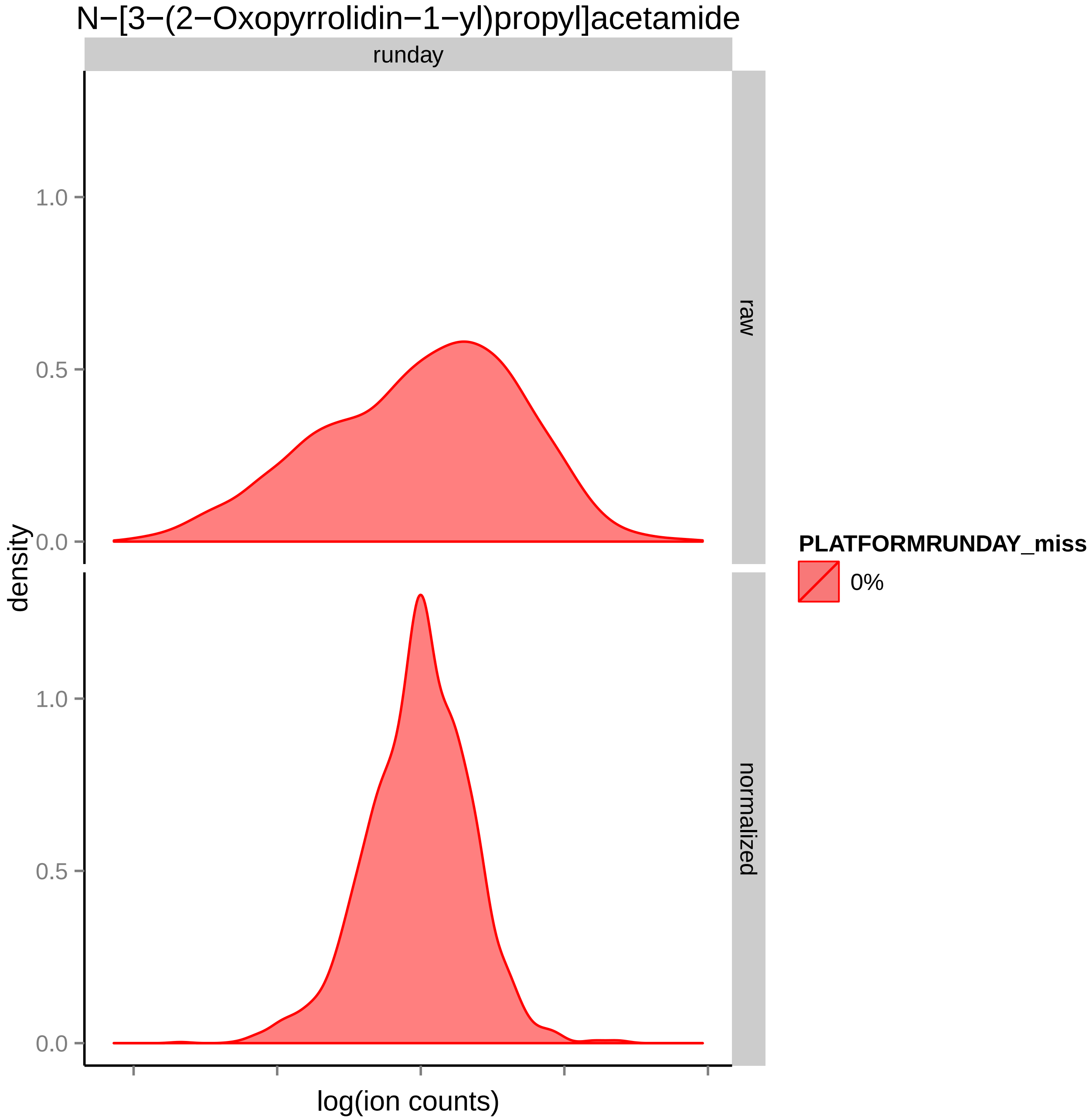
N−[3−(2−Oxopyrrolidin−1−yl)propyl]acetamide
runday
raw
normalized
density
1.0
0.5
0.0
log(ion counts)
PLATFORMRUNDAY_miss
0%

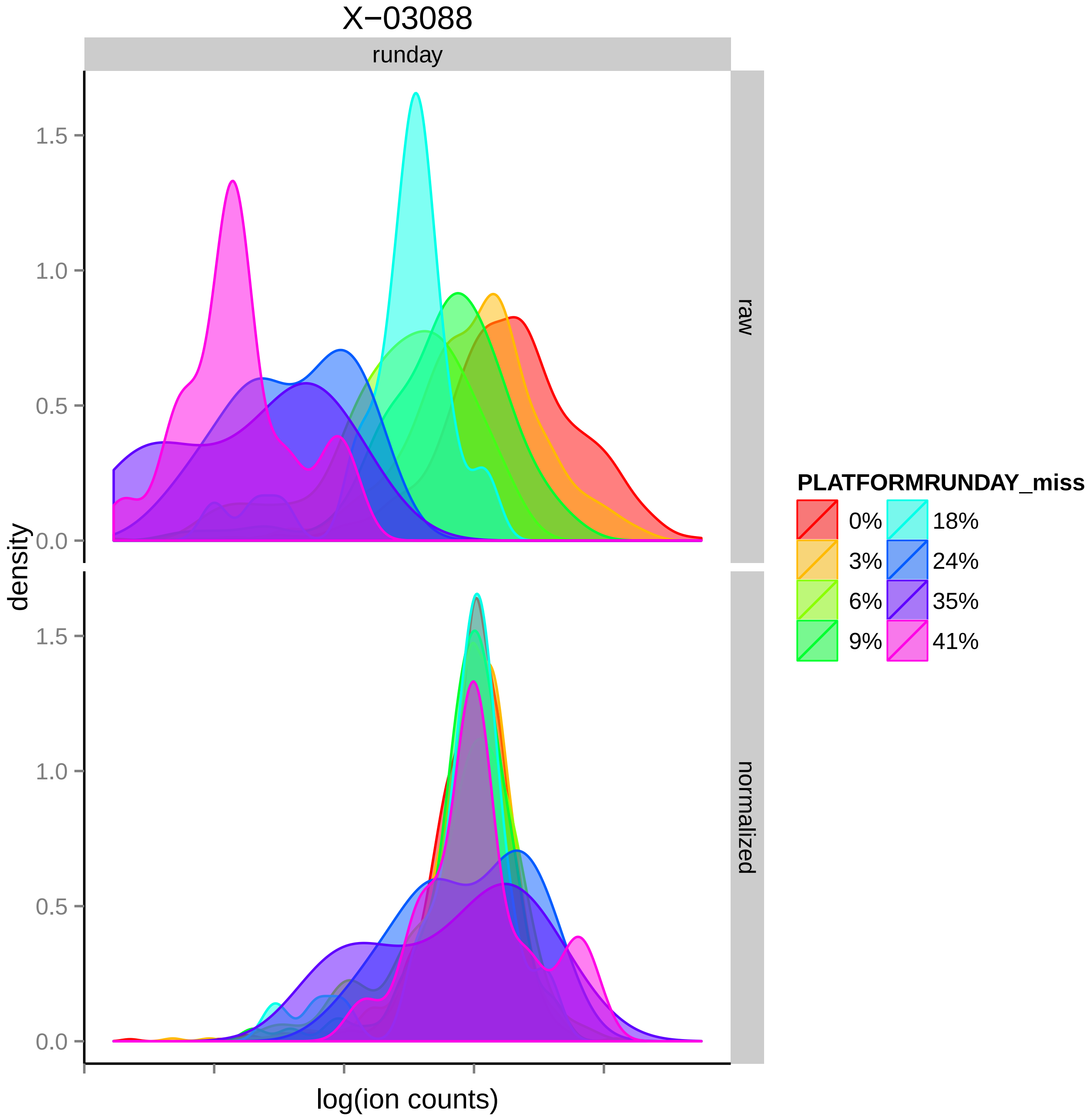
X−03088
runday
raw
normalized
density
log(ion counts)
1.5
1.0
0.5
0.0
PLATFORMRUNDAY_miss
0%
3%
6%
9%
18%
24%
35%
41%

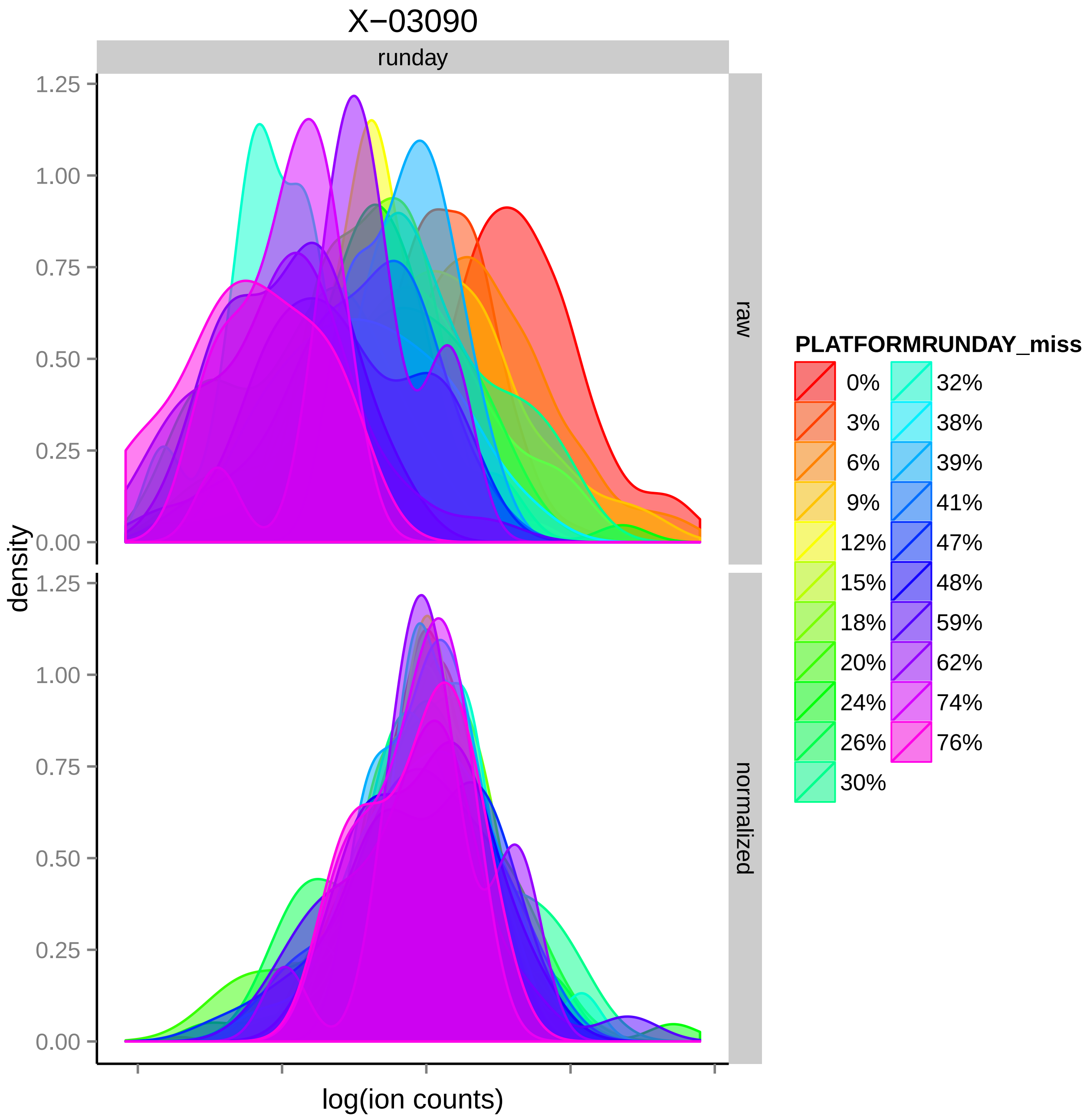

X−03090
runday
raw
normalized
density
log(ion counts)
1.25
1.00
0.75
0.50
0.25
0.00
PLATFORMRUNDAY_miss
0%
3%
6%
9%
12%
15%
18%
20%
24%
26%
30%
32%
38%
39%
41%
47%
48%
59%
62%
74%
76%

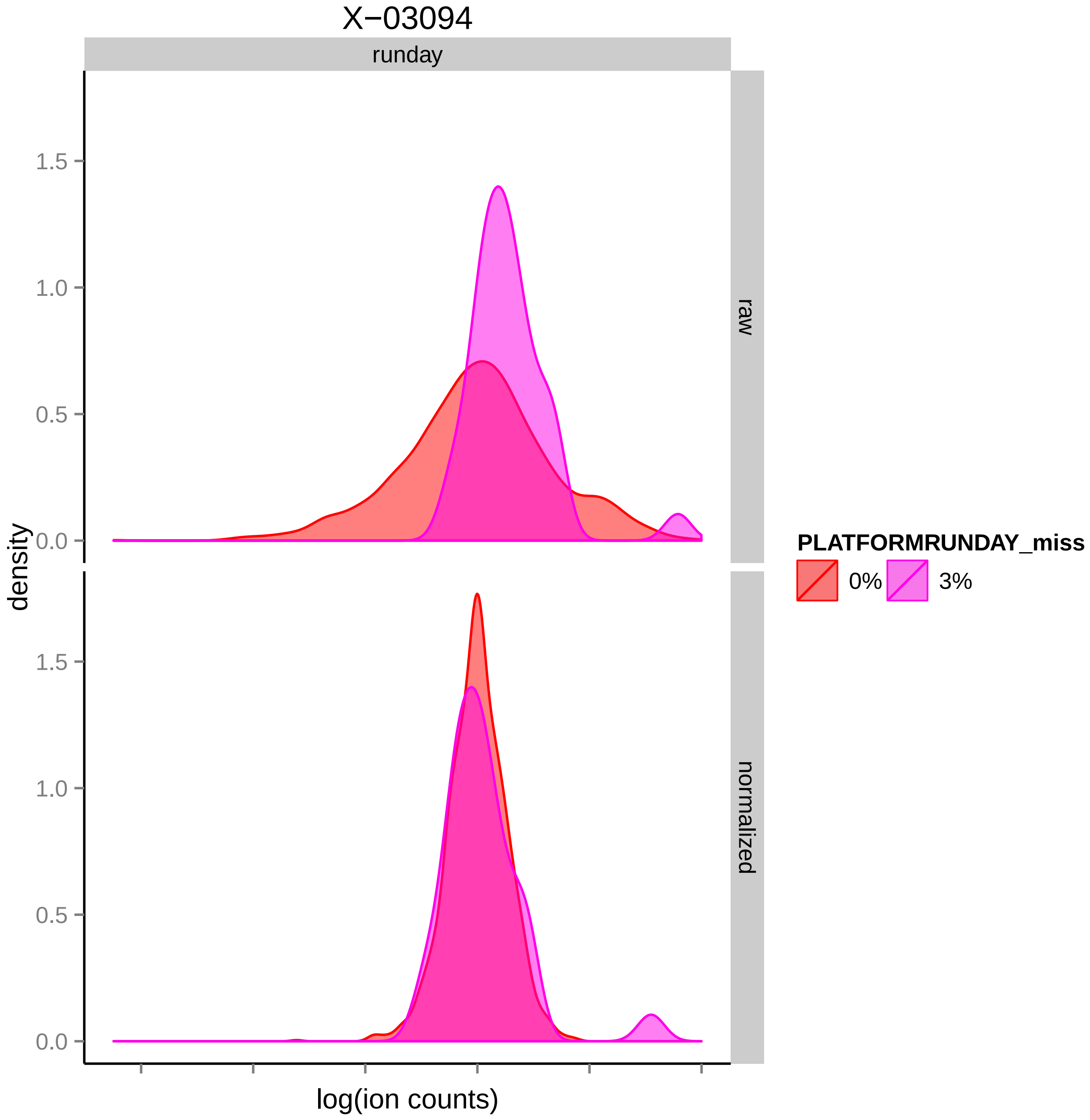

X−03094
runday
raw
normalized
density
1.5
1.0
0.5
0.0
log(ion counts)
PLATFORMRUNDAY_miss
0%
3%

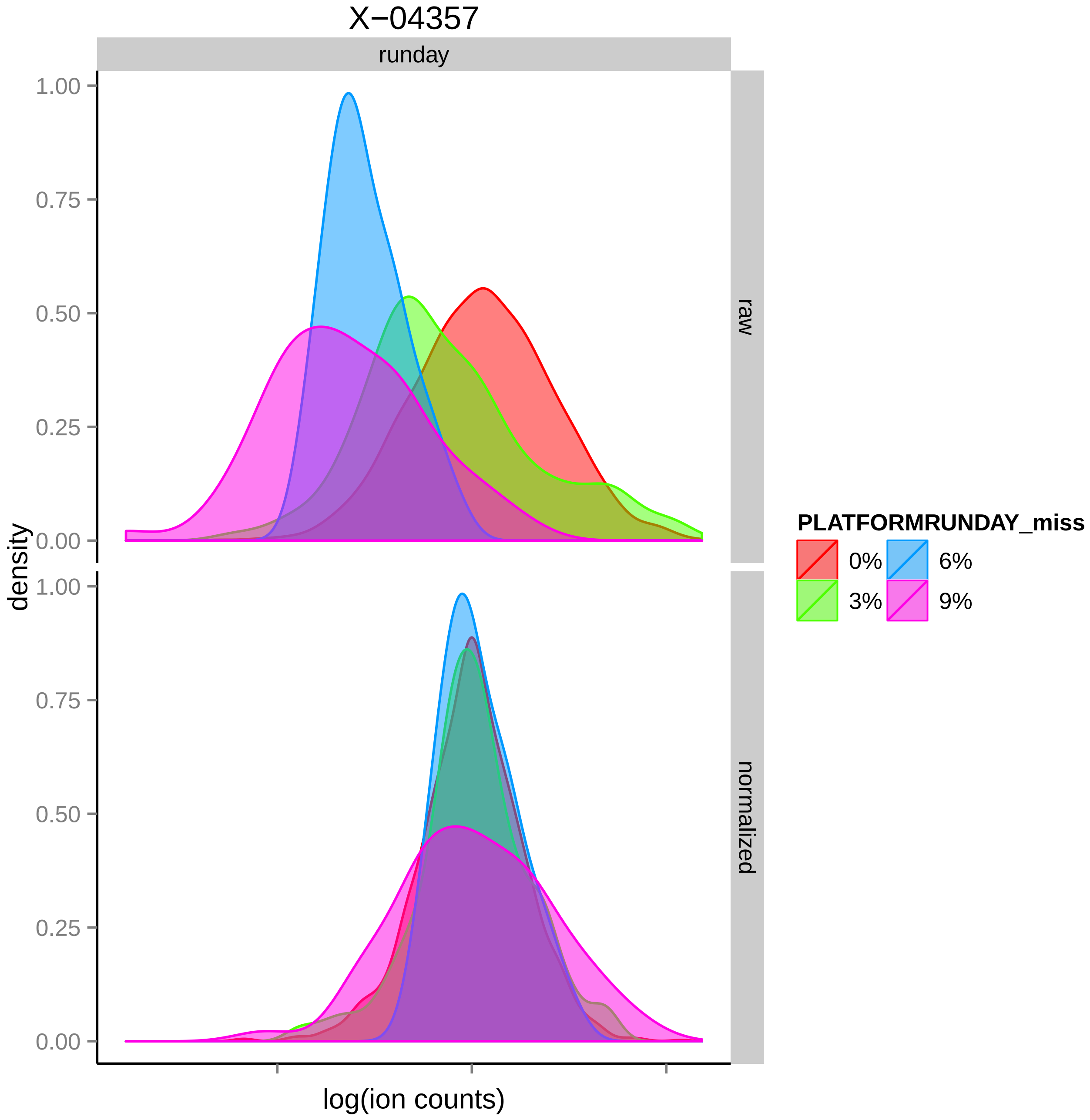

X−04357
runday
1.00
0.75
0.50
0.25
0.00
raw
normalized
density
log(ion counts)
PLATFORMRUNDAY_miss
0%
6%
3%
9%

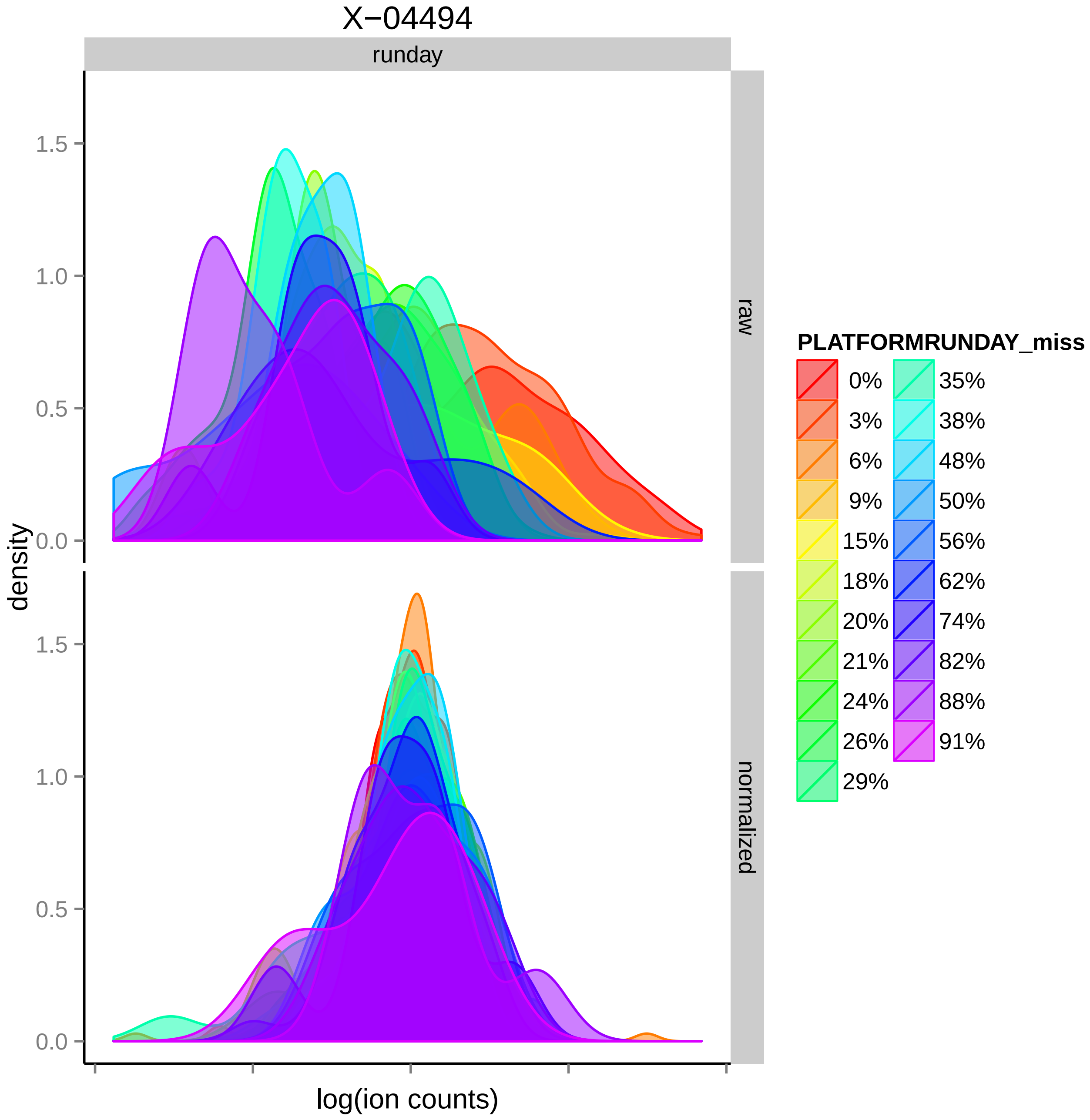

X−04494
runday
raw
normalized
density
log(ion counts)
1.5
1.0
0.5
0.0
PLATFORMRUNDAY_miss
0%
3%
6%
9%
15%
18%
20%
21%
24%
26%
29%
35%
38%
48%
50%
56%
62%
74%
82%
88%
91%

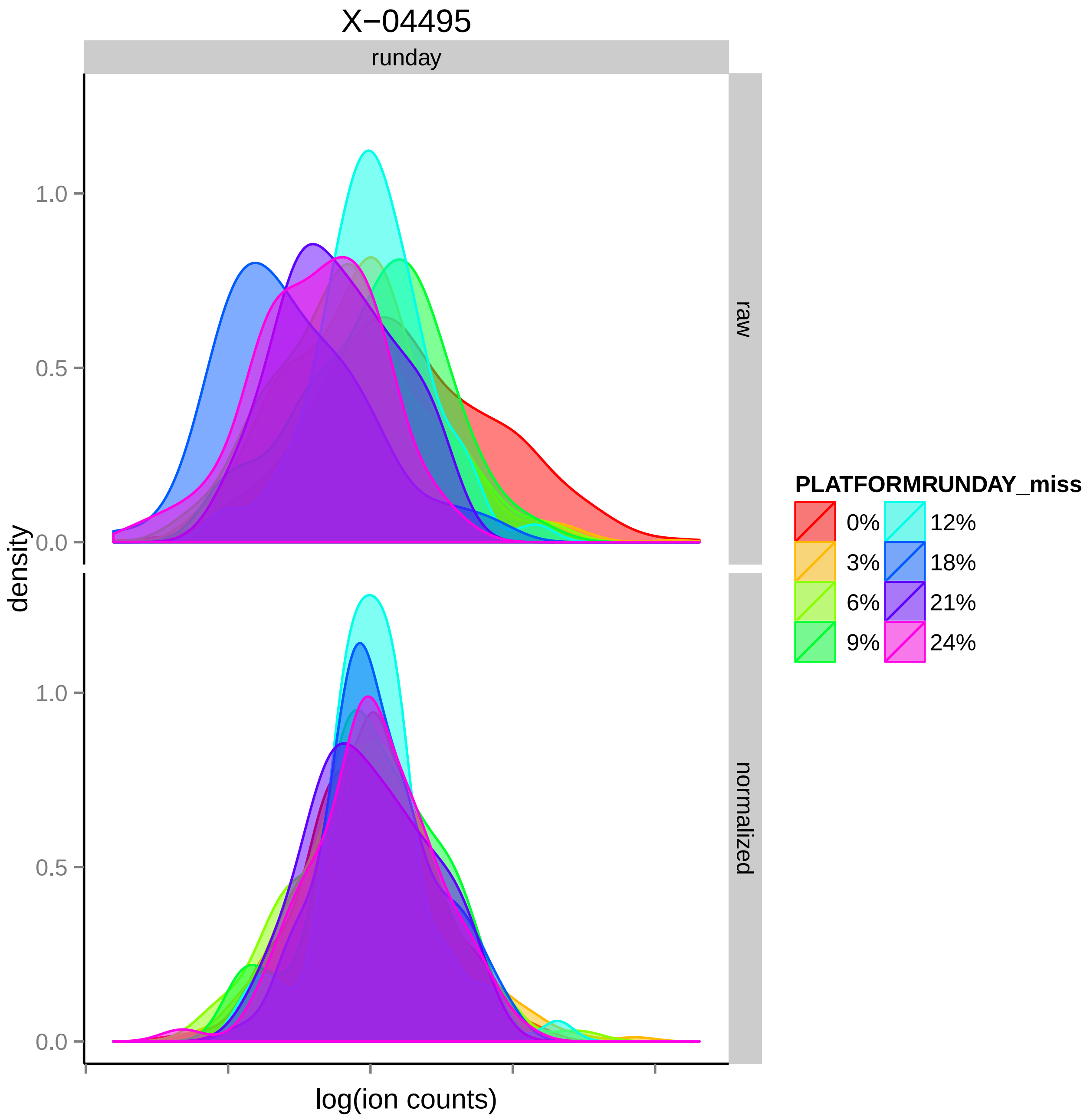

X−04495
runday
raw
normalized
density
1.0
0.5
0.0
log(ion counts)
PLATFORMRUNDAY_miss
0%
3%
6%
9%
12%
18%
21%
24%

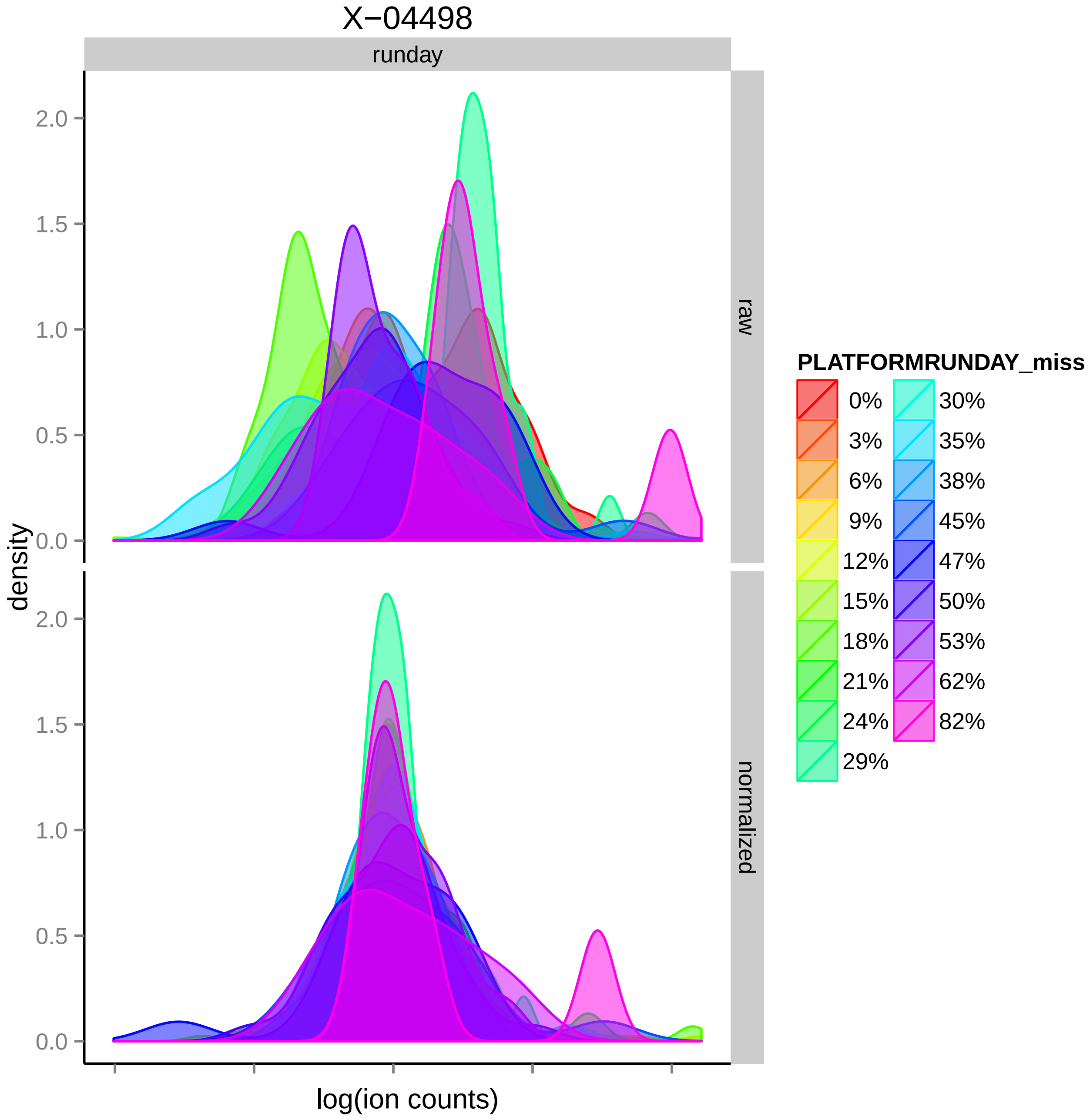

X−04498
runday
raw
normalized
density
2.0
1.5
1.0
0.5
0.0
log(ion counts)
PLATFORMRUNDAY_miss
0%
3%
6%
9%
12%
15%
18%
21%
24%
29%
30%
35%
38%
45%
47%
50%
53%
62%
82%

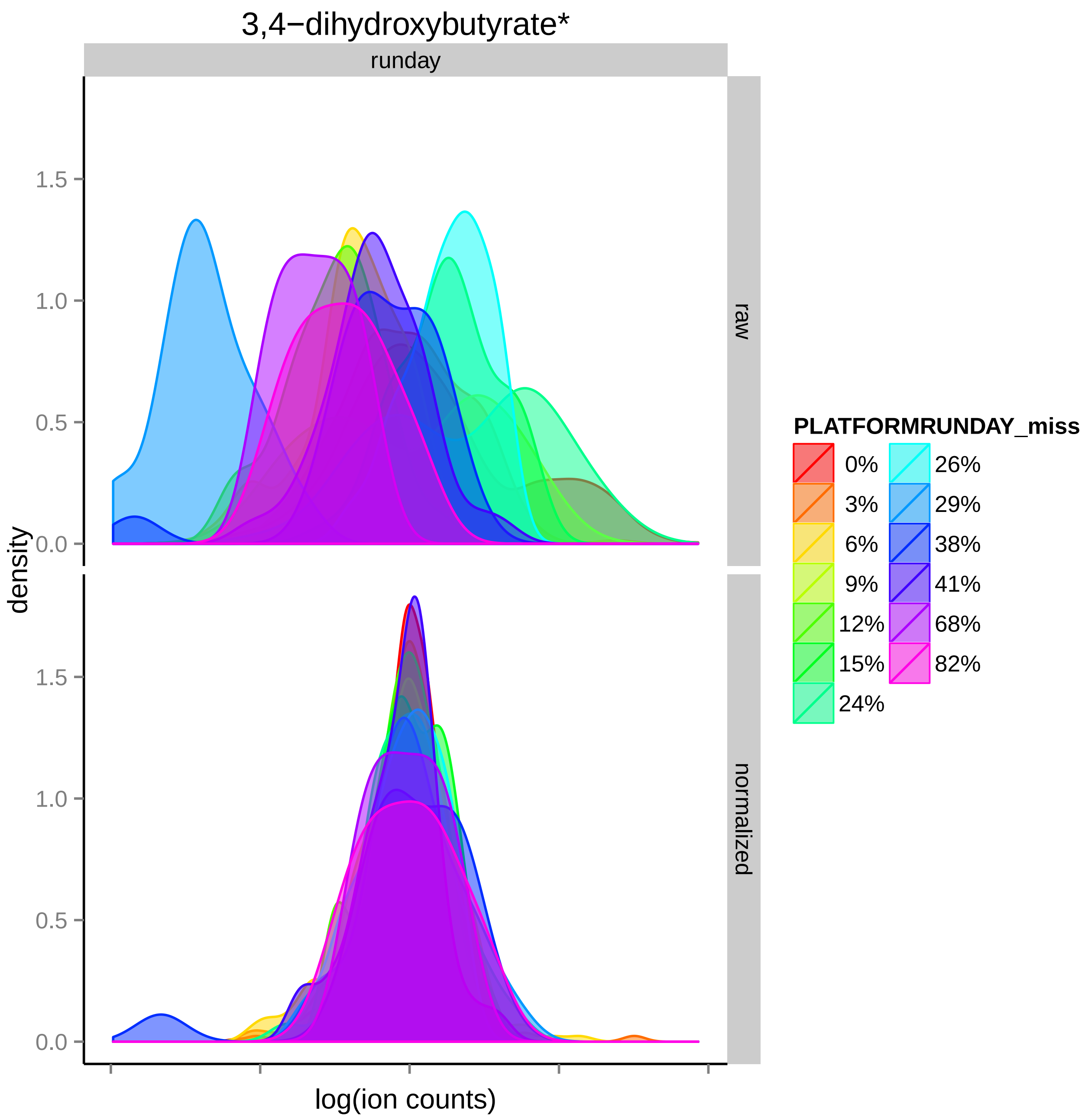

3,4−dihydroxybutyrate*
runday
raw
normalized
density
log(ion counts)
0.0
0.5
1.0
1.5
PLATFORMRUNDAY_miss
0%
3%
6%
9%
12%
15%
24%
26%
29%
38%
41%
68%
82%

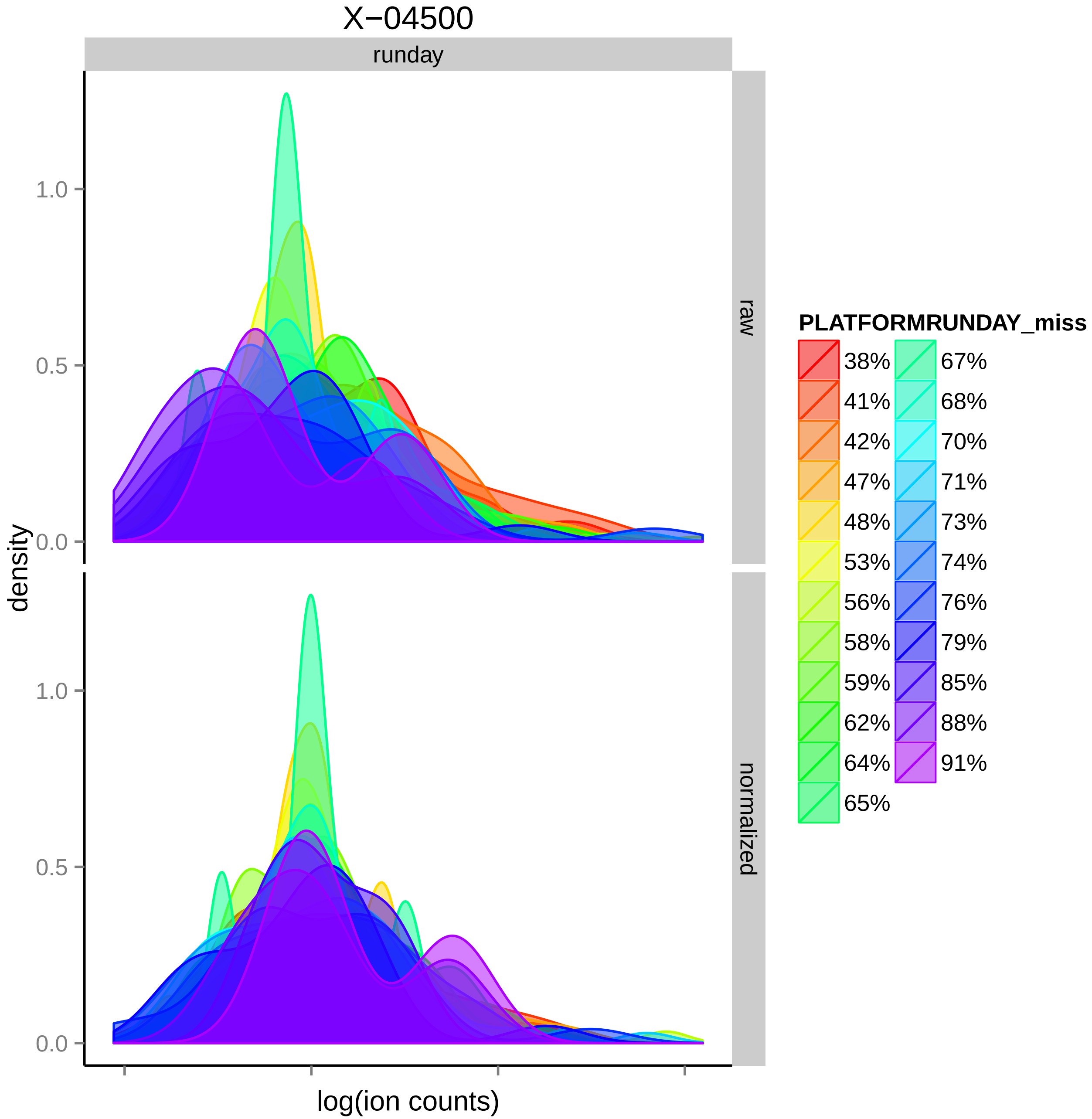
X−04500
runday
raw
normalized
density
1.0
0.5
0.0
log(ion counts)
PLATFORMRUNDAY_miss
38%
41%
42%
47%
48%
53%
56%
58%
59%
62%
64%
65%
67%
68%
70%
71%
73%
74%
76%
79%
85%
88%
91%

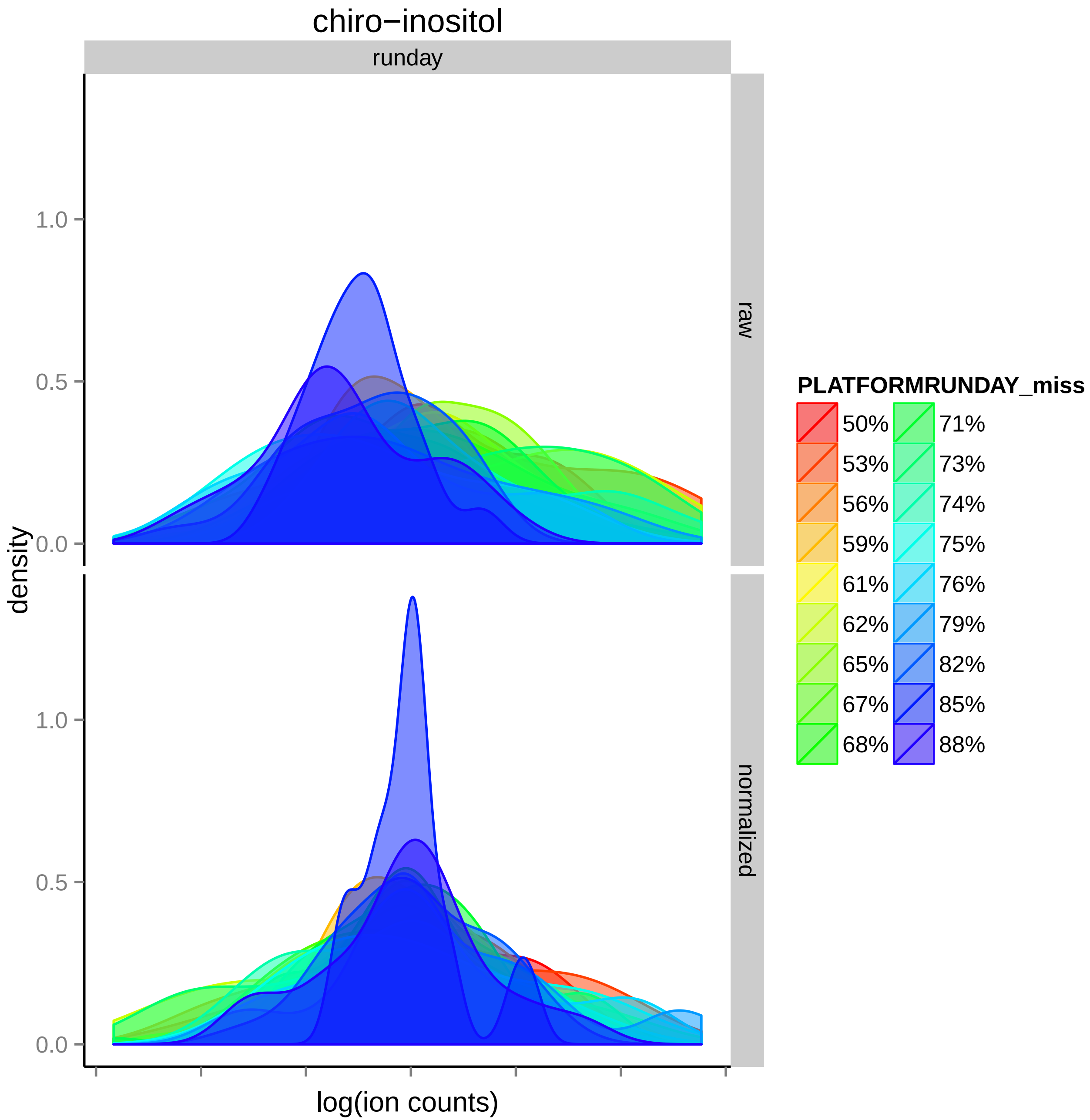
chiro−inositol
runday
raw
normalized
density
log(ion counts)
1.0
0.5
0.0
PLATFORMRUNDAY_miss
50%
53%
56%
59%
61%
62%
65%
67%
68%
71%
73%
74%
75%
76%
79%
82%
85%
88%

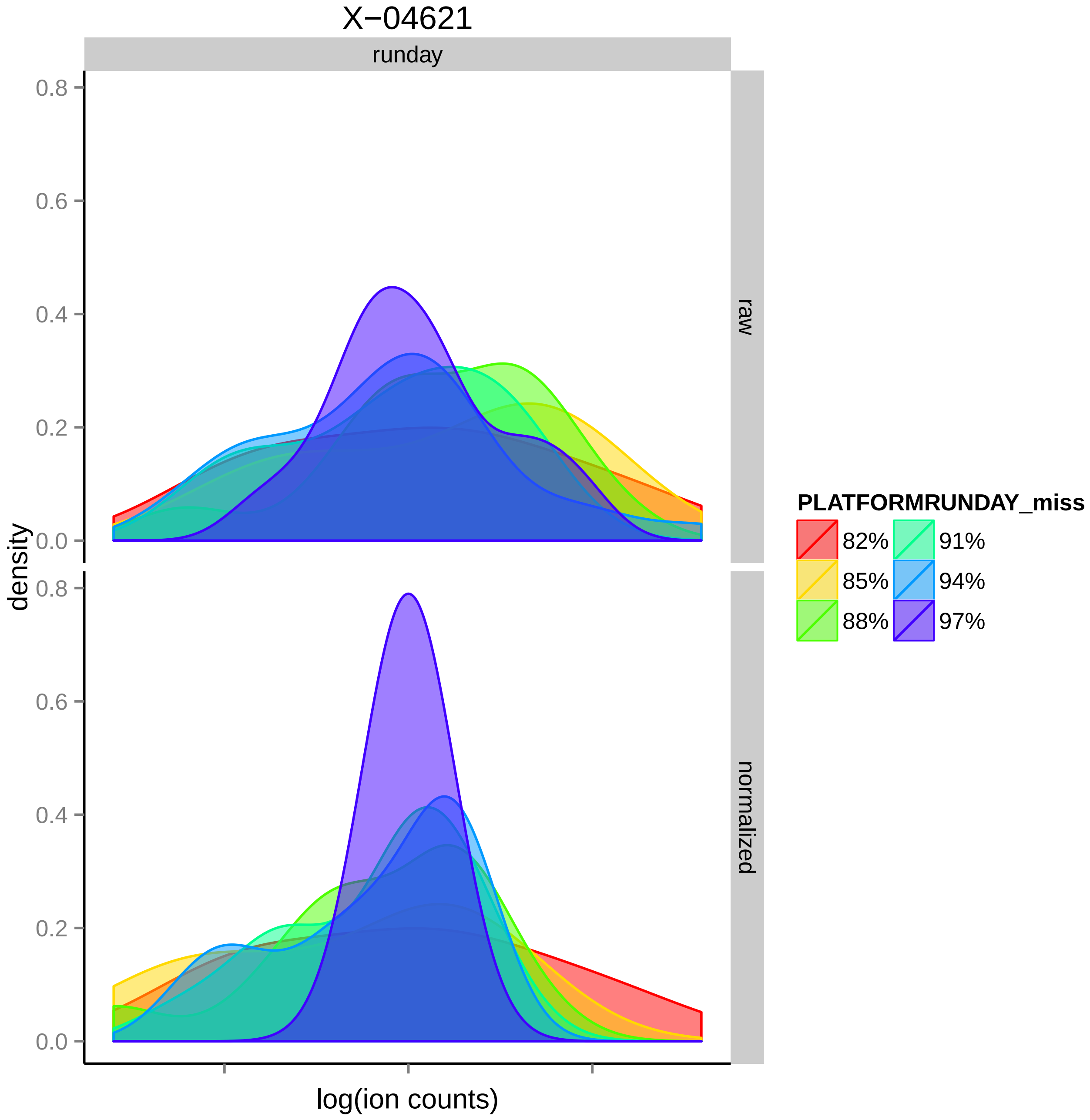

X−04621
runday
raw
normalized
0.8
0.6
0.4
0.2
0.0
density
log(ion counts)
PLATFORMRUNDAY_miss
82%
85%
88%
91%
94%
97%

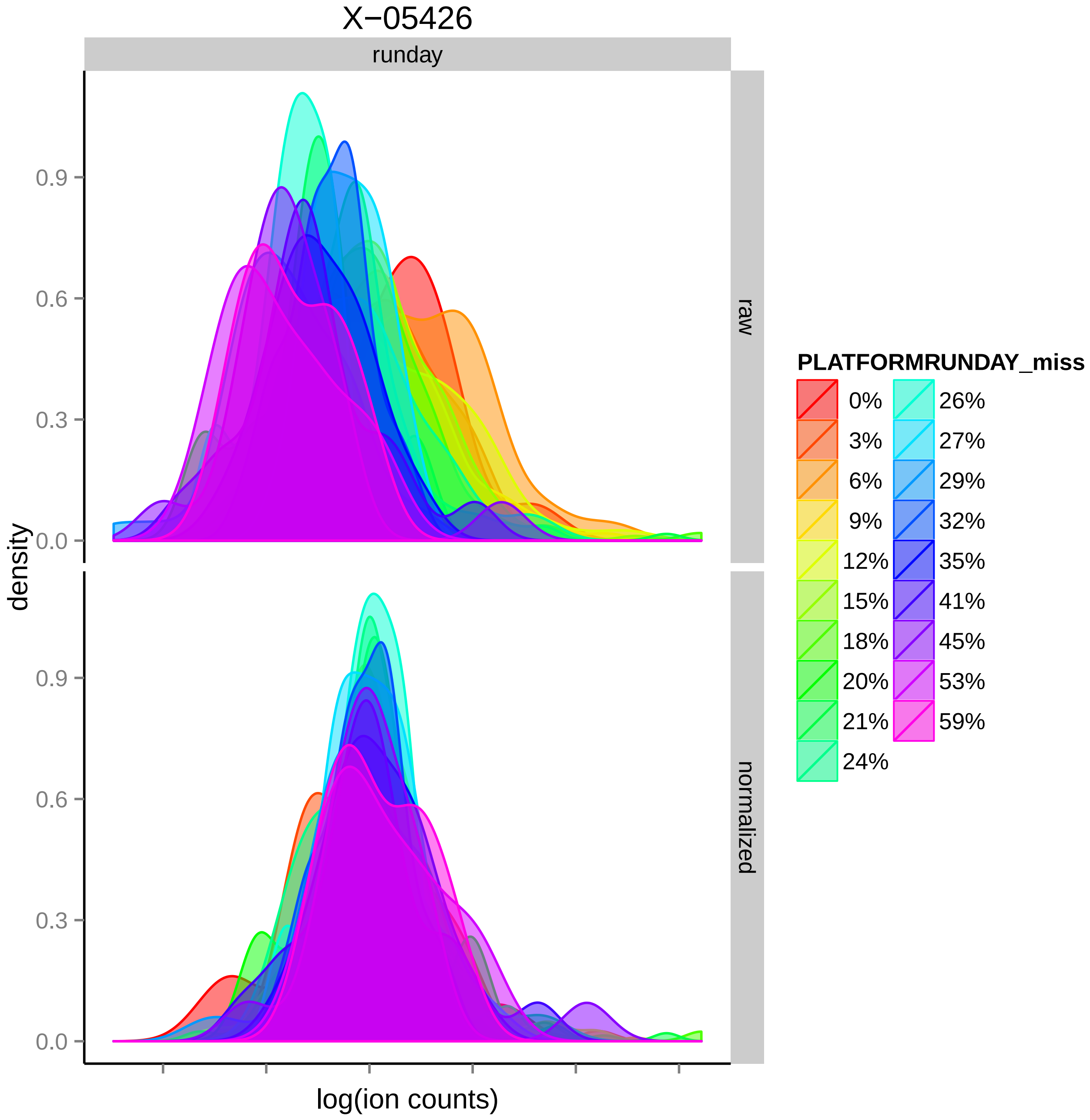

X−05426
runday
raw
normalized
density
0.0
0.3
0.6
0.9
log(ion counts)
PLATFORMRUNDAY_miss
0%
3%
6%
9%
12%
15%
18%
20%
21%
24%
26%
27%
29%
32%
35%
41%
45%
53%
59%

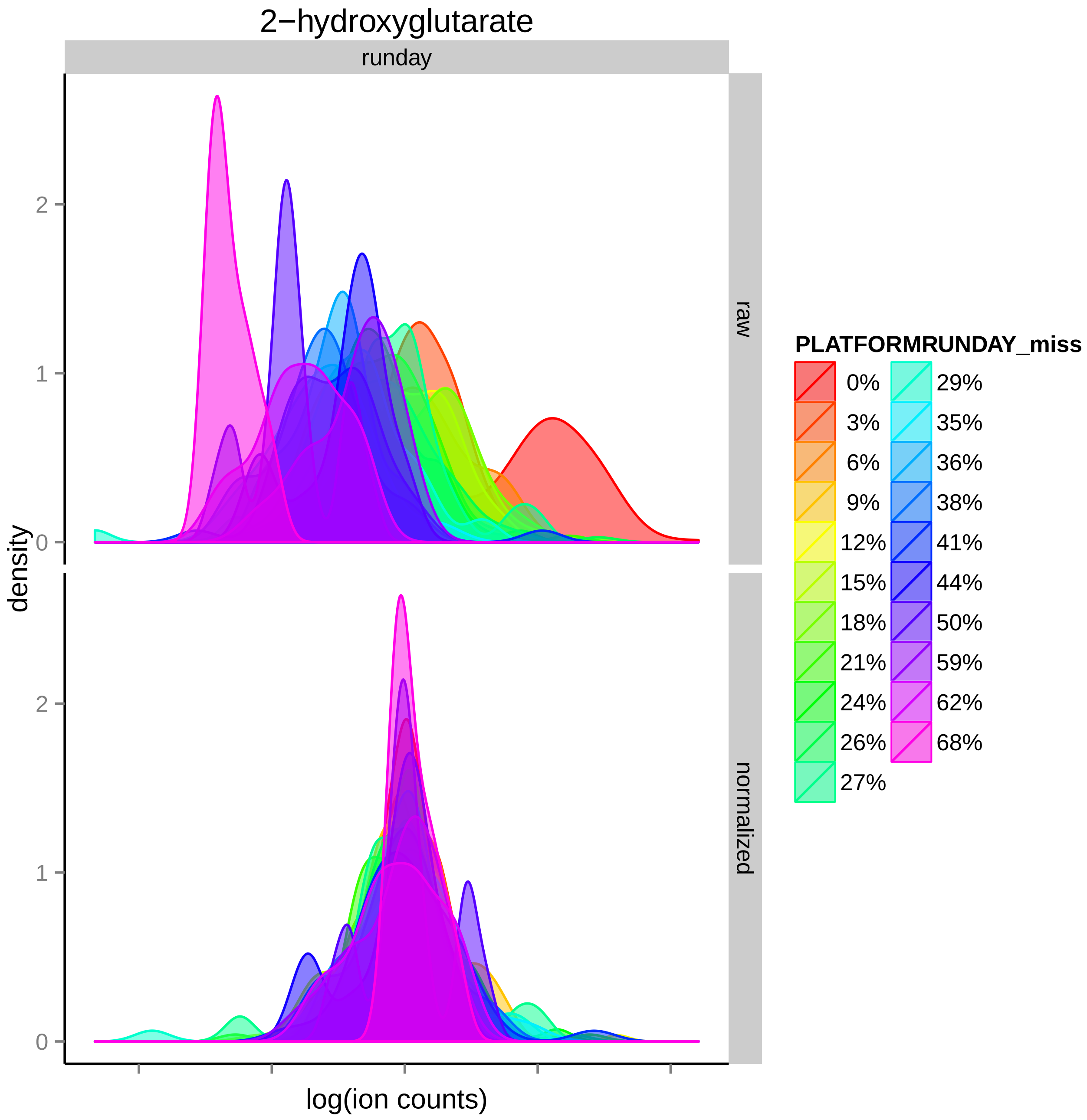
2−hydroxyglutarate
runday
raw
normalized
density
log(ion counts)
0
1
2
PLATFORMRUNDAY_miss
0%
3%
6%
9%
12%
15%
18%
21%
24%
26%
27%
29%
35%
36%
38%
41%
44%
50%
59%
62%
68%

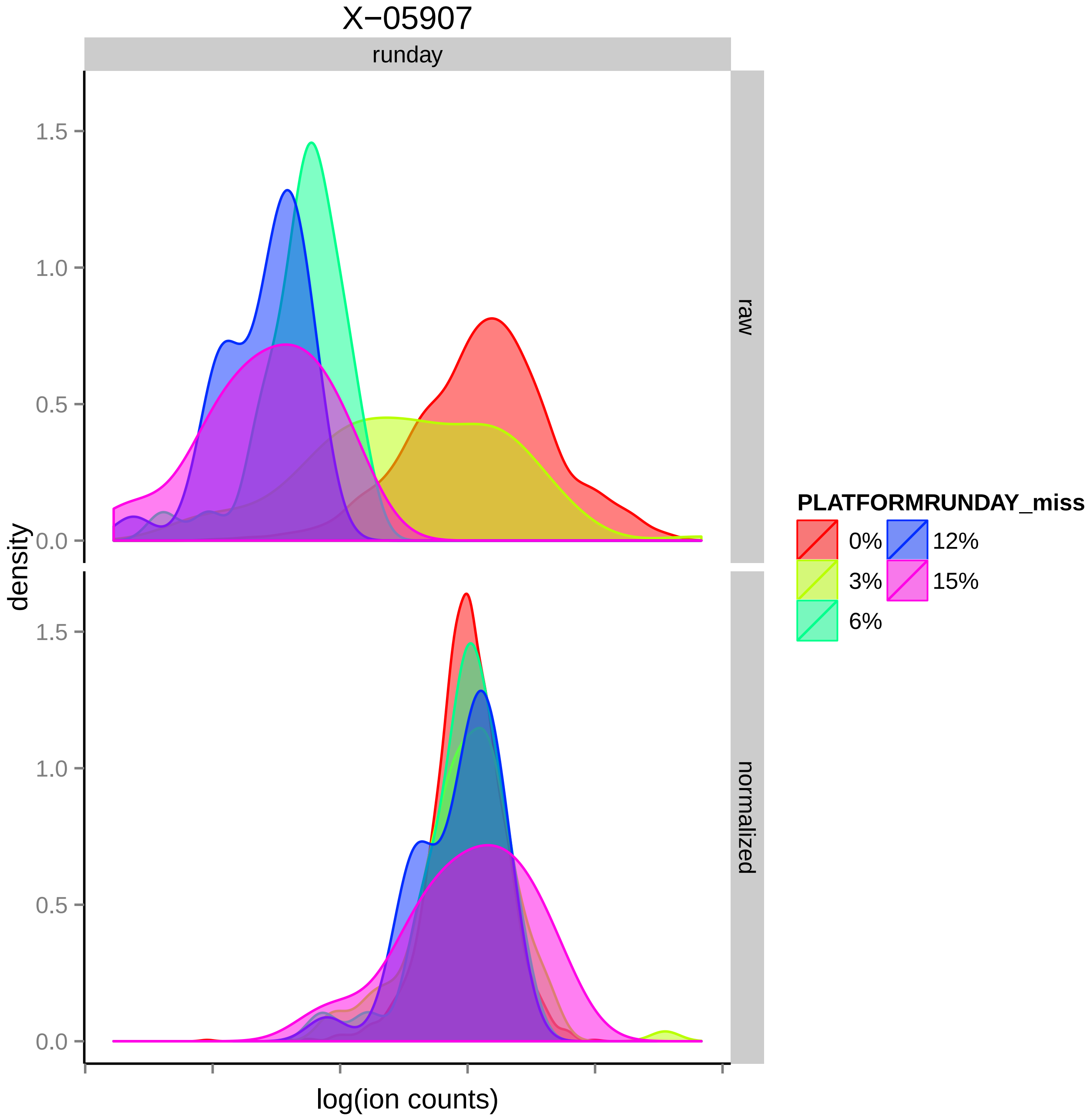

X−05907
runday
raw
normalized
density
0.0
0.5
1.0
1.5
log(ion counts)
PLATFORMRUNDAY_miss
0%
3%
6%
12%
15%

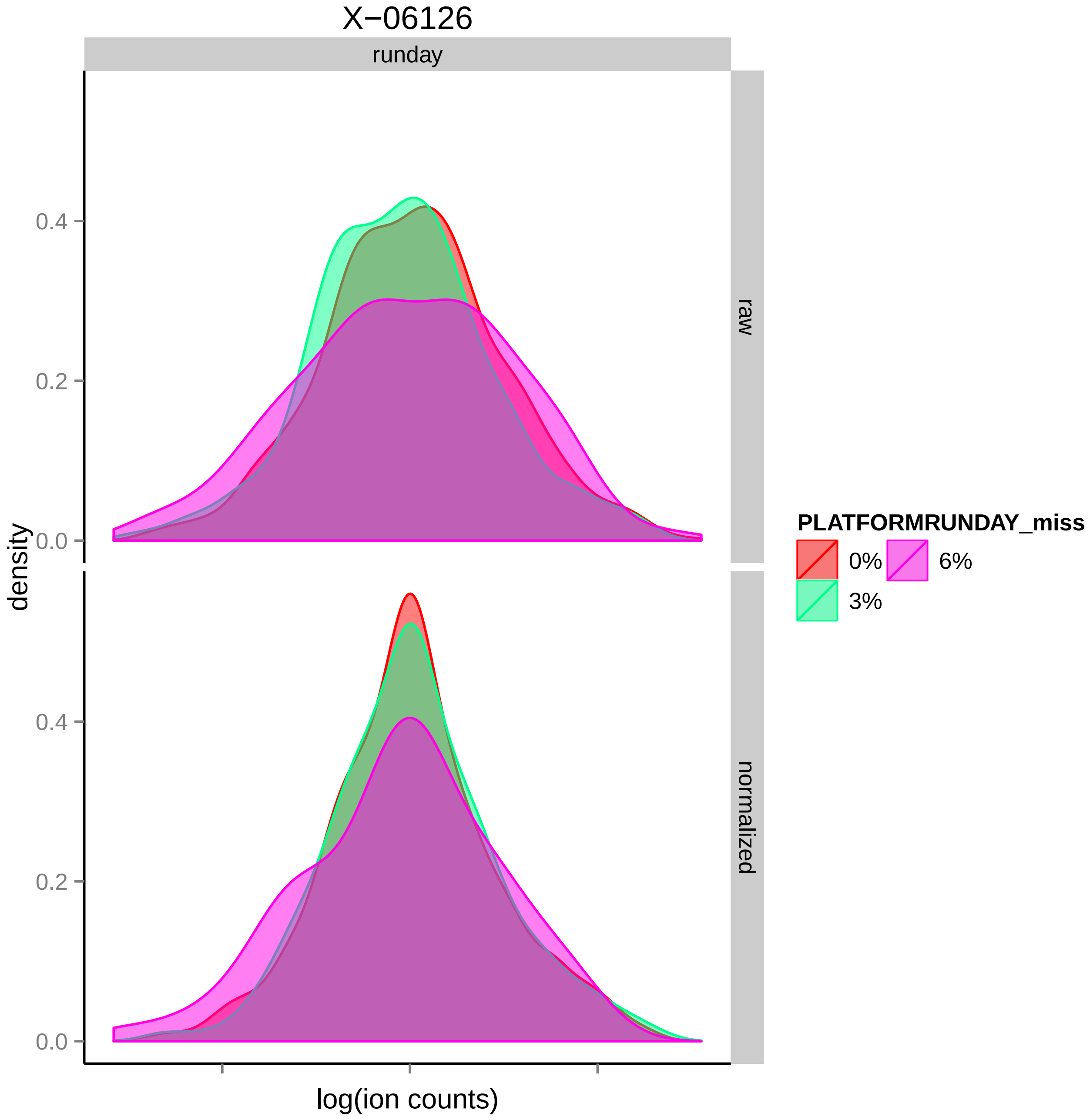
X−06126
runday
raw
normalized
density
0.0
0.2
0.4
log(ion counts)
PLATFORMRUNDAY_miss
0%
3%
6%

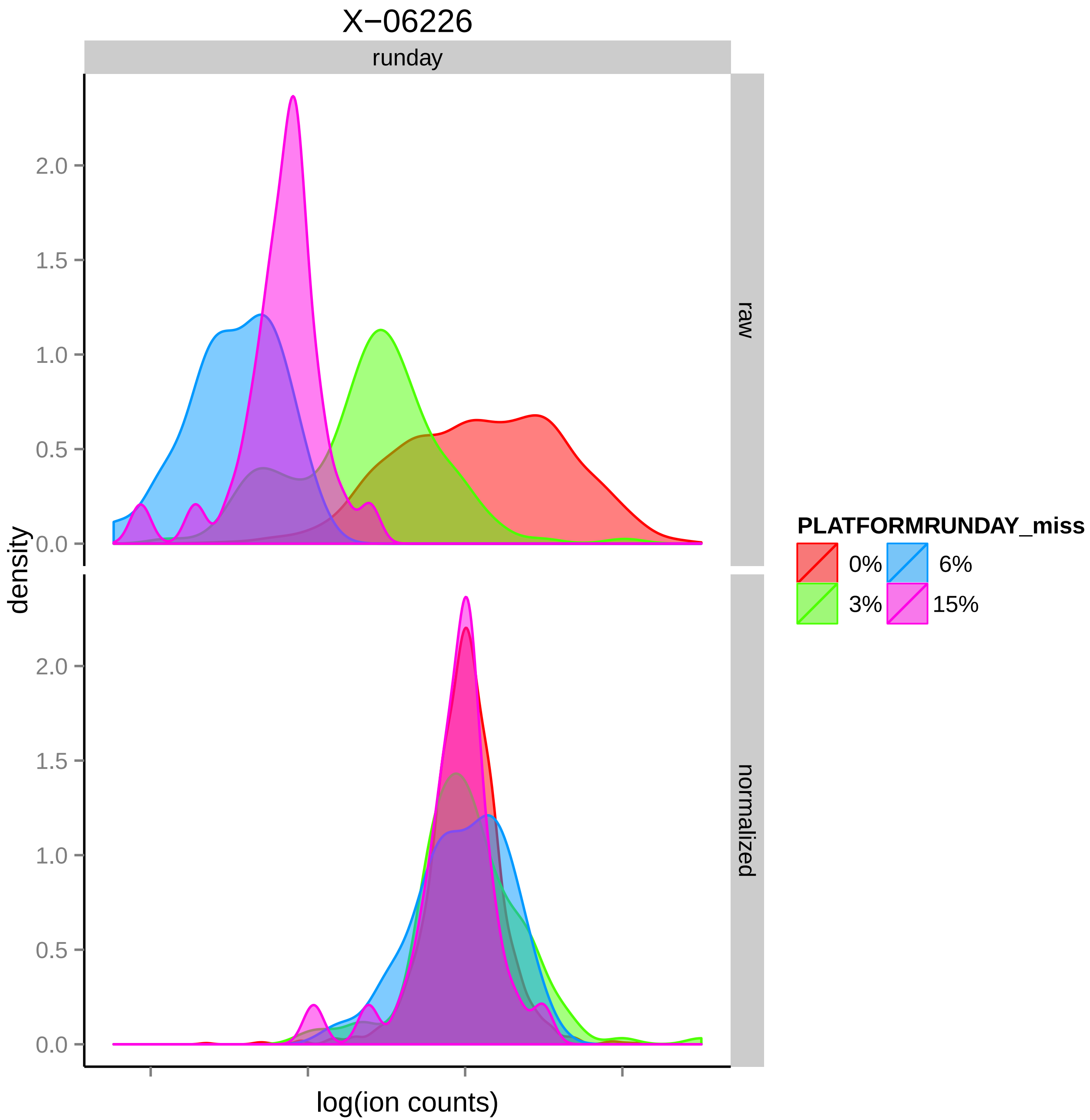

X−06226
runday
raw
normalized
density
log(ion counts)
2.0
1.5
1.0
0.5
0.0
PLATFORMRUNDAY_miss
0%
6%
3%
15%

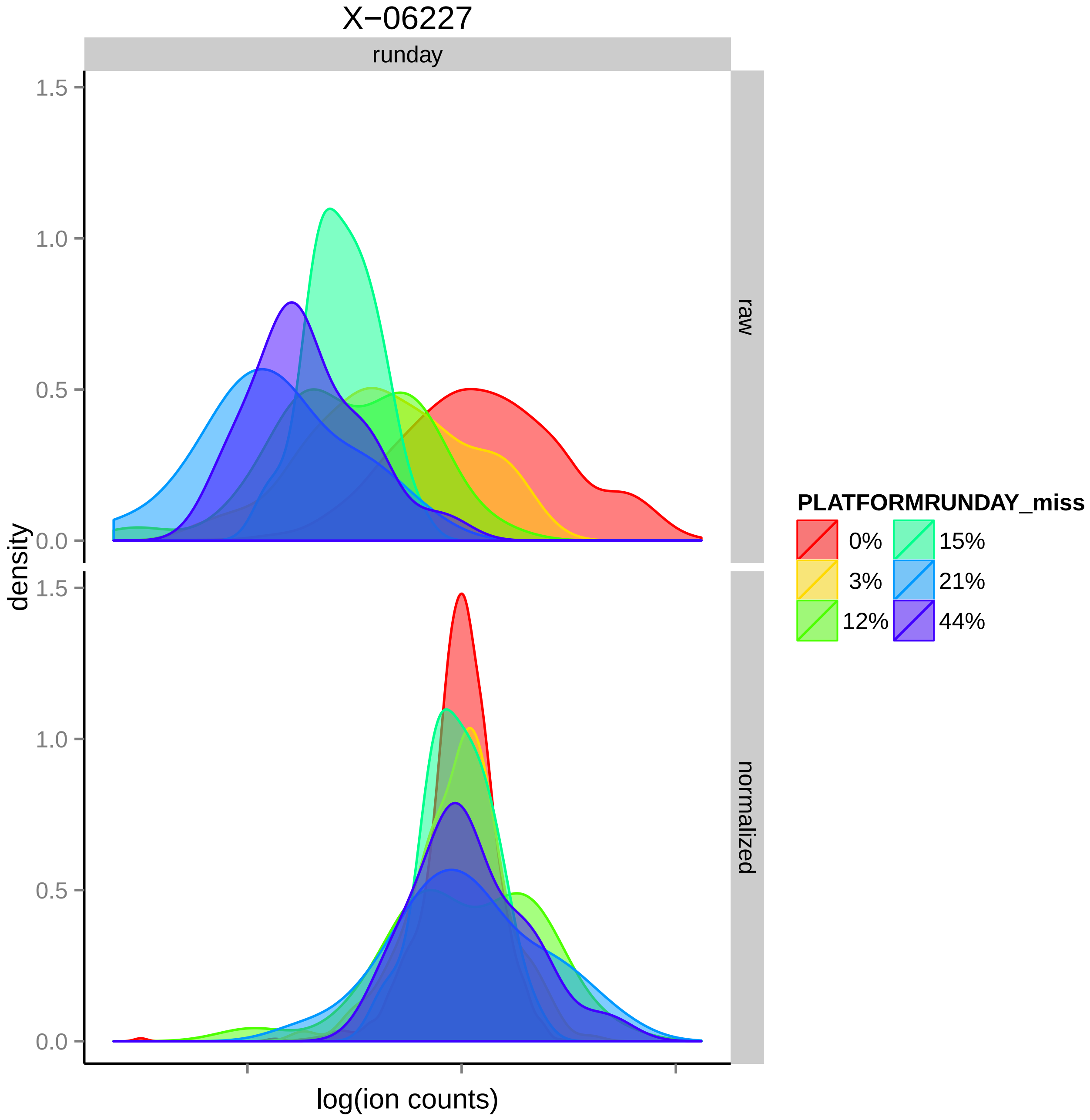

X−06227
runday
raw
normalized
density
log(ion counts)
1.5
1.0
0.5
0.0
PLATFORMRUNDAY_miss
0%
3%
12%
15%
21%
44%

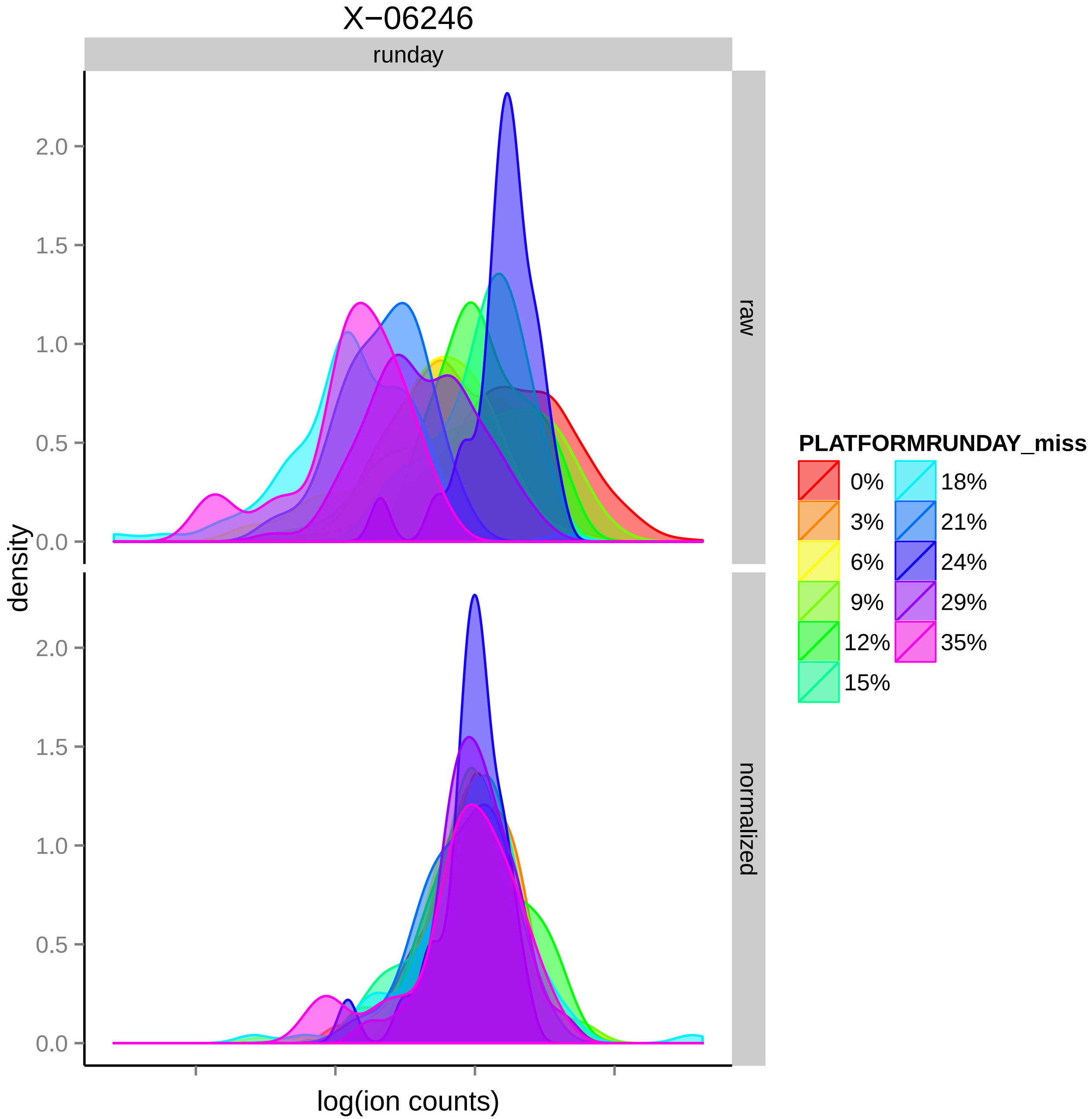
X−06246
runday
raw
normalized
density
log(ion counts)
2.0
1.5
1.0
0.5
0.0
PLATFORMRUNDAY_miss
0%
3%
6%
9%
12%
15%
18%
21%
24%
29%
35%

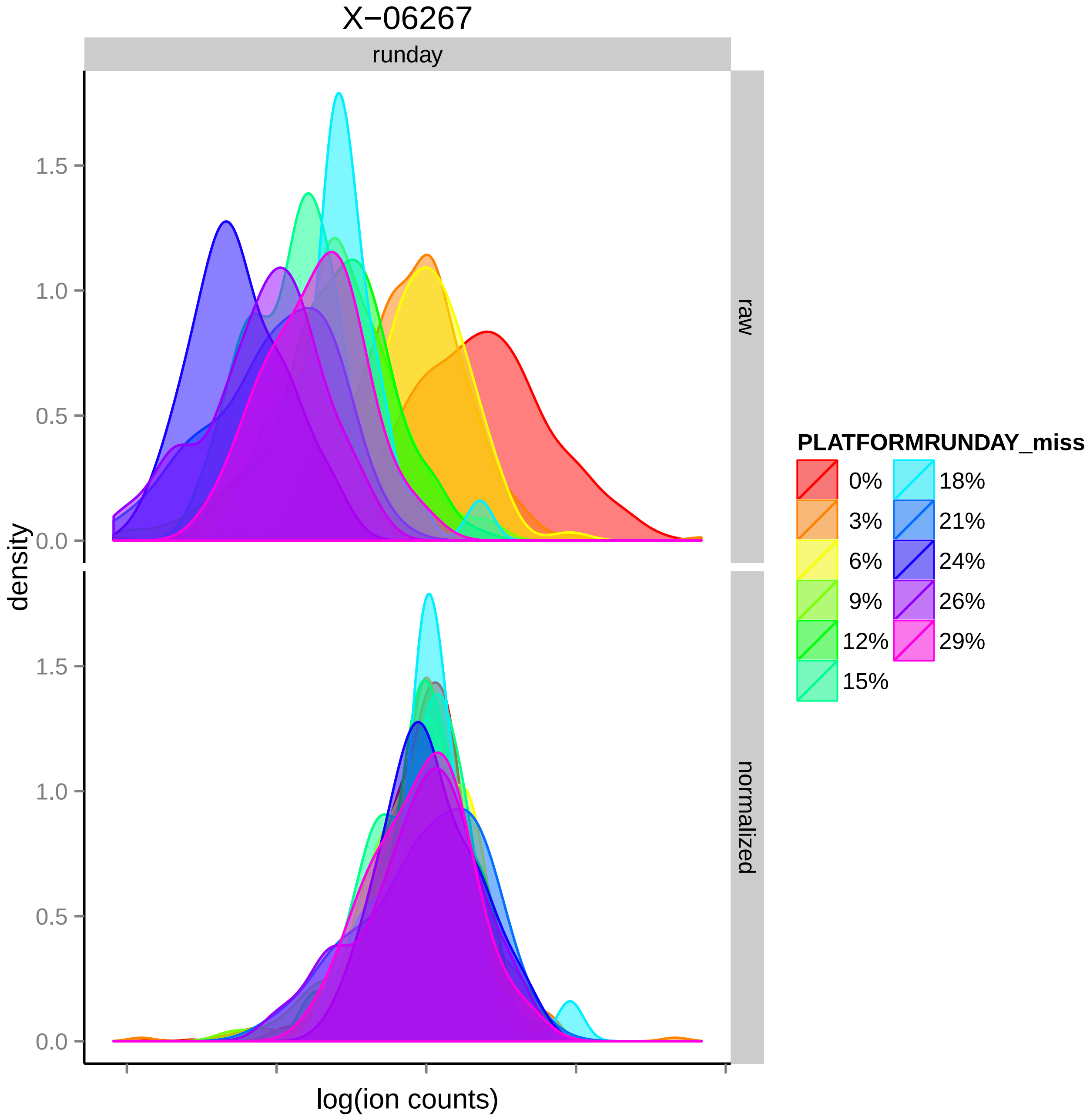
X−06267
runday
raw
normalized
density
log(ion counts)
1.5
1.0
0.5
0.0
PLATFORMRUNDAY_miss
0%
3%
6%
9%
12%
15%
18%
21%
24%
26%
29%

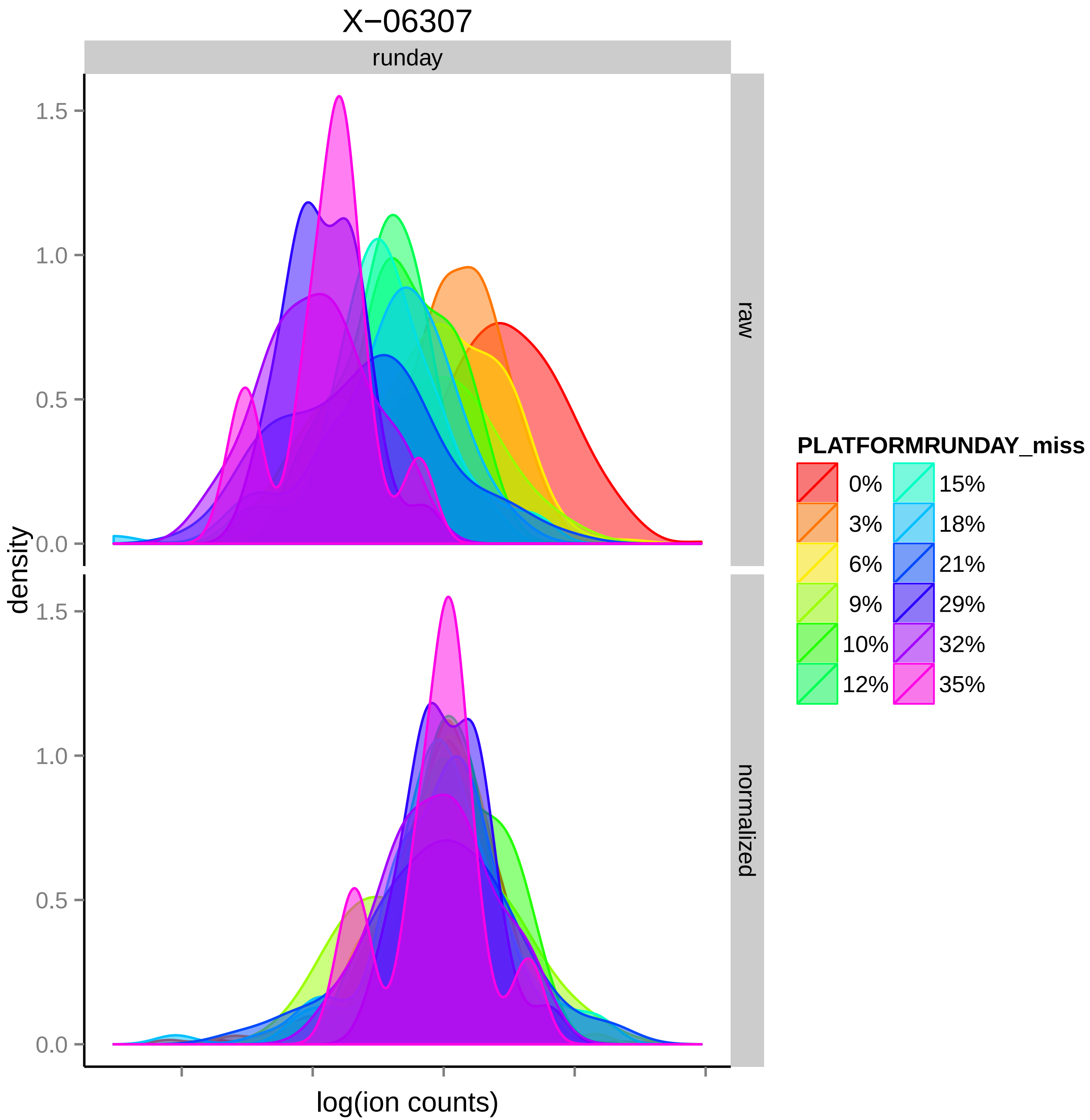
X−06307
runday
raw
normalized
density
log(ion counts)
1.5
1.0
0.5
0.0
1.5
1.0
0.5
0.0
PLATFORMRUNDAY_miss
0%
3%
6%
9%
10%
12%
15%
18%
21%
29%
32%
35%

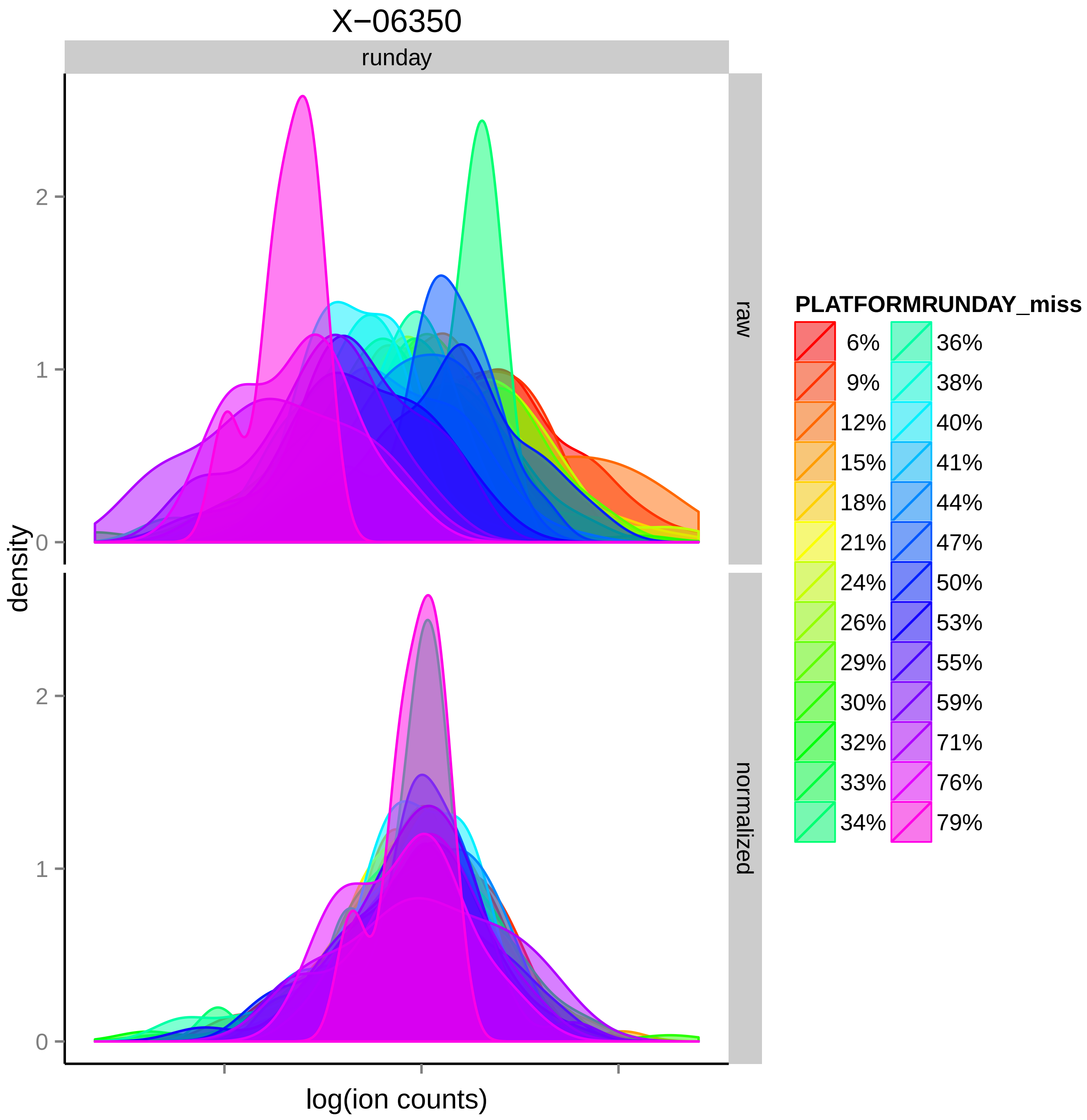
X−06350
runday
raw
normalized
density
log(ion counts)
2
1
0
2
1
0
PLATFORMRUNDAY_miss
6%
9%
12%
15%
18%
21%
24%
26%
29%
30%
32%
33%
34%
36%
38%
40%
41%
44%
47%
50%
53%
55%
59%
71%
76%
79%

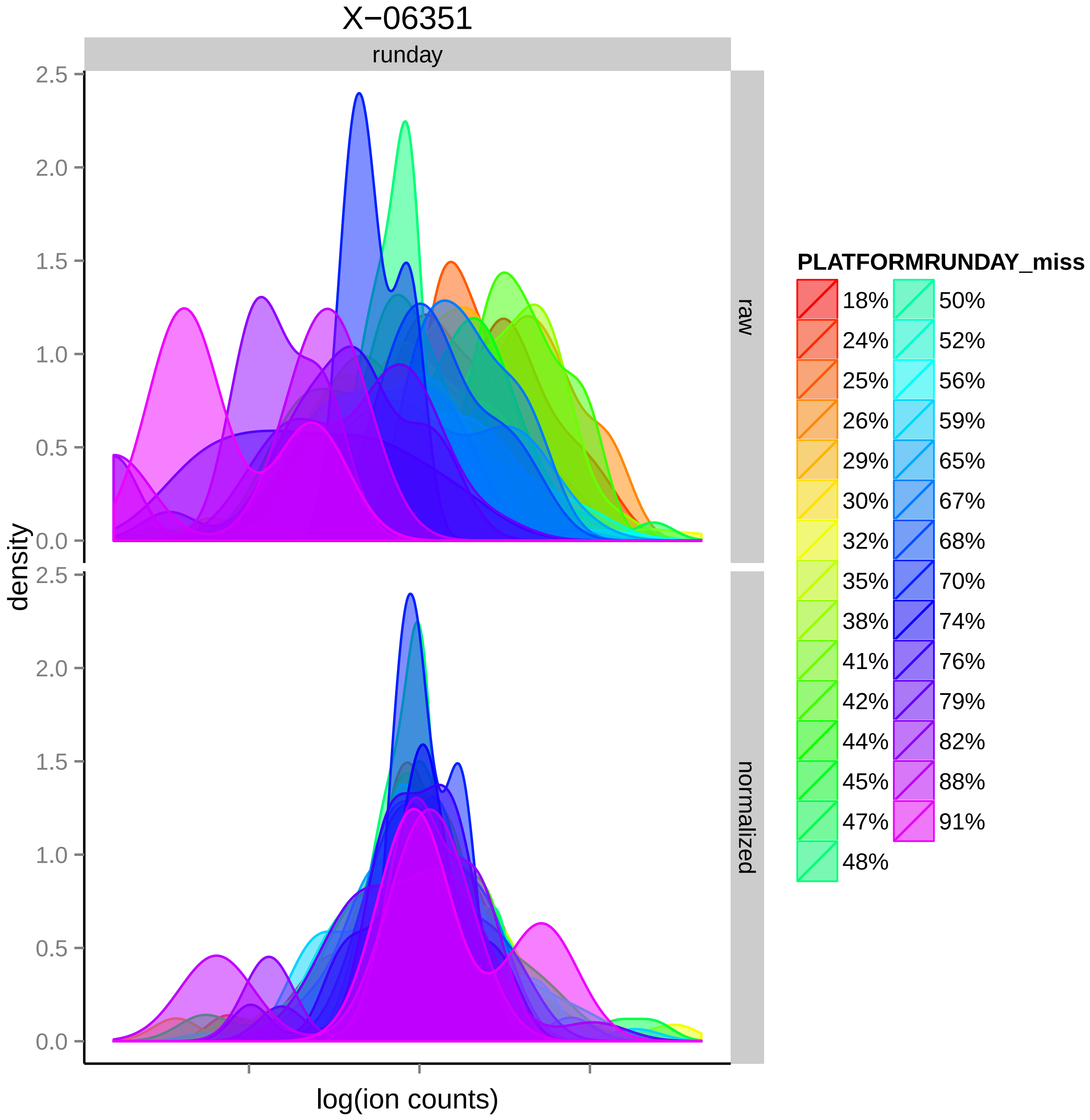

X−06351
runday
raw
normalized
density
log(ion counts)
2.5
2.0
1.5
1.0
0.5
0.0
PLATFORMRUNDAY_miss
18%
24%
25%
26%
29%
30%
32%
35%
38%
41%
42%
44%
45%
47%
48%
50%
52%
56%
59%
65%
67%
68%
70%
74%
76%
79%
82%
88%
91%

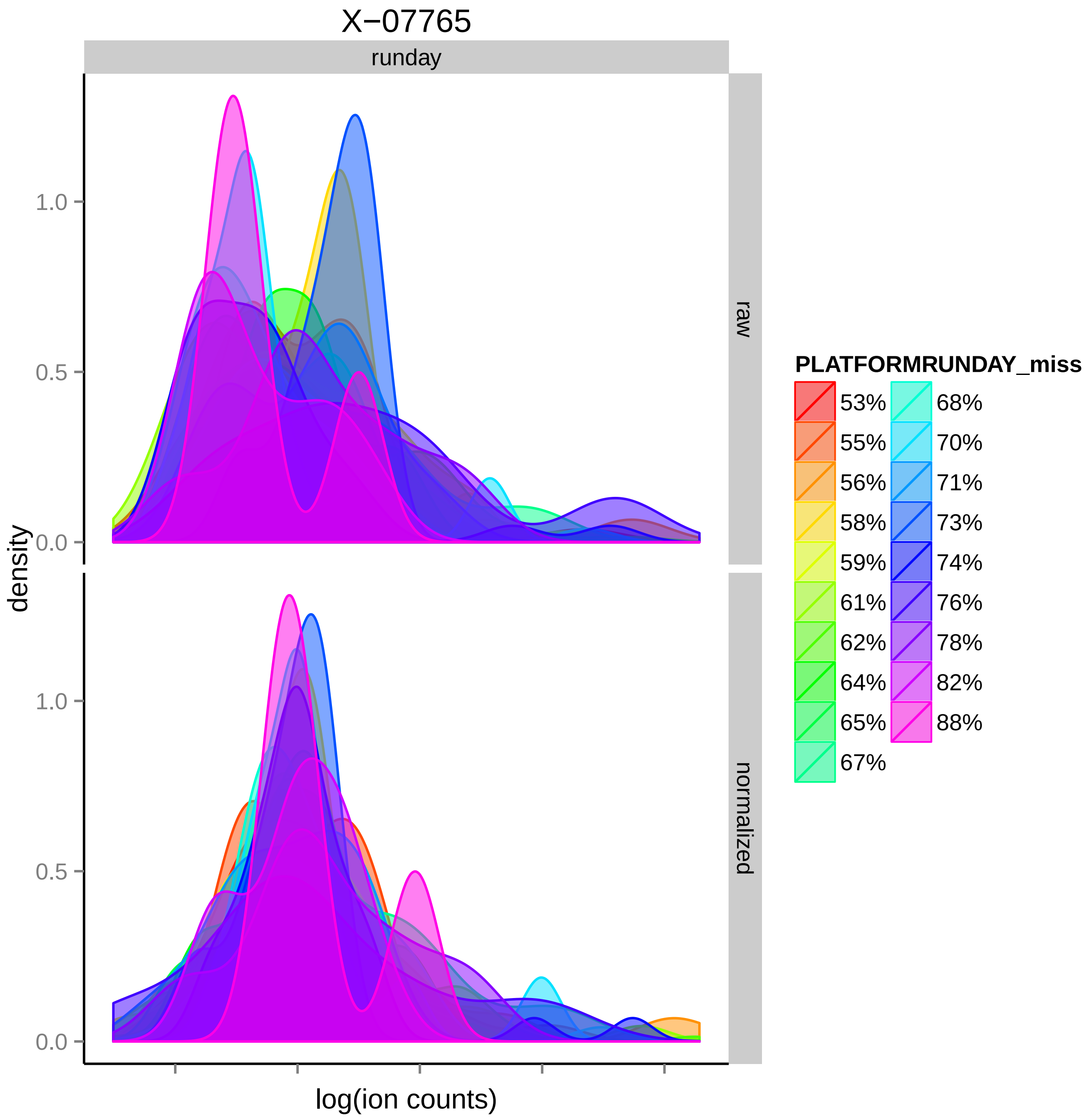
X−07765
runday
raw
normalized
density
log(ion counts)
1.0
0.5
0.0
PLATFORMRUNDAY_miss
53%
55%
56%
58%
59%
61%
62%
64%
65%
67%
68%
70%
71%
73%
74%
76%
78%
82%
88%

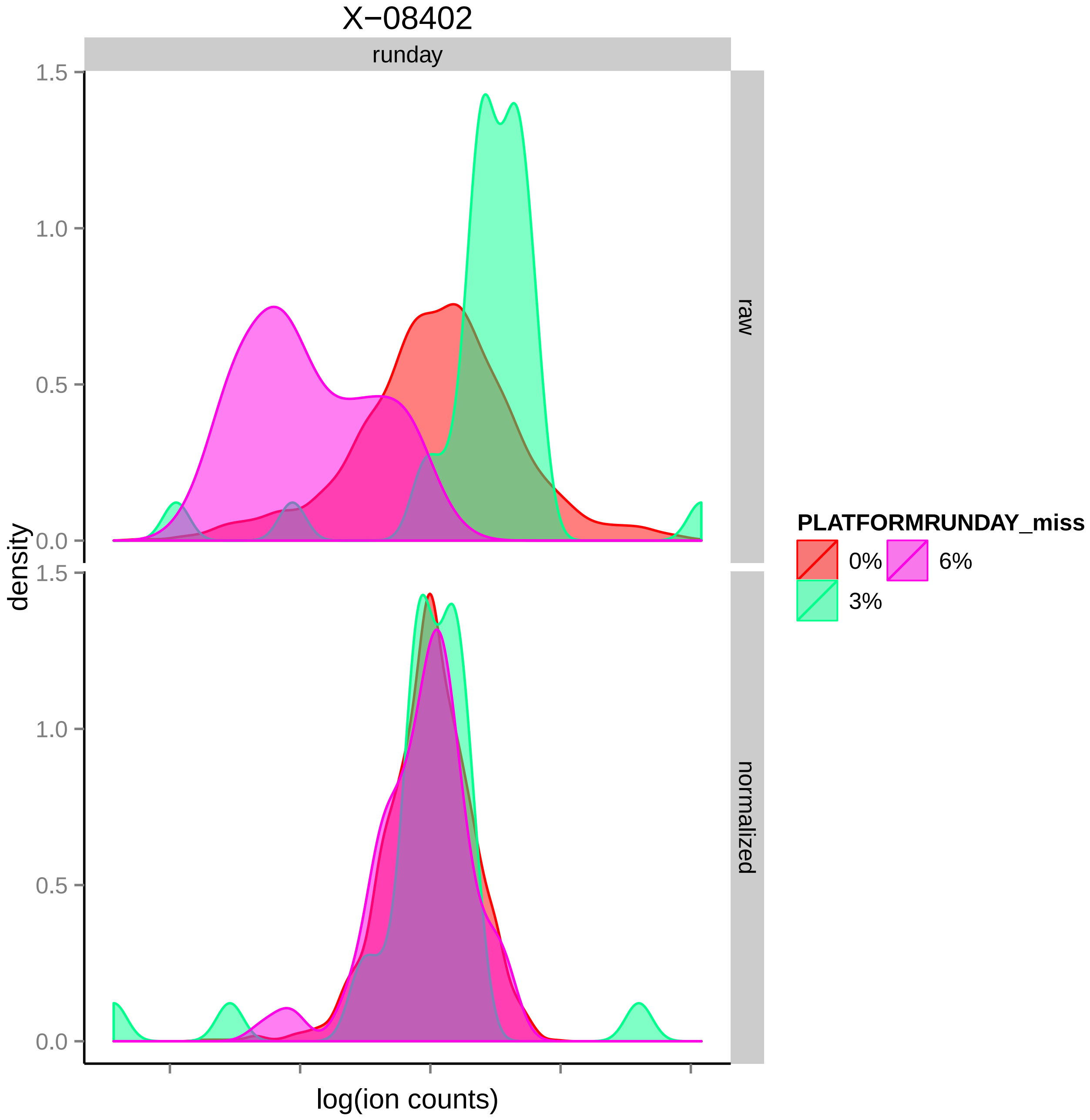

X−08402
runday
raw
normalized
density
log(ion counts)
1.5
1.0
0.5
0.0
PLATFORMRUNDAY_miss
0%
3%
6%

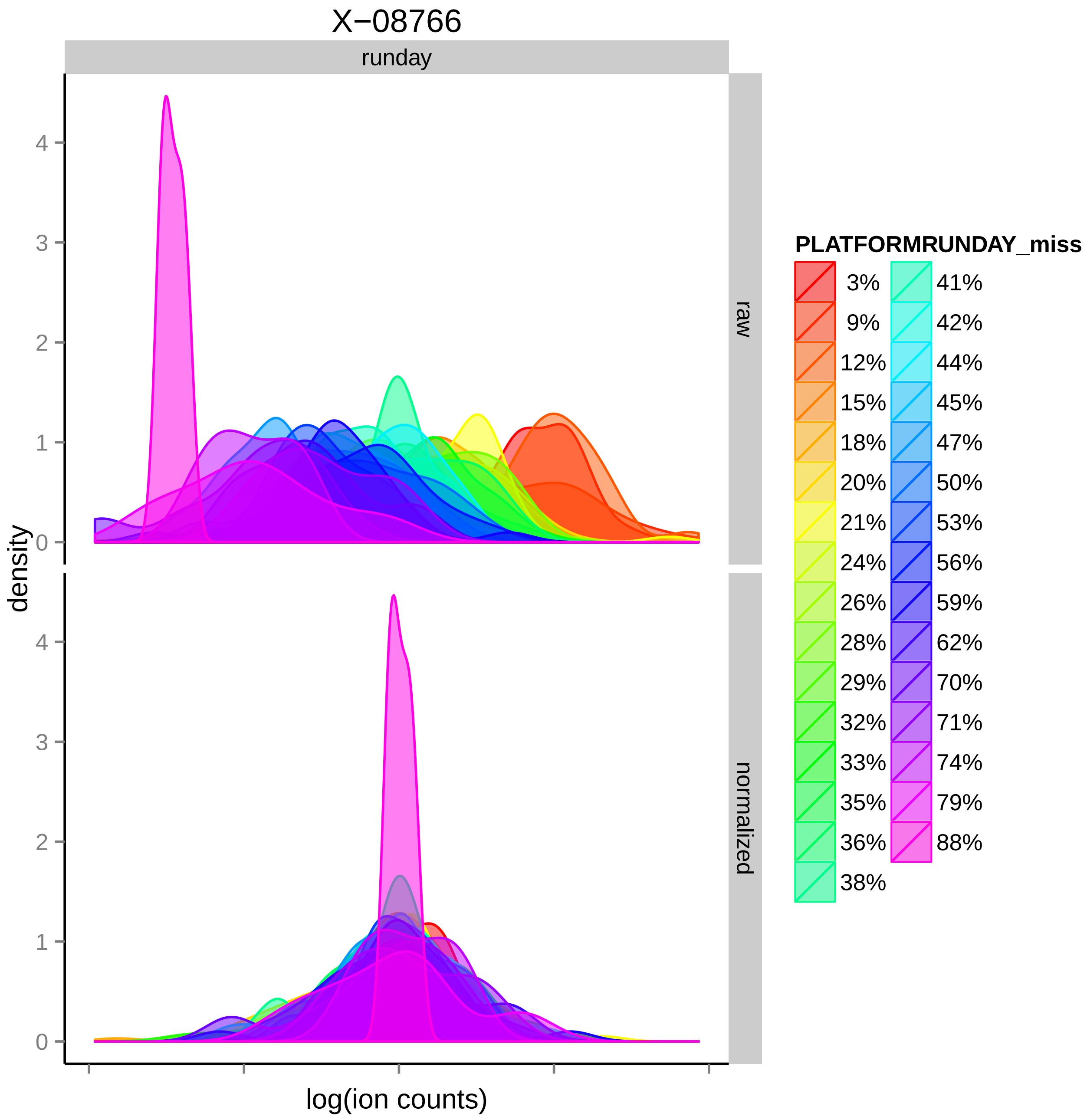
X−08766
runday
raw
normalized
density
log(ion counts)
0
1
2
3
4
PLATFORMRUNDAY_miss
3%
9%
12%
15%
18%
20%
21%
24%
26%
28%
29%
32%
33%
35%
36%
38%
41%
42%
44%
45%
47%
50%
53%
56%
59%
62%
70%
71%
74%
79%
88%

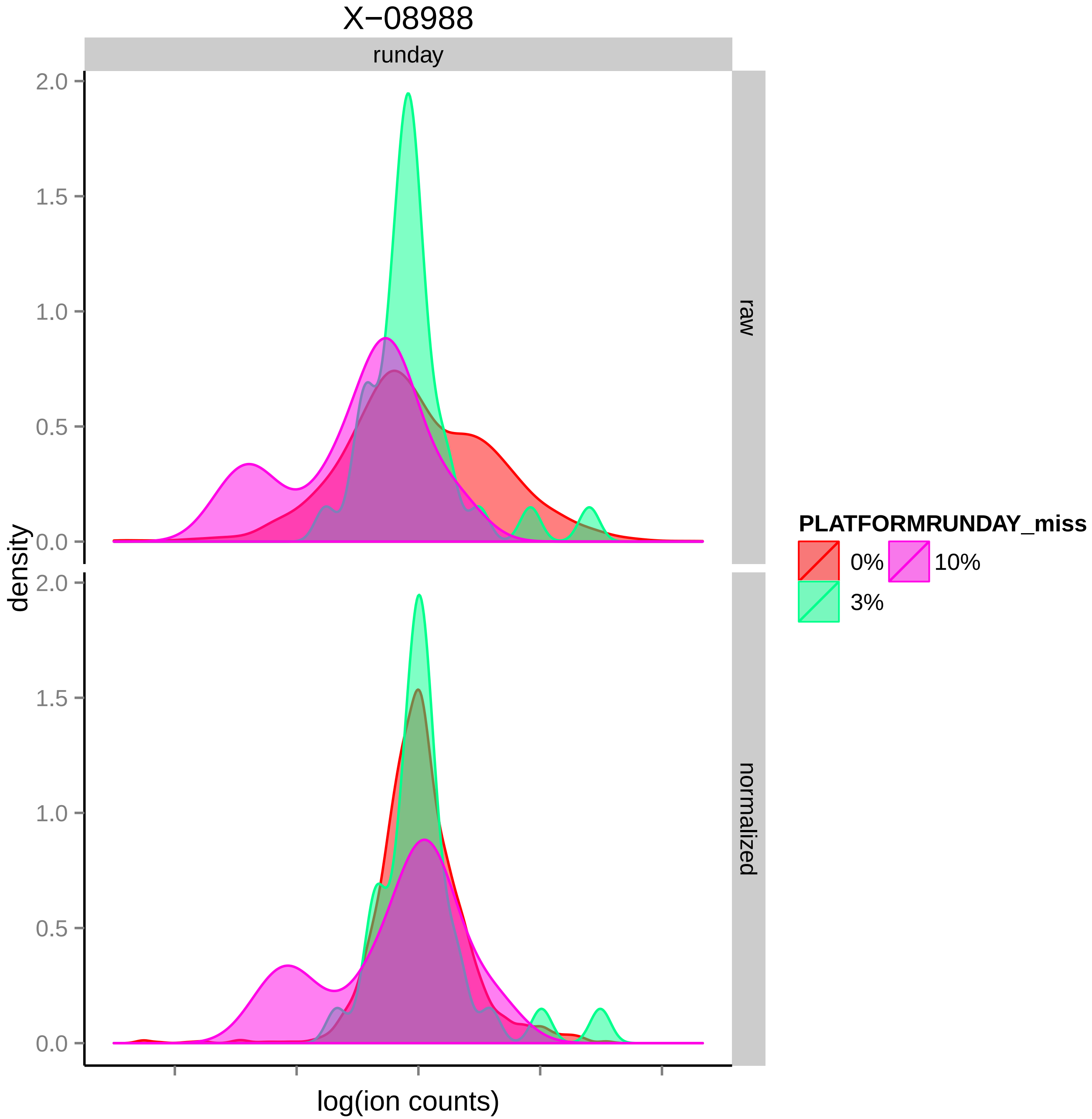

X−08988
runday
raw
normalized
density
2.0
1.5
1.0
0.5
0.0
log(ion counts)
PLATFORMRUNDAY_miss
0%
10%
3%

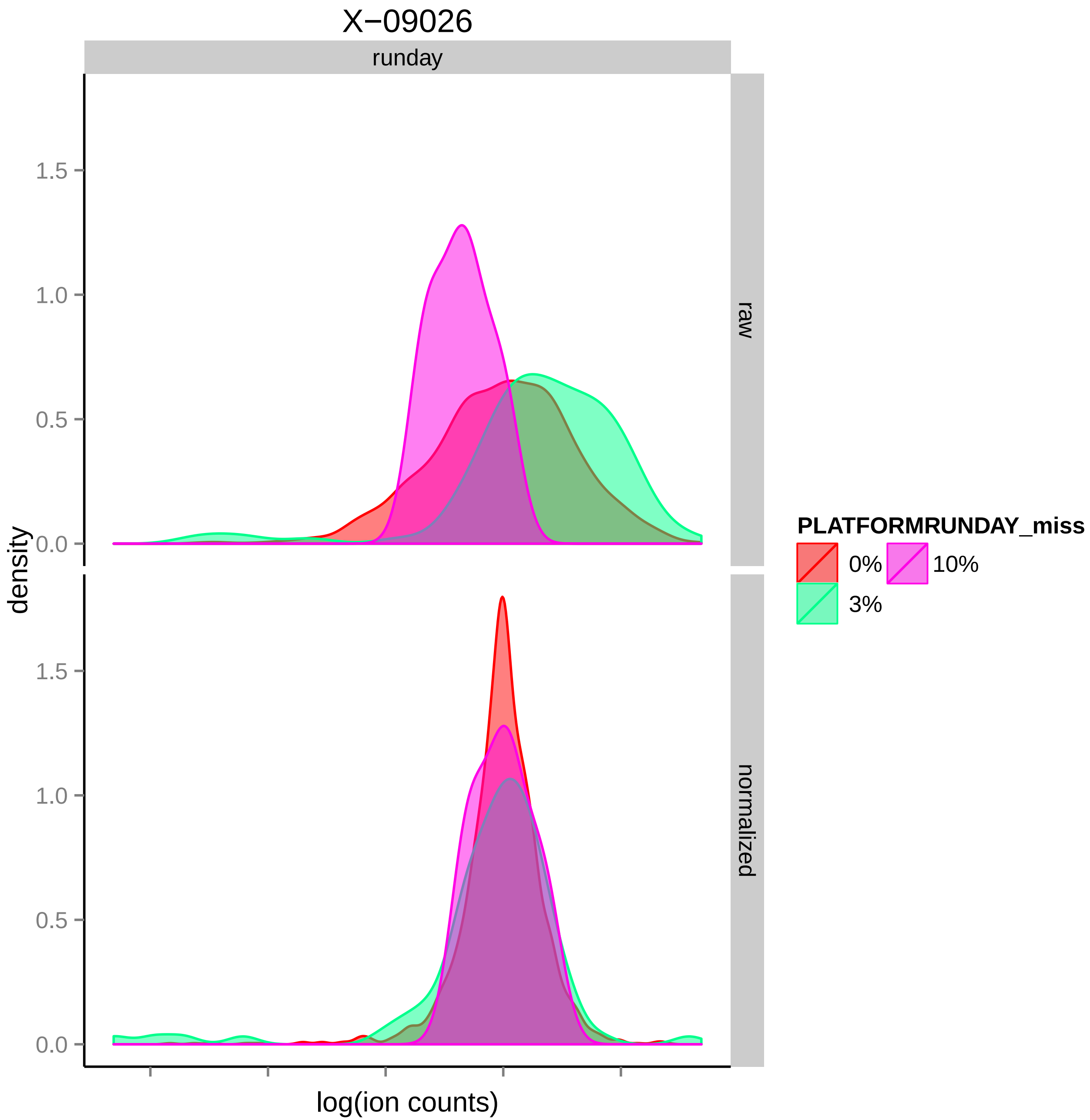

X−09026
runday
raw
normalized
density
log(ion counts)
1.5
1.0
0.5
0.0
PLATFORMRUNDAY_miss
0%
10%
3%

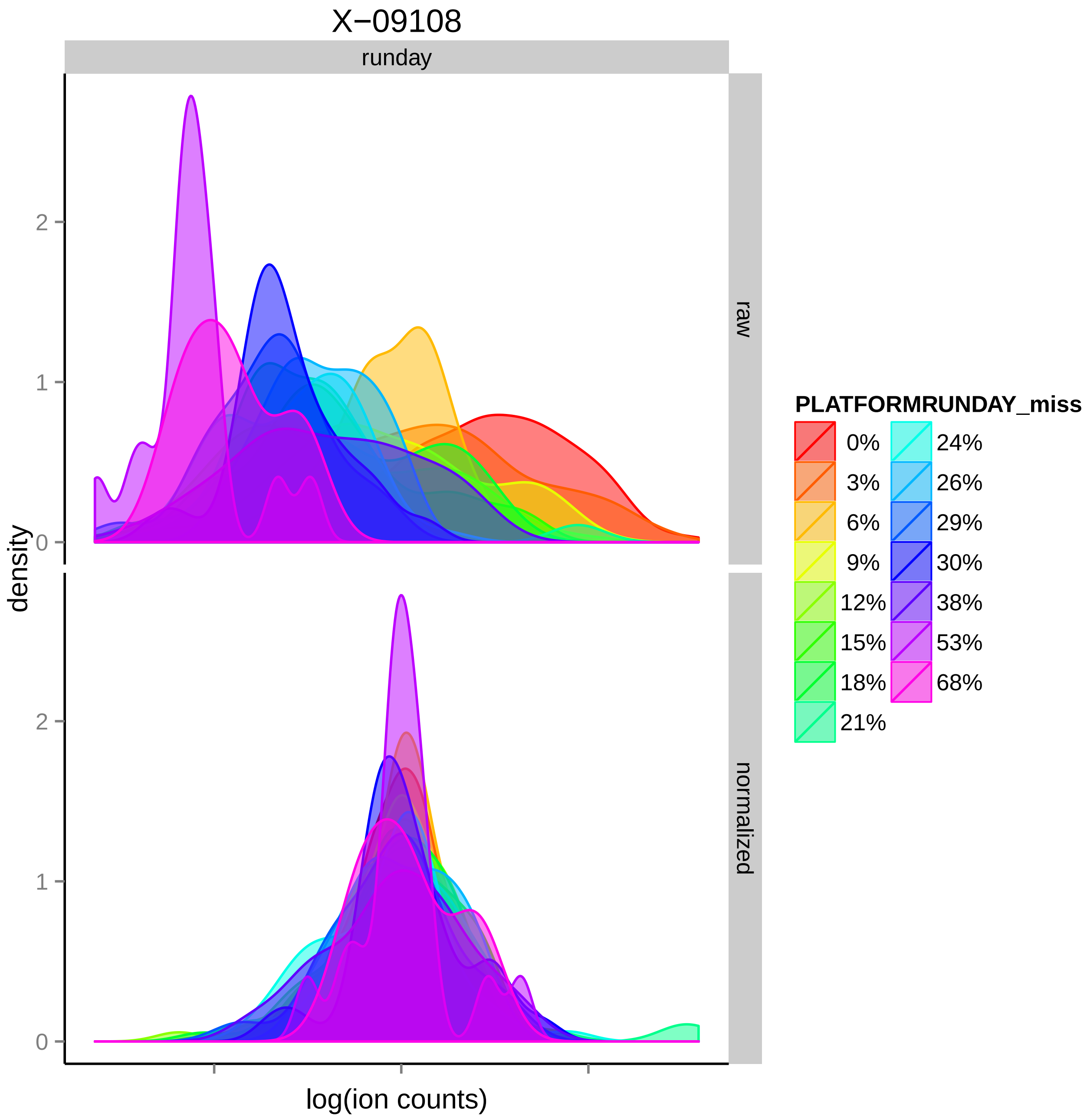

X−09108
runday
raw
normalized
density
log(ion counts)
0
1
2
PLATFORMRUNDAY_miss
0%
3%
6%
9%
12%
15%
18%
21%
24%
26%
29%
30%
38%
53%
68%

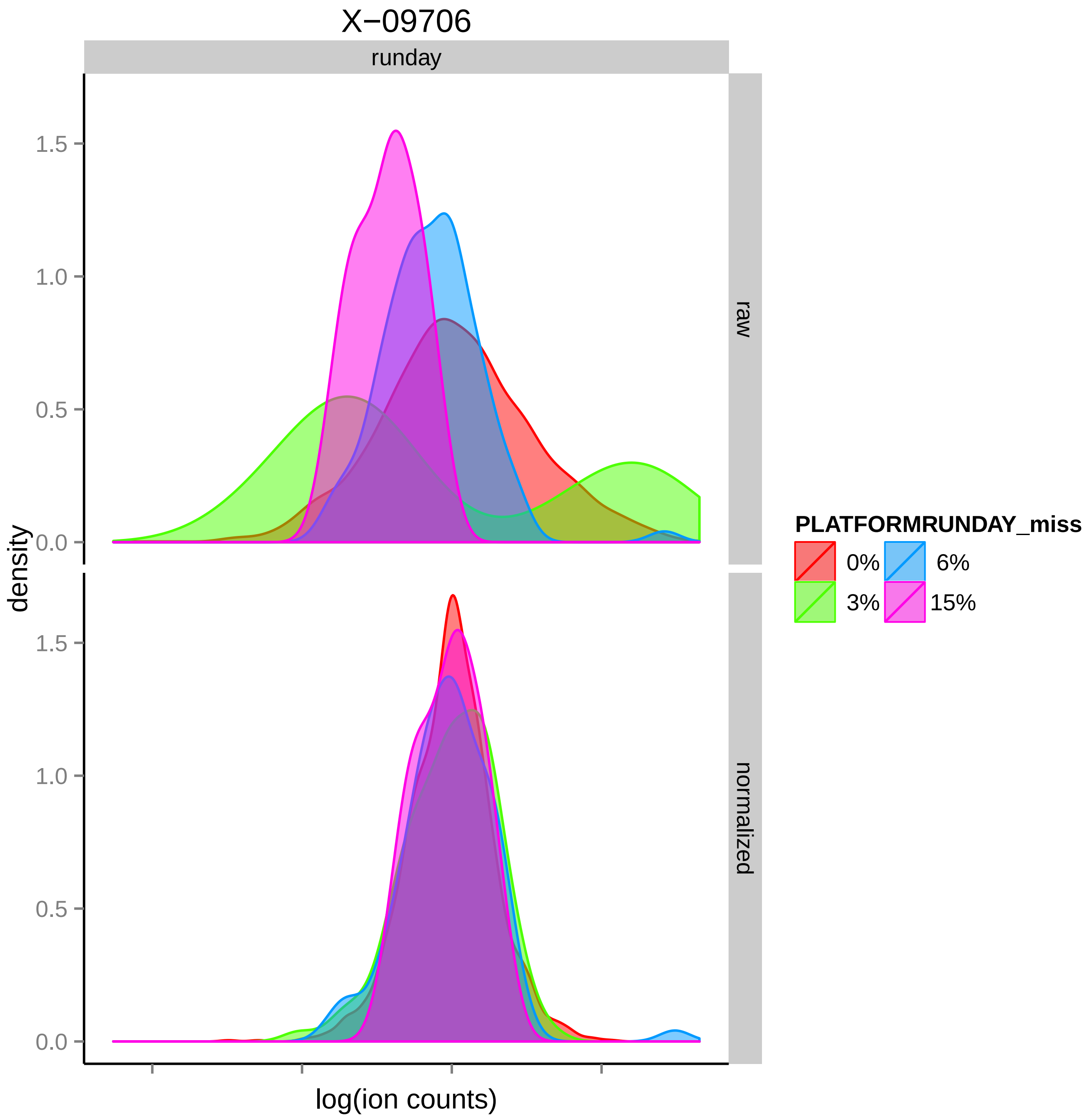

X−09706
runday
raw
normalized
density
1.5
1.0
0.5
0.0
1.5
1.0
0.5
0.0
log(ion counts)
PLATFORMRUNDAY_miss
0%
6%
3%
15%

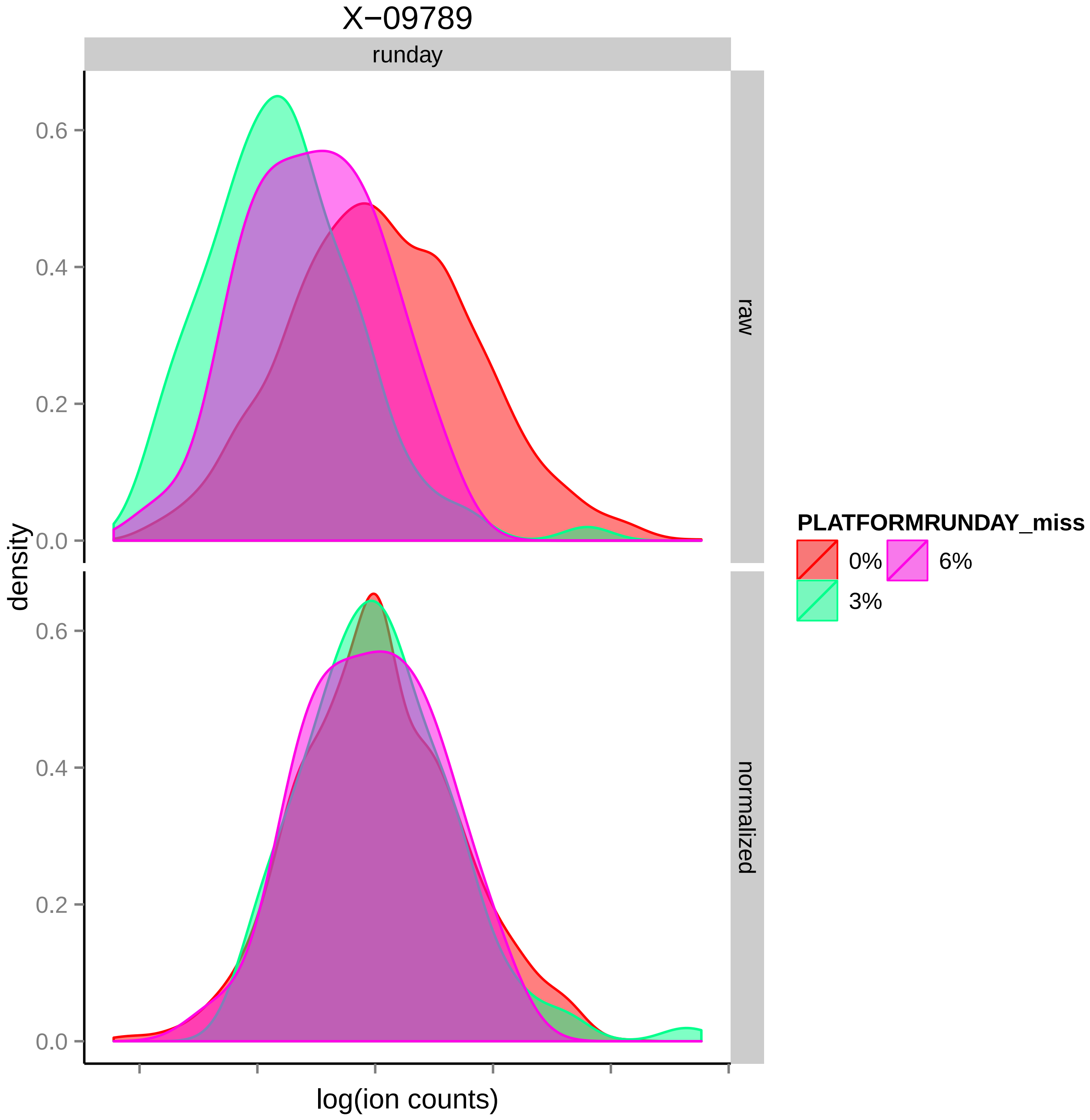
X−09789
runday
raw
normalized
density
0.0
0.2
0.4
0.6
log(ion counts)
PLATFORMRUNDAY_miss
0%
3%
6%

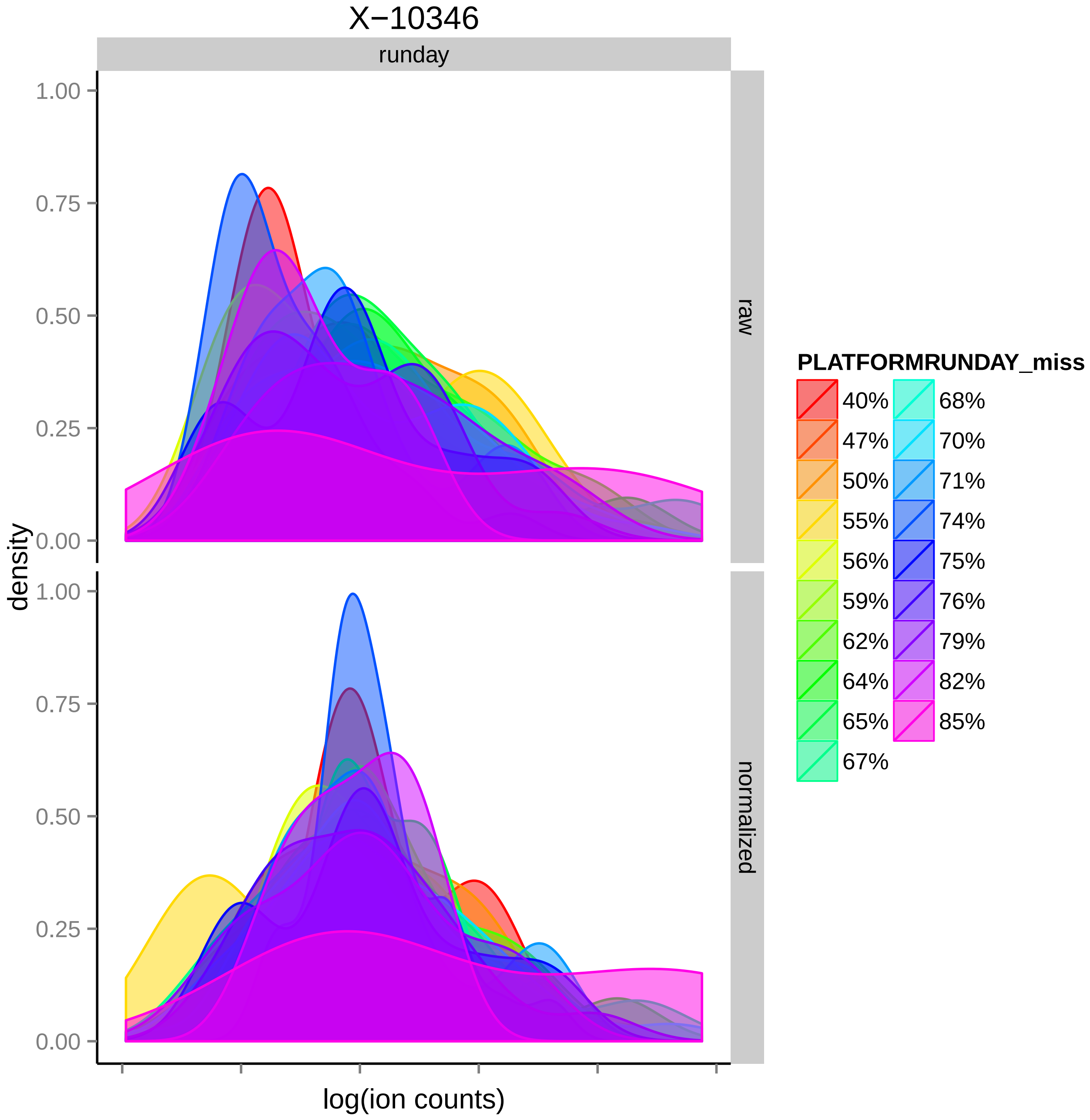

X−10346
runday
raw
normalized
density
1.00
0.75
0.50
0.25
0.00
log(ion counts)
PLATFORMRUNDAY_miss
40%
47%
50%
55%
56%
59%
62%
64%
65%
67%
68%
70%
71%
74%
75%
76%
79%
82%
85%

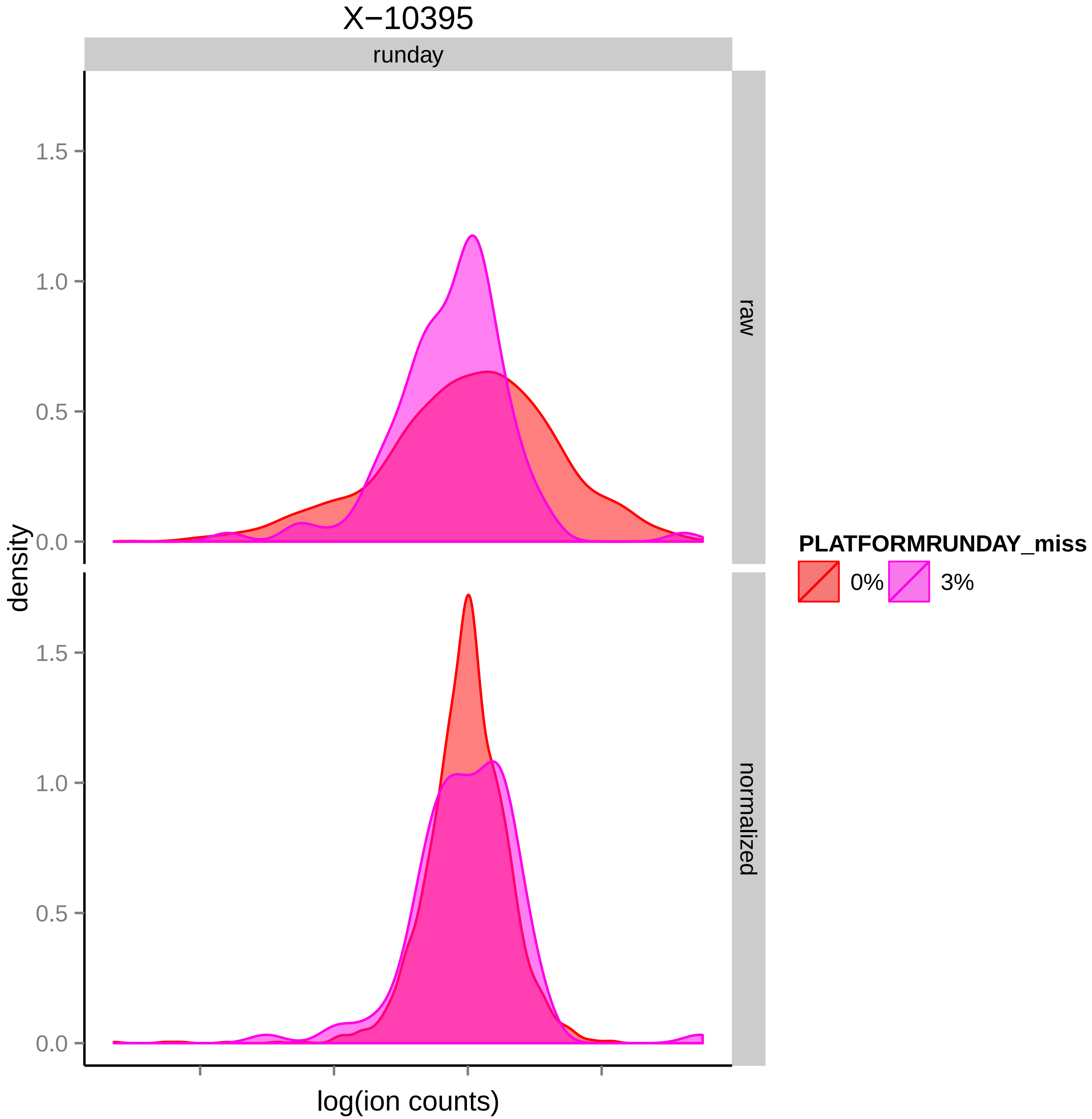

X−10395
runday
raw
normalized
density
1.5
1.0
0.5
0.0
log(ion counts)
PLATFORMRUNDAY_miss
0%
3%

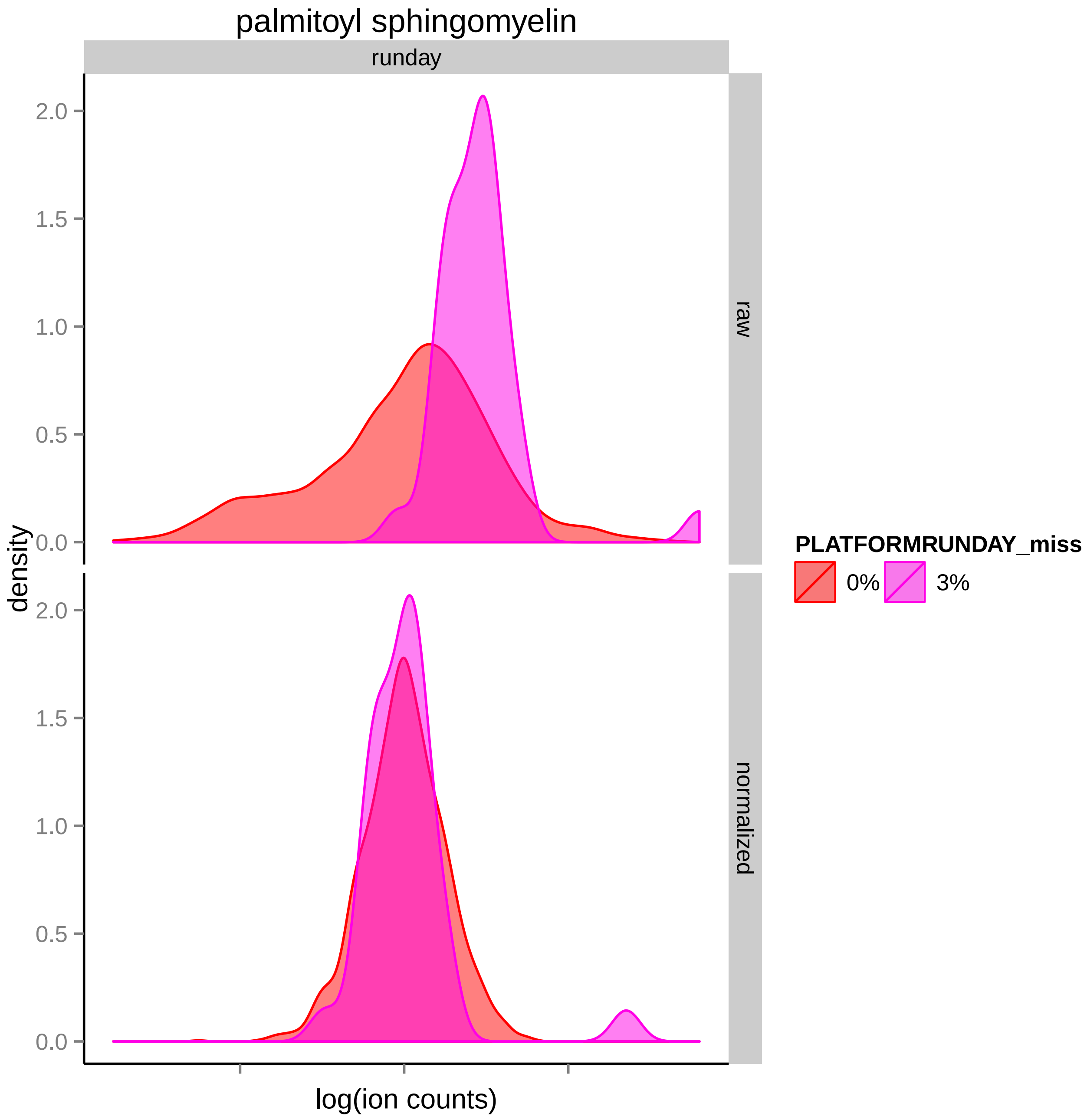
palmitoyl sphingomyelin
runday
raw
normalized
density
log(ion counts)
0.0
0.5
1.0
1.5
2.0
PLATFORMRUNDAY_miss
0%
3%

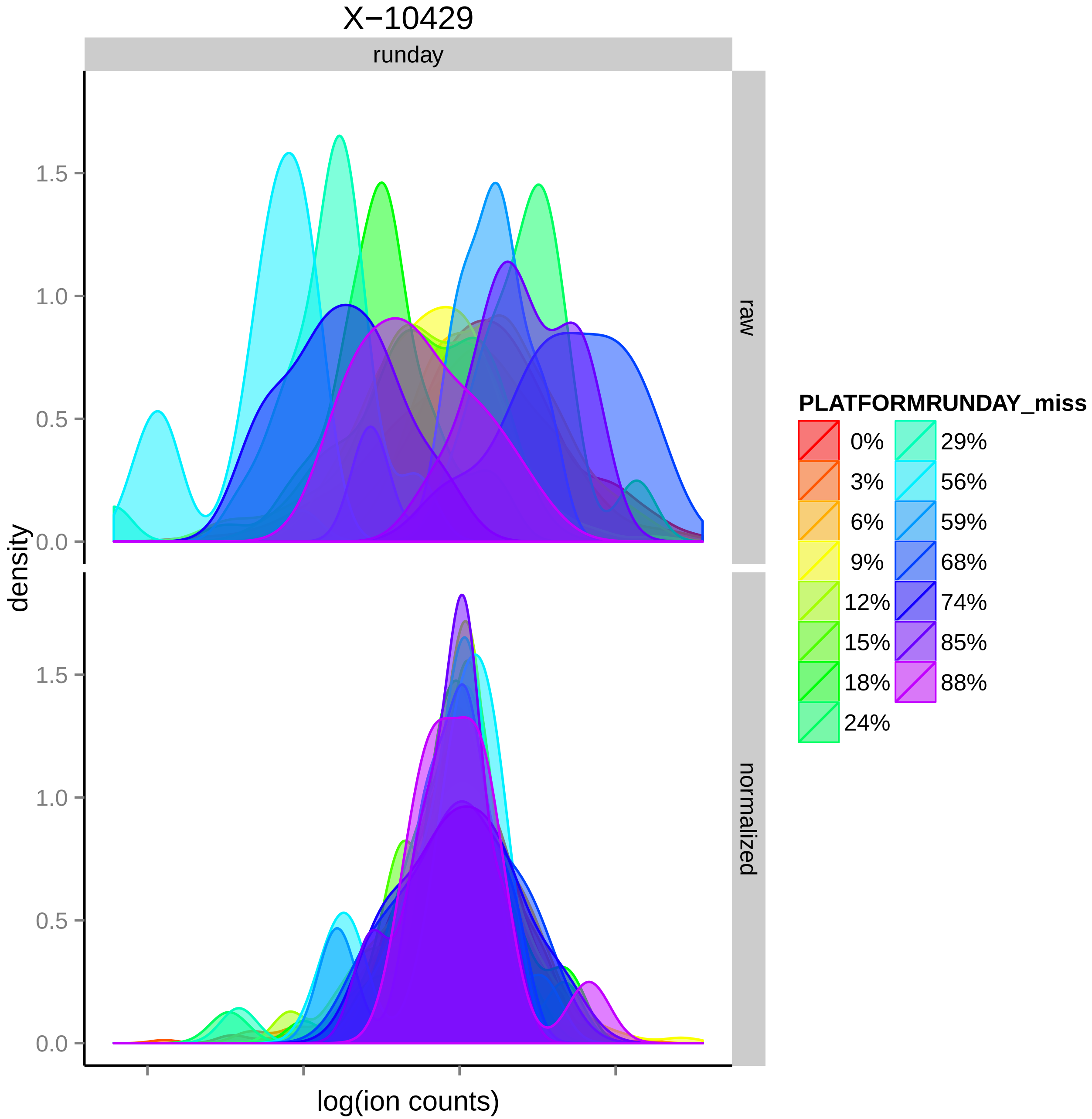

X−10429
runday
raw
normalized
density
log(ion counts)
0.0
0.5
1.0
1.5
PLATFORMRUNDAY_miss
0%
3%
6%
9%
12%
15%
18%
24%
29%
56%
59%
68%
74%
85%
88%

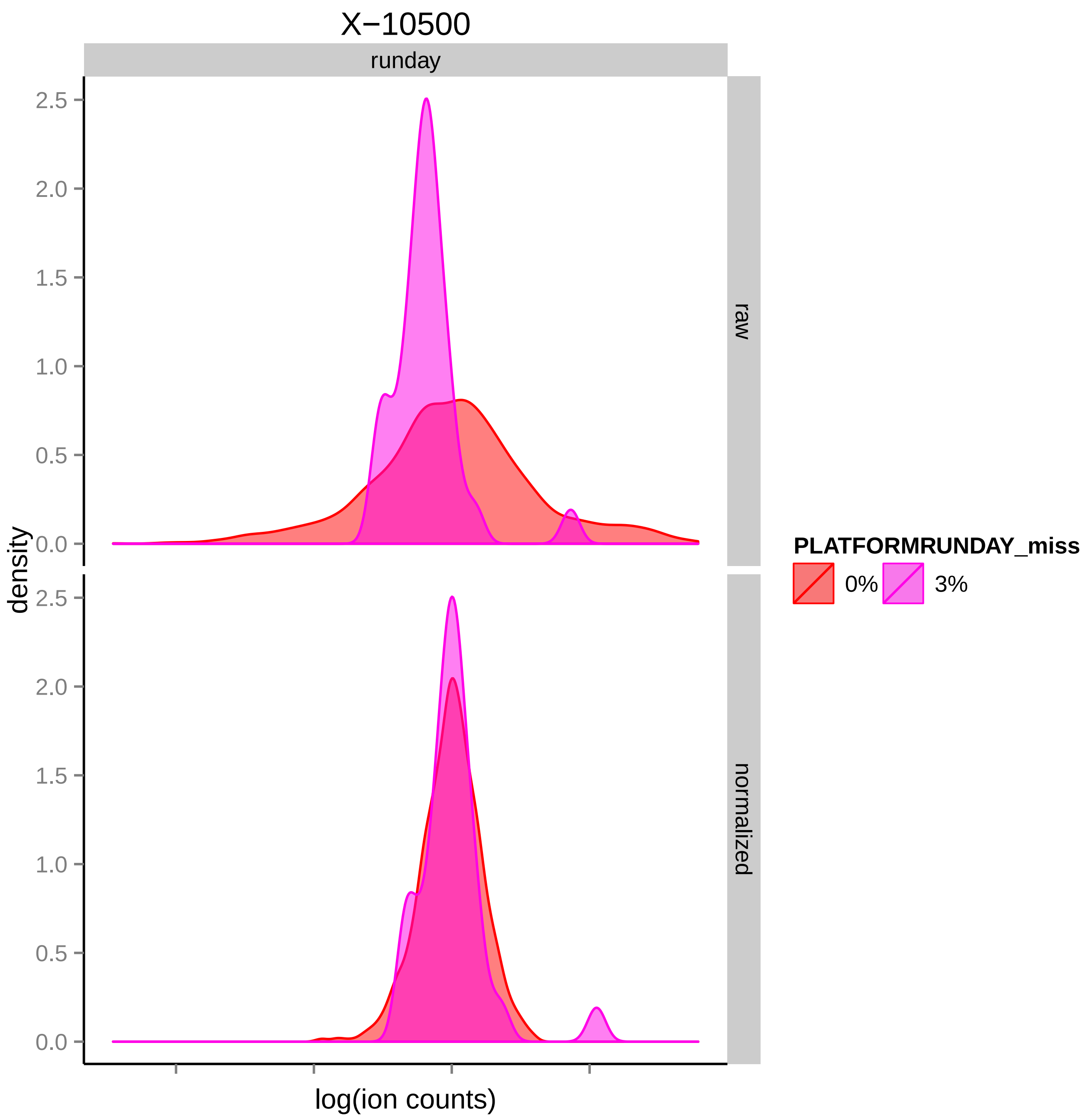
X−10500
runday
raw
normalized
density
2.5
2.0
1.5
1.0
0.5
0.0
log(ion counts)
PLATFORMRUNDAY_miss
0%
3%

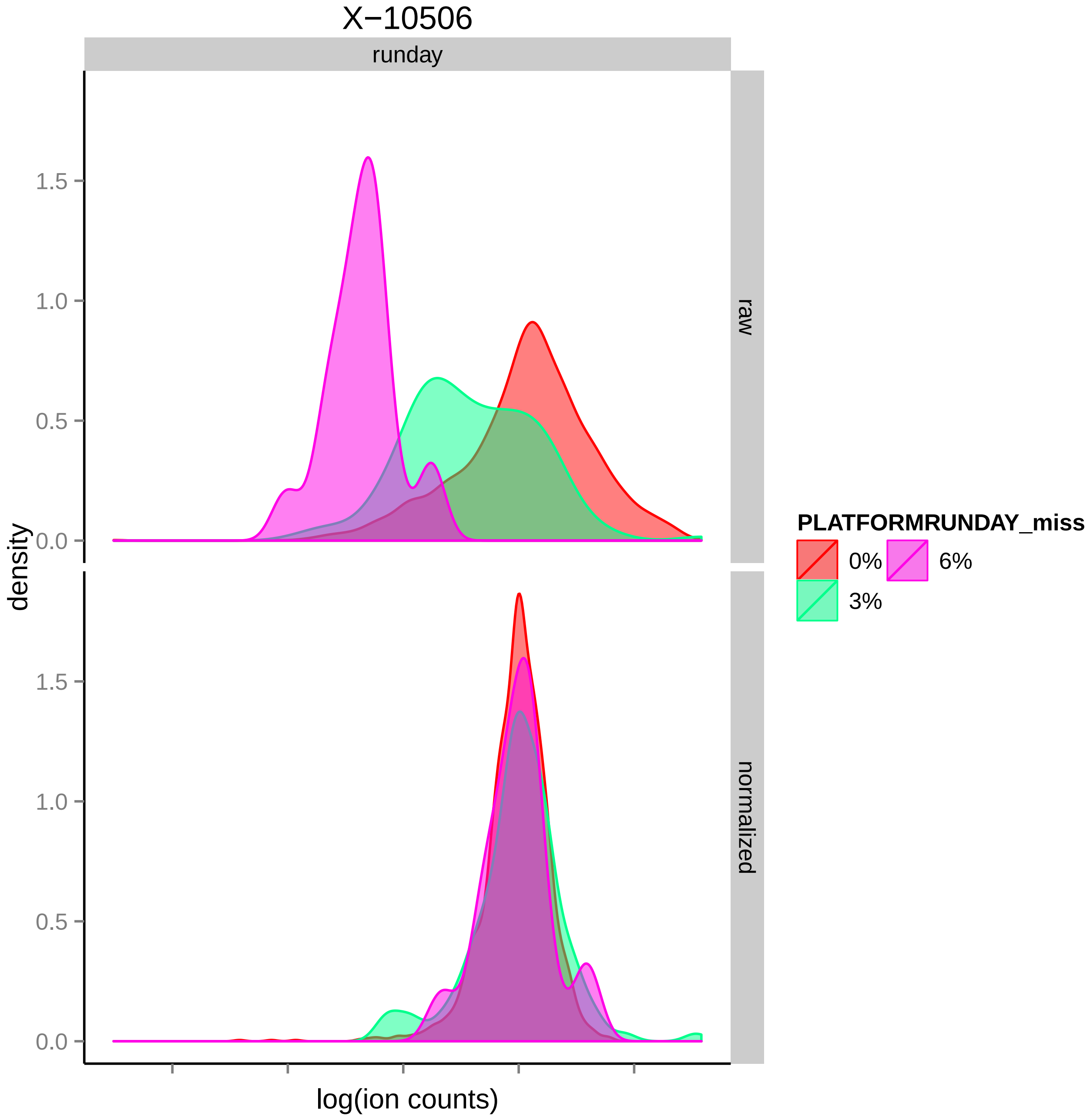
X−10506
runday
raw
normalized
density
log(ion counts)
1.5
1.0
0.5
0.0
PLATFORMRUNDAY_miss
0%
6%
3%

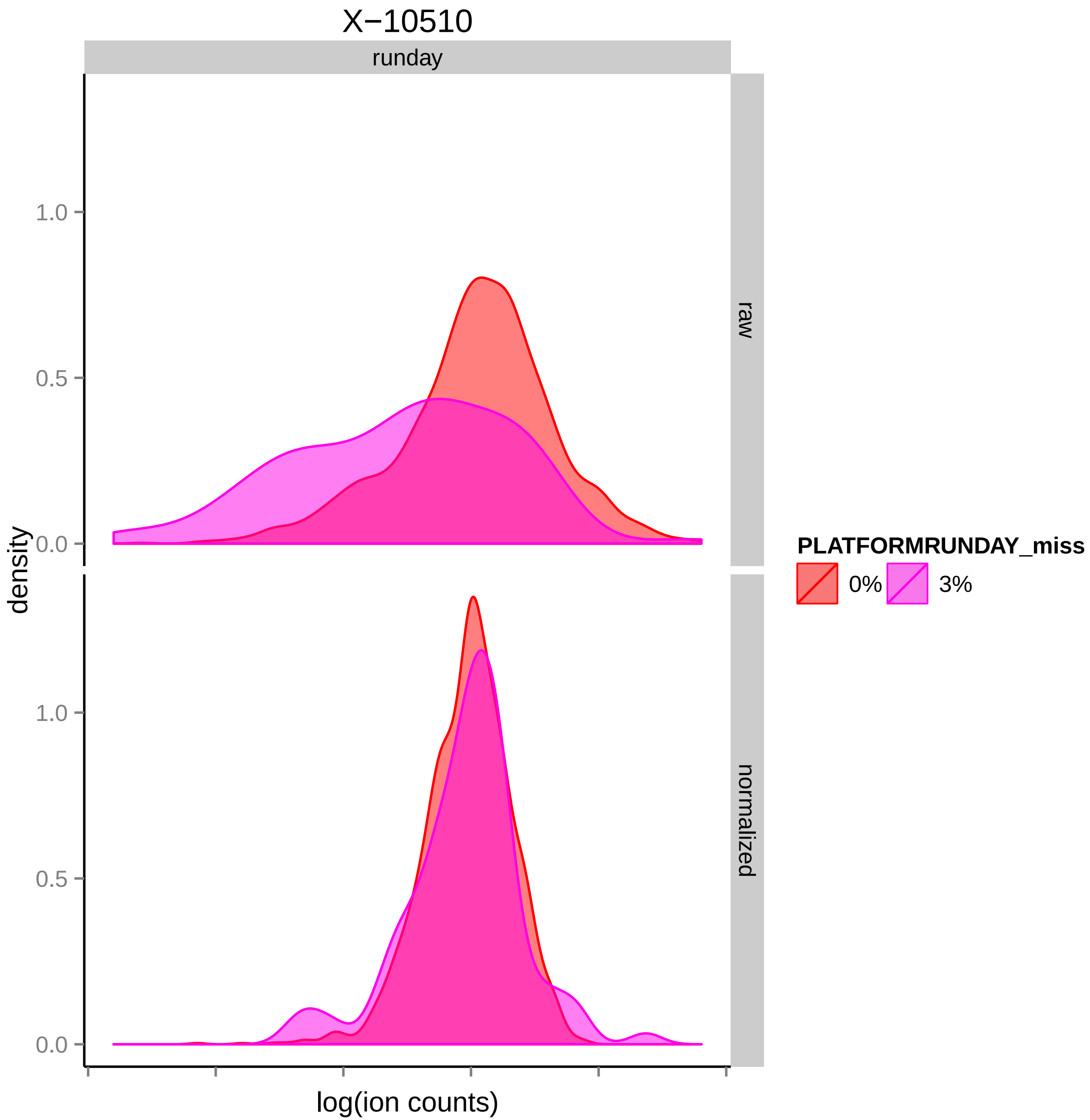

X−10510
runday
raw
normalized
density
1.0
0.5
0.0
1.0
0.5
0.0
log(ion counts)
PLATFORMRUNDAY_miss
0%
3%

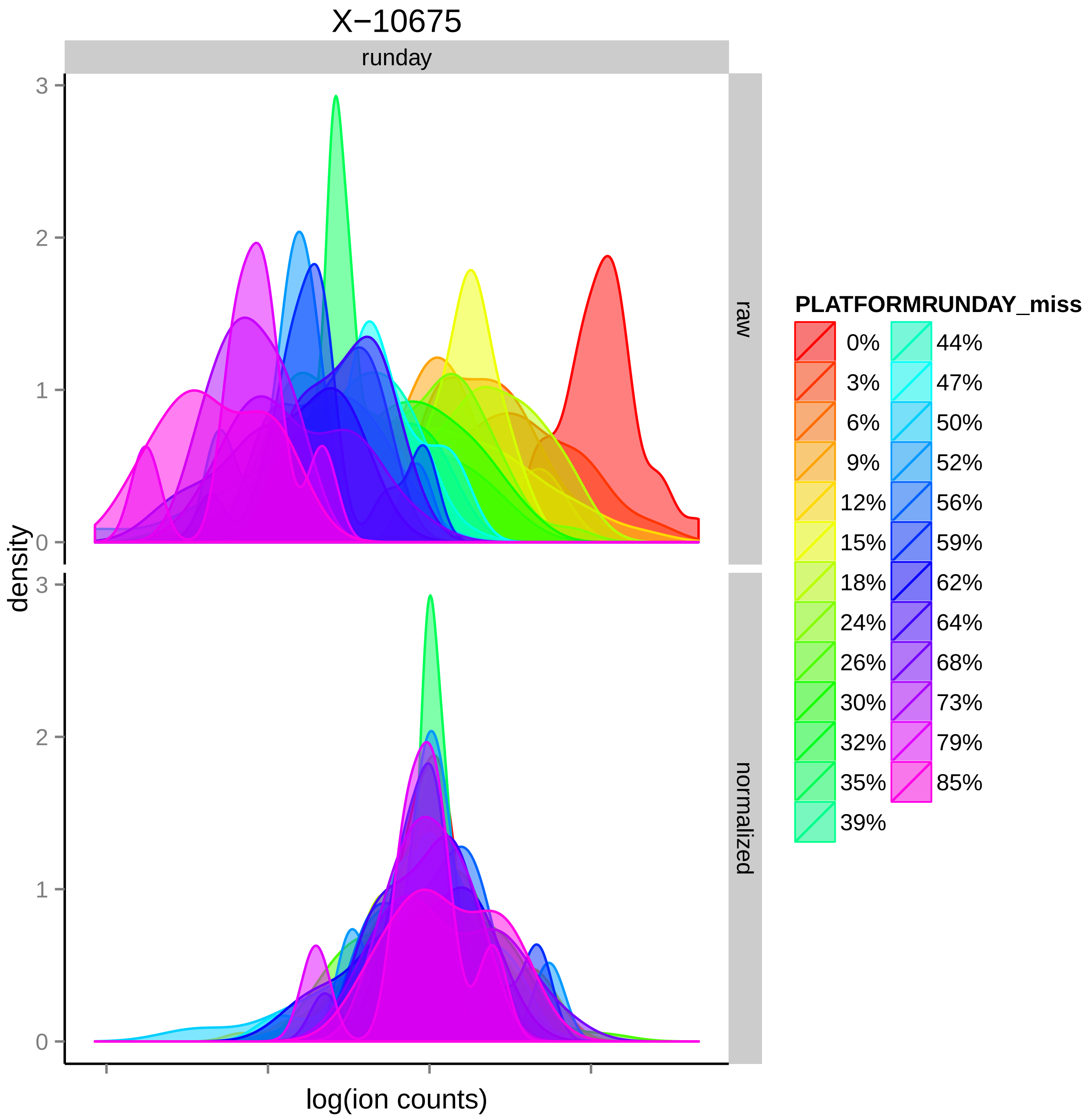

X−10675
runday
raw
normalized
density
log(ion counts)
0
1
2
3
PLATFORMRUNDAY_miss
0%
3%
6%
9%
12%
15%
18%
24%
26%
30%
32%
35%
39%
44%
47%
50%
52%
56%
59%
62%
64%
68%
73%
79%
85%

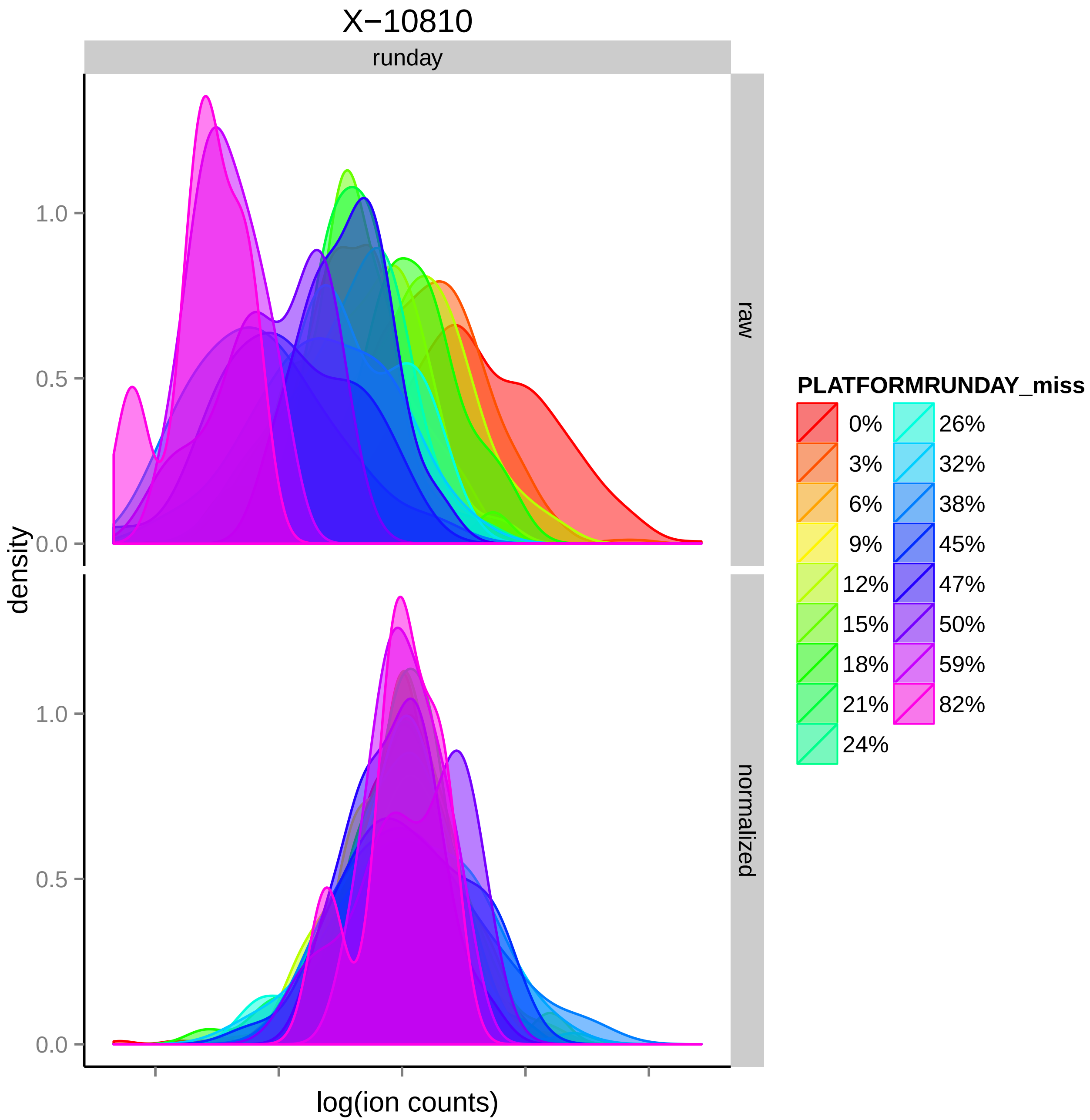

X−10810
runday
raw
normalized
density
1.0
0.5
0.0
1.0
0.5
0.0
log(ion counts)
PLATFORMRUNDAY_miss
0%
3%
6%
9%
12%
15%
18%
21%
24%
26%
32%
38%
45%
47%
50%
59%
82%

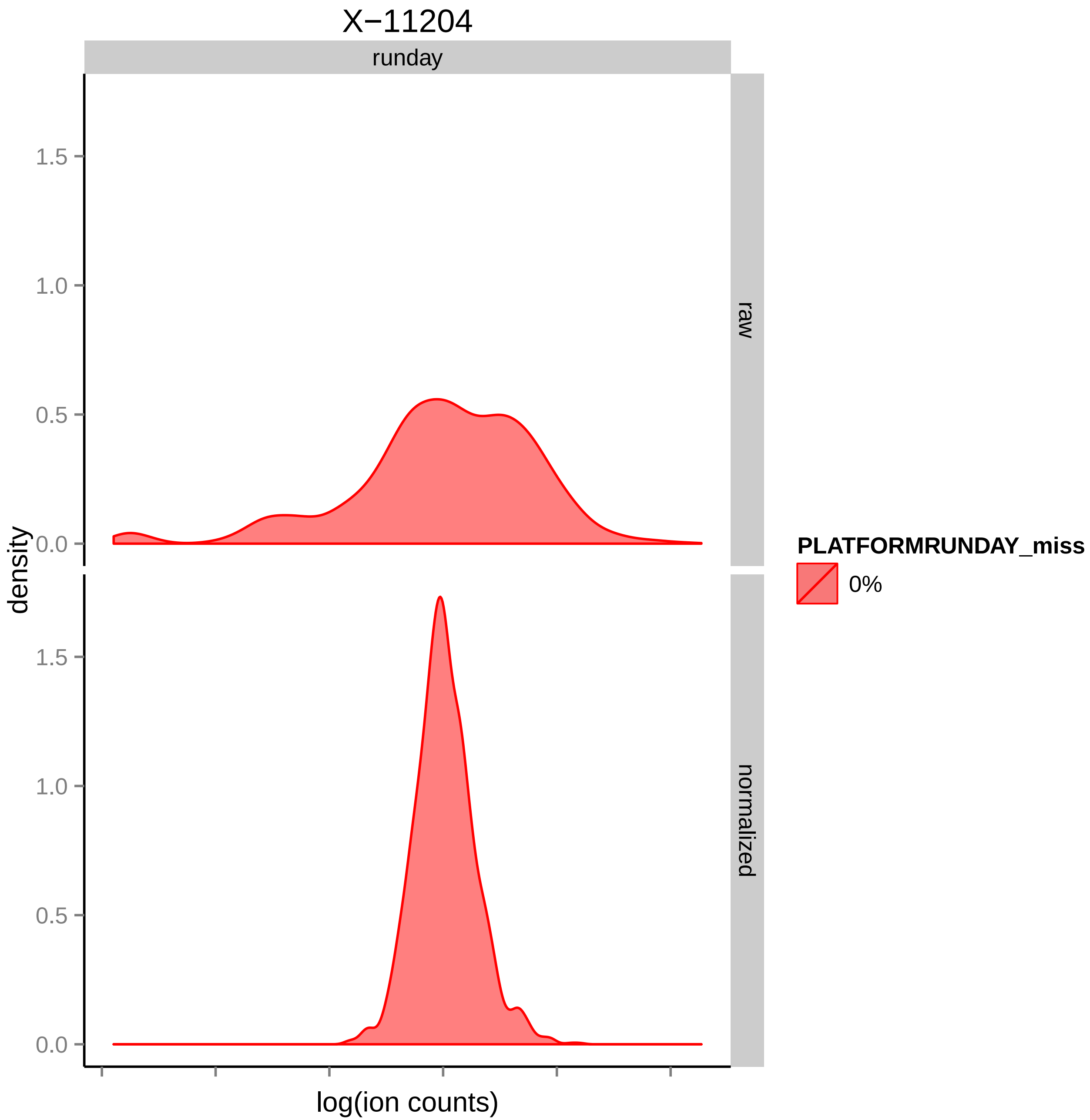
X−11204
runday
raw
normalized
density
1.5
1.0
0.5
0.0
1.5
1.0
0.5
0.0
log(ion counts)
PLATFORMRUNDAY_miss
0%

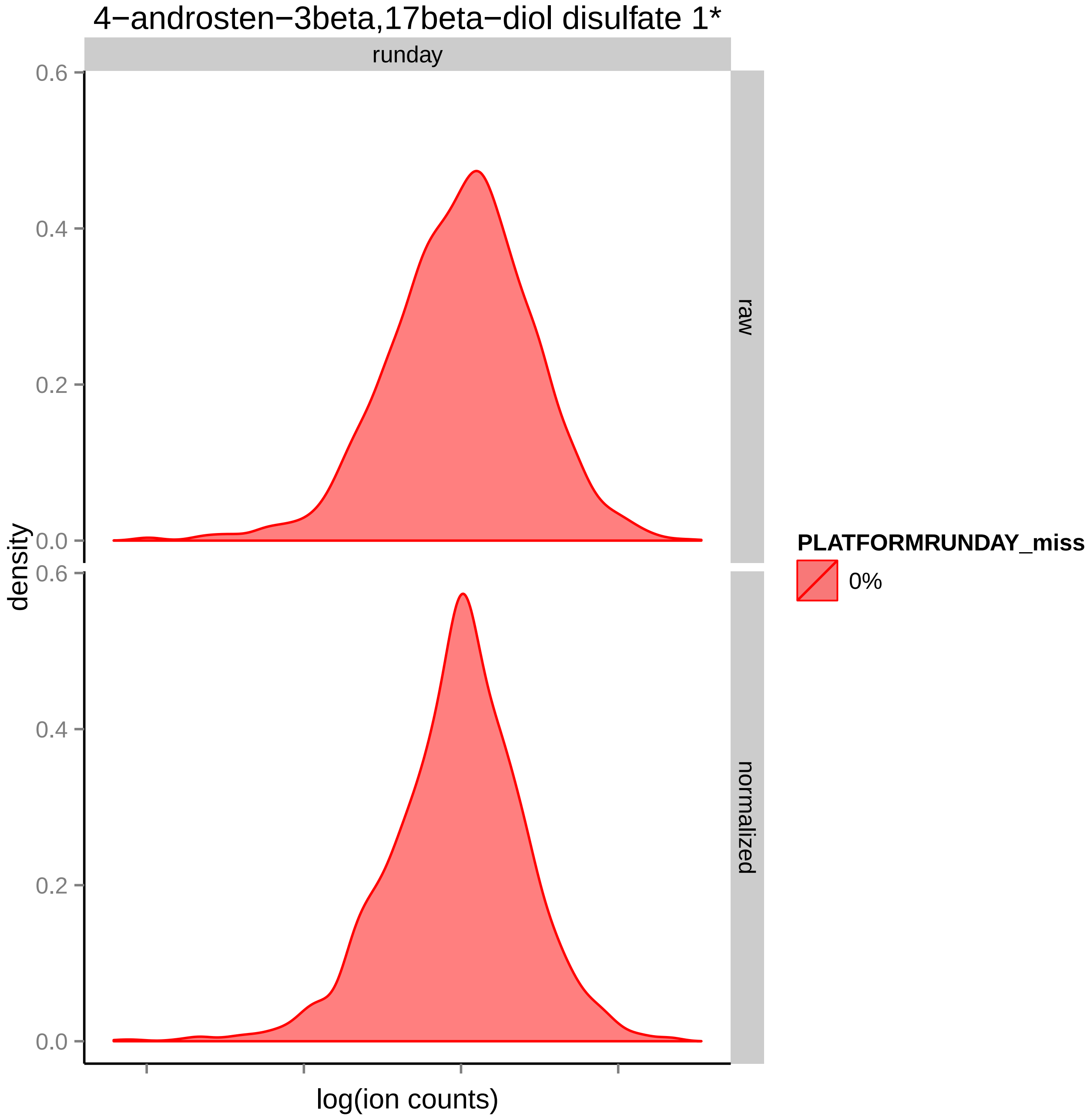

4−androsten−3beta,17beta−diol disulfate 1*
runday
raw
normalized
density
0.6
0.4
0.2
0.0
log(ion counts)
PLATFORMRUNDAY_miss
0%

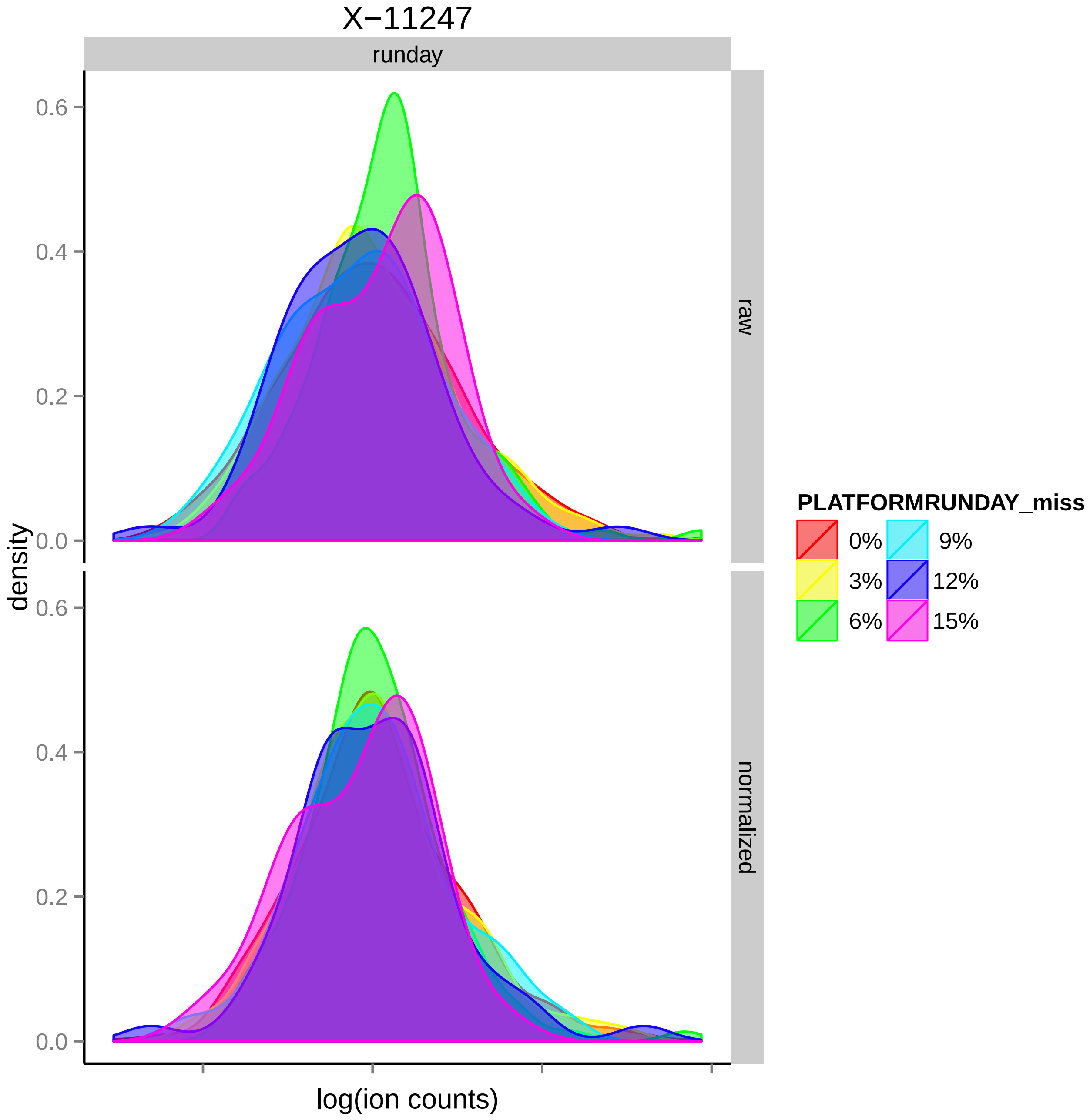

X−11247
runday
raw
normalized
density
0.0
0.2
0.4
0.6
log(ion counts)
PLATFORMRUNDAY_miss
0%
3%
6%
9%
12%
15%

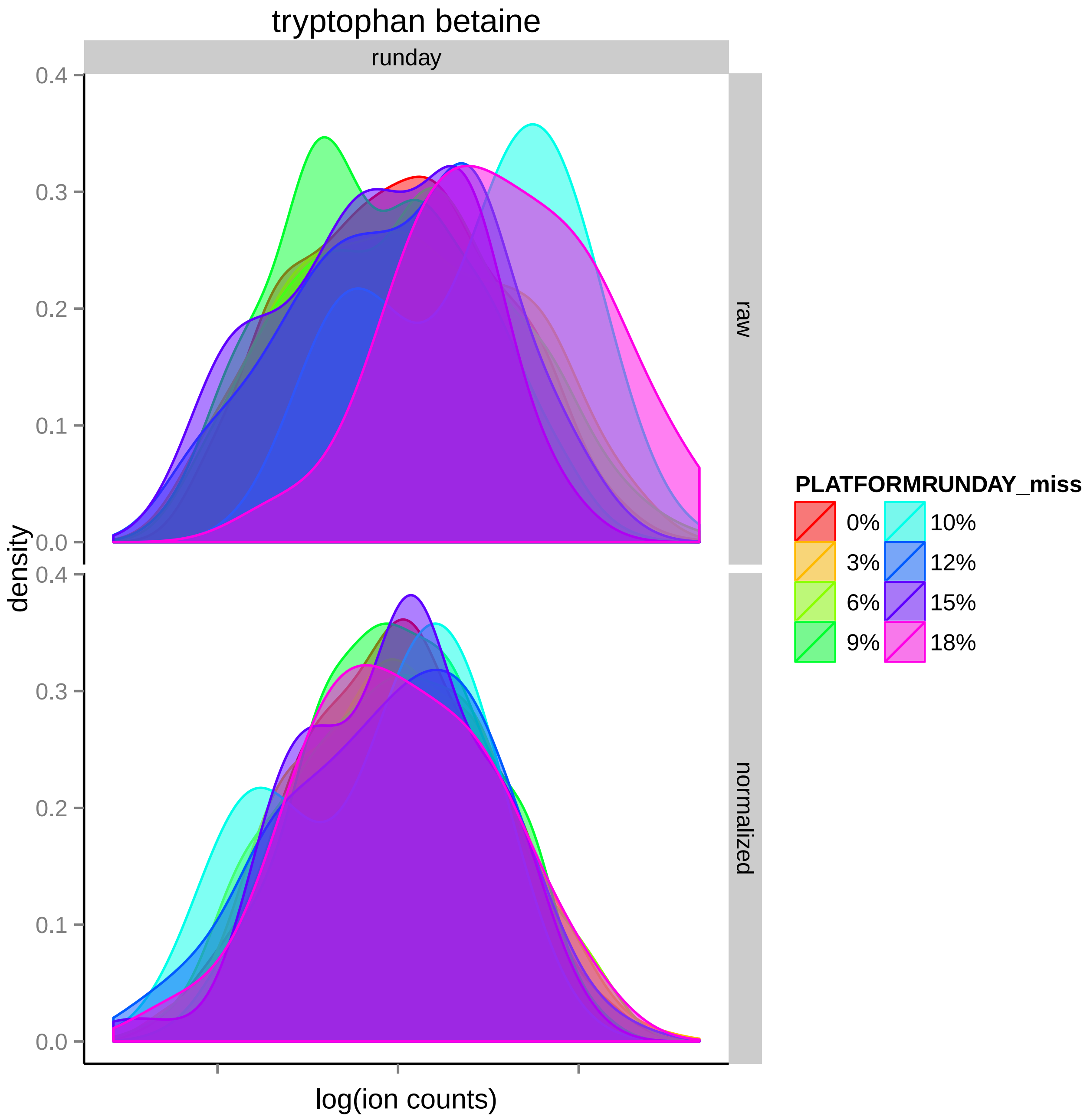
tryptophan betaine
runday
raw
normalized
density
0.0
0.1
0.2
0.3
0.4
log(ion counts)
PLATFORMRUNDAY_miss
0%
3%
6%
9%
10%
12%
15%
18%

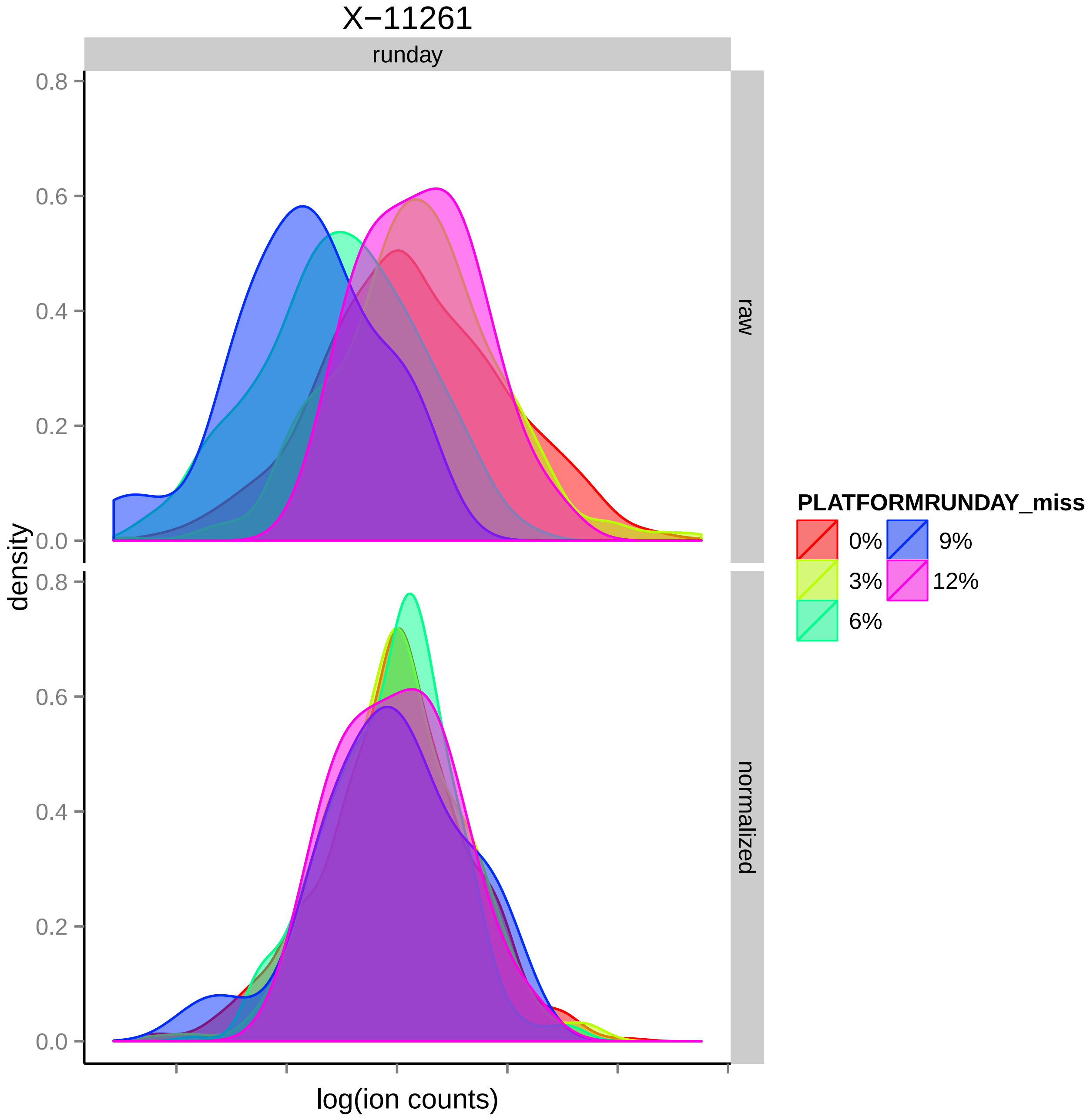

X−11261
runday
raw
normalized
density
0.0
0.2
0.4
0.6
0.8
log(ion counts)
PLATFORMRUNDAY_miss
0%
3%
6%
9%
12%

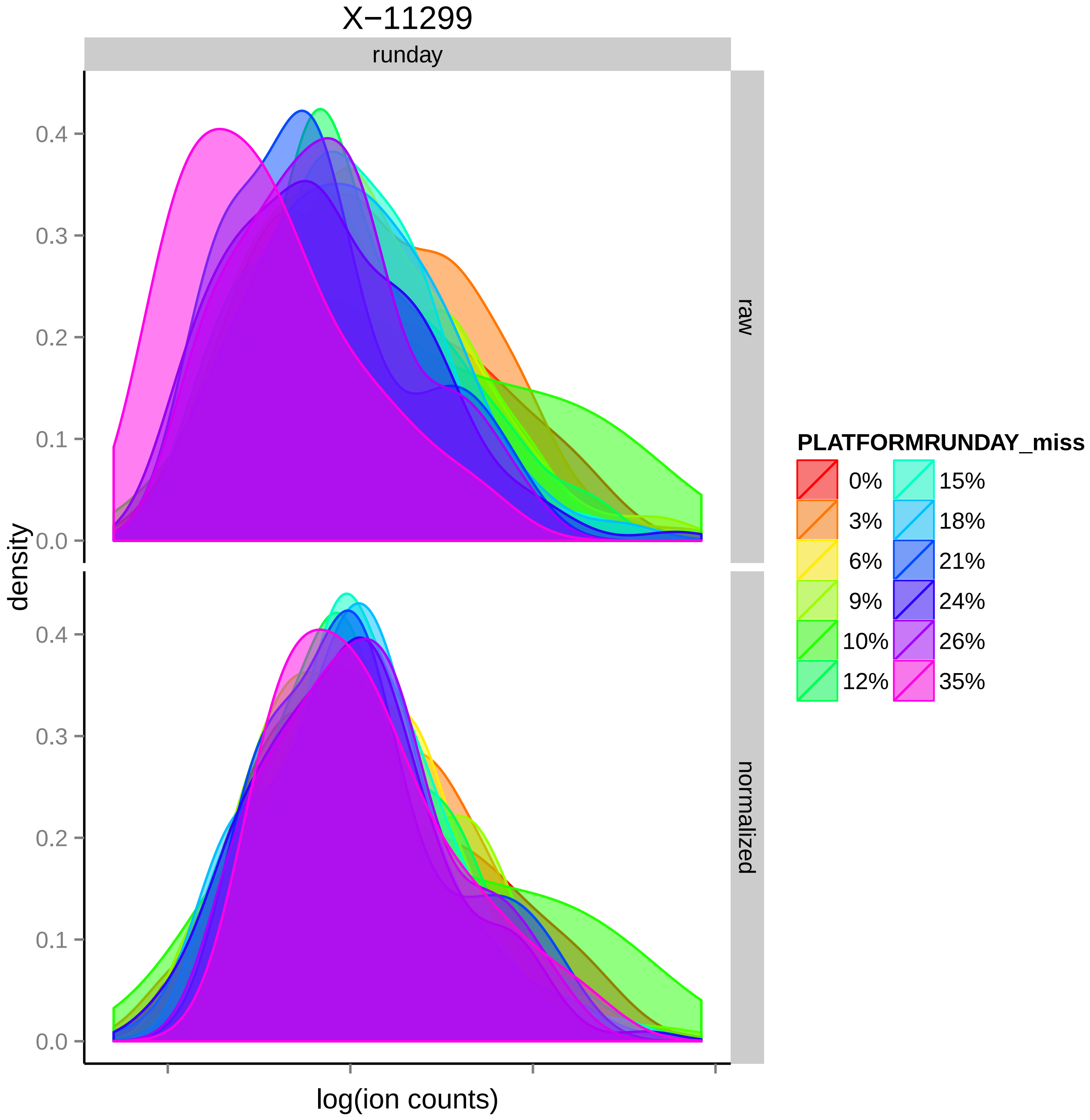

X−11299
runday
raw
normalized
density
0.0
0.1
0.2
0.3
0.4
log(ion counts)
PLATFORMRUNDAY_miss
0%
3%
6%
9%
10%
12%
15%
18%
21%
24%
26%
35%

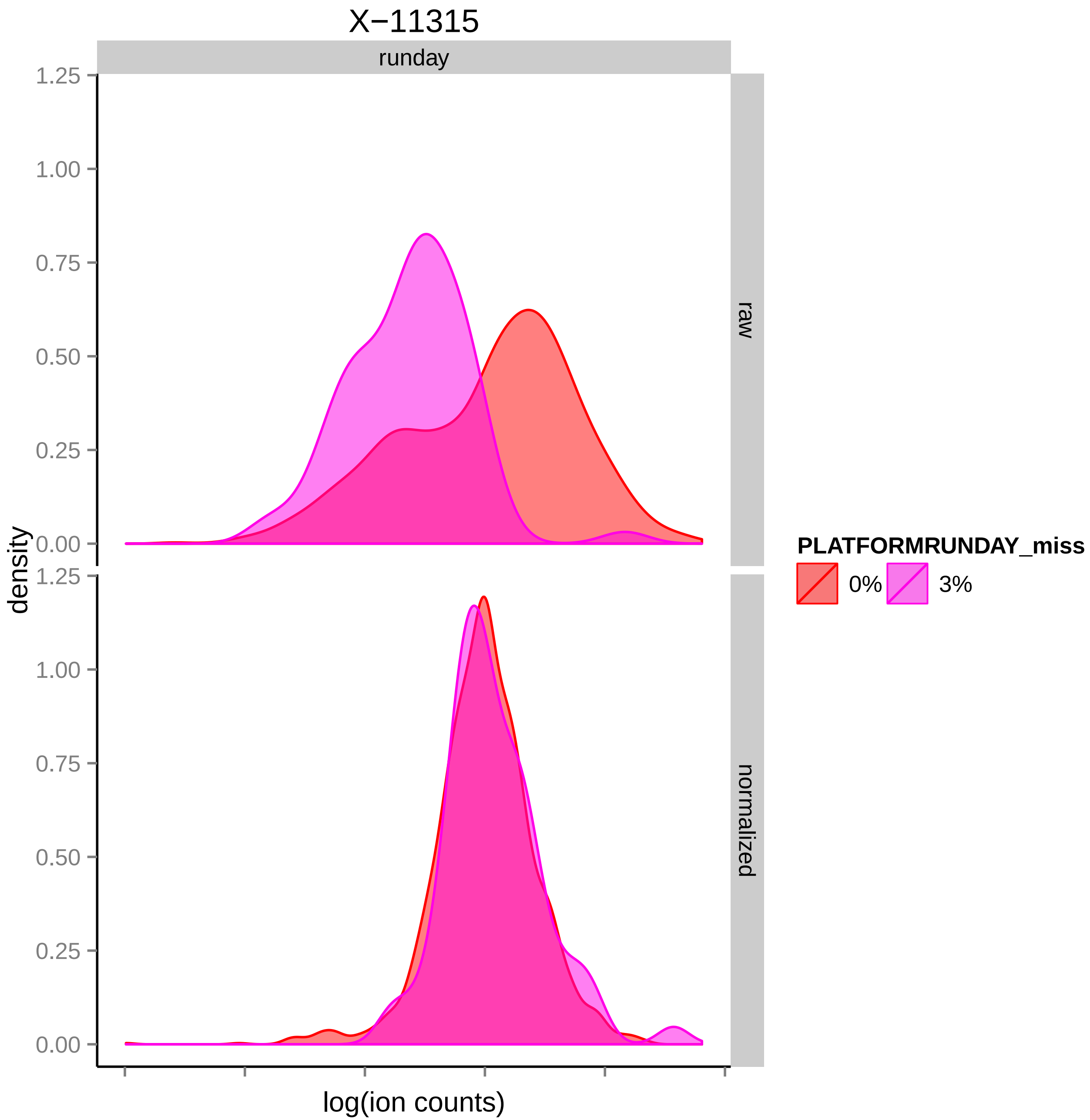
X−11315
runday
raw
normalized
density
log(ion counts)
1.25
1.00
0.75
0.50
0.25
0.00
PLATFORMRUNDAY_miss
0%
3%

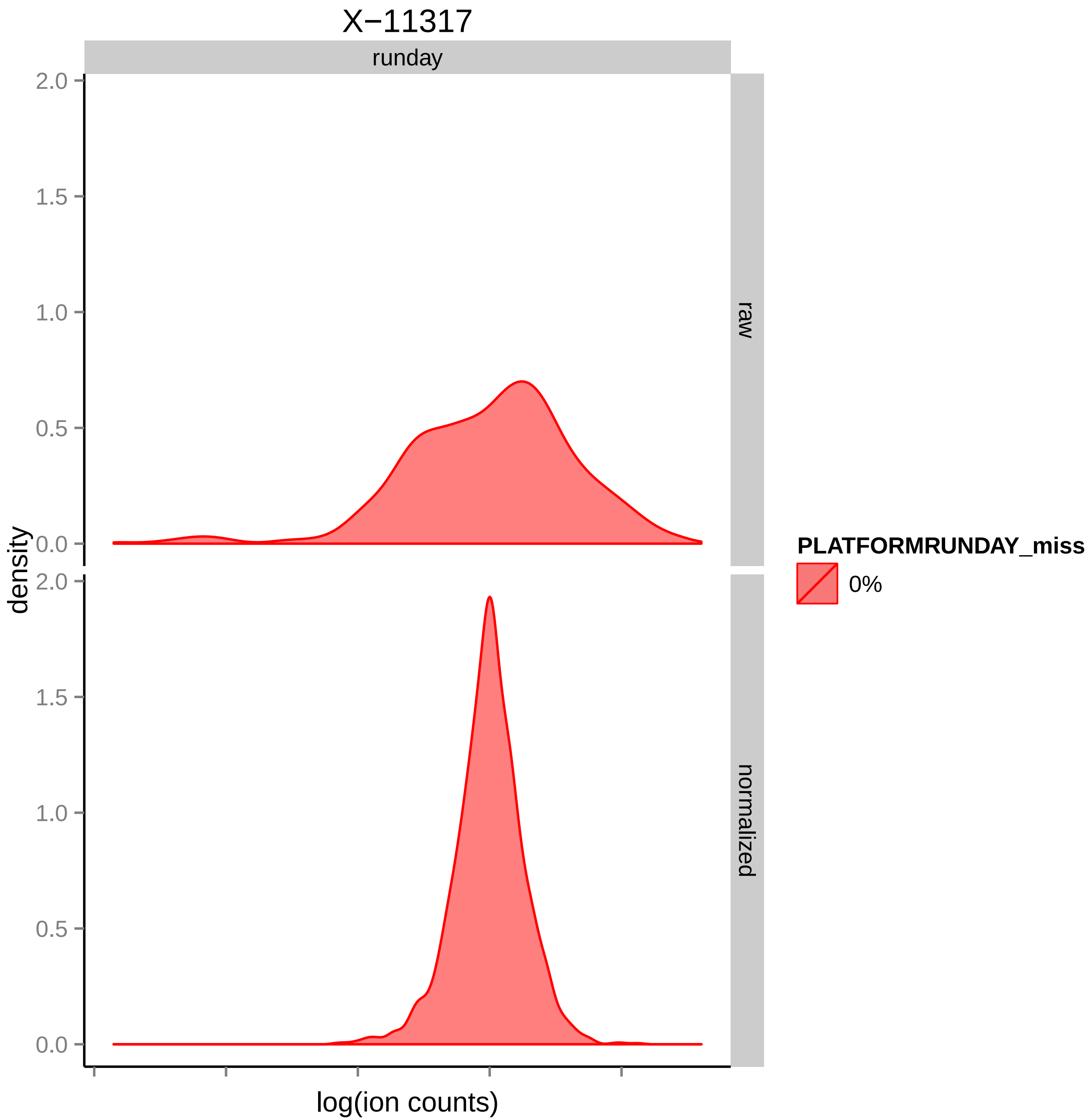
X−11317
runday
raw
normalized
density
log(ion counts)
2.0
1.5
1.0
0.5
0.0
PLATFORMRUNDAY_miss
0%

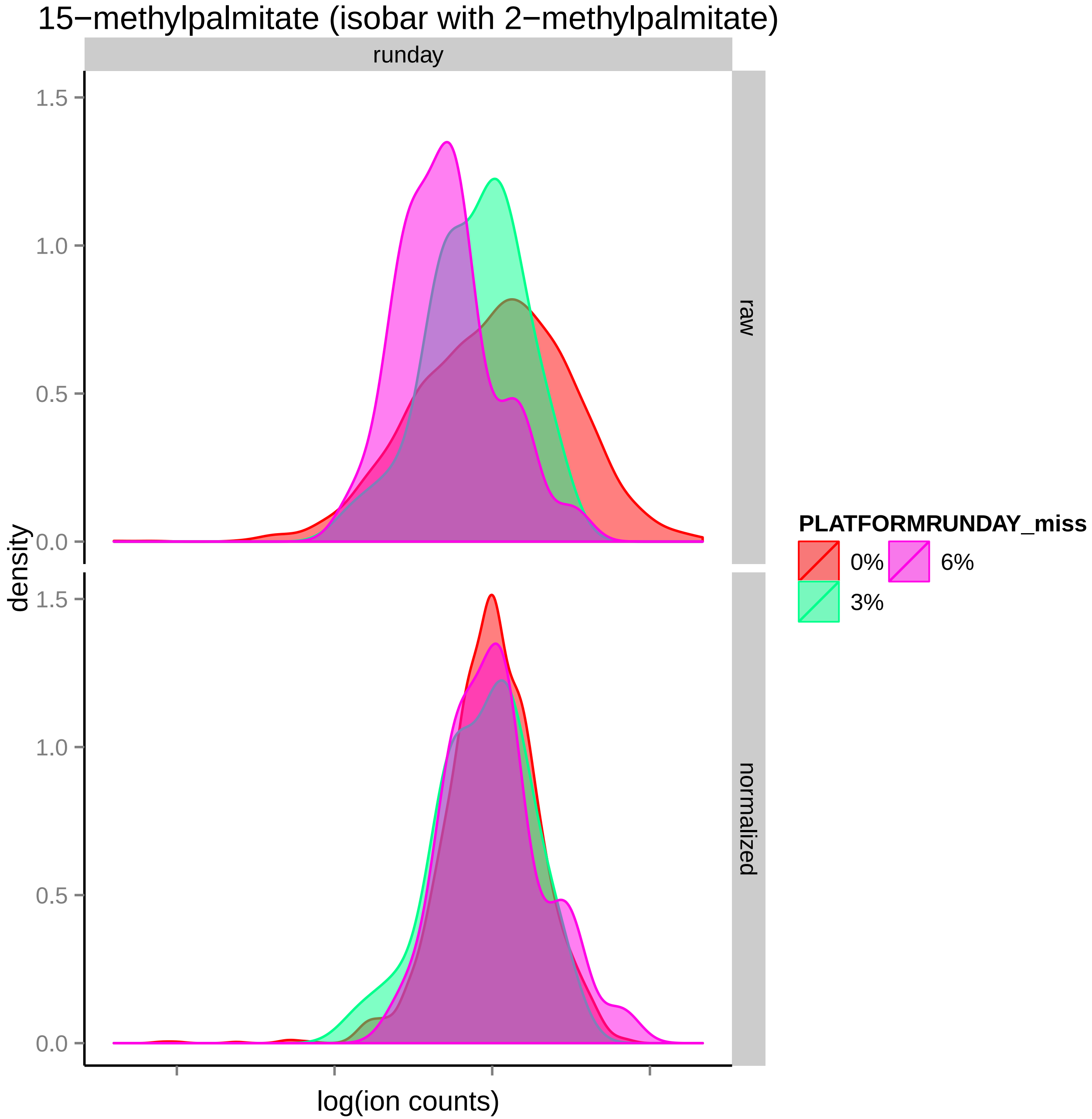
15−methylpalmitate (isobar with 2−methylpalmitate)
runday
raw
normalized
density
log(ion counts)
PLATFORMRUNDAY_miss
0%
3%
6%
0.0
0.5
1.0
1.5

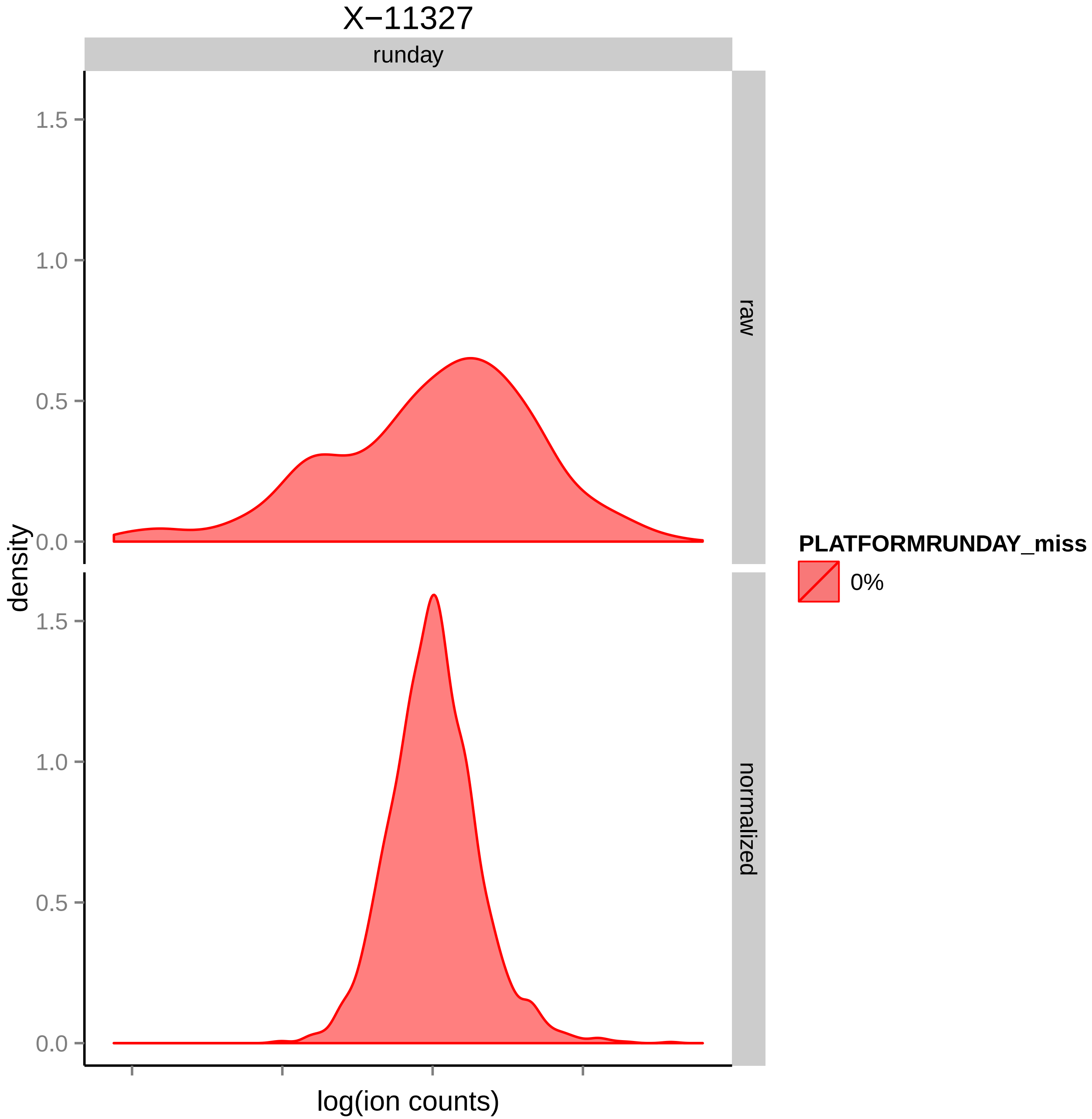
X−11327
runday
raw
normalized
density
log(ion counts)
0.0
0.5
1.0
1.5
PLATFORMRUNDAY_miss
0%

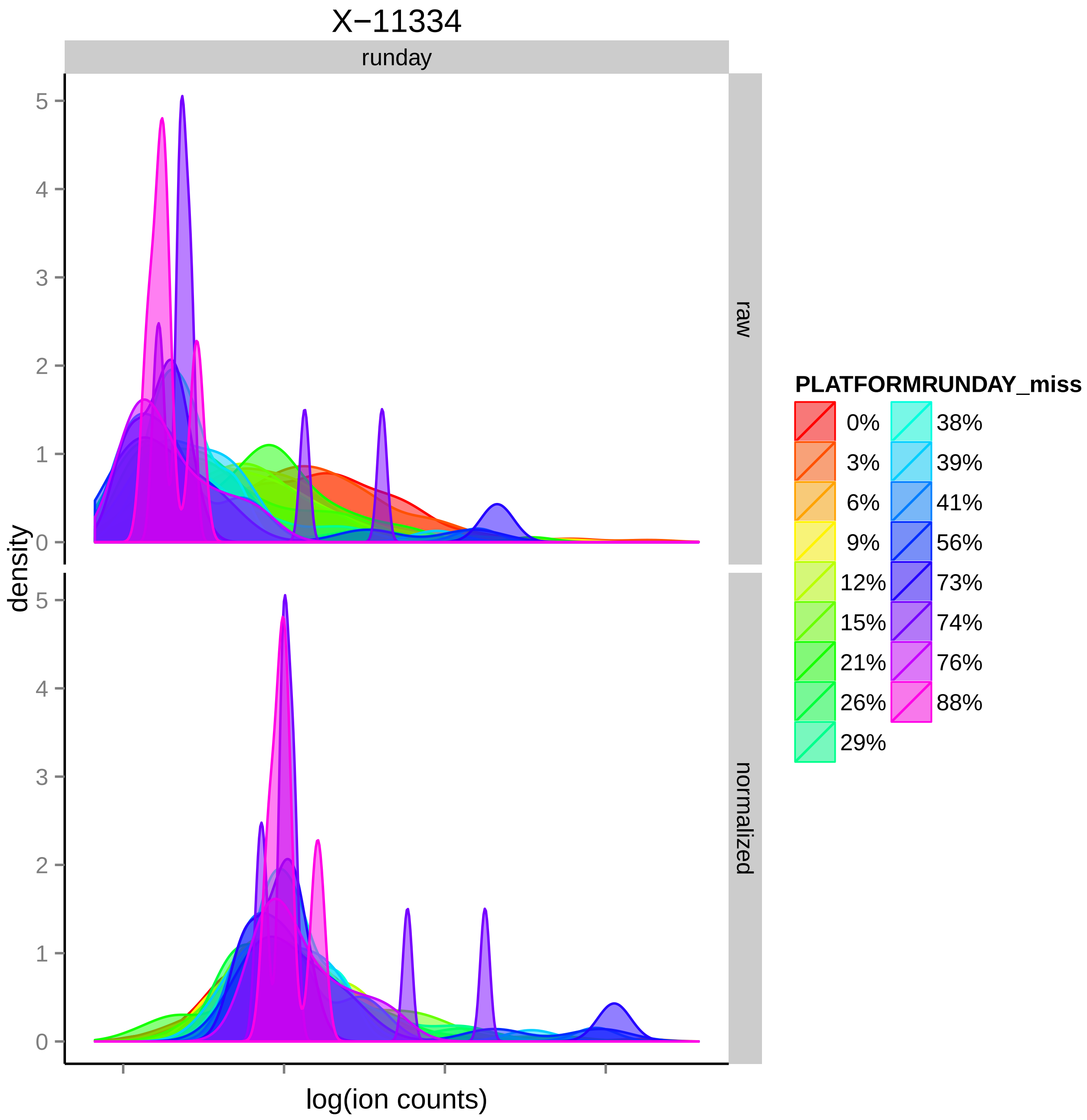
X−11334
runday
raw
normalized
density
log(ion counts)
0
1
2
3
4
5
PLATFORMRUNDAY_miss
0%
3%
6%
9%
12%
15%
21%
26%
29%
38%
39%
41%
56%
73%
74%
76%
88%

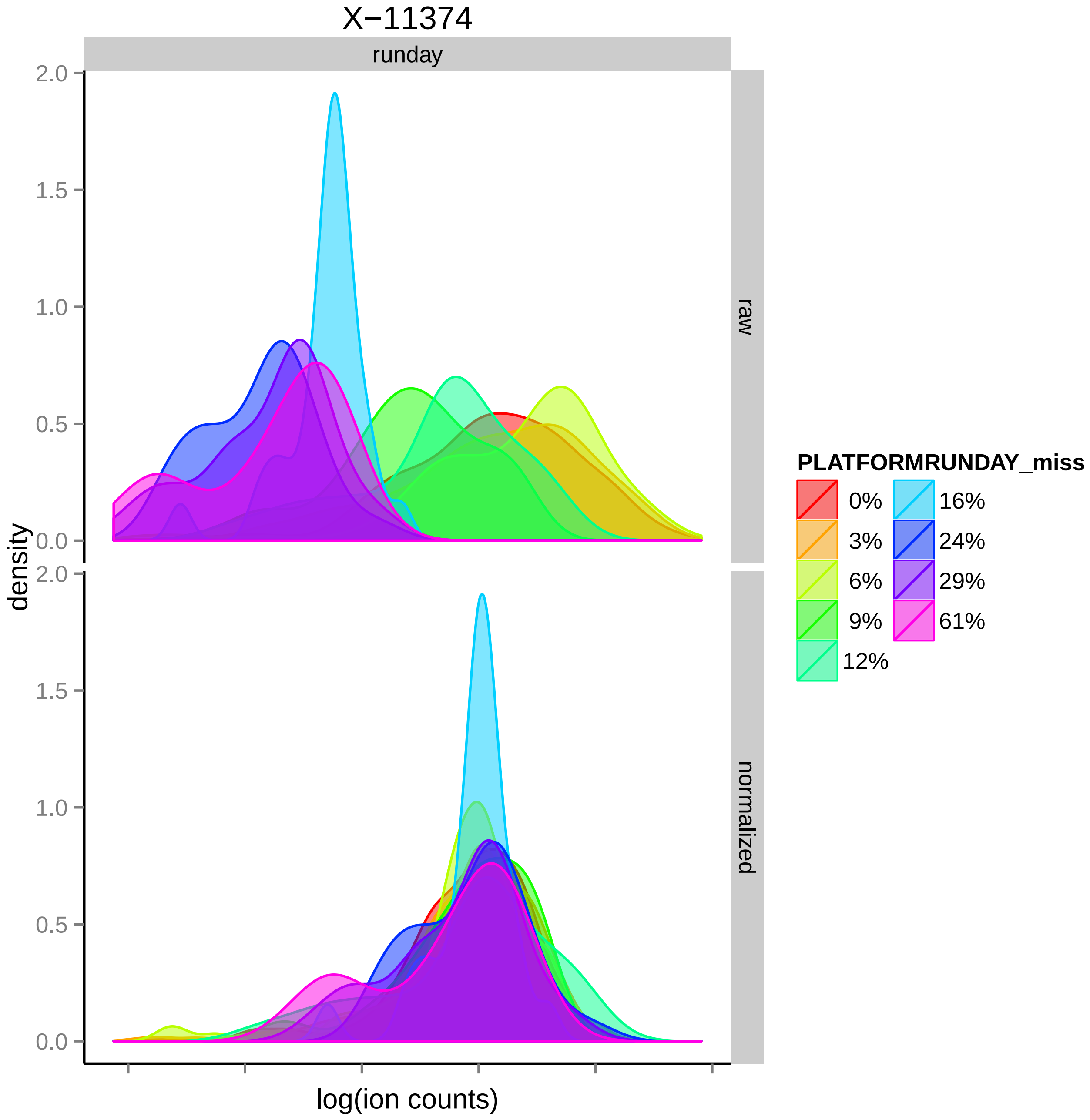
X−11374
runday
raw
normalized
density
log(ion counts)
2.0
1.5
1.0
0.5
0.0
PLATFORMRUNDAY_miss
0%
3%
6%
9%
12%
16%
24%
29%
61%

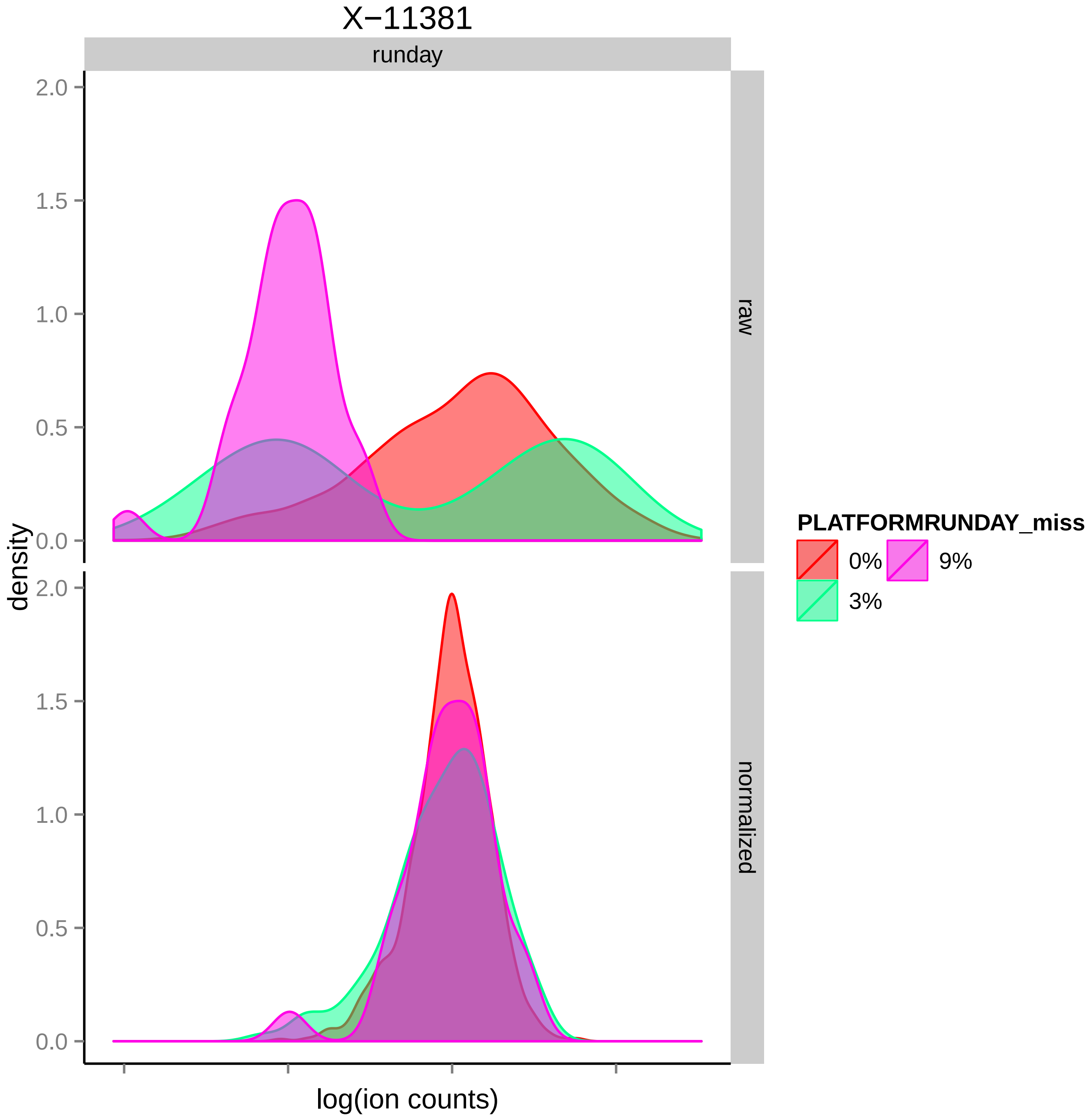
X−11381
runday
raw
normalized
density
log(ion counts)
2.0
1.5
1.0
0.5
0.0
PLATFORMRUNDAY_miss
0%
3%
9%

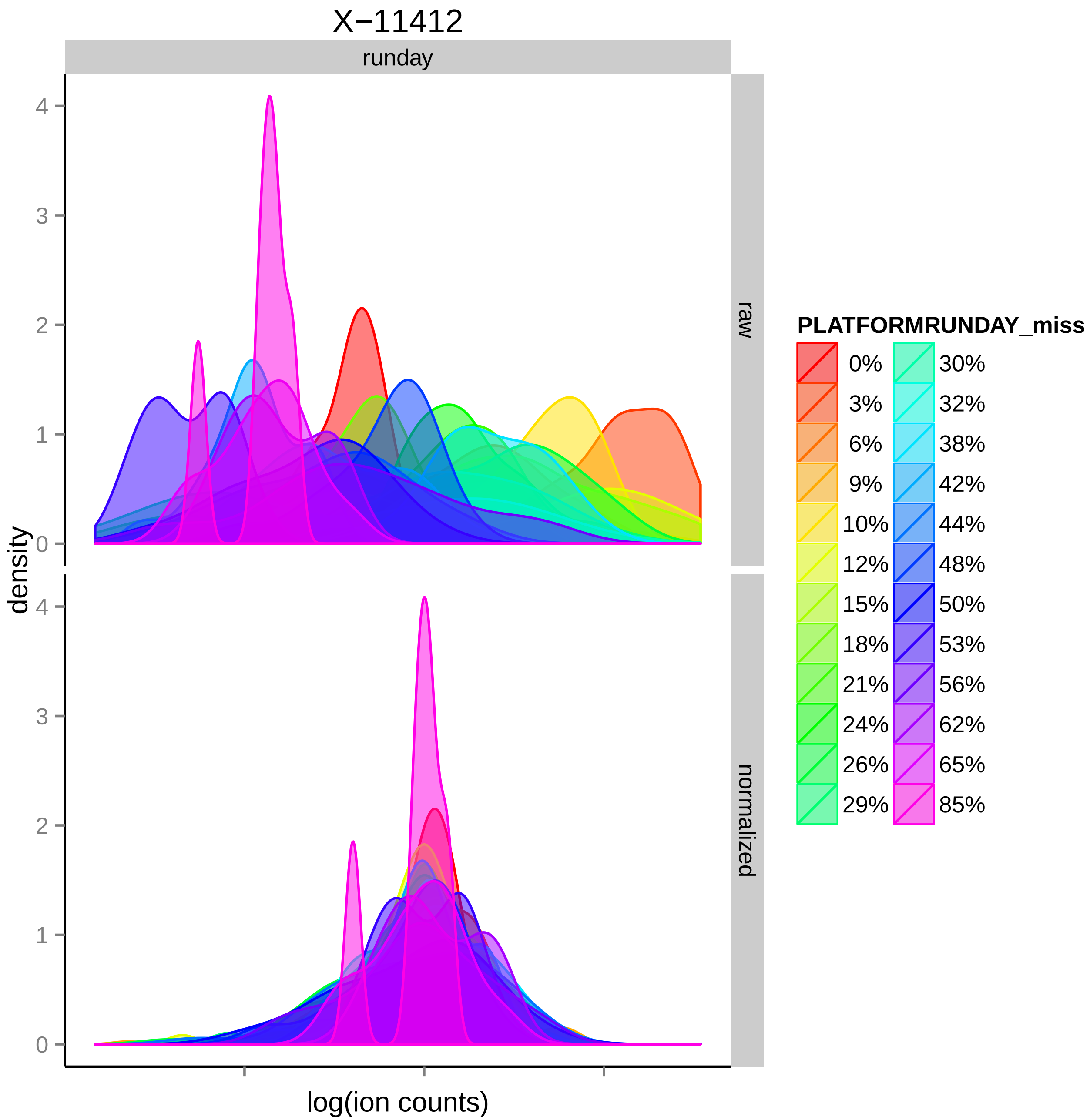

X−11412
runday
raw
normalized
density
log(ion counts)
4
3
2
1
0
4
3
2
1
0
PLATFORMRUNDAY_miss
0%
3%
6%
9%
10%
12%
15%
18%
21%
24%
26%
29%
30%
32%
38%
42%
44%
48%
50%
53%
56%
62%
65%
85%

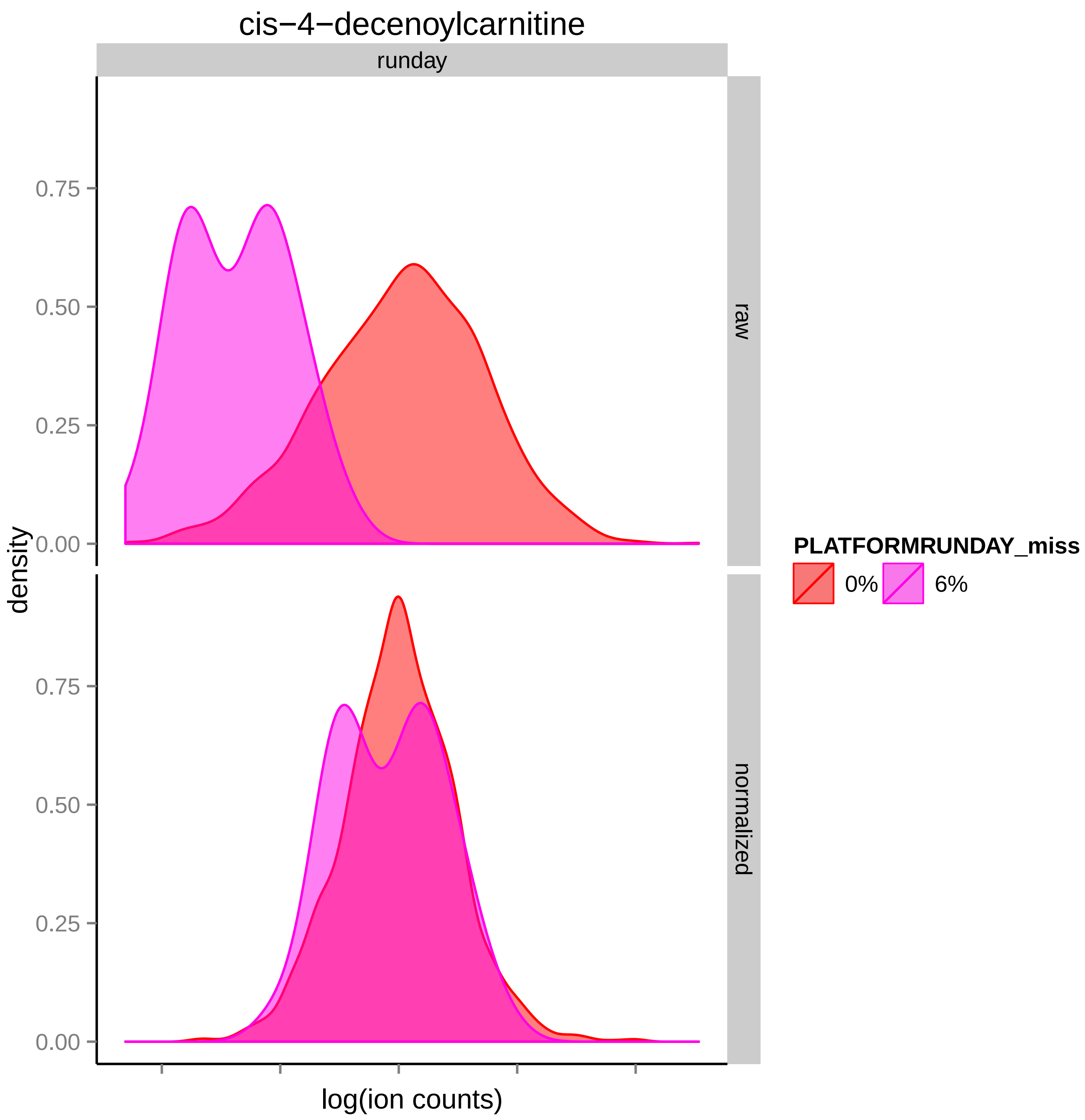
cis−4−decenoylcarnitine
runday
raw
normalized
density
0.75
0.50
0.25
0.00
log(ion counts)
PLATFORMRUNDAY_miss
0%
6%

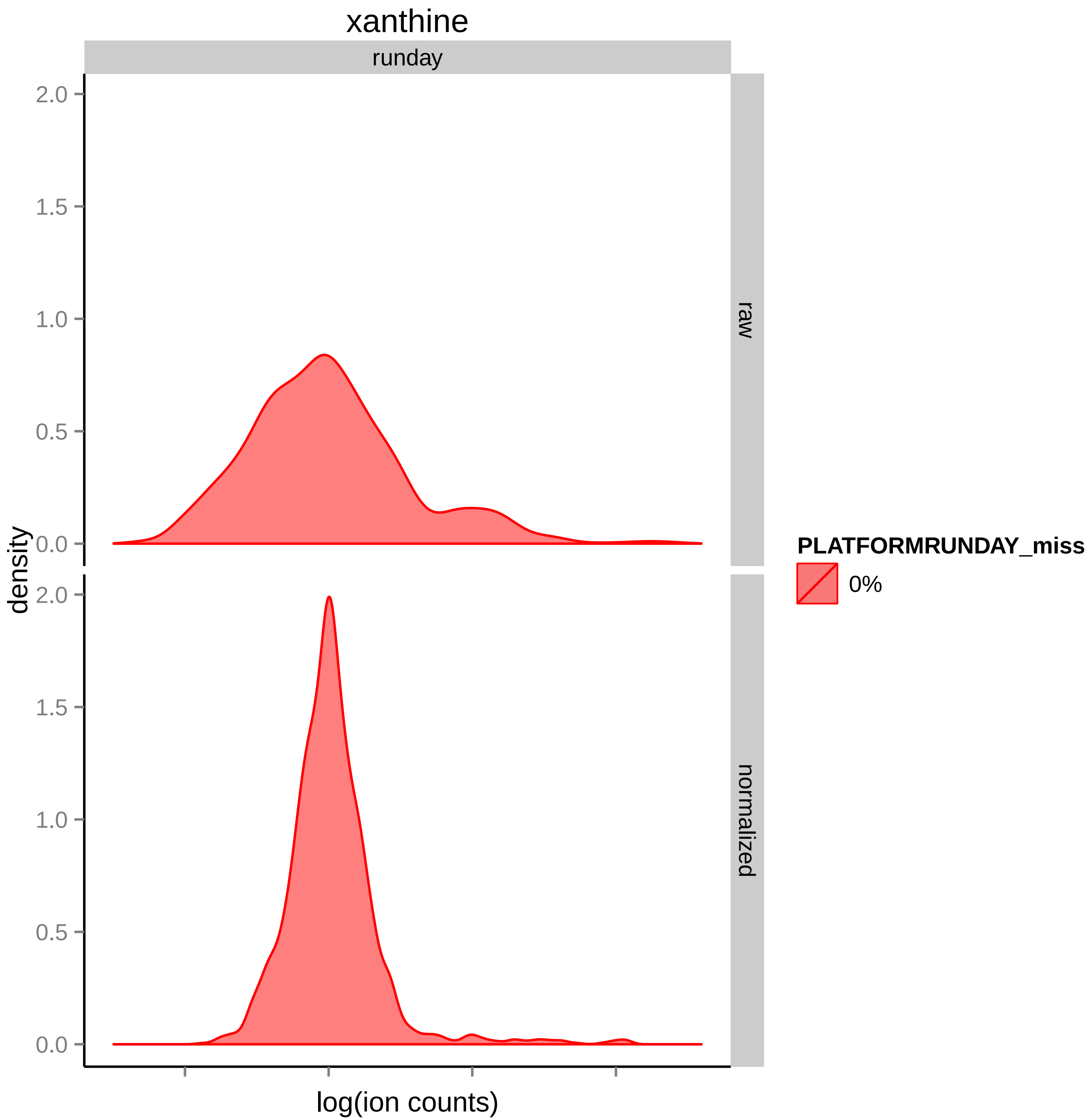

xanthine
runday
raw
normalized
density
log(ion counts)
2.0
1.5
1.0
0.5
0.0
PLATFORMRUNDAY_miss
0%

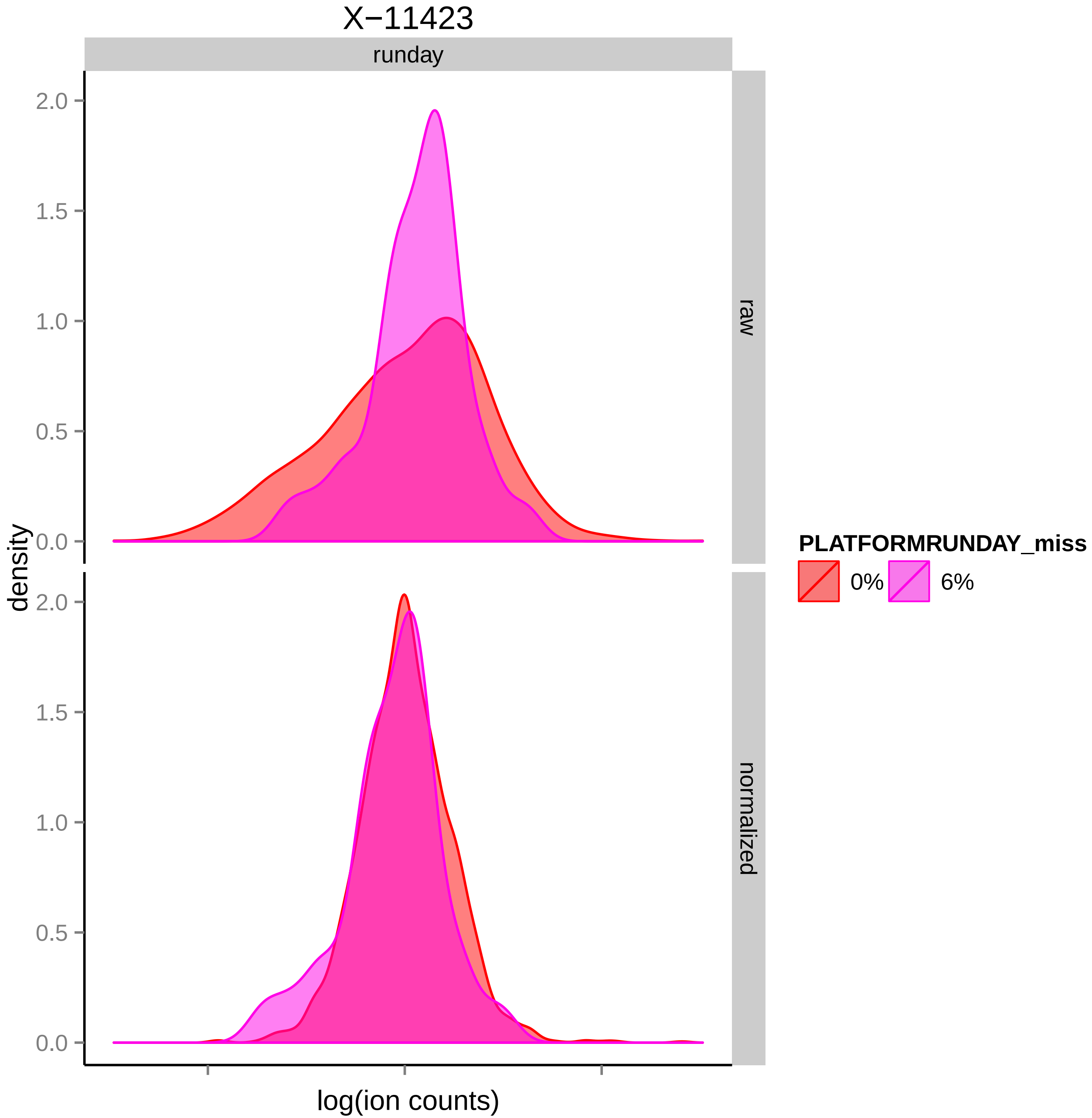
X−11423
runday
raw
normalized
density
2.0
1.5
1.0
0.5
0.0
log(ion counts)
PLATFORMRUNDAY_miss
0%
6%

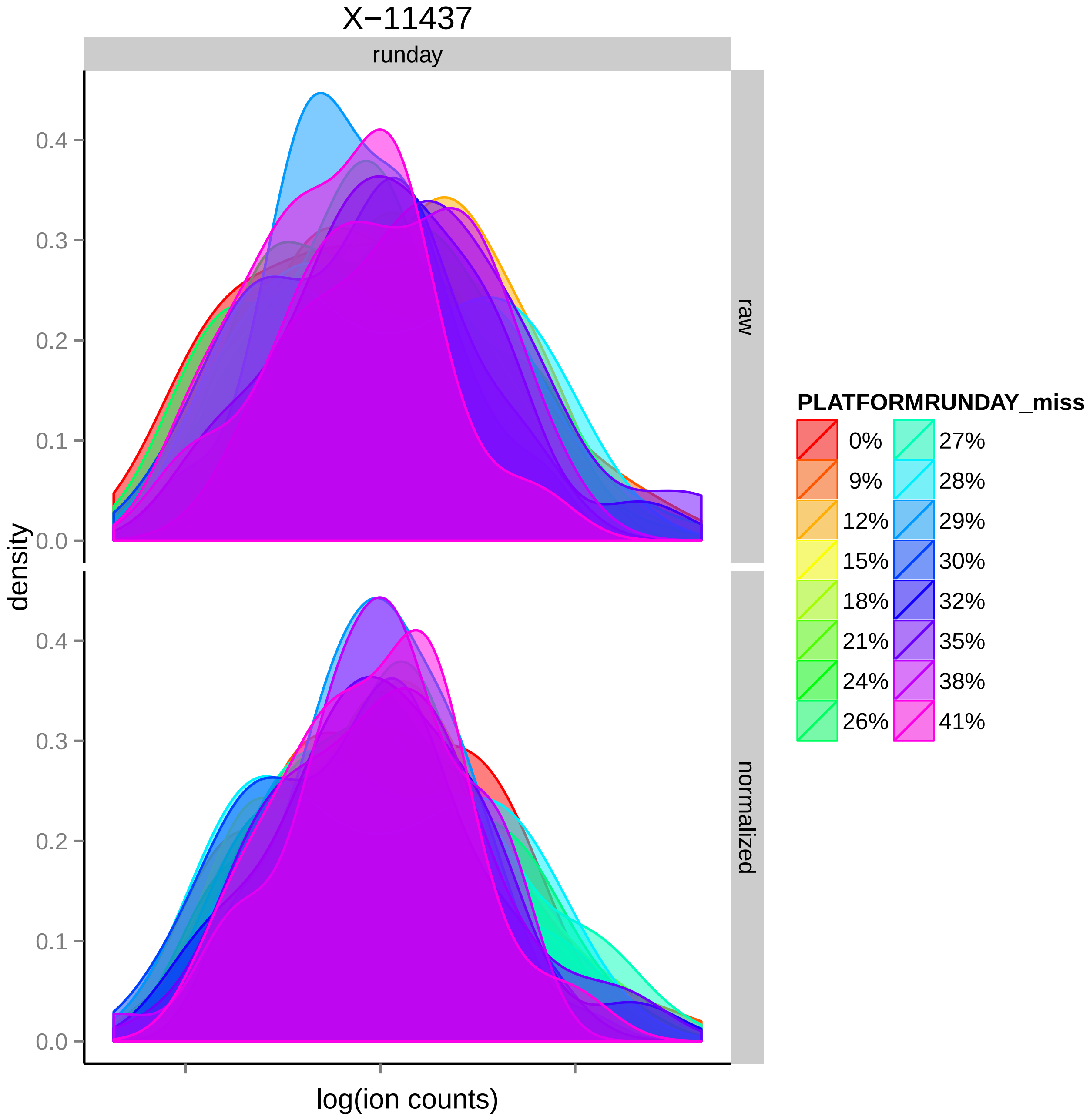

X−11437
runday
raw
normalized
density
0.0
0.1
0.2
0.3
0.4
log(ion counts)
PLATFORMRUNDAY_miss
0%
9%
12%
15%
18%
21%
24%
26%
27%
28%
29%
30%
32%
35%
38%
41%

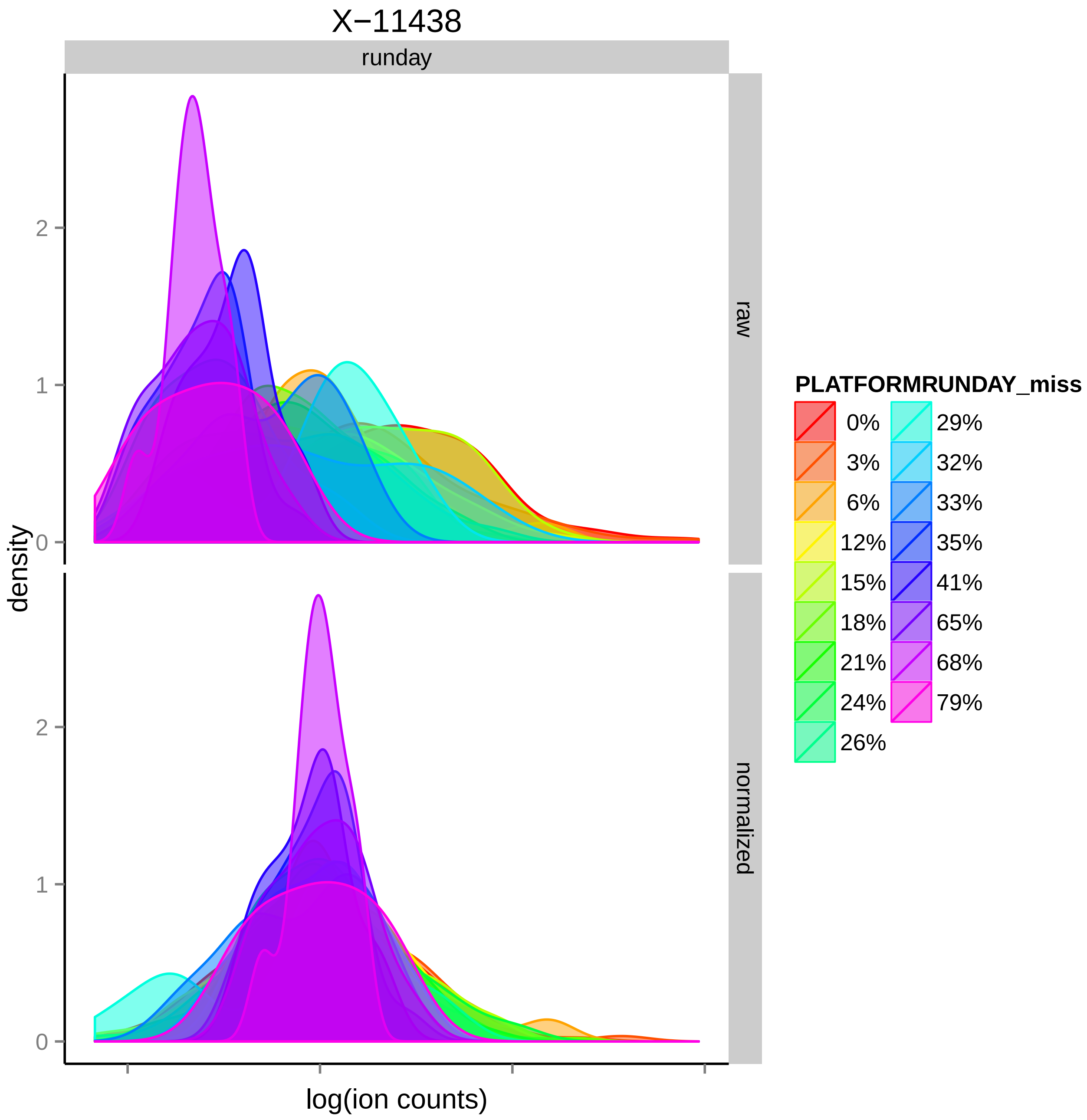
X−11438
runday
raw
normalized
density
log(ion counts)
2
1
0
2
1
0
PLATFORMRUNDAY_miss
0%
3%
6%
12%
15%
18%
21%
24%
26%
29%
32%
33%
35%
41%
65%
68%
79%

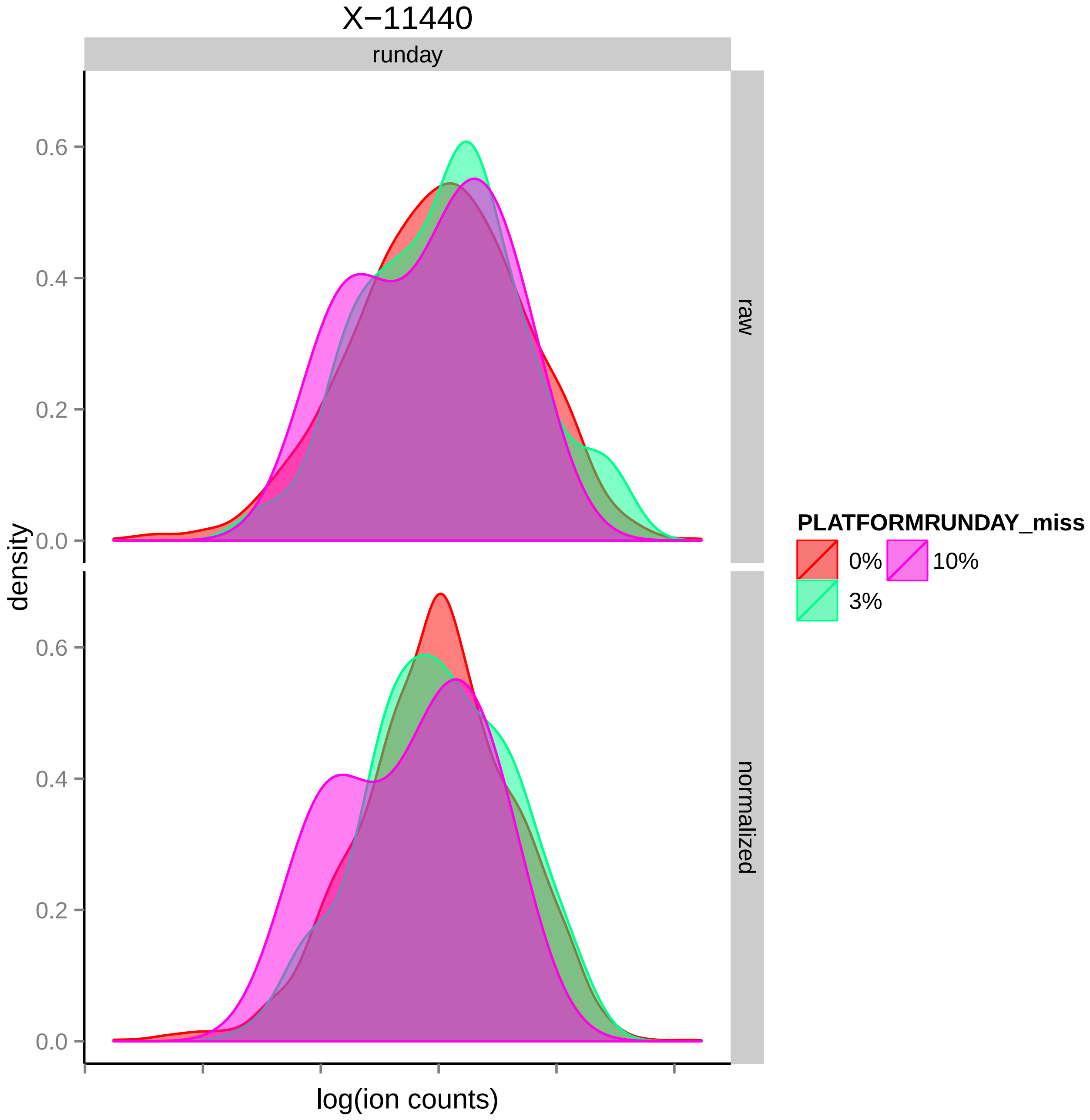

X−11440
runday
raw
normalized
density
0.0
0.2
0.4
0.6
log(ion counts)
PLATFORMRUNDAY_miss
0%
3%
10%

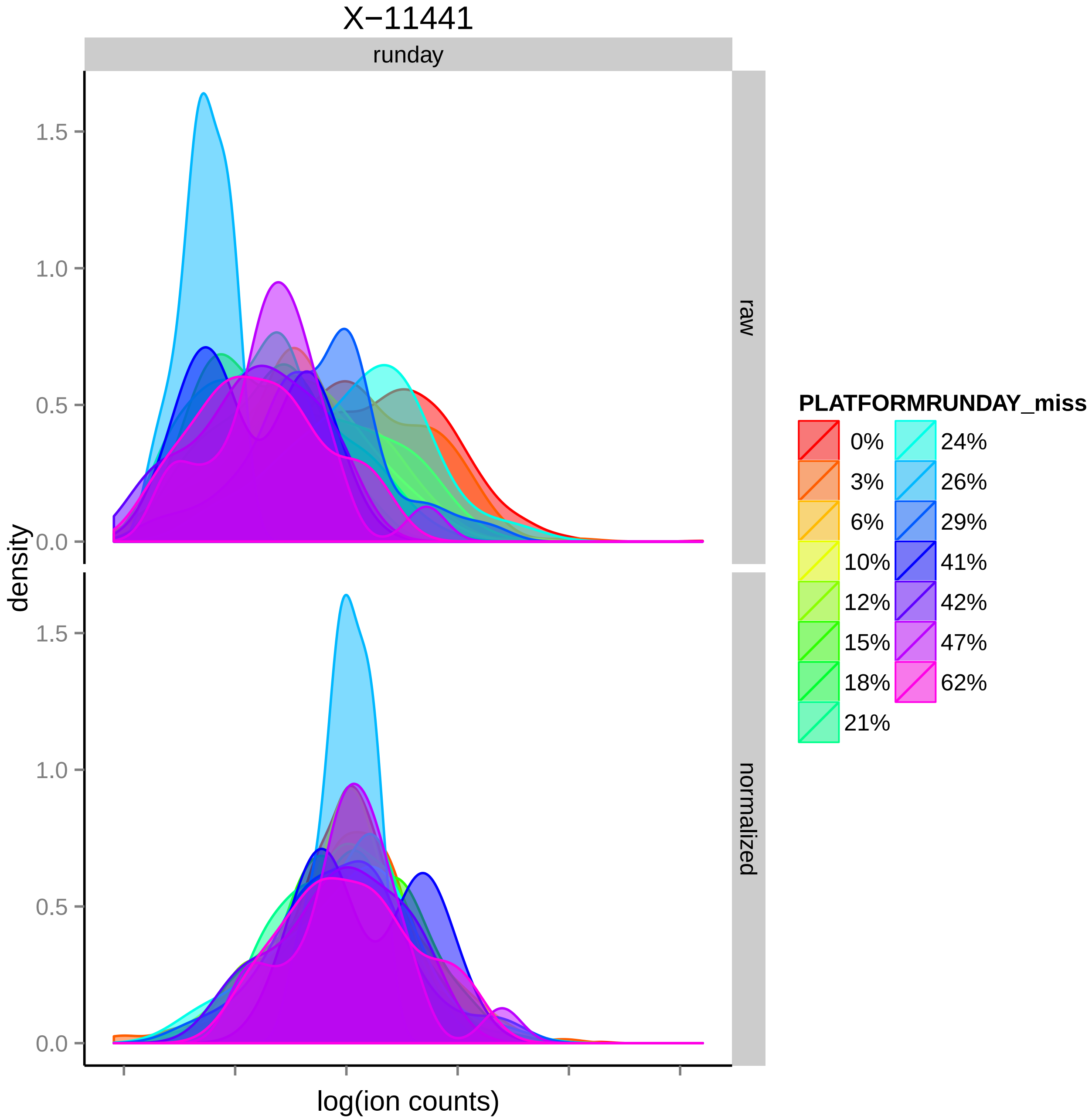
X−11441
runday
raw
normalized
density
log(ion counts)
1.5
1.0
0.5
0.0
PLATFORMRUNDAY_miss
0%
3%
6%
10%
12%
15%
18%
21%
24%
26%
29%
41%
42%
47%
62%

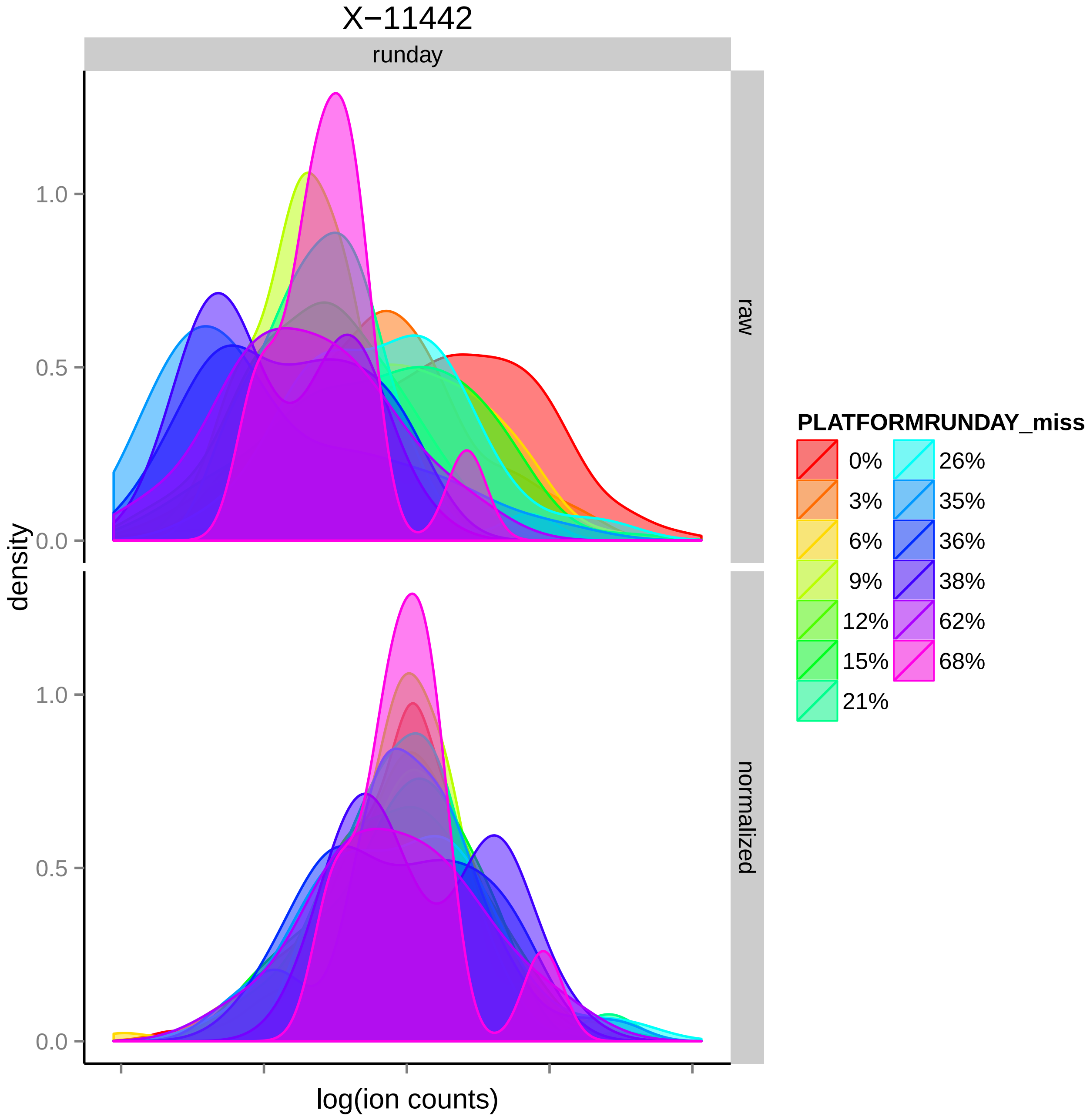
X−11442
runday
raw
normalized
density
log(ion counts)
1.0
0.5
0.0
PLATFORMRUNDAY_miss
0%
3%
6%
9%
12%
15%
21%
26%
35%
36%
38%
62%
68%

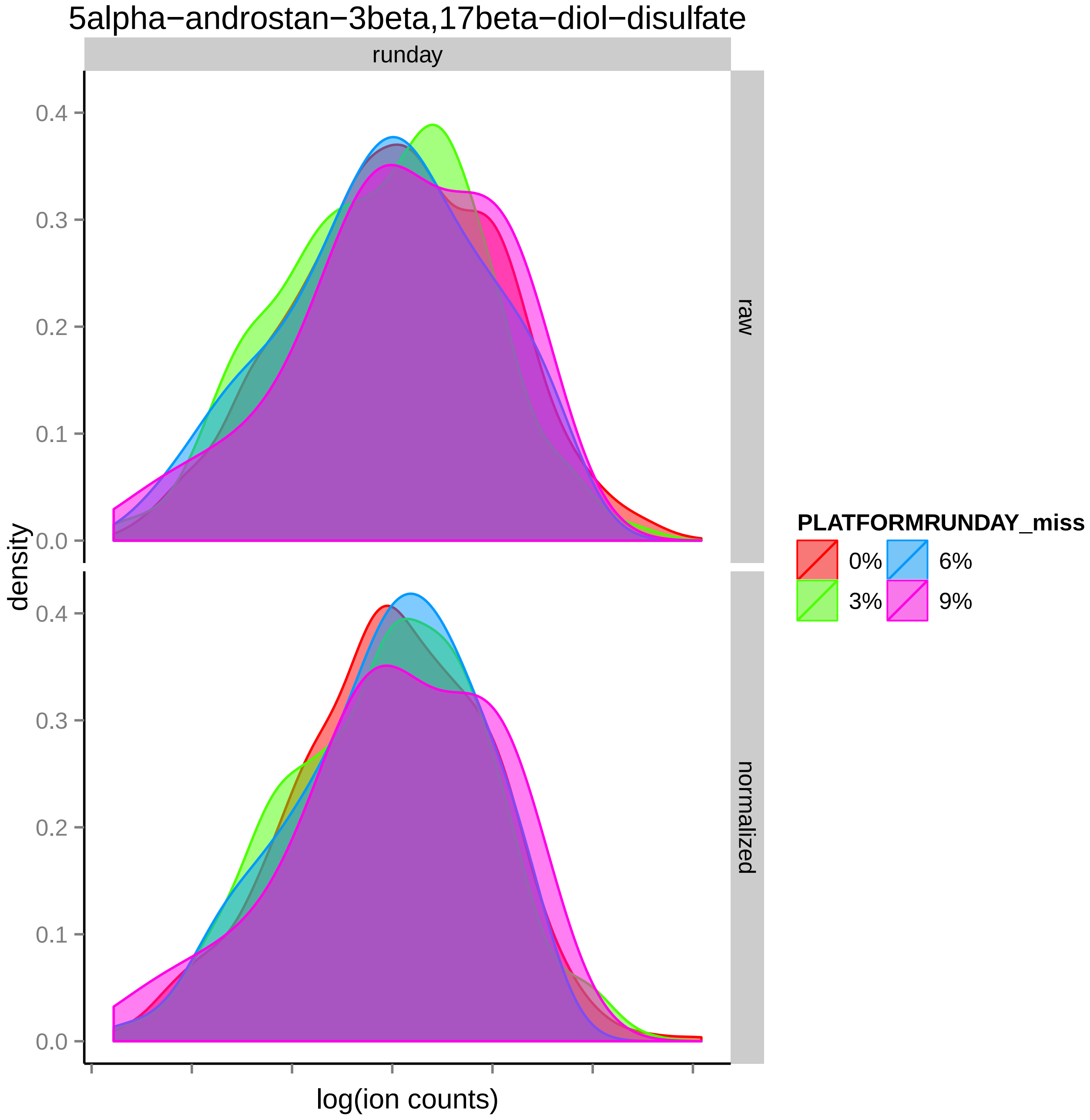

5alpha−androstan−3beta,17beta−diol−disulfate
runday
raw
normalized
density
0.0
0.1
0.2
0.3
0.4
log(ion counts)
PLATFORMRUNDAY_miss
0%
3%
6%
9%

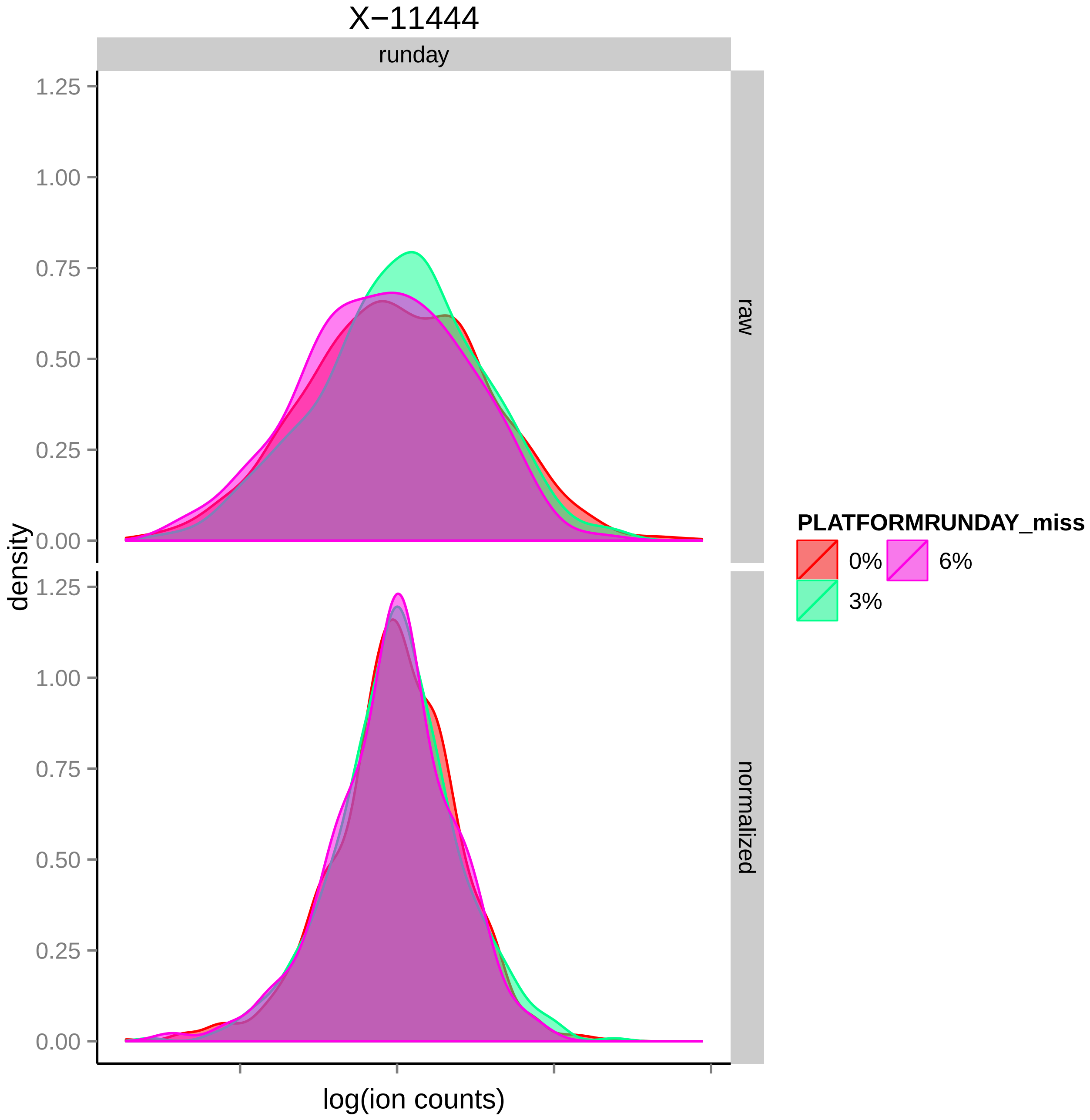
X−11444
runday
raw
normalized
density
log(ion counts)
1.25
1.00
0.75
0.50
0.25
0.00
PLATFORMRUNDAY_miss
0%
3%
6%

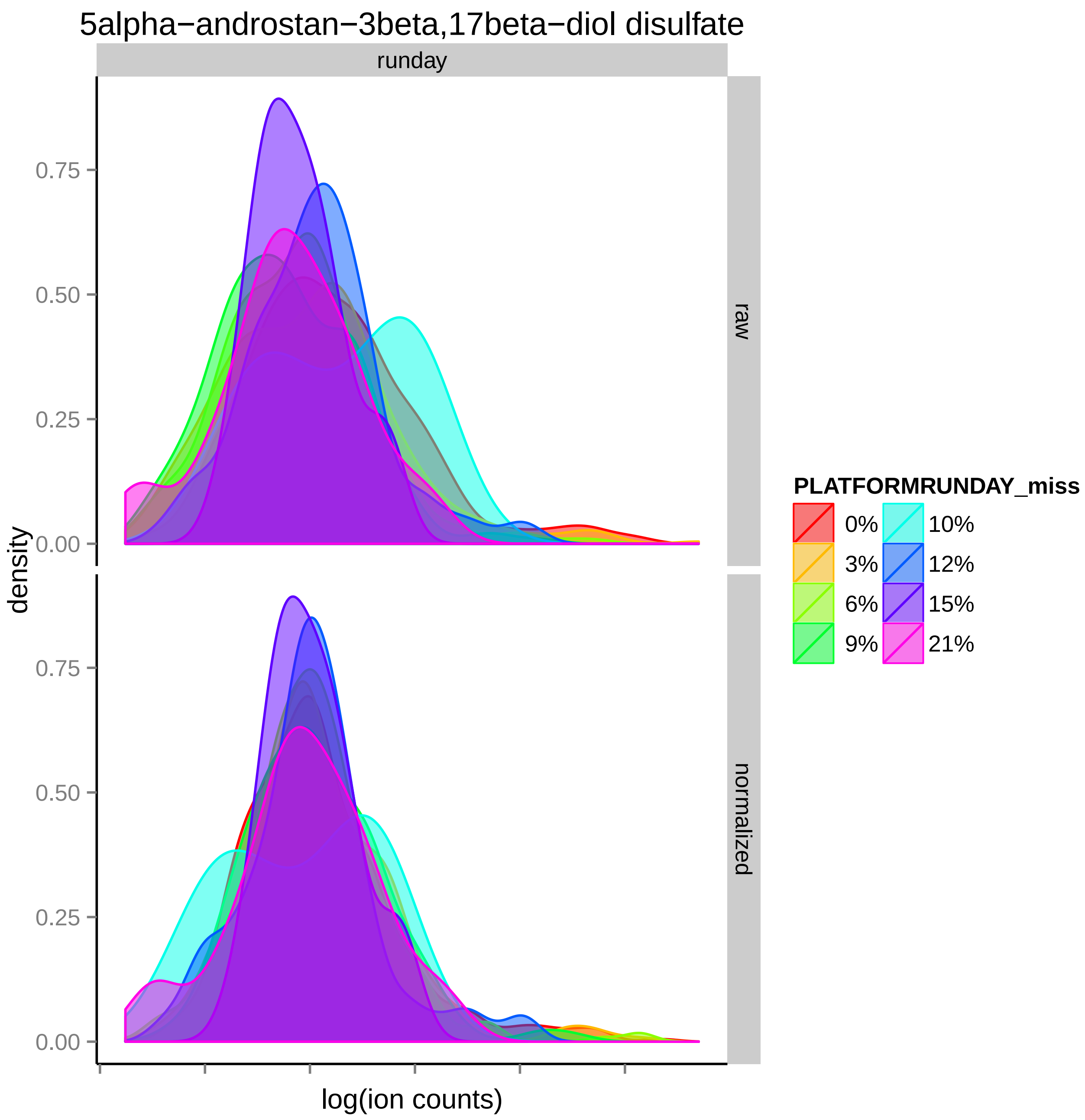
5alpha−androstan−3beta,17beta−diol disulfate
runday
raw
normalized
density
0.00
0.25
0.50
0.75
log(ion counts)
PLATFORMRUNDAY_miss
0%
3%
6%
9%
10%
12%
15%
21%

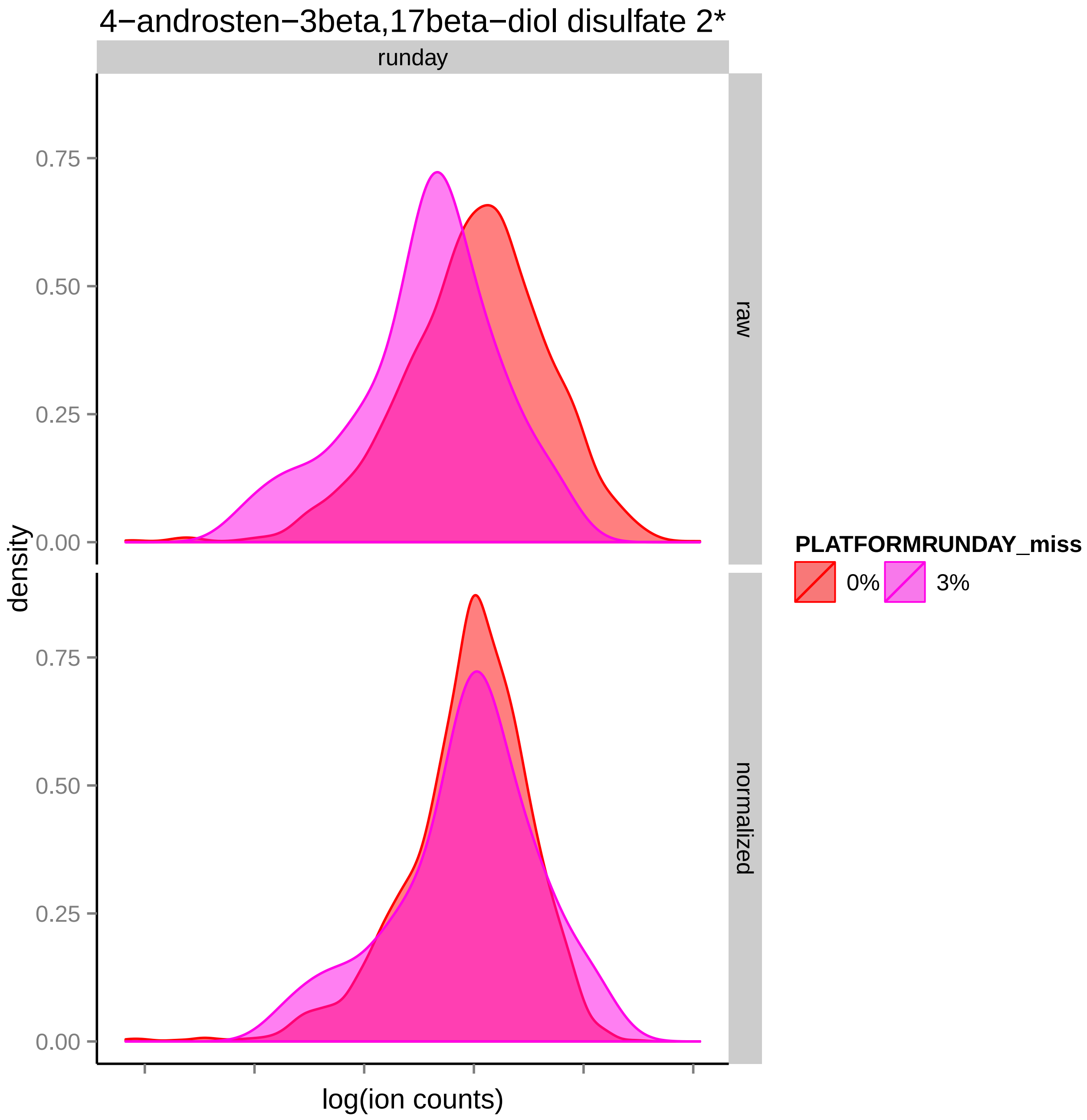
4−androsten−3beta,17beta−diol disulfate 2*
runday
raw
normalized
0.00
0.25
0.50
0.75
density
log(ion counts)
PLATFORMRUNDAY_miss
0%
3%

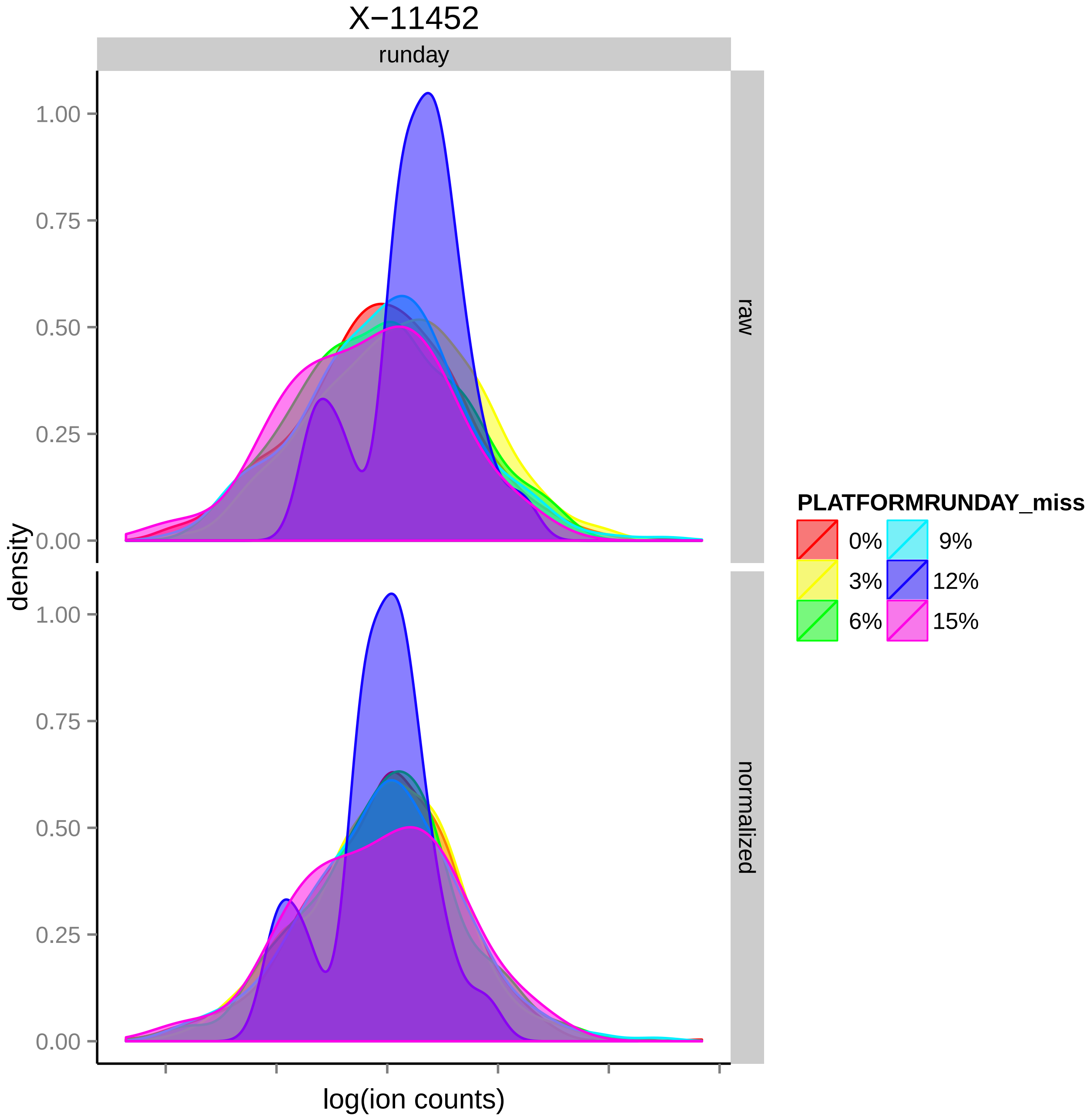
X−11452
runday
raw
normalized
density
1.00
0.75
0.50
0.25
0.00
1.00
0.75
0.50
0.25
0.00
log(ion counts)
PLATFORMRUNDAY_miss
0%
3%
6%
9%
12%
15%

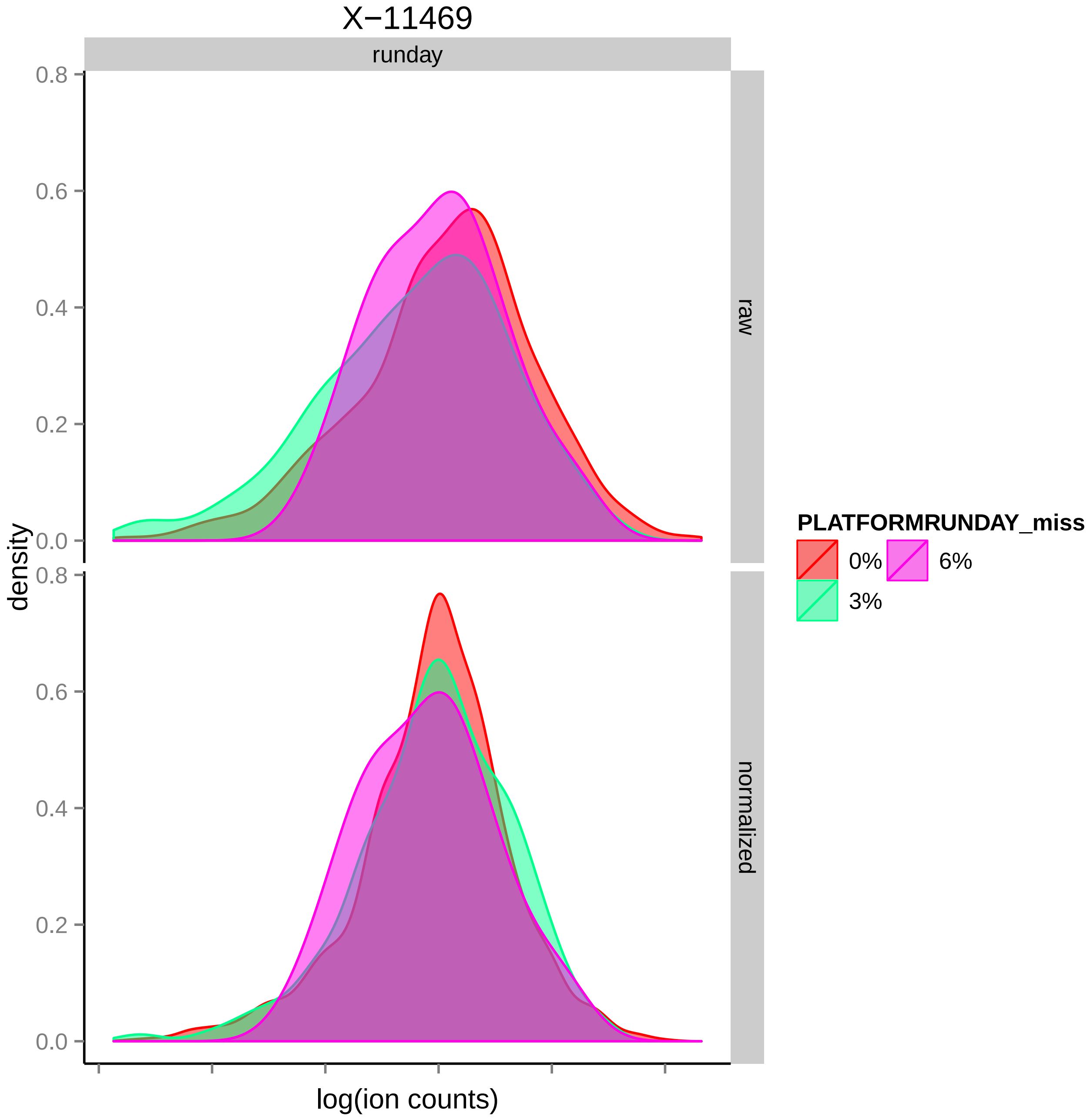

X−11469
runday
raw
normalized
density
log(ion counts)
0.0
0.2
0.4
0.6
0.8
PLATFORMRUNDAY_miss
0%
3%
6%

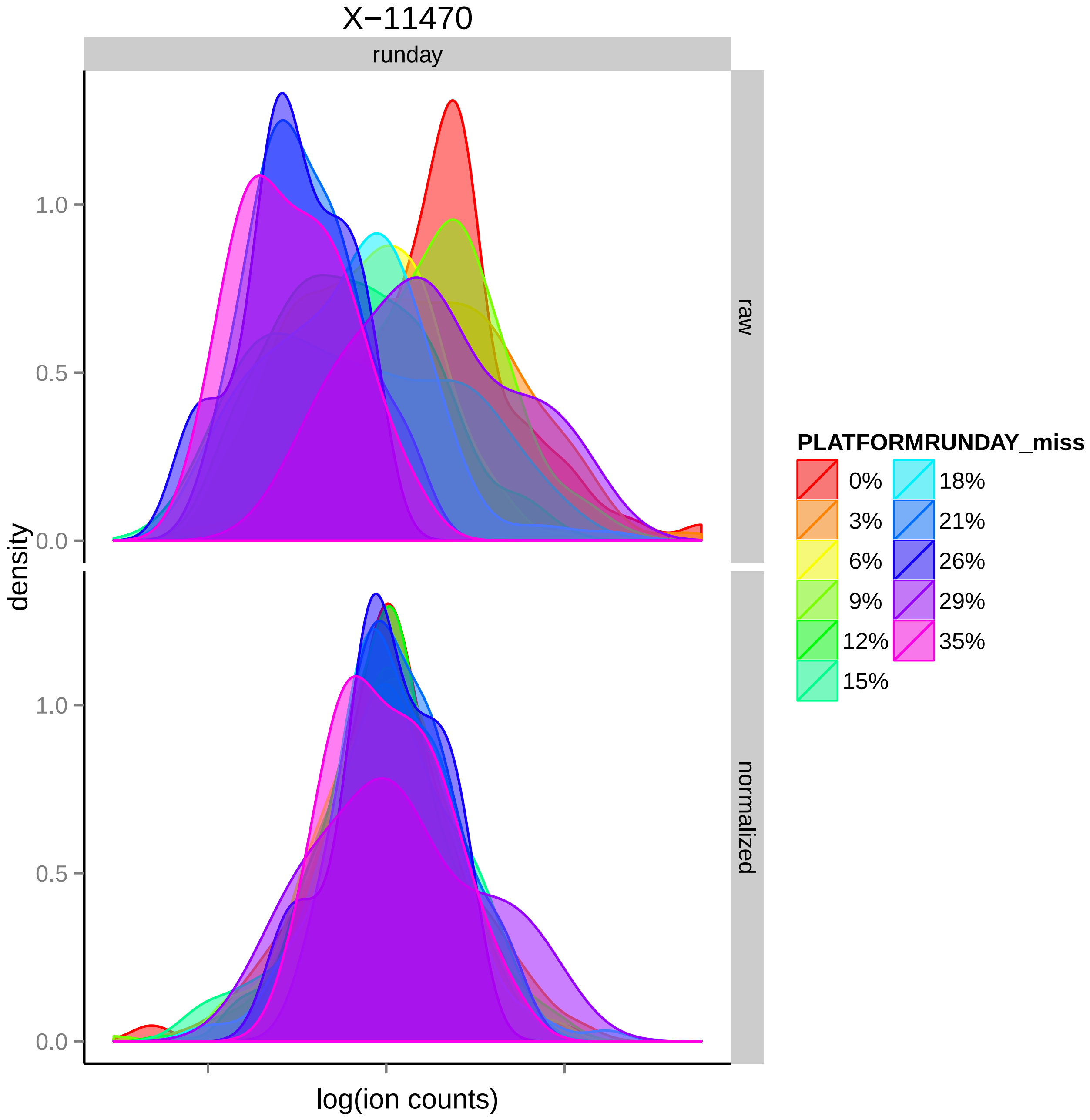

X−11470
runday
raw
normalized
density
log(ion counts)
1.0
0.5
0.0
1.0
0.5
0.0
PLATFORMRUNDAY_miss
0%
3%
6%
9%
12%
15%
18%
21%
26%
29%
35%

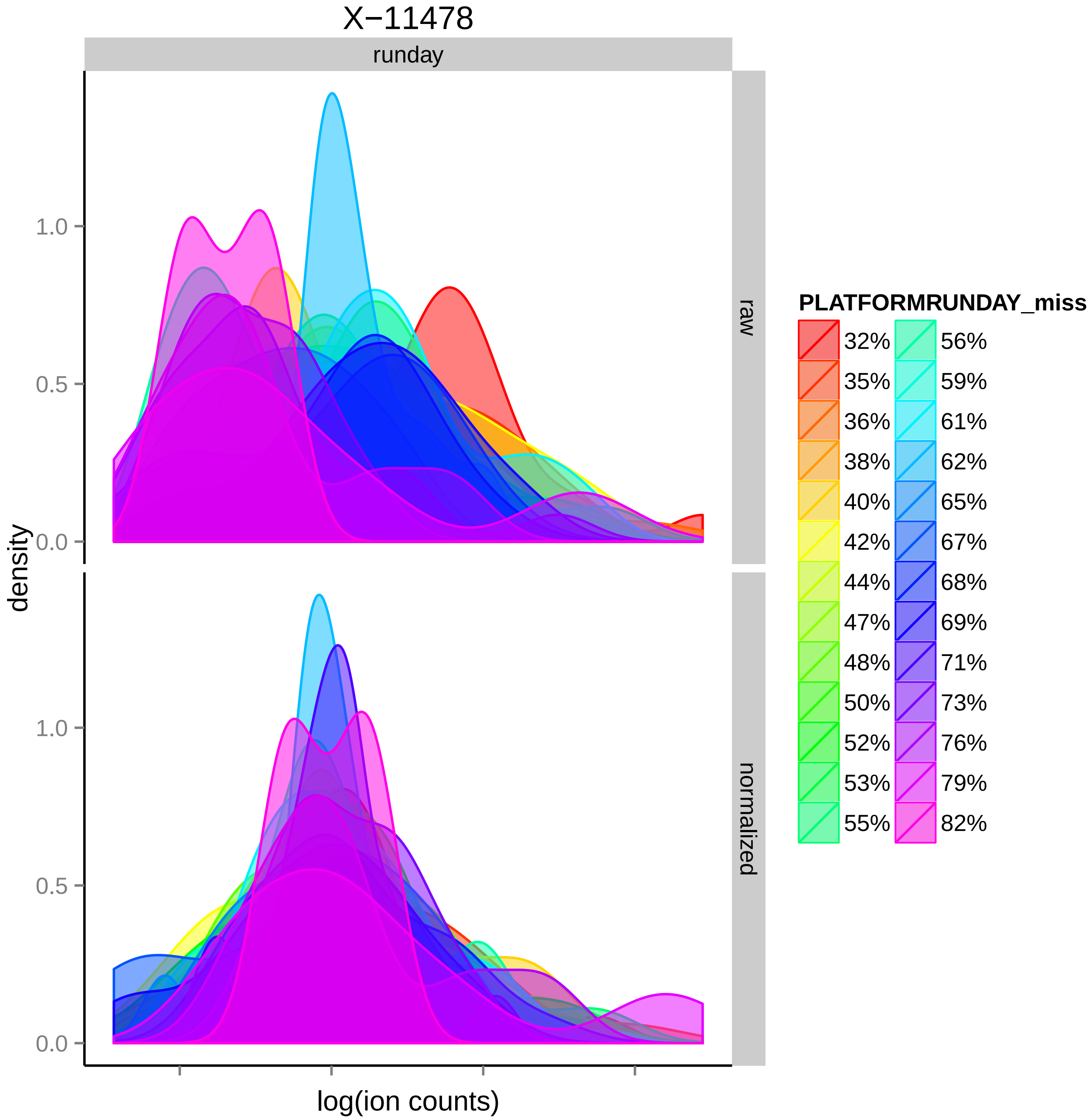

X−11478
runday
raw
normalized
density
log(ion counts)
1.0
0.5
0.0
1.0
0.5
0.0
PLATFORMRUNDAY_miss
32%
35%
36%
38%
40%
42%
44%
47%
48%
50%
52%
53%
55%
56%
59%
61%
62%
65%
67%
68%
69%
71%
73%
76%
79%
82%

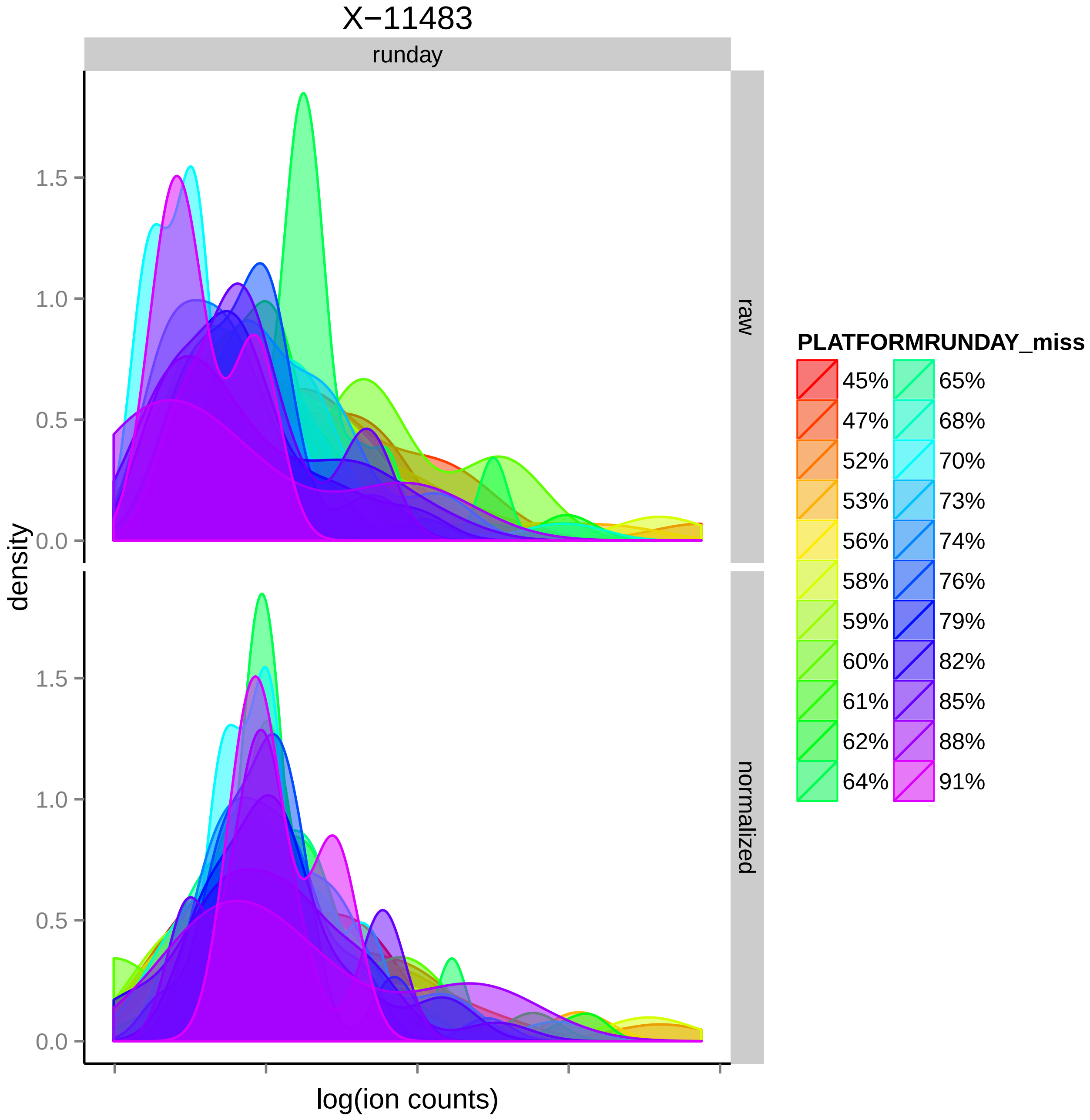

X−11483
runday
raw
normalized
density
log(ion counts)
1.5
1.0
0.5
0.0
PLATFORMRUNDAY_miss
45%
47%
52%
53%
56%
58%
59%
60%
61%
62%
64%
65%
68%
70%
73%
74%
76%
79%
82%
85%
88%
91%

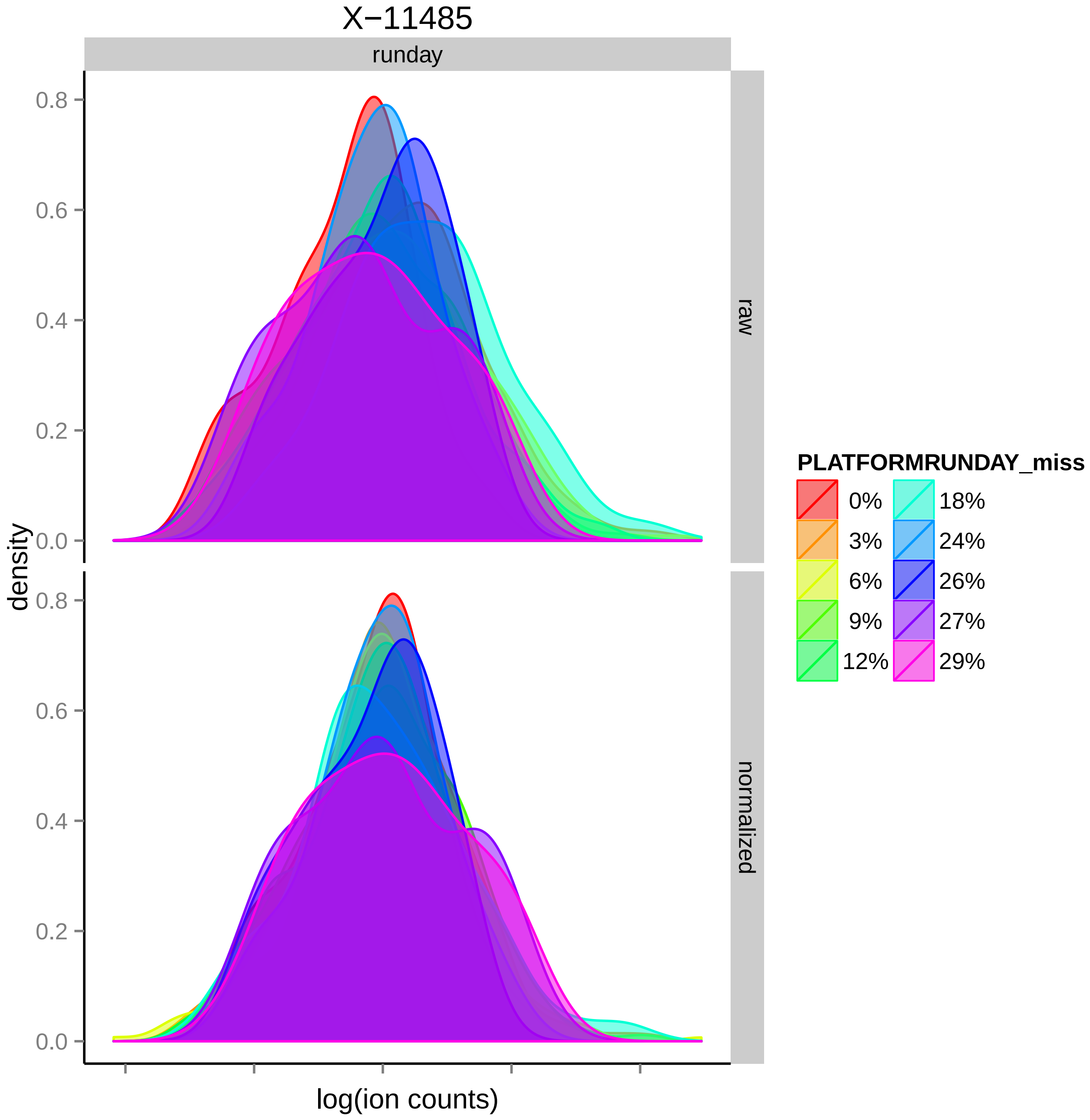

X−11485
runday
raw
normalized
density
0.0
0.2
0.4
0.6
0.8
log(ion counts)
PLATFORMRUNDAY_miss
0%
3%
6%
9%
12%
18%
24%
26%
27%
29%

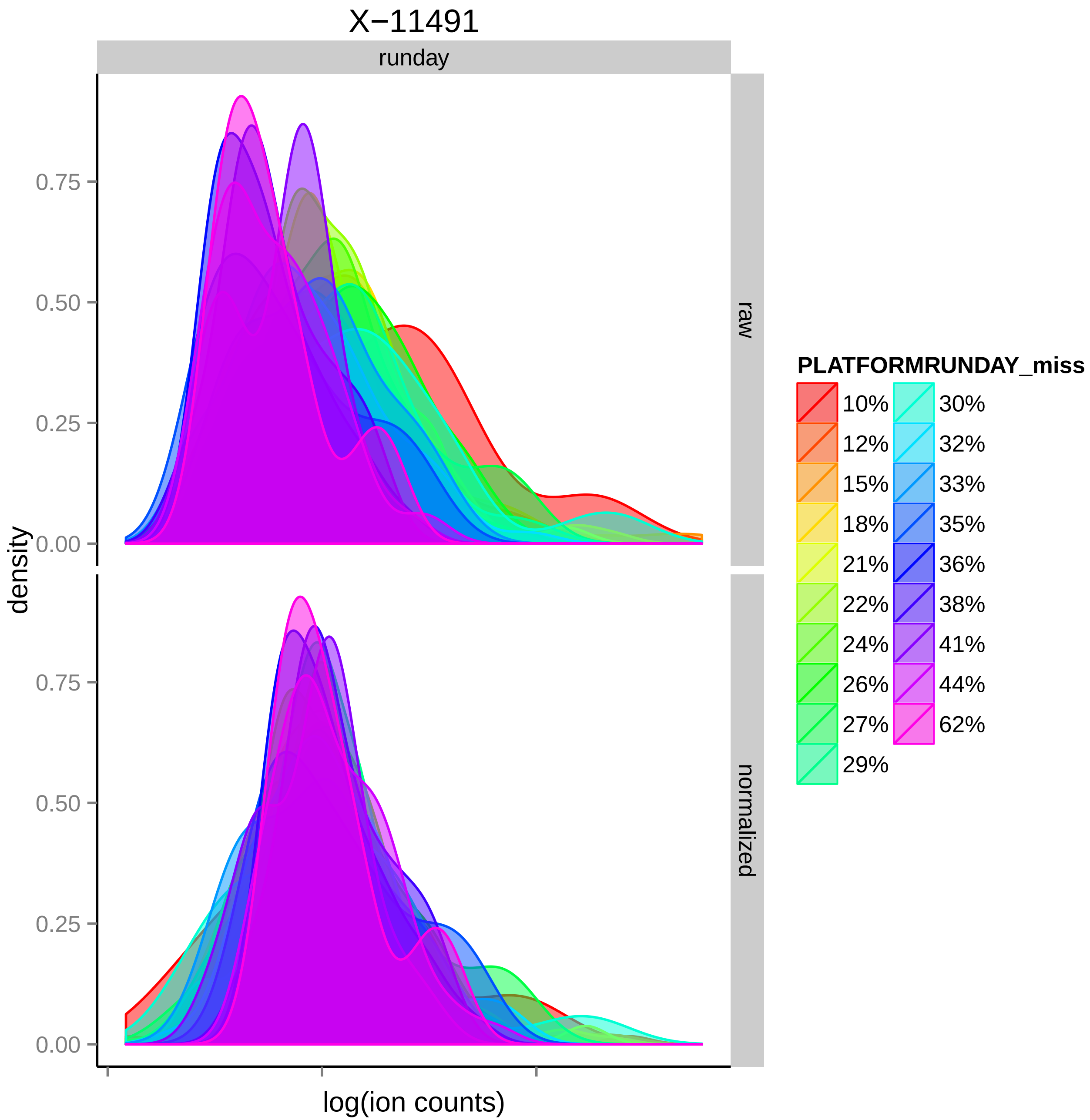

X−11491
runday
raw
normalized
density
0.00
0.25
0.50
0.75
log(ion counts)
PLATFORMRUNDAY_miss
10%
12%
15%
18%
21%
22%
24%
26%
27%
29%
30%
32%
33%
35%
36%
38%
41%
44%
62%

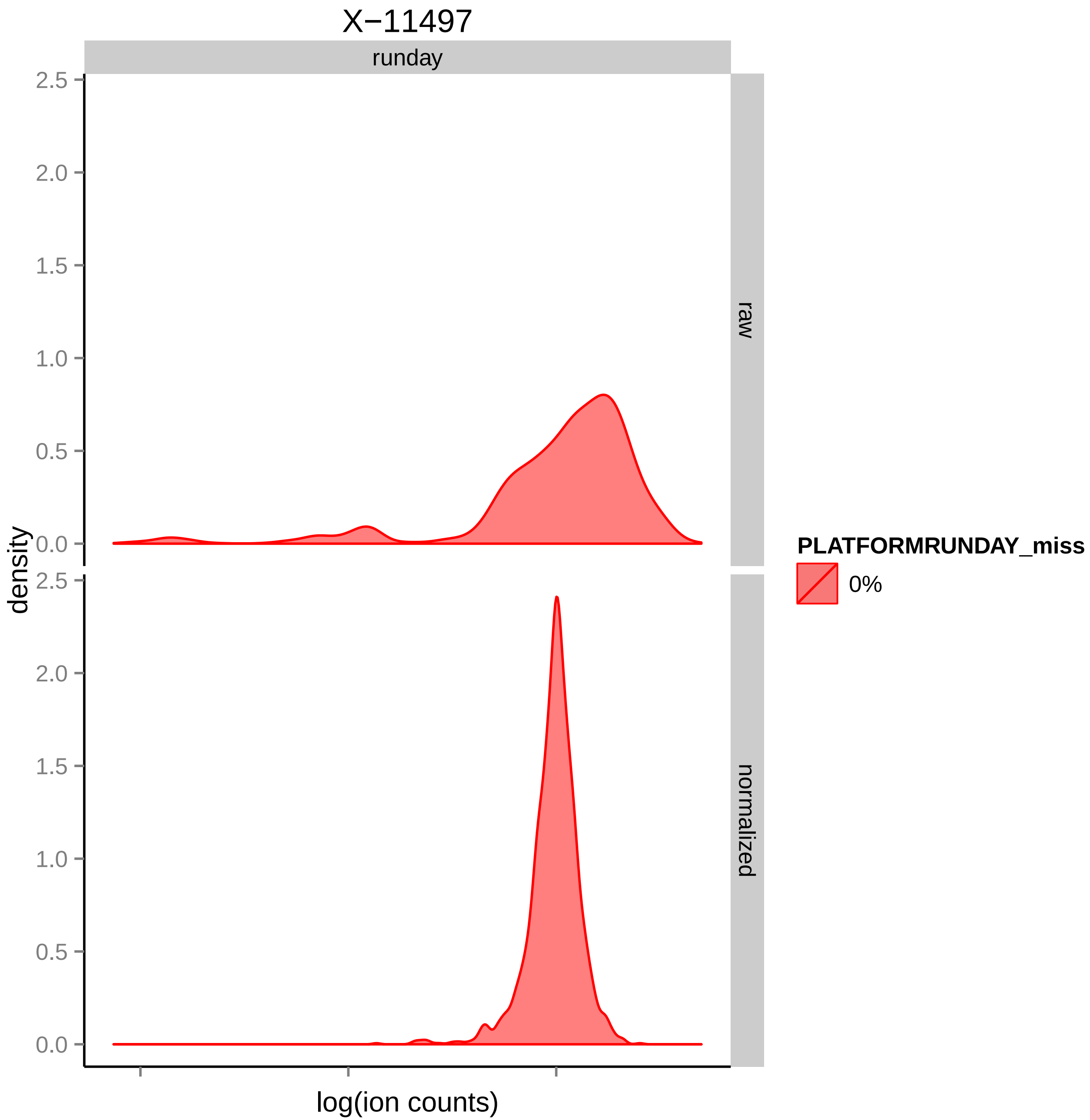
X−11497
runday
raw
normalized
density
log(ion counts)
2.5
2.0
1.5
1.0
0.5
0.0
PLATFORMRUNDAY_miss
0%

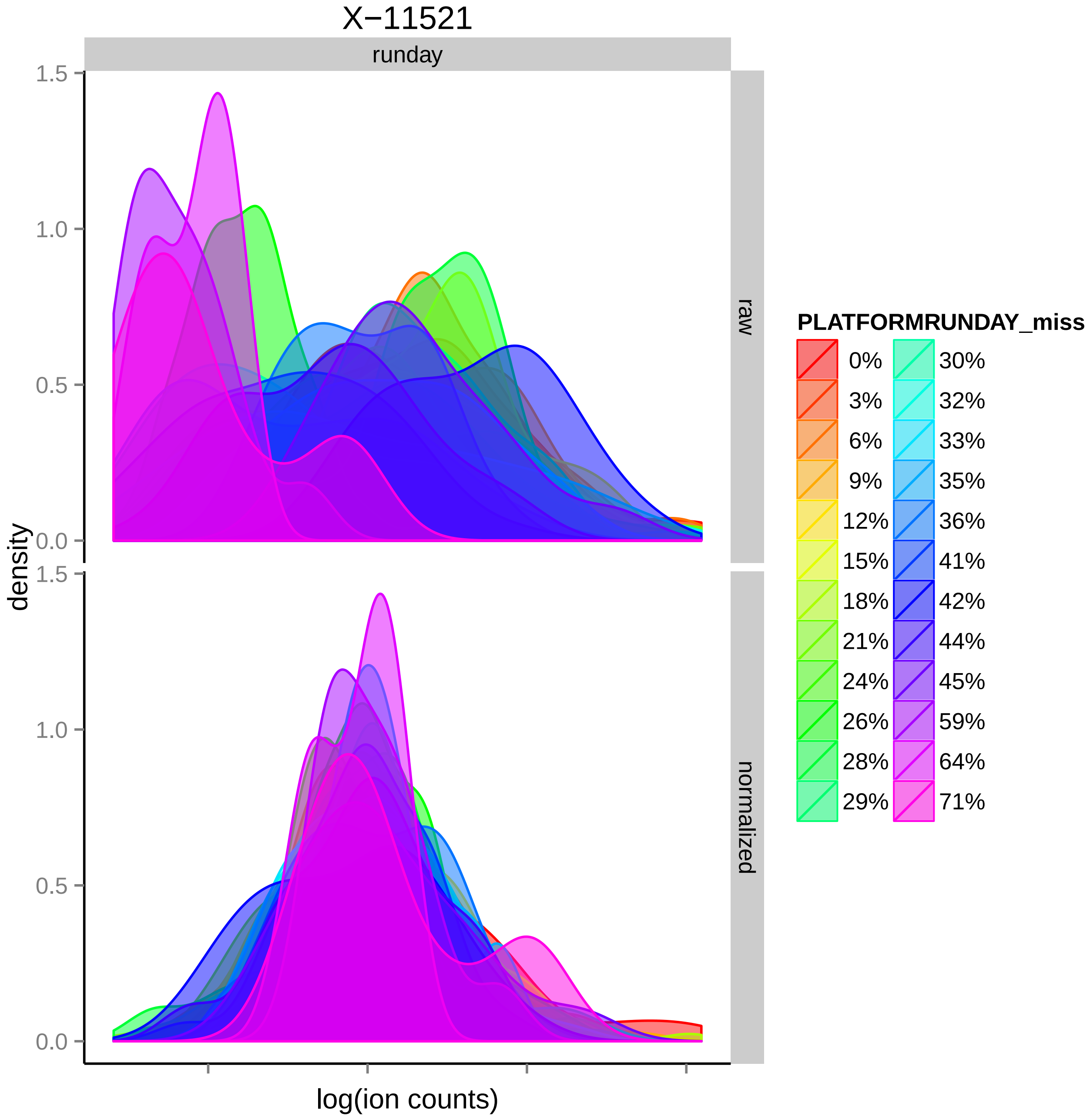

X−11521
runday
raw
normalized
density
1.5
1.0
0.5
0.0
log(ion counts)
PLATFORMRUNDAY_miss
0%
3%
6%
9%
12%
15%
18%
21%
24%
26%
28%
29%
30%
32%
33%
35%
36%
41%
42%
44%
45%
59%
64%
71%

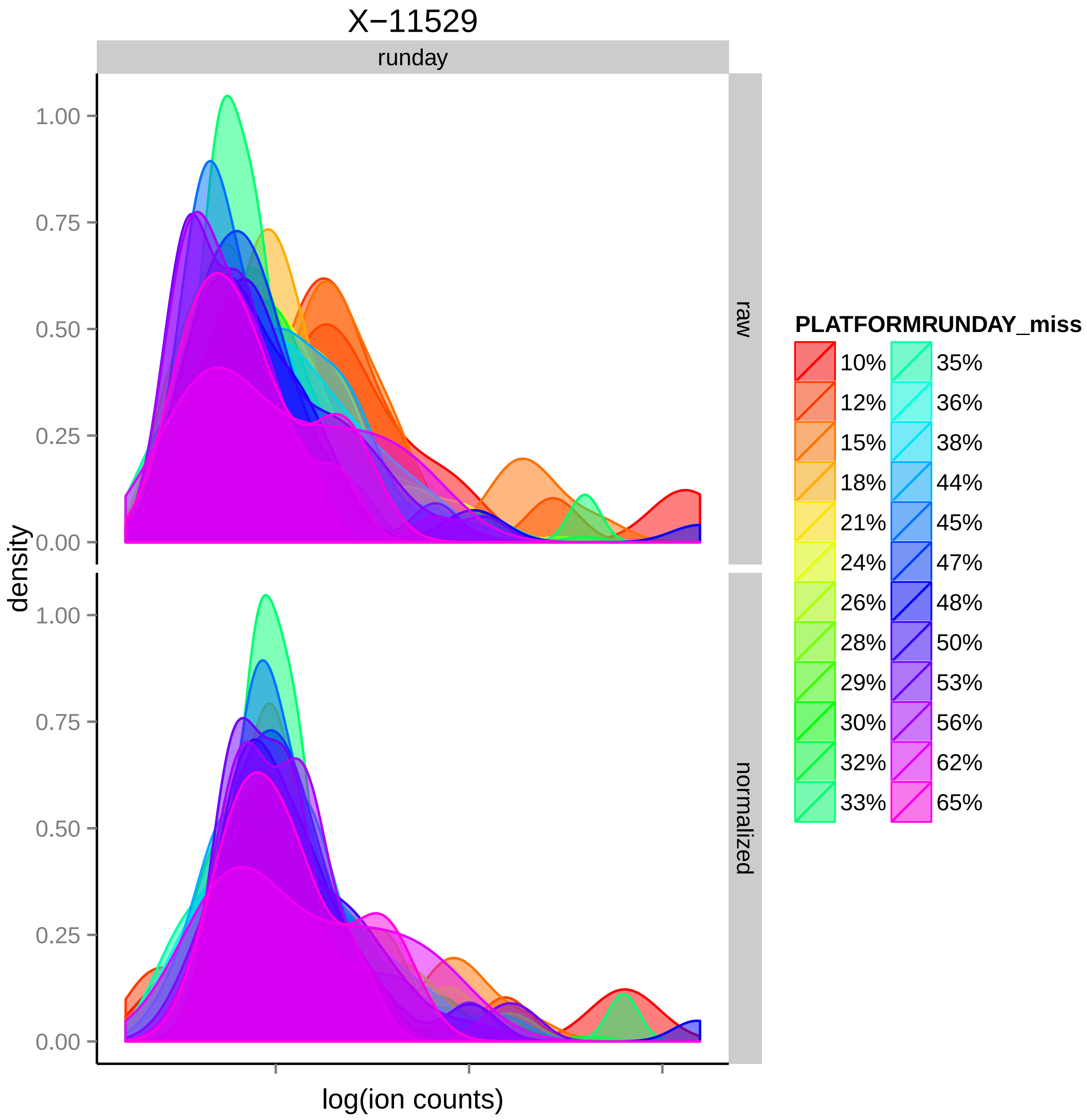
X−11529
runday
raw
normalized
density
log(ion counts)
1.00
0.75
0.50
0.25
0.00
PLATFORMRUNDAY_miss
10%
12%
15%
18%
21%
24%
26%
28%
29%
30%
32%
33%
35%
36%
38%
44%
45%
47%
48%
50%
53%
56%
62%
65%

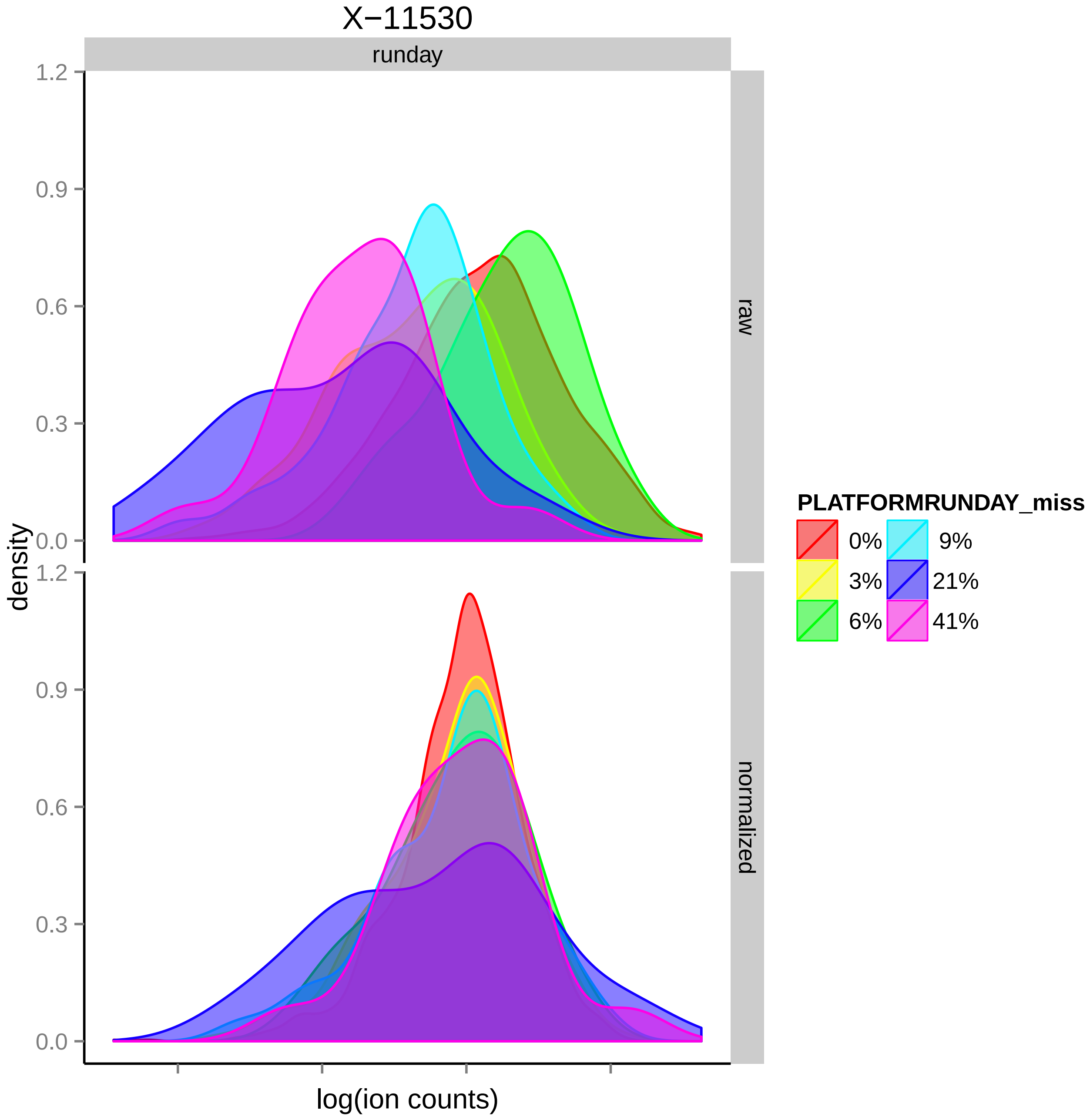
X−11530
runday
raw
normalized
density
log(ion counts)
1.2
0.9
0.6
0.3
0.0
1.2
0.9
0.6
0.3
0.0
PLATFORMRUNDAY_miss
0%
3%
6%
9%
21%
41%

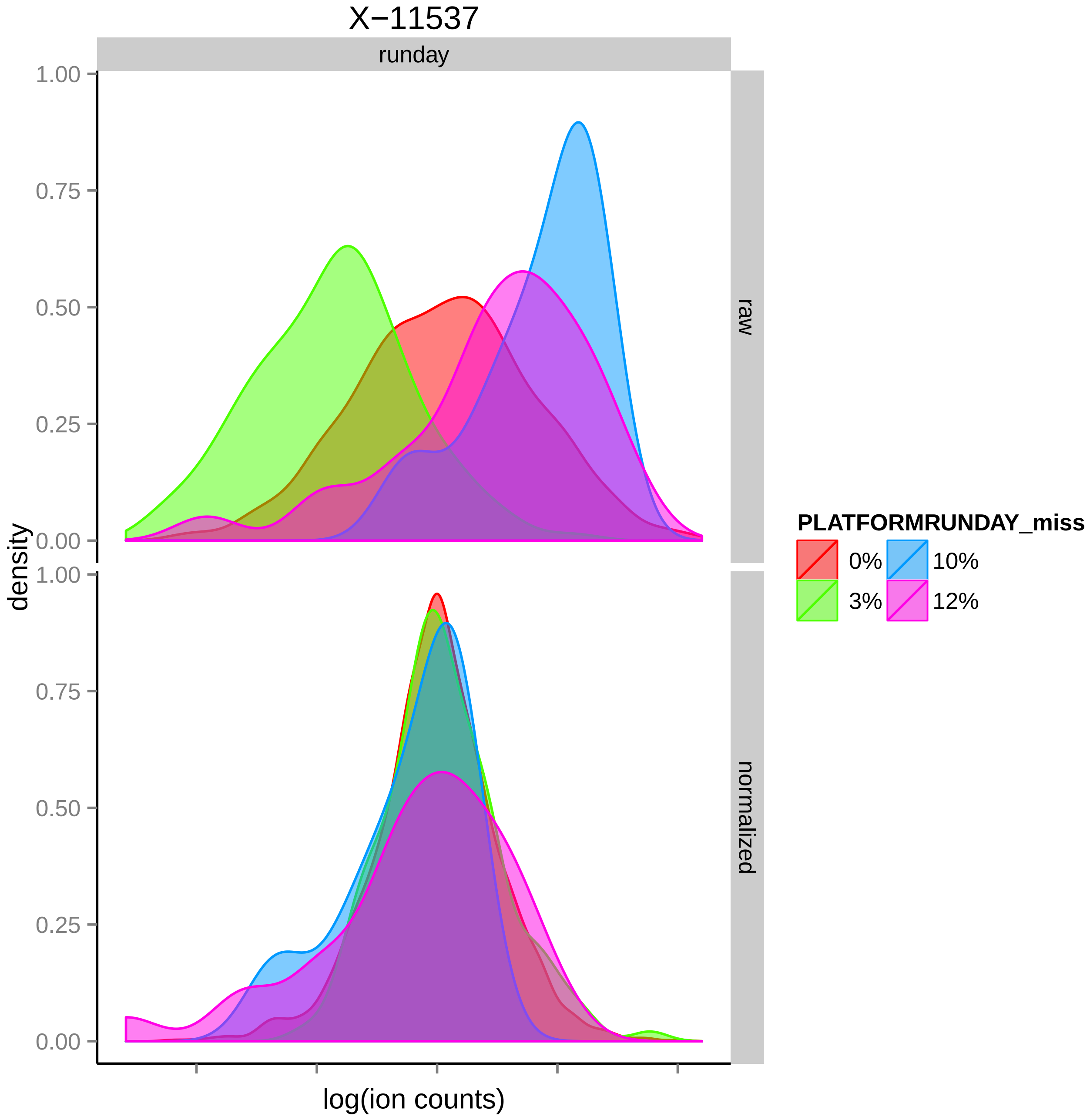

X−11537
runday
raw
normalized
density
1.00
0.75
0.50
0.25
0.00
log(ion counts)
PLATFORMRUNDAY_miss
0%
3%
10%
12%

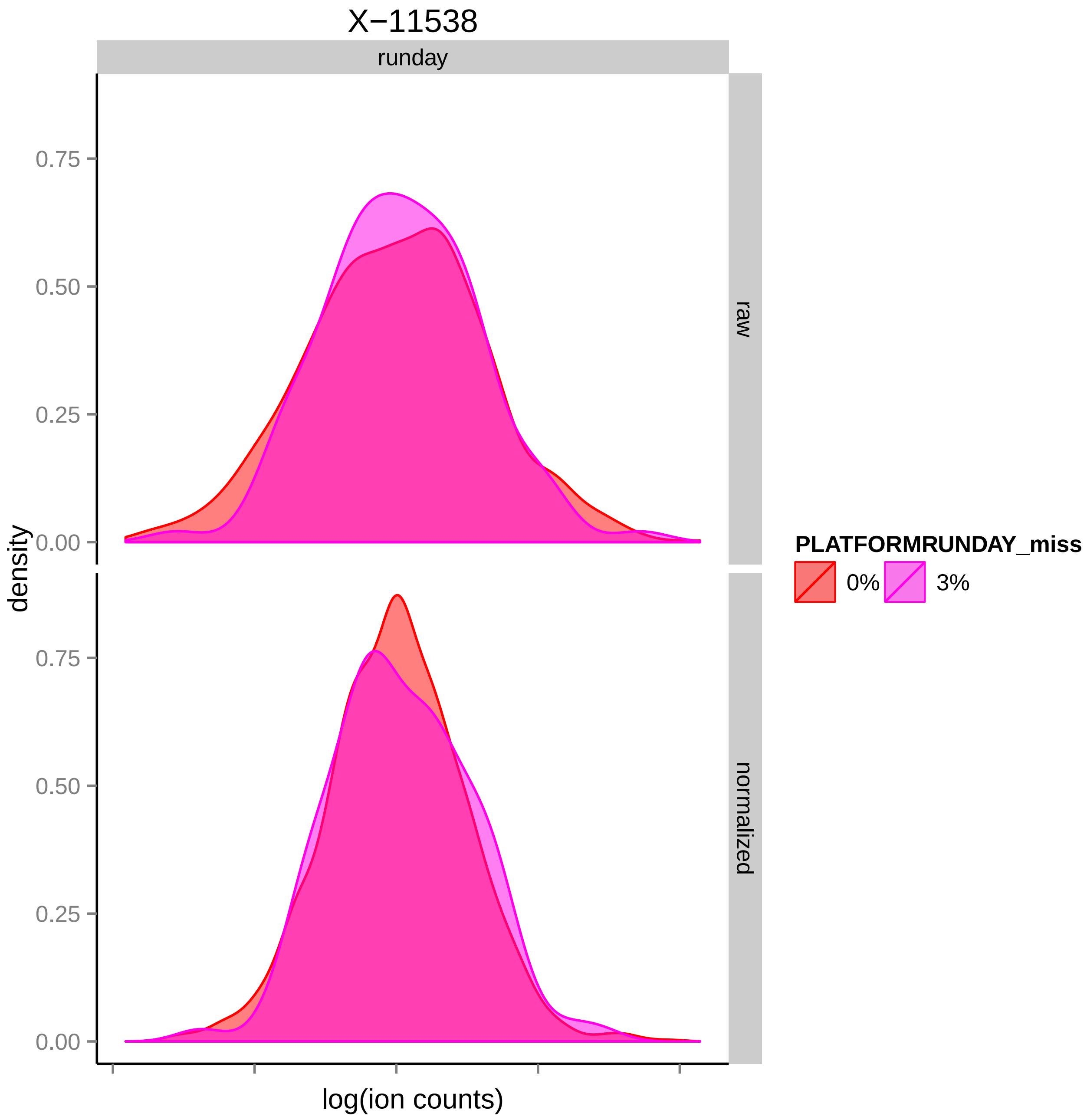

X−11538
runday
raw
normalized
density
0.75
0.50
0.25
0.00
0.75
0.50
0.25
0.00
log(ion counts)
PLATFORMRUNDAY_miss
0%
3%

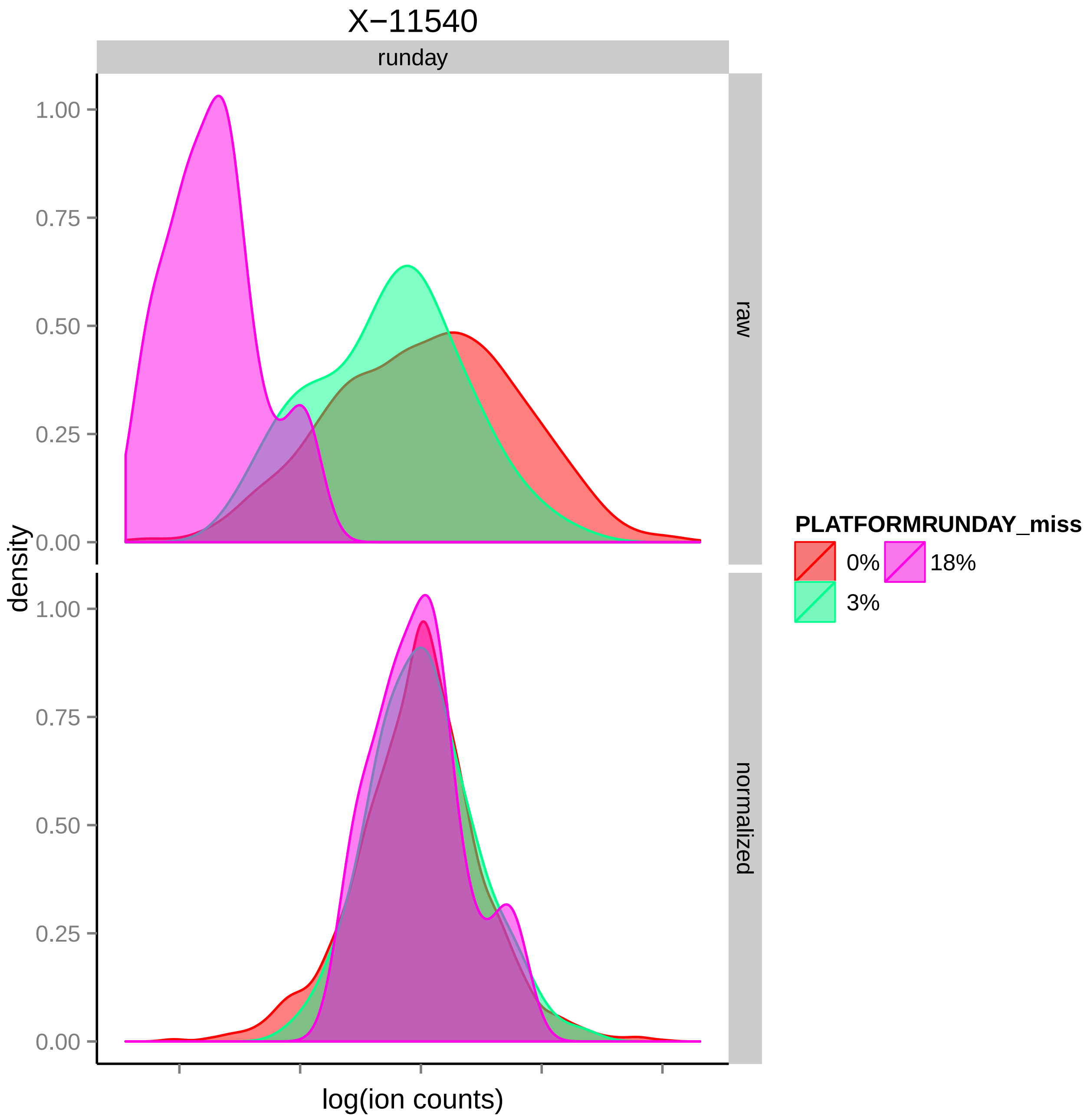
X−11540
runday
raw
normalized
density
1.00
0.75
0.50
0.25
0.00
log(ion counts)
PLATFORMRUNDAY_miss
0%
18%
3%

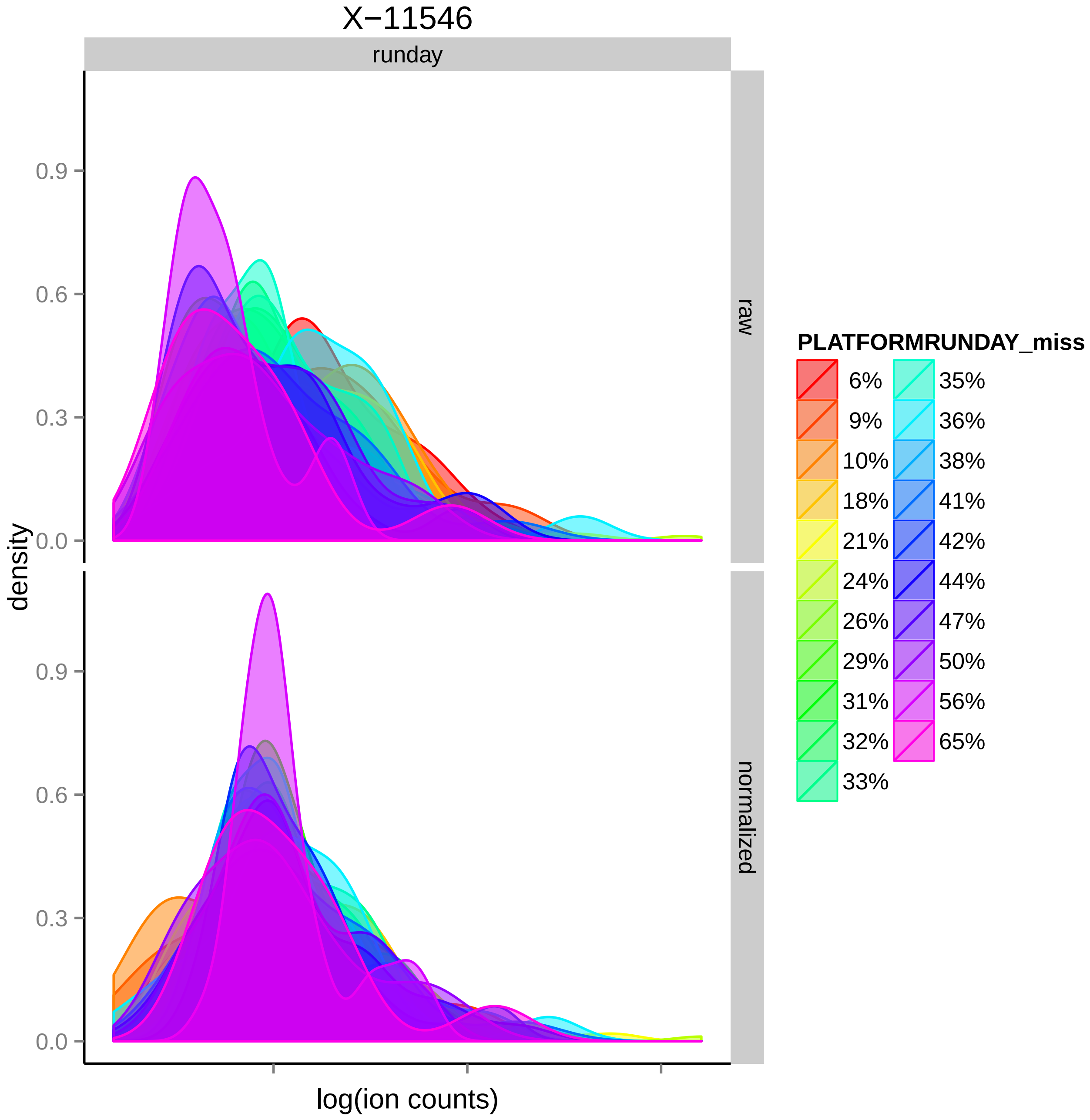
X−11546
runday
raw
normalized
density
log(ion counts)
0.0
0.3
0.6
0.9
PLATFORMRUNDAY_miss
6%
9%
10%
18%
21%
24%
26%
29%
31%
32%
33%
35%
36%
38%
41%
42%
44%
47%
50%
56%
65%

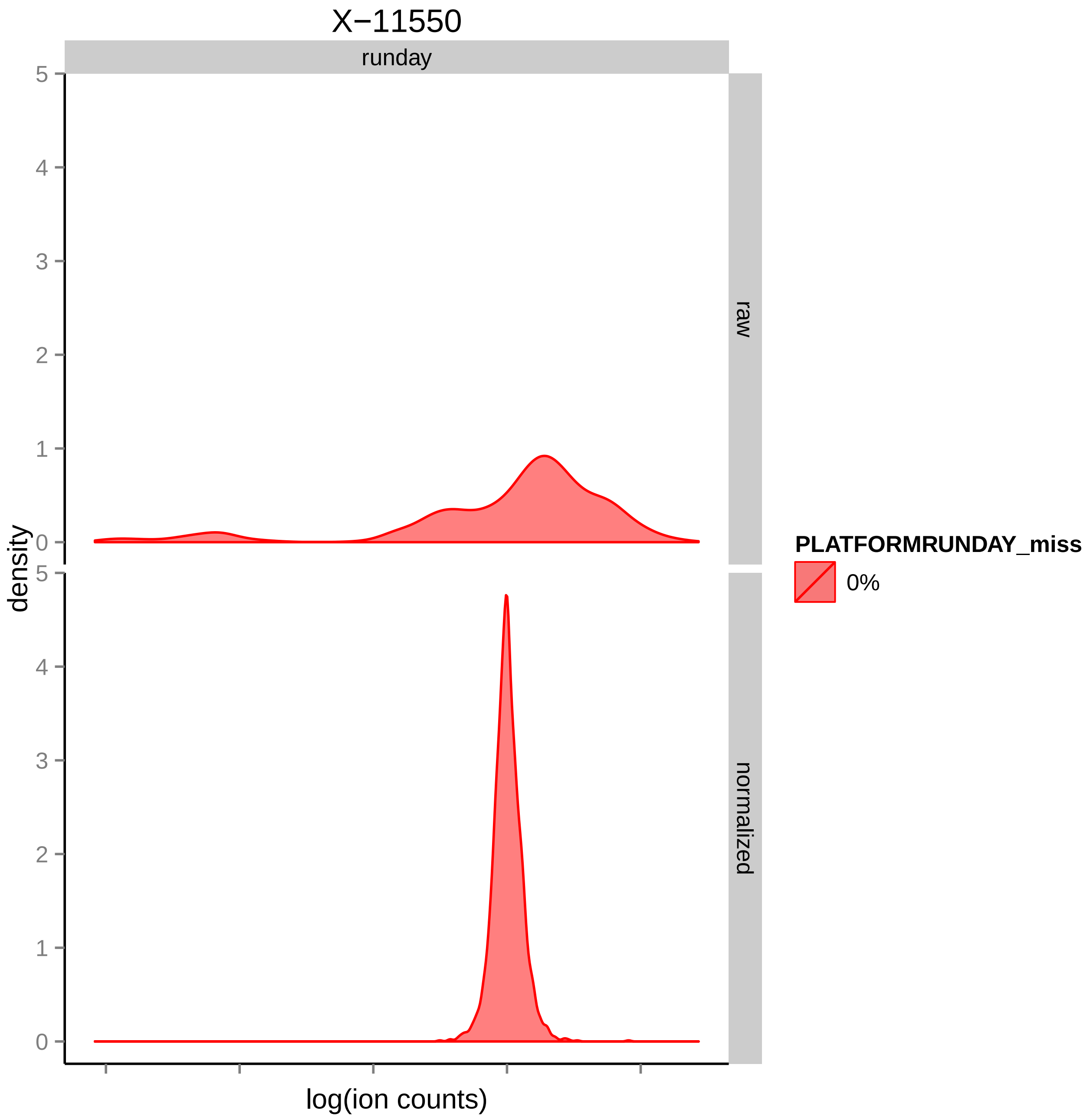

X−11550
runday
raw
normalized
density
log(ion counts)
0
1
2
3
4
5
PLATFORMRUNDAY_miss
0%

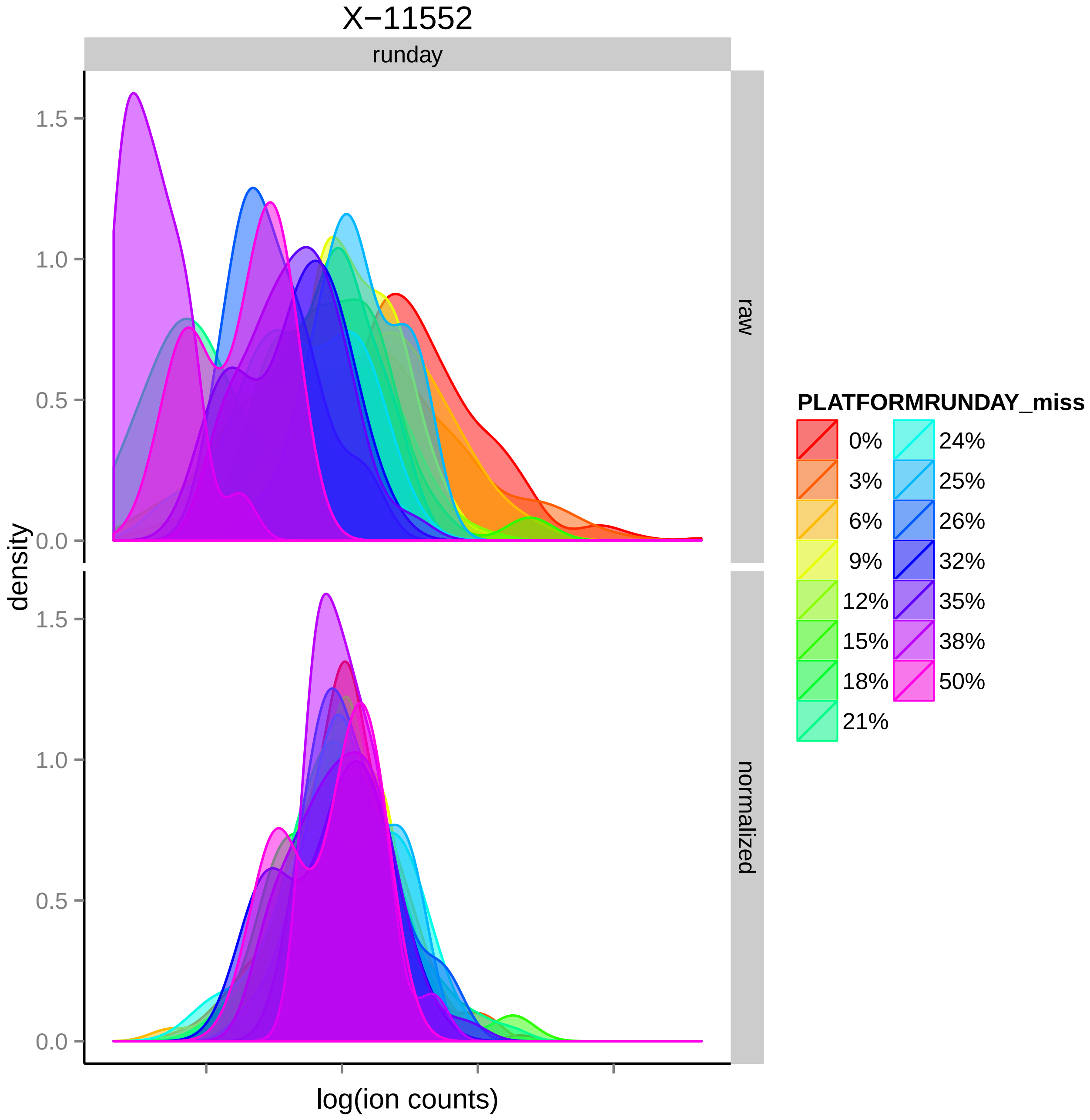
X−11552
runday
raw
normalized
density
log(ion counts)
1.5
1.0
0.5
0.0
1.5
1.0
0.5
0.0
PLATFORMRUNDAY_miss
0%
3%
6%
9%
12%
15%
18%
21%
24%
25%
26%
32%
35%
38%
50%

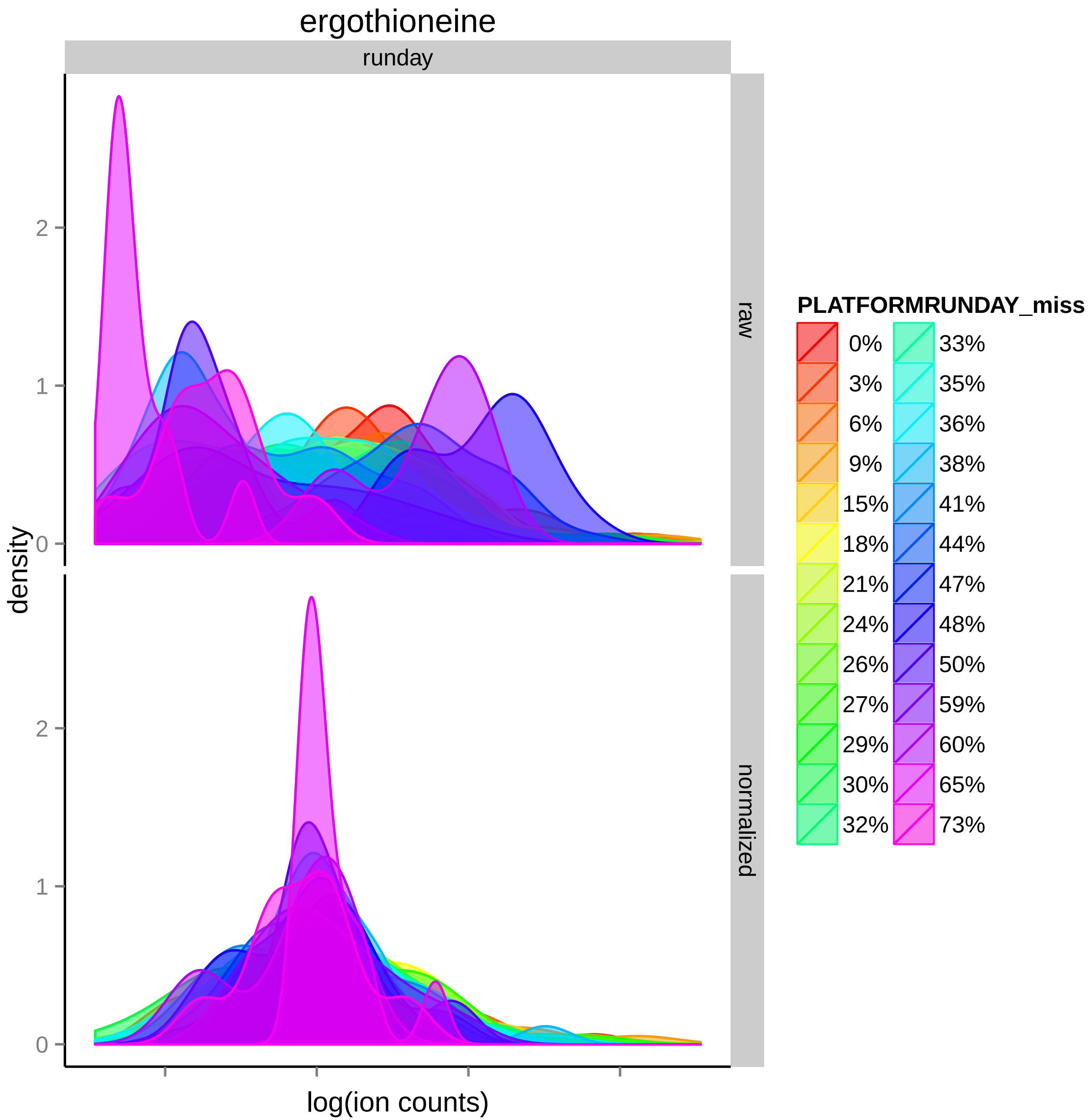
ergothioneine
runday
raw
normalized
density
log(ion counts)
0
1
2
0
1
2
PLATFORMRUNDAY_miss
0%
3%
6%
9%
15%
18%
21%
24%
26%
27%
29%
30%
32%
33%
35%
36%
38%
41%
44%
47%
48%
50%
59%
60%
65%
73%

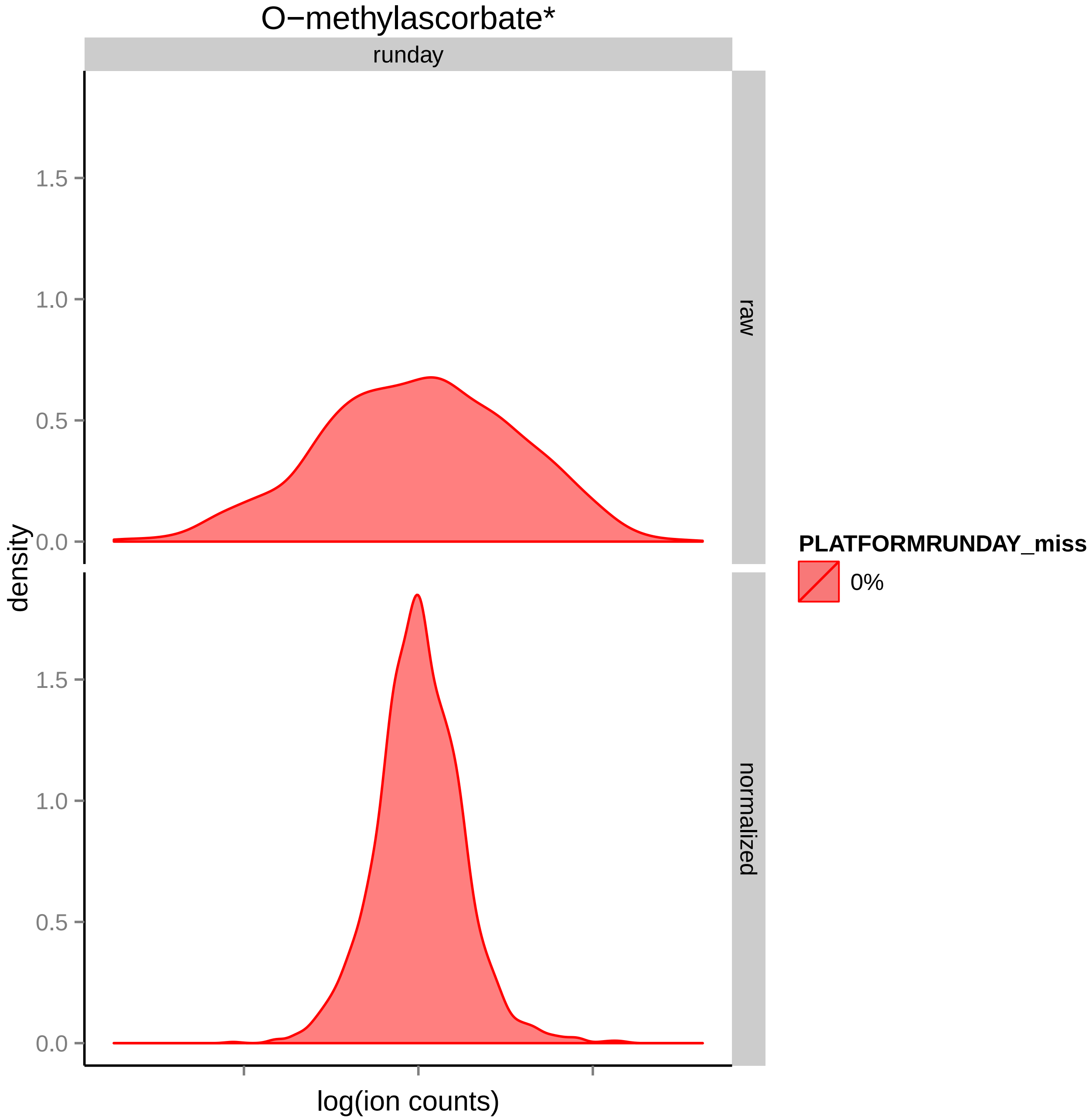
O−methylascorbate*
runday
raw
normalized
density
log(ion counts)
0.0
0.5
1.0
1.5
PLATFORMRUNDAY_miss
0%

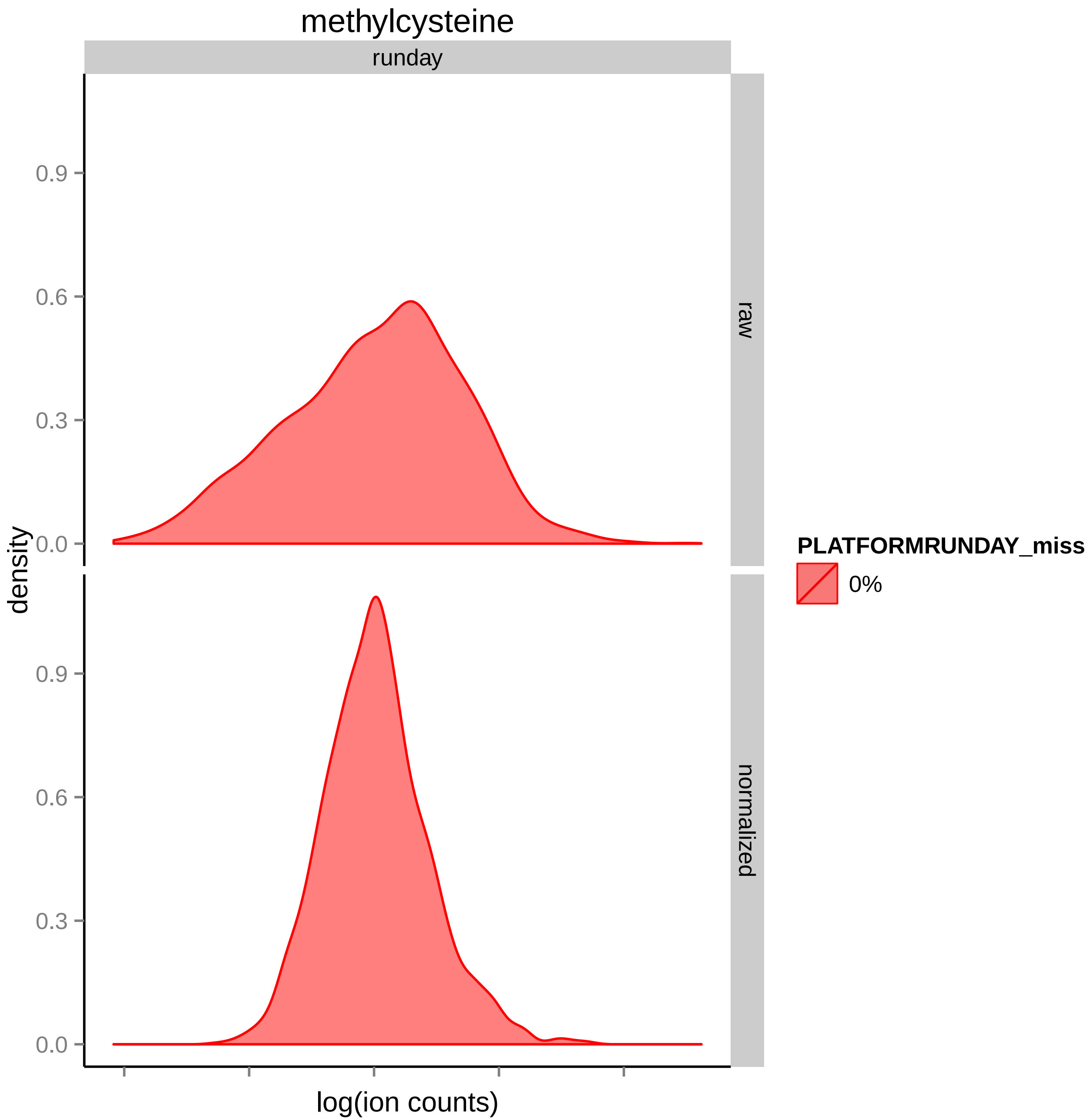

methylcysteine
runday
raw
normalized
density
0.9
0.6
0.3
0.0
0.9
0.6
0.3
0.0
log(ion counts)
PLATFORMRUNDAY_miss
0%

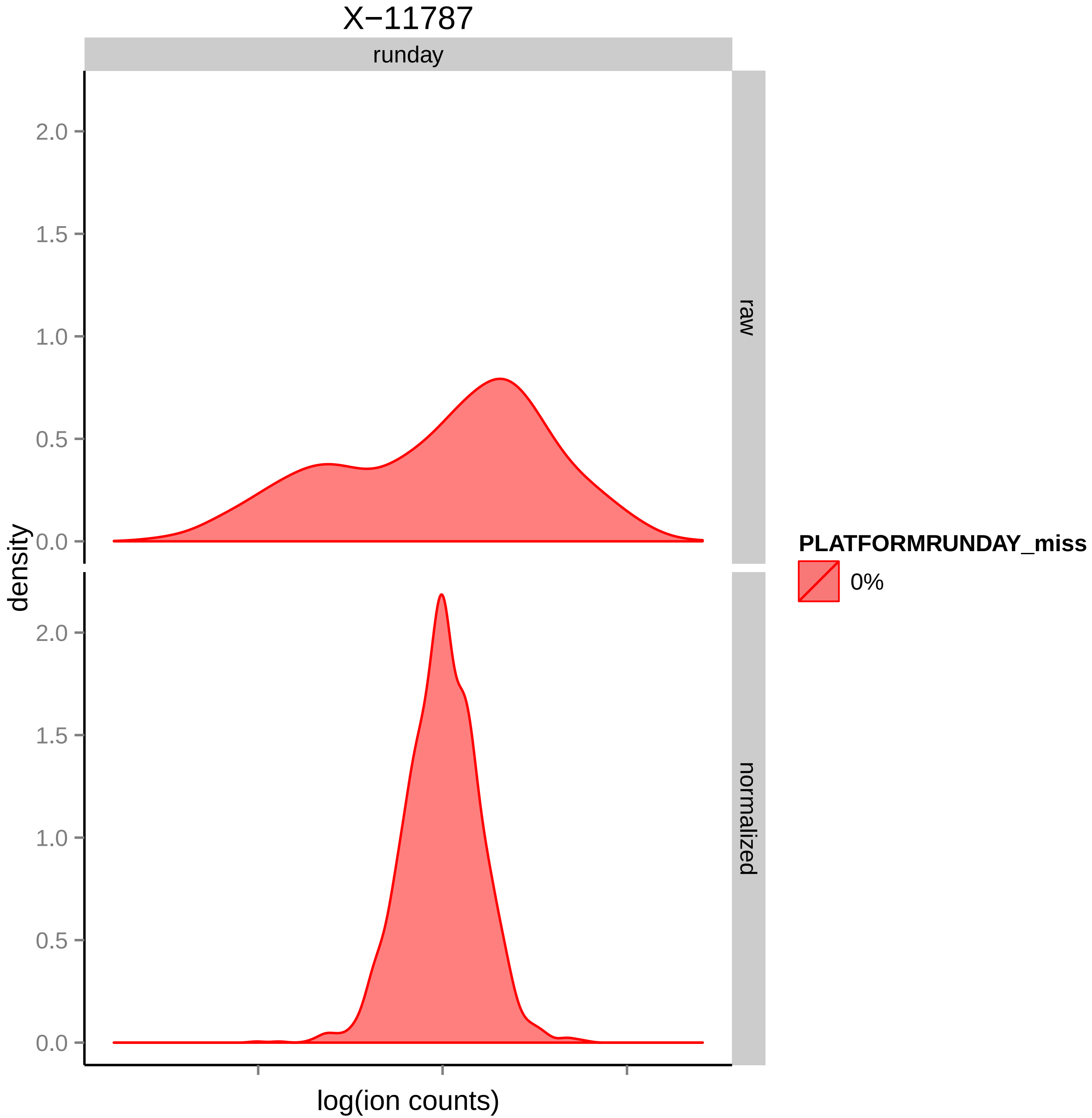

X−11787
runday
raw
normalized
density
log(ion counts)
2.0
1.5
1.0
0.5
0.0
PLATFORMRUNDAY_miss
0%

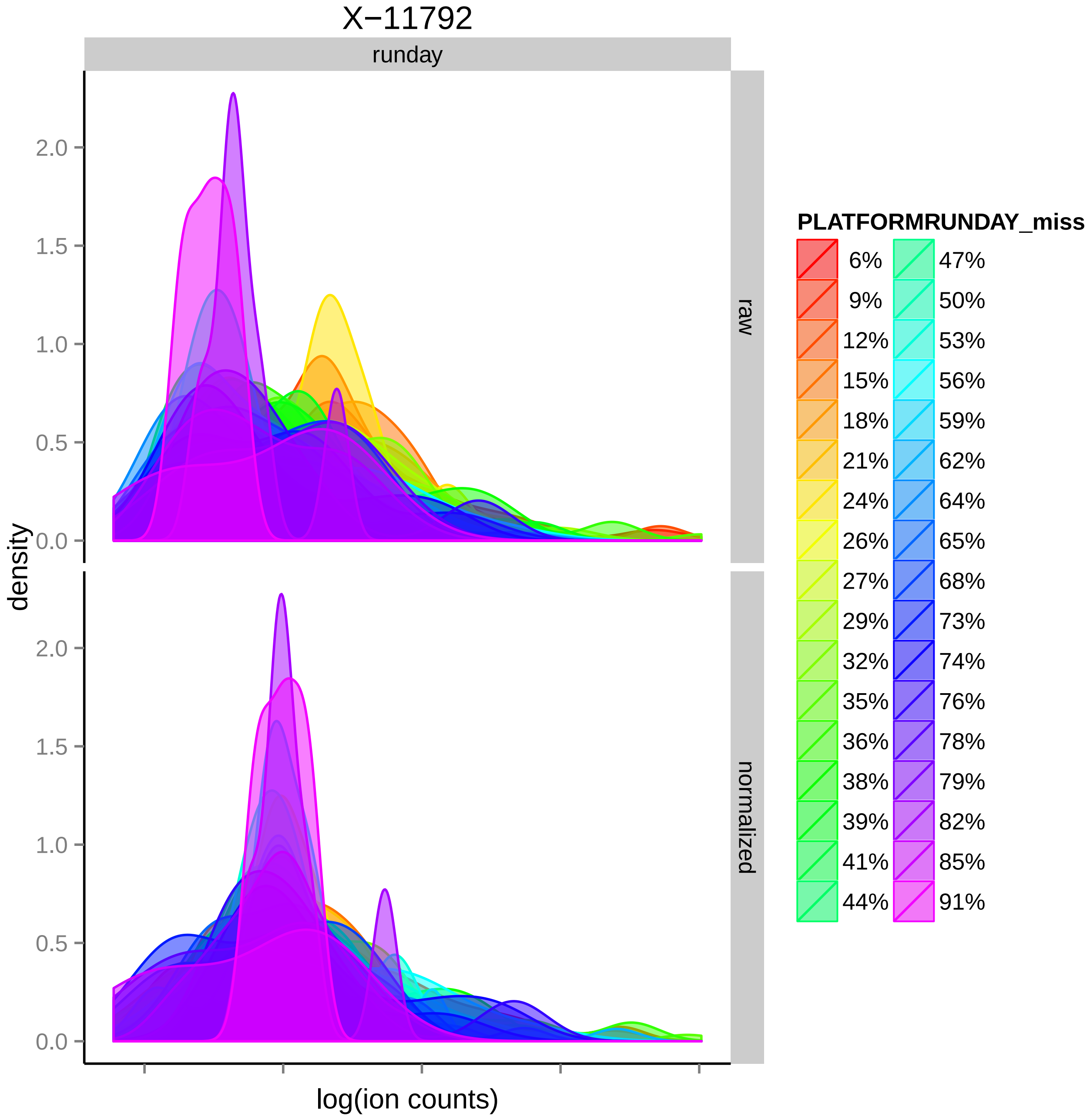

X−11792
runday
raw
normalized
density
log(ion counts)
2.0
1.5
1.0
0.5
0.0
PLATFORMRUNDAY_miss
6%
9%
12%
15%
18%
21%
24%
26%
27%
29%
32%
35%
36%
38%
39%
41%
44%
47%
50%
53%
56%
59%
62%
64%
65%
68%
73%
74%
76%
78%
79%
82%
85%
91%

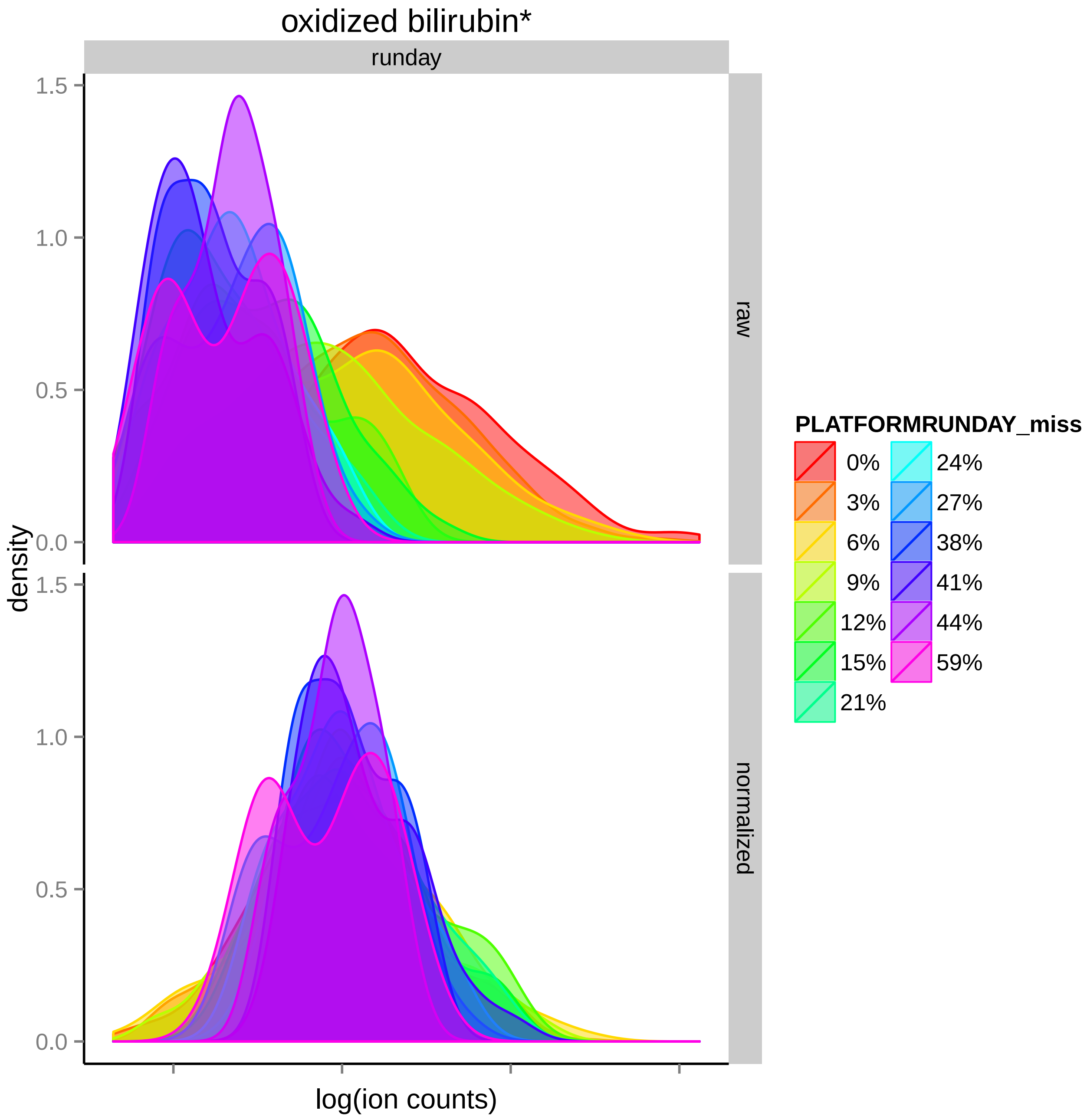

oxidized bilirubin*
runday
raw
normalized
density
log(ion counts)
1.5
1.0
0.5
0.0
PLATFORMRUNDAY_miss
0%
3%
6%
9%
12%
15%
21%
24%
27%
38%
41%
44%
59%

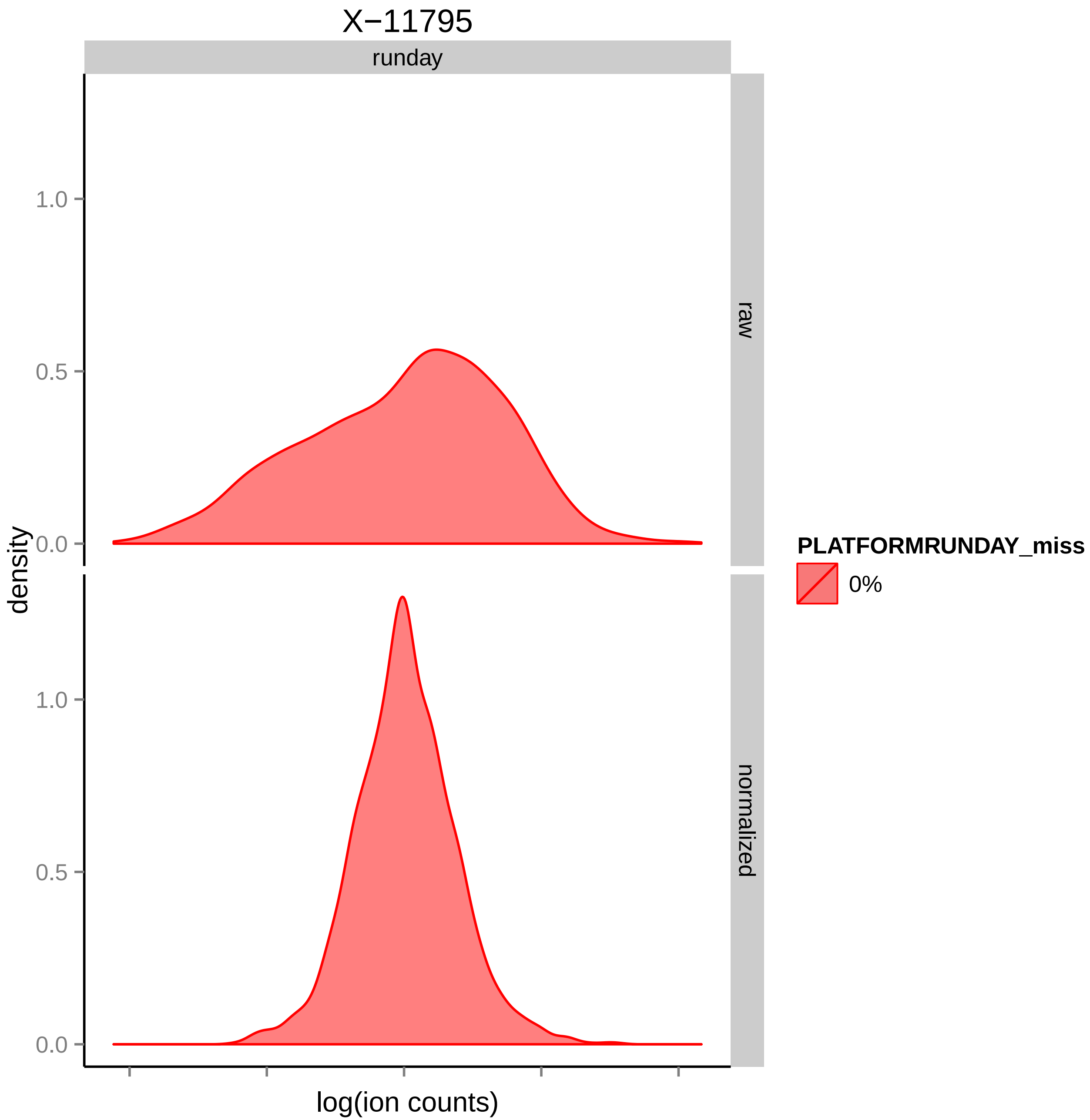

X−11795
runday
raw
normalized
density
1.0
0.5
0.0
1.0
0.5
0.0
log(ion counts)
PLATFORMRUNDAY_miss
0%

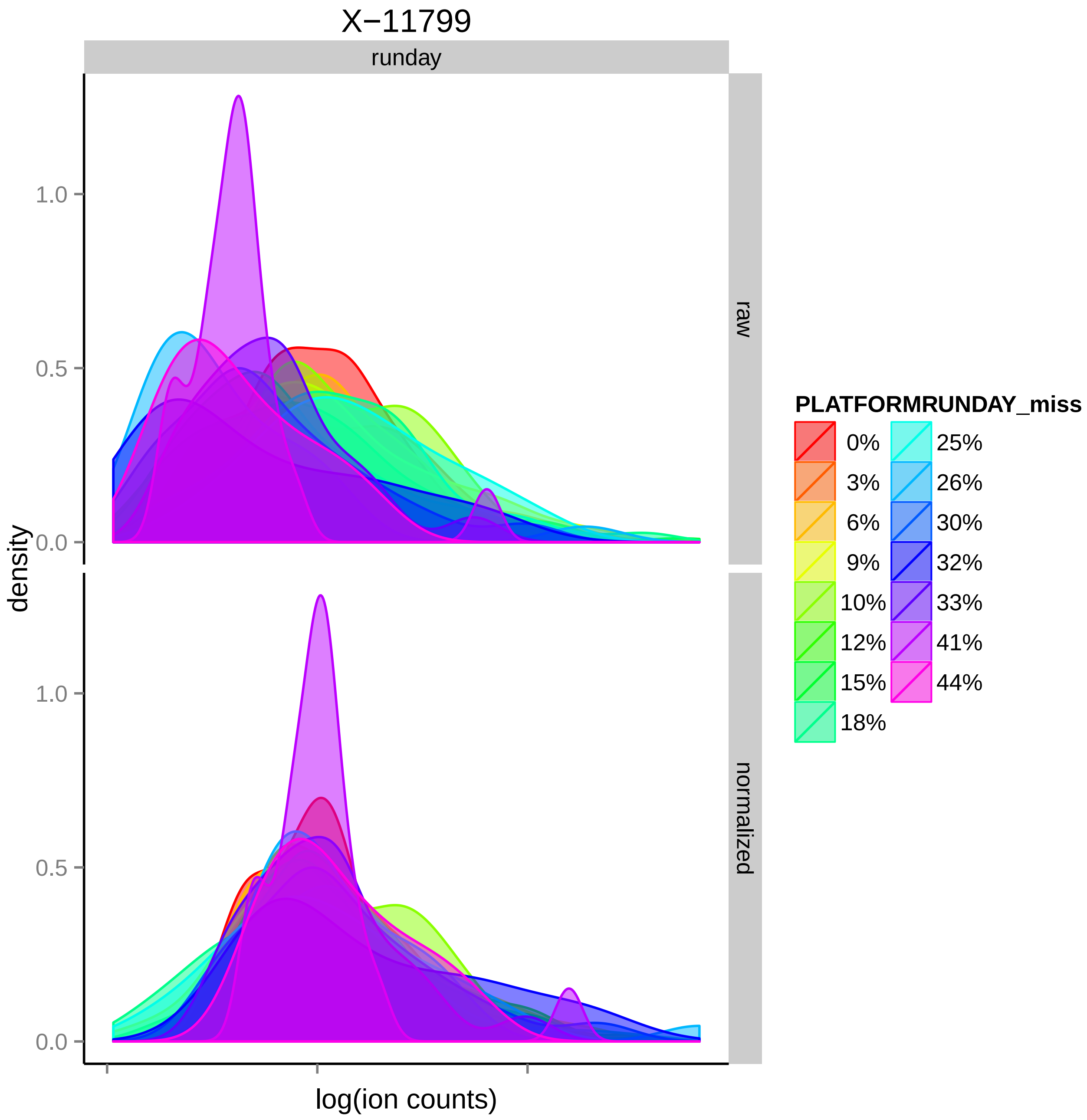
X−11799
runday
raw
normalized
density
1.0
0.5
0.0
1.0
0.5
0.0
log(ion counts)
PLATFORMRUNDAY_miss
0%
3%
6%
9%
10%
12%
15%
18%
25%
26%
30%
32%
33%
41%
44%

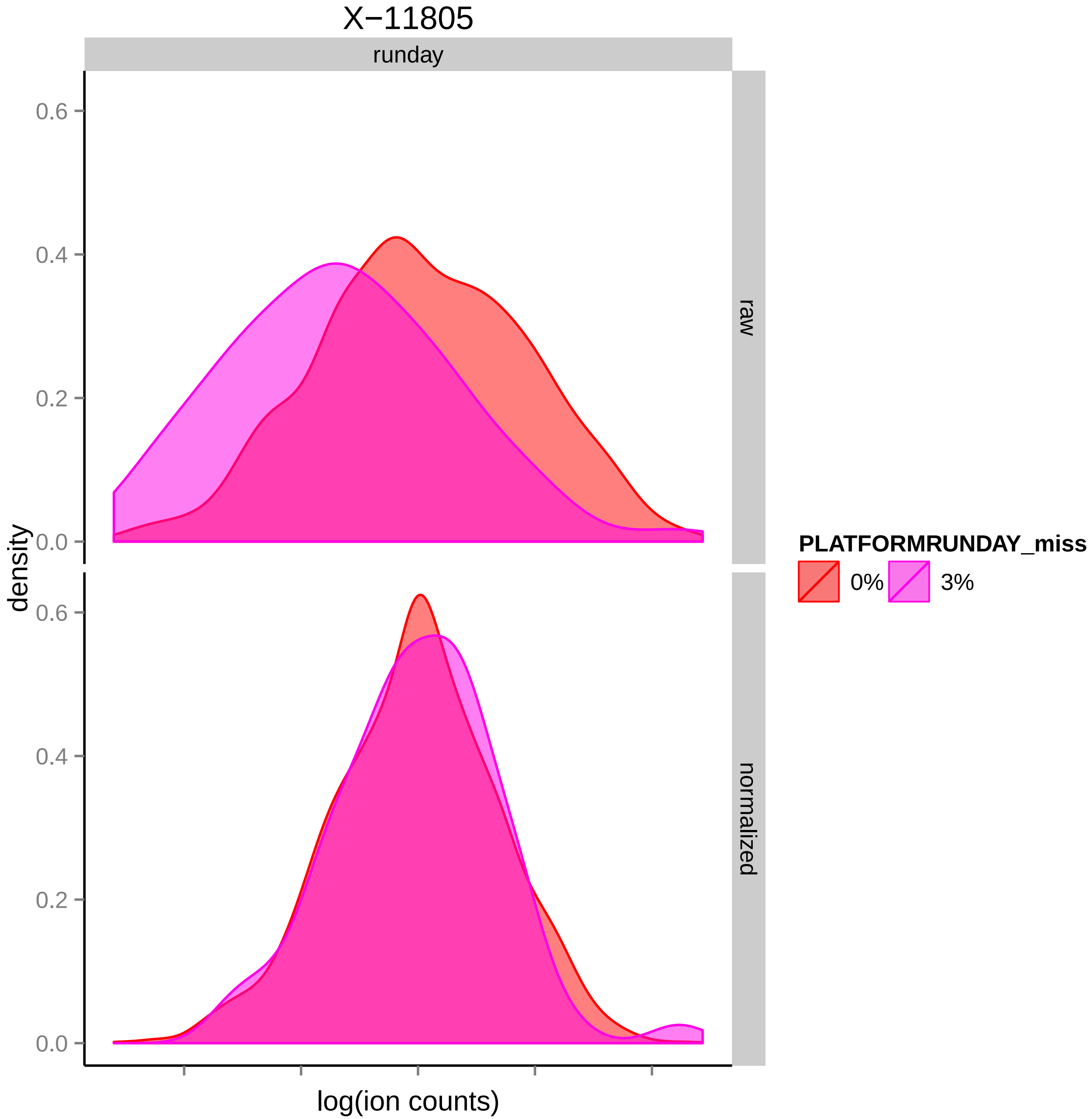

X−11805
runday
raw
normalized
density
log(ion counts)
0.0
0.2
0.4
0.6
PLATFORMRUNDAY_miss
0%
3%

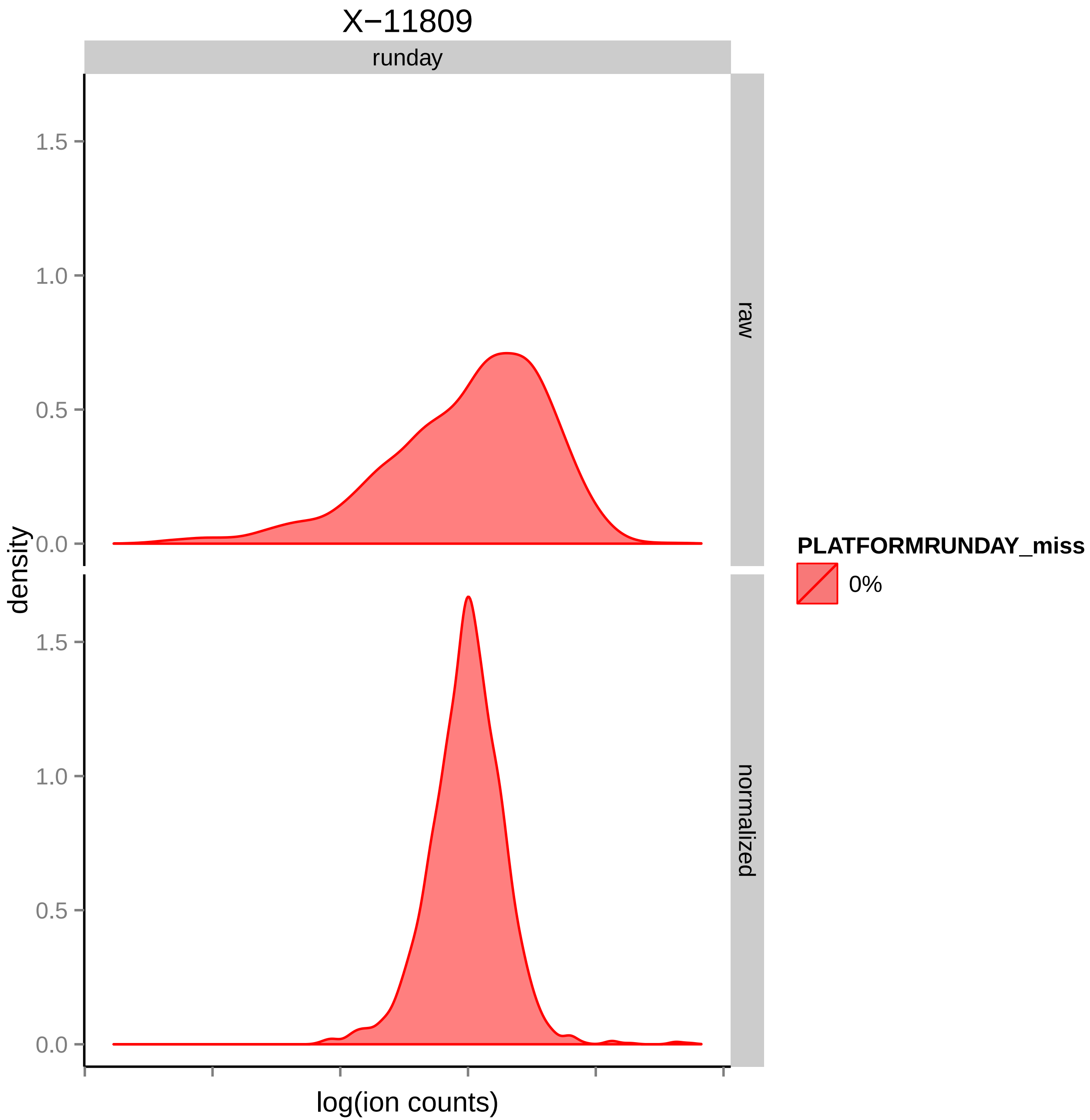

X−11809
runday
raw
normalized
1.5
1.0
0.5
0.0
density
log(ion counts)
PLATFORMRUNDAY_miss
0%

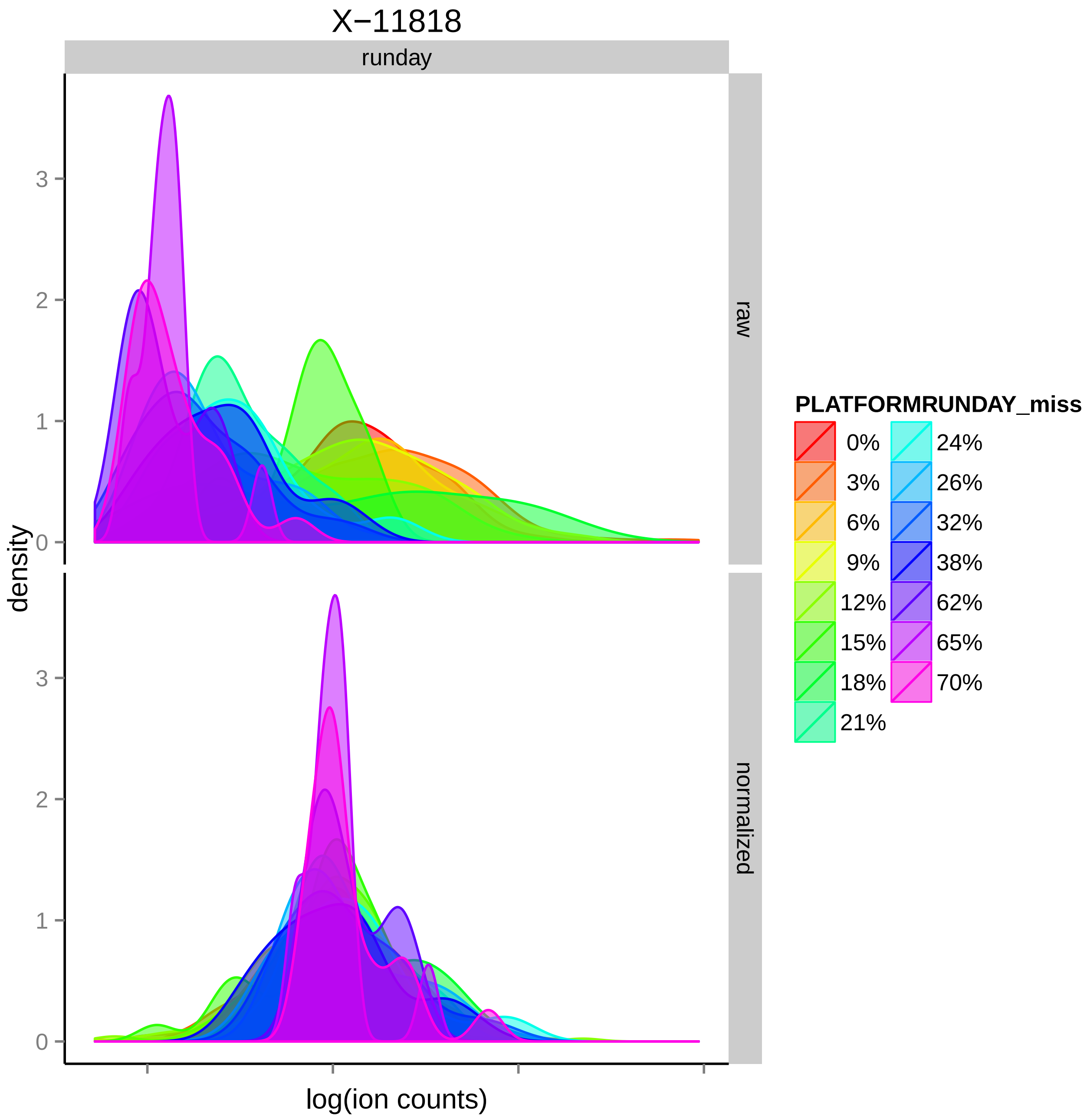
X−11818
runday
raw
normalized
density
log(ion counts)
3
2
1
0
3
2
1
0
PLATFORMRUNDAY_miss
0%
3%
6%
9%
12%
15%
18%
21%
24%
26%
32%
38%
62%
65%
70%

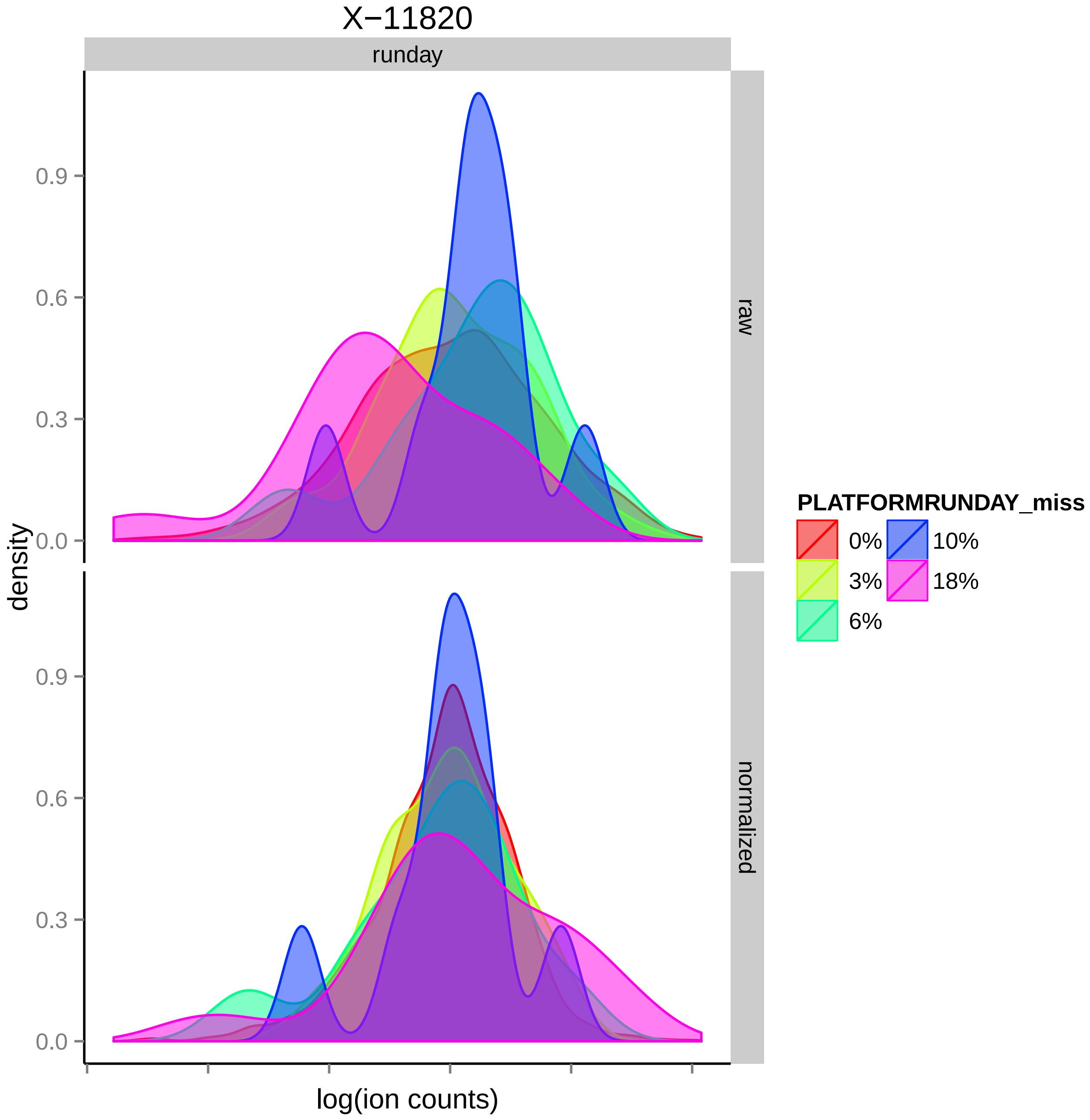
X−11820
runday
raw
normalized
density
0.0
0.3
0.6
0.9
log(ion counts)
PLATFORMRUNDAY_miss
0%
3%
6%
10%
18%

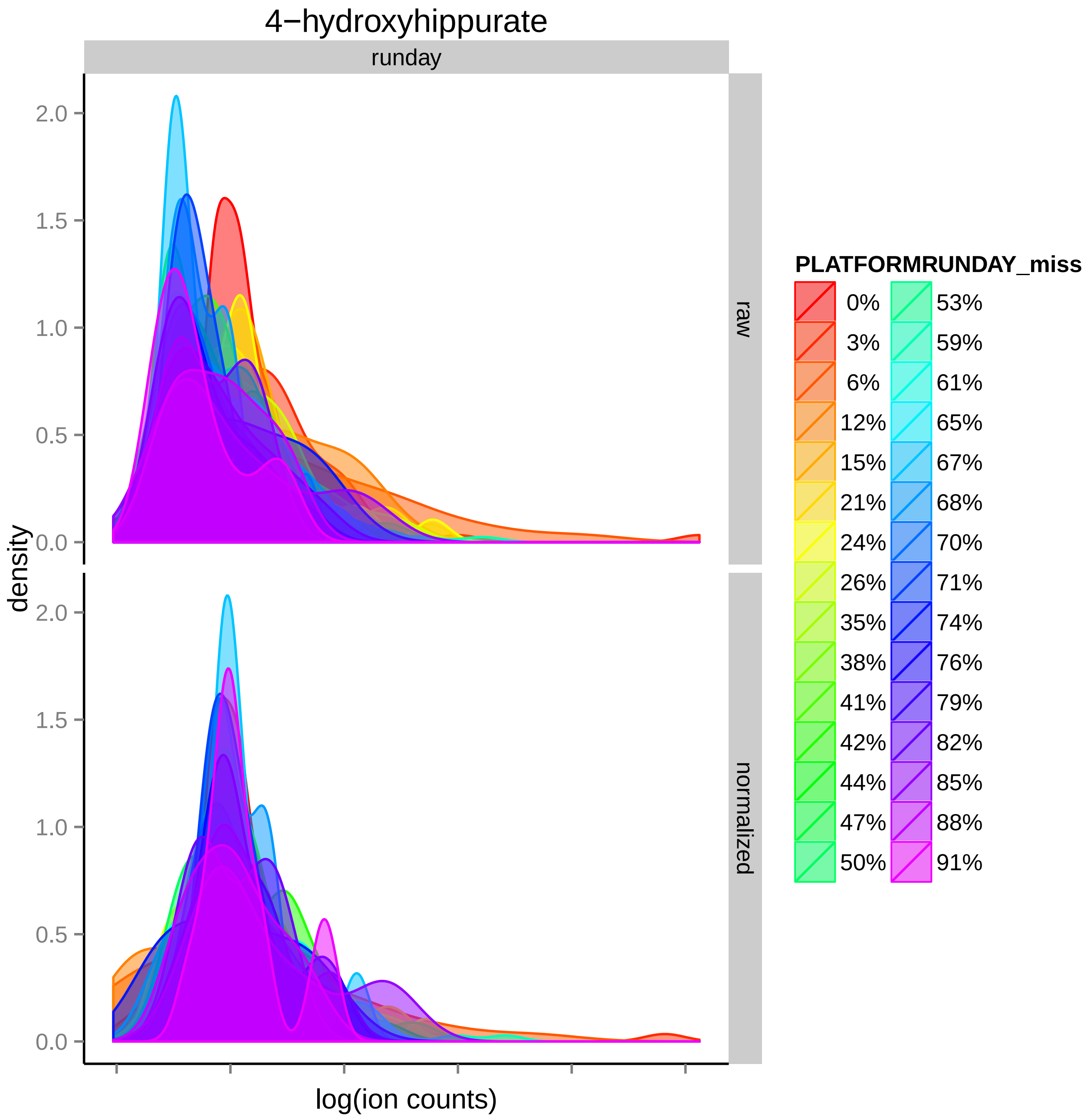
4−hydroxyhippurate
runday
raw
normalized
density
log(ion counts)
2.0
1.5
1.0
0.5
0.0
PLATFORMRUNDAY_miss
0%
3%
6%
12%
15%
21%
24%
26%
35%
38%
41%
42%
44%
47%
50%
53%
59%
61%
65%
67%
68%
70%
71%
74%
76%
79%
82%
85%
88%
91%

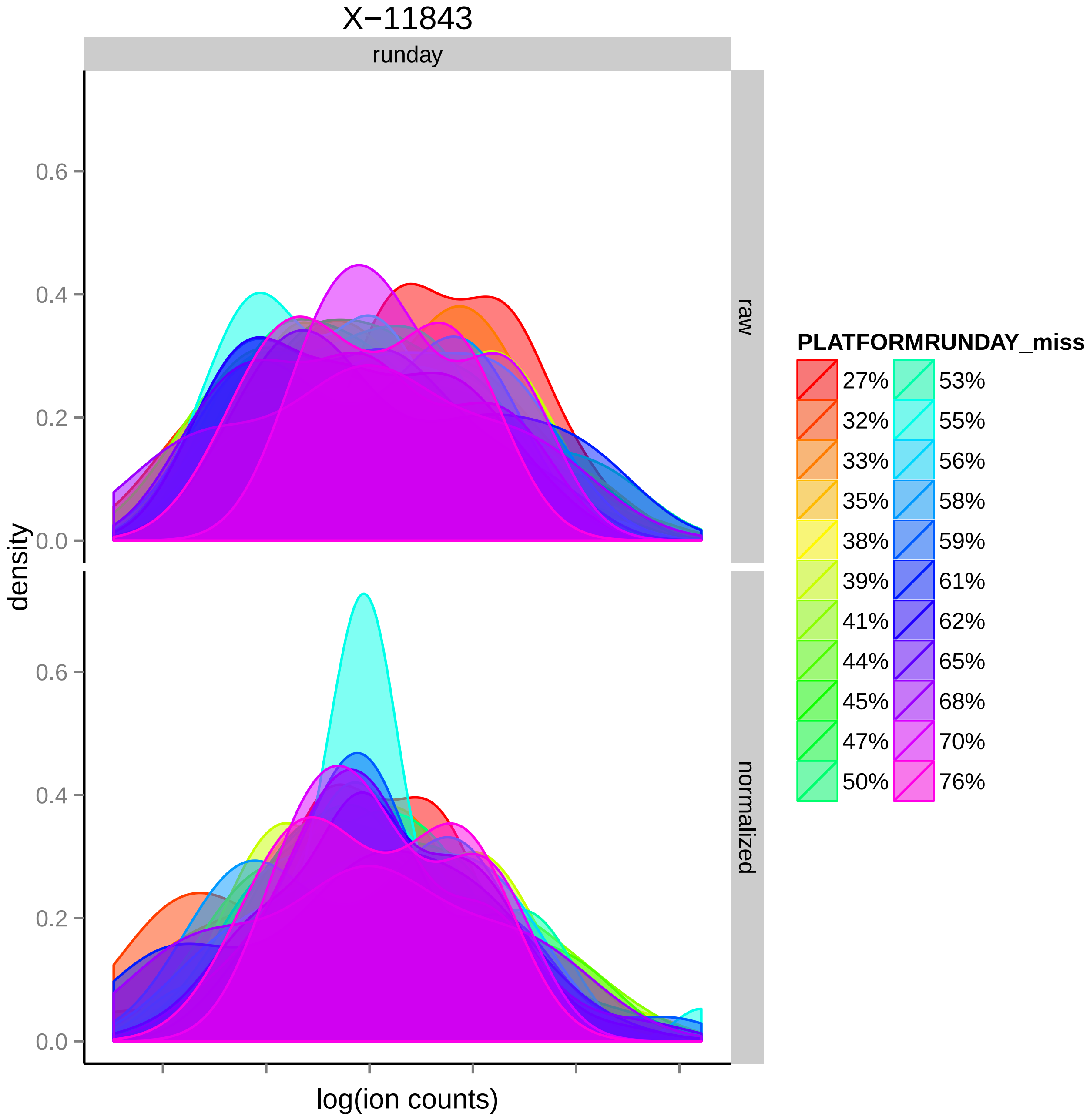

X−11843
runday
raw
normalized
density
log(ion counts)
0.0
0.2
0.4
0.6
PLATFORMRUNDAY_miss
27%
32%
33%
35%
38%
39%
41%
44%
45%
47%
50%
53%
55%
56%
58%
59%
61%
62%
65%
68%
70%
76%

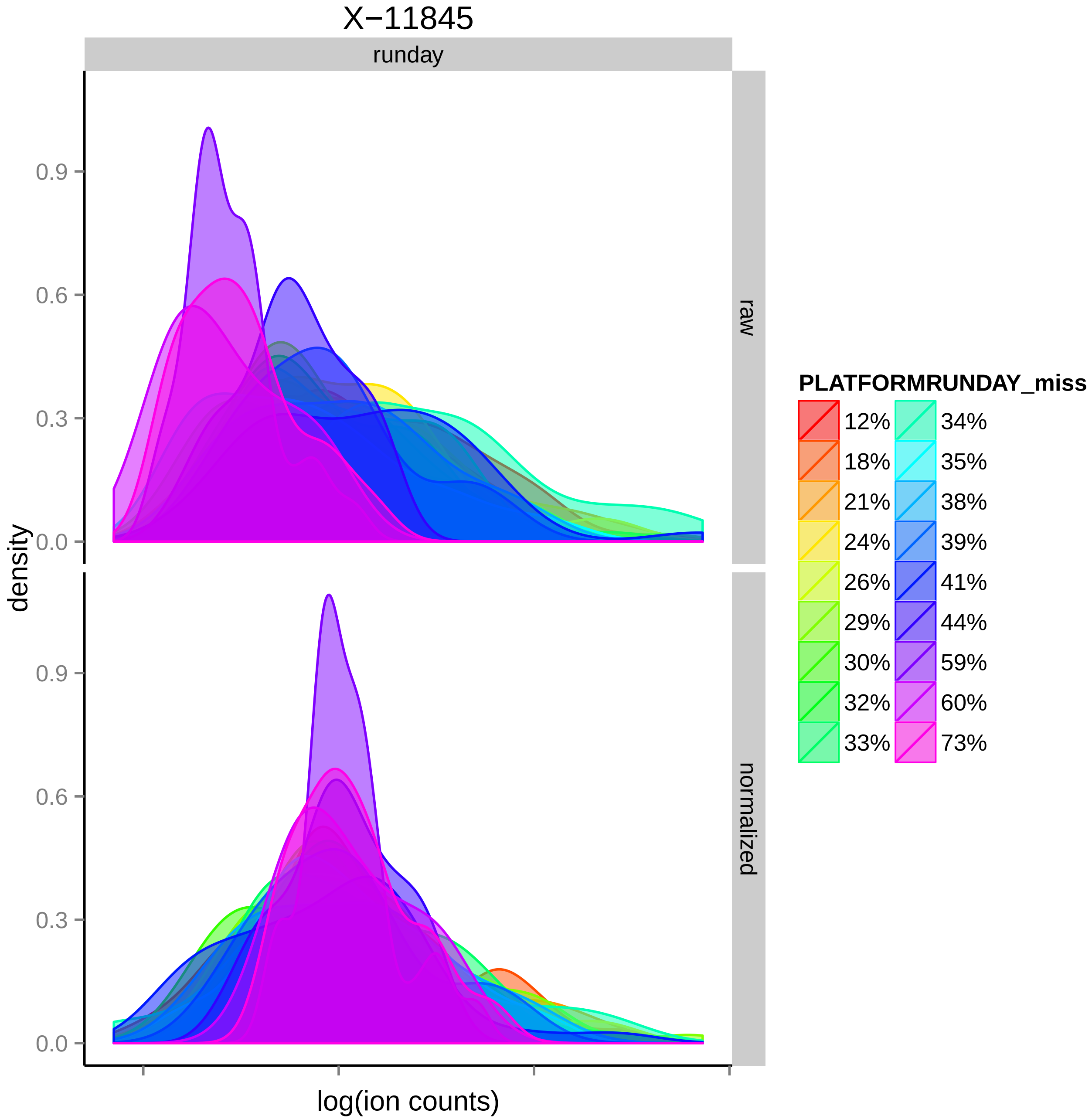

X−11845
runday
raw
normalized
density
0.9
0.6
0.3
0.0
log(ion counts)
PLATFORMRUNDAY_miss
12%
18%
21%
24%
26%
29%
30%
32%
33%
34%
35%
38%
39%
41%
44%
59%
60%
73%

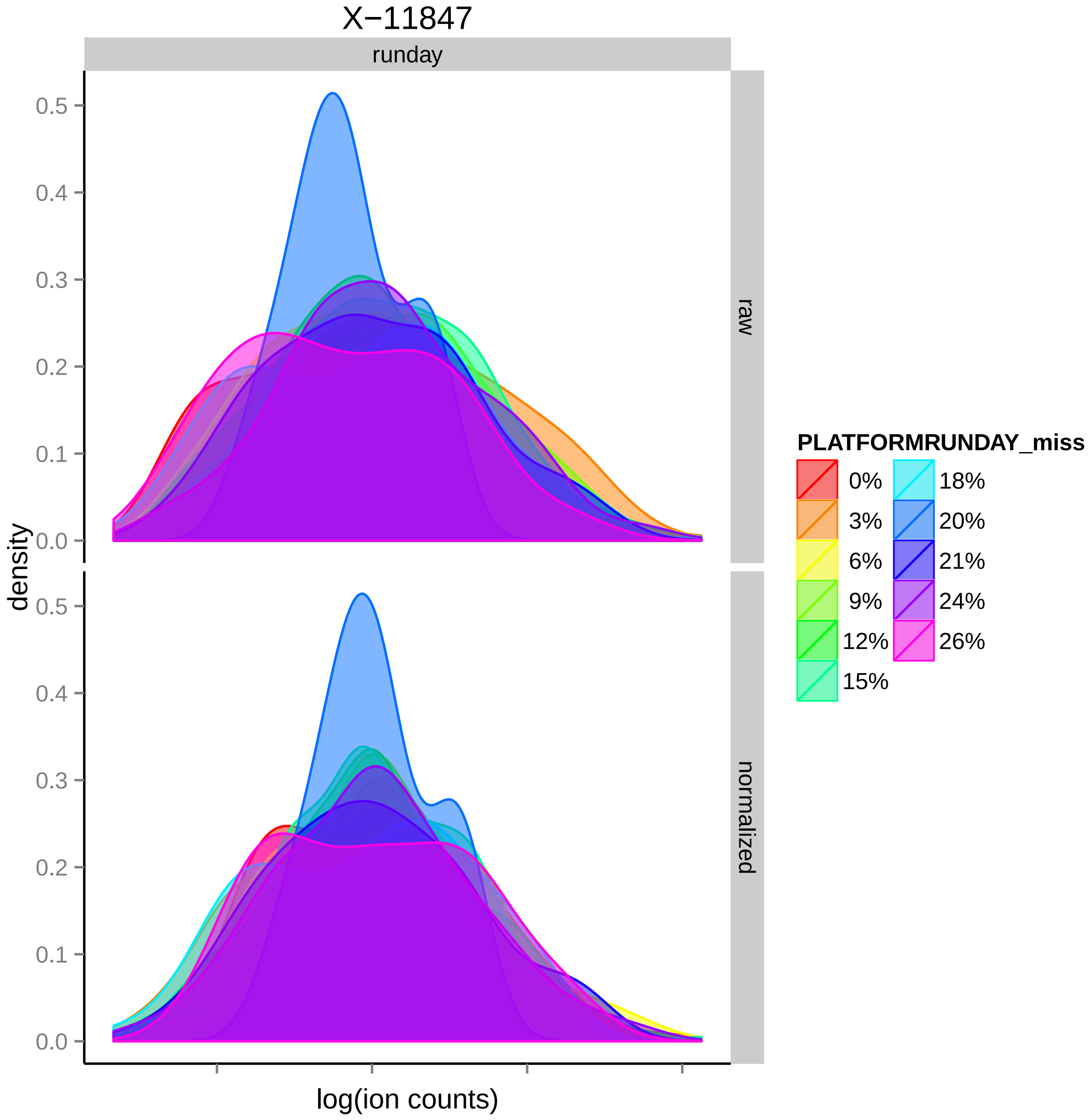

X−11847
runday
raw
normalized
density
0.5
0.4
0.3
0.2
0.1
0.0
log(ion counts)
PLATFORMRUNDAY_miss
0%
3%
6%
9%
12%
15%
18%
20%
21%
24%
26%

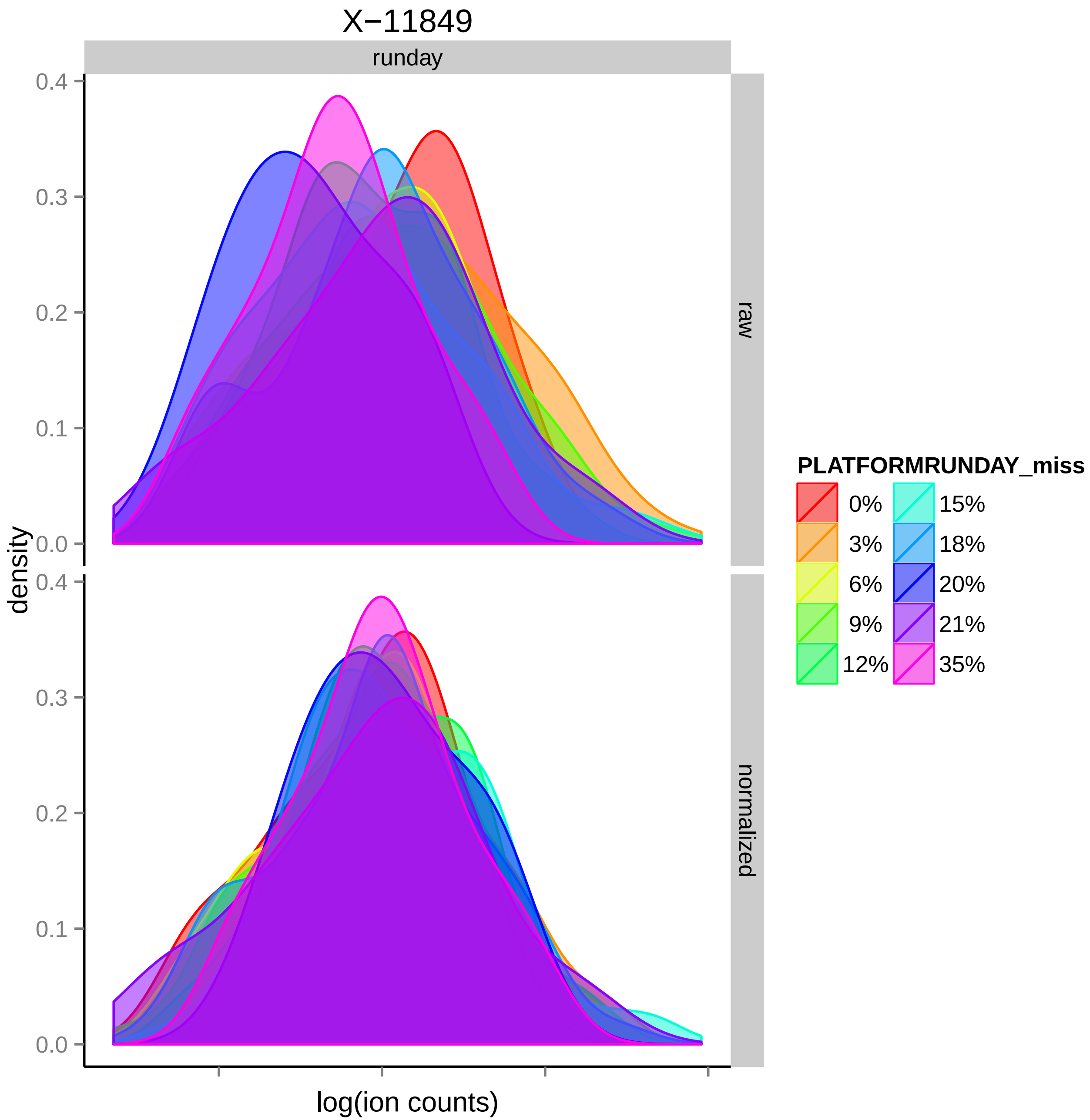
X−11849
runday
raw
normalized
density
0.0
0.1
0.2
0.3
0.4
log(ion counts)
PLATFORMRUNDAY_miss
0%
3%
6%
9%
12%
15%
18%
20%
21%
35%

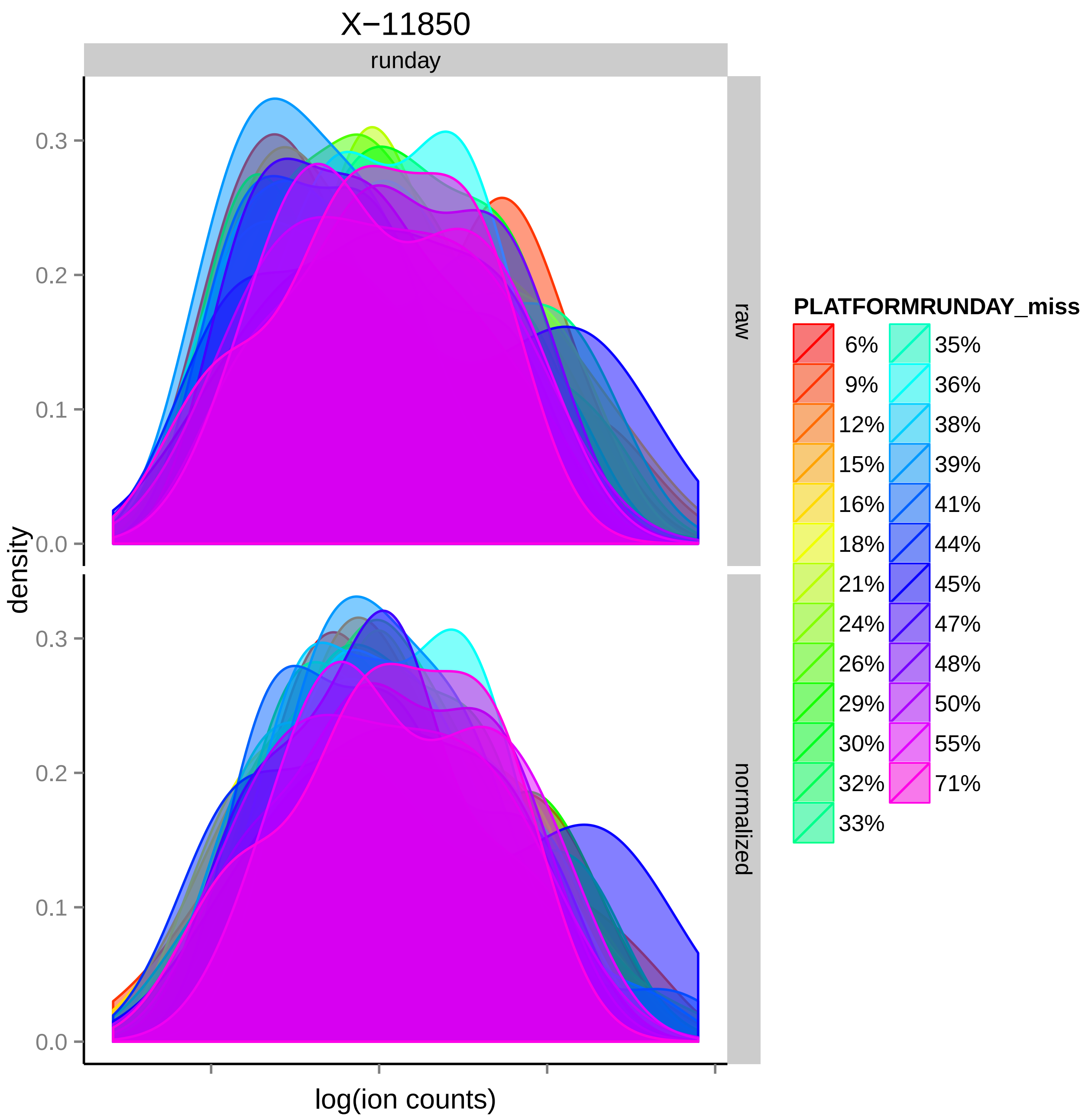

X−11850
runday
raw
normalized
density
0.0
0.1
0.2
0.3
log(ion counts)
PLATFORMRUNDAY_miss
6%
9%
12%
15%
16%
18%
21%
24%
26%
29%
30%
32%
33%
35%
36%
38%
39%
41%
44%
45%
47%
48%
50%
55%
71%

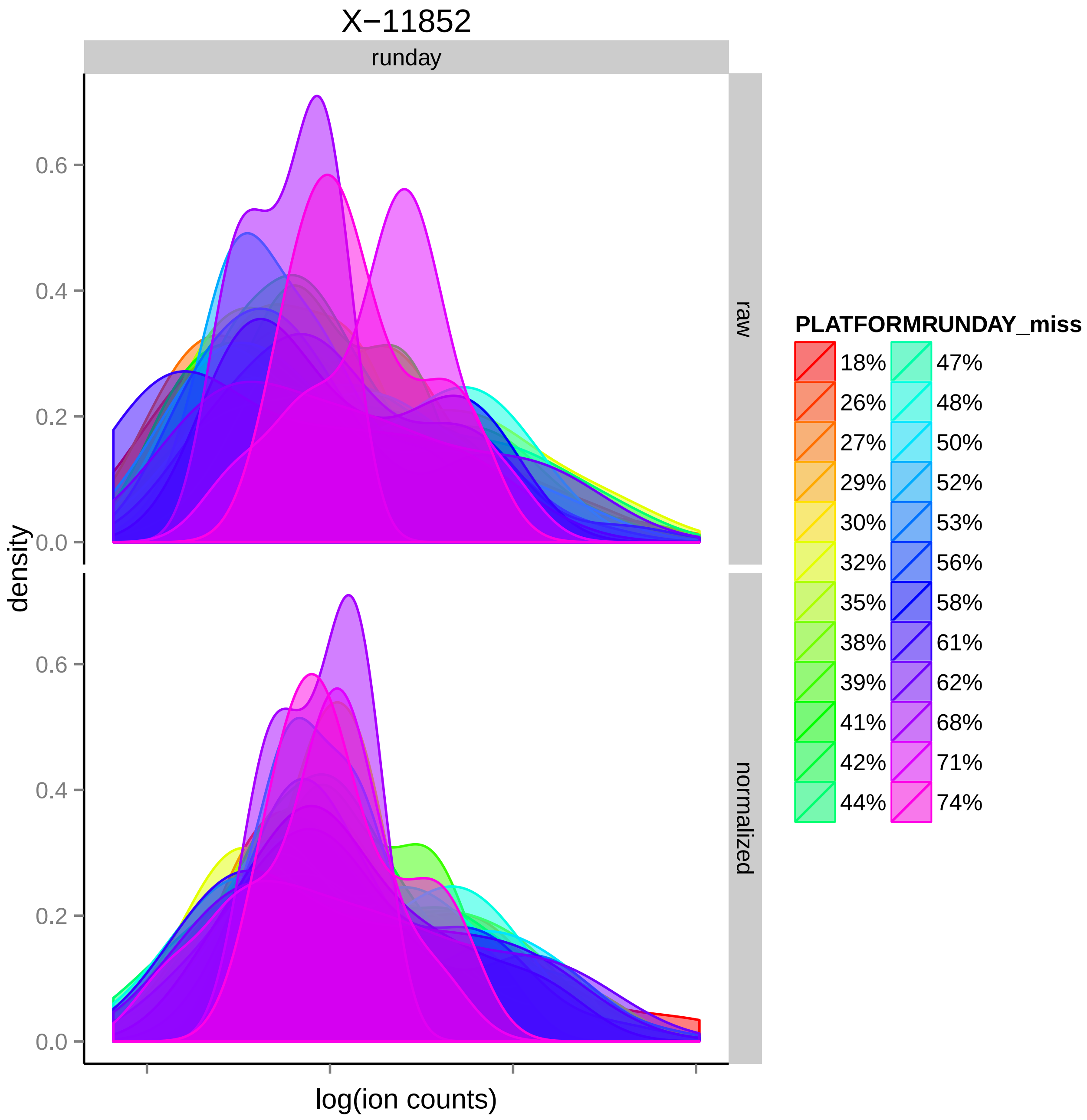

X−11852
runday
raw
normalized
density
0.0
0.2
0.4
0.6
log(ion counts)
PLATFORMRUNDAY_miss
18%
26%
27%
29%
30%
32%
35%
38%
39%
41%
42%
44%
47%
48%
50%
52%
53%
56%
58%
61%
62%
68%
71%
74%

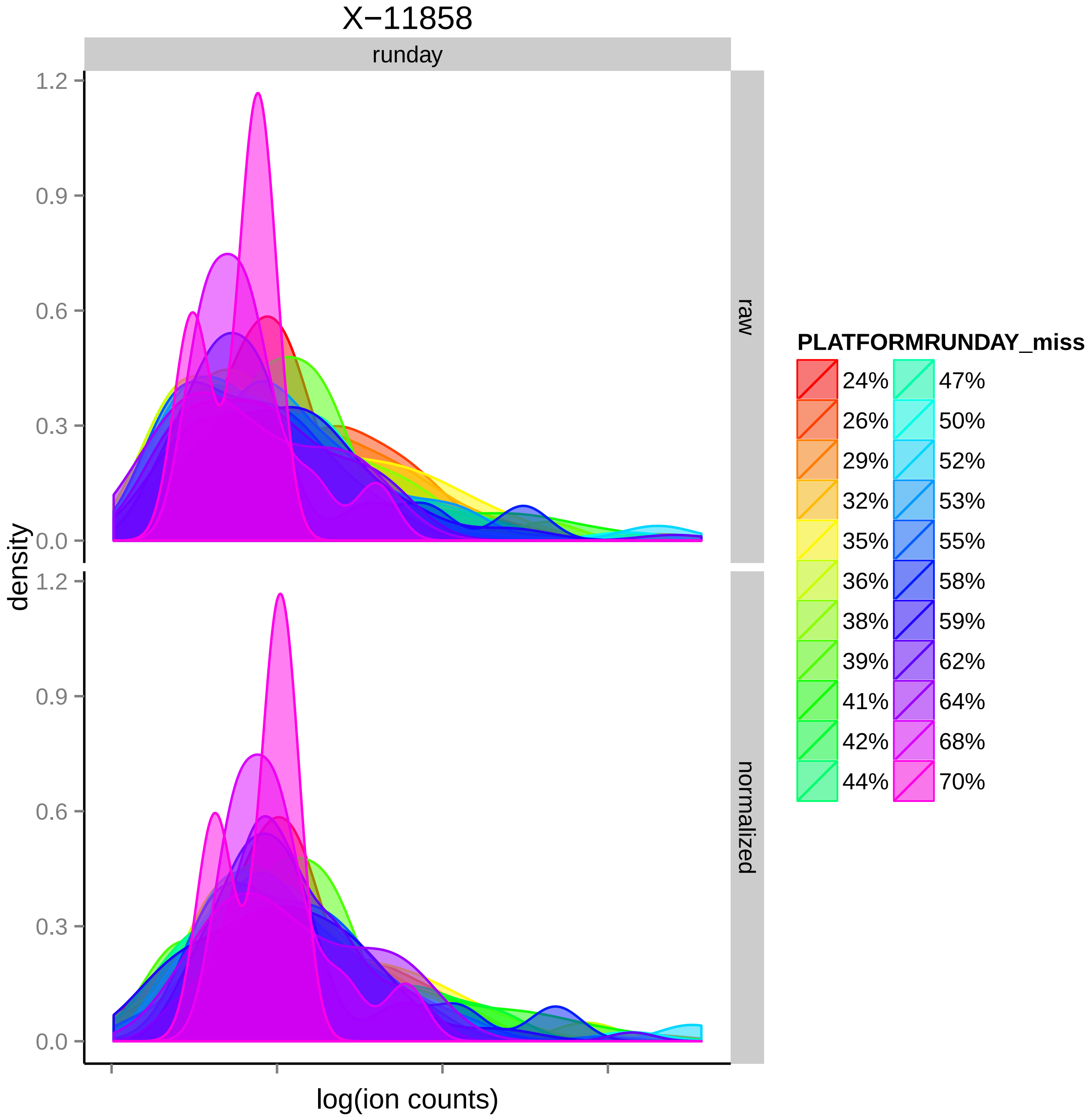
X−11858
runday
raw
normalized
density
log(ion counts)
1.2
0.9
0.6
0.3
0.0
PLATFORMRUNDAY_miss
24%
26%
29%
32%
35%
36%
38%
39%
41%
42%
44%
47%
50%
52%
53%
55%
58%
59%
62%
64%
68%
70%

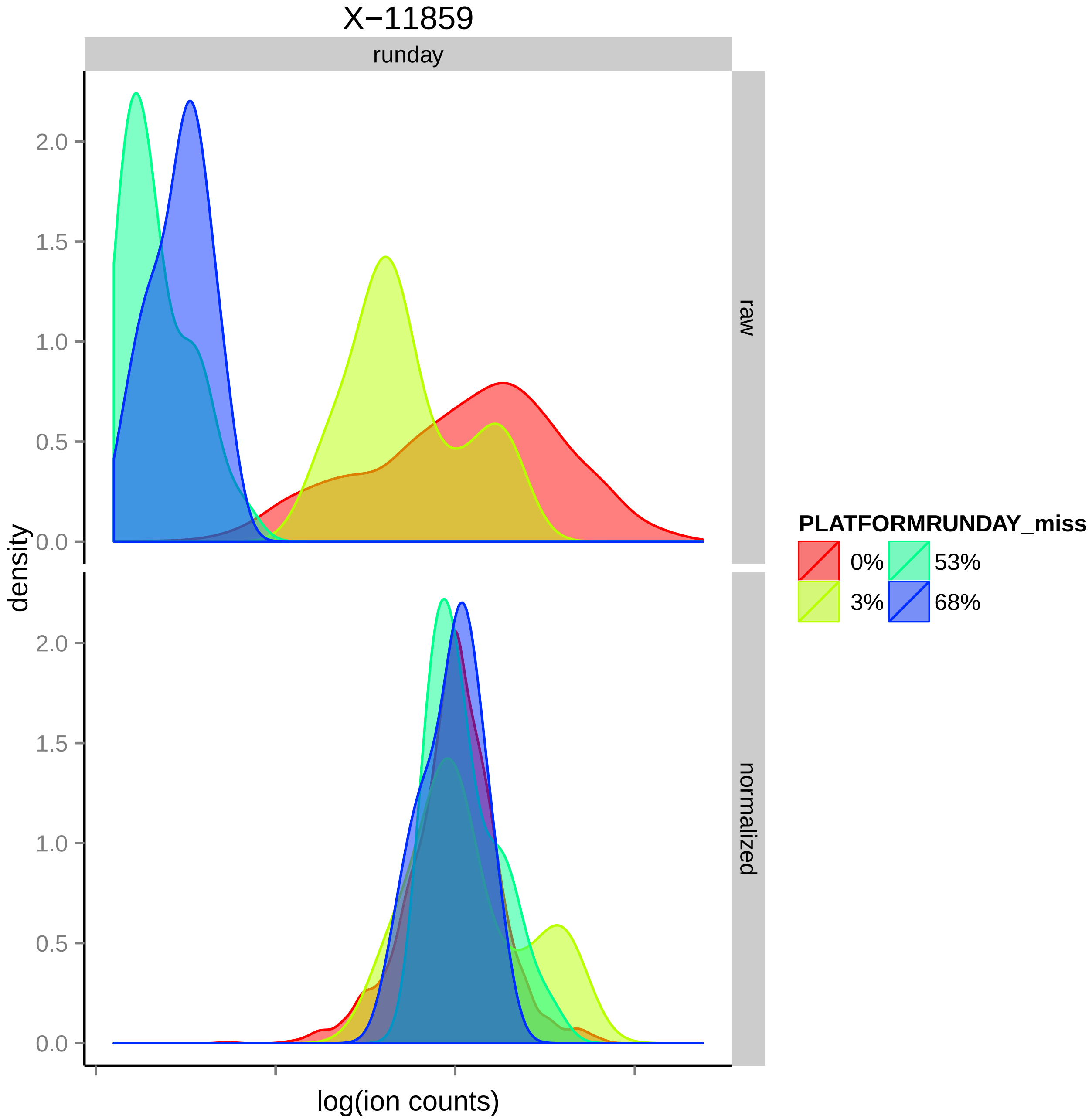
X−11859
runday
raw
normalized
density
log(ion counts)
2.0
1.5
1.0
0.5
0.0
PLATFORMRUNDAY_miss
0%
3%
53%
68%

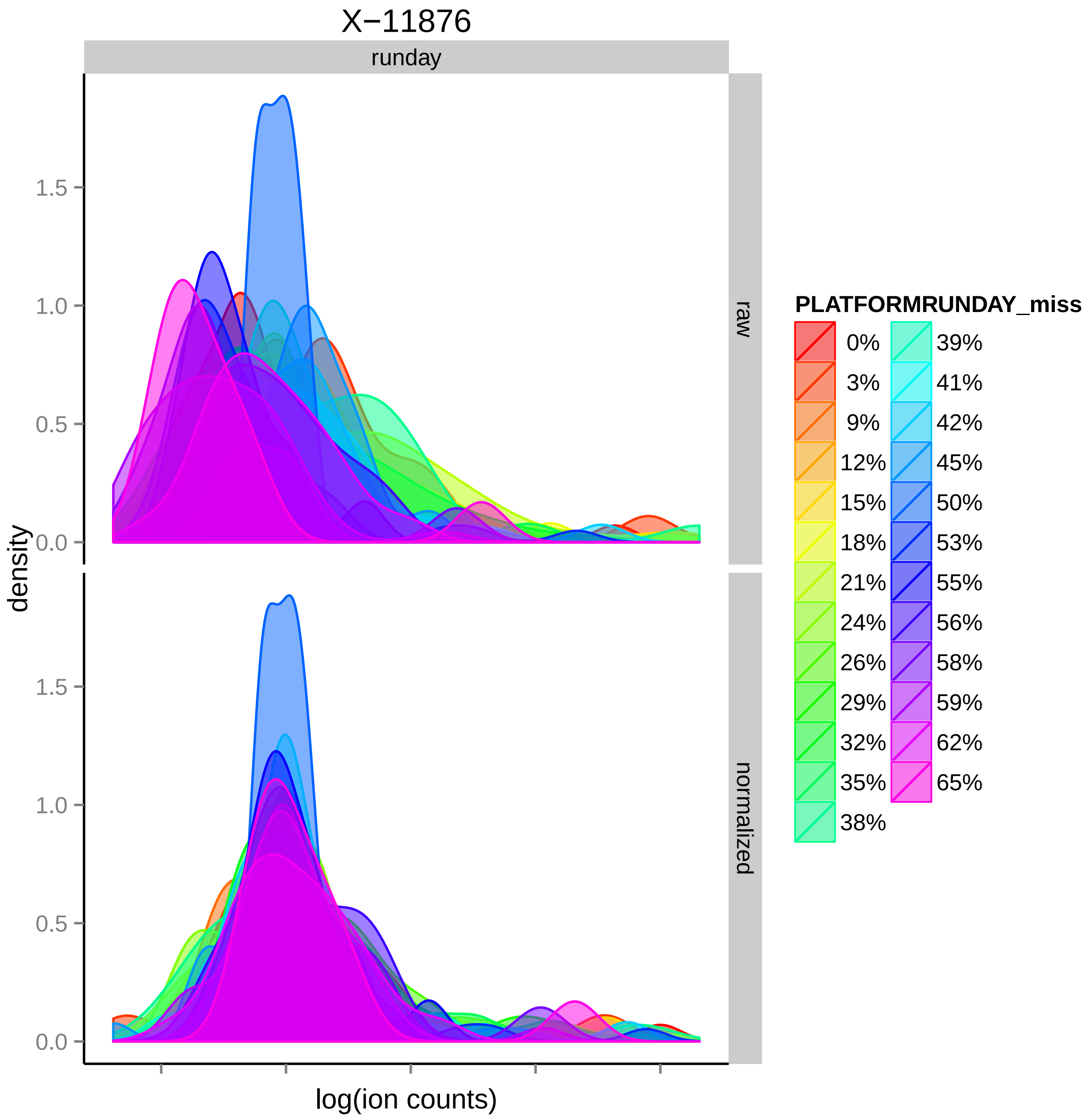

X−11876
runday
raw
normalized
density
log(ion counts)
1.5
1.0
0.5
0.0
1.5
1.0
0.5
0.0
PLATFORMRUNDAY_miss
0%
3%
9%
12%
15%
18%
21%
24%
26%
29%
32%
35%
38%
39%
41%
42%
45%
50%
53%
55%
56%
58%
59%
62%
65%

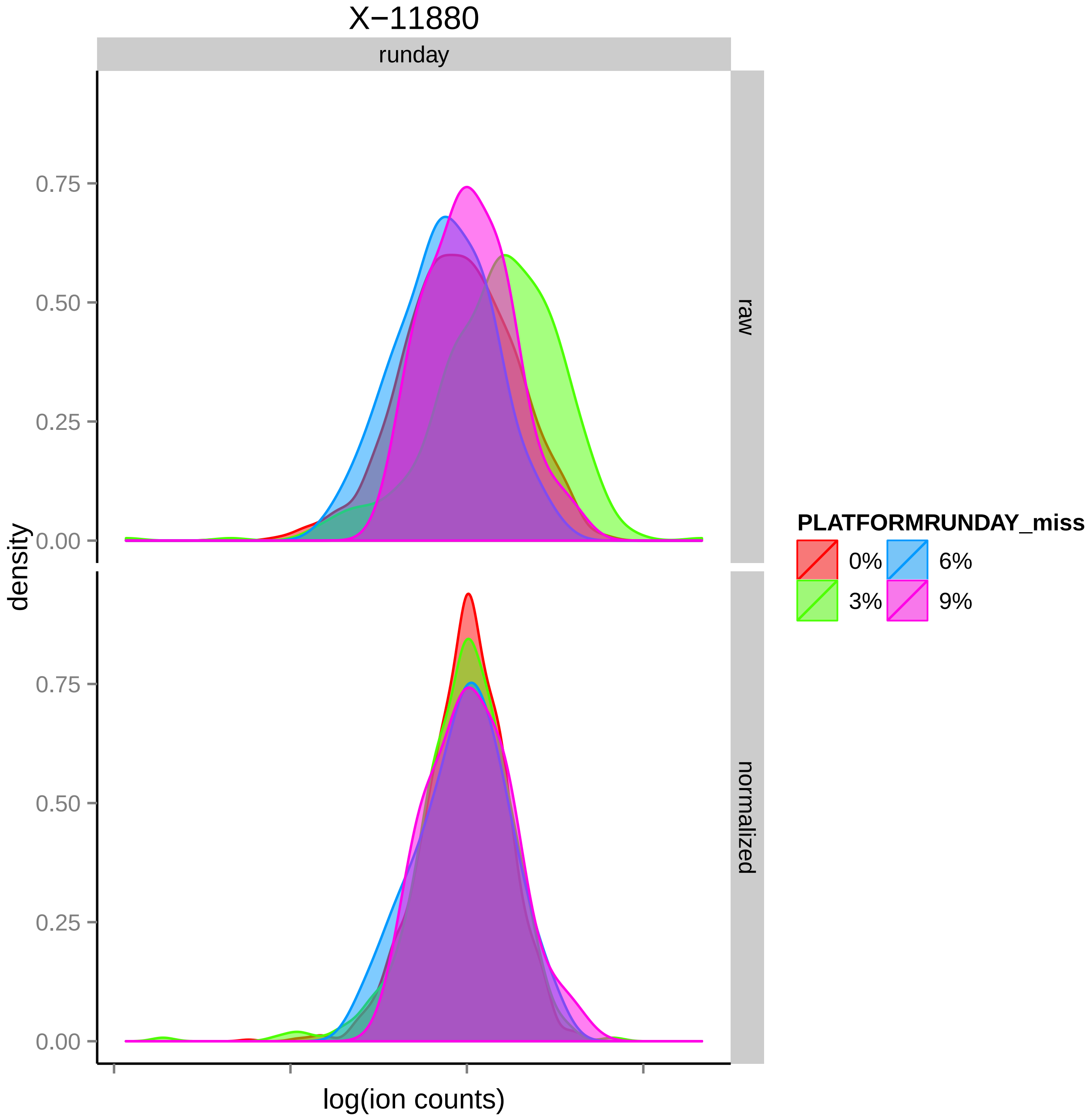

X−11880
runday
raw
normalized
density
log(ion counts)
0.00
0.25
0.50
0.75
PLATFORMRUNDAY_miss
0%
3%
6%
9%

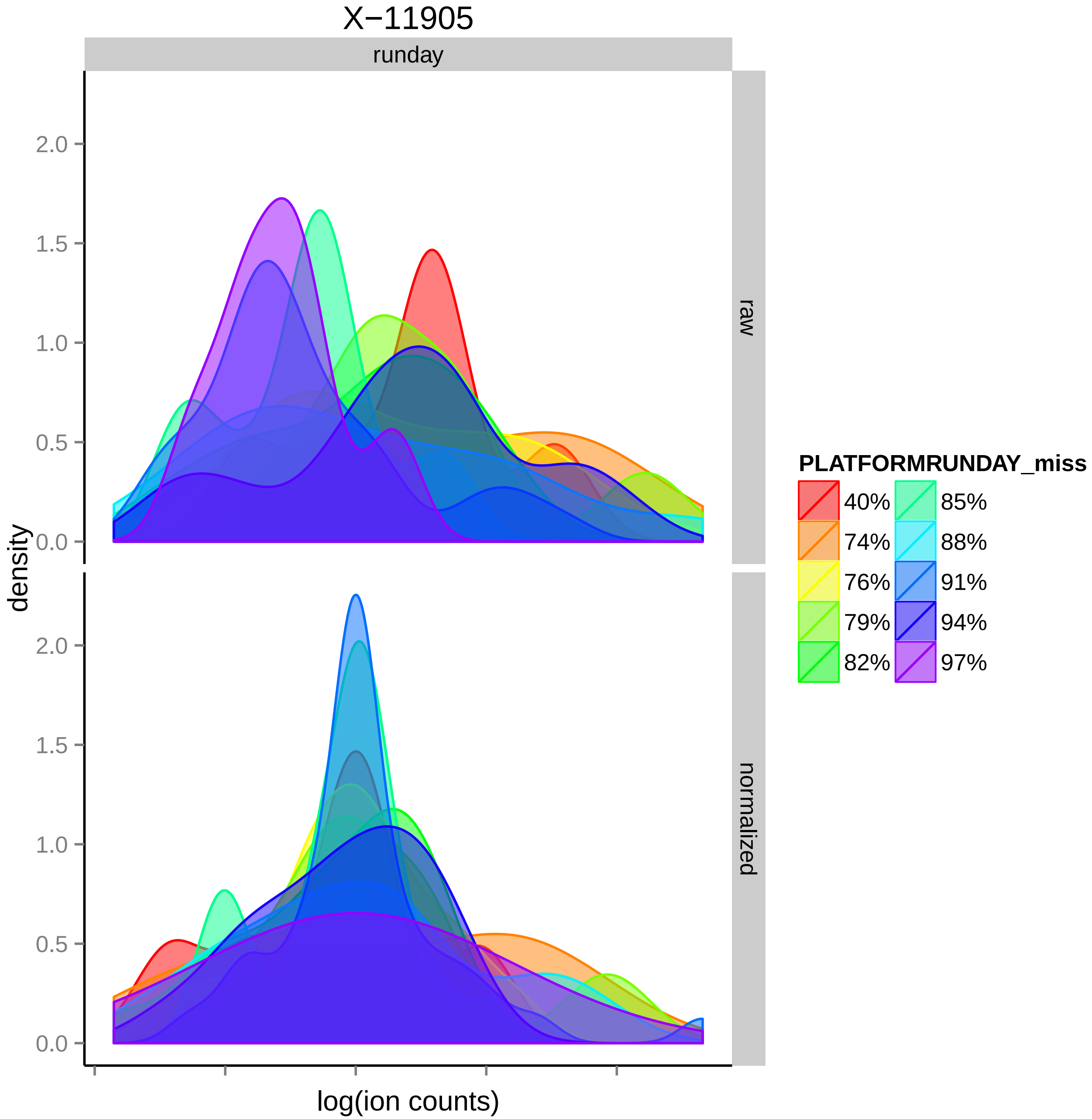
X−11905
runday
raw
normalized
density
log(ion counts)
2.0
1.5
1.0
0.5
0.0
PLATFORMRUNDAY_miss
40%
74%
76%
79%
82%
85%
88%
91%
94%
97%

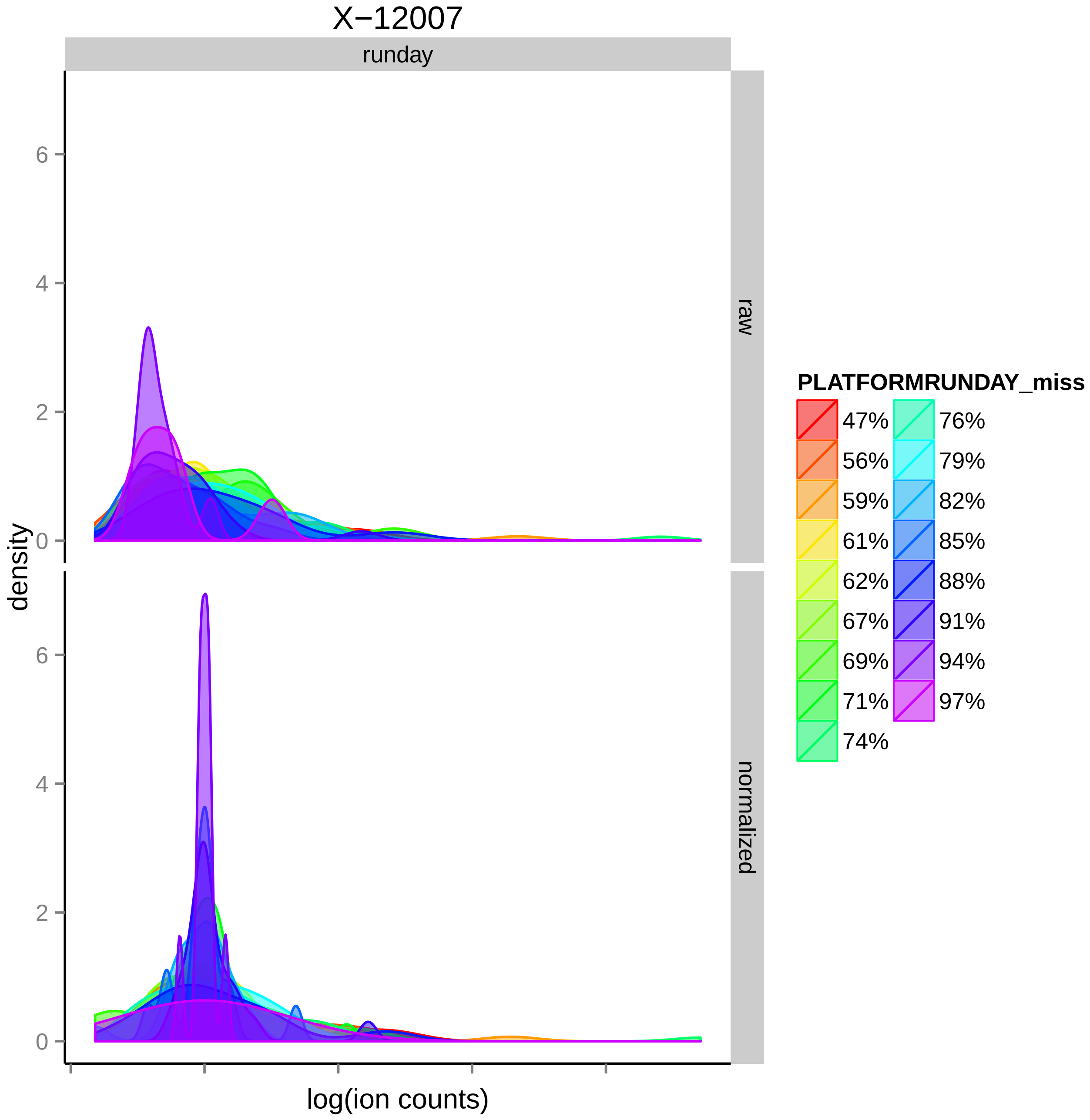
X−12007
runday
raw
normalized
density
log(ion counts)
6
4
2
0
PLATFORMRUNDAY_miss
47%
56%
59%
61%
62%
67%
69%
71%
74%
76%
79%
82%
85%
88%
91%
94%
97%

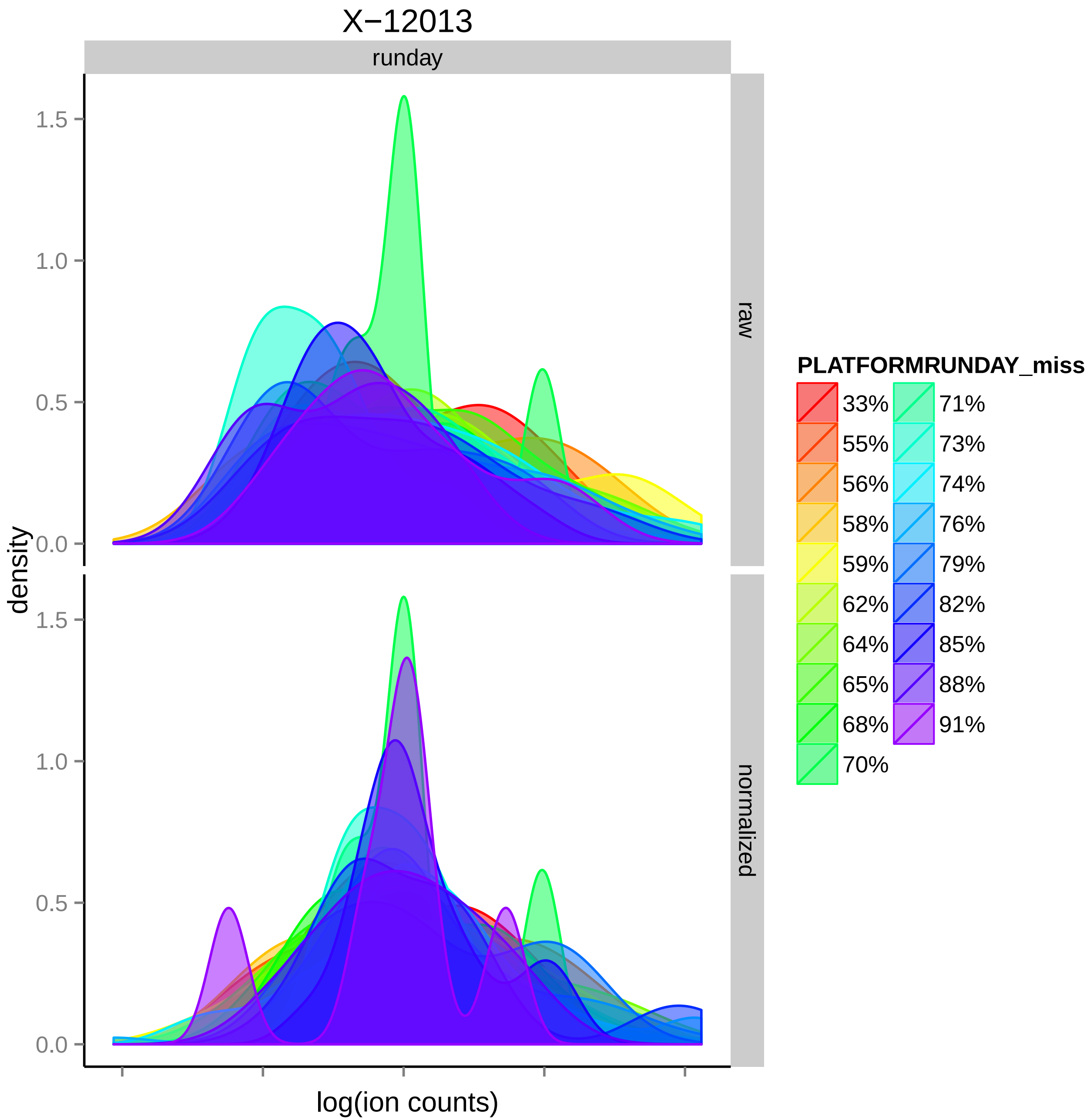
X−12013
runday
raw
normalized
density
1.5
1.0
0.5
0.0
log(ion counts)
PLATFORMRUNDAY_miss
33%
55%
56%
58%
59%
62%
64%
65%
68%
70%
71%
73%
74%
76%
79%
82%
85%
88%
91%

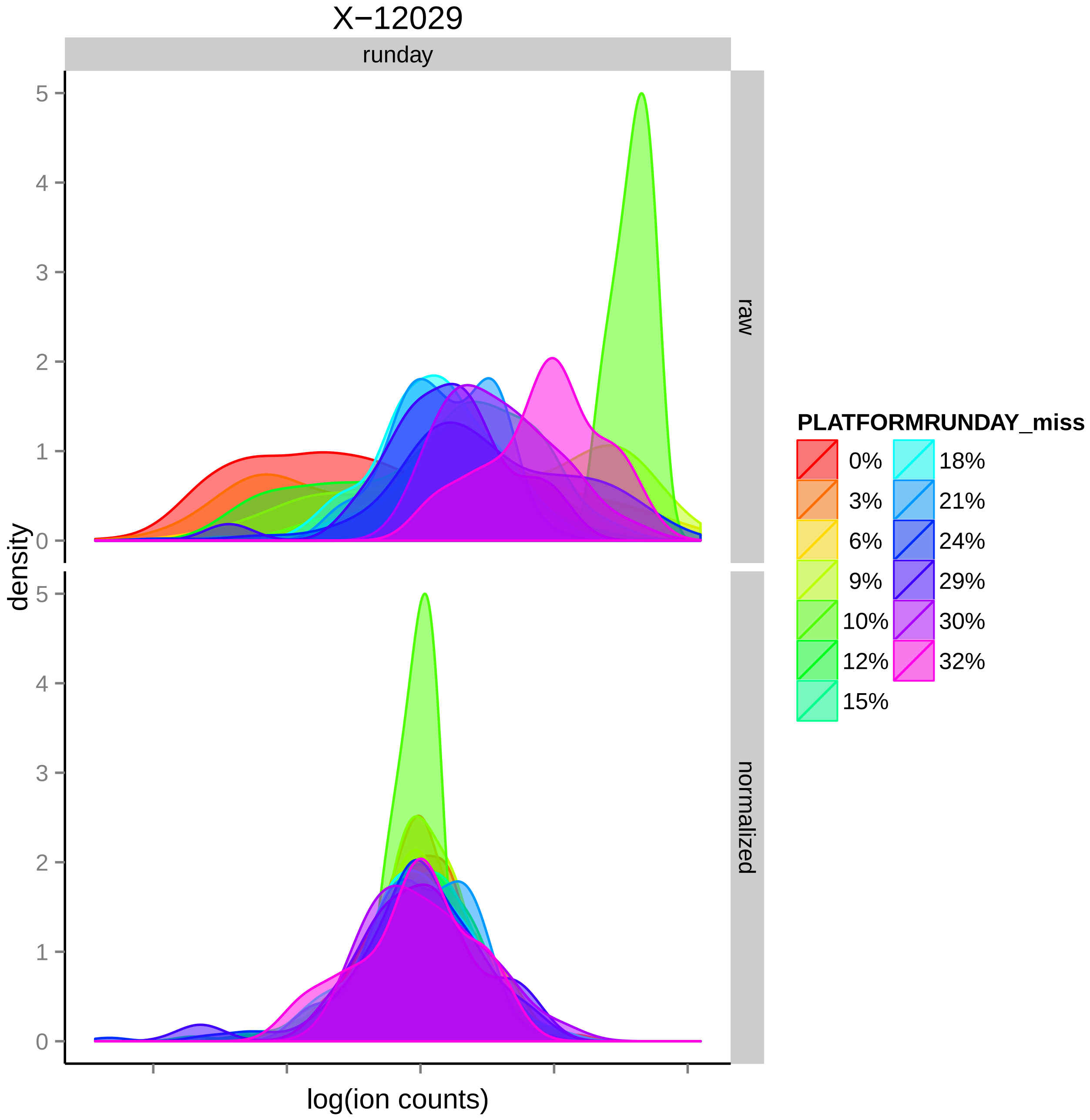

X−12029
runday
raw
normalized
density
log(ion counts)
0
1
2
3
4
5
PLATFORMRUNDAY_miss
0%
3%
6%
9%
10%
12%
15%
18%
21%
24%
29%
30%
32%

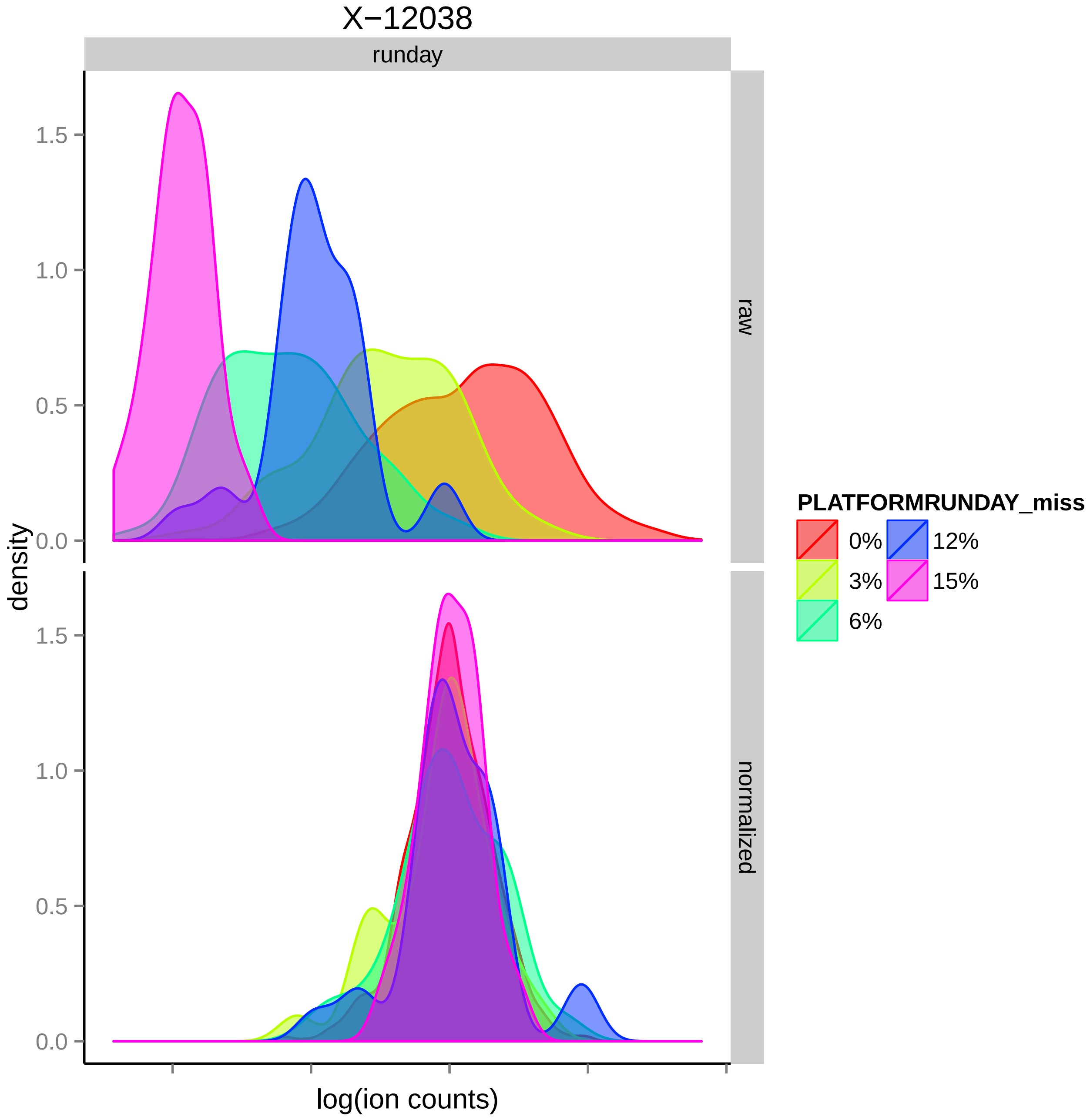
X−12038
runday
raw
normalized
density
1.5
1.0
0.5
0.0
1.5
1.0
0.5
0.0
log(ion counts)
PLATFORMRUNDAY_miss
0%
3%
6%
12%
15%

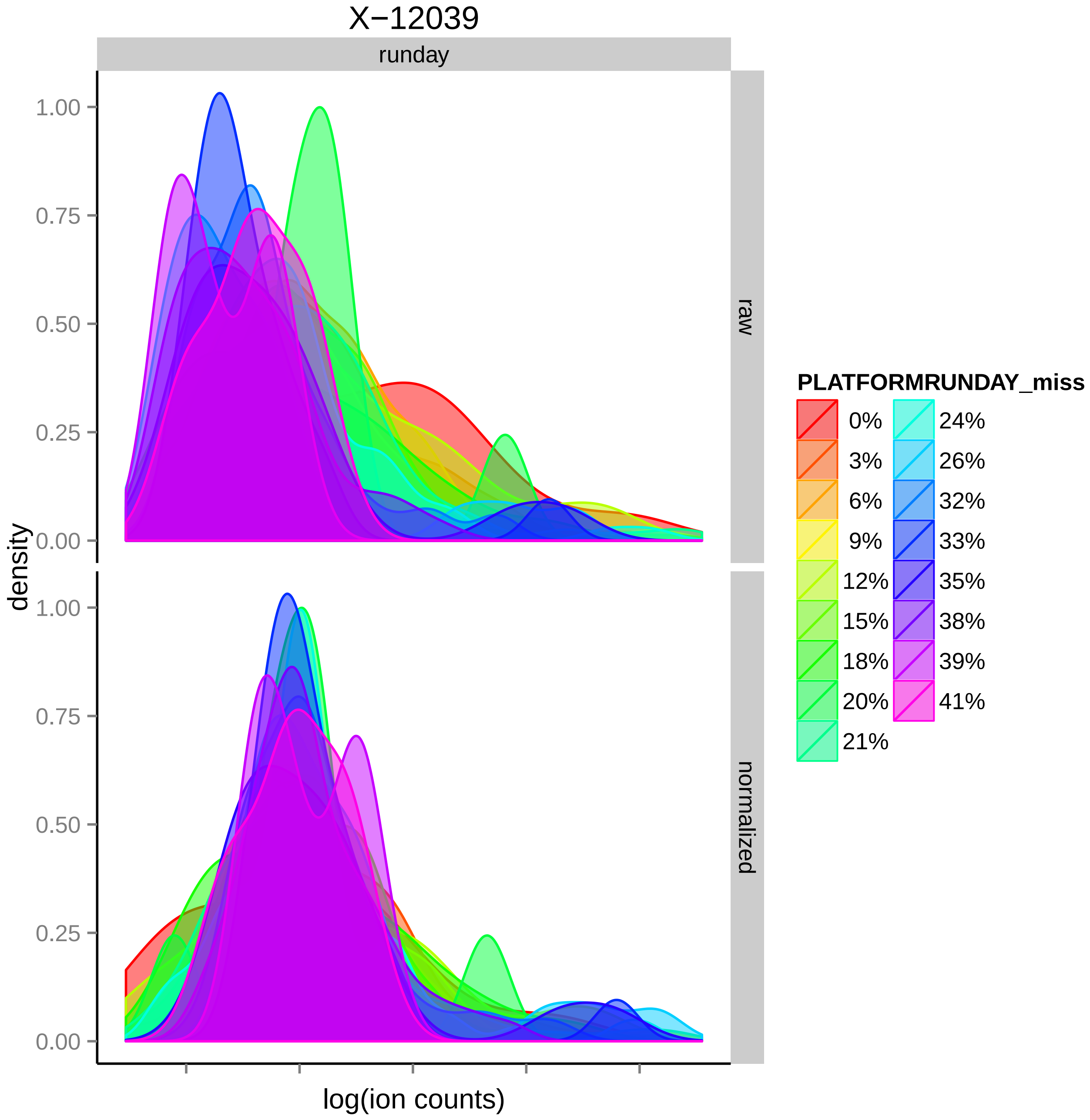

X−12039
runday
raw
normalized
density
log(ion counts)
1.00
0.75
0.50
0.25
0.00
PLATFORMRUNDAY_miss
0%
3%
6%
9%
12%
15%
18%
20%
21%
24%
26%
32%
33%
35%
38%
39%
41%

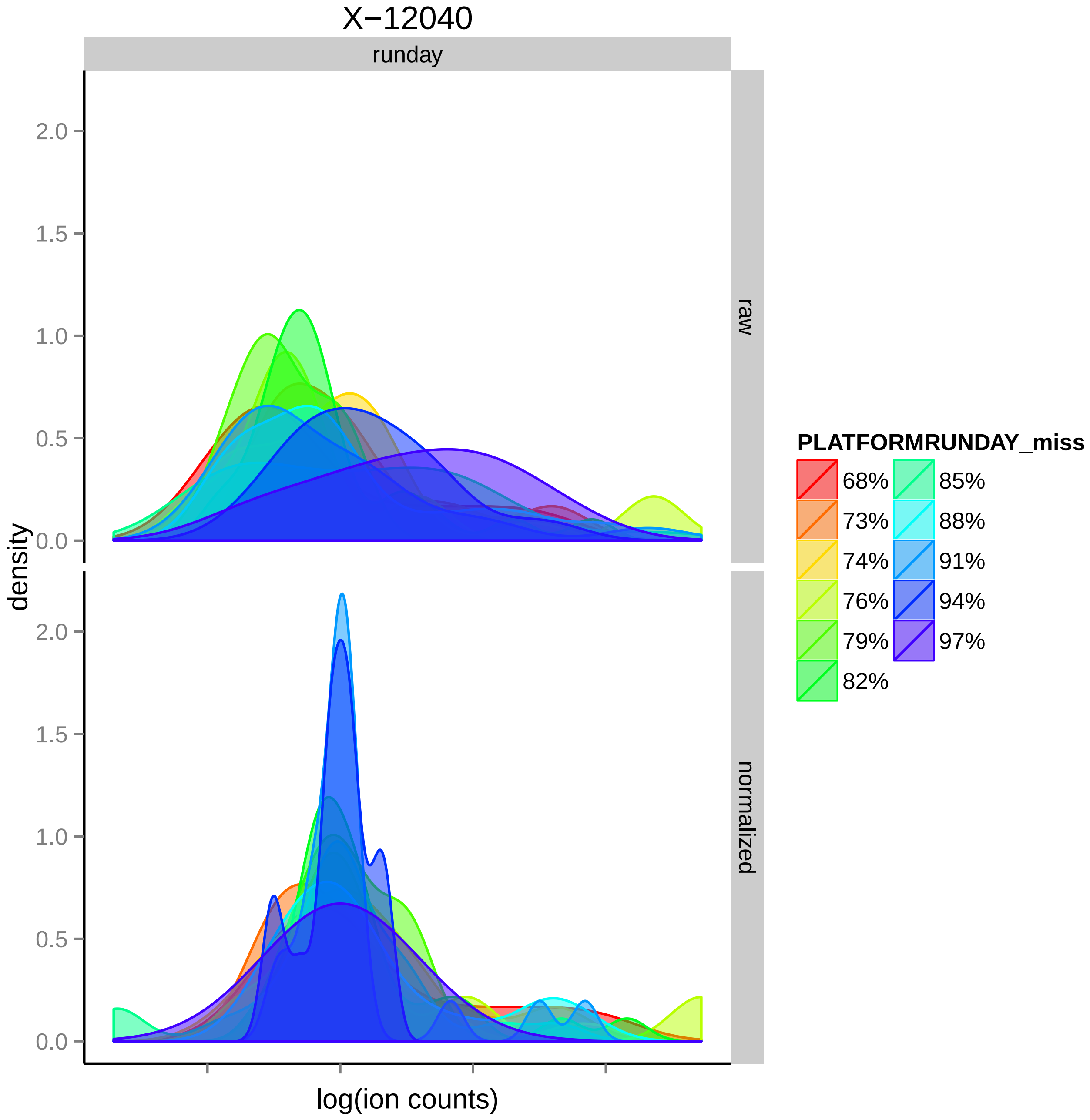
X−12040
runday
raw
normalized
density
log(ion counts)
0.0
0.5
1.0
1.5
2.0
PLATFORMRUNDAY_miss
68%
73%
74%
76%
79%
82%
85%
88%
91%
94%
97%

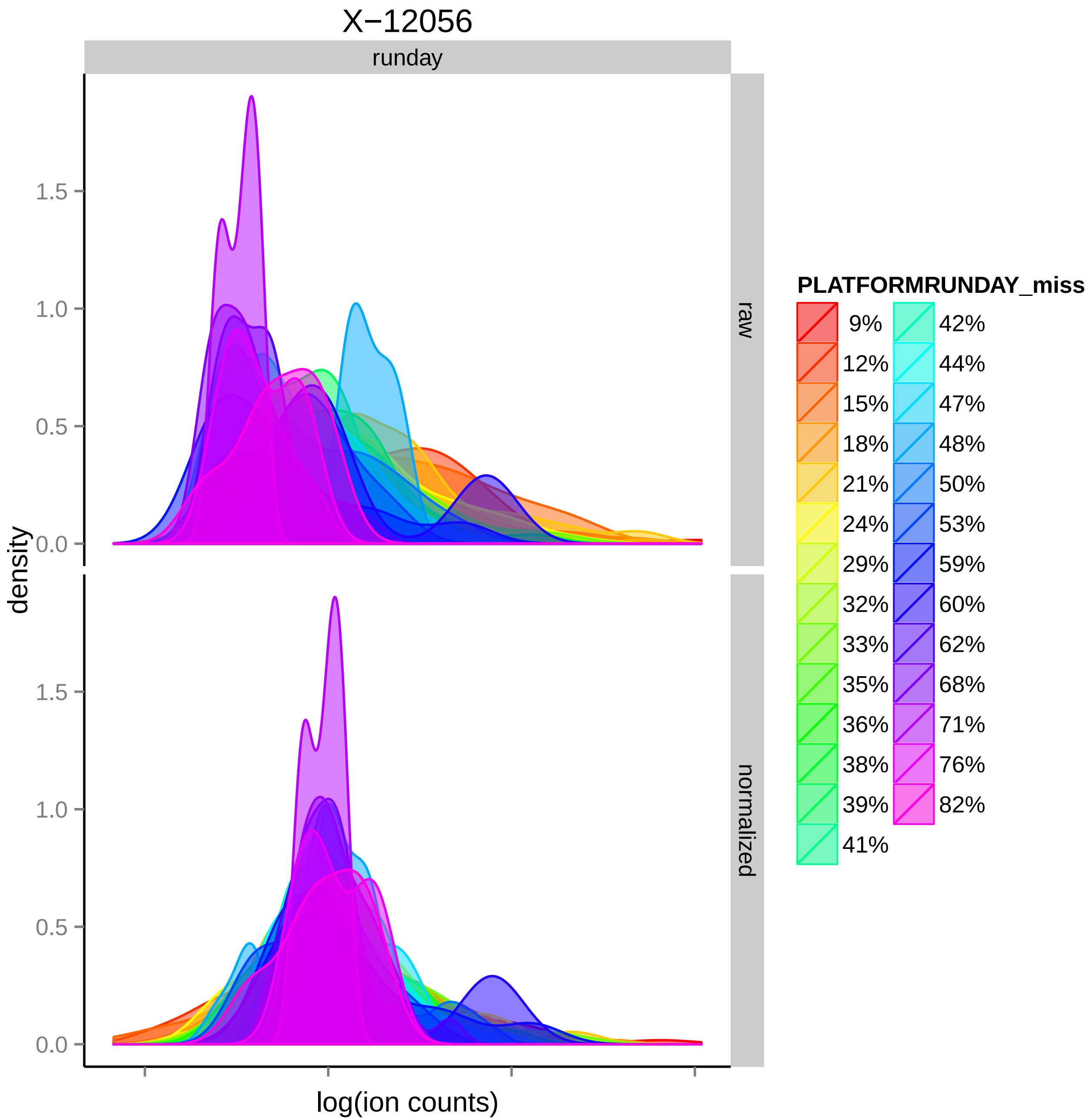
X−12056
runday
raw
normalized
density
1.5
1.0
0.5
0.0
log(ion counts)
PLATFORMRUNDAY_miss
9%
12%
15%
18%
21%
24%
29%
32%
33%
35%
36%
38%
39%
41%
42%
44%
47%
48%
50%
53%
59%
60%
62%
68%
71%
76%
82%

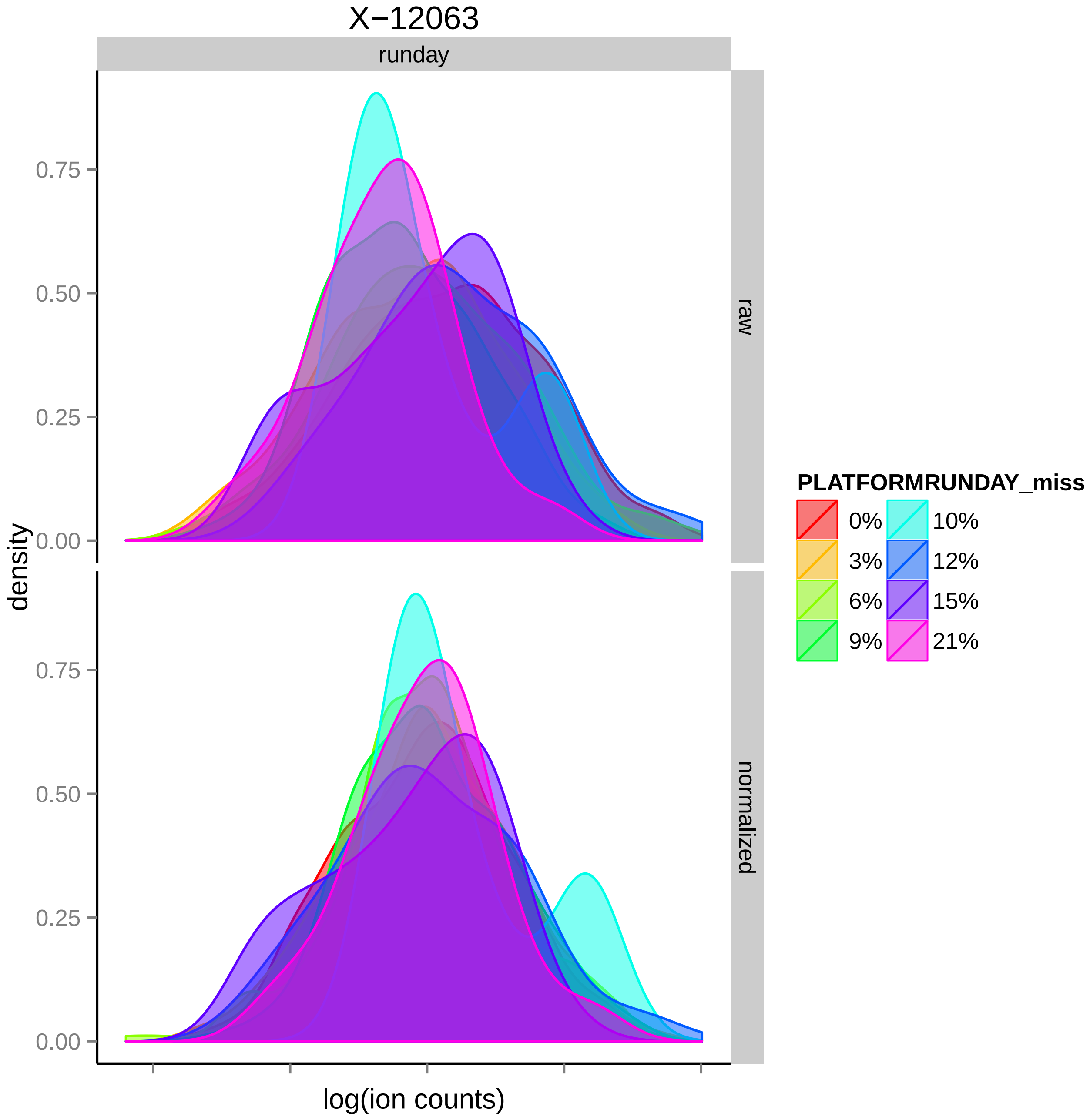

X−12063
runday
raw
normalized
density
log(ion counts)
0.00
0.25
0.50
0.75
PLATFORMRUNDAY_miss
0%
3%
6%
9%
10%
12%
15%
21%

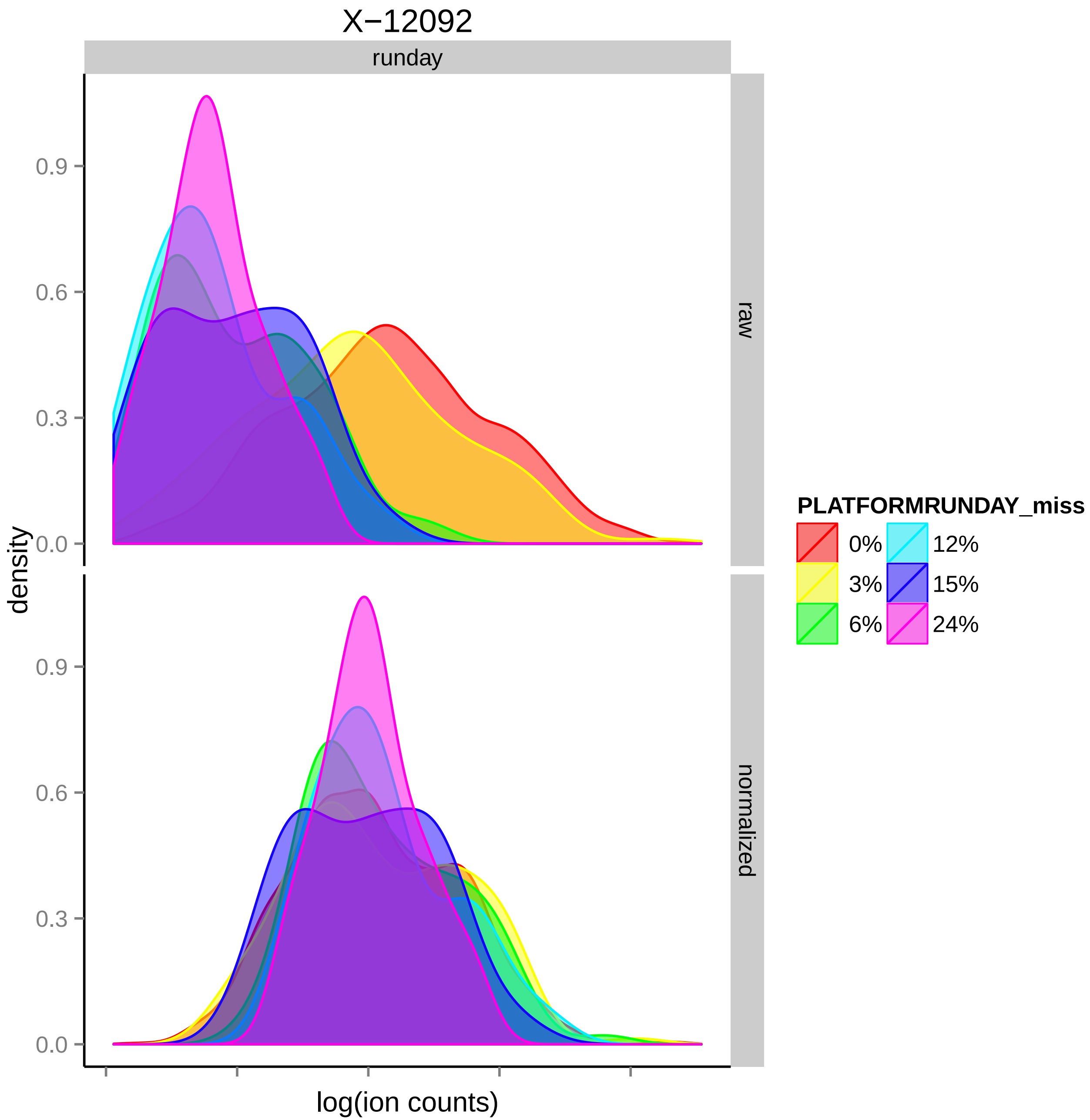
X−12092
runday
raw
normalized
density
0.0
0.3
0.6
0.9
log(ion counts)
PLATFORMRUNDAY_miss
0%
3%
6%
12%
15%
24%

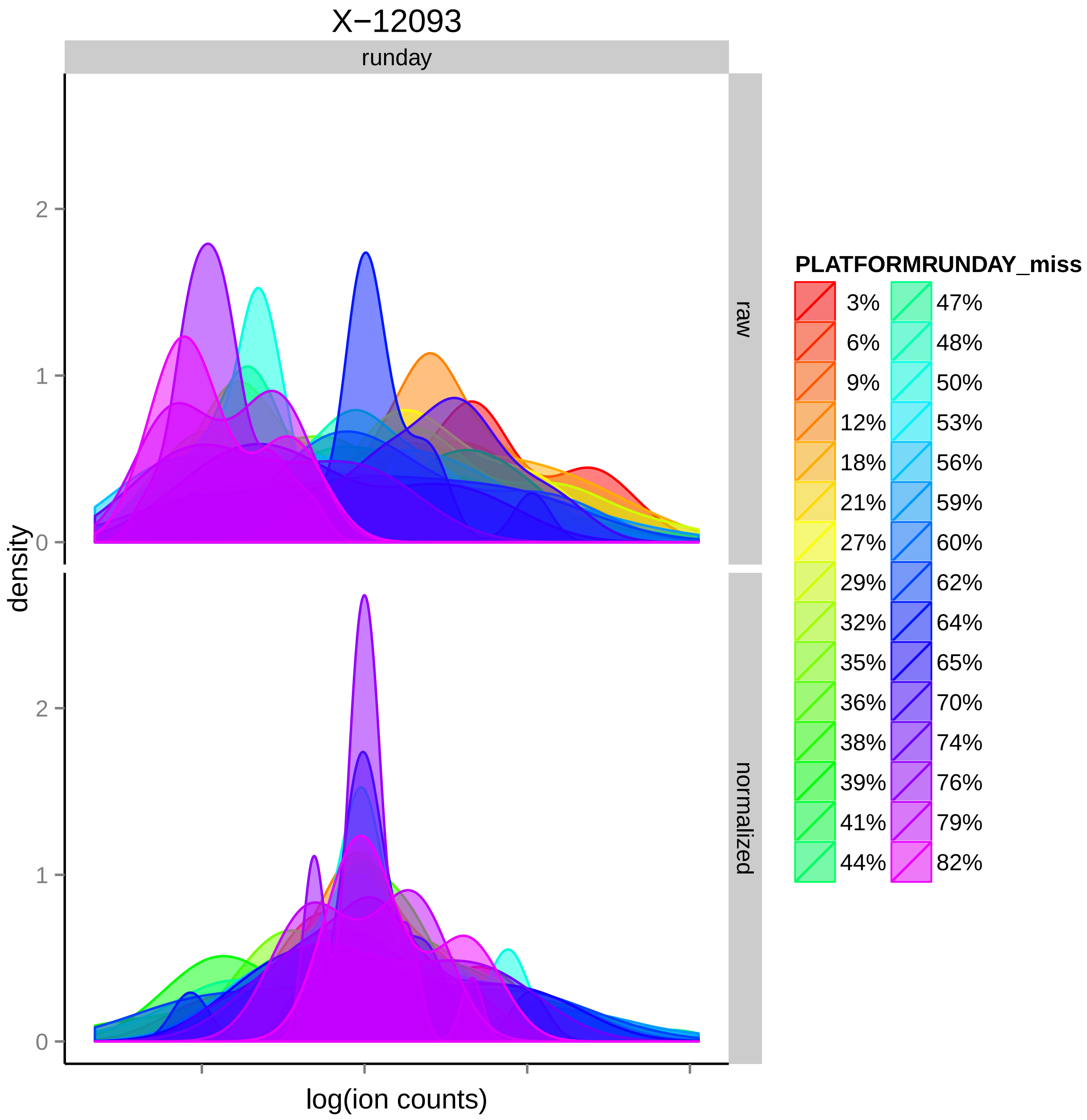

X−12093
runday
raw
normalized
density
log(ion counts)
0
1
2
0
1
2
PLATFORMRUNDAY_miss
3%
6%
9%
12%
18%
21%
27%
29%
32%
35%
36%
38%
39%
41%
44%
47%
48%
50%
53%
56%
59%
60%
62%
64%
65%
70%
74%
76%
79%
82%

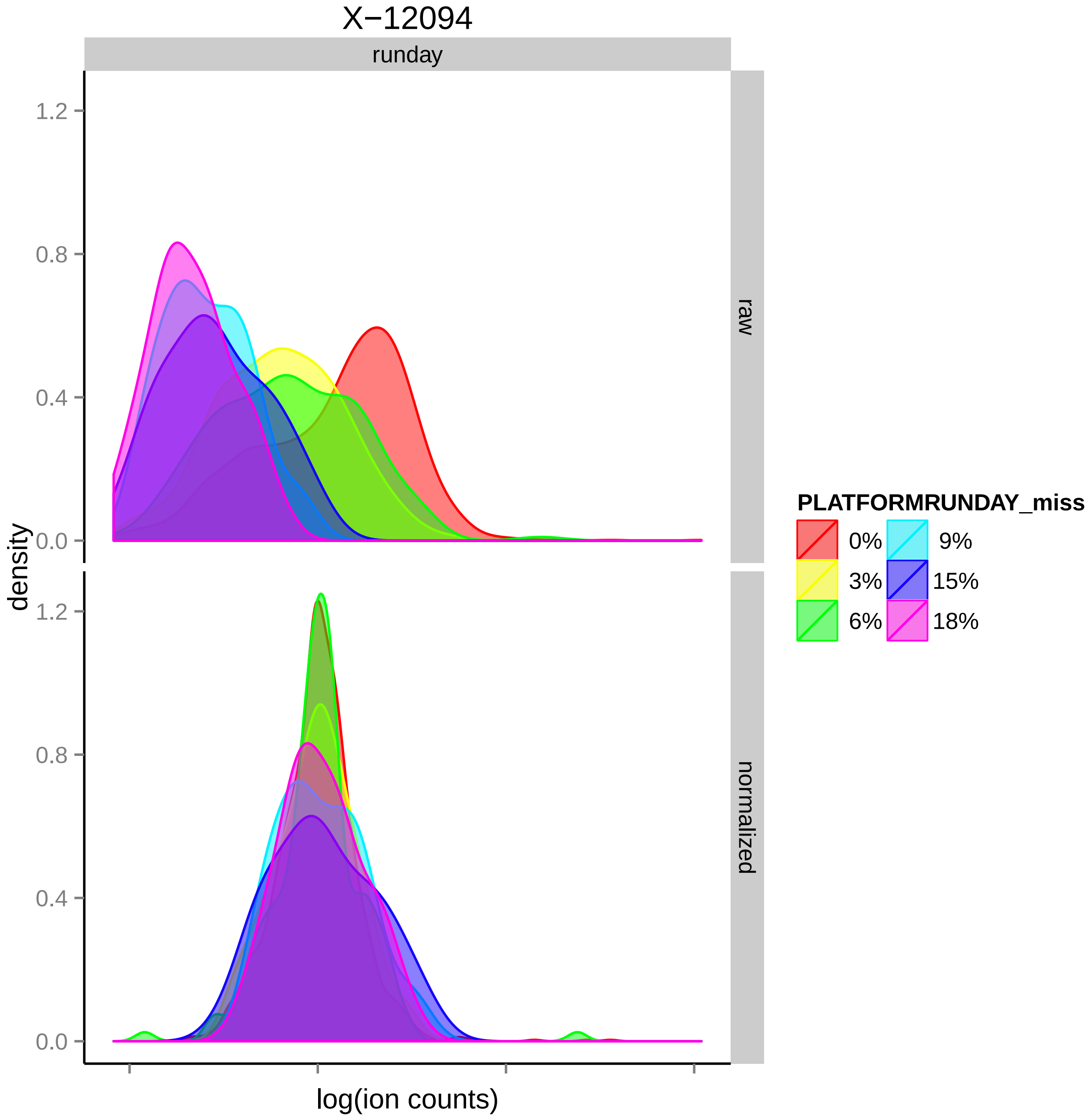

X−12094
runday
raw
normalized
density
log(ion counts)
1.2
0.8
0.4
0.0
PLATFORMRUNDAY_miss
0%
3%
6%
9%
15%
18%

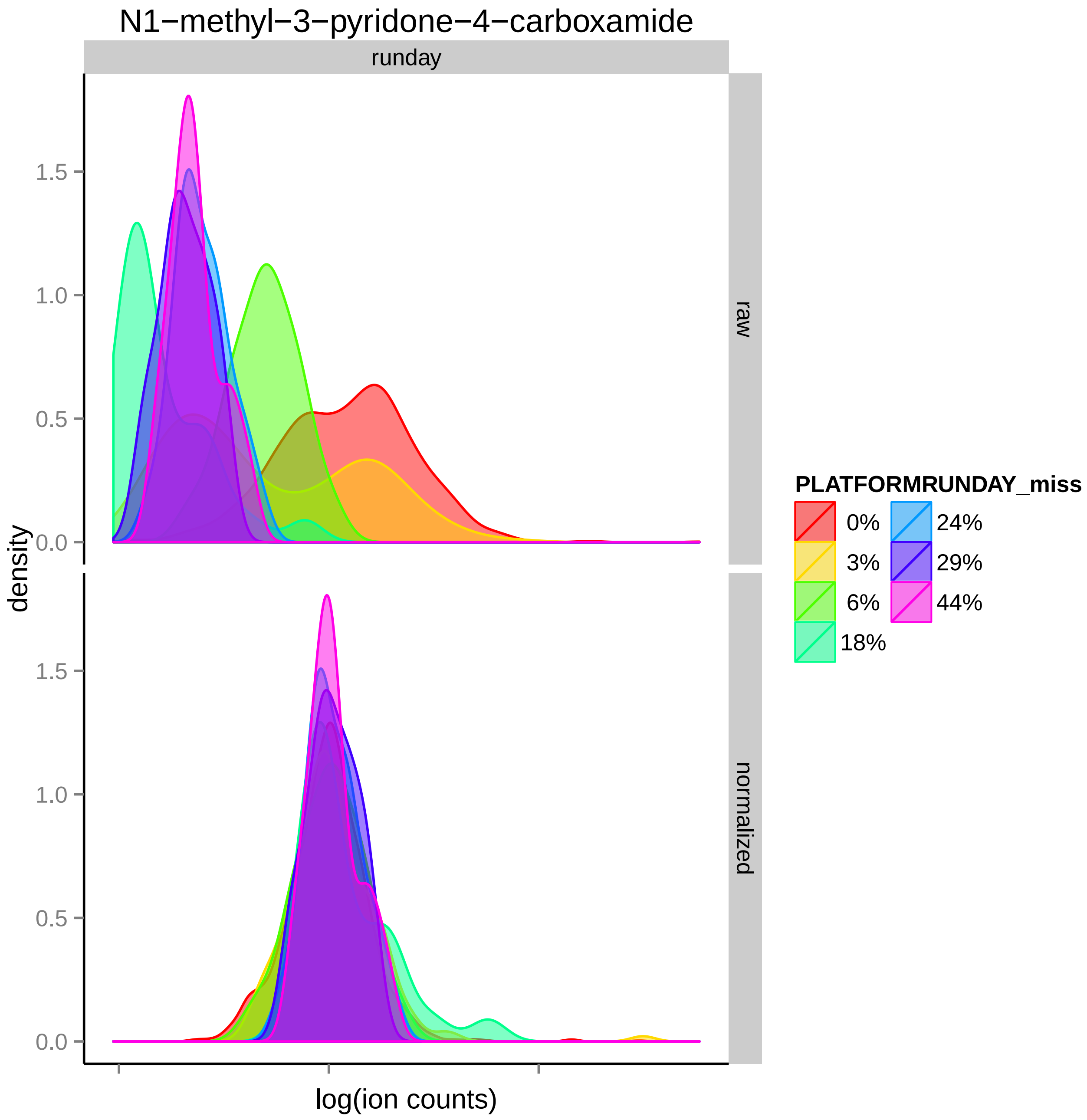
N1−methyl−3−pyridone−4−carboxamide
runday
raw
normalized
density
log(ion counts)
1.5
1.0
0.5
0.0
PLATFORMRUNDAY_miss
0%
3%
6%
18%
24%
29%
44%

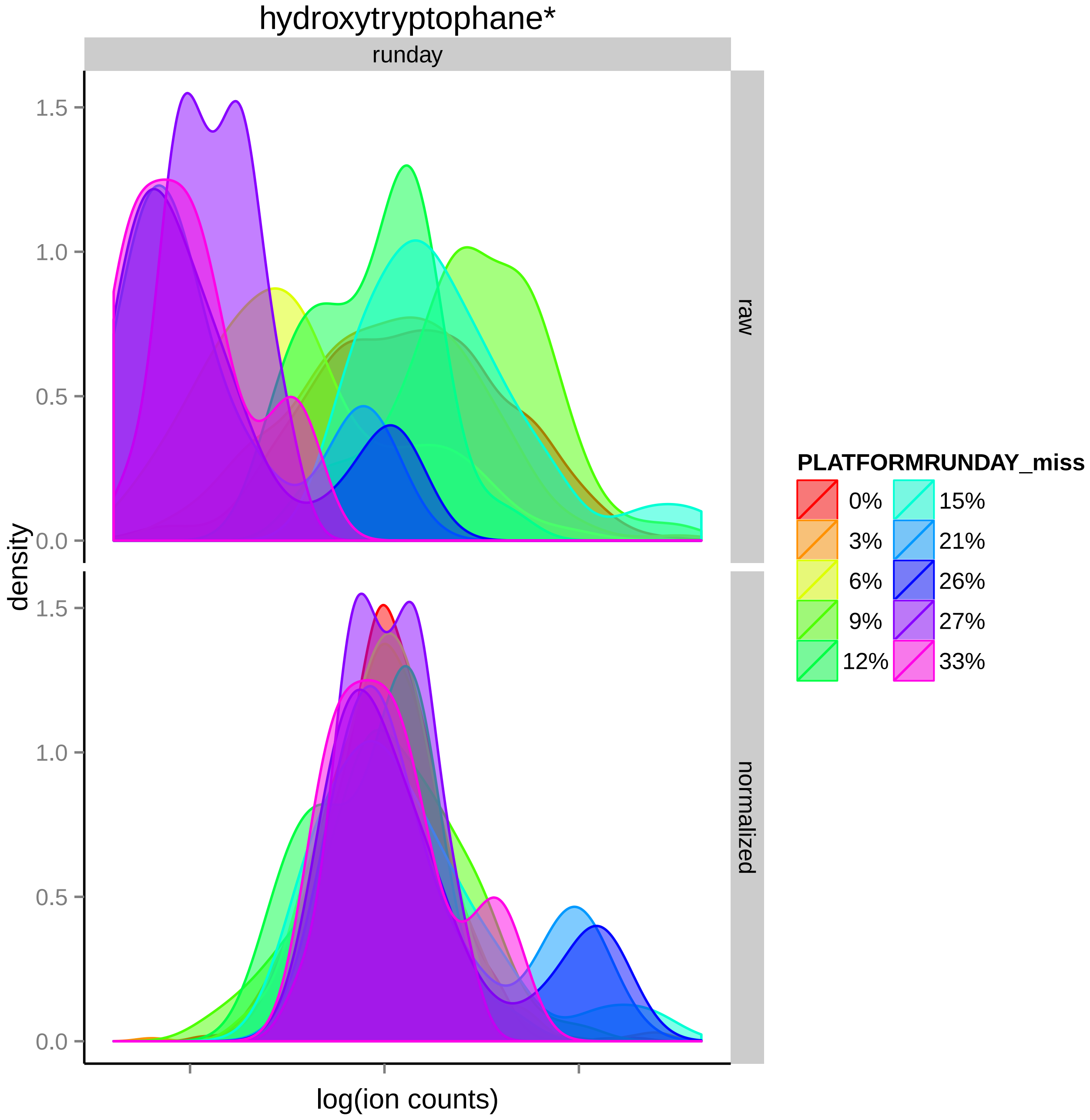

hydroxytryptophane*
runday
raw
normalized
density
1.5
1.0
0.5
0.0
log(ion counts)
PLATFORMRUNDAY_miss
0%
3%
6%
9%
12%
15%
21%
26%
27%
33%

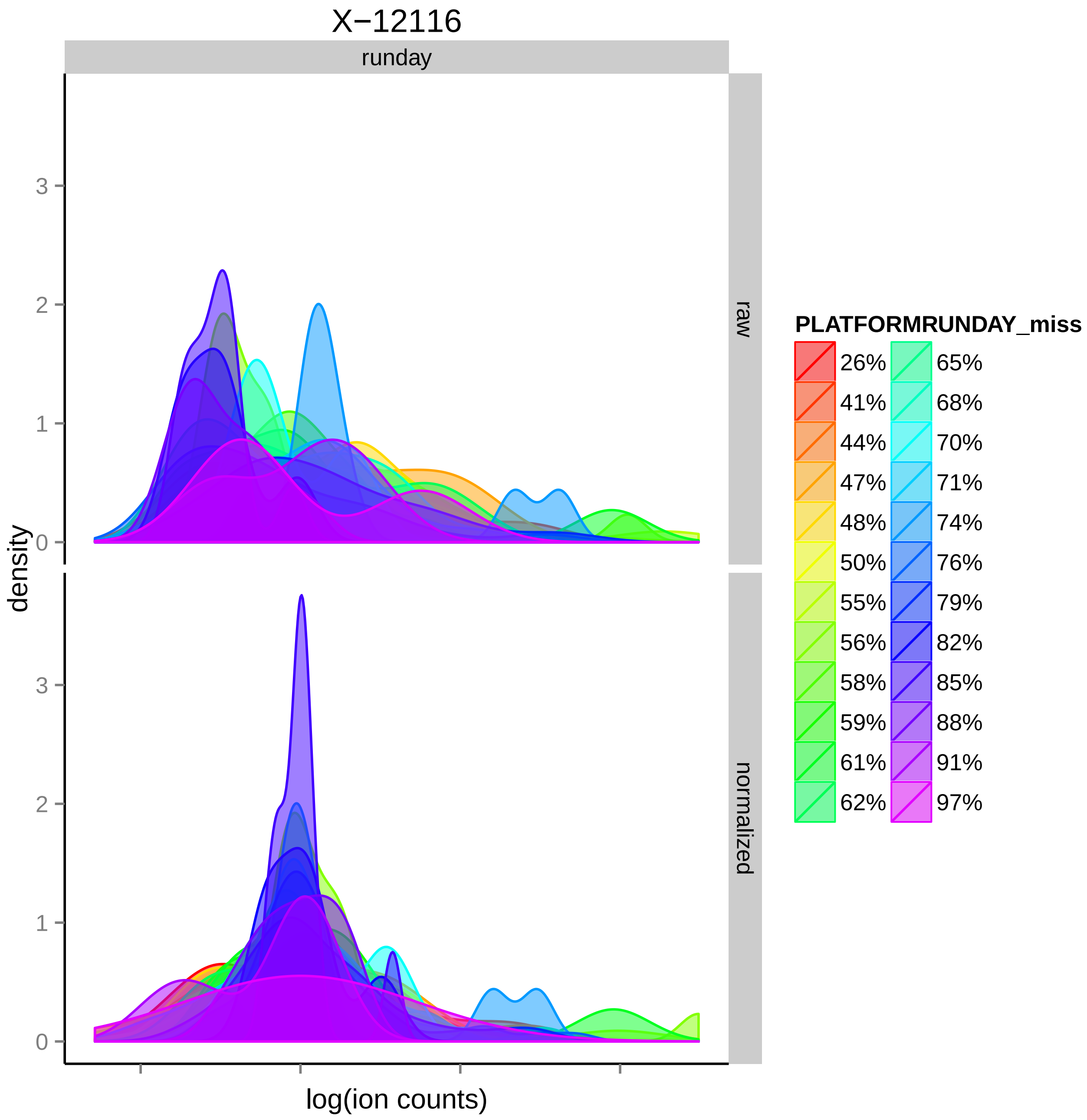
X−12116
runday
raw
normalized
density
log(ion counts)
0
1
2
3
PLATFORMRUNDAY_miss
26%
41%
44%
47%
48%
50%
55%
56%
58%
59%
61%
62%
65%
68%
70%
71%
74%
76%
79%
82%
85%
88%
91%
97%

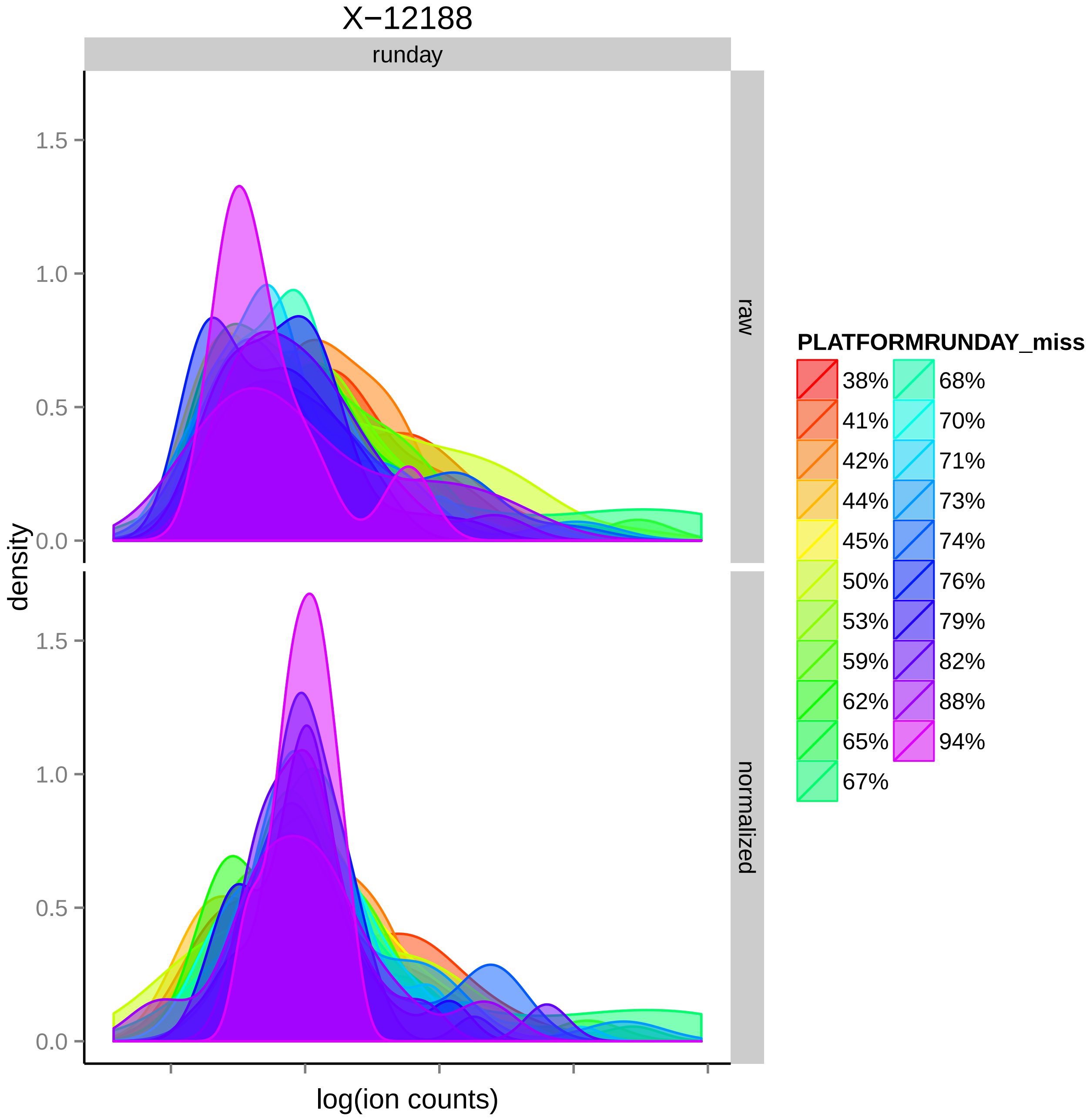

X−12188
runday
raw
normalized
density
log(ion counts)
1.5
1.0
0.5
0.0
PLATFORMRUNDAY_miss
38%
41%
42%
44%
45%
50%
53%
59%
62%
65%
67%
68%
70%
71%
73%
74%
76%
79%
82%
88%
94%

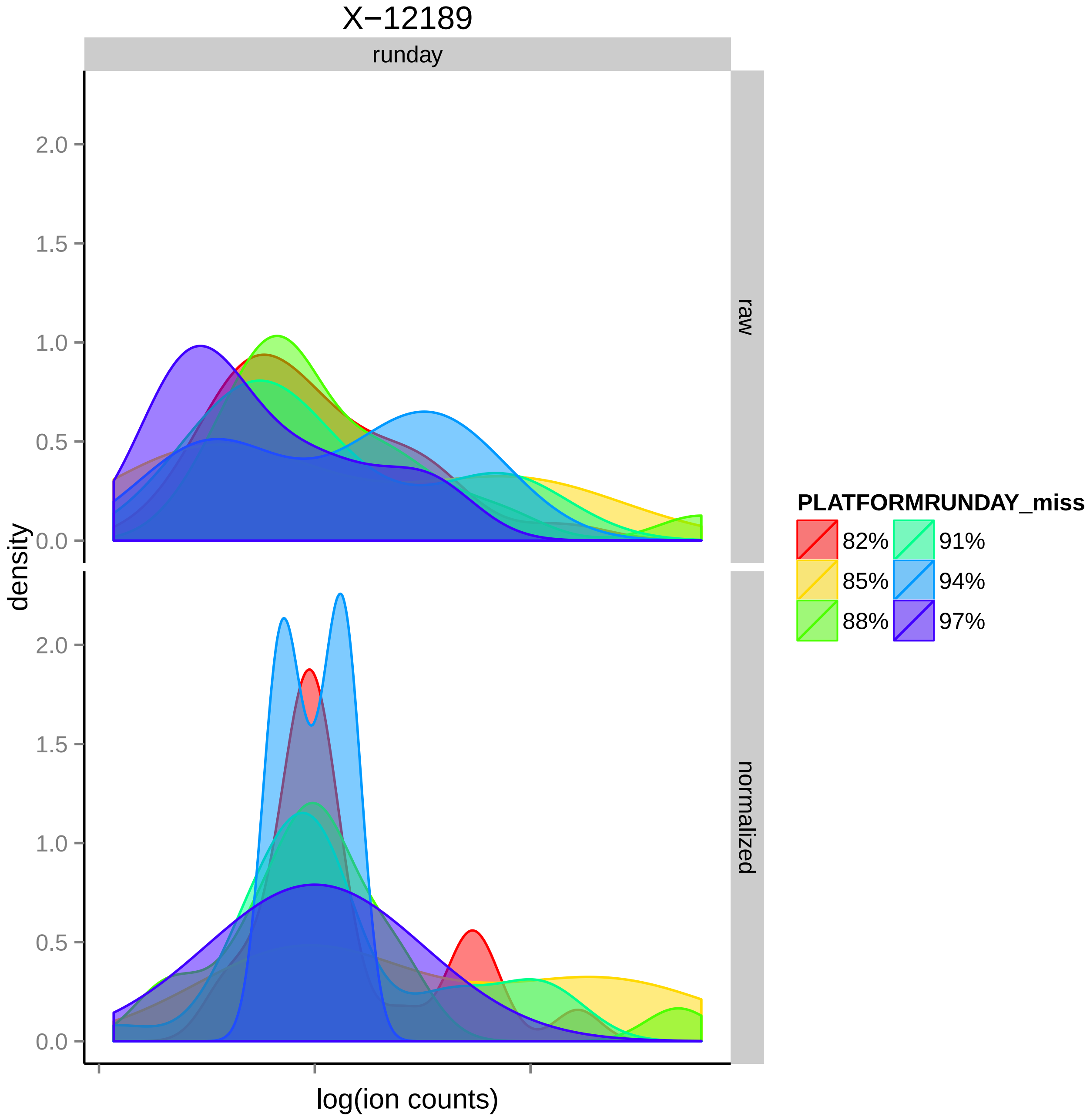

X−12189
runday
raw
normalized
density
2.0
1.5
1.0
0.5
0.0
log(ion counts)
PLATFORMRUNDAY_miss
82%
85%
88%
91%
94%
97%

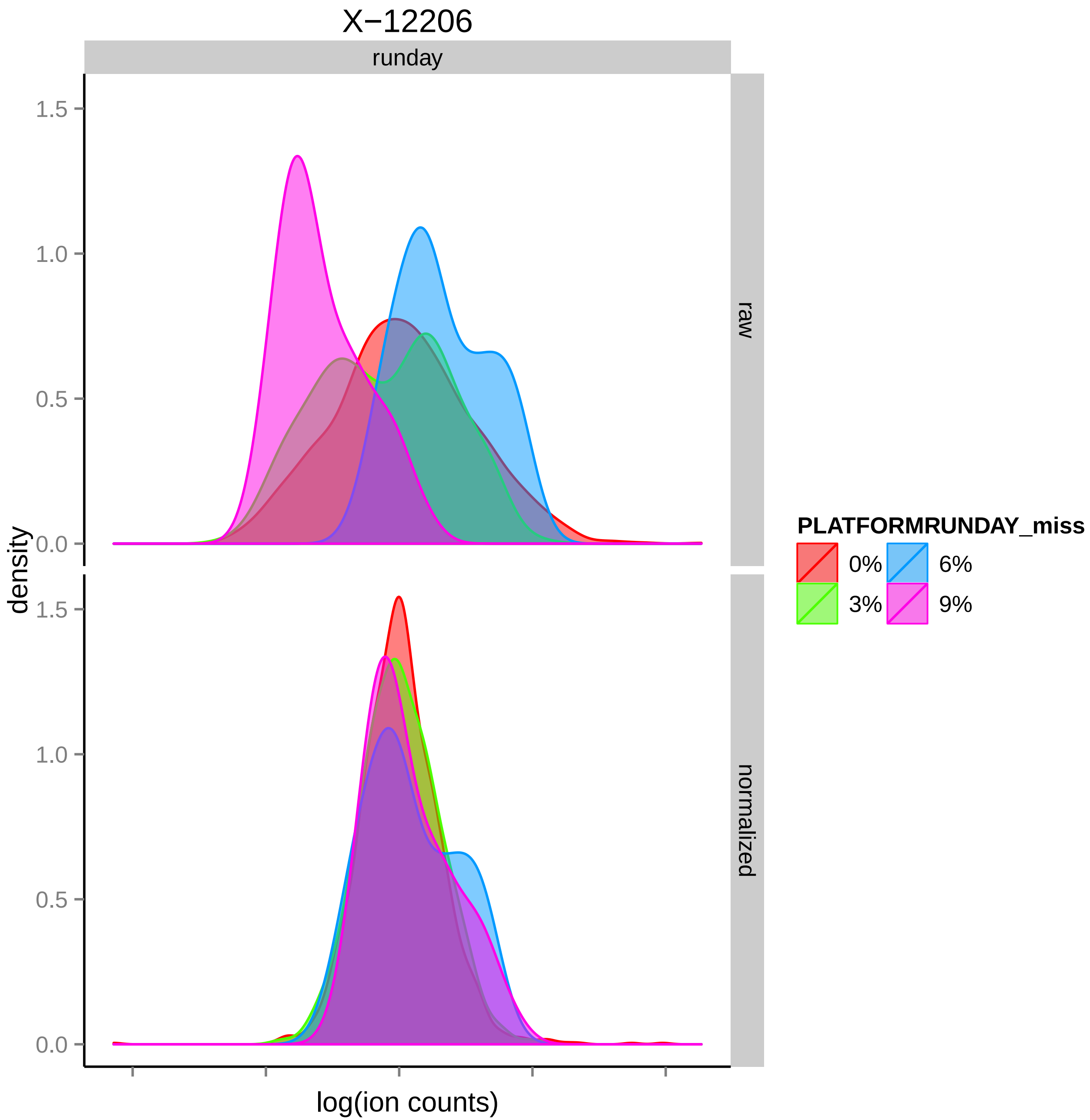

X−12206
runday
raw
normalized
density
1.5
1.0
0.5
0.0
1.5
1.0
0.5
0.0
log(ion counts)
PLATFORMRUNDAY_miss
0%
6%
3%
9%

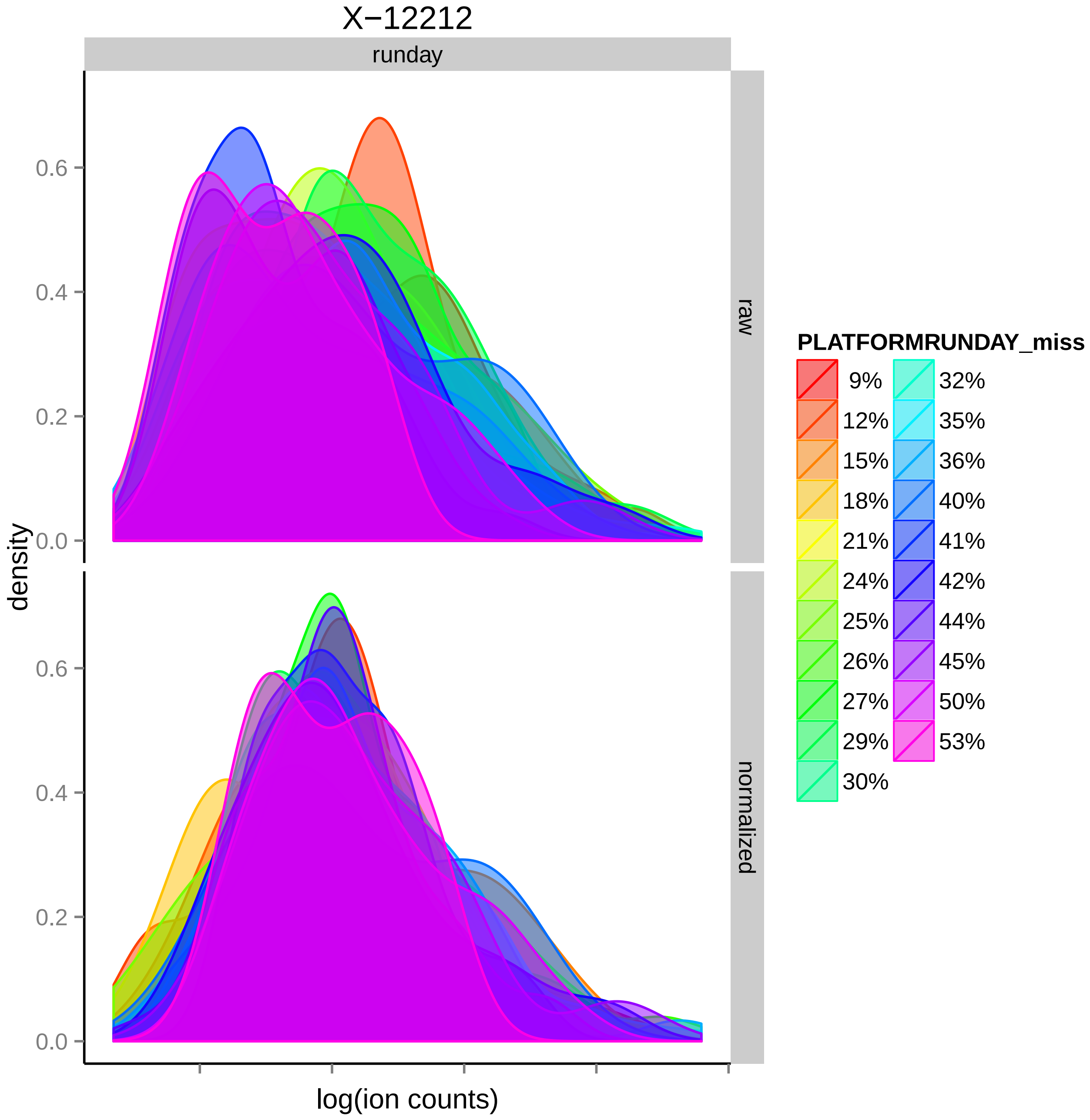

X−12212
runday
raw
normalized
density
0.0
0.2
0.4
0.6
log(ion counts)
PLATFORMRUNDAY_miss
9%
12%
15%
18%
21%
24%
25%
26%
27%
29%
30%
32%
35%
36%
40%
41%
42%
44%
45%
50%
53%

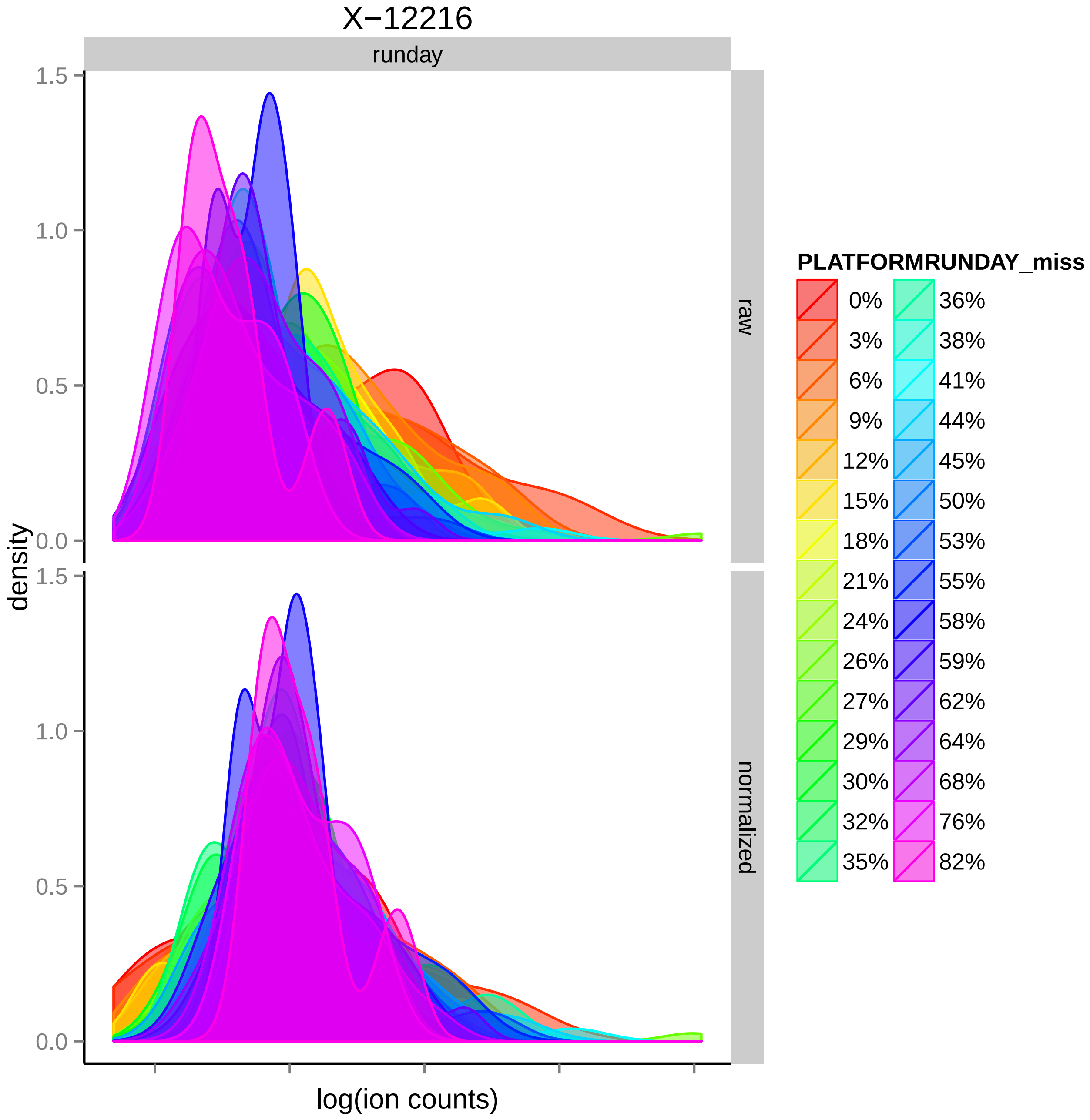

X−12216
runday
raw
normalized
density
log(ion counts)
1.5
1.0
0.5
0.0
PLATFORMRUNDAY_miss
0%
3%
6%
9%
12%
15%
18%
21%
24%
26%
27%
29%
30%
32%
35%
36%
38%
41%
44%
45%
50%
53%
55%
58%
59%
62%
64%
68%
76%
82%

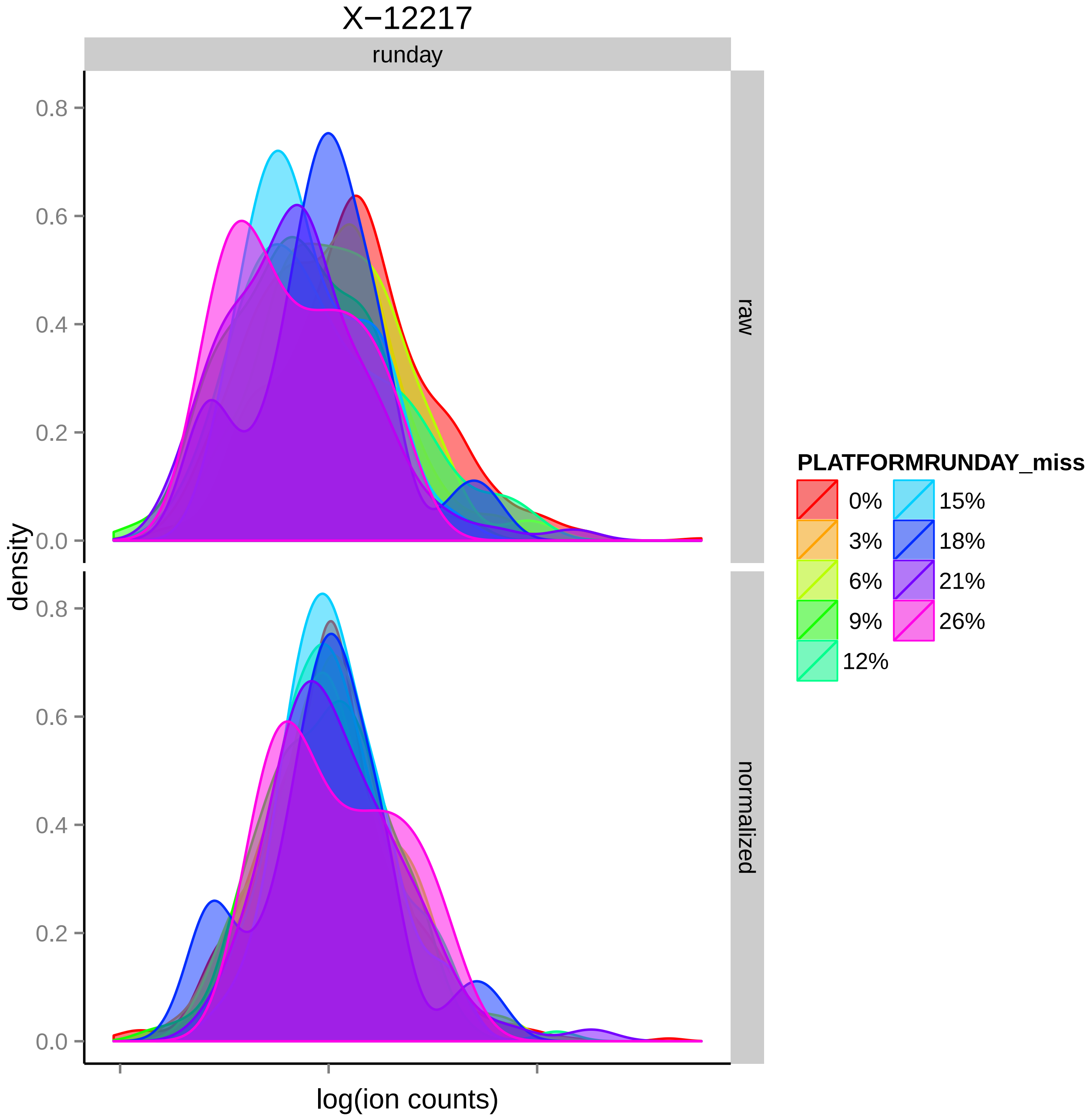

X−12217
runday
raw
normalized
density
log(ion counts)
0.0
0.2
0.4
0.6
0.8
0.0
0.2
0.4
0.6
0.8
PLATFORMRUNDAY_miss
0%
3%
6%
9%
12%
15%
18%
21%
26%

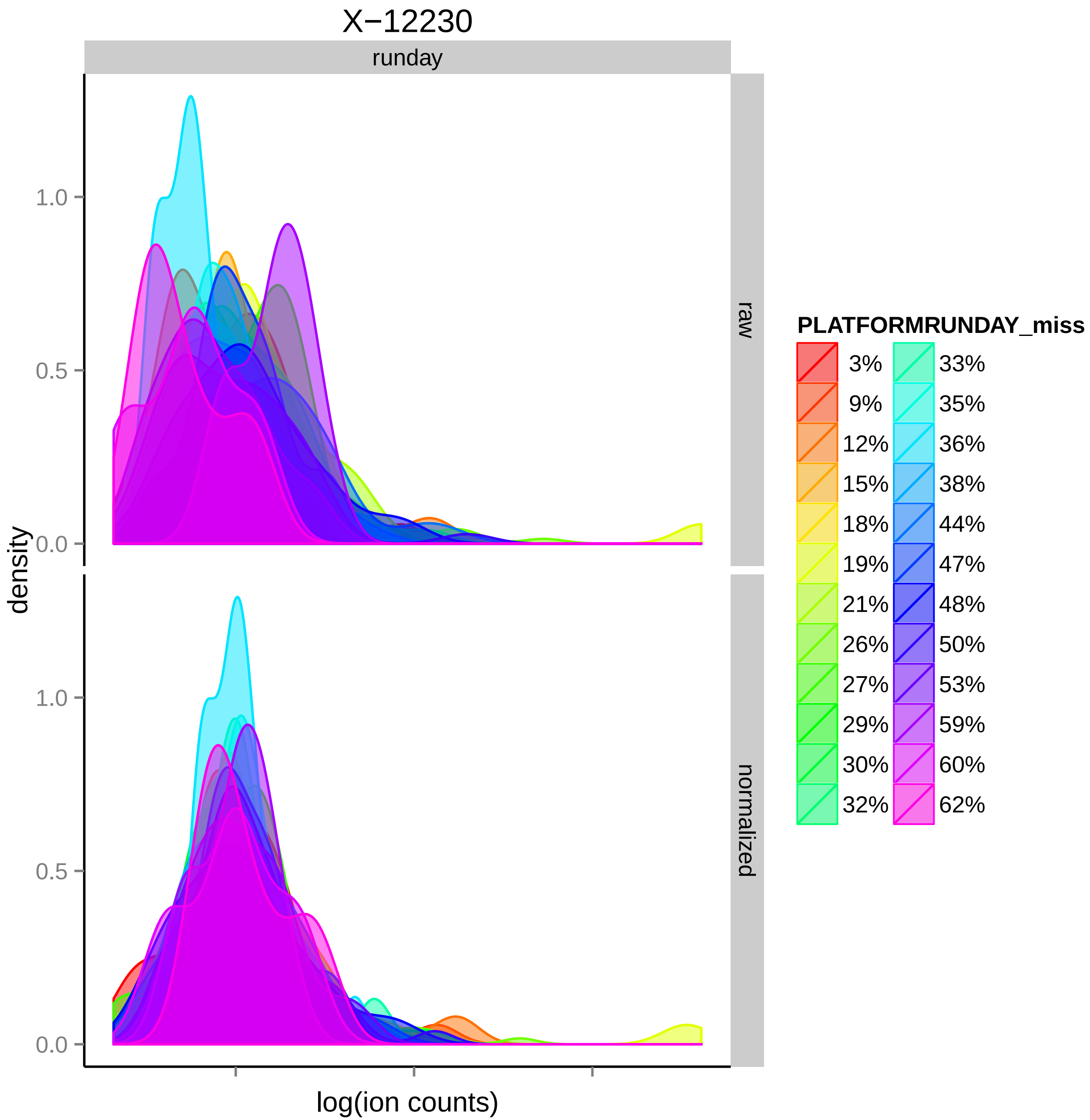
X−12230
runday
raw
normalized
density
log(ion counts)
1.0
0.5
0.0
1.0
0.5
0.0
PLATFORMRUNDAY_miss
3%
9%
12%
15%
18%
19%
21%
26%
27%
29%
30%
32%
33%
35%
36%
38%
44%
47%
48%
50%
53%
59%
60%
62%

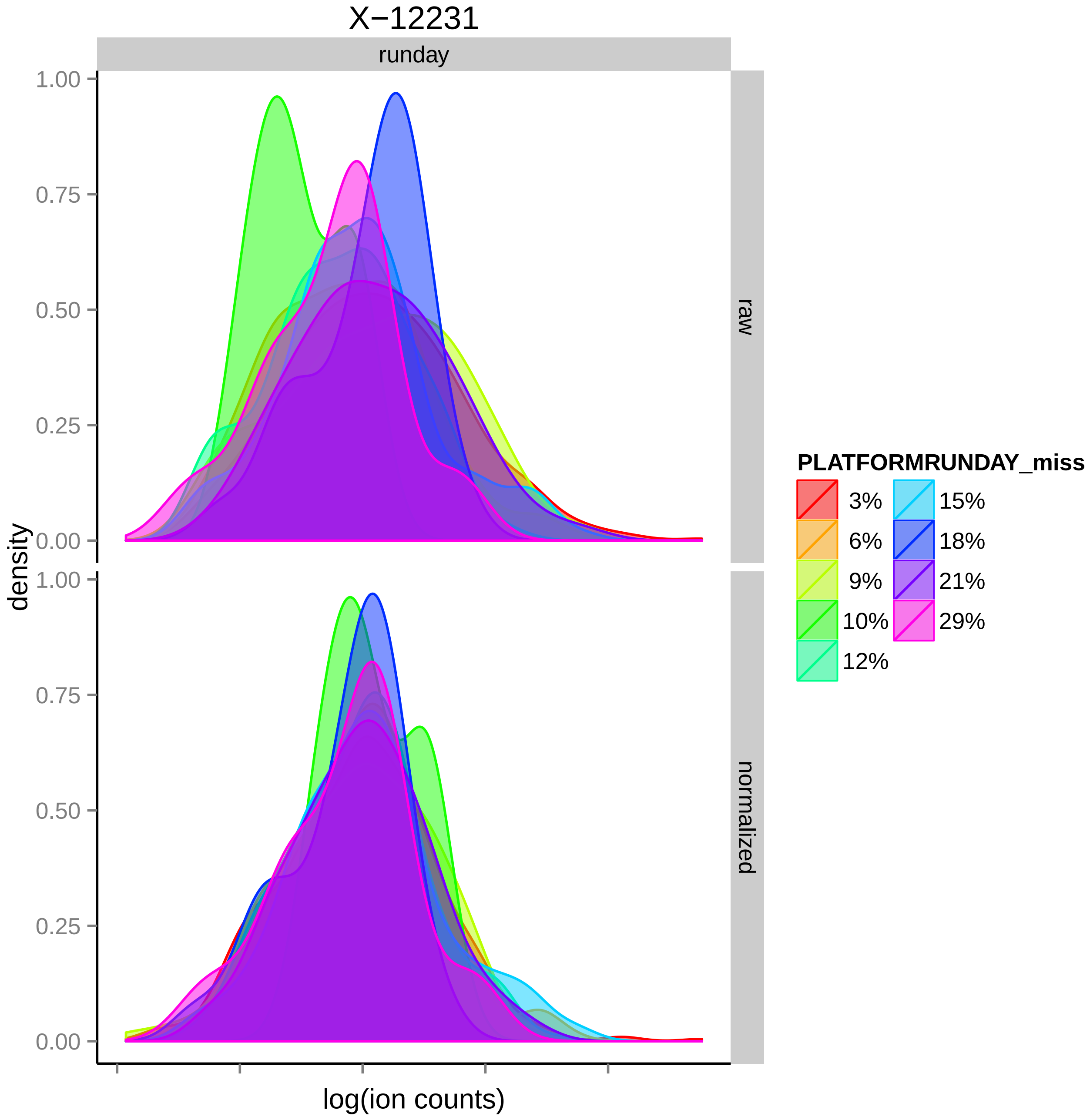

X−12231
runday
raw
normalized
density
log(ion counts)
1.00
0.75
0.50
0.25
0.00
PLATFORMRUNDAY_miss
3%
6%
9%
10%
12%
15%
18%
21%
29%

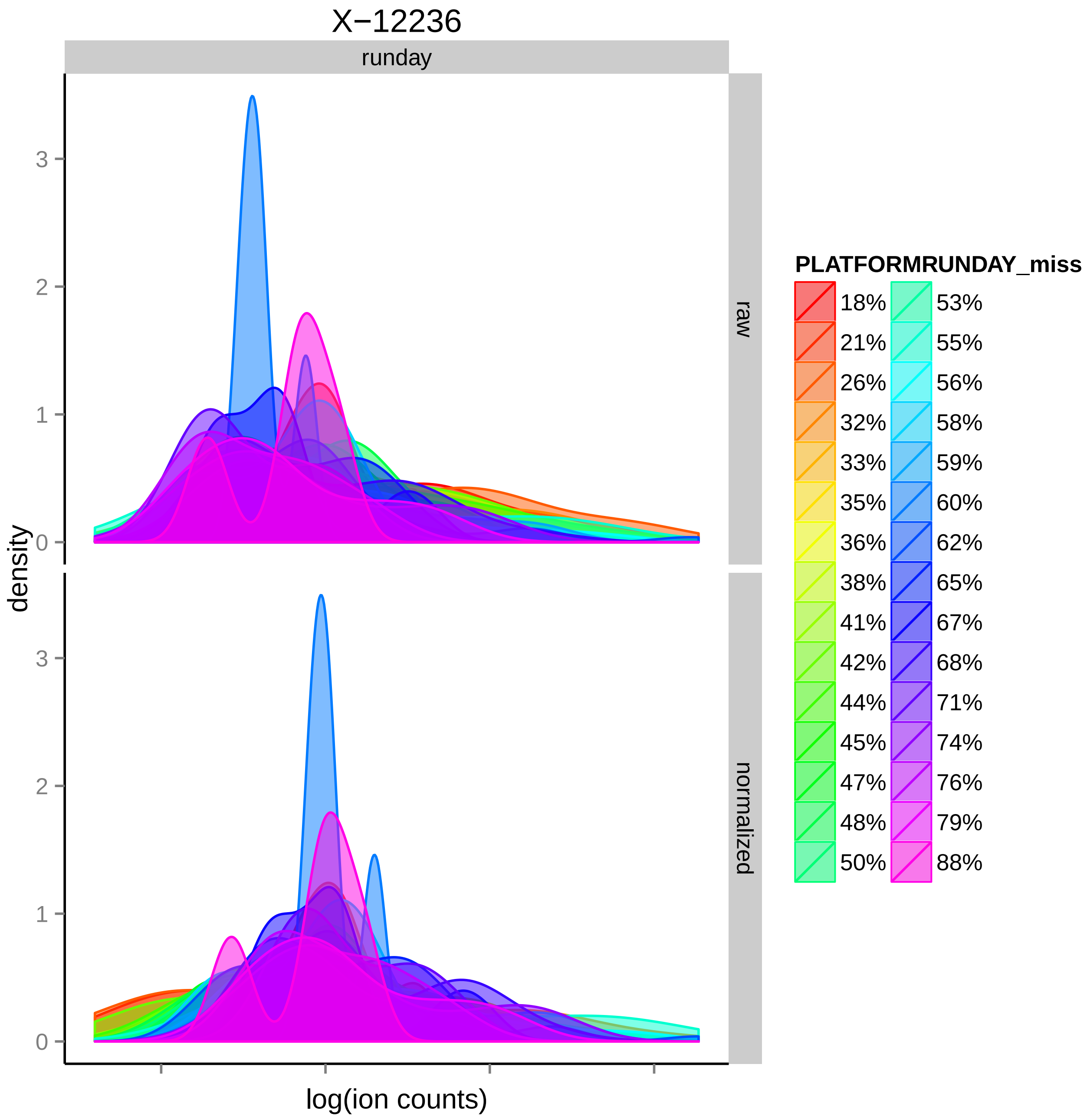
X−12236
runday
raw
normalized
density
log(ion counts)
0
1
2
3
0
1
2
3
PLATFORMRUNDAY_miss
18%
21%
26%
32%
33%
35%
36%
38%
41%
42%
44%
45%
47%
48%
50%
53%
55%
56%
58%
59%
60%
62%
65%
67%
68%
71%
74%
76%
79%
88%

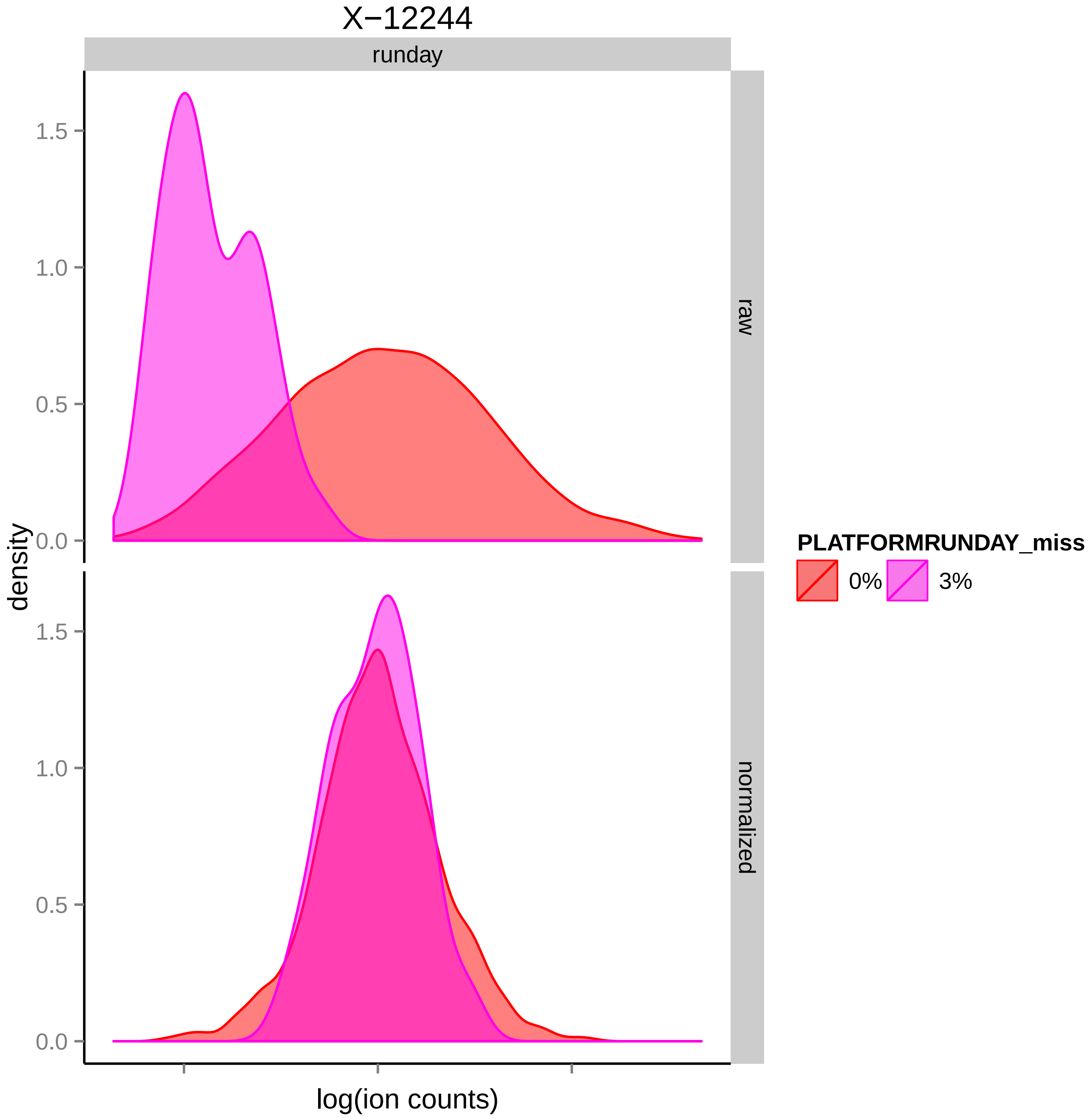
X−12244
runday
raw
normalized
density
log(ion counts)
1.5
1.0
0.5
0.0
PLATFORMRUNDAY_miss
0%
3%

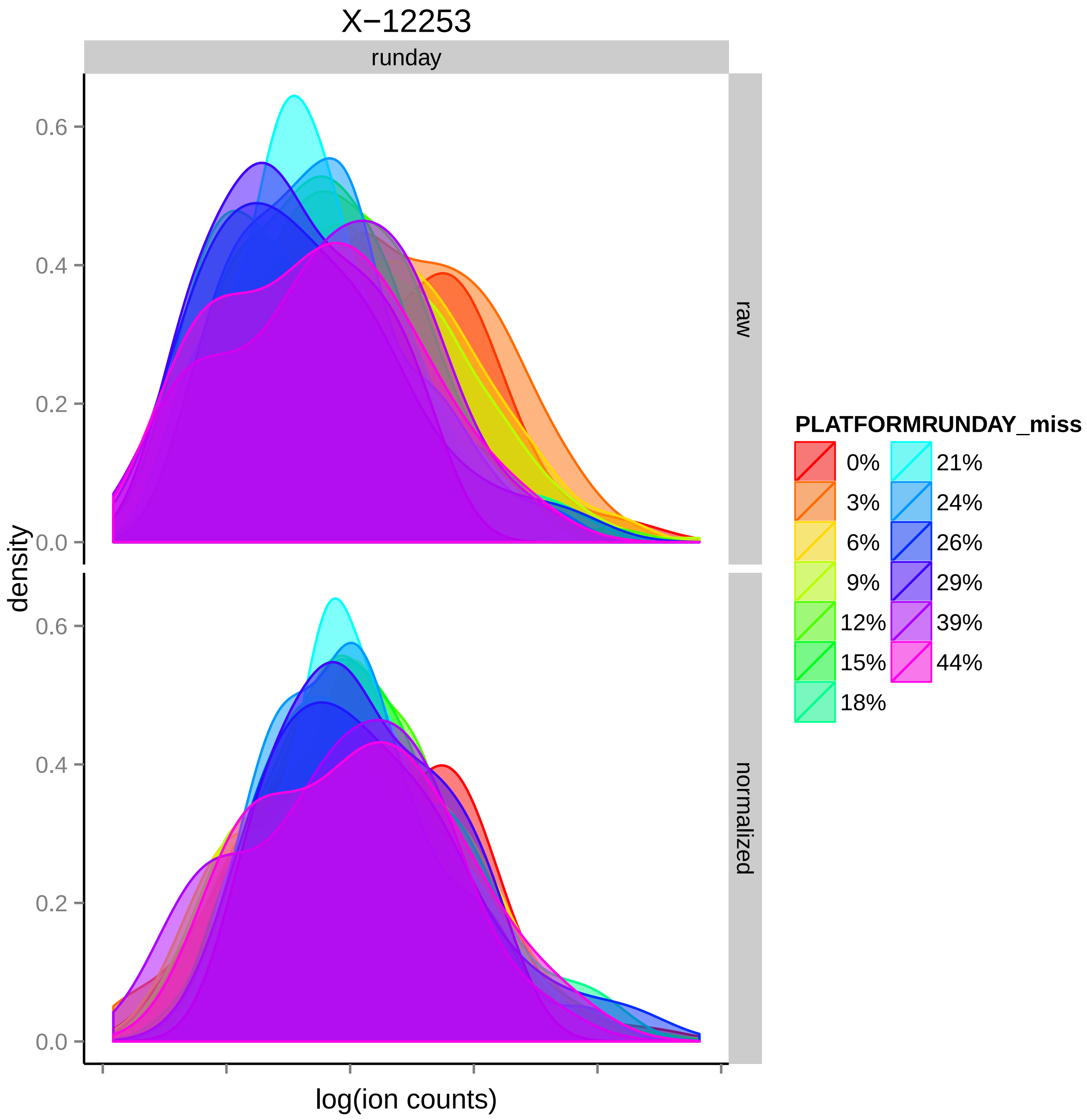

X−12253
runday
raw
normalized
density
0.0
0.2
0.4
0.6
log(ion counts)
PLATFORMRUNDAY_miss
0%
3%
6%
9%
12%
15%
18%
21%
24%
26%
29%
39%
44%

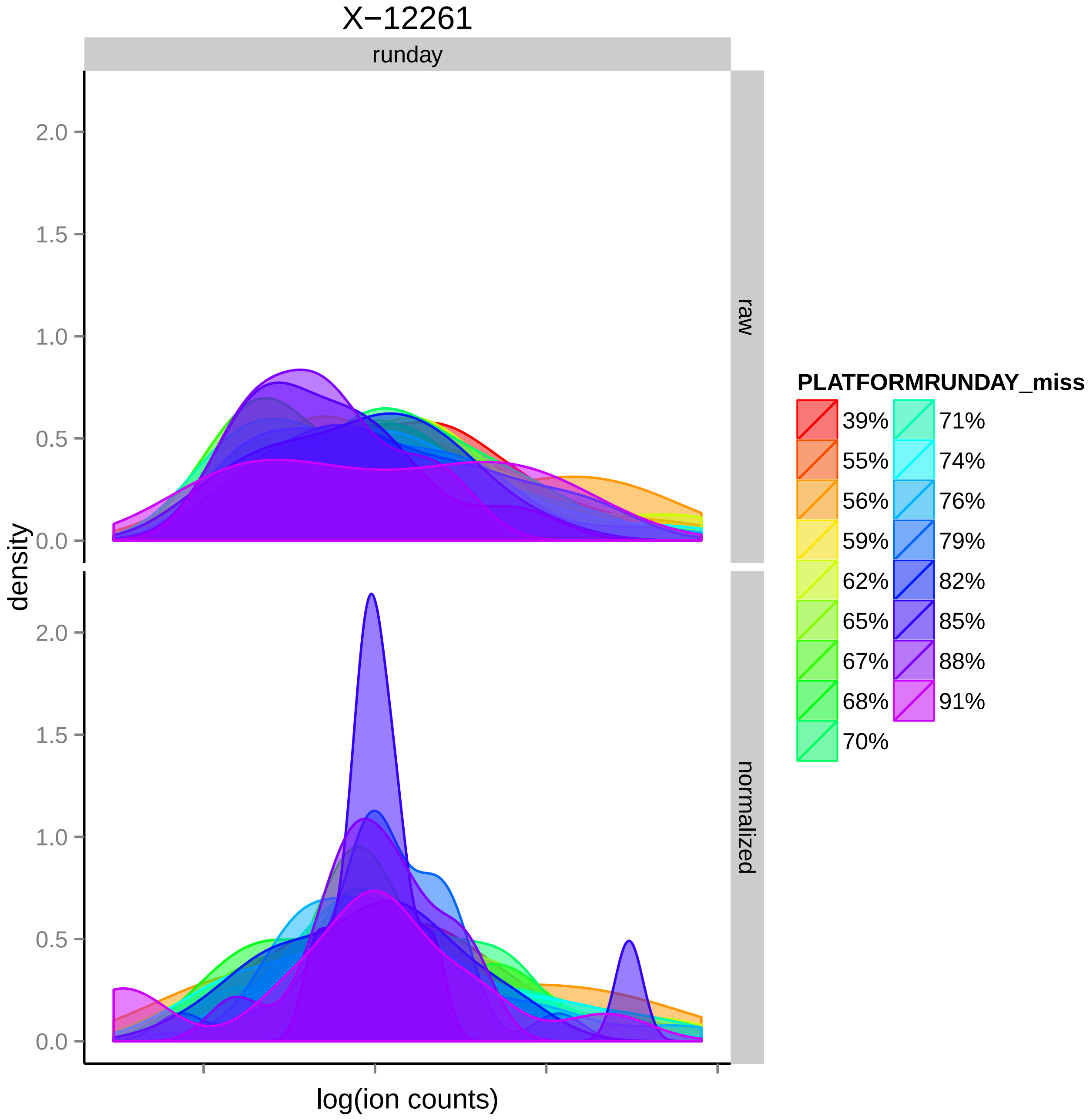
X−12261
runday
raw
normalized
density
log(ion counts)
2.0
1.5
1.0
0.5
0.0
PLATFORMRUNDAY_miss
39%
55%
56%
59%
62%
65%
67%
68%
70%
71%
74%
76%
79%
82%
85%
88%
91%

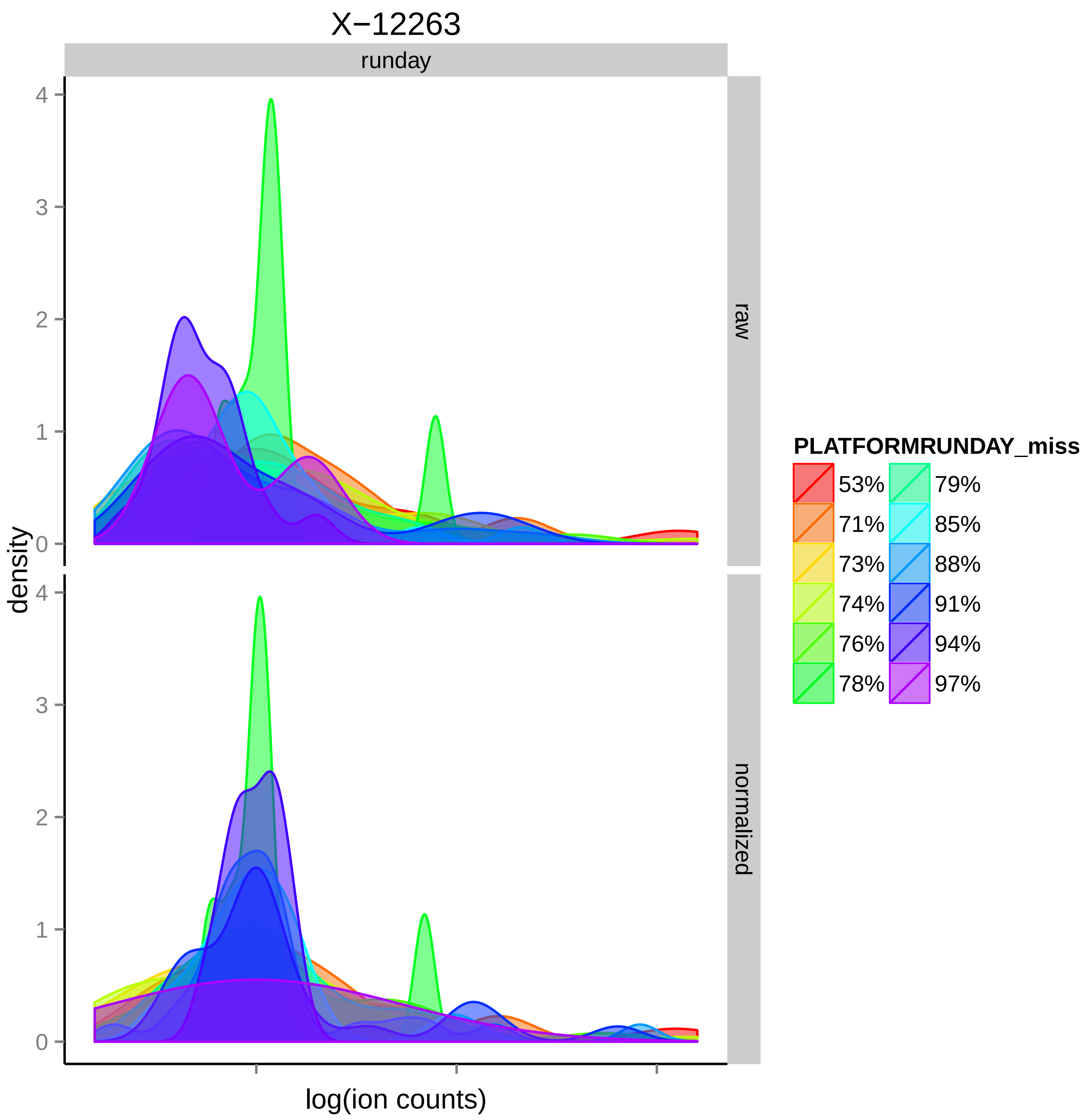
X−12263
runday
raw
normalized
density
log(ion counts)
0
1
2
3
4
PLATFORMRUNDAY_miss
53%
71%
73%
74%
76%
78%
79%
85%
88%
91%
94%
97%

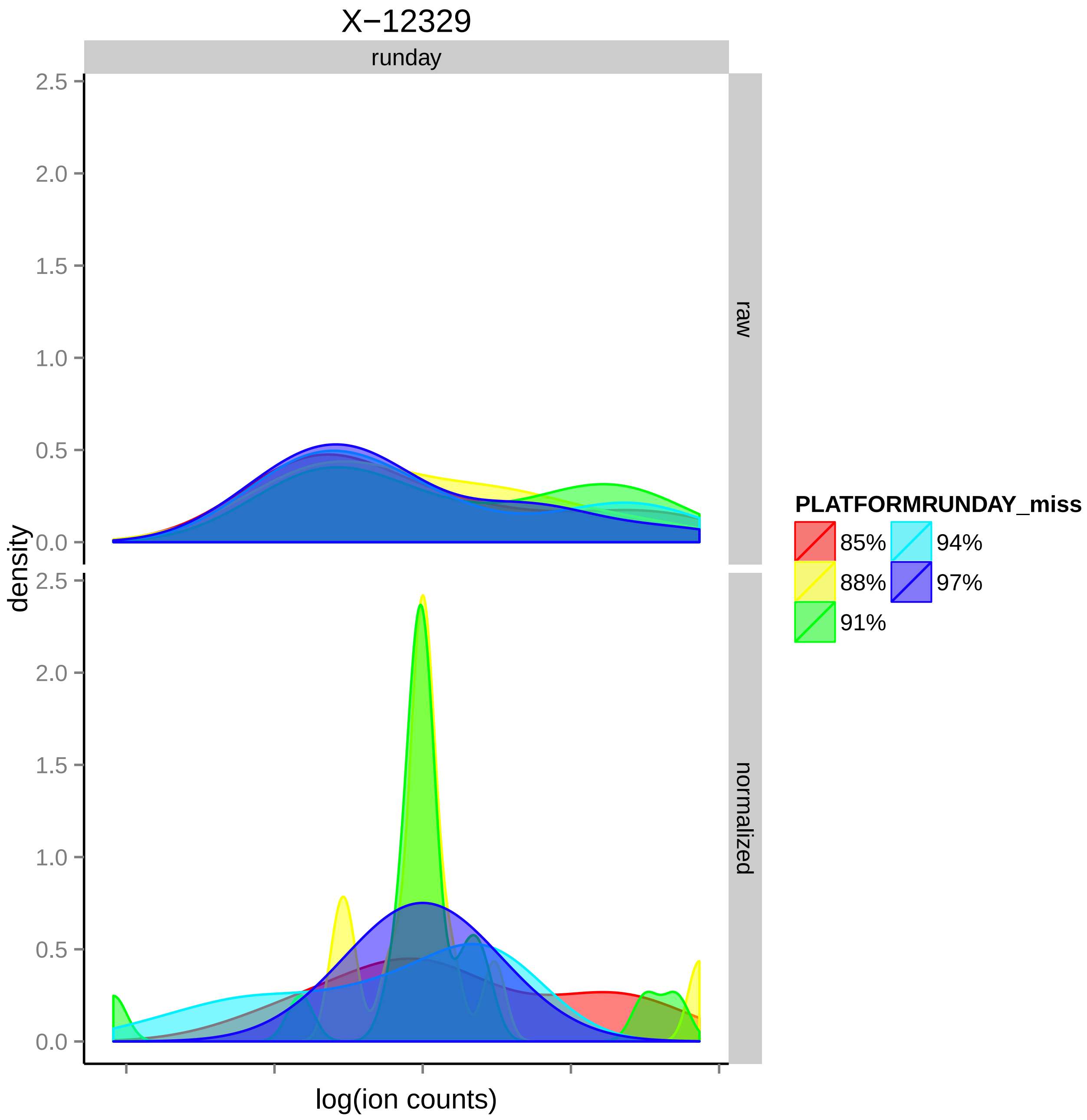
X−12329
runday
raw
normalized
density
log(ion counts)
2.5
2.0
1.5
1.0
0.5
0.0
PLATFORMRUNDAY_miss
85%
88%
91%
94%
97%

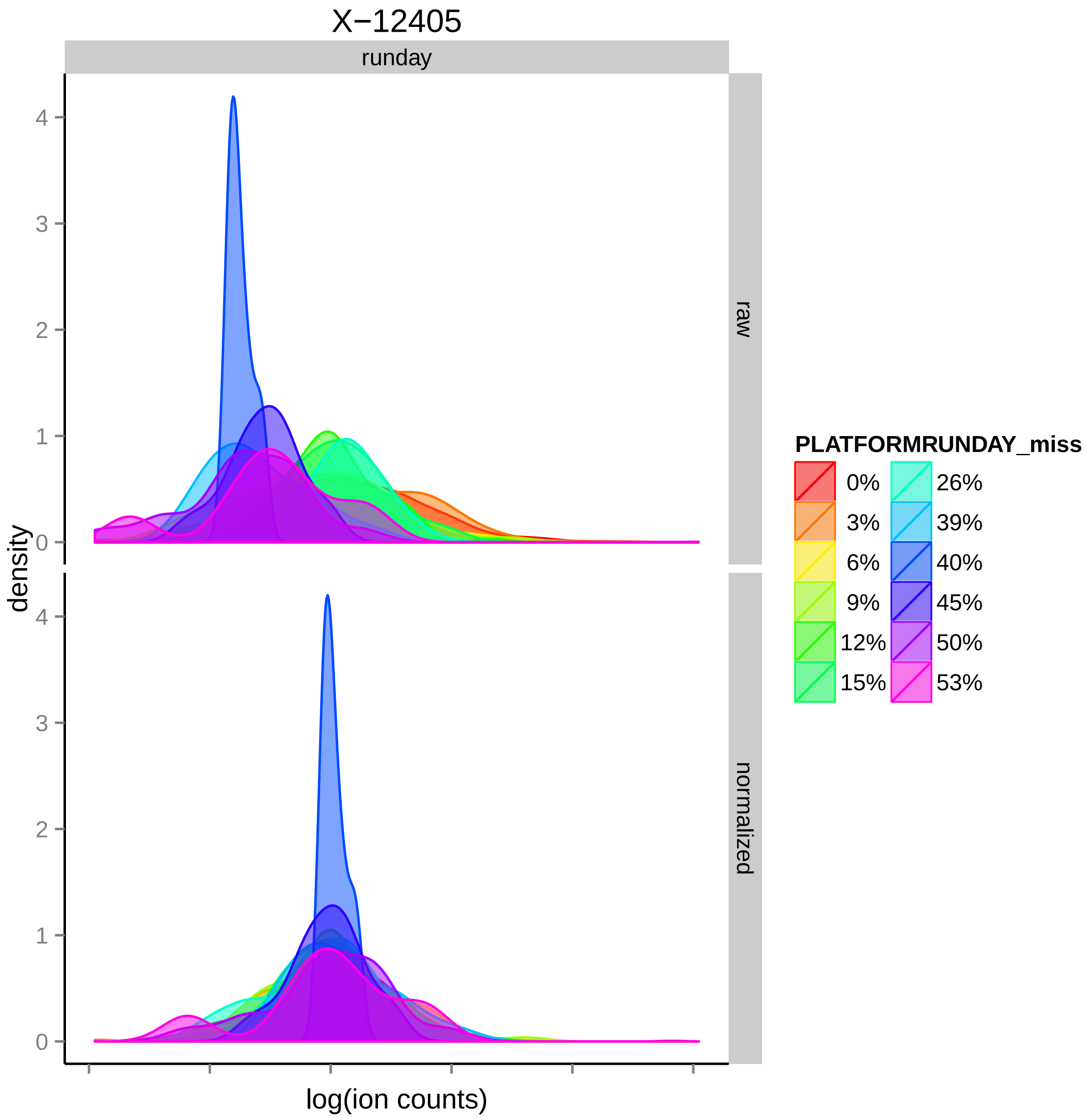
X−12405
runday
raw
normalized
density
log(ion counts)
0
1
2
3
4
0
1
2
3
4
PLATFORMRUNDAY_miss
0%
3%
6%
9%
12%
15%
26%
39%
40%
45%
50%
53%

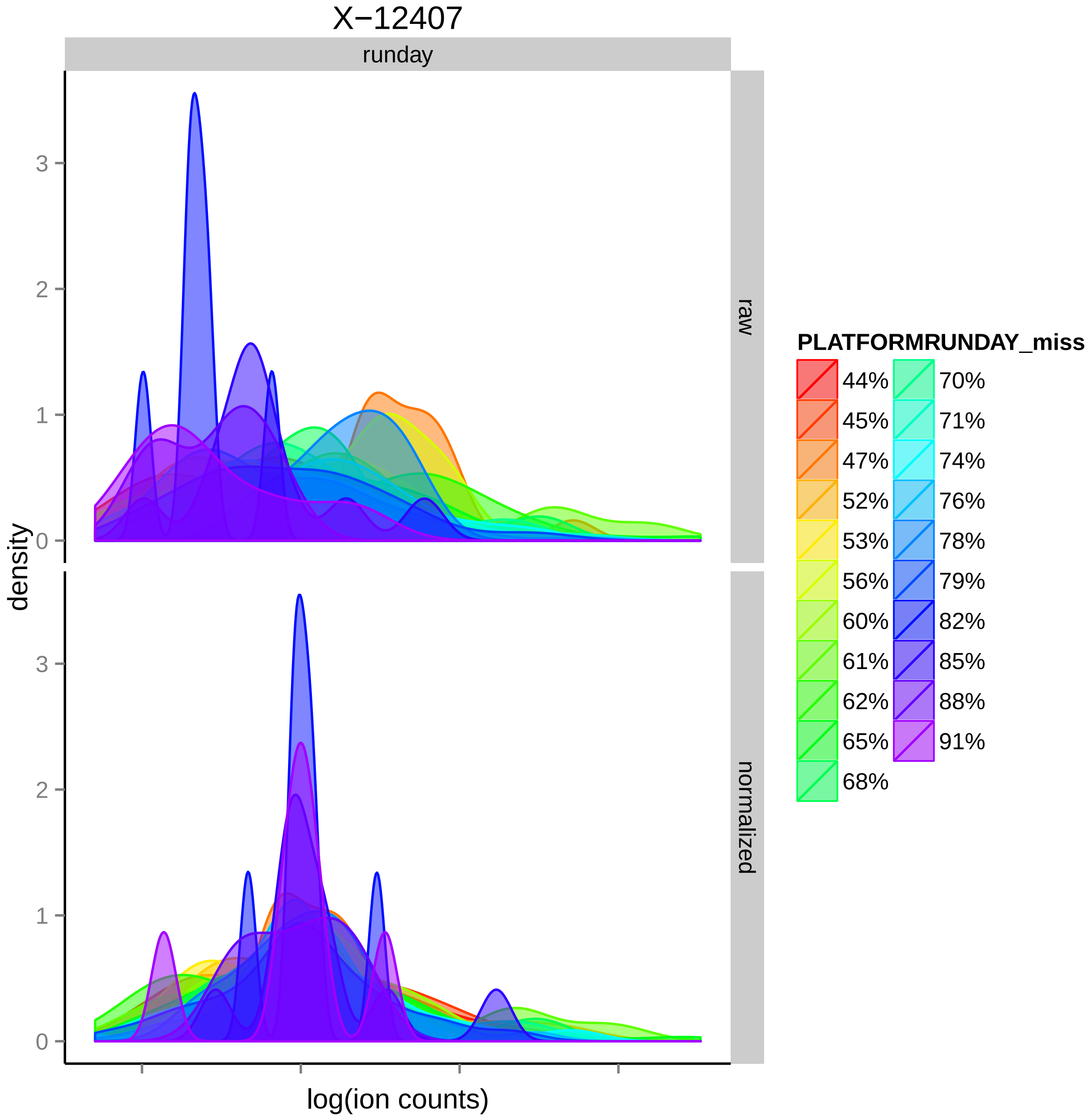

X−12407
runday
raw
normalized
density
log(ion counts)
0
1
2
3
PLATFORMRUNDAY_miss
44%
45%
47%
52%
53%
56%
60%
61%
62%
65%
68%
70%
71%
74%
76%
78%
79%
82%
85%
88%
91%

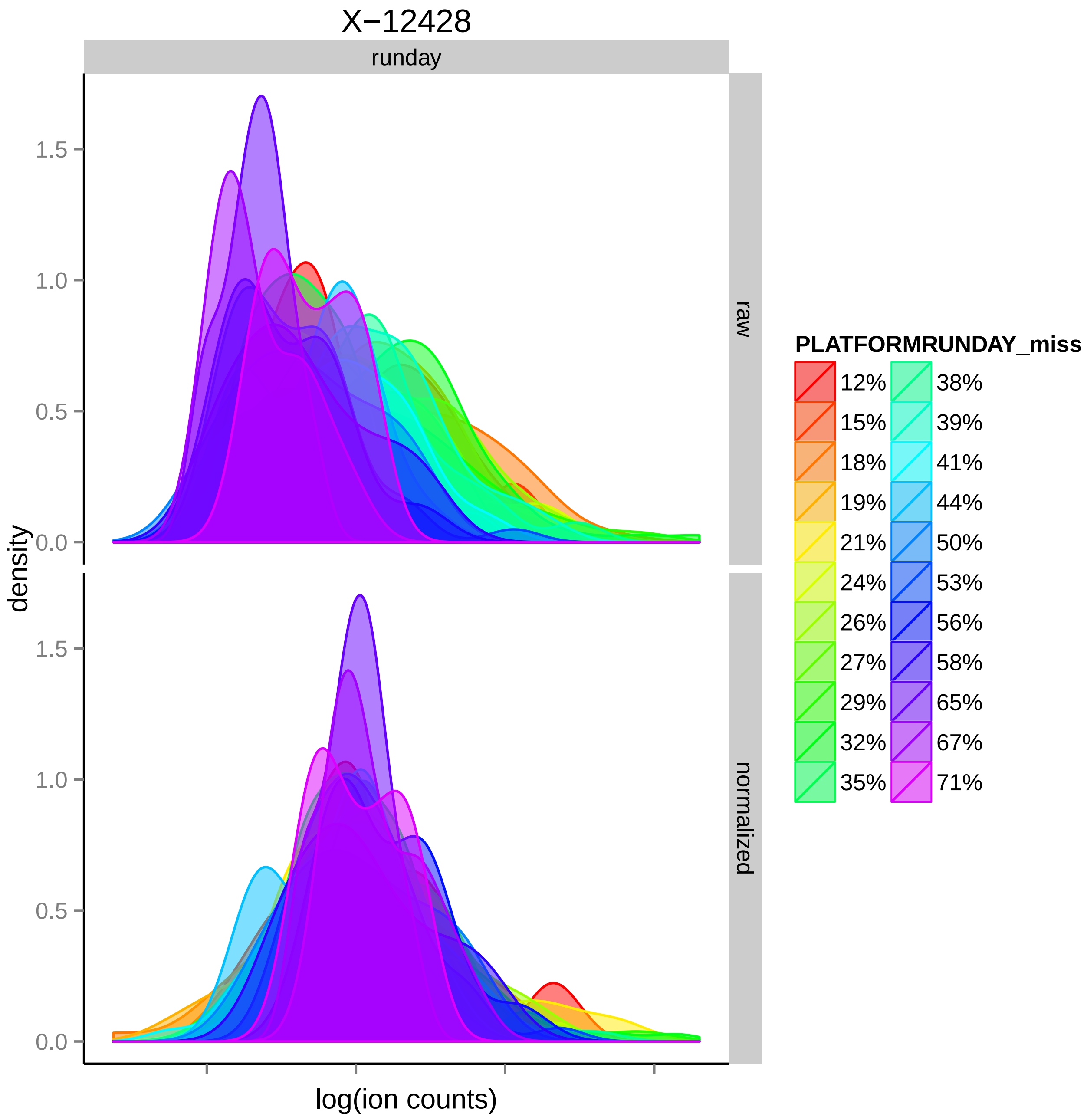

X−12428
runday
raw
normalized
density
1.5
1.0
0.5
0.0
1.5
1.0
0.5
0.0
log(ion counts)
PLATFORMRUNDAY_miss
12%
15%
18%
19%
21%
24%
26%
27%
29%
32%
35%
38%
39%
41%
44%
50%
53%
56%
58%
65%
67%
71%

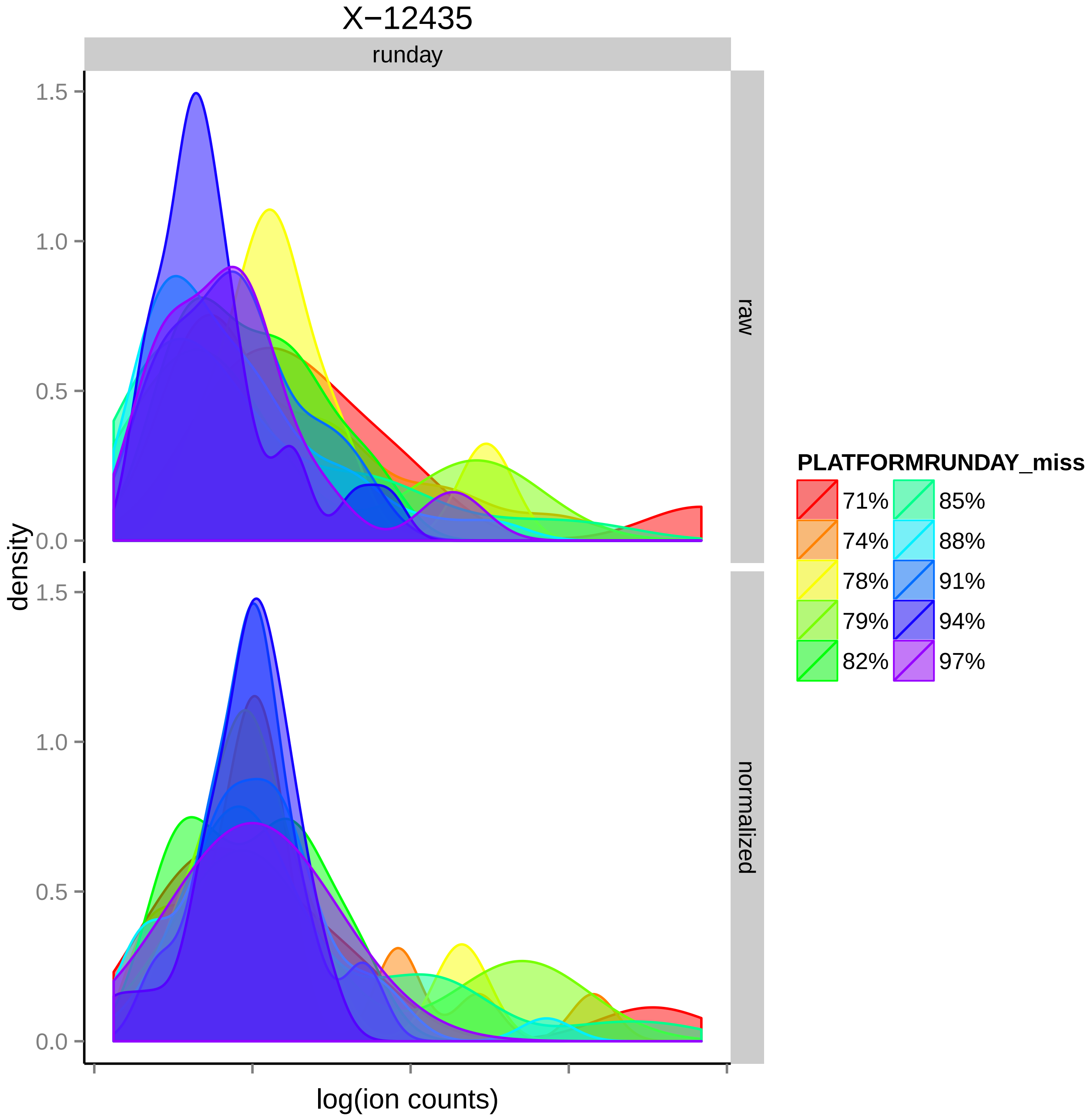
X−12435
runday
raw
normalized
density
log(ion counts)
1.5
1.0
0.5
0.0
PLATFORMRUNDAY_miss
71%
74%
78%
79%
82%
85%
88%
91%
94%
97%

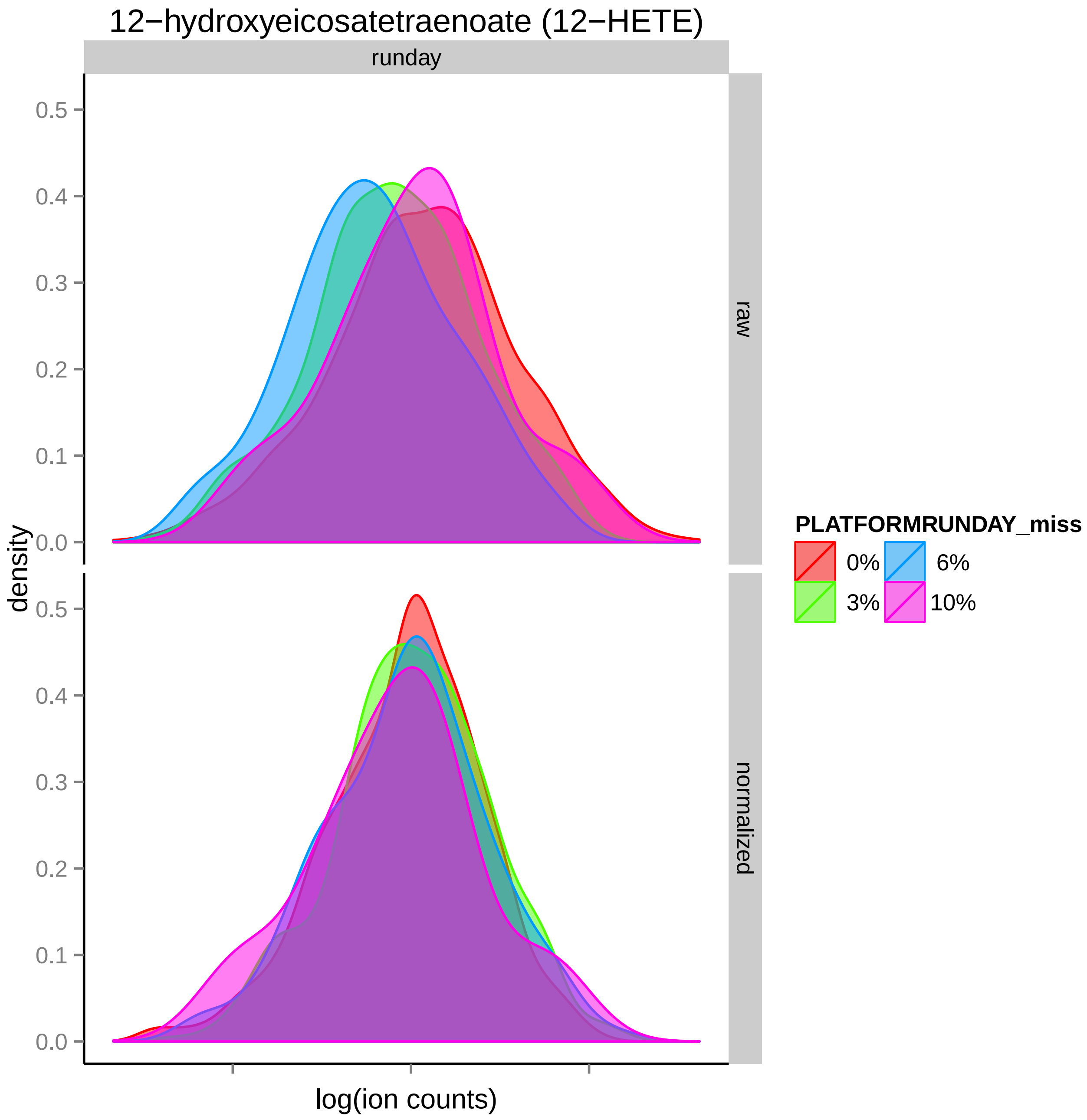

12−hydroxyeicosatetraenoate (12−HETE)
runday
raw
normalized
density
log(ion counts)
0.0
0.1
0.2
0.3
0.4
0.5
PLATFORMRUNDAY_miss
0%
3%
6%
10%

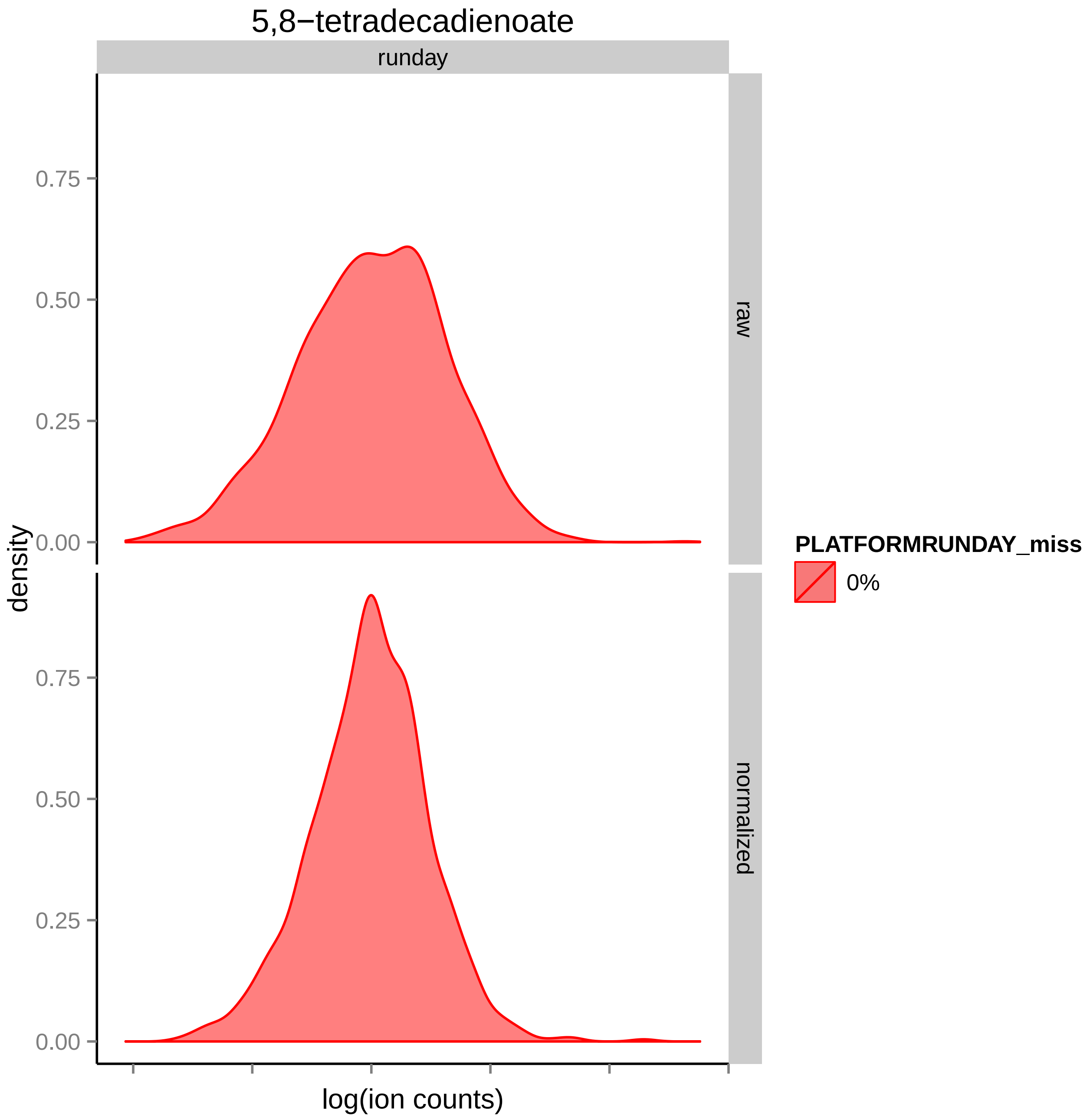
5,8−tetradecadienoate
runday
raw
normalized
density
0.00
0.25
0.50
0.75
log(ion counts)
PLATFORMRUNDAY_miss
0%

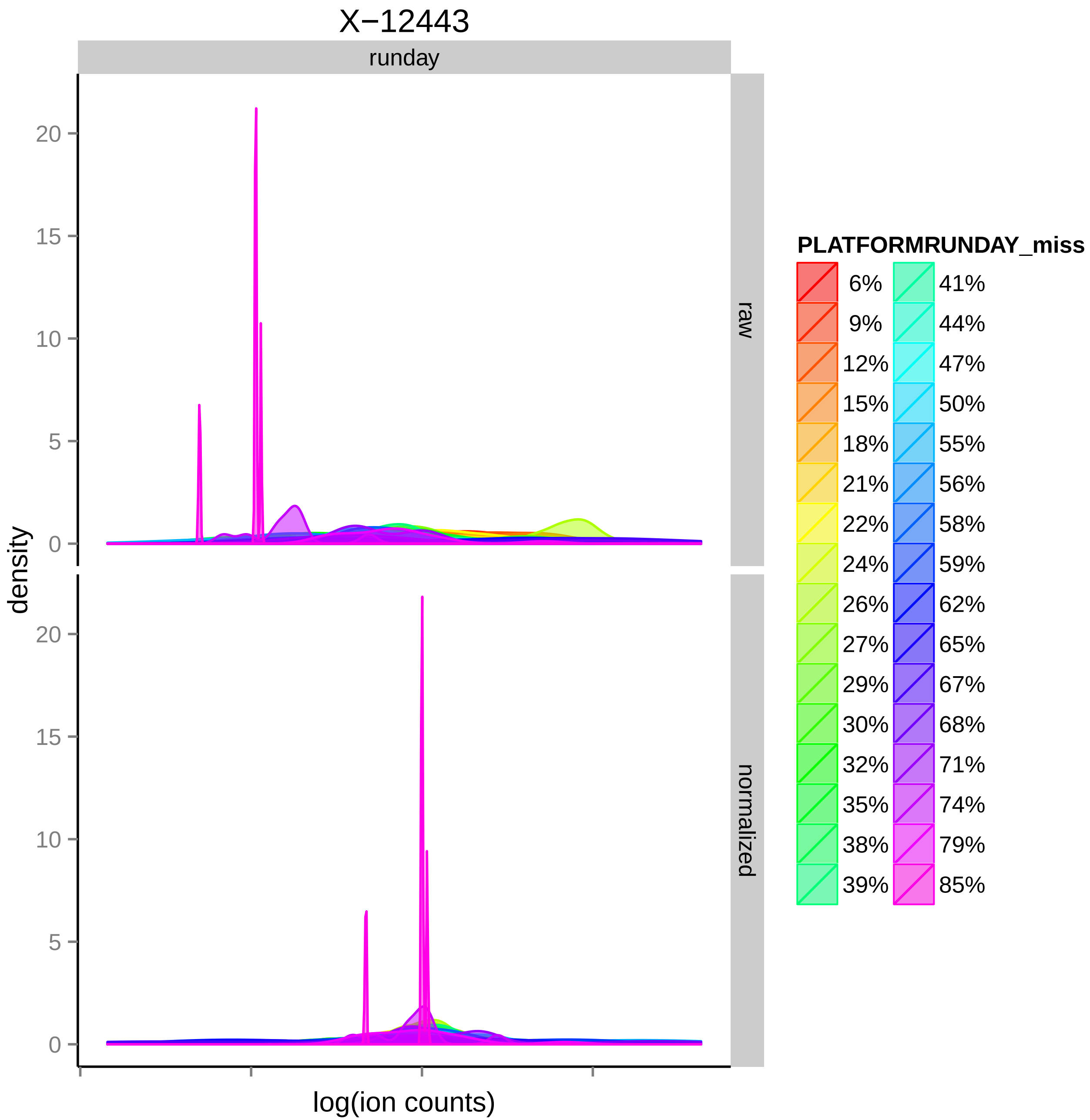

X−12443
runday
raw
normalized
density
log(ion counts)
0
5
10
15
20
PLATFORMRUNDAY_miss
6%
9%
12%
15%
18%
21%
22%
24%
26%
27%
29%
30%
32%
35%
38%
39%
41%
44%
47%
50%
55%
56%
58%
59%
62%
65%
67%
68%
71%
74%
79%
85%

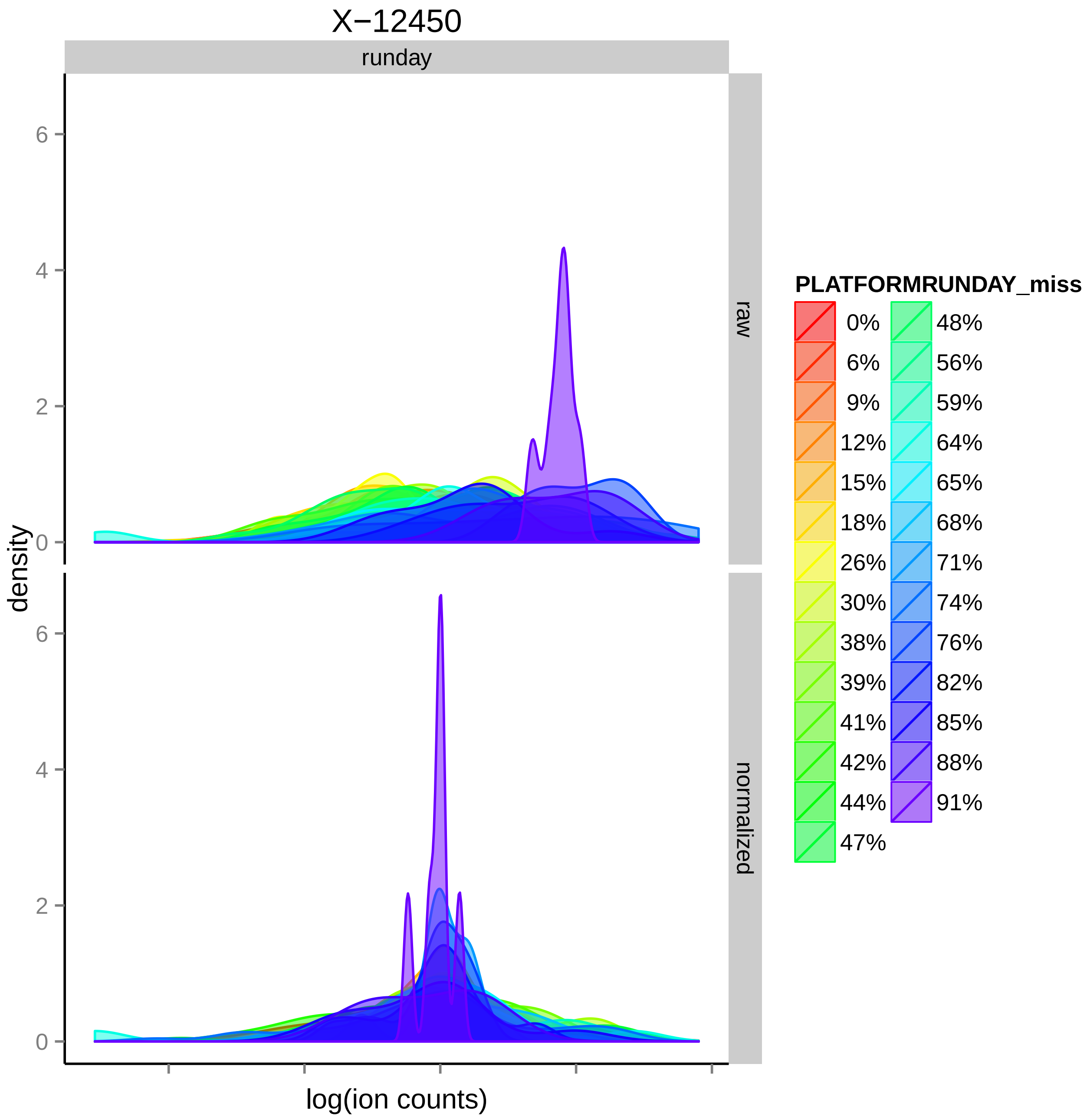
X−12450
runday
raw
normalized
density
log(ion counts)
0
2
4
6
PLATFORMRUNDAY_miss
0%
6%
9%
12%
15%
18%
26%
30%
38%
39%
41%
42%
44%
47%
48%
56%
59%
64%
65%
68%
71%
74%
76%
82%
85%
88%
91%

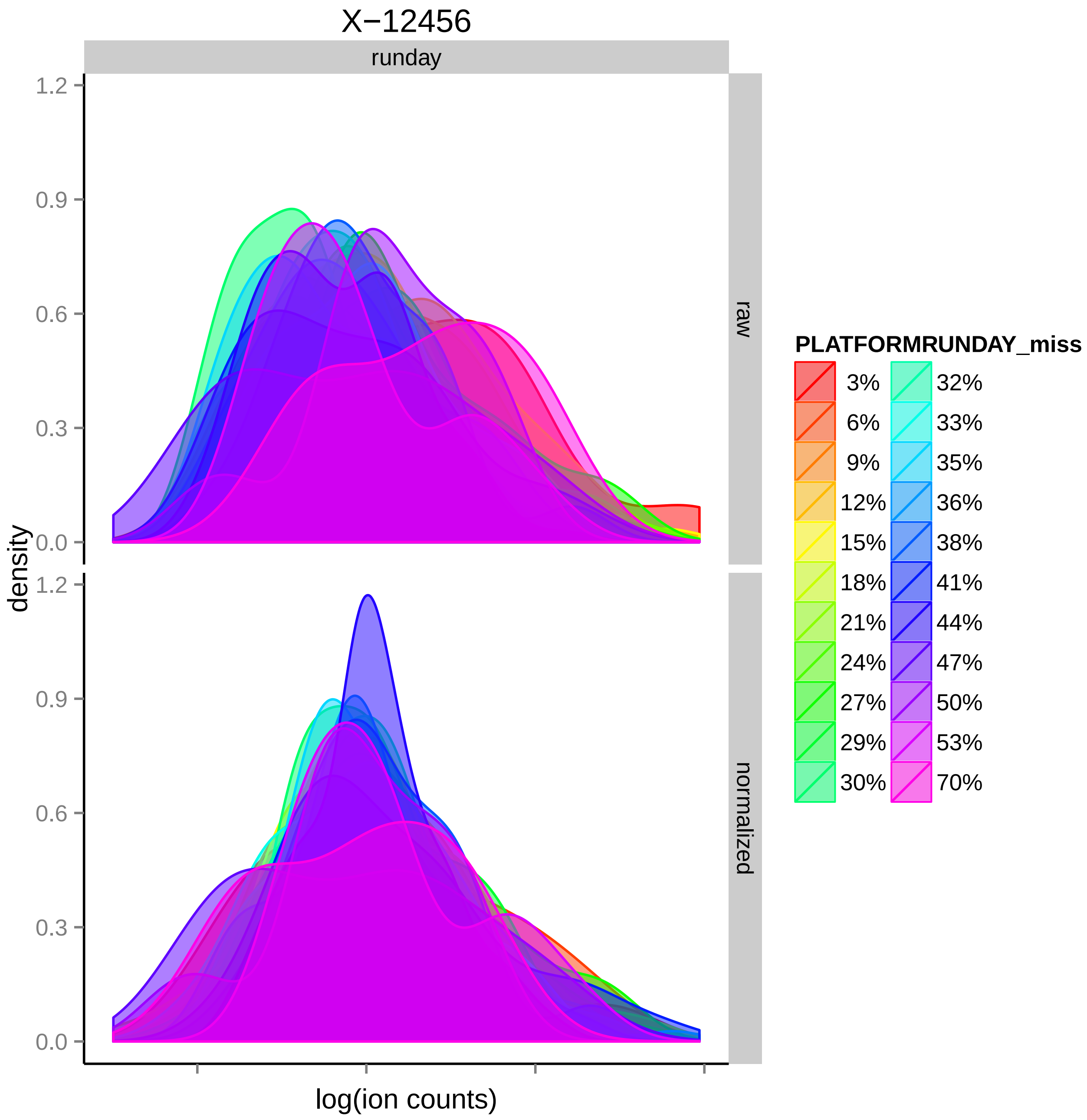

X−12456
runday
raw
normalized
density
log(ion counts)
1.2
0.9
0.6
0.3
0.0
PLATFORMRUNDAY_miss
3%
6%
9%
12%
15%
18%
21%
24%
27%
29%
30%
32%
33%
35%
36%
38%
41%
44%
47%
50%
53%
70%

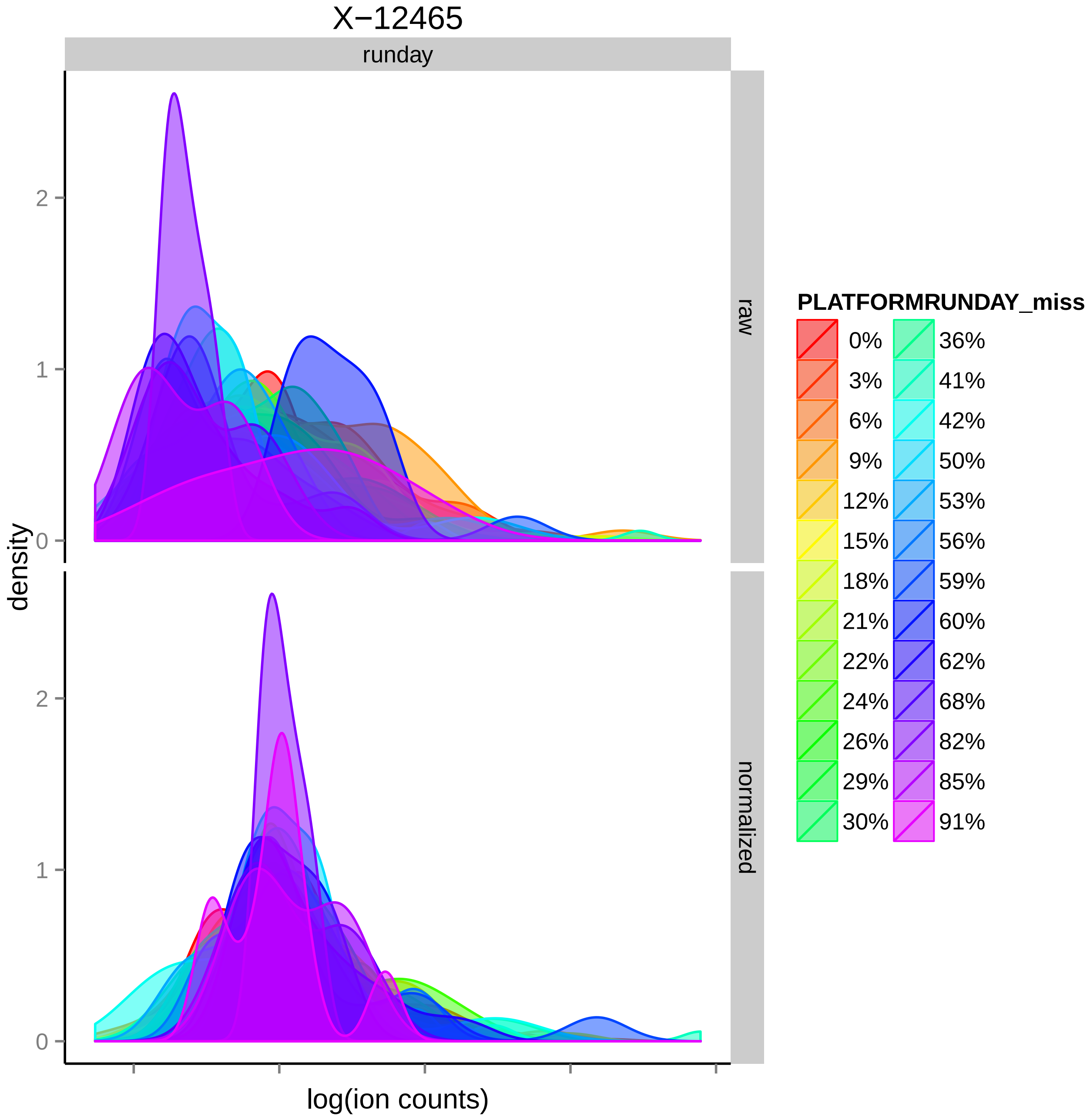

X−12465
runday
raw
normalized
density
log(ion counts)
0
1
2
0
1
2
PLATFORMRUNDAY_miss
0%
3%
6%
9%
12%
15%
18%
21%
22%
24%
26%
29%
30%
36%
41%
42%
50%
53%
56%
59%
60%
62%
68%
82%
85%
91%

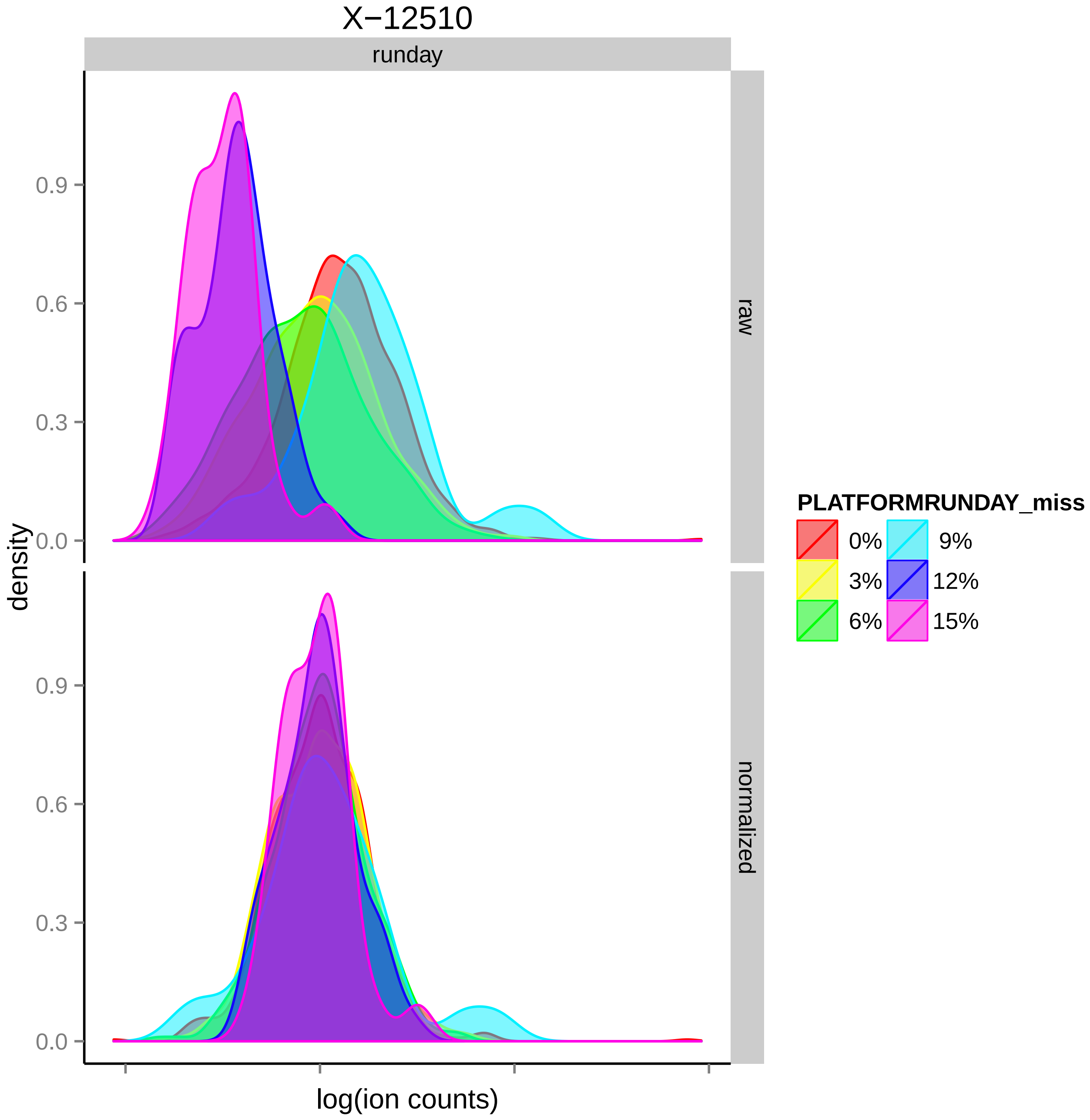
X−12510
runday
raw
normalized
density
0.9
0.6
0.3
0.0
log(ion counts)
PLATFORMRUNDAY_miss
0%
3%
6%
9%
12%
15%

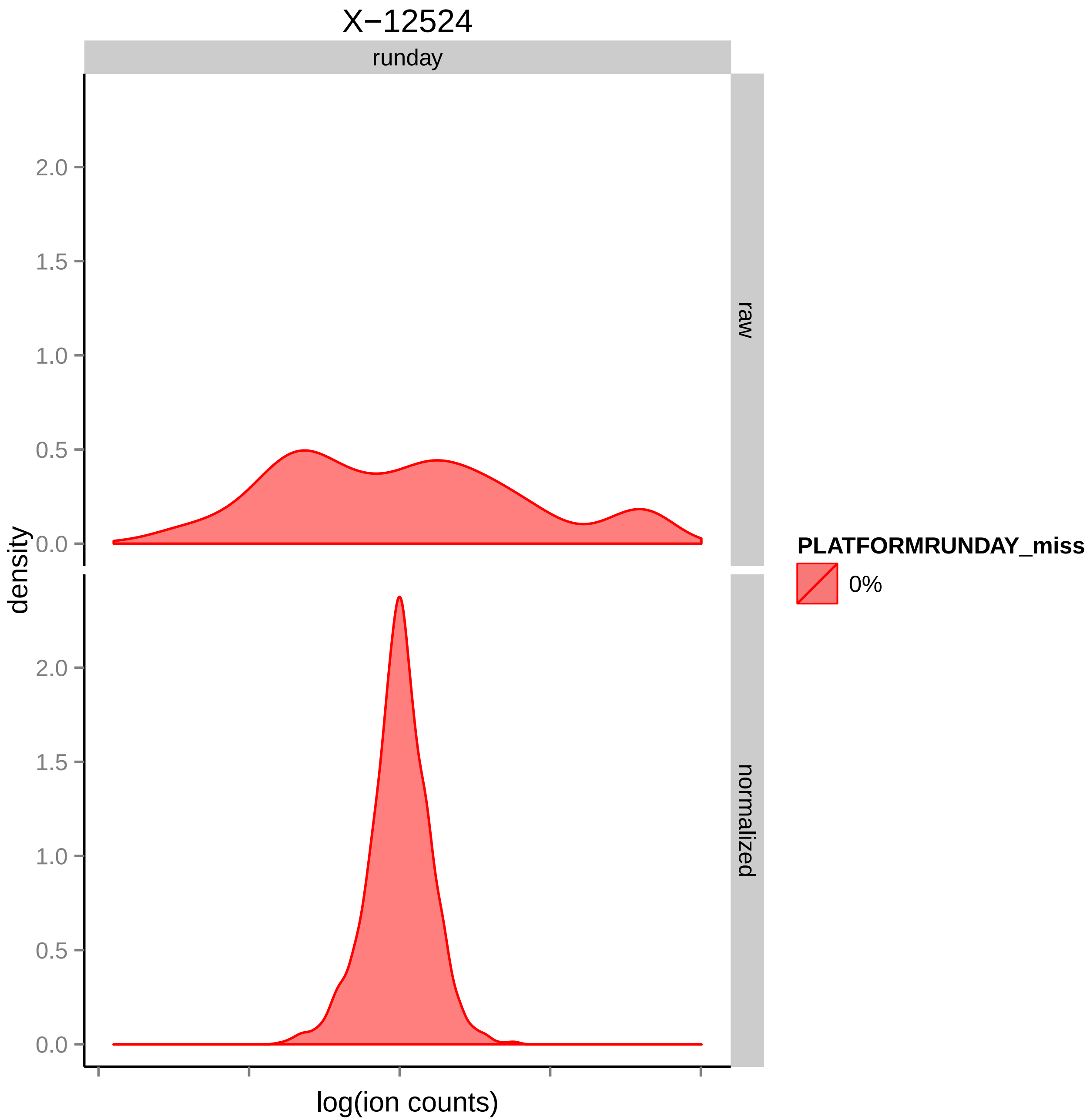

X−12524
runday
raw
normalized
density
log(ion counts)
2.0
1.5
1.0
0.5
0.0
PLATFORMRUNDAY_miss
0%

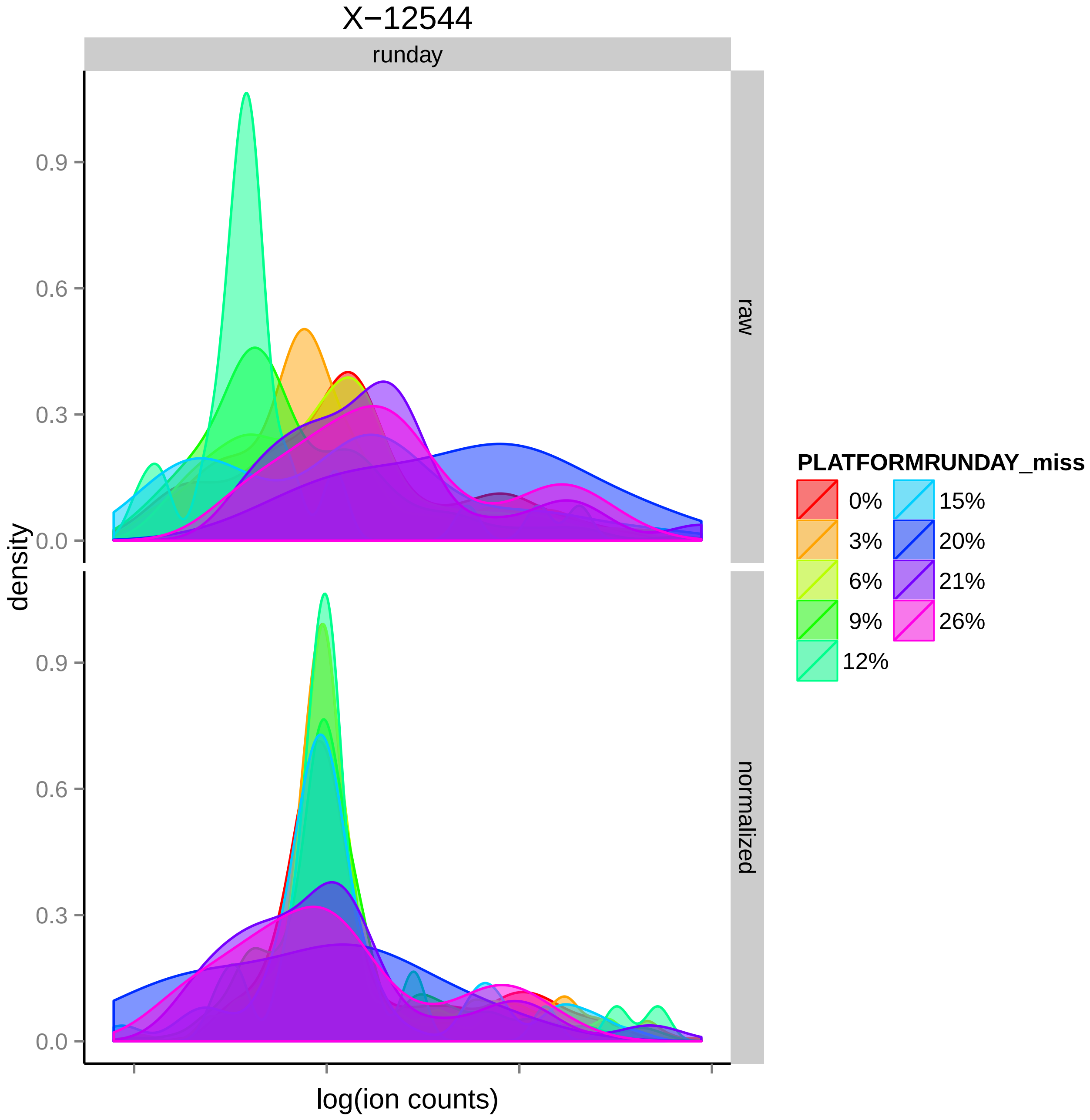
X−12544
runday
raw
normalized
density
0.0
0.3
0.6
0.9
log(ion counts)
PLATFORMRUNDAY_miss
0%
3%
6%
9%
12%
15%
20%
21%
26%

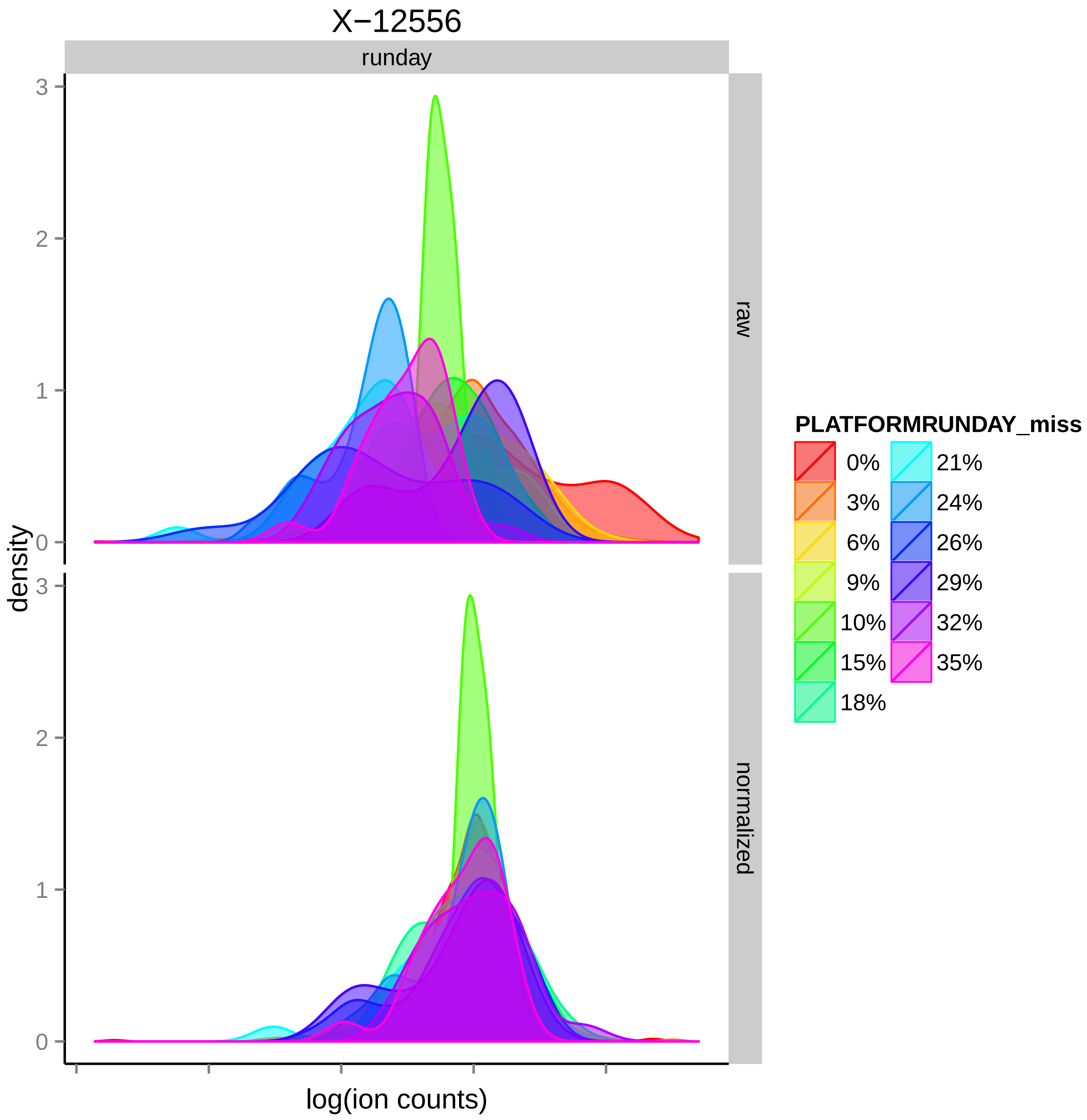

X−12556
runday
raw
normalized
density
log(ion counts)
3
2
1
0
3
2
1
0
PLATFORMRUNDAY_miss
0%
3%
6%
9%
10%
15%
18%
21%
24%
26%
29%
32%
35%

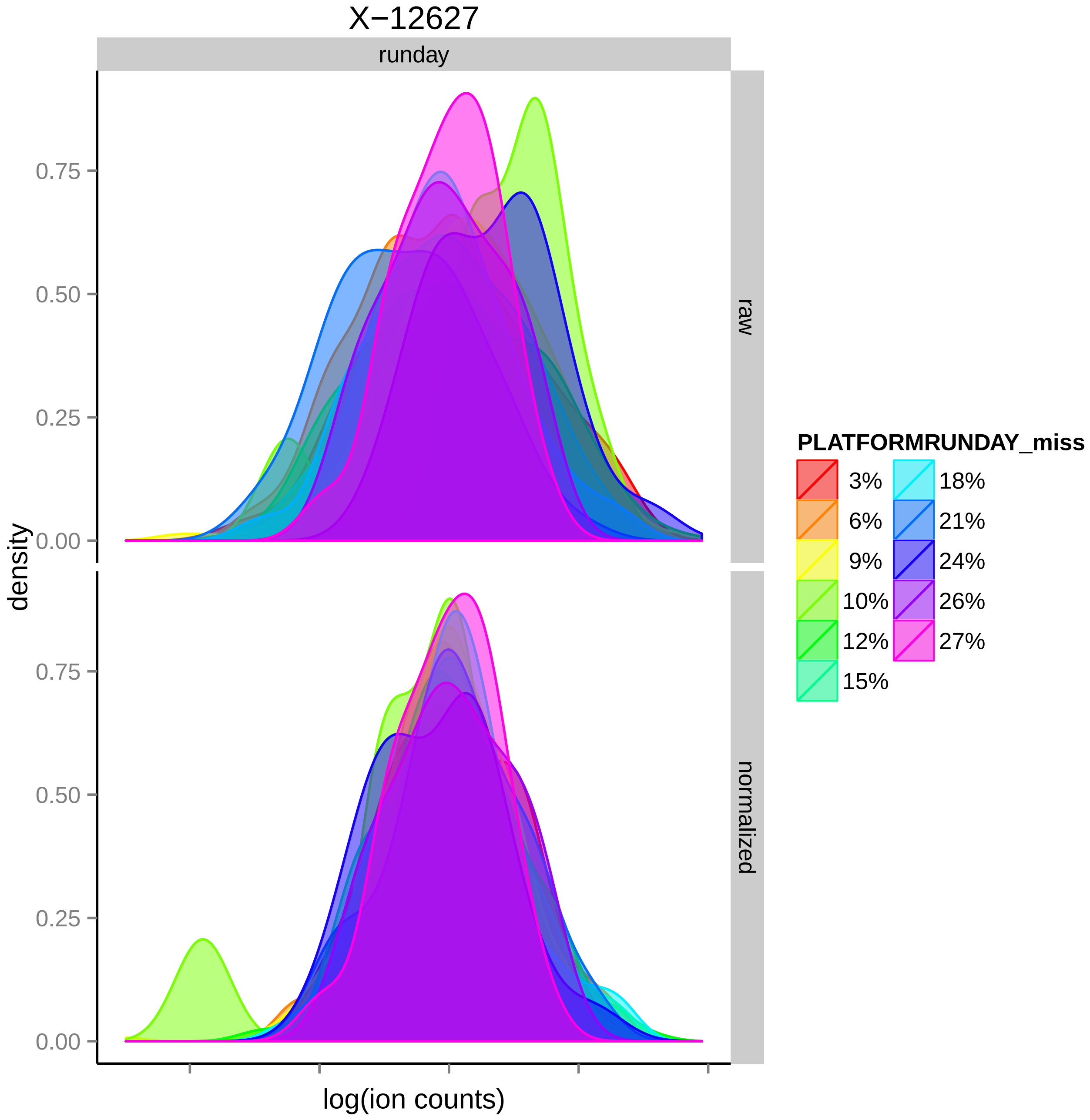
X−12627
runday
raw
normalized
density
0.00
0.25
0.50
0.75
log(ion counts)
PLATFORMRUNDAY_miss
3%
6%
9%
10%
12%
15%
18%
21%
24%
26%
27%

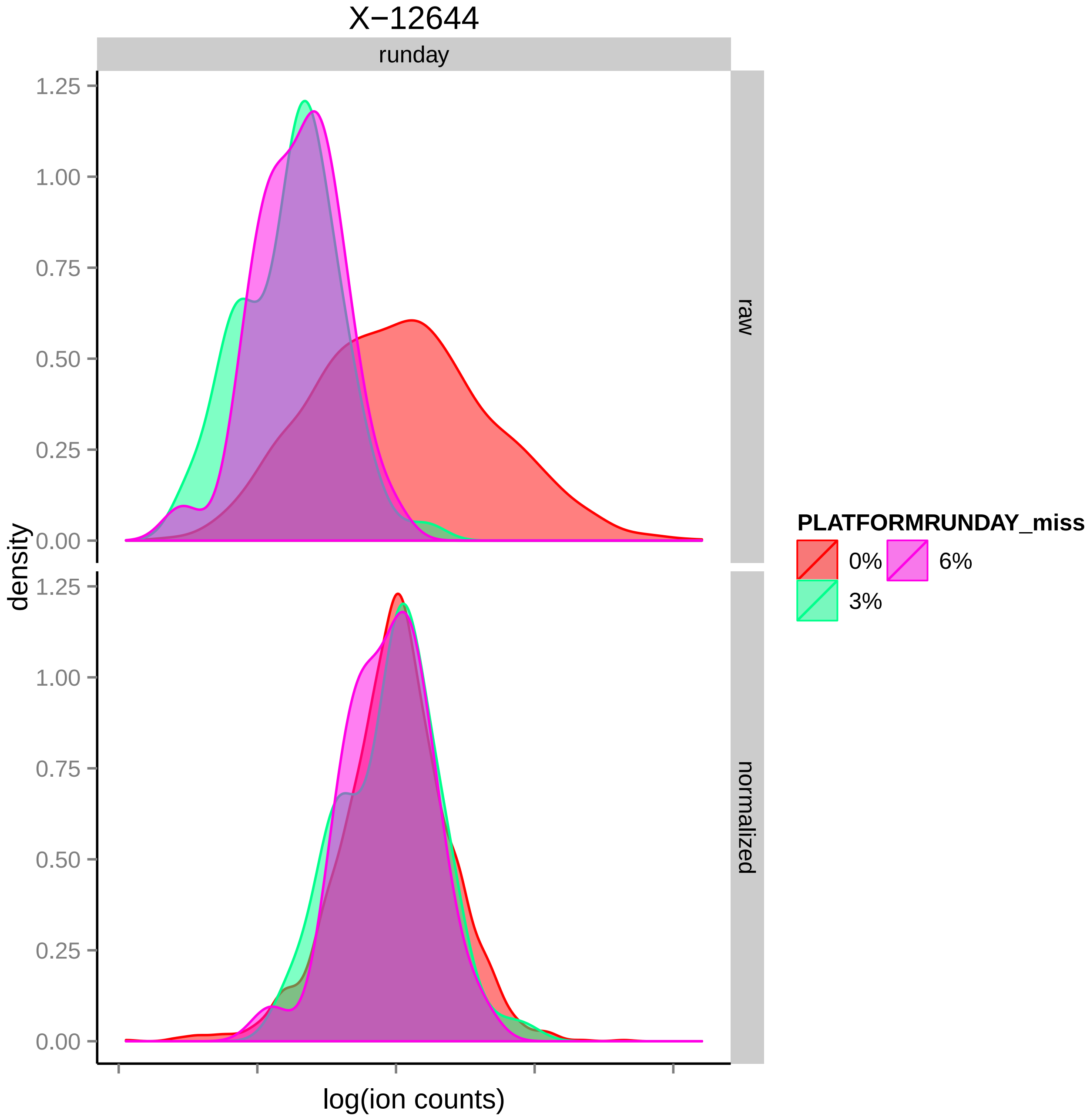

X−12644
runday
raw
normalized
density
log(ion counts)
1.25
1.00
0.75
0.50
0.25
0.00
PLATFORMRUNDAY_miss
0%
3%
6%

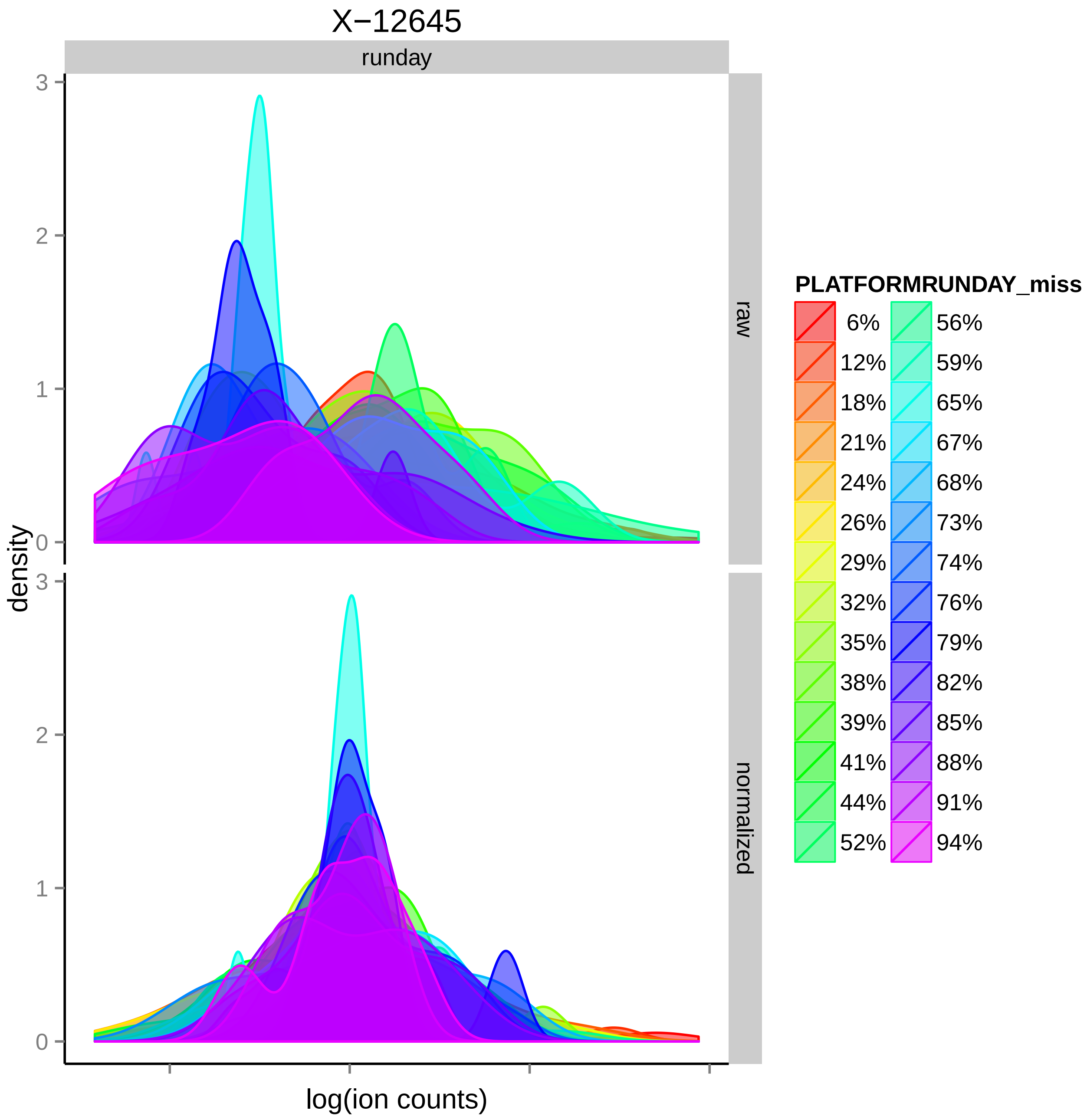
X−12645
runday
raw
normalized
density
log(ion counts)
3
2
1
0
3
2
1
0
PLATFORMRUNDAY_miss
6%
12%
18%
21%
24%
26%
29%
32%
35%
38%
39%
41%
44%
52%
56%
59%
65%
67%
68%
73%
74%
76%
79%
82%
85%
88%
91%
94%

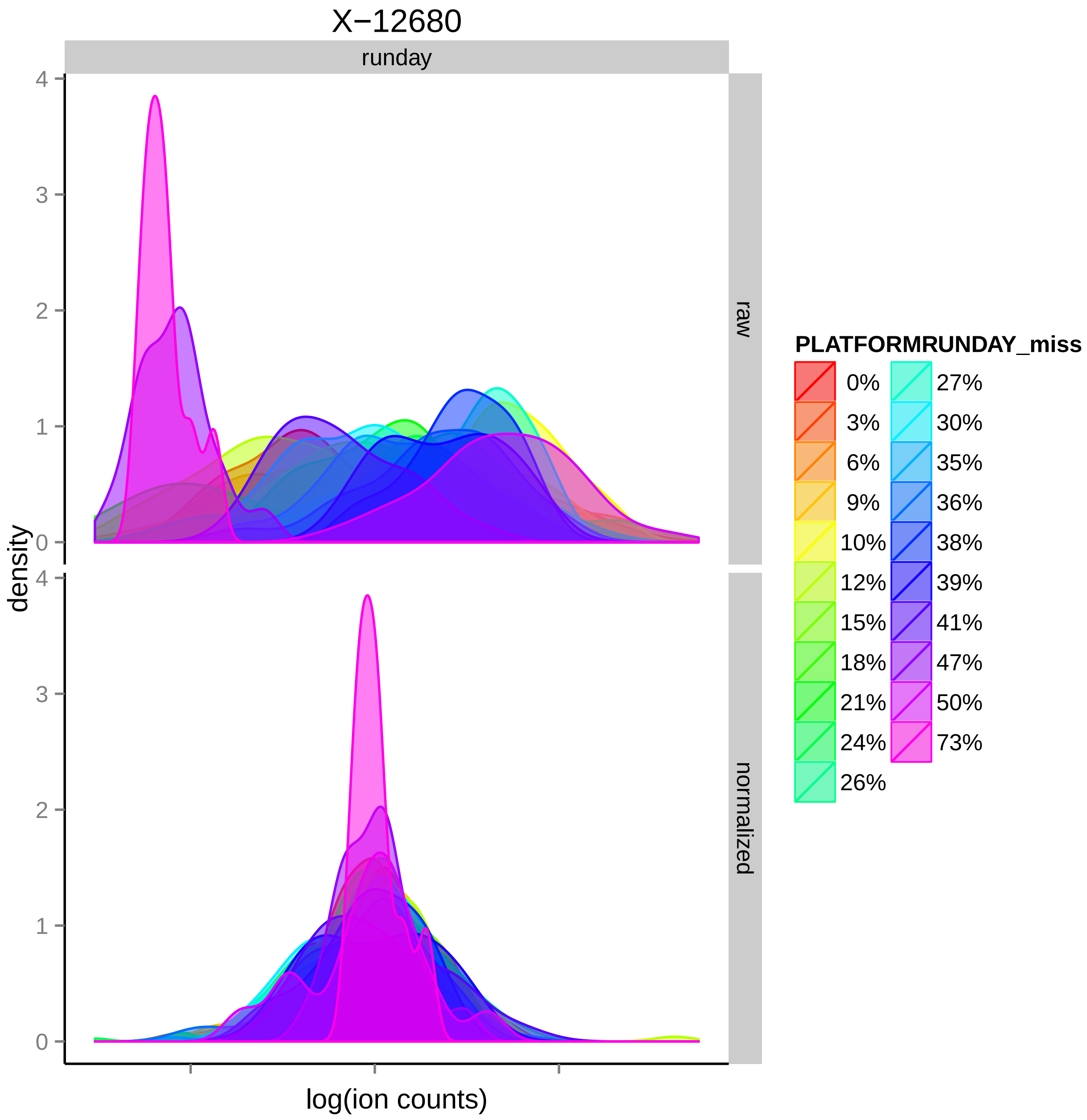
X−12680
runday
raw
normalized
density
log(ion counts)
0
1
2
3
4
PLATFORMRUNDAY_miss
0%
3%
6%
9%
10%
12%
15%
18%
21%
24%
26%
27%
30%
35%
36%
38%
39%
41%
47%
50%
73%

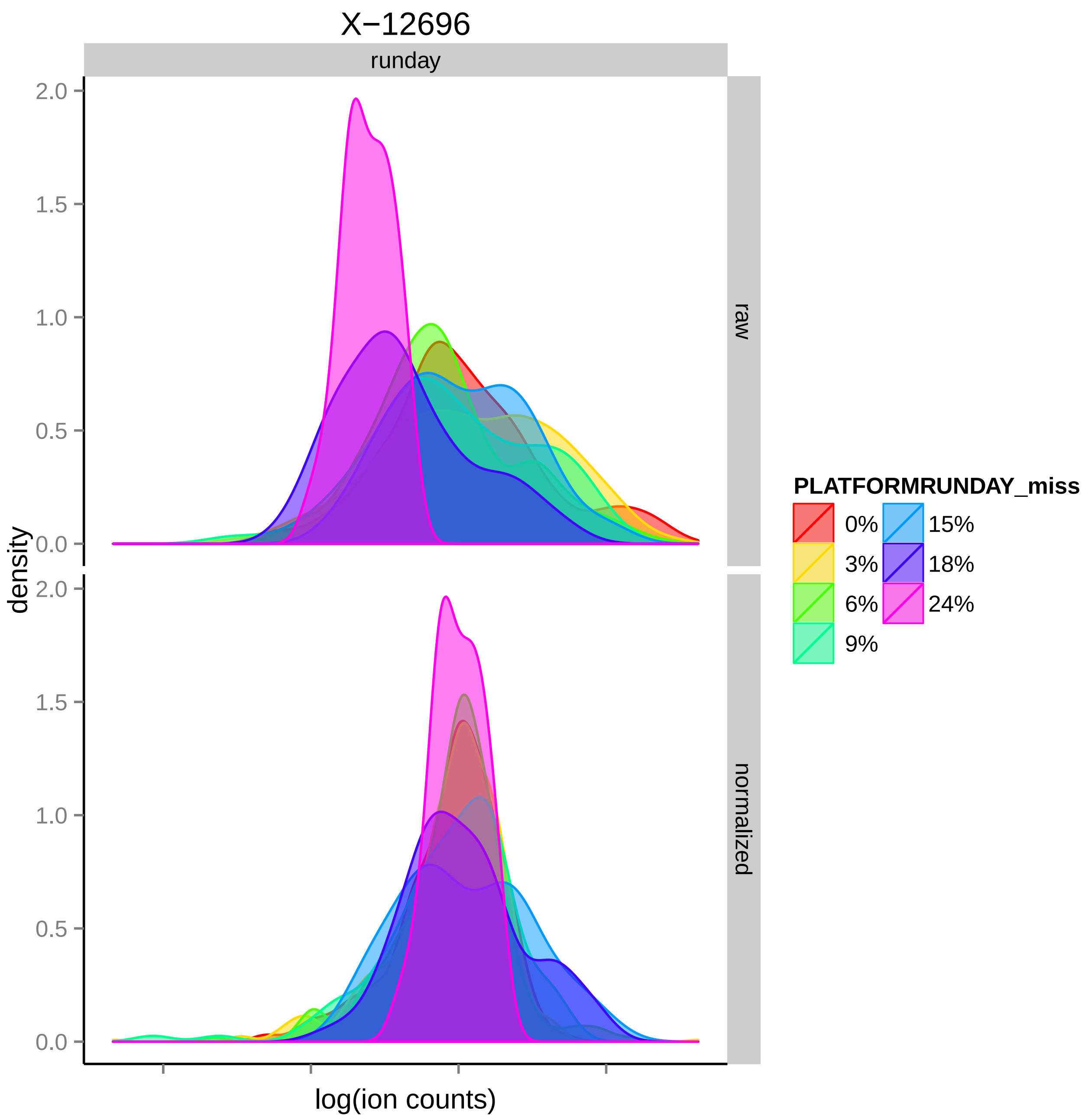

X−12696
runday
raw
normalized
density
log(ion counts)
2.0
1.5
1.0
0.5
0.0
PLATFORMRUNDAY_miss
0%
3%
6%
9%
15%
18%
24%

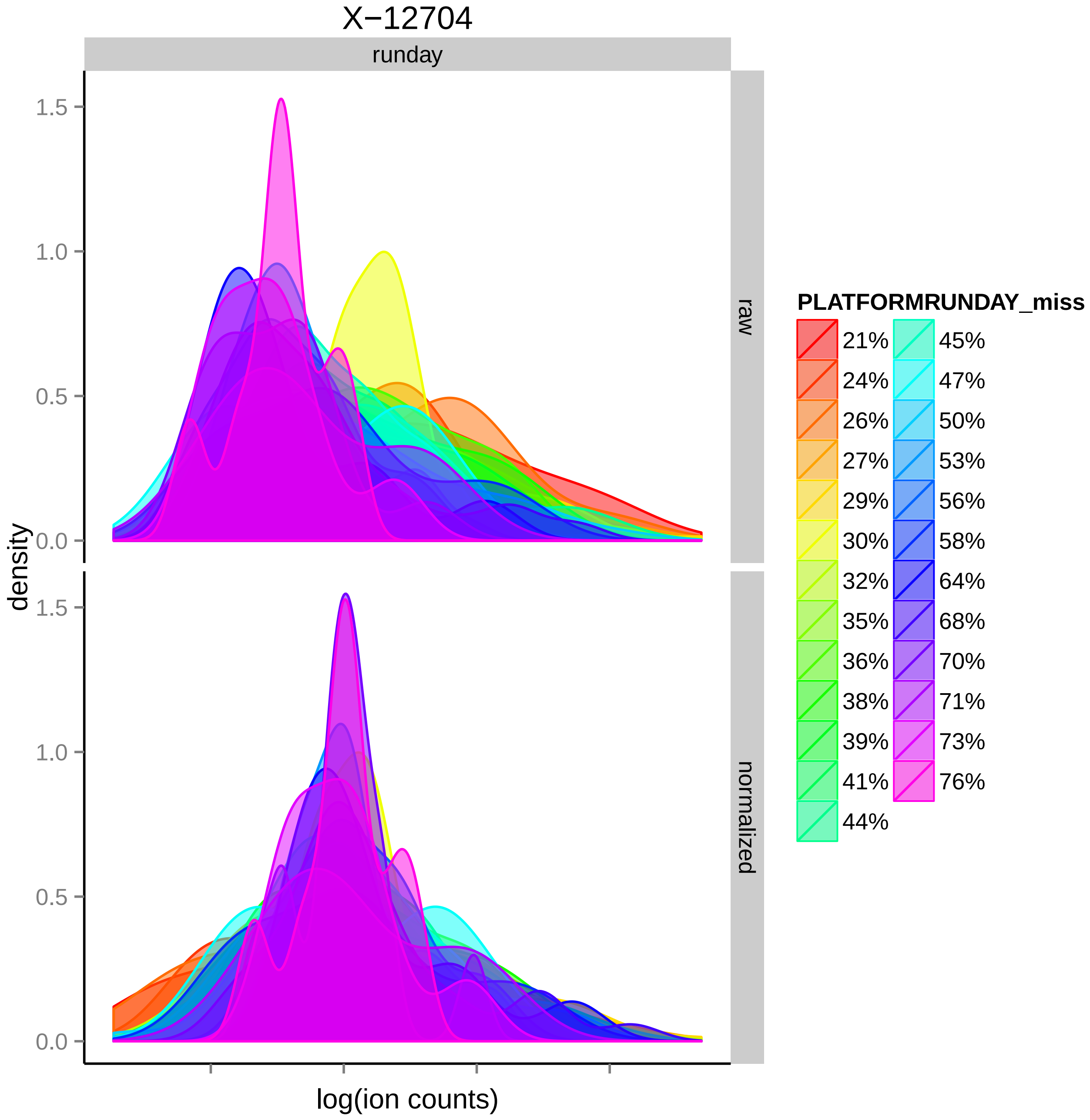

X−12704
runday
raw
normalized
density
log(ion counts)
1.5
1.0
0.5
0.0
PLATFORMRUNDAY_miss
21%
24%
26%
27%
29%
30%
32%
35%
36%
38%
39%
41%
44%
45%
47%
50%
53%
56%
58%
64%
68%
70%
71%
73%
76%

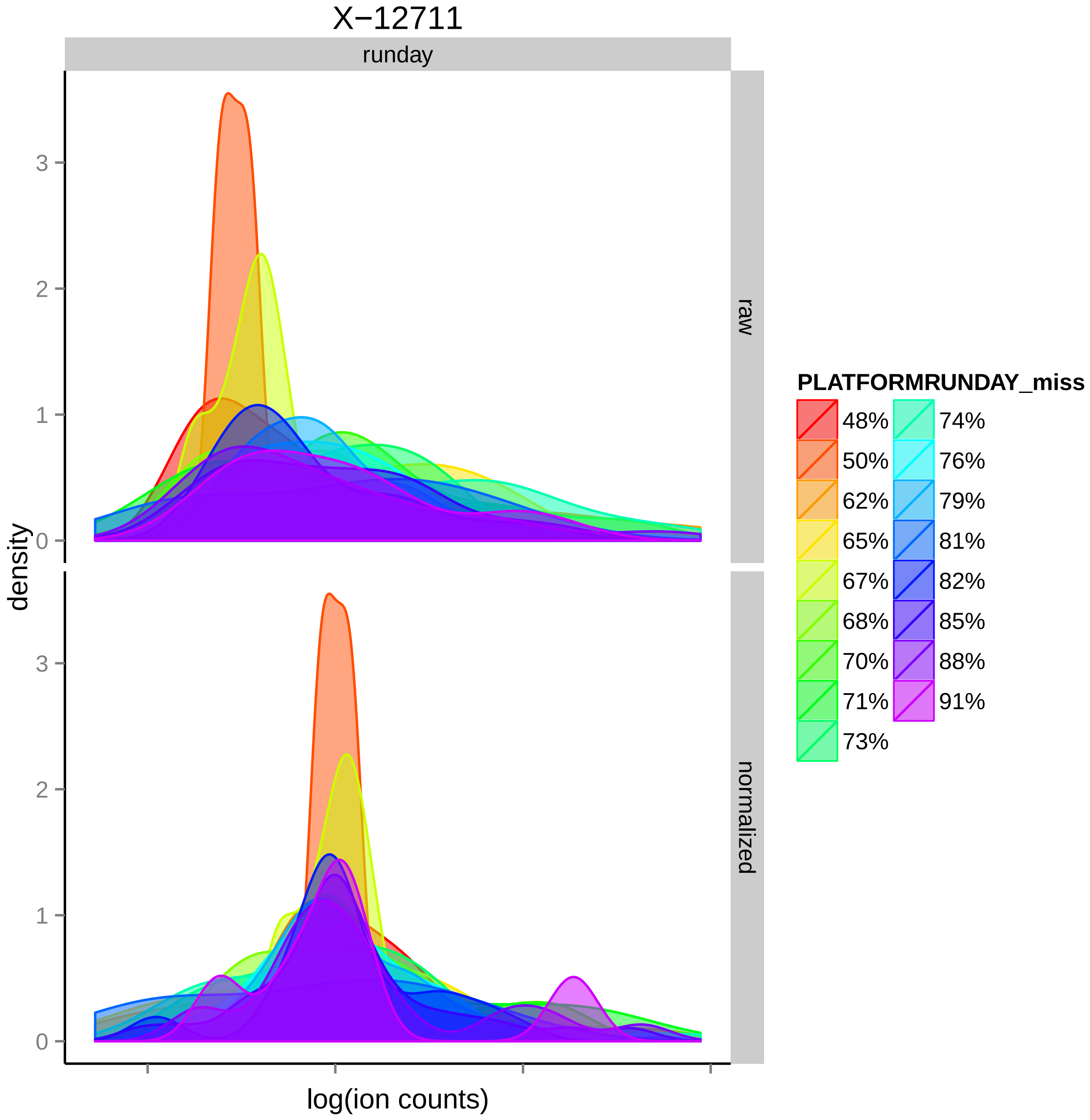
X−12711
runday
raw
normalized
density
log(ion counts)
0
1
2
3
PLATFORMRUNDAY_miss
48%
50%
62%
65%
67%
68%
70%
71%
73%
74%
76%
79%
81%
82%
85%
88%
91%

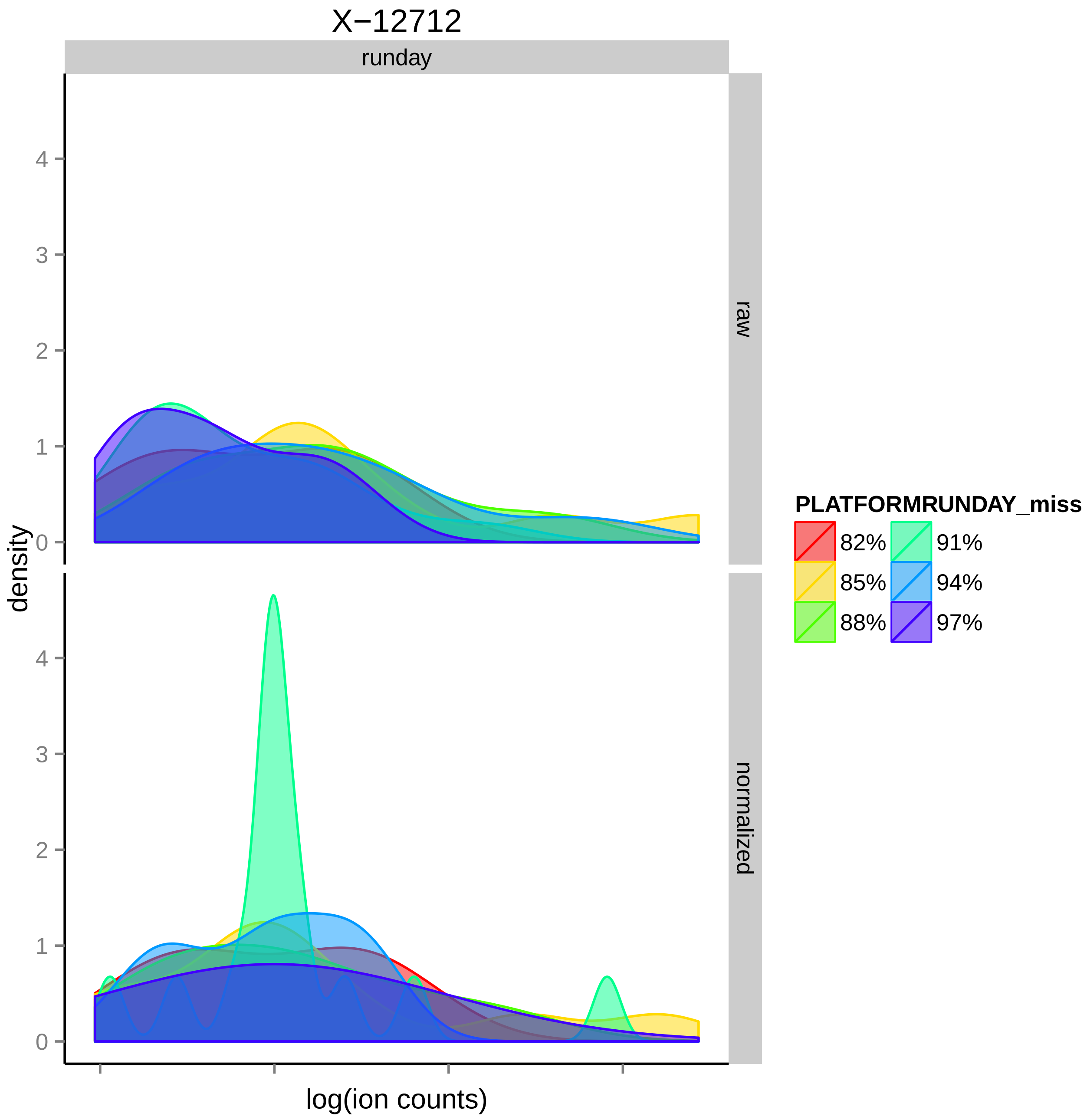

X−12712
runday
raw
normalized
density
log(ion counts)
0
1
2
3
4
PLATFORMRUNDAY_miss
82%
85%
88%
91%
94%
97%

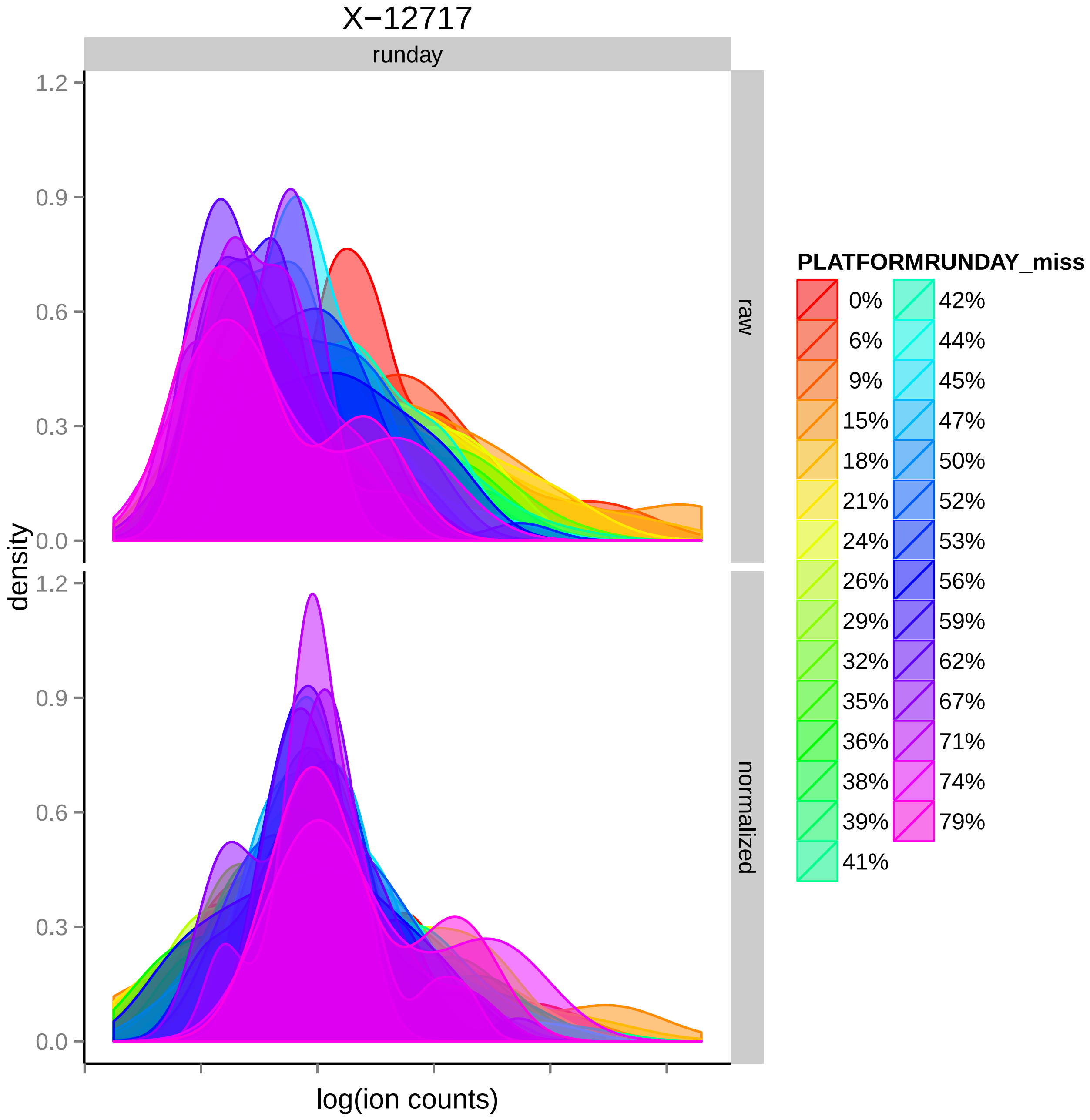

X−12717
runday
raw
normalized
density
1.2
0.9
0.6
0.3
0.0
log(ion counts)
PLATFORMRUNDAY_miss
0%
6%
9%
15%
18%
21%
24%
26%
29%
32%
35%
36%
38%
39%
41%
42%
44%
45%
47%
50%
52%
53%
56%
59%
62%
67%
71%
74%
79%

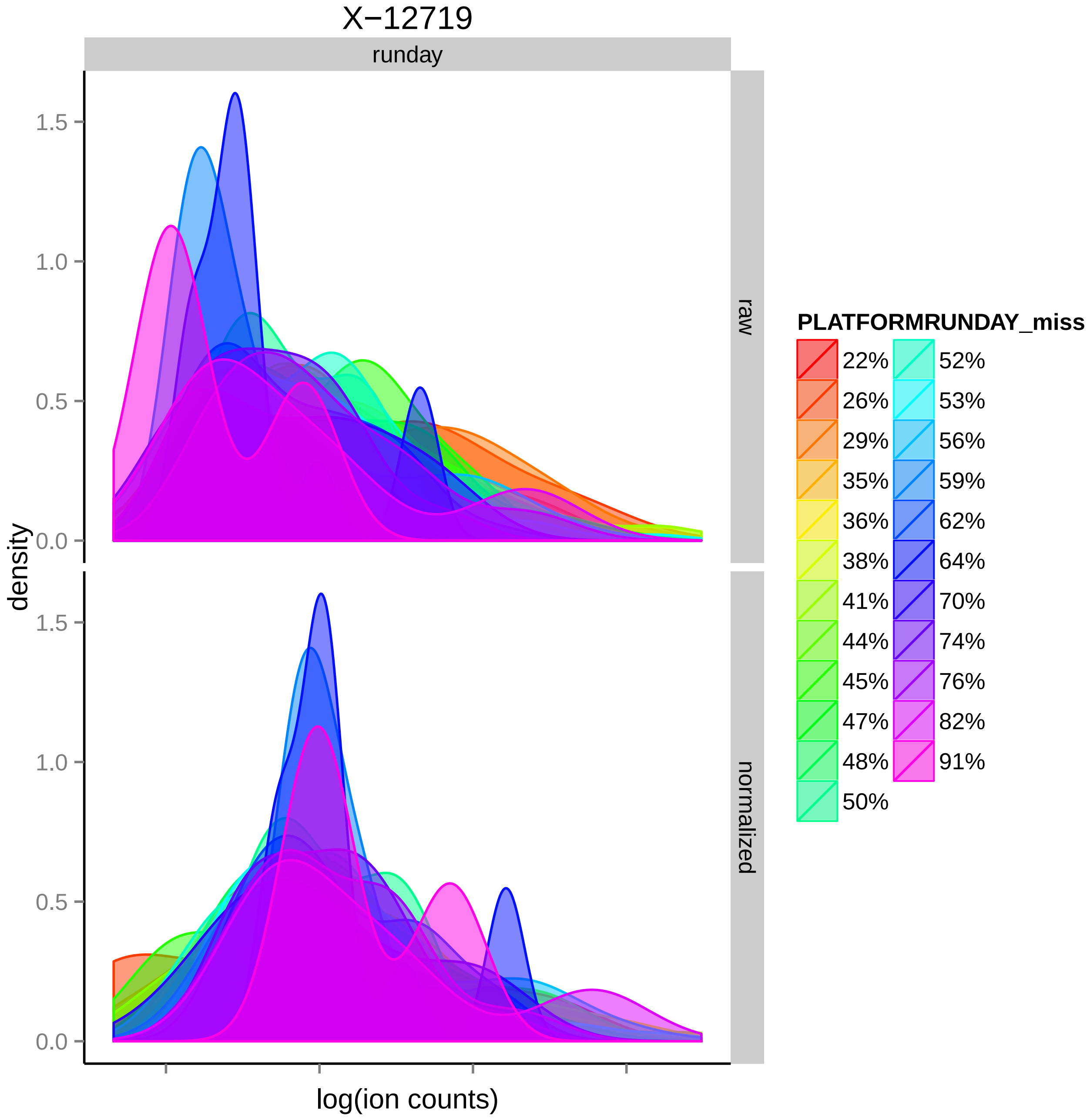
X−12719
runday
raw
normalized
density
1.5
1.0
0.5
0.0
log(ion counts)
PLATFORMRUNDAY_miss
22%
26%
29%
35%
36%
38%
41%
44%
45%
47%
48%
50%
52%
53%
56%
59%
62%
64%
70%
74%
76%
82%
91%

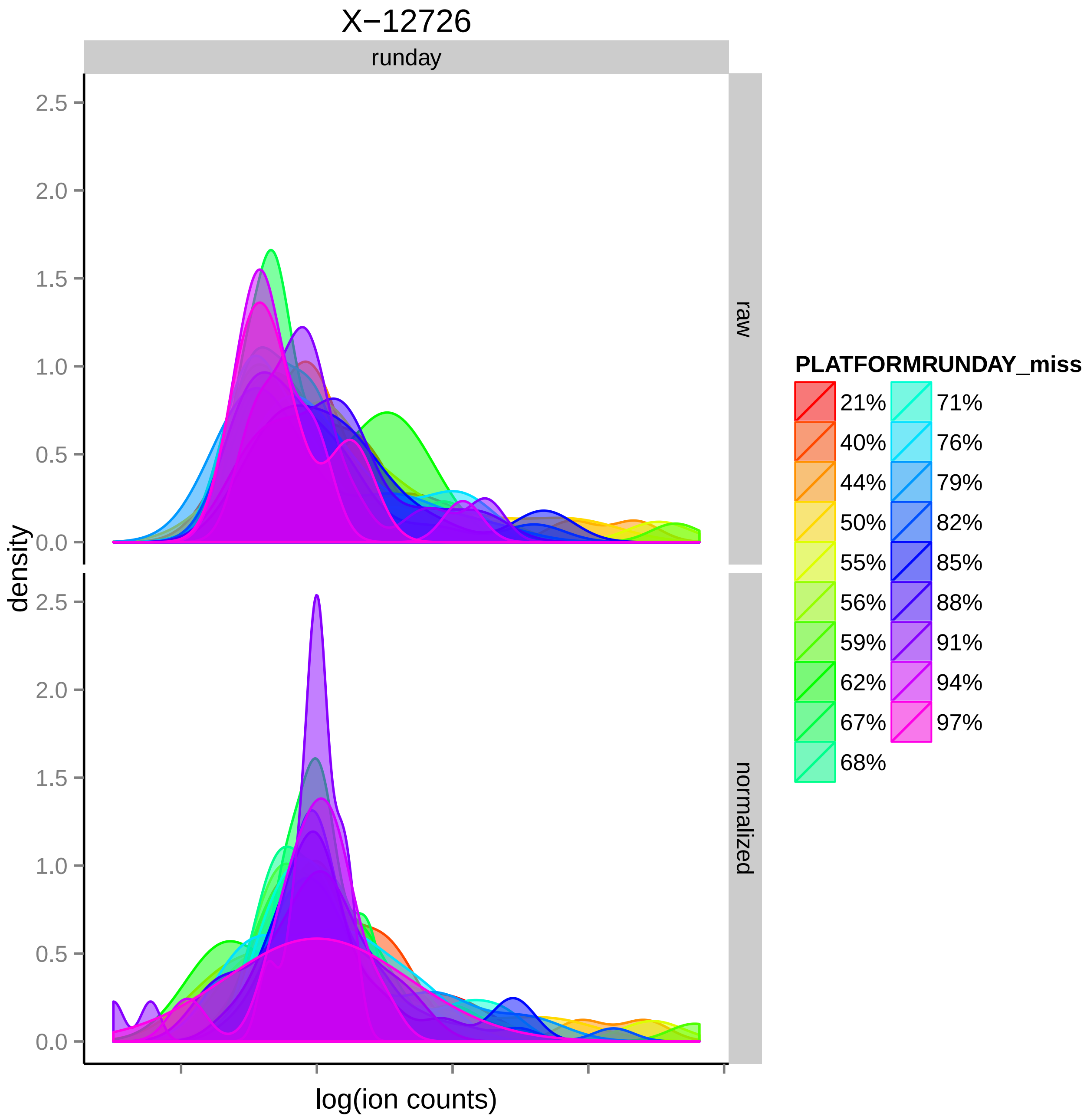
X−12726
runday
raw
normalized
density
log(ion counts)
2.5
2.0
1.5
1.0
0.5
0.0
PLATFORMRUNDAY_miss
21%
40%
44%
50%
55%
56%
59%
62%
67%
68%
71%
76%
79%
82%
85%
88%
91%
94%
97%

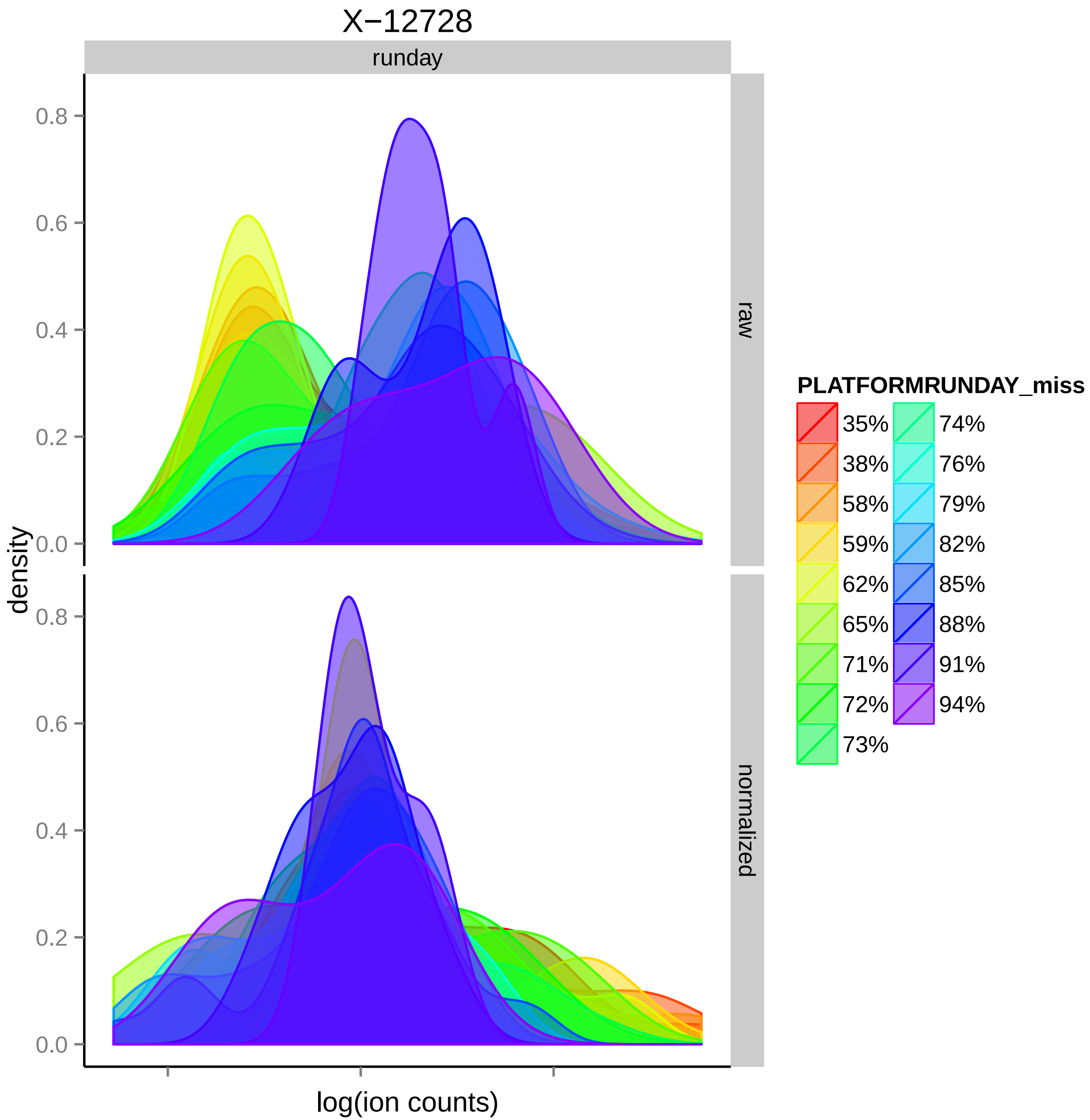

X−12728
runday
raw
normalized
density
log(ion counts)
0.0
0.2
0.4
0.6
0.8
PLATFORMRUNDAY_miss
35%
38%
58%
59%
62%
65%
71%
72%
73%
74%
76%
79%
82%
85%
88%
91%
94%

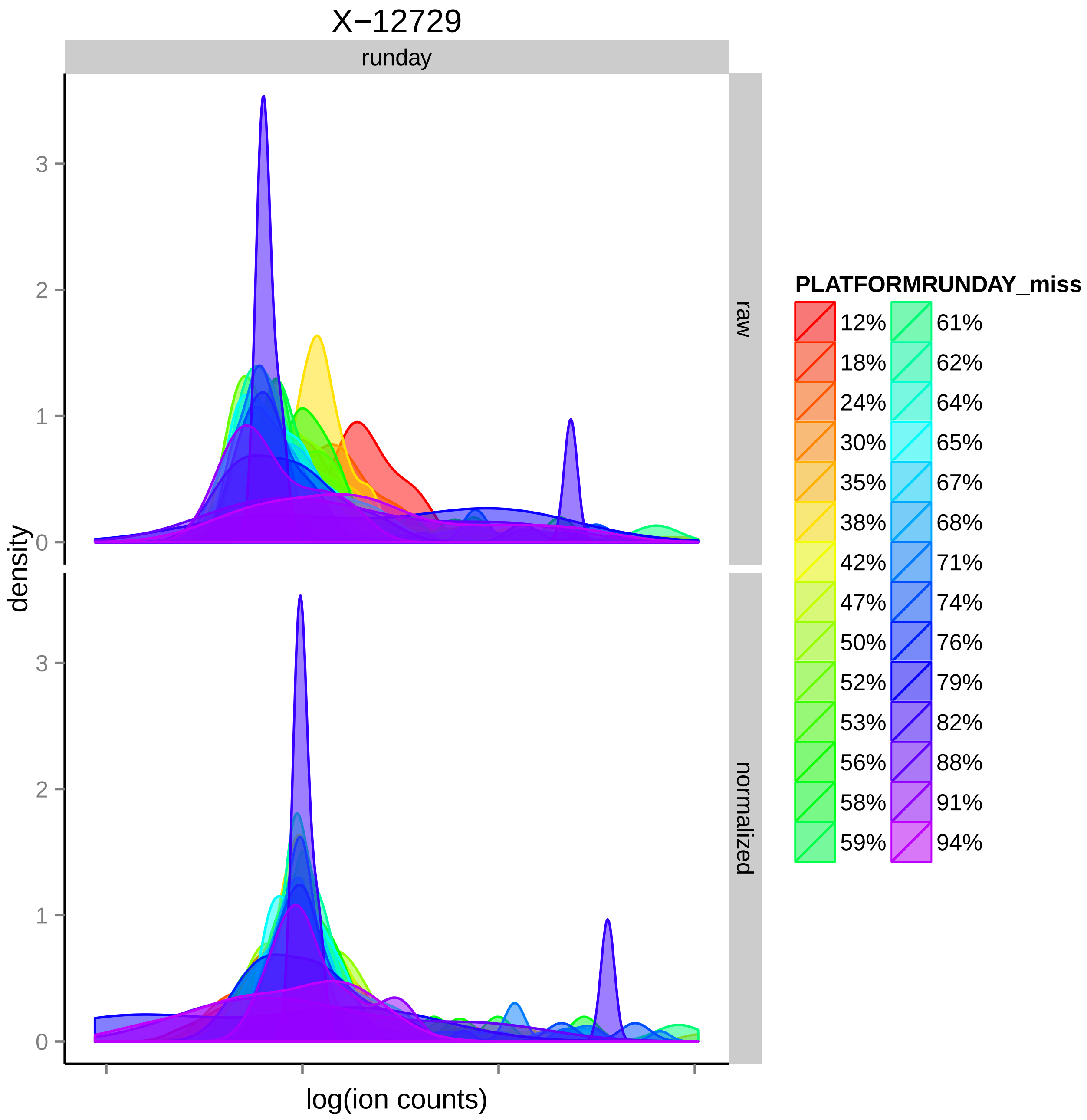

X−12729
runday
raw
normalized
density
log(ion counts)
3
2
1
0
PLATFORMRUNDAY_miss
12%
18%
24%
30%
35%
38%
42%
47%
50%
52%
53%
56%
58%
59%
61%
62%
64%
65%
67%
68%
71%
74%
76%
79%
82%
88%
91%
94%

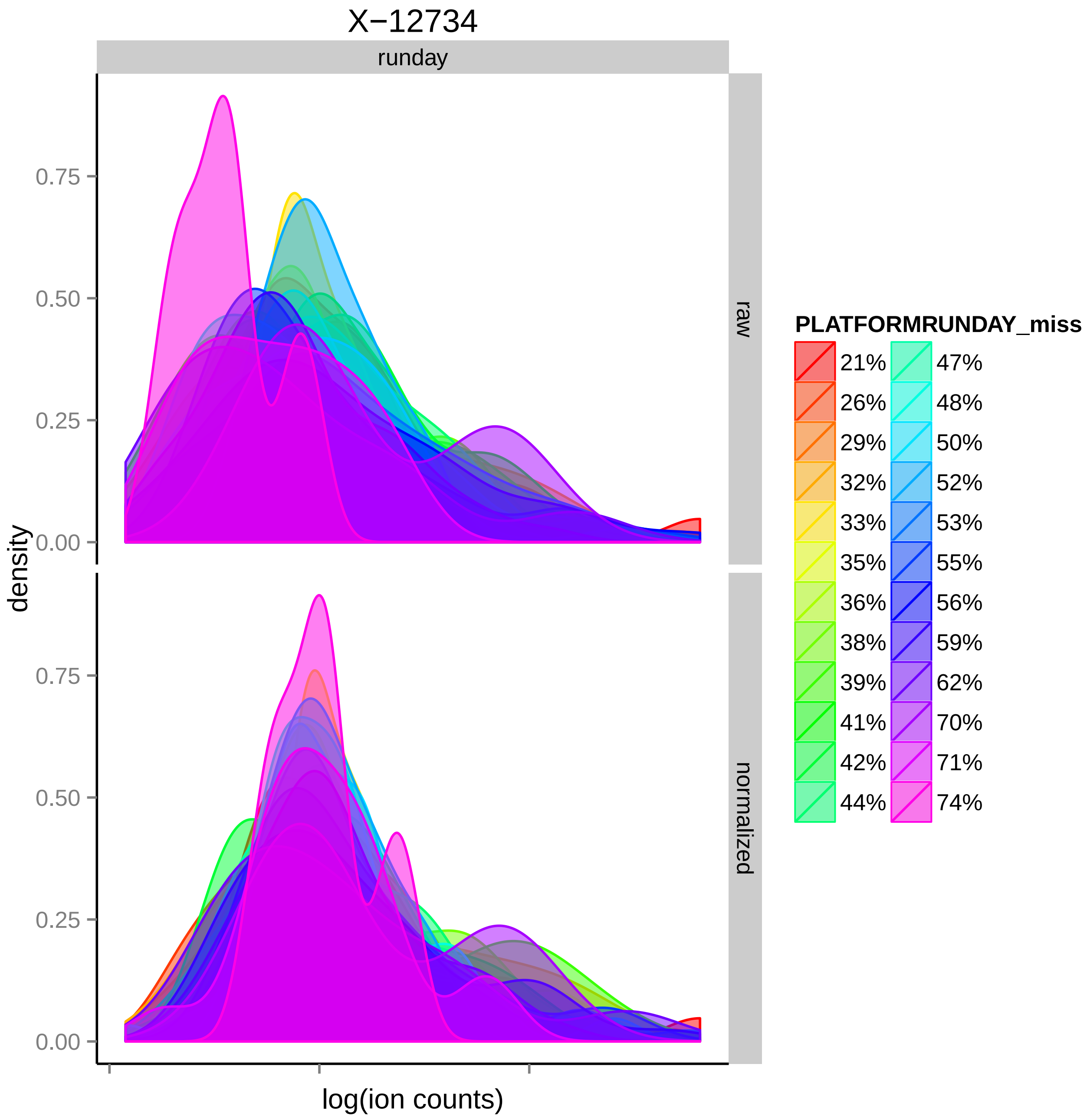

X−12734
runday
raw
normalized
density
0.75
0.50
0.25
0.00
log(ion counts)
PLATFORMRUNDAY_miss
21%
26%
29%
32%
33%
35%
36%
38%
39%
41%
42%
44%
47%
48%
50%
52%
53%
55%
56%
59%
62%
70%
71%
74%

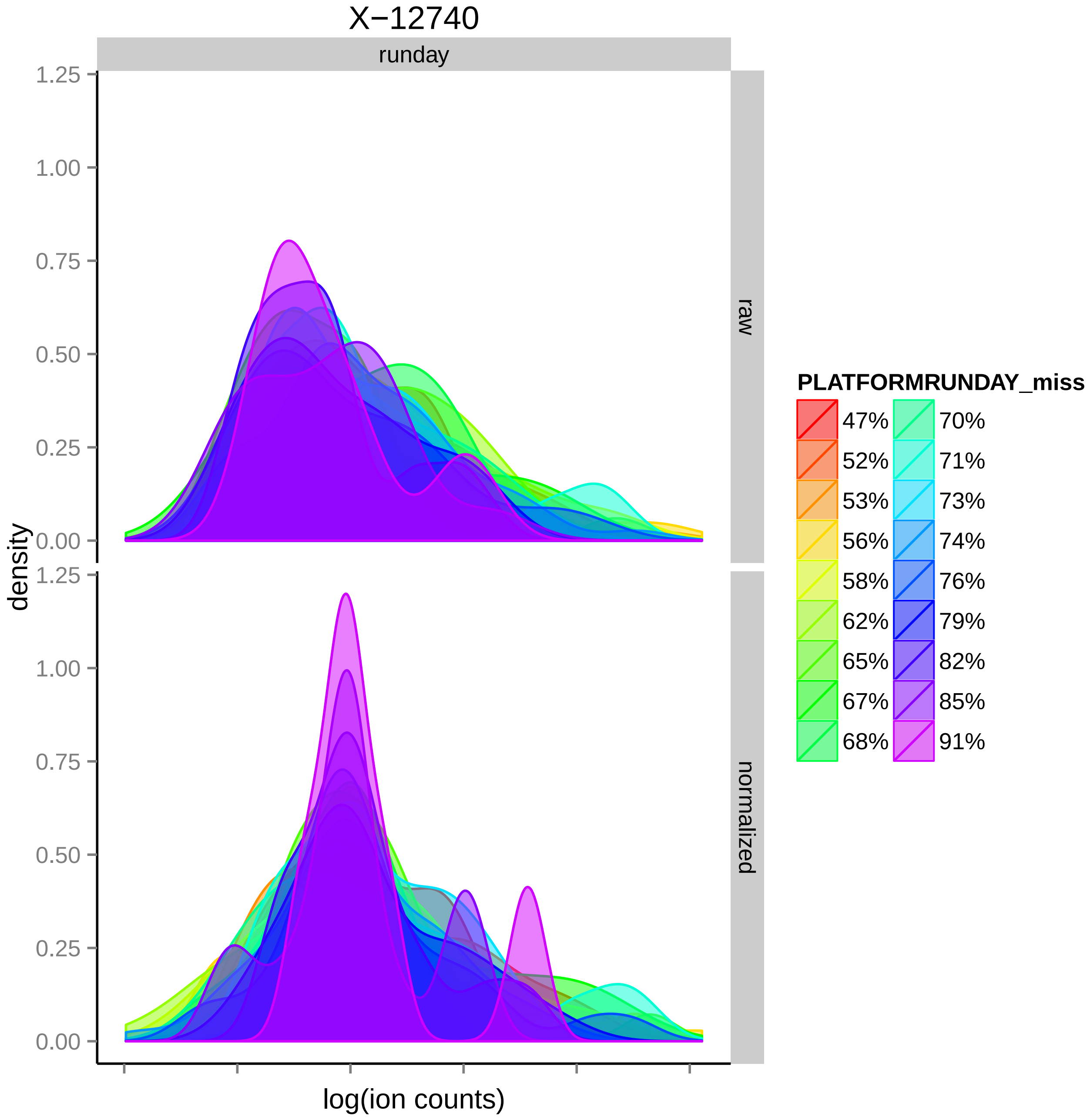
X−12740
runday
raw
normalized
density
log(ion counts)
1.25
1.00
0.75
0.50
0.25
0.00
PLATFORMRUNDAY_miss
47%
52%
53%
56%
58%
62%
65%
67%
68%
70%
71%
73%
74%
76%
79%
82%
85%
91%

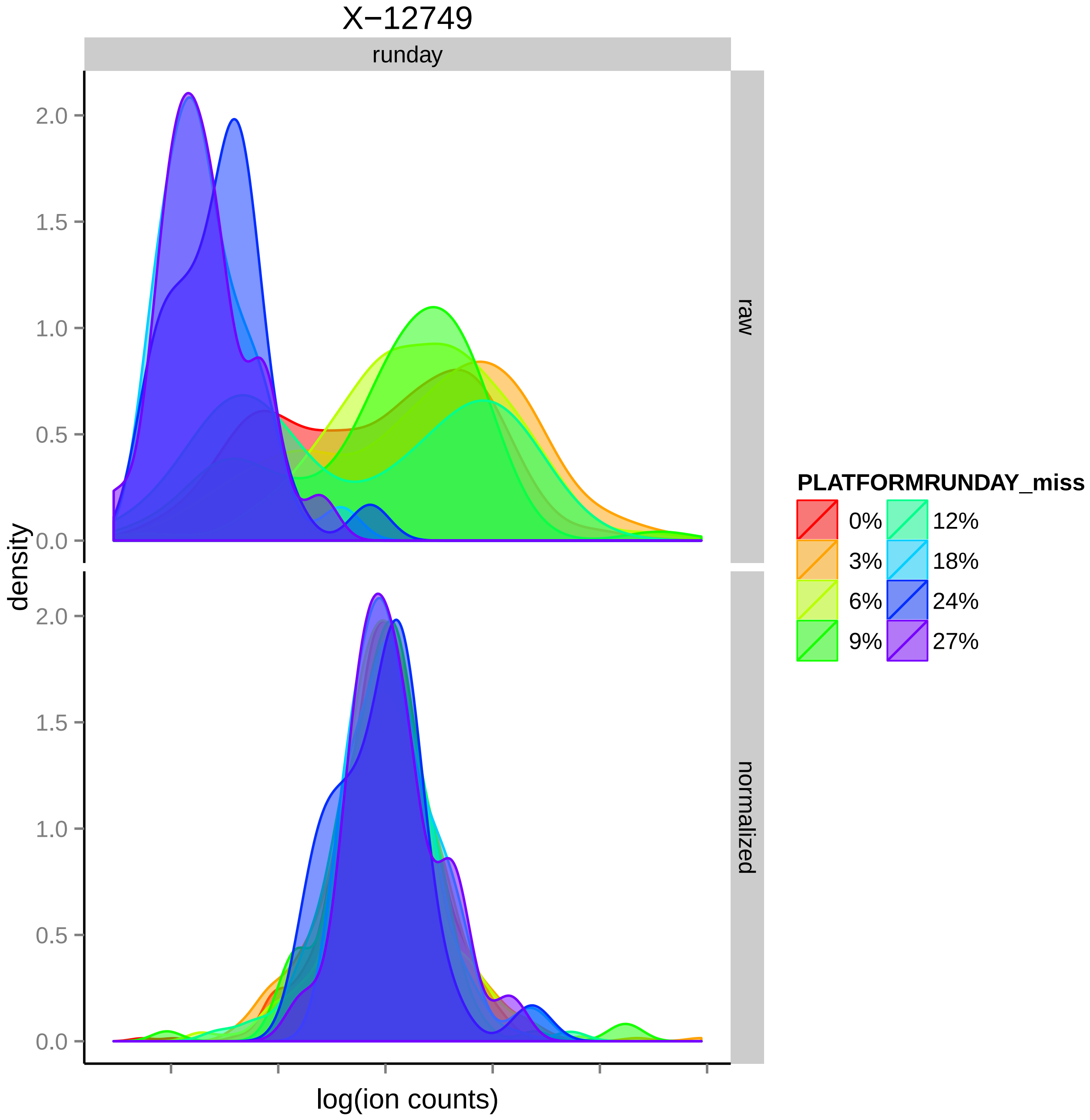
X−12749
runday
raw
normalized
density
2.0
1.5
1.0
0.5
0.0
log(ion counts)
PLATFORMRUNDAY_miss
0%
3%
6%
9%
12%
18%
24%
27%

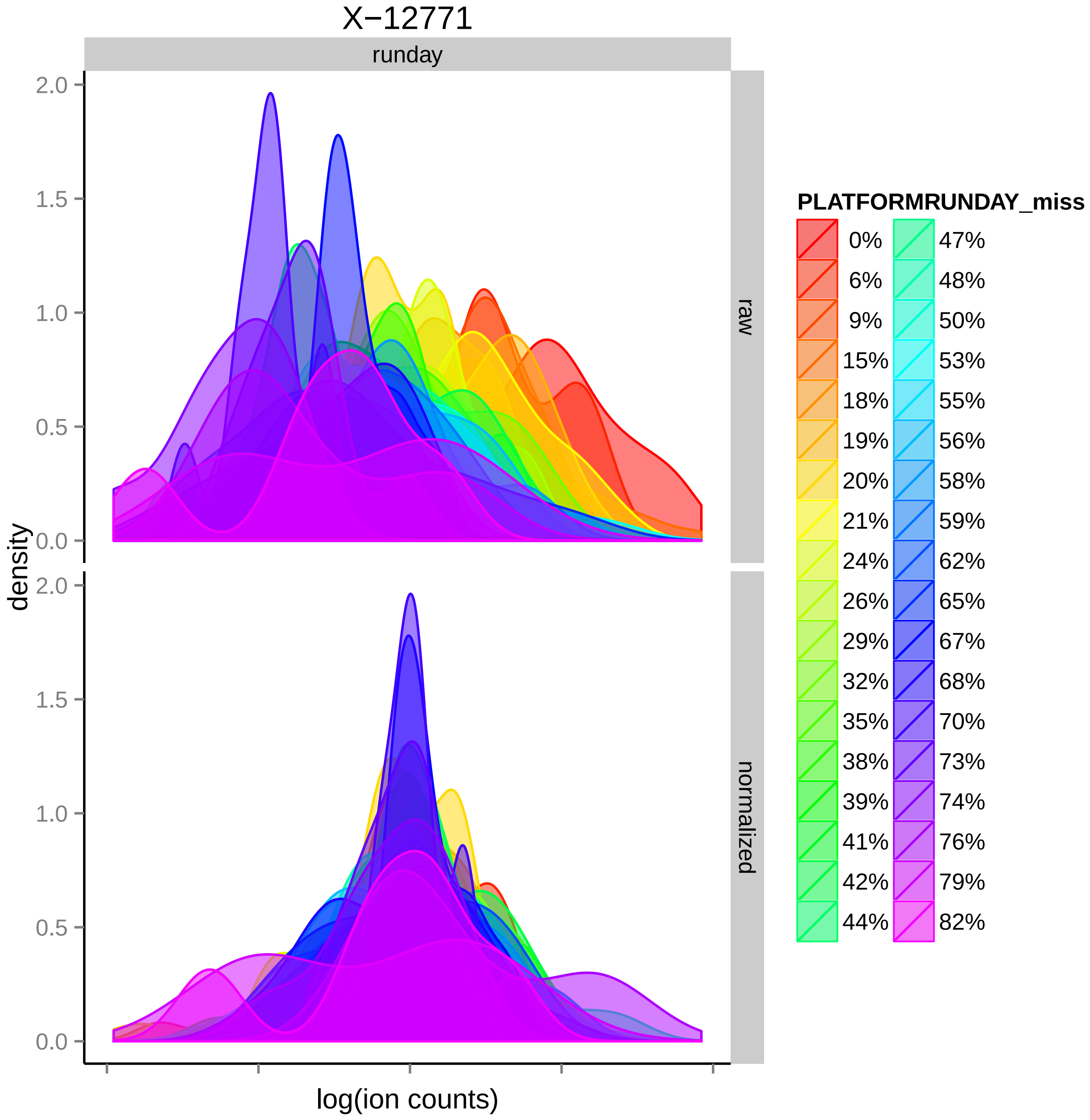
X−12771
runday
raw
normalized
density
log(ion counts)
2.0
1.5
1.0
0.5
0.0
PLATFORMRUNDAY_miss
0%
6%
9%
15%
18%
19%
20%
21%
24%
26%
29%
32%
35%
38%
39%
41%
42%
44%
47%
48%
50%
53%
55%
56%
58%
59%
62%
65%
67%
68%
70%
73%
74%
76%
79%
82%

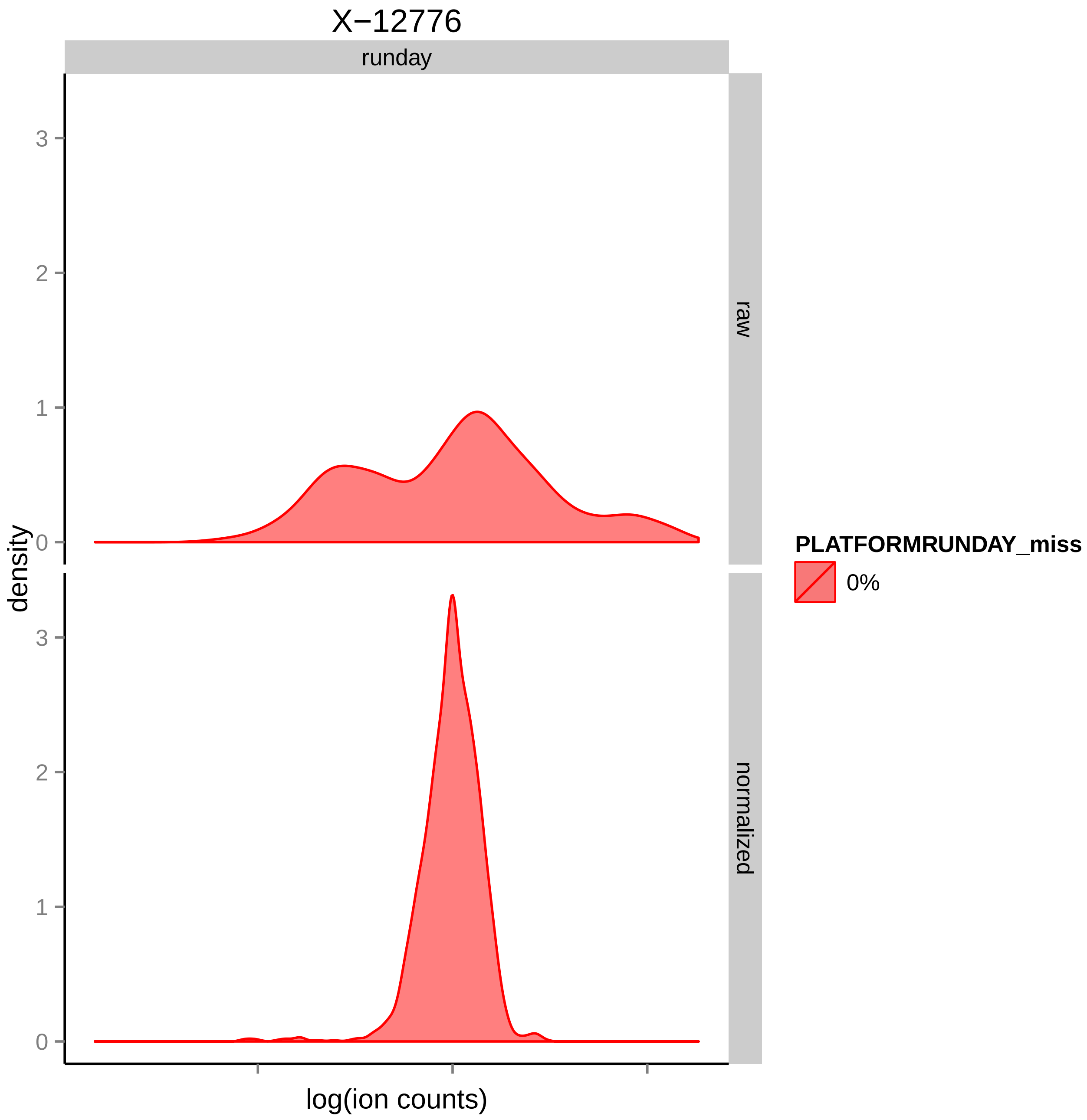
X−12776
runday
raw
normalized
density
log(ion counts)
0
1
2
3
0
1
2
3
PLATFORMRUNDAY_miss
0%

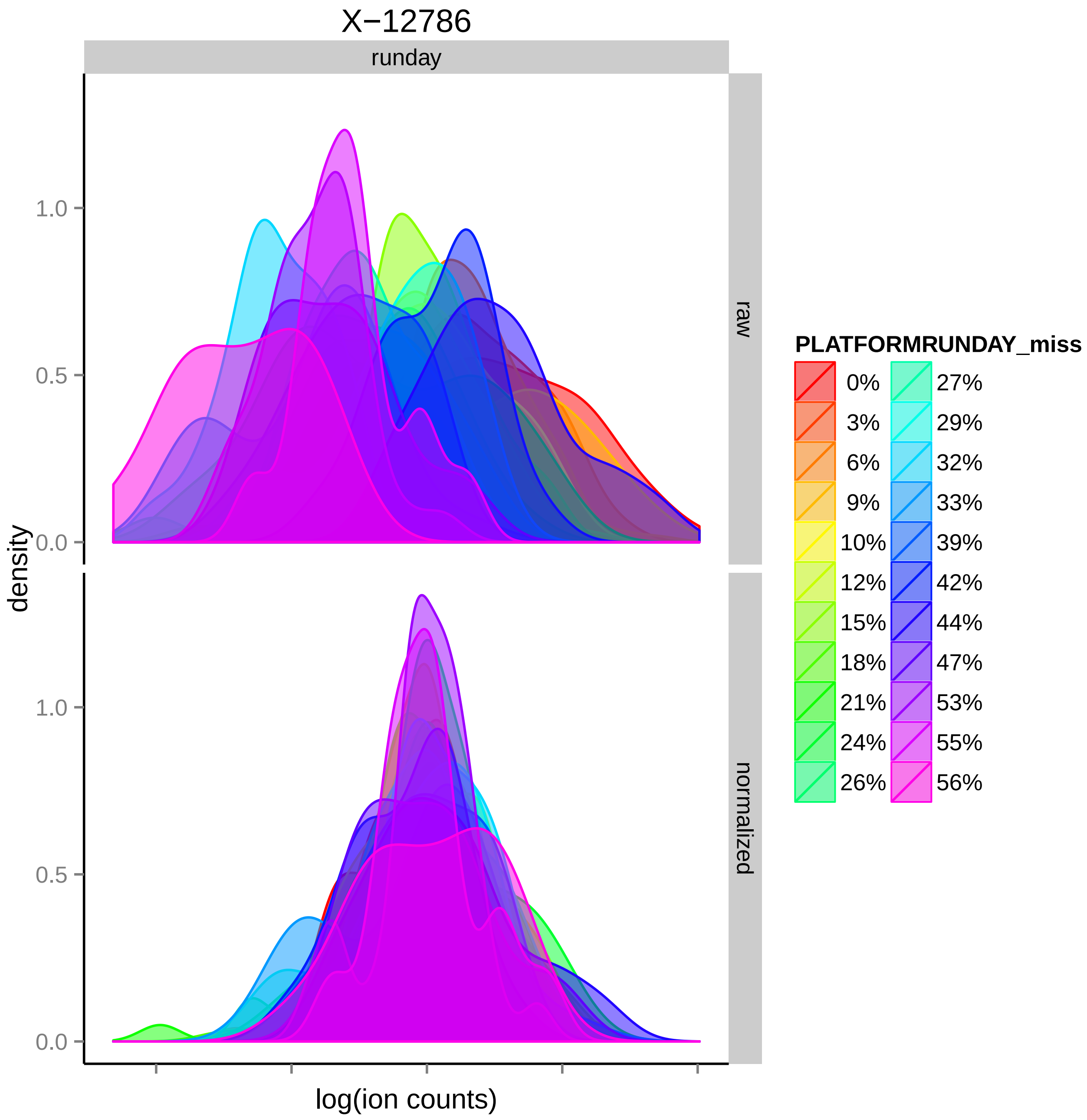
X−12786
runday
raw
normalized
density
1.0
0.5
0.0
log(ion counts)
PLATFORMRUNDAY_miss
0%
3%
6%
9%
10%
12%
15%
18%
21%
24%
26%
27%
29%
32%
33%
39%
42%
44%
47%
53%
55%
56%

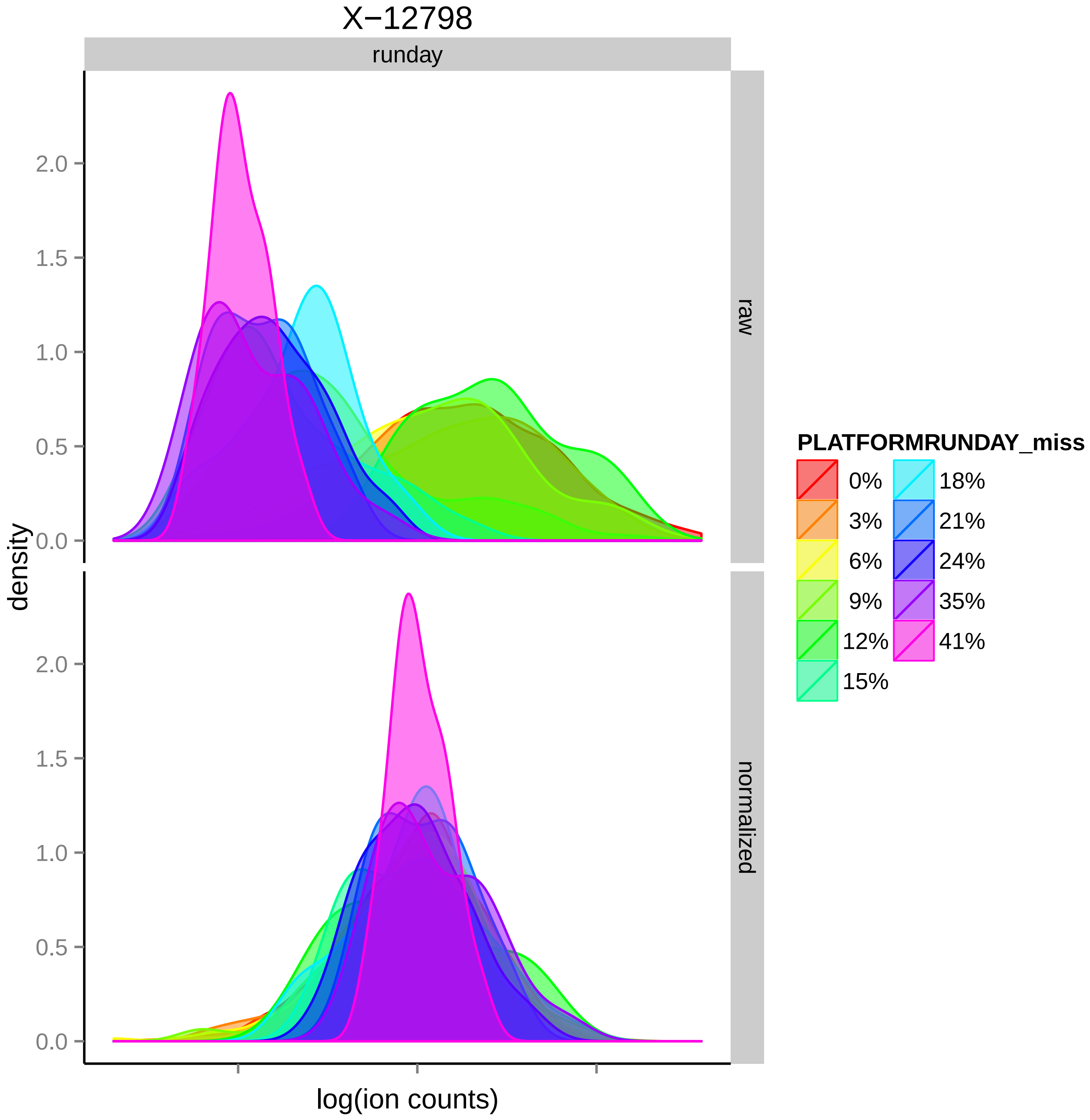
X−12798
runday
raw
normalized
density
log(ion counts)
0.0
0.5
1.0
1.5
2.0
PLATFORMRUNDAY_miss
0%
3%
6%
9%
12%
15%
18%
21%
24%
35%
41%

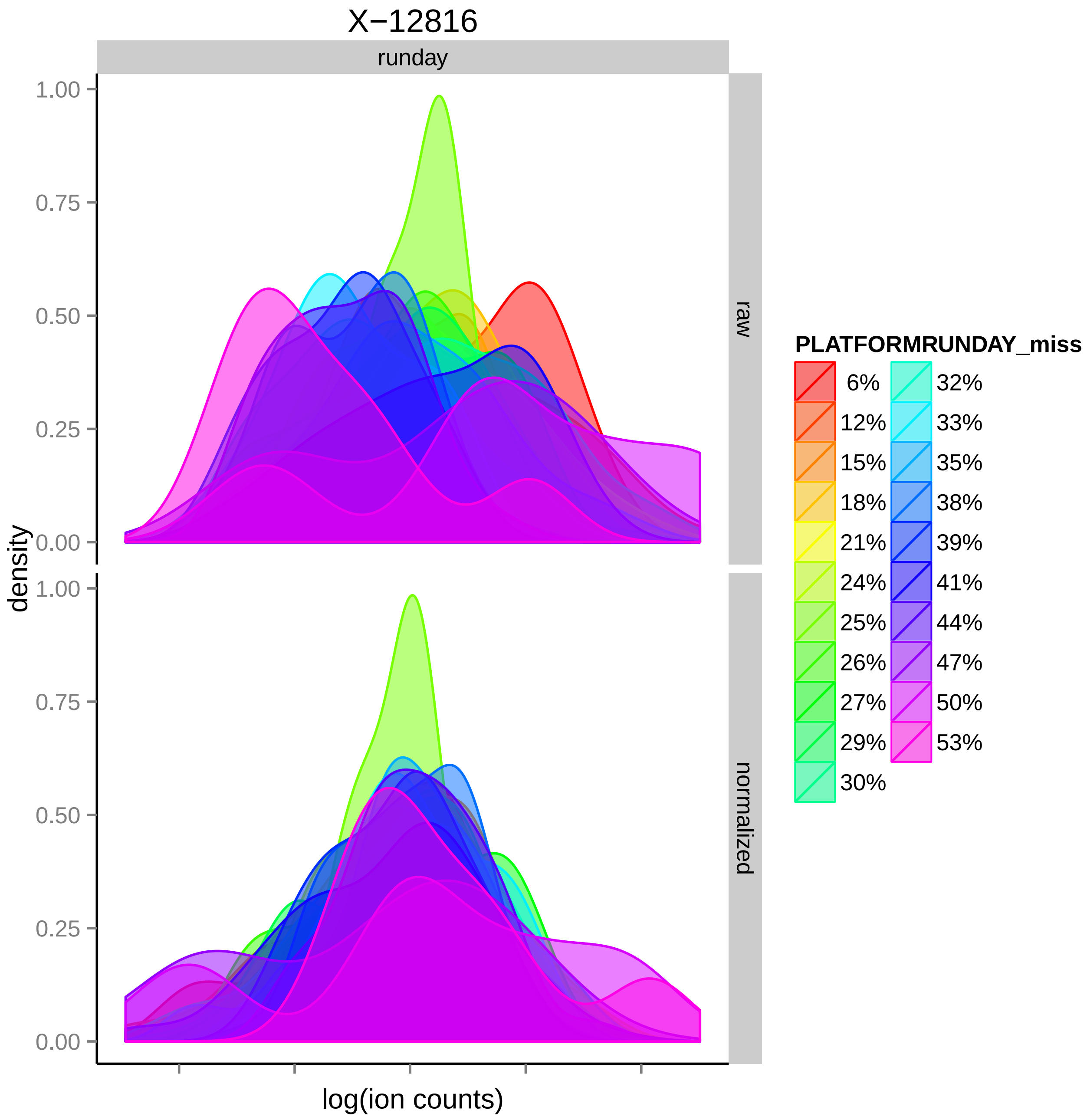

X−12816
runday
raw
normalized
density
log(ion counts)
1.00
0.75
0.50
0.25
0.00
PLATFORMRUNDAY_miss
6%
12%
15%
18%
21%
24%
25%
26%
27%
29%
30%
32%
33%
35%
38%
39%
41%
44%
47%
50%
53%

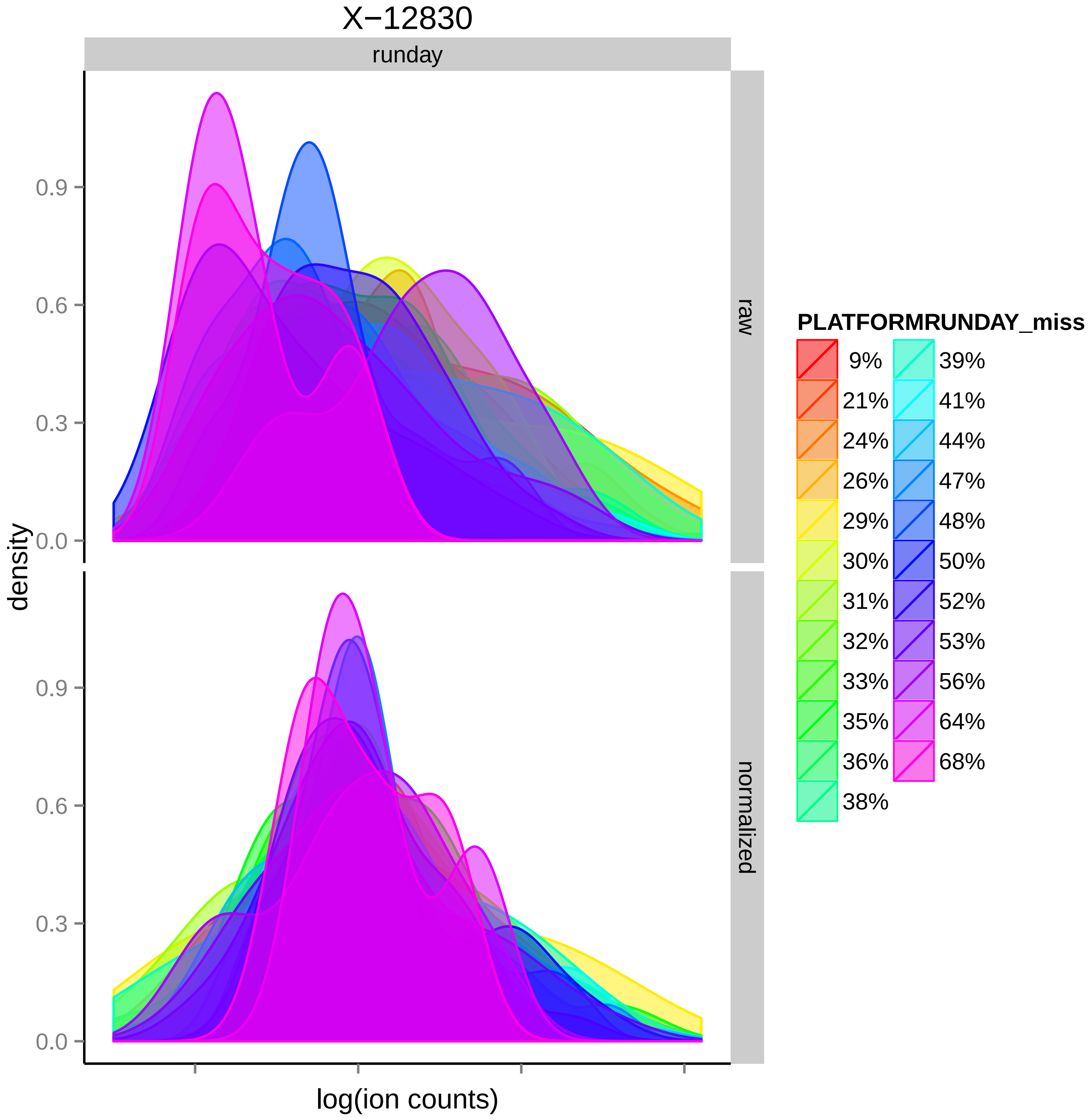
X−12830
runday
raw
normalized
density
log(ion counts)
0.0
0.3
0.6
0.9
PLATFORMRUNDAY_miss
9%
21%
24%
26%
29%
30%
31%
32%
33%
35%
36%
38%
39%
41%
44%
47%
48%
50%
52%
53%
56%
64%
68%

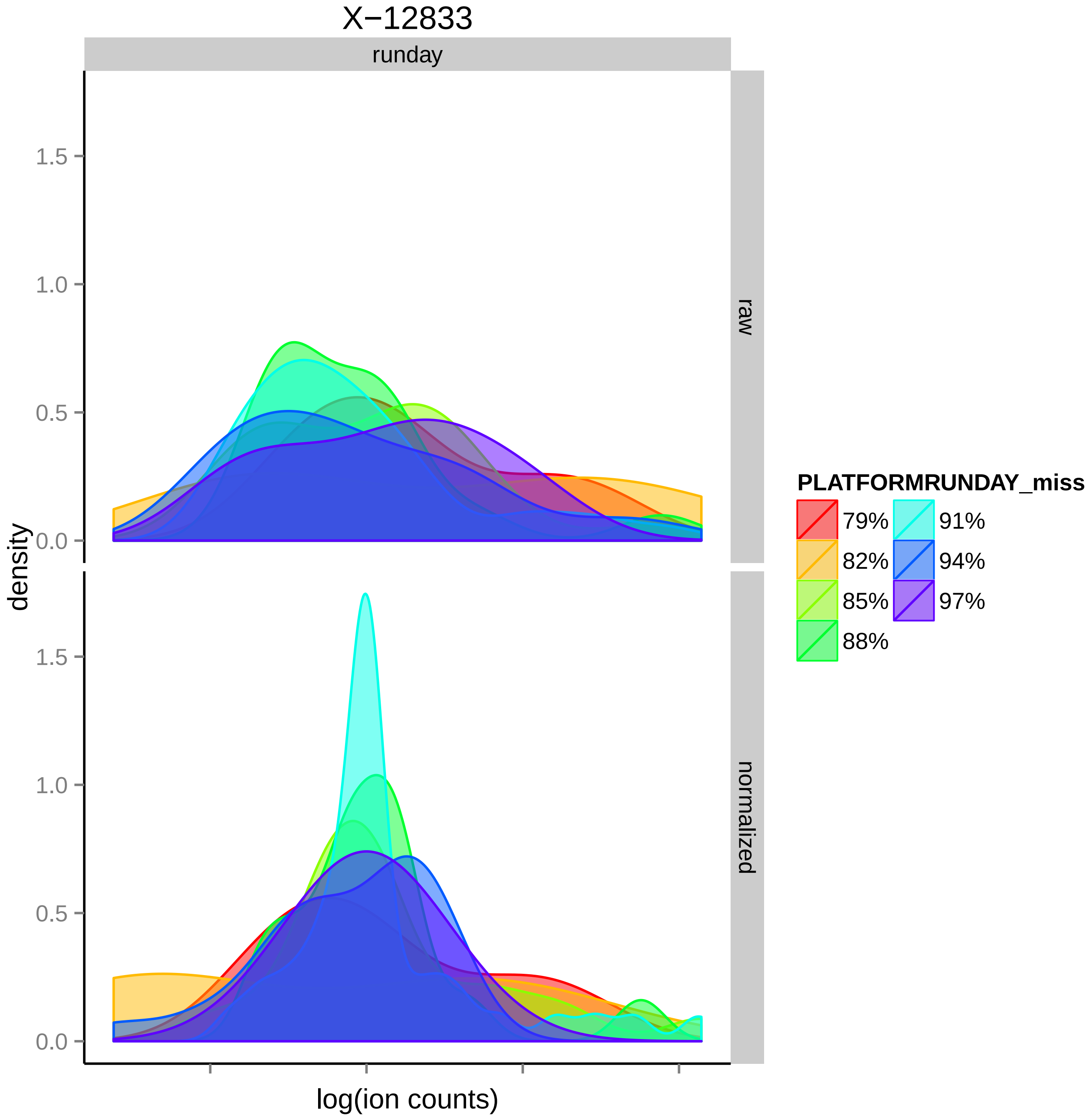
X−12833
runday
raw
normalized
density
log(ion counts)
0.0
0.5
1.0
1.5
PLATFORMRUNDAY_miss
79%
82%
85%
88%
91%
94%
97%

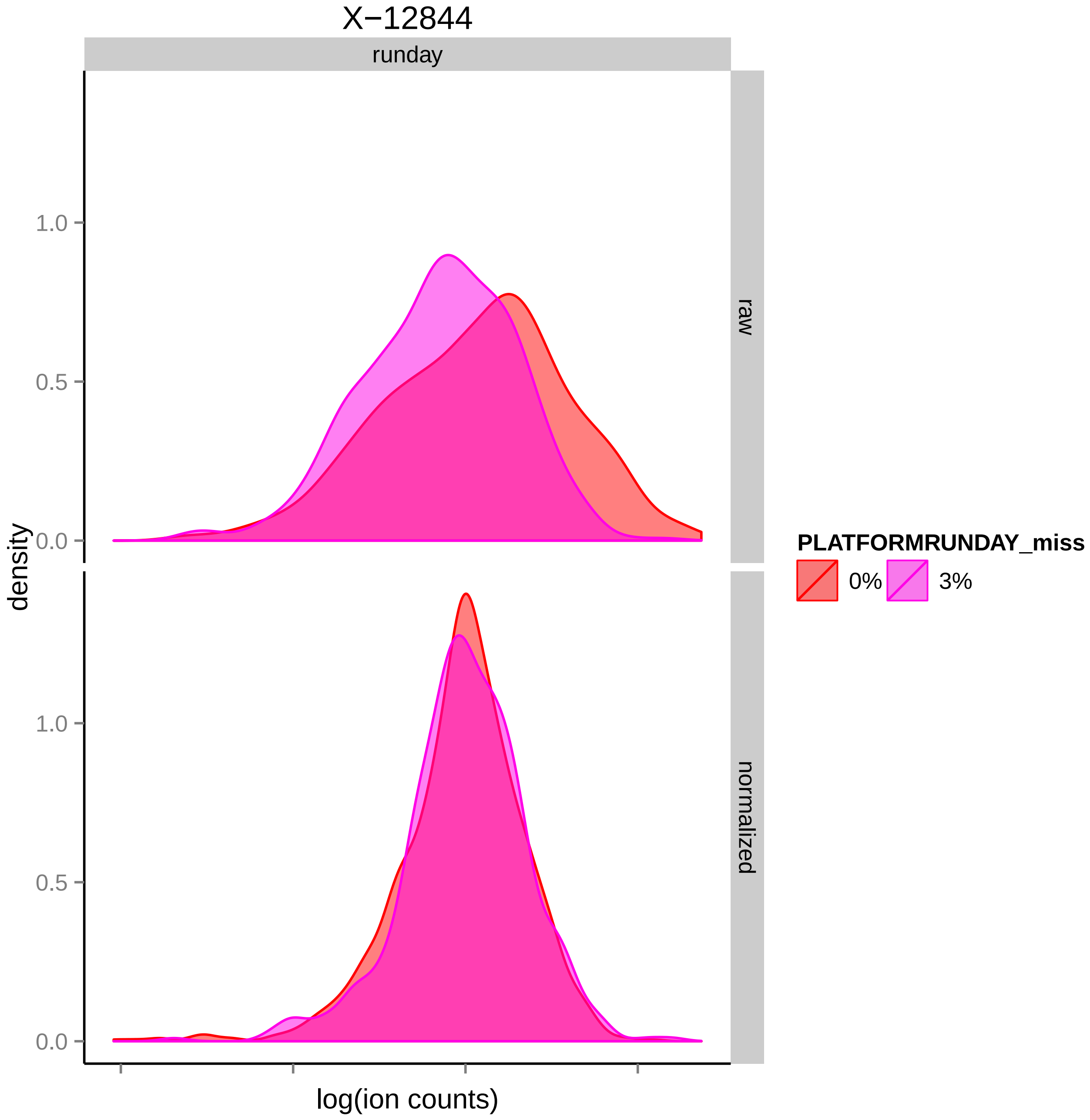
X−12844
runday
raw
normalized
1.0
0.5
0.0
1.0
0.5
0.0
density
log(ion counts)
PLATFORMRUNDAY_miss
0%
3%

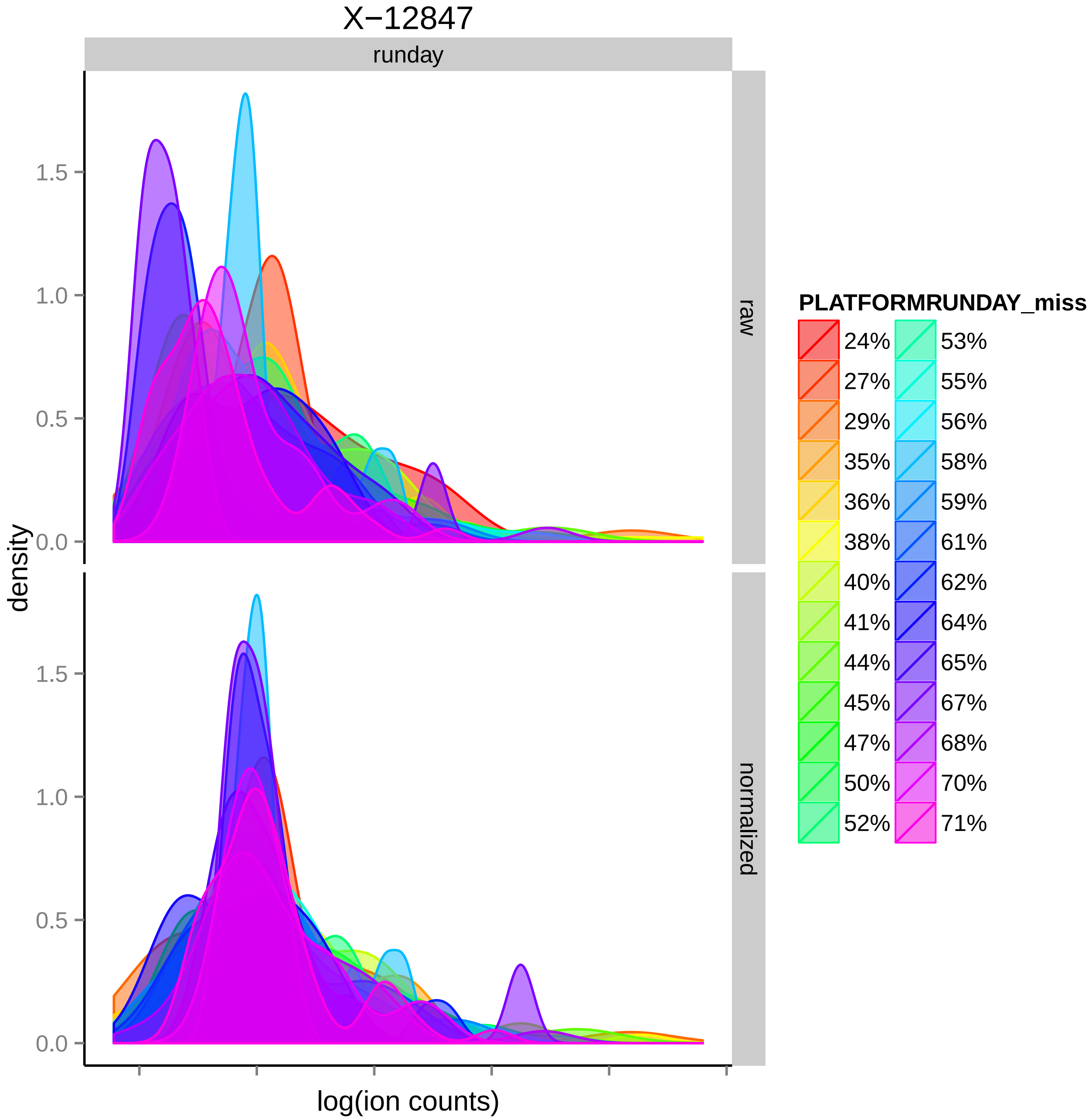
X−12847
runday
raw
normalized
density
log(ion counts)
0.0
0.5
1.0
1.5
0.0
0.5
1.0
1.5
PLATFORMRUNDAY_miss
24%
27%
29%
35%
36%
38%
40%
41%
44%
45%
47%
50%
52%
53%
55%
56%
58%
59%
61%
62%
64%
65%
67%
68%
70%
71%

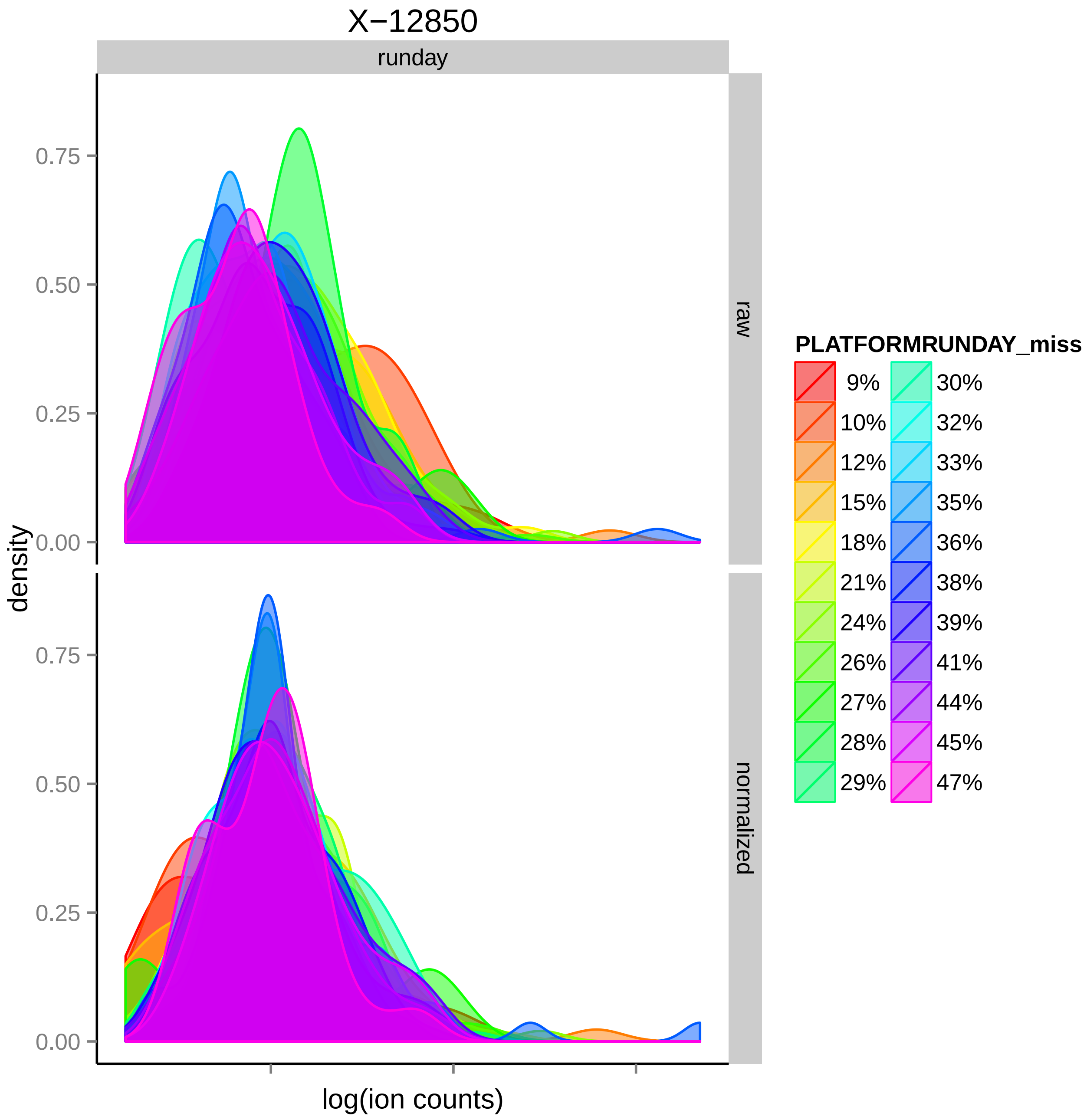
X−12850
runday
raw
normalized
density
log(ion counts)
0.75
0.50
0.25
0.00
0.75
0.50
0.25
0.00
PLATFORMRUNDAY_miss
9%
10%
12%
15%
18%
21%
24%
26%
27%
28%
29%
30%
32%
33%
35%
36%
38%
39%
41%
44%
45%
47%

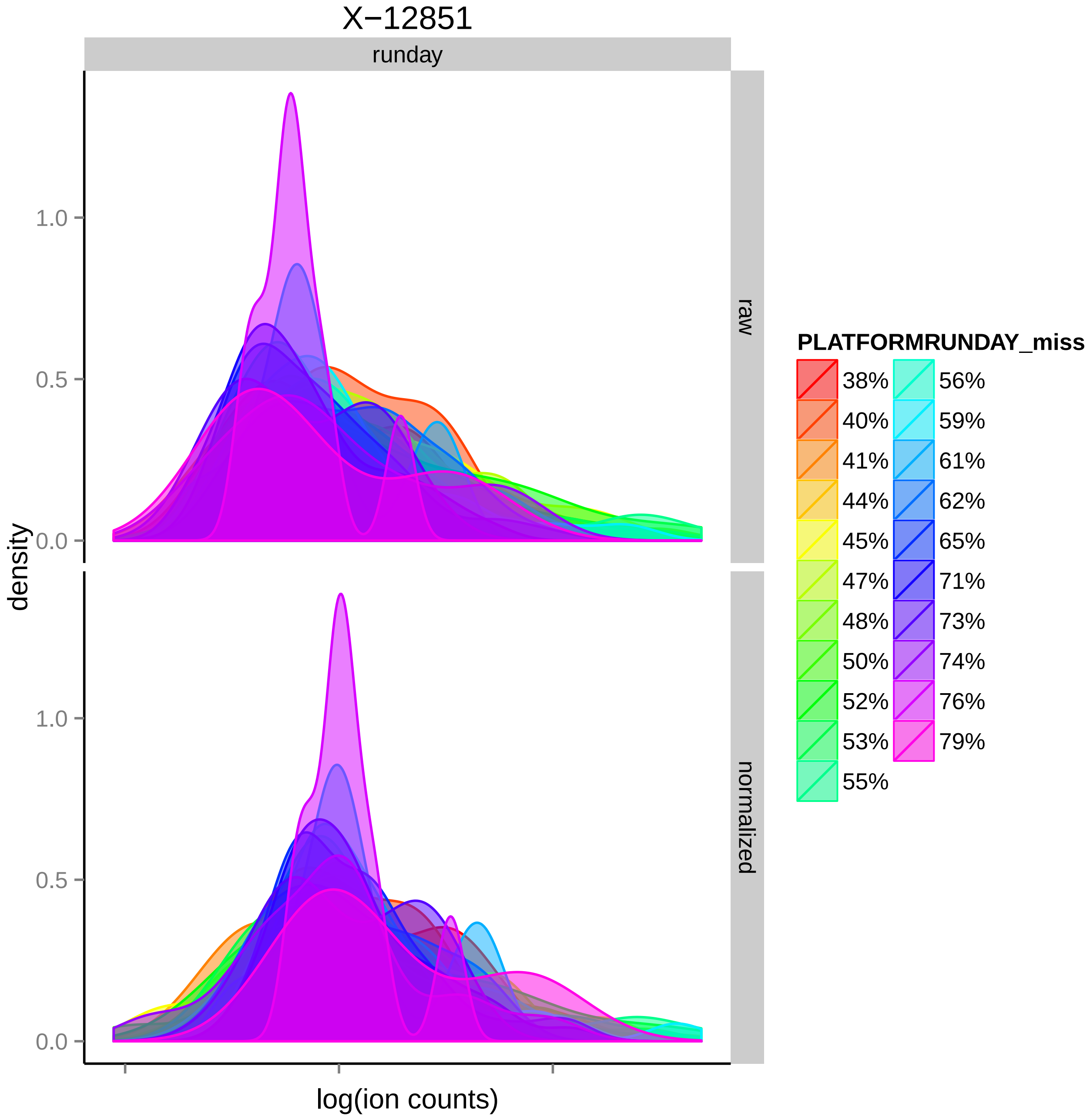
X−12851
runday
raw
normalized
density
1.0
0.5
0.0
1.0
0.5
0.0
log(ion counts)
PLATFORMRUNDAY_miss
38%
40%
41%
44%
45%
47%
48%
50%
52%
53%
55%
56%
59%
61%
62%
65%
71%
73%
74%
76%
79%

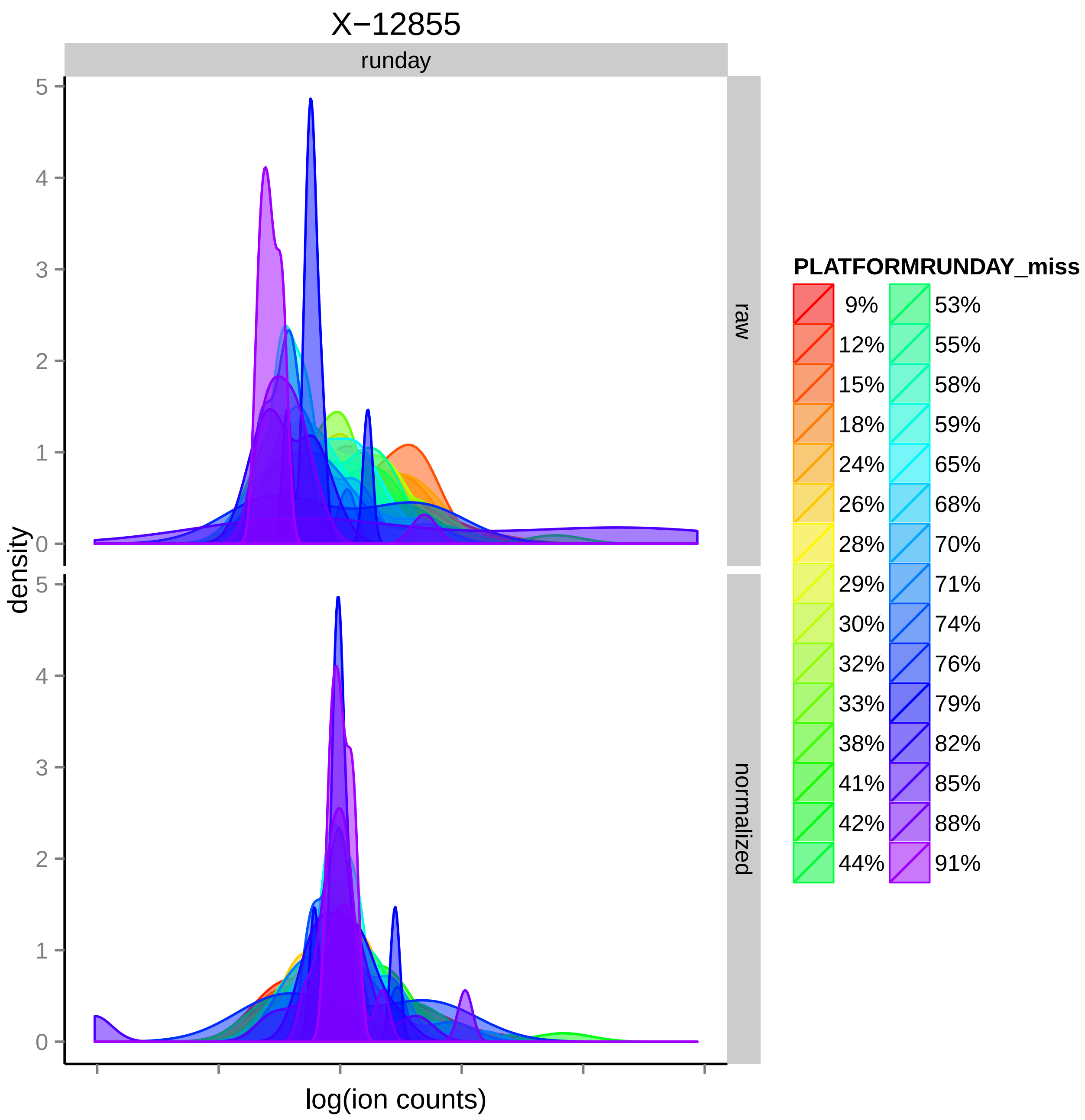

X−12855
runday
raw
normalized
density
log(ion counts)
0
1
2
3
4
5
PLATFORMRUNDAY_miss
9%
12%
15%
18%
24%
26%
28%
29%
30%
32%
33%
38%
41%
42%
44%
53%
55%
58%
59%
65%
68%
70%
71%
74%
76%
79%
82%
85%
88%
91%

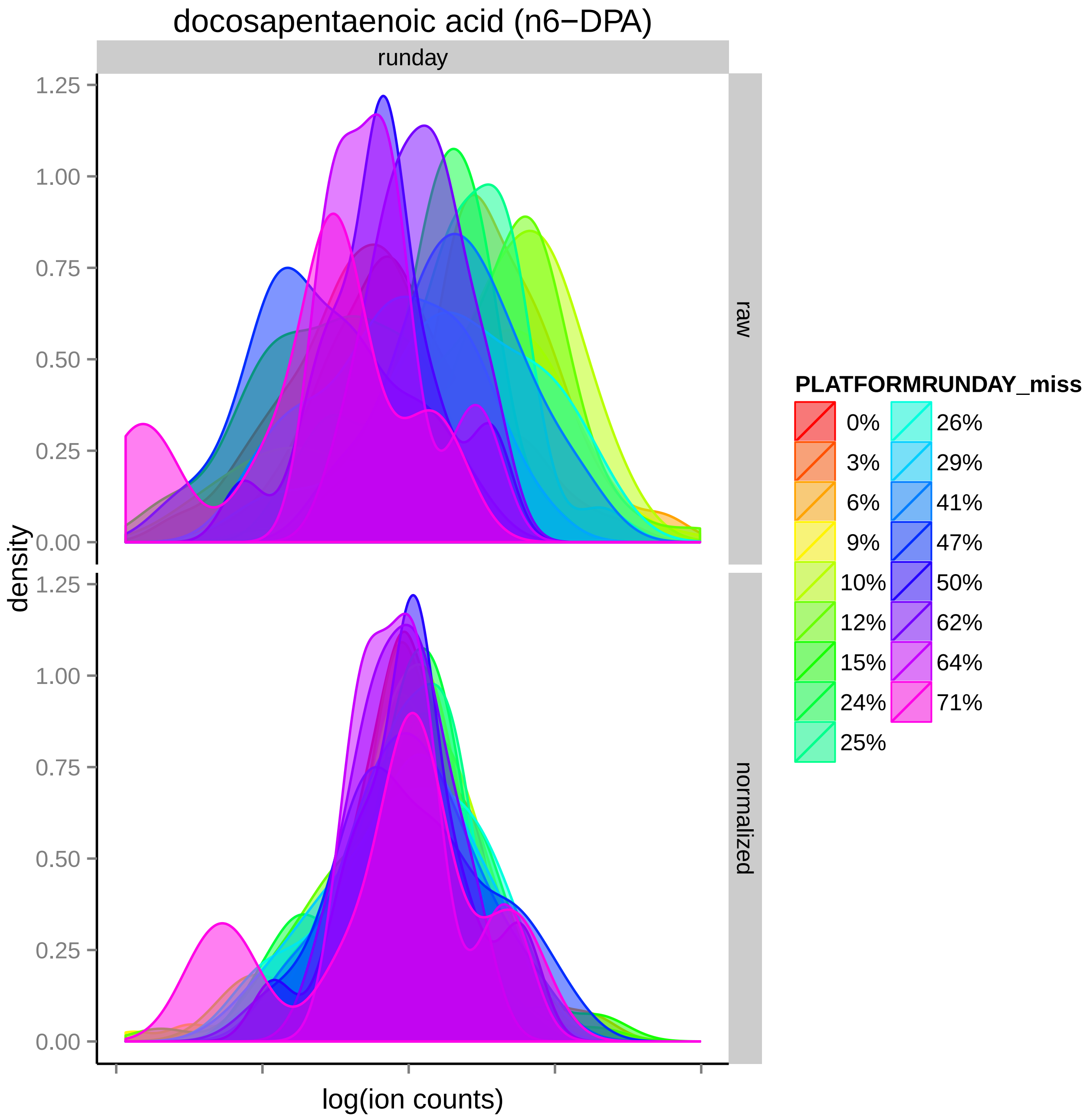
docosapentaenoic acid (n6−DPA)
runday
raw
normalized
density
log(ion counts)
0.00
0.25
0.50
0.75
1.00
1.25
PLATFORMRUNDAY_miss
0%
3%
6%
9%
10%
12%
15%
24%
25%
26%
29%
41%
47%
50%
62%
64%
71%

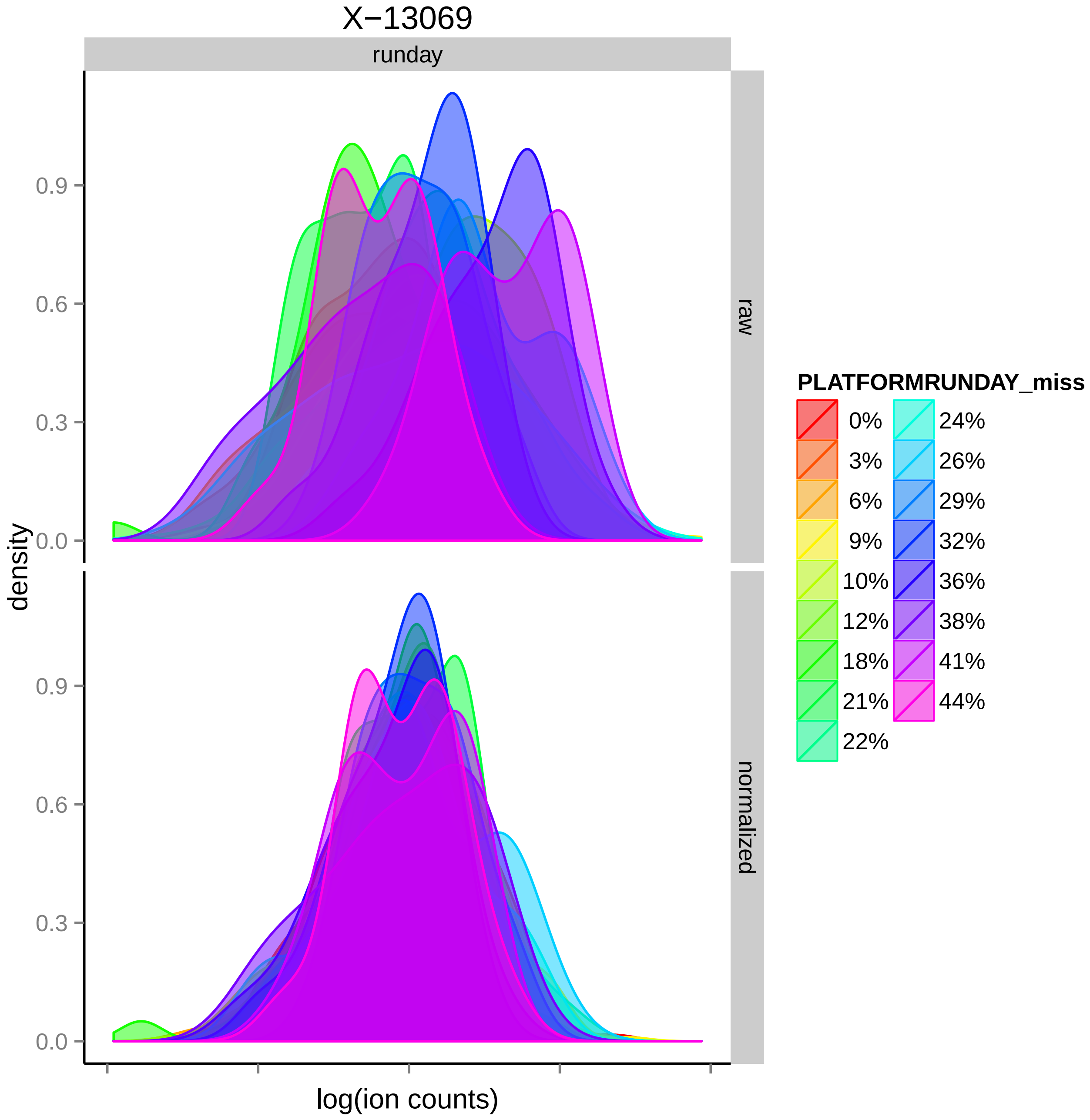
X−13069
runday
raw
normalized
density
log(ion counts)
0.0
0.3
0.6
0.9
PLATFORMRUNDAY_miss
0%
3%
6%
9%
10%
12%
18%
21%
22%
24%
26%
29%
32%
36%
38%
41%
44%

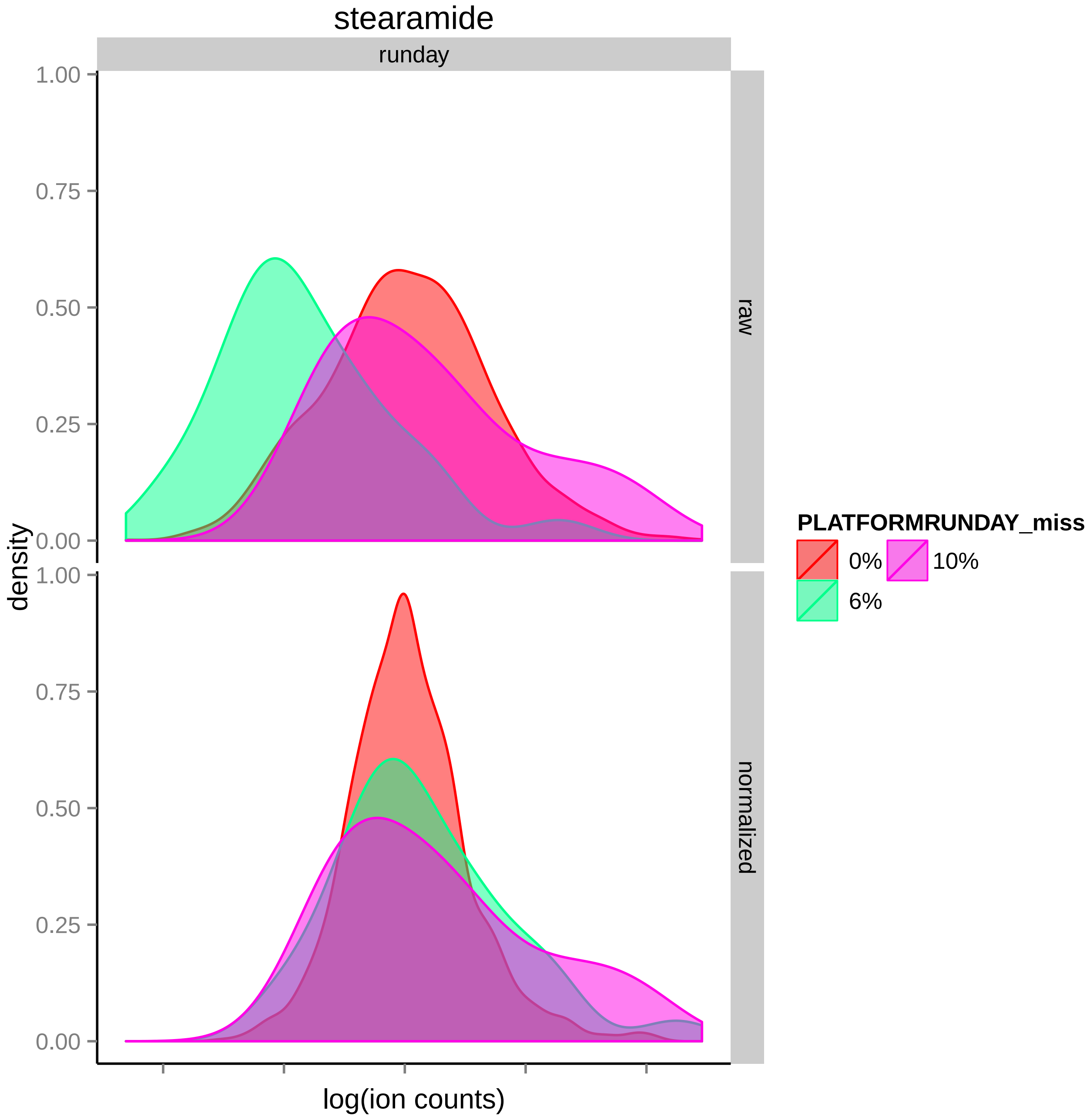

stearamide
runday
raw
normalized
density
log(ion counts)
1.00
0.75
0.50
0.25
0.00
PLATFORMRUNDAY_miss
0%
6%
10%

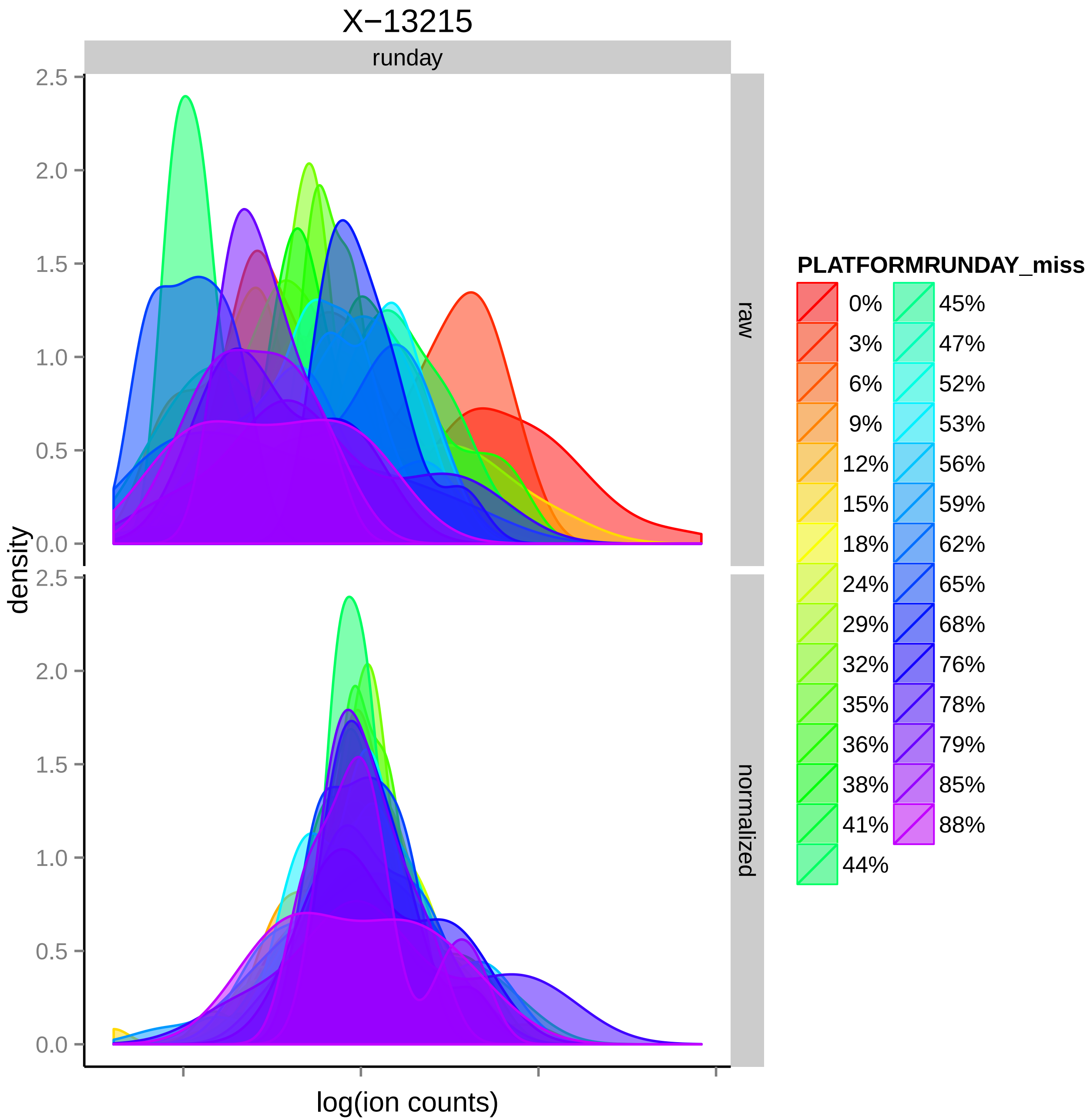
X−13215
runday
raw
normalized
density
log(ion counts)
2.5
2.0
1.5
1.0
0.5
0.0
PLATFORMRUNDAY_miss
0%
3%
6%
9%
12%
15%
18%
24%
29%
32%
35%
36%
38%
41%
44%
45%
47%
52%
53%
56%
59%
62%
65%
68%
76%
78%
79%
85%
88%

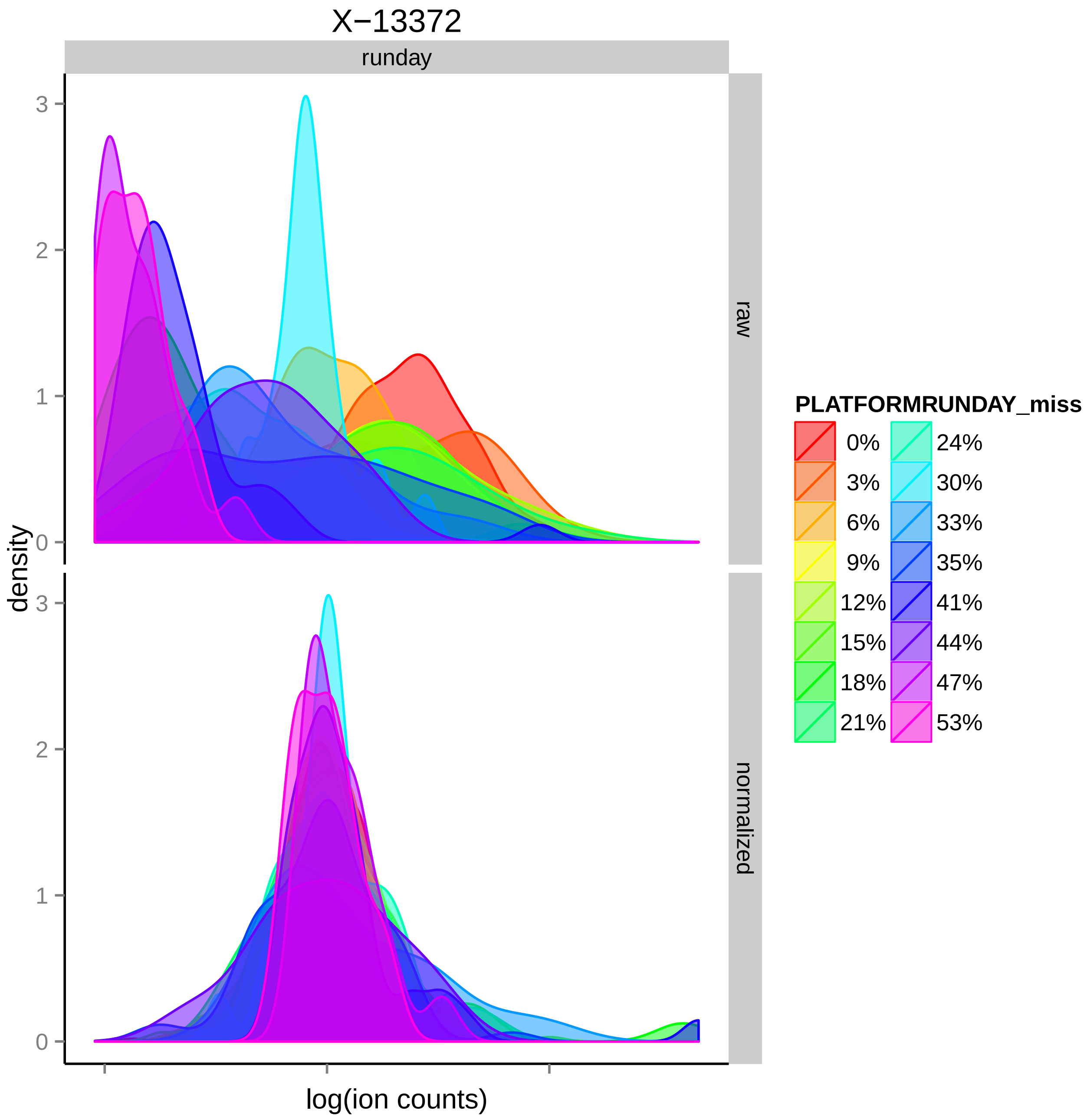

X−13372
runday
raw
normalized
density
log(ion counts)
0
1
2
3
PLATFORMRUNDAY_miss
0%
3%
6%
9%
12%
15%
18%
21%
24%
30%
33%
35%
41%
44%
47%
53%

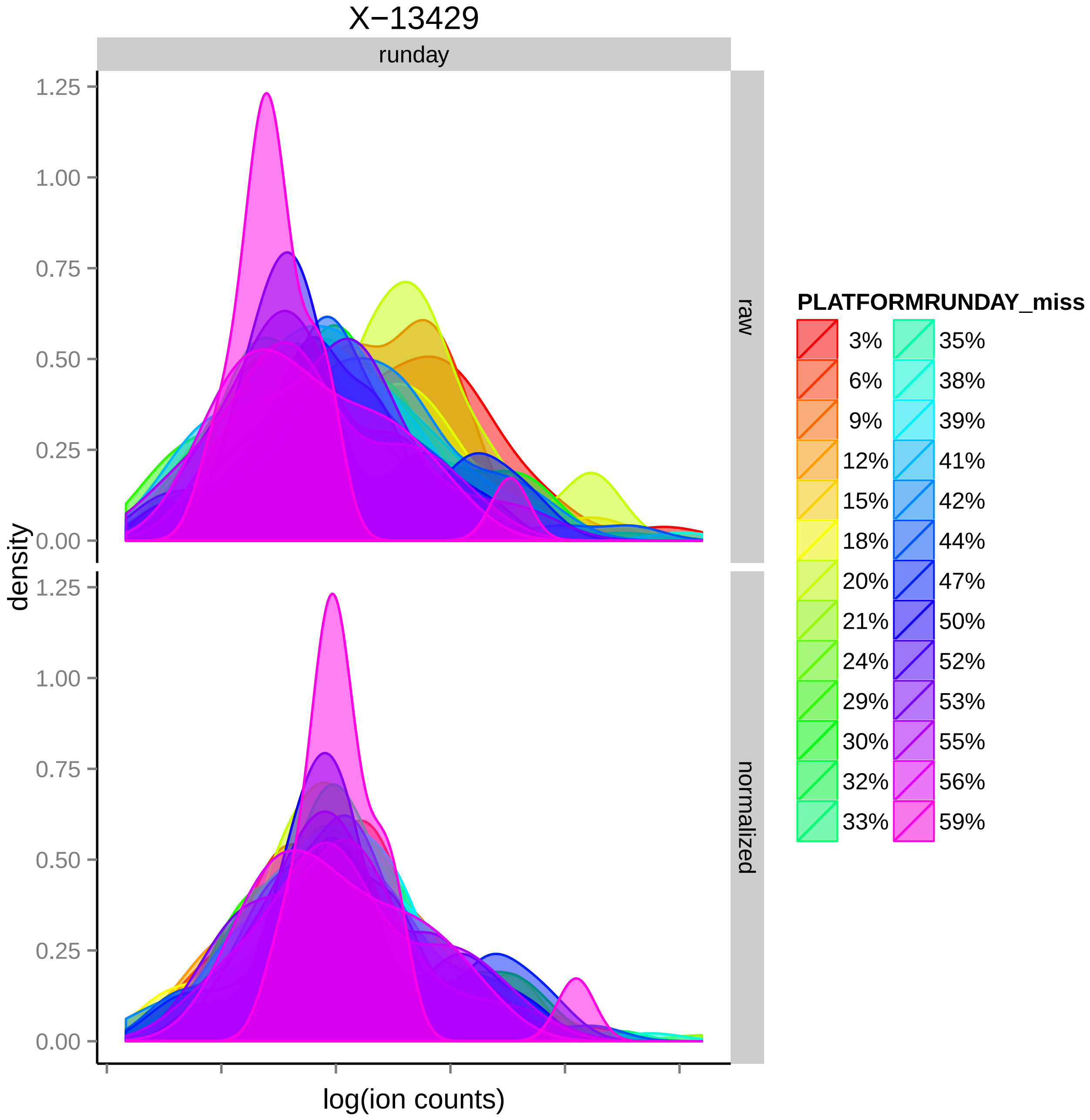
X−13429
runday
raw
normalized
density
log(ion counts)
1.25
1.00
0.75
0.50
0.25
0.00
PLATFORMRUNDAY_miss
3%
6%
9%
12%
15%
18%
20%
21%
24%
29%
30%
32%
33%
35%
38%
39%
41%
42%
44%
47%
50%
52%
53%
55%
56%
59%

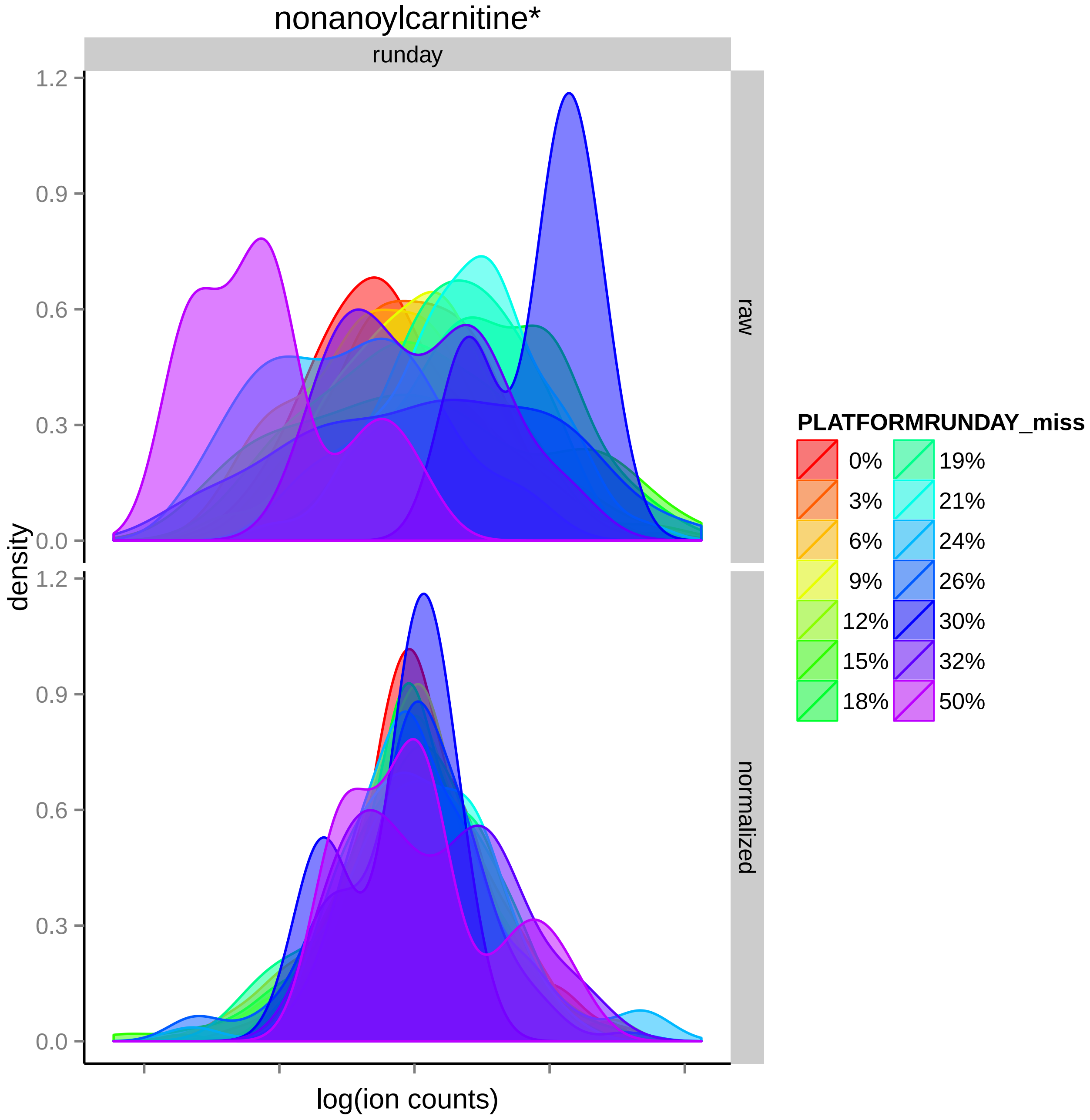

nonanoylcarnitine*
runday
raw
normalized
density
1.2
0.9
0.6
0.3
0.0
log(ion counts)
PLATFORMRUNDAY_miss
0%
3%
6%
9%
12%
15%
18%
19%
21%
24%
26%
30%
32%
50%

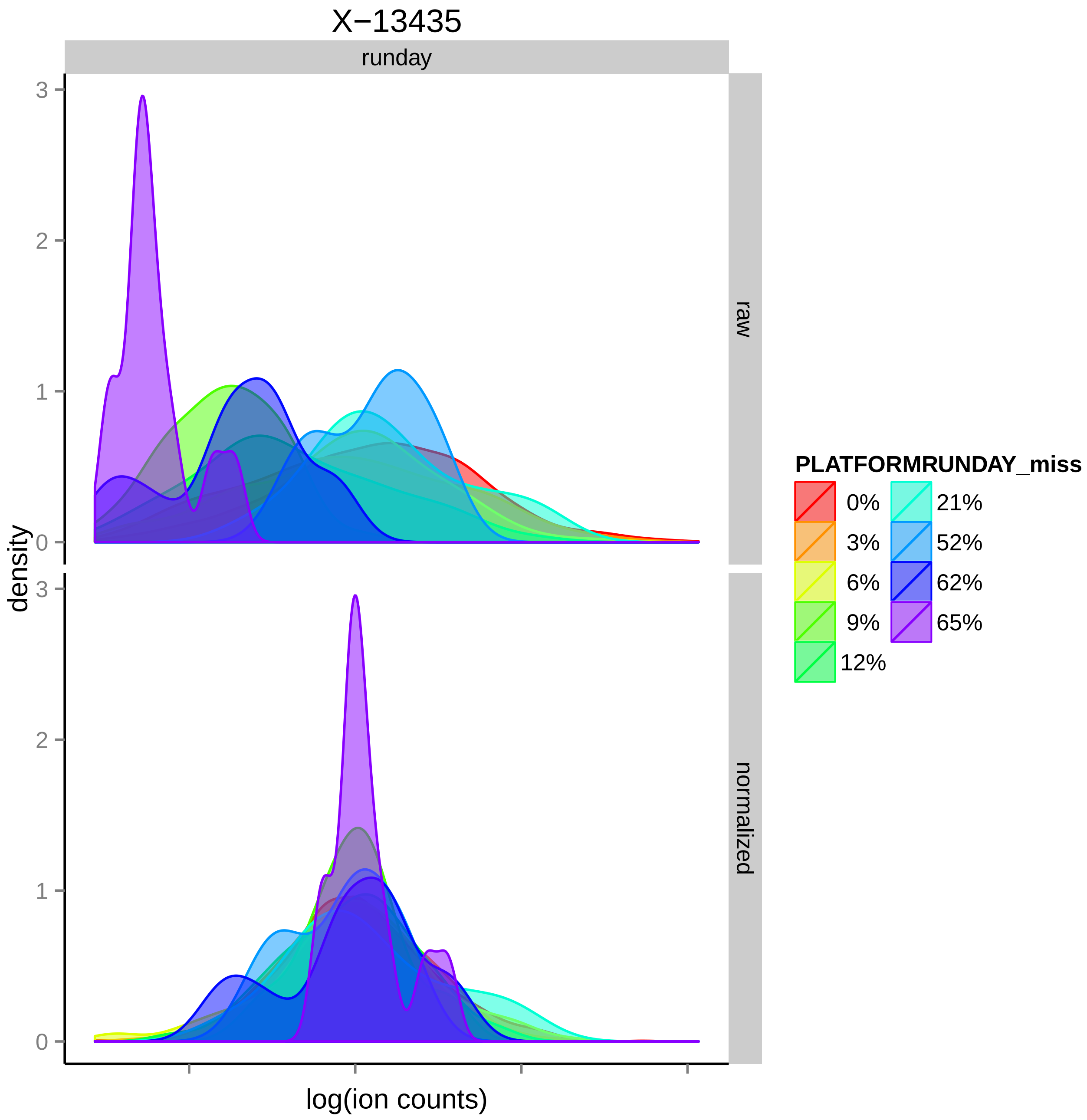

X−13435
runday
raw
normalized
density
log(ion counts)
3
2
1
0
PLATFORMRUNDAY_miss
0%
3%
6%
9%
12%
21%
52%
62%
65%

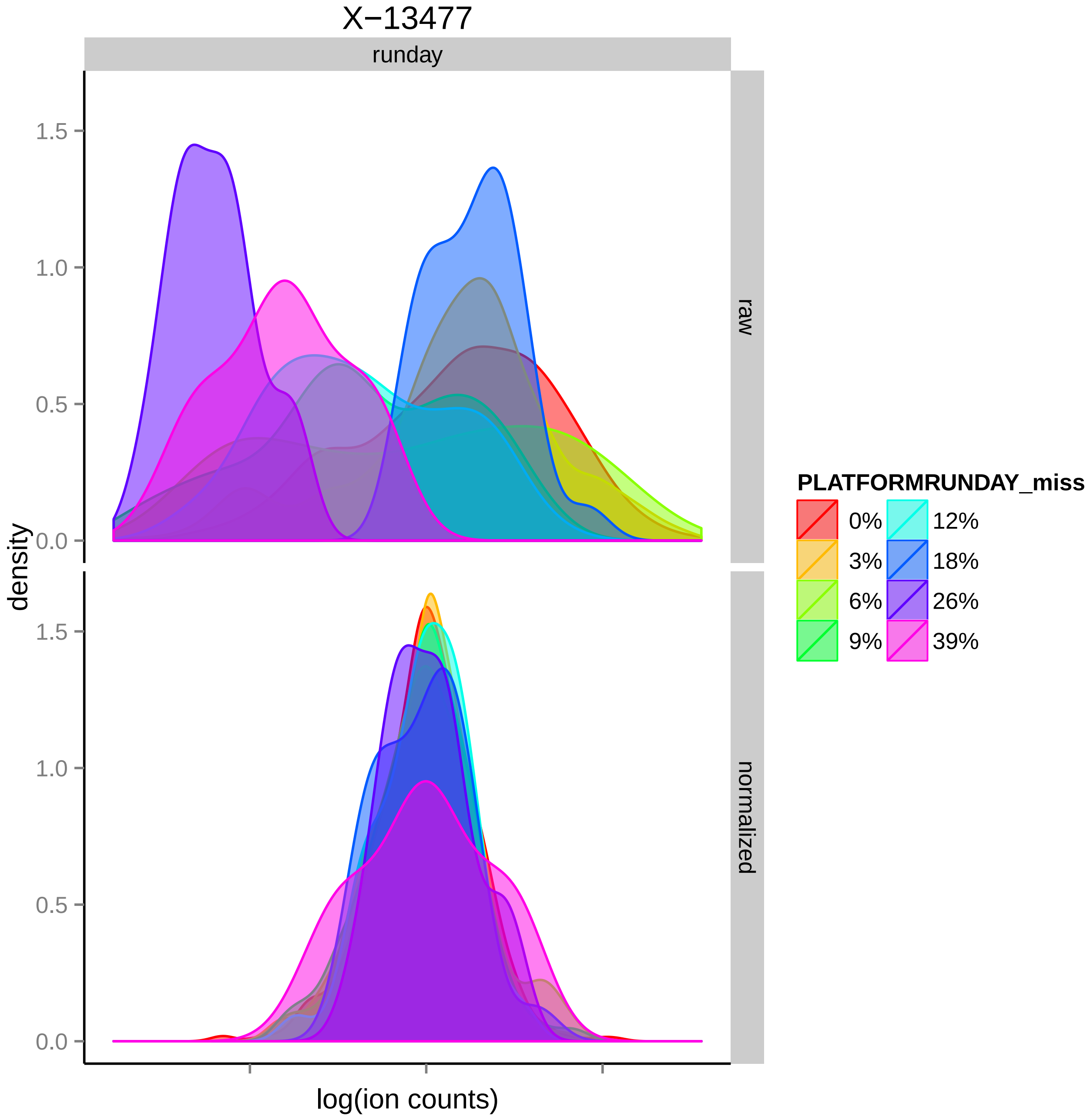

X−13477
runday
raw
normalized
density
1.5
1.0
0.5
0.0
log(ion counts)
PLATFORMRUNDAY_miss
0%
3%
6%
9%
12%
18%
26%
39%

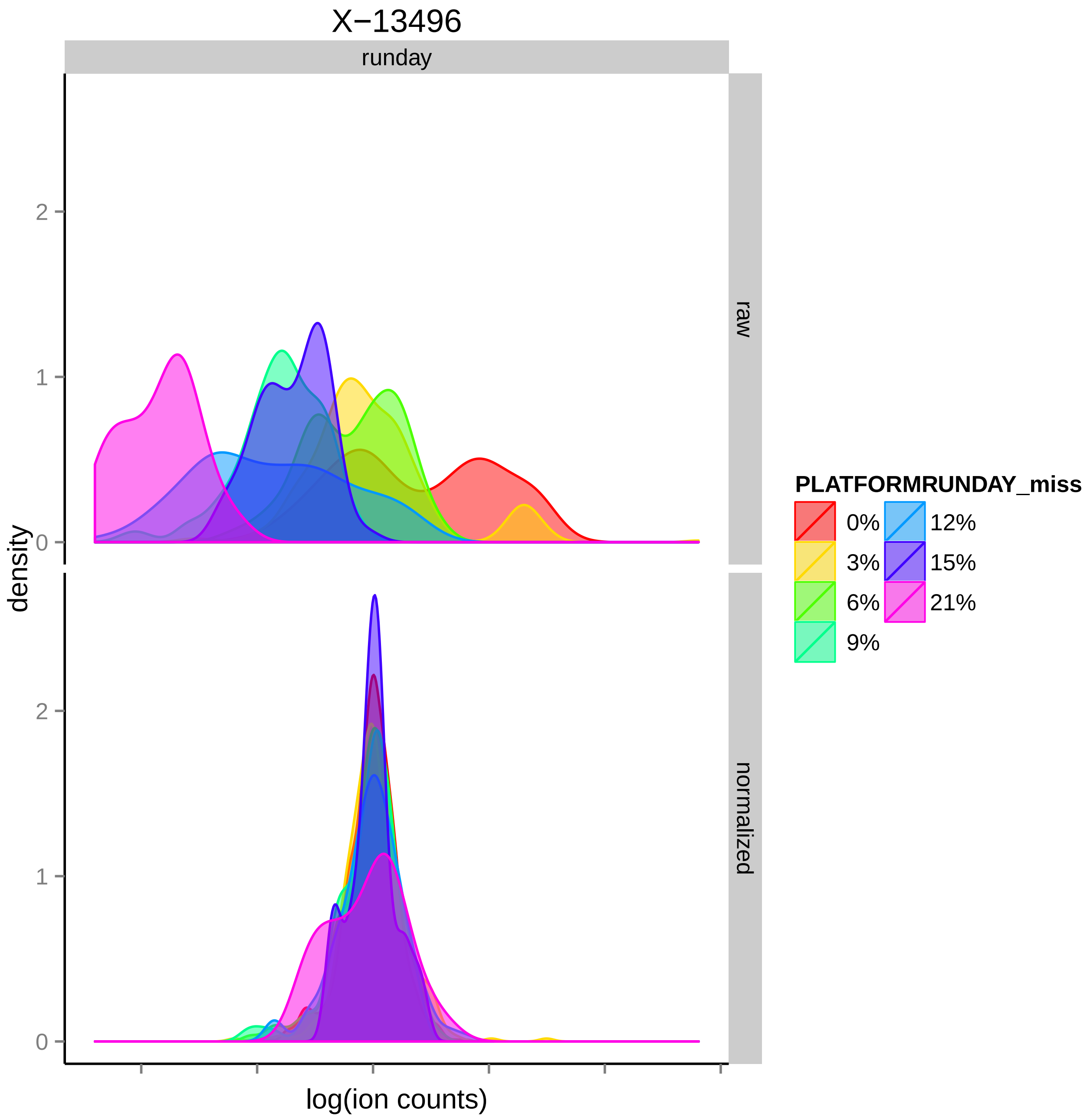
X−13496
runday
raw
normalized
density
log(ion counts)
0
1
2
0
1
2
PLATFORMRUNDAY_miss
0%
3%
6%
9%
12%
15%
21%

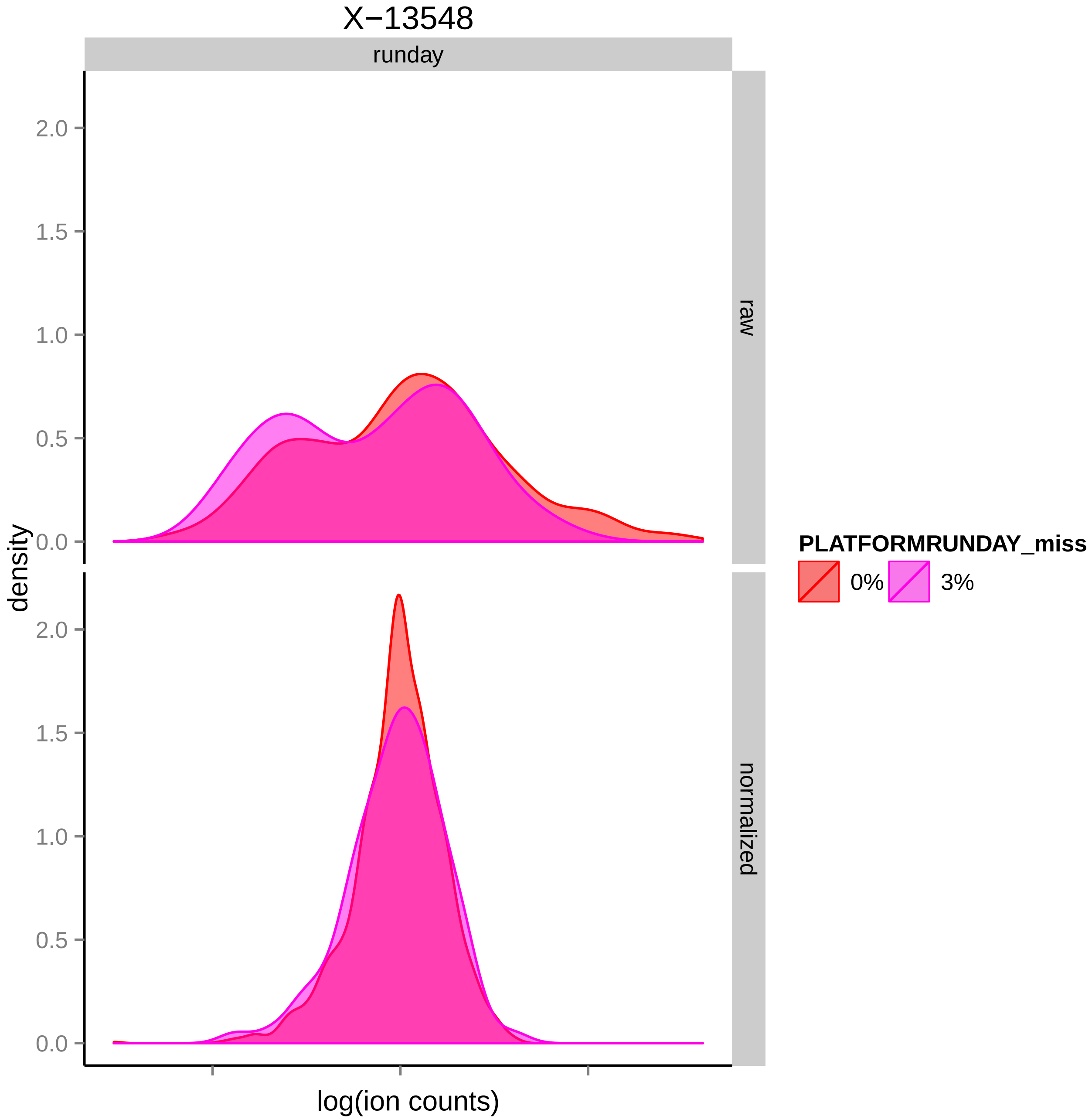
X−13548
runday
raw
normalized
density
2.0
1.5
1.0
0.5
0.0
log(ion counts)
PLATFORMRUNDAY_miss
0%
3%

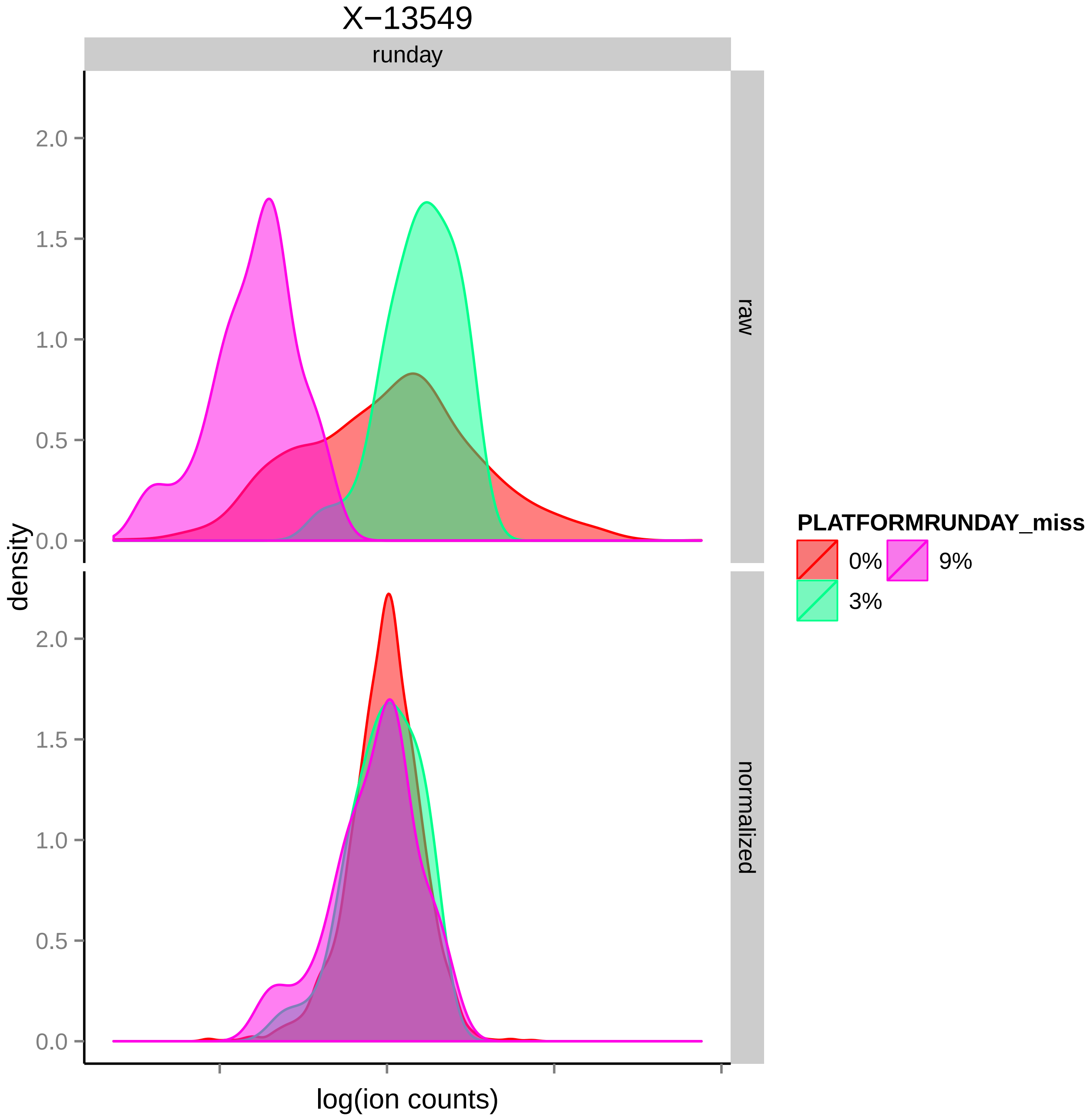

X−13549
runday
raw
normalized
density
2.0
1.5
1.0
0.5
0.0
log(ion counts)
PLATFORMRUNDAY_miss
0%
3%
9%

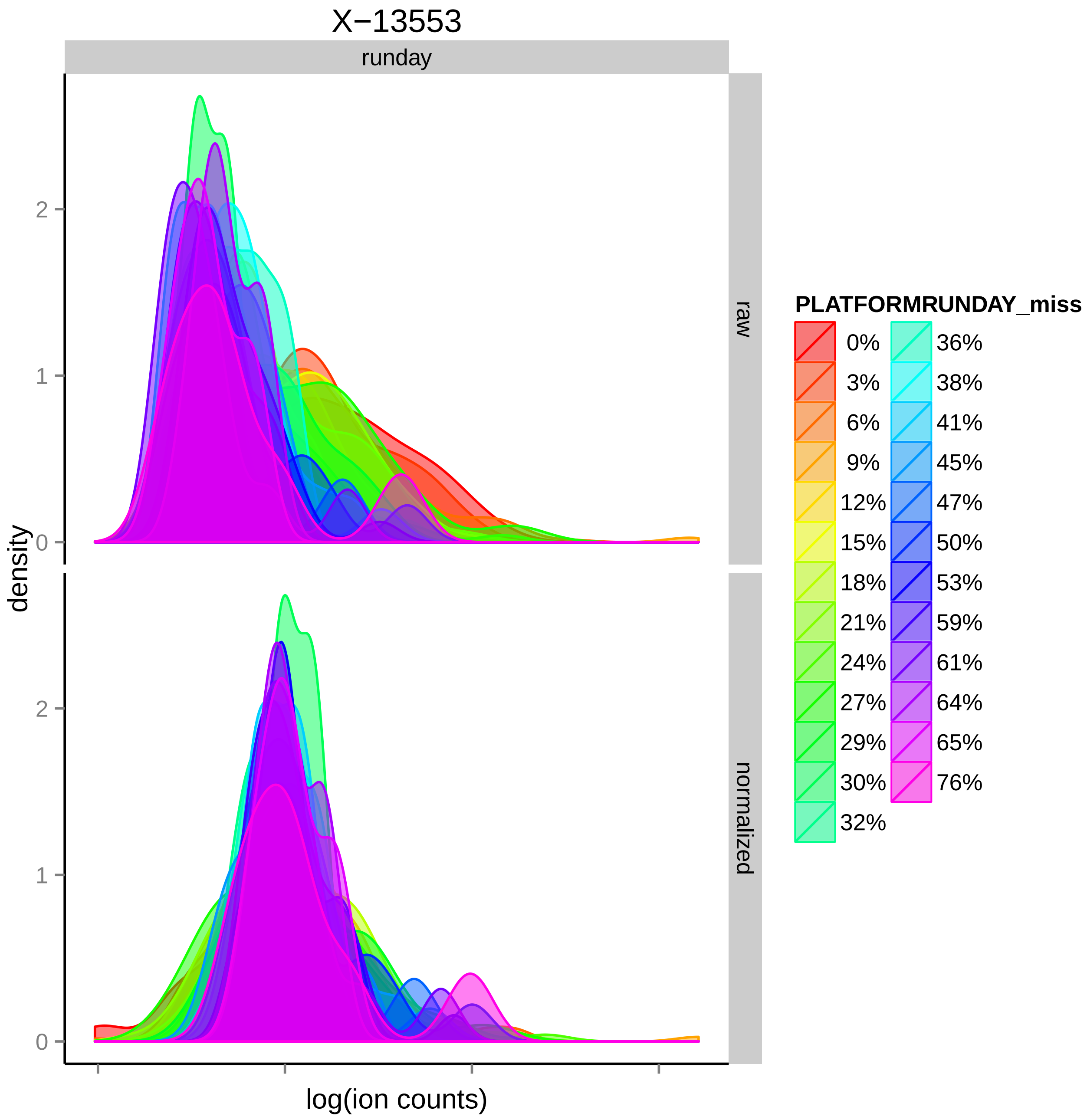

X−13553
runday
raw
normalized
density
log(ion counts)
0
1
2
0
1
2
PLATFORMRUNDAY_miss
0%
3%
6%
9%
12%
15%
18%
21%
24%
27%
29%
30%
32%
36%
38%
41%
45%
47%
50%
53%
59%
61%
64%
65%
76%

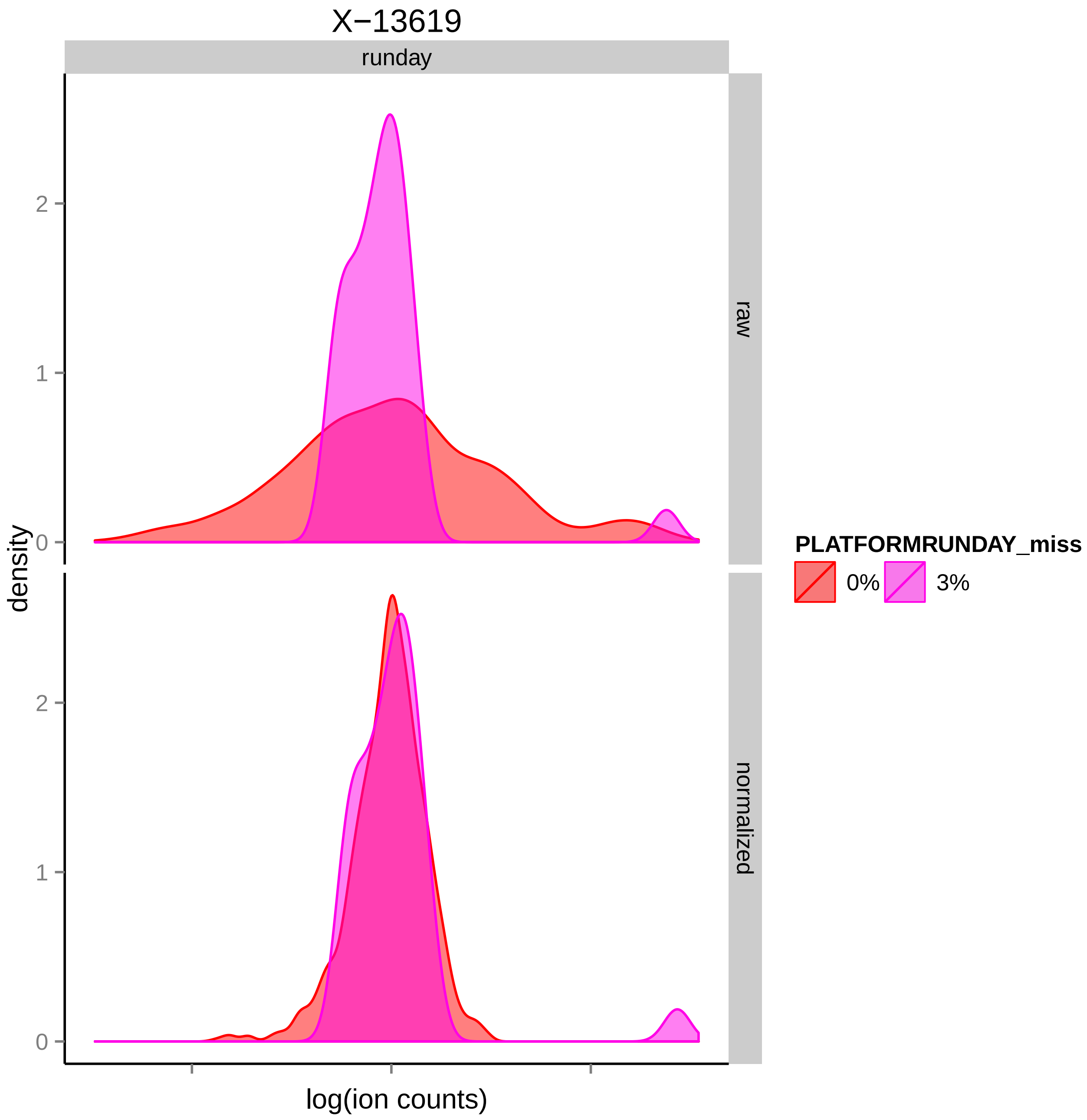
X−13619
runday
raw
normalized
density
2
1
0
2
1
0
log(ion counts)
PLATFORMRUNDAY_miss
0%
3%

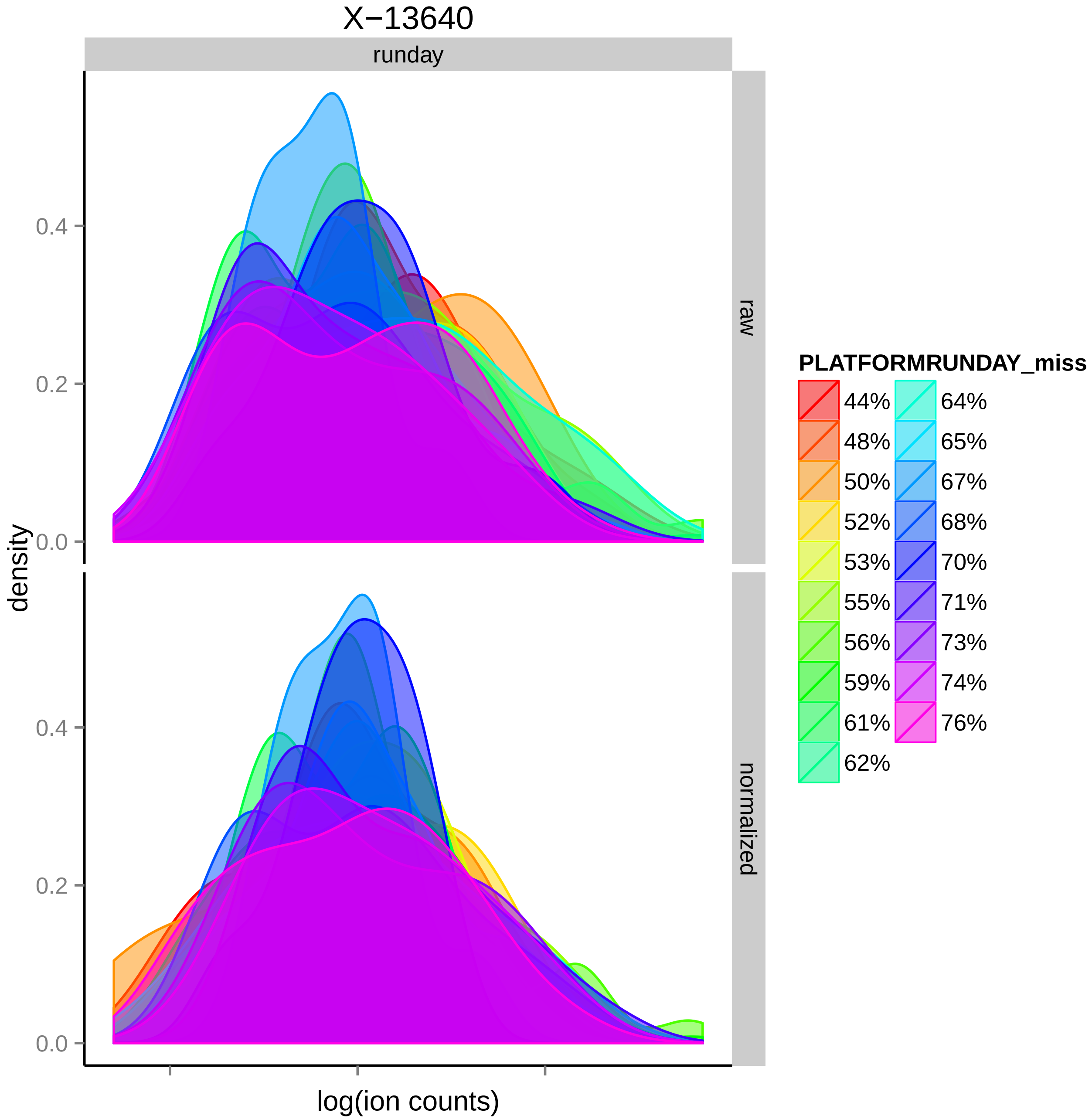
X−13640
runday
raw
normalized
density
0.0
0.2
0.4
log(ion counts)
PLATFORMRUNDAY_miss
44%
48%
50%
52%
53%
55%
56%
59%
61%
62%
64%
65%
67%
68%
70%
71%
73%
74%
76%

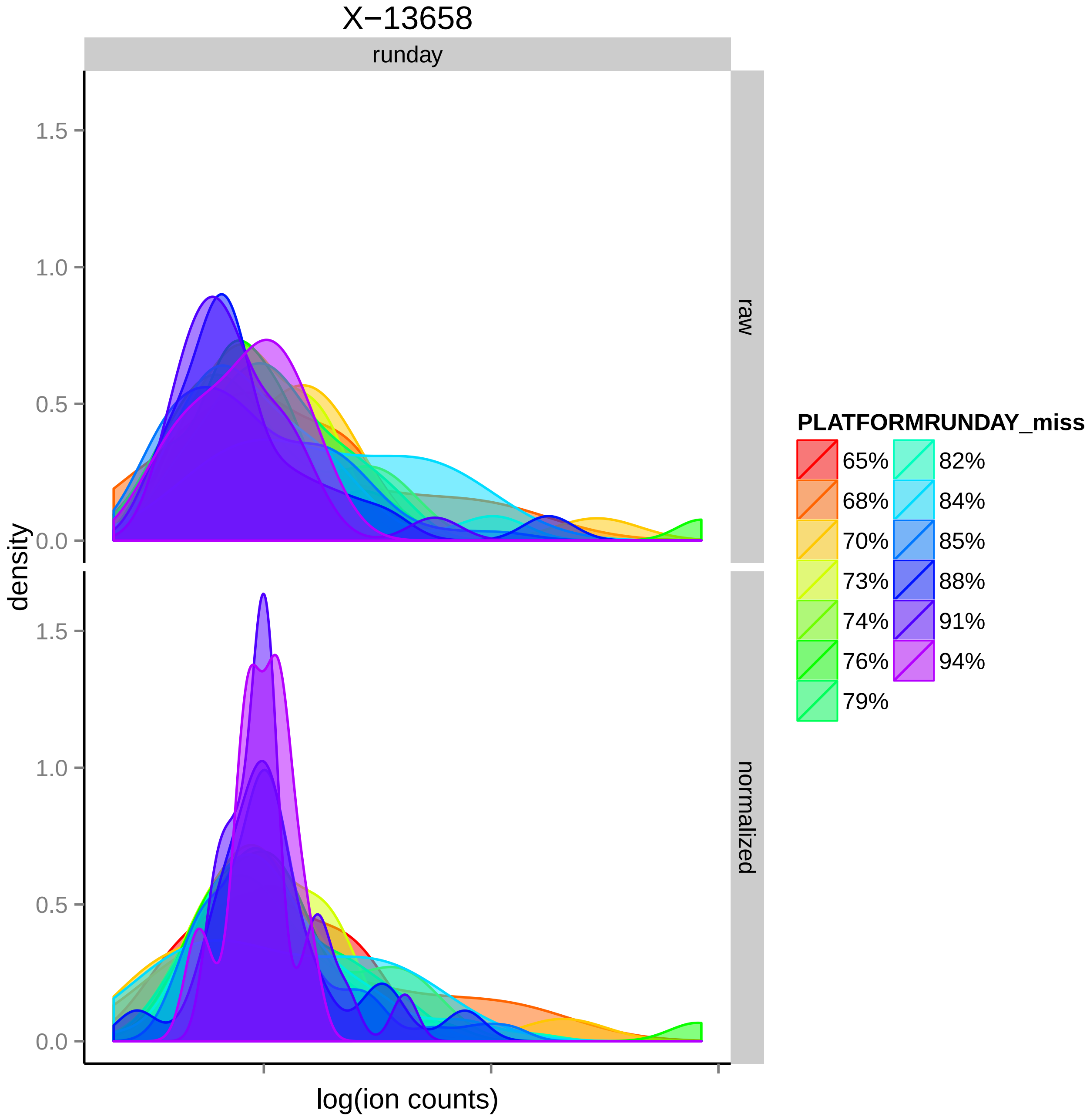

X−13658
runday
raw
normalized
density
log(ion counts)
1.5
1.0
0.5
0.0
PLATFORMRUNDAY_miss
65%
68%
70%
73%
74%
76%
79%
82%
84%
85%
88%
91%
94%

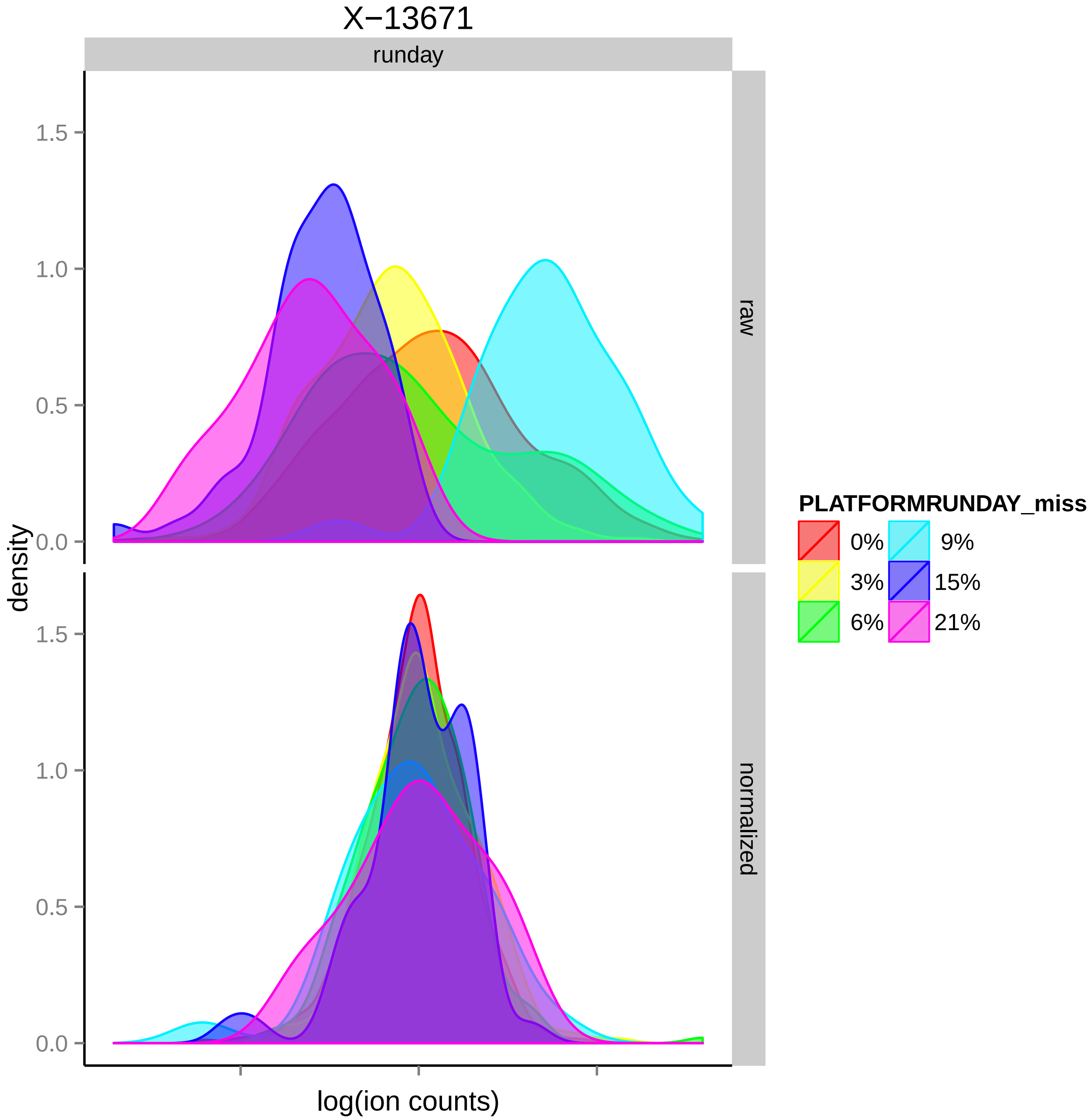
X−13671
runday
raw
normalized
density
1.5
1.0
0.5
0.0
log(ion counts)
PLATFORMRUNDAY_miss
0%
3%
6%
9%
15%
21%

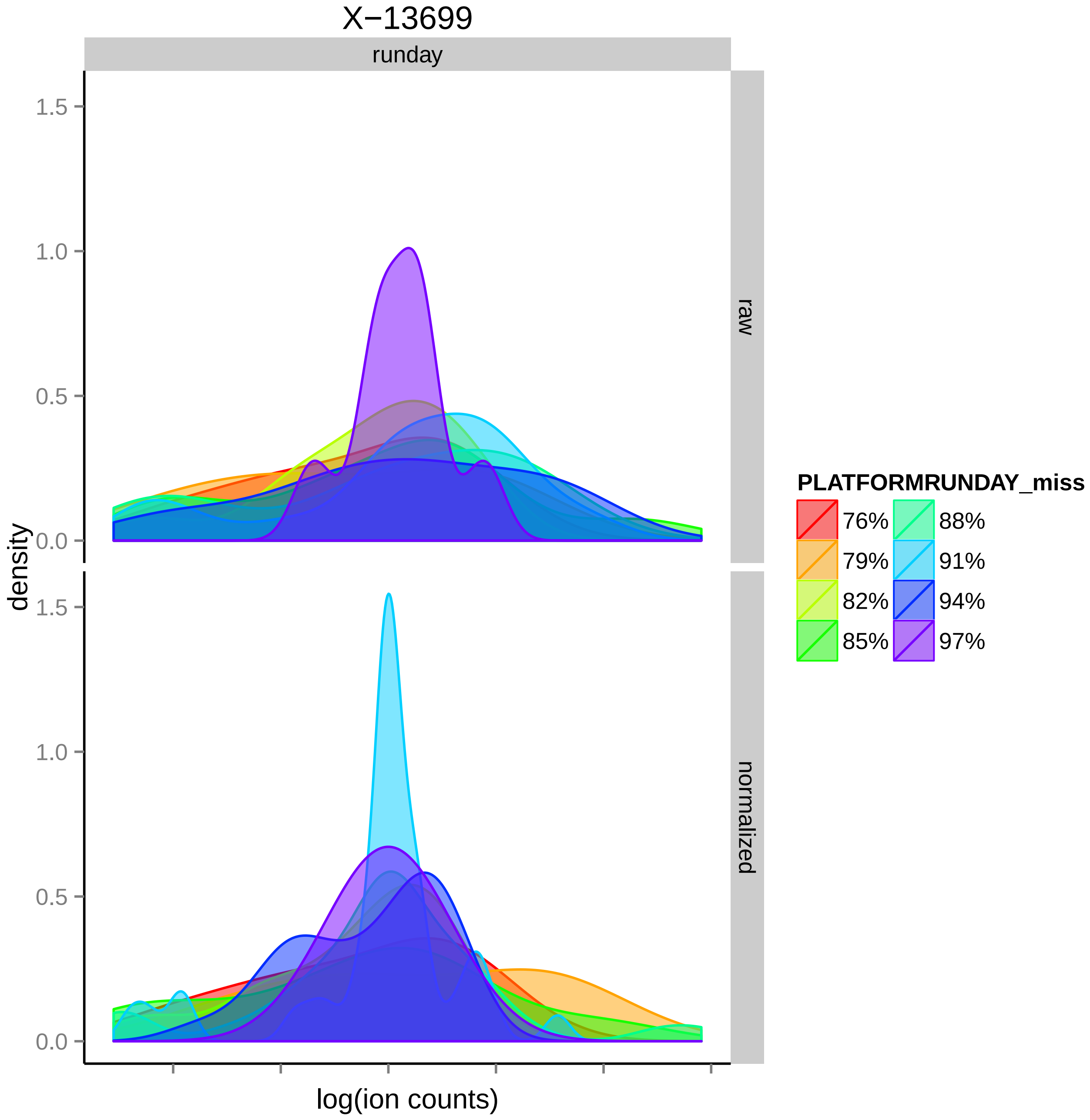

X−13699
runday
raw
normalized
density
log(ion counts)
1.5
1.0
0.5
0.0
PLATFORMRUNDAY_miss
76%
79%
82%
85%
88%
91%
94%
97%

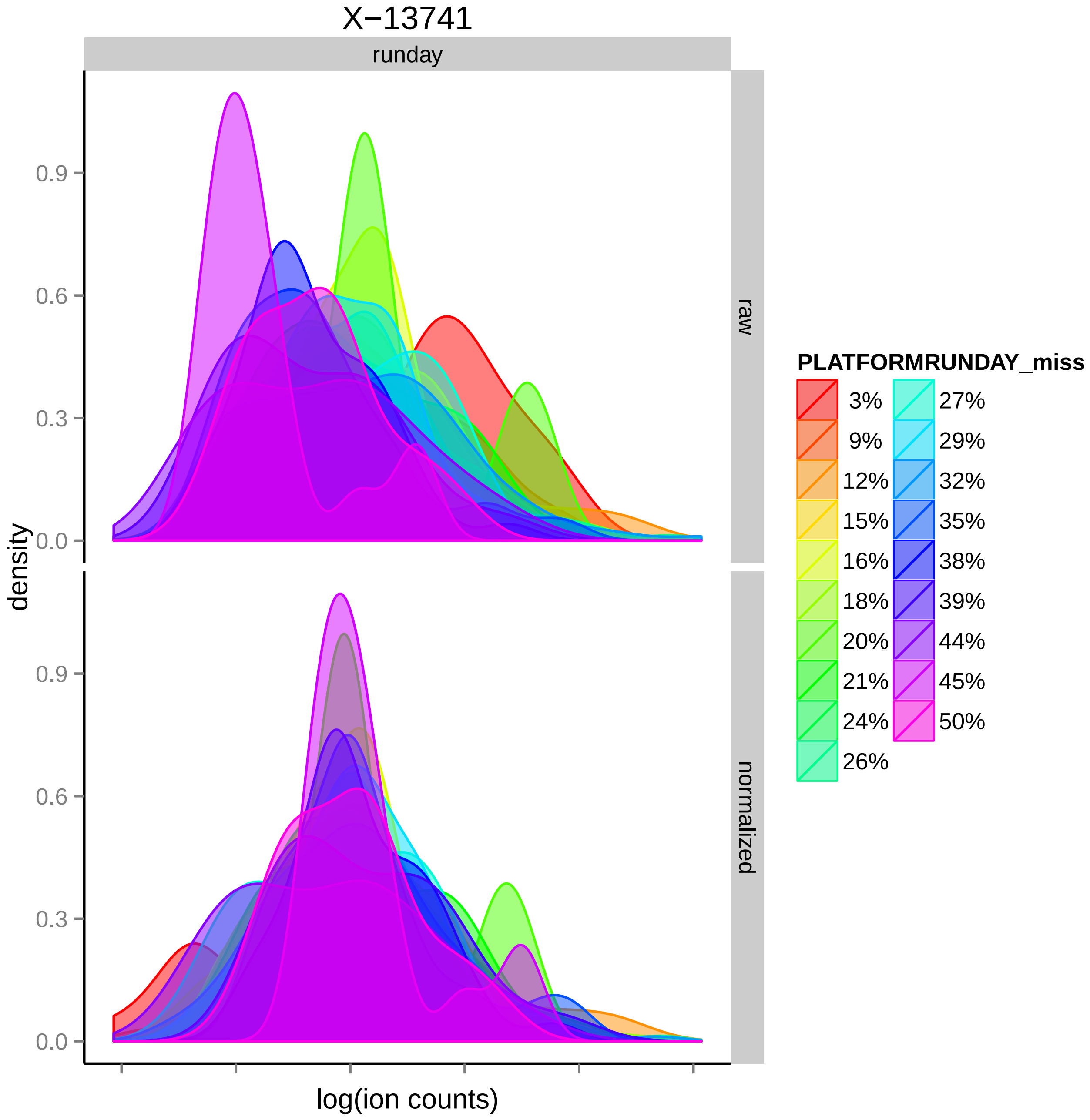

X−13741
runday
raw
normalized
density
0.9
0.6
0.3
0.0
log(ion counts)
PLATFORMRUNDAY_miss
3%
9%
12%
15%
16%
18%
20%
21%
24%
26%
27%
29%
32%
35%
38%
39%
44%
45%
50%

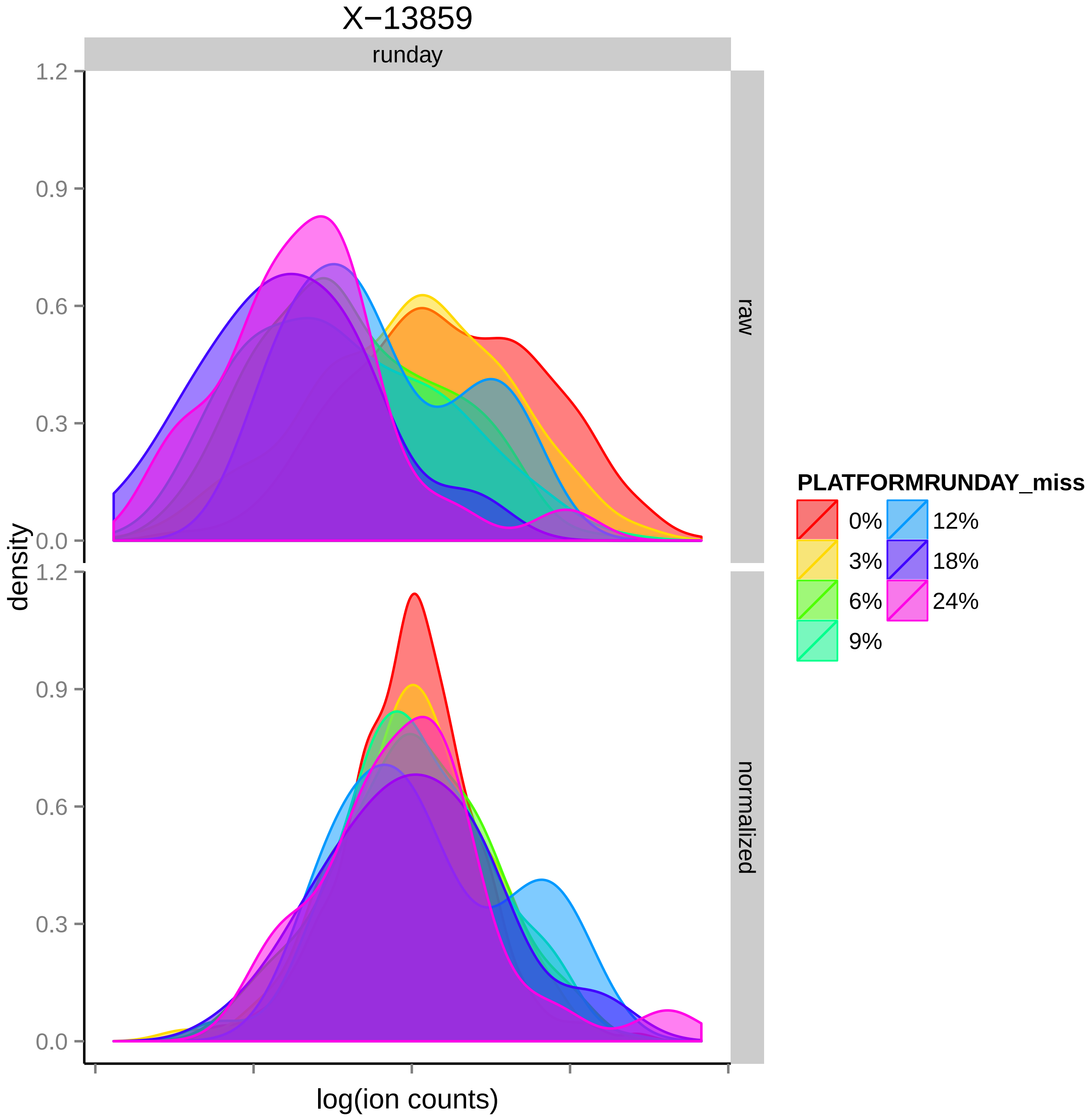

X−13859
runday
raw
normalized
density
1.2
0.9
0.6
0.3
0.0
1.2
0.9
0.6
0.3
0.0
log(ion counts)
PLATFORMRUNDAY_miss
0%
3%
6%
9%
12%
18%
24%

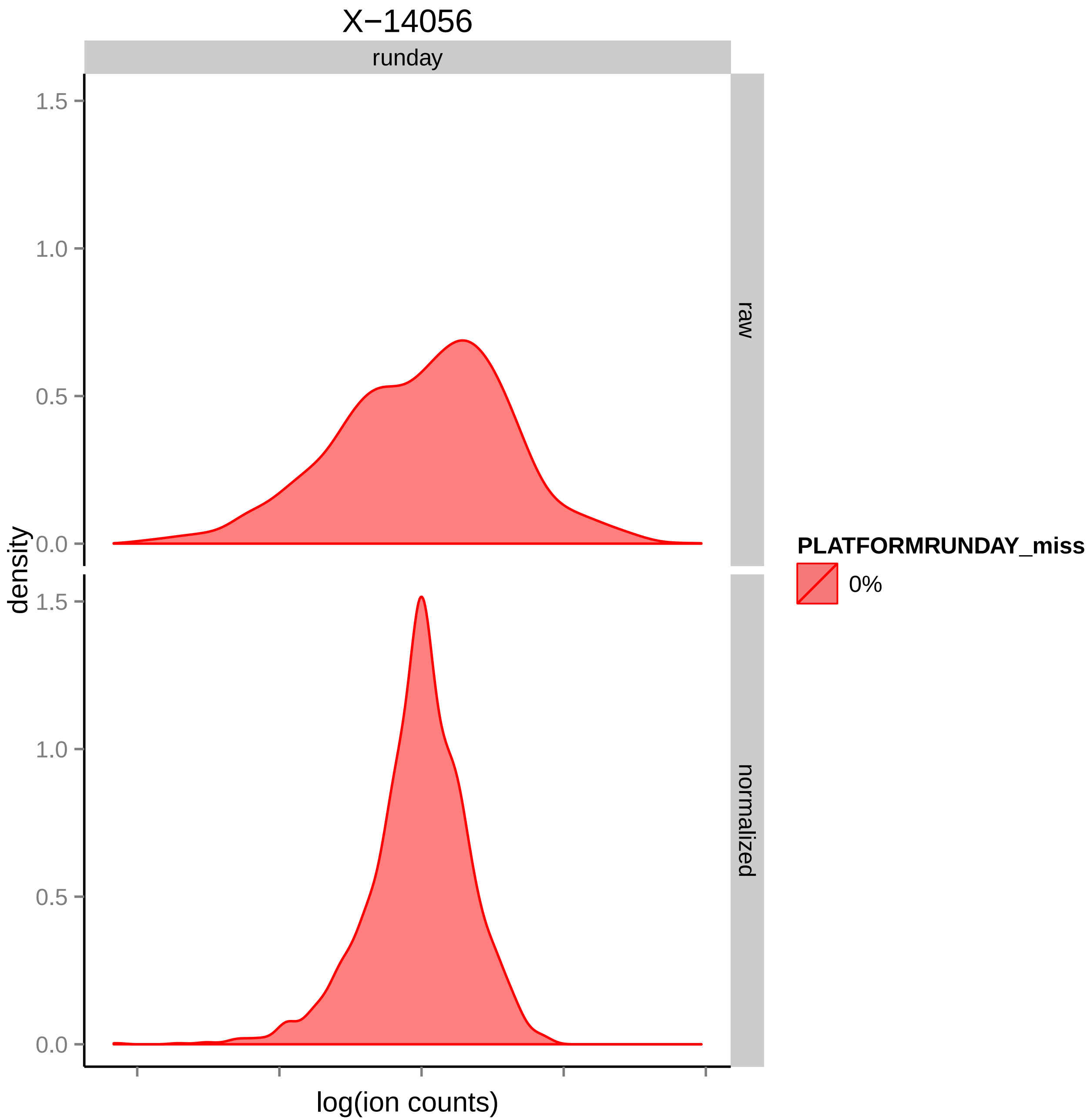
X−14056
runday
raw
normalized
density
log(ion counts)
1.5
1.0
0.5
0.0
PLATFORMRUNDAY_miss
0%

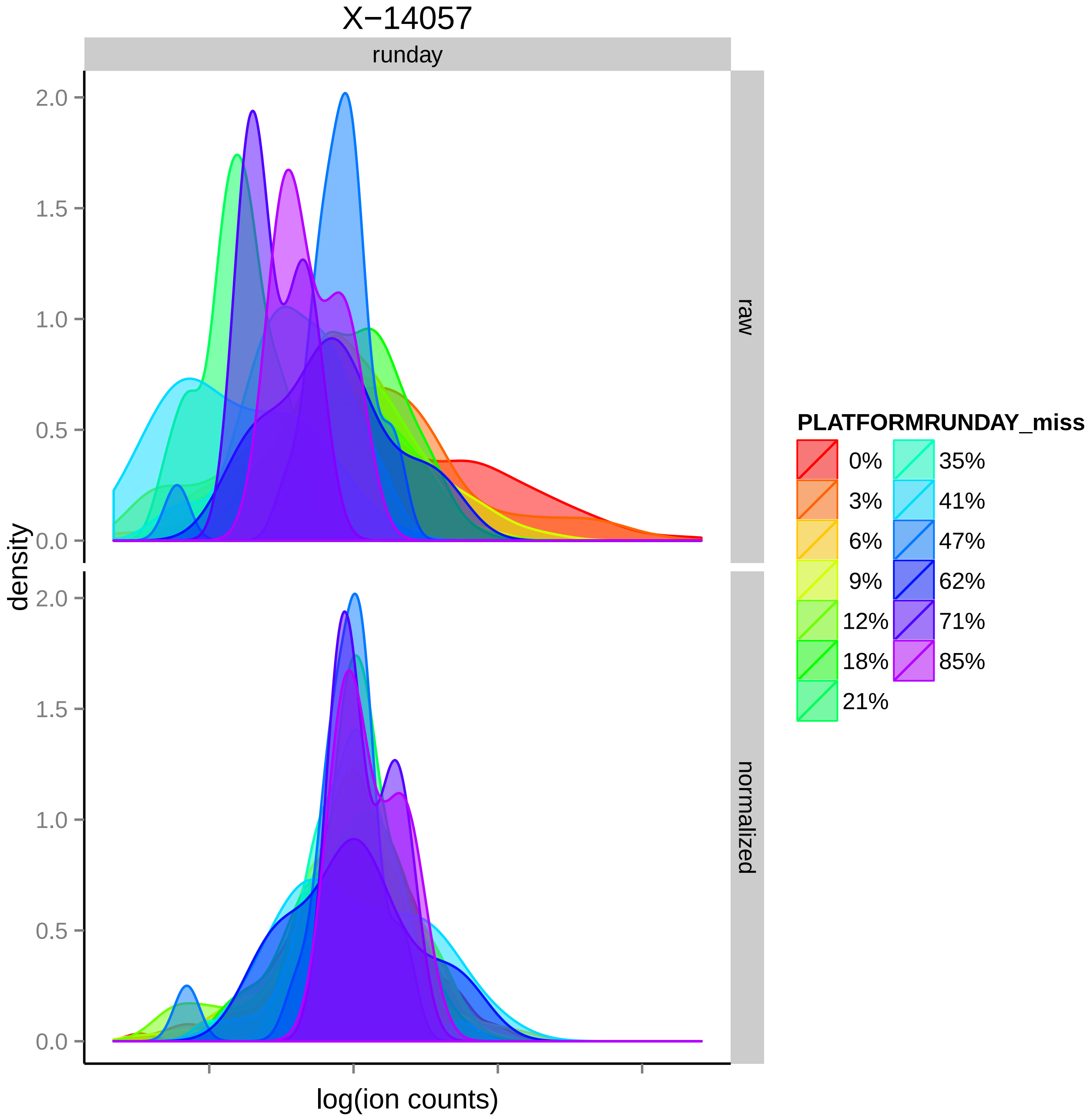
X−14057
runday
raw
normalized
density
log(ion counts)
2.0
1.5
1.0
0.5
0.0
2.0
1.5
1.0
0.5
0.0
PLATFORMRUNDAY_miss
0%
3%
6%
9%
12%
18%
21%
35%
41%
47%
62%
71%
85%

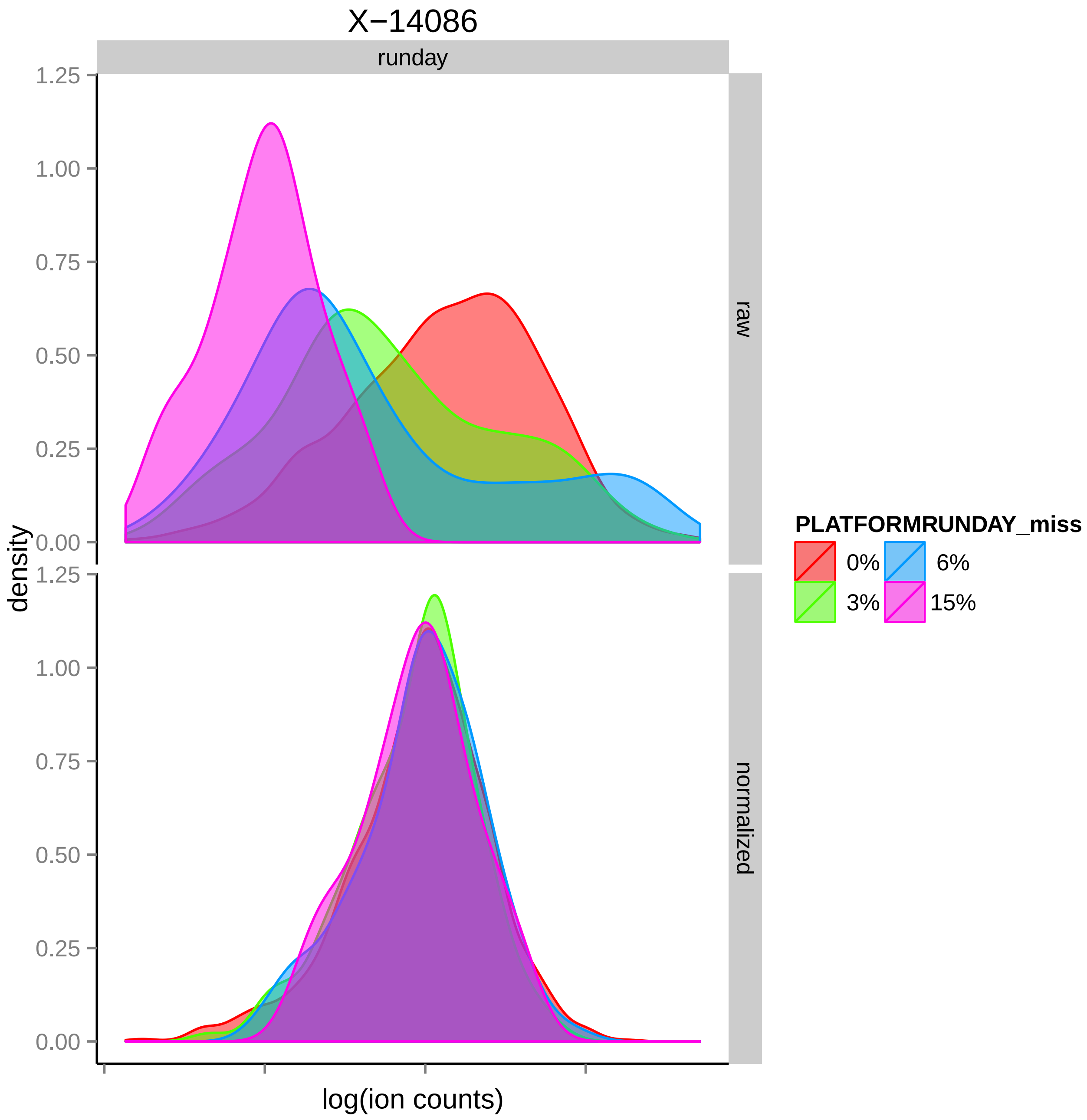
X−14086
runday
raw
normalized
density
log(ion counts)
1.25
1.00
0.75
0.50
0.25
0.00
PLATFORMRUNDAY_miss
0%
6%
3%
15%

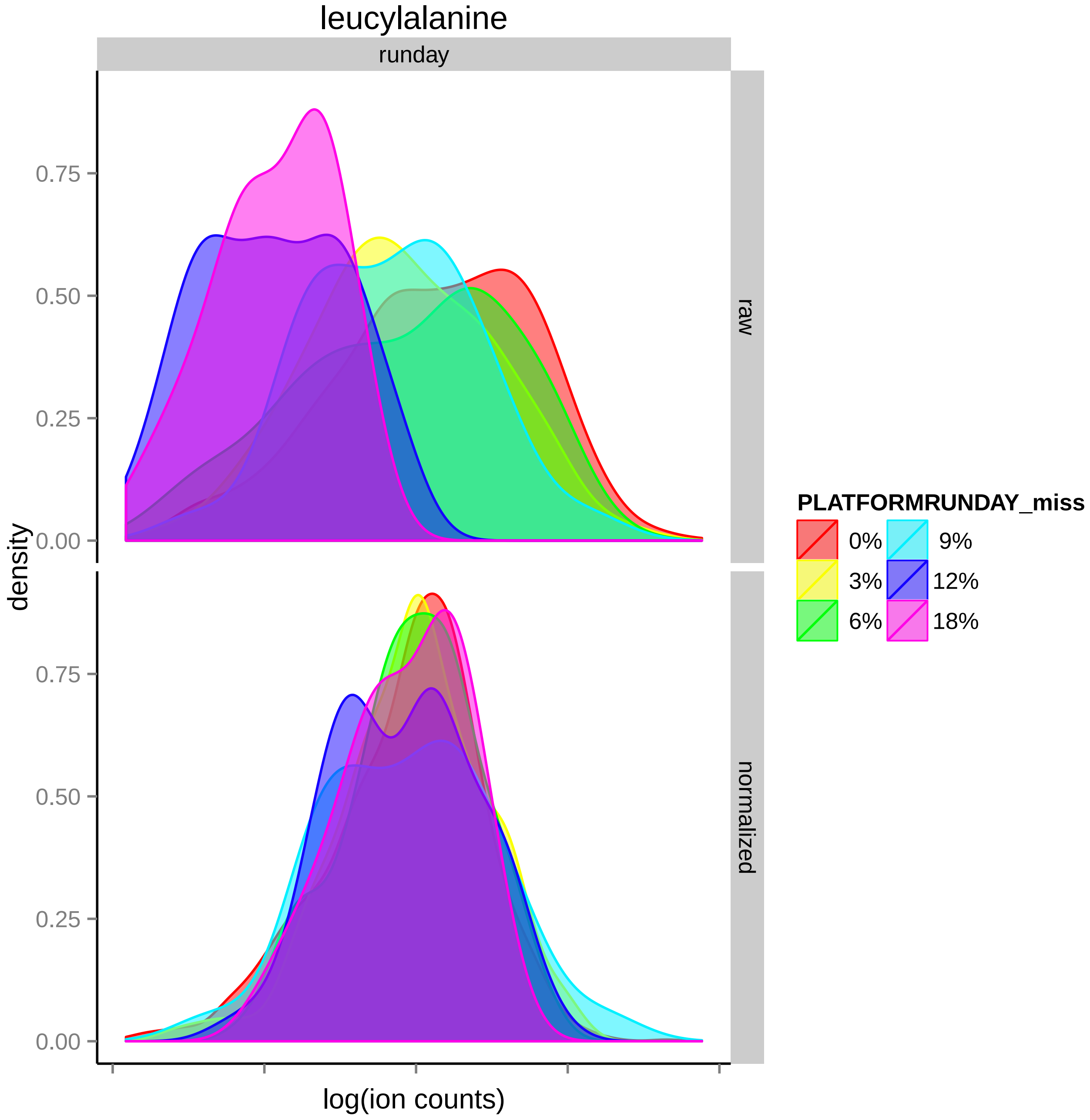
leucylalanine
runday
raw
normalized
density
0.00
0.25
0.50
0.75
log(ion counts)
PLATFORMRUNDAY_miss
0%
3%
6%
9%
12%
18%

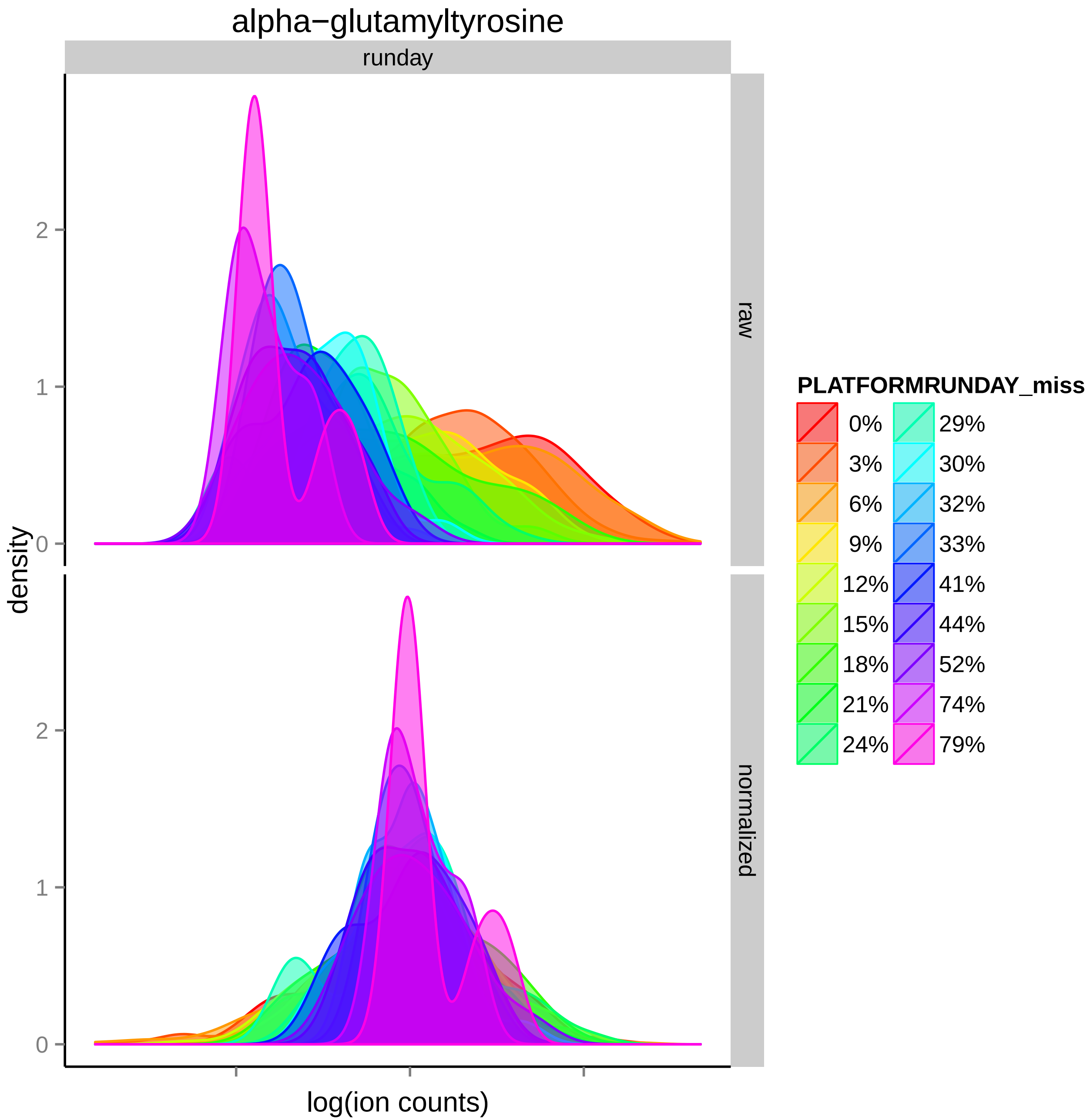

alpha−glutamyltyrosine
runday
raw
normalized
density
log(ion counts)
0
1
2
0
1
2
PLATFORMRUNDAY_miss
0%
3%
6%
9%
12%
15%
18%
21%
24%
29%
30%
32%
33%
41%
44%
52%
74%
79%

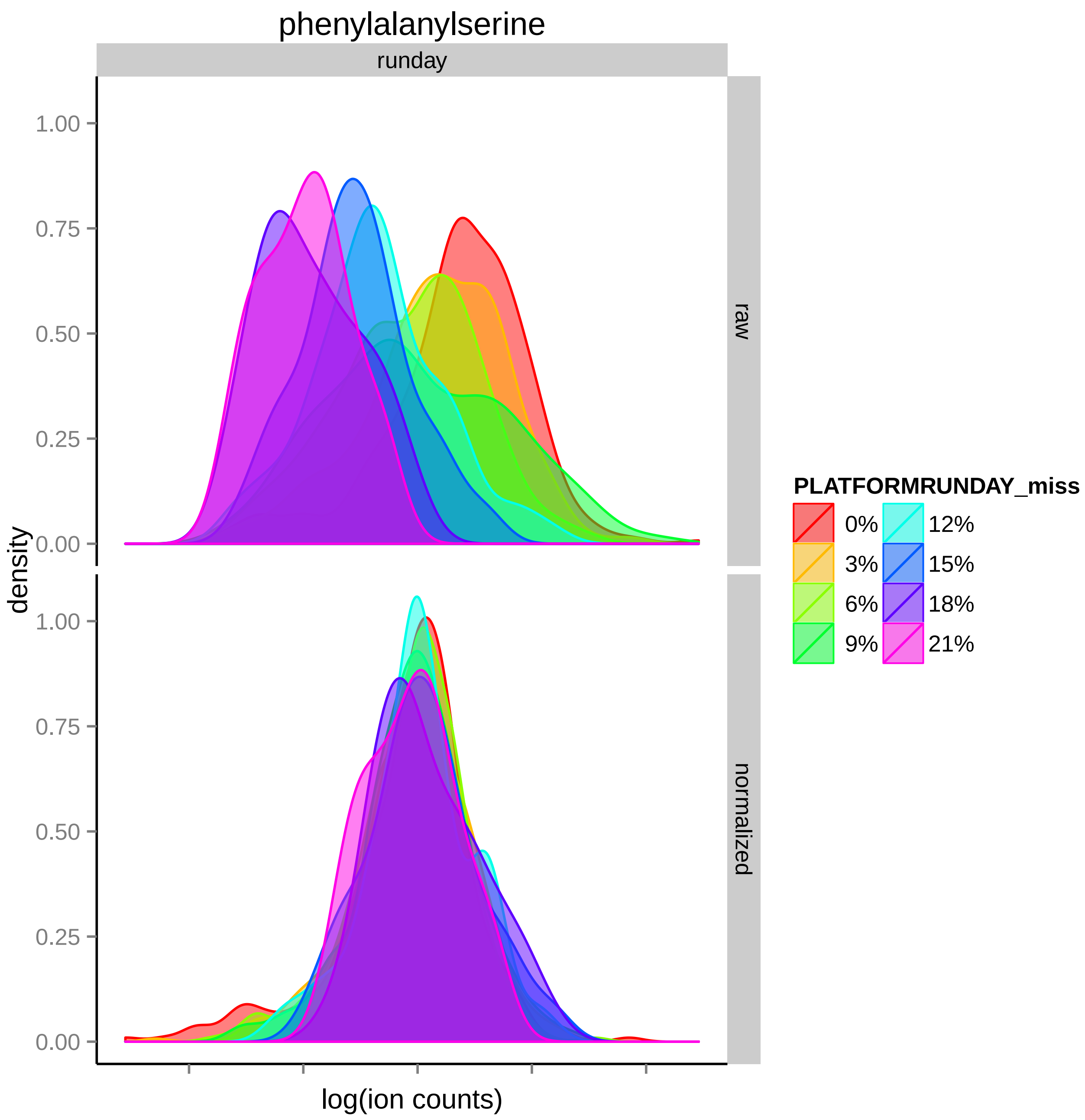
phenylalanylserine
runday
raw
normalized
density
log(ion counts)
1.00
0.75
0.50
0.25
0.00
PLATFORMRUNDAY_miss
0%
3%
6%
9%
12%
15%
18%
21%

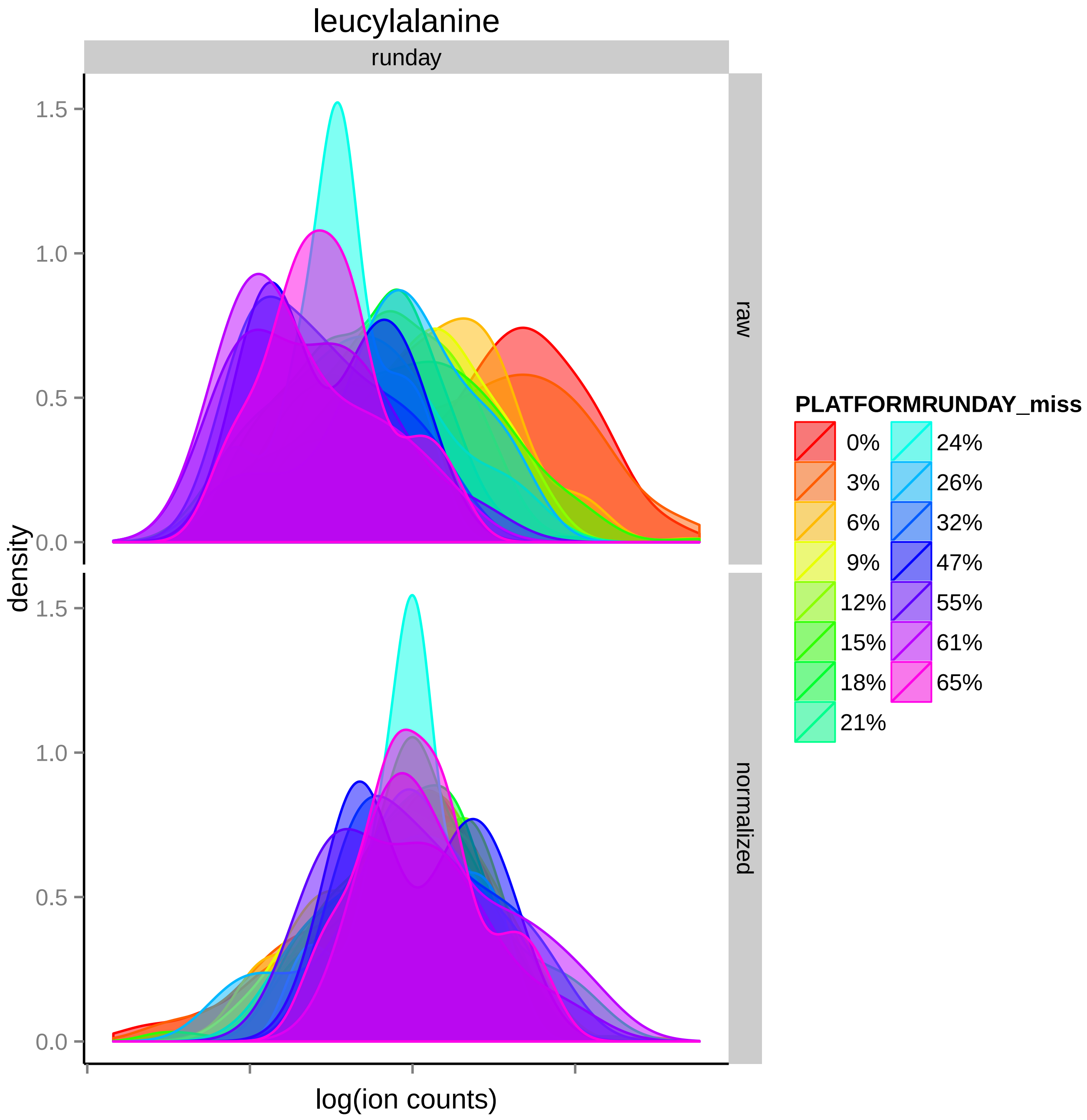

leucylalanine
runday
raw
normalized
density
1.5
1.0
0.5
0.0
log(ion counts)
PLATFORMRUNDAY_miss
0%
3%
6%
9%
12%
15%
18%
21%
24%
26%
32%
47%
55%
61%
65%

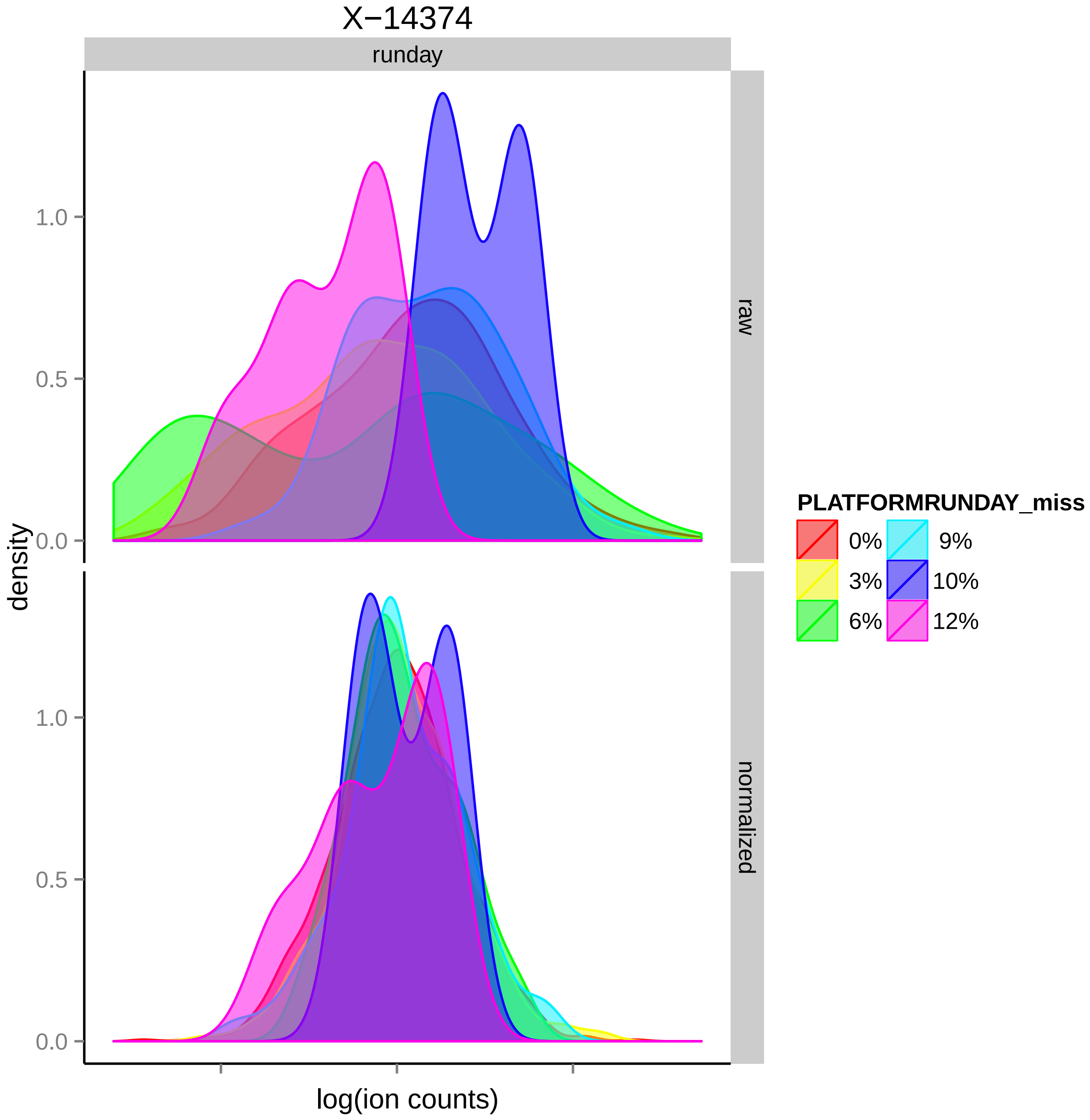
X−14374
runday
raw
normalized
density
1.0
0.5
0.0
1.0
0.5
0.0
log(ion counts)
PLATFORMRUNDAY_miss
0%
3%
6%
9%
10%
12%

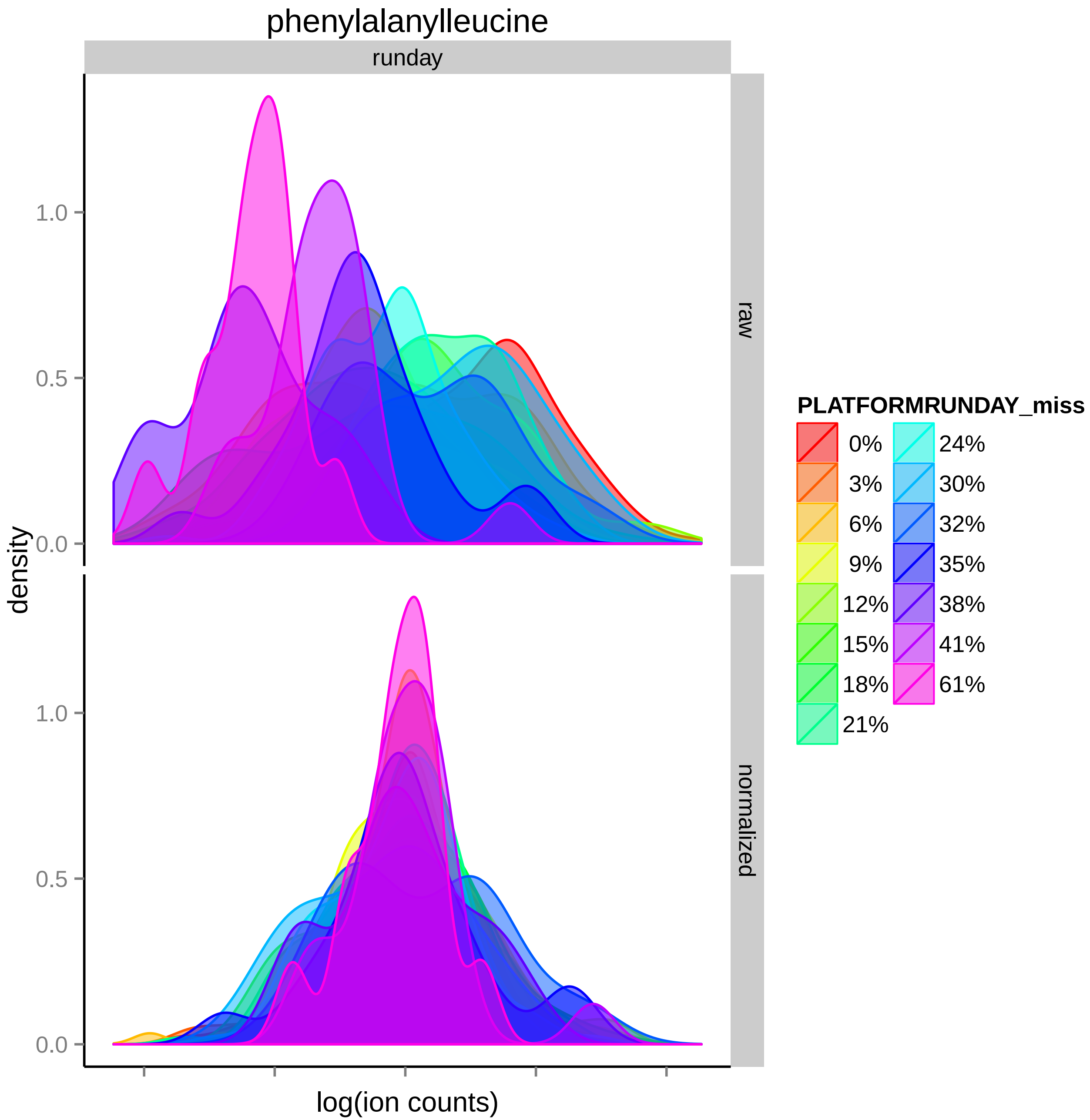

phenylalanylleucine
runday
raw
normalized
density
log(ion counts)
0.0
0.5
1.0
PLATFORMRUNDAY_miss
0%
3%
6%
9%
12%
15%
18%
21%
24%
30%
32%
35%
38%
41%
61%

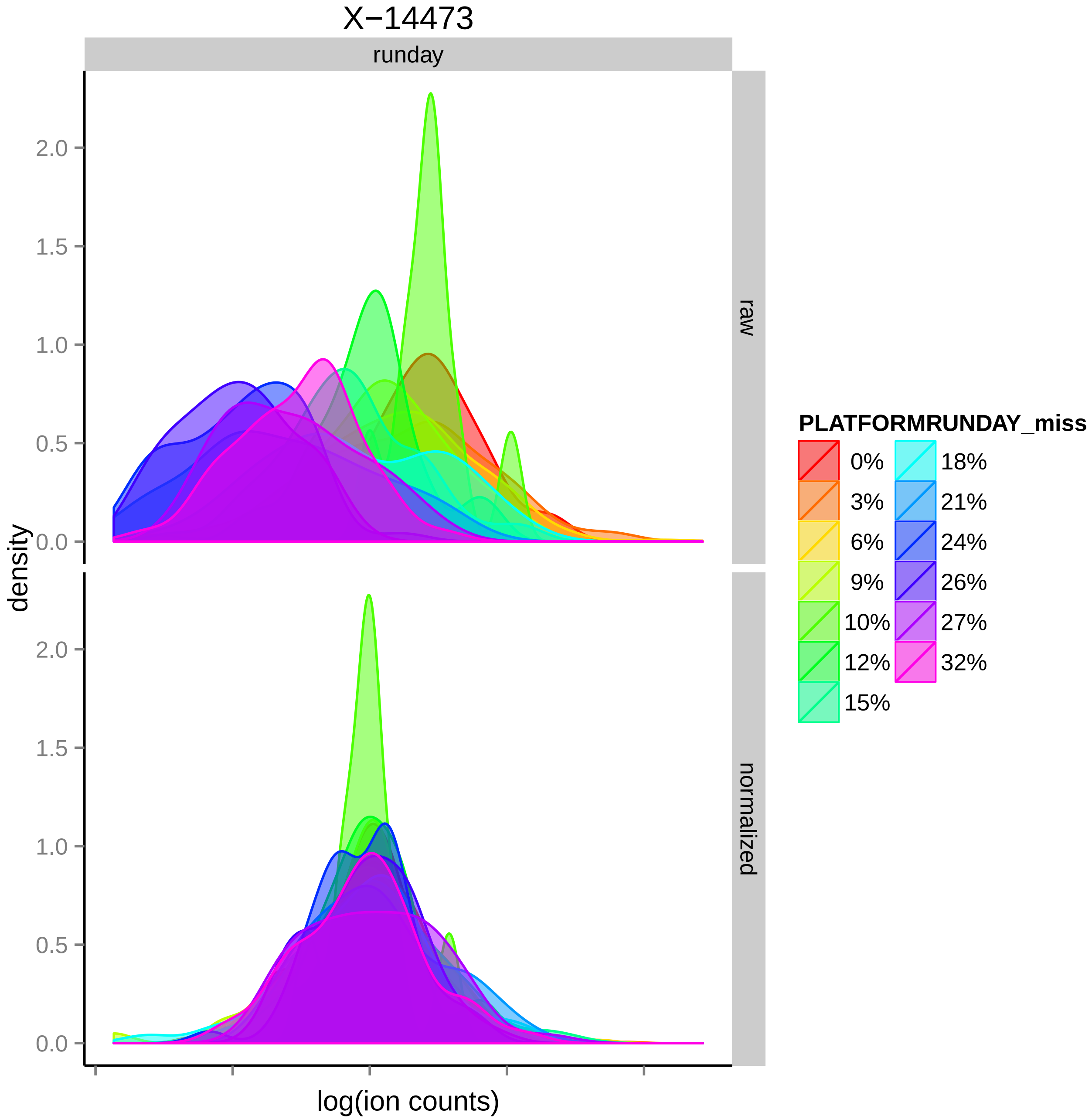

X−14473
runday
raw
normalized
density
log(ion counts)
2.0
1.5
1.0
0.5
0.0
PLATFORMRUNDAY_miss
0%
3%
6%
9%
10%
12%
15%
18%
21%
24%
26%
27%
32%

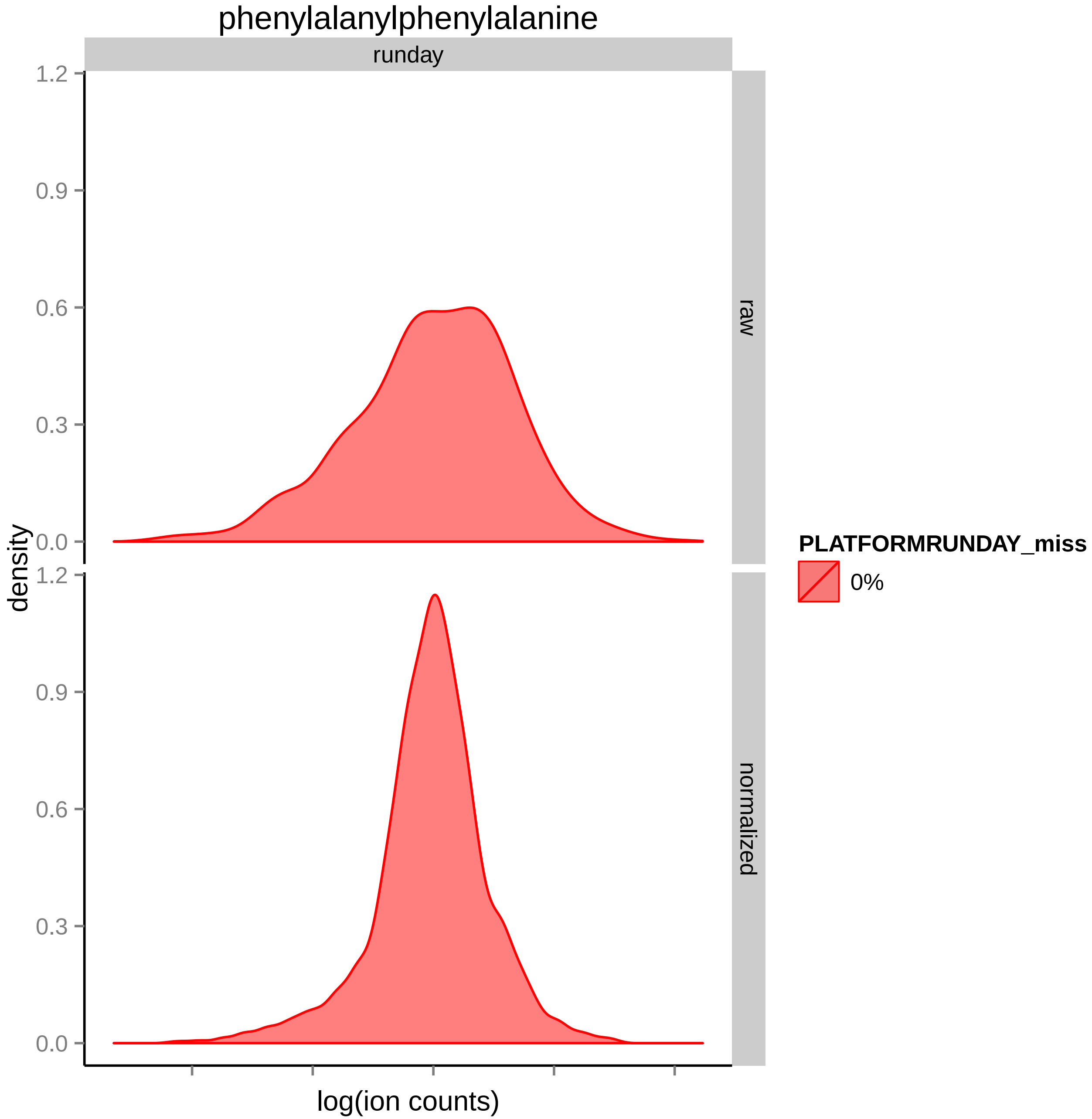

phenylalanylphenylalanine
runday
raw
normalized
density
1.2
0.9
0.6
0.3
0.0
log(ion counts)
PLATFORMRUNDAY_miss
0%

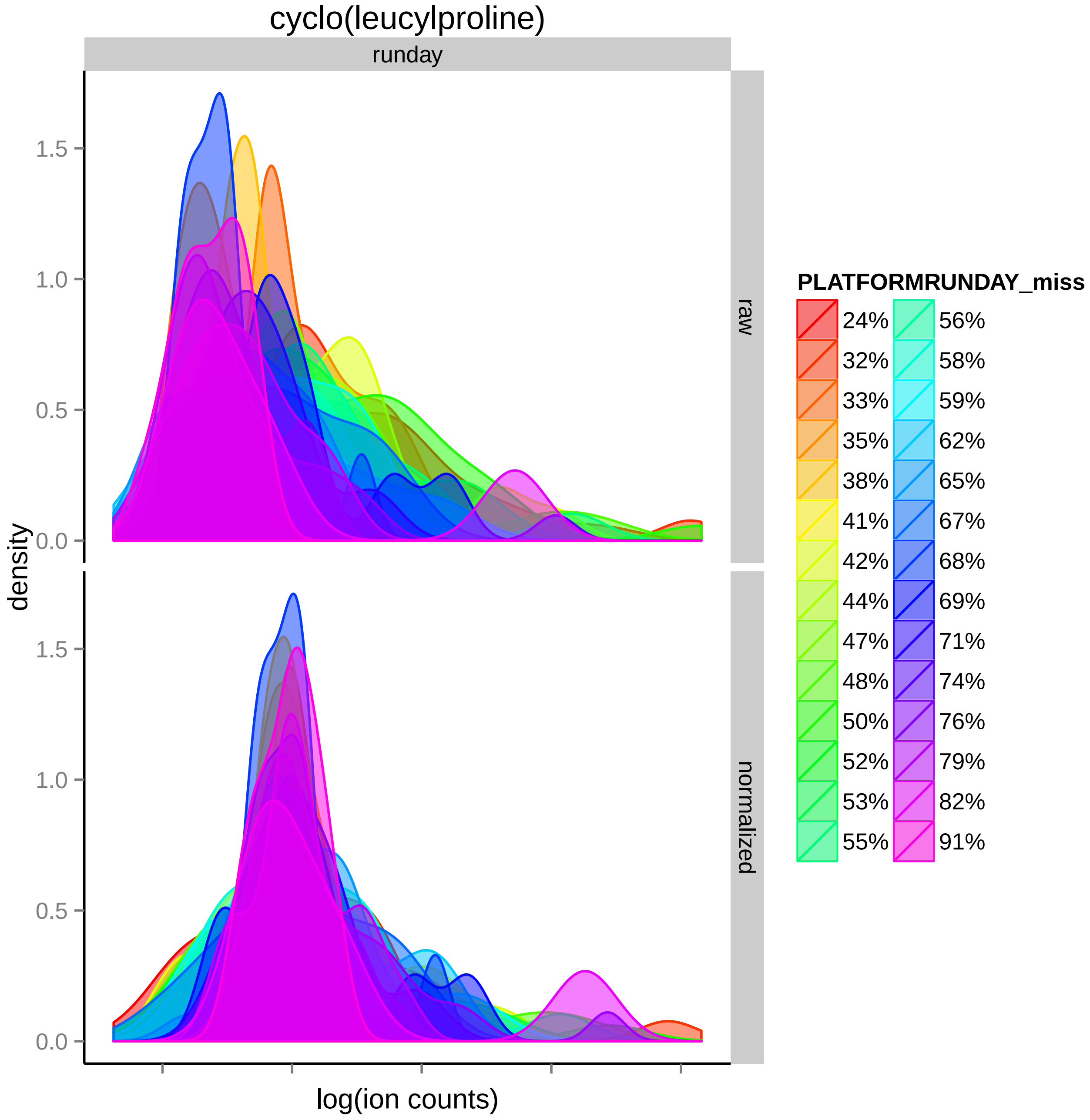
cyclo(leucylproline)
runday
raw
normalized
density
1.5
1.0
0.5
0.0
1.5
1.0
0.5
0.0
log(ion counts)
PLATFORMRUNDAY_miss
24%
32%
33%
35%
38%
41%
42%
44%
47%
48%
50%
52%
53%
55%
56%
58%
59%
62%
65%
67%
68%
69%
71%
74%
76%
79%
82%
91%

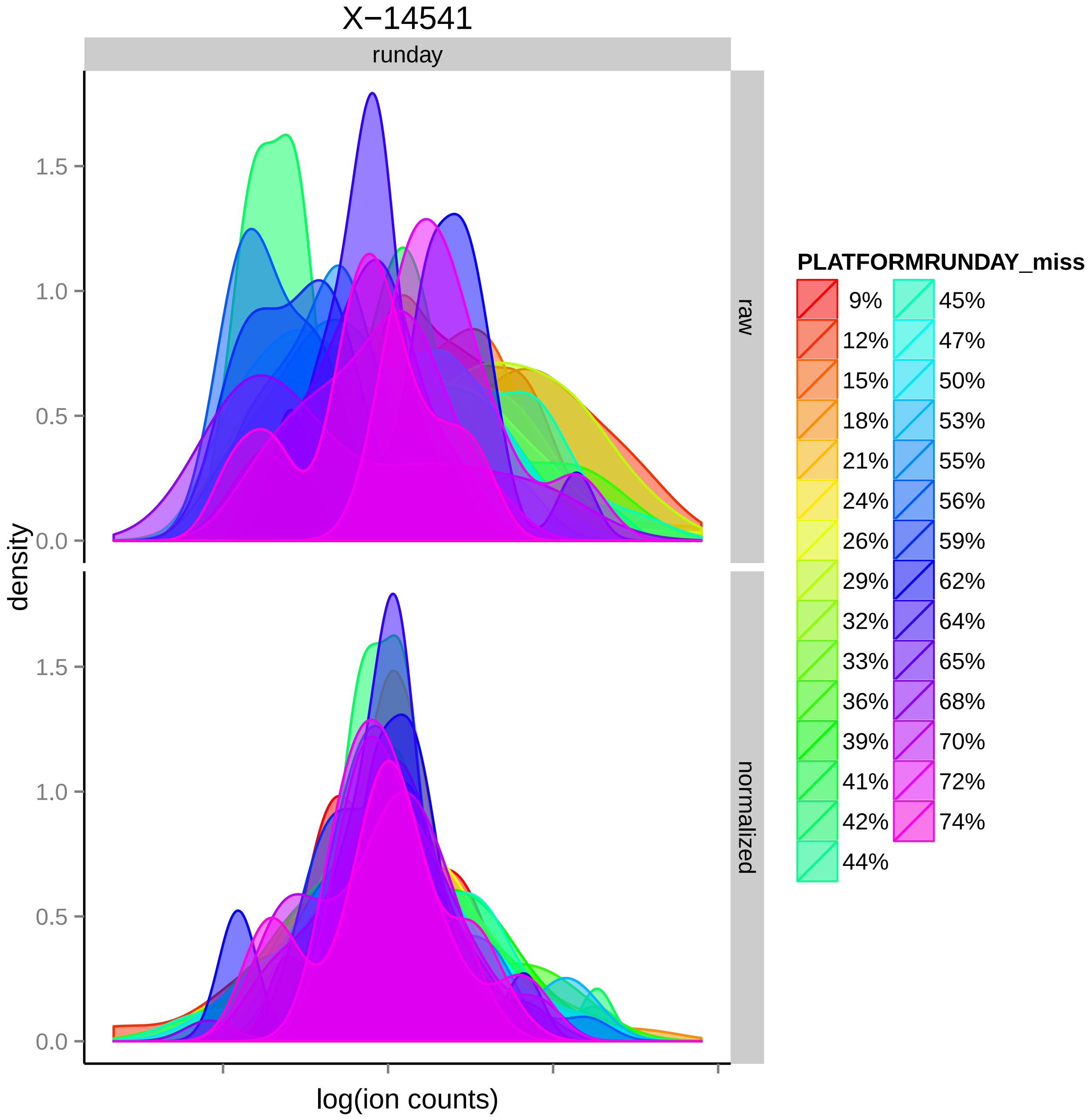
X−14541
runday
raw
normalized
density
log(ion counts)
1.5
1.0
0.5
0.0
1.5
1.0
0.5
0.0
PLATFORMRUNDAY_miss
9%
12%
15%
18%
21%
24%
26%
29%
32%
33%
36%
39%
41%
42%
44%
45%
47%
50%
53%
55%
56%
59%
62%
64%
65%
68%
70%
72%
74%

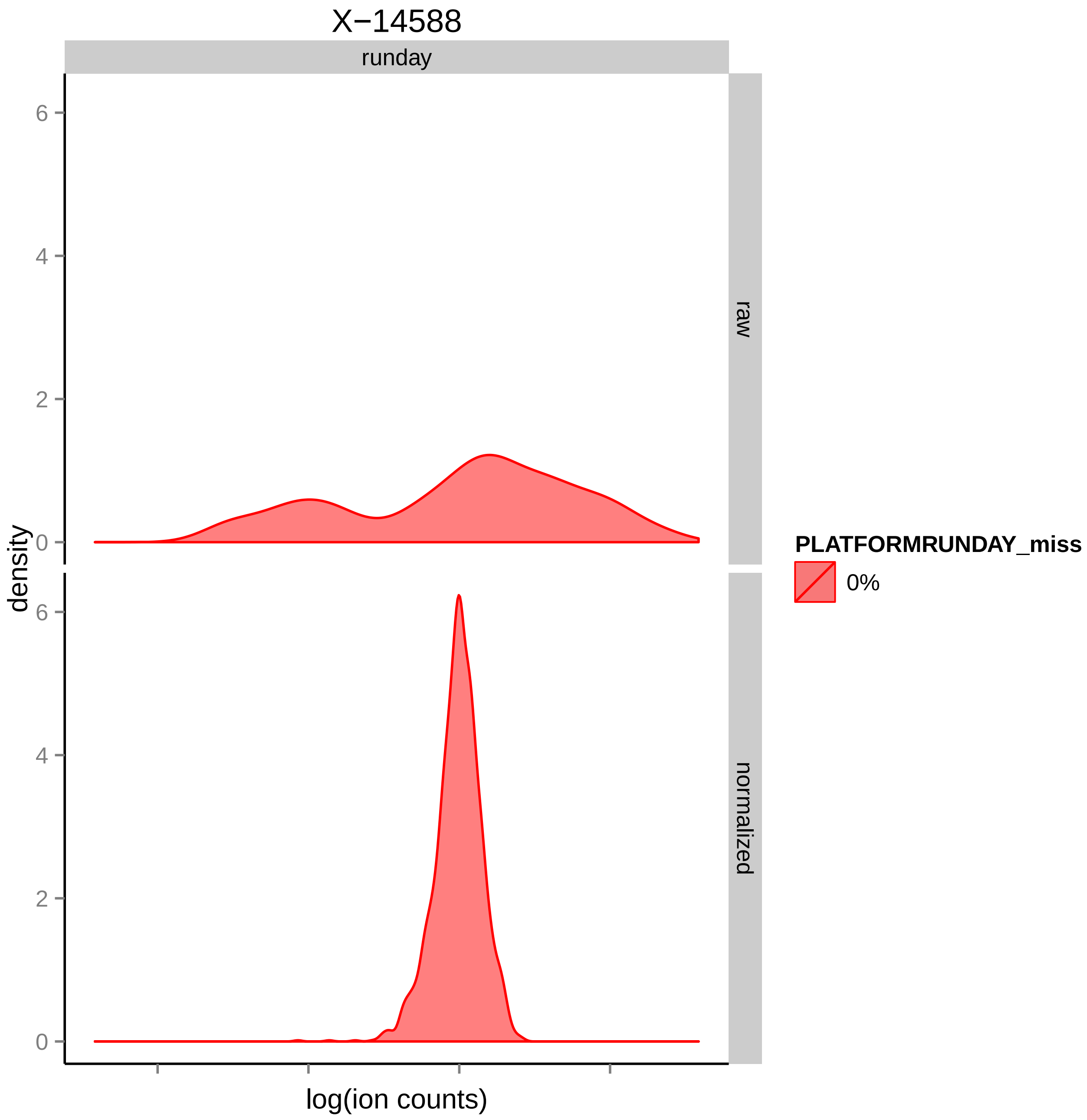

X−14588
runday
raw
normalized
density
log(ion counts)
0
2
4
6
PLATFORMRUNDAY_miss
0%

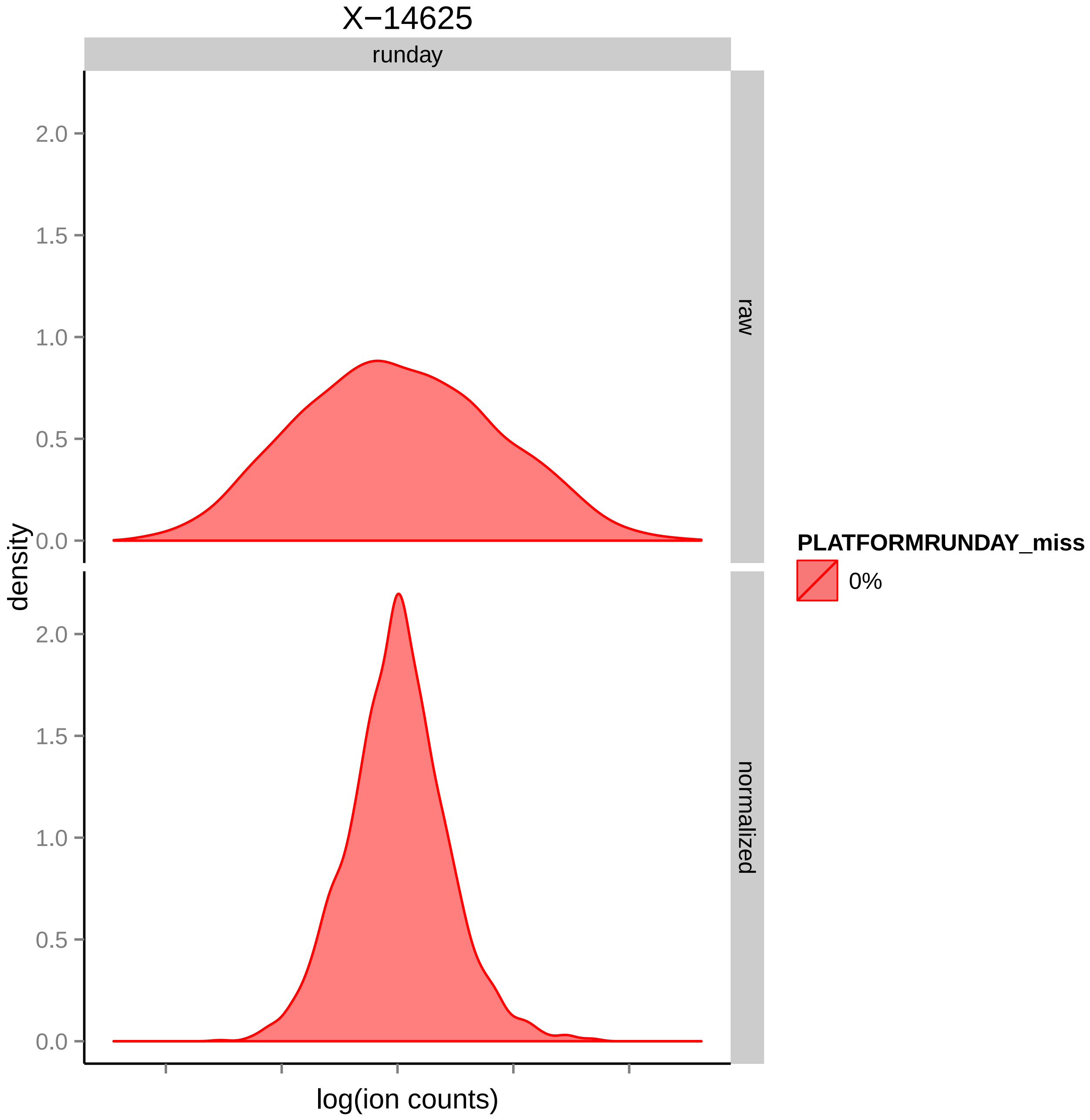

X−14625
runday
raw
normalized
density
2.0
1.5
1.0
0.5
0.0
log(ion counts)
PLATFORMRUNDAY_miss
0%

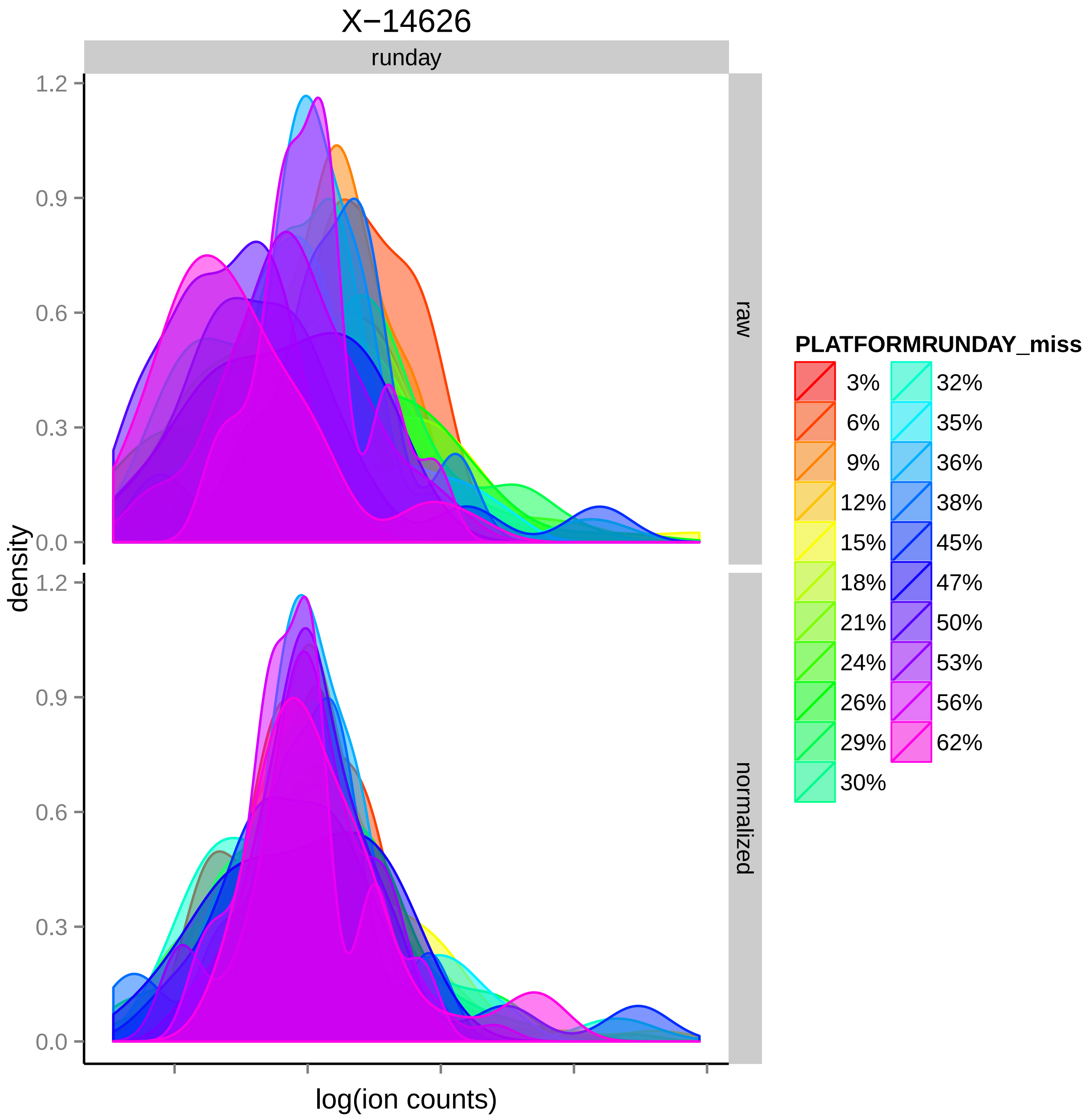

X−14626
runday
raw
normalized
density
log(ion counts)
1.2
0.9
0.6
0.3
0.0
PLATFORMRUNDAY_miss
3%
6%
9%
12%
15%
18%
21%
24%
26%
29%
30%
32%
35%
36%
38%
45%
47%
50%
53%
56%
62%

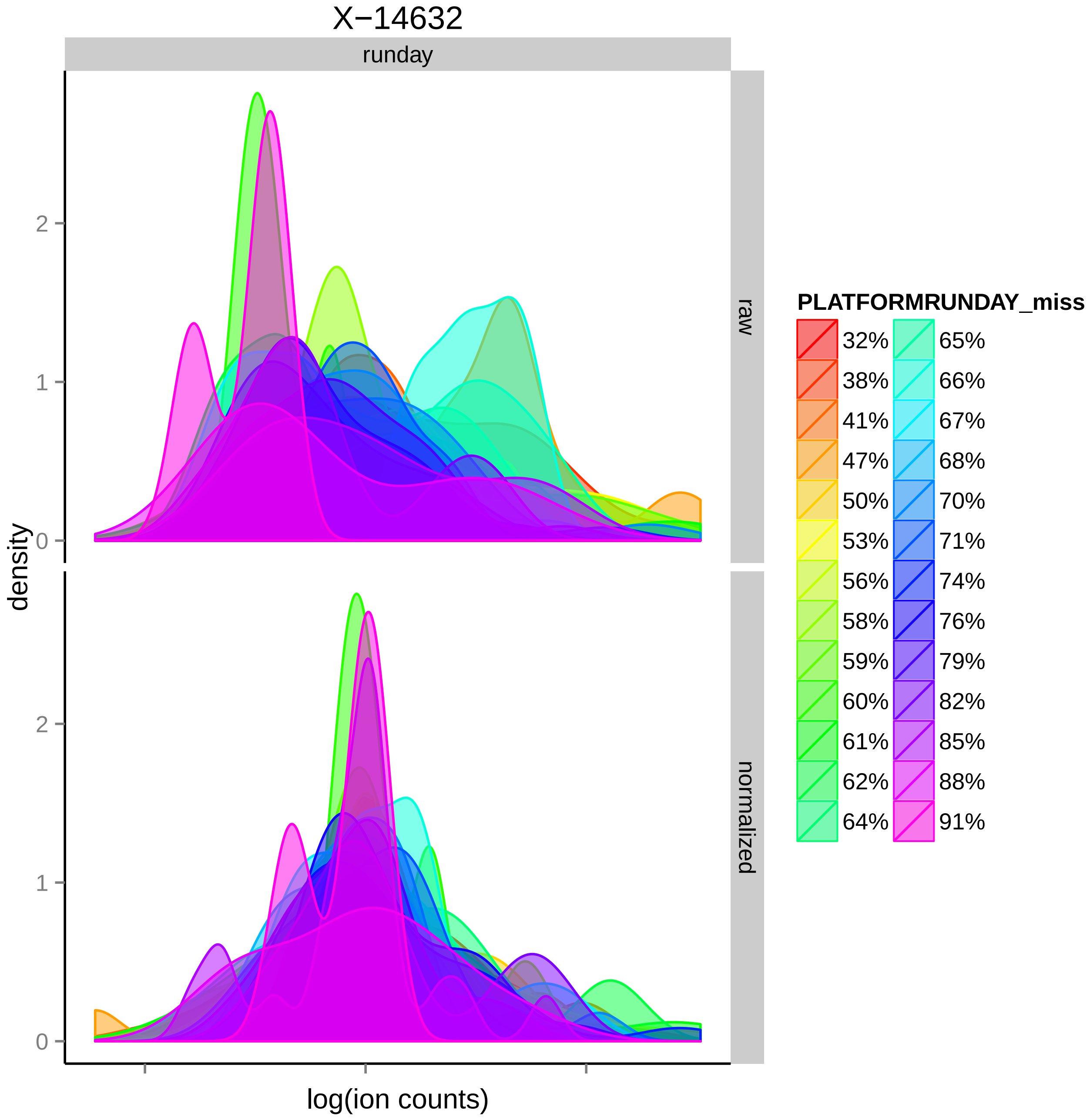

X−14632
runday
raw
normalized
density
log(ion counts)
2
1
0
2
1
0
PLATFORMRUNDAY_miss
32%
38%
41%
47%
50%
53%
56%
58%
59%
60%
61%
62%
64%
65%
66%
67%
68%
70%
71%
74%
76%
79%
82%
85%
88%
91%

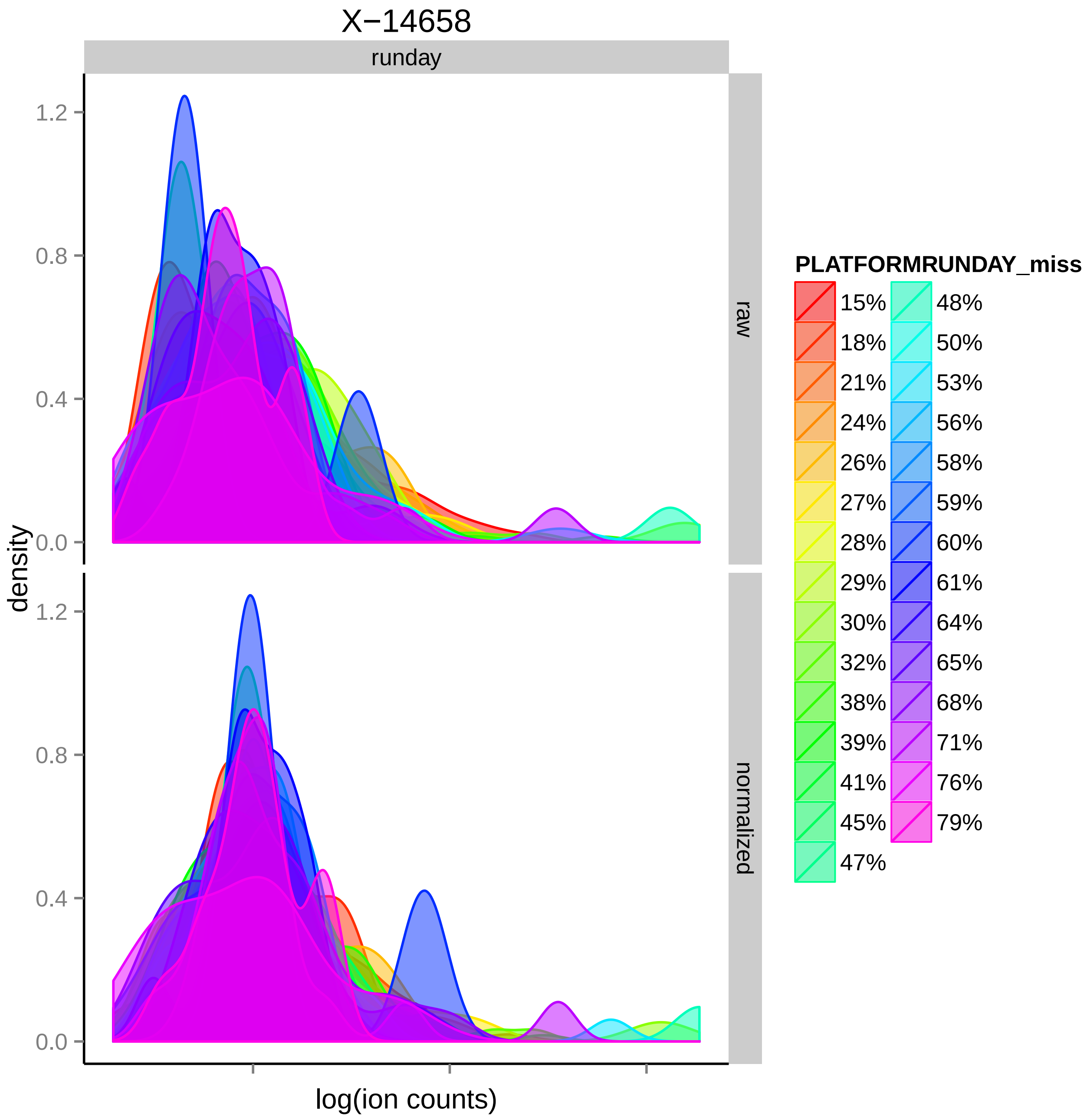
X−14658
runday
raw
normalized
density
log(ion counts)
1.2
0.8
0.4
0.0
PLATFORMRUNDAY_miss
15%
18%
21%
24%
26%
27%
28%
29%
30%
32%
38%
39%
41%
45%
47%
48%
50%
53%
56%
58%
59%
60%
61%
64%
65%
68%
71%
76%
79%

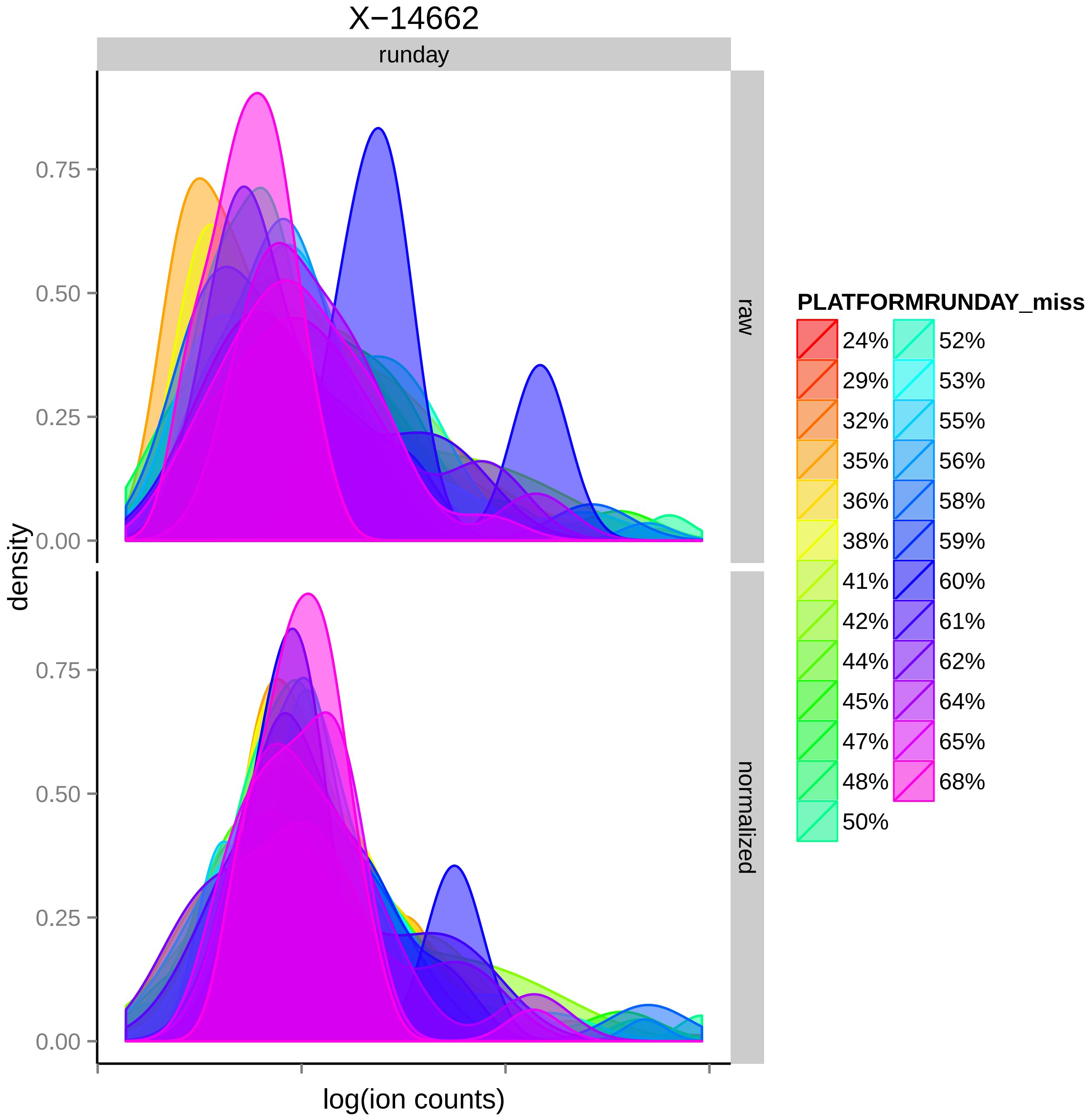
X−14662
runday
raw
normalized
density
log(ion counts)
0.00
0.25
0.50
0.75
PLATFORMRUNDAY_miss
24%
29%
32%
35%
36%
38%
41%
42%
44%
45%
47%
48%
50%
52%
53%
55%
56%
58%
59%
60%
61%
62%
64%
65%
68%

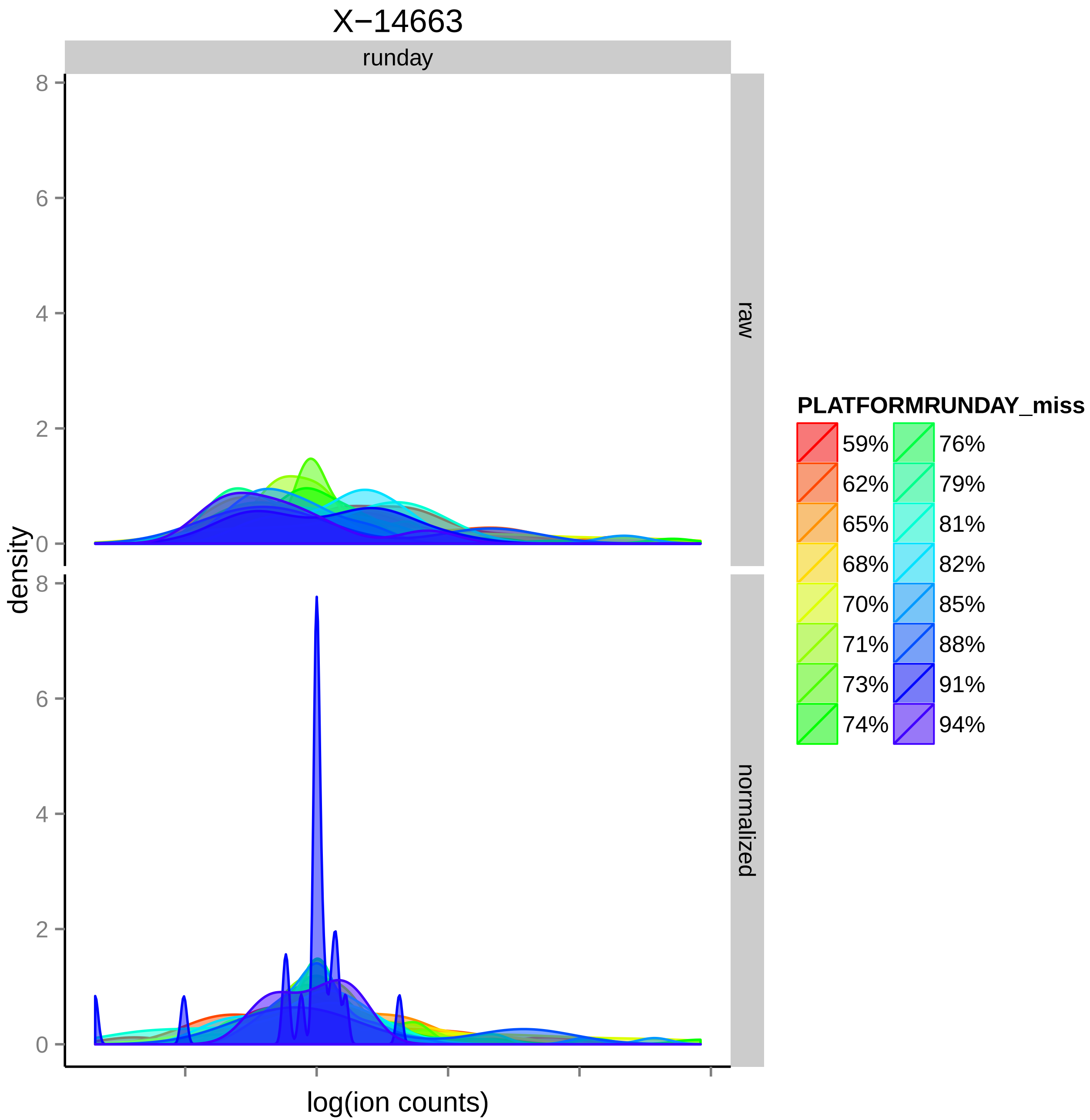
X−14663
runday
raw
normalized
density
log(ion counts)
0
2
4
6
8
PLATFORMRUNDAY_miss
59%
62%
65%
68%
70%
71%
73%
74%
76%
79%
81%
82%
85%
88%
91%
94%

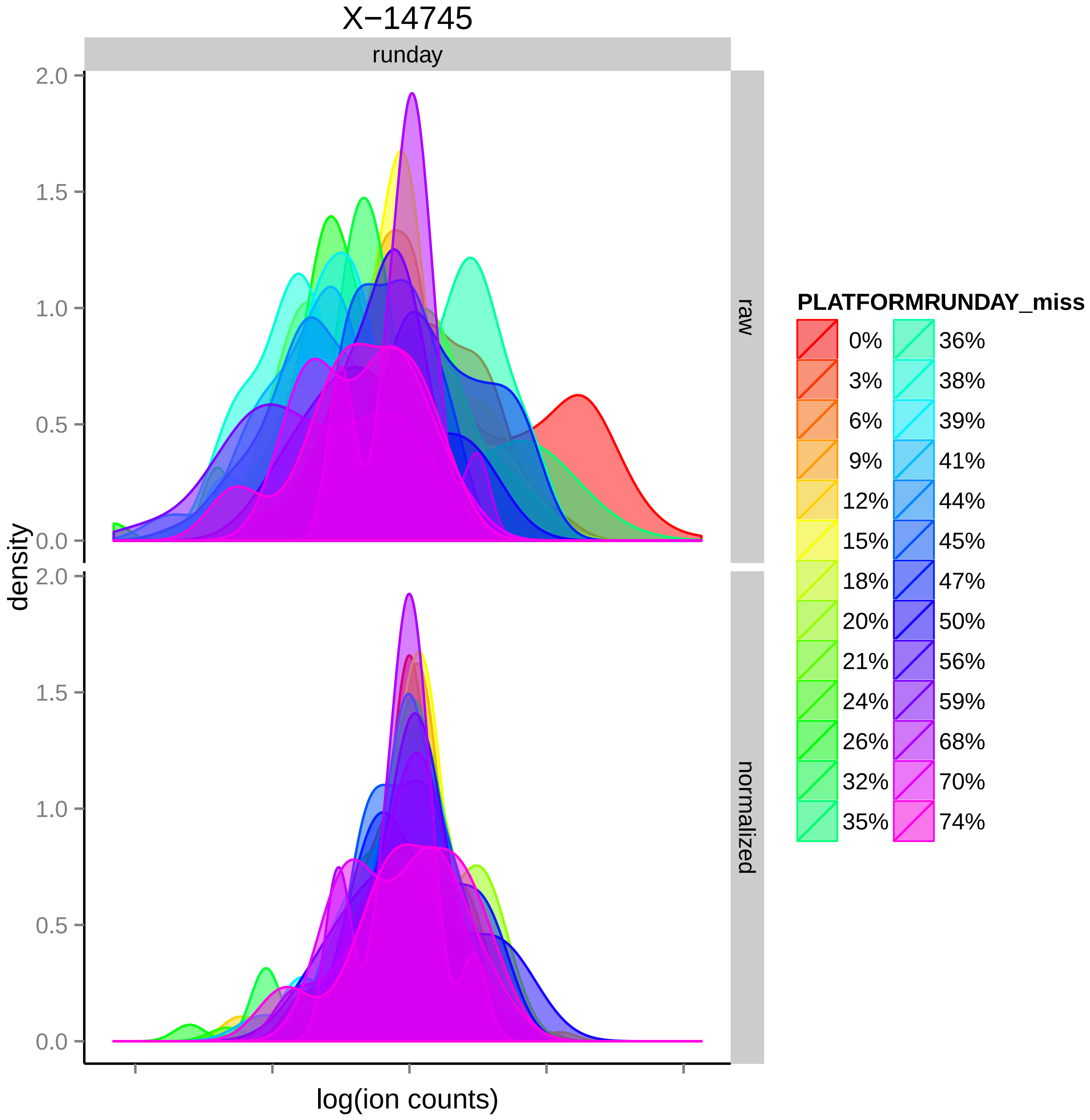

X−14745
runday
raw
normalized
density
log(ion counts)
2.0
1.5
1.0
0.5
0.0
PLATFORMRUNDAY_miss
0%
3%
6%
9%
12%
15%
18%
20%
21%
24%
26%
32%
35%
36%
38%
39%
41%
44%
45%
47%
50%
56%
59%
68%
70%
74%

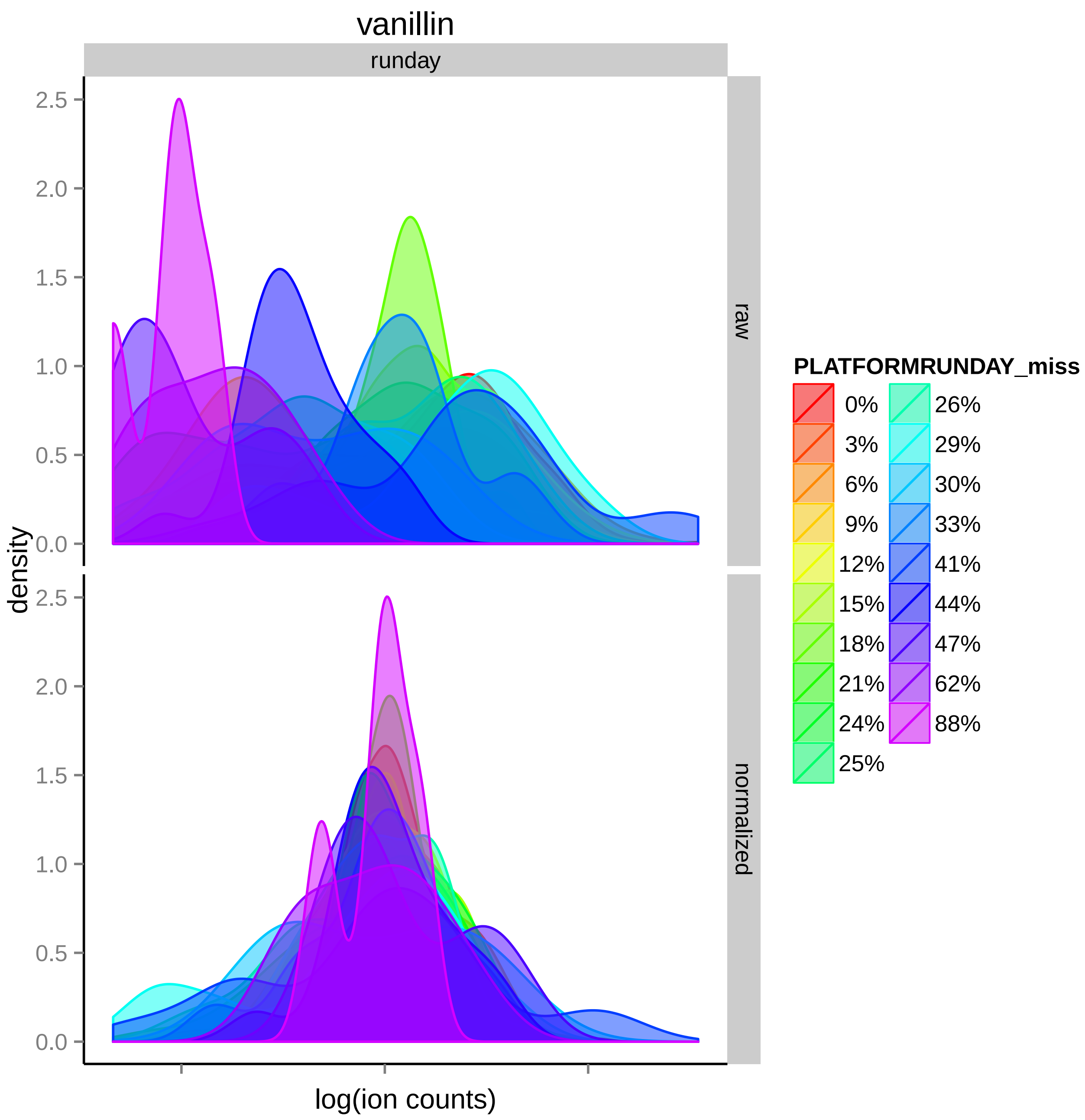
vanillin
runday
raw
normalized
density
log(ion counts)
0.0
0.5
1.0
1.5
2.0
2.5
PLATFORMRUNDAY_miss
0%
3%
6%
9%
12%
15%
18%
21%
24%
25%
26%
29%
30%
33%
41%
44%
47%
62%
88%
