# Supplementary material for: Characterization of missing values in untargeted MS-based metabolomics data and evaluation of missing data handling strategies
Source: Metabolomics. 2018 Sep 20;14(10):128. doi: 10.1007/s11306-018-1420-2 (PMC6153696; doi:10.1007/s11306-018-1420-2)
Supplement: Supplementary file 3 — Supplementary material 3 (DOCX 223 KB) [file 11306_2018_1420_MOESM3_ESM.docx]

# Characterization of missingness in untargeted MS-based metabolomics data sets and evaluation of missing data handling strategies

*Kieu Trinh Do^¶^, Simone Wahl^¶^, Johannes Raffler, Sophie Molnos, Michael Laimighofer, Jerzy Adamski, Karsten Suhre, Konstantin Strauch, Annette Peters, Christian Gieger, Claudia Langenberg, Isobel D. Stewart, Fabian J. Theis, Harald Grallert, Gabi Kastenmüller****^#^****, Jan Krumsiek****^#^***

## Supporting Information File S3: Simulation framework

### Data generation

We generated 250 random data sets for each set of parameters corresponding to a certain data situation. Each data set consisted of a pair of variables, which were generated by drawing from a multivariate normal distribution with sample size $n$ = 100, 250 or 1000. The means of the variables were equal to zero and covariance chosen such that variances were equal to one, i.e., representing scaled variables. The Pearson correlation of the two variables was set to$cor$ = 0.1, 0.2, or 0.4, according to correlations observed in the real data (Figure S2A).


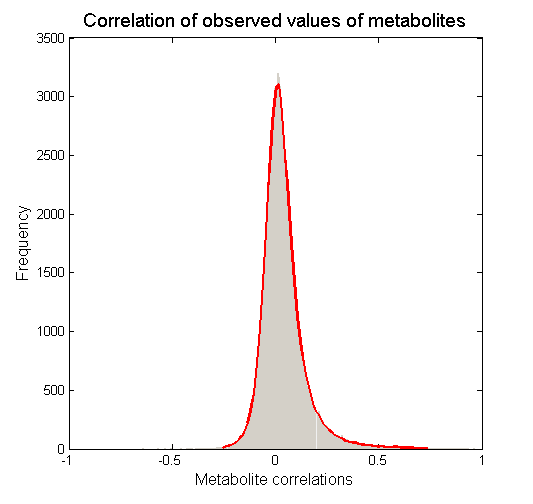


Figure S2A. Distribution of Pearson correlation coefficients in KORA F4.

In addition, since some of the considered imputation methods utilize the data covariance structure and one of our evaluation criteria for imputation methods was based on partial correlation, we included auxiliary variables correlated with the two (main) variables. Their number and correlation strength were chosen such that the real-data situation was reflected: For each metabolite in the real data set we selected its five most correlated (Pearson correlation) partners. Then we computed the adjusted R^2^ criterion of the linear model with the metabolite in question as outcome and the five selected metabolites as covariates. Adjusted R^2^ was 45.5% on average, ranging from 2.9 to 94.1%. We adapted this data situation to the simulation framework by including five auxiliary variables for each of the two main variables to the simulation with partial correlation 0 and 0.317 to the respective variable. These values were numerically chosen such that an adjusted R^2^ of 0 and 50% was achieved on average. The former resembles the worst case and the latter the average situation observed in the KORA data. The overall correlation matrix for the simulation of the twelve variables (2 metabolites + 10 auxiliary variables) was computed from the partial correlation matrix using R package *corpcor*, version 1.6.7.

We introduced “rundays” into the data by randomly assigning the observations to groups such that each group comprised 34 observations, according to the average number of observations in rundays of the real KORA F4 cohort. Note that the simulation scheme does not consider the fact that rundays do not only differ in the number of missing values, but also in metabolite concentrations (Figure 4C). This is justified by the fact that in reality, runday correction is generally performed prior to analysis, aligning the metabolite concentrations across rundays. In our study, we do not include the fact that such runday correction might be biased.

### Imposing missingness

A proportion of missing values $miss=$ 0.1, 0.3, 0.5 or 0.7 was introduced into the main variable pair (or only into one variable for logistic regression analysis where a dichotomized variable serves as outcome) according to diﬀerent mechanisms derived from our observations in the KORA Metabolon data (Figure 5, Table 1).

The *fixed LOD* mechanism assumes that values below a ﬁxed limit of detection ($\mathrm{LOD}$), deﬁned as $\mathrm{LOD}=x_{(round\left( n*miss) \right)}$, where $x_{(i)}$denotes entry $i$ in the vector of ordered concentrations, $n$ is the number of samples, and $round()$ a probabilistic rounding function. The *probabilistic LOD*mechanism assumes that $\mathrm{LOD}$is not ﬁxed but the probability that a value is missing inversely depends on itself. Therefore, the probability of missingness for each observation $i, i=1,\ldots,n$ of a variable $x$ was modeled as a function of $x$:

$$P\left( x_{i} \mathrm{missing} \right)=logistic\left( \beta_{0}+\beta_{1}x_{i} \right),$$

with $logistic\left( a \right)=\frac{exp\left( a \right)}{1+exp\left( a \right)}$ the logistic function and coefficient $\beta_{1}$ set to -1 or -10 to resemble moderate or strong dependence of the probability of missingness from variable value, respectively. This can also be interpreted as ‘steepness’ of the truncated distribution. The intercept $\beta_{0}$ was estimated by numerically solving the equation

$$\frac{1}{n}\sum_{i=1}^{n} P\left( x_{i} \mathrm{missing} \right)=miss.$$

To achieve the proportion of missing values $miss$ exactly, values were set to missing by drawing $n\times miss$ times from a multinomial distribution with probability vector $\left( P\left( x_{i} missing \right) \right)_{i=1,\ldots,n}$.

Since missingness was found to vary between rundays for many metabolites in the KORA data, and LOD-related mechanisms were observed within rundays, we implemented two further mechanisms: *runday-specific fixed LOD* and *runday-specific probabilistic LOD*. Here, we let runday-specific LOD vary around a global LOD (defined as described above) using the normal distribution as a simple approximation:

$$\left( \begin{matrix} {miss}_{1r} \\ {miss}_{2r} \end{matrix} \right)\sim N\left( \left( \begin{matrix} {miss}_{1} \\ {miss}_{2} \end{matrix} \right), \left( \begin{matrix} \sigma_{miss,1}^{2} & \rho_{miss}{\cdot\sigma}_{miss,1}{\cdot\sigma}_{miss,2} \\ \rho_{miss}{\cdot\sigma}_{miss,1}{\cdot\sigma}_{miss,2} & \sigma_{miss,2}^{2} \end{matrix} \right) \right),$$

where ${miss}_{1}$ and ${miss}_{2}$ denote the global, and ${miss}_{1r}$ and ${miss}_{2r}$ the runday-specific proportions of missingness for variables 1 and 2, respectively. The parameters $\sigma_{miss,1}^{2}$ and $\sigma_{miss,2}^{2}$ specify the variation of the proportion of missing values across rundays for the two variables. Their selection was guided by the real KORA data, where we observed that the variability of missingness proportion across rundays differs between metabolites, ranging from moderate (i.e. covering approximately a third to half of the 0-100% range of missingness, Fig 3B) to strong (i.e. covering most of the 0-100% range of missingness, Fig 3C) for most metabolites. We are aware that the distribution of missingness across rundays does not always follow a symmetric distribution (S3 File) and is bounded within 0-100%. Still, we considered the normal distribution as a convenient approximation and achieved moderate or strong variation by setting the variance $\sigma_{miss}^{2}$ such that the runday-specific proportion of missingness ranged approximately from $miss/2$ to $3*miss/2$ (moderate setting, with $miss<0.5$), or from $0$ to $2*miss$ (strong setting, with $miss<0.5$), whereby $miss$ denotes ${miss}_{1}$ or ${miss}_{2}$ for variable 1 and 2, respectively. This can approximately be achieved with

$$\sigma_{miss}^{2}=\left( \frac{min\left( miss,1-miss \right)}{s} \right)^{2},$$

with $s=4$ for the moderate setting, and $s=2$ for the strong setting. Values occurring outside $\left[ 0,1 \right]$ were set to the respective border. The parameter $\rho_{miss}$ specifies the correlation of missingness across rundays between the two variables, and was set to 0 or 0.2, corresponding to the median correlation observed for metabolite pairs of the same or different platform (Figure S2B).


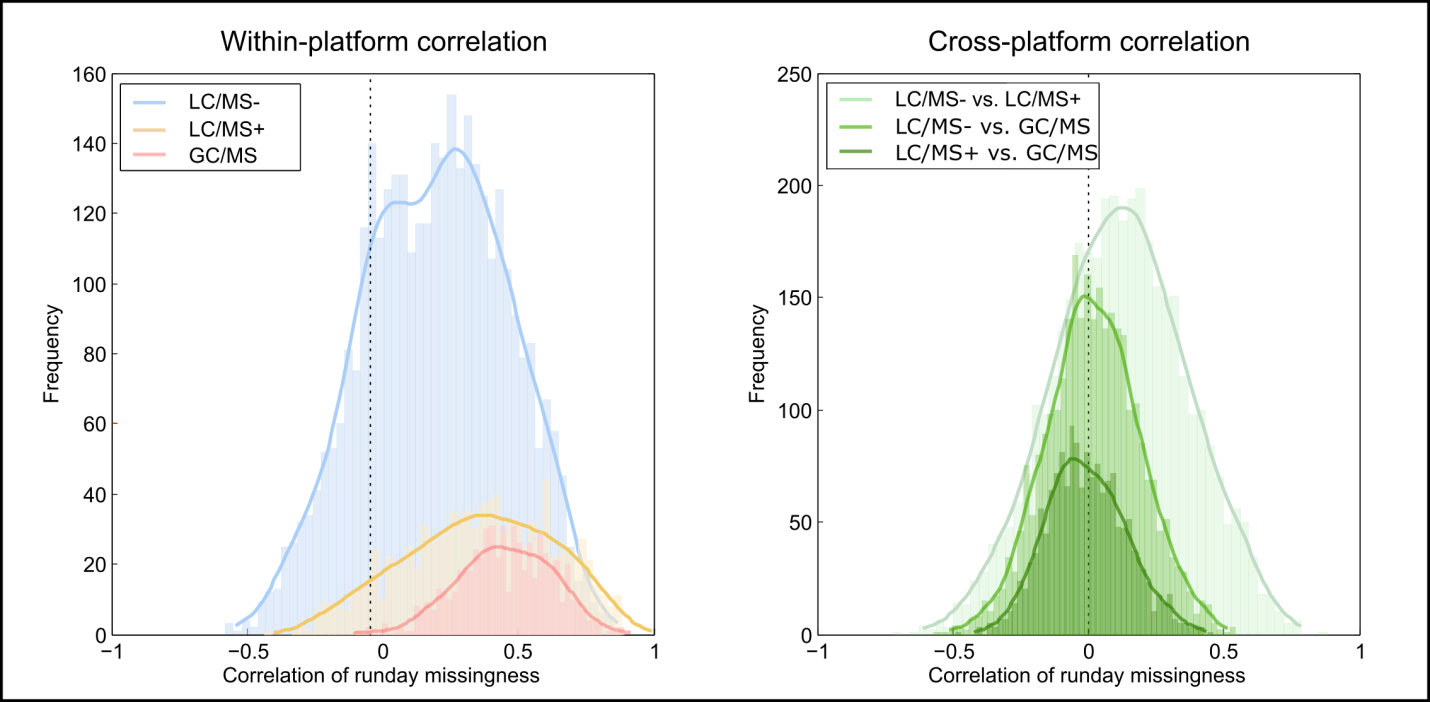


Figure S2B. Distribution of Pearson correlation coefficients of runday missingness between metabolites measured on the same and on different platforms, respectively.

For the probabilistic method, we either kept $\beta_{1}$ for each variable constant across the rundays or let it vary similarly to $miss$, with mean at the global $\beta_{1}$ values, correlation set to $\rho_{miss}$, and variance $\sigma_{\beta_{1}}^{2}=\left( \frac{\beta_{1}}{2} \right)^{2}$ denoting strong variation of $\beta_{1}$ across rundays. Otherwise we proceeded as described for *probabilistic LOD* above*,* stratiﬁed by runday.

Finally, we also simulated missing values completely randomly to acknowledge *unsystematic missingness*, and created mixtures of LOD-related mechanisms and unsystematic missingness by introducing $miss/2$ missingness using the LOD-related mechanism, and another $miss/2$ completely randomly (*mixtures LOD/unsystematic*) on top.

| **Parameter** | **Notation** | **Values** | **Evidence from real data set** |
| --- | --- | --- | --- |
| Correlation of metabolite pair | $cor$ | 0, 0.1, 0.2, 0.4 | Figure S2A |
| Sample size | $n$ | 100, 250, 1000 | Table S9 |
| Proportion of variance explained by 5 auxiliary metabolites | ${var}_{aux}$ | 0, 0.5 |  |
| Total proportion of missing values | ${miss}_{1}$*,* ${miss}_{2}$ | 0.1, 0.3, 0.5, 0.7 | Figure 2 |
| Missingness mechanism | *Fixed LOD* | | Figure 2, S1 File |
|  | *Probabilistic LOD* | | Figure 2, S1 File |
|  | *Runday-specific fixed LOD* | | Figure 3, Figure 4, S4 File |
|  | *Runday-specific probabilistic LOD* | | Figure 3, Figure 4, S4 File |
|  | *Unsystematic missingness* | | Figure 2, S1 File |
|  | *Mixtures LOD / unsystematic* | | Figure 2, S1 File |
| **Additional parameters for the mechanisms *(runday-specific) probabilistic LOD*** | | | |
| Strength of relation between metabolite concentration and missingness | $\beta_{1,1}$, $\beta_{1,2}$ | -1, -10 | Figure 3, Figure 4, S4 File |
| **Additional parameters for the mechanisms *runday-specific fixed/probabilistic LOD*** | | | |
| Variation of missingness across rundays | $\sigma_{miss,1}^{2}$, $\sigma_{miss,2}^{2}$ | $\sigma_{miss}^{2}=\left( \frac{min\left( miss,1-miss \right)}{s} \right)^{2}$ $s=4$ for moderate setting  $s=2$ for strong setting | Figure 3 |
| Correlation of missingness across rundays | $\rho_{miss}$ | 0, 0.2 | Figure 4, Figure 2SB |
| **Additional parameters for the mechanism *runday-specific probabilistic LOD*** | | | |
| Variation of $\beta_{1,1}$ and $\beta_{1,2}$across rundays | $\sigma_{\beta_{1,1}}^{2}$, $\sigma_{\beta_{1,2}}^{2}$ | $\sigma_{\beta_{1}}^{2}=0, \left( \frac{\beta_{1}}{2} \right)^{2}$ | Figure 3 |

Table S8. Overview of simulation parameters.

### Statistical evaluation

We applied the 31 (variations of) imputation methods to the simulated data. First, basic imputation diagnostics were performed. We compared histograms and bivariate scatter plots of the complete, observed and imputed data to visually assess how close the imputed values resembled the true complete values, and how well the relationship between the two variables was preserved after imputation (Supporting Information S5). In addition, we determined the mean squared deviation of the imputed versus original complete entries of the data (Supporting Information S5).

Next, we evaluated the ability of the imputation methods to retain the Pearson correlation, partial correlation, linear regression, and logistic regression estimates. For the latter analysis, we additionally simulated a dichotomized variable for each artificial data set by setting all values of one continuous variable in the data set that were above the median to one, and below the median to zero. This dichotomized variable was used as response in the logistic regression model, and the remaining continuous variable was used as predictor.

All statistical analyses were applied on the imputed data and compared to the true estimates. To this end, we calculated the type 1 error as the proportion of simulations with significant statistical estimate (p-value <0.05) in the absence of a true effect. Power was determined as the proportion of simulations with statistical estimate (p-value<0.05) in the presence of a true effect. Note that power = 1 - type 2 error.

Results for all data scenarios, all analyses, all imputation methods, and all parameter variations can be found in Supporting Information S5. The results shown in the main manuscript (Figure 6) contains the following parameter settings for all scenarios: moderate variability of missingness across rundays (see Table S8), uncorrelated runday-specific missingness of the metabolite pair ($\rho_{miss}$ = 0, see Table S8), varying steepness of the inverse relation between metabolite concentration and missingness ($\beta_{1}=-10, \sigma_{\beta_{1}}^{2}=25$), $n=250$, and presence of informative auxiliary metabolites (${var}_{aux}=0.5$). Both main variables had the same degree and mechanism of missingness.

### References

1. Richardson DB, Ciampi A. Effects of exposure measurement error when an exposure variable is constrained by a lower limit. Am J Epidemiol. 2003 Feb 15;157(4):355–63.

2. Nie L, Chu H, Liu C, Cole SR, Vexler A, Schisterman EF. Linear Regression with an Independent Variable Subject to a Detection Limit. Epidemiol Camb Mass. 2010 Jul;21(Suppl 4):S17–24.

3. Handbook of Statistics: Epidemiology and Medical Statistics. Elsevier; 2007. 871 p.

4. Rubin DB. Introduction. In: Multiple Imputation for Nonresponse in Surveys [Internet]. John Wiley & Sons, Inc.; 1987 [cited 2016 Feb 1]. p. 1–26. Available from: http://onlinelibrary.wiley.com/doi/10.1002/9780470316696.ch1/summary

5. Marshall A, Altman DG, Holder RL, Royston P. Combining estimates of interest in prognostic modelling studies after multiple imputation: current practice and guidelines. BMC Med Res Methodol. 2009 Jul 28;9:57.

6. D’Angelo GM, Luo J, Xiong C. Missing Data Methods for Partial Correlations. J Biom Biostat [Internet]. 2012 Dec [cited 2016 Feb 28];3(8). Available from: http://www.ncbi.nlm.nih.gov/pmc/articles/PMC3772686/

7. Helsel DR. Less than obvious - statistical treatment of data below the detection limit. Environ Sci Technol. 1990 Dezember;24(12):1766–74.

8. van Buuren S, Boshuizen HC, Knook DL. Multiple imputation of missing blood pressure covariates in survival analysis. Stat Med. 1999 Mar 30;18(6):681–94.

9. Van Hoewyk J, Lepkowski JM, Solenberger P, Raghunathan TE. A multivariate technique for multiply imputing missing values using a sequence of regression models. Surv Methodol. 2001 Aug 22;27(1):85–95.

10. van Buuren S, Groothuis-Oudshoorn K. mice: Multivariate Imputation by Chained Equations in R | van Buuren | Journal of Statistical Software. J Stat Softw [Internet]. 2011 Dec 12 [cited 2016 Feb 28];45(3). Available from: https://www.jstatsoft.org/article/view/v045i03

11. Yuan Y. Multiple Imputation Using SAS Software | Yuan | Journal of Statistical Software. J Stat Softw [Internet]. 2011 Dec 12 [cited 2016 Feb 28];45(6). Available from: https://www.jstatsoft.org/article/view/v045i06

12. Troyanskaya O, Cantor M, Sherlock G, Brown P, Hastie T, Tibshirani R, et al. Missing value estimation methods for DNA microarrays. Bioinforma Oxf Engl. 2001 Jun;17(6):520–5.

13. Hrydziuszko O, Viant MR. Missing values in mass spectrometry based metabolomics: an undervalued step in the data processing pipeline. Metabolomics. 2011 Oct 8;8(1):161–74.

14. Gromski PS, Xu Y, Kotze HL, Correa E, Ellis DI, Armitage EG, et al. Influence of Missing Values Substitutes on Multivariate Analysis of Metabolomics Data. Metabolites. 2014 Jun 16;4(2):433–52.

15. Armitage EG, Godzien J, Alonso-Herranz V, López-Gonzálvez Á, Barbas C. Missing value imputation strategies for metabolomics data. Electrophoresis. 2015 Dec;36(24):3050–60.

16. Tutz G, Ramzan S. Improved methods for the imputation of missing data by nearest neighbor methods. Comput Stat Data Anal. 2015 Oktober;90:84–99.
