# Supplementary material for: Characterization of missing values in untargeted MS-based metabolomics data and evaluation of missing data handling strategies
Source: Metabolomics. 2018 Sep 20;14(10):128. doi: 10.1007/s11306-018-1420-2 (PMC6153696; doi:10.1007/s11306-018-1420-2)
Supplement: Supplementary file 4 — Supplementary material 4 (DOCX 149 KB) [file 11306_2018_1420_MOESM4_ESM.docx]

# Characterization of missingness in untargeted MS-based metabolomics data sets and evaluation of missing data handling strategies

*Kieu Trinh Do^¶^, Simone Wahl^¶^, Johannes Raffler, Sophie Molnos, Michael Laimighofer, Jerzy Adamski, Karsten Suhre, Konstantin Strauch, Annette Peters, Christian Gieger, Claudia Langenberg, Isobel D. Stewart, Fabian J. Theis, Harald Grallert, Gabi Kastenmüller****^#^****, Jan Krumsiek****^#^***

## Supporting Information File S4: Imputation methods

***Richardson & Ciampi (RC):***

Assuming that the observed metabolite values – after log-transformation – follow a left-truncated normal distribution, Richardson and Ciampi (1) proposed to estimate this distribution and to replace missing values by the expectation value $E(x|x\leq LOD)$ of the censored part of the distribution. We estimated a truncated normal distribution with maximum likelihood estimation (MLE) with the function mle.tmvnorm from the R package tmvtnorm, version 1.4-9, using the smallest observed value as truncation point and the complete data estimates of mean and variance as start values. $E(x|x\leq LOD)$ was calculated as ${E\left( x | x\leq LOD \right)=\hat{\mu}}_{x}-\frac{{\hat{\sigma}_{x}}^{\left[ -\frac{\left( LOD-\hat{\mu}_{x} \right)^{2}}{2{\hat{\sigma}_{x}}^{2}} \right]}}{\Phi\left( \frac{LOD-\hat{\mu}_{x}}{\hat{\sigma}_{x}} \right)}$, with $\hat{\mu}_{x}$ and $\hat{\sigma}_{x}$ are the estimated parameters by MLE, and $\Phi(x)$ is the cumulative Gaussian distribution function. RC outperformed simple substitution methods (including replacement by the estimated LOD (i.e., min), by LOD/2 or by LOD/$\sqrt{2}$) in a recent investigation (2), for which reason we did not, besides min and RC, further include such methods in the investigation.

***Imputation by truncated sampling (ITS):***

Provided that we rely on the assumption of a truncated normal distribution, an extension of the *RC* method is to draw randomly from the censored part of the estimated truncated normal distribution. This was implemented with the function *rtmvnorm* from the R package *tmvtnorm*, using Gibbs sampling with 100 burn-in iterations.

***Multiple imputation by truncated sampling (MITS):***

Independent on the type of imputation, the variance estimates obtained from data generated by single imputation followed by applying complete-data methods generally tend to be too low, since the additional uncertainty about the missing values is ignored (3). It is widely acknowledged that multiple imputation (MI) can improve the performance by reducing the variance. Following the procedure established by Rubin (4), we (i) imputed the data sets $m$ times, (ii) analyzed each of the $m$ completed data sets separately, and (iii) combined the $m$ resulting estimates using established procedures (4–6), thereby including the missingness-related uncertainty as the inter-imputation variance component. Our implementation used the R package *mice* (version 2.25) and *miceadds* (version 1.5-0) for combination of regression and correlation coefficients, respectively. We set the number of imputations to $m=20$ for all methods, assuming this to be a sufficient number to assure accuracy of the obtained estimates in a simulation study.

***RC/ITS/MITS within rundays (RC-R / ITS-R / MITS-R):***

In MS-based metabolomics data the LOD varies between rundays, for which reason we extended *RC, ITS,* and *MITS* to be applied within each runday. In previous analyses MLE was found to perform well for larger sample sizes ($n$ = 25, 50) (7). However, the majority of missing values in the KORA data set occurred in rundays with less than 25 values observed, so that we relaxed that rule of thumb and set the required number of observed values to 17, i.e. half of the number of samples in a typical Metabolon runday. For *RC-R*, the remaining missing values were set to the mean of the expected values across all rundays with a sufficient number of values. For *ITS-R* and *MITS-R*, the remaining missing values were replaced using *ICE-norm*.

***Imputation by chained equations (ICE-norm / -pmm / -adjR):***

The principle is a repeated chain of equations through the incomplete variables, where in each imputation model, the respective incomplete variable is modeled as a function of the remaining variables, optionally after variable selection (8–10) . A popular type of imputation model for continuous incomplete variables is Bayesian linear regression (*ICE-norm*), where after modeling, the posterior predictive distribution of the data is specified. Missing entries of the target variable are then replaced by draws from this distribution. In *ICE-pmm*, instead of directly using the draws from the posterior predictive distribution, values are replaced by a random draw of actually observed values from other observations with the closed predicted values. Here, the default of 5 closest values were used. This procedures are described in detail by Yuan 2011 (11). Additionally, a two-level normal model (*ICE-adjR*) was specified with random intercept per runday (method 2l.pan), aiming to better utilize runday information. This model assumes that variable values (i.e., metabolite concentrations) have a runday-specific component, which varies randomly following a normal distribution.

We used the function *mice* (R package mice) with $m=1$ imputation and 5 iterations (i.e., equation chains). For the higher-dimensional KORA data, we reduced the covariates for each model prior to modeling using the *quickpred* function, requiring a minimum correlation of 0.1 for a variable to be included as a covariate. Moreover, variables were required to have observed values on at least 25% of the missing observations of the target variable. In addition to the matrix of metabolomics data, age, sex and BMI were available as covariates.

***MICE vs. MICE-avg:***

The original *MICE* approach consists of a pooling step of the statistical results (see above). For correlation and regression analysis, Rubin’s rules can be applied (4), however, for more sophisticated statistical approaches (e.g., PCA), pooling of the results is not straightforward.

Due to this pooling complexity, a nowadays widely used alternative was developed (*MICE-avg*). Here, the imputed data is pooled by calculating the average of the imputed values from the different imputations (see Figure S4 below). Although *MICE-avg* is simpler in application, it forfeits in performance.


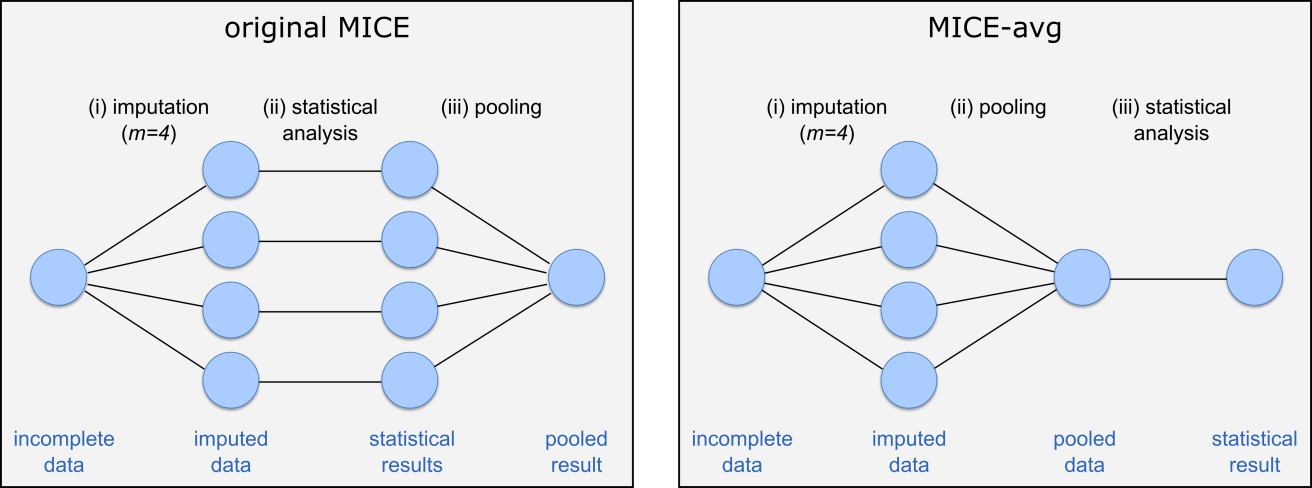


Figure S4. Strategies of the original *MICE* and *MICE-avg* approach.

***K-nearest neighbor imputation (KNN-var(K) / KNN-obs(K) / KNN-obs-sel(K)):***

*KNN* is another class of multivariate imputation methods that has been used for omics data (gene expression; metabolomics) before (12–15). It replaces missing values of each variable by the (weighted) average of (pre-specified) *K* nearest variables (*KNN-var*) or observations (*KNN-obs*), whereby “near” is defined through some distance measure. We used the Euclidean distance, i.e. the mean squared difference between observed entries of two variables or observations, and chose weights as $e^{-dist}$, where $dist$ defines the distances between the two variables or observations. Since metabolites are not necessarily on the same scale, we scaled the data matrix prior to applying imputation, and rescaled the imputed entries.

In medium- to high-dimensional settings, traditional *KNN-obs* using all variables in order to determine distances between observations can be much improved by including, for each incomplete variable, only the most strongly correlated variable in the calculation of the distances between observations (16). Thus, we implemented an *ad hoc* strategy selecting the 5 to 10 strongest correlated variables at $\left| \rho\right| \geq0.2$ (*KNN-obs-sel*). For all *KNN* versions, *K* was chosen to be 3, 5, 10, and 20.

### References

1. Richardson DB, Ciampi A. Effects of exposure measurement error when an exposure variable is constrained by a lower limit. Am J Epidemiol. 2003 Feb 15;157(4):355–63.

2. Nie L, Chu H, Liu C, Cole SR, Vexler A, Schisterman EF. Linear Regression with an Independent Variable Subject to a Detection Limit. Epidemiol Camb Mass. 2010 Jul;21(Suppl 4):S17–24.

3. Handbook of Statistics: Epidemiology and Medical Statistics. Elsevier; 2007. 871 p.

4. Rubin DB. Introduction. In: Multiple Imputation for Nonresponse in Surveys [Internet]. John Wiley & Sons, Inc.; 1987 [cited 2016 Feb 1]. p. 1–26. Available from: http://onlinelibrary.wiley.com/doi/10.1002/9780470316696.ch1/summary

5. Marshall A, Altman DG, Holder RL, Royston P. Combining estimates of interest in prognostic modelling studies after multiple imputation: current practice and guidelines. BMC Med Res Methodol. 2009 Jul 28;9:57.

6. D’Angelo GM, Luo J, Xiong C. Missing Data Methods for Partial Correlations. J Biom Biostat [Internet]. 2012 Dec [cited 2016 Feb 28];3(8). Available from: http://www.ncbi.nlm.nih.gov/pmc/articles/PMC3772686/

7. Helsel DR. Less than obvious - statistical treatment of data below the detection limit. Environ Sci Technol. 1990 Dezember;24(12):1766–74.

8. van Buuren S, Boshuizen HC, Knook DL. Multiple imputation of missing blood pressure covariates in survival analysis. Stat Med. 1999 Mar 30;18(6):681–94.

9. Van Hoewyk J, Lepkowski JM, Solenberger P, Raghunathan TE. A multivariate technique for multiply imputing missing values using a sequence of regression models. Surv Methodol. 2001 Aug 22;27(1):85–95.

10. van Buuren S, Groothuis-Oudshoorn K. mice: Multivariate Imputation by Chained Equations in R | van Buuren | Journal of Statistical Software. J Stat Softw [Internet]. 2011 Dec 12 [cited 2016 Feb 28];45(3). Available from: https://www.jstatsoft.org/article/view/v045i03

11. Yuan Y. Multiple Imputation Using SAS Software | Yuan | Journal of Statistical Software. J Stat Softw [Internet]. 2011 Dec 12 [cited 2016 Feb 28];45(6). Available from: https://www.jstatsoft.org/article/view/v045i06

12. Troyanskaya O, Cantor M, Sherlock G, Brown P, Hastie T, Tibshirani R, et al. Missing value estimation methods for DNA microarrays. Bioinforma Oxf Engl. 2001 Jun;17(6):520–5.

13. Hrydziuszko O, Viant MR. Missing values in mass spectrometry based metabolomics: an undervalued step in the data processing pipeline. Metabolomics. 2011 Oct 8;8(1):161–74.

14. Gromski PS, Xu Y, Kotze HL, Correa E, Ellis DI, Armitage EG, et al. Influence of Missing Values Substitutes on Multivariate Analysis of Metabolomics Data. Metabolites. 2014 Jun 16;4(2):433–52.

15. Armitage EG, Godzien J, Alonso-Herranz V, López-Gonzálvez Á, Barbas C. Missing value imputation strategies for metabolomics data. Electrophoresis. 2015 Dec;36(24):3050–60.

16. Tutz G, Ramzan S. Improved methods for the imputation of missing data by nearest neighbor methods. Comput Stat Data Anal. 2015 Oktober;90:84–99.
