# Supplementary material for: Characterization of missing values in untargeted MS-based metabolomics data and evaluation of missing data handling strategies
Source: Metabolomics. 2018 Sep 20;14(10):128. doi: 10.1007/s11306-018-1420-2 (PMC6153696; doi:10.1007/s11306-018-1420-2)
Supplement: Supplementary file 5 — Supplementary material 5 (PDF 27221 KB) [file 11306_2018_1420_MOESM5_ESM.pdf]

Characterization of missingness in untargeted MS-based metabolomics data sets and evaluation of missing data handling strategies

Kieu Trinh Do[¶], Simone Wahl[¶], Johannes Raffler, Sophie Molnos, Michael Laimighofer, Jerzy Adamski, Karsten Suhre, Konstantin Strauch, Annette Peters, Christian Gieger, Claudia Langenberg, Isobel D. Stewart, Fabian J. Theis, Harald Grallert, Gabi Kastenmüller[#], Jan Krumsiek[#]

Supporting Information File S5: Evaluation results of the simulation study

Shown are evaluation results for different simulated data scenarios. (A) Kernel density plots of original complete data of metabolites with histograms of observed (blue), imputed data points in one metabolite (violet), and imputed data points in both metabolites (red). (B) Scatterplots of metabolite 1 and metabolite 2. (C) Mean squared deviation (MSD) for different amount of missing values with increasing true effect. (D) Average absolute bias of Pearson correlation (cor), partial correlation (pcor), linear regression (lm), and logistic regression estimates (logistic regr.) for different amount of missing values with increasing true effect. Bias is defined as the estimate obtained from the respective imputation method minus true correlation, with mean ± 1 SD across 250 simulations. (D) Power curves of hypothesis test for Pearson correlation, partial correlation, linear regression, and logistic regression analyses for different amount of missing values with increasing true effect. The y-axis shows the proportion of significant estimates among the 250 simulations; at null hypothesis correlation = 0, it shows the type 1 error, while power is reflected when true correlation is >0.

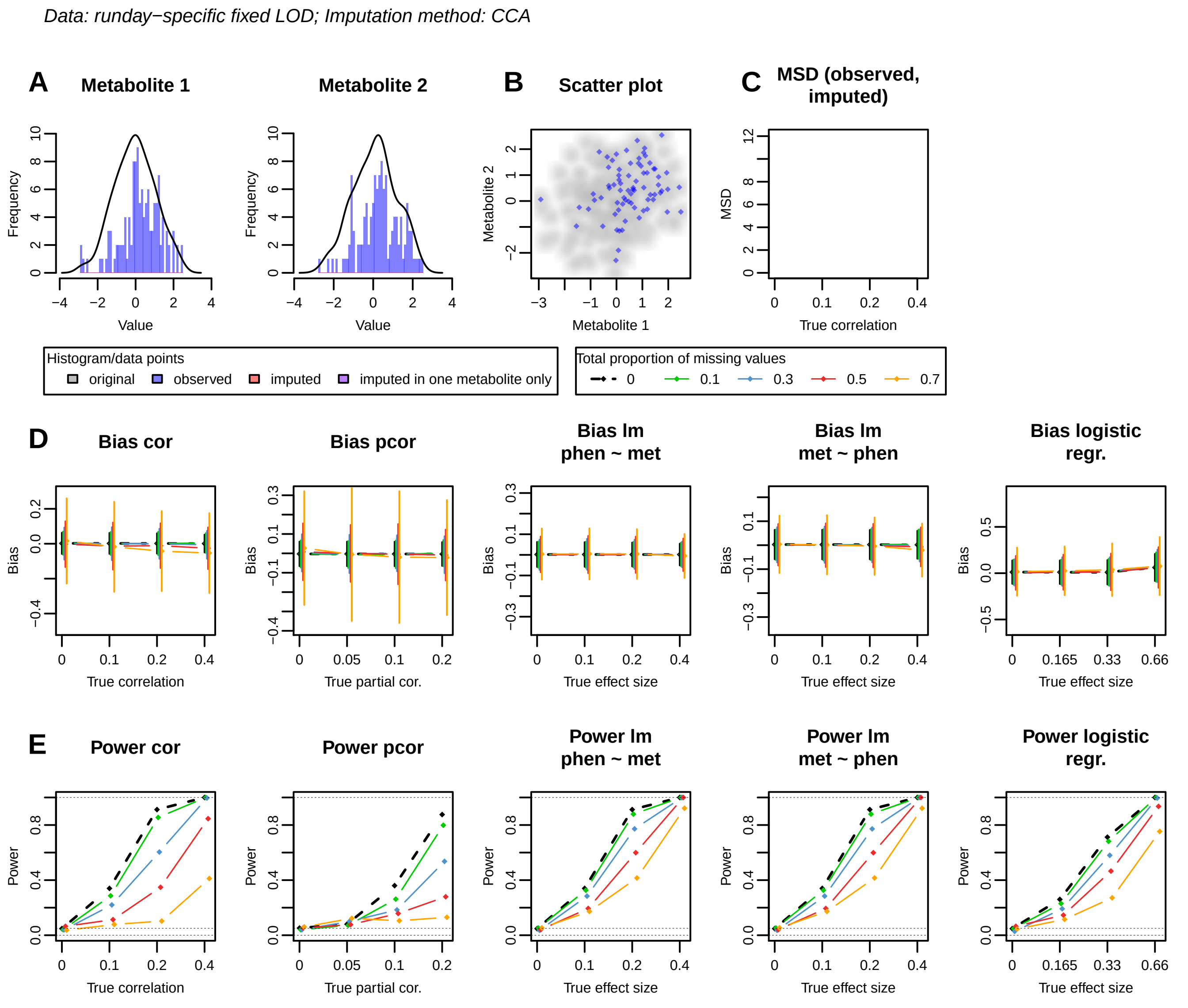
Data: runday−specific fixed LOD; Imputation method: CCA
A
Metabolite 1
Metabolite 2
Frequency
Value
B
Scatter plot
Metabolite 2
Metabolite 1
C
MSD (observed, imputed)
MSD
True correlation
Histogram/data points
original
observed
imputed
imputed in one metabolite only
Total proportion of missing values
0
0.1
0.3
0.5
0.7
D
Bias cor
Bias pcor
Bias lm phen ~ met
Bias lm met ~ phen
Bias logistic regr.
Bias
True correlation
True partial cor.
True effect size
E
Power cor
Power pcor
Power lm phen ~ met
Power lm met ~ phen
Power logistic regr.
Power
True correlation
True partial cor.
True effect size

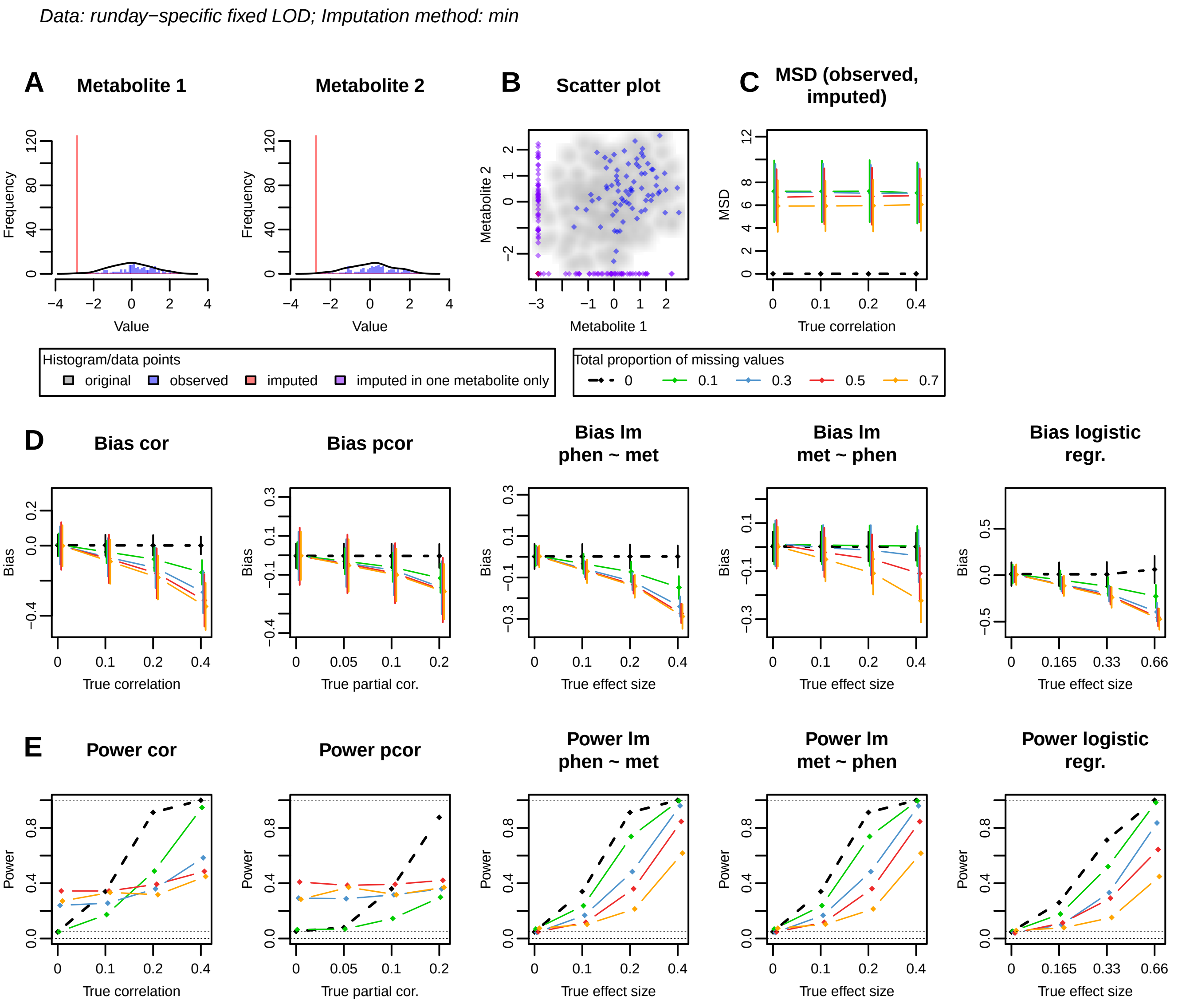
Data: runday−specific fixed LOD; Imputation method: min
A
Metabolite 1
Metabolite 2
Frequency
Value
B
Scatter plot
Metabolite 1
Metabolite 2
C
MSD (observed, imputed)
MSD
True correlation
Histogram/data points
original
observed
imputed
imputed in one metabolite only
Total proportion of missing values
0
0.1
0.3
0.5
0.7
D
Bias cor
Bias pcor
Bias lm phen ~ met
Bias lm met ~ phen
Bias logistic regr.
Bias
True correlation
True partial cor.
True effect size
E
Power cor
Power pcor
Power lm phen ~ met
Power lm met ~ phen
Power logistic regr.
Power
True correlation
True partial cor.
True effect size

Data: runday−specific fixed LOD; Imputation method: RC

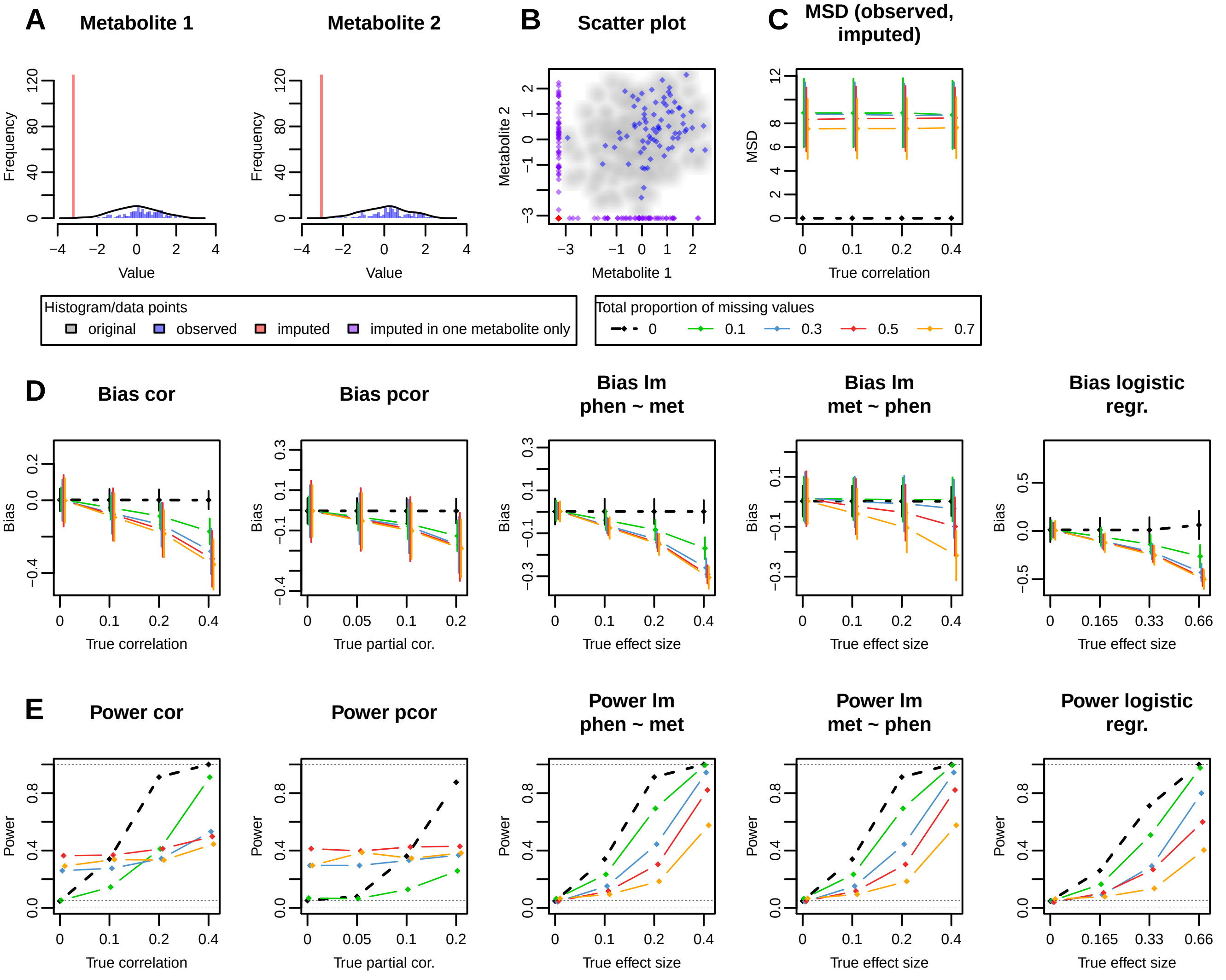

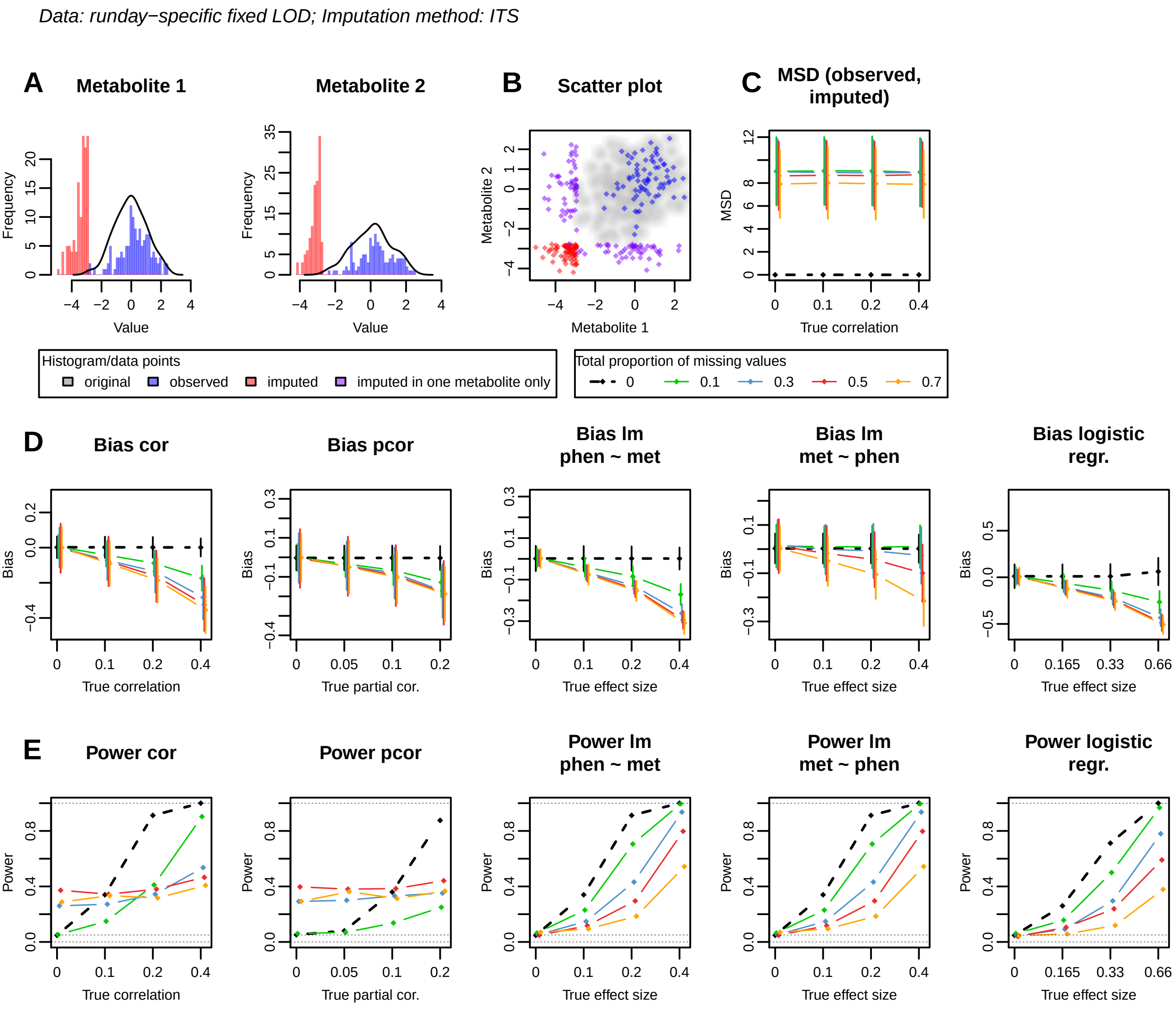
Data: runday−specific fixed LOD; Imputation method: ITS
A
Metabolite 1
Metabolite 2
Frequency
Value
B
Scatter plot
Metabolite 1
Metabolite 2
C
MSD (observed, imputed)
MSD
True correlation
Histogram/data points
original
observed
imputed
imputed in one metabolite only
Total proportion of missing values
0
0.1
0.3
0.5
0.7
D
Bias cor
Bias pcor
Bias lm phen ~ met
Bias lm met ~ phen
Bias logistic regr.
Bias
True correlation
True partial cor.
True effect size
E
Power cor
Power pcor
Power lm phen ~ met
Power lm met ~ phen
Power logistic regr.
Power
True correlation
True partial cor.
True effect size

Data: runday−specific fixed LOD; Imputation method: MITS

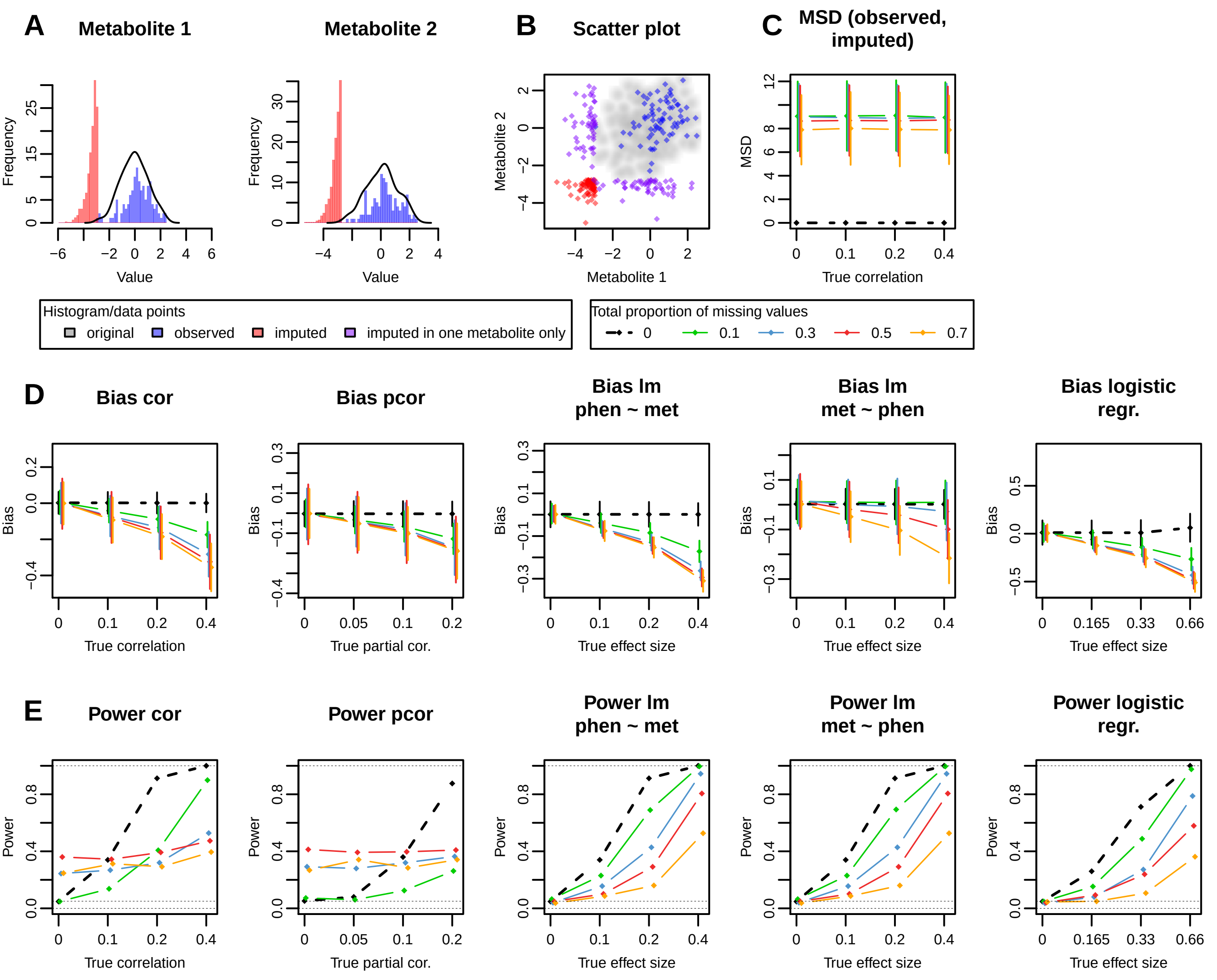

Data: runday−specific fixed LOD; Imputation method: RC−R

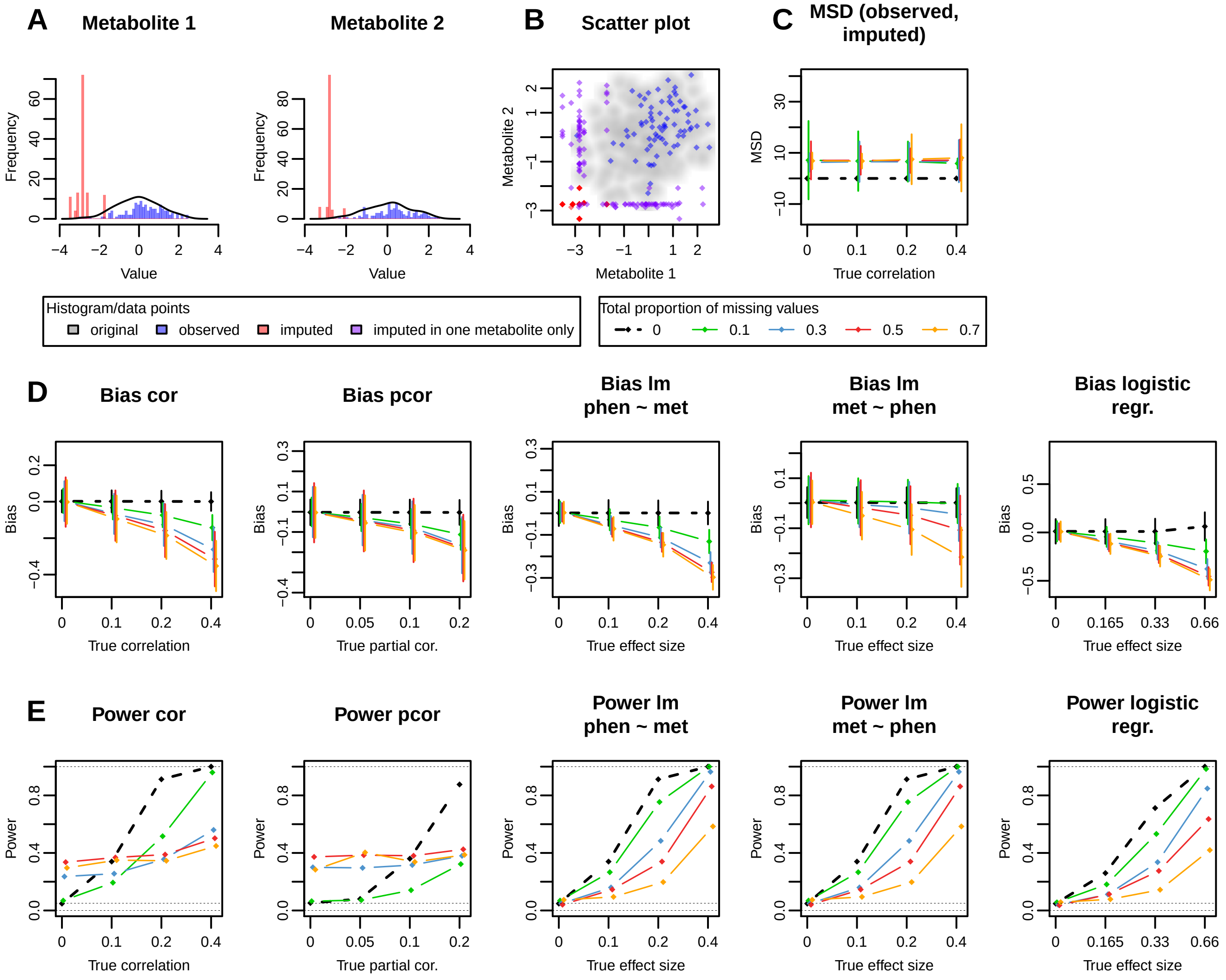

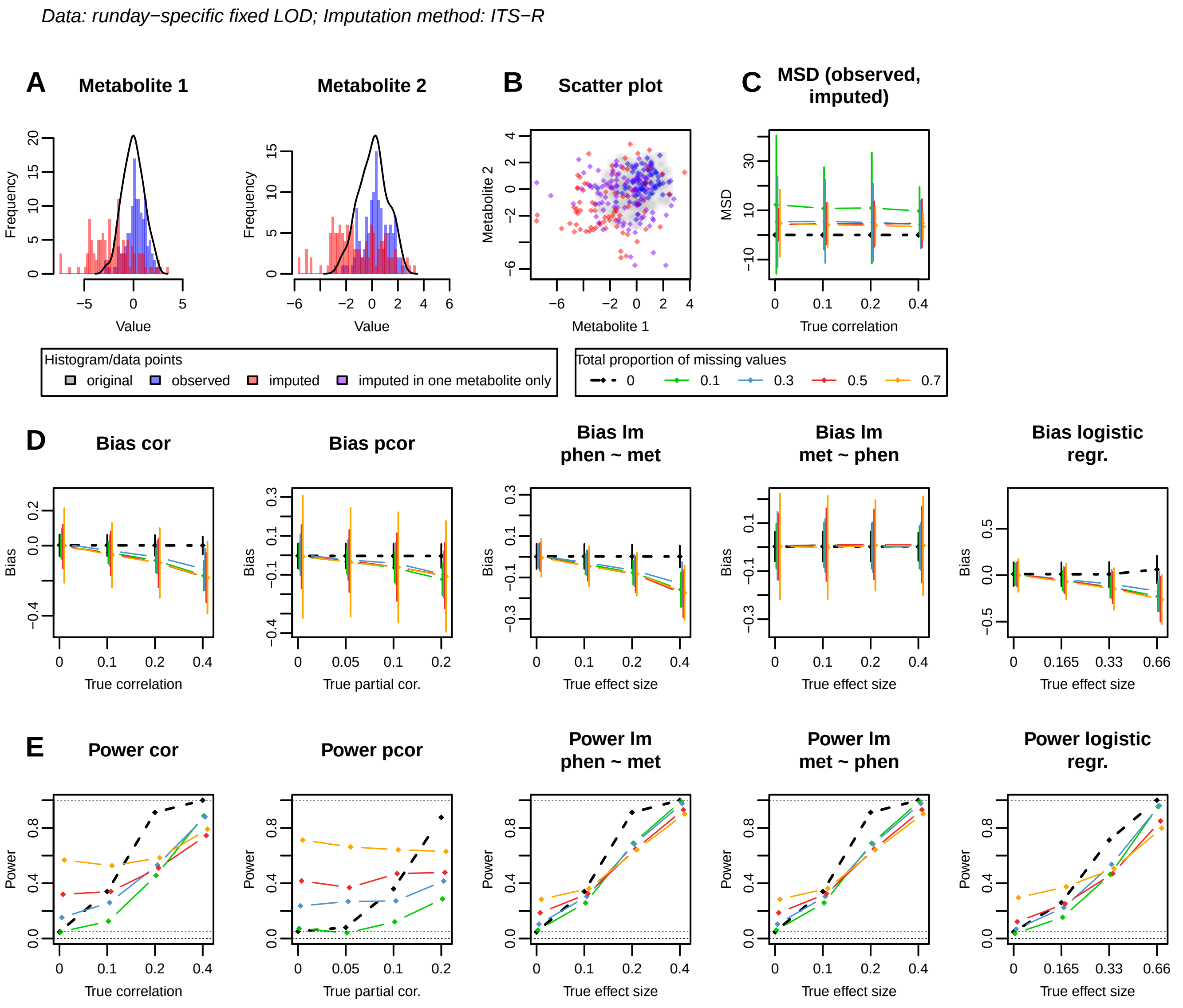

Data: runday−specific fixed LOD; Imputation method: ITS−R
A
Metabolite 1
Metabolite 2
Frequency
Value
B
Scatter plot
Metabolite 1
Metabolite 2
C
MSD (observed, imputed)
MSD
True correlation
Histogram/data points
original
observed
imputed
imputed in one metabolite only
Total proportion of missing values
0
0.1
0.3
0.5
0.7
D
Bias cor
Bias pcor
Bias lm phen ~ met
Bias lm met ~ phen
Bias logistic regr.
Bias
True correlation
True partial cor.
True effect size
E
Power cor
Power pcor
Power lm phen ~ met
Power lm met ~ phen
Power logistic regr.
Power
True correlation
True partial cor.
True effect size

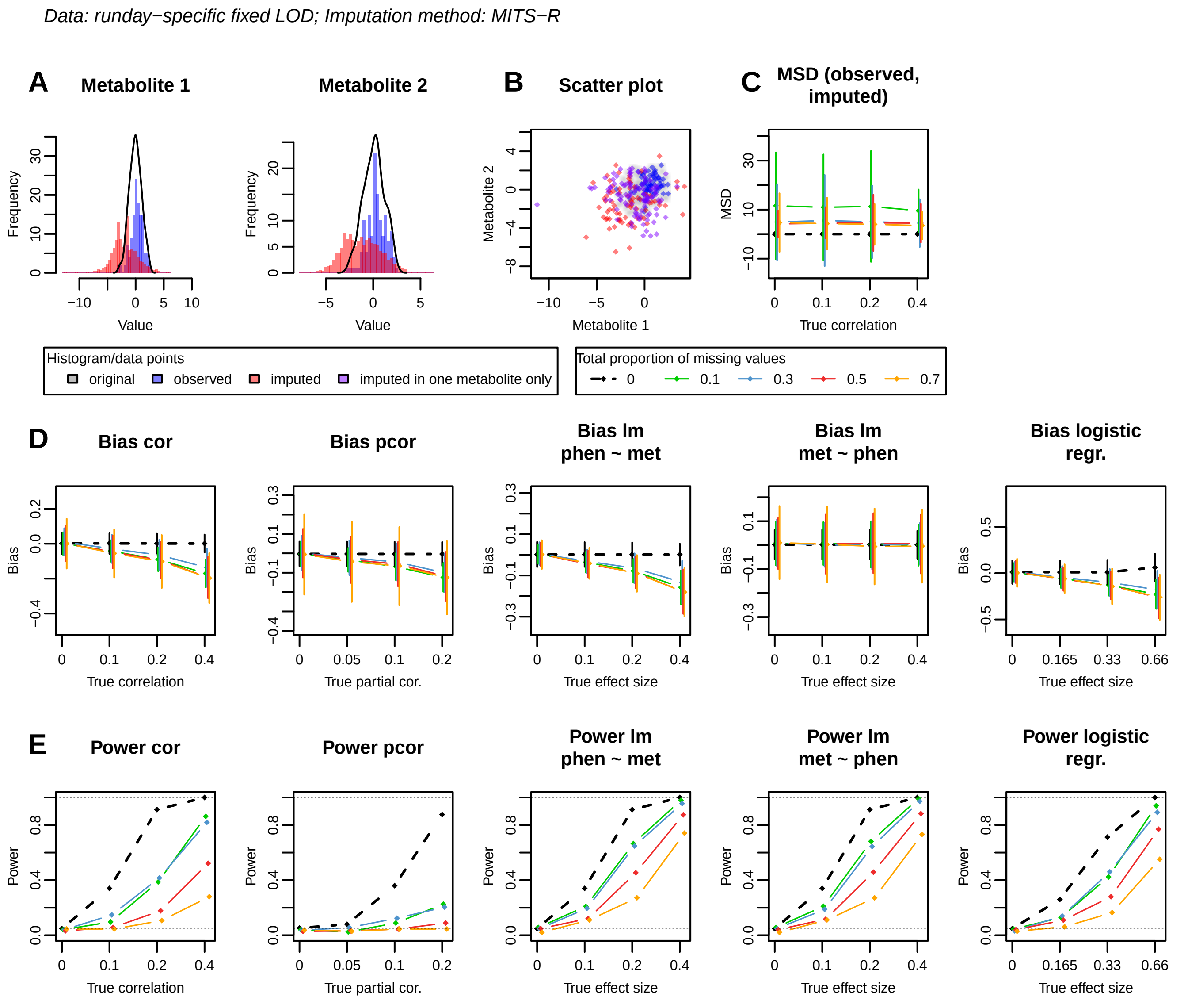
Data: runday−specific fixed LOD; Imputation method: MITS−R
A
Metabolite 1
Metabolite 2
B
Scatter plot
C
MSD (observed, imputed)
Frequency
Value
Metabolite 2
Metabolite 1
MSD
True correlation
Histogram/data points
original
observed
imputed
imputed in one metabolite only
Total proportion of missing values
0
0.1
0.3
0.5
0.7
D
Bias cor
Bias pcor
Bias lm phen ~ met
Bias lm met ~ phen
Bias logistic regr.
Bias
True correlation
True partial cor.
True effect size
E
Power cor
Power pcor
Power lm phen ~ met
Power lm met ~ phen
Power logistic regr.
Power
True correlation
True partial cor.
True effect size

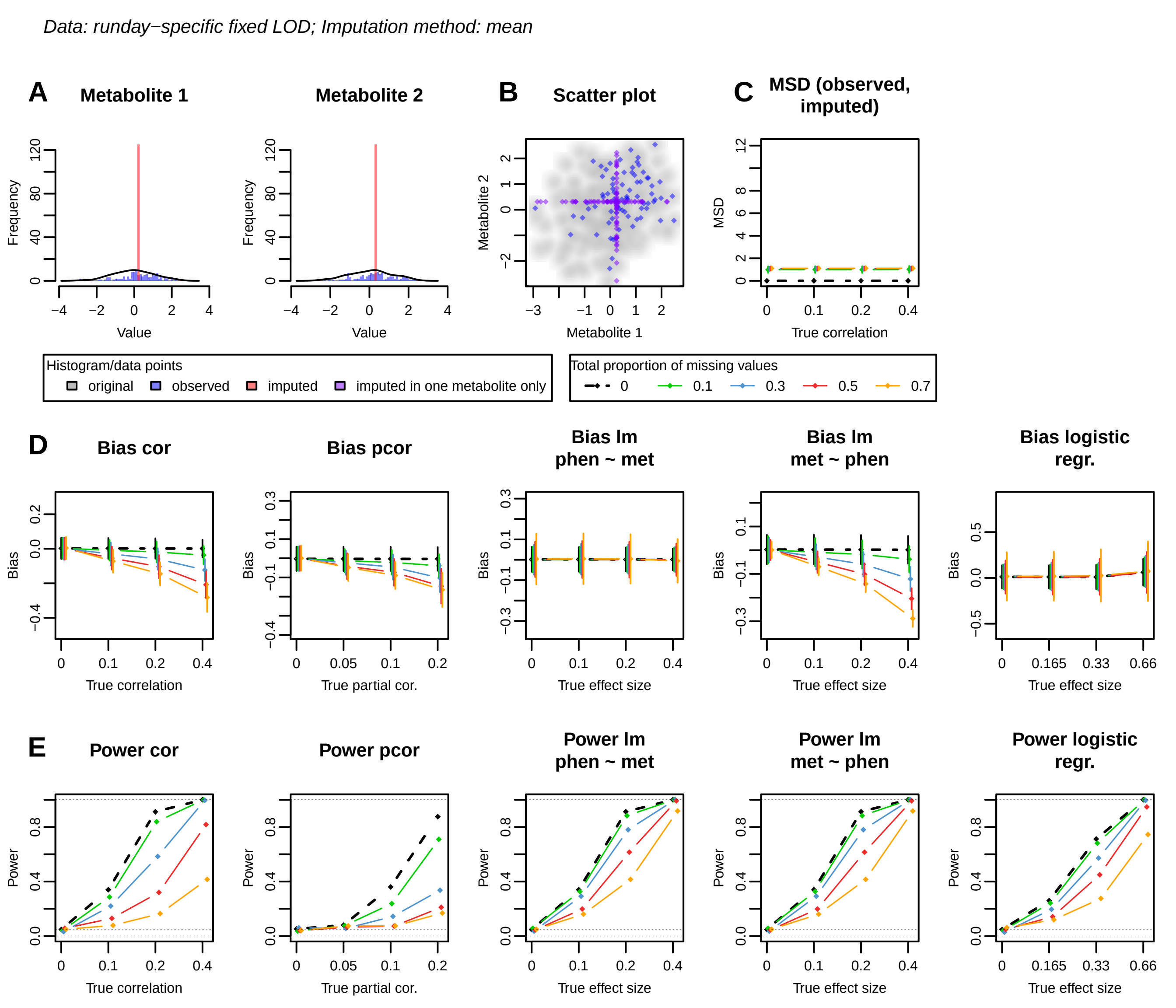
Data: runday−specific fixed LOD; Imputation method: mean
A
Metabolite 1
Metabolite 2
Frequency
Value
B
Scatter plot
Metabolite 1
Metabolite 2
C
MSD (observed, imputed)
MSD
True correlation
Histogram/data points
original
observed
imputed
imputed in one metabolite only
Total proportion of missing values
0
0.1
0.3
0.5
0.7
D
Bias cor
Bias pcor
Bias lm phen ~ met
Bias lm met ~ phen
Bias logistic regr.
Bias
True correlation
True partial cor.
True effect size
E
Power cor
Power pcor
Power lm phen ~ met
Power lm met ~ phen
Power logistic regr.
Power
True correlation
True partial cor.
True effect size

Data: runday−specific fixed LOD; Imputation method: ICE−norm

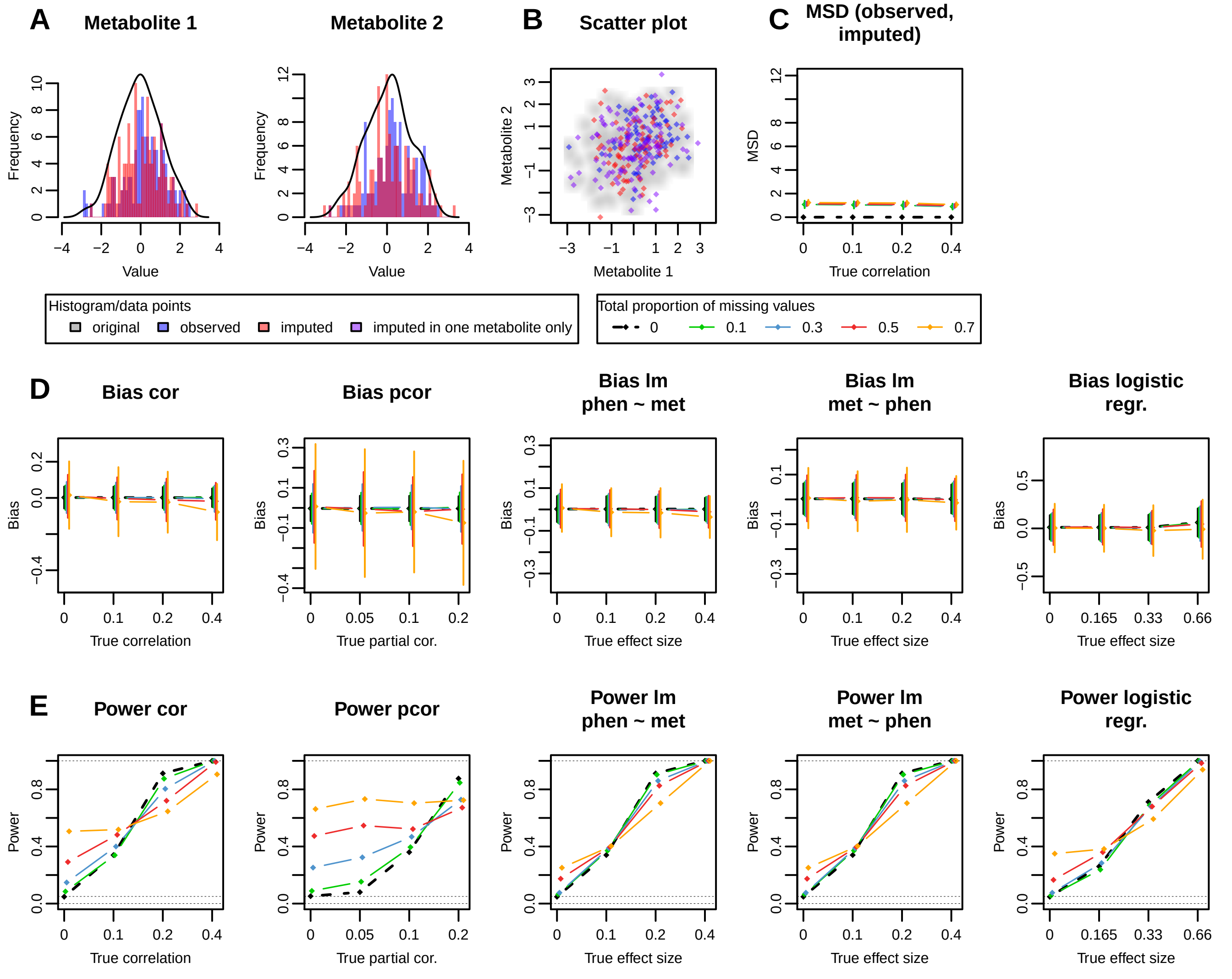

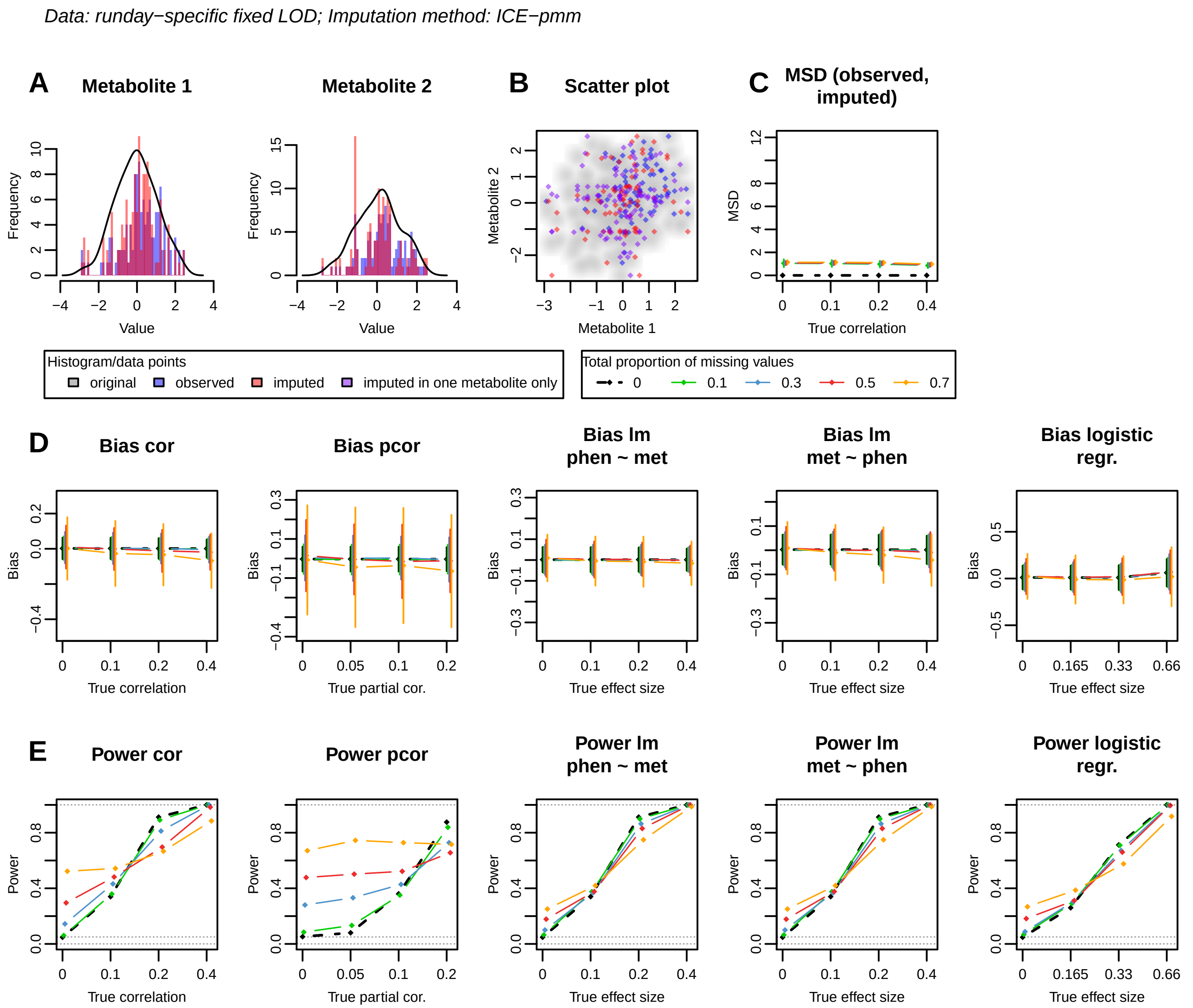

Data: runday−specific fixed LOD; Imputation method: ICE−pmm
A
Metabolite 1
Metabolite 2
Frequency
Value
B
Scatter plot
Metabolite 1
Metabolite 2
C
MSD (observed, imputed)
MSD
True correlation
Histogram/data points
original
observed
imputed
imputed in one metabolite only
Total proportion of missing values
0
0.1
0.3
0.5
0.7
D
Bias cor
Bias pcor
Bias lm phen ~ met
Bias lm met ~ phen
Bias logistic regr.
Bias
True correlation
True partial cor.
True effect size
E
Power cor
Power pcor
Power lm phen ~ met
Power lm met ~ phen
Power logistic regr.
Power
True correlation
True partial cor.
True effect size

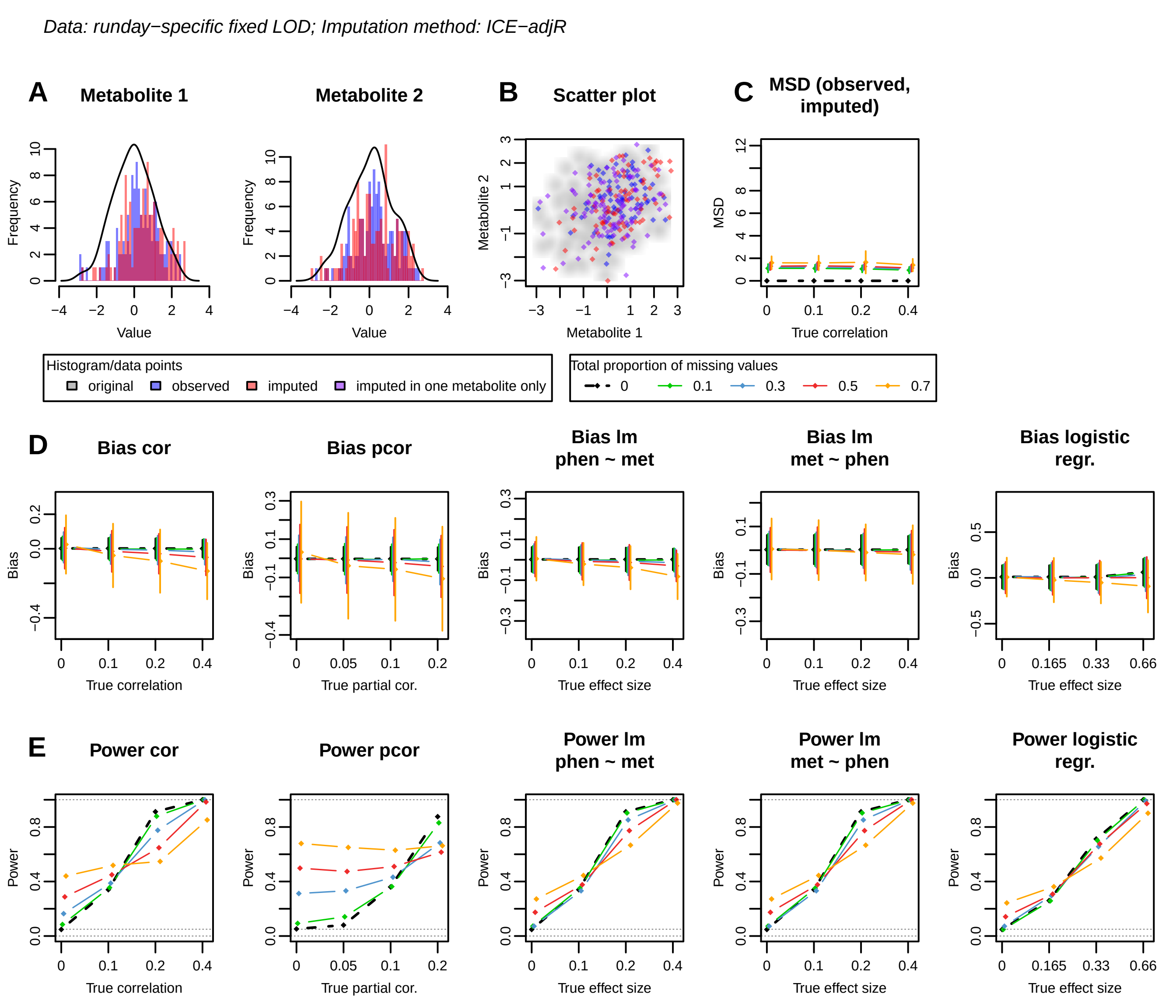
Data: runday−specific fixed LOD; Imputation method: ICE−adjR
A
Metabolite 1
Metabolite 2
Frequency
Value
B
Scatter plot
Metabolite 1
Metabolite 2
C
MSD (observed, imputed)
MSD
True correlation
Histogram/data points
original
observed
imputed
imputed in one metabolite only
Total proportion of missing values
0
0.1
0.3
0.5
0.7
D
Bias cor
Bias pcor
Bias lm phen ~ met
Bias lm met ~ phen
Bias logistic regr.
Bias
True correlation
True partial cor.
True effect size
E
Power cor
Power pcor
Power lm phen ~ met
Power lm met ~ phen
Power logistic regr.
Power
True correlation
True partial cor.
True effect size

Data: runday−specific fixed LOD; Imputation method: MICE−norm

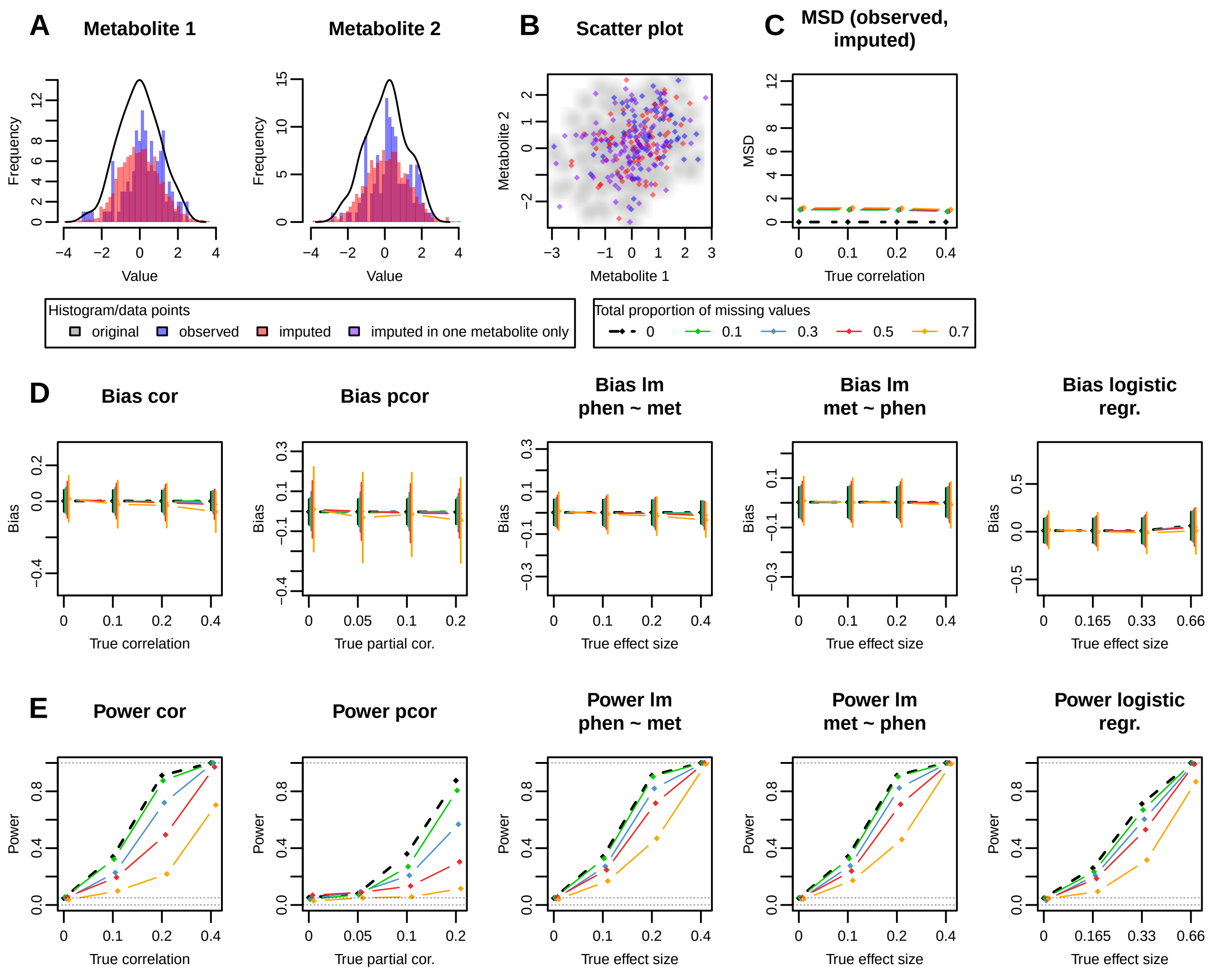

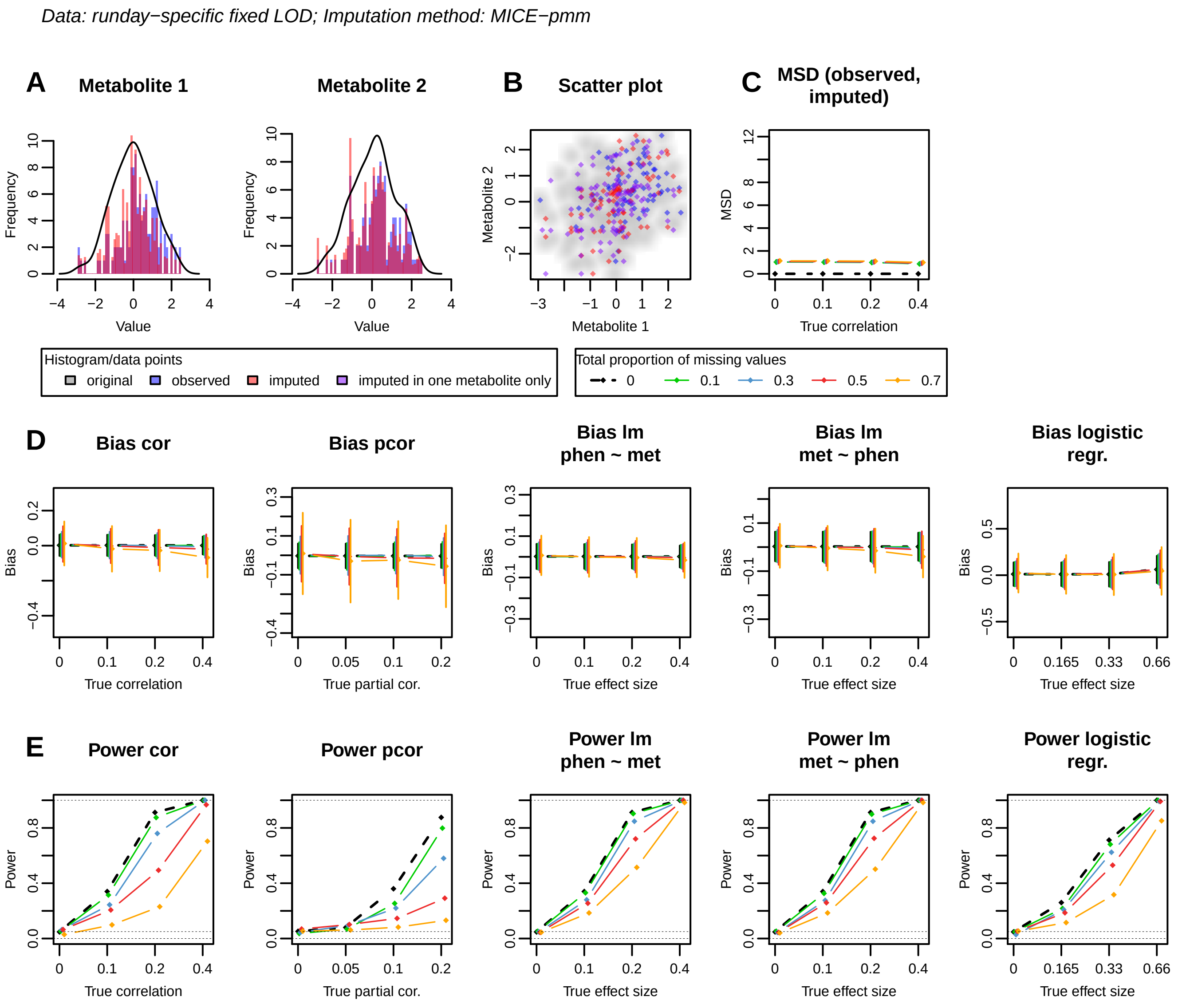

Data: runday−specific fixed LOD; Imputation method: MICE−pmm
A
Metabolite 1
Metabolite 2
Frequency
Value
B
Scatter plot
Metabolite 2
Metabolite 1
C
MSD (observed, imputed)
MSD
True correlation
Histogram/data points
original
observed
imputed
imputed in one metabolite only
Total proportion of missing values
0
0.1
0.3
0.5
0.7
D
Bias cor
Bias pcor
Bias lm phen ~ met
Bias lm met ~ phen
Bias logistic regr.
Bias
True correlation
True partial cor.
True effect size
E
Power cor
Power pcor
Power lm phen ~ met
Power lm met ~ phen
Power logistic regr.
Power

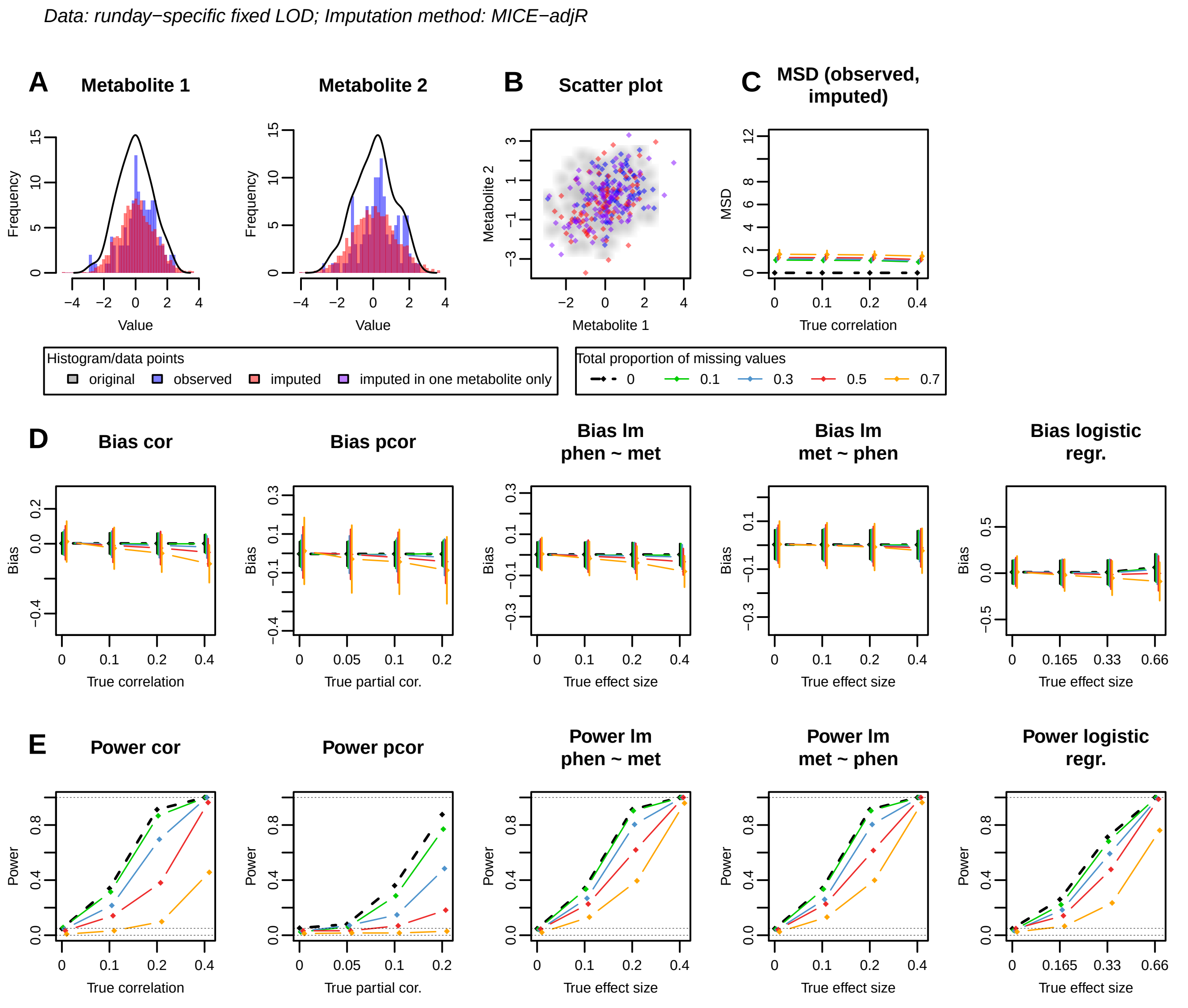
Data: runday−specific fixed LOD; Imputation method: MICE−adjR
A
Metabolite 1
Metabolite 2
B
Scatter plot
C
MSD (observed, imputed)
Frequency
Value
Metabolite 2
Metabolite 1
MSD
True correlation
Histogram/data points
original
observed
imputed
imputed in one metabolite only
Total proportion of missing values
0
0.1
0.3
0.5
0.7
D
Bias cor
Bias pcor
Bias lm phen ~ met
Bias lm met ~ phen
Bias logistic regr.
Bias
True correlation
True partial cor.
True effect size
E
Power cor
Power pcor
Power lm phen ~ met
Power lm met ~ phen
Power logistic regr.
Power

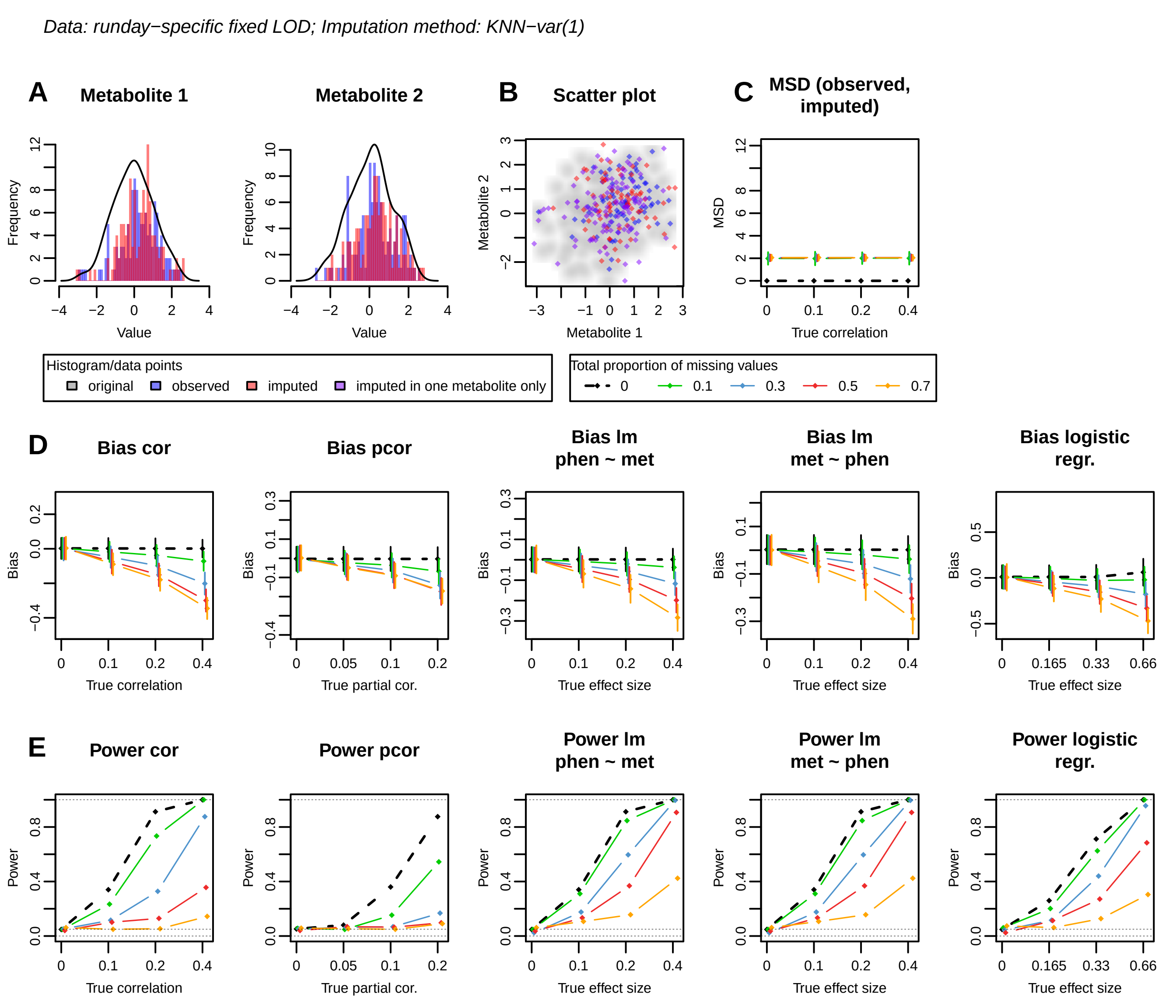

Data: runday−specific fixed LOD; Imputation method: KNN−var(1)
A
Metabolite 1
Metabolite 2
B
Scatter plot
C
MSD (observed, imputed)
Frequency
Value
Metabolite 1
Metabolite 2
MSD
True correlation
Histogram/data points
original
observed
imputed
imputed in one metabolite only
Total proportion of missing values
0
0.1
0.3
0.5
0.7
D
Bias cor
Bias pcor
Bias lm phen ~ met
Bias lm met ~ phen
Bias logistic regr.
Bias
True correlation
True partial cor.
True effect size
E
Power cor
Power pcor
Power lm phen ~ met
Power lm met ~ phen
Power logistic regr.
Power

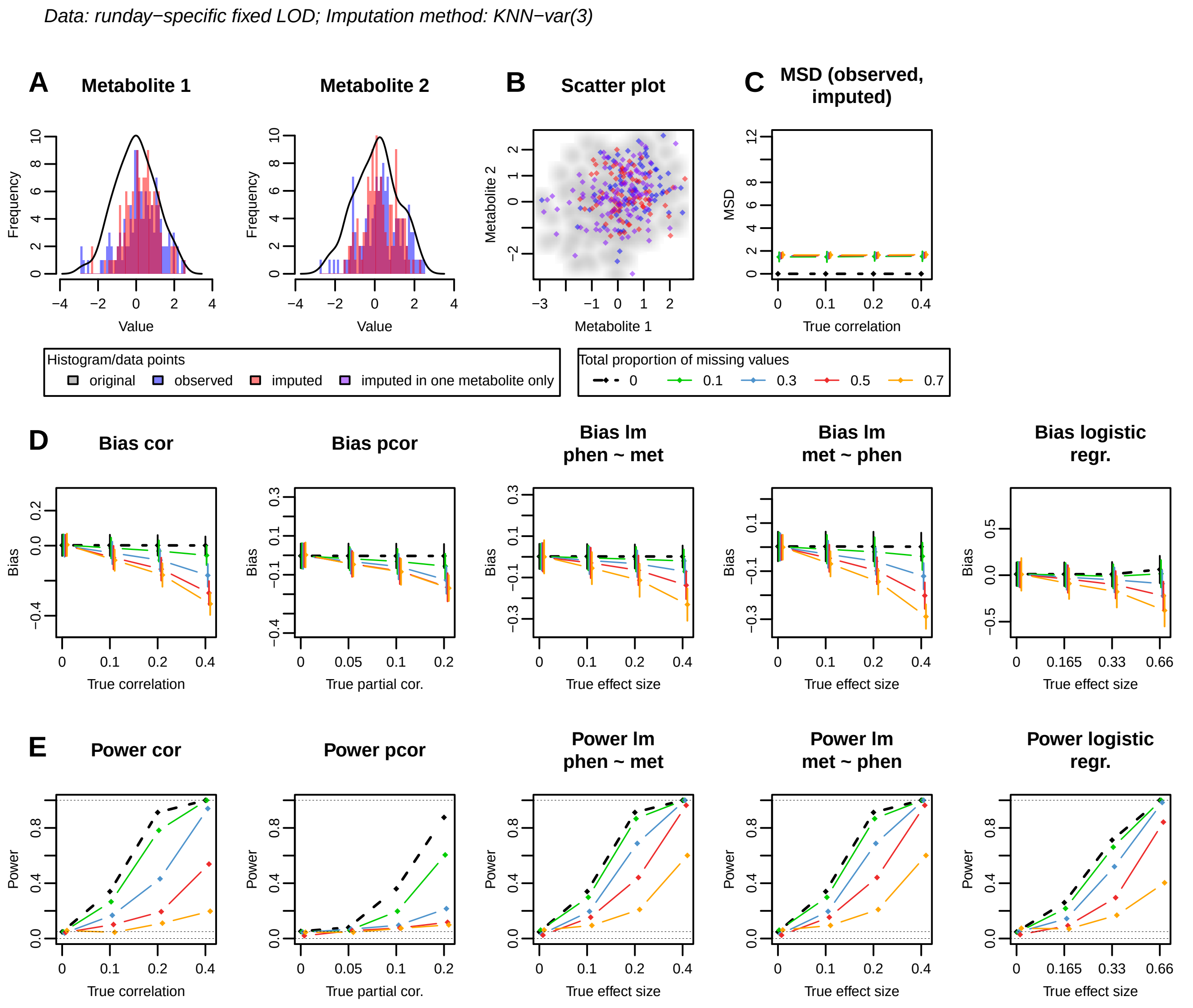
Data: runday−specific fixed LOD; Imputation method: KNN−var(3)
A
Metabolite 1
Metabolite 2
Frequency
Value
B
Scatter plot
Metabolite 1
Metabolite 2
C
MSD (observed, imputed)
MSD
True correlation
Histogram/data points
original
observed
imputed
imputed in one metabolite only
Total proportion of missing values
0
0.1
0.3
0.5
0.7
D
Bias cor
Bias pcor
Bias lm phen ~ met
Bias lm met ~ phen
Bias logistic regr.
Bias
True correlation
True partial cor.
True effect size
E
Power cor
Power pcor
Power lm phen ~ met
Power lm met ~ phen
Power logistic regr.
Power
True correlation
True partial cor.
True effect size

Data: runday−specific fixed LOD; Imputation method: KNN−var(5)

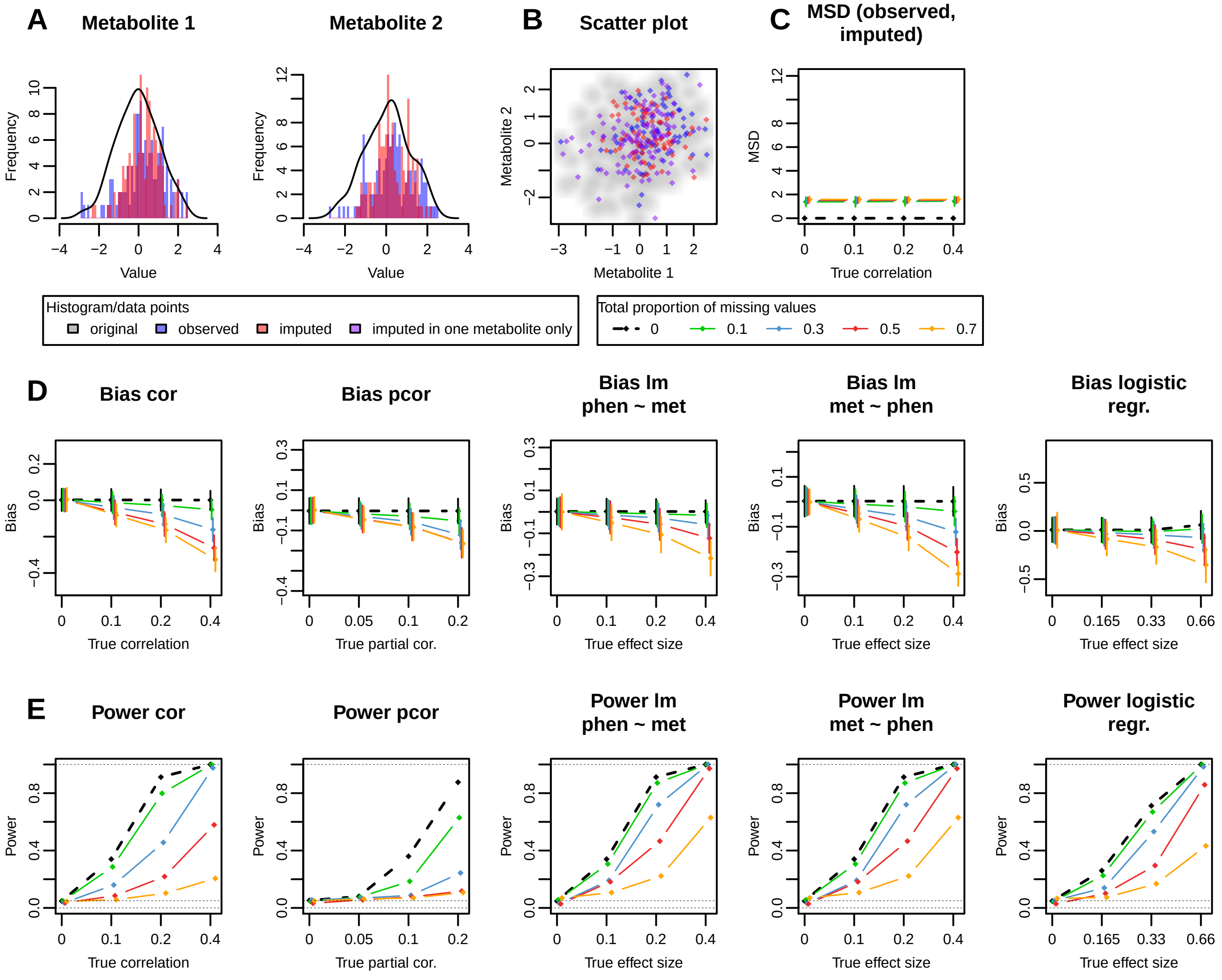

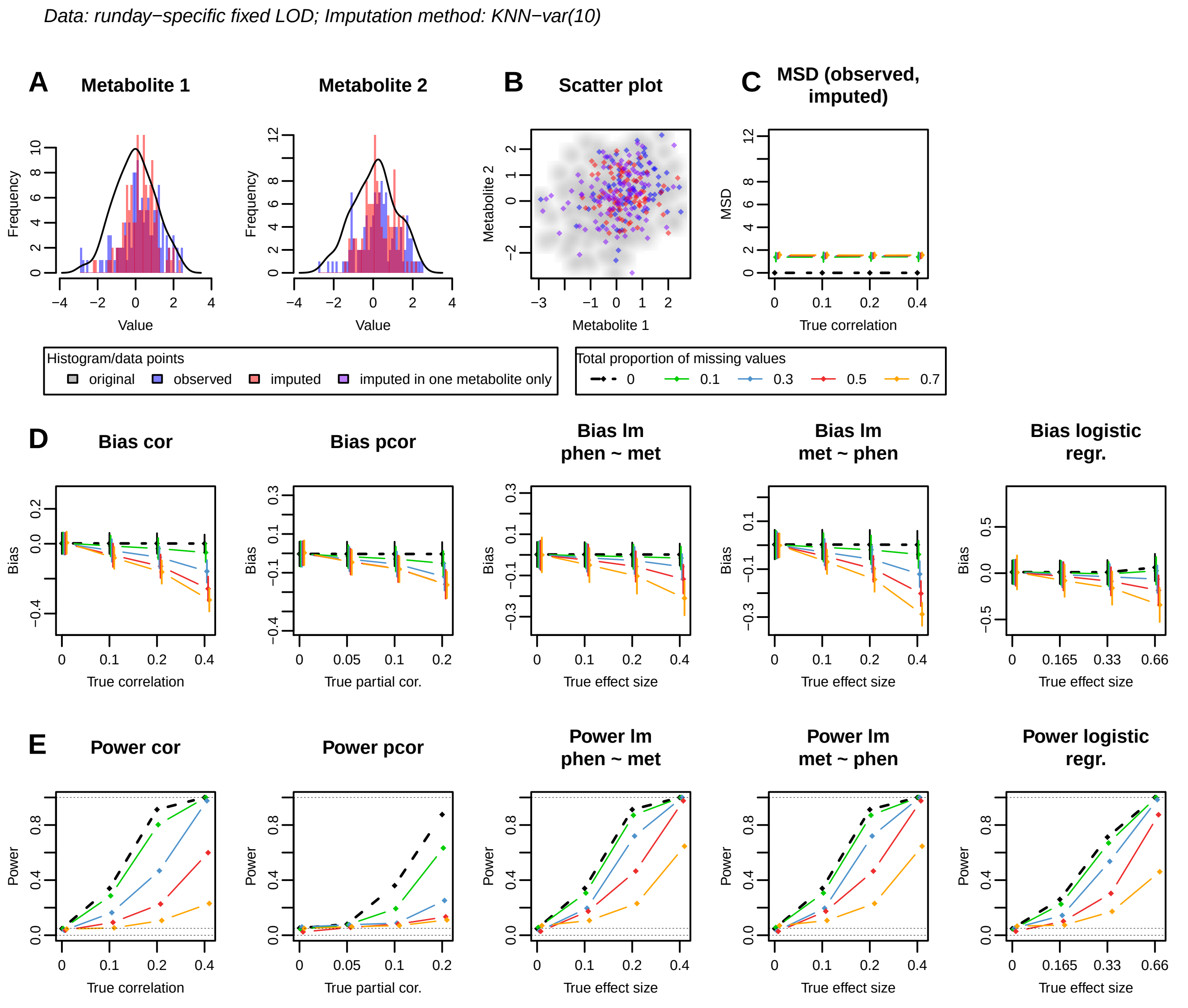
Data: runday−specific fixed LOD; Imputation method: KNN−var(10)
A
Metabolite 1
Metabolite 2
B
Scatter plot
C
MSD (observed, imputed)
Frequency
Value
Metabolite 1
Metabolite 2
MSD
True correlation
Histogram/data points
original
observed
imputed
imputed in one metabolite only
Total proportion of missing values
0
0.1
0.3
0.5
0.7
D
Bias cor
Bias pcor
Bias lm phen ~ met
Bias lm met ~ phen
Bias logistic regr.
Bias
True correlation
True partial cor.
True effect size
E
Power cor
Power pcor
Power lm phen ~ met
Power lm met ~ phen
Power logistic regr.
Power

Data: runday−specific fixed LOD; Imputation method: KNN−var(20)

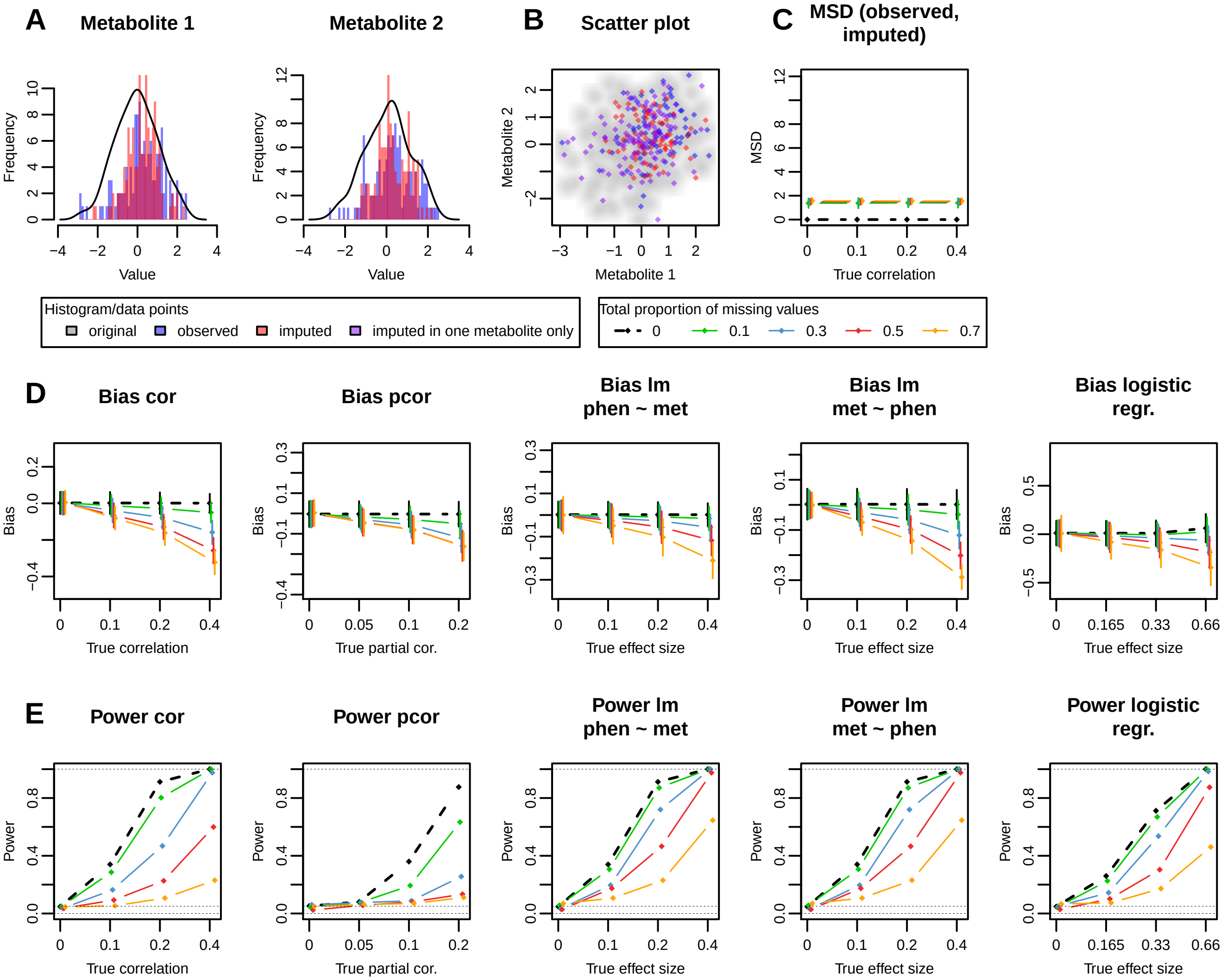

Data: runday−specific fixed LOD; Imputation method: KNN−obs(1)

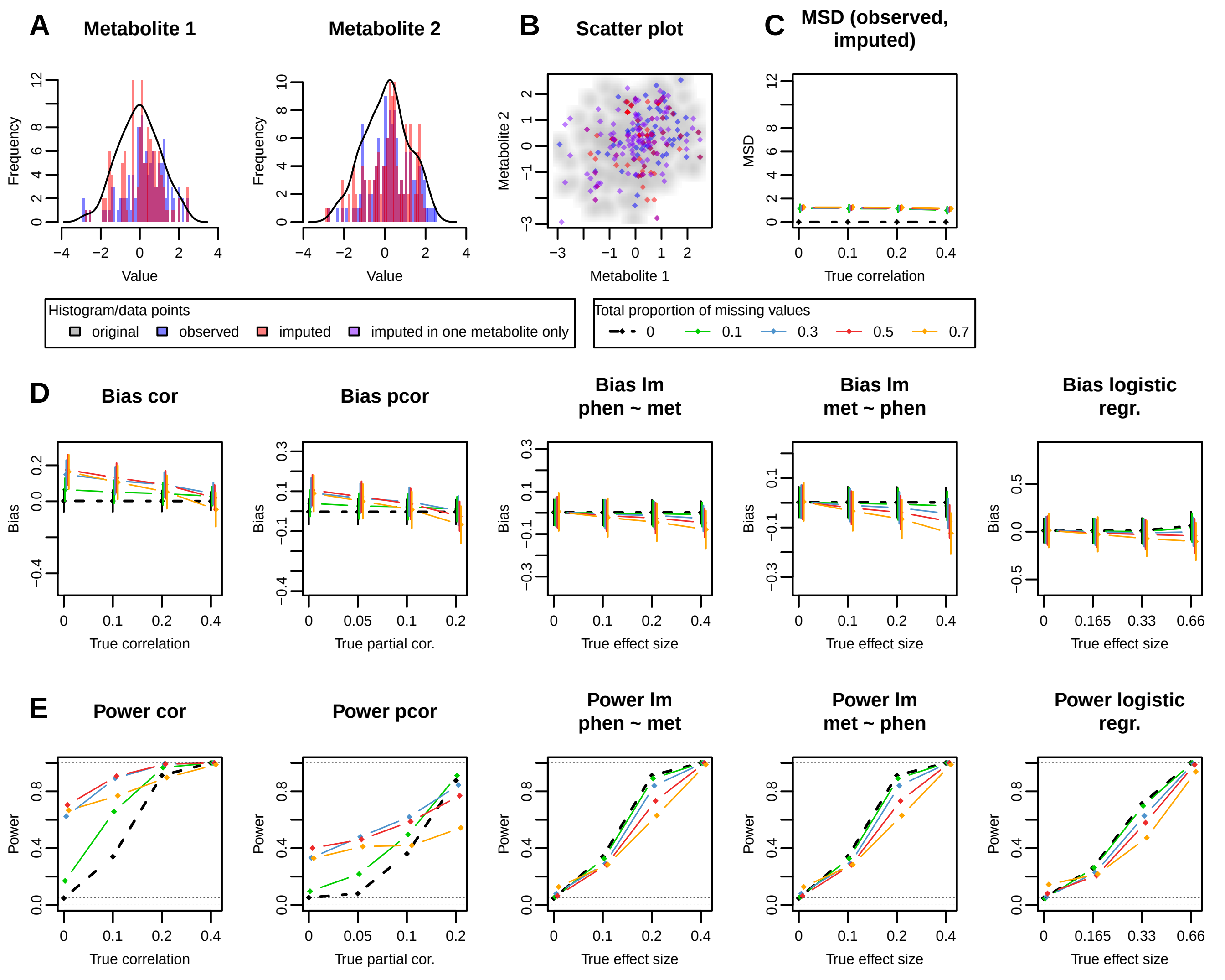

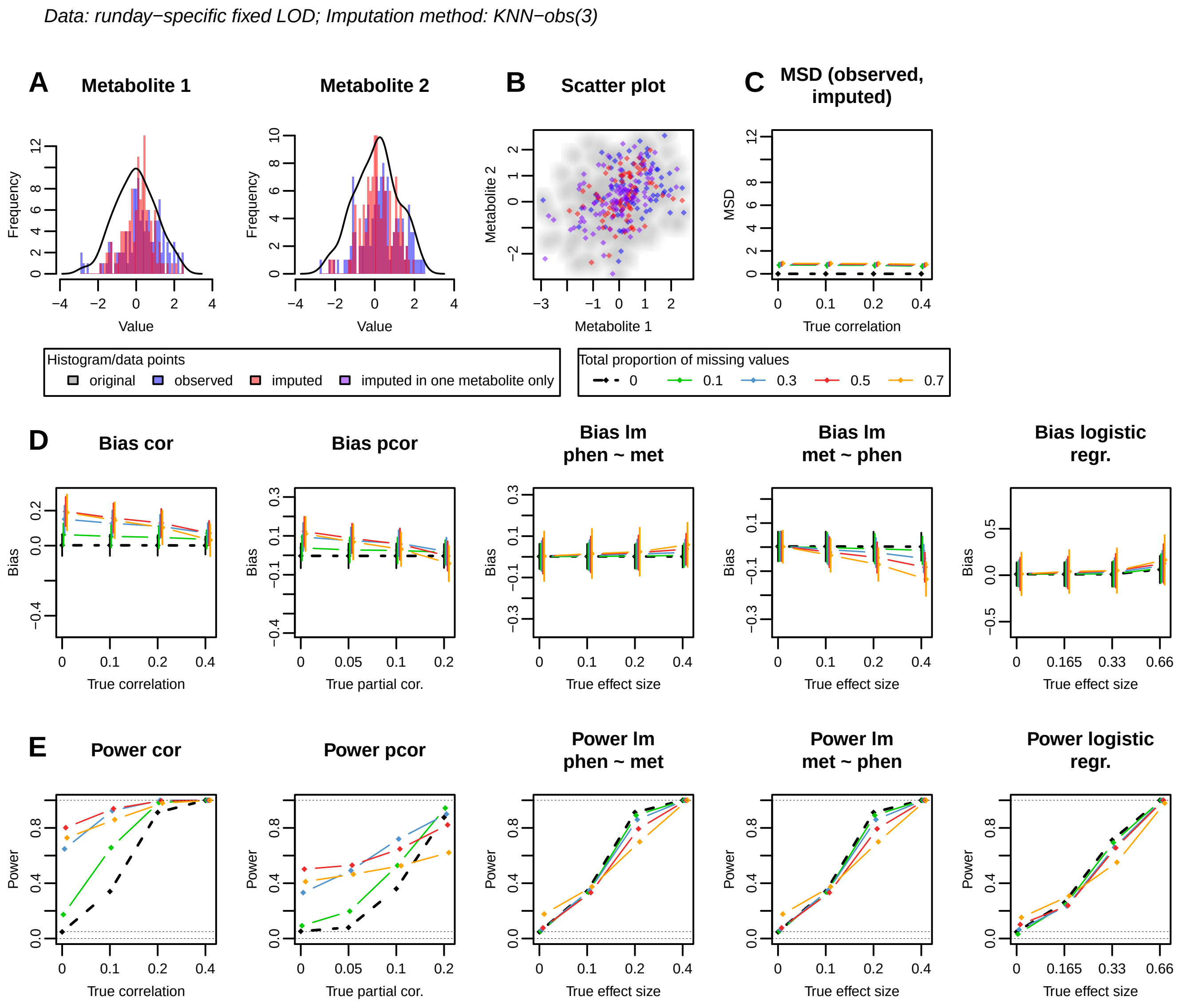
Data: runday−specific fixed LOD; Imputation method: KNN−obs(3)
A
Metabolite 1
Metabolite 2
B
Scatter plot
C
MSD (observed, imputed)
Frequency
Value
Metabolite 2
Metabolite 1
MSD
True correlation
Histogram/data points
original
observed
imputed
imputed in one metabolite only
Total proportion of missing values
0
0.1
0.3
0.5
0.7
D
Bias cor
Bias pcor
Bias lm phen ~ met
Bias lm met ~ phen
Bias logistic regr.
Bias
True correlation
True partial cor.
True effect size
E
Power cor
Power pcor
Power lm phen ~ met
Power lm met ~ phen
Power logistic regr.
Power

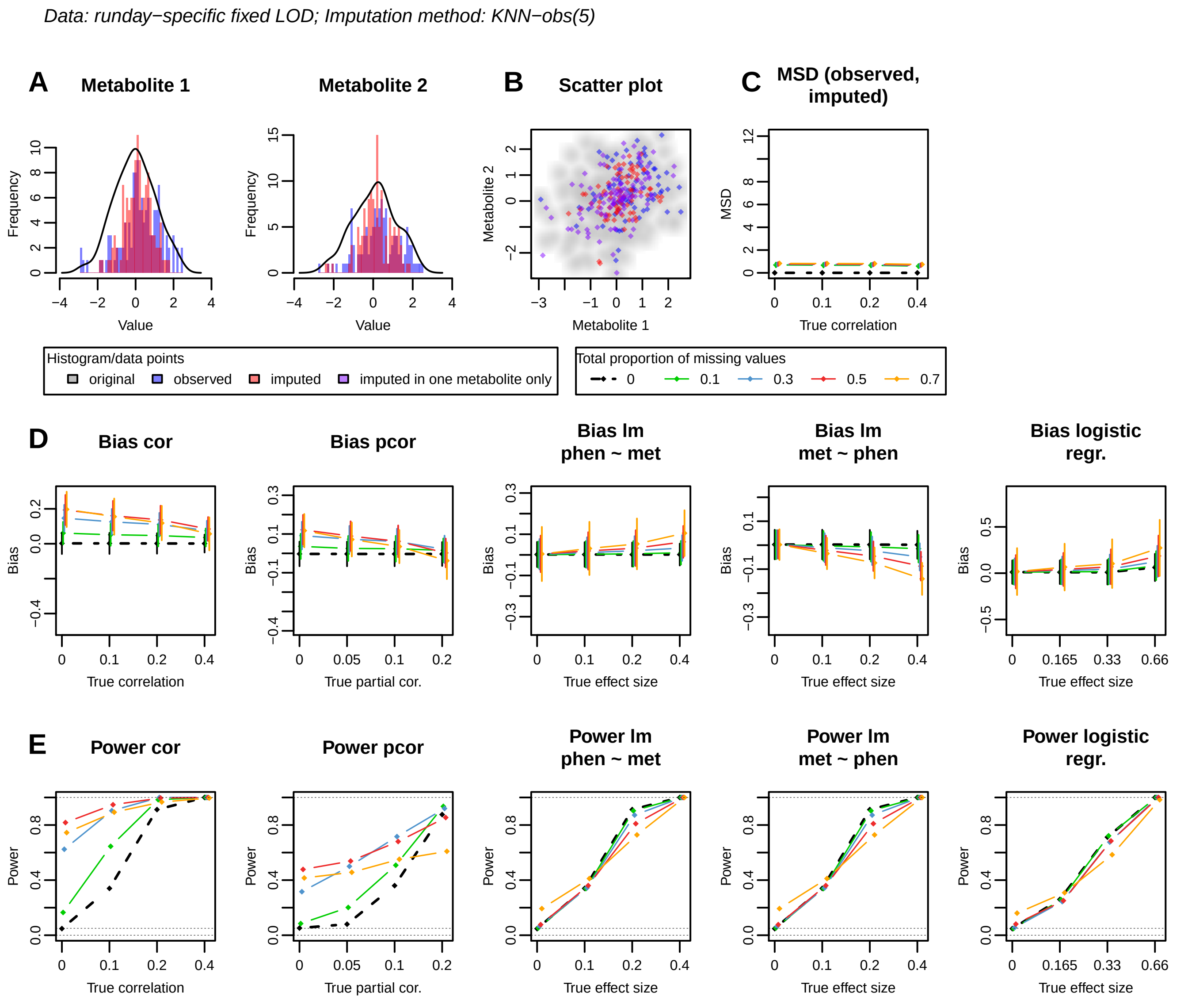
Data: runday−specific fixed LOD; Imputation method: KNN−obs(5)
A
Metabolite 1
Metabolite 2
B
Scatter plot
C
MSD (observed, imputed)
Frequency
Value
Metabolite 2
Metabolite 1
MSD
True correlation
Histogram/data points
original
observed
imputed
imputed in one metabolite only
Total proportion of missing values
0
0.1
0.3
0.5
0.7
D
Bias cor
Bias pcor
Bias lm phen ~ met
Bias lm met ~ phen
Bias logistic regr.
Bias
True correlation
True partial cor.
True effect size
E
Power cor
Power pcor
Power lm phen ~ met
Power lm met ~ phen
Power logistic regr.
Power
True correlation
True partial cor.
True effect size

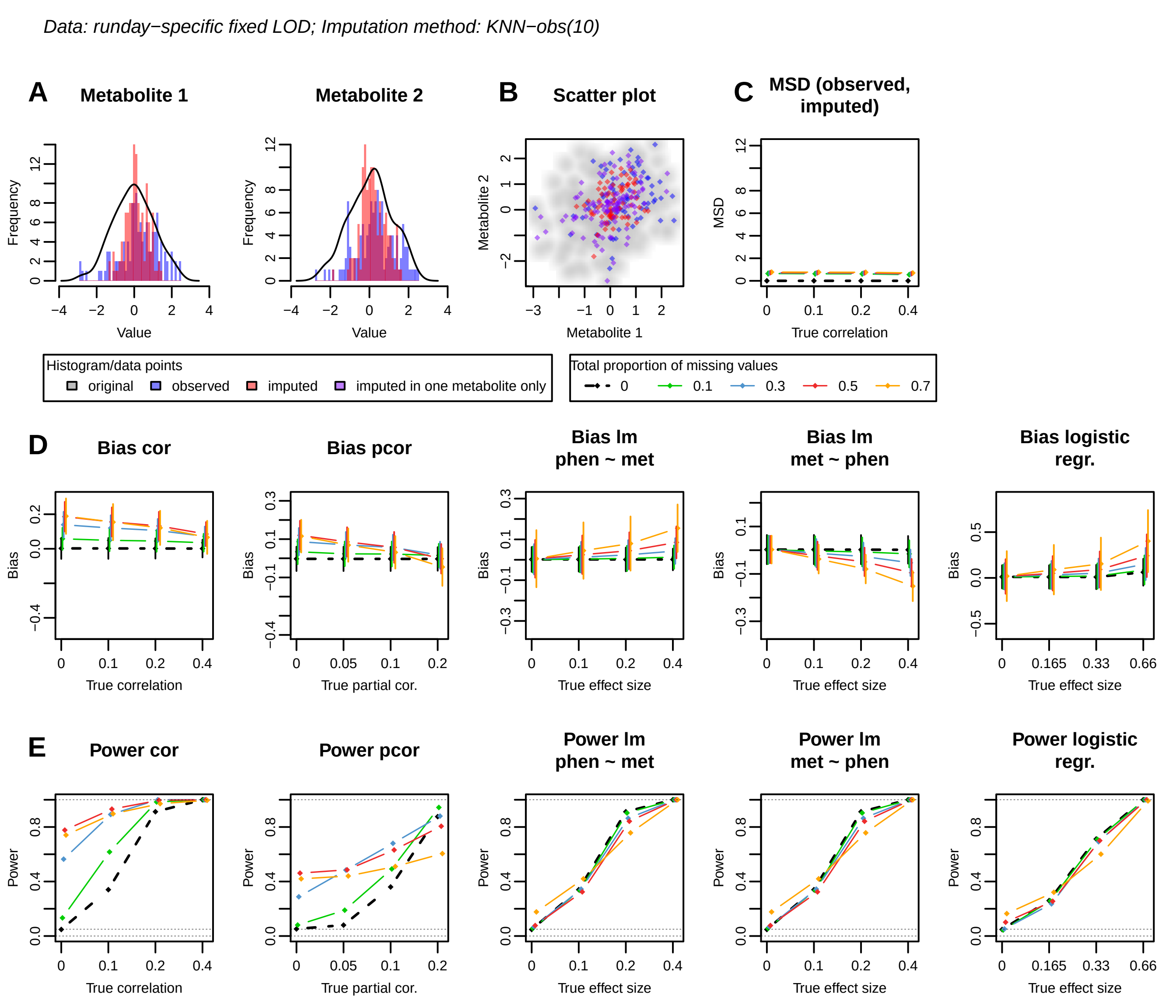

Data: runday−specific fixed LOD; Imputation method: KNN−obs(10)
A
Metabolite 1
Metabolite 2
B
Scatter plot
C
MSD (observed, imputed)
Frequency
Value
Metabolite 2
Metabolite 1
MSD
True correlation
Histogram/data points
original
observed
imputed
imputed in one metabolite only
Total proportion of missing values
0
0.1
0.3
0.5
0.7
D
Bias cor
Bias pcor
Bias lm phen ~ met
Bias lm met ~ phen
Bias logistic regr.
Bias
True correlation
True partial cor.
True effect size
E
Power cor
Power pcor
Power lm phen ~ met
Power lm met ~ phen
Power logistic regr.
Power

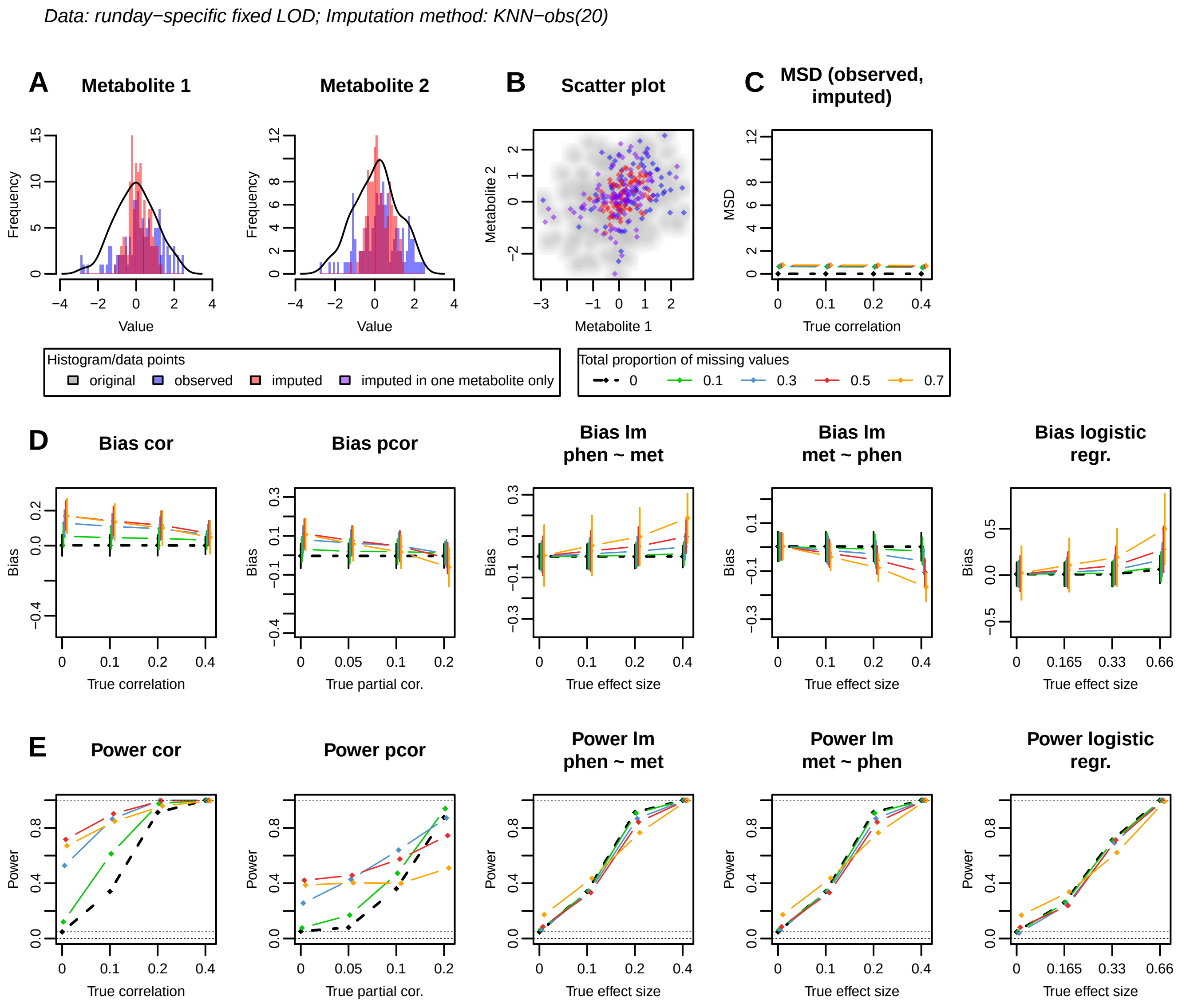

Data: runday−specific fixed LOD; Imputation method: KNN−obs(20)
A
Metabolite 1
Metabolite 2
B
Scatter plot
C
MSD (observed, imputed)
Frequency
Value
Metabolite 2
Metabolite 1
MSD
True correlation
Histogram/data points
original
observed
imputed
imputed in one metabolite only
Total proportion of missing values
0
0.1
0.3
0.5
0.7
D
Bias cor
Bias pcor
Bias lm phen ~ met
Bias lm met ~ phen
Bias logistic regr.
Bias
True correlation
True partial cor.
True effect size
E
Power cor
Power pcor
Power lm phen ~ met
Power lm met ~ phen
Power logistic regr.
Power

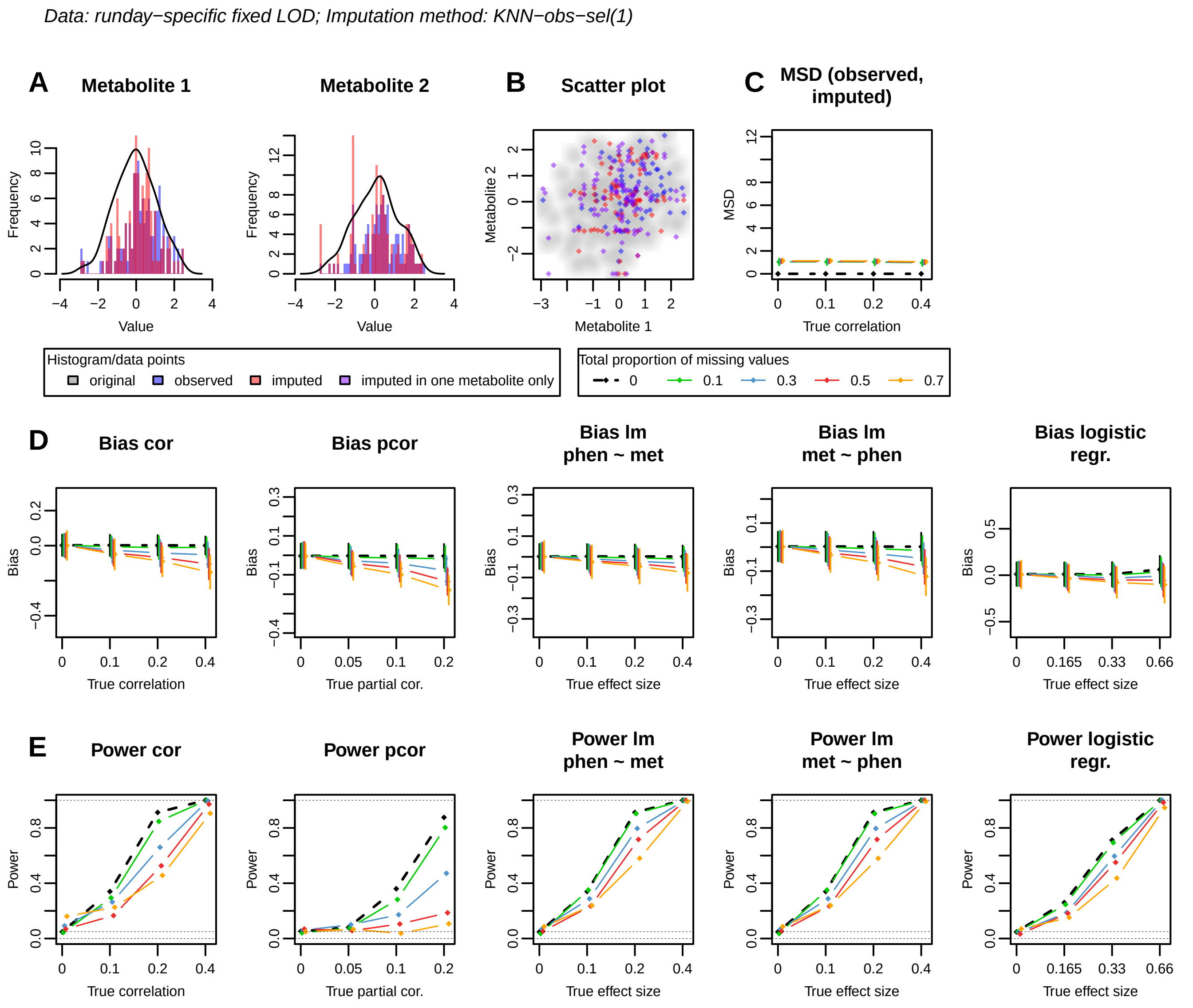

Data: runday−specific fixed LOD; Imputation method: KNN−obs−sel(1)
A
Metabolite 1
Metabolite 2
B
Scatter plot
C
MSD (observed, imputed)
Frequency
Value
Metabolite 2
Metabolite 1
MSD
True correlation
Histogram/data points
original
observed
imputed
imputed in one metabolite only
Total proportion of missing values
0
0.1
0.3
0.5
0.7
D
Bias cor
Bias pcor
Bias lm phen ~ met
Bias lm met ~ phen
Bias logistic regr.
Bias
True correlation
True partial cor.
True effect size
E
Power cor
Power pcor
Power lm phen ~ met
Power lm met ~ phen
Power logistic regr.
Power

Data: runday−specific fixed LOD; Imputation method: KNN−obs−sel(3)

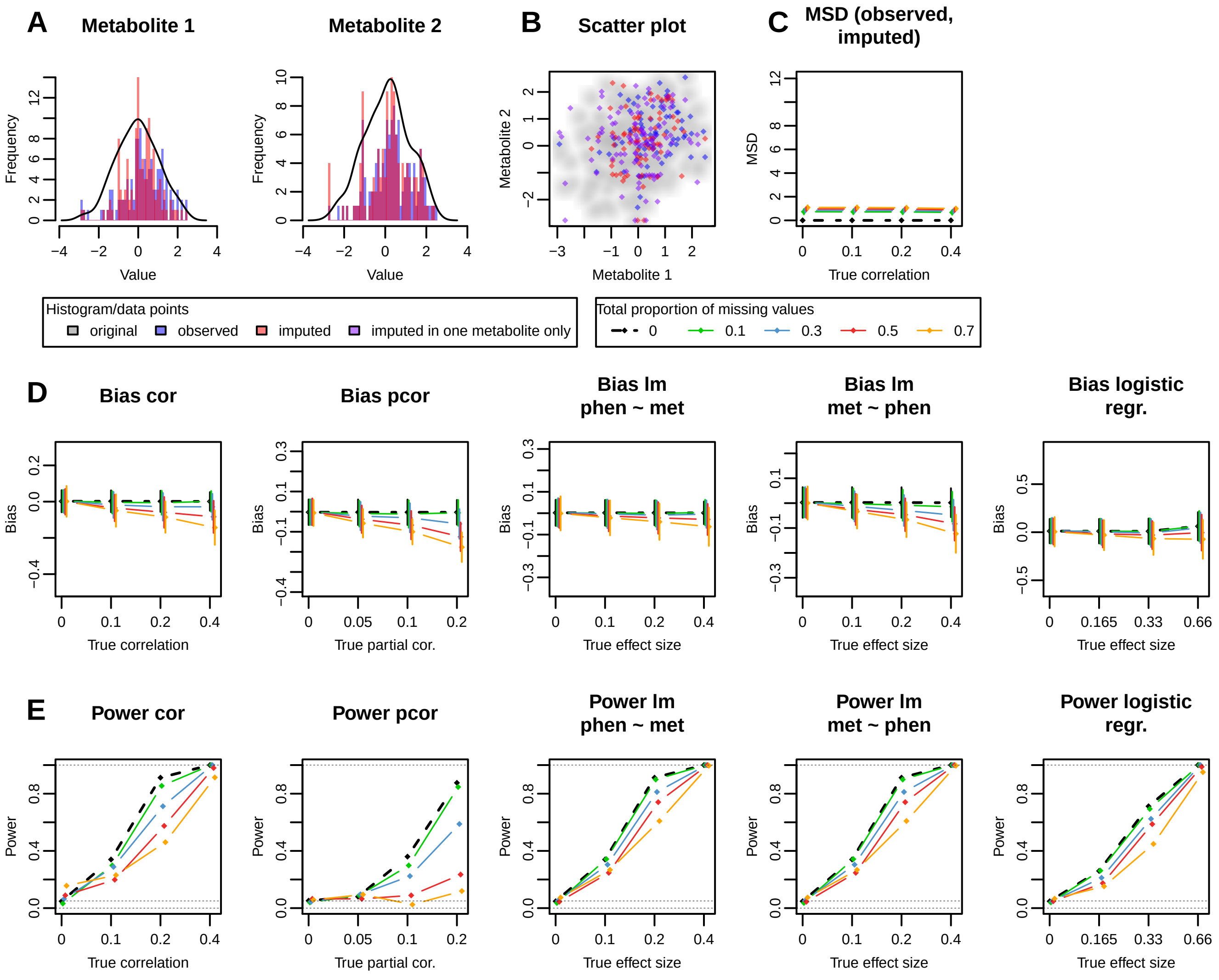

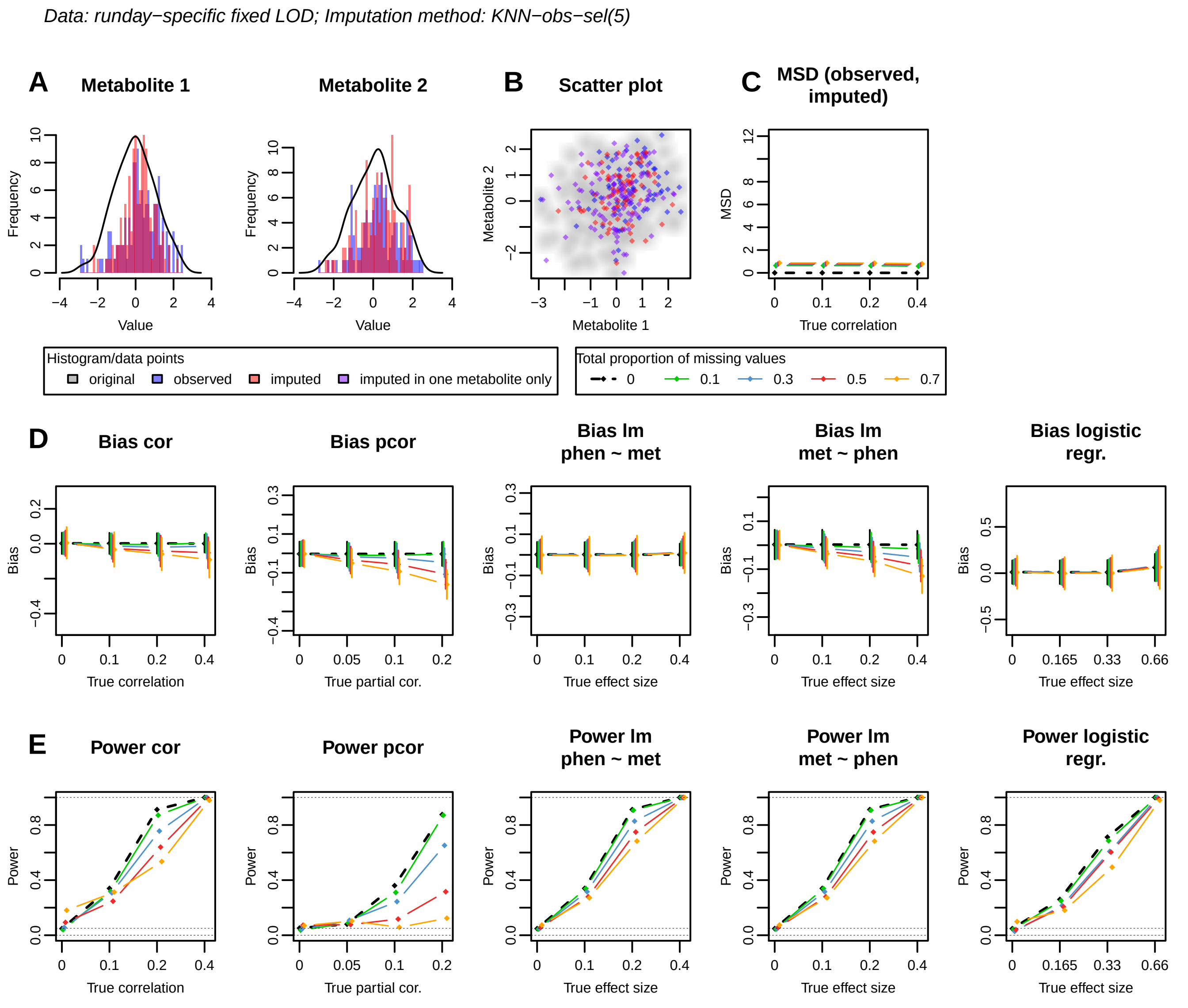
Data: runday−specific fixed LOD; Imputation method: KNN−obs−sel(5)
A
Metabolite 1
Metabolite 2
Frequency
Value
B
Scatter plot
Metabolite 1
Metabolite 2
C
MSD (observed, imputed)
MSD
True correlation
Histogram/data points
original
observed
imputed
imputed in one metabolite only
Total proportion of missing values
0
0.1
0.3
0.5
0.7
D
Bias cor
Bias pcor
Bias lm phen ~ met
Bias lm met ~ phen
Bias logistic regr.
Bias
True correlation
True partial cor.
True effect size
E
Power cor
Power pcor
Power lm phen ~ met
Power lm met ~ phen
Power logistic regr.
Power
True correlation
True partial cor.
True effect size

Data: runday−specific fixed LOD; Imputation method: KNN−obs−sel(10)

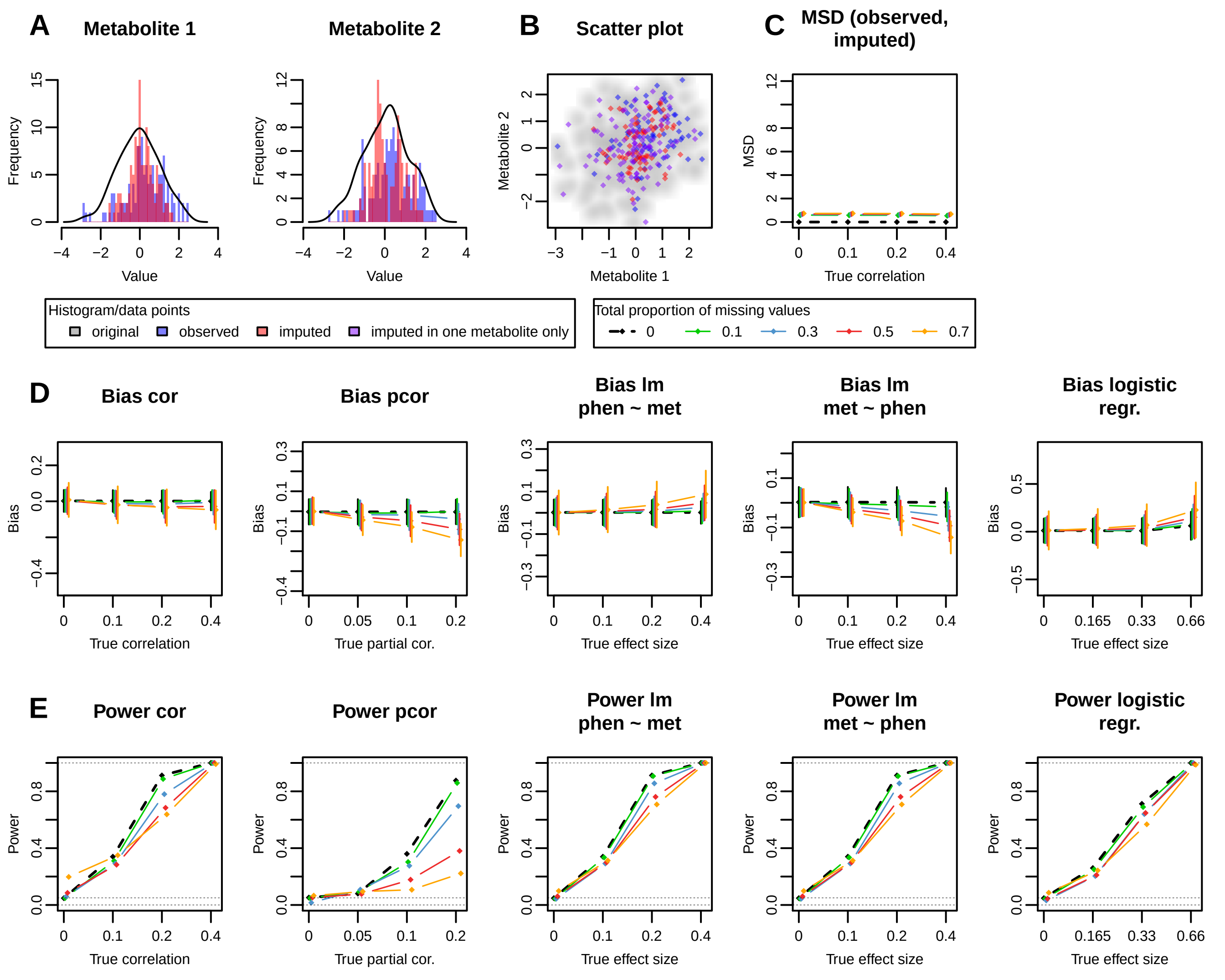

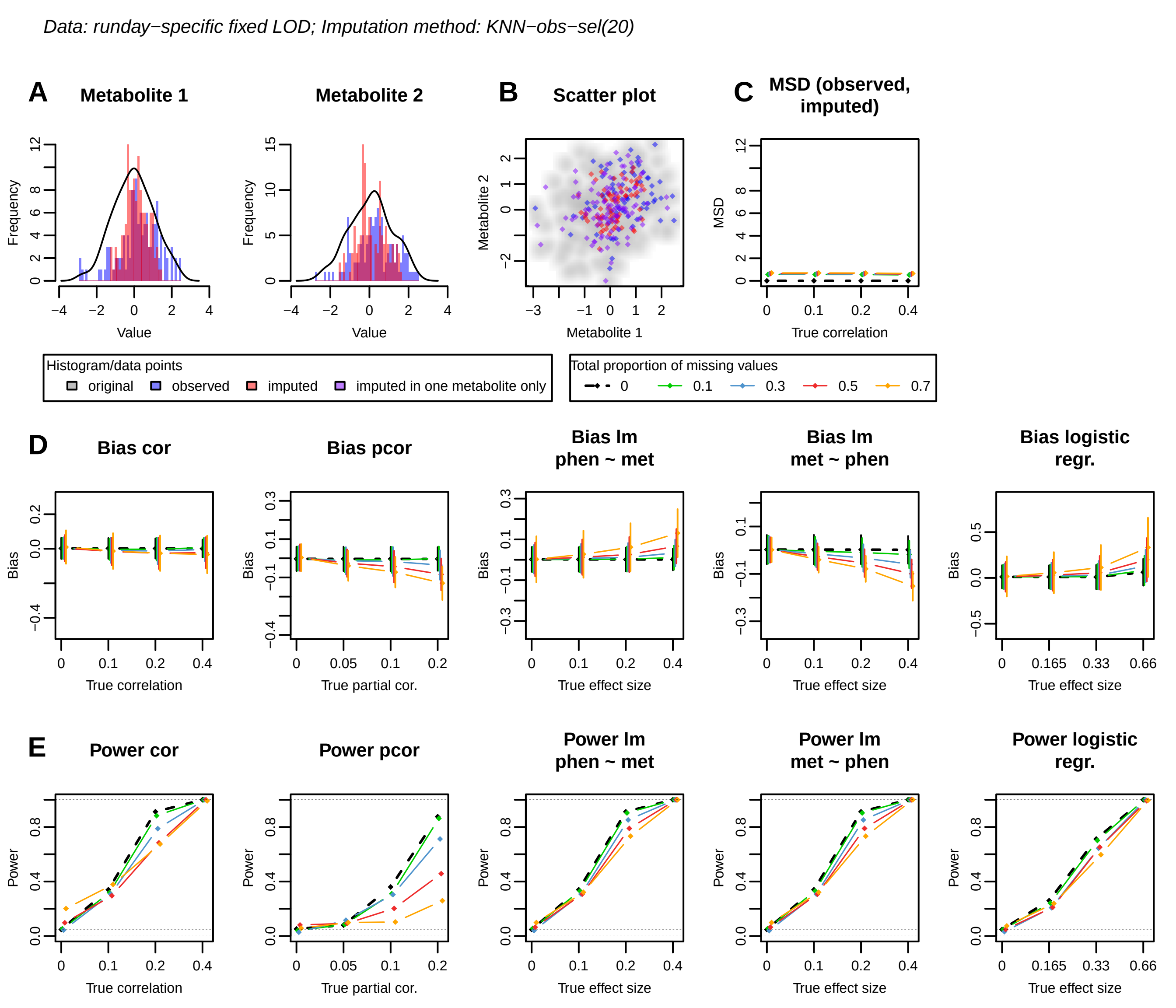
Data: runday−specific fixed LOD; Imputation method: KNN−obs−sel(20)
A
Metabolite 1
Metabolite 2
B
Scatter plot
C
MSD (observed, imputed)
Frequency
Value
Metabolite 2
Metabolite 1
MSD
True correlation
Histogram/data points
original
observed
imputed
imputed in one metabolite only
Total proportion of missing values
0
0.1
0.3
0.5
0.7
D
Bias cor
Bias pcor
Bias lm phen ~ met
Bias lm met ~ phen
Bias logistic regr.
Bias
True correlation
True partial cor.
True effect size
E
Power cor
Power pcor
Power lm phen ~ met
Power lm met ~ phen
Power logistic regr.
Power
True correlation
True partial cor.
True effect size

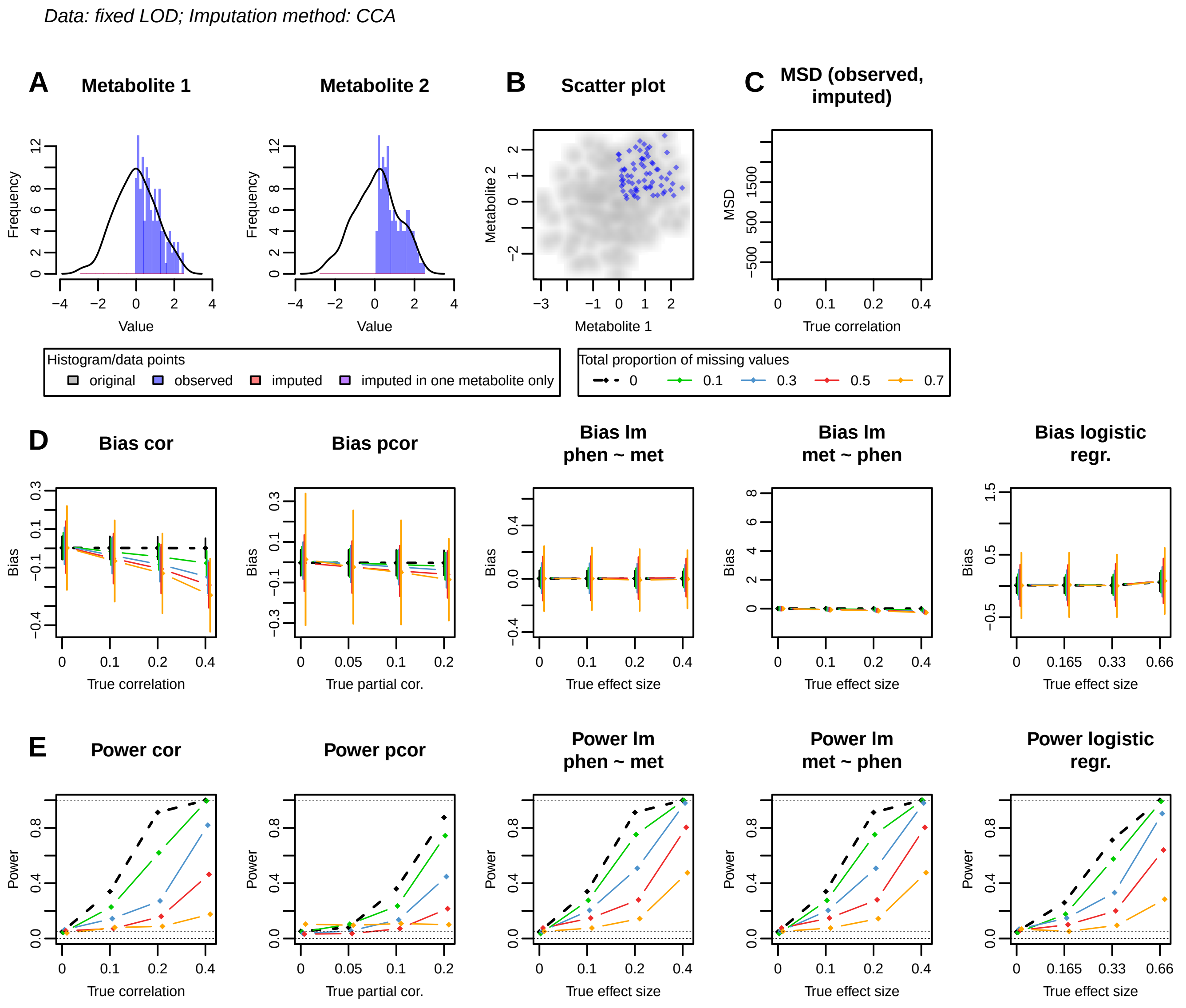

Data: fixed LOD; Imputation method: CCA
A
Metabolite 1
Metabolite 2
Frequency
Value
B
Scatter plot
Metabolite 1
Metabolite 2
C
MSD (observed, imputed)
MSD
True correlation
Histogram/data points
original
observed
imputed
imputed in one metabolite only
Total proportion of missing values
0
0.1
0.3
0.5
0.7
D
Bias cor
Bias pcor
Bias lm phen ~ met
Bias lm met ~ phen
Bias logistic regr.
Bias
True correlation
True partial cor.
True effect size
E
Power cor
Power pcor
Power lm phen ~ met
Power lm met ~ phen
Power logistic regr.
Power
True correlation
True partial cor.
True effect size

Data: fixed LOD; Imputation method: min

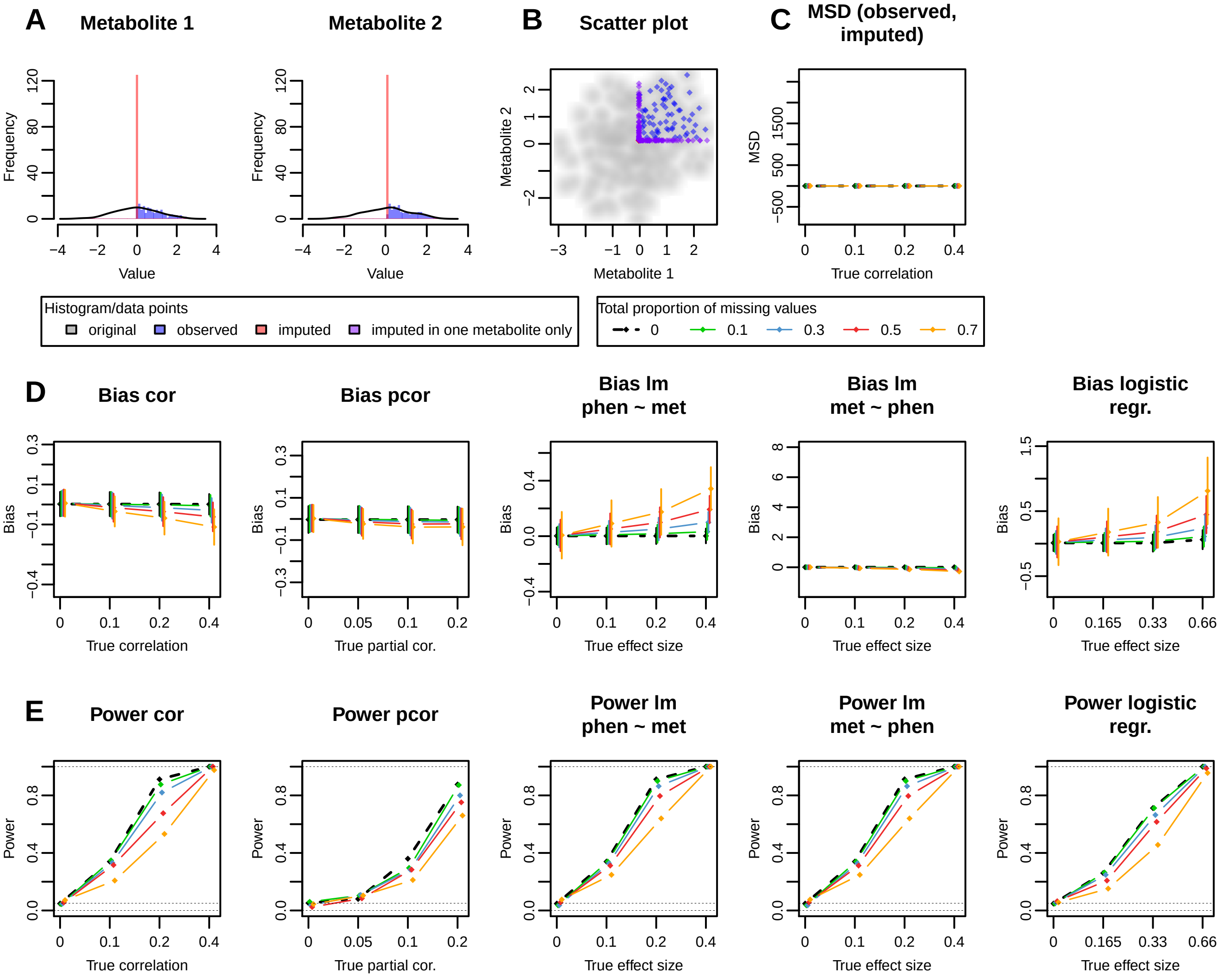

Data: fixed LOD; Imputation method: RC

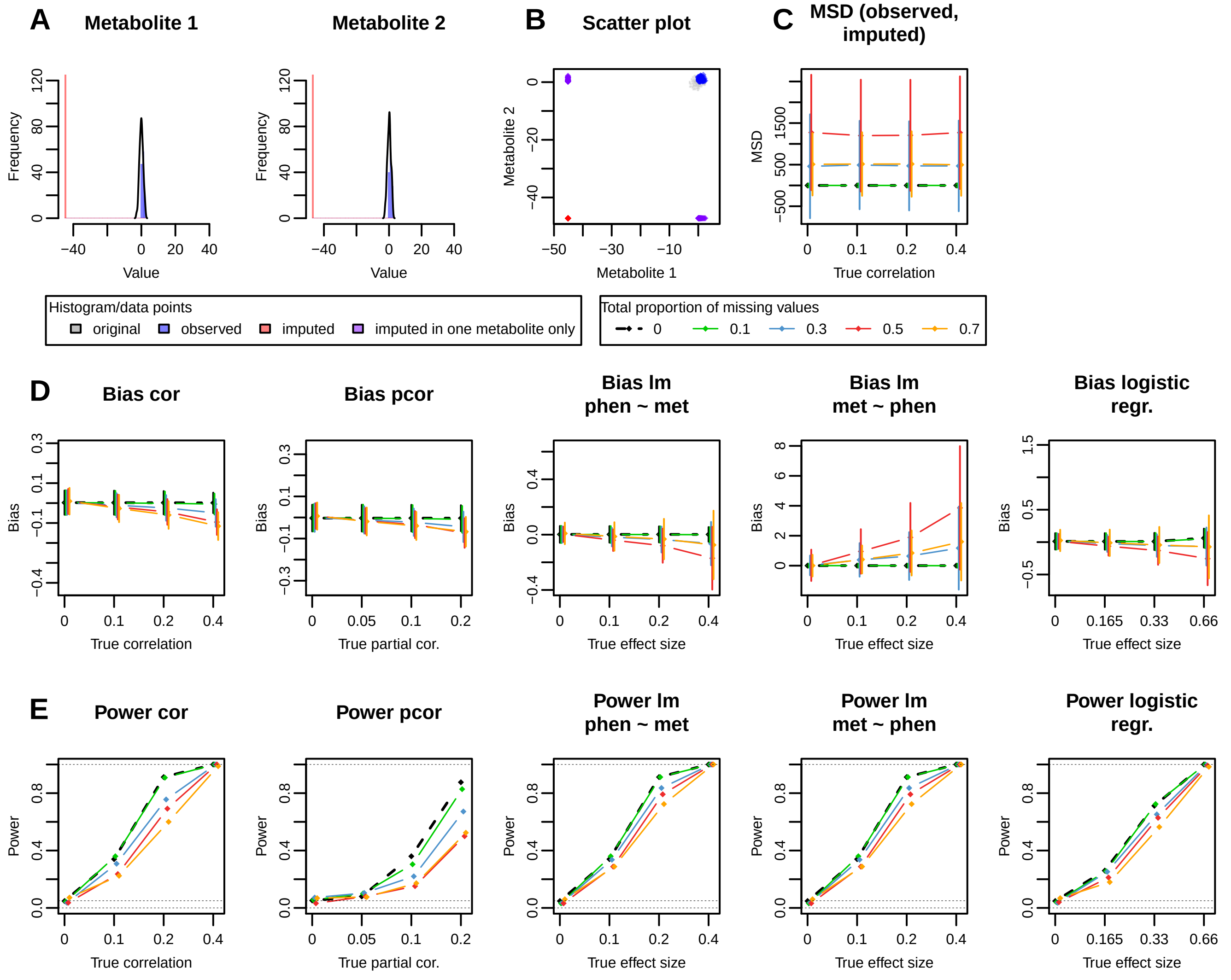

Data: fixed LOD; Imputation method: ITS

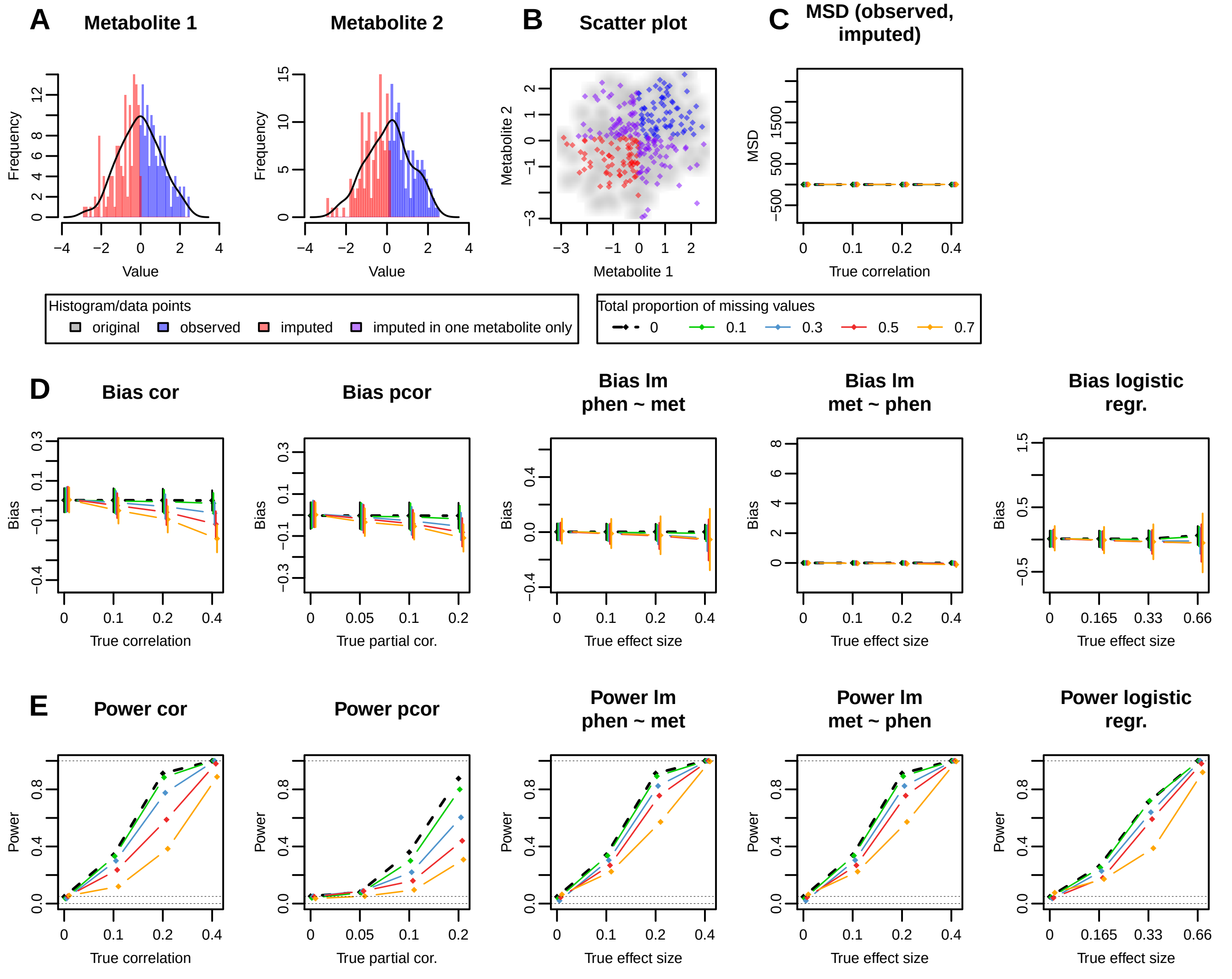

Data: fixed LOD; Imputation method: MITS

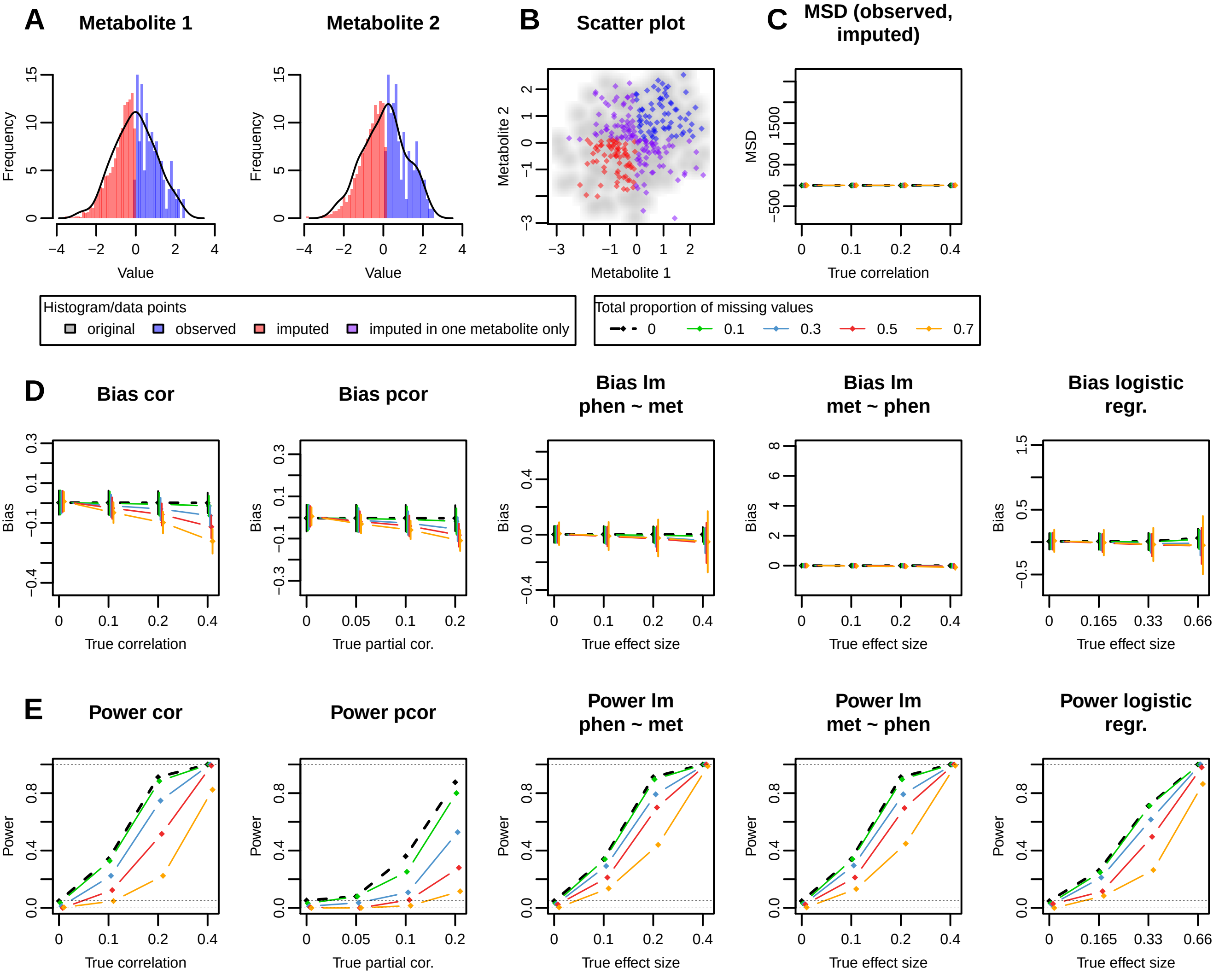

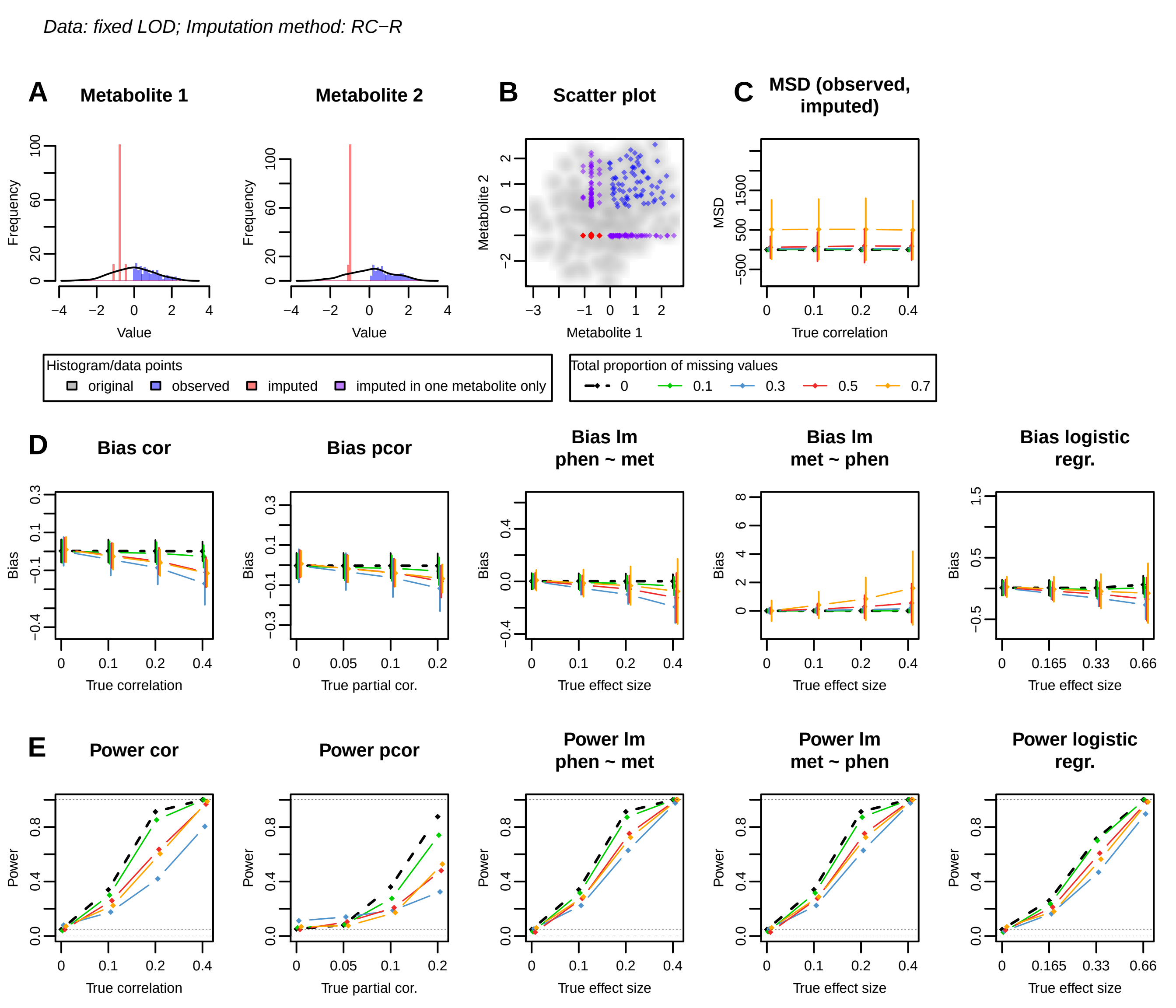

Data: fixed LOD; Imputation method: RC−R
A
Metabolite 1
Metabolite 2
Frequency
Value
B
Scatter plot
Metabolite 1
Metabolite 2
C
MSD (observed, imputed)
MSD
True correlation
Histogram/data points
original
observed
imputed
imputed in one metabolite only
Total proportion of missing values
0
0.1
0.3
0.5
0.7
D
Bias cor
Bias pcor
Bias lm phen ~ met
Bias lm met ~ phen
Bias logistic regr.
Bias
True correlation
True partial cor.
True effect size
E
Power cor
Power pcor
Power lm phen ~ met
Power lm met ~ phen
Power logistic regr.
Power
True correlation
True partial cor.
True effect size

Data: fixed LOD; Imputation method: ITS−R

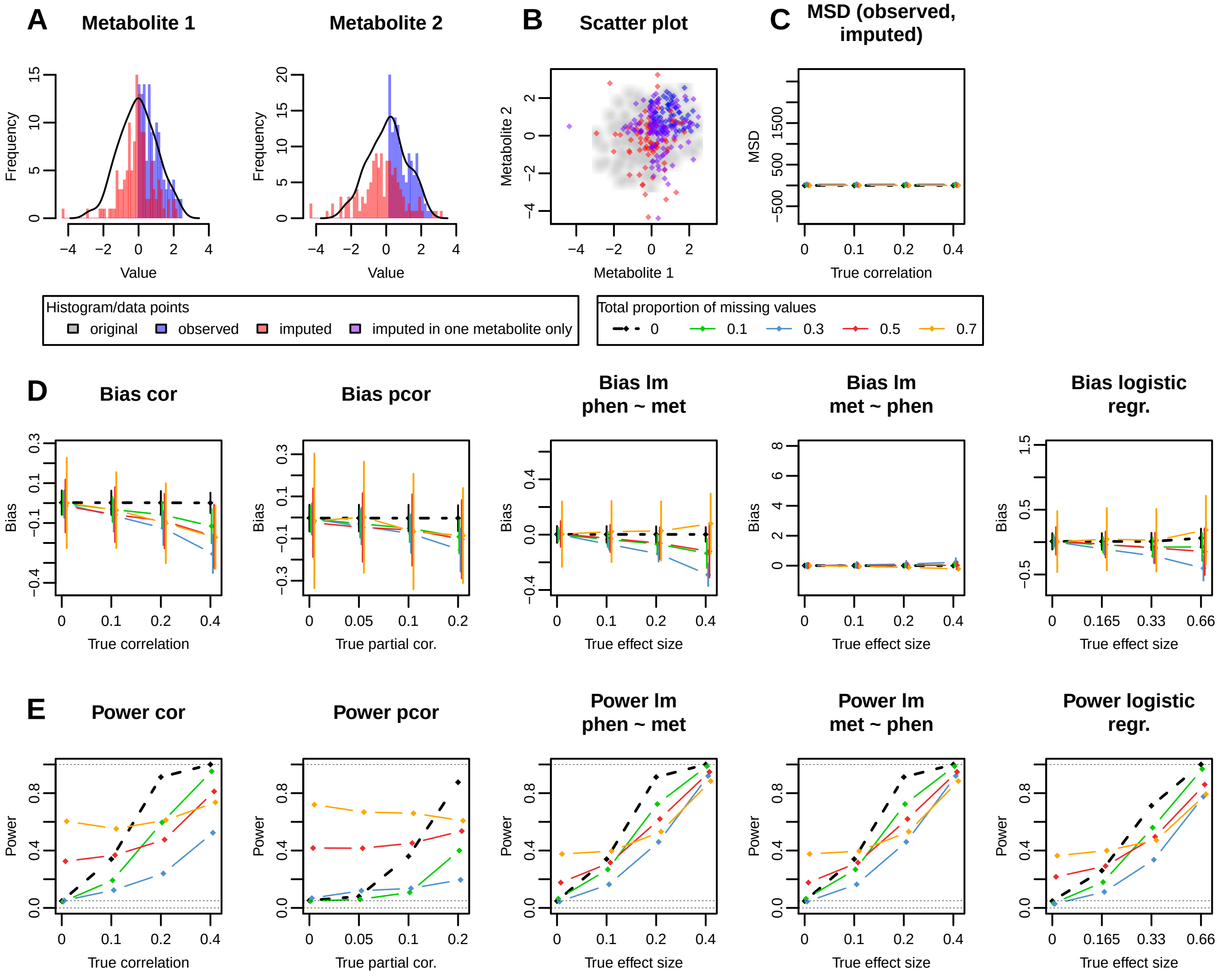

Data: fixed LOD; Imputation method: MITS−R

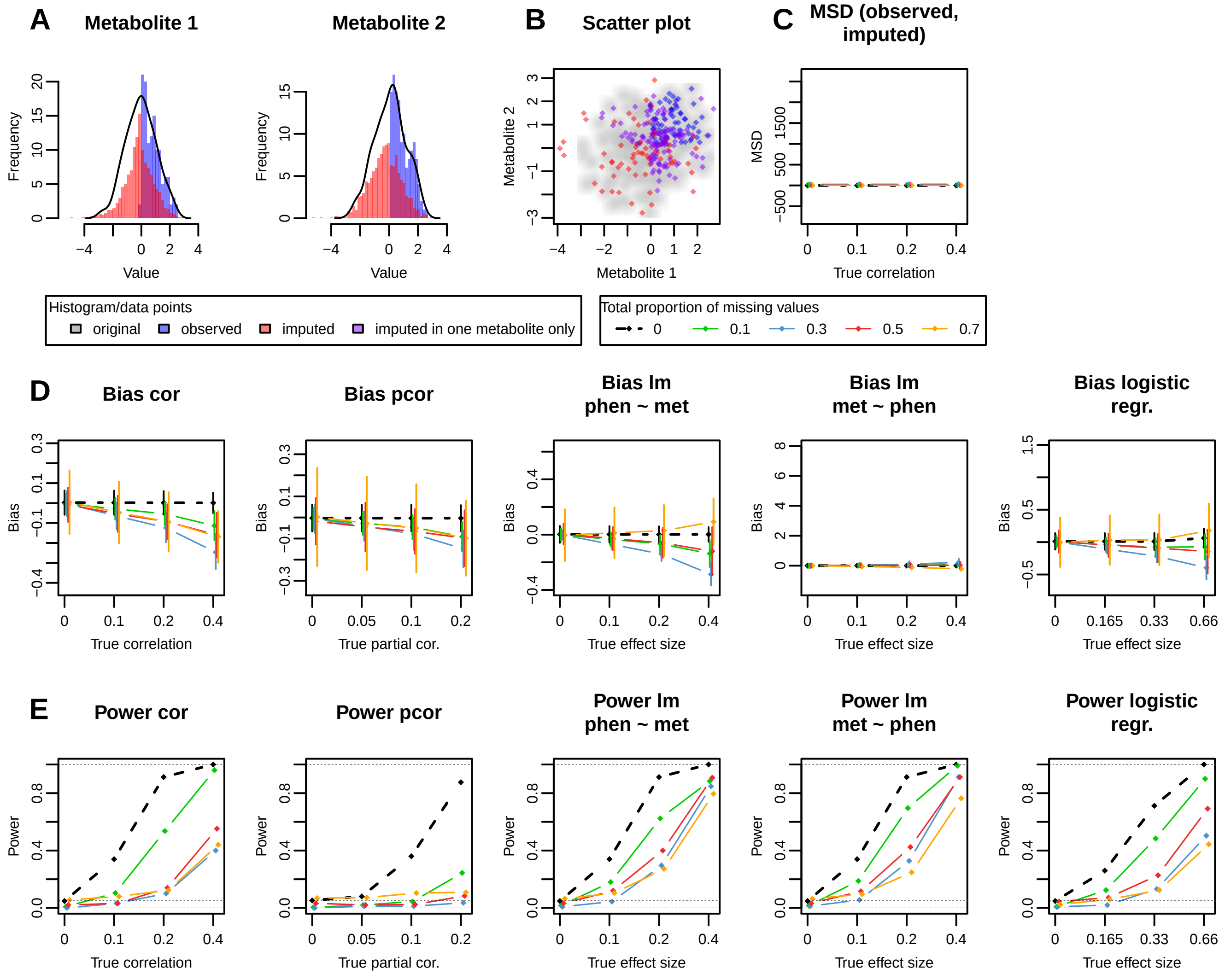

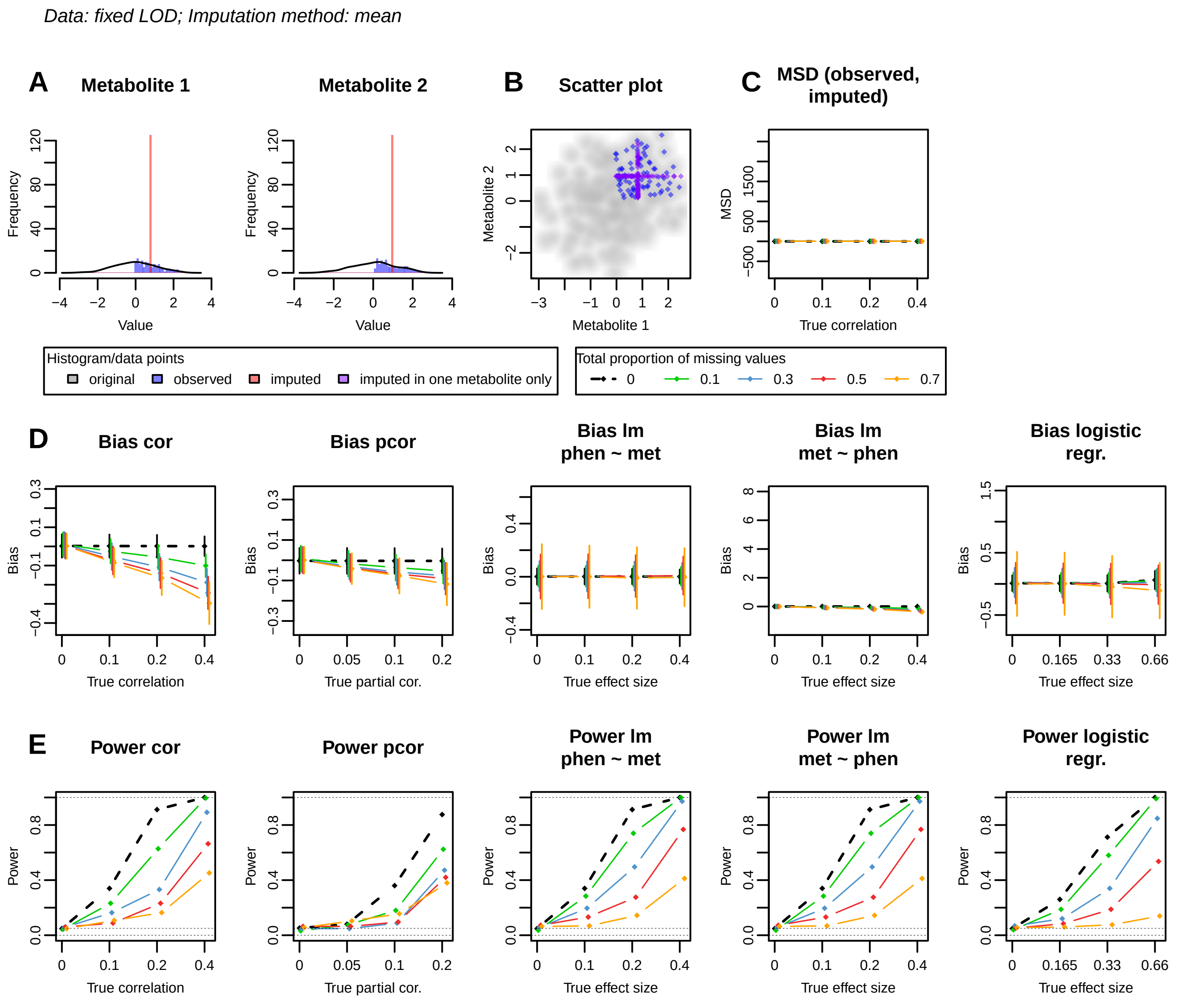

Data: fixed LOD; Imputation method: mean
A
Metabolite 1
Metabolite 2
Frequency
Value
B
Scatter plot
Metabolite 2
Metabolite 1
C
MSD (observed, imputed)
MSD
True correlation
Histogram/data points
original
observed
imputed
imputed in one metabolite only
Total proportion of missing values
0
0.1
0.3
0.5
0.7
D
Bias cor
Bias pcor
Bias lm phen ~ met
Bias lm met ~ phen
Bias logistic regr.
Bias
True correlation
True partial cor.
True effect size
E
Power cor
Power pcor
Power lm phen ~ met
Power lm met ~ phen
Power logistic regr.
Power
True correlation
True partial cor.
True effect size

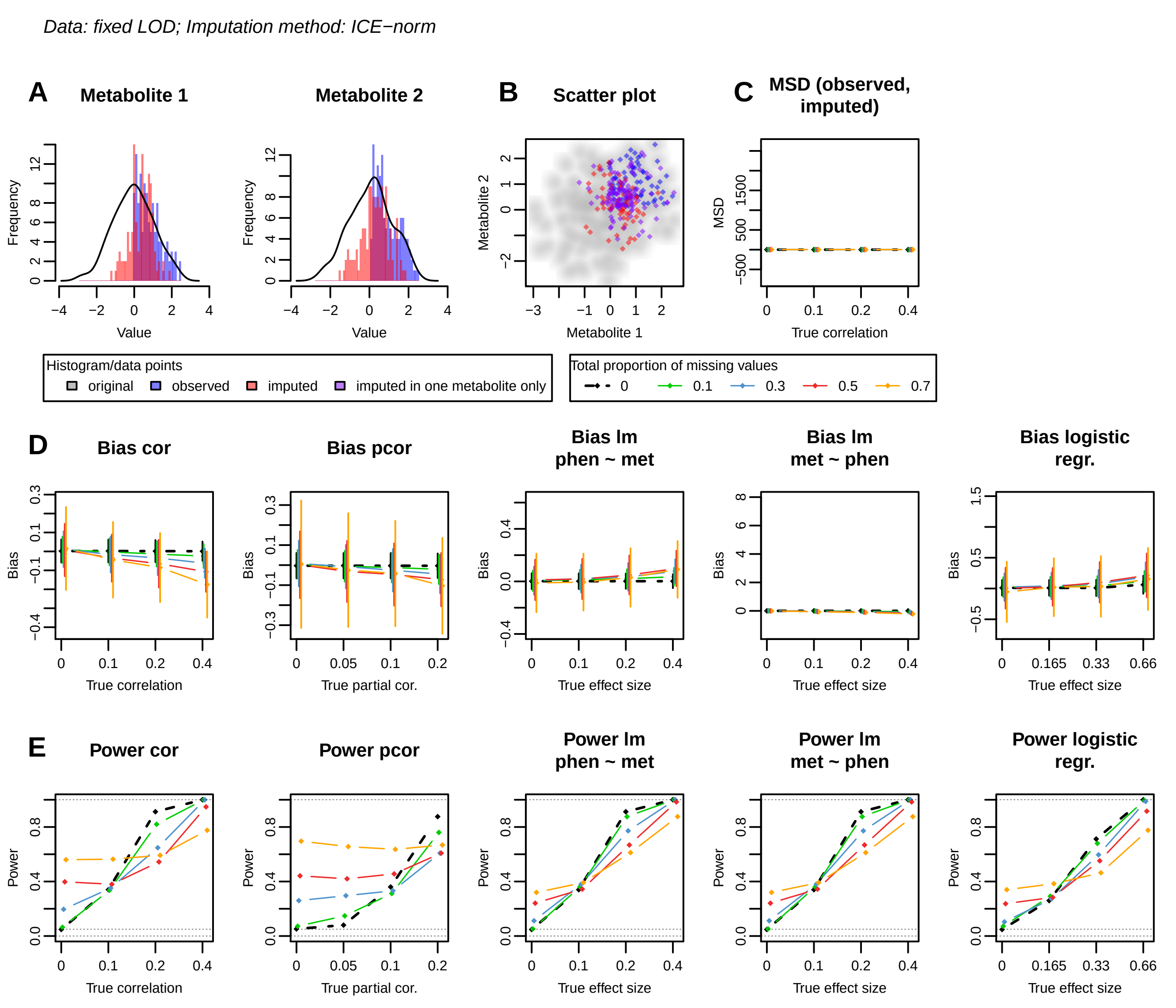
Data: fixed LOD; Imputation method: ICE−norm
A
Metabolite 1
Metabolite 2
B
Scatter plot
C
MSD (observed, imputed)
Frequency
Value
Metabolite 2
Metabolite 1
MSD
True correlation
Histogram/data points
original
observed
imputed
imputed in one metabolite only
Total proportion of missing values
0
0.1
0.3
0.5
0.7
D
Bias cor
Bias pcor
Bias lm phen ~ met
Bias lm met ~ phen
Bias logistic regr.
Bias
True correlation
True partial cor.
True effect size
E
Power cor
Power pcor
Power lm phen ~ met
Power lm met ~ phen
Power logistic regr.
Power
True correlation
True partial cor.
True effect size

Data: fixed LOD; Imputation method: ICE−pmm

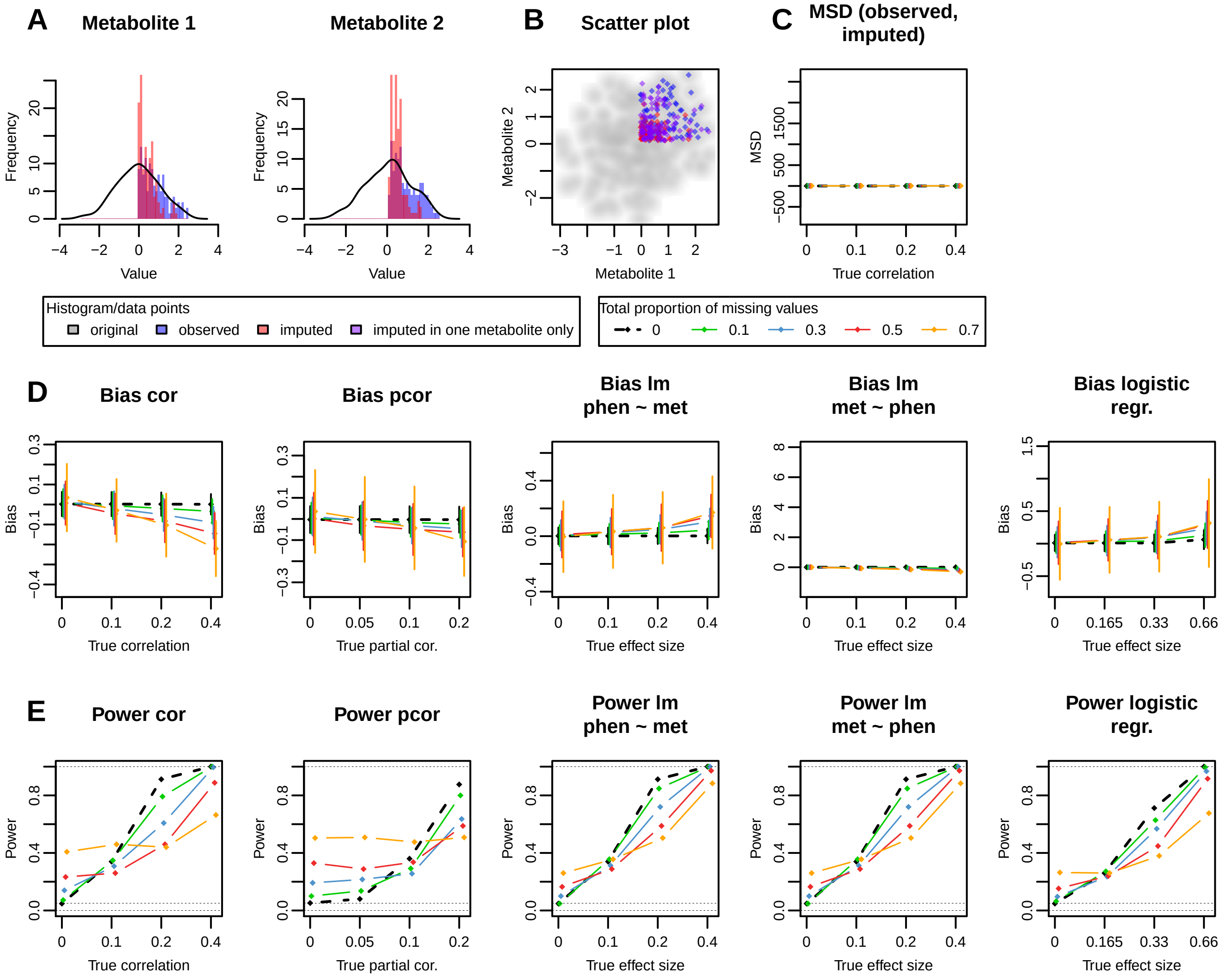

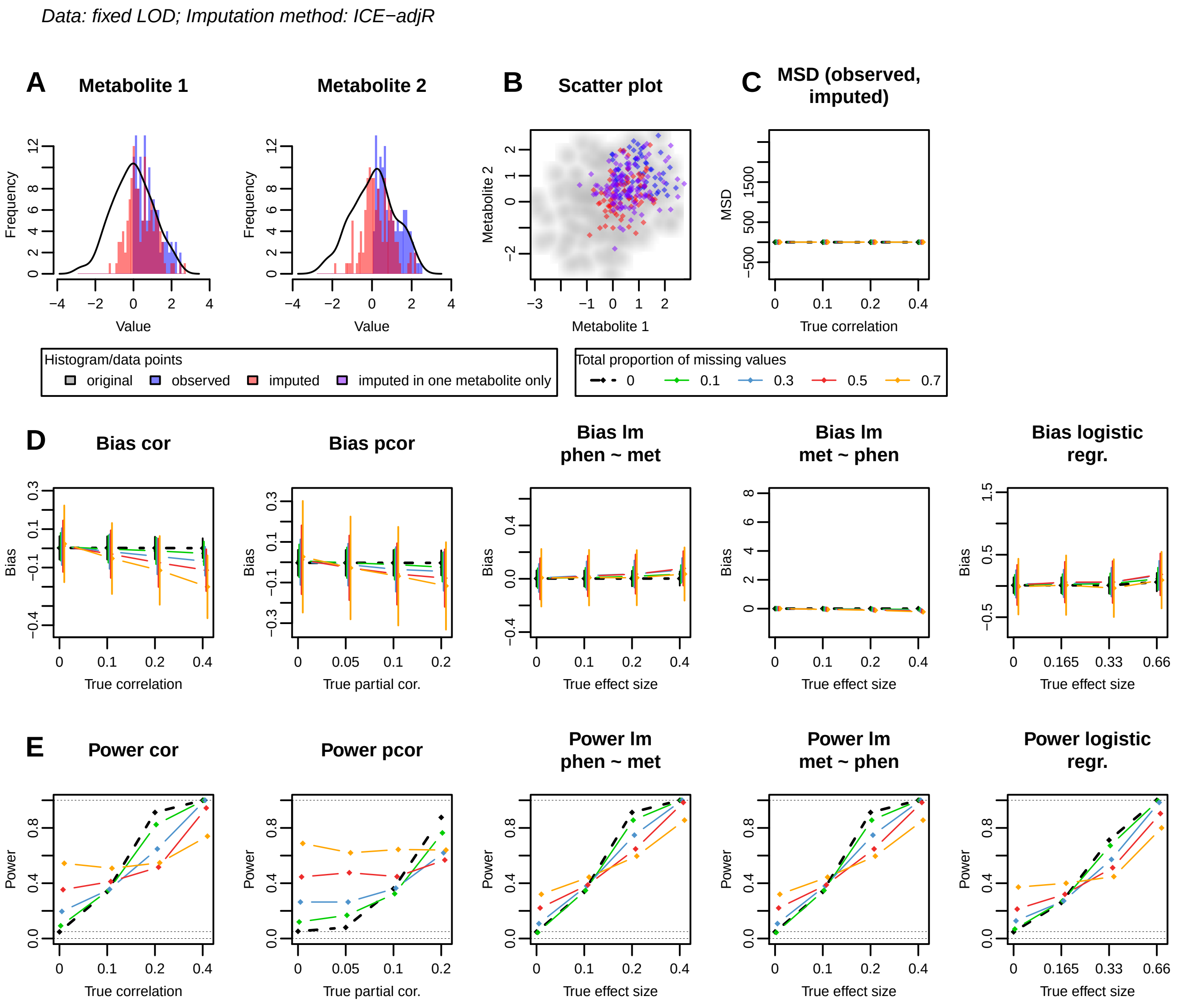

Data: fixed LOD; Imputation method: ICE−adjR
A
Metabolite 1
Metabolite 2
Frequency
Value
B
Scatter plot
Metabolite 1
Metabolite 2
C
MSD (observed, imputed)
MSD
True correlation
Histogram/data points
original
observed
imputed
imputed in one metabolite only
Total proportion of missing values
0
0.1
0.3
0.5
0.7
D
Bias cor
Bias pcor
Bias lm phen ~ met
Bias lm met ~ phen
Bias logistic regr.
Bias
True correlation
True partial cor.
True effect size
E
Power cor
Power pcor
Power lm phen ~ met
Power lm met ~ phen
Power logistic regr.
Power
True correlation
True partial cor.
True effect size

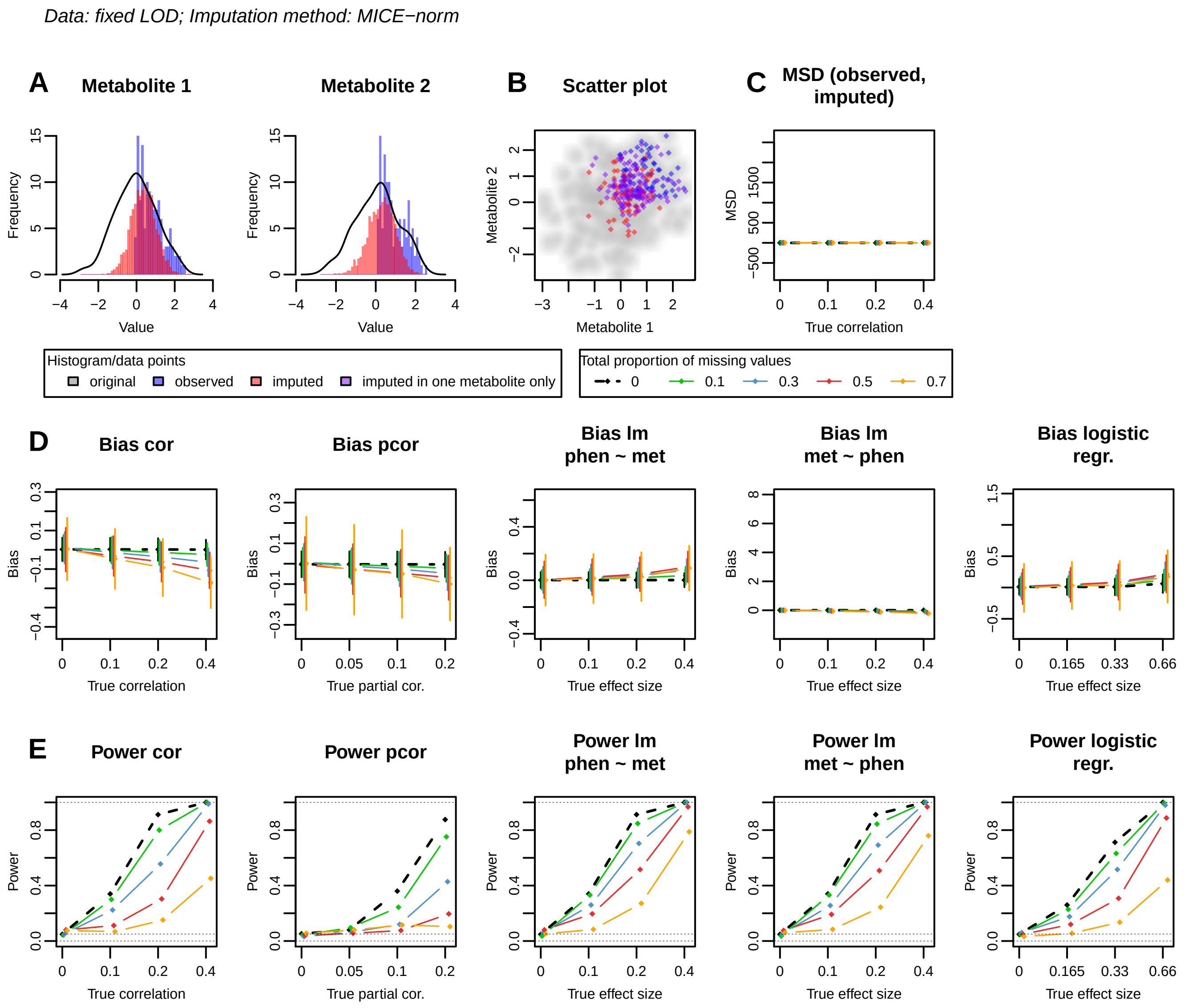
Data: fixed LOD; Imputation method: MICE−norm
A
Metabolite 1
Metabolite 2
Frequency
Value
B
Scatter plot
Metabolite 2
Metabolite 1
C
MSD (observed, imputed)
MSD
True correlation
Histogram/data points
original
observed
imputed
imputed in one metabolite only
Total proportion of missing values
0
0.1
0.3
0.5
0.7
D
Bias cor
Bias pcor
Bias lm phen ~ met
Bias lm met ~ phen
Bias logistic regr.
Bias
True correlation
True partial cor.
True effect size
E
Power cor
Power pcor
Power lm phen ~ met
Power lm met ~ phen
Power logistic regr.
Power

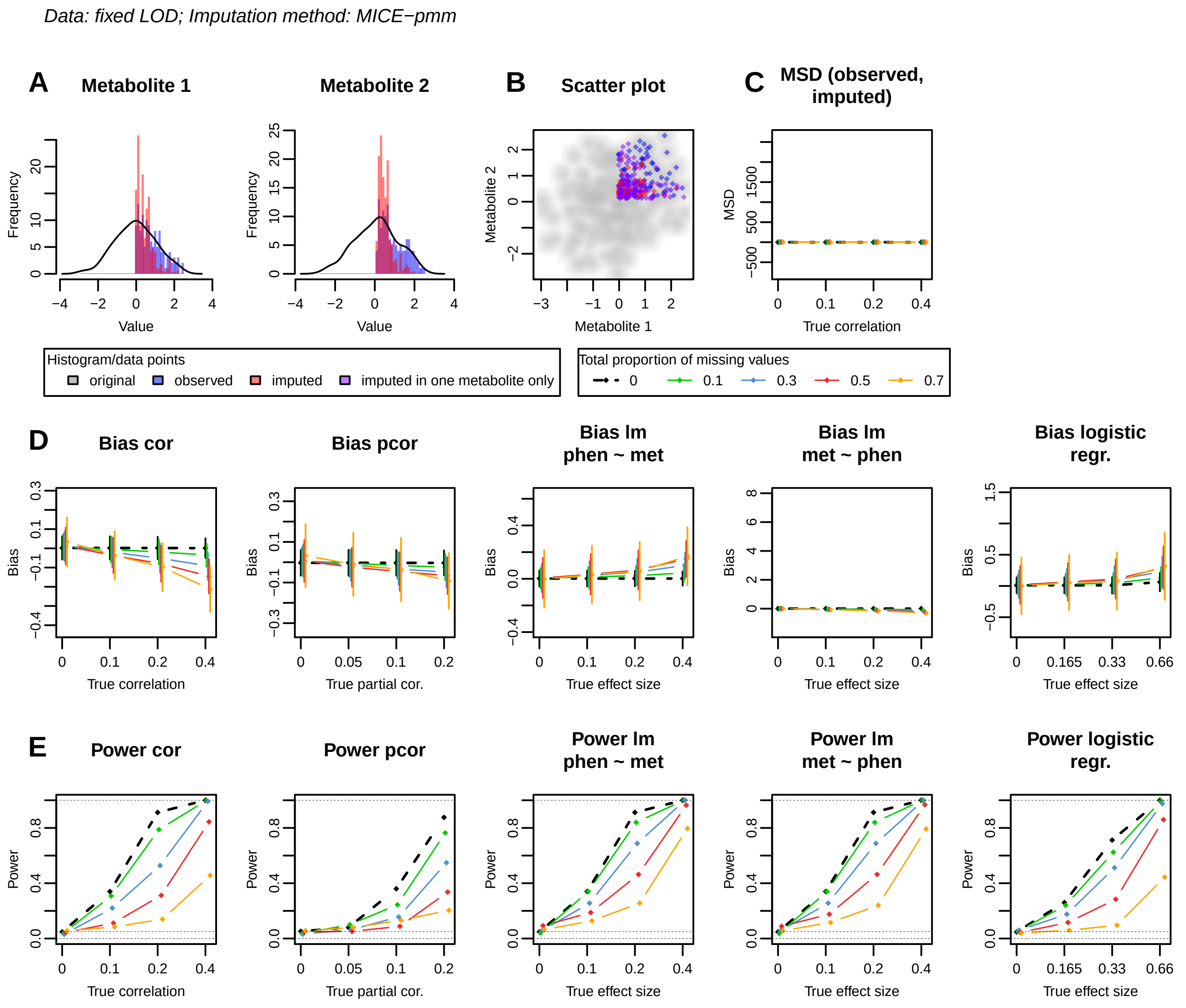

Data: fixed LOD; Imputation method: MICE−pmm
A
Metabolite 1
Metabolite 2
B
Scatter plot
C
MSD (observed, imputed)
Frequency
Value
Metabolite 2
Metabolite 1
MSD
True correlation
Histogram/data points
original
observed
imputed
imputed in one metabolite only
Total proportion of missing values
0
0.1
0.3
0.5
0.7
D
Bias cor
Bias pcor
Bias lm phen ~ met
Bias lm met ~ phen
Bias logistic regr.
Bias
True correlation
True partial cor.
True effect size
E
Power cor
Power pcor
Power lm phen ~ met
Power lm met ~ phen
Power logistic regr.
Power

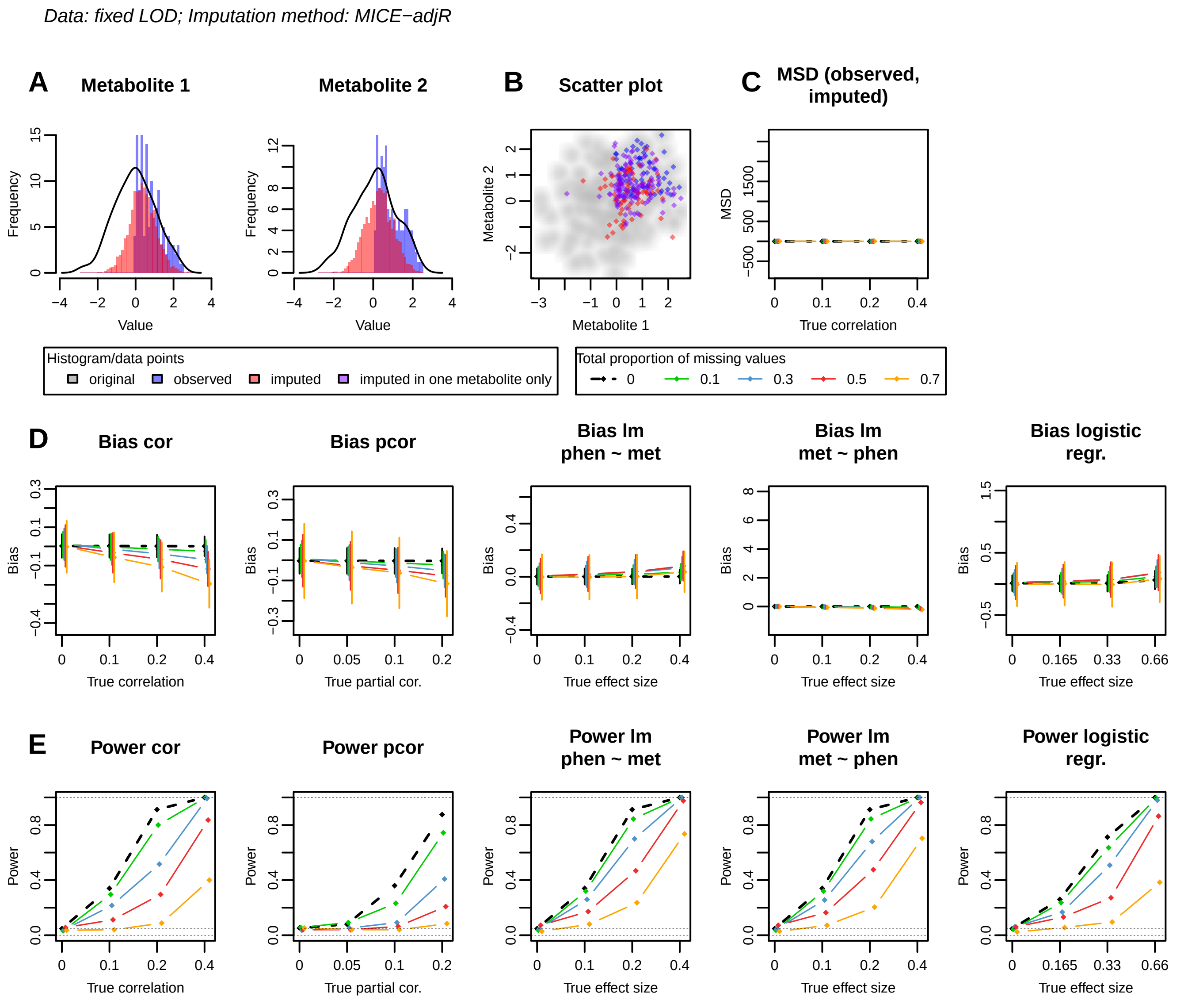

Data: fixed LOD; Imputation method: MICE−adjR
A
Metabolite 1
Metabolite 2
B
Scatter plot
C
MSD (observed, imputed)
Frequency
Value
Metabolite 2
Metabolite 1
MSD
True correlation
Histogram/data points
original
observed
imputed
imputed in one metabolite only
Total proportion of missing values
0
0.1
0.3
0.5
0.7
D
Bias cor
Bias pcor
Bias lm phen ~ met
Bias lm met ~ phen
Bias logistic regr.
Bias
True correlation
True partial cor.
True effect size
E
Power cor
Power pcor
Power lm phen ~ met
Power lm met ~ phen
Power logistic regr.
Power

Data: fixed LOD; Imputation method: KNN−var(1)

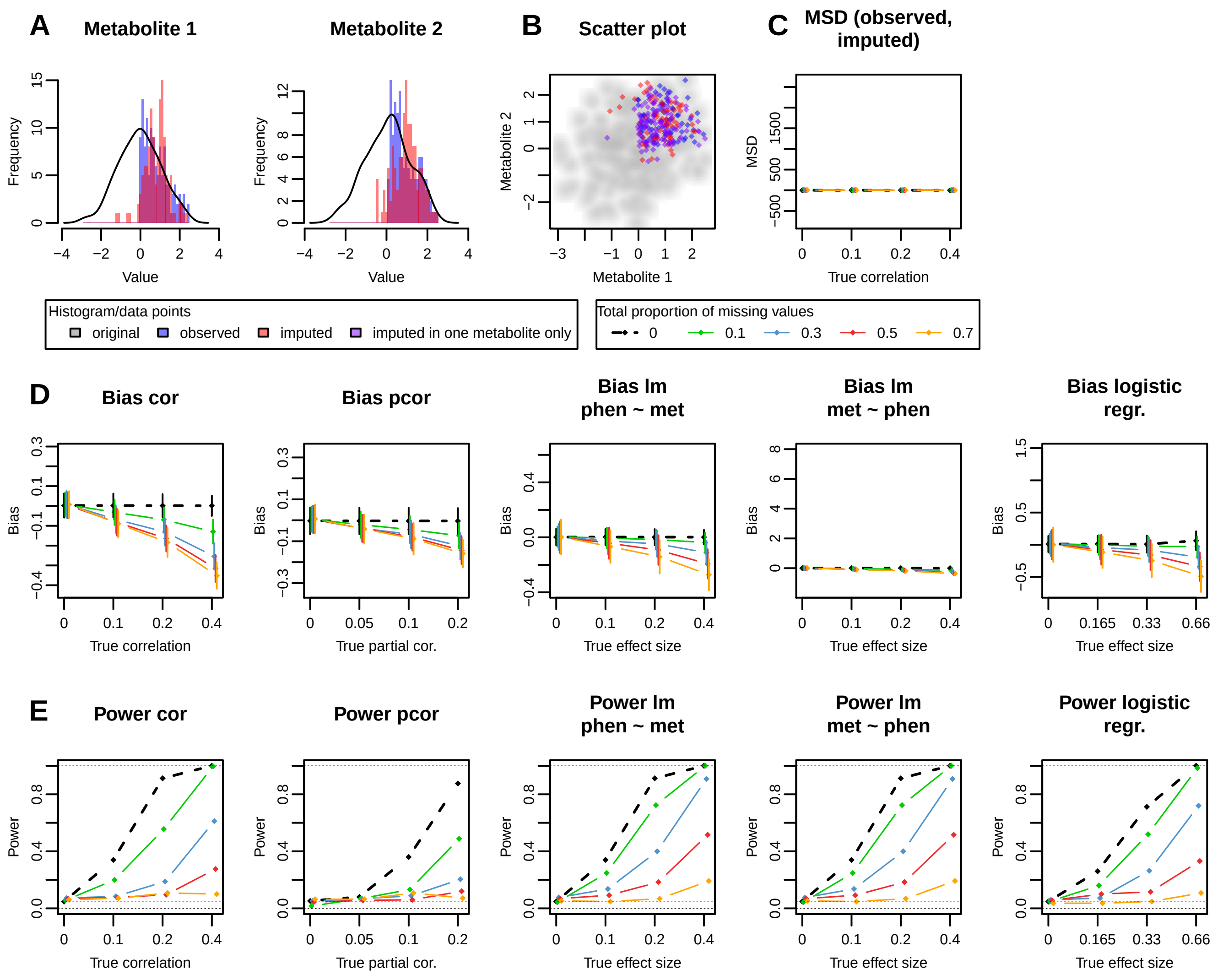

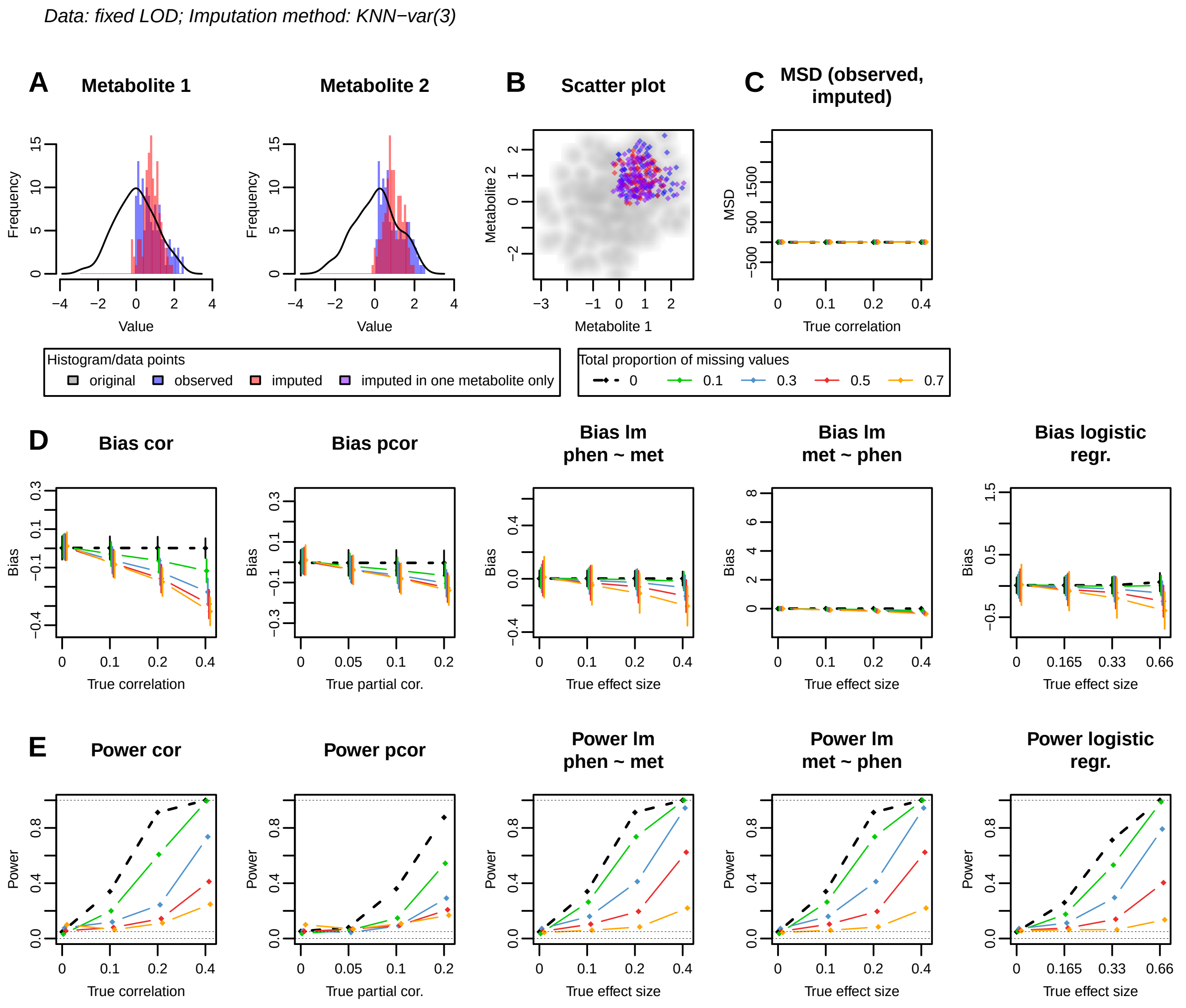
Data: fixed LOD; Imputation method: KNN−var(3)
A
Metabolite 1
Metabolite 2
B
Scatter plot
C
MSD (observed, imputed)
Frequency
Value
Metabolite 2
Metabolite 1
MSD
True correlation
Histogram/data points
original
observed
imputed
imputed in one metabolite only
Total proportion of missing values
0
0.1
0.3
0.5
0.7
D
Bias cor
Bias pcor
Bias lm phen ~ met
Bias lm met ~ phen
Bias logistic regr.
Bias
True correlation
True partial cor.
True effect size
E
Power cor
Power pcor
Power lm phen ~ met
Power lm met ~ phen
Power logistic regr.
Power

Data: fixed LOD; Imputation method: KNN−var(5)

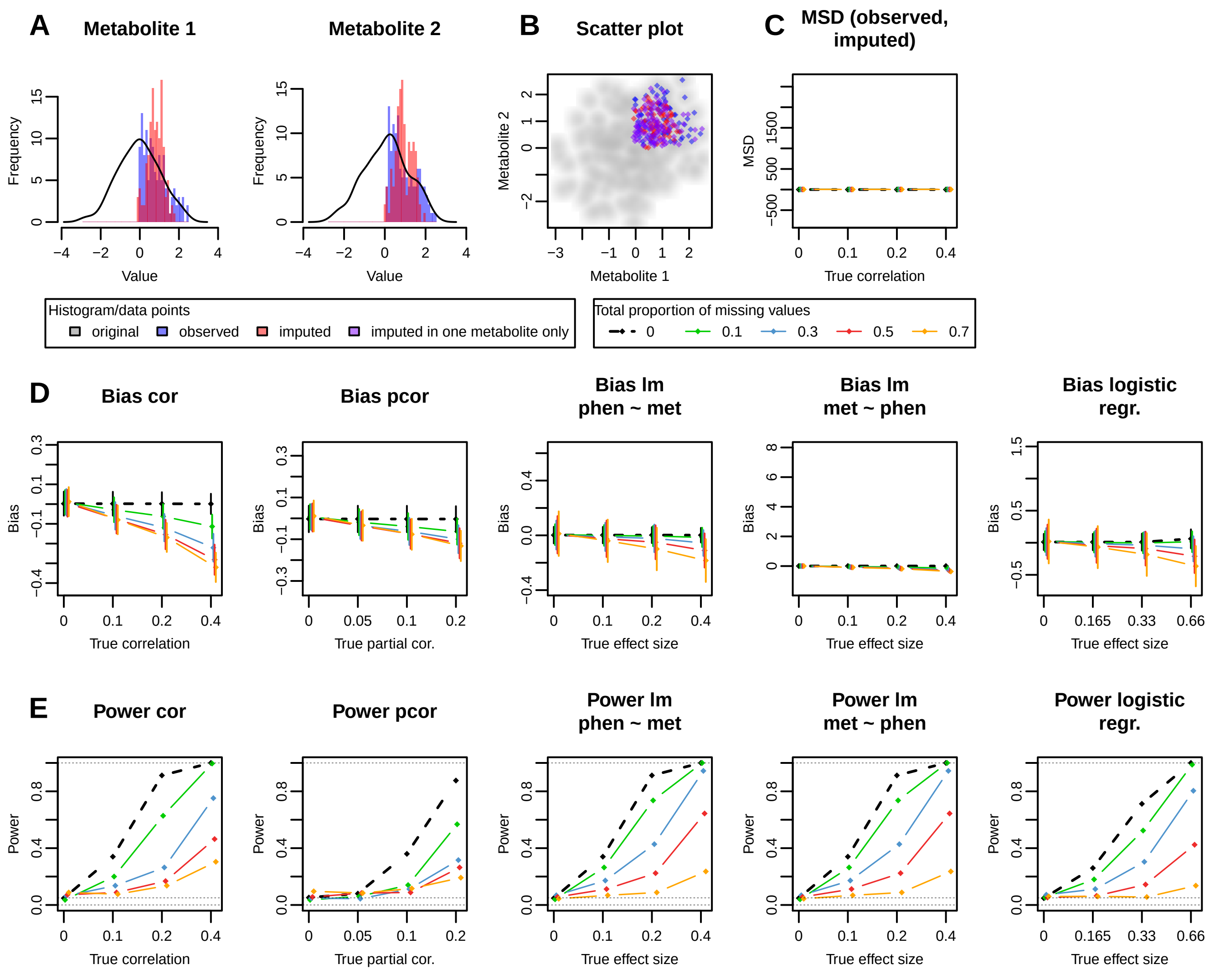

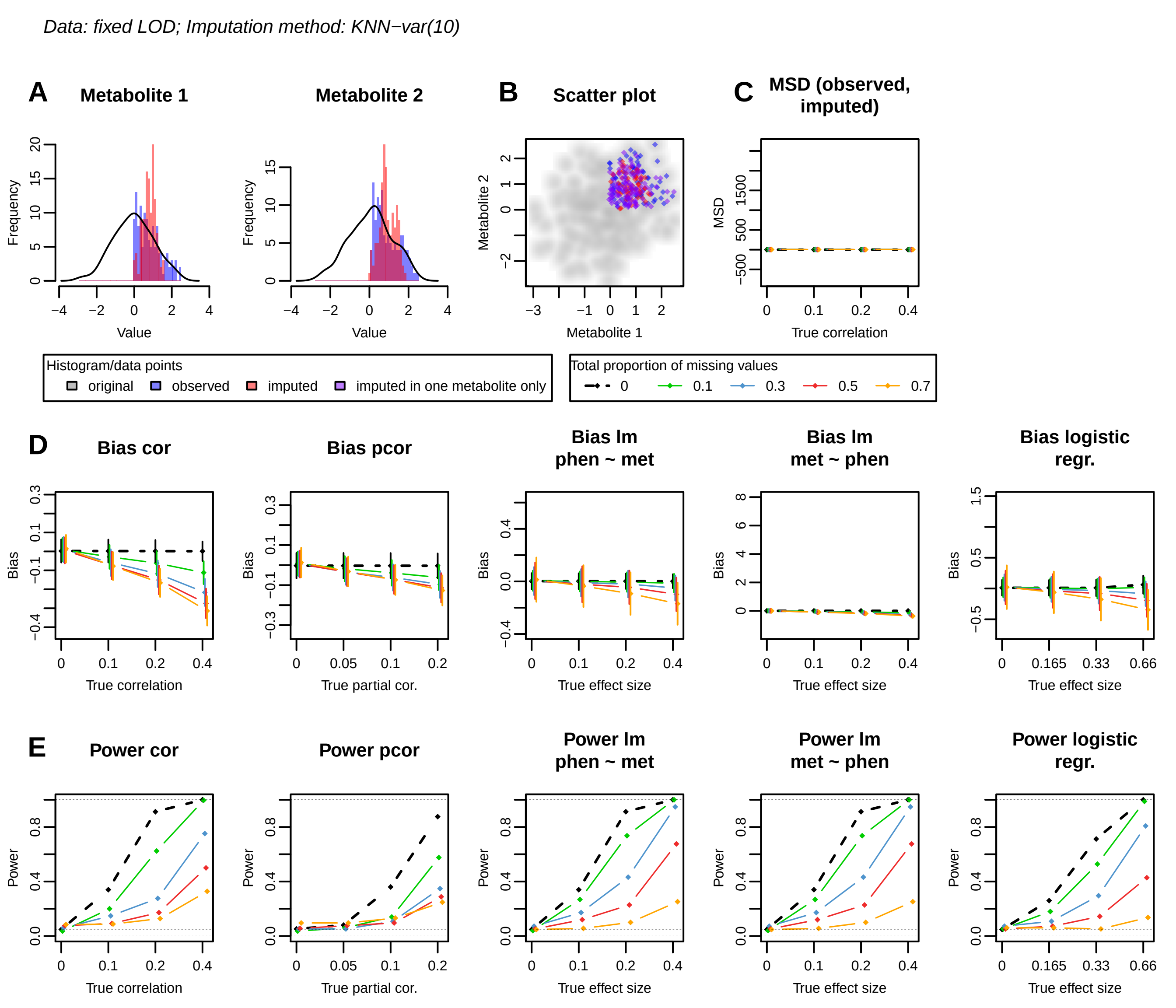

Data: fixed LOD; Imputation method: KNN−var(10)
A
Metabolite 1
Metabolite 2
B
Scatter plot
C
MSD (observed, imputed)
Frequency
Value
Metabolite 2
Metabolite 1
MSD
True correlation
Histogram/data points
original
observed
imputed
imputed in one metabolite only
Total proportion of missing values
0
0.1
0.3
0.5
0.7
D
Bias cor
Bias pcor
Bias lm phen ~ met
Bias lm met ~ phen
Bias logistic regr.
Bias
True correlation
True partial cor.
True effect size
E
Power cor
Power pcor
Power lm phen ~ met
Power lm met ~ phen
Power logistic regr.
Power

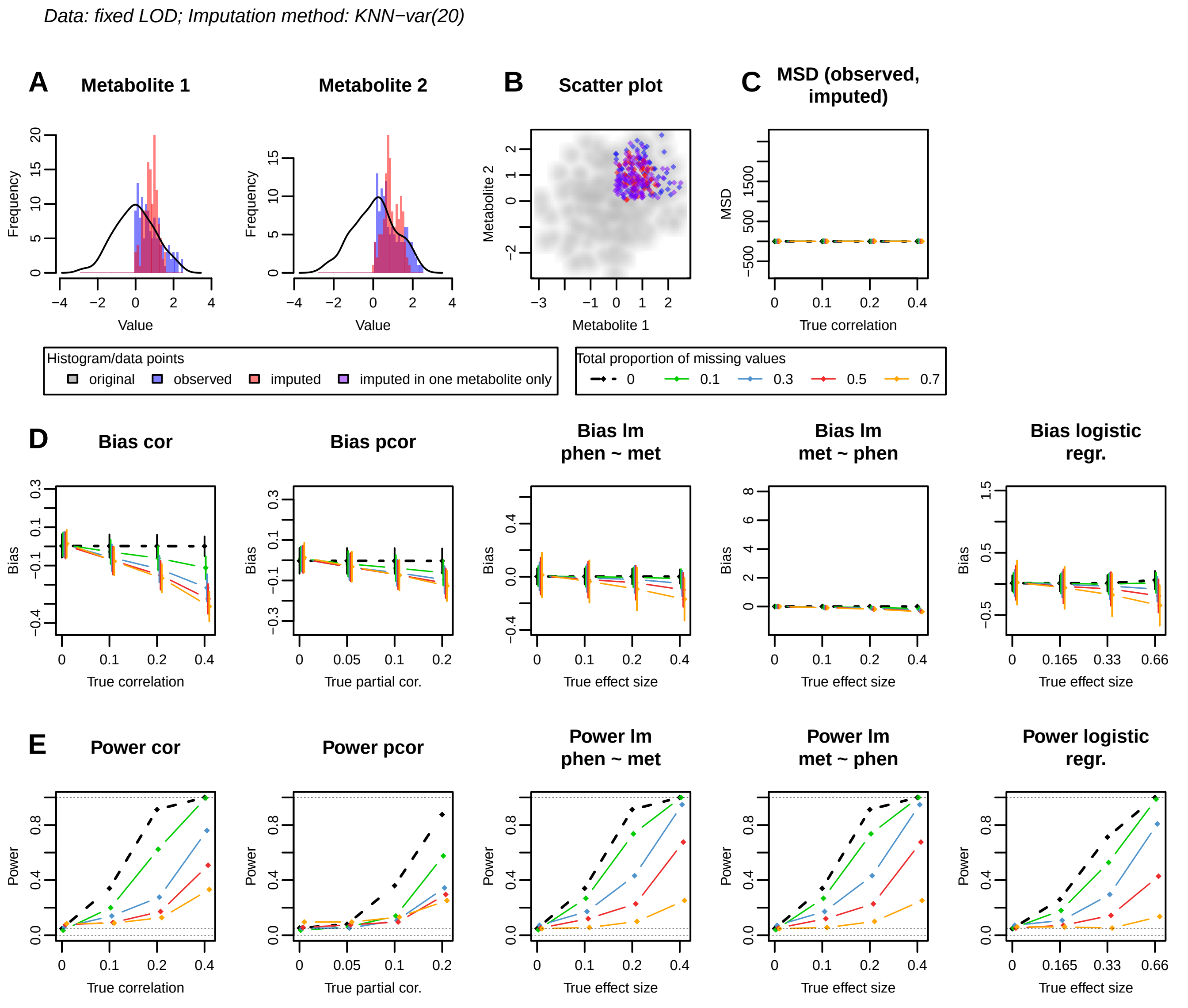
Data: fixed LOD; Imputation method: KNN−var(20)
A
Metabolite 1
Metabolite 2
Frequency
Value
B
Scatter plot
Metabolite 1
Metabolite 2
C
MSD (observed, imputed)
MSD
True correlation
Histogram/data points
original
observed
imputed
imputed in one metabolite only
Total proportion of missing values
0
0.1
0.3
0.5
0.7
D
Bias cor
Bias pcor
Bias lm phen ~ met
Bias lm met ~ phen
Bias logistic regr.
Bias
True correlation
True partial cor.
True effect size
E
Power cor
Power pcor
Power lm phen ~ met
Power lm met ~ phen
Power logistic regr.
Power
True correlation
True partial cor.
True effect size

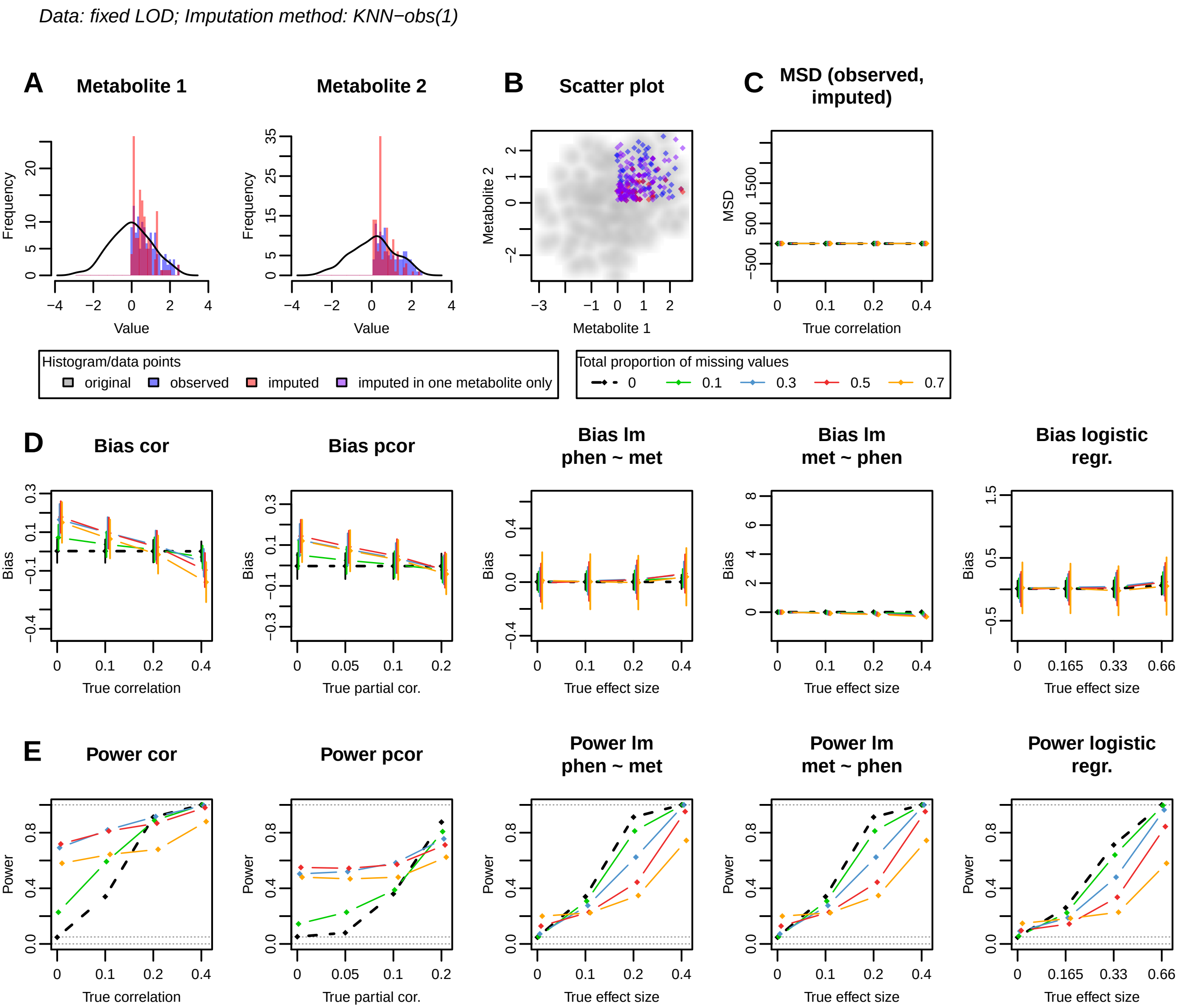
Data: fixed LOD; Imputation method: KNN−obs(1)
A
Metabolite 1
Metabolite 2
Frequency
Value
B
Scatter plot
Metabolite 2
Metabolite 1
C
MSD (observed, imputed)
MSD
True correlation
Histogram/data points
original
observed
imputed
imputed in one metabolite only
Total proportion of missing values
0
0.1
0.3
0.5
0.7
D
Bias cor
Bias pcor
Bias lm phen ~ met
Bias lm met ~ phen
Bias logistic regr.
Bias
True correlation
True partial cor.
True effect size
E
Power cor
Power pcor
Power lm phen ~ met
Power lm met ~ phen
Power logistic regr.
Power
True correlation
True partial cor.
True effect size

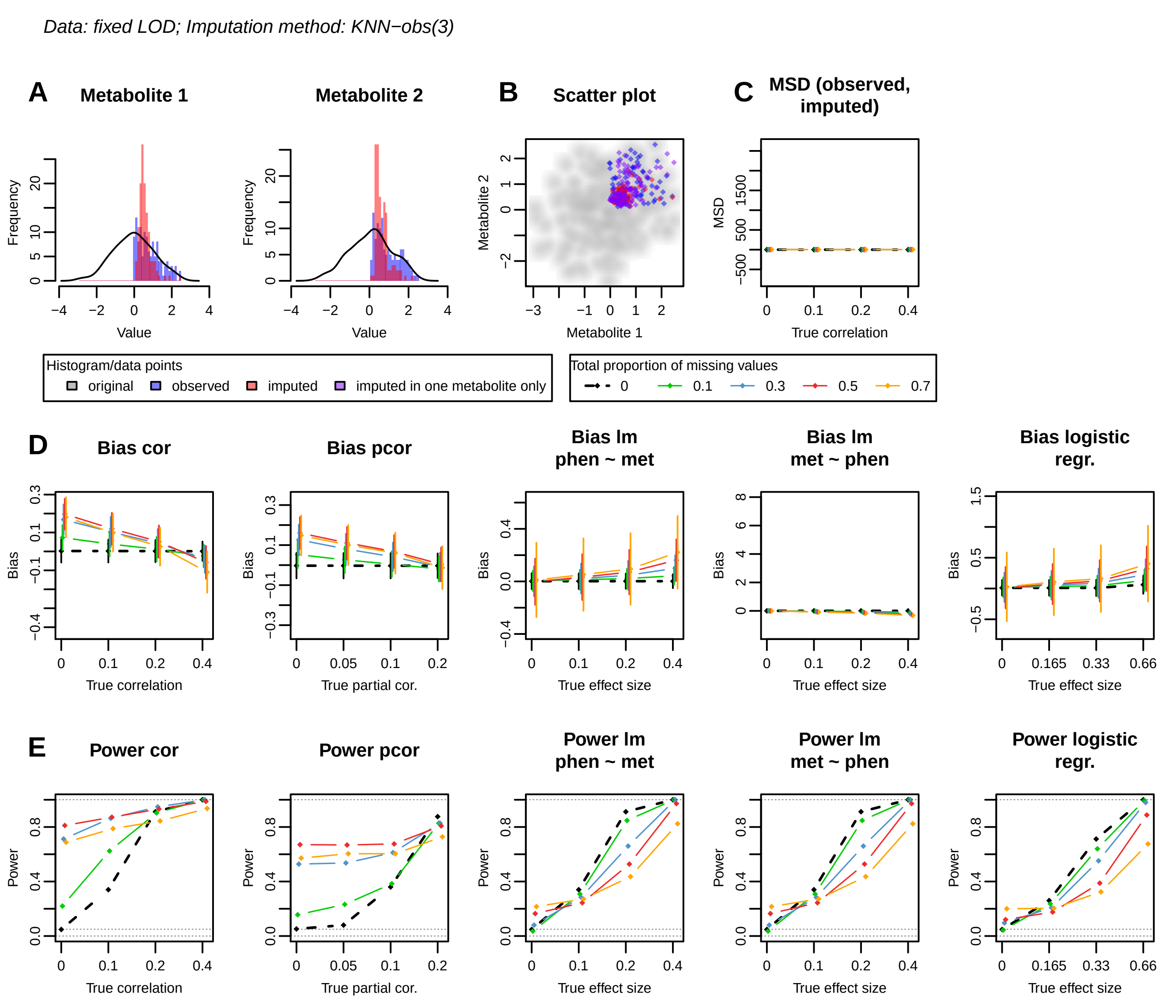
Data: fixed LOD; Imputation method: KNN−obs(3)
A
Metabolite 1
Metabolite 2
Frequency
Value
B
Scatter plot
Metabolite 2
Metabolite 1
C
MSD (observed, imputed)
MSD
True correlation
Histogram/data points
original
observed
imputed
imputed in one metabolite only
Total proportion of missing values
0
0.1
0.3
0.5
0.7
D
Bias cor
Bias pcor
Bias lm phen ~ met
Bias lm met ~ phen
Bias logistic regr.
Bias
True correlation
True partial cor.
True effect size
E
Power cor
Power pcor
Power lm phen ~ met
Power lm met ~ phen
Power logistic regr.
Power
True correlation
True partial cor.
True effect size

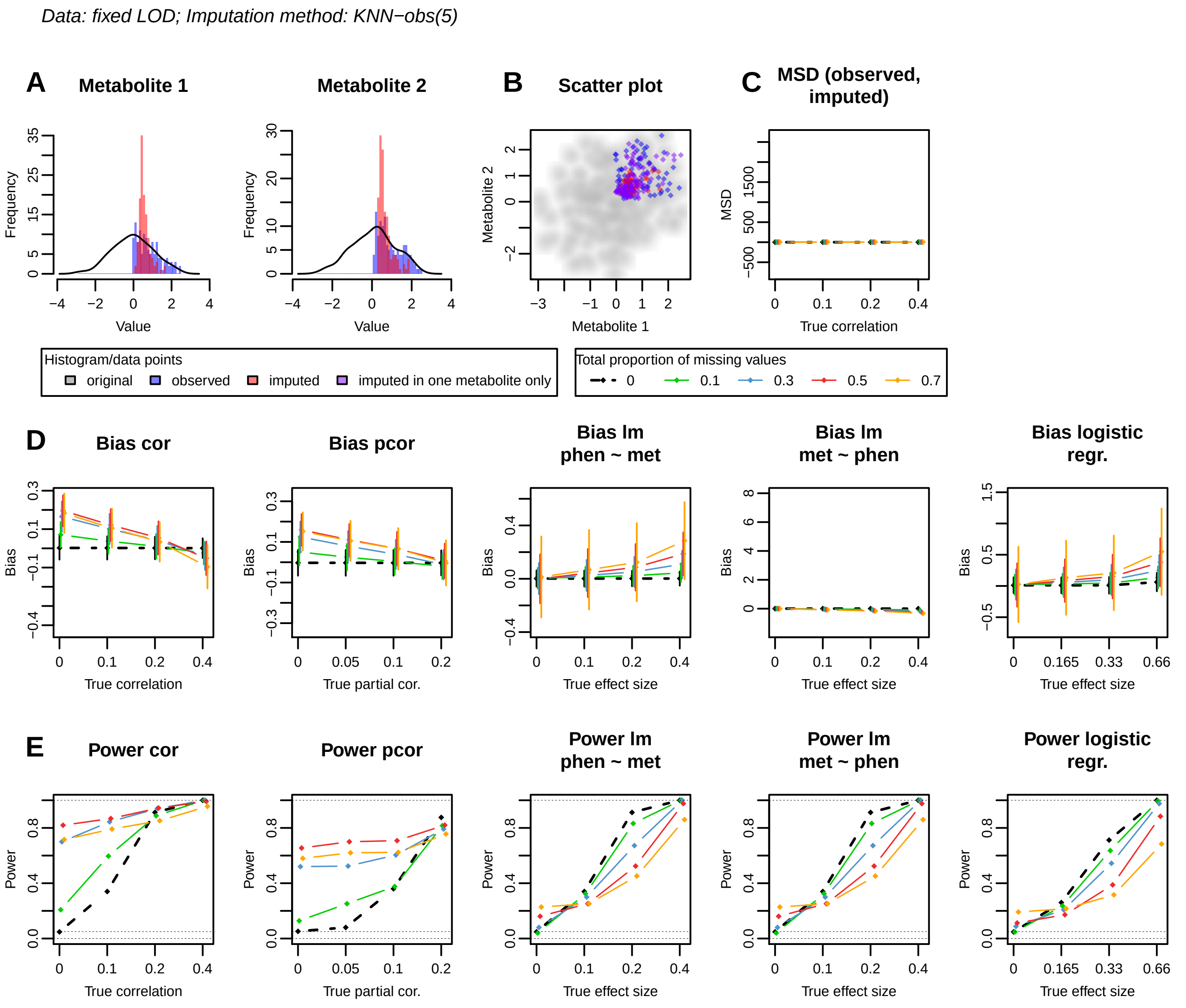
Data: fixed LOD; Imputation method: KNN−obs(5)
A
Metabolite 1
Metabolite 2
Frequency
Value
B
Scatter plot
Metabolite 1
Metabolite 2
C
MSD (observed, imputed)
MSD
True correlation
Histogram/data points
original
observed
imputed
imputed in one metabolite only
Total proportion of missing values
0
0.1
0.3
0.5
0.7
D
Bias cor
Bias pcor
Bias lm phen ~ met
Bias lm met ~ phen
Bias logistic regr.
Bias
True correlation
True partial cor.
True effect size
E
Power cor
Power pcor
Power lm phen ~ met
Power lm met ~ phen
Power logistic regr.
Power
True correlation
True partial cor.
True effect size

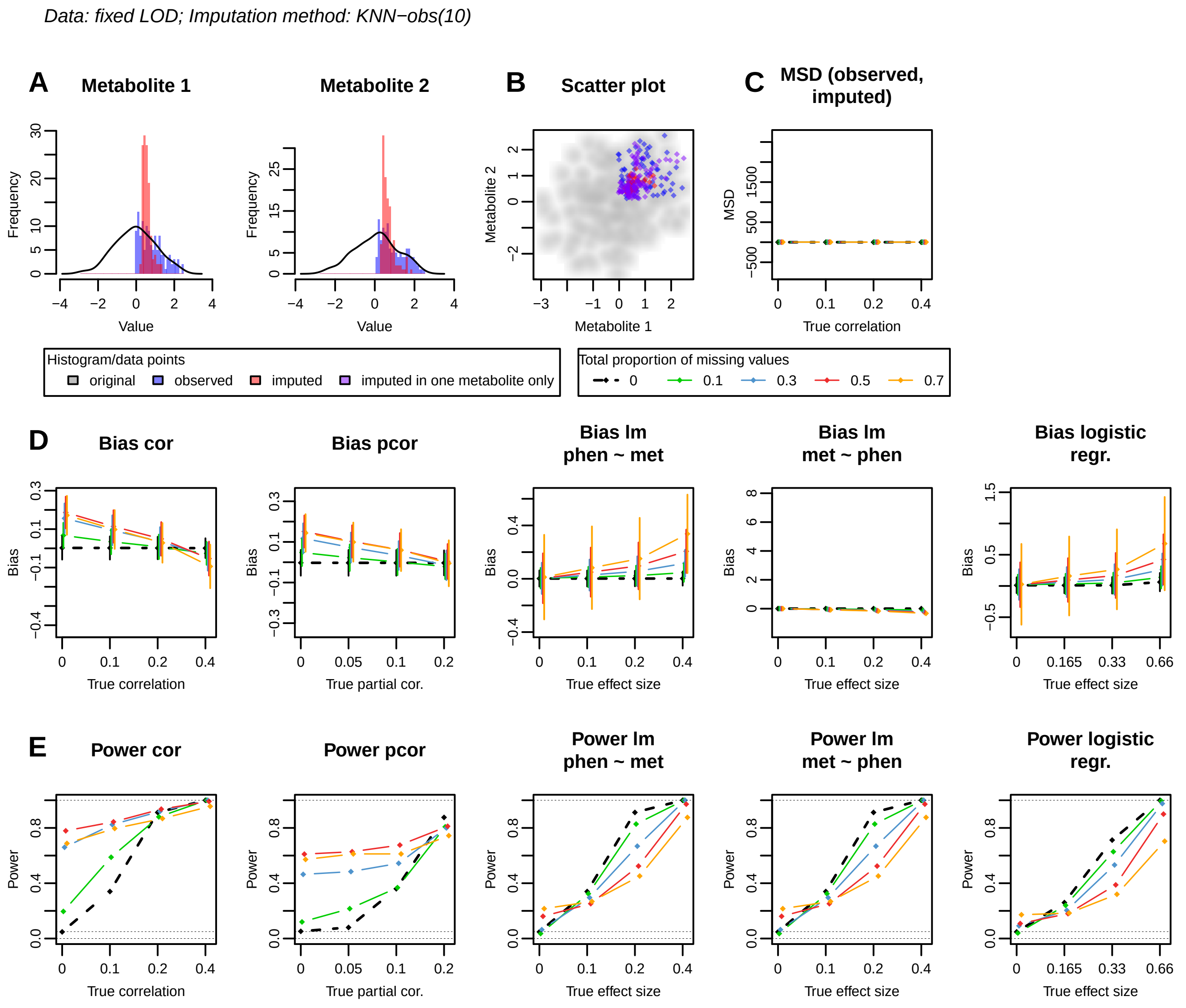
Data: fixed LOD; Imputation method: KNN−obs(10)
A
Metabolite 1
Metabolite 2
B
Scatter plot
C
MSD (observed, imputed)
Frequency
Value
Metabolite 2
Metabolite 1
MSD
True correlation
Histogram/data points
original
observed
imputed
imputed in one metabolite only
Total proportion of missing values
0
0.1
0.3
0.5
0.7
D
Bias cor
Bias pcor
Bias lm phen ~ met
Bias lm met ~ phen
Bias logistic regr.
Bias
True correlation
True partial cor.
True effect size
E
Power cor
Power pcor
Power lm phen ~ met
Power lm met ~ phen
Power logistic regr.
Power

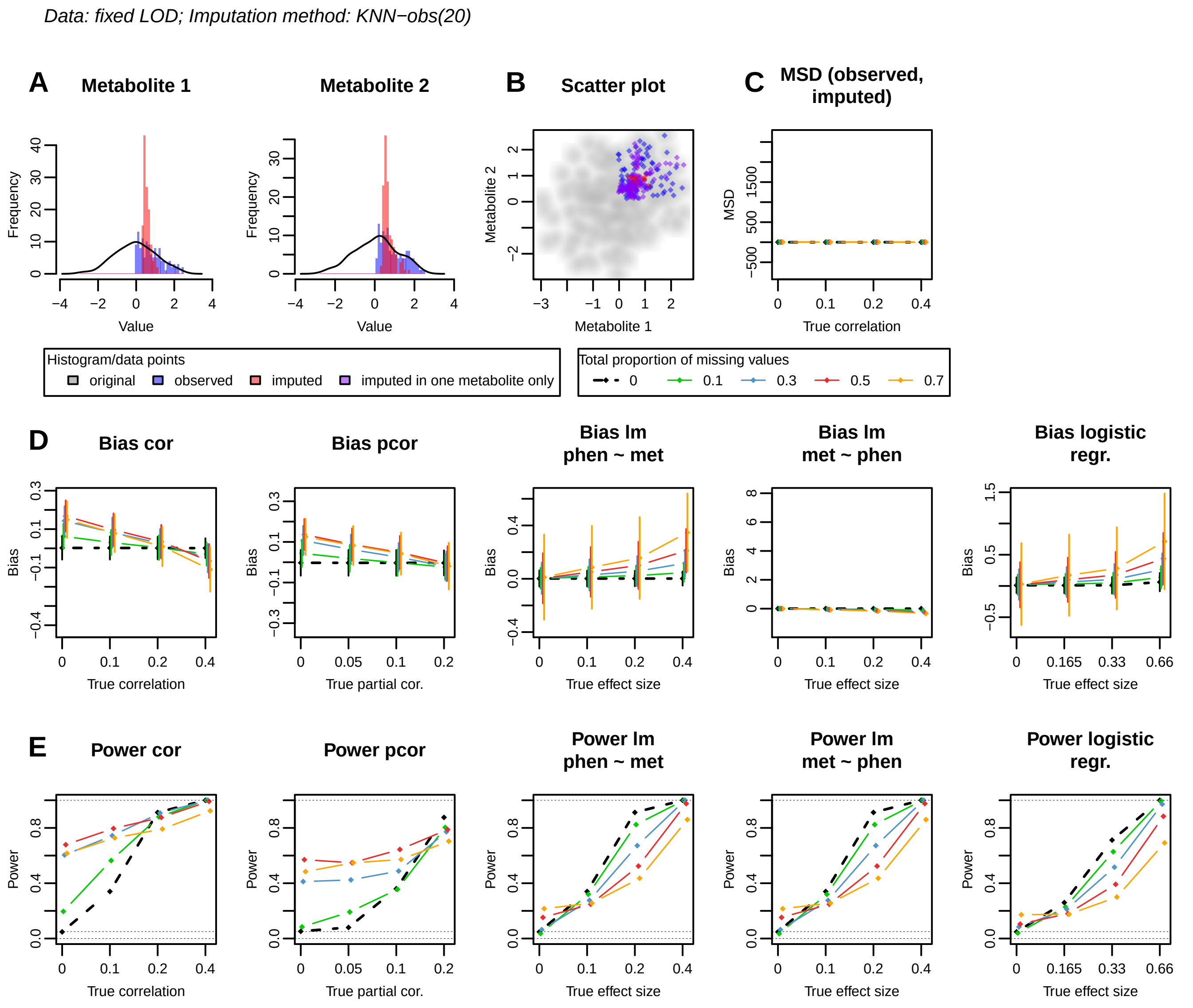

Data: fixed LOD; Imputation method: KNN−obs(20)
A
Metabolite 1
Metabolite 2
Frequency
Value
B
Scatter plot
Metabolite 2
Metabolite 1
C
MSD (observed, imputed)
MSD
True correlation
Histogram/data points
original
observed
imputed
imputed in one metabolite only
Total proportion of missing values
0
0.1
0.3
0.5
0.7
D
Bias cor
Bias pcor
Bias lm phen ~ met
Bias lm met ~ phen
Bias logistic regr.
Bias
True correlation
True partial cor.
True effect size
E
Power cor
Power pcor
Power lm phen ~ met
Power lm met ~ phen
Power logistic regr.
Power
True correlation
True partial cor.
True effect size

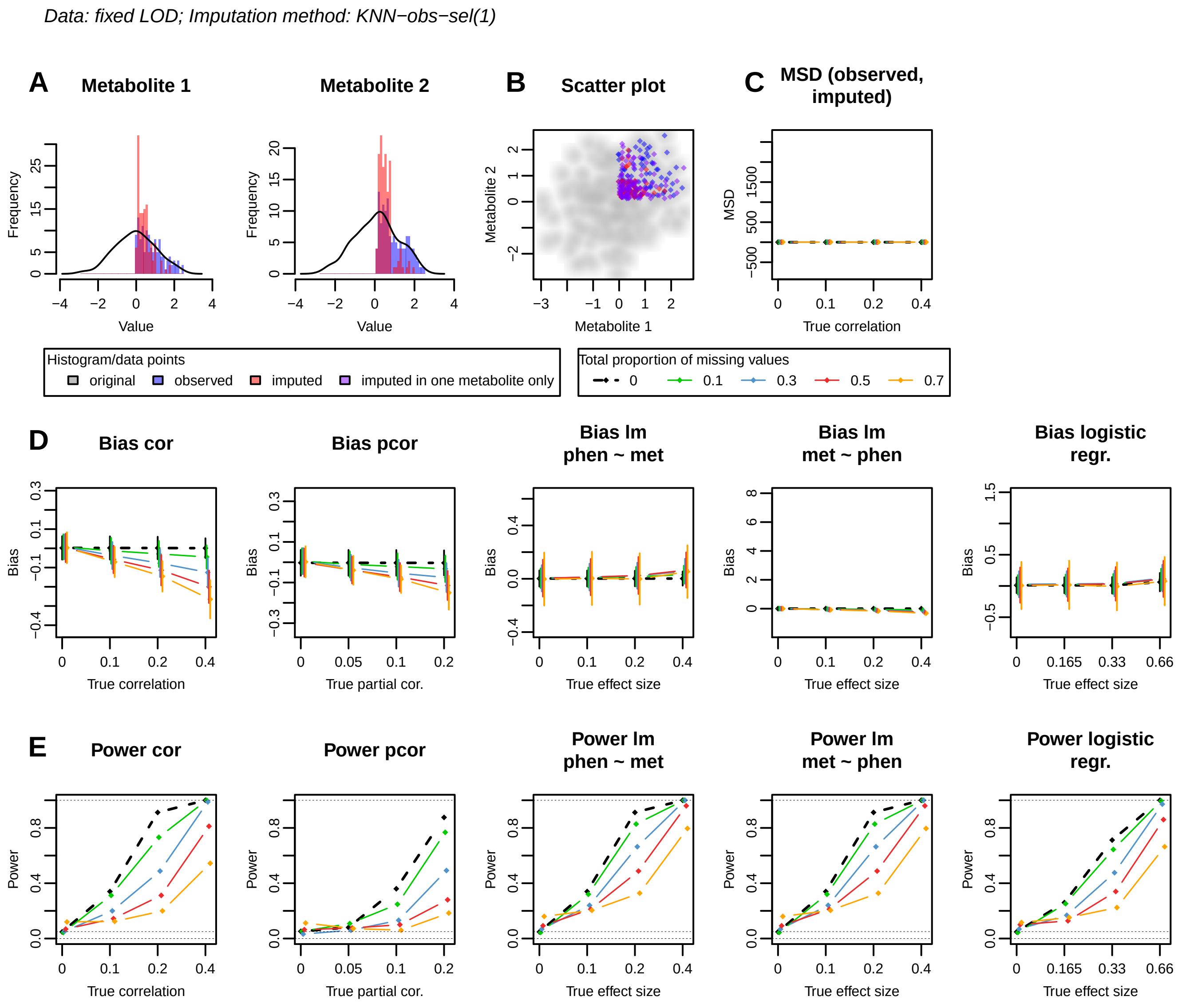
Data: fixed LOD; Imputation method: KNN−obs−sel(1)
A
Metabolite 1
Metabolite 2
B
Scatter plot
C
MSD (observed, imputed)
Frequency
Value
Metabolite 1
Metabolite 2
MSD
True correlation
Histogram/data points
original
observed
imputed
imputed in one metabolite only
Total proportion of missing values
0
0.1
0.3
0.5
0.7
D
Bias cor
Bias pcor
Bias lm phen ~ met
Bias lm met ~ phen
Bias logistic regr.
Bias
True correlation
True partial cor.
True effect size
E
Power cor
Power pcor
Power lm phen ~ met
Power lm met ~ phen
Power logistic regr.
Power

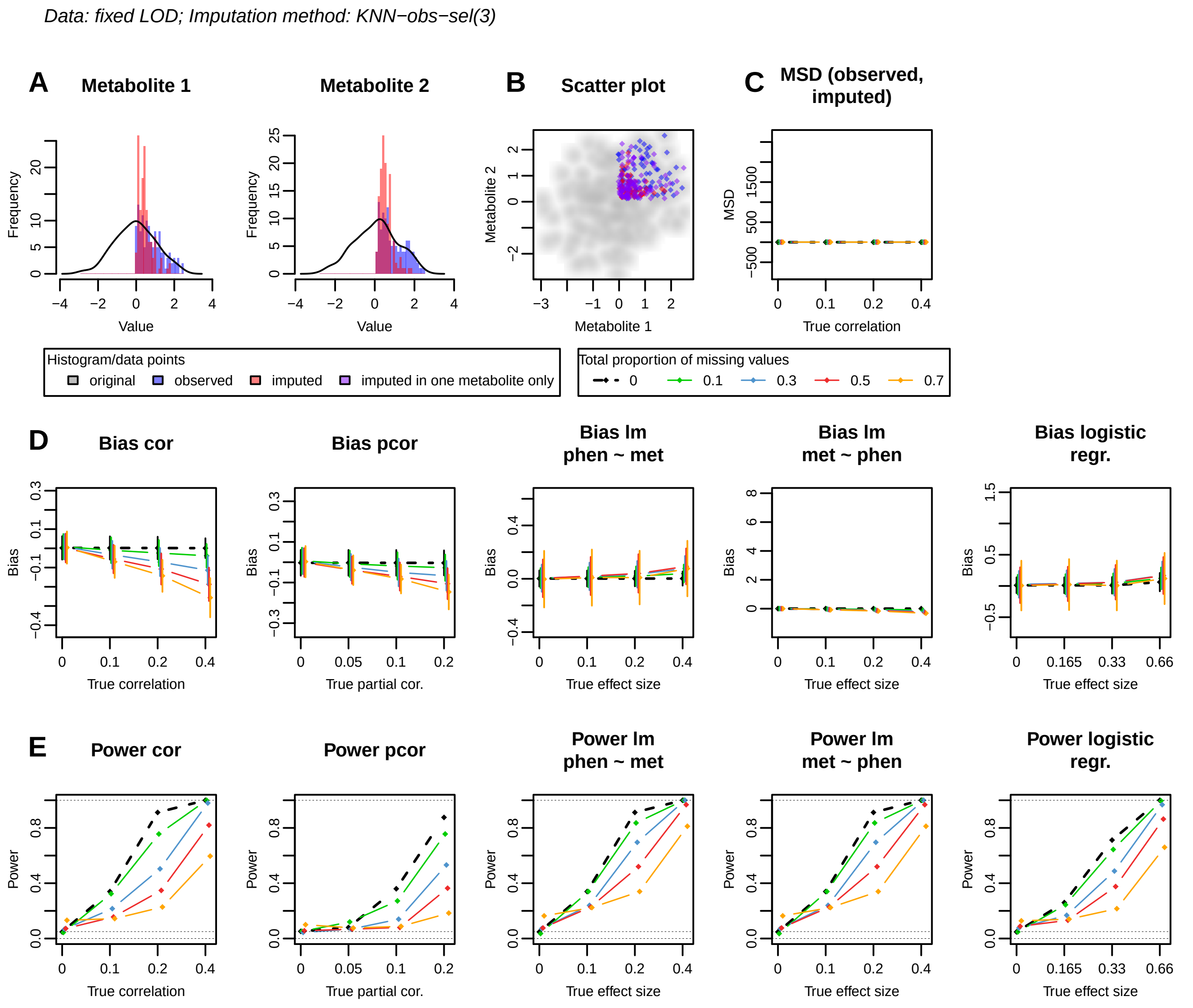

Data: fixed LOD; Imputation method: KNN−obs−sel(3)
A
Metabolite 1
Metabolite 2
B
Scatter plot
C
MSD (observed, imputed)
Frequency
Value
Metabolite 2
Metabolite 1
MSD
True correlation
Histogram/data points
original
observed
imputed
imputed in one metabolite only
Total proportion of missing values
0
0.1
0.3
0.5
0.7
D
Bias cor
Bias pcor
Bias lm phen ~ met
Bias lm met ~ phen
Bias logistic regr.
Bias
True correlation
True partial cor.
True effect size
E
Power cor
Power pcor
Power lm phen ~ met
Power lm met ~ phen
Power logistic regr.
Power

Data: fixed LOD; Imputation method: KNN−obs−sel(5)

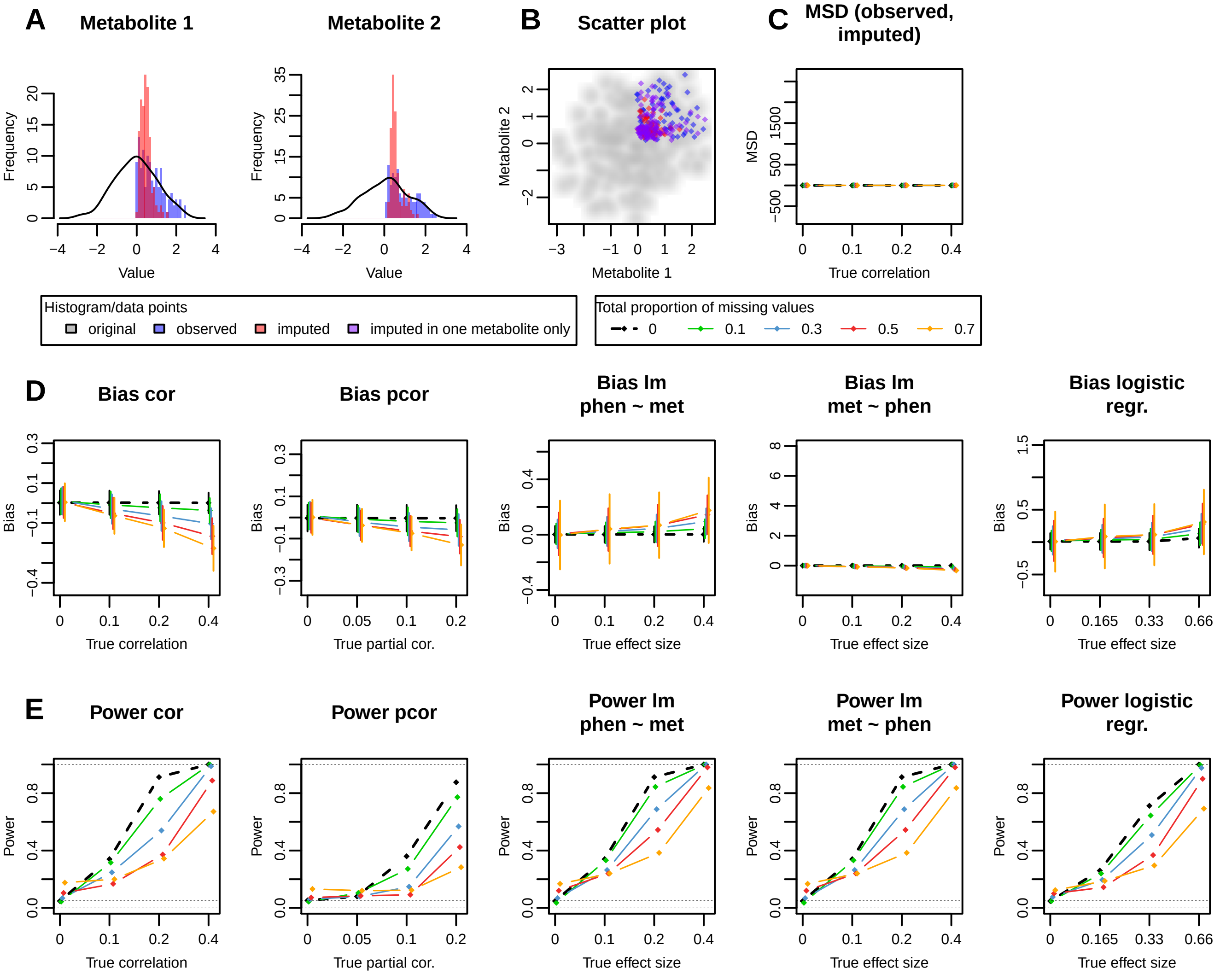

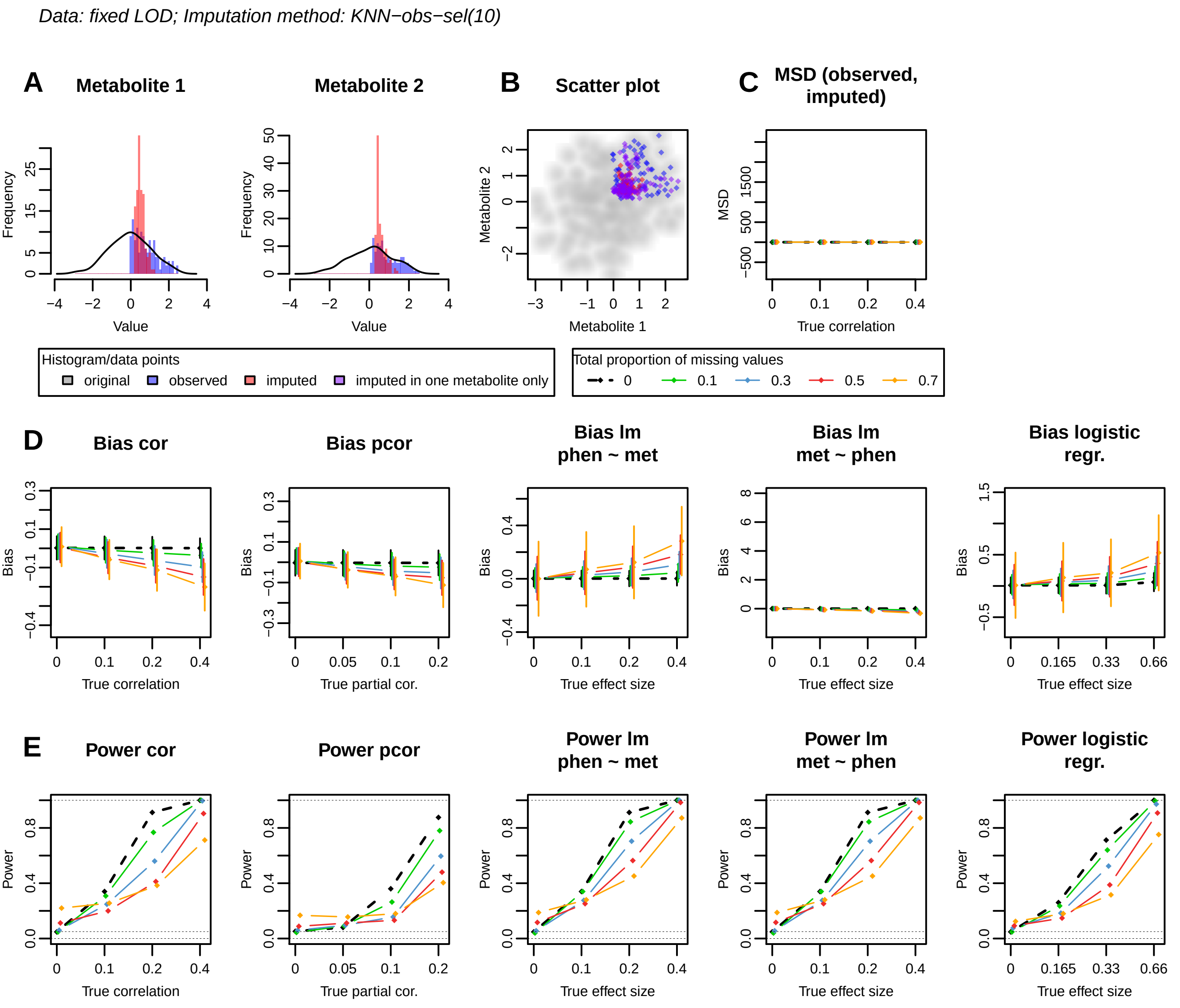
Data: fixed LOD; Imputation method: KNN−obs−sel(10)
A
Metabolite 1
Metabolite 2
Frequency
Value
B
Scatter plot
Metabolite 1
Metabolite 2
C
MSD (observed, imputed)
MSD
True correlation
Histogram/data points
original
observed
imputed
imputed in one metabolite only
Total proportion of missing values
0
0.1
0.3
0.5
0.7
D
Bias cor
Bias pcor
Bias lm phen ~ met
Bias lm met ~ phen
Bias logistic regr.
Bias
True correlation
True partial cor.
True effect size
E
Power cor
Power pcor
Power lm phen ~ met
Power lm met ~ phen
Power logistic regr.
Power
True correlation
True partial cor.
True effect size

Data: fixed LOD; Imputation method: KNN−obs−sel(20)

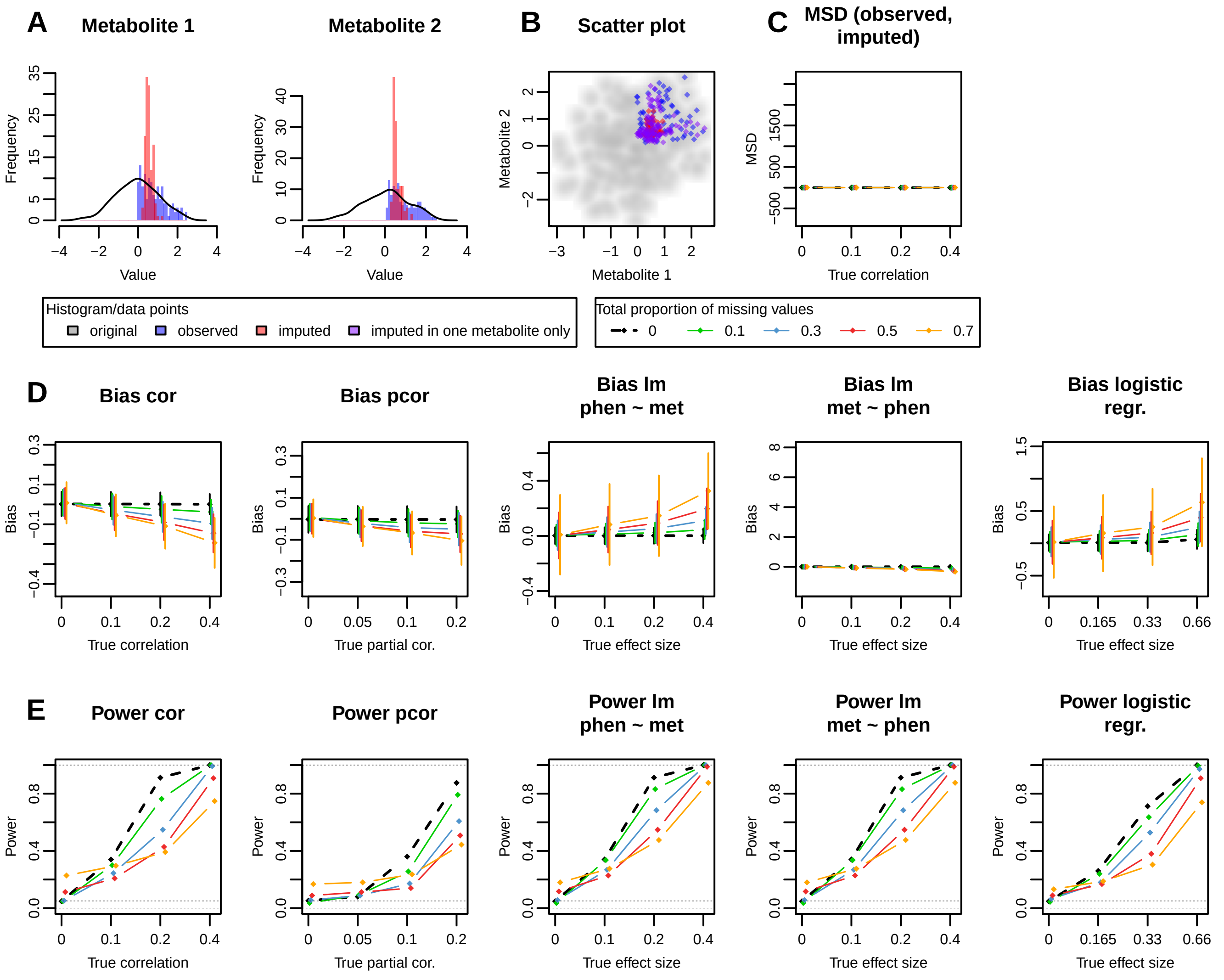

Data: Unsystematic missingness; Imputation method: CCA

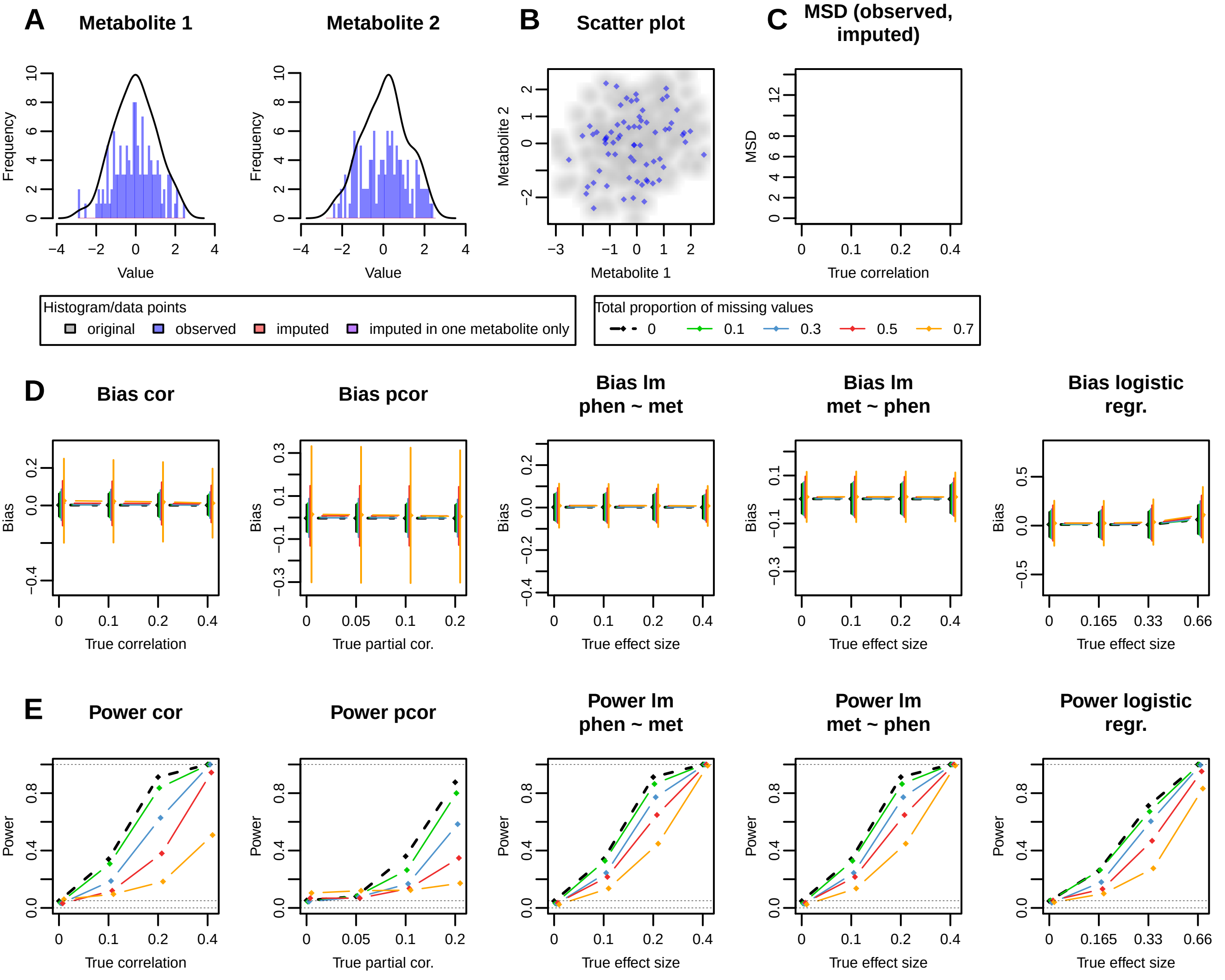

Data: Unsystematic missingness; Imputation method: min

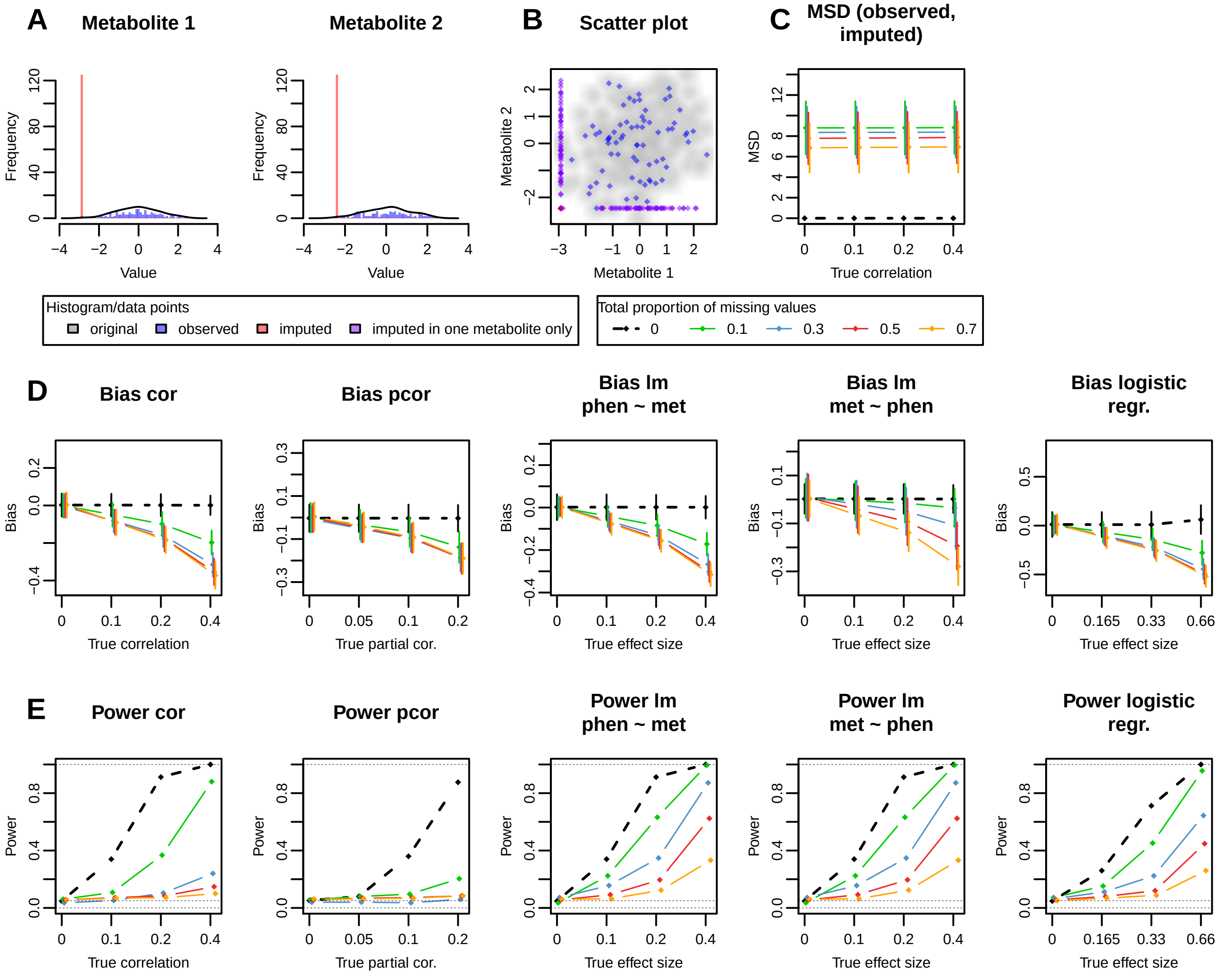

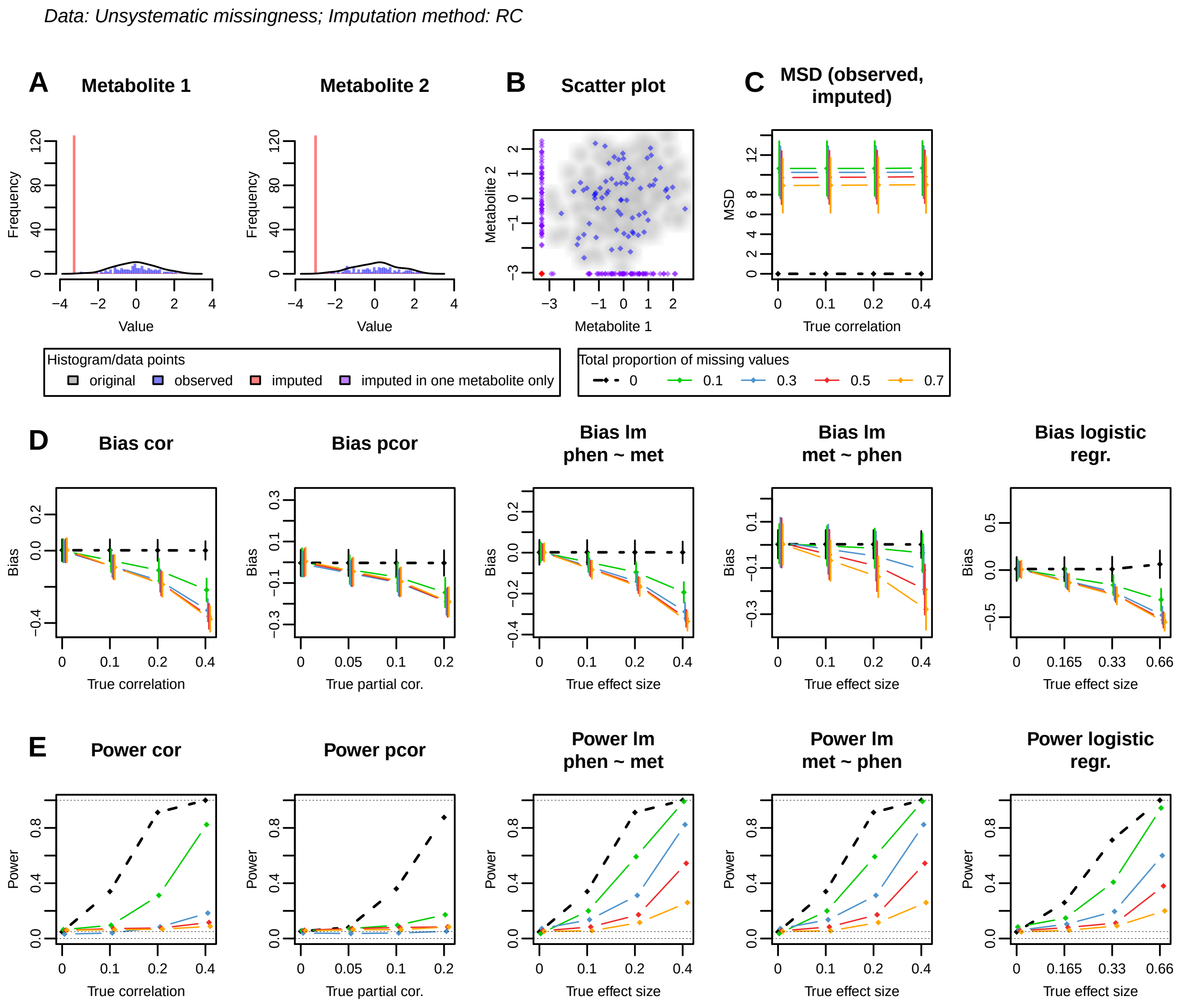

Data: Unsystematic missingness; Imputation method: RC
A
Metabolite 1
Metabolite 2
Frequency
Value
B
Scatter plot
Metabolite 2
Metabolite 1
C
MSD (observed, imputed)
MSD
True correlation
Histogram/data points
original
observed
imputed
imputed in one metabolite only
Total proportion of missing values
0
0.1
0.3
0.5
0.7
D
Bias cor
Bias pcor
Bias lm phen ~ met
Bias lm met ~ phen
Bias logistic regr.
Bias
True correlation
True partial cor.
True effect size
E
Power cor
Power pcor
Power lm phen ~ met
Power lm met ~ phen
Power logistic regr.
Power
True correlation
True partial cor.
True effect size

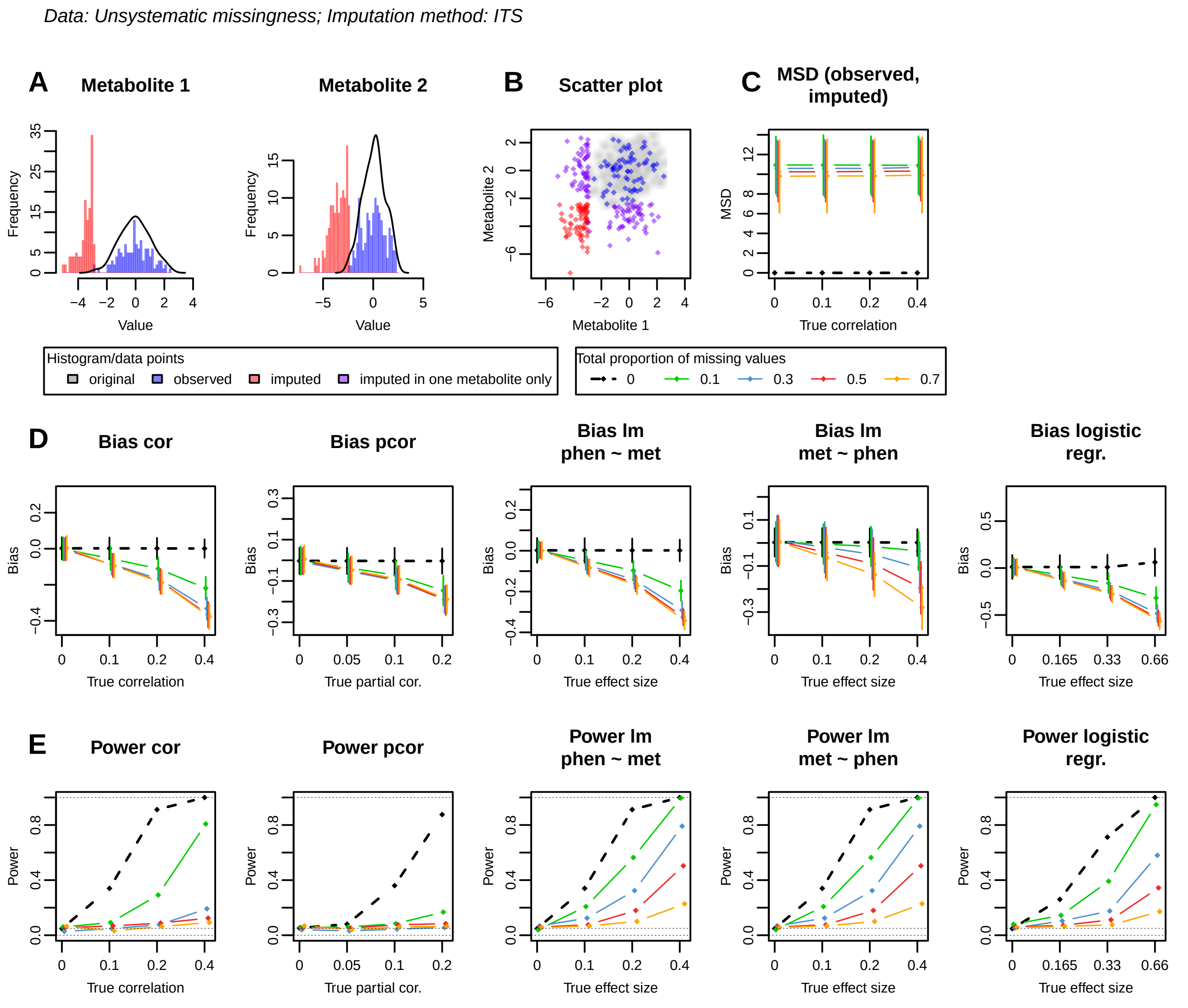
Data: Unsystematic missingness; Imputation method: ITS
A
Metabolite 1
Metabolite 2
Frequency
Value
B
Scatter plot
Metabolite 1
Metabolite 2
C
MSD (observed, imputed)
MSD
True correlation
Histogram/data points
original
observed
imputed
imputed in one metabolite only
Total proportion of missing values
0
0.1
0.3
0.5
0.7
D
Bias cor
Bias pcor
Bias lm phen ~ met
Bias lm met ~ phen
Bias logistic regr.
Bias
True correlation
True partial cor.
True effect size
E
Power cor
Power pcor
Power lm phen ~ met
Power lm met ~ phen
Power logistic regr.
Power
True correlation
True partial cor.
True effect size

Data: Unsystematic missingness; Imputation method: MITS

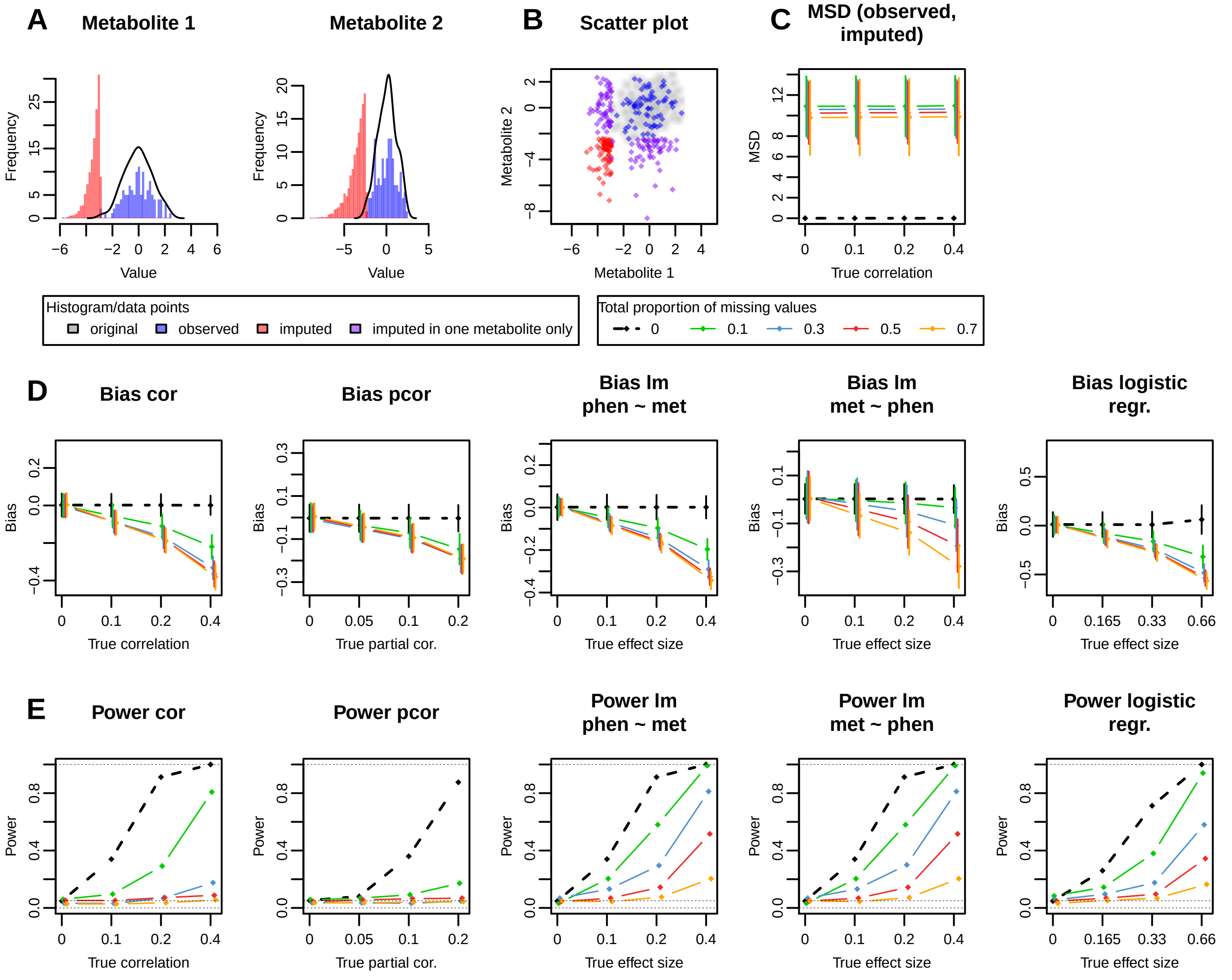

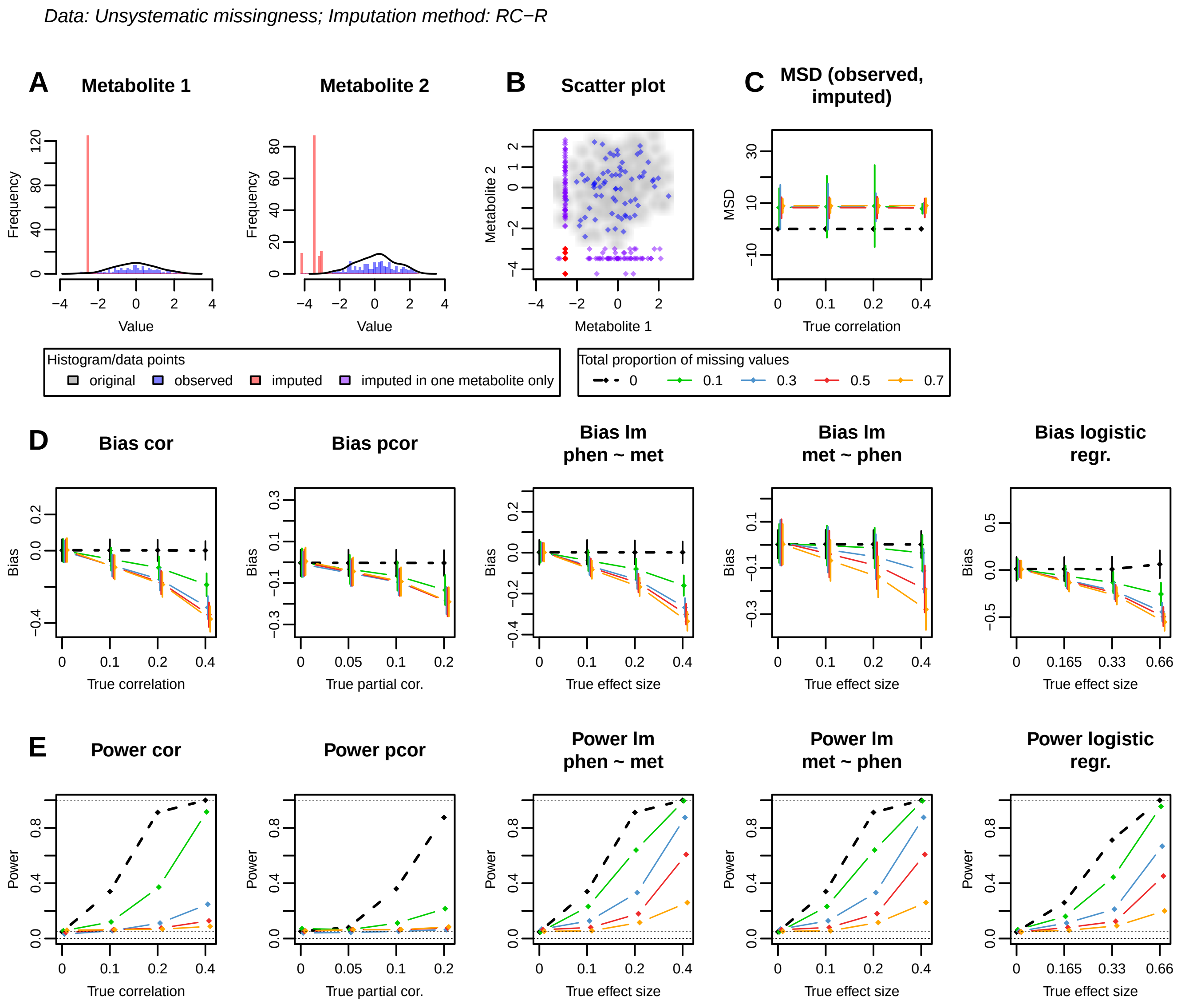
Data: Unsystematic missingness; Imputation method: RC−R
A
Metabolite 1
Metabolite 2
B
Scatter plot
C
MSD (observed, imputed)
Frequency
Value
Metabolite 2
Metabolite 1
MSD
True correlation
Histogram/data points
original
observed
imputed
imputed in one metabolite only
Total proportion of missing values
0
0.1
0.3
0.5
0.7
D
Bias cor
Bias pcor
Bias lm phen ~ met
Bias lm met ~ phen
Bias logistic regr.
Bias
True correlation
True partial cor.
True effect size
E
Power cor
Power pcor
Power lm phen ~ met
Power lm met ~ phen
Power logistic regr.
Power

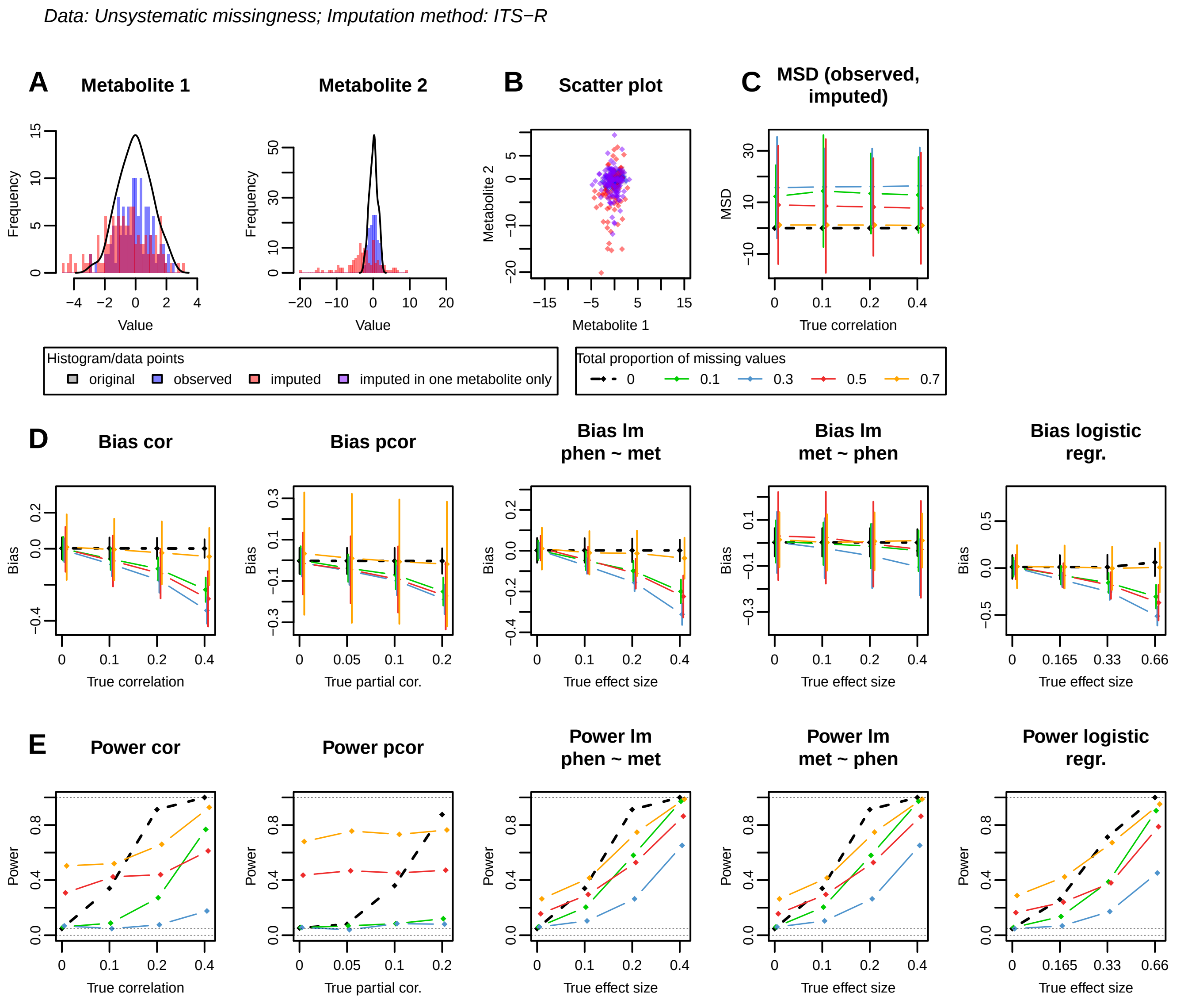
Data: Unsystematic missingness; Imputation method: ITS−R
A
Metabolite 1
Metabolite 2
Frequency
Value
B
Scatter plot
Metabolite 2
Metabolite 1
C
MSD (observed, imputed)
MSD
True correlation
Histogram/data points
original
observed
imputed
imputed in one metabolite only
Total proportion of missing values
0
0.1
0.3
0.5
0.7
D
Bias cor
Bias pcor
Bias lm phen ~ met
Bias lm met ~ phen
Bias logistic regr.
Bias
True correlation
True partial cor.
True effect size
E
Power cor
Power pcor
Power lm phen ~ met
Power lm met ~ phen
Power logistic regr.
Power
True correlation
True partial cor.
True effect size

Data: Unsystematic missingness; Imputation method: MITS−R

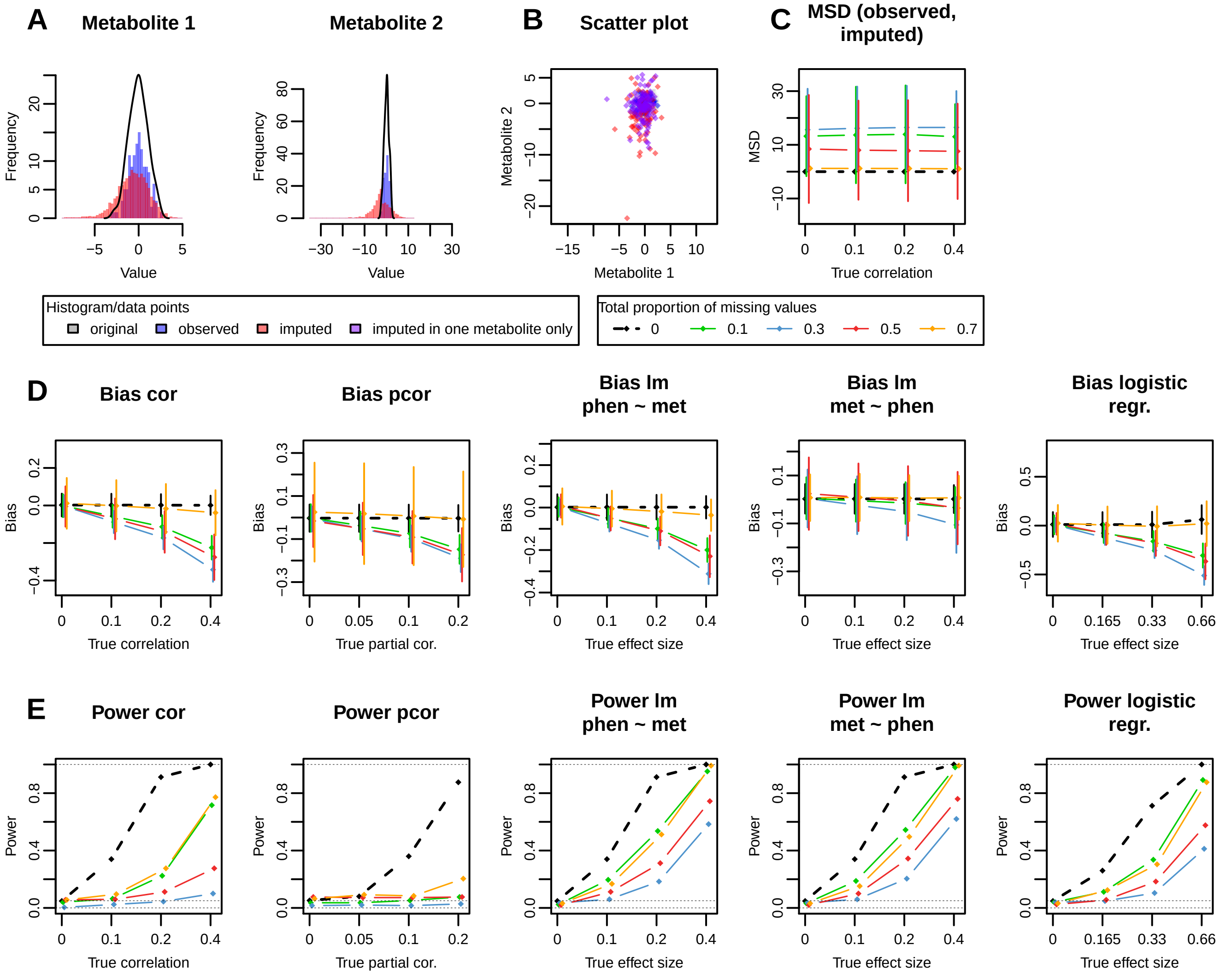

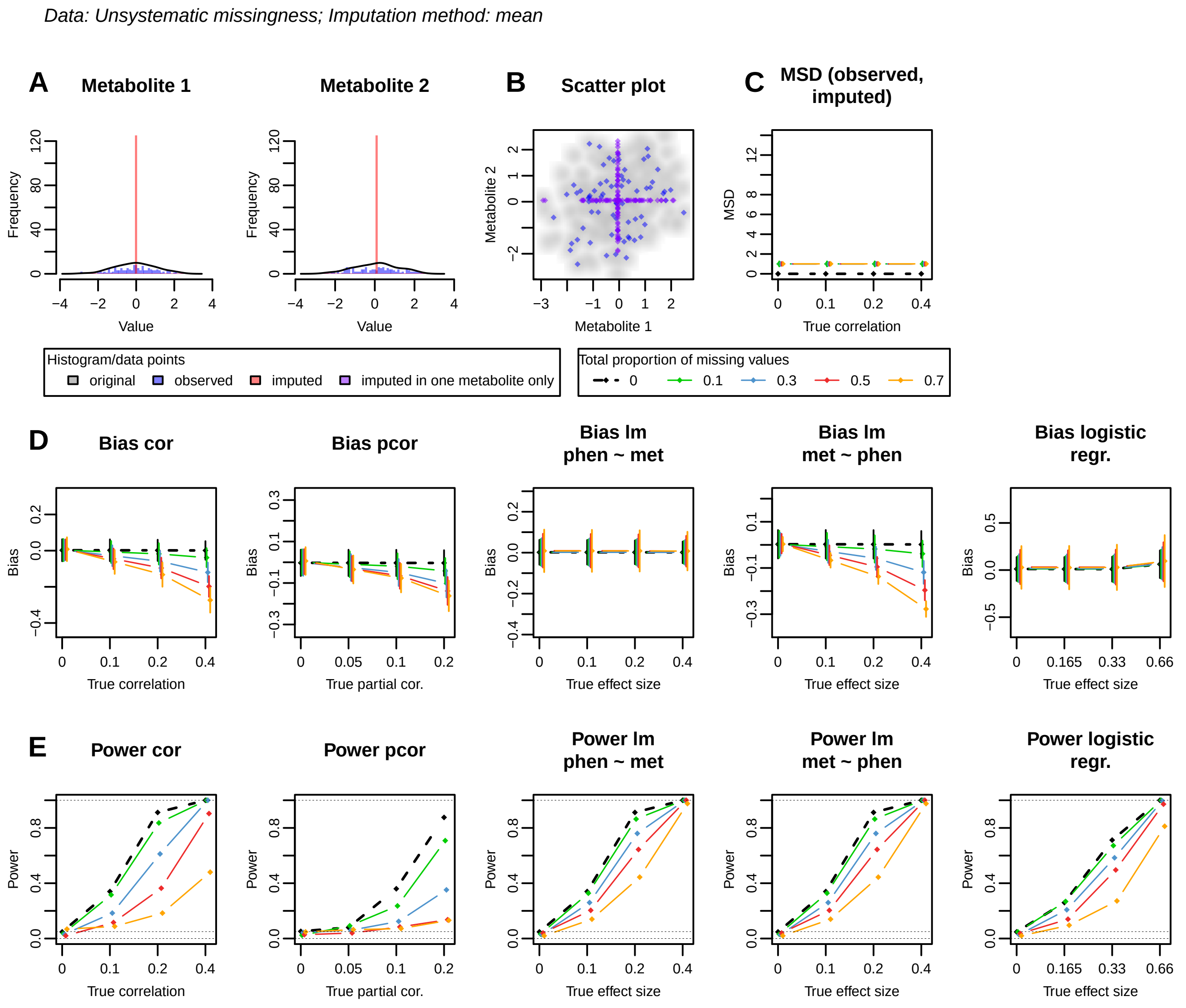
Data: Unsystematic missingness; Imputation method: mean
A
Metabolite 1
Metabolite 2
Frequency
Value
B
Scatter plot
Metabolite 1
Metabolite 2
C
MSD (observed, imputed)
MSD
True correlation
Histogram/data points
original
observed
imputed
imputed in one metabolite only
Total proportion of missing values
0
0.1
0.3
0.5
0.7
D
Bias cor
Bias pcor
Bias lm phen ~ met
Bias lm met ~ phen
Bias logistic regr.
Bias
True correlation
True partial cor.
True effect size
E
Power cor
Power pcor
Power lm phen ~ met
Power lm met ~ phen
Power logistic regr.
Power
True correlation
True partial cor.
True effect size

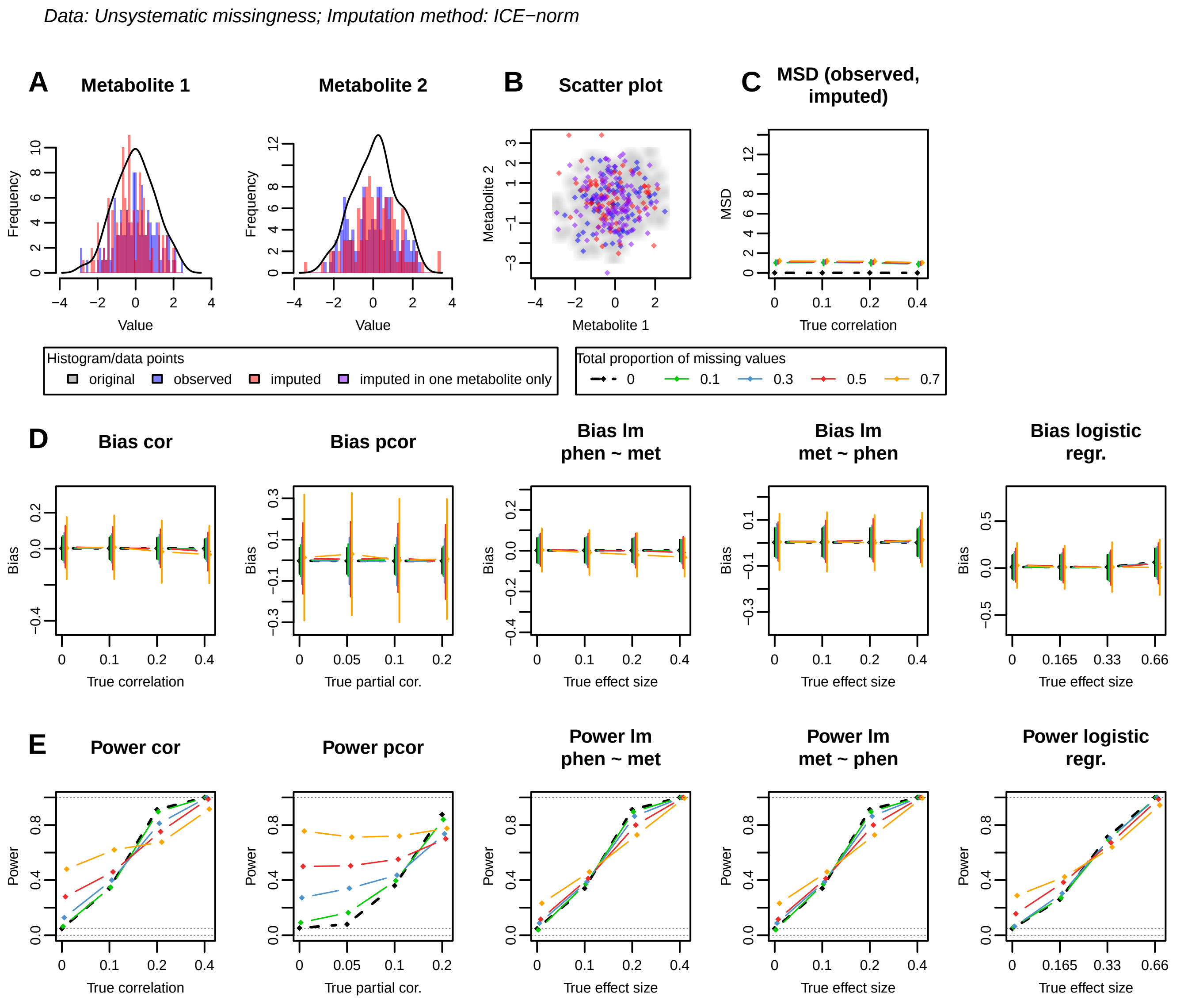
Data: Unsystematic missingness; Imputation method: ICE−norm
A
Metabolite 1
Metabolite 2
Frequency
Value
B
Scatter plot
Metabolite 2
Metabolite 1
C
MSD (observed, imputed)
MSD
True correlation
Histogram/data points
original
observed
imputed
imputed in one metabolite only
Total proportion of missing values
0
0.1
0.3
0.5
0.7
D
Bias cor
Bias pcor
Bias lm phen ~ met
Bias lm met ~ phen
Bias logistic regr.
Bias
True correlation
True partial cor.
True effect size
E
Power cor
Power pcor
Power lm phen ~ met
Power lm met ~ phen
Power logistic regr.
Power
True correlation
True partial cor.
True effect size

Data: Unsystematic missingness; Imputation method: ICE−pmm

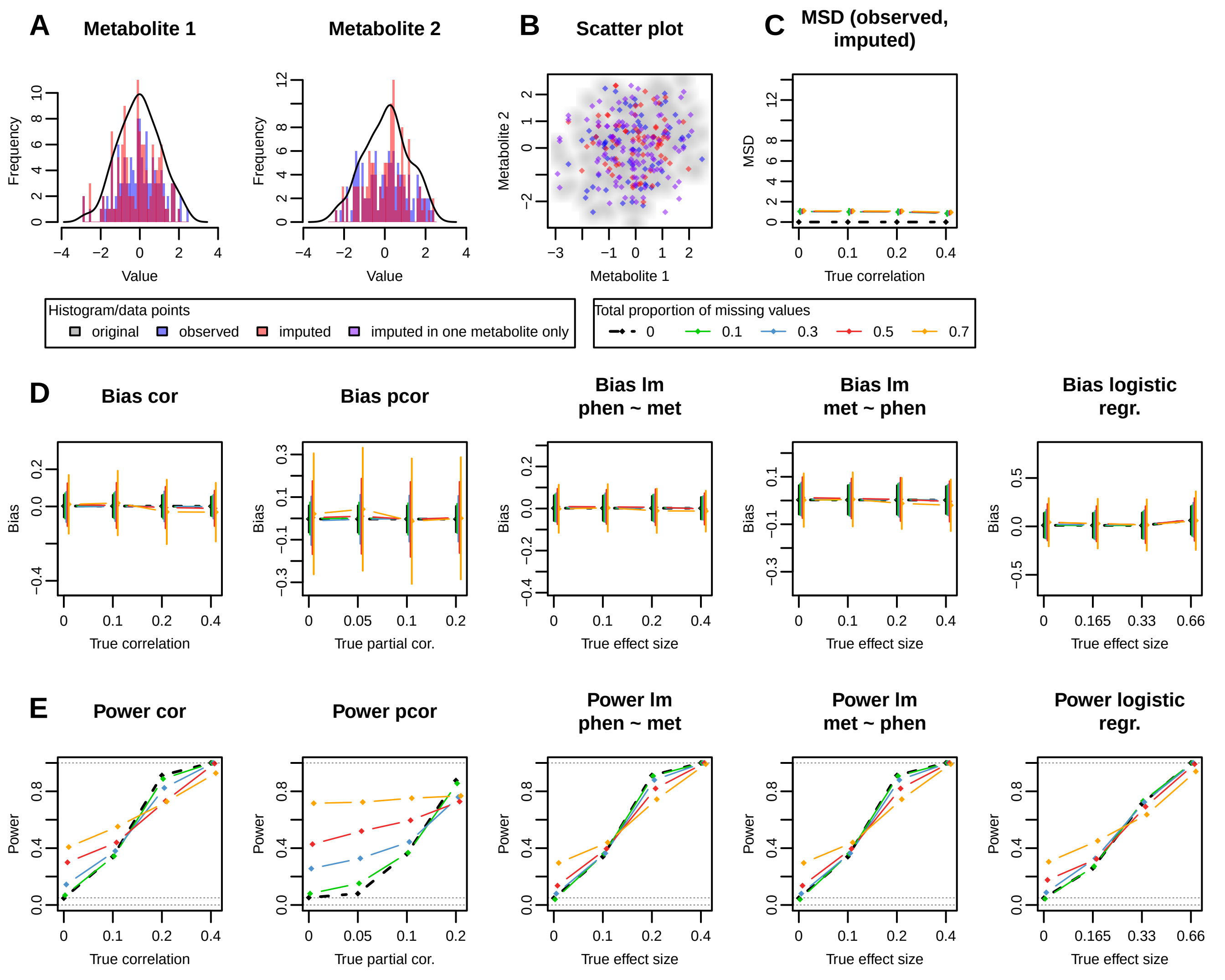

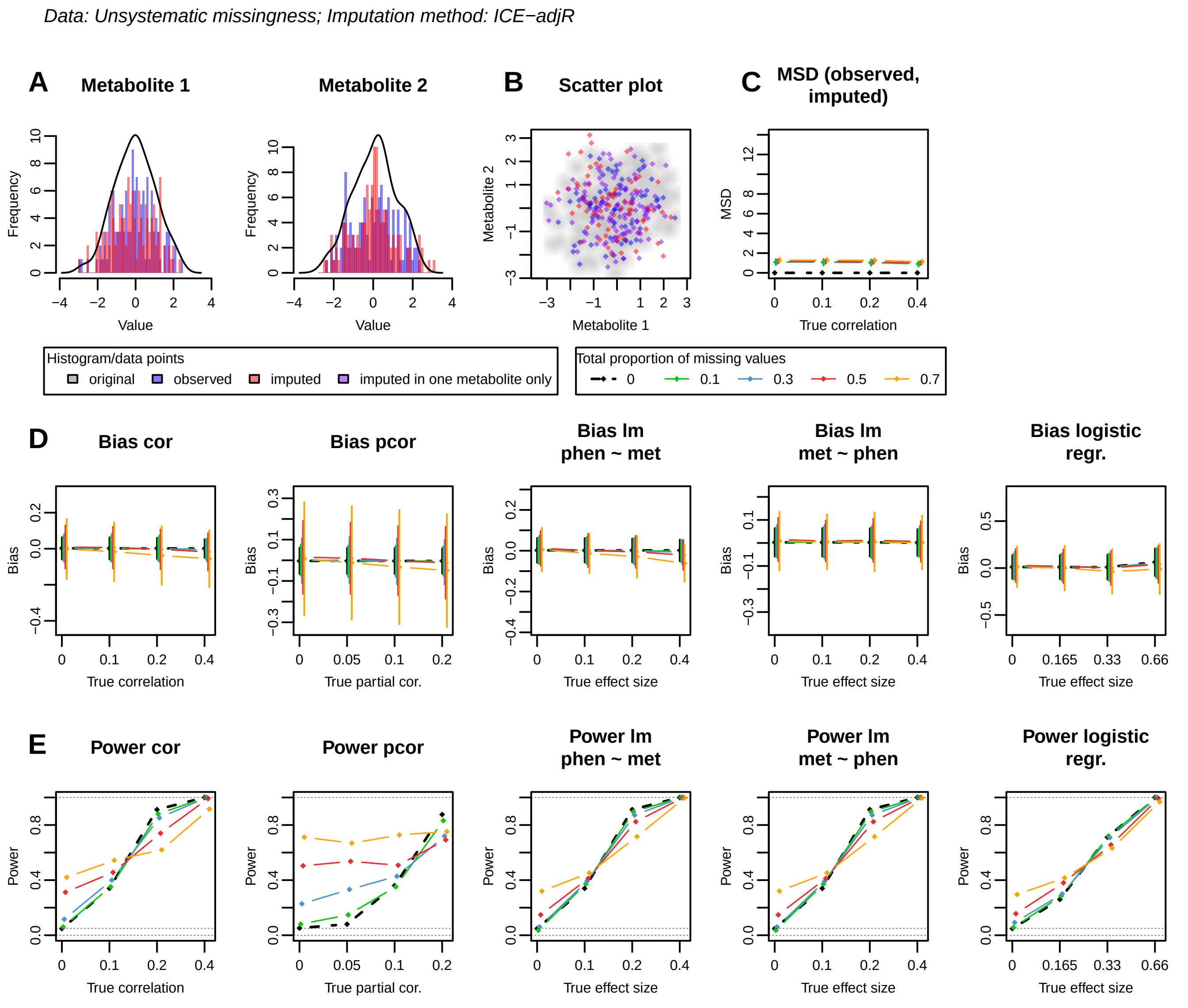

Data: Unsystematic missingness; Imputation method: ICE−adjR
A
Metabolite 1
Metabolite 2
B
Scatter plot
C
MSD (observed, imputed)
Frequency
Value
Metabolite 2
Metabolite 1
MSD
True correlation
Histogram/data points
original
observed
imputed
imputed in one metabolite only
Total proportion of missing values
0
0.1
0.3
0.5
0.7
D
Bias cor
Bias pcor
Bias lm phen ~ met
Bias lm met ~ phen
Bias logistic regr.
Bias
True correlation
True partial cor.
True effect size
E
Power cor
Power pcor
Power lm phen ~ met
Power lm met ~ phen
Power logistic regr.
Power

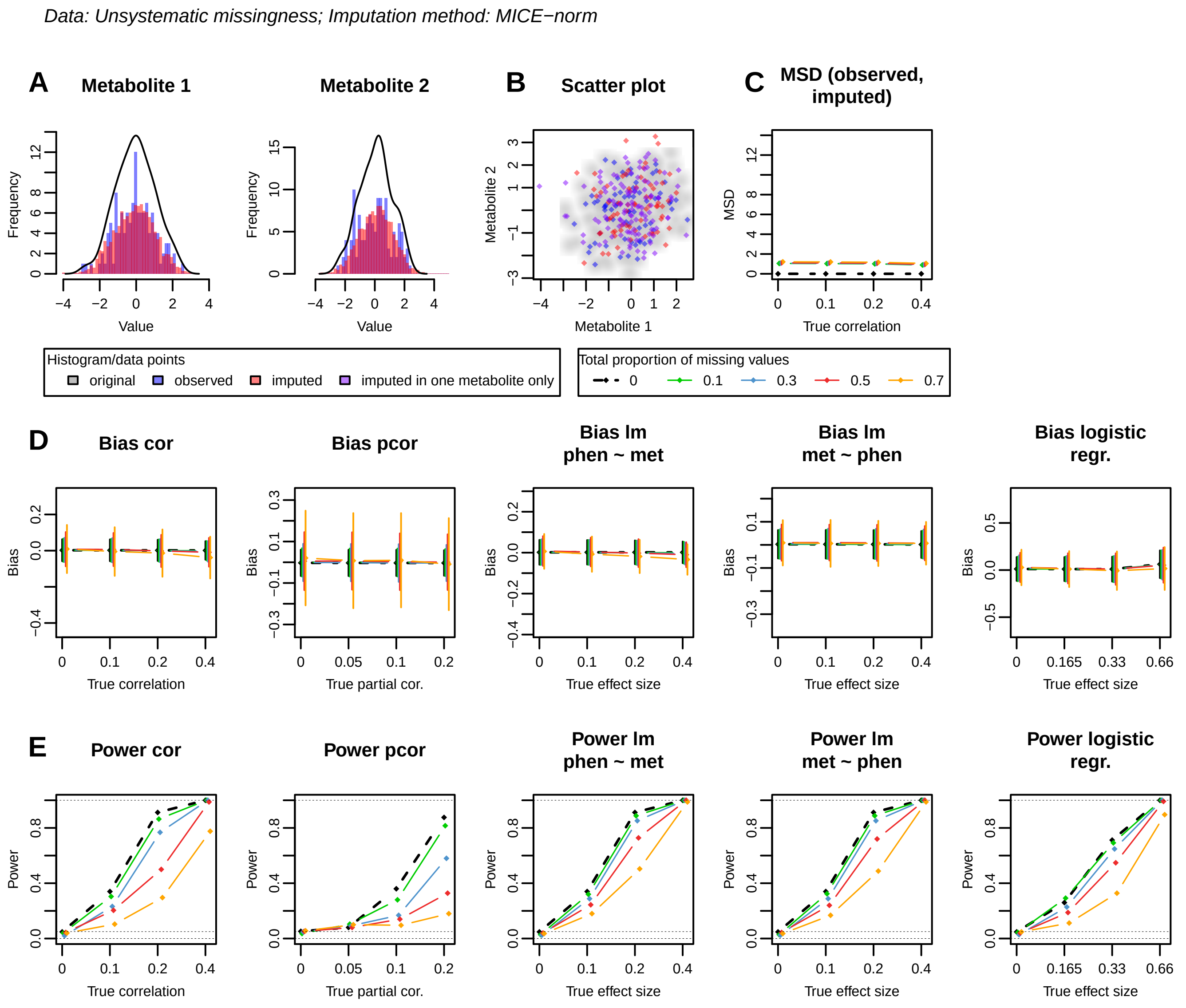

Data: Unsystematic missingness; Imputation method: MICE−norm
A
Metabolite 1
Metabolite 2
B
Scatter plot
C
MSD (observed, imputed)
Frequency
Value
Metabolite 2
Metabolite 1
MSD
True correlation
Histogram/data points
original
observed
imputed
imputed in one metabolite only
Total proportion of missing values
0
0.1
0.3
0.5
0.7
D
Bias cor
Bias pcor
Bias lm phen ~ met
Bias lm met ~ phen
Bias logistic regr.
Bias
True correlation
True partial cor.
True effect size
E
Power cor
Power pcor
Power lm phen ~ met
Power lm met ~ phen
Power logistic regr.
Power

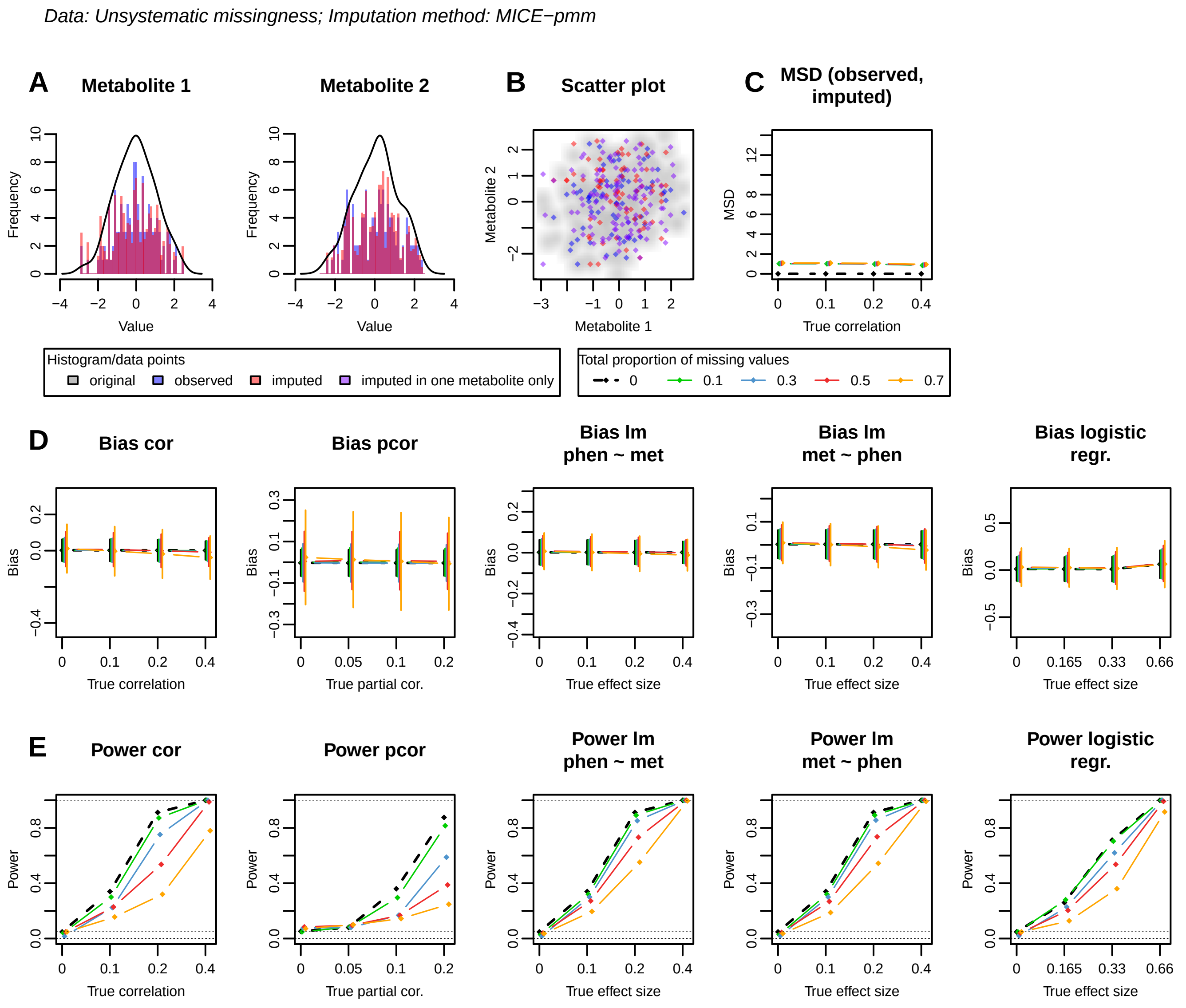

Data: Unsystematic missingness; Imputation method: MICE−pmm
A
Metabolite 1
Metabolite 2
Frequency
Value
B
Scatter plot
Metabolite 1
Metabolite 2
C
MSD (observed, imputed)
MSD
True correlation
Histogram/data points
original
observed
imputed
imputed in one metabolite only
Total proportion of missing values
0
0.1
0.3
0.5
0.7
D
Bias cor
Bias pcor
Bias lm phen ~ met
Bias lm met ~ phen
Bias logistic regr.
Bias
True correlation
True partial cor.
True effect size
E
Power cor
Power pcor
Power lm phen ~ met
Power lm met ~ phen
Power logistic regr.
Power
True correlation
True partial cor.
True effect size

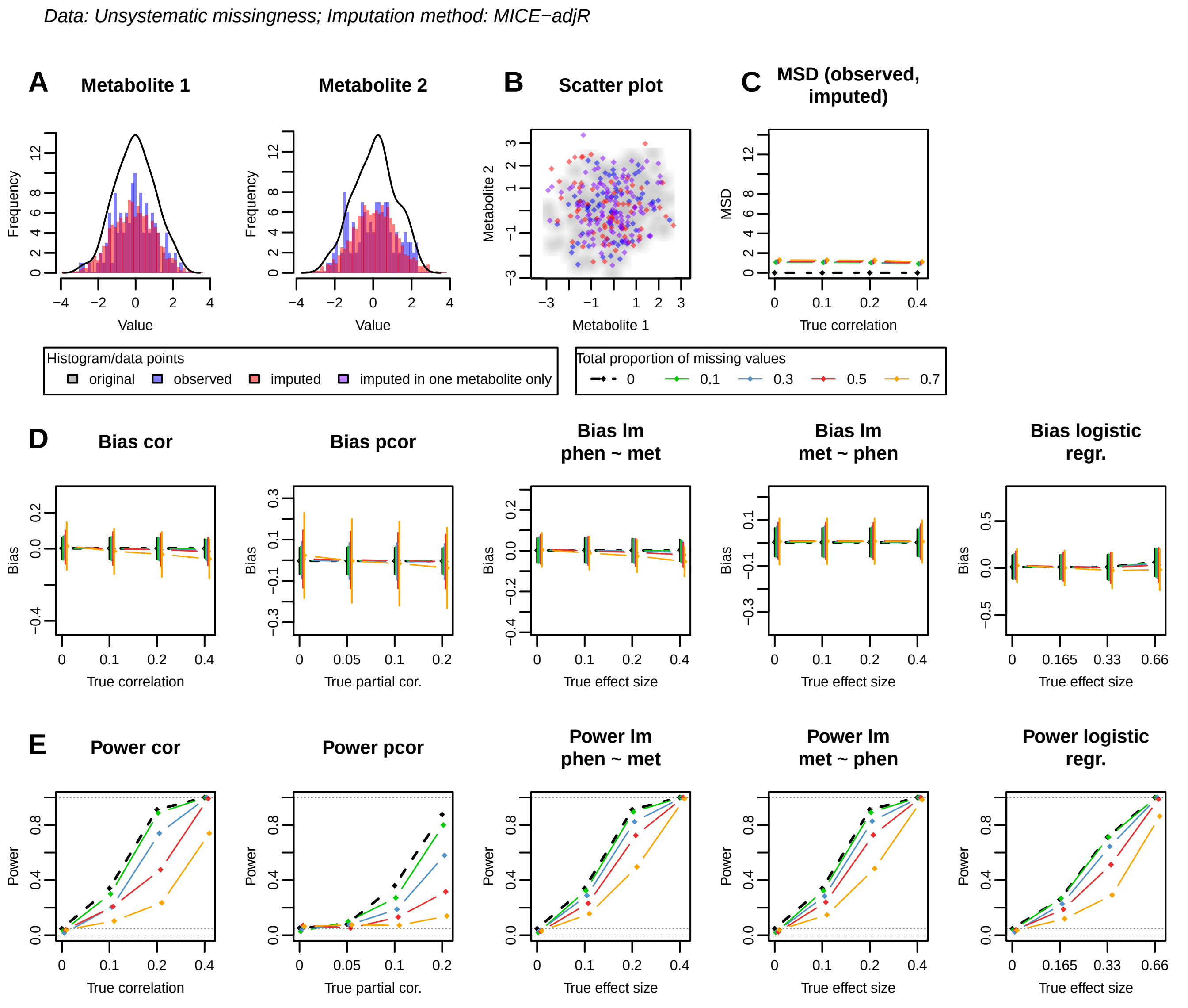
Data: Unsystematic missingness; Imputation method: MICE−adjR
A
Metabolite 1
Metabolite 2
B
Scatter plot
C
MSD (observed, imputed)
Frequency
Value
Metabolite 2
Metabolite 1
MSD
True correlation
Histogram/data points
original
observed
imputed
imputed in one metabolite only
Total proportion of missing values
0
0.1
0.3
0.5
0.7
D
Bias cor
Bias pcor
Bias lm phen ~ met
Bias lm met ~ phen
Bias logistic regr.
Bias
True correlation
True partial cor.
True effect size
E
Power cor
Power pcor
Power lm phen ~ met
Power lm met ~ phen
Power logistic regr.
Power
True correlation
True partial cor.
True effect size

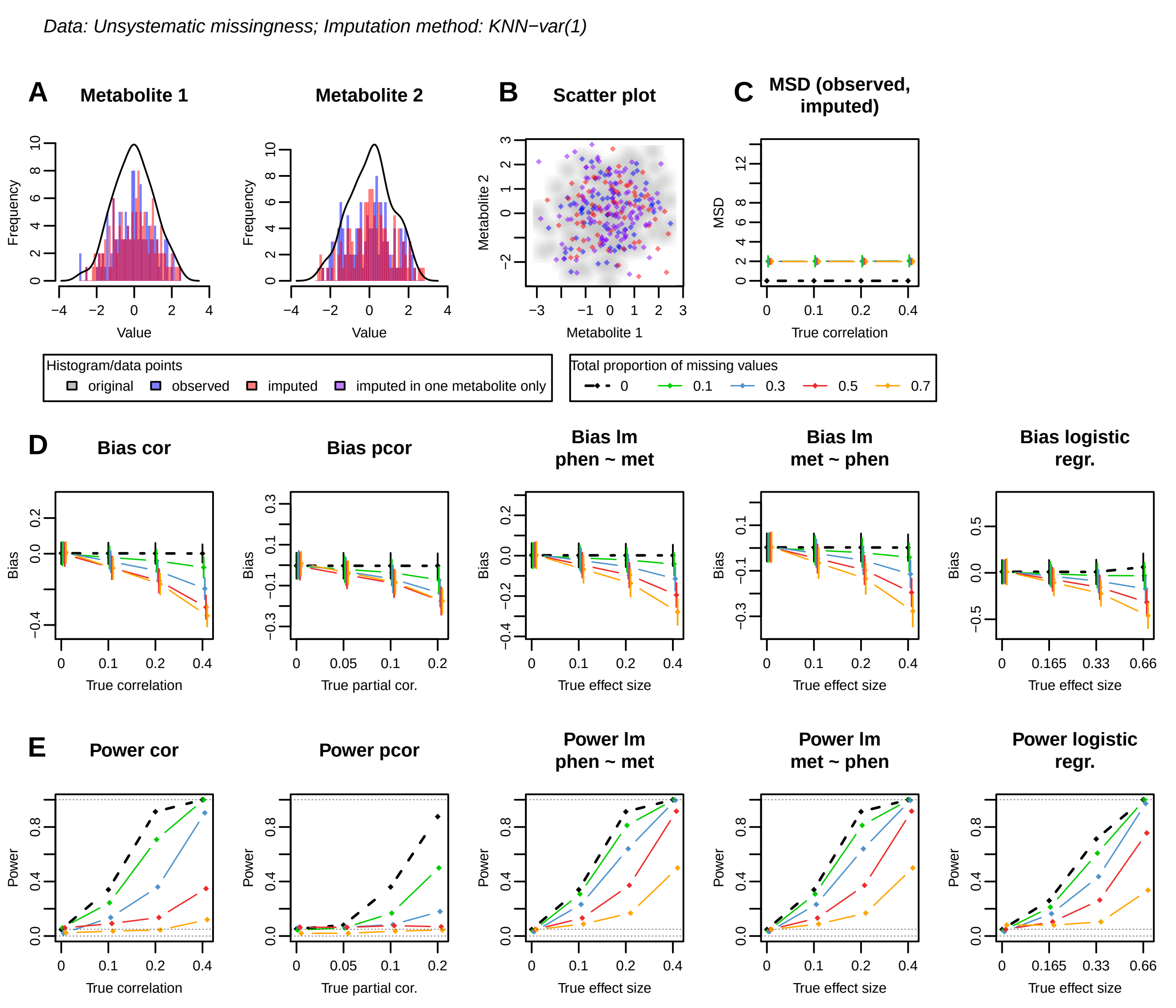

Data: Unsystematic missingness; Imputation method: KNN−var(1)
A
Metabolite 1
Metabolite 2
Frequency
Value
B
Scatter plot
Metabolite 2
Metabolite 1
C
MSD (observed, imputed)
MSD
True correlation
Histogram/data points
original
observed
imputed
imputed in one metabolite only
Total proportion of missing values
0
0.1
0.3
0.5
0.7
D
Bias cor
Bias pcor
Bias lm phen ~ met
Bias lm met ~ phen
Bias logistic regr.
Bias
True correlation
True partial cor.
True effect size
E
Power cor
Power pcor
Power lm phen ~ met
Power lm met ~ phen
Power logistic regr.
Power
True correlation
True partial cor.
True effect size

Data: Unsystematic missingness; Imputation method: KNN−var(3)

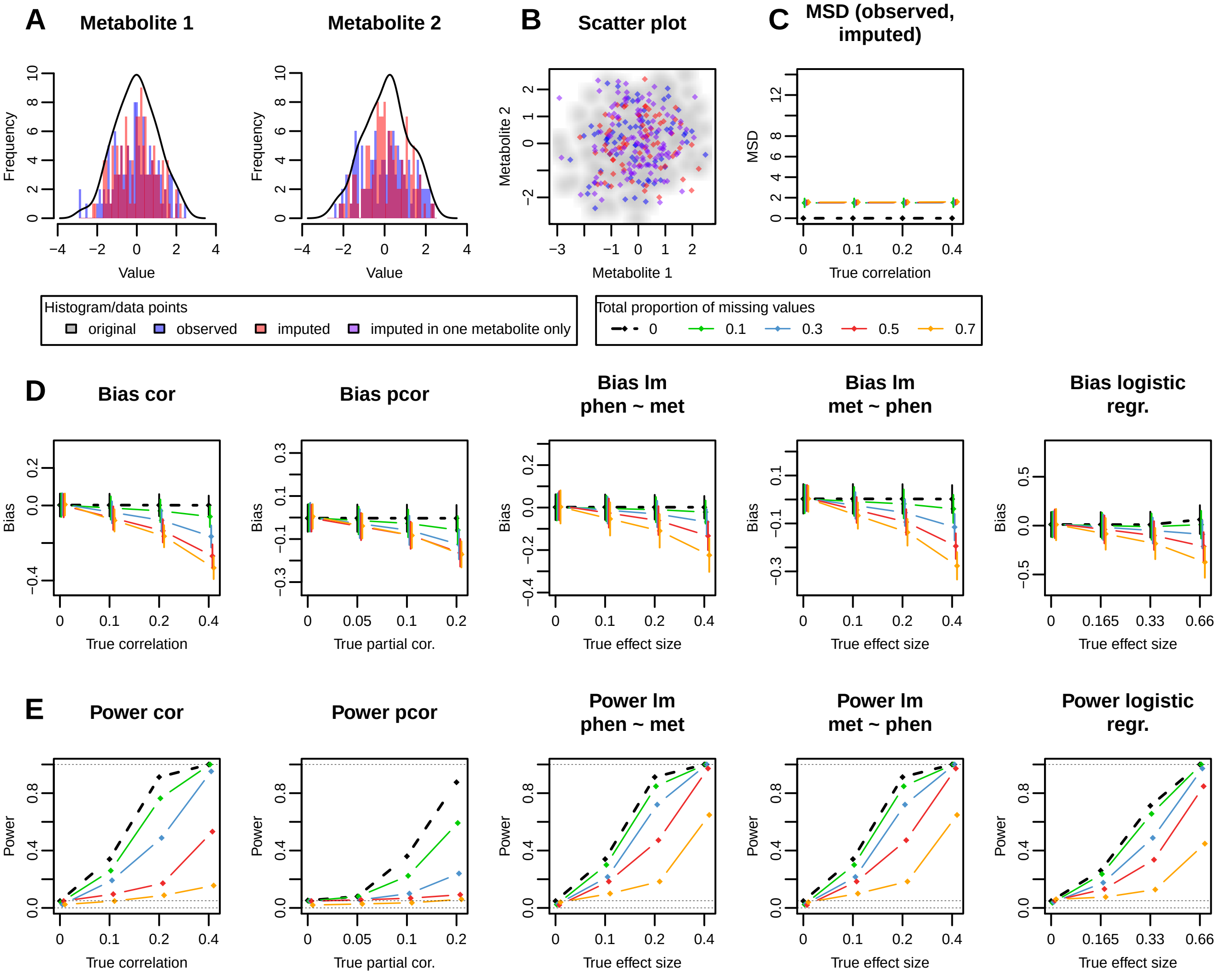

Data: Unsystematic missingness; Imputation method: KNN−var(5)

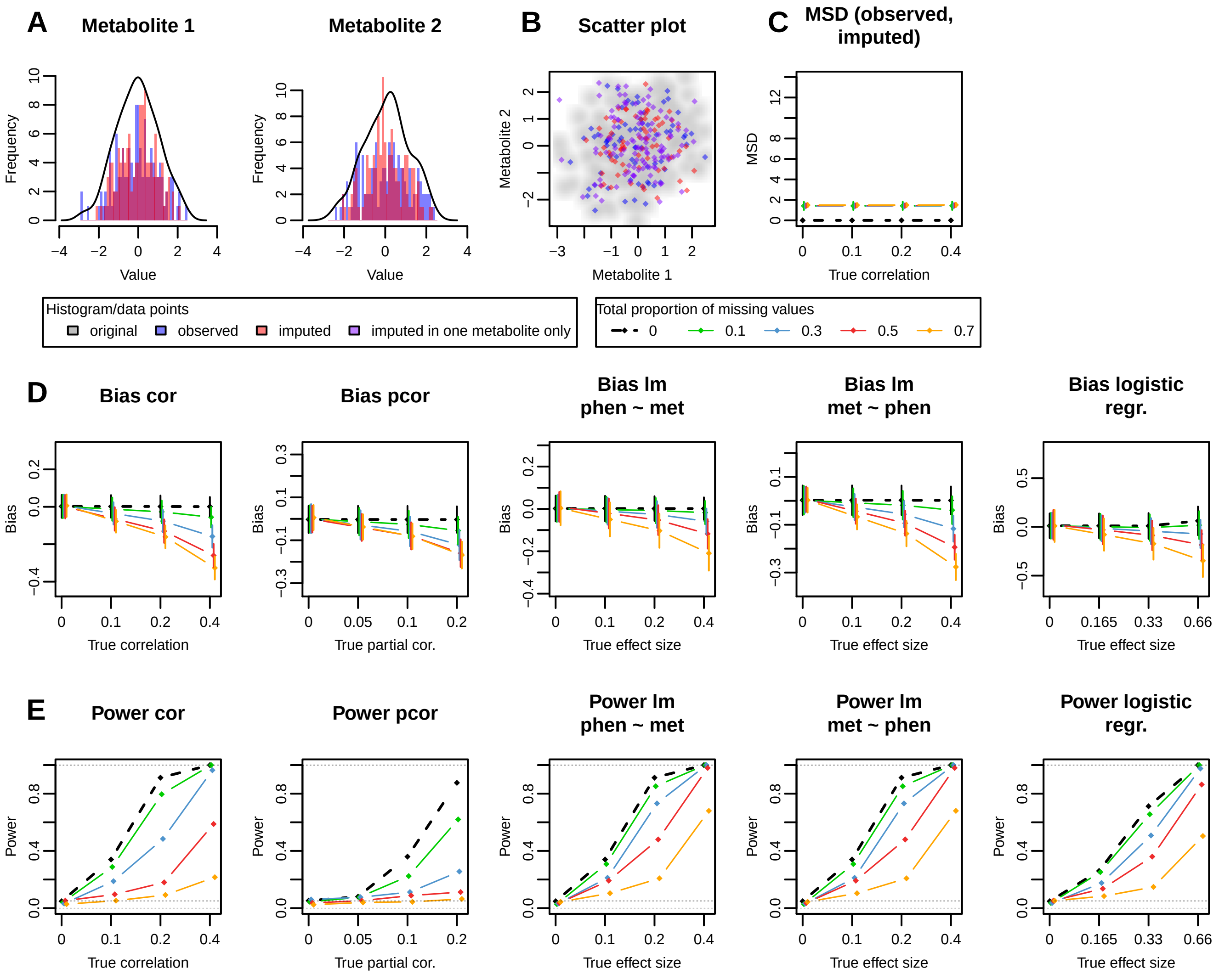

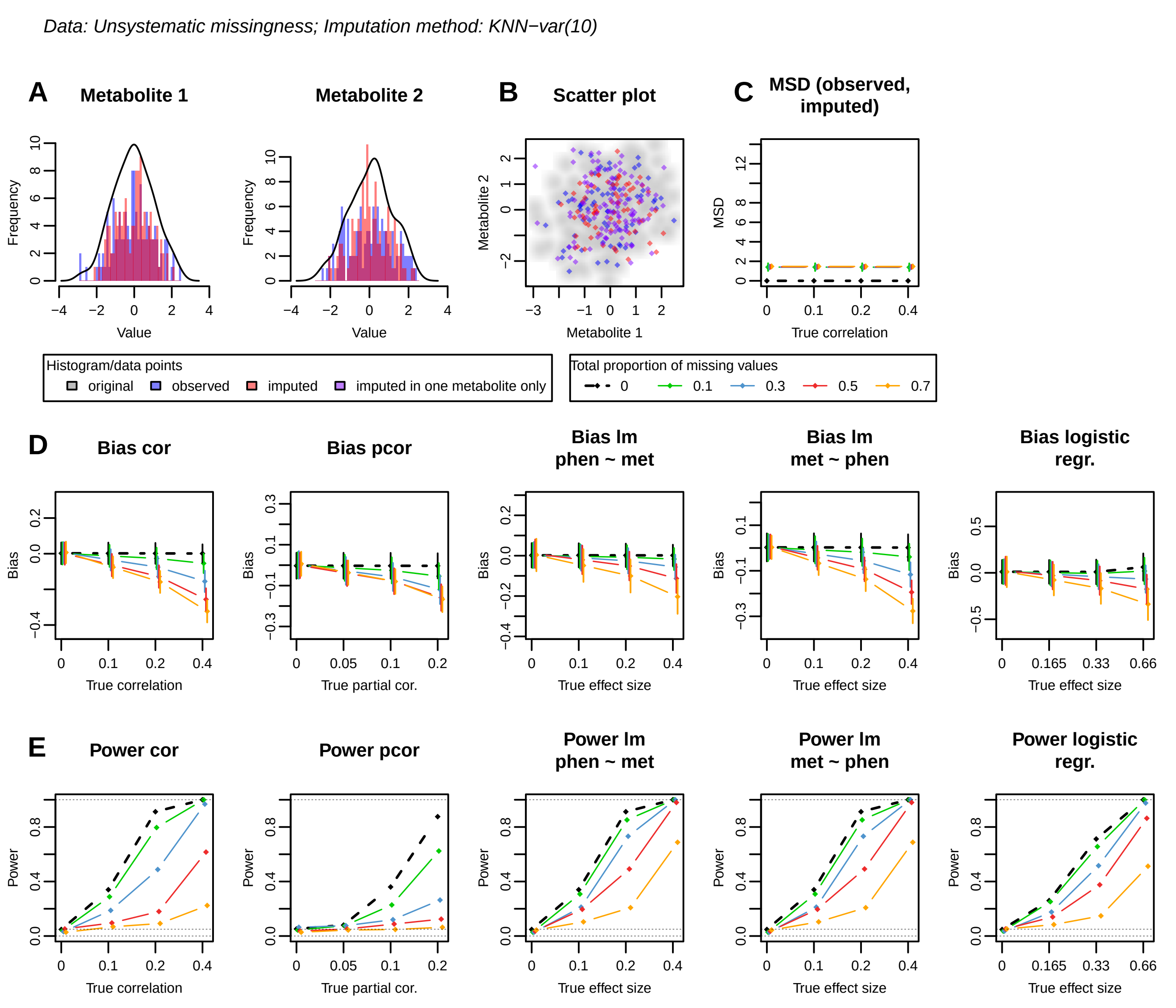
Data: Unsystematic missingness; Imputation method: KNN−var(10)
A
Metabolite 1
Metabolite 2
Frequency
Value
B
Scatter plot
Metabolite 1
Metabolite 2
C
MSD (observed, imputed)
MSD
True correlation
Histogram/data points
original
observed
imputed
imputed in one metabolite only
Total proportion of missing values
0
0.1
0.3
0.5
0.7
D
Bias cor
Bias pcor
Bias lm phen ~ met
Bias lm met ~ phen
Bias logistic regr.
Bias
True correlation
True partial cor.
True effect size
E
Power cor
Power pcor
Power lm phen ~ met
Power lm met ~ phen
Power logistic regr.
Power
True correlation
True partial cor.
True effect size

Data: Unsystematic missingness; Imputation method: KNN−var(20)

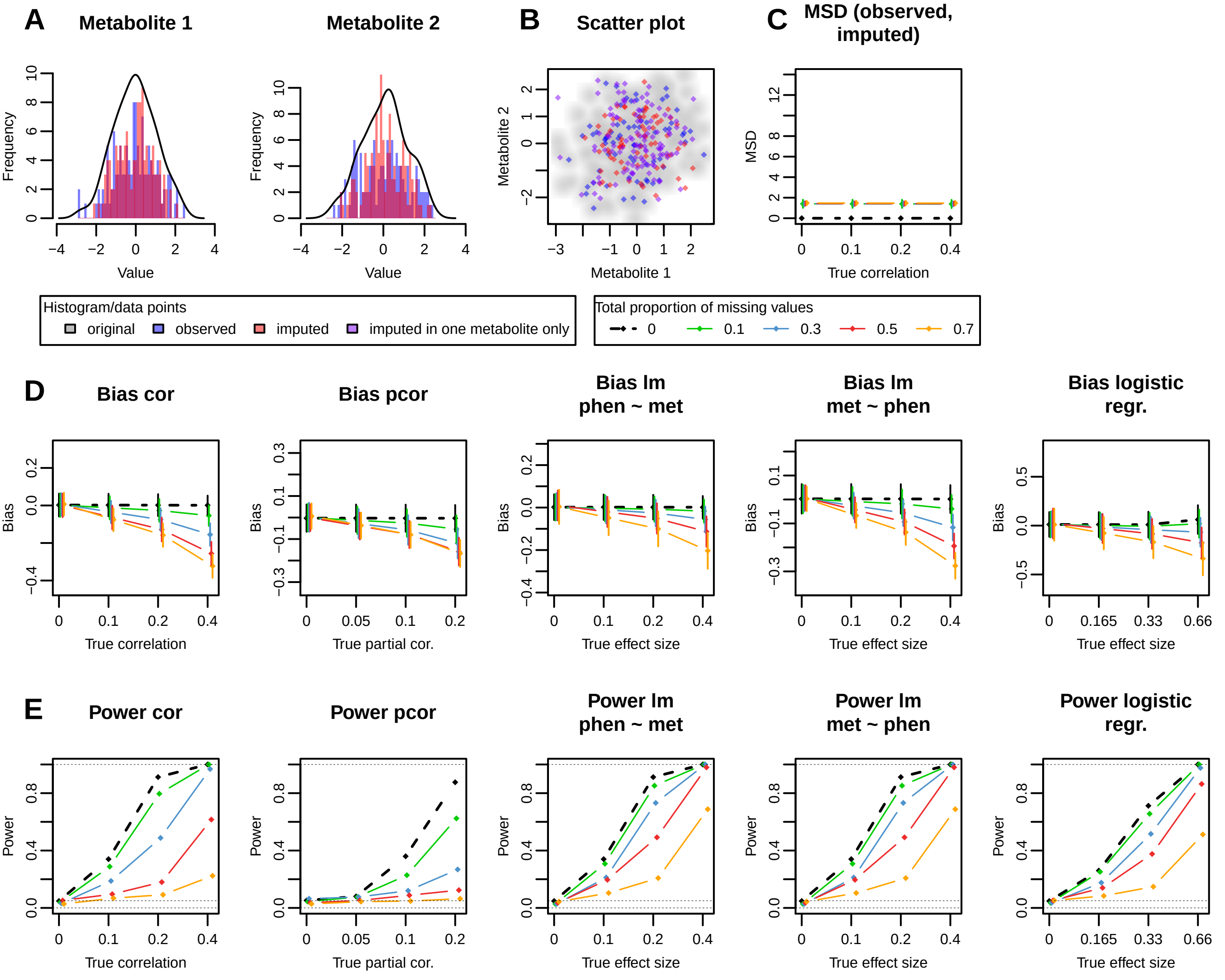

Data: Unsystematic missingness; Imputation method: KNN−obs(1)

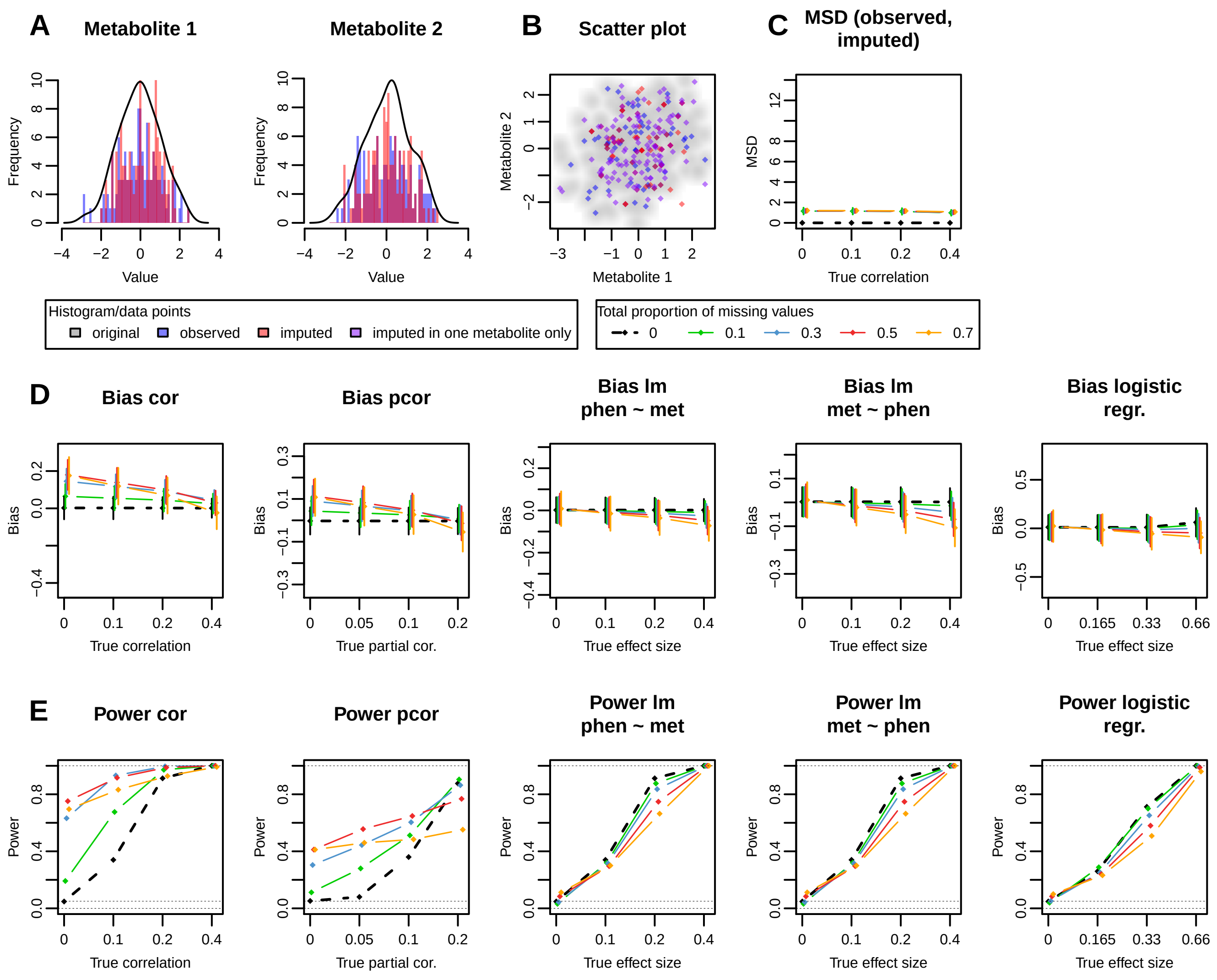

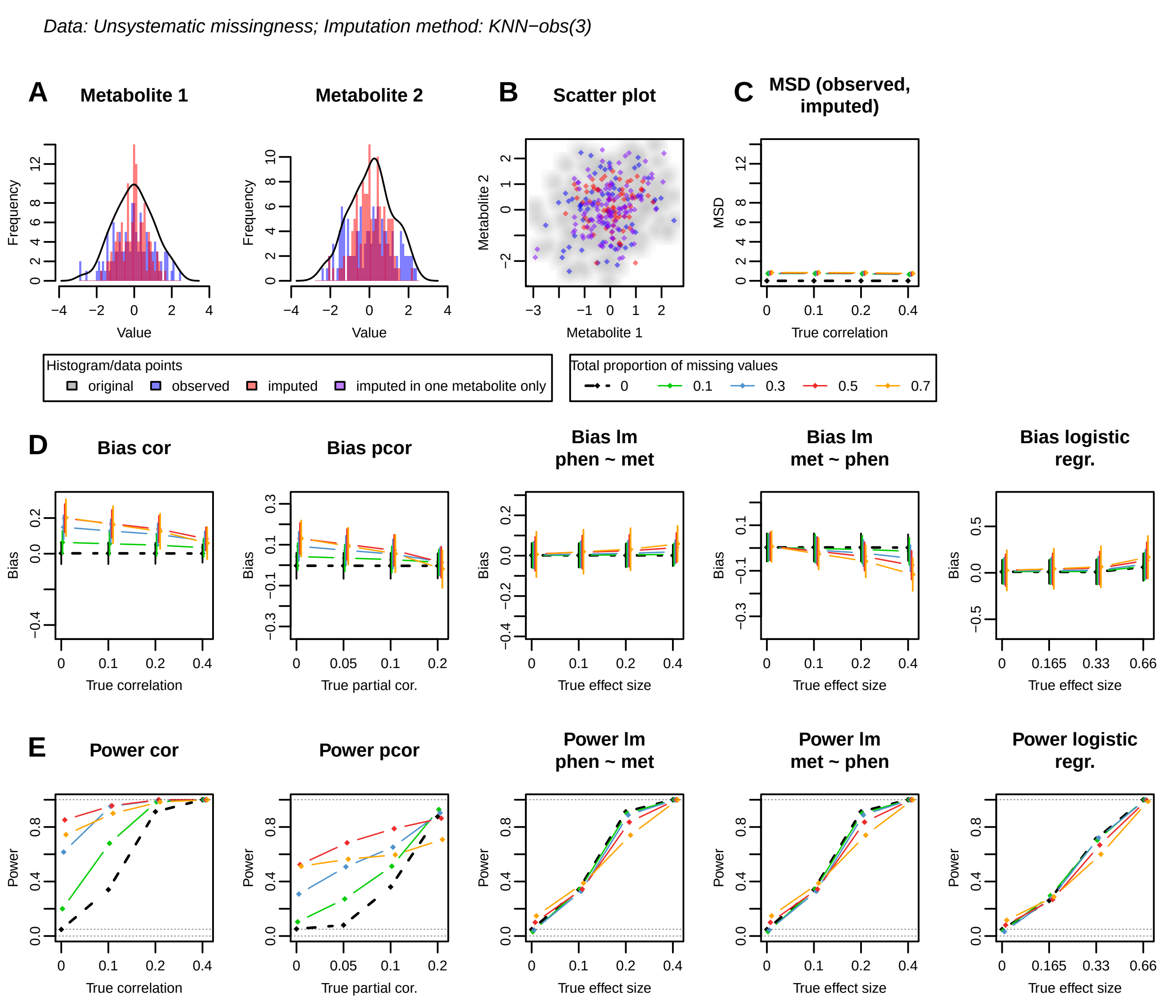
Data: Unsystematic missingness; Imputation method: KNN−obs(3)
A
Metabolite 1
Metabolite 2
B
Scatter plot
C
MSD (observed, imputed)
Frequency
Value
Metabolite 2
Metabolite 1
MSD
True correlation
Histogram/data points
original
observed
imputed
imputed in one metabolite only
Total proportion of missing values
0
0.1
0.3
0.5
0.7
D
Bias cor
Bias pcor
Bias lm phen ~ met
Bias lm met ~ phen
Bias logistic regr.
Bias
True correlation
True partial cor.
True effect size
E
Power cor
Power pcor
Power lm phen ~ met
Power lm met ~ phen
Power logistic regr.
Power

Data: Unsystematic missingness; Imputation method: KNN−obs(5)

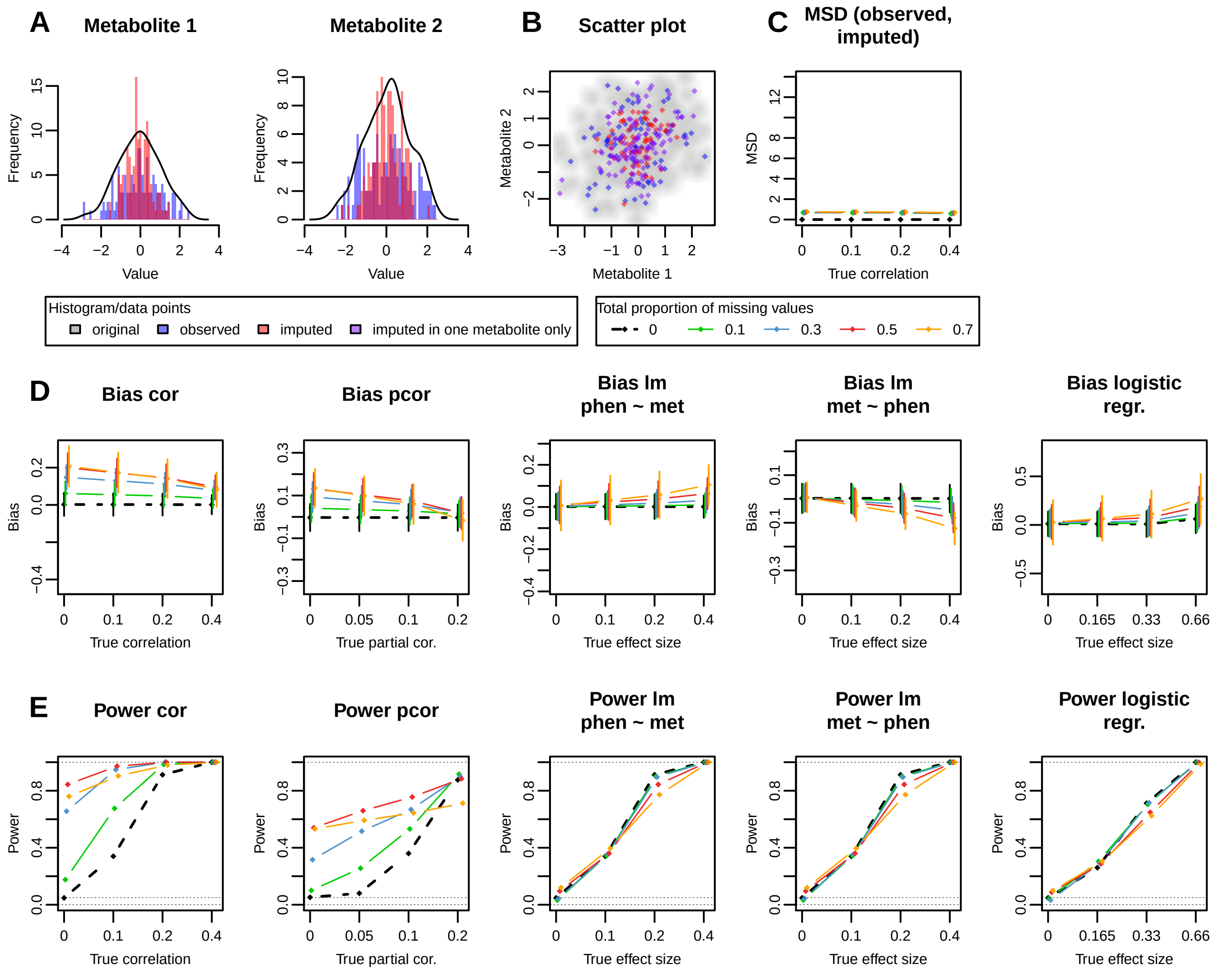

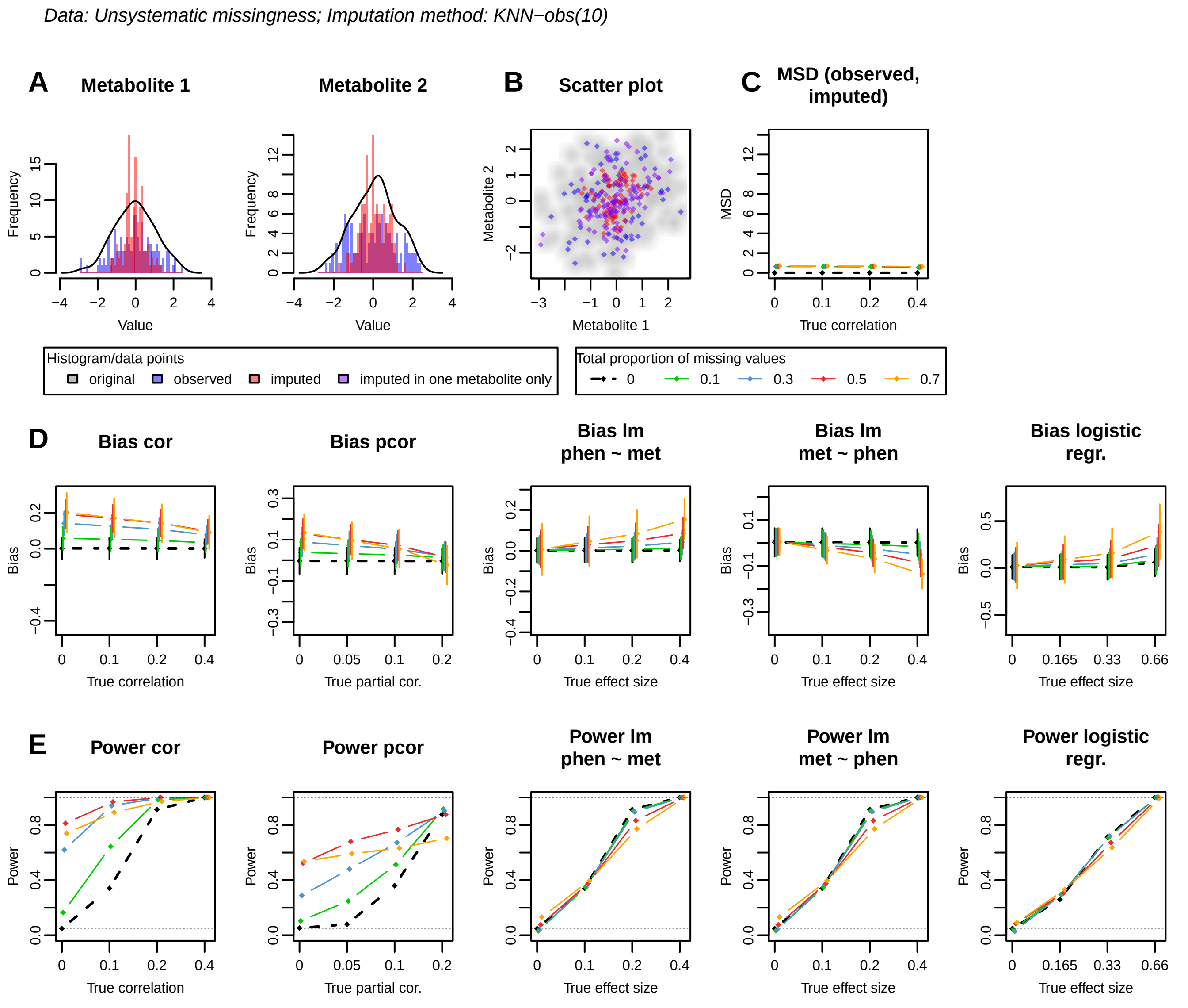

Data: Unsystematic missingness; Imputation method: KNN−obs(10)
A
Metabolite 1
Metabolite 2
B
Scatter plot
C
MSD (observed, imputed)
Frequency
Value
Metabolite 2
Metabolite 1
MSD
True correlation
Histogram/data points
original
observed
imputed
imputed in one metabolite only
Total proportion of missing values
0
0.1
0.3
0.5
0.7
D
Bias cor
Bias pcor
Bias lm phen ~ met
Bias lm met ~ phen
Bias logistic regr.
Bias
True correlation
True partial cor.
True effect size
E
Power cor
Power pcor
Power lm phen ~ met
Power lm met ~ phen
Power logistic regr.
Power

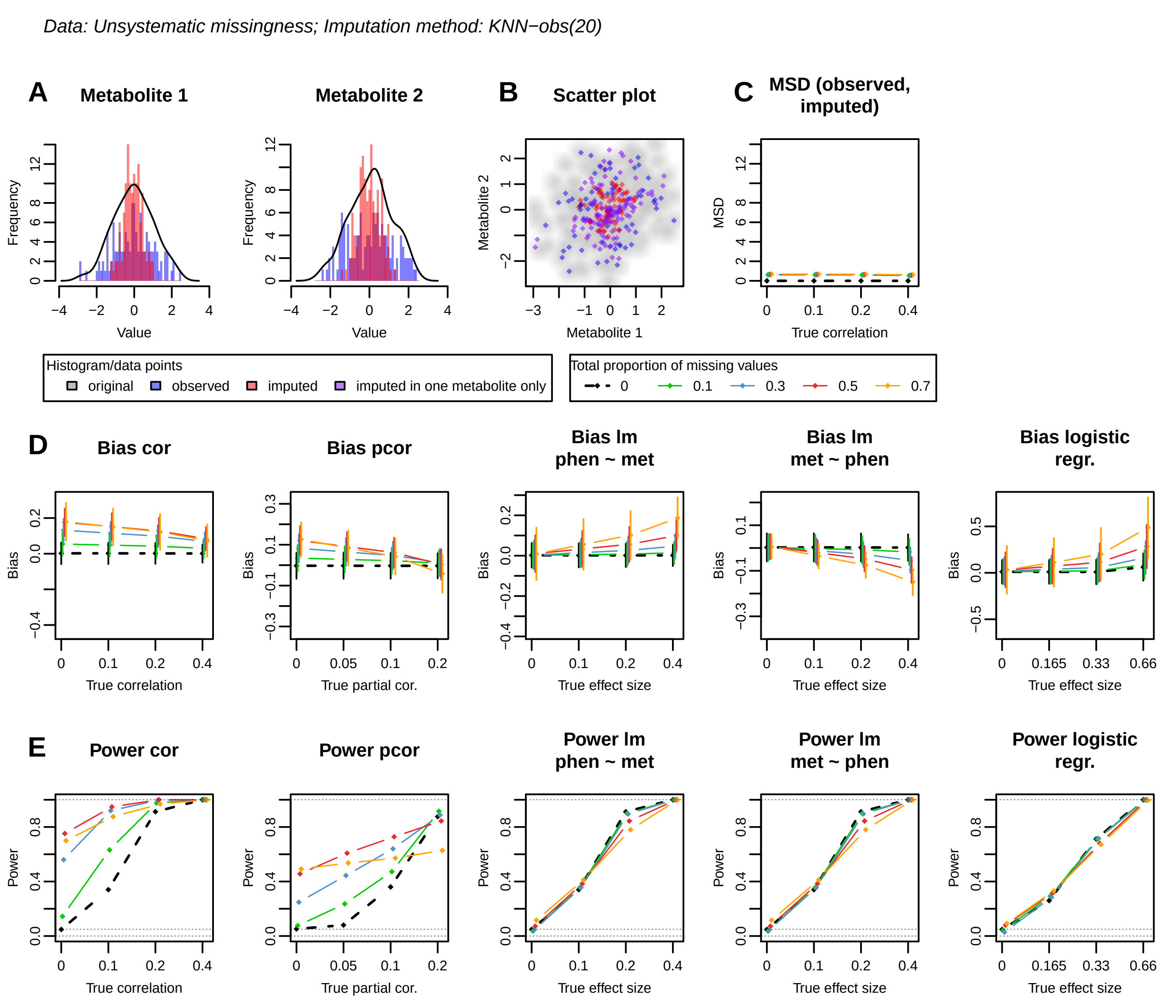

Data: Unsystematic missingness; Imputation method: KNN−obs(20)
A
Metabolite 1
Metabolite 2
Frequency
Value
B
Scatter plot
Metabolite 2
Metabolite 1
C
MSD (observed, imputed)
MSD
True correlation
Histogram/data points
original
observed
imputed
imputed in one metabolite only
Total proportion of missing values
0
0.1
0.3
0.5
0.7
D
Bias cor
Bias pcor
Bias lm phen ~ met
Bias lm met ~ phen
Bias logistic regr.
Bias
True correlation
True partial cor.
True effect size
E
Power cor
Power pcor
Power lm phen ~ met
Power lm met ~ phen
Power logistic regr.
Power
True correlation
True partial cor.
True effect size

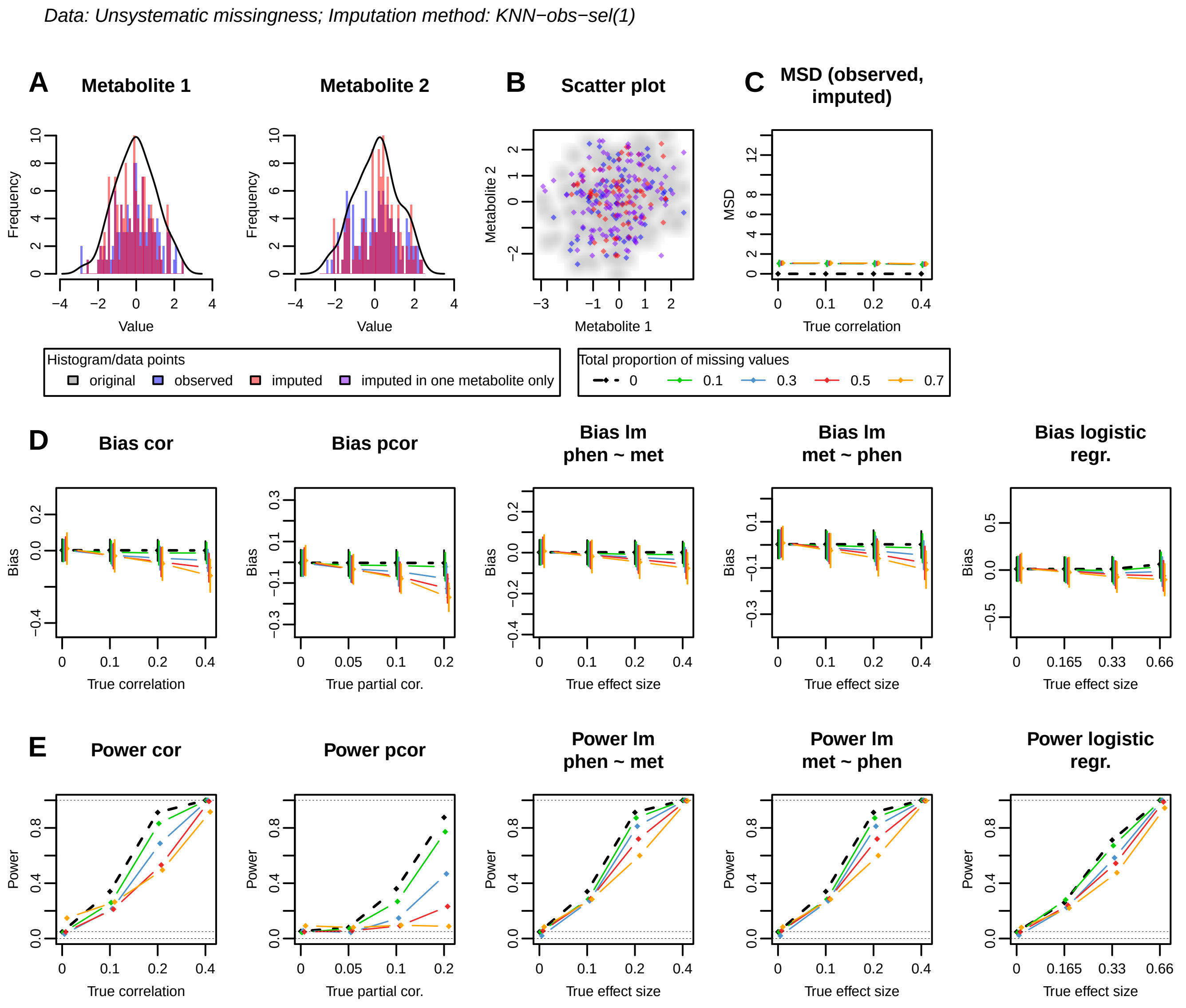

Data: Unsystematic missingness; Imputation method: KNN−obs−sel(1)
A
Metabolite 1
Metabolite 2
Frequency
Value
B
Scatter plot
Metabolite 1
Metabolite 2
C
MSD (observed, imputed)
MSD
True correlation
Histogram/data points
original
observed
imputed
imputed in one metabolite only
Total proportion of missing values
0
0.1
0.3
0.5
0.7
D
Bias cor
Bias pcor
Bias lm phen ~ met
Bias lm met ~ phen
Bias logistic regr.
Bias
True correlation
True partial cor.
True effect size
E
Power cor
Power pcor
Power lm phen ~ met
Power lm met ~ phen
Power logistic regr.
Power
True correlation
True partial cor.
True effect size

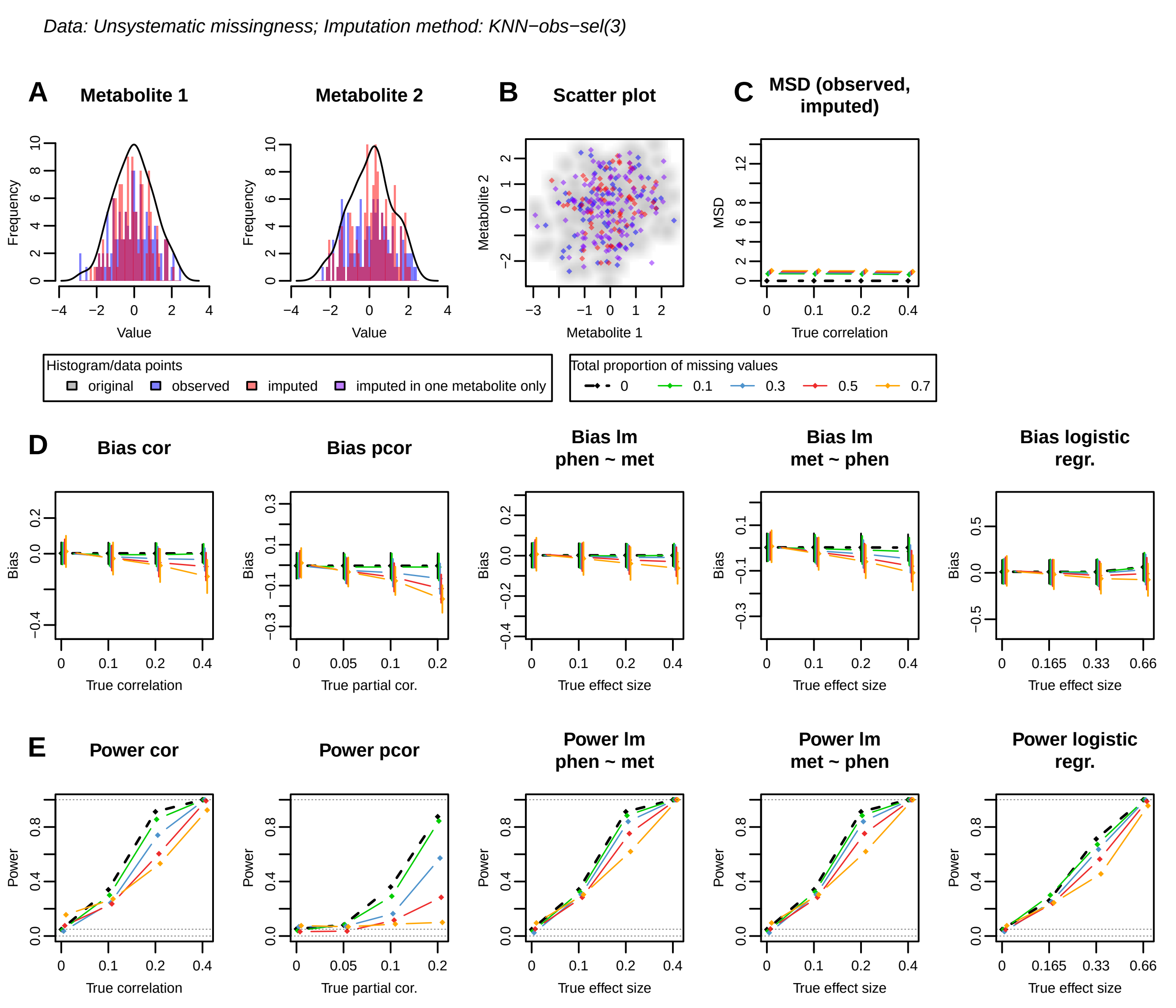
Data: Unsystematic missingness; Imputation method: KNN−obs−sel(3)
A
Metabolite 1
Metabolite 2
Frequency
Value
B
Scatter plot
Metabolite 1
Metabolite 2
C
MSD (observed, imputed)
MSD
True correlation
Histogram/data points
original
observed
imputed
imputed in one metabolite only
Total proportion of missing values
0
0.1
0.3
0.5
0.7
D
Bias cor
Bias pcor
Bias lm phen ~ met
Bias lm met ~ phen
Bias logistic regr.
Bias
True correlation
True partial cor.
True effect size
E
Power cor
Power pcor
Power lm phen ~ met
Power lm met ~ phen
Power logistic regr.
Power
True correlation
True partial cor.
True effect size

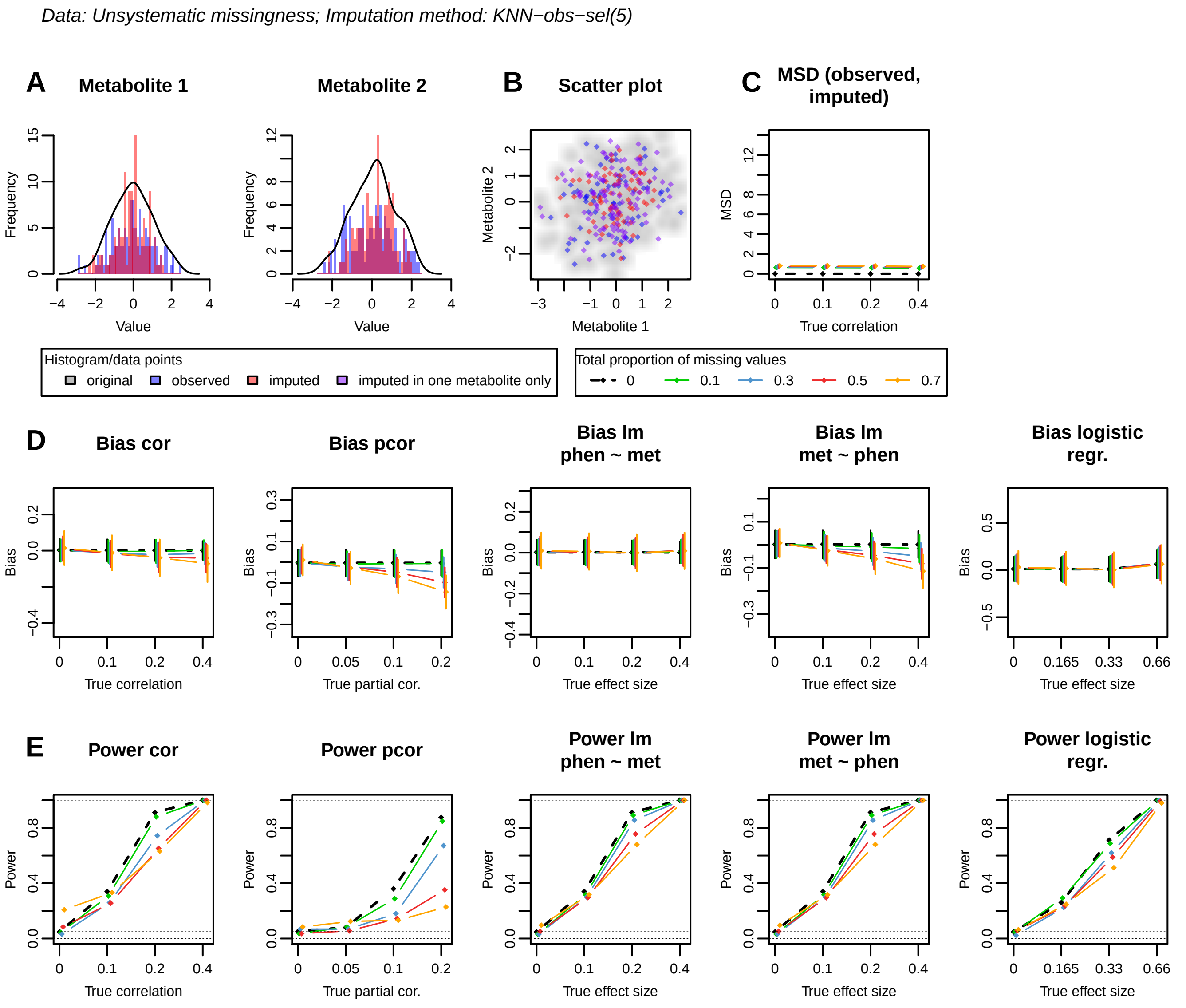
Data: Unsystematic missingness; Imputation method: KNN−obs−sel(5)
A
Metabolite 1
Metabolite 2
B
Scatter plot
C
MSD (observed, imputed)
Frequency
Value
Metabolite 2
Metabolite 1
MSD
True correlation
Histogram/data points
original
observed
imputed
imputed in one metabolite only
Total proportion of missing values
0
0.1
0.3
0.5
0.7
D
Bias cor
Bias pcor
Bias lm phen ~ met
Bias lm met ~ phen
Bias logistic regr.
Bias
True correlation
True partial cor.
True effect size
E
Power cor
Power pcor
Power lm phen ~ met
Power lm met ~ phen
Power logistic regr.
Power
True correlation
True partial cor.
True effect size

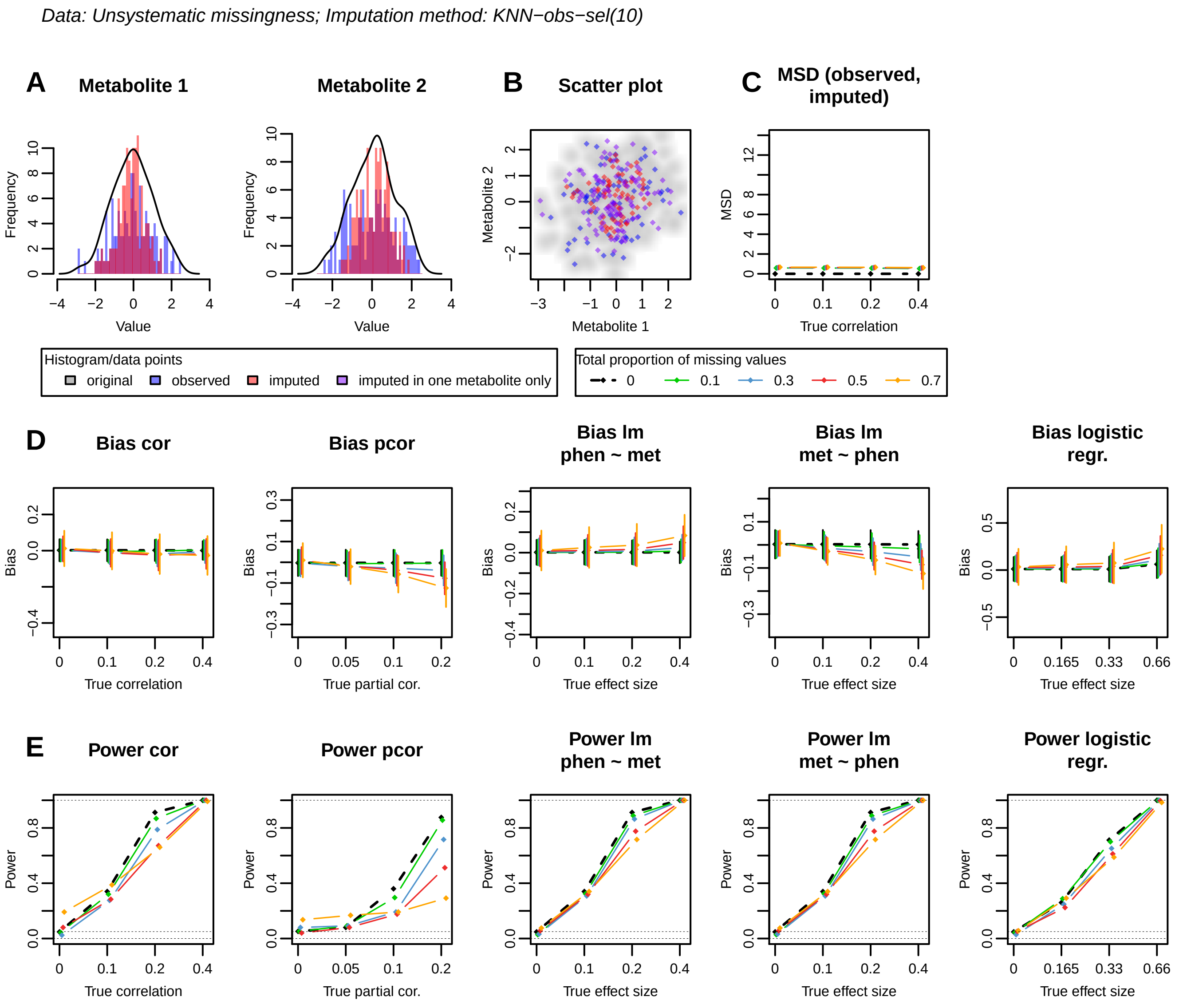

Data: Unsystematic missingness; Imputation method: KNN−obs−sel(10)
A
Metabolite 1
Metabolite 2
B
Scatter plot
C
MSD (observed, imputed)
Frequency
Value
Metabolite 2
Metabolite 1
MSD
True correlation
Histogram/data points
original
observed
imputed
imputed in one metabolite only
Total proportion of missing values
0
0.1
0.3
0.5
0.7
D
Bias cor
Bias pcor
Bias lm phen ~ met
Bias lm met ~ phen
Bias logistic regr.
Bias
True correlation
True partial cor.
True effect size
E
Power cor
Power pcor
Power lm phen ~ met
Power lm met ~ phen
Power logistic regr.
Power
True correlation
True partial cor.
True effect size

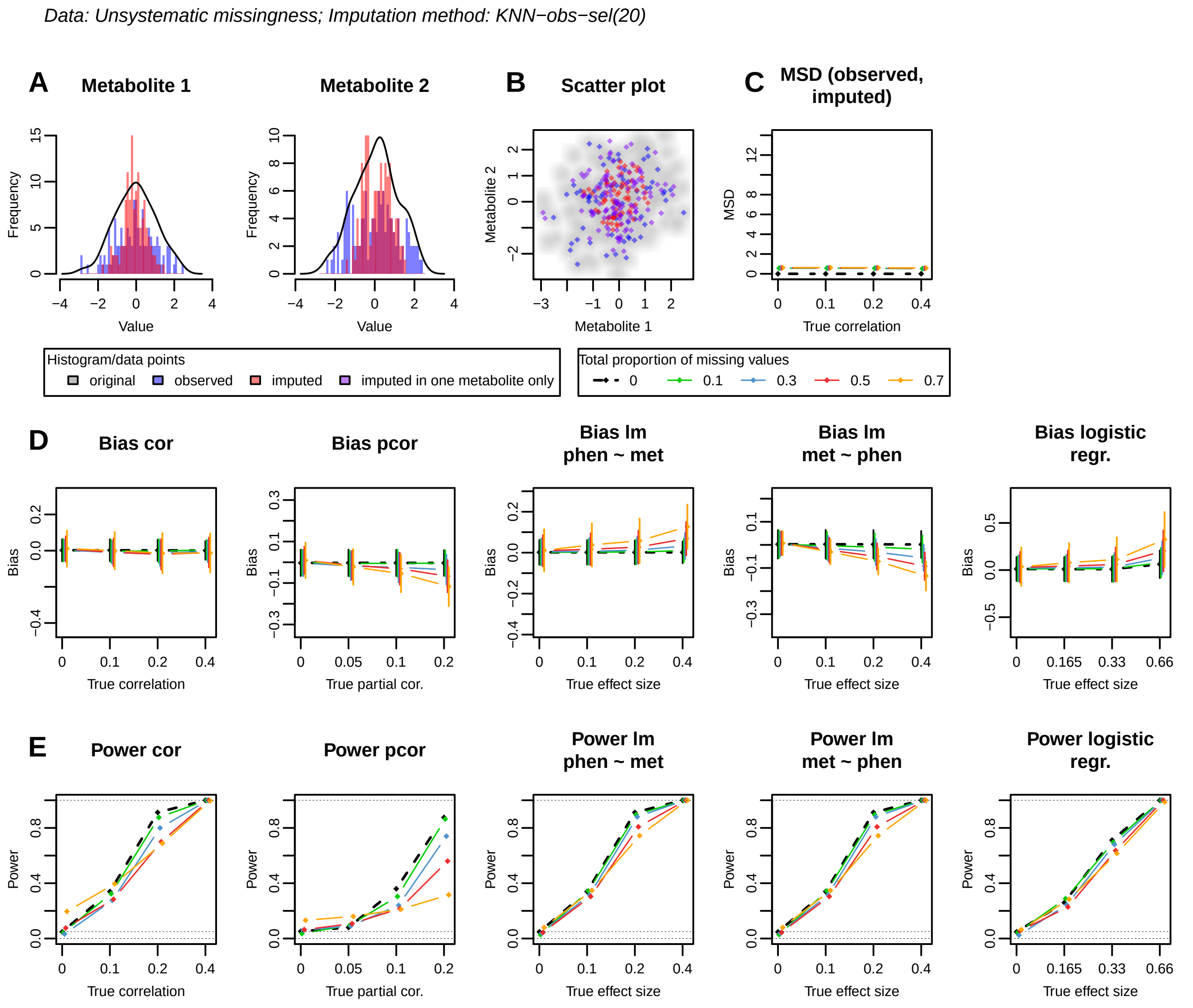

Data: Unsystematic missingness; Imputation method: KNN−obs−sel(20)
A
Metabolite 1
Metabolite 2
B
Scatter plot
C
MSD (observed, imputed)
Frequency
Value
Metabolite 2
Metabolite 1
MSD
True correlation
Histogram/data points
original
observed
imputed
imputed in one metabolite only
Total proportion of missing values
0
0.1
0.3
0.5
0.7
D
Bias cor
Bias pcor
Bias lm phen ~ met
Bias lm met ~ phen
Bias logistic regr.
Bias
True correlation
True partial cor.
True effect size
E
Power cor
Power pcor
Power lm phen ~ met
Power lm met ~ phen
Power logistic regr.
Power

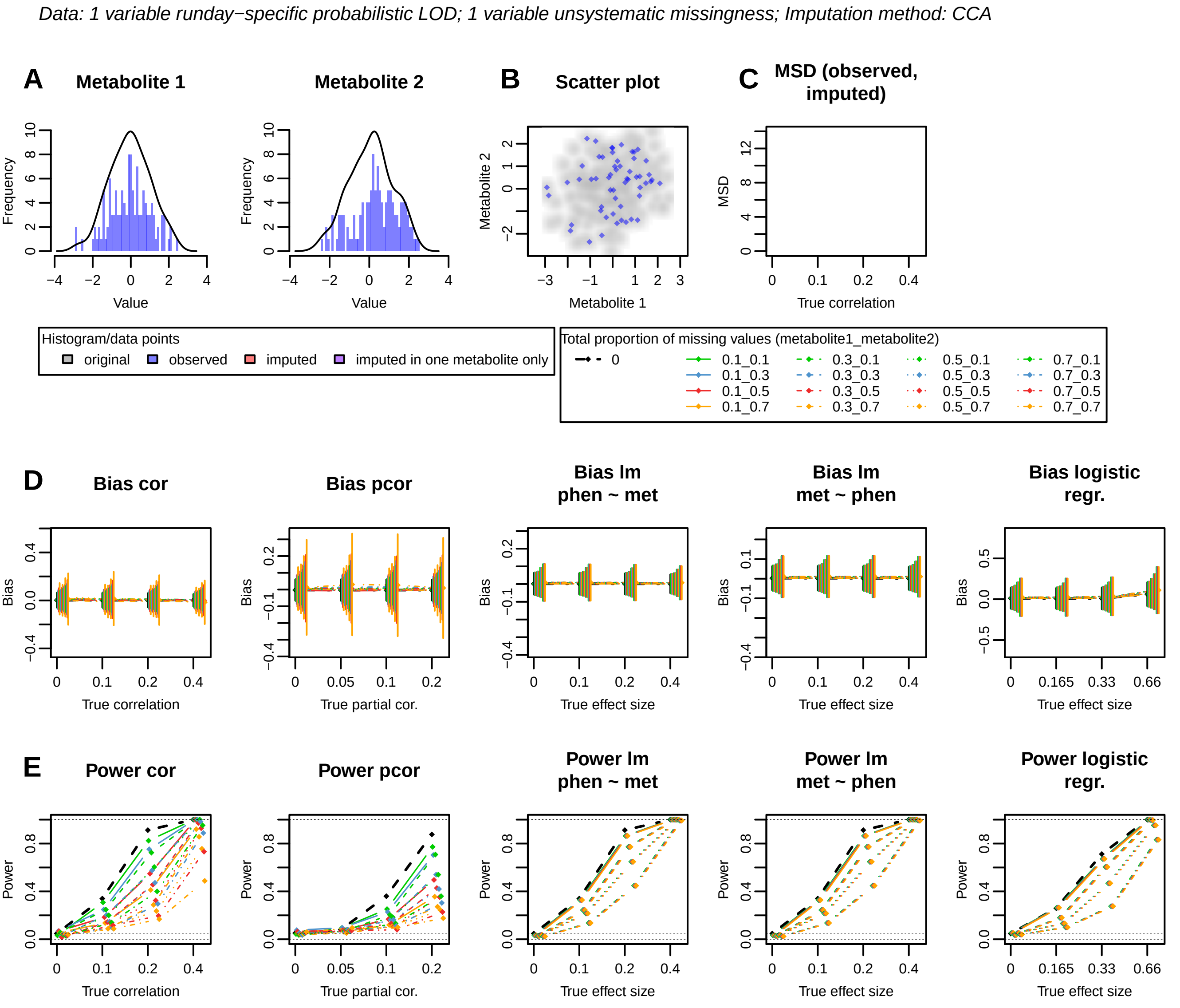

Data: 1 variable runday−specific probabilistic LOD; 1 variable unsystematic missingness; Imputation method: CCA
A
Metabolite 1
Metabolite 2
B
Scatter plot
C
MSD (observed, imputed)
Frequency
Value
Metabolite 1
Metabolite 2
MSD
True correlation
Histogram/data points
original
observed
imputed
imputed in one metabolite only
Total proportion of missing values (metabolite1_metabolite2)
0
0.1_0.1
0.1_0.3
0.1_0.5
0.1_0.7
0.3_0.1
0.3_0.3
0.3_0.5
0.3_0.7
0.5_0.1
0.5_0.3
0.5_0.5
0.5_0.7
0.7_0.1
0.7_0.3
0.7_0.5
0.7_0.7
D
Bias cor
Bias pcor
Bias lm phen ~ met
Bias lm met ~ phen
Bias logistic regr.
Bias
True correlation
True partial cor.
True effect size
E
Power cor
Power pcor
Power lm phen ~ met
Power lm met ~ phen
Power logistic regr.
Power
True correlation
True partial cor.
True effect size

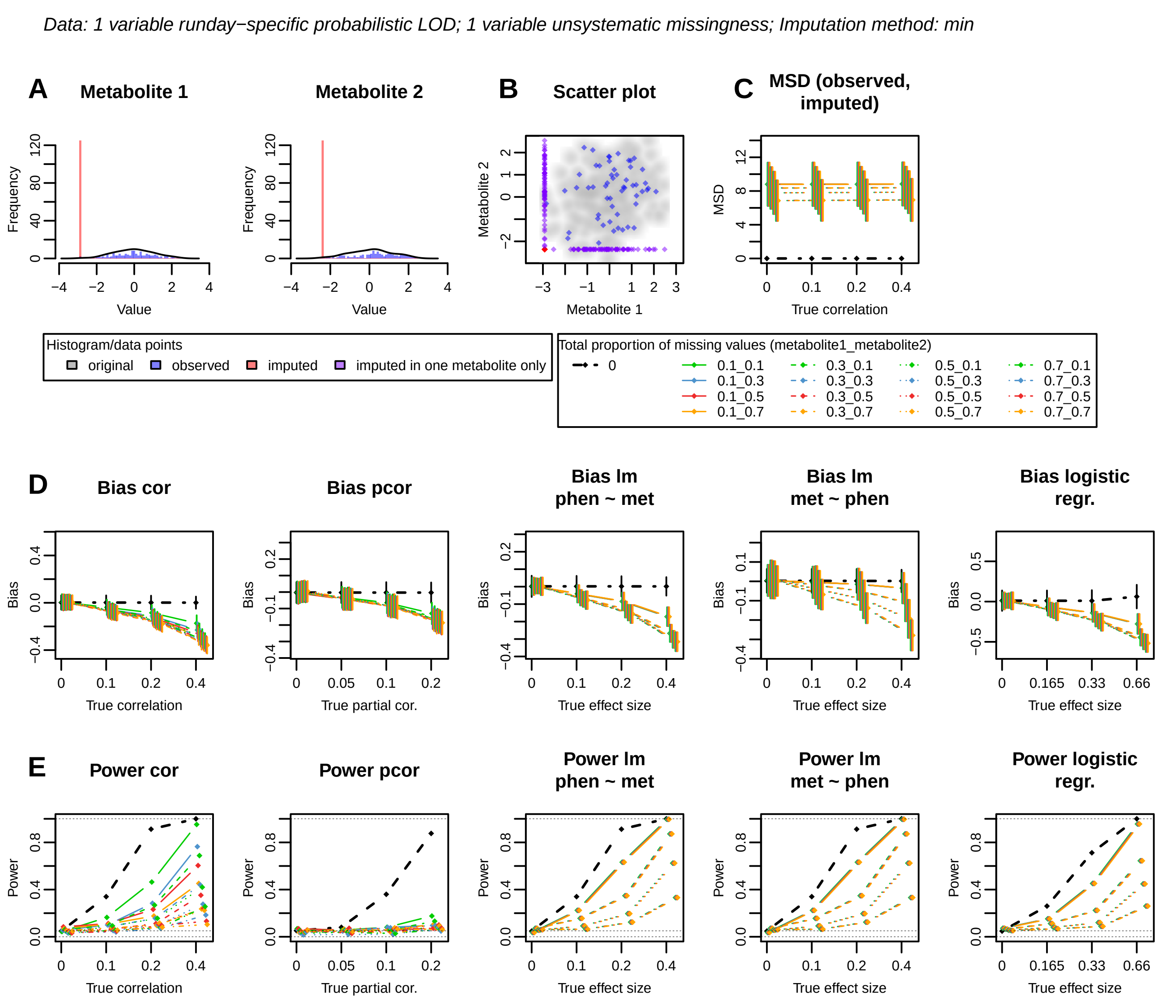
Data: 1 variable runday−specific probabilistic LOD; 1 variable unsystematic missingness; Imputation method: min
A
Metabolite 1
Metabolite 2
B
Scatter plot
C
MSD (observed, imputed)
Frequency
Value
Metabolite 2
Metabolite 1
MSD
True correlation
Histogram/data points
original
observed
imputed
imputed in one metabolite only
Total proportion of missing values (metabolite1_metabolite2)
0
0.1_0.1
0.1_0.3
0.1_0.5
0.1_0.7
0.3_0.1
0.3_0.3
0.3_0.5
0.3_0.7
0.5_0.1
0.5_0.3
0.5_0.5
0.5_0.7
0.7_0.1
0.7_0.3
0.7_0.5
0.7_0.7
D
Bias cor
Bias pcor
Bias lm phen ~ met
Bias lm met ~ phen
Bias logistic regr.
Bias
True correlation
True partial cor.
True effect size
E
Power cor
Power pcor
Power lm phen ~ met
Power lm met ~ phen
Power logistic regr.
Power

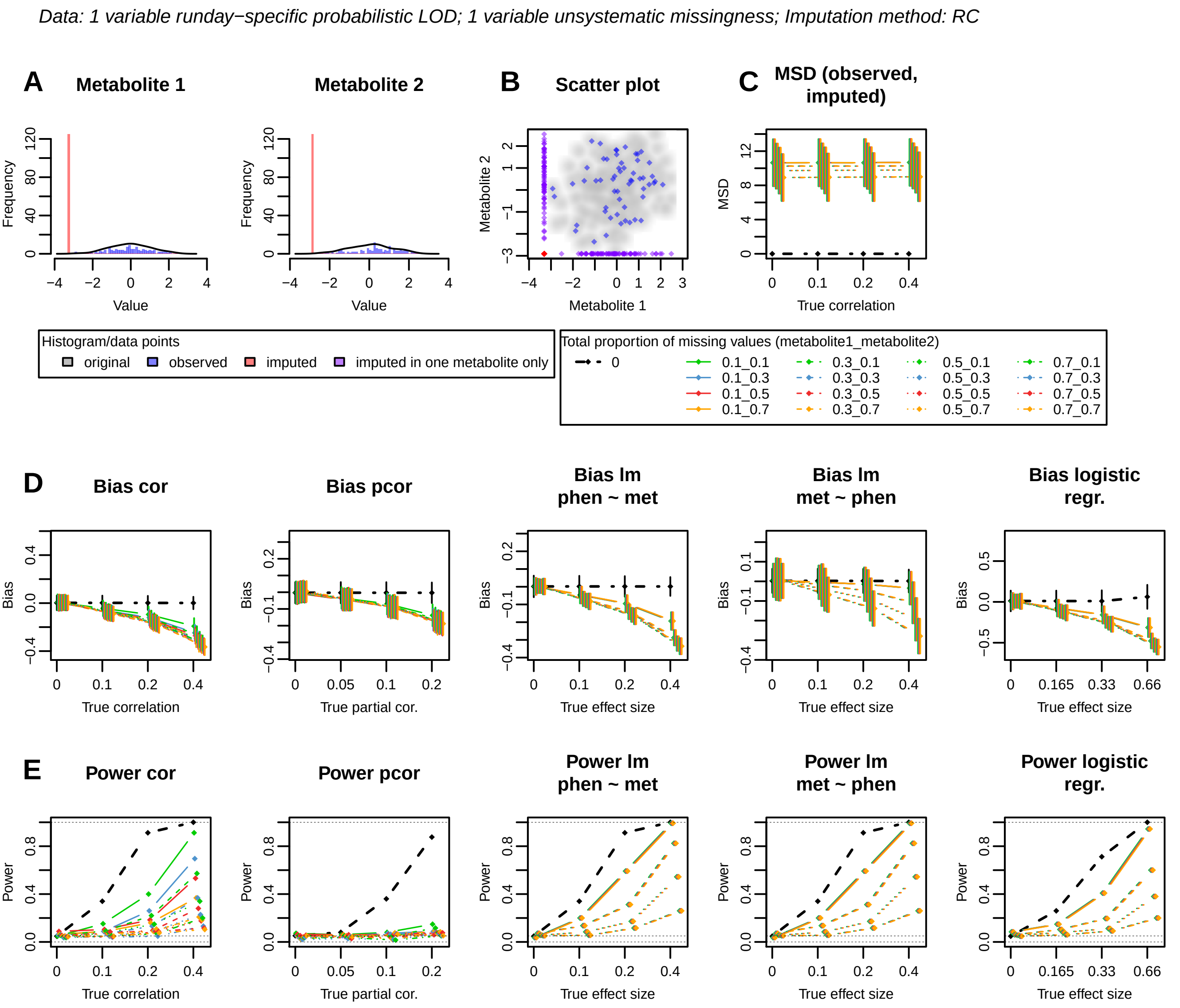
Data: 1 variable runday−specific probabilistic LOD; 1 variable unsystematic missingness; Imputation method: RC
A
Metabolite 1
Metabolite 2
Frequency
Value
B
Scatter plot
Metabolite 2
Metabolite 1
C
MSD (observed, imputed)
MSD
True correlation
Histogram/data points
original
observed
imputed
imputed in one metabolite only
Total proportion of missing values (metabolite1_metabolite2)
0
0.1_0.1
0.1_0.3
0.1_0.5
0.1_0.7
0.3_0.1
0.3_0.3
0.3_0.5
0.3_0.7
0.5_0.1
0.5_0.3
0.5_0.5
0.5_0.7
0.7_0.1
0.7_0.3
0.7_0.5
0.7_0.7
D
Bias cor
Bias pcor
Bias lm phen ~ met
Bias lm met ~ phen
Bias logistic regr.
Bias
True correlation
True partial cor.
True effect size
E
Power cor
Power pcor
Power lm phen ~ met
Power lm met ~ phen
Power logistic regr.
Power
True correlation
True partial cor.
True effect size

Data: 1 variable runday−specific probabilistic LOD; 1 variable unsystematic missingness; Imputation method: ITS

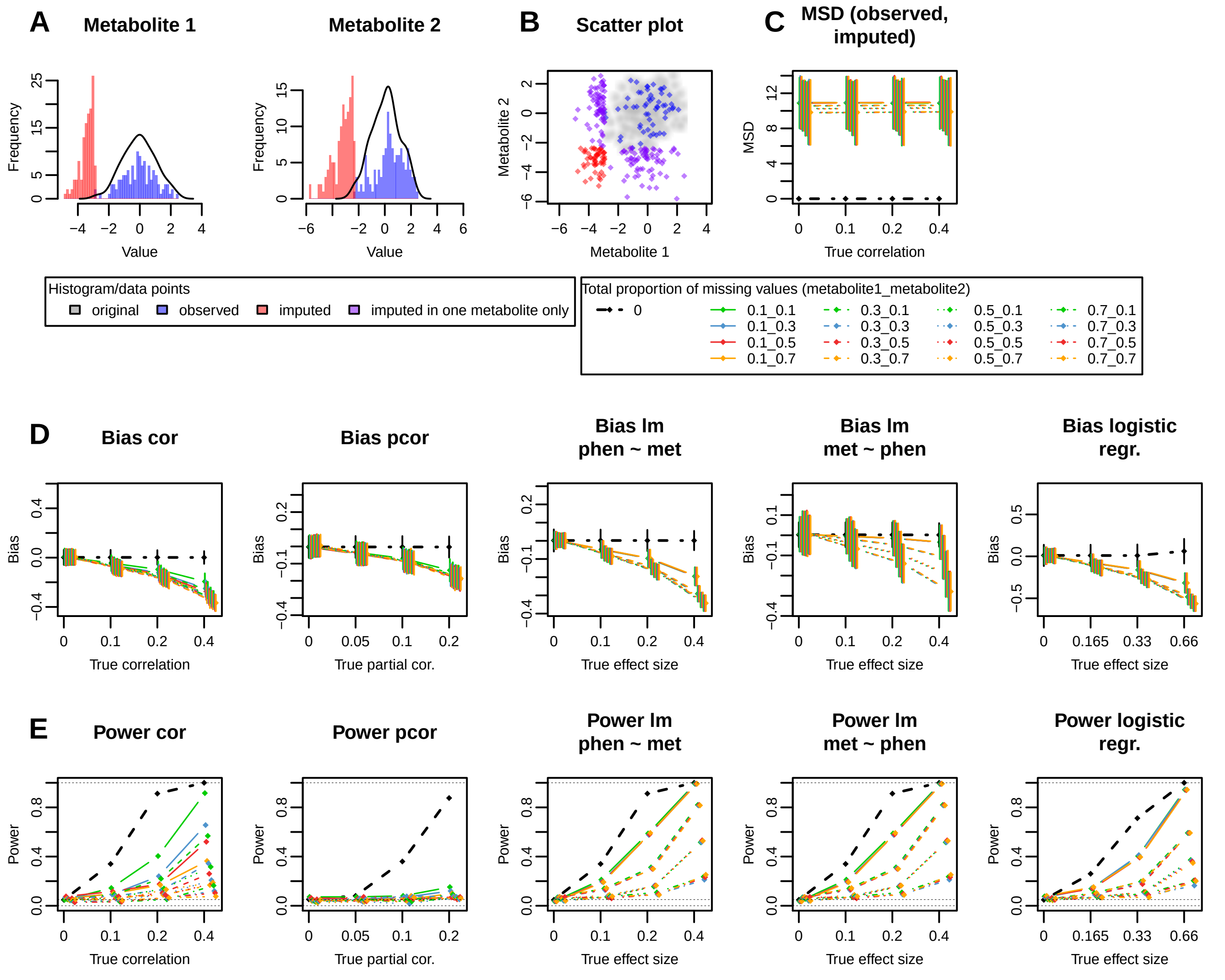

Data: 1 variable runday−specific probabilistic LOD; 1 variable unsystematic missingness; Imputation method: MITS

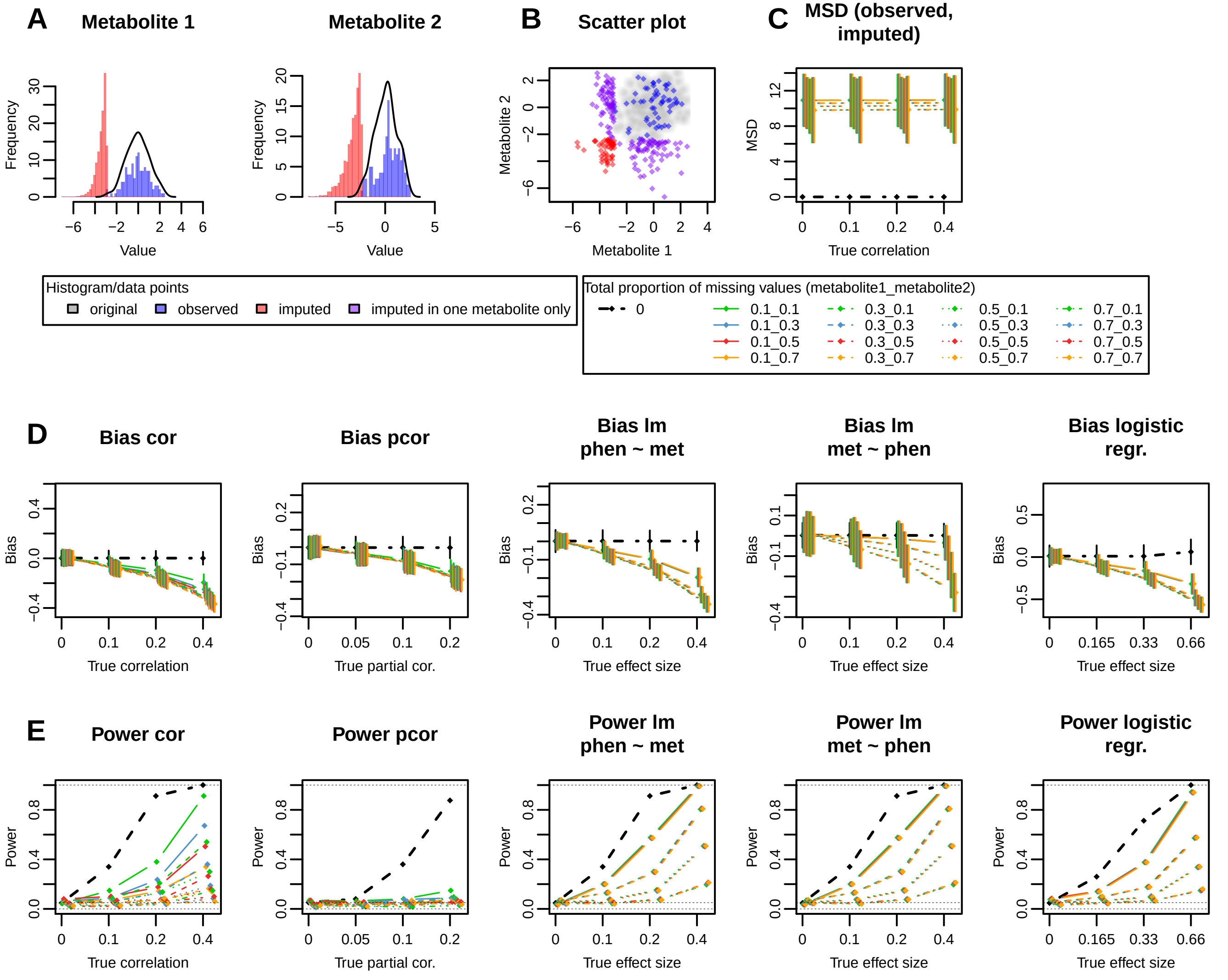

Data: 1 variable runday−specific probabilistic LOD; 1 variable unsystematic missingness; Imputation method: RC−R

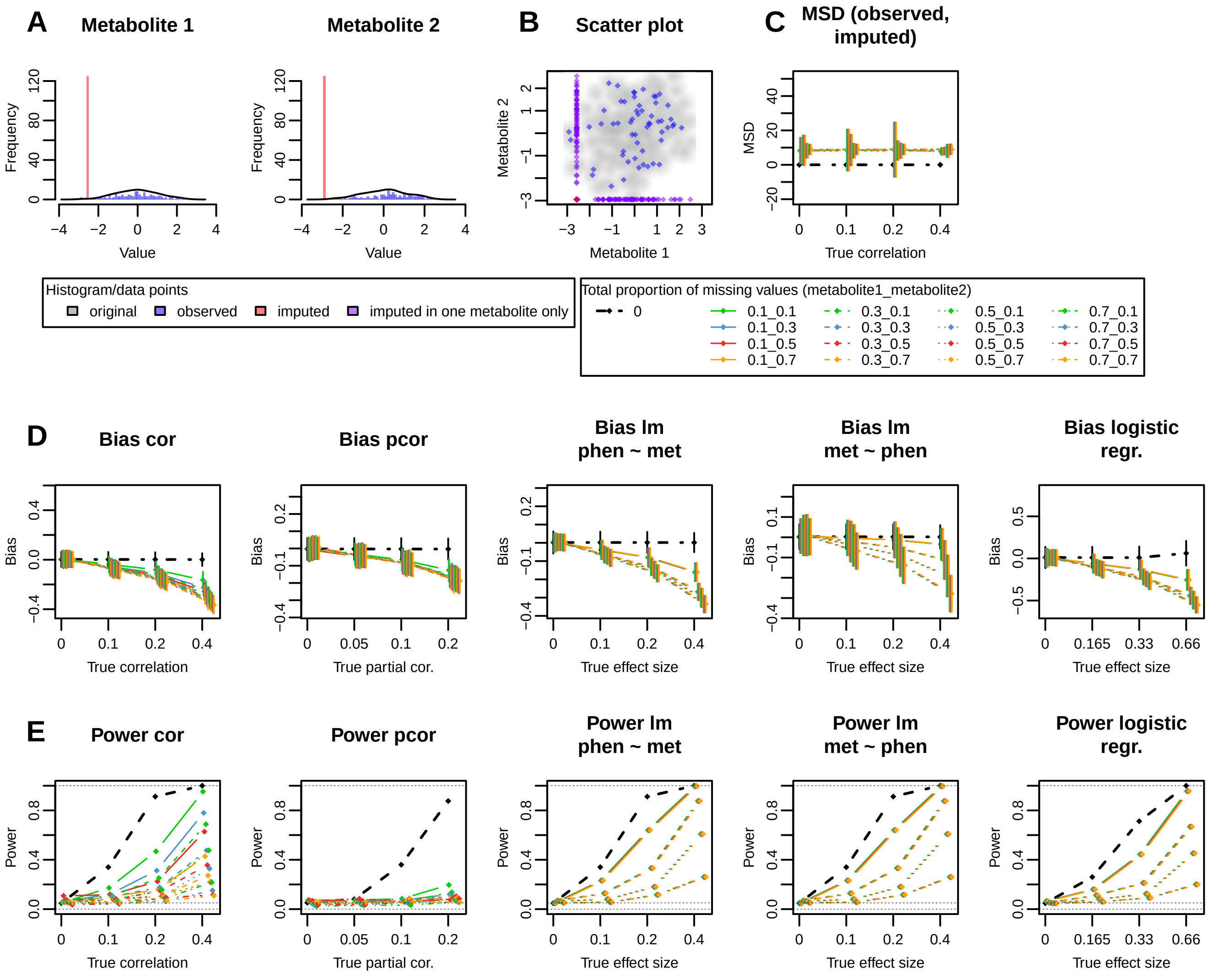

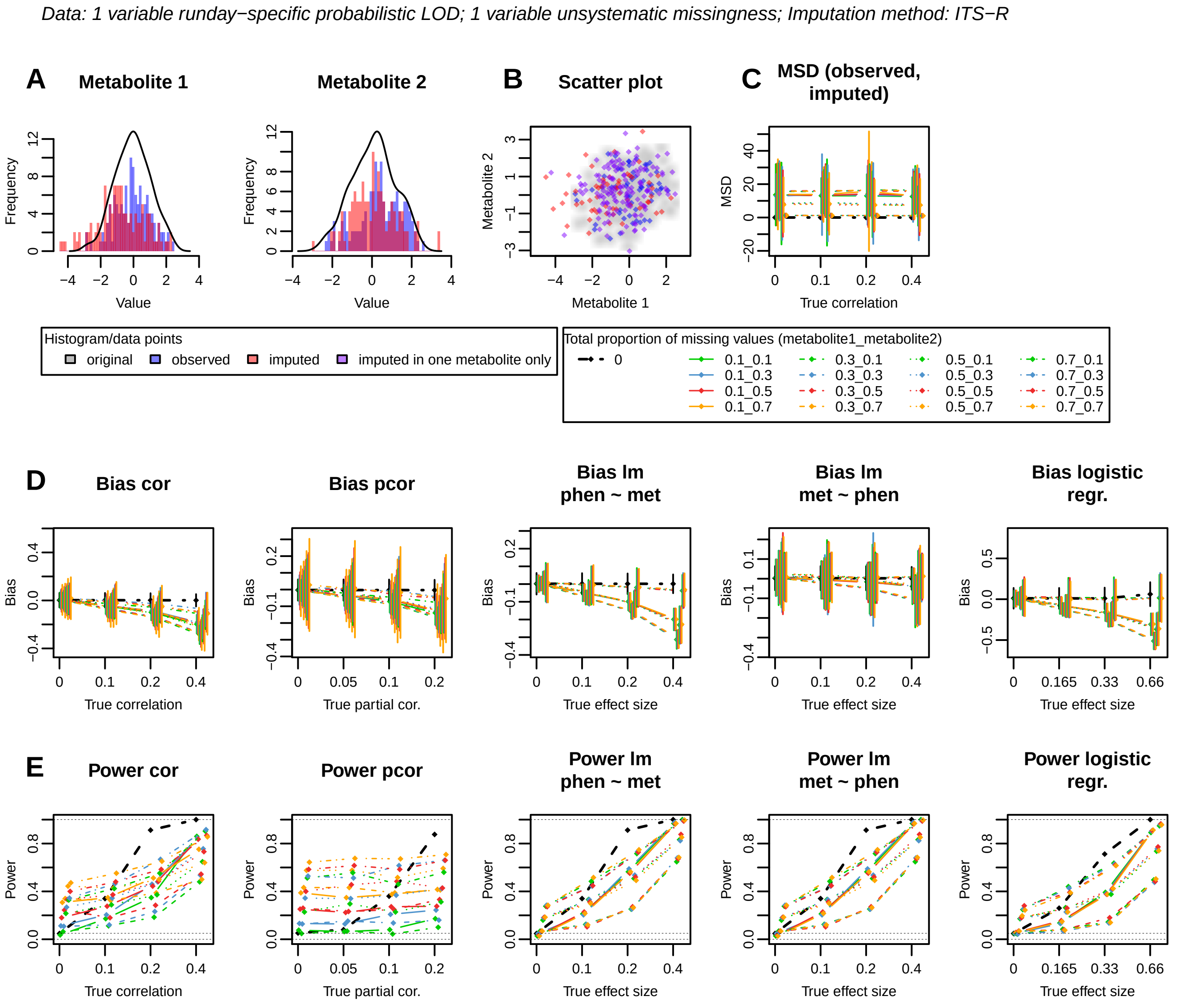
Data: 1 variable runday−specific probabilistic LOD; 1 variable unsystematic missingness; Imputation method: ITS−R
A
Metabolite 1
Metabolite 2
Frequency
Value
B
Scatter plot
Metabolite 2
Metabolite 1
C
MSD (observed, imputed)
MSD
True correlation
Histogram/data points
original
observed
imputed
imputed in one metabolite only
Total proportion of missing values (metabolite1_metabolite2)
0
0.1_0.1
0.1_0.3
0.1_0.5
0.1_0.7
0.3_0.1
0.3_0.3
0.3_0.5
0.3_0.7
0.5_0.1
0.5_0.3
0.5_0.5
0.5_0.7
0.7_0.1
0.7_0.3
0.7_0.5
0.7_0.7
D
Bias cor
Bias pcor
Bias lm phen ~ met
Bias lm met ~ phen
Bias logistic regr.
Bias
True correlation
True partial cor.
True effect size
E
Power cor
Power pcor
Power lm phen ~ met
Power lm met ~ phen
Power logistic regr.
Power
True correlation
True partial cor.
True effect size

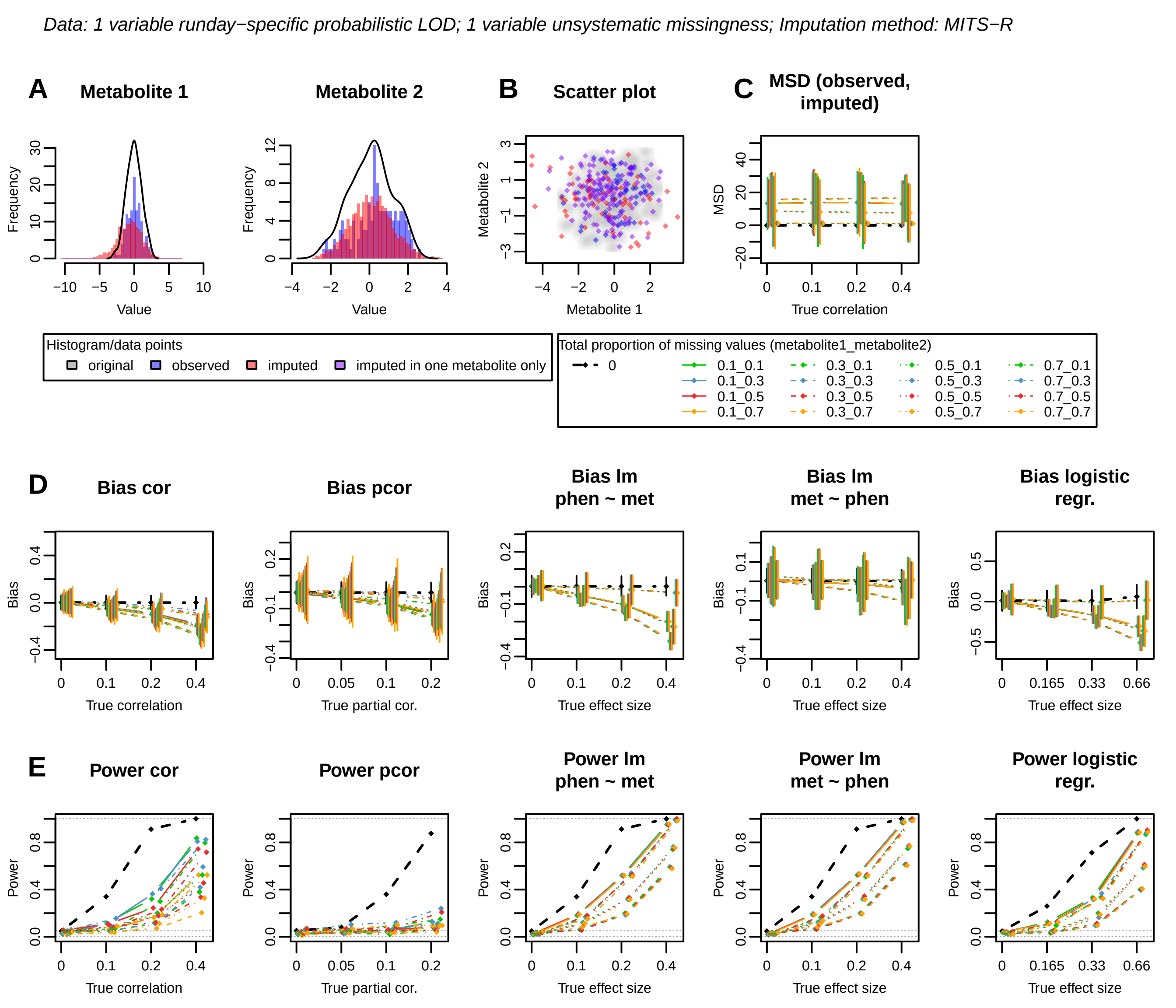
Data: 1 variable runday−specific probabilistic LOD; 1 variable unsystematic missingness; Imputation method: MITS−R
A
Metabolite 1
Metabolite 2
Frequency
Value
B
Scatter plot
Metabolite 1
Metabolite 2
C
MSD (observed, imputed)
MSD
True correlation
Histogram/data points
original
observed
imputed
imputed in one metabolite only
Total proportion of missing values (metabolite1_metabolite2)
0
0.1_0.1
0.1_0.3
0.1_0.5
0.1_0.7
0.3_0.1
0.3_0.3
0.3_0.5
0.3_0.7
0.5_0.1
0.5_0.3
0.5_0.5
0.5_0.7
0.7_0.1
0.7_0.3
0.7_0.5
0.7_0.7
D
Bias cor
Bias pcor
Bias lm phen ~ met
Bias lm met ~ phen
Bias logistic regr.
Bias
True correlation
True partial cor.
True effect size
E
Power cor
Power pcor
Power lm phen ~ met
Power lm met ~ phen
Power logistic regr.
Power
True correlation
True partial cor.
True effect size

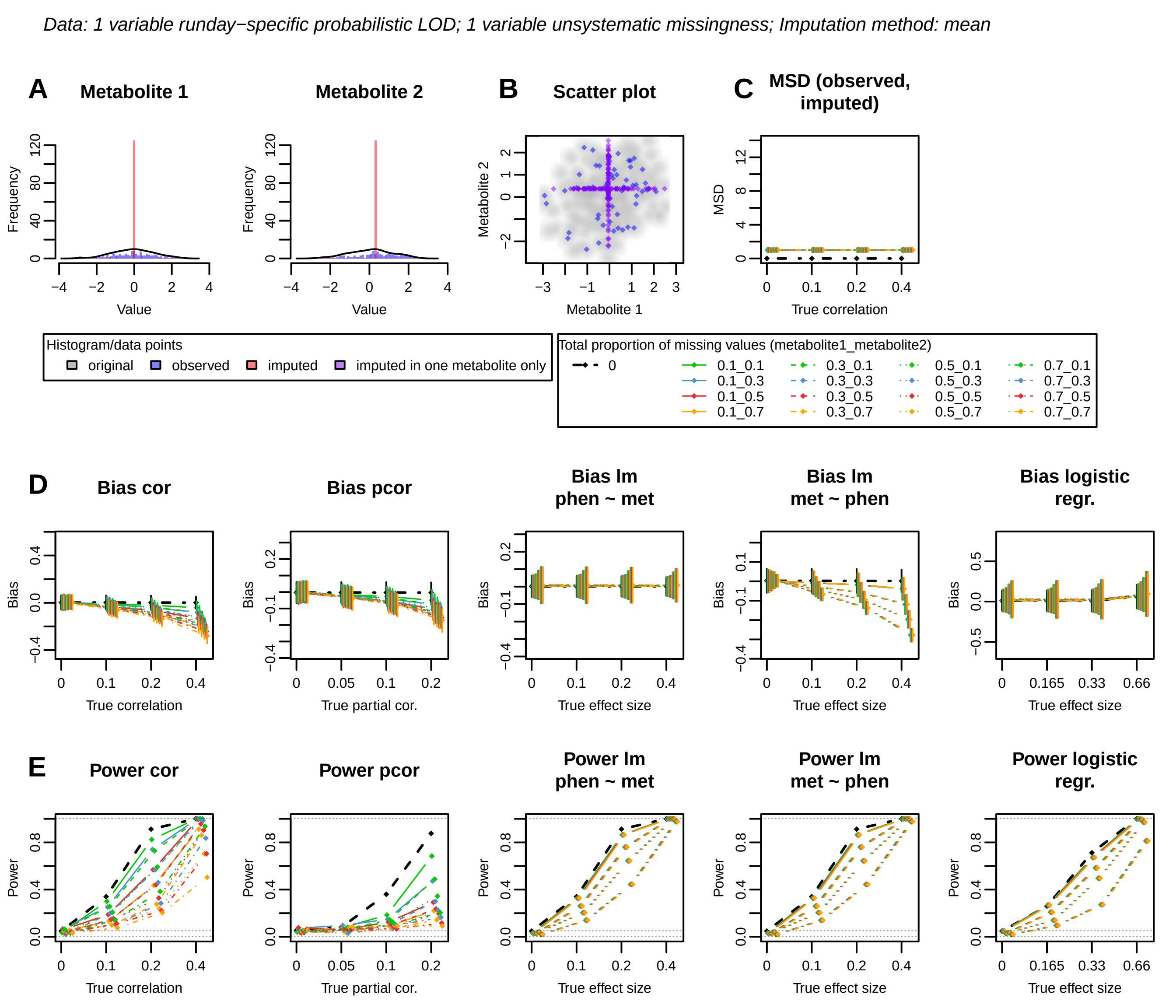
Data: 1 variable runday−specific probabilistic LOD; 1 variable unsystematic missingness; Imputation method: mean
A
Metabolite 1
Metabolite 2
Frequency
Value
B
Scatter plot
Metabolite 2
Metabolite 1
C
MSD (observed, imputed)
MSD
True correlation
Histogram/data points
original
observed
imputed
imputed in one metabolite only
Total proportion of missing values (metabolite1_metabolite2)
0
0.1_0.1
0.1_0.3
0.1_0.5
0.1_0.7
0.3_0.1
0.3_0.3
0.3_0.5
0.3_0.7
0.5_0.1
0.5_0.3
0.5_0.5
0.5_0.7
0.7_0.1
0.7_0.3
0.7_0.5
0.7_0.7
D
Bias cor
Bias pcor
Bias lm phen ~ met
Bias lm met ~ phen
Bias logistic regr.
Bias
True correlation
True partial cor.
True effect size
E
Power cor
Power pcor
Power lm phen ~ met
Power lm met ~ phen
Power logistic regr.
Power
True correlation
True partial cor.
True effect size

Data: 1 variable runday−specific probabilistic LOD; 1 variable unsystematic missingness; Imputation method: ICE−norm

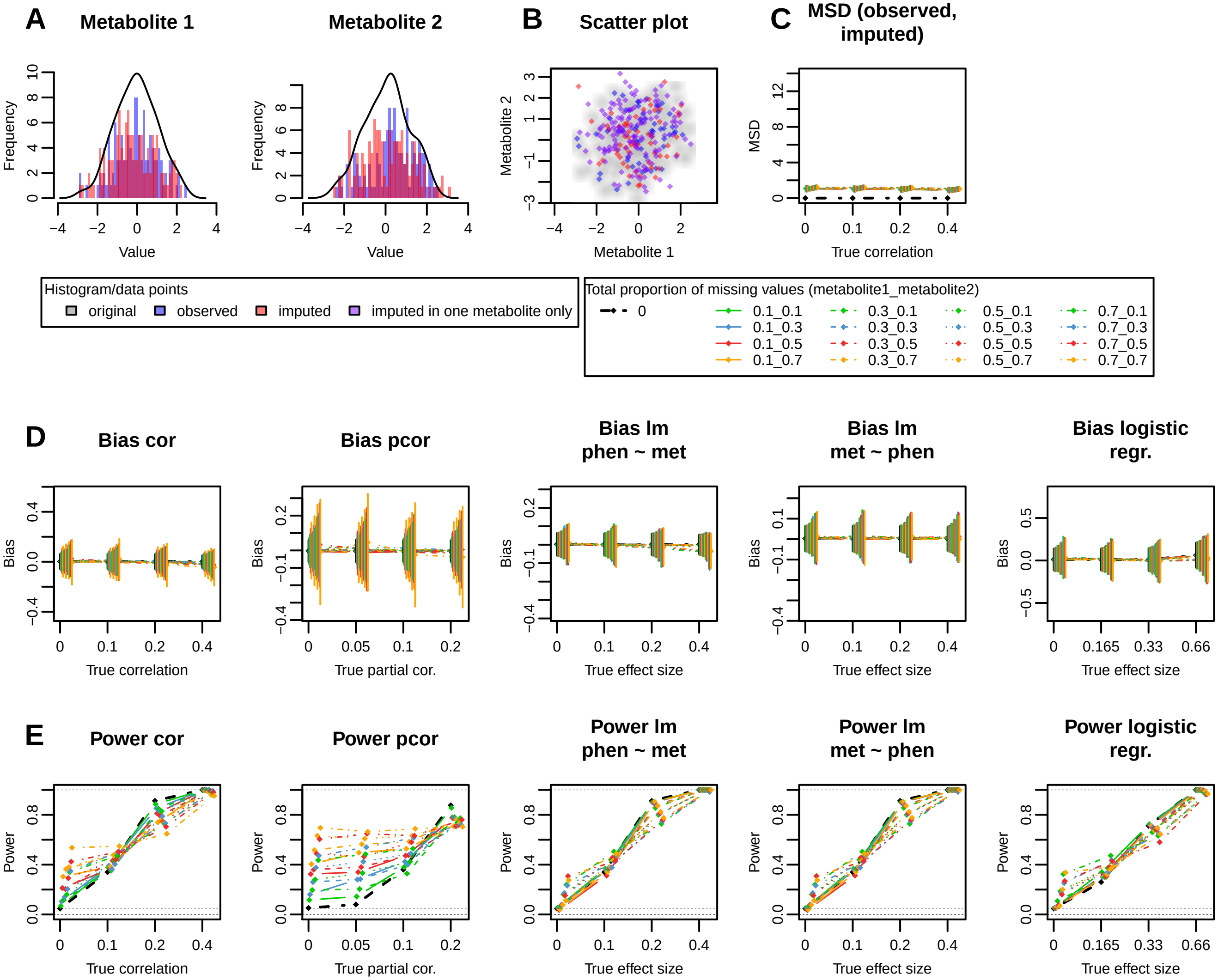

Data: 1 variable runday−specific probabilistic LOD; 1 variable unsystematic missingness; Imputation method: ICE−pmm

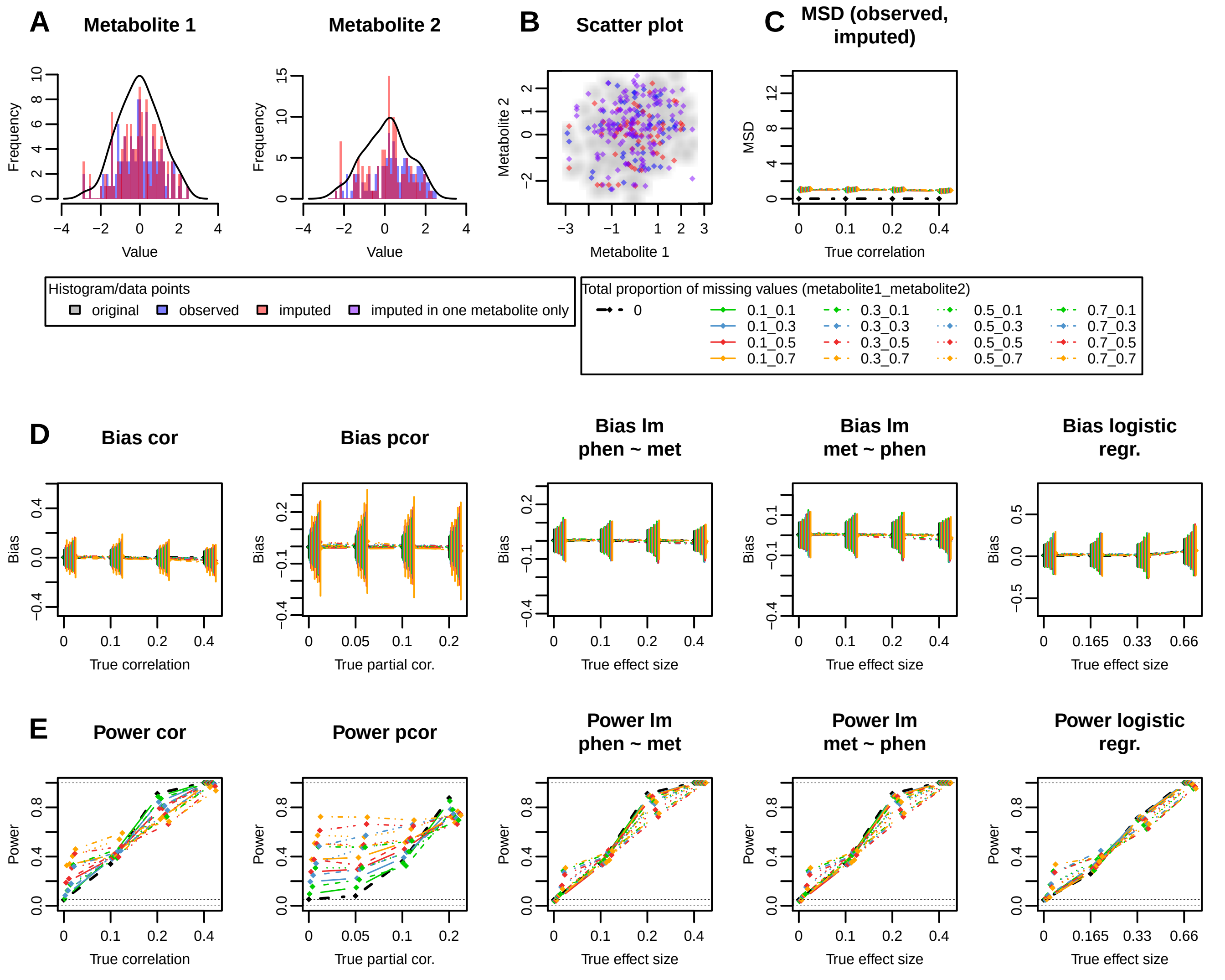

Data: 1 variable runday−specific probabilistic LOD; 1 variable unsystematic missingness; Imputation method: ICE−adjR

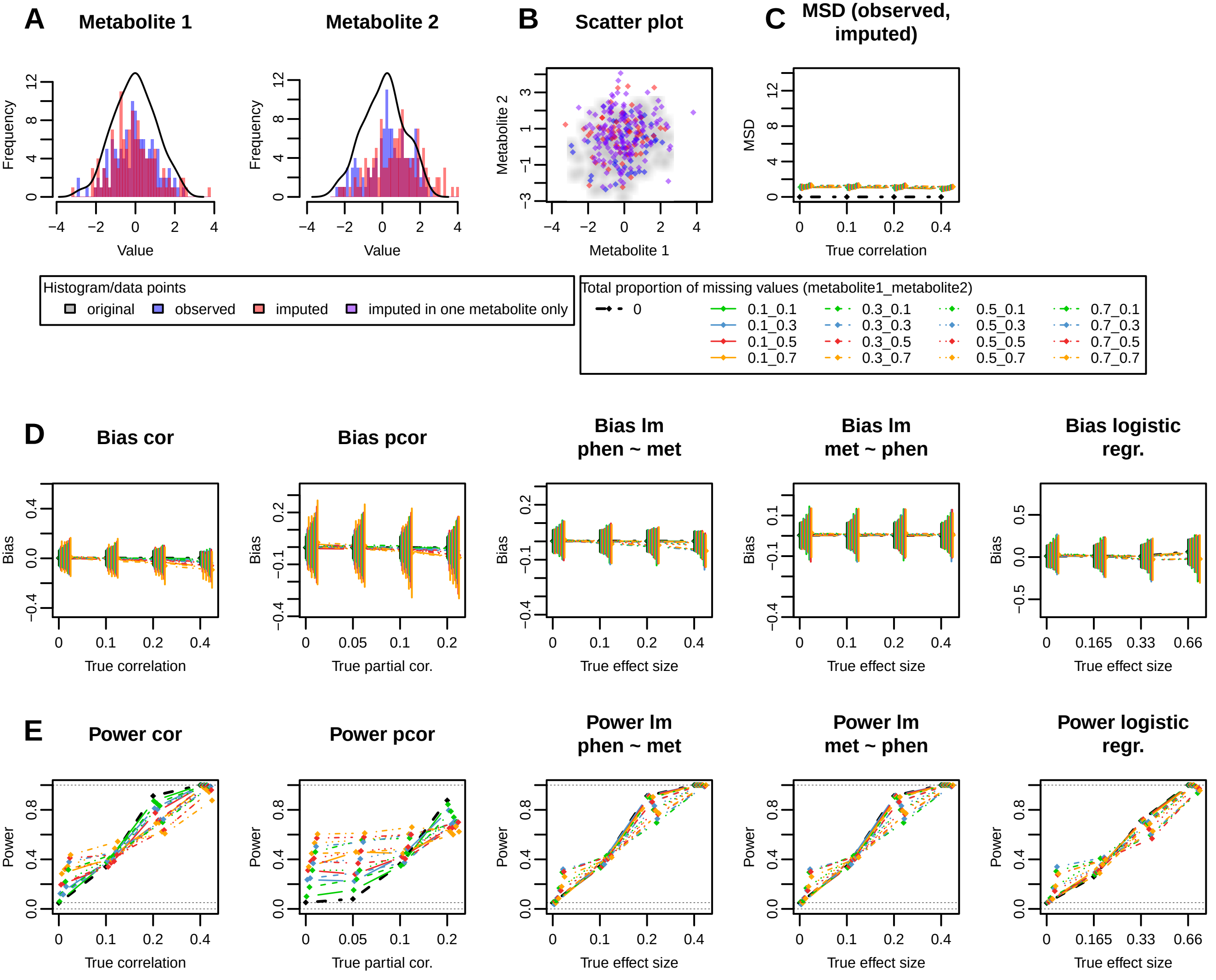

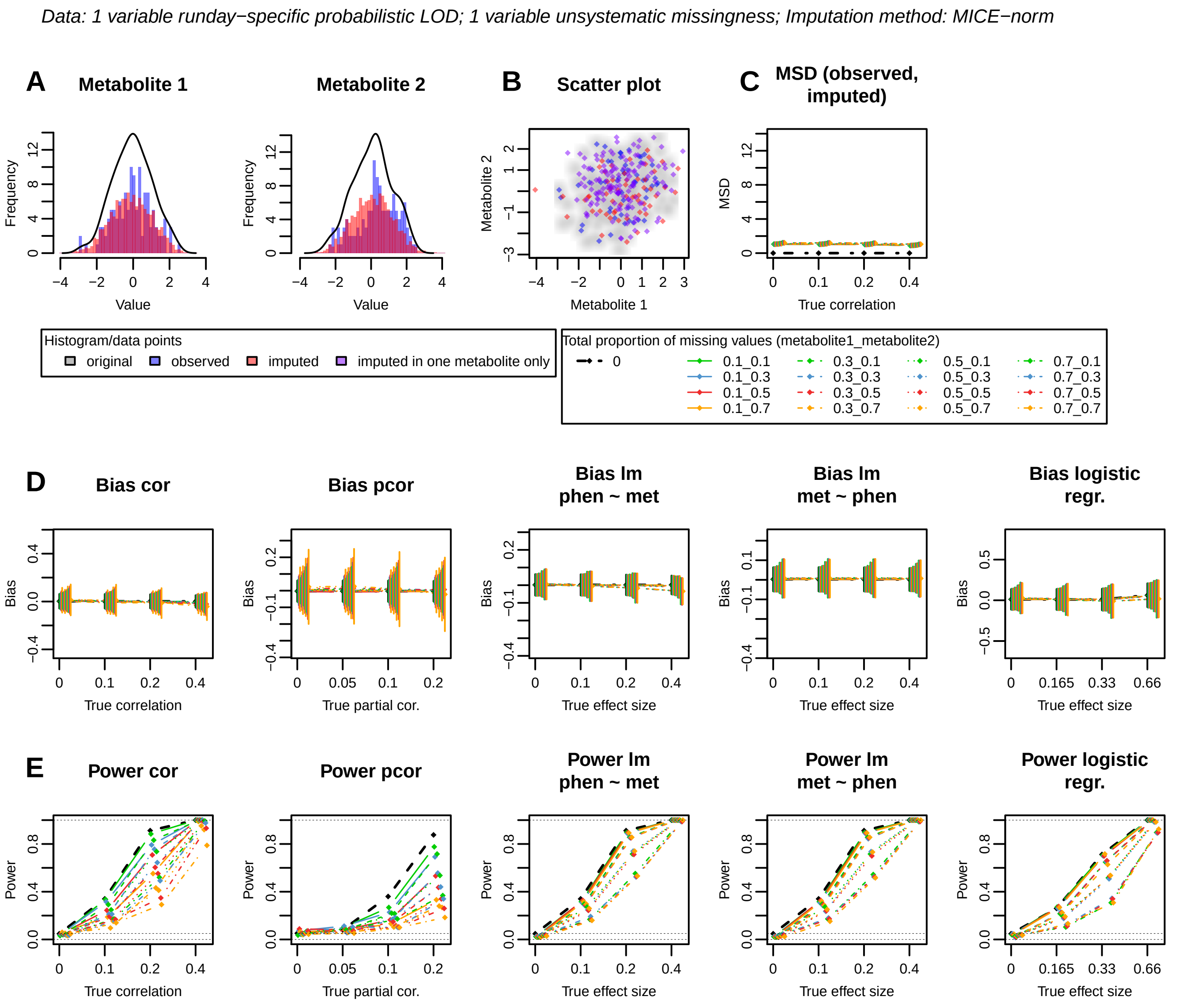

Data: 1 variable runday−specific probabilistic LOD; 1 variable unsystematic missingness; Imputation method: MICE−norm
A
Metabolite 1
Metabolite 2
Frequency
Value
B
Scatter plot
Metabolite 2
Metabolite 1
C
MSD (observed, imputed)
MSD
True correlation
Histogram/data points
original
observed
imputed
imputed in one metabolite only
Total proportion of missing values (metabolite1_metabolite2)
0
0.1_0.1
0.1_0.3
0.1_0.5
0.1_0.7
0.3_0.1
0.3_0.3
0.3_0.5
0.3_0.7
0.5_0.1
0.5_0.3
0.5_0.5
0.5_0.7
0.7_0.1
0.7_0.3
0.7_0.5
0.7_0.7
D
Bias cor
Bias pcor
Bias lm phen ~ met
Bias lm met ~ phen
Bias logistic regr.
Bias
True correlation
True partial cor.
True effect size
E
Power cor
Power pcor
Power lm phen ~ met
Power lm met ~ phen
Power logistic regr.
Power

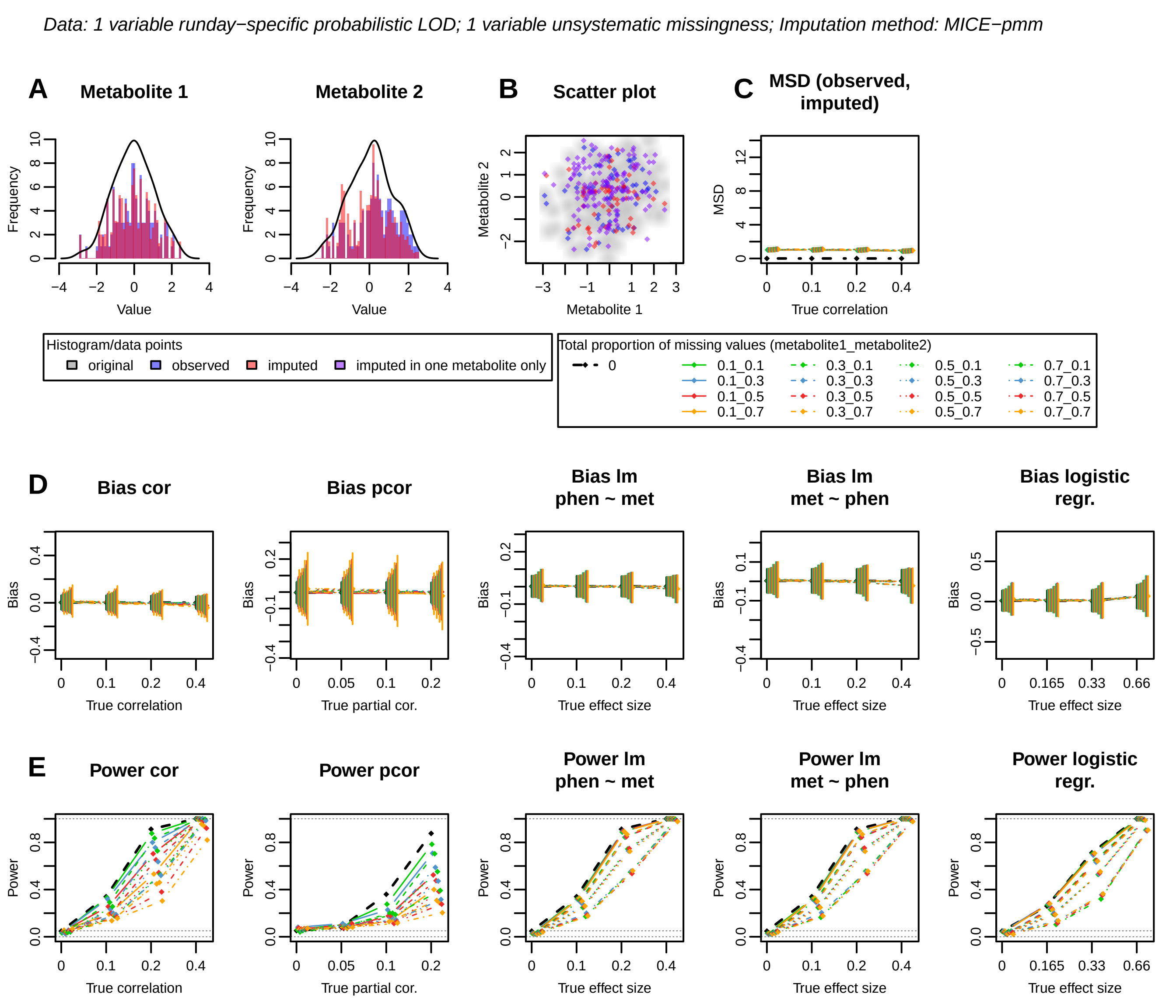
Data: 1 variable runday−specific probabilistic LOD; 1 variable unsystematic missingness; Imputation method: MICE−pmm
A
Metabolite 1
Metabolite 2
Frequency
Value
B
Scatter plot
Metabolite 2
Metabolite 1
C
MSD (observed, imputed)
MSD
True correlation
Histogram/data points
original
observed
imputed
imputed in one metabolite only
Total proportion of missing values (metabolite1_metabolite2)
0
0.1_0.1
0.1_0.3
0.1_0.5
0.1_0.7
0.3_0.1
0.3_0.3
0.3_0.5
0.3_0.7
0.5_0.1
0.5_0.3
0.5_0.5
0.5_0.7
0.7_0.1
0.7_0.3
0.7_0.5
0.7_0.7
D
Bias cor
Bias pcor
Bias lm phen ~ met
Bias lm met ~ phen
Bias logistic regr.
Bias
True correlation
True partial cor.
True effect size
E
Power cor
Power pcor
Power lm phen ~ met
Power lm met ~ phen
Power logistic regr.
Power
True correlation
True partial cor.
True effect size

Data: 1 variable runday−specific probabilistic LOD; 1 variable unsystematic missingness; Imputation method: MICE−adjR

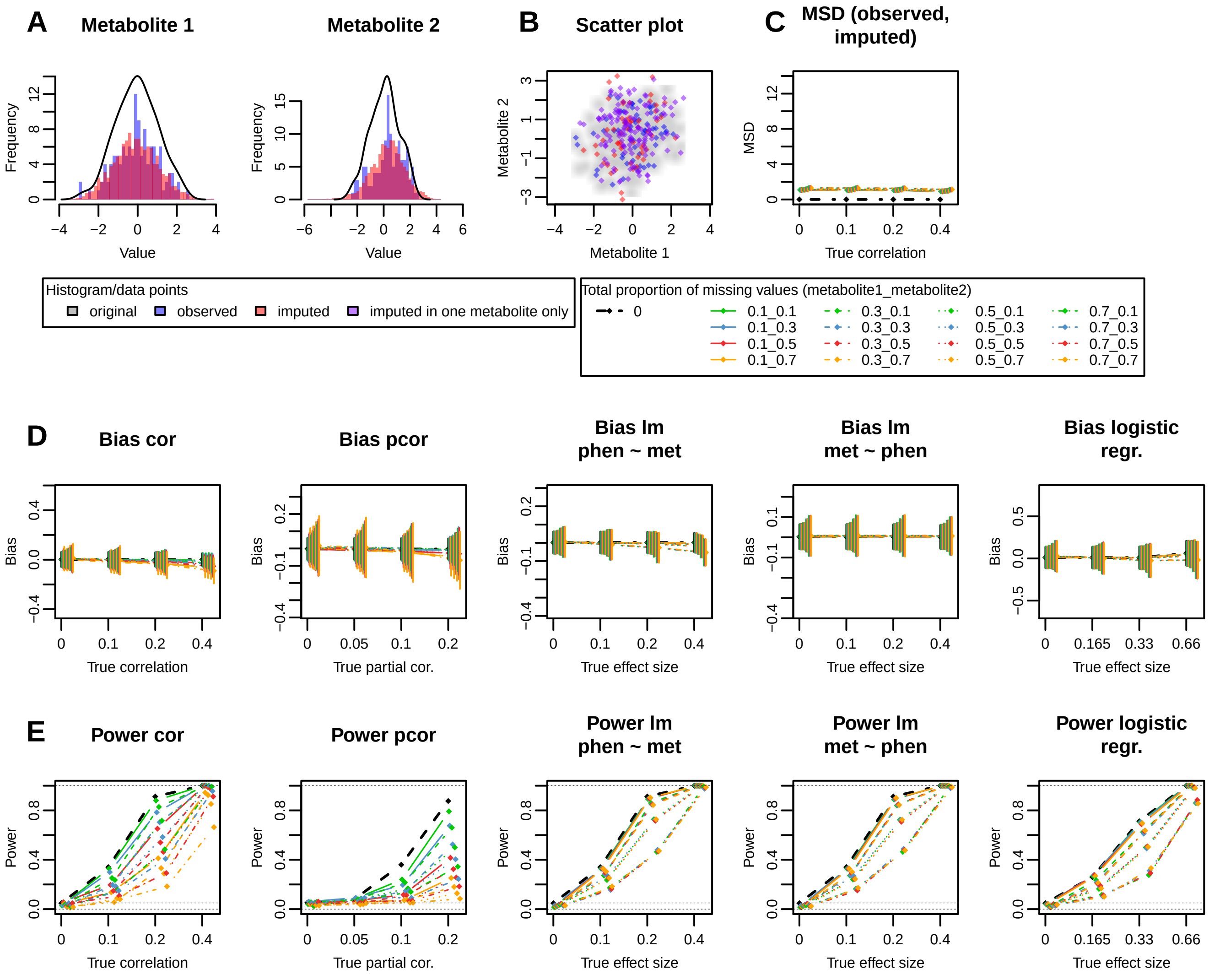

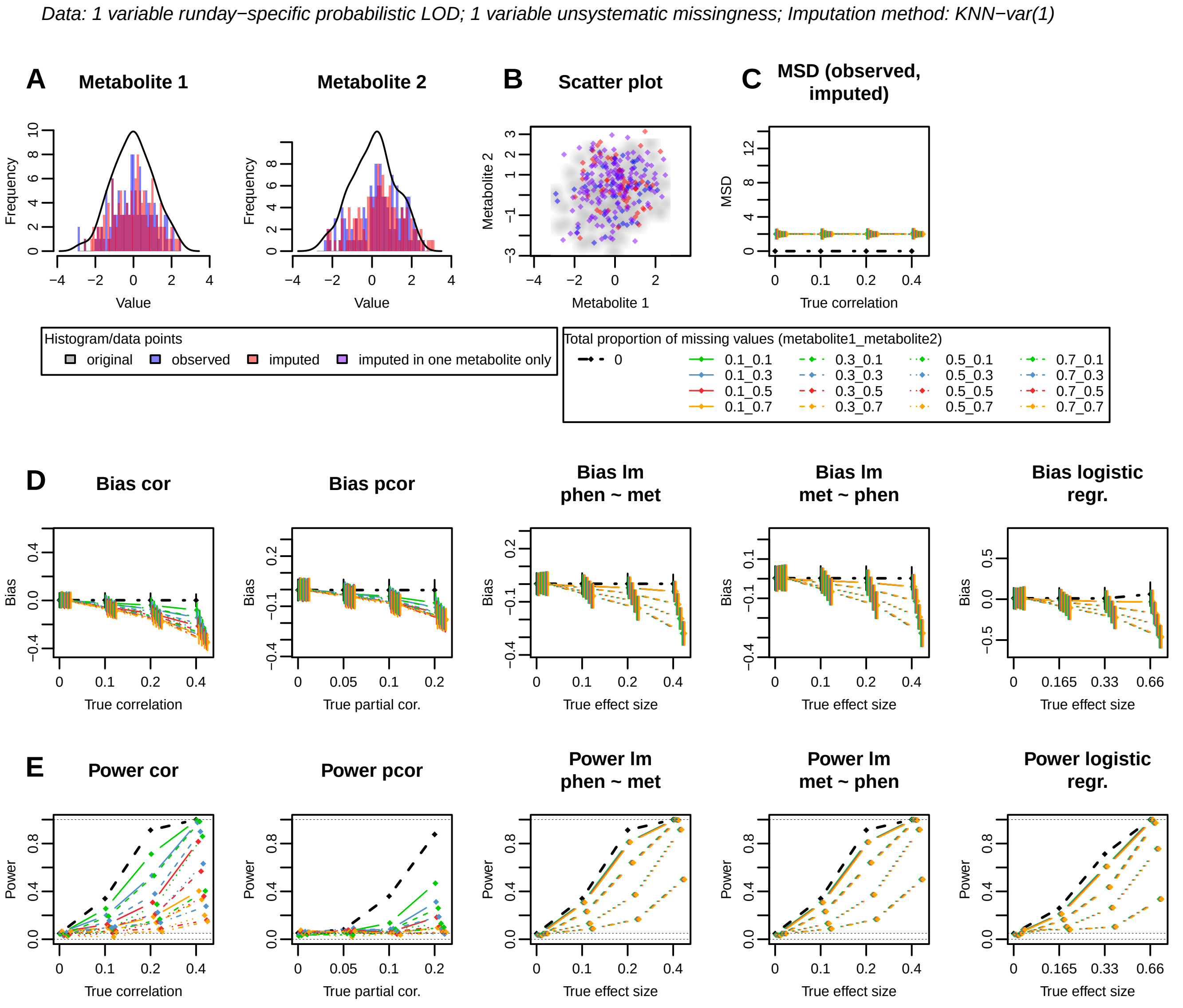
Data: 1 variable runday−specific probabilistic LOD; 1 variable unsystematic missingness; Imputation method: KNN−var(1)
A
Metabolite 1
Metabolite 2
Frequency
Value
B
Scatter plot
Metabolite 2
Metabolite 1
C
MSD (observed, imputed)
MSD
True correlation
Histogram/data points
original
observed
imputed
imputed in one metabolite only
Total proportion of missing values (metabolite1_metabolite2)
0
0.1_0.1
0.1_0.3
0.1_0.5
0.1_0.7
0.3_0.1
0.3_0.3
0.3_0.5
0.3_0.7
0.5_0.1
0.5_0.3
0.5_0.5
0.5_0.7
0.7_0.1
0.7_0.3
0.7_0.5
0.7_0.7
D
Bias cor
Bias pcor
Bias lm phen ~ met
Bias lm met ~ phen
Bias logistic regr.
Bias
True correlation
True partial cor.
True effect size
E
Power cor
Power pcor
Power lm phen ~ met
Power lm met ~ phen
Power logistic regr.
Power
True correlation
True partial cor.
True effect size

Data: 1 variable runday−specific probabilistic LOD; 1 variable unsystematic missingness; Imputation method: KNN−var(3)

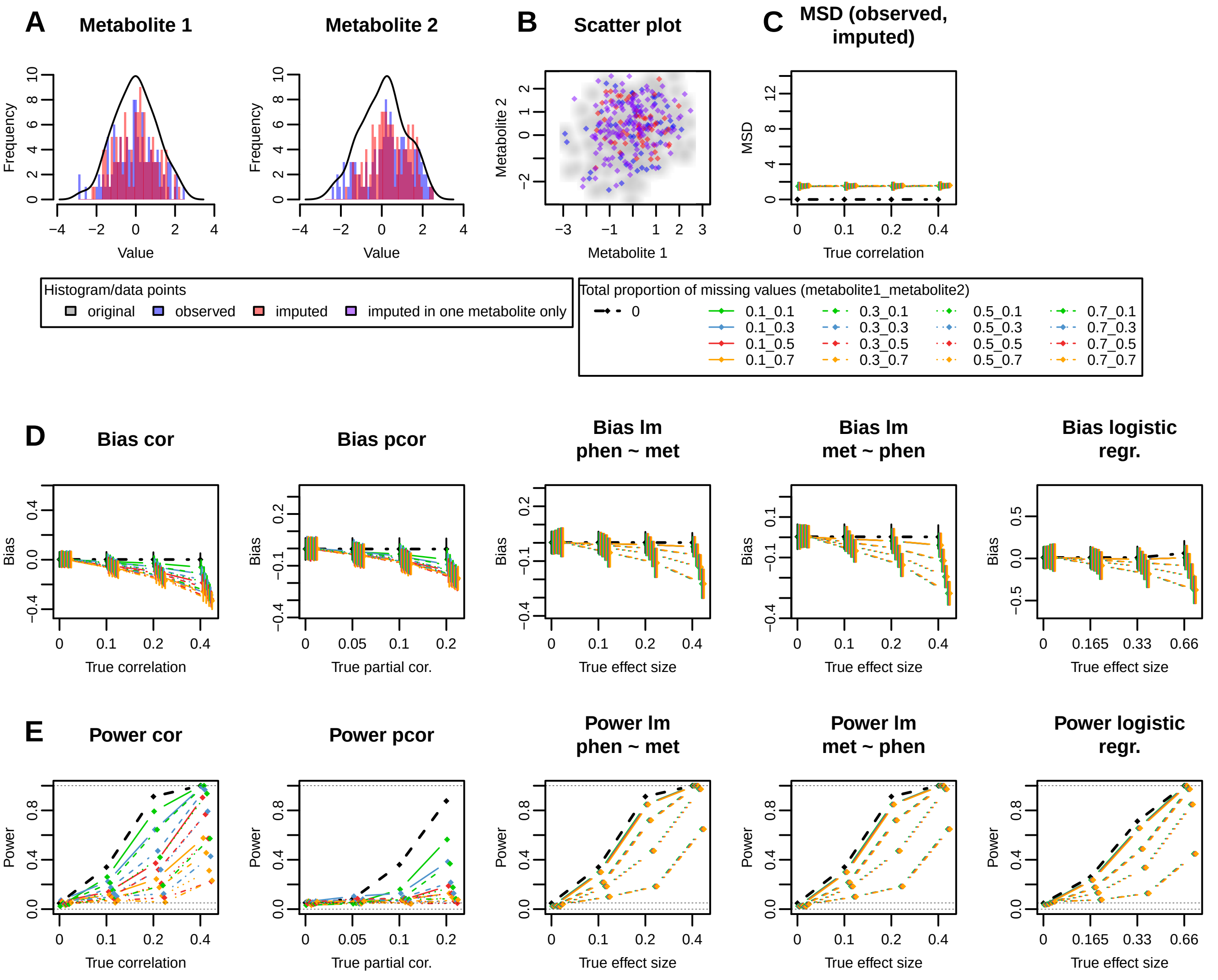

Data: 1 variable runday−specific probabilistic LOD; 1 variable unsystematic missingness; Imputation method: KNN−var(5)

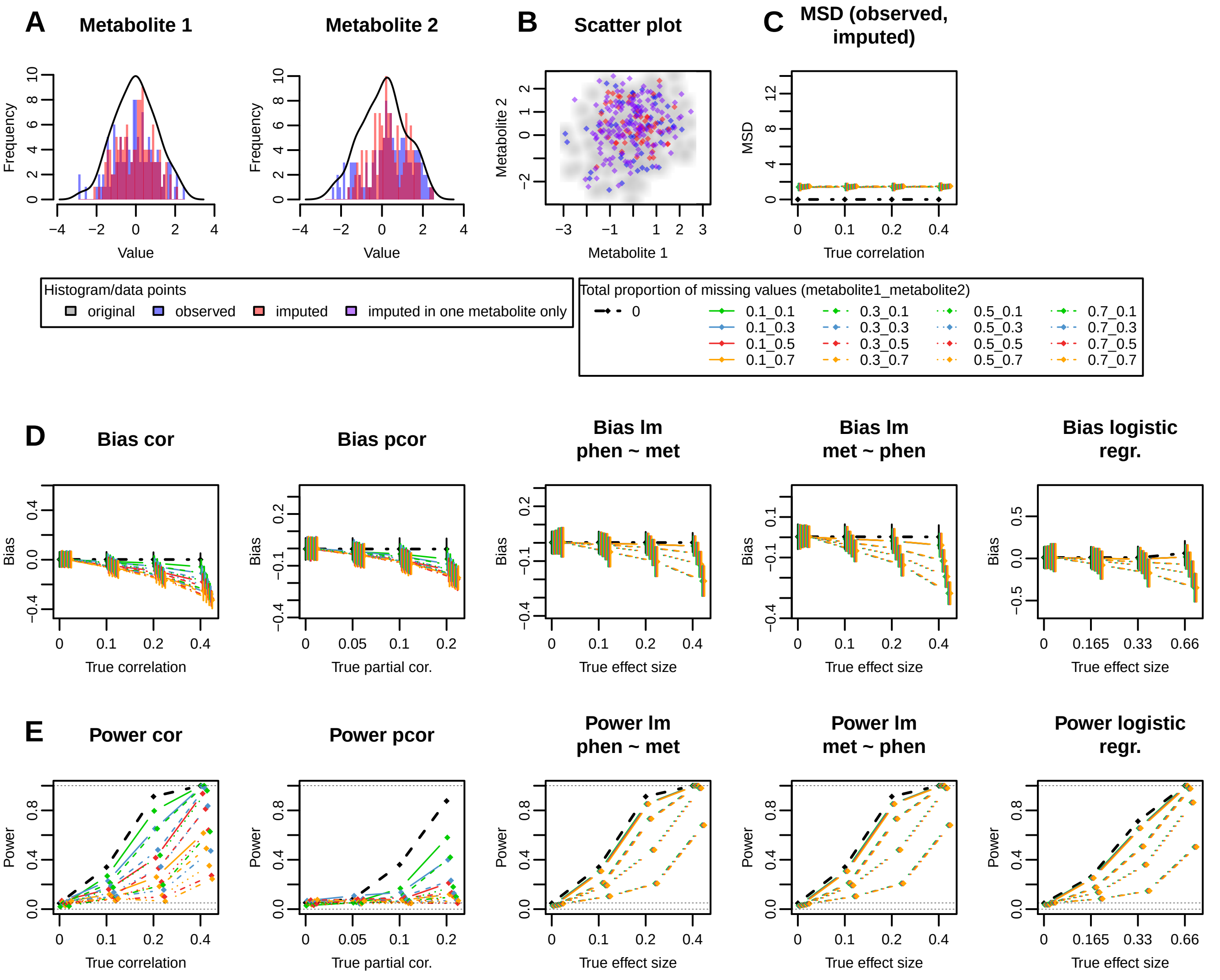

Data: 1 variable runday−specific probabilistic LOD; 1 variable unsystematic missingness; Imputation method: KNN−var(10)

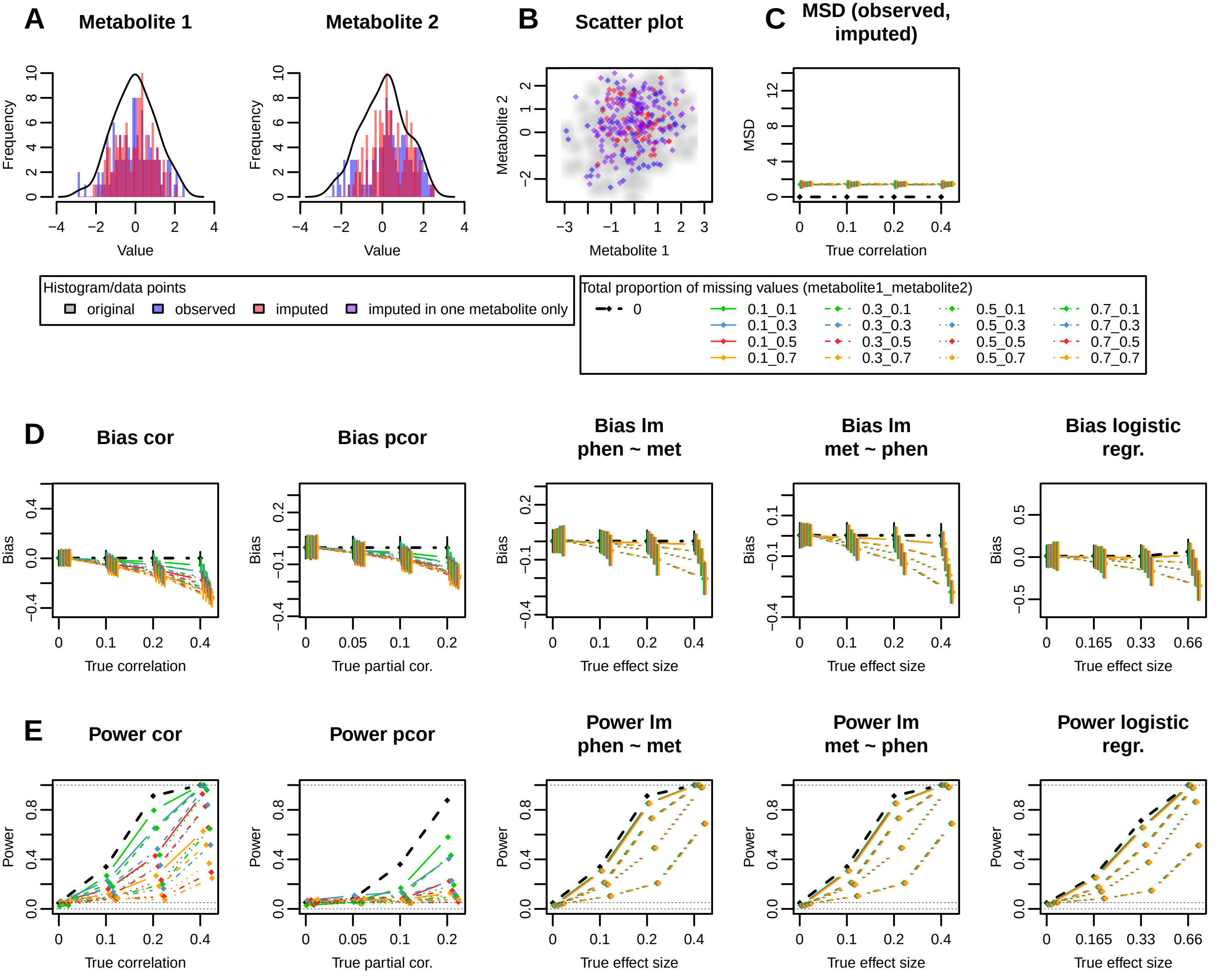

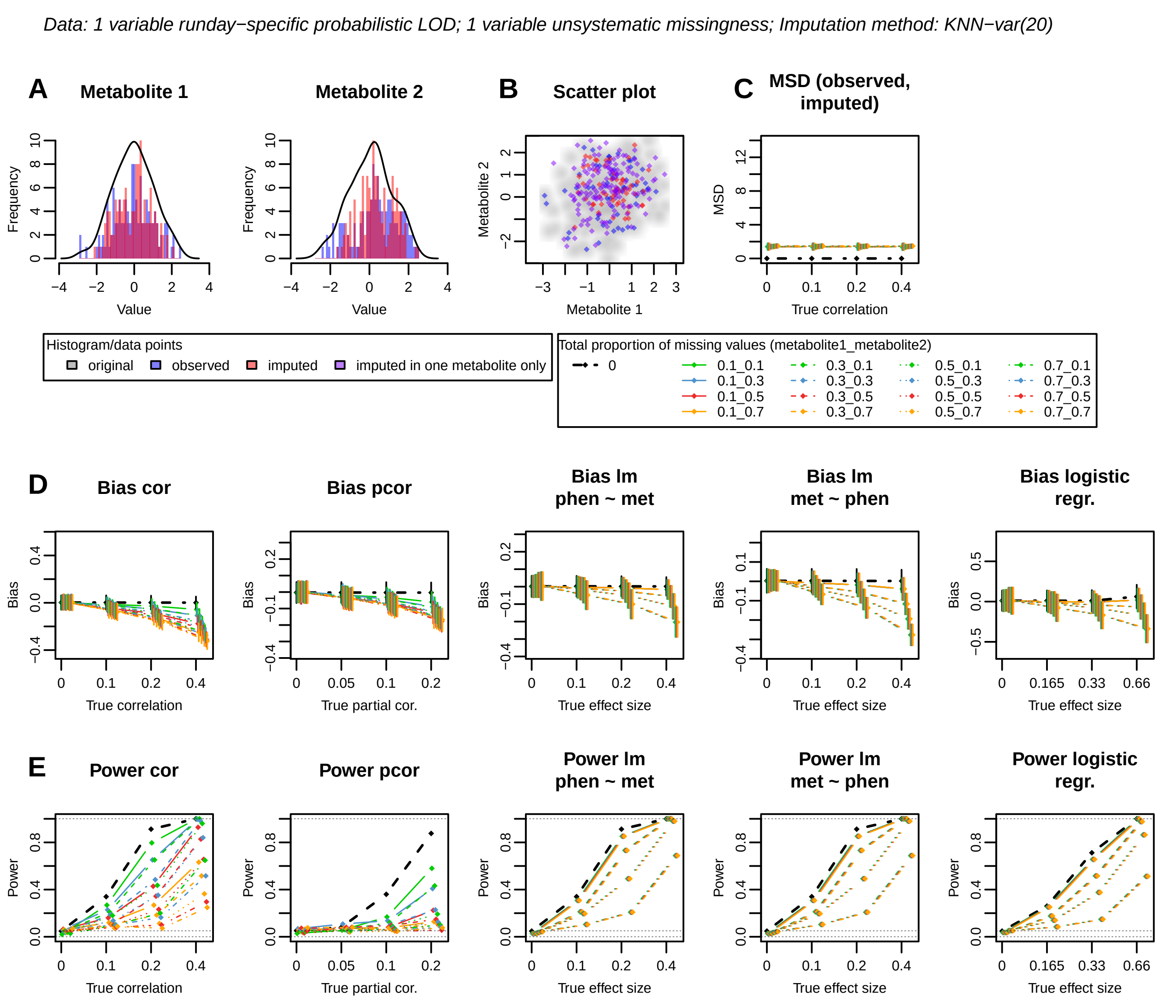

Data: 1 variable runday−specific probabilistic LOD; 1 variable unsystematic missingness; Imputation method: KNN−var(20)
A
Metabolite 1
Metabolite 2
B
Scatter plot
C
MSD (observed, imputed)
Frequency
Value
Metabolite 2
Metabolite 1
MSD
True correlation
Histogram/data points
original
observed
imputed
imputed in one metabolite only
Total proportion of missing values (metabolite1_metabolite2)
0
0.1_0.1
0.1_0.3
0.1_0.5
0.1_0.7
0.3_0.1
0.3_0.3
0.3_0.5
0.3_0.7
0.5_0.1
0.5_0.3
0.5_0.5
0.5_0.7
0.7_0.1
0.7_0.3
0.7_0.5
0.7_0.7
D
Bias cor
Bias pcor
Bias lm phen ~ met
Bias lm met ~ phen
Bias logistic regr.
Bias
True correlation
True partial cor.
True effect size
E
Power cor
Power pcor
Power lm phen ~ met
Power lm met ~ phen
Power logistic regr.
Power

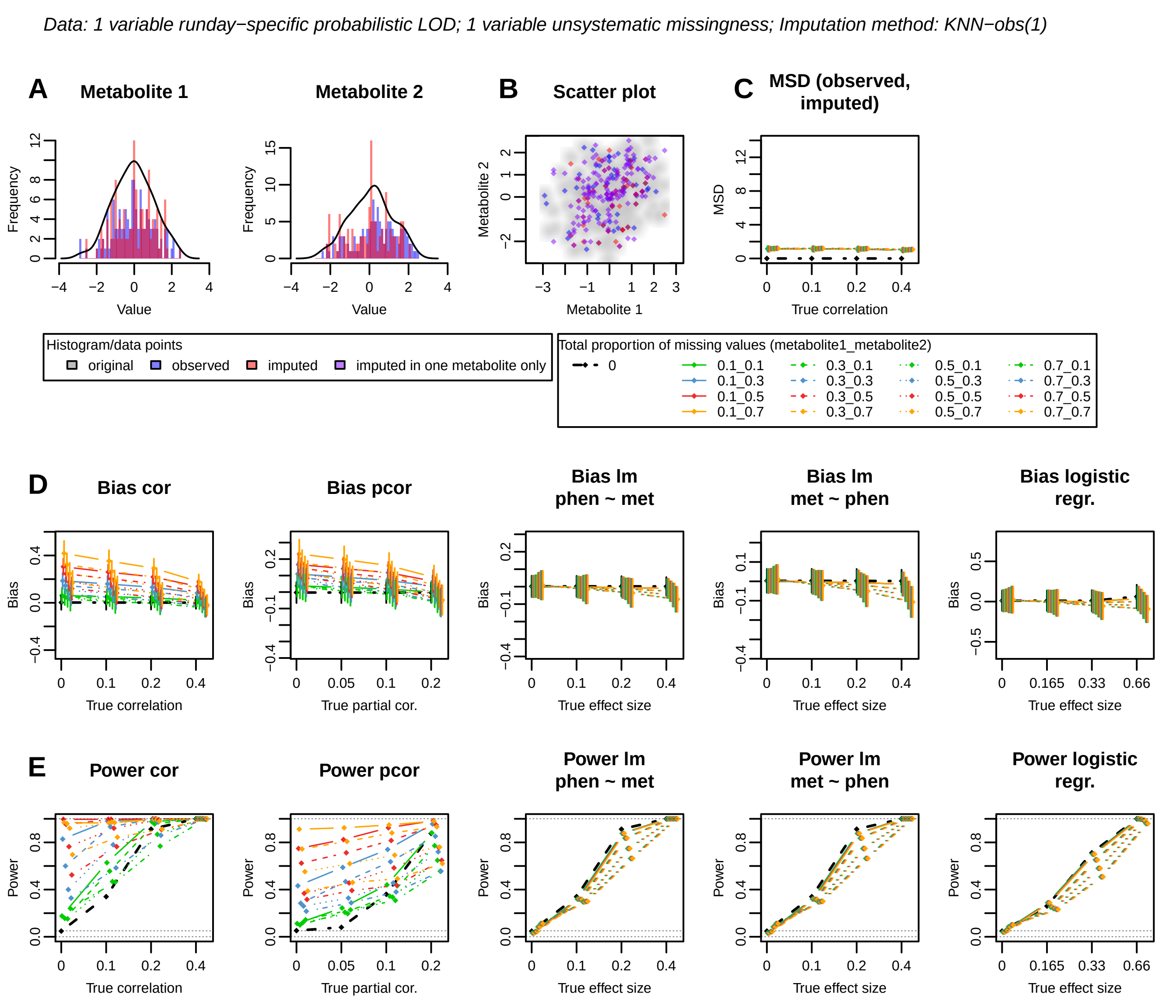
Data: 1 variable runday−specific probabilistic LOD; 1 variable unsystematic missingness; Imputation method: KNN−obs(1)
A
Metabolite 1
Metabolite 2
Frequency
Value
B
Scatter plot
Metabolite 2
Metabolite 1
C
MSD (observed, imputed)
MSD
True correlation
Histogram/data points
original
observed
imputed
imputed in one metabolite only
Total proportion of missing values (metabolite1_metabolite2)
0
0.1_0.1
0.1_0.3
0.1_0.5
0.1_0.7
0.3_0.1
0.3_0.3
0.3_0.5
0.3_0.7
0.5_0.1
0.5_0.3
0.5_0.5
0.5_0.7
0.7_0.1
0.7_0.3
0.7_0.5
0.7_0.7
D
Bias cor
Bias pcor
Bias lm phen ~ met
Bias lm met ~ phen
Bias logistic regr.
Bias
True correlation
True partial cor.
True effect size
E
Power cor
Power pcor
Power lm phen ~ met
Power lm met ~ phen
Power logistic regr.
Power
True correlation
True partial cor.
True effect size

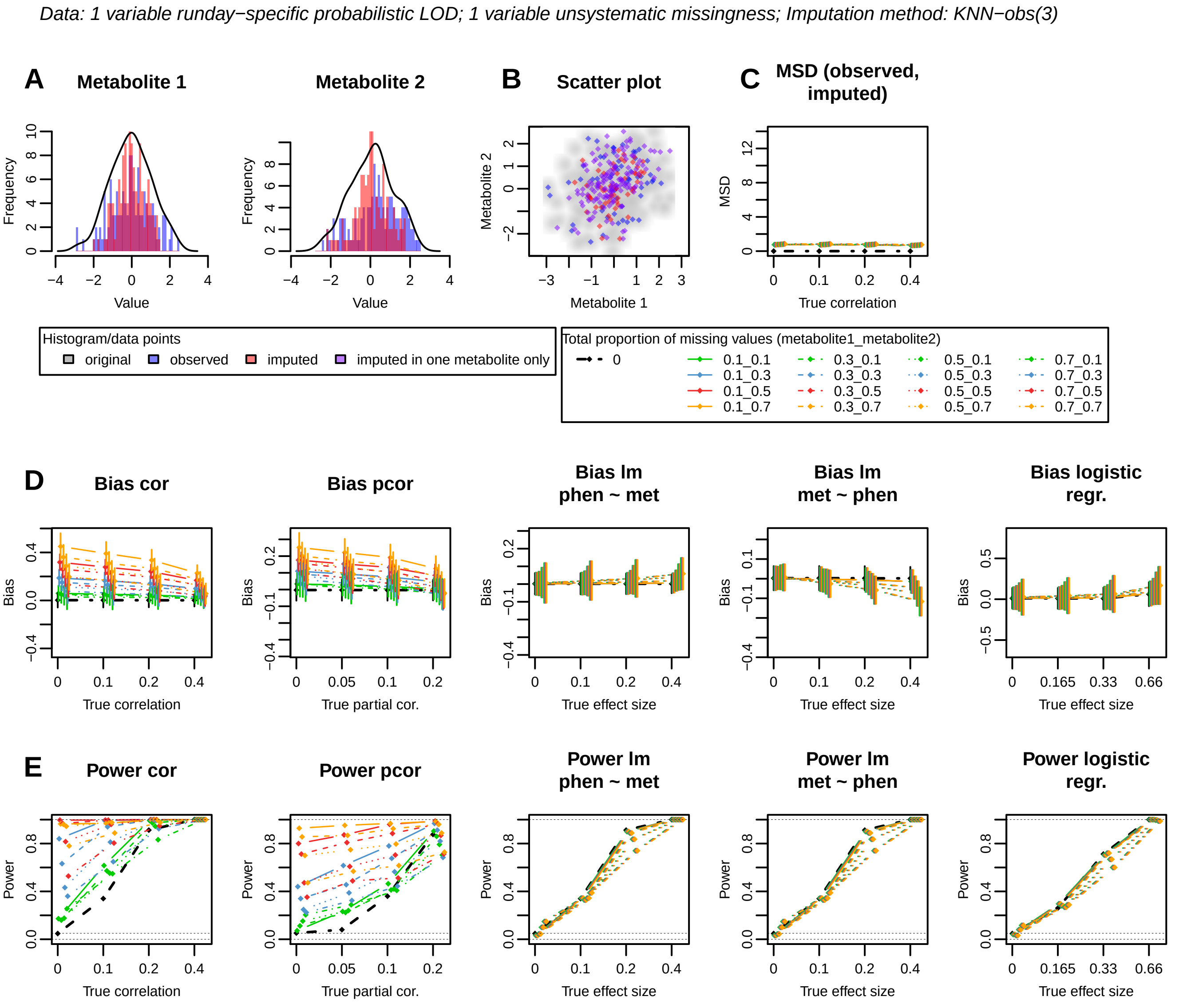
Data: 1 variable runday−specific probabilistic LOD; 1 variable unsystematic missingness; Imputation method: KNN−obs(3)
A
Metabolite 1
Metabolite 2
Frequency
Value
B
Scatter plot
Metabolite 1
Metabolite 2
C
MSD (observed, imputed)
MSD
True correlation
Histogram/data points
original
observed
imputed
imputed in one metabolite only
Total proportion of missing values (metabolite1_metabolite2)
0
0.1_0.1
0.1_0.3
0.1_0.5
0.1_0.7
0.3_0.1
0.3_0.3
0.3_0.5
0.3_0.7
0.5_0.1
0.5_0.3
0.5_0.5
0.5_0.7
0.7_0.1
0.7_0.3
0.7_0.5
0.7_0.7
D
Bias cor
Bias pcor
Bias lm phen ~ met
Bias lm met ~ phen
Bias logistic regr.
Bias
True correlation
True partial cor.
True effect size
E
Power cor
Power pcor
Power lm phen ~ met
Power lm met ~ phen
Power logistic regr.
Power
True correlation
True partial cor.
True effect size

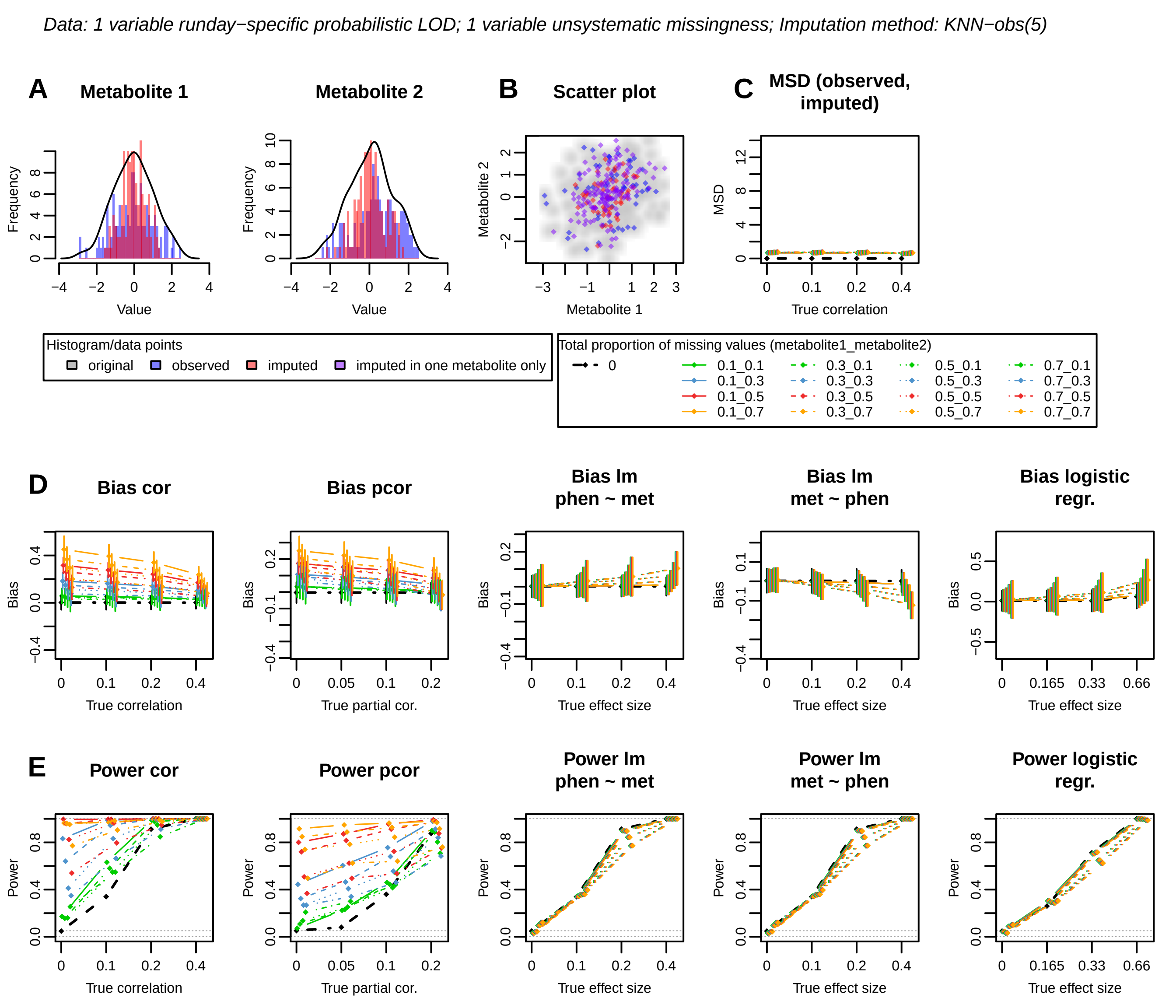

Data: 1 variable runday−specific probabilistic LOD; 1 variable unsystematic missingness; Imputation method: KNN−obs(5)
A
Metabolite 1
Metabolite 2
Frequency
Value
B
Scatter plot
Metabolite 1
Metabolite 2
C
MSD (observed, imputed)
MSD
True correlation
Histogram/data points
original
observed
imputed
imputed in one metabolite only
Total proportion of missing values (metabolite1_metabolite2)
0
0.1_0.1
0.1_0.3
0.1_0.5
0.1_0.7
0.3_0.1
0.3_0.3
0.3_0.5
0.3_0.7
0.5_0.1
0.5_0.3
0.5_0.5
0.5_0.7
0.7_0.1
0.7_0.3
0.7_0.5
0.7_0.7
D
Bias cor
Bias pcor
Bias lm phen ~ met
Bias lm met ~ phen
Bias logistic regr.
Bias
True correlation
True partial cor.
True effect size
E
Power cor
Power pcor
Power lm phen ~ met
Power lm met ~ phen
Power logistic regr.
Power
True correlation
True partial cor.
True effect size

Data: 1 variable runday−specific probabilistic LOD; 1 variable unsystematic missingness; Imputation method: KNN−obs(10)

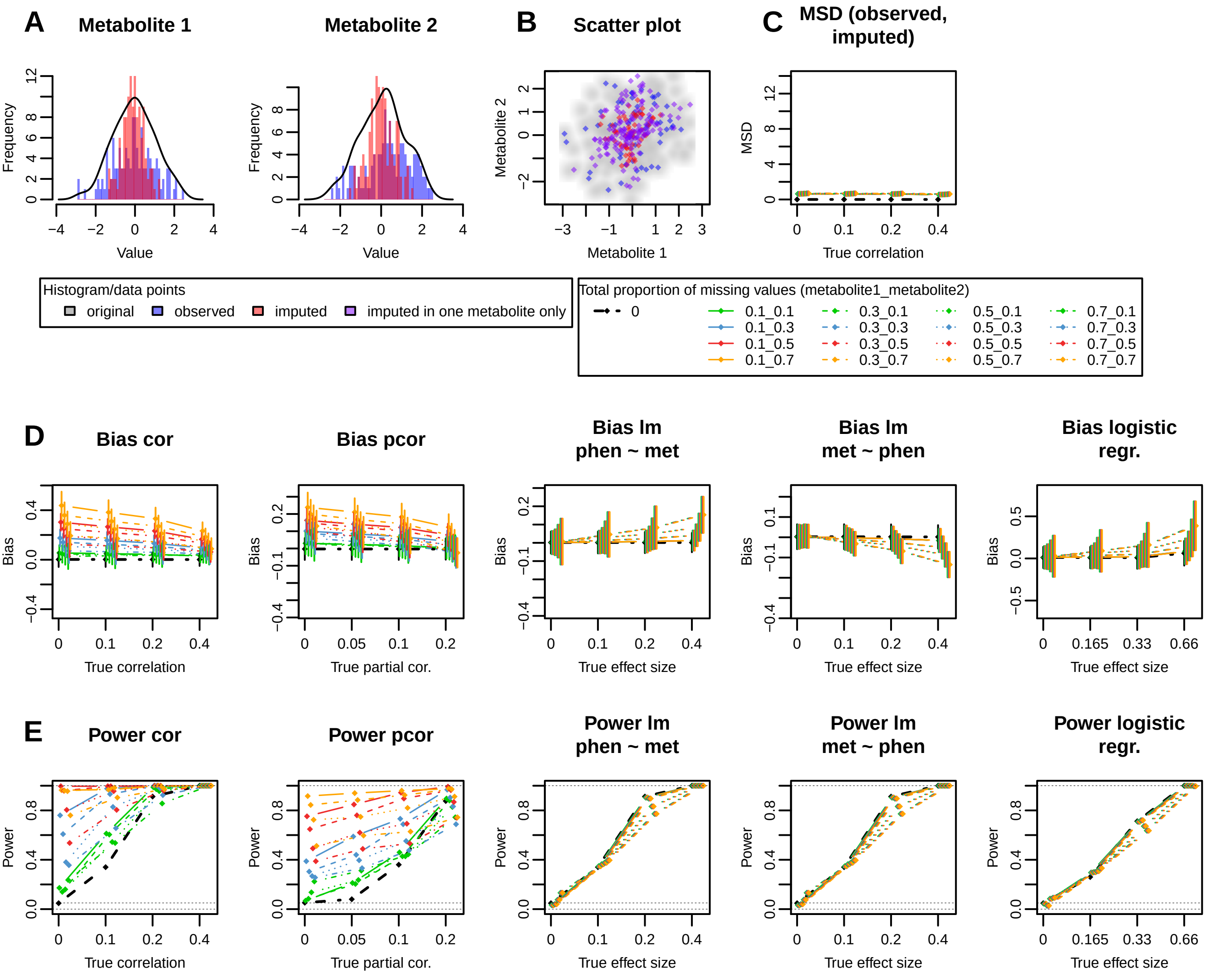

Data: 1 variable runday−specific probabilistic LOD; 1 variable unsystematic missingness; Imputation method: KNN−obs(20)

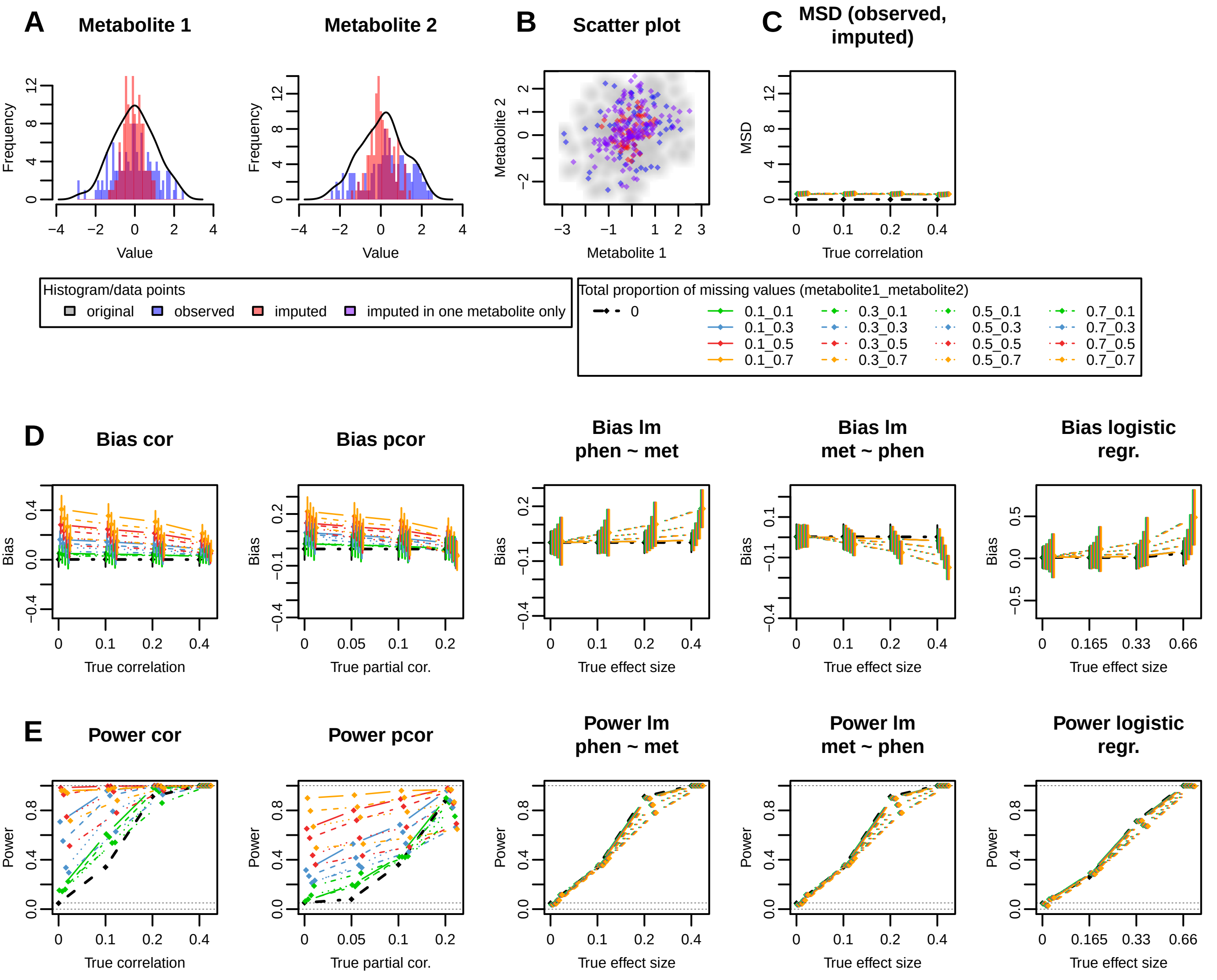

Data: 1 variable runday−specific probabilistic LOD; 1 variable unsystematic missingness; Imputation method: KNN−obs−sel(1)

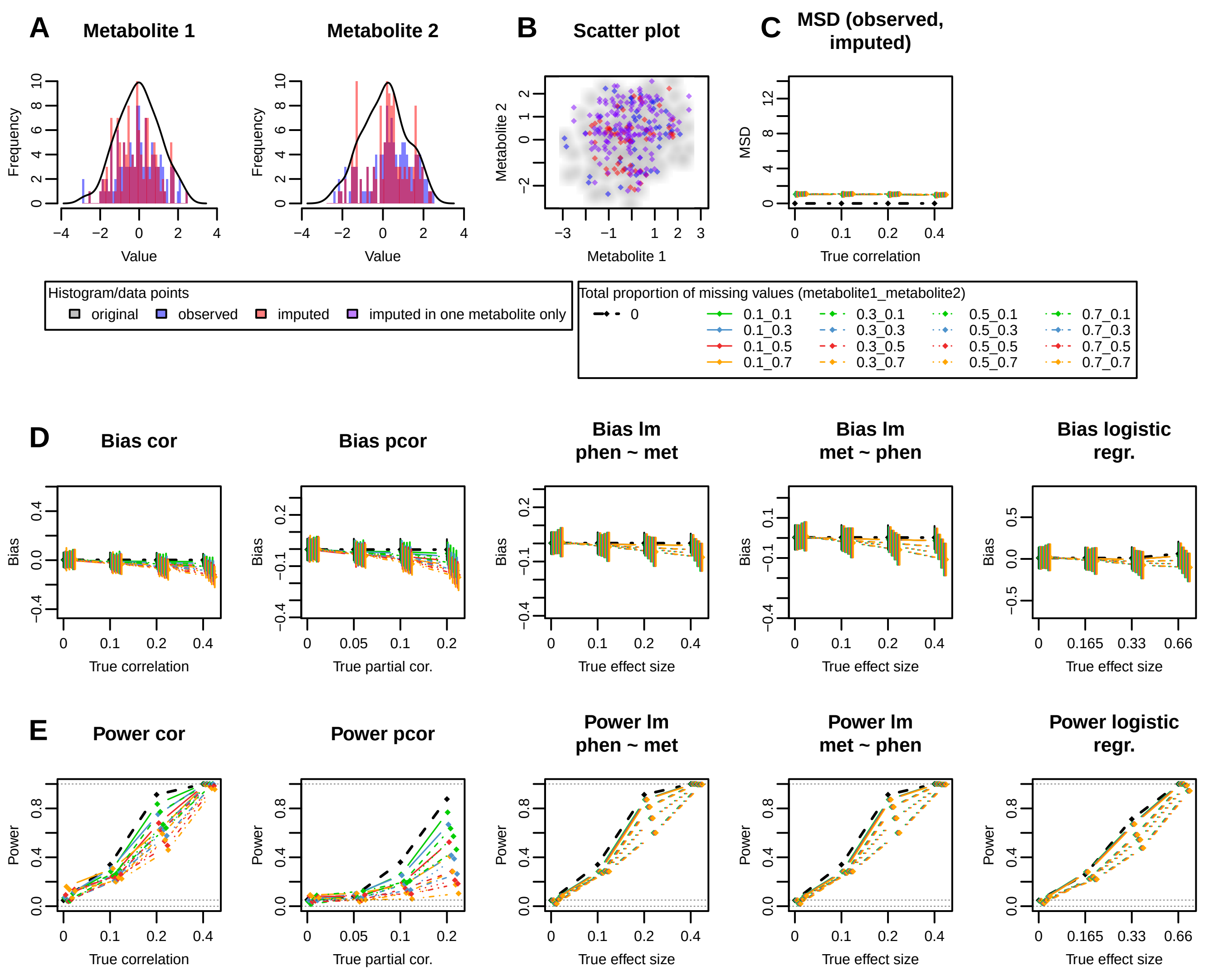

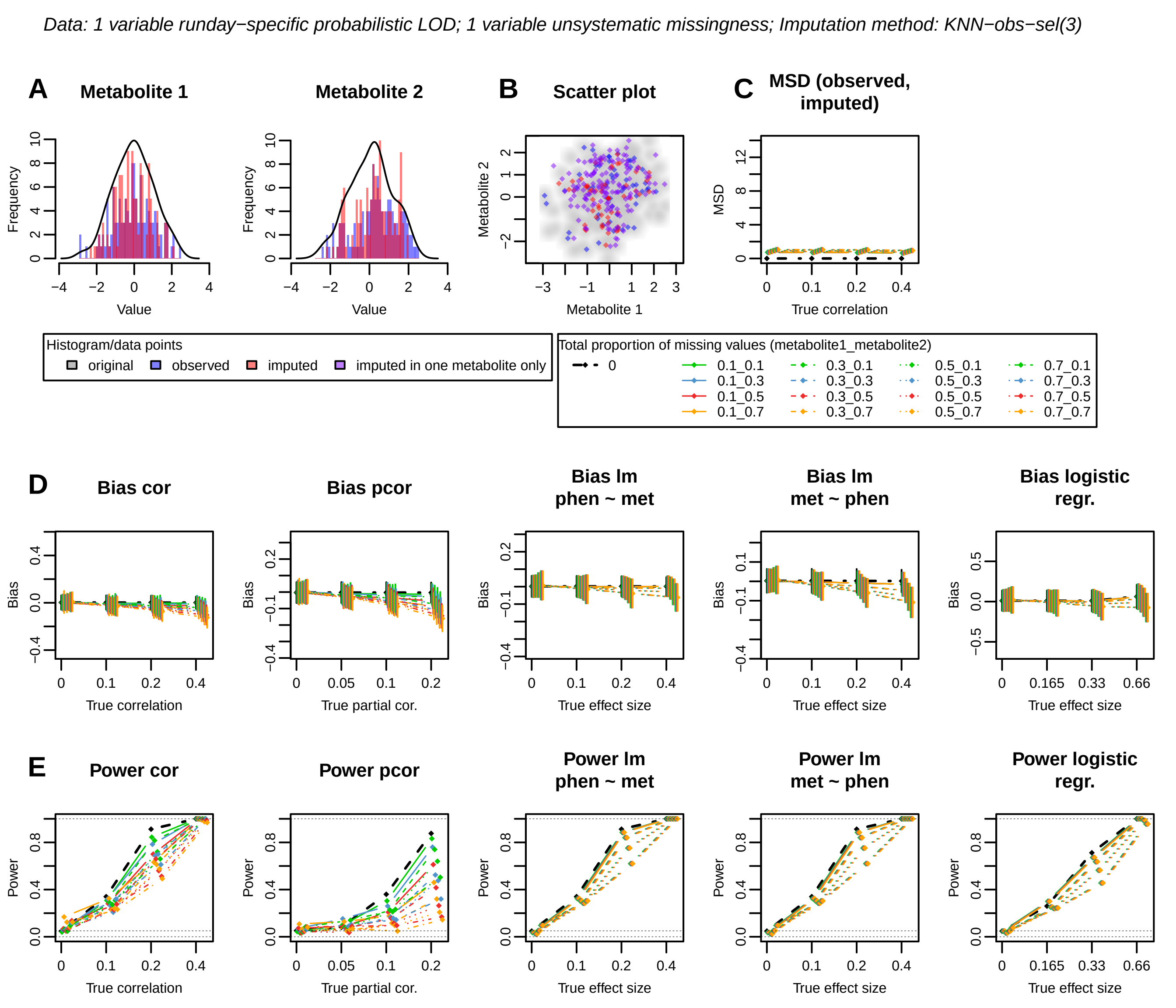
Data: 1 variable runday−specific probabilistic LOD; 1 variable unsystematic missingness; Imputation method: KNN−obs−sel(3)
A
Metabolite 1
Metabolite 2
Frequency
Value
B
Scatter plot
Metabolite 2
Metabolite 1
C
MSD (observed, imputed)
MSD
True correlation
Histogram/data points
original
observed
imputed
imputed in one metabolite only
Total proportion of missing values (metabolite1_metabolite2)
0
0.1_0.1
0.1_0.3
0.1_0.5
0.1_0.7
0.3_0.1
0.3_0.3
0.3_0.5
0.3_0.7
0.5_0.1
0.5_0.3
0.5_0.5
0.5_0.7
0.7_0.1
0.7_0.3
0.7_0.5
0.7_0.7
D
Bias cor
Bias pcor
Bias lm phen ~ met
Bias lm met ~ phen
Bias logistic regr.
Bias
True correlation
True partial cor.
True effect size
E
Power cor
Power pcor
Power lm phen ~ met
Power lm met ~ phen
Power logistic regr.
Power
True correlation
True partial cor.
True effect size

Data: 1 variable runday−specific probabilistic LOD; 1 variable unsystematic missingness; Imputation method: KNN−obs−sel(5)

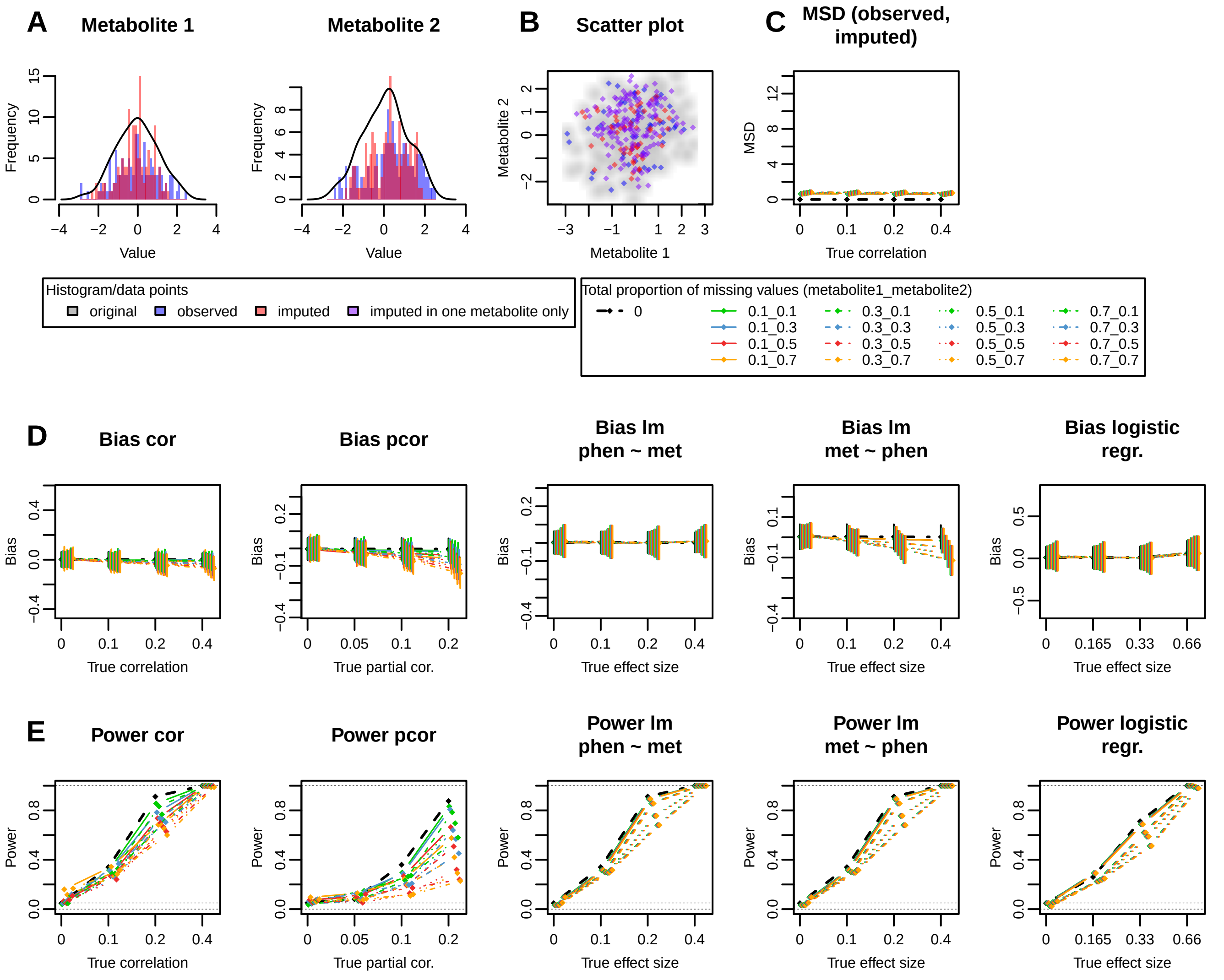

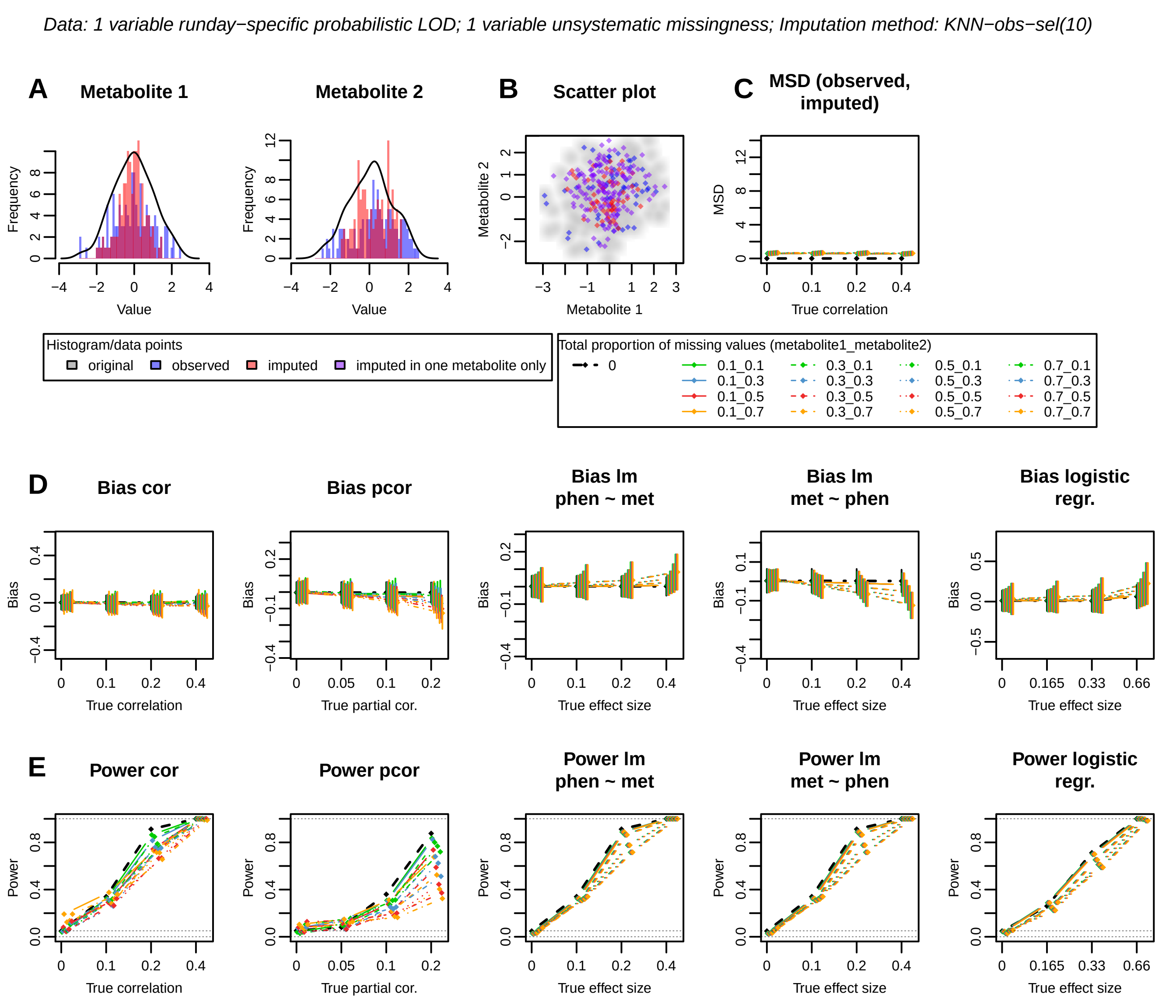
Data: 1 variable runday−specific probabilistic LOD; 1 variable unsystematic missingness; Imputation method: KNN−obs−sel(10)
A
Metabolite 1
Metabolite 2
Frequency
Value
B
Scatter plot
Metabolite 1
Metabolite 2
C
MSD (observed, imputed)
MSD
True correlation
Histogram/data points
original
observed
imputed
imputed in one metabolite only
Total proportion of missing values (metabolite1_metabolite2)
0
0.1_0.1
0.1_0.3
0.1_0.5
0.1_0.7
0.3_0.1
0.3_0.3
0.3_0.5
0.3_0.7
0.5_0.1
0.5_0.3
0.5_0.5
0.5_0.7
0.7_0.1
0.7_0.3
0.7_0.5
0.7_0.7
D
Bias cor
Bias pcor
Bias lm phen ~ met
Bias lm met ~ phen
Bias logistic regr.
Bias
True correlation
True partial cor.
True effect size
E
Power cor
Power pcor
Power lm phen ~ met
Power lm met ~ phen
Power logistic regr.
Power
True correlation
True partial cor.
True effect size

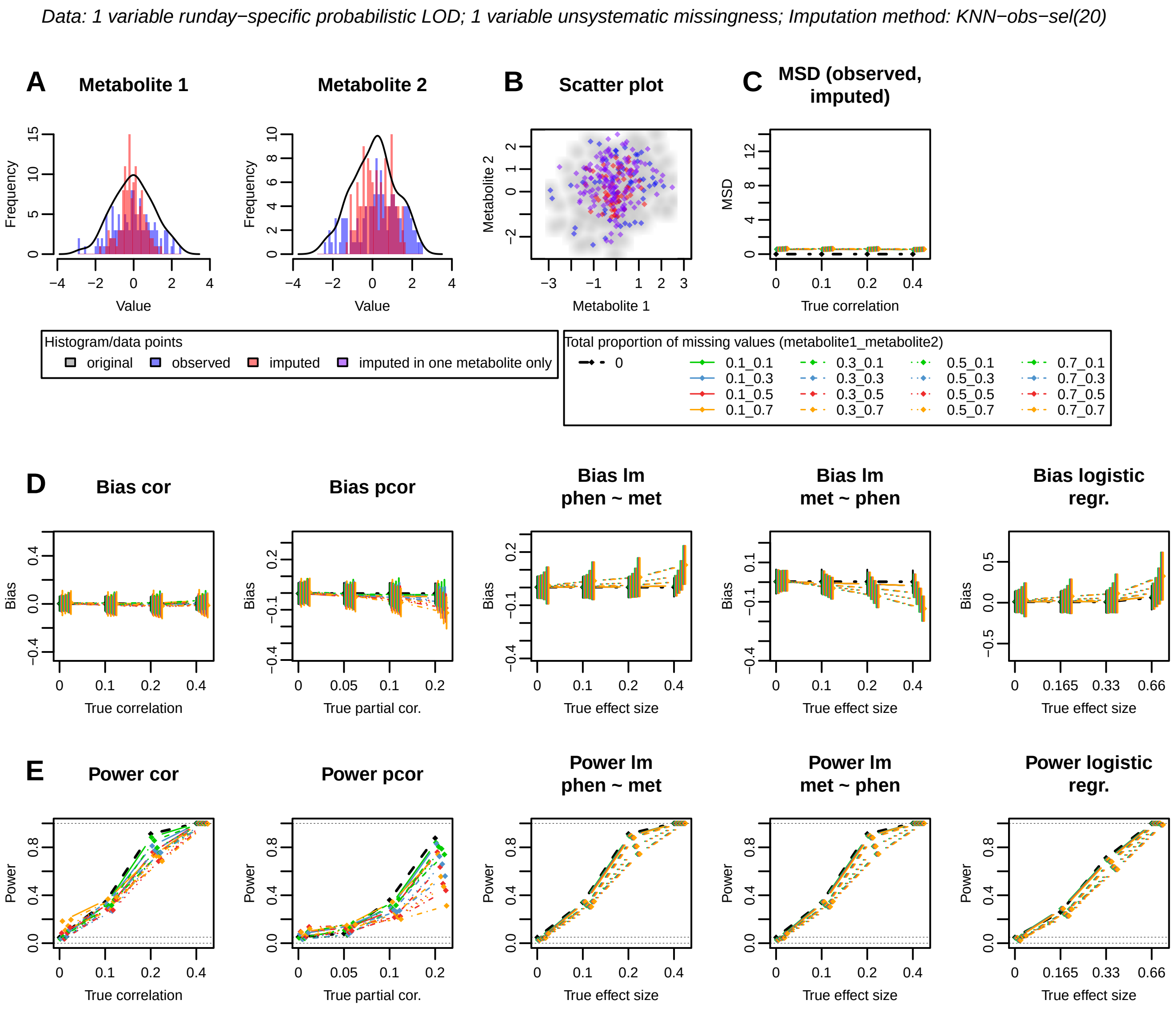
Data: 1 variable runday−specific probabilistic LOD; 1 variable unsystematic missingness; Imputation method: KNN−obs−sel(20)
A
Metabolite 1
Metabolite 2
Frequency
Value
B
Scatter plot
Metabolite 2
Metabolite 1
C
MSD (observed, imputed)
MSD
True correlation
Histogram/data points
original
observed
imputed
imputed in one metabolite only
Total proportion of missing values (metabolite1_metabolite2)
0
0.1_0.1
0.1_0.3
0.1_0.5
0.1_0.7
0.3_0.1
0.3_0.3
0.3_0.5
0.3_0.7
0.5_0.1
0.5_0.3
0.5_0.5
0.5_0.7
0.7_0.1
0.7_0.3
0.7_0.5
0.7_0.7
D
Bias cor
Bias pcor
Bias lm phen ~ met
Bias lm met ~ phen
Bias logistic regr.
Bias
True correlation
True partial cor.
True effect size
E
Power cor
Power pcor
Power lm phen ~ met
Power lm met ~ phen
Power logistic regr.
Power
True correlation
True partial cor.
True effect size

Data: 1:1 mixture of runday−specific probabilistic LOD with unsystematic missingness; Imputation method: CCA

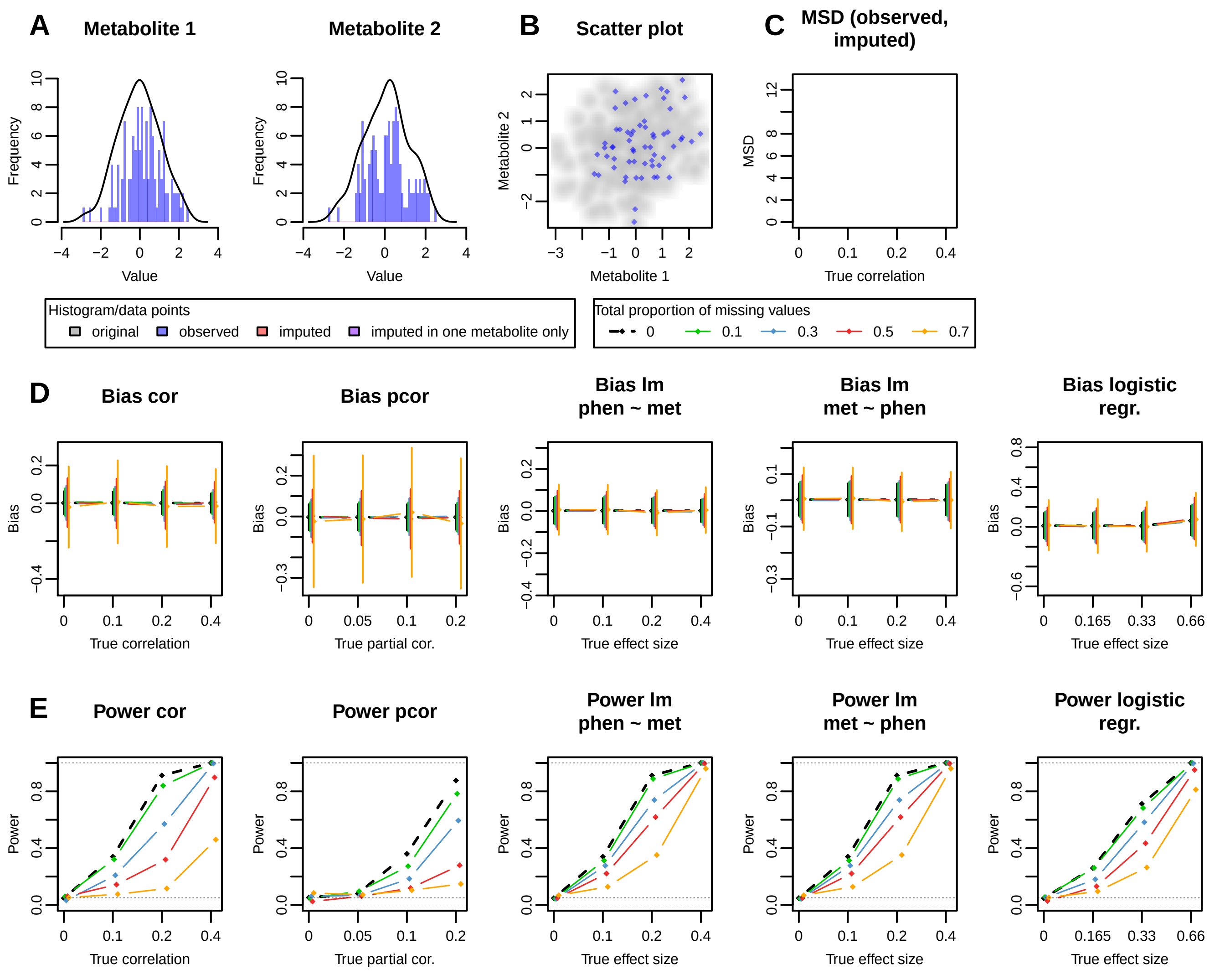

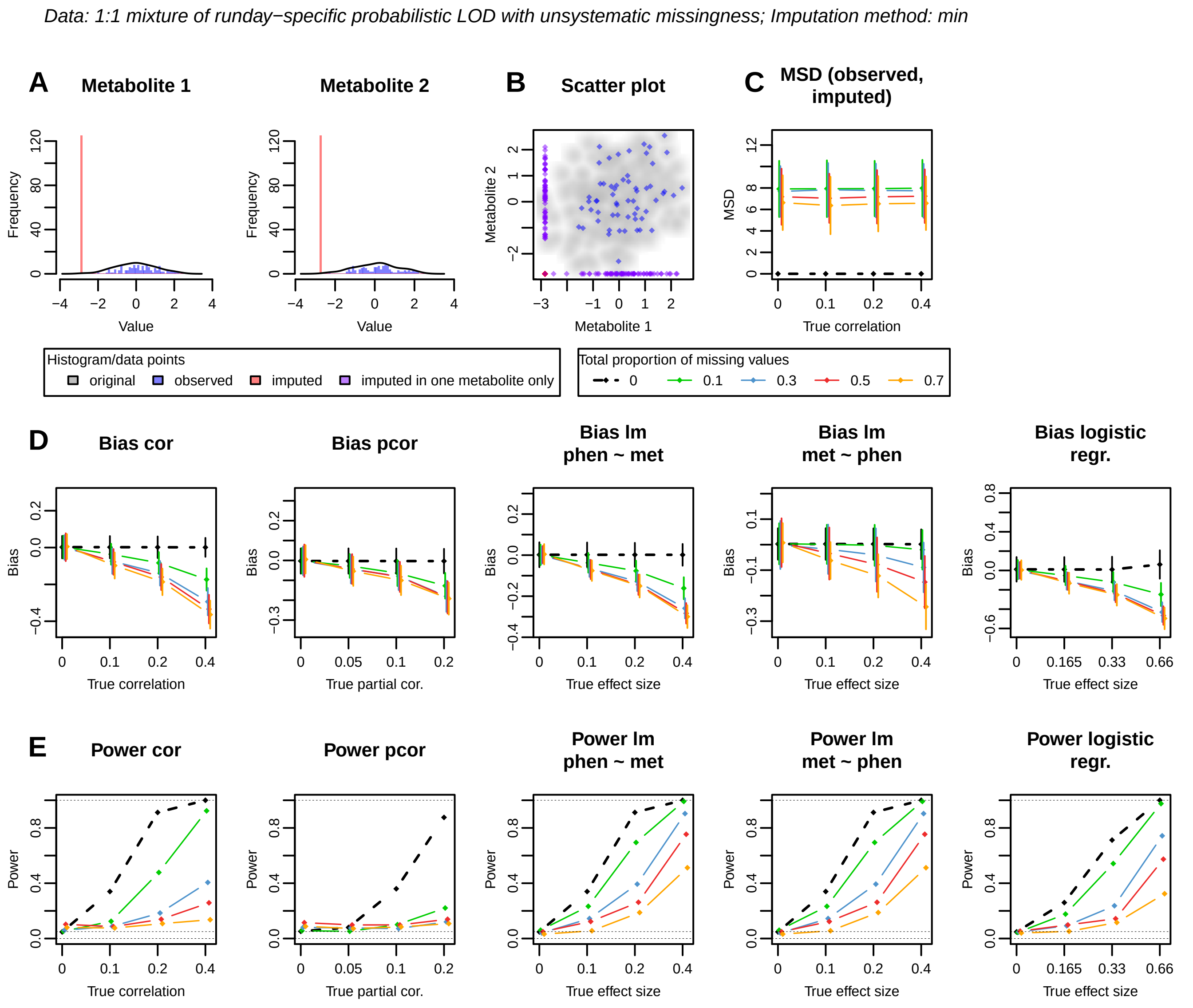

Data: 1:1 mixture of runday−specific probabilistic LOD with unsystematic missingness; Imputation method: min
A
Metabolite 1
Metabolite 2
B
Scatter plot
C
MSD (observed, imputed)
Frequency
Value
Metabolite 2
Metabolite 1
MSD
True correlation
Histogram/data points
original
observed
imputed
imputed in one metabolite only
Total proportion of missing values
0
0.1
0.3
0.5
0.7
D
Bias cor
Bias pcor
Bias lm phen ~ met
Bias lm met ~ phen
Bias logistic regr.
Bias
True correlation
True partial cor.
True effect size
E
Power cor
Power pcor
Power lm phen ~ met
Power lm met ~ phen
Power logistic regr.
Power

Data: 1:1 mixture of runday−specific probabilistic LOD with unsystematic missingness; Imputation method: RC

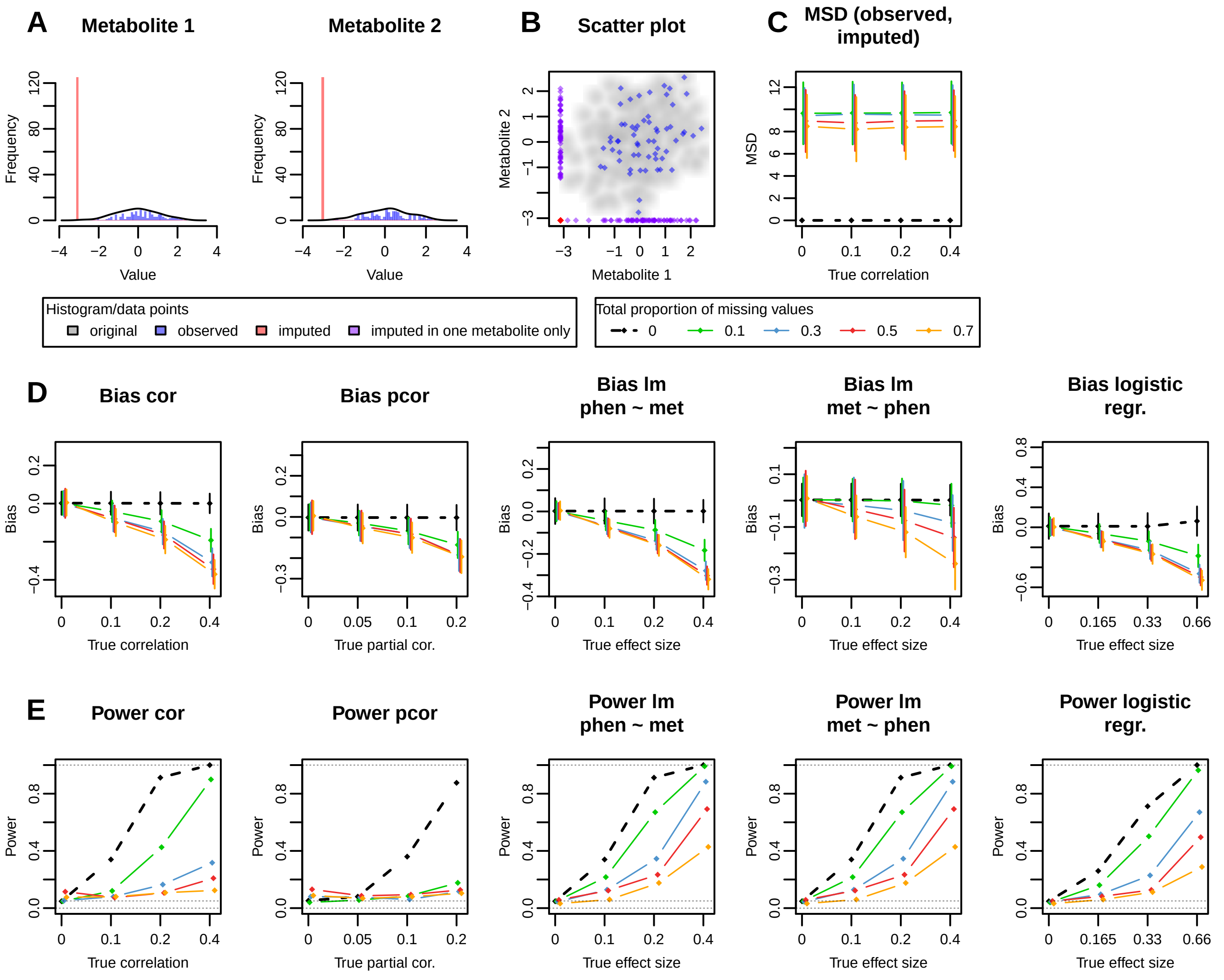

Data: 1:1 mixture of runday−specific probabilistic LOD with unsystematic missingness; Imputation method: ITS

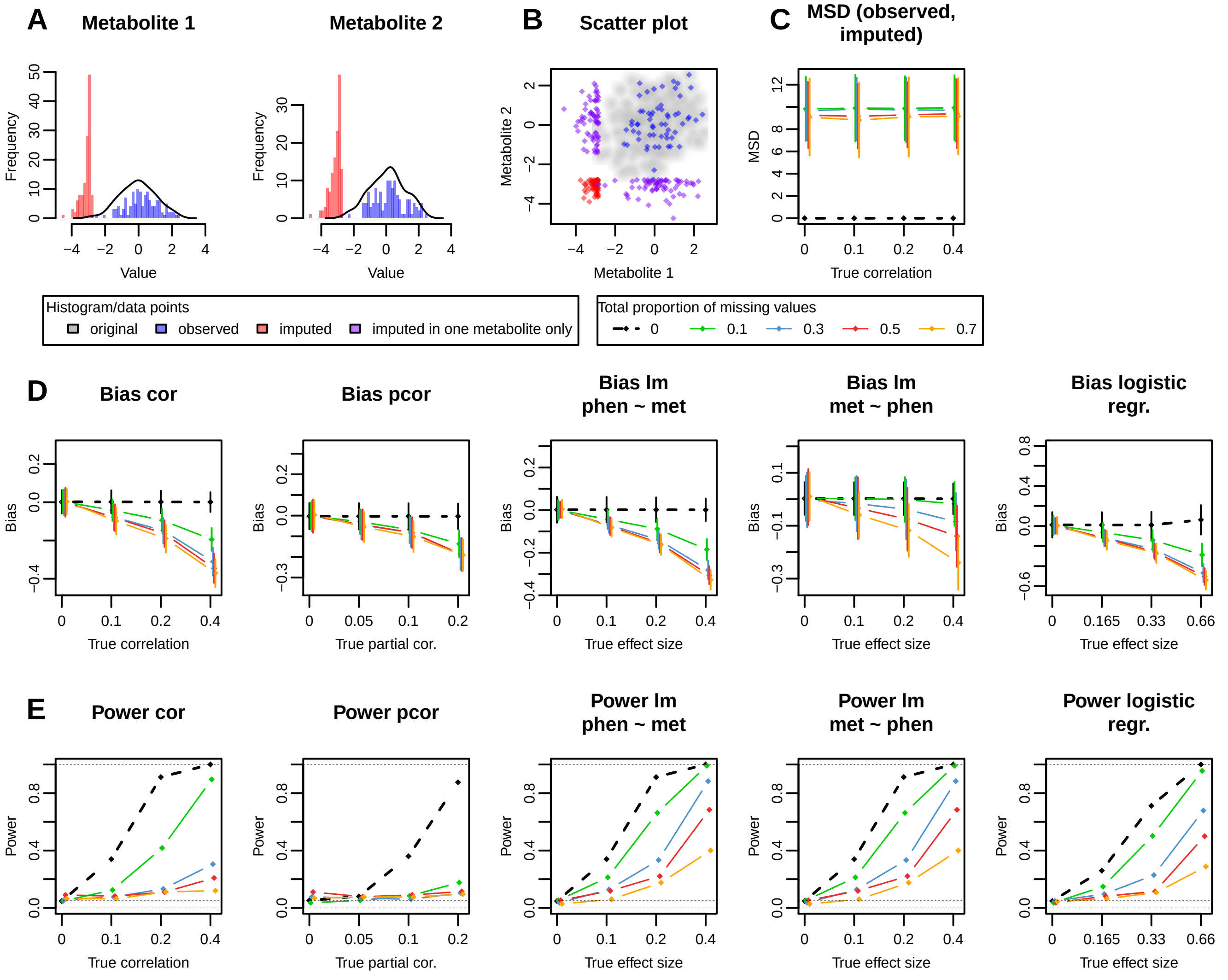

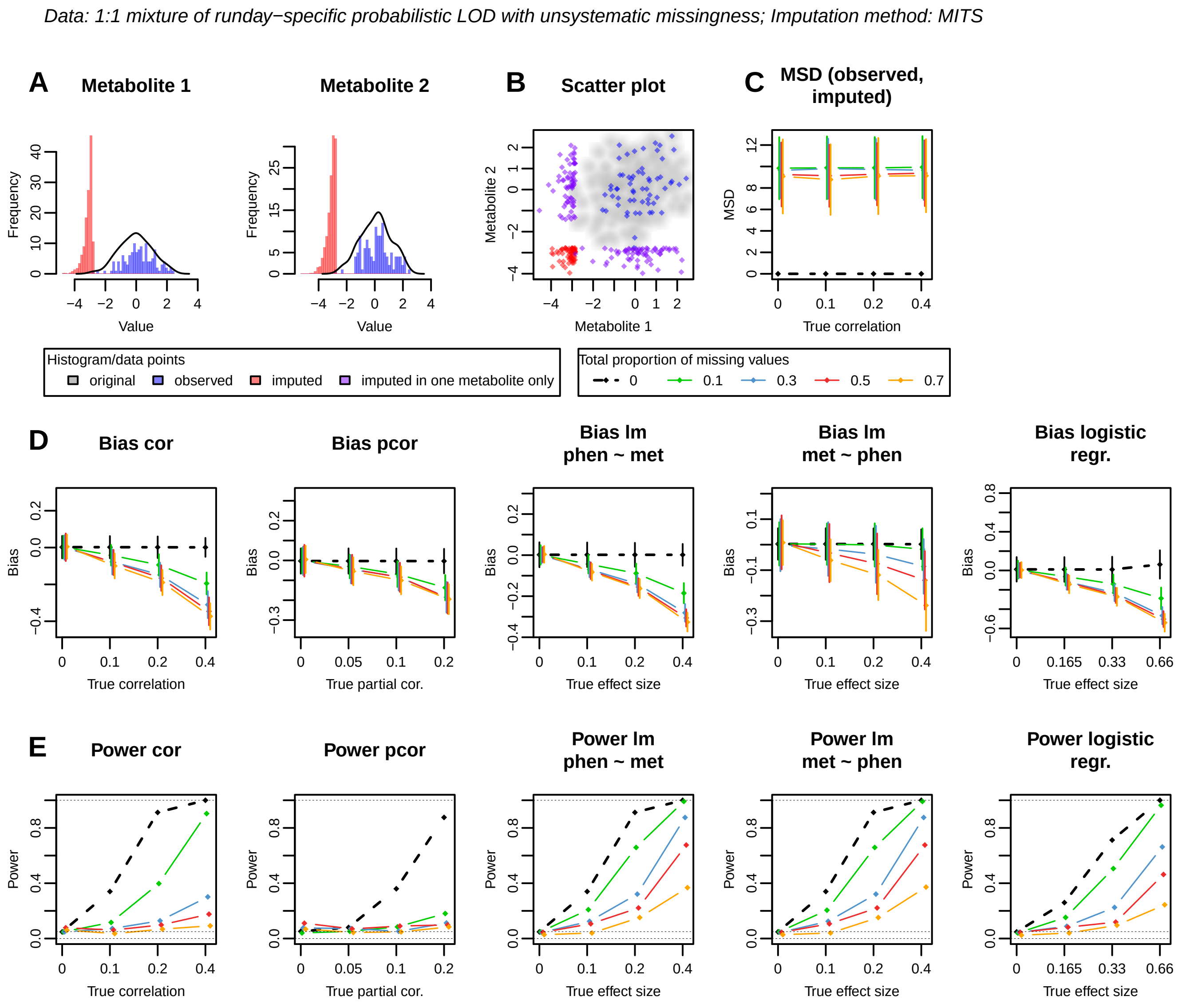
Data: 1:1 mixture of runday−specific probabilistic LOD with unsystematic missingness; Imputation method: MITS
A
Metabolite 1
Metabolite 2
Frequency
Value
B
Scatter plot
Metabolite 2
Metabolite 1
C
MSD (observed, imputed)
MSD
True correlation
Histogram/data points
original
observed
imputed
imputed in one metabolite only
Total proportion of missing values
0
0.1
0.3
0.5
0.7
D
Bias cor
Bias pcor
Bias lm phen ~ met
Bias lm met ~ phen
Bias logistic regr.
Bias
True correlation
True partial cor.
True effect size
E
Power cor
Power pcor
Power lm phen ~ met
Power lm met ~ phen
Power logistic regr.
Power
True correlation
True partial cor.
True effect size

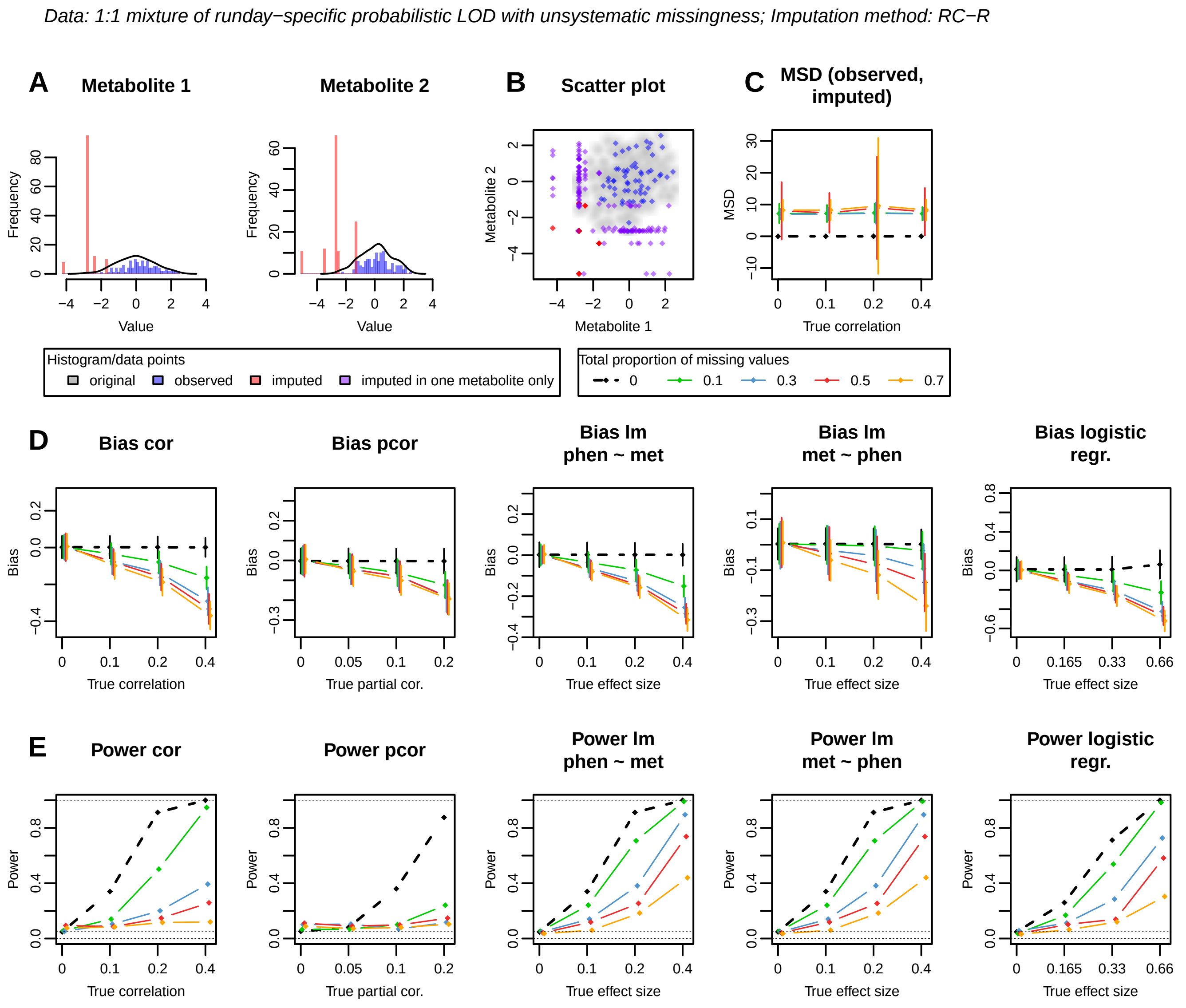

Data: 1:1 mixture of runday−specific probabilistic LOD with unsystematic missingness; Imputation method: RC−R
A
Metabolite 1
Metabolite 2
B
Scatter plot
C
MSD (observed, imputed)
Frequency
Value
Metabolite 2
Metabolite 1
MSD
True correlation
Histogram/data points
original
observed
imputed
imputed in one metabolite only
Total proportion of missing values
0
0.1
0.3
0.5
0.7
D
Bias cor
Bias pcor
Bias lm phen ~ met
Bias lm met ~ phen
Bias logistic regr.
Bias
True correlation
True partial cor.
True effect size
E
Power cor
Power pcor
Power lm phen ~ met
Power lm met ~ phen
Power logistic regr.
Power

Data: 1:1 mixture of runday−specific probabilistic LOD with unsystematic missingness; Imputation method: ITS−R

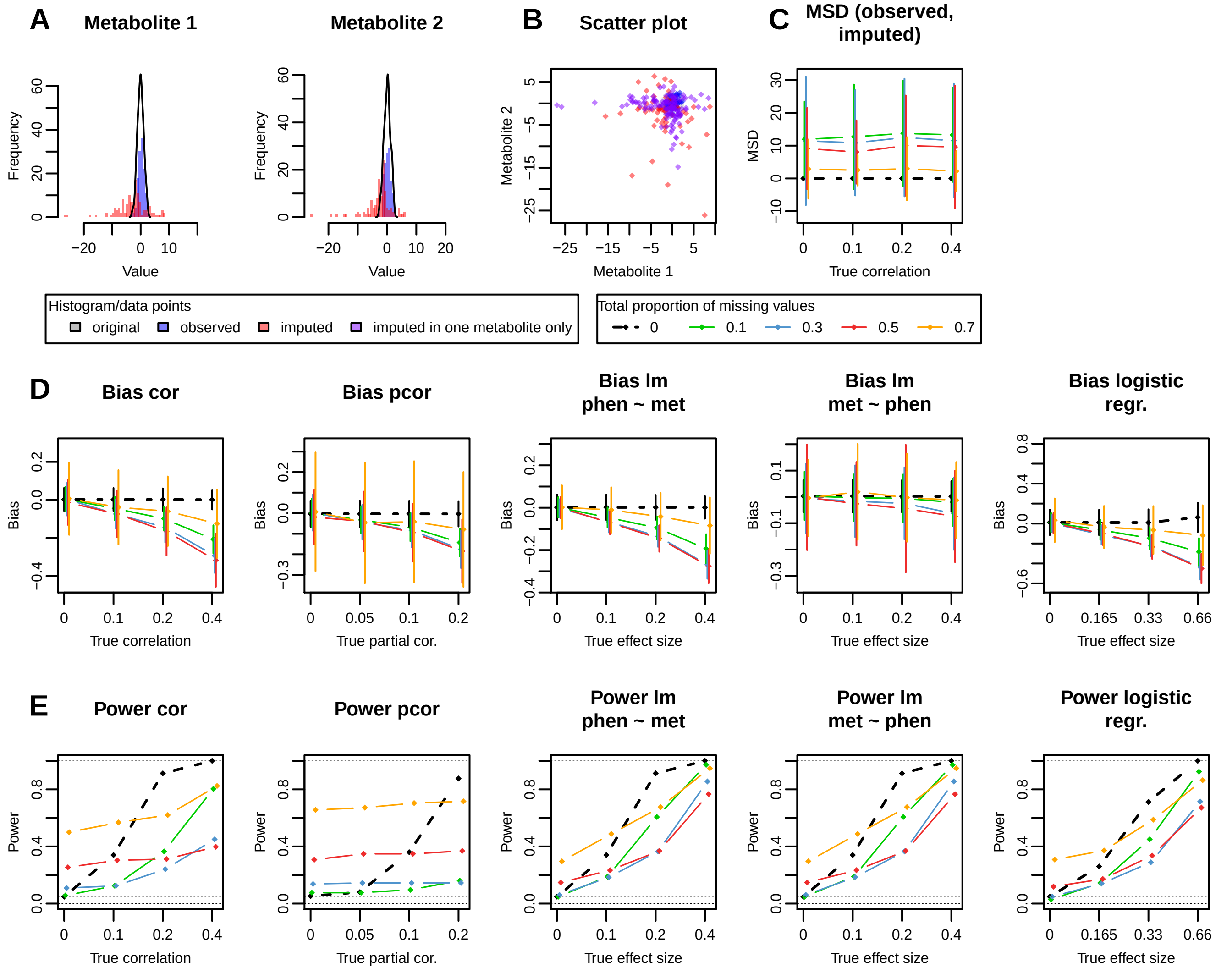

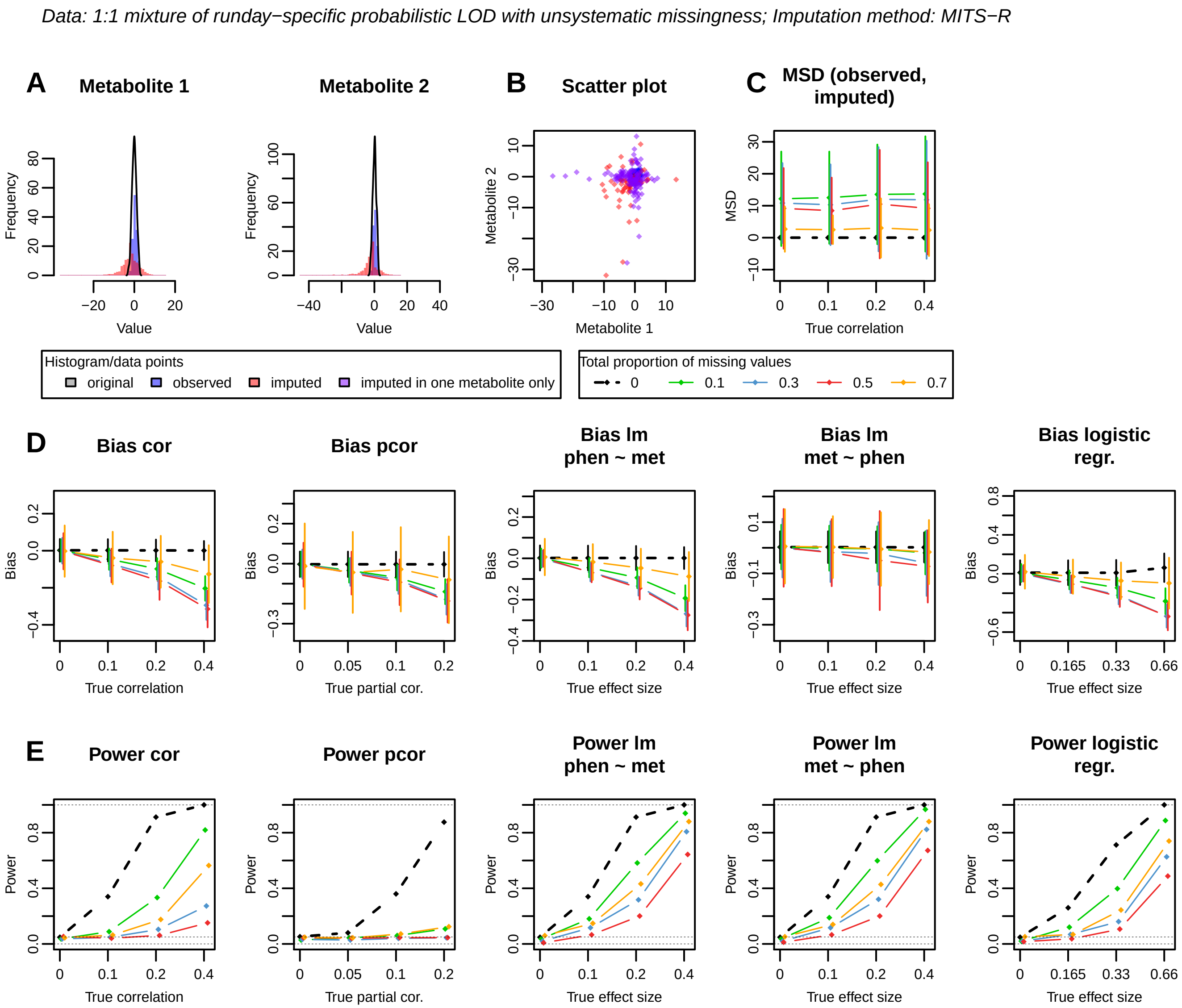
Data: 1:1 mixture of runday−specific probabilistic LOD with unsystematic missingness; Imputation method: MITS−R
A
Metabolite 1
Metabolite 2
B
Scatter plot
C
MSD (observed, imputed)
Frequency
Value
Metabolite 1
Metabolite 2
MSD
True correlation
Histogram/data points
original
observed
imputed
imputed in one metabolite only
Total proportion of missing values
0
0.1
0.3
0.5
0.7
D
Bias cor
Bias pcor
Bias lm phen ~ met
Bias lm met ~ phen
Bias logistic regr.
Bias
True correlation
True partial cor.
True effect size
E
Power cor
Power pcor
Power lm phen ~ met
Power lm met ~ phen
Power logistic regr.
Power

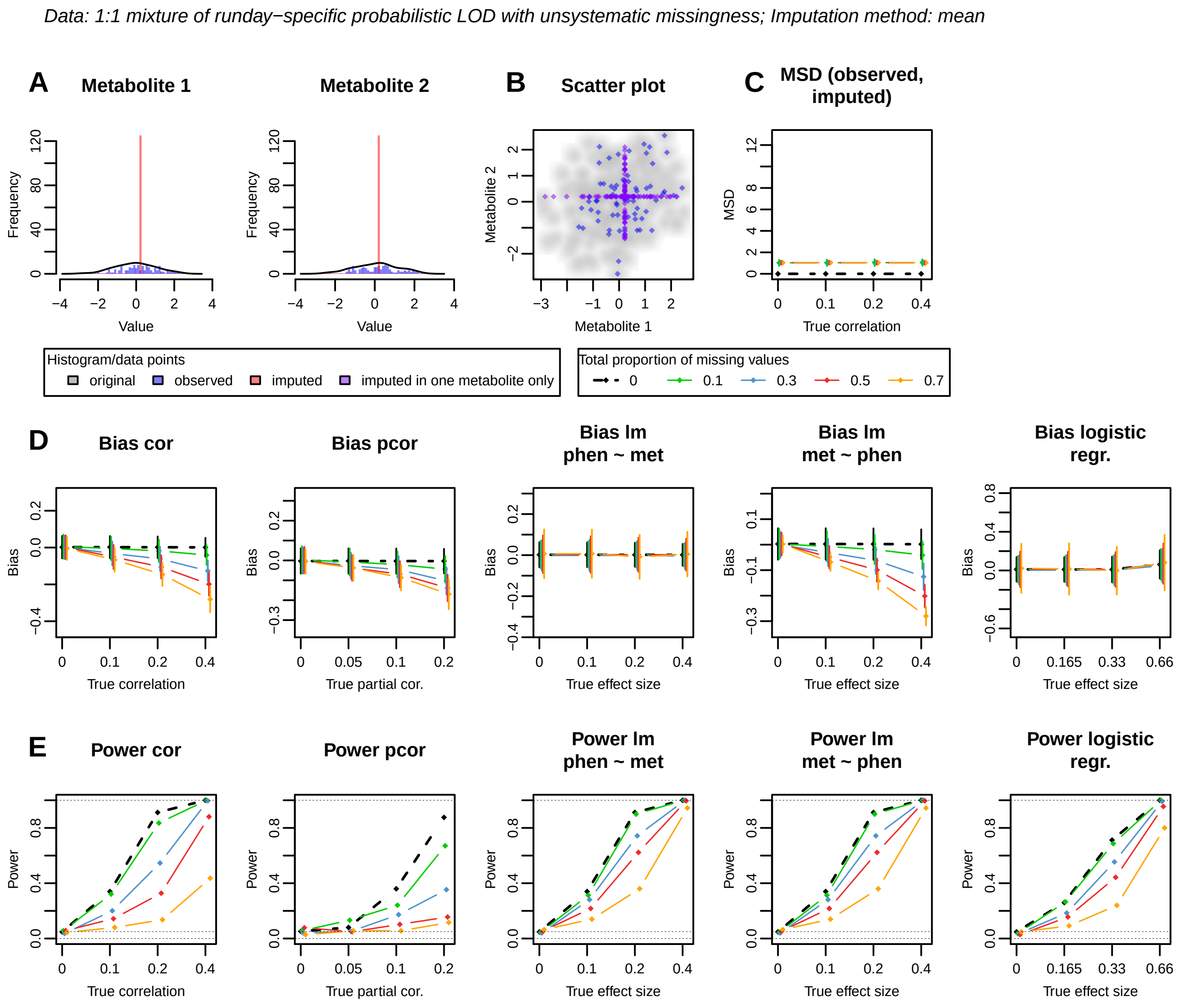

Data: 1:1 mixture of runday−specific probabilistic LOD with unsystematic missingness; Imputation method: mean
A
Metabolite 1
Metabolite 2
Frequency
Value
B
Scatter plot
Metabolite 2
Metabolite 1
C
MSD (observed, imputed)
MSD
True correlation
Histogram/data points
original
observed
imputed
imputed in one metabolite only
Total proportion of missing values
0
0.1
0.3
0.5
0.7
D
Bias cor
Bias pcor
Bias lm phen ~ met
Bias lm met ~ phen
Bias logistic regr.
Bias
True correlation
True partial cor.
True effect size
E
Power cor
Power pcor
Power lm phen ~ met
Power lm met ~ phen
Power logistic regr.
Power

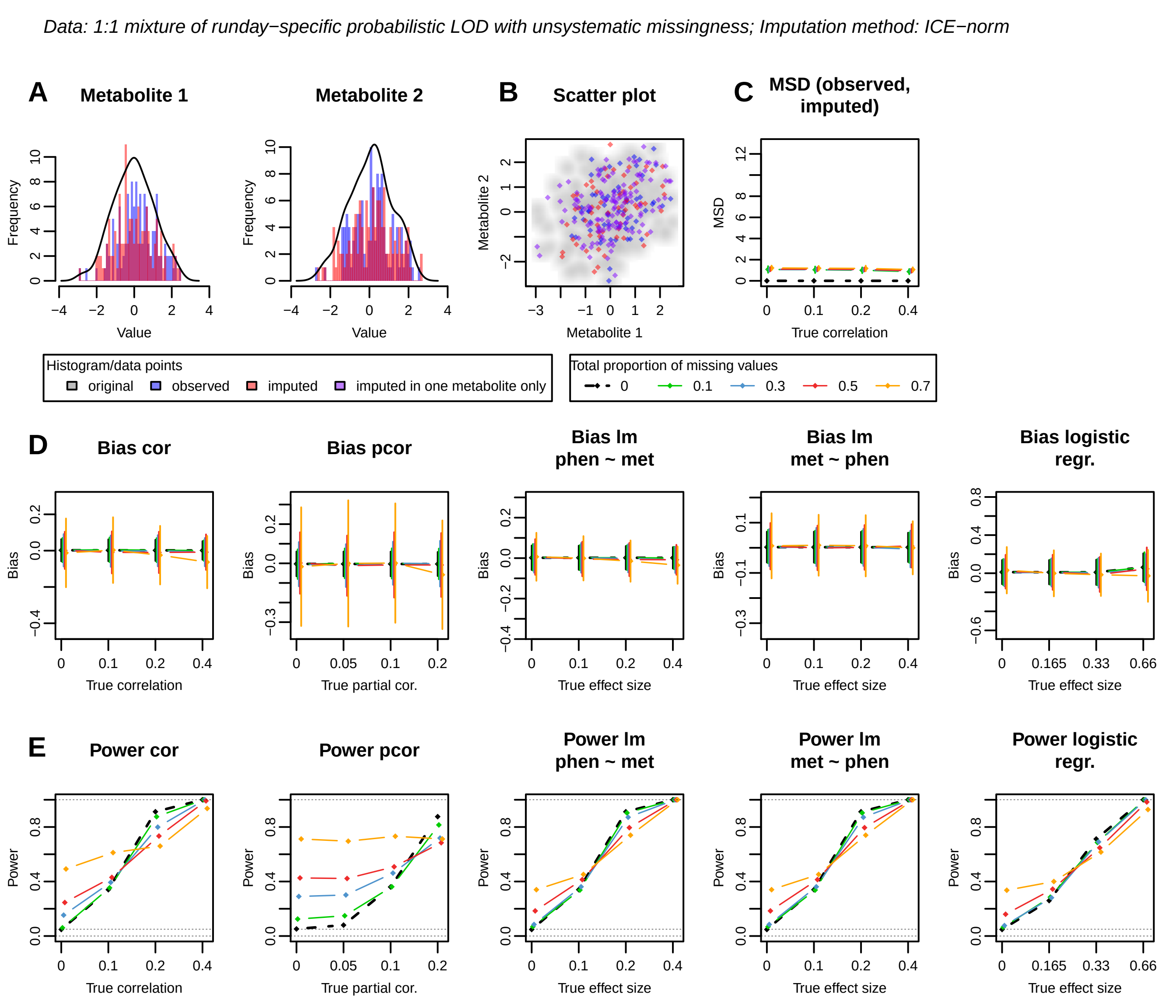
Data: 1:1 mixture of runday−specific probabilistic LOD with unsystematic missingness; Imputation method: ICE−norm
A
Metabolite 1
Metabolite 2
B
Scatter plot
C
MSD (observed, imputed)
Frequency
Value
Metabolite 2
Metabolite 1
MSD
True correlation
Histogram/data points
original
observed
imputed
imputed in one metabolite only
Total proportion of missing values
0
0.1
0.3
0.5
0.7
D
Bias cor
Bias pcor
Bias lm phen ~ met
Bias lm met ~ phen
Bias logistic regr.
Bias
True correlation
True partial cor.
True effect size
E
Power cor
Power pcor
Power lm phen ~ met
Power lm met ~ phen
Power logistic regr.
Power

Data: 1:1 mixture of runday−specific probabilistic LOD with unsystematic missingness; Imputation method: ICE−pmm

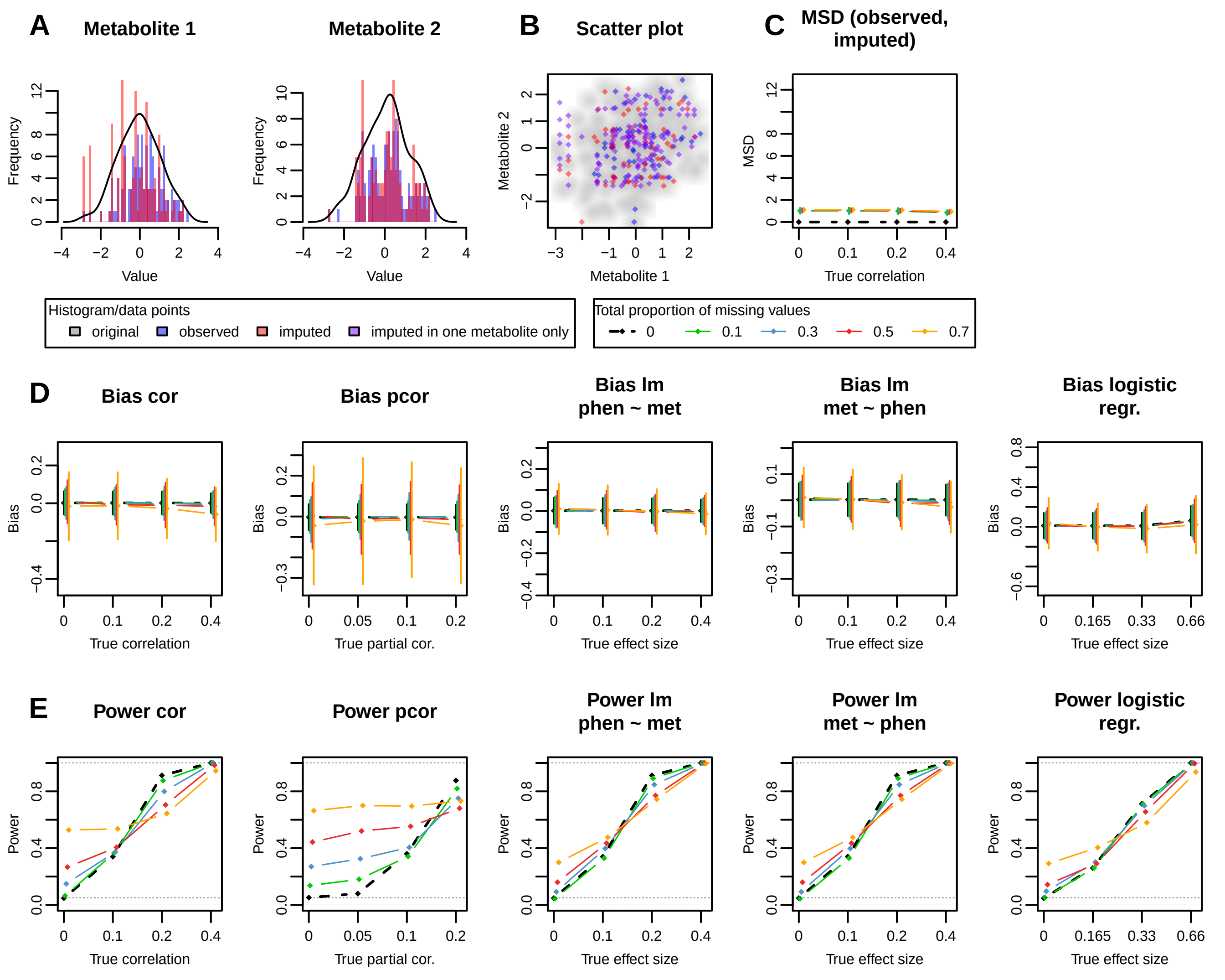

Data: 1:1 mixture of runday−specific probabilistic LOD with unsystematic missingness; Imputation method: ICE−adjR

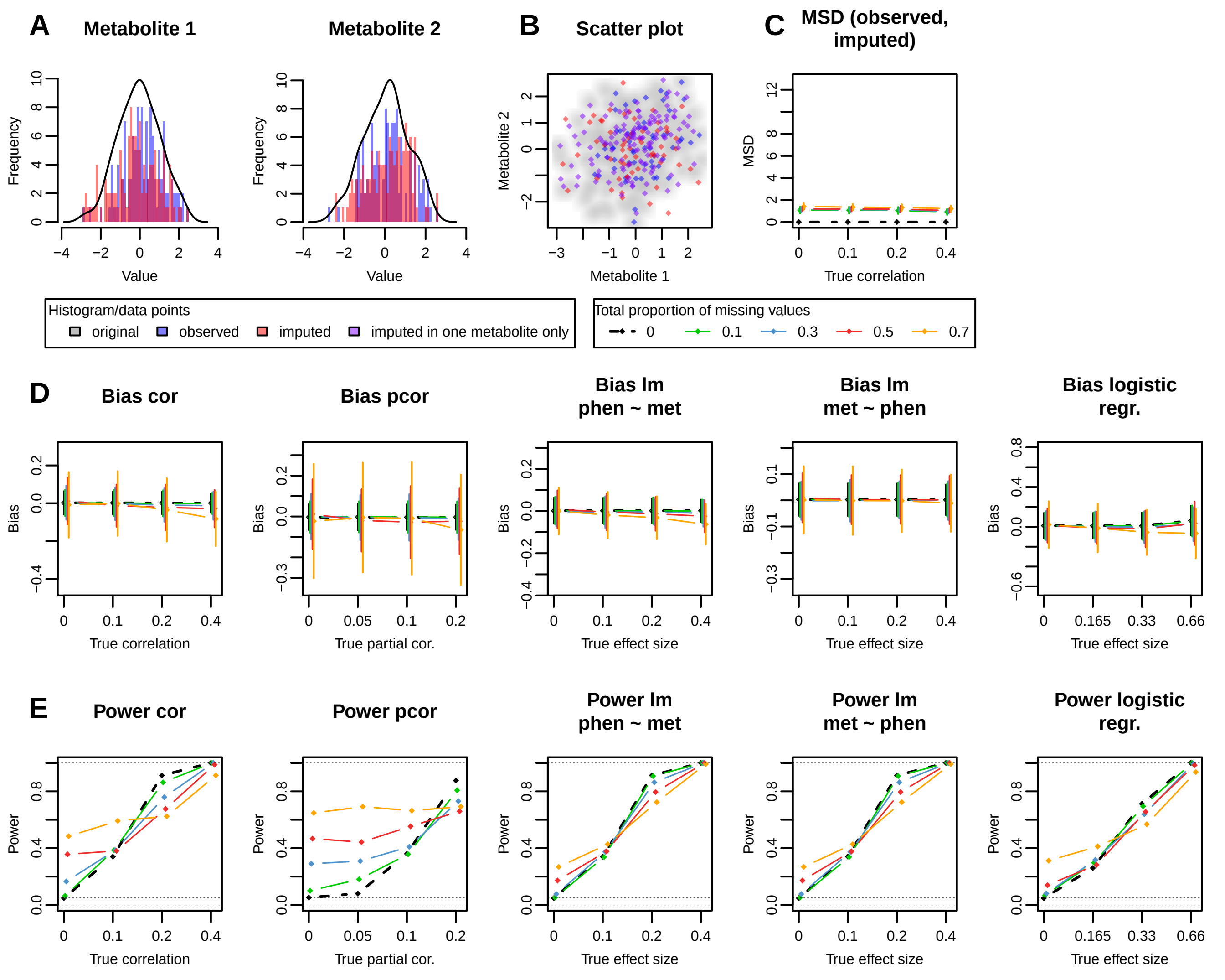

Data: 1:1 mixture of runday−specific probabilistic LOD with unsystematic missingness; Imputation method: MICE−norm

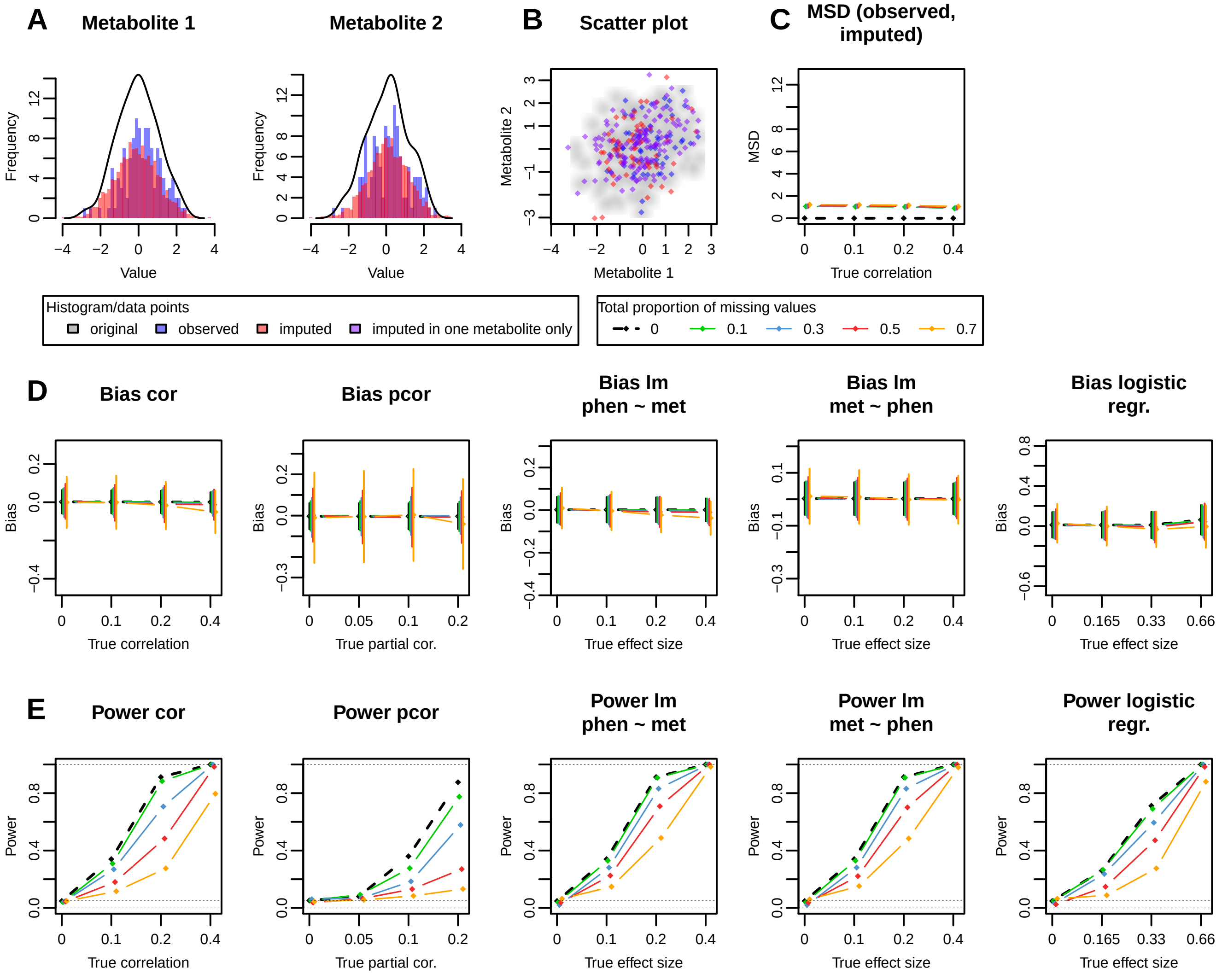

Data: 1:1 mixture of runday−specific probabilistic LOD with unsystematic missingness; Imputation method: MICE−pmm

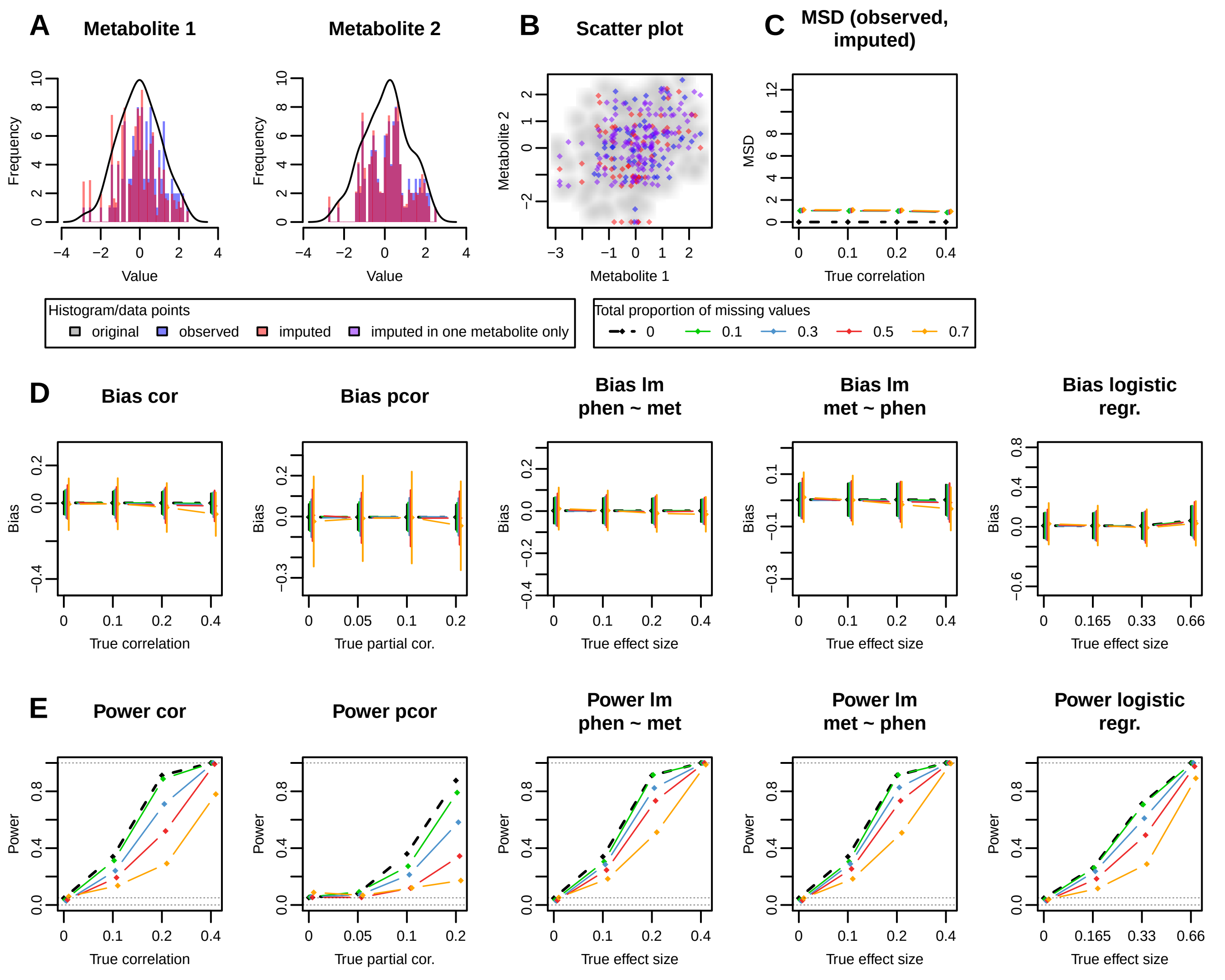

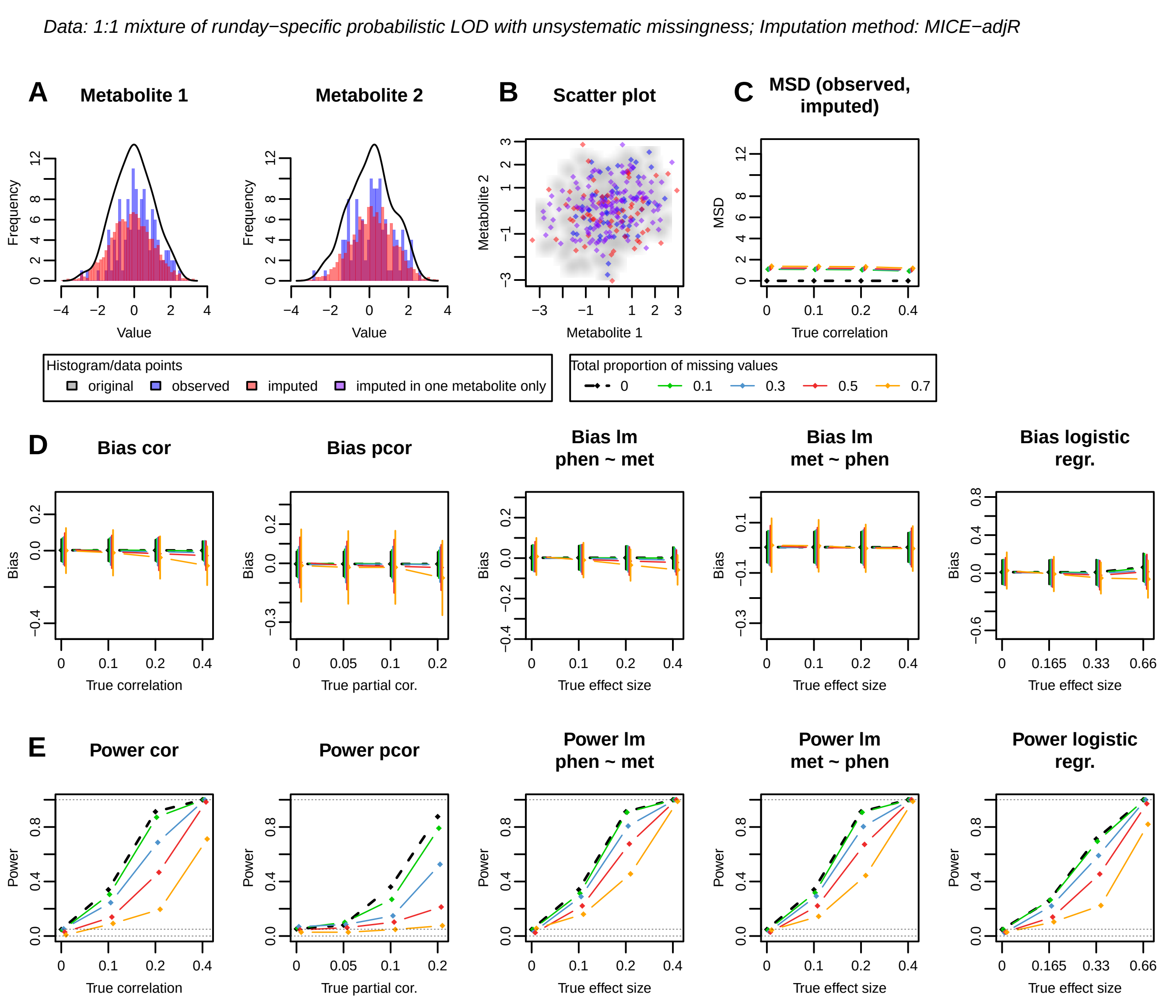
Data: 1:1 mixture of runday−specific probabilistic LOD with unsystematic missingness; Imputation method: MICE−adjR
A
Metabolite 1
Metabolite 2
Frequency
Value
B
Scatter plot
Metabolite 2
Metabolite 1
C
MSD (observed, imputed)
MSD
True correlation
Histogram/data points
original
observed
imputed
imputed in one metabolite only
Total proportion of missing values
0
0.1
0.3
0.5
0.7
D
Bias cor
Bias pcor
Bias lm phen ~ met
Bias lm met ~ phen
Bias logistic regr.
Bias
True correlation
True partial cor.
True effect size
E
Power cor
Power pcor
Power lm phen ~ met
Power lm met ~ phen
Power logistic regr.
Power
True correlation
True partial cor.
True effect size

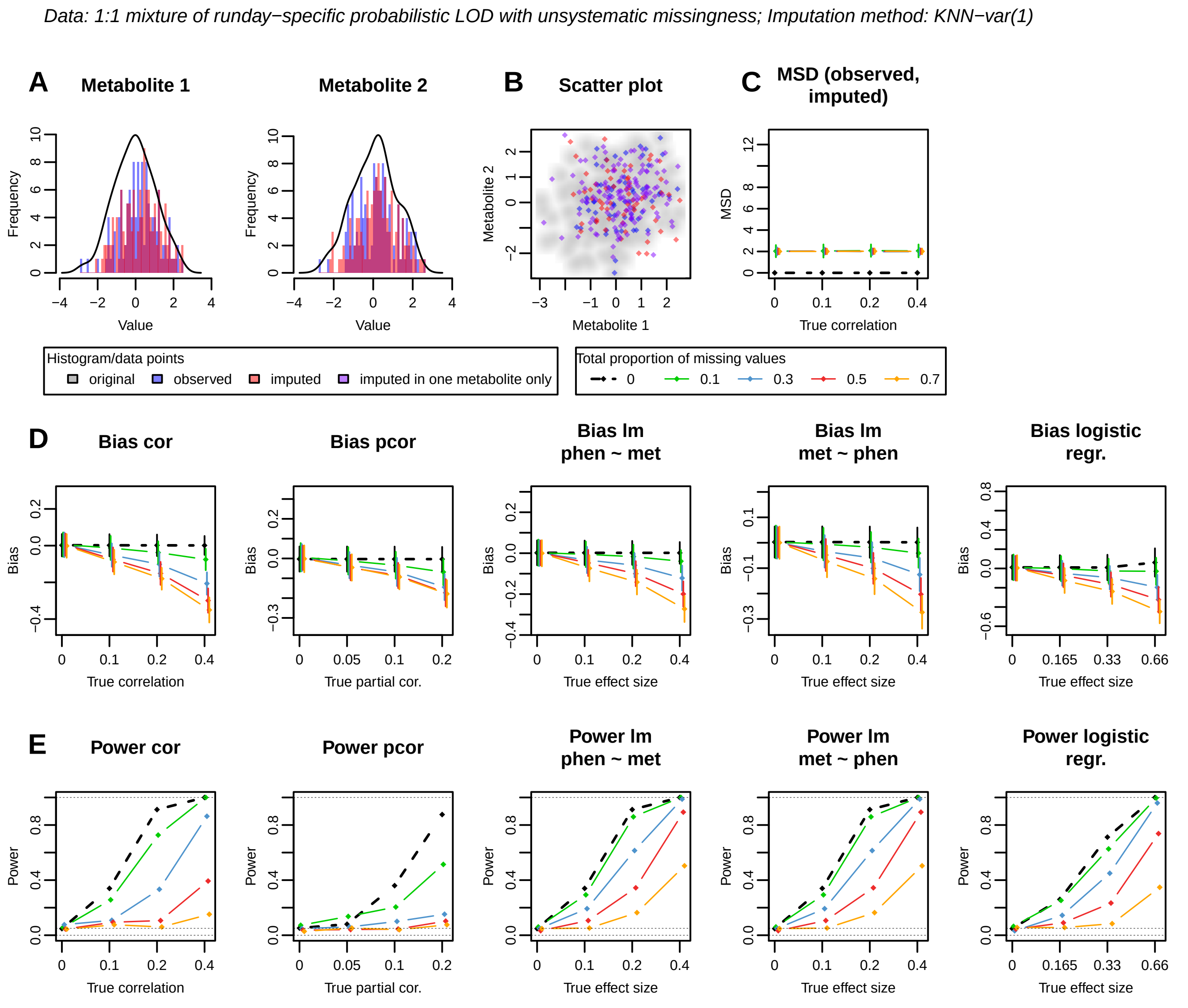

Data: 1:1 mixture of runday−specific probabilistic LOD with unsystematic missingness; Imputation method: KNN−var(1)
A
Metabolite 1
Metabolite 2
B
Scatter plot
C
MSD (observed, imputed)
Frequency
Value
Metabolite 2
Metabolite 1
MSD
True correlation
Histogram/data points
original
observed
imputed
imputed in one metabolite only
Total proportion of missing values
0
0.1
0.3
0.5
0.7
D
Bias cor
Bias pcor
Bias lm phen ~ met
Bias lm met ~ phen
Bias logistic regr.
Bias
True correlation
True partial cor.
True effect size
E
Power cor
Power pcor
Power lm phen ~ met
Power lm met ~ phen
Power logistic regr.
Power

Data: 1:1 mixture of runday−specific probabilistic LOD with unsystematic missingness; Imputation method: KNN−var(3)

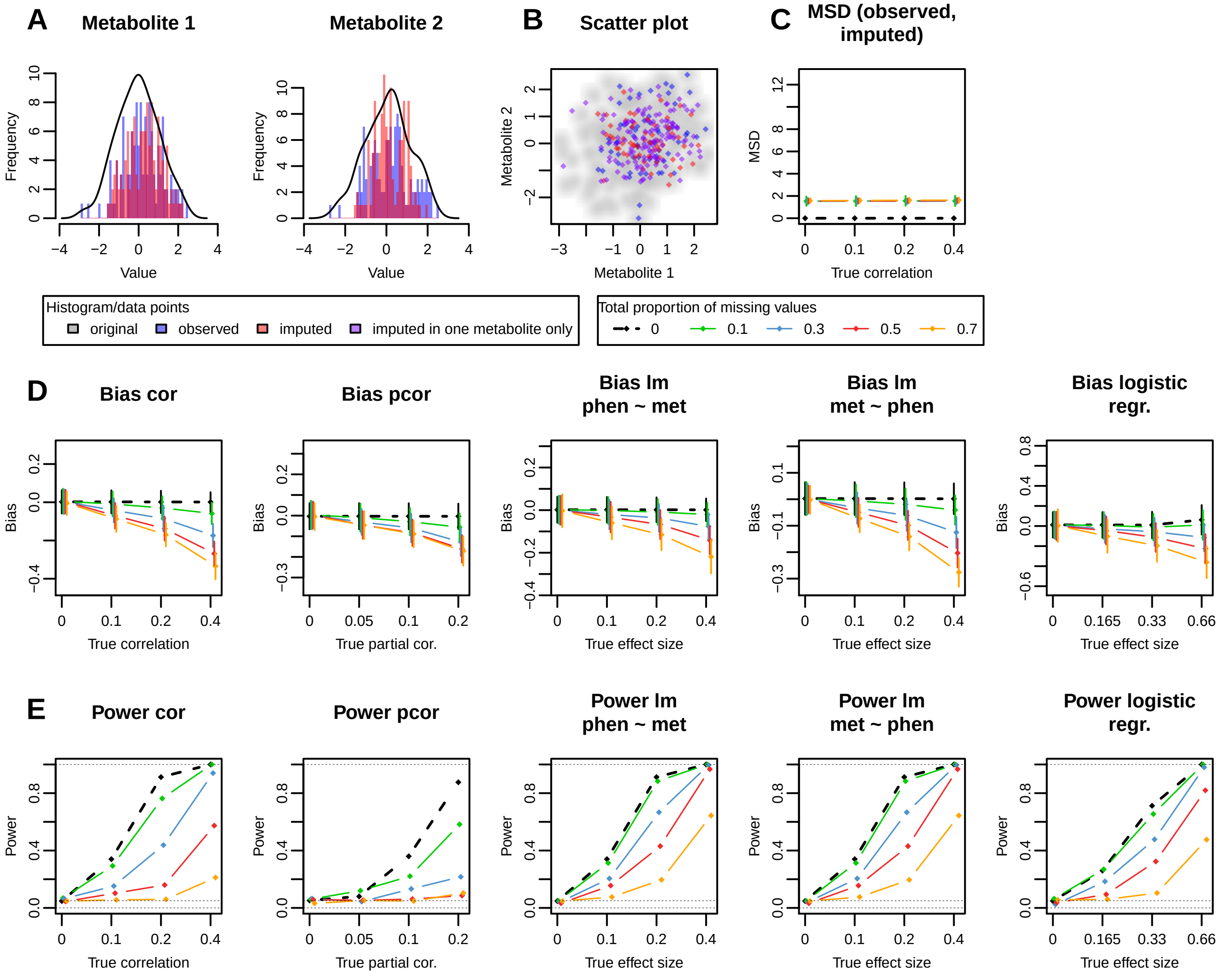

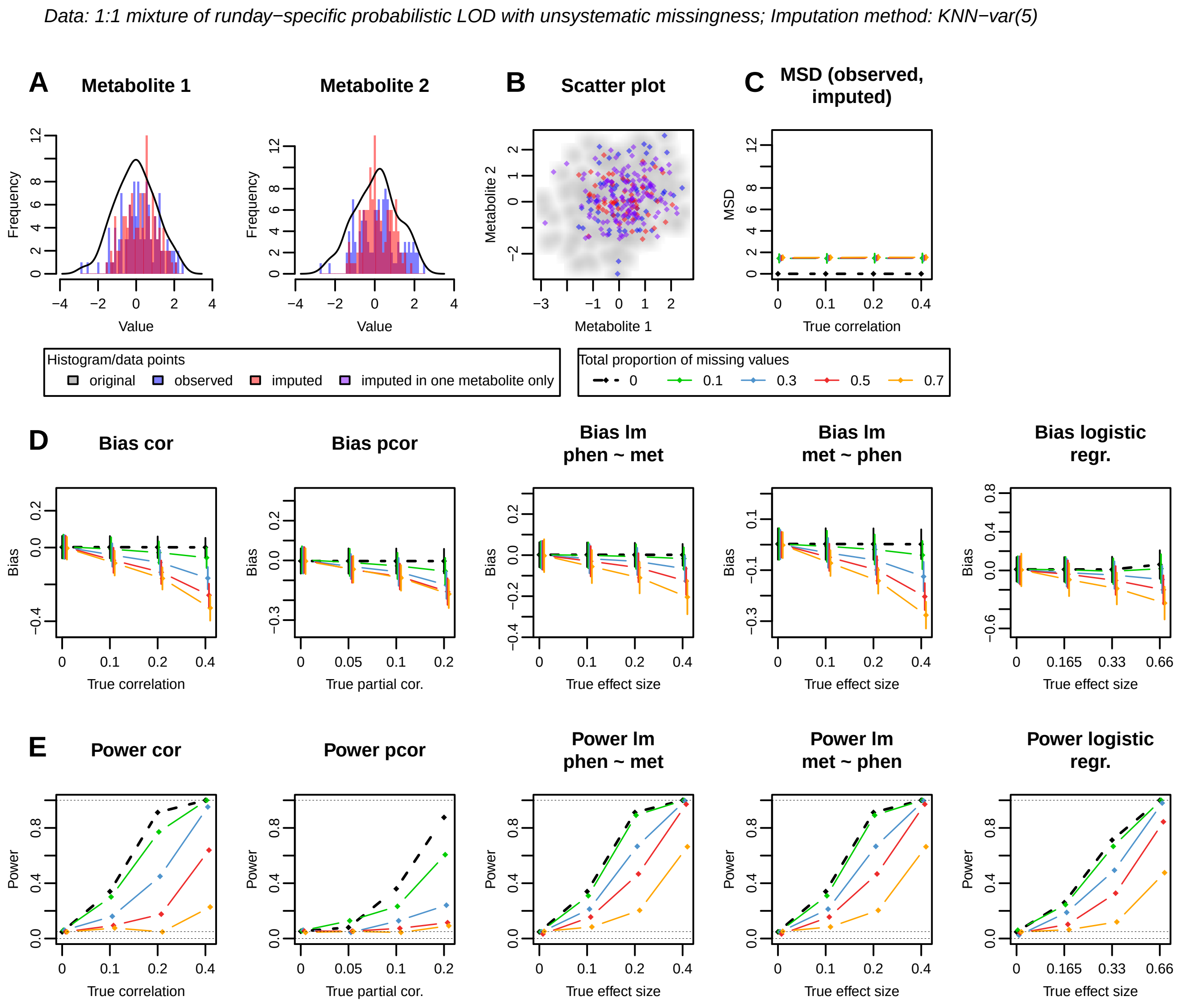

Data: 1:1 mixture of runday−specific probabilistic LOD with unsystematic missingness; Imputation method: KNN−var(5)
A
Metabolite 1
Metabolite 2
B
Scatter plot
C
MSD (observed, imputed)
Frequency
Value
Metabolite 2
Metabolite 1
MSD
True correlation
Histogram/data points
original
observed
imputed
imputed in one metabolite only
Total proportion of missing values
0
0.1
0.3
0.5
0.7
D
Bias cor
Bias pcor
Bias lm phen ~ met
Bias lm met ~ phen
Bias logistic regr.
Bias
True correlation
True partial cor.
True effect size
E
Power cor
Power pcor
Power lm phen ~ met
Power lm met ~ phen
Power logistic regr.
Power

Data: 1:1 mixture of runday−specific probabilistic LOD with unsystematic missingness; Imputation method: KNN−var(10)

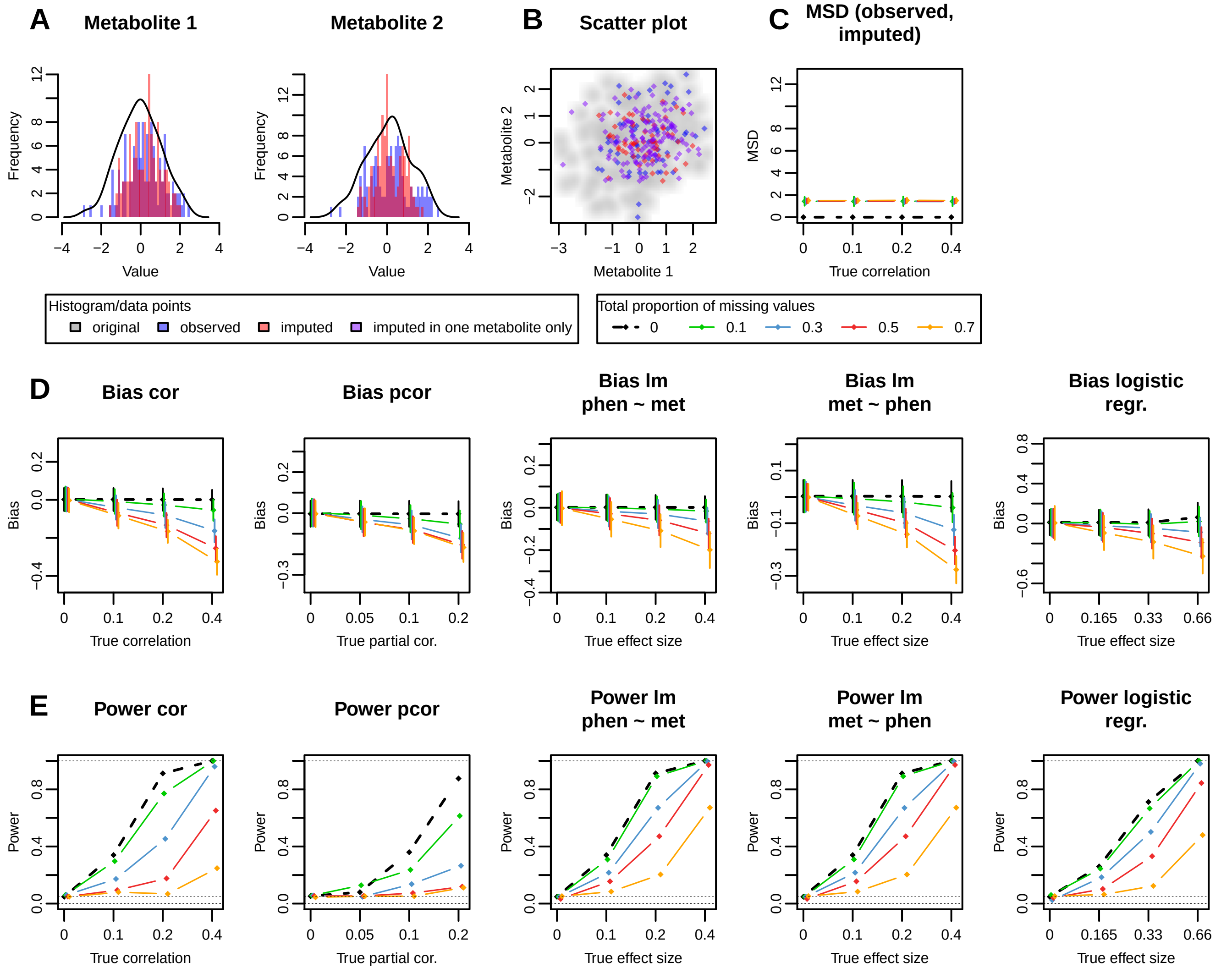

Data: 1:1 mixture of runday−specific probabilistic LOD with unsystematic missingness; Imputation method: KNN−var(20)

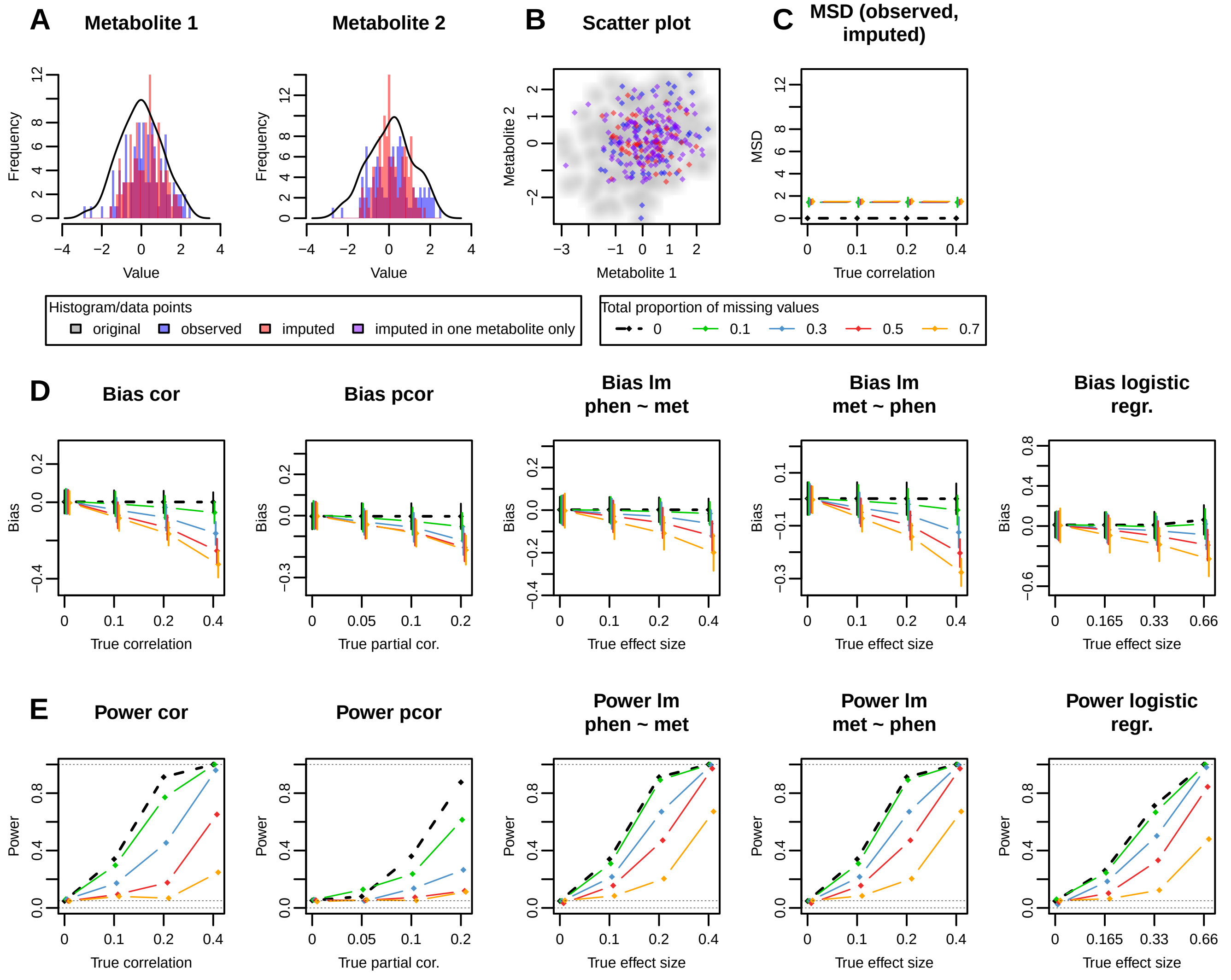

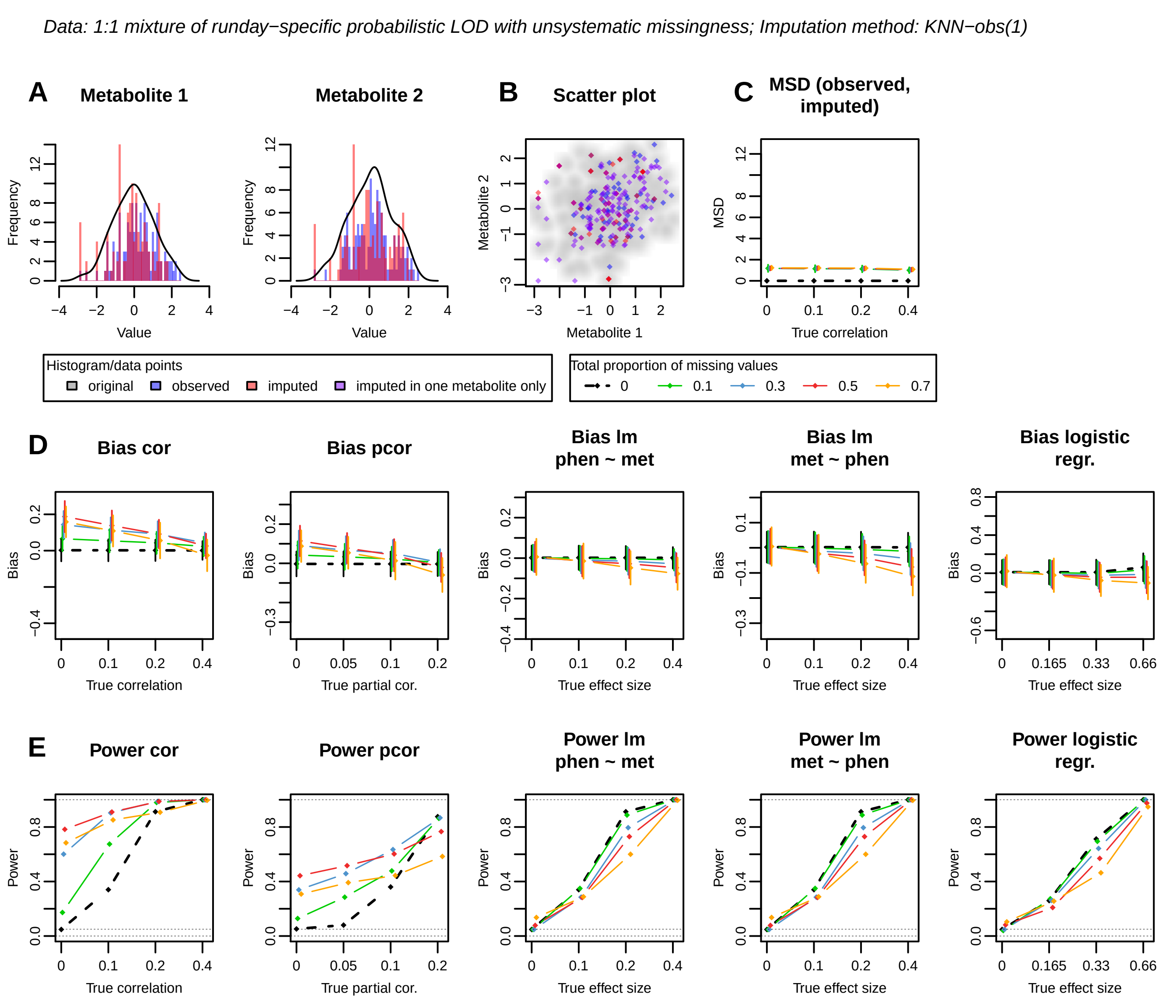
Data: 1:1 mixture of runday−specific probabilistic LOD with unsystematic missingness; Imputation method: KNN−obs(1)
A
Metabolite 1
Metabolite 2
Frequency
Value
B
Scatter plot
Metabolite 1
Metabolite 2
C
MSD (observed, imputed)
MSD
True correlation
Histogram/data points
original
observed
imputed
imputed in one metabolite only
Total proportion of missing values
0
0.1
0.3
0.5
0.7
D
Bias cor
Bias pcor
Bias lm phen ~ met
Bias lm met ~ phen
Bias logistic regr.
Bias
True correlation
True partial cor.
True effect size
E
Power cor
Power pcor
Power lm phen ~ met
Power lm met ~ phen
Power logistic regr.
Power
True correlation
True partial cor.
True effect size

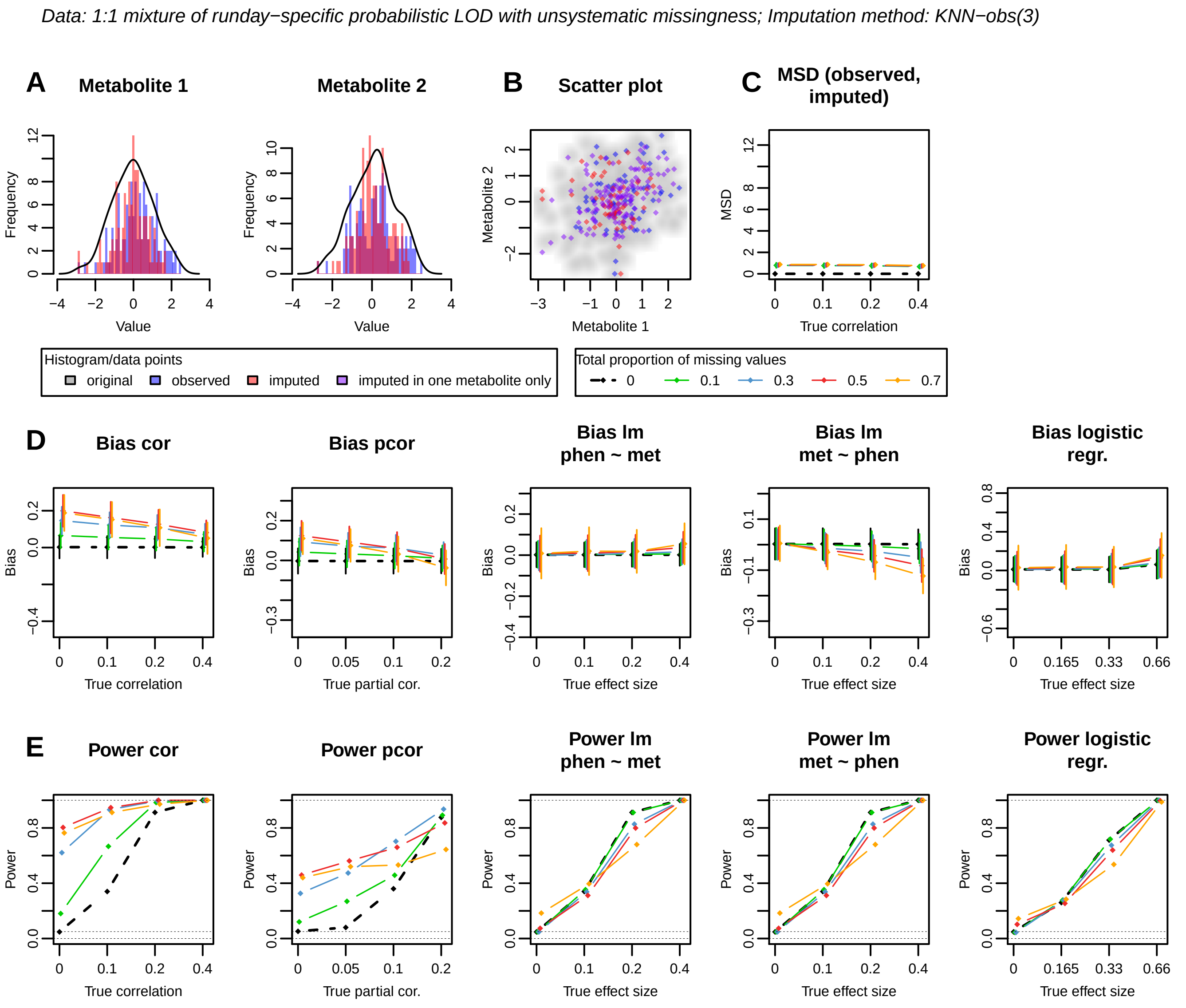
Data: 1:1 mixture of runday−specific probabilistic LOD with unsystematic missingness; Imputation method: KNN−obs(3)
A
Metabolite 1
Metabolite 2
B
Scatter plot
C
MSD (observed, imputed)
Frequency
Value
Metabolite 2
Metabolite 1
MSD
True correlation
Histogram/data points
original
observed
imputed
imputed in one metabolite only
Total proportion of missing values
0
0.1
0.3
0.5
0.7
D
Bias cor
Bias pcor
Bias lm phen ~ met
Bias lm met ~ phen
Bias logistic regr.
Bias
True correlation
True partial cor.
True effect size
E
Power cor
Power pcor
Power lm phen ~ met
Power lm met ~ phen
Power logistic regr.
Power
True correlation
True partial cor.
True effect size

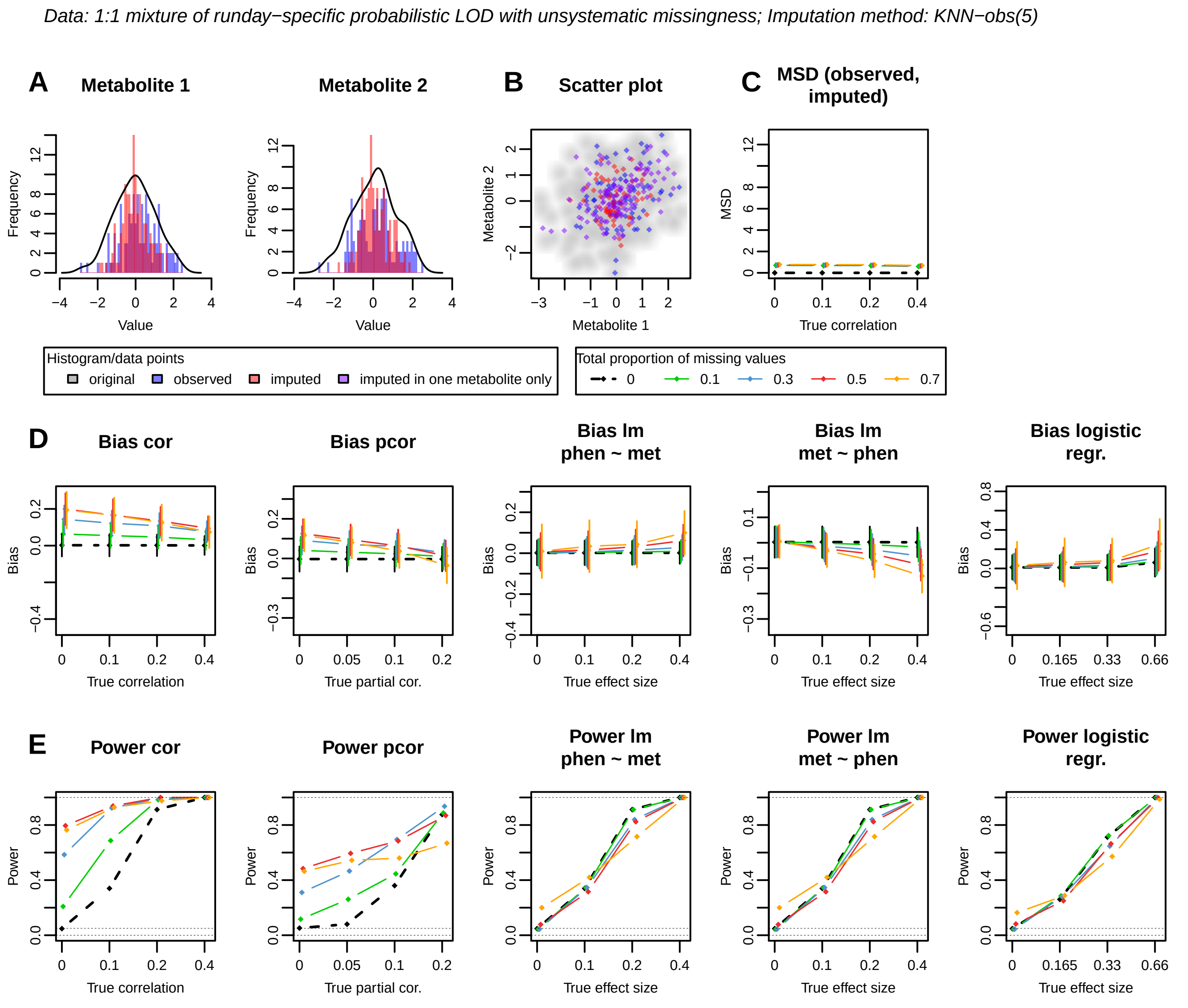

Data: 1:1 mixture of runday−specific probabilistic LOD with unsystematic missingness; Imputation method: KNN−obs(5)
A
Metabolite 1
Metabolite 2
B
Scatter plot
C
MSD (observed, imputed)
Frequency
Value
Metabolite 2
Metabolite 1
MSD
True correlation
Histogram/data points
original
observed
imputed
imputed in one metabolite only
Total proportion of missing values
0
0.1
0.3
0.5
0.7
D
Bias cor
Bias pcor
Bias lm phen ~ met
Bias lm met ~ phen
Bias logistic regr.
Bias
True correlation
True partial cor.
True effect size
E
Power cor
Power pcor
Power lm phen ~ met
Power lm met ~ phen
Power logistic regr.
Power

Data: 1:1 mixture of runday−specific probabilistic LOD with unsystematic missingness; Imputation method: KNN−obs(10)

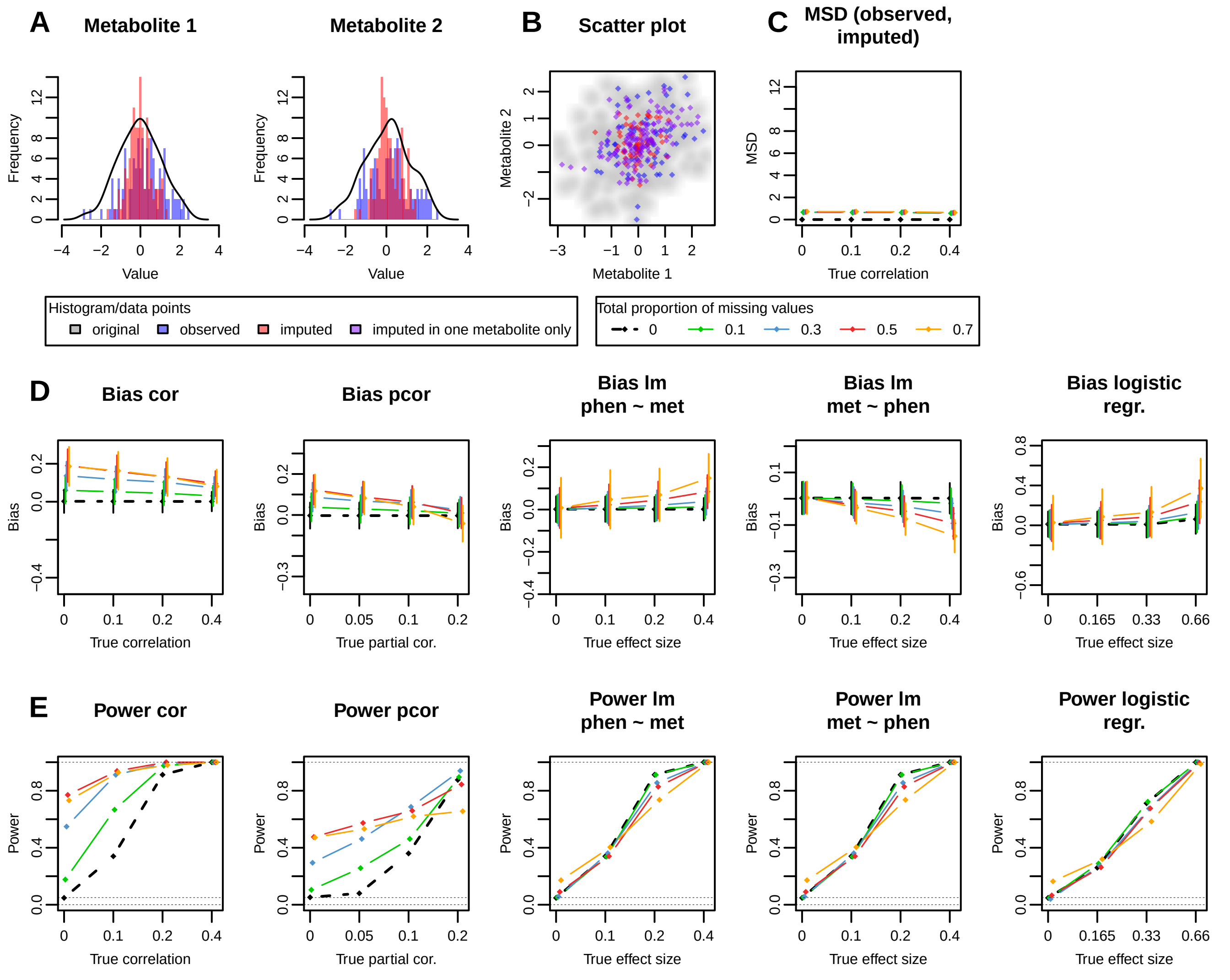

Data: 1:1 mixture of runday−specific probabilistic LOD with unsystematic missingness; Imputation method: KNN−obs(20)

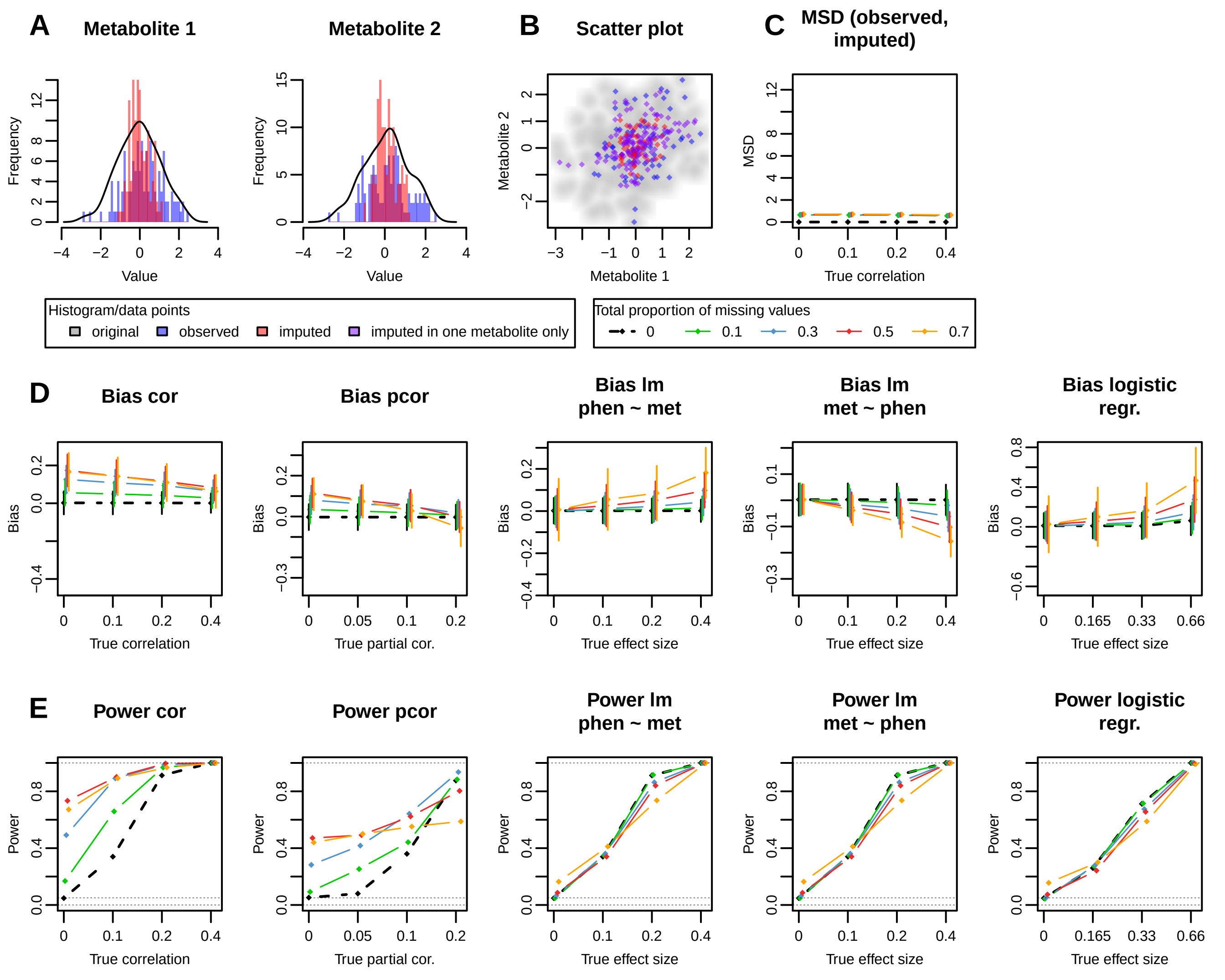

Data: 1:1 mixture of runday−specific probabilistic LOD with unsystematic missingness; Imputation method: KNN−obs−sel(1)

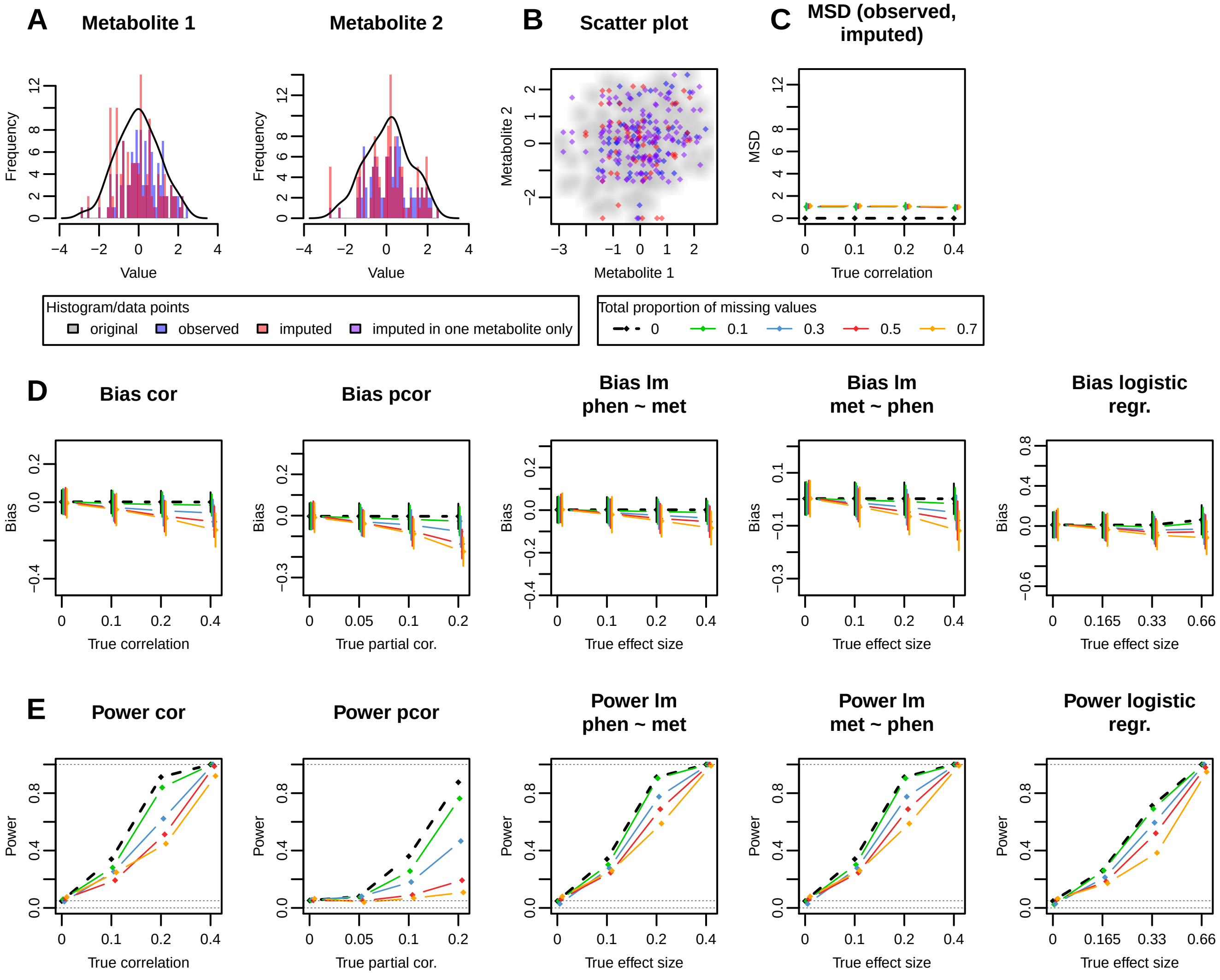

Data: 1:1 mixture of runday−specific probabilistic LOD with unsystematic missingness; Imputation method: KNN−obs−sel(3)

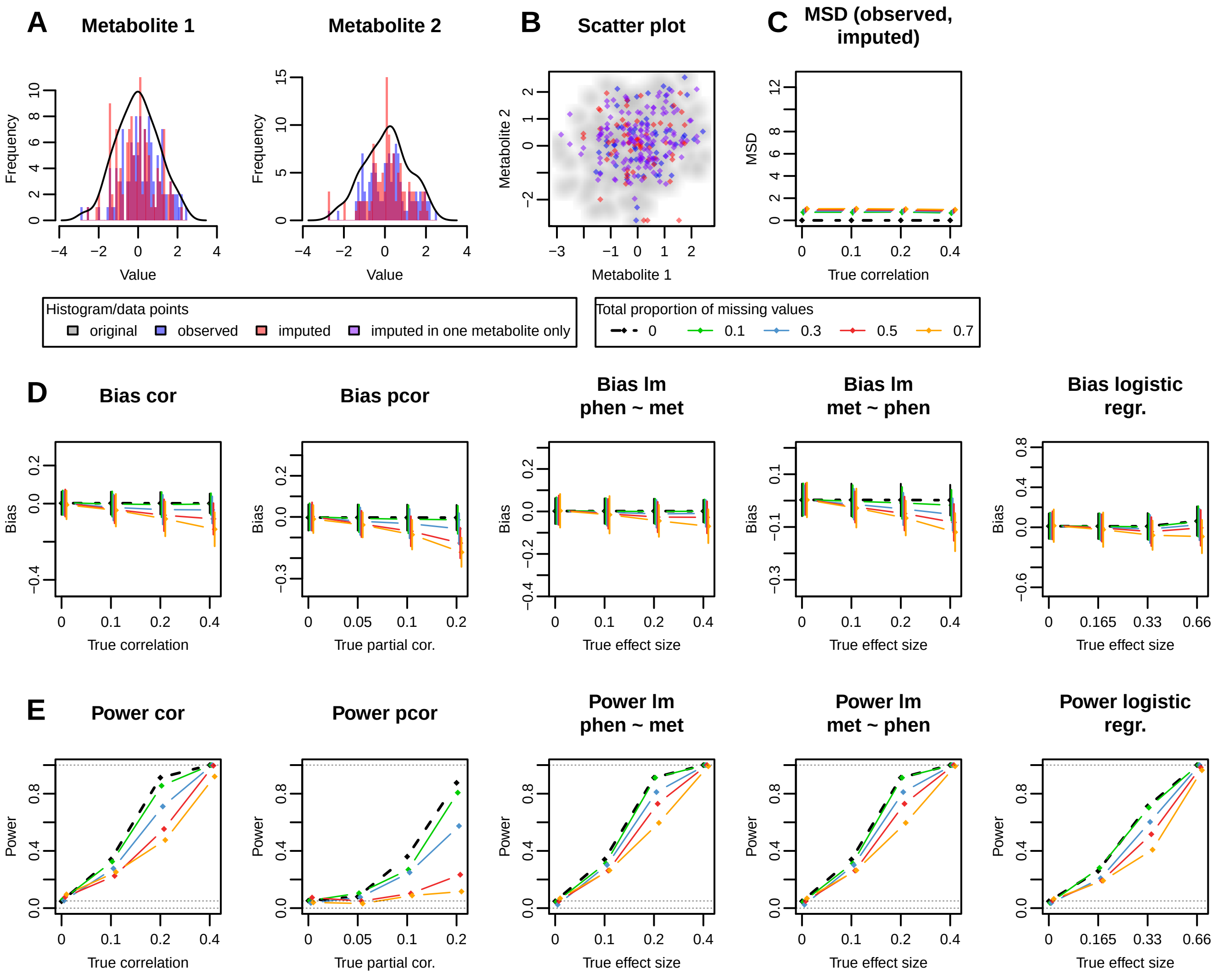

Data: 1:1 mixture of runday−specific probabilistic LOD with unsystematic missingness; Imputation method: KNN−obs−sel(5)

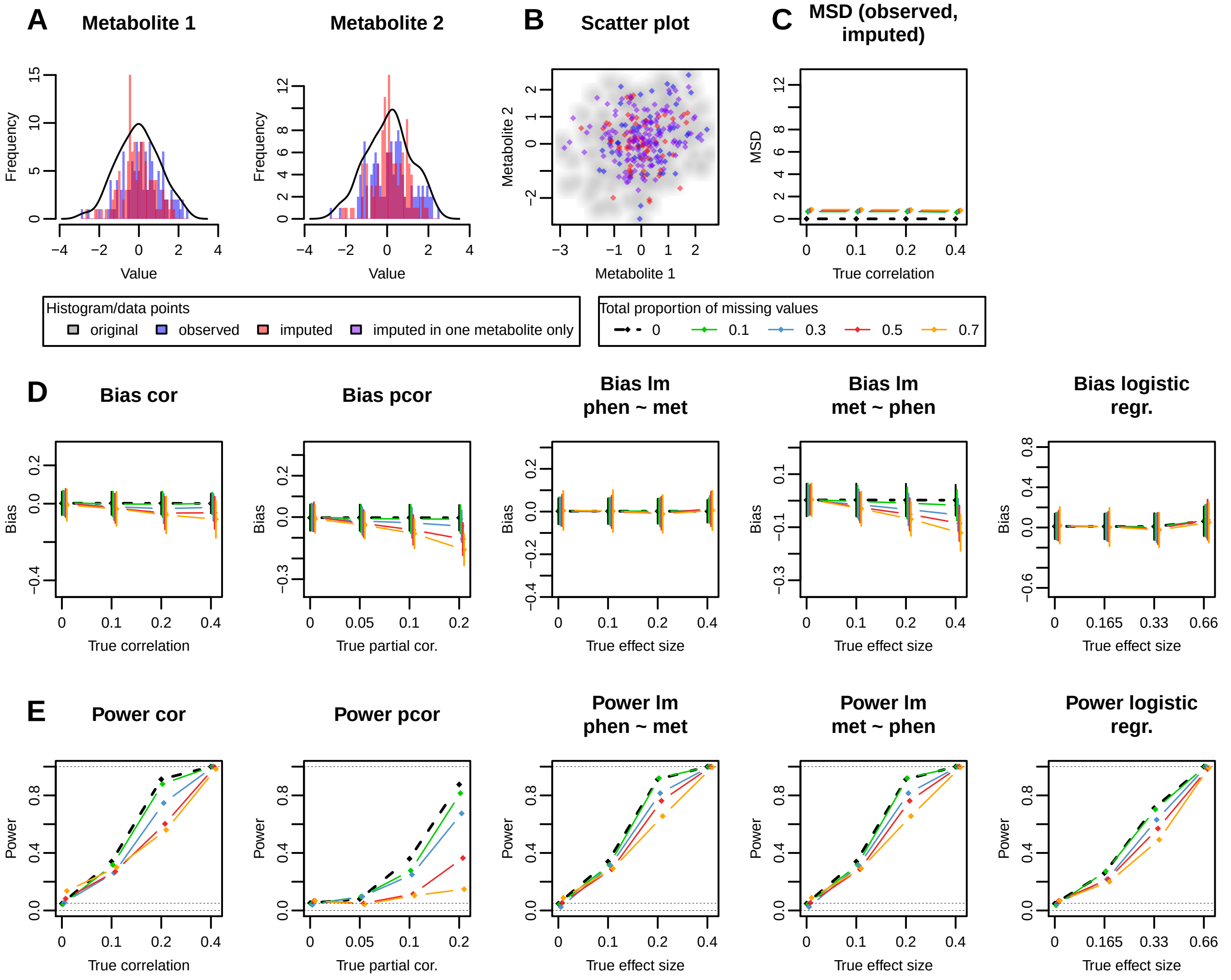

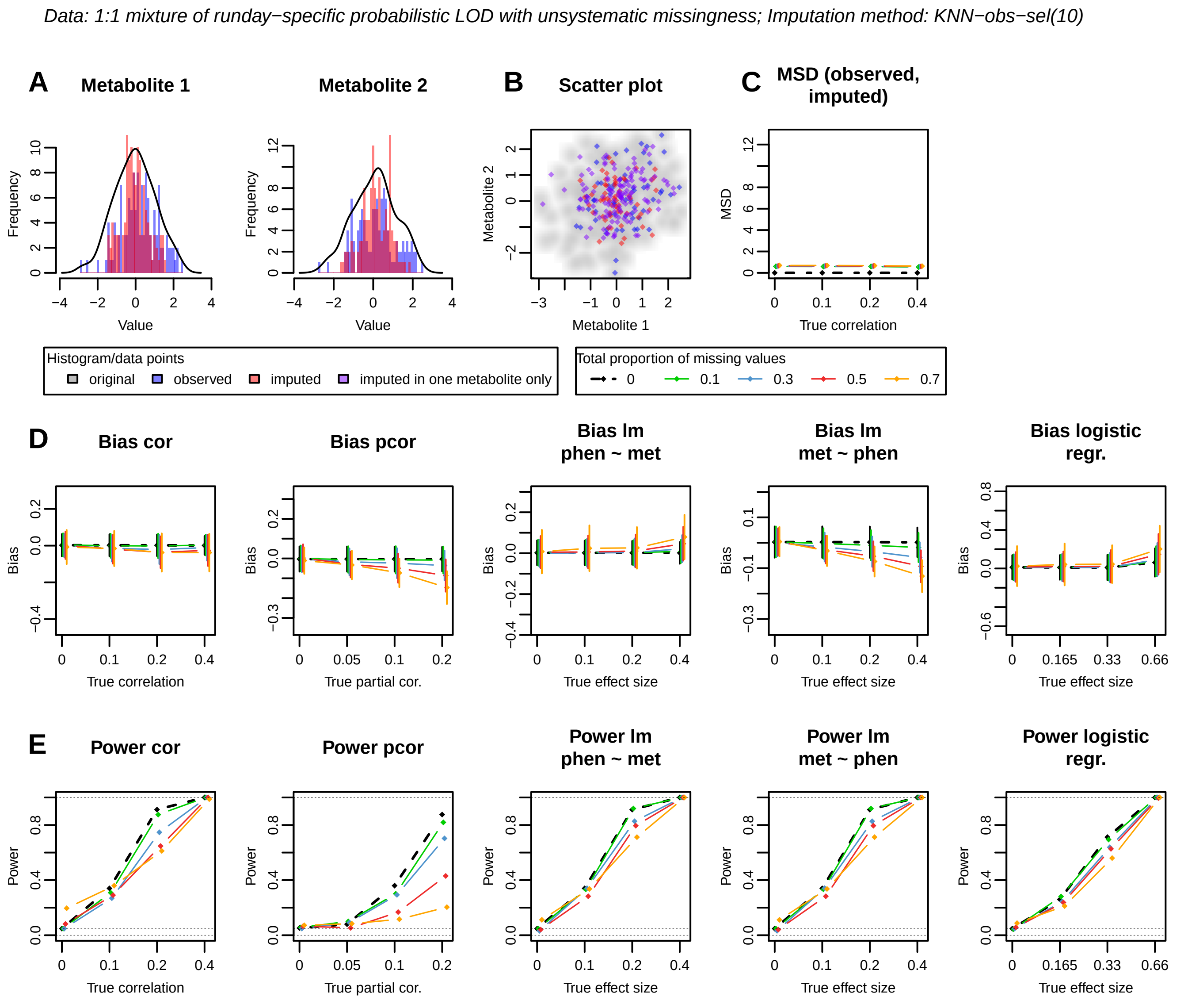
Data: 1:1 mixture of runday−specific probabilistic LOD with unsystematic missingness; Imputation method: KNN−obs−sel(10)
A
Metabolite 1
Metabolite 2
Frequency
Value
B
Scatter plot
Metabolite 1
Metabolite 2
C
MSD (observed, imputed)
MSD
True correlation
Histogram/data points
original
observed
imputed
imputed in one metabolite only
Total proportion of missing values
0
0.1
0.3
0.5
0.7
D
Bias cor
Bias pcor
Bias lm phen ~ met
Bias lm met ~ phen
Bias logistic regr.
Bias
True correlation
True partial cor.
True effect size
E
Power cor
Power pcor
Power lm phen ~ met
Power lm met ~ phen
Power logistic regr.
Power

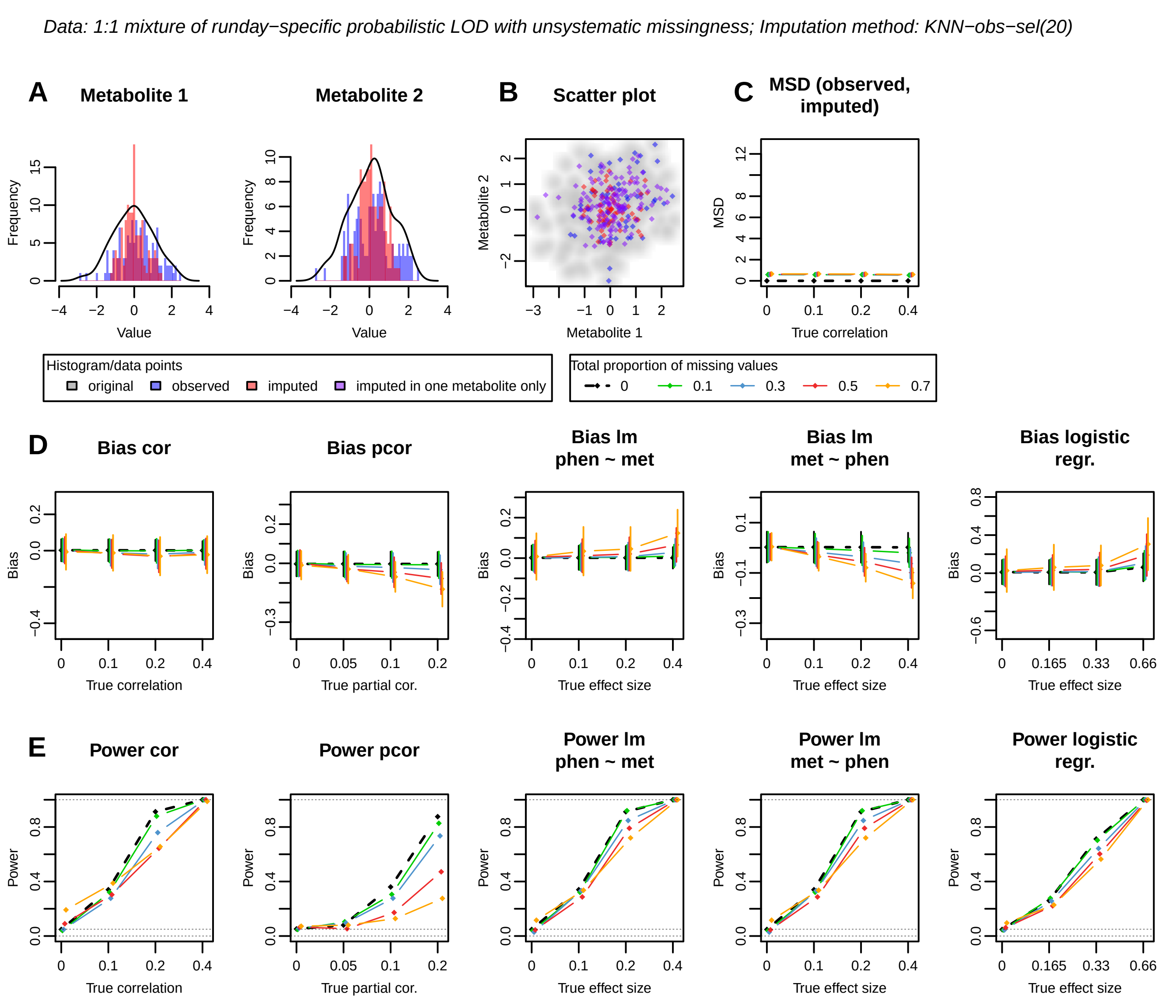

Data: 1:1 mixture of runday−specific probabilistic LOD with unsystematic missingness; Imputation method: KNN−obs−sel(20)
A
Metabolite 1
Metabolite 2
Frequency
Value
B
Scatter plot
Metabolite 2
Metabolite 1
C
MSD (observed, imputed)
MSD
True correlation
Histogram/data points
original
observed
imputed
imputed in one metabolite only
Total proportion of missing values
0
0.1
0.3
0.5
0.7
D
Bias cor
Bias pcor
Bias lm phen ~ met
Bias lm met ~ phen
Bias logistic regr.
Bias
True correlation
True partial cor.
True effect size
E
Power cor
Power pcor
Power lm phen ~ met
Power lm met ~ phen
Power logistic regr.
Power
True correlation
True partial cor.
True effect size

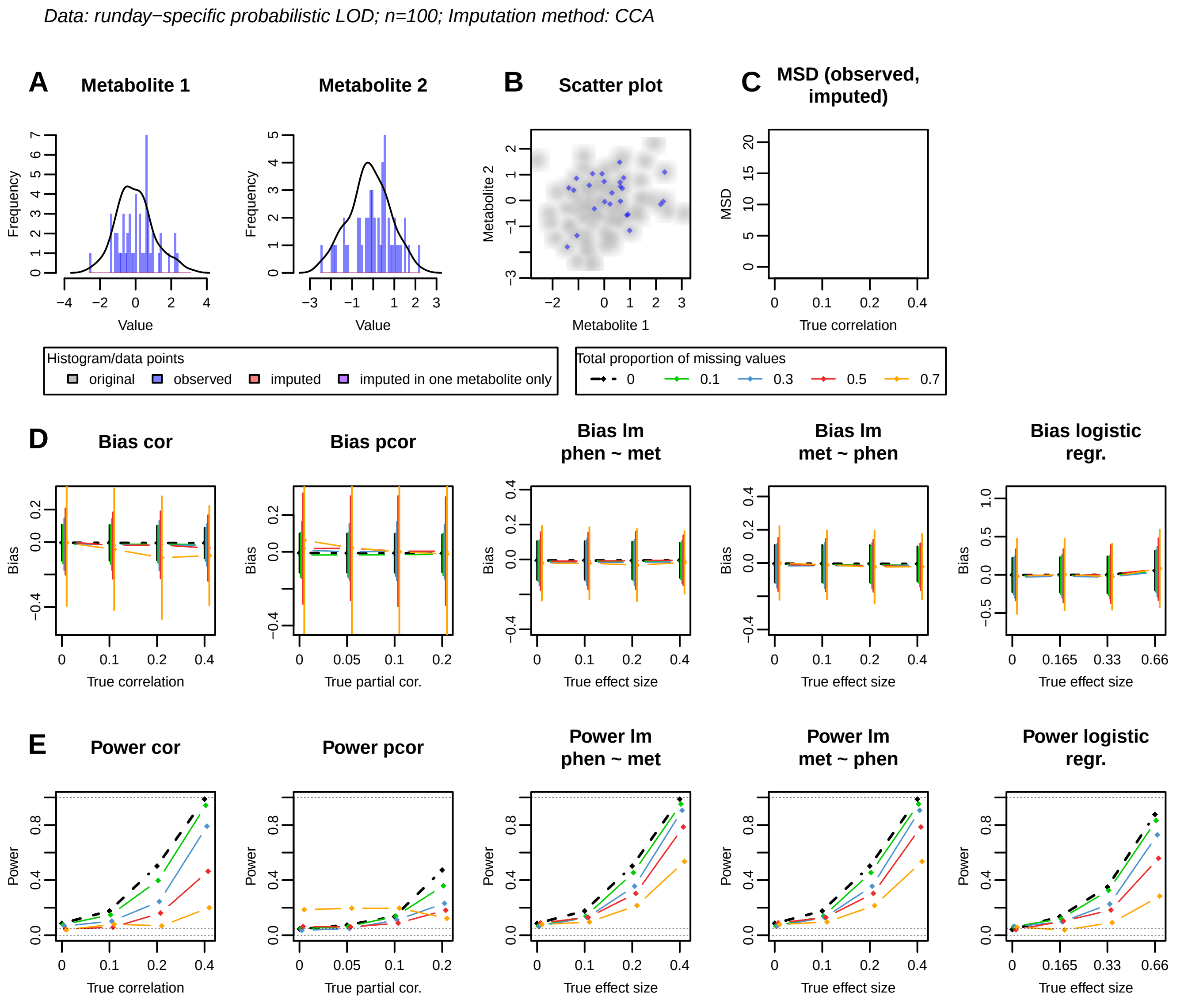
Data: runday−specific probabilistic LOD; n=100; Imputation method: CCA
A
Metabolite 1
Metabolite 2
B
Scatter plot
C
MSD (observed, imputed)
Frequency
Value
Metabolite 2
Metabolite 1
MSD
True correlation
Histogram/data points
original
observed
imputed
imputed in one metabolite only
Total proportion of missing values
0
0.1
0.3
0.5
0.7
D
Bias cor
Bias pcor
Bias lm phen ~ met
Bias lm met ~ phen
Bias logistic regr.
Bias
True correlation
True partial cor.
True effect size
E
Power cor
Power pcor
Power lm phen ~ met
Power lm met ~ phen
Power logistic regr.
Power
True correlation
True partial cor.
True effect size

Data: runday−specific probabilistic LOD; n=100; Imputation method: min

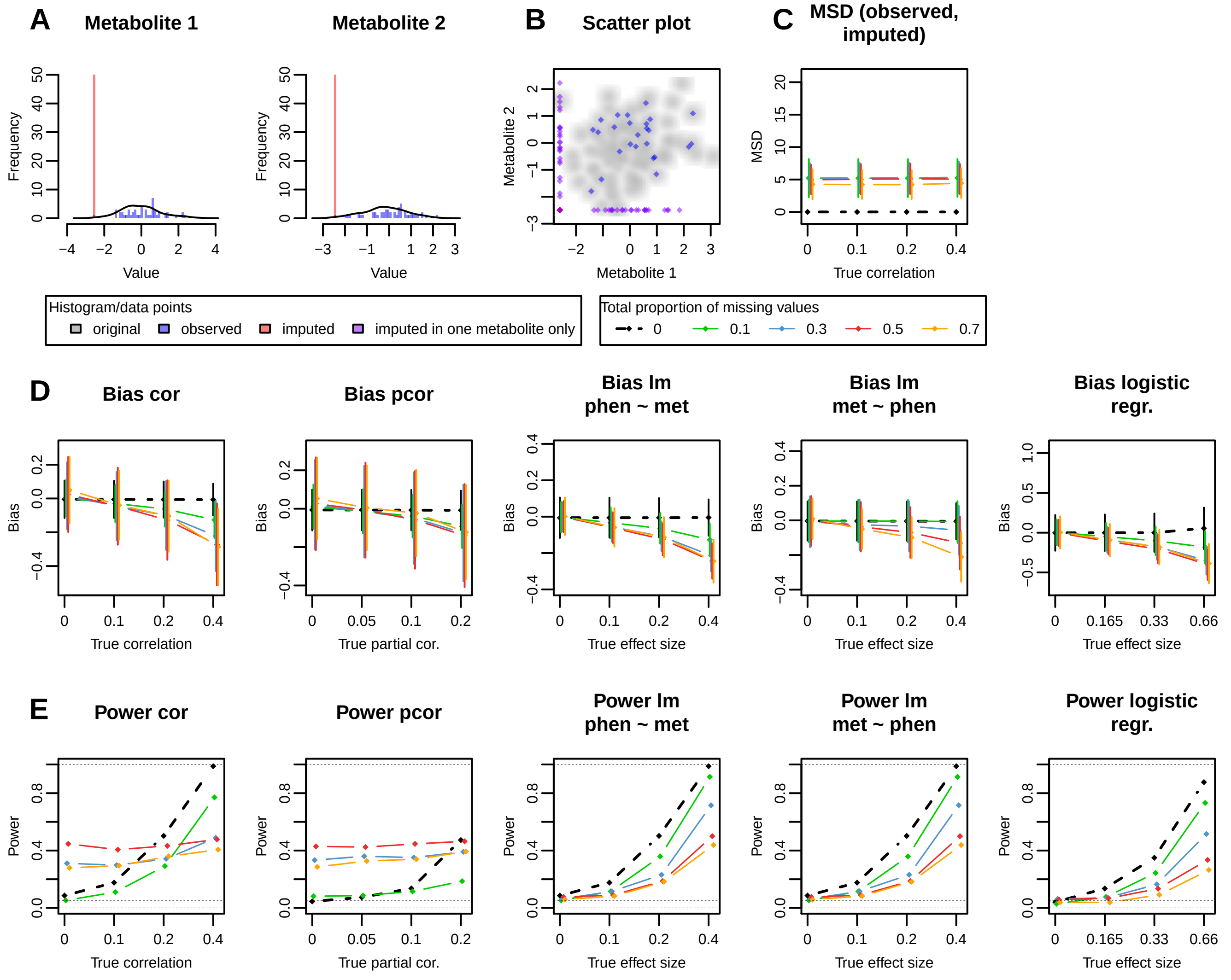

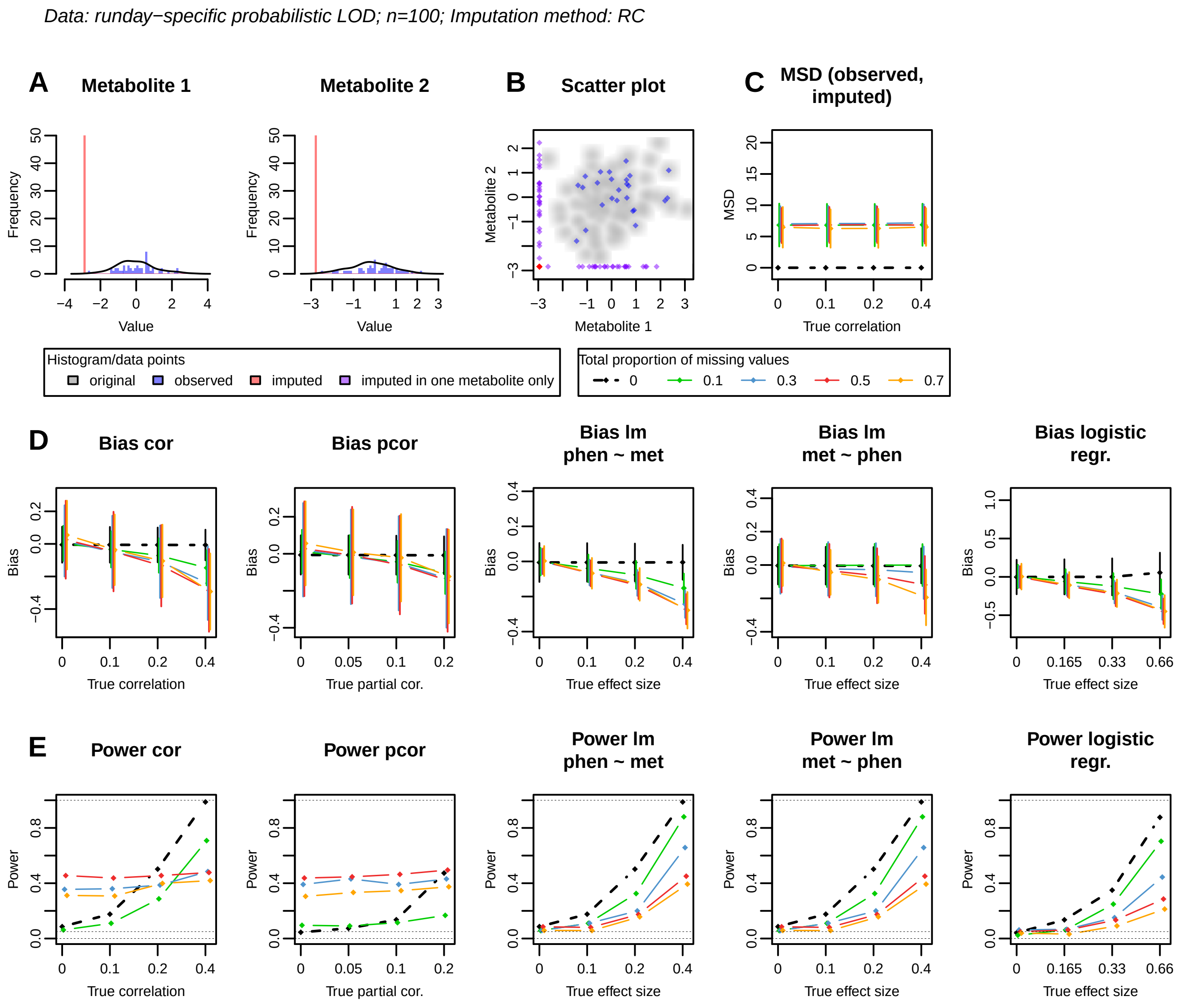
Data: runday−specific probabilistic LOD; n=100; Imputation method: RC
A
Metabolite 1
Metabolite 2
Frequency
Value
B
Scatter plot
Metabolite 1
Metabolite 2
C
MSD (observed, imputed)
MSD
True correlation
Histogram/data points
original
observed
imputed
imputed in one metabolite only
Total proportion of missing values
0
0.1
0.3
0.5
0.7
D
Bias cor
Bias pcor
Bias lm phen ~ met
Bias lm met ~ phen
Bias logistic regr.
Bias
True correlation
True partial cor.
True effect size
E
Power cor
Power pcor
Power lm phen ~ met
Power lm met ~ phen
Power logistic regr.
Power
True correlation
True partial cor.
True effect size

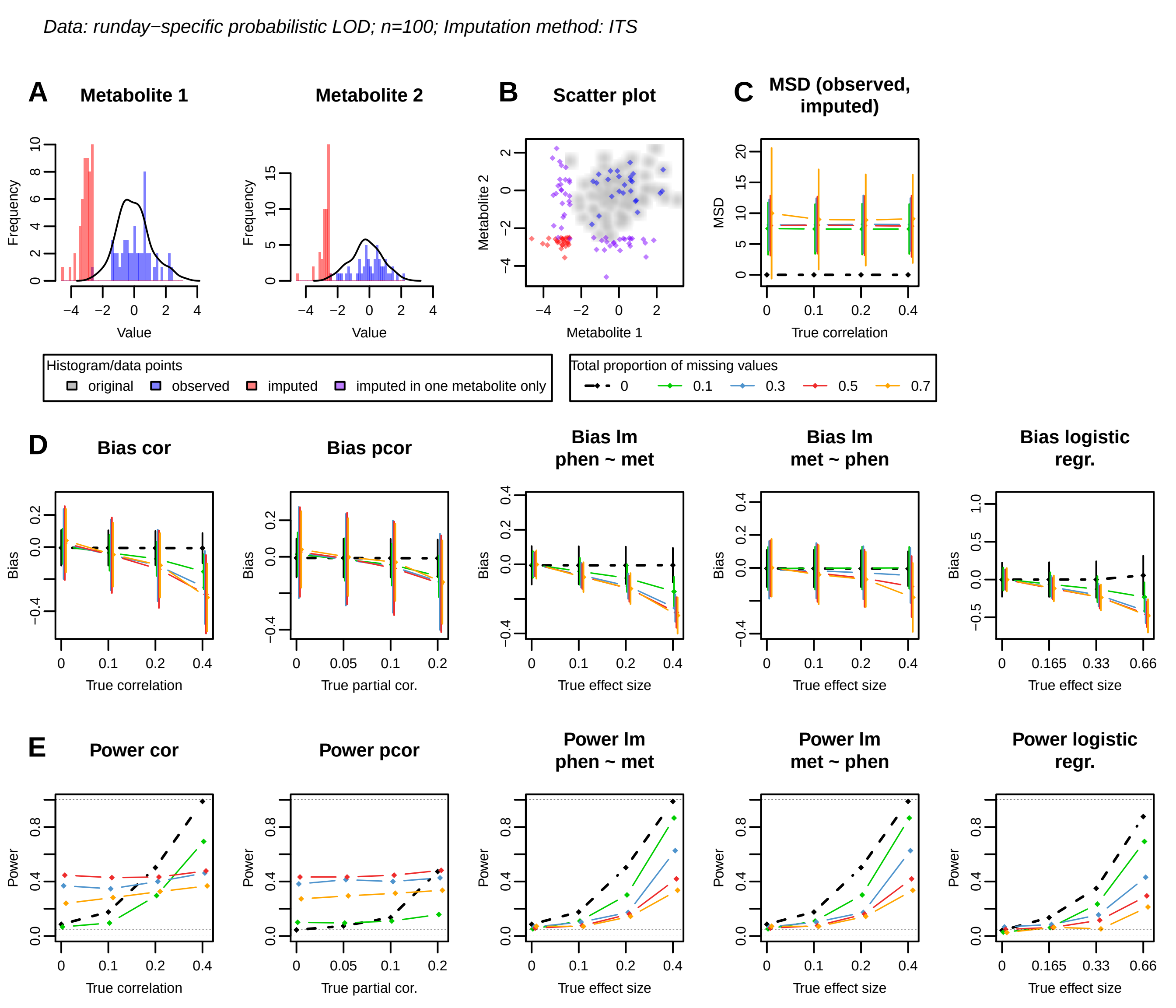
Data: runday−specific probabilistic LOD; n=100; Imputation method: ITS
A
Metabolite 1
Metabolite 2
Frequency
Value
B
Scatter plot
Metabolite 2
Metabolite 1
C
MSD (observed, imputed)
MSD
True correlation
Histogram/data points
original
observed
imputed
imputed in one metabolite only
Total proportion of missing values
0
0.1
0.3
0.5
0.7
D
Bias cor
Bias pcor
Bias lm phen ~ met
Bias lm met ~ phen
Bias logistic regr.
Bias
True correlation
True partial cor.
True effect size
E
Power cor
Power pcor
Power lm phen ~ met
Power lm met ~ phen
Power logistic regr.
Power

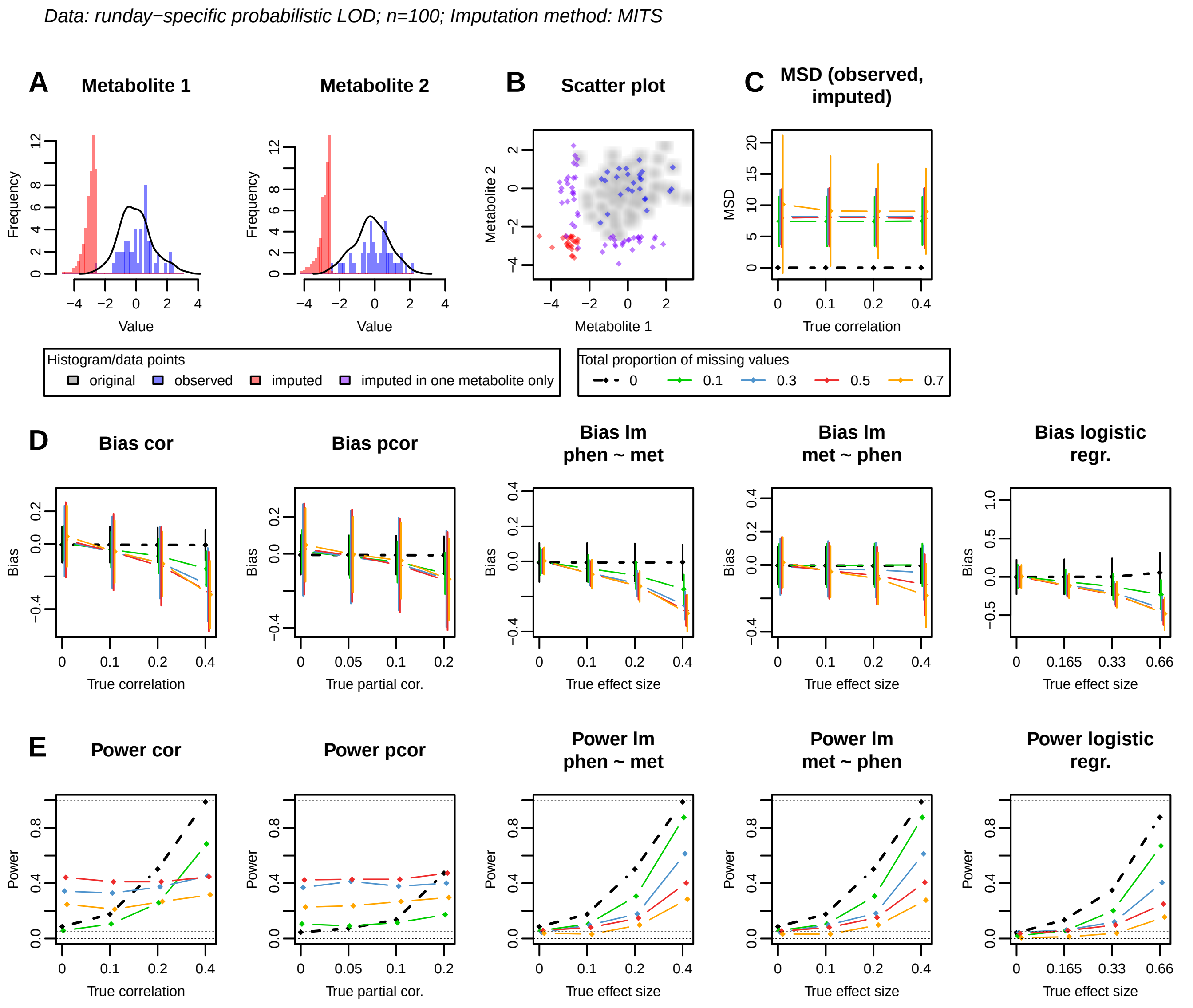

Data: runday−specific probabilistic LOD; n=100; Imputation method: MITS
A
Metabolite 1
Metabolite 2
B
Scatter plot
C
MSD (observed, imputed)
Frequency
Value
Metabolite 2
Metabolite 1
MSD
True correlation
Histogram/data points
original
observed
imputed
imputed in one metabolite only
Total proportion of missing values
0
0.1
0.3
0.5
0.7
D
Bias cor
Bias pcor
Bias lm phen ~ met
Bias lm met ~ phen
Bias logistic regr.
Bias
True correlation
True partial cor.
True effect size
E
Power cor
Power pcor
Power lm phen ~ met
Power lm met ~ phen
Power logistic regr.
Power
True correlation
True partial cor.
True effect size

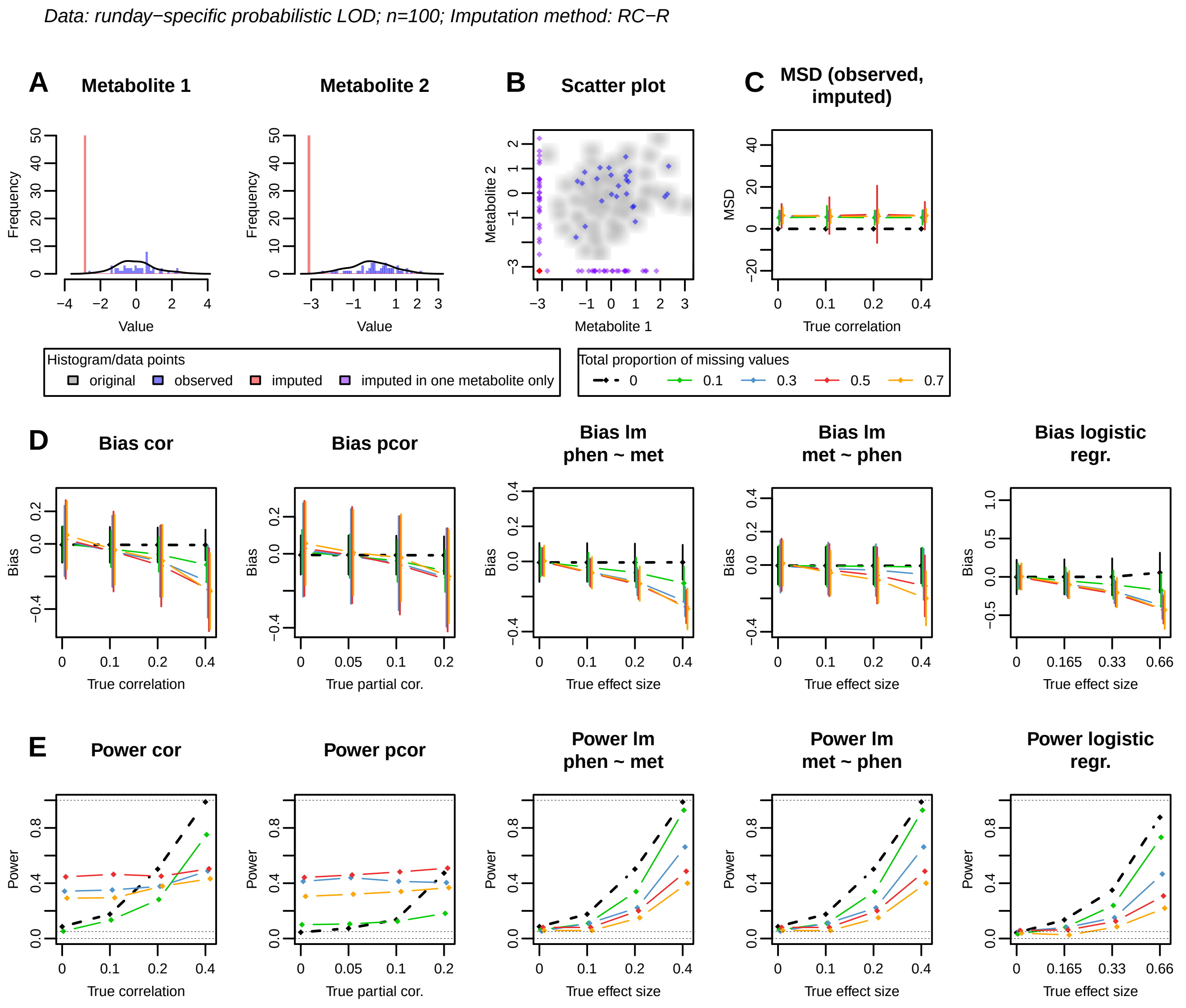

Data: runday−specific probabilistic LOD; n=100; Imputation method: RC−R
A
Metabolite 1
Metabolite 2
B
Scatter plot
C
MSD (observed, imputed)
Frequency
Value
Metabolite 2
Metabolite 1
MSD
True correlation
Histogram/data points
original
observed
imputed
imputed in one metabolite only
Total proportion of missing values
0
0.1
0.3
0.5
0.7
D
Bias cor
Bias pcor
Bias lm phen ~ met
Bias lm met ~ phen
Bias logistic regr.
Bias
True correlation
True partial cor.
True effect size
E
Power cor
Power pcor
Power lm phen ~ met
Power lm met ~ phen
Power logistic regr.
Power
True correlation
True partial cor.
True effect size

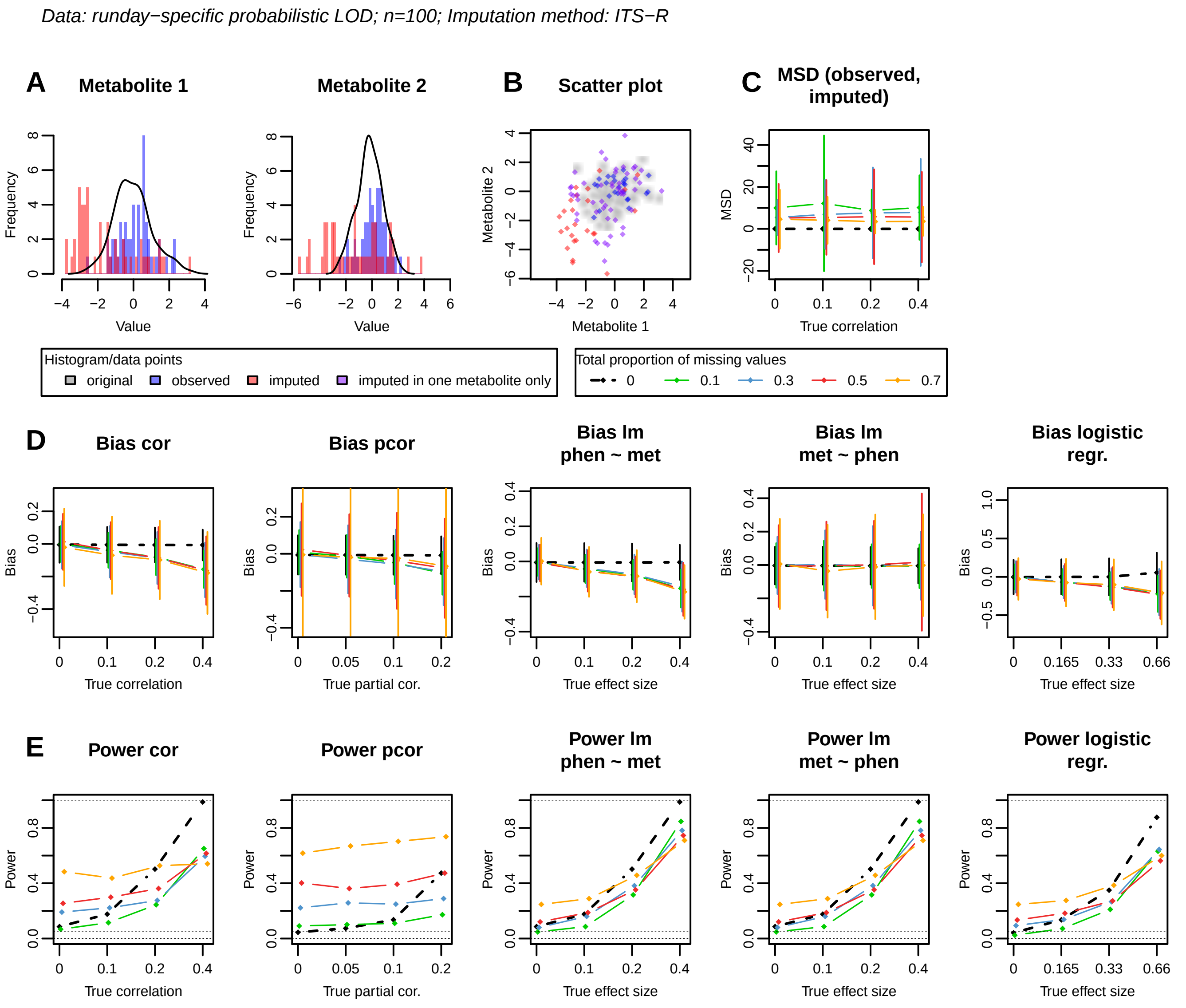
Data: runday−specific probabilistic LOD; n=100; Imputation method: ITS−R
A
Metabolite 1
Metabolite 2
B
Scatter plot
C
MSD (observed, imputed)
Frequency
Value
Metabolite 2
Metabolite 1
MSD
True correlation
Histogram/data points
original
observed
imputed
imputed in one metabolite only
Total proportion of missing values
0
0.1
0.3
0.5
0.7
D
Bias cor
Bias pcor
Bias lm phen ~ met
Bias lm met ~ phen
Bias logistic regr.
Bias
True correlation
True partial cor.
True effect size
E
Power cor
Power pcor
Power lm phen ~ met
Power lm met ~ phen
Power logistic regr.
Power

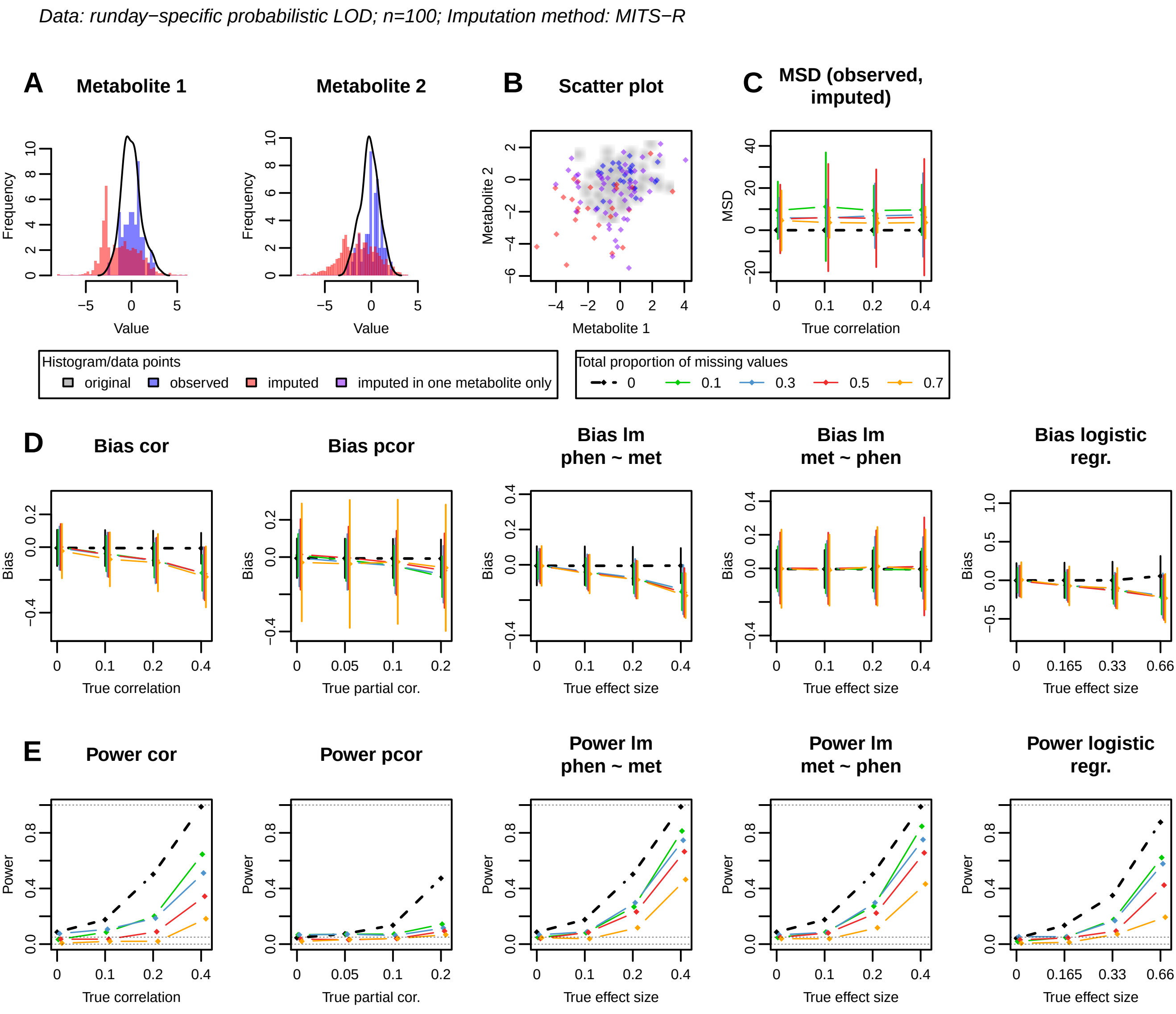
Data: runday−specific probabilistic LOD; n=100; Imputation method: MITS−R
A
Metabolite 1
Metabolite 2
Frequency
Value
B
Scatter plot
Metabolite 2
Metabolite 1
C
MSD (observed, imputed)
MSD
True correlation
Histogram/data points
original
observed
imputed
imputed in one metabolite only
Total proportion of missing values
0
0.1
0.3
0.5
0.7
D
Bias cor
Bias pcor
Bias lm phen ~ met
Bias lm met ~ phen
Bias logistic regr.
Bias
True correlation
True partial cor.
True effect size
E
Power cor
Power pcor
Power lm phen ~ met
Power lm met ~ phen
Power logistic regr.
Power
True correlation
True partial cor.
True effect size

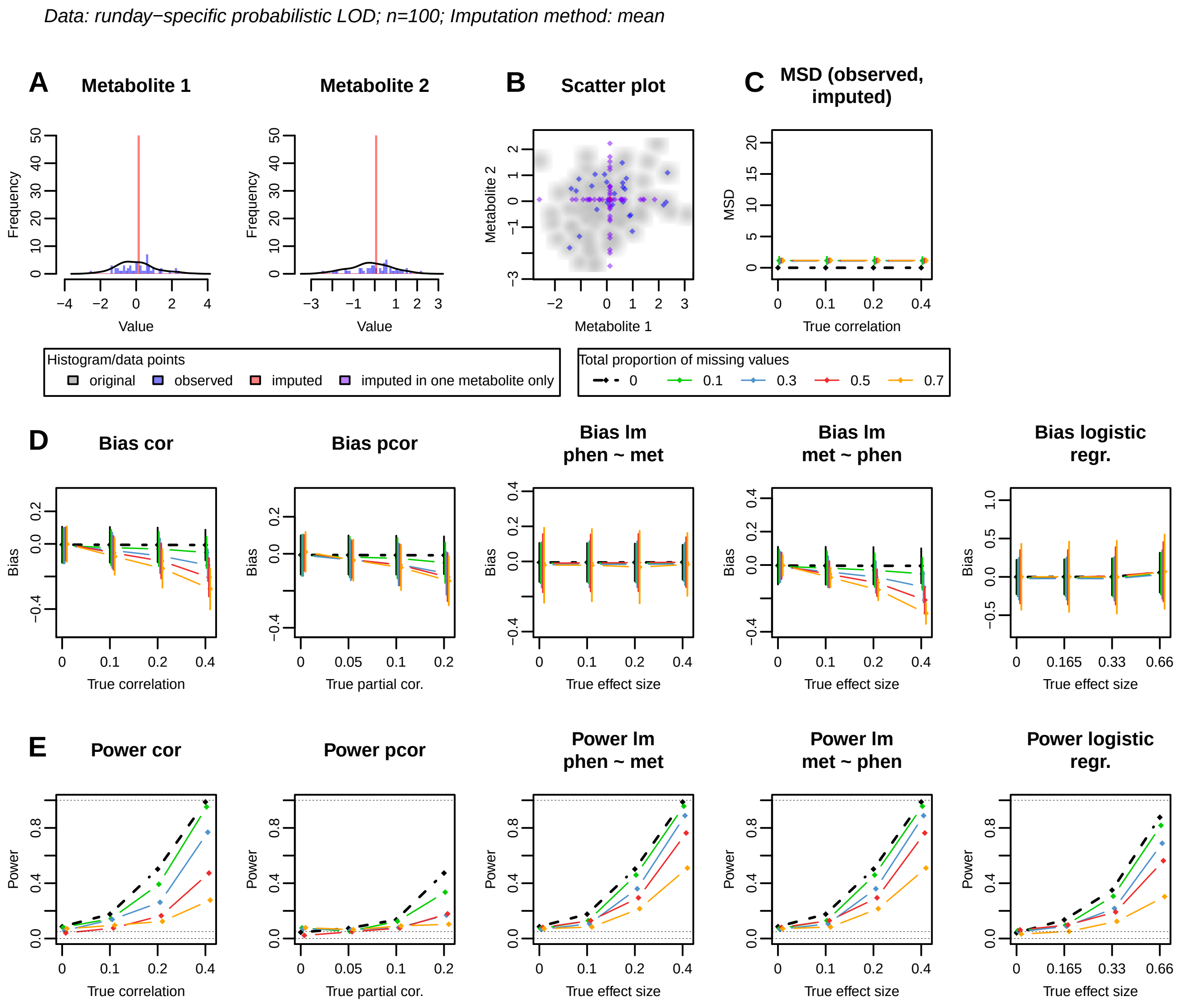
Data: runday−specific probabilistic LOD; n=100; Imputation method: mean
A
Metabolite 1
Metabolite 2
Frequency
Value
B
Scatter plot
Metabolite 1
Metabolite 2
C
MSD (observed, imputed)
MSD
True correlation
Histogram/data points
original
observed
imputed
imputed in one metabolite only
Total proportion of missing values
0
0.1
0.3
0.5
0.7
D
Bias cor
Bias pcor
Bias lm phen ~ met
Bias lm met ~ phen
Bias logistic regr.
Bias
True correlation
True partial cor.
True effect size
E
Power cor
Power pcor
Power lm phen ~ met
Power lm met ~ phen
Power logistic regr.
Power
True correlation
True partial cor.
True effect size

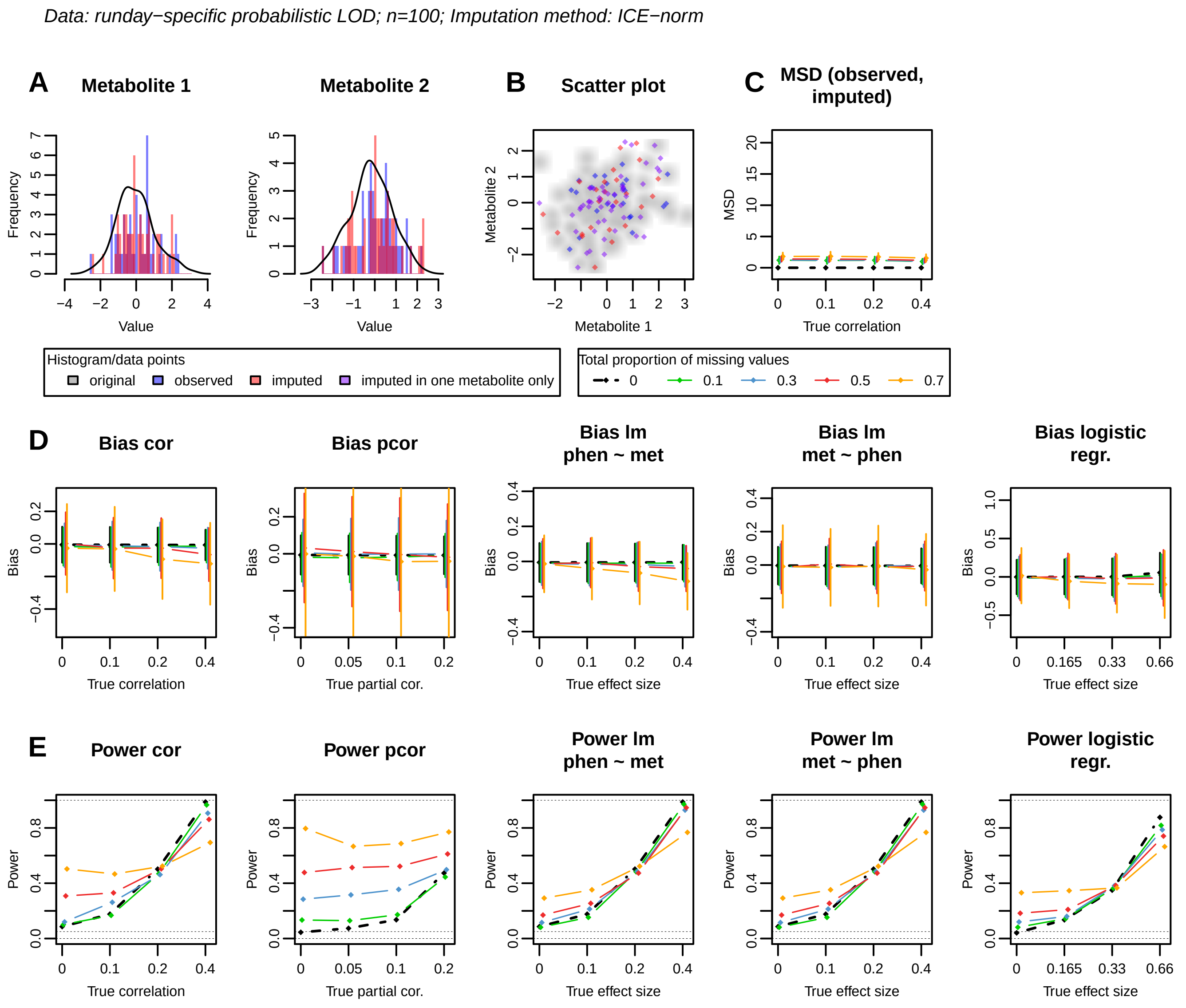

Data: runday−specific probabilistic LOD; n=100; Imputation method: ICE−norm
A
Metabolite 1
Metabolite 2
B
Scatter plot
C
MSD (observed, imputed)
Frequency
Value
Metabolite 2
Metabolite 1
MSD
True correlation
Histogram/data points
original
observed
imputed
imputed in one metabolite only
Total proportion of missing values
0
0.1
0.3
0.5
0.7
D
Bias cor
Bias pcor
Bias lm phen ~ met
Bias lm met ~ phen
Bias logistic regr.
Bias
True correlation
True partial cor.
True effect size
E
Power cor
Power pcor
Power lm phen ~ met
Power lm met ~ phen
Power logistic regr.
Power

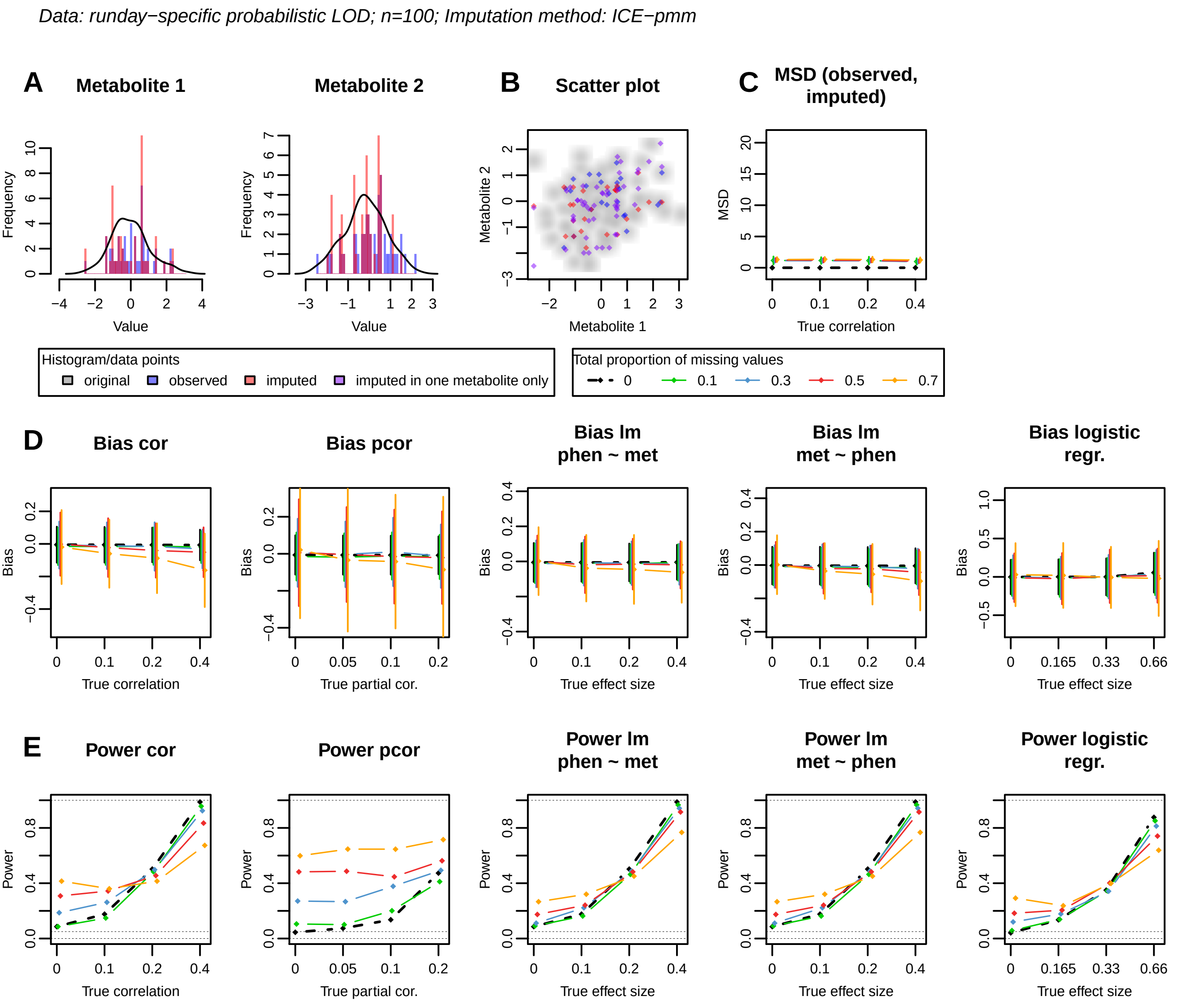
Data: runday−specific probabilistic LOD; n=100; Imputation method: ICE−pmm
A
Metabolite 1
Metabolite 2
Frequency
Value
B
Scatter plot
Metabolite 2
Metabolite 1
C
MSD (observed, imputed)
MSD
True correlation
Histogram/data points
original
observed
imputed
imputed in one metabolite only
Total proportion of missing values
0
0.1
0.3
0.5
0.7
D
Bias cor
Bias pcor
Bias lm phen ~ met
Bias lm met ~ phen
Bias logistic regr.
Bias
True correlation
True partial cor.
True effect size
E
Power cor
Power pcor
Power lm phen ~ met
Power lm met ~ phen
Power logistic regr.
Power

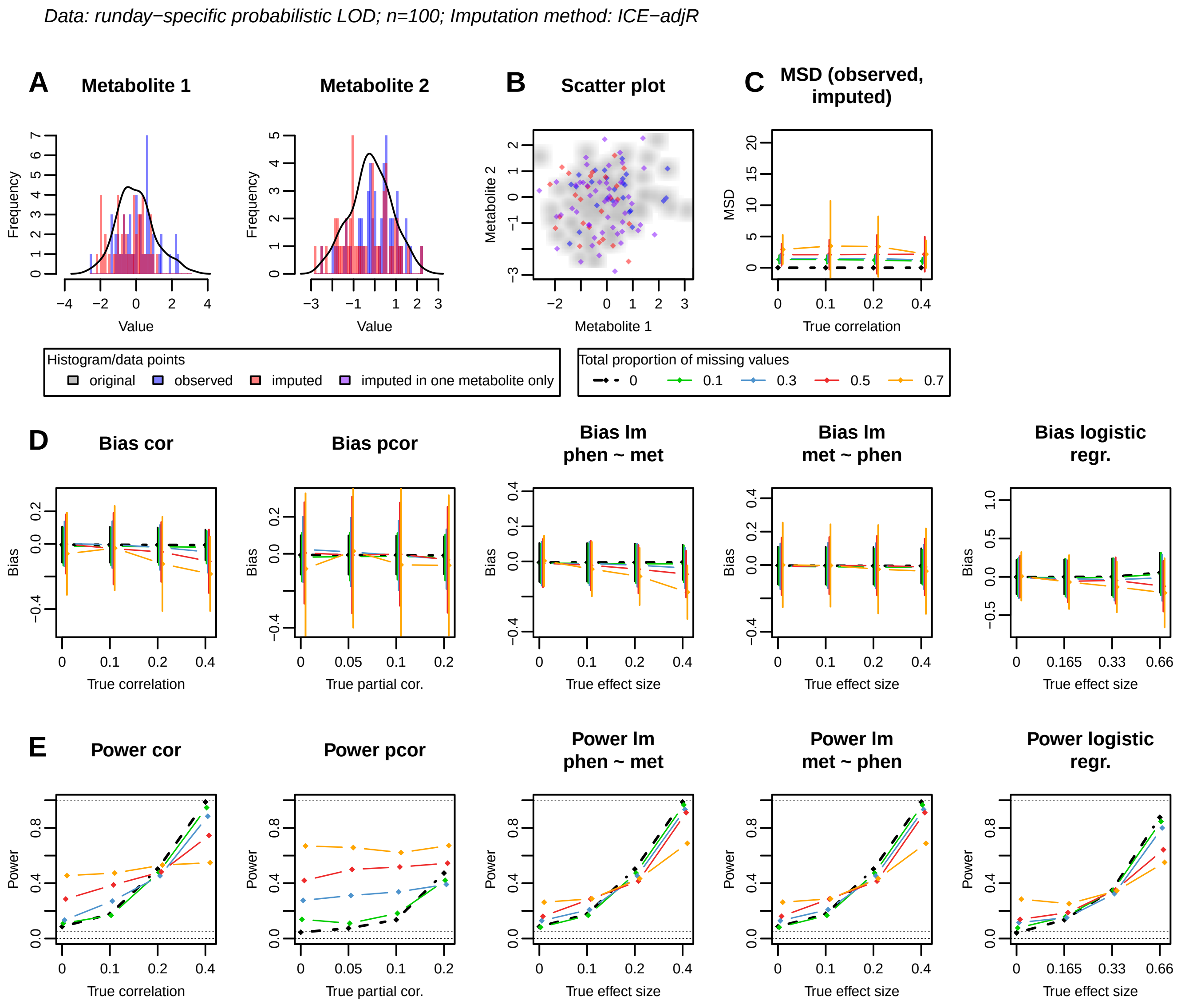

Data: runday−specific probabilistic LOD; n=100; Imputation method: ICE−adjR
A
Metabolite 1
Metabolite 2
B
Scatter plot
C
MSD (observed, imputed)
Frequency
Value
Metabolite 2
Metabolite 1
MSD
True correlation
Histogram/data points
original
observed
imputed
imputed in one metabolite only
Total proportion of missing values
0
0.1
0.3
0.5
0.7
D
Bias cor
Bias pcor
Bias lm phen ~ met
Bias lm met ~ phen
Bias logistic regr.
Bias
True correlation
True partial cor.
True effect size
E
Power cor
Power pcor
Power lm phen ~ met
Power lm met ~ phen
Power logistic regr.
Power
True correlation
True partial cor.
True effect size

Data: runday−specific probabilistic LOD; n=100; Imputation method: MICE−norm

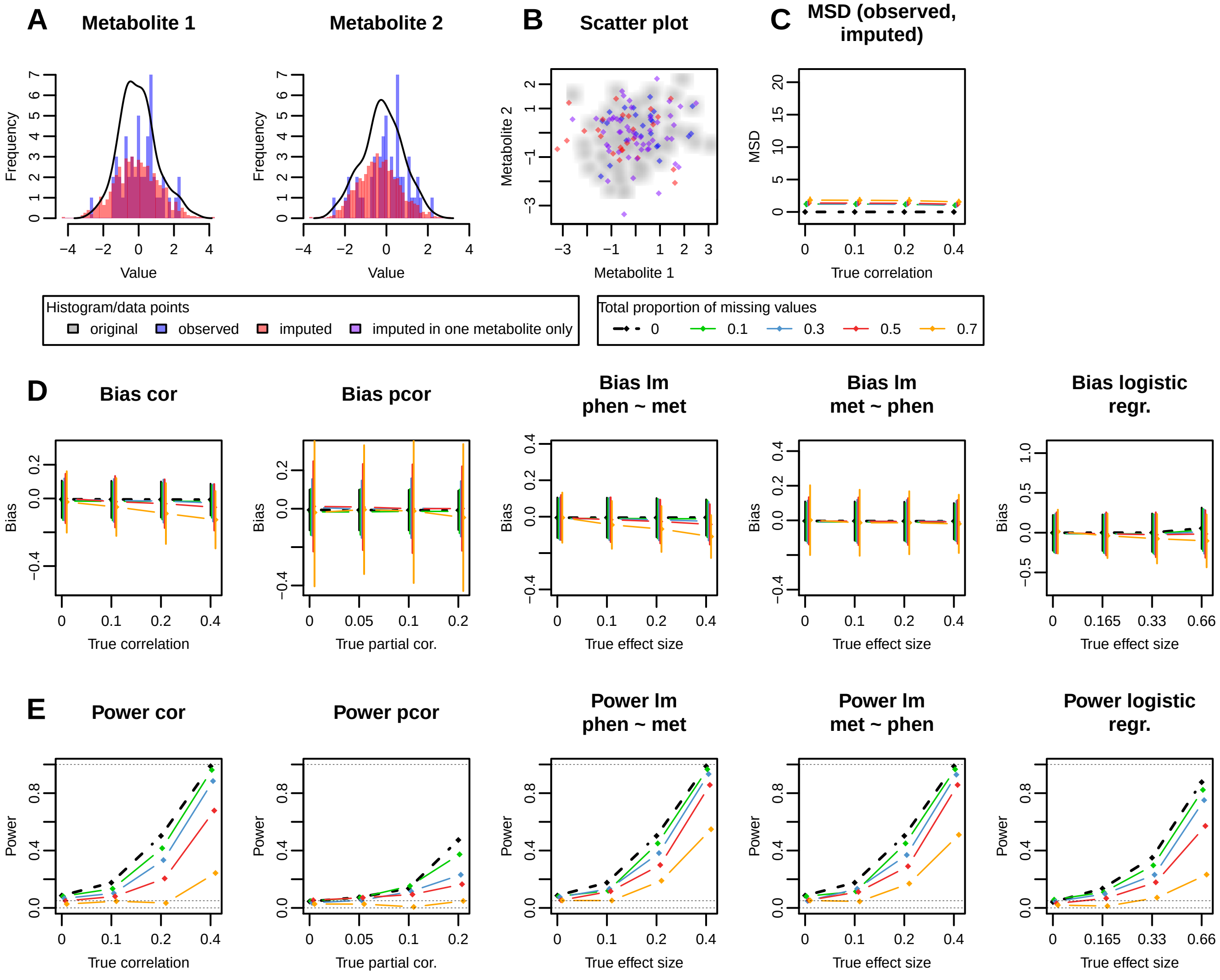

Data: runday−specific probabilistic LOD; n=100; Imputation method: MICE−pmm

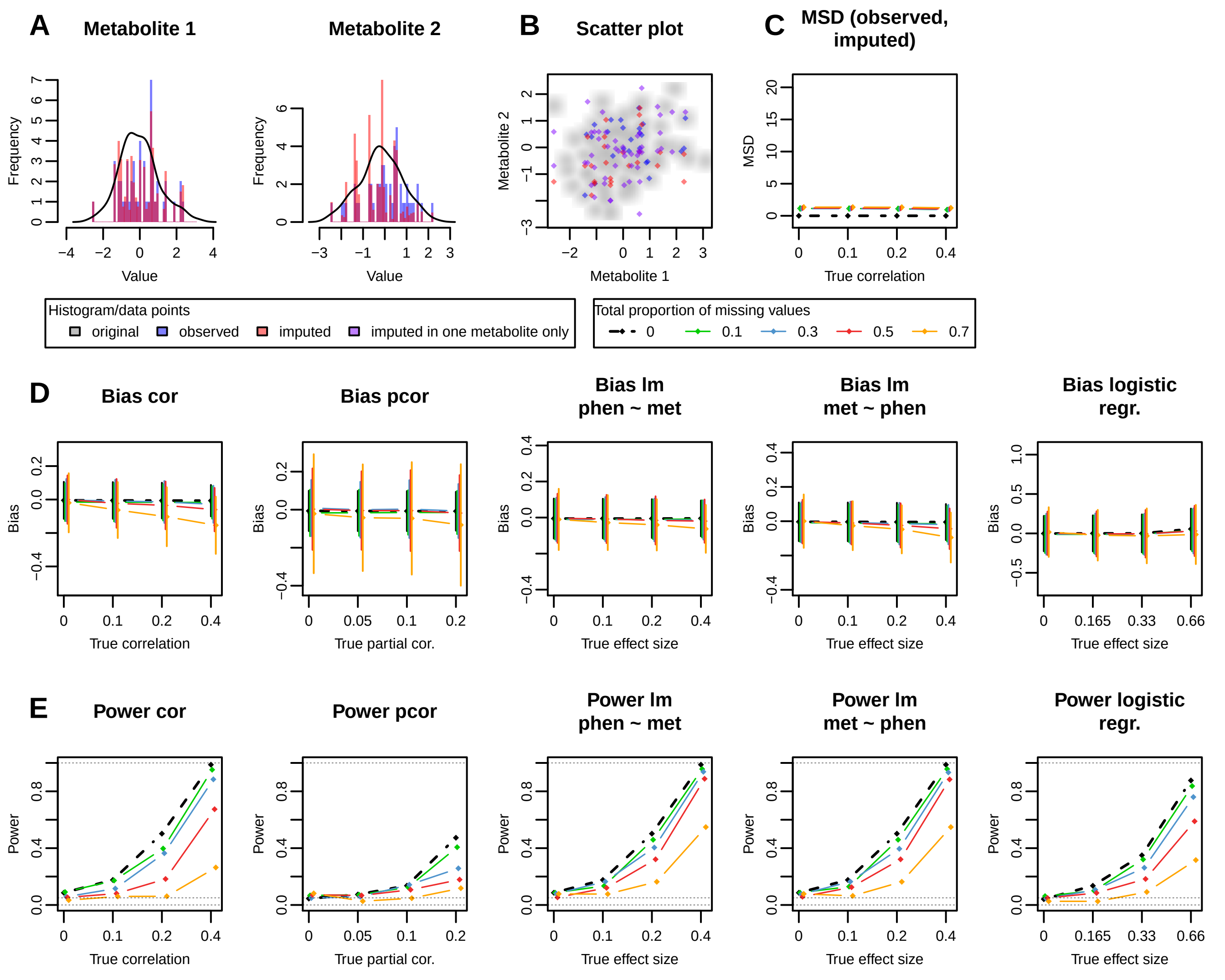

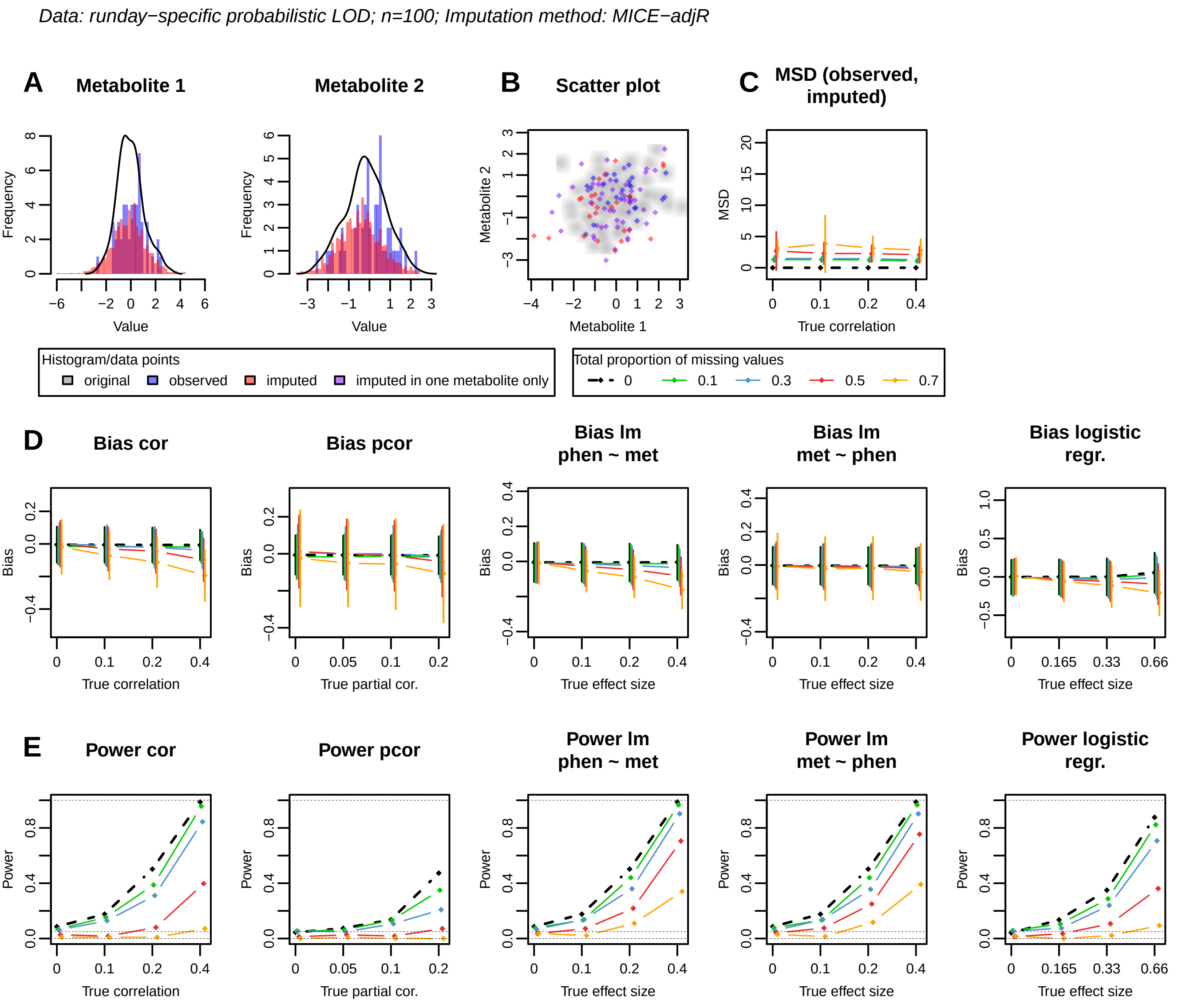
Data: runday−specific probabilistic LOD; n=100; Imputation method: MICE−adjR
A
Metabolite 1
Metabolite 2
B
Scatter plot
C
MSD (observed, imputed)
Frequency
Value
Metabolite 2
Metabolite 1
MSD
True correlation
Histogram/data points
original
observed
imputed
imputed in one metabolite only
Total proportion of missing values
0
0.1
0.3
0.5
0.7
D
Bias cor
Bias pcor
Bias lm phen ~ met
Bias lm met ~ phen
Bias logistic regr.
Bias
True correlation
True partial cor.
True effect size
E
Power cor
Power pcor
Power lm phen ~ met
Power lm met ~ phen
Power logistic regr.
Power
True correlation
True partial cor.
True effect size

Data: runday−specific probabilistic LOD; n=100; Imputation method: KNN−var(1)

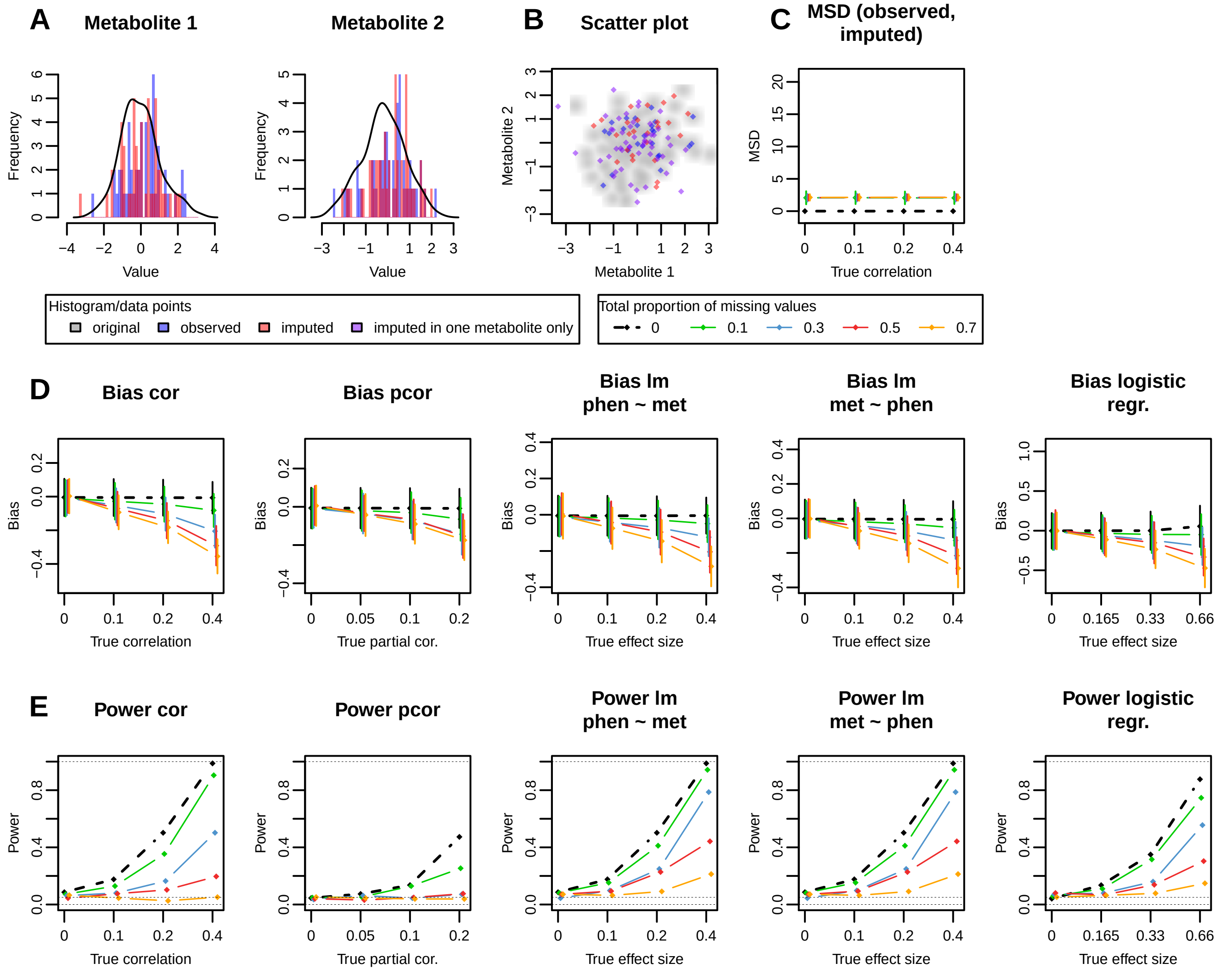

Data: runday−specific probabilistic LOD; n=100; Imputation method: KNN−var(3)

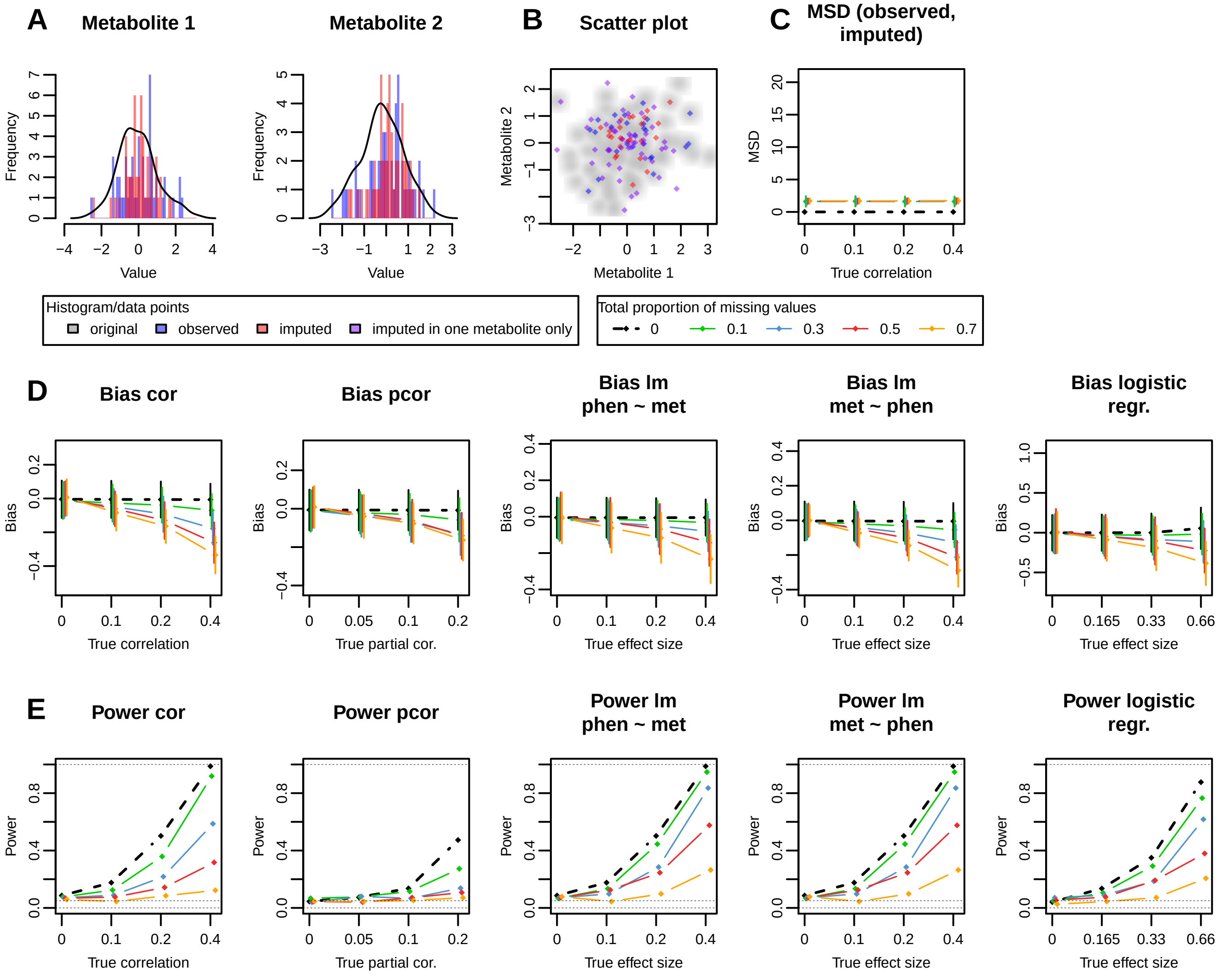

Data: runday−specific probabilistic LOD; n=100; Imputation method: KNN−var(5)

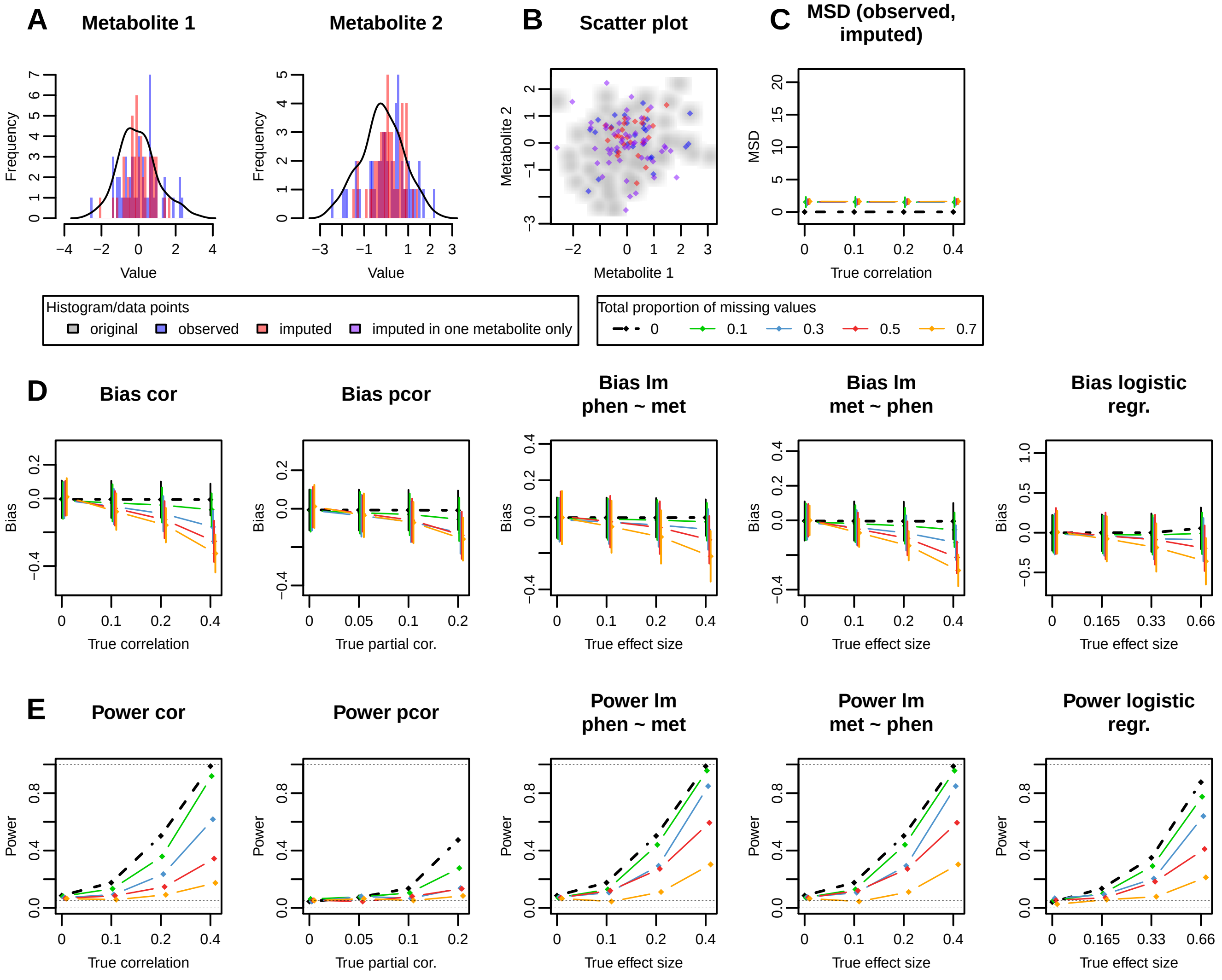

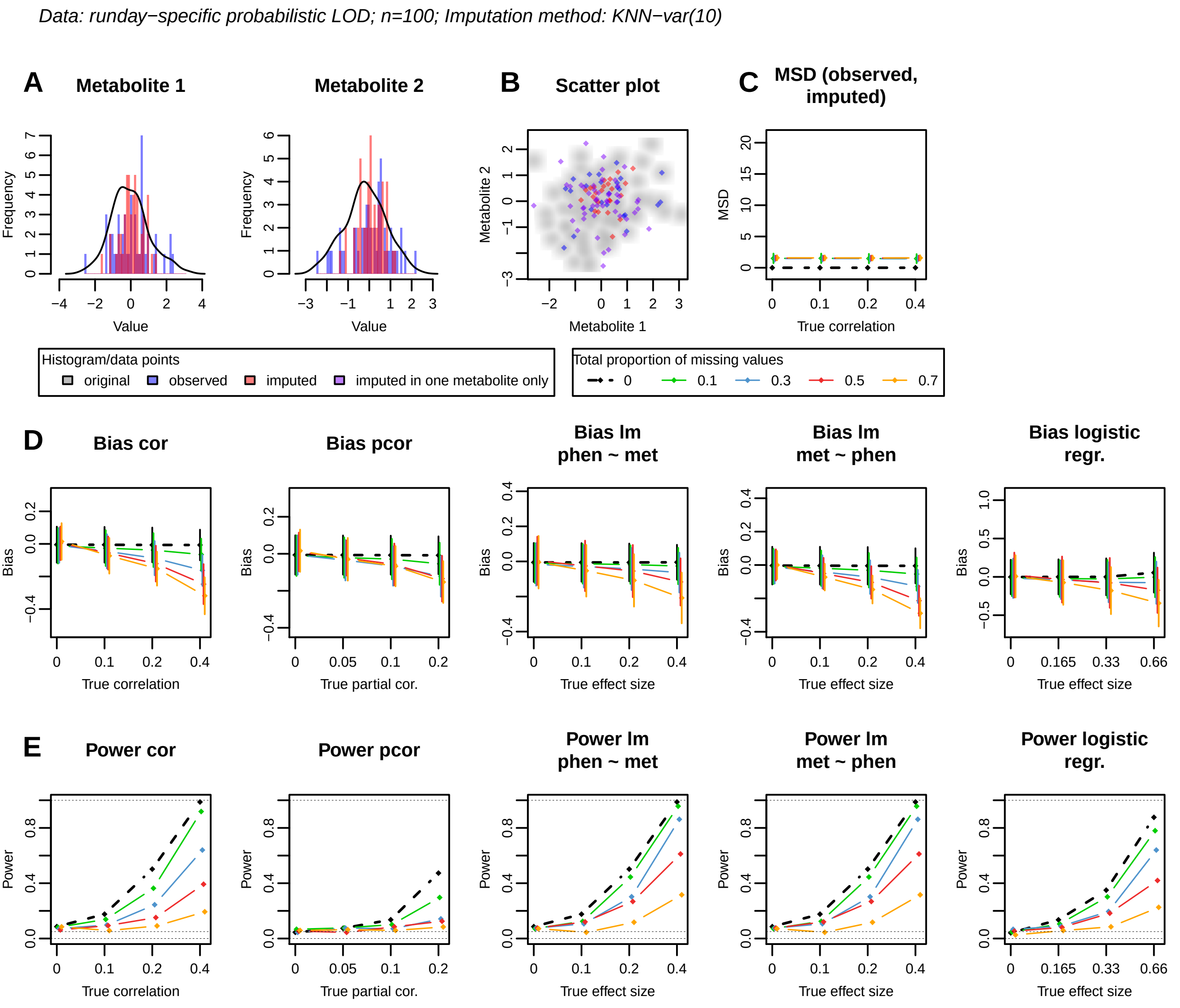
Data: runday−specific probabilistic LOD; n=100; Imputation method: KNN−var(10)
A
Metabolite 1
Metabolite 2
B
Scatter plot
C
MSD (observed, imputed)
Frequency
Value
Metabolite 2
Metabolite 1
MSD
True correlation
Histogram/data points
original
observed
imputed
imputed in one metabolite only
Total proportion of missing values
0
0.1
0.3
0.5
0.7
D
Bias cor
Bias pcor
Bias lm phen ~ met
Bias lm met ~ phen
Bias logistic regr.
Bias
True correlation
True partial cor.
True effect size
E
Power cor
Power pcor
Power lm phen ~ met
Power lm met ~ phen
Power logistic regr.
Power

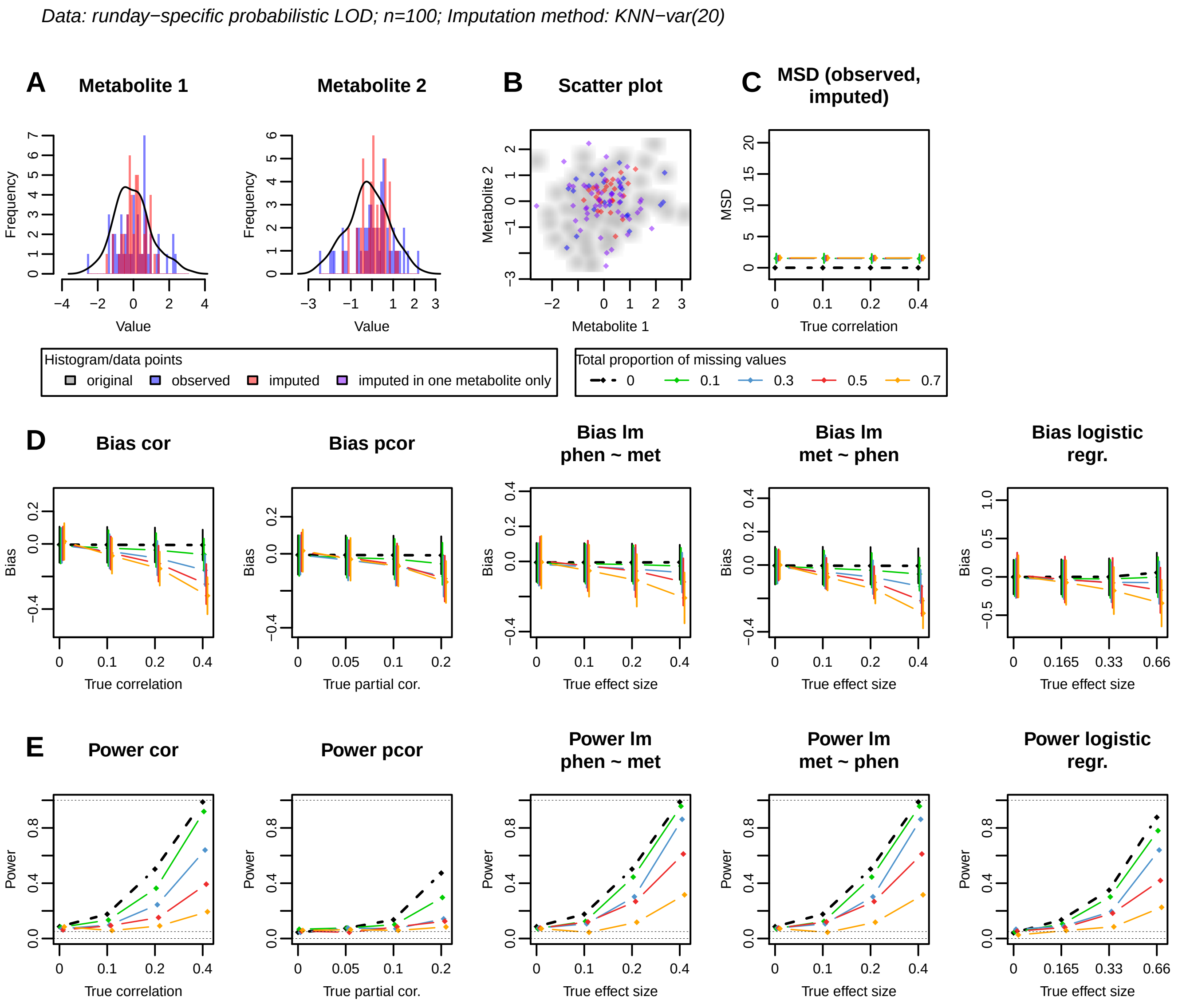

Data: runday−specific probabilistic LOD; n=100; Imputation method: KNN−var(20)
A
Metabolite 1
Metabolite 2
Frequency
Value
B
Scatter plot
Metabolite 1
Metabolite 2
C
MSD (observed, imputed)
MSD
True correlation
Histogram/data points
original
observed
imputed
imputed in one metabolite only
Total proportion of missing values
0
0.1
0.3
0.5
0.7
D
Bias cor
Bias pcor
Bias lm phen ~ met
Bias lm met ~ phen
Bias logistic regr.
Bias
True correlation
True partial cor.
True effect size
E
Power cor
Power pcor
Power lm phen ~ met
Power lm met ~ phen
Power logistic regr.
Power
True correlation
True partial cor.
True effect size

Data: runday−specific probabilistic LOD; n=100; Imputation method: KNN−obs(1)

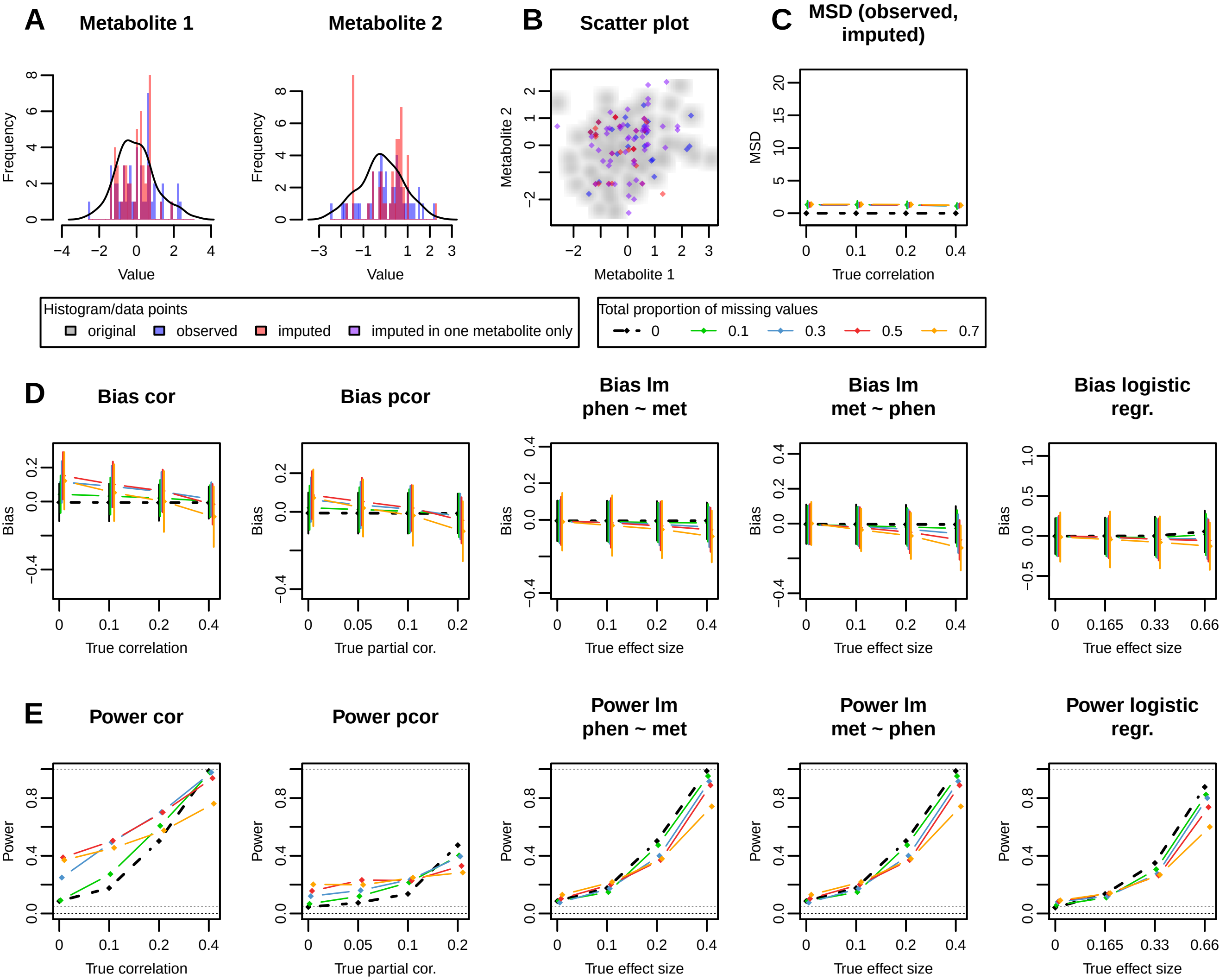

Data: runday−specific probabilistic LOD; n=100; Imputation method: KNN−obs(3)

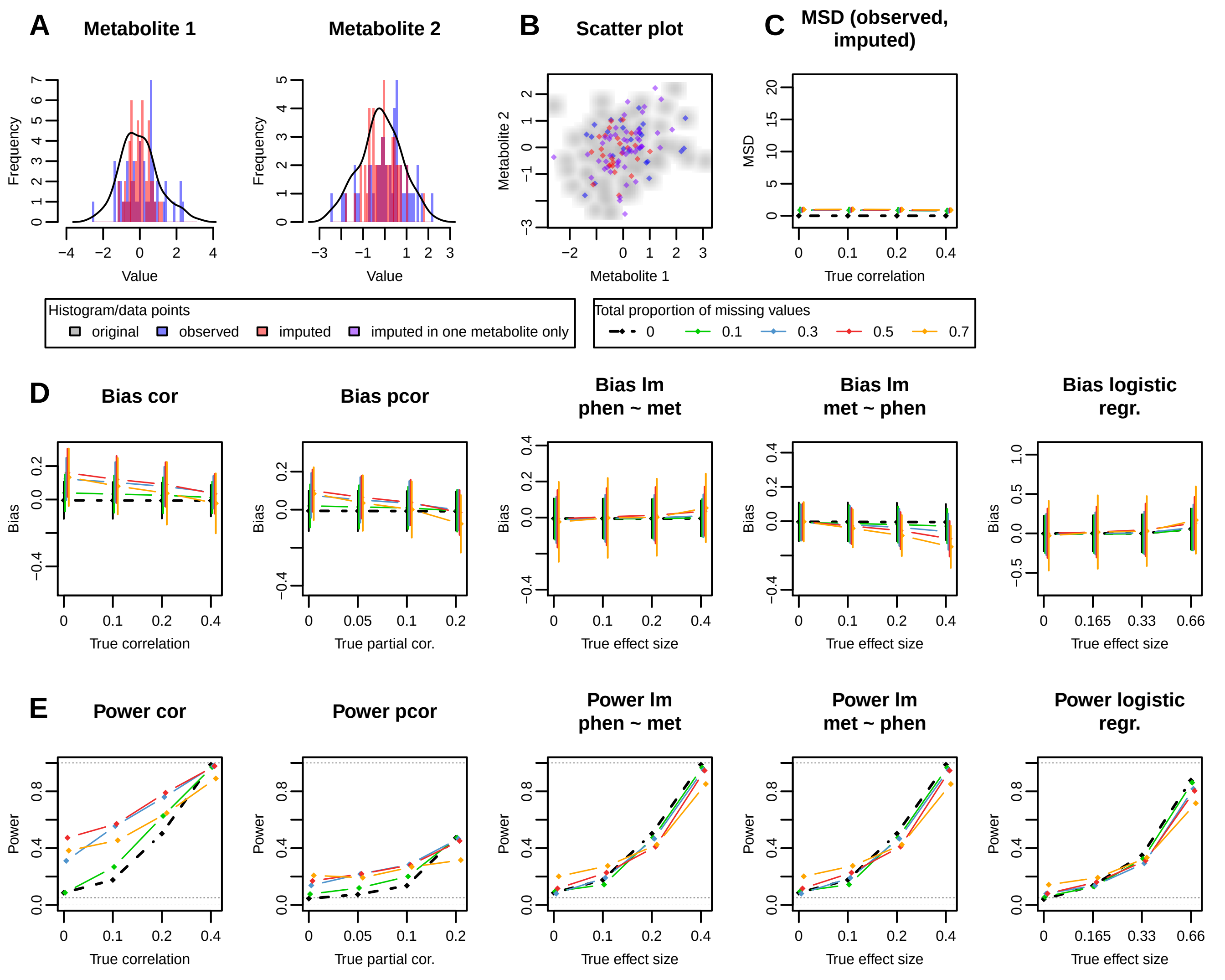

Data: runday−specific probabilistic LOD; n=100; Imputation method: KNN−obs(5)

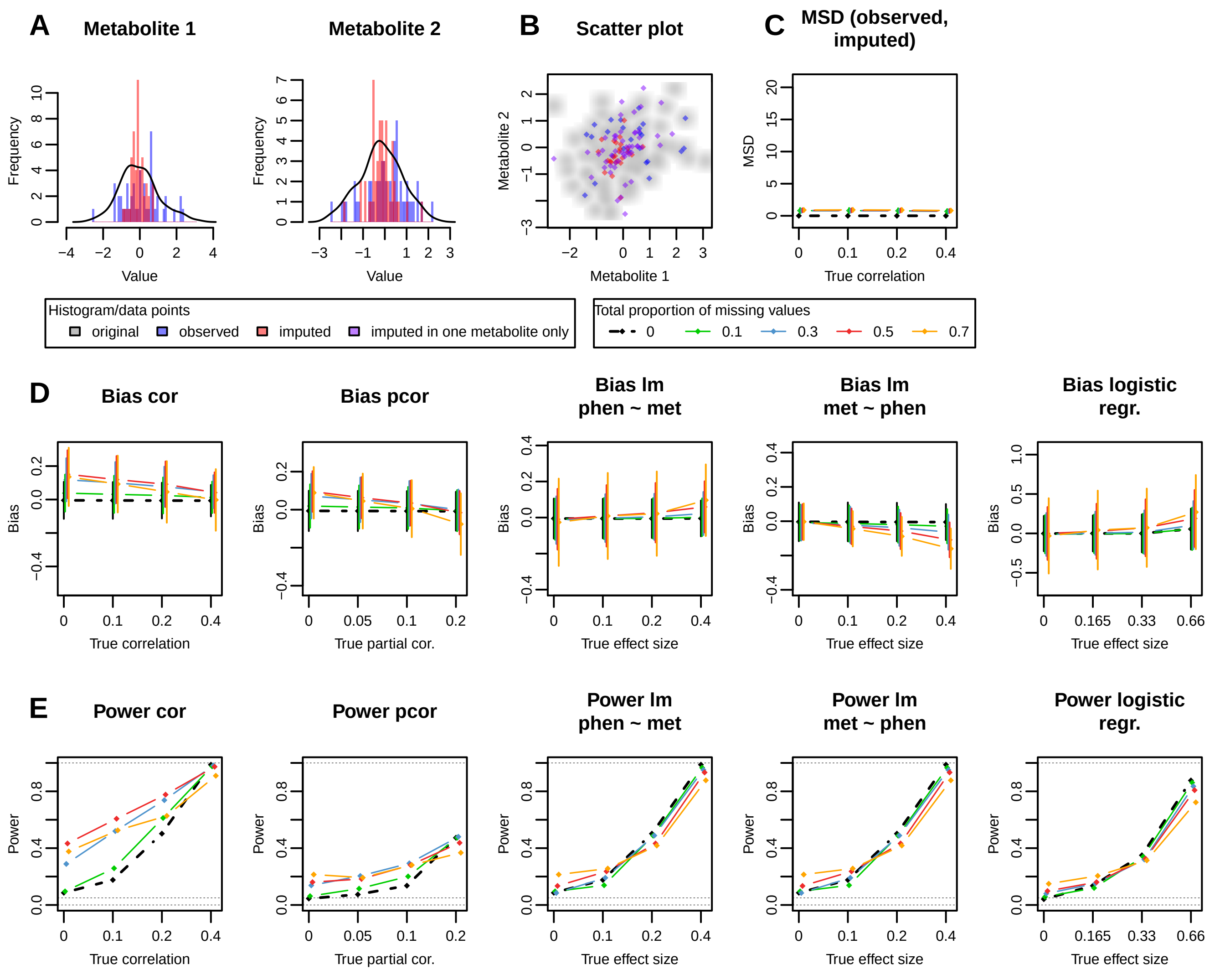

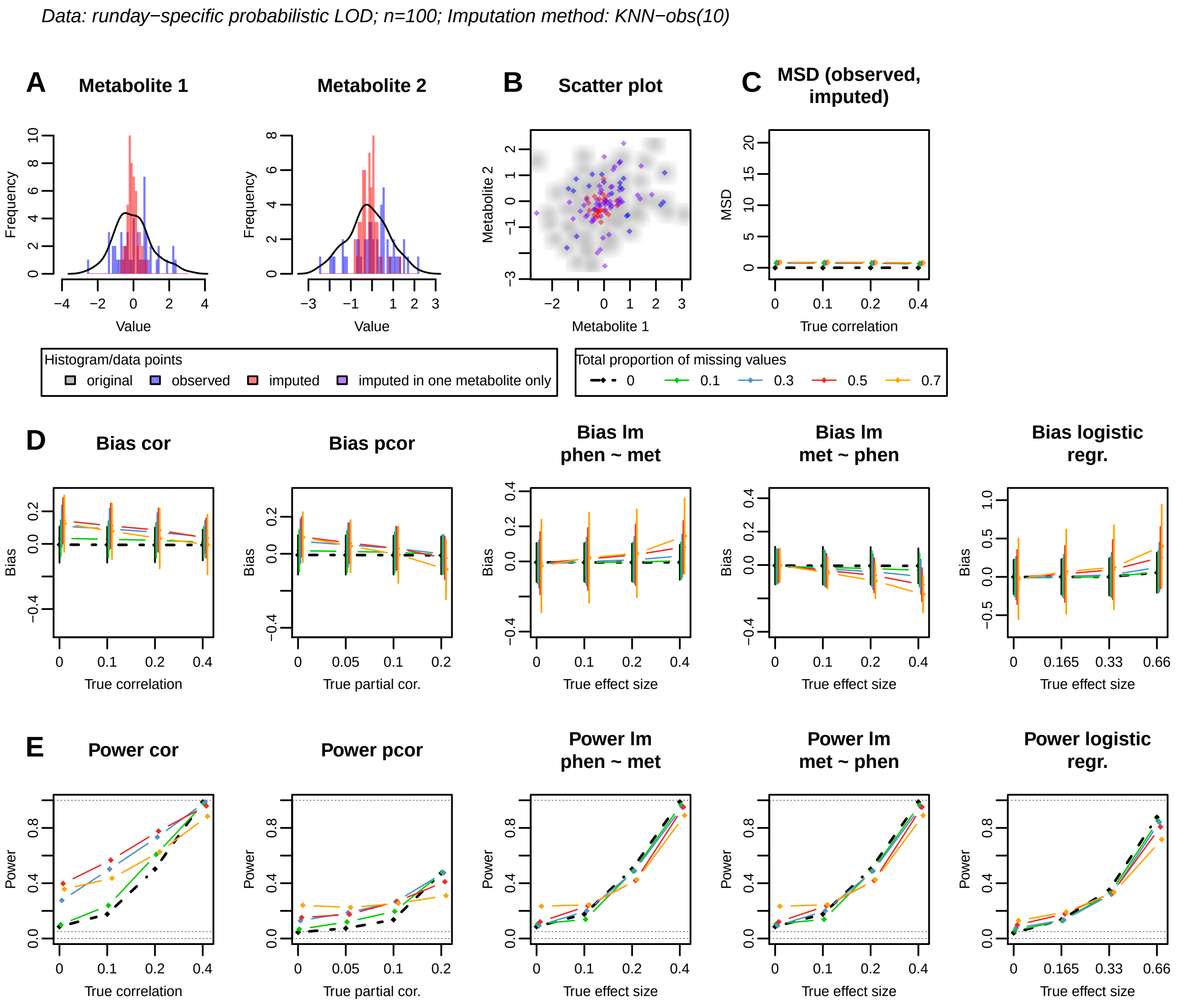
Data: runday−specific probabilistic LOD; n=100; Imputation method: KNN−obs(10)
A
Metabolite 1
Metabolite 2
B
Scatter plot
C
MSD (observed, imputed)
Frequency
Value
Metabolite 2
Metabolite 1
MSD
True correlation
Histogram/data points
original
observed
imputed
imputed in one metabolite only
Total proportion of missing values
0
0.1
0.3
0.5
0.7
D
Bias cor
Bias pcor
Bias lm phen ~ met
Bias lm met ~ phen
Bias logistic regr.
Bias
True correlation
True partial cor.
True effect size
E
Power cor
Power pcor
Power lm phen ~ met
Power lm met ~ phen
Power logistic regr.
Power
True correlation
True partial cor.
True effect size

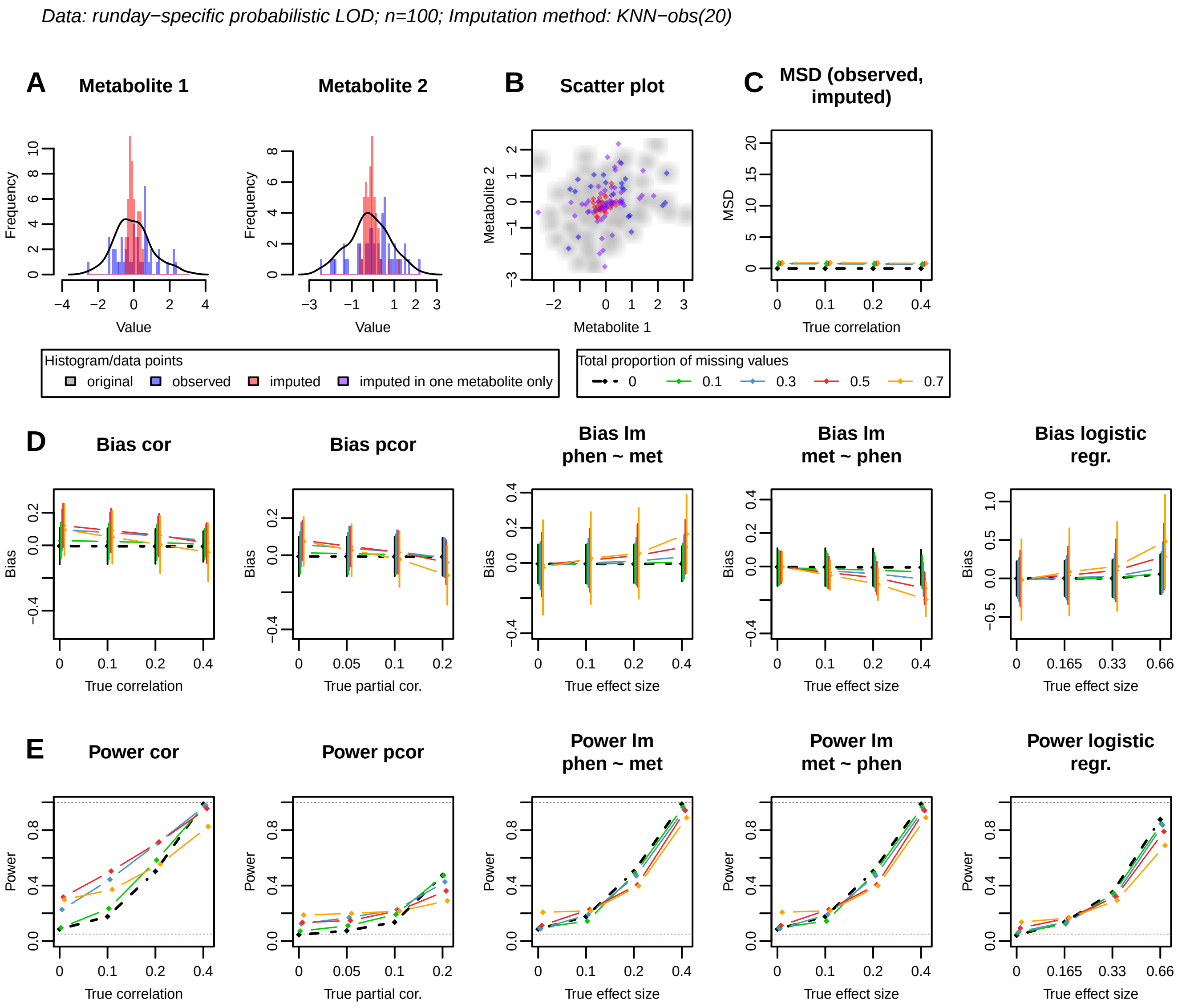

Data: runday−specific probabilistic LOD; n=100; Imputation method: KNN−obs(20)
A
Metabolite 1
Metabolite 2
Frequency
Value
B
Scatter plot
Metabolite 1
Metabolite 2
C
MSD (observed, imputed)
MSD
True correlation
Histogram/data points
original
observed
imputed
imputed in one metabolite only
Total proportion of missing values
0
0.1
0.3
0.5
0.7
D
Bias cor
Bias pcor
Bias lm phen ~ met
Bias lm met ~ phen
Bias logistic regr.
Bias
True correlation
True partial cor.
True effect size
E
Power cor
Power pcor
Power lm phen ~ met
Power lm met ~ phen
Power logistic regr.
Power

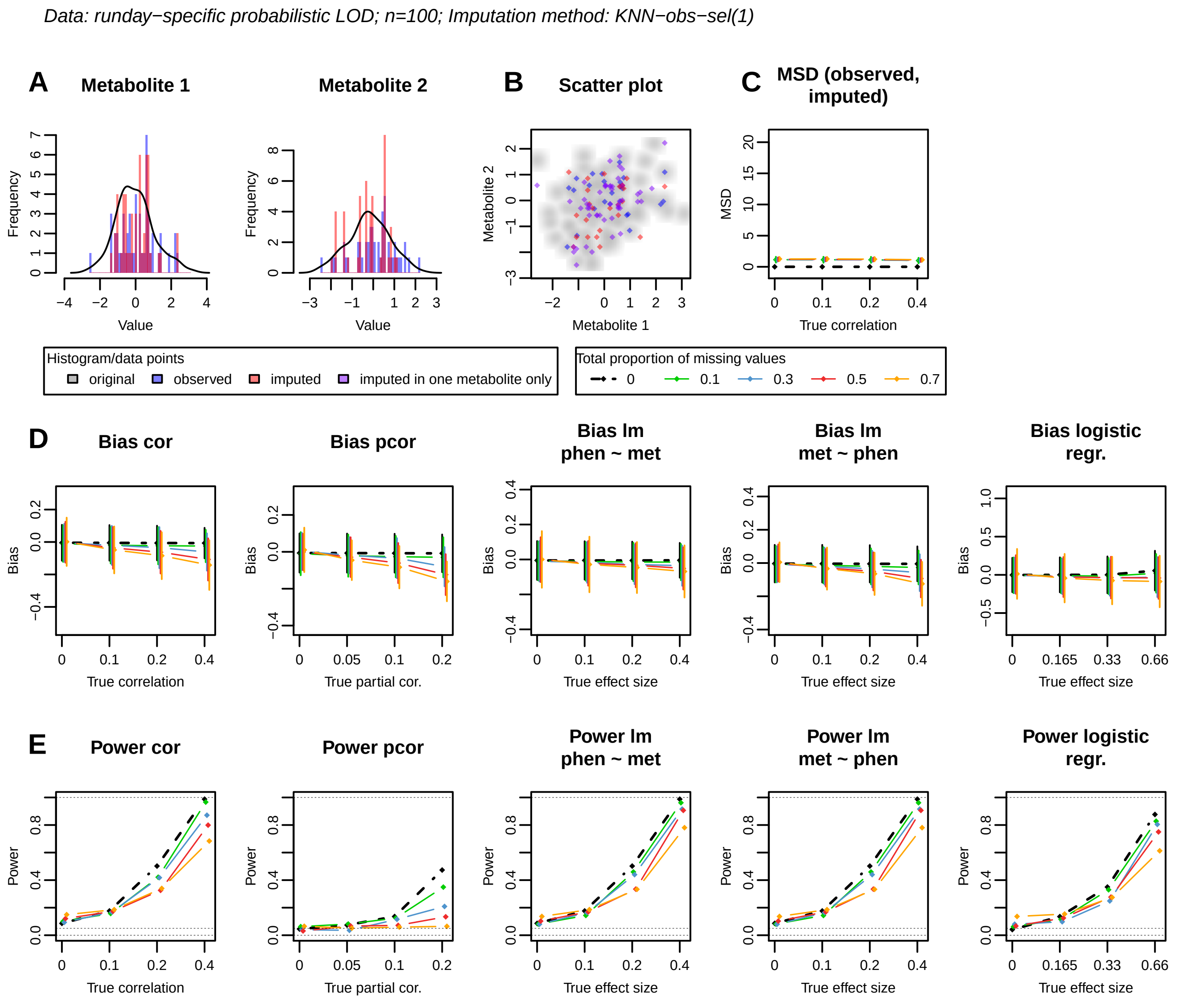

Data: runday−specific probabilistic LOD; n=100; Imputation method: KNN−obs−sel(1)
A
Metabolite 1
Metabolite 2
B
Scatter plot
C
MSD (observed, imputed)
Frequency
Value
Metabolite 2
Metabolite 1
MSD
True correlation
Histogram/data points
original
observed
imputed
imputed in one metabolite only
Total proportion of missing values
0
0.1
0.3
0.5
0.7
D
Bias cor
Bias pcor
Bias lm phen ~ met
Bias lm met ~ phen
Bias logistic regr.
Bias
True correlation
True partial cor.
True effect size
E
Power cor
Power pcor
Power lm phen ~ met
Power lm met ~ phen
Power logistic regr.
Power

Data: runday−specific probabilistic LOD; n=100; Imputation method: KNN−obs−sel(3)

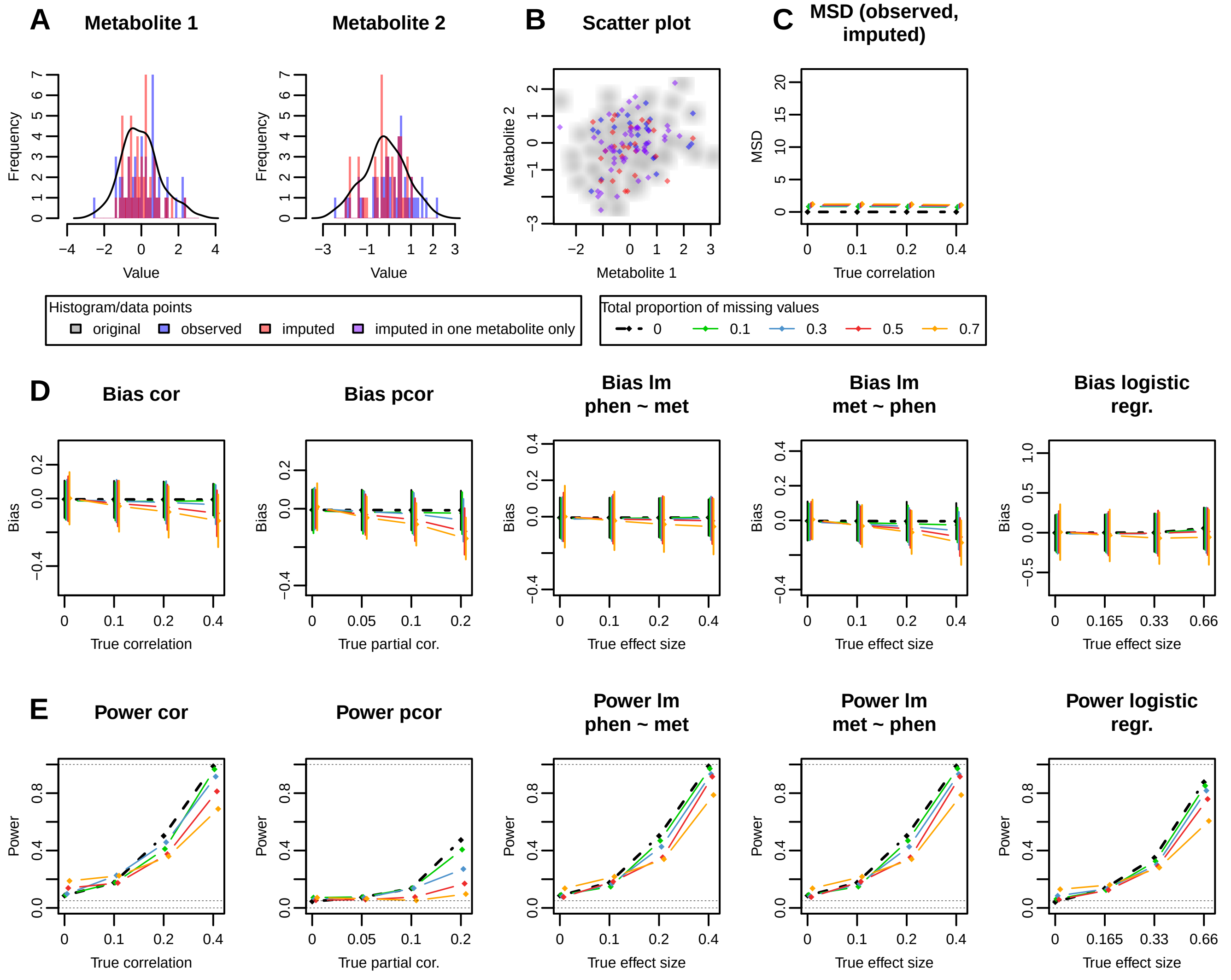

Data: runday−specific probabilistic LOD; n=100; Imputation method: KNN−obs−sel(5)

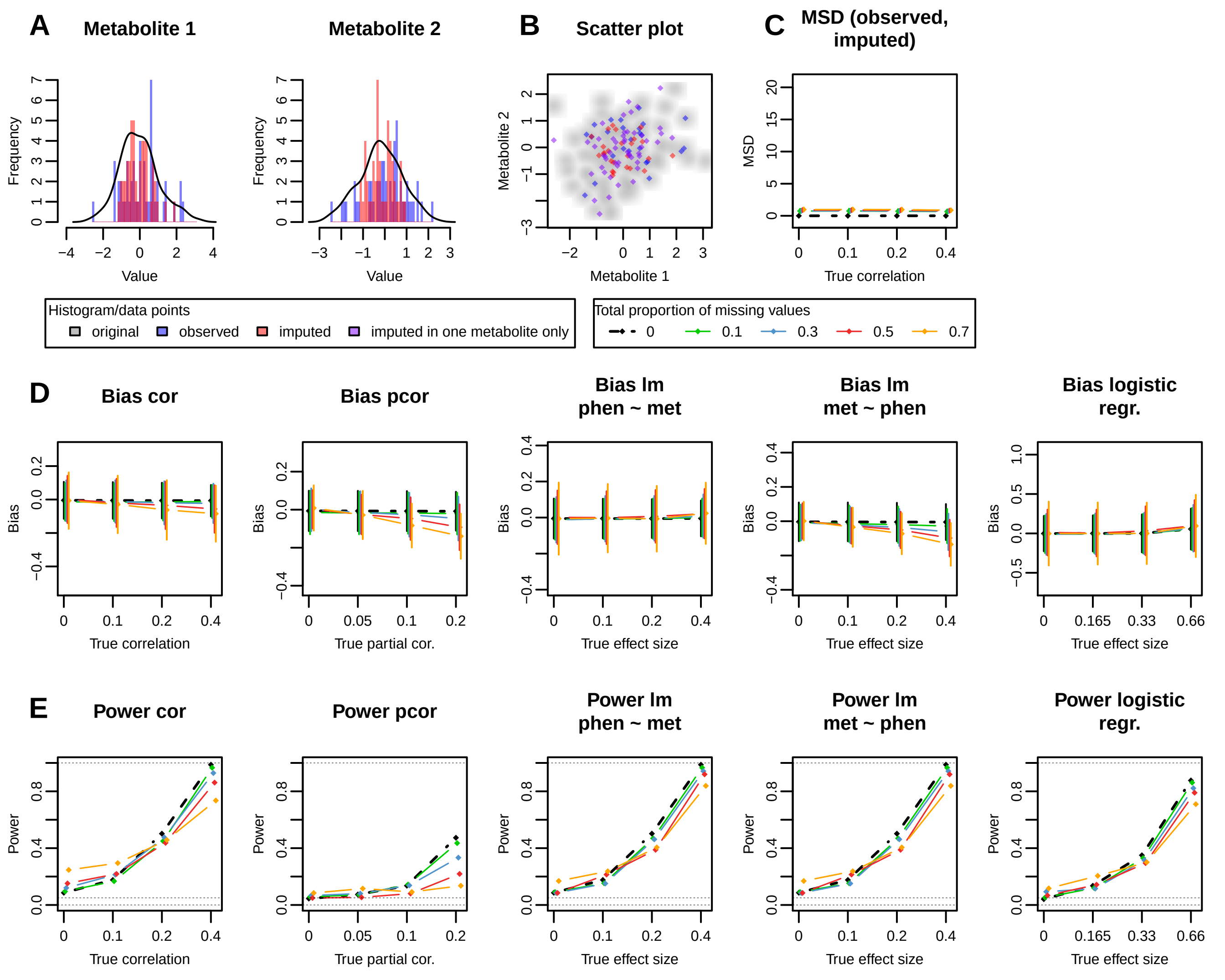

Data: runday−specific probabilistic LOD; n=100; Imputation method: KNN−obs−sel(10)

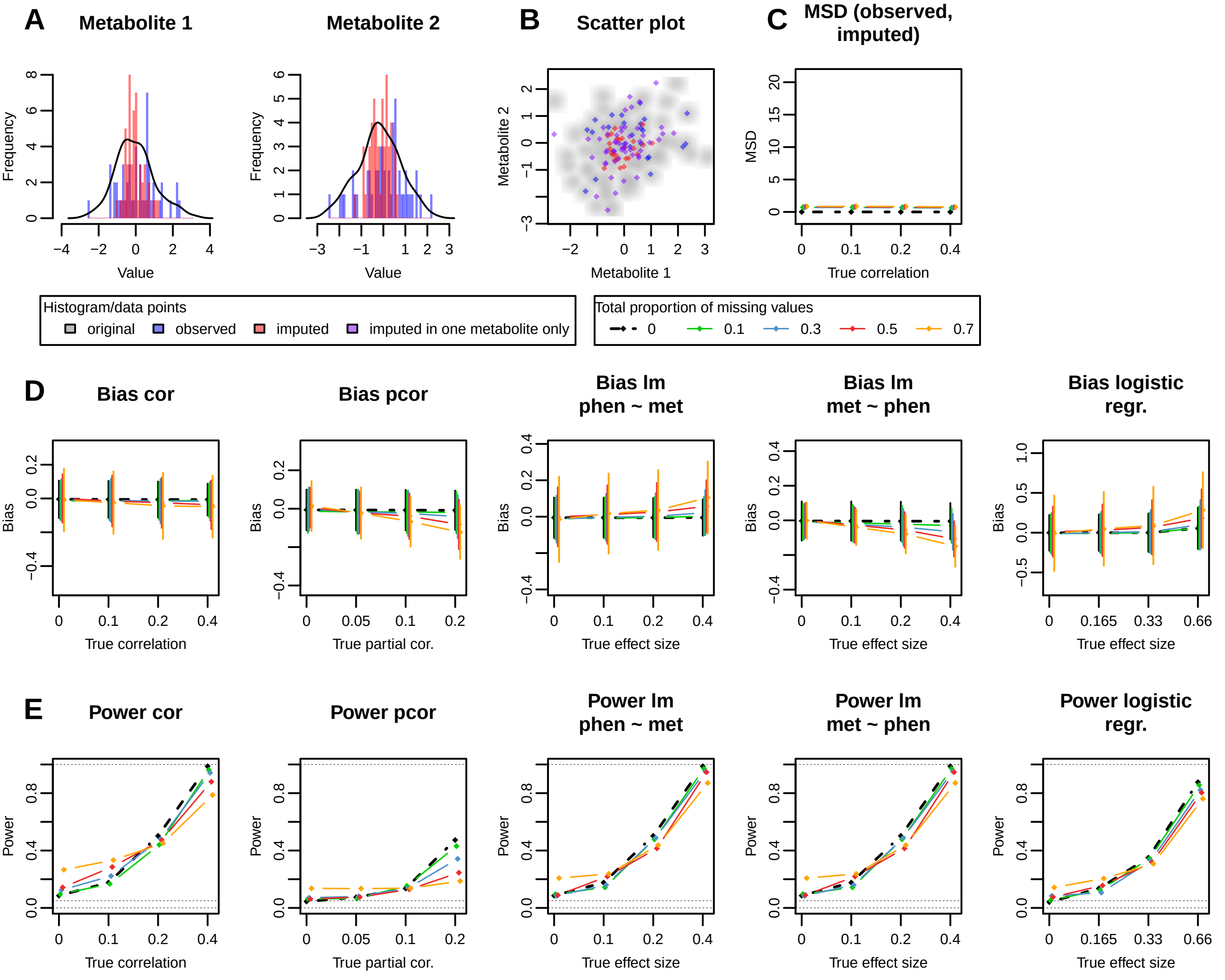

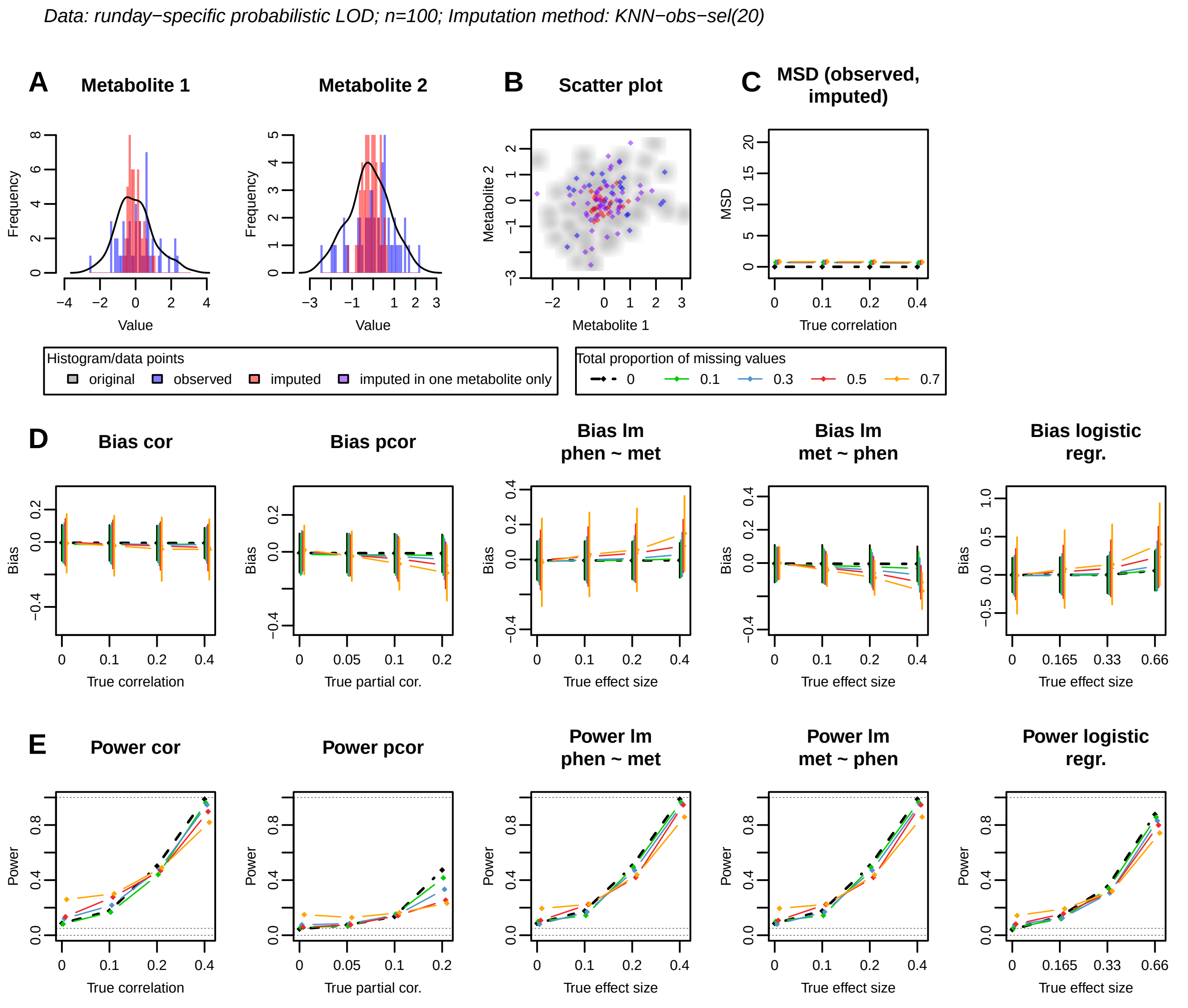

Data: runday−specific probabilistic LOD; n=100; Imputation method: KNN−obs−sel(20)
A
Metabolite 1
Metabolite 2
B
Scatter plot
C
MSD (observed, imputed)
Frequency
Value
Metabolite 1
Metabolite 2
MSD
True correlation
Histogram/data points
original
observed
imputed
imputed in one metabolite only
Total proportion of missing values
0
0.1
0.3
0.5
0.7
D
Bias cor
Bias pcor
Bias lm phen ~ met
Bias lm met ~ phen
Bias logistic regr.
Bias
True correlation
True partial cor.
True effect size
E
Power cor
Power pcor
Power lm phen ~ met
Power lm met ~ phen
Power logistic regr.
Power

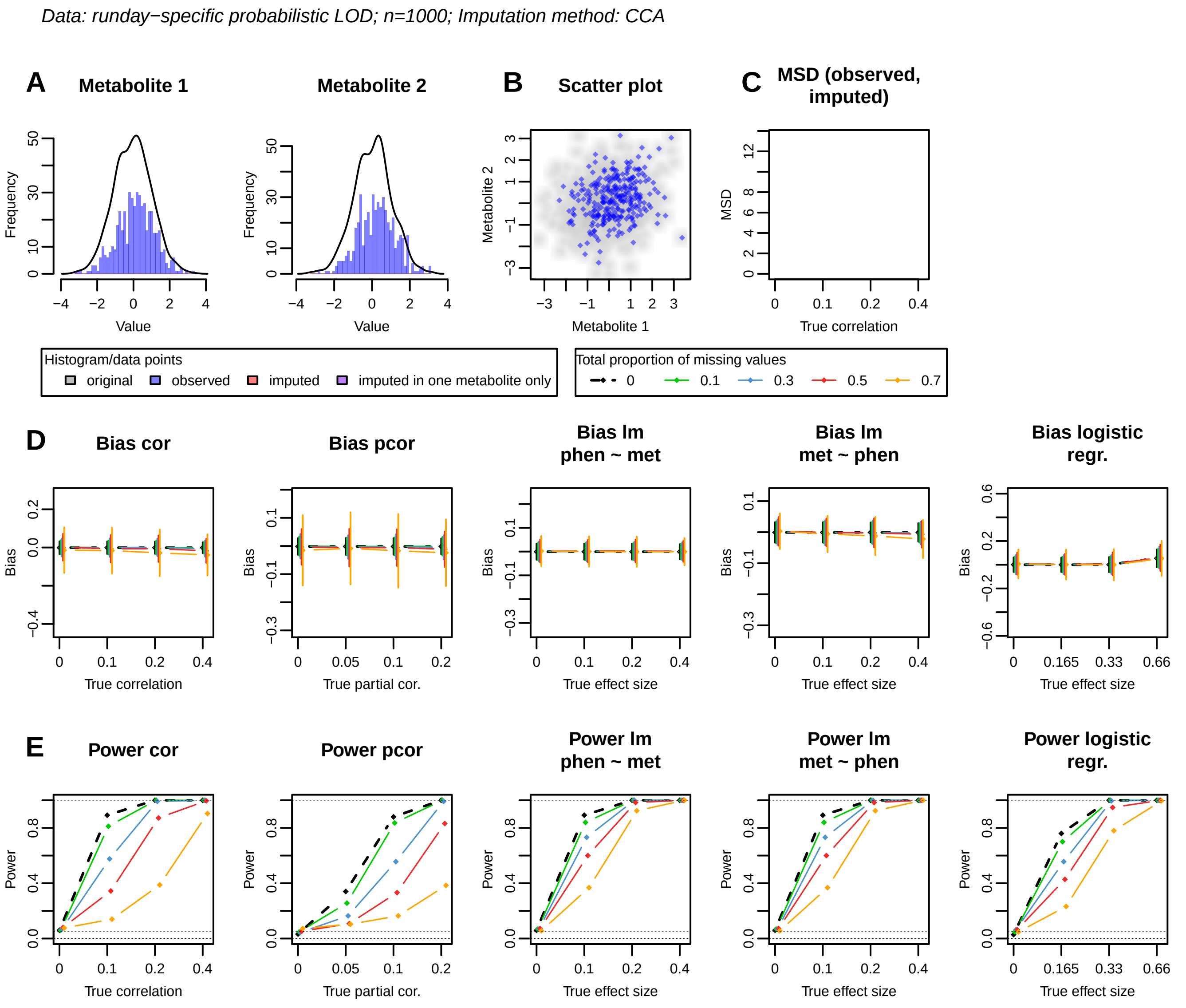
Data: runday−specific probabilistic LOD; n=1000; Imputation method: CCA
A
Metabolite 1
Metabolite 2
Frequency
Value
B
Scatter plot
Metabolite 1
Metabolite 2
C
MSD (observed, imputed)
MSD
True correlation
Histogram/data points
original
observed
imputed
imputed in one metabolite only
Total proportion of missing values
0
0.1
0.3
0.5
0.7
D
Bias cor
Bias pcor
Bias lm phen ~ met
Bias lm met ~ phen
Bias logistic regr.
Bias
True correlation
True partial cor.
True effect size
E
Power cor
Power pcor
Power lm phen ~ met
Power lm met ~ phen
Power logistic regr.
Power
True correlation
True partial cor.
True effect size

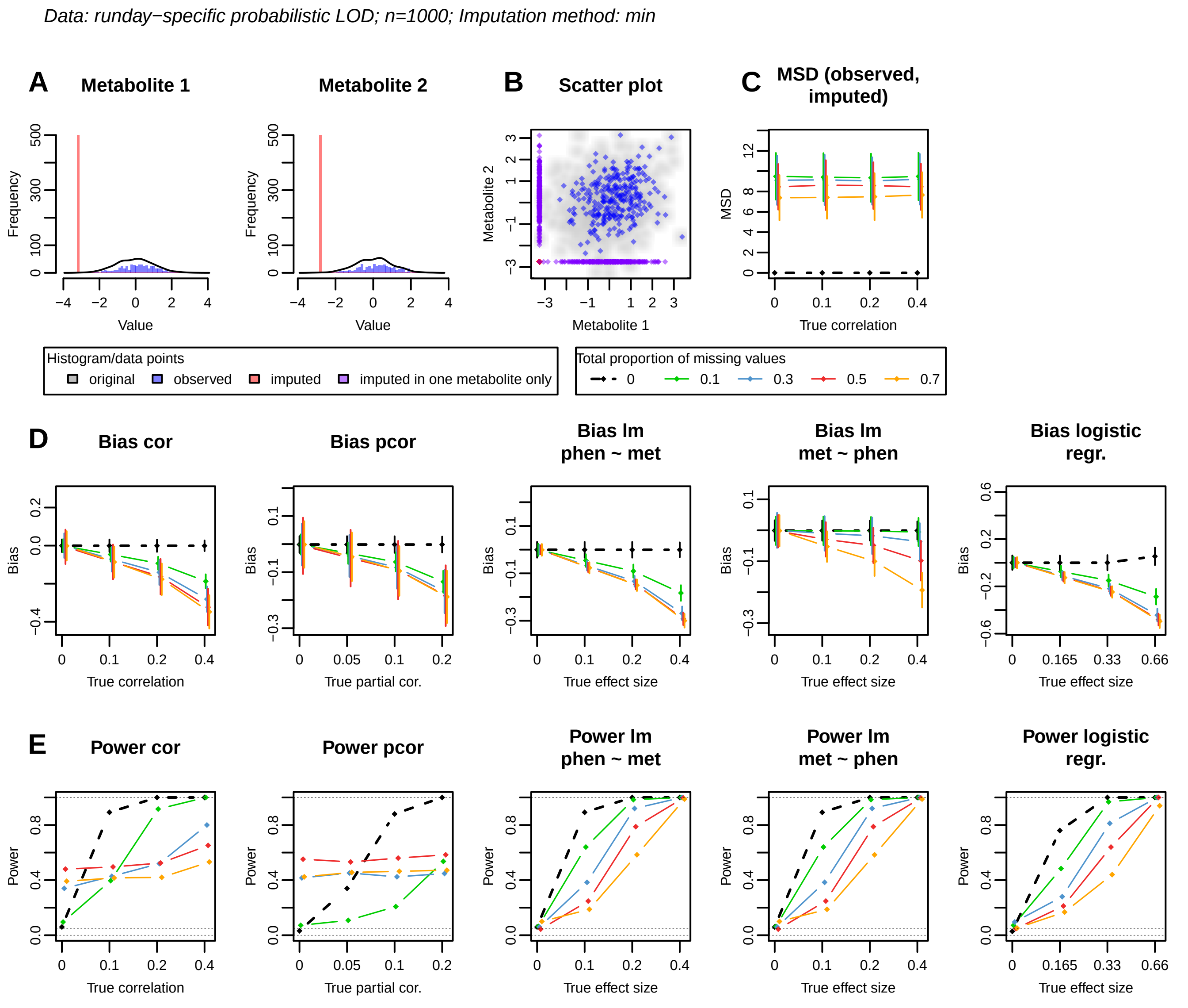
Data: runday−specific probabilistic LOD; n=1000; Imputation method: min
A
Metabolite 1
Metabolite 2
Frequency
Value
B
Scatter plot
Metabolite 1
Metabolite 2
C
MSD (observed, imputed)
MSD
True correlation
Histogram/data points
original
observed
imputed
imputed in one metabolite only
Total proportion of missing values
0
0.1
0.3
0.5
0.7
D
Bias cor
Bias pcor
Bias lm phen ~ met
Bias lm met ~ phen
Bias logistic regr.
Bias
True correlation
True partial cor.
True effect size
E
Power cor
Power pcor
Power lm phen ~ met
Power lm met ~ phen
Power logistic regr.
Power
True correlation
True partial cor.
True effect size

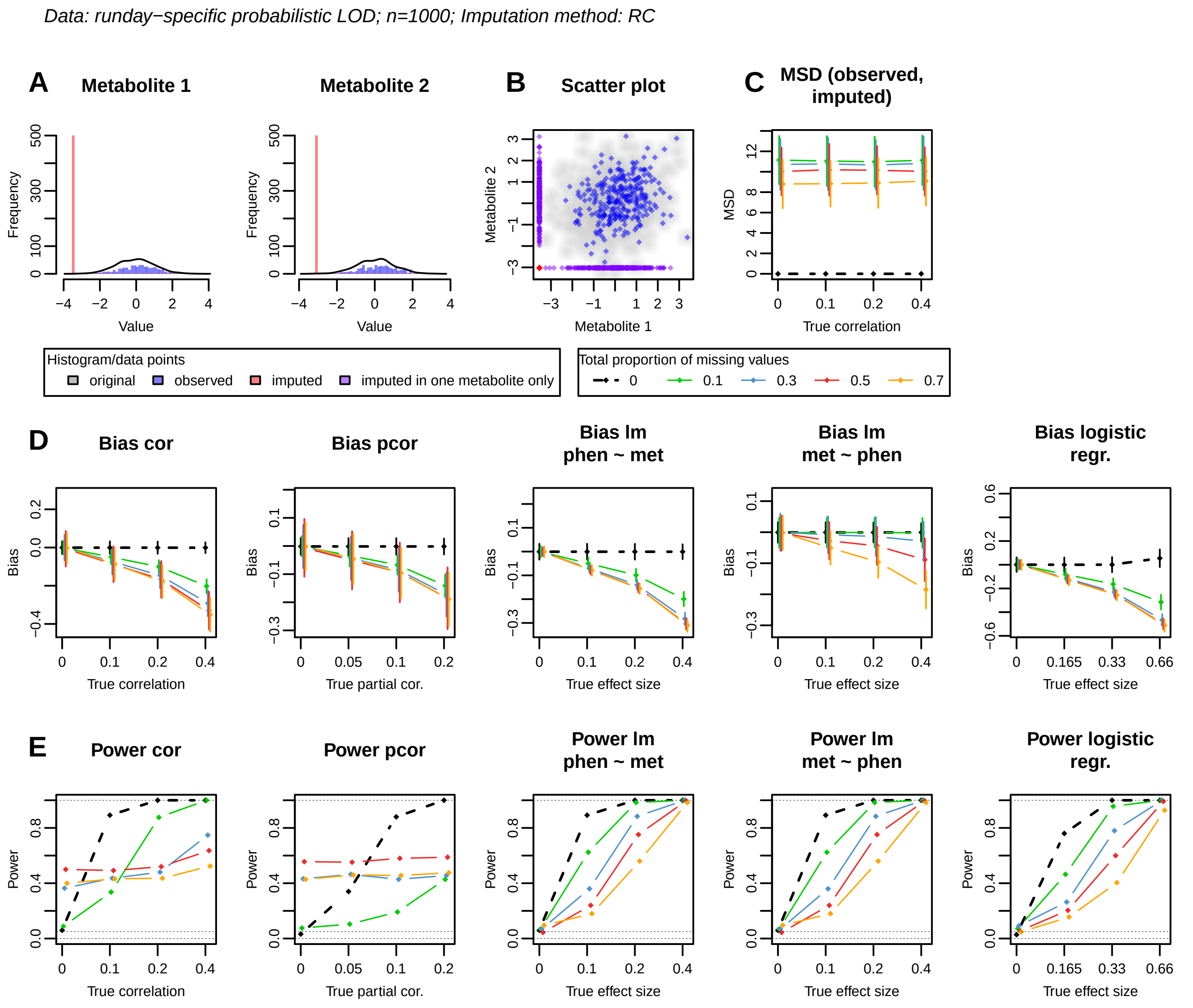
Data: runday−specific probabilistic LOD; n=1000; Imputation method: RC
A
Metabolite 1
Metabolite 2
B
Scatter plot
C
MSD (observed, imputed)
Frequency
Value
Metabolite 1
Metabolite 2
MSD
True correlation
Histogram/data points
original
observed
imputed
imputed in one metabolite only
Total proportion of missing values
0
0.1
0.3
0.5
0.7
D
Bias cor
Bias pcor
Bias lm phen ~ met
Bias lm met ~ phen
Bias logistic regr.
Bias
True correlation
True partial cor.
True effect size
E
Power cor
Power pcor
Power lm phen ~ met
Power lm met ~ phen
Power logistic regr.
Power
True correlation
True partial cor.
True effect size

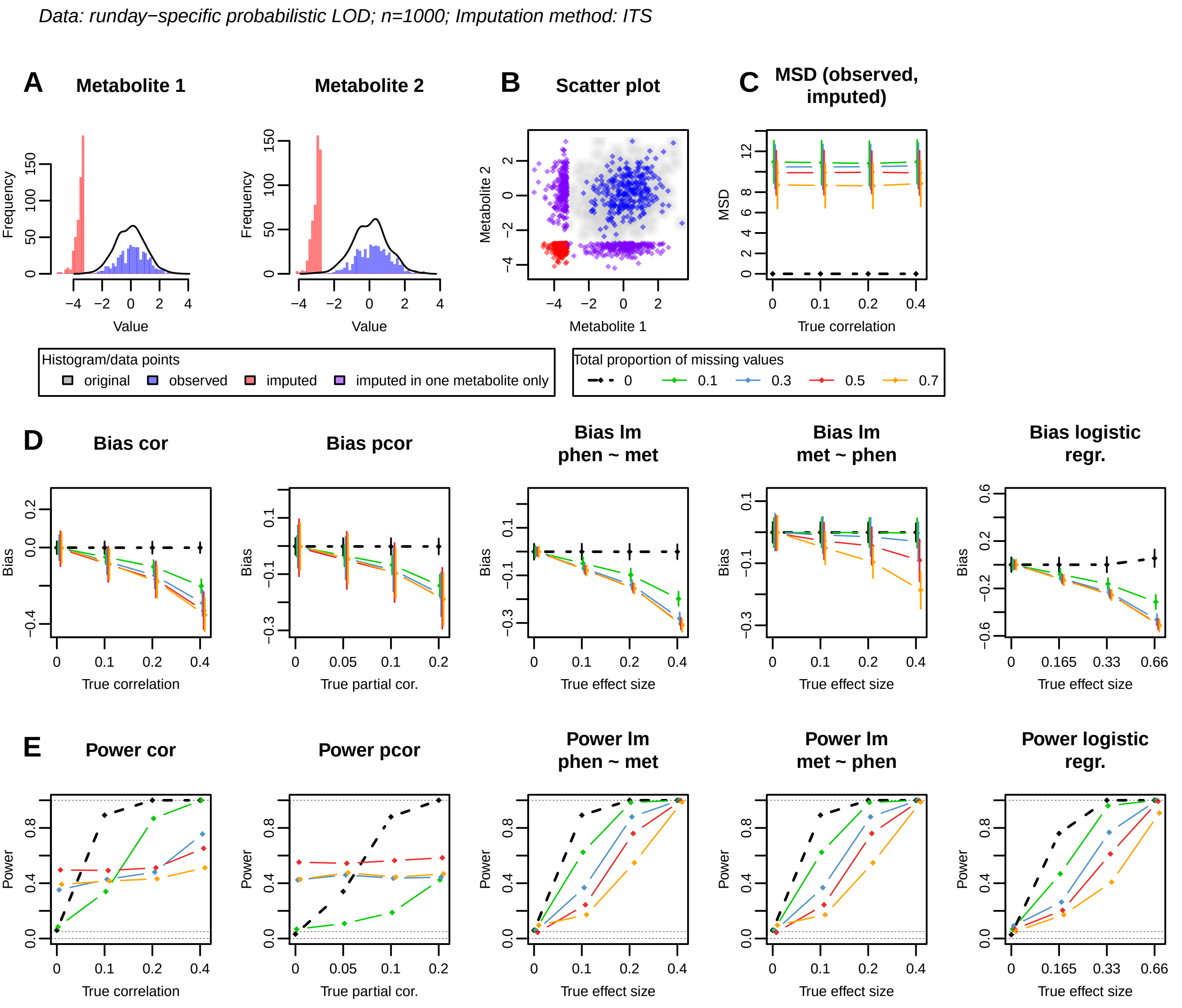

Data: runday−specific probabilistic LOD; n=1000; Imputation method: ITS
A
Metabolite 1
Metabolite 2
Frequency
Value
B
Scatter plot
Metabolite 2
Metabolite 1
C
MSD (observed, imputed)
MSD
True correlation
Histogram/data points
original
observed
imputed
imputed in one metabolite only
Total proportion of missing values
0
0.1
0.3
0.5
0.7
D
Bias cor
Bias pcor
Bias lm phen ~ met
Bias lm met ~ phen
Bias logistic regr.
Bias
True correlation
True partial cor.
True effect size
E
Power cor
Power pcor
Power lm phen ~ met
Power lm met ~ phen
Power logistic regr.
Power

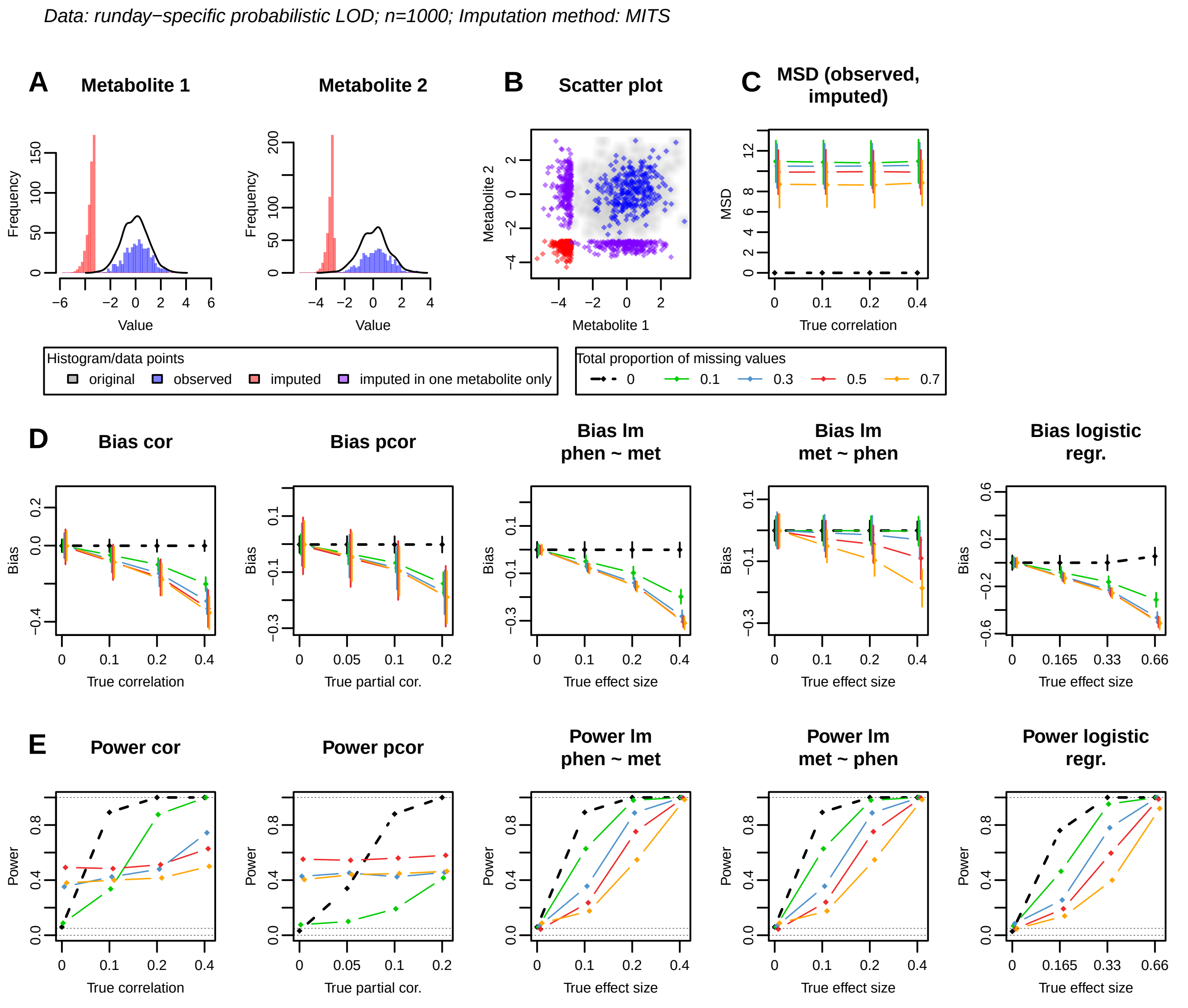

Data: runday−specific probabilistic LOD; n=1000; Imputation method: MITS
A
Metabolite 1
Metabolite 2
B
Scatter plot
C
MSD (observed, imputed)
Frequency
Value
Metabolite 1
Metabolite 2
MSD
True correlation
Histogram/data points
original
observed
imputed
imputed in one metabolite only
Total proportion of missing values
0
0.1
0.3
0.5
0.7
D
Bias cor
Bias pcor
Bias lm phen ~ met
Bias lm met ~ phen
Bias logistic regr.
Bias
True correlation
True partial cor.
True effect size
E
Power cor
Power pcor
Power lm phen ~ met
Power lm met ~ phen
Power logistic regr.
Power

Data: runday−specific probabilistic LOD; n=1000; Imputation method: RC−R

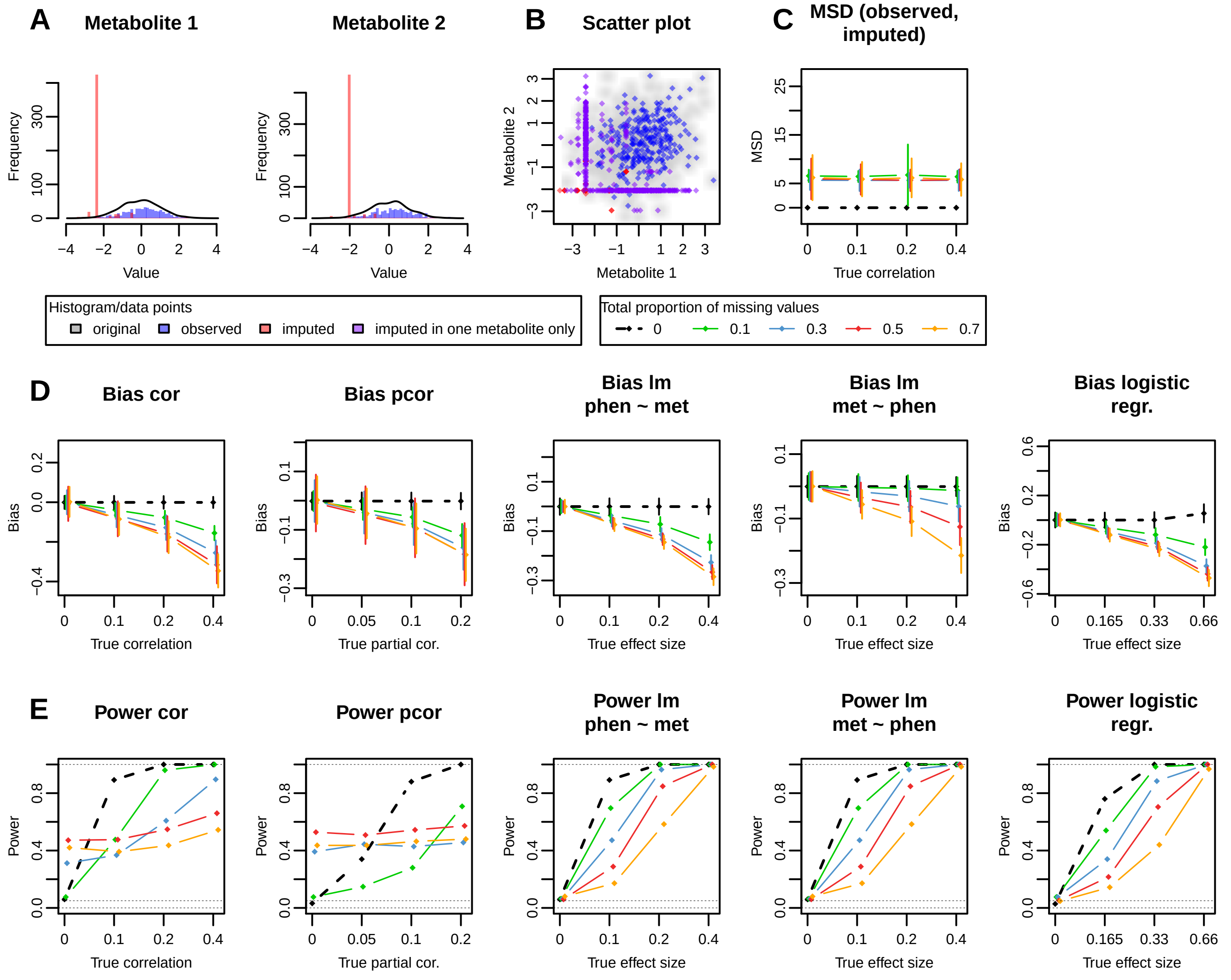

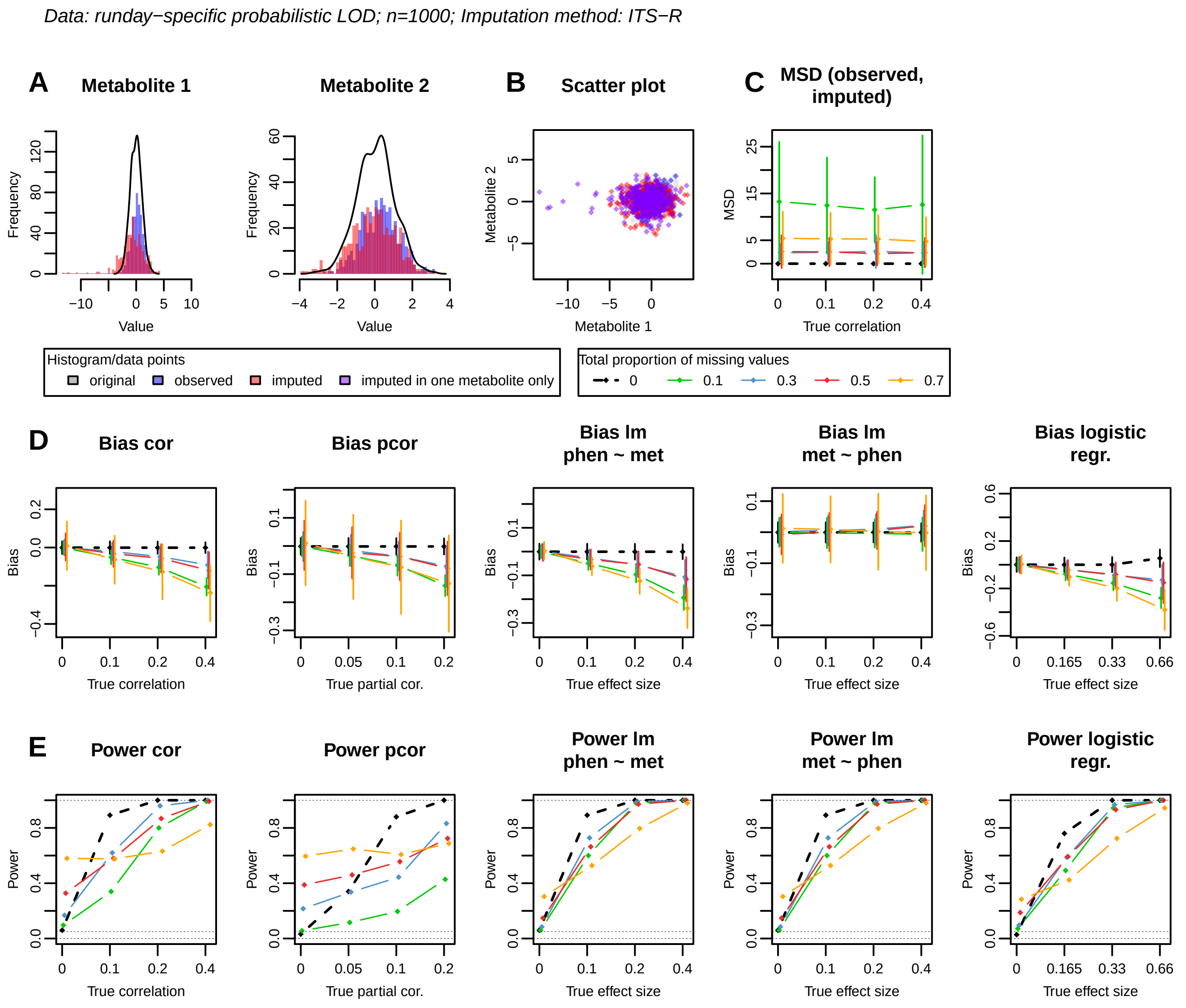
Data: runday−specific probabilistic LOD; n=1000; Imputation method: ITS−R
A
Metabolite 1
Metabolite 2
B
Scatter plot
C
MSD (observed, imputed)
Frequency
Value
Metabolite 2
Metabolite 1
MSD
True correlation
Histogram/data points
original
observed
imputed
imputed in one metabolite only
Total proportion of missing values
0
0.1
0.3
0.5
0.7
D
Bias cor
Bias pcor
Bias lm phen ~ met
Bias lm met ~ phen
Bias logistic regr.
Bias
True correlation
True partial cor.
True effect size
E
Power cor
Power pcor
Power lm phen ~ met
Power lm met ~ phen
Power logistic regr.
Power
True correlation
True partial cor.
True effect size

Data: runday−specific probabilistic LOD; n=1000; Imputation method: MITS−R

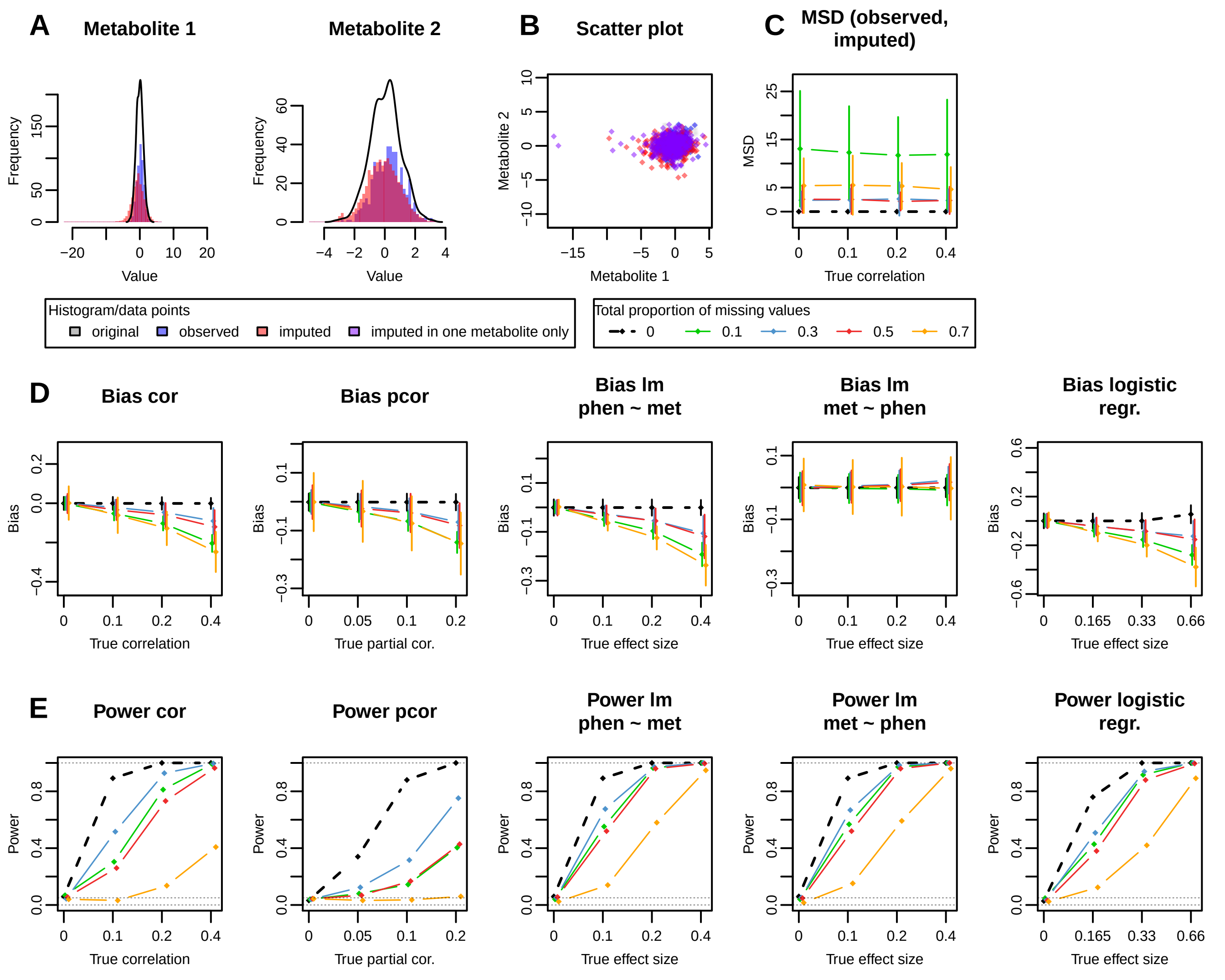

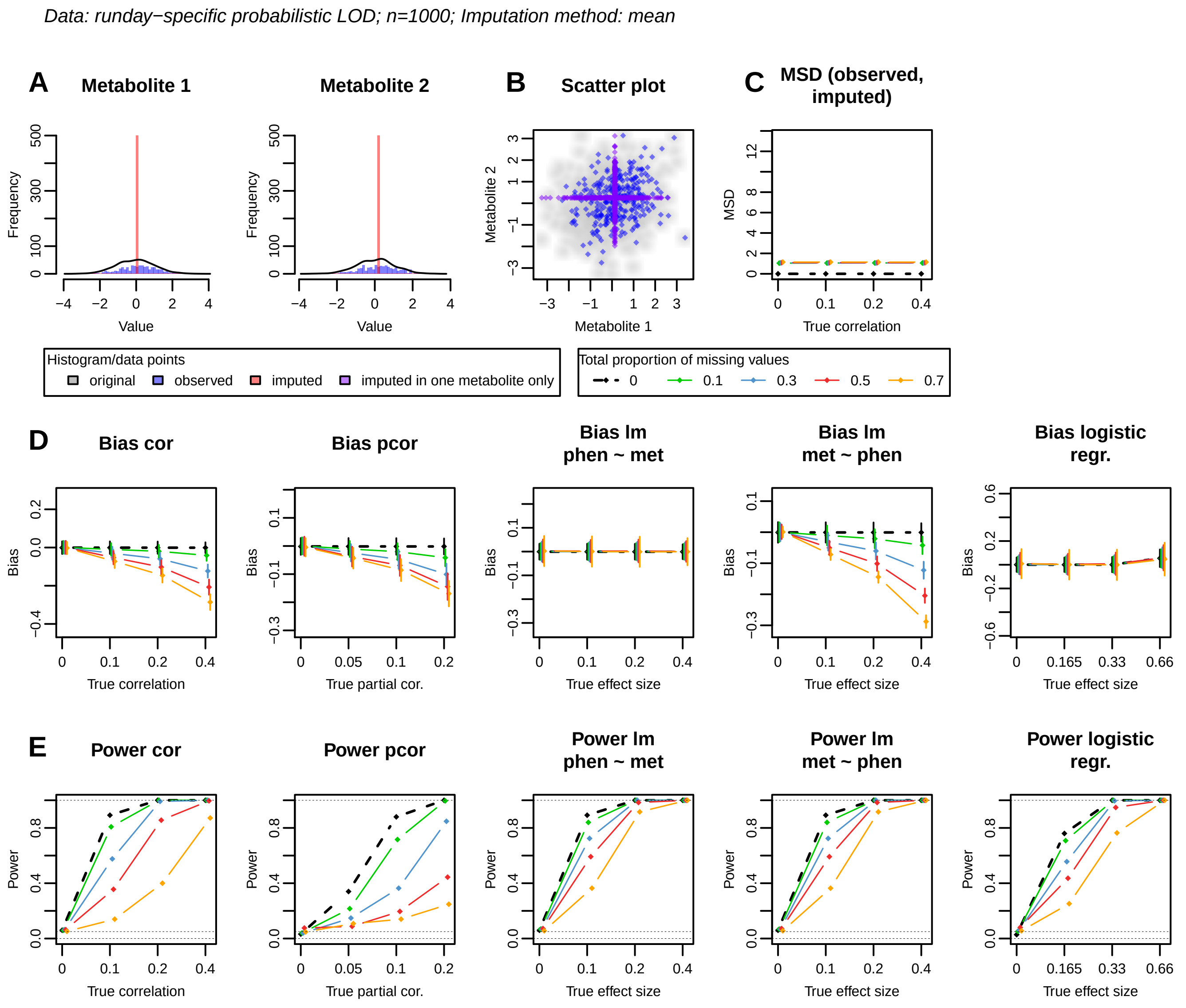
Data: runday−specific probabilistic LOD; n=1000; Imputation method: mean
A
Metabolite 1
Metabolite 2
Frequency
Value
B
Scatter plot
Metabolite 1
Metabolite 2
C
MSD (observed, imputed)
MSD
True correlation
Histogram/data points
original
observed
imputed
imputed in one metabolite only
Total proportion of missing values
0
0.1
0.3
0.5
0.7
D
Bias cor
Bias pcor
Bias lm phen ~ met
Bias lm met ~ phen
Bias logistic regr.
Bias
True correlation
True partial cor.
True effect size
E
Power cor
Power pcor
Power lm phen ~ met
Power lm met ~ phen
Power logistic regr.
Power
True correlation
True partial cor.
True effect size

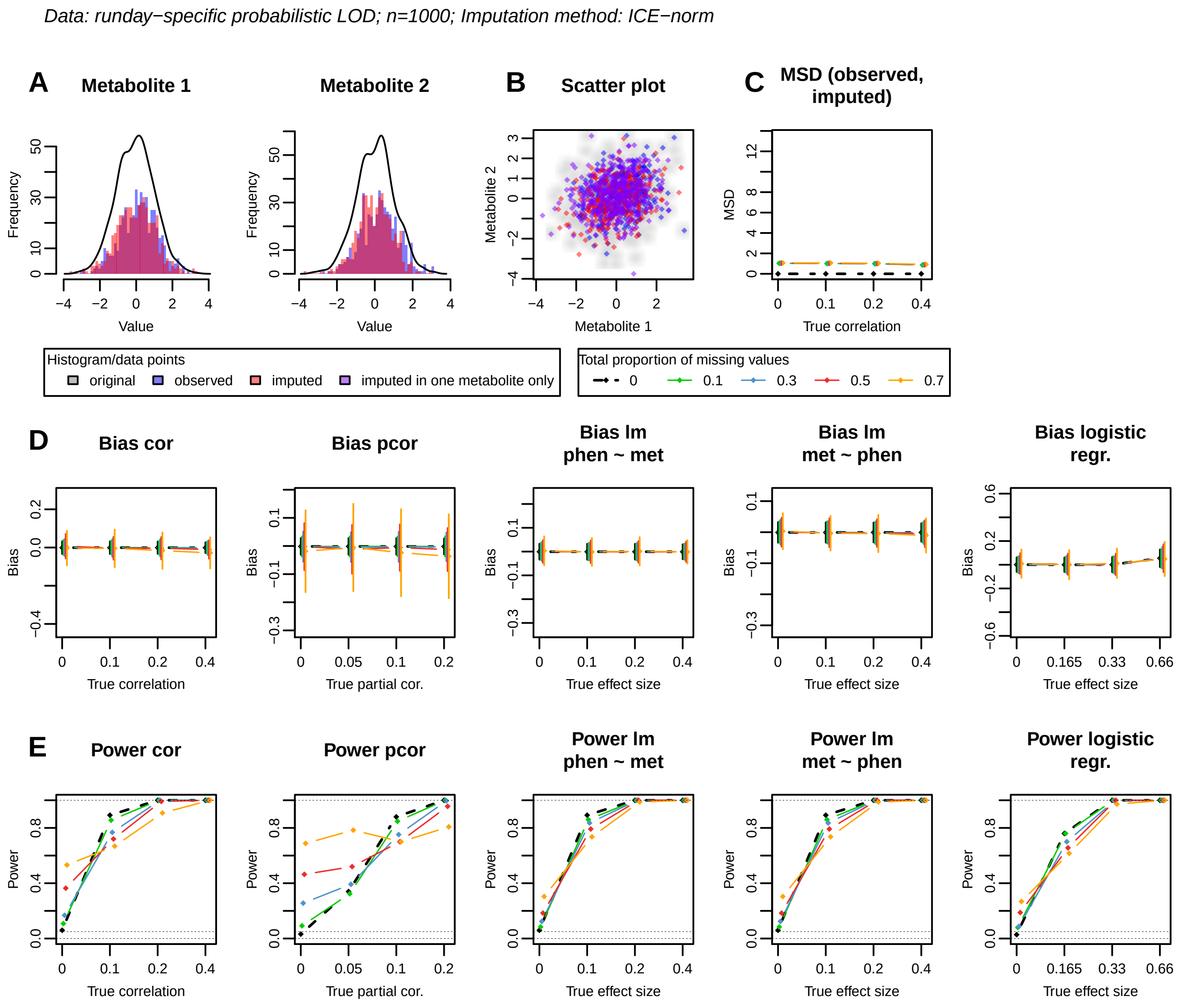
Data: runday−specific probabilistic LOD; n=1000; Imputation method: ICE−norm
A
Metabolite 1
Metabolite 2
B
Scatter plot
C
MSD (observed, imputed)
Frequency
Value
Metabolite 2
Metabolite 1
MSD
True correlation
Histogram/data points
original
observed
imputed
imputed in one metabolite only
Total proportion of missing values
0
0.1
0.3
0.5
0.7
D
Bias cor
Bias pcor
Bias lm phen ~ met
Bias lm met ~ phen
Bias logistic regr.
Bias
True correlation
True partial cor.
True effect size
E
Power cor
Power pcor
Power lm phen ~ met
Power lm met ~ phen
Power logistic regr.
Power

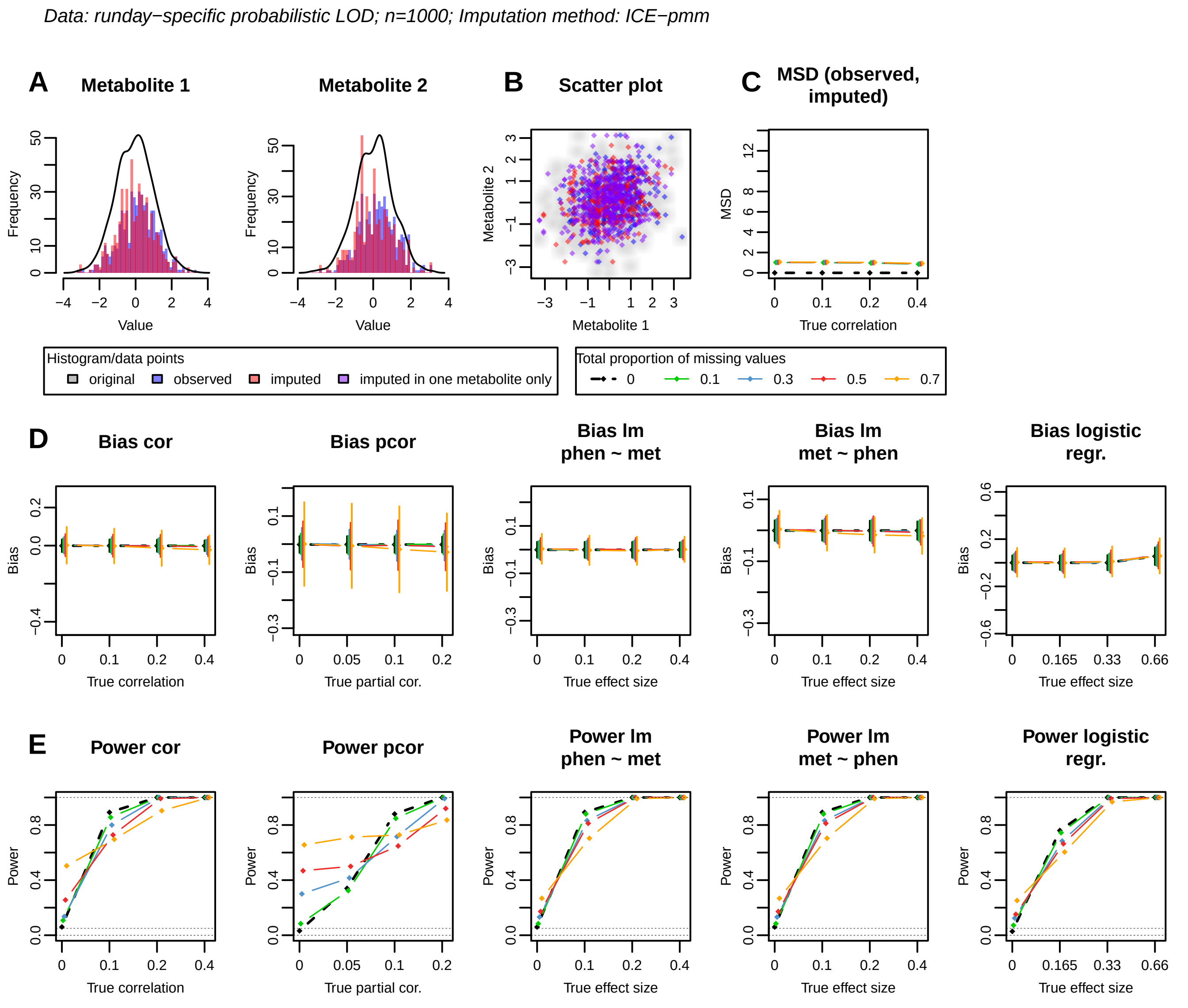
Data: runday−specific probabilistic LOD; n=1000; Imputation method: ICE−pmm
A
Metabolite 1
Metabolite 2
Frequency
Value
B
Scatter plot
Metabolite 1
Metabolite 2
C
MSD (observed, imputed)
MSD
True correlation
Histogram/data points
original
observed
imputed
imputed in one metabolite only
Total proportion of missing values
0
0.1
0.3
0.5
0.7
D
Bias cor
Bias pcor
Bias lm phen ~ met
Bias lm met ~ phen
Bias logistic regr.
Bias
True correlation
True partial cor.
True effect size
E
Power cor
Power pcor
Power lm phen ~ met
Power lm met ~ phen
Power logistic regr.
Power
True correlation
True partial cor.
True effect size

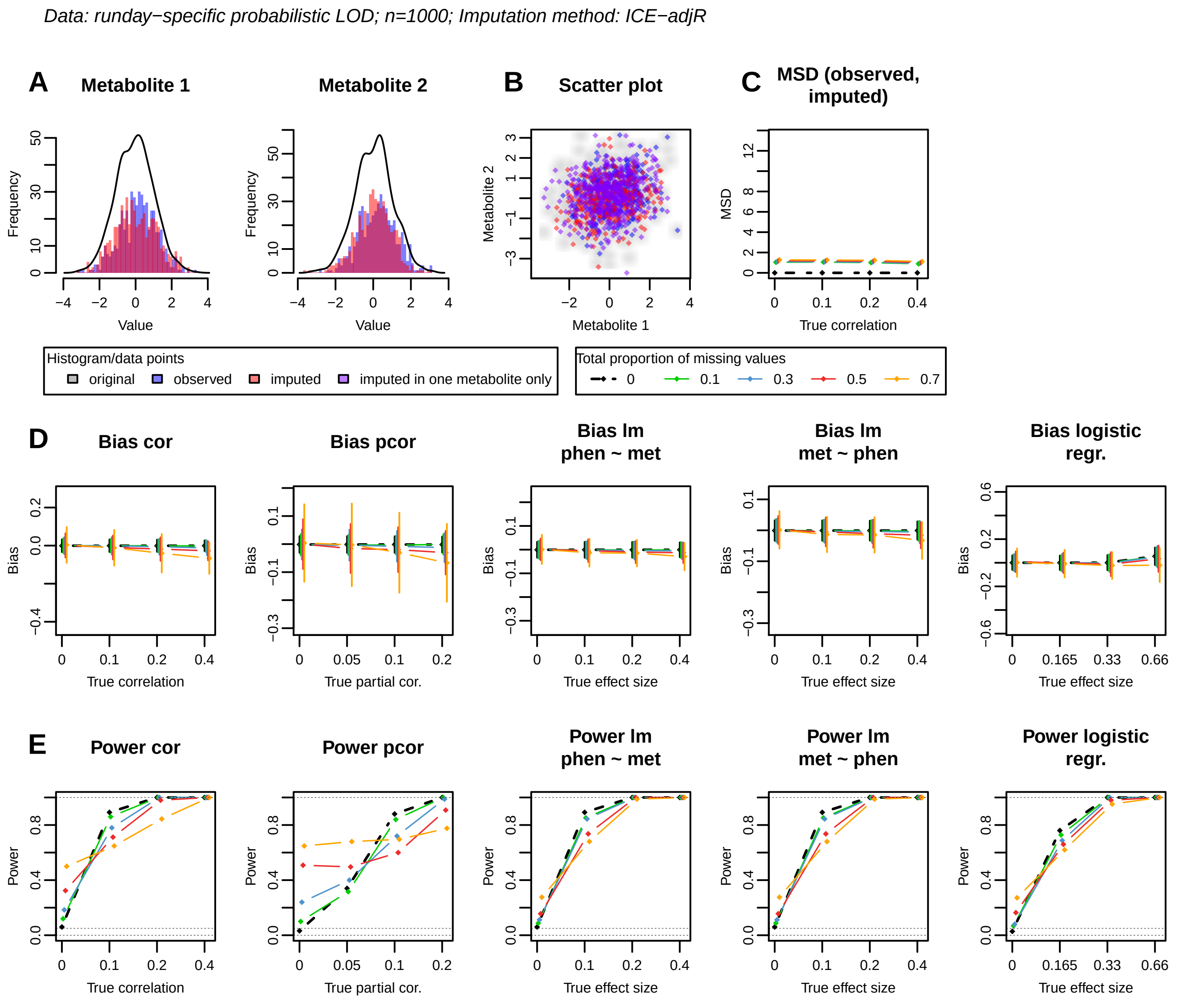

Data: runday−specific probabilistic LOD; n=1000; Imputation method: ICE−adjR
A
Metabolite 1
Metabolite 2
B
Scatter plot
C
MSD (observed, imputed)
Frequency
Value
Metabolite 2
Metabolite 1
MSD
True correlation
Histogram/data points
original
observed
imputed
imputed in one metabolite only
Total proportion of missing values
0
0.1
0.3
0.5
0.7
D
Bias cor
Bias pcor
Bias lm phen ~ met
Bias lm met ~ phen
Bias logistic regr.
Bias
True correlation
True partial cor.
True effect size
E
Power cor
Power pcor
Power lm phen ~ met
Power lm met ~ phen
Power logistic regr.
Power

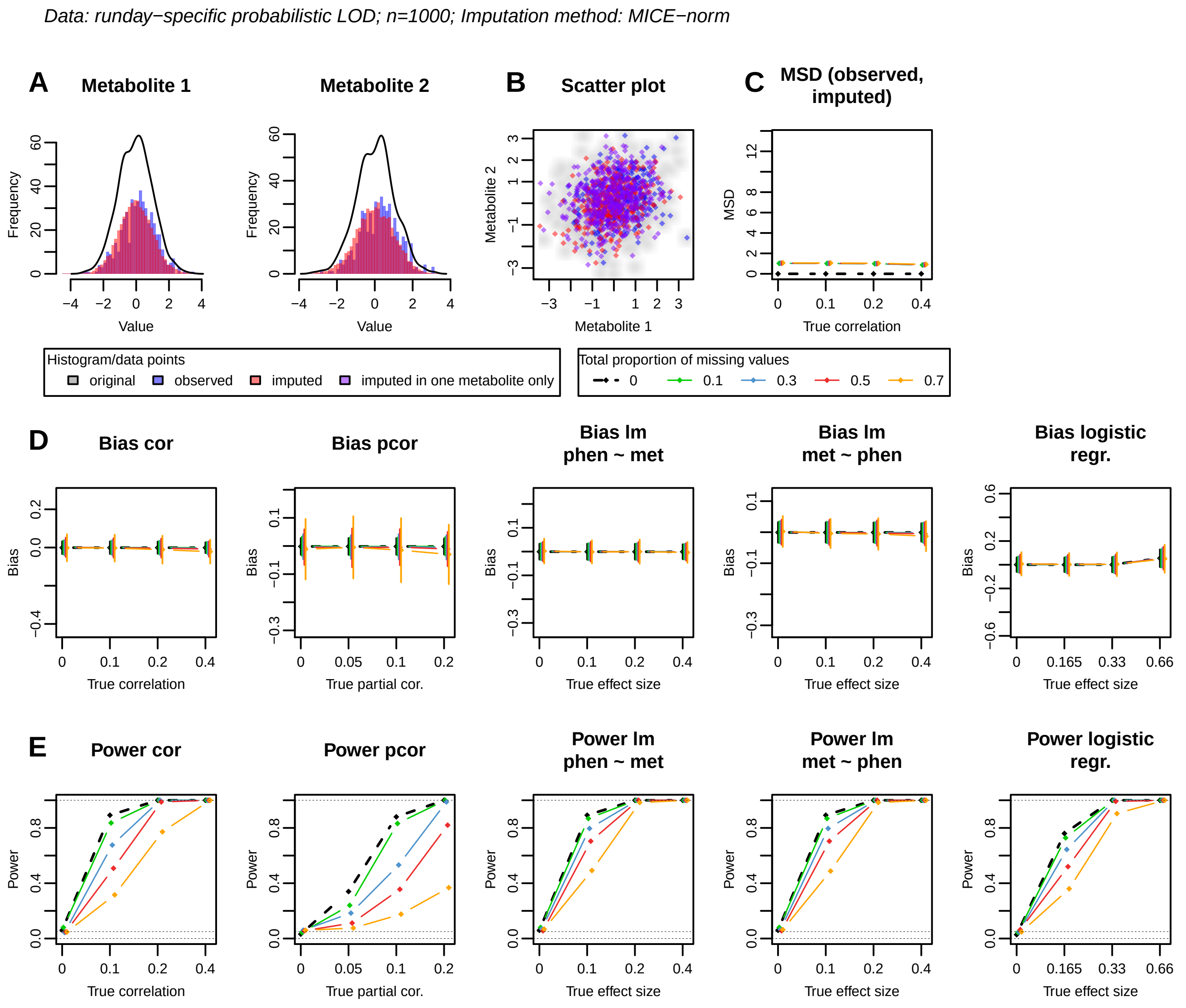
Data: runday−specific probabilistic LOD; n=1000; Imputation method: MICE−norm
A
Metabolite 1
Metabolite 2
B
Scatter plot
C
MSD (observed, imputed)
Frequency
Value
Metabolite 2
Metabolite 1
MSD
True correlation
Histogram/data points
original
observed
imputed
imputed in one metabolite only
Total proportion of missing values
0
0.1
0.3
0.5
0.7
D
Bias cor
Bias pcor
Bias lm phen ~ met
Bias lm met ~ phen
Bias logistic regr.
Bias
True correlation
True partial cor.
True effect size
E
Power cor
Power pcor
Power lm phen ~ met
Power lm met ~ phen
Power logistic regr.
Power

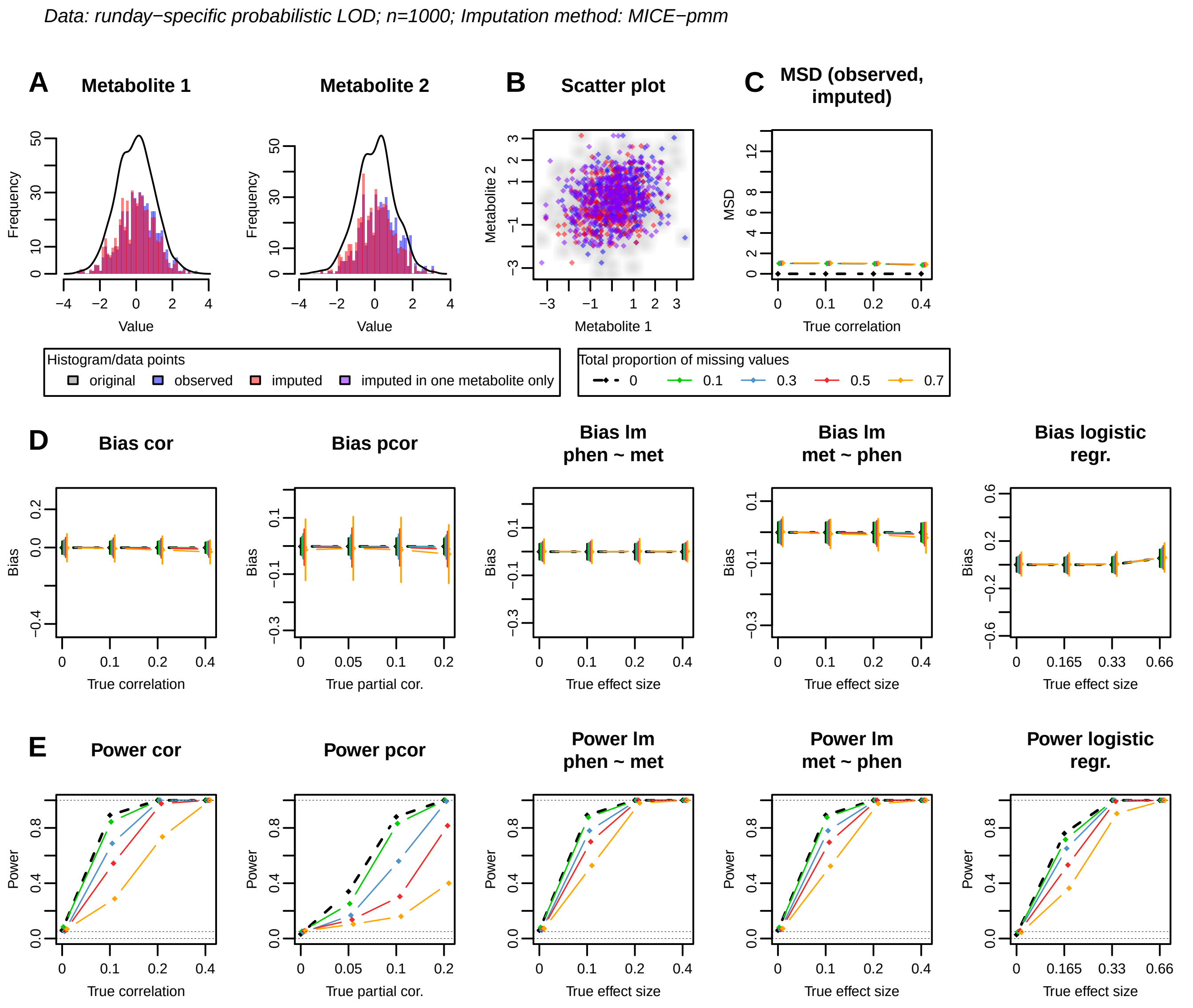
Data: runday−specific probabilistic LOD; n=1000; Imputation method: MICE−pmm
A
Metabolite 1
Metabolite 2
B
Scatter plot
C
MSD (observed, imputed)
Frequency
Value
Metabolite 2
Metabolite 1
MSD
True correlation
Histogram/data points
original
observed
imputed
imputed in one metabolite only
Total proportion of missing values
0
0.1
0.3
0.5
0.7
D
Bias cor
Bias pcor
Bias lm phen ~ met
Bias lm met ~ phen
Bias logistic regr.
Bias
True correlation
True partial cor.
True effect size
E
Power cor
Power pcor
Power lm phen ~ met
Power lm met ~ phen
Power logistic regr.
Power

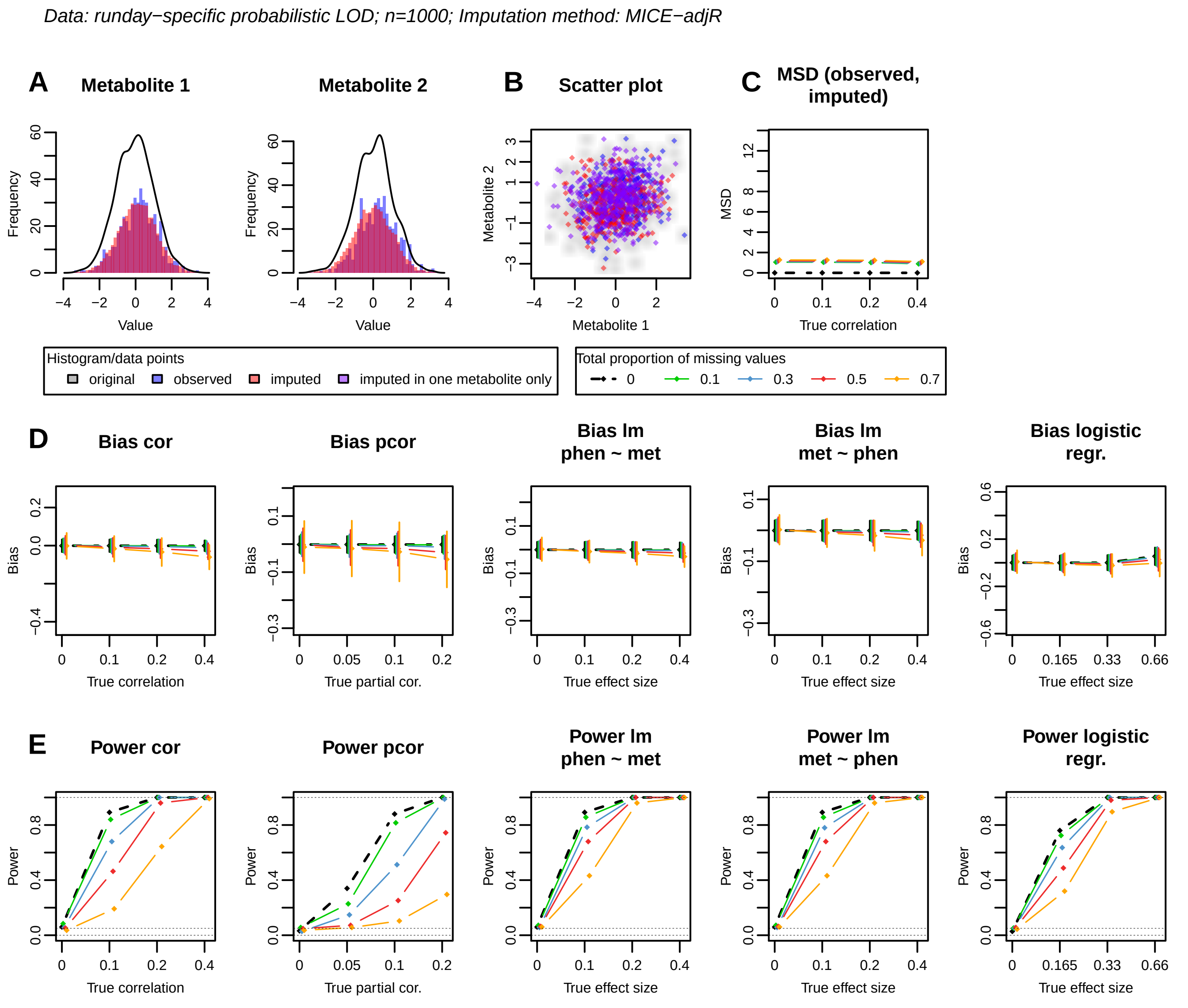
Data: runday−specific probabilistic LOD; n=1000; Imputation method: MICE−adjR
A
Metabolite 1
Metabolite 2
B
Scatter plot
C
MSD (observed, imputed)
Frequency
Value
Metabolite 2
Metabolite 1
MSD
True correlation
Histogram/data points
original
observed
imputed
imputed in one metabolite only
Total proportion of missing values
0
0.1
0.3
0.5
0.7
D
Bias cor
Bias pcor
Bias lm phen ~ met
Bias lm met ~ phen
Bias logistic regr.
Bias
True correlation
True partial cor.
True effect size
E
Power cor
Power pcor
Power lm phen ~ met
Power lm met ~ phen
Power logistic regr.
Power

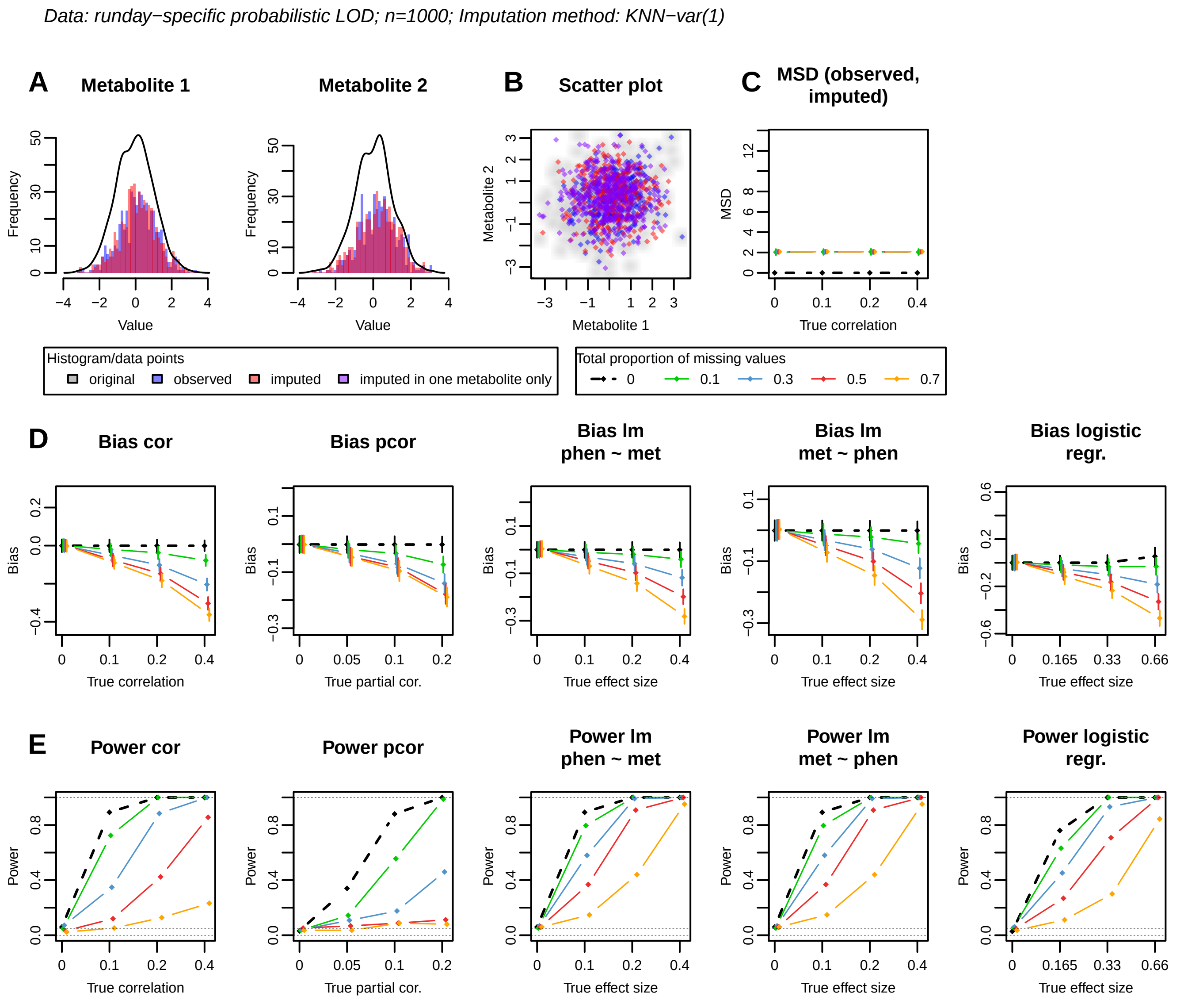
Data: runday−specific probabilistic LOD; n=1000; Imputation method: KNN−var(1)
A
Metabolite 1
Metabolite 2
Frequency
Value
B
Scatter plot
Metabolite 2
Metabolite 1
C
MSD (observed, imputed)
MSD
True correlation
Histogram/data points
original
observed
imputed
imputed in one metabolite only
Total proportion of missing values
0
0.1
0.3
0.5
0.7
D
Bias cor
Bias pcor
Bias lm phen ~ met
Bias lm met ~ phen
Bias logistic regr.
Bias
True correlation
True partial cor.
True effect size
E
Power cor
Power pcor
Power lm phen ~ met
Power lm met ~ phen
Power logistic regr.
Power
True correlation
True partial cor.
True effect size

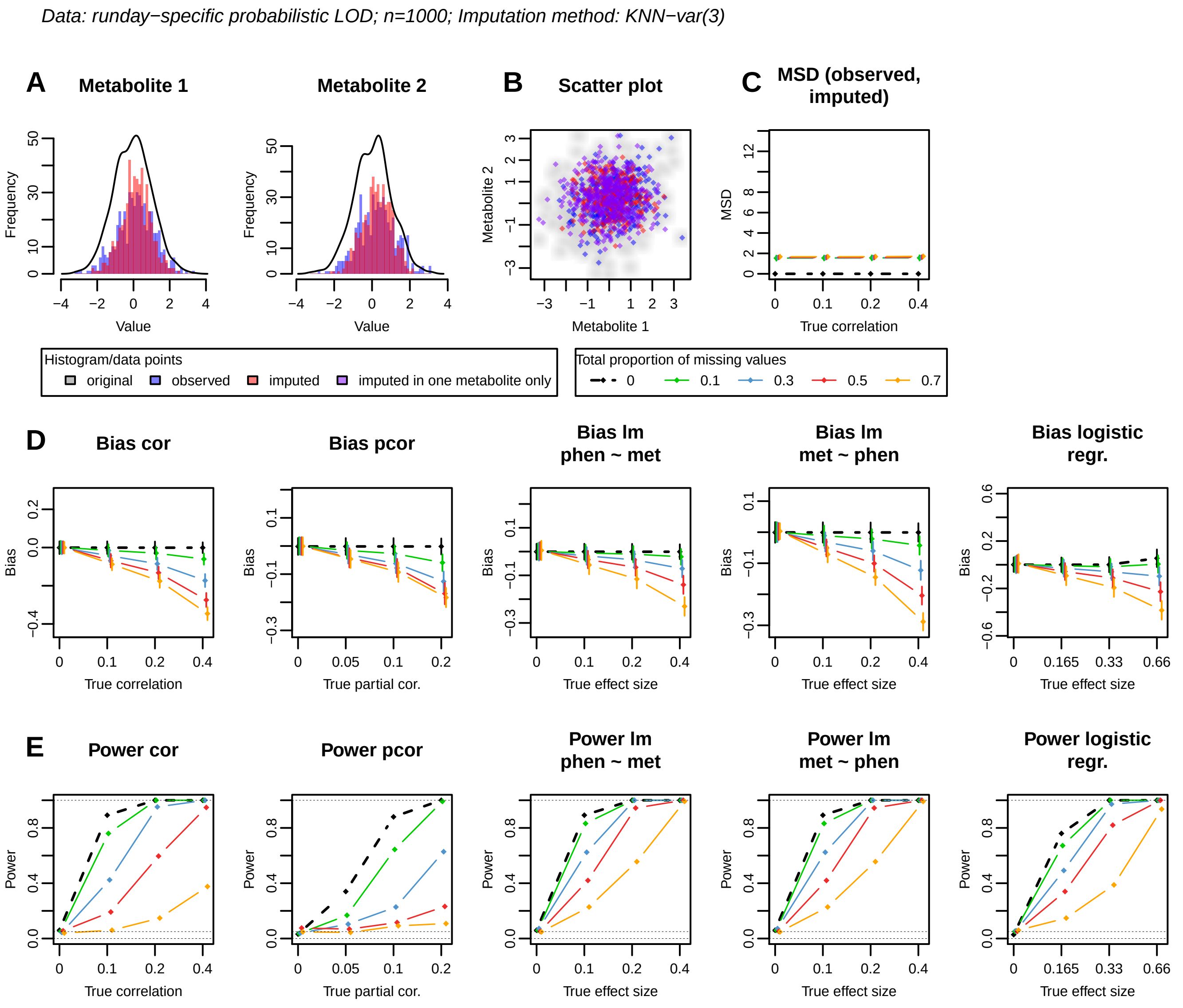

Data: runday−specific probabilistic LOD; n=1000; Imputation method: KNN−var(3)
A
Metabolite 1
Metabolite 2
B
Scatter plot
C
MSD (observed, imputed)
Frequency
Value
Metabolite 2
Metabolite 1
MSD
True correlation
Histogram/data points
original
observed
imputed
imputed in one metabolite only
Total proportion of missing values
0
0.1
0.3
0.5
0.7
D
Bias cor
Bias pcor
Bias lm phen ~ met
Bias lm met ~ phen
Bias logistic regr.
Bias
True correlation
True partial cor.
True effect size
E
Power cor
Power pcor
Power lm phen ~ met
Power lm met ~ phen
Power logistic regr.
Power

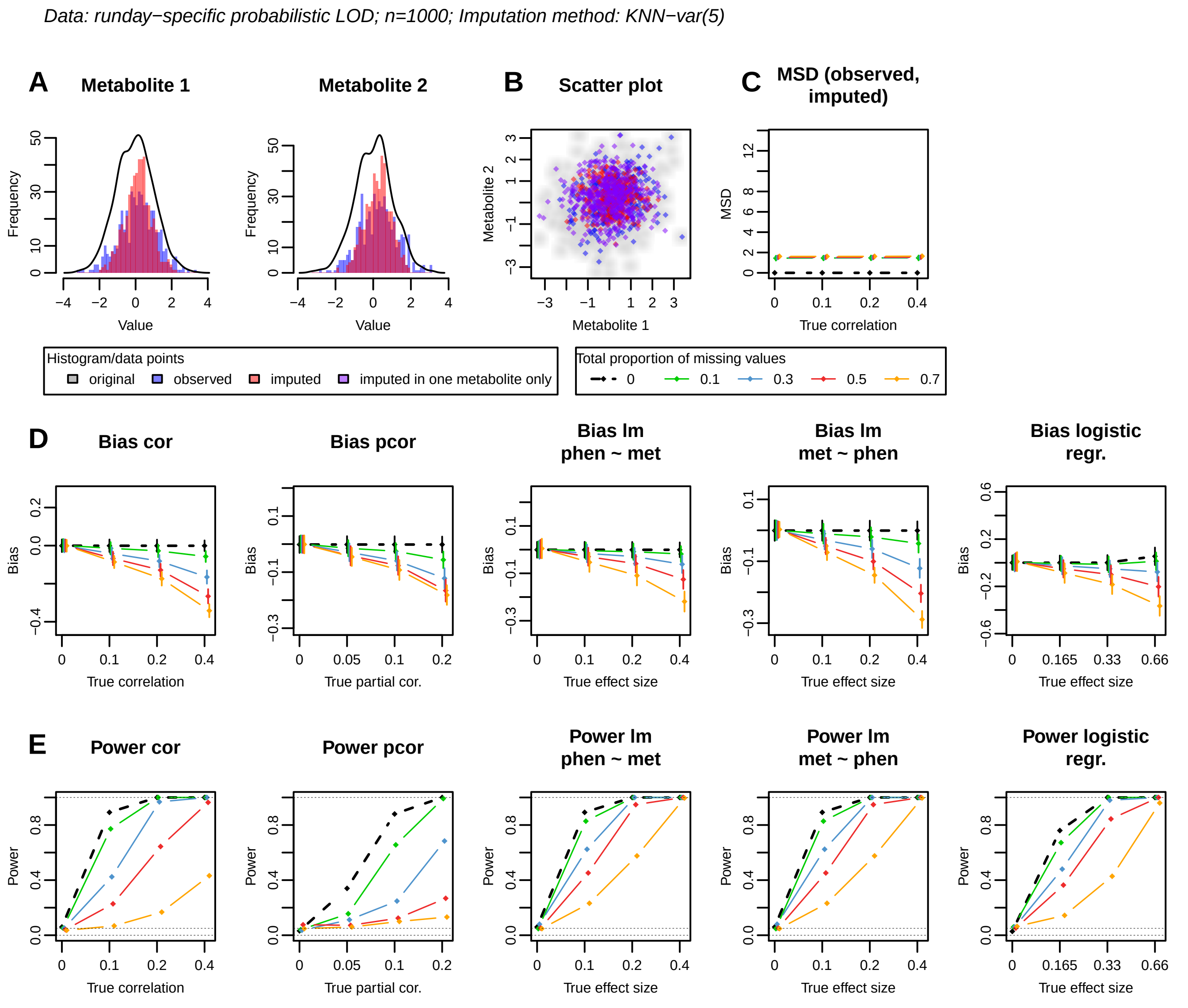

Data: runday−specific probabilistic LOD; n=1000; Imputation method: KNN−var(5)
A
Metabolite 1
Metabolite 2
B
Scatter plot
C
MSD (observed, imputed)
Frequency
Value
Metabolite 2
Metabolite 1
MSD
True correlation
Histogram/data points
original
observed
imputed
imputed in one metabolite only
Total proportion of missing values
0
0.1
0.3
0.5
0.7
D
Bias cor
Bias pcor
Bias lm phen ~ met
Bias lm met ~ phen
Bias logistic regr.
Bias
True correlation
True partial cor.
True effect size
E
Power cor
Power pcor
Power lm phen ~ met
Power lm met ~ phen
Power logistic regr.
Power

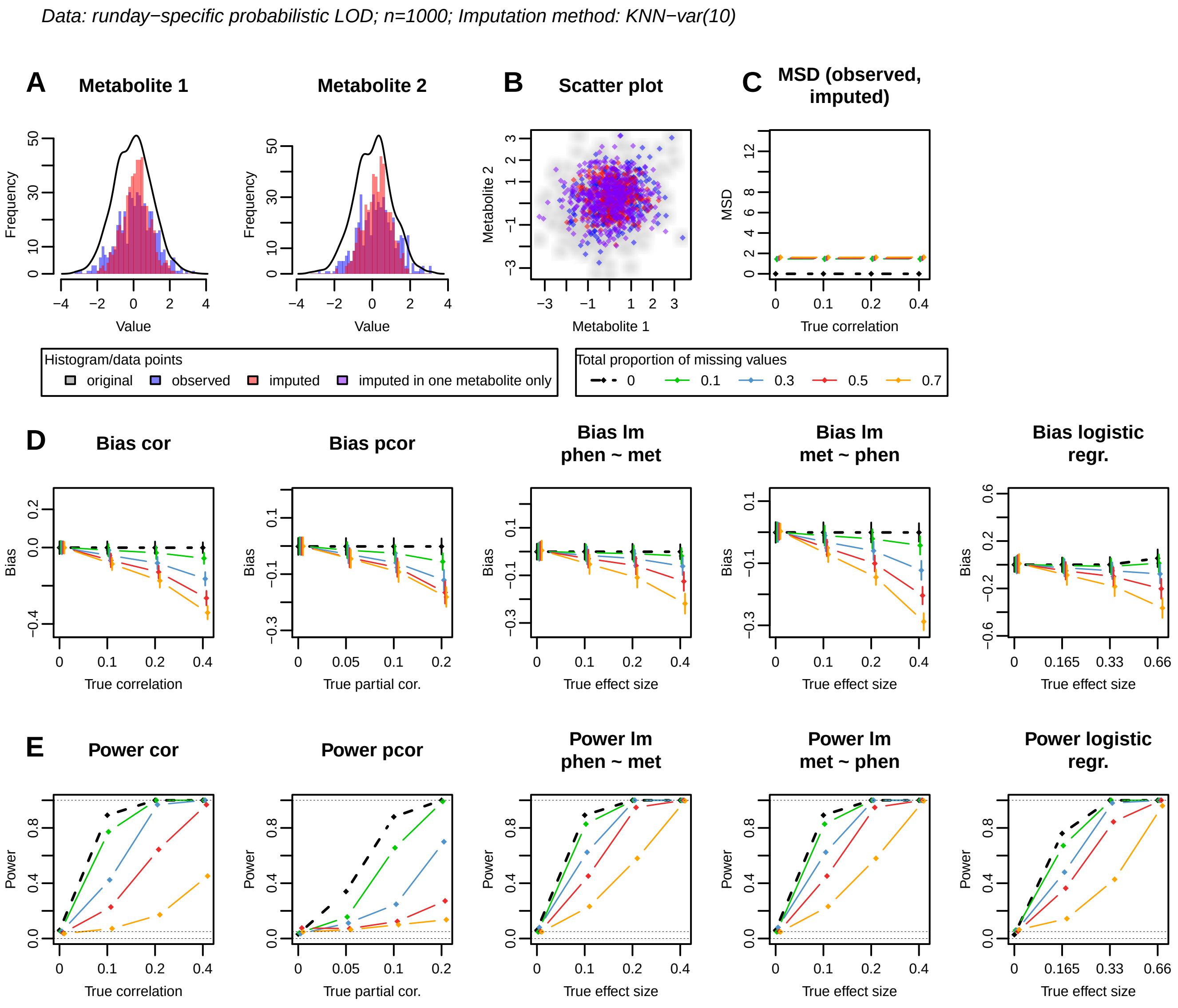

Data: runday−specific probabilistic LOD; n=1000; Imputation method: KNN−var(10)
A
Metabolite 1
Metabolite 2
B
Scatter plot
C
MSD (observed, imputed)
Frequency
Value
Metabolite 1
Metabolite 2
MSD
True correlation
Histogram/data points
original
observed
imputed
imputed in one metabolite only
Total proportion of missing values
0
0.1
0.3
0.5
0.7
D
Bias cor
Bias pcor
Bias lm phen ~ met
Bias lm met ~ phen
Bias logistic regr.
Bias
True correlation
True partial cor.
True effect size
E
Power cor
Power pcor
Power lm phen ~ met
Power lm met ~ phen
Power logistic regr.
Power

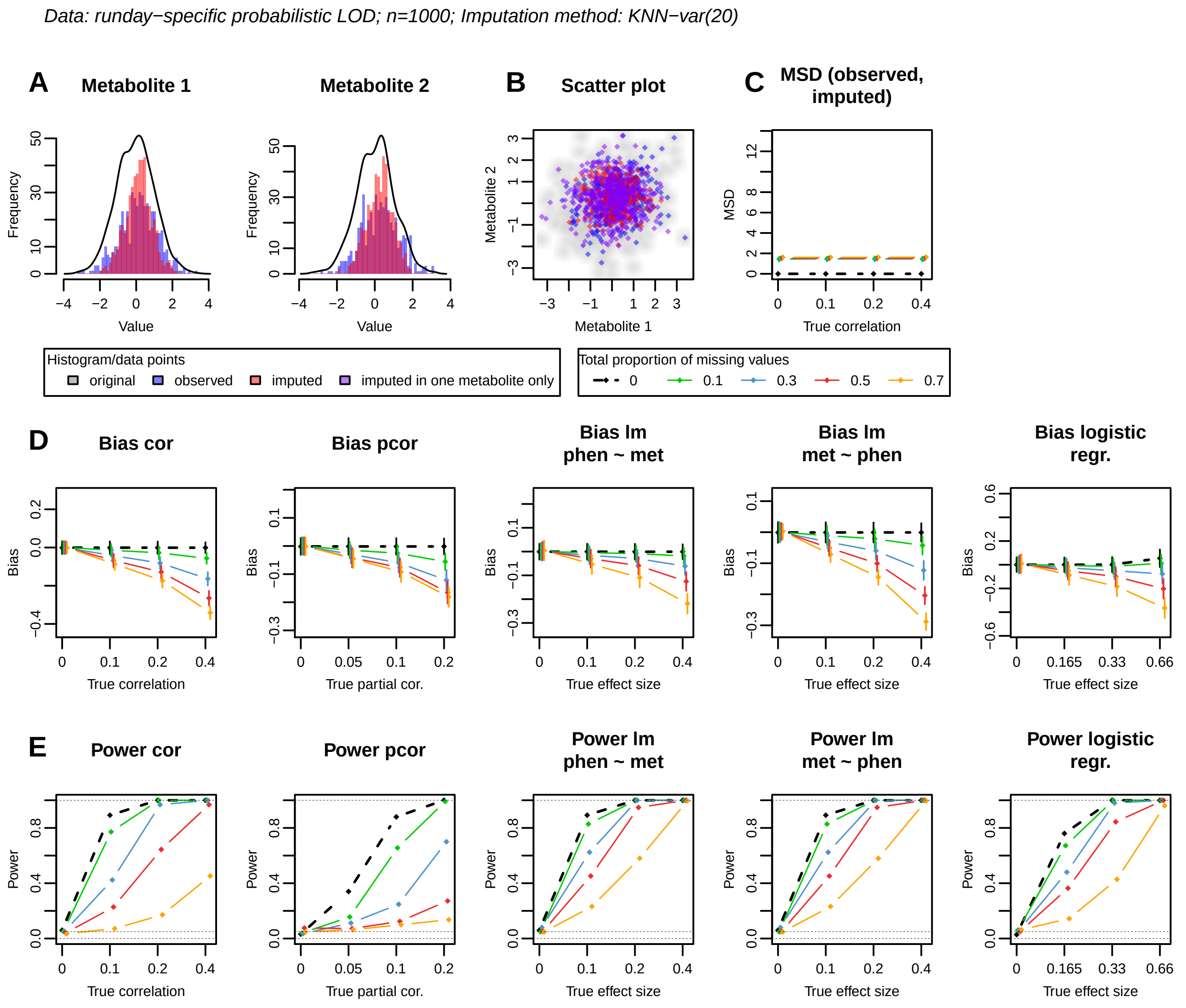
Data: runday−specific probabilistic LOD; n=1000; Imputation method: KNN−var(20)
A
Metabolite 1
Metabolite 2
Frequency
Value
B
Scatter plot
Metabolite 2
Metabolite 1
C
MSD (observed, imputed)
MSD
True correlation
Histogram/data points
original
observed
imputed
imputed in one metabolite only
Total proportion of missing values
0
0.1
0.3
0.5
0.7
D
Bias cor
Bias pcor
Bias lm phen ~ met
Bias lm met ~ phen
Bias logistic regr.
Bias
True correlation
True partial cor.
True effect size
E
Power cor
Power pcor
Power lm phen ~ met
Power lm met ~ phen
Power logistic regr.
Power
True correlation
True partial cor.
True effect size

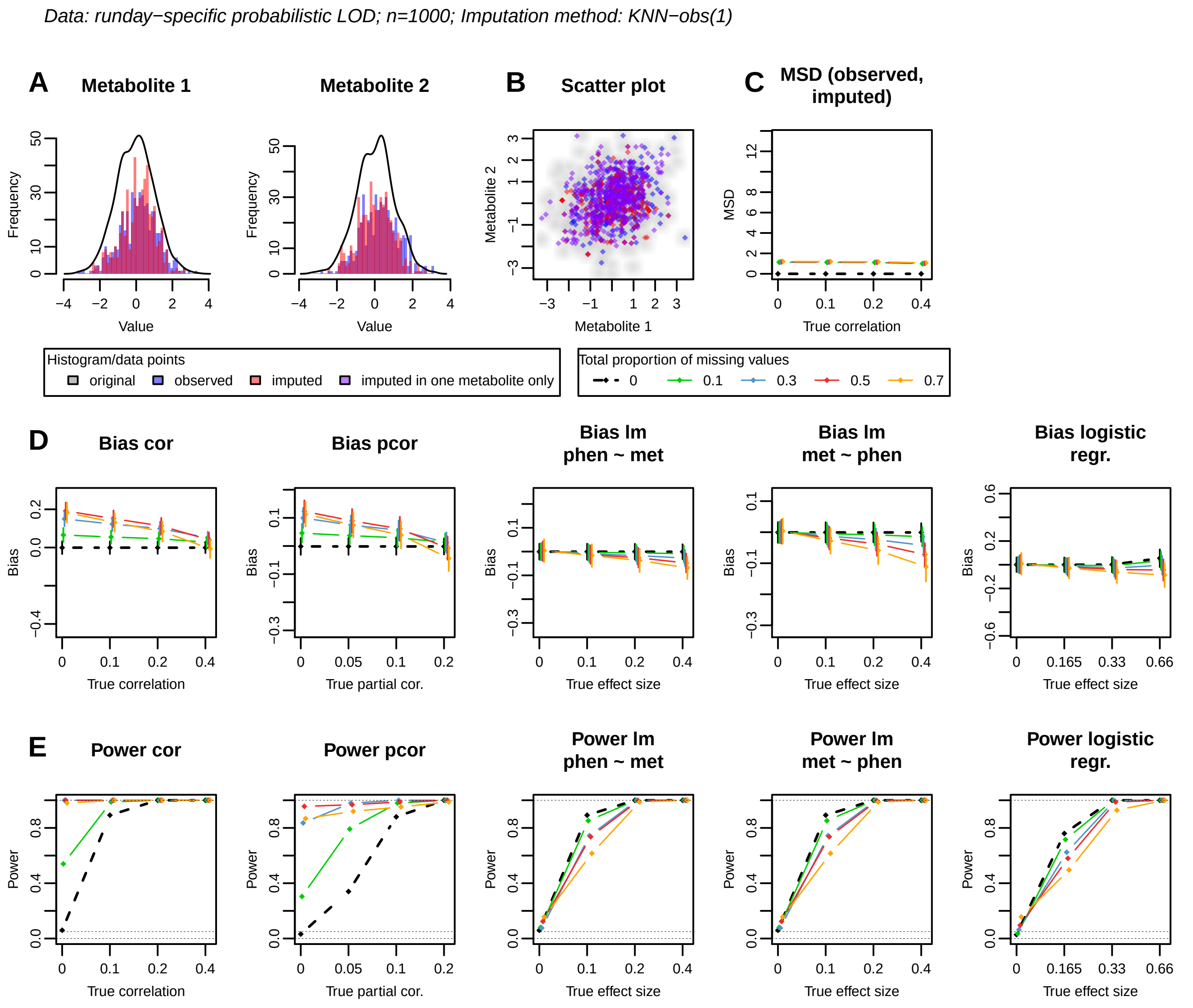

Data: runday−specific probabilistic LOD; n=1000; Imputation method: KNN−obs(1)
A
Metabolite 1
Metabolite 2
B
Scatter plot
C
MSD (observed, imputed)
Frequency
Value
Metabolite 2
Metabolite 1
MSD
True correlation
Histogram/data points
original
observed
imputed
imputed in one metabolite only
Total proportion of missing values
0
0.1
0.3
0.5
0.7
D
Bias cor
Bias pcor
Bias lm phen ~ met
Bias lm met ~ phen
Bias logistic regr.
Bias
True correlation
True partial cor.
True effect size
E
Power cor
Power pcor
Power lm phen ~ met
Power lm met ~ phen
Power logistic regr.
Power

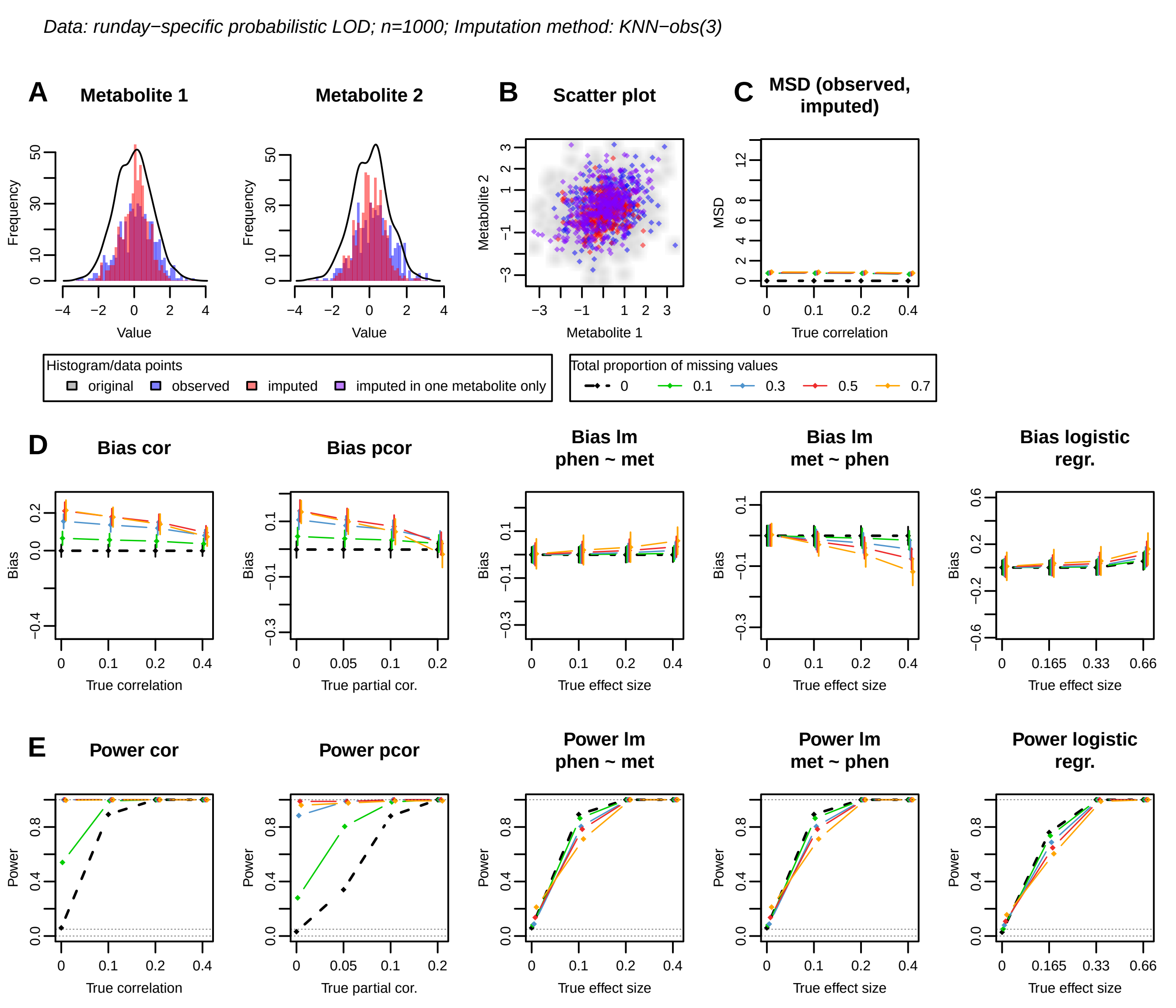

Data: runday−specific probabilistic LOD; n=1000; Imputation method: KNN−obs(3)
A
Metabolite 1
Metabolite 2
B
Scatter plot
C
MSD (observed, imputed)
Frequency
Value
Metabolite 2
Metabolite 1
MSD
True correlation
Histogram/data points
original
observed
imputed
imputed in one metabolite only
Total proportion of missing values
0
0.1
0.3
0.5
0.7
D
Bias cor
Bias pcor
Bias lm phen ~ met
Bias lm met ~ phen
Bias logistic regr.
Bias
True correlation
True partial cor.
True effect size
E
Power cor
Power pcor
Power lm phen ~ met
Power lm met ~ phen
Power logistic regr.
Power
True correlation
True partial cor.
True effect size

Data: runday−specific probabilistic LOD; n=1000; Imputation method: KNN−obs(5)

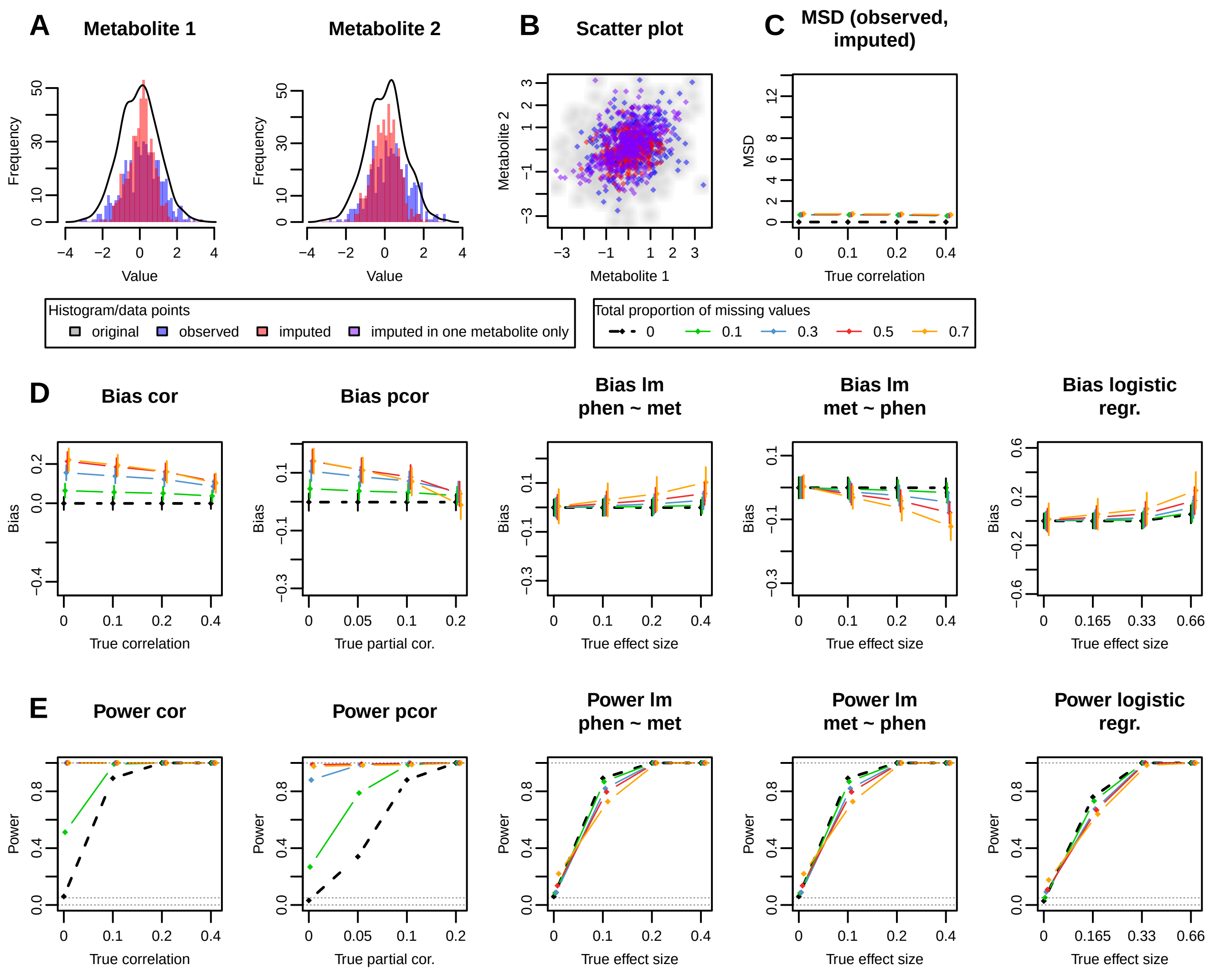

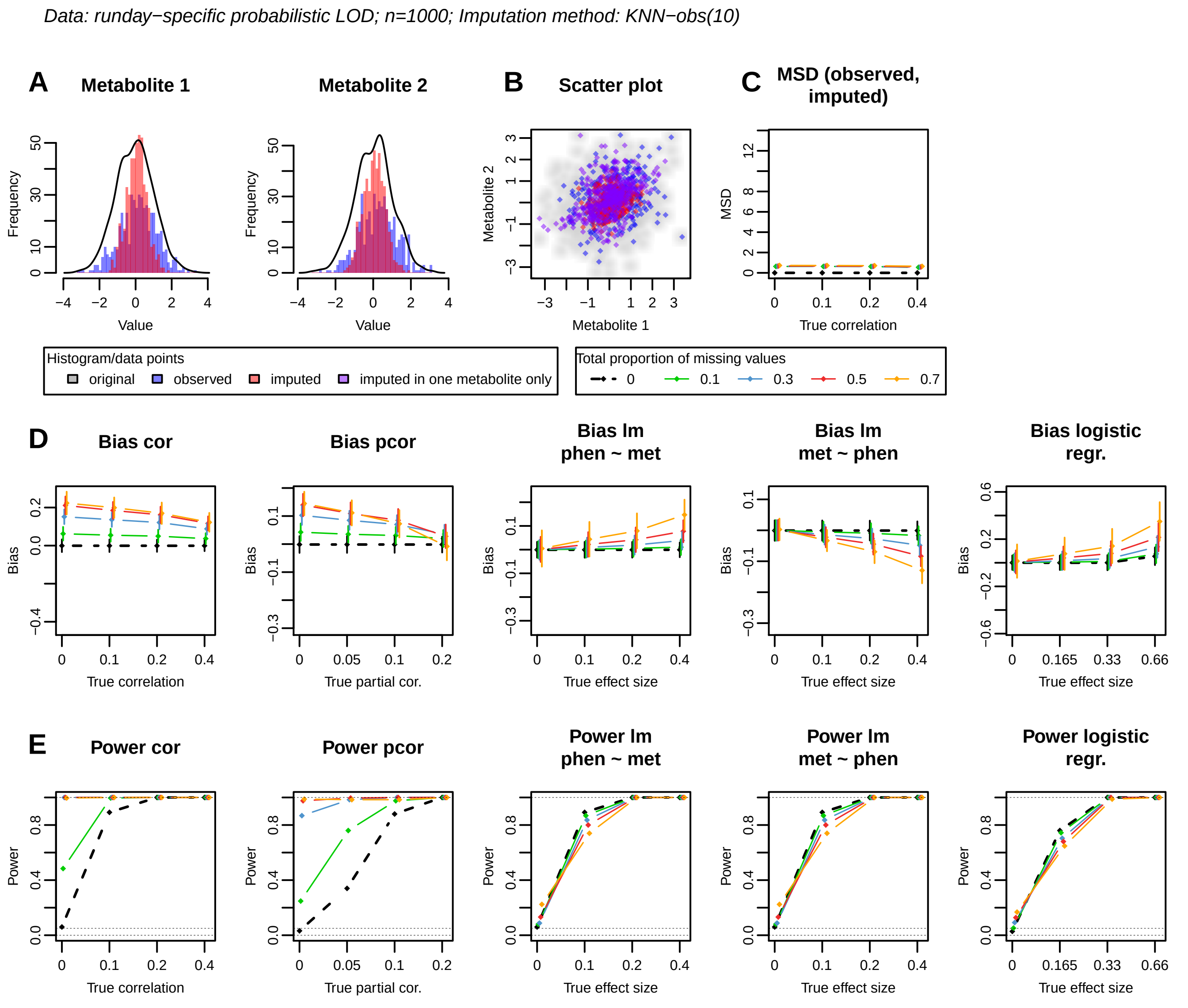

Data: runday−specific probabilistic LOD; n=1000; Imputation method: KNN−obs(10)
A
Metabolite 1
Metabolite 2
B
Scatter plot
C
MSD (observed, imputed)
Frequency
Value
Metabolite 2
Metabolite 1
MSD
True correlation
Histogram/data points
original
observed
imputed
imputed in one metabolite only
Total proportion of missing values
0
0.1
0.3
0.5
0.7
D
Bias cor
Bias pcor
Bias lm phen ~ met
Bias lm met ~ phen
Bias logistic regr.
Bias
True correlation
True partial cor.
True effect size
E
Power cor
Power pcor
Power lm phen ~ met
Power lm met ~ phen
Power logistic regr.
Power
True correlation
True partial cor.
True effect size

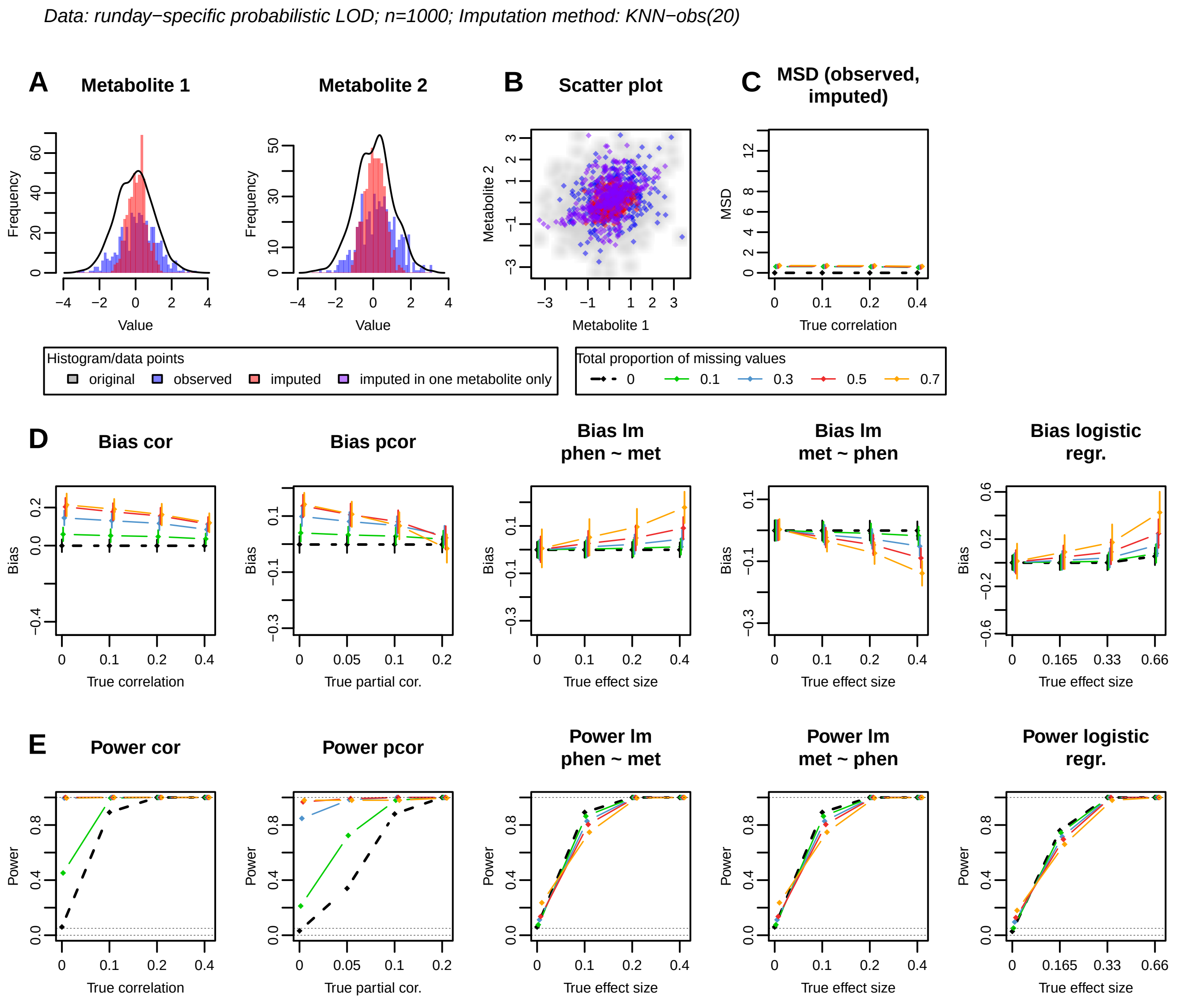
Data: runday−specific probabilistic LOD; n=1000; Imputation method: KNN−obs(20)
A
Metabolite 1
Metabolite 2
Frequency
Value
B
Scatter plot
Metabolite 1
Metabolite 2
C
MSD (observed, imputed)
MSD
True correlation
Histogram/data points
original
observed
imputed
imputed in one metabolite only
Total proportion of missing values
0
0.1
0.3
0.5
0.7
D
Bias cor
Bias pcor
Bias lm phen ~ met
Bias lm met ~ phen
Bias logistic regr.
Bias
True correlation
True partial cor.
True effect size
E
Power cor
Power pcor
Power lm phen ~ met
Power lm met ~ phen
Power logistic regr.
Power
True correlation
True partial cor.
True effect size

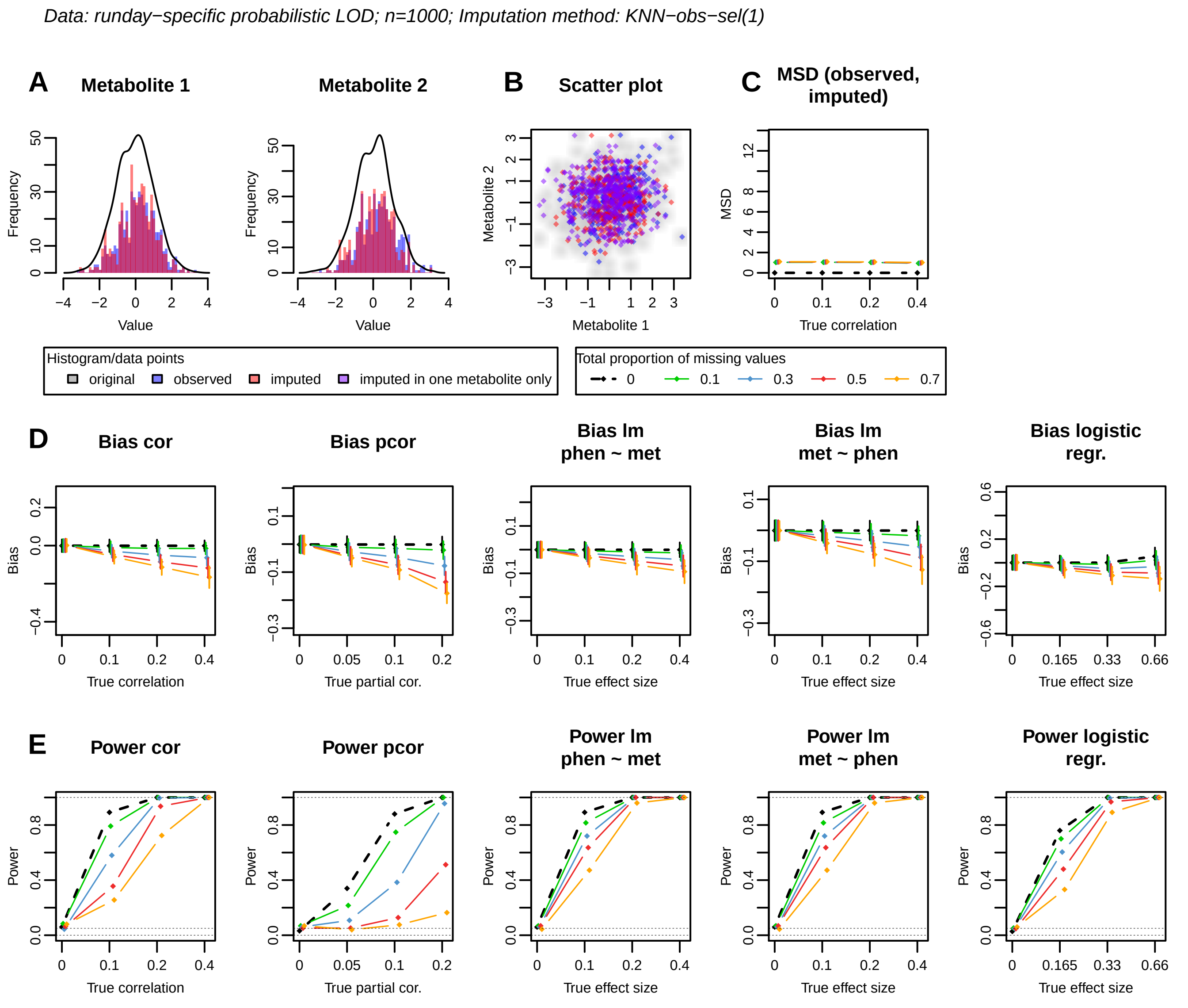
Data: runday−specific probabilistic LOD; n=1000; Imputation method: KNN−obs−sel(1)
A
Metabolite 1
Metabolite 2
B
Scatter plot
C
MSD (observed, imputed)
Frequency
Value
Metabolite 2
Metabolite 1
MSD
True correlation
Histogram/data points
original
observed
imputed
imputed in one metabolite only
Total proportion of missing values
0
0.1
0.3
0.5
0.7
D
Bias cor
Bias pcor
Bias lm phen ~ met
Bias lm met ~ phen
Bias logistic regr.
Bias
True correlation
True partial cor.
True effect size
E
Power cor
Power pcor
Power lm phen ~ met
Power lm met ~ phen
Power logistic regr.
Power
True correlation
True partial cor.
True effect size

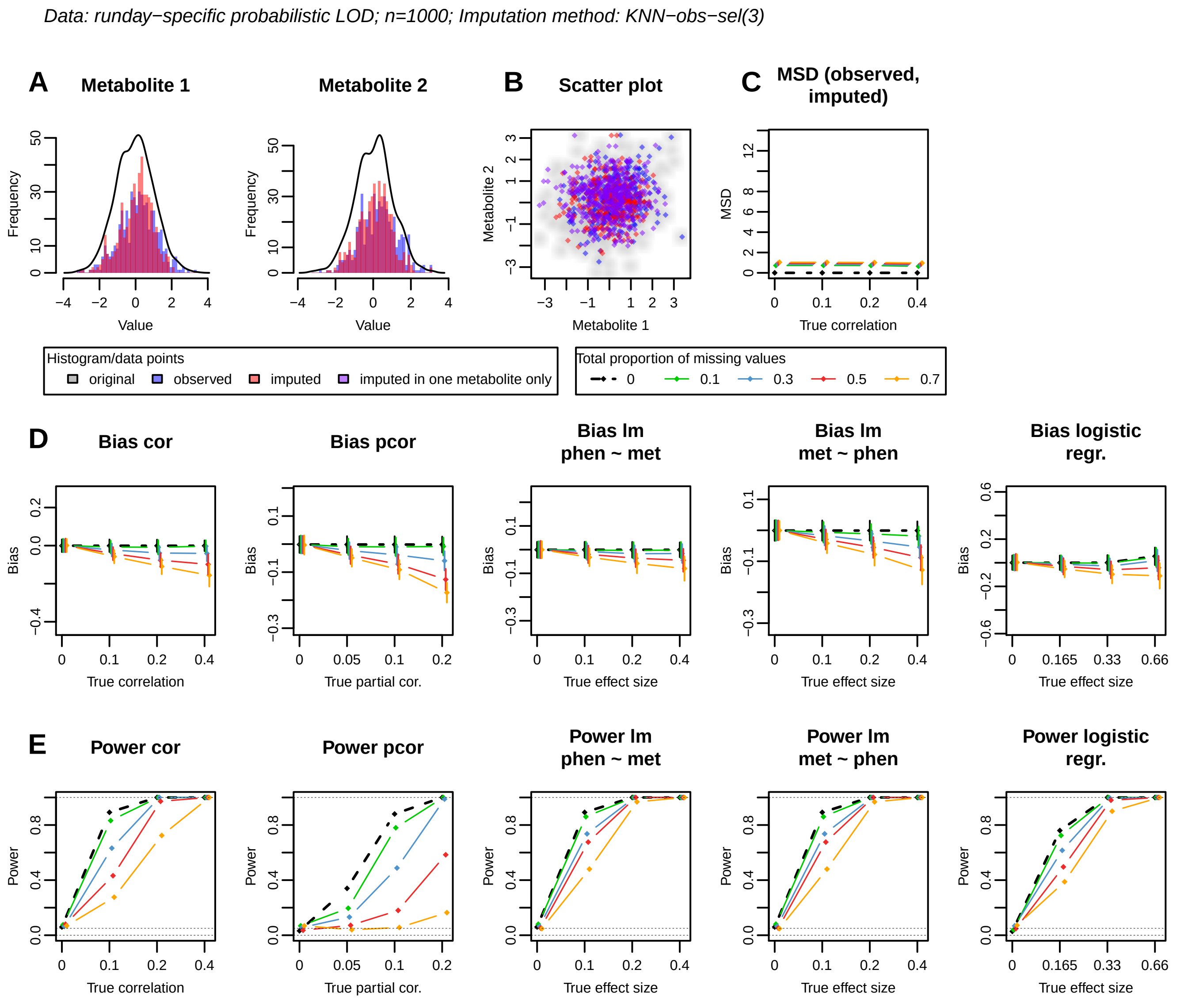
Data: runday−specific probabilistic LOD; n=1000; Imputation method: KNN−obs−sel(3)
A
Metabolite 1
Metabolite 2
Frequency
Value
B
Scatter plot
Metabolite 2
Metabolite 1
C
MSD (observed, imputed)
MSD
True correlation
Histogram/data points
original
observed
imputed
imputed in one metabolite only
Total proportion of missing values
0
0.1
0.3
0.5
0.7
D
Bias cor
Bias pcor
Bias lm phen ~ met
Bias lm met ~ phen
Bias logistic regr.
Bias
True correlation
True partial cor.
True effect size
E
Power cor
Power pcor
Power lm phen ~ met
Power lm met ~ phen
Power logistic regr.
Power

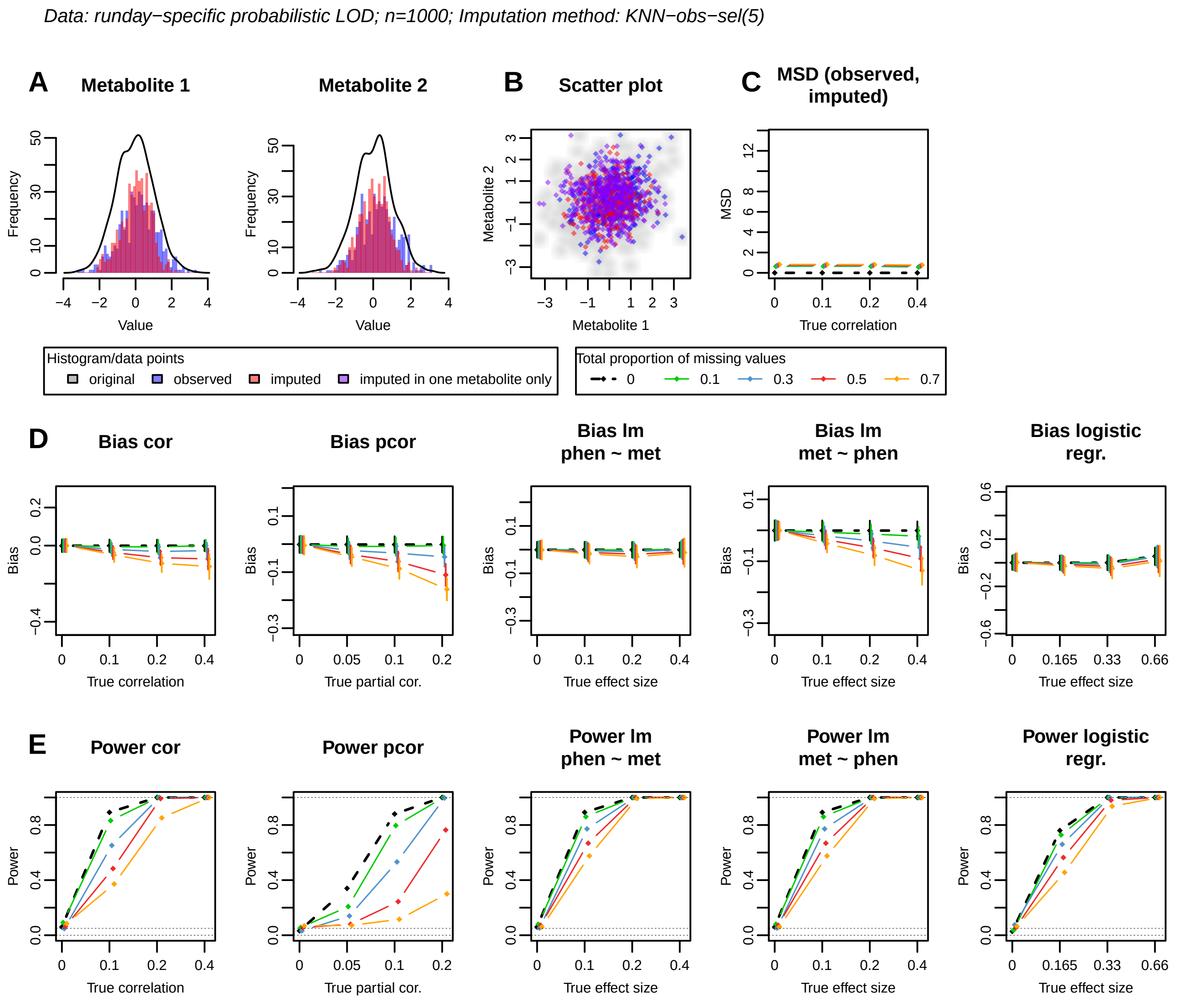

Data: runday−specific probabilistic LOD; n=1000; Imputation method: KNN−obs−sel(5)
A
Metabolite 1
Metabolite 2
B
Scatter plot
C
MSD (observed, imputed)
Frequency
Value
Metabolite 2
Metabolite 1
MSD
True correlation
Histogram/data points
original
observed
imputed
imputed in one metabolite only
Total proportion of missing values
0
0.1
0.3
0.5
0.7
D
Bias cor
Bias pcor
Bias lm phen ~ met
Bias lm met ~ phen
Bias logistic regr.
Bias
True correlation
True partial cor.
True effect size
E
Power cor
Power pcor
Power lm phen ~ met
Power lm met ~ phen
Power logistic regr.
Power
True correlation
True partial cor.
True effect size

Data: runday−specific probabilistic LOD; n=1000; Imputation method: KNN−obs−sel(10)

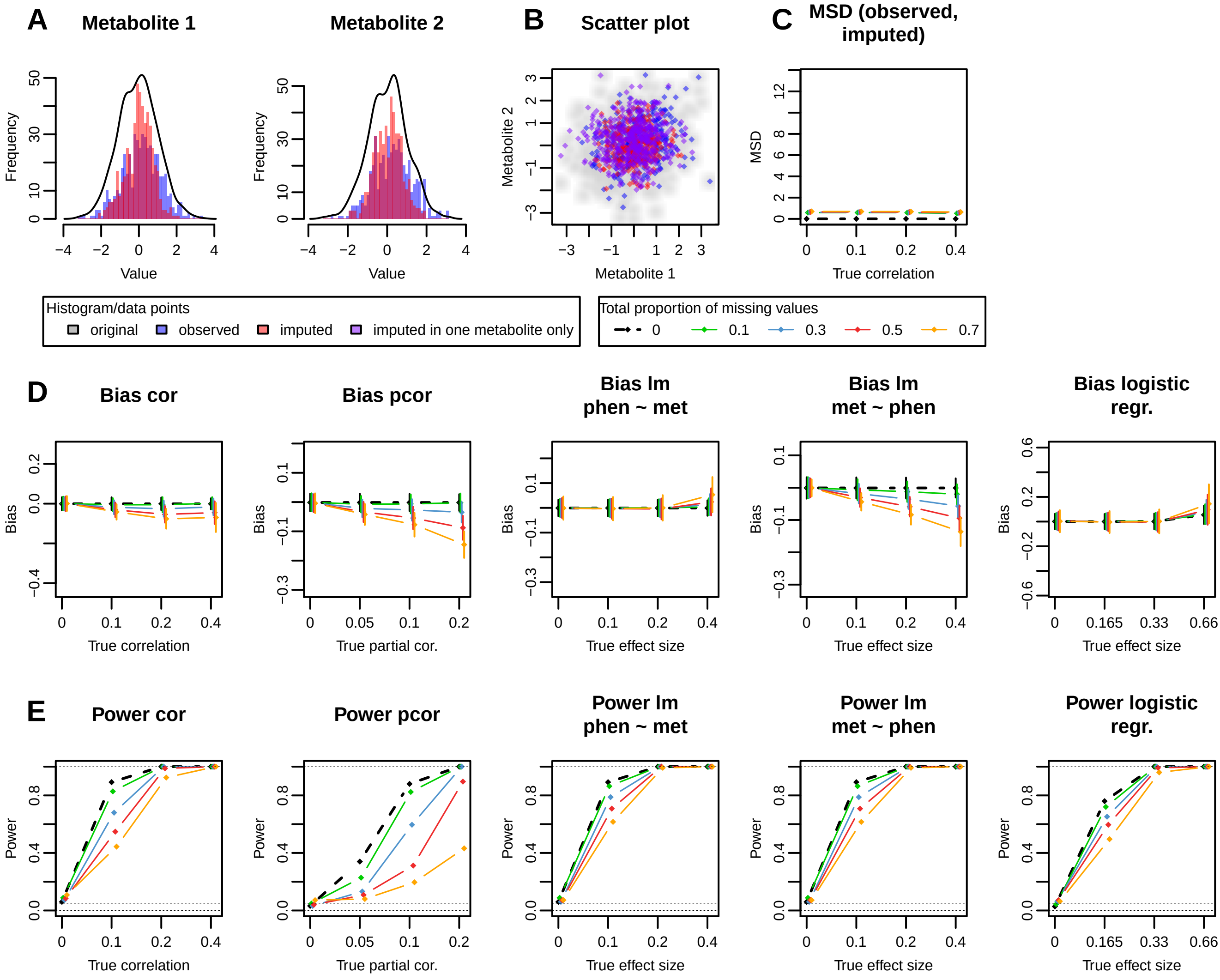

Data: runday−specific probabilistic LOD; n=1000; Imputation method: KNN−obs−sel(20)

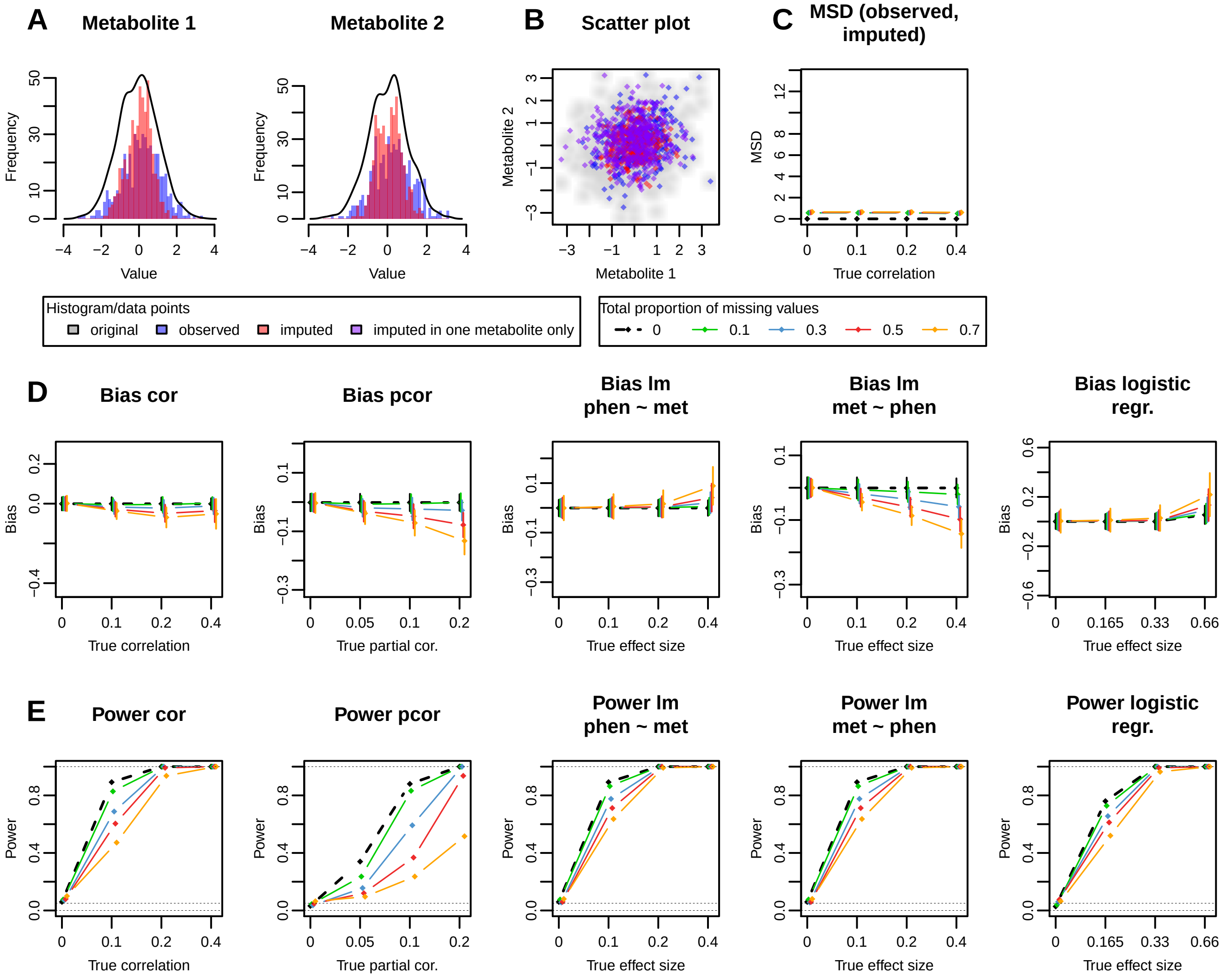

Data: runday−specific probabilistic LOD; without auxiliary variables; Imputation method: CCA

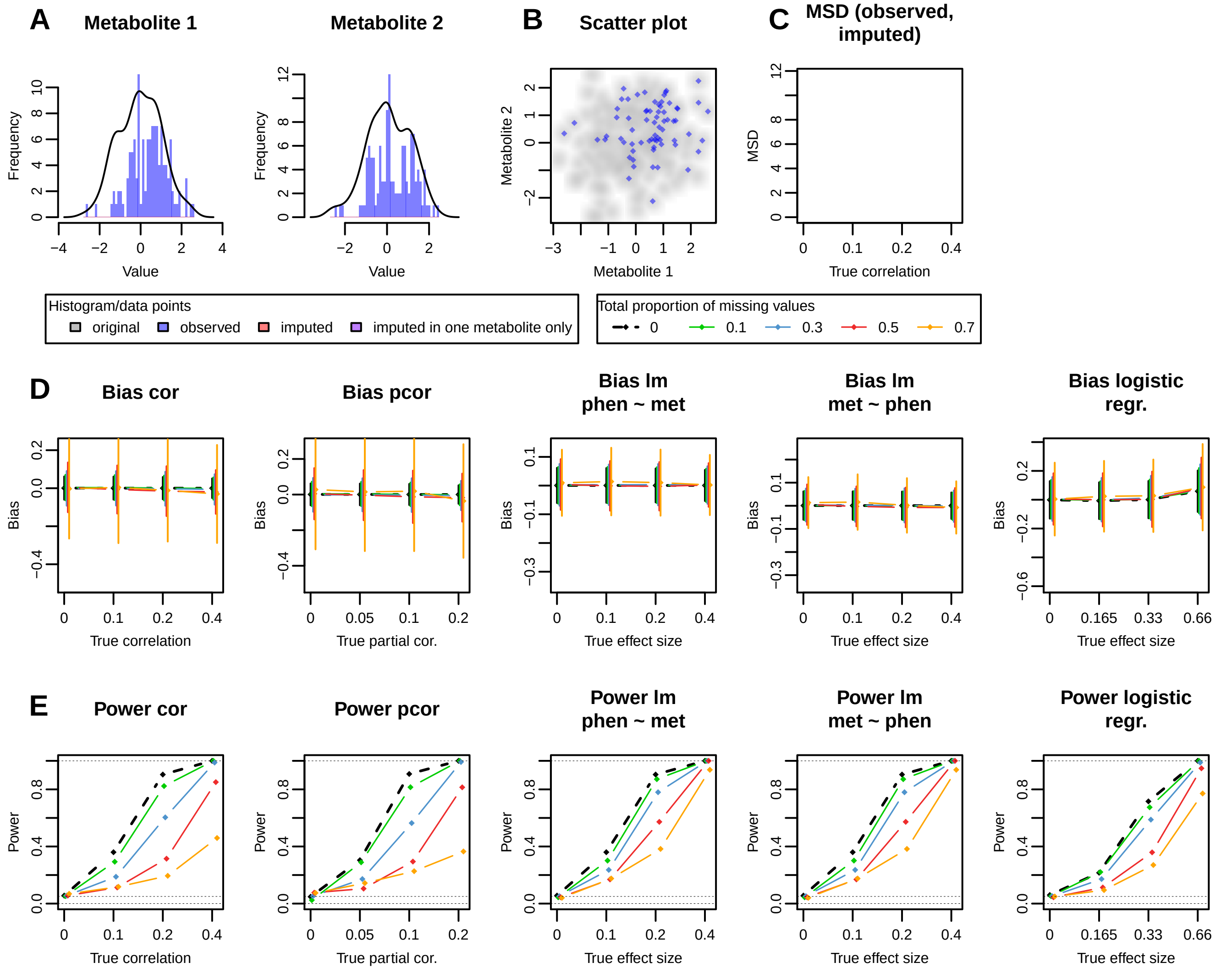

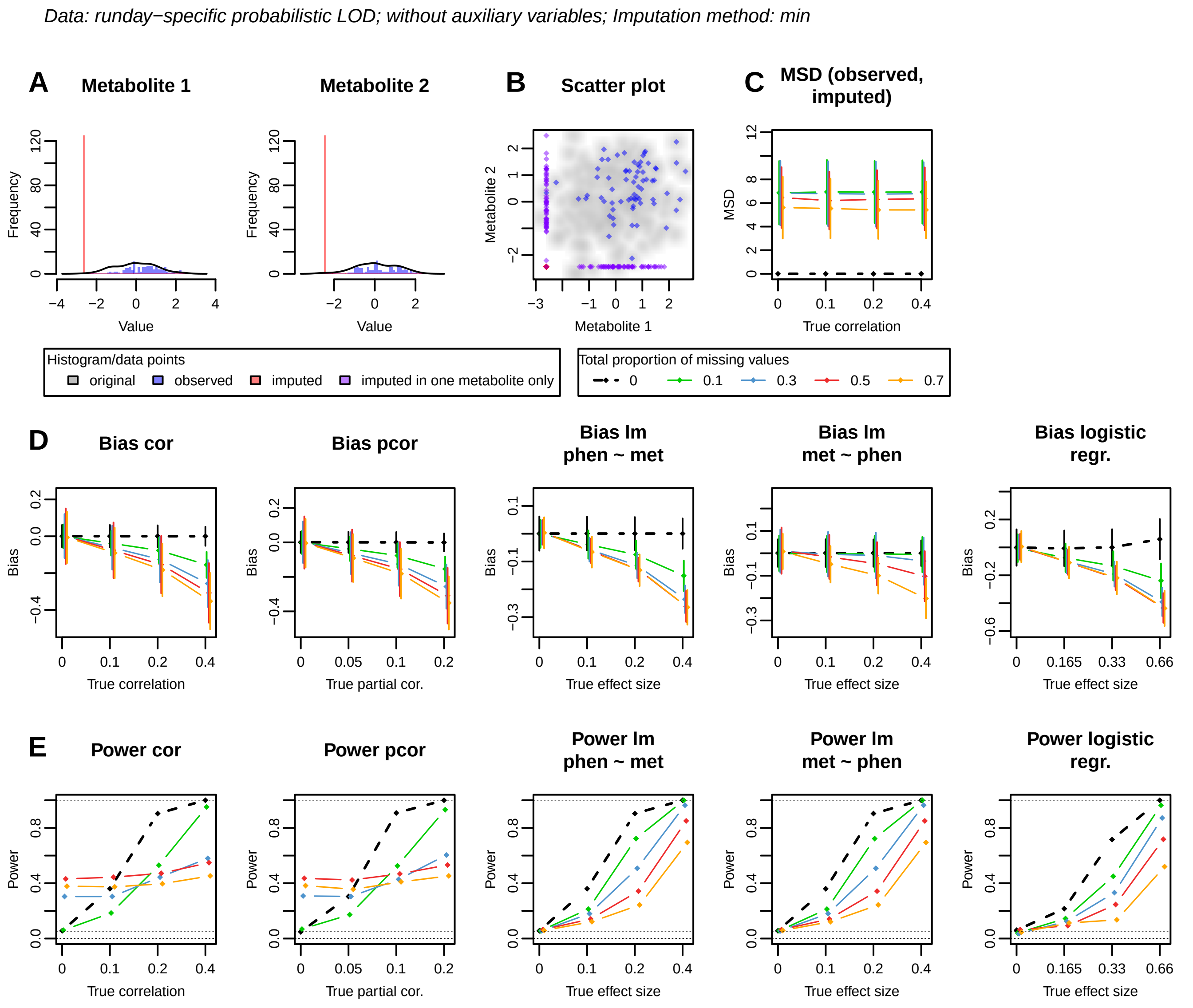

Data: runday−specific probabilistic LOD; without auxiliary variables; Imputation method: min
A
Metabolite 1
Metabolite 2
Frequency
Value
B
Scatter plot
Metabolite 1
Metabolite 2
C
MSD (observed, imputed)
MSD
True correlation
Histogram/data points
original
observed
imputed
imputed in one metabolite only
Total proportion of missing values
0
0.1
0.3
0.5
0.7
D
Bias cor
Bias pcor
Bias lm phen ~ met
Bias lm met ~ phen
Bias logistic regr.
Bias
True correlation
True partial cor.
True effect size
E
Power cor
Power pcor
Power lm phen ~ met
Power lm met ~ phen
Power logistic regr.
Power

Data: runday−specific probabilistic LOD; without auxiliary variables; Imputation method: RC

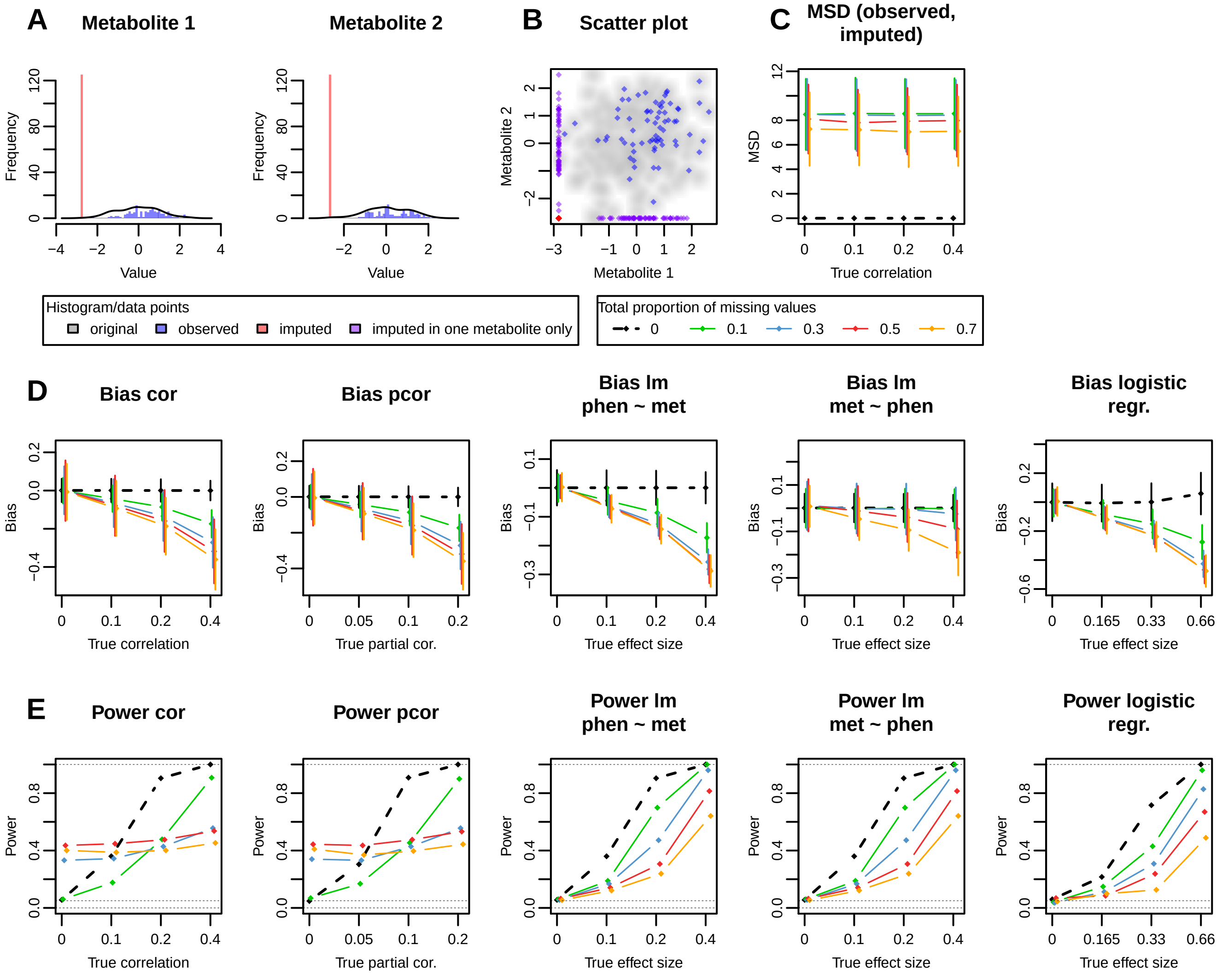

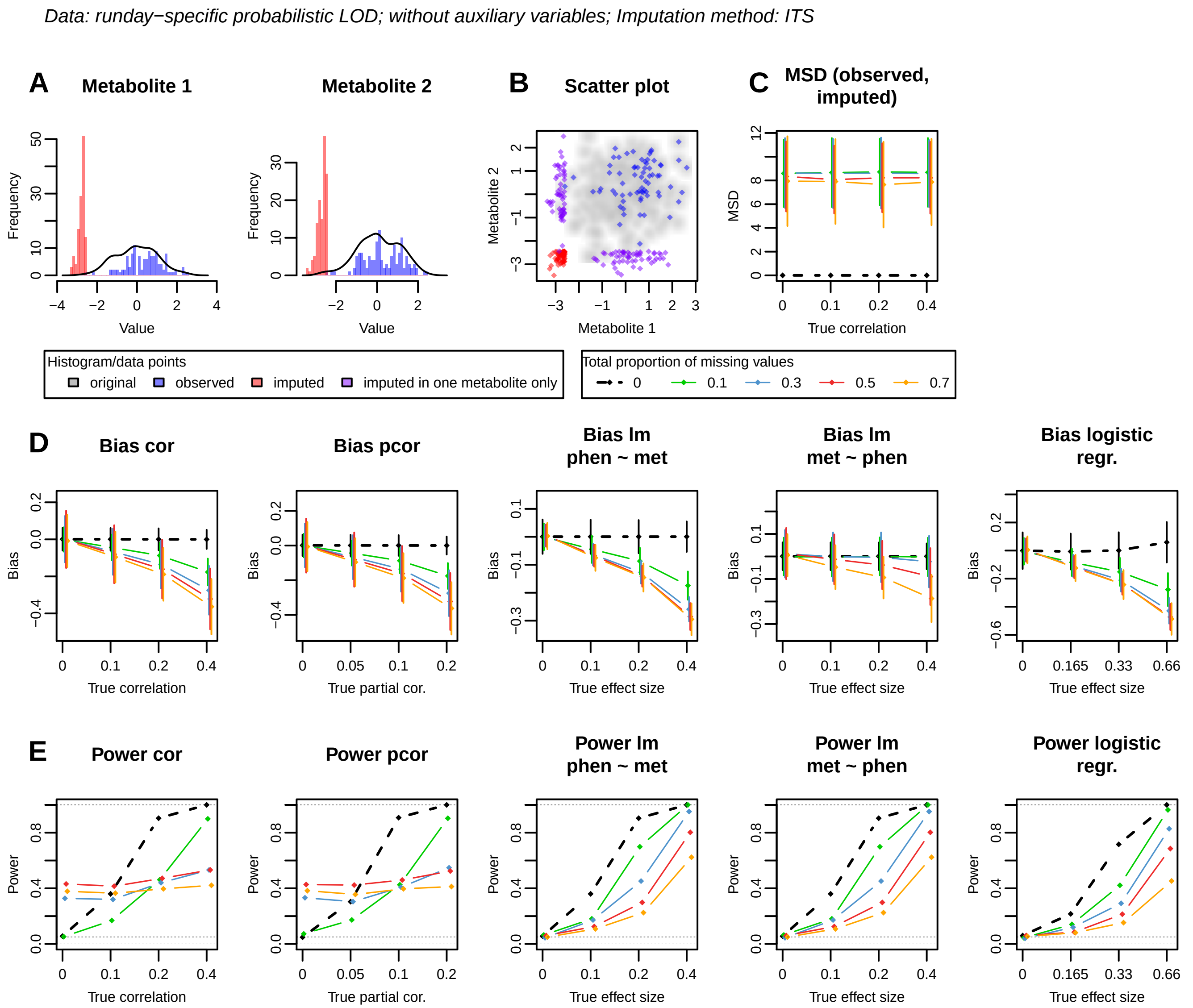
Data: runday−specific probabilistic LOD; without auxiliary variables; Imputation method: ITS
A
Metabolite 1
Metabolite 2
Frequency
Value
B
Scatter plot
Metabolite 1
Metabolite 2
C
MSD (observed, imputed)
MSD
True correlation
Histogram/data points
original
observed
imputed
imputed in one metabolite only
Total proportion of missing values
0
0.1
0.3
0.5
0.7
D
Bias cor
Bias pcor
Bias lm phen ~ met
Bias lm met ~ phen
Bias logistic regr.
Bias
True correlation
True partial cor.
True effect size
E
Power cor
Power pcor
Power lm phen ~ met
Power lm met ~ phen
Power logistic regr.
Power

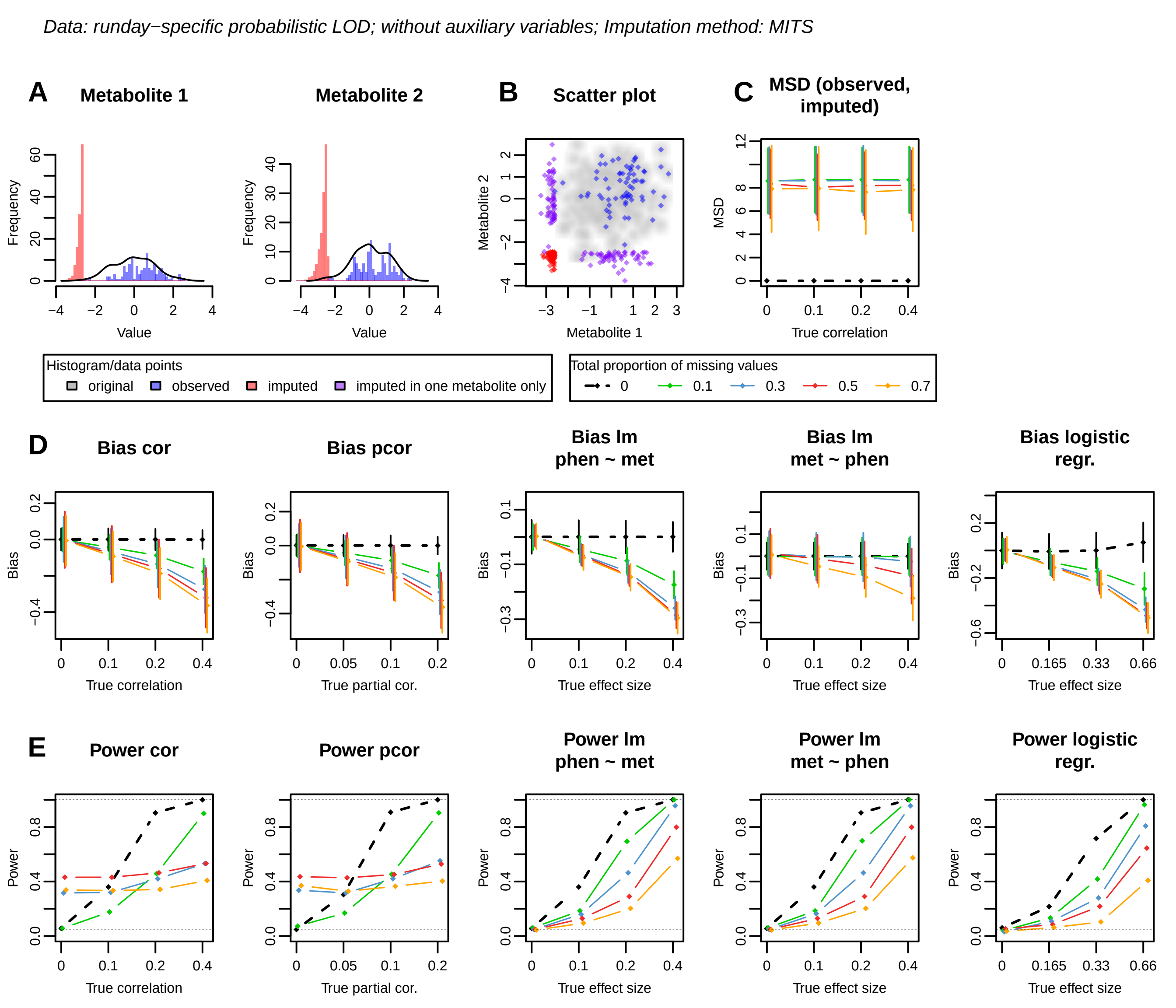
Data: runday−specific probabilistic LOD; without auxiliary variables; Imputation method: MITS
A
Metabolite 1
Metabolite 2
B
Scatter plot
C
MSD (observed, imputed)
Frequency
Value
Metabolite 2
Metabolite 1
MSD
True correlation
Histogram/data points
original
observed
imputed
imputed in one metabolite only
Total proportion of missing values
0
0.1
0.3
0.5
0.7
D
Bias cor
Bias pcor
Bias lm phen ~ met
Bias lm met ~ phen
Bias logistic regr.
Bias
True correlation
True partial cor.
True effect size
E
Power cor
Power pcor
Power lm phen ~ met
Power lm met ~ phen
Power logistic regr.
Power

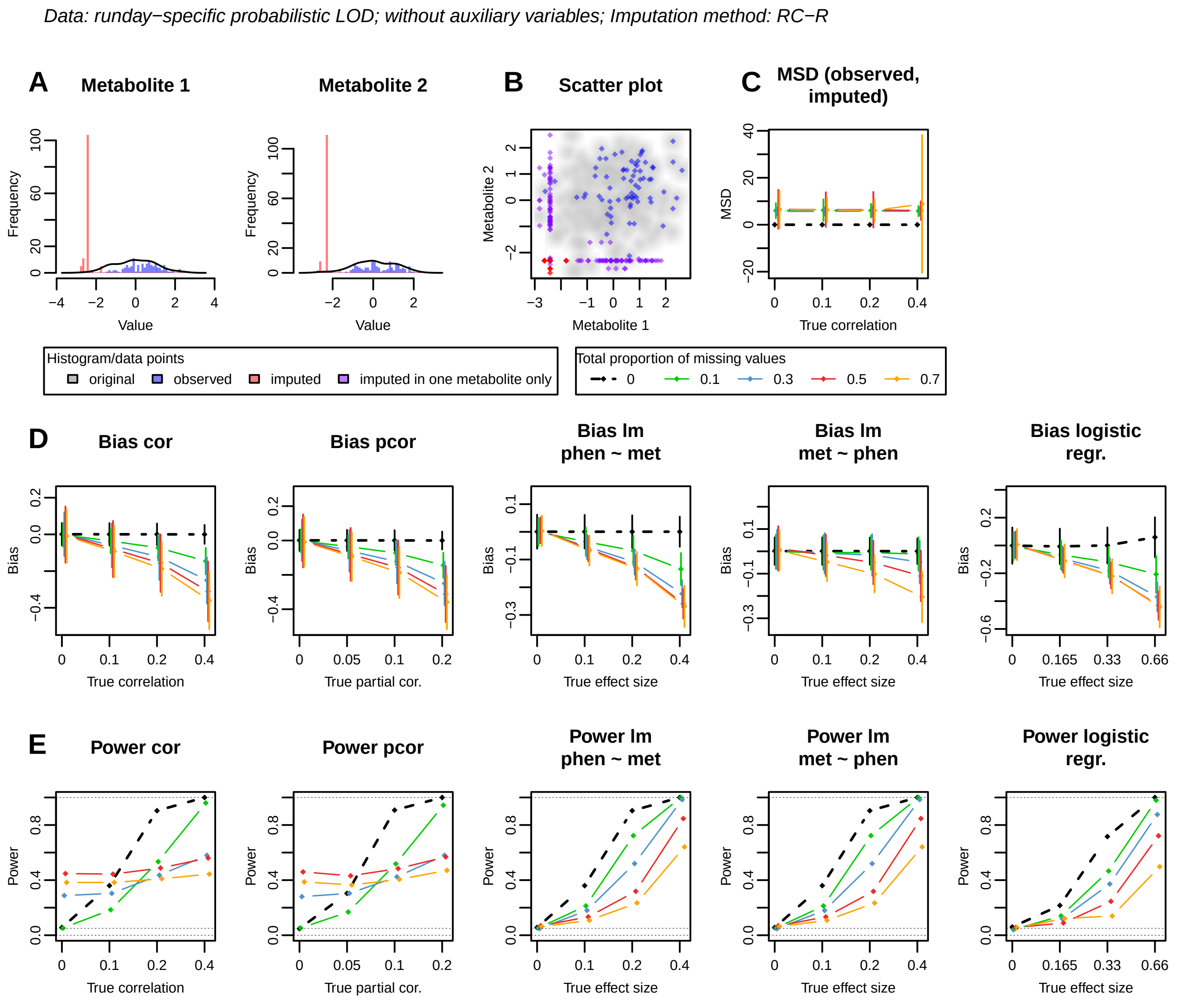
Data: runday−specific probabilistic LOD; without auxiliary variables; Imputation method: RC−R
A
Metabolite 1
Metabolite 2
Frequency
Value
B
Scatter plot
Metabolite 2
Metabolite 1
C
MSD (observed, imputed)
MSD
True correlation
Histogram/data points
original
observed
imputed
imputed in one metabolite only
Total proportion of missing values
0
0.1
0.3
0.5
0.7
D
Bias cor
Bias pcor
Bias lm phen ~ met
Bias lm met ~ phen
Bias logistic regr.
Bias
True correlation
True partial cor.
True effect size
E
Power cor
Power pcor
Power lm phen ~ met
Power lm met ~ phen
Power logistic regr.
Power
True correlation
True partial cor.
True effect size

Data: runday−specific probabilistic LOD; without auxiliary variables; Imputation method: ITS−R

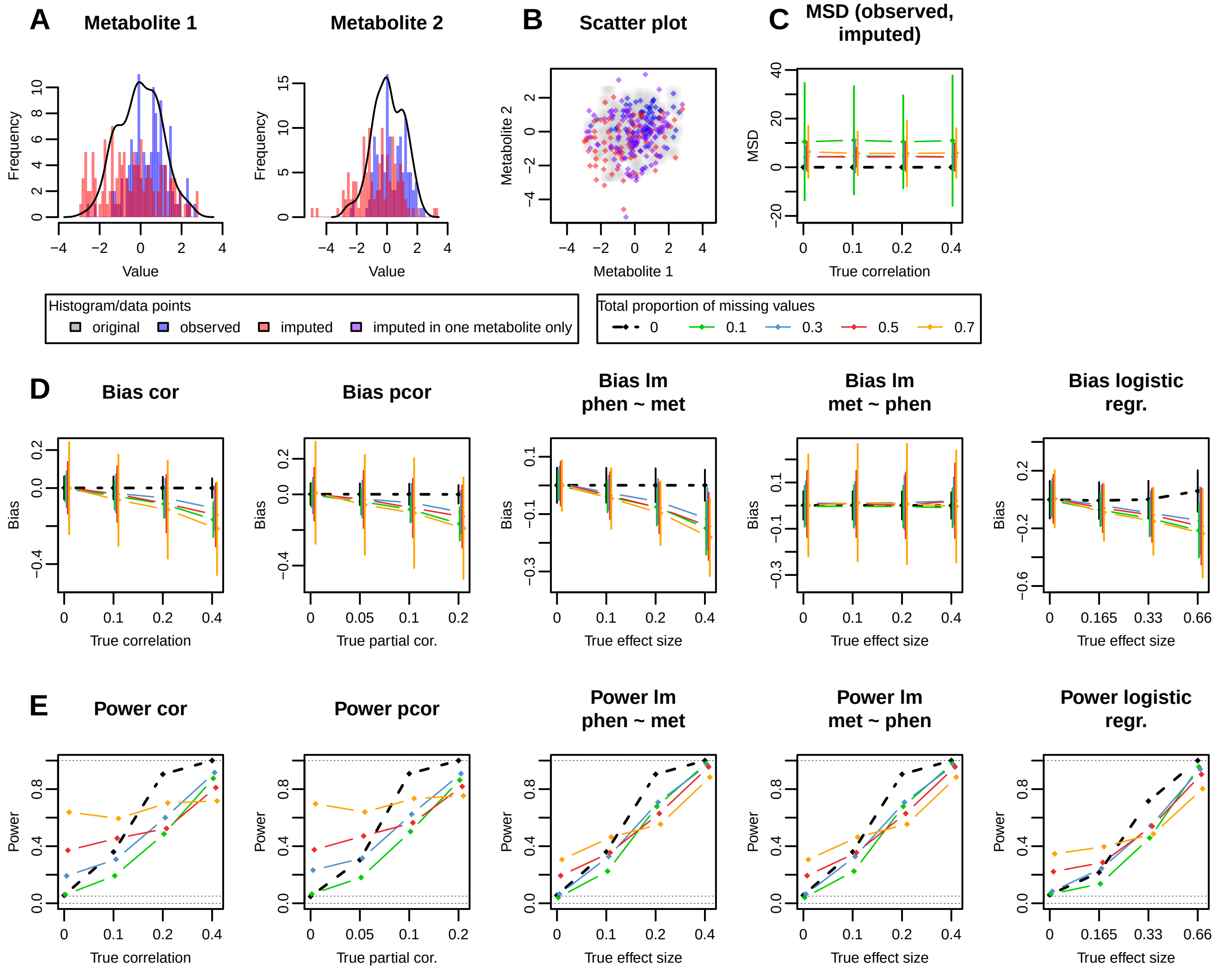

Data: runday−specific probabilistic LOD; without auxiliary variables; Imputation method: MITS−R

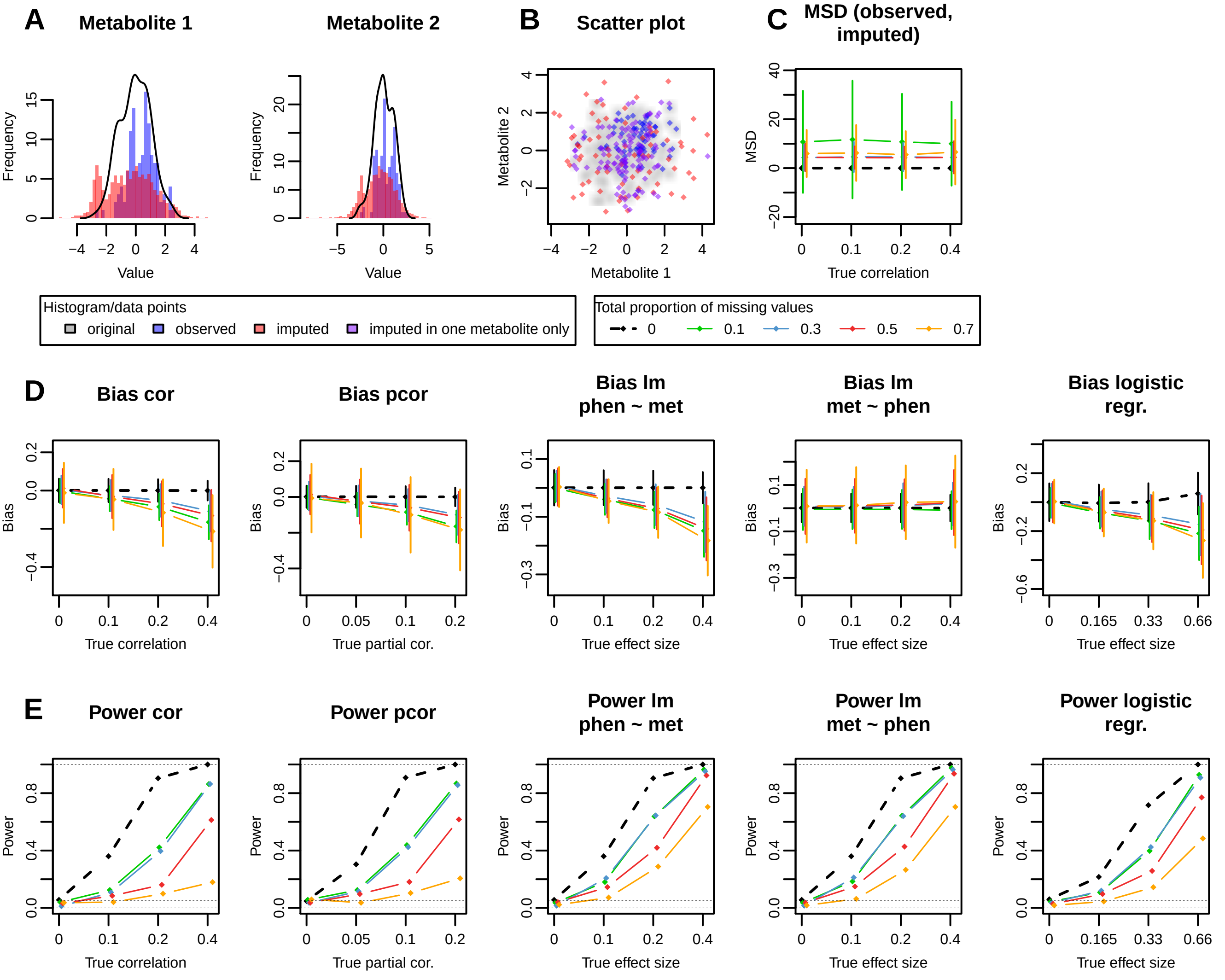

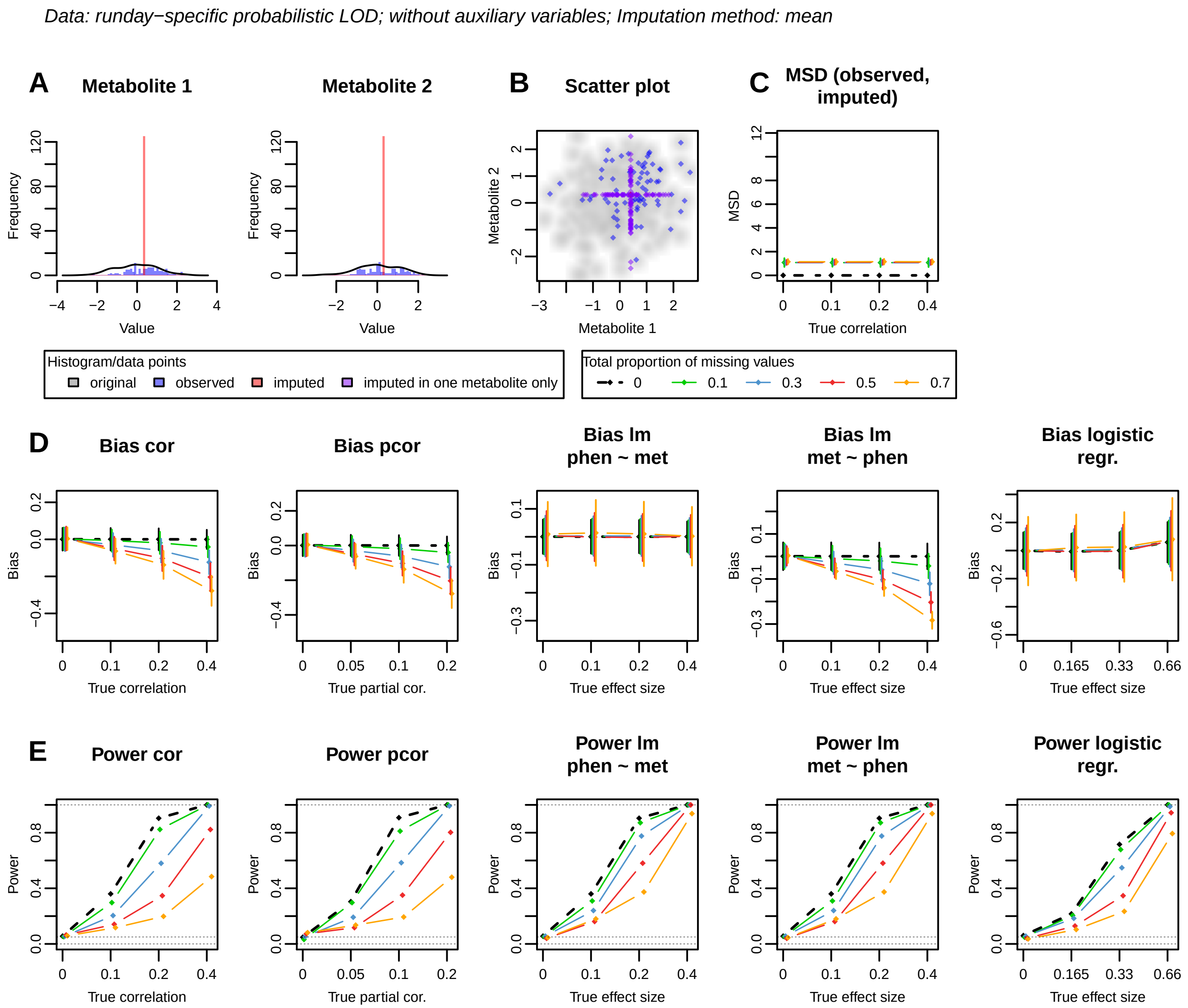
Data: runday−specific probabilistic LOD; without auxiliary variables; Imputation method: mean
A
Metabolite 1
Metabolite 2
Frequency
Value
B
Scatter plot
Metabolite 2
Metabolite 1
C
MSD (observed, imputed)
MSD
True correlation
Histogram/data points
original
observed
imputed
imputed in one metabolite only
Total proportion of missing values
0
0.1
0.3
0.5
0.7
D
Bias cor
Bias pcor
Bias lm phen ~ met
Bias lm met ~ phen
Bias logistic regr.
Bias
True correlation
True partial cor.
True effect size
E
Power cor
Power pcor
Power lm phen ~ met
Power lm met ~ phen
Power logistic regr.
Power

Data: runday−specific probabilistic LOD; without auxiliary variables; Imputation method: ICE−norm

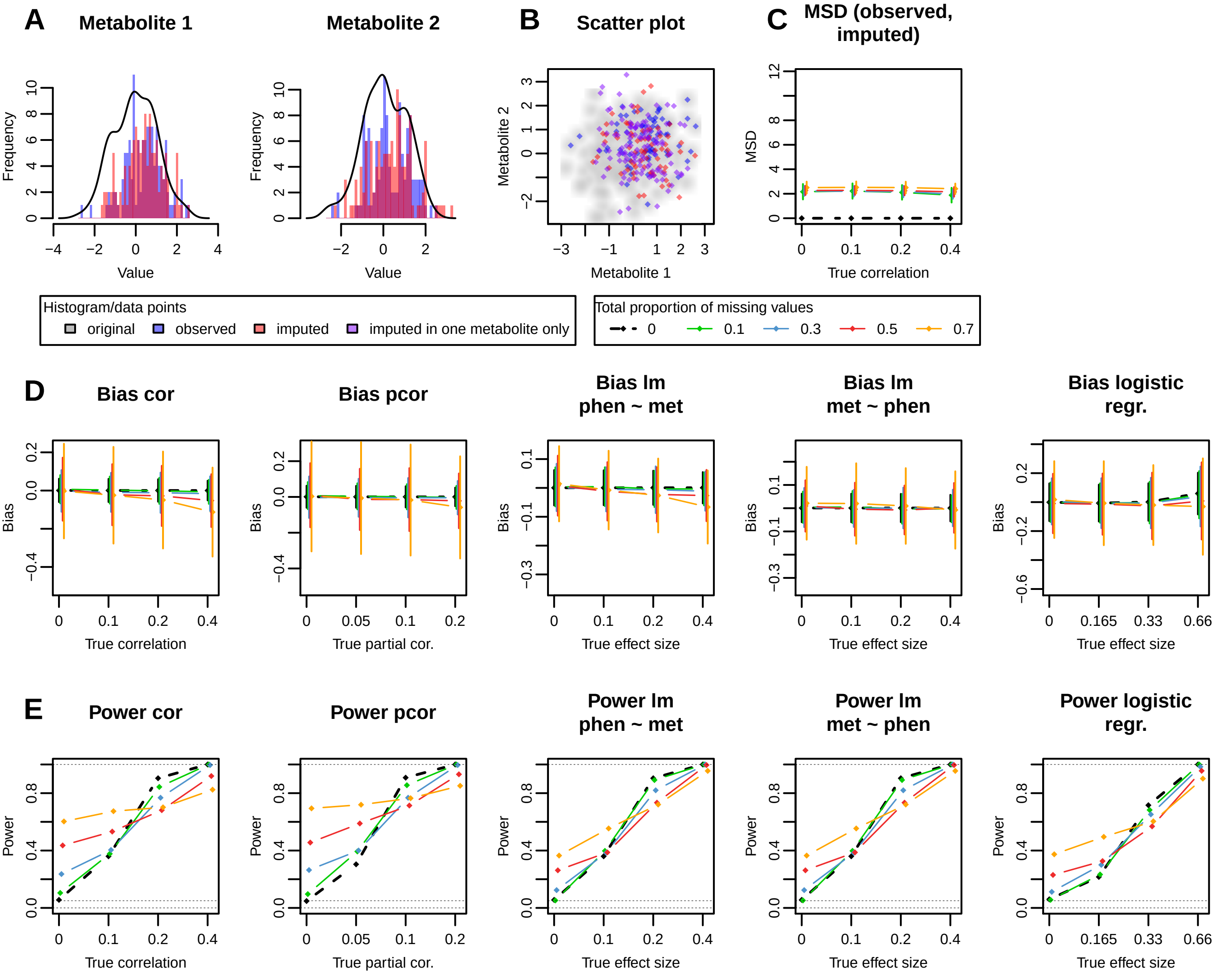

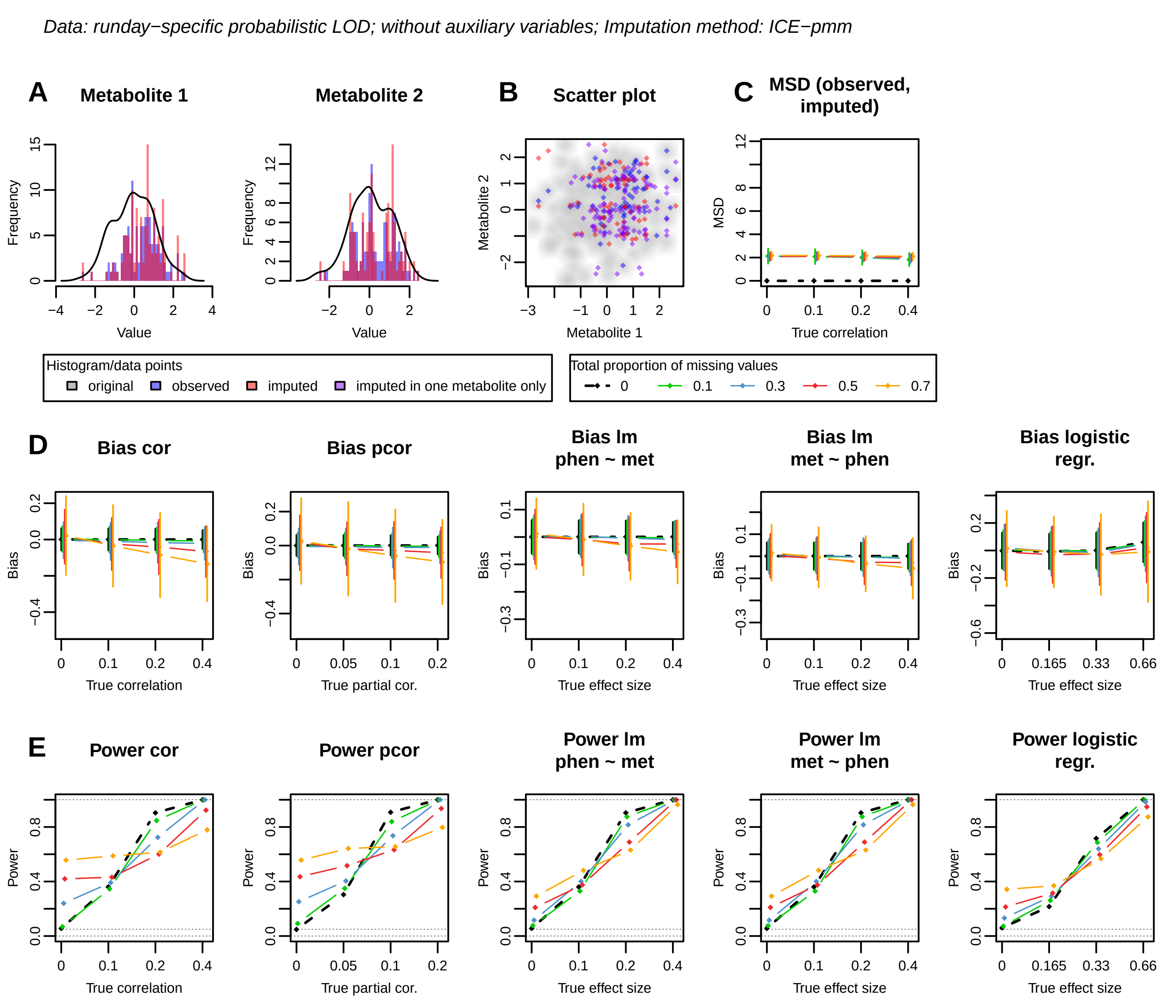
Data: runday−specific probabilistic LOD; without auxiliary variables; Imputation method: ICE−pmm
A
Metabolite 1
Metabolite 2
B
Scatter plot
C
MSD (observed, imputed)
Frequency
Value
Metabolite 2
Metabolite 1
MSD
True correlation
Histogram/data points
original
observed
imputed
imputed in one metabolite only
Total proportion of missing values
0
0.1
0.3
0.5
0.7
D
Bias cor
Bias pcor
Bias lm phen ~ met
Bias lm met ~ phen
Bias logistic regr.
Bias
True correlation
True partial cor.
True effect size
E
Power cor
Power pcor
Power lm phen ~ met
Power lm met ~ phen
Power logistic regr.
Power

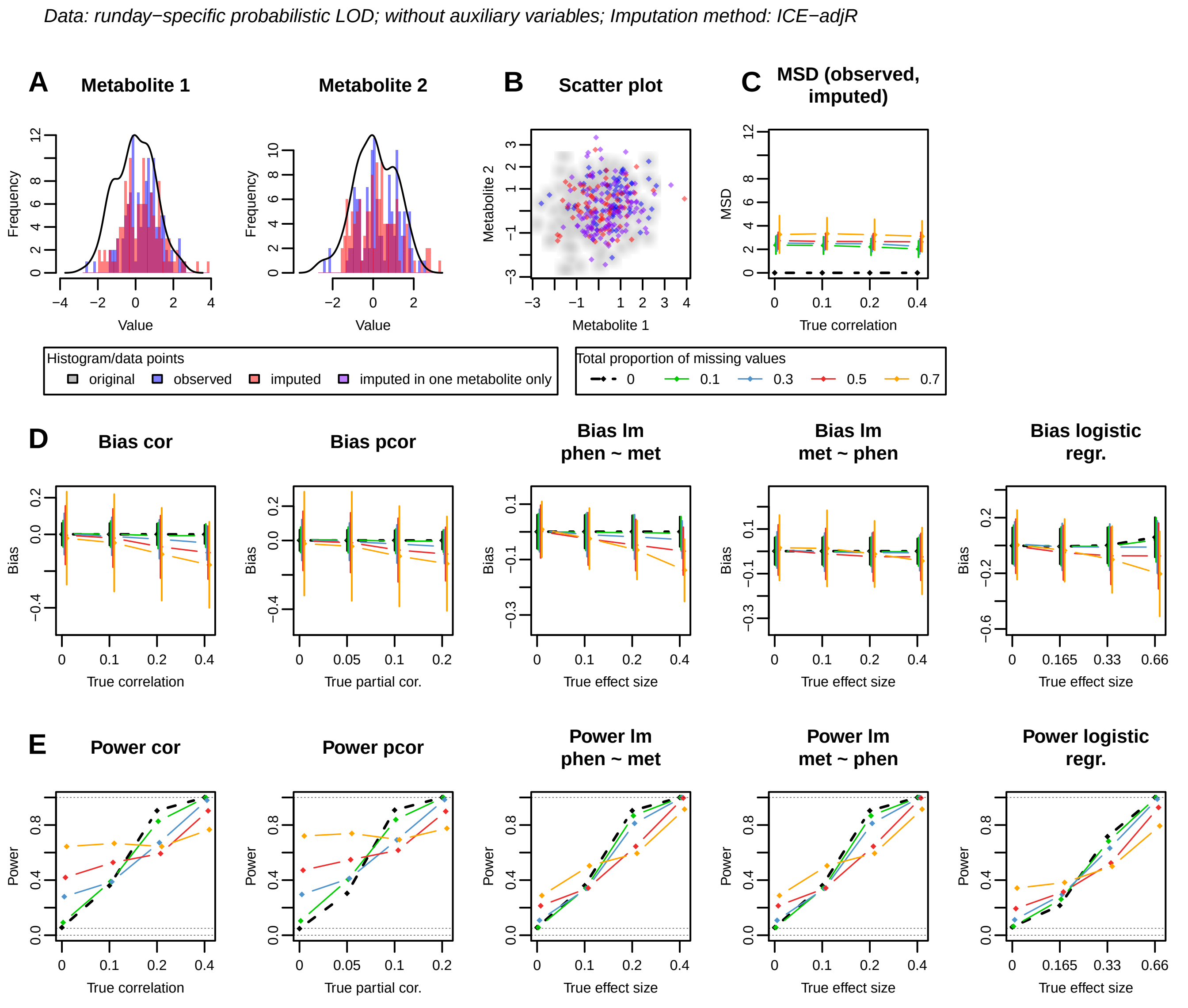
Data: runday−specific probabilistic LOD; without auxiliary variables; Imputation method: ICE−adjR
A
Metabolite 1
Metabolite 2
B
Scatter plot
C
MSD (observed, imputed)
Frequency
Value
Metabolite 2
Metabolite 1
MSD
True correlation
Histogram/data points
original
observed
imputed
imputed in one metabolite only
Total proportion of missing values
0
0.1
0.3
0.5
0.7
D
Bias cor
Bias pcor
Bias lm phen ~ met
Bias lm met ~ phen
Bias logistic regr.
Bias
True correlation
True partial cor.
True effect size
E
Power cor
Power pcor
Power lm phen ~ met
Power lm met ~ phen
Power logistic regr.
Power

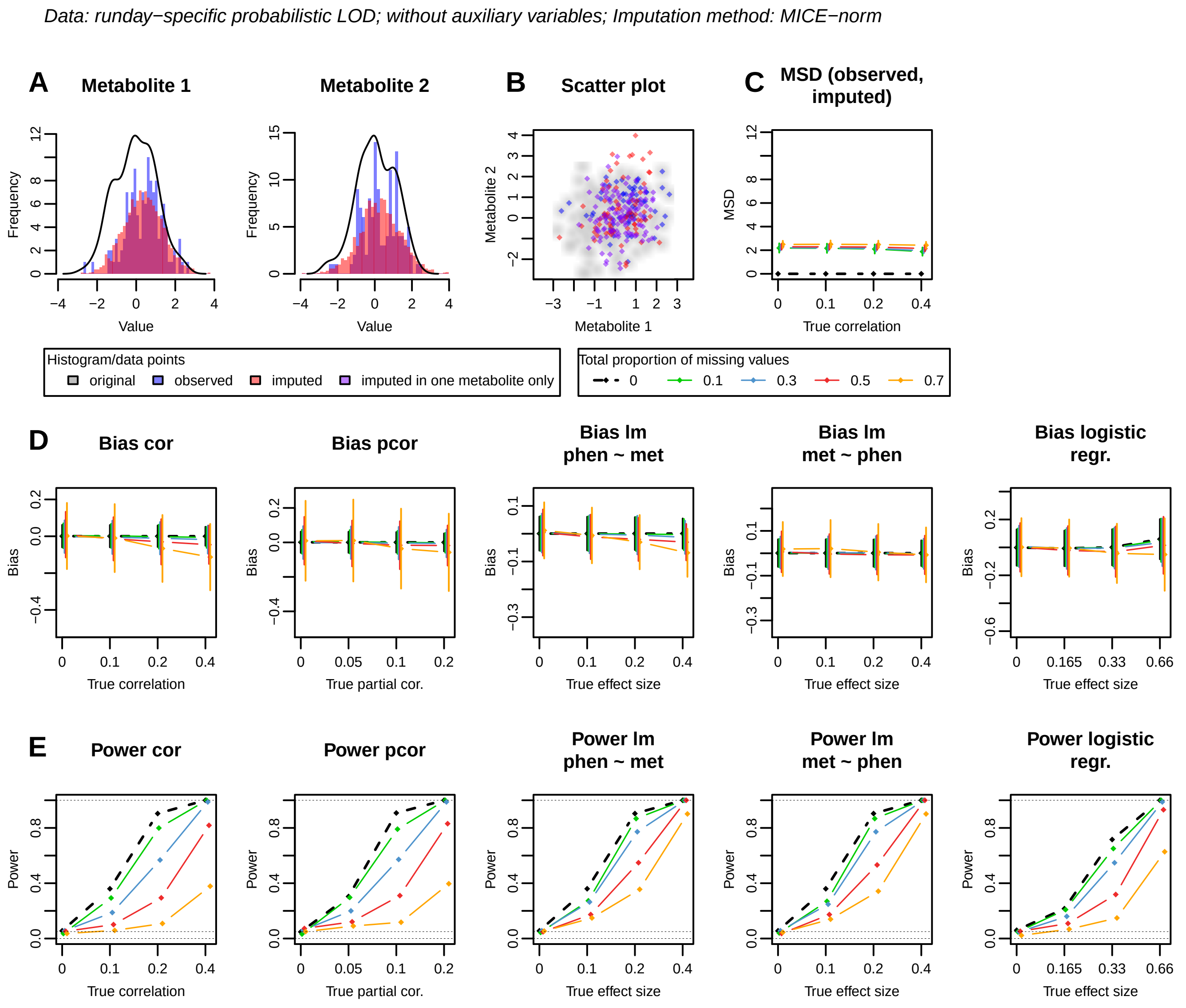

Data: runday−specific probabilistic LOD; without auxiliary variables; Imputation method: MICE−norm
A
Metabolite 1
Metabolite 2
B
Scatter plot
C
MSD (observed, imputed)
Frequency
Value
Metabolite 2
Metabolite 1
MSD
True correlation
Histogram/data points
original
observed
imputed
imputed in one metabolite only
Total proportion of missing values
0
0.1
0.3
0.5
0.7
D
Bias cor
Bias pcor
Bias lm phen ~ met
Bias lm met ~ phen
Bias logistic regr.
Bias
True correlation
True partial cor.
True effect size
E
Power cor
Power pcor
Power lm phen ~ met
Power lm met ~ phen
Power logistic regr.
Power
True correlation
True partial cor.
True effect size

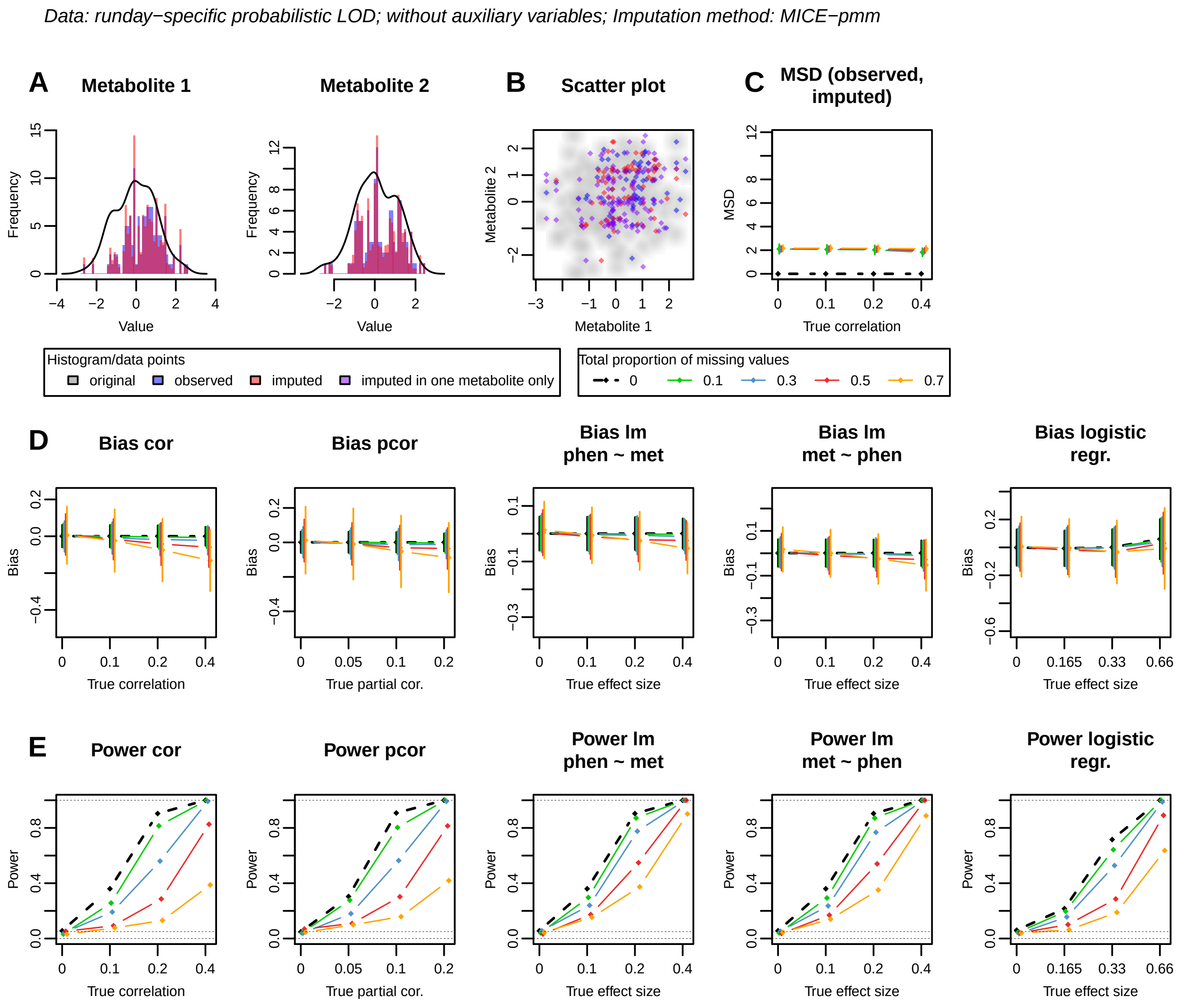
Data: runday−specific probabilistic LOD; without auxiliary variables; Imputation method: MICE−pmm
A
Metabolite 1
Metabolite 2
B
Scatter plot
C
MSD (observed, imputed)
Frequency
Value
Metabolite 2
Metabolite 1
MSD
True correlation
Histogram/data points
original
observed
imputed
imputed in one metabolite only
Total proportion of missing values
0
0.1
0.3
0.5
0.7
D
Bias cor
Bias pcor
Bias lm phen ~ met
Bias lm met ~ phen
Bias logistic regr.
Bias
True correlation
True partial cor.
True effect size
E
Power cor
Power pcor
Power lm phen ~ met
Power lm met ~ phen
Power logistic regr.
Power

Data: runday−specific probabilistic LOD; without auxiliary variables; Imputation method: MICE−adjR

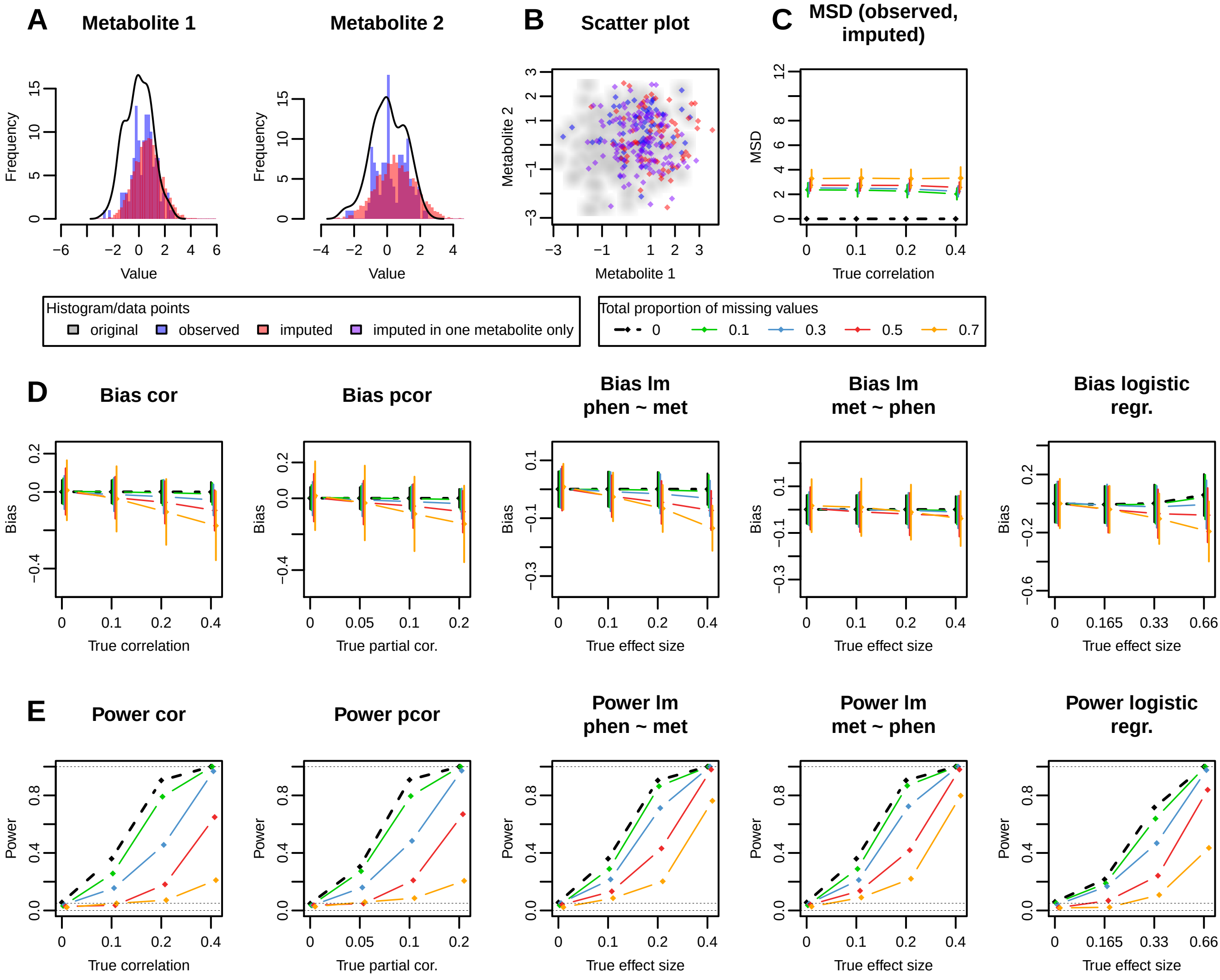

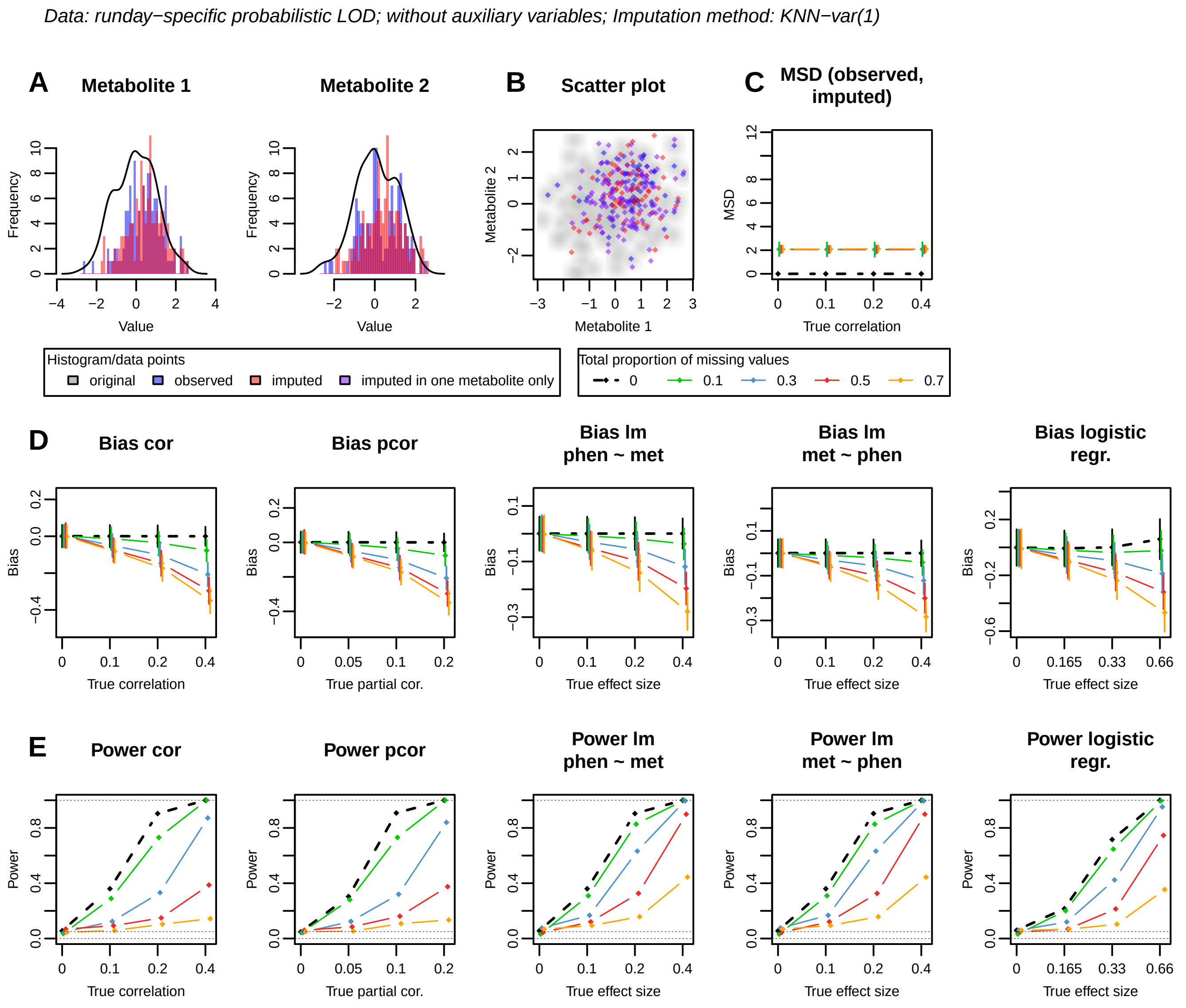
Data: runday−specific probabilistic LOD; without auxiliary variables; Imputation method: KNN−var(1)
A
Metabolite 1
Metabolite 2
B
Scatter plot
C
MSD (observed, imputed)
Frequency
Value
Metabolite 2
Metabolite 1
MSD
True correlation
Histogram/data points
original
observed
imputed
imputed in one metabolite only
Total proportion of missing values
0
0.1
0.3
0.5
0.7
D
Bias cor
Bias pcor
Bias lm phen ~ met
Bias lm met ~ phen
Bias logistic regr.
Bias
True correlation
True partial cor.
True effect size
E
Power cor
Power pcor
Power lm phen ~ met
Power lm met ~ phen
Power logistic regr.
Power

Data: runday−specific probabilistic LOD; without auxiliary variables; Imputation method: KNN−var(3)

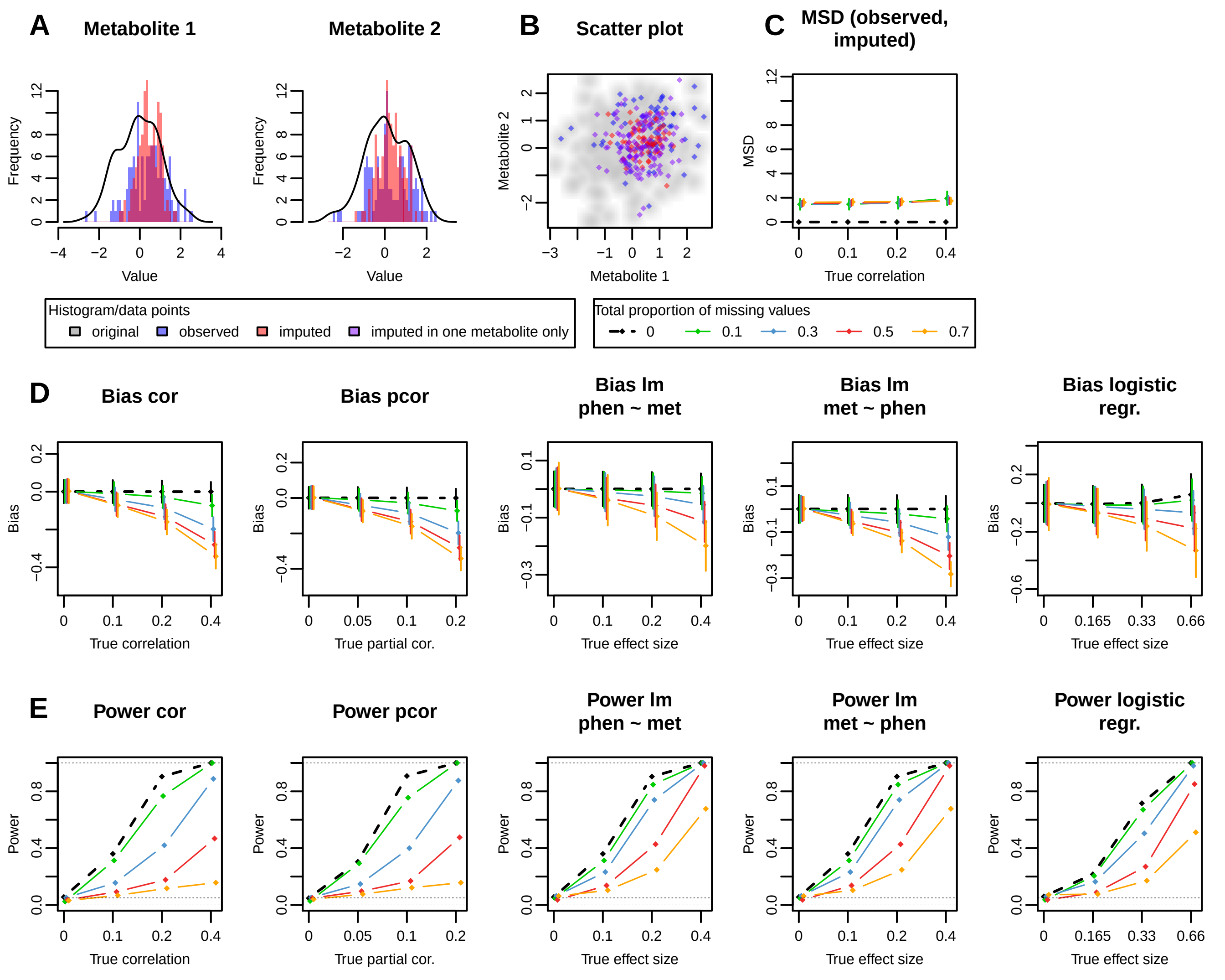

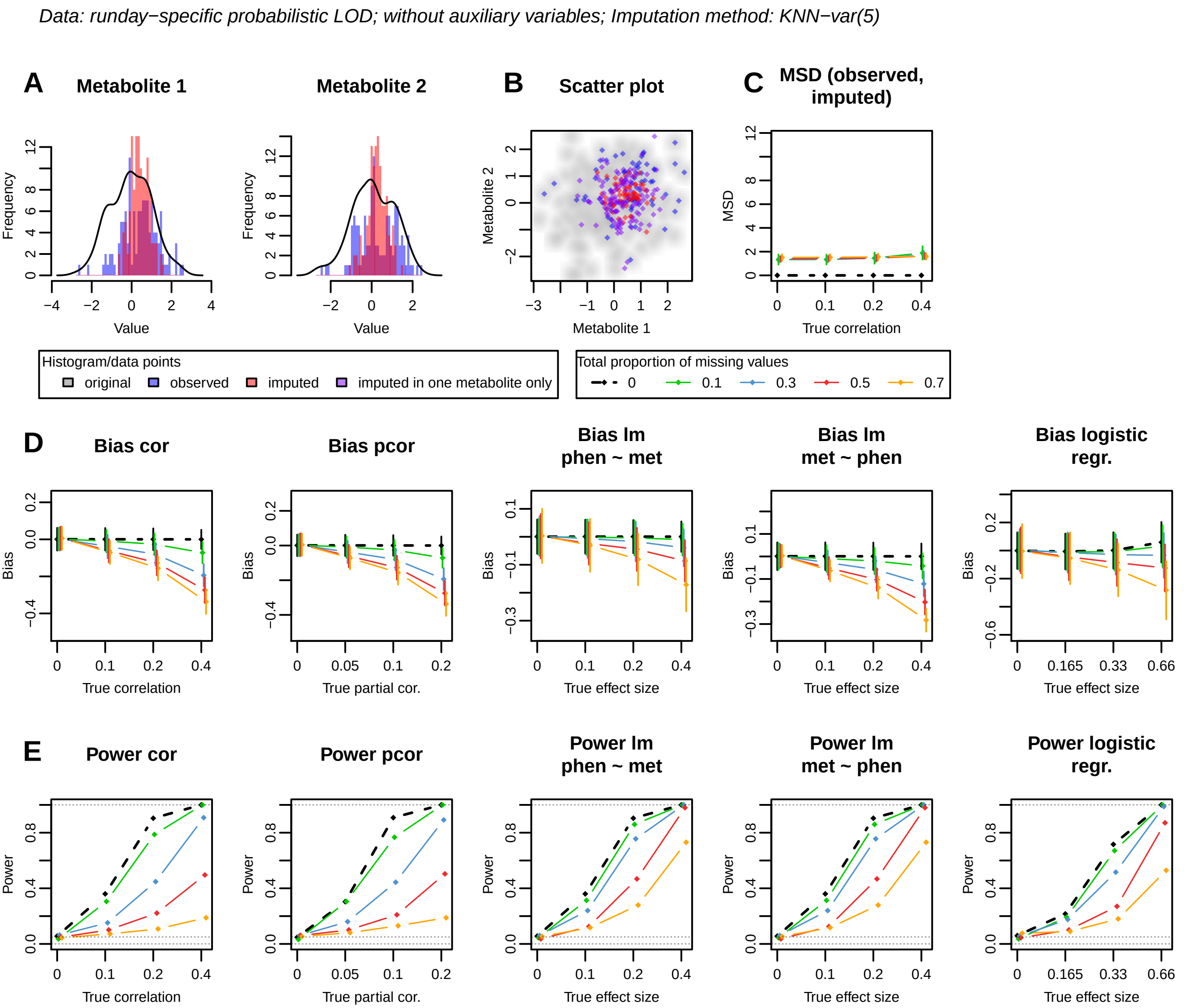

Data: runday−specific probabilistic LOD; without auxiliary variables; Imputation method: KNN−var(5)
A
Metabolite 1
Metabolite 2
B
Scatter plot
C
MSD (observed, imputed)
Frequency
Value
Metabolite 2
Metabolite 1
MSD
True correlation
Histogram/data points
original
observed
imputed
imputed in one metabolite only
Total proportion of missing values
0
0.1
0.3
0.5
0.7
D
Bias cor
Bias pcor
Bias lm phen ~ met
Bias lm met ~ phen
Bias logistic regr.
Bias
True correlation
True partial cor.
True effect size
E
Power cor
Power pcor
Power lm phen ~ met
Power lm met ~ phen
Power logistic regr.
Power
True correlation
True partial cor.
True effect size

Data: runday−specific probabilistic LOD; without auxiliary variables; Imputation method: KNN−var(10)

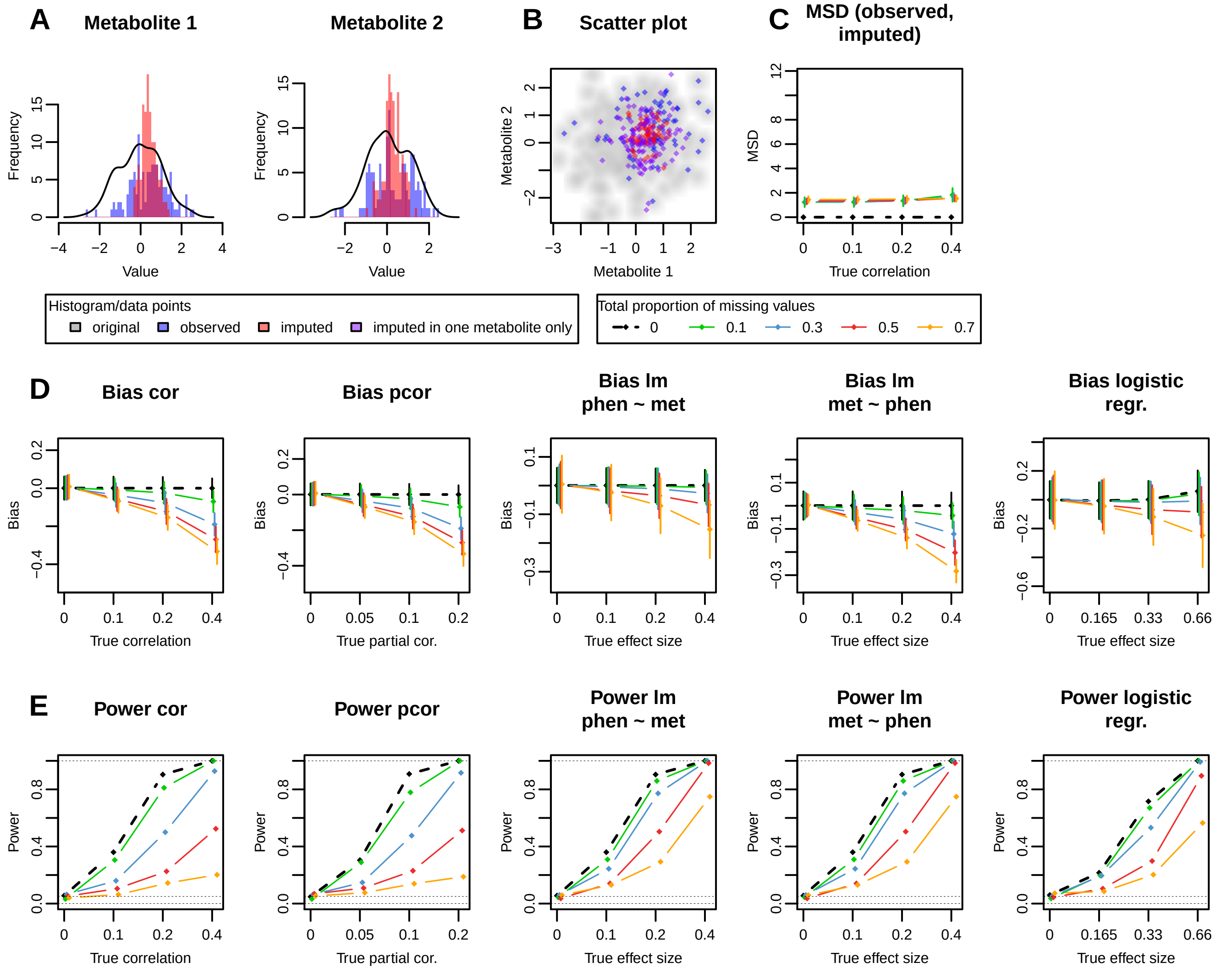

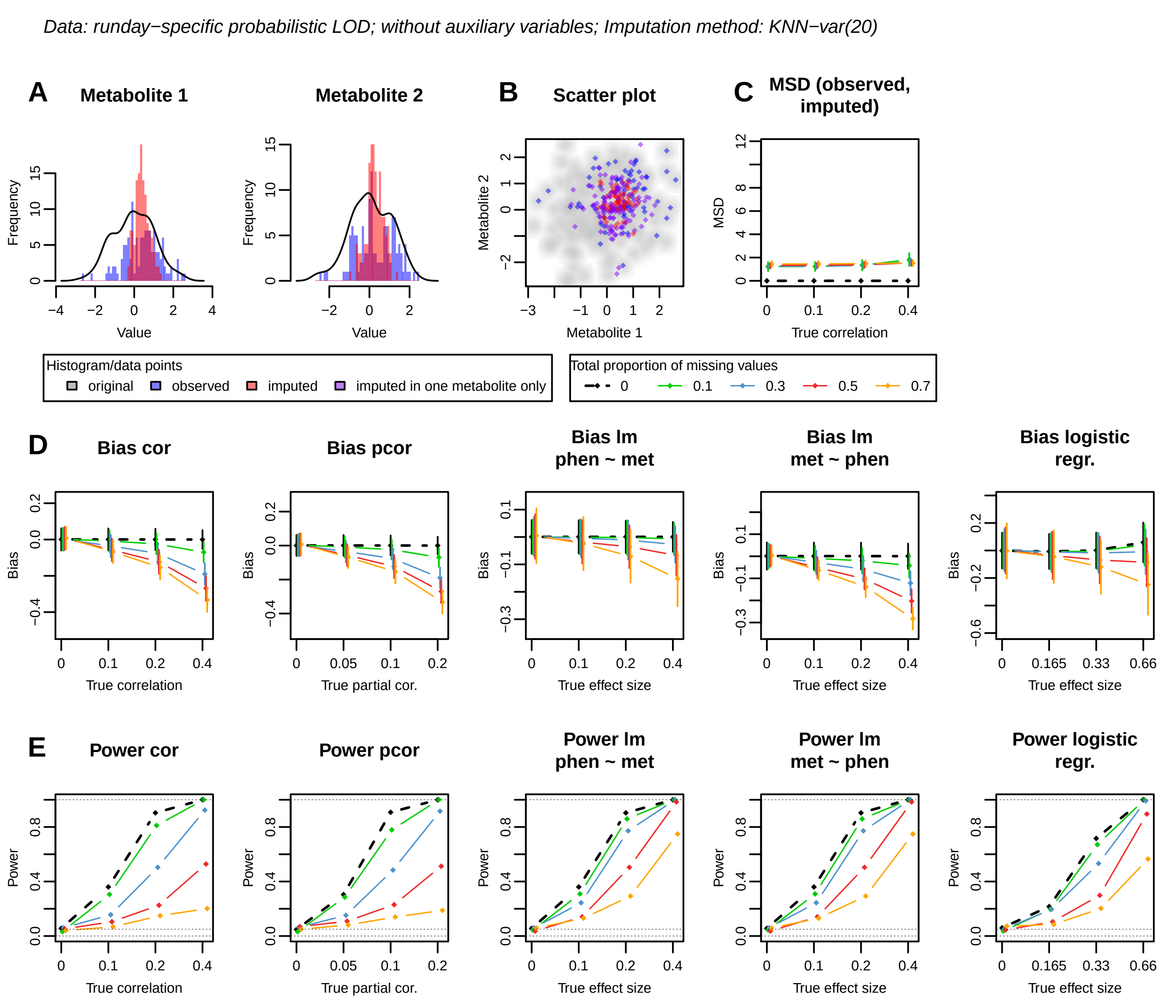

Data: runday−specific probabilistic LOD; without auxiliary variables; Imputation method: KNN−var(20)
A
Metabolite 1
Metabolite 2
B
Scatter plot
C
MSD (observed, imputed)
Frequency
Value
Metabolite 2
Metabolite 1
MSD
True correlation
Histogram/data points
original
observed
imputed
imputed in one metabolite only
Total proportion of missing values
0
0.1
0.3
0.5
0.7
D
Bias cor
Bias pcor
Bias lm phen ~ met
Bias lm met ~ phen
Bias logistic regr.
Bias
True correlation
True partial cor.
True effect size
E
Power cor
Power pcor
Power lm phen ~ met
Power lm met ~ phen
Power logistic regr.
Power
True correlation
True partial cor.
True effect size

Data: runday−specific probabilistic LOD; without auxiliary variables; Imputation method: KNN−obs(1)

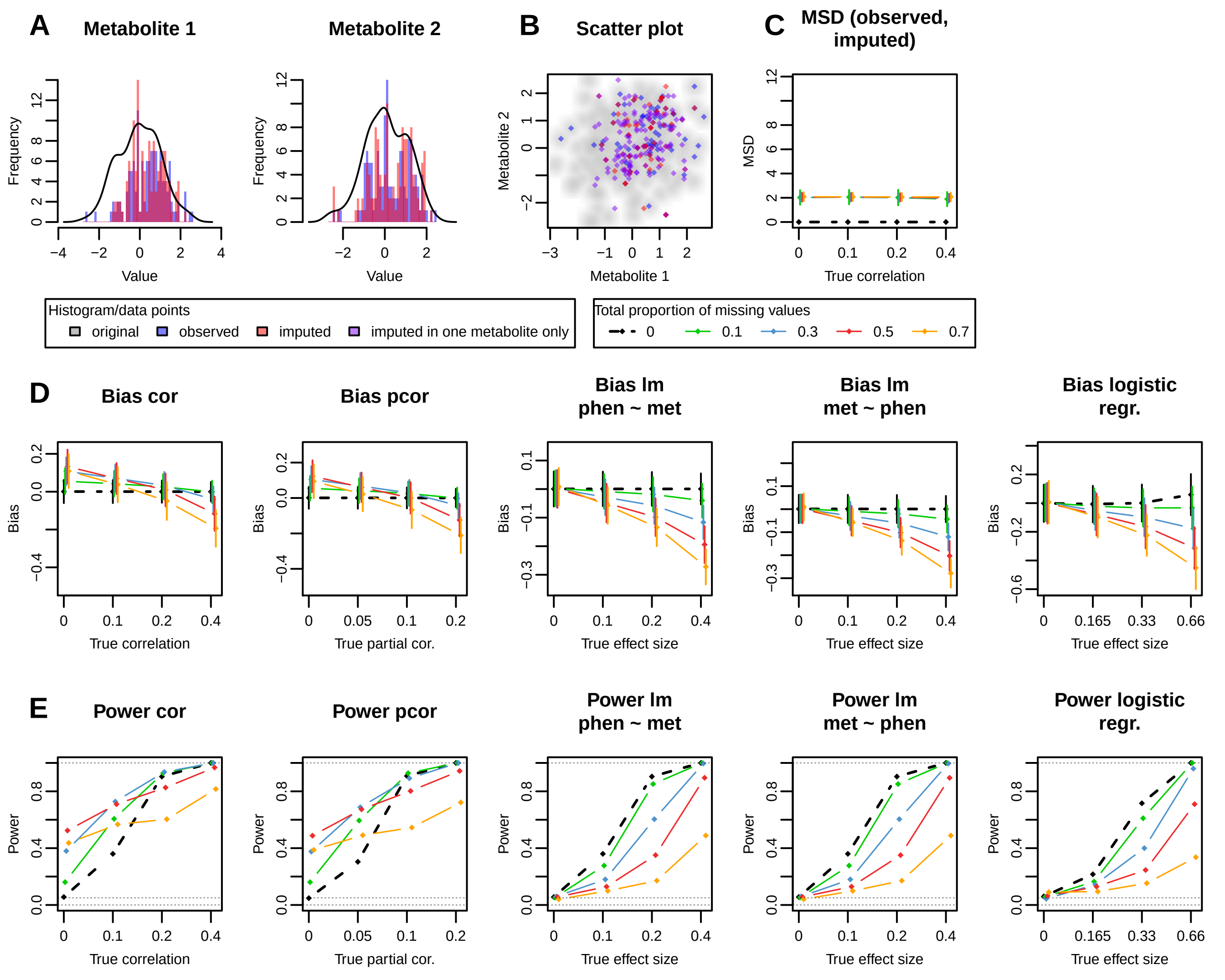

Data: runday−specific probabilistic LOD; without auxiliary variables; Imputation method: KNN−obs(3)

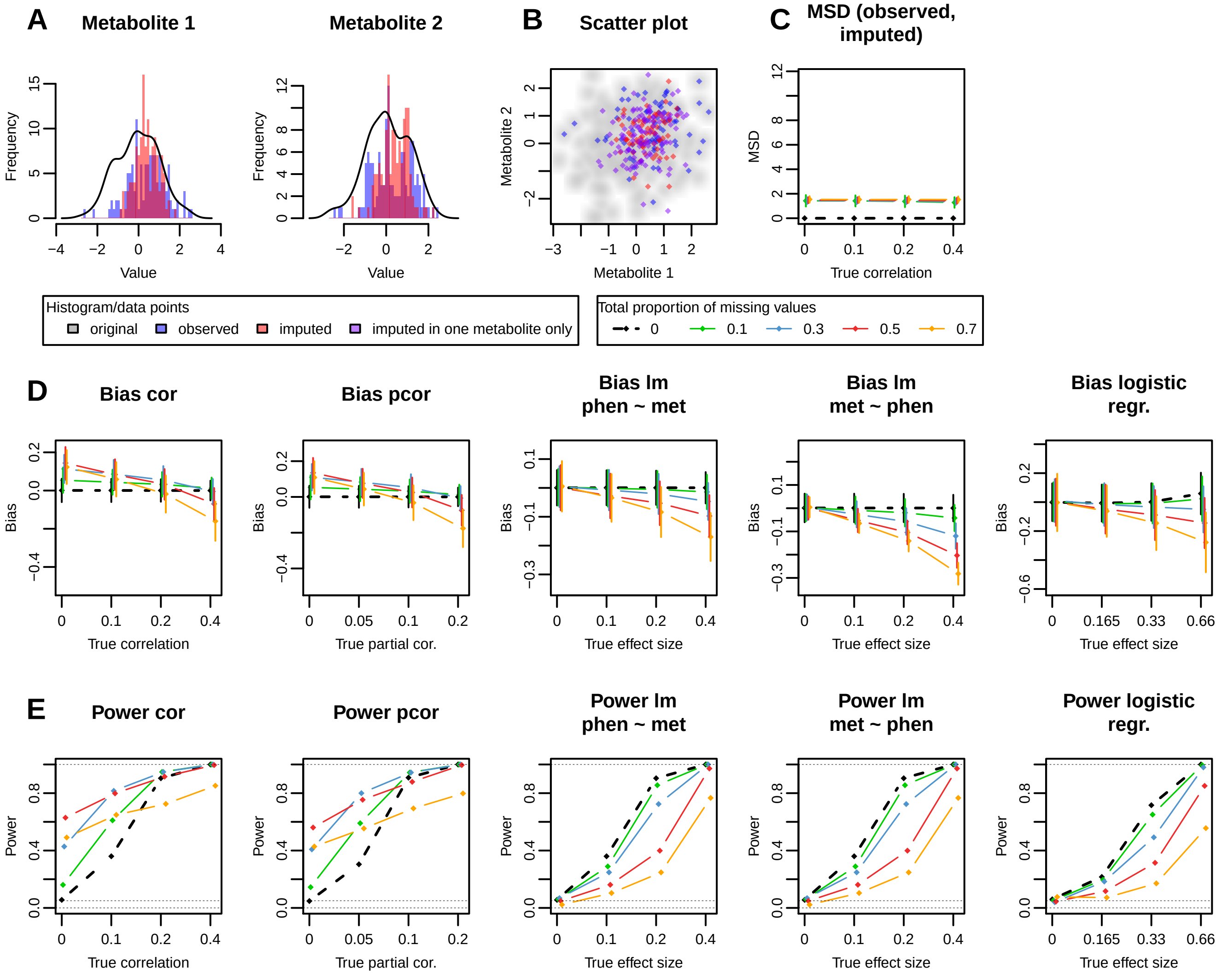

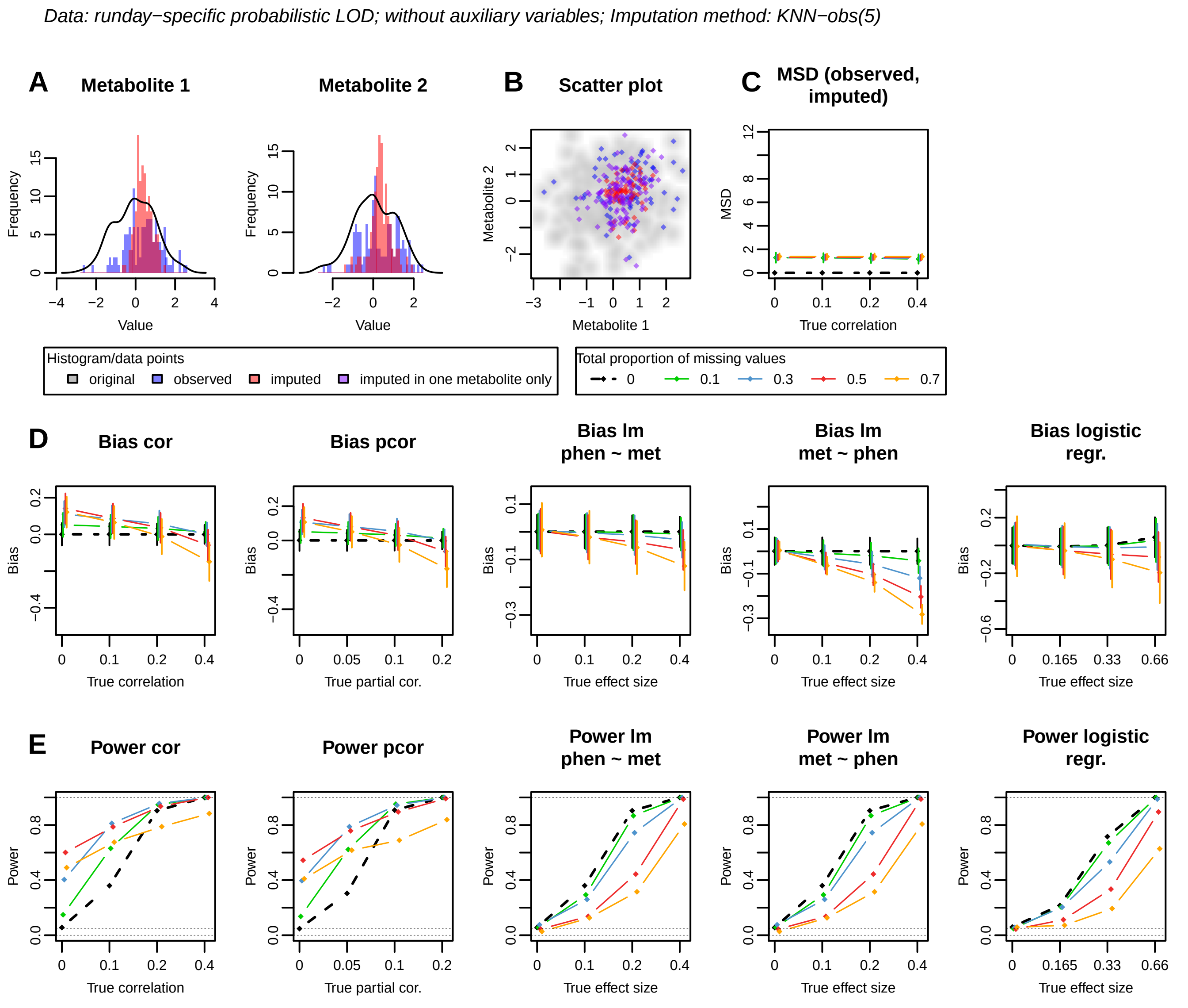
Data: runday−specific probabilistic LOD; without auxiliary variables; Imputation method: KNN−obs(5)
A
Metabolite 1
Metabolite 2
Frequency
Value
B
Scatter plot
Metabolite 2
Metabolite 1
C
MSD (observed, imputed)
MSD
True correlation
Histogram/data points
original
observed
imputed
imputed in one metabolite only
Total proportion of missing values
0
0.1
0.3
0.5
0.7
D
Bias cor
Bias pcor
Bias lm phen ~ met
Bias lm met ~ phen
Bias logistic regr.
Bias
True correlation
True partial cor.
True effect size
E
Power cor
Power pcor
Power lm phen ~ met
Power lm met ~ phen
Power logistic regr.
Power
True correlation
True partial cor.
True effect size

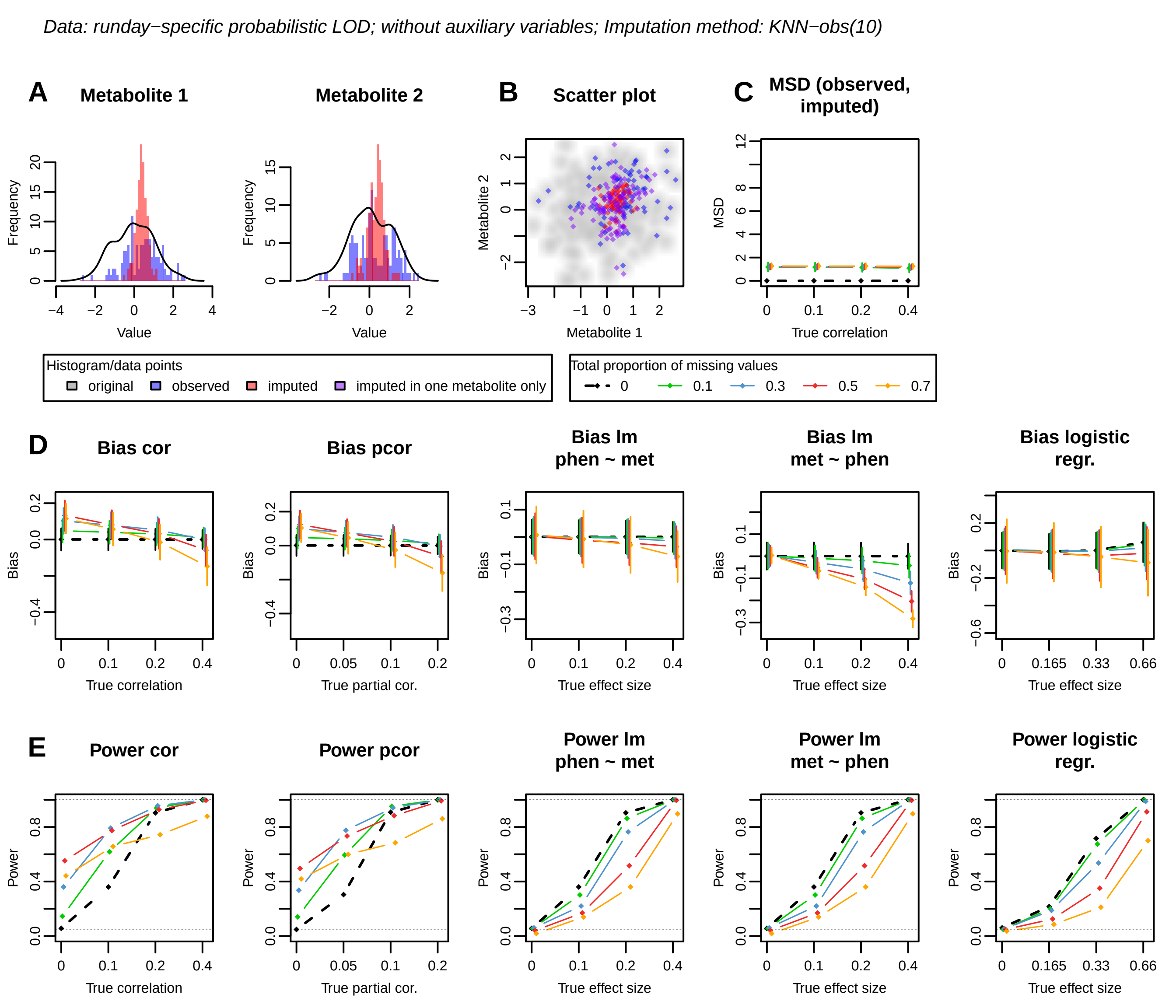
Data: runday−specific probabilistic LOD; without auxiliary variables; Imputation method: KNN−obs(10)
A
Metabolite 1
Metabolite 2
B
Scatter plot
C
MSD (observed, imputed)
Frequency
Value
Metabolite 1
Metabolite 2
MSD
True correlation
Histogram/data points
original
observed
imputed
imputed in one metabolite only
Total proportion of missing values
0
0.1
0.3
0.5
0.7
D
Bias cor
Bias pcor
Bias lm phen ~ met
Bias lm met ~ phen
Bias logistic regr.
Bias
True correlation
True partial cor.
True effect size
E
Power cor
Power pcor
Power lm phen ~ met
Power lm met ~ phen
Power logistic regr.
Power

Data: runday−specific probabilistic LOD; without auxiliary variables; Imputation method: KNN−obs(20)

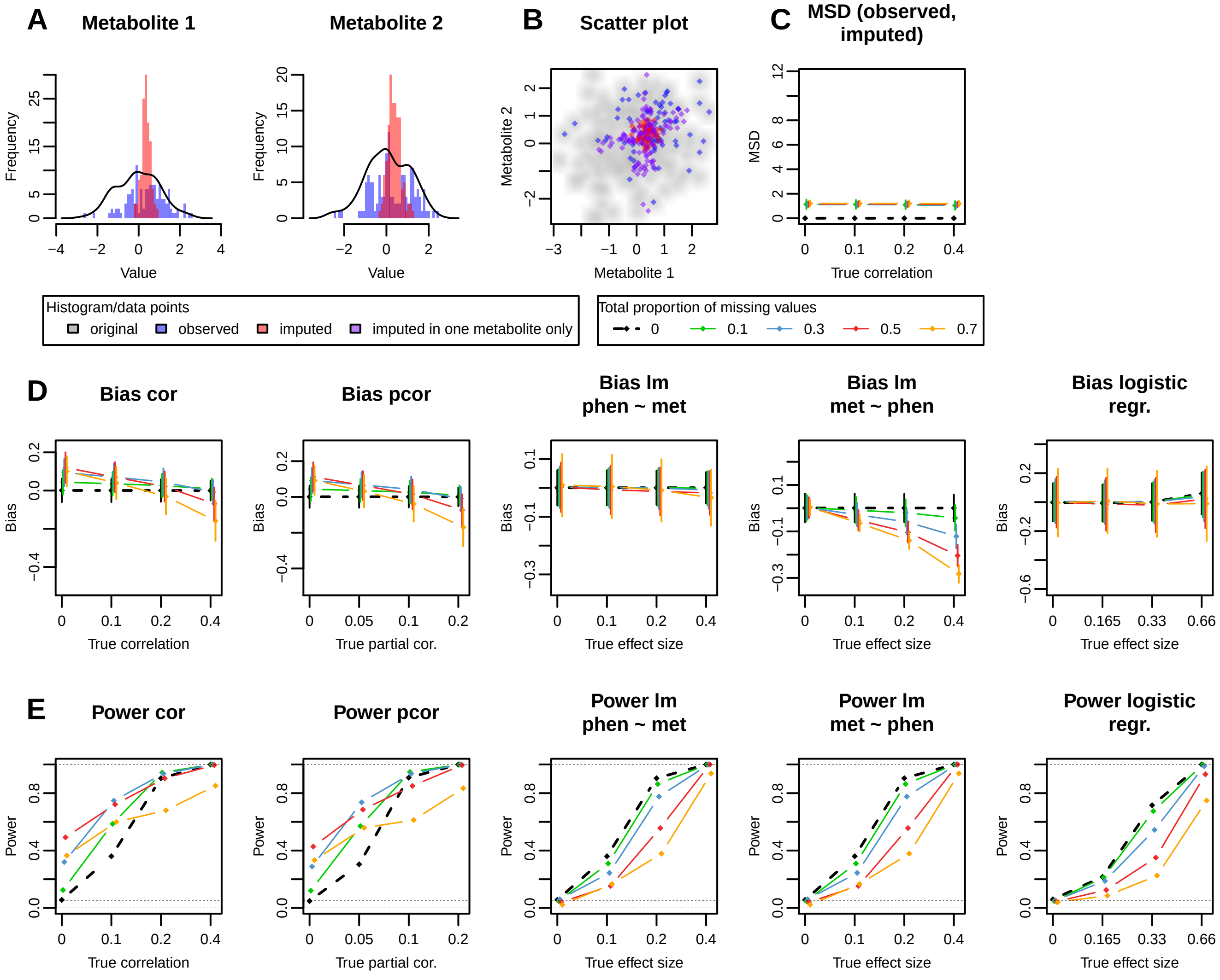

Data: runday−specific probabilistic LOD; without auxiliary variables; Imputation method: KNN−obs−sel(1)

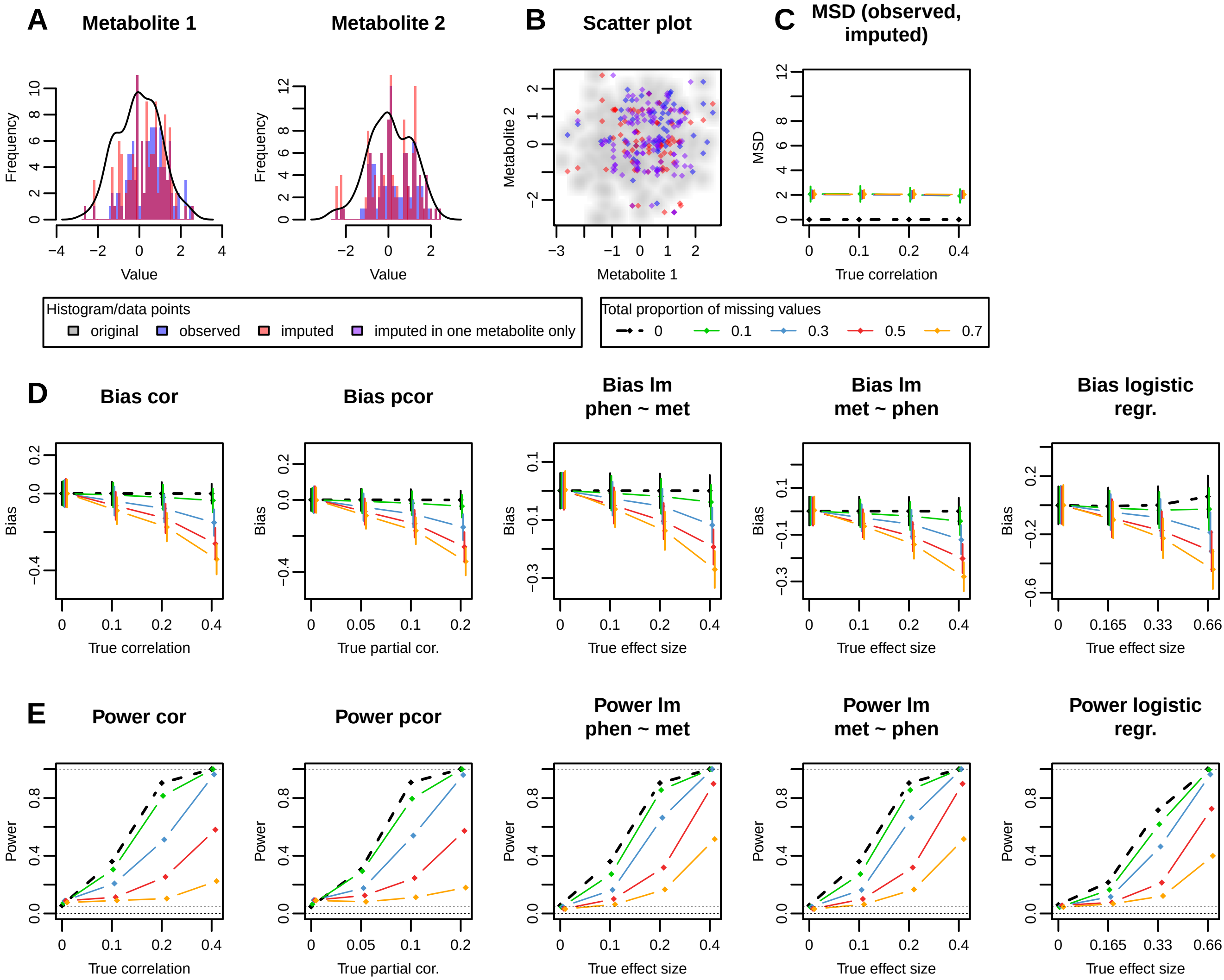

Data: runday−specific probabilistic LOD; without auxiliary variables; Imputation method: KNN−obs−sel(3)

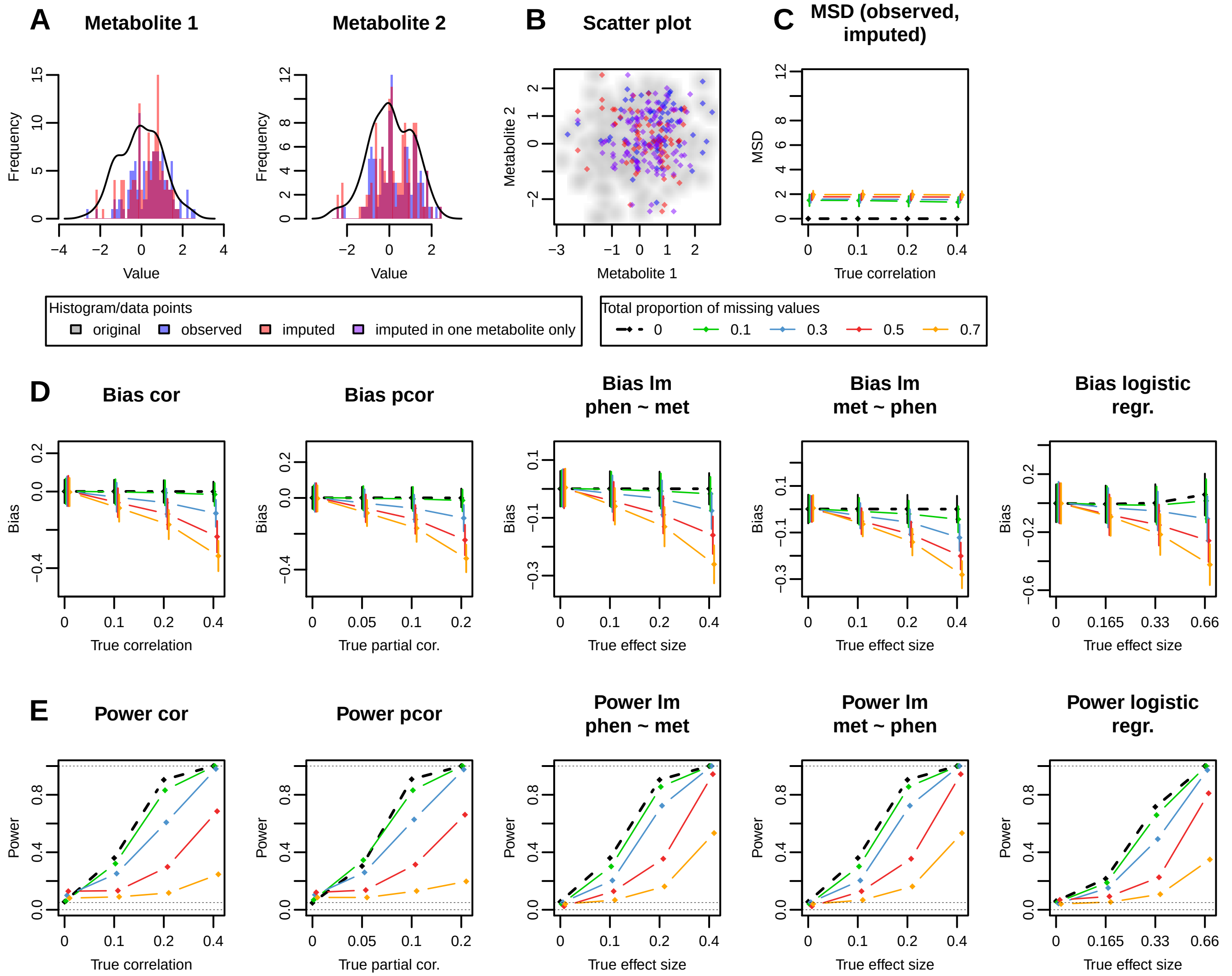

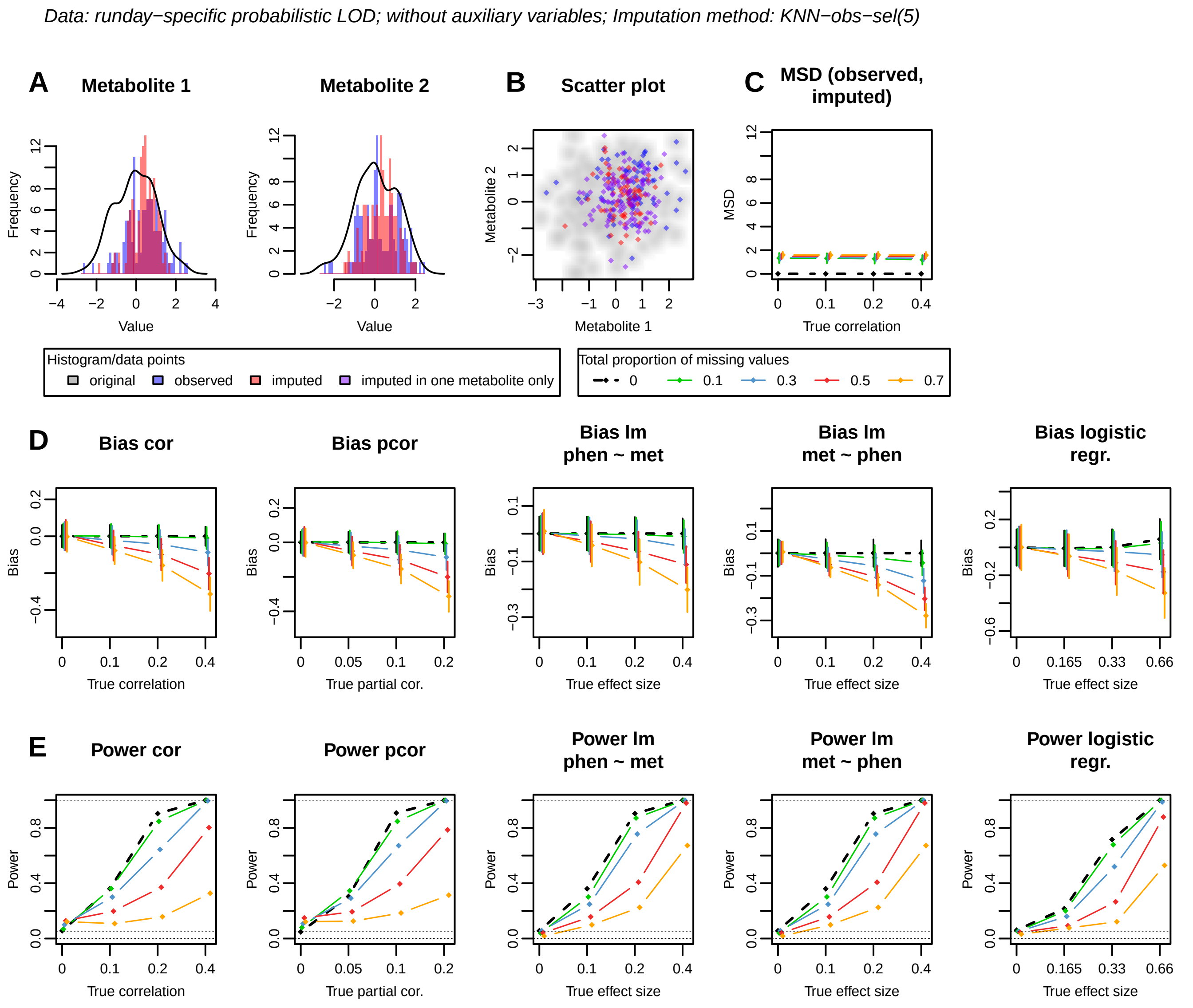
Data: runday−specific probabilistic LOD; without auxiliary variables; Imputation method: KNN−obs−sel(5)
A
Metabolite 1
Metabolite 2
B
Scatter plot
C
MSD (observed, imputed)
Frequency
Value
Metabolite 2
Metabolite 1
MSD
True correlation
Histogram/data points
original
observed
imputed
imputed in one metabolite only
Total proportion of missing values
0
0.1
0.3
0.5
0.7
D
Bias cor
Bias pcor
Bias lm phen ~ met
Bias lm met ~ phen
Bias logistic regr.
Bias
True correlation
True partial cor.
True effect size
E
Power cor
Power pcor
Power lm phen ~ met
Power lm met ~ phen
Power logistic regr.
Power
True correlation
True partial cor.
True effect size

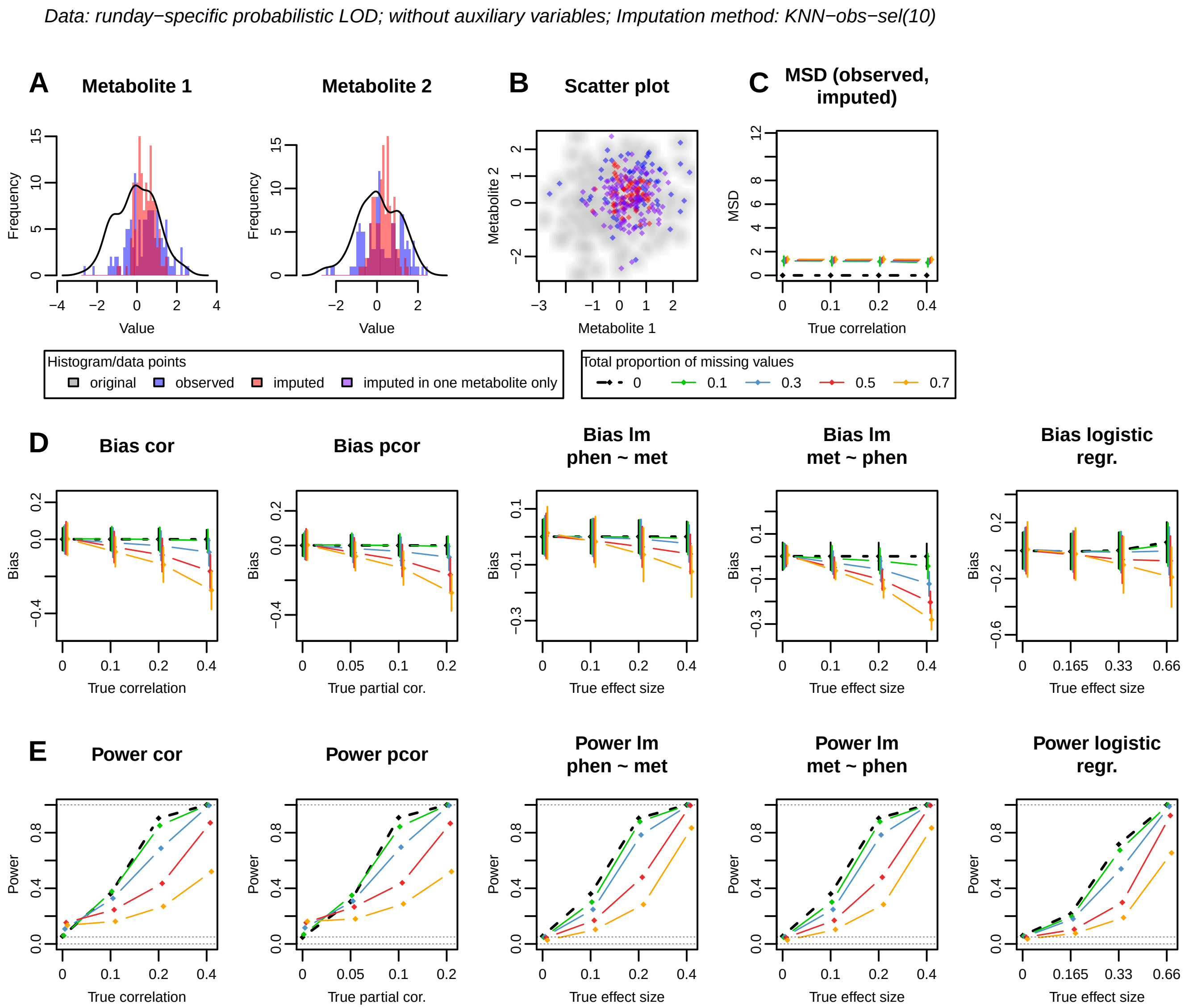

Data: runday−specific probabilistic LOD; without auxiliary variables; Imputation method: KNN−obs−sel(10)
A
Metabolite 1
Metabolite 2
B
Scatter plot
C
MSD (observed, imputed)
Frequency
Value
Metabolite 2
Metabolite 1
MSD
True correlation
Histogram/data points
original
observed
imputed
imputed in one metabolite only
Total proportion of missing values
0
0.1
0.3
0.5
0.7
D
Bias cor
Bias pcor
Bias lm phen ~ met
Bias lm met ~ phen
Bias logistic regr.
Bias
True correlation
True partial cor.
True effect size
E
Power cor
Power pcor
Power lm phen ~ met
Power lm met ~ phen
Power logistic regr.
Power

Data: runday−specific probabilistic LOD; without auxiliary variables; Imputation method: KNN−obs−sel(20)

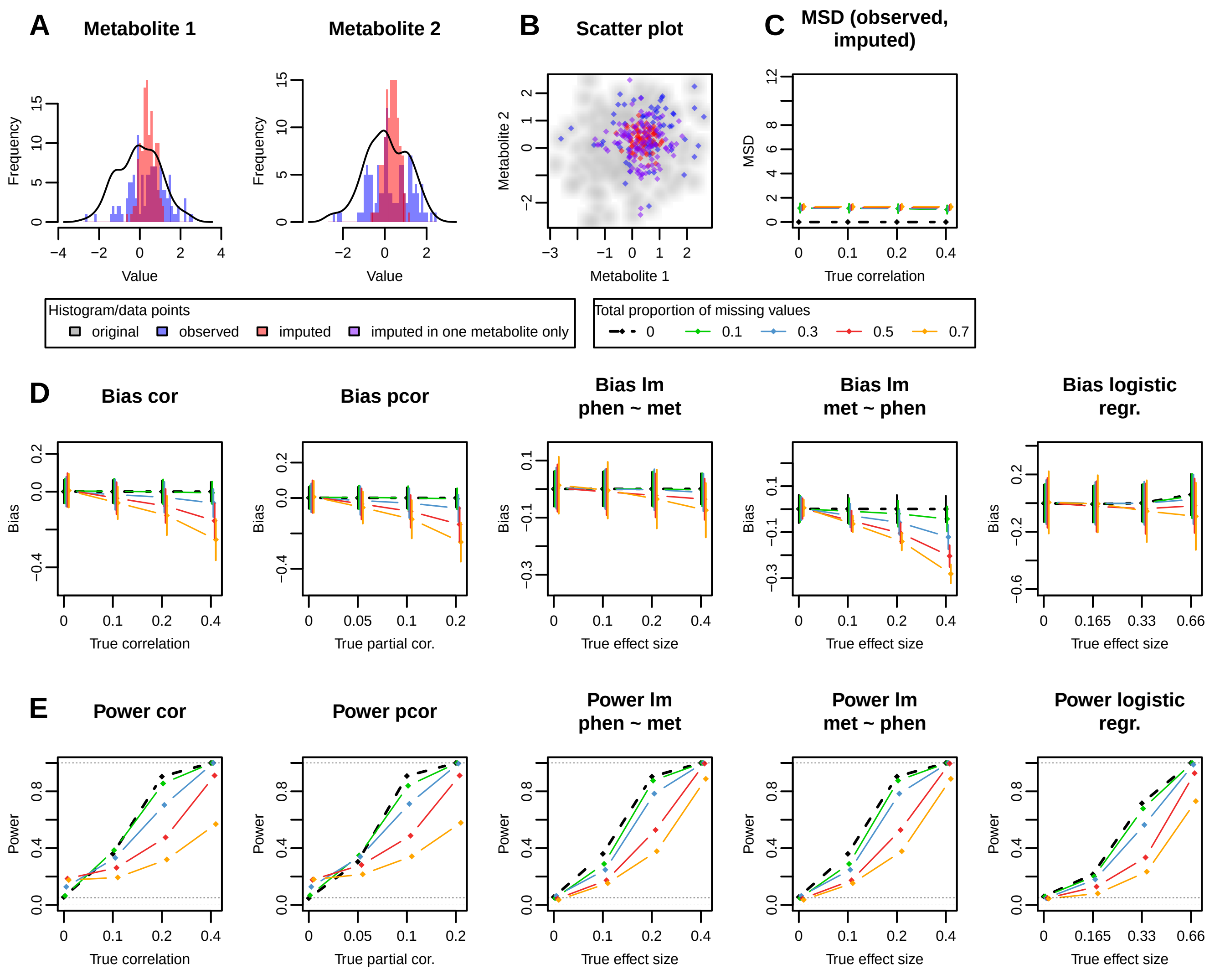

Data: runday−specific probabilistic LOD; correlated runday missingness; Imputation method: CCA

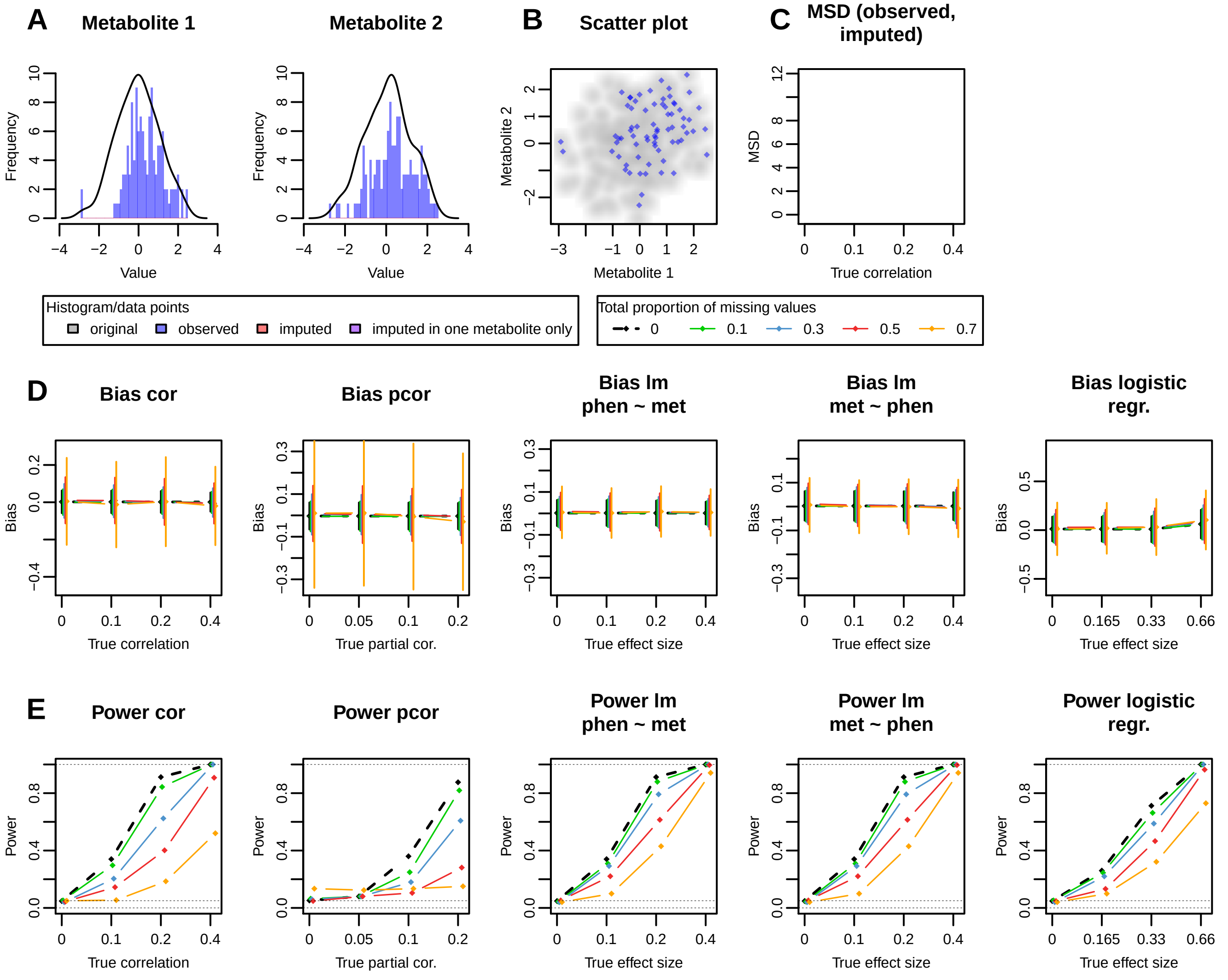

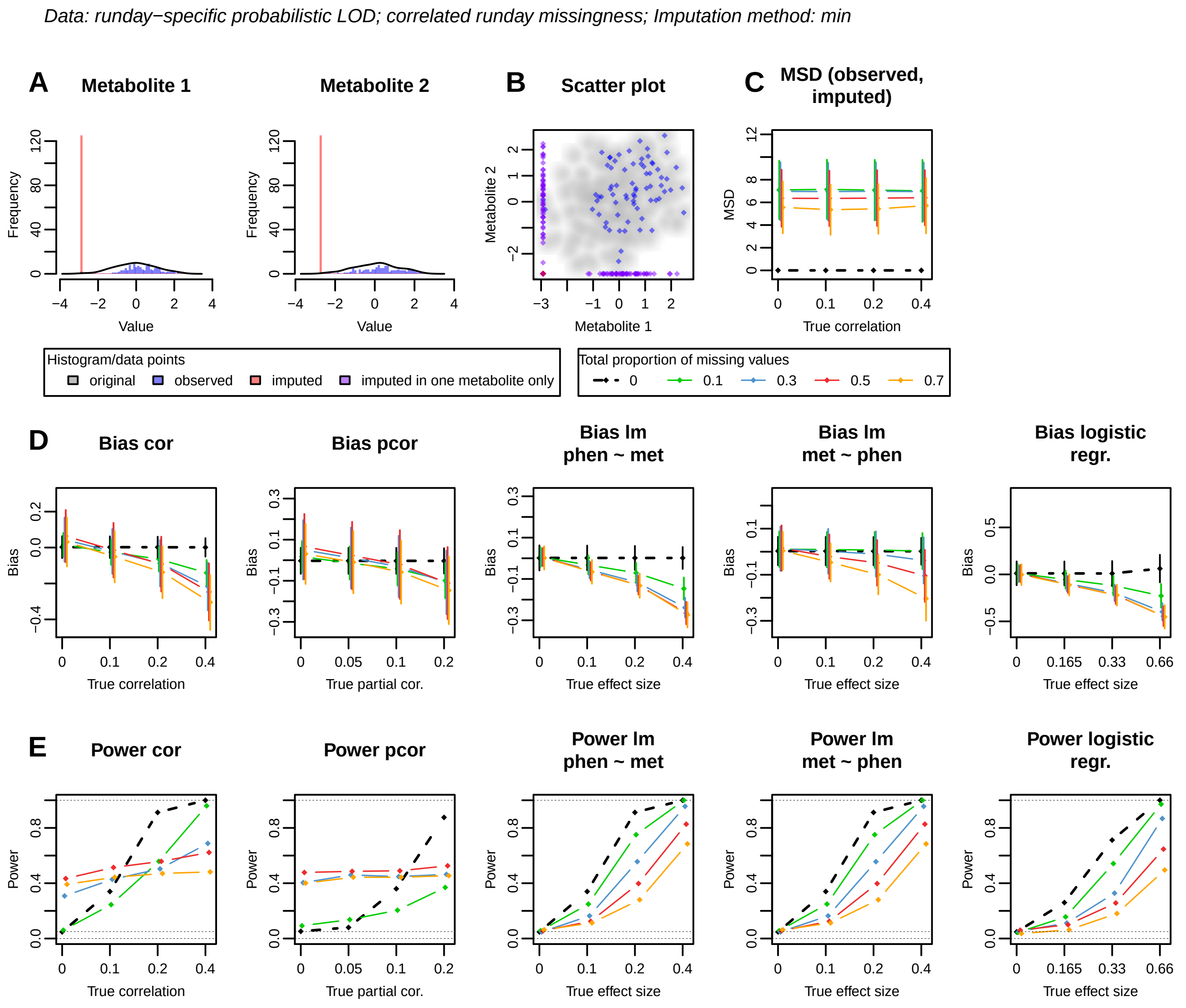

Data: runday−specific probabilistic LOD; correlated runday missingness; Imputation method: min
A
Metabolite 1
Metabolite 2
B
Scatter plot
C
MSD (observed, imputed)
Frequency
Value
Metabolite 2
Metabolite 1
MSD
True correlation
Histogram/data points
original
observed
imputed
imputed in one metabolite only
Total proportion of missing values
0
0.1
0.3
0.5
0.7
D
Bias cor
Bias pcor
Bias lm phen ~ met
Bias lm met ~ phen
Bias logistic regr.
Bias
True correlation
True partial cor.
True effect size
E
Power cor
Power pcor
Power lm phen ~ met
Power lm met ~ phen
Power logistic regr.
Power
True correlation
True partial cor.
True effect size

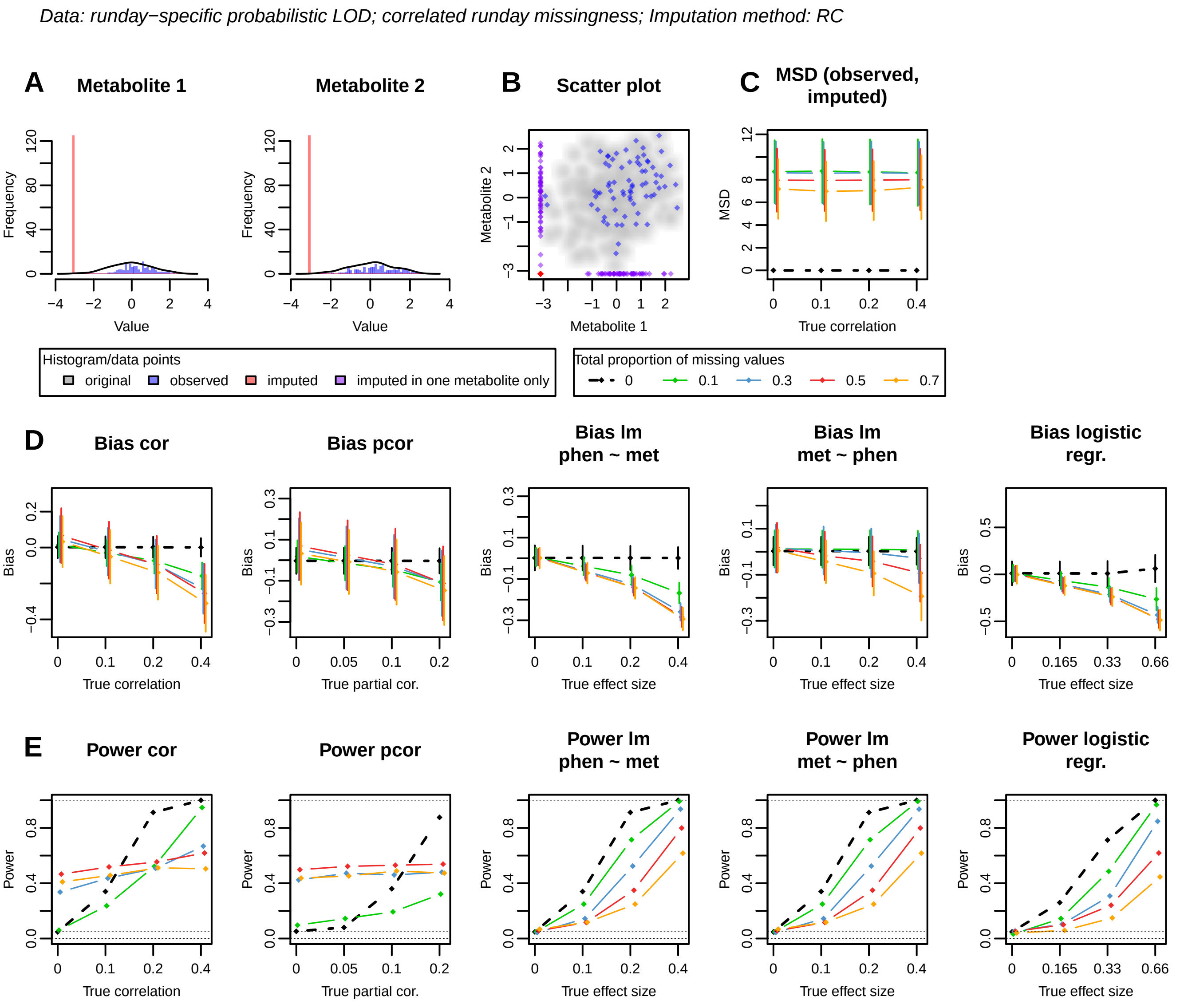
Data: runday−specific probabilistic LOD; correlated runday missingness; Imputation method: RC
A
Metabolite 1
Metabolite 2
Frequency
Value
B
Scatter plot
Metabolite 2
Metabolite 1
C
MSD (observed, imputed)
MSD
True correlation
Histogram/data points
original
observed
imputed
imputed in one metabolite only
Total proportion of missing values
0
0.1
0.3
0.5
0.7
D
Bias cor
Bias pcor
Bias lm phen ~ met
Bias lm met ~ phen
Bias logistic regr.
Bias
True correlation
True partial cor.
True effect size
E
Power cor
Power pcor
Power lm phen ~ met
Power lm met ~ phen
Power logistic regr.
Power
True correlation
True partial cor.
True effect size

Data: runday−specific probabilistic LOD; correlated runday missingness; Imputation method: ITS

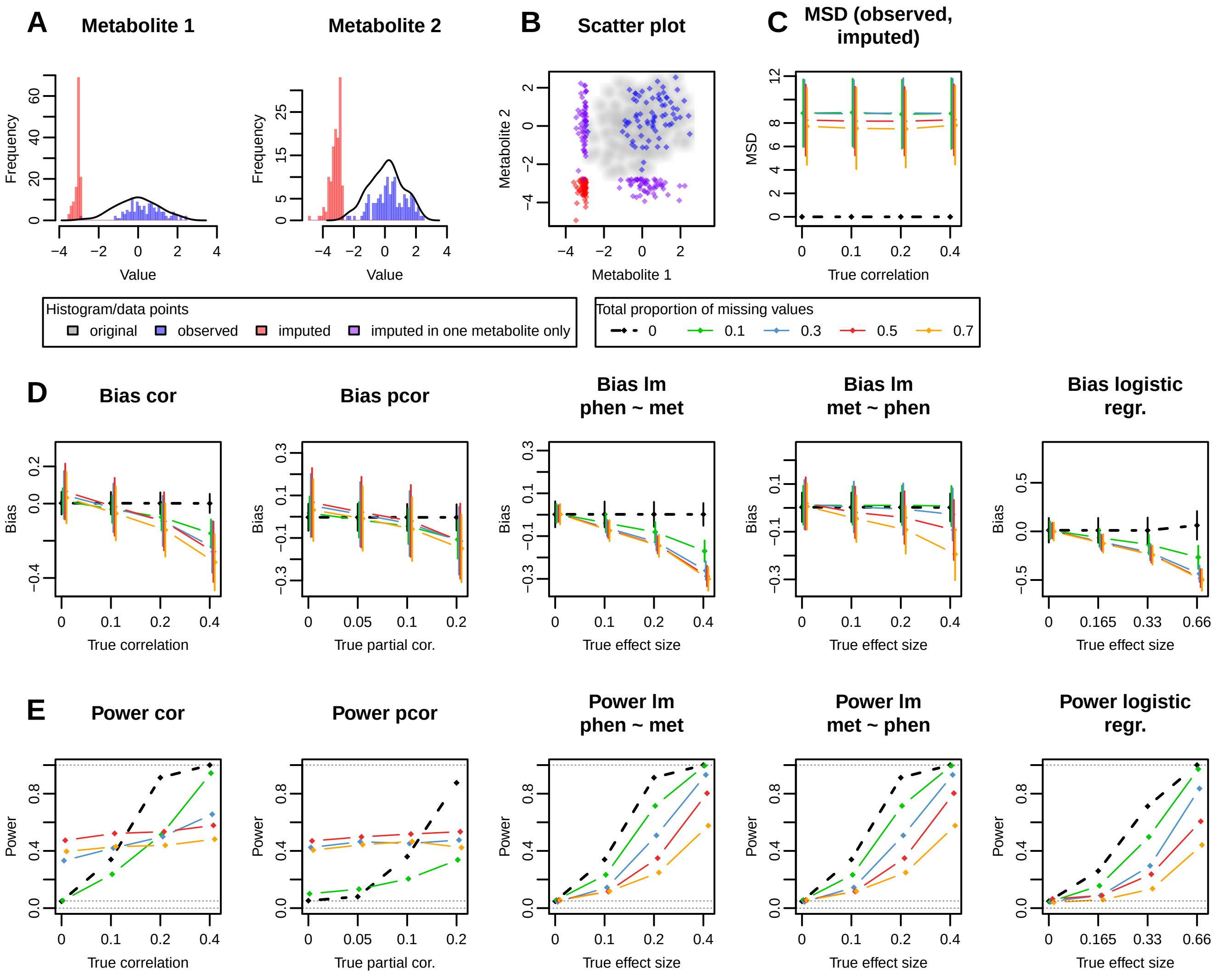

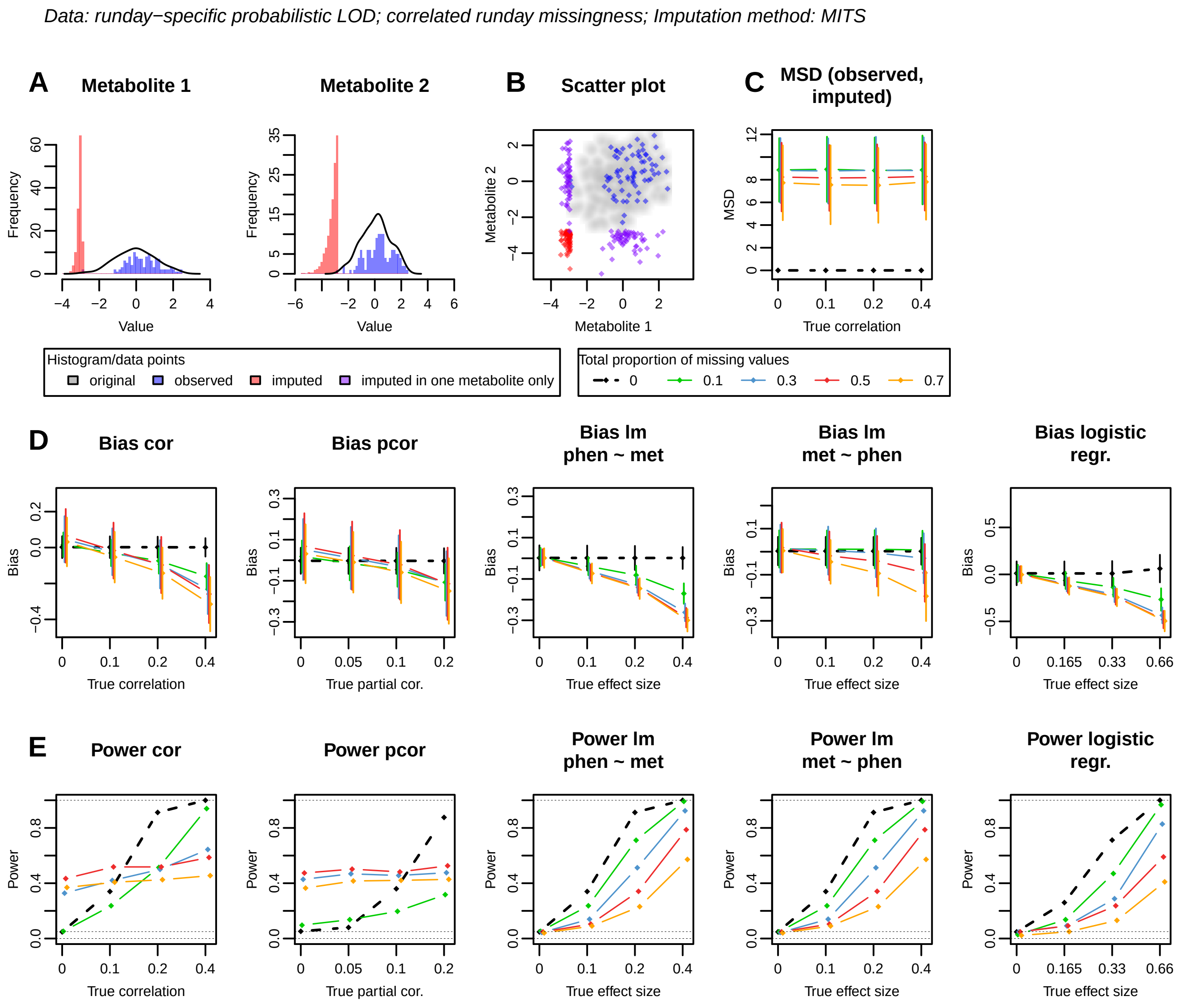
Data: runday−specific probabilistic LOD; correlated runday missingness; Imputation method: MITS
A
Metabolite 1
Metabolite 2
B
Scatter plot
C
MSD (observed, imputed)
Frequency
Value
Metabolite 2
Metabolite 1
MSD
True correlation
Histogram/data points
original
observed
imputed
imputed in one metabolite only
Total proportion of missing values
0
0.1
0.3
0.5
0.7
D
Bias cor
Bias pcor
Bias lm phen ~ met
Bias lm met ~ phen
Bias logistic regr.
Bias
True correlation
True partial cor.
True effect size
E
Power cor
Power pcor
Power lm phen ~ met
Power lm met ~ phen
Power logistic regr.
Power
True correlation
True partial cor.
True effect size

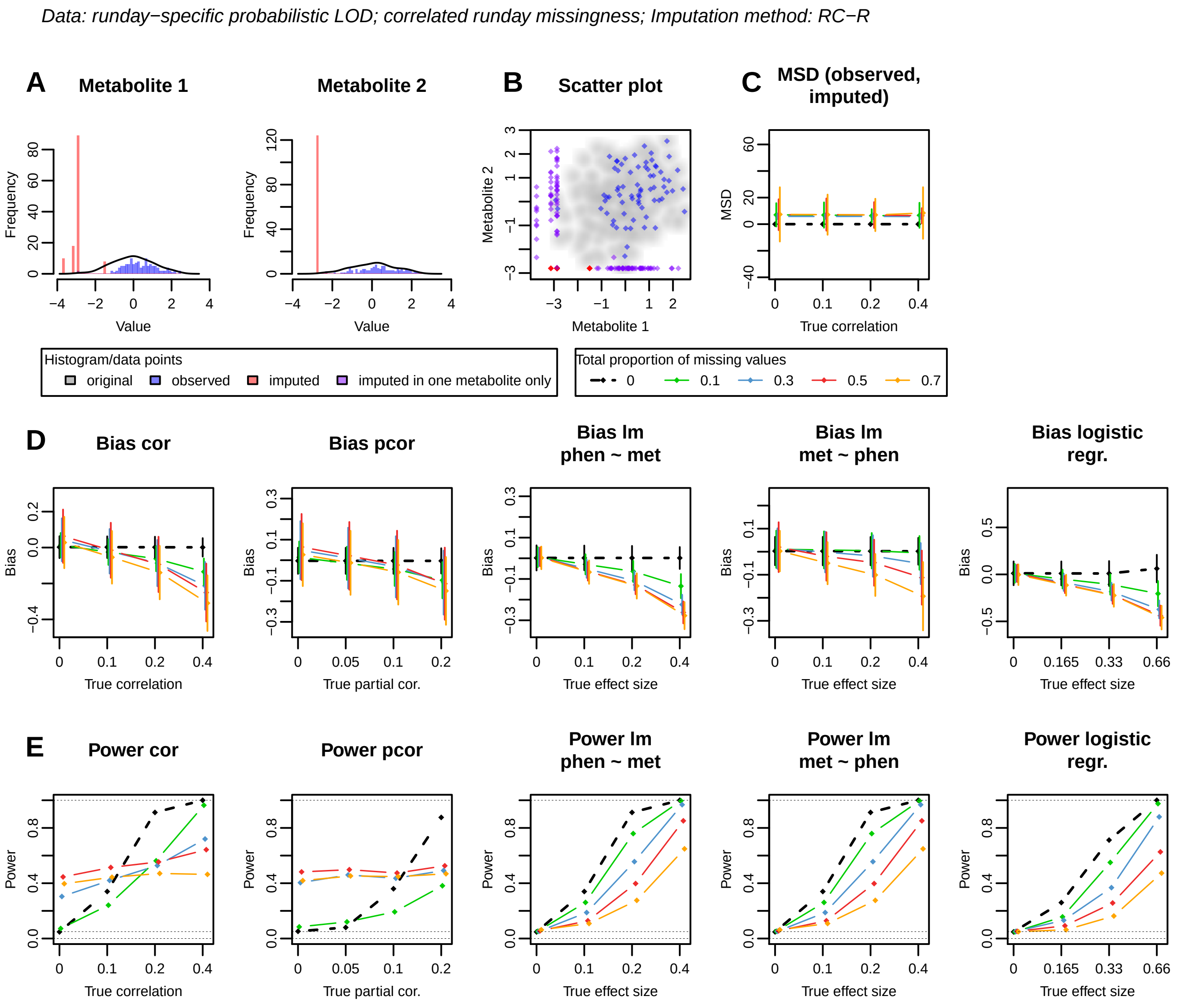

Data: runday−specific probabilistic LOD; correlated runday missingness; Imputation method: RC−R
A
Metabolite 1
Metabolite 2
Frequency
Value
B
Scatter plot
Metabolite 1
Metabolite 2
C
MSD (observed, imputed)
MSD
True correlation
Histogram/data points
original
observed
imputed
imputed in one metabolite only
Total proportion of missing values
0
0.1
0.3
0.5
0.7
D
Bias cor
Bias pcor
Bias lm phen ~ met
Bias lm met ~ phen
Bias logistic regr.
Bias
True correlation
True partial cor.
True effect size
E
Power cor
Power pcor
Power lm phen ~ met
Power lm met ~ phen
Power logistic regr.
Power
True correlation
True partial cor.
True effect size

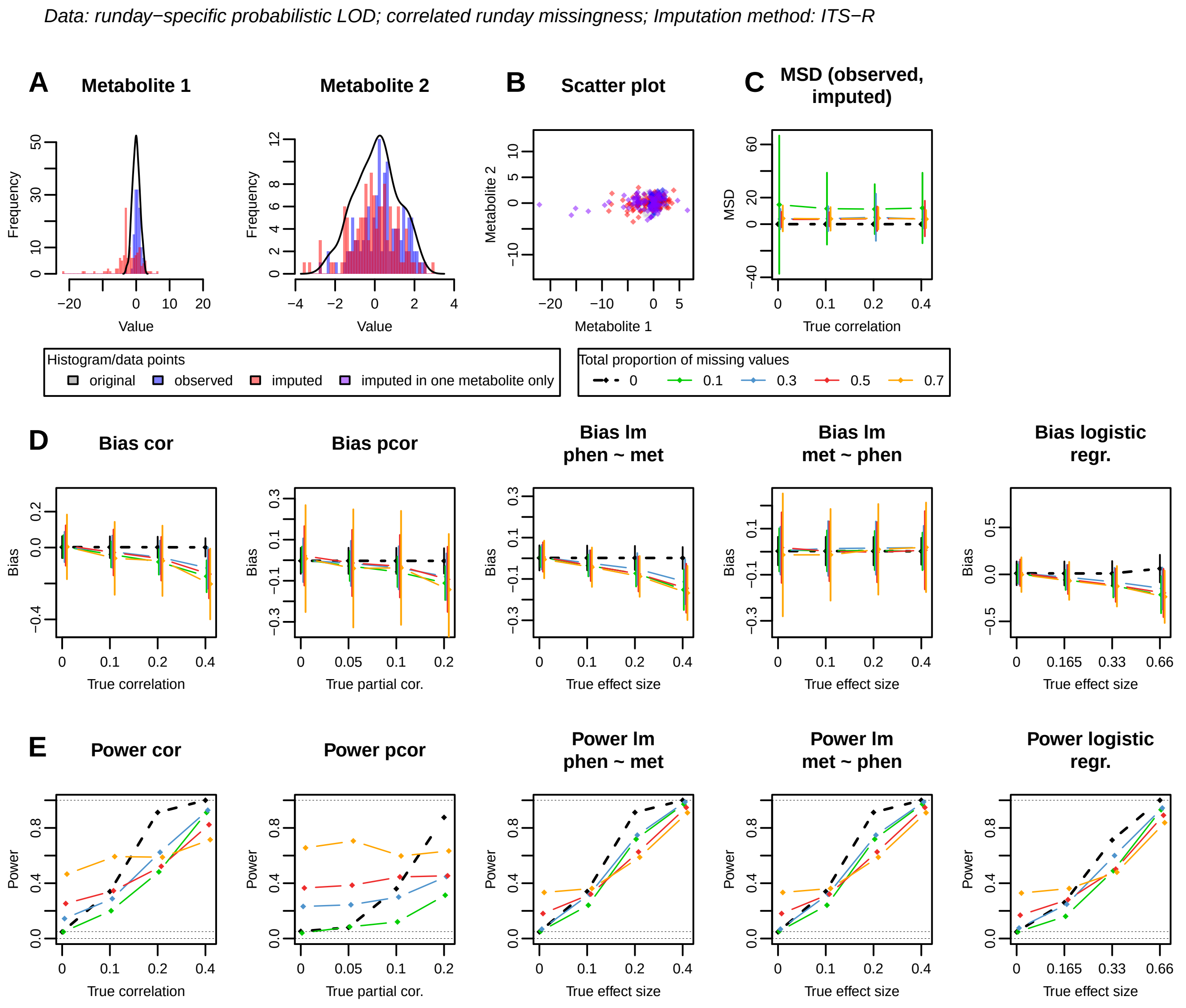
Data: runday−specific probabilistic LOD; correlated runday missingness; Imputation method: ITS−R
A
Metabolite 1
Metabolite 2
Frequency
Value
B
Scatter plot
Metabolite 2
Metabolite 1
C
MSD (observed, imputed)
MSD
True correlation
Histogram/data points
original
observed
imputed
imputed in one metabolite only
Total proportion of missing values
0
0.1
0.3
0.5
0.7
D
Bias cor
Bias pcor
Bias lm phen ~ met
Bias lm met ~ phen
Bias logistic regr.
Bias
True correlation
True partial cor.
True effect size
E
Power cor
Power pcor
Power lm phen ~ met
Power lm met ~ phen
Power logistic regr.
Power
True correlation
True partial cor.
True effect size

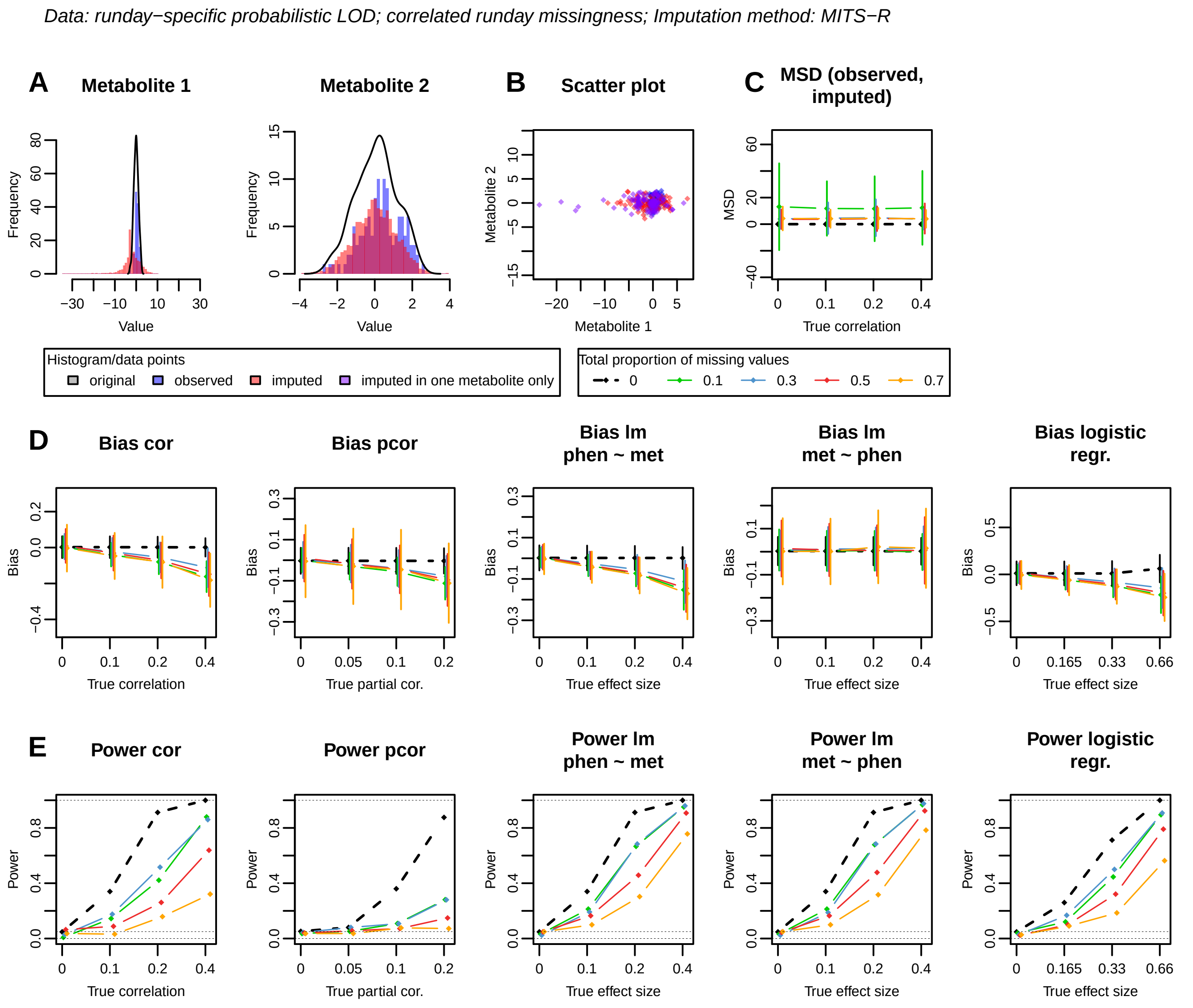

Data: runday−specific probabilistic LOD; correlated runday missingness; Imputation method: MITS−R
A
Metabolite 1
Metabolite 2
B
Scatter plot
C
MSD (observed, imputed)
Frequency
Value
Metabolite 2
Metabolite 1
MSD
True correlation
Histogram/data points
original
observed
imputed
imputed in one metabolite only
Total proportion of missing values
0
0.1
0.3
0.5
0.7
D
Bias cor
Bias pcor
Bias lm phen ~ met
Bias lm met ~ phen
Bias logistic regr.
Bias
True correlation
True partial cor.
True effect size
E
Power cor
Power pcor
Power lm phen ~ met
Power lm met ~ phen
Power logistic regr.
Power
True correlation
True partial cor.
True effect size

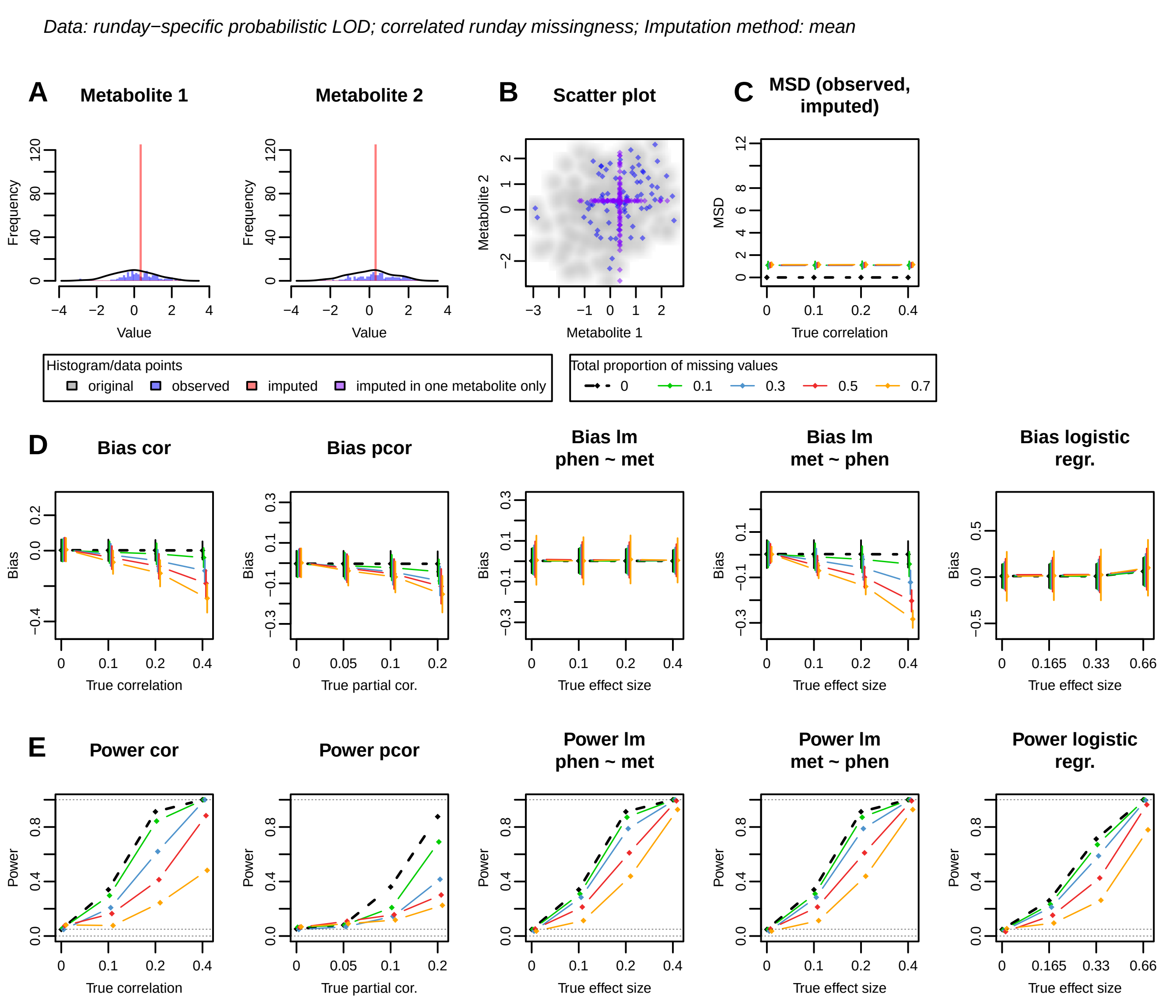
Data: runday−specific probabilistic LOD; correlated runday missingness; Imputation method: mean
A
Metabolite 1
Metabolite 2
B
Scatter plot
C
MSD (observed, imputed)
Frequency
Value
Metabolite 2
Metabolite 1
MSD
True correlation
Histogram/data points
original
observed
imputed
imputed in one metabolite only
Total proportion of missing values
0
0.1
0.3
0.5
0.7
D
Bias cor
Bias pcor
Bias lm phen ~ met
Bias lm met ~ phen
Bias logistic regr.
Bias
True correlation
True partial cor.
True effect size
E
Power cor
Power pcor
Power lm phen ~ met
Power lm met ~ phen
Power logistic regr.
Power

Data: runday−specific probabilistic LOD; correlated runday missingness; Imputation method: ICE−norm

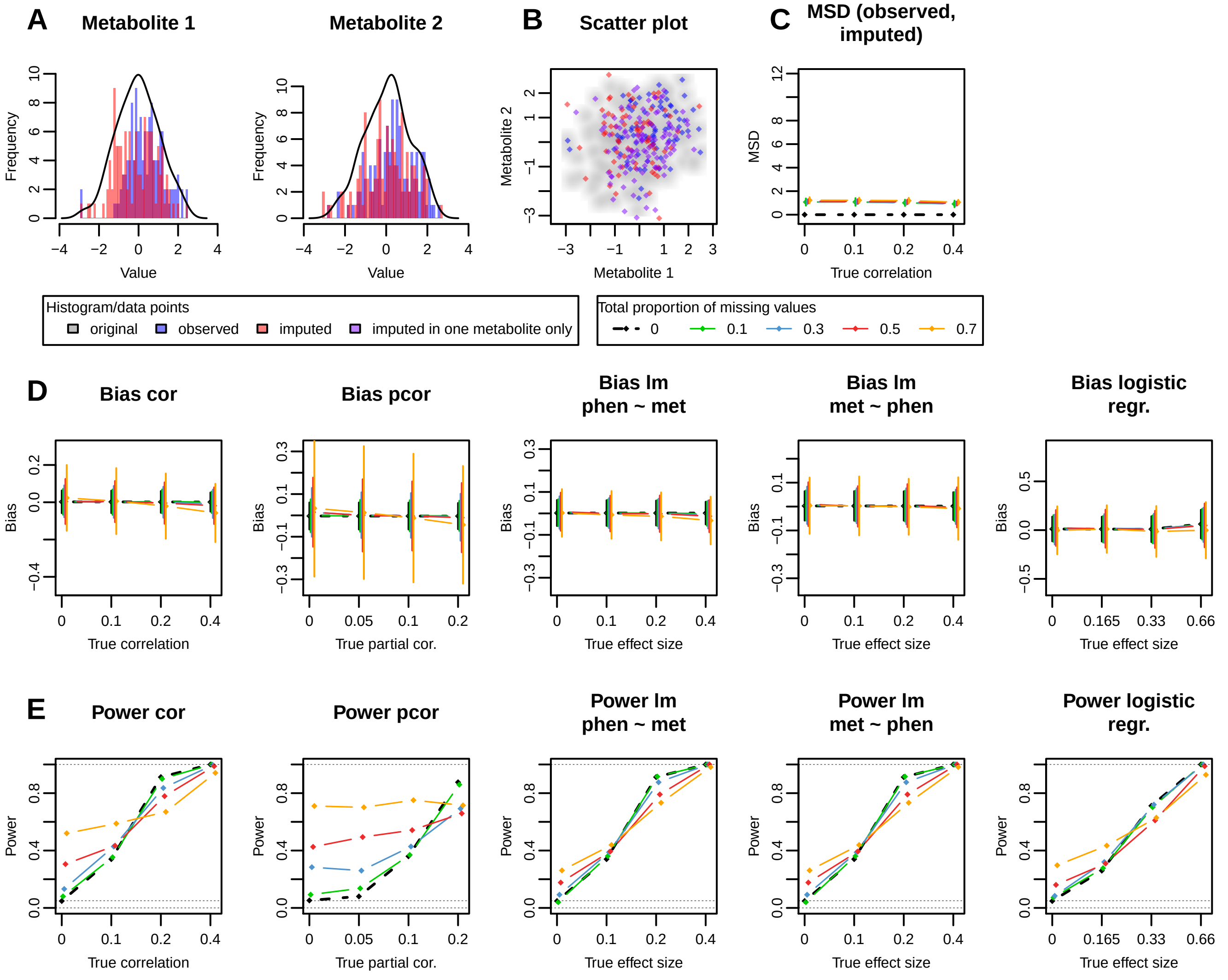

Data: runday−specific probabilistic LOD; correlated runday missingness; Imputation method: ICE−pmm

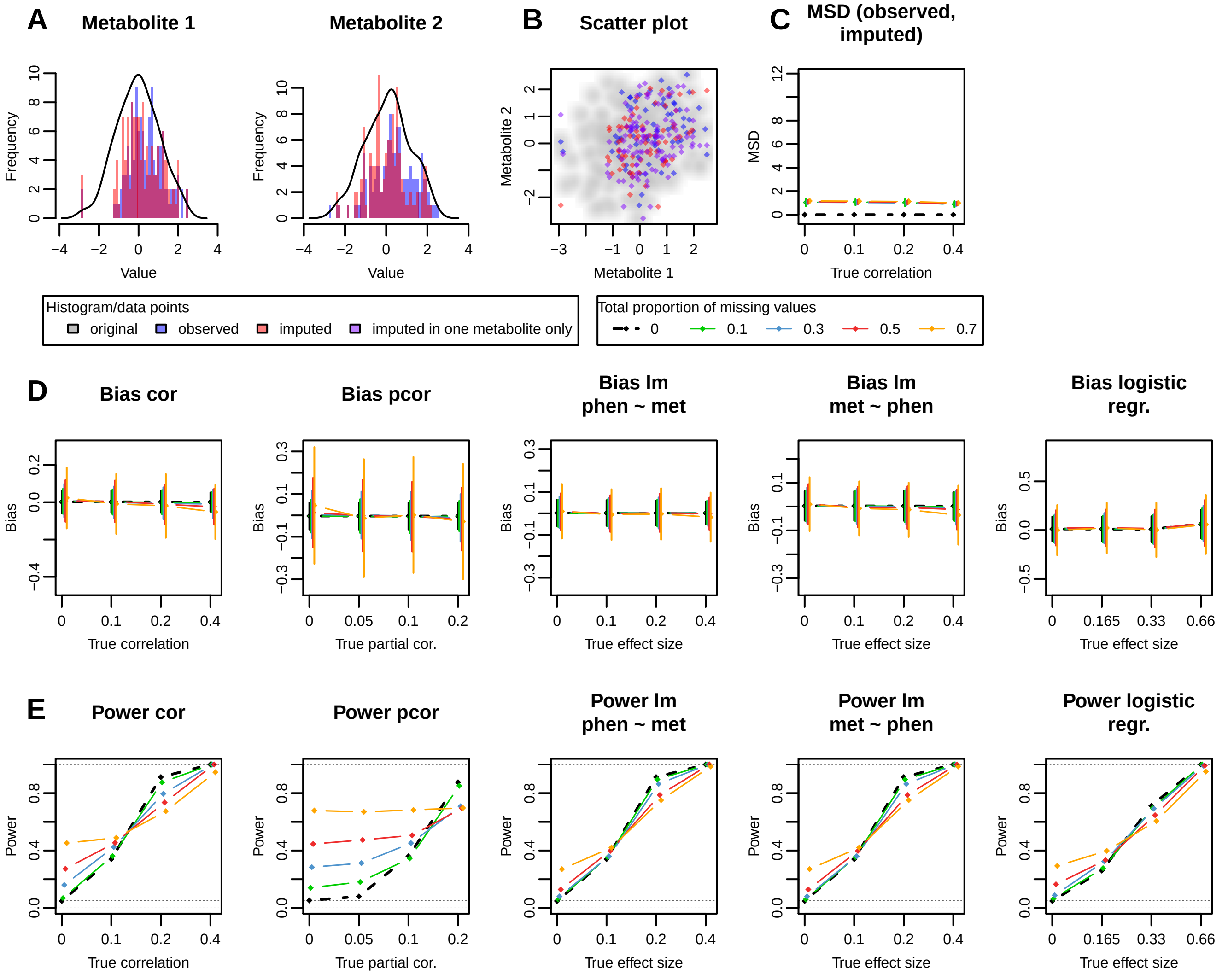

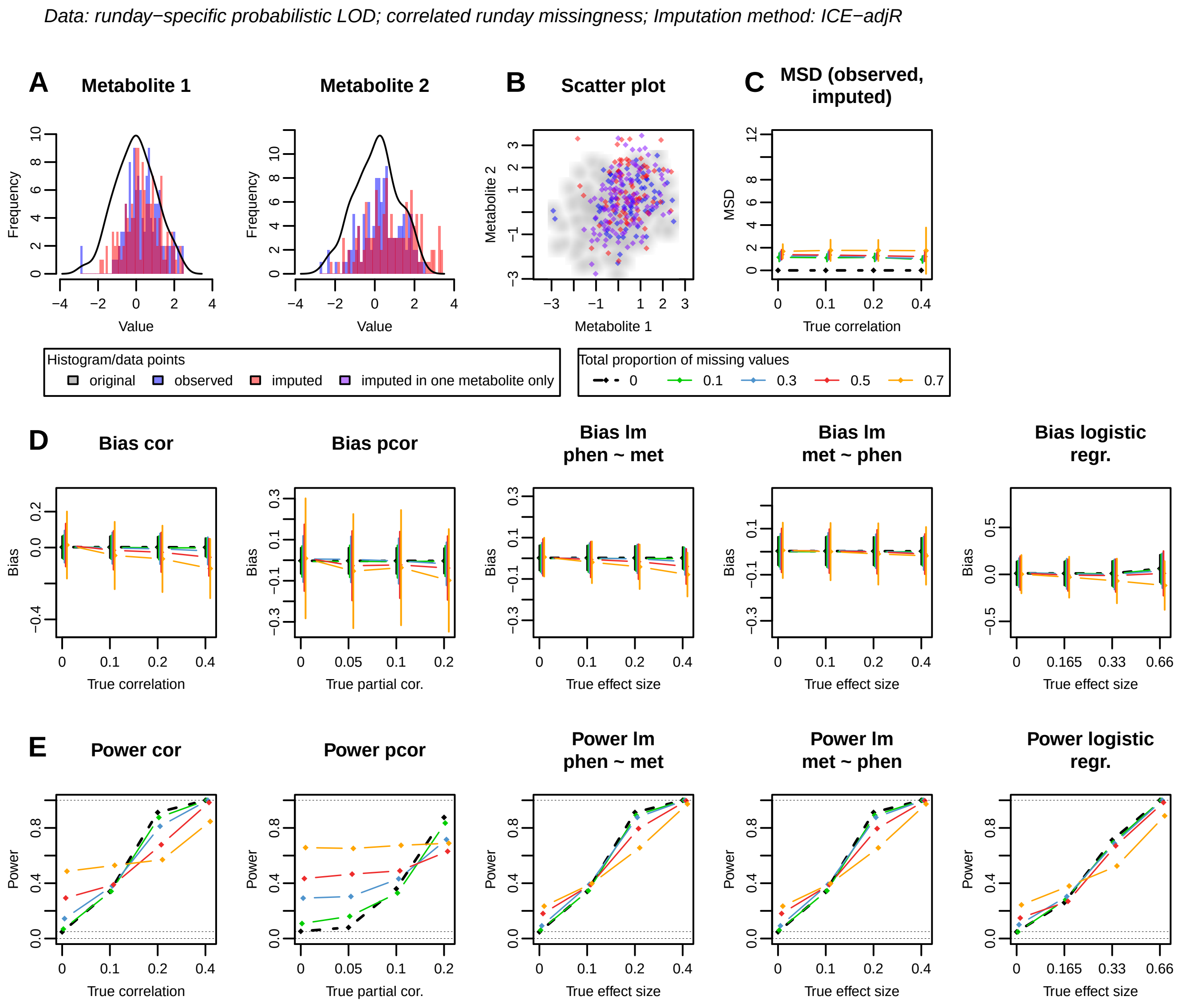

Data: runday−specific probabilistic LOD; correlated runday missingness; Imputation method: ICE−adjR
A
Metabolite 1
Metabolite 2
B
Scatter plot
C
MSD (observed, imputed)
Frequency
Value
Metabolite 2
Metabolite 1
MSD
True correlation
Histogram/data points
original
observed
imputed
imputed in one metabolite only
Total proportion of missing values
0
0.1
0.3
0.5
0.7
D
Bias cor
Bias pcor
Bias lm phen ~ met
Bias lm met ~ phen
Bias logistic regr.
Bias
True correlation
True partial cor.
True effect size
E
Power cor
Power pcor
Power lm phen ~ met
Power lm met ~ phen
Power logistic regr.
Power
True correlation
True partial cor.
True effect size

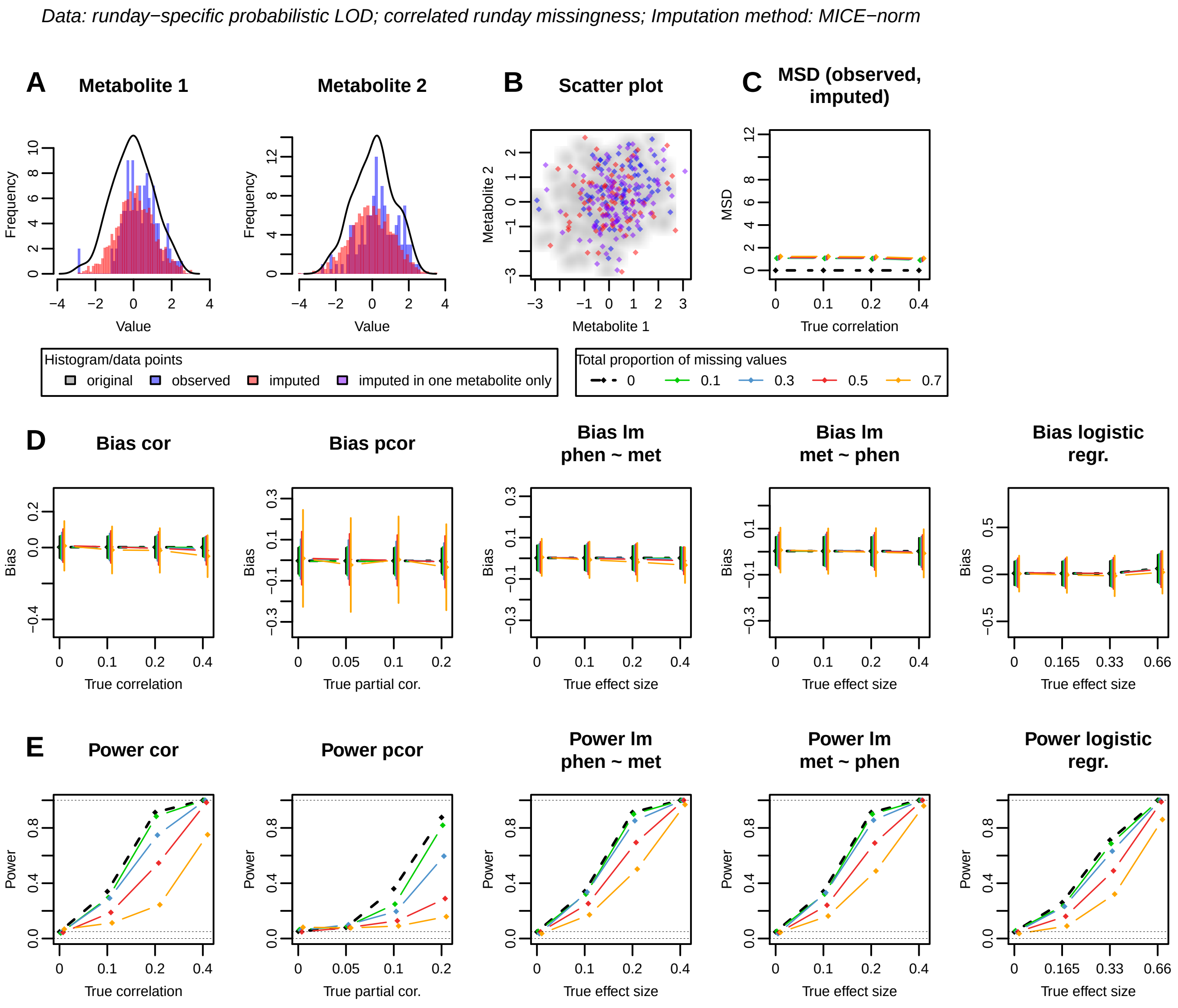
Data: runday−specific probabilistic LOD; correlated runday missingness; Imputation method: MICE−norm
A
Metabolite 1
Metabolite 2
Frequency
Value
B
Scatter plot
Metabolite 1
Metabolite 2
C
MSD (observed, imputed)
MSD
True correlation
Histogram/data points
original
observed
imputed
imputed in one metabolite only
Total proportion of missing values
0
0.1
0.3
0.5
0.7
D
Bias cor
Bias pcor
Bias lm phen ~ met
Bias lm met ~ phen
Bias logistic regr.
Bias
True correlation
True partial cor.
True effect size
E
Power cor
Power pcor
Power lm phen ~ met
Power lm met ~ phen
Power logistic regr.
Power
True correlation
True partial cor.
True effect size

Data: runday−specific probabilistic LOD; correlated runday missingness; Imputation method: MICE−pmm

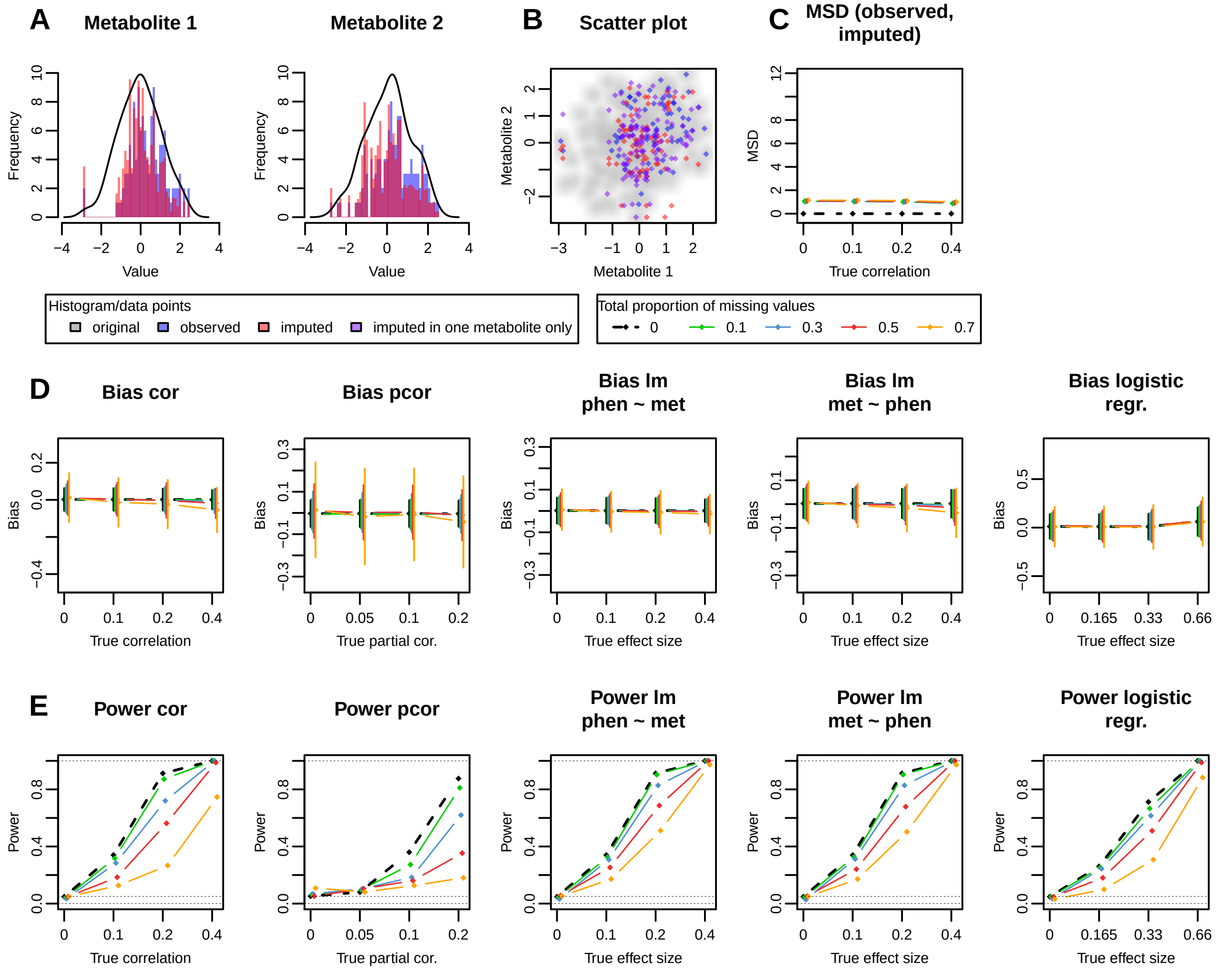

Data: runday−specific probabilistic LOD; correlated runday missingness; Imputation method: MICE−adjR

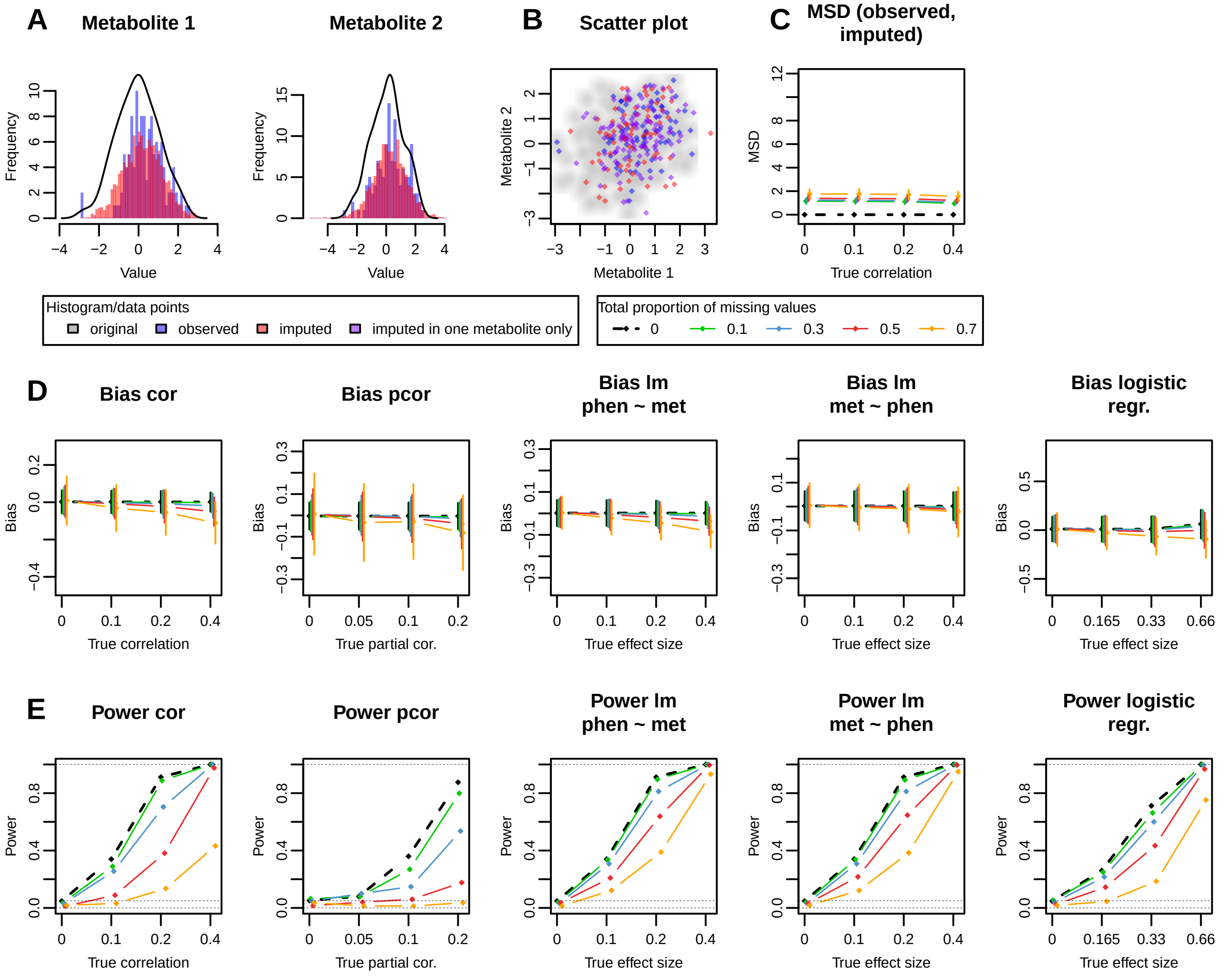

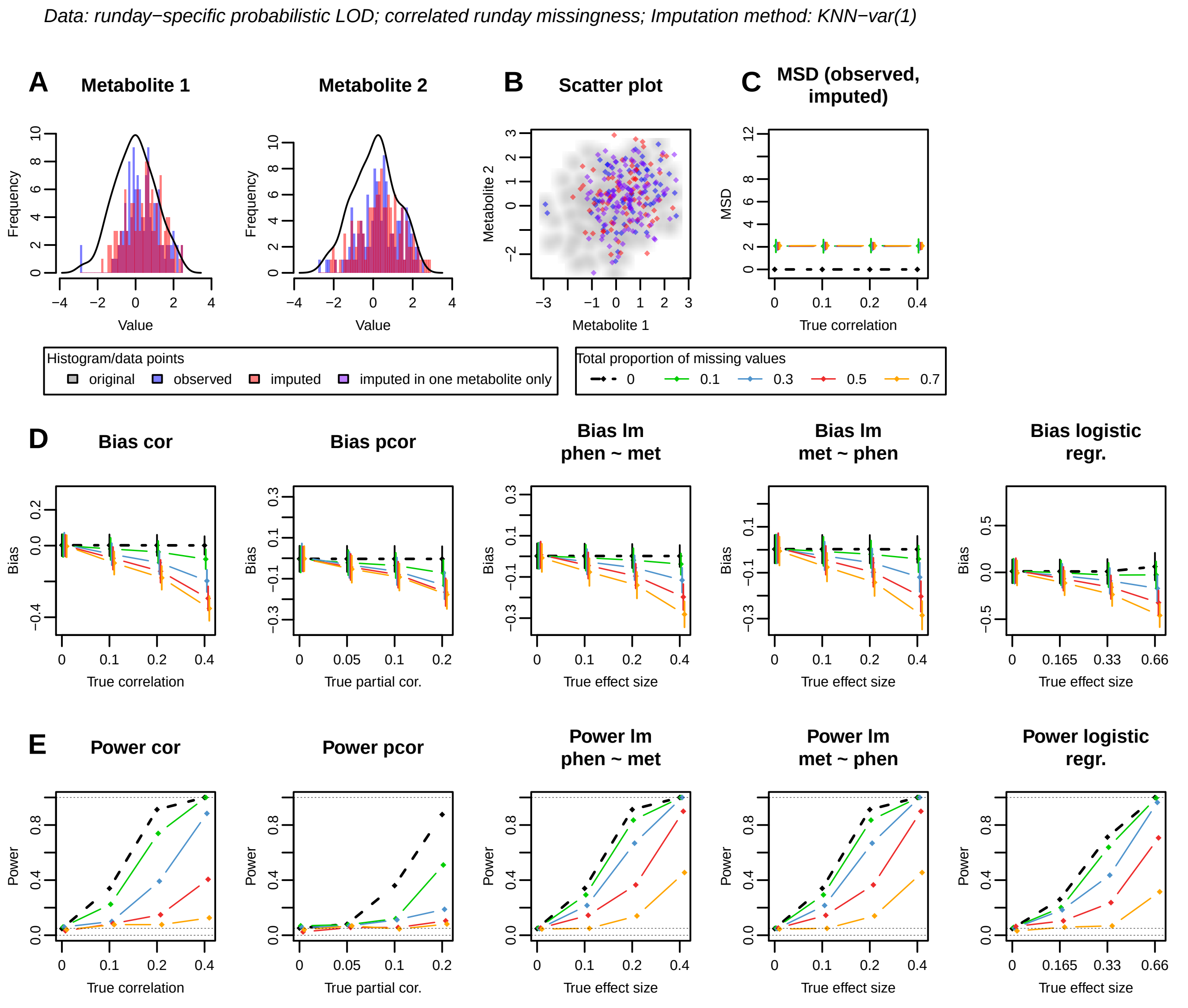
Data: runday−specific probabilistic LOD; correlated runday missingness; Imputation method: KNN−var(1)
A
Metabolite 1
Metabolite 2
Frequency
Value
B
Scatter plot
Metabolite 1
Metabolite 2
C
MSD (observed, imputed)
MSD
True correlation
Histogram/data points
original
observed
imputed
imputed in one metabolite only
Total proportion of missing values
0
0.1
0.3
0.5
0.7
D
Bias cor
Bias pcor
Bias lm phen ~ met
Bias lm met ~ phen
Bias logistic regr.
Bias
True correlation
True partial cor.
True effect size
E
Power cor
Power pcor
Power lm phen ~ met
Power lm met ~ phen
Power logistic regr.
Power
True correlation
True partial cor.
True effect size

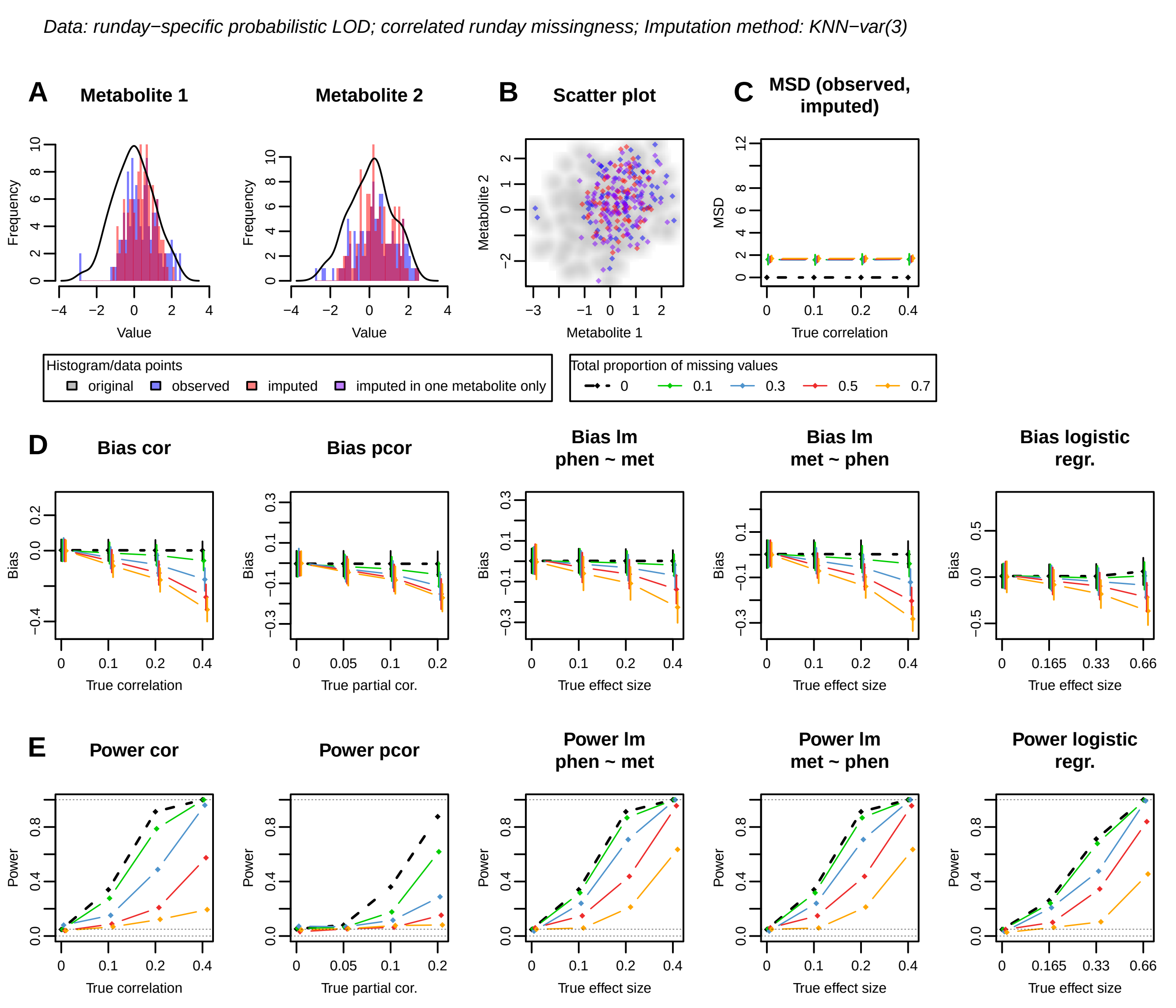

Data: runday−specific probabilistic LOD; correlated runday missingness; Imputation method: KNN−var(3)
A
Metabolite 1
Metabolite 2
Frequency
Value
B
Scatter plot
Metabolite 2
Metabolite 1
C
MSD (observed, imputed)
MSD
True correlation
Histogram/data points
original
observed
imputed
imputed in one metabolite only
Total proportion of missing values
0
0.1
0.3
0.5
0.7
D
Bias cor
Bias pcor
Bias lm phen ~ met
Bias lm met ~ phen
Bias logistic regr.
Bias
True correlation
True partial cor.
True effect size
E
Power cor
Power pcor
Power lm phen ~ met
Power lm met ~ phen
Power logistic regr.
Power
True correlation
True partial cor.
True effect size

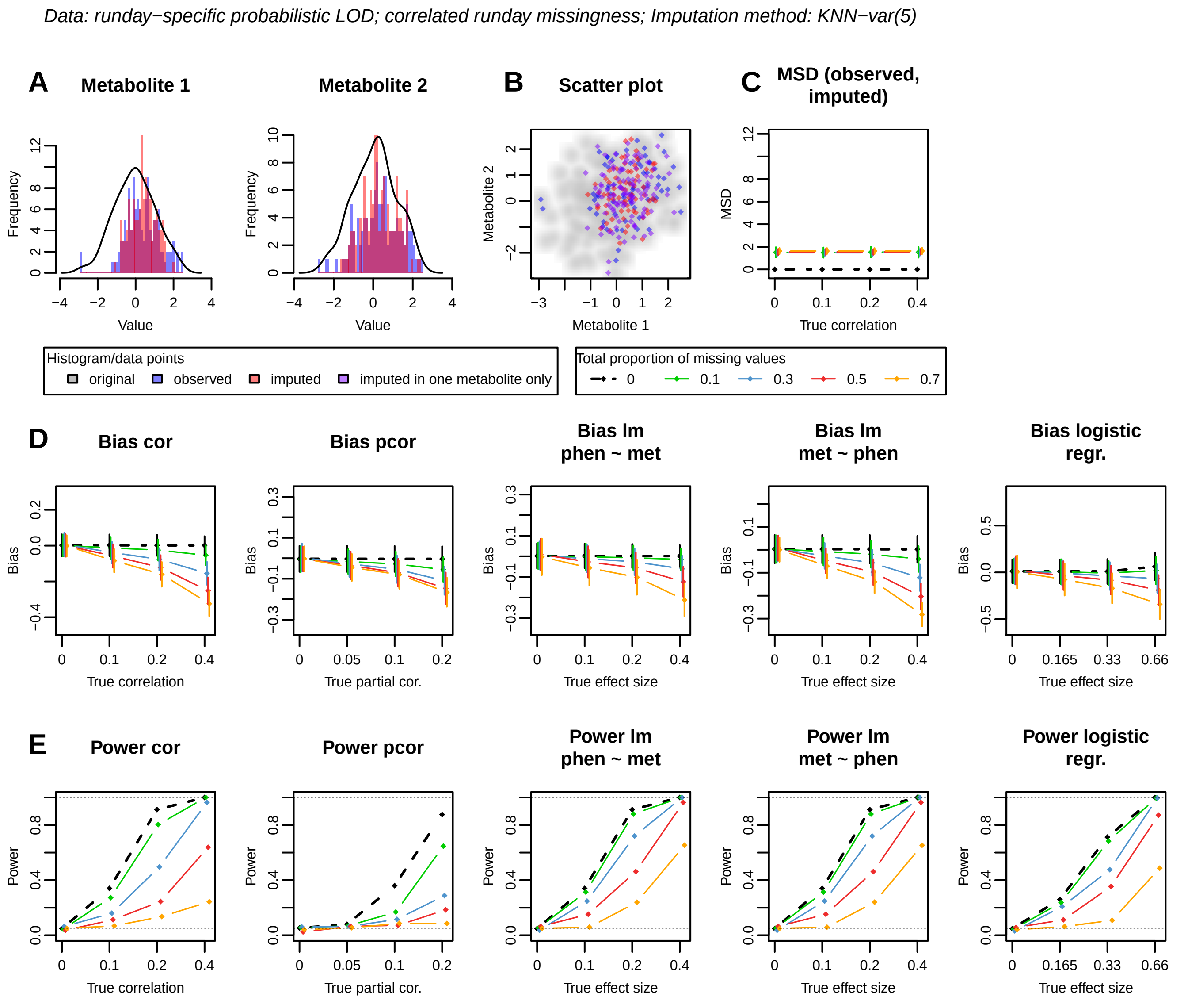
Data: runday−specific probabilistic LOD; correlated runday missingness; Imputation method: KNN−var(5)
A
Metabolite 1
Metabolite 2
Frequency
Value
B
Scatter plot
Metabolite 2
Metabolite 1
C
MSD (observed, imputed)
MSD
True correlation
Histogram/data points
original
observed
imputed
imputed in one metabolite only
Total proportion of missing values
0
0.1
0.3
0.5
0.7
D
Bias cor
Bias pcor
Bias lm phen ~ met
Bias lm met ~ phen
Bias logistic regr.
Bias
True correlation
True partial cor.
True effect size
E
Power cor
Power pcor
Power lm phen ~ met
Power lm met ~ phen
Power logistic regr.
Power
True correlation
True partial cor.
True effect size

Data: runday−specific probabilistic LOD; correlated runday missingness; Imputation method: KNN−var(10)

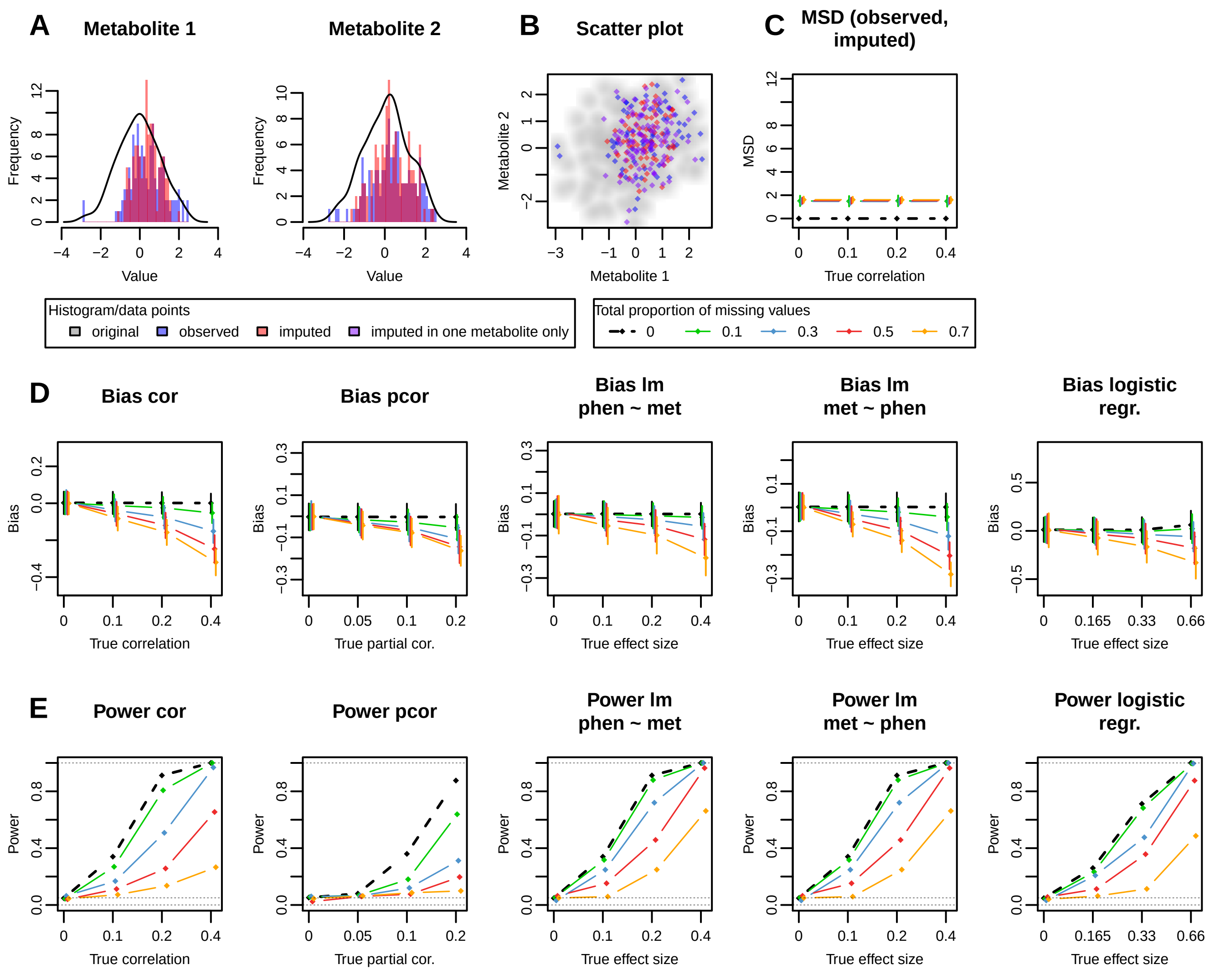

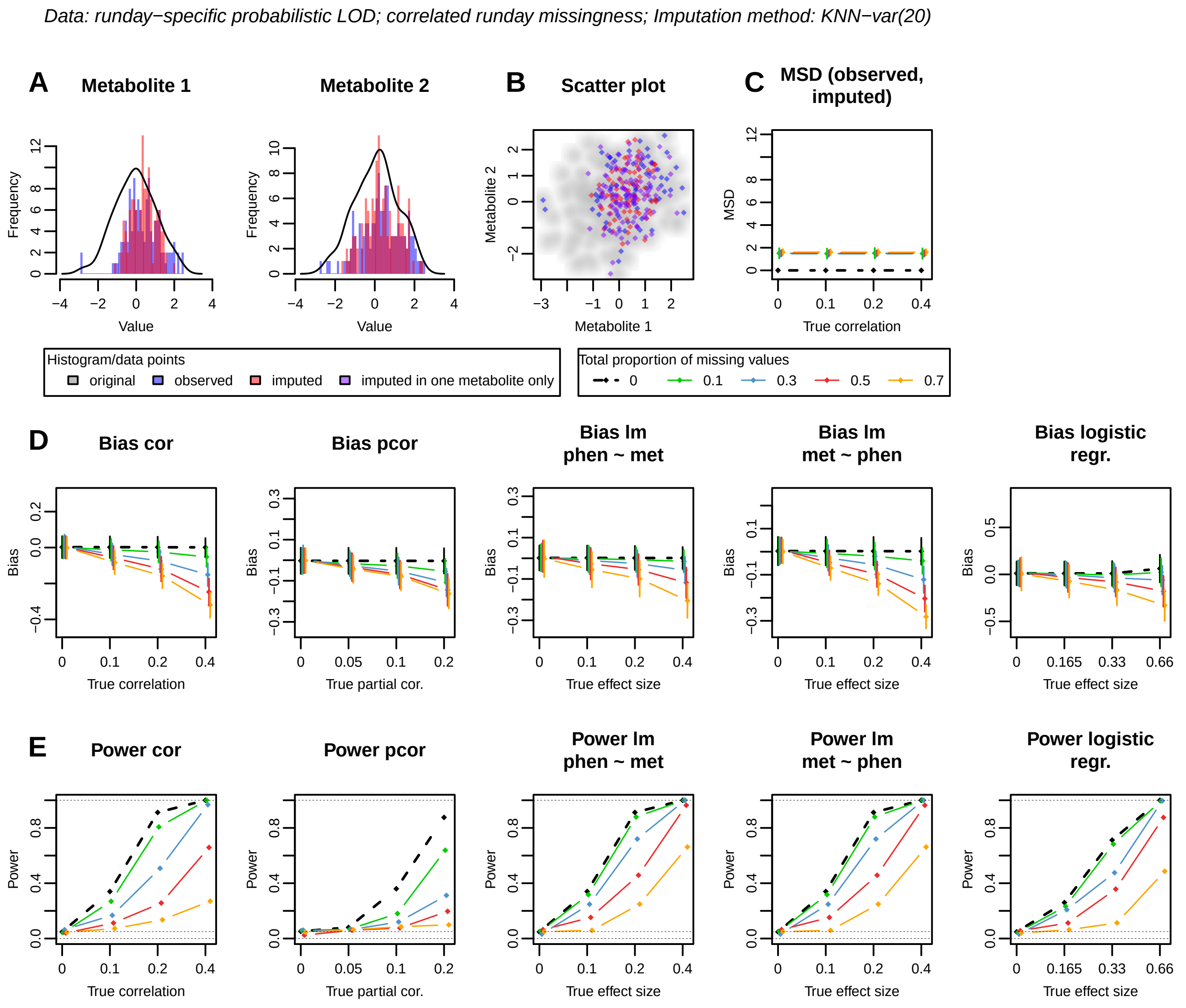

Data: runday−specific probabilistic LOD; correlated runday missingness; Imputation method: KNN−var(20)
A
Metabolite 1
Metabolite 2
B
Scatter plot
C
MSD (observed, imputed)
Frequency
Value
Metabolite 2
Metabolite 1
MSD
True correlation
Histogram/data points
original
observed
imputed
imputed in one metabolite only
Total proportion of missing values
0
0.1
0.3
0.5
0.7
D
Bias cor
Bias pcor
Bias lm phen ~ met
Bias lm met ~ phen
Bias logistic regr.
Bias
True correlation
True partial cor.
True effect size
E
Power cor
Power pcor
Power lm phen ~ met
Power lm met ~ phen
Power logistic regr.
Power

Data: runday−specific probabilistic LOD; correlated runday missingness; Imputation method: KNN−obs(1)

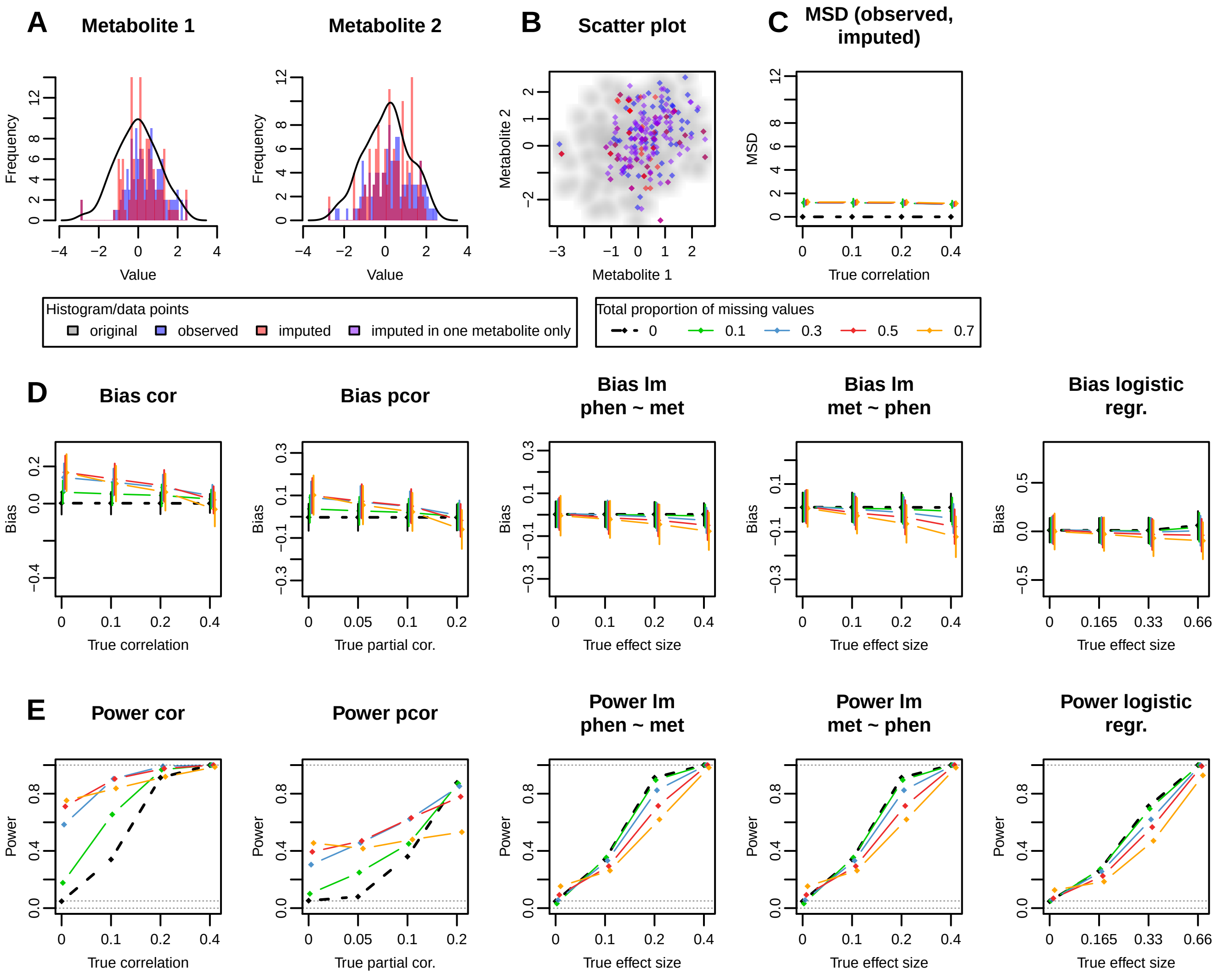

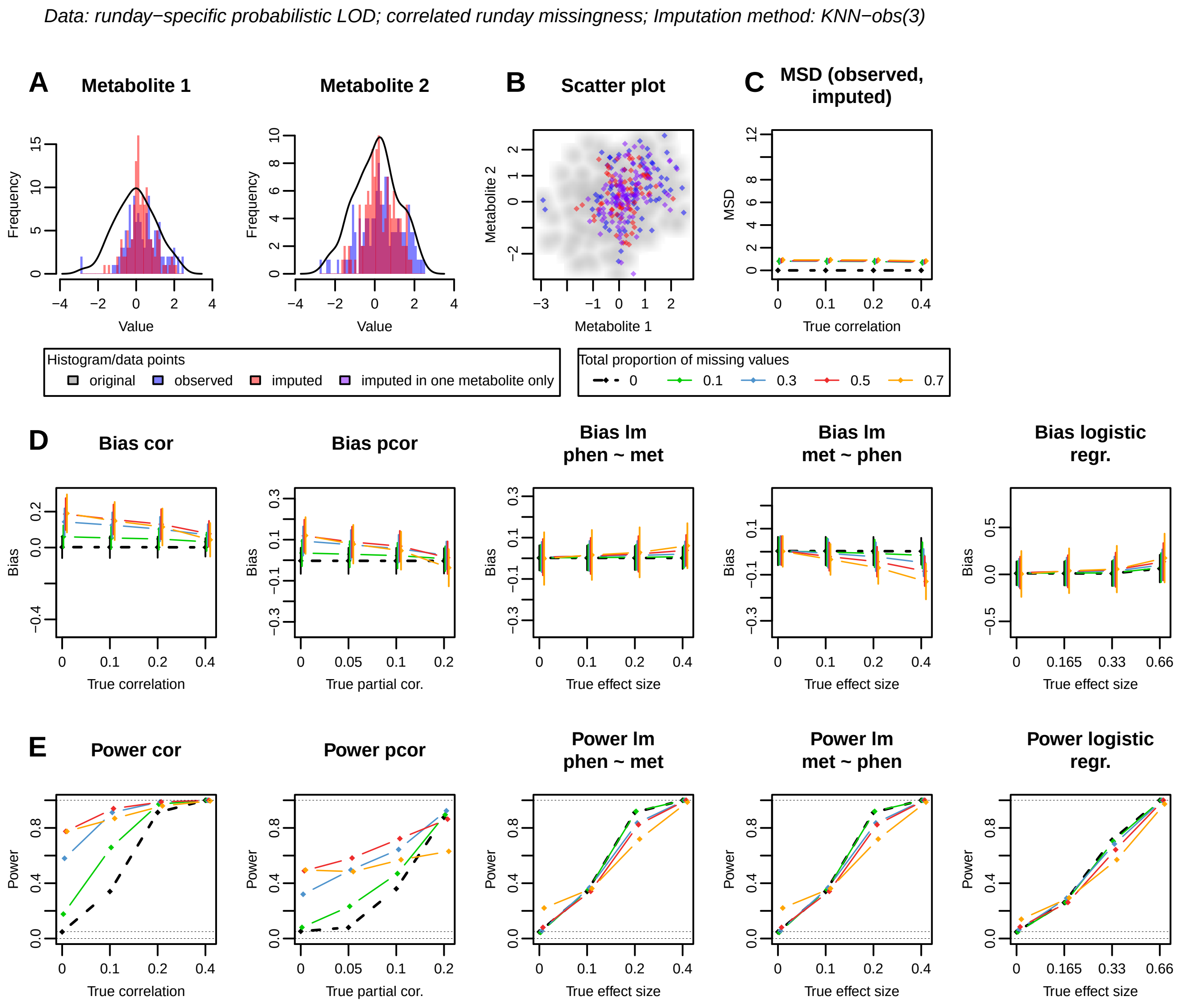
Data: runday−specific probabilistic LOD; correlated runday missingness; Imputation method: KNN−obs(3)
A
Metabolite 1
Metabolite 2
B
Scatter plot
C
MSD (observed, imputed)
Frequency
Value
Metabolite 2
Metabolite 1
MSD
True correlation
Histogram/data points
original
observed
imputed
imputed in one metabolite only
Total proportion of missing values
0
0.1
0.3
0.5
0.7
D
Bias cor
Bias pcor
Bias lm phen ~ met
Bias lm met ~ phen
Bias logistic regr.
Bias
True correlation
True partial cor.
True effect size
E
Power cor
Power pcor
Power lm phen ~ met
Power lm met ~ phen
Power logistic regr.
Power

Data: runday−specific probabilistic LOD; correlated runday missingness; Imputation method: KNN−obs(5)

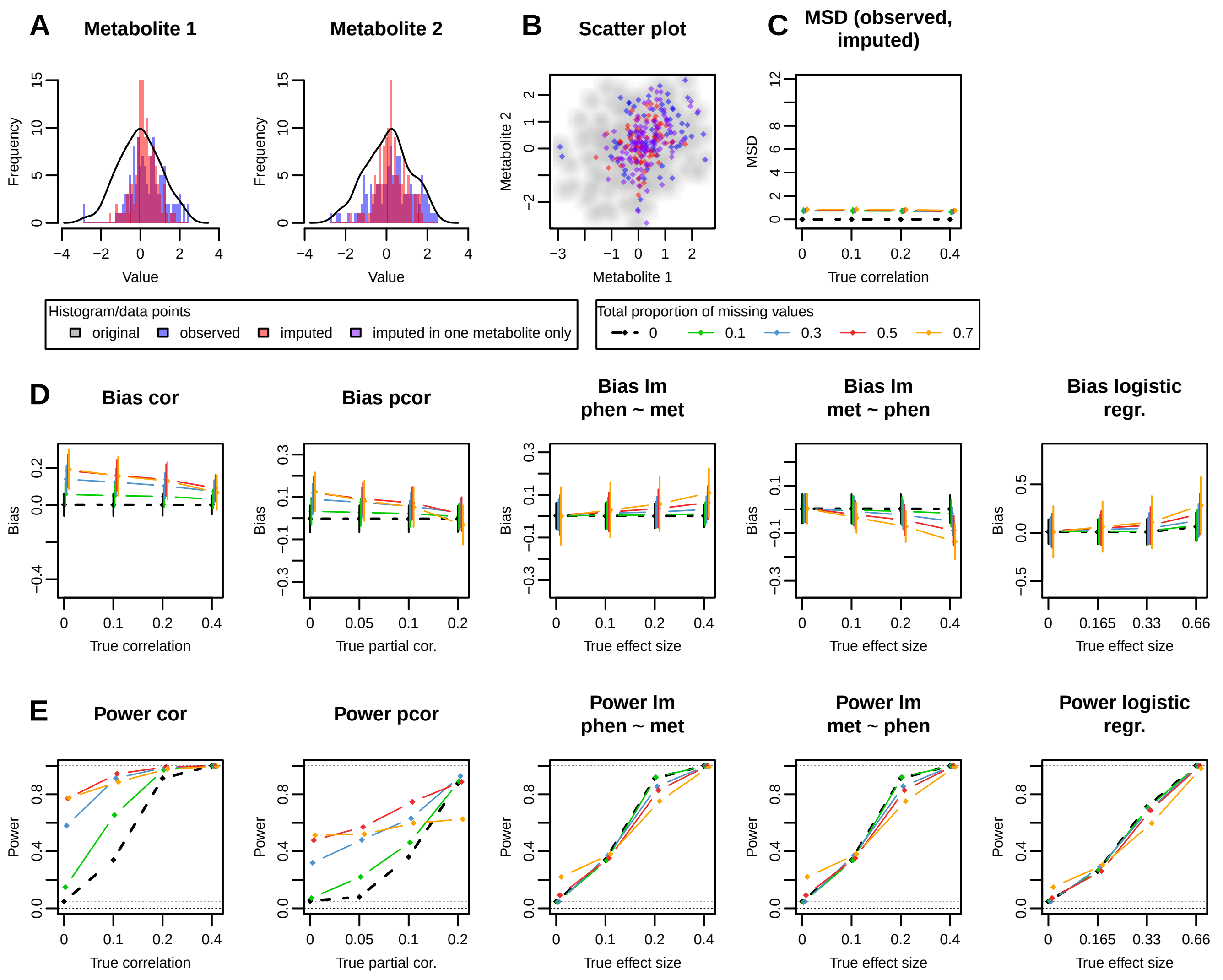

Data: runday−specific probabilistic LOD; correlated runday missingness; Imputation method: KNN−obs(10)

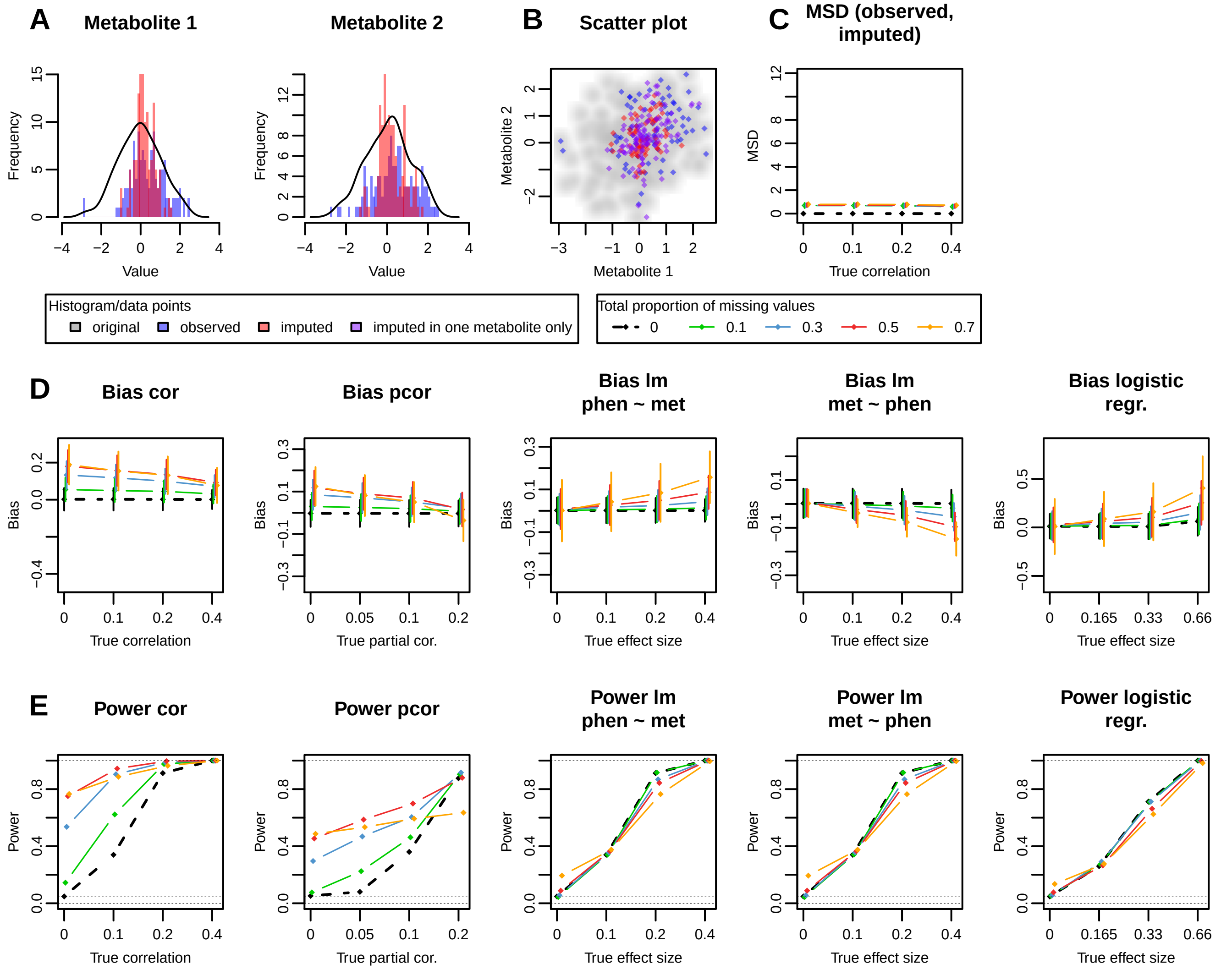

Data: runday−specific probabilistic LOD; correlated runday missingness; Imputation method: KNN−obs(20)

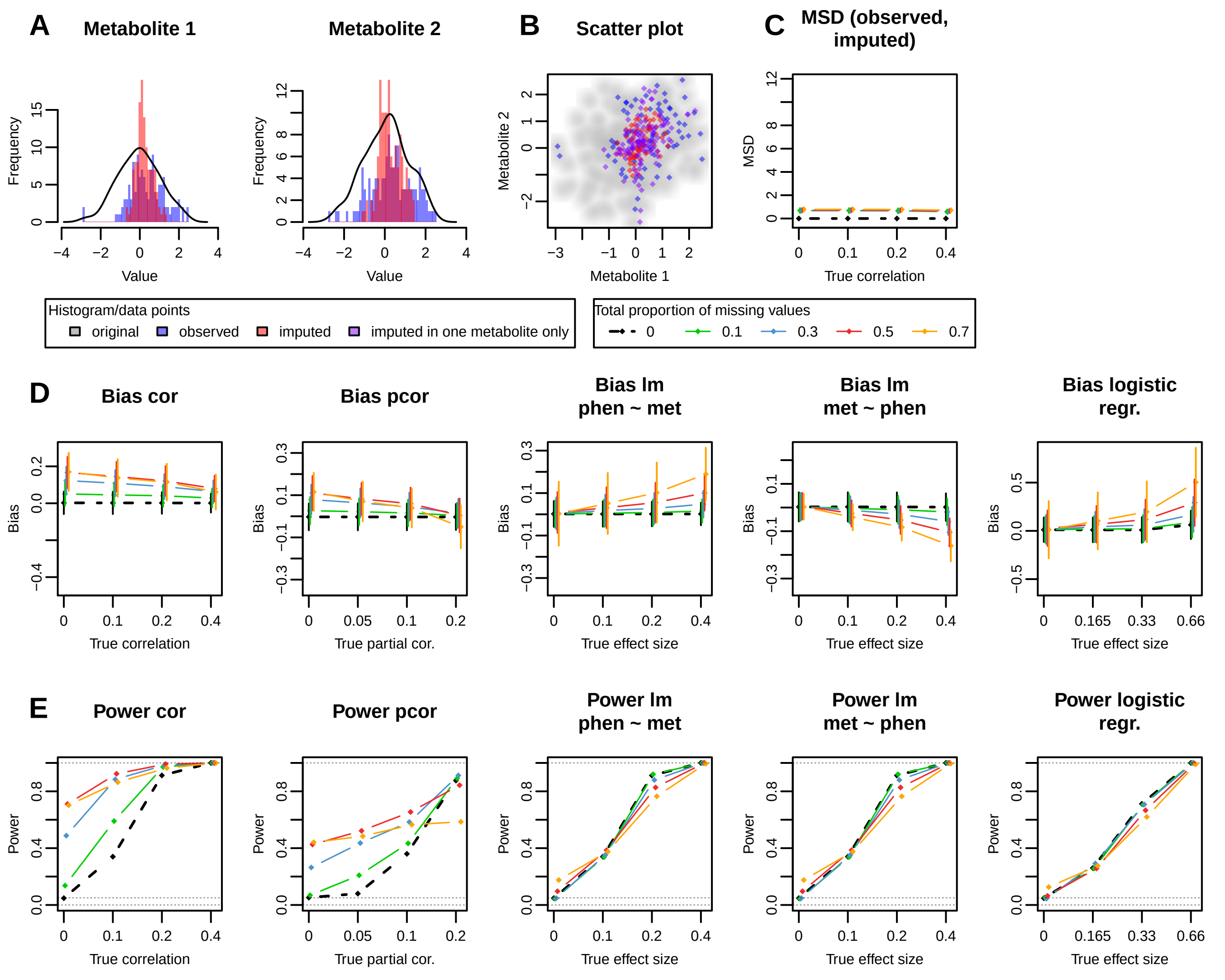

Data: runday−specific probabilistic LOD; correlated runday missingness; Imputation method: KNN−obs−sel(1)

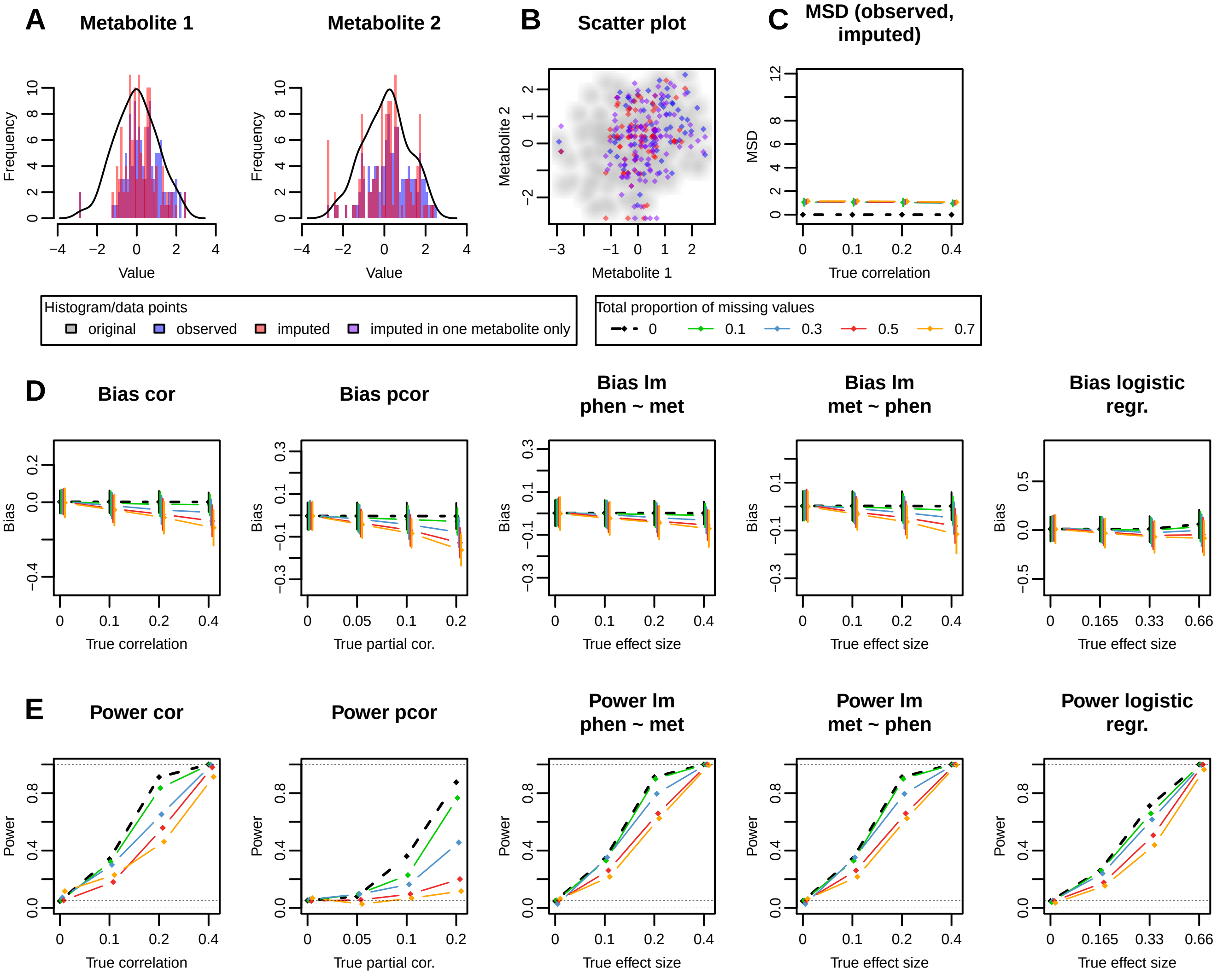

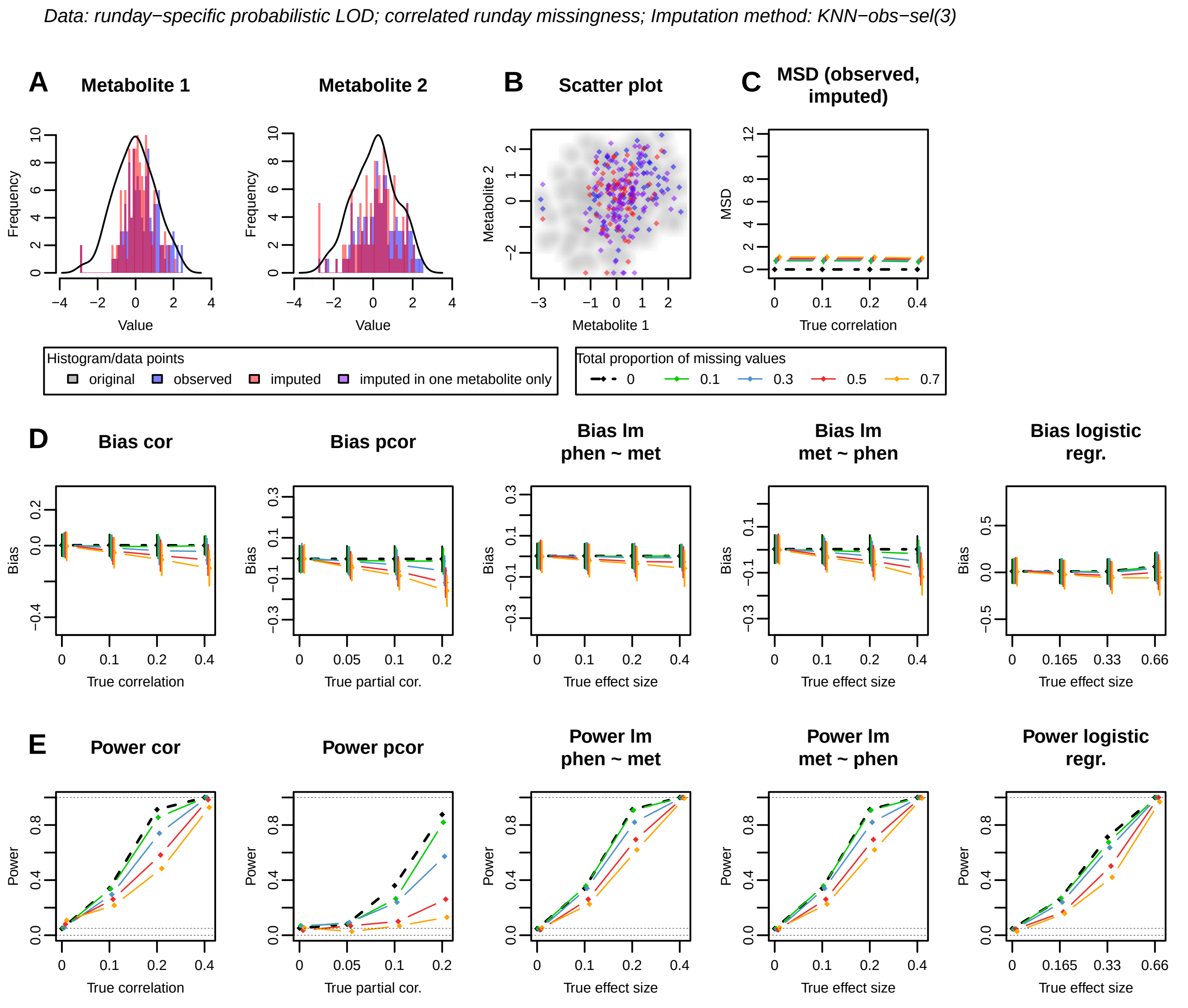
Data: runday−specific probabilistic LOD; correlated runday missingness; Imputation method: KNN−obs−sel(3)
A
Metabolite 1
Metabolite 2
Frequency
Value
B
Scatter plot
Metabolite 1
Metabolite 2
C
MSD (observed, imputed)
MSD
True correlation
Histogram/data points
original
observed
imputed
imputed in one metabolite only
Total proportion of missing values
0
0.1
0.3
0.5
0.7
D
Bias cor
Bias pcor
Bias lm phen ~ met
Bias lm met ~ phen
Bias logistic regr.
Bias
True correlation
True partial cor.
True effect size
E
Power cor
Power pcor
Power lm phen ~ met
Power lm met ~ phen
Power logistic regr.
Power
True correlation
True partial cor.
True effect size

Data: runday−specific probabilistic LOD; correlated runday missingness; Imputation method: KNN−obs−sel(5)

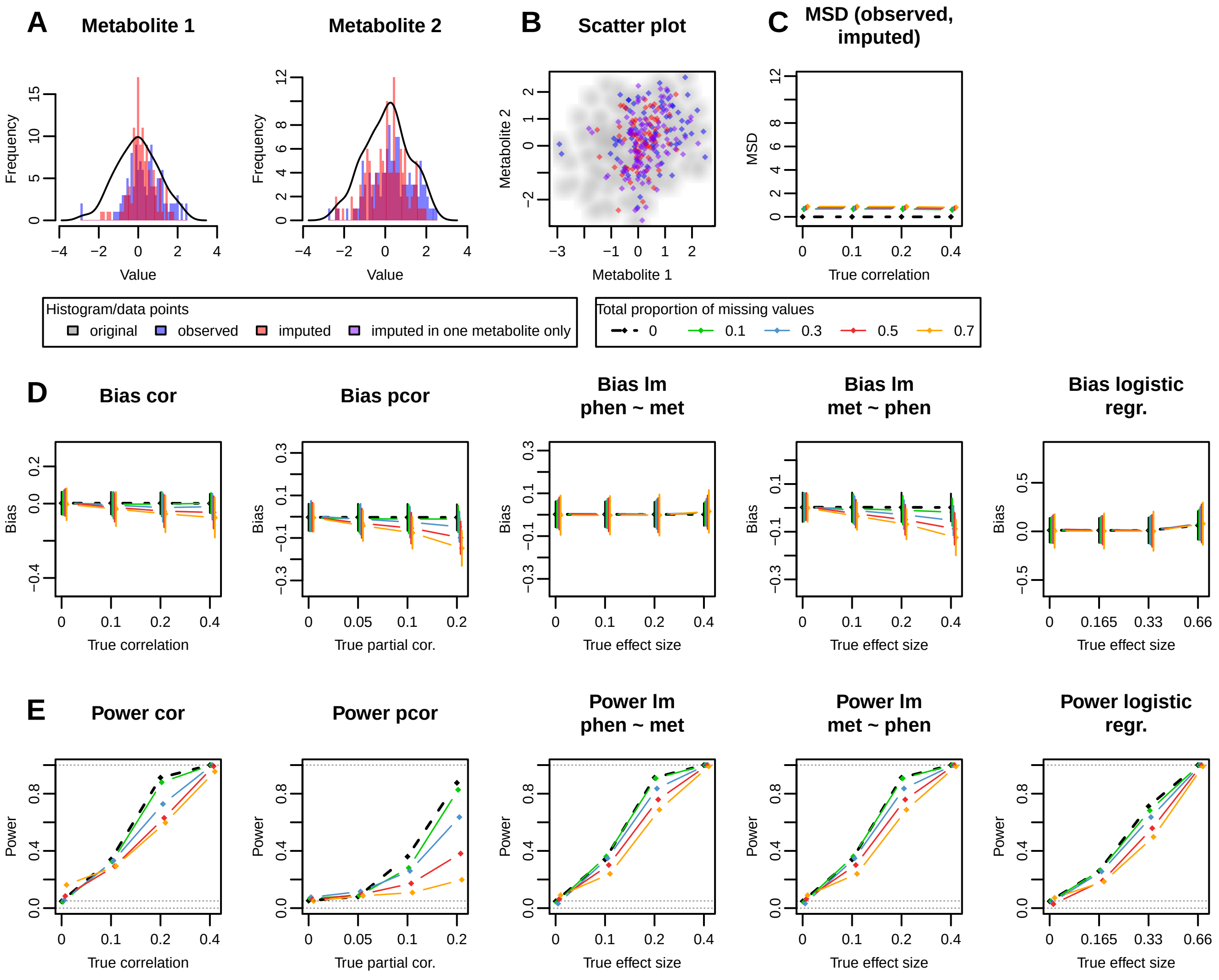

Data: runday−specific probabilistic LOD; correlated runday missingness; Imputation method: KNN−obs−sel(10)

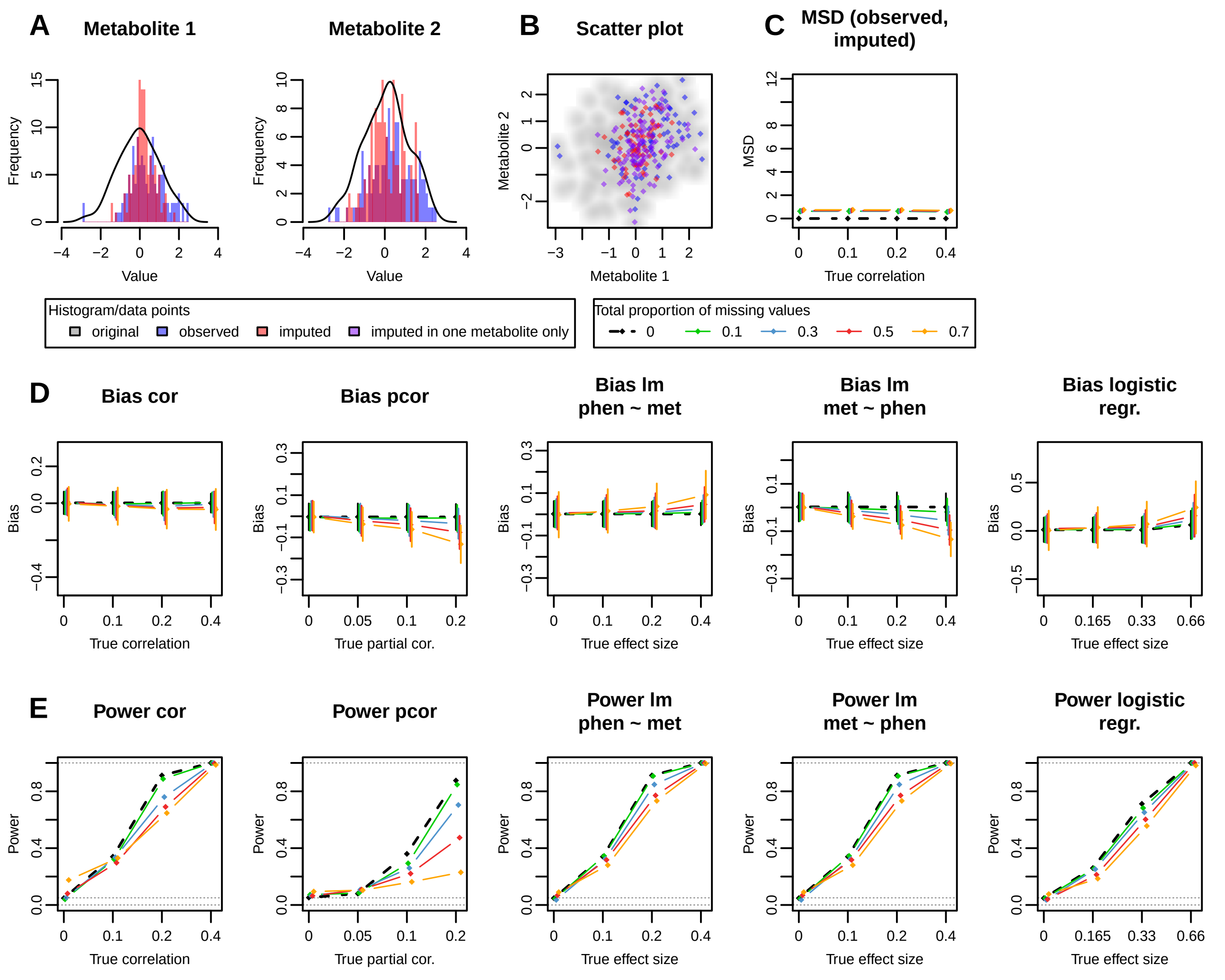

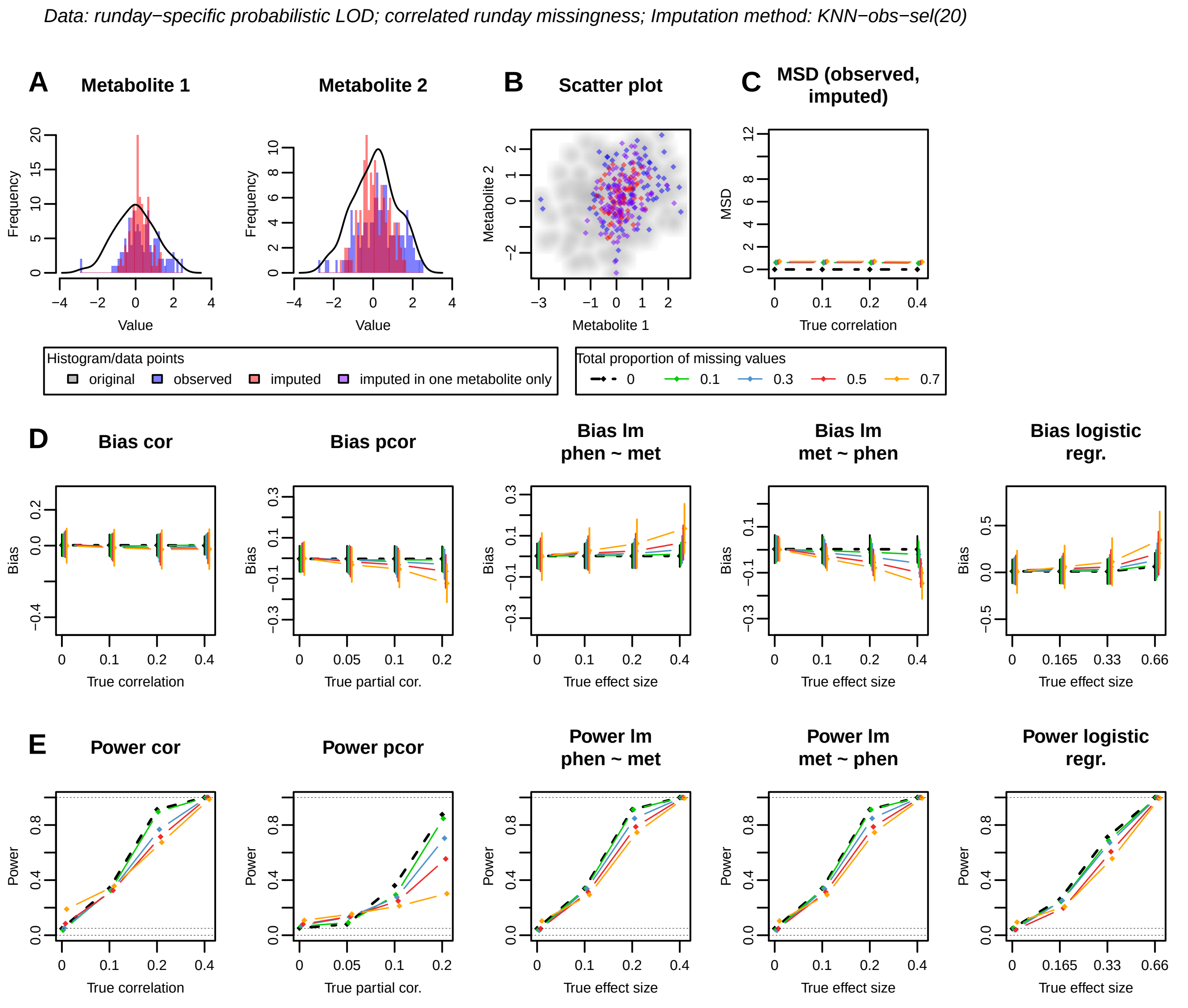

Data: runday−specific probabilistic LOD; correlated runday missingness; Imputation method: KNN−obs−sel(20)
A
Metabolite 1
Metabolite 2
Frequency
Value
B
Scatter plot
Metabolite 1
Metabolite 2
C
MSD (observed, imputed)
MSD
True correlation
Histogram/data points
original
observed
imputed
imputed in one metabolite only
Total proportion of missing values
0
0.1
0.3
0.5
0.7
D
Bias cor
Bias pcor
Bias lm phen ~ met
Bias lm met ~ phen
Bias logistic regr.
Bias
True correlation
True partial cor.
True effect size
E
Power cor
Power pcor
Power lm phen ~ met
Power lm met ~ phen
Power logistic regr.
Power
True correlation
True partial cor.
True effect size

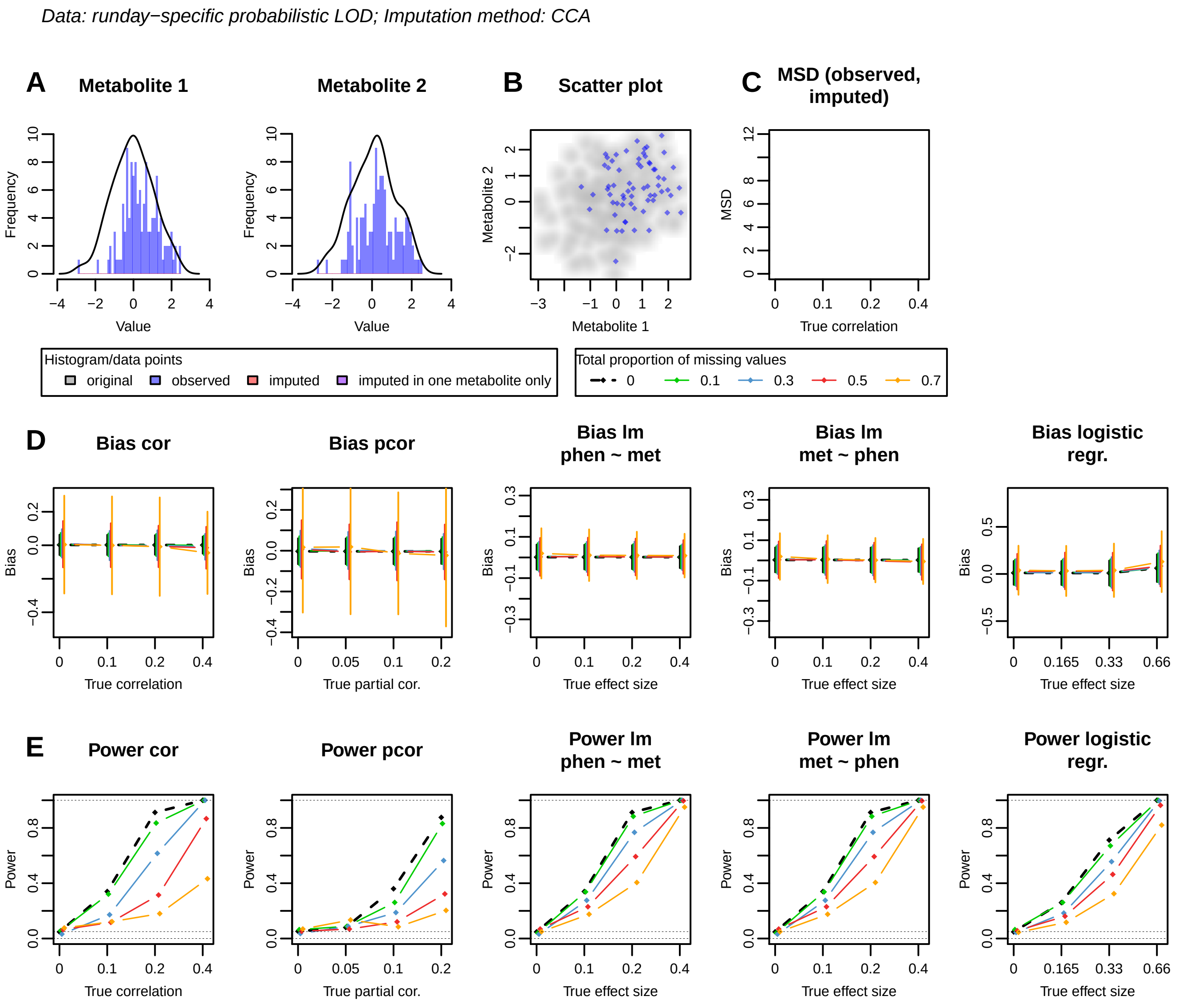
Data: runday−specific probabilistic LOD; Imputation method: CCA
A
Metabolite 1
Metabolite 2
B
Scatter plot
C
MSD (observed, imputed)
Frequency
Value
Metabolite 2
Metabolite 1
MSD
True correlation
Histogram/data points
original
observed
imputed
imputed in one metabolite only
Total proportion of missing values
0
0.1
0.3
0.5
0.7
D
Bias cor
Bias pcor
Bias lm phen ~ met
Bias lm met ~ phen
Bias logistic regr.
Bias
True correlation
True partial cor.
True effect size
E
Power cor
Power pcor
Power lm phen ~ met
Power lm met ~ phen
Power logistic regr.
Power

Data: runday−specific probabilistic LOD; Imputation method: min

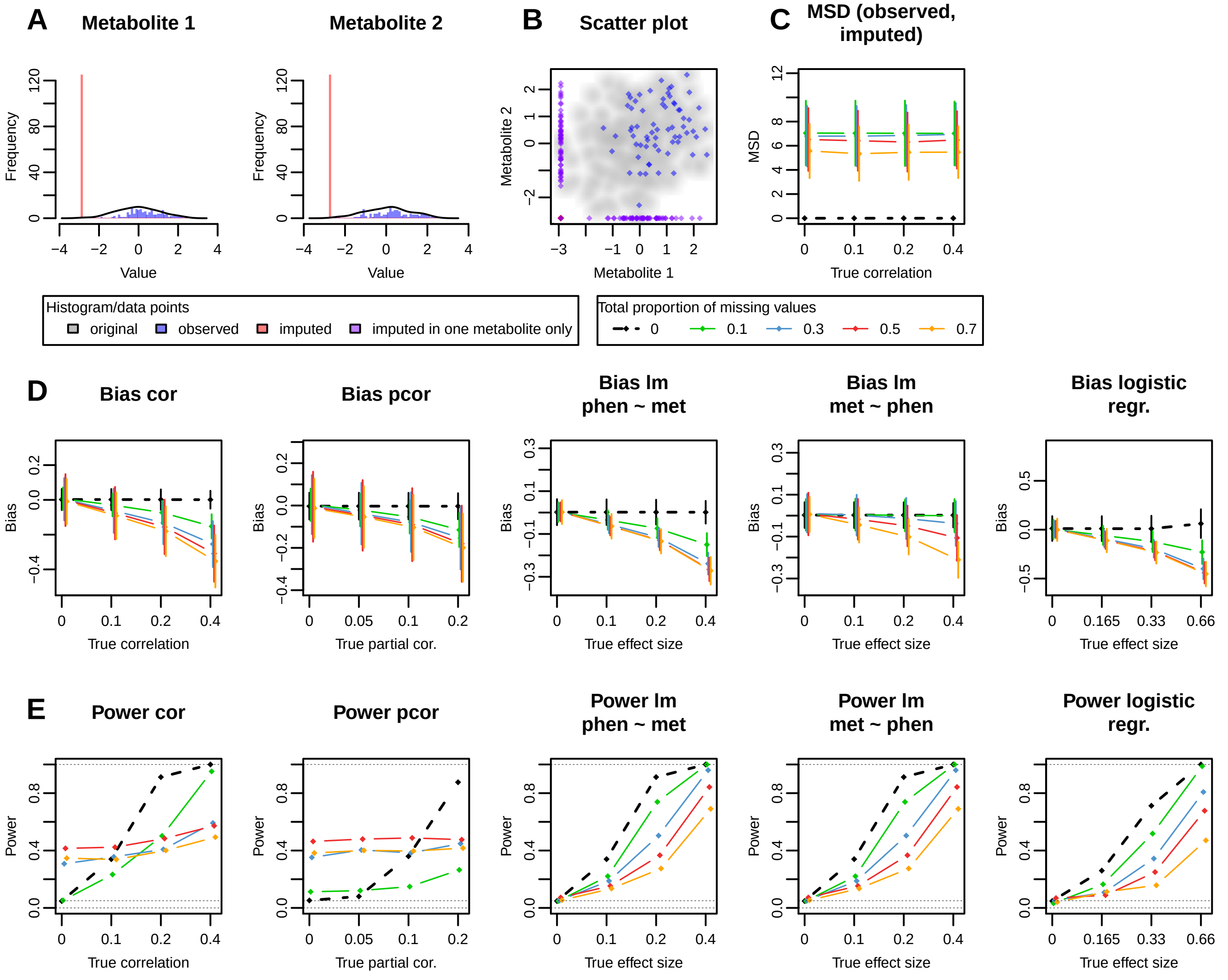

Data: runday−specific probabilistic LOD; Imputation method: RC

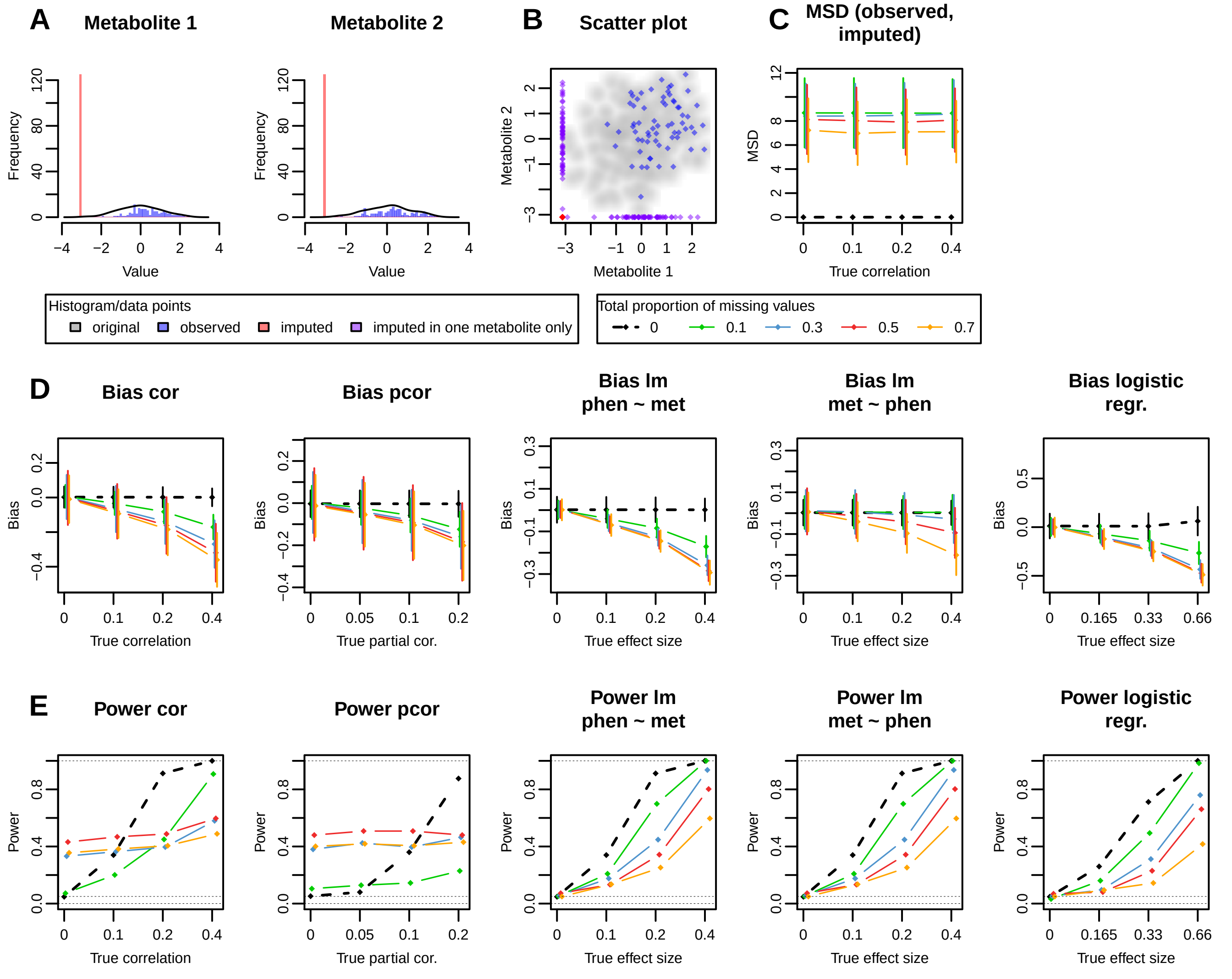

Data: runday−specific probabilistic LOD; Imputation method: ITS

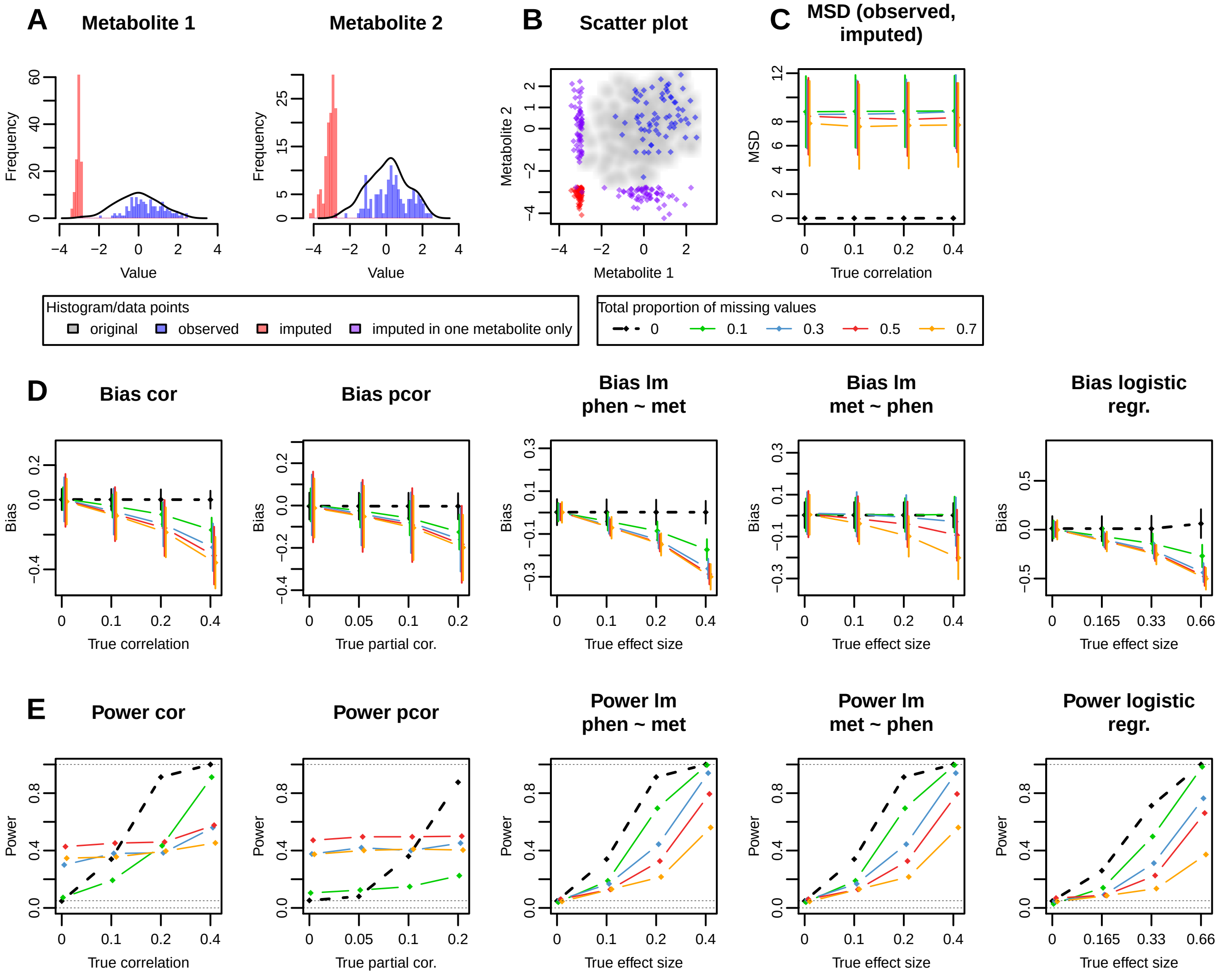

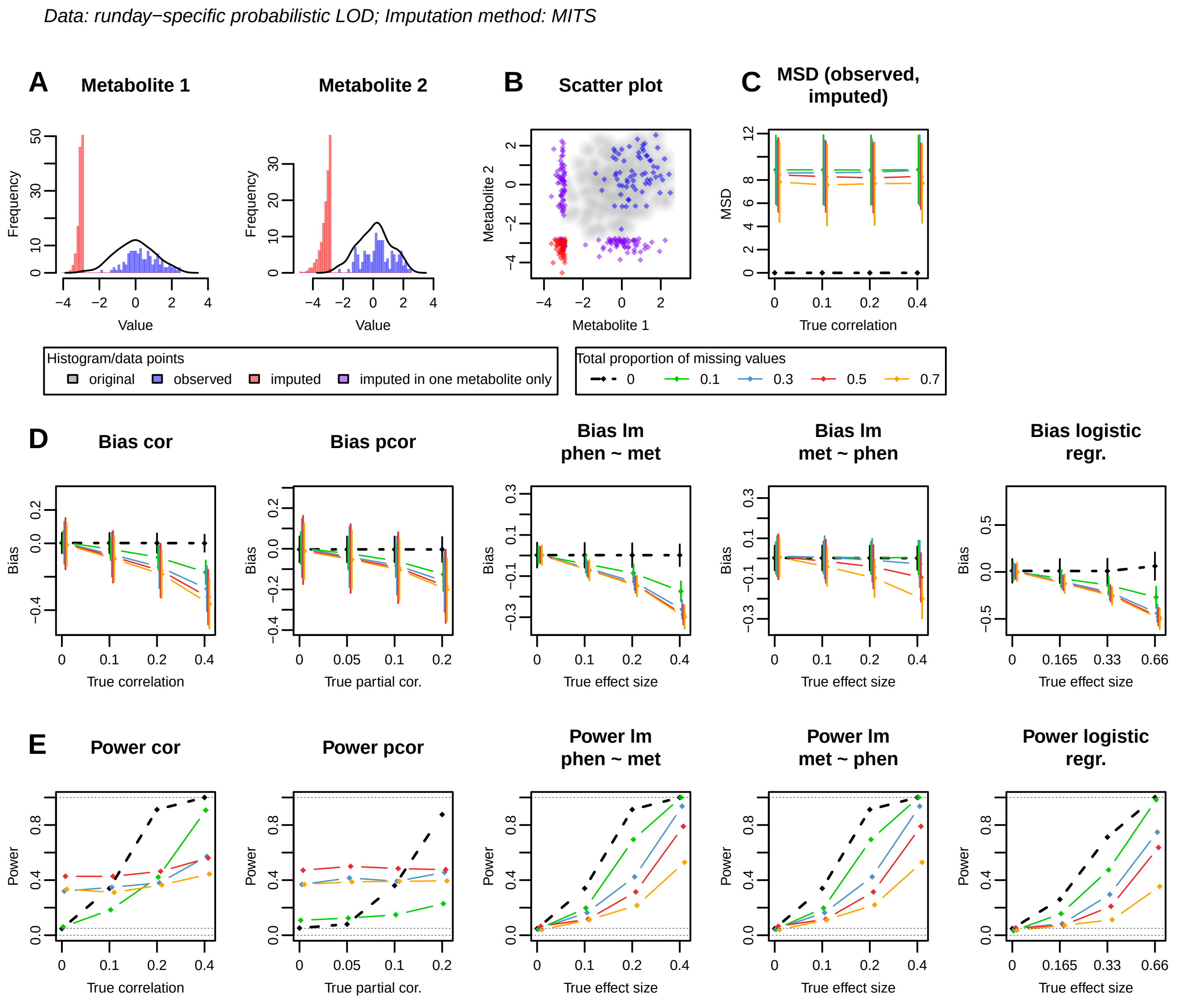
Data: runday−specific probabilistic LOD; Imputation method: MITS
A
Metabolite 1
Metabolite 2
Frequency
Value
B
Scatter plot
Metabolite 2
Metabolite 1
C
MSD (observed, imputed)
MSD
True correlation
Histogram/data points
original
observed
imputed
imputed in one metabolite only
Total proportion of missing values
0
0.1
0.3
0.5
0.7
D
Bias cor
Bias pcor
Bias lm phen ~ met
Bias lm met ~ phen
Bias logistic regr.
Bias
True correlation
True partial cor.
True effect size
E
Power cor
Power pcor
Power lm phen ~ met
Power lm met ~ phen
Power logistic regr.
Power
True correlation
True partial cor.
True effect size

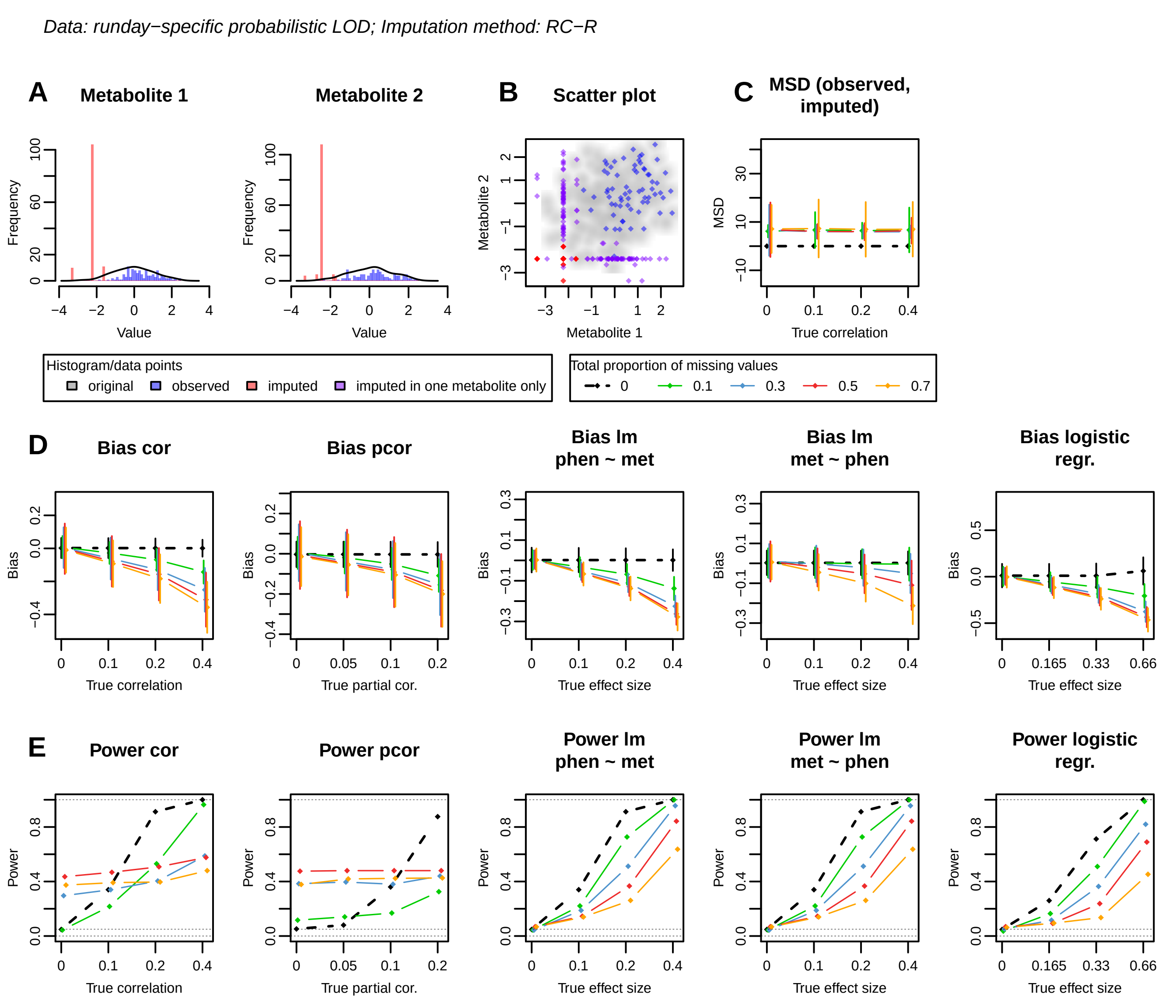

Data: runday−specific probabilistic LOD; Imputation method: RC−R
A
Metabolite 1
Metabolite 2
B
Scatter plot
C
MSD (observed, imputed)
Frequency
Value
Metabolite 2
Metabolite 1
MSD
True correlation
Histogram/data points
original
observed
imputed
imputed in one metabolite only
Total proportion of missing values
0
0.1
0.3
0.5
0.7
D
Bias cor
Bias pcor
Bias lm phen ~ met
Bias lm met ~ phen
Bias logistic regr.
Bias
True correlation
True partial cor.
True effect size
E
Power cor
Power pcor
Power lm phen ~ met
Power lm met ~ phen
Power logistic regr.
Power

Data: runday−specific probabilistic LOD; Imputation method: ITS−R

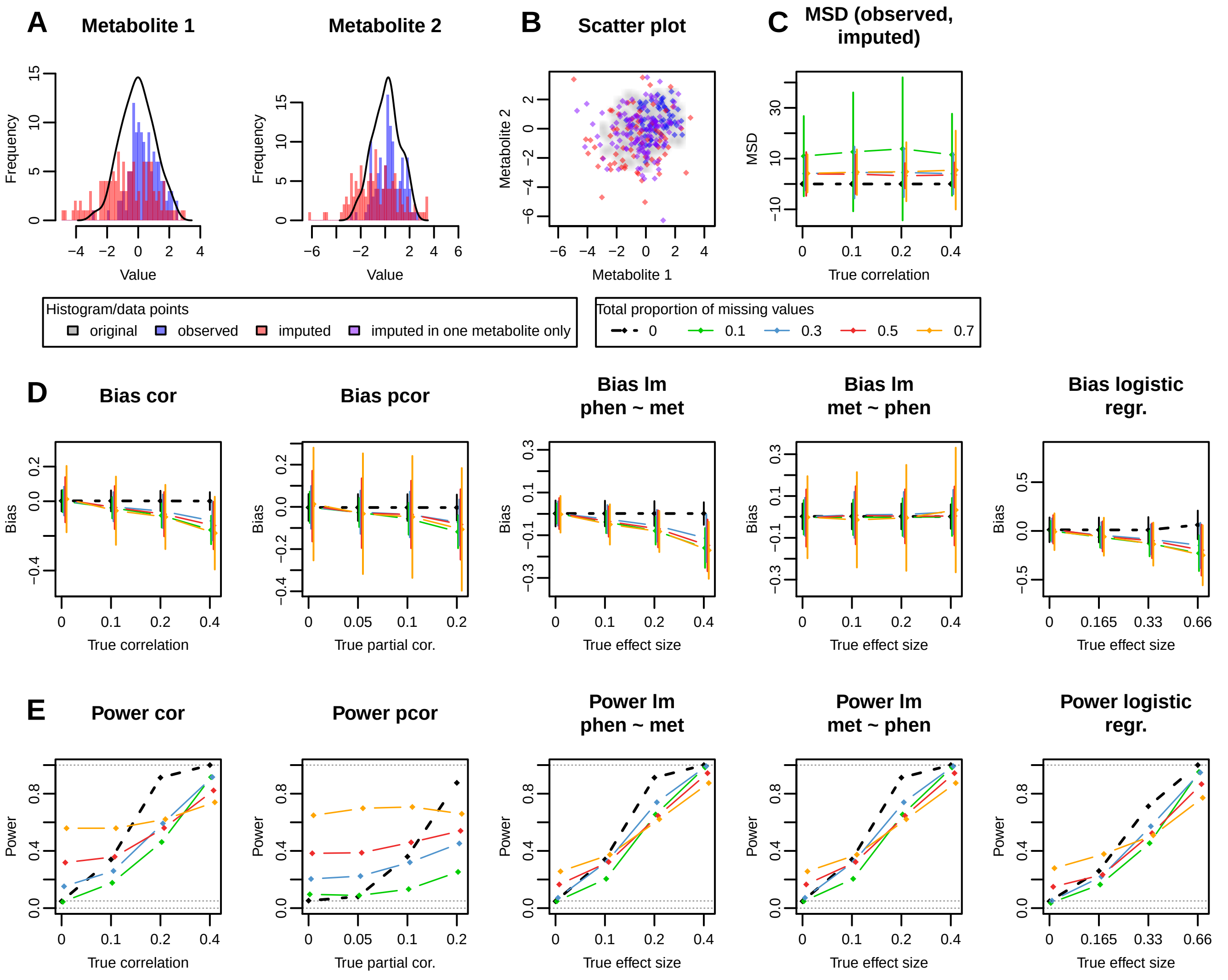

Data: runday−specific probabilistic LOD; Imputation method: MITS−R

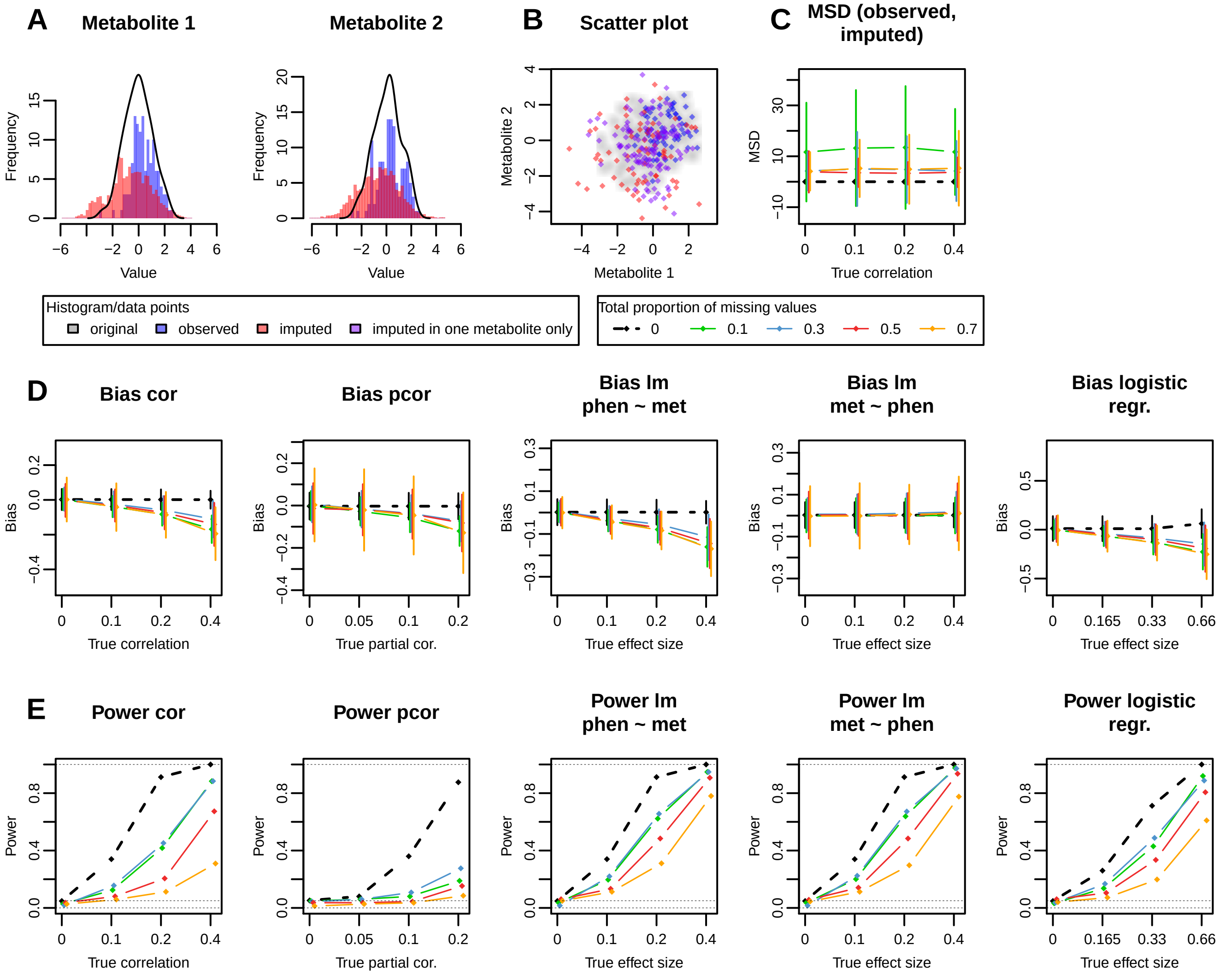

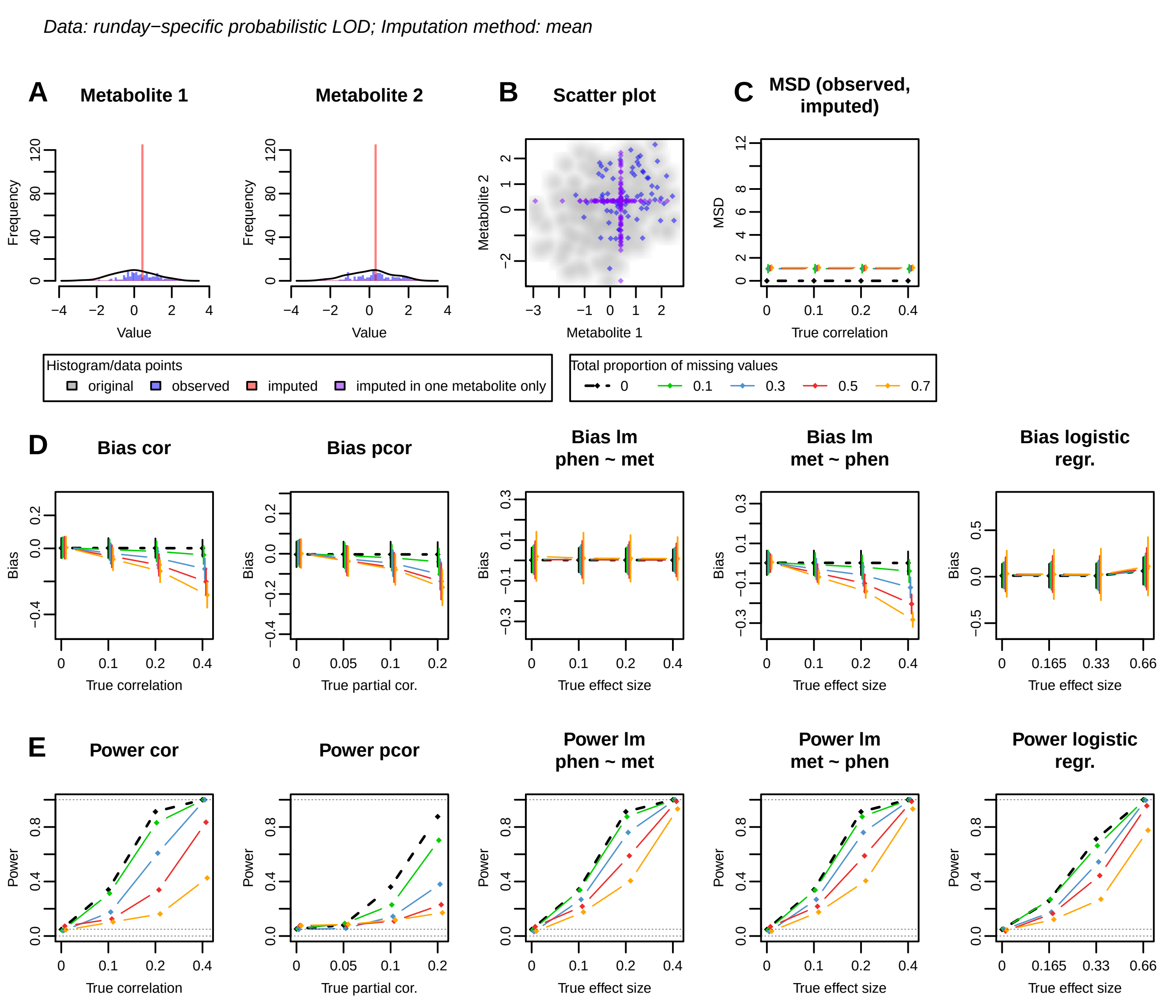
Data: runday−specific probabilistic LOD; Imputation method: mean
A
Metabolite 1
Metabolite 2
B
Scatter plot
C
MSD (observed, imputed)
Frequency
Value
Metabolite 1
Metabolite 2
MSD
True correlation
Histogram/data points
original
observed
imputed
imputed in one metabolite only
Total proportion of missing values
0
0.1
0.3
0.5
0.7
D
Bias cor
Bias pcor
Bias lm phen ~ met
Bias lm met ~ phen
Bias logistic regr.
Bias
True correlation
True partial cor.
True effect size
E
Power cor
Power pcor
Power lm phen ~ met
Power lm met ~ phen
Power logistic regr.
Power
True correlation
True partial cor.
True effect size

Data: runday−specific probabilistic LOD; Imputation method: ICE−norm

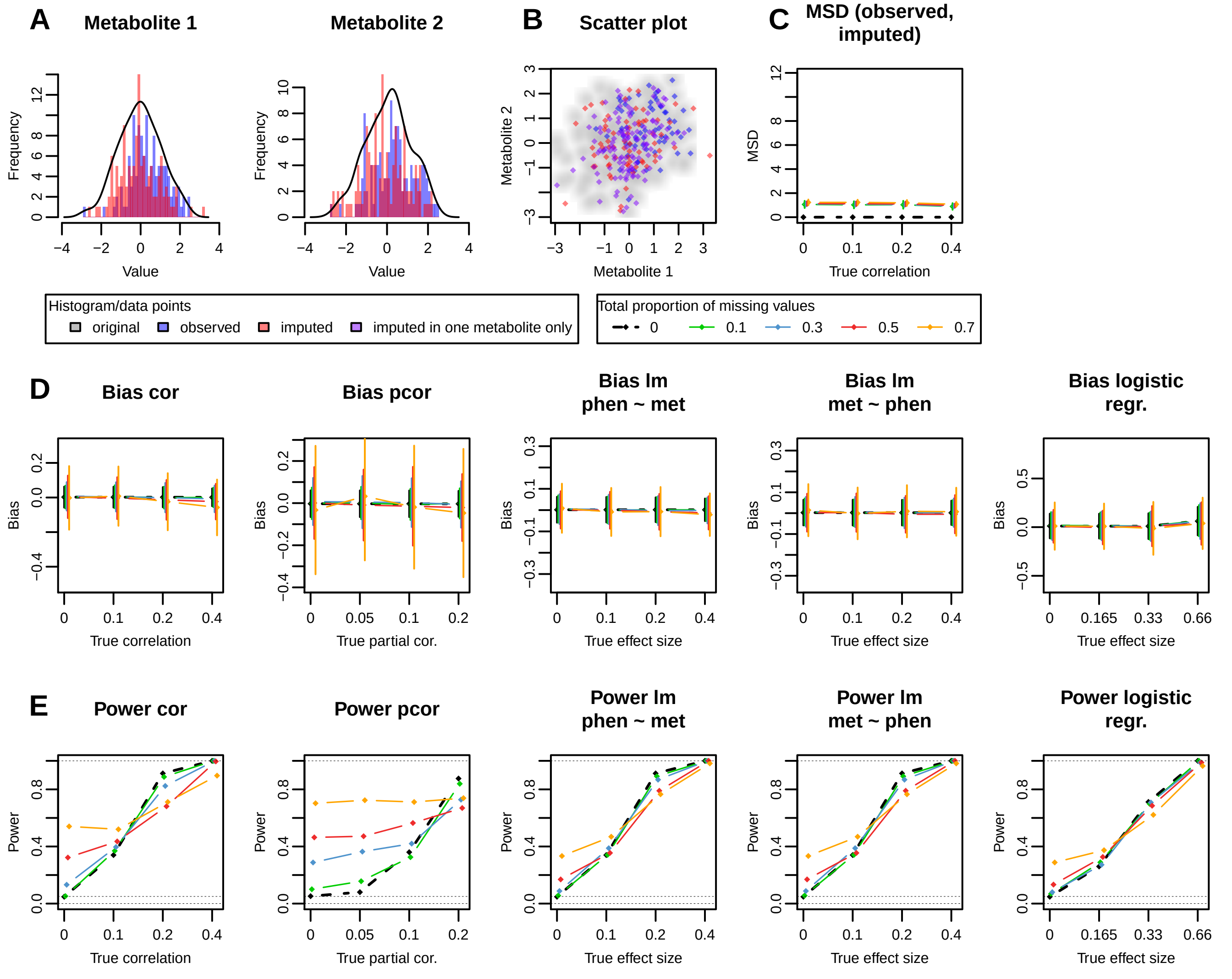

Data: runday−specific probabilistic LOD; Imputation method: ICE−pmm

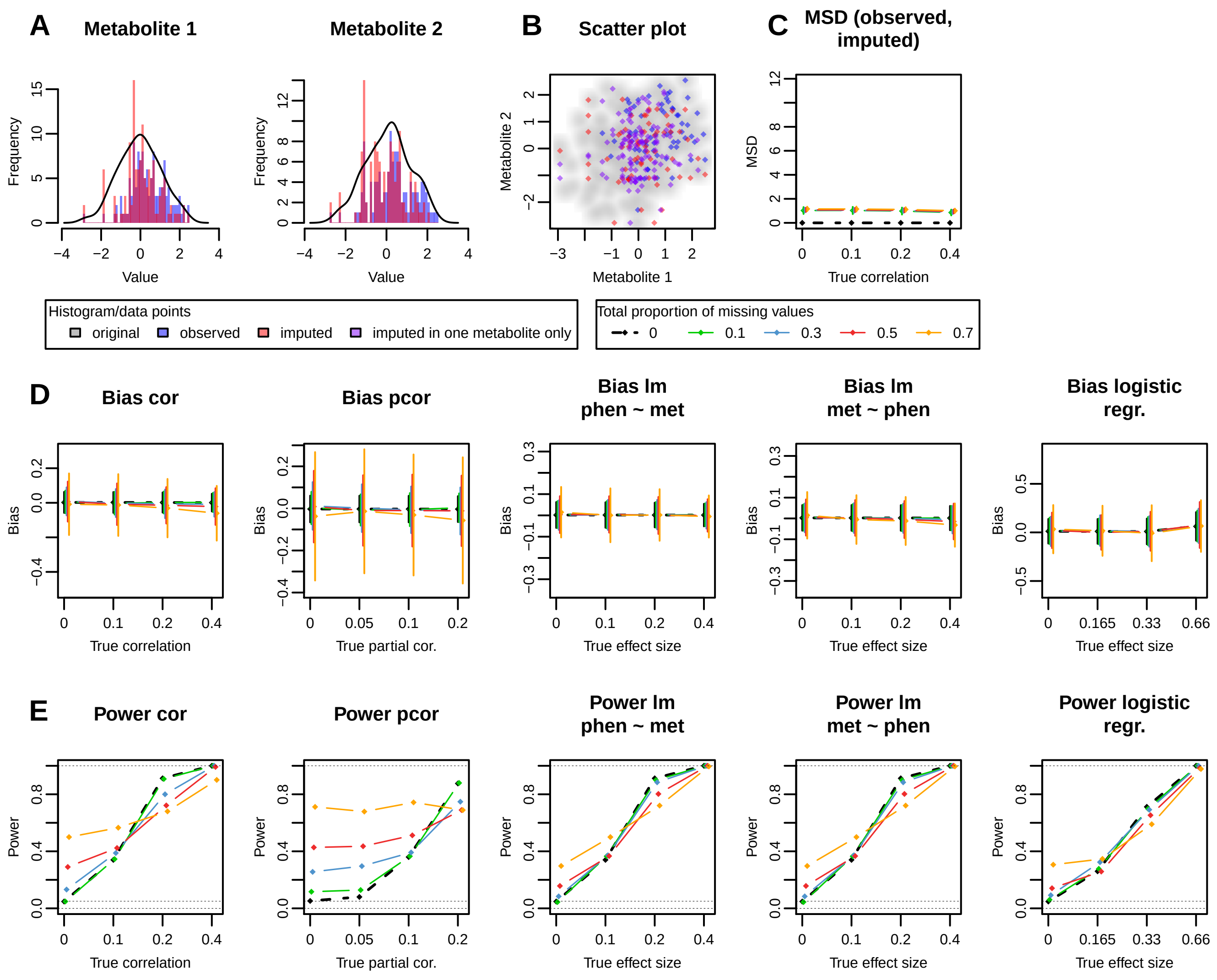

Data: runday−specific probabilistic LOD; Imputation method: ICE−adjR

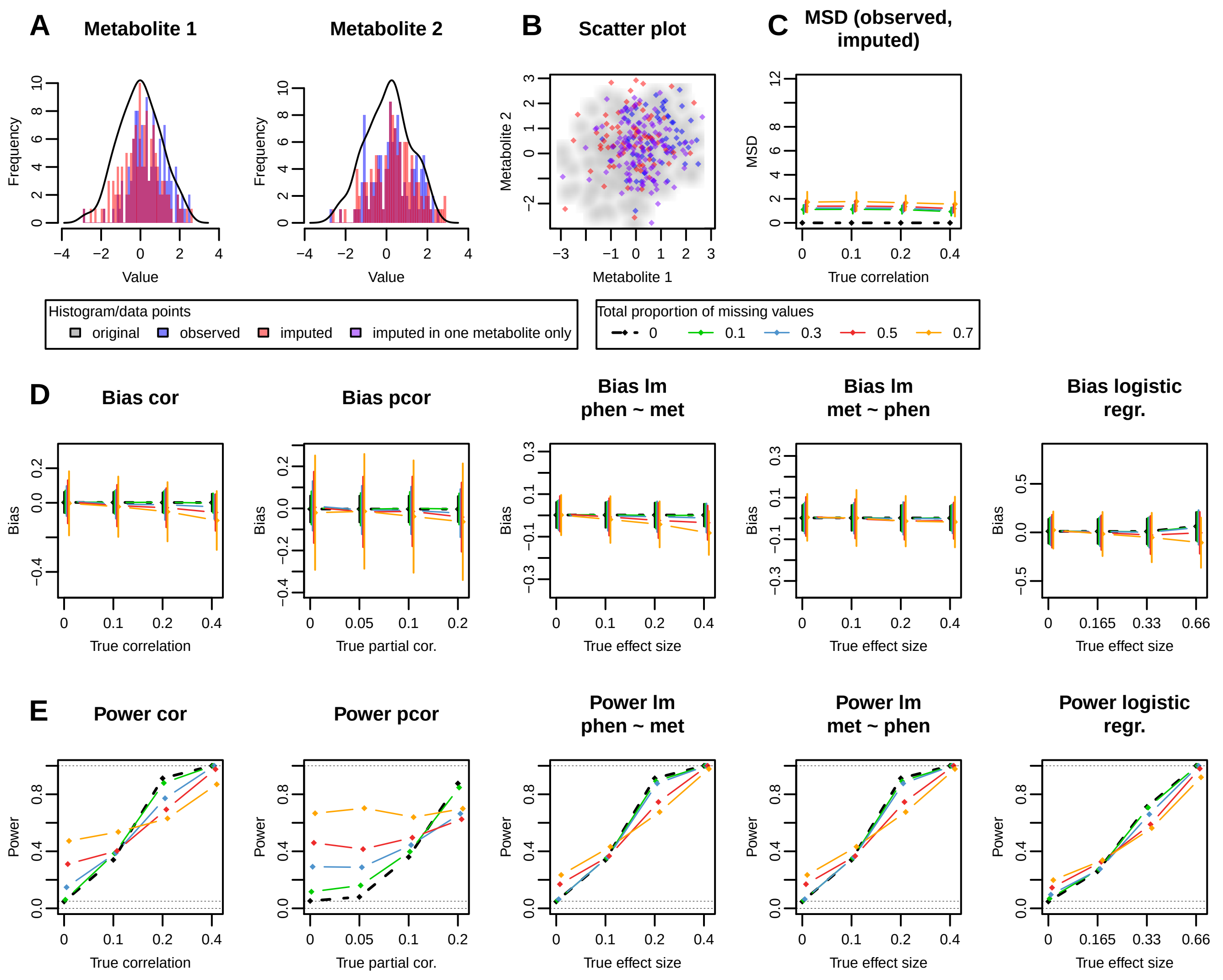

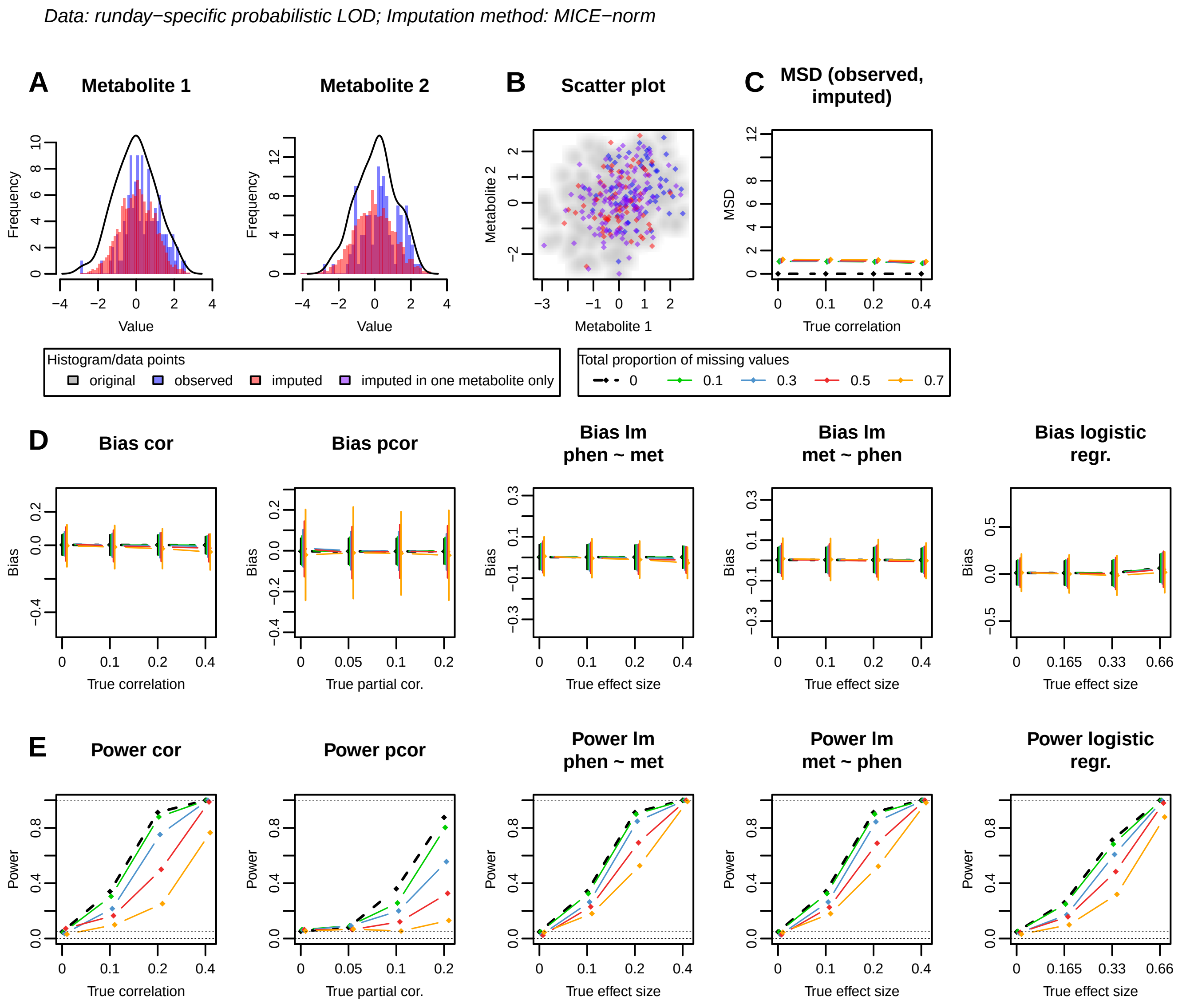

Data: runday−specific probabilistic LOD; Imputation method: MICE−norm
A
Metabolite 1
Metabolite 2
B
Scatter plot
C
MSD (observed, imputed)
Frequency
Value
Metabolite 2
Metabolite 1
MSD
True correlation
Histogram/data points
original
observed
imputed
imputed in one metabolite only
Total proportion of missing values
0
0.1
0.3
0.5
0.7
D
Bias cor
Bias pcor
Bias lm phen ~ met
Bias lm met ~ phen
Bias logistic regr.
Bias
True correlation
True partial cor.
True effect size
E
Power cor
Power pcor
Power lm phen ~ met
Power lm met ~ phen
Power logistic regr.
Power
True correlation
True partial cor.
True effect size

Data: runday−specific probabilistic LOD; Imputation method: MICE−pmm

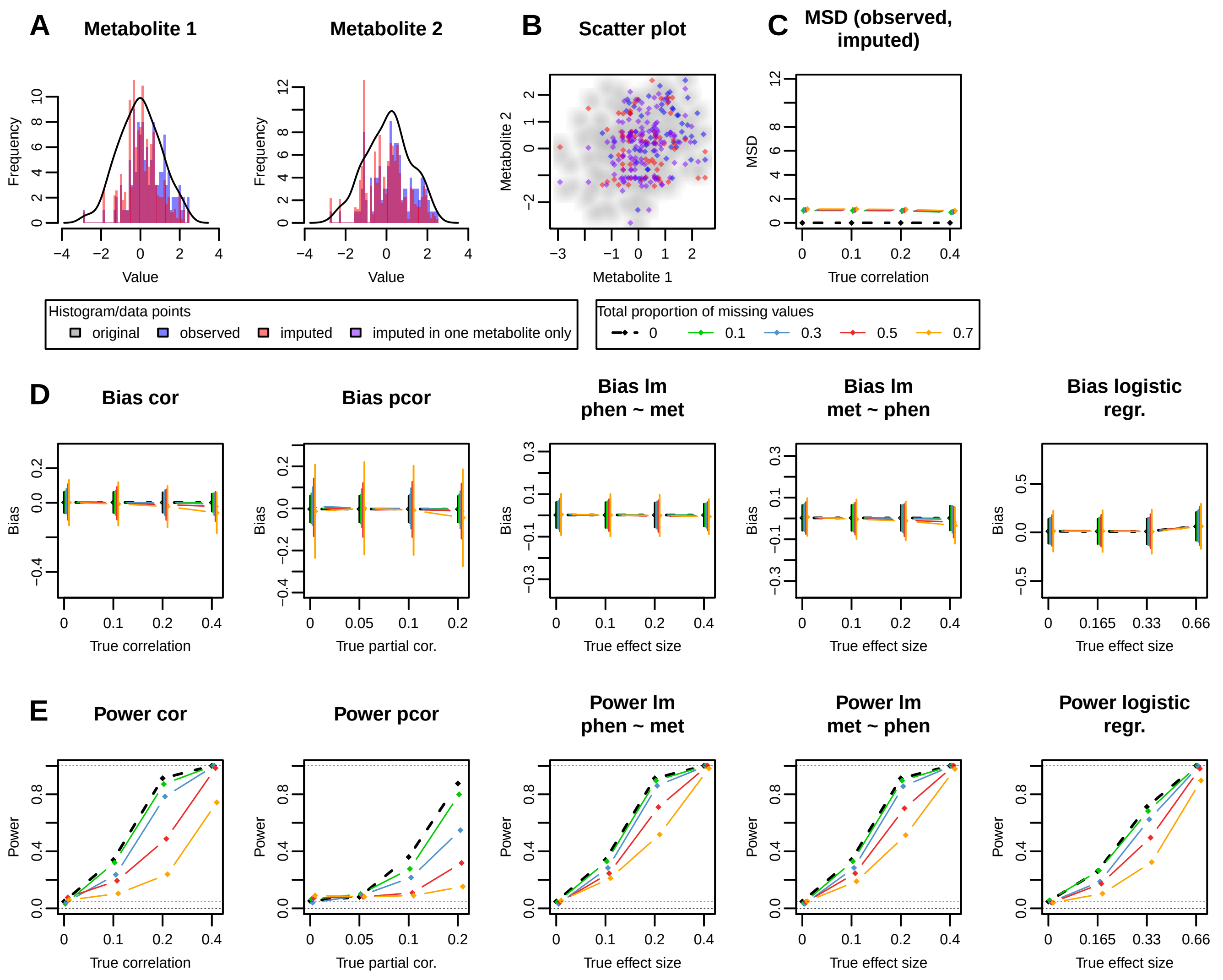

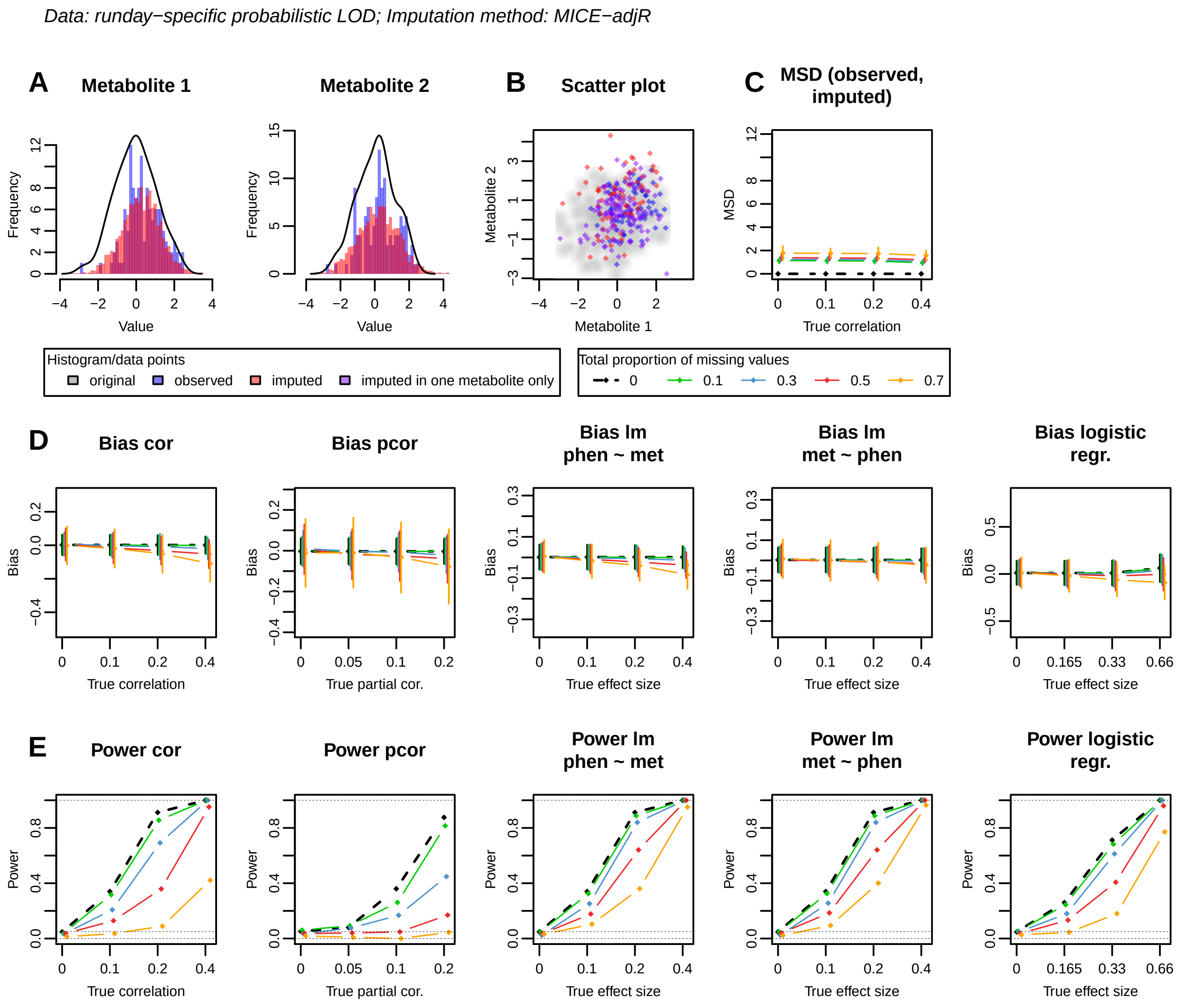

Data: runday−specific probabilistic LOD; Imputation method: MICE−adjR
A
Metabolite 1
Metabolite 2
B
Scatter plot
C
MSD (observed, imputed)
Frequency
Value
Metabolite 1
Metabolite 2
MSD
True correlation
Histogram/data points
original
observed
imputed
imputed in one metabolite only
Total proportion of missing values
0
0.1
0.3
0.5
0.7
D
Bias cor
Bias pcor
Bias lm phen ~ met
Bias lm met ~ phen
Bias logistic regr.
Bias
True correlation
True partial cor.
True effect size
E
Power cor
Power pcor
Power lm phen ~ met
Power lm met ~ phen
Power logistic regr.
Power

Data: runday−specific probabilistic LOD; Imputation method: KNN−var(1)

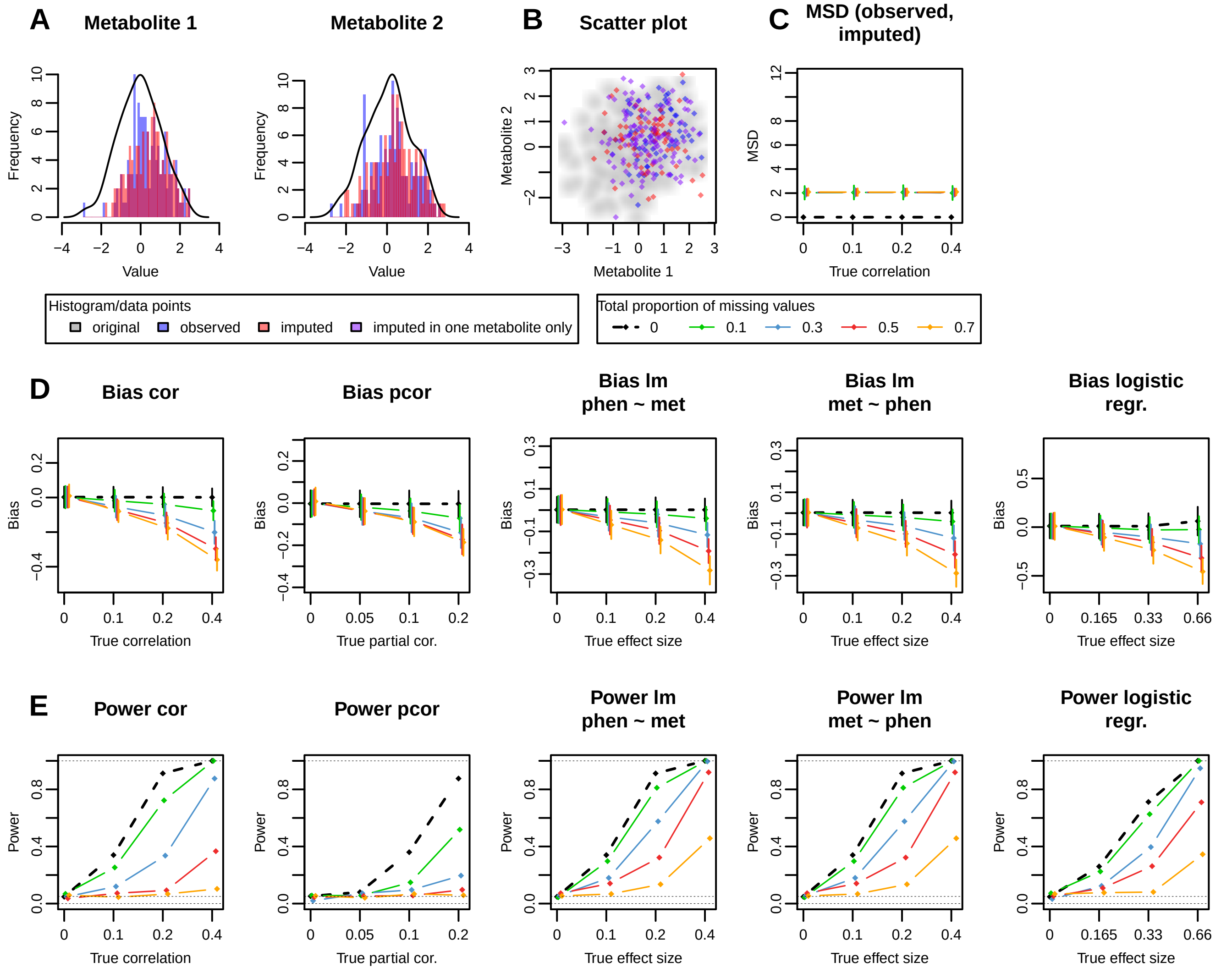

Data: runday−specific probabilistic LOD; Imputation method: KNN−var(3)

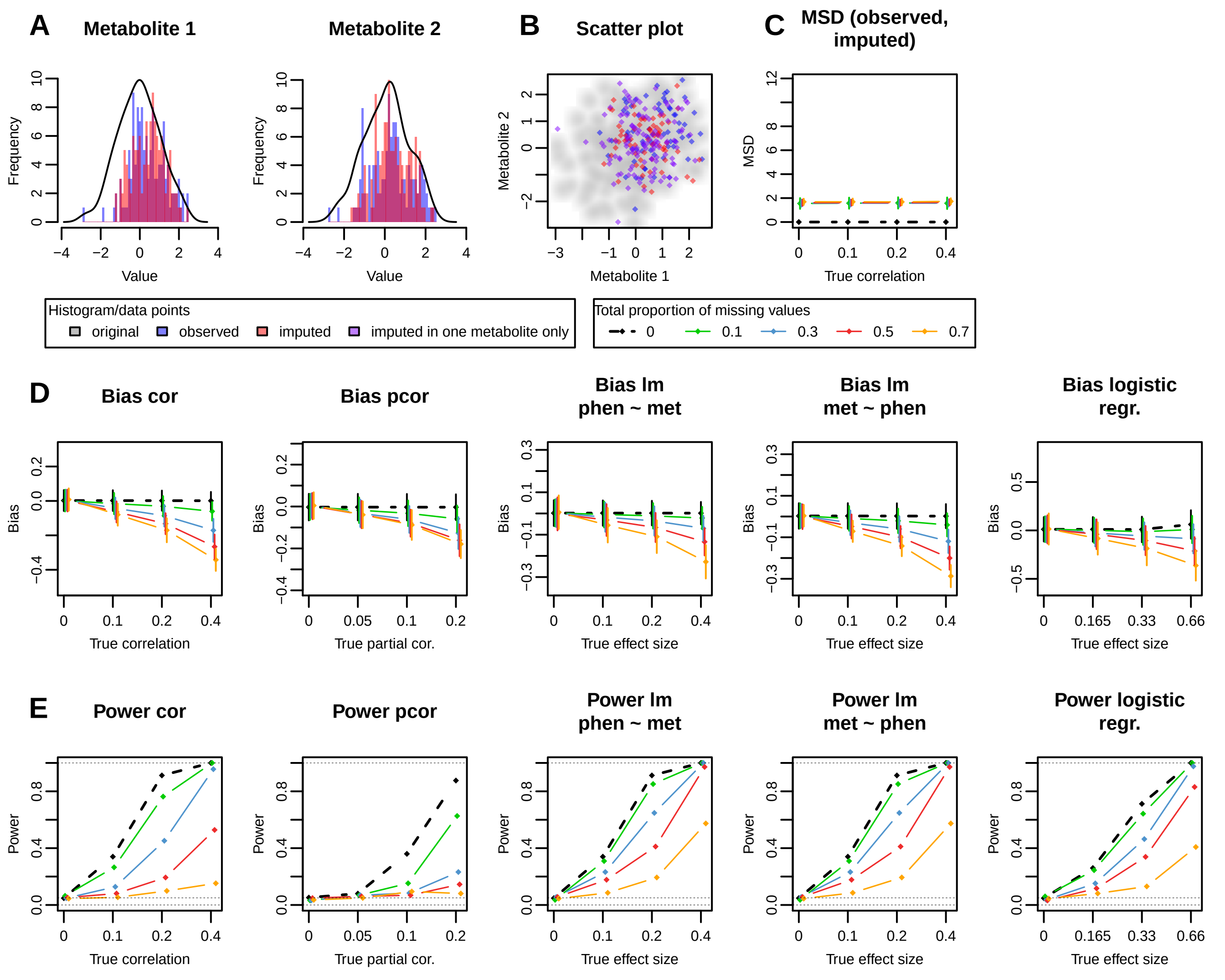

Data: runday−specific probabilistic LOD; Imputation method: KNN−var(5)

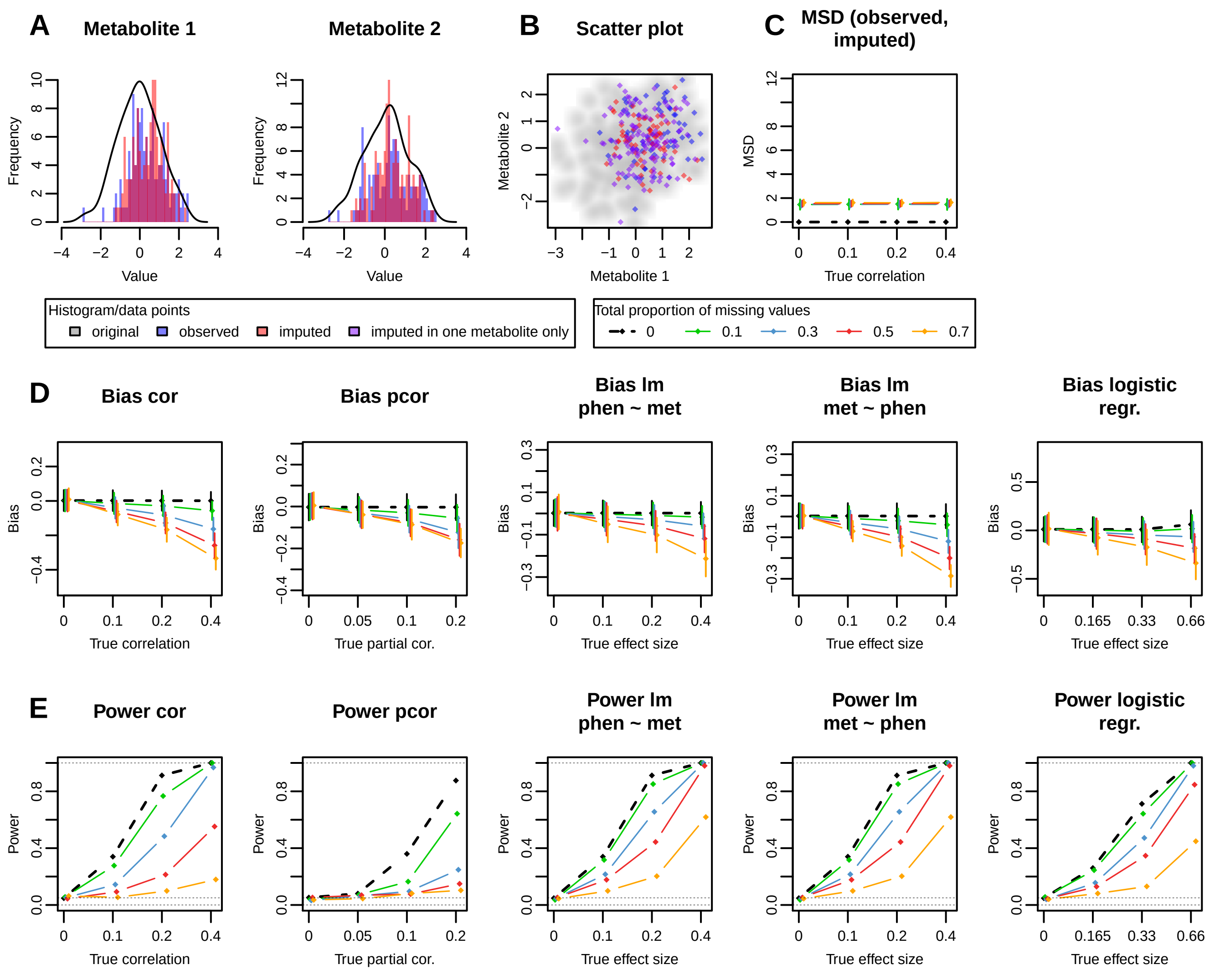

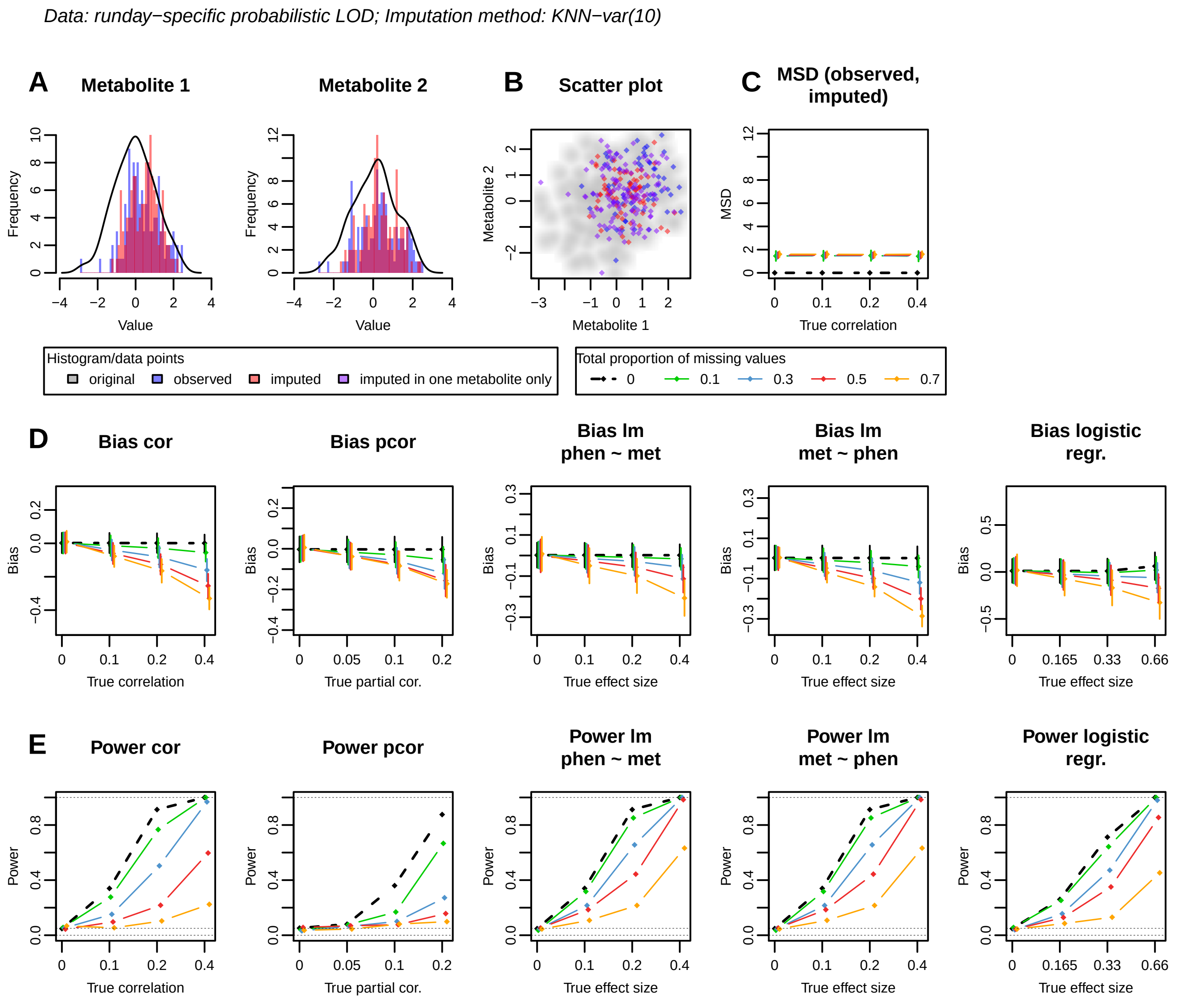
Data: runday−specific probabilistic LOD; Imputation method: KNN−var(10)
A
Metabolite 1
Metabolite 2
Frequency
Value
B
Scatter plot
Metabolite 1
Metabolite 2
C
MSD (observed, imputed)
MSD
True correlation
Histogram/data points
original
observed
imputed
imputed in one metabolite only
Total proportion of missing values
0
0.1
0.3
0.5
0.7
D
Bias cor
Bias pcor
Bias lm phen ~ met
Bias lm met ~ phen
Bias logistic regr.
Bias
True correlation
True partial cor.
True effect size
E
Power cor
Power pcor
Power lm phen ~ met
Power lm met ~ phen
Power logistic regr.
Power

Data: runday−specific probabilistic LOD; Imputation method: KNN−var(20)

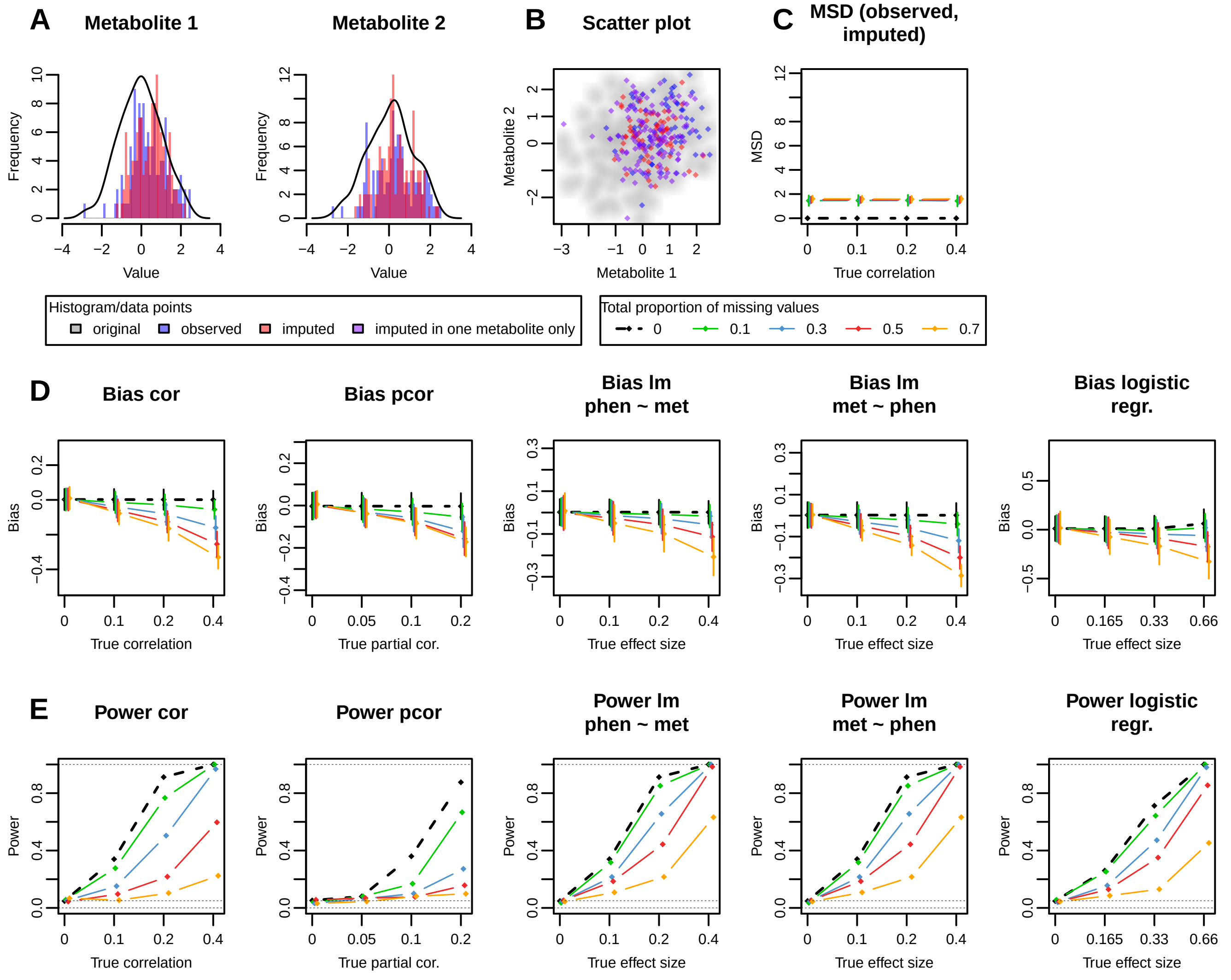

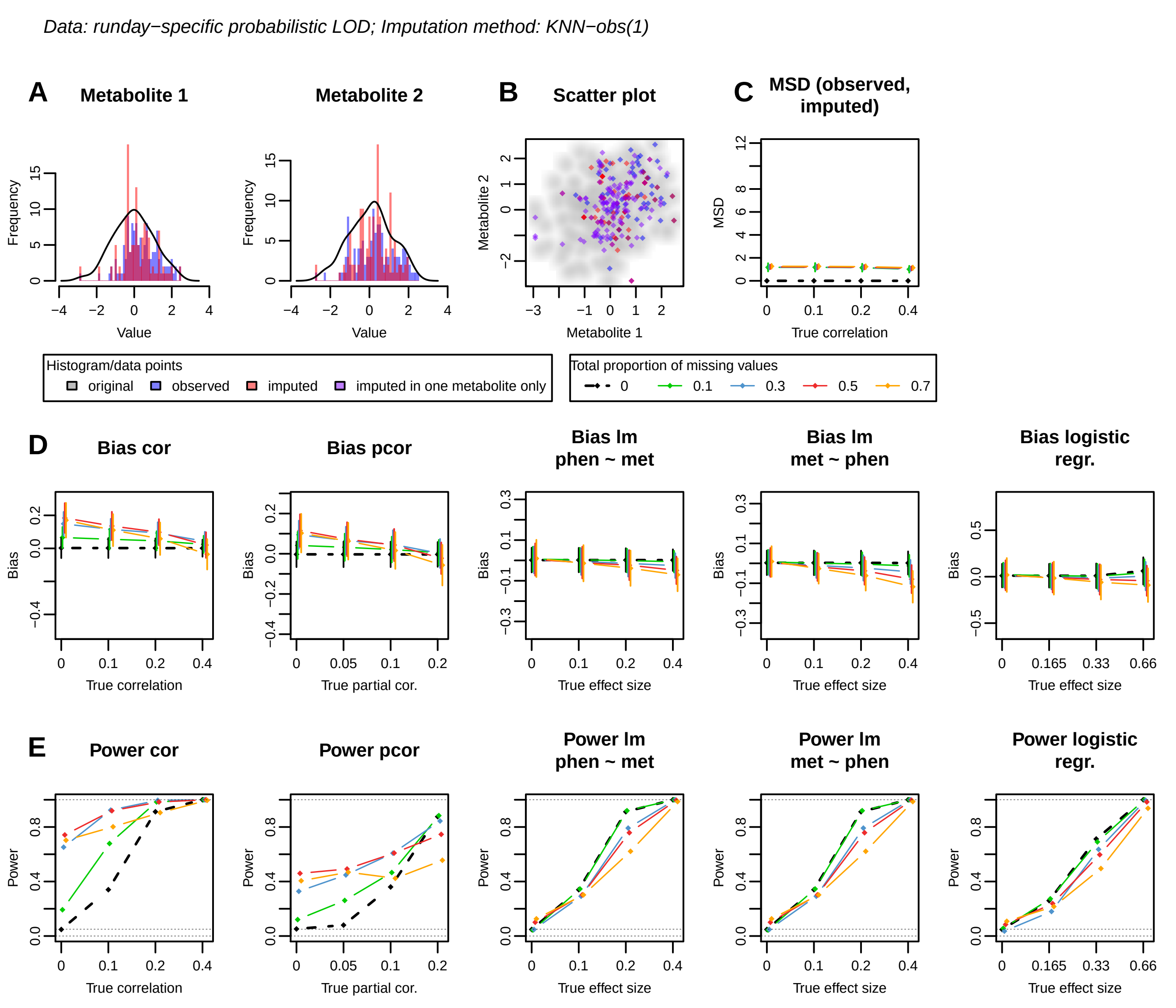

Data: runday−specific probabilistic LOD; Imputation method: KNN−obs(1)
A
Metabolite 1
Metabolite 2
Frequency
Value
B
Scatter plot
Metabolite 2
Metabolite 1
C
MSD (observed, imputed)
MSD
True correlation
Histogram/data points
original
observed
imputed
imputed in one metabolite only
Total proportion of missing values
0
0.1
0.3
0.5
0.7
D
Bias cor
Bias pcor
Bias lm phen ~ met
Bias lm met ~ phen
Bias logistic regr.
Bias
True correlation
True partial cor.
True effect size
E
Power cor
Power pcor
Power lm phen ~ met
Power lm met ~ phen
Power logistic regr.
Power
True correlation
True partial cor.
True effect size

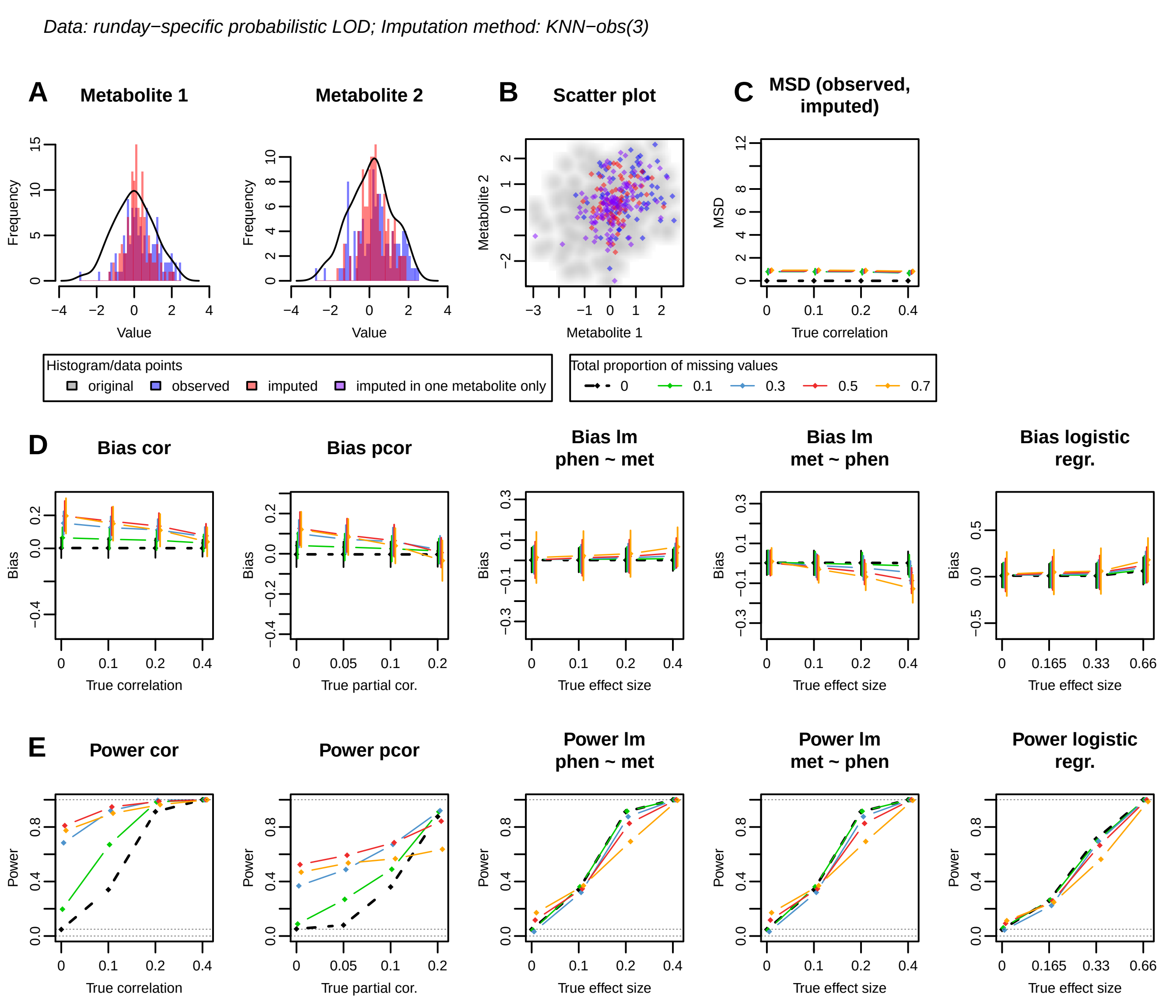

Data: runday−specific probabilistic LOD; Imputation method: KNN−obs(3)
A
Metabolite 1
Metabolite 2
B
Scatter plot
C
MSD (observed, imputed)
Frequency
Value
Metabolite 2
Metabolite 1
MSD
True correlation
Histogram/data points
original
observed
imputed
imputed in one metabolite only
Total proportion of missing values
0
0.1
0.3
0.5
0.7
D
Bias cor
Bias pcor
Bias lm phen ~ met
Bias lm met ~ phen
Bias logistic regr.
Bias
True correlation
True partial cor.
True effect size
E
Power cor
Power pcor
Power lm phen ~ met
Power lm met ~ phen
Power logistic regr.
Power
True correlation
True partial cor.
True effect size

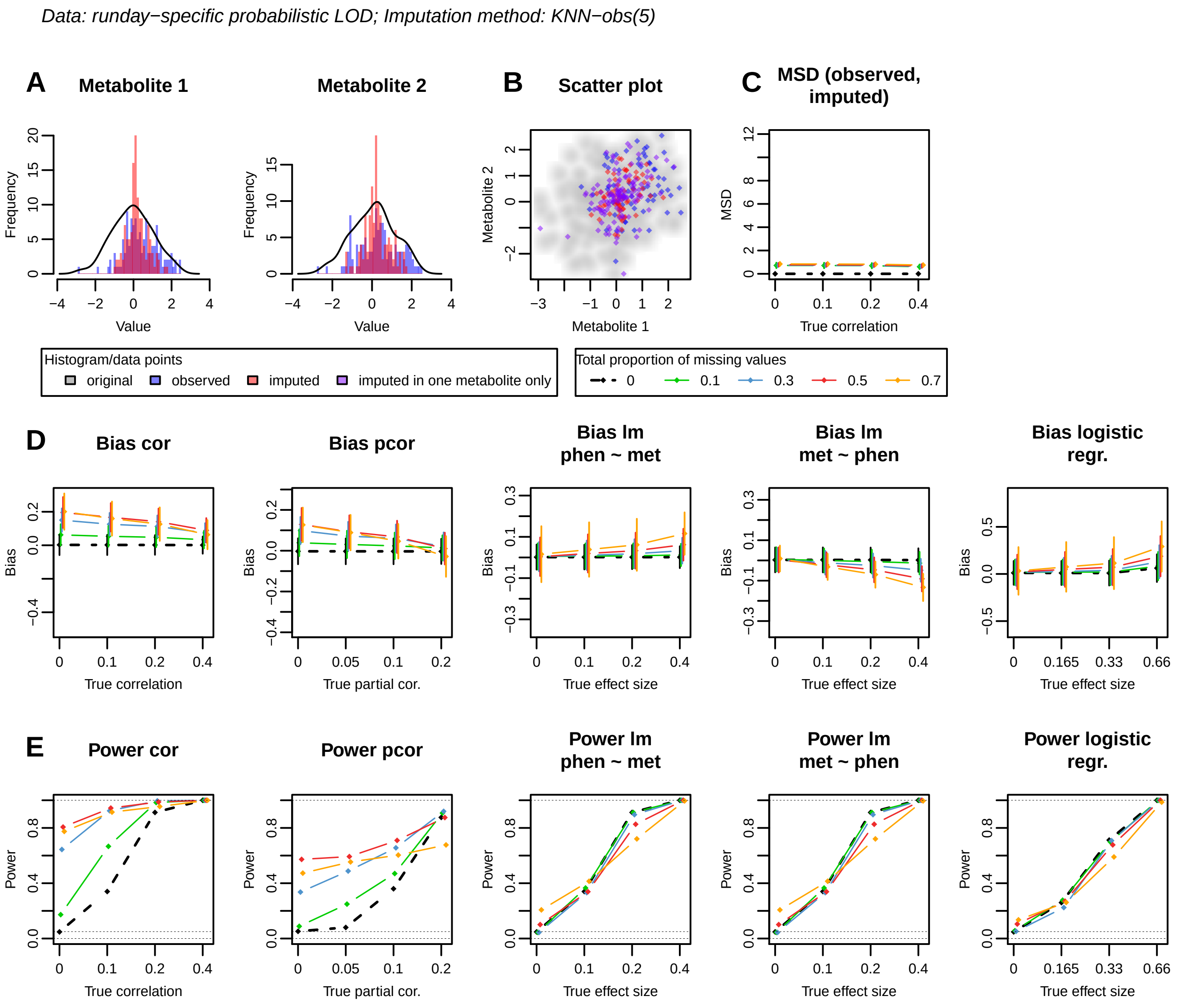
Data: runday−specific probabilistic LOD; Imputation method: KNN−obs(5)
A
Metabolite 1
Metabolite 2
Frequency
Value
B
Scatter plot
Metabolite 2
Metabolite 1
C
MSD (observed, imputed)
MSD
True correlation
Histogram/data points
original
observed
imputed
imputed in one metabolite only
Total proportion of missing values
0
0.1
0.3
0.5
0.7
D
Bias cor
Bias pcor
Bias lm phen ~ met
Bias lm met ~ phen
Bias logistic regr.
Bias
True correlation
True partial cor.
True effect size
E
Power cor
Power pcor
Power lm phen ~ met
Power lm met ~ phen
Power logistic regr.
Power

Data: runday−specific probabilistic LOD; Imputation method: KNN−obs(10)

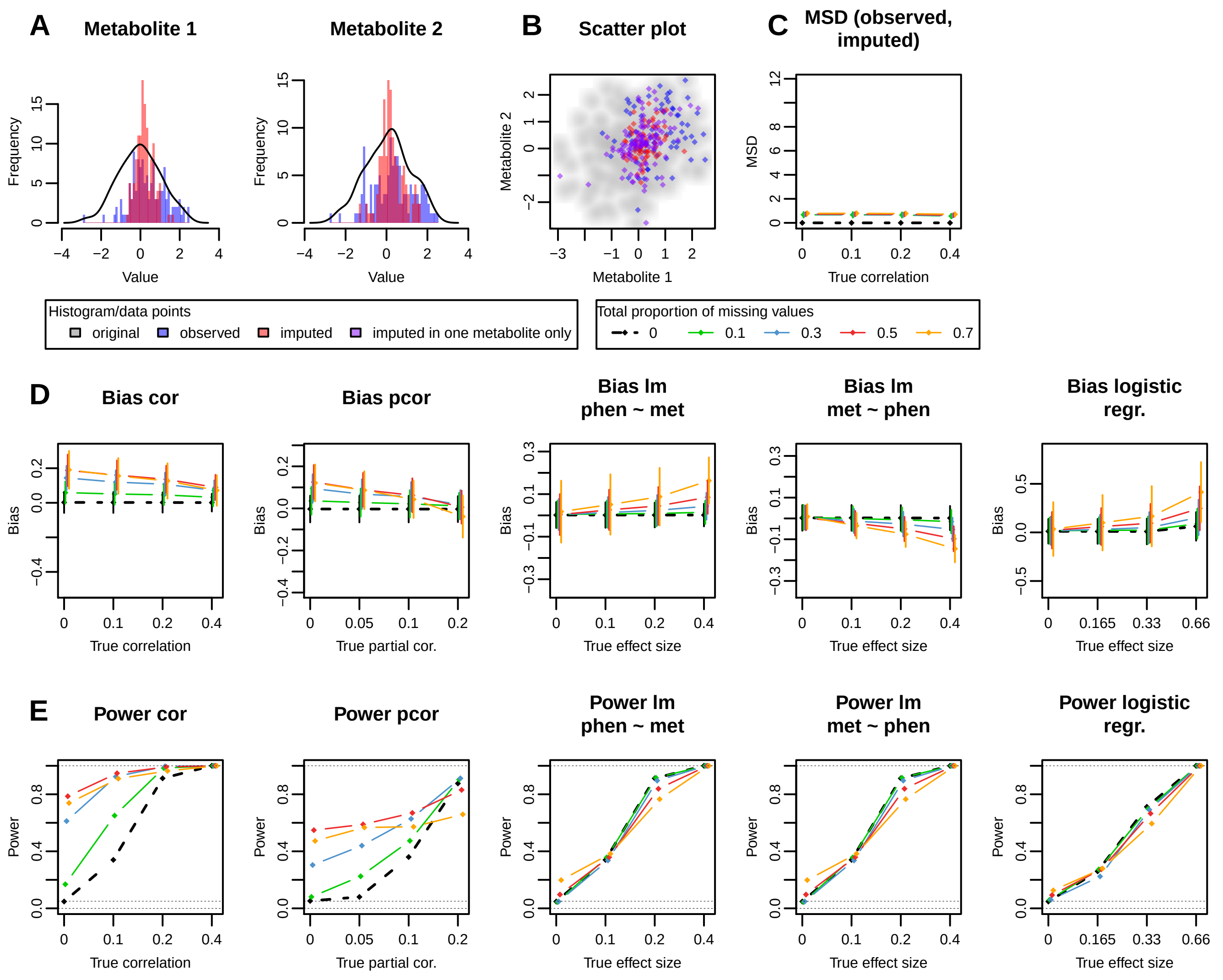

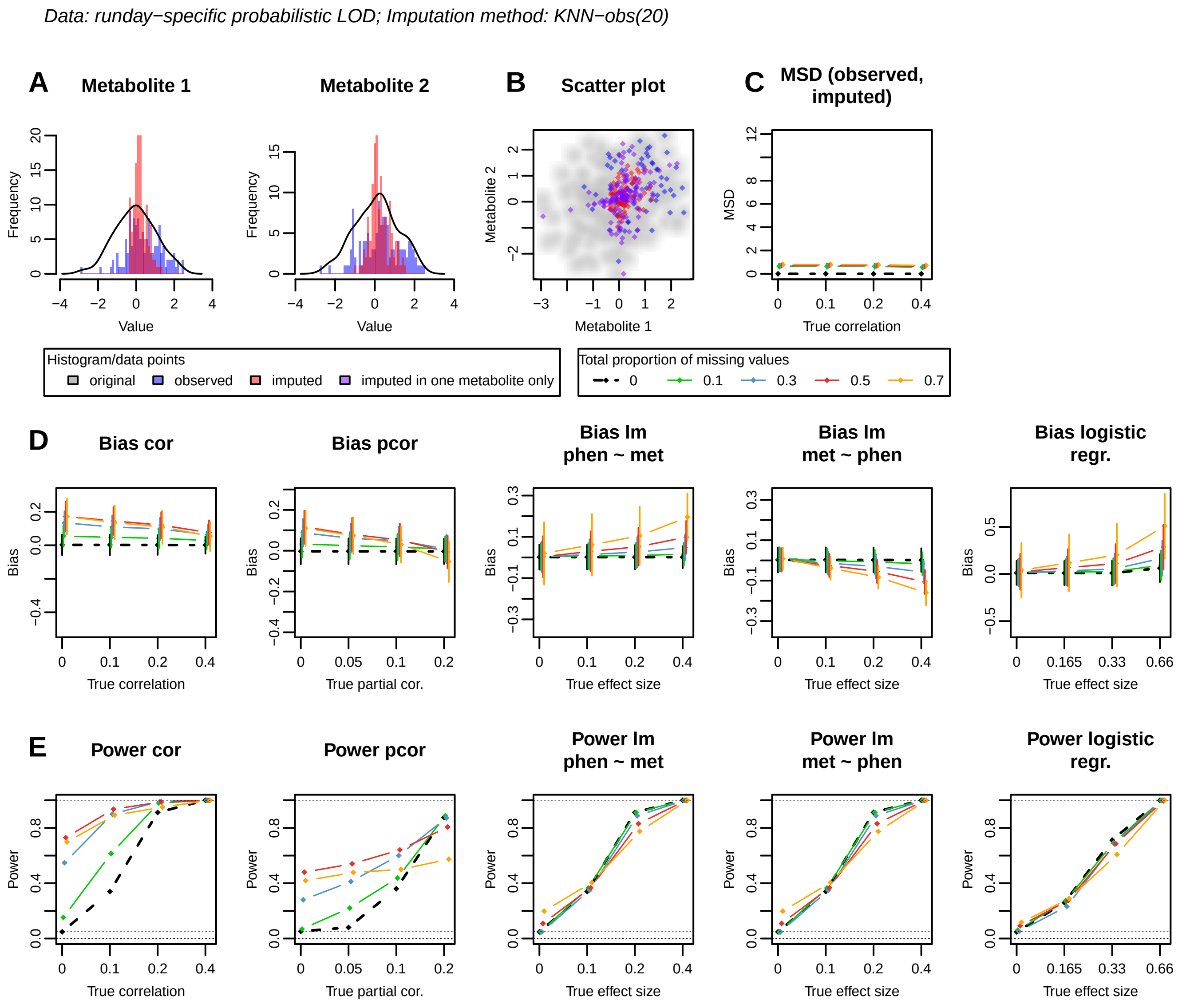
Data: runday−specific probabilistic LOD; Imputation method: KNN−obs(20)
A
Metabolite 1
Metabolite 2
Frequency
Value
B
Scatter plot
Metabolite 2
Metabolite 1
C
MSD (observed, imputed)
MSD
True correlation
Histogram/data points
original
observed
imputed
imputed in one metabolite only
Total proportion of missing values
0
0.1
0.3
0.5
0.7
D
Bias cor
Bias pcor
Bias lm phen ~ met
Bias lm met ~ phen
Bias logistic regr.
Bias
True correlation
True partial cor.
True effect size
E
Power cor
Power pcor
Power lm phen ~ met
Power lm met ~ phen
Power logistic regr.
Power
True correlation
True partial cor.
True effect size

Data: runday−specific probabilistic LOD; Imputation method: KNN−obs−sel(1)

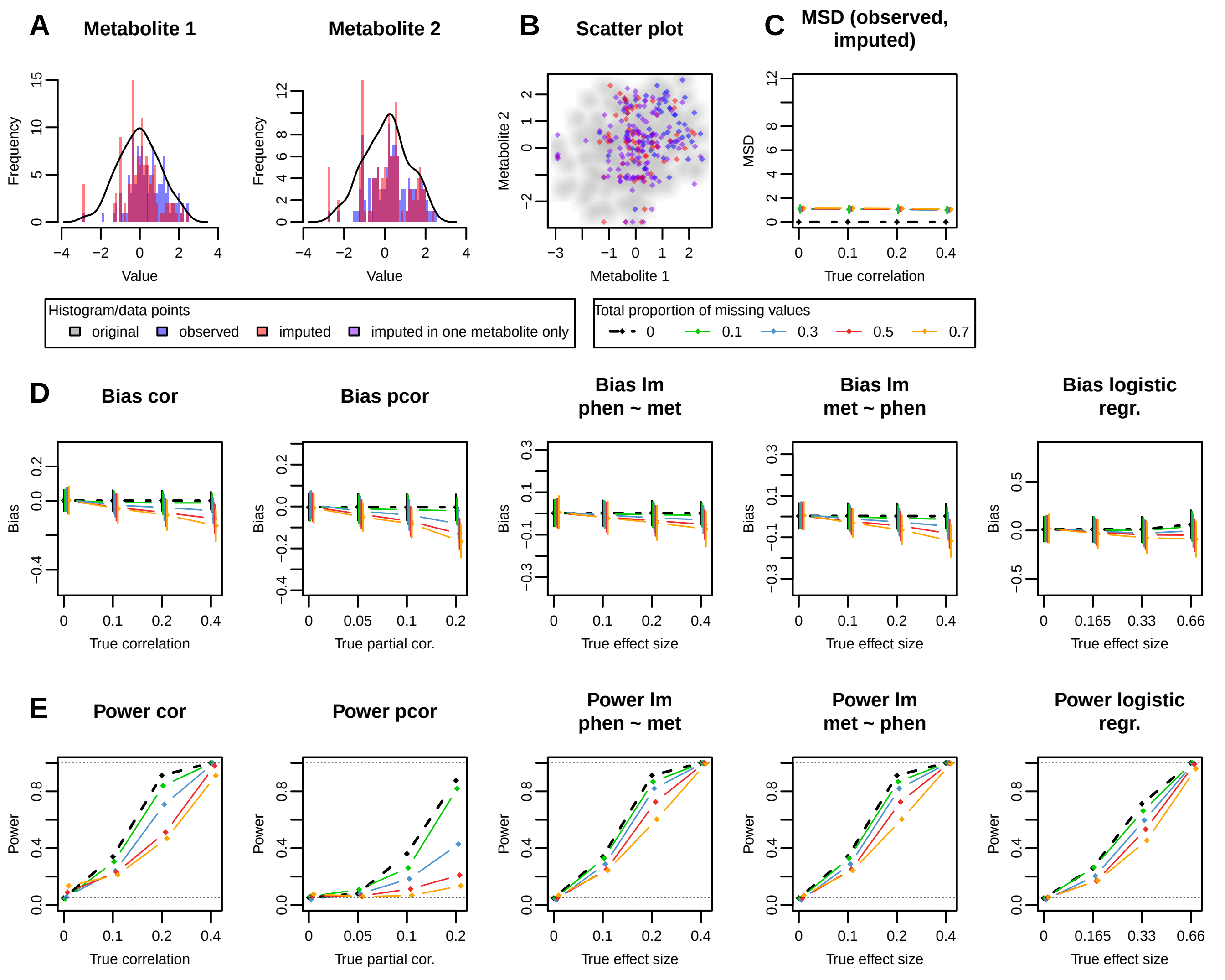

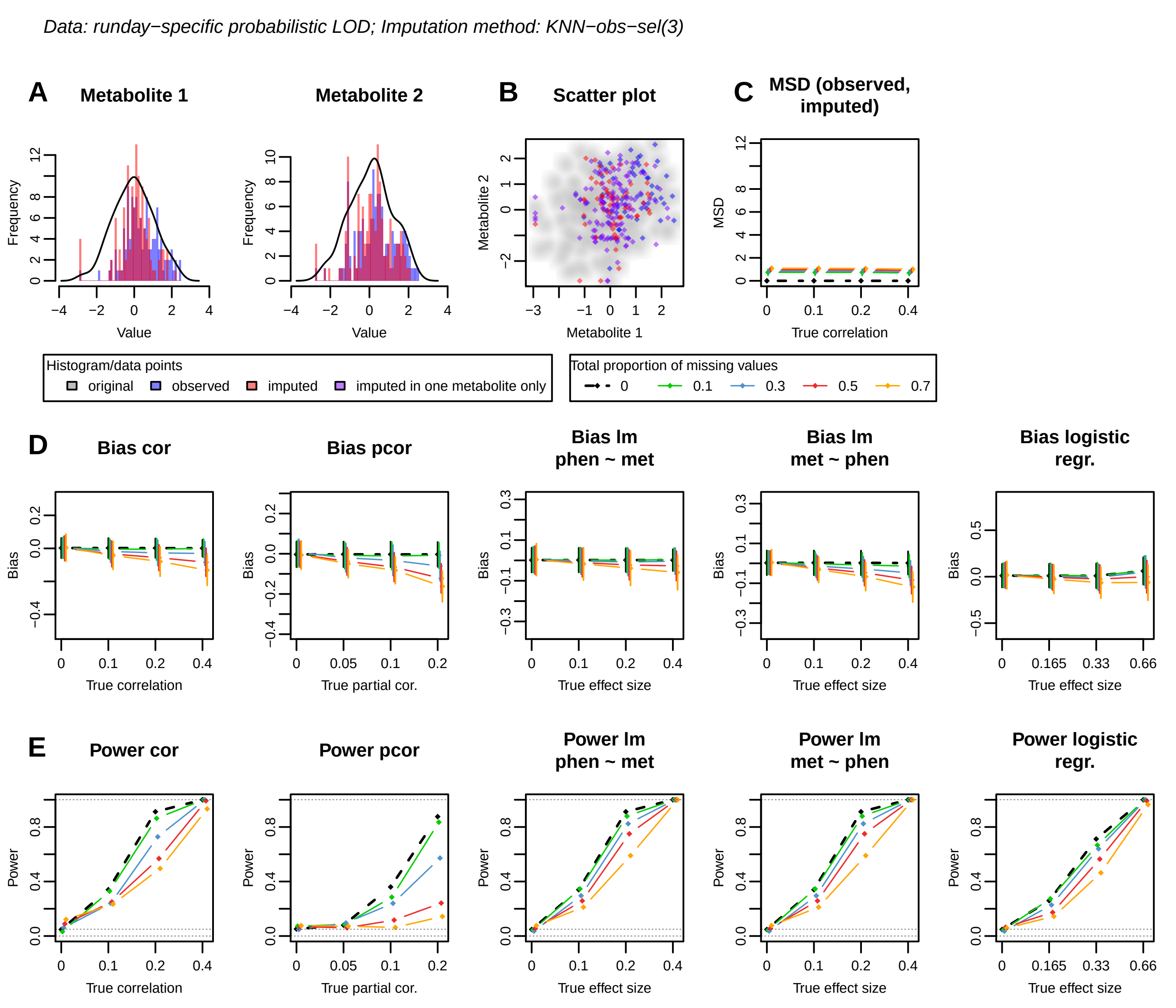
Data: runday−specific probabilistic LOD; Imputation method: KNN−obs−sel(3)
A
Metabolite 1
Metabolite 2
Frequency
Value
B
Scatter plot
Metabolite 1
Metabolite 2
C
MSD (observed, imputed)
MSD
True correlation
Histogram/data points
original
observed
imputed
imputed in one metabolite only
Total proportion of missing values
0
0.1
0.3
0.5
0.7
D
Bias cor
Bias pcor
Bias lm phen ~ met
Bias lm met ~ phen
Bias logistic regr.
Bias
True correlation
True partial cor.
True effect size
E
Power cor
Power pcor
Power lm phen ~ met
Power lm met ~ phen
Power logistic regr.
Power
True correlation
True partial cor.
True effect size

Data: runday−specific probabilistic LOD; Imputation method: KNN−obs−sel(5)

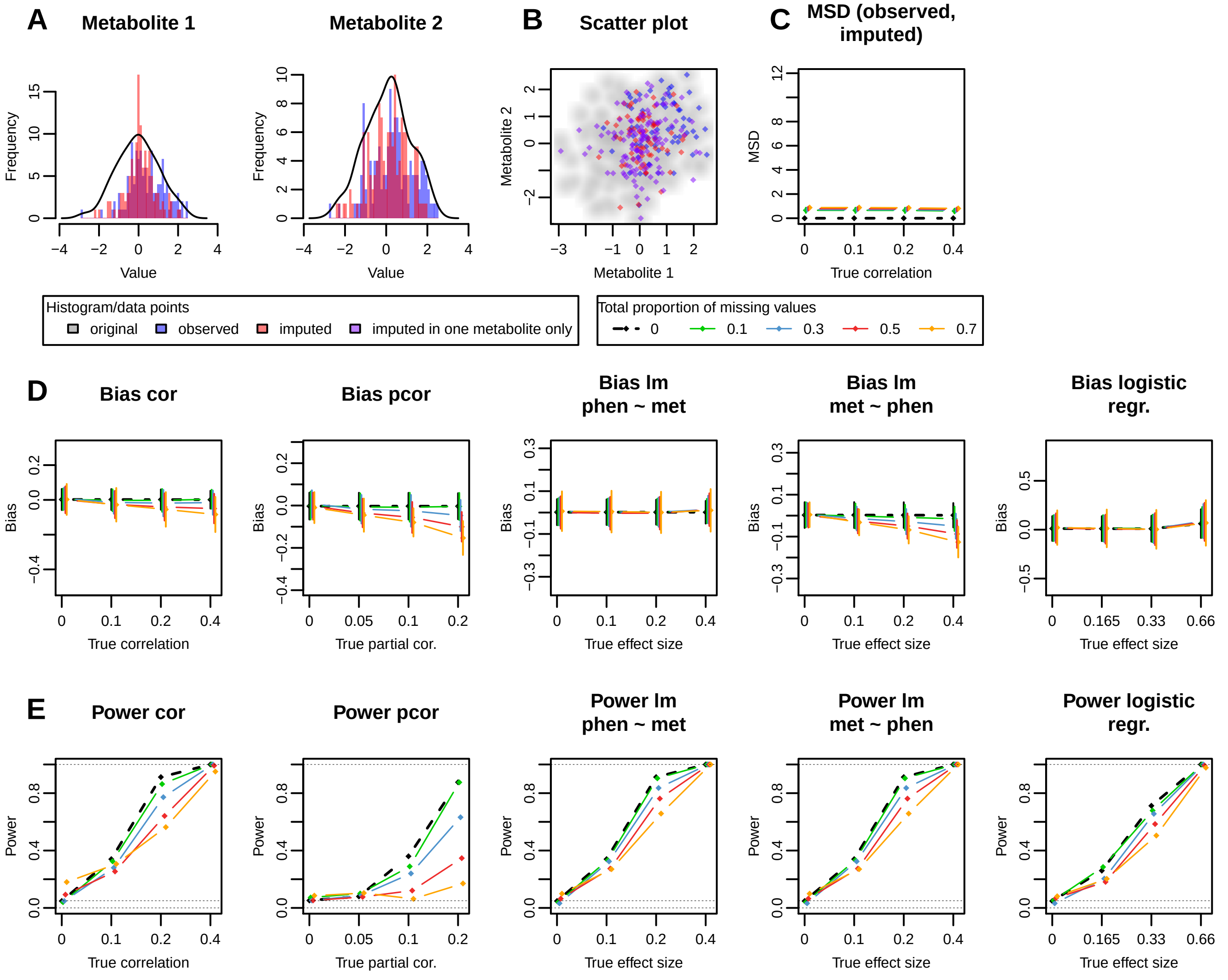

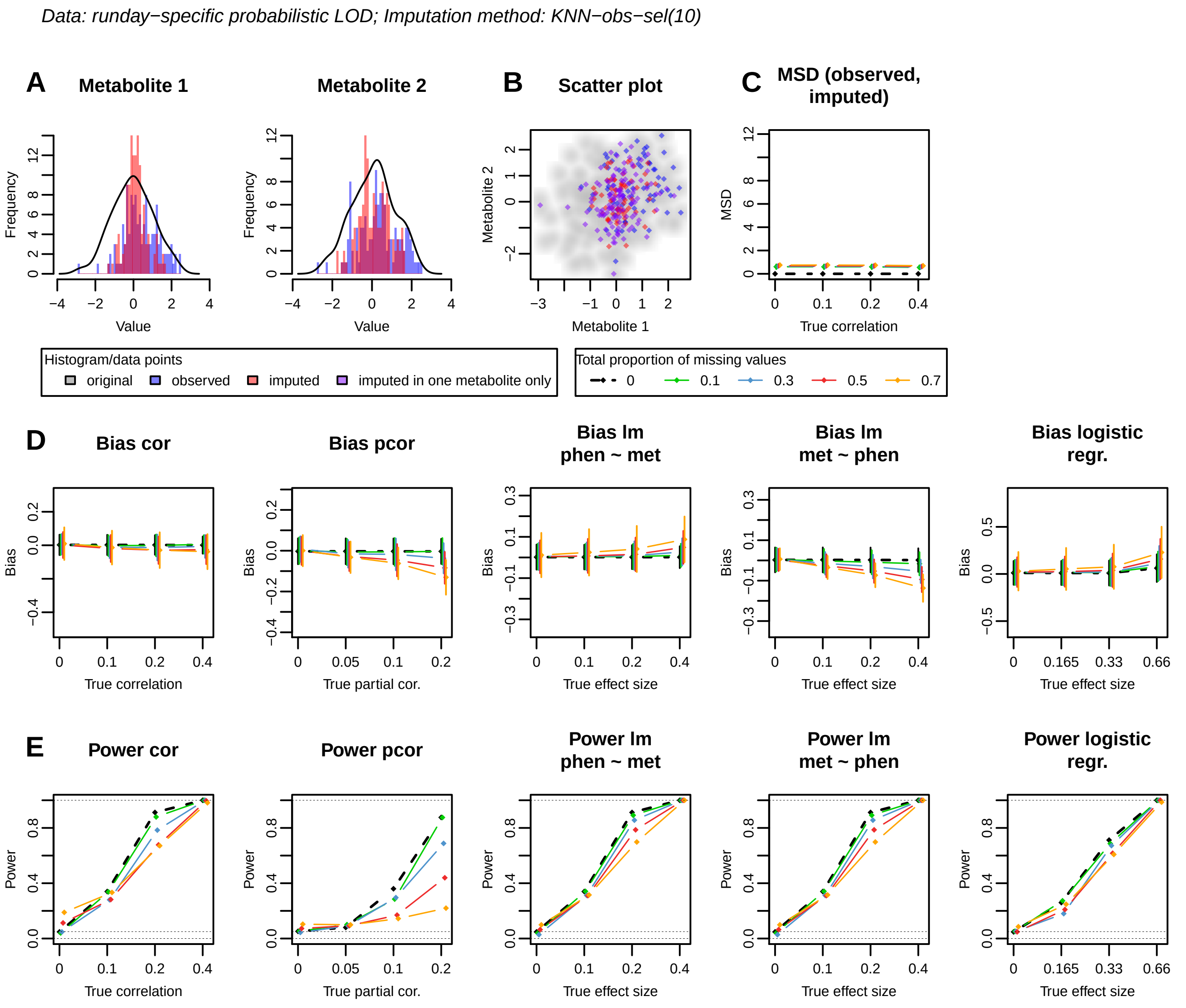

Data: runday−specific probabilistic LOD; Imputation method: KNN−obs−sel(10)
A
Metabolite 1
Metabolite 2
B
Scatter plot
C
MSD (observed, imputed)
Frequency
Value
Metabolite 2
Metabolite 1
MSD
True correlation
Histogram/data points
original
observed
imputed
imputed in one metabolite only
Total proportion of missing values
0
0.1
0.3
0.5
0.7
D
Bias cor
Bias pcor
Bias lm phen ~ met
Bias lm met ~ phen
Bias logistic regr.
Bias
True correlation
True partial cor.
True effect size
E
Power cor
Power pcor
Power lm phen ~ met
Power lm met ~ phen
Power logistic regr.
Power
True correlation
True partial cor.
True effect size

Data: runday−specific probabilistic LOD; Imputation method: KNN−obs−sel(20)

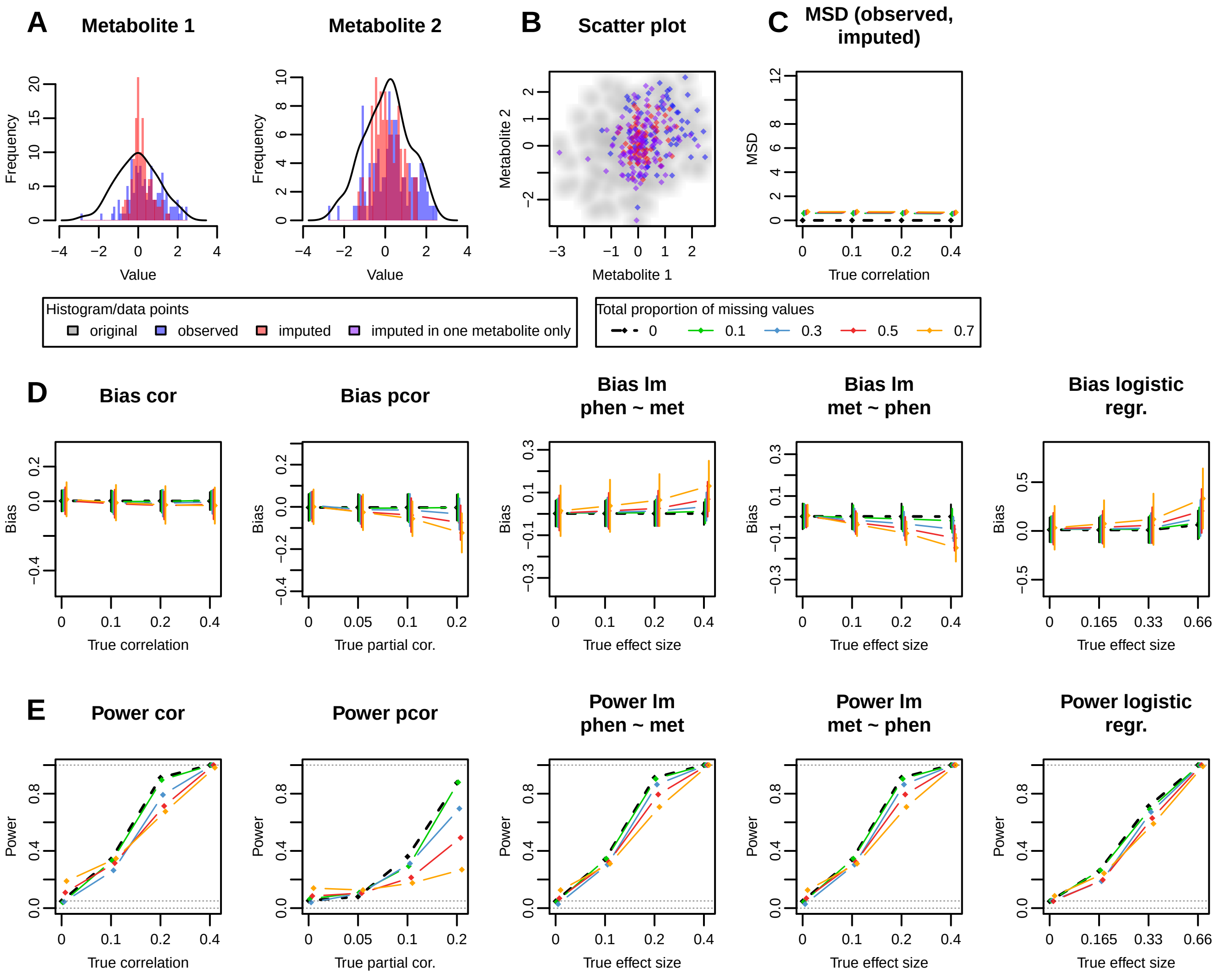

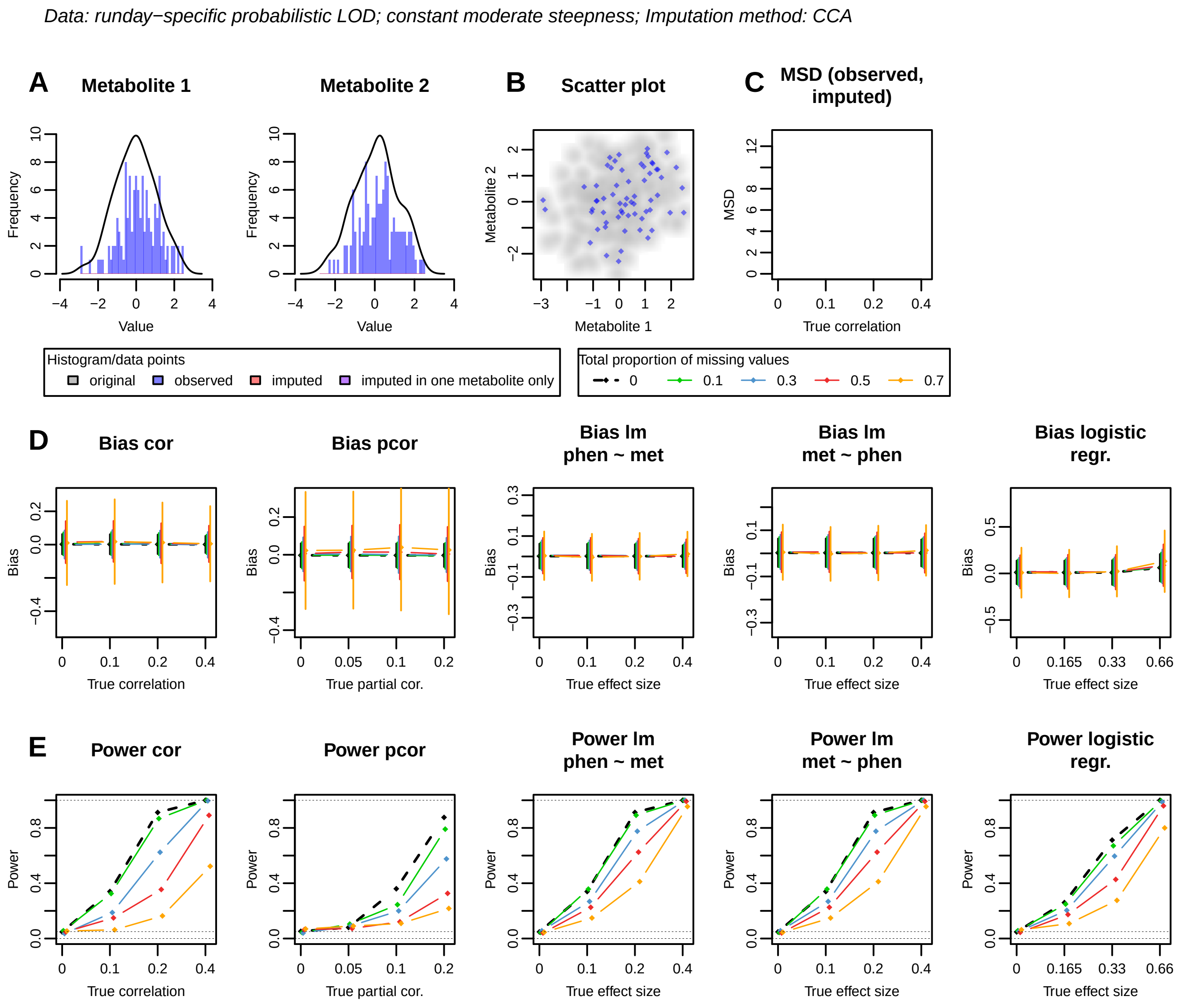

Data: runday−specific probabilistic LOD; constant moderate steepness; Imputation method: CCA
A
Metabolite 1
Metabolite 2
Frequency
Value
B
Scatter plot
Metabolite 2
Metabolite 1
C
MSD (observed, imputed)
MSD
True correlation
Histogram/data points
original
observed
imputed
imputed in one metabolite only
Total proportion of missing values
0
0.1
0.3
0.5
0.7
D
Bias cor
Bias pcor
Bias lm phen ~ met
Bias lm met ~ phen
Bias logistic regr.
Bias
True correlation
True partial cor.
True effect size
E
Power cor
Power pcor
Power lm phen ~ met
Power lm met ~ phen
Power logistic regr.
Power
True correlation
True partial cor.
True effect size

Data: runday−specific probabilistic LOD; constant moderate steepness; Imputation method: min

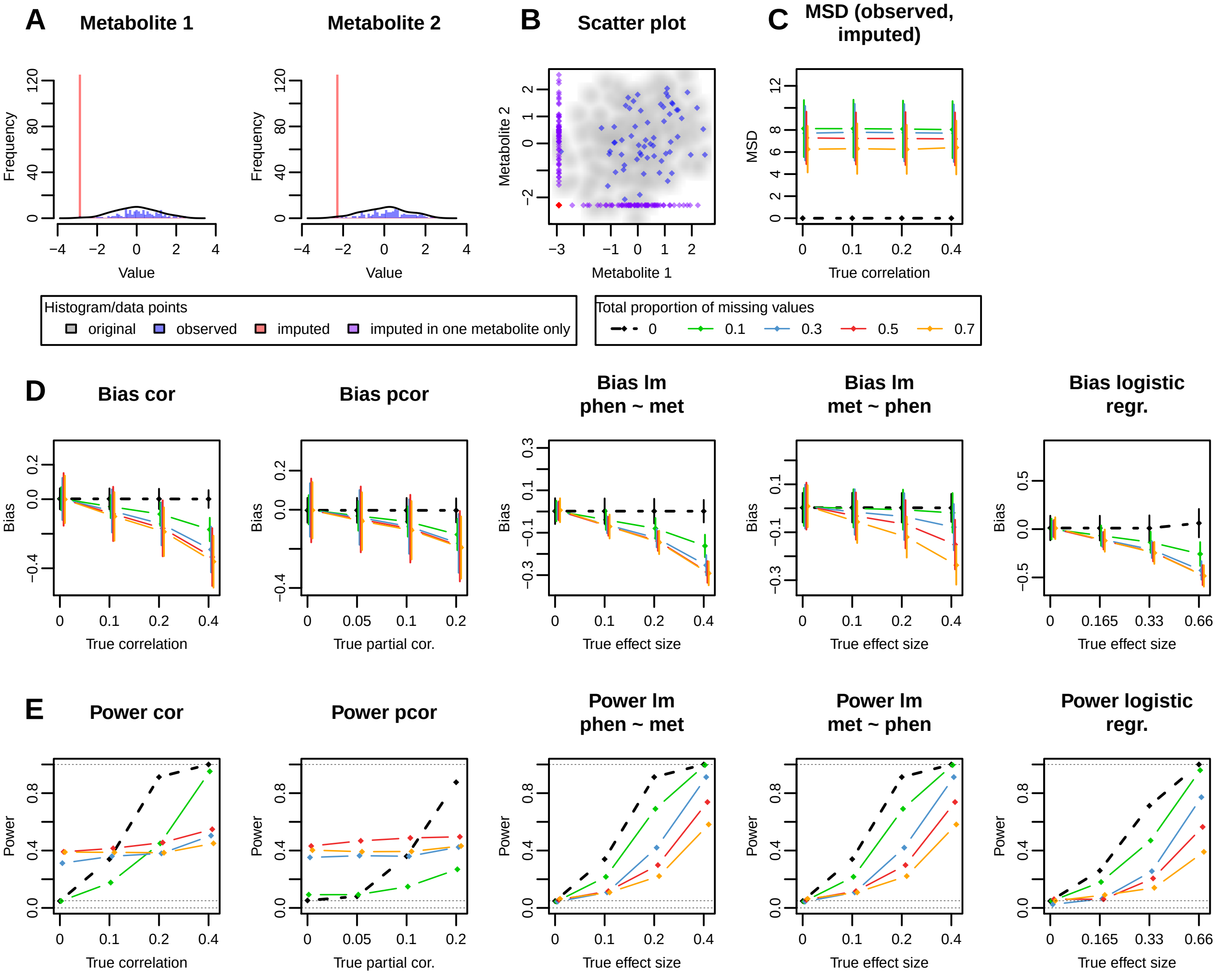

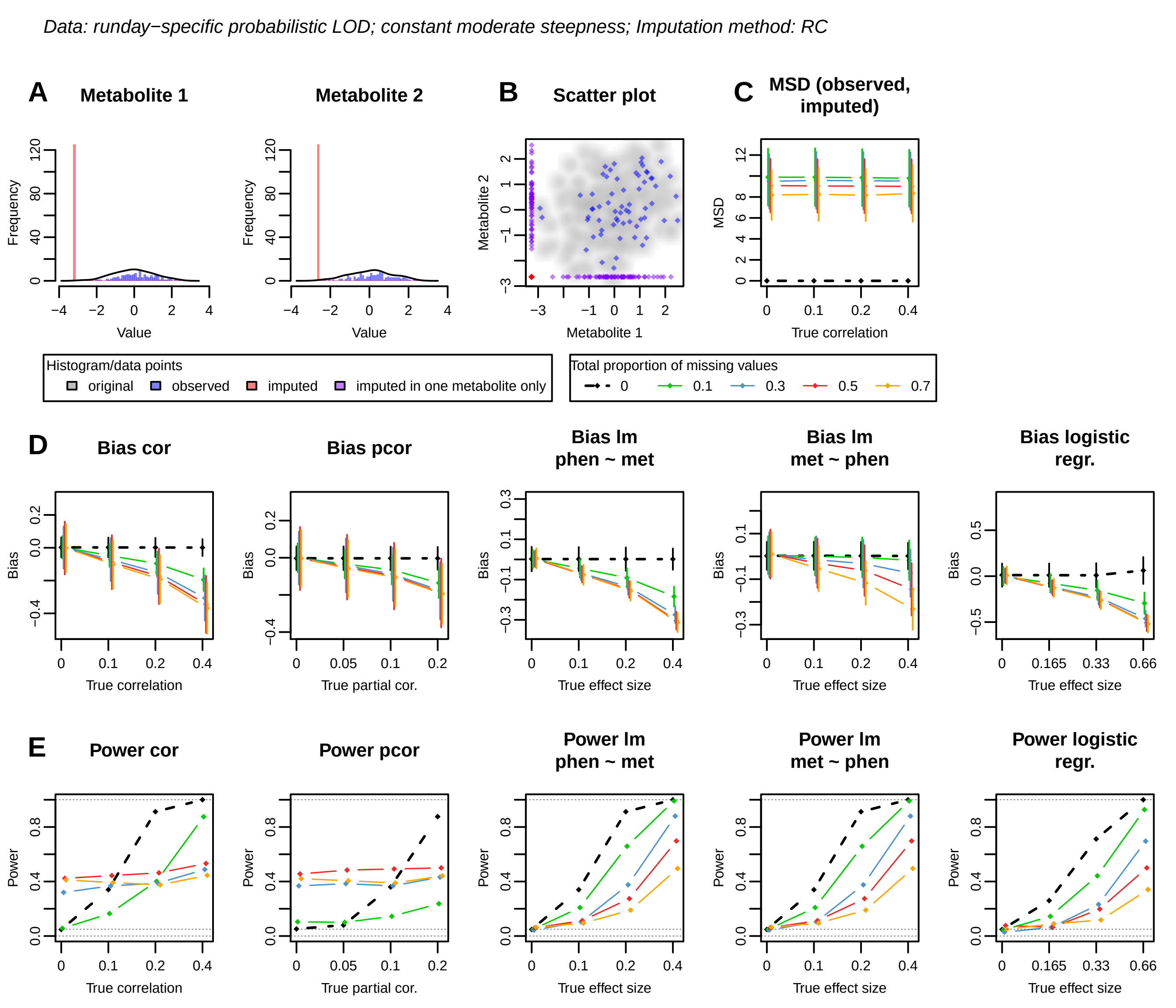

Data: runday−specific probabilistic LOD; constant moderate steepness; Imputation method: RC
A
Metabolite 1
Metabolite 2
Frequency
Value
B
Scatter plot
Metabolite 1
Metabolite 2
C
MSD (observed, imputed)
MSD
True correlation
Histogram/data points
original
observed
imputed
imputed in one metabolite only
Total proportion of missing values
0
0.1
0.3
0.5
0.7
D
Bias cor
Bias pcor
Bias lm phen ~ met
Bias lm met ~ phen
Bias logistic regr.
Bias
True correlation
True partial cor.
True effect size
E
Power cor
Power pcor
Power lm phen ~ met
Power lm met ~ phen
Power logistic regr.
Power
True correlation
True partial cor.
True effect size

Data: runday−specific probabilistic LOD; constant moderate steepness; Imputation method: ITS

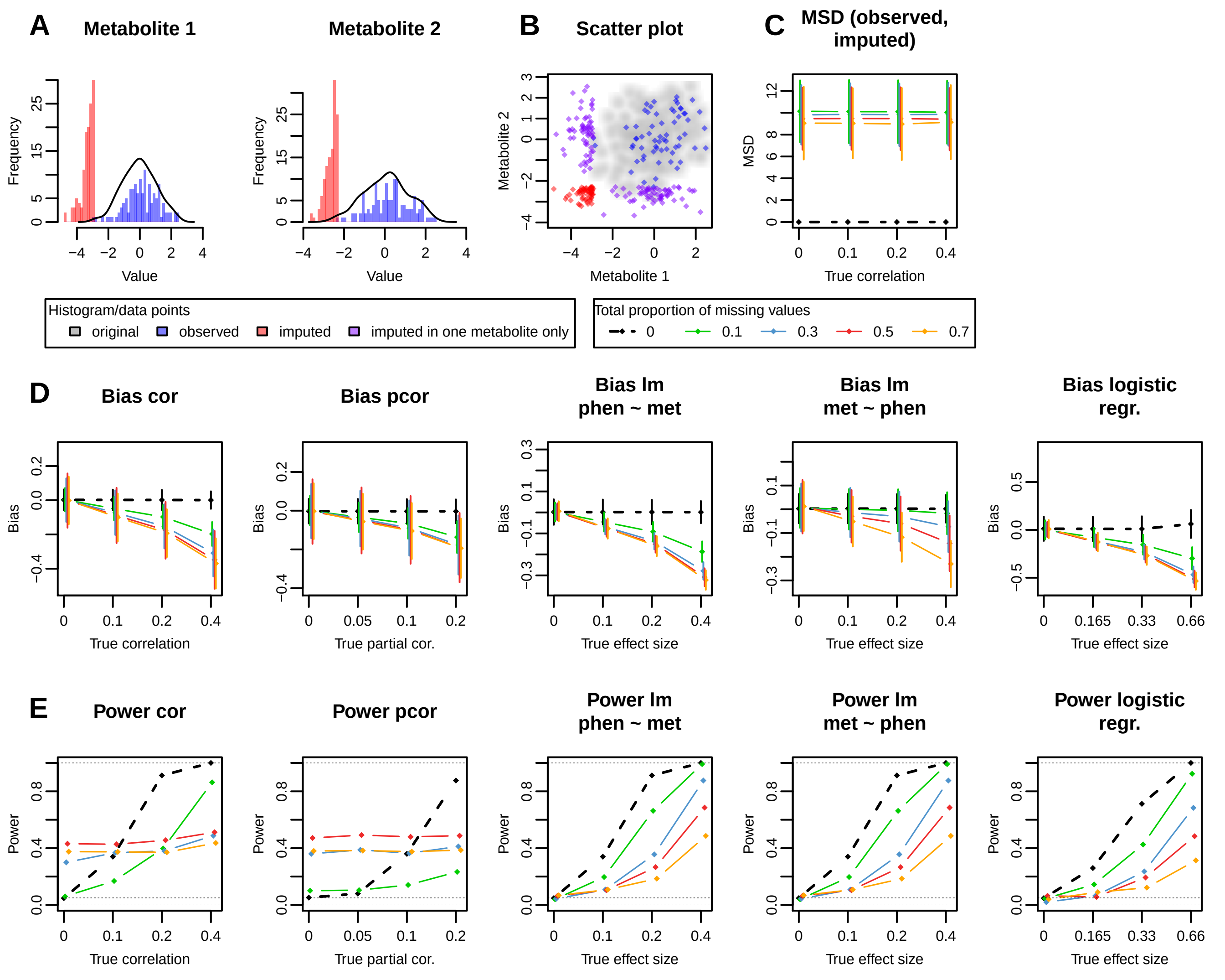

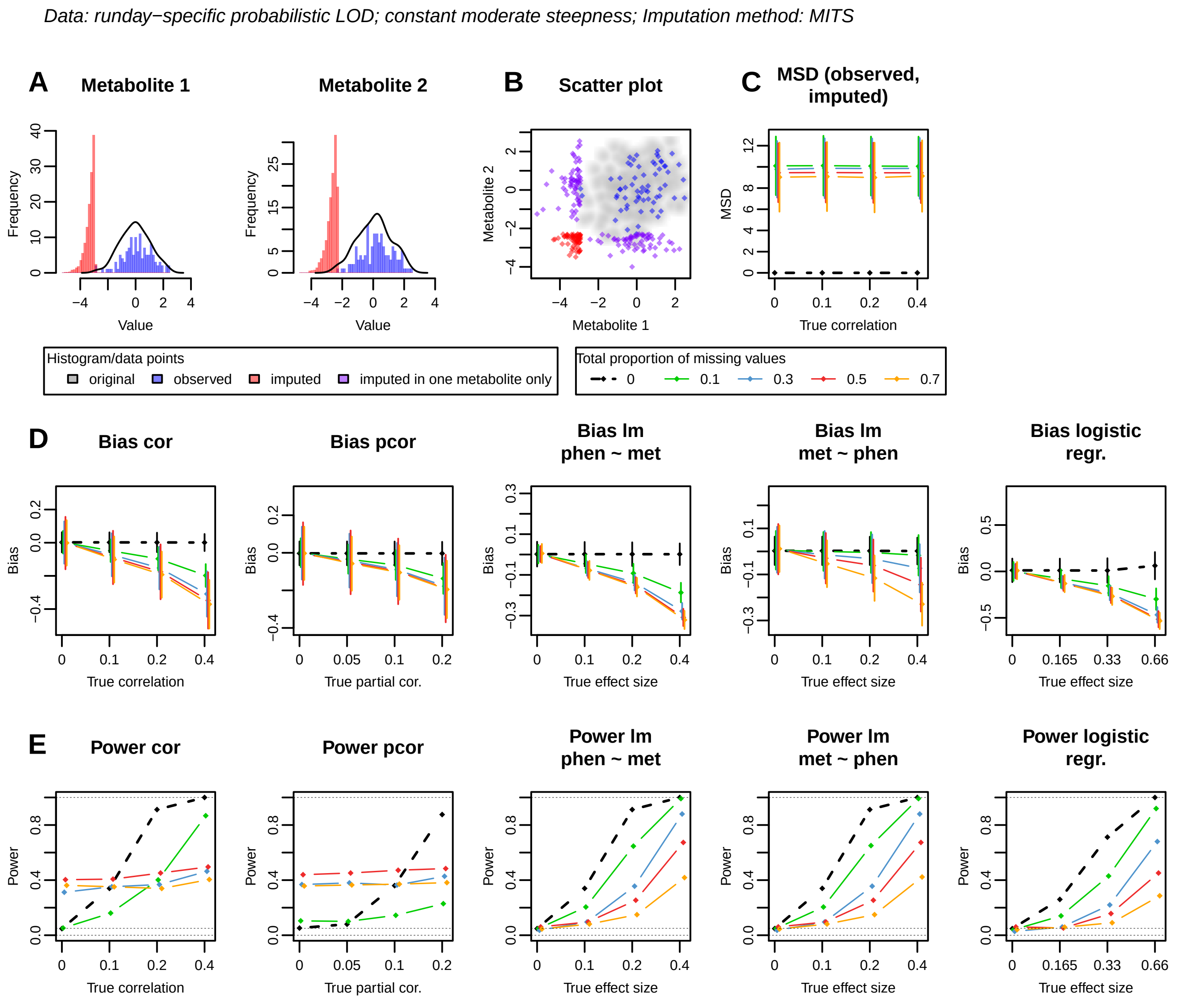
Data: runday−specific probabilistic LOD; constant moderate steepness; Imputation method: MITS
A
Metabolite 1
Metabolite 2
B
Scatter plot
C
MSD (observed, imputed)
Frequency
Value
Metabolite 2
Metabolite 1
MSD
True correlation
Histogram/data points
original
observed
imputed
imputed in one metabolite only
Total proportion of missing values
0
0.1
0.3
0.5
0.7
D
Bias cor
Bias pcor
Bias lm phen ~ met
Bias lm met ~ phen
Bias logistic regr.
Bias
True correlation
True partial cor.
True effect size
E
Power cor
Power pcor
Power lm phen ~ met
Power lm met ~ phen
Power logistic regr.
Power
True correlation
True partial cor.
True effect size

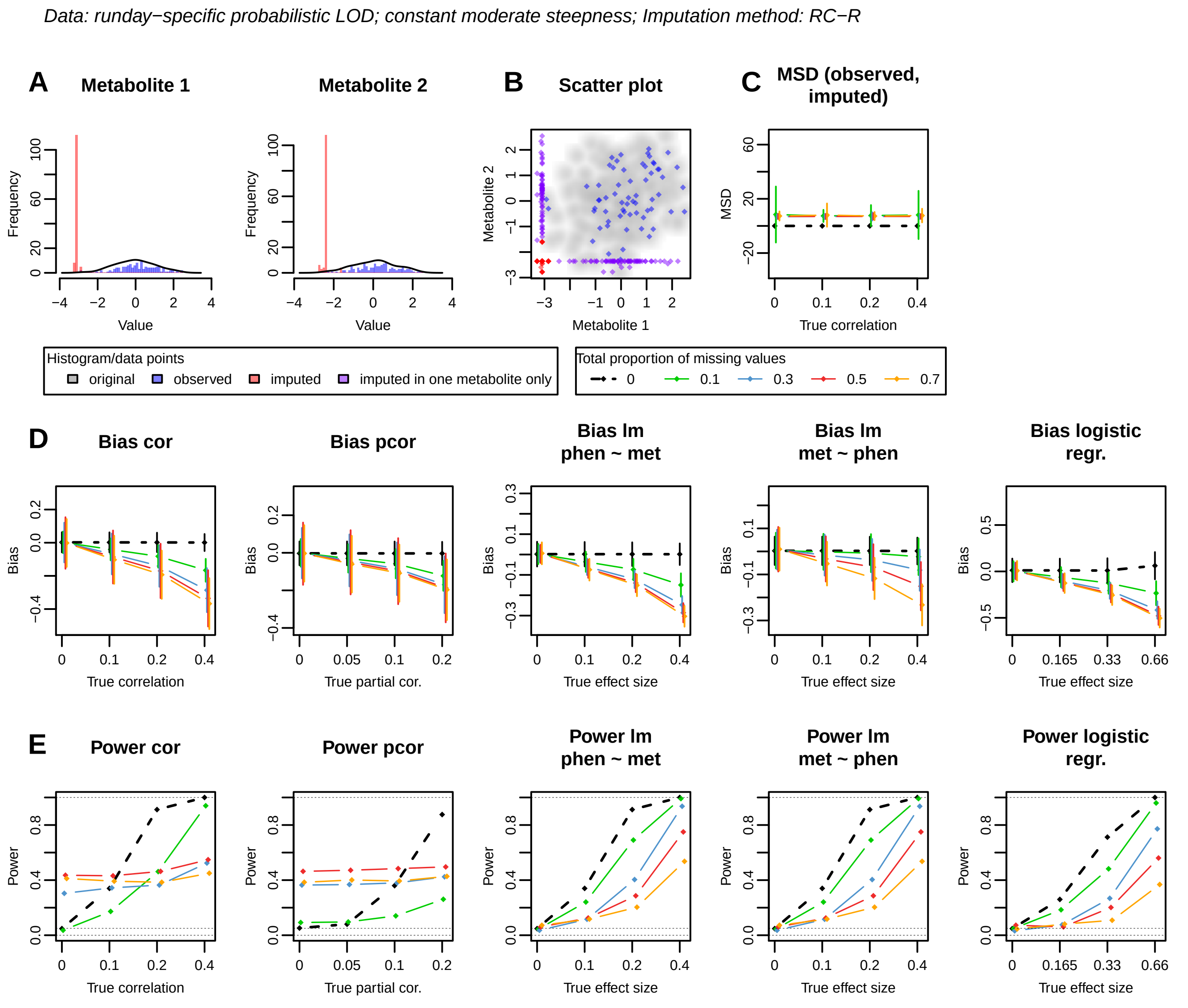
Data: runday−specific probabilistic LOD; constant moderate steepness; Imputation method: RC−R
A
Metabolite 1
Metabolite 2
B
Scatter plot
C
MSD (observed, imputed)
Frequency
Value
Metabolite 2
Metabolite 1
MSD
True correlation
Histogram/data points
original
observed
imputed
imputed in one metabolite only
Total proportion of missing values
0
0.1
0.3
0.5
0.7
D
Bias cor
Bias pcor
Bias lm phen ~ met
Bias lm met ~ phen
Bias logistic regr.
Bias
True correlation
True partial cor.
True effect size
E
Power cor
Power pcor
Power lm phen ~ met
Power lm met ~ phen
Power logistic regr.
Power

Data: runday−specific probabilistic LOD; constant moderate steepness; Imputation method: ITS−R

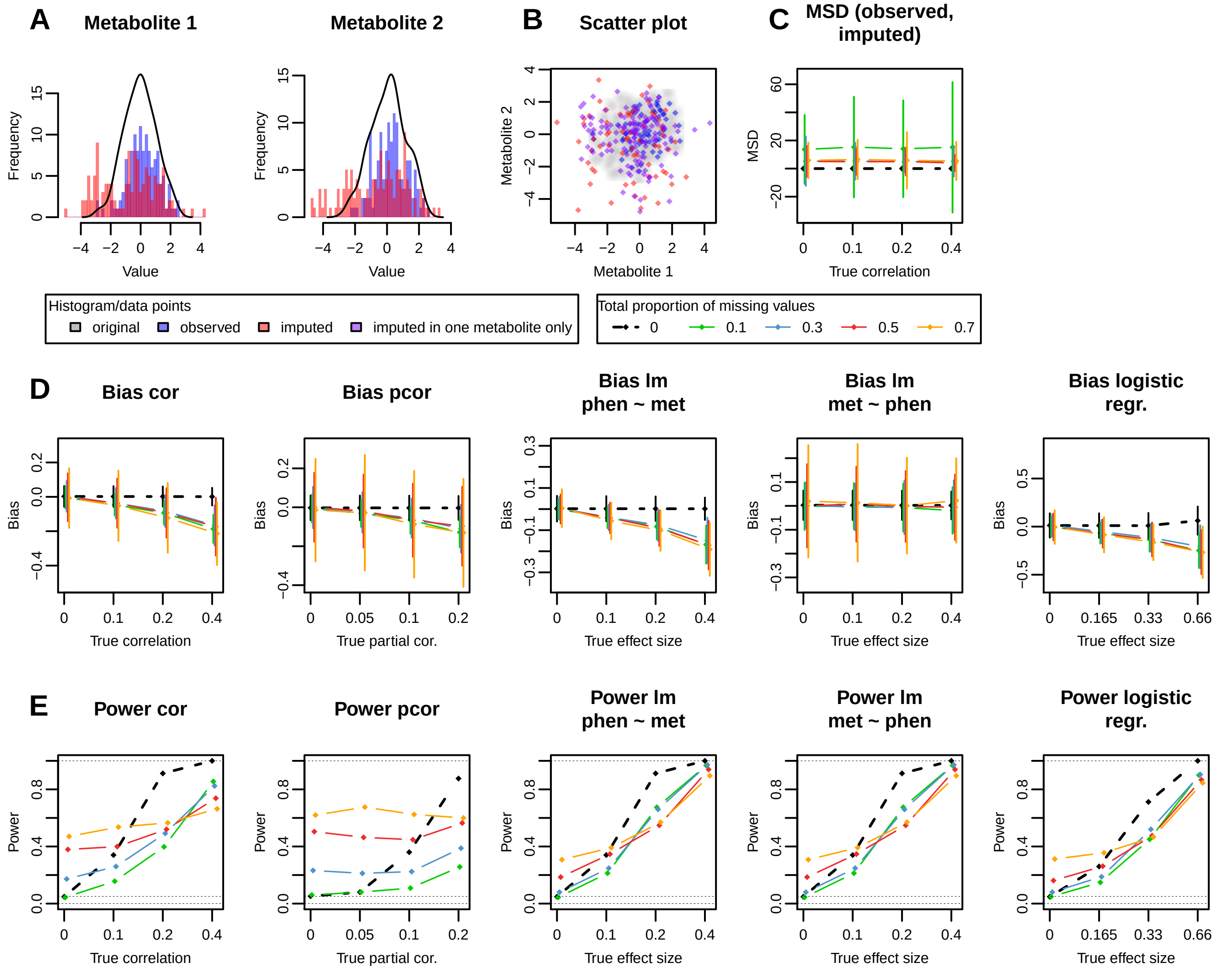

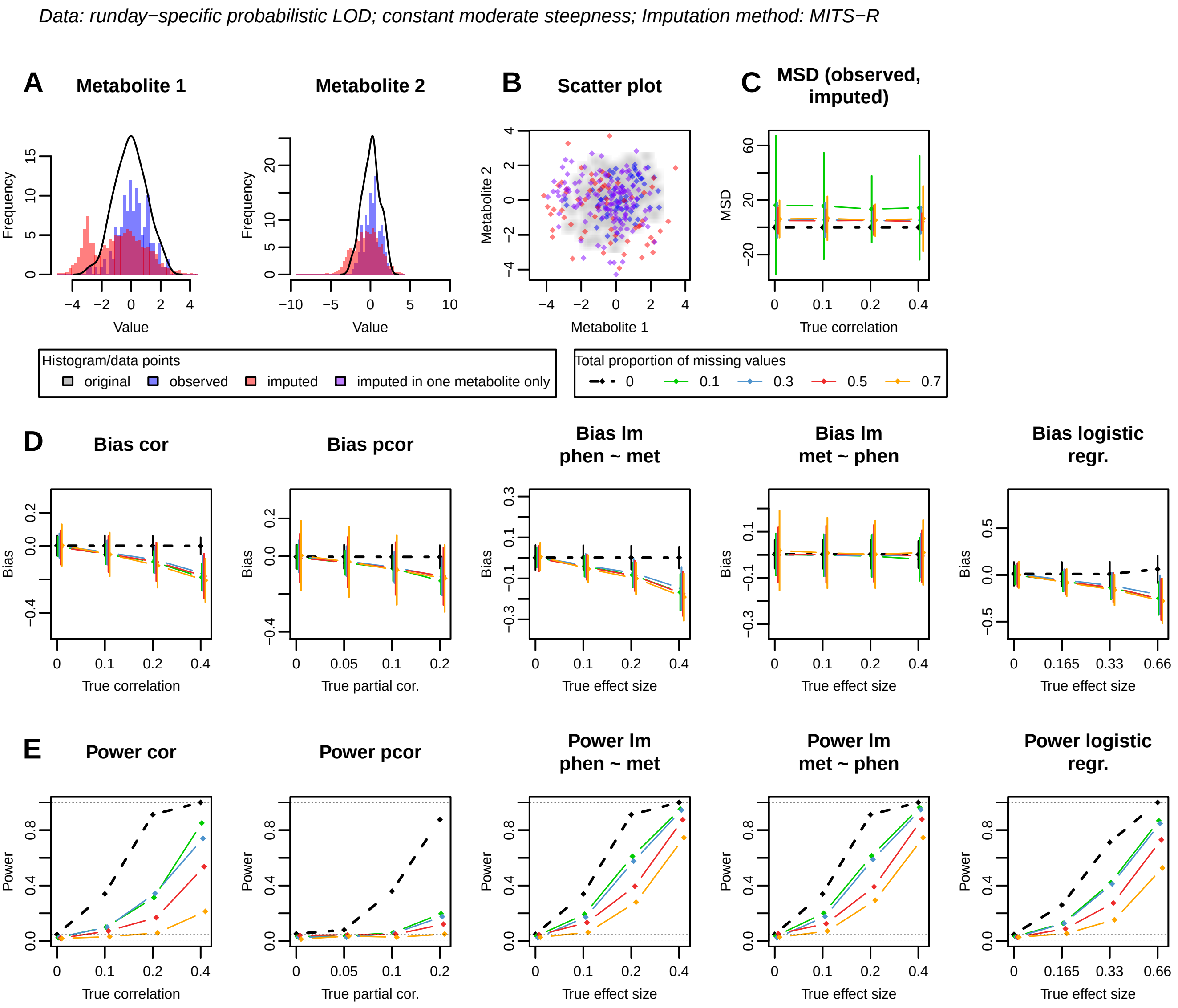
Data: runday−specific probabilistic LOD; constant moderate steepness; Imputation method: MITS−R
A
Metabolite 1
Metabolite 2
Frequency
Value
B
Scatter plot
Metabolite 1
Metabolite 2
C
MSD (observed, imputed)
MSD
True correlation
Histogram/data points
original
observed
imputed
imputed in one metabolite only
Total proportion of missing values
0
0.1
0.3
0.5
0.7
D
Bias cor
Bias pcor
Bias lm phen ~ met
Bias lm met ~ phen
Bias logistic regr.
Bias
True correlation
True partial cor.
True effect size
E
Power cor
Power pcor
Power lm phen ~ met
Power lm met ~ phen
Power logistic regr.
Power
True correlation
True partial cor.
True effect size

Data: runday−specific probabilistic LOD; constant moderate steepness; Imputation method: mean

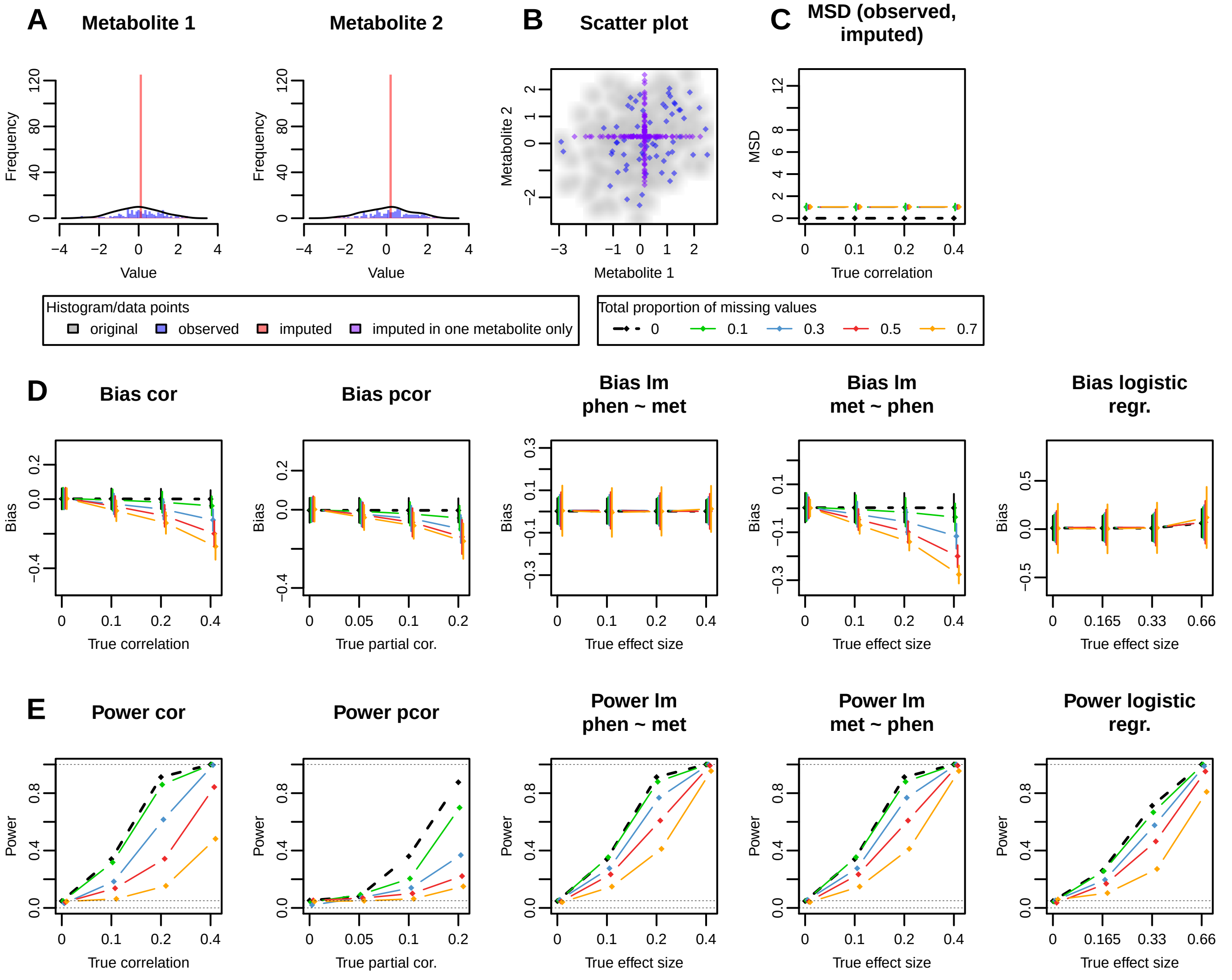

Data: runday−specific probabilistic LOD; constant moderate steepness; Imputation method: ICE−norm

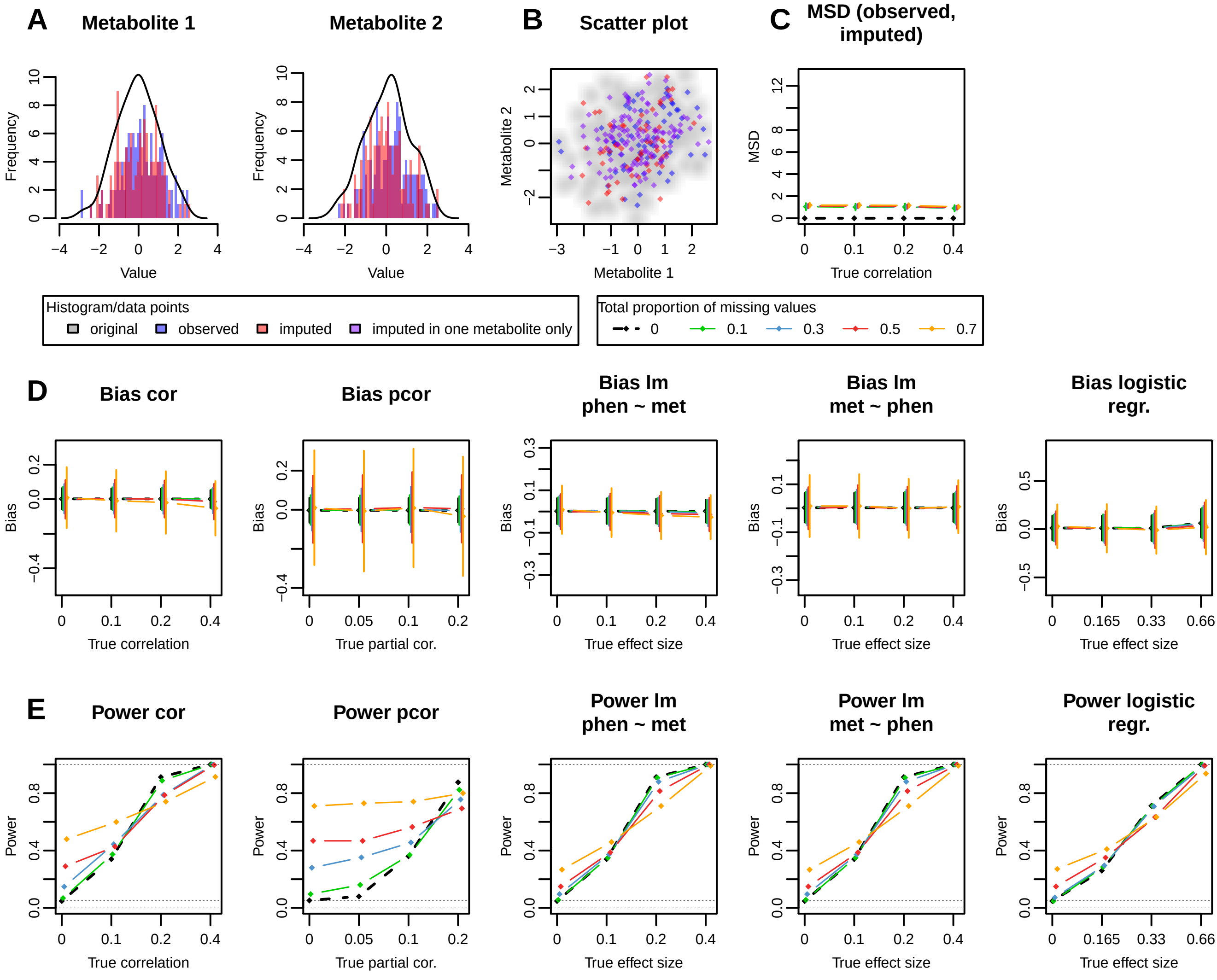

Data: runday−specific probabilistic LOD; constant moderate steepness; Imputation method: ICE−pmm

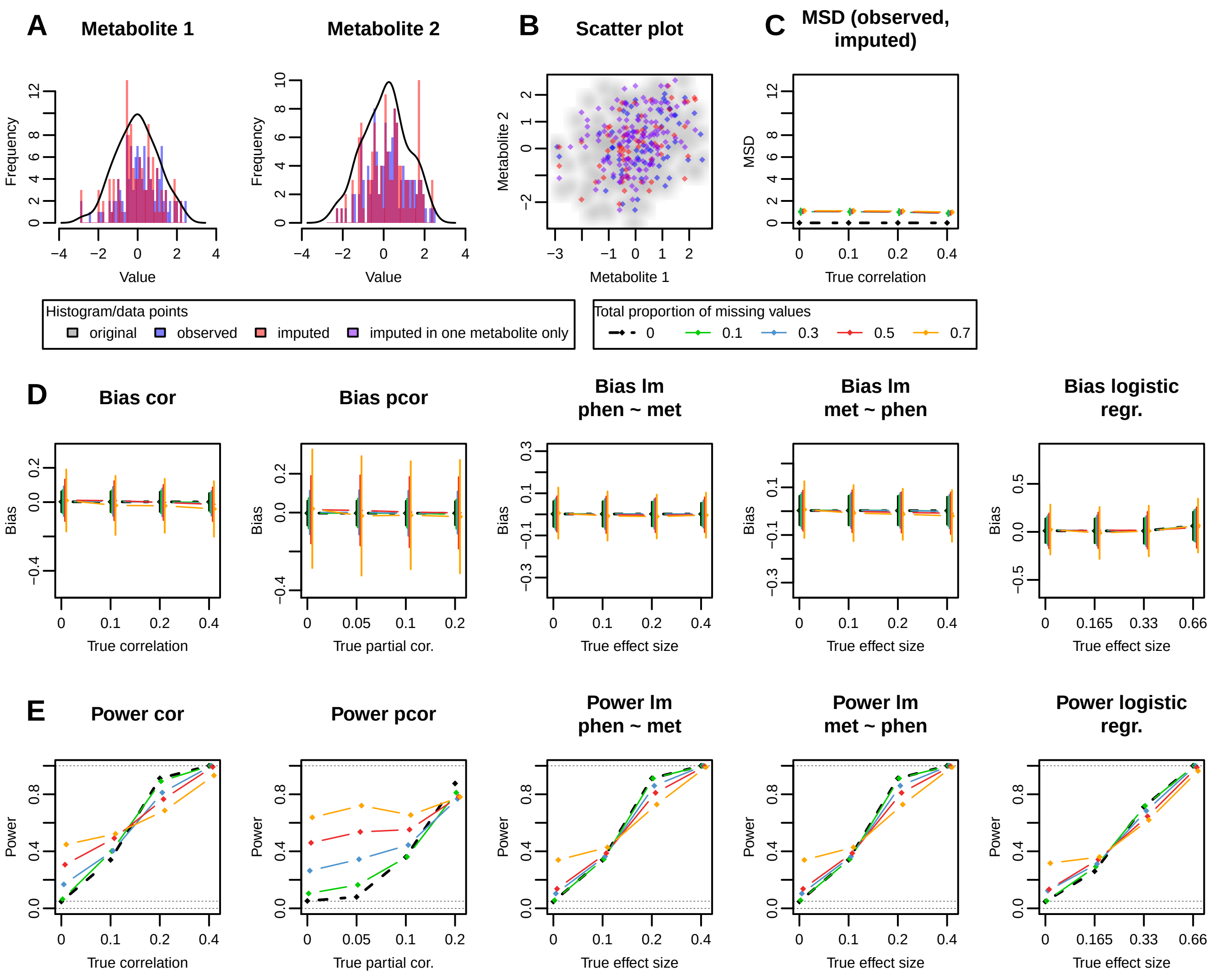

Data: runday−specific probabilistic LOD; constant moderate steepness; Imputation method: ICE−adjR

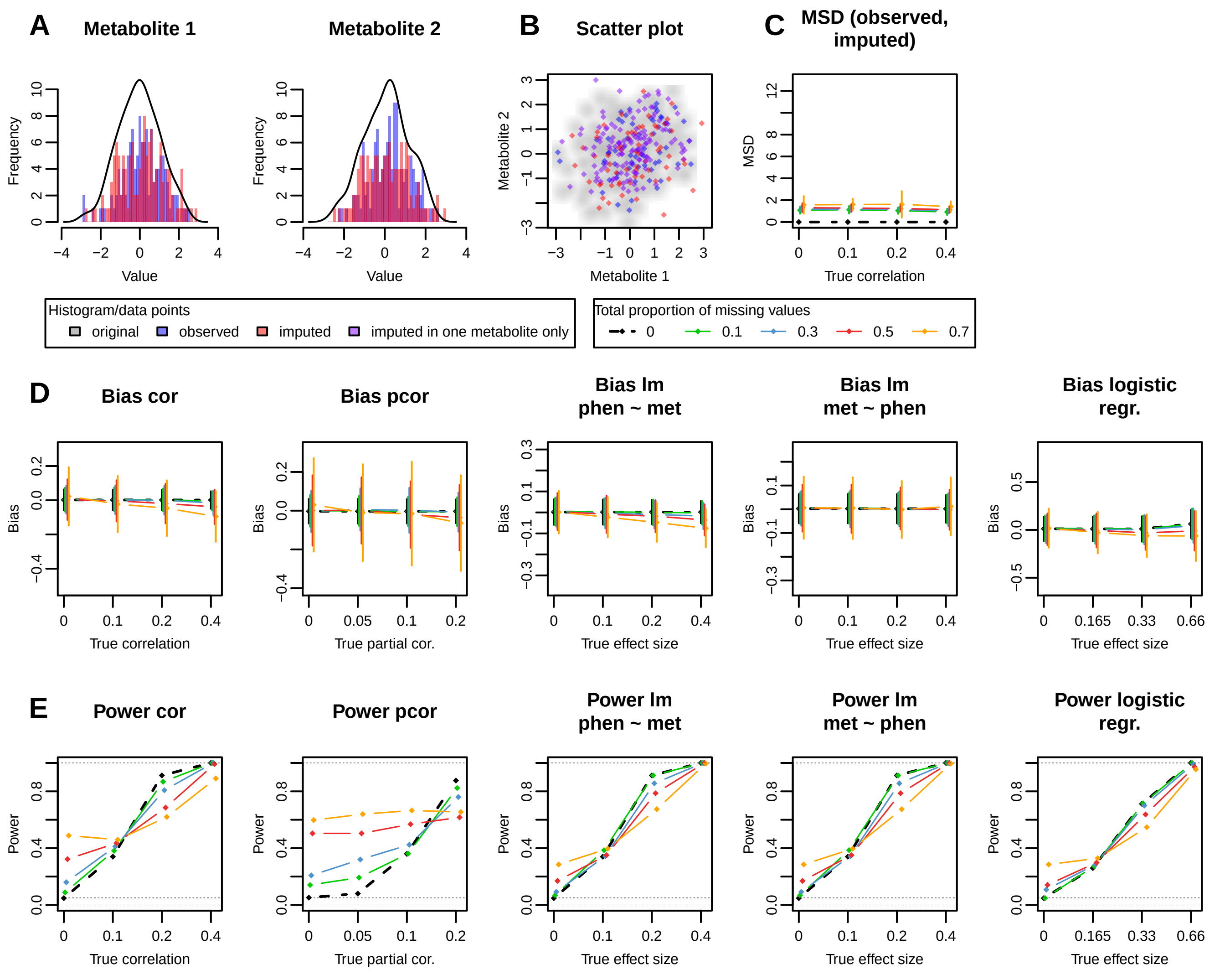

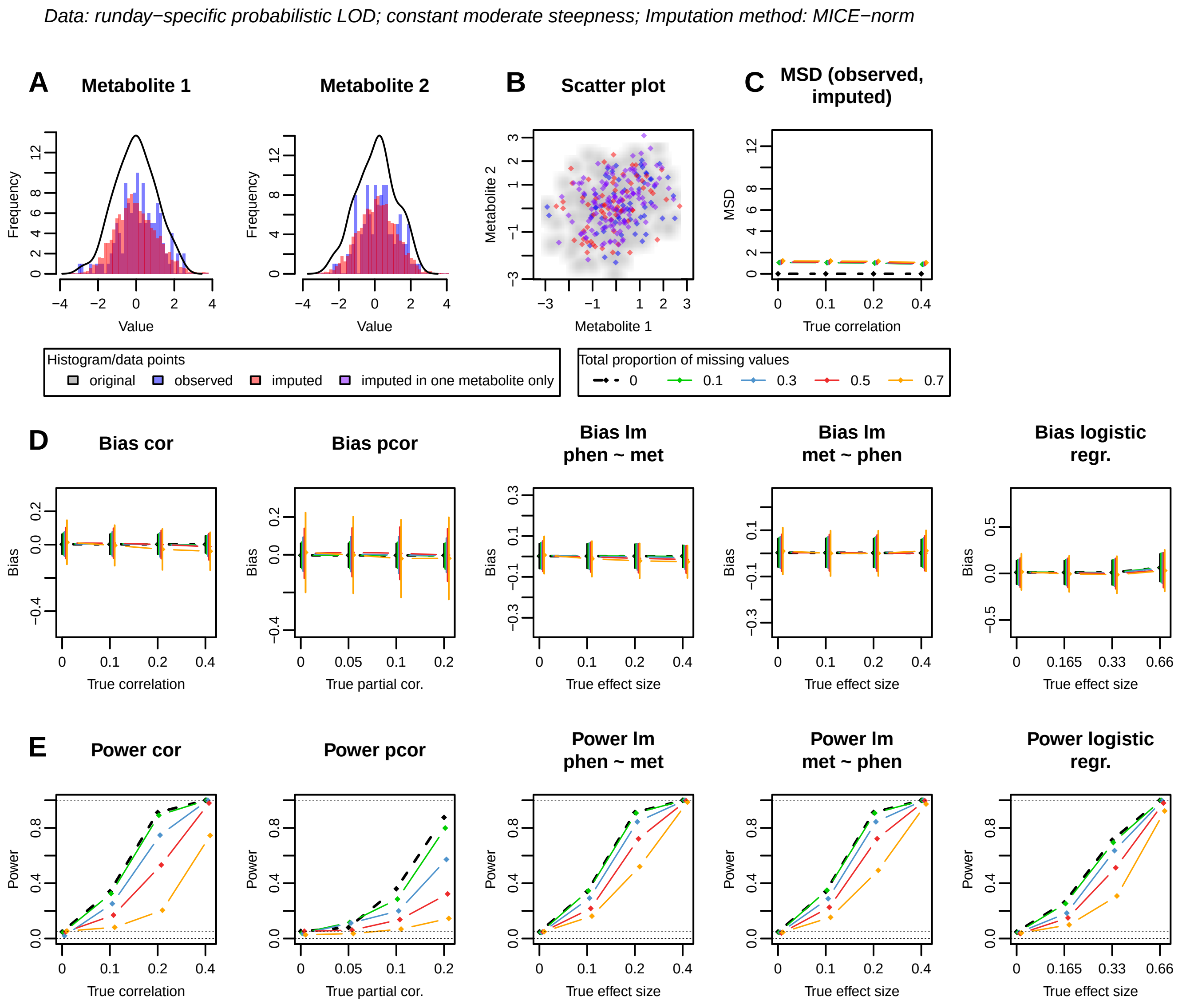

Data: runday−specific probabilistic LOD; constant moderate steepness; Imputation method: MICE−norm
A
Metabolite 1
Metabolite 2
B
Scatter plot
C
MSD (observed, imputed)
Frequency
Value
Metabolite 1
Metabolite 2
MSD
True correlation
Histogram/data points
original
observed
imputed
imputed in one metabolite only
Total proportion of missing values
0
0.1
0.3
0.5
0.7
D
Bias cor
Bias pcor
Bias lm phen ~ met
Bias lm met ~ phen
Bias logistic regr.
Bias
True correlation
True partial cor.
True effect size
E
Power cor
Power pcor
Power lm phen ~ met
Power lm met ~ phen
Power logistic regr.
Power

Data: runday−specific probabilistic LOD; constant moderate steepness; Imputation method: MICE−pmm

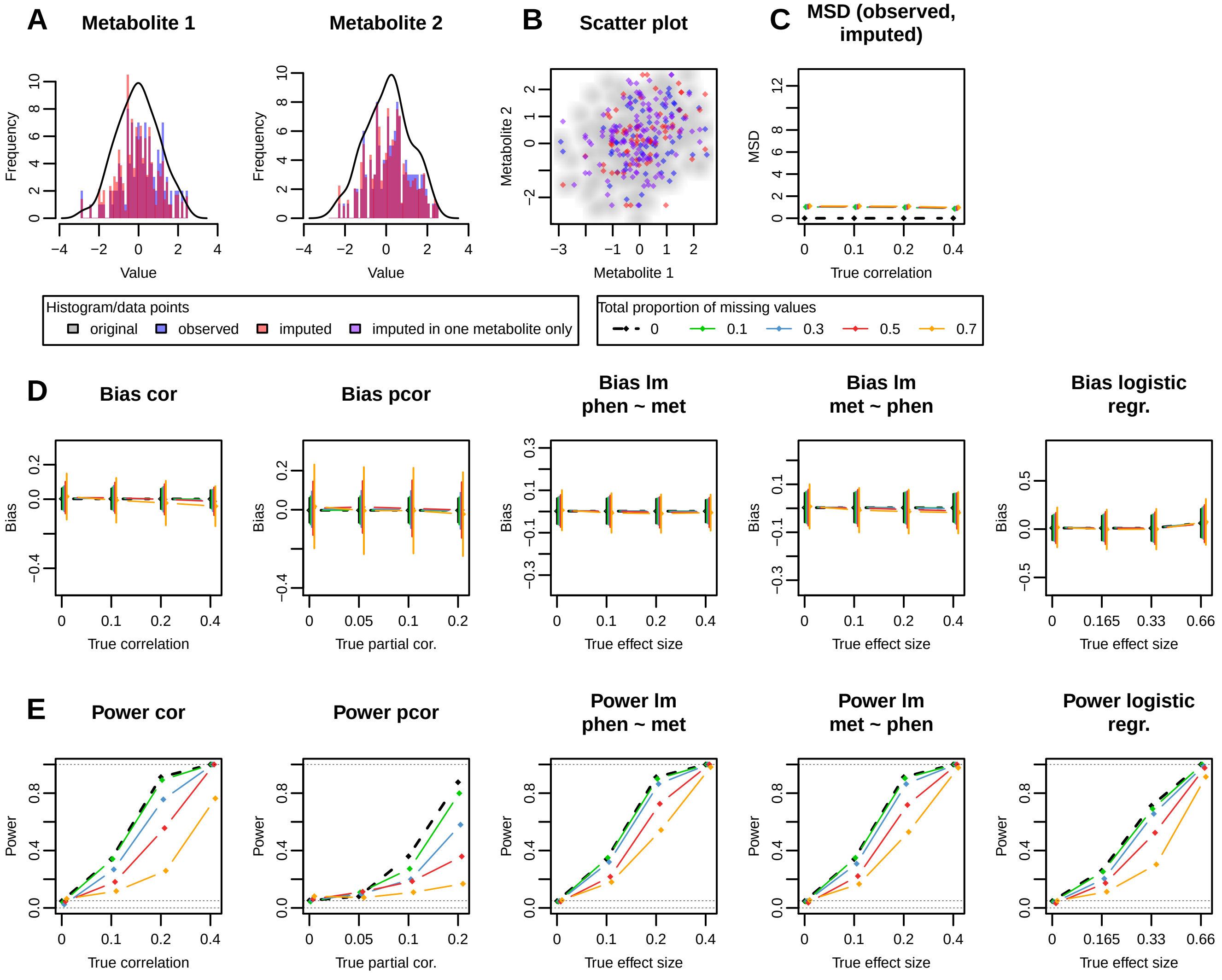

Data: runday−specific probabilistic LOD; constant moderate steepness; Imputation method: MICE−adjR

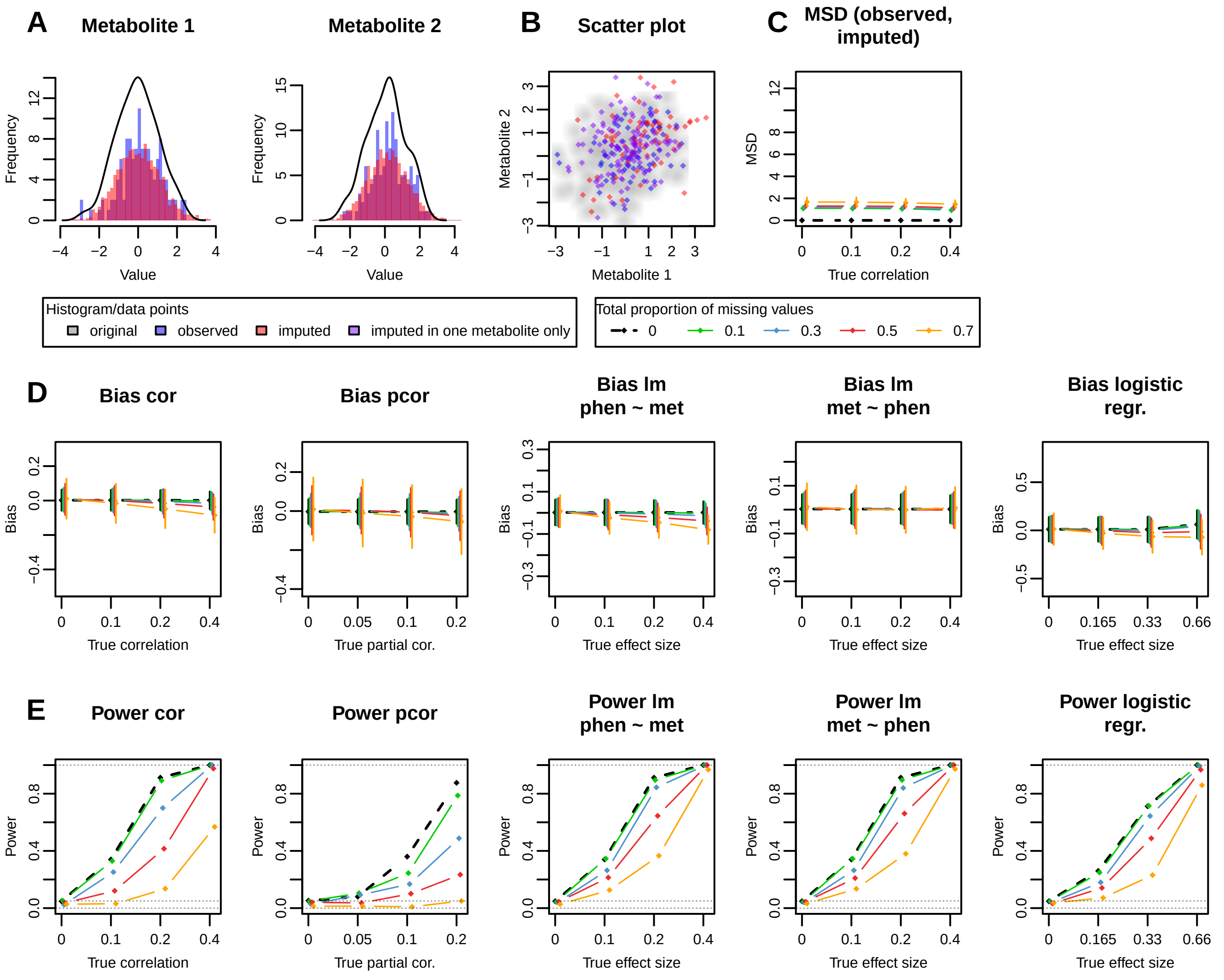

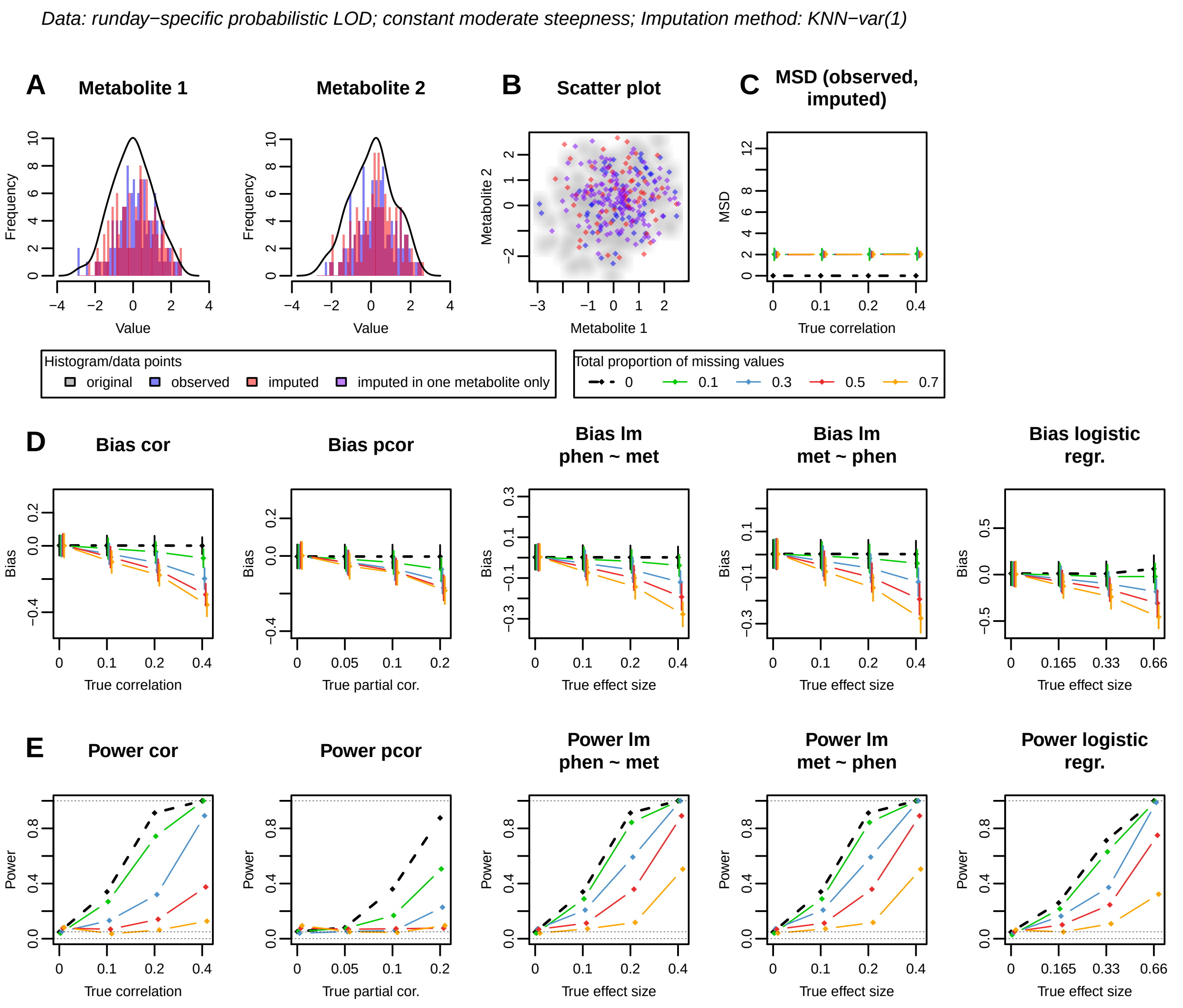
Data: runday−specific probabilistic LOD; constant moderate steepness; Imputation method: KNN−var(1)
A
Metabolite 1
Metabolite 2
Frequency
Value
B
Scatter plot
Metabolite 1
Metabolite 2
C
MSD (observed, imputed)
MSD
True correlation
Histogram/data points
original
observed
imputed
imputed in one metabolite only
Total proportion of missing values
0
0.1
0.3
0.5
0.7
D
Bias cor
Bias pcor
Bias lm phen ~ met
Bias lm met ~ phen
Bias logistic regr.
Bias
True correlation
True partial cor.
True effect size
E
Power cor
Power pcor
Power lm phen ~ met
Power lm met ~ phen
Power logistic regr.
Power
True correlation
True partial cor.
True effect size

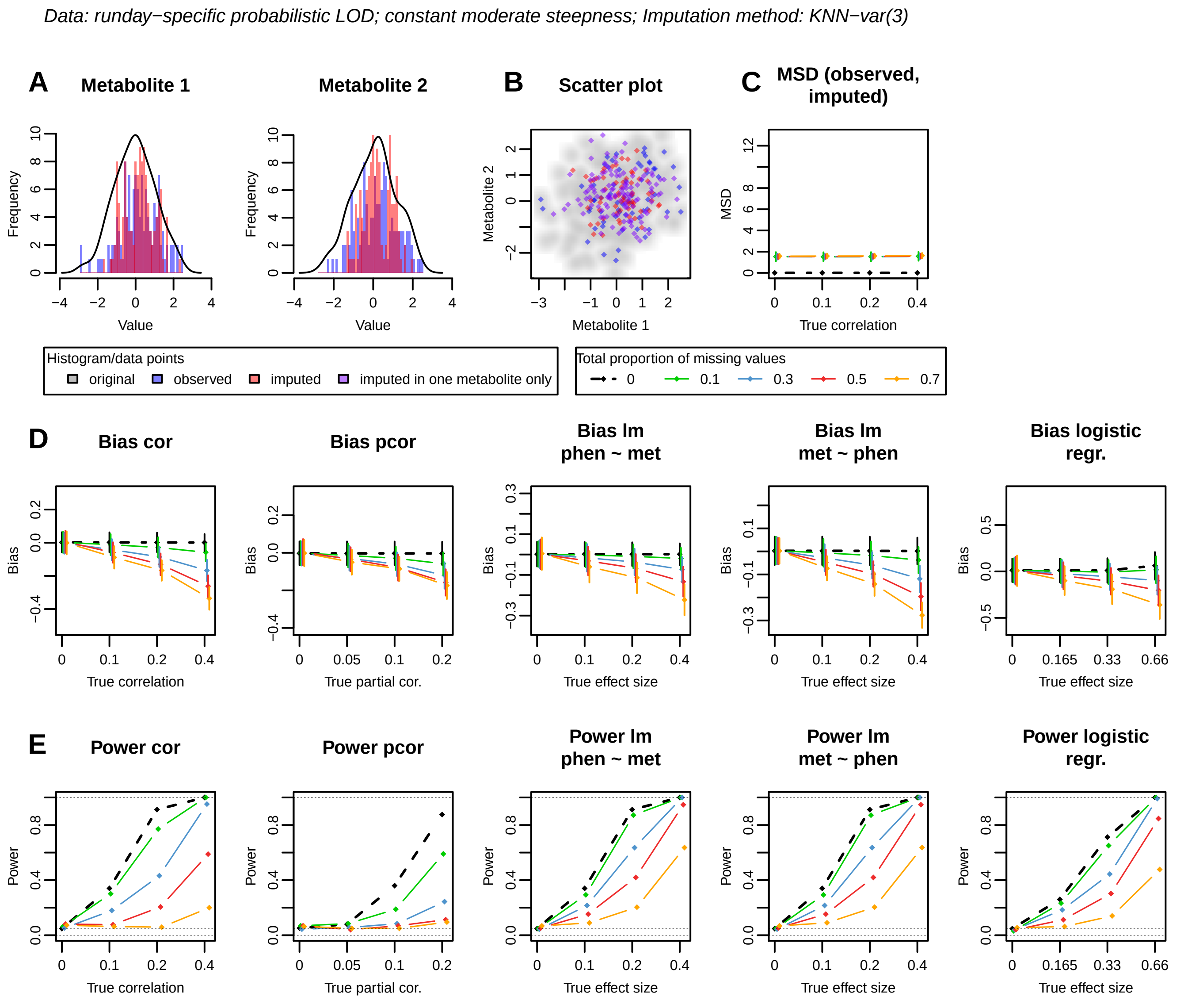
Data: runday−specific probabilistic LOD; constant moderate steepness; Imputation method: KNN−var(3)
A
Metabolite 1
Metabolite 2
Frequency
Value
B
Scatter plot
Metabolite 1
Metabolite 2
C
MSD (observed, imputed)
MSD
True correlation
Histogram/data points
original
observed
imputed
imputed in one metabolite only
Total proportion of missing values
0
0.1
0.3
0.5
0.7
D
Bias cor
Bias pcor
Bias lm phen ~ met
Bias lm met ~ phen
Bias logistic regr.
Bias
True correlation
True partial cor.
True effect size
E
Power cor
Power pcor
Power lm phen ~ met
Power lm met ~ phen
Power logistic regr.
Power

Data: runday−specific probabilistic LOD; constant moderate steepness; Imputation method: KNN−var(5)

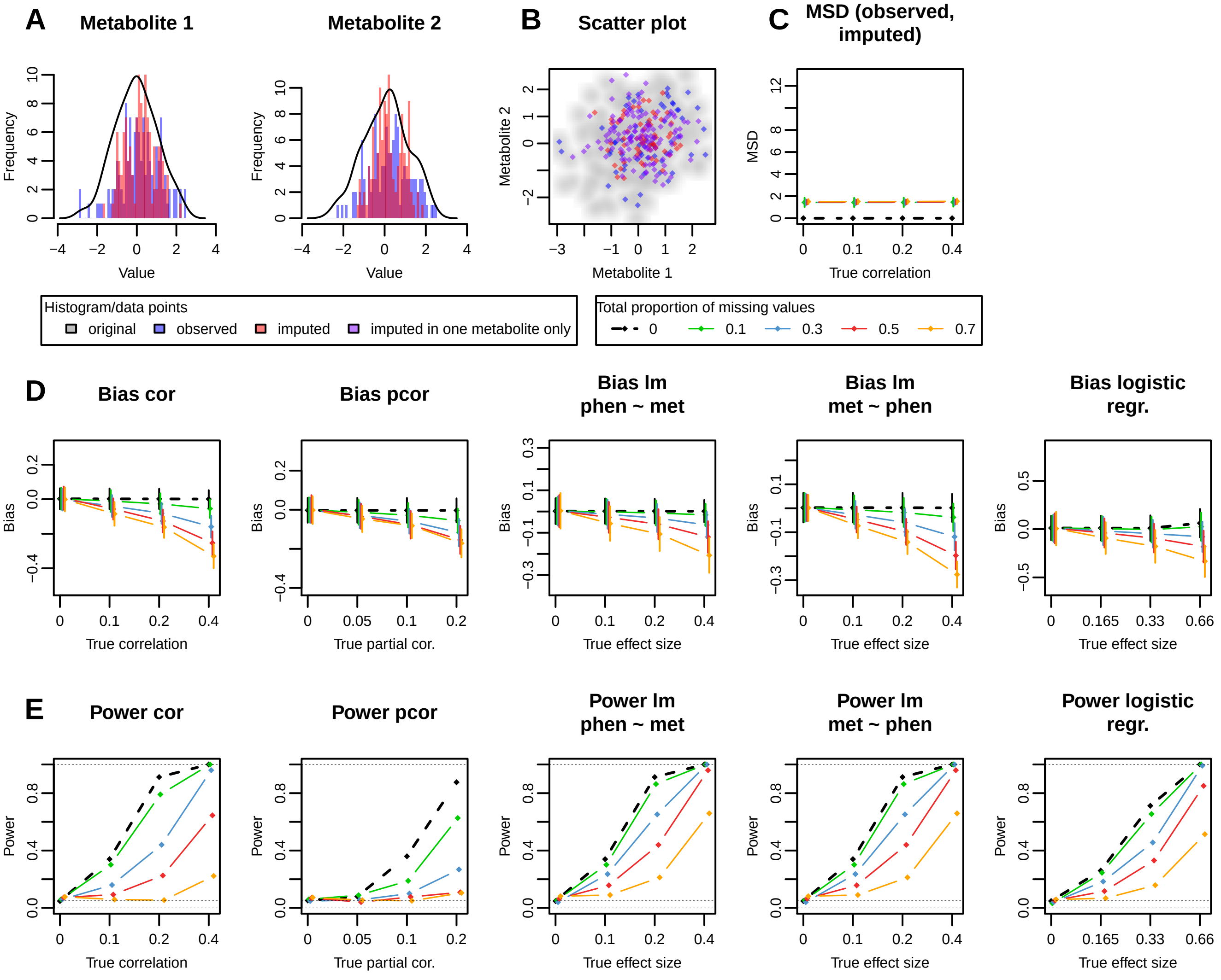

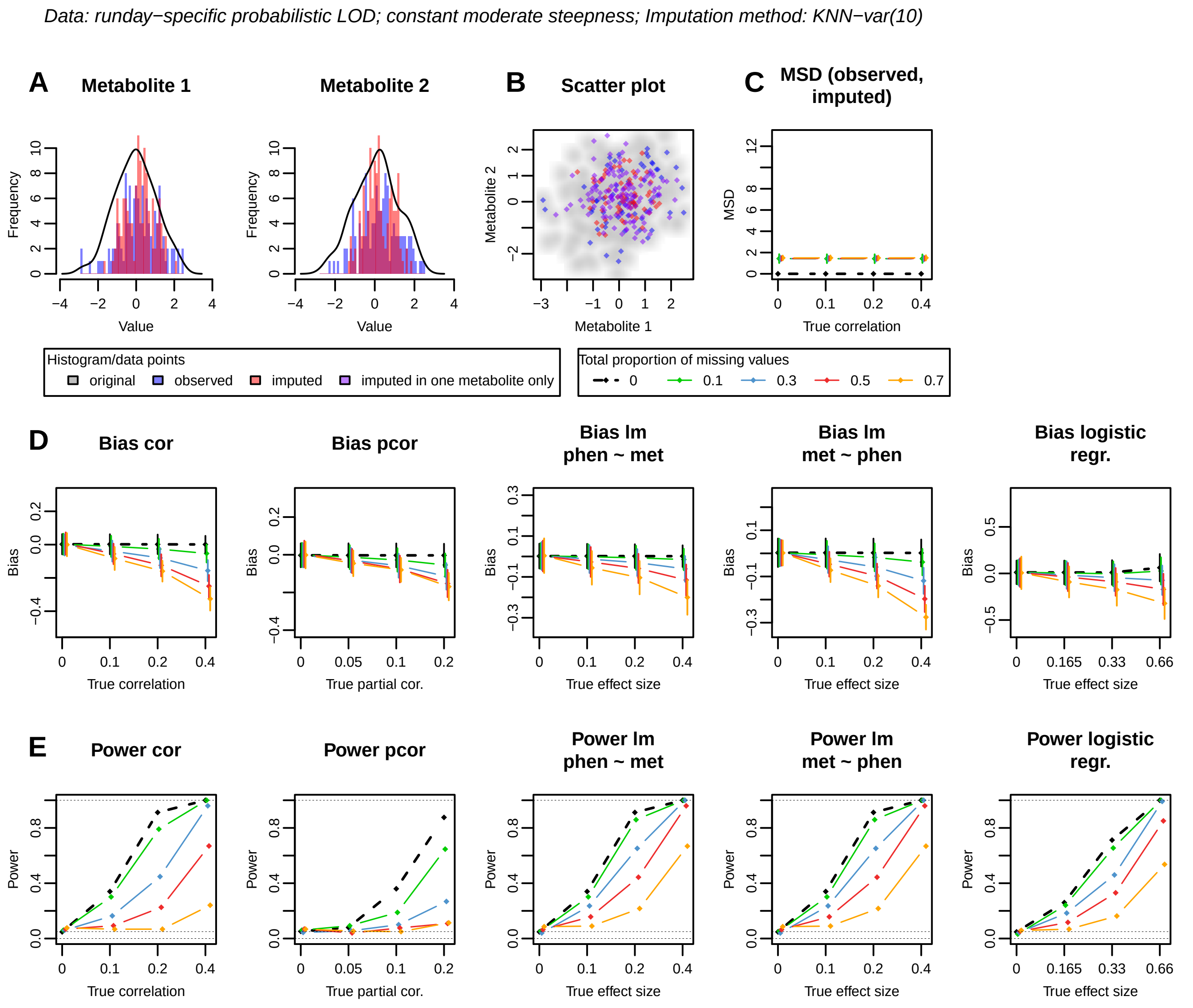

Data: runday−specific probabilistic LOD; constant moderate steepness; Imputation method: KNN−var(10)
A
Metabolite 1
Metabolite 2
B
Scatter plot
C
MSD (observed, imputed)
Frequency
Value
Metabolite 2
Metabolite 1
MSD
True correlation
Histogram/data points
original
observed
imputed
imputed in one metabolite only
Total proportion of missing values
0
0.1
0.3
0.5
0.7
D
Bias cor
Bias pcor
Bias lm phen ~ met
Bias lm met ~ phen
Bias logistic regr.
Bias
True correlation
True partial cor.
True effect size
E
Power cor
Power pcor
Power lm phen ~ met
Power lm met ~ phen
Power logistic regr.
Power

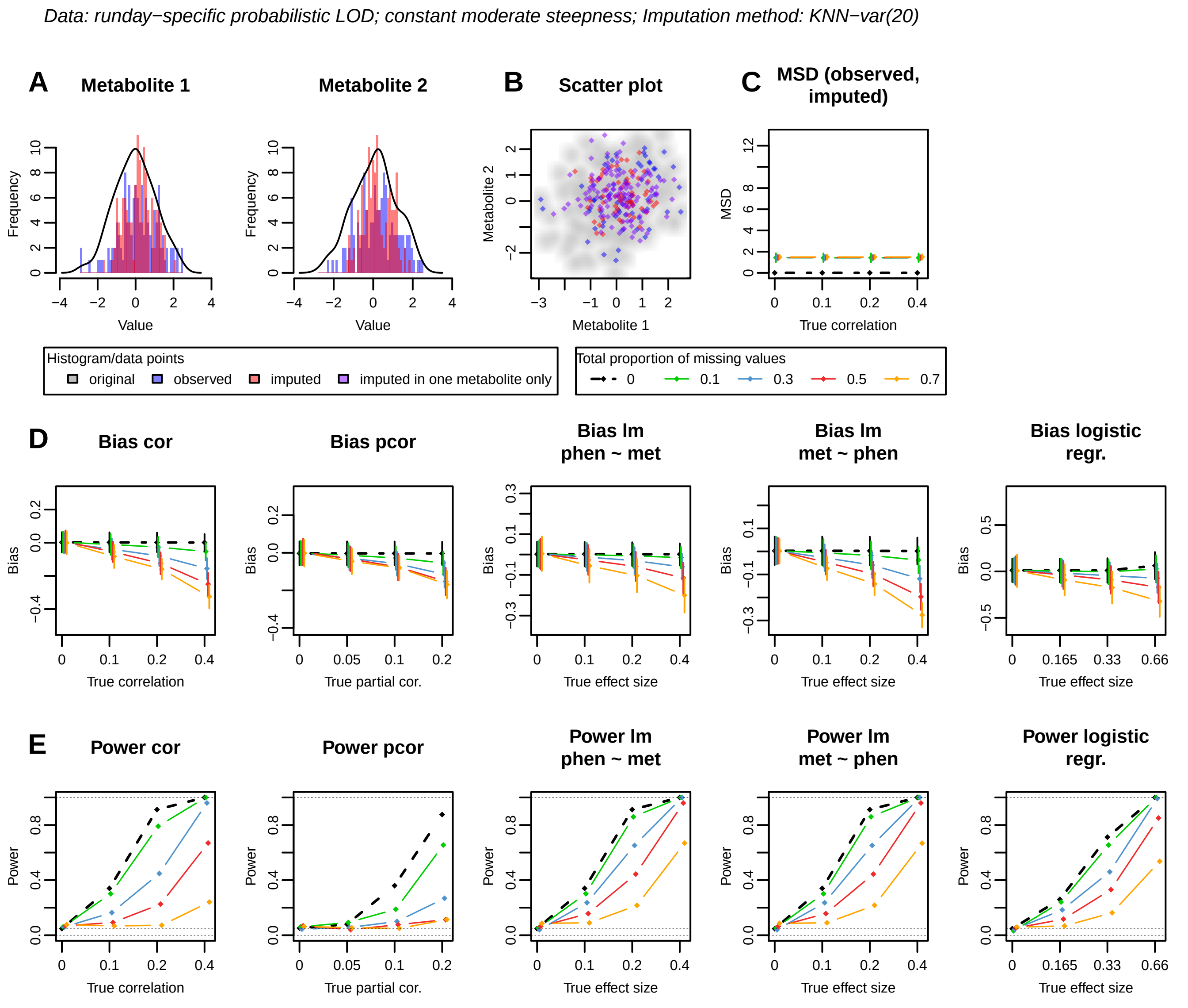

Data: runday−specific probabilistic LOD; constant moderate steepness; Imputation method: KNN−var(20)
A
Metabolite 1
Metabolite 2
Frequency
Value
B
Scatter plot
Metabolite 2
Metabolite 1
C
MSD (observed, imputed)
MSD
True correlation
Histogram/data points
original
observed
imputed
imputed in one metabolite only
Total proportion of missing values
0
0.1
0.3
0.5
0.7
D
Bias cor
Bias pcor
Bias lm phen ~ met
Bias lm met ~ phen
Bias logistic regr.
Bias
True correlation
True partial cor.
True effect size
E
Power cor
Power pcor
Power lm phen ~ met
Power lm met ~ phen
Power logistic regr.
Power
True correlation
True partial cor.
True effect size

Data: runday−specific probabilistic LOD; constant moderate steepness; Imputation method: KNN−obs(1)

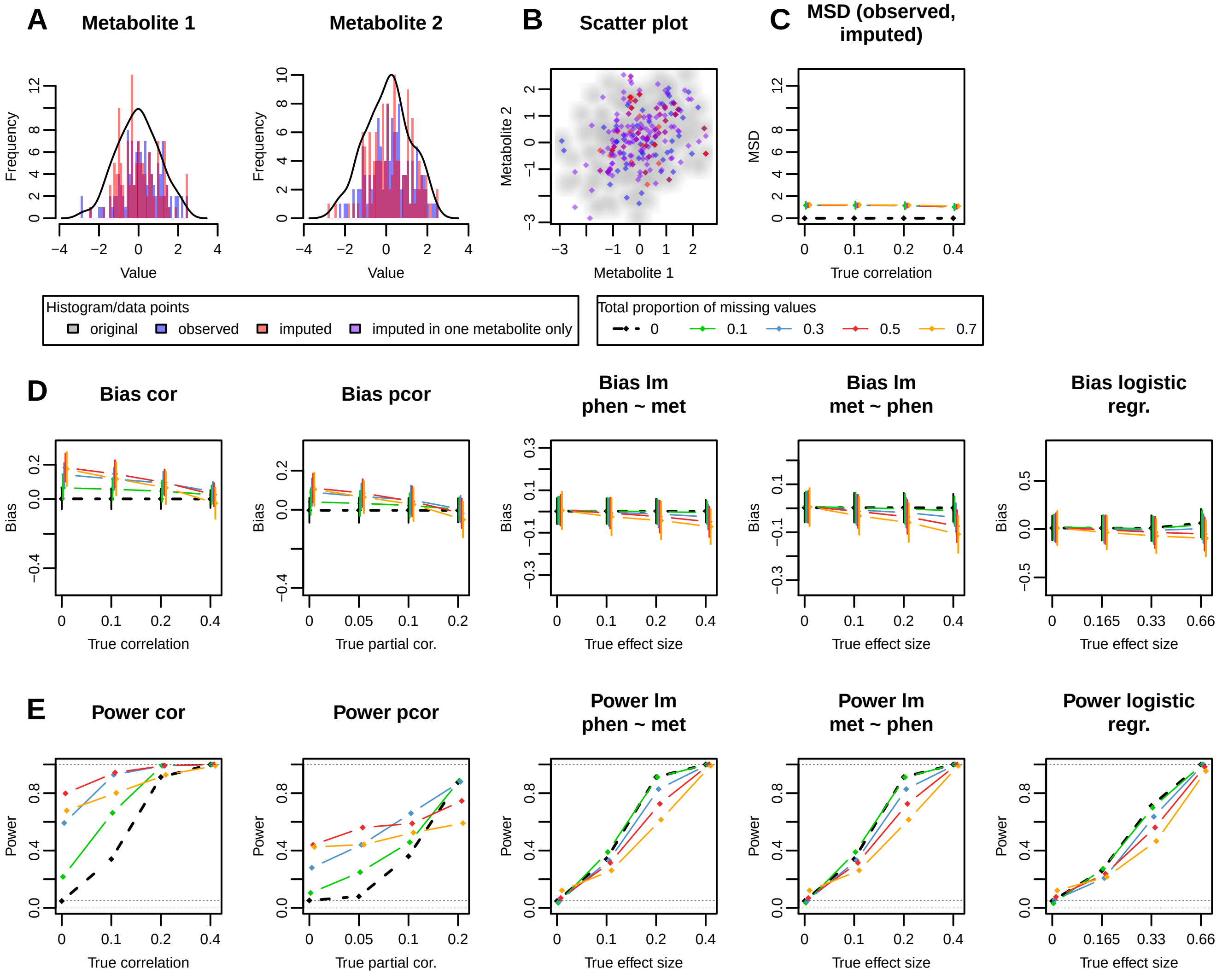

Data: runday−specific probabilistic LOD; constant moderate steepness; Imputation method: KNN−obs(3)

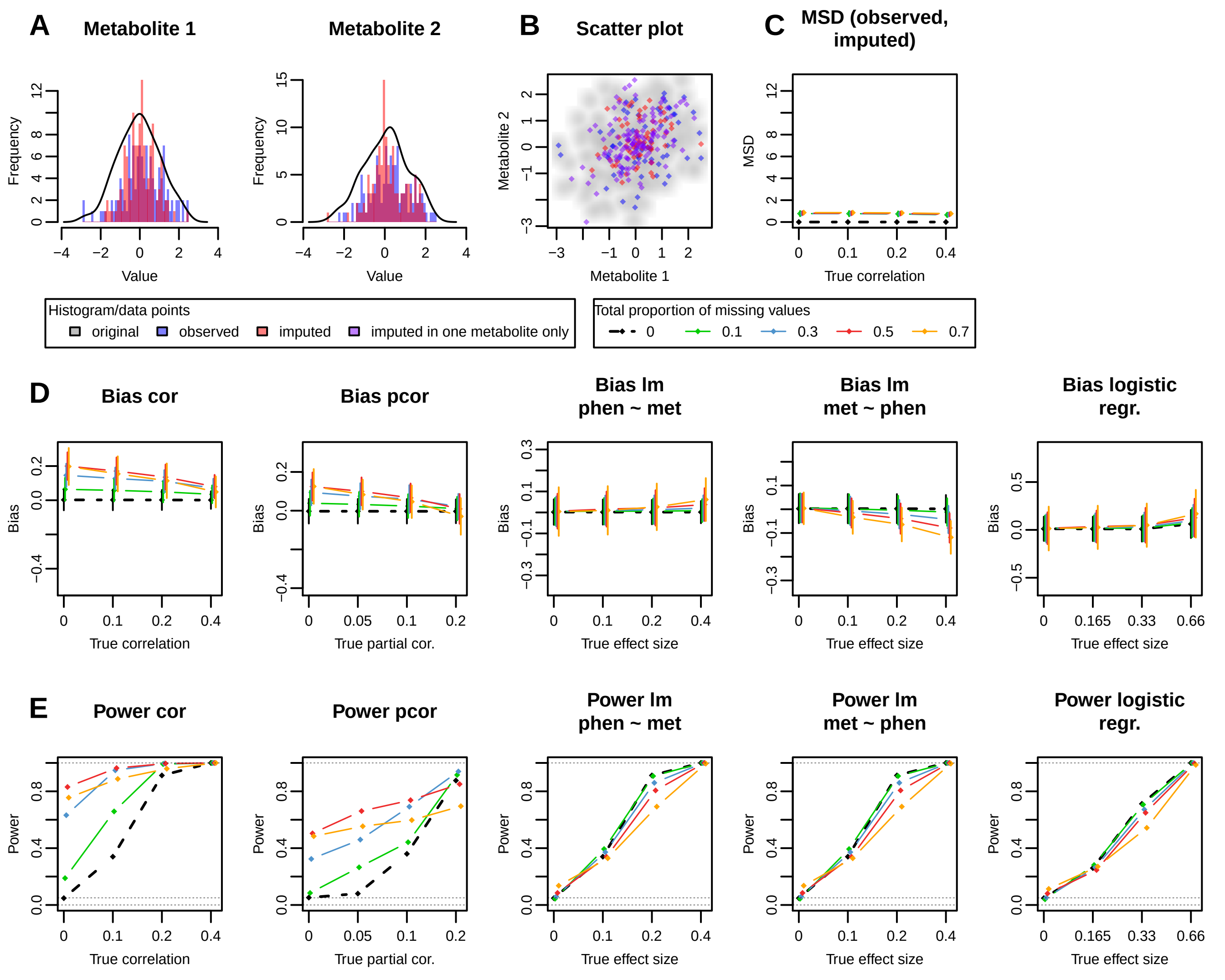

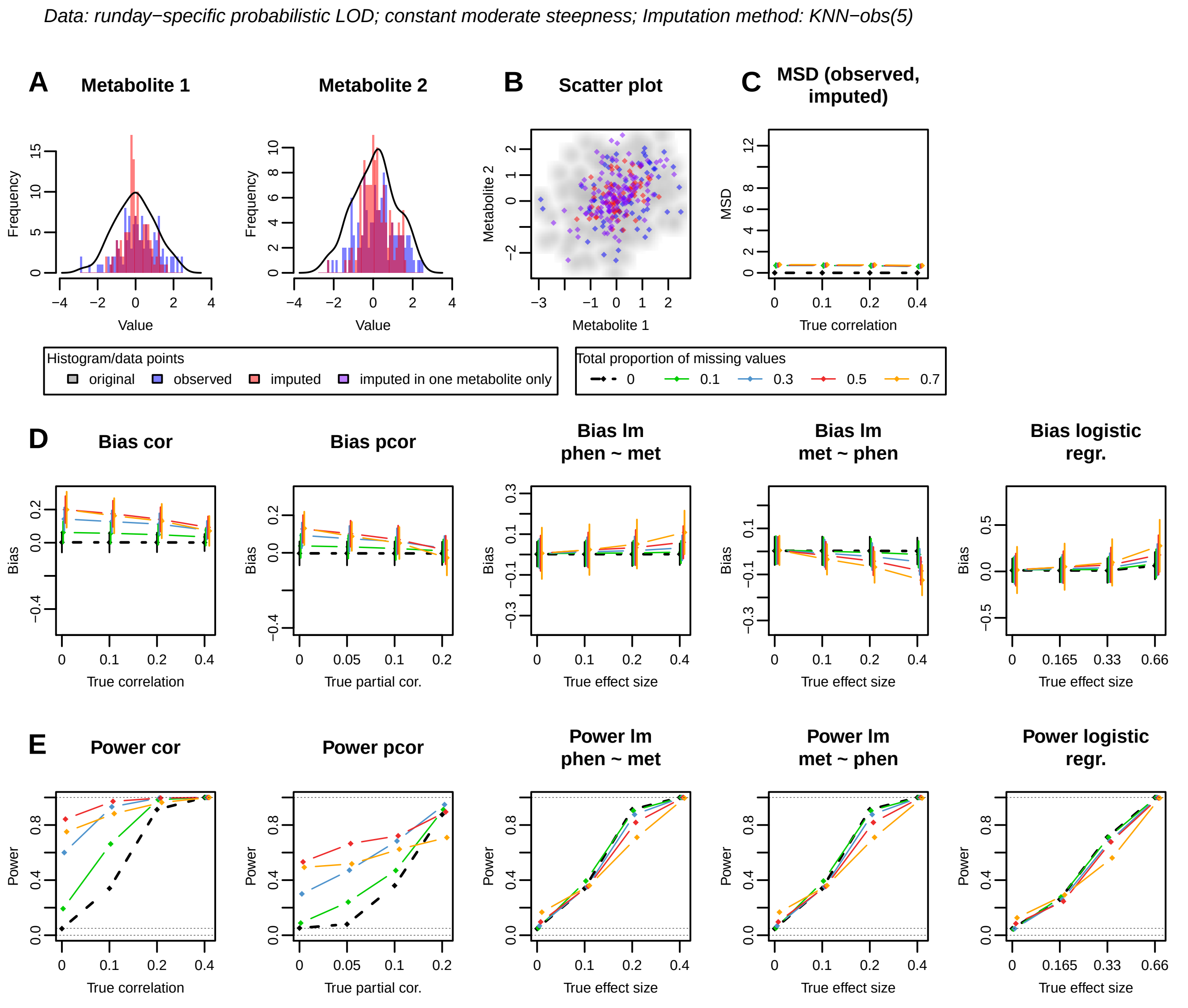

Data: runday−specific probabilistic LOD; constant moderate steepness; Imputation method: KNN−obs(5)
A
Metabolite 1
Metabolite 2
B
Scatter plot
C
MSD (observed, imputed)
Frequency
Value
Metabolite 2
Metabolite 1
MSD
True correlation
Histogram/data points
original
observed
imputed
imputed in one metabolite only
Total proportion of missing values
0
0.1
0.3
0.5
0.7
D
Bias cor
Bias pcor
Bias lm phen ~ met
Bias lm met ~ phen
Bias logistic regr.
Bias
True correlation
True partial cor.
True effect size
E
Power cor
Power pcor
Power lm phen ~ met
Power lm met ~ phen
Power logistic regr.
Power

Data: runday−specific probabilistic LOD; constant moderate steepness; Imputation method: KNN−obs(10)

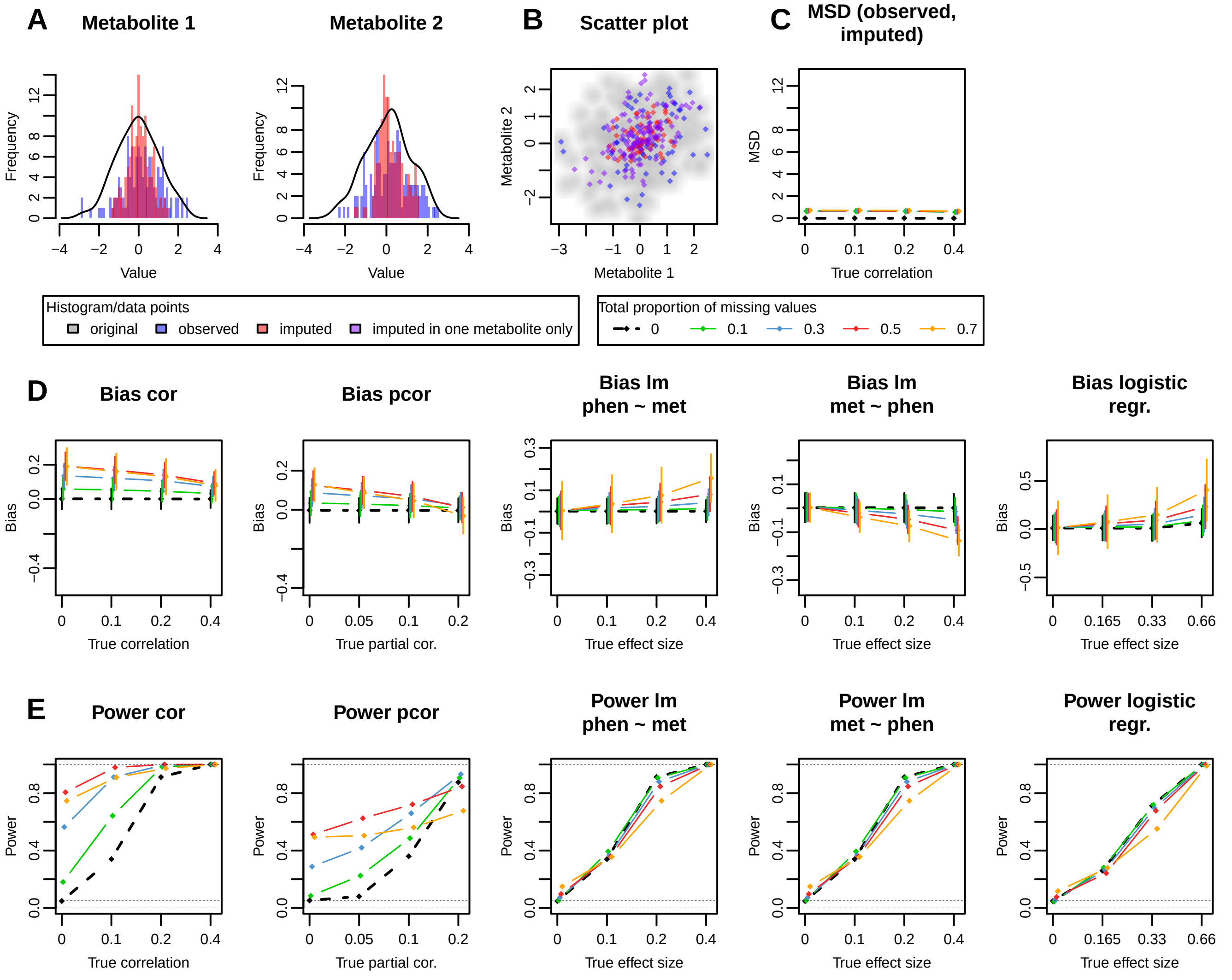

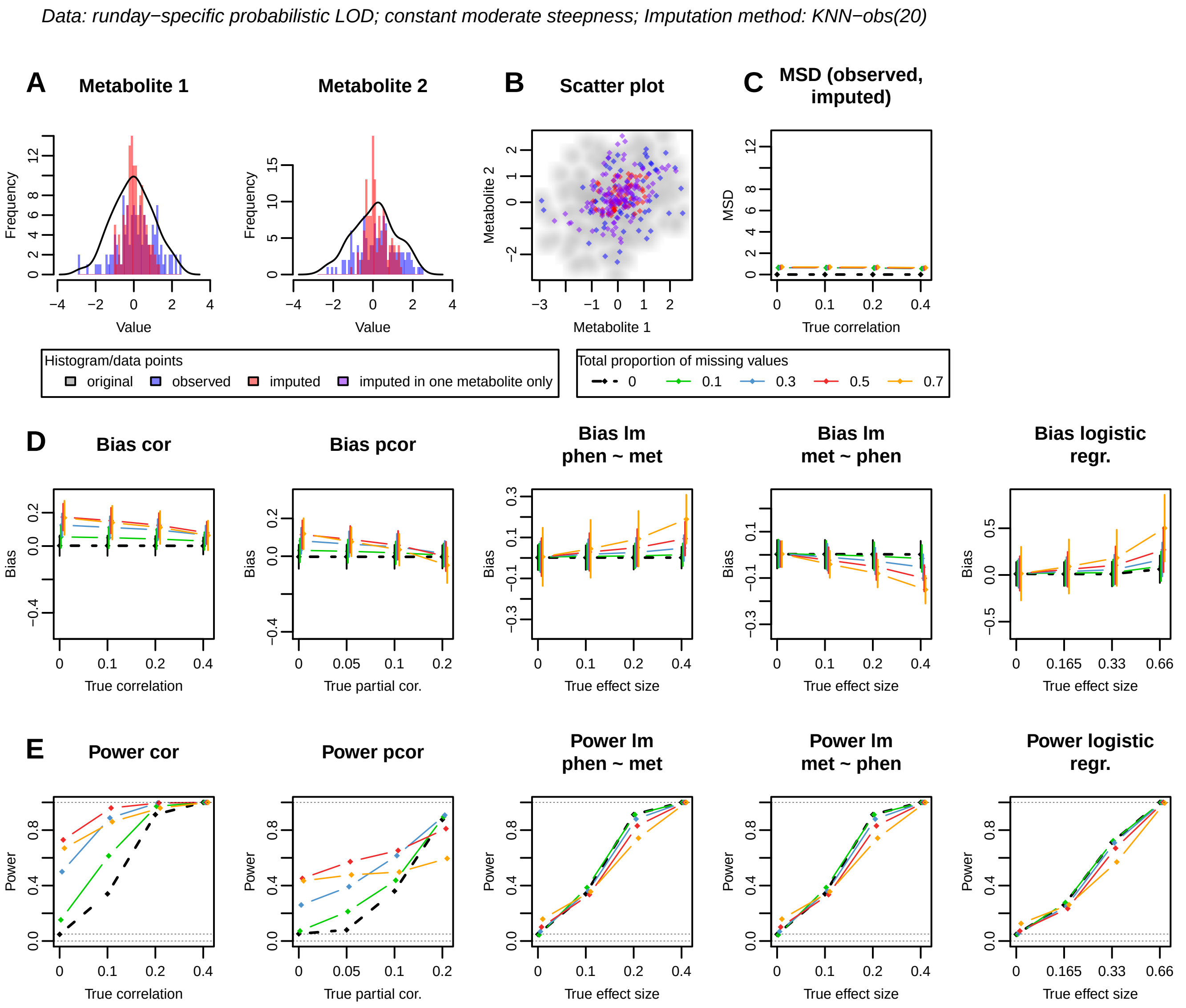
Data: runday−specific probabilistic LOD; constant moderate steepness; Imputation method: KNN−obs(20)
A
Metabolite 1
Metabolite 2
Frequency
Value
B
Scatter plot
Metabolite 1
Metabolite 2
C
MSD (observed, imputed)
MSD
True correlation
Histogram/data points
original
observed
imputed
imputed in one metabolite only
Total proportion of missing values
0
0.1
0.3
0.5
0.7
D
Bias cor
Bias pcor
Bias lm phen ~ met
Bias lm met ~ phen
Bias logistic regr.
Bias
True correlation
True partial cor.
True effect size
E
Power cor
Power pcor
Power lm phen ~ met
Power lm met ~ phen
Power logistic regr.
Power
True correlation
True partial cor.
True effect size

Data: runday−specific probabilistic LOD; constant moderate steepness; Imputation method: KNN−obs−sel(1)

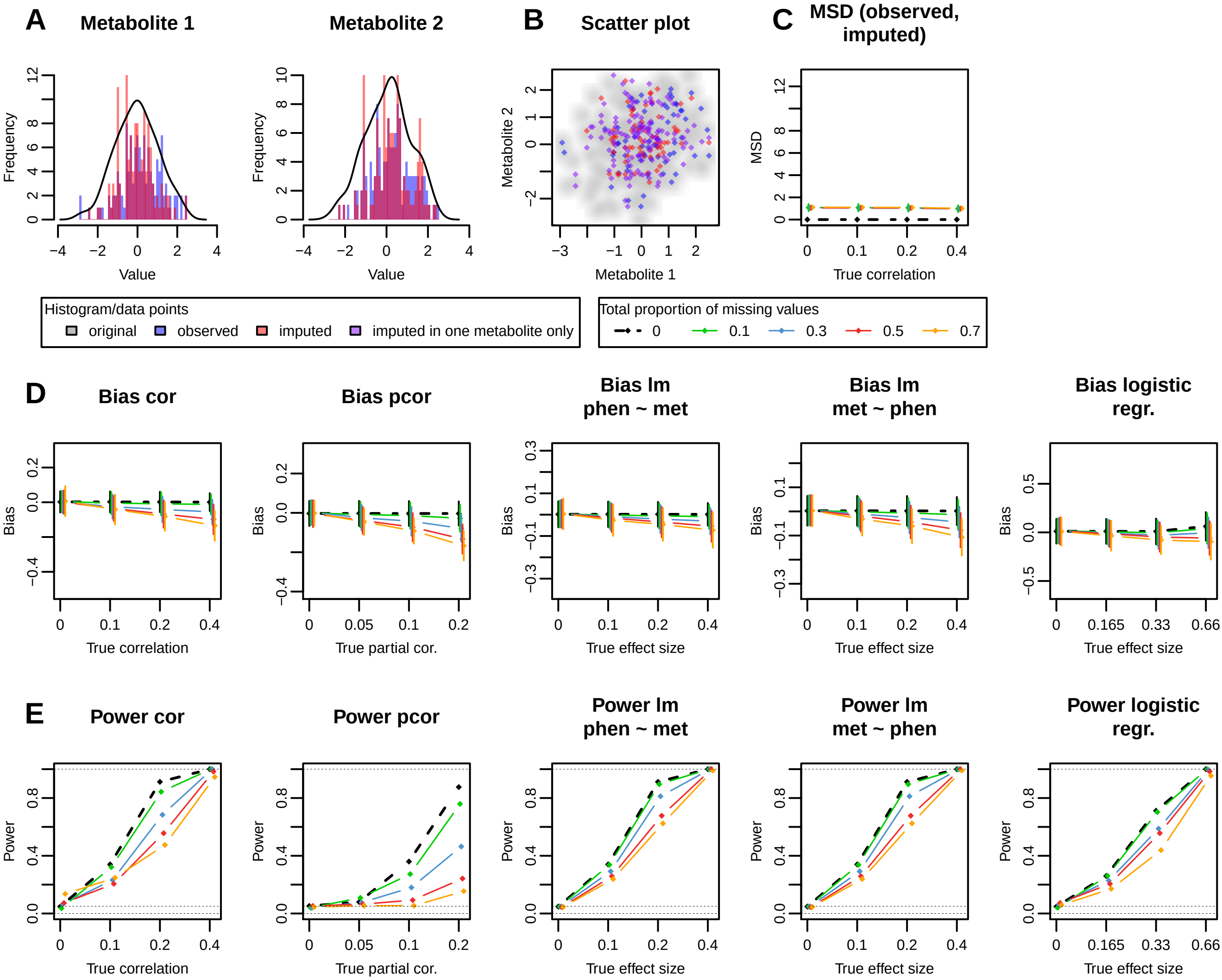

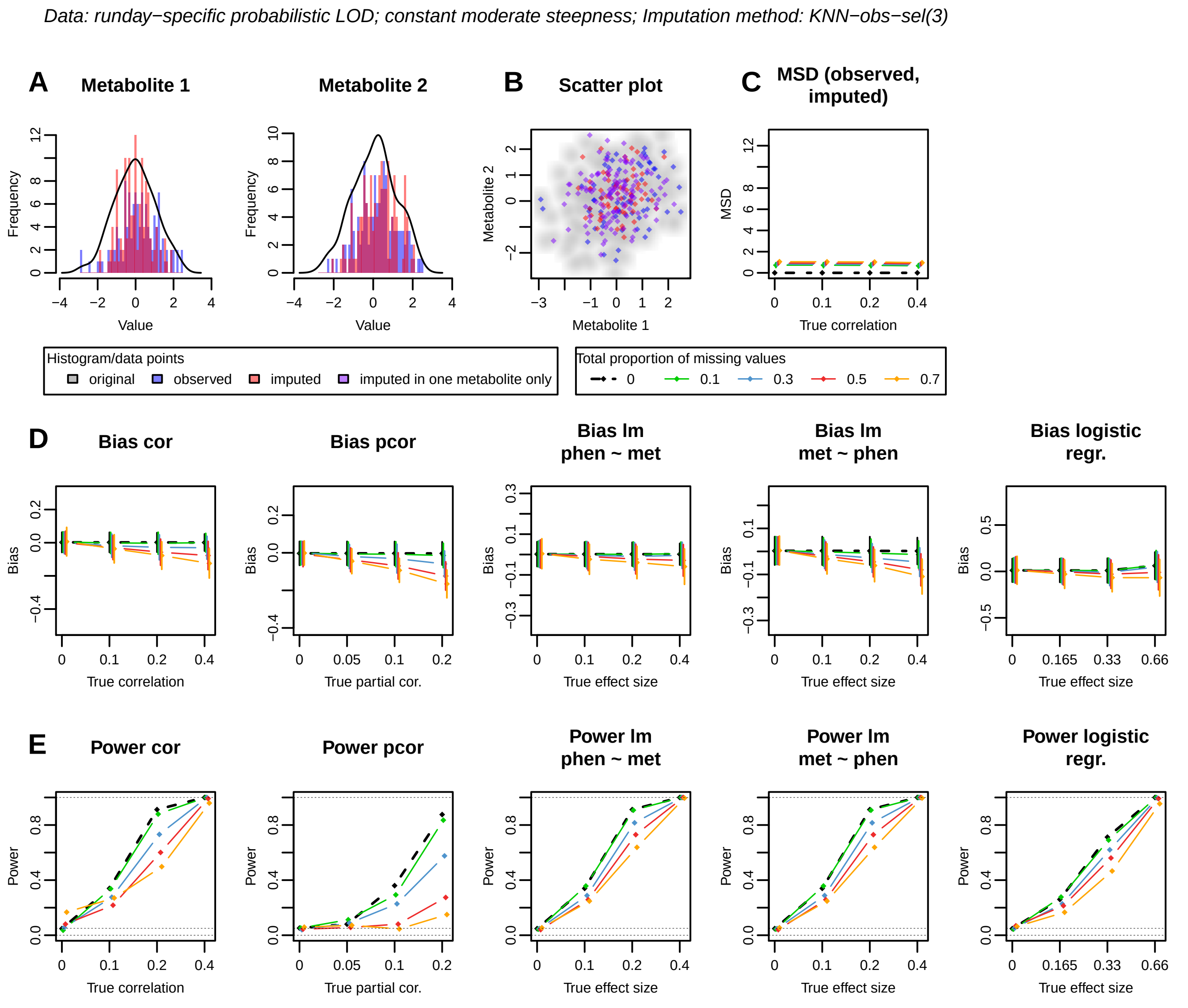

Data: runday−specific probabilistic LOD; constant moderate steepness; Imputation method: KNN−obs−sel(3)
A
Metabolite 1
Metabolite 2
B
Scatter plot
C
MSD (observed, imputed)
Frequency
Value
Metabolite 2
Metabolite 1
MSD
True correlation
Histogram/data points
original
observed
imputed
imputed in one metabolite only
Total proportion of missing values
0
0.1
0.3
0.5
0.7
D
Bias cor
Bias pcor
Bias lm phen ~ met
Bias lm met ~ phen
Bias logistic regr.
Bias
True correlation
True partial cor.
True effect size
E
Power cor
Power pcor
Power lm phen ~ met
Power lm met ~ phen
Power logistic regr.
Power

Data: runday−specific probabilistic LOD; constant moderate steepness; Imputation method: KNN−obs−sel(5)

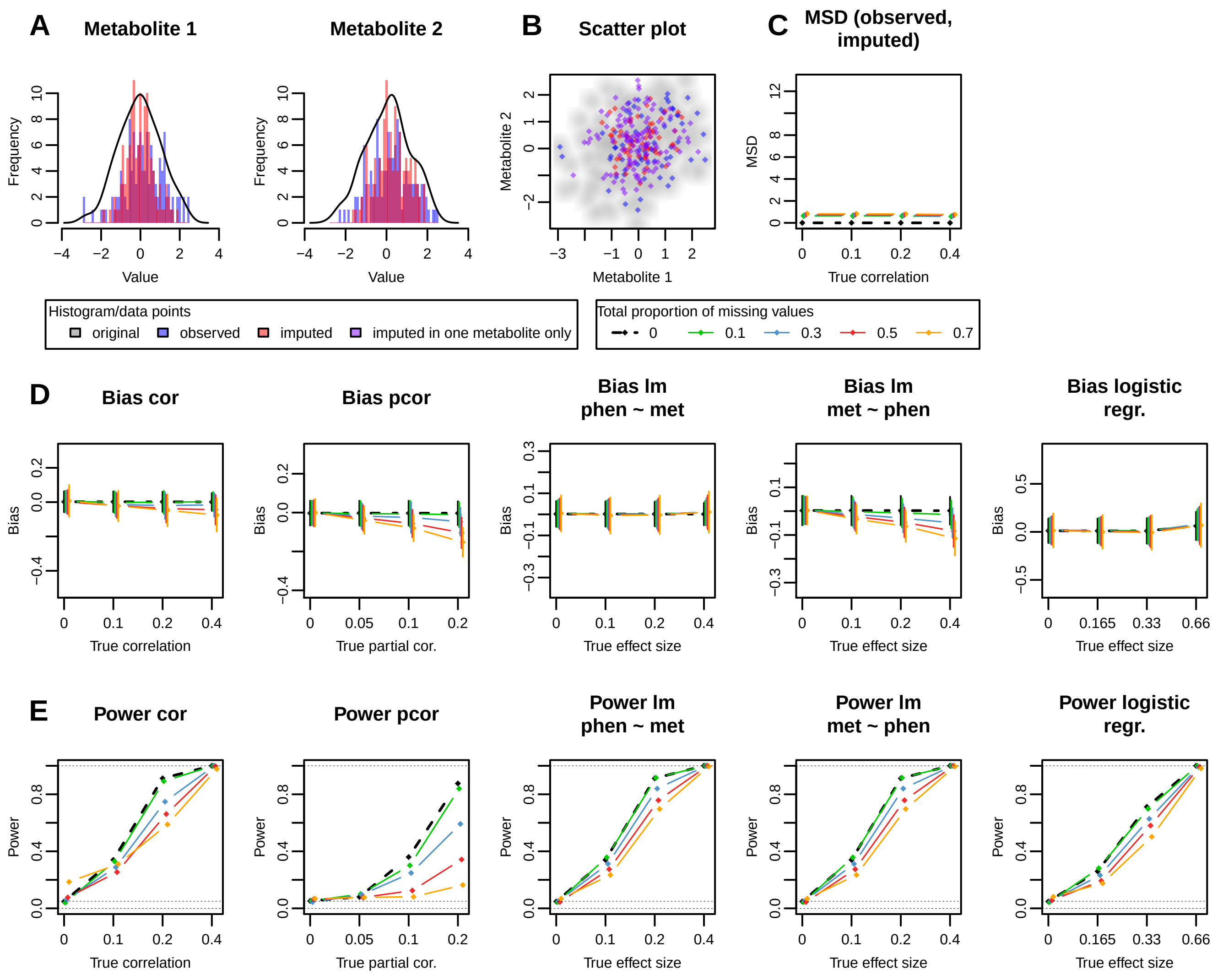

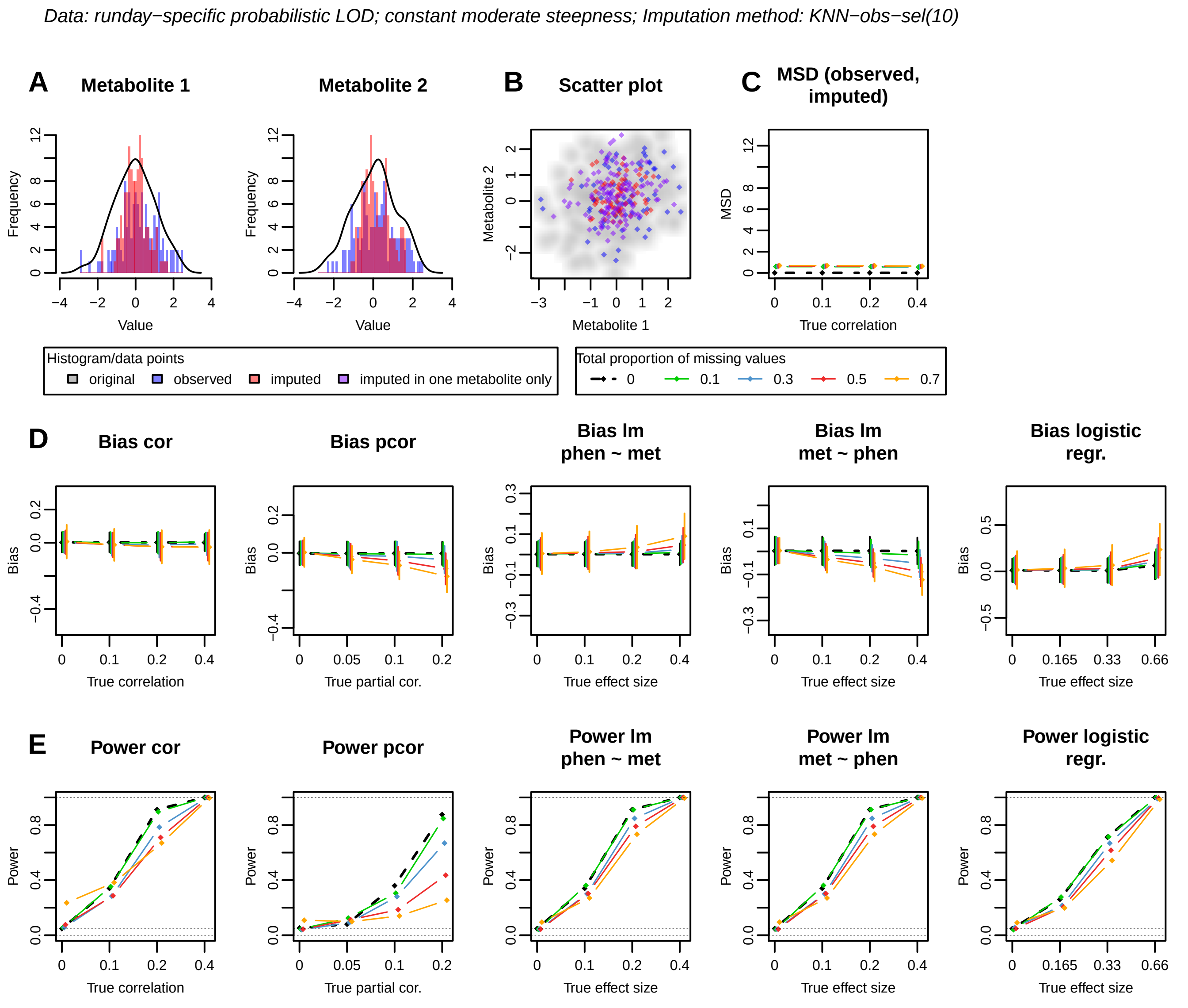
Data: runday−specific probabilistic LOD; constant moderate steepness; Imputation method: KNN−obs−sel(10)
A
Metabolite 1
Metabolite 2
Frequency
Value
B
Scatter plot
Metabolite 1
Metabolite 2
C
MSD (observed, imputed)
MSD
True correlation
Histogram/data points
original
observed
imputed
imputed in one metabolite only
Total proportion of missing values
0
0.1
0.3
0.5
0.7
D
Bias cor
Bias pcor
Bias lm phen ~ met
Bias lm met ~ phen
Bias logistic regr.
Bias
True correlation
True partial cor.
True effect size
E
Power cor
Power pcor
Power lm phen ~ met
Power lm met ~ phen
Power logistic regr.
Power
True correlation
True partial cor.
True effect size

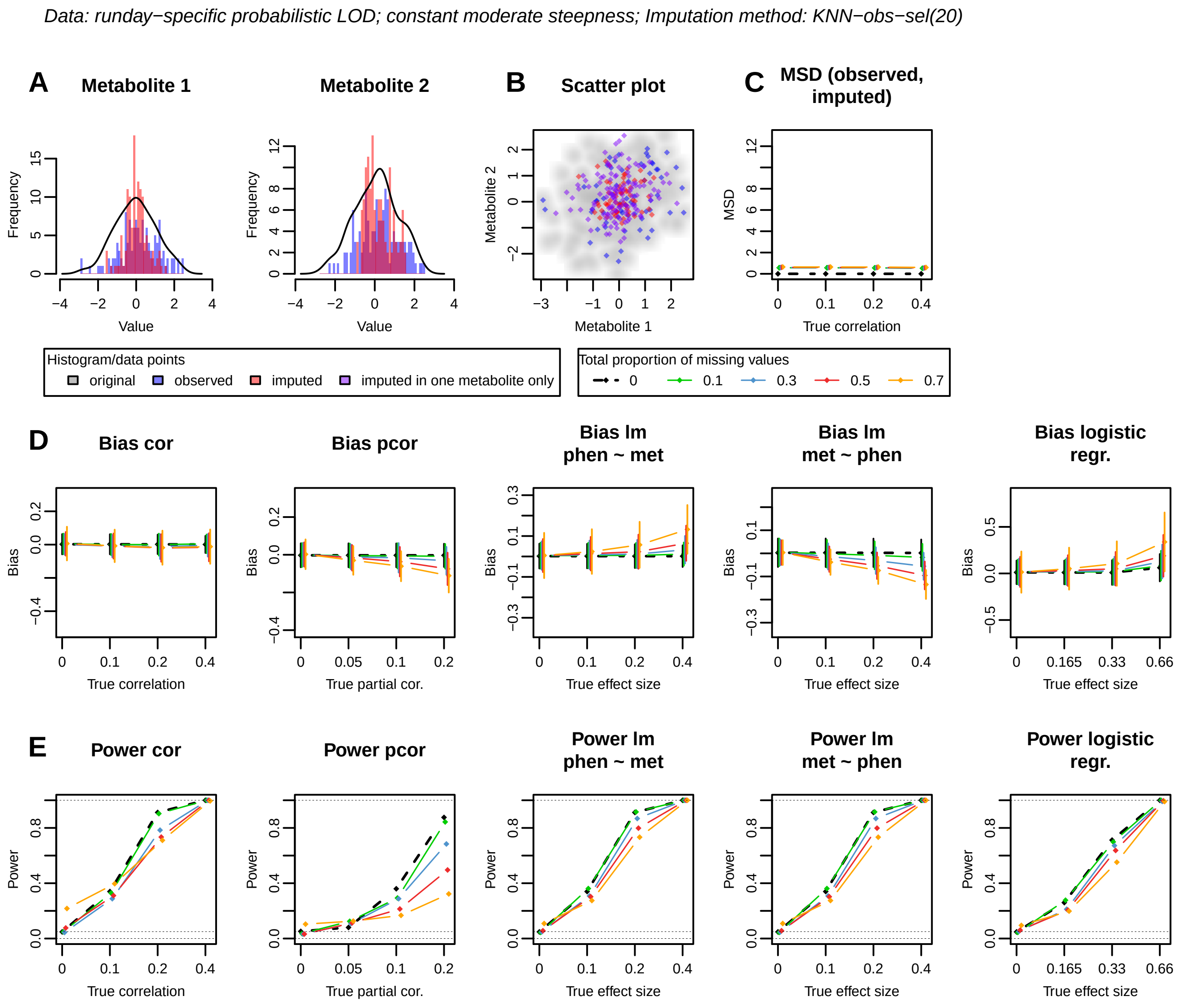

Data: runday−specific probabilistic LOD; constant moderate steepness; Imputation method: KNN−obs−sel(20)
A
Metabolite 1
Metabolite 2
B
Scatter plot
C
MSD (observed, imputed)
Frequency
Value
Metabolite 2
Metabolite 1
MSD
True correlation
Histogram/data points
original
observed
imputed
imputed in one metabolite only
Total proportion of missing values
0
0.1
0.3
0.5
0.7
D
Bias cor
Bias pcor
Bias lm phen ~ met
Bias lm met ~ phen
Bias logistic regr.
Bias
True correlation
True partial cor.
True effect size
E
Power cor
Power pcor
Power lm phen ~ met
Power lm met ~ phen
Power logistic regr.
Power

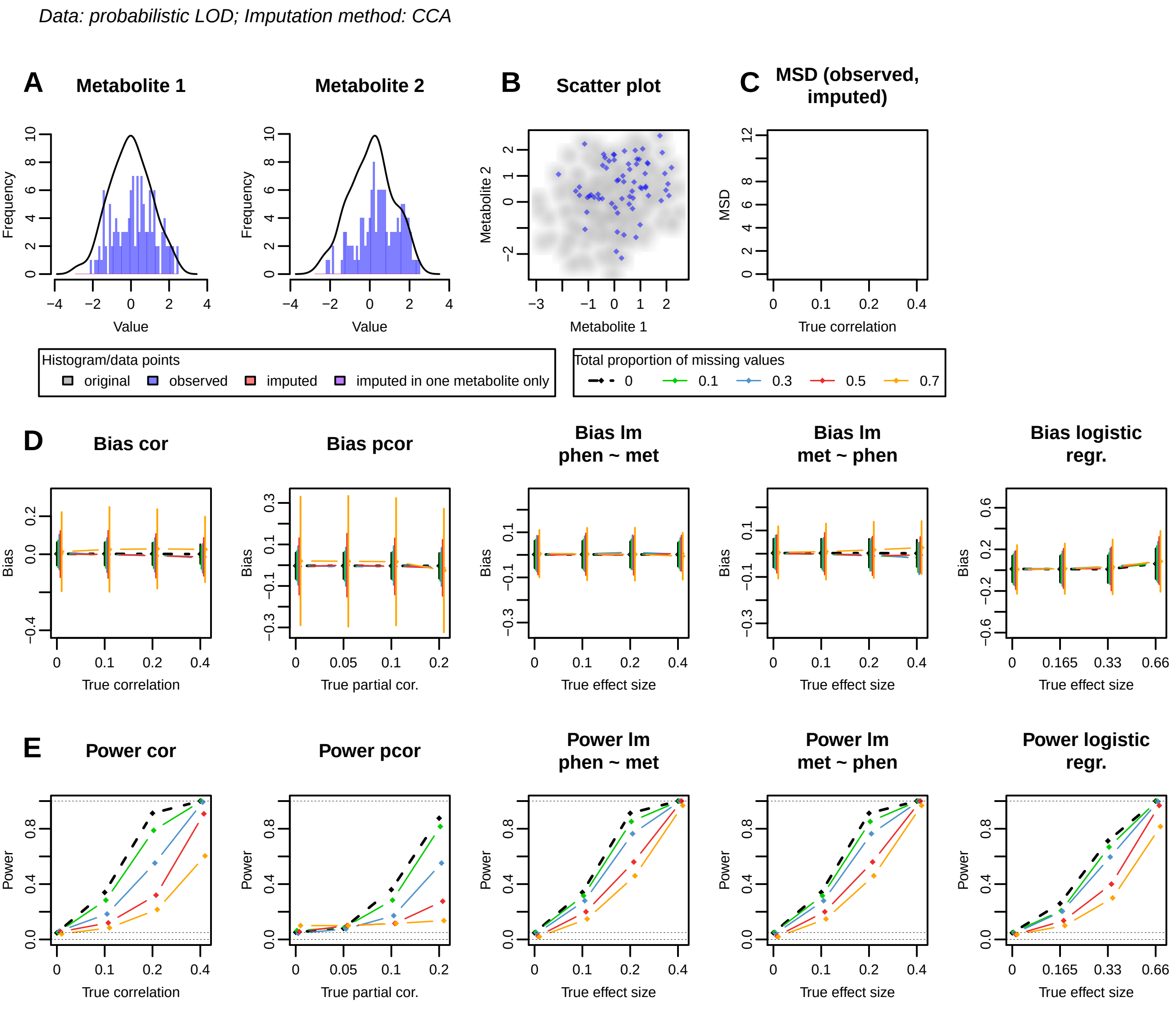
Data: probabilistic LOD; Imputation method: CCA
A
Metabolite 1
Metabolite 2
Frequency
Value
B
Scatter plot
Metabolite 1
Metabolite 2
C
MSD (observed, imputed)
MSD
True correlation
Histogram/data points
original
observed
imputed
imputed in one metabolite only
Total proportion of missing values
0
0.1
0.3
0.5
0.7
D
Bias cor
Bias pcor
Bias lm phen ~ met
Bias lm met ~ phen
Bias logistic regr.
Bias
True correlation
True partial cor.
True effect size
E
Power cor
Power pcor
Power lm phen ~ met
Power lm met ~ phen
Power logistic regr.
Power
True correlation
True partial cor.
True effect size

Data: probabilistic LOD; Imputation method: min

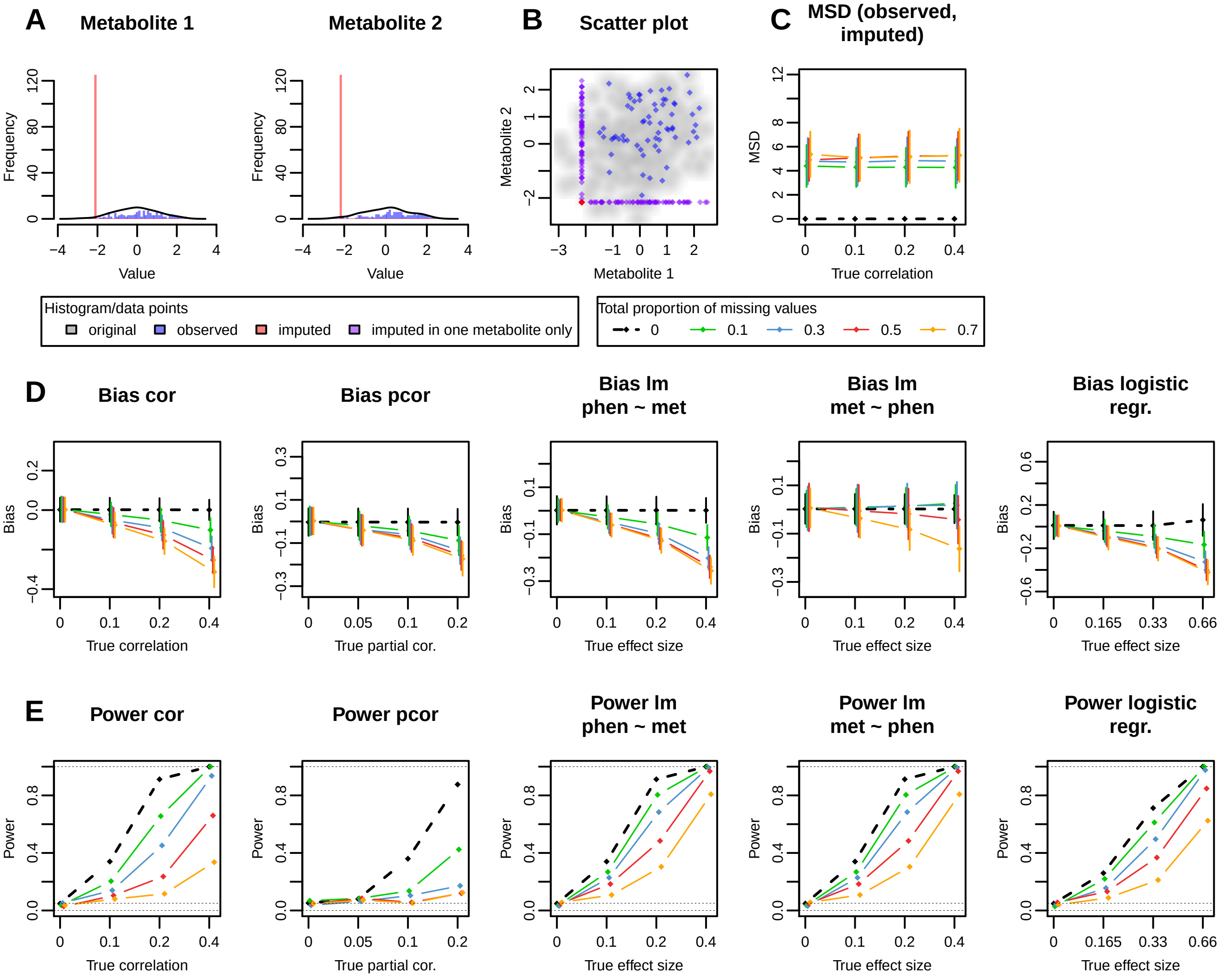

Data: probabilistic LOD; Imputation method: RC

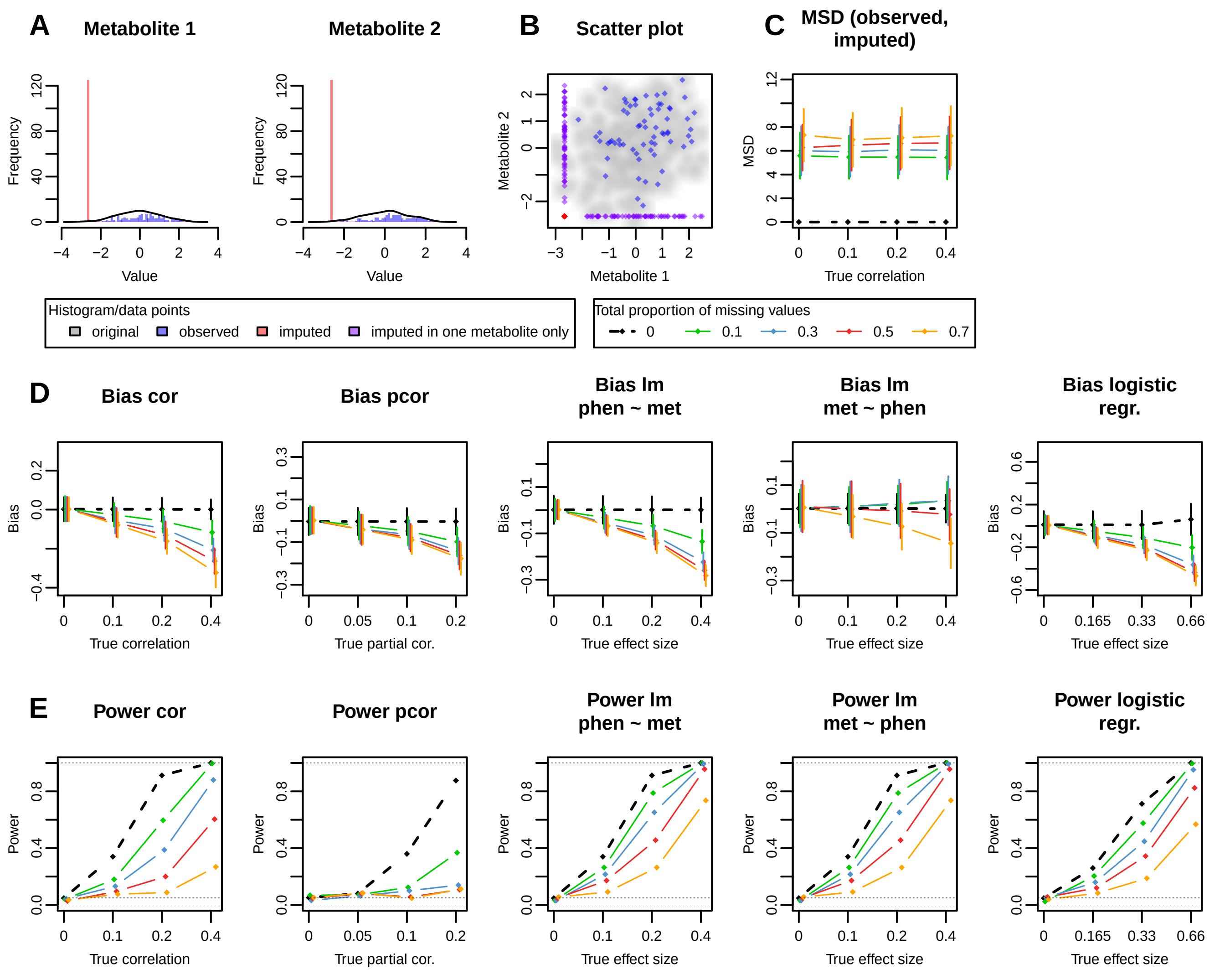

Data: probabilistic LOD; Imputation method: ITS

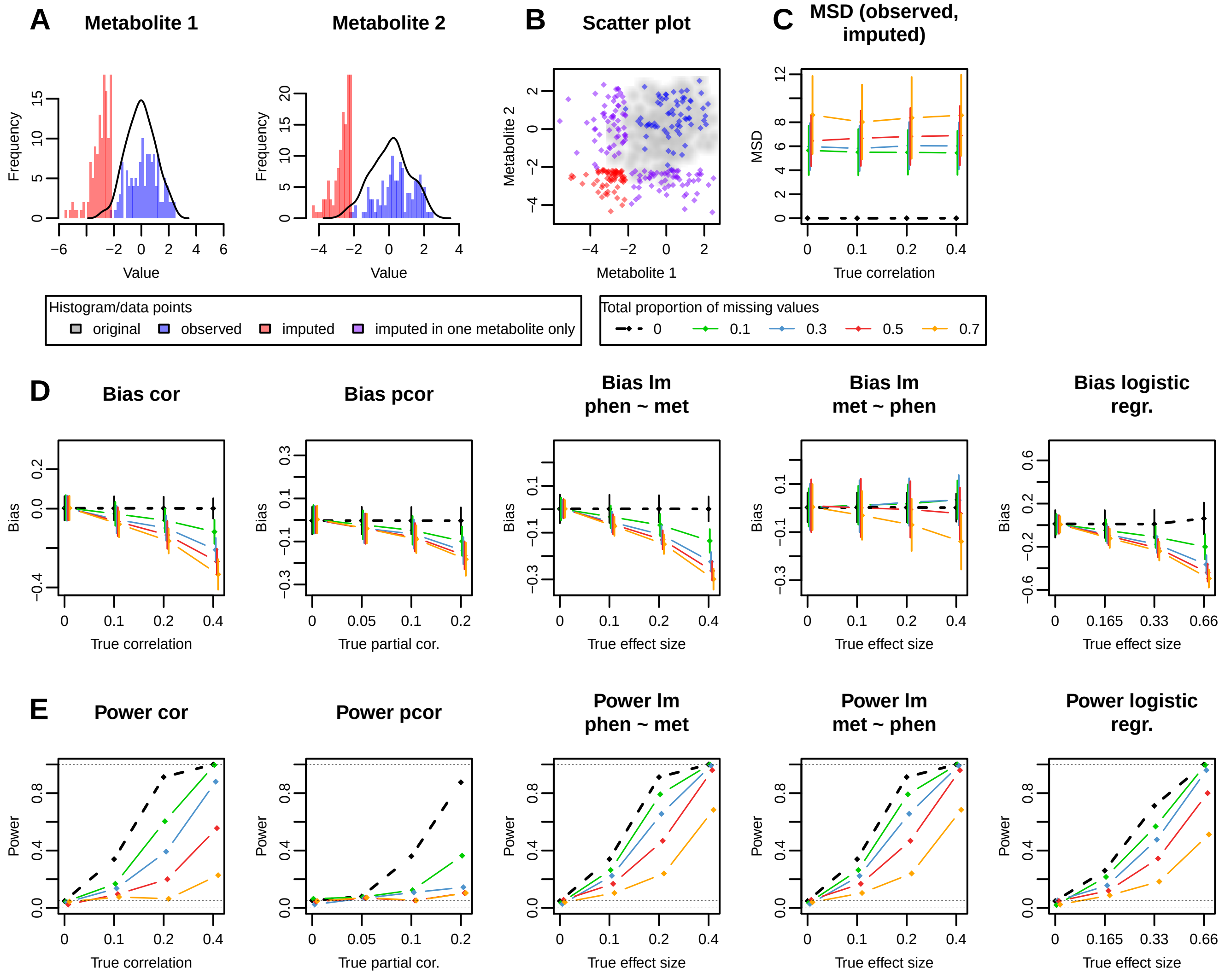

Data: probabilistic LOD; Imputation method: MITS

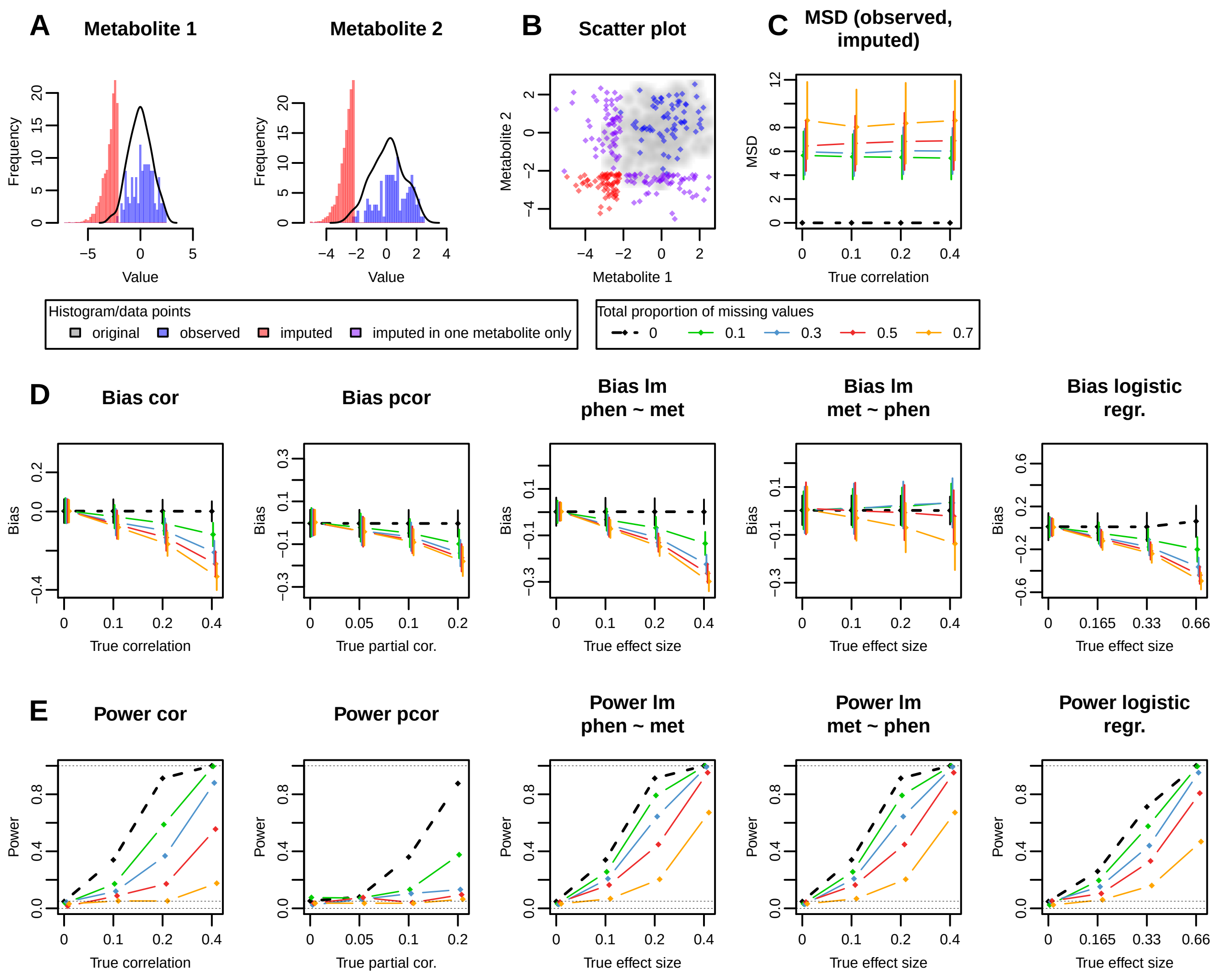

Data: probabilistic LOD; Imputation method: RC−R

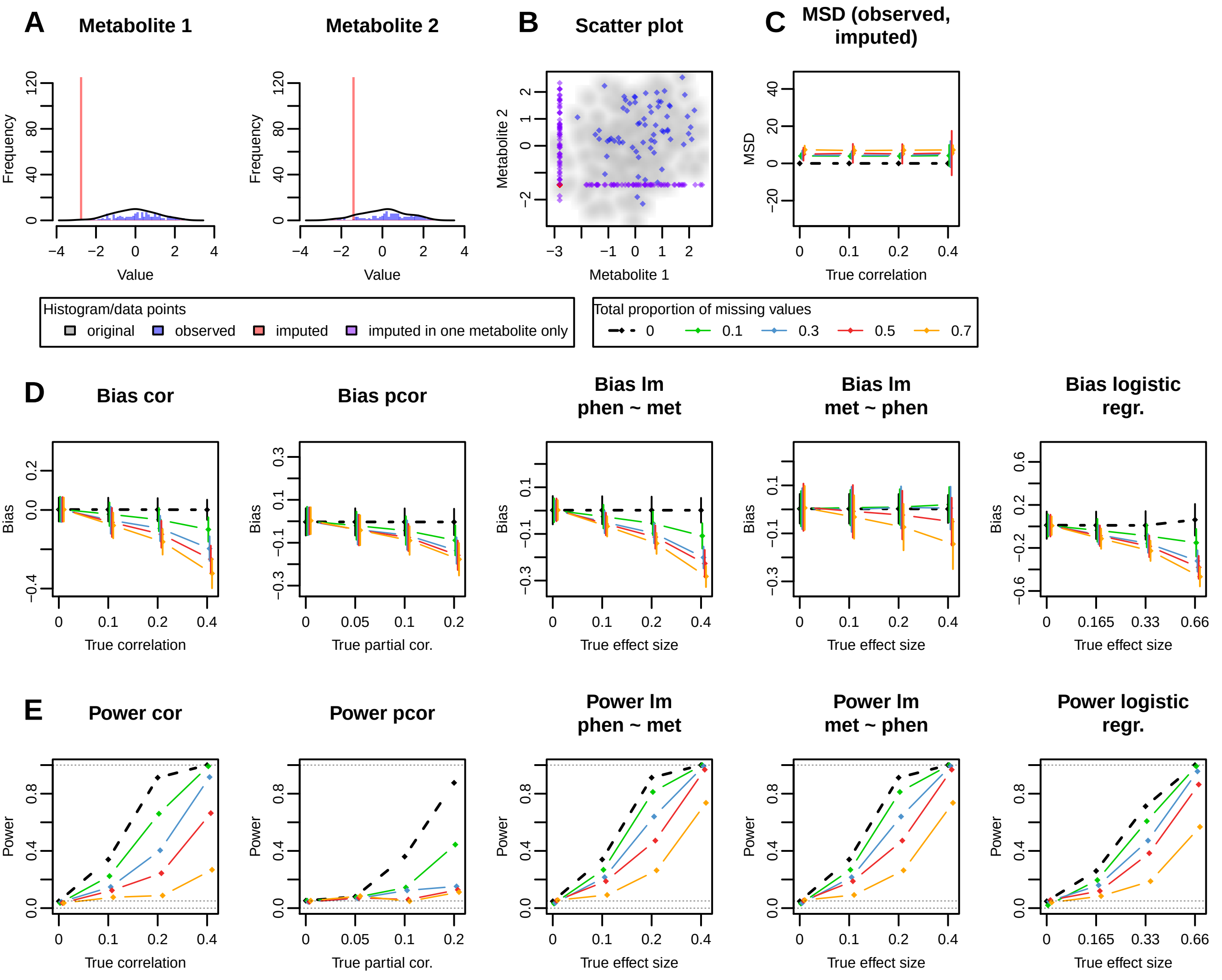

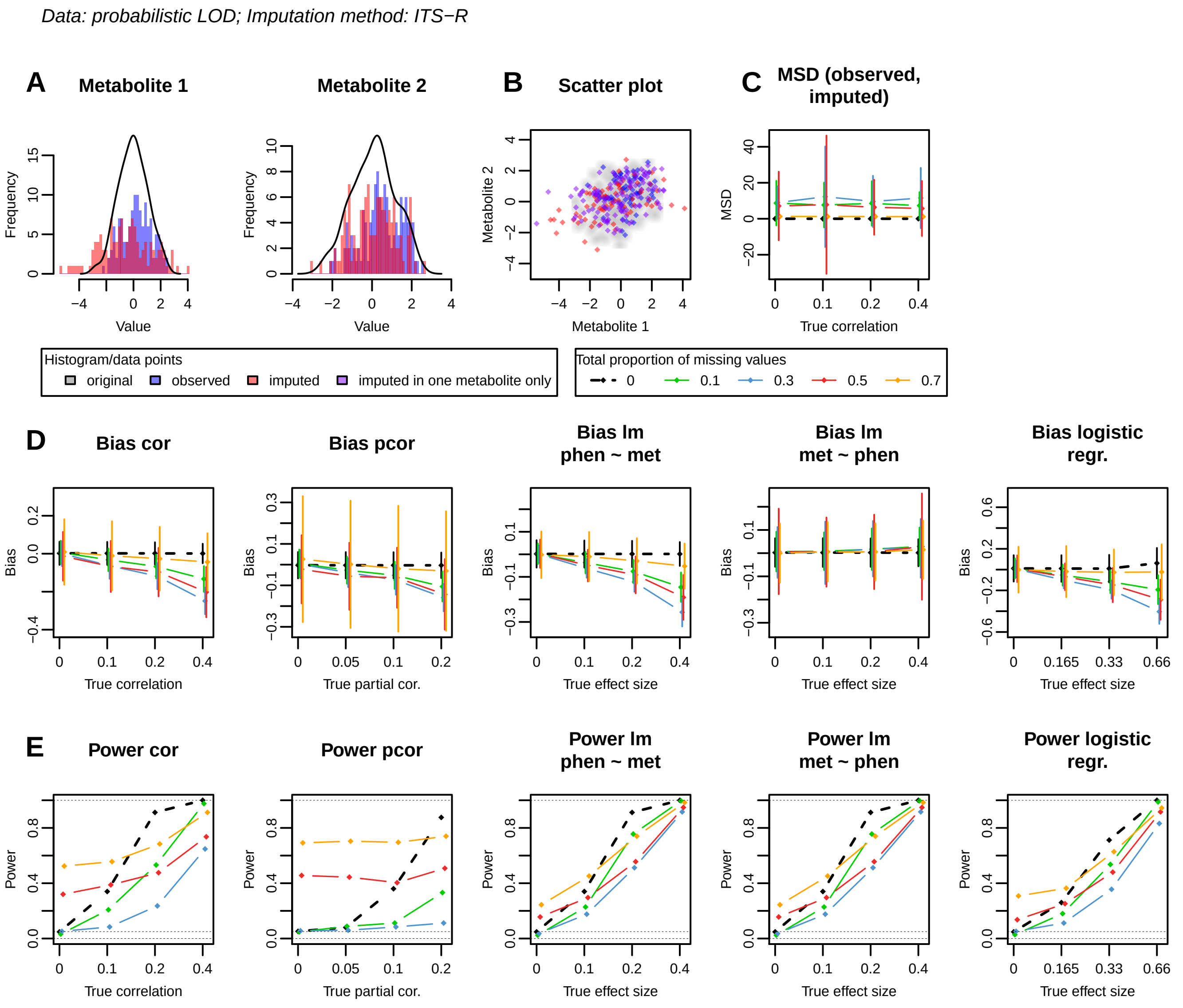

Data: probabilistic LOD; Imputation method: ITS−R
A
Metabolite 1
Metabolite 2
B
Scatter plot
C
MSD (observed, imputed)
Frequency
Value
Metabolite 2
Metabolite 1
MSD
True correlation
Histogram/data points
original
observed
imputed
imputed in one metabolite only
Total proportion of missing values
0
0.1
0.3
0.5
0.7
D
Bias cor
Bias pcor
Bias lm phen ~ met
Bias lm met ~ phen
Bias logistic regr.
Bias
True correlation
True partial cor.
True effect size
E
Power cor
Power pcor
Power lm phen ~ met
Power lm met ~ phen
Power logistic regr.
Power
True correlation
True partial cor.
True effect size

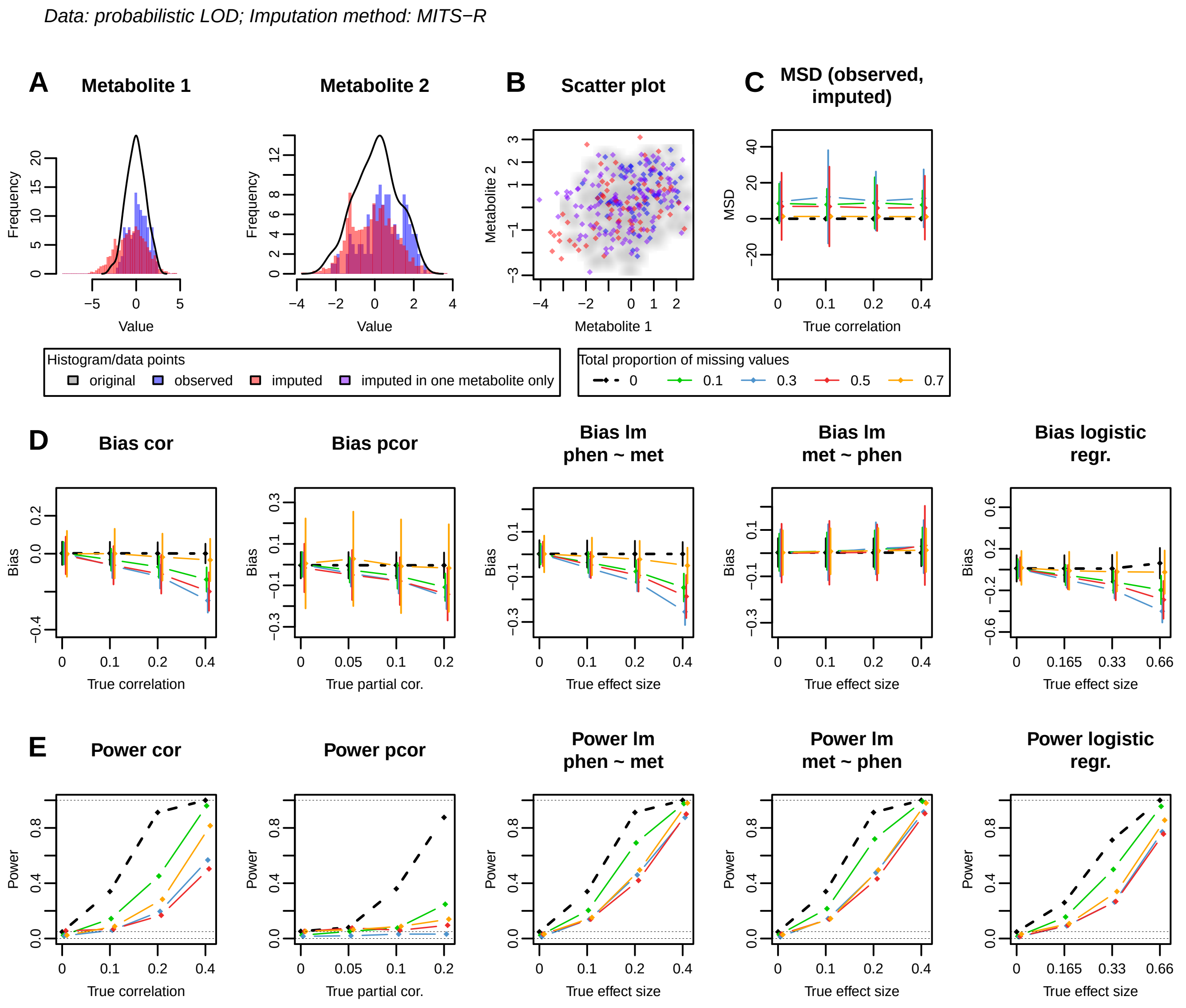
Data: probabilistic LOD; Imputation method: MITS−R
A
Metabolite 1
Metabolite 2
Frequency
Value
B
Scatter plot
Metabolite 2
Metabolite 1
C
MSD (observed, imputed)
MSD
True correlation
Histogram/data points
original
observed
imputed
imputed in one metabolite only
Total proportion of missing values
0
0.1
0.3
0.5
0.7
D
Bias cor
Bias pcor
Bias lm phen ~ met
Bias lm met ~ phen
Bias logistic regr.
Bias
True correlation
True partial cor.
True effect size
E
Power cor
Power pcor
Power lm phen ~ met
Power lm met ~ phen
Power logistic regr.
Power
True correlation
True partial cor.
True effect size

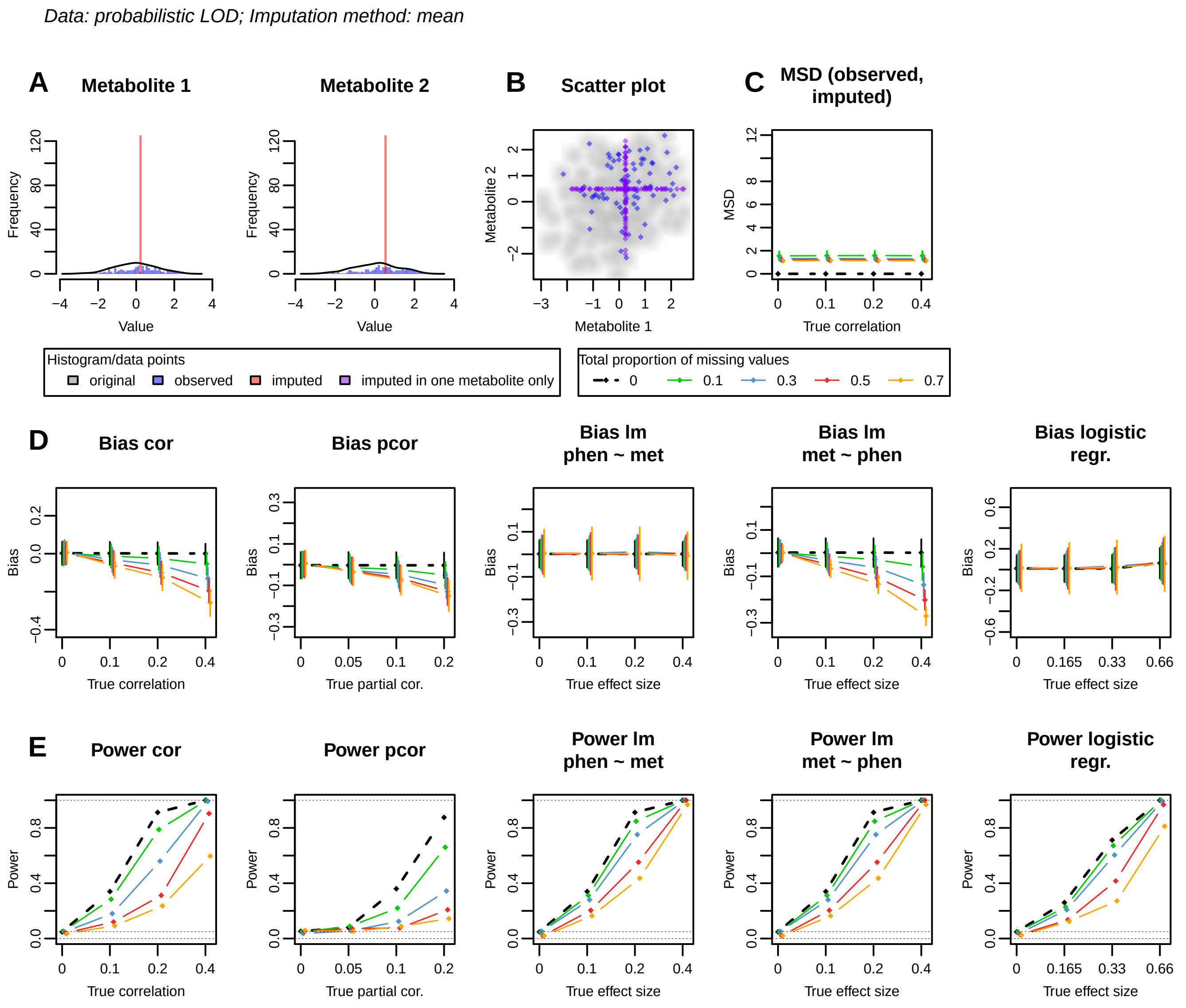
Data: probabilistic LOD; Imputation method: mean
A
Metabolite 1
Metabolite 2
Frequency
Value
B
Scatter plot
Metabolite 1
Metabolite 2
C
MSD (observed, imputed)
MSD
True correlation
Histogram/data points
original
observed
imputed
imputed in one metabolite only
Total proportion of missing values
0
0.1
0.3
0.5
0.7
D
Bias cor
Bias pcor
Bias lm phen ~ met
Bias lm met ~ phen
Bias logistic regr.
Bias
True correlation
True partial cor.
True effect size
E
Power cor
Power pcor
Power lm phen ~ met
Power lm met ~ phen
Power logistic regr.
Power
True correlation
True partial cor.
True effect size

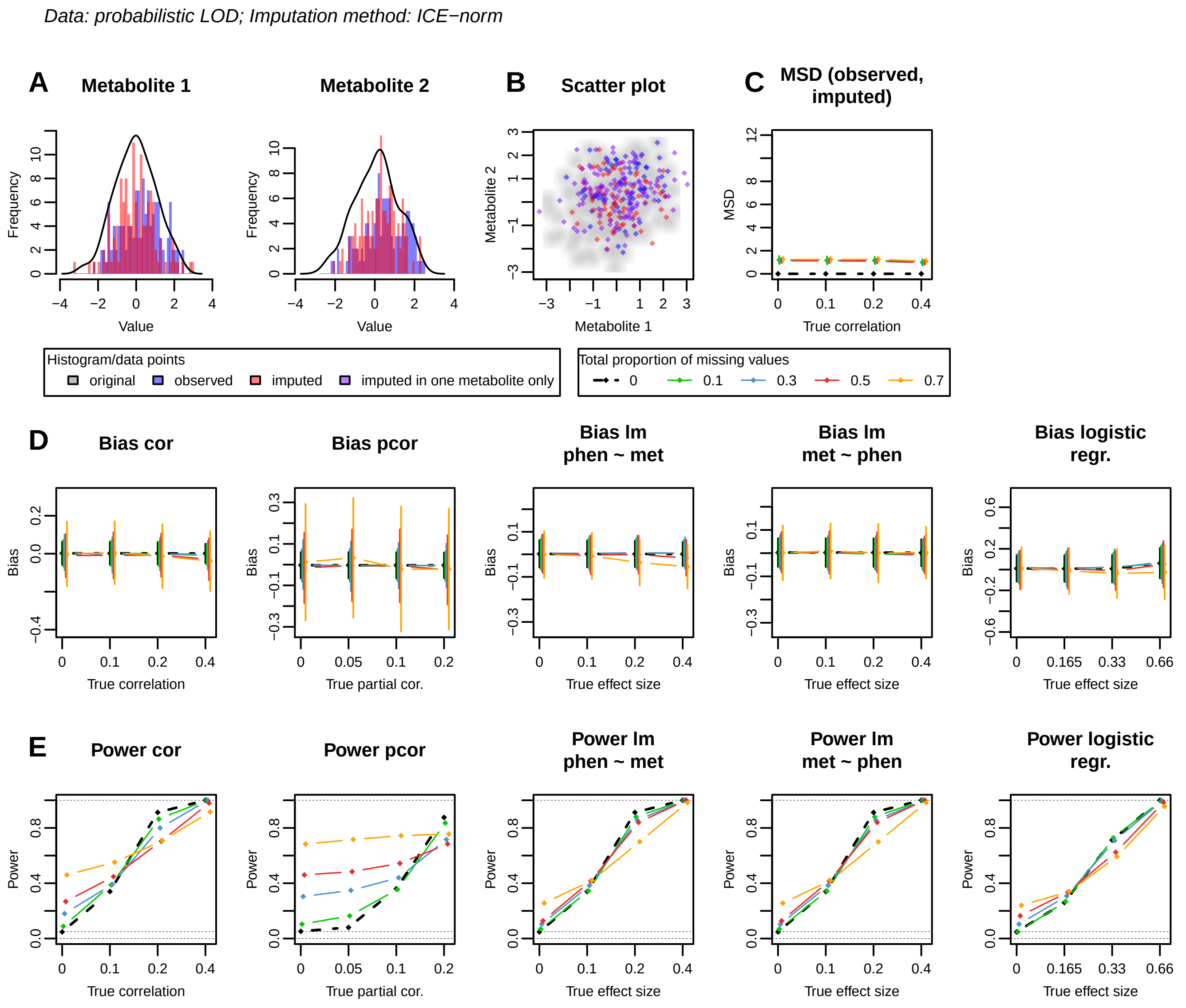

Data: probabilistic LOD; Imputation method: ICE−norm
A
Metabolite 1
Metabolite 2
B
Scatter plot
C
MSD (observed, imputed)
Frequency
Value
Metabolite 2
Metabolite 1
MSD
True correlation
Histogram/data points
original
observed
imputed
imputed in one metabolite only
Total proportion of missing values
0
0.1
0.3
0.5
0.7
D
Bias cor
Bias pcor
Bias lm phen ~ met
Bias lm met ~ phen
Bias logistic regr.
Bias
True correlation
True partial cor.
True effect size
E
Power cor
Power pcor
Power lm phen ~ met
Power lm met ~ phen
Power logistic regr.
Power
True correlation
True partial cor.
True effect size

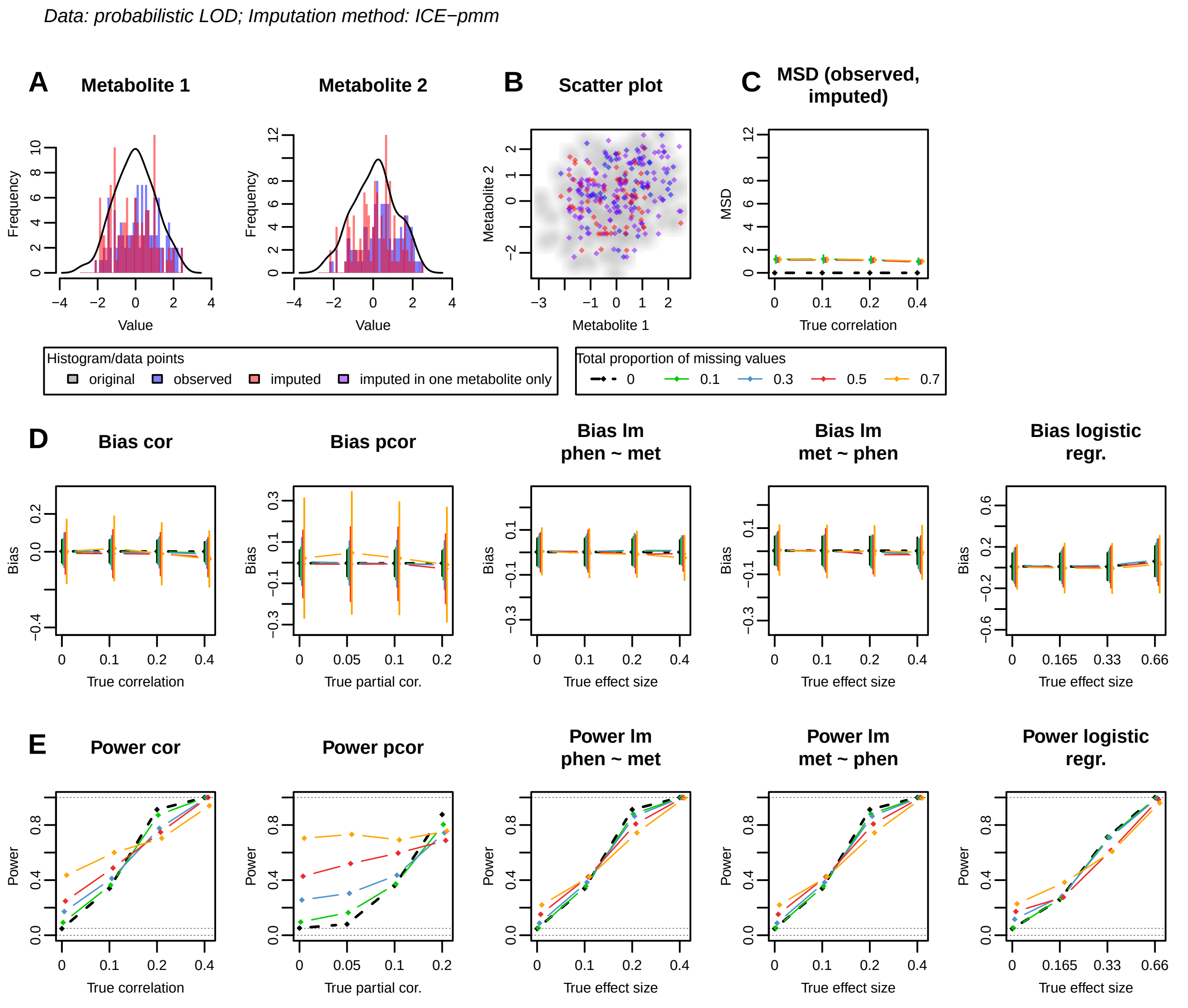
Data: probabilistic LOD; Imputation method: ICE−pmm
A
Metabolite 1
Metabolite 2
B
Scatter plot
C
MSD (observed, imputed)
Frequency
Value
Metabolite 2
Metabolite 1
MSD
True correlation
Histogram/data points
original
observed
imputed
imputed in one metabolite only
Total proportion of missing values
0
0.1
0.3
0.5
0.7
D
Bias cor
Bias pcor
Bias lm phen ~ met
Bias lm met ~ phen
Bias logistic regr.
Bias
True correlation
True partial cor.
True effect size
E
Power cor
Power pcor
Power lm phen ~ met
Power lm met ~ phen
Power logistic regr.
Power

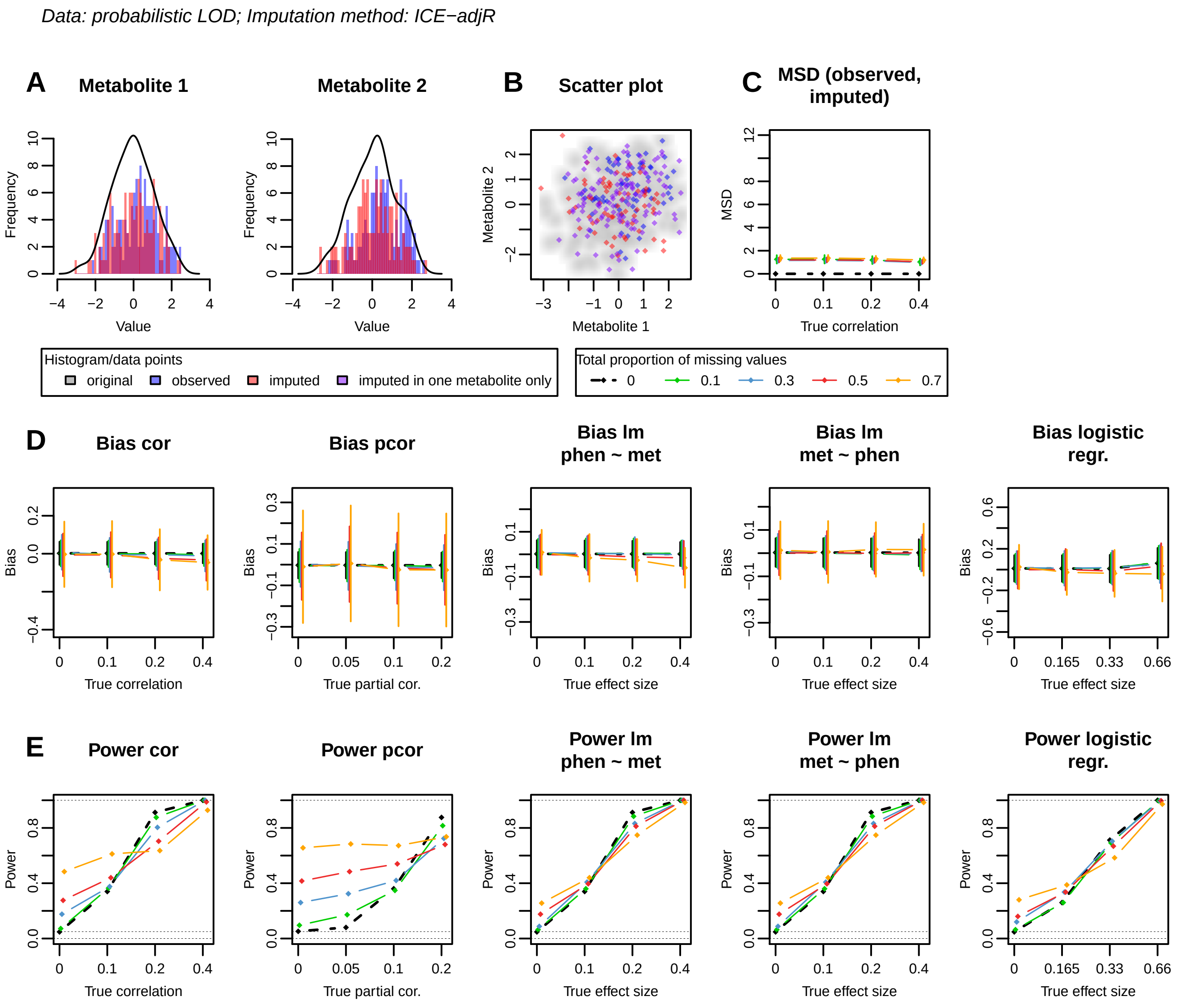
Data: probabilistic LOD; Imputation method: ICE−adjR
A
Metabolite 1
Metabolite 2
Frequency
Value
B
Scatter plot
Metabolite 2
Metabolite 1
C
MSD (observed, imputed)
MSD
True correlation
Histogram/data points
original
observed
imputed
imputed in one metabolite only
Total proportion of missing values
0
0.1
0.3
0.5
0.7
D
Bias cor
Bias pcor
Bias lm phen ~ met
Bias lm met ~ phen
Bias logistic regr.
Bias
True correlation
True partial cor.
True effect size
E
Power cor
Power pcor
Power lm phen ~ met
Power lm met ~ phen
Power logistic regr.
Power
True correlation
True partial cor.
True effect size

Data: probabilistic LOD; Imputation method: MICE−norm

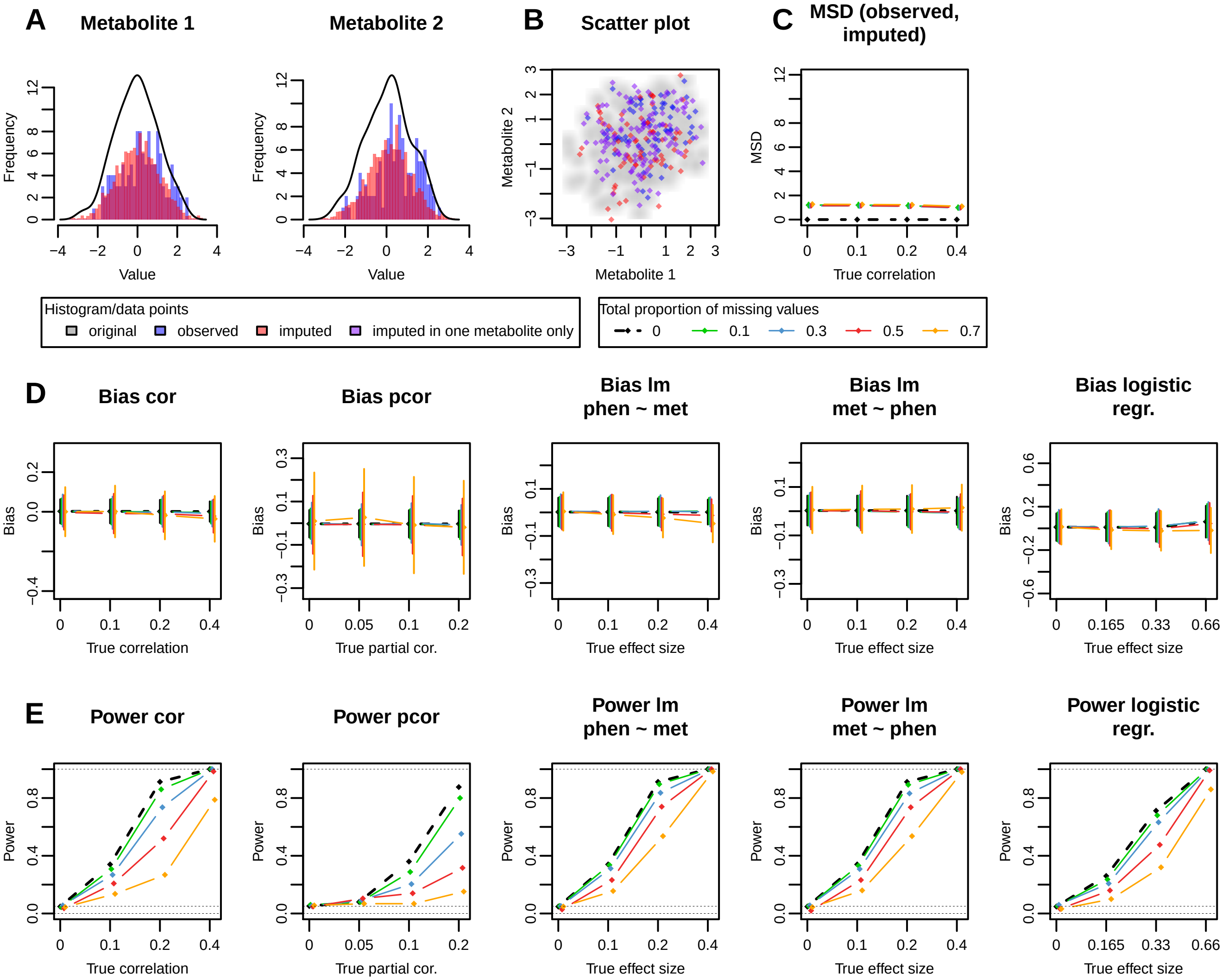

Data: probabilistic LOD; Imputation method: MICE−pmm

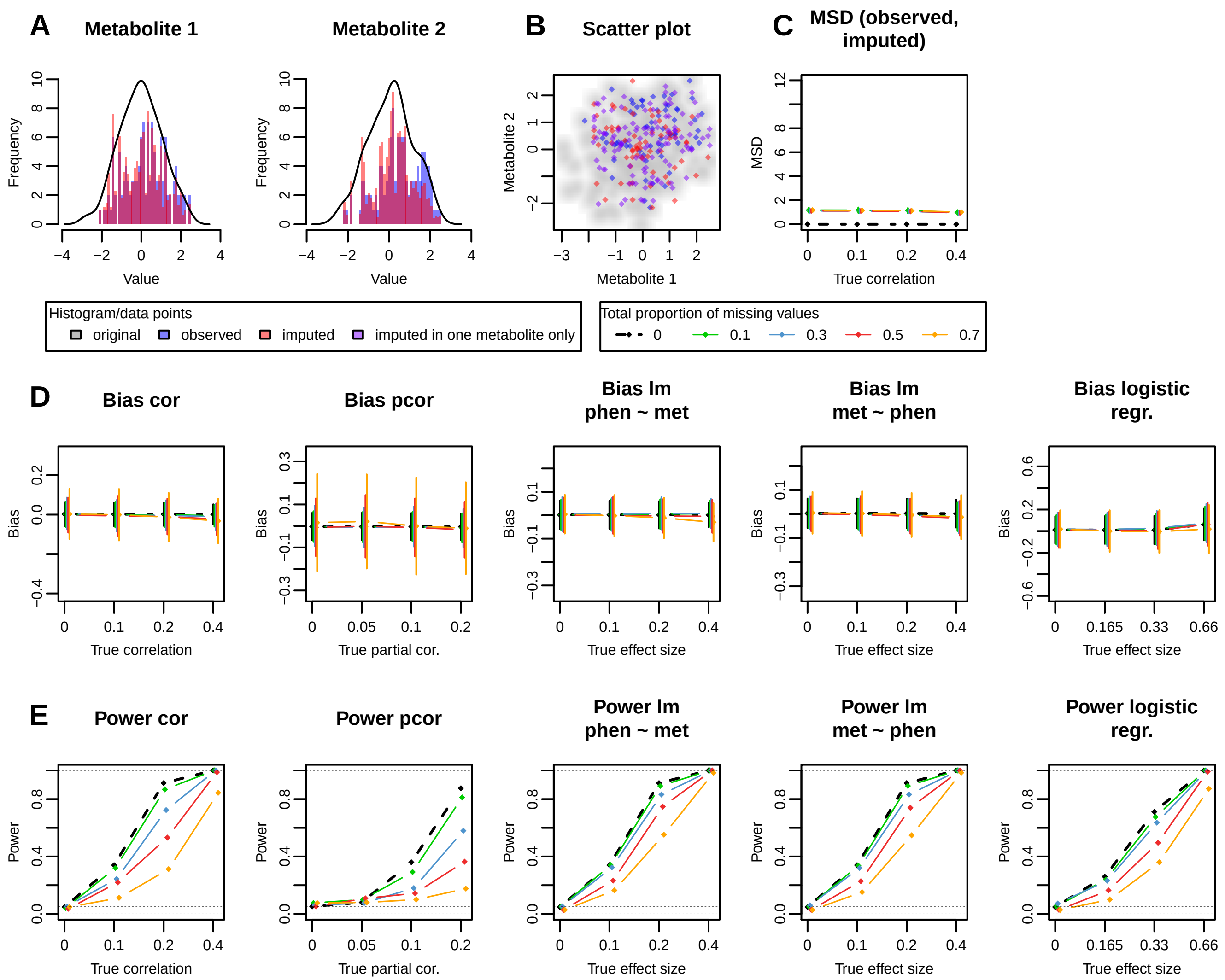

Data: probabilistic LOD; Imputation method: MICE−adjR

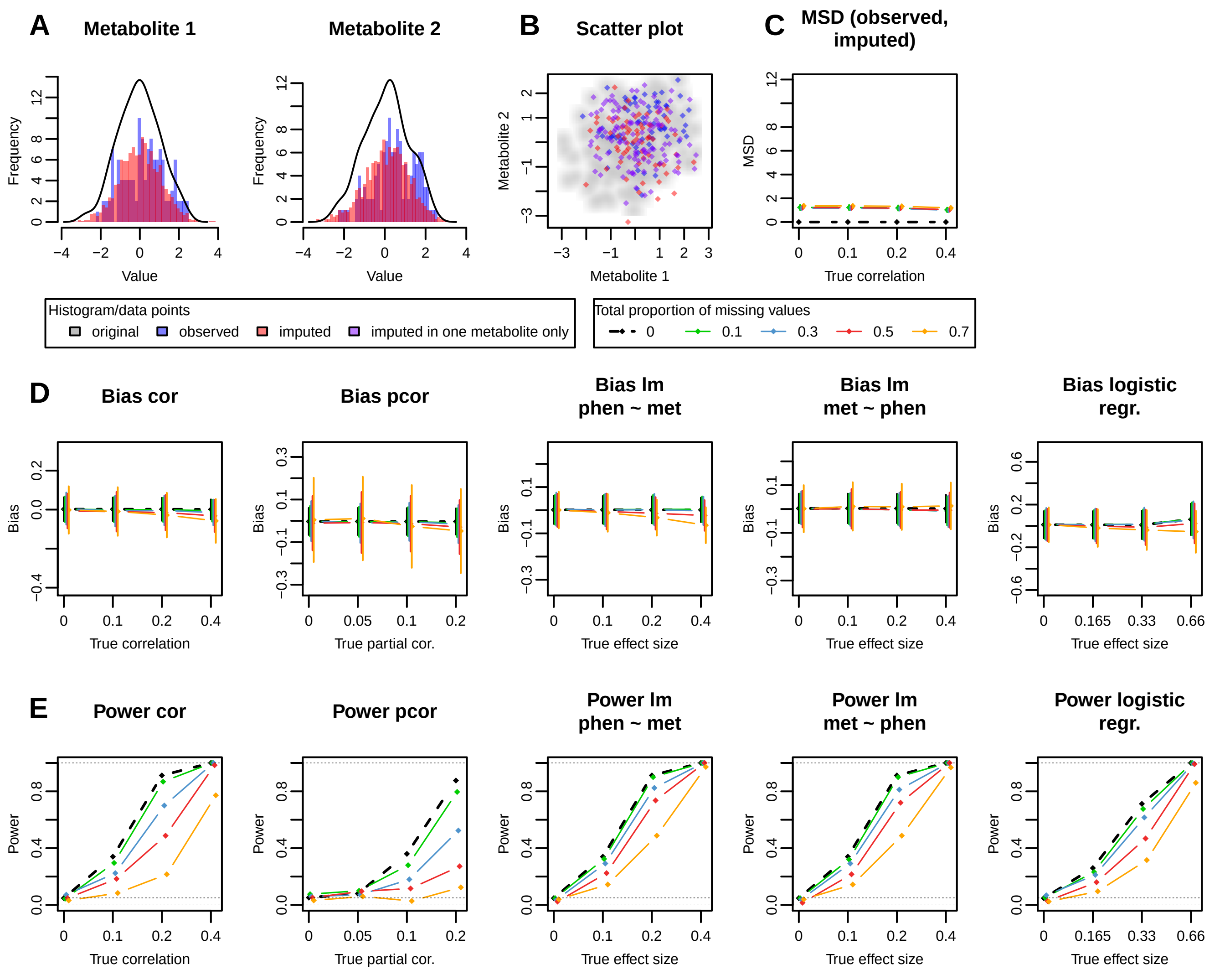

Data: probabilistic LOD; Imputation method: KNN−var(1)

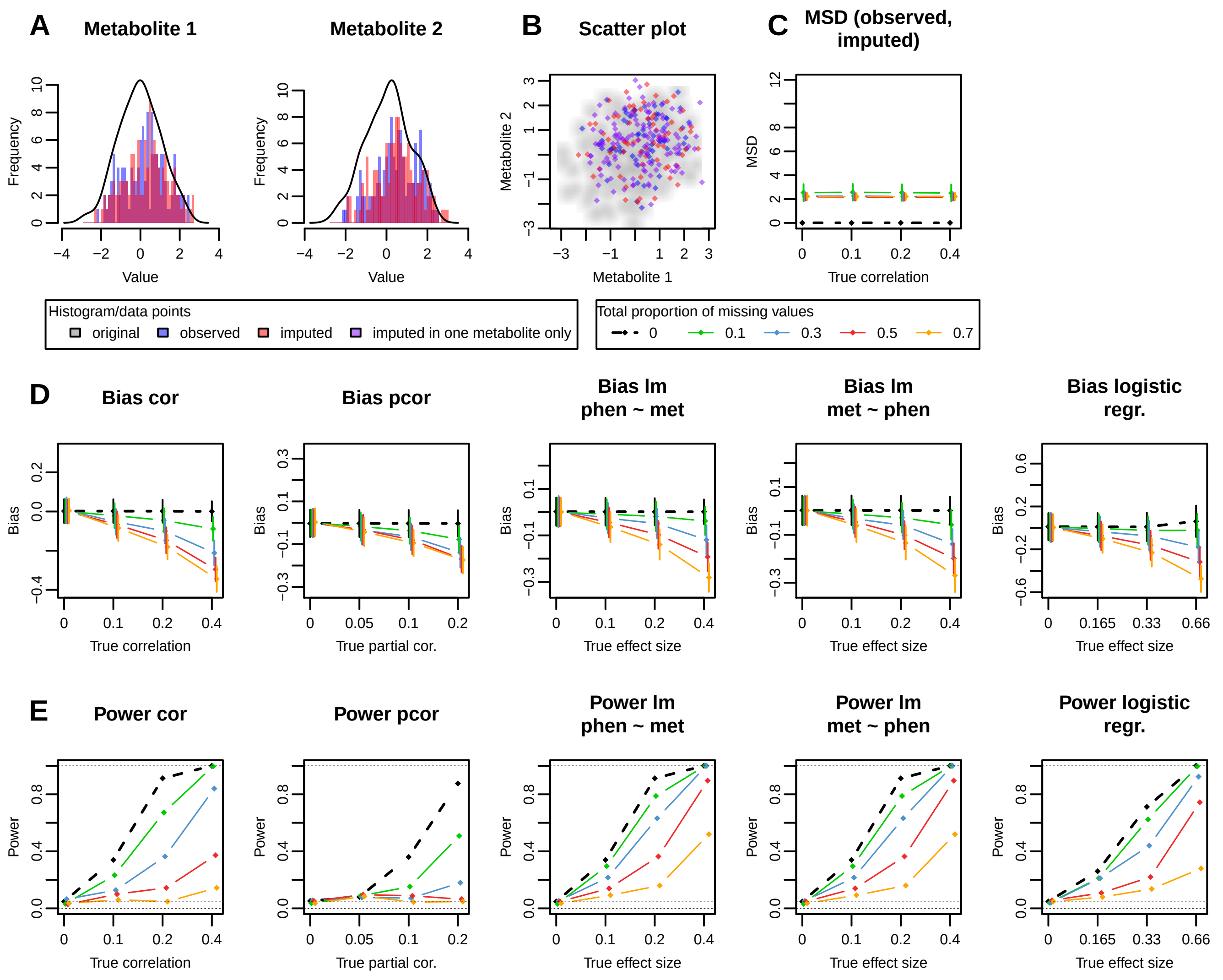

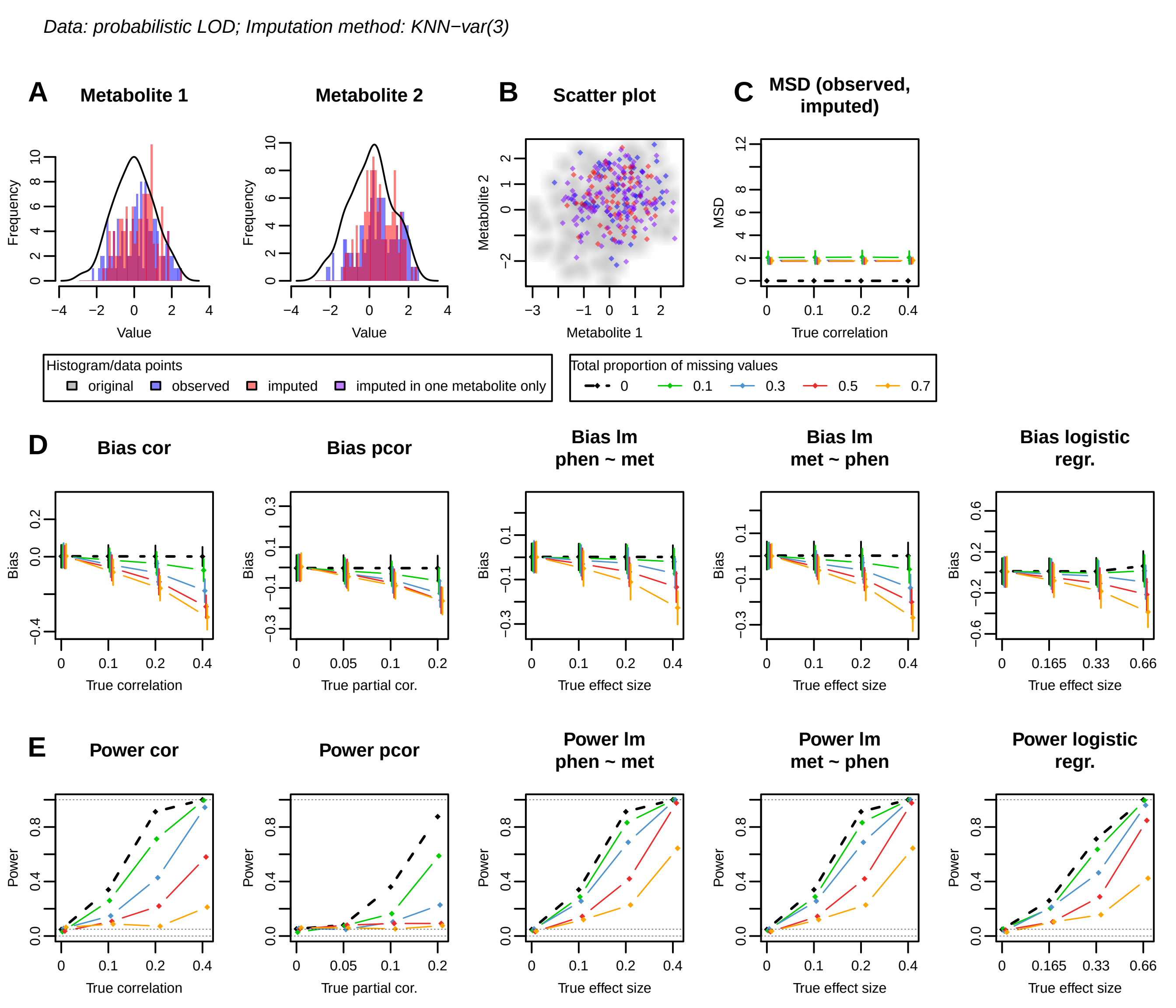
Data: probabilistic LOD; Imputation method: KNN−var(3)
A
Metabolite 1
Metabolite 2
B
Scatter plot
C
MSD (observed, imputed)
Frequency
Value
Metabolite 2
Metabolite 1
MSD
True correlation
Histogram/data points
original
observed
imputed
imputed in one metabolite only
Total proportion of missing values
0
0.1
0.3
0.5
0.7
D
Bias cor
Bias pcor
Bias lm phen ~ met
Bias lm met ~ phen
Bias logistic regr.
Bias
True correlation
True partial cor.
True effect size
E
Power cor
Power pcor
Power lm phen ~ met
Power lm met ~ phen
Power logistic regr.
Power

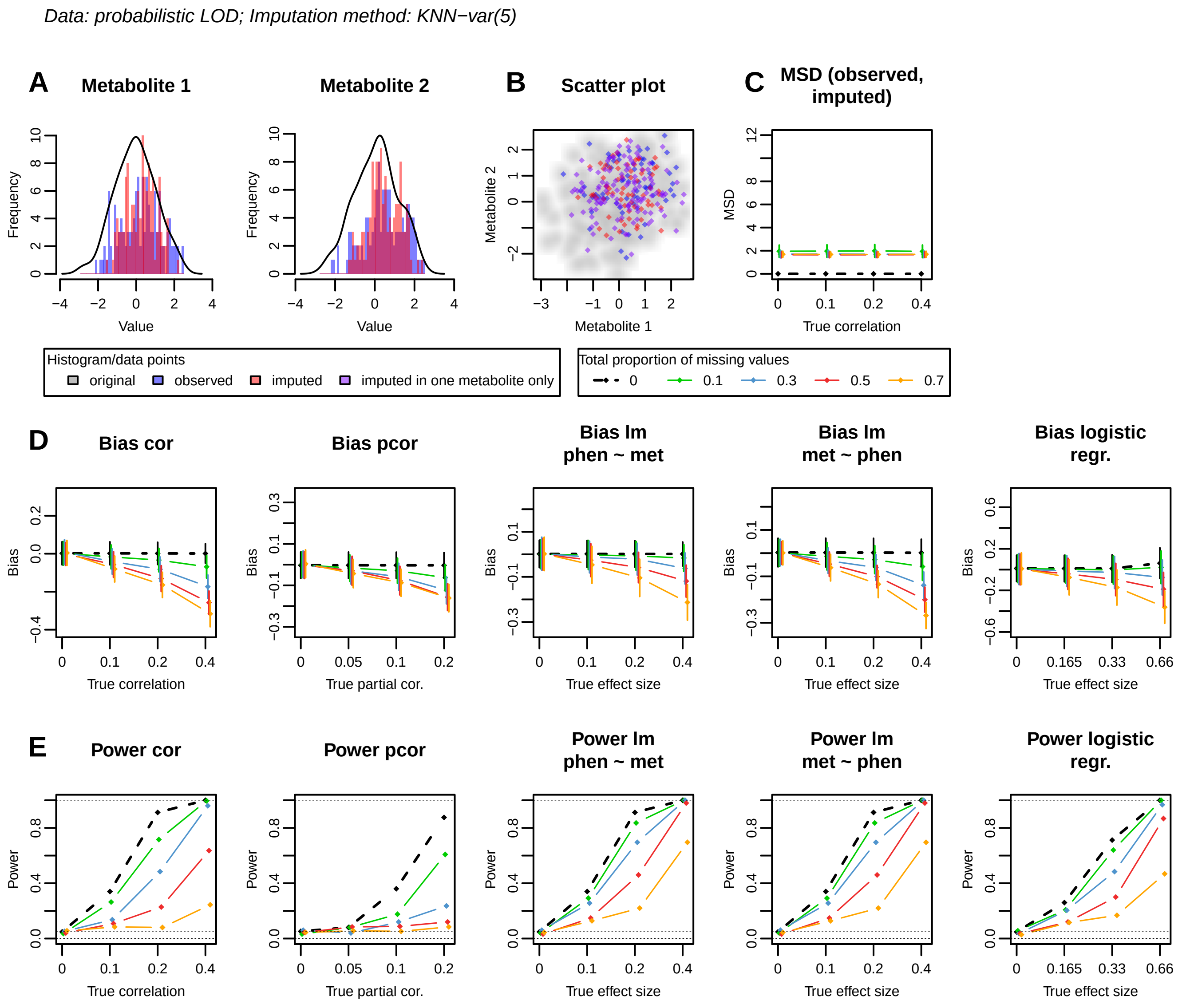

Data: probabilistic LOD; Imputation method: KNN−var(5)
A
Metabolite 1
Metabolite 2
Frequency
Value
B
Scatter plot
Metabolite 1
Metabolite 2
C
MSD (observed, imputed)
MSD
True correlation
Histogram/data points
original
observed
imputed
imputed in one metabolite only
Total proportion of missing values
0
0.1
0.3
0.5
0.7
D
Bias cor
Bias pcor
Bias lm phen ~ met
Bias lm met ~ phen
Bias logistic regr.
Bias
True correlation
True partial cor.
True effect size
E
Power cor
Power pcor
Power lm phen ~ met
Power lm met ~ phen
Power logistic regr.
Power
True correlation
True partial cor.
True effect size

Data: probabilistic LOD; Imputation method: KNN−var(10)

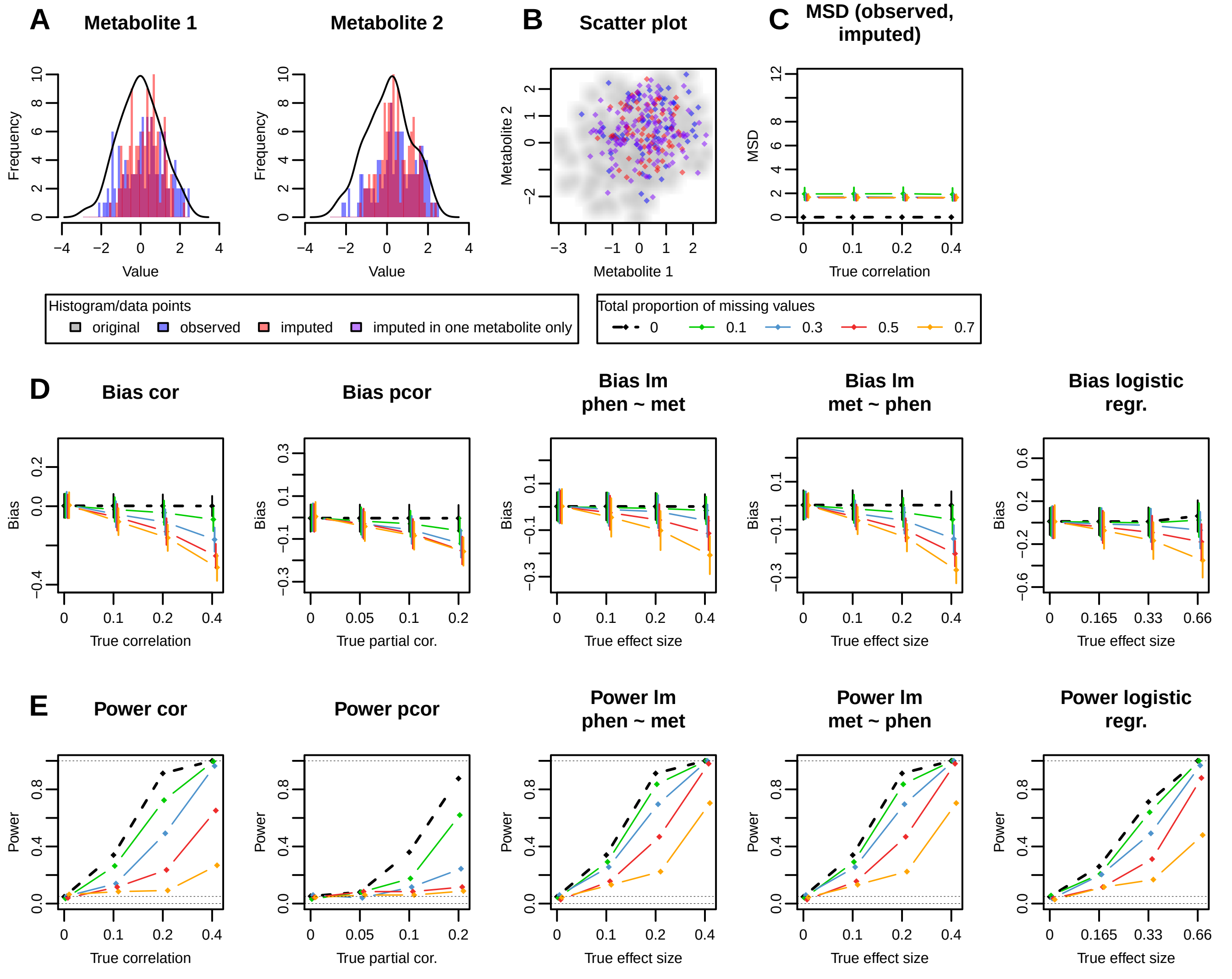

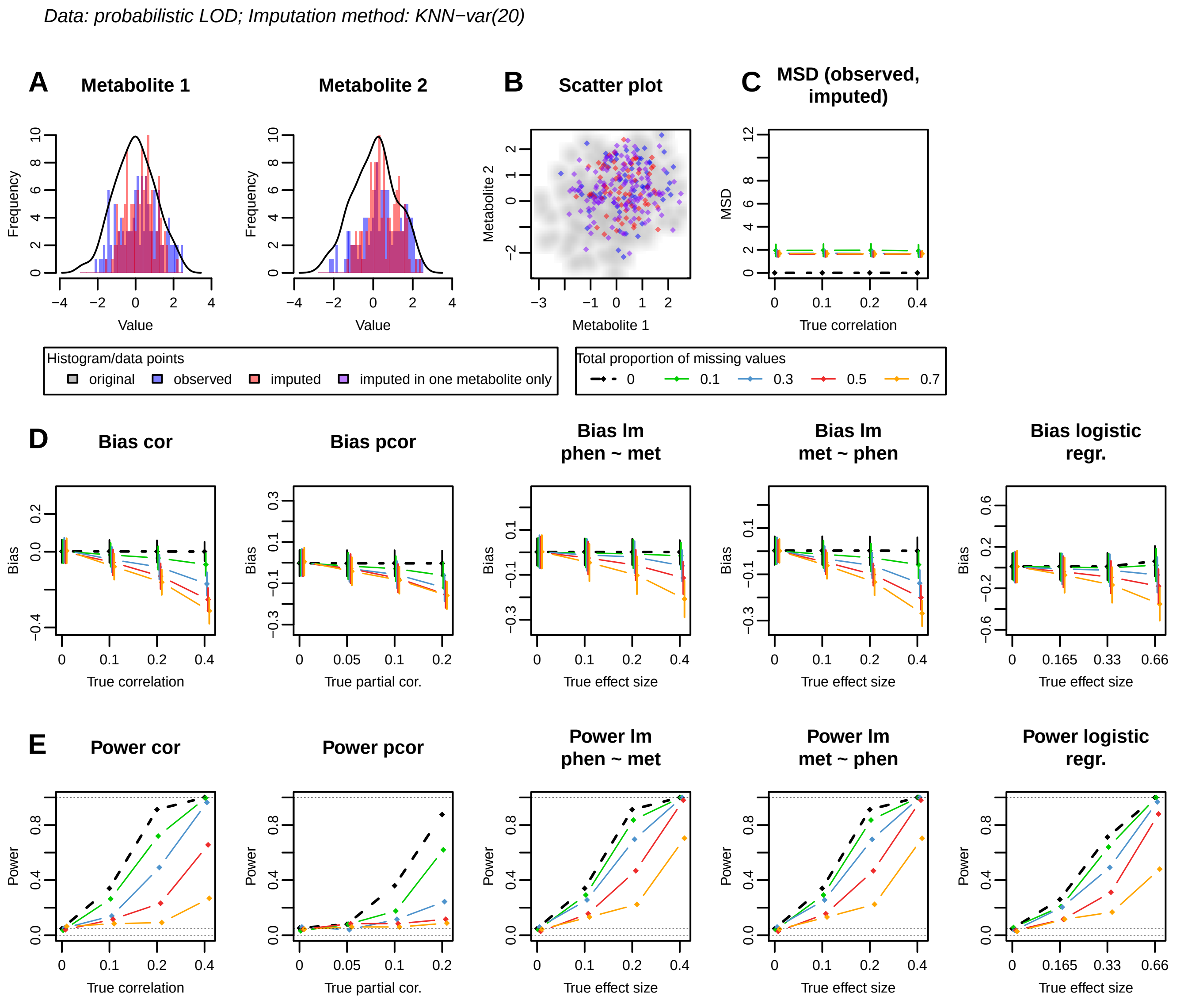

Data: probabilistic LOD; Imputation method: KNN−var(20)
A
Metabolite 1
Metabolite 2
Frequency
Value
B
Scatter plot
Metabolite 1
Metabolite 2
C
MSD (observed, imputed)
MSD
True correlation
Histogram/data points
original
observed
imputed
imputed in one metabolite only
Total proportion of missing values
0
0.1
0.3
0.5
0.7
D
Bias cor
Bias pcor
Bias lm phen ~ met
Bias lm met ~ phen
Bias logistic regr.
Bias
True correlation
True partial cor.
True effect size
E
Power cor
Power pcor
Power lm phen ~ met
Power lm met ~ phen
Power logistic regr.
Power

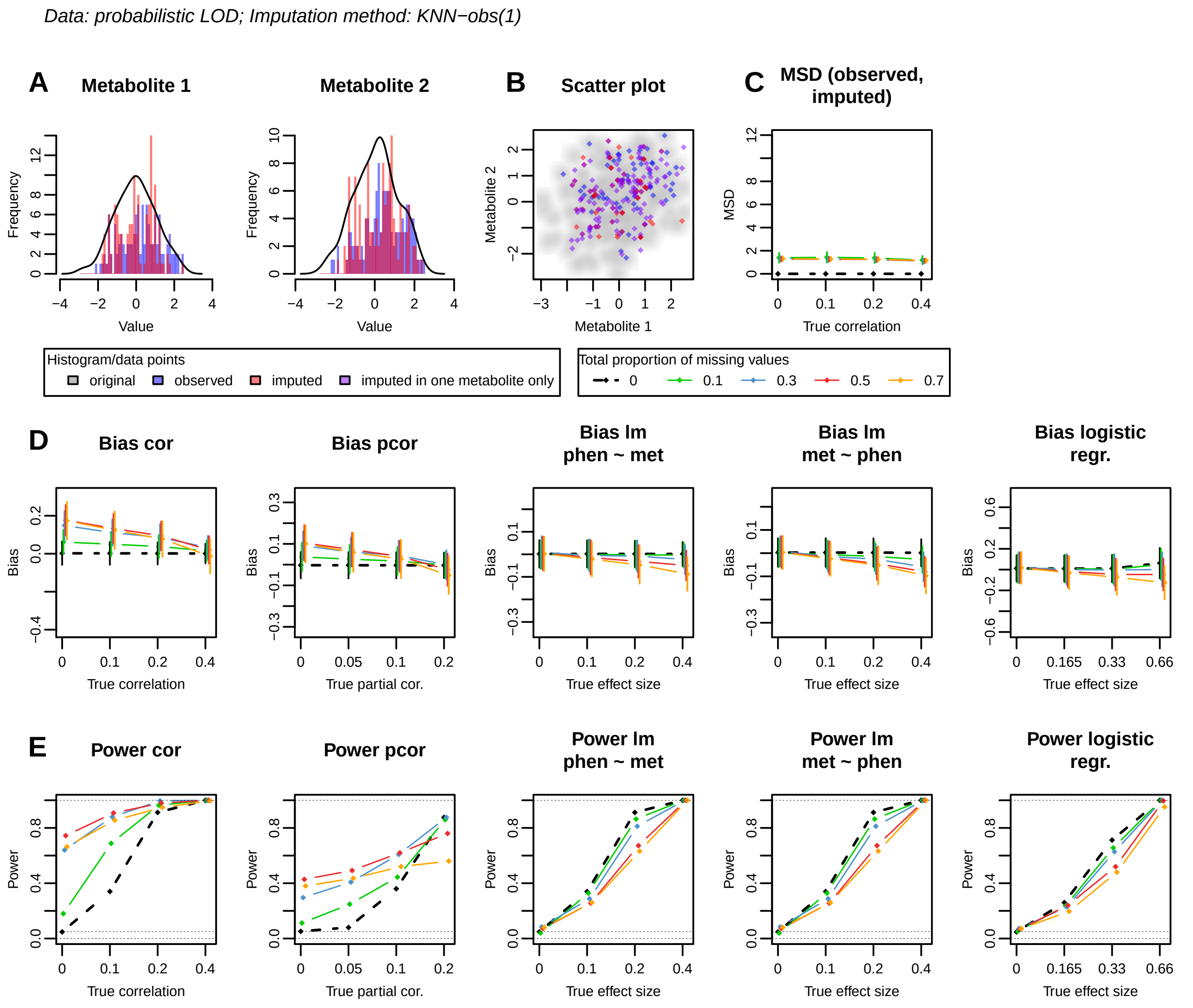

Data: probabilistic LOD; Imputation method: KNN−obs(1)
A
Metabolite 1
Metabolite 2
B
Scatter plot
C
MSD (observed, imputed)
Frequency
Value
Metabolite 2
Metabolite 1
MSD
True correlation
Histogram/data points
original
observed
imputed
imputed in one metabolite only
Total proportion of missing values
0
0.1
0.3
0.5
0.7
D
Bias cor
Bias pcor
Bias lm phen ~ met
Bias lm met ~ phen
Bias logistic regr.
Bias
True correlation
True partial cor.
True effect size
E
Power cor
Power pcor
Power lm phen ~ met
Power lm met ~ phen
Power logistic regr.
Power

Data: probabilistic LOD; Imputation method: KNN−obs(3)

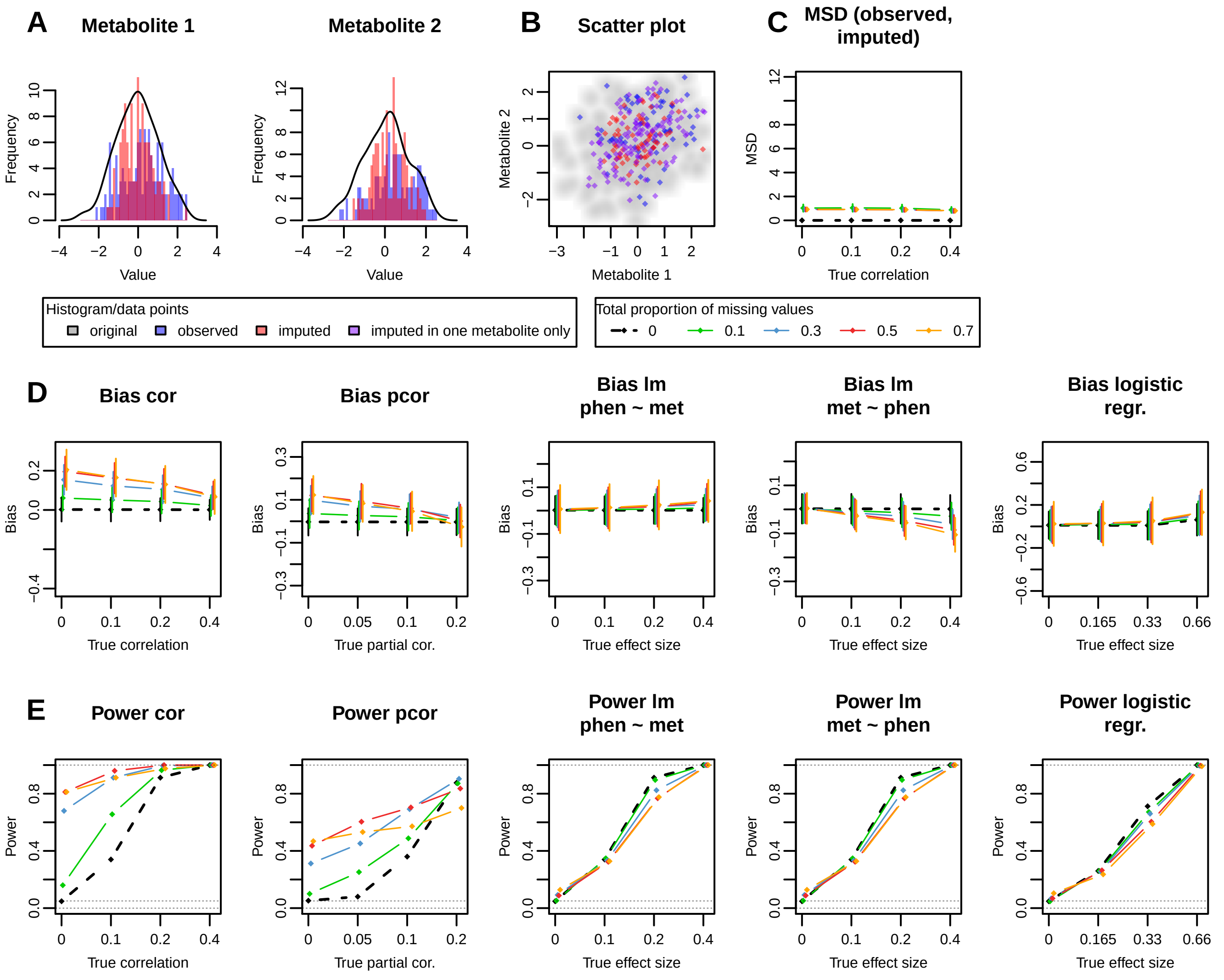

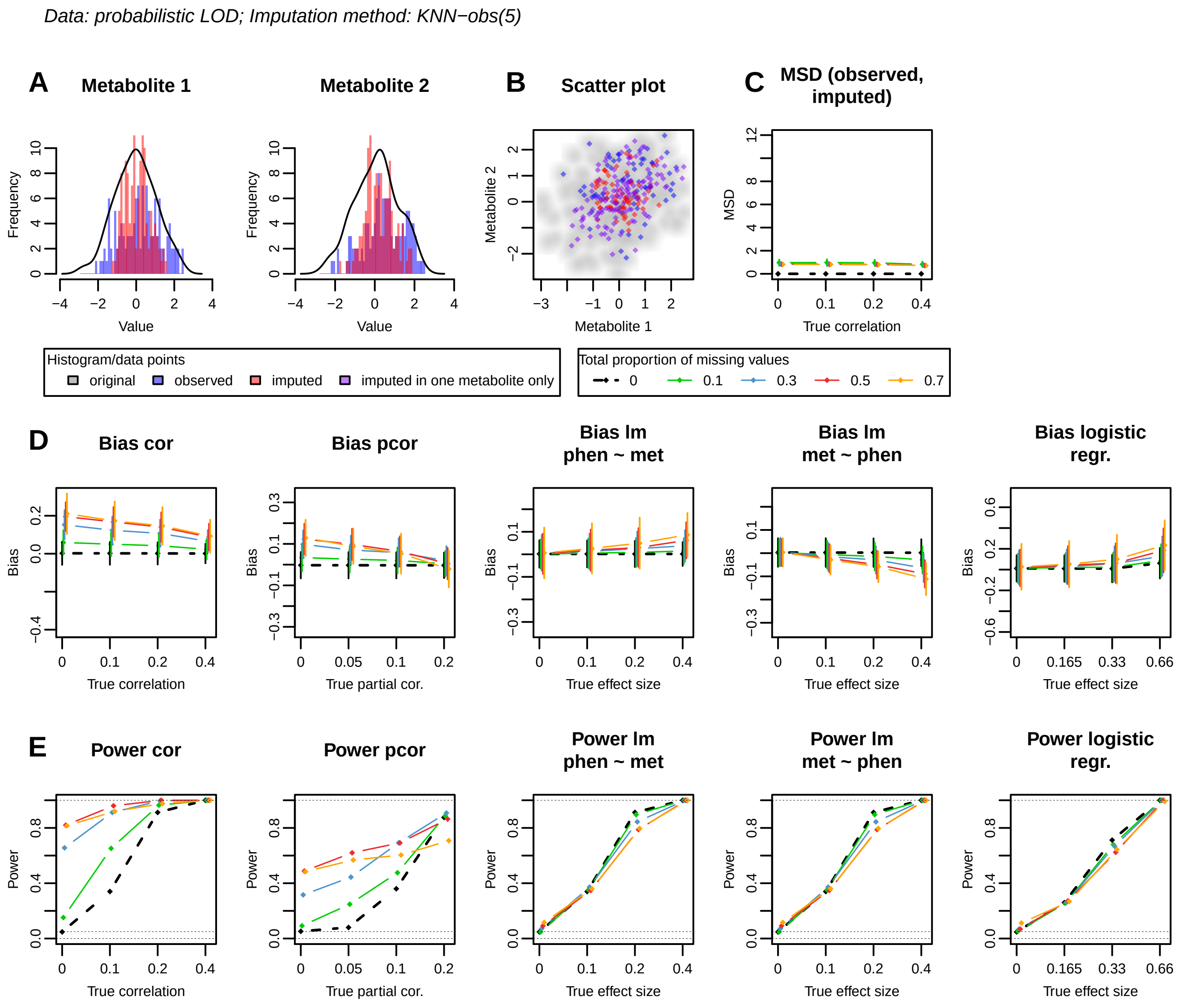
Data: probabilistic LOD; Imputation method: KNN−obs(5)
A
Metabolite 1
Metabolite 2
Frequency
Value
B
Scatter plot
Metabolite 1
Metabolite 2
C
MSD (observed, imputed)
MSD
True correlation
Histogram/data points
original
observed
imputed
imputed in one metabolite only
Total proportion of missing values
0
0.1
0.3
0.5
0.7
D
Bias cor
Bias pcor
Bias lm phen ~ met
Bias lm met ~ phen
Bias logistic regr.
Bias
True correlation
True partial cor.
True effect size
E
Power cor
Power pcor
Power lm phen ~ met
Power lm met ~ phen
Power logistic regr.
Power

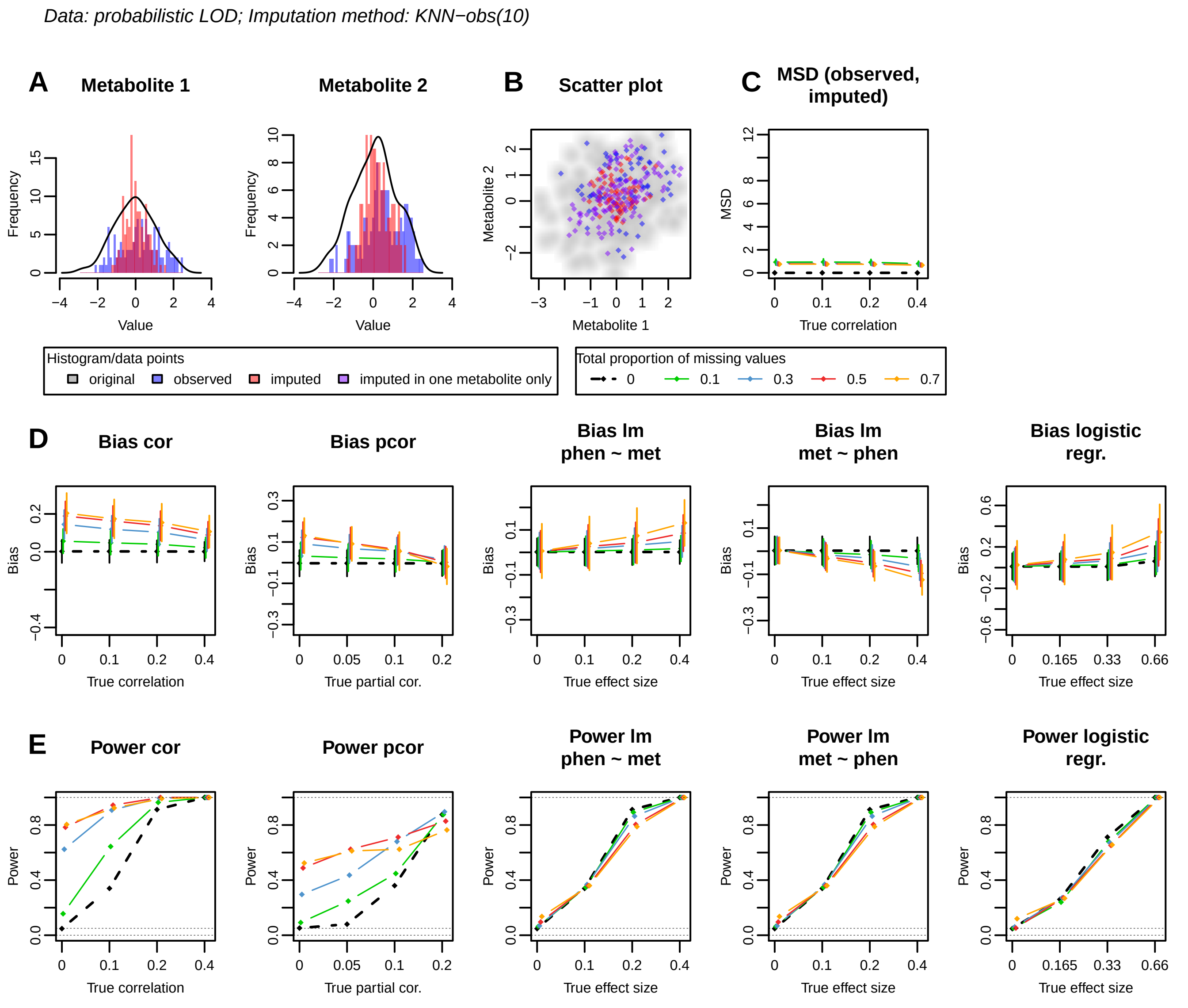

Data: probabilistic LOD; Imputation method: KNN−obs(10)
A
Metabolite 1
Metabolite 2
B
Scatter plot
C
MSD (observed, imputed)
Frequency
Value
Metabolite 2
Metabolite 1
MSD
True correlation
Histogram/data points
original
observed
imputed
imputed in one metabolite only
Total proportion of missing values
0
0.1
0.3
0.5
0.7
D
Bias cor
Bias pcor
Bias lm phen ~ met
Bias lm met ~ phen
Bias logistic regr.
Bias
True correlation
True partial cor.
True effect size
E
Power cor
Power pcor
Power lm phen ~ met
Power lm met ~ phen
Power logistic regr.
Power
True correlation
True partial cor.
True effect size

Data: probabilistic LOD; Imputation method: KNN−obs(20)

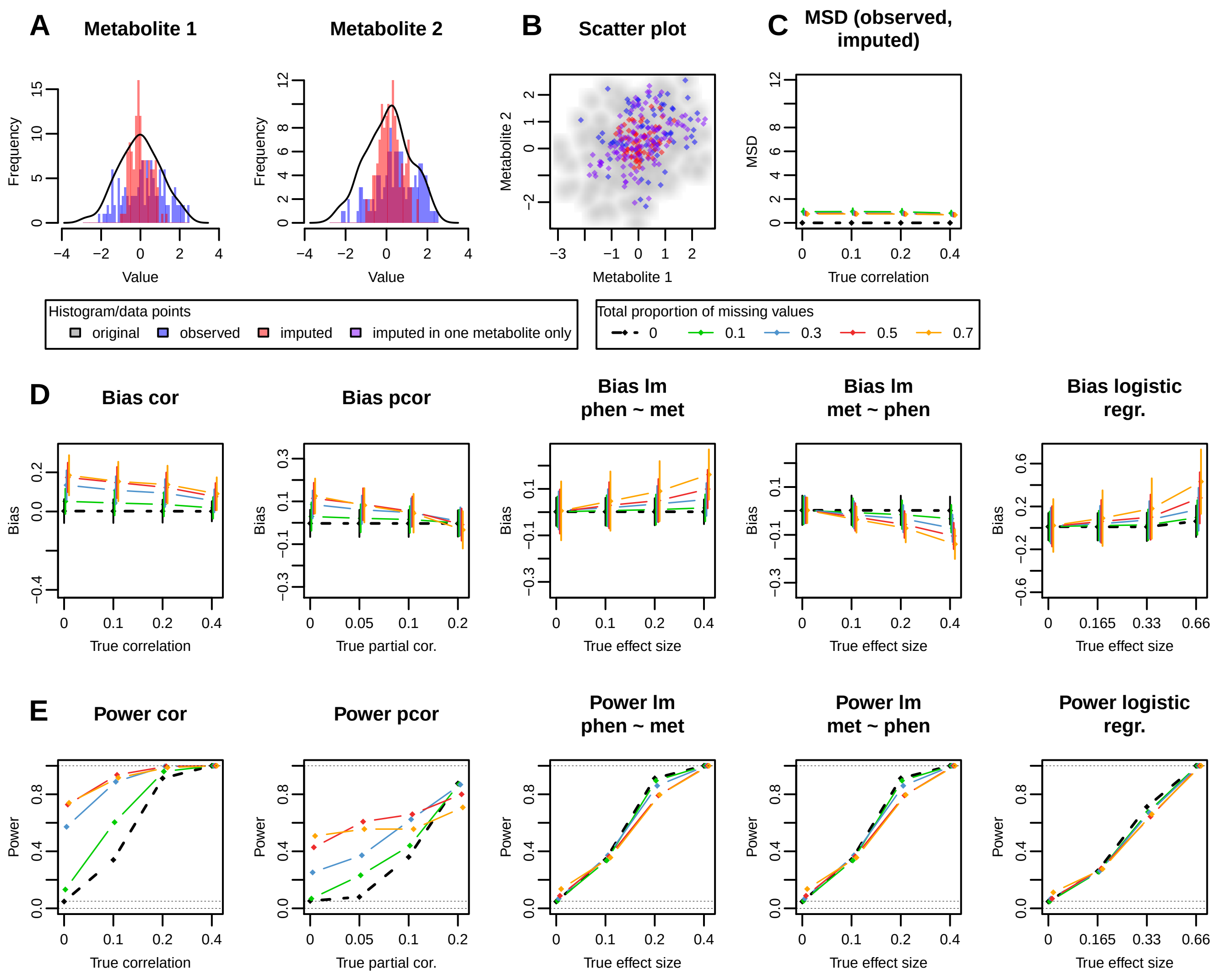

Data: probabilistic LOD; Imputation method: KNN−obs−sel(1)

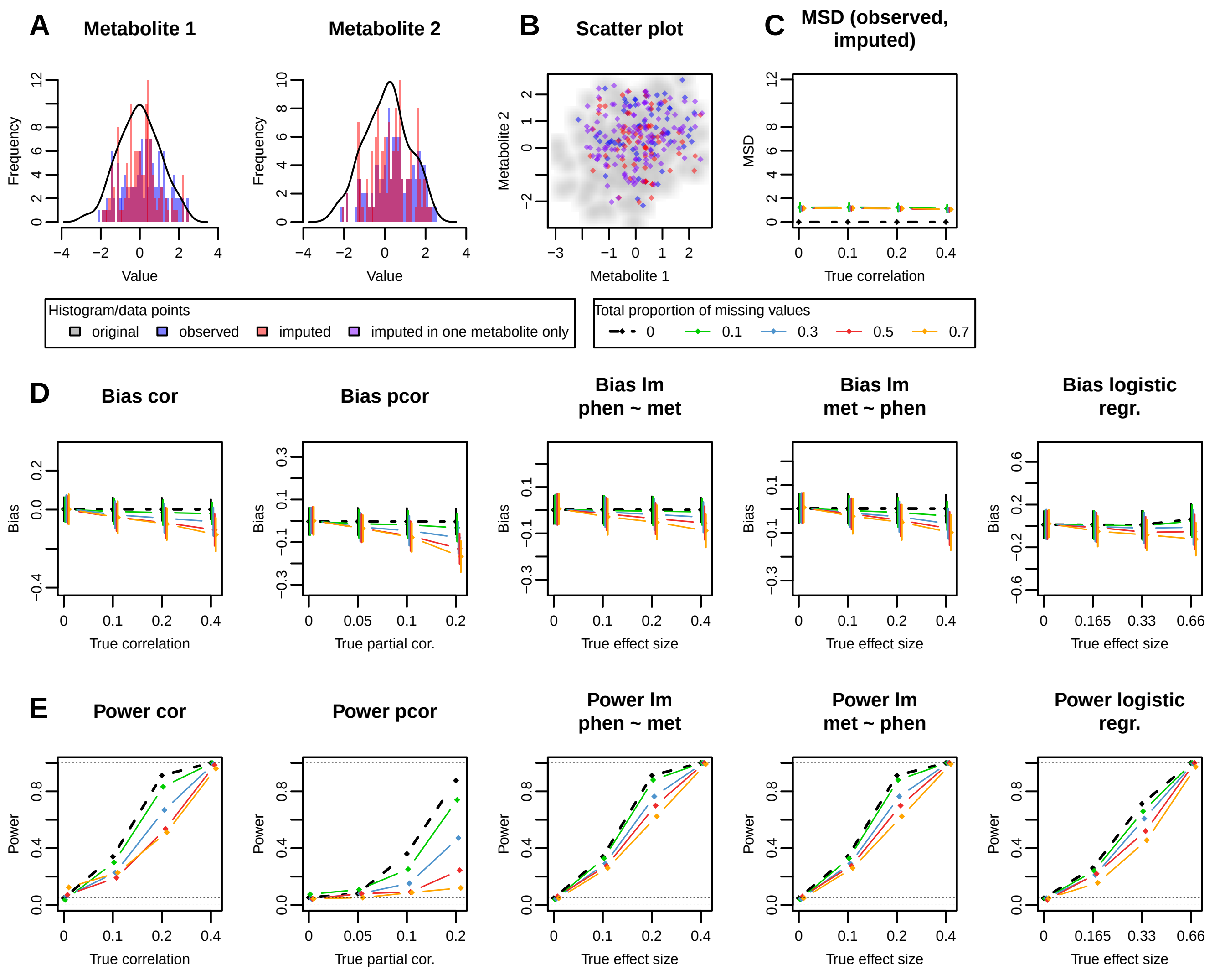

Data: probabilistic LOD; Imputation method: KNN−obs−sel(3)

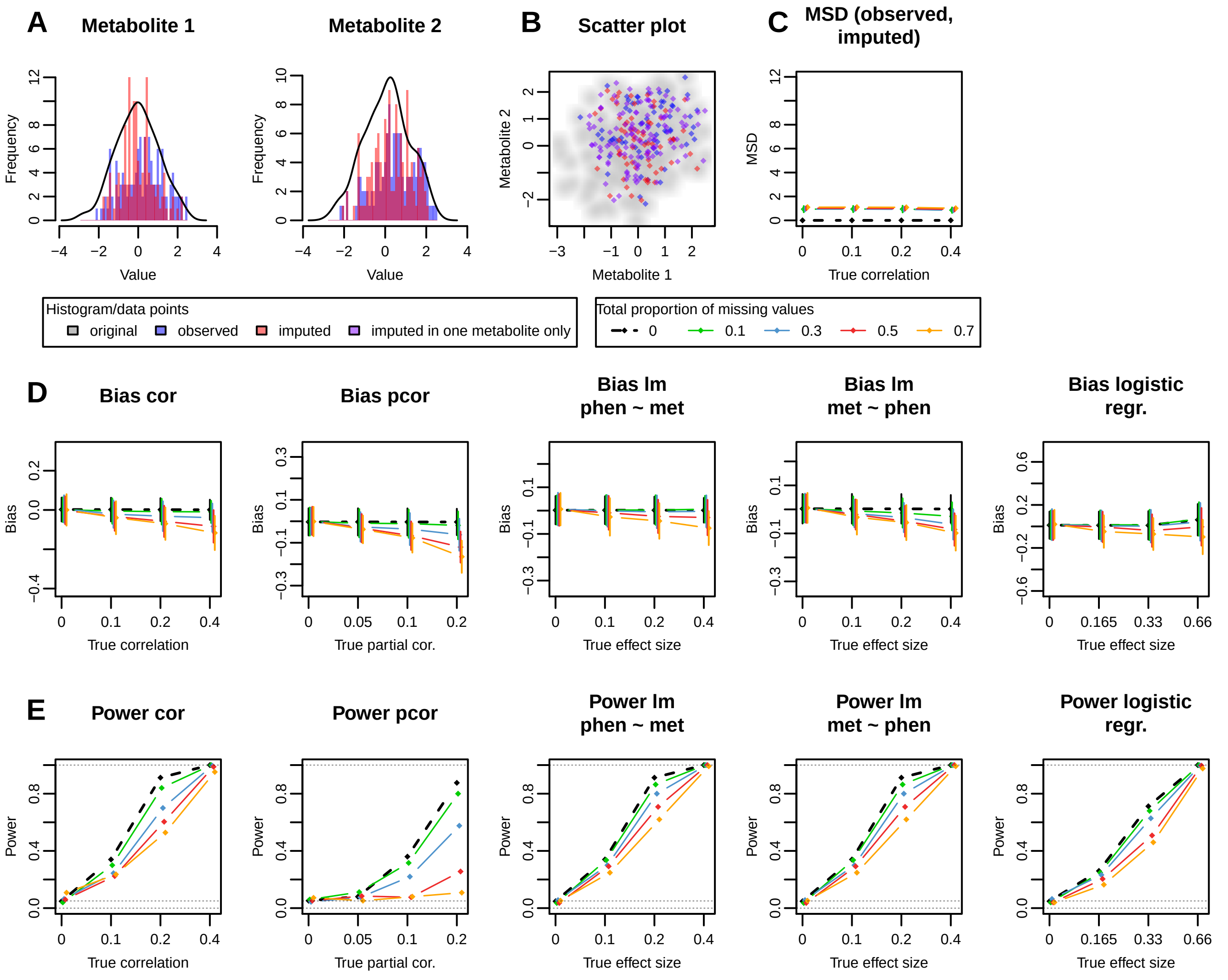

Data: probabilistic LOD; Imputation method: KNN−obs−sel(5)

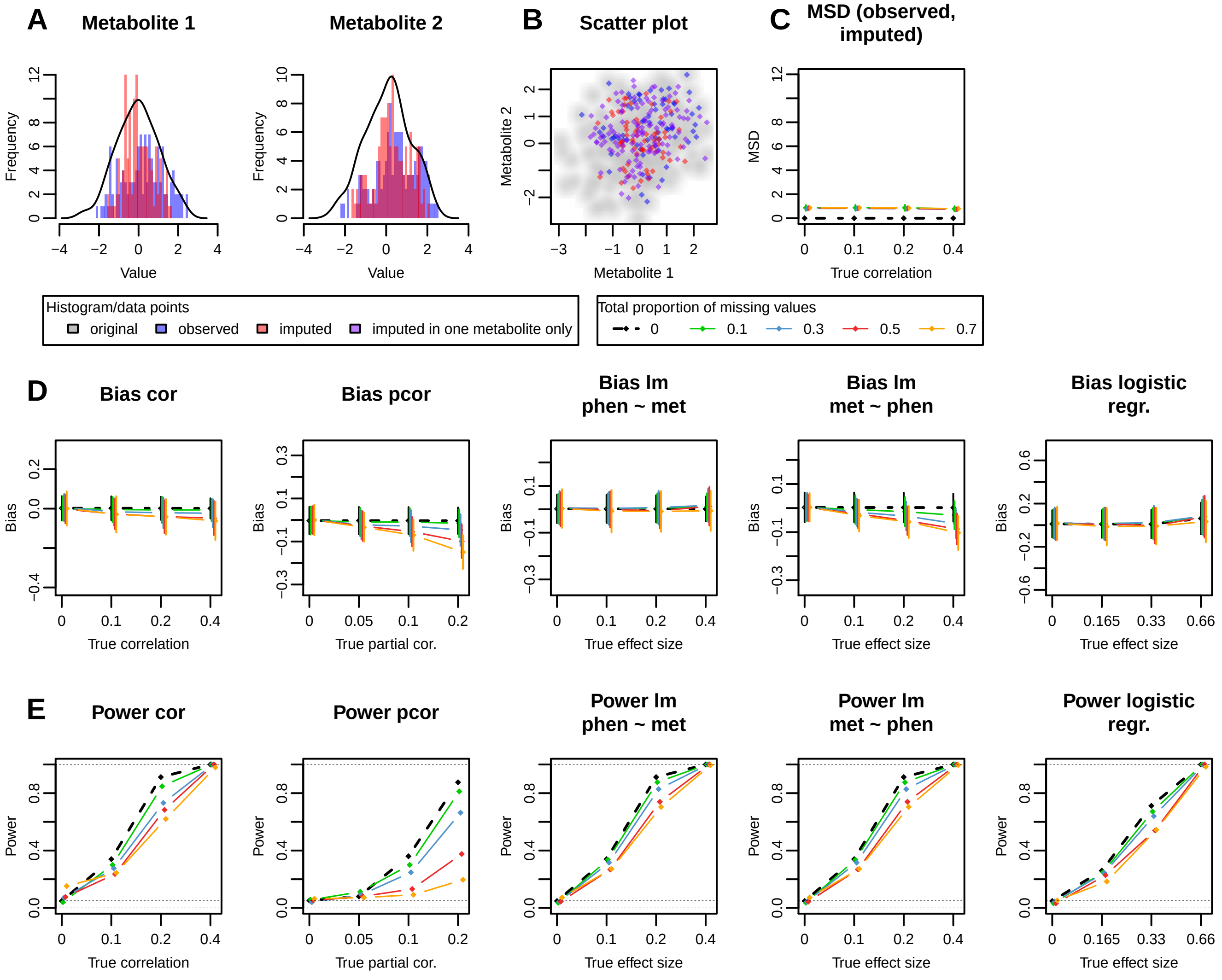

Data: probabilistic LOD; Imputation method: KNN−obs−sel(10)

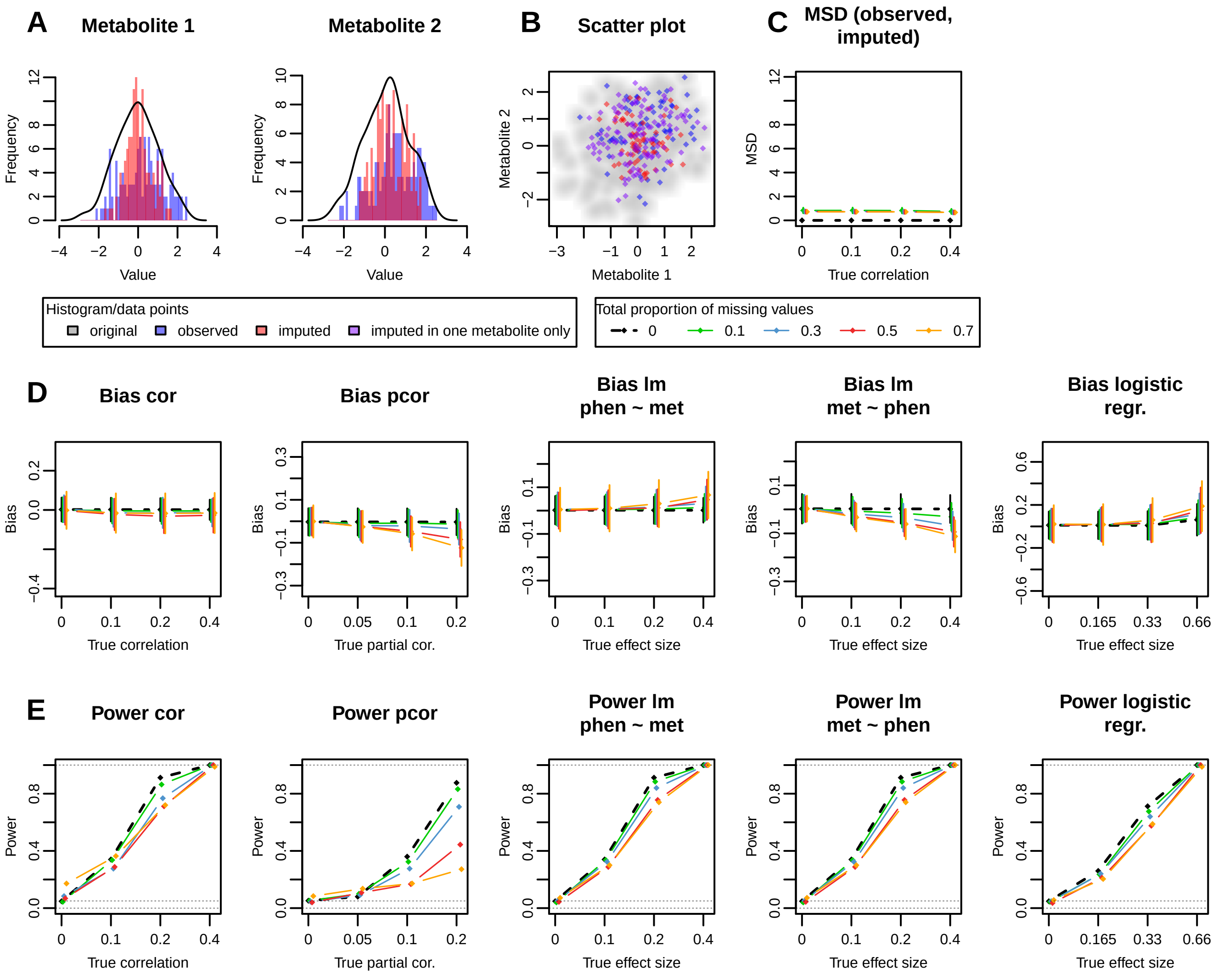

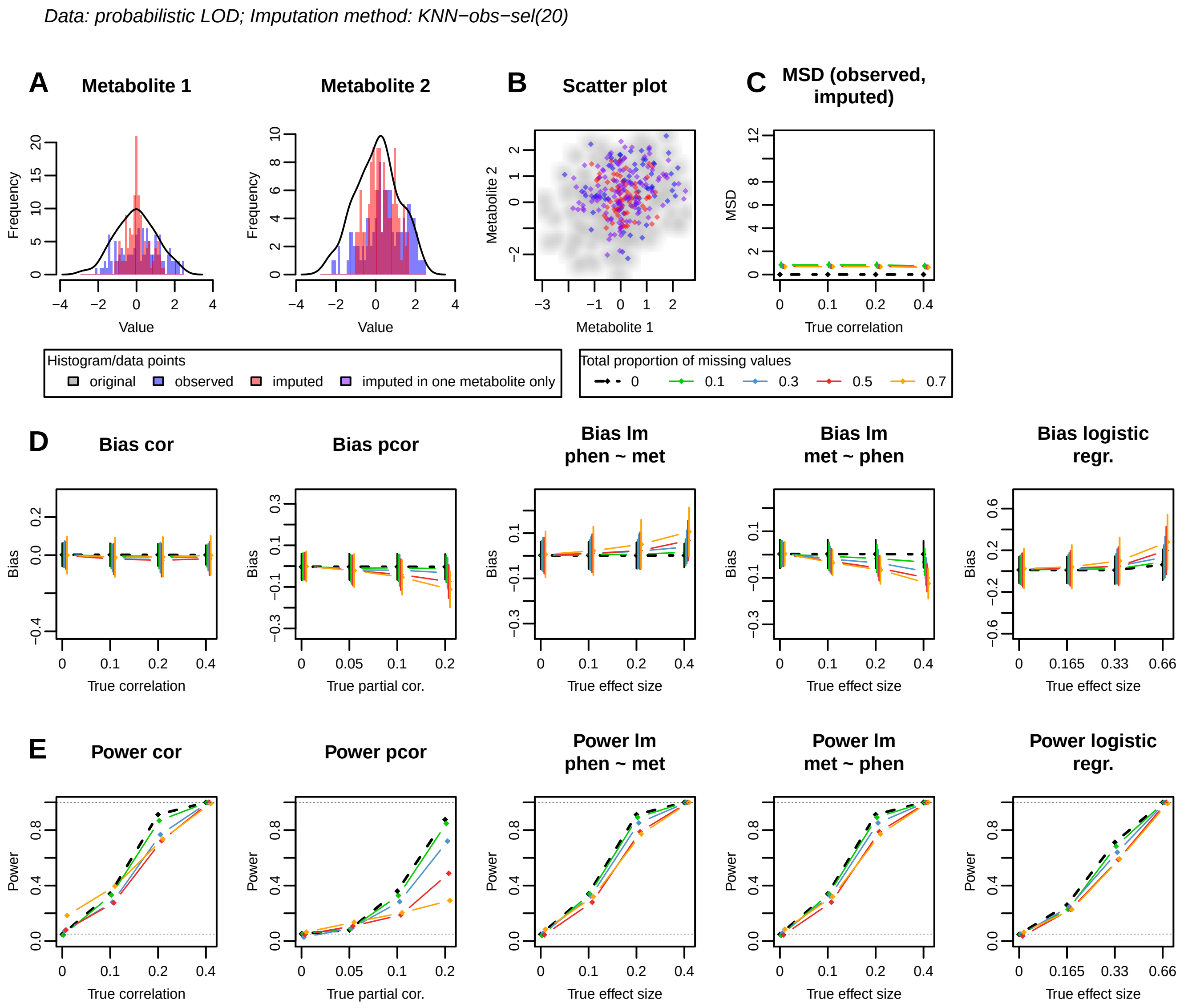
Data: probabilistic LOD; Imputation method: KNN−obs−sel(20)
A
Metabolite 1
Metabolite 2
Frequency
Value
B
Scatter plot
Metabolite 1
Metabolite 2
C
MSD (observed, imputed)
MSD
True correlation
Histogram/data points
original
observed
imputed
imputed in one metabolite only
Total proportion of missing values
0
0.1
0.3
0.5
0.7
D
Bias cor
Bias pcor
Bias lm phen ~ met
Bias lm met ~ phen
Bias logistic regr.
Bias
True correlation
True partial cor.
True effect size
E
Power cor
Power pcor
Power lm phen ~ met
Power lm met ~ phen
Power logistic regr.
Power
True correlation
True partial cor.
True effect size
