# Supplementary material for: Characterization of missing values in untargeted MS-based metabolomics data and evaluation of missing data handling strategies
Source: Metabolomics. 2018 Sep 20;14(10):128. doi: 10.1007/s11306-018-1420-2 (PMC6153696; doi:10.1007/s11306-018-1420-2)

# Characterization of missingness in untargeted MS-based metabolomics data sets and evaluation of missing data handling strategies

*Kieu Trinh Do^¶^, Simone Wahl^¶^, Johannes Raffler, Sophie Molnos, Michael Laimighofer, Jerzy Adamski, Karsten Suhre, Konstantin Strauch, Annette Peters, Christian Gieger, Claudia Langenberg, Isobel D. Stewart, Fabian J. Theis, Harald Grallert, Gabi Kastenmüller****^#^****, Jan Krumsiek****^#^***

## Supporting Information File S7: Metabolite-SNP associations compared to EPIC-Norfolk

### Metabolite-SNP associations compared to EPIC-Norfolk

Additional to the comparison of KORA F4 imputed data with KORA F4 *CCA* with respect to effects of genetic variants on metabolite levels, we performed a second analysis comparing effect sizes estimated from KORA F4 imputed data to effect sizes estimated from metabolomics and genotype data of n=10,634 subjects from the EPIC-Norfolk cohort (1). Here the hypothesis was that effect sizes can be more accurately estimated in the more than six times larger data set.

The EPIC-Norfolk study includes 30,446 men and women who were aged 40-79 years and resident in Norfolk, a county in East Anglia, United Kingdom, when they were recruited at baseline in 1993-1997 (http://www.srl.cam.ac.uk/epic). A total of 25,639 participants attended a clinic examination at which blood samples were taken. Participants were genotyped using the UK Biobank Axiom^TM^ array (n=21,448) and genotypes were imputed using the Haplotype Reference Consortium (HRC, release 1) and the combined UK10K+1000 Genomes Phase 3 reference panels. For SNPs existing in both imputation panels, the HRC imputation was used. Plasma metabolites were measured in 2016-2017 for 11,972 individuals (separated in 2 batches of 5,992 and5,980 samples), using Metabolon's DiscoveryHD4 platform on samples collected at baseline and stored in liquid nitrogen. Metabolite measure transformations and sample exclusions based on these were performed within batch. Measures median normalized for runday were log-transformed. The following samples were excluded: samples with extreme missingness (>70%) of metabolite measures (n=6), outlier samples (n=52) identified based on the Mahalanobis distance (calculated on complete metabolite data) greater than four standard deviations from the mean, samples with missing age, sex or BMI (n=15), samples without genotype data available (n=1,162), and samples that were ancestry outliers (n=103). This resulted in 10,634 samples to contribute to the current analysis.

Linear regression models under the assumption of an additive genetic model were estimated for the18 metabolite-SNP pairs for which matching metabolites and SNPs were available in EPIC-Norfolk:

$$metabolite \sim\beta_{0}+\beta_{1}\times SNP+ \beta_{2}\times age+ \beta_{3}\times sex+\sum_{\left\{ i=1 \right\}}^{4} \beta_{3+i}\times PC_{i}$$

where $\beta_{0}$ is the intercept, $\beta_{j}$ with$j>0$ are the regression coefficients, and $PC_{i}$ is the *i-th* genetic principal component of EPIC-Norfolk.

To avoid spurious correlations, metabolic data points with more than four standard deviations away from the mean were removed prior to computation of linear models. Similar to the comparison analysis with KORA F4 CCA as reference, the ability of imputation methods to preserve EPIC-Norfolk reference effect sizes was assessed by calculating the ratio $r_{\beta}=\log_{2} (|\frac{\beta_{imp}}{\beta_{EPIC}}|)$, where $\beta_{imp}$and $\beta_{EPIC}$ are the estimated regression coefficients for the KORA F4 imputed data and EPIC-Norfolk CCA for the SNP, respectively. $r_{\beta}$ is then visualized as circle color in the overview plot below. Note that circle size still reflects the ratio of p-values ($r_{p}= \frac{-\log_{10} \left( \frac{P_{imp}}{P_{CCA}} \right)}{-\log_{10} (P_{CCA})}$, where $P_{imp}$ and $P_{CCA}$ are the p-values obtained for KORA F4 imputed data and KORA F4 CCA) from the analysis with KORA F4 CCA as reference since comparing p-values of two different cohorts with substantial differences in sample size is not meaningful.


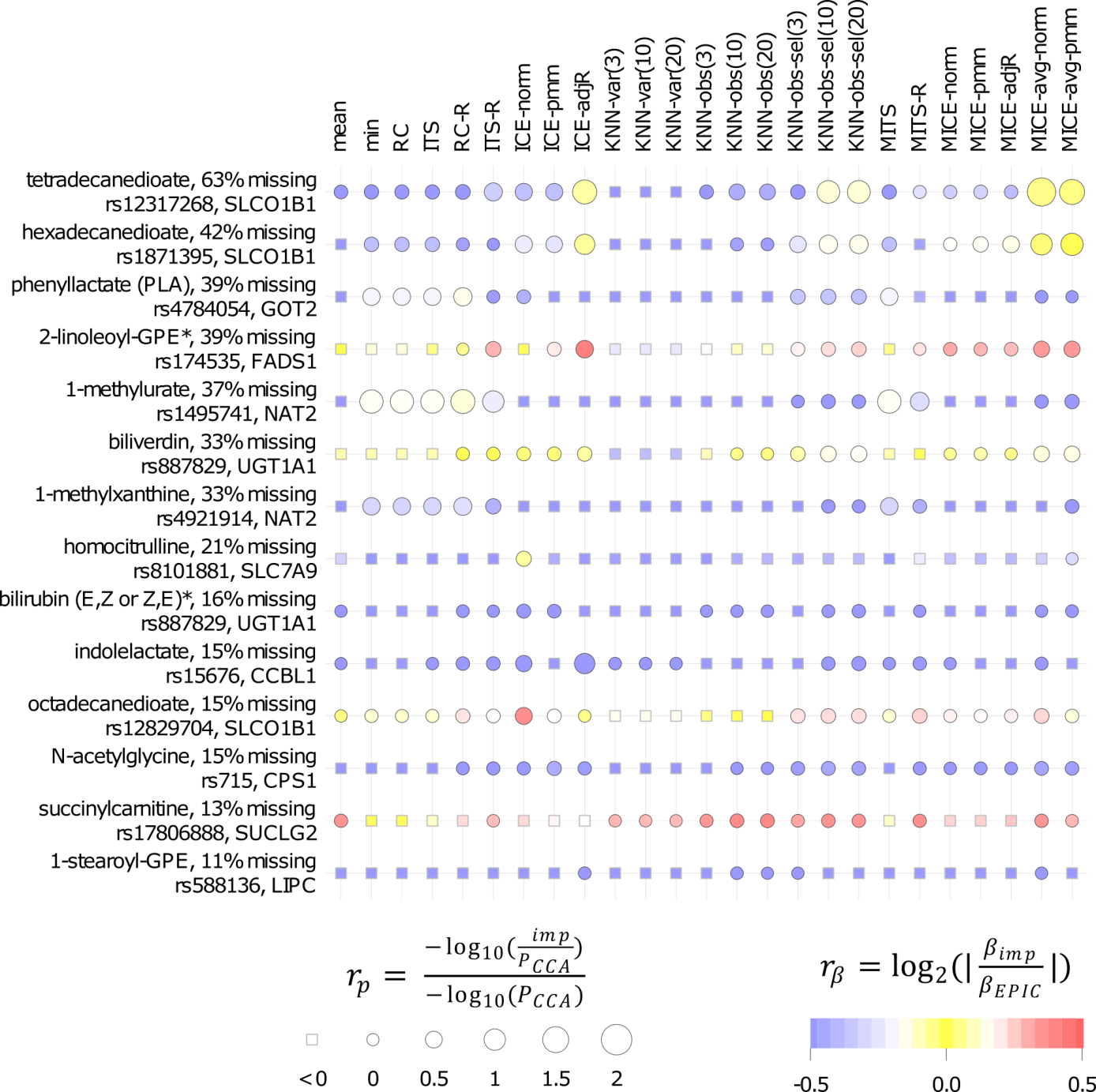

Supplement: Supplementary file 7 — Supplementary material 7 (DOCX 466 KB) [file 11306_2018_1420_MOESM7_ESM.docx]
