# Supplementary material for: Characterization of missing values in untargeted MS-based metabolomics data and evaluation of missing data handling strategies
Source: Metabolomics. 2018 Sep 20;14(10):128. doi: 10.1007/s11306-018-1420-2 (PMC6153696; doi:10.1007/s11306-018-1420-2)
Supplement: Supplementary file 10 — Supplementary material 10 (DOCX 25 KB) [file 11306_2018_1420_MOESM10_ESM.docx]

**Characterization of missingness in untargeted MS-based metabolomics data sets and evaluation of missing data handling strategies**

*Kieu Trinh Do^¶^, Simone Wahl^¶^, Johannes Raffler, Sophie Molnos, Michael Laimighofer, Jerzy Adamski, Karsten Suhre, Konstantin Strauch, Annette Peters, Christian Gieger, Claudia Langenberg, Isobel D. Stewart, Fabian J. Theis, Harald Grallert, Gabi Kastenmüller^#^, Jan Krumsiek^#^*

**Supporting Information File S10: Experimental procedures for KORA F4**

The KORA F4 study comprises serum samples from 1768 participants. After thawing on ice, samples (100 µl) were extracted with methanol (400 µl) containing recovery standards by shaking for 2 min. After centrifugation, the supernatant was split into four aliquots. Solvent was removed on a TurboVap (Zymark), and the extracts were frozen and dried under vacuum. Two aliquots were reconstituted in 0.1% formic acid in water for LC/MS positive ion mode, and in 6.5 mM ammonium bicarbonate (pH 8.0) in water for negative ion mode. For chromatographic separation, a 2.1 mm × 100 mm Waters BEH C18 column with a 1.7 μm particle-size, and a gradient elution from water to 98% methanol, each containing 0.1% formic acid for positive ion mode and 6.5 mM ammonium bicarbonate for negative ion mode was used at a flow rate of 350 μL/min for a run time of 11 minutes. The eluent was connected directly to the electrospray ionization source of the mass spectrometer. Full MS scans were recorded from 99 to 1000 m/z, alternating with data-dependent MS/MS fragmentation scans with dynamic exclusion. LC/MS analyses were performed on an LTQ XL mass spectrometer (Thermo Fisher Scientific Inc., Waltham, MA, USA) coupled to a Waters Acquity UPLC system (Waters Corporation, Milford, MA, USA). The aliquot destined for GC/MS analysis was derivatized at 60°C with N,O-bistrimethylsilyl-trifluoroacetamide under dried nitrogen for 1h in a solvent mixture of acetonitrile/dichloromethane/cyclohexane (5:4:1), containing 5% triethylamine and retention time markers. The gas chromatography analysis was performed using a temperature gradient from 60°C to 340°C, with helium as the carrier gas over a 16 min period. MS scans with electron impact ionization (70 eV) and a 50–750 m/z scan range were used. GC-MS analyses were performed on a Finnigan Trace DSQ single quadrupole mass spectrometer (Thermo Fisher Scientific Inc., Waltham, MA, USA) equipped with a GC column (20 m × 0.18 mm, 1.8 μm film phase consisting of 5% phenyldimethyl silicone). More details on experimental procedures can be found in (1,2).

Metabolite identification was performed at Metabolon, Inc. by comparing chromatographic properties and mass spectra of identified and aligned peaks to entries in a proprietary spectra library of purified standards and recurrent analytes. Levels of metabolite identification for all analytes are provided in Table S9. Following the guidelines of the Metabolomics Standards Initiative (MSI), metabolite identification (3) level 1 was assigned if retention index, mass spectrum, and, in case of LC/MS, the fragmentation spectrum of at least one parent mass matched the spectrum of the respective authentic compound measured under identical conditions on the same platform. For metabolite identification at levels 2 and 3, also retention time, m/z, and fragmentation patterns were used; for these analytes, identification was either based on comparison with publicly available spectra or on spectral similarities to related compounds in the proprietary library.

1. Suhre K, Shin S-Y, Petersen A-K, Mohney RP, Meredith D, Wägele B, et al. Human metabolic individuality in biomedical and pharmaceutical research. Nature [Internet]. 2011 Aug 31 [cited 2016 Jan 4];477(7362). Available from: http://www.ncbi.nlm.nih.gov/pmc/articles/PMC3832838/

2. Sekula P, Goek O-N, Quaye L, Barrios C, Levey AS, Römisch-Margl W, et al. A Metabolome-Wide Association Study of Kidney Function and Disease in the General Population. J Am Soc Nephrol. 2016 Apr 1;27(4):1175–88.

3. Sumner LW, Amberg A, Barrett D, Beale MH, Beger R, Daykin CA, et al. Proposed minimum reporting standards for chemical analysis Chemical Analysis Working Group (CAWG) Metabolomics Standards Initiative (MSI). Metabolomics Off J Metabolomic Soc. 2007 Sep;3(3):211–21.
