# Supplementary material for: Characterization of missing values in untargeted MS-based metabolomics data and evaluation of missing data handling strategies
Source: Metabolomics. 2018 Sep 20;14(10):128. doi: 10.1007/s11306-018-1420-2 (PMC6153696; doi:10.1007/s11306-018-1420-2)
Supplement: Supplementary file 6 — Supplementary material 6 (PDF 74632 KB) [file 11306_2018_1420_MOESM6_ESM.pdf]

Characterization of missingness in untargeted MS-based metabolomics data sets and evaluation of missing data handling strategies

Kieu Trinh Do¶, Simone Wahl¶, Johannes Raffler, Sophie Molnos, Michael Laimighofer, Jerzy Adamski, Karsten Suhre, Konstantin Strauch, Annette Peters, Christian Gieger, Claudia Langenberg, Isobel D. Stewart, Fabian J. Theis, Harald Grallert, Gabi Kastenmüller#, Jan Krumsiek#

Supporting Information File S6: Metabolite-SNP associations beeswarm plots

The residuals of the linear regression model

$$metabolite \sim \beta_0 + \beta_1 \times age + \beta_2 \times sex + \epsilon$$

are plotted as circles and represent the (normalized and scaled) metabolite value for each study participant, corrected for the effects of age and sex. The study participants are grouped by the genotype of the tested SNP. In each group, the residuals are shown for the un-imputed KORA F4 data set (*CCA*; left) and the imputed data set (right). Red circles indicate values that are present in the imputed data set only. Boxplots display the distribution of the residuals in each group (left: *CCA*, right: imputed). The lines represent the regression coefficient estimates of the linear model

$$residuals \sim \beta_0 + \beta_1 \times SNP + \epsilon$$

for the *CCA* data set (black) and the imputed data set (red). The values for β and P shown in the top left corner are the standardized estimate and the corresponding p-value for SNP of the linear regression model

$$metabolite \sim \beta_0 + \beta_1 \times SNP + \beta_2 \times age + \beta_3 \times sex + \epsilon$$

when using the *CCA* data set and the imputed data set, respectively (S7 File).

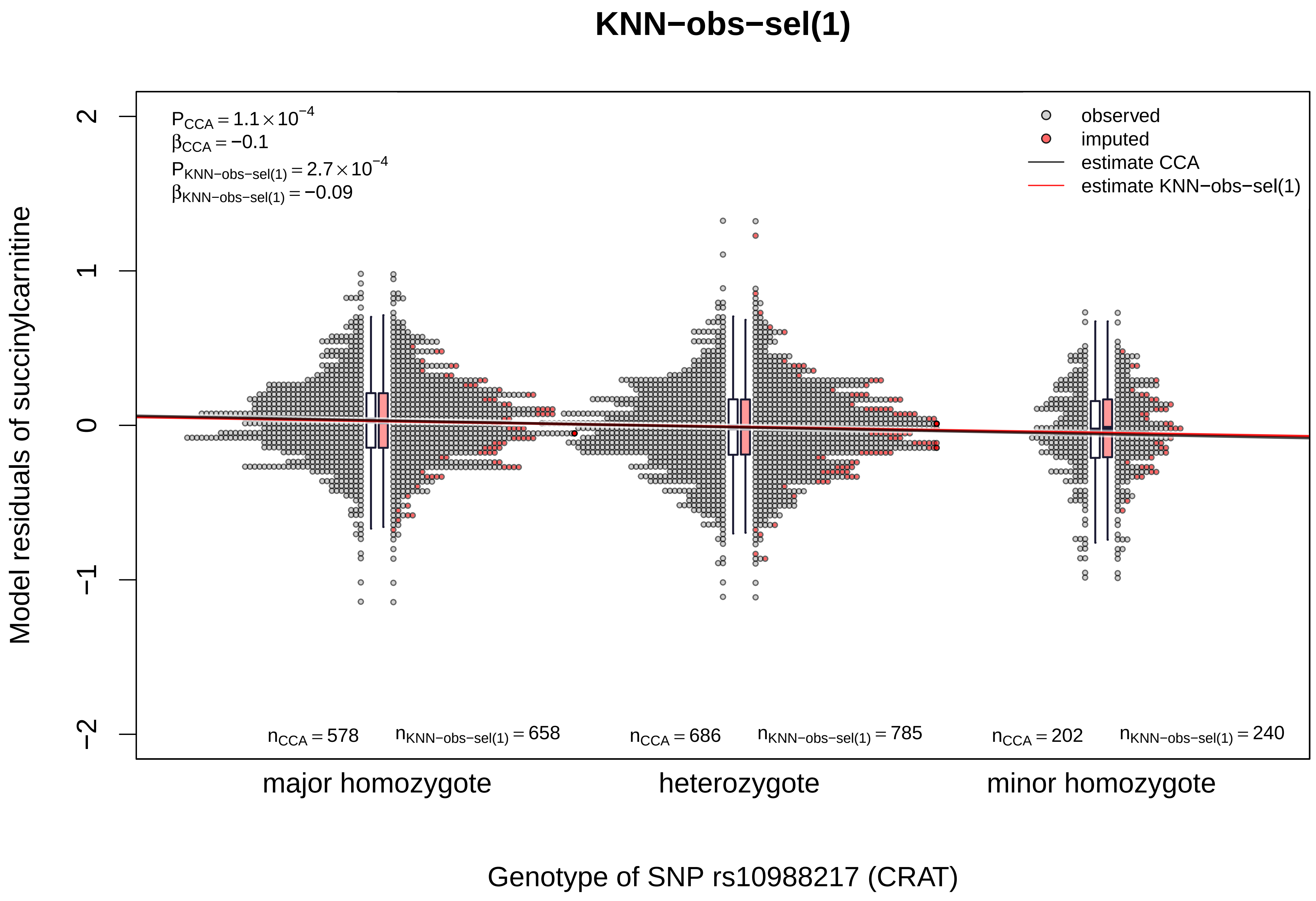

KNN−obs−sel(1)
$P_{CCA} = 1.1 \times 10^{-4}$
$\beta_{CCA} = -0.1$
$P_{KNN-obs-sel(1)} = 2.7 \times 10^{-4}$
$\beta_{KNN-obs-sel(1)} = -0.09$
observed
imputed
estimate CCA
estimate KNN−obs−sel(1)
Model residuals of succinylcarnitine
2
1
0
−1
−2
$n_{CCA} = 578$
$n_{KNN-obs-sel(1)} = 658$
$n_{CCA} = 686$
$n_{KNN-obs-sel(1)} = 785$
$n_{CCA} = 202$
$n_{KNN-obs-sel(1)} = 240$
major homozygote
heterozygote
minor homozygote
Genotype of SNP rs10988217 (CRAT)

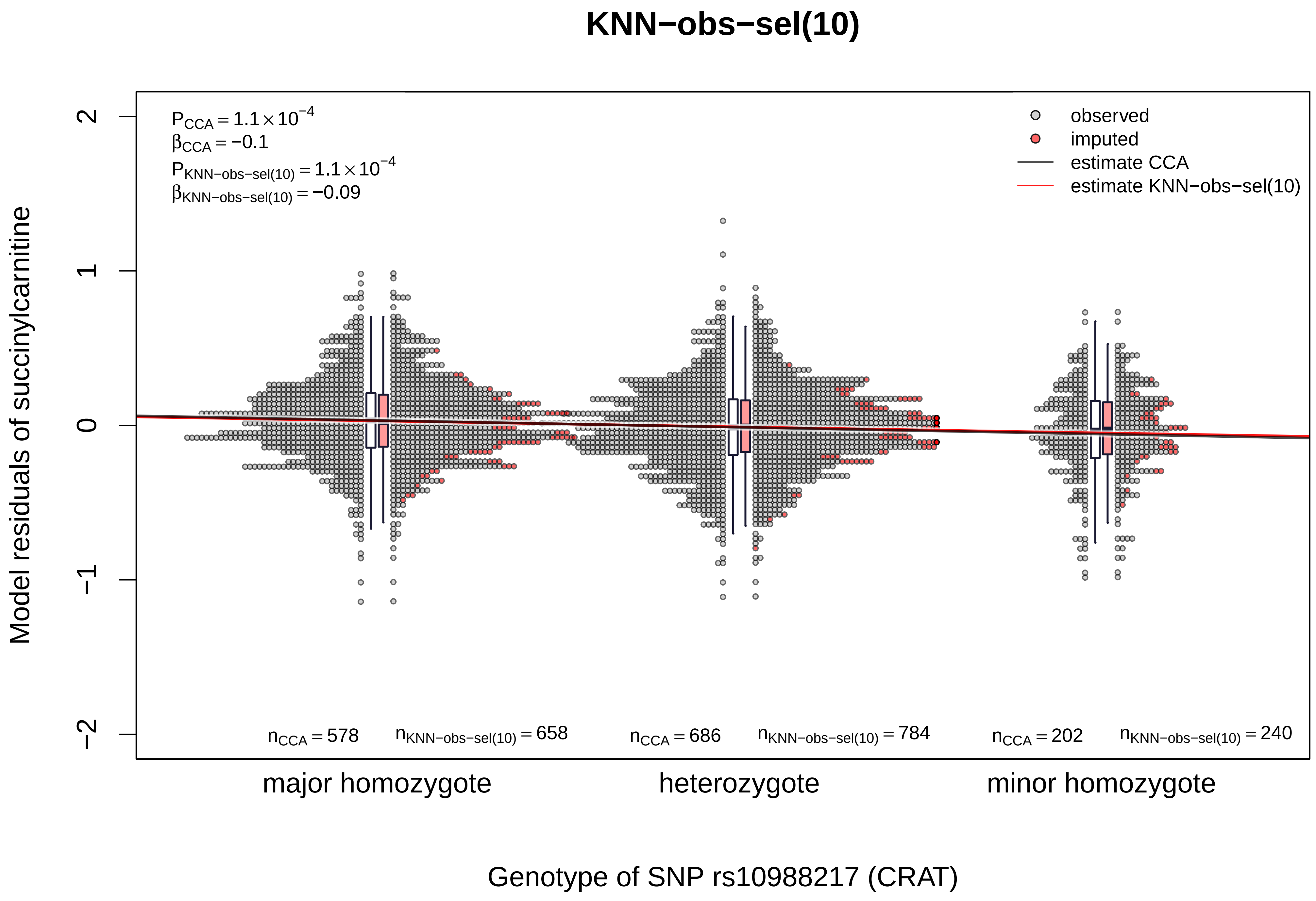
KNN−obs−sel(10)
$P_{CCA} = 1.1 \times 10^{-4}$
$\beta_{CCA} = -0.1$
$P_{KNN-obs-sel(10)} = 1.1 \times 10^{-4}$
$\beta_{KNN-obs-sel(10)} = -0.09$
observed
imputed
estimate CCA
estimate KNN−obs−sel(10)
Model residuals of succinylcarnitine
2
1
0
−1
−2
$n_{CCA} = 578$
$n_{KNN-obs-sel(10)} = 658$
$n_{CCA} = 686$
$n_{KNN-obs-sel(10)} = 784$
$n_{CCA} = 202$
$n_{KNN-obs-sel(10)} = 240$
major homozygote
heterozygote
minor homozygote
Genotype of SNP rs10988217 (CRAT)

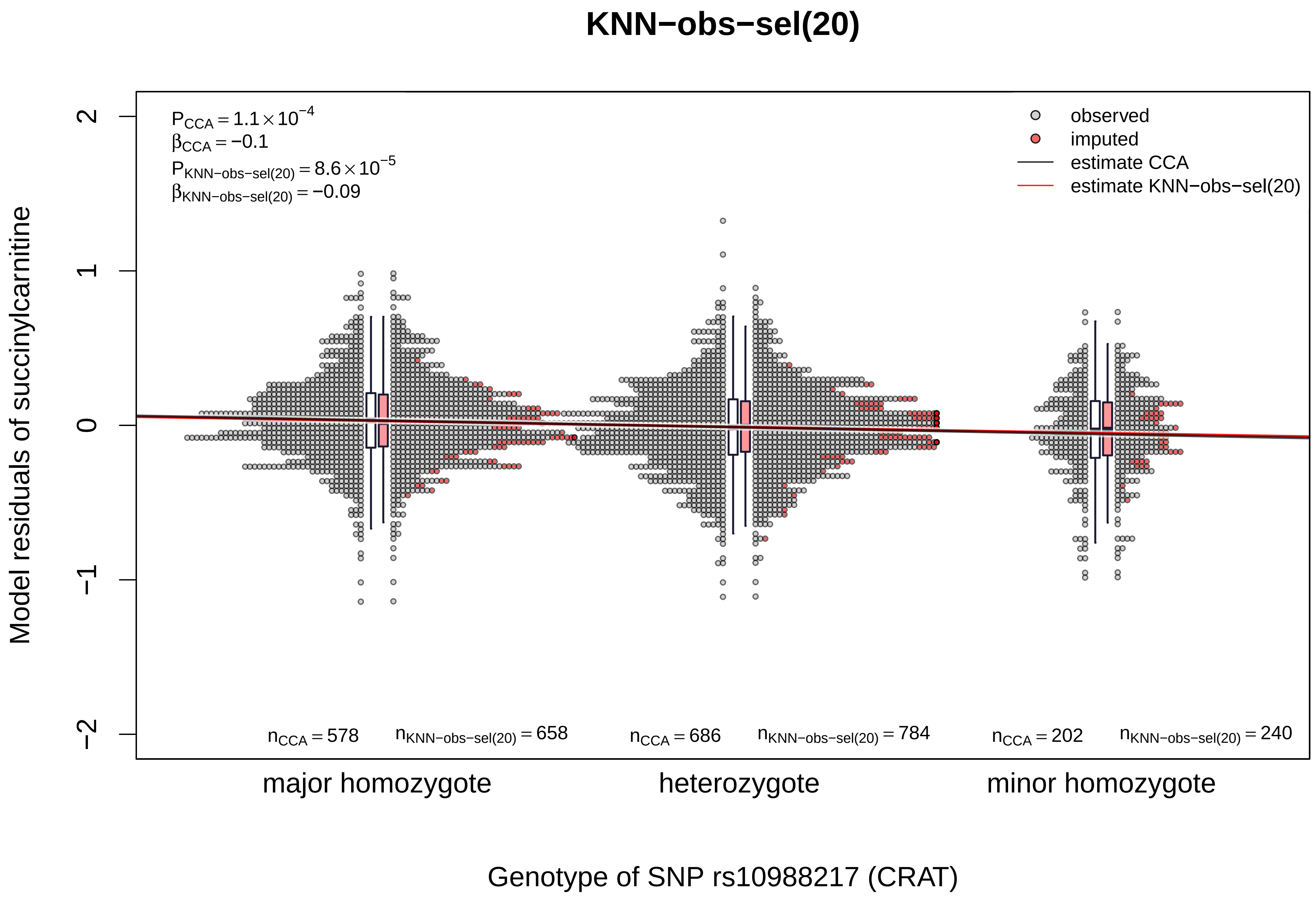

KNN−obs−sel(20)
$P_{CCA} = 1.1 \times 10^{-4}$
$\beta_{CCA} = -0.1$
$P_{KNN-obs-sel(20)} = 8.6 \times 10^{-5}$
$\beta_{KNN-obs-sel(20)} = -0.09$
observed
imputed
estimate CCA
estimate KNN−obs−sel(20)
Model residuals of succinylcarnitine
2
1
0
−1
−2
$n_{CCA} = 578$
$n_{KNN-obs-sel(20)} = 658$
$n_{CCA} = 686$
$n_{KNN-obs-sel(20)} = 784$
$n_{CCA} = 202$
$n_{KNN-obs-sel(20)} = 240$
major homozygote
heterozygote
minor homozygote
Genotype of SNP rs10988217 (CRAT)

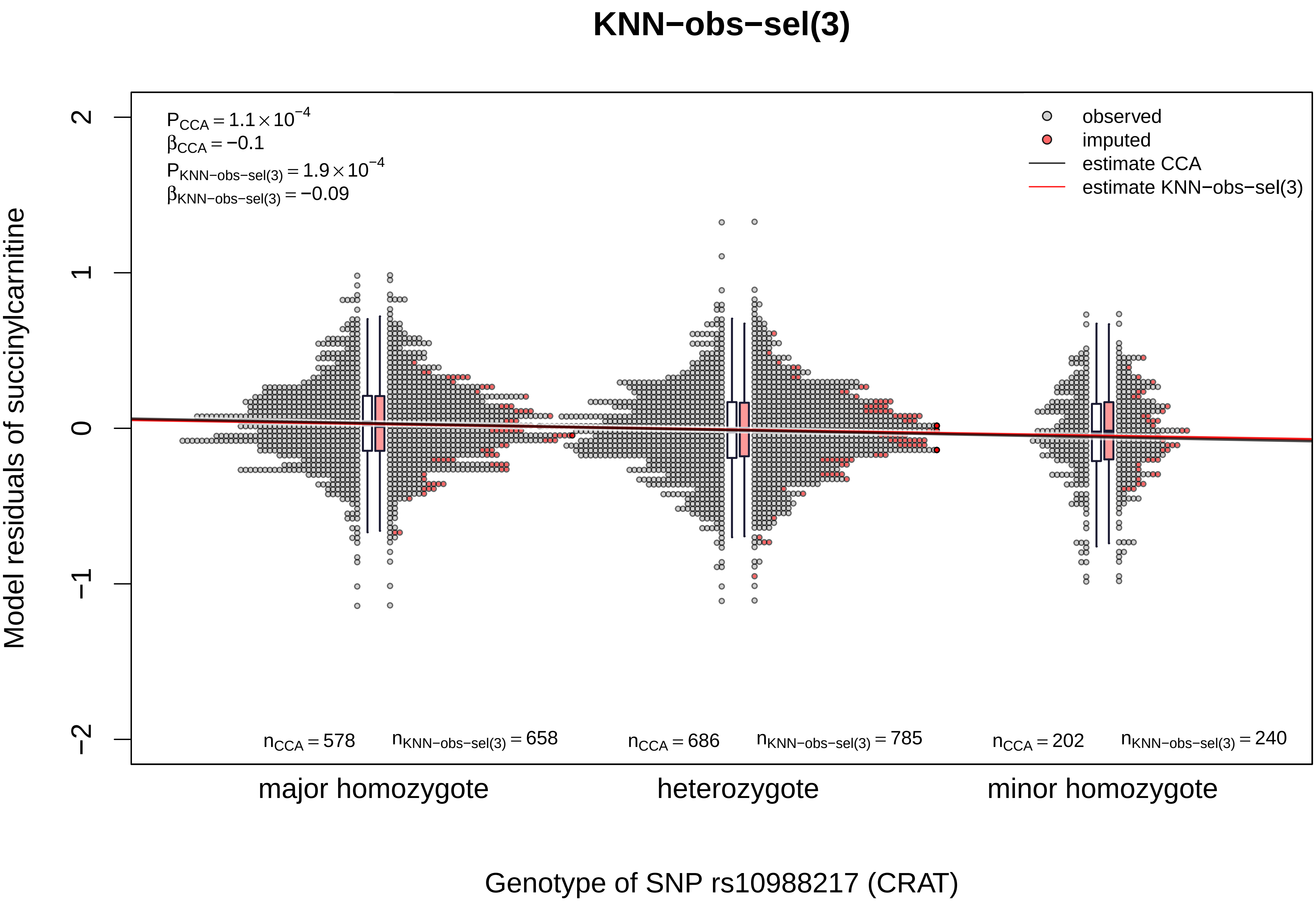

KNN−obs−sel(3)
$P_{CCA} = 1.1 \times 10^{-4}$
$\beta_{CCA} = -0.1$
$P_{KNN-obs-sel(3)} = 1.9 \times 10^{-4}$
$\beta_{KNN-obs-sel(3)} = -0.09$
observed
imputed
estimate CCA
estimate KNN−obs−sel(3)
Model residuals of succinylcarnitine
2
1
0
−1
−2
$n_{CCA} = 578$
$n_{KNN-obs-sel(3)} = 658$
$n_{CCA} = 686$
$n_{KNN-obs-sel(3)} = 785$
$n_{CCA} = 202$
$n_{KNN-obs-sel(3)} = 240$
major homozygote
heterozygote
minor homozygote
Genotype of SNP rs10988217 (CRAT)

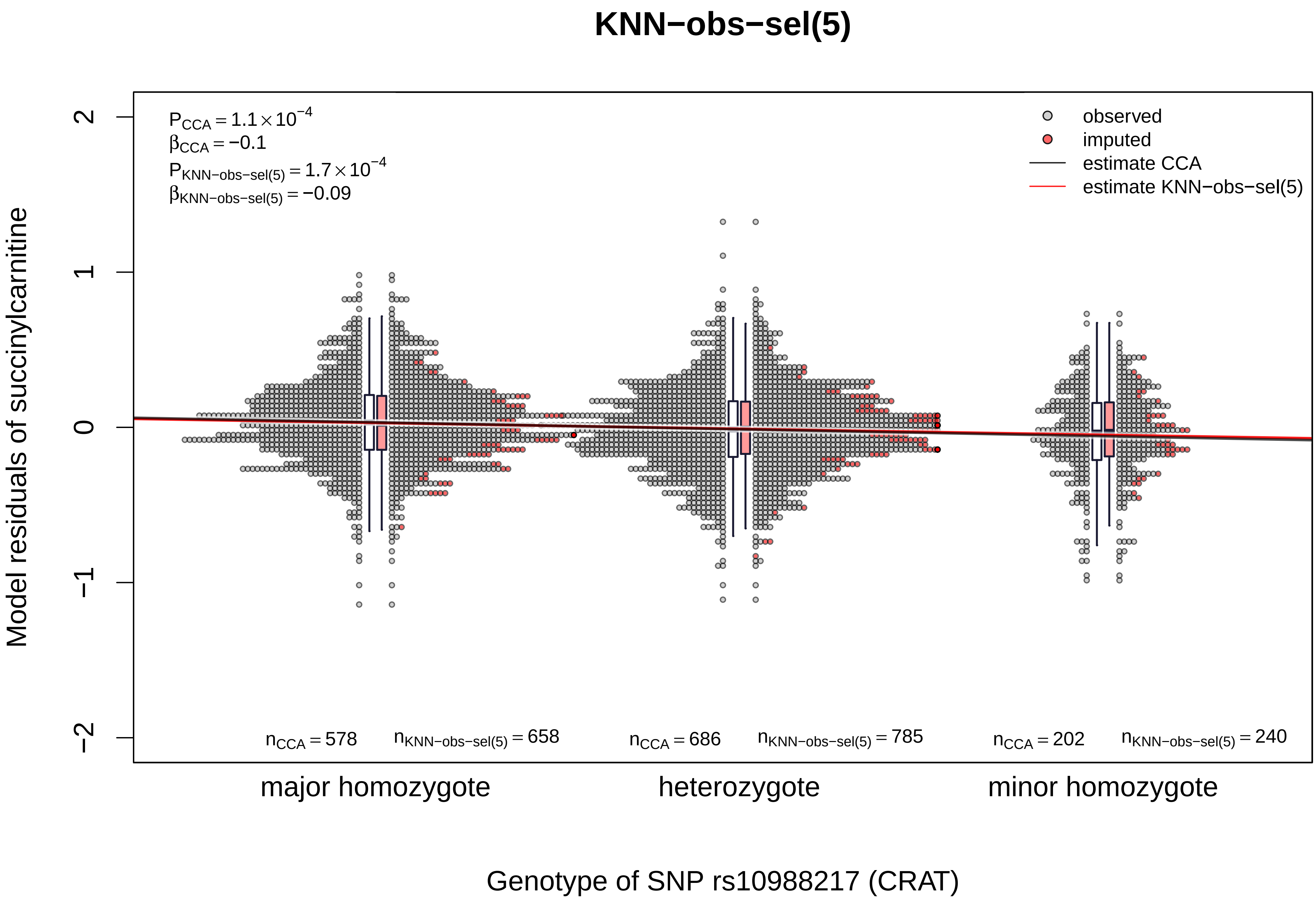
KNN−obs−sel(5)
$P_{CCA} = 1.1 \times 10^{-4}$
$\beta_{CCA} = -0.1$
$P_{KNN-obs-sel(5)} = 1.7 \times 10^{-4}$
$\beta_{KNN-obs-sel(5)} = -0.09$
observed
imputed
estimate CCA
estimate KNN−obs−sel(5)
Model residuals of succinylcarnitine
2
1
0
−1
−2
$n_{CCA} = 578$
$n_{KNN-obs-sel(5)} = 658$
$n_{CCA} = 686$
$n_{KNN-obs-sel(5)} = 785$
$n_{CCA} = 202$
$n_{KNN-obs-sel(5)} = 240$
major homozygote
heterozygote
minor homozygote
Genotype of SNP rs10988217 (CRAT)

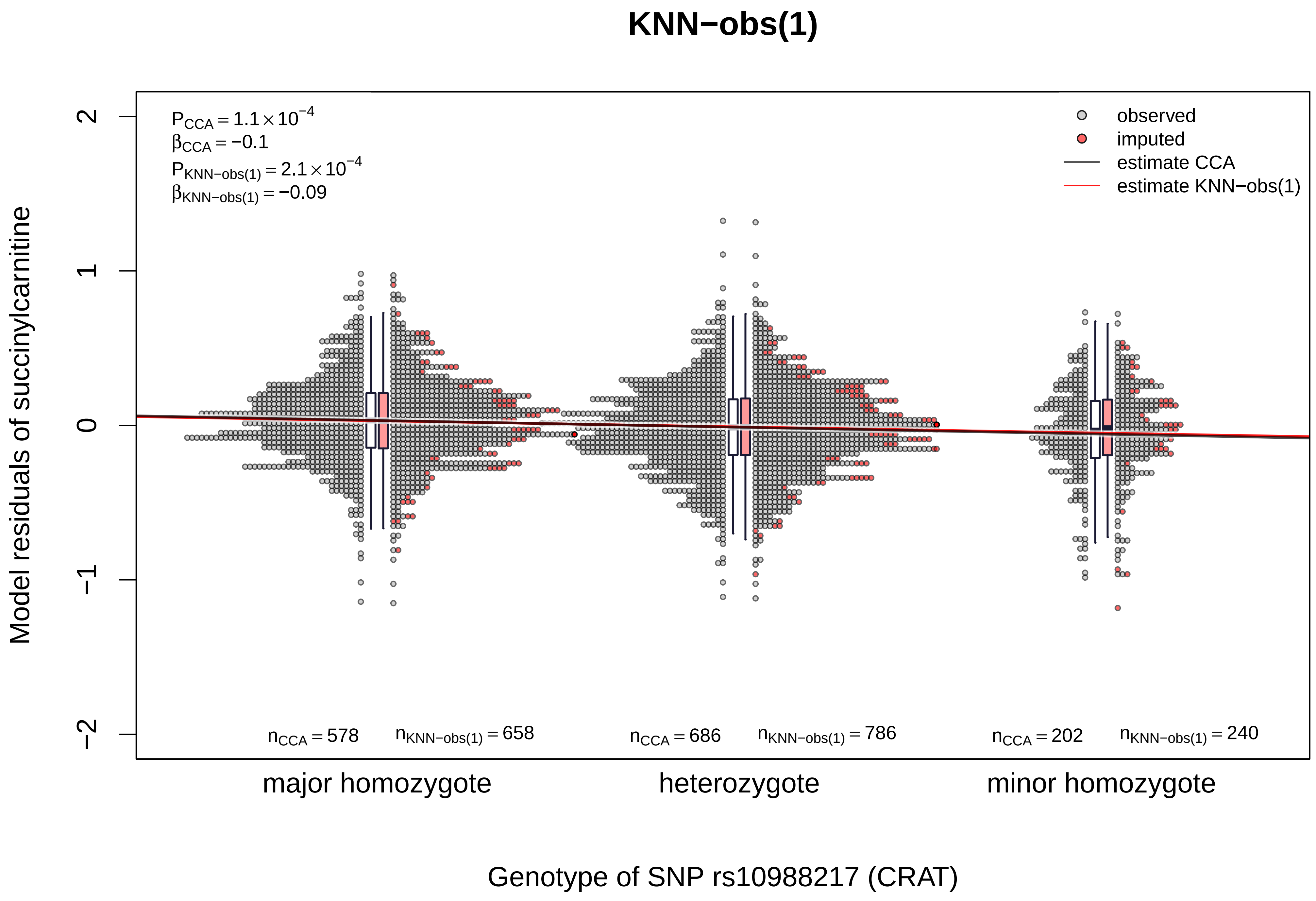

KNN−obs(1)
$P_{CCA} = 1.1 \times 10^{-4}$
$\beta_{CCA} = -0.1$
$P_{KNN-obs(1)} = 2.1 \times 10^{-4}$
$\beta_{KNN-obs(1)} = -0.09$
observed
imputed
estimate CCA
estimate KNN−obs(1)
Model residuals of succinylcarnitine
2
1
0
−1
−2
$n_{CCA} = 578$
$n_{KNN-obs(1)} = 658$
$n_{CCA} = 686$
$n_{KNN-obs(1)} = 786$
$n_{CCA} = 202$
$n_{KNN-obs(1)} = 240$
major homozygote
heterozygote
minor homozygote
Genotype of SNP rs10988217 (CRAT)

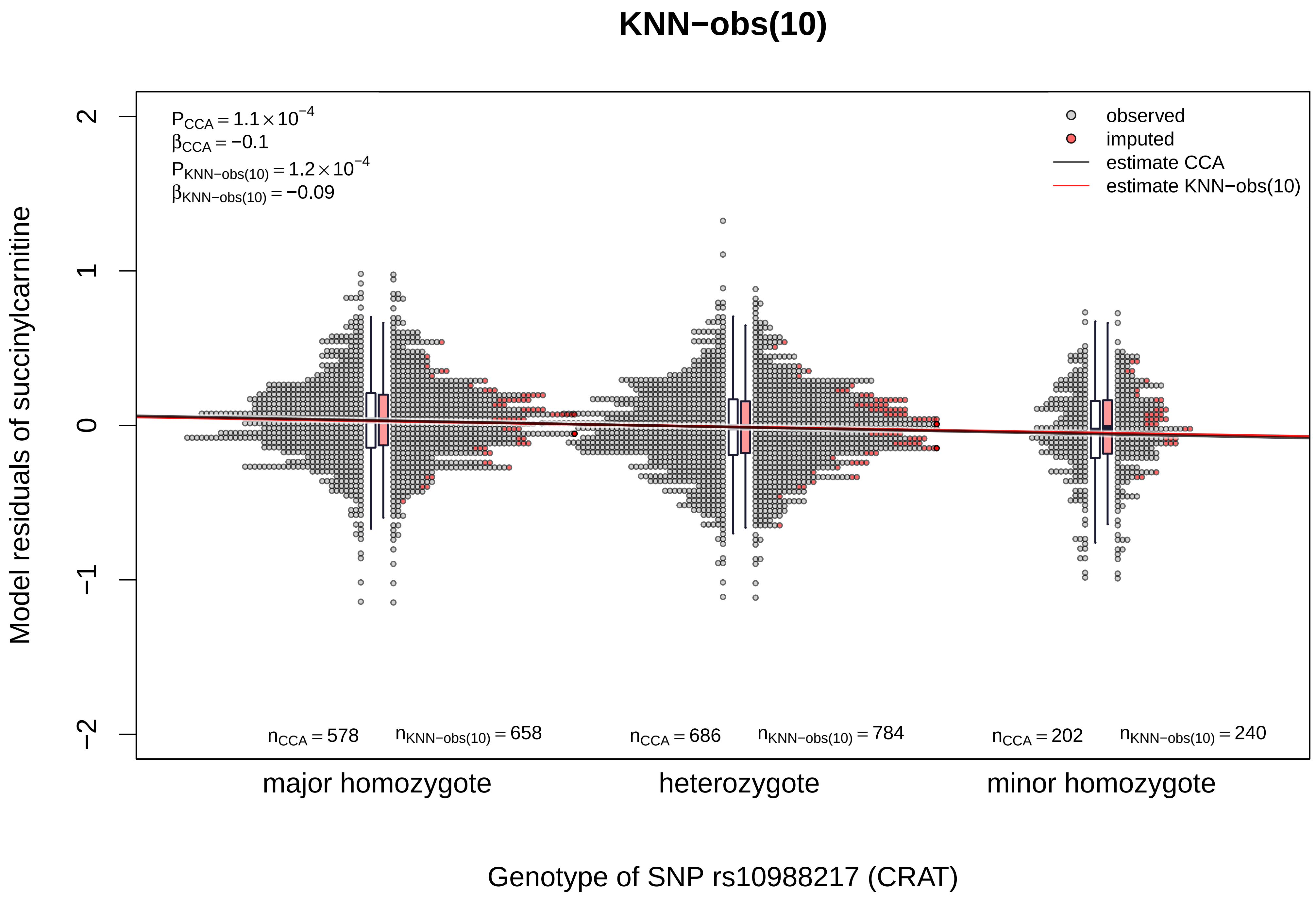

KNN−obs(10)
$P_{CCA} = 1.1 \times 10^{-4}$
$\beta_{CCA} = -0.1$
$P_{KNN-obs(10)} = 1.2 \times 10^{-4}$
$\beta_{KNN-obs(10)} = -0.09$
observed
imputed
estimate CCA
estimate KNN−obs(10)
2
1
0
−1
−2
Model residuals of succinylcarnitine
$n_{CCA} = 578$
$n_{KNN-obs(10)} = 658$
$n_{CCA} = 686$
$n_{KNN-obs(10)} = 784$
$n_{CCA} = 202$
$n_{KNN-obs(10)} = 240$
major homozygote
heterozygote
minor homozygote
Genotype of SNP rs10988217 (CRAT)

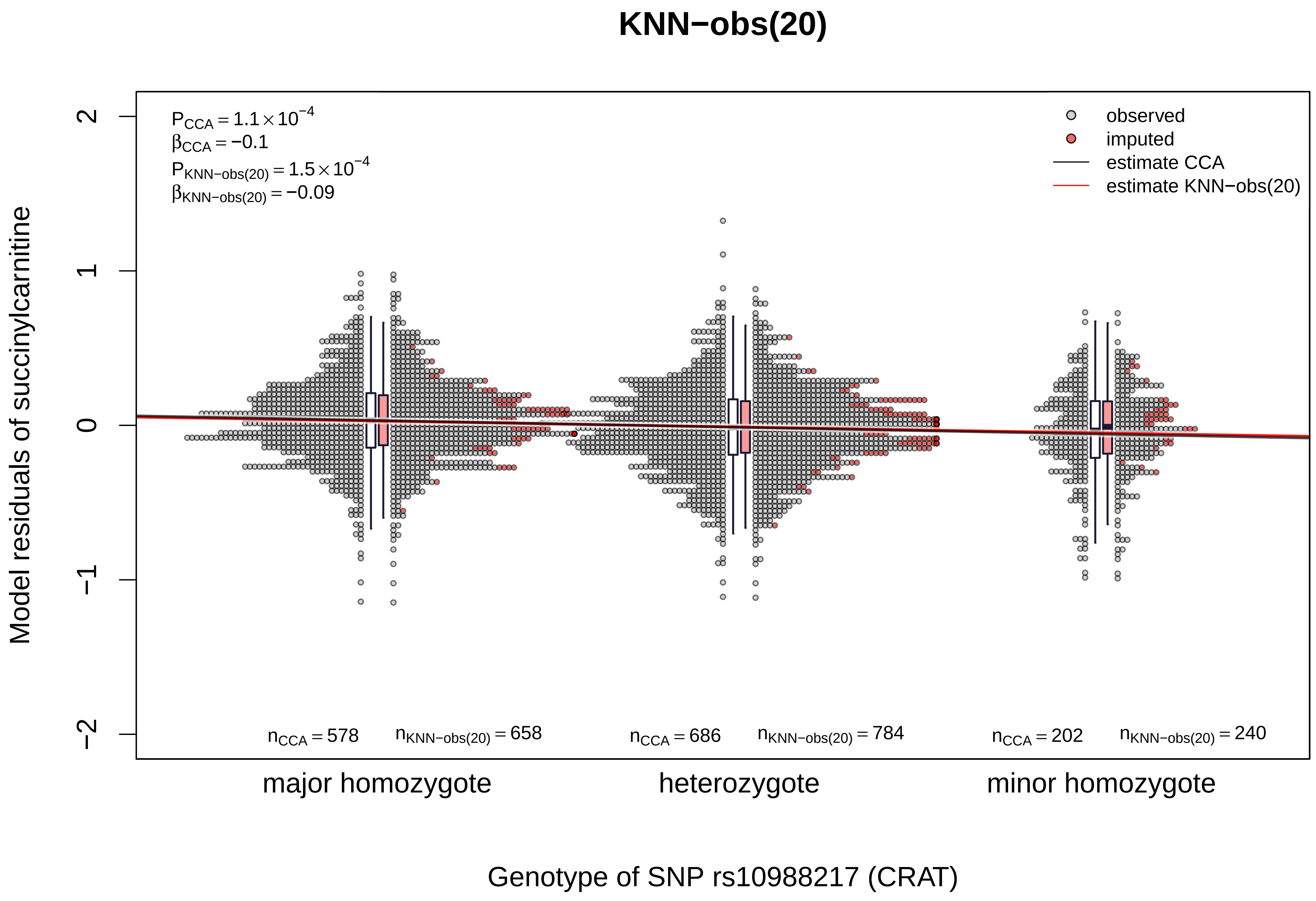

KNN−obs(20)
$P_{CCA} = 1.1 \times 10^{-4}$
$\beta_{CCA} = -0.1$
$P_{KNN-obs(20)} = 1.5 \times 10^{-4}$
$\beta_{KNN-obs(20)} = -0.09$
observed
imputed
estimate CCA
estimate KNN−obs(20)
Model residuals of succinylcarnitine
2
1
0
−1
−2
$n_{CCA} = 578$
$n_{KNN-obs(20)} = 658$
$n_{CCA} = 686$
$n_{KNN-obs(20)} = 784$
$n_{CCA} = 202$
$n_{KNN-obs(20)} = 240$
major homozygote
heterozygote
minor homozygote
Genotype of SNP rs10988217 (CRAT)

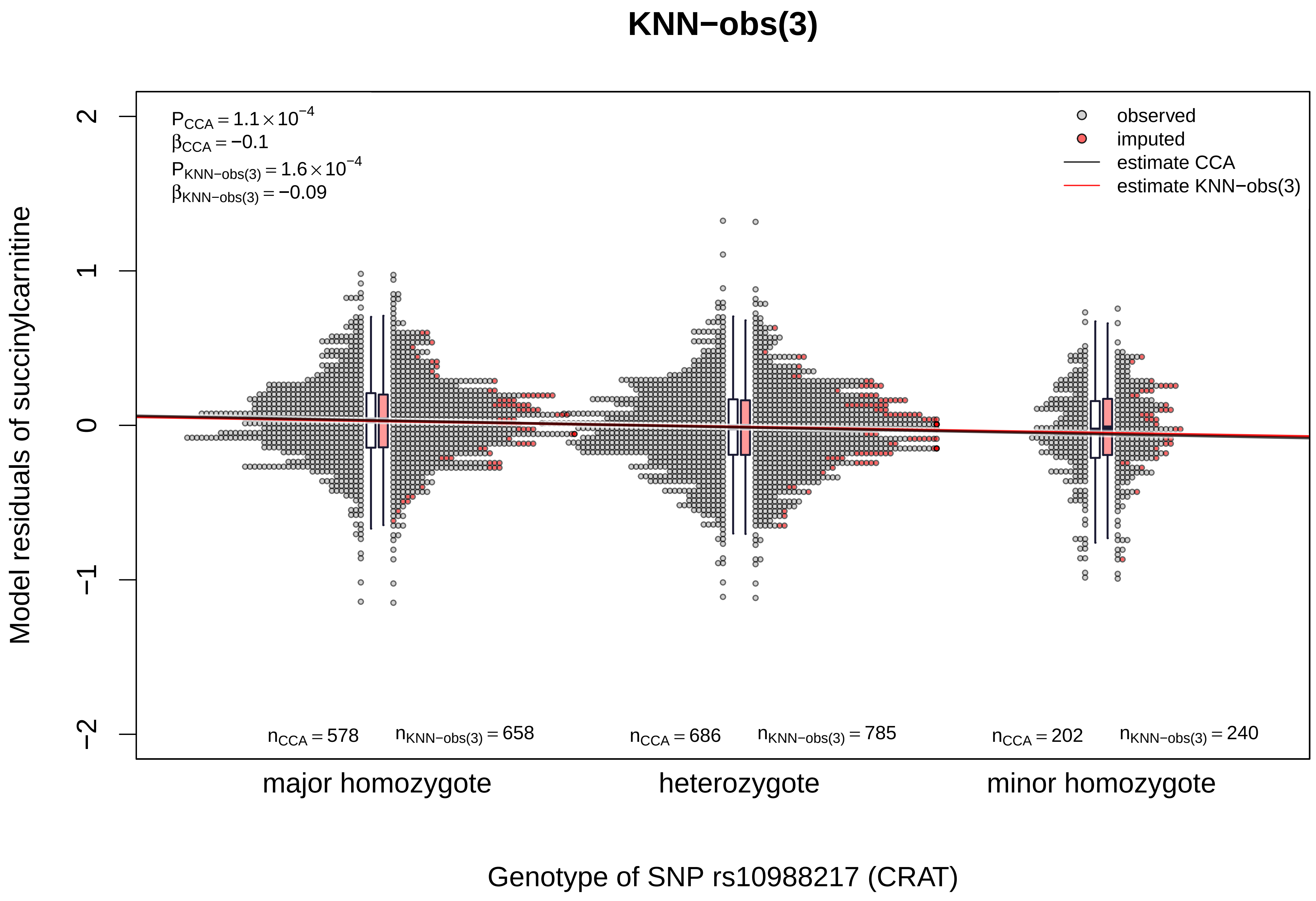

KNN−obs(3)
$P_{CCA} = 1.1 \times 10^{-4}$
$\beta_{CCA} = -0.1$
$P_{KNN-obs(3)} = 1.6 \times 10^{-4}$
$\beta_{KNN-obs(3)} = -0.09$
observed
imputed
estimate CCA
estimate KNN−obs(3)
Model residuals of succinylcarnitine
2
1
0
−1
−2
$n_{CCA} = 578$
$n_{KNN-obs(3)} = 658$
$n_{CCA} = 686$
$n_{KNN-obs(3)} = 785$
$n_{CCA} = 202$
$n_{KNN-obs(3)} = 240$
major homozygote
heterozygote
minor homozygote
Genotype of SNP rs10988217 (CRAT)

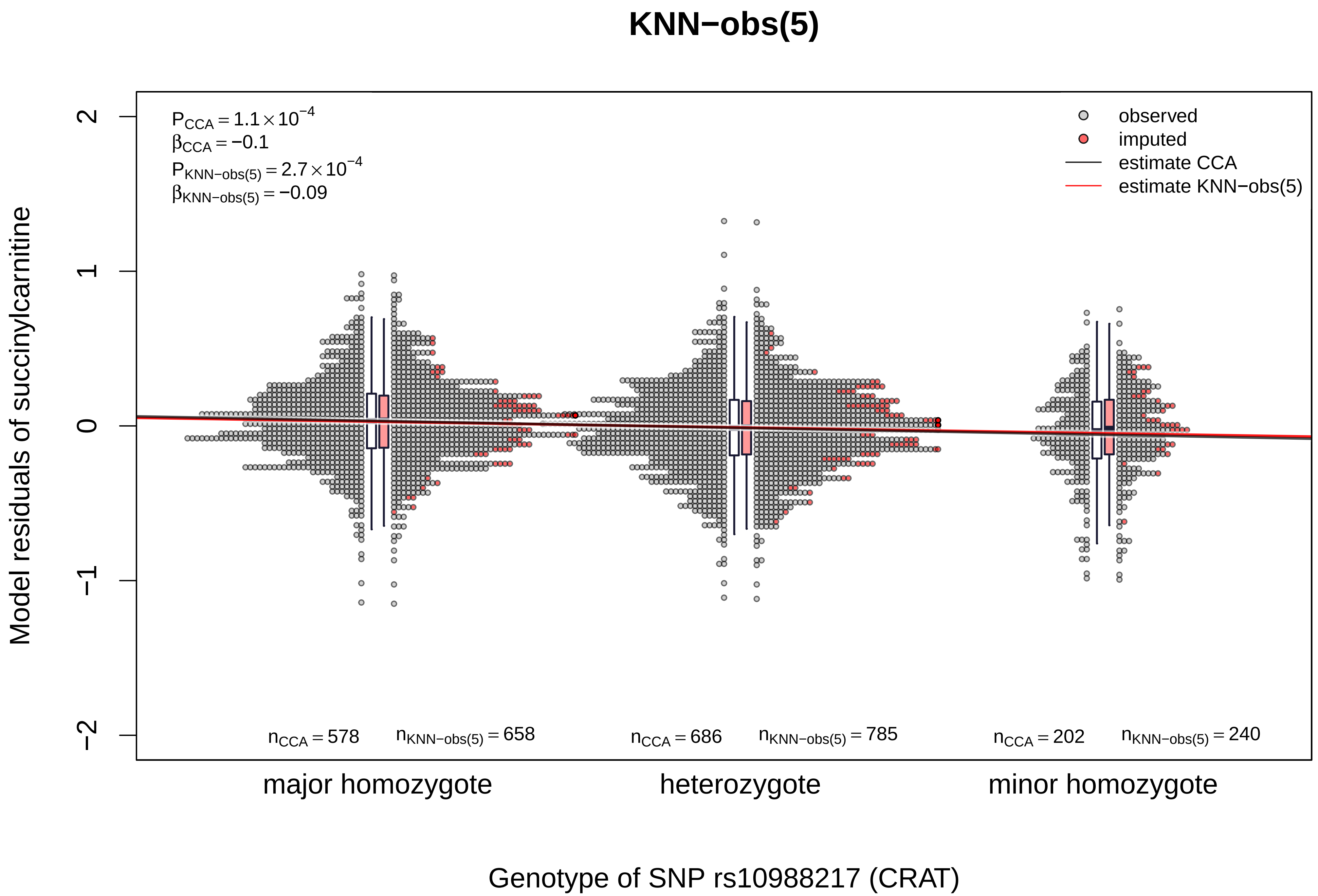
KNN−obs(5)
$P_{CCA} = 1.1 \times 10^{-4}$
$\beta_{CCA} = -0.1$
$P_{KNN-obs(5)} = 2.7 \times 10^{-4}$
$\beta_{KNN-obs(5)} = -0.09$
observed
imputed
estimate CCA
estimate KNN−obs(5)
Model residuals of succinylcarnitine
2
1
0
−1
−2
$n_{CCA} = 578$
$n_{KNN-obs(5)} = 658$
$n_{CCA} = 686$
$n_{KNN-obs(5)} = 785$
$n_{CCA} = 202$
$n_{KNN-obs(5)} = 240$
major homozygote
heterozygote
minor homozygote
Genotype of SNP rs10988217 (CRAT)

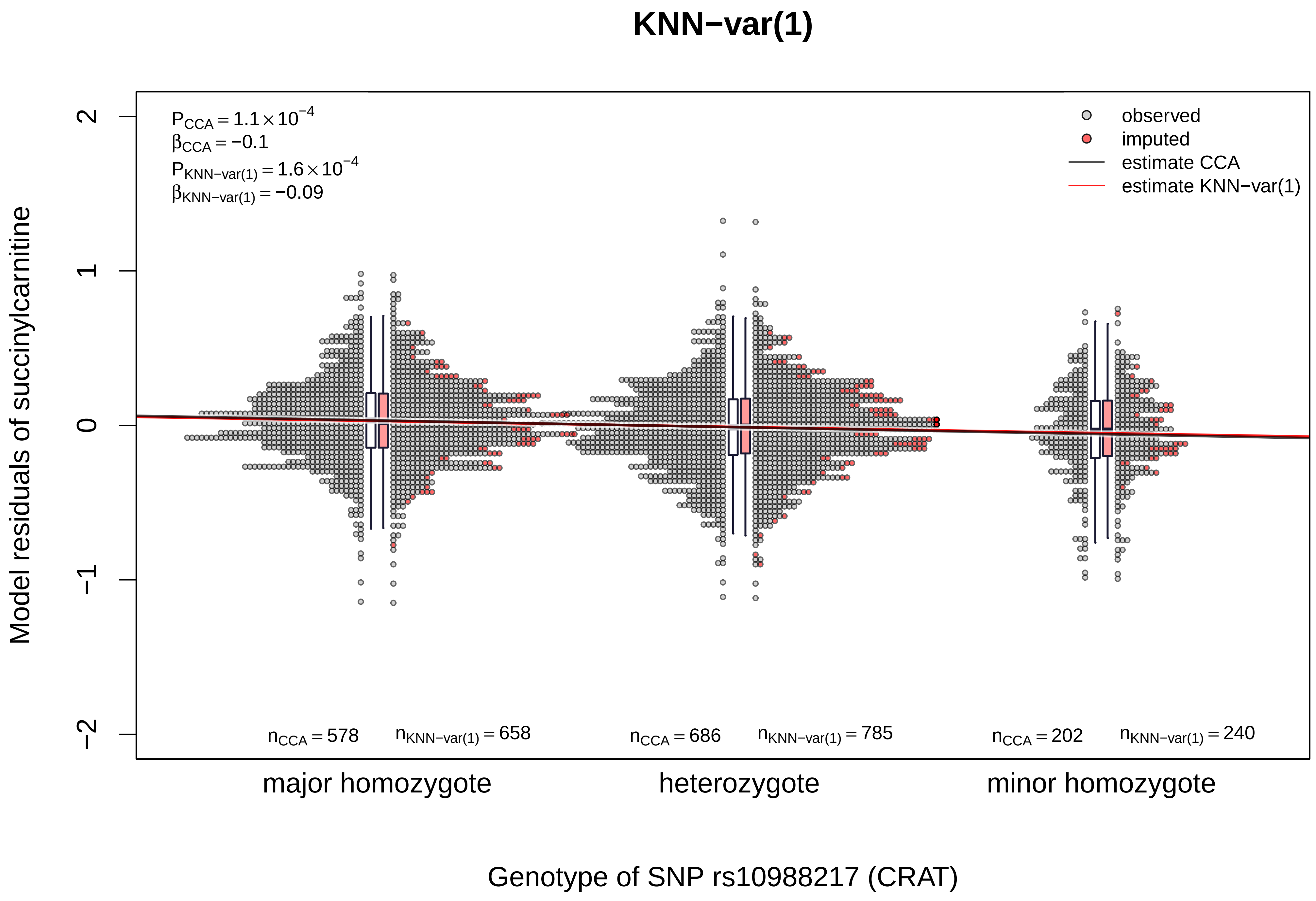
KNN−var(1)
$P_{CCA} = 1.1 \times 10^{-4}$
$\beta_{CCA} = -0.1$
$P_{KNN-var(1)} = 1.6 \times 10^{-4}$
$\beta_{KNN-var(1)} = -0.09$
observed
imputed
estimate CCA
estimate KNN−var(1)
Model residuals of succinylcarnitine
2
1
0
−1
−2
$n_{CCA} = 578$
$n_{KNN-var(1)} = 658$
$n_{CCA} = 686$
$n_{KNN-var(1)} = 785$
$n_{CCA} = 202$
$n_{KNN-var(1)} = 240$
major homozygote
heterozygote
minor homozygote
Genotype of SNP rs10988217 (CRAT)

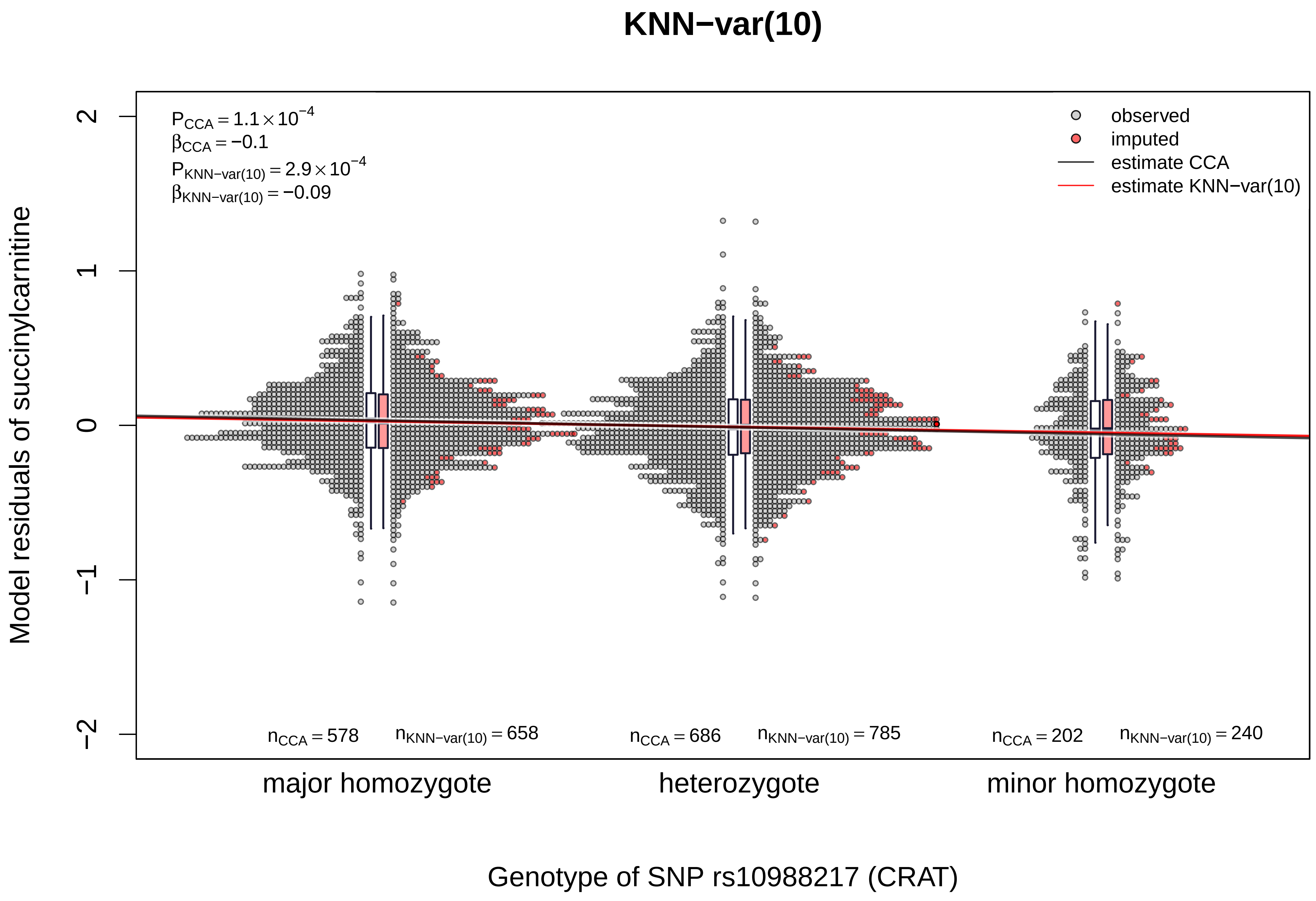

KNN−var(10)
$P_{CCA} = 1.1 \times 10^{-4}$
$\beta_{CCA} = -0.1$
$P_{KNN-var(10)} = 2.9 \times 10^{-4}$
$\beta_{KNN-var(10)} = -0.09$
observed
imputed
estimate CCA
estimate KNN−var(10)
Model residuals of succinylcarnitine
2
1
0
−1
−2
$n_{CCA} = 578$
$n_{KNN-var(10)} = 658$
$n_{CCA} = 686$
$n_{KNN-var(10)} = 785$
$n_{CCA} = 202$
$n_{KNN-var(10)} = 240$
major homozygote
heterozygote
minor homozygote
Genotype of SNP rs10988217 (CRAT)

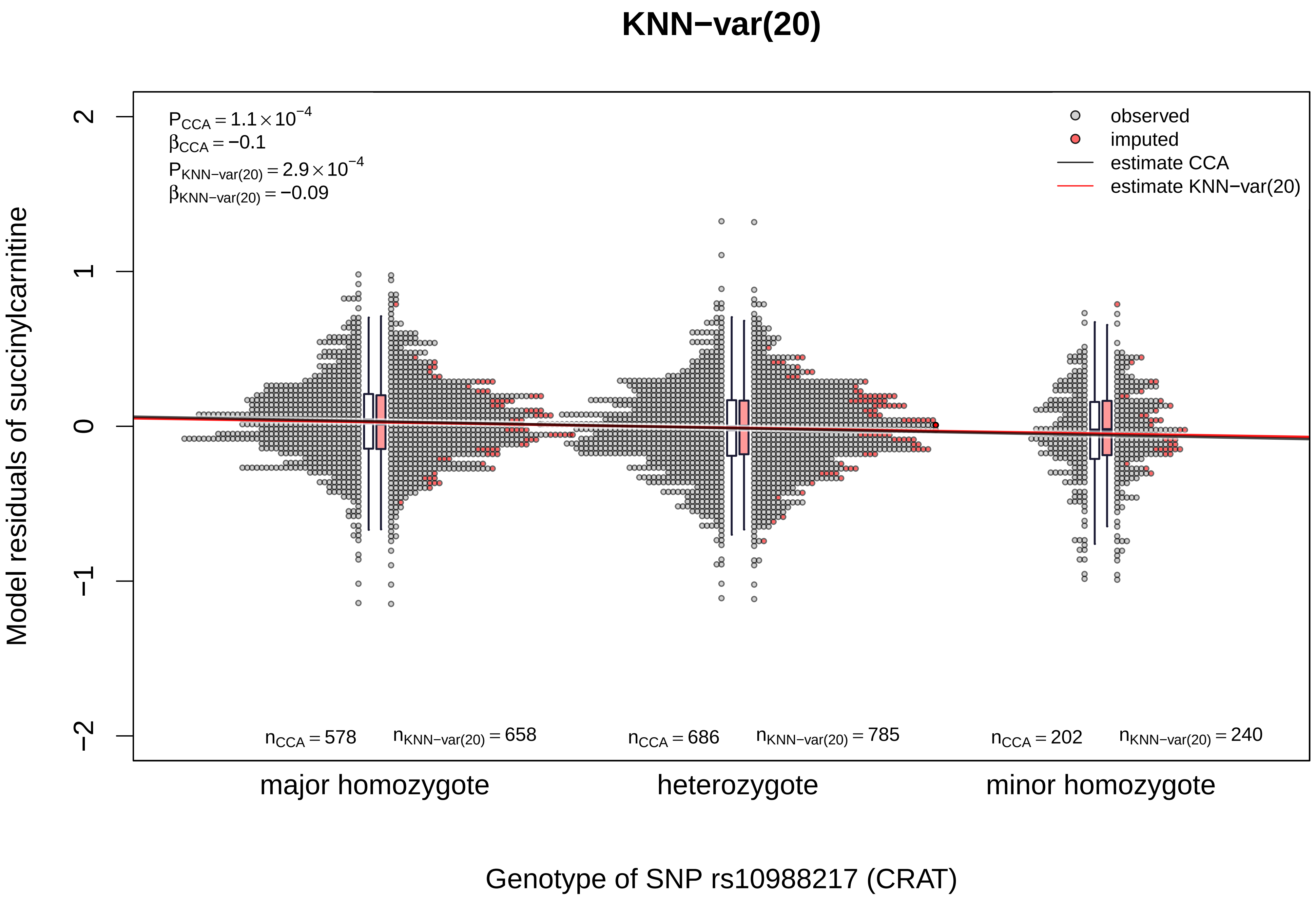
KNN−var(20)
$P_{CCA} = 1.1 \times 10^{-4}$
$\beta_{CCA} = -0.1$
$P_{KNN-var(20)} = 2.9 \times 10^{-4}$
$\beta_{KNN-var(20)} = -0.09$
observed
imputed
estimate CCA
estimate KNN−var(20)
2
1
0
−1
−2
Model residuals of succinylcarnitine
$n_{CCA} = 578$
$n_{KNN-var(20)} = 658$
$n_{CCA} = 686$
$n_{KNN-var(20)} = 785$
$n_{CCA} = 202$
$n_{KNN-var(20)} = 240$
major homozygote
heterozygote
minor homozygote
Genotype of SNP rs10988217 (CRAT)

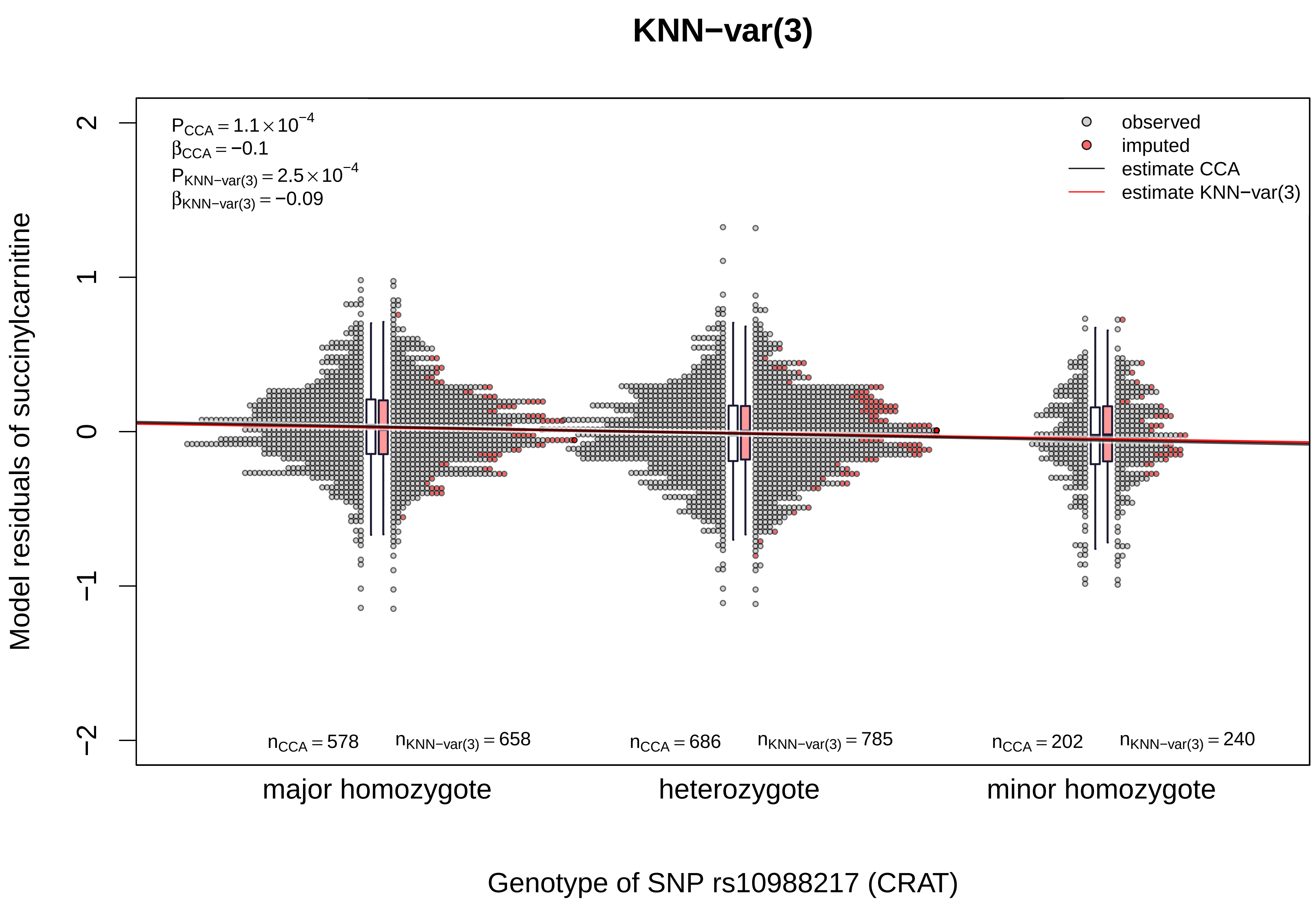

KNN−var(3)
$P_{CCA} = 1.1 \times 10^{-4}$
$\beta_{CCA} = -0.1$
$P_{KNN-var(3)} = 2.5 \times 10^{-4}$
$\beta_{KNN-var(3)} = -0.09$
observed
imputed
estimate CCA
estimate KNN−var(3)
2
1
0
−1
−2
Model residuals of succinylcarnitine
$n_{CCA} = 578$
$n_{KNN-var(3)} = 658$
$n_{CCA} = 686$
$n_{KNN-var(3)} = 785$
$n_{CCA} = 202$
$n_{KNN-var(3)} = 240$
major homozygote
heterozygote
minor homozygote
Genotype of SNP rs10988217 (CRAT)

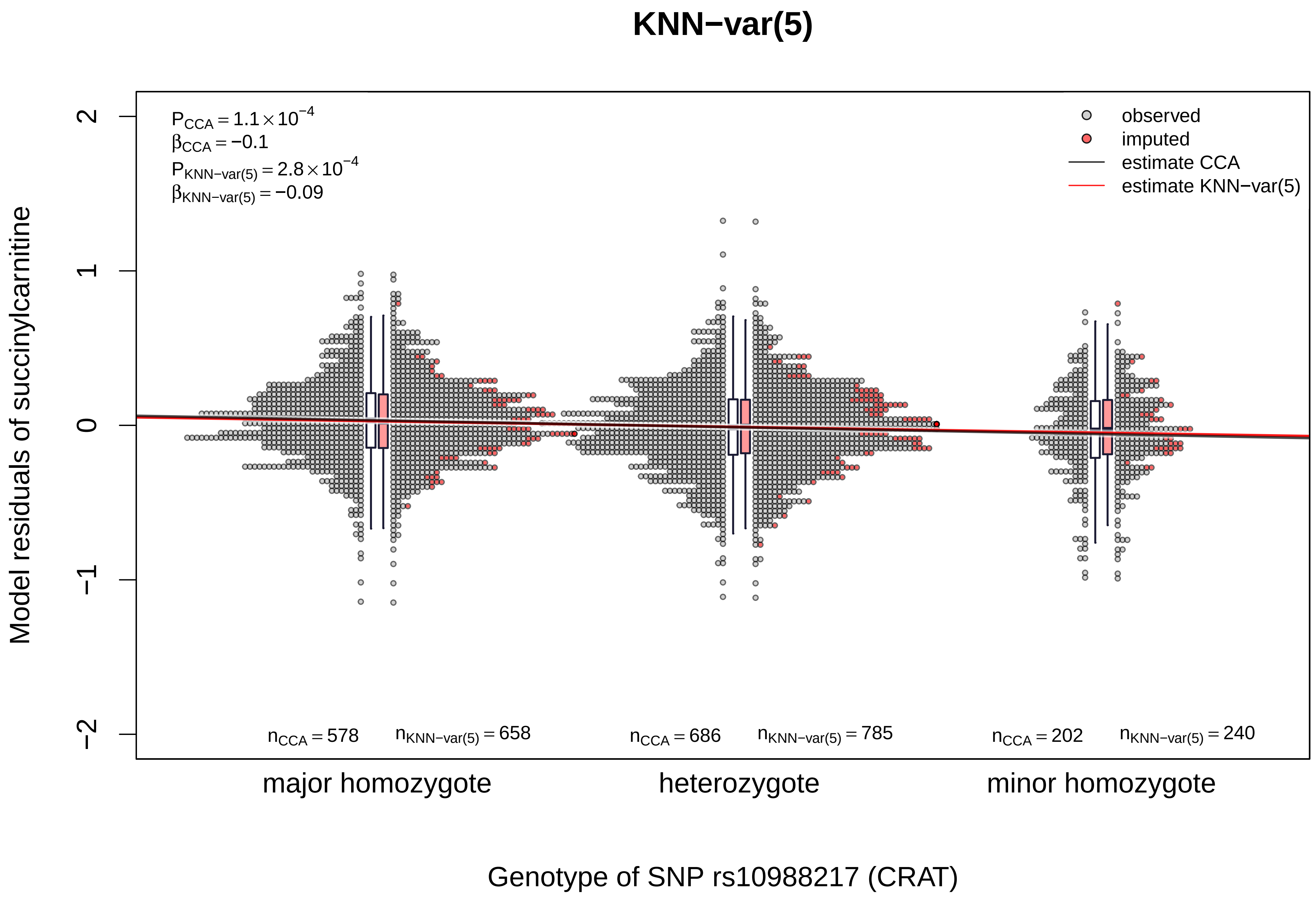

KNN−var(5)
$P_{CCA} = 1.1 \times 10^{-4}$
$\beta_{CCA} = -0.1$
$P_{KNN-var(5)} = 2.8 \times 10^{-4}$
$\beta_{KNN-var(5)} = -0.09$
observed
imputed
estimate CCA
estimate KNN−var(5)
Model residuals of succinylcarnitine
2
1
0
−1
−2
$n_{CCA} = 578$
$n_{KNN-var(5)} = 658$
$n_{CCA} = 686$
$n_{KNN-var(5)} = 785$
$n_{CCA} = 202$
$n_{KNN-var(5)} = 240$
major homozygote
heterozygote
minor homozygote
Genotype of SNP rs10988217 (CRAT)

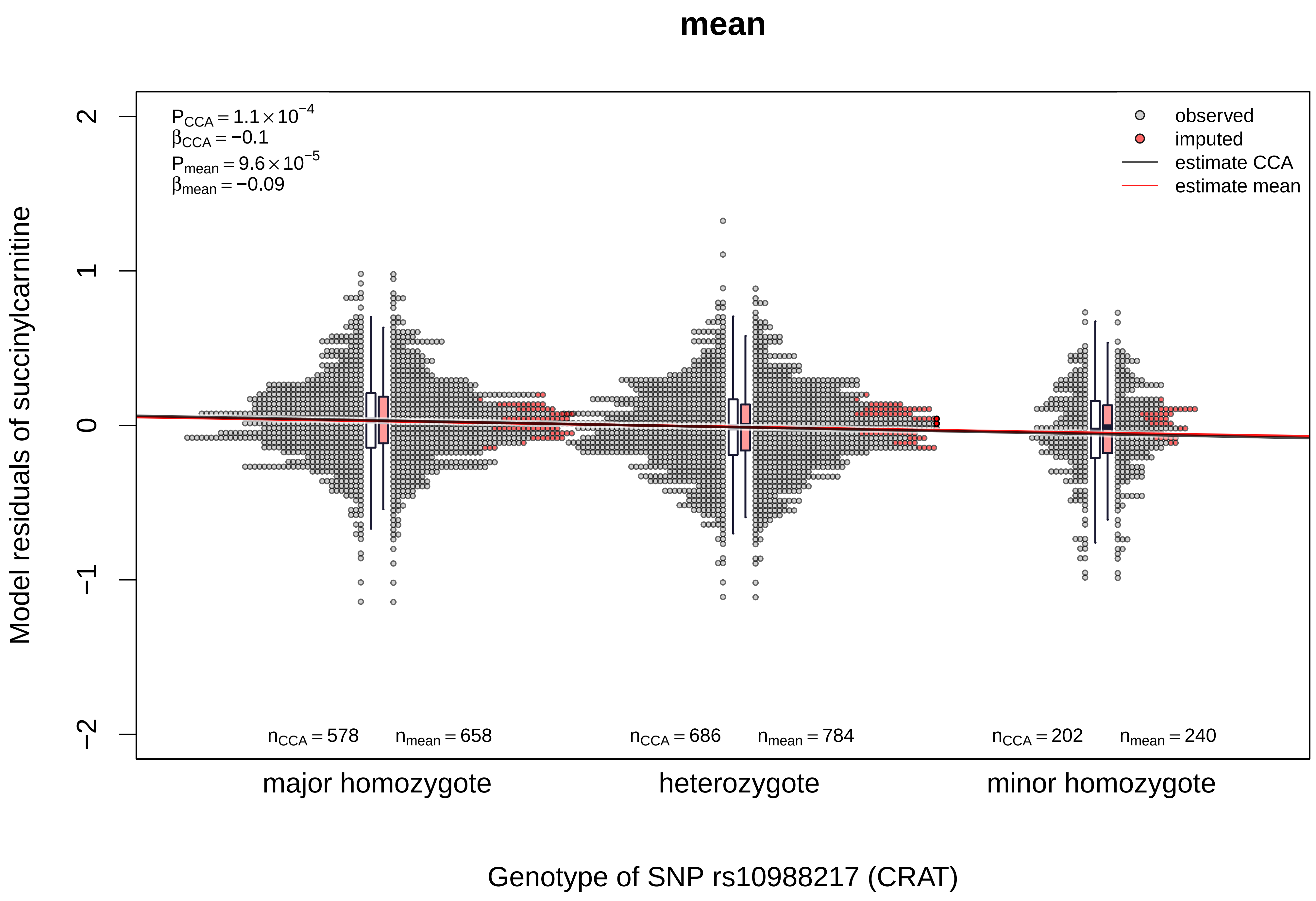
mean
$P_{CCA} = 1.1 \times 10^{-4}$
$\beta_{CCA} = -0.1$
$P_{mean} = 9.6 \times 10^{-5}$
$\beta_{mean} = -0.09$
observed
imputed
estimate CCA
estimate mean
2
1
0
−1
−2
Model residuals of succinylcarnitine
$n_{CCA} = 578$
$n_{mean} = 658$
$n_{CCA} = 686$
$n_{mean} = 784$
$n_{CCA} = 202$
$n_{mean} = 240$
major homozygote
heterozygote
minor homozygote
Genotype of SNP rs10988217 (CRAT)

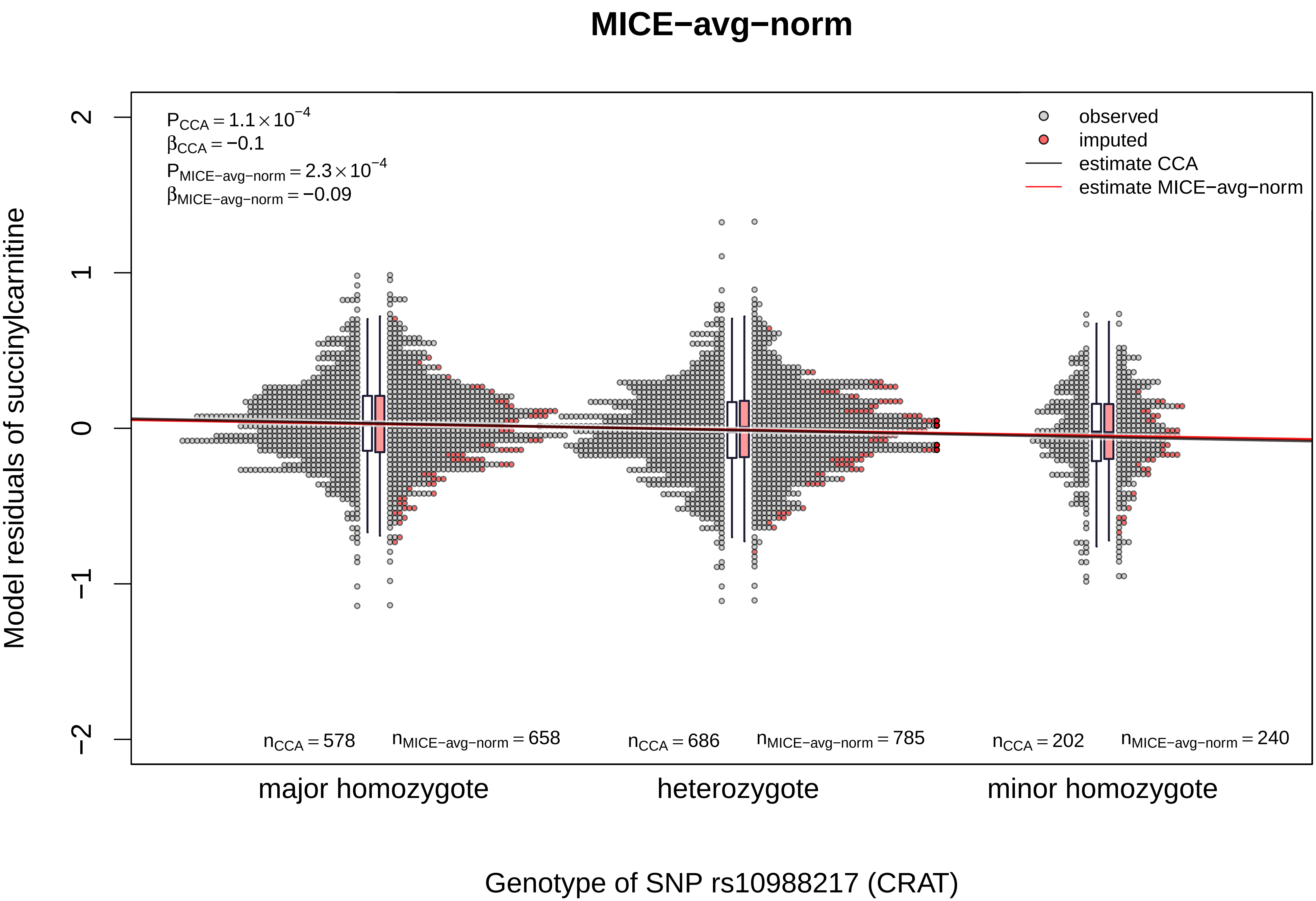

MICE−avg−norm
$P_{CCA} = 1.1 \times 10^{-4}$
$\beta_{CCA} = -0.1$
$P_{MICE-avg-norm} = 2.3 \times 10^{-4}$
$\beta_{MICE-avg-norm} = -0.09$
observed
imputed
estimate CCA
estimate MICE−avg−norm
2
1
0
−1
−2
Model residuals of succinylcarnitine
$n_{CCA} = 578$
$n_{MICE-avg-norm} = 658$
$n_{CCA} = 686$
$n_{MICE-avg-norm} = 785$
$n_{CCA} = 202$
$n_{MICE-avg-norm} = 240$
major homozygote
heterozygote
minor homozygote
Genotype of SNP rs10988217 (CRAT)

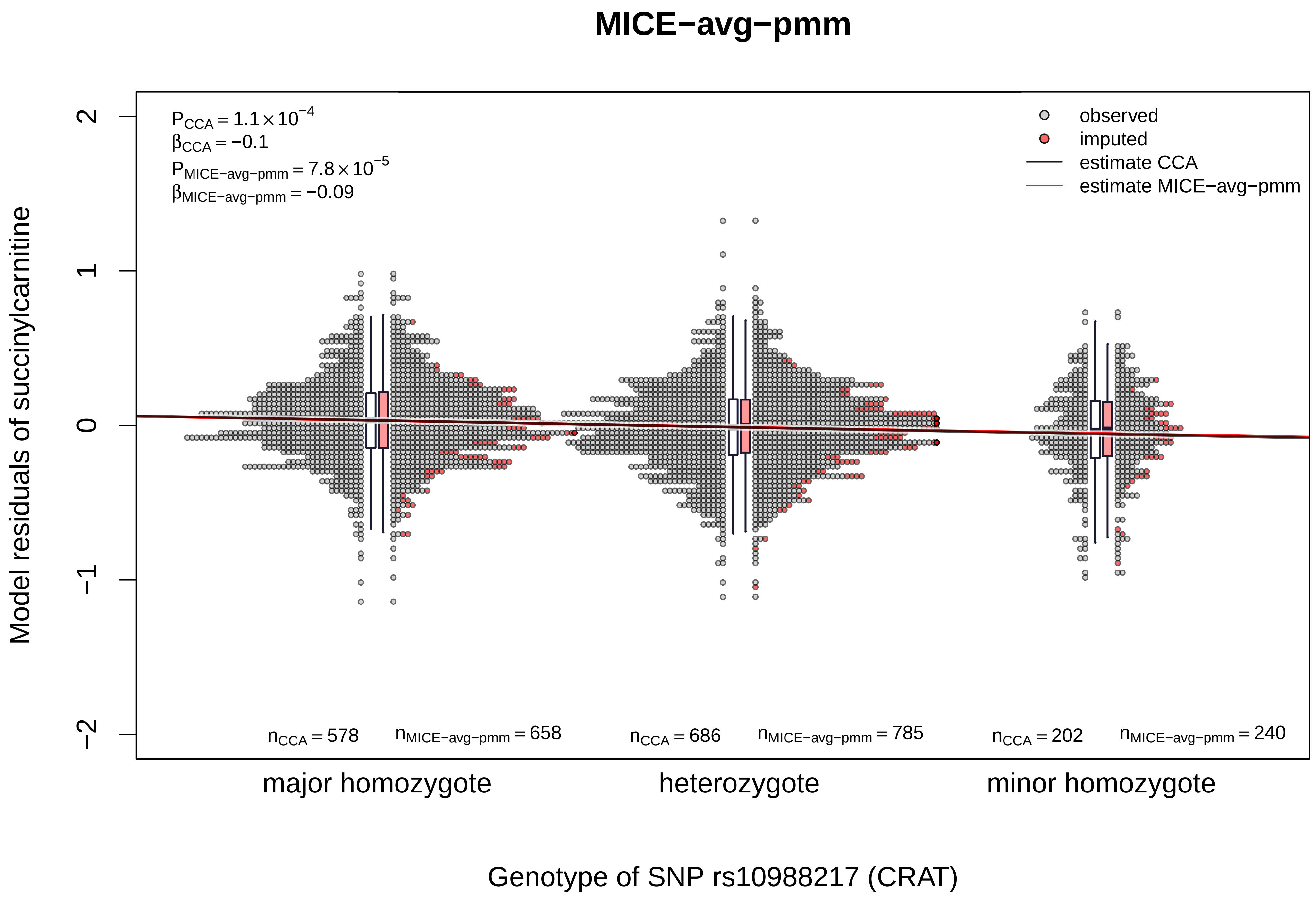

MICE−avg−pmm
$P_{CCA} = 1.1 \times 10^{-4}$
$\beta_{CCA} = -0.1$
$P_{MICE-avg-pmm} = 7.8 \times 10^{-5}$
$\beta_{MICE-avg-pmm} = -0.09$
observed
imputed
estimate CCA
estimate MICE−avg−pmm
2
1
0
−1
−2
Model residuals of succinylcarnitine
$n_{CCA} = 578$
$n_{MICE-avg-pmm} = 658$
$n_{CCA} = 686$
$n_{MICE-avg-pmm} = 785$
$n_{CCA} = 202$
$n_{MICE-avg-pmm} = 240$
major homozygote
heterozygote
minor homozygote
Genotype of SNP rs10988217 (CRAT)

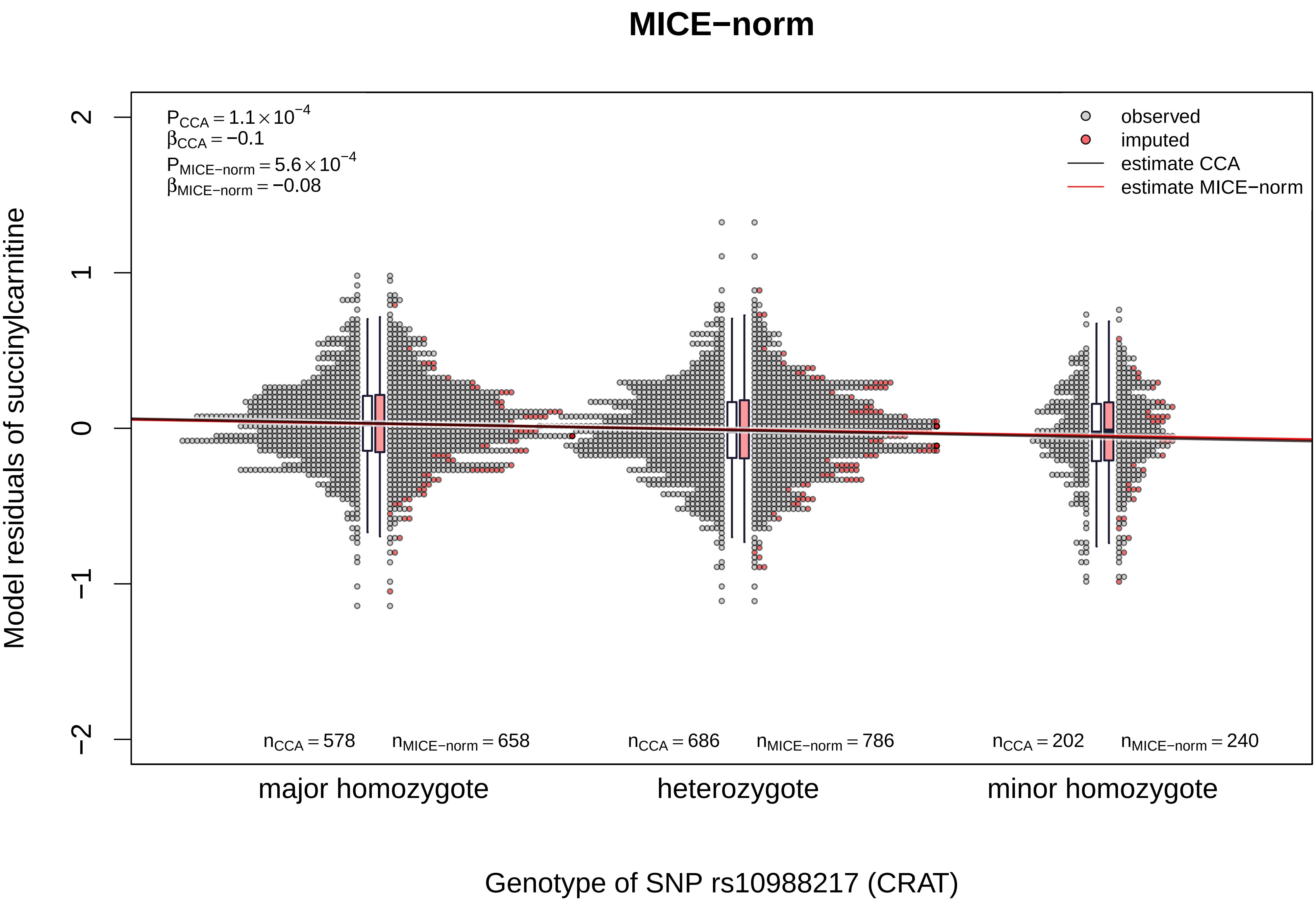

MICE−norm
$P_{CCA} = 1.1 \times 10^{-4}$
$\beta_{CCA} = -0.1$
$P_{MICE-norm} = 5.6 \times 10^{-4}$
$\beta_{MICE-norm} = -0.08$
observed
imputed
estimate CCA
estimate MICE−norm
Model residuals of succinylcarnitine
2
1
0
−1
−2
$n_{CCA} = 578$
$n_{MICE-norm} = 658$
$n_{CCA} = 686$
$n_{MICE-norm} = 786$
$n_{CCA} = 202$
$n_{MICE-norm} = 240$
major homozygote
heterozygote
minor homozygote
Genotype of SNP rs10988217 (CRAT)

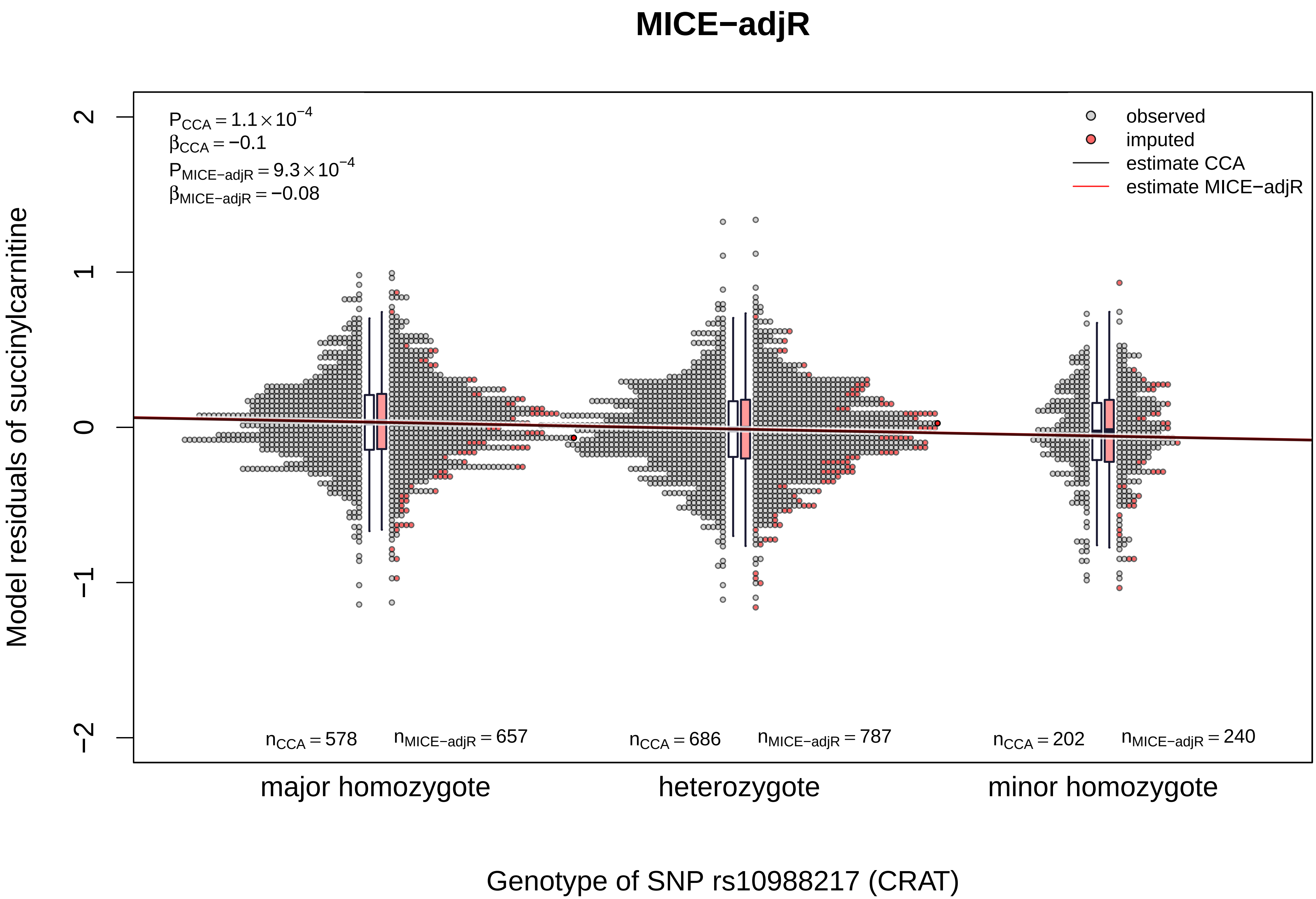
MICE−adjR
$P_{CCA} = 1.1 \times 10^{-4}$
$\beta_{CCA} = -0.1$
$P_{MICE-adjR} = 9.3 \times 10^{-4}$
$\beta_{MICE-adjR} = -0.08$
observed
imputed
estimate CCA
estimate MICE−adjR
Model residuals of succinylcarnitine
2
1
0
−1
−2
$n_{CCA} = 578$
$n_{MICE-adjR} = 657$
$n_{CCA} = 686$
$n_{MICE-adjR} = 787$
$n_{CCA} = 202$
$n_{MICE-adjR} = 240$
major homozygote
heterozygote
minor homozygote
Genotype of SNP rs10988217 (CRAT)

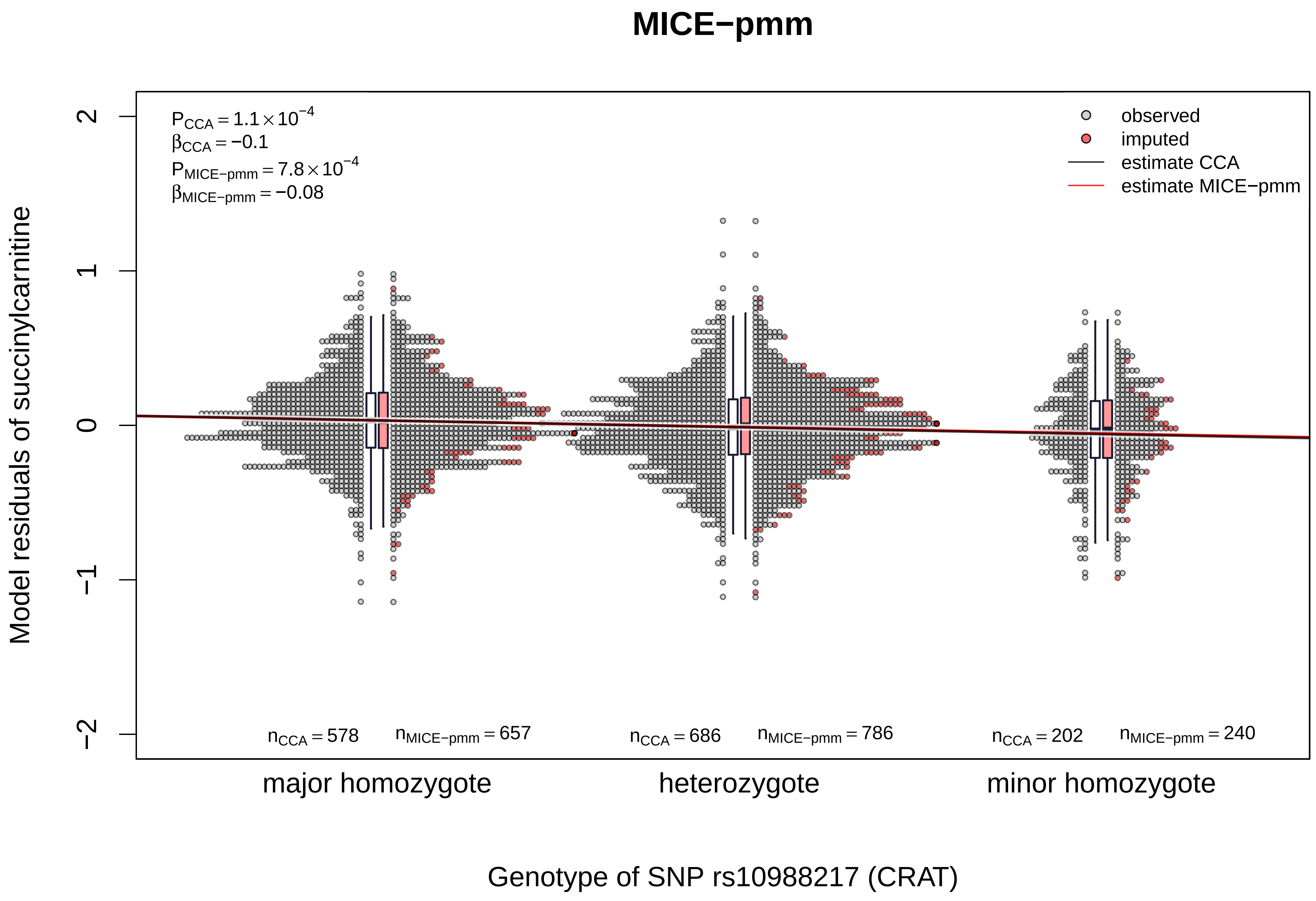

MICE−pmm
$P_{CCA} = 1.1 \times 10^{-4}$
$\beta_{CCA} = -0.1$
$P_{MICE-pmm} = 7.8 \times 10^{-4}$
$\beta_{MICE-pmm} = -0.08$
observed
imputed
estimate CCA
estimate MICE−pmm
2
1
0
−1
−2
Model residuals of succinylcarnitine
$n_{CCA} = 578$
$n_{MICE-pmm} = 657$
$n_{CCA} = 686$
$n_{MICE-pmm} = 786$
$n_{CCA} = 202$
$n_{MICE-pmm} = 240$
major homozygote
heterozygote
minor homozygote
Genotype of SNP rs10988217 (CRAT)

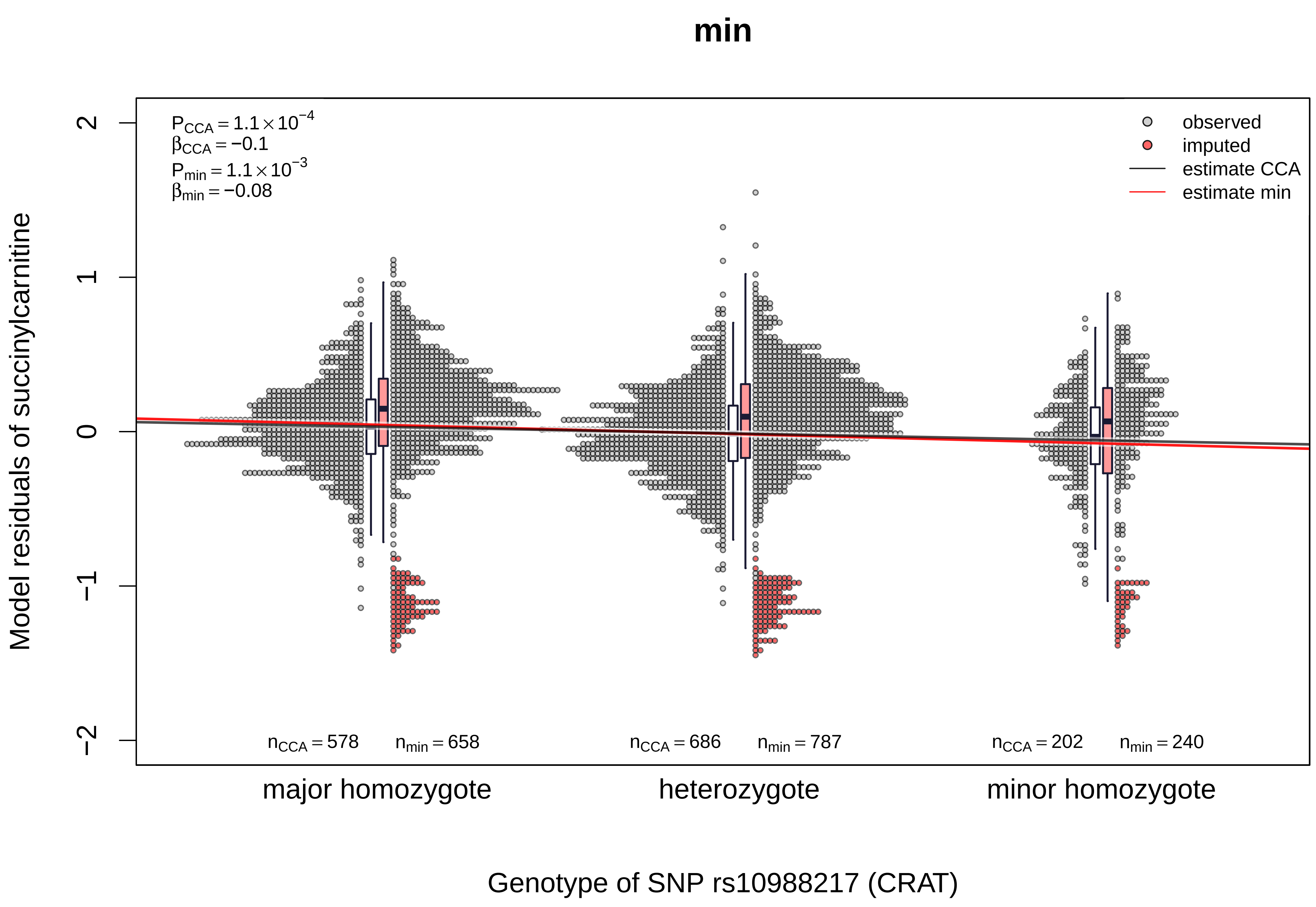

min
$P_{CCA} = 1.1 \times 10^{-4}$
$\beta_{CCA} = -0.1$
$P_{min} = 1.1 \times 10^{-3}$
$\beta_{min} = -0.08$
observed
imputed
estimate CCA
estimate min
Model residuals of succinylcarnitine
2
1
0
−1
−2
$n_{CCA} = 578$
$n_{min} = 658$
$n_{CCA} = 686$
$n_{min} = 787$
$n_{CCA} = 202$
$n_{min} = 240$
major homozygote
heterozygote
minor homozygote
Genotype of SNP rs10988217 (CRAT)

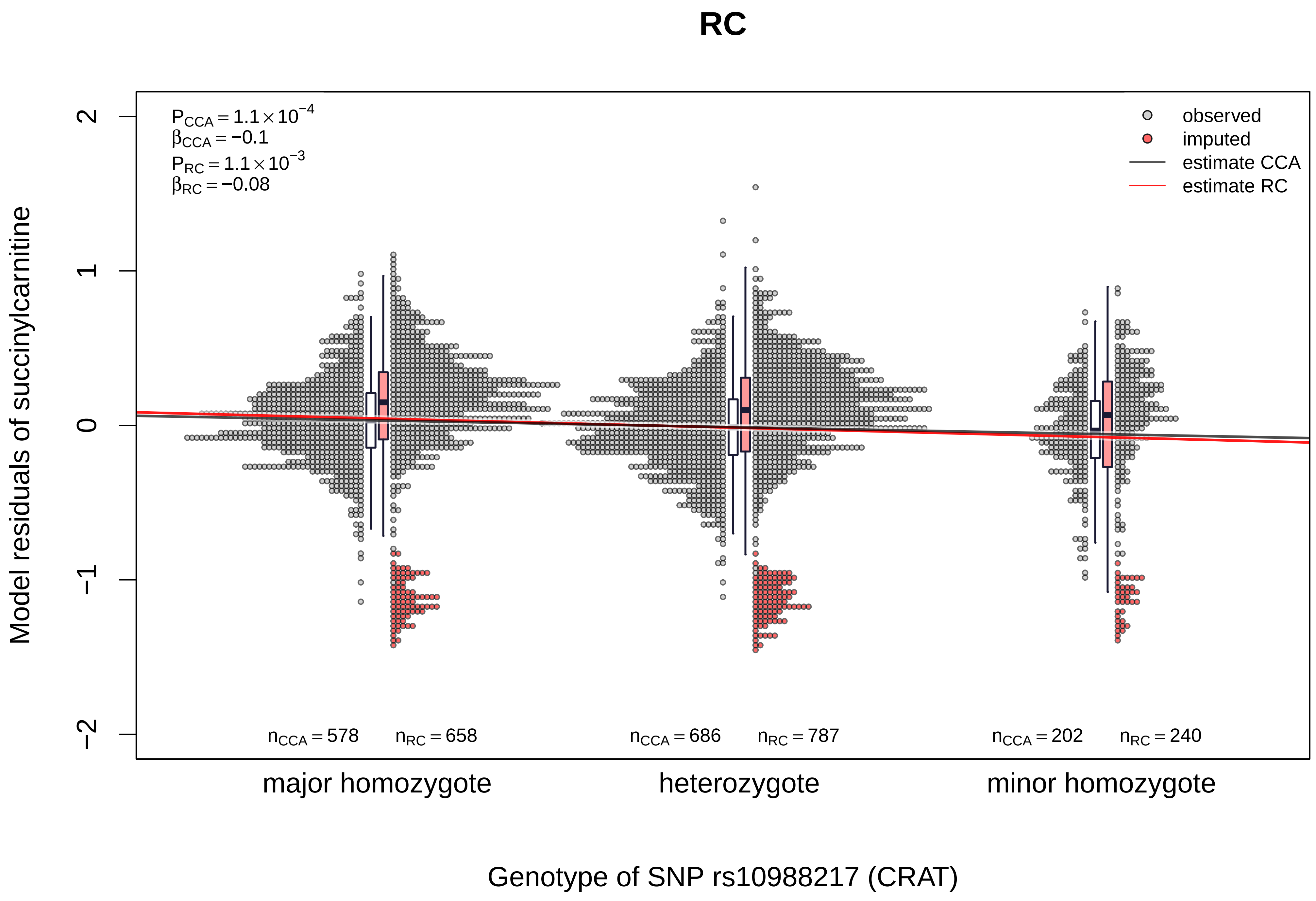
RC
$P_{CCA} = 1.1 \times 10^{-4}$
$\beta_{CCA} = -0.1$
$P_{RC} = 1.1 \times 10^{-3}$
$\beta_{RC} = -0.08$
observed
imputed
estimate CCA
estimate RC
Model residuals of succinylcarnitine
2
1
0
−1
−2
$n_{CCA} = 578$
$n_{RC} = 658$
$n_{CCA} = 686$
$n_{RC} = 787$
$n_{CCA} = 202$
$n_{RC} = 240$
major homozygote
heterozygote
minor homozygote
Genotype of SNP rs10988217 (CRAT)

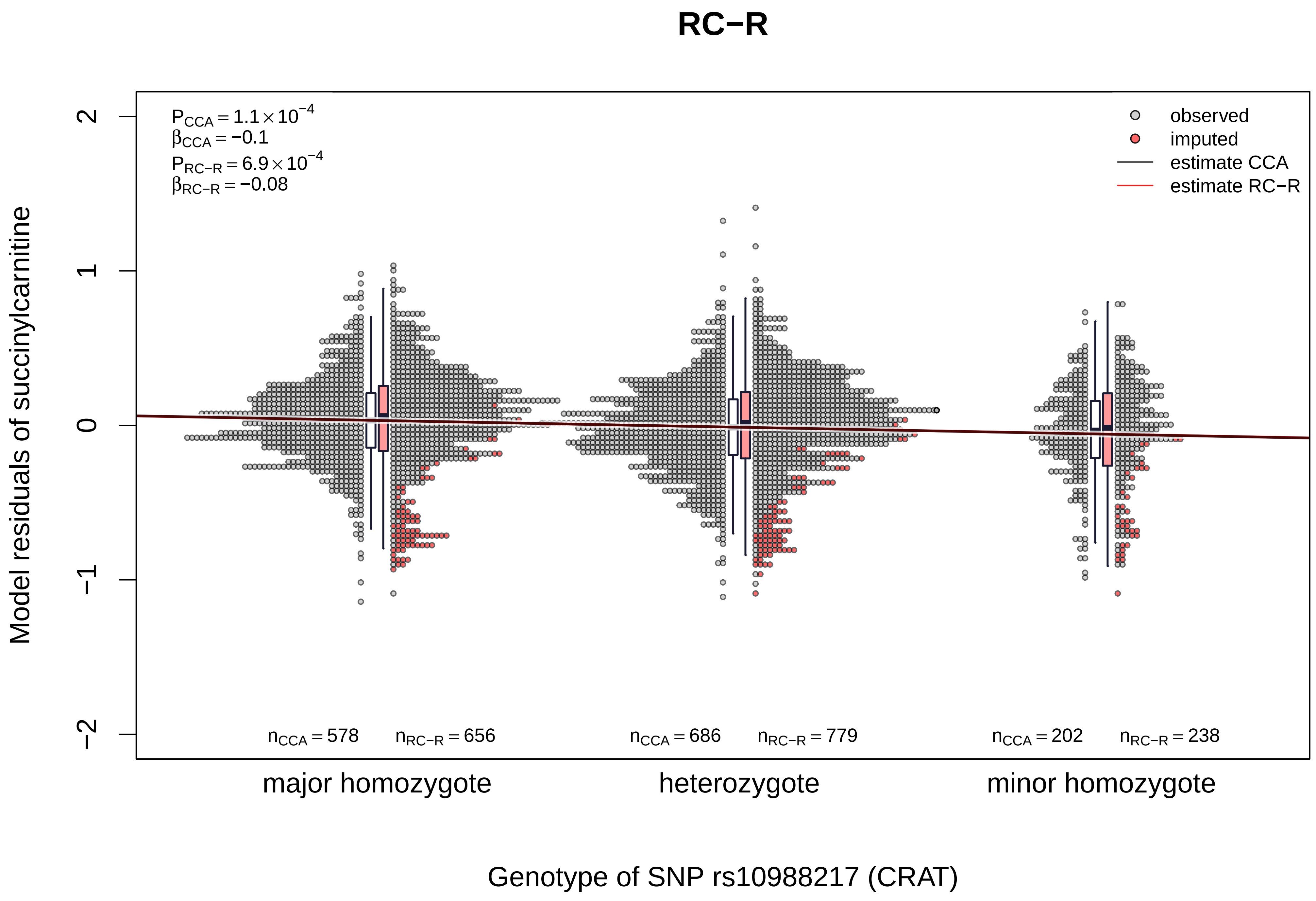

RC−R
$P_{CCA} = 1.1 \times 10^{-4}$
$\beta_{CCA} = -0.1$
$P_{RC-R} = 6.9 \times 10^{-4}$
$\beta_{RC-R} = -0.08$
observed
imputed
estimate CCA
estimate RC−R
Model residuals of succinylcarnitine
2
1
0
−1
−2
$n_{CCA} = 578$
$n_{RC-R} = 656$
$n_{CCA} = 686$
$n_{RC-R} = 779$
$n_{CCA} = 202$
$n_{RC-R} = 238$
major homozygote
heterozygote
minor homozygote
Genotype of SNP rs10988217 (CRAT)

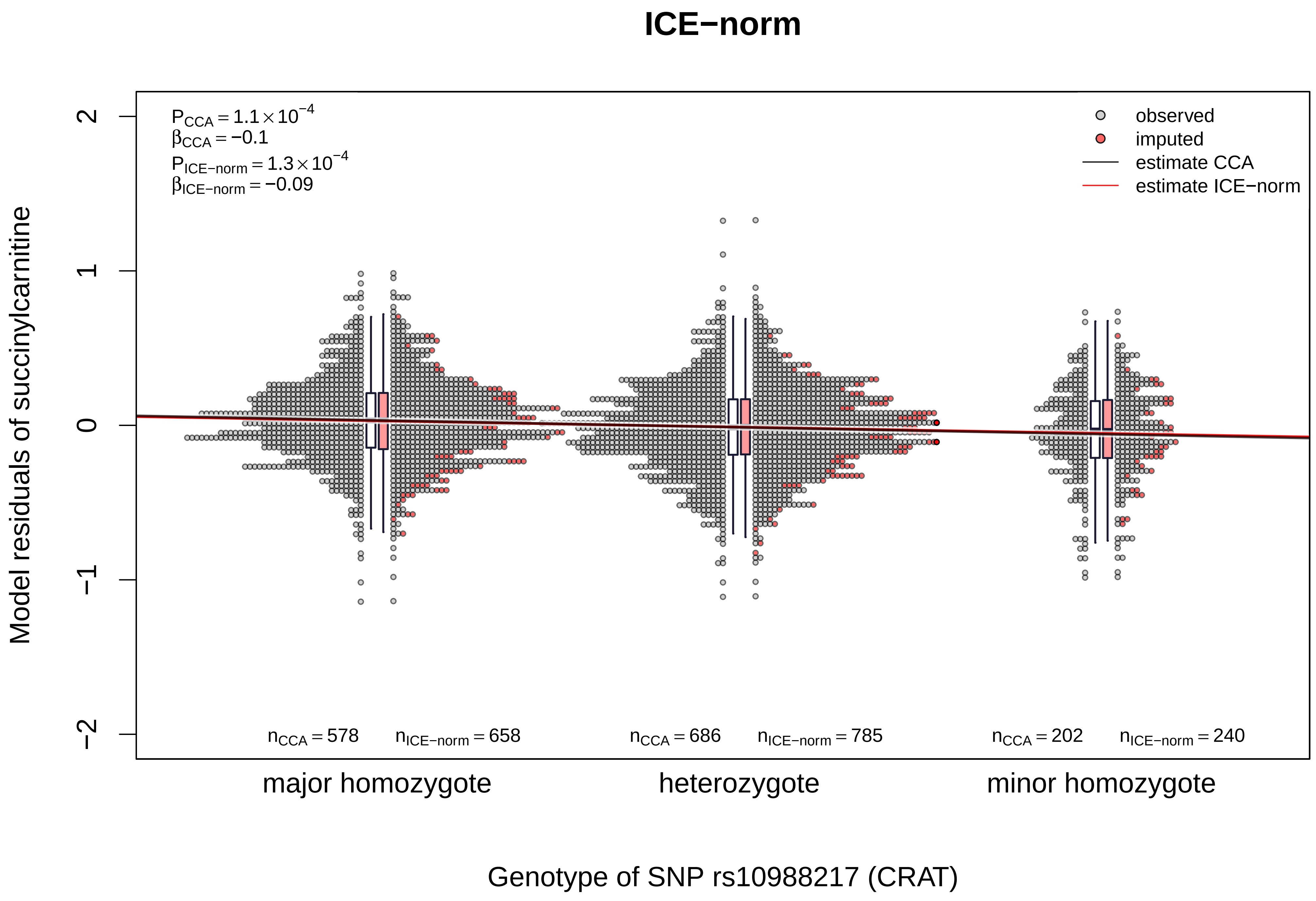

ICE−norm
$P_{CCA} = 1.1 \times 10^{-4}$
$\beta_{CCA} = -0.1$
$P_{ICE-norm} = 1.3 \times 10^{-4}$
$\beta_{ICE-norm} = -0.09$
observed
imputed
estimate CCA
estimate ICE−norm
Model residuals of succinylcarnitine
2
1
0
−1
−2
$n_{CCA} = 578$
$n_{ICE-norm} = 658$
$n_{CCA} = 686$
$n_{ICE-norm} = 785$
$n_{CCA} = 202$
$n_{ICE-norm} = 240$
major homozygote
heterozygote
minor homozygote
Genotype of SNP rs10988217 (CRAT)

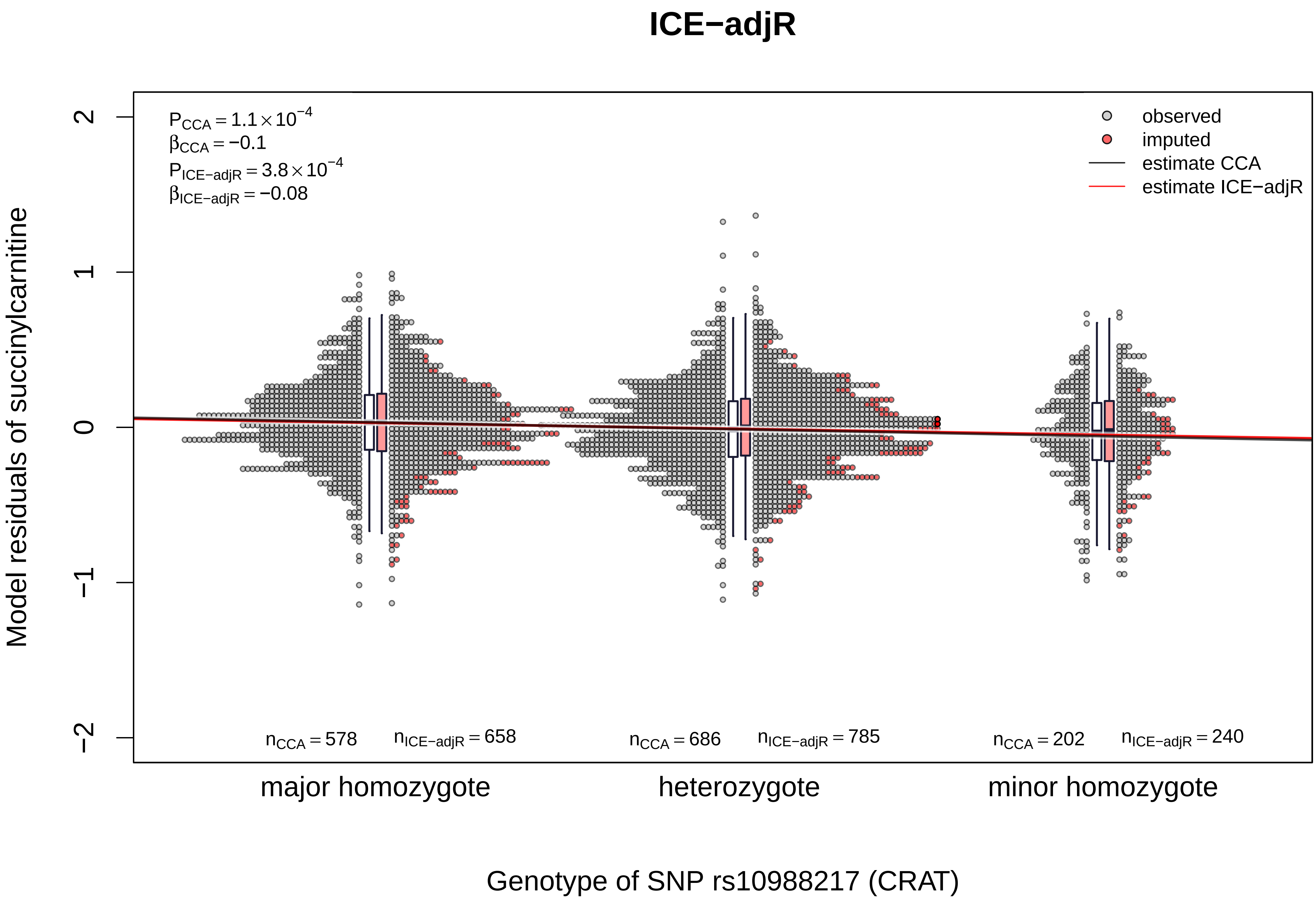
ICE−adjR
$P_{CCA} = 1.1 \times 10^{-4}$
$\beta_{CCA} = -0.1$
$P_{ICE-adjR} = 3.8 \times 10^{-4}$
$\beta_{ICE-adjR} = -0.08$
observed
imputed
estimate CCA
estimate ICE−adjR
Model residuals of succinylcarnitine
2
1
0
−1
−2
$n_{CCA} = 578$
$n_{ICE-adjR} = 658$
$n_{CCA} = 686$
$n_{ICE-adjR} = 785$
$n_{CCA} = 202$
$n_{ICE-adjR} = 240$
major homozygote
heterozygote
minor homozygote
Genotype of SNP rs10988217 (CRAT)

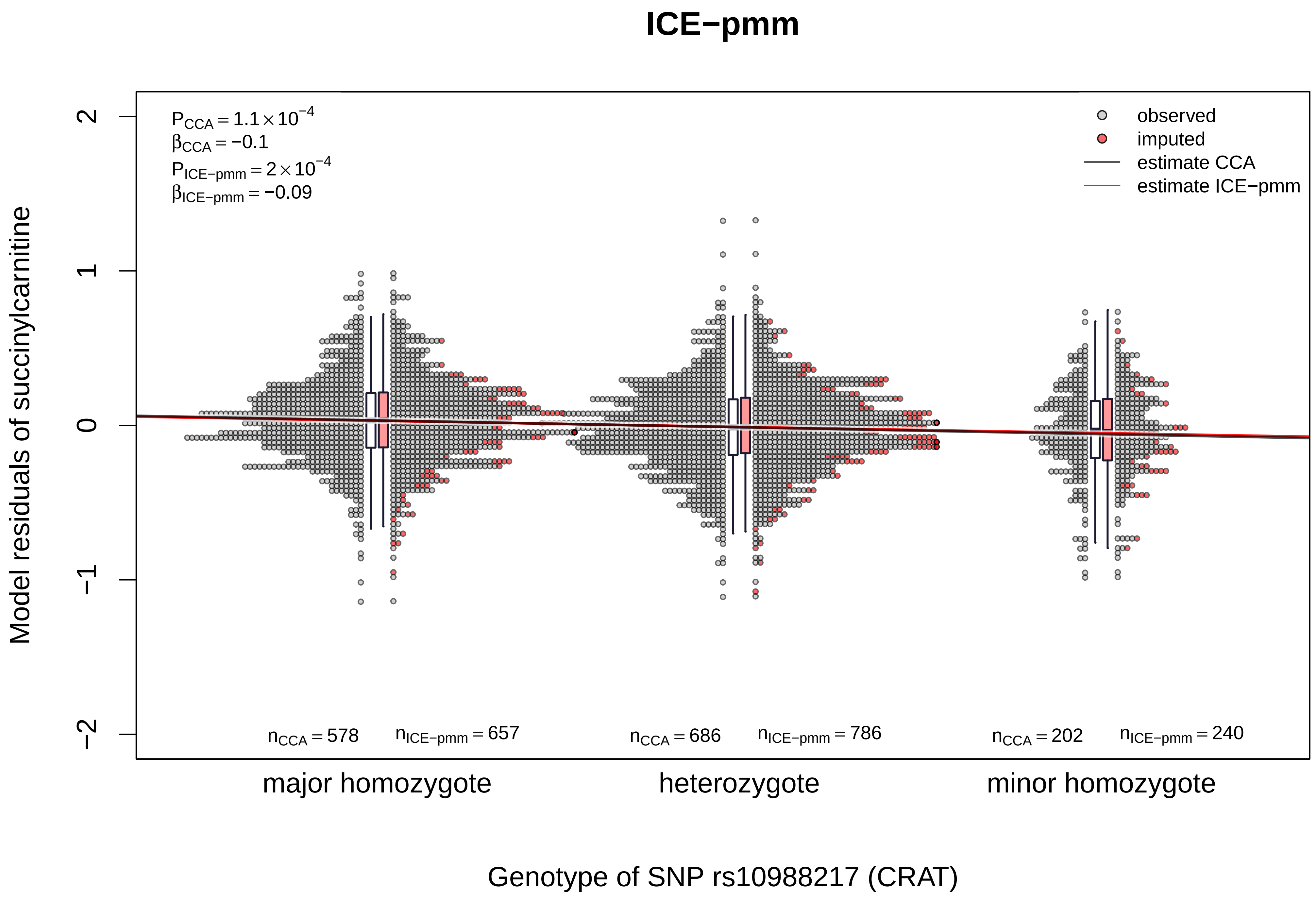
ICE−pmm
$P_{CCA} = 1.1 \times 10^{-4}$
$\beta_{CCA} = -0.1$
$P_{ICE-pmm} = 2 \times 10^{-4}$
$\beta_{ICE-pmm} = -0.09$
observed
imputed
estimate CCA
estimate ICE−pmm
Model residuals of succinylcarnitine
2
1
0
−1
−2
$n_{CCA} = 578$
$n_{ICE-pmm} = 657$
$n_{CCA} = 686$
$n_{ICE-pmm} = 786$
$n_{CCA} = 202$
$n_{ICE-pmm} = 240$
major homozygote
heterozygote
minor homozygote
Genotype of SNP rs10988217 (CRAT)

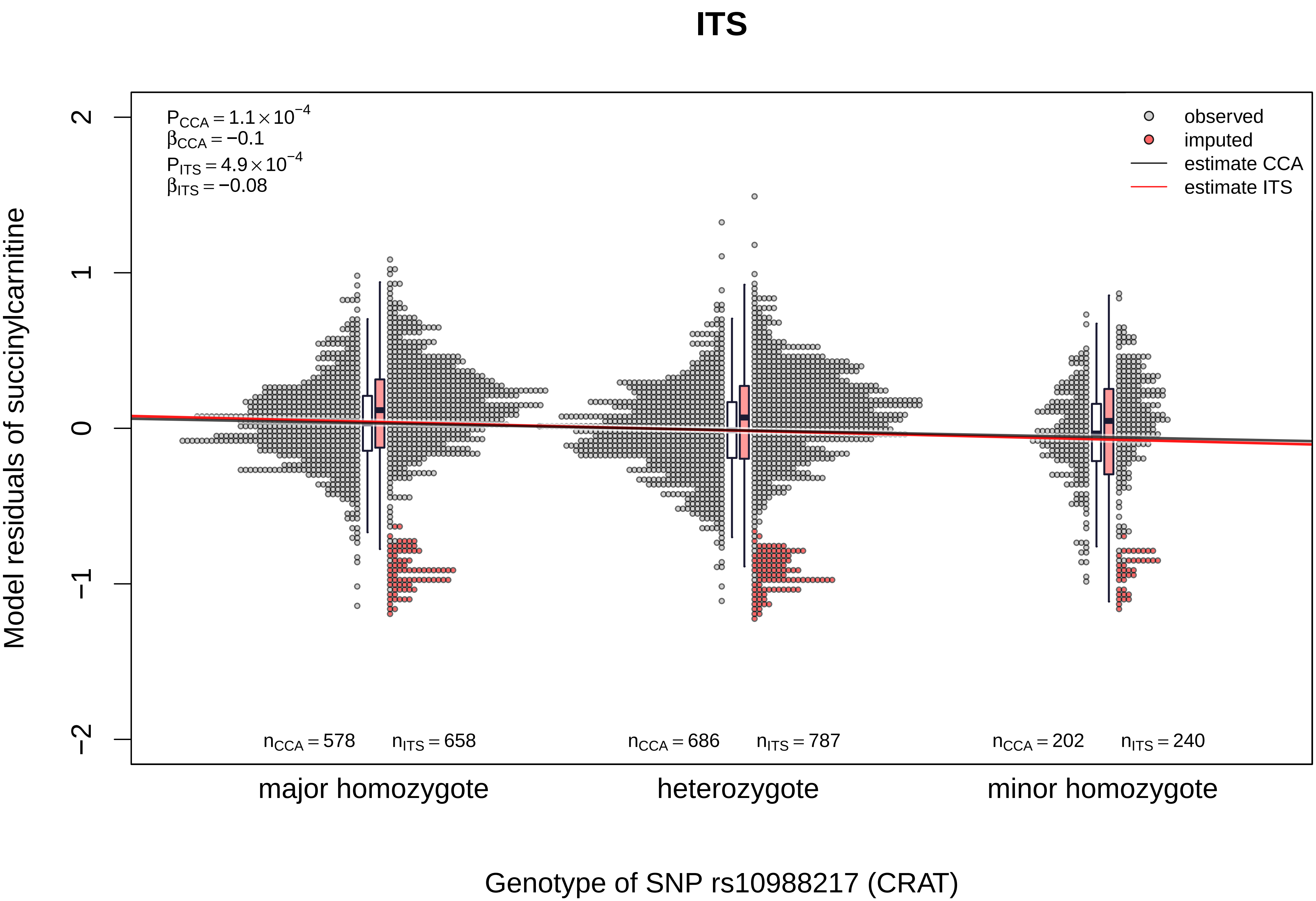

ITS
$P_{CCA} = 1.1 \times 10^{-4}$
$\beta_{CCA} = -0.1$
$P_{ITS} = 4.9 \times 10^{-4}$
$\beta_{ITS} = -0.08$
observed
imputed
estimate CCA
estimate ITS
2
1
0
−1
−2
Model residuals of succinylcarnitine
$n_{CCA} = 578$
$n_{ITS} = 658$
$n_{CCA} = 686$
$n_{ITS} = 787$
$n_{CCA} = 202$
$n_{ITS} = 240$
major homozygote
heterozygote
minor homozygote
Genotype of SNP rs10988217 (CRAT)

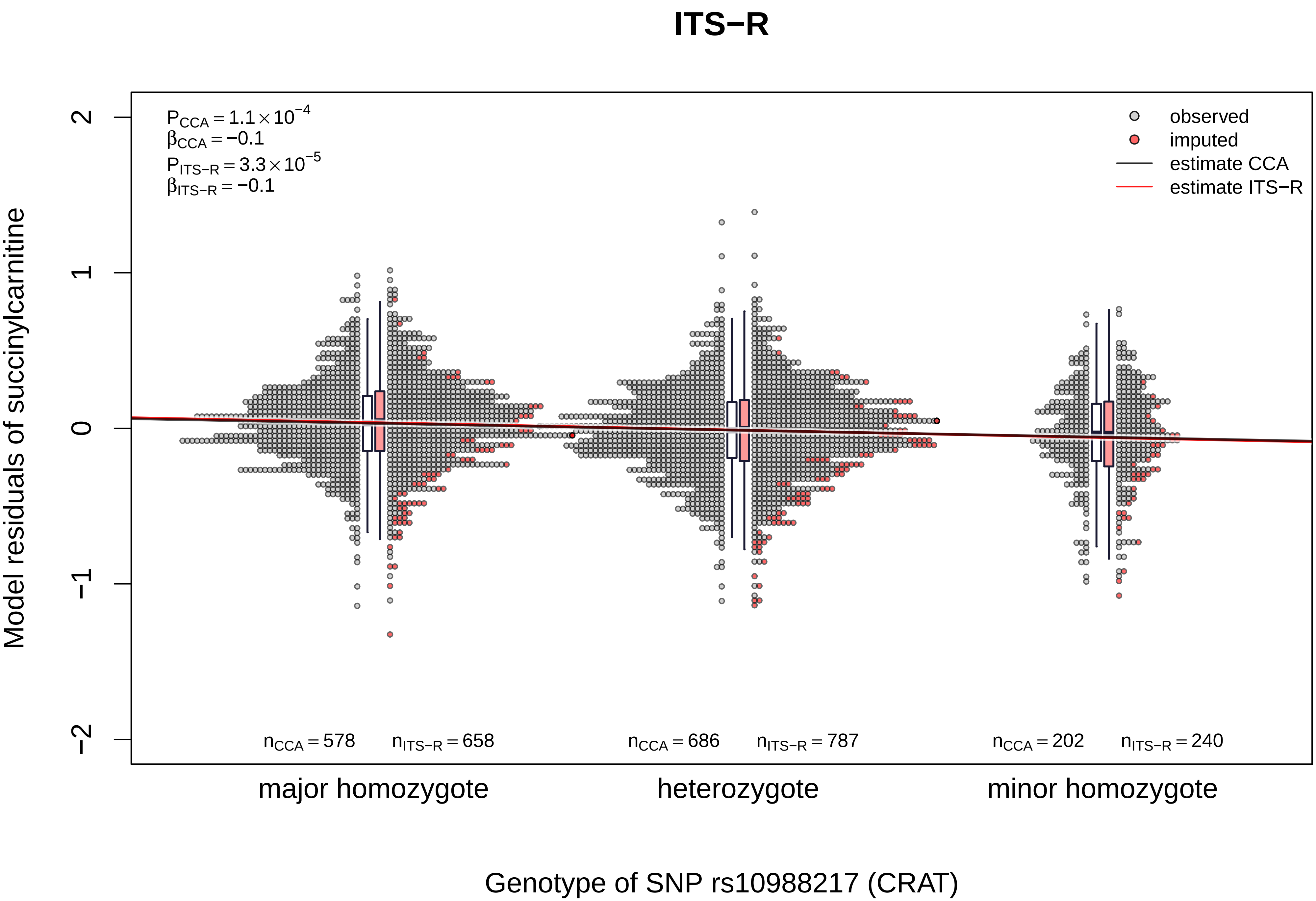
ITS−R
$P_{CCA} = 1.1 \times 10^{-4}$
$\beta_{CCA} = -0.1$
$P_{ITS-R} = 3.3 \times 10^{-5}$
$\beta_{ITS-R} = -0.1$
observed
imputed
estimate CCA
estimate ITS−R
Model residuals of succinylcarnitine
2
1
0
−1
−2
$n_{CCA} = 578$
$n_{ITS-R} = 658$
$n_{CCA} = 686$
$n_{ITS-R} = 787$
$n_{CCA} = 202$
$n_{ITS-R} = 240$
major homozygote
heterozygote
minor homozygote
Genotype of SNP rs10988217 (CRAT)

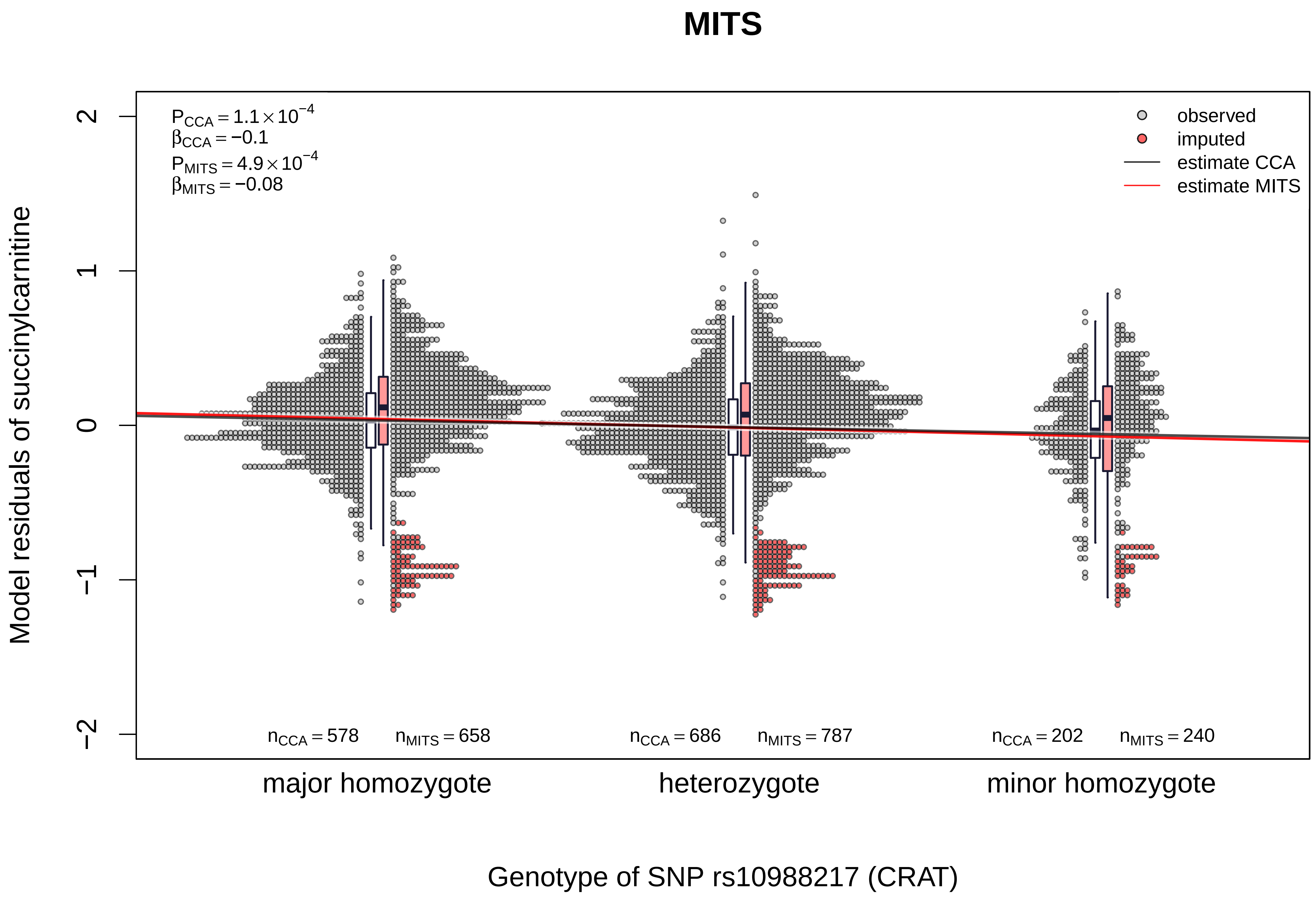

MITS
$P_{CCA} = 1.1 \times 10^{-4}$
$\beta_{CCA} = -0.1$
$P_{MITS} = 4.9 \times 10^{-4}$
$\beta_{MITS} = -0.08$
observed
imputed
estimate CCA
estimate MITS
Model residuals of succinylcarnitine
2
1
0
−1
−2
$n_{CCA} = 578$
$n_{MITS} = 658$
$n_{CCA} = 686$
$n_{MITS} = 787$
$n_{CCA} = 202$
$n_{MITS} = 240$
major homozygote
heterozygote
minor homozygote
Genotype of SNP rs10988217 (CRAT)

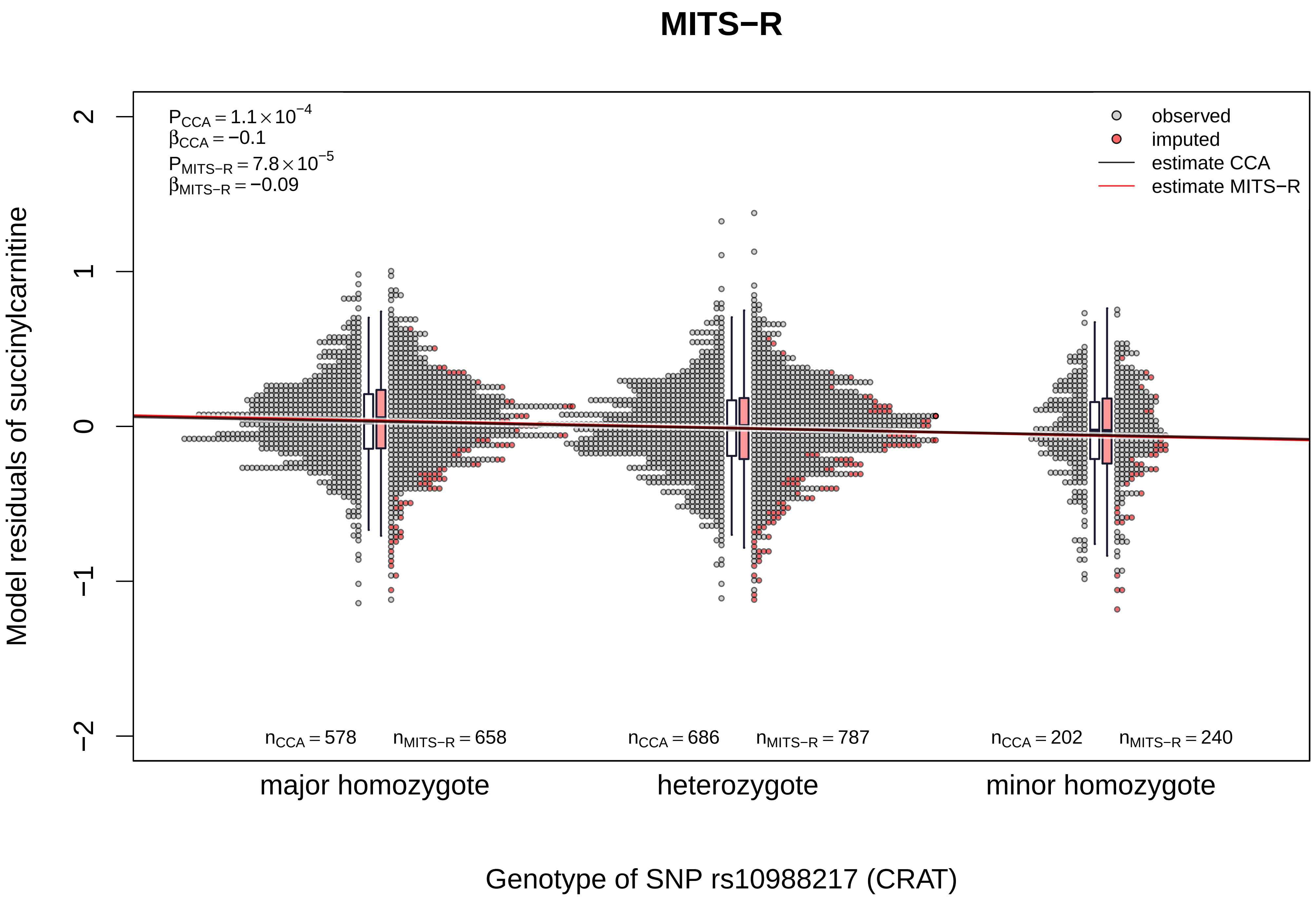

MITS−R
$P_{CCA} = 1.1 \times 10^{-4}$
$\beta_{CCA} = -0.1$
$P_{MITS-R} = 7.8 \times 10^{-5}$
$\beta_{MITS-R} = -0.09$
observed
imputed
estimate CCA
estimate MITS−R
Model residuals of succinylcarnitine
2
1
0
−1
−2
$n_{CCA} = 578$
$n_{MITS-R} = 658$
$n_{CCA} = 686$
$n_{MITS-R} = 787$
$n_{CCA} = 202$
$n_{MITS-R} = 240$
major homozygote
heterozygote
minor homozygote
Genotype of SNP rs10988217 (CRAT)

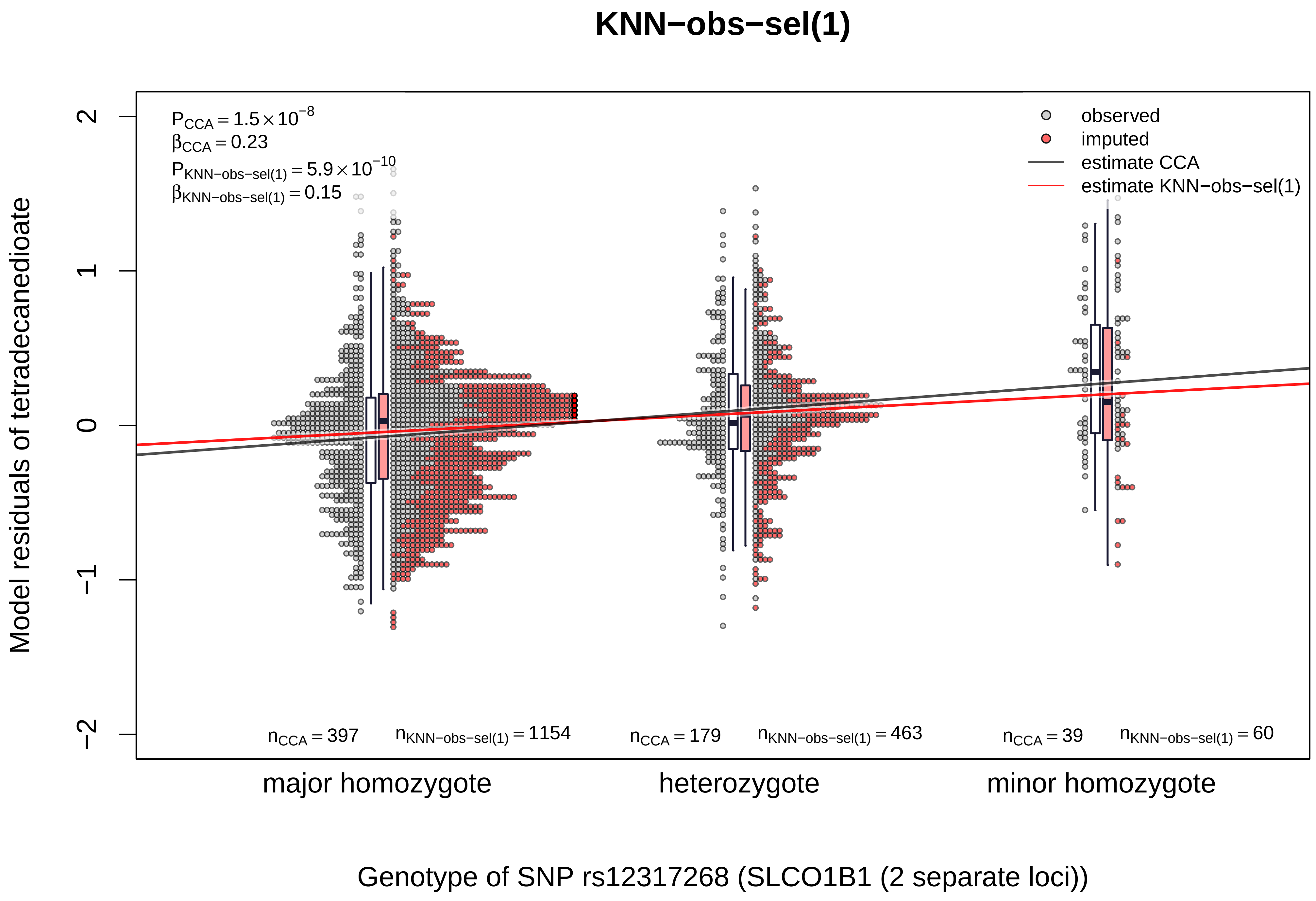
KNN−obs−sel(1)
$P_{CCA} = 1.5 \times 10^{-8}$
$\beta_{CCA} = 0.23$
$P_{KNN-obs-sel(1)} = 5.9 \times 10^{-10}$
$\beta_{KNN-obs-sel(1)} = 0.15$
observed
imputed
estimate CCA
estimate KNN−obs−sel(1)
Model residuals of tetradecanedioate
2
1
0
−1
−2
$n_{CCA} = 397$
$n_{KNN-obs-sel(1)} = 1154$
$n_{CCA} = 179$
$n_{KNN-obs-sel(1)} = 463$
$n_{CCA} = 39$
$n_{KNN-obs-sel(1)} = 60$
major homozygote
heterozygote
minor homozygote
Genotype of SNP rs12317268 (SLCO1B1 (2 separate loci))

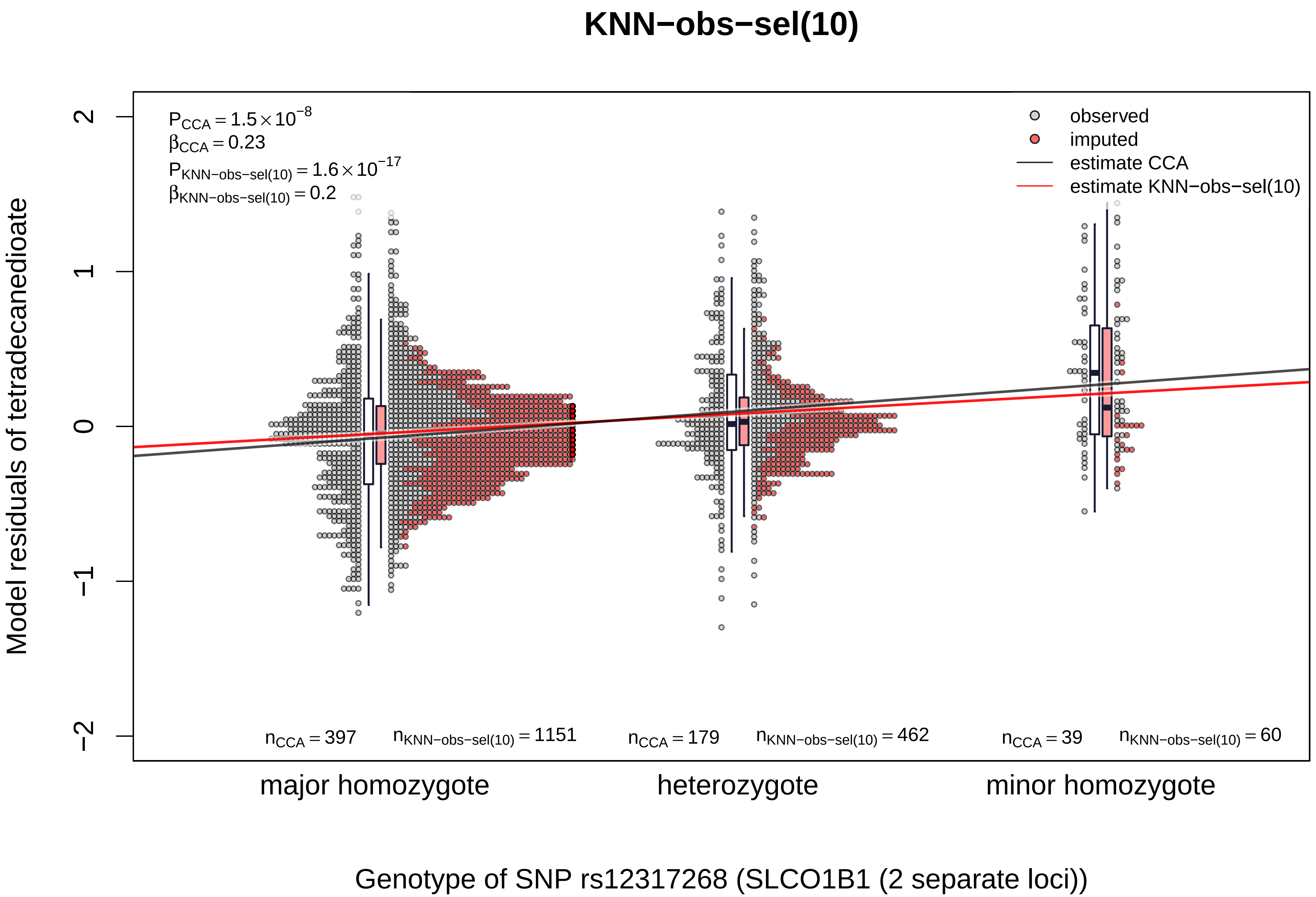
KNN−obs−sel(10)
$P_{CCA} = 1.5 \times 10^{-8}$
$\beta_{CCA} = 0.23$
$P_{KNN-obs-sel(10)} = 1.6 \times 10^{-17}$
$\beta_{KNN-obs-sel(10)} = 0.2$
observed
imputed
estimate CCA
estimate KNN−obs−sel(10)
Model residuals of tetradecanedioate
2
1
0
−1
−2
$n_{CCA} = 397$
$n_{KNN-obs-sel(10)} = 1151$
$n_{CCA} = 179$
$n_{KNN-obs-sel(10)} = 462$
$n_{CCA} = 39$
$n_{KNN-obs-sel(10)} = 60$
major homozygote
heterozygote
minor homozygote
Genotype of SNP rs12317268 (SLCO1B1 (2 separate loci))

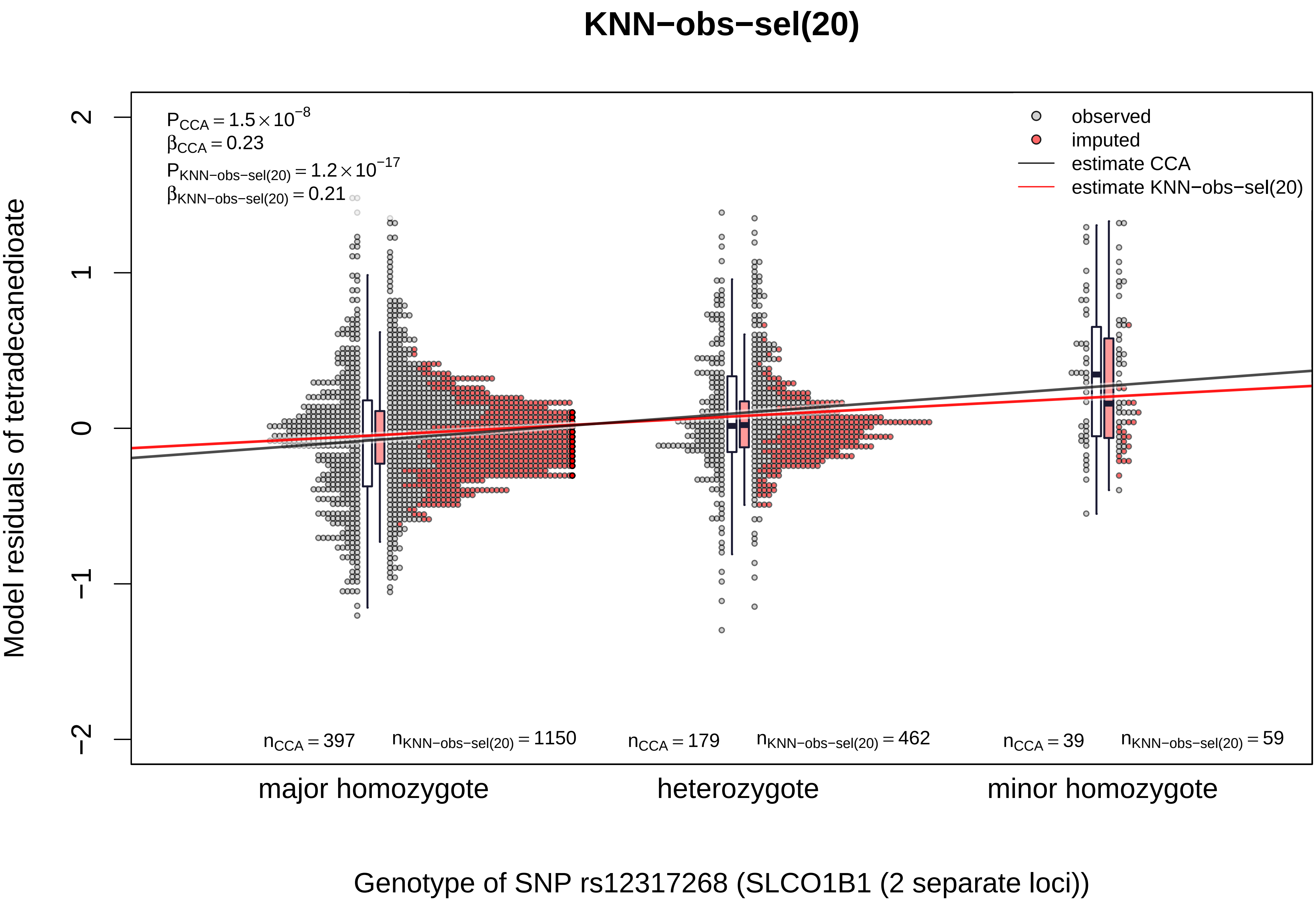

KNN−obs−sel(20)
$P_{CCA} = 1.5 \times 10^{-8}$
$\beta_{CCA} = 0.23$
$P_{KNN-obs-sel(20)} = 1.2 \times 10^{-17}$
$\beta_{KNN-obs-sel(20)} = 0.21$
observed
imputed
estimate CCA
estimate KNN−obs−sel(20)
Model residuals of tetradecanedioate
2
1
0
−1
−2
$n_{CCA} = 397$
$n_{KNN-obs-sel(20)} = 1150$
$n_{CCA} = 179$
$n_{KNN-obs-sel(20)} = 462$
$n_{CCA} = 39$
$n_{KNN-obs-sel(20)} = 59$
major homozygote
heterozygote
minor homozygote
Genotype of SNP rs12317268 (SLCO1B1 (2 separate loci))

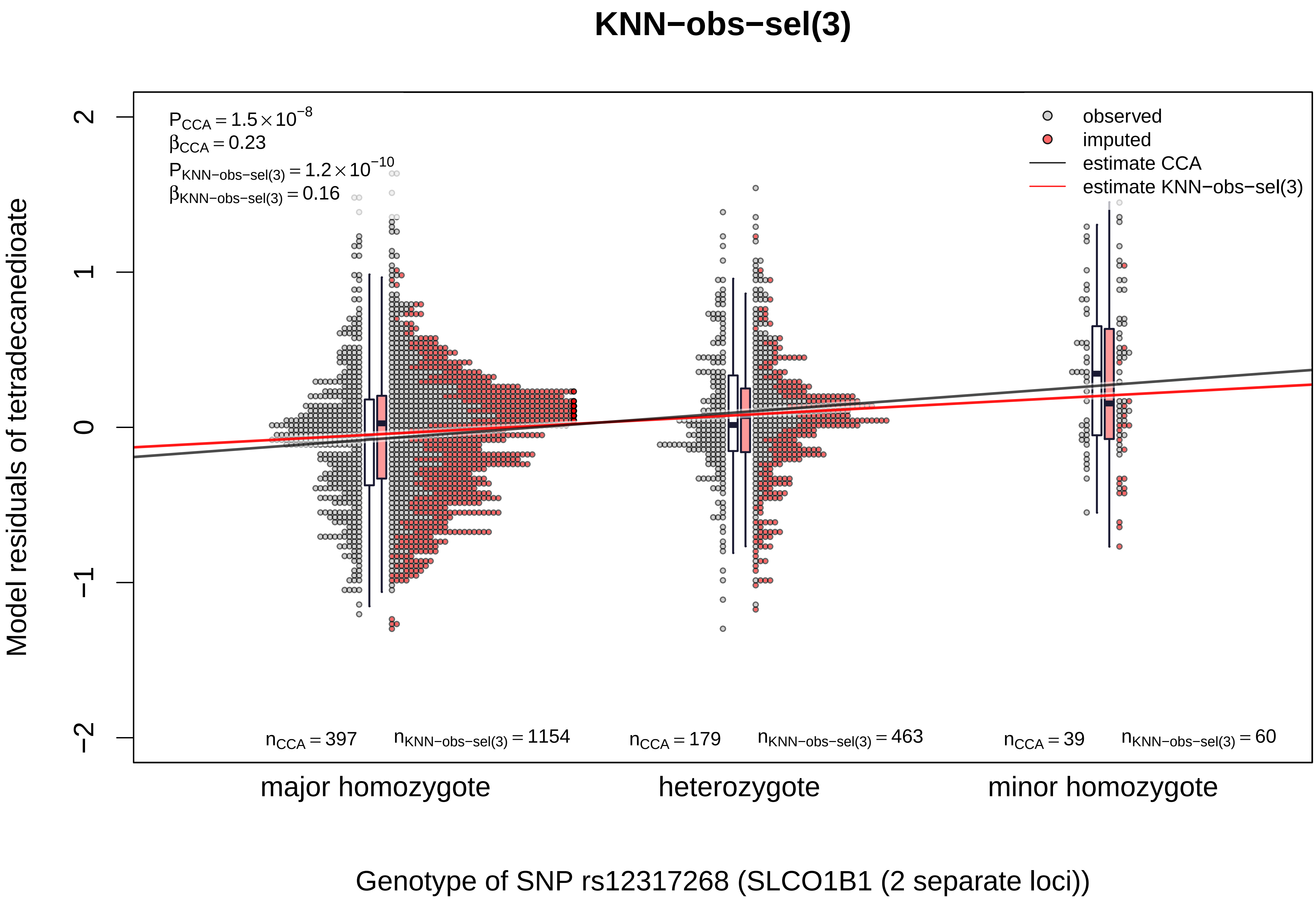

KNN−obs−sel(3)
$P_{CCA} = 1.5 \times 10^{-8}$
$\beta_{CCA} = 0.23$
$P_{KNN-obs-sel(3)} = 1.2 \times 10^{-10}$
$\beta_{KNN-obs-sel(3)} = 0.16$
observed
imputed
estimate CCA
estimate KNN−obs−sel(3)
Model residuals of tetradecanedioate
2
1
0
−1
−2
$n_{CCA} = 397$
$n_{KNN-obs-sel(3)} = 1154$
$n_{CCA} = 179$
$n_{KNN-obs-sel(3)} = 463$
$n_{CCA} = 39$
$n_{KNN-obs-sel(3)} = 60$
major homozygote
heterozygote
minor homozygote
Genotype of SNP rs12317268 (SLCO1B1 (2 separate loci))

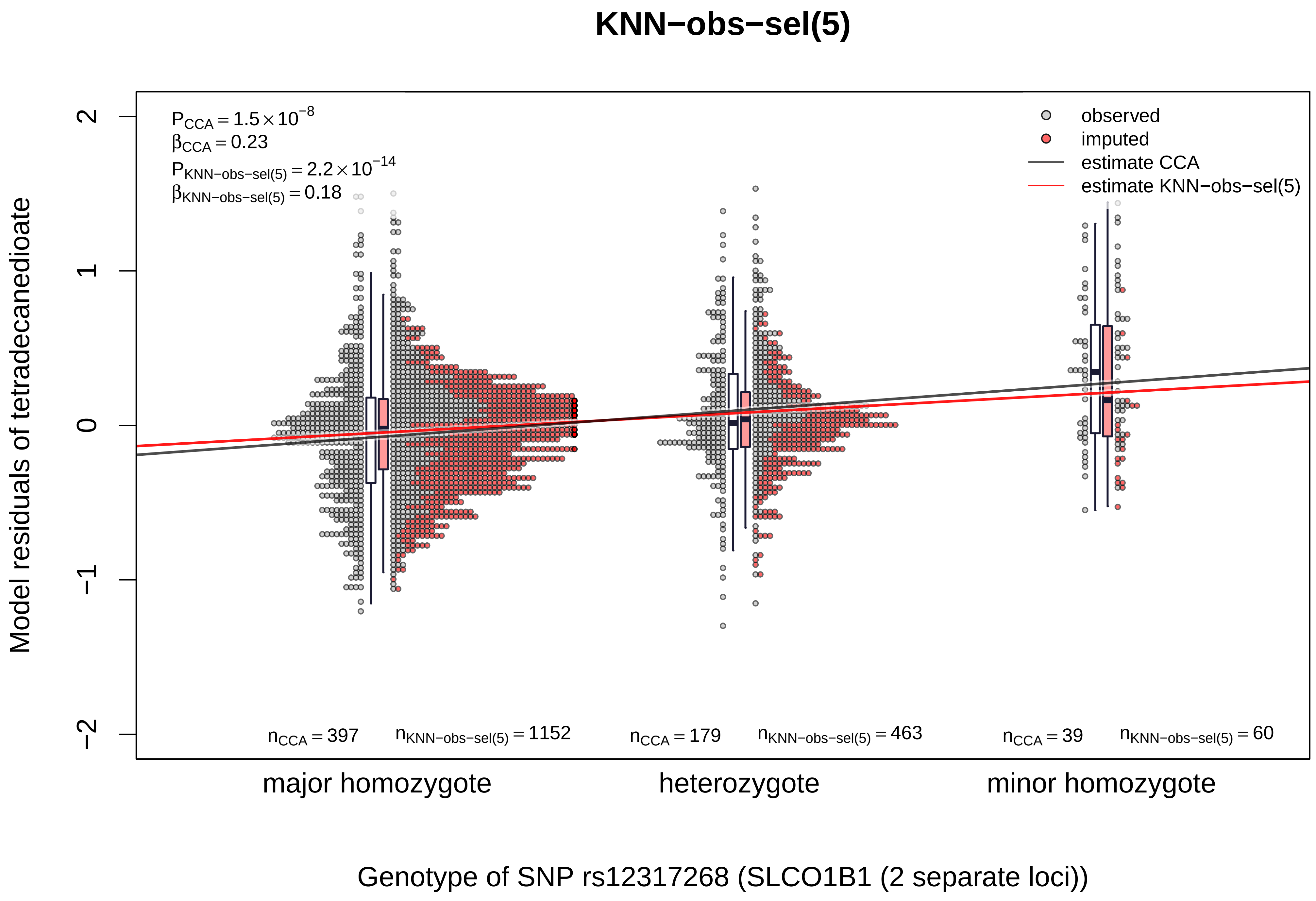

KNN−obs−sel(5)
$P_{CCA} = 1.5 \times 10^{-8}$
$\beta_{CCA} = 0.23$
$P_{KNN-obs-sel(5)} = 2.2 \times 10^{-14}$
$\beta_{KNN-obs-sel(5)} = 0.18$
observed
imputed
estimate CCA
estimate KNN−obs−sel(5)
Model residuals of tetradecanedioate
2
1
0
−1
−2
$n_{CCA} = 397$
$n_{KNN-obs-sel(5)} = 1152$
$n_{CCA} = 179$
$n_{KNN-obs-sel(5)} = 463$
$n_{CCA} = 39$
$n_{KNN-obs-sel(5)} = 60$
major homozygote
heterozygote
minor homozygote
Genotype of SNP rs12317268 (SLCO1B1 (2 separate loci))

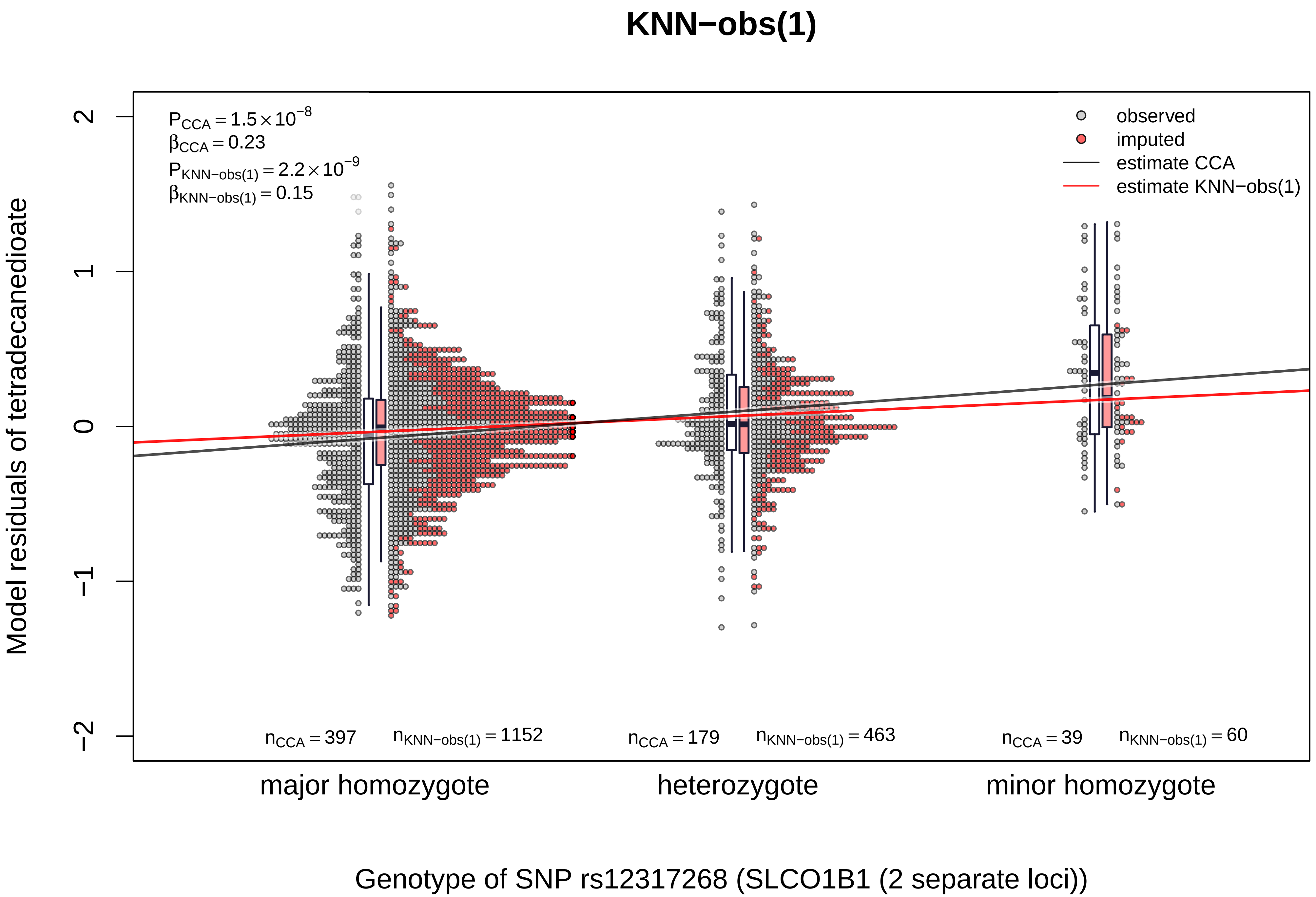

KNN−obs(1)
$P_{CCA} = 1.5 \times 10^{-8}$
$\beta_{CCA} = 0.23$
$P_{KNN-obs(1)} = 2.2 \times 10^{-9}$
$\beta_{KNN-obs(1)} = 0.15$
observed
imputed
estimate CCA
estimate KNN−obs(1)
Model residuals of tetradecanedioate
2
1
0
−1
−2
$n_{CCA} = 397$
$n_{KNN-obs(1)} = 1152$
$n_{CCA} = 179$
$n_{KNN-obs(1)} = 463$
$n_{CCA} = 39$
$n_{KNN-obs(1)} = 60$
major homozygote
heterozygote
minor homozygote
Genotype of SNP rs12317268 (SLCO1B1 (2 separate loci))

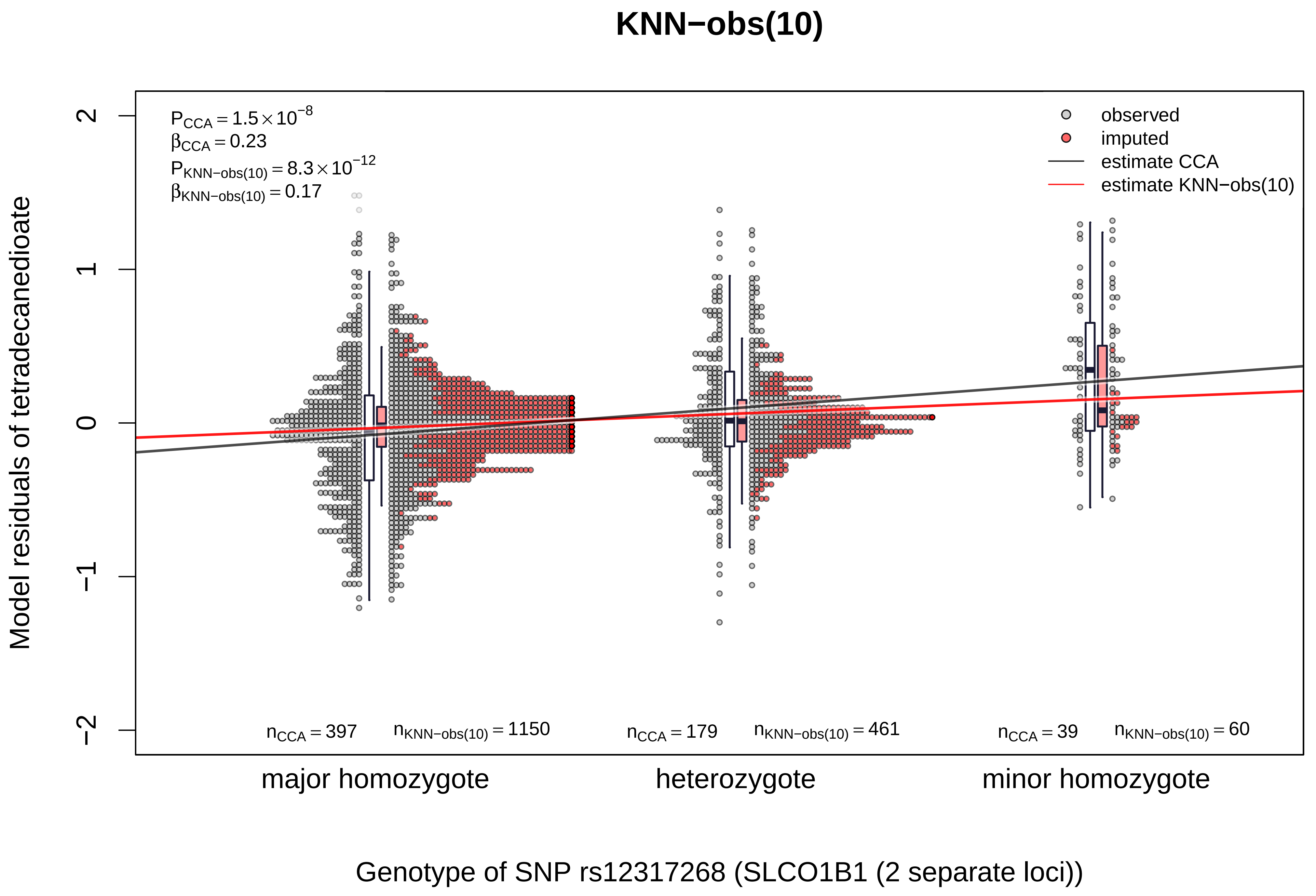
KNN−obs(10)
$P_{CCA} = 1.5 \times 10^{-8}$
$\beta_{CCA} = 0.23$
$P_{KNN-obs(10)} = 8.3 \times 10^{-12}$
$\beta_{KNN-obs(10)} = 0.17$
observed
imputed
estimate CCA
estimate KNN−obs(10)
Model residuals of tetradecanedioate
2
1
0
−1
−2
$n_{CCA} = 397$
$n_{KNN-obs(10)} = 1150$
$n_{CCA} = 179$
$n_{KNN-obs(10)} = 461$
$n_{CCA} = 39$
$n_{KNN-obs(10)} = 60$
major homozygote
heterozygote
minor homozygote
Genotype of SNP rs12317268 (SLCO1B1 (2 separate loci))

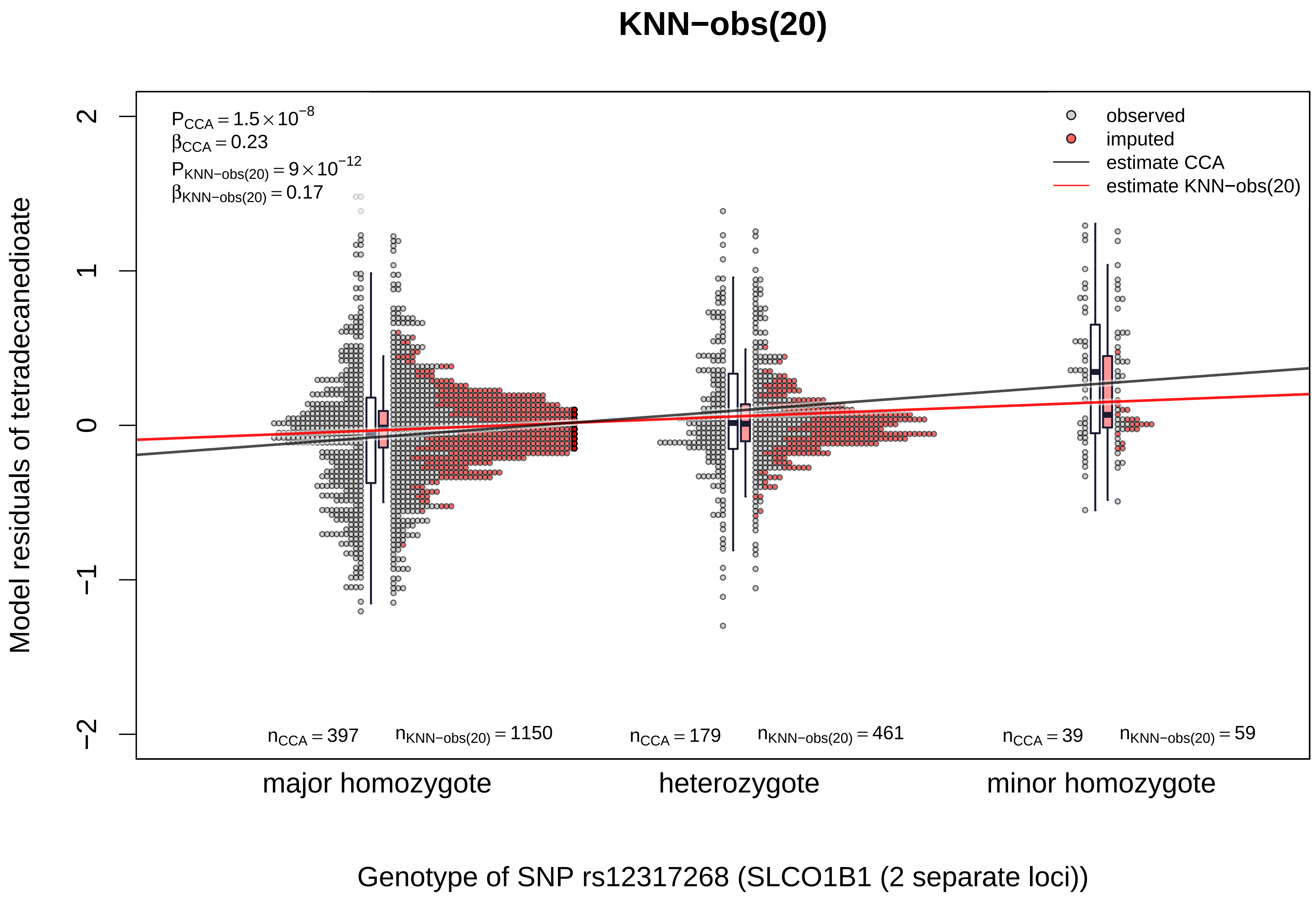

KNN−obs(20)
$P_{CCA} = 1.5 \times 10^{-8}$
$\beta_{CCA} = 0.23$
$P_{KNN-obs(20)} = 9 \times 10^{-12}$
$\beta_{KNN-obs(20)} = 0.17$
observed
imputed
estimate CCA
estimate KNN−obs(20)
Model residuals of tetradecanedioate
2
1
0
−1
−2
$n_{CCA} = 397$
$n_{KNN-obs(20)} = 1150$
$n_{CCA} = 179$
$n_{KNN-obs(20)} = 461$
$n_{CCA} = 39$
$n_{KNN-obs(20)} = 59$
major homozygote
heterozygote
minor homozygote
Genotype of SNP rs12317268 (SLCO1B1 (2 separate loci))

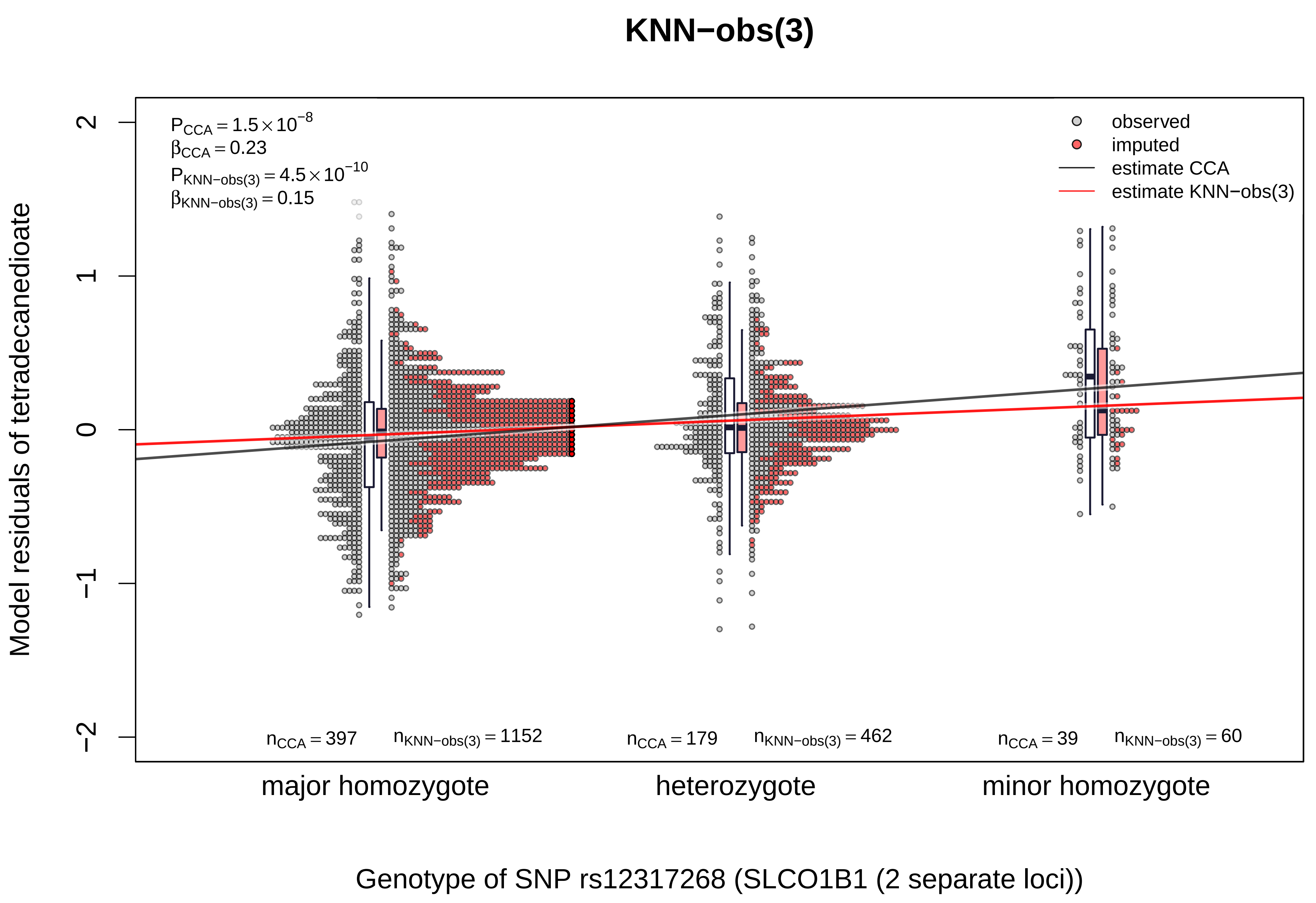

KNN−obs(3)
$P_{CCA} = 1.5 \times 10^{-8}$
$\beta_{CCA} = 0.23$
$P_{KNN-obs(3)} = 4.5 \times 10^{-10}$
$\beta_{KNN-obs(3)} = 0.15$
observed
imputed
estimate CCA
estimate KNN−obs(3)
Model residuals of tetradecanedioate
2
1
0
−1
−2
$n_{CCA} = 397$
$n_{KNN-obs(3)} = 1152$
$n_{CCA} = 179$
$n_{KNN-obs(3)} = 462$
$n_{CCA} = 39$
$n_{KNN-obs(3)} = 60$
major homozygote
heterozygote
minor homozygote
Genotype of SNP rs12317268 (SLCO1B1 (2 separate loci))

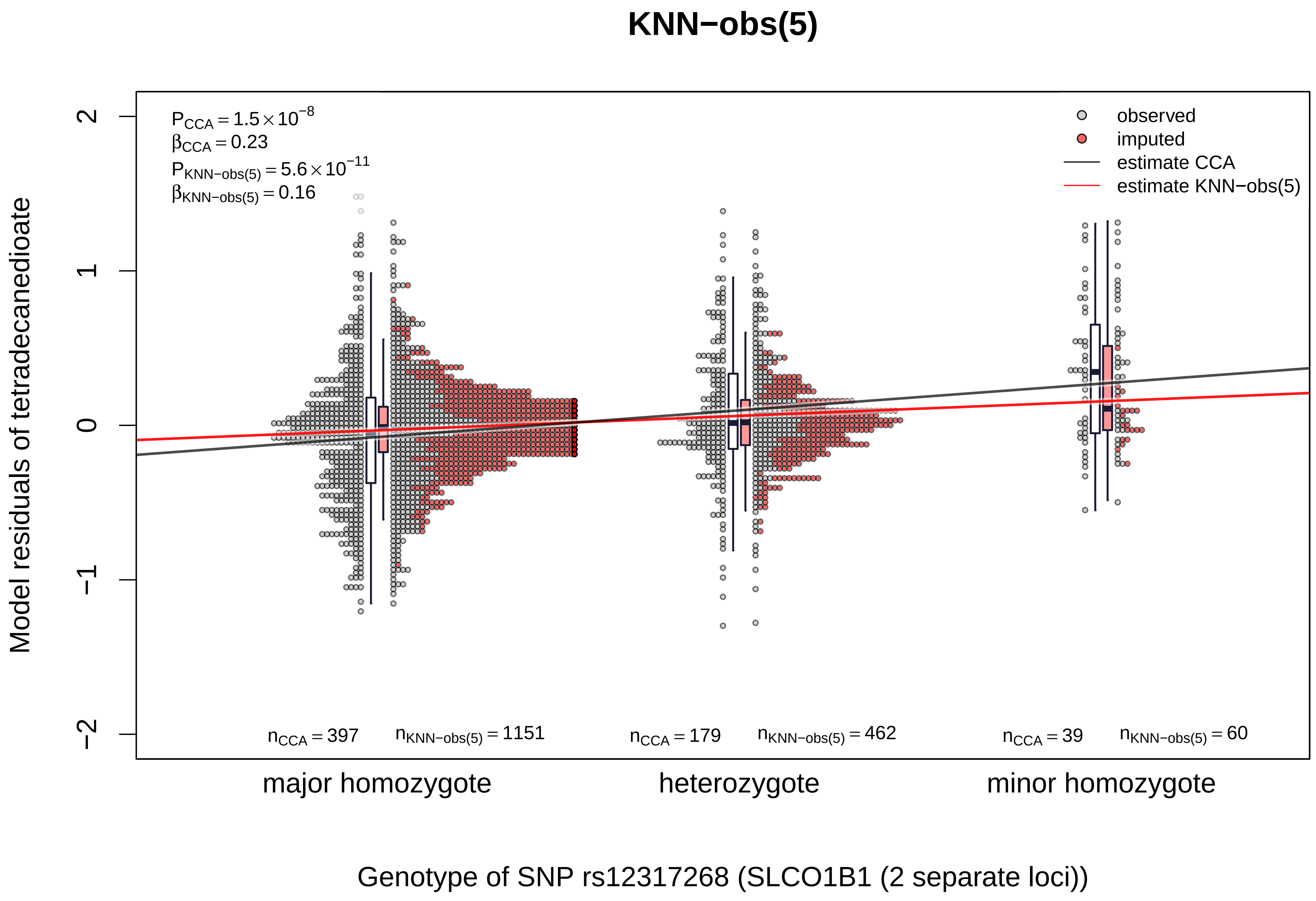
KNN−obs(5)
$P_{CCA} = 1.5 \times 10^{-8}$
$\beta_{CCA} = 0.23$
$P_{KNN-obs(5)} = 5.6 \times 10^{-11}$
$\beta_{KNN-obs(5)} = 0.16$
observed
imputed
estimate CCA
estimate KNN−obs(5)
Model residuals of tetradecanedioate
2
1
0
−1
−2
$n_{CCA} = 397$
$n_{KNN-obs(5)} = 1151$
$n_{CCA} = 179$
$n_{KNN-obs(5)} = 462$
$n_{CCA} = 39$
$n_{KNN-obs(5)} = 60$
major homozygote
heterozygote
minor homozygote
Genotype of SNP rs12317268 (SLCO1B1 (2 separate loci))

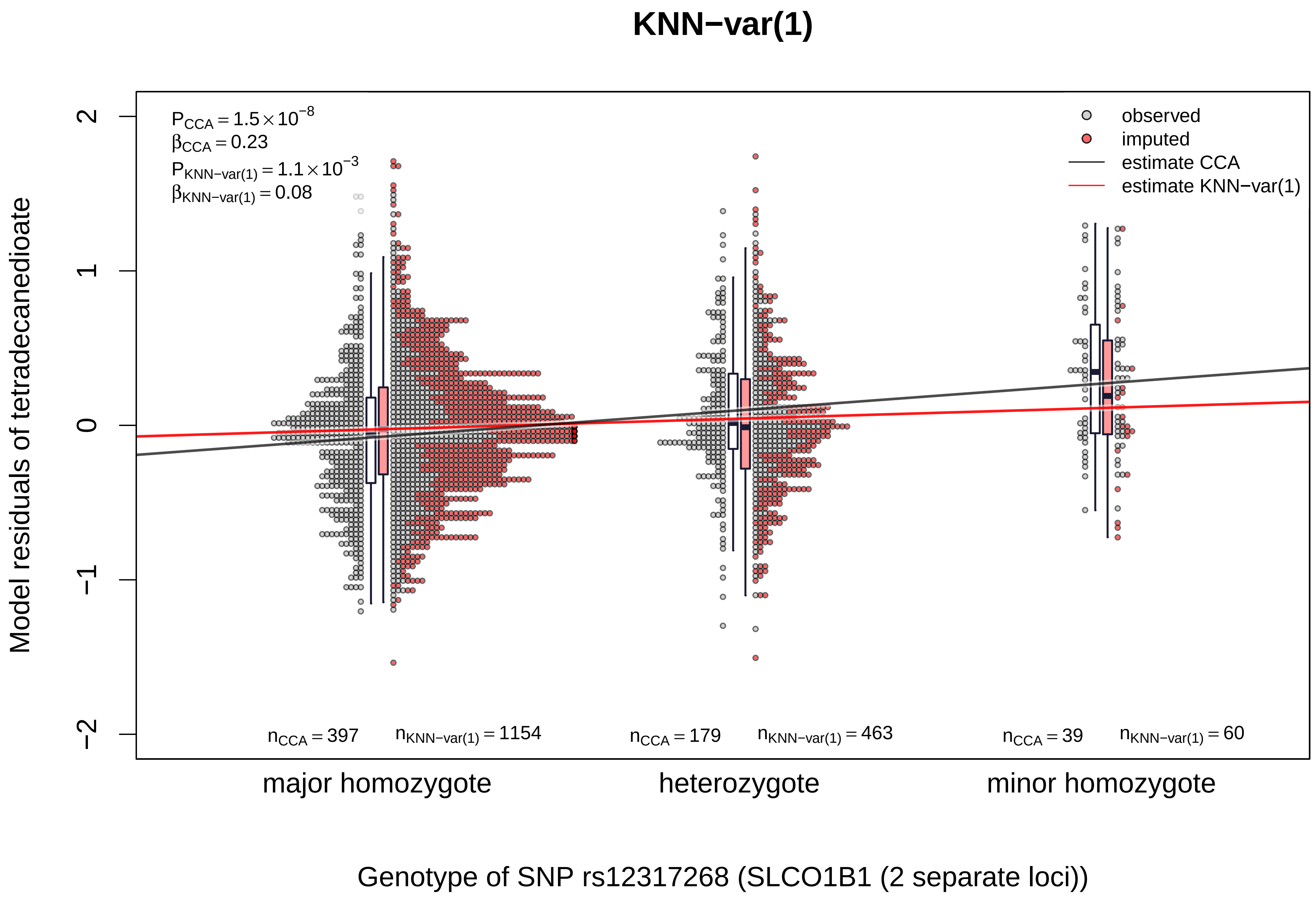

KNN−var(1)
$P_{CCA} = 1.5 \times 10^{-8}$
$\beta_{CCA} = 0.23$
$P_{KNN-var(1)} = 1.1 \times 10^{-3}$
$\beta_{KNN-var(1)} = 0.08$
observed
imputed
estimate CCA
estimate KNN−var(1)
$n_{CCA} = 397$
$n_{KNN-var(1)} = 1154$
$n_{CCA} = 179$
$n_{KNN-var(1)} = 463$
$n_{CCA} = 39$
$n_{KNN-var(1)} = 60$
2
1
0
−1
−2
Model residuals of tetradecanedioate
major homozygote
heterozygote
minor homozygote
Genotype of SNP rs12317268 (SLCO1B1 (2 separate loci))

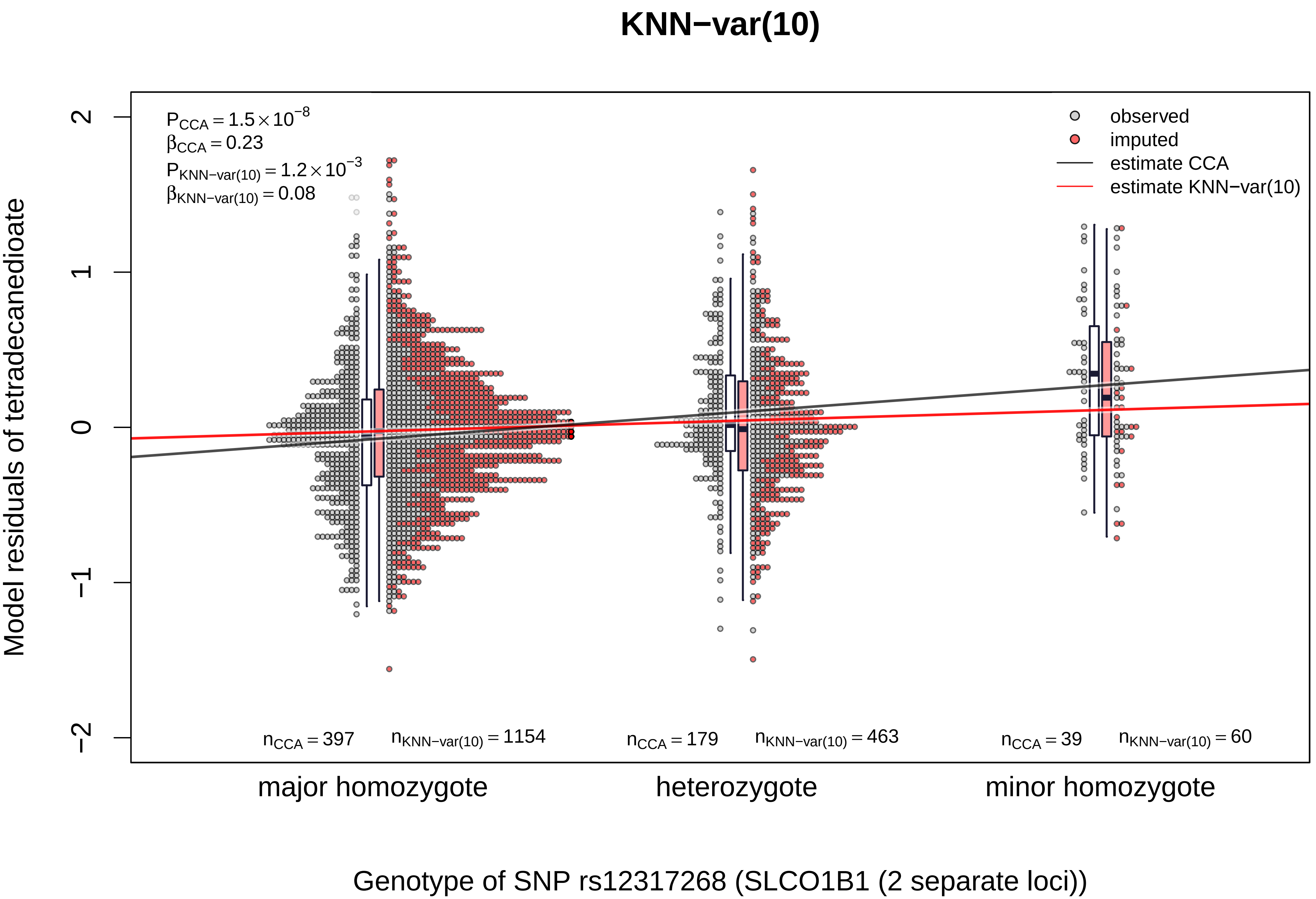

KNN−var(10)
$P_{CCA} = 1.5 \times 10^{-8}$
$\beta_{CCA} = 0.23$
$P_{KNN-var(10)} = 1.2 \times 10^{-3}$
$\beta_{KNN-var(10)} = 0.08$
observed
imputed
estimate CCA
estimate KNN−var(10)
Model residuals of tetradecanedioate
2
1
0
−1
−2
$n_{CCA} = 397$
$n_{KNN-var(10)} = 1154$
$n_{CCA} = 179$
$n_{KNN-var(10)} = 463$
$n_{CCA} = 39$
$n_{KNN-var(10)} = 60$
major homozygote
heterozygote
minor homozygote
Genotype of SNP rs12317268 (SLCO1B1 (2 separate loci))

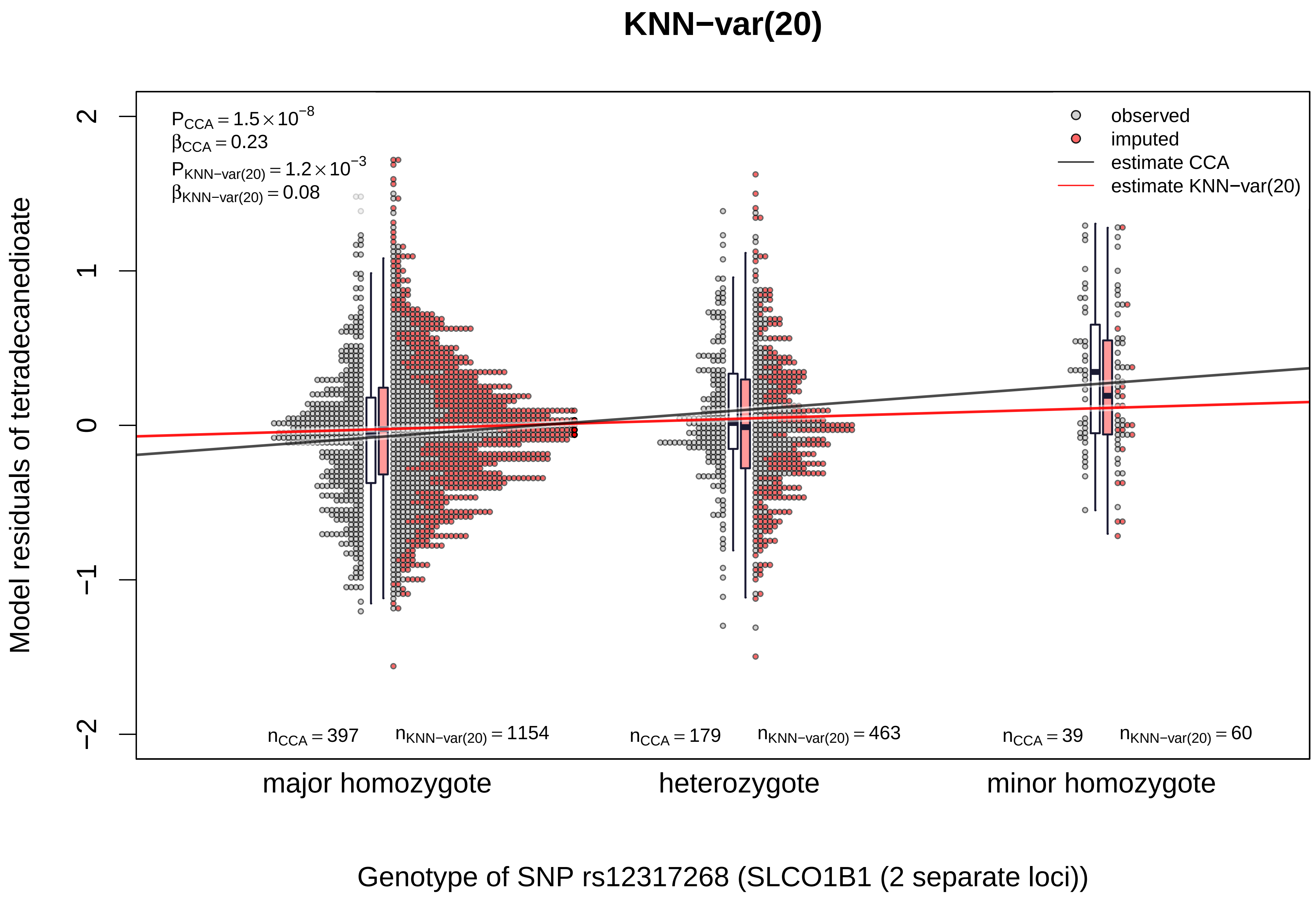
KNN−var(20)
$P_{CCA} = 1.5 \times 10^{-8}$
$\beta_{CCA} = 0.23$
$P_{KNN-var(20)} = 1.2 \times 10^{-3}$
$\beta_{KNN-var(20)} = 0.08$
observed
imputed
estimate CCA
estimate KNN−var(20)
Model residuals of tetradecanedioate
2
1
0
−1
−2
$n_{CCA} = 397$
$n_{KNN-var(20)} = 1154$
$n_{CCA} = 179$
$n_{KNN-var(20)} = 463$
$n_{CCA} = 39$
$n_{KNN-var(20)} = 60$
major homozygote
heterozygote
minor homozygote
Genotype of SNP rs12317268 (SLCO1B1 (2 separate loci))

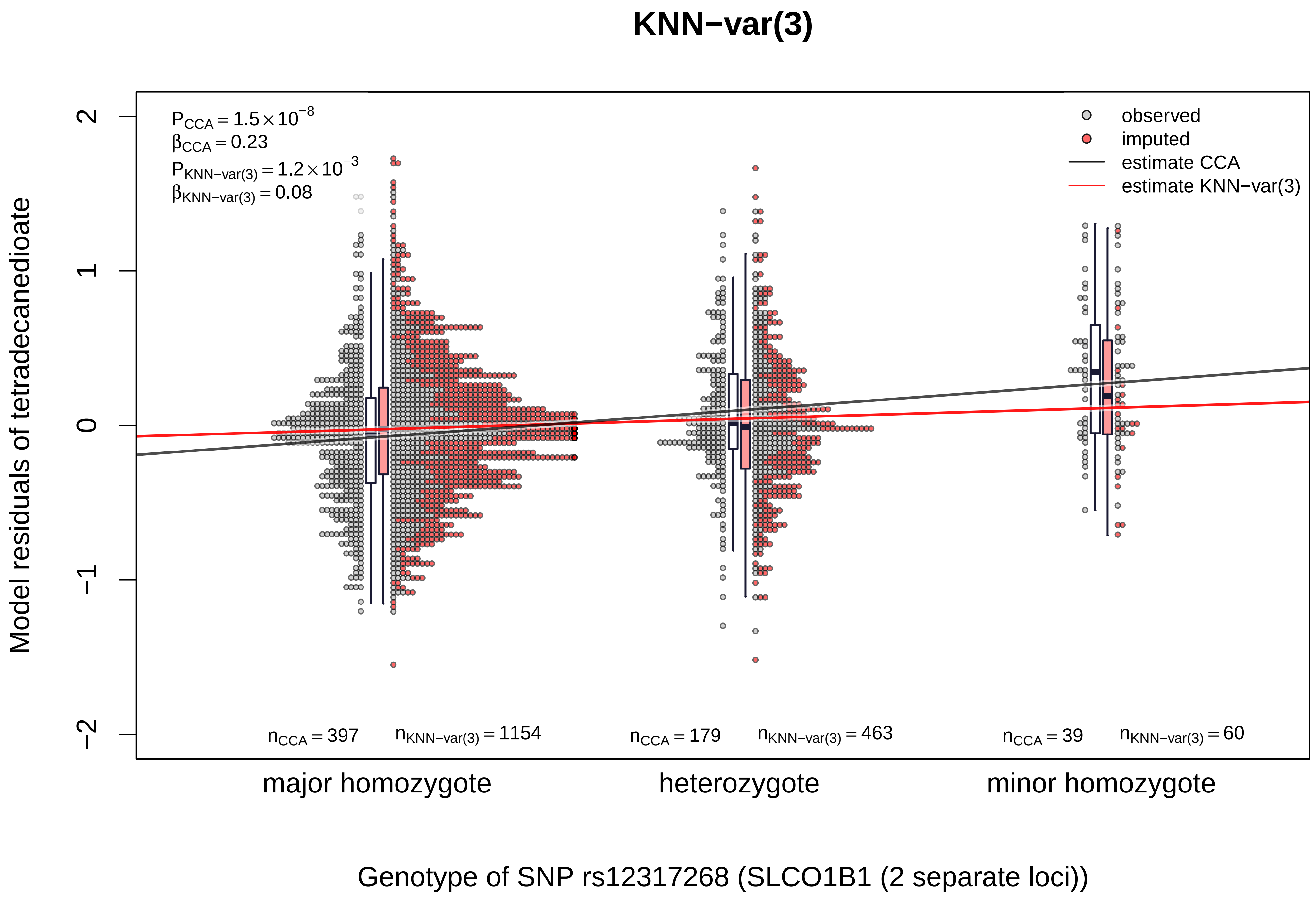
KNN−var(3)
$P_{CCA} = 1.5 \times 10^{-8}$
$\beta_{CCA} = 0.23$
$P_{KNN-var(3)} = 1.2 \times 10^{-3}$
$\beta_{KNN-var(3)} = 0.08$
observed
imputed
estimate CCA
estimate KNN−var(3)
Model residuals of tetradecanedioate
2
1
0
−1
−2
$n_{CCA} = 397$
$n_{KNN-var(3)} = 1154$
$n_{CCA} = 179$
$n_{KNN-var(3)} = 463$
$n_{CCA} = 39$
$n_{KNN-var(3)} = 60$
major homozygote
heterozygote
minor homozygote
Genotype of SNP rs12317268 (SLCO1B1 (2 separate loci))

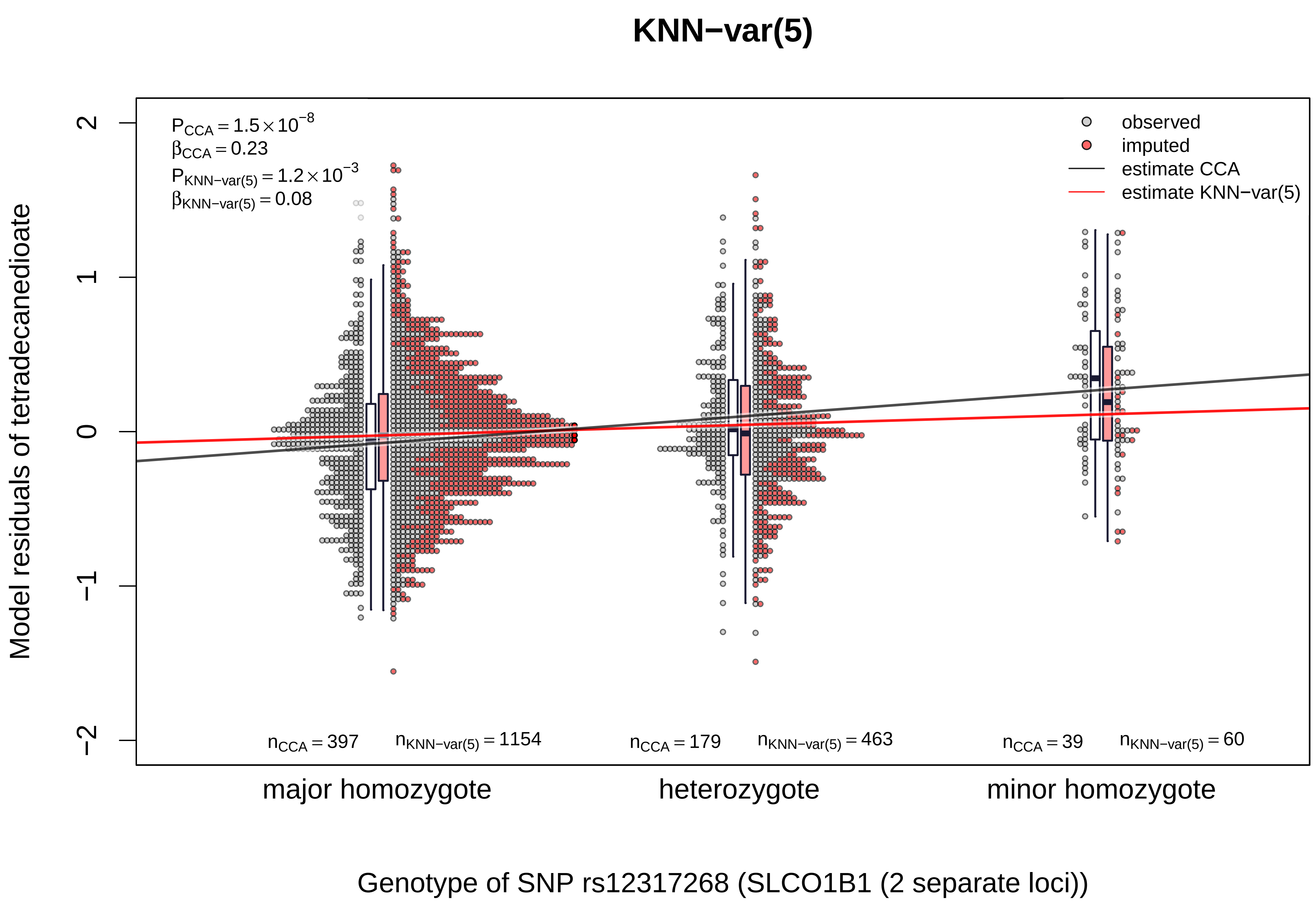

KNN−var(5)
$P_{CCA} = 1.5 \times 10^{-8}$
$\beta_{CCA} = 0.23$
$P_{KNN-var(5)} = 1.2 \times 10^{-3}$
$\beta_{KNN-var(5)} = 0.08$
observed
imputed
estimate CCA
estimate KNN−var(5)
$n_{CCA} = 397$
$n_{KNN-var(5)} = 1154$
$n_{CCA} = 179$
$n_{KNN-var(5)} = 463$
$n_{CCA} = 39$
$n_{KNN-var(5)} = 60$
2
1
0
−1
−2
Model residuals of tetradecanedioate
major homozygote
heterozygote
minor homozygote
Genotype of SNP rs12317268 (SLCO1B1 (2 separate loci))

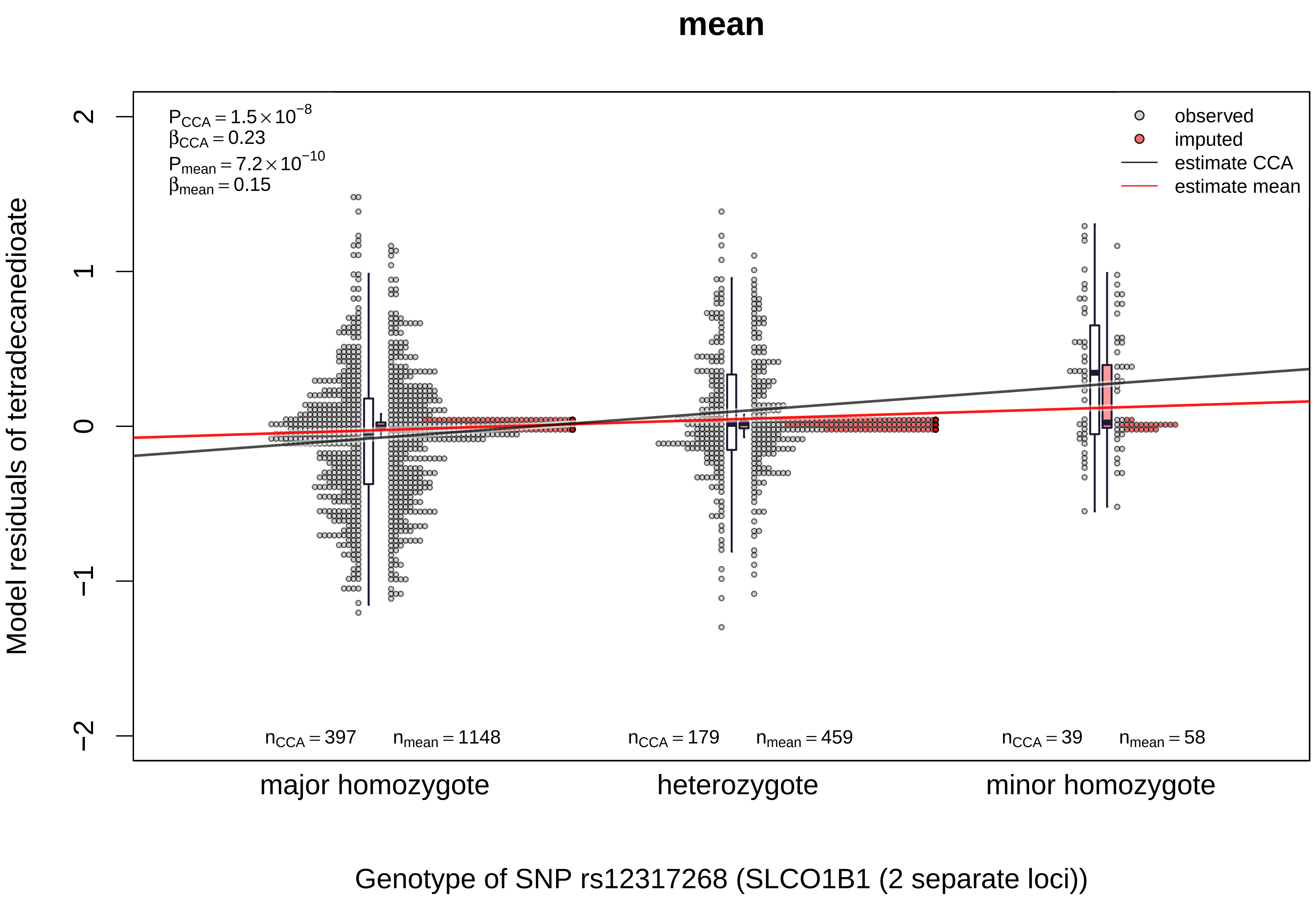

mean
$P_{CCA} = 1.5 \times 10^{-8}$
$\beta_{CCA} = 0.23$
$P_{mean} = 7.2 \times 10^{-10}$
$\beta_{mean} = 0.15$
observed
imputed
estimate CCA
estimate mean
2
1
0
−1
−2
Model residuals of tetradecanedioate
$n_{CCA} = 397$
$n_{mean} = 1148$
$n_{CCA} = 179$
$n_{mean} = 459$
$n_{CCA} = 39$
$n_{mean} = 58$
major homozygote
heterozygote
minor homozygote
Genotype of SNP rs12317268 (SLCO1B1 (2 separate loci))

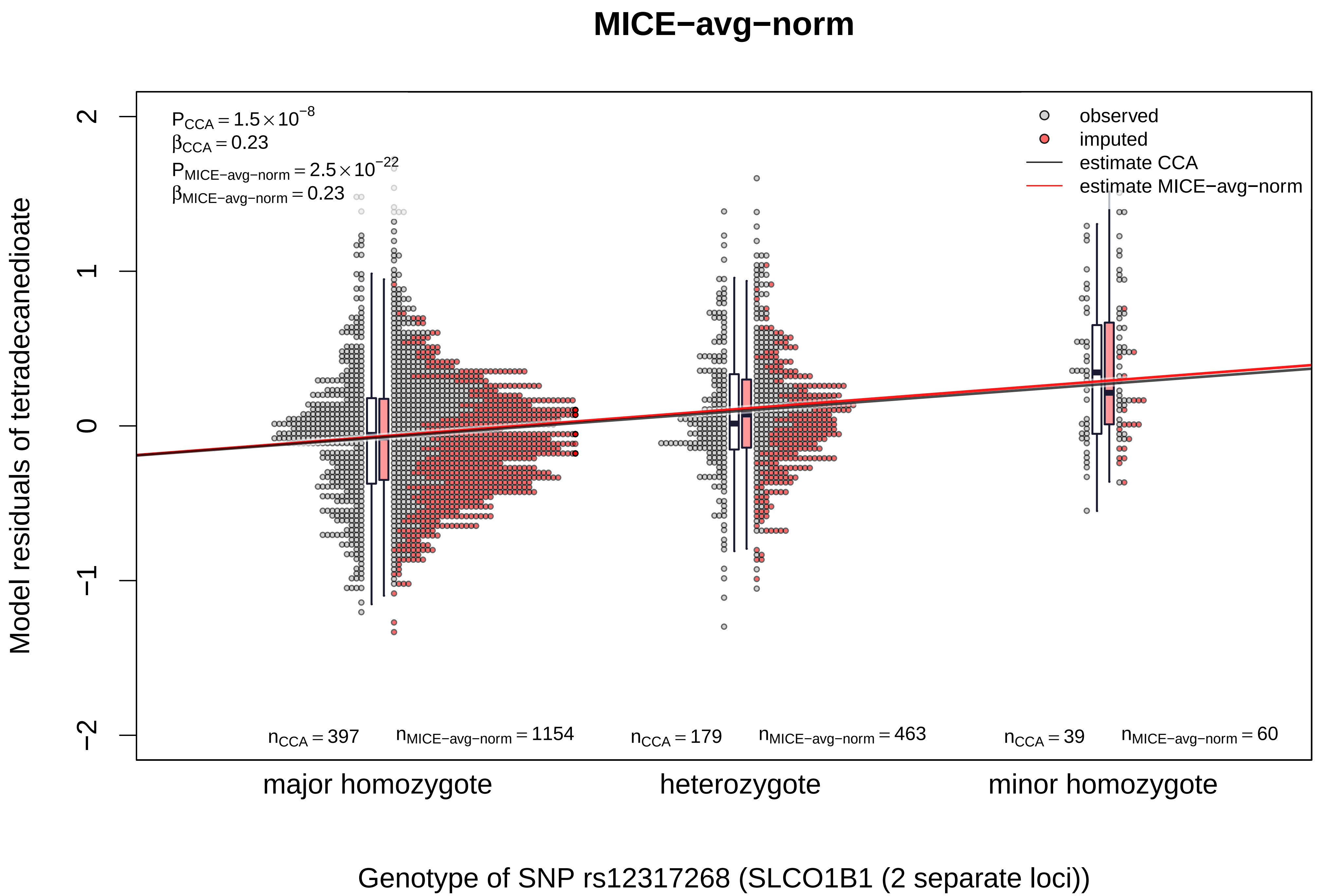
MICE−avg−norm
$P_{CCA} = 1.5 \times 10^{-8}$
$\beta_{CCA} = 0.23$
$P_{MICE-avg-norm} = 2.5 \times 10^{-22}$
$\beta_{MICE-avg-norm} = 0.23$
observed
imputed
estimate CCA
estimate MICE−avg−norm
2
1
0
−1
−2
Model residuals of tetradecanedioate
$n_{CCA} = 397$
$n_{MICE-avg-norm} = 1154$
$n_{CCA} = 179$
$n_{MICE-avg-norm} = 463$
$n_{CCA} = 39$
$n_{MICE-avg-norm} = 60$
major homozygote
heterozygote
minor homozygote
Genotype of SNP rs12317268 (SLCO1B1 (2 separate loci))

MICE−avg−pmm

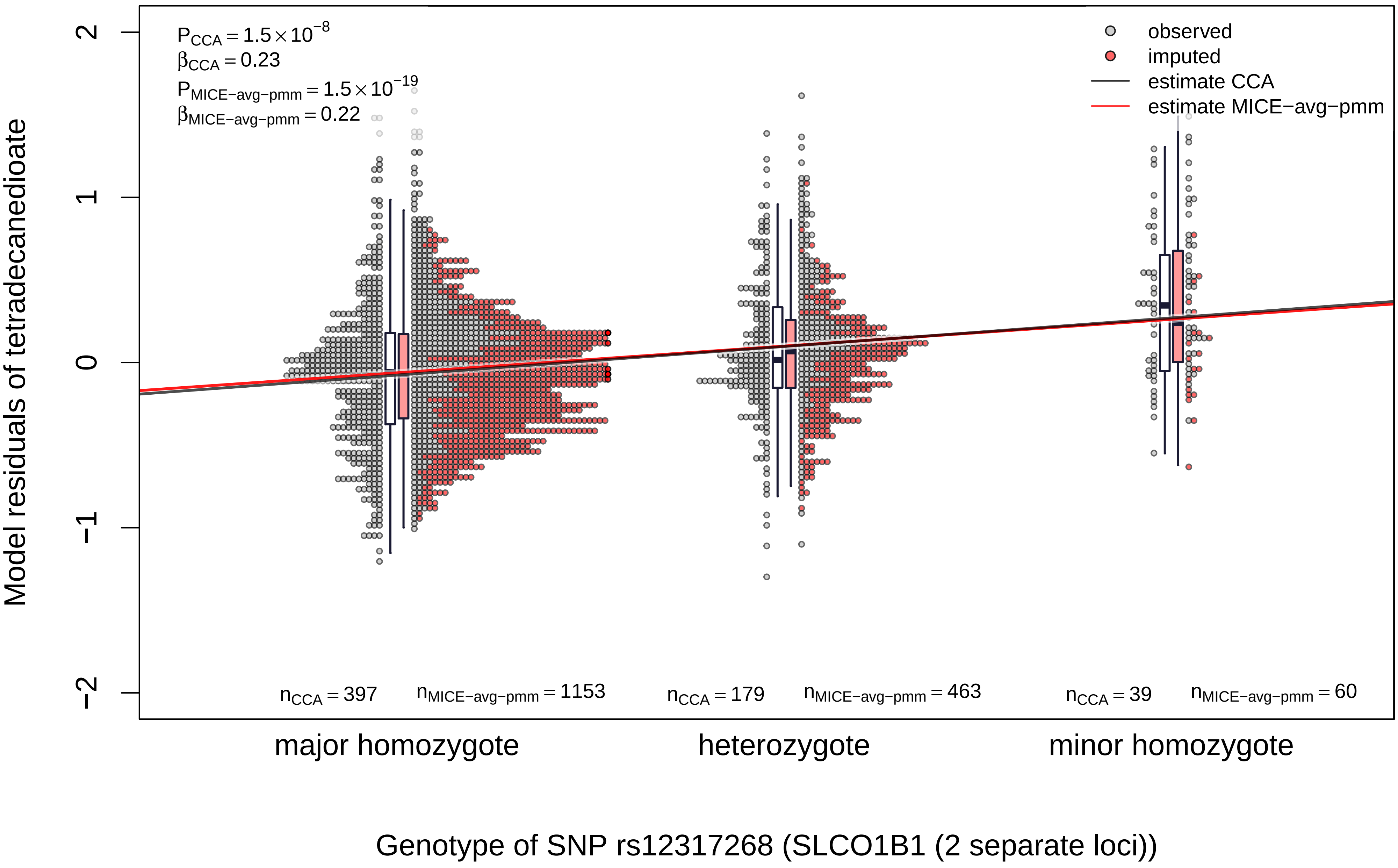

$P_{CCA} = 1.5 \times 10^{-8}$
$\beta_{CCA} = 0.23$
$P_{MICE-avg-pmm} = 1.5 \times 10^{-19}$
$\beta_{MICE-avg-pmm} = 0.22$
observed
imputed
estimate CCA
estimate MICE−avg−pmm
Model residuals of tetradecanedioate
2
1
0
−1
−2
$n_{CCA} = 397$
$n_{MICE-avg-pmm} = 1153$
$n_{CCA} = 179$
$n_{MICE-avg-pmm} = 463$
$n_{CCA} = 39$
$n_{MICE-avg-pmm} = 60$
major homozygote
heterozygote
minor homozygote
Genotype of SNP rs12317268 (SLCO1B1 (2 separate loci))

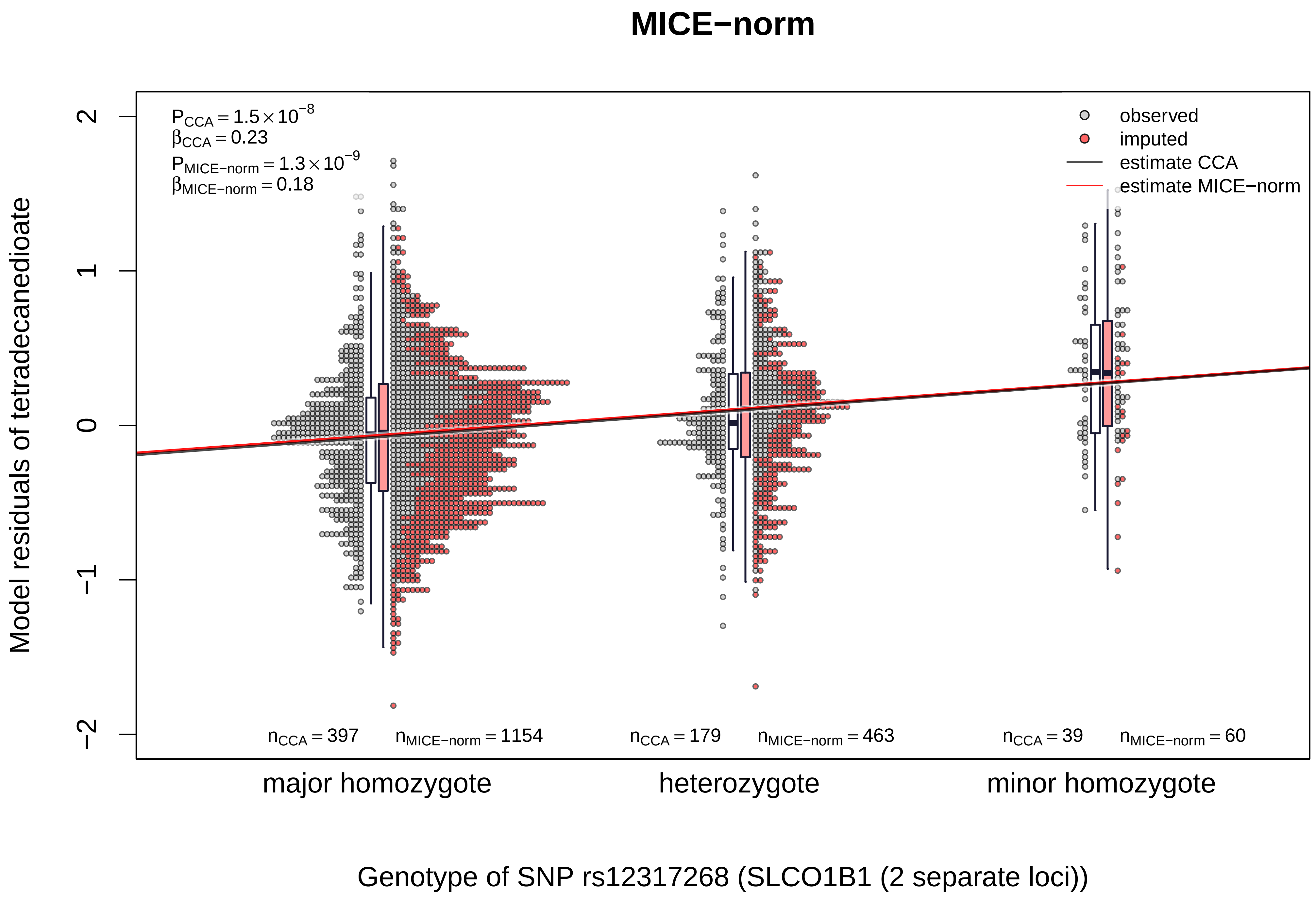
MICE−norm
$P_{CCA} = 1.5 \times 10^{-8}$
$\beta_{CCA} = 0.23$
$P_{MICE-norm} = 1.3 \times 10^{-9}$
$\beta_{MICE-norm} = 0.18$
observed
imputed
estimate CCA
estimate MICE−norm
Model residuals of tetradecanedioate
2
1
0
−1
−2
$n_{CCA} = 397$
$n_{MICE-norm} = 1154$
$n_{CCA} = 179$
$n_{MICE-norm} = 463$
$n_{CCA} = 39$
$n_{MICE-norm} = 60$
major homozygote
heterozygote
minor homozygote
Genotype of SNP rs12317268 (SLCO1B1 (2 separate loci))

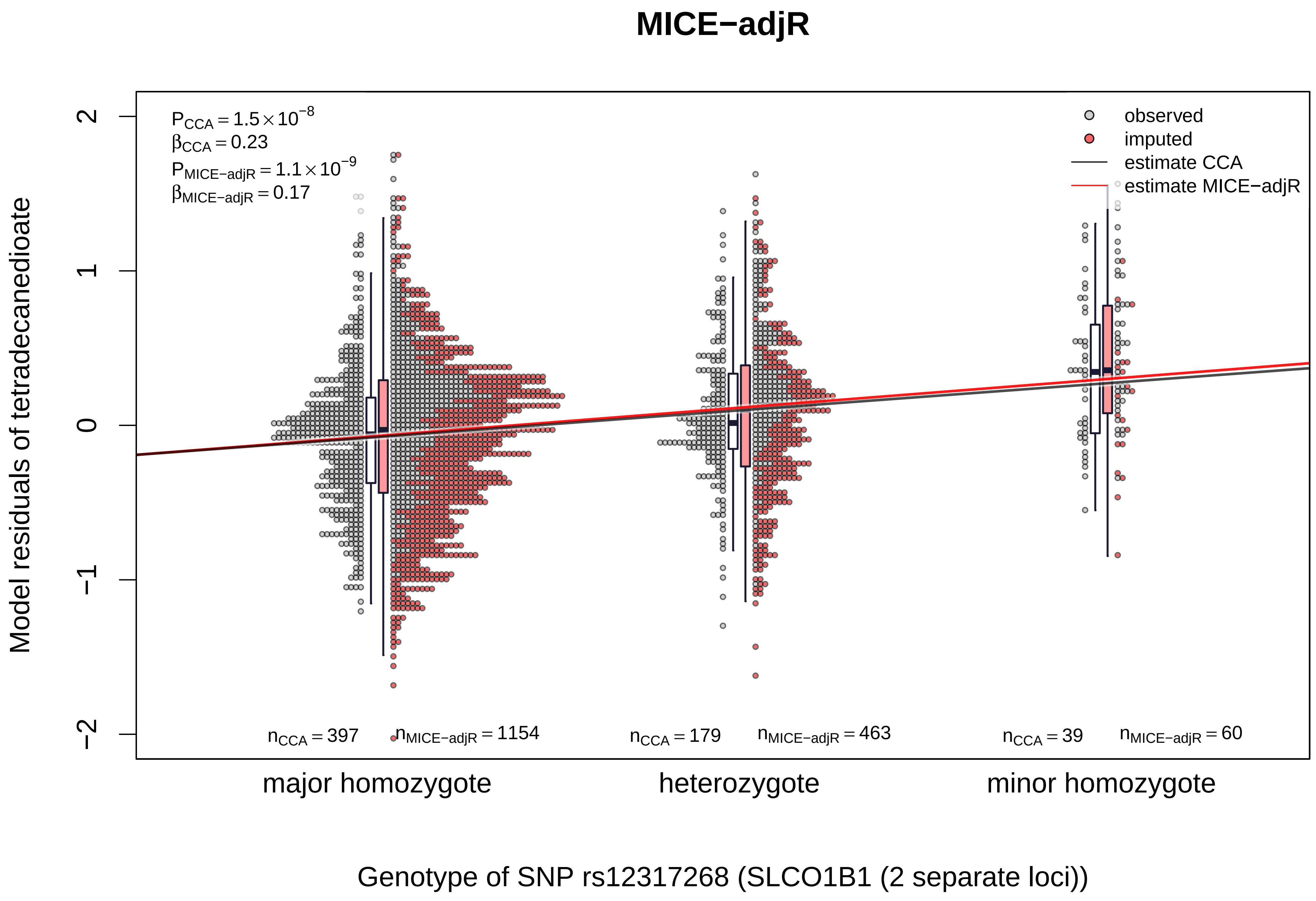

MICE−adjR
$P_{CCA} = 1.5 \times 10^{-8}$
$\beta_{CCA} = 0.23$
$P_{MICE-adjR} = 1.1 \times 10^{-9}$
$\beta_{MICE-adjR} = 0.17$
observed
imputed
estimate CCA
estimate MICE−adjR
2
1
0
−1
−2
Model residuals of tetradecanedioate
$n_{CCA} = 397$
$n_{MICE-adjR} = 1154$
$n_{CCA} = 179$
$n_{MICE-adjR} = 463$
$n_{CCA} = 39$
$n_{MICE-adjR} = 60$
major homozygote
heterozygote
minor homozygote
Genotype of SNP rs12317268 (SLCO1B1 (2 separate loci))

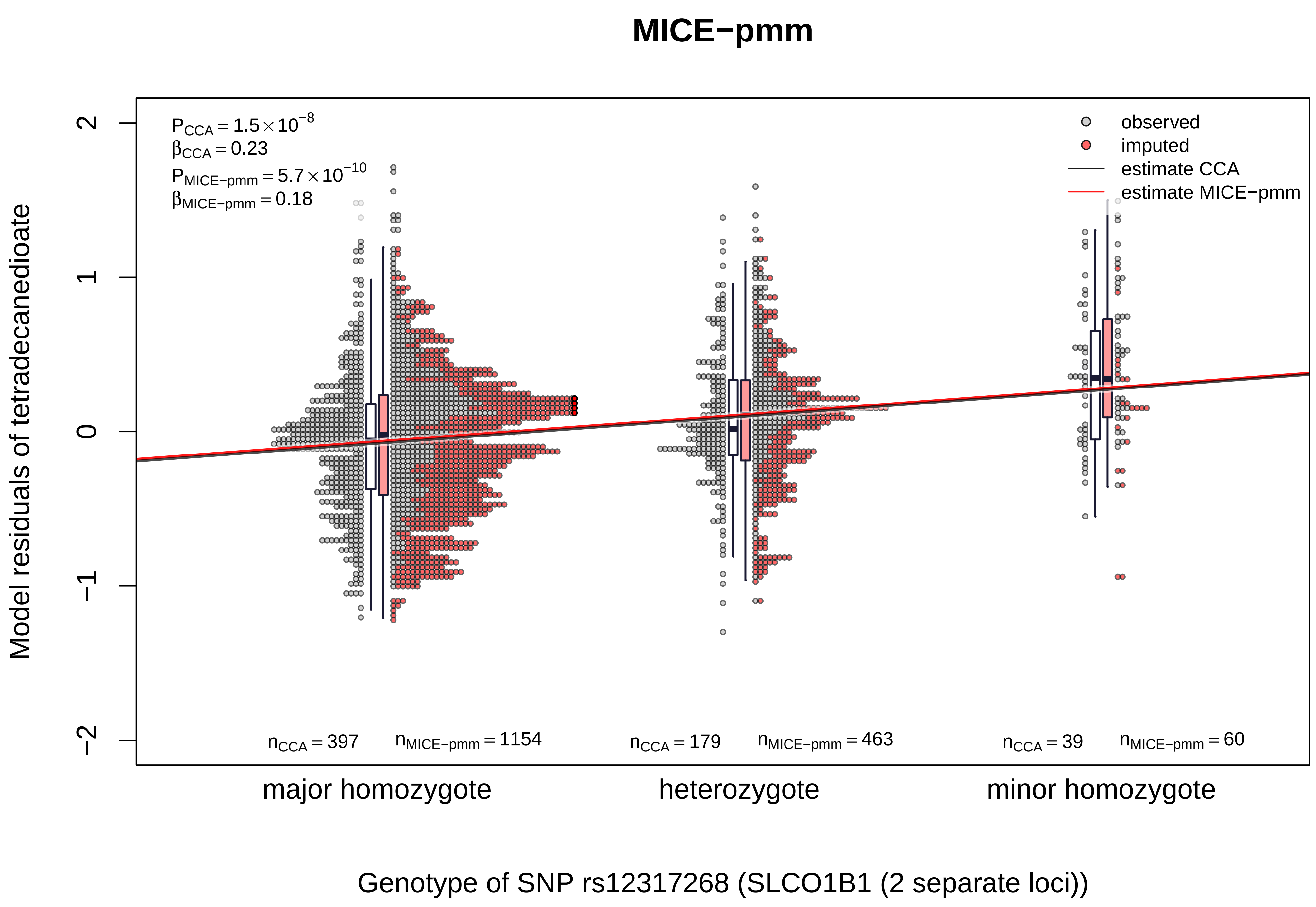

MICE−pmm
$P_{CCA} = 1.5 \times 10^{-8}$
$\beta_{CCA} = 0.23$
$P_{MICE-pmm} = 5.7 \times 10^{-10}$
$\beta_{MICE-pmm} = 0.18$
observed
imputed
estimate CCA
estimate MICE−pmm
Model residuals of tetradecanedioate
2
1
0
−1
−2
$n_{CCA} = 397$
$n_{MICE-pmm} = 1154$
$n_{CCA} = 179$
$n_{MICE-pmm} = 463$
$n_{CCA} = 39$
$n_{MICE-pmm} = 60$
major homozygote
heterozygote
minor homozygote
Genotype of SNP rs12317268 (SLCO1B1 (2 separate loci))

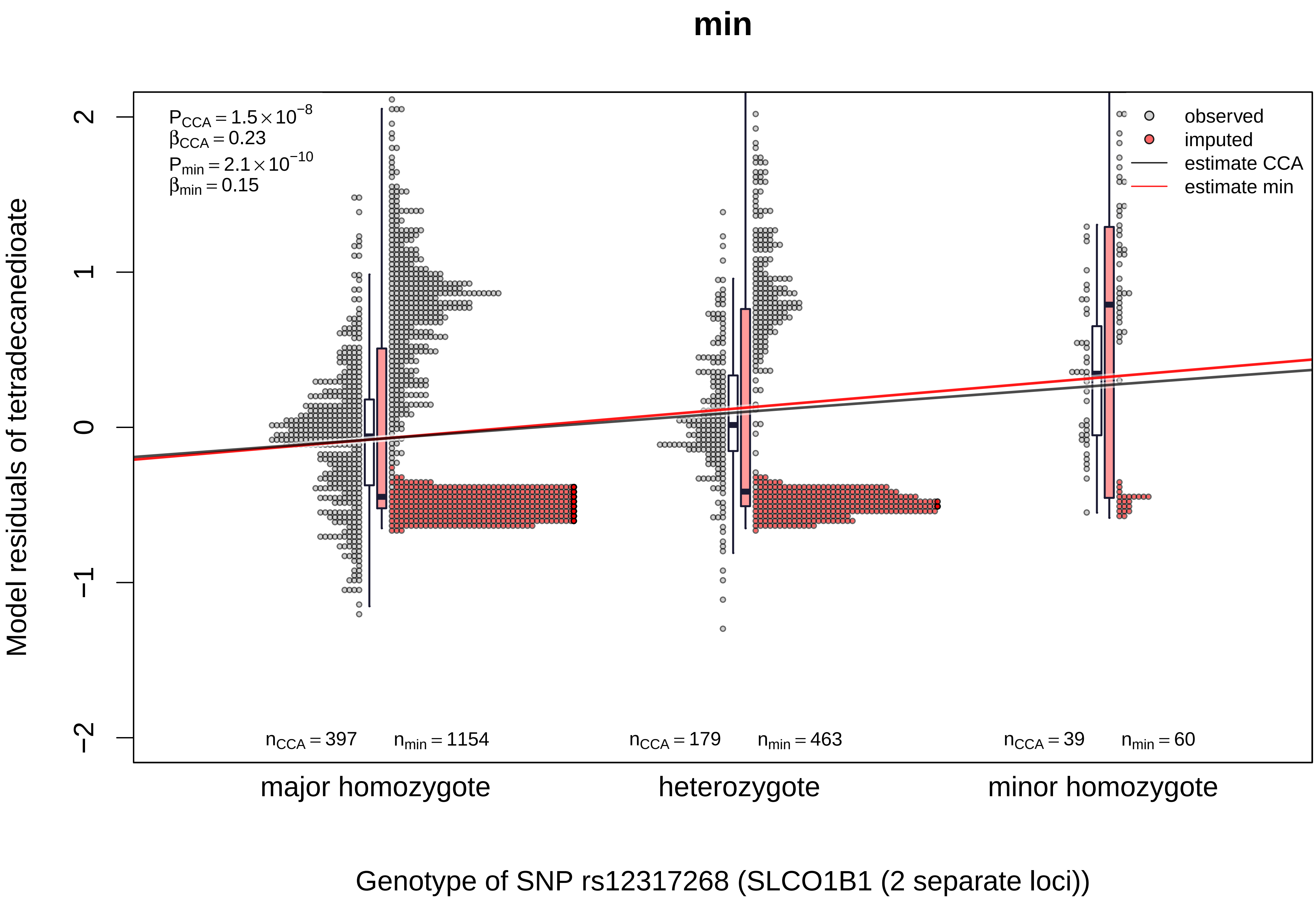
min
$P_{CCA} = 1.5 \times 10^{-8}$
$\beta_{CCA} = 0.23$
$P_{min} = 2.1 \times 10^{-10}$
$\beta_{min} = 0.15$
observed
imputed
estimate CCA
estimate min
Model residuals of tetradecanedioate
2
1
0
−1
−2
$n_{CCA} = 397$
$n_{min} = 1154$
$n_{CCA} = 179$
$n_{min} = 463$
$n_{CCA} = 39$
$n_{min} = 60$
major homozygote
heterozygote
minor homozygote
Genotype of SNP rs12317268 (SLCO1B1 (2 separate loci))

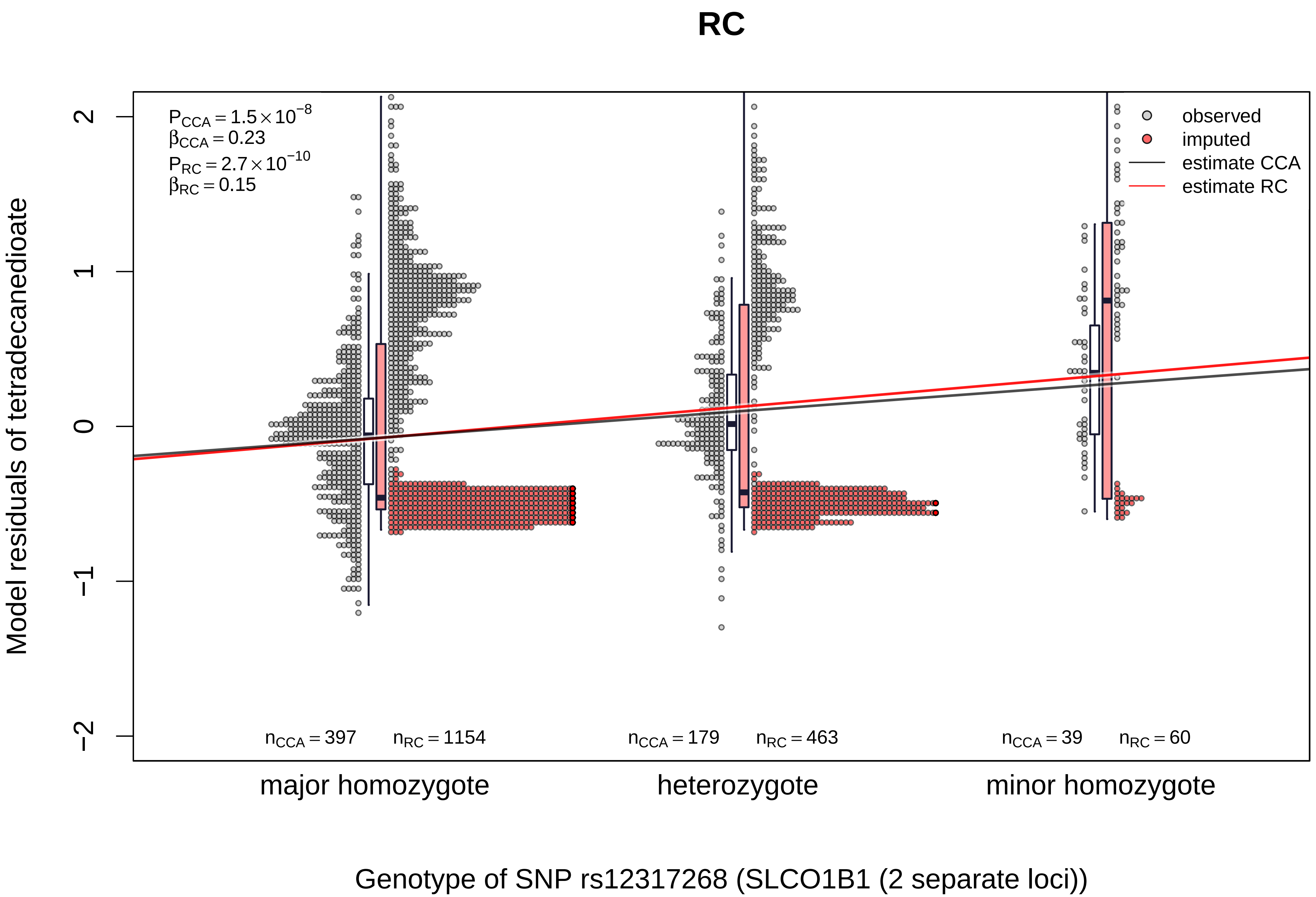

RC
$P_{CCA} = 1.5 \times 10^{-8}$
$\beta_{CCA} = 0.23$
$P_{RC} = 2.7 \times 10^{-10}$
$\beta_{RC} = 0.15$
observed
imputed
estimate CCA
estimate RC
Model residuals of tetradecanedioate
2
1
0
−1
−2
$n_{CCA} = 397$
$n_{RC} = 1154$
$n_{CCA} = 179$
$n_{RC} = 463$
$n_{CCA} = 39$
$n_{RC} = 60$
major homozygote
heterozygote
minor homozygote
Genotype of SNP rs12317268 (SLCO1B1 (2 separate loci))

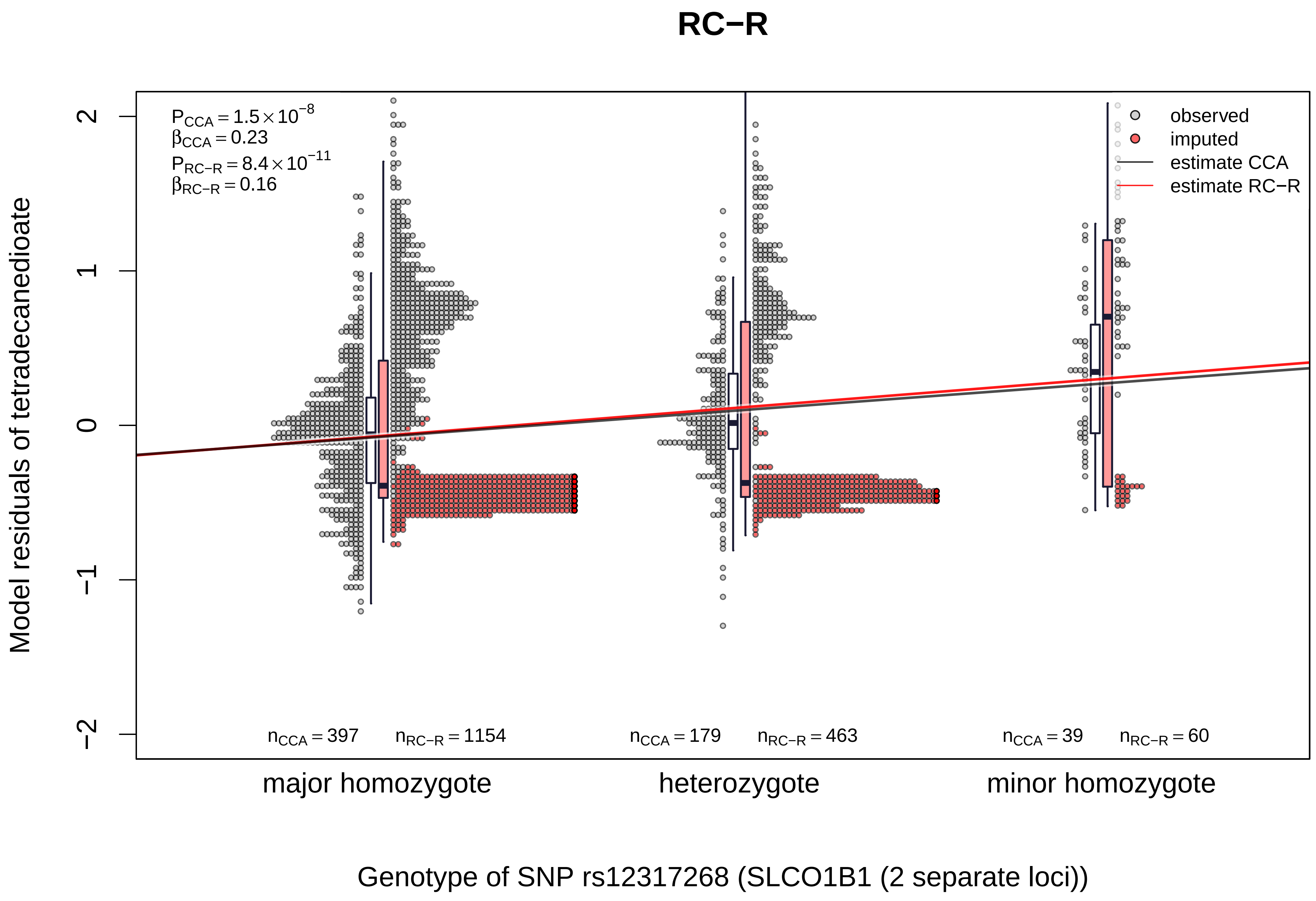

RC−R
$P_{CCA} = 1.5 \times 10^{-8}$
$\beta_{CCA} = 0.23$
$P_{RC-R} = 8.4 \times 10^{-11}$
$\beta_{RC-R} = 0.16$
observed
imputed
estimate CCA
estimate RC−R
Model residuals of tetradecanedioate
2
1
0
−1
−2
$n_{CCA} = 397$
$n_{RC-R} = 1154$
$n_{CCA} = 179$
$n_{RC-R} = 463$
$n_{CCA} = 39$
$n_{RC-R} = 60$
major homozygote
heterozygote
minor homozygote
Genotype of SNP rs12317268 (SLCO1B1 (2 separate loci))

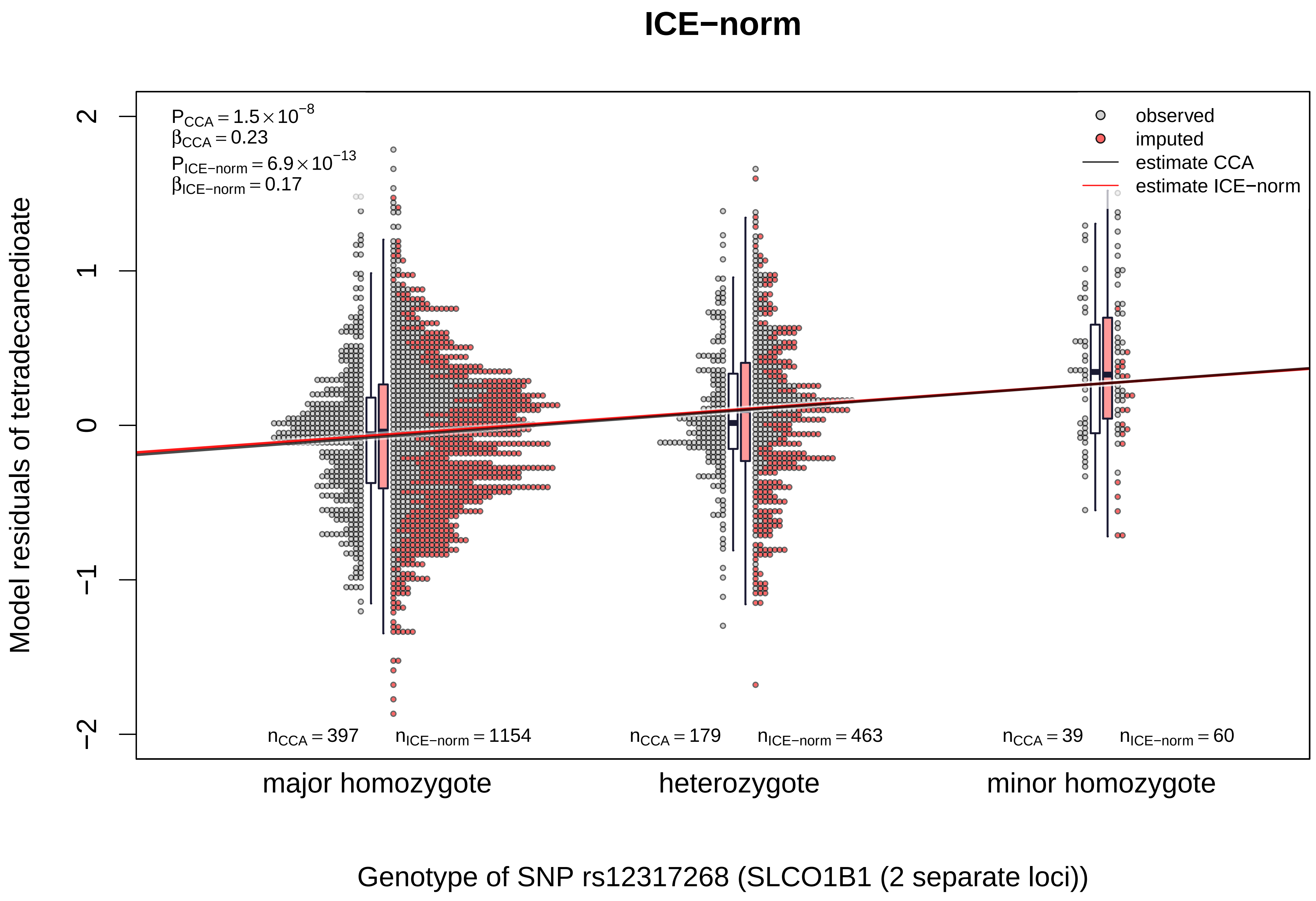

ICE−norm
$P_{CCA} = 1.5 \times 10^{-8}$
$\beta_{CCA} = 0.23$
$P_{ICE-norm} = 6.9 \times 10^{-13}$
$\beta_{ICE-norm} = 0.17$
observed
imputed
estimate CCA
estimate ICE−norm
Model residuals of tetradecanedioate
2
1
0
−1
−2
$n_{CCA} = 397$
$n_{ICE-norm} = 1154$
$n_{CCA} = 179$
$n_{ICE-norm} = 463$
$n_{CCA} = 39$
$n_{ICE-norm} = 60$
major homozygote
heterozygote
minor homozygote
Genotype of SNP rs12317268 (SLCO1B1 (2 separate loci))

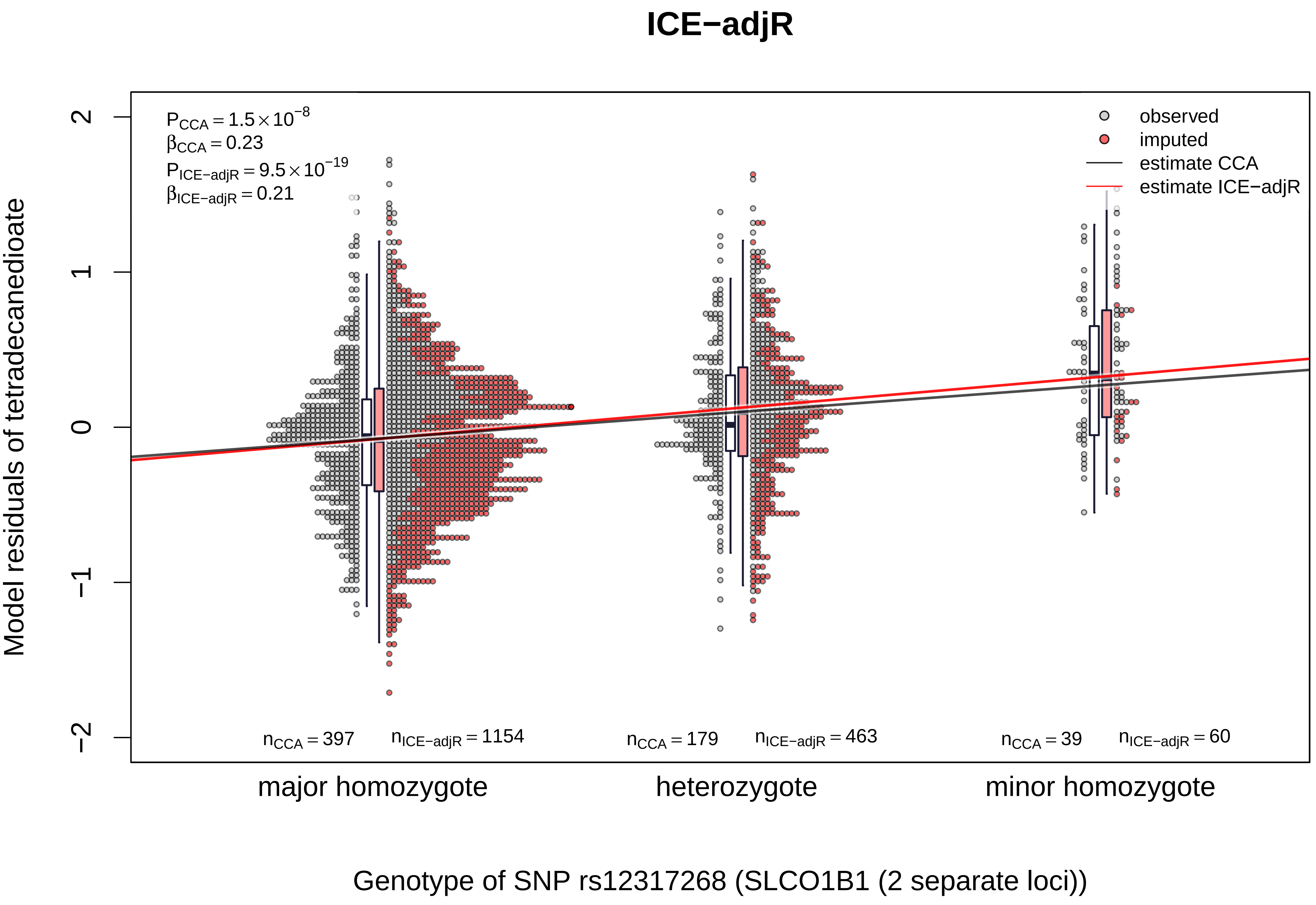
ICE−adjR
$P_{CCA} = 1.5 \times 10^{-8}$
$\beta_{CCA} = 0.23$
$P_{ICE-adjR} = 9.5 \times 10^{-19}$
$\beta_{ICE-adjR} = 0.21$
observed
imputed
estimate CCA
estimate ICE−adjR
Model residuals of tetradecanedioate
2
1
0
−1
−2
$n_{CCA} = 397$
$n_{ICE-adjR} = 1154$
$n_{CCA} = 179$
$n_{ICE-adjR} = 463$
$n_{CCA} = 39$
$n_{ICE-adjR} = 60$
major homozygote
heterozygote
minor homozygote
Genotype of SNP rs12317268 (SLCO1B1 (2 separate loci))

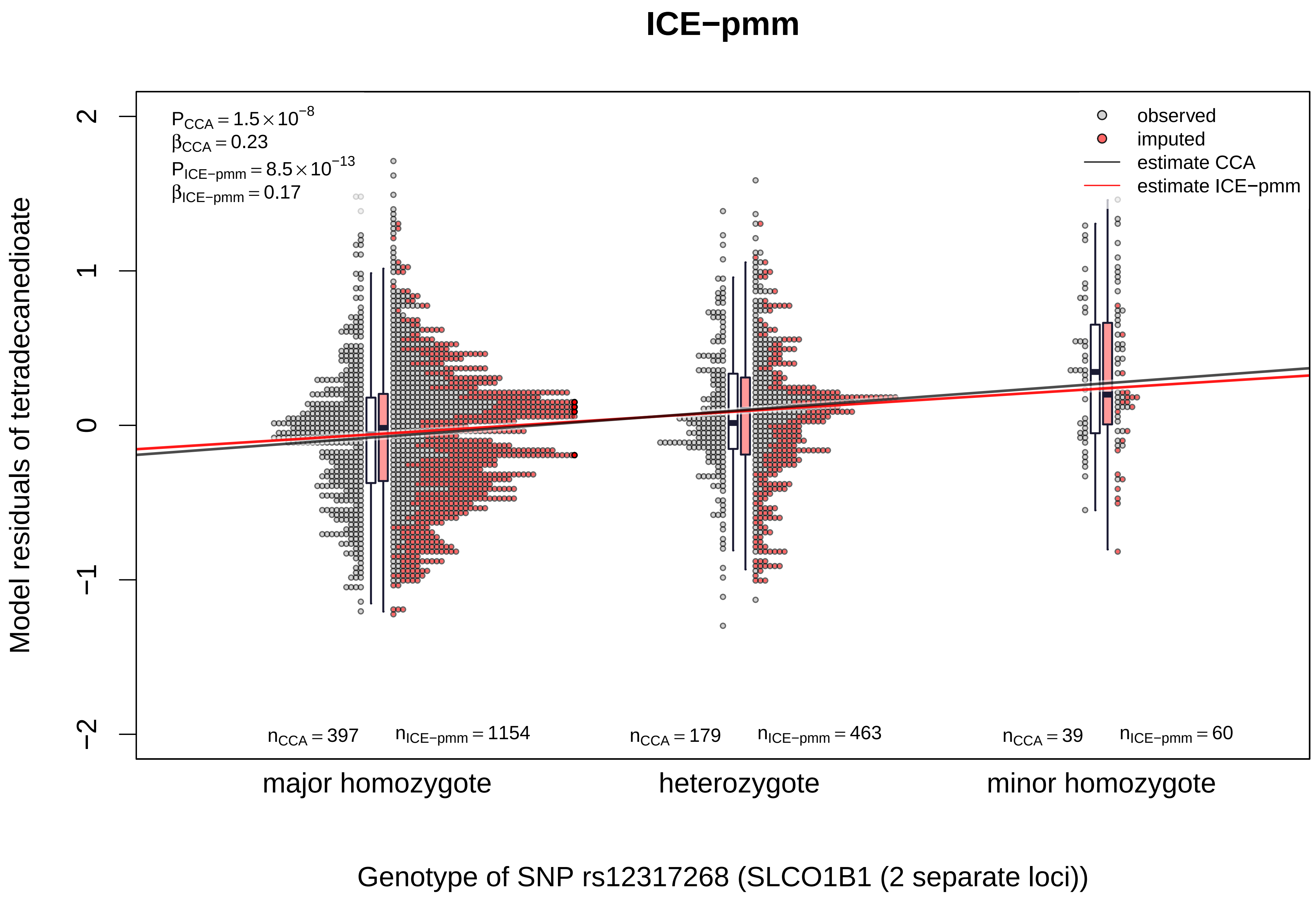

ICE−pmm
$P_{CCA} = 1.5 \times 10^{-8}$
$\beta_{CCA} = 0.23$
$P_{ICE-pmm} = 8.5 \times 10^{-13}$
$\beta_{ICE-pmm} = 0.17$
observed
imputed
estimate CCA
estimate ICE−pmm
Model residuals of tetradecanedioate
2
1
0
−1
−2
$n_{CCA} = 397$
$n_{ICE-pmm} = 1154$
$n_{CCA} = 179$
$n_{ICE-pmm} = 463$
$n_{CCA} = 39$
$n_{ICE-pmm} = 60$
major homozygote
heterozygote
minor homozygote
Genotype of SNP rs12317268 (SLCO1B1 (2 separate loci))

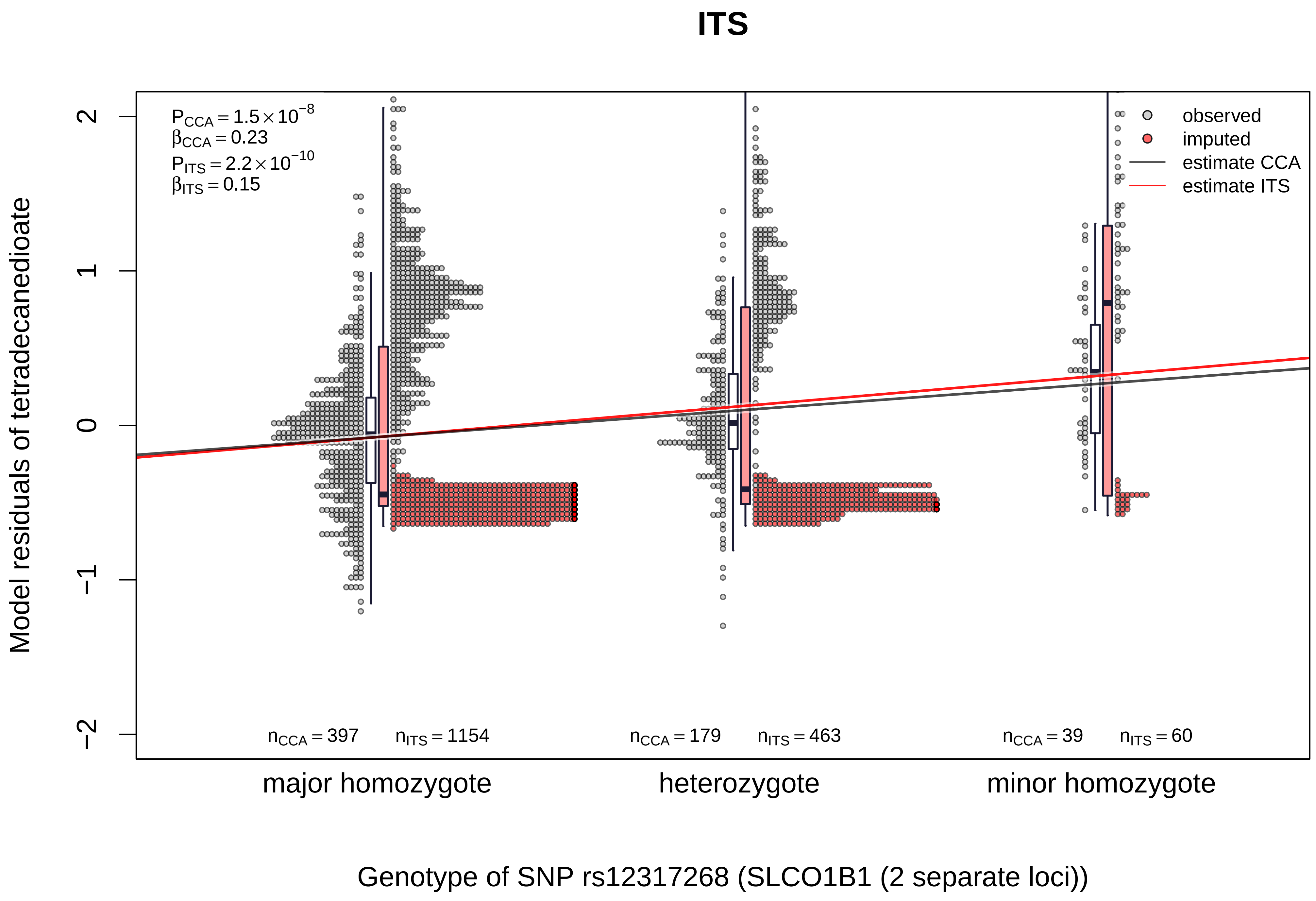

ITS
$P_{CCA} = 1.5 \times 10^{-8}$
$\beta_{CCA} = 0.23$
$P_{ITS} = 2.2 \times 10^{-10}$
$\beta_{ITS} = 0.15$
observed
imputed
estimate CCA
estimate ITS
Model residuals of tetradecanedioate
2
1
0
−1
−2
$n_{CCA} = 397$
$n_{ITS} = 1154$
$n_{CCA} = 179$
$n_{ITS} = 463$
$n_{CCA} = 39$
$n_{ITS} = 60$
major homozygote
heterozygote
minor homozygote
Genotype of SNP rs12317268 (SLCO1B1 (2 separate loci))

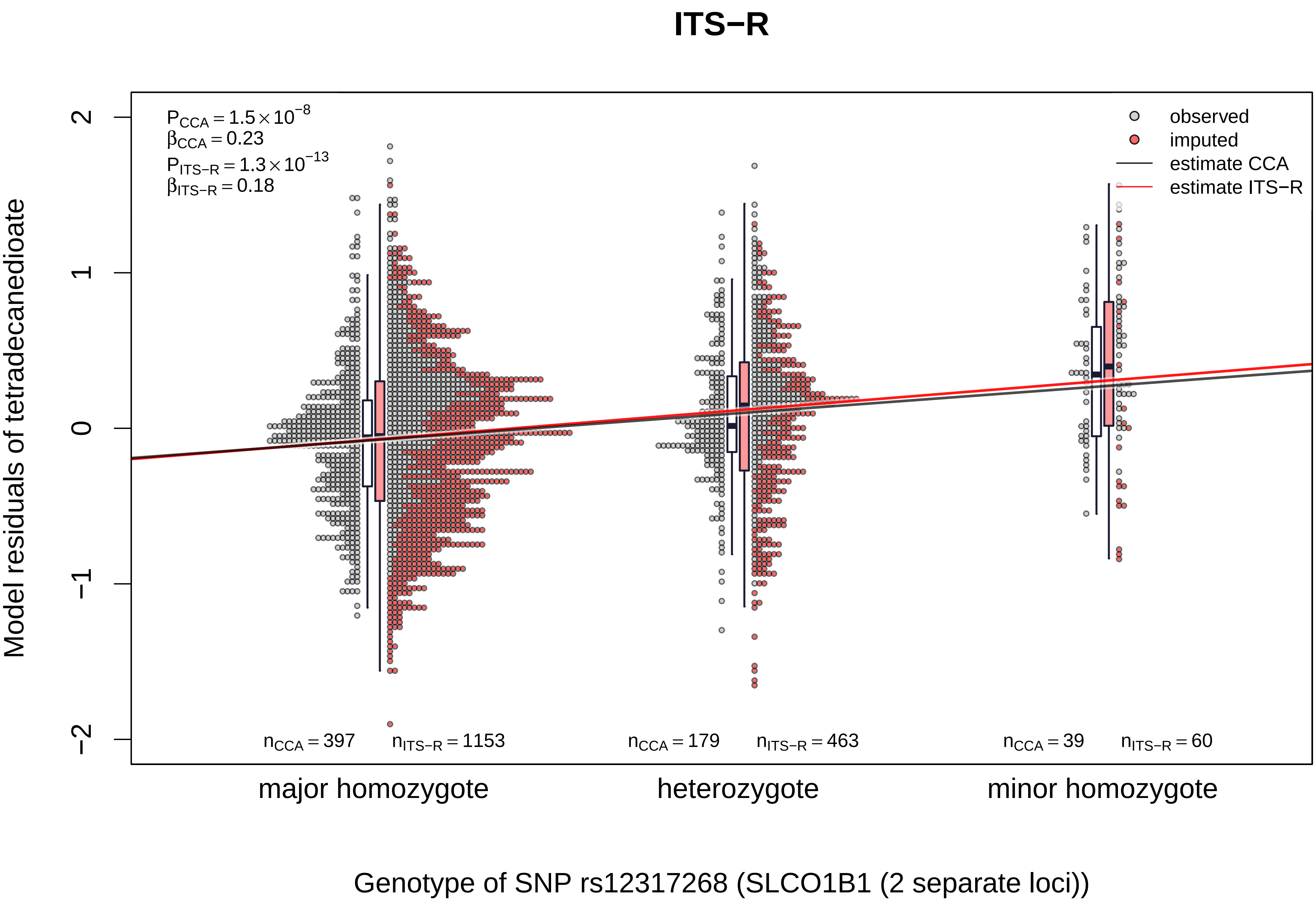

ITS−R
$P_{CCA} = 1.5 \times 10^{-8}$
$\beta_{CCA} = 0.23$
$P_{ITS-R} = 1.3 \times 10^{-13}$
$\beta_{ITS-R} = 0.18$
observed
imputed
estimate CCA
estimate ITS−R
Model residuals of tetradecanediaote
2
1
0
−1
−2
$n_{CCA} = 397$
$n_{ITS-R} = 1153$
$n_{CCA} = 179$
$n_{ITS-R} = 463$
$n_{CCA} = 39$
$n_{ITS-R} = 60$
major homozygote
heterozygote
minor homozygote
Genotype of SNP rs12317268 (SLCO1B1 (2 separate loci))

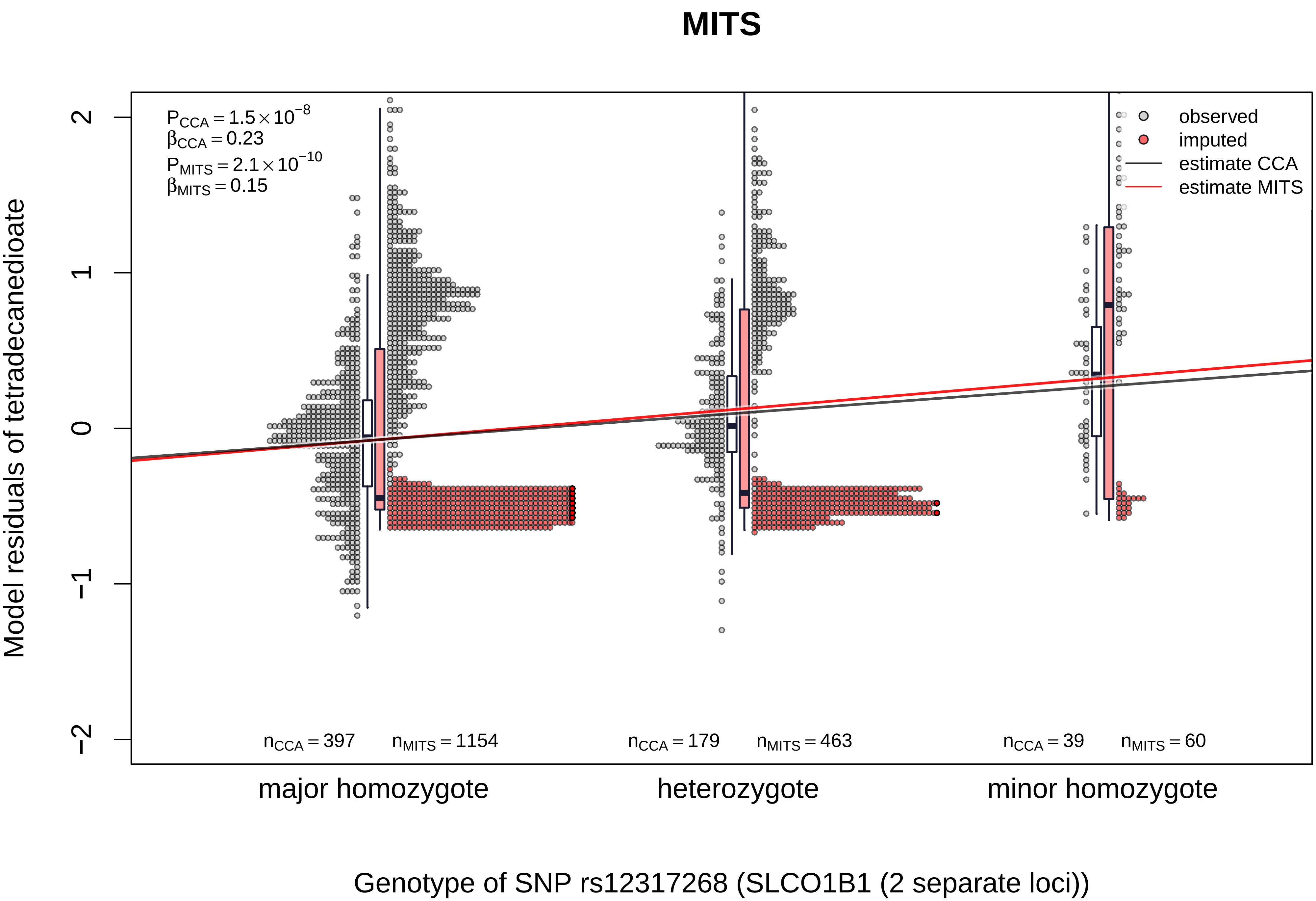

MITS
$P_{CCA} = 1.5 \times 10^{-8}$
$\beta_{CCA} = 0.23$
$P_{MITS} = 2.1 \times 10^{-10}$
$\beta_{MITS} = 0.15$
observed
imputed
estimate CCA
estimate MITS
Model residuals of tetradecanedioate
2
1
0
−1
−2
$n_{CCA} = 397$
$n_{MITS} = 1154$
$n_{CCA} = 179$
$n_{MITS} = 463$
$n_{CCA} = 39$
$n_{MITS} = 60$
major homozygote
heterozygote
minor homozygote
Genotype of SNP rs12317268 (SLCO1B1 (2 separate loci))

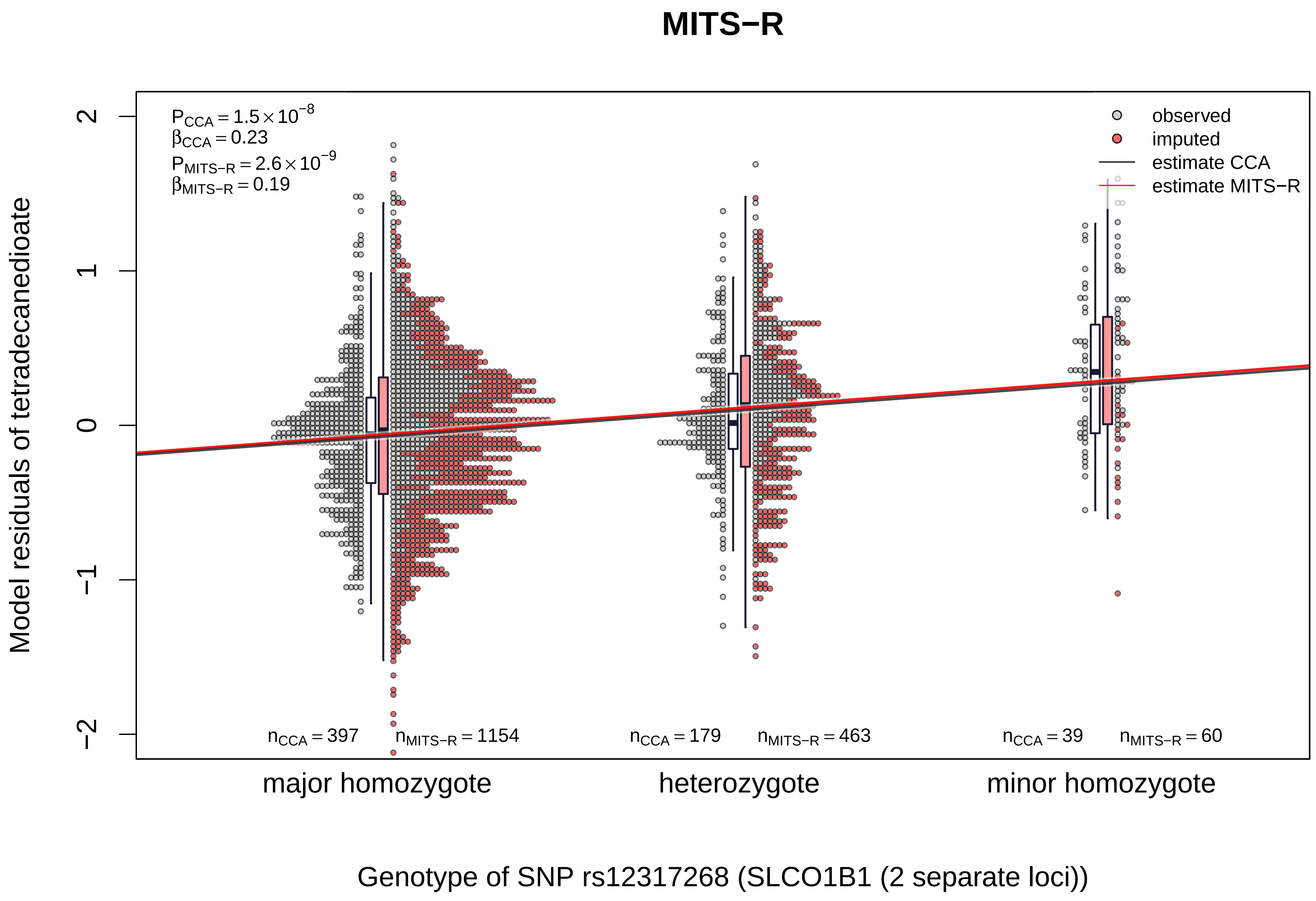

MITS−R
$P_{CCA} = 1.5 \times 10^{-8}$
$\beta_{CCA} = 0.23$
$P_{MITS-R} = 2.6 \times 10^{-9}$
$\beta_{MITS-R} = 0.19$
observed
imputed
estimate CCA
estimate MITS−R
Model residuals of tetradecanediote
2
1
0
−1
−2
$n_{CCA} = 397$
$n_{MITS-R} = 1154$
$n_{CCA} = 179$
$n_{MITS-R} = 463$
$n_{CCA} = 39$
$n_{MITS-R} = 60$
major homozygote
heterozygote
minor homozygote
Genotype of SNP rs12317268 (SLCO1B1 (2 separate loci))

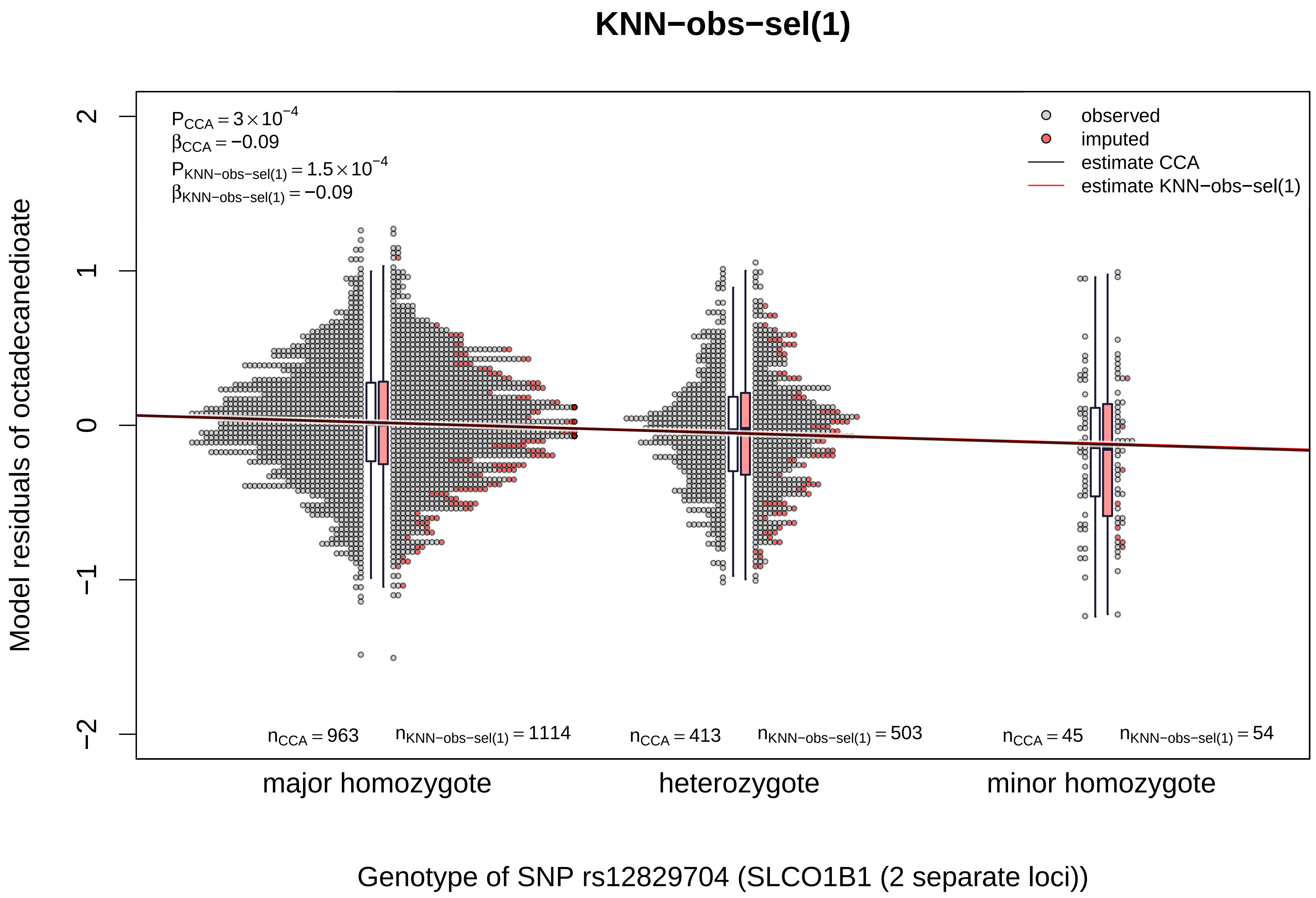

KNN−obs−sel(1)
P_CCA = 3×10^−4
β_CCA = −0.09
P_KNN−obs−sel(1) = 1.5×10^−4
β_KNN−obs−sel(1) = −0.09
observed
imputed
estimate CCA
estimate KNN−obs−sel(1)
Model residuals of octadecanedioate
2
1
0
−1
−2
n_CCA = 963
n_KNN−obs−sel(1) = 1114
n_CCA = 413
n_KNN−obs−sel(1) = 503
n_CCA = 45
n_KNN−obs−sel(1) = 54
major homozygote
heterozygote
minor homozygote
Genotype of SNP rs12829704 (SLCO1B1 (2 separate loci))

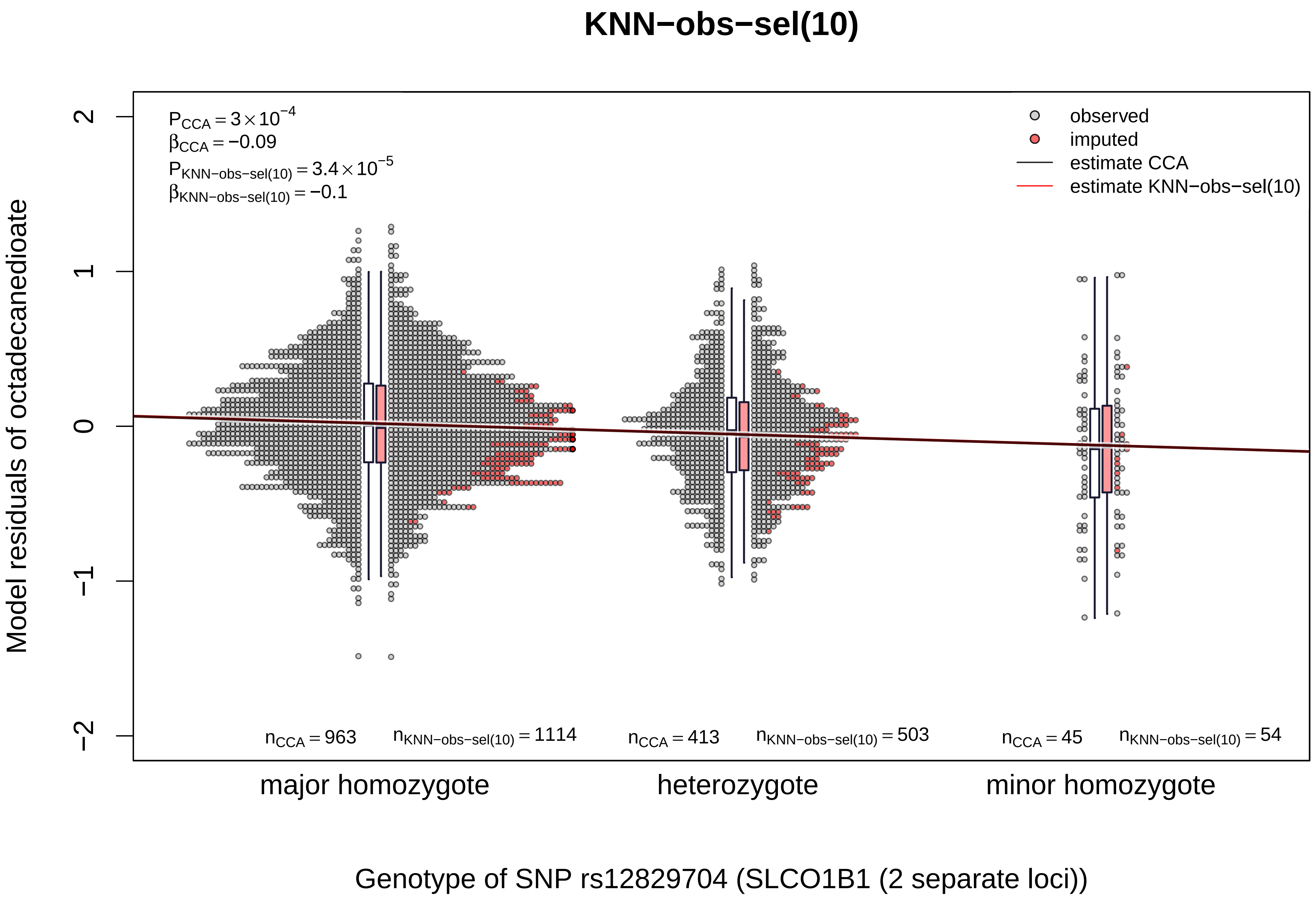

KNN−obs−sel(10)
P_CCA = 3 × 10^−4
β_CCA = −0.09
P_KNN−obs−sel(10) = 3.4 × 10^−5
β_KNN−obs−sel(10) = −0.1
observed
imputed
estimate CCA
estimate KNN−obs−sel(10)
Model residuals of octadecanediolate
2
1
0
−1
−2
n_CCA = 963
n_KNN−obs−sel(10) = 1114
n_CCA = 413
n_KNN−obs−sel(10) = 503
n_CCA = 45
n_KNN−obs−sel(10) = 54
major homozygote
heterozygote
minor homozygote
Genotype of SNP rs12829704 (SLCO1B1 (2 separate loci))

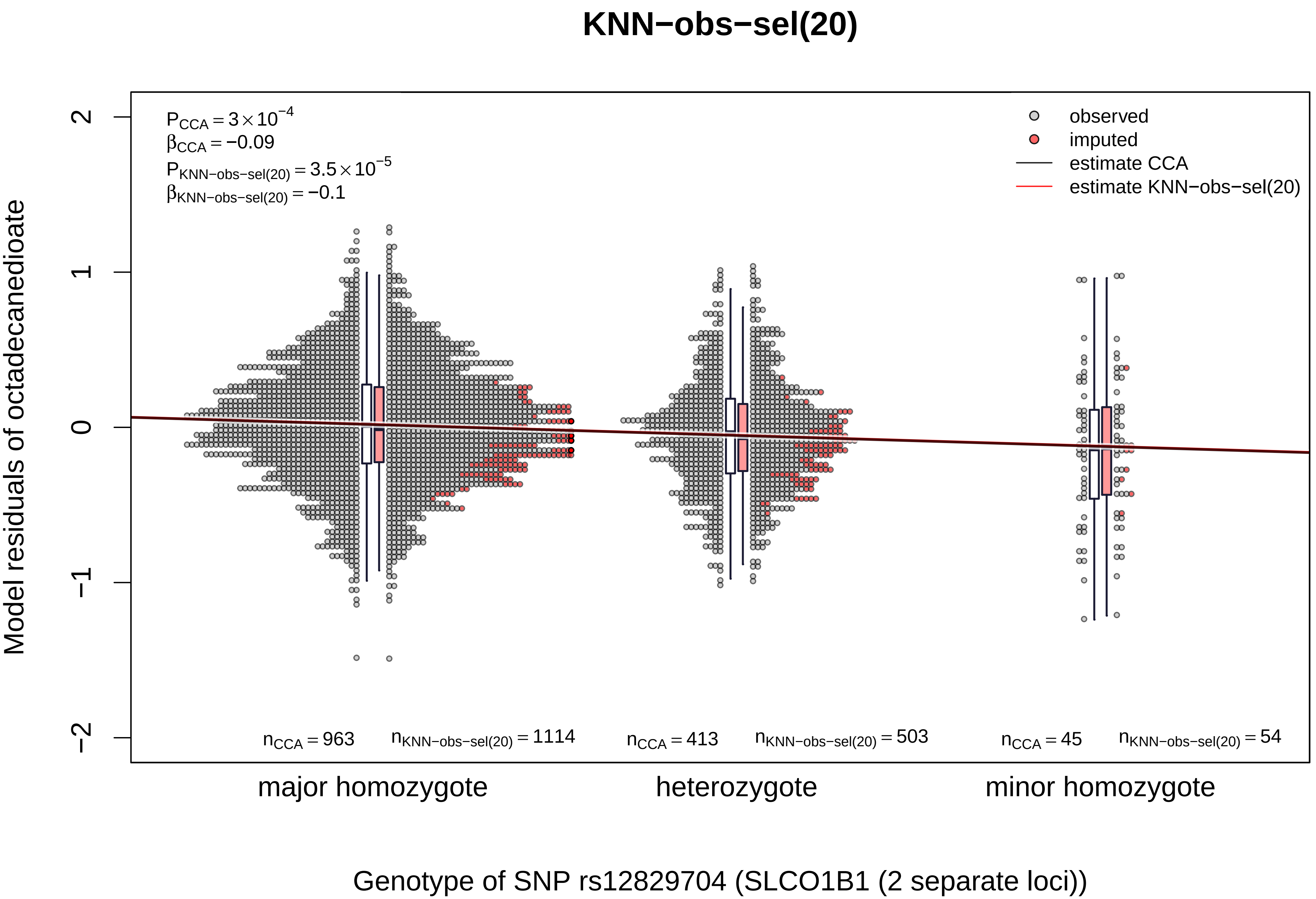
KNN−obs−sel(20)
$P_{CCA} = 3 \times 10^{-4}$
$\beta_{CCA} = -0.09$
$P_{KNN-obs-sel(20)} = 3.5 \times 10^{-5}$
$\beta_{KNN-obs-sel(20)} = -0.1$
observed
imputed
estimate CCA
estimate KNN−obs−sel(20)
Model residuals of octadecanedioate
2
1
0
−1
−2
$n_{CCA} = 963$
$n_{KNN-obs-sel(20)} = 1114$
$n_{CCA} = 413$
$n_{KNN-obs-sel(20)} = 503$
$n_{CCA} = 45$
$n_{KNN-obs-sel(20)} = 54$
major homozygote
heterozygote
minor homozygote
Genotype of SNP rs12829704 (SLCO1B1 (2 separate loci))

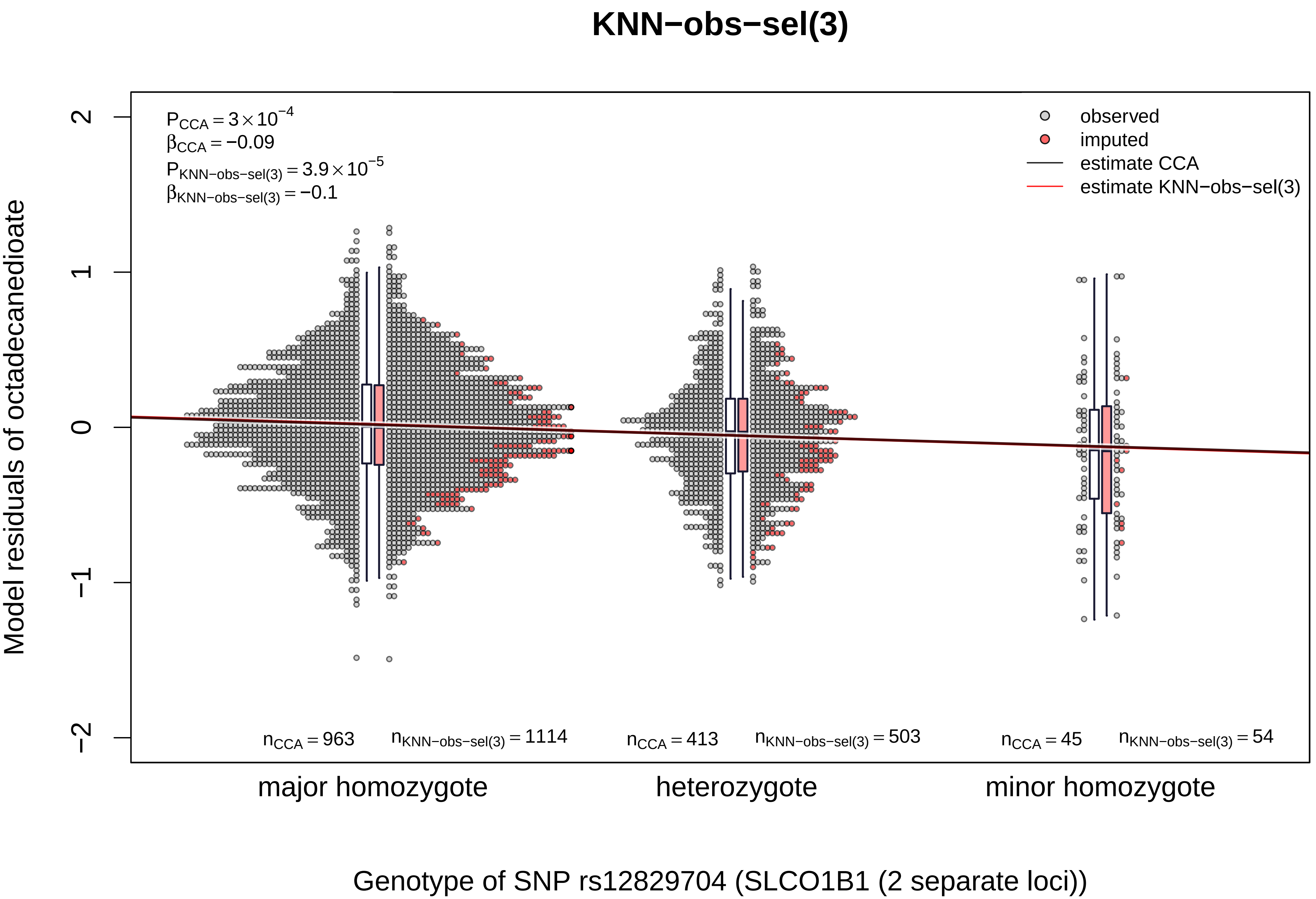

KNN−obs−sel(3)
$P_{CCA} = 3 \times 10^{-4}$
$\beta_{CCA} = -0.09$
$P_{KNN-obs-sel(3)} = 3.9 \times 10^{-5}$
$\beta_{KNN-obs-sel(3)} = -0.1$
observed
imputed
estimate CCA
estimate KNN−obs−sel(3)
Model residuals of octadecanediodate
2
1
0
−1
−2
$n_{CCA} = 963$
$n_{KNN-obs-sel(3)} = 1114$
$n_{CCA} = 413$
$n_{KNN-obs-sel(3)} = 503$
$n_{CCA} = 45$
$n_{KNN-obs-sel(3)} = 54$
major homozygote
heterozygote
minor homozygote
Genotype of SNP rs12829704 (SLCO1B1 (2 separate loci))

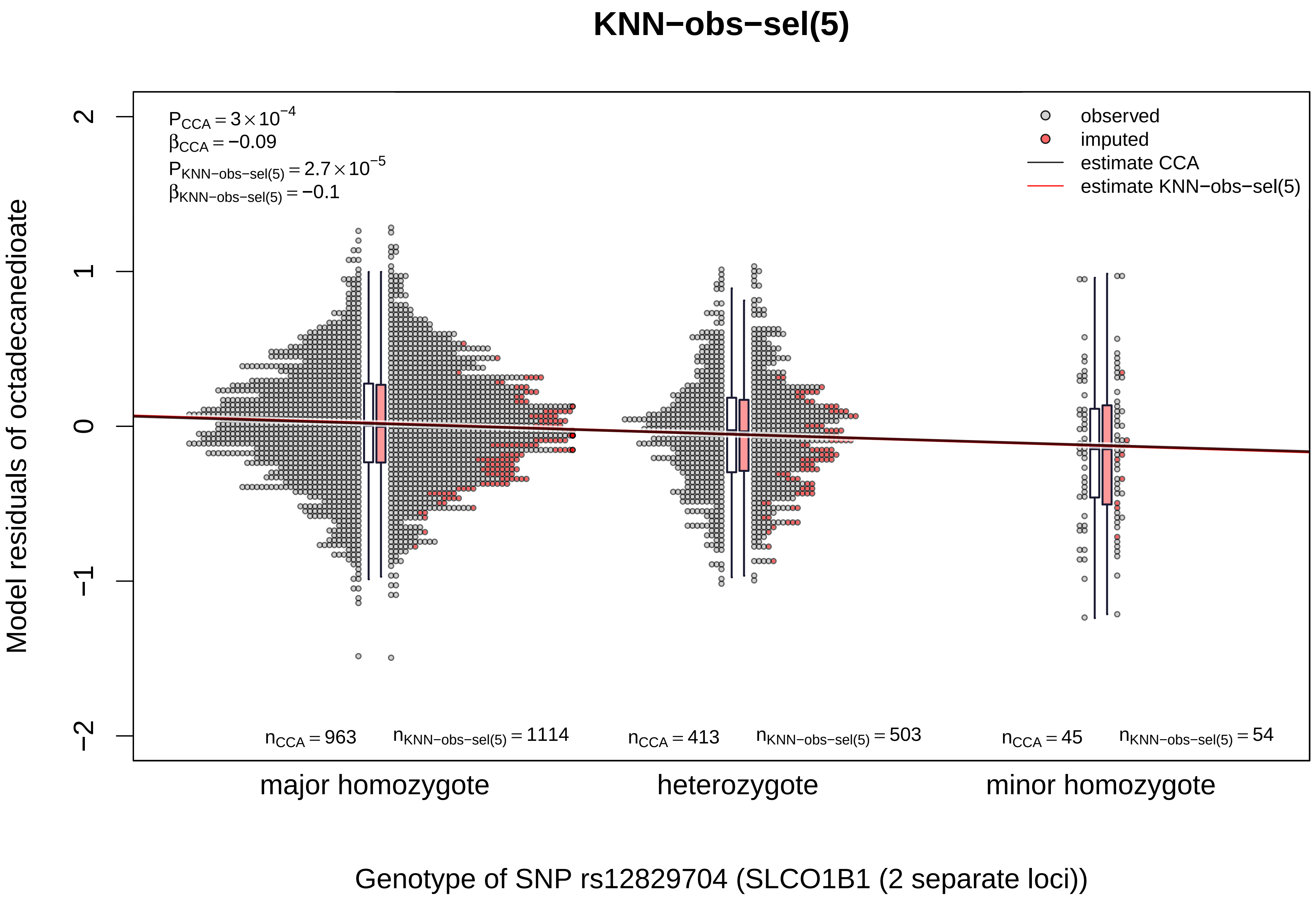

KNN−obs−sel(5)
$P_{CCA} = 3 \times 10^{-4}$
$\beta_{CCA} = -0.09$
$P_{KNN-obs-sel(5)} = 2.7 \times 10^{-5}$
$\beta_{KNN-obs-sel(5)} = -0.1$
observed
imputed
estimate CCA
estimate KNN−obs−sel(5)
Model residuals of octadecanediodate
2
1
0
−1
−2
$n_{CCA} = 963$
$n_{KNN-obs-sel(5)} = 1114$
$n_{CCA} = 413$
$n_{KNN-obs-sel(5)} = 503$
$n_{CCA} = 45$
$n_{KNN-obs-sel(5)} = 54$
major homozygote
heterozygote
minor homozygote
Genotype of SNP rs12829704 (SLCO1B1 (2 separate loci))

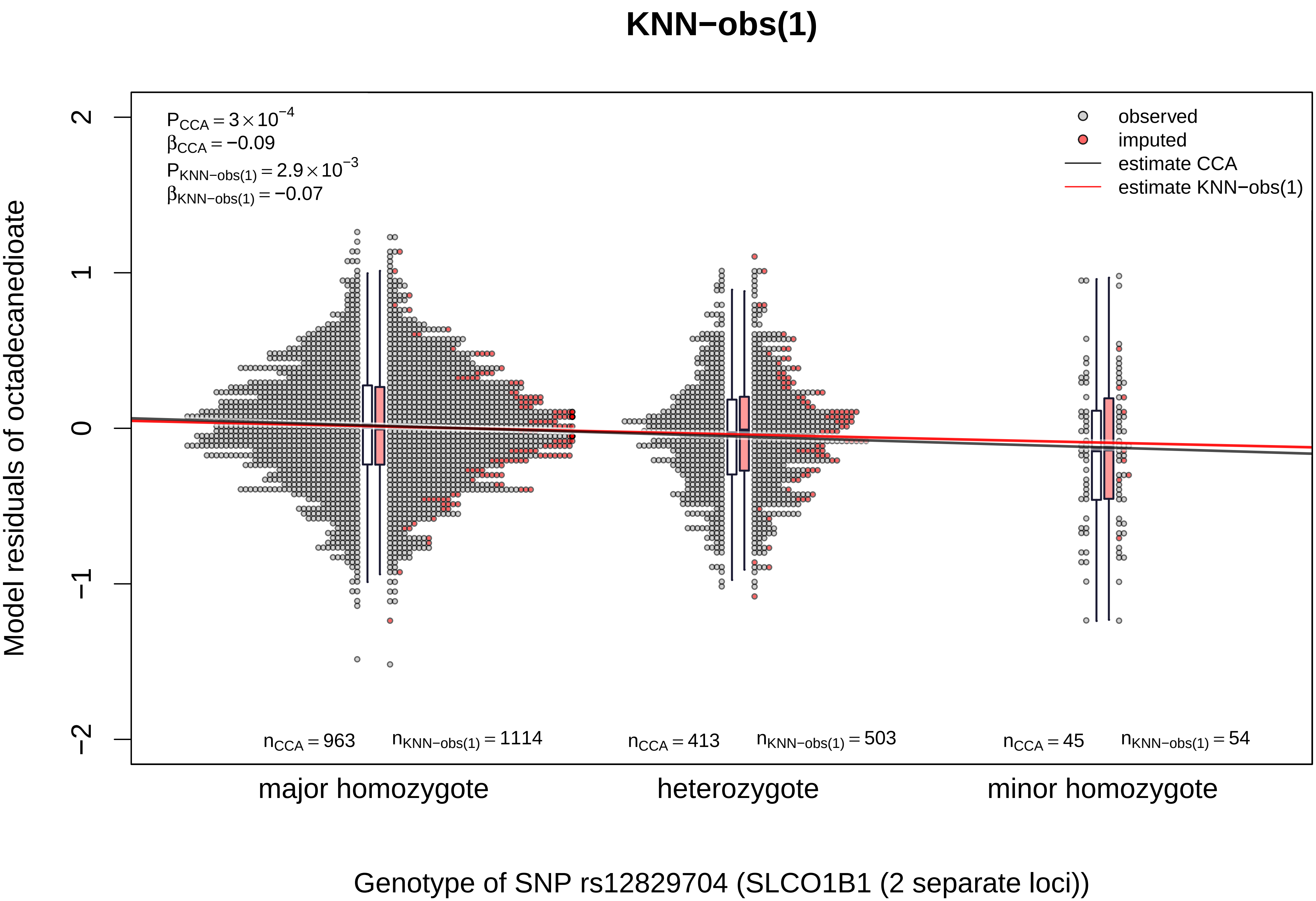

KNN−obs(1)
$P_{CCA} = 3 \times 10^{-4}$
$\beta_{CCA} = -0.09$
$P_{KNN-obs(1)} = 2.9 \times 10^{-3}$
$\beta_{KNN-obs(1)} = -0.07$
observed
imputed
estimate CCA
estimate KNN−obs(1)
Model residuals of octadecanedioate
2
1
0
−1
−2
$n_{CCA} = 963$
$n_{KNN-obs(1)} = 1114$
$n_{CCA} = 413$
$n_{KNN-obs(1)} = 503$
$n_{CCA} = 45$
$n_{KNN-obs(1)} = 54$
major homozygote
heterozygote
minor homozygote
Genotype of SNP rs12829704 (SLCO1B1 (2 separate loci))

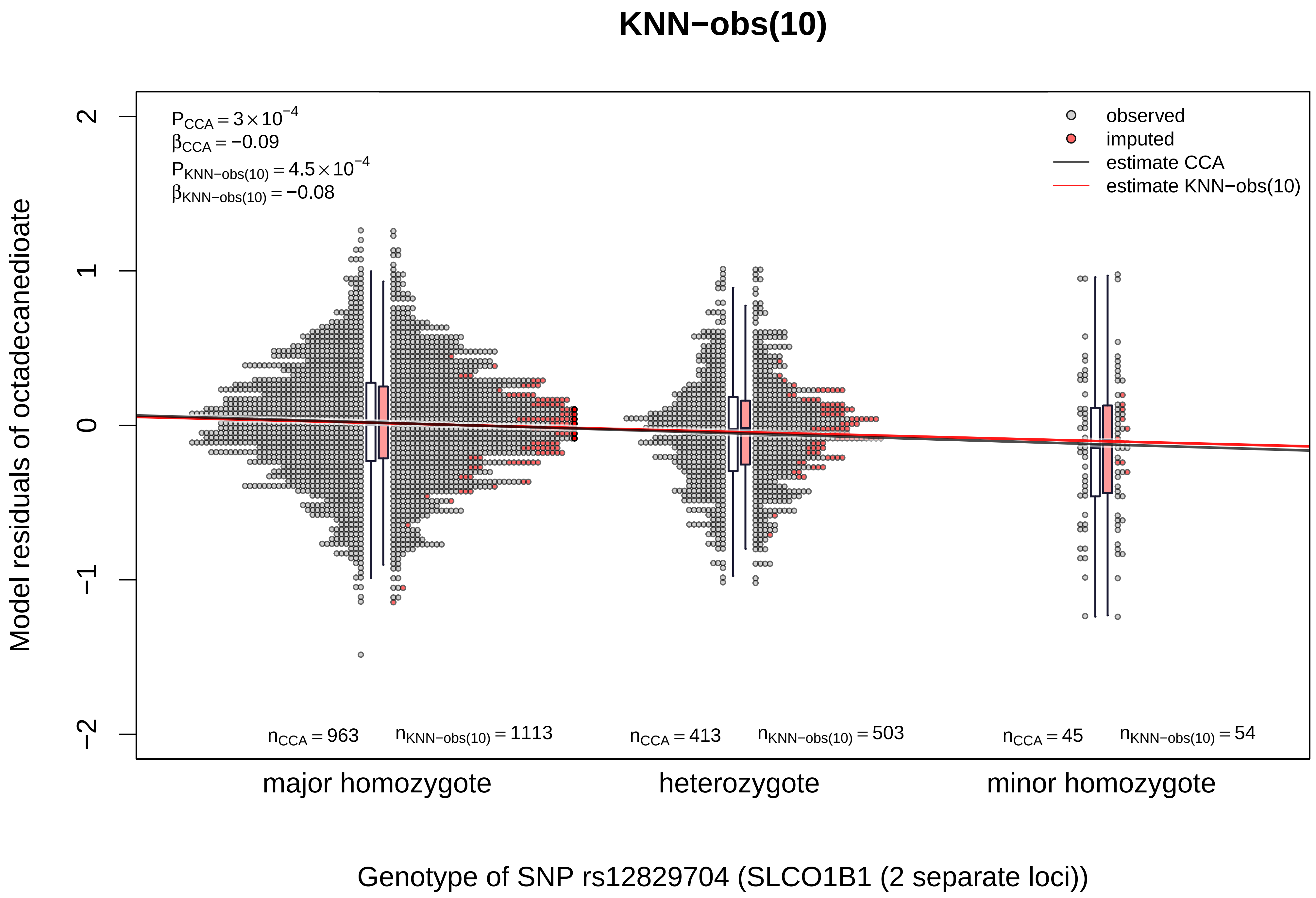

KNN−obs(10)
$P_{CCA} = 3 \times 10^{-4}$
$\beta_{CCA} = -0.09$
$P_{KNN-obs(10)} = 4.5 \times 10^{-4}$
$\beta_{KNN-obs(10)} = -0.08$
observed
imputed
estimate CCA
estimate KNN−obs(10)
Model residuals of octadecanedioate
2
1
0
−1
−2
$n_{CCA} = 963$
$n_{KNN-obs(10)} = 1113$
$n_{CCA} = 413$
$n_{KNN-obs(10)} = 503$
$n_{CCA} = 45$
$n_{KNN-obs(10)} = 54$
major homozygote
heterozygote
minor homozygote
Genotype of SNP rs12829704 (SLCO1B1 (2 separate loci))

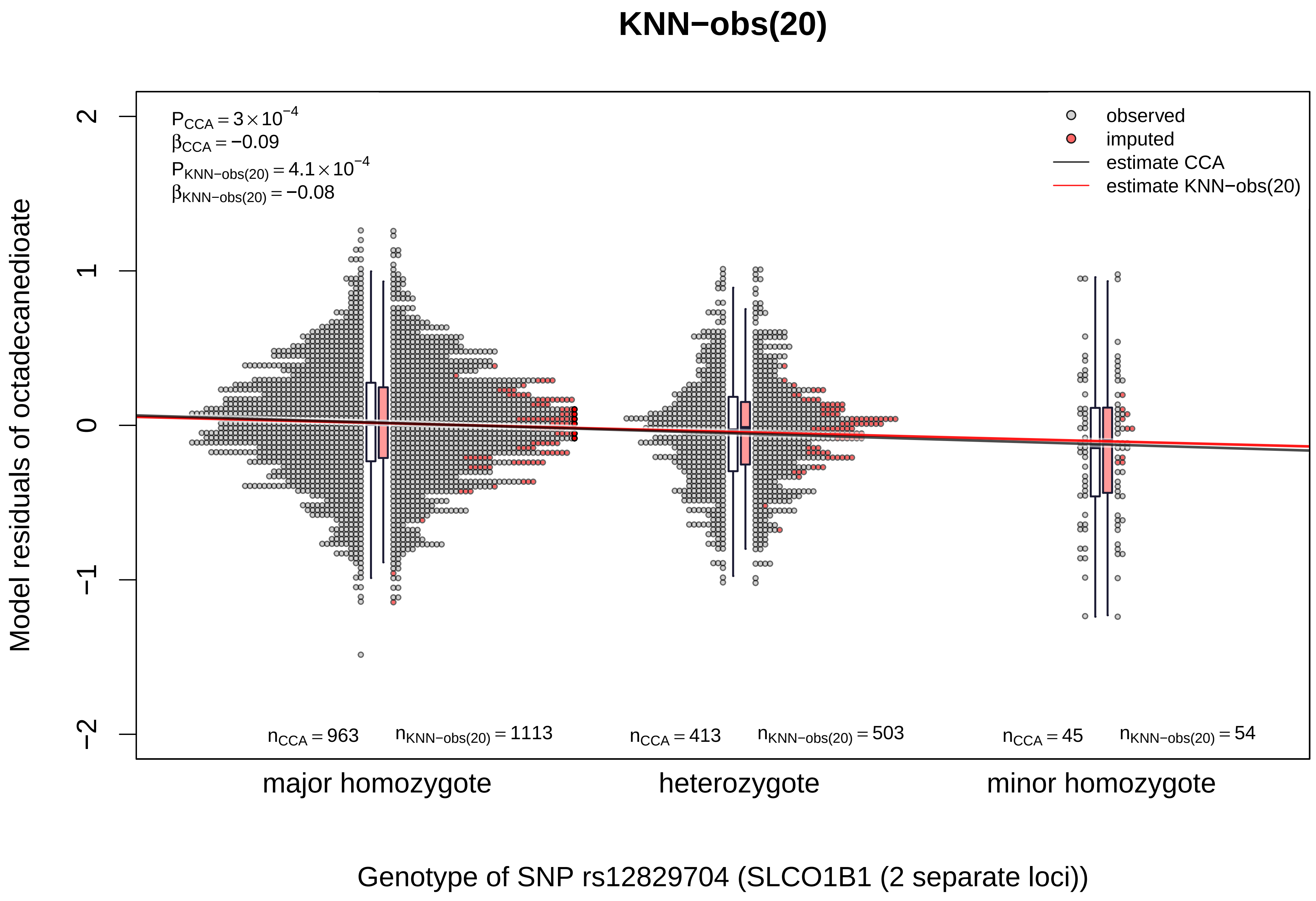
KNN−obs(20)
$P_{CCA} = 3 \times 10^{-4}$
$\beta_{CCA} = -0.09$
$P_{KNN-obs(20)} = 4.1 \times 10^{-4}$
$\beta_{KNN-obs(20)} = -0.08$
observed
imputed
estimate CCA
estimate KNN−obs(20)
Model residuals of octadecanedioate
2
1
0
−1
−2
$n_{CCA} = 963$
$n_{KNN-obs(20)} = 1113$
$n_{CCA} = 413$
$n_{KNN-obs(20)} = 503$
$n_{CCA} = 45$
$n_{KNN-obs(20)} = 54$
major homozygote
heterozygote
minor homozygote
Genotype of SNP rs12829704 (SLCO1B1 (2 separate loci))

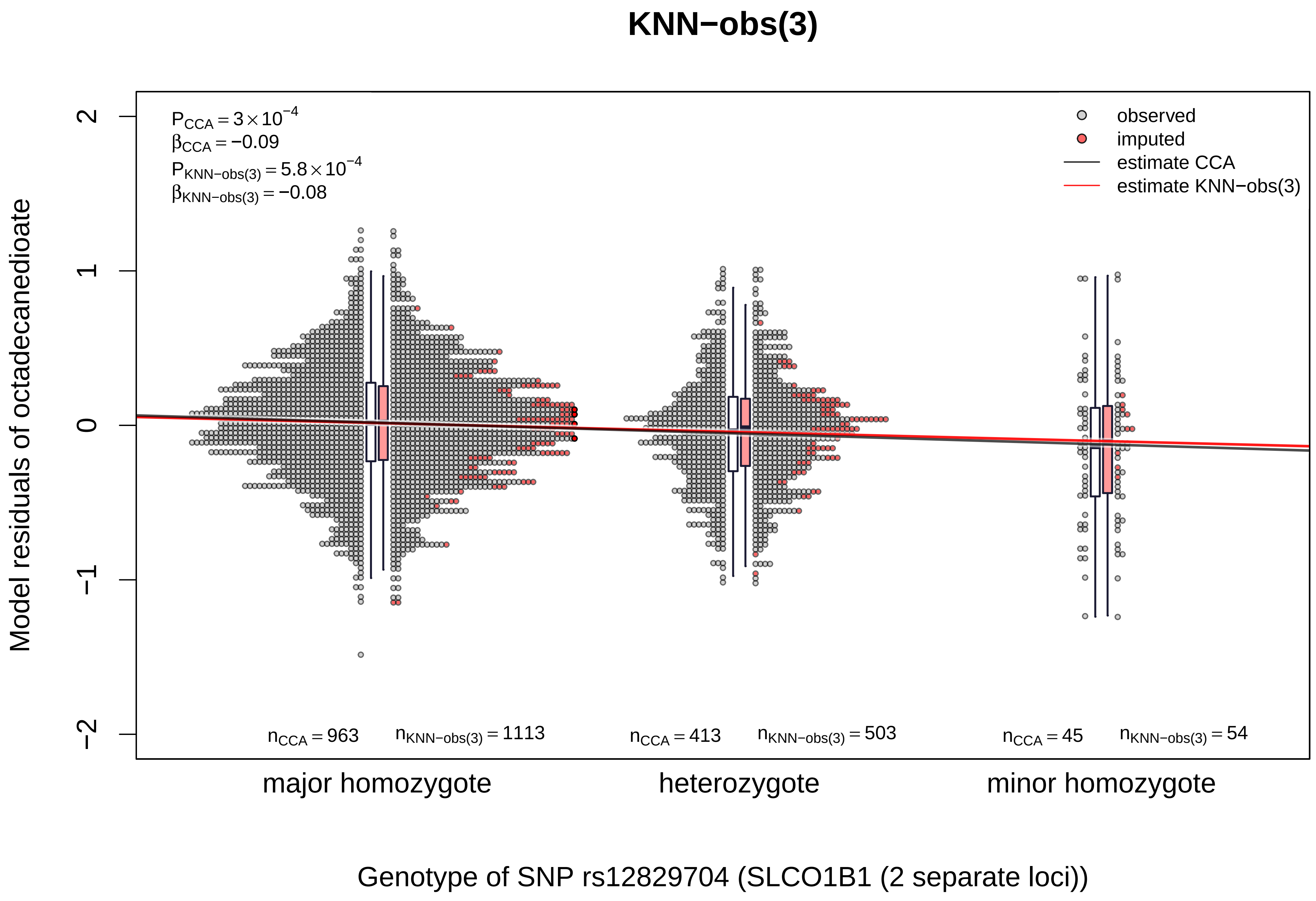

KNN−obs(3)
$P_{CCA} = 3 \times 10^{-4}$
$\beta_{CCA} = -0.09$
$P_{KNN-obs(3)} = 5.8 \times 10^{-4}$
$\beta_{KNN-obs(3)} = -0.08$
observed
imputed
estimate CCA
estimate KNN−obs(3)
Model residuals of octadecanedioate
2
1
0
−1
−2
$n_{CCA} = 963$
$n_{KNN-obs(3)} = 1113$
$n_{CCA} = 413$
$n_{KNN-obs(3)} = 503$
$n_{CCA} = 45$
$n_{KNN-obs(3)} = 54$
major homozygote
heterozygote
minor homozygote
Genotype of SNP rs12829704 (SLCO1B1 (2 separate loci))

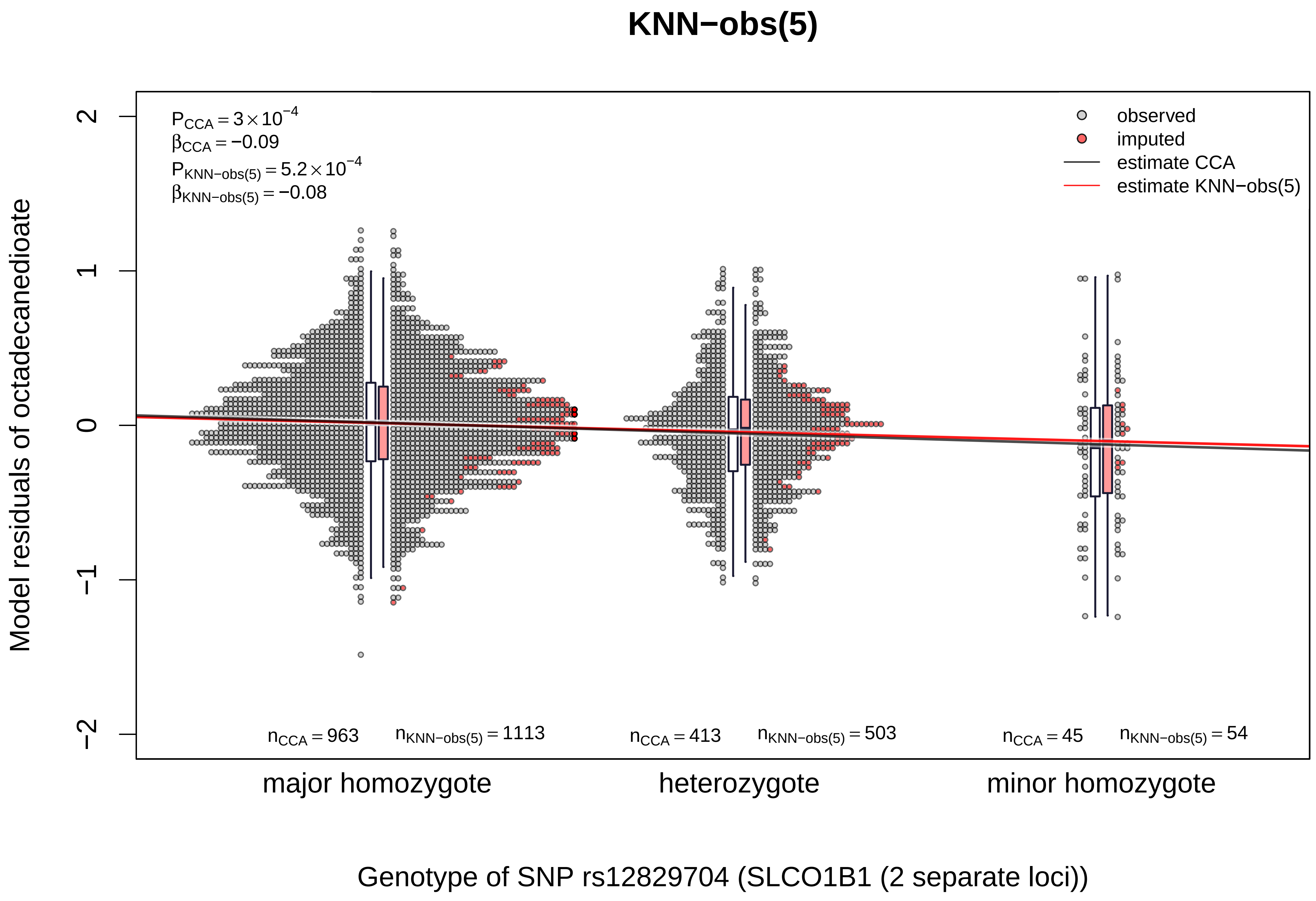

KNN−obs(5)
$P_{CCA} = 3 \times 10^{-4}$
$\beta_{CCA} = -0.09$
$P_{KNN-obs(5)} = 5.2 \times 10^{-4}$
$\beta_{KNN-obs(5)} = -0.08$
observed
imputed
estimate CCA
estimate KNN−obs(5)
Model residuals of octadecanedioate
2
1
0
−1
−2
$n_{CCA} = 963$
$n_{KNN-obs(5)} = 1113$
$n_{CCA} = 413$
$n_{KNN-obs(5)} = 503$
$n_{CCA} = 45$
$n_{KNN-obs(5)} = 54$
major homozygote
heterozygote
minor homozygote
Genotype of SNP rs12829704 (SLCO1B1 (2 separate loci))

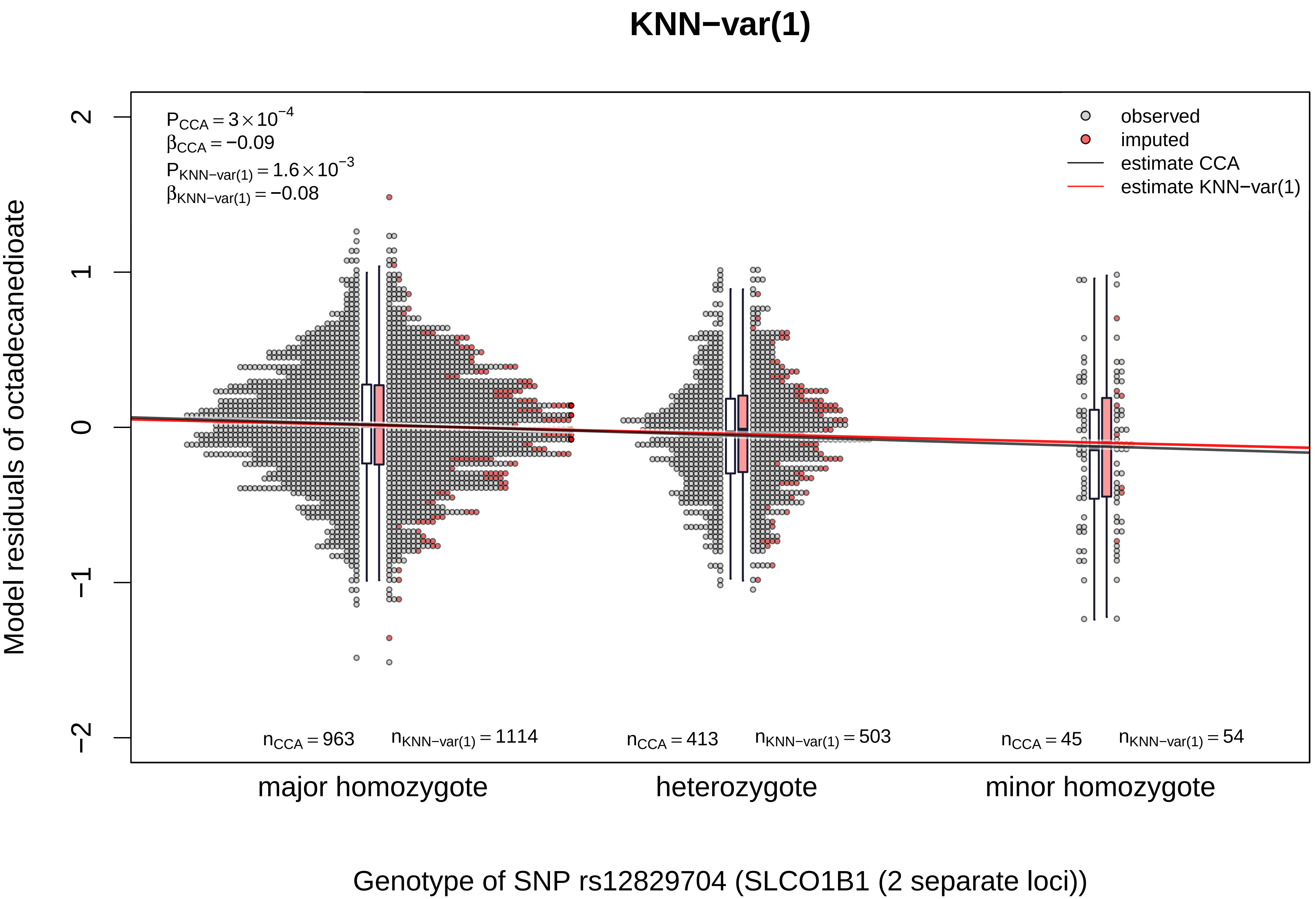
KNN−var(1)
$P_{CCA} = 3 \times 10^{-4}$
$\beta_{CCA} = -0.09$
$P_{KNN-var(1)} = 1.6 \times 10^{-3}$
$\beta_{KNN-var(1)} = -0.08$
observed
imputed
estimate CCA
estimate KNN−var(1)
Model residuals of octadecanediolate
$n_{CCA} = 963$
$n_{KNN-var(1)} = 1114$
$n_{CCA} = 413$
$n_{KNN-var(1)} = 503$
$n_{CCA} = 45$
$n_{KNN-var(1)} = 54$
major homozygote
heterozygote
minor homozygote
Genotype of SNP rs12829704 (SLCO1B1 (2 separate loci))

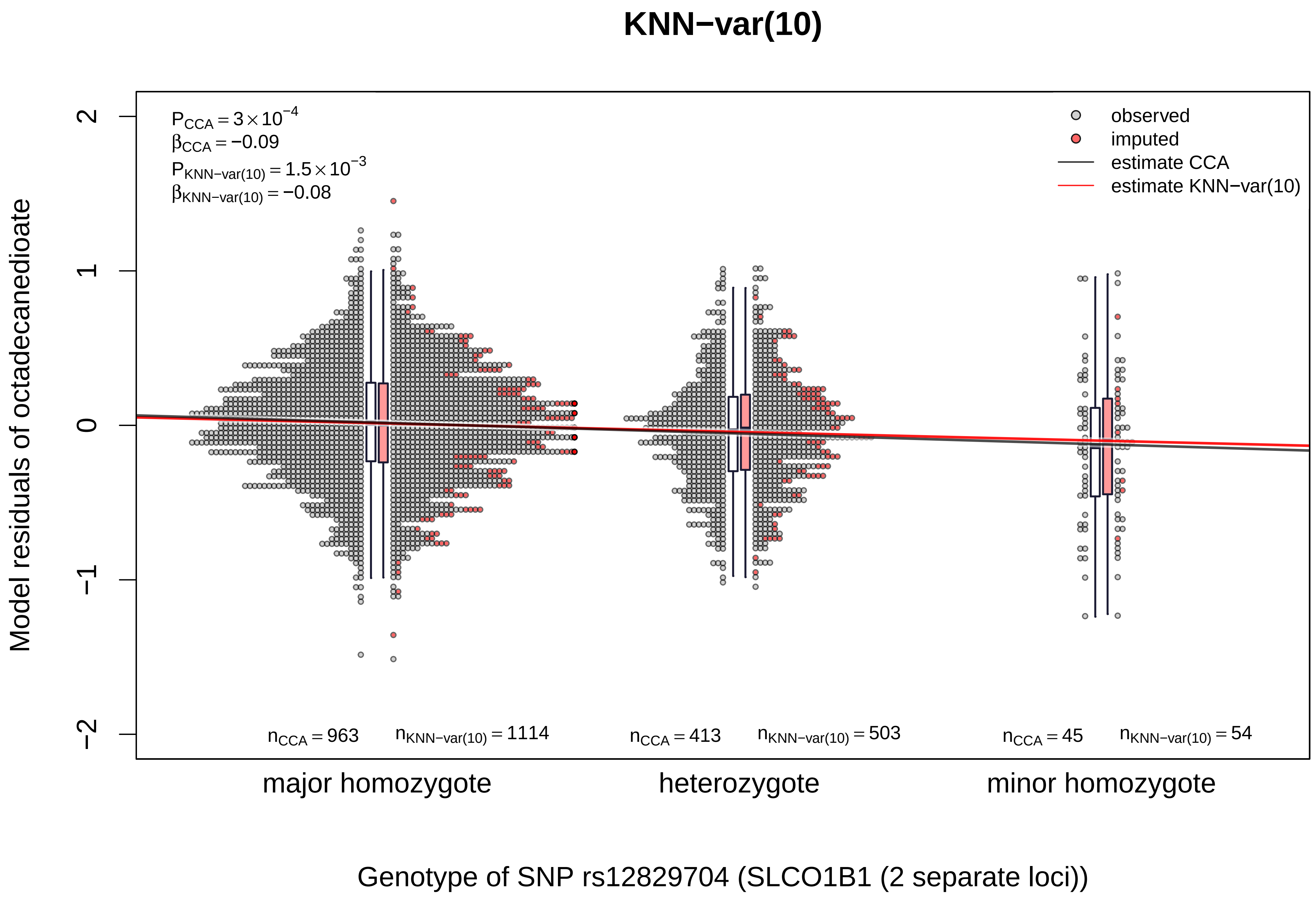
KNN−var(10)
$P_{CCA} = 3 \times 10^{-4}$
$\beta_{CCA} = -0.09$
$P_{KNN-var(10)} = 1.5 \times 10^{-3}$
$\beta_{KNN-var(10)} = -0.08$
observed
imputed
estimate CCA
estimate KNN−var(10)
Model residuals of octadecanedioate
2
1
0
−1
−2
$n_{CCA} = 963$
$n_{KNN-var(10)} = 1114$
$n_{CCA} = 413$
$n_{KNN-var(10)} = 503$
$n_{CCA} = 45$
$n_{KNN-var(10)} = 54$
major homozygote
heterozygote
minor homozygote
Genotype of SNP rs12829704 (SLCO1B1 (2 separate loci))

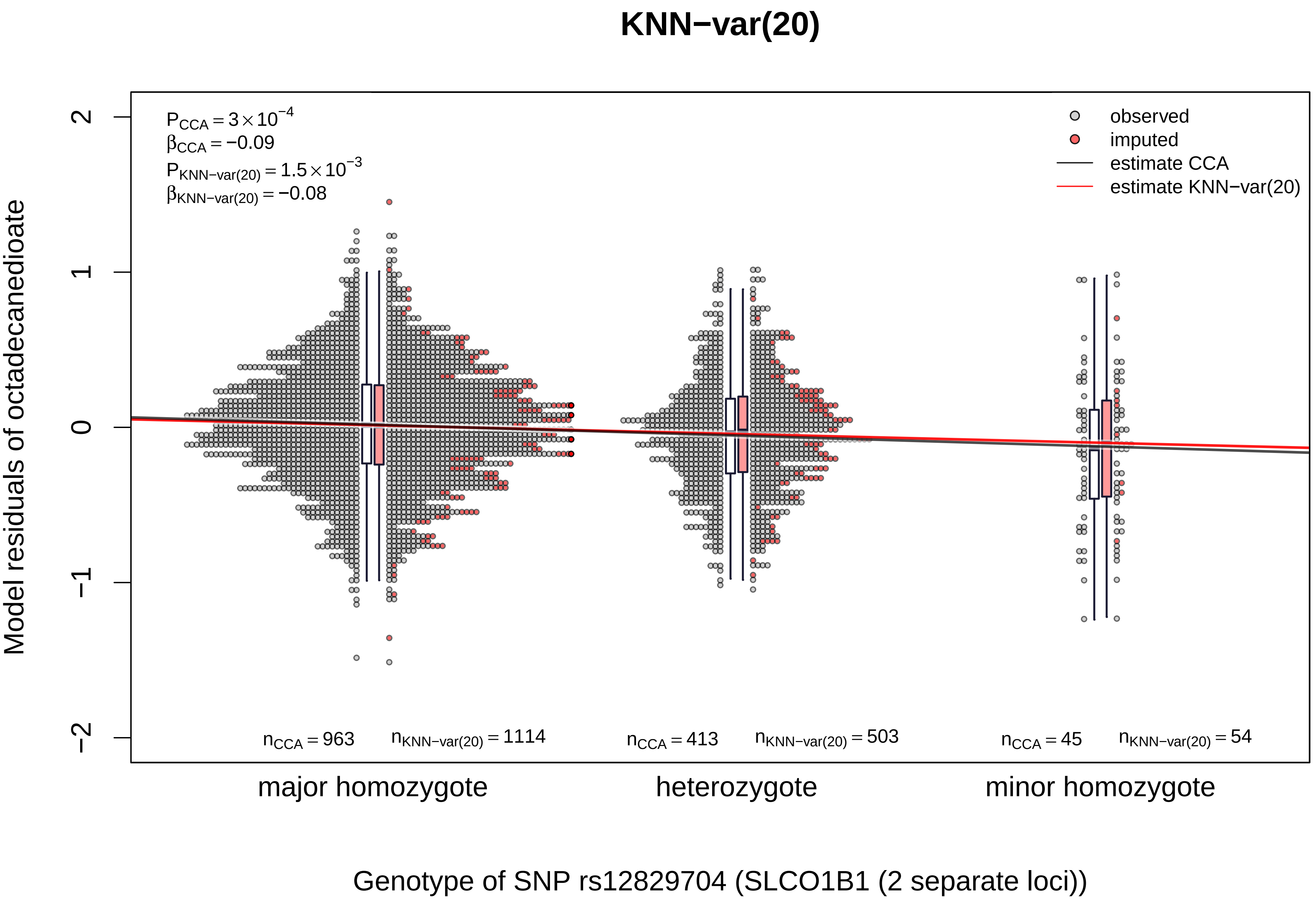

KNN−var(20)
$P_{CCA} = 3 \times 10^{-4}$
$\beta_{CCA} = -0.09$
$P_{KNN-var(20)} = 1.5 \times 10^{-3}$
$\beta_{KNN-var(20)} = -0.08$
observed
imputed
estimate CCA
estimate KNN−var(20)
2
1
0
−1
−2
Model residuals of octadecanediaote
$n_{CCA} = 963$
$n_{KNN-var(20)} = 1114$
$n_{CCA} = 413$
$n_{KNN-var(20)} = 503$
$n_{CCA} = 45$
$n_{KNN-var(20)} = 54$
major homozygote
heterozygote
minor homozygote
Genotype of SNP rs12829704 (SLCO1B1 (2 separate loci))

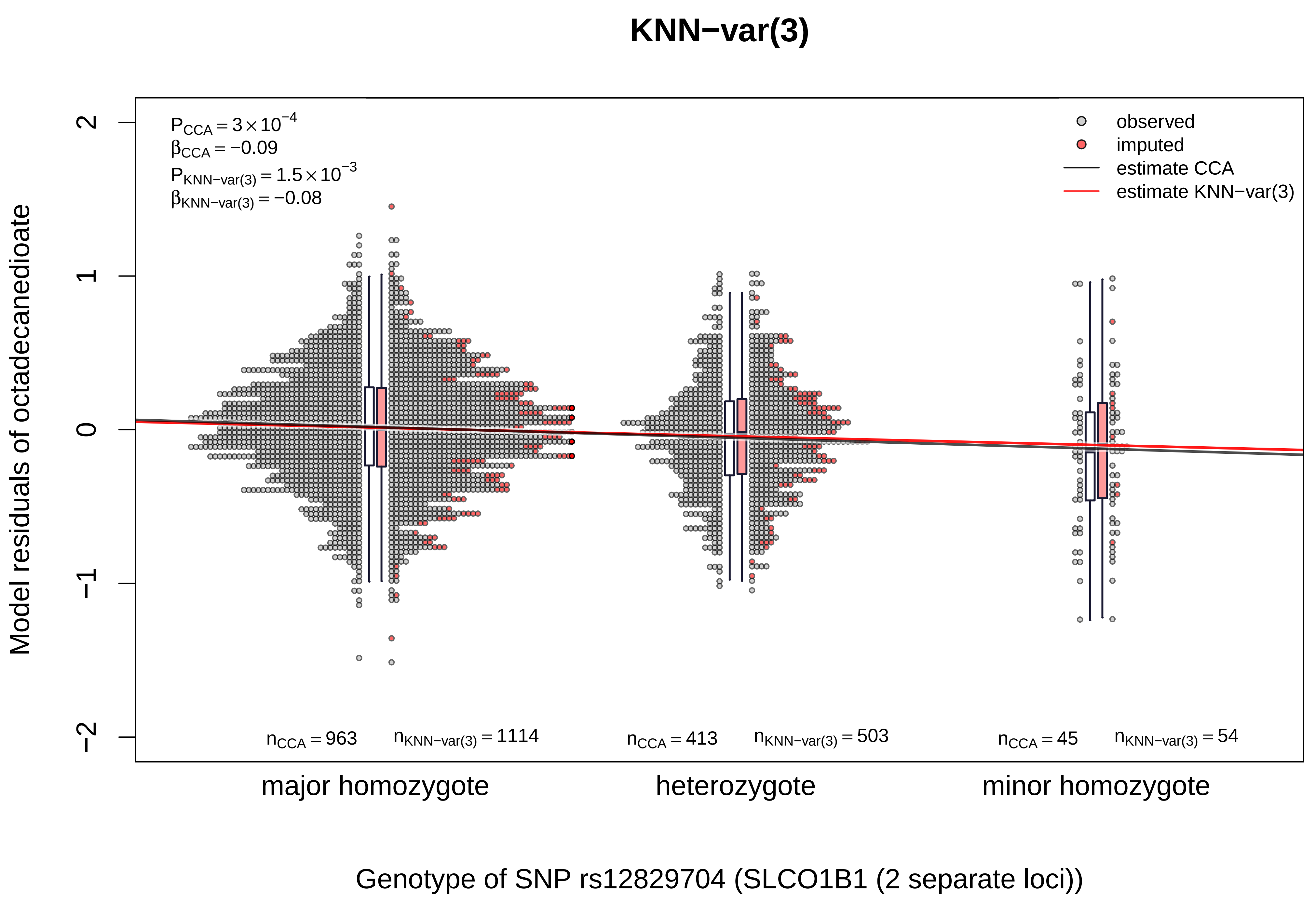

KNN−var(3)
$P_{CCA} = 3 \times 10^{-4}$
$\beta_{CCA} = -0.09$
$P_{KNN-var(3)} = 1.5 \times 10^{-3}$
$\beta_{KNN-var(3)} = -0.08$
observed
imputed
estimate CCA
estimate KNN−var(3)
Model residuals of octadecanedioate
2
1
0
−1
−2
$n_{CCA} = 963$
$n_{KNN-var(3)} = 1114$
$n_{CCA} = 413$
$n_{KNN-var(3)} = 503$
$n_{CCA} = 45$
$n_{KNN-var(3)} = 54$
major homozygote
heterozygote
minor homozygote
Genotype of SNP rs12829704 (SLCO1B1 (2 separate loci))

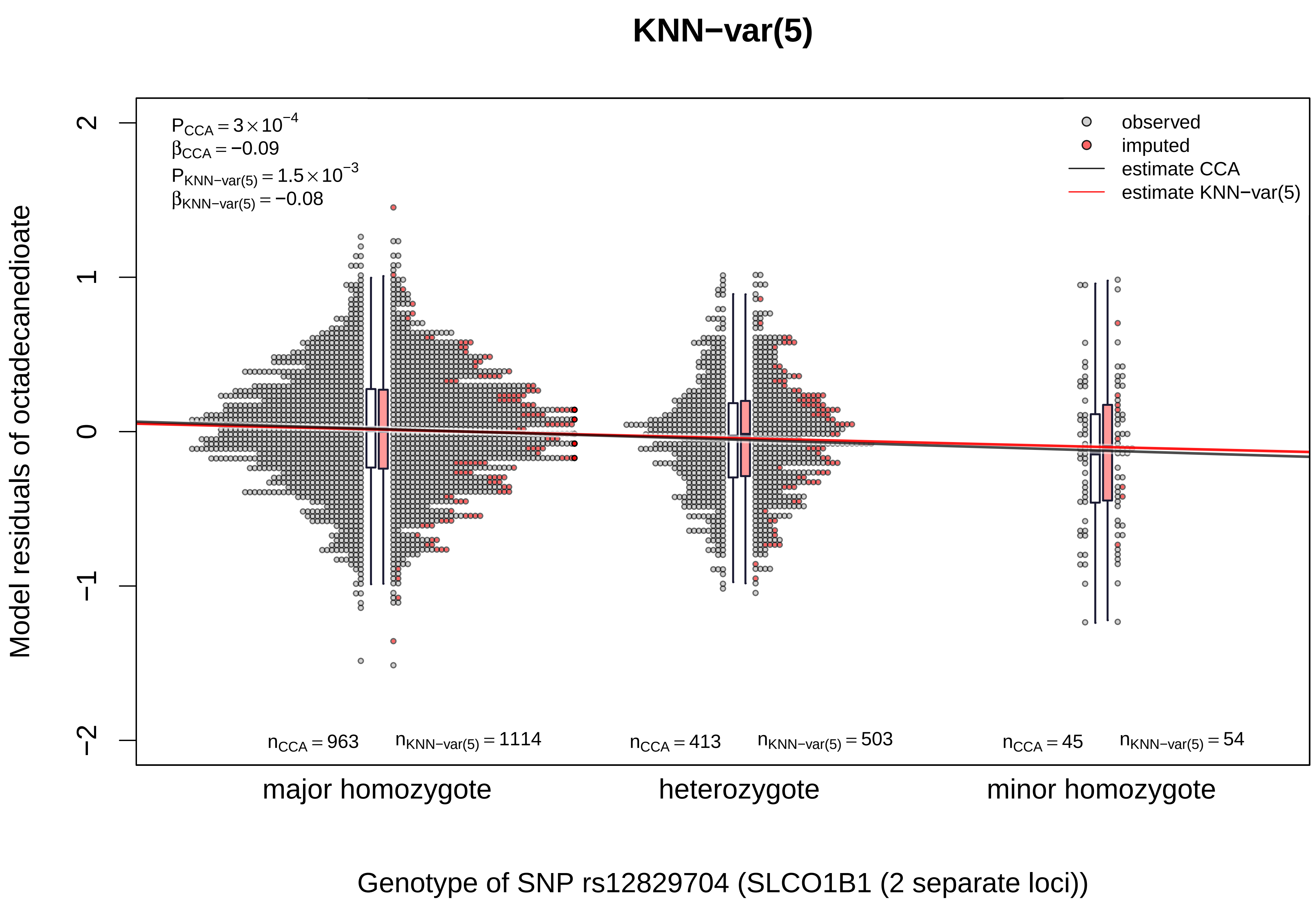

KNN−var(5)
$P_{CCA} = 3 \times 10^{-4}$
$\beta_{CCA} = -0.09$
$P_{KNN-var(5)} = 1.5 \times 10^{-3}$
$\beta_{KNN-var(5)} = -0.08$
observed
imputed
estimate CCA
estimate KNN−var(5)
Model residuals of octadecanedioate
2
1
0
−1
−2
$n_{CCA} = 963$
$n_{KNN-var(5)} = 1114$
$n_{CCA} = 413$
$n_{KNN-var(5)} = 503$
$n_{CCA} = 45$
$n_{KNN-var(5)} = 54$
major homozygote
heterozygote
minor homozygote
Genotype of SNP rs12829704 (SLCO1B1 (2 separate loci))

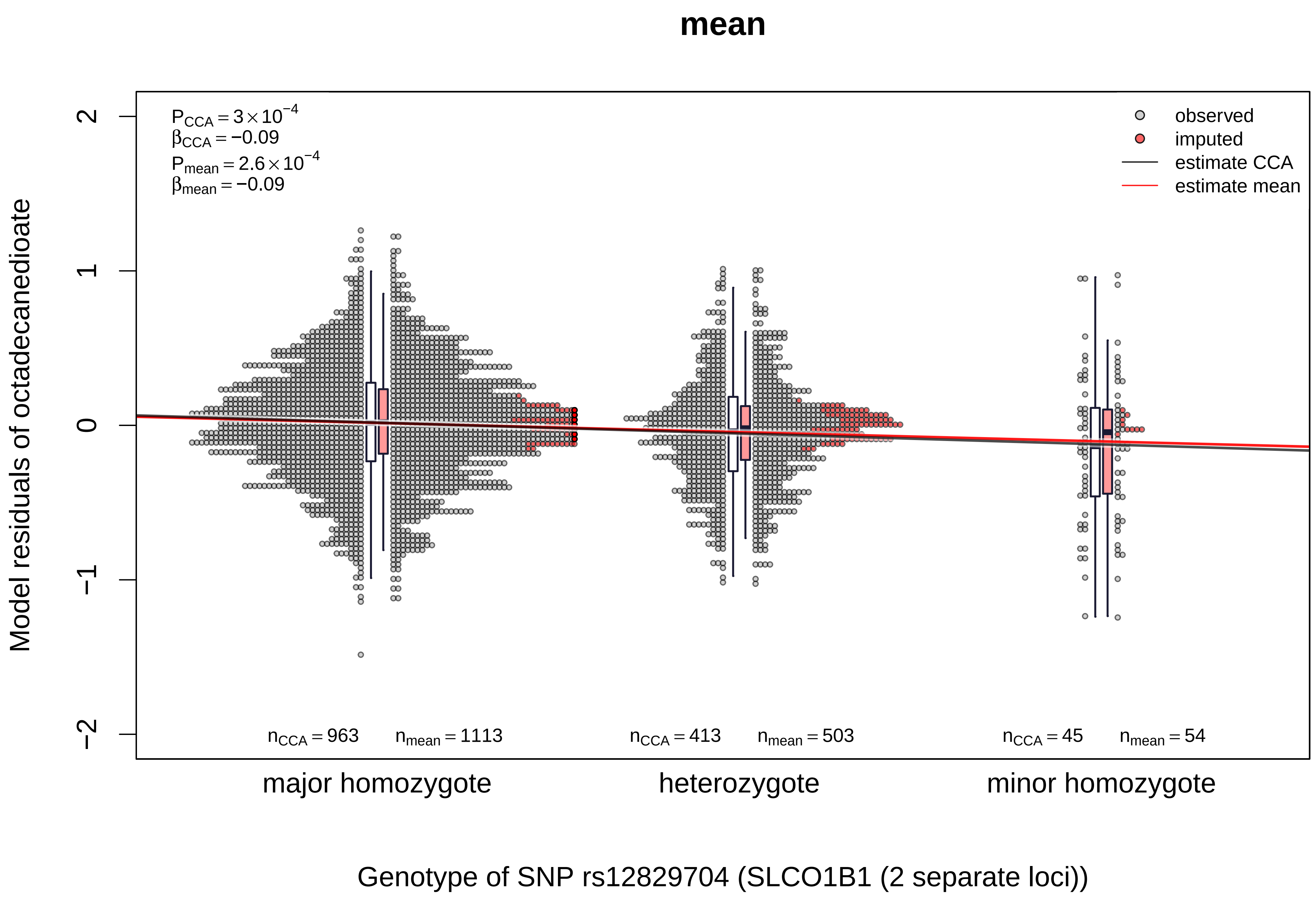

mean
$P_{CCA} = 3 \times 10^{-4}$
$\beta_{CCA} = -0.09$
$P_{mean} = 2.6 \times 10^{-4}$
$\beta_{mean} = -0.09$
observed
imputed
estimate CCA
estimate mean
Model residuals of octadecanediodate
2
1
0
−1
−2
$n_{CCA} = 963$
$n_{mean} = 1113$
$n_{CCA} = 413$
$n_{mean} = 503$
$n_{CCA} = 45$
$n_{mean} = 54$
major homozygote
heterozygote
minor homozygote
Genotype of SNP rs12829704 (SLCO1B1 (2 separate loci))

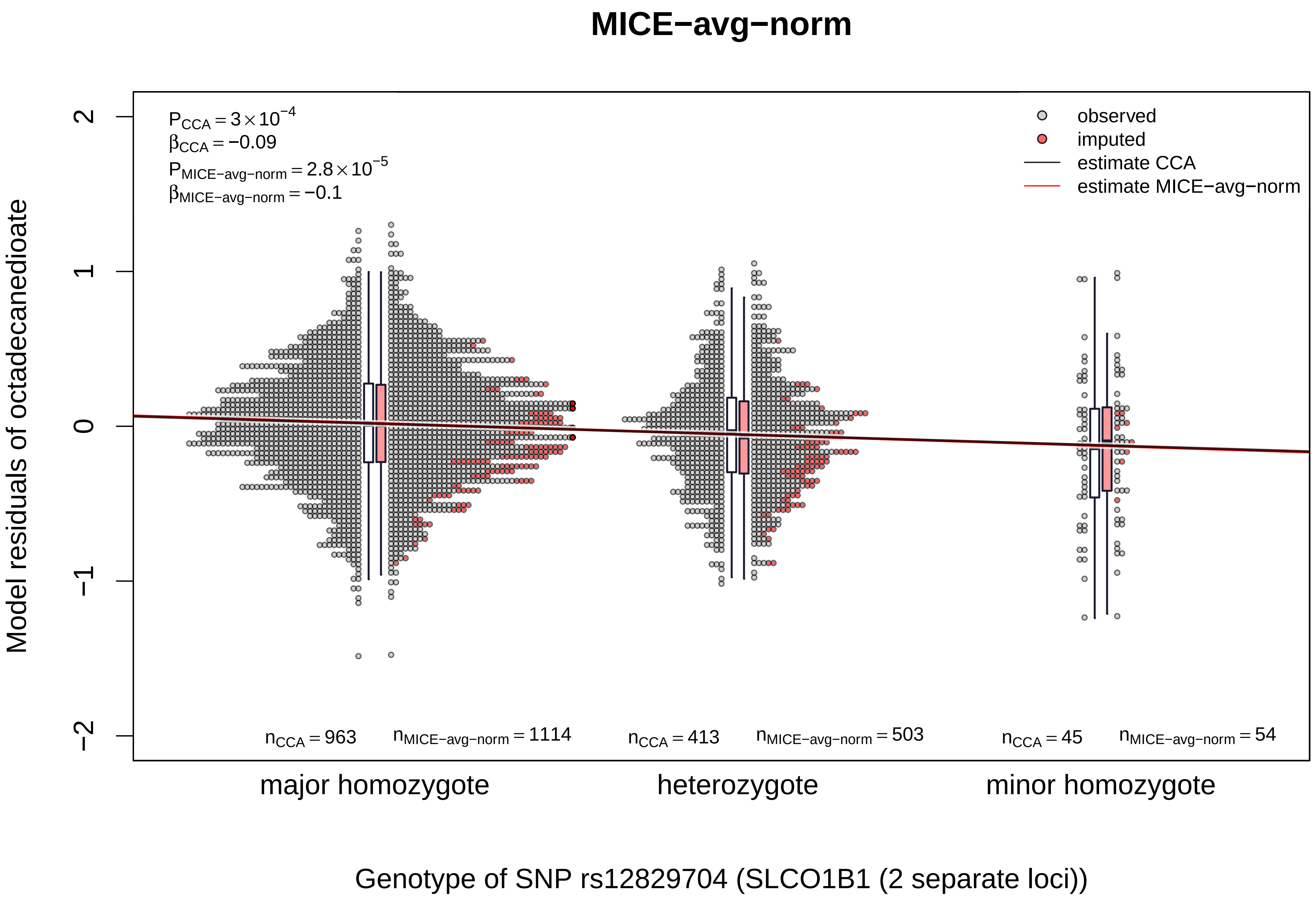

MICE−avg−norm
$P_{CCA} = 3 \times 10^{-4}$
$\beta_{CCA} = -0.09$
$P_{MICE-avg-norm} = 2.8 \times 10^{-5}$
$\beta_{MICE-avg-norm} = -0.1$
observed
imputed
estimate CCA
estimate MICE−avg−norm
Model residuals of octadecanedioate
2
1
0
−1
−2
$n_{CCA} = 963$
$n_{MICE-avg-norm} = 1114$
$n_{CCA} = 413$
$n_{MICE-avg-norm} = 503$
$n_{CCA} = 45$
$n_{MICE-avg-norm} = 54$
major homozygote
heterozygote
minor homozygote
Genotype of SNP rs12829704 (SLCO1B1 (2 separate loci))

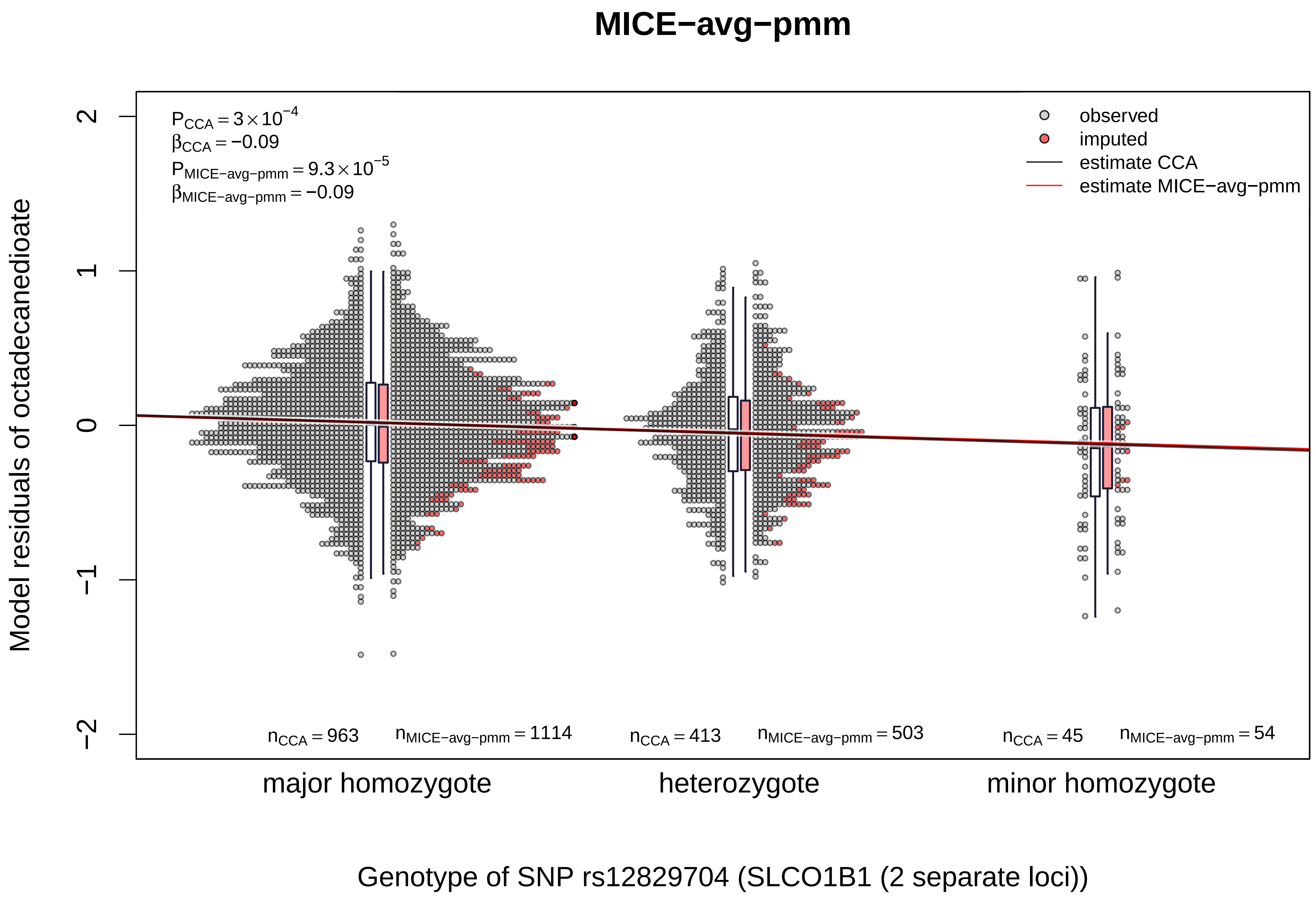

MICE−avg−pmm
$P_{CCA} = 3 \times 10^{-4}$
$\beta_{CCA} = -0.09$
$P_{MICE-avg-pmm} = 9.3 \times 10^{-5}$
$\beta_{MICE-avg-pmm} = -0.09$
observed
imputed
estimate CCA
estimate MICE−avg−pmm
Model residuals of octadecanedioate
2
1
0
−1
−2
$n_{CCA} = 963$
$n_{MICE-avg-pmm} = 1114$
$n_{CCA} = 413$
$n_{MICE-avg-pmm} = 503$
$n_{CCA} = 45$
$n_{MICE-avg-pmm} = 54$
major homozygote
heterozygote
minor homozygote
Genotype of SNP rs12829704 (SLCO1B1 (2 separate loci))

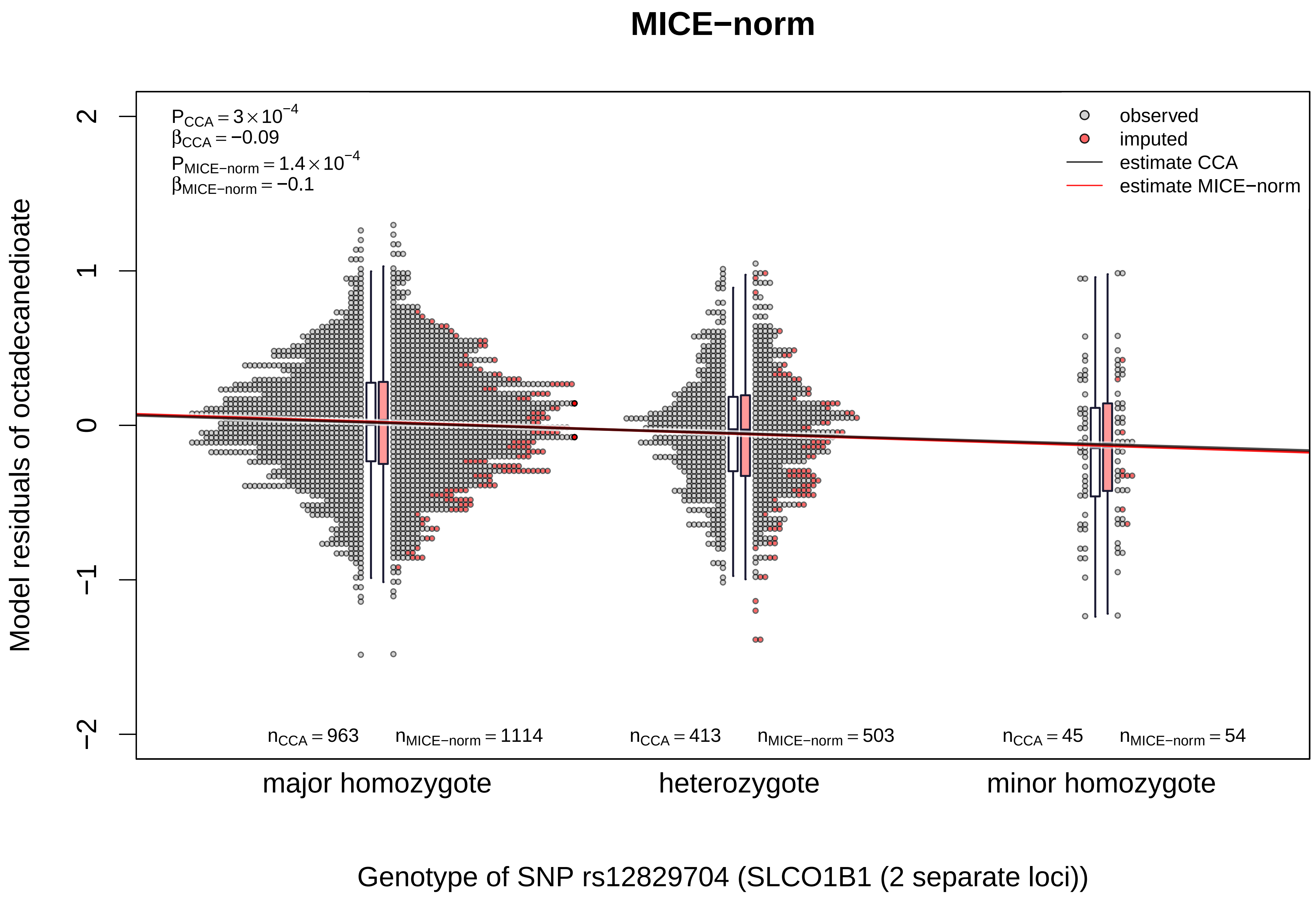

MICE−norm
$P_{CCA} = 3 \times 10^{-4}$
$\beta_{CCA} = -0.09$
$P_{MICE-norm} = 1.4 \times 10^{-4}$
$\beta_{MICE-norm} = -0.1$
observed
imputed
estimate CCA
estimate MICE−norm
2
1
0
−1
−2
Model residuals of octadecanediate
$n_{CCA} = 963$
$n_{MICE-norm} = 1114$
$n_{CCA} = 413$
$n_{MICE-norm} = 503$
$n_{CCA} = 45$
$n_{MICE-norm} = 54$
major homozygote
heterozygote
minor homozygote
Genotype of SNP rs12829704 (SLCO1B1 (2 separate loci))

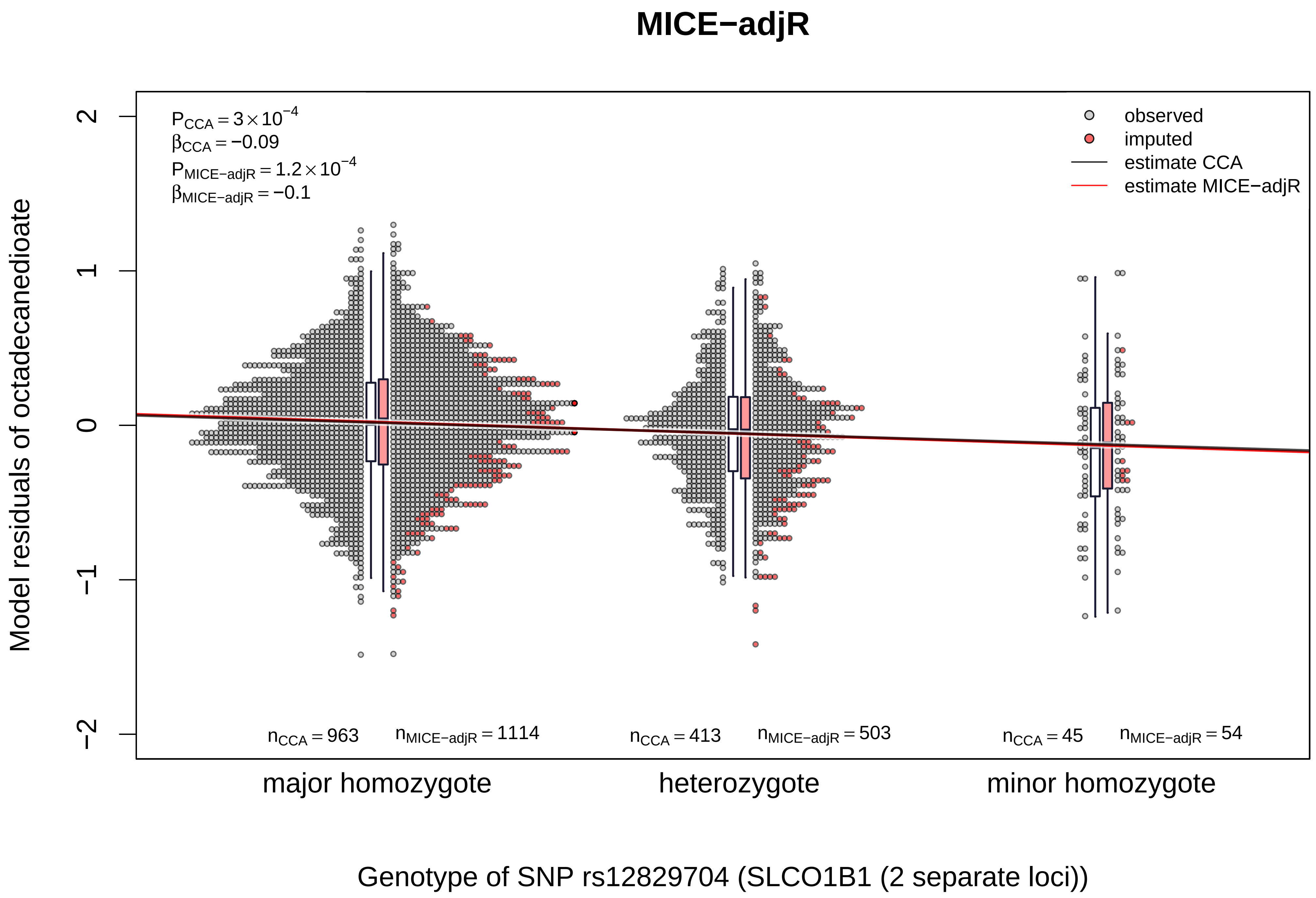

MICE−adjR
$P_{CCA} = 3 \times 10^{-4}$
$\beta_{CCA} = -0.09$
$P_{MICE-adjR} = 1.2 \times 10^{-4}$
$\beta_{MICE-adjR} = -0.1$
observed
imputed
estimate CCA
estimate MICE−adjR
Model residuals of octadecanedioate
2
1
0
−1
−2
$n_{CCA} = 963$
$n_{MICE-adjR} = 1114$
$n_{CCA} = 413$
$n_{MICE-adjR} = 503$
$n_{CCA} = 45$
$n_{MICE-adjR} = 54$
major homozygote
heterozygote
minor homozygote
Genotype of SNP rs12829704 (SLCO1B1 (2 separate loci))

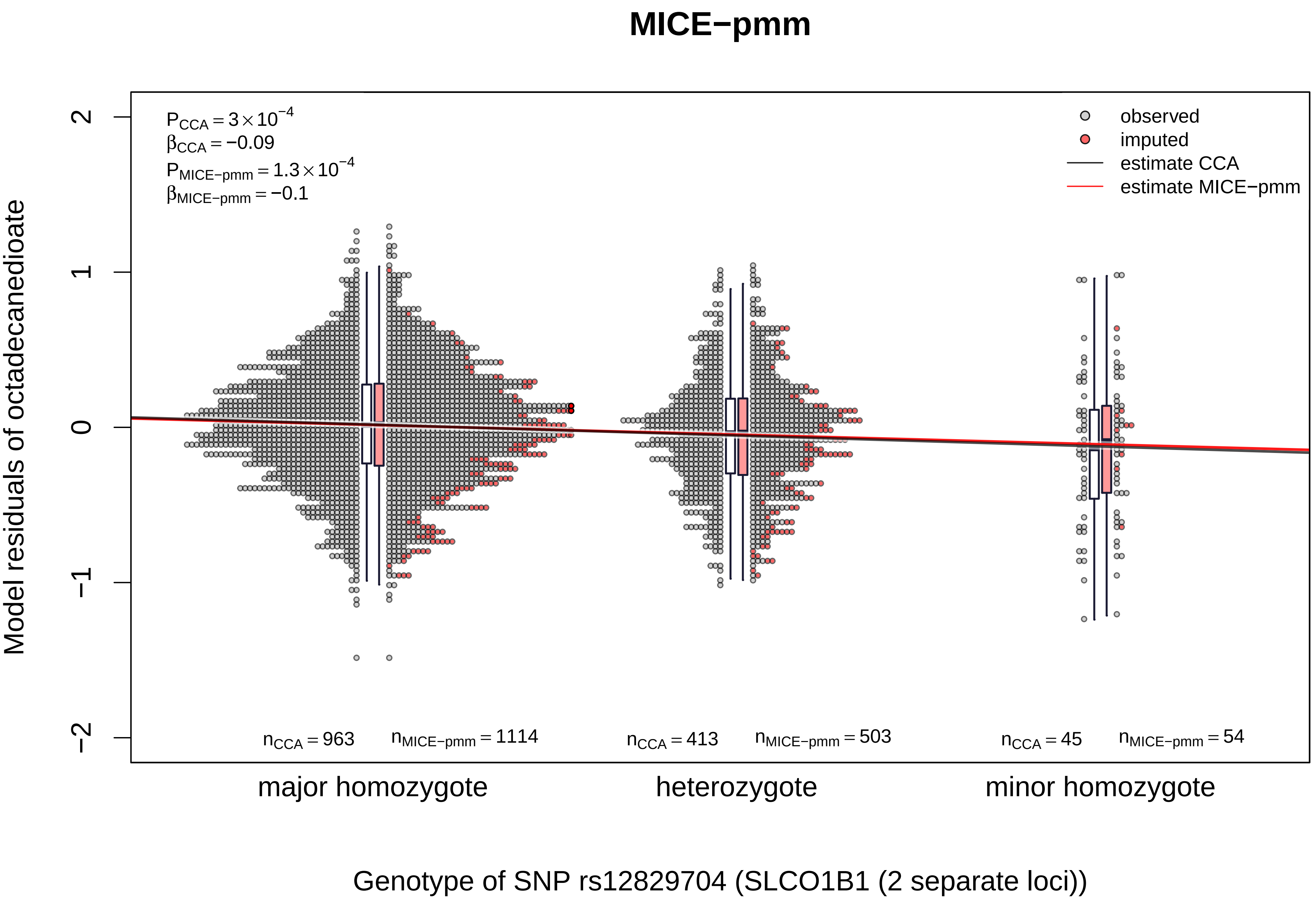
MICE−pmm
$P_{CCA} = 3 \times 10^{-4}$
$\beta_{CCA} = -0.09$
$P_{MICE-pmm} = 1.3 \times 10^{-4}$
$\beta_{MICE-pmm} = -0.1$
observed
imputed
estimate CCA
estimate MICE−pmm
Model residuals of octadecanedioate
2
1
0
−1
−2
$n_{CCA} = 963$
$n_{MICE-pmm} = 1114$
$n_{CCA} = 413$
$n_{MICE-pmm} = 503$
$n_{CCA} = 45$
$n_{MICE-pmm} = 54$
major homozygote
heterozygote
minor homozygote
Genotype of SNP rs12829704 (SLCO1B1 (2 separate loci))

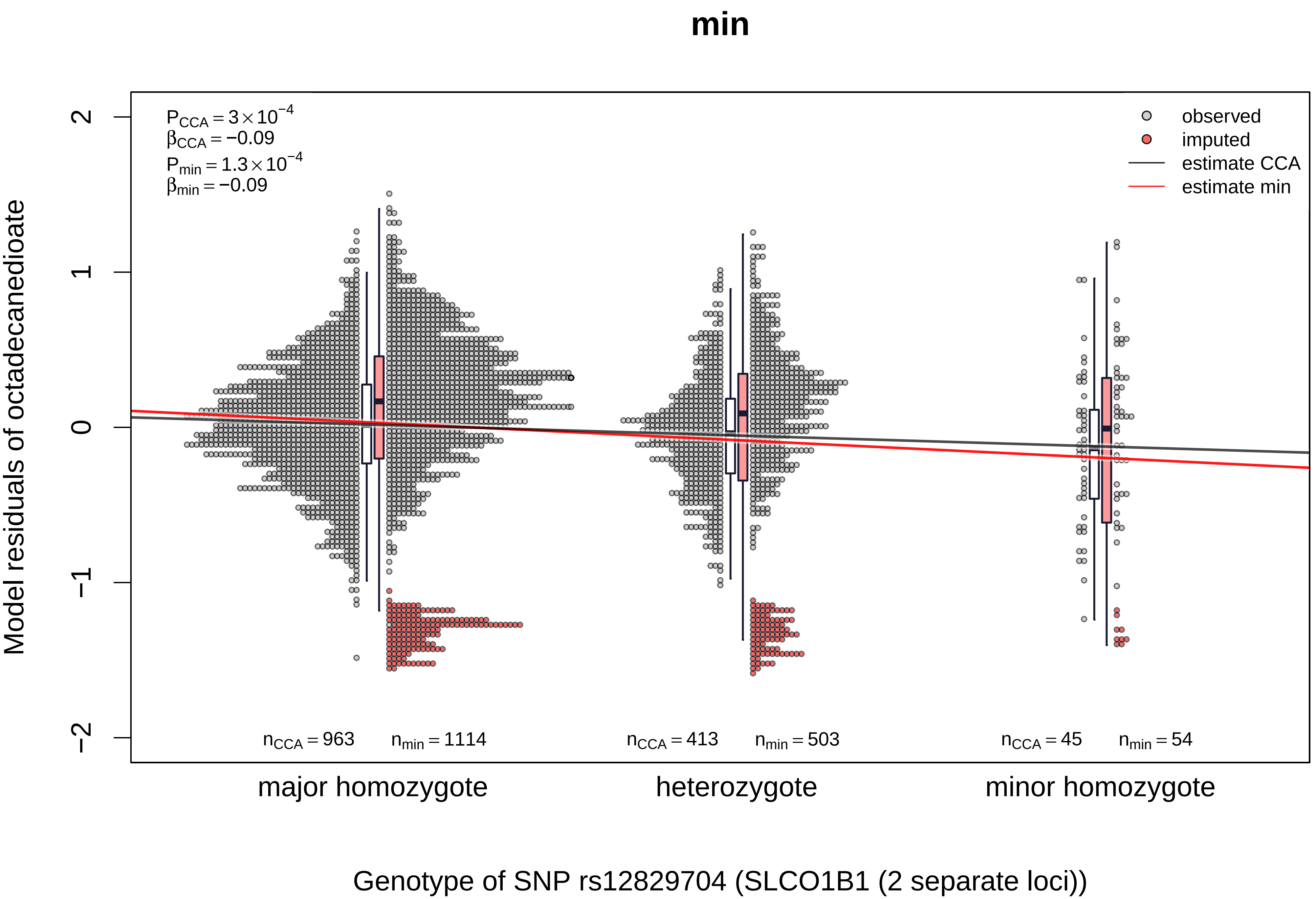

min
$P_{CCA} = 3 \times 10^{-4}$
$\beta_{CCA} = -0.09$
$P_{min} = 1.3 \times 10^{-4}$
$\beta_{min} = -0.09$
observed
imputed
estimate CCA
estimate min
Model residuals of octadecanedioate
2
1
0
−1
−2
$n_{CCA} = 963$
$n_{min} = 1114$
$n_{CCA} = 413$
$n_{min} = 503$
$n_{CCA} = 45$
$n_{min} = 54$
major homozygote
heterozygote
minor homozygote
Genotype of SNP rs12829704 (SLCO1B1 (2 separate loci))

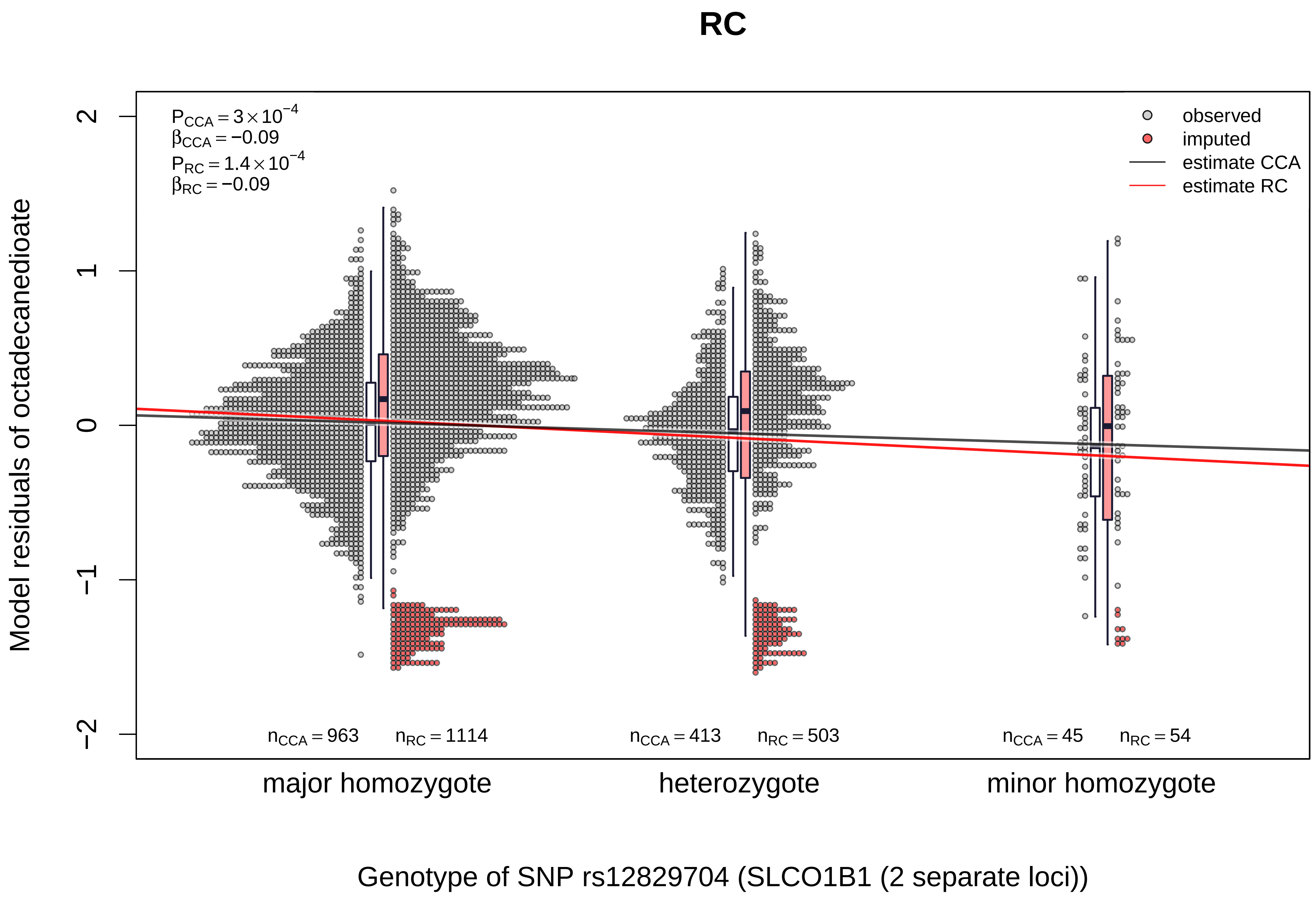
RC
$P_{CCA} = 3 \times 10^{-4}$
$\beta_{CCA} = -0.09$
$P_{RC} = 1.4 \times 10^{-4}$
$\beta_{RC} = -0.09$
observed
imputed
estimate CCA
estimate RC
Model residuals of octadecanedioate
2
1
0
−1
−2
$n_{CCA} = 963$
$n_{RC} = 1114$
$n_{CCA} = 413$
$n_{RC} = 503$
$n_{CCA} = 45$
$n_{RC} = 54$
major homozygote
heterozygote
minor homozygote
Genotype of SNP rs12829704 (SLCO1B1 (2 separate loci))

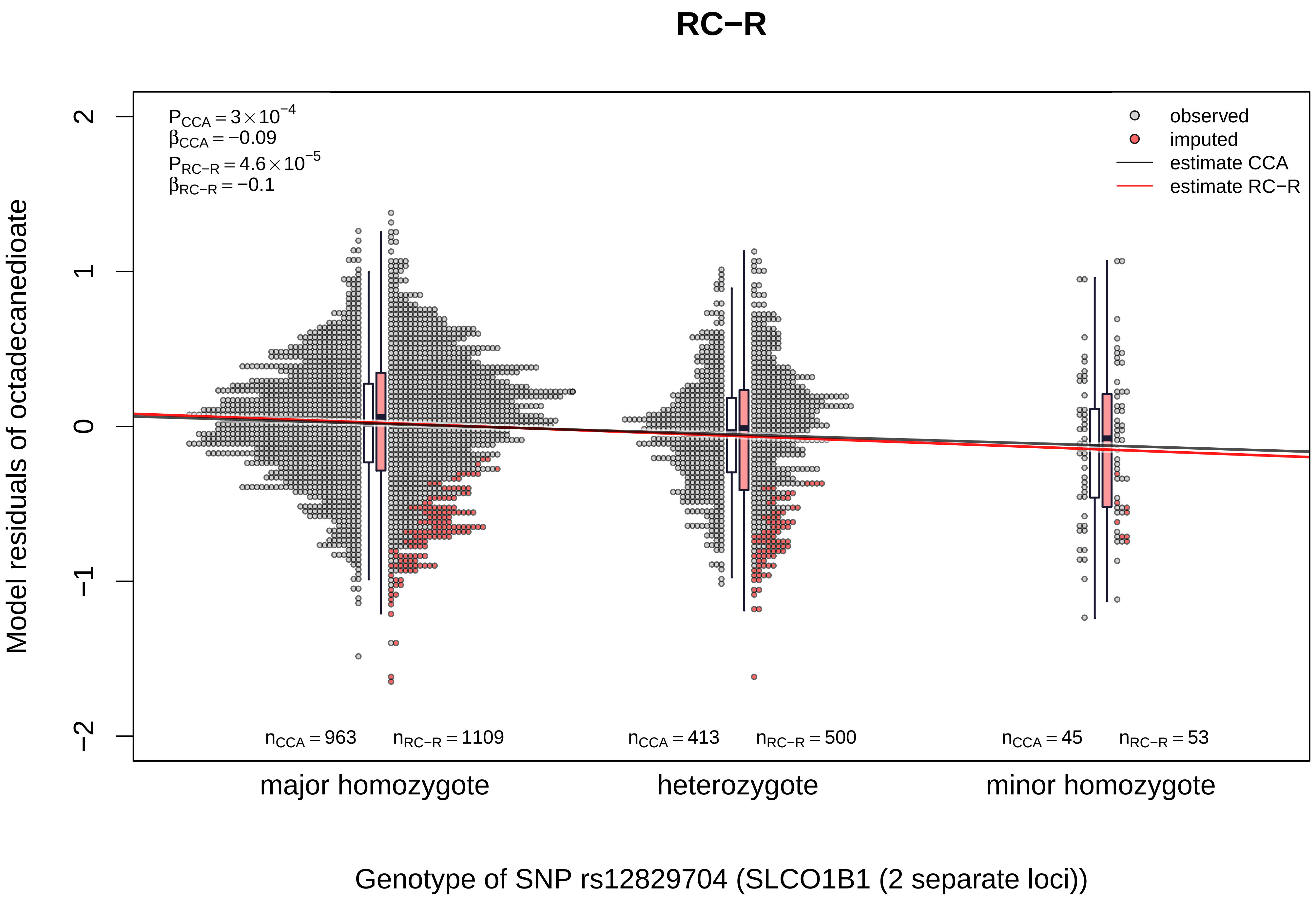

RC−R
$P_{CCA} = 3 \times 10^{-4}$
$\beta_{CCA} = -0.09$
$P_{RC-R} = 4.6 \times 10^{-5}$
$\beta_{RC-R} = -0.1$
observed
imputed
estimate CCA
estimate RC−R
Model residuals of octadecanedioate
2
1
0
−1
−2
$n_{CCA} = 963$
$n_{RC-R} = 1109$
$n_{CCA} = 413$
$n_{RC-R} = 500$
$n_{CCA} = 45$
$n_{RC-R} = 53$
major homozygote
heterozygote
minor homozygote
Genotype of SNP rs12829704 (SLCO1B1 (2 separate loci))

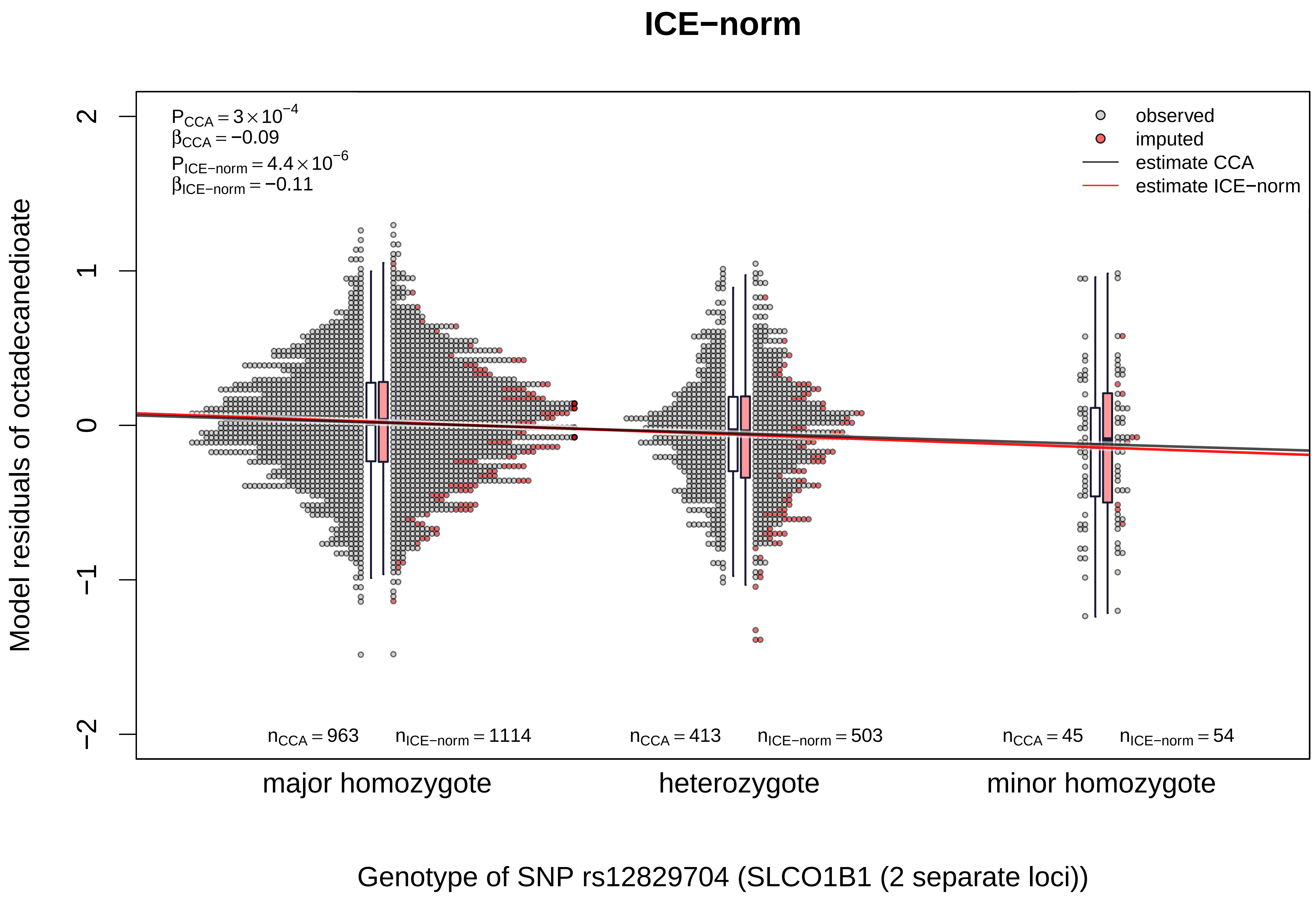
ICE−norm
$P_{CCA} = 3 \times 10^{-4}$
$\beta_{CCA} = -0.09$
$P_{ICE-norm} = 4.4 \times 10^{-6}$
$\beta_{ICE-norm} = -0.11$
observed
imputed
estimate CCA
estimate ICE−norm
2
1
0
−1
−2
Model residuals of octadecanedioate
$n_{CCA} = 963$
$n_{ICE-norm} = 1114$
$n_{CCA} = 413$
$n_{ICE-norm} = 503$
$n_{CCA} = 45$
$n_{ICE-norm} = 54$
major homozygote
heterozygote
minor homozygote
Genotype of SNP rs12829704 (SLCO1B1 (2 separate loci))

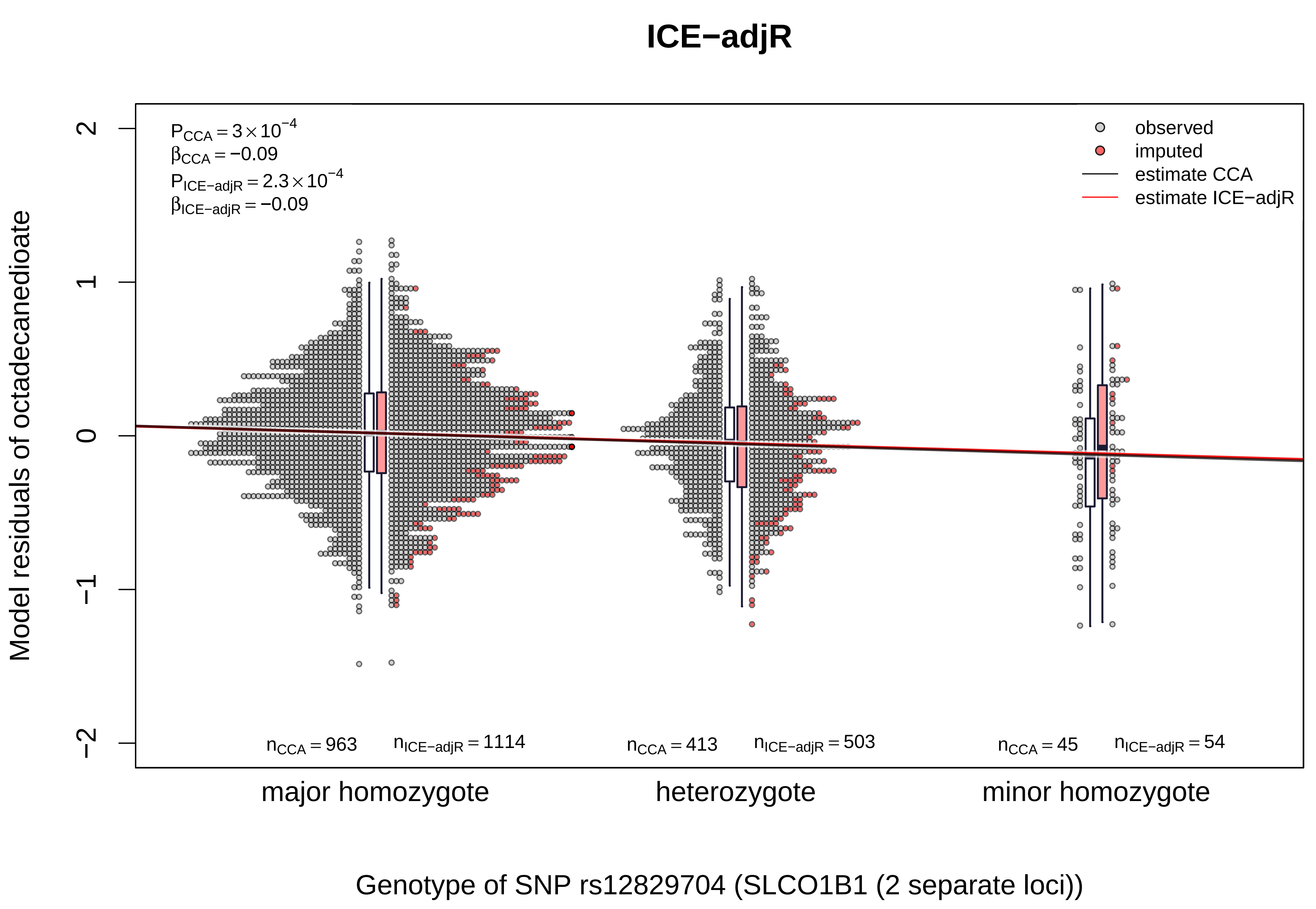

ICE−adjR
$P_{CCA} = 3 \times 10^{-4}$
$\beta_{CCA} = -0.09$
$P_{ICE-adjR} = 2.3 \times 10^{-4}$
$\beta_{ICE-adjR} = -0.09$
observed
imputed
estimate CCA
estimate ICE−adjR
Model residuals of octadecanedioate
2
1
0
−1
−2
$n_{CCA} = 963$
$n_{ICE-adjR} = 1114$
$n_{CCA} = 413$
$n_{ICE-adjR} = 503$
$n_{CCA} = 45$
$n_{ICE-adjR} = 54$
major homozygote
heterozygote
minor homozygote
Genotype of SNP rs12829704 (SLCO1B1 (2 separate loci))

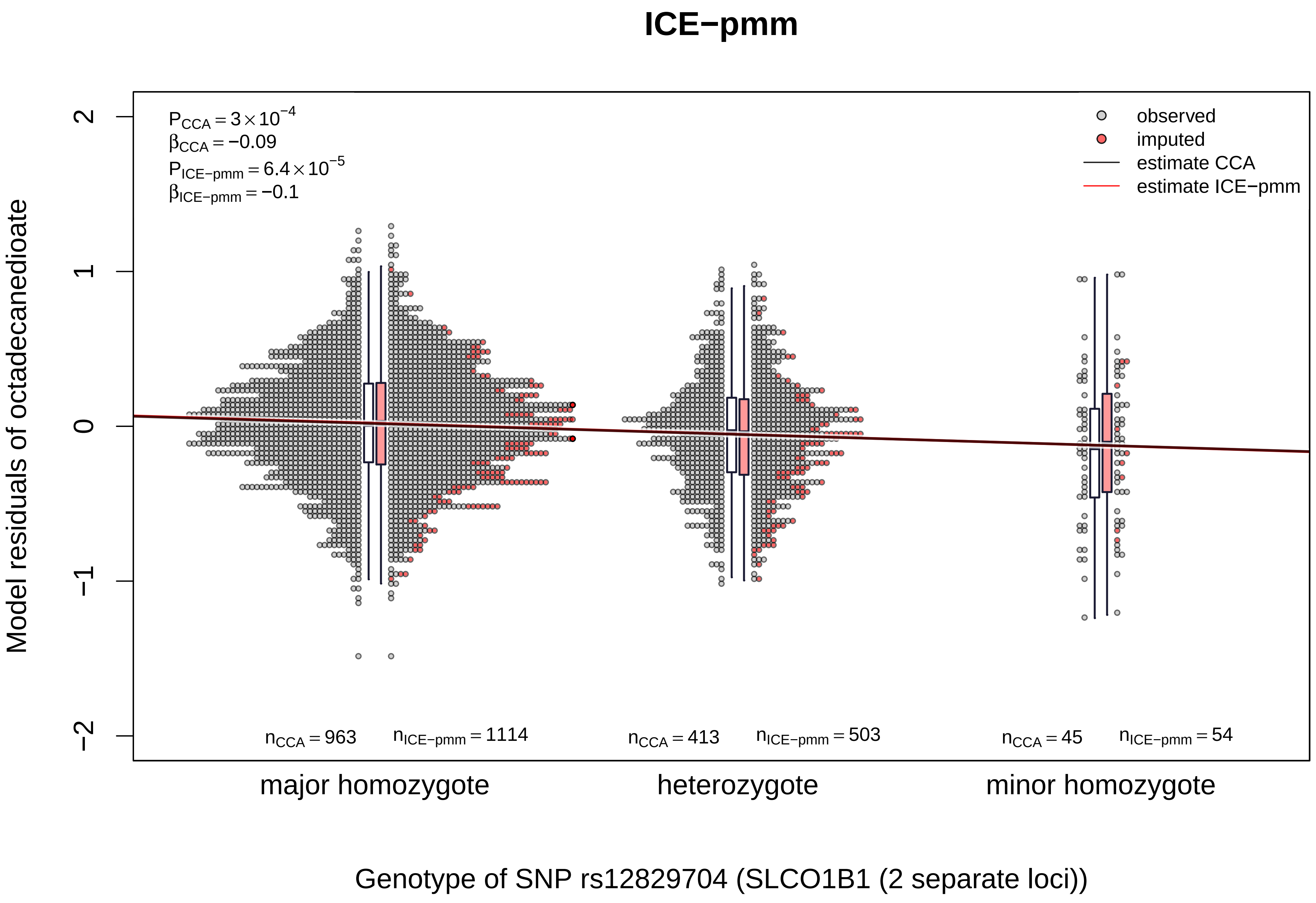

ICE−pmm
$P_{CCA} = 3 \times 10^{-4}$
$\beta_{CCA} = -0.09$
$P_{ICE-pmm} = 6.4 \times 10^{-5}$
$\beta_{ICE-pmm} = -0.1$
observed
imputed
estimate CCA
estimate ICE−pmm
Model residuals of octadecanedioate
2
1
0
−1
−2
$n_{CCA} = 963$
$n_{ICE-pmm} = 1114$
$n_{CCA} = 413$
$n_{ICE-pmm} = 503$
$n_{CCA} = 45$
$n_{ICE-pmm} = 54$
major homozygote
heterozygote
minor homozygote
Genotype of SNP rs12829704 (SLCO1B1 (2 separate loci))

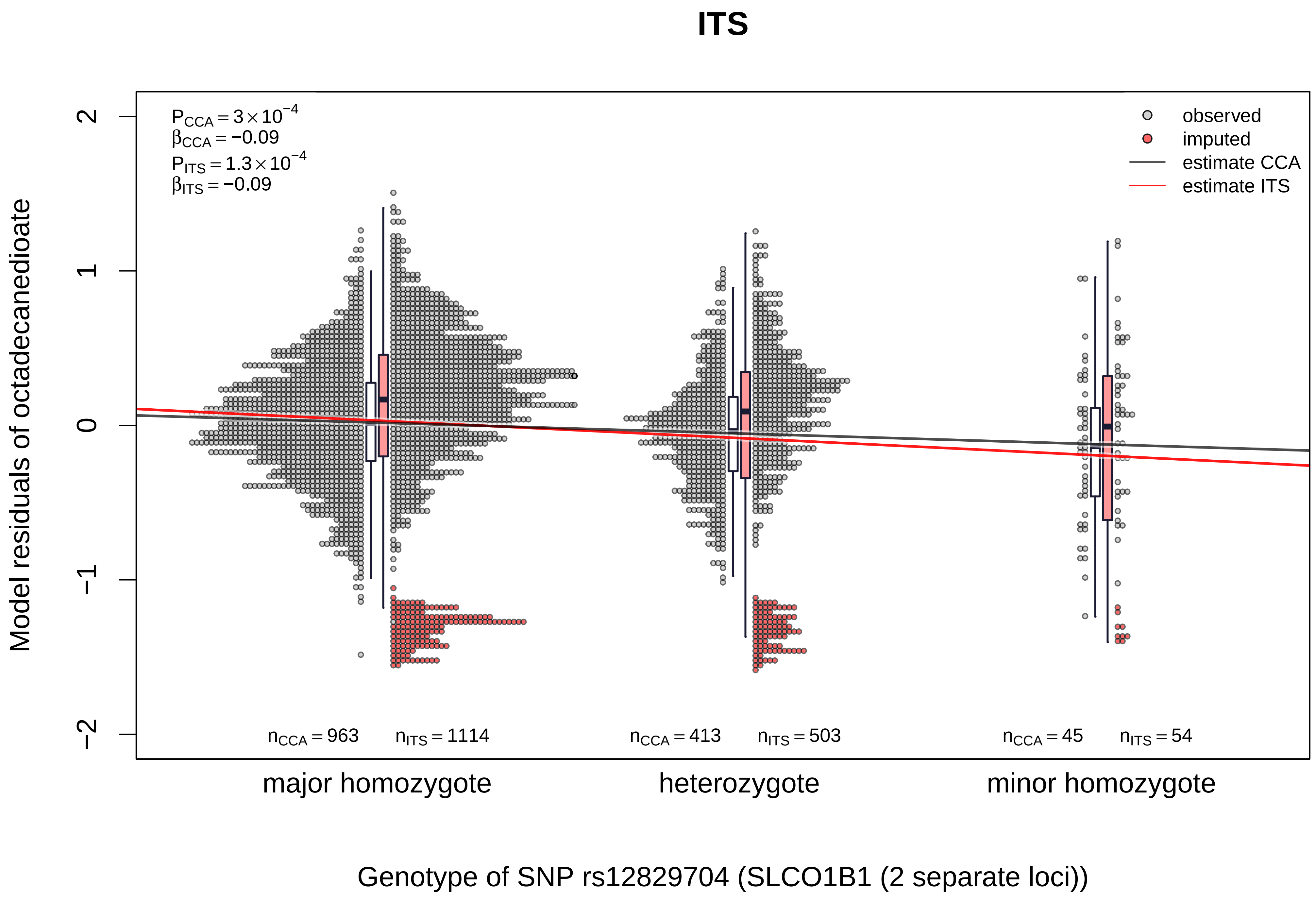

ITS
$P_{CCA} = 3 \times 10^{-4}$
$\beta_{CCA} = -0.09$
$P_{ITS} = 1.3 \times 10^{-4}$
$\beta_{ITS} = -0.09$
observed
imputed
estimate CCA
estimate ITS
2
1
0
−1
−2
Model residuals of octadecanedioate
$n_{CCA} = 963$
$n_{ITS} = 1114$
$n_{CCA} = 413$
$n_{ITS} = 503$
$n_{CCA} = 45$
$n_{ITS} = 54$
major homozygote
heterozygote
minor homozygote
Genotype of SNP rs12829704 (SLCO1B1 (2 separate loci))

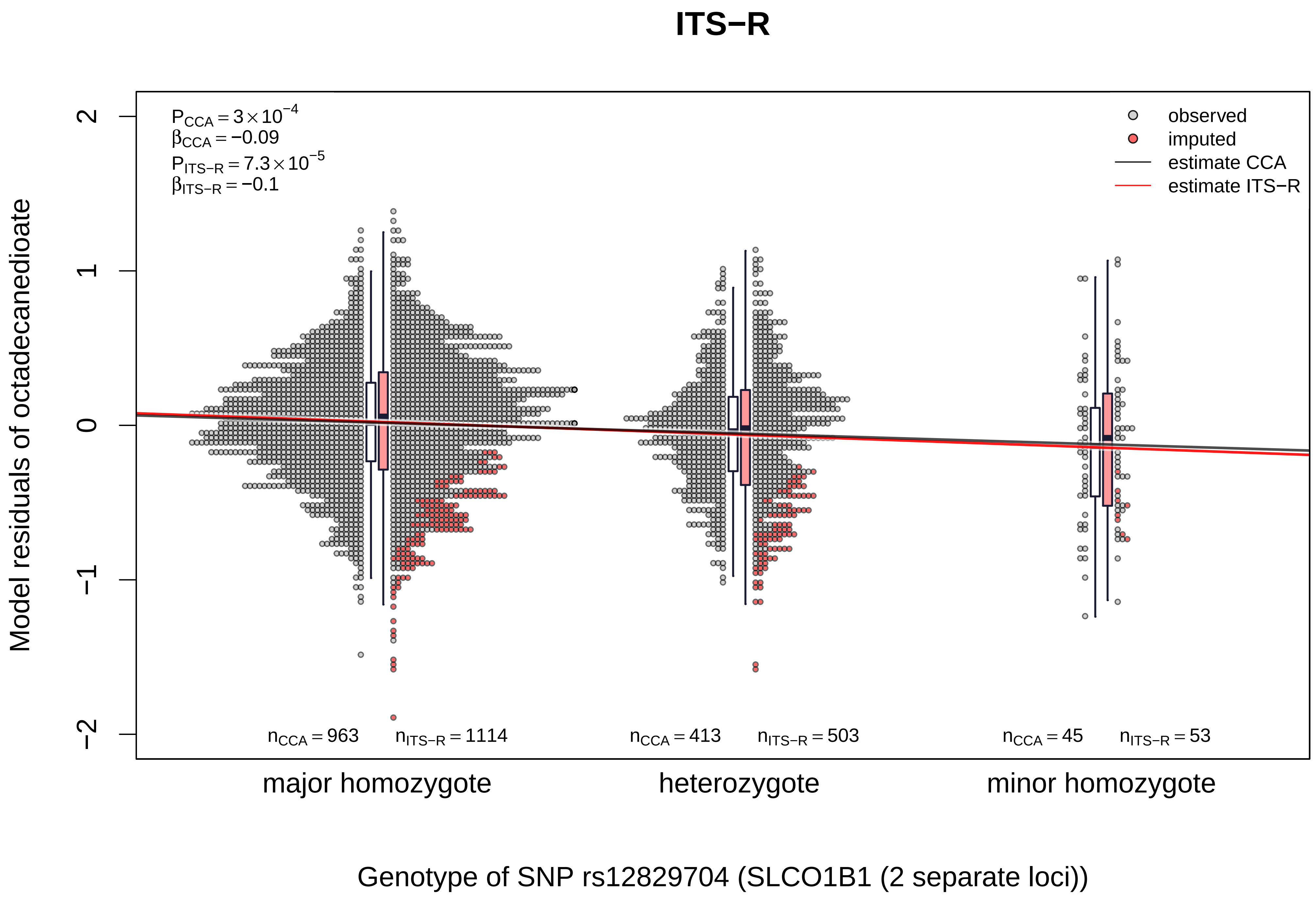

ITS−R
$P_{CCA} = 3 \times 10^{-4}$
$\beta_{CCA} = -0.09$
$P_{ITS-R} = 7.3 \times 10^{-5}$
$\beta_{ITS-R} = -0.1$
observed
imputed
estimate CCA
estimate ITS−R
Model residuals of octadecanedioate
2
1
0
−1
−2
$n_{CCA} = 963$
$n_{ITS-R} = 1114$
$n_{CCA} = 413$
$n_{ITS-R} = 503$
$n_{CCA} = 45$
$n_{ITS-R} = 53$
major homozygote
heterozygote
minor homozygote
Genotype of SNP rs12829704 (SLCO1B1 (2 separate loci))

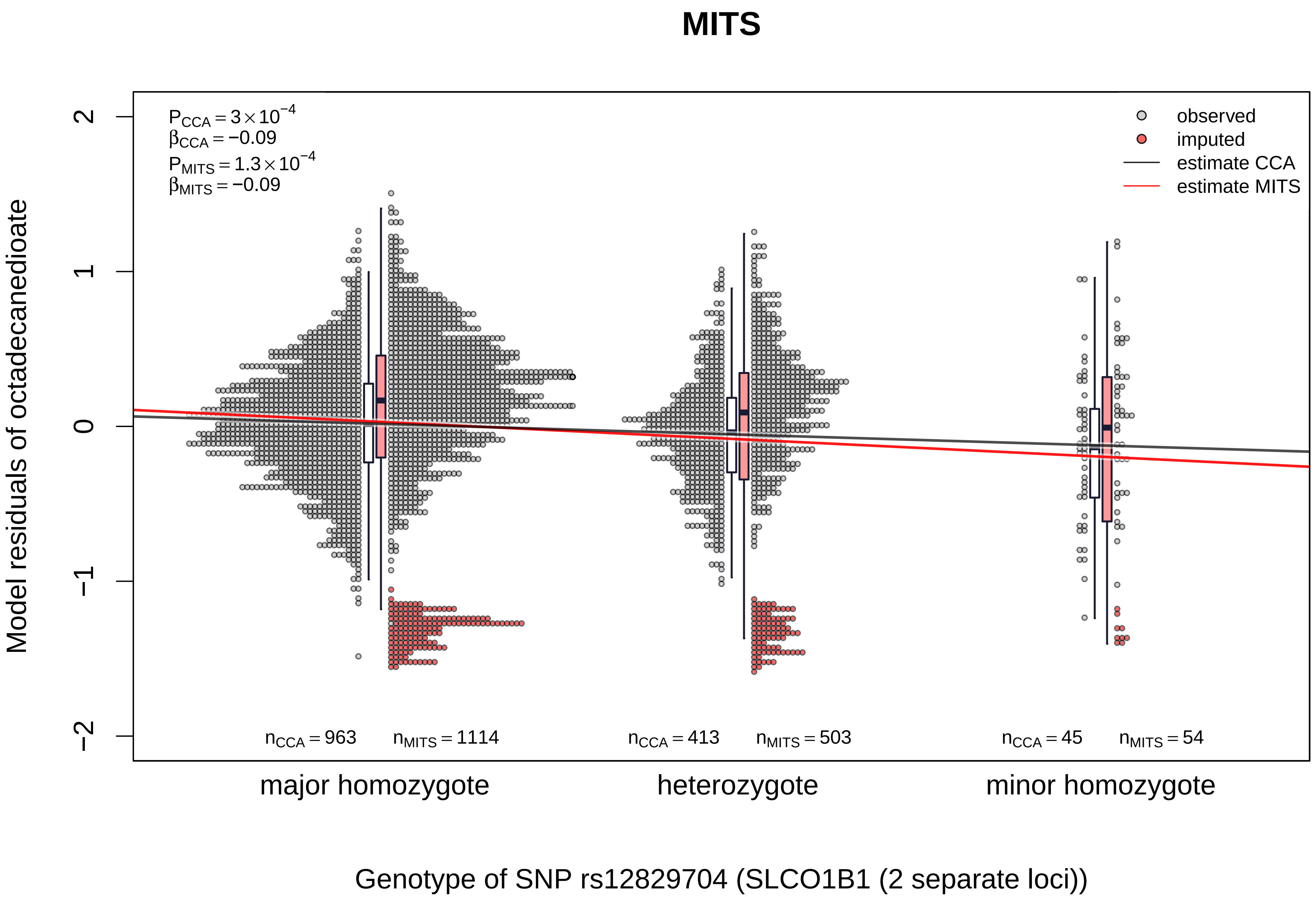
MITS
$P_{CCA} = 3 \times 10^{-4}$
$\beta_{CCA} = -0.09$
$P_{MITS} = 1.3 \times 10^{-4}$
$\beta_{MITS} = -0.09$
observed
imputed
estimate CCA
estimate MITS
Model residuals of octadecanedioate
2
1
0
−1
−2
$n_{CCA} = 963$
$n_{MITS} = 1114$
$n_{CCA} = 413$
$n_{MITS} = 503$
$n_{CCA} = 45$
$n_{MITS} = 54$
major homozygote
heterozygote
minor homozygote
Genotype of SNP rs12829704 (SLCO1B1 (2 separate loci))

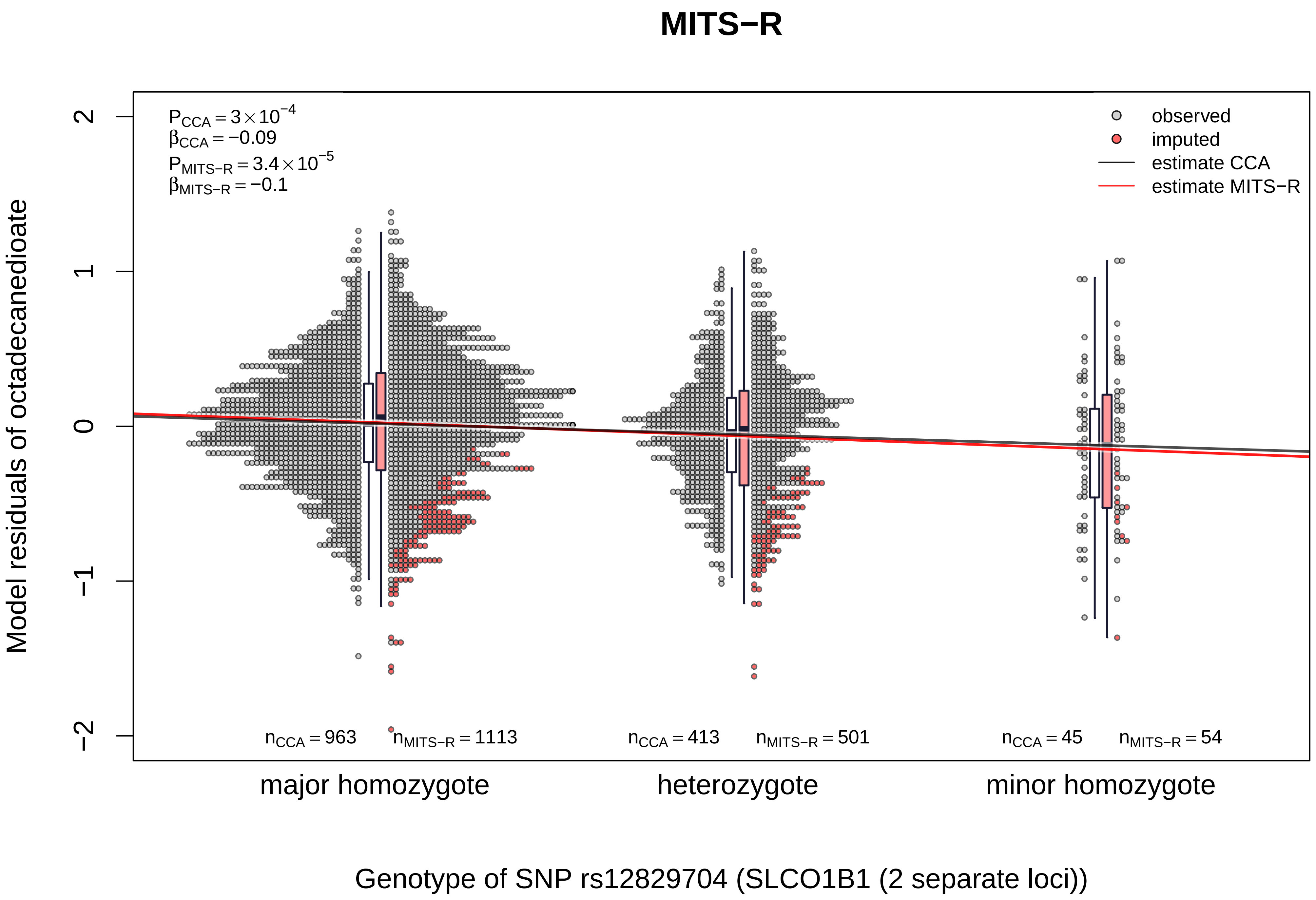
MITS−R
$P_{CCA} = 3 \times 10^{-4}$
$\beta_{CCA} = -0.09$
$P_{MITS-R} = 3.4 \times 10^{-5}$
$\beta_{MITS-R} = -0.1$
observed
imputed
estimate CCA
estimate MITS−R
Model residuals of octadecanedioate
2
1
0
−1
−2
$n_{CCA} = 963$
$n_{MITS-R} = 1113$
$n_{CCA} = 413$
$n_{MITS-R} = 501$
$n_{CCA} = 45$
$n_{MITS-R} = 54$
major homozygote
heterozygote
minor homozygote
Genotype of SNP rs12829704 (SLCO1B1 (2 separate loci))

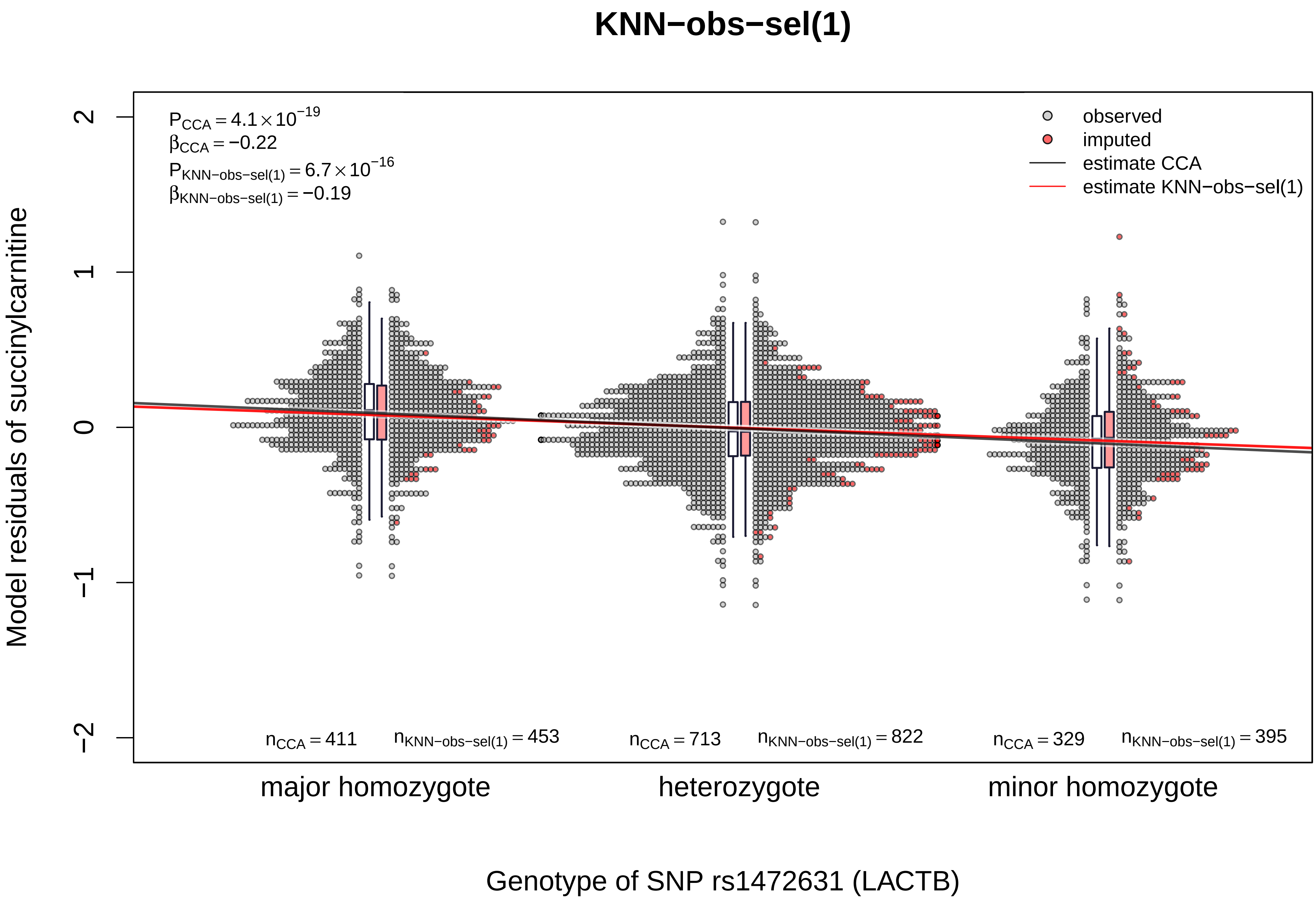

KNN−obs−sel(1)
$P_{CCA} = 4.1 \times 10^{-19}$
$\beta_{CCA} = -0.22$
$P_{KNN-obs-sel(1)} = 6.7 \times 10^{-16}$
$\beta_{KNN-obs-sel(1)} = -0.19$
observed
imputed
estimate CCA
estimate KNN−obs−sel(1)
Model residuals of succinylcarnitine
2
1
0
−1
−2
$n_{CCA} = 411$
$n_{KNN-obs-sel(1)} = 453$
$n_{CCA} = 713$
$n_{KNN-obs-sel(1)} = 822$
$n_{CCA} = 329$
$n_{KNN-obs-sel(1)} = 395$
major homozygote
heterozygote
minor homozygote
Genotype of SNP rs1472631 (LACTB)

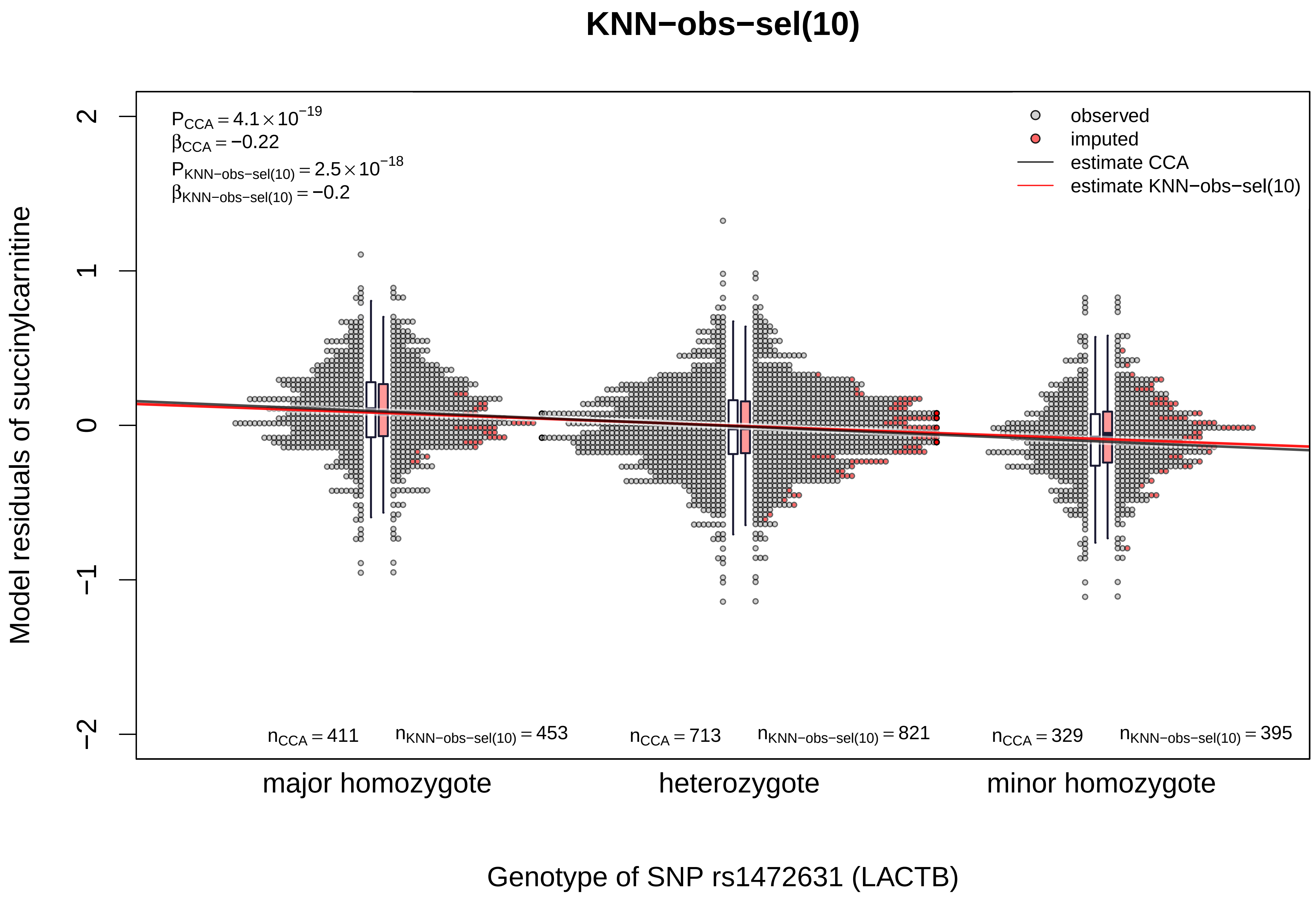

KNN−obs−sel(10)
$P_{CCA} = 4.1 \times 10^{-19}$
$\beta_{CCA} = -0.22$
$P_{KNN-obs-sel(10)} = 2.5 \times 10^{-18}$
$\beta_{KNN-obs-sel(10)} = -0.2$
observed
imputed
estimate CCA
estimate KNN−obs−sel(10)
2
1
0
−1
−2
Model residuals of succinylcarnitine
$n_{CCA} = 411$
$n_{KNN-obs-sel(10)} = 453$
$n_{CCA} = 713$
$n_{KNN-obs-sel(10)} = 821$
$n_{CCA} = 329$
$n_{KNN-obs-sel(10)} = 395$
major homozygote
heterozygote
minor homozygote
Genotype of SNP rs1472631 (LACTB)

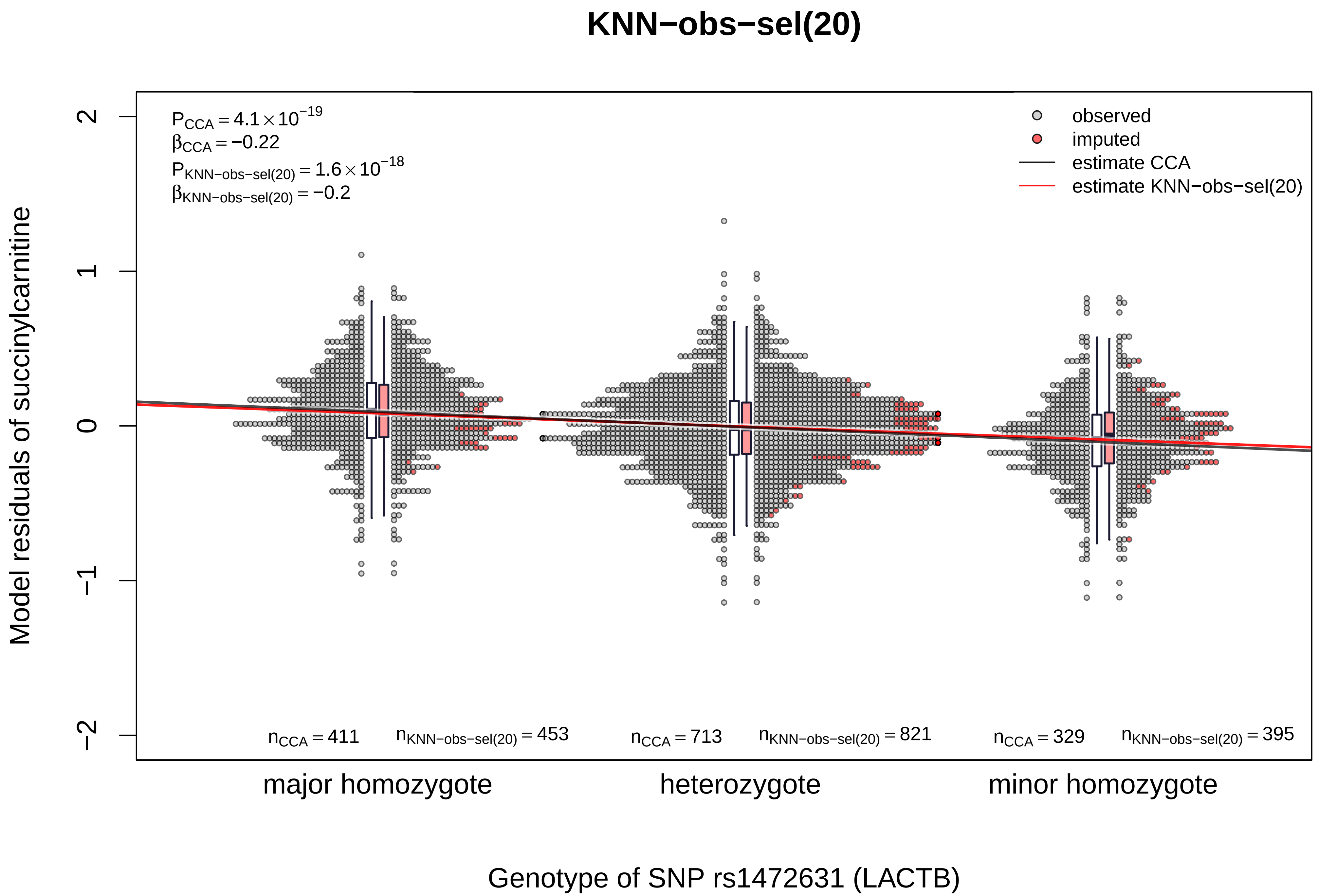

KNN−obs−sel(20)
$P_{CCA} = 4.1 \times 10^{-19}$
$\beta_{CCA} = -0.22$
$P_{KNN-obs-sel(20)} = 1.6 \times 10^{-18}$
$\beta_{KNN-obs-sel(20)} = -0.2$
observed
imputed
estimate CCA
estimate KNN−obs−sel(20)
Model residuals of succinylcarnitine
2
1
0
−1
−2
$n_{CCA} = 411$
$n_{KNN-obs-sel(20)} = 453$
$n_{CCA} = 713$
$n_{KNN-obs-sel(20)} = 821$
$n_{CCA} = 329$
$n_{KNN-obs-sel(20)} = 395$
major homozygote
heterozygote
minor homozygote
Genotype of SNP rs1472631 (LACTB)

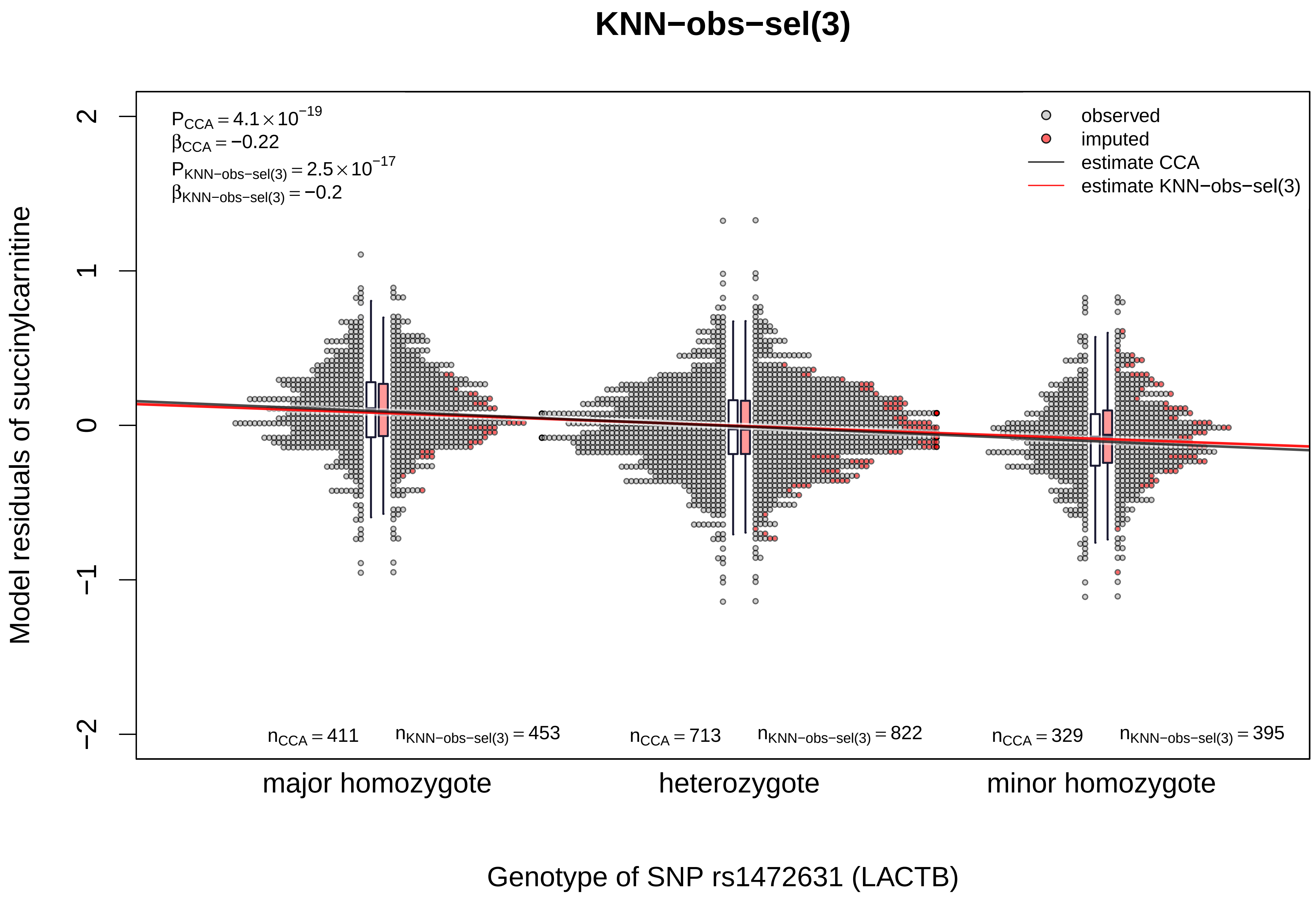

KNN−obs−sel(3)
$P_{CCA} = 4.1 \times 10^{-19}$
$\beta_{CCA} = -0.22$
$P_{KNN-obs-sel(3)} = 2.5 \times 10^{-17}$
$\beta_{KNN-obs-sel(3)} = -0.2$
observed
imputed
estimate CCA
estimate KNN−obs−sel(3)
2
1
0
−1
−2
Model residuals of succinylcarnitine
$n_{CCA} = 411$
$n_{KNN-obs-sel(3)} = 453$
$n_{CCA} = 713$
$n_{KNN-obs-sel(3)} = 822$
$n_{CCA} = 329$
$n_{KNN-obs-sel(3)} = 395$
major homozygote
heterozygote
minor homozygote
Genotype of SNP rs1472631 (LACTB)

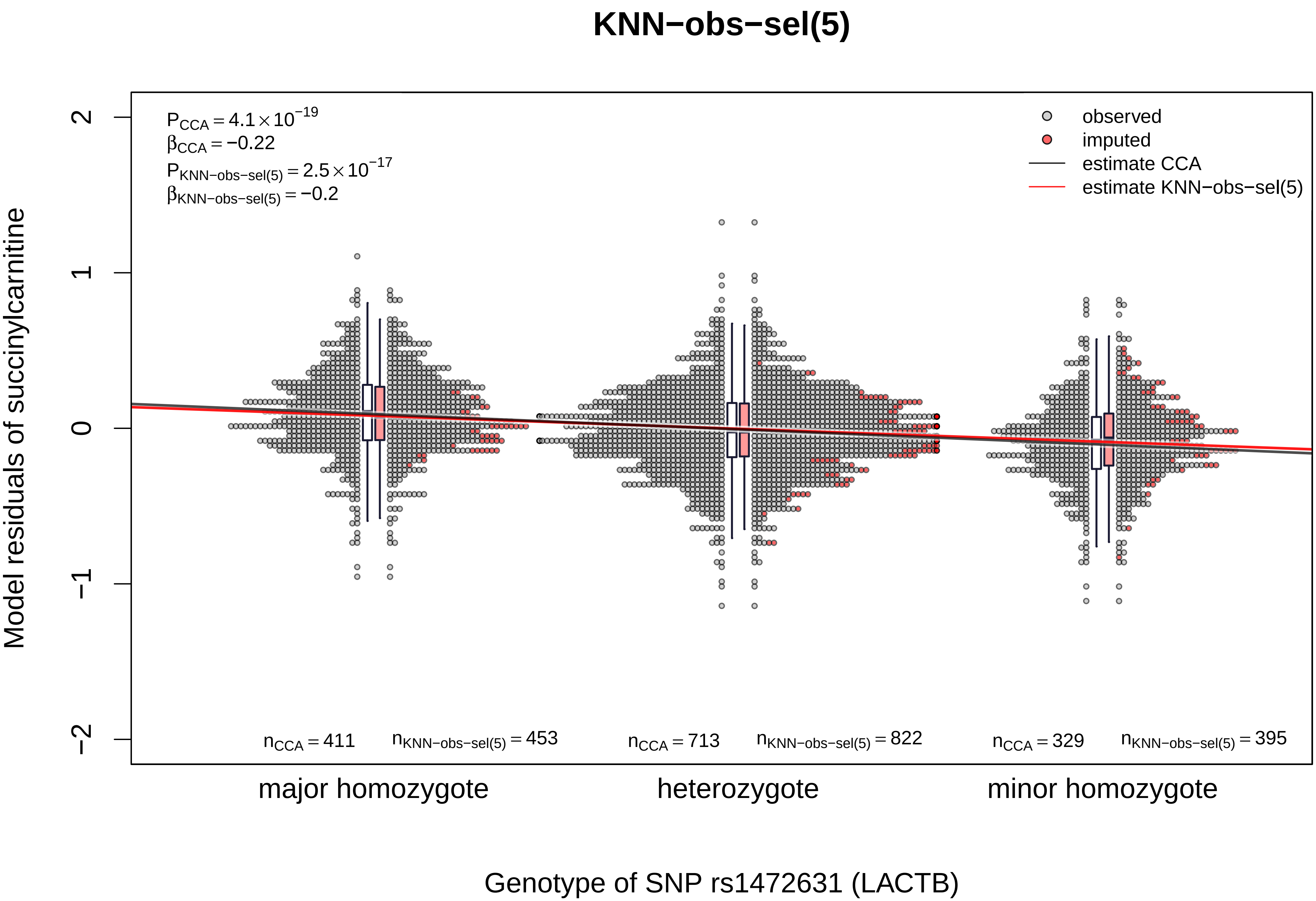

KNN−obs−sel(5)
$P_{CCA} = 4.1 \times 10^{-19}$
$\beta_{CCA} = -0.22$
$P_{KNN-obs-sel(5)} = 2.5 \times 10^{-17}$
$\beta_{KNN-obs-sel(5)} = -0.2$
observed
imputed
estimate CCA
estimate KNN−obs−sel(5)
Model residuals of succinylcarnitine
2
1
0
−1
−2
$n_{CCA} = 411$
$n_{KNN-obs-sel(5)} = 453$
$n_{CCA} = 713$
$n_{KNN-obs-sel(5)} = 822$
$n_{CCA} = 329$
$n_{KNN-obs-sel(5)} = 395$
major homozygote
heterozygote
minor homozygote
Genotype of SNP rs1472631 (LACTB)

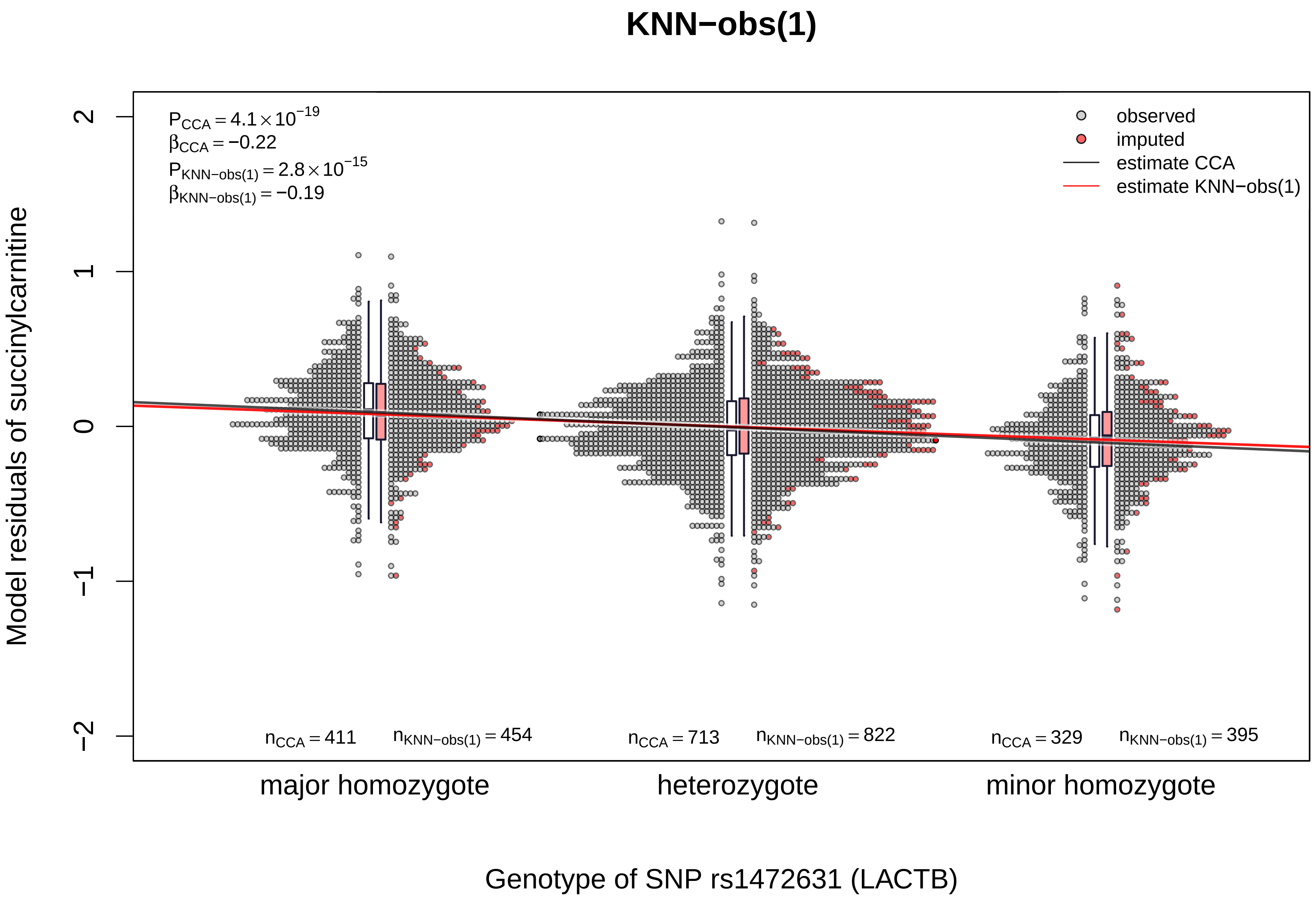

KNN−obs(1)
$P_{CCA} = 4.1 \times 10^{-19}$
$\beta_{CCA} = -0.22$
$P_{KNN-obs(1)} = 2.8 \times 10^{-15}$
$\beta_{KNN-obs(1)} = -0.19$
observed
imputed
estimate CCA
estimate KNN−obs(1)
Model residuals of succinylcarnitine
2
1
0
−1
−2
$n_{CCA} = 411$
$n_{KNN-obs(1)} = 454$
$n_{CCA} = 713$
$n_{KNN-obs(1)} = 822$
$n_{CCA} = 329$
$n_{KNN-obs(1)} = 395$
major homozygote
heterozygote
minor homozygote
Genotype of SNP rs1472631 (LACTB)

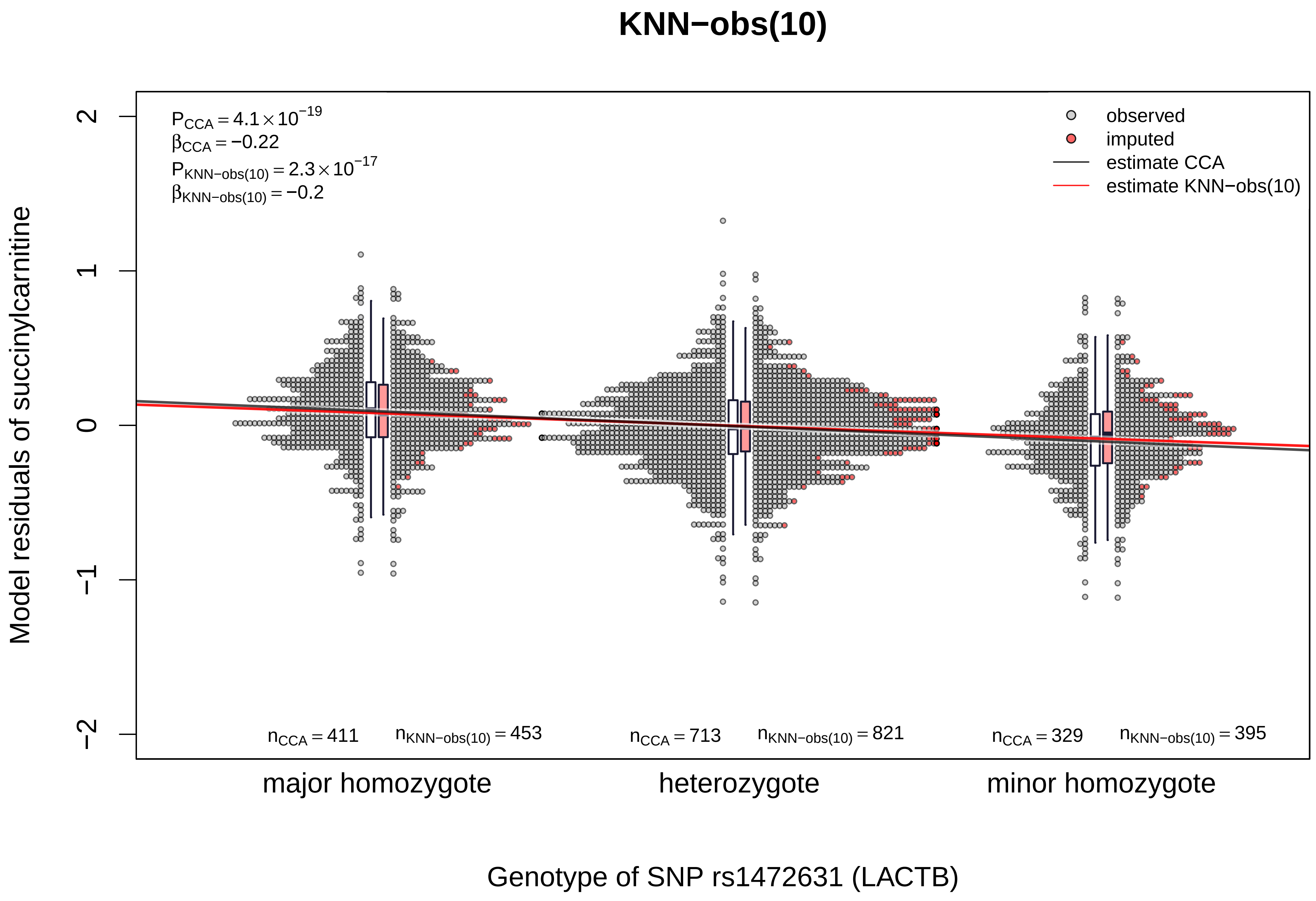

KNN−obs(10)
$P_{CCA} = 4.1 \times 10^{-19}$
$\beta_{CCA} = -0.22$
$P_{KNN-obs(10)} = 2.3 \times 10^{-17}$
$\beta_{KNN-obs(10)} = -0.2$
observed
imputed
estimate CCA
estimate KNN−obs(10)
2
1
0
−1
−2
Model residuals of succinylcarnitine
$n_{CCA} = 411$
$n_{KNN-obs(10)} = 453$
$n_{CCA} = 713$
$n_{KNN-obs(10)} = 821$
$n_{CCA} = 329$
$n_{KNN-obs(10)} = 395$
major homozygote
heterozygote
minor homozygote
Genotype of SNP rs1472631 (LACTB)

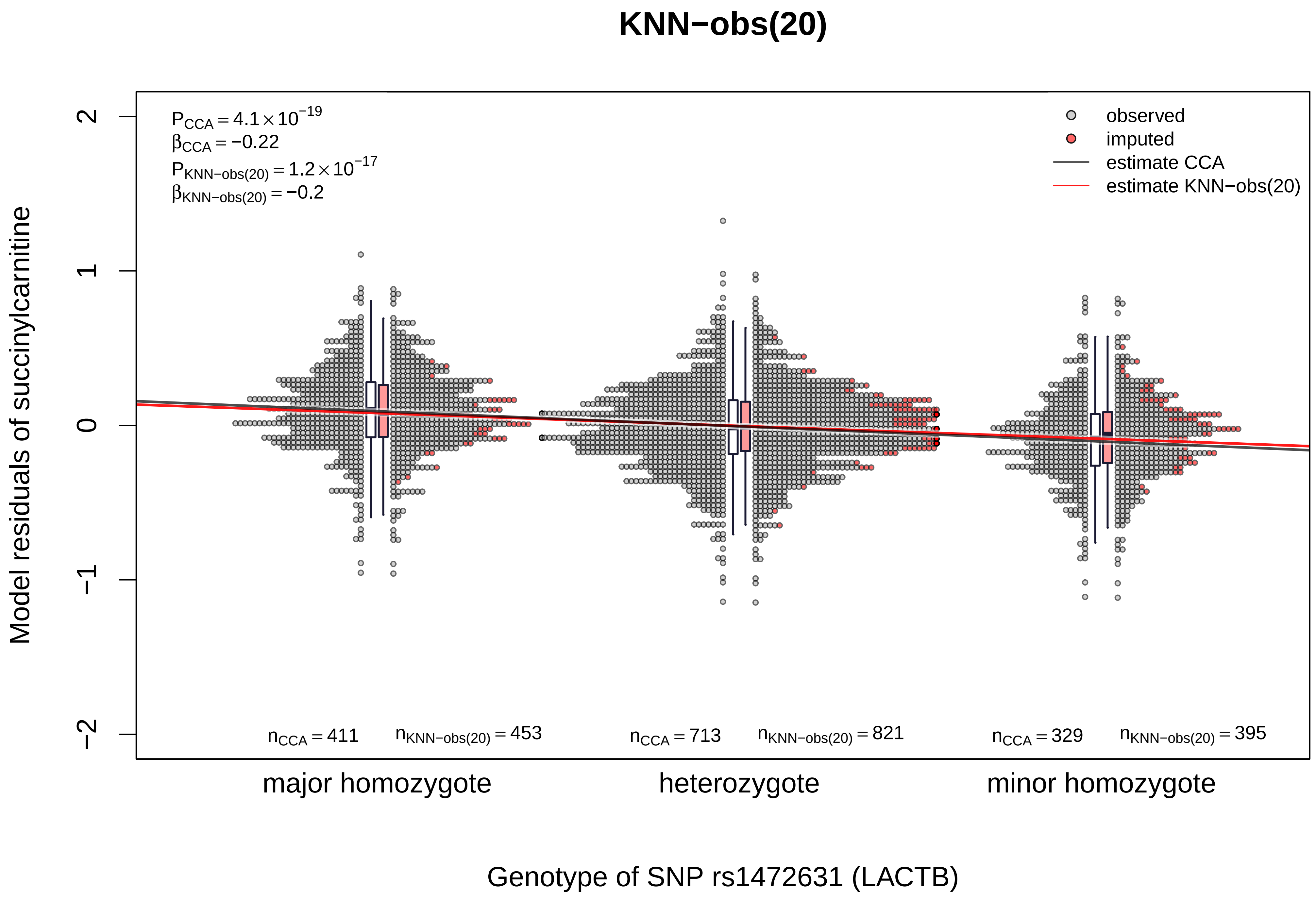

KNN−obs(20)
$P_{CCA} = 4.1 \times 10^{-19}$
$\beta_{CCA} = -0.22$
$P_{KNN-obs(20)} = 1.2 \times 10^{-17}$
$\beta_{KNN-obs(20)} = -0.2$
observed
imputed
estimate CCA
estimate KNN−obs(20)
2
1
0
−1
−2
Model residuals of succinylcarnitine
$n_{CCA} = 411$
$n_{KNN-obs(20)} = 453$
$n_{CCA} = 713$
$n_{KNN-obs(20)} = 821$
$n_{CCA} = 329$
$n_{KNN-obs(20)} = 395$
major homozygote
heterozygote
minor homozygote
Genotype of SNP rs1472631 (LACTB)

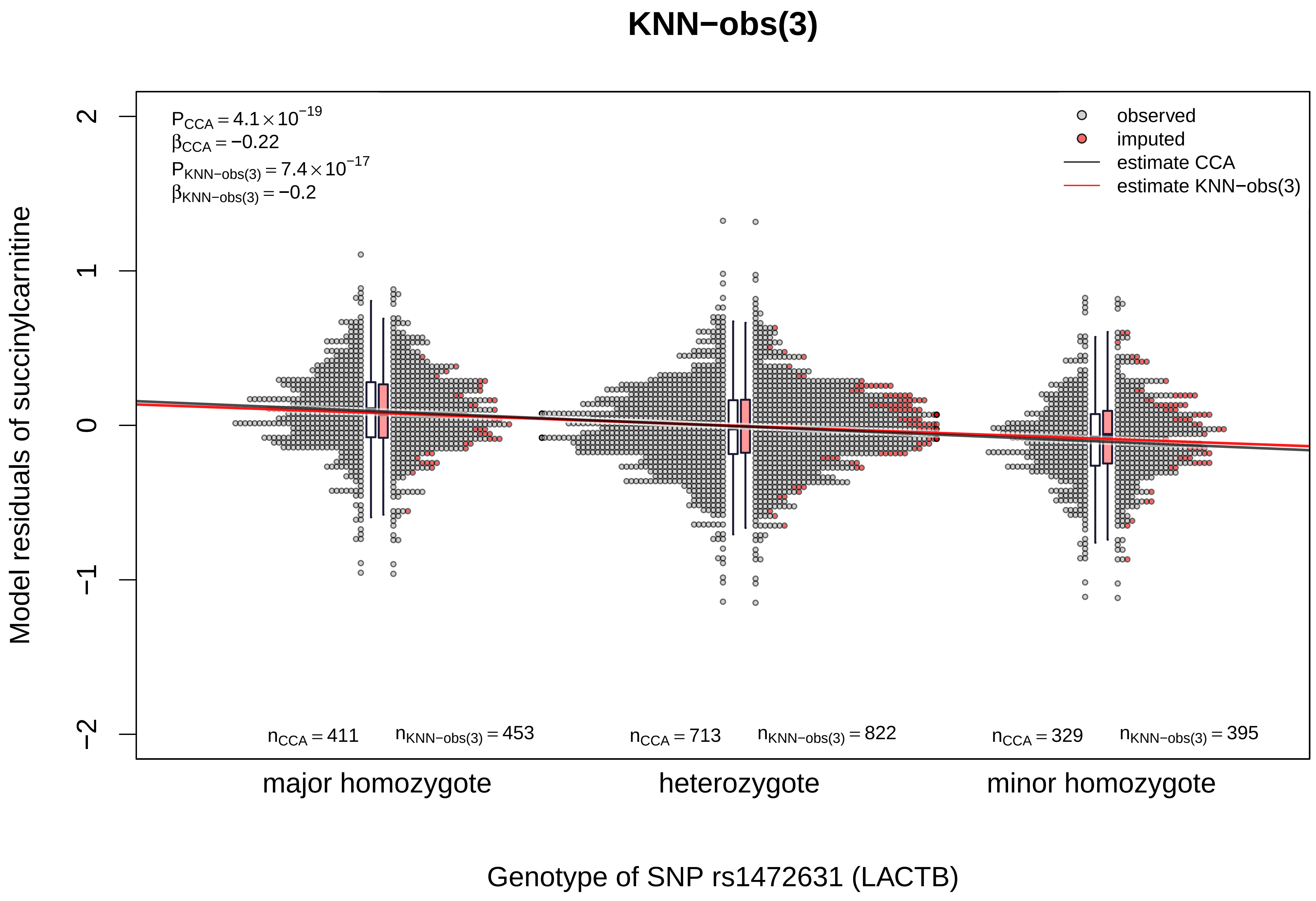

KNN−obs(3)
$P_{CCA} = 4.1 \times 10^{-19}$
$\beta_{CCA} = -0.22$
$P_{KNN-obs(3)} = 7.4 \times 10^{-17}$
$\beta_{KNN-obs(3)} = -0.2$
observed
imputed
estimate CCA
estimate KNN−obs(3)
Model residuals of succinylcarnitine
2
1
0
−1
−2
$n_{CCA} = 411$
$n_{KNN-obs(3)} = 453$
$n_{CCA} = 713$
$n_{KNN-obs(3)} = 822$
$n_{CCA} = 329$
$n_{KNN-obs(3)} = 395$
major homozygote
heterozygote
minor homozygote
Genotype of SNP rs1472631 (LACTB)

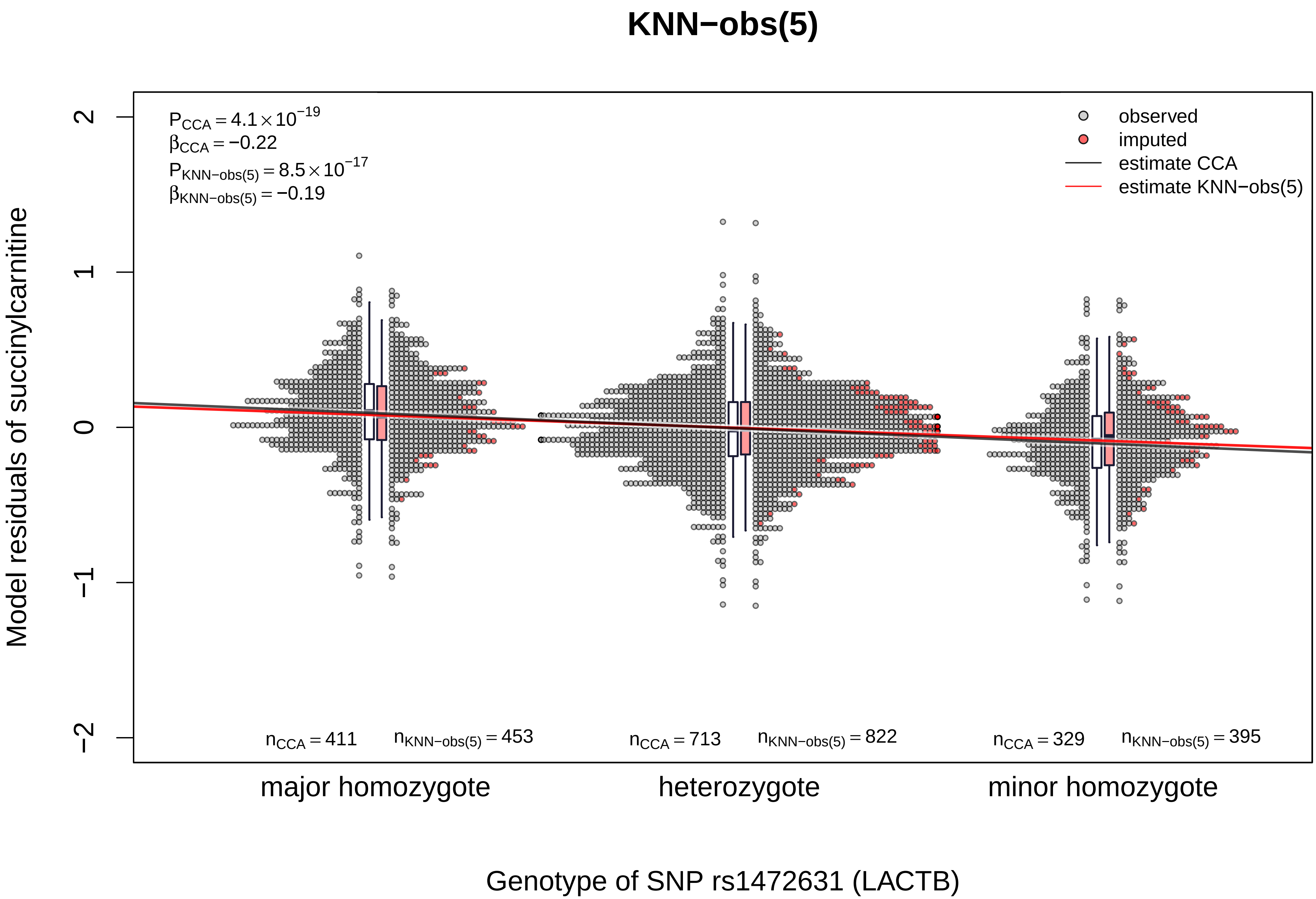

KNN−obs(5)
$P_{CCA} = 4.1 \times 10^{-19}$
$\beta_{CCA} = -0.22$
$P_{KNN-obs(5)} = 8.5 \times 10^{-17}$
$\beta_{KNN-obs(5)} = -0.19$
observed
imputed
estimate CCA
estimate KNN−obs(5)
Model residuals of succinylcarnitine
2
1
0
−1
−2
$n_{CCA} = 411$
$n_{KNN-obs(5)} = 453$
$n_{CCA} = 713$
$n_{KNN-obs(5)} = 822$
$n_{CCA} = 329$
$n_{KNN-obs(5)} = 395$
major homozygote
heterozygote
minor homozygote
Genotype of SNP rs1472631 (LACTB)

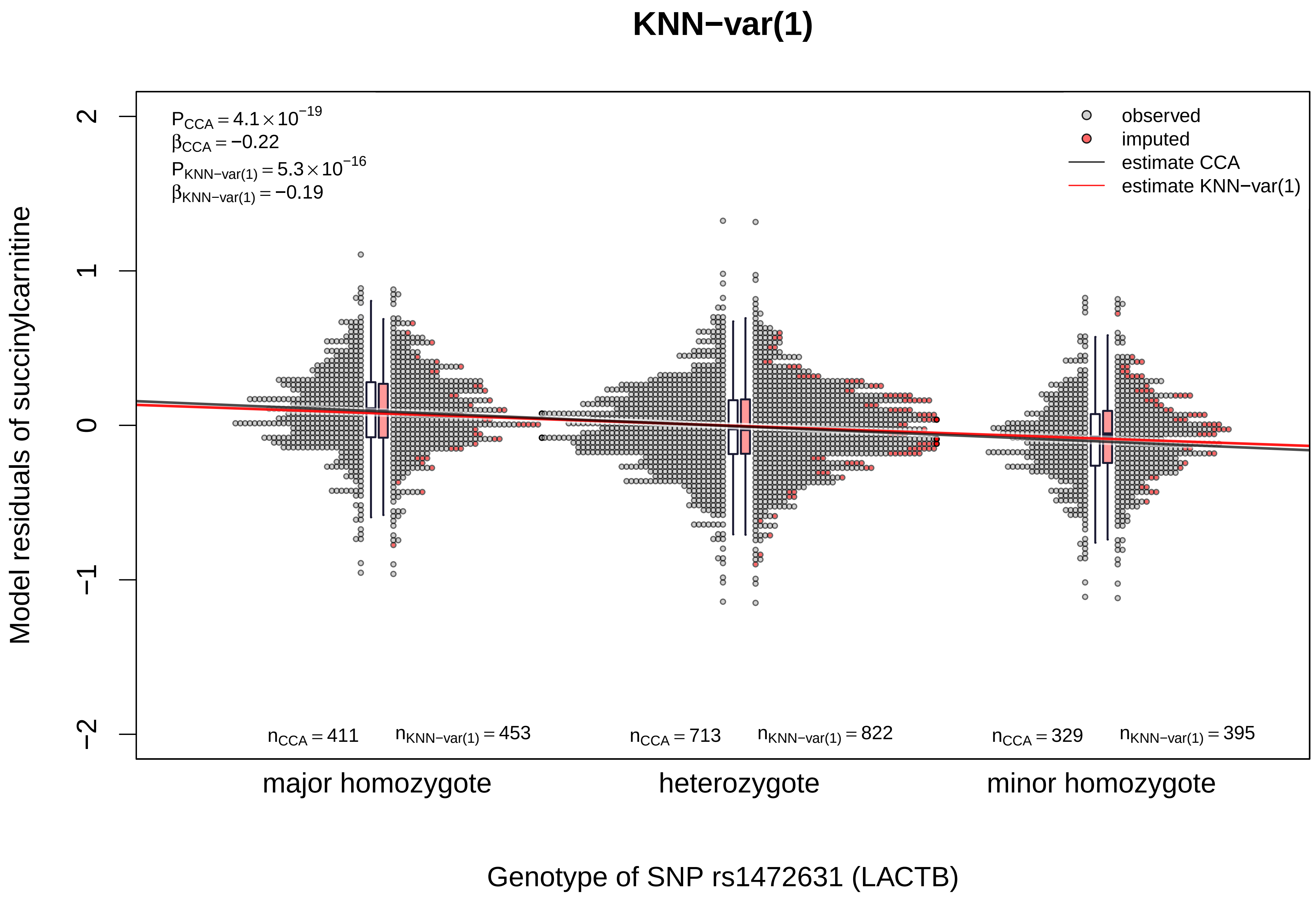
KNN−var(1)
$P_{CCA} = 4.1 \times 10^{-19}$
$\beta_{CCA} = -0.22$
$P_{KNN-var(1)} = 5.3 \times 10^{-16}$
$\beta_{KNN-var(1)} = -0.19$
observed
imputed
estimate CCA
estimate KNN−var(1)
Model residuals of succinylcarnitine
2
1
0
−1
−2
$n_{CCA} = 411$
$n_{KNN-var(1)} = 453$
$n_{CCA} = 713$
$n_{KNN-var(1)} = 822$
$n_{CCA} = 329$
$n_{KNN-var(1)} = 395$
major homozygote
heterozygote
minor homozygote
Genotype of SNP rs1472631 (LACTB)

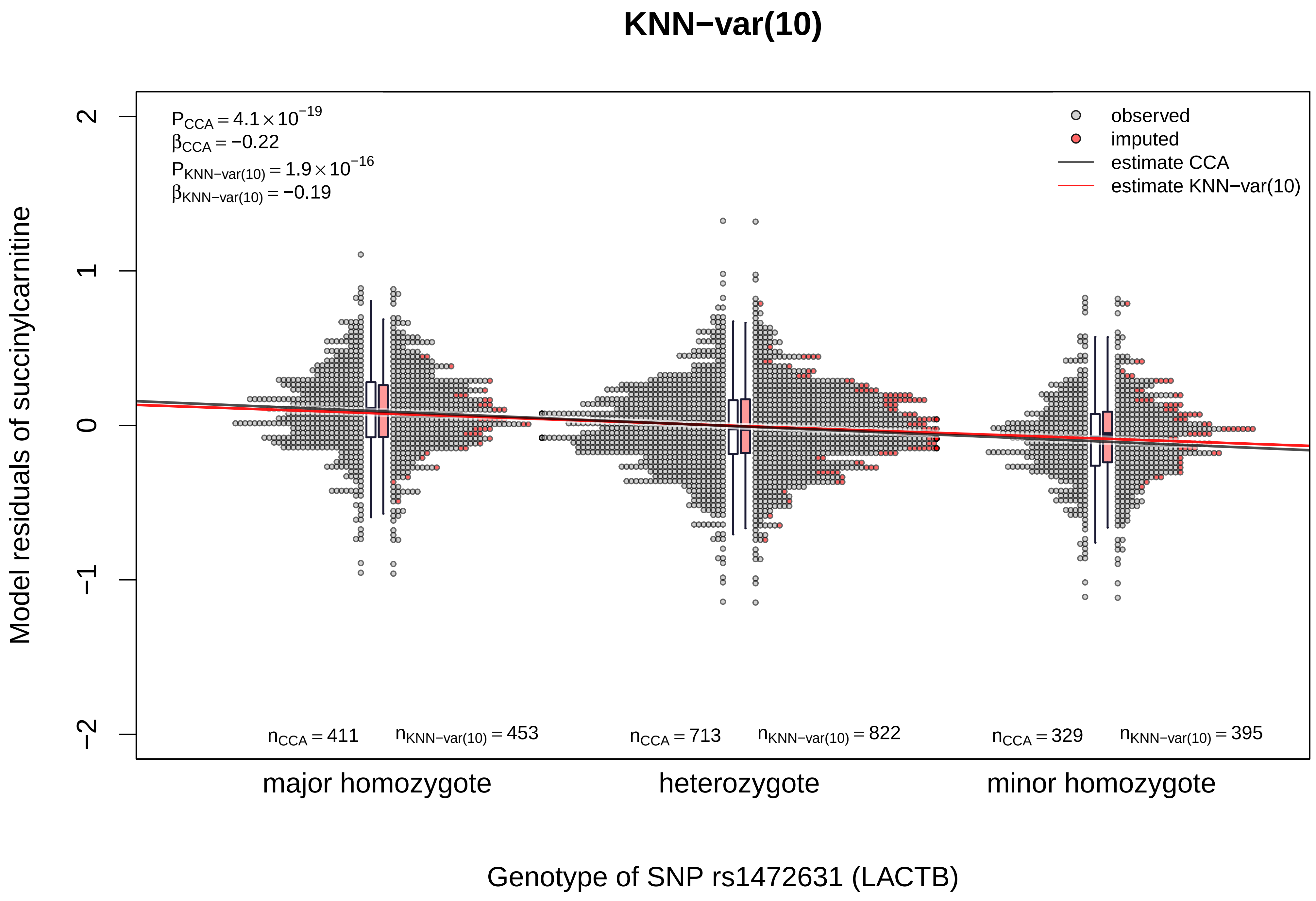

KNN−var(10)
$P_{CCA} = 4.1 \times 10^{-19}$
$\beta_{CCA} = -0.22$
$P_{KNN-var(10)} = 1.9 \times 10^{-16}$
$\beta_{KNN-var(10)} = -0.19$
observed
imputed
estimate CCA
estimate KNN−var(10)
Model residuals of succinylcarnitine
2
1
0
−1
−2
$n_{CCA} = 411$
$n_{KNN-var(10)} = 453$
$n_{CCA} = 713$
$n_{KNN-var(10)} = 822$
$n_{CCA} = 329$
$n_{KNN-var(10)} = 395$
major homozygote
heterozygote
minor homozygote
Genotype of SNP rs1472631 (LACTB)

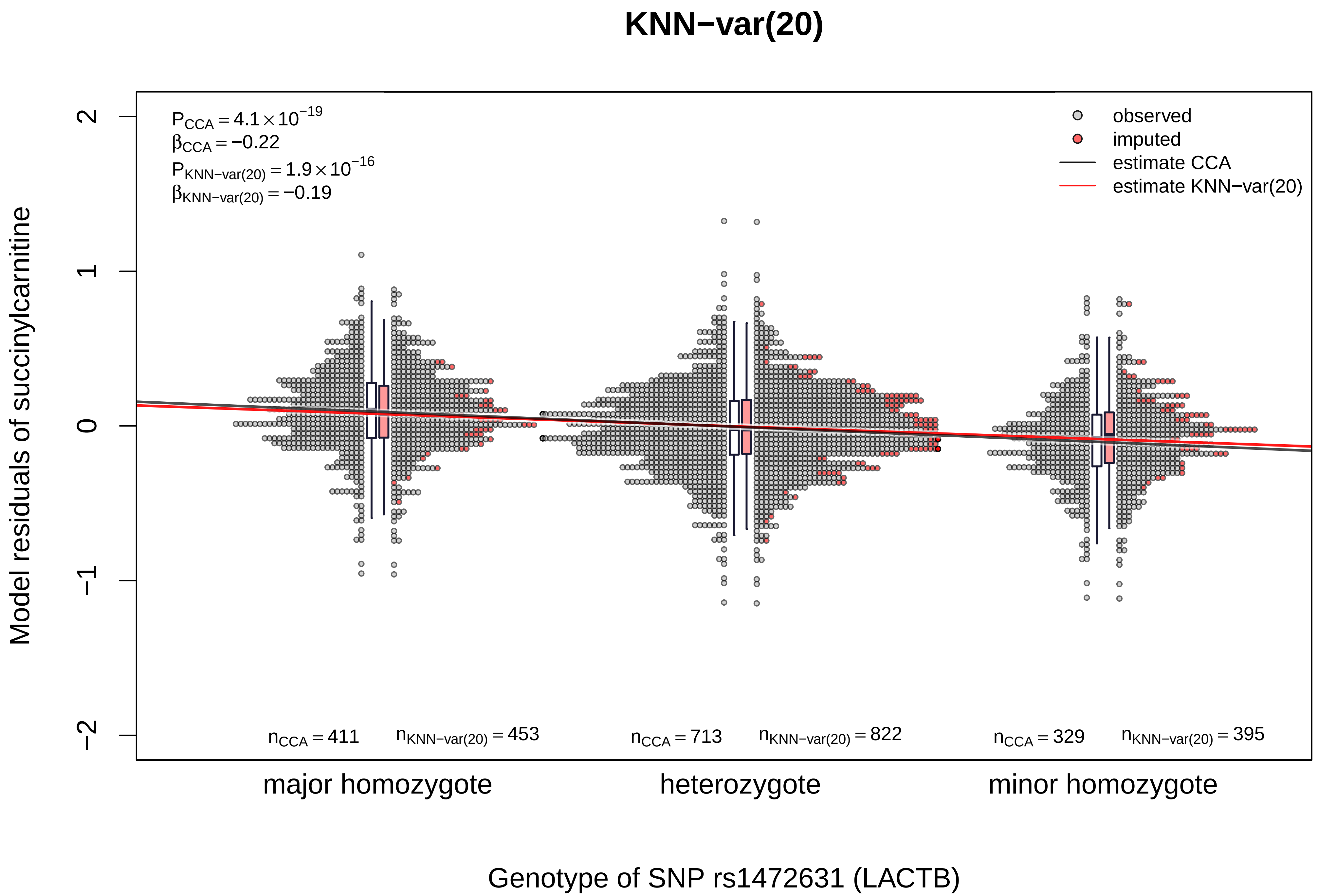

KNN−var(20)
$P_{CCA} = 4.1 \times 10^{-19}$
$\beta_{CCA} = -0.22$
$P_{KNN-var(20)} = 1.9 \times 10^{-16}$
$\beta_{KNN-var(20)} = -0.19$
observed
imputed
estimate CCA
estimate KNN−var(20)
2
1
0
−1
−2
Model residuals of succinylcarnitine
$n_{CCA} = 411$
$n_{KNN-var(20)} = 453$
$n_{CCA} = 713$
$n_{KNN-var(20)} = 822$
$n_{CCA} = 329$
$n_{KNN-var(20)} = 395$
major homozygote
heterozygote
minor homozygote
Genotype of SNP rs1472631 (LACTB)

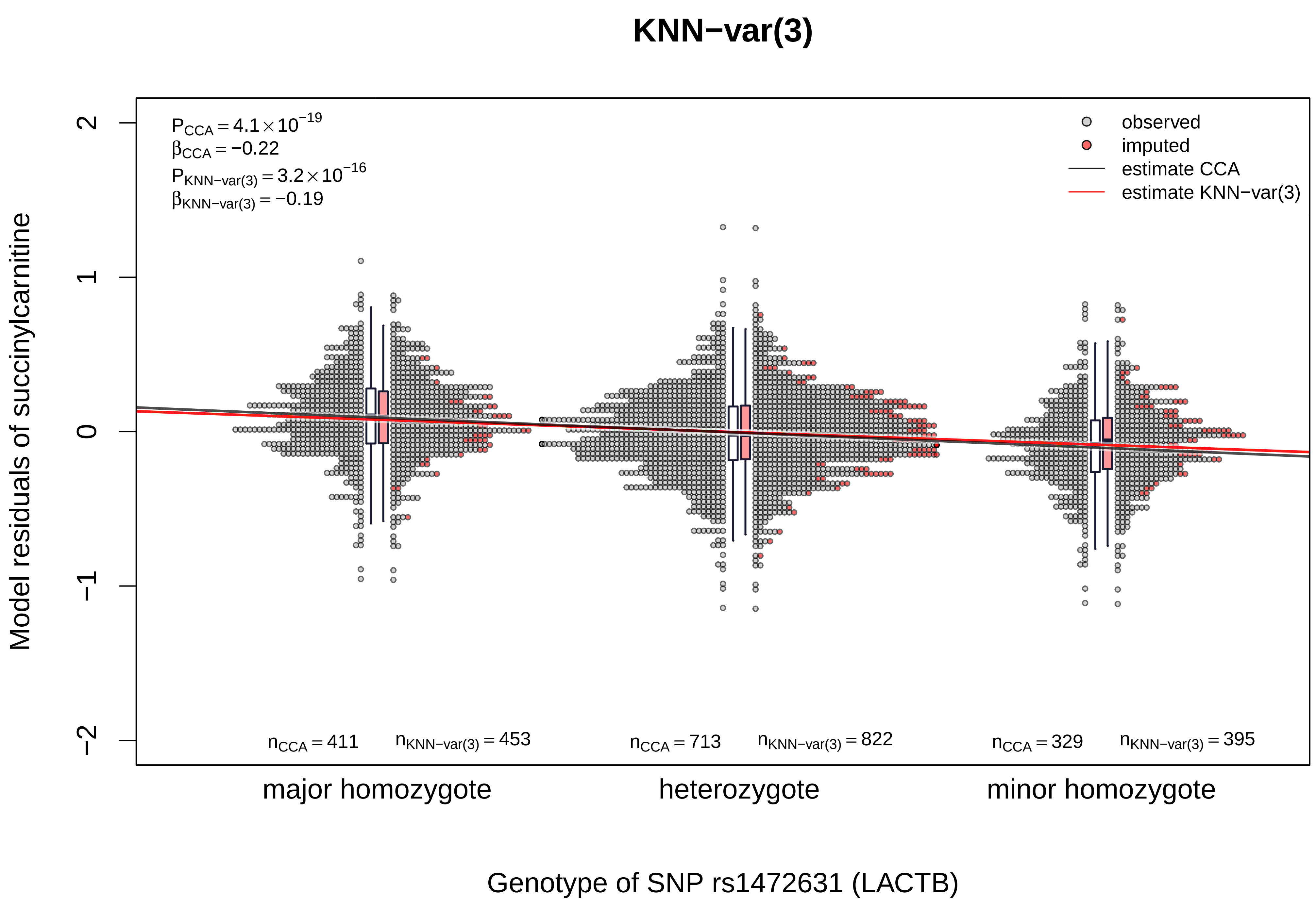

KNN−var(3)
$P_{CCA} = 4.1 \times 10^{-19}$
$\beta_{CCA} = -0.22$
$P_{KNN-var(3)} = 3.2 \times 10^{-16}$
$\beta_{KNN-var(3)} = -0.19$
observed
imputed
estimate CCA
estimate KNN−var(3)
Model residuals of succinylcarnitine
2
1
0
−1
−2
$n_{CCA} = 411$
$n_{KNN-var(3)} = 453$
$n_{CCA} = 713$
$n_{KNN-var(3)} = 822$
$n_{CCA} = 329$
$n_{KNN-var(3)} = 395$
major homozygote
heterozygote
minor homozygote
Genotype of SNP rs1472631 (LACTB)

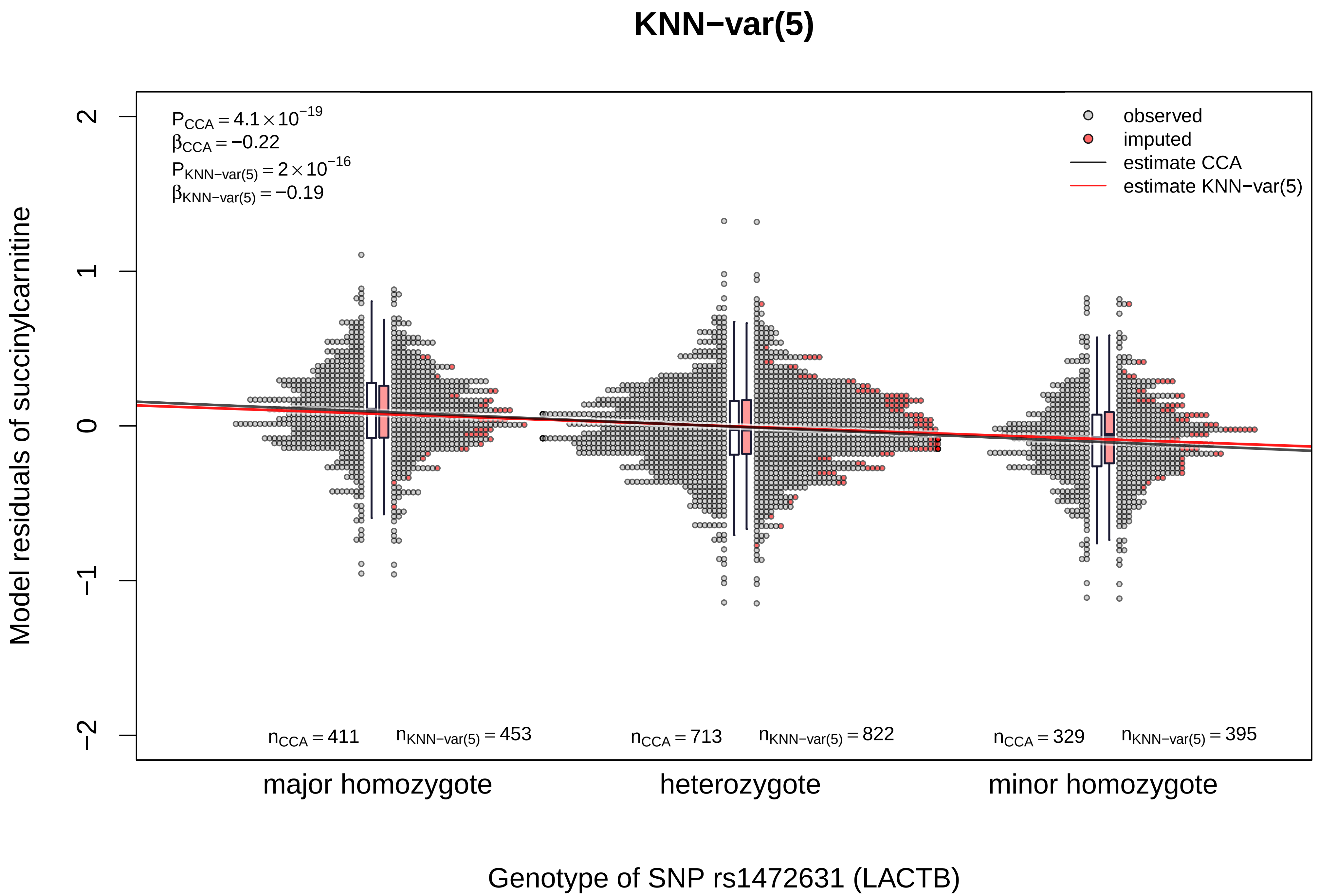

KNN−var(5)
$P_{CCA} = 4.1 \times 10^{-19}$
$\beta_{CCA} = -0.22$
$P_{KNN-var(5)} = 2 \times 10^{-16}$
$\beta_{KNN-var(5)} = -0.19$
observed
imputed
estimate CCA
estimate KNN−var(5)
$n_{CCA} = 411$
$n_{KNN-var(5)} = 453$
$n_{CCA} = 713$
$n_{KNN-var(5)} = 822$
$n_{CCA} = 329$
$n_{KNN-var(5)} = 395$
Model residuals of succinylcarnitine
2
1
0
−1
−2
major homozygote
heterozygote
minor homozygote
Genotype of SNP rs1472631 (LACTB)

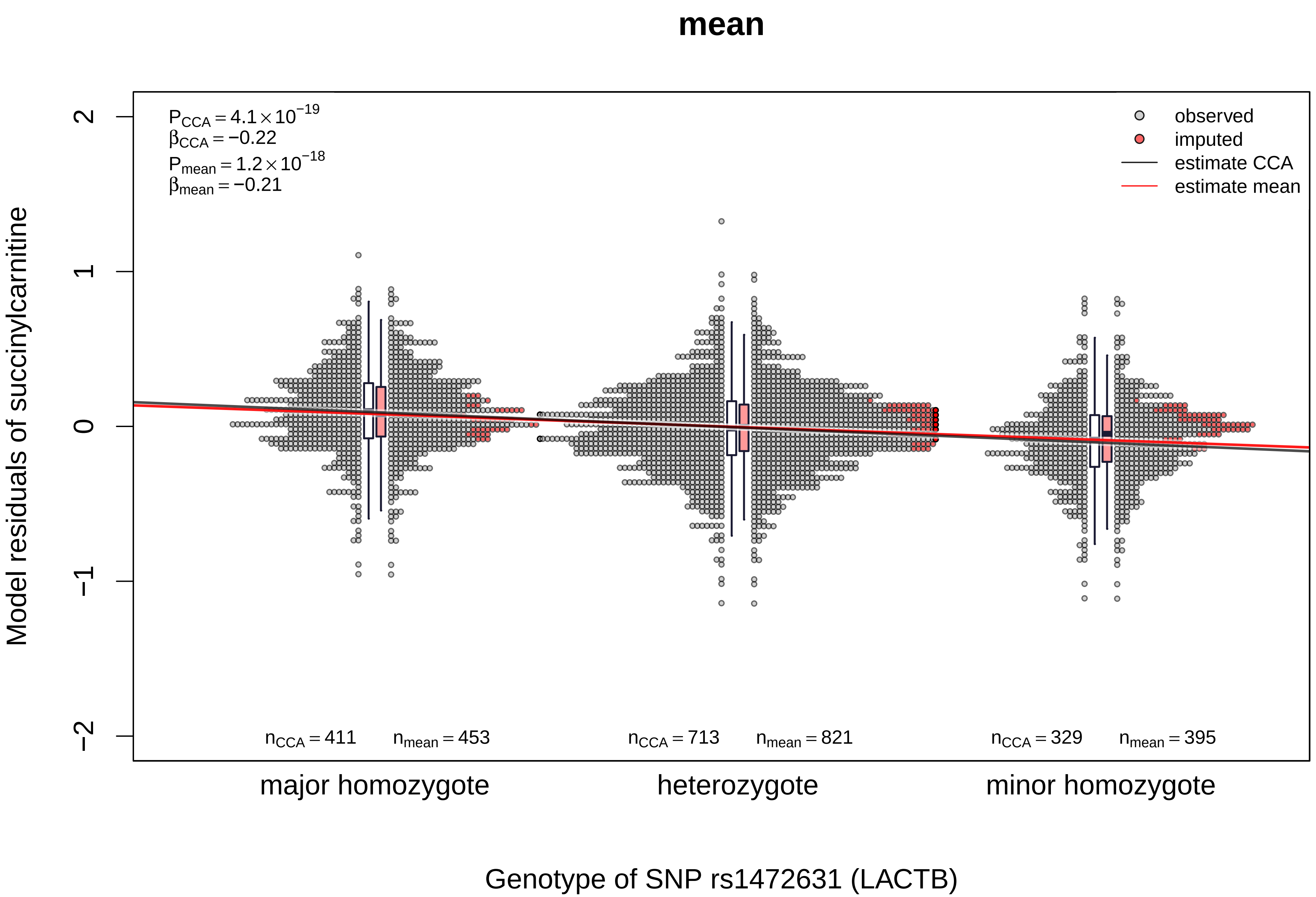
mean
$P_{CCA} = 4.1 \times 10^{-19}$
$\beta_{CCA} = -0.22$
$P_{mean} = 1.2 \times 10^{-18}$
$\beta_{mean} = -0.21$
observed
imputed
estimate CCA
estimate mean
2
1
0
−1
−2
Model residuals of succinylcarnitine
$n_{CCA} = 411$
$n_{mean} = 453$
$n_{CCA} = 713$
$n_{mean} = 821$
$n_{CCA} = 329$
$n_{mean} = 395$
major homozygote
heterozygote
minor homozygote
Genotype of SNP rs1472631 (LACTB)

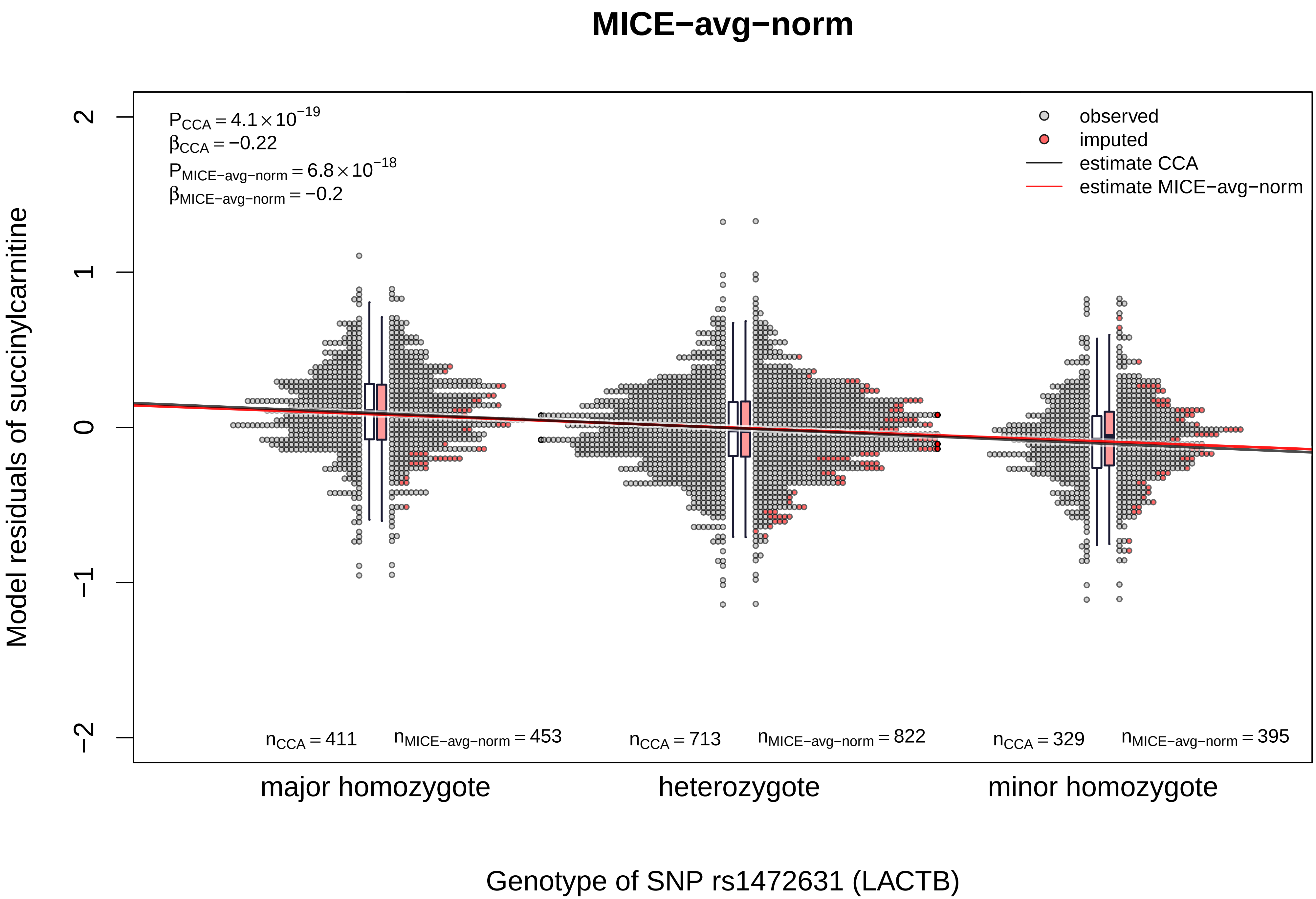

MICE−avg−norm
$P_{CCA} = 4.1 \times 10^{-19}$
$\beta_{CCA} = -0.22$
$P_{MICE-avg-norm} = 6.8 \times 10^{-18}$
$\beta_{MICE-avg-norm} = -0.2$
observed
imputed
estimate CCA
estimate MICE−avg−norm
2
1
0
−1
−2
Model residuals of succinylcarnitine
$n_{CCA} = 411$
$n_{MICE-avg-norm} = 453$
$n_{CCA} = 713$
$n_{MICE-avg-norm} = 822$
$n_{CCA} = 329$
$n_{MICE-avg-norm} = 395$
major homozygote
heterozygote
minor homozygote
Genotype of SNP rs1472631 (LACTB)

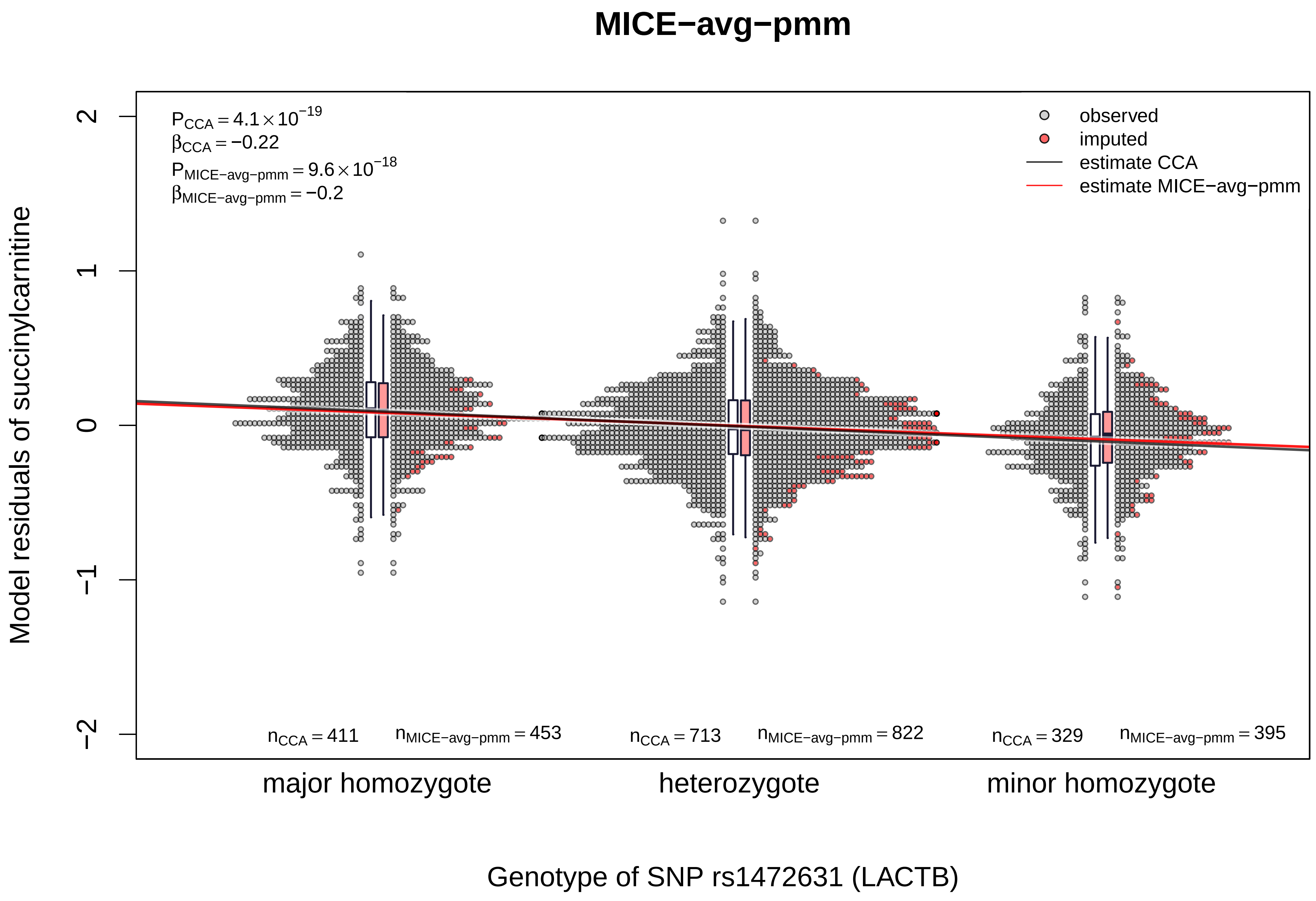
MICE−avg−pmm
$P_{CCA} = 4.1 \times 10^{-19}$
$\beta_{CCA} = -0.22$
$P_{MICE-avg-pmm} = 9.6 \times 10^{-18}$
$\beta_{MICE-avg-pmm} = -0.2$
observed
imputed
estimate CCA
estimate MICE−avg−pmm
2
1
0
−1
−2
Model residuals of succinylcarnitine
$n_{CCA} = 411$
$n_{MICE-avg-pmm} = 453$
$n_{CCA} = 713$
$n_{MICE-avg-pmm} = 822$
$n_{CCA} = 329$
$n_{MICE-avg-pmm} = 395$
major homozygote
heterozygote
minor homozygote
Genotype of SNP rs1472631 (LACTB)

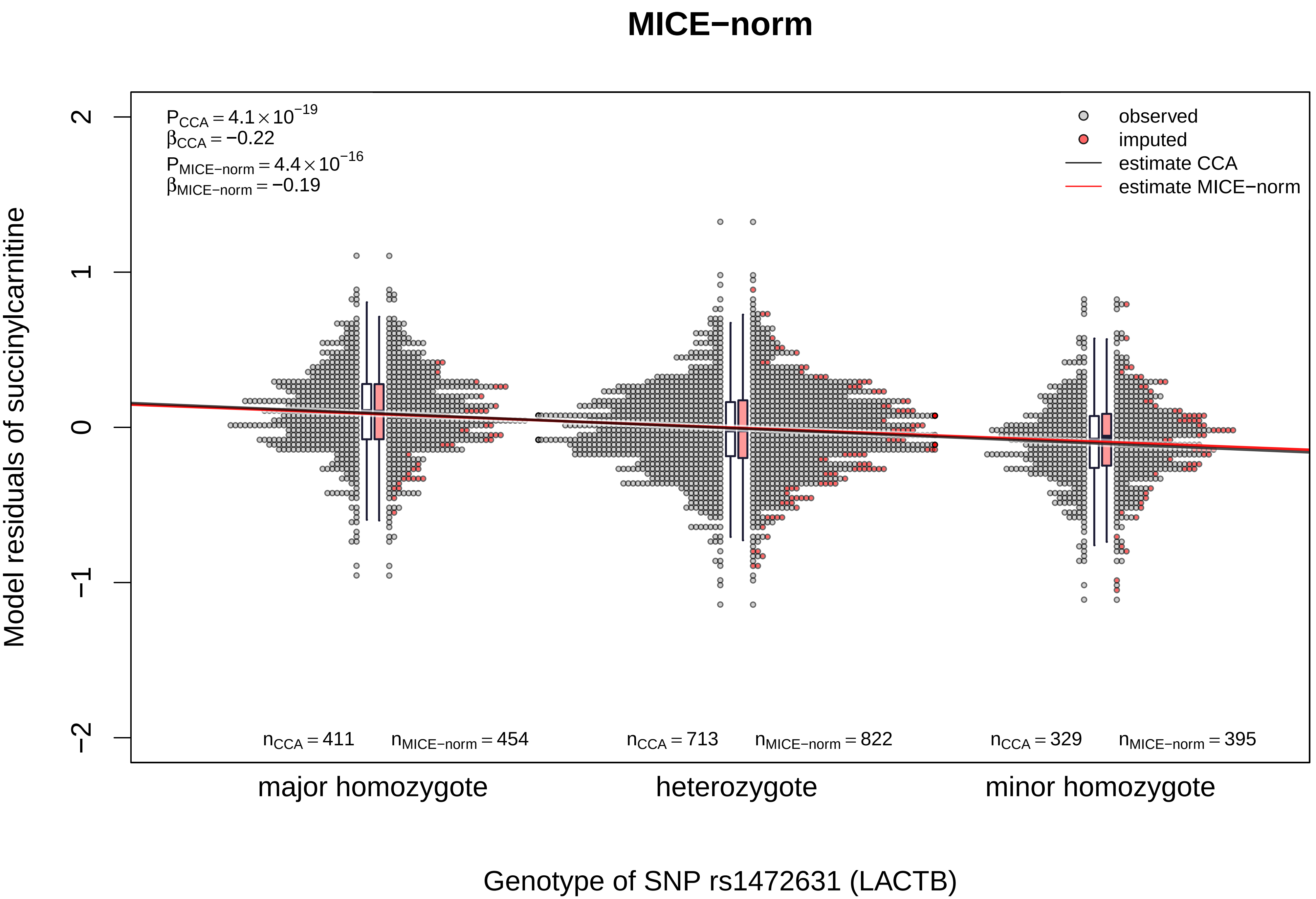

MICE−norm
$P_{CCA} = 4.1 \times 10^{-19}$
$\beta_{CCA} = -0.22$
$P_{MICE-norm} = 4.4 \times 10^{-16}$
$\beta_{MICE-norm} = -0.19$
observed
imputed
estimate CCA
estimate MICE−norm
$n_{CCA} = 411$
$n_{MICE-norm} = 454$
$n_{CCA} = 713$
$n_{MICE-norm} = 822$
$n_{CCA} = 329$
$n_{MICE-norm} = 395$
Model residuals of succinylcarnitine
2
1
0
−1
−2
major homozygote
heterozygote
minor homozygote
Genotype of SNP rs1472631 (LACTB)

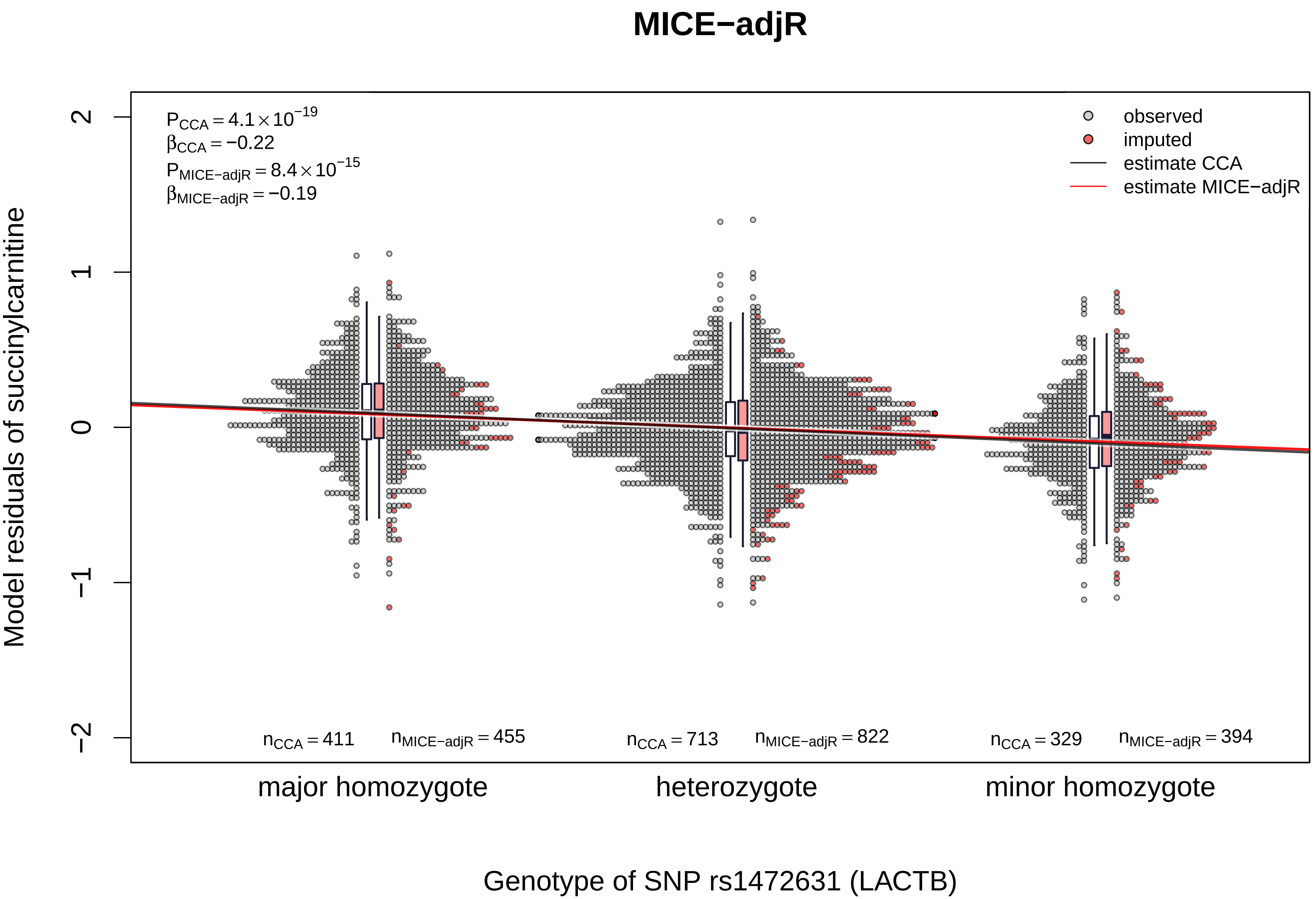

MICE−adjR
$P_{CCA} = 4.1 \times 10^{-19}$
$\beta_{CCA} = -0.22$
$P_{MICE-adjR} = 8.4 \times 10^{-15}$
$\beta_{MICE-adjR} = -0.19$
observed
imputed
estimate CCA
estimate MICE−adjR
$n_{CCA} = 411$
$n_{MICE-adjR} = 455$
$n_{CCA} = 713$
$n_{MICE-adjR} = 822$
$n_{CCA} = 329$
$n_{MICE-adjR} = 394$
2
1
0
−1
−2
Model residuals of succinylcarnitine
major homozygote
heterozygote
minor homozygote
Genotype of SNP rs1472631 (LACTB)

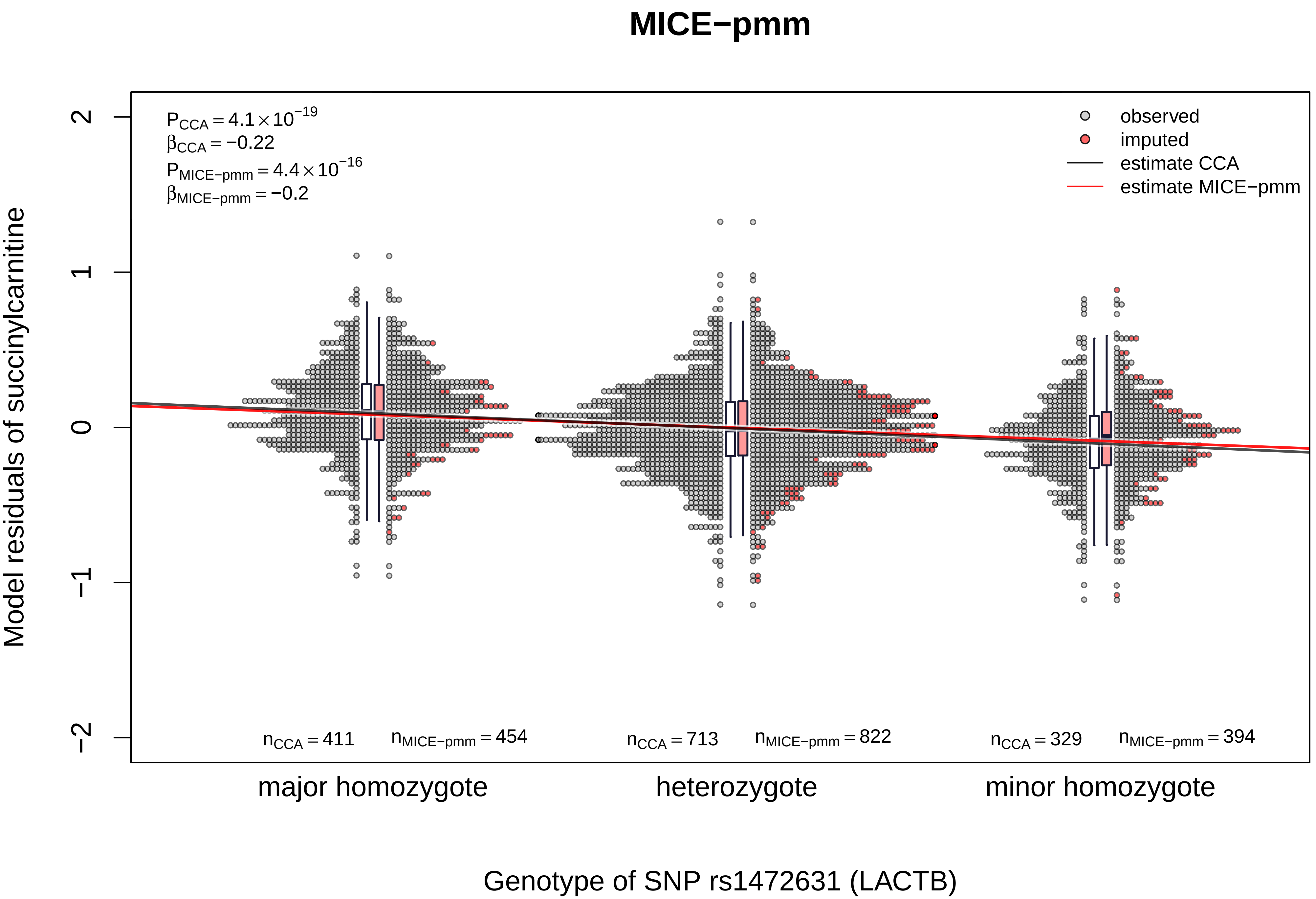
MICE−pmm
$P_{CCA} = 4.1 \times 10^{-19}$
$\beta_{CCA} = -0.22$
$P_{MICE-pmm} = 4.4 \times 10^{-16}$
$\beta_{MICE-pmm} = -0.2$
observed
imputed
estimate CCA
estimate MICE−pmm
$n_{CCA} = 411$
$n_{MICE-pmm} = 454$
$n_{CCA} = 713$
$n_{MICE-pmm} = 822$
$n_{CCA} = 329$
$n_{MICE-pmm} = 394$
Model residuals of succinylcarnitine
2
1
0
−1
−2
major homozygote
heterozygote
minor homozygote
Genotype of SNP rs1472631 (LACTB)

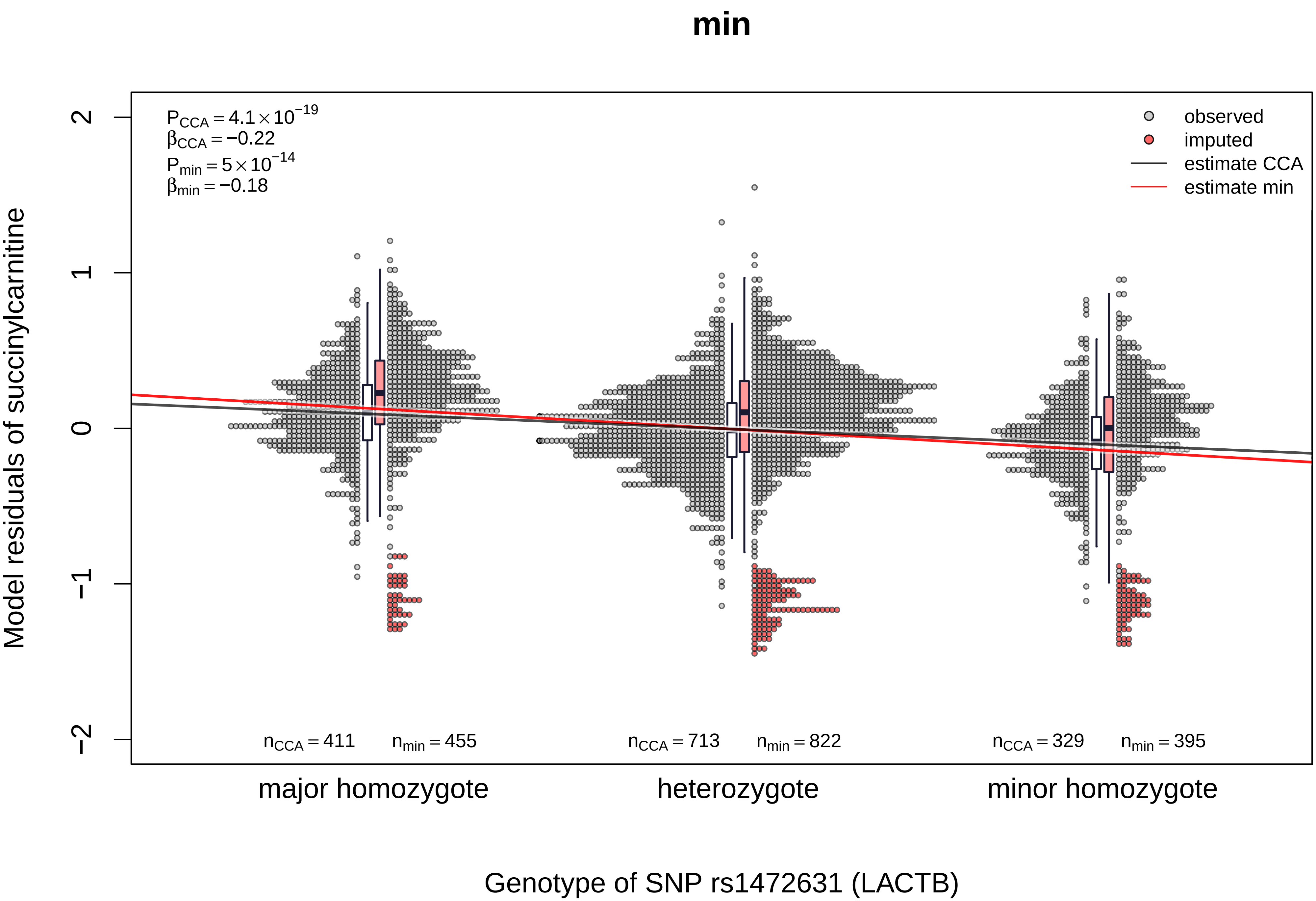

min
$P_{CCA} = 4.1 \times 10^{-19}$
$\beta_{CCA} = -0.22$
$P_{min} = 5 \times 10^{-14}$
$\beta_{min} = -0.18$
observed
imputed
estimate CCA
estimate min
Model residuals of succinylcarnitine
2
1
0
−1
−2
$n_{CCA} = 411$
$n_{min} = 455$
$n_{CCA} = 713$
$n_{min} = 822$
$n_{CCA} = 329$
$n_{min} = 395$
major homozygote
heterozygote
minor homozygote
Genotype of SNP rs1472631 (LACTB)

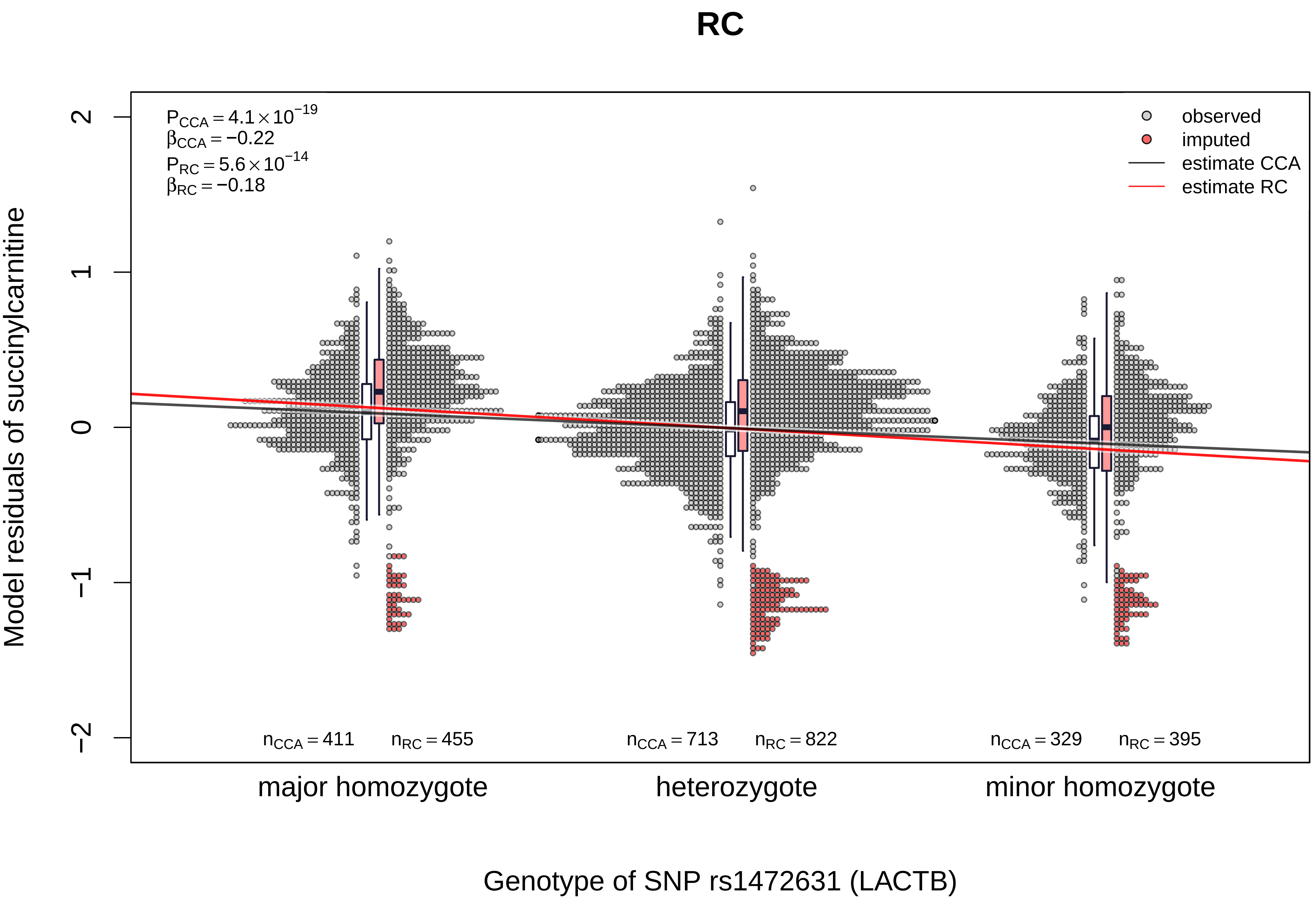
RC
$P_{CCA} = 4.1 \times 10^{-19}$
$\beta_{CCA} = -0.22$
$P_{RC} = 5.6 \times 10^{-14}$
$\beta_{RC} = -0.18$
observed
imputed
estimate CCA
estimate RC
2
1
0
−1
−2
Model residuals of succinylcarnitine
$n_{CCA} = 411$
$n_{RC} = 455$
$n_{CCA} = 713$
$n_{RC} = 822$
$n_{CCA} = 329$
$n_{RC} = 395$
major homozygote
heterozygote
minor homozygote
Genotype of SNP rs1472631 (LACTB)

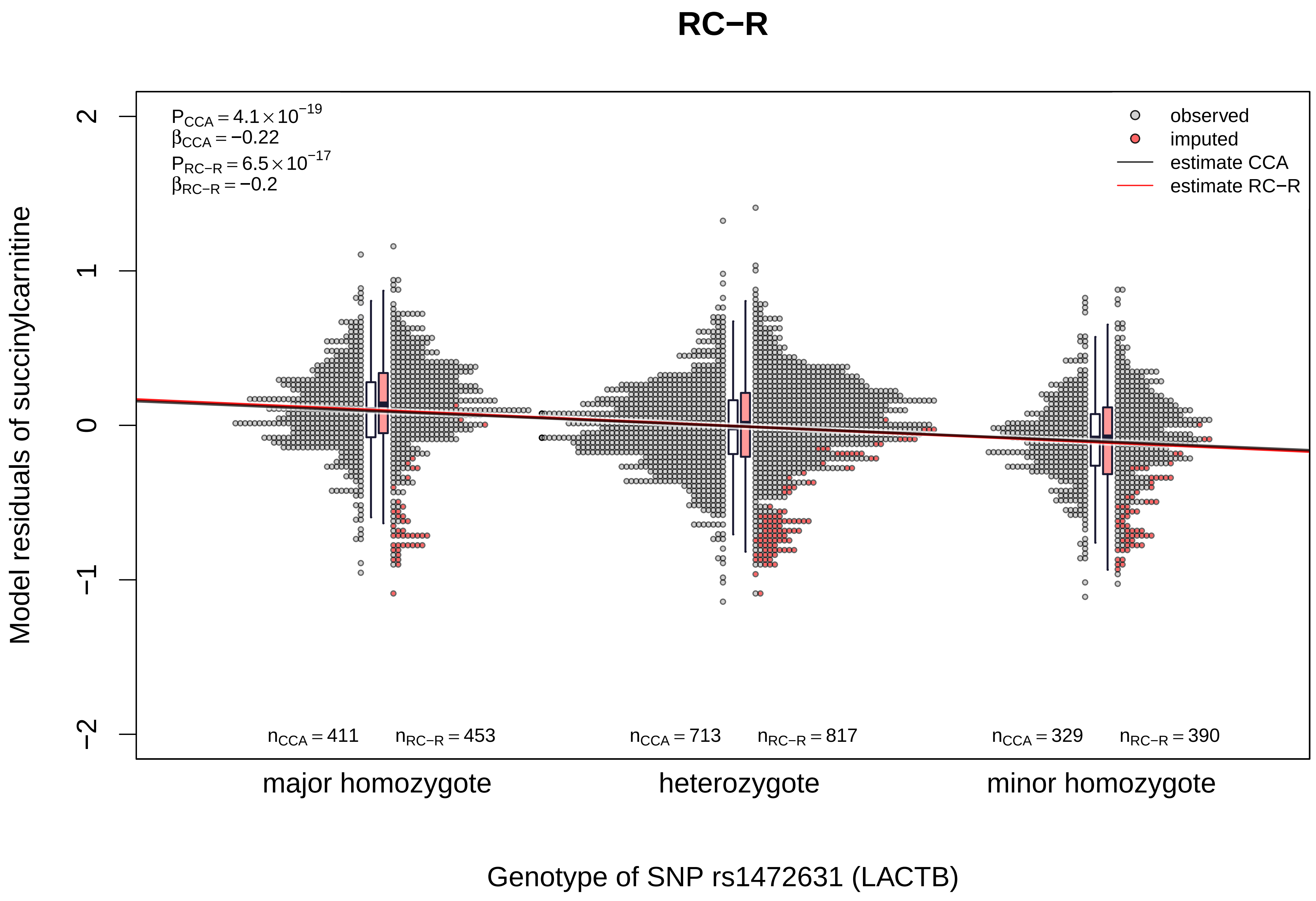
RC−R
$P_{CCA} = 4.1 \times 10^{-19}$
$\beta_{CCA} = -0.22$
$P_{RC-R} = 6.5 \times 10^{-17}$
$\beta_{RC-R} = -0.2$
observed
imputed
estimate CCA
estimate RC−R
Model residuals of succinylcarnitine
2
1
0
−1
−2
$n_{CCA} = 411$
$n_{RC-R} = 453$
$n_{CCA} = 713$
$n_{RC-R} = 817$
$n_{CCA} = 329$
$n_{RC-R} = 390$
major homozygote
heterozygote
minor homozygote
Genotype of SNP rs1472631 (LACTB)

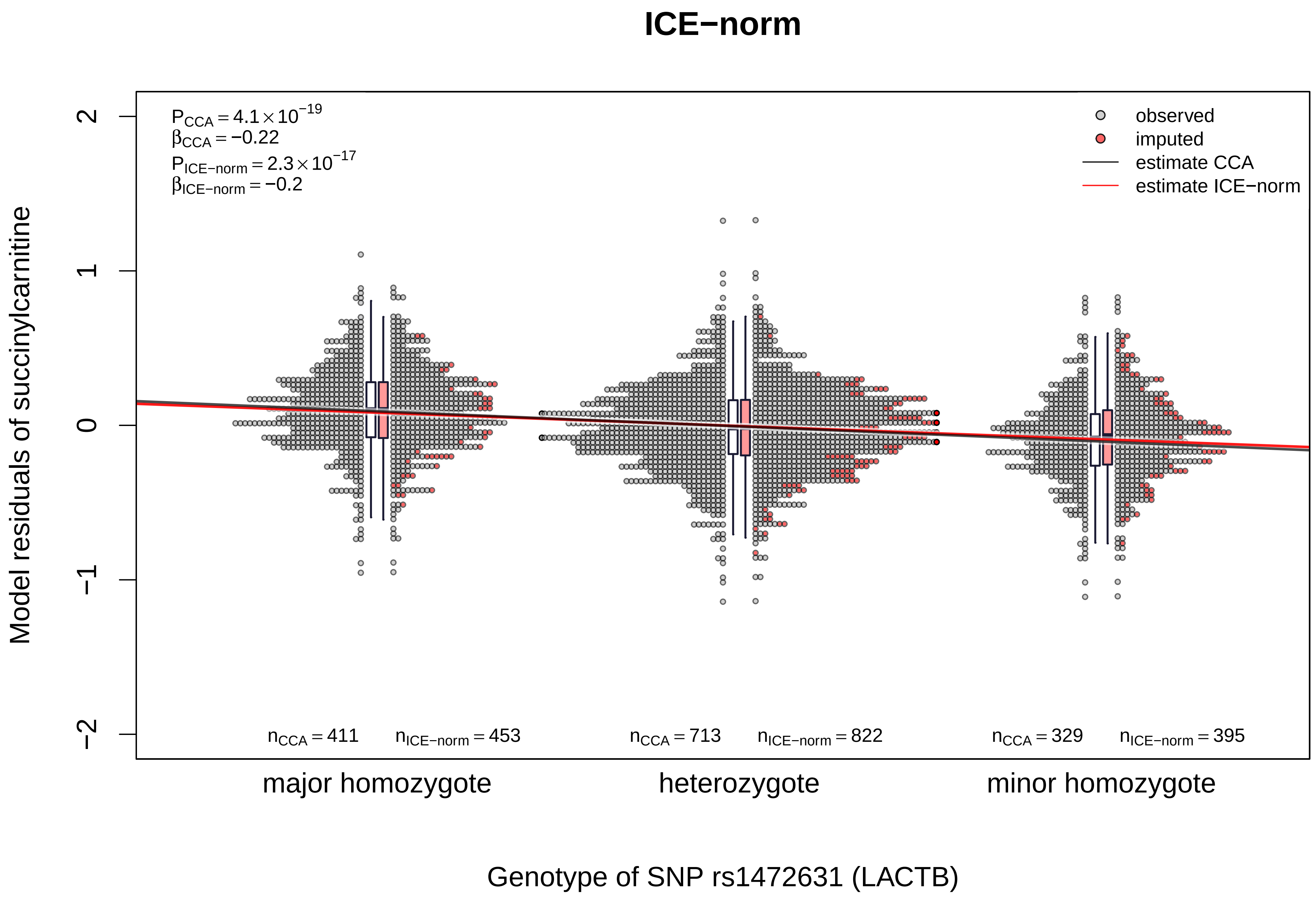

ICE−norm
$P_{CCA} = 4.1 \times 10^{-19}$
$\beta_{CCA} = -0.22$
$P_{ICE-norm} = 2.3 \times 10^{-17}$
$\beta_{ICE-norm} = -0.2$
observed
imputed
estimate CCA
estimate ICE−norm
Model residuals of succinylcarnitine
2
1
0
−1
−2
$n_{CCA} = 411$
$n_{ICE-norm} = 453$
$n_{CCA} = 713$
$n_{ICE-norm} = 822$
$n_{CCA} = 329$
$n_{ICE-norm} = 395$
major homozygote
heterozygote
minor homozygote
Genotype of SNP rs1472631 (LACTB)

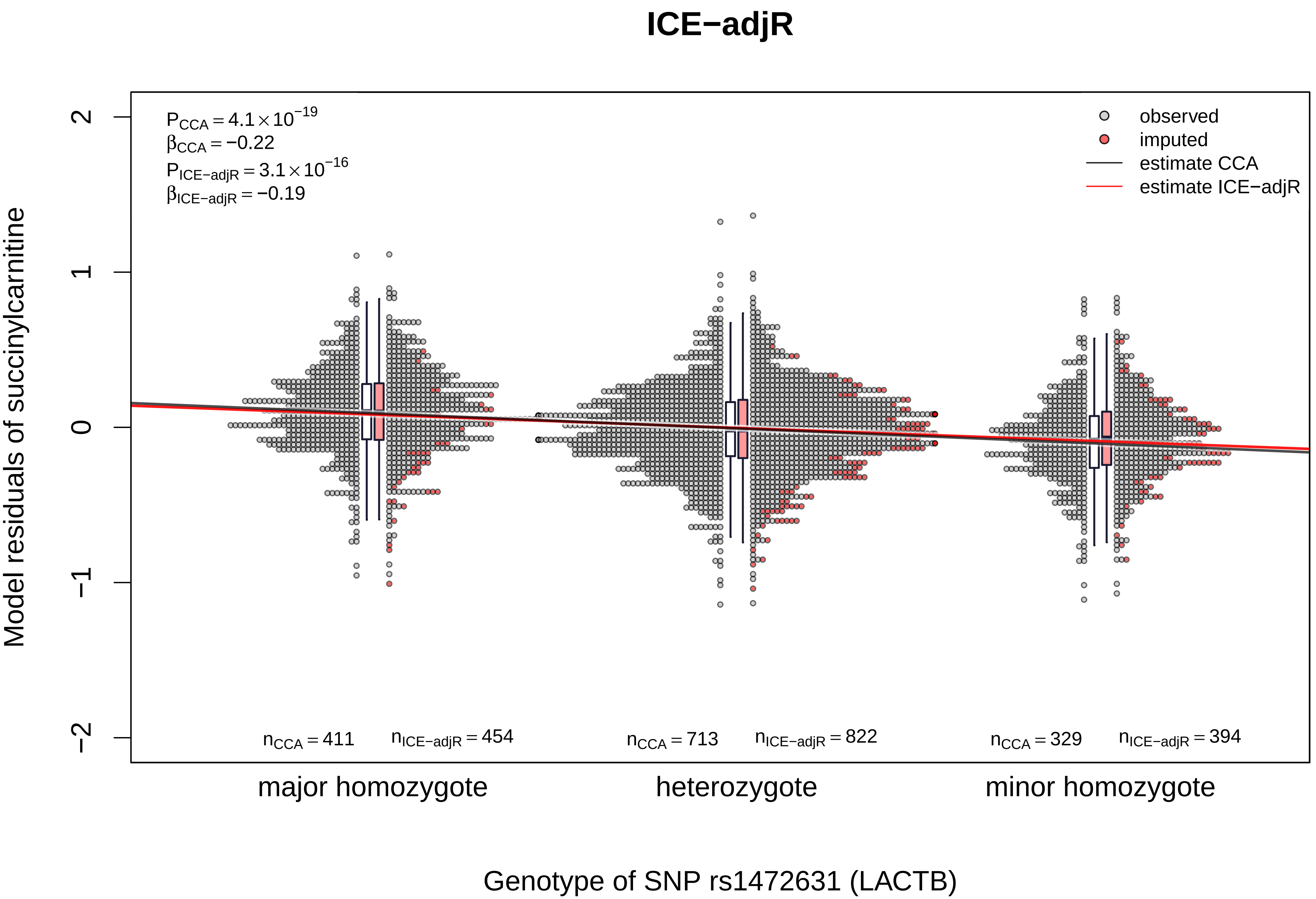
ICE−adjR
$P_{CCA} = 4.1 \times 10^{-19}$
$\beta_{CCA} = -0.22$
$P_{ICE-adjR} = 3.1 \times 10^{-16}$
$\beta_{ICE-adjR} = -0.19$
observed
imputed
estimate CCA
estimate ICE−adjR
Model residuals of succinylcarnitine
2
1
0
−1
−2
$n_{CCA} = 411$
$n_{ICE-adjR} = 454$
$n_{CCA} = 713$
$n_{ICE-adjR} = 822$
$n_{CCA} = 329$
$n_{ICE-adjR} = 394$
major homozygote
heterozygote
minor homozygote
Genotype of SNP rs1472631 (LACTB)

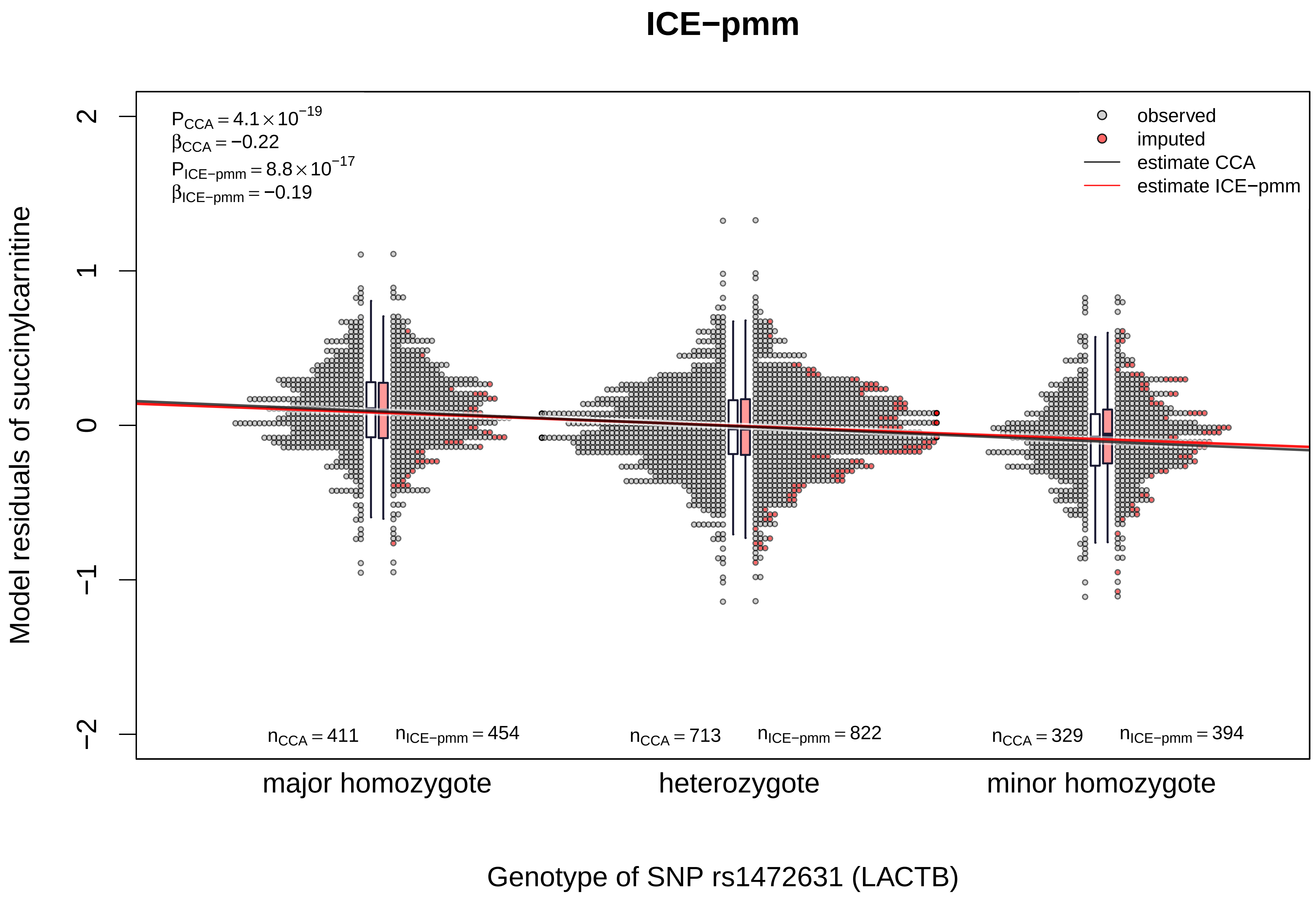
ICE−pmm
$P_{CCA} = 4.1 \times 10^{-19}$
$\beta_{CCA} = -0.22$
$P_{ICE-pmm} = 8.8 \times 10^{-17}$
$\beta_{ICE-pmm} = -0.19$
observed
imputed
estimate CCA
estimate ICE−pmm
Model residuals of succinylcarnitine
2
1
0
−1
−2
$n_{CCA} = 411$
$n_{ICE-pmm} = 454$
$n_{CCA} = 713$
$n_{ICE-pmm} = 822$
$n_{CCA} = 329$
$n_{ICE-pmm} = 394$
major homozygote
heterozygote
minor homozygote
Genotype of SNP rs1472631 (LACTB)

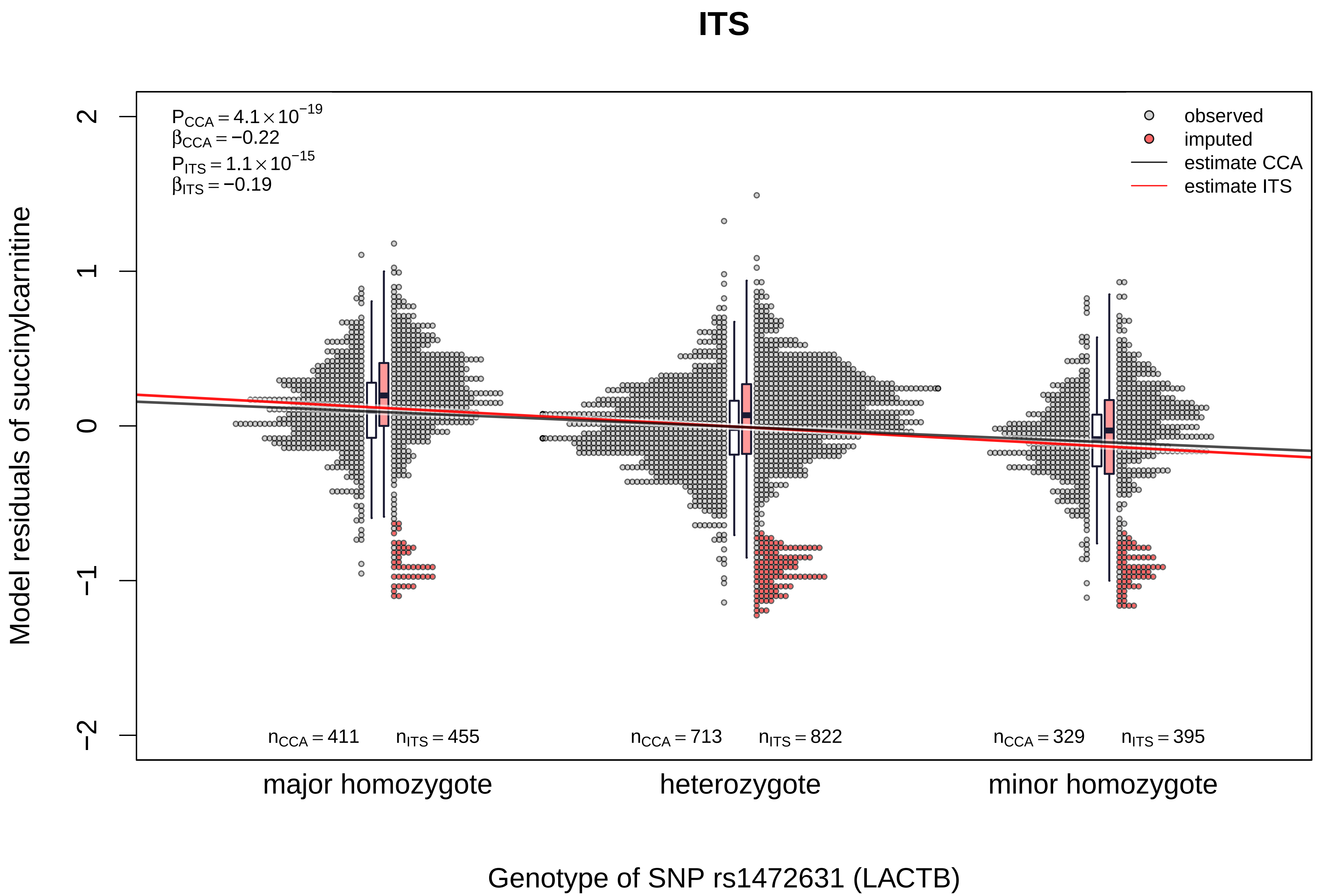
ITS
$P_{CCA} = 4.1 \times 10^{-19}$
$\beta_{CCA} = -0.22$
$P_{ITS} = 1.1 \times 10^{-15}$
$\beta_{ITS} = -0.19$
observed
imputed
estimate CCA
estimate ITS
Model residuals of succinylcarnitine
2
1
0
−1
−2
$n_{CCA} = 411$
$n_{ITS} = 455$
$n_{CCA} = 713$
$n_{ITS} = 822$
$n_{CCA} = 329$
$n_{ITS} = 395$
major homozygote
heterozygote
minor homozygote
Genotype of SNP rs1472631 (LACTB)

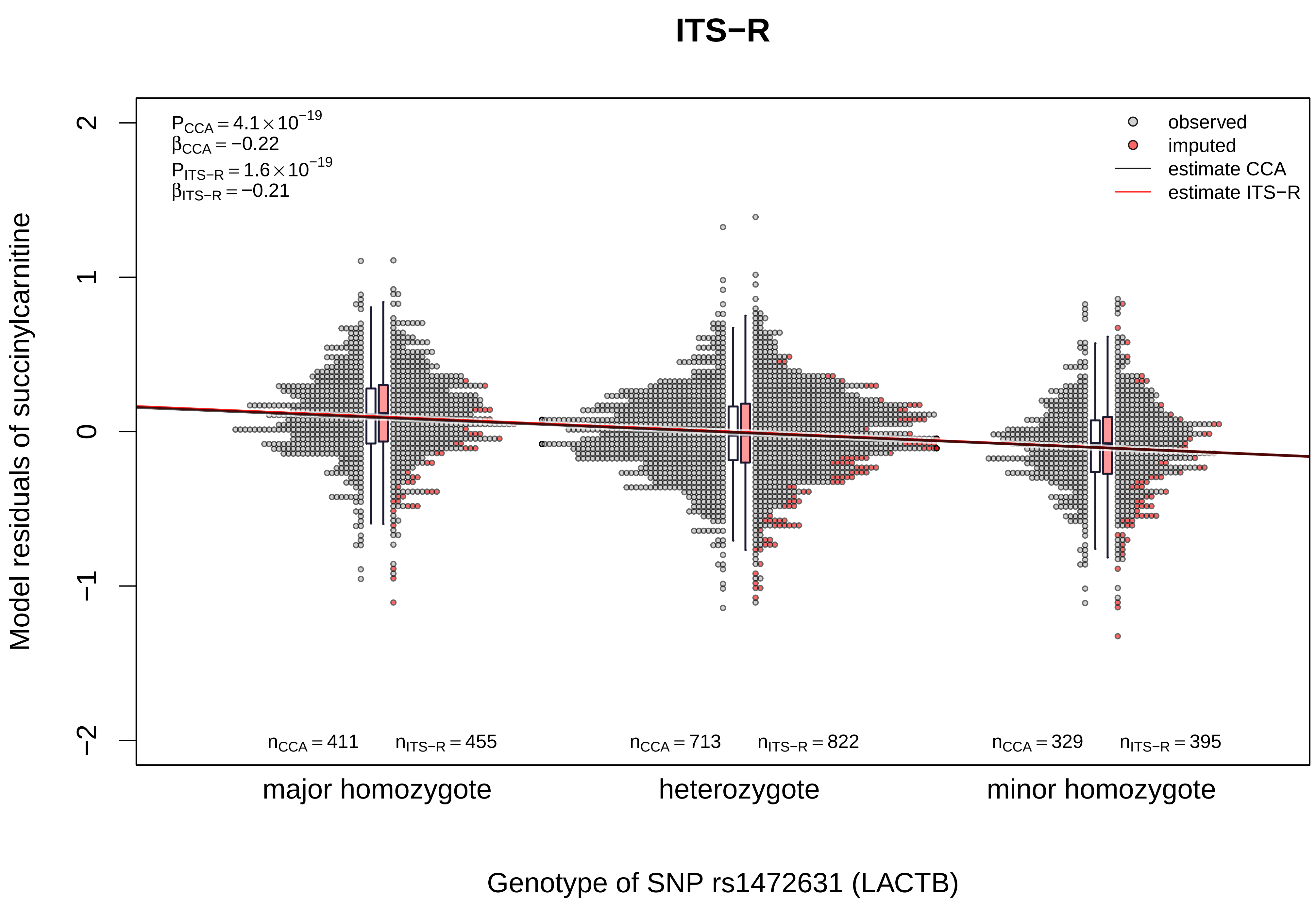

ITS−R
$P_{CCA} = 4.1 \times 10^{-19}$
$\beta_{CCA} = -0.22$
$P_{ITS-R} = 1.6 \times 10^{-19}$
$\beta_{ITS-R} = -0.21$
observed
imputed
estimate CCA
estimate ITS−R
Model residuals of succinylcarnitine
2
1
0
−1
−2
$n_{CCA} = 411$
$n_{ITS-R} = 455$
$n_{CCA} = 713$
$n_{ITS-R} = 822$
$n_{CCA} = 329$
$n_{ITS-R} = 395$
major homozygote
heterozygote
minor homozygote
Genotype of SNP rs1472631 (LACTB)

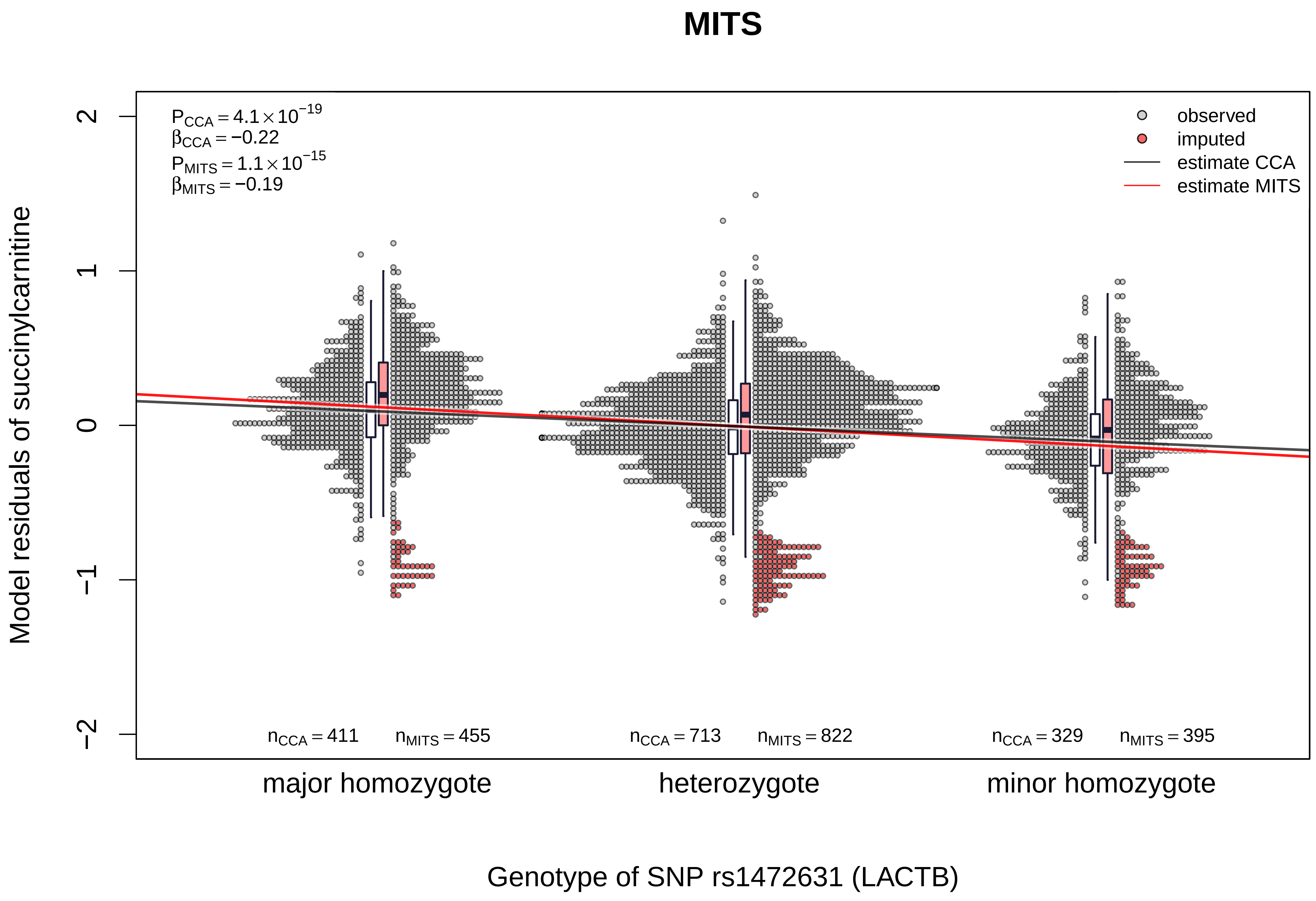

MITS
$P_{CCA} = 4.1 \times 10^{-19}$
$\beta_{CCA} = -0.22$
$P_{MITS} = 1.1 \times 10^{-15}$
$\beta_{MITS} = -0.19$
observed
imputed
estimate CCA
estimate MITS
2
1
0
−1
−2
Model residuals of succinylcarnitine
$n_{CCA} = 411$
$n_{MITS} = 455$
$n_{CCA} = 713$
$n_{MITS} = 822$
$n_{CCA} = 329$
$n_{MITS} = 395$
major homozygote
heterozygote
minor homozygote
Genotype of SNP rs1472631 (LACTB)

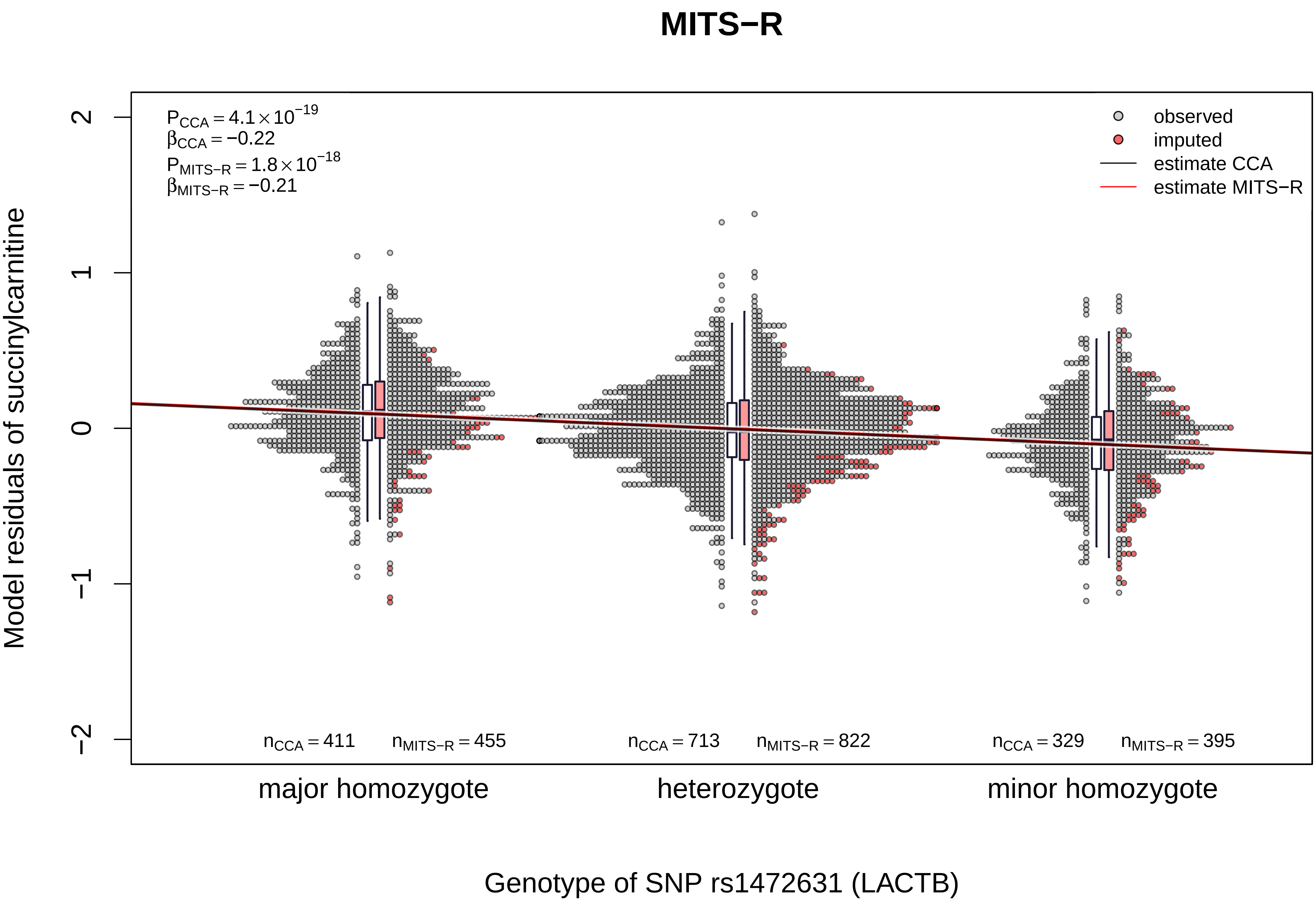

MITS−R
P_CCA = 4.1 × 10^−19
β_CCA = −0.22
P_MITS−R = 1.8 × 10^−18
β_MITS−R = −0.21
observed
imputed
estimate CCA
estimate MITS−R
Model residuals of succinylcarnitine
2
1
0
−1
−2
n_CCA = 411
n_MITS−R = 455
n_CCA = 713
n_MITS−R = 822
n_CCA = 329
n_MITS−R = 395
major homozygote
heterozygote
minor homozygote
Genotype of SNP rs1472631 (LACTB)

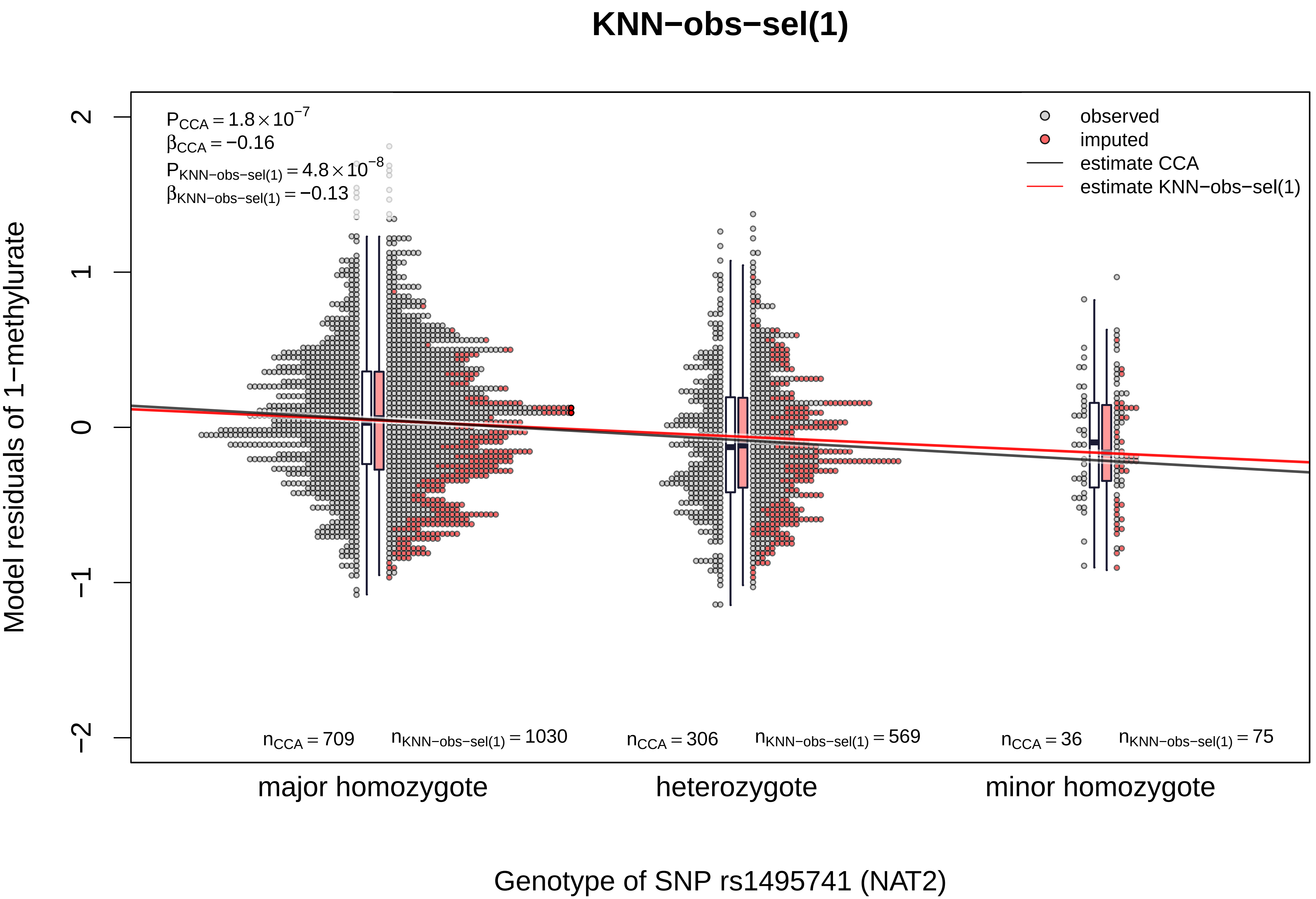

KNN−obs−sel(1)
$P_{CCA} = 1.8 \times 10^{-7}$
$\beta_{CCA} = -0.16$
$P_{KNN-obs-sel(1)} = 4.8 \times 10^{-8}$
$\beta_{KNN-obs-sel(1)} = -0.13$
observed
imputed
estimate CCA
estimate KNN−obs−sel(1)
Model residuals of 1−methylurate
2
1
0
−1
−2
$n_{CCA} = 709$
$n_{KNN-obs-sel(1)} = 1030$
$n_{CCA} = 306$
$n_{KNN-obs-sel(1)} = 569$
$n_{CCA} = 36$
$n_{KNN-obs-sel(1)} = 75$
major homozygote
heterozygote
minor homozygote
Genotype of SNP rs1495741 (NAT2)

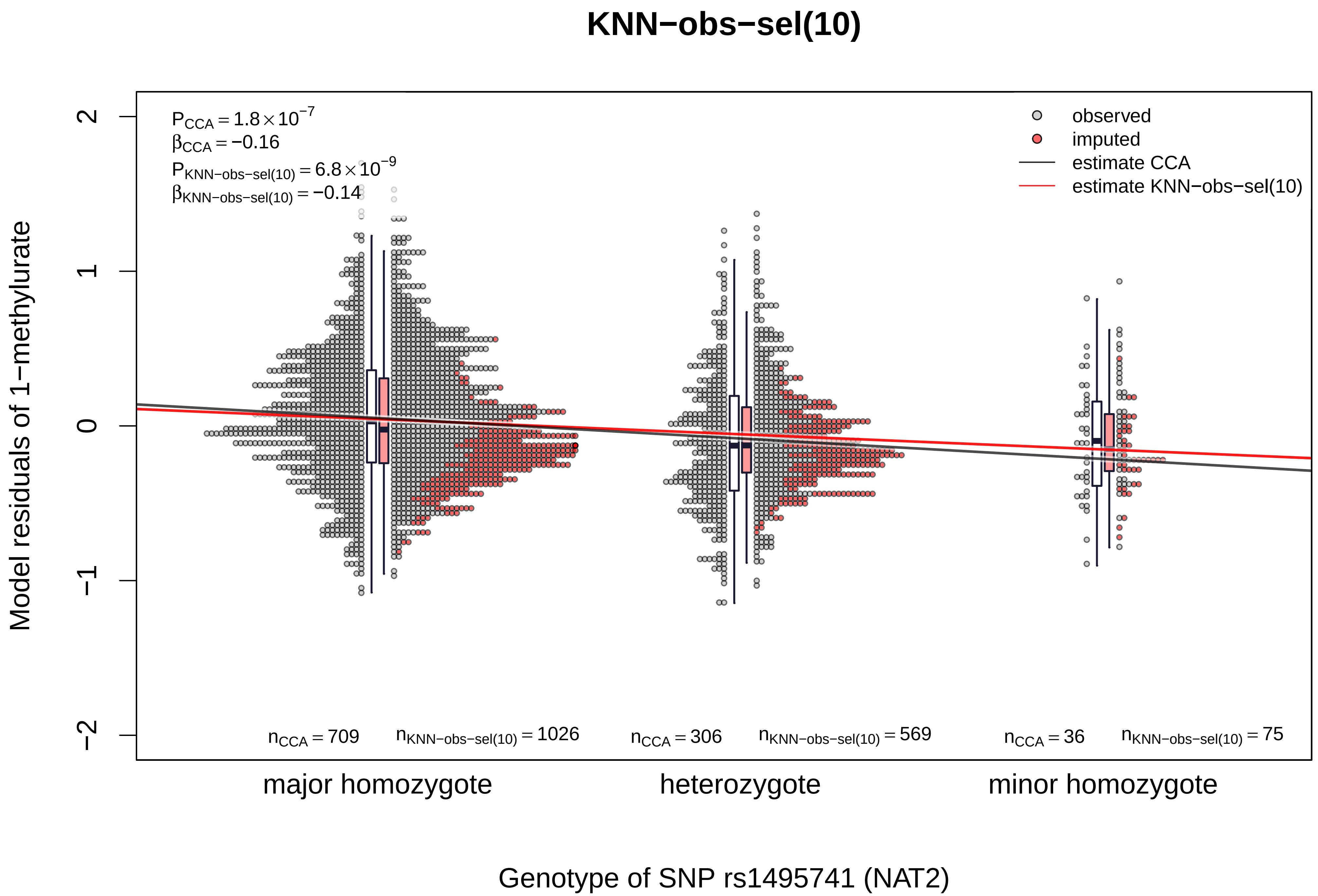
KNN−obs−sel(10)
$P_{CCA} = 1.8 \times 10^{-7}$
$\beta_{CCA} = -0.16$
$P_{KNN-obs-sel(10)} = 6.8 \times 10^{-9}$
$\beta_{KNN-obs-sel(10)} = -0.14$
observed
imputed
estimate CCA
estimate KNN−obs−sel(10)
2
1
0
−1
−2
Model residuals of 1−methylurate
$n_{CCA} = 709$
$n_{KNN-obs-sel(10)} = 1026$
$n_{CCA} = 306$
$n_{KNN-obs-sel(10)} = 569$
$n_{CCA} = 36$
$n_{KNN-obs-sel(10)} = 75$
major homozygote
heterozygote
minor homozygote
Genotype of SNP rs1495741 (NAT2)

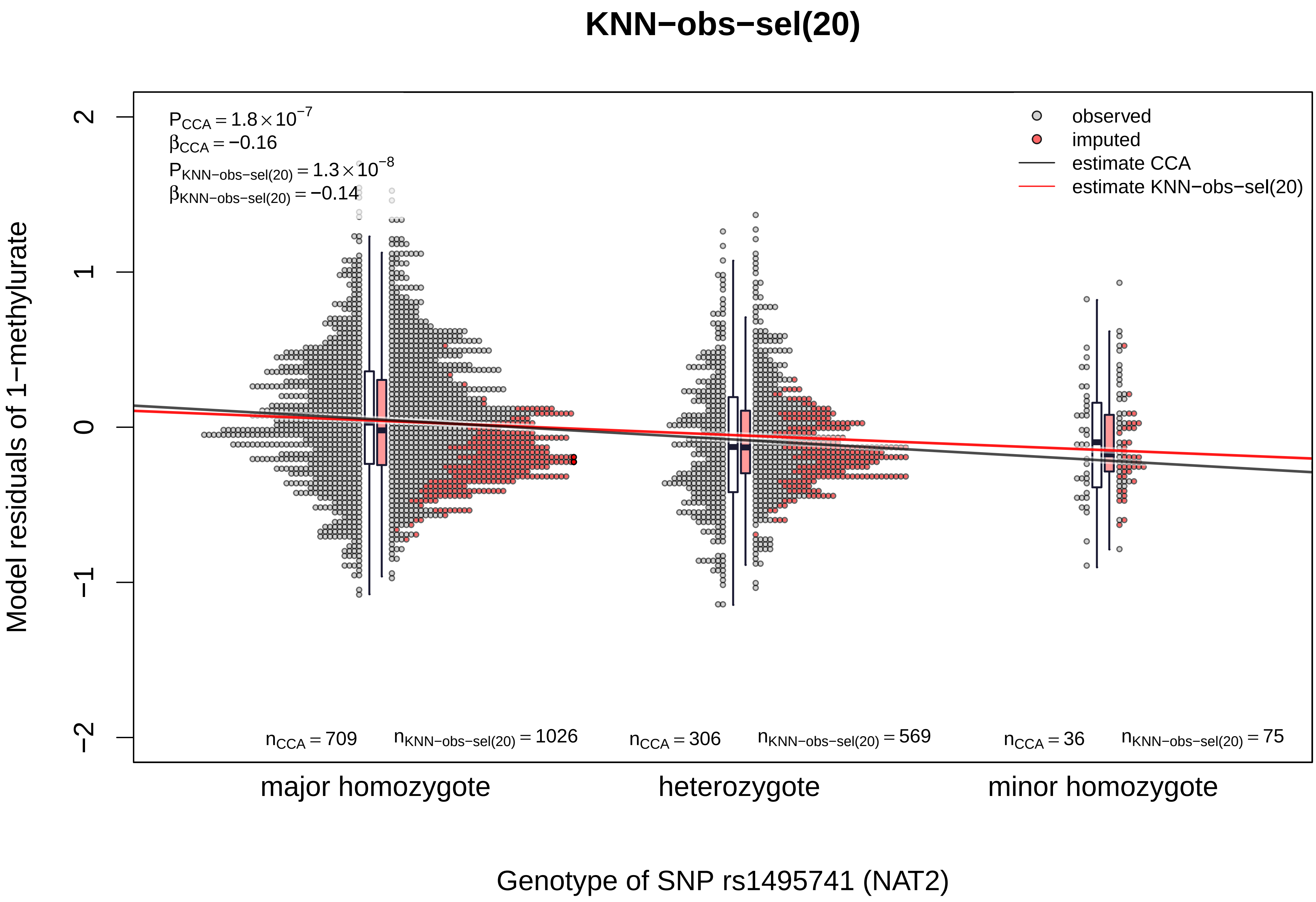

KNN−obs−sel(20)
$P_{CCA} = 1.8 \times 10^{-7}$
$\beta_{CCA} = -0.16$
$P_{KNN-obs-sel(20)} = 1.3 \times 10^{-8}$
$\beta_{KNN-obs-sel(20)} = -0.14$
observed
imputed
estimate CCA
estimate KNN−obs−sel(20)
Model residuals of 1−methylurate
2
1
0
−1
−2
$n_{CCA} = 709$
$n_{KNN-obs-sel(20)} = 1026$
$n_{CCA} = 306$
$n_{KNN-obs-sel(20)} = 569$
$n_{CCA} = 36$
$n_{KNN-obs-sel(20)} = 75$
major homozygote
heterozygote
minor homozygote
Genotype of SNP rs1495741 (NAT2)

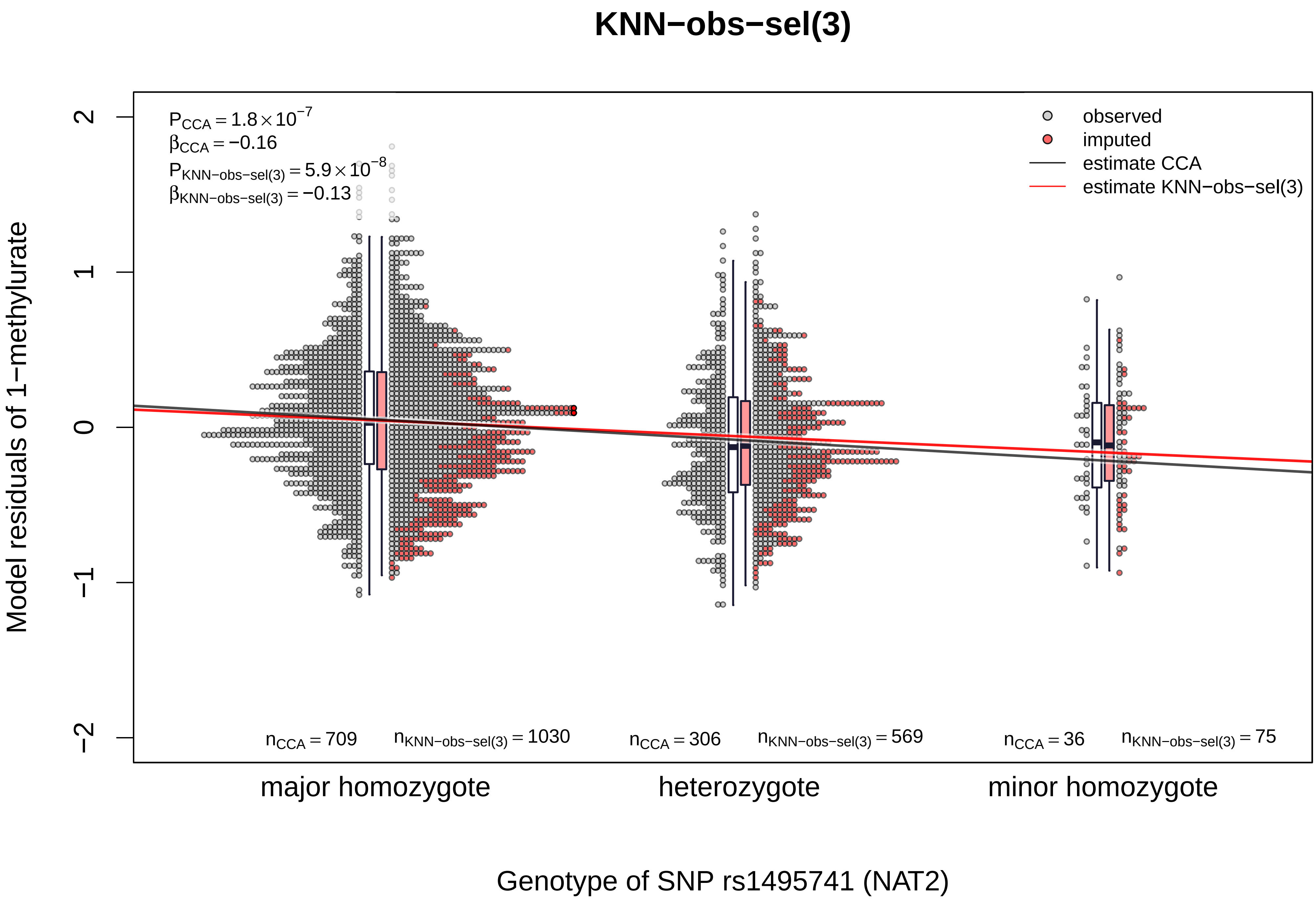

KNN−obs−sel(3)
$P_{CCA} = 1.8 \times 10^{-7}$
$\beta_{CCA} = -0.16$
$P_{KNN-obs-sel(3)} = 5.9 \times 10^{-8}$
$\beta_{KNN-obs-sel(3)} = -0.13$
observed
imputed
estimate CCA
estimate KNN−obs−sel(3)
Model residuals of 1−methylurate
2
1
0
−1
−2
$n_{CCA} = 709$
$n_{KNN-obs-sel(3)} = 1030$
$n_{CCA} = 306$
$n_{KNN-obs-sel(3)} = 569$
$n_{CCA} = 36$
$n_{KNN-obs-sel(3)} = 75$
major homozygote
heterozygote
minor homozygote
Genotype of SNP rs1495741 (NAT2)

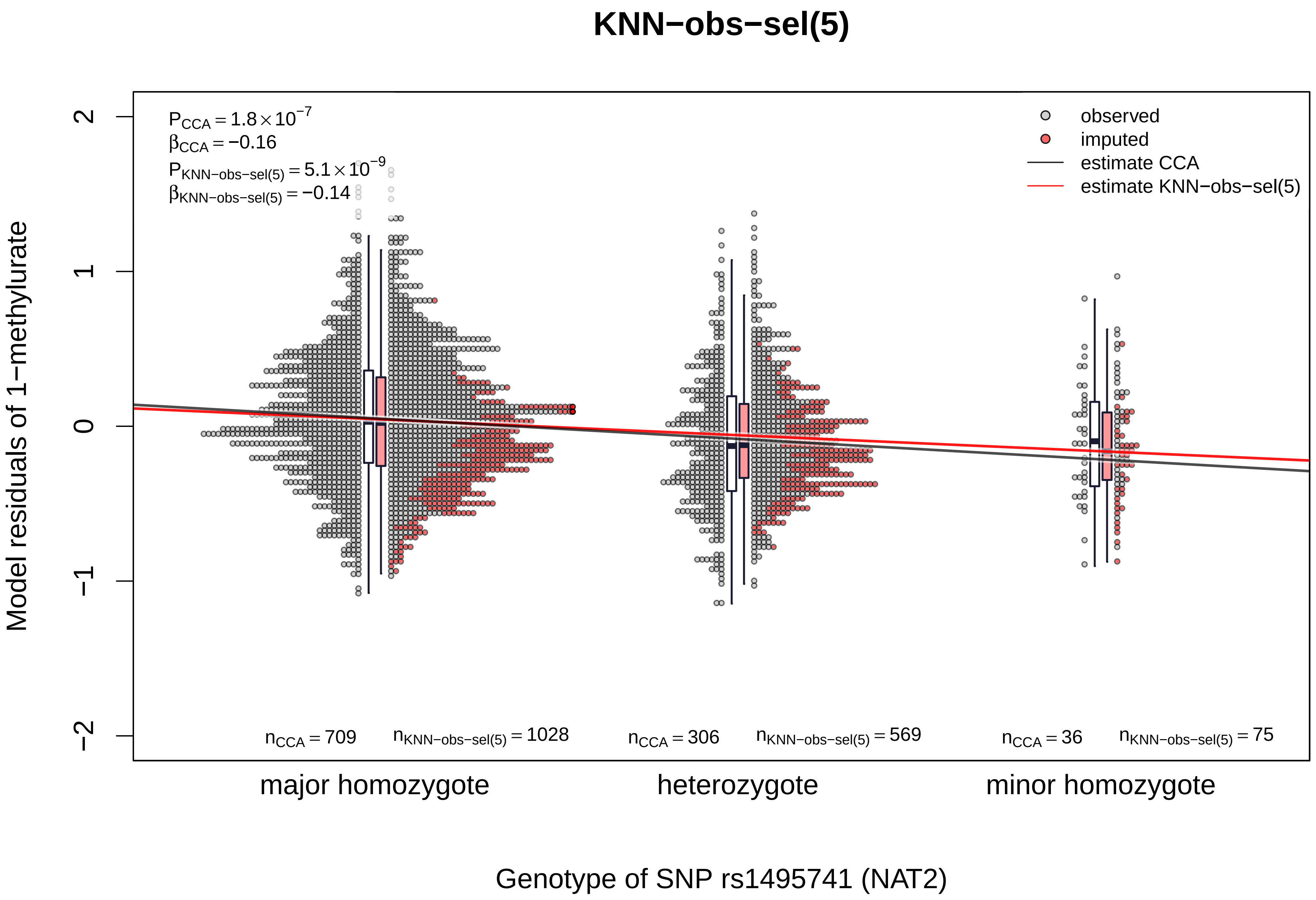

KNN−obs−sel(5)
P_CCA = 1.8 × 10^−7
β_CCA = −0.16
P_KNN−obs−sel(5) = 5.1 × 10^−9
β_KNN−obs−sel(5) = −0.14
observed
imputed
estimate CCA
estimate KNN−obs−sel(5)
2
1
0
−1
−2
Model residuals of 1−methylurate
n_CCA = 709
n_KNN−obs−sel(5) = 1028
n_CCA = 306
n_KNN−obs−sel(5) = 569
n_CCA = 36
n_KNN−obs−sel(5) = 75
major homozygote
heterozygote
minor homozygote
Genotype of SNP rs1495741 (NAT2)

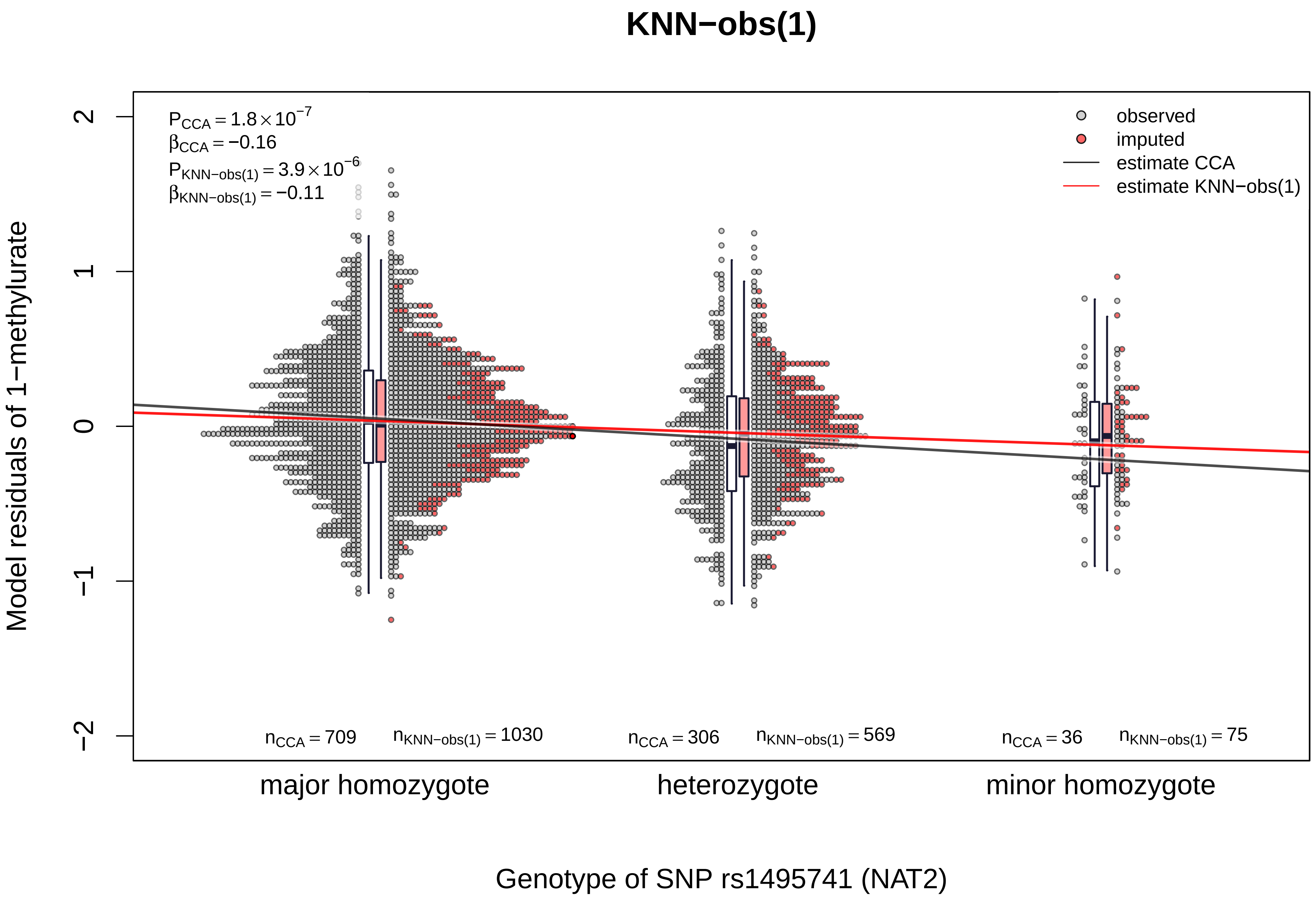

KNN−obs(1)
$P_{CCA} = 1.8 \times 10^{-7}$
$\beta_{CCA} = -0.16$
$P_{KNN-obs(1)} = 3.9 \times 10^{-6}$
$\beta_{KNN-obs(1)} = -0.11$
observed
imputed
estimate CCA
estimate KNN−obs(1)
Model residuals of 1−methylurate
2
1
0
−1
−2
$n_{CCA} = 709$
$n_{KNN-obs(1)} = 1030$
$n_{CCA} = 306$
$n_{KNN-obs(1)} = 569$
$n_{CCA} = 36$
$n_{KNN-obs(1)} = 75$
major homozygote
heterozygote
minor homozygote
Genotype of SNP rs1495741 (NAT2)

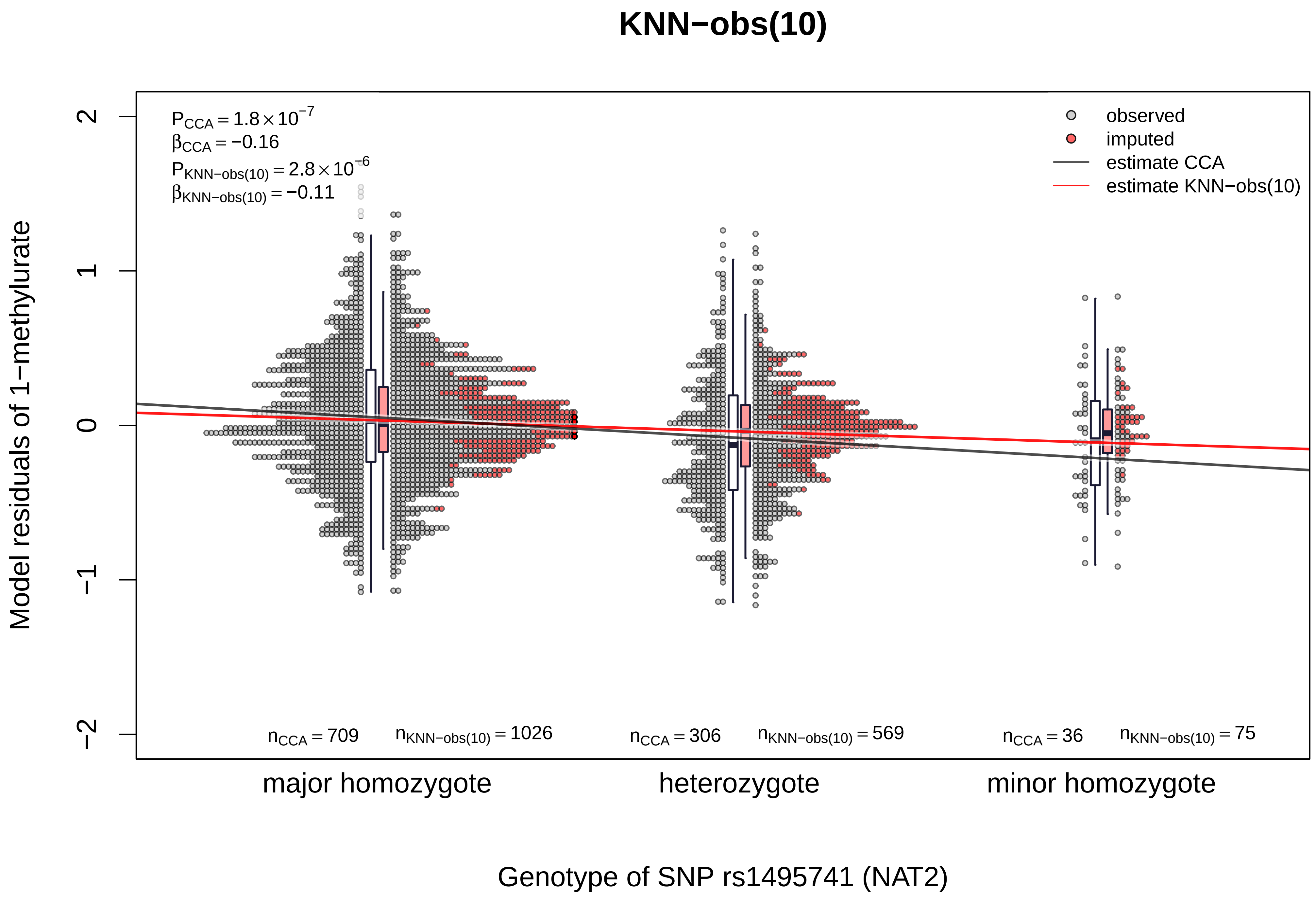

KNN−obs(10)
$P_{CCA} = 1.8 \times 10^{-7}$
$\beta_{CCA} = -0.16$
$P_{KNN-obs(10)} = 2.8 \times 10^{-6}$
$\beta_{KNN-obs(10)} = -0.11$
observed
imputed
estimate CCA
estimate KNN−obs(10)
Model residuals of 1−methylurate
2
1
0
−1
−2
$n_{CCA} = 709$
$n_{KNN-obs(10)} = 1026$
$n_{CCA} = 306$
$n_{KNN-obs(10)} = 569$
$n_{CCA} = 36$
$n_{KNN-obs(10)} = 75$
major homozygote
heterozygote
minor homozygote
Genotype of SNP rs1495741 (NAT2)

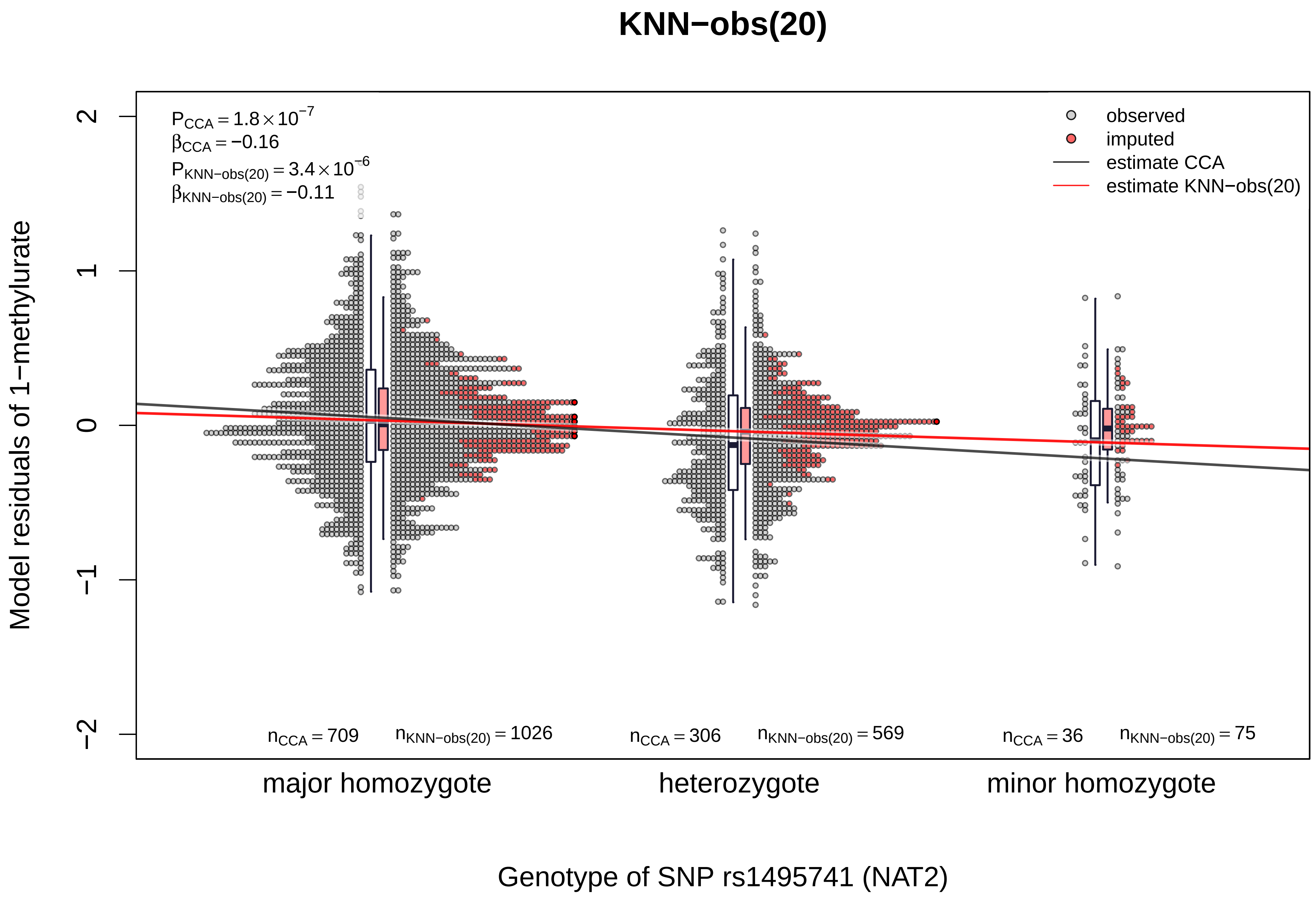
KNN−obs(20)
$P_{CCA} = 1.8 \times 10^{-7}$
$\beta_{CCA} = -0.16$
$P_{KNN-obs(20)} = 3.4 \times 10^{-6}$
$\beta_{KNN-obs(20)} = -0.11$
observed
imputed
estimate CCA
estimate KNN−obs(20)
Model residuals of 1−methylurate
2
1
0
−1
−2
$n_{CCA} = 709$
$n_{KNN-obs(20)} = 1026$
$n_{CCA} = 306$
$n_{KNN-obs(20)} = 569$
$n_{CCA} = 36$
$n_{KNN-obs(20)} = 75$
major homozygote
heterozygote
minor homozygote
Genotype of SNP rs1495741 (NAT2)

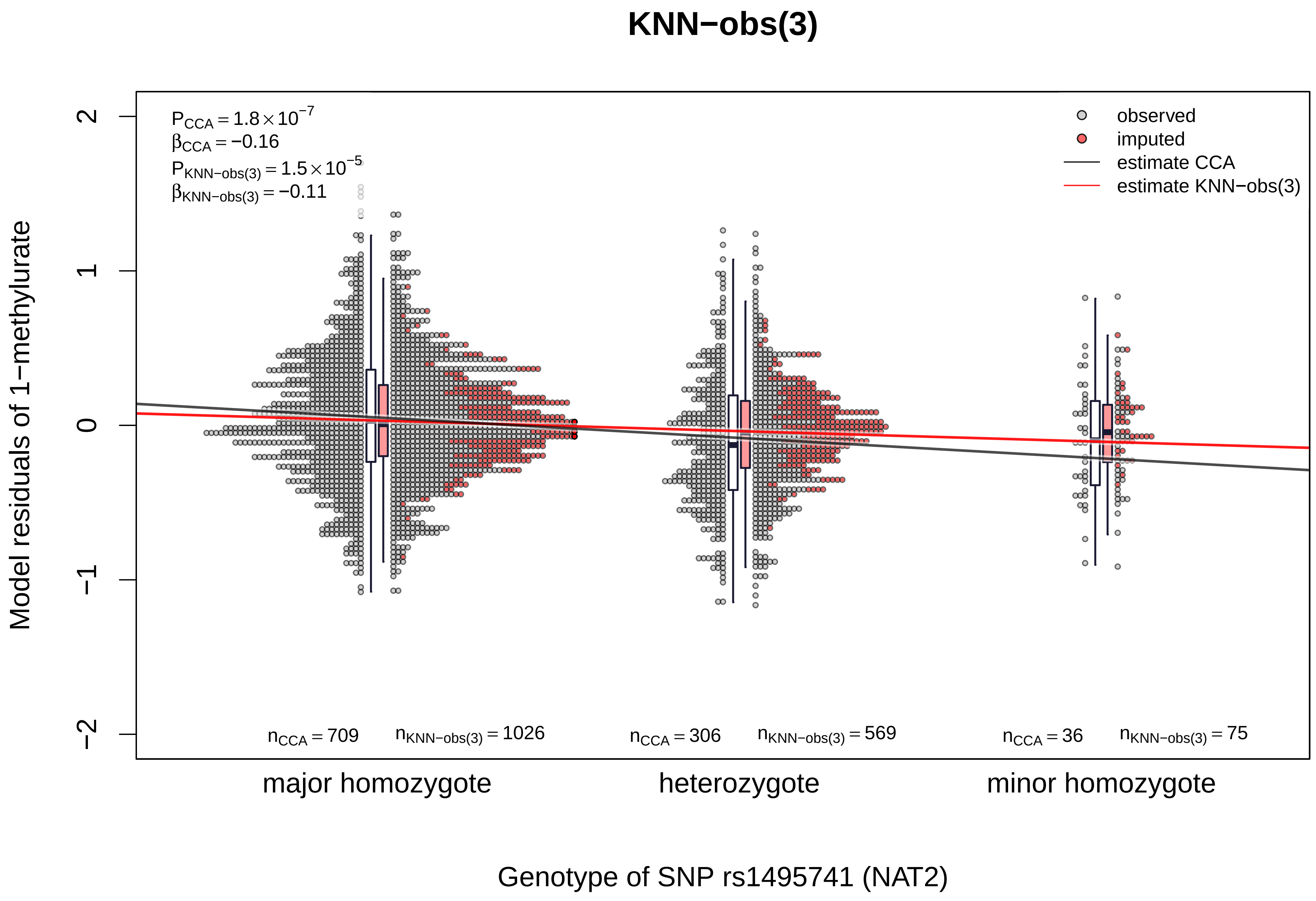

KNN−obs(3)
$P_{CCA} = 1.8 \times 10^{-7}$
$\beta_{CCA} = -0.16$
$P_{KNN-obs(3)} = 1.5 \times 10^{-5}$
$\beta_{KNN-obs(3)} = -0.11$
observed
imputed
estimate CCA
estimate KNN−obs(3)
Model residuals of 1−methylurate
2
1
0
−1
−2
$n_{CCA} = 709$
$n_{KNN-obs(3)} = 1026$
$n_{CCA} = 306$
$n_{KNN-obs(3)} = 569$
$n_{CCA} = 36$
$n_{KNN-obs(3)} = 75$
major homozygote
heterozygote
minor homozygote
Genotype of SNP rs1495741 (NAT2)

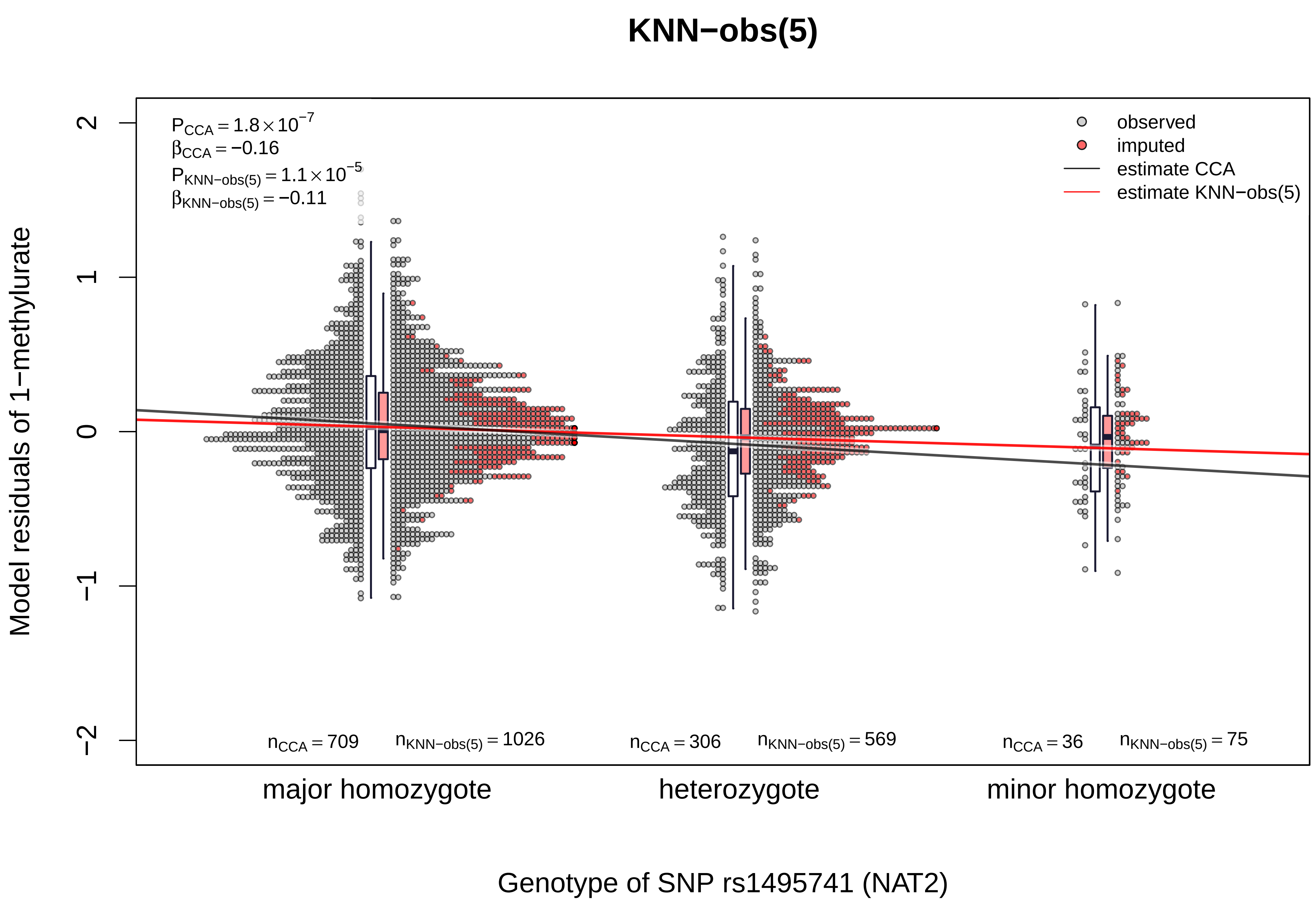

KNN−obs(5)
$P_{CCA} = 1.8 \times 10^{-7}$
$\beta_{CCA} = -0.16$
$P_{KNN-obs(5)} = 1.1 \times 10^{-5}$
$\beta_{KNN-obs(5)} = -0.11$
observed
imputed
estimate CCA
estimate KNN−obs(5)
Model residuals of 1−methylurate
2
1
0
−1
−2
$n_{CCA} = 709$
$n_{KNN-obs(5)} = 1026$
$n_{CCA} = 306$
$n_{KNN-obs(5)} = 569$
$n_{CCA} = 36$
$n_{KNN-obs(5)} = 75$
major homozygote
heterozygote
minor homozygote
Genotype of SNP rs1495741 (NAT2)

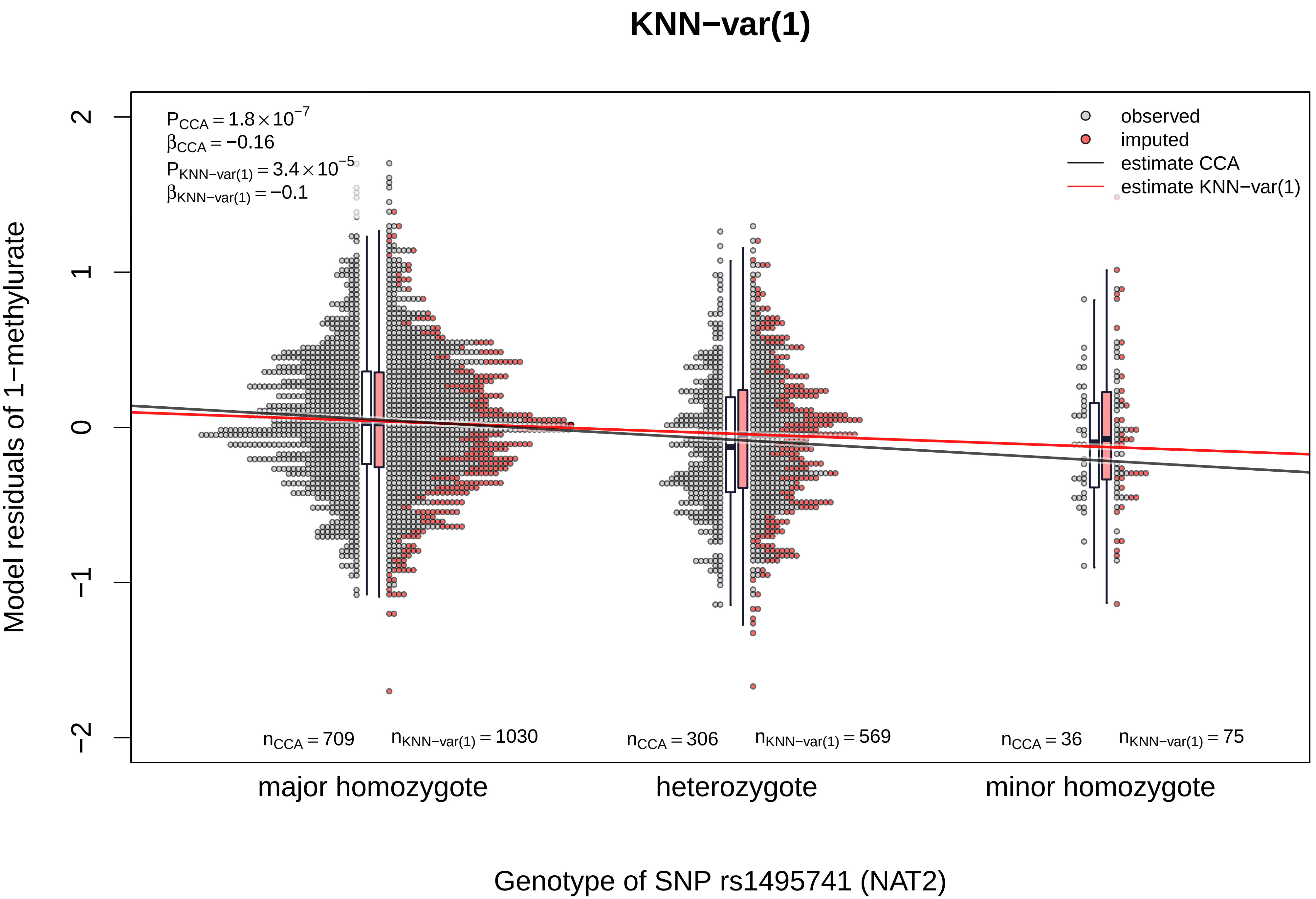

KNN−var(1)
$P_{CCA} = 1.8 \times 10^{-7}$
$\beta_{CCA} = -0.16$
$P_{KNN-var(1)} = 3.4 \times 10^{-5}$
$\beta_{KNN-var(1)} = -0.1$
observed
imputed
estimate CCA
estimate KNN−var(1)
2
1
0
−1
−2
Model residuals of 1−methylurate
$n_{CCA} = 709$
$n_{KNN-var(1)} = 1030$
$n_{CCA} = 306$
$n_{KNN-var(1)} = 569$
$n_{CCA} = 36$
$n_{KNN-var(1)} = 75$
major homozygote
heterozygote
minor homozygote
Genotype of SNP rs1495741 (NAT2)

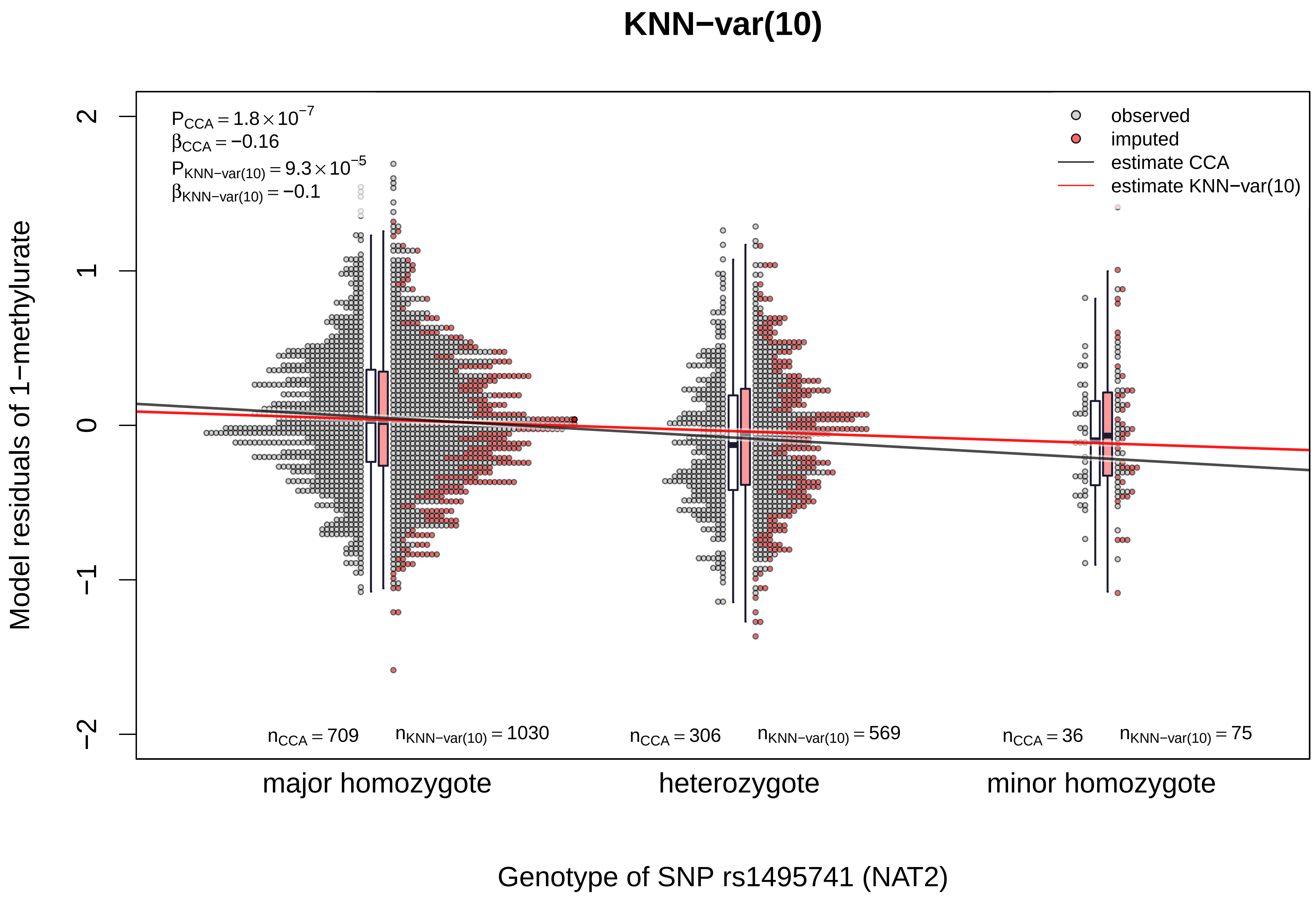

KNN−var(10)
$P_{CCA} = 1.8 \times 10^{-7}$
$\beta_{CCA} = -0.16$
$P_{KNN-var(10)} = 9.3 \times 10^{-5}$
$\beta_{KNN-var(10)} = -0.1$
observed
imputed
estimate CCA
estimate KNN−var(10)
2
1
0
−1
−2
Model residuals of 1−methylurate
$n_{CCA} = 709$
$n_{KNN-var(10)} = 1030$
$n_{CCA} = 306$
$n_{KNN-var(10)} = 569$
$n_{CCA} = 36$
$n_{KNN-var(10)} = 75$
major homozygote
heterozygote
minor homozygote
Genotype of SNP rs1495741 (NAT2)

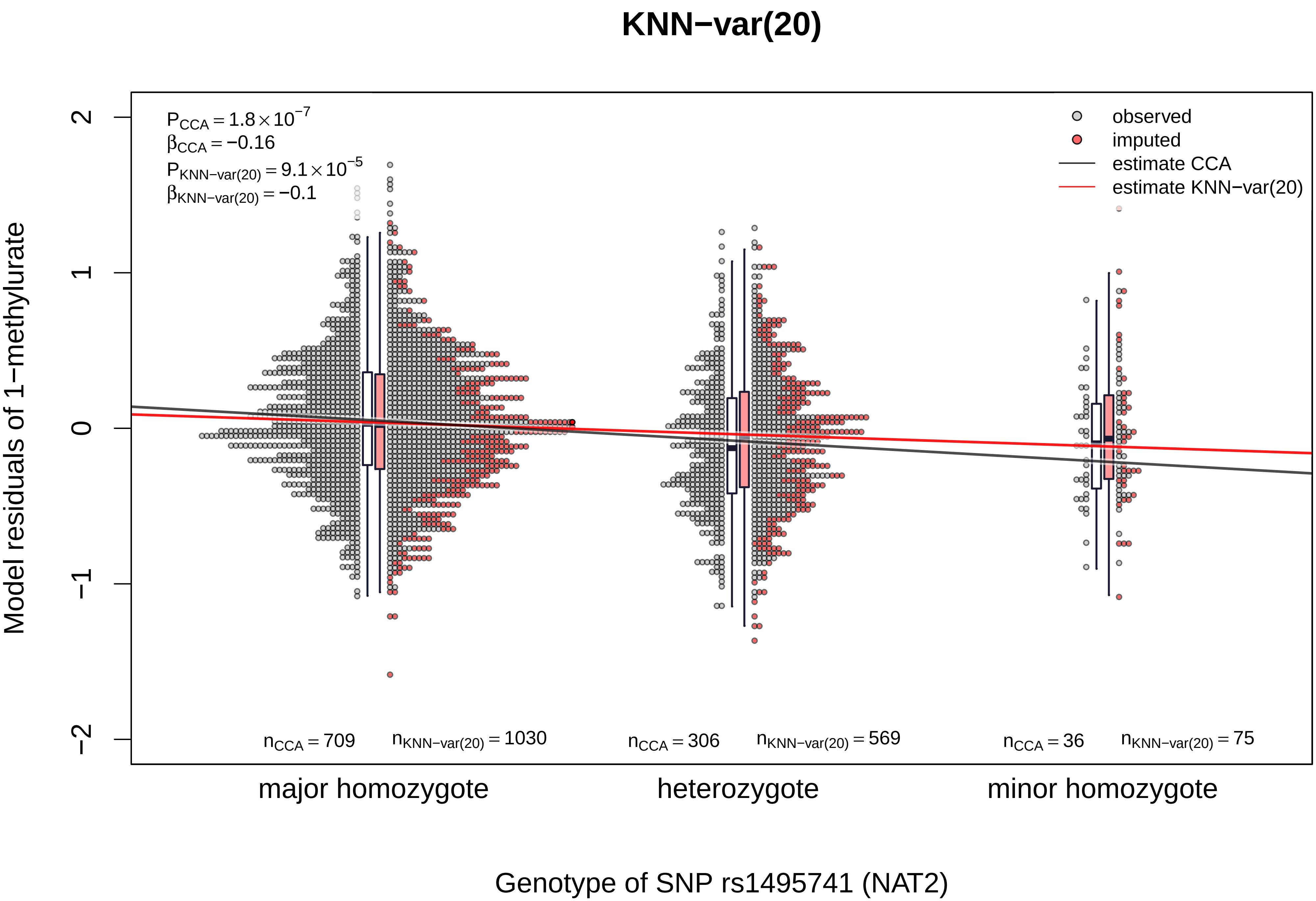

KNN−var(20)
$P_{CCA} = 1.8 \times 10^{-7}$
$\beta_{CCA} = -0.16$
$P_{KNN-var(20)} = 9.1 \times 10^{-5}$
$\beta_{KNN-var(20)} = -0.1$
observed
imputed
estimate CCA
estimate KNN−var(20)
2
1
0
−1
−2
Model residuals of 1−methylurate
$n_{CCA} = 709$
$n_{KNN-var(20)} = 1030$
$n_{CCA} = 306$
$n_{KNN-var(20)} = 569$
$n_{CCA} = 36$
$n_{KNN-var(20)} = 75$
major homozygote
heterozygote
minor homozygote
Genotype of SNP rs1495741 (NAT2)

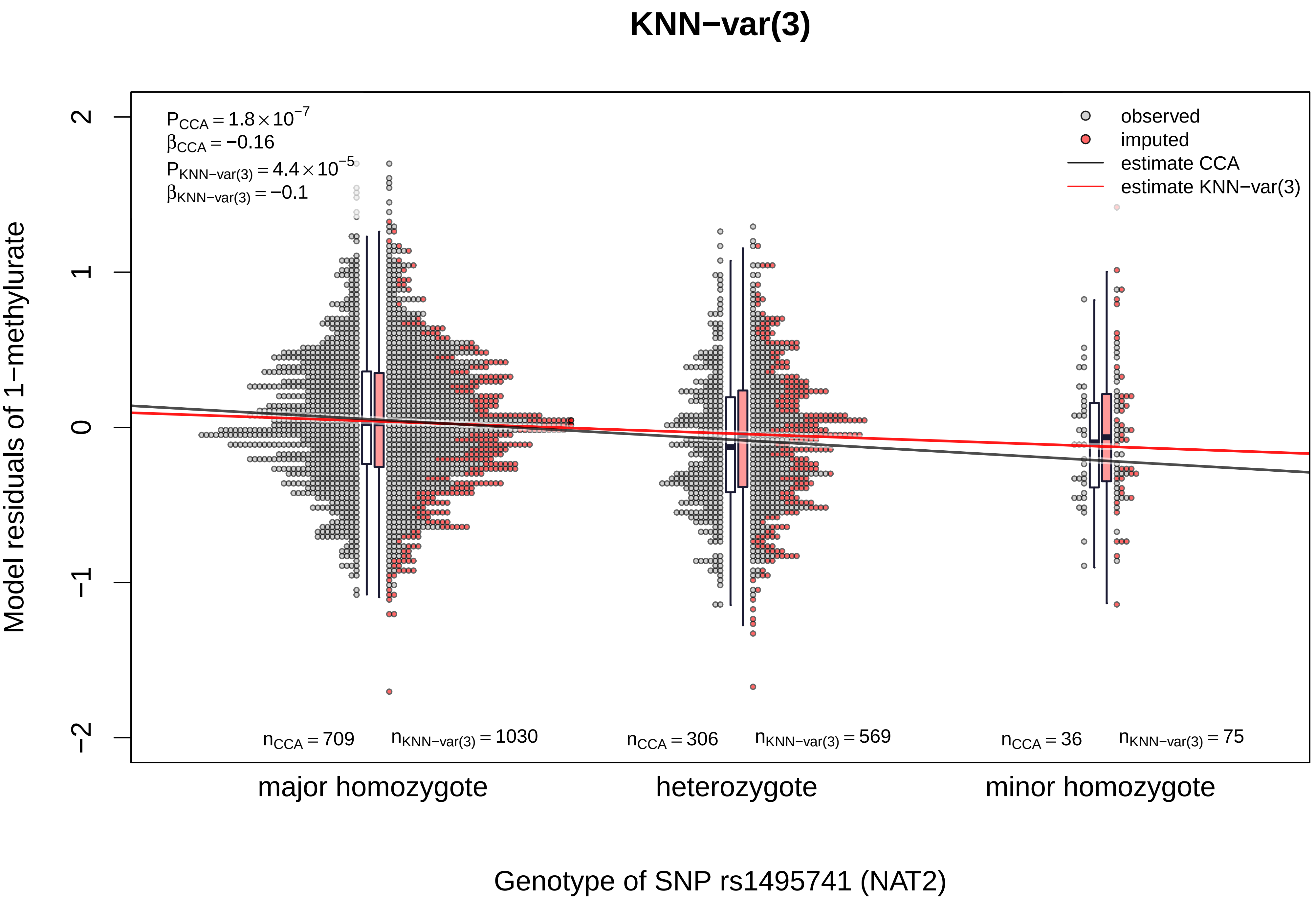

KNN−var(3)
$P_{CCA} = 1.8 \times 10^{-7}$
$\beta_{CCA} = -0.16$
$P_{KNN-var(3)} = 4.4 \times 10^{-5}$
$\beta_{KNN-var(3)} = -0.1$
observed
imputed
estimate CCA
estimate KNN−var(3)
Model residuals of 1−methylurate
2
1
0
−1
−2
$n_{CCA} = 709$
$n_{KNN-var(3)} = 1030$
$n_{CCA} = 306$
$n_{KNN-var(3)} = 569$
$n_{CCA} = 36$
$n_{KNN-var(3)} = 75$
major homozygote
heterozygote
minor homozygote
Genotype of SNP rs1495741 (NAT2)

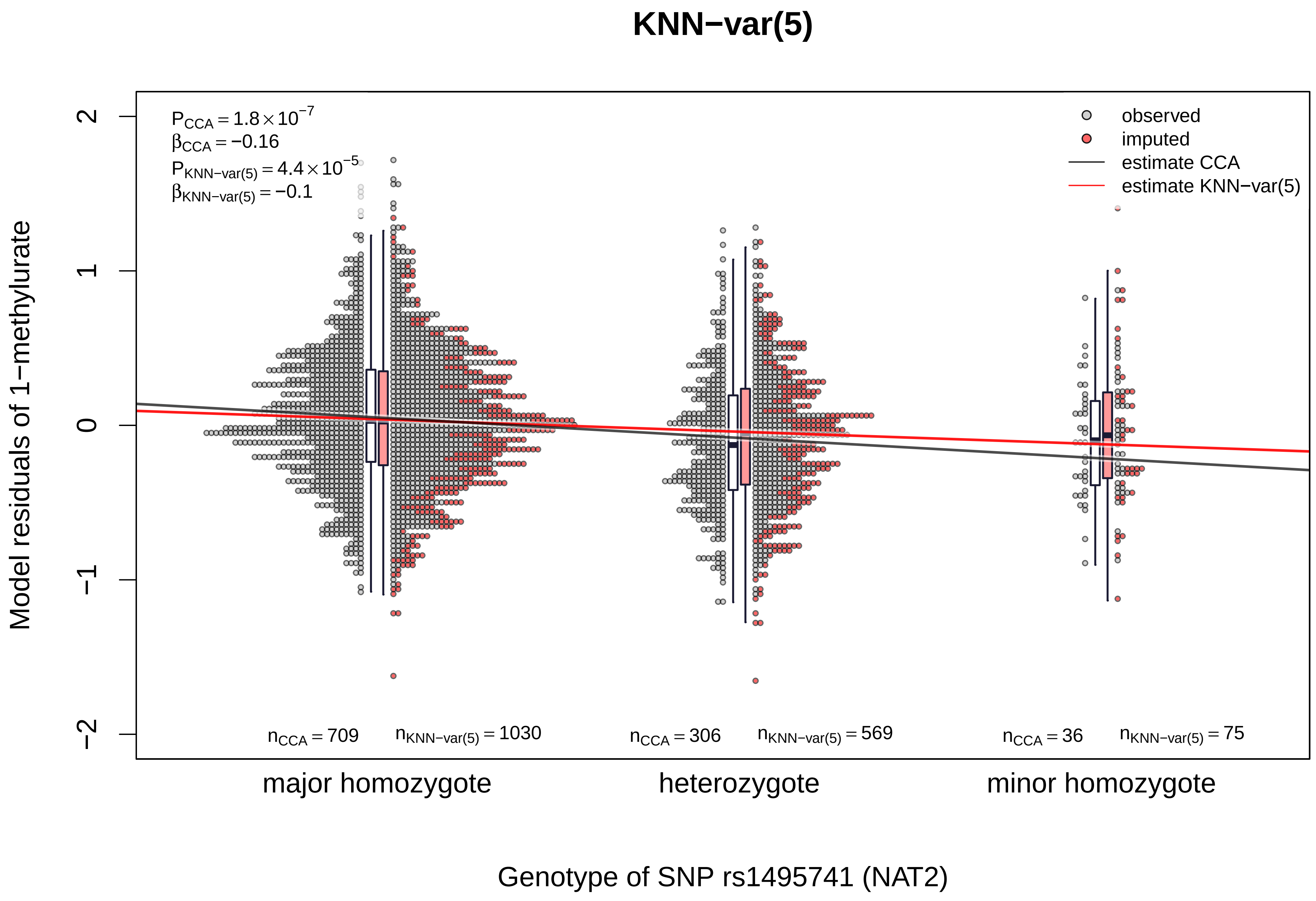
KNN−var(5)
$P_{CCA} = 1.8 \times 10^{-7}$
$\beta_{CCA} = -0.16$
$P_{KNN-var(5)} = 4.4 \times 10^{-5}$
$\beta_{KNN-var(5)} = -0.1$
observed
imputed
estimate CCA
estimate KNN−var(5)
2
1
0
−1
−2
Model residuals of 1−methylurate
$n_{CCA} = 709$
$n_{KNN-var(5)} = 1030$
$n_{CCA} = 306$
$n_{KNN-var(5)} = 569$
$n_{CCA} = 36$
$n_{KNN-var(5)} = 75$
major homozygote
heterozygote
minor homozygote
Genotype of SNP rs1495741 (NAT2)

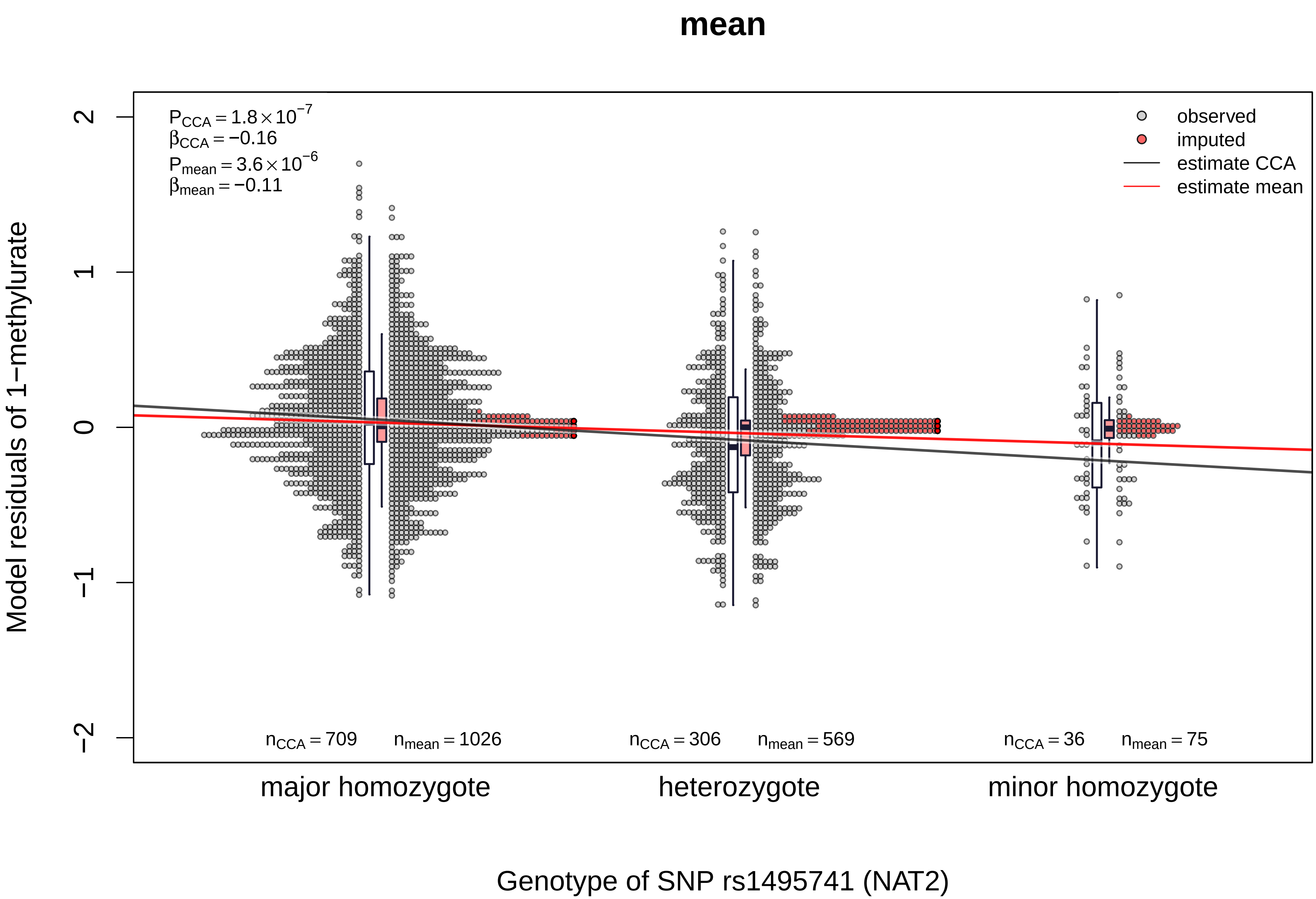
mean
$P_{CCA} = 1.8 \times 10^{-7}$
$\beta_{CCA} = -0.16$
$P_{mean} = 3.6 \times 10^{-6}$
$\beta_{mean} = -0.11$
observed
imputed
estimate CCA
estimate mean
2
1
0
−1
−2
Model residuals of 1−methylurate
$n_{CCA} = 709$
$n_{mean} = 1026$
$n_{CCA} = 306$
$n_{mean} = 569$
$n_{CCA} = 36$
$n_{mean} = 75$
major homozygote
heterozygote
minor homozygote
Genotype of SNP rs1495741 (NAT2)

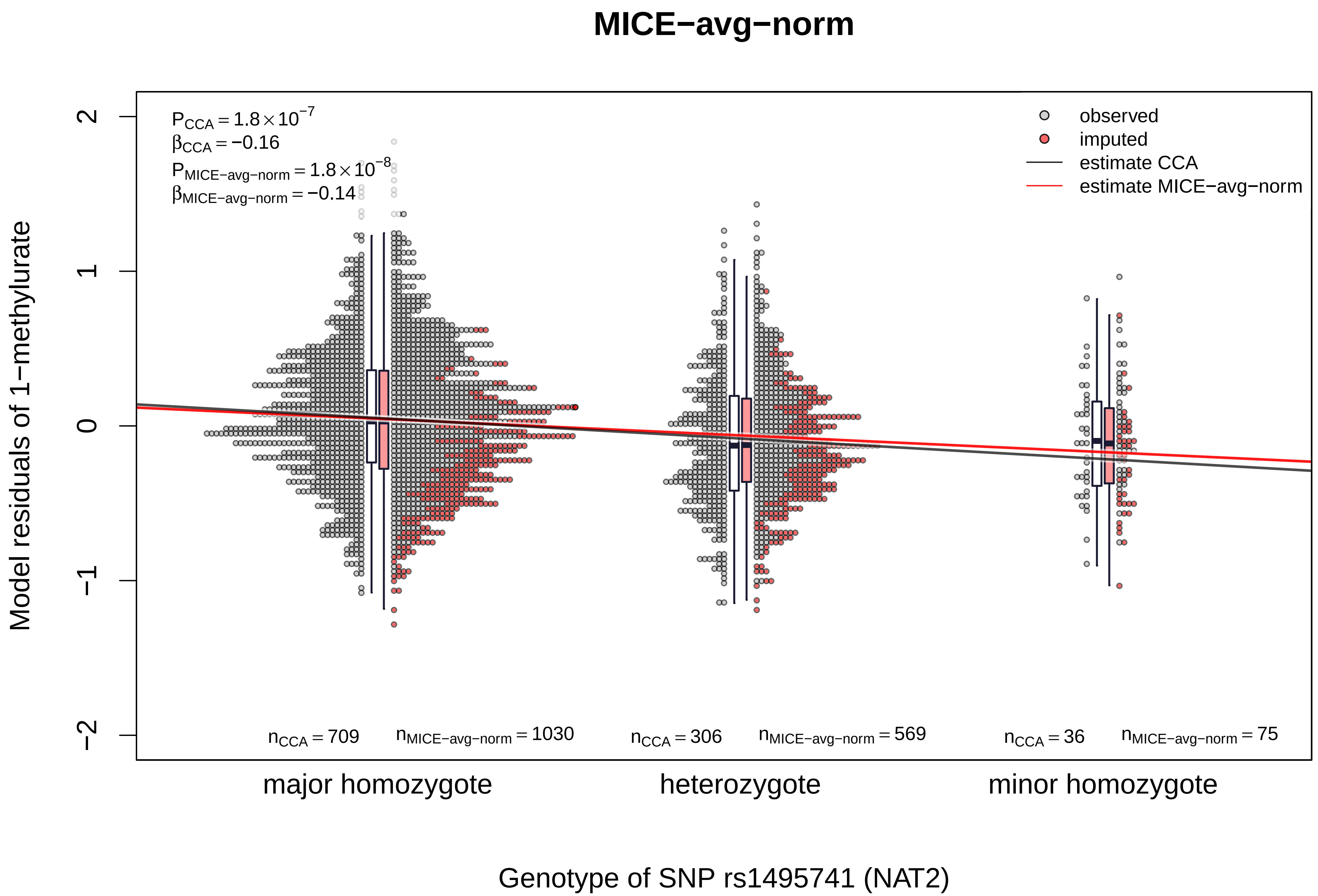

MICE−avg−norm
$P_{CCA} = 1.8 \times 10^{-7}$
$\beta_{CCA} = -0.16$
$P_{MICE-avg-norm} = 1.8 \times 10^{-8}$
$\beta_{MICE-avg-norm} = -0.14$
observed
imputed
estimate CCA
estimate MICE−avg−norm
Model residuals of 1−methylurate
2
1
0
−1
−2
$n_{CCA} = 709$
$n_{MICE-avg-norm} = 1030$
$n_{CCA} = 306$
$n_{MICE-avg-norm} = 569$
$n_{CCA} = 36$
$n_{MICE-avg-norm} = 75$
major homozygote
heterozygote
minor homozygote
Genotype of SNP rs1495741 (NAT2)

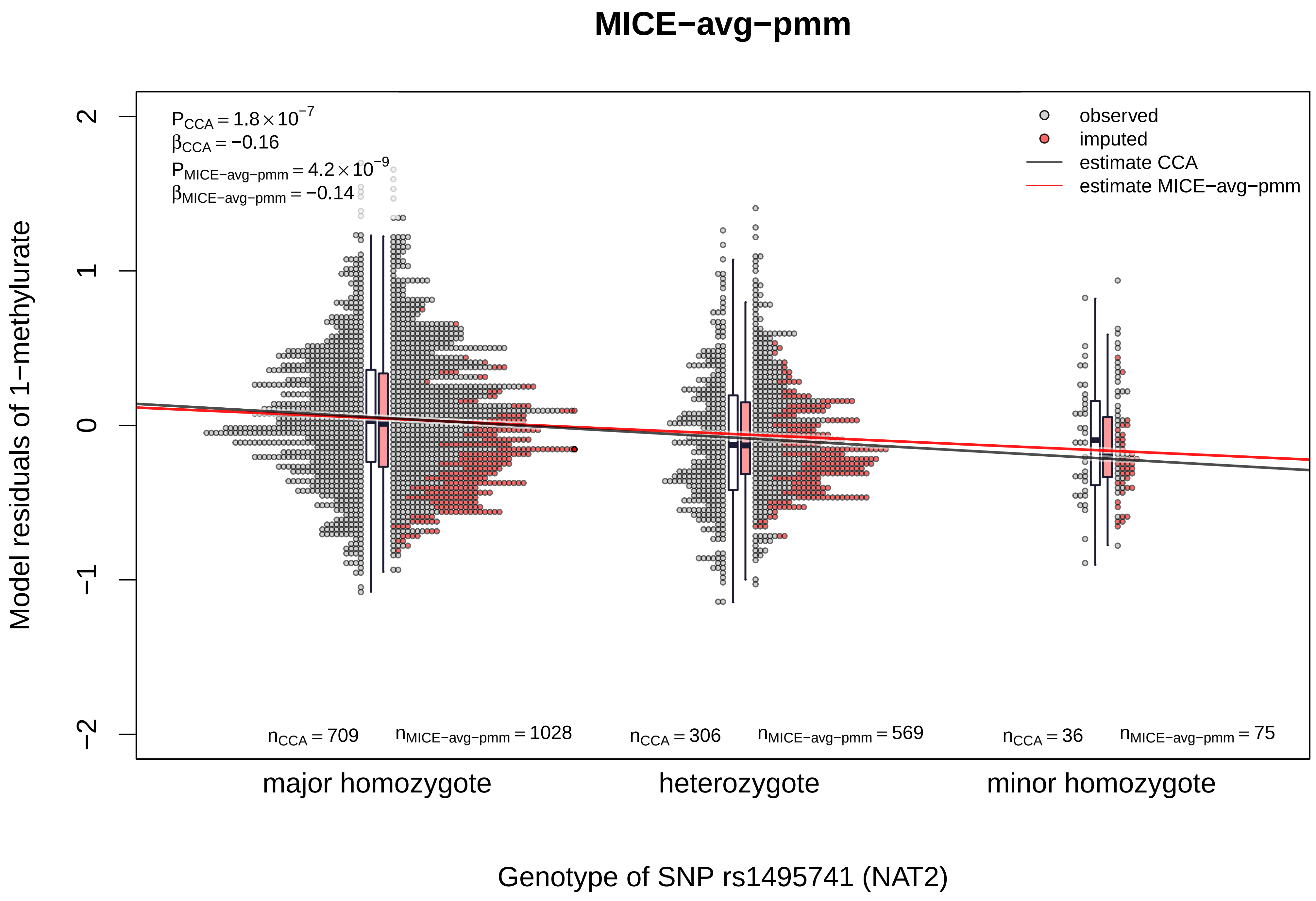

MICE−avg−pmm
$P_{CCA} = 1.8 \times 10^{-7}$
$\beta_{CCA} = -0.16$
$P_{MICE-avg-pmm} = 4.2 \times 10^{-9}$
$\beta_{MICE-avg-pmm} = -0.14$
observed
imputed
estimate CCA
estimate MICE−avg−pmm
$n_{CCA} = 709$
$n_{MICE-avg-pmm} = 1028$
$n_{CCA} = 306$
$n_{MICE-avg-pmm} = 569$
$n_{CCA} = 36$
$n_{MICE-avg-pmm} = 75$
2
1
0
−1
−2
Model residuals of 1−methylurate
major homozygote
heterozygote
minor homozygote
Genotype of SNP rs1495741 (NAT2)

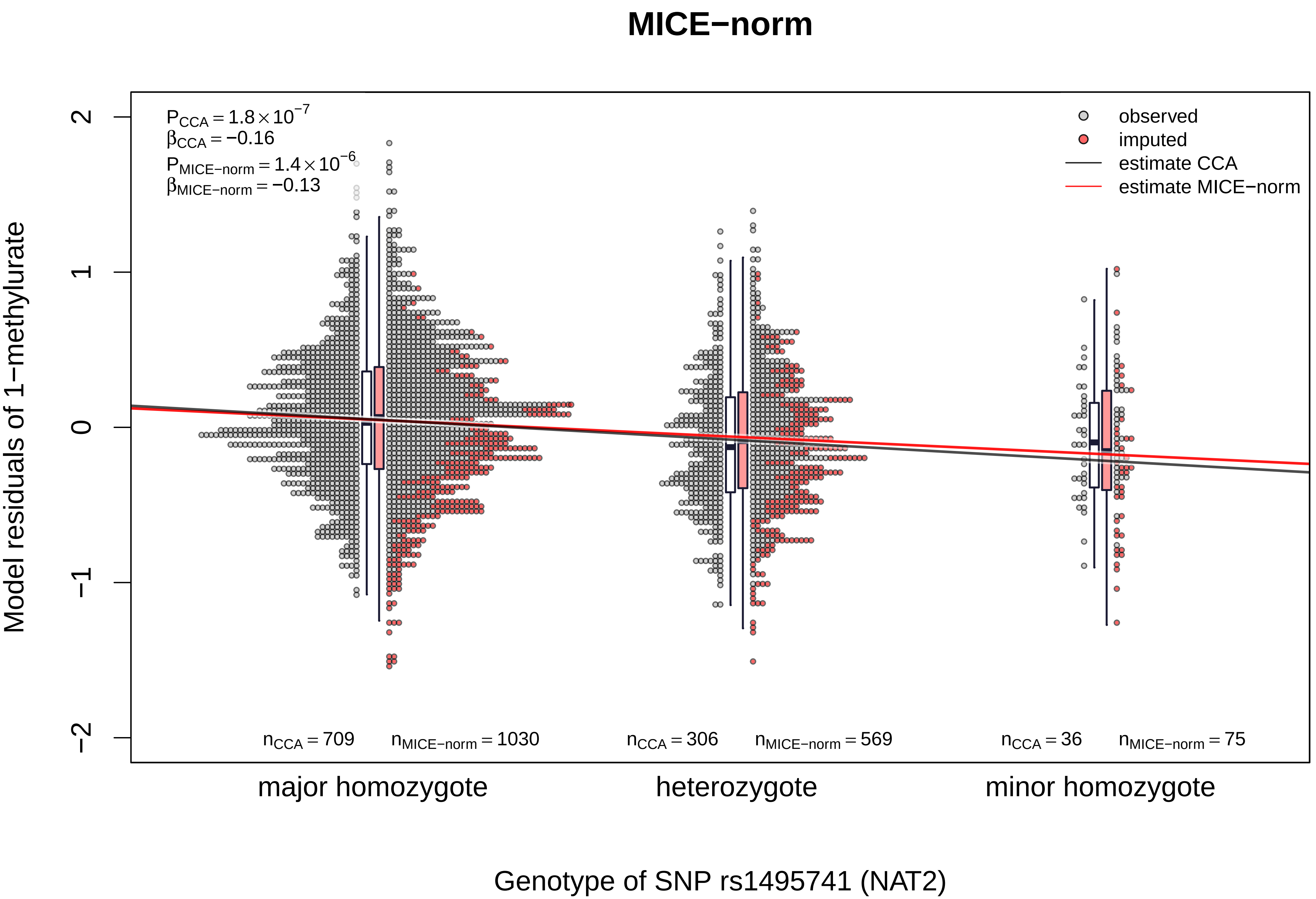
MICE−norm
$P_{CCA} = 1.8 \times 10^{-7}$
$\beta_{CCA} = -0.16$
$P_{MICE-norm} = 1.4 \times 10^{-6}$
$\beta_{MICE-norm} = -0.13$
observed
imputed
estimate CCA
estimate MICE−norm
Model residuals of 1−methylurate
2
1
0
−1
−2
$n_{CCA} = 709$
$n_{MICE-norm} = 1030$
$n_{CCA} = 306$
$n_{MICE-norm} = 569$
$n_{CCA} = 36$
$n_{MICE-norm} = 75$
major homozygote
heterozygote
minor homozygote
Genotype of SNP rs1495741 (NAT2)

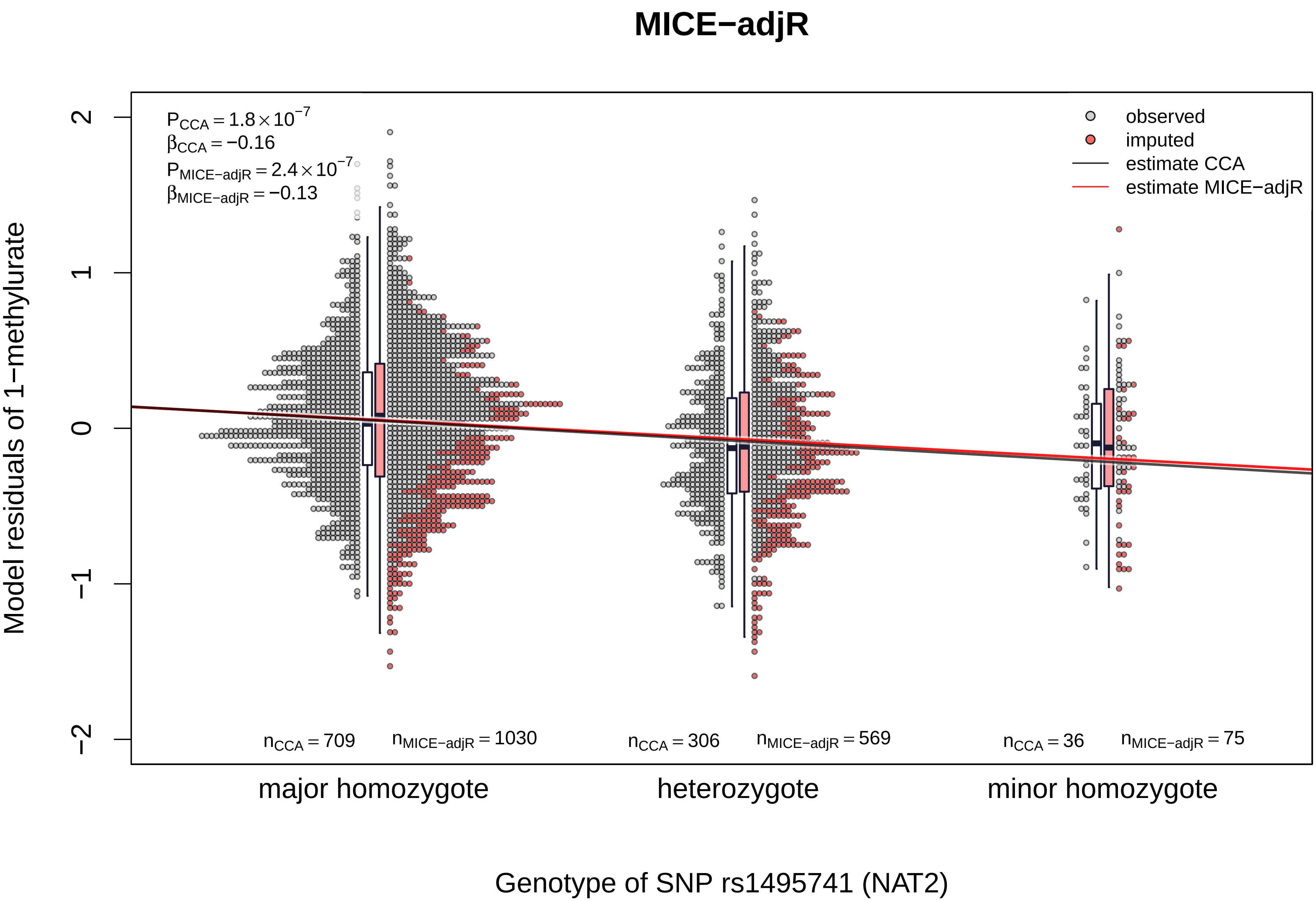
MICE−adjR
$P_{CCA} = 1.8 \times 10^{-7}$
$\beta_{CCA} = -0.16$
$P_{MICE-adjR} = 2.4 \times 10^{-7}$
$\beta_{MICE-adjR} = -0.13$
observed
imputed
estimate CCA
estimate MICE−adjR
Model residuals of 1−methylurate
2
1
0
−1
−2
$n_{CCA} = 709$
$n_{MICE-adjR} = 1030$
$n_{CCA} = 306$
$n_{MICE-adjR} = 569$
$n_{CCA} = 36$
$n_{MICE-adjR} = 75$
major homozygote
heterozygote
minor homozygote
Genotype of SNP rs1495741 (NAT2)

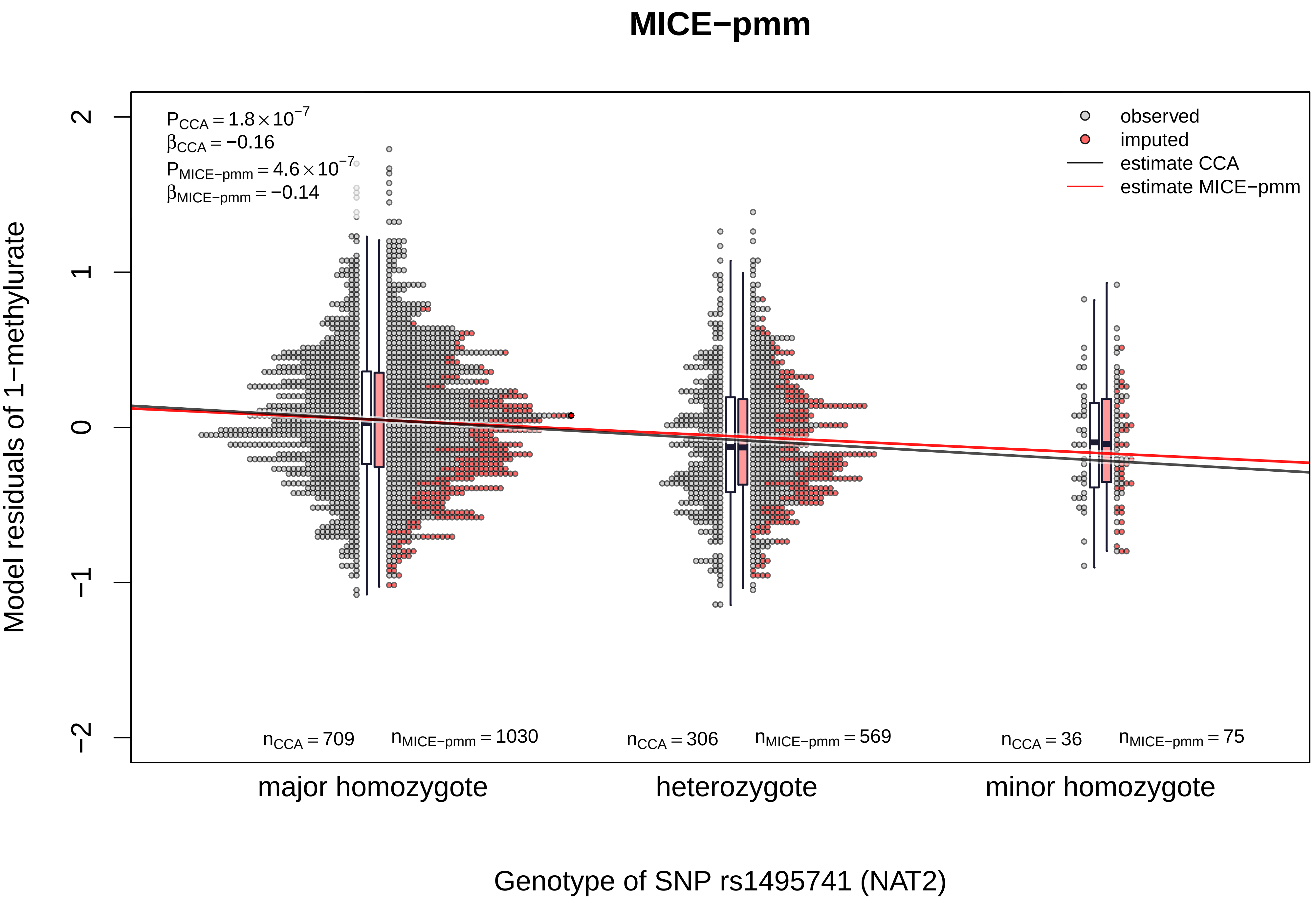

MICE−pmm
$P_{CCA} = 1.8 \times 10^{-7}$
$\beta_{CCA} = -0.16$
$P_{MICE-pmm} = 4.6 \times 10^{-7}$
$\beta_{MICE-pmm} = -0.14$
observed
imputed
estimate CCA
estimate MICE−pmm
Model residuals of 1−methylurate
2
1
0
−1
−2
$n_{CCA} = 709$
$n_{MICE-pmm} = 1030$
$n_{CCA} = 306$
$n_{MICE-pmm} = 569$
$n_{CCA} = 36$
$n_{MICE-pmm} = 75$
major homozygote
heterozygote
minor homozygote
Genotype of SNP rs1495741 (NAT2)

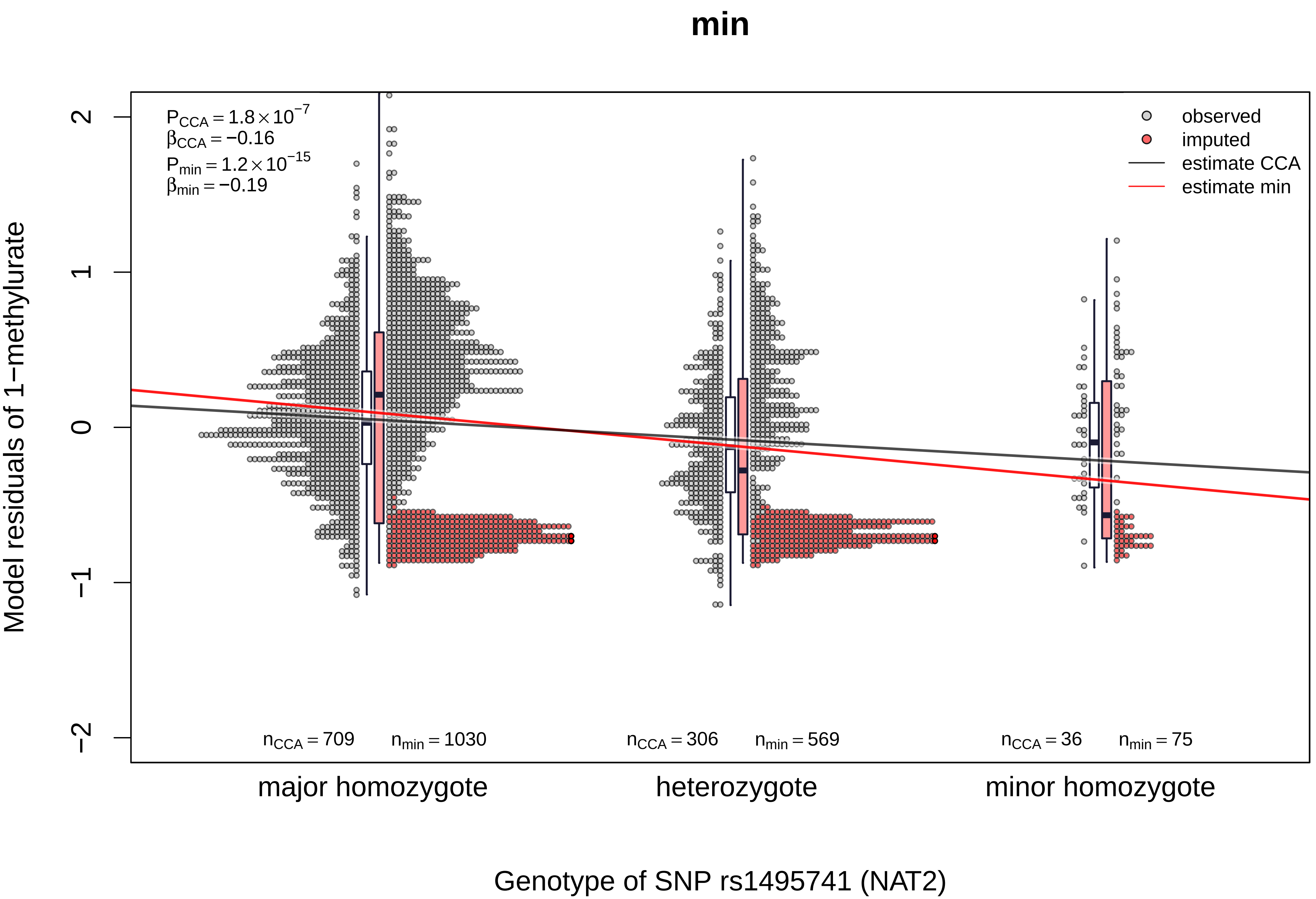
min
$P_{CCA} = 1.8 \times 10^{-7}$
$\beta_{CCA} = -0.16$
$P_{min} = 1.2 \times 10^{-15}$
$\beta_{min} = -0.19$
observed
imputed
estimate CCA
estimate min
Model residuals of 1−methylurate
2
1
0
−1
−2
$n_{CCA} = 709$
$n_{min} = 1030$
$n_{CCA} = 306$
$n_{min} = 569$
$n_{CCA} = 36$
$n_{min} = 75$
major homozygote
heterozygote
minor homozygote
Genotype of SNP rs1495741 (NAT2)

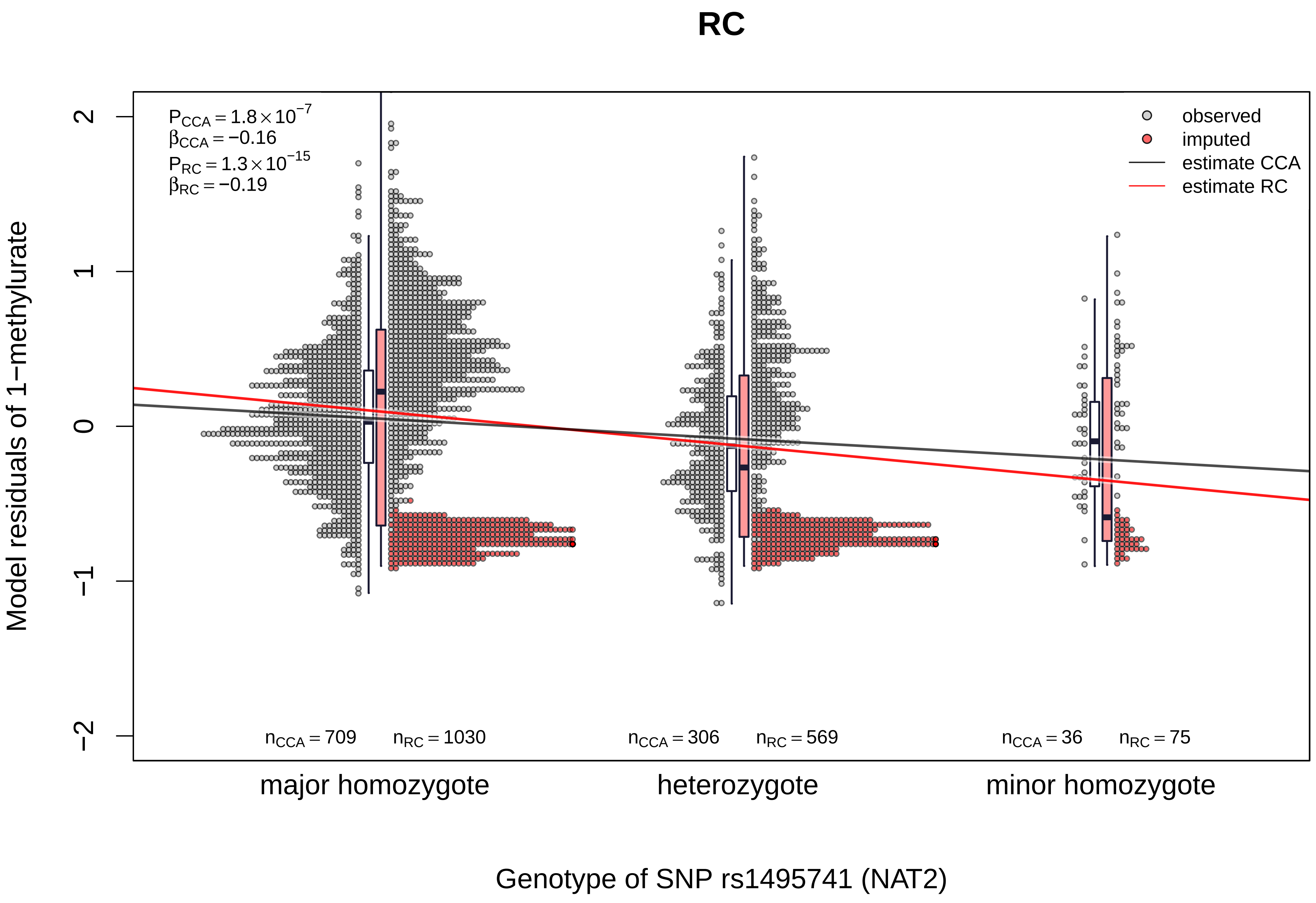

RC
$P_{CCA} = 1.8 \times 10^{-7}$
$\beta_{CCA} = -0.16$
$P_{RC} = 1.3 \times 10^{-15}$
$\beta_{RC} = -0.19$
observed
imputed
estimate CCA
estimate RC
Model residuals of 1−methylurate
2
1
0
−1
−2
$n_{CCA} = 709$
$n_{RC} = 1030$
$n_{CCA} = 306$
$n_{RC} = 569$
$n_{CCA} = 36$
$n_{RC} = 75$
major homozygote
heterozygote
minor homozygote
Genotype of SNP rs1495741 (NAT2)

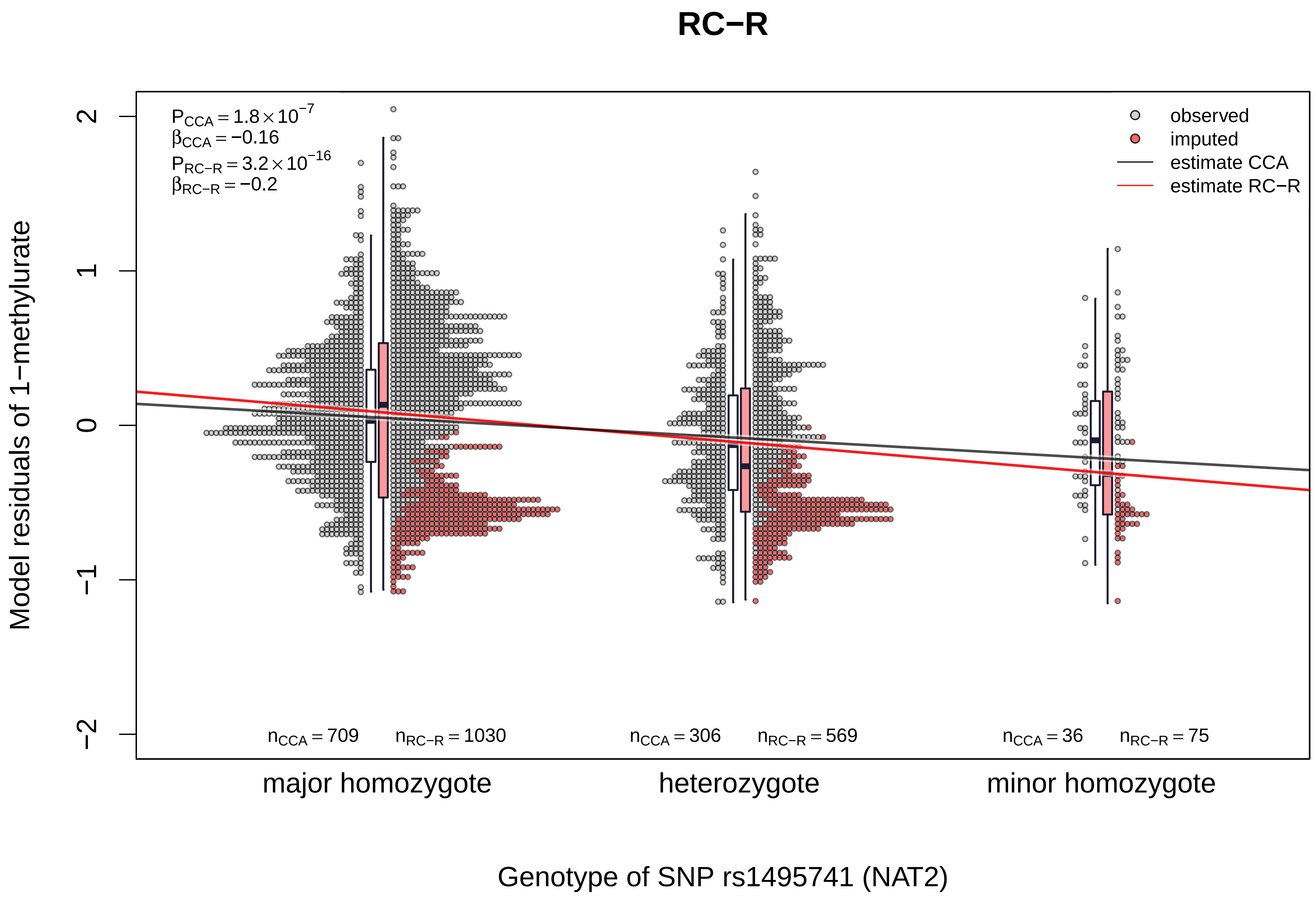

RC−R
$P_{CCA} = 1.8 \times 10^{-7}$
$\beta_{CCA} = -0.16$
$P_{RC-R} = 3.2 \times 10^{-16}$
$\beta_{RC-R} = -0.2$
observed
imputed
estimate CCA
estimate RC−R
Model residuals of 1−methylurate
2
1
0
−1
−2
$n_{CCA} = 709$
$n_{RC-R} = 1030$
$n_{CCA} = 306$
$n_{RC-R} = 569$
$n_{CCA} = 36$
$n_{RC-R} = 75$
major homozygote
heterozygote
minor homozygote
Genotype of SNP rs1495741 (NAT2)

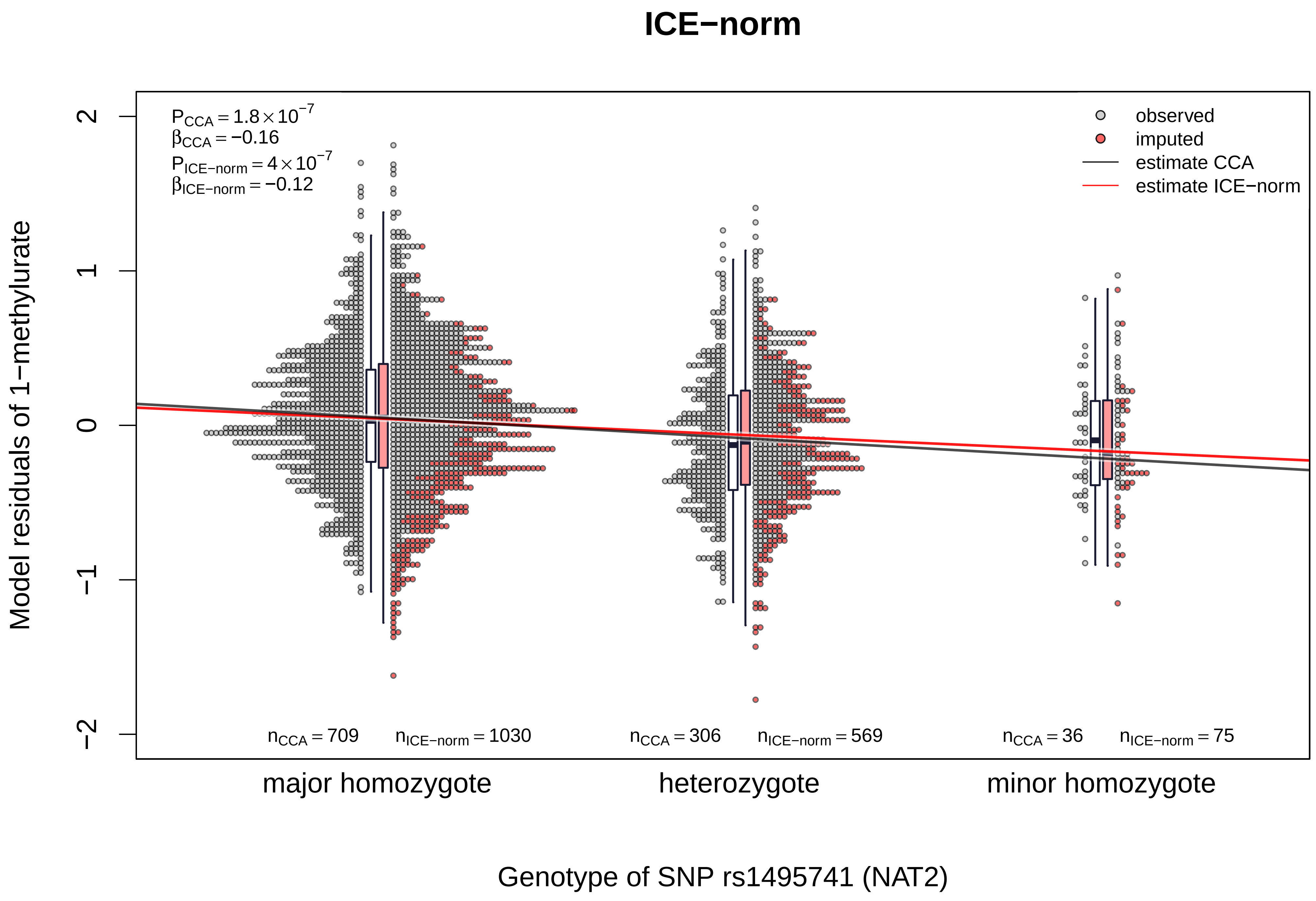

ICE−norm
$P_{CCA} = 1.8 \times 10^{-7}$
$\beta_{CCA} = -0.16$
$P_{ICE-norm} = 4 \times 10^{-7}$
$\beta_{ICE-norm} = -0.12$
observed
imputed
estimate CCA
estimate ICE−norm
Model residuals of 1−methylurate
2
1
0
−1
−2
$n_{CCA} = 709$
$n_{ICE-norm} = 1030$
$n_{CCA} = 306$
$n_{ICE-norm} = 569$
$n_{CCA} = 36$
$n_{ICE-norm} = 75$
major homozygote
heterozygote
minor homozygote
Genotype of SNP rs1495741 (NAT2)

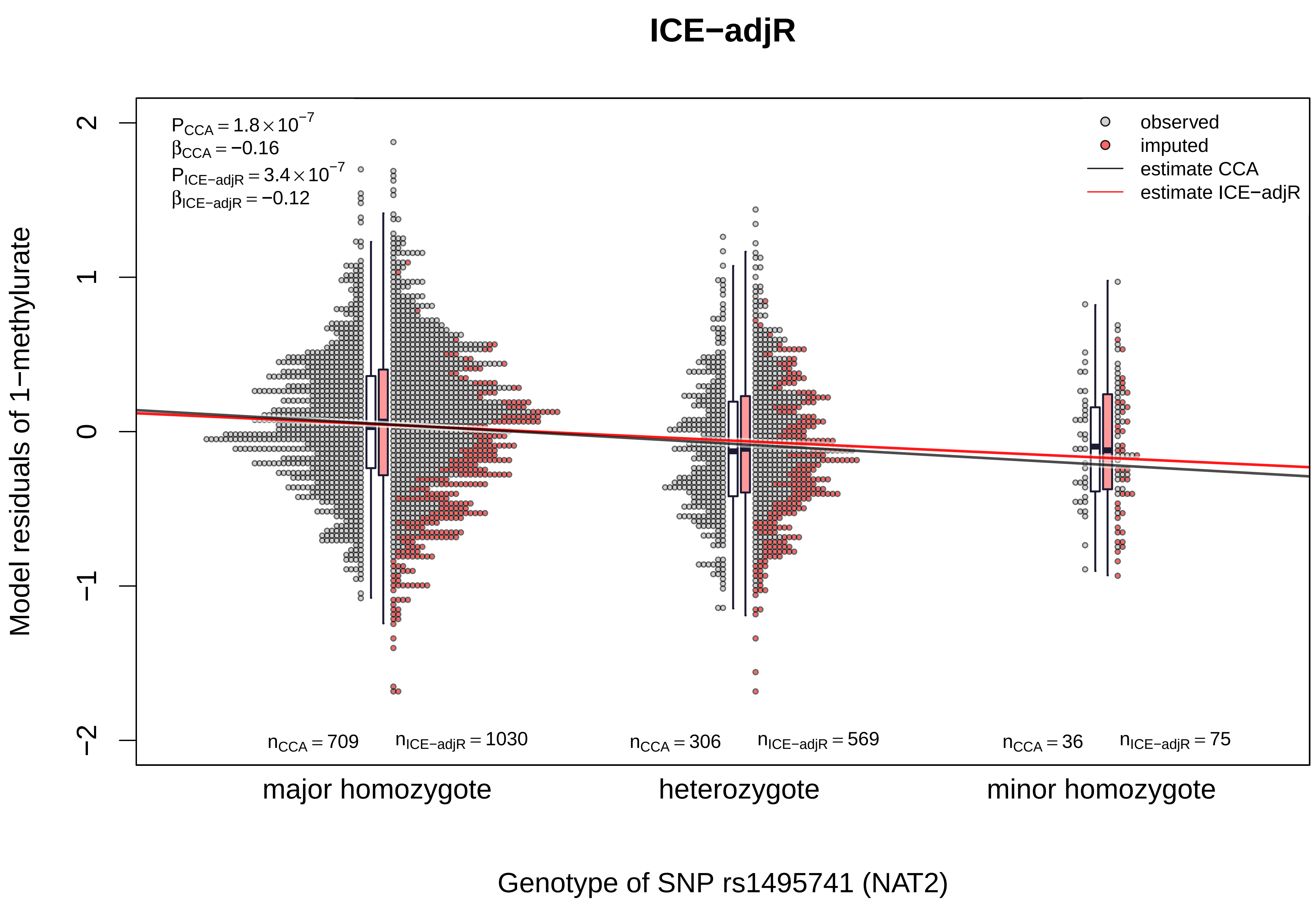

ICE−adjR
$P_{CCA} = 1.8 \times 10^{-7}$
$\beta_{CCA} = -0.16$
$P_{ICE-adjR} = 3.4 \times 10^{-7}$
$\beta_{ICE-adjR} = -0.12$
observed
imputed
estimate CCA
estimate ICE−adjR
Model residuals of 1−methylurate
2
1
0
−1
−2
$n_{CCA} = 709$
$n_{ICE-adjR} = 1030$
$n_{CCA} = 306$
$n_{ICE-adjR} = 569$
$n_{CCA} = 36$
$n_{ICE-adjR} = 75$
major homozygote
heterozygote
minor homozygote
Genotype of SNP rs1495741 (NAT2)

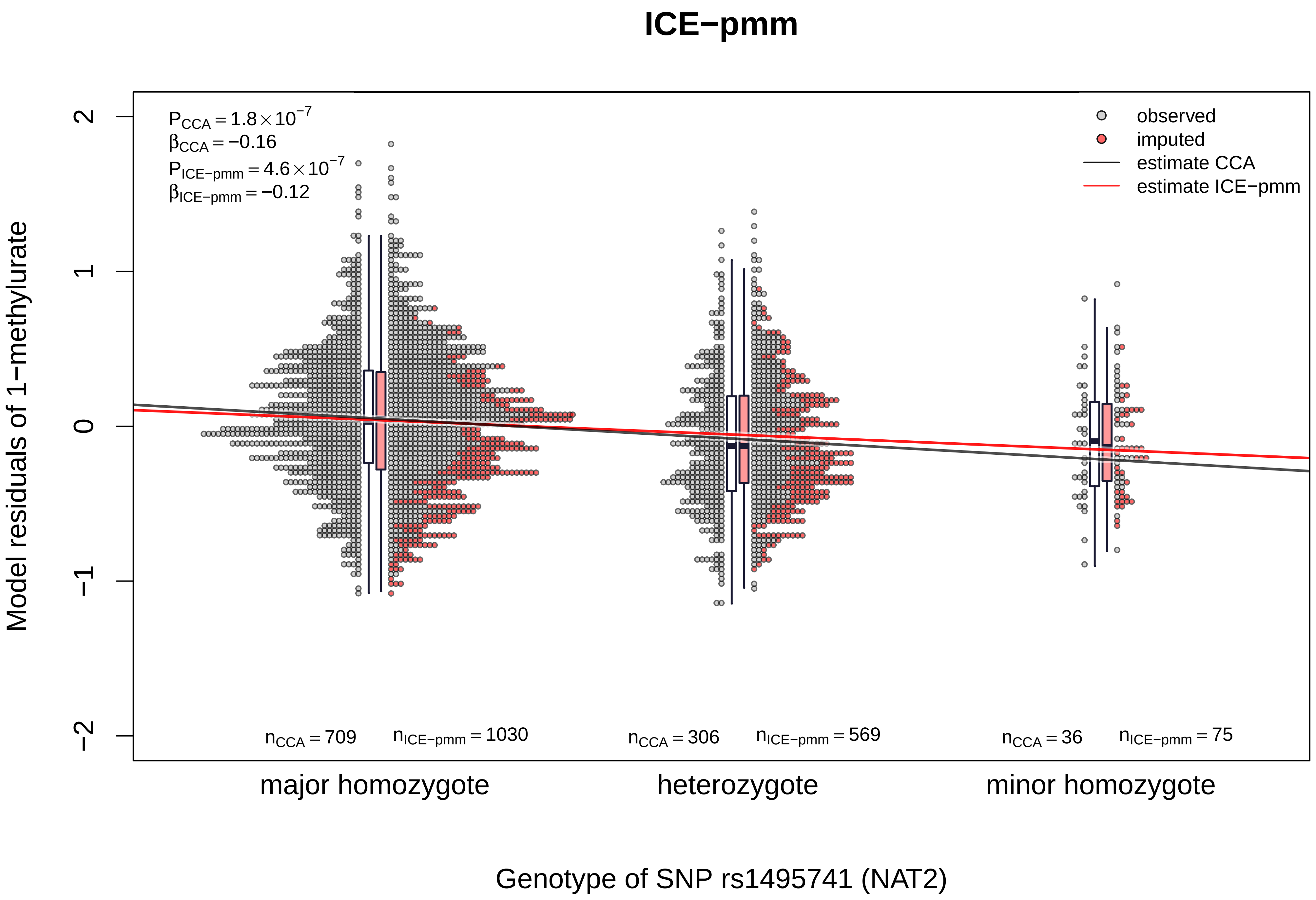

ICE−pmm
$P_{CCA} = 1.8 \times 10^{-7}$
$\beta_{CCA} = -0.16$
$P_{ICE-pmm} = 4.6 \times 10^{-7}$
$\beta_{ICE-pmm} = -0.12$
observed
imputed
estimate CCA
estimate ICE−pmm
Model residuals of 1−methylurate
2
1
0
−1
−2
$n_{CCA} = 709$
$n_{ICE-pmm} = 1030$
$n_{CCA} = 306$
$n_{ICE-pmm} = 569$
$n_{CCA} = 36$
$n_{ICE-pmm} = 75$
major homozygote
heterozygote
minor homozygote
Genotype of SNP rs1495741 (NAT2)

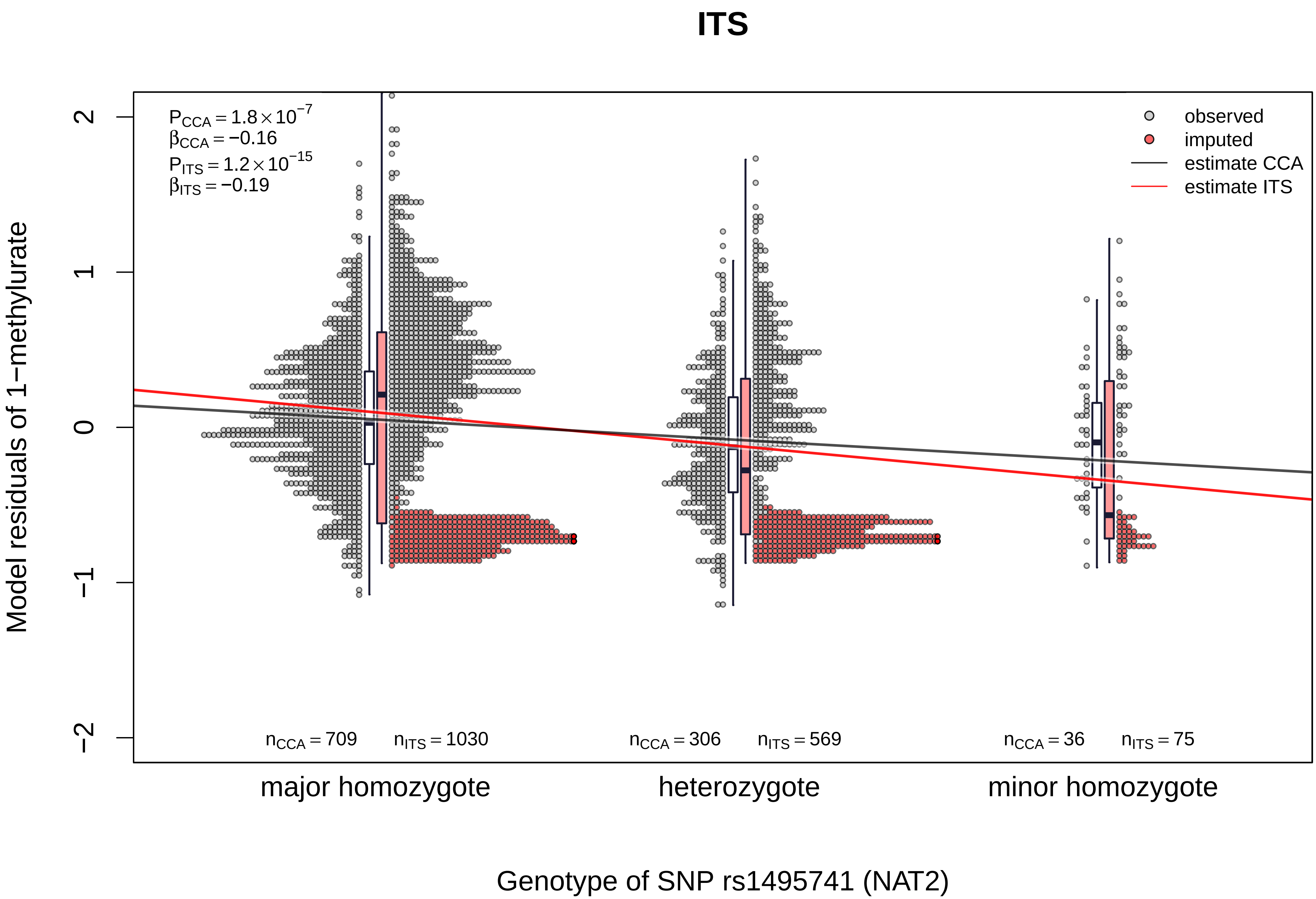
ITS
$P_{CCA} = 1.8 \times 10^{-7}$
$\beta_{CCA} = -0.16$
$P_{ITS} = 1.2 \times 10^{-15}$
$\beta_{ITS} = -0.19$
observed
imputed
estimate CCA
estimate ITS
Model residuals of 1−methylurate
2
1
0
−1
−2
$n_{CCA} = 709$
$n_{ITS} = 1030$
$n_{CCA} = 306$
$n_{ITS} = 569$
$n_{CCA} = 36$
$n_{ITS} = 75$
major homozygote
heterozygote
minor homozygote
Genotype of SNP rs1495741 (NAT2)

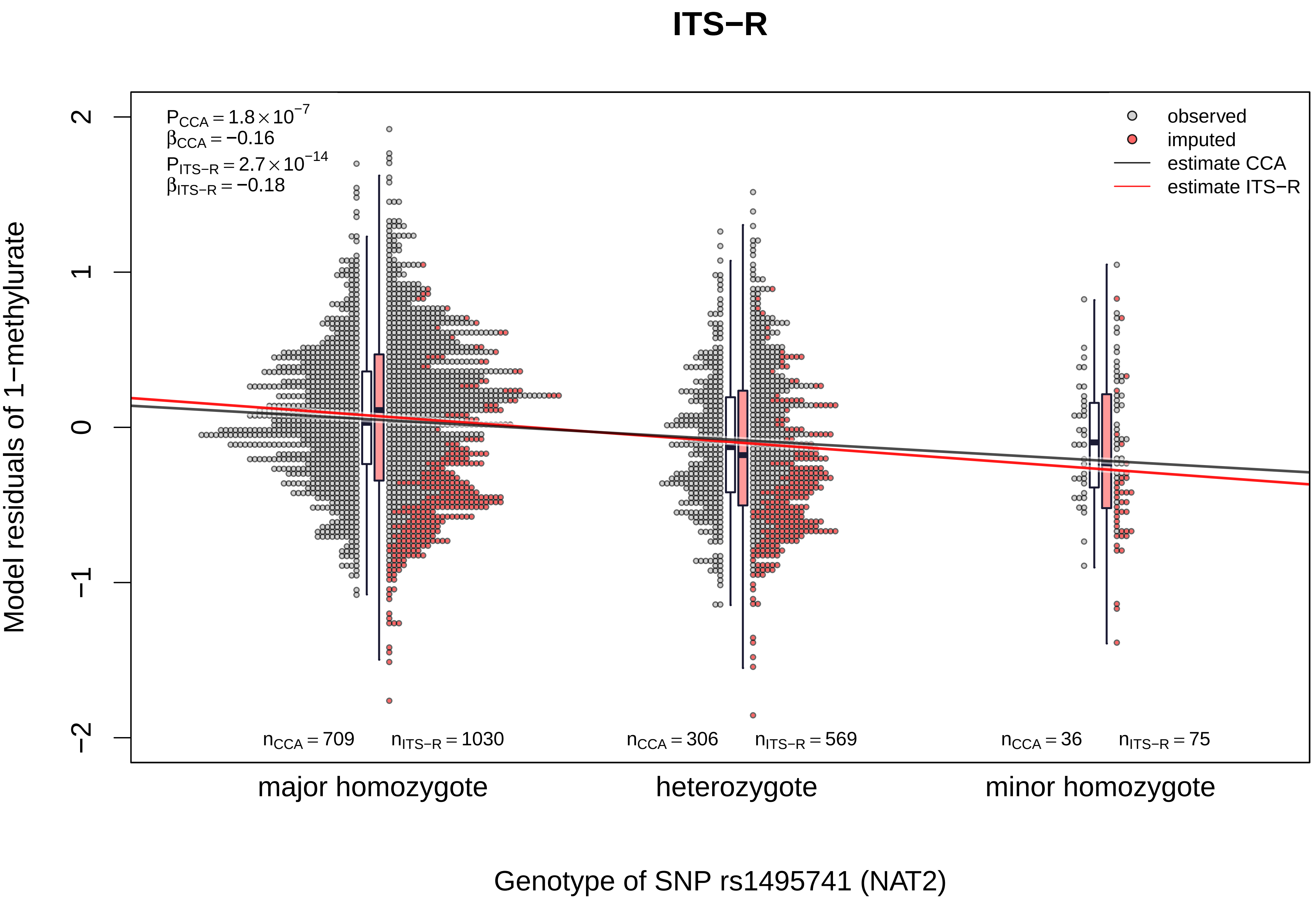

ITS−R
$P_{CCA} = 1.8 \times 10^{-7}$
$\beta_{CCA} = -0.16$
$P_{ITS-R} = 2.7 \times 10^{-14}$
$\beta_{ITS-R} = -0.18$
observed
imputed
estimate CCA
estimate ITS−R
Model residuals of 1−methylurate
2
1
0
−1
−2
$n_{CCA} = 709$
$n_{ITS-R} = 1030$
$n_{CCA} = 306$
$n_{ITS-R} = 569$
$n_{CCA} = 36$
$n_{ITS-R} = 75$
major homozygote
heterozygote
minor homozygote
Genotype of SNP rs1495741 (NAT2)

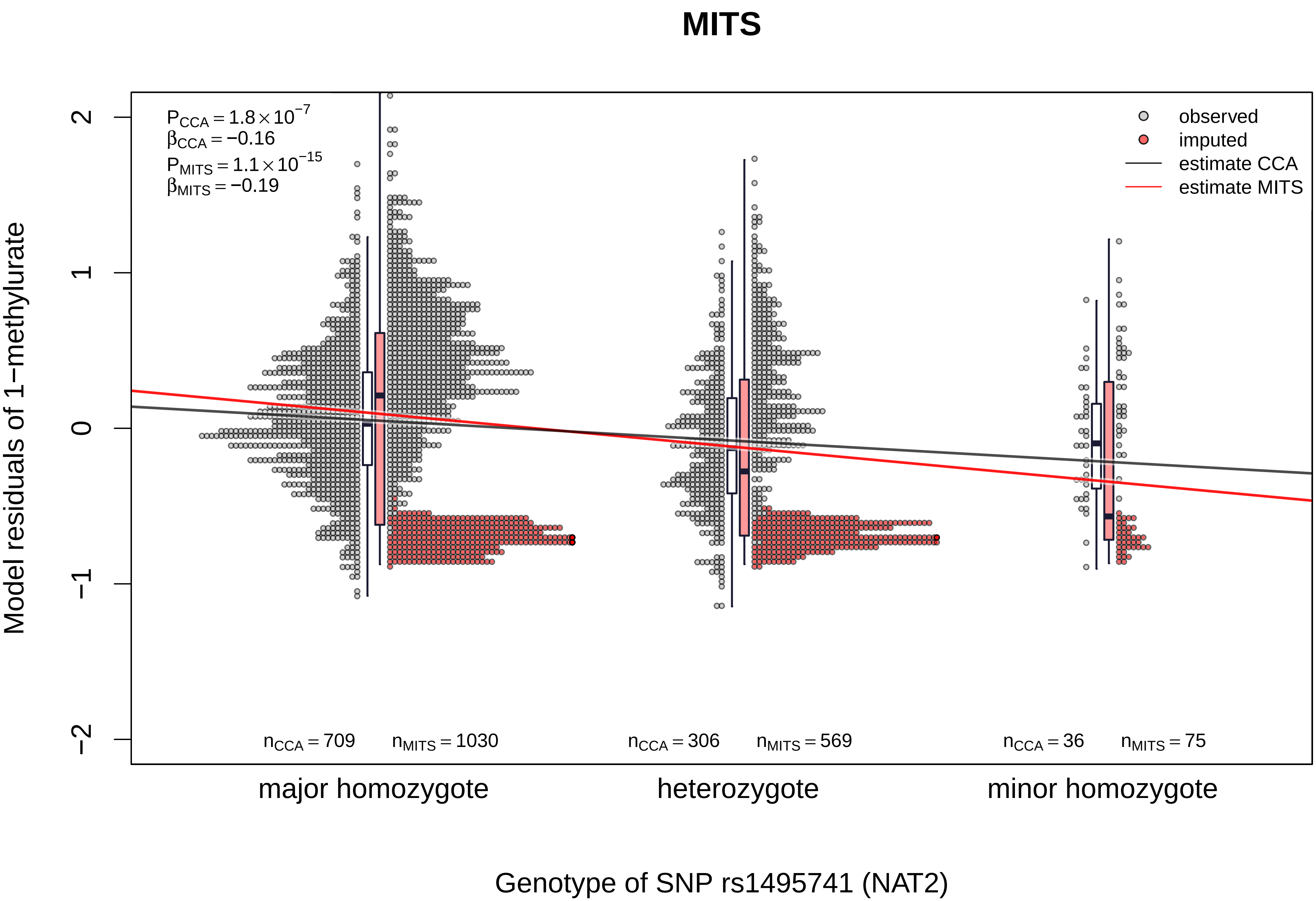

MITS
$P_{CCA} = 1.8 \times 10^{-7}$
$\beta_{CCA} = -0.16$
$P_{MITS} = 1.1 \times 10^{-15}$
$\beta_{MITS} = -0.19$
observed
imputed
estimate CCA
estimate MITS
Model residuals of 1−methylurate
2
1
0
−1
−2
$n_{CCA} = 709$
$n_{MITS} = 1030$
$n_{CCA} = 306$
$n_{MITS} = 569$
$n_{CCA} = 36$
$n_{MITS} = 75$
major homozygote
heterozygote
minor homozygote
Genotype of SNP rs1495741 (NAT2)

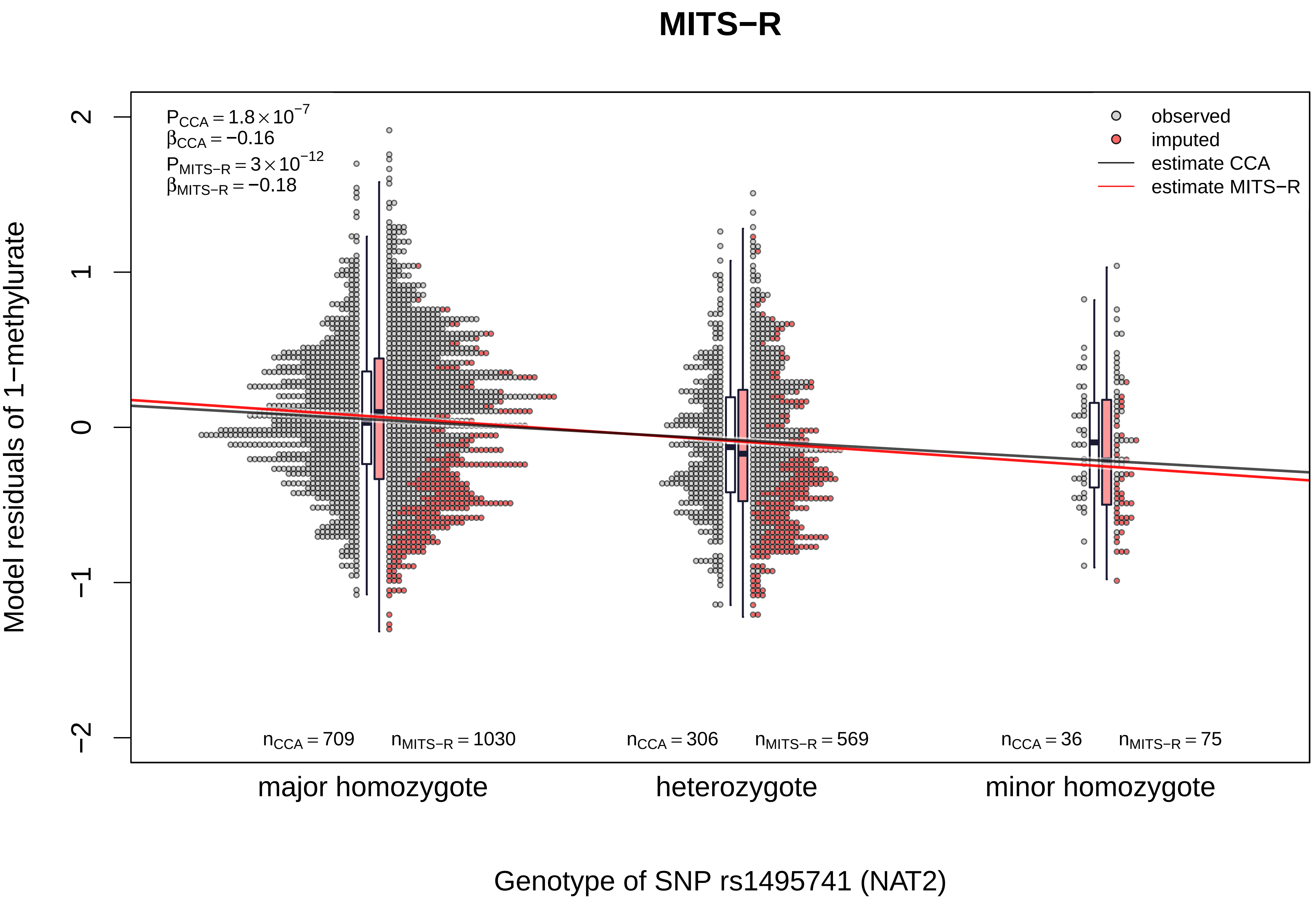
MITS−R
$P_{CCA} = 1.8 \times 10^{-7}$
$\beta_{CCA} = -0.16$
$P_{MITS-R} = 3 \times 10^{-12}$
$\beta_{MITS-R} = -0.18$
observed
imputed
estimate CCA
estimate MITS−R
2
1
0
−1
−2
Model residuals of 1−methylurate
$n_{CCA} = 709$
$n_{MITS-R} = 1030$
$n_{CCA} = 306$
$n_{MITS-R} = 569$
$n_{CCA} = 36$
$n_{MITS-R} = 75$
major homozygote
heterozygote
minor homozygote
Genotype of SNP rs1495741 (NAT2)

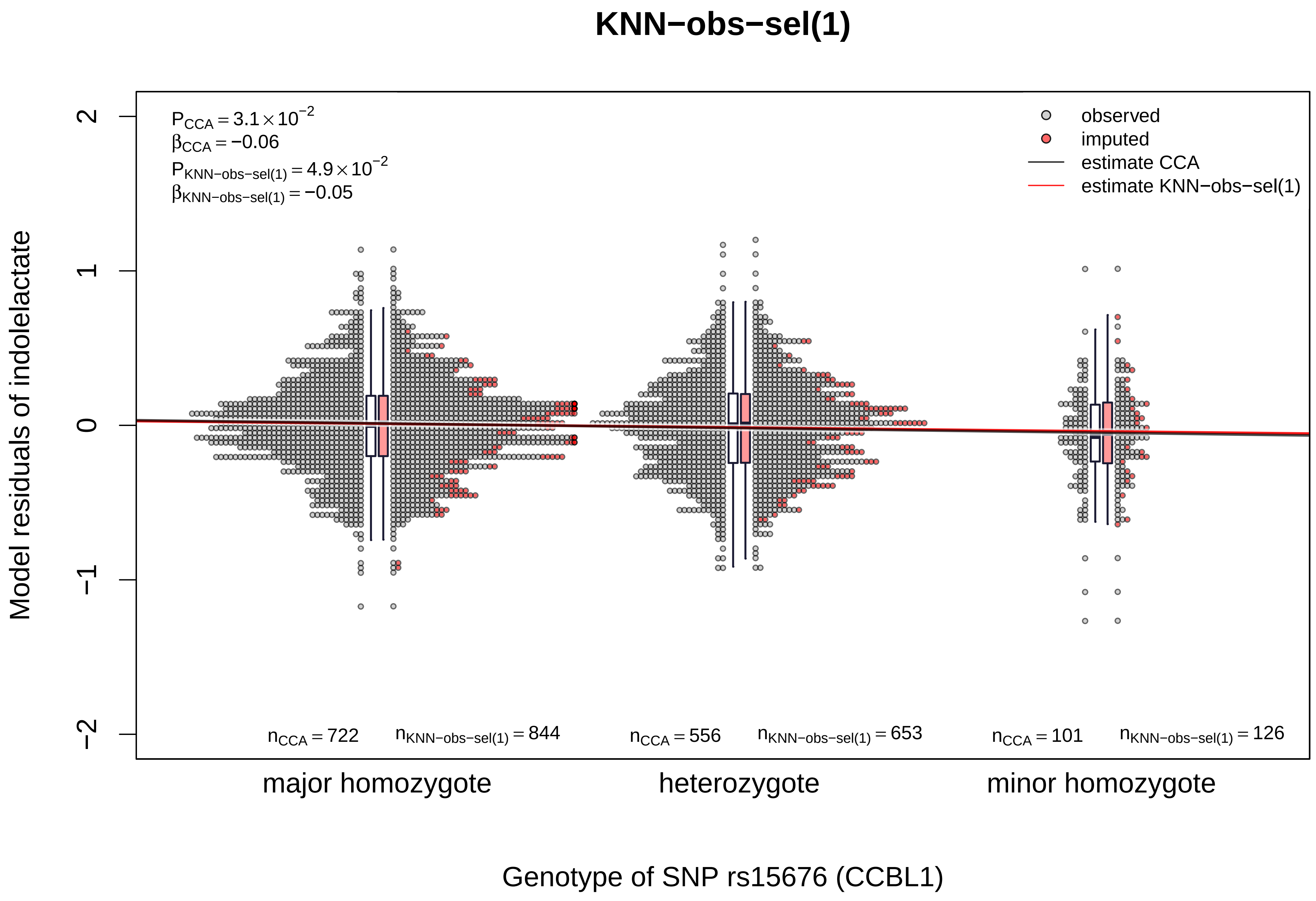
KNN−obs−sel(1)
$P_{CCA} = 3.1 \times 10^{-2}$
$\beta_{CCA} = -0.06$
$P_{KNN-obs-sel(1)} = 4.9 \times 10^{-2}$
$\beta_{KNN-obs-sel(1)} = -0.05$
observed
imputed
estimate CCA
estimate KNN−obs−sel(1)
Model residuals of indolelactate
2
1
0
−1
−2
$n_{CCA} = 722$
$n_{KNN-obs-sel(1)} = 844$
$n_{CCA} = 556$
$n_{KNN-obs-sel(1)} = 653$
$n_{CCA} = 101$
$n_{KNN-obs-sel(1)} = 126$
major homozygote
heterozygote
minor homozygote
Genotype of SNP rs15676 (CCBL1)

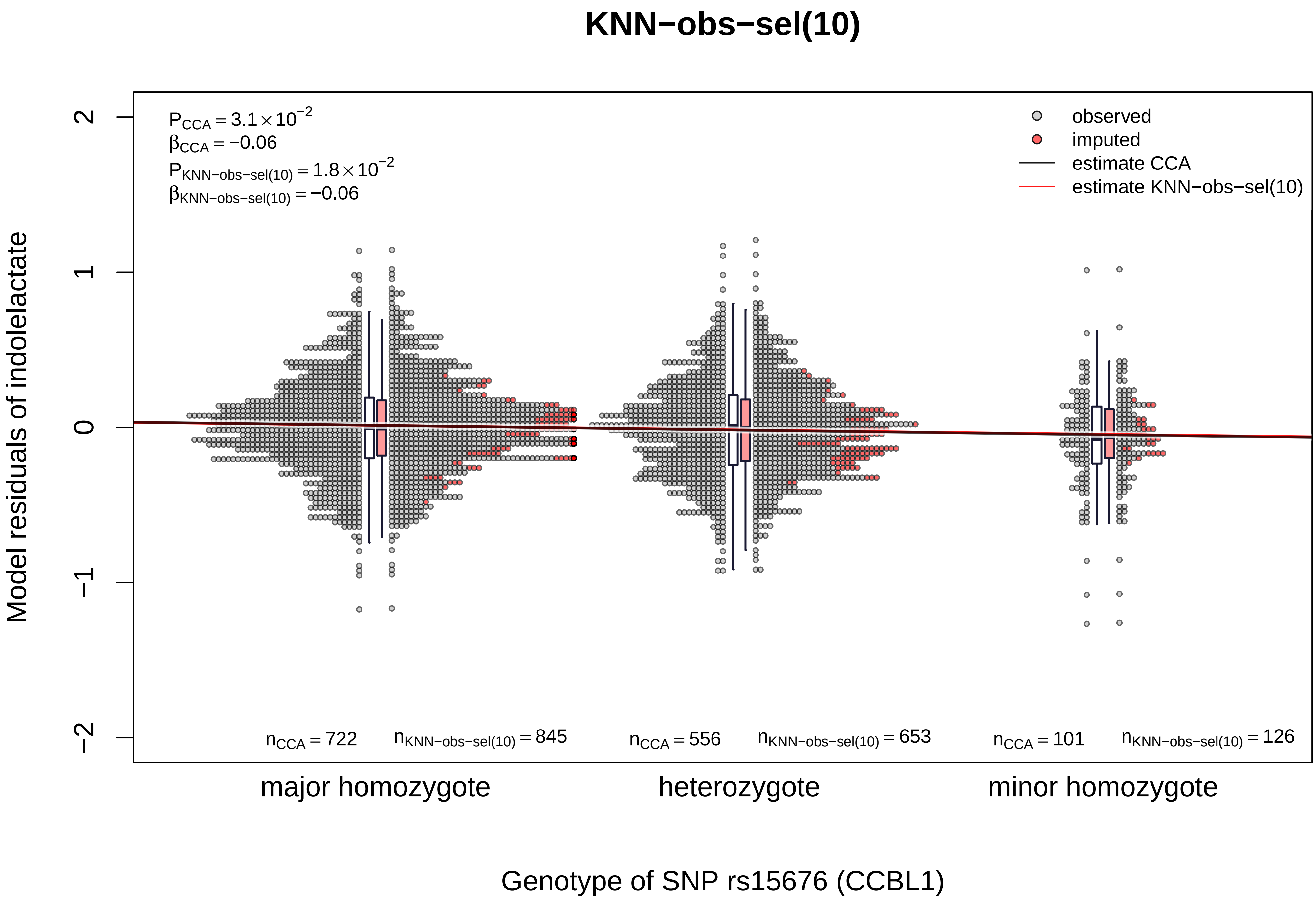
KNN−obs−sel(10)
$P_{CCA} = 3.1 \times 10^{-2}$
$\beta_{CCA} = -0.06$
$P_{KNN-obs-sel(10)} = 1.8 \times 10^{-2}$
$\beta_{KNN-obs-sel(10)} = -0.06$
observed
imputed
estimate CCA
estimate KNN−obs−sel(10)
Model residuals of indolelactate
2
1
0
−1
−2
$n_{CCA} = 722$
$n_{KNN-obs-sel(10)} = 845$
$n_{CCA} = 556$
$n_{KNN-obs-sel(10)} = 653$
$n_{CCA} = 101$
$n_{KNN-obs-sel(10)} = 126$
major homozygote
heterozygote
minor homozygote
Genotype of SNP rs15676 (CCBL1)

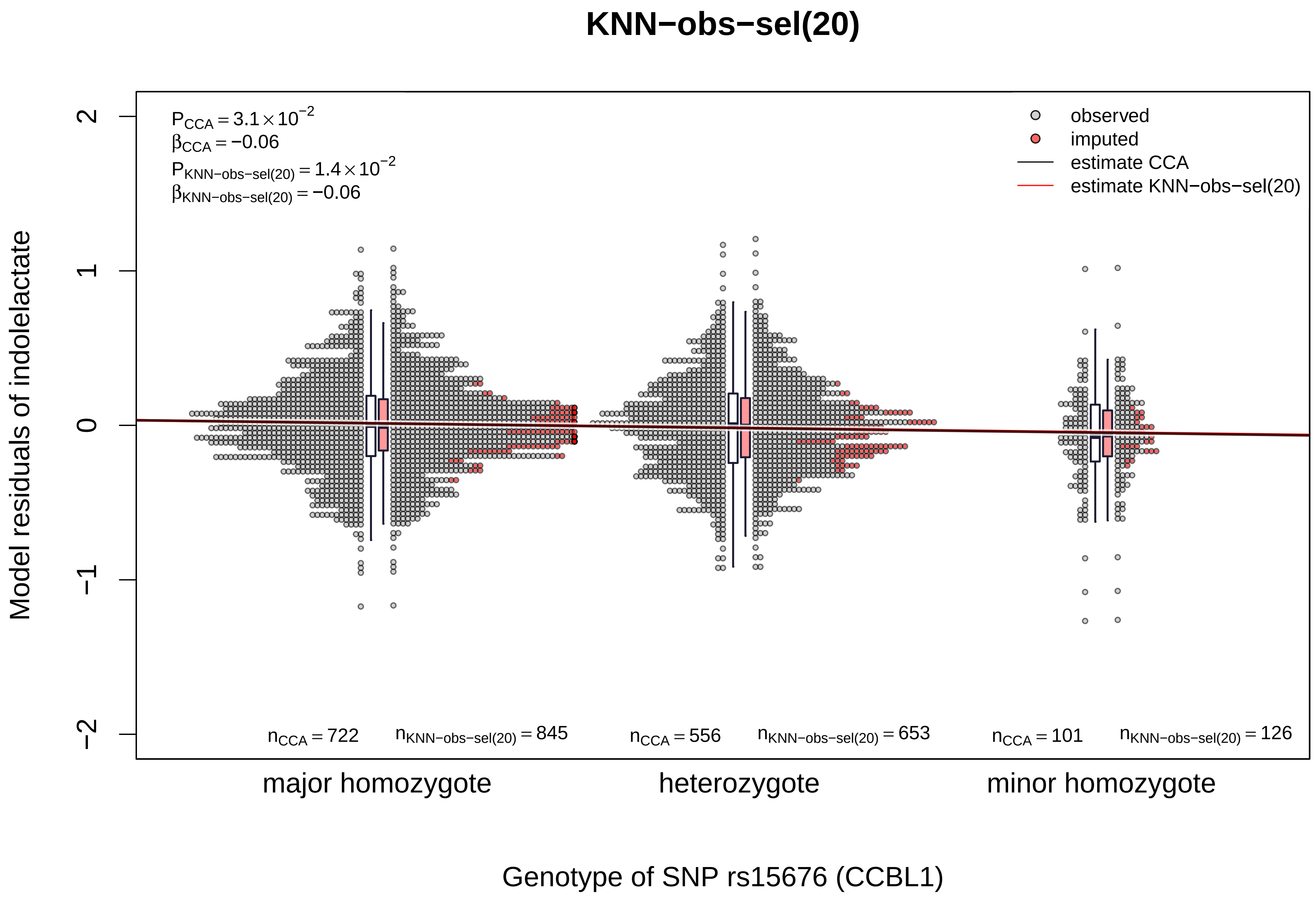
KNN−obs−sel(20)
$P_{CCA} = 3.1 \times 10^{-2}$
$\beta_{CCA} = -0.06$
$P_{KNN-obs-sel(20)} = 1.4 \times 10^{-2}$
$\beta_{KNN-obs-sel(20)} = -0.06$
observed
imputed
estimate CCA
estimate KNN−obs−sel(20)
Model residuals of indolelactate
$n_{CCA} = 722$
$n_{KNN-obs-sel(20)} = 845$
$n_{CCA} = 556$
$n_{KNN-obs-sel(20)} = 653$
$n_{CCA} = 101$
$n_{KNN-obs-sel(20)} = 126$
major homozygote
heterozygote
minor homozygote
Genotype of SNP rs15676 (CCBL1)

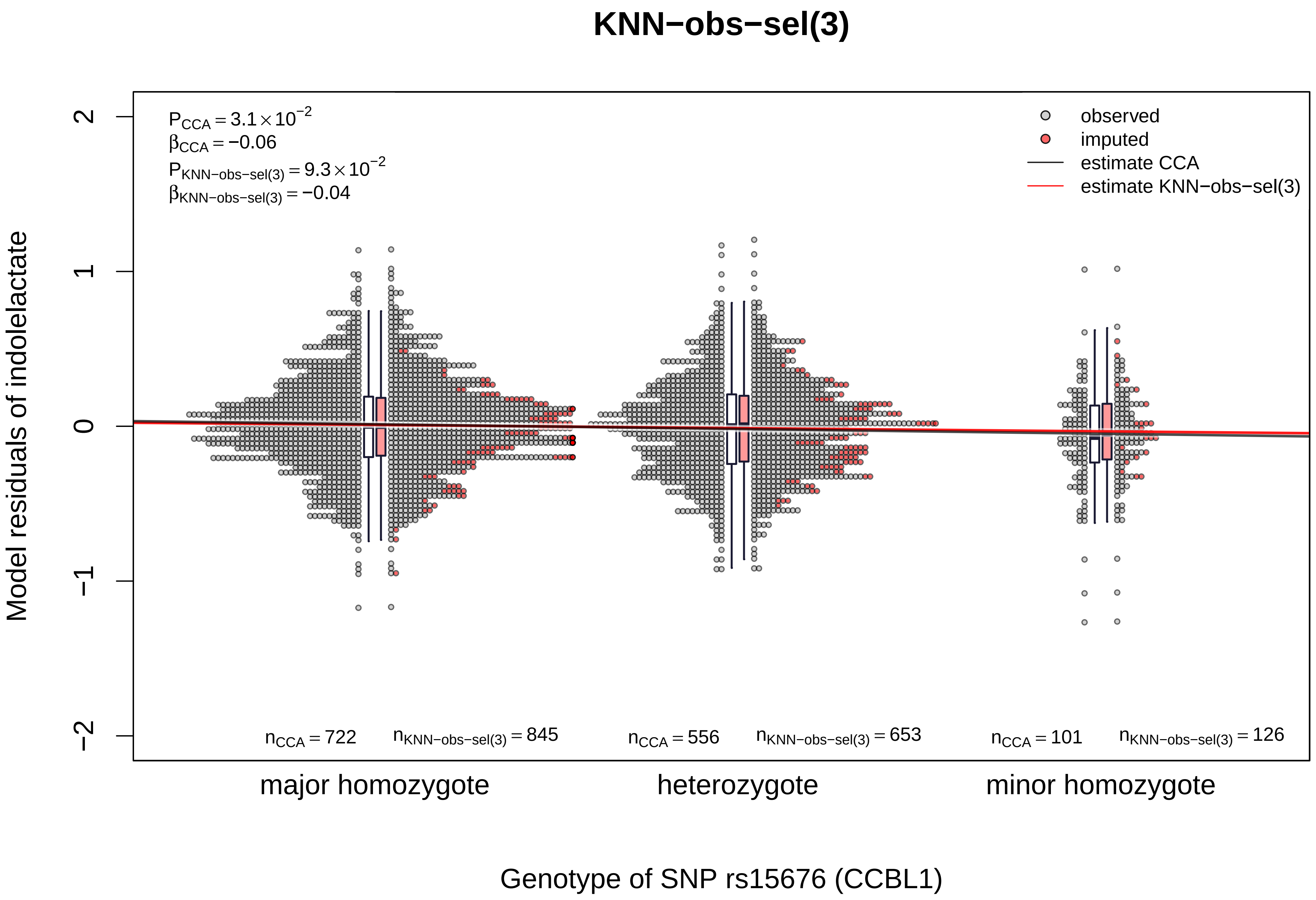

KNN−obs−sel(3)
$P_{CCA} = 3.1 \times 10^{-2}$
$\beta_{CCA} = -0.06$
$P_{KNN-obs-sel(3)} = 9.3 \times 10^{-2}$
$\beta_{KNN-obs-sel(3)} = -0.04$
observed
imputed
estimate CCA
estimate KNN−obs−sel(3)
2
1
0
−1
−2
Model residuals of indolelactate
$n_{CCA} = 722$
$n_{KNN-obs-sel(3)} = 845$
$n_{CCA} = 556$
$n_{KNN-obs-sel(3)} = 653$
$n_{CCA} = 101$
$n_{KNN-obs-sel(3)} = 126$
major homozygote
heterozygote
minor homozygote
Genotype of SNP rs15676 (CCBL1)

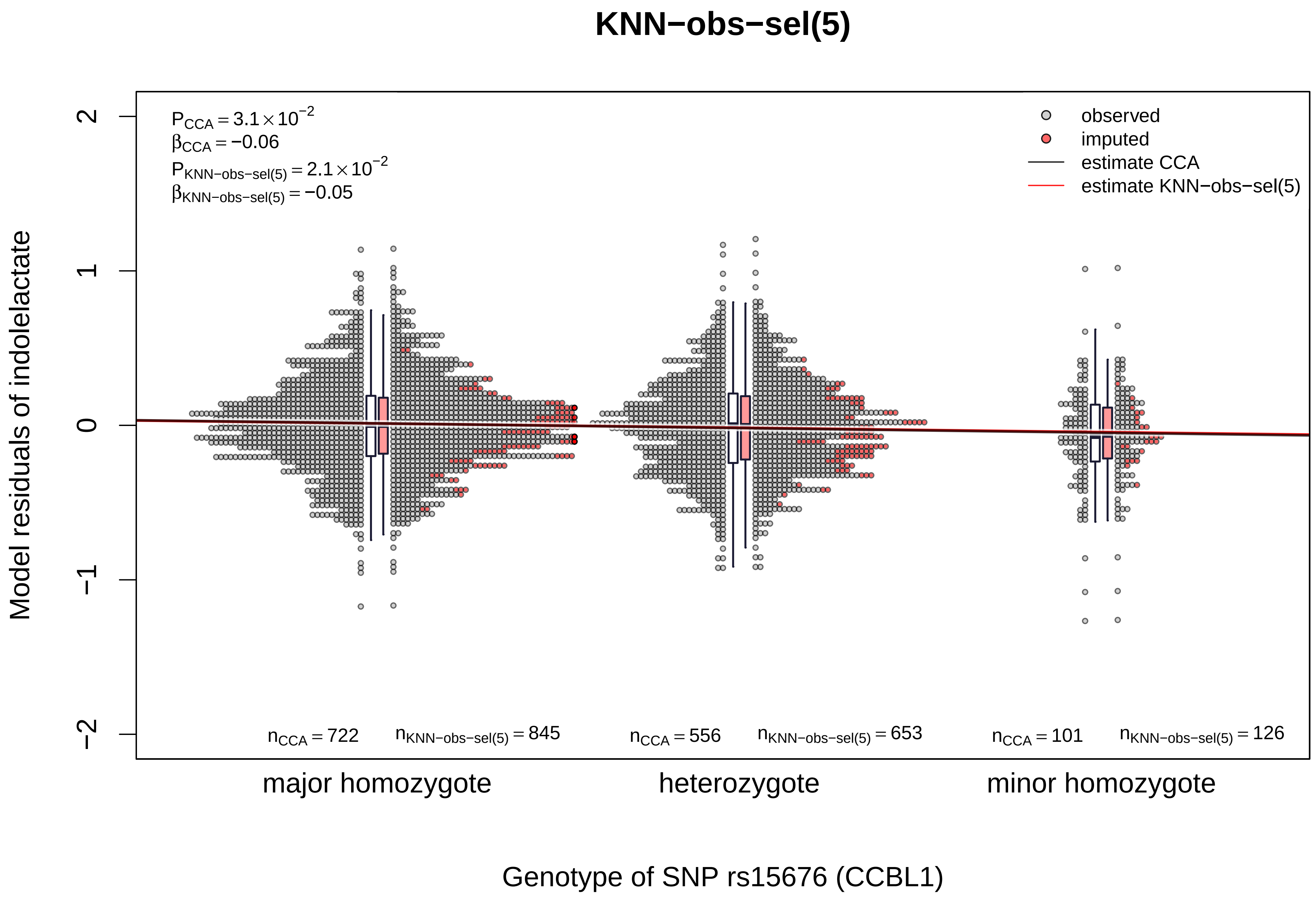

KNN−obs−sel(5)
$P_{CCA} = 3.1 \times 10^{-2}$
$\beta_{CCA} = -0.06$
$P_{KNN-obs-sel(5)} = 2.1 \times 10^{-2}$
$\beta_{KNN-obs-sel(5)} = -0.05$
observed
imputed
estimate CCA
estimate KNN−obs−sel(5)
Model residuals of indolelactate
2
1
0
−1
−2
$n_{CCA} = 722$
$n_{KNN-obs-sel(5)} = 845$
$n_{CCA} = 556$
$n_{KNN-obs-sel(5)} = 653$
$n_{CCA} = 101$
$n_{KNN-obs-sel(5)} = 126$
major homozygote
heterozygote
minor homozygote
Genotype of SNP rs15676 (CCBL1)

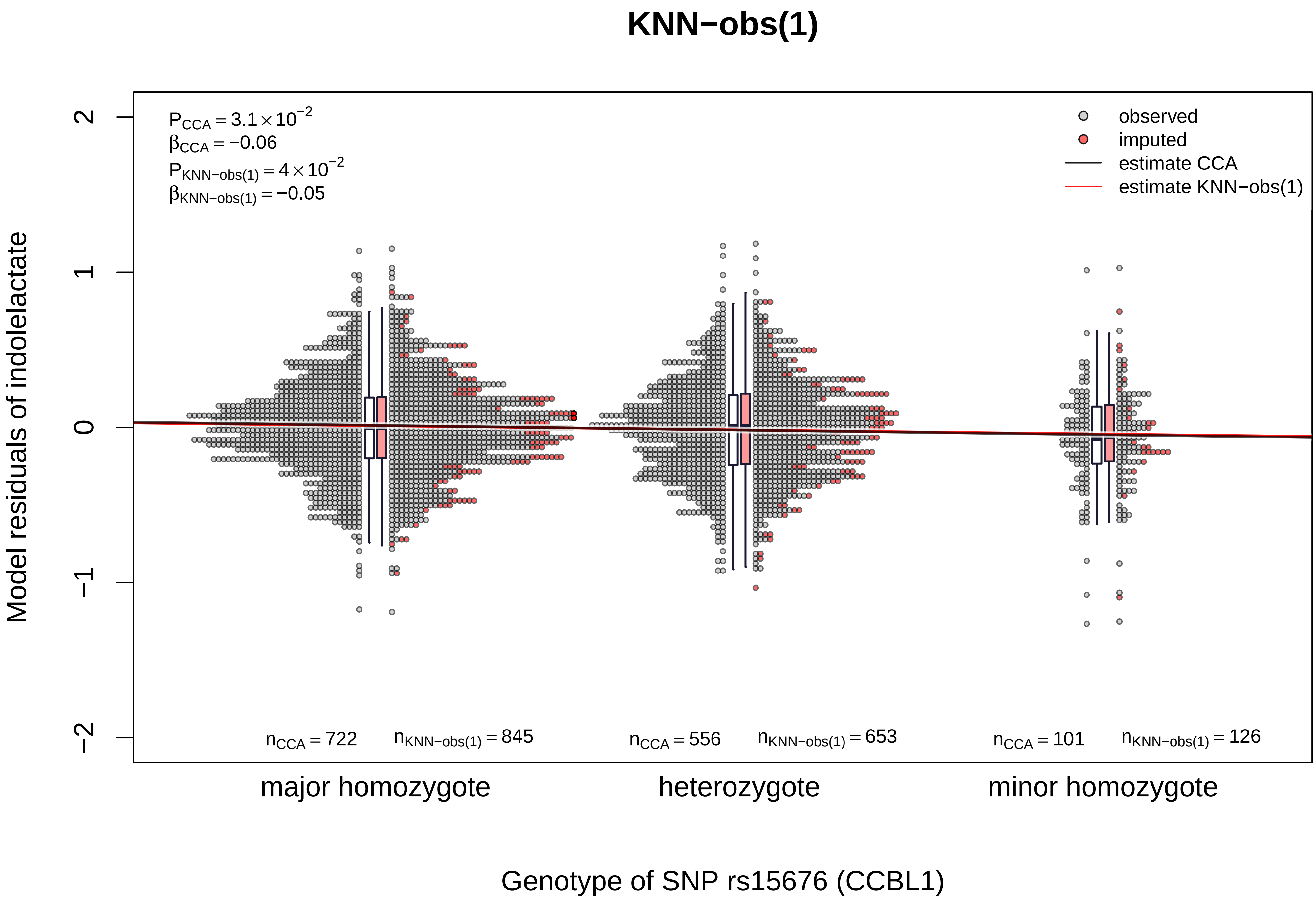

KNN−obs(1)
$P_{CCA} = 3.1 \times 10^{-2}$
$\beta_{CCA} = -0.06$
$P_{KNN-obs(1)} = 4 \times 10^{-2}$
$\beta_{KNN-obs(1)} = -0.05$
observed
imputed
estimate CCA
estimate KNN−obs(1)
Model residuals of indolelactate
2
1
0
−1
−2
$n_{CCA} = 722$
$n_{KNN-obs(1)} = 845$
$n_{CCA} = 556$
$n_{KNN-obs(1)} = 653$
$n_{CCA} = 101$
$n_{KNN-obs(1)} = 126$
major homozygote
heterozygote
minor homozygote
Genotype of SNP rs15676 (CCBL1)

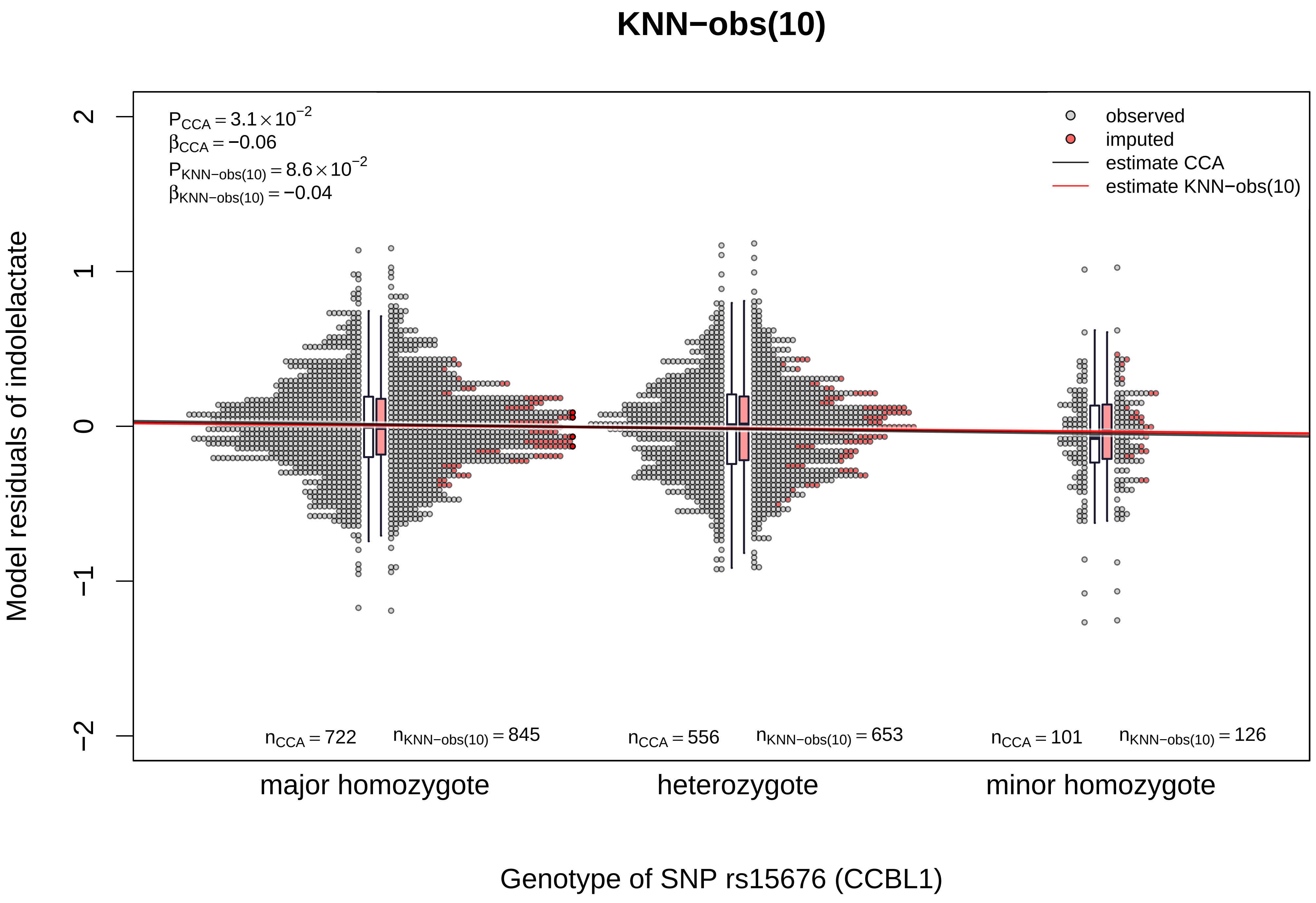
KNN−obs(10)
$P_{CCA} = 3.1 \times 10^{-2}$
$\beta_{CCA} = -0.06$
$P_{KNN-obs(10)} = 8.6 \times 10^{-2}$
$\beta_{KNN-obs(10)} = -0.04$
observed
imputed
estimate CCA
estimate KNN−obs(10)
Model residuals of indolelactate
2
1
0
−1
−2
$n_{CCA} = 722$
$n_{KNN-obs(10)} = 845$
$n_{CCA} = 556$
$n_{KNN-obs(10)} = 653$
$n_{CCA} = 101$
$n_{KNN-obs(10)} = 126$
major homozygote
heterozygote
minor homozygote
Genotype of SNP rs15676 (CCBL1)

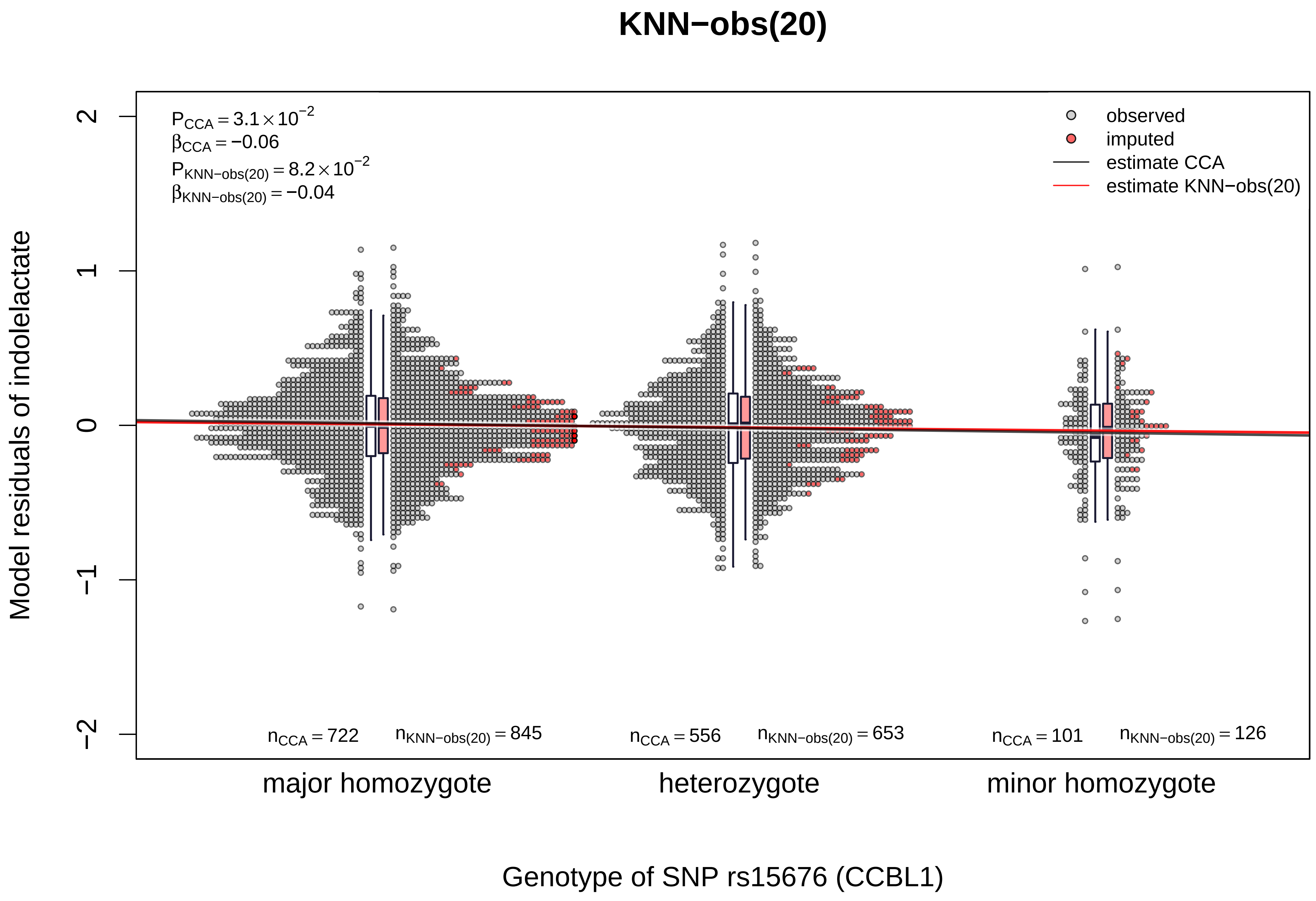

KNN−obs(20)
$P_{CCA} = 3.1 \times 10^{-2}$
$\beta_{CCA} = -0.06$
$P_{KNN-obs(20)} = 8.2 \times 10^{-2}$
$\beta_{KNN-obs(20)} = -0.04$
observed
imputed
estimate CCA
estimate KNN−obs(20)
2
1
0
−1
−2
Model residuals of indolelactate
$n_{CCA} = 722$
$n_{KNN-obs(20)} = 845$
$n_{CCA} = 556$
$n_{KNN-obs(20)} = 653$
$n_{CCA} = 101$
$n_{KNN-obs(20)} = 126$
major homozygote
heterozygote
minor homozygote
Genotype of SNP rs15676 (CCBL1)

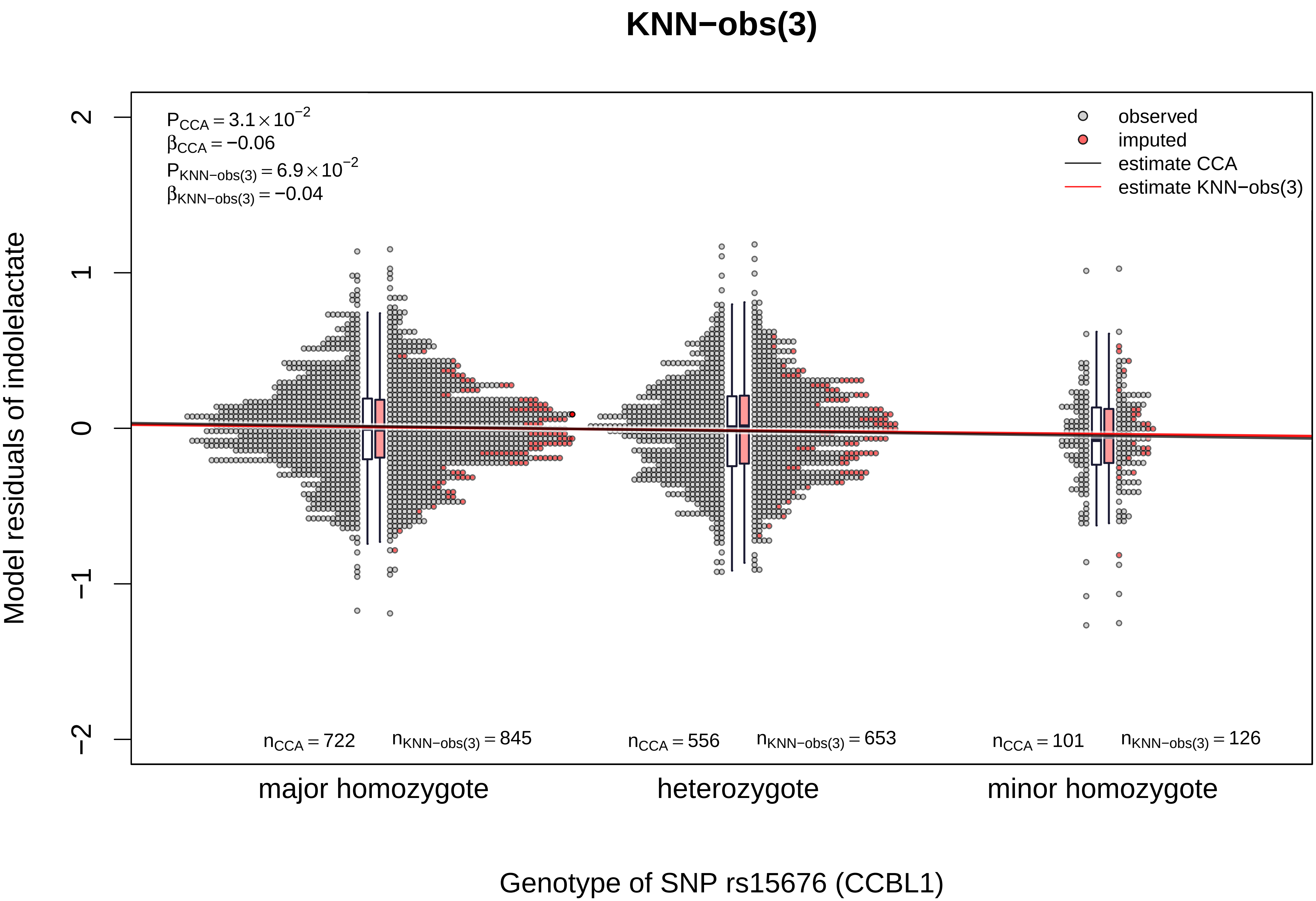
KNN−obs(3)
$P_{CCA} = 3.1 \times 10^{-2}$
$\beta_{CCA} = -0.06$
$P_{KNN-obs(3)} = 6.9 \times 10^{-2}$
$\beta_{KNN-obs(3)} = -0.04$
observed
imputed
estimate CCA
estimate KNN−obs(3)
Model residuals of indolelactate
2
1
0
−1
−2
$n_{CCA} = 722$
$n_{KNN-obs(3)} = 845$
$n_{CCA} = 556$
$n_{KNN-obs(3)} = 653$
$n_{CCA} = 101$
$n_{KNN-obs(3)} = 126$
major homozygote
heterozygote
minor homozygote
Genotype of SNP rs15676 (CCBL1)

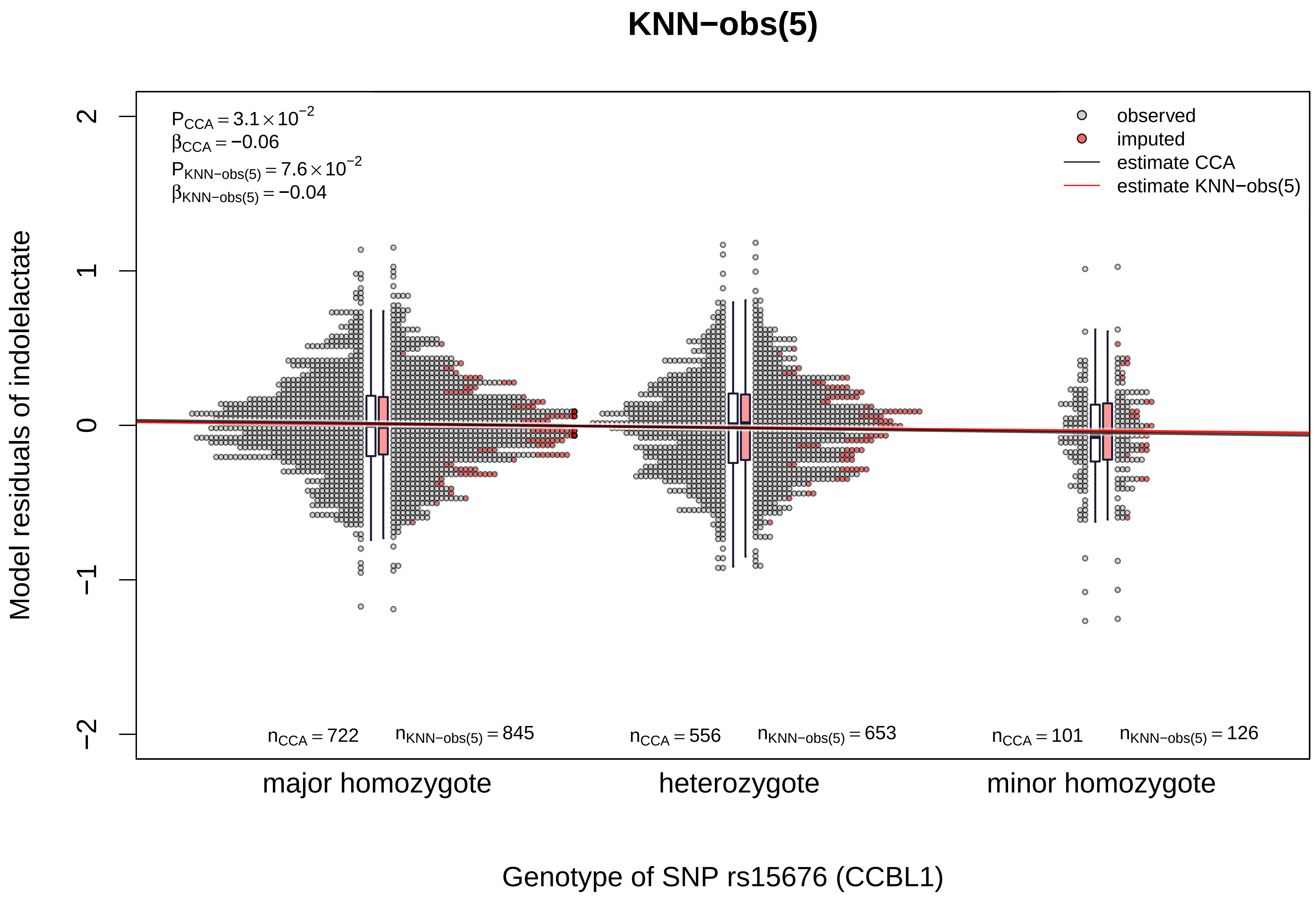

KNN−obs(5)
$P_{CCA} = 3.1 \times 10^{-2}$
$\beta_{CCA} = -0.06$
$P_{KNN-obs(5)} = 7.6 \times 10^{-2}$
$\beta_{KNN-obs(5)} = -0.04$
observed
imputed
estimate CCA
estimate KNN−obs(5)
Model residuals of indolelactate
2
1
0
−1
−2
$n_{CCA} = 722$
$n_{KNN-obs(5)} = 845$
$n_{CCA} = 556$
$n_{KNN-obs(5)} = 653$
$n_{CCA} = 101$
$n_{KNN-obs(5)} = 126$
major homozygote
heterozygote
minor homozygote
Genotype of SNP rs15676 (CCBL1)

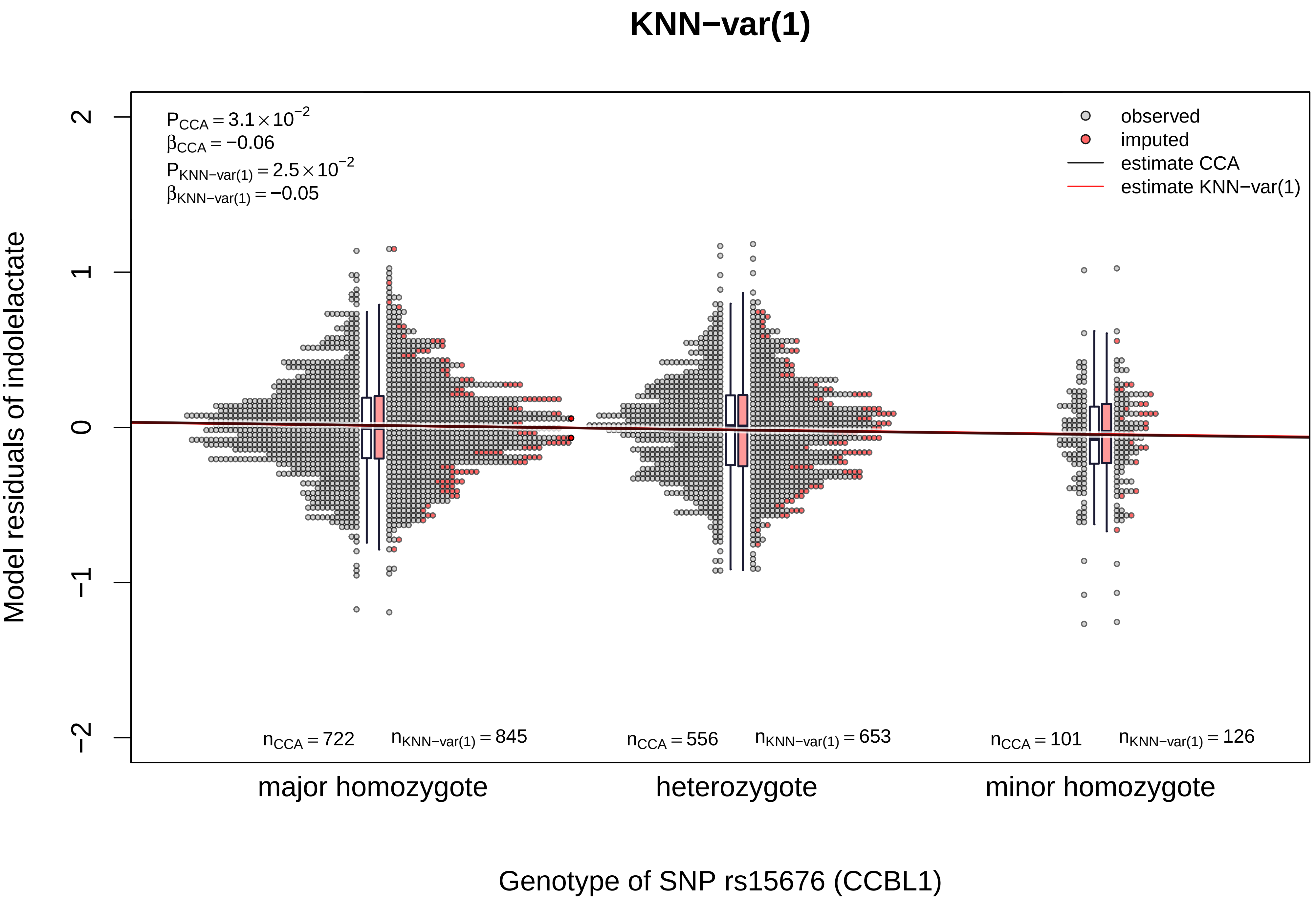

KNN−var(1)
$P_{CCA} = 3.1 \times 10^{-2}$
$\beta_{CCA} = -0.06$
$P_{KNN-var(1)} = 2.5 \times 10^{-2}$
$\beta_{KNN-var(1)} = -0.05$
observed
imputed
estimate CCA
estimate KNN−var(1)
Model residuals of indolelactate
2
1
0
−1
−2
$n_{CCA} = 722$
$n_{KNN-var(1)} = 845$
$n_{CCA} = 556$
$n_{KNN-var(1)} = 653$
$n_{CCA} = 101$
$n_{KNN-var(1)} = 126$
major homozygote
heterozygote
minor homozygote
Genotype of SNP rs15676 (CCBL1)

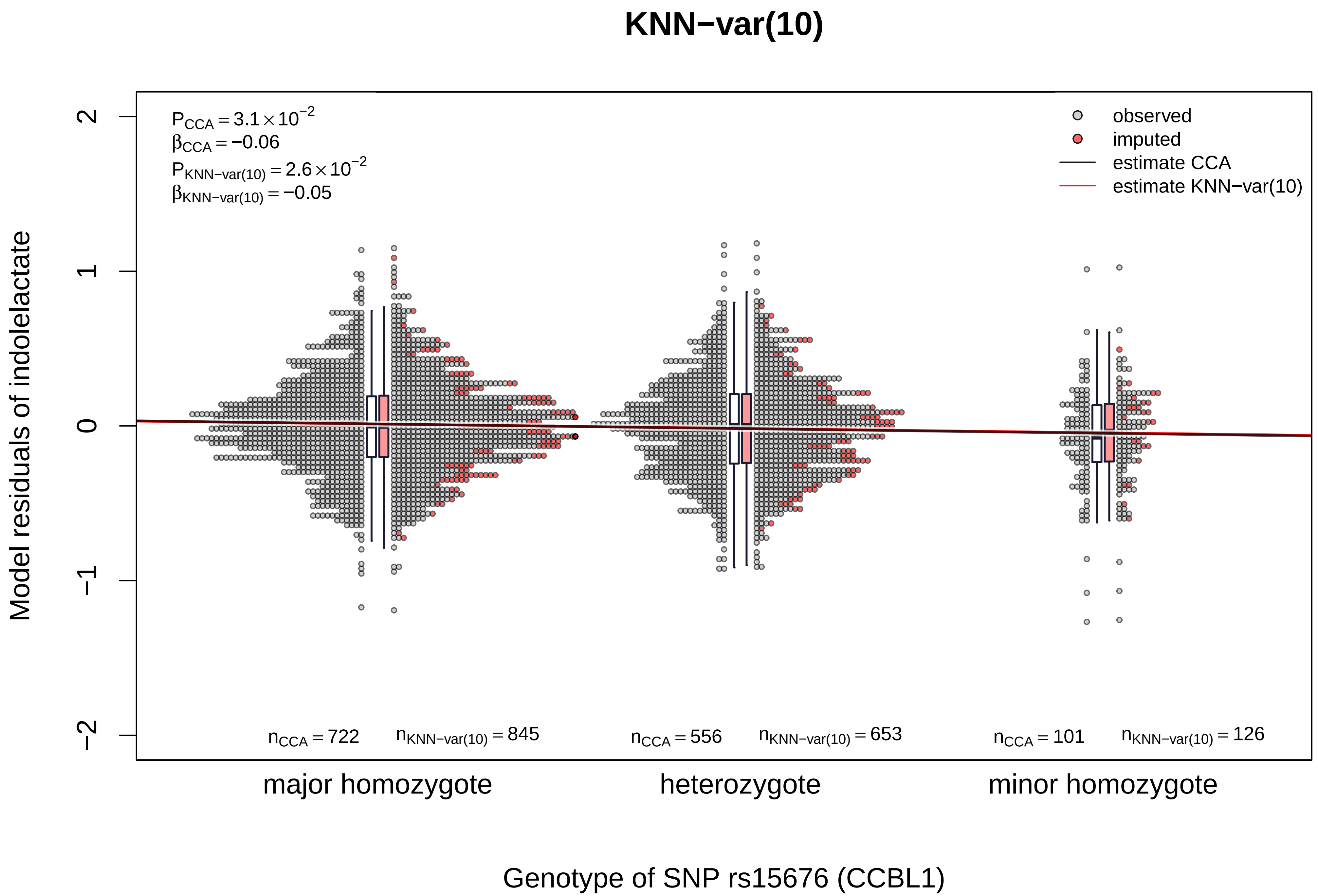

KNN−var(10)
$P_{CCA} = 3.1 \times 10^{-2}$
$\beta_{CCA} = -0.06$
$P_{KNN-var(10)} = 2.6 \times 10^{-2}$
$\beta_{KNN-var(10)} = -0.05$
observed
imputed
estimate CCA
estimate KNN−var(10)
2
1
0
−1
−2
Model residuals of indolelactate
$n_{CCA} = 722$
$n_{KNN-var(10)} = 845$
$n_{CCA} = 556$
$n_{KNN-var(10)} = 653$
$n_{CCA} = 101$
$n_{KNN-var(10)} = 126$
major homozygote
heterozygote
minor homozygote
Genotype of SNP rs15676 (CCBL1)

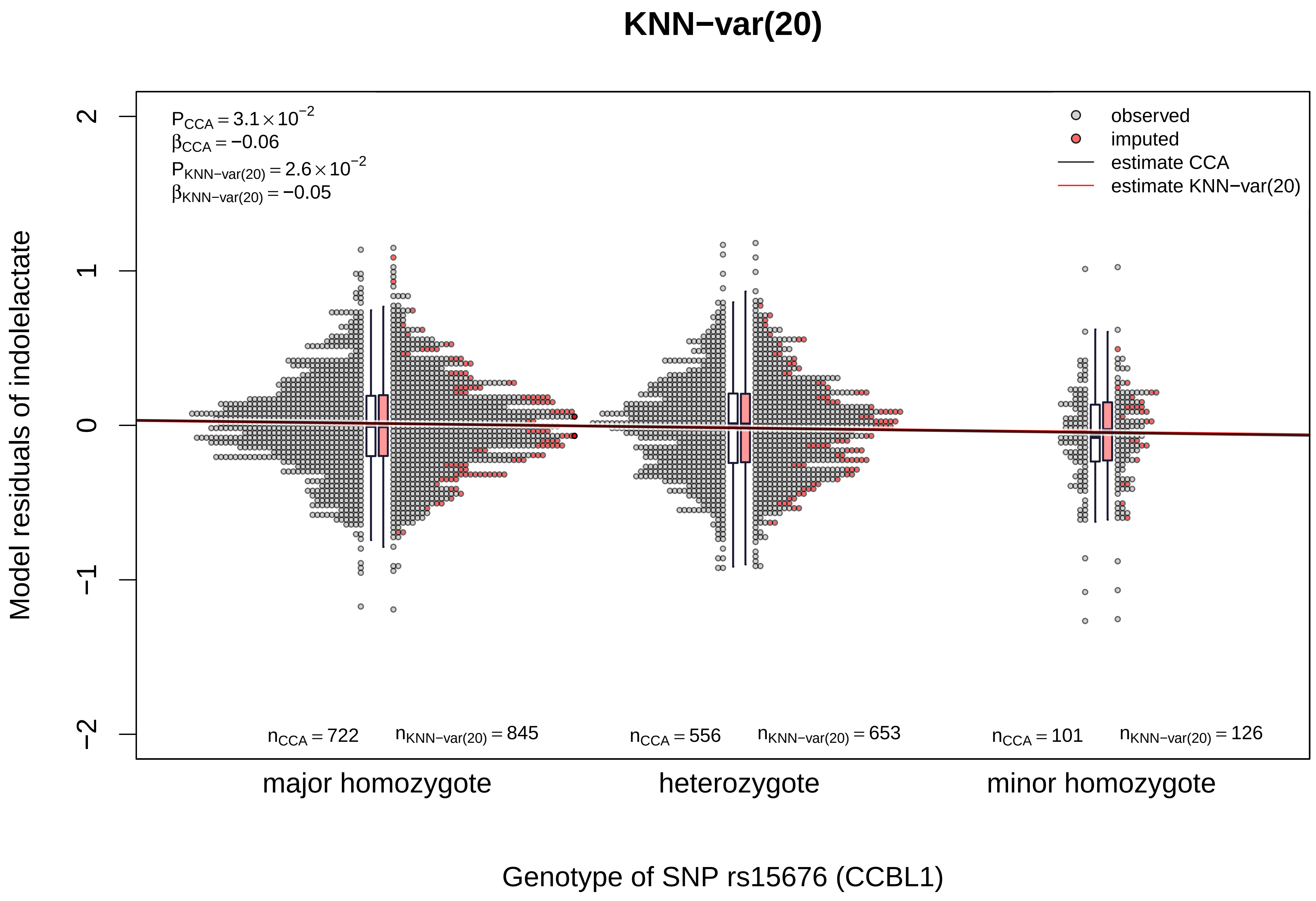

KNN−var(20)
$P_{CCA} = 3.1 \times 10^{-2}$
$\beta_{CCA} = -0.06$
$P_{KNN-var(20)} = 2.6 \times 10^{-2}$
$\beta_{KNN-var(20)} = -0.05$
observed
imputed
estimate CCA
estimate KNN−var(20)
Model residuals of indolelactate
2
1
0
−1
−2
$n_{CCA} = 722$
$n_{KNN-var(20)} = 845$
$n_{CCA} = 556$
$n_{KNN-var(20)} = 653$
$n_{CCA} = 101$
$n_{KNN-var(20)} = 126$
major homozygote
heterozygote
minor homozygote
Genotype of SNP rs15676 (CCBL1)

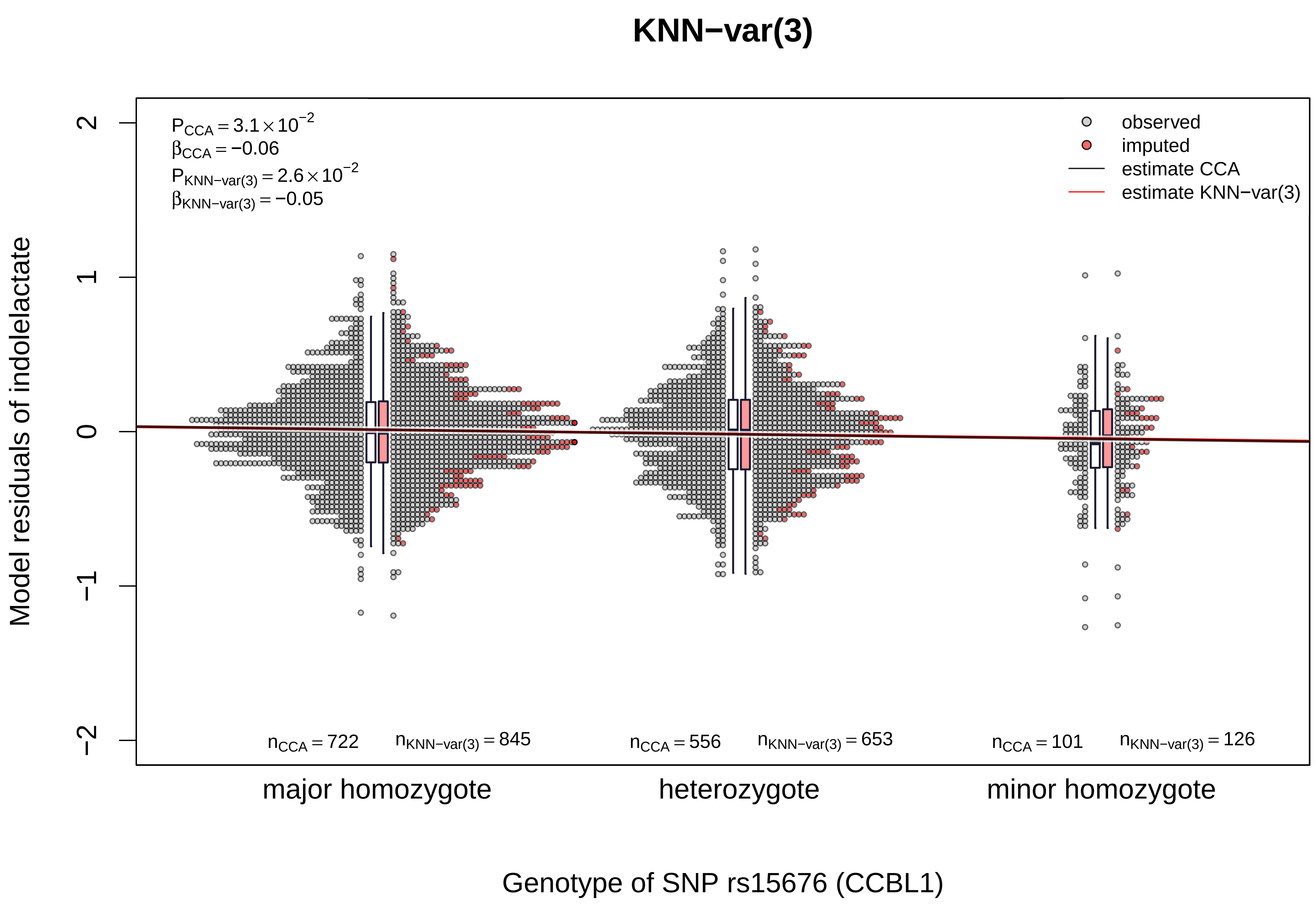

KNN−var(3)
$P_{CCA} = 3.1 \times 10^{-2}$
$\beta_{CCA} = -0.06$
$P_{KNN-var(3)} = 2.6 \times 10^{-2}$
$\beta_{KNN-var(3)} = -0.05$
observed
imputed
estimate CCA
estimate KNN−var(3)
Model residuals of indolelactate
2
1
0
−1
−2
$n_{CCA} = 722$
$n_{KNN-var(3)} = 845$
$n_{CCA} = 556$
$n_{KNN-var(3)} = 653$
$n_{CCA} = 101$
$n_{KNN-var(3)} = 126$
major homozygote
heterozygote
minor homozygote
Genotype of SNP rs15676 (CCBL1)

KNN−var(5)

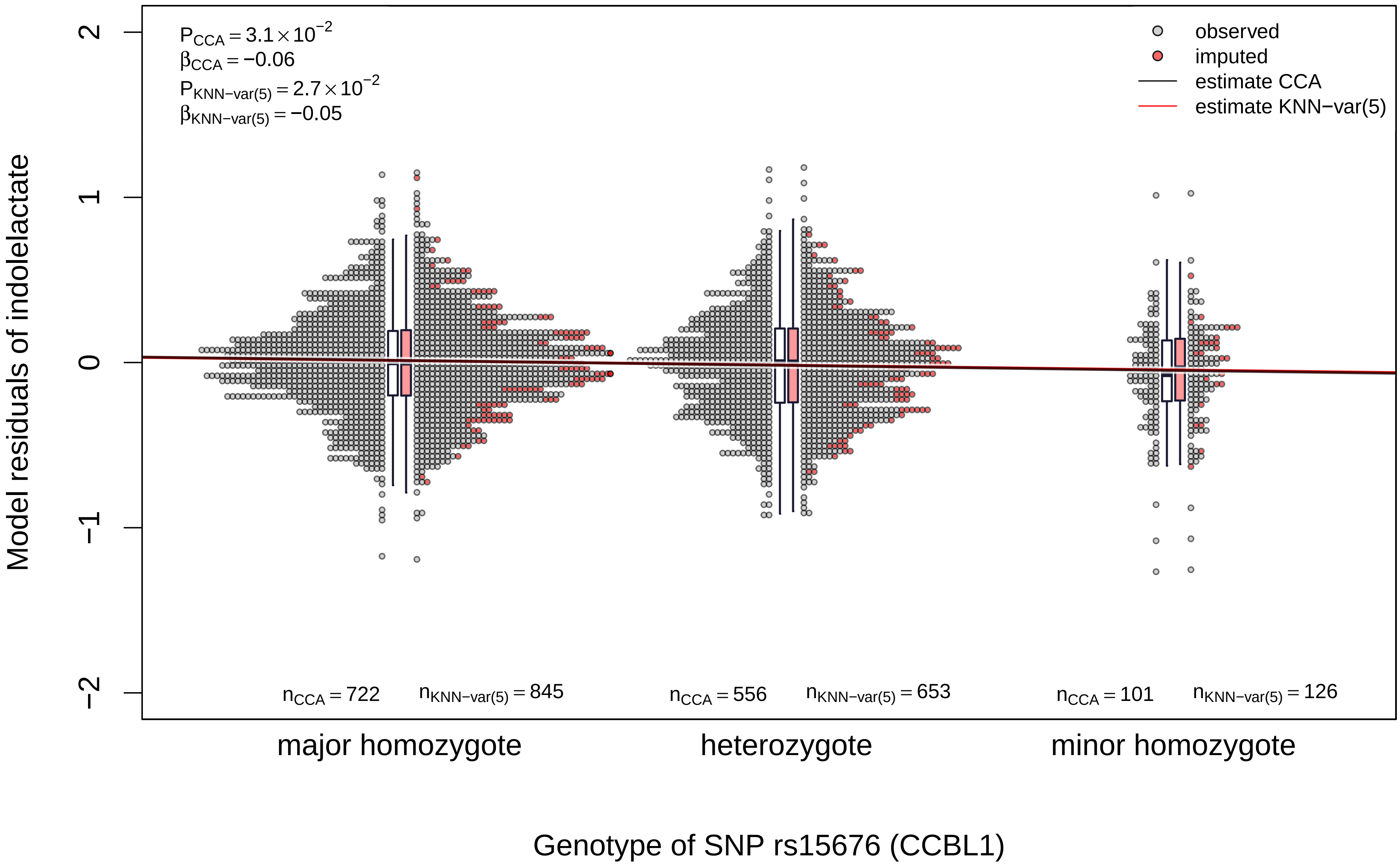

$P_{CCA} = 3.1 \times 10^{-2}$
$\beta_{CCA} = -0.06$
$P_{KNN-var(5)} = 2.7 \times 10^{-2}$
$\beta_{KNN-var(5)} = -0.05$
observed
imputed
estimate CCA
estimate KNN−var(5)
Model residuals of indolelactate
2
1
0
−1
−2
$n_{CCA} = 722$
$n_{KNN-var(5)} = 845$
$n_{CCA} = 556$
$n_{KNN-var(5)} = 653$
$n_{CCA} = 101$
$n_{KNN-var(5)} = 126$
major homozygote
heterozygote
minor homozygote
Genotype of SNP rs15676 (CCBL1)

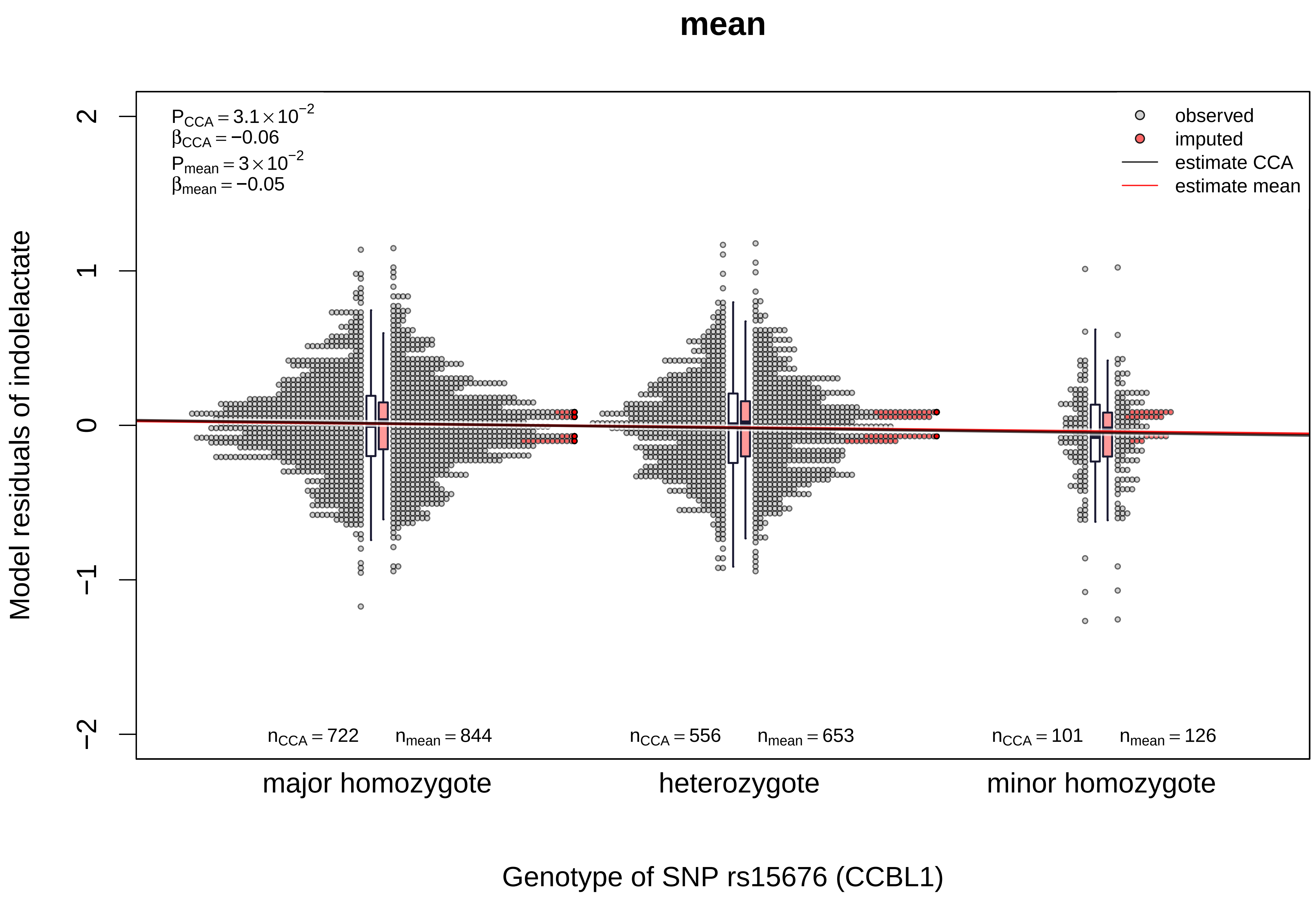

mean
$P_{CCA} = 3.1 \times 10^{-2}$
$\beta_{CCA} = -0.06$
$P_{mean} = 3 \times 10^{-2}$
$\beta_{mean} = -0.05$
observed
imputed
estimate CCA
estimate mean
Model residuals of indolelactate
2
1
0
−1
−2
$n_{CCA} = 722$
$n_{mean} = 844$
$n_{CCA} = 556$
$n_{mean} = 653$
$n_{CCA} = 101$
$n_{mean} = 126$
major homozygote
heterozygote
minor homozygote
Genotype of SNP rs15676 (CCBL1)

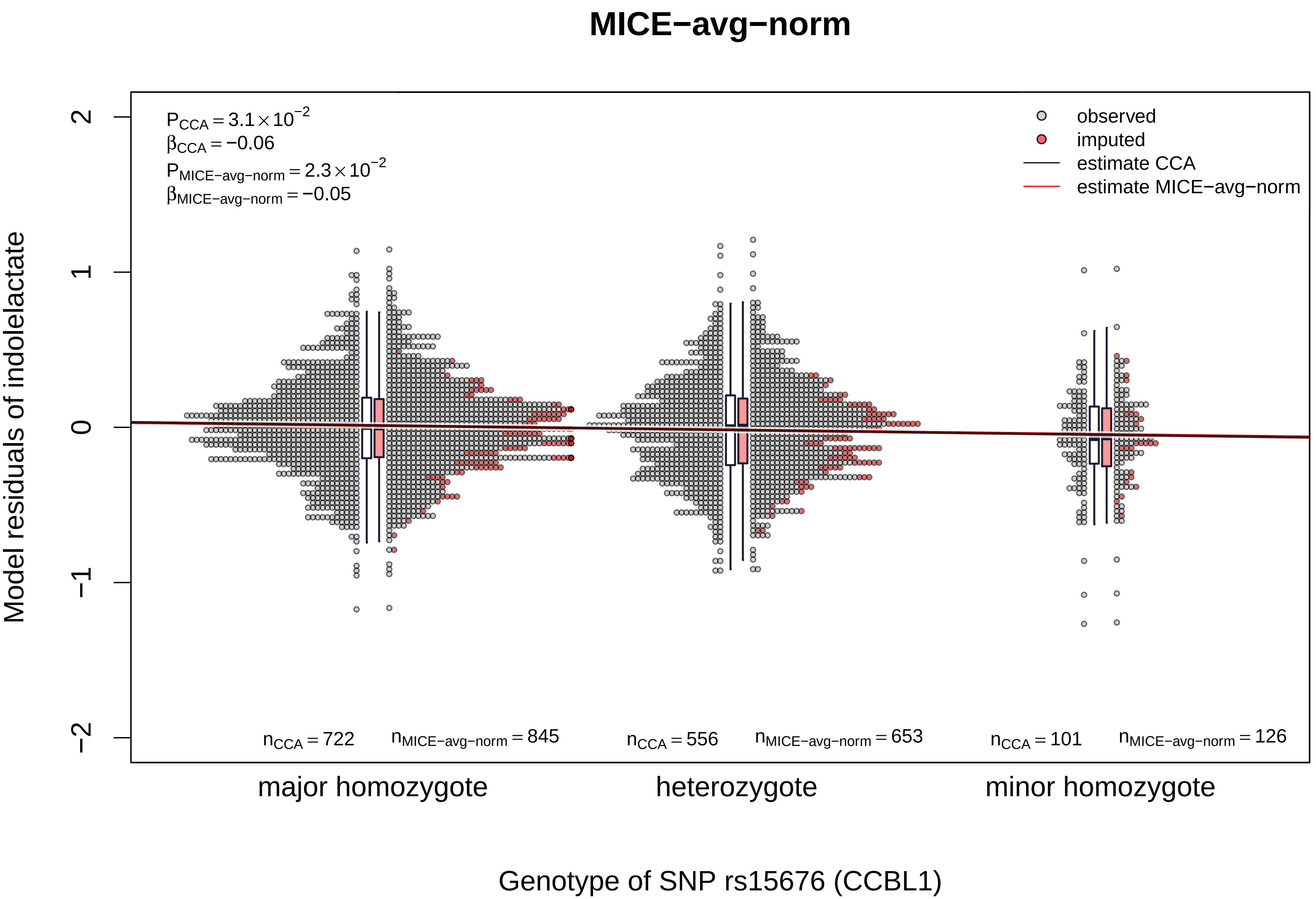

MICE−avg−norm
$P_{CCA} = 3.1 \times 10^{-2}$
$\beta_{CCA} = -0.06$
$P_{MICE-avg-norm} = 2.3 \times 10^{-2}$
$\beta_{MICE-avg-norm} = -0.05$
observed
imputed
estimate CCA
estimate MICE−avg−norm
Model residuals of indolelactate
2
1
0
−1
−2
$n_{CCA} = 722$
$n_{MICE-avg-norm} = 845$
$n_{CCA} = 556$
$n_{MICE-avg-norm} = 653$
$n_{CCA} = 101$
$n_{MICE-avg-norm} = 126$
major homozygote
heterozygote
minor homozygote
Genotype of SNP rs15676 (CCBL1)

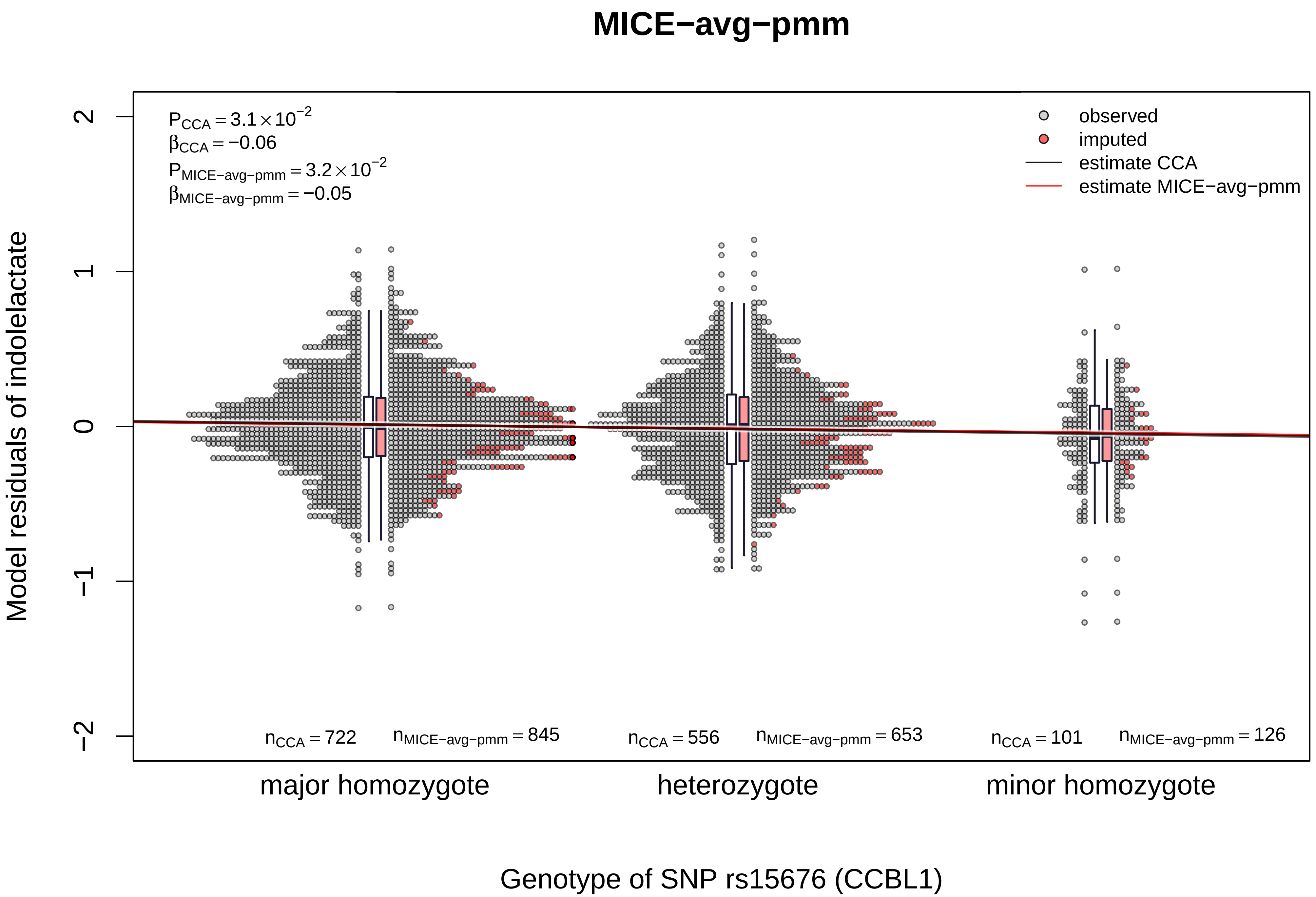

MICE−avg−pmm
$P_{CCA} = 3.1 \times 10^{-2}$
$\beta_{CCA} = -0.06$
$P_{MICE-avg-pmm} = 3.2 \times 10^{-2}$
$\beta_{MICE-avg-pmm} = -0.05$
observed
imputed
estimate CCA
estimate MICE−avg−pmm
Model residuals of indolelactate
2
1
0
−1
−2
$n_{CCA} = 722$
$n_{MICE-avg-pmm} = 845$
$n_{CCA} = 556$
$n_{MICE-avg-pmm} = 653$
$n_{CCA} = 101$
$n_{MICE-avg-pmm} = 126$
major homozygote
heterozygote
minor homozygote
Genotype of SNP rs15676 (CCBL1)

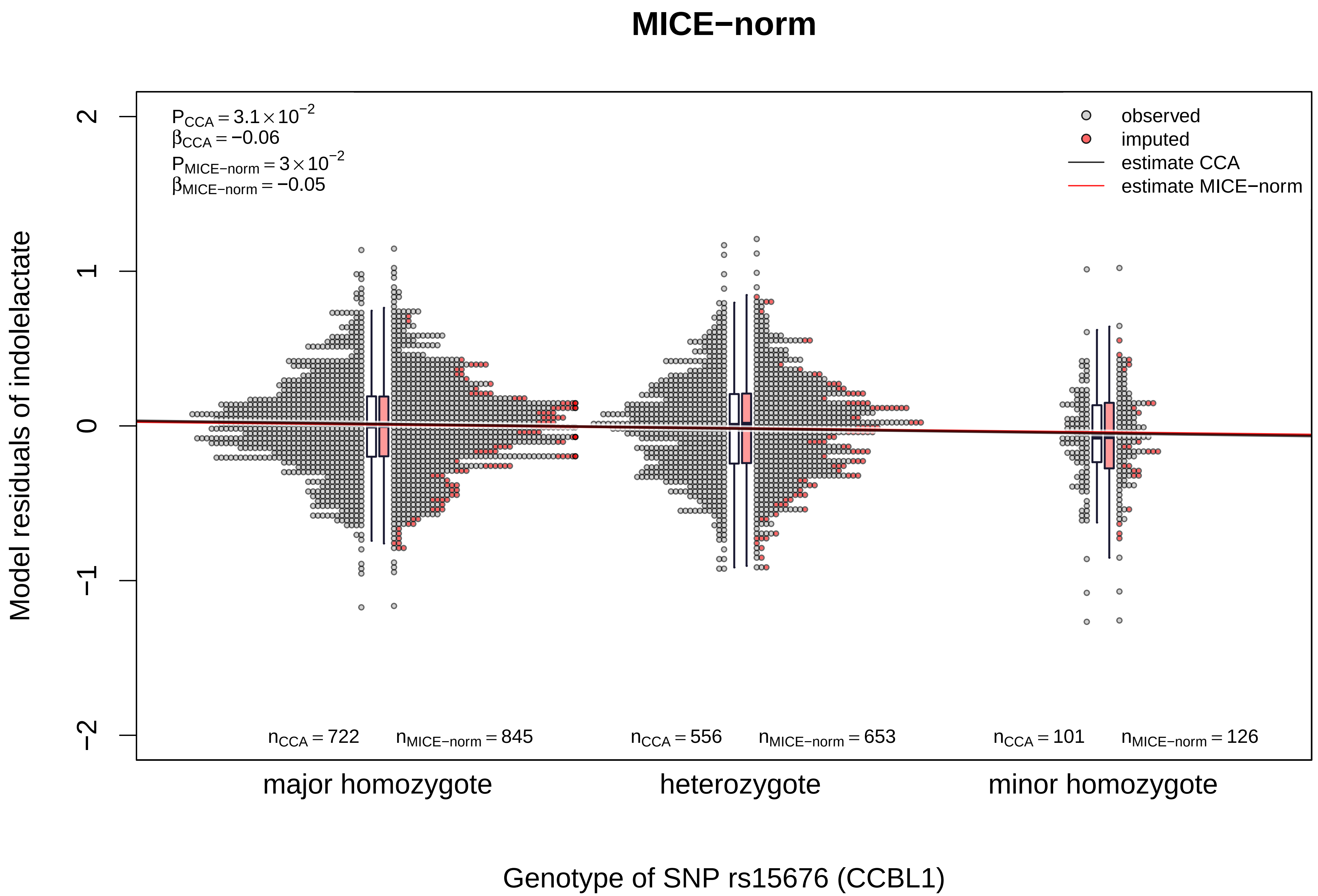
MICE−norm
$P_{CCA} = 3.1 \times 10^{-2}$
$\beta_{CCA} = -0.06$
$P_{MICE-norm} = 3 \times 10^{-2}$
$\beta_{MICE-norm} = -0.05$
observed
imputed
estimate CCA
estimate MICE−norm
Model residuals of indolelactate
2
1
0
−1
−2
$n_{CCA} = 722$
$n_{MICE-norm} = 845$
$n_{CCA} = 556$
$n_{MICE-norm} = 653$
$n_{CCA} = 101$
$n_{MICE-norm} = 126$
major homozygote
heterozygote
minor homozygote
Genotype of SNP rs15676 (CCBL1)

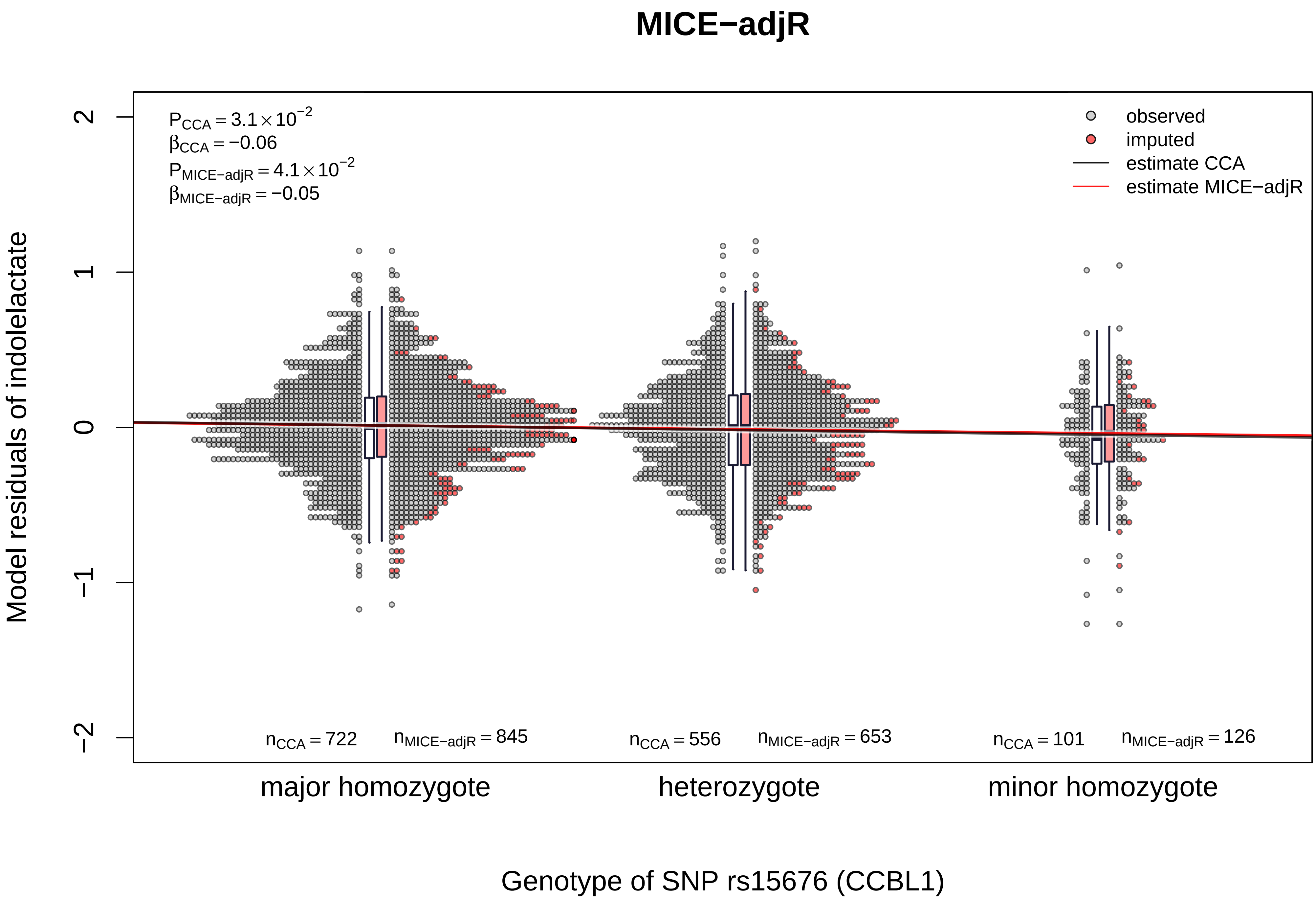

MICE−adjR
$P_{CCA} = 3.1 \times 10^{-2}$
$\beta_{CCA} = -0.06$
$P_{MICE-adjR} = 4.1 \times 10^{-2}$
$\beta_{MICE-adjR} = -0.05$
observed
imputed
estimate CCA
estimate MICE−adjR
Model residuals of indolelactate
2
1
0
−1
−2
$n_{CCA} = 722$
$n_{MICE-adjR} = 845$
$n_{CCA} = 556$
$n_{MICE-adjR} = 653$
$n_{CCA} = 101$
$n_{MICE-adjR} = 126$
major homozygote
heterozygote
minor homozygote
Genotype of SNP rs15676 (CCBL1)

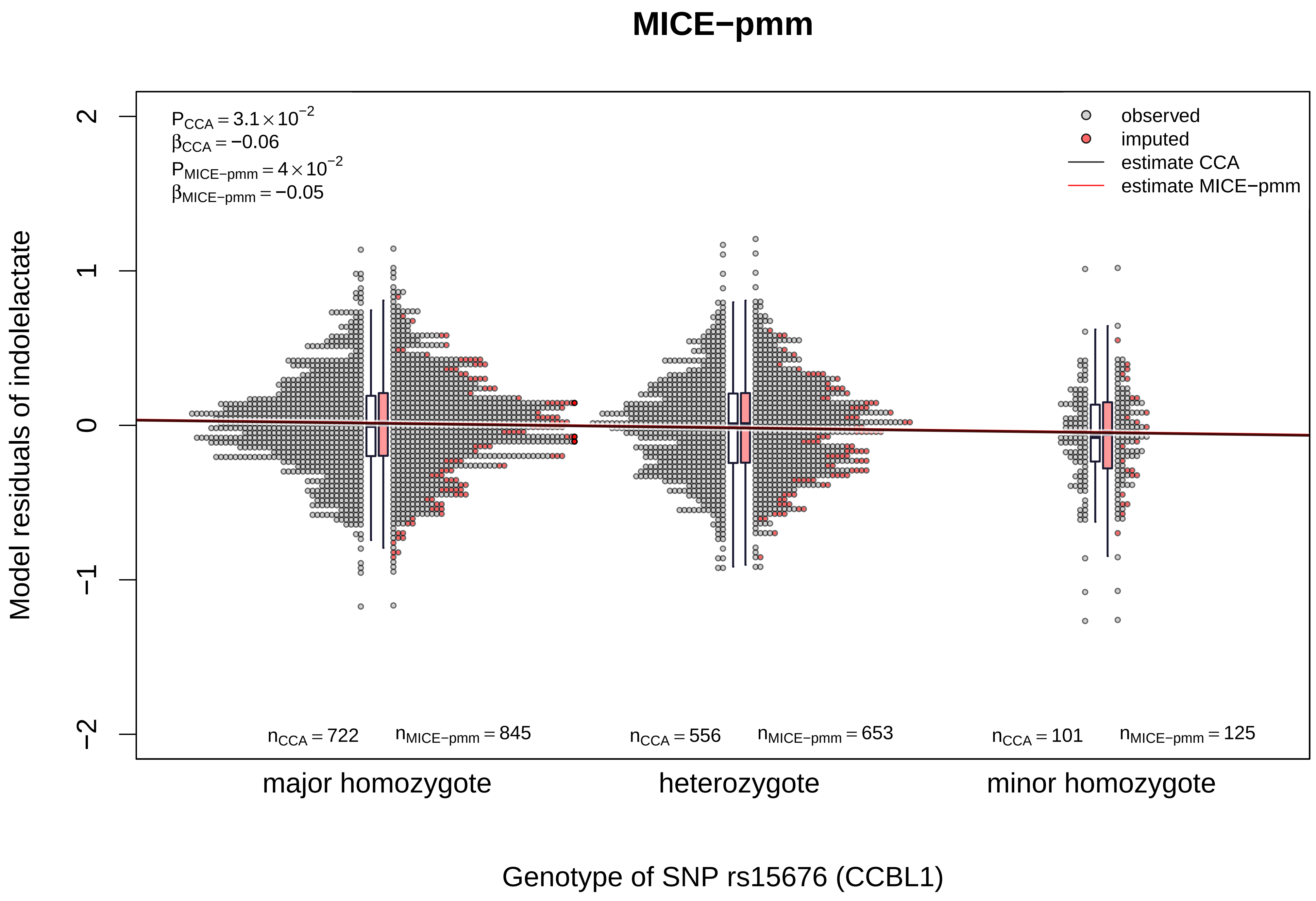

MICE−pmm
$P_{CCA} = 3.1 \times 10^{-2}$
$\beta_{CCA} = -0.06$
$P_{MICE-pmm} = 4 \times 10^{-2}$
$\beta_{MICE-pmm} = -0.05$
observed
imputed
estimate CCA
estimate MICE−pmm
Model residuals of indolelactate
2
1
0
−1
−2
$n_{CCA} = 722$
$n_{MICE-pmm} = 845$
$n_{CCA} = 556$
$n_{MICE-pmm} = 653$
$n_{CCA} = 101$
$n_{MICE-pmm} = 125$
major homozygote
heterozygote
minor homozygote
Genotype of SNP rs15676 (CCBL1)

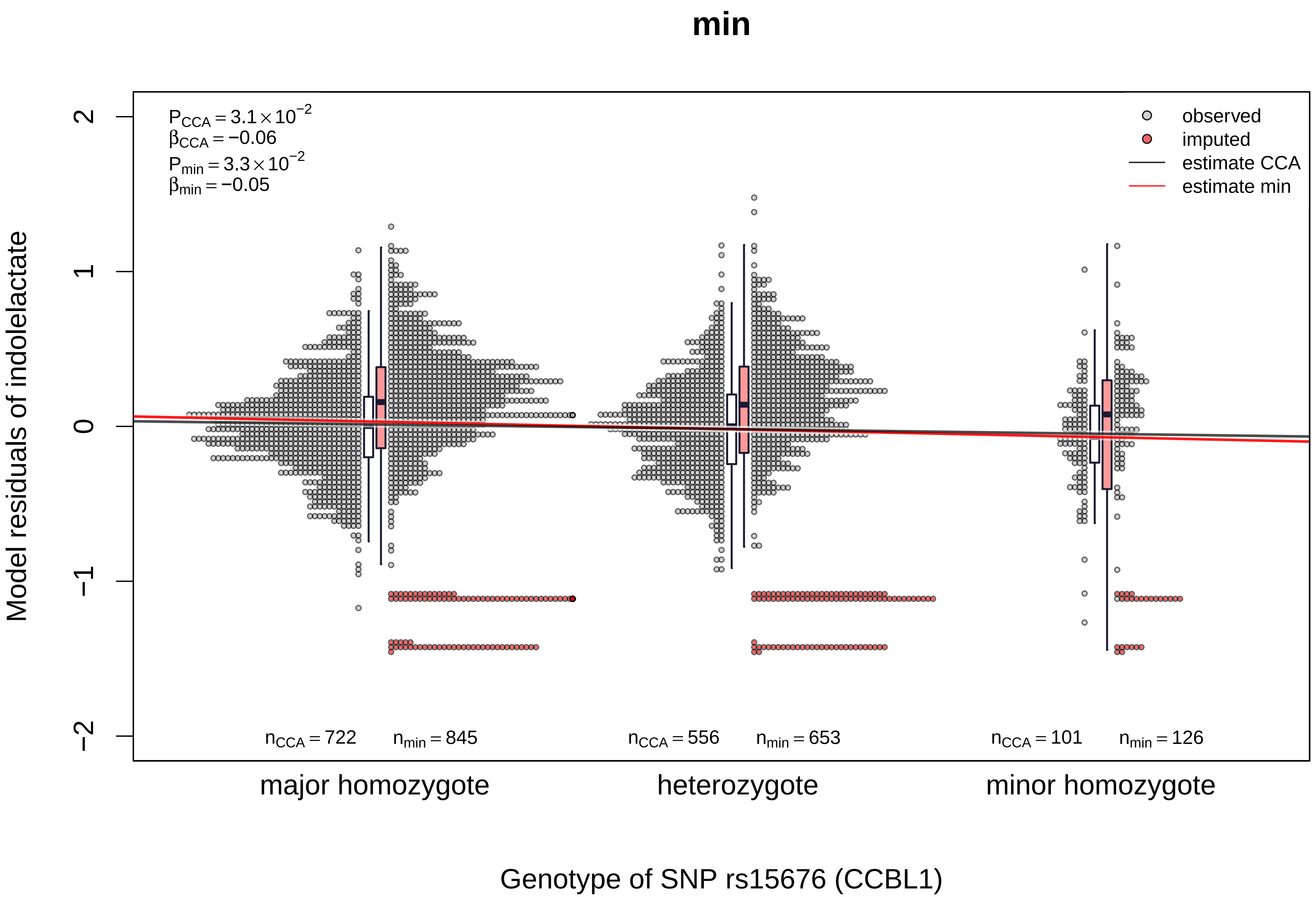

min
$P_{CCA} = 3.1 \times 10^{-2}$
$\beta_{CCA} = -0.06$
$P_{min} = 3.3 \times 10^{-2}$
$\beta_{min} = -0.05$
observed
imputed
estimate CCA
estimate min
Model residuals of indolelactate
2
1
0
−1
−2
$n_{CCA} = 722$
$n_{min} = 845$
$n_{CCA} = 556$
$n_{min} = 653$
$n_{CCA} = 101$
$n_{min} = 126$
major homozygote
heterozygote
minor homozygote
Genotype of SNP rs15676 (CCBL1)

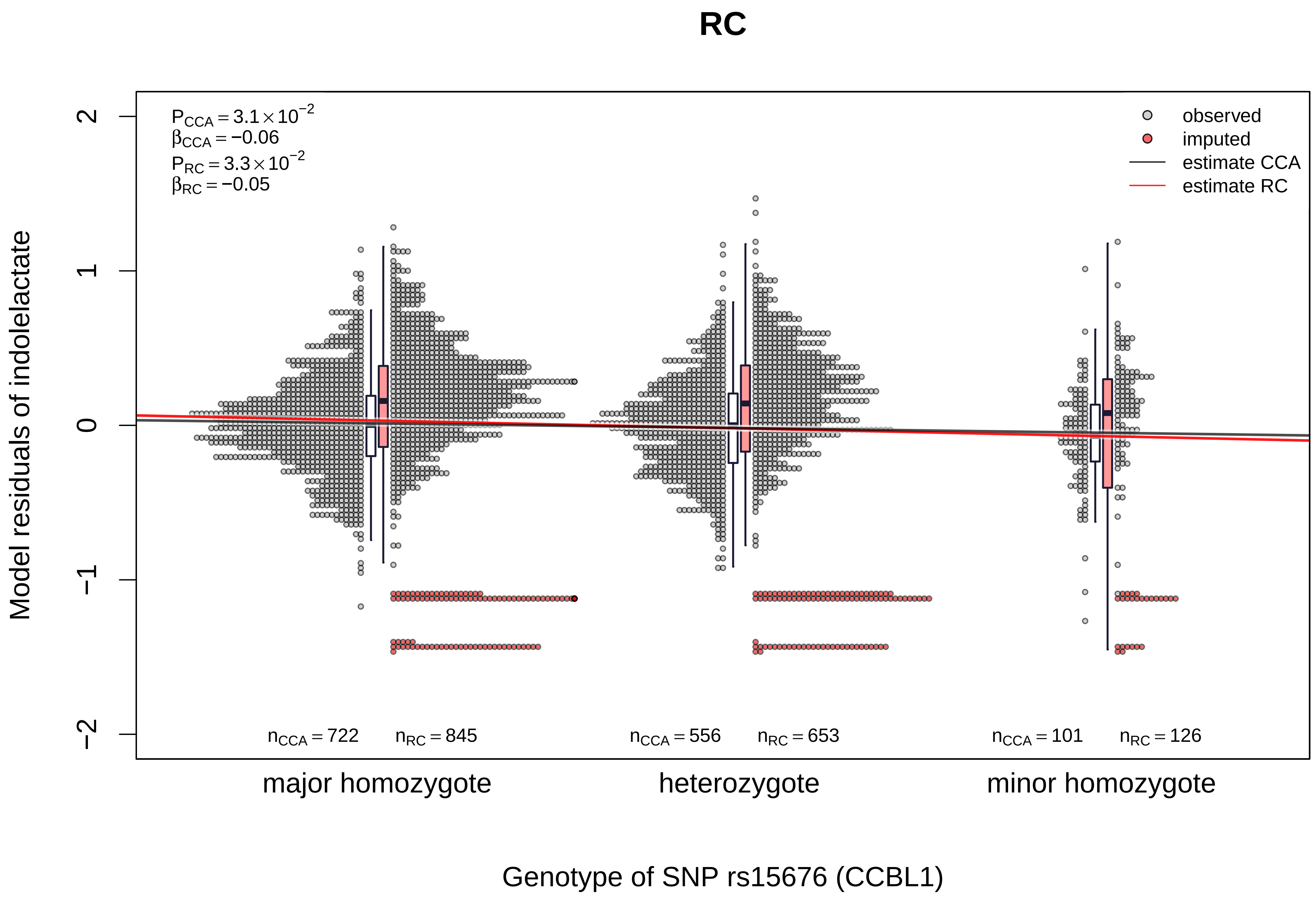

RC
$P_{CCA} = 3.1 \times 10^{-2}$
$\beta_{CCA} = -0.06$
$P_{RC} = 3.3 \times 10^{-2}$
$\beta_{RC} = -0.05$
observed
imputed
estimate CCA
estimate RC
Model residuals of indolelactate
2
1
0
−1
−2
$n_{CCA} = 722$
$n_{RC} = 845$
$n_{CCA} = 556$
$n_{RC} = 653$
$n_{CCA} = 101$
$n_{RC} = 126$
major homozygote
heterozygote
minor homozygote
Genotype of SNP rs15676 (CCBL1)

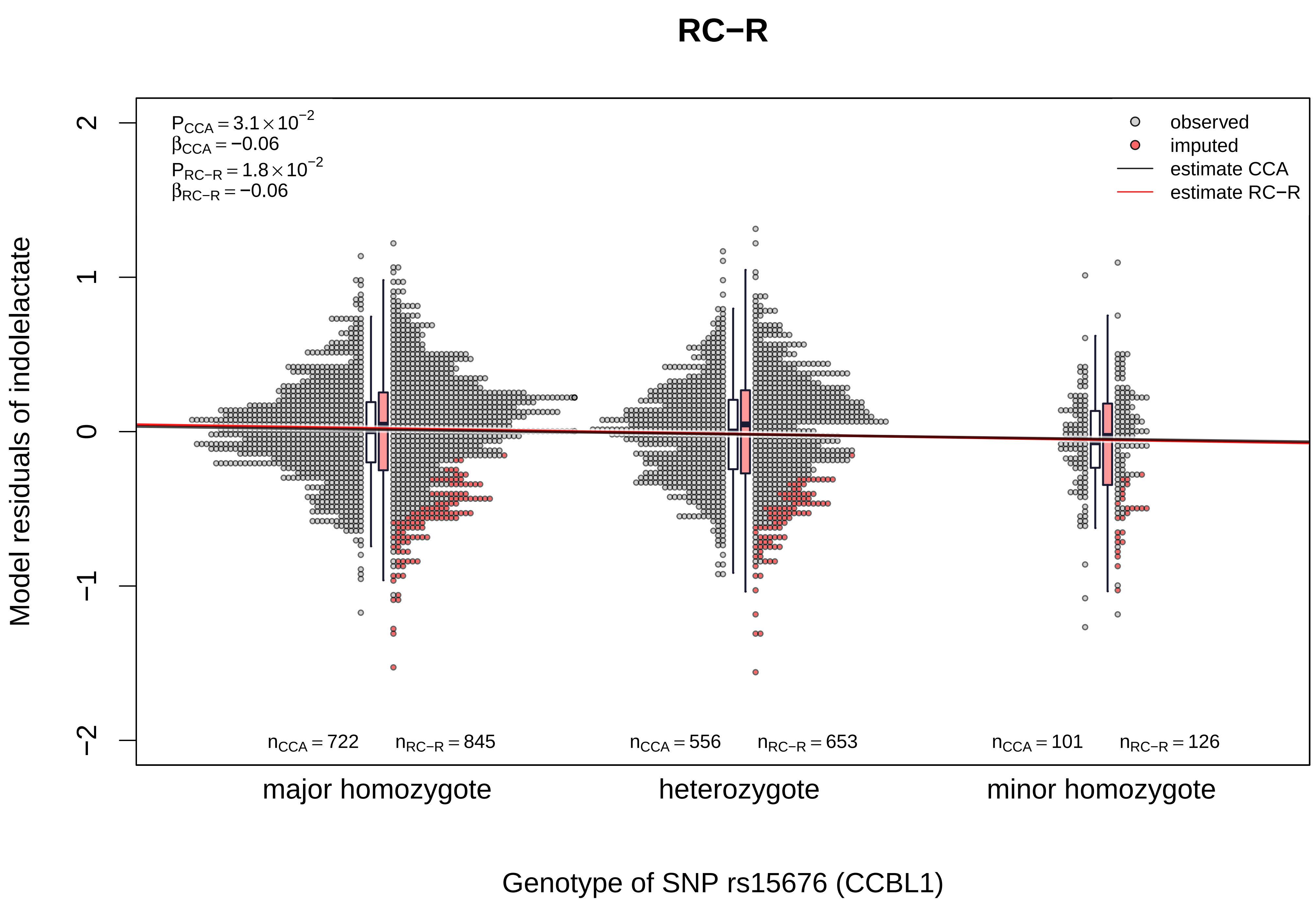

RC−R
$P_{CCA} = 3.1 \times 10^{-2}$
$\beta_{CCA} = -0.06$
$P_{RC-R} = 1.8 \times 10^{-2}$
$\beta_{RC-R} = -0.06$
observed
imputed
estimate CCA
estimate RC−R
Model residuals of indolelactate
2
1
0
−1
−2
$n_{CCA} = 722$
$n_{RC-R} = 845$
$n_{CCA} = 556$
$n_{RC-R} = 653$
$n_{CCA} = 101$
$n_{RC-R} = 126$
major homozygote
heterozygote
minor homozygote
Genotype of SNP rs15676 (CCBL1)

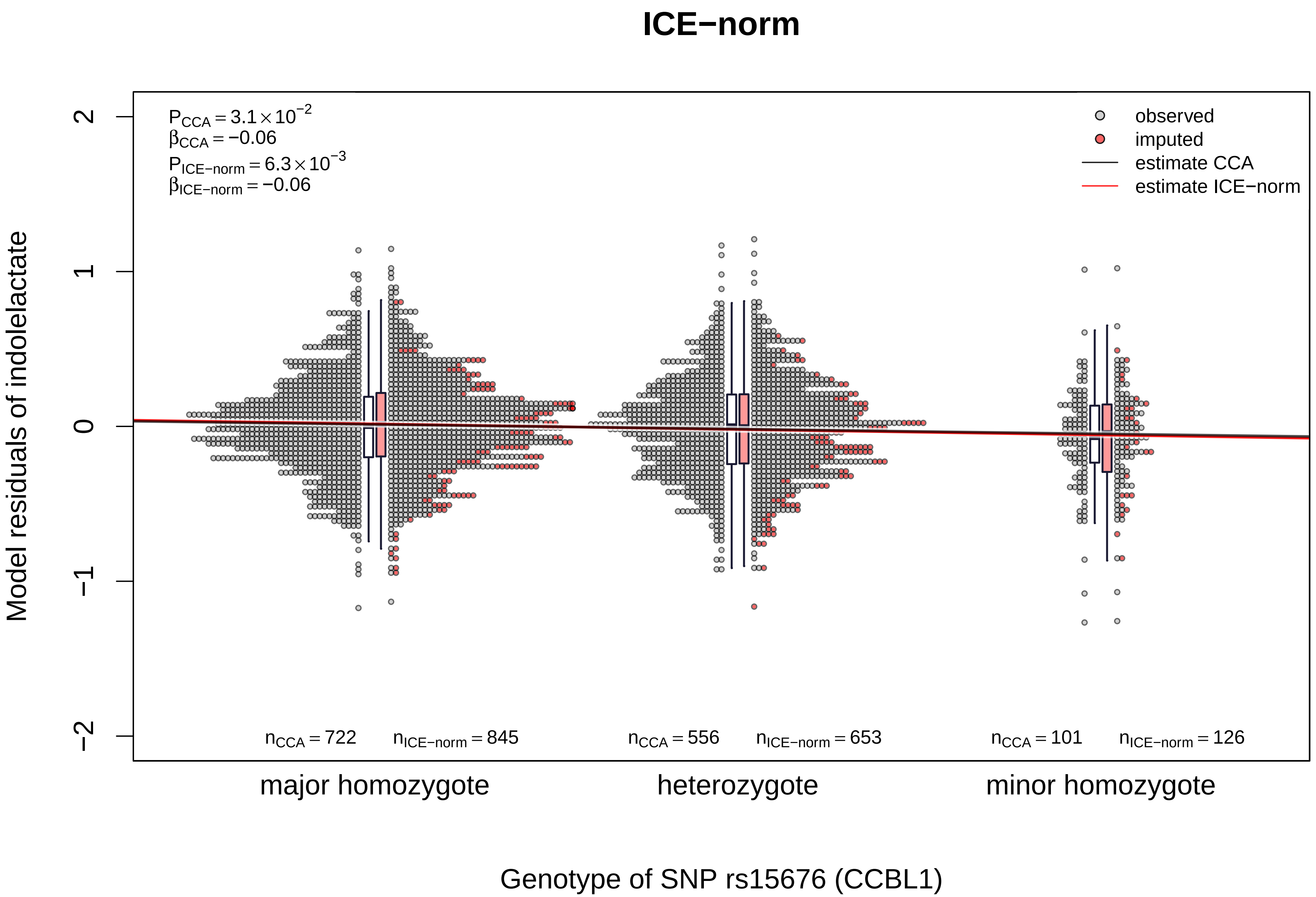

ICE−norm
$P_{CCA} = 3.1 \times 10^{-2}$
$\beta_{CCA} = -0.06$
$P_{ICE-norm} = 6.3 \times 10^{-3}$
$\beta_{ICE-norm} = -0.06$
observed
imputed
estimate CCA
estimate ICE−norm
Model residuals of indolelactate
2
1
0
−1
−2
$n_{CCA} = 722$
$n_{ICE-norm} = 845$
$n_{CCA} = 556$
$n_{ICE-norm} = 653$
$n_{CCA} = 101$
$n_{ICE-norm} = 126$
major homozygote
heterozygote
minor homozygote
Genotype of SNP rs15676 (CCBL1)

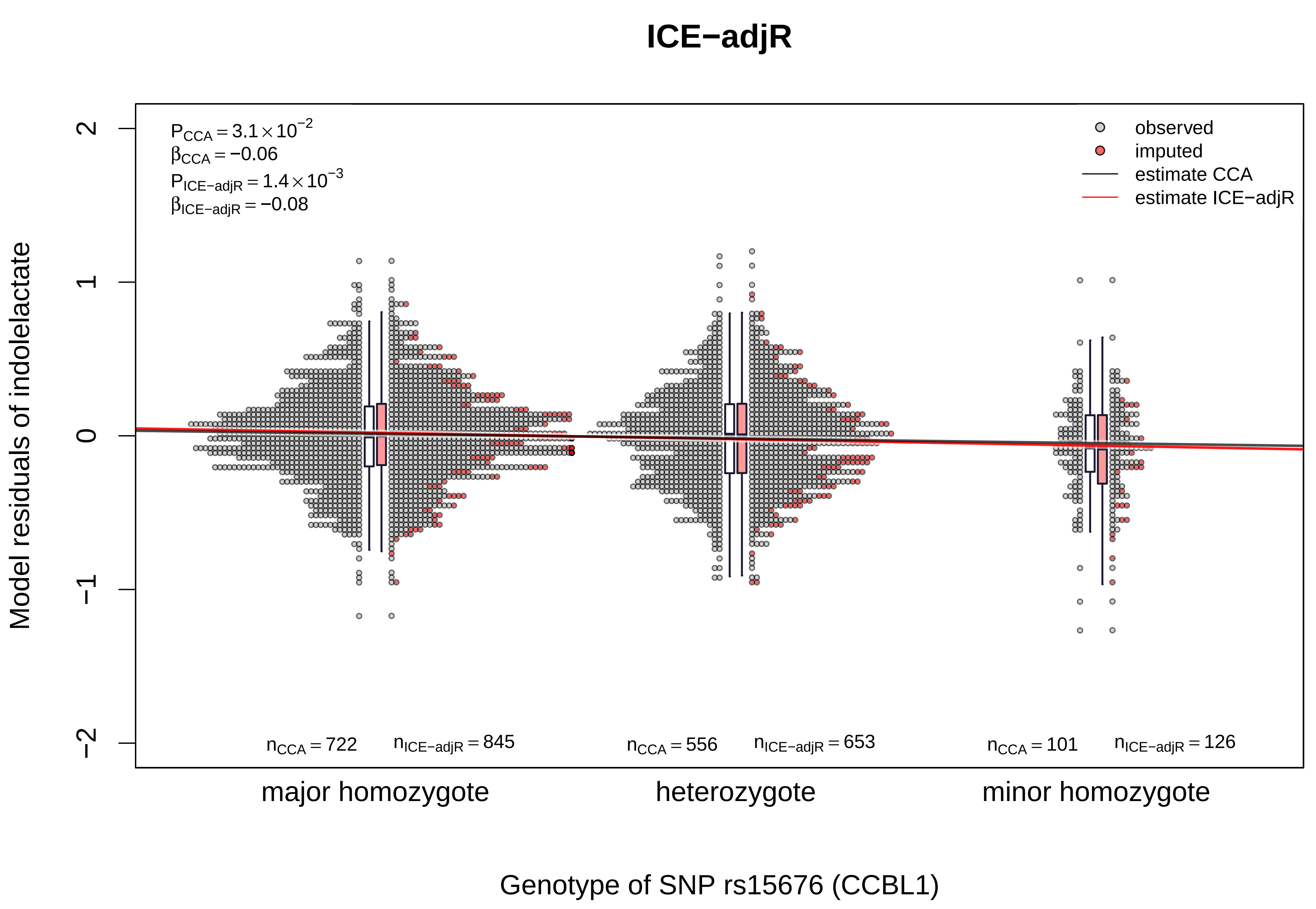
ICE−adjR
$P_{CCA} = 3.1 \times 10^{-2}$
$\beta_{CCA} = -0.06$
$P_{ICE-adjR} = 1.4 \times 10^{-3}$
$\beta_{ICE-adjR} = -0.08$
observed
imputed
estimate CCA
estimate ICE−adjR
Model residuals of indolelactate
2
1
0
−1
−2
$n_{CCA} = 722$
$n_{ICE-adjR} = 845$
$n_{CCA} = 556$
$n_{ICE-adjR} = 653$
$n_{CCA} = 101$
$n_{ICE-adjR} = 126$
major homozygote
heterozygote
minor homozygote
Genotype of SNP rs15676 (CCBL1)

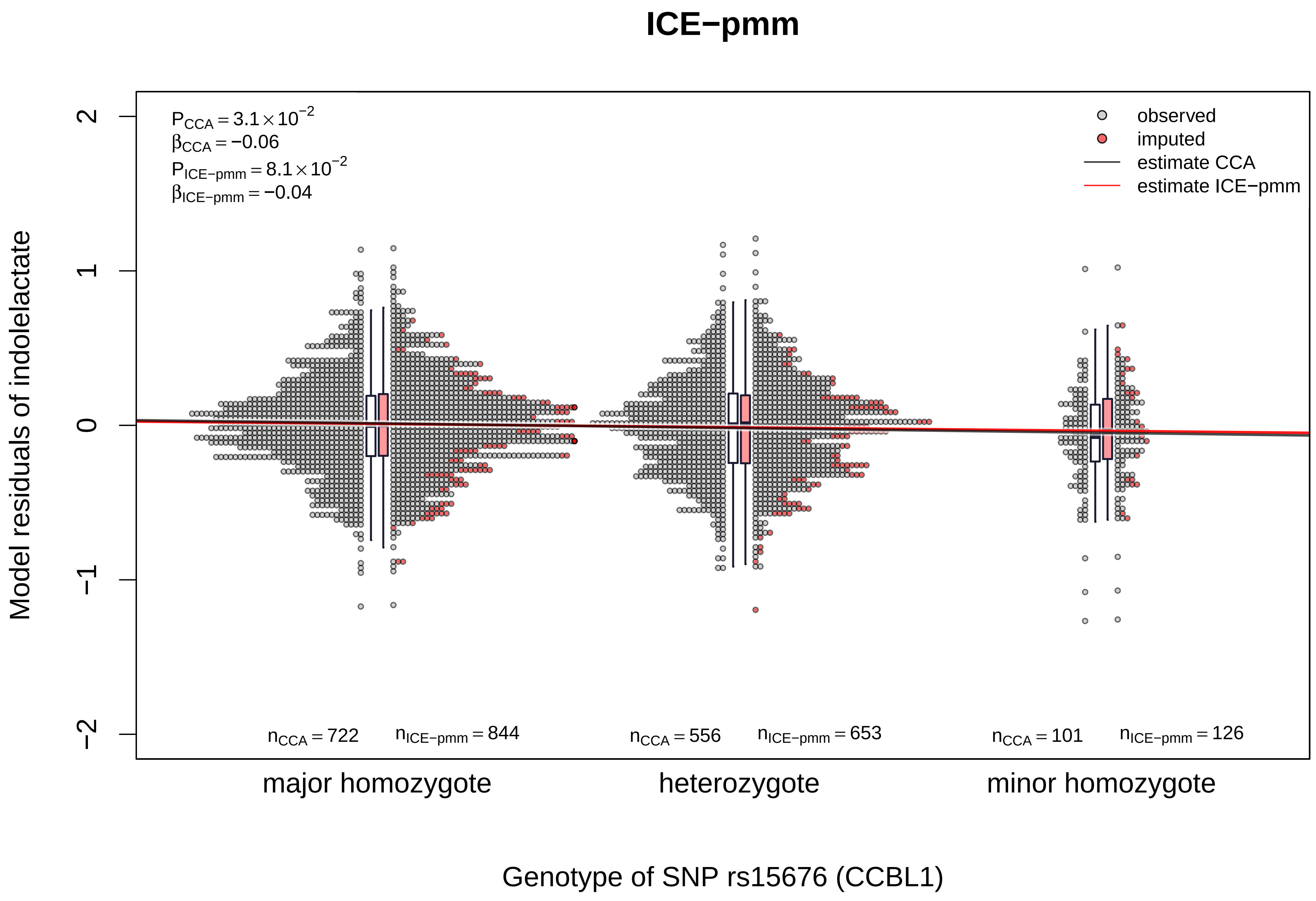
ICE−pmm
$P_{CCA} = 3.1 \times 10^{-2}$
$\beta_{CCA} = -0.06$
$P_{ICE-pmm} = 8.1 \times 10^{-2}$
$\beta_{ICE-pmm} = -0.04$
observed
imputed
estimate CCA
estimate ICE−pmm
Model residuals of indolelactate
2
1
0
−1
−2
$n_{CCA} = 722$
$n_{ICE-pmm} = 844$
$n_{CCA} = 556$
$n_{ICE-pmm} = 653$
$n_{CCA} = 101$
$n_{ICE-pmm} = 126$
major homozygote
heterozygote
minor homozygote
Genotype of SNP rs15676 (CCBL1)

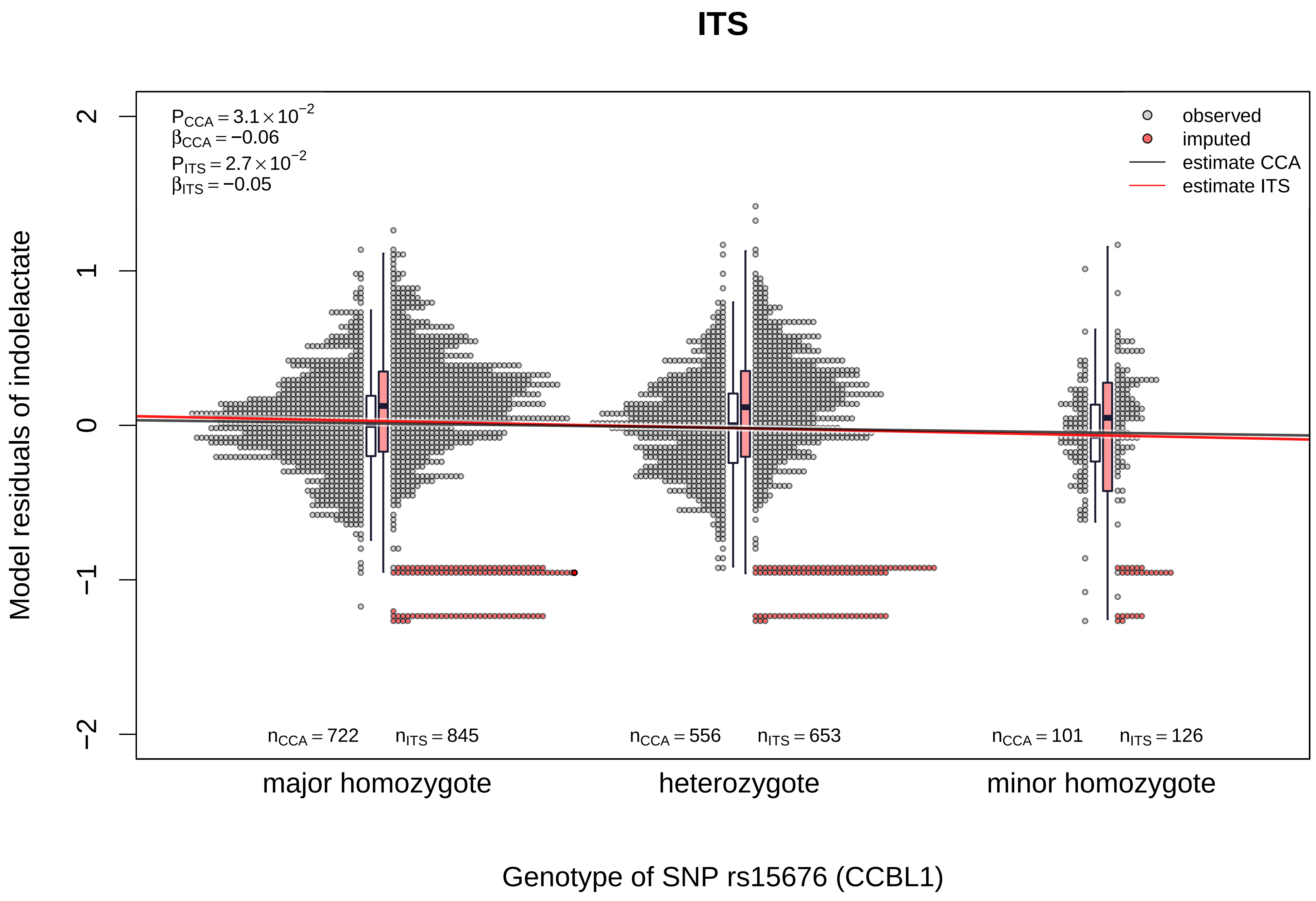
ITS
$P_{CCA} = 3.1 \times 10^{-2}$
$\beta_{CCA} = -0.06$
$P_{ITS} = 2.7 \times 10^{-2}$
$\beta_{ITS} = -0.05$
observed
imputed
estimate CCA
estimate ITS
Model residuals of indolelactate
2
1
0
−1
−2
$n_{CCA} = 722$
$n_{ITS} = 845$
$n_{CCA} = 556$
$n_{ITS} = 653$
$n_{CCA} = 101$
$n_{ITS} = 126$
major homozygote
heterozygote
minor homozygote
Genotype of SNP rs15676 (CCBL1)

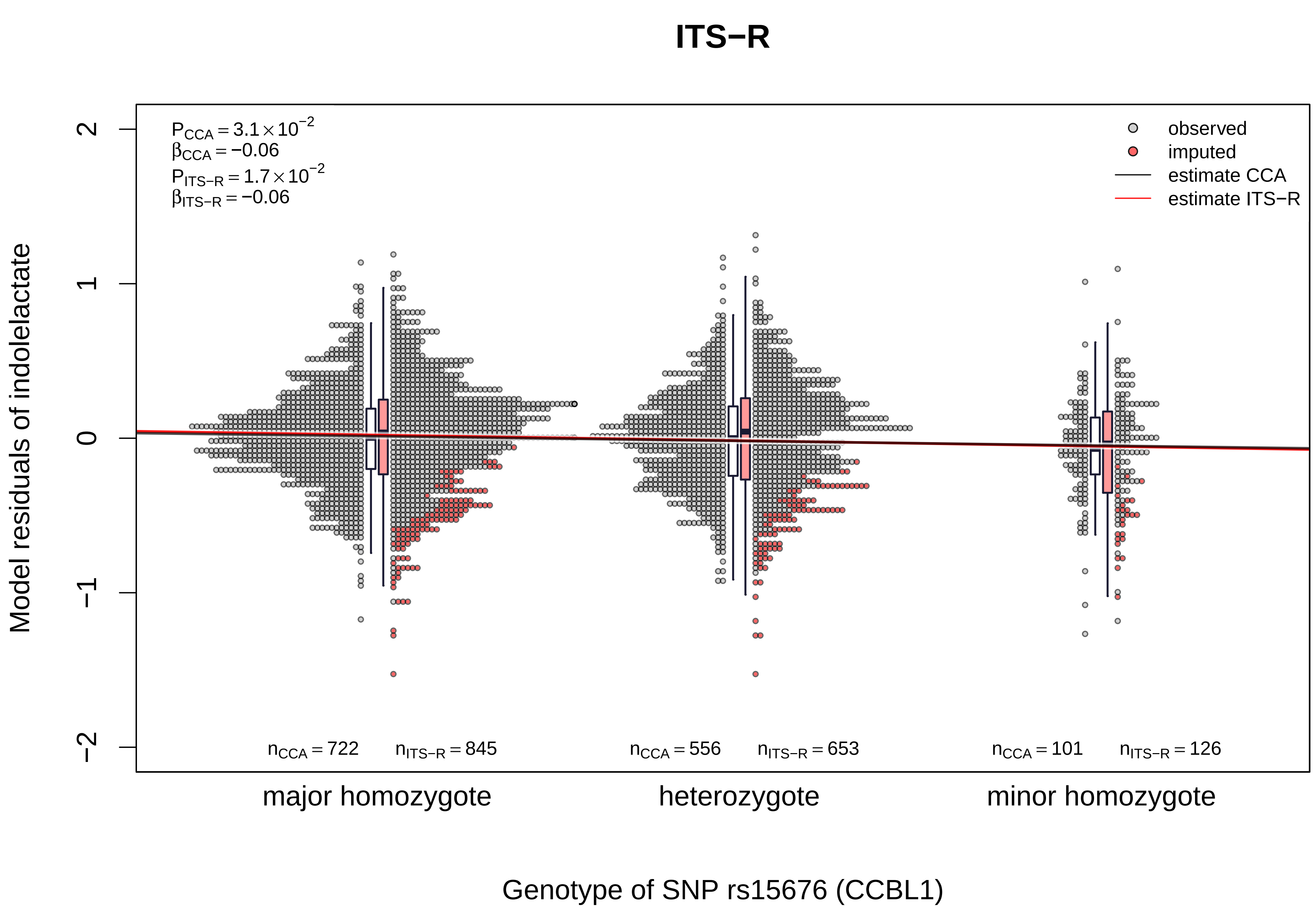

ITS−R
$P_{CCA} = 3.1 \times 10^{-2}$
$\beta_{CCA} = -0.06$
$P_{ITS-R} = 1.7 \times 10^{-2}$
$\beta_{ITS-R} = -0.06$
observed
imputed
estimate CCA
estimate ITS−R
Model residuals of indolelactate
2
1
0
−1
−2
$n_{CCA} = 722$
$n_{ITS-R} = 845$
$n_{CCA} = 556$
$n_{ITS-R} = 653$
$n_{CCA} = 101$
$n_{ITS-R} = 126$
major homozygote
heterozygote
minor homozygote
Genotype of SNP rs15676 (CCBL1)

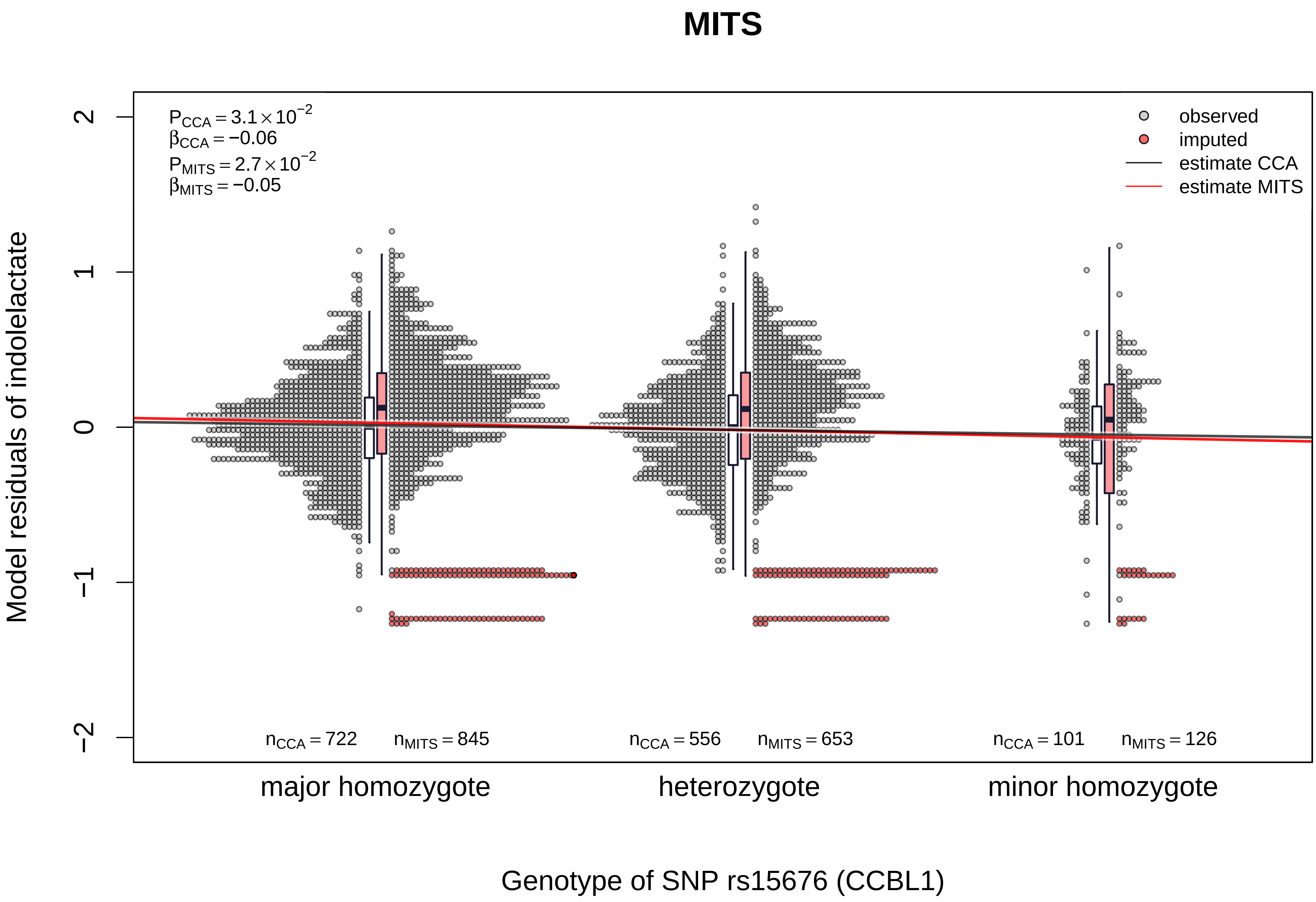
MITS
$P_{CCA} = 3.1 \times 10^{-2}$
$\beta_{CCA} = -0.06$
$P_{MITS} = 2.7 \times 10^{-2}$
$\beta_{MITS} = -0.05$
observed
imputed
estimate CCA
estimate MITS
$n_{CCA} = 722$
$n_{MITS} = 845$
$n_{CCA} = 556$
$n_{MITS} = 653$
$n_{CCA} = 101$
$n_{MITS} = 126$
Model residuals of indolelactate
2
1
0
−1
−2
major homozygote
heterozygote
minor homozygote
Genotype of SNP rs15676 (CCBL1)

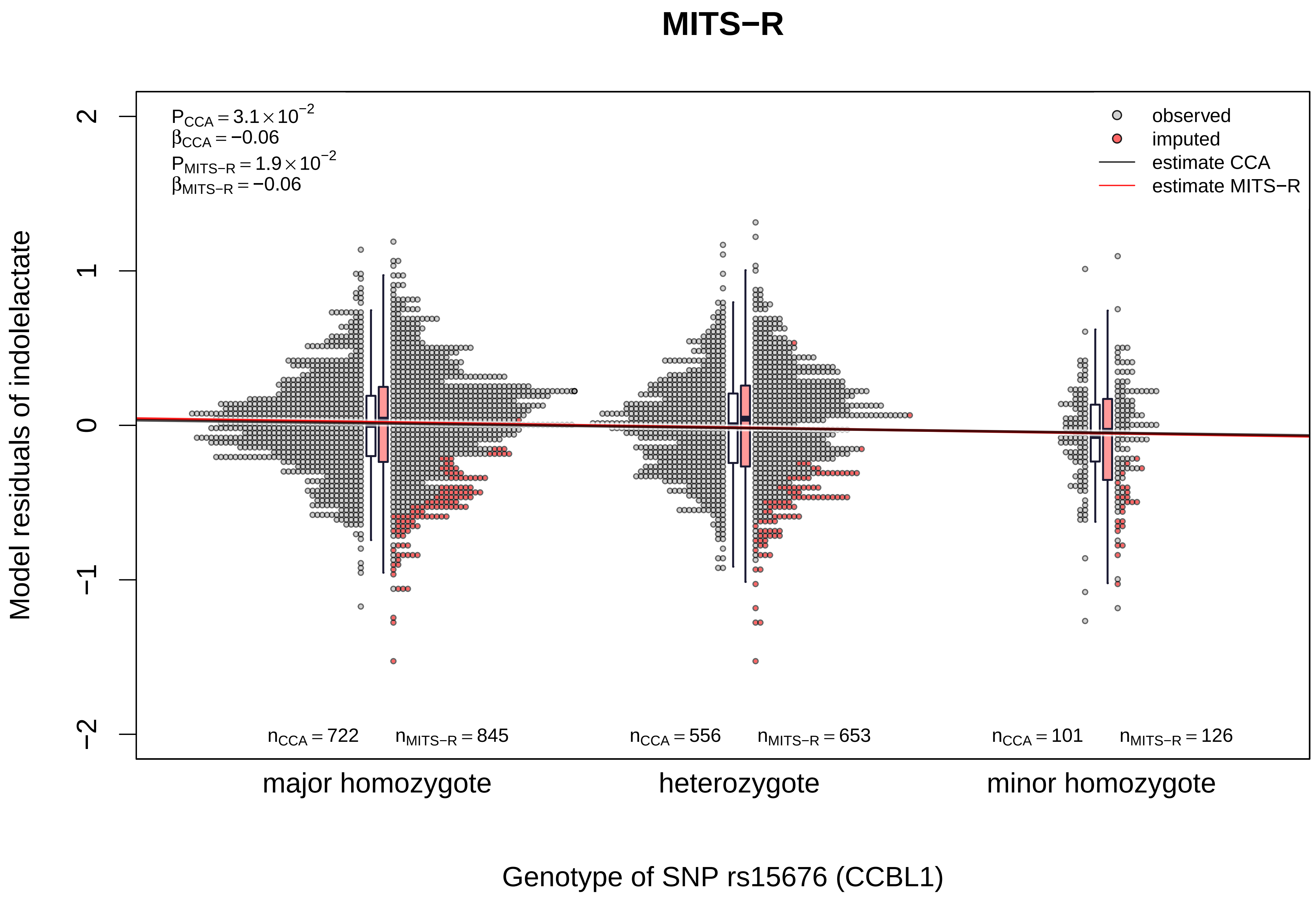
MITS−R
$P_{CCA} = 3.1 \times 10^{-2}$
$\beta_{CCA} = -0.06$
$P_{MITS-R} = 1.9 \times 10^{-2}$
$\beta_{MITS-R} = -0.06$
observed
imputed
estimate CCA
estimate MITS−R
Model residuals of indolelactate
2
1
0
−1
−2
$n_{CCA} = 722$
$n_{MITS-R} = 845$
$n_{CCA} = 556$
$n_{MITS-R} = 653$
$n_{CCA} = 101$
$n_{MITS-R} = 126$
major homozygote
heterozygote
minor homozygote
Genotype of SNP rs15676 (CCBL1)

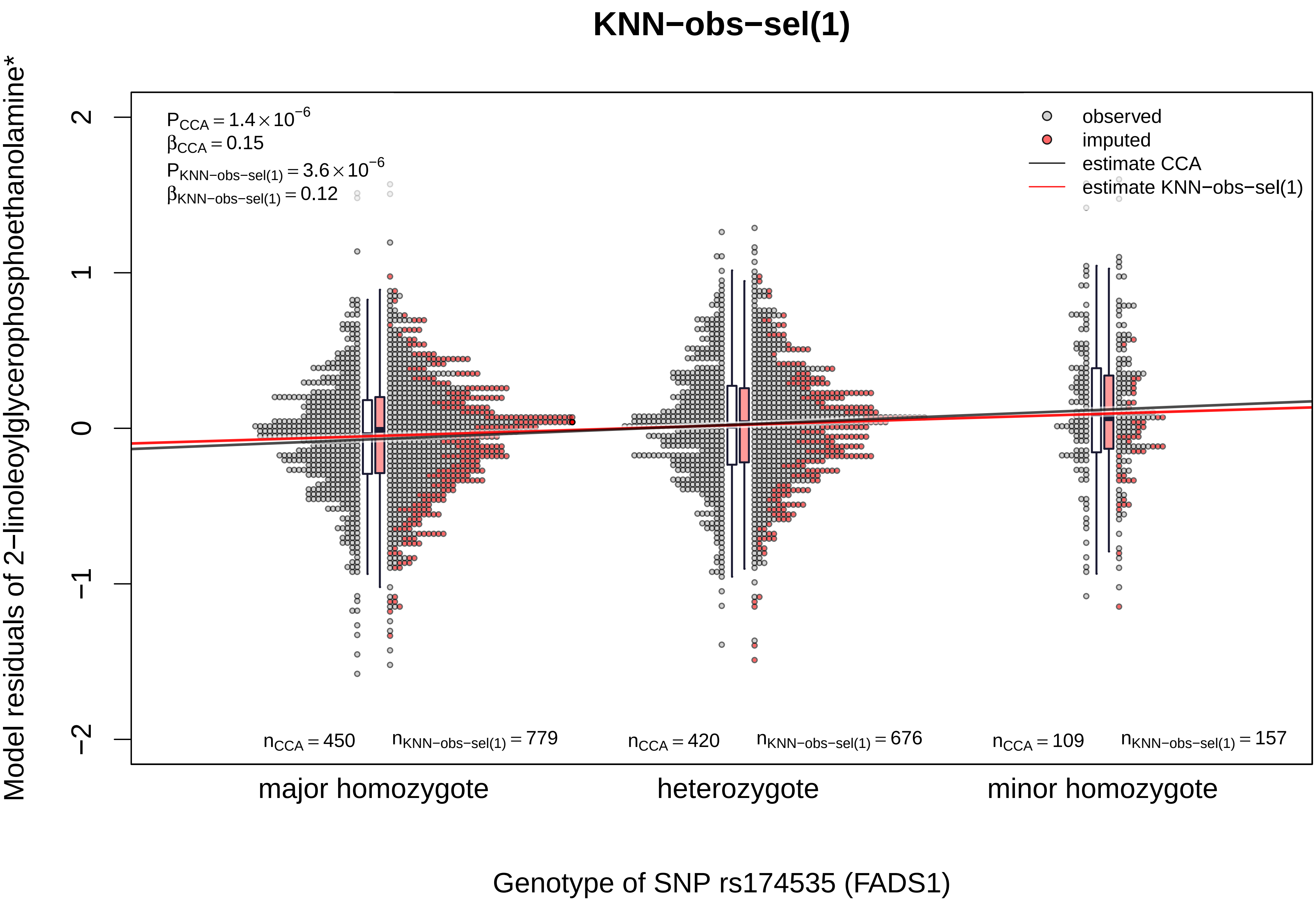
KNN−obs−sel(1)
$P_{CCA} = 1.4 \times 10^{-6}$
$\beta_{CCA} = 0.15$
$P_{KNN-obs-sel(1)} = 3.6 \times 10^{-6}$
$\beta_{KNN-obs-sel(1)} = 0.12$
observed
imputed
estimate CCA
estimate KNN−obs−sel(1)
$n_{CCA} = 450$
$n_{KNN-obs-sel(1)} = 779$
$n_{CCA} = 420$
$n_{KNN-obs-sel(1)} = 676$
$n_{CCA} = 109$
$n_{KNN-obs-sel(1)} = 157$
2
1
0
−1
−2
major homozygote
heterozygote
minor homozygote
Model residuals of 2−linoleoylglycerophosphoethanolamine*
Genotype of SNP rs174535 (FADS1)

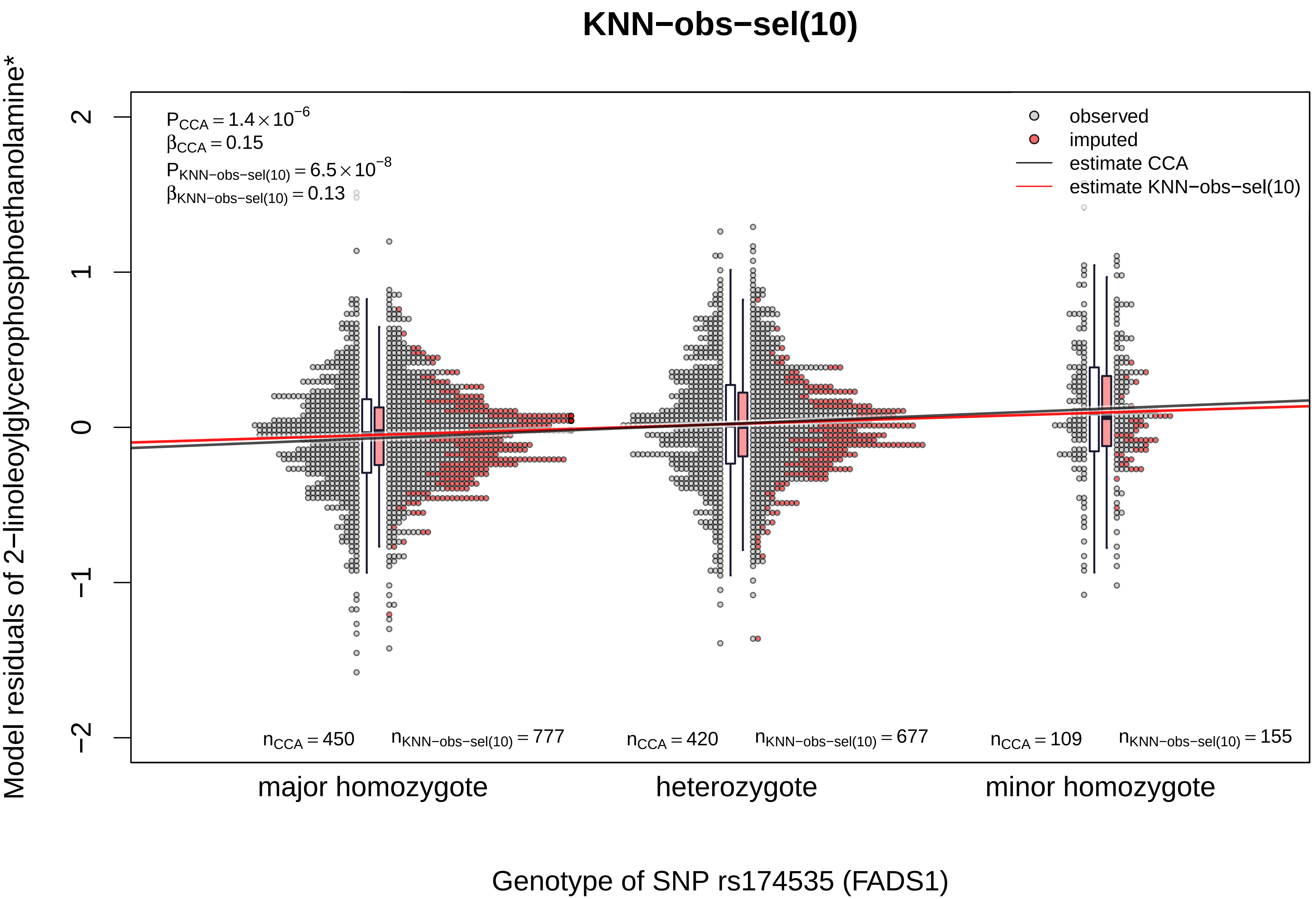

KNN−obs−sel(10)
$P_{CCA} = 1.4 \times 10^{-6}$
$\beta_{CCA} = 0.15$
$P_{KNN-obs-sel(10)} = 6.5 \times 10^{-8}$
$\beta_{KNN-obs-sel(10)} = 0.13$
observed
imputed
estimate CCA
estimate KNN−obs−sel(10)
$n_{CCA} = 450$
$n_{KNN-obs-sel(10)} = 777$
$n_{CCA} = 420$
$n_{KNN-obs-sel(10)} = 677$
$n_{CCA} = 109$
$n_{KNN-obs-sel(10)} = 155$
2
1
0
−1
−2
major homozygote
heterozygote
minor homozygote
Model residuals of 2−linoleoylglycerophosphoethanolamine*
Genotype of SNP rs174535 (FADS1)

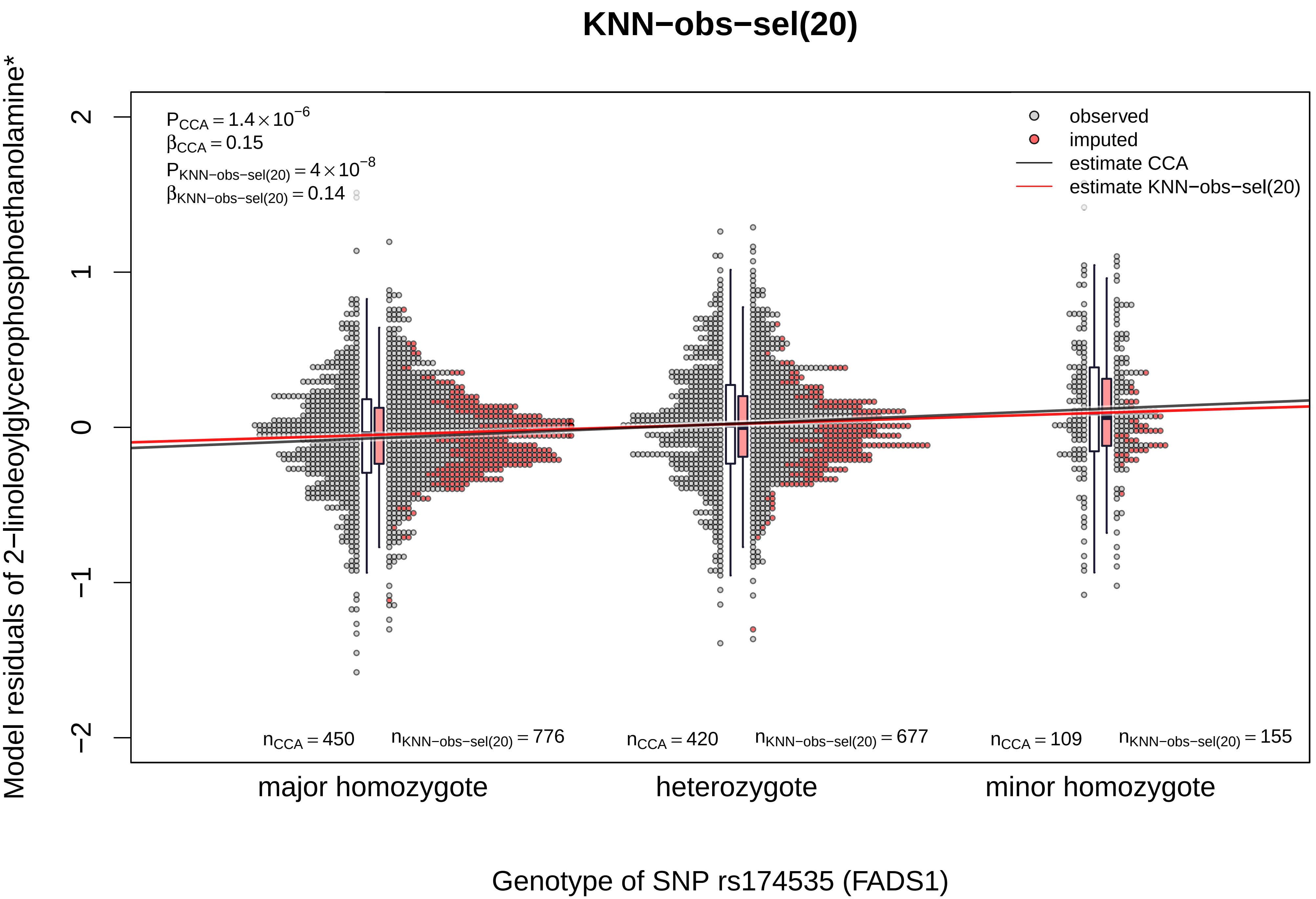
KNN−obs−sel(20)
$P_{CCA} = 1.4 \times 10^{-6}$
$\beta_{CCA} = 0.15$
$P_{KNN-obs-sel(20)} = 4 \times 10^{-8}$
$\beta_{KNN-obs-sel(20)} = 0.14$
observed
imputed
estimate CCA
estimate KNN−obs−sel(20)
Model residuals of 2−linoleoylglycerophosphoethanolamine*
2
1
0
−1
−2
$n_{CCA} = 450$
$n_{KNN-obs-sel(20)} = 776$
$n_{CCA} = 420$
$n_{KNN-obs-sel(20)} = 677$
$n_{CCA} = 109$
$n_{KNN-obs-sel(20)} = 155$
major homozygote
heterozygote
minor homozygote
Genotype of SNP rs174535 (FADS1)

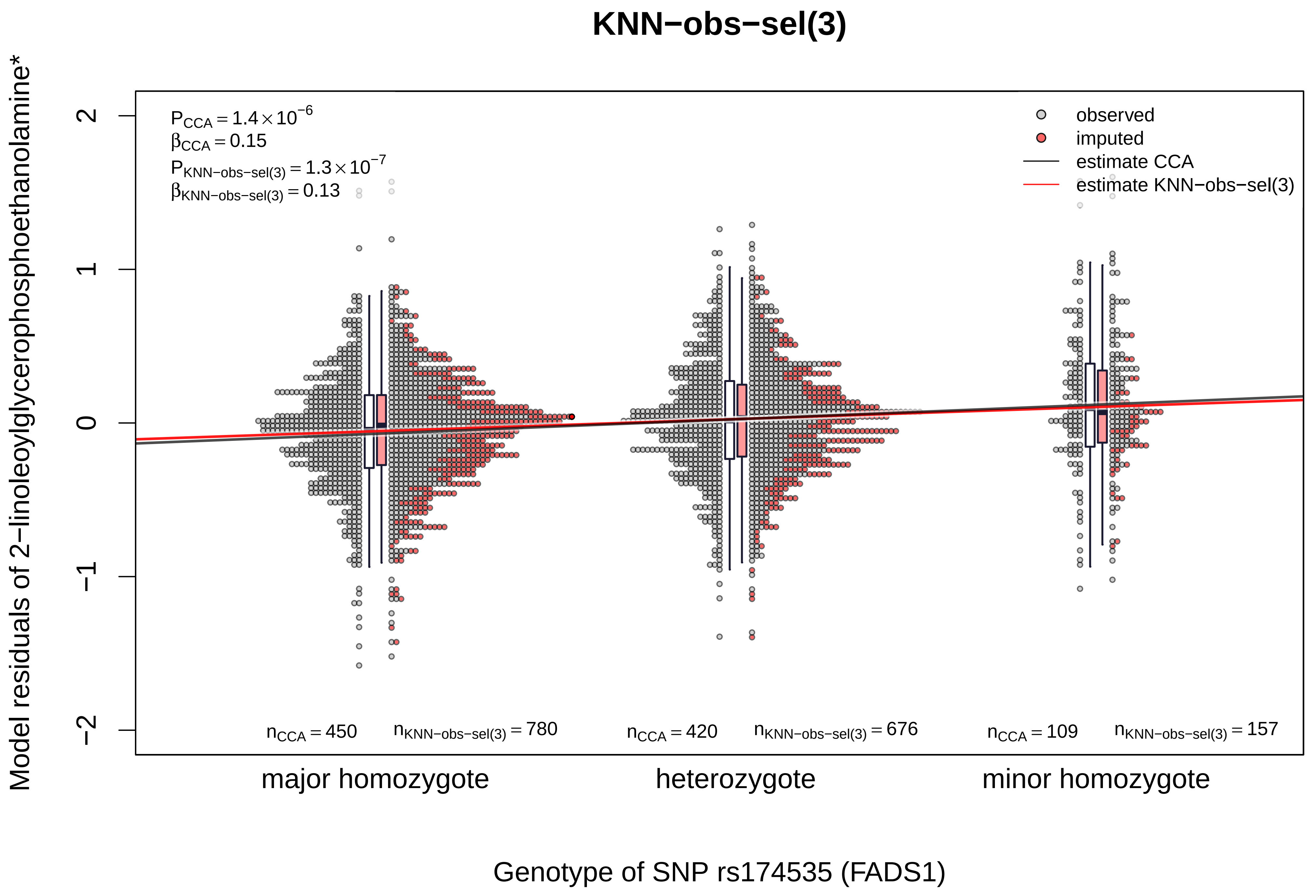

KNN−obs−sel(3)
$P_{CCA} = 1.4 \times 10^{-6}$
$\beta_{CCA} = 0.15$
$P_{KNN-obs-sel(3)} = 1.3 \times 10^{-7}$
$\beta_{KNN-obs-sel(3)} = 0.13$
observed
imputed
estimate CCA
estimate KNN−obs−sel(3)
2
1
0
−1
−2
$n_{CCA} = 450$
$n_{KNN-obs-sel(3)} = 780$
$n_{CCA} = 420$
$n_{KNN-obs-sel(3)} = 676$
$n_{CCA} = 109$
$n_{KNN-obs-sel(3)} = 157$
major homozygote
heterozygote
minor homozygote
Model residuals of 2−linoleoylglycerophosphoethanolamine*
Genotype of SNP rs174535 (FADS1)

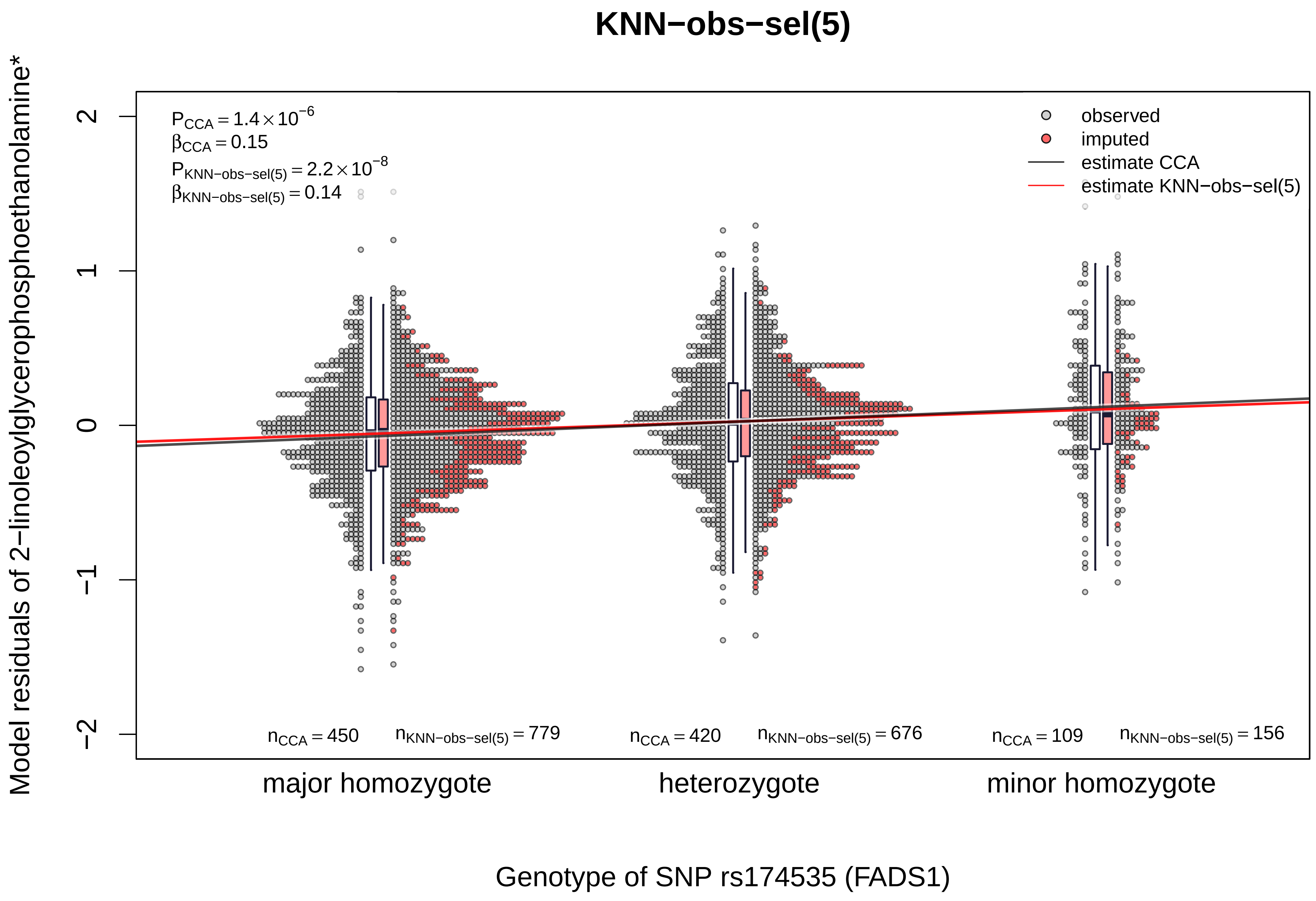

KNN−obs−sel(5)
$P_{CCA} = 1.4 \times 10^{-6}$
$\beta_{CCA} = 0.15$
$P_{KNN-obs-sel(5)} = 2.2 \times 10^{-8}$
$\beta_{KNN-obs-sel(5)} = 0.14$
observed
imputed
estimate CCA
estimate KNN−obs−sel(5)
$n_{CCA} = 450$
$n_{KNN-obs-sel(5)} = 779$
$n_{CCA} = 420$
$n_{KNN-obs-sel(5)} = 676$
$n_{CCA} = 109$
$n_{KNN-obs-sel(5)} = 156$
2
1
0
−1
−2
major homozygote
heterozygote
minor homozygote
Model residuals of 2−linoleoylglycerophosphoethanolamine*
Genotype of SNP rs174535 (FADS1)

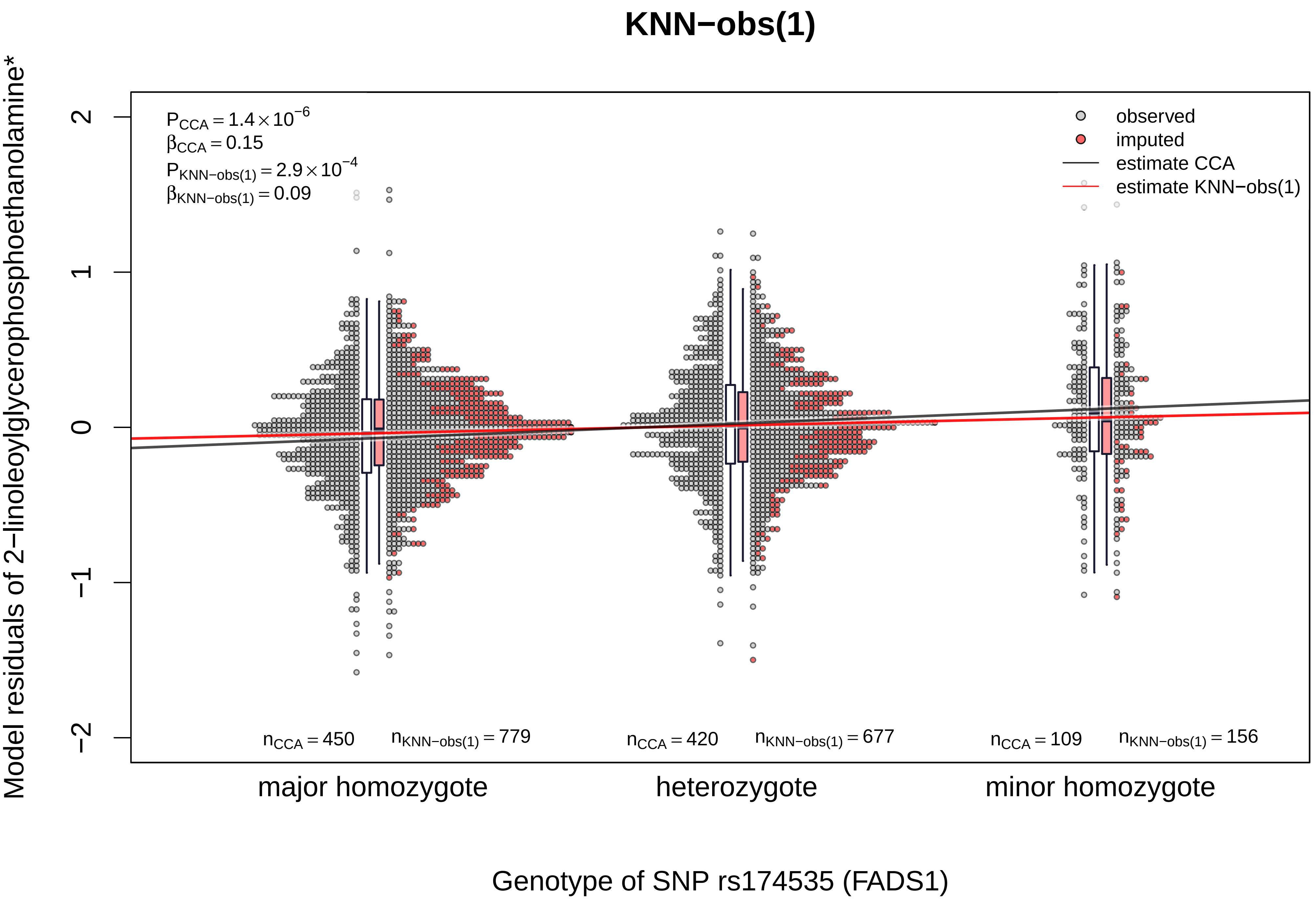
KNN−obs(1)
$P_{CCA} = 1.4 \times 10^{-6}$
$\beta_{CCA} = 0.15$
$P_{KNN-obs(1)} = 2.9 \times 10^{-4}$
$\beta_{KNN-obs(1)} = 0.09$
observed
imputed
estimate CCA
estimate KNN−obs(1)
Model residuals of 2−linoleoylglycerophosphoethanolamine*
2
1
0
−1
−2
$n_{CCA} = 450$
$n_{KNN-obs(1)} = 779$
$n_{CCA} = 420$
$n_{KNN-obs(1)} = 677$
$n_{CCA} = 109$
$n_{KNN-obs(1)} = 156$
major homozygote
heterozygote
minor homozygote
Genotype of SNP rs174535 (FADS1)

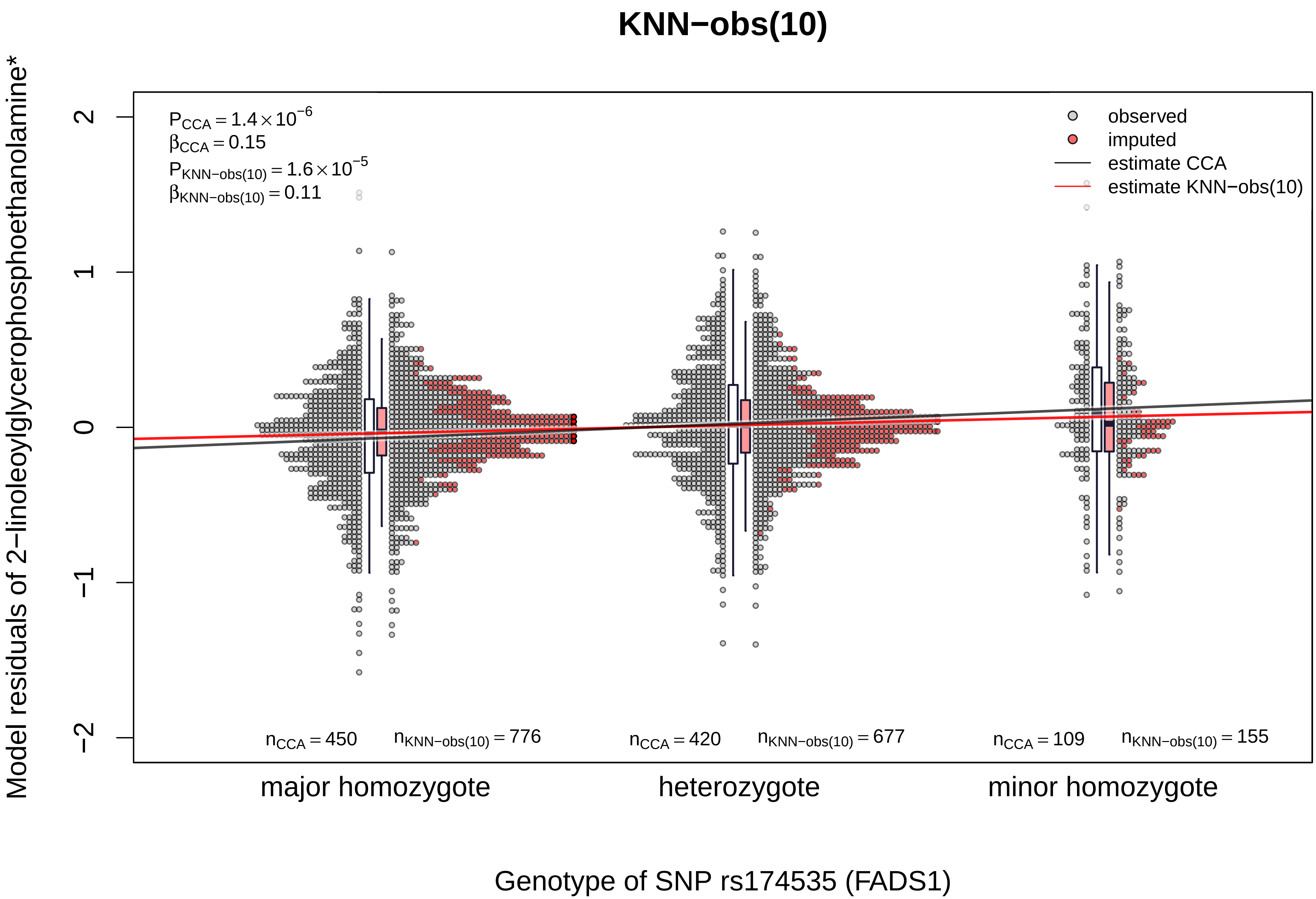
KNN−obs(10)
$P_{CCA} = 1.4 \times 10^{-6}$
$\beta_{CCA} = 0.15$
$P_{KNN-obs(10)} = 1.6 \times 10^{-5}$
$\beta_{KNN-obs(10)} = 0.11$
observed
imputed
estimate CCA
estimate KNN−obs(10)
2
1
0
−1
−2
Model residuals of 2−linoleoylglycerophosphoethanolamine*
$n_{CCA} = 450$
$n_{KNN-obs(10)} = 776$
$n_{CCA} = 420$
$n_{KNN-obs(10)} = 677$
$n_{CCA} = 109$
$n_{KNN-obs(10)} = 155$
major homozygote
heterozygote
minor homozygote
Genotype of SNP rs174535 (FADS1)

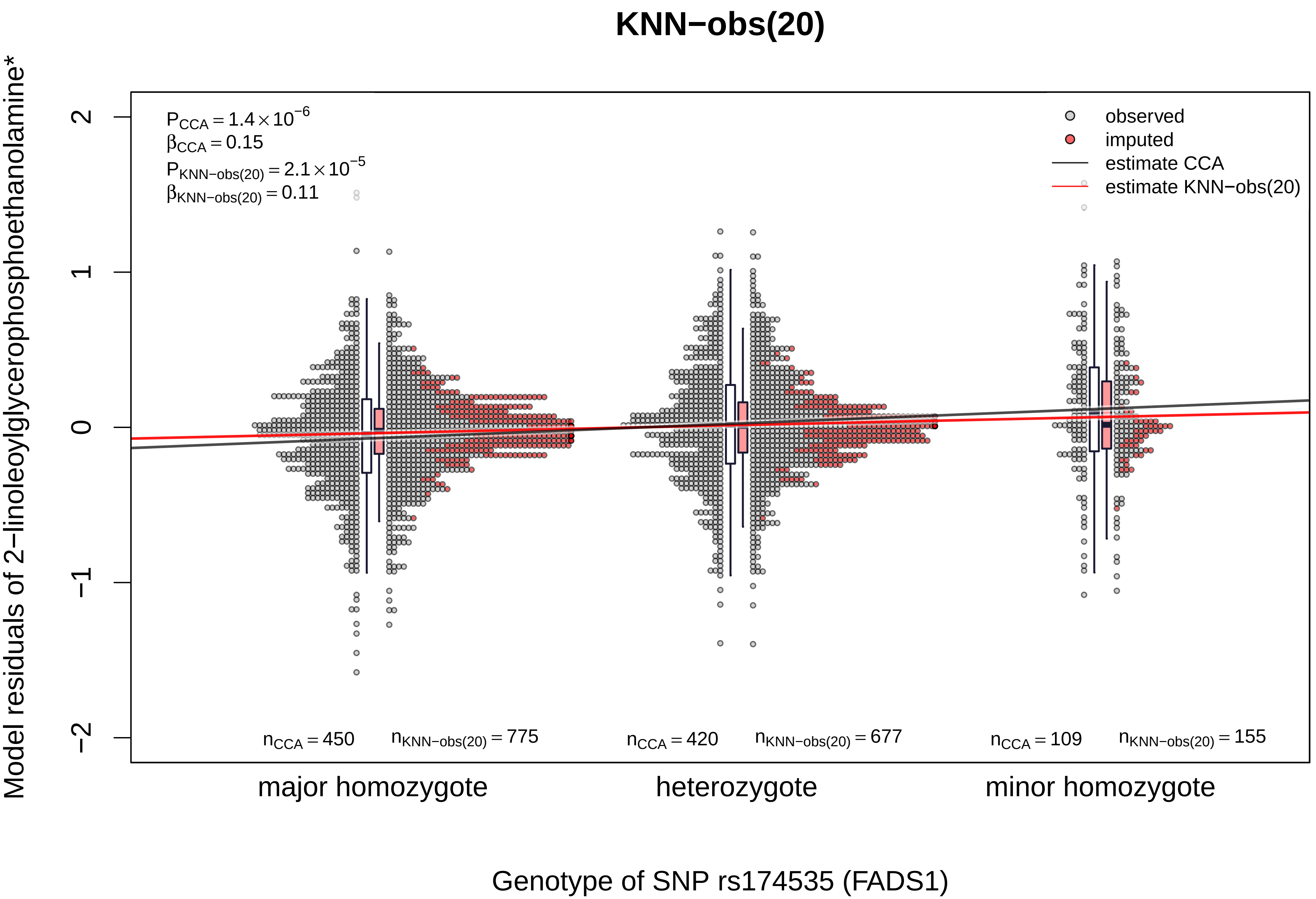

KNN−obs(20)
$P_{CCA} = 1.4 \times 10^{-6}$
$\beta_{CCA} = 0.15$
$P_{KNN-obs(20)} = 2.1 \times 10^{-5}$
$\beta_{KNN-obs(20)} = 0.11$
observed
imputed
estimate CCA
estimate KNN−obs(20)
Model residuals of 2−linoleoylglycerophosphoethanolamine*
2
1
0
−1
−2
$n_{CCA} = 450$
$n_{KNN-obs(20)} = 775$
$n_{CCA} = 420$
$n_{KNN-obs(20)} = 677$
$n_{CCA} = 109$
$n_{KNN-obs(20)} = 155$
major homozygote
heterozygote
minor homozygote
Genotype of SNP rs174535 (FADS1)

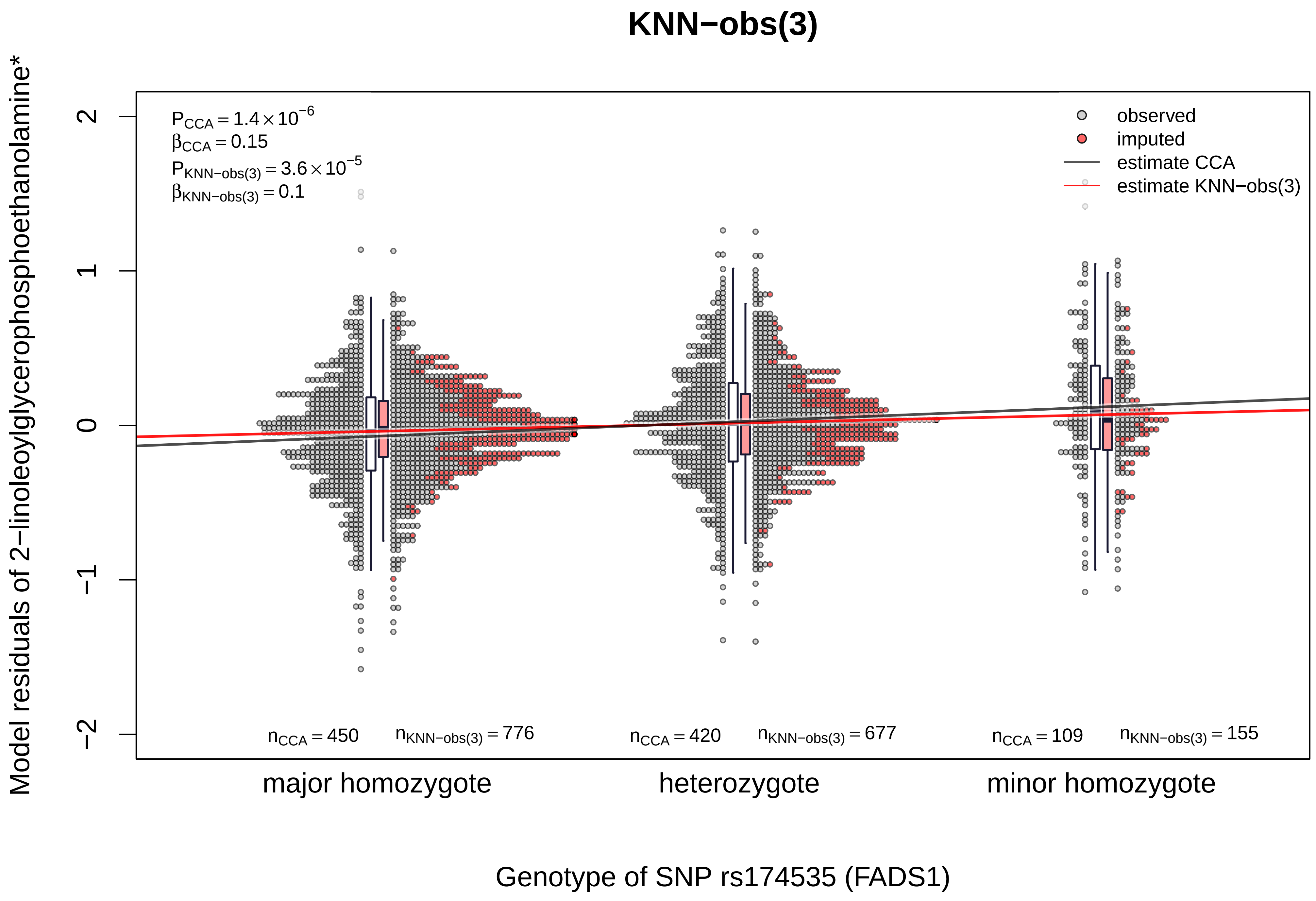
KNN−obs(3)
$P_{CCA} = 1.4 \times 10^{-6}$
$\beta_{CCA} = 0.15$
$P_{KNN-obs(3)} = 3.6 \times 10^{-5}$
$\beta_{KNN-obs(3)} = 0.1$
observed
imputed
estimate CCA
estimate KNN−obs(3)
Model residuals of 2−linoleoylglycerophosphoethanolamine*
2
1
0
−1
−2
$n_{CCA} = 450$
$n_{KNN-obs(3)} = 776$
$n_{CCA} = 420$
$n_{KNN-obs(3)} = 677$
$n_{CCA} = 109$
$n_{KNN-obs(3)} = 155$
major homozygote
heterozygote
minor homozygote
Genotype of SNP rs174535 (FADS1)

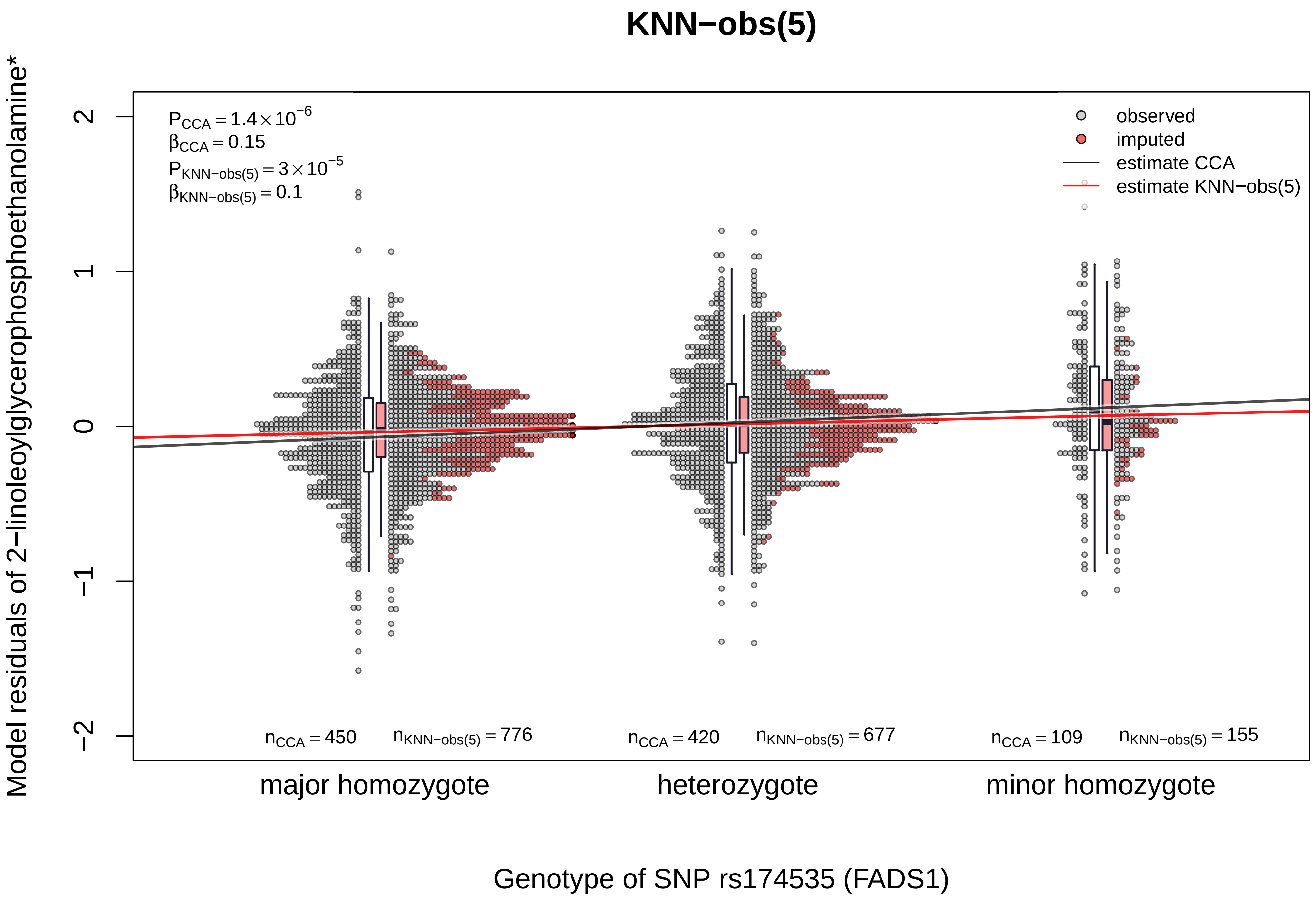
KNN−obs(5)
$P_{CCA} = 1.4 \times 10^{-6}$
$\beta_{CCA} = 0.15$
$P_{KNN-obs(5)} = 3 \times 10^{-5}$
$\beta_{KNN-obs(5)} = 0.1$
observed
imputed
estimate CCA
estimate KNN−obs(5)
Model residuals of 2−linoleoylglycerophosphoethanolamine*
2
1
0
−1
−2
$n_{CCA} = 450$
$n_{KNN-obs(5)} = 776$
$n_{CCA} = 420$
$n_{KNN-obs(5)} = 677$
$n_{CCA} = 109$
$n_{KNN-obs(5)} = 155$
major homozygote
heterozygote
minor homozygote
Genotype of SNP rs174535 (FADS1)

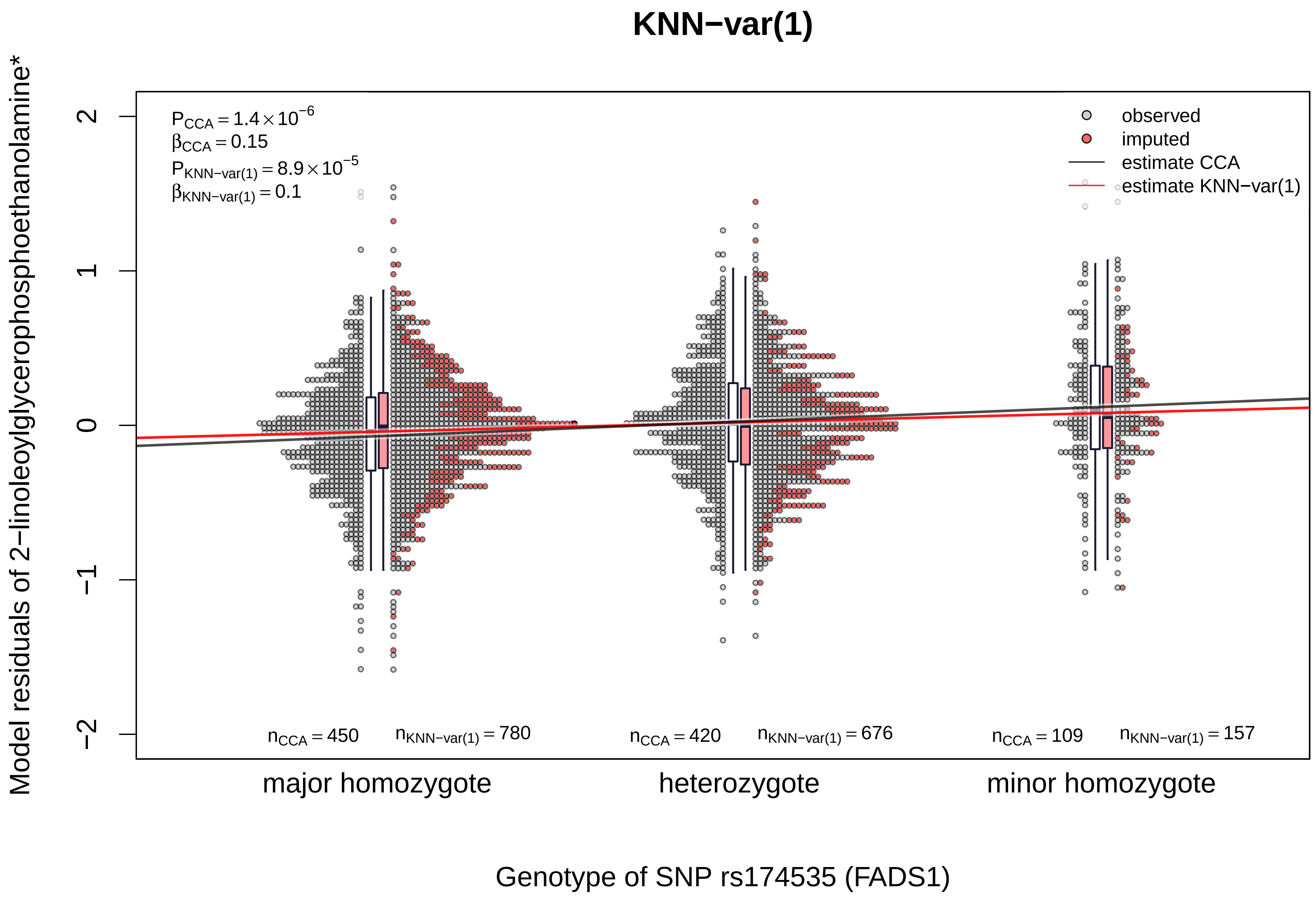

KNN−var(1)
$P_{CCA} = 1.4 \times 10^{-6}$
$\beta_{CCA} = 0.15$
$P_{KNN-var(1)} = 8.9 \times 10^{-5}$
$\beta_{KNN-var(1)} = 0.1$
observed
imputed
estimate CCA
estimate KNN−var(1)
Model residuals of 2−linoleoylglycerophosphoethanolamine*
2
1
0
−1
−2
$n_{CCA} = 450$
$n_{KNN-var(1)} = 780$
$n_{CCA} = 420$
$n_{KNN-var(1)} = 676$
$n_{CCA} = 109$
$n_{KNN-var(1)} = 157$
major homozygote
heterozygote
minor homozygote
Genotype of SNP rs174535 (FADS1)

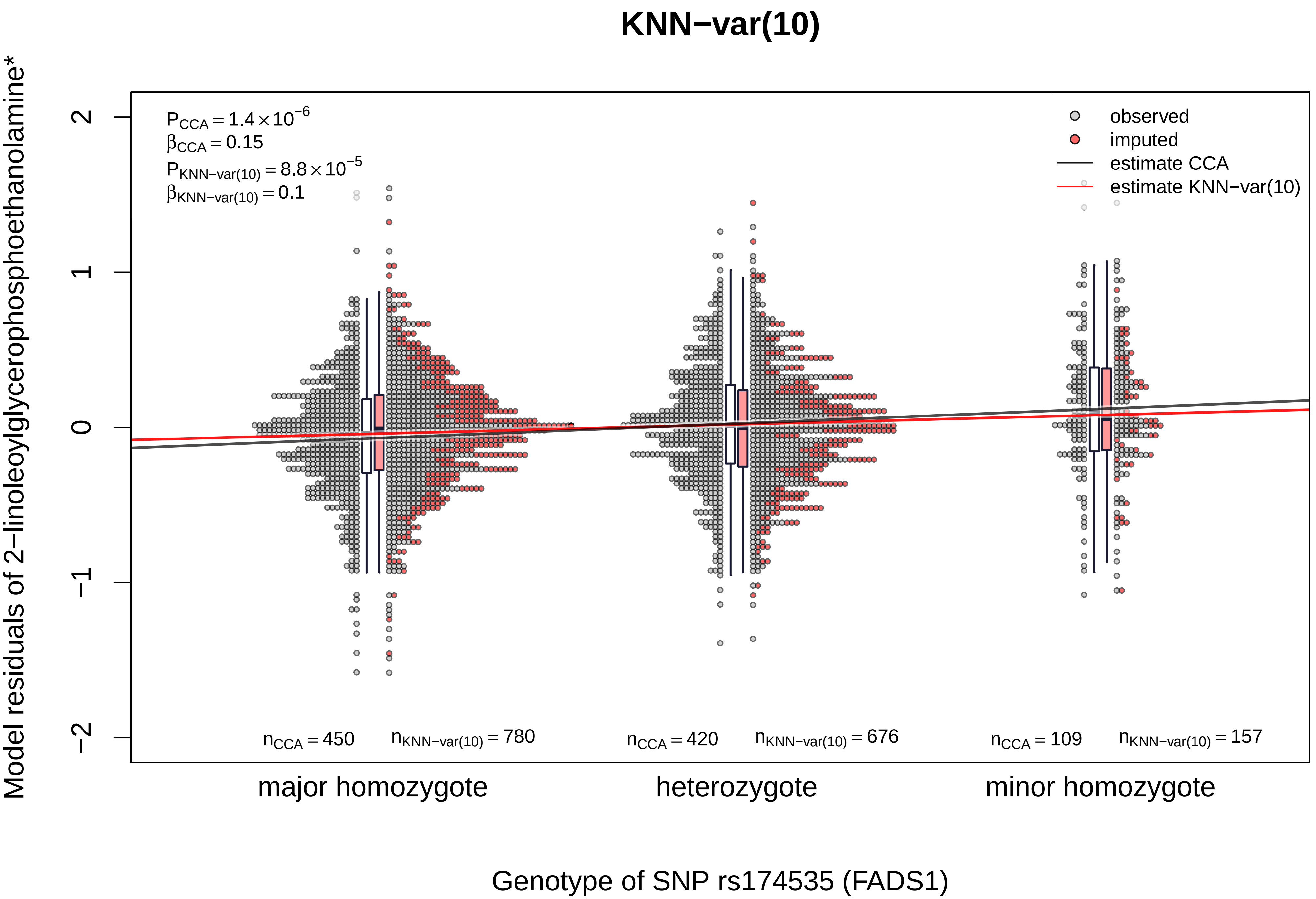

KNN−var(10)
P_CCA = 1.4 × 10^−6
β_CCA = 0.15
P_KNN−var(10) = 8.8 × 10^−5
β_KNN−var(10) = 0.1
observed
imputed
estimate CCA
estimate KNN−var(10)
2
1
0
−1
−2
n_CCA = 450
n_KNN−var(10) = 780
n_CCA = 420
n_KNN−var(10) = 676
n_CCA = 109
n_KNN−var(10) = 157
major homozygote
heterozygote
minor homozygote
Genotype of SNP rs174535 (FADS1)
Model residuals of 2−linoleoylglycerophosphoethanolamine*

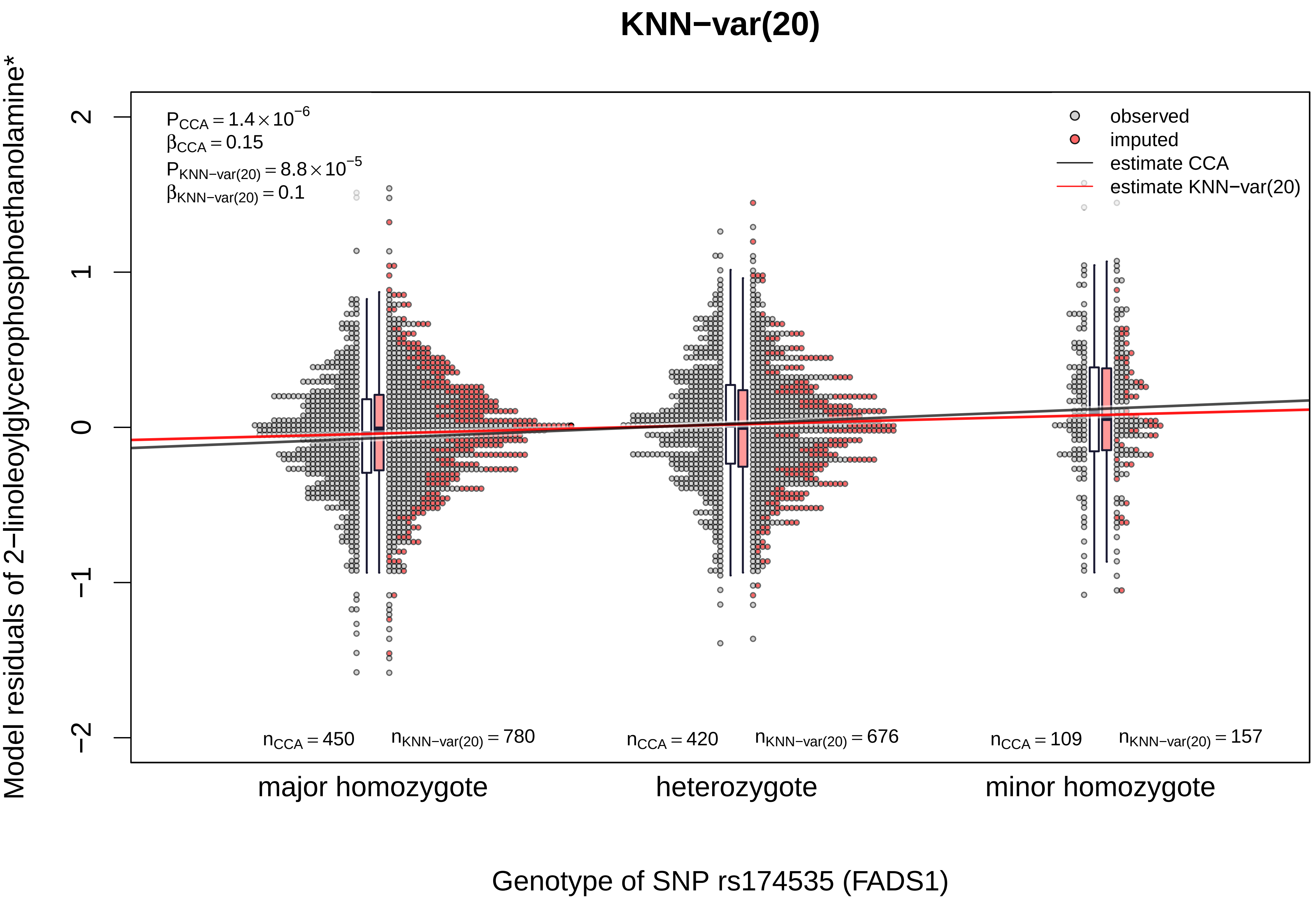

KNN−var(20)
$P_{CCA} = 1.4 \times 10^{-6}$
$\beta_{CCA} = 0.15$
$P_{KNN-var(20)} = 8.8 \times 10^{-5}$
$\beta_{KNN-var(20)} = 0.1$
observed
imputed
estimate CCA
estimate KNN−var(20)
$n_{CCA} = 450$
$n_{KNN-var(20)} = 780$
$n_{CCA} = 420$
$n_{KNN-var(20)} = 676$
$n_{CCA} = 109$
$n_{KNN-var(20)} = 157$
2
1
0
−1
−2
major homozygote
heterozygote
minor homozygote
Model residuals of 2−linoleoylglycerophosphoethanolamine*
Genotype of SNP rs174535 (FADS1)

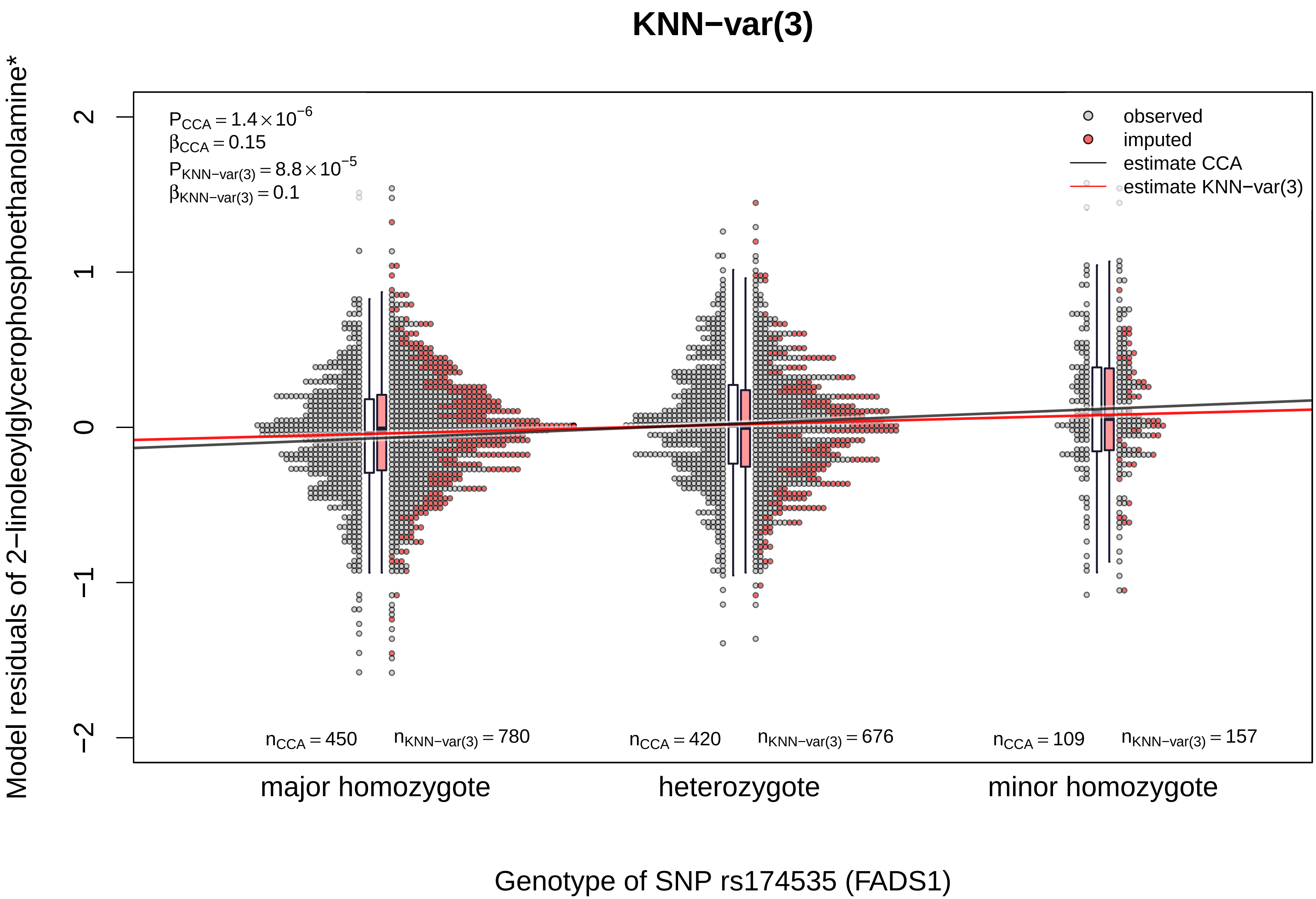

KNN−var(3)
$P_{CCA} = 1.4 \times 10^{-6}$
$\beta_{CCA} = 0.15$
$P_{KNN-var(3)} = 8.8 \times 10^{-5}$
$\beta_{KNN-var(3)} = 0.1$
observed
imputed
estimate CCA
estimate KNN−var(3)
Model residuals of 2−linoleoylglycerophosphoethanolamine*
2
1
0
−1
−2
$n_{CCA} = 450$
$n_{KNN-var(3)} = 780$
$n_{CCA} = 420$
$n_{KNN-var(3)} = 676$
$n_{CCA} = 109$
$n_{KNN-var(3)} = 157$
major homozygote
heterozygote
minor homozygote
Genotype of SNP rs174535 (FADS1)

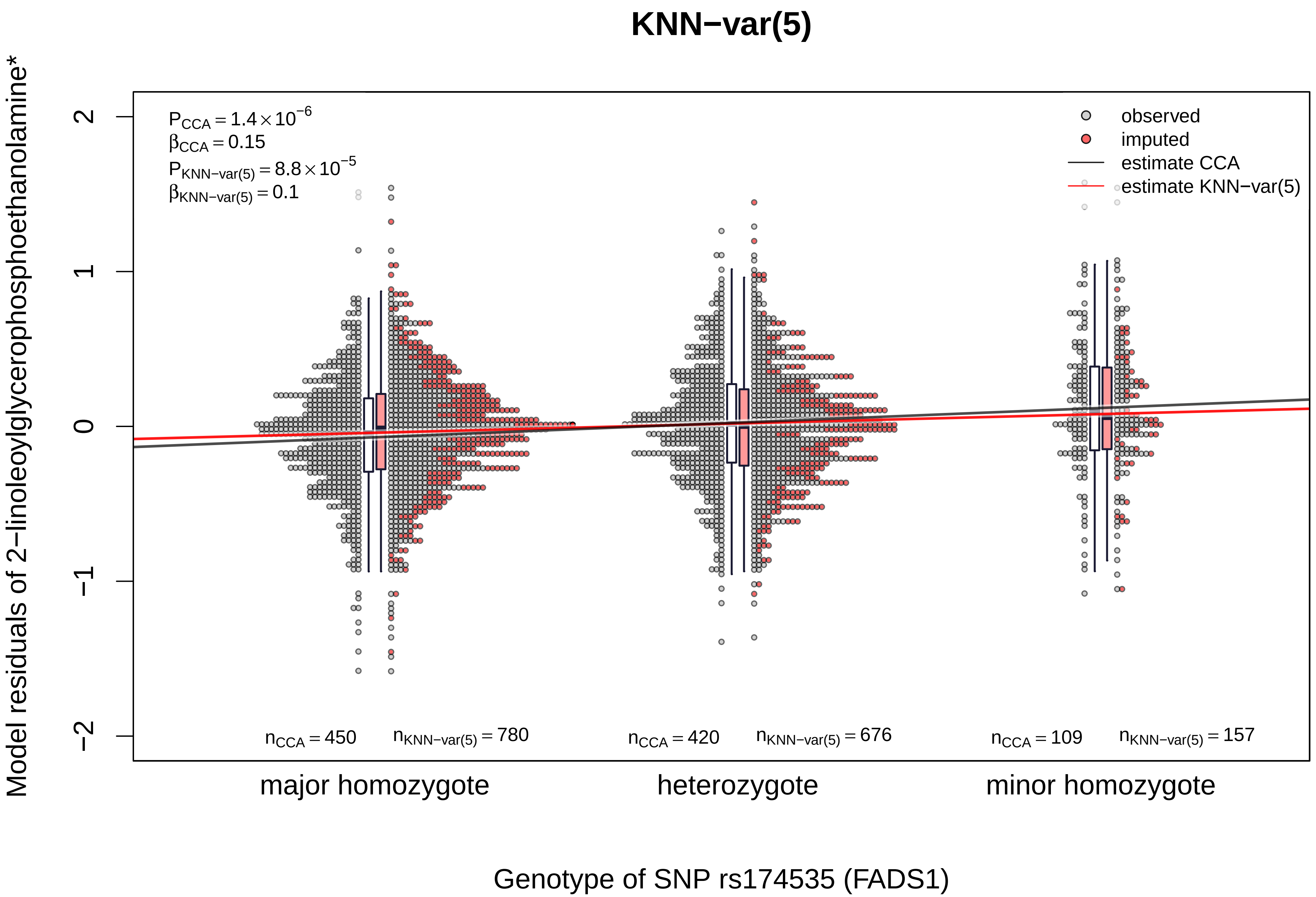
KNN−var(5)
$P_{CCA} = 1.4 \times 10^{-6}$
$\beta_{CCA} = 0.15$
$P_{KNN-var(5)} = 8.8 \times 10^{-5}$
$\beta_{KNN-var(5)} = 0.1$
observed
imputed
estimate CCA
estimate KNN−var(5)
$n_{CCA} = 450$
$n_{KNN-var(5)} = 780$
$n_{CCA} = 420$
$n_{KNN-var(5)} = 676$
$n_{CCA} = 109$
$n_{KNN-var(5)} = 157$
2
1
0
−1
−2
major homozygote
heterozygote
minor homozygote
Model residuals of 2−linoleoylglycerophosphoethanolamine*
Genotype of SNP rs174535 (FADS1)

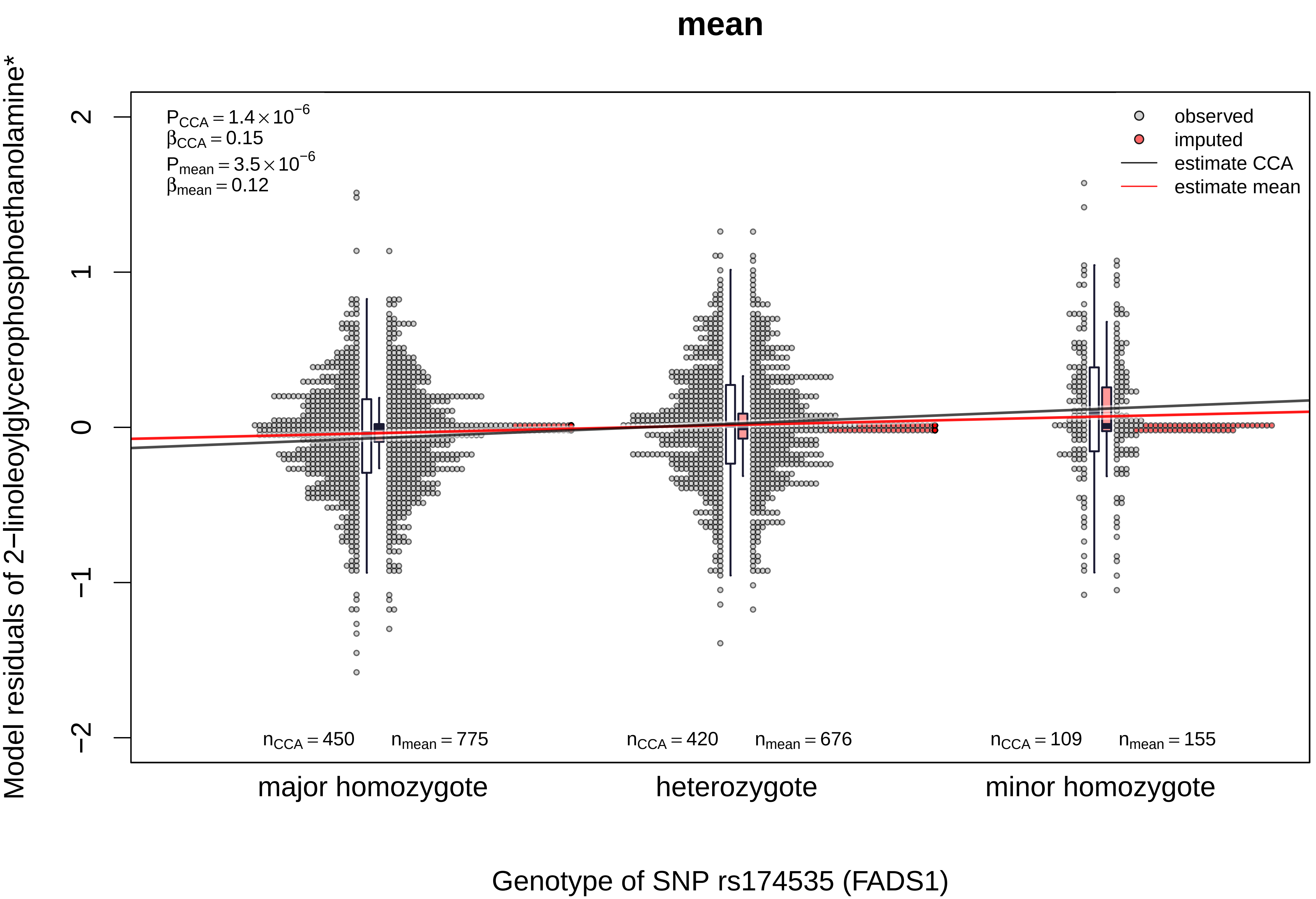

mean
$P_{CCA} = 1.4 \times 10^{-6}$
$\beta_{CCA} = 0.15$
$P_{mean} = 3.5 \times 10^{-6}$
$\beta_{mean} = 0.12$
observed
imputed
estimate CCA
estimate mean
$n_{CCA} = 450$
$n_{mean} = 775$
$n_{CCA} = 420$
$n_{mean} = 676$
$n_{CCA} = 109$
$n_{mean} = 155$
Model residuals of 2−linoleoylglycerophosphoethanolamine*
2
1
0
−1
−2
major homozygote
heterozygote
minor homozygote
Genotype of SNP rs174535 (FADS1)

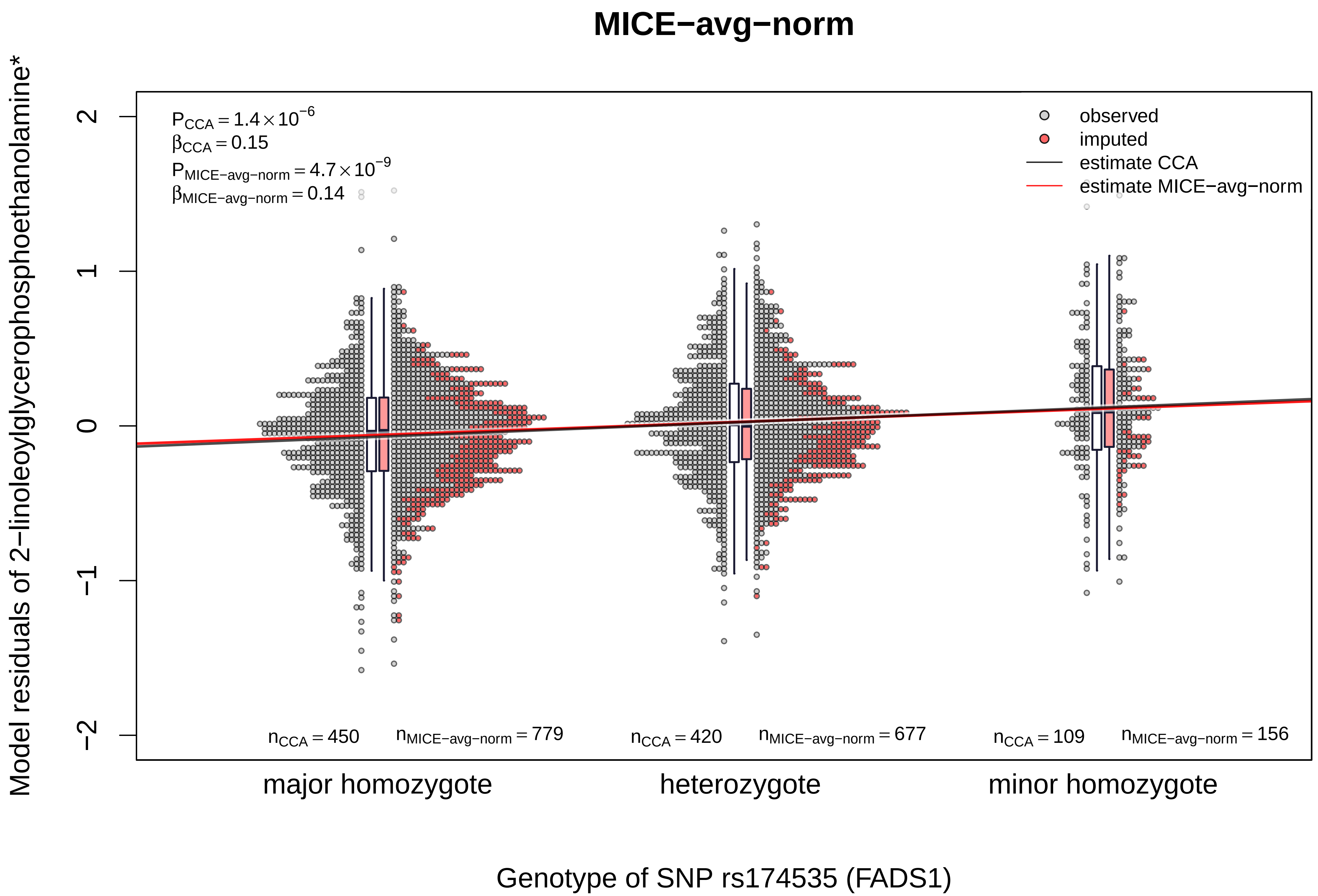

MICE−avg−norm
$P_{CCA} = 1.4 \times 10^{-6}$
$\beta_{CCA} = 0.15$
$P_{MICE-avg-norm} = 4.7 \times 10^{-9}$
$\beta_{MICE-avg-norm} = 0.14$
observed
imputed
estimate CCA
estimate MICE−avg−norm
$n_{CCA} = 450$
$n_{MICE-avg-norm} = 779$
$n_{CCA} = 420$
$n_{MICE-avg-norm} = 677$
$n_{CCA} = 109$
$n_{MICE-avg-norm} = 156$
2
1
0
−1
−2
major homozygote
heterozygote
minor homozygote
Model residuals of 2−linoleoylglycerophosphoethanolamine*
Genotype of SNP rs174535 (FADS1)

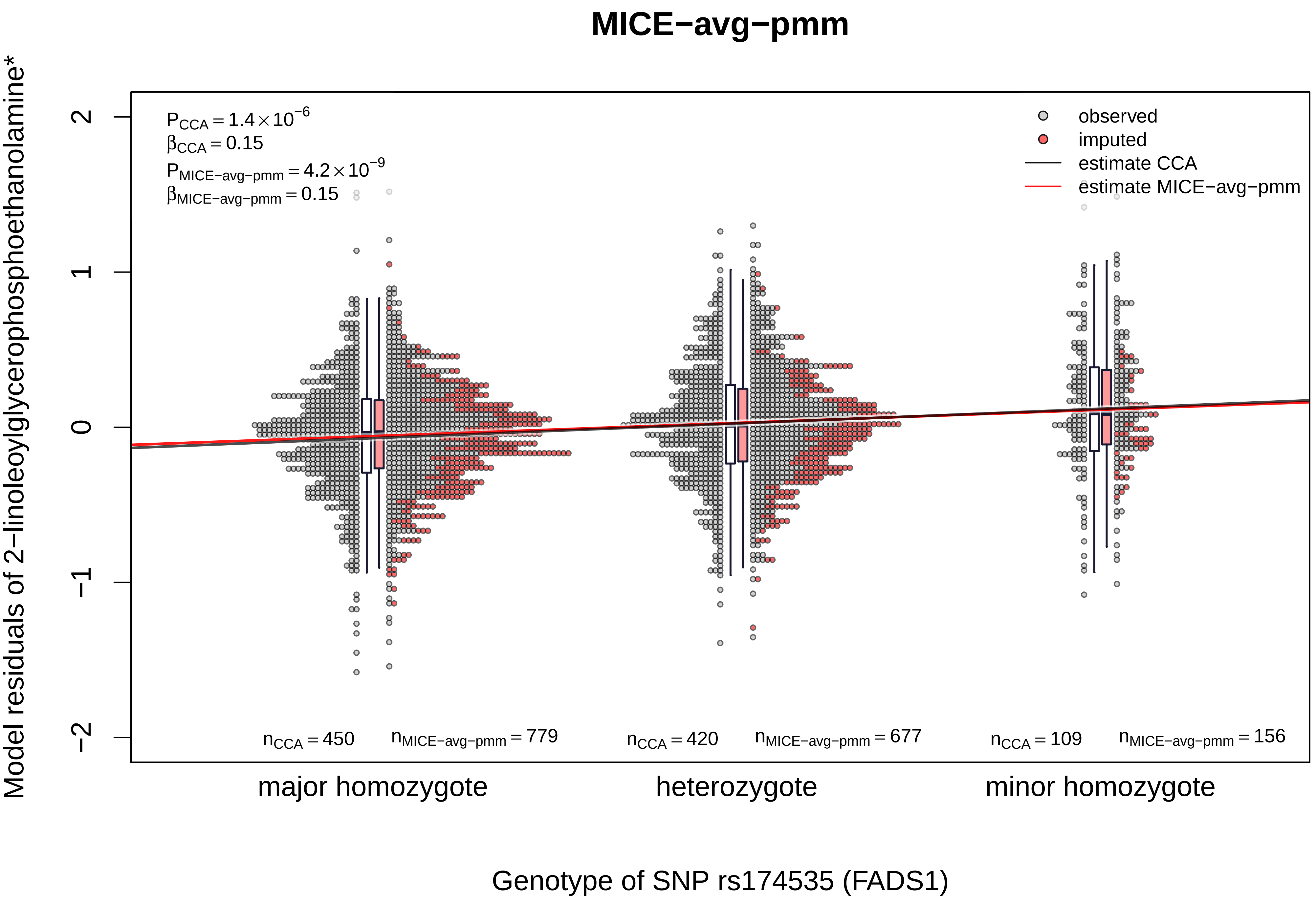

MICE−avg−pmm
Model residuals of 2−linoleoylglycerophosphoethanolamine*
2
1
0
−1
−2
$P_{CCA} = 1.4 \times 10^{-6}$
$\beta_{CCA} = 0.15$
$P_{MICE-avg-pmm} = 4.2 \times 10^{-9}$
$\beta_{MICE-avg-pmm} = 0.15$
observed
imputed
estimate CCA
estimate MICE−avg−pmm
$n_{CCA} = 450$
$n_{MICE-avg-pmm} = 779$
$n_{CCA} = 420$
$n_{MICE-avg-pmm} = 677$
$n_{CCA} = 109$
$n_{MICE-avg-pmm} = 156$
major homozygote
heterozygote
minor homozygote
Genotype of SNP rs174535 (FADS1)

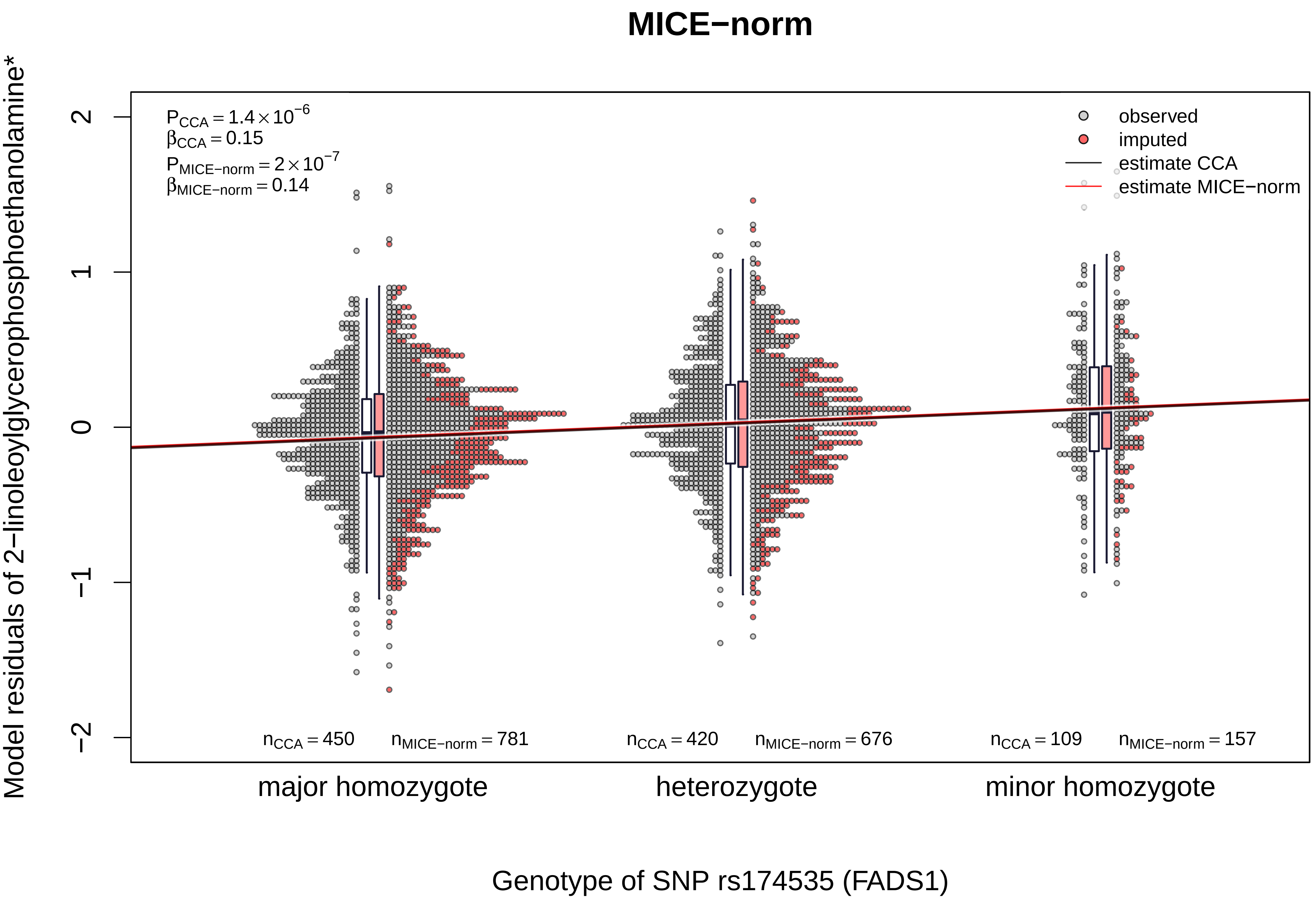

MICE−norm
$P_{CCA} = 1.4 \times 10^{-6}$
$\beta_{CCA} = 0.15$
$P_{MICE-norm} = 2 \times 10^{-7}$
$\beta_{MICE-norm} = 0.14$
observed
imputed
estimate CCA
estimate MICE−norm
2
1
0
−1
−2
$n_{CCA} = 450$
$n_{MICE-norm} = 781$
$n_{CCA} = 420$
$n_{MICE-norm} = 676$
$n_{CCA} = 109$
$n_{MICE-norm} = 157$
major homozygote
heterozygote
minor homozygote
Model residuals of 2−linoleoylglycerophosphoethanolamine*
Genotype of SNP rs174535 (FADS1)

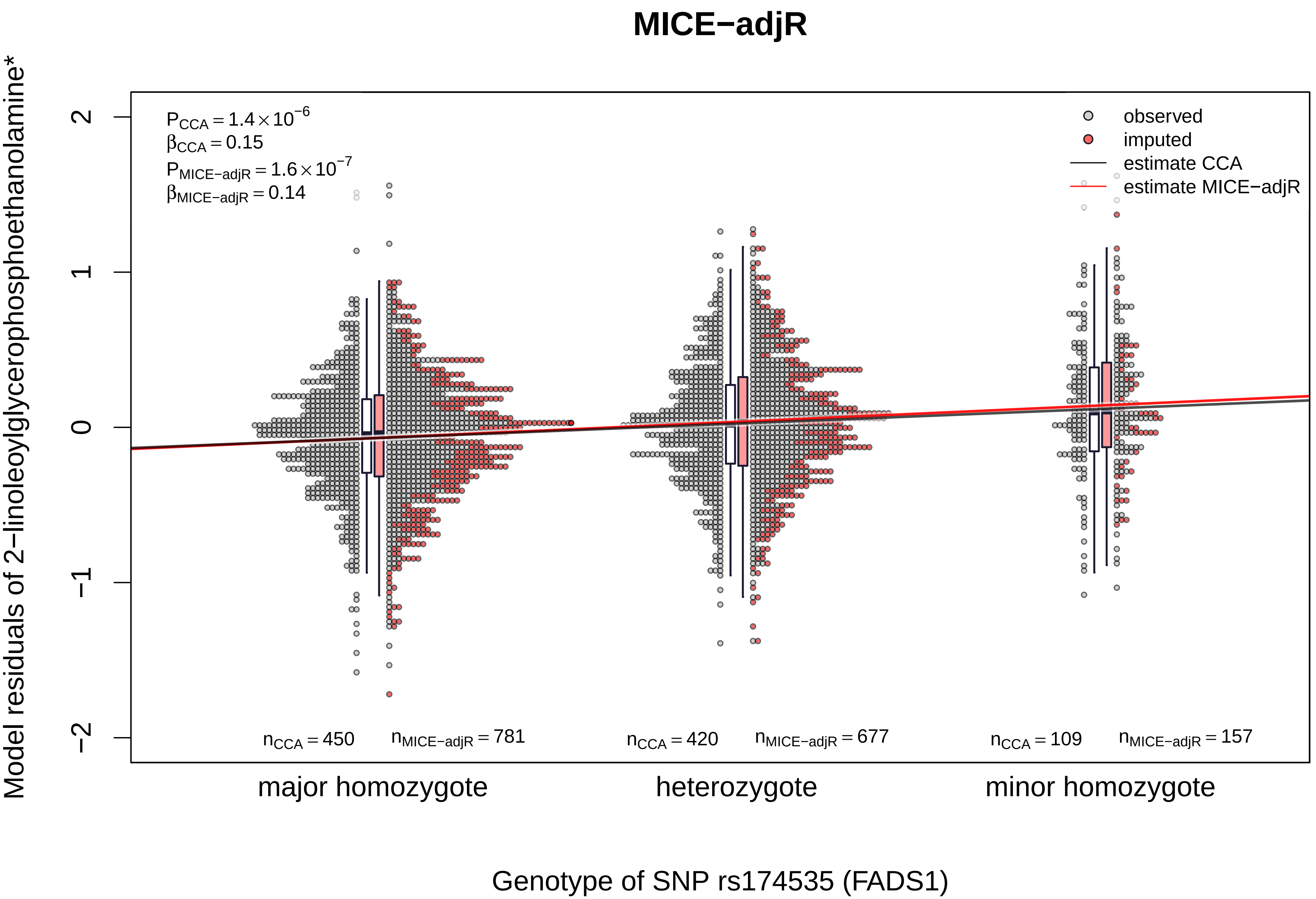

MICE−adjR
$P_{CCA} = 1.4 \times 10^{-6}$
$\beta_{CCA} = 0.15$
$P_{MICE-adjR} = 1.6 \times 10^{-7}$
$\beta_{MICE-adjR} = 0.14$
observed
imputed
estimate CCA
estimate MICE−adjR
$n_{CCA} = 450$
$n_{MICE-adjR} = 781$
$n_{CCA} = 420$
$n_{MICE-adjR} = 677$
$n_{CCA} = 109$
$n_{MICE-adjR} = 157$
2
1
0
−1
−2
major homozygote
heterozygote
minor homozygote
Model residuals of 2−linoleoylglycerophosphoethanolamine*
Genotype of SNP rs174535 (FADS1)

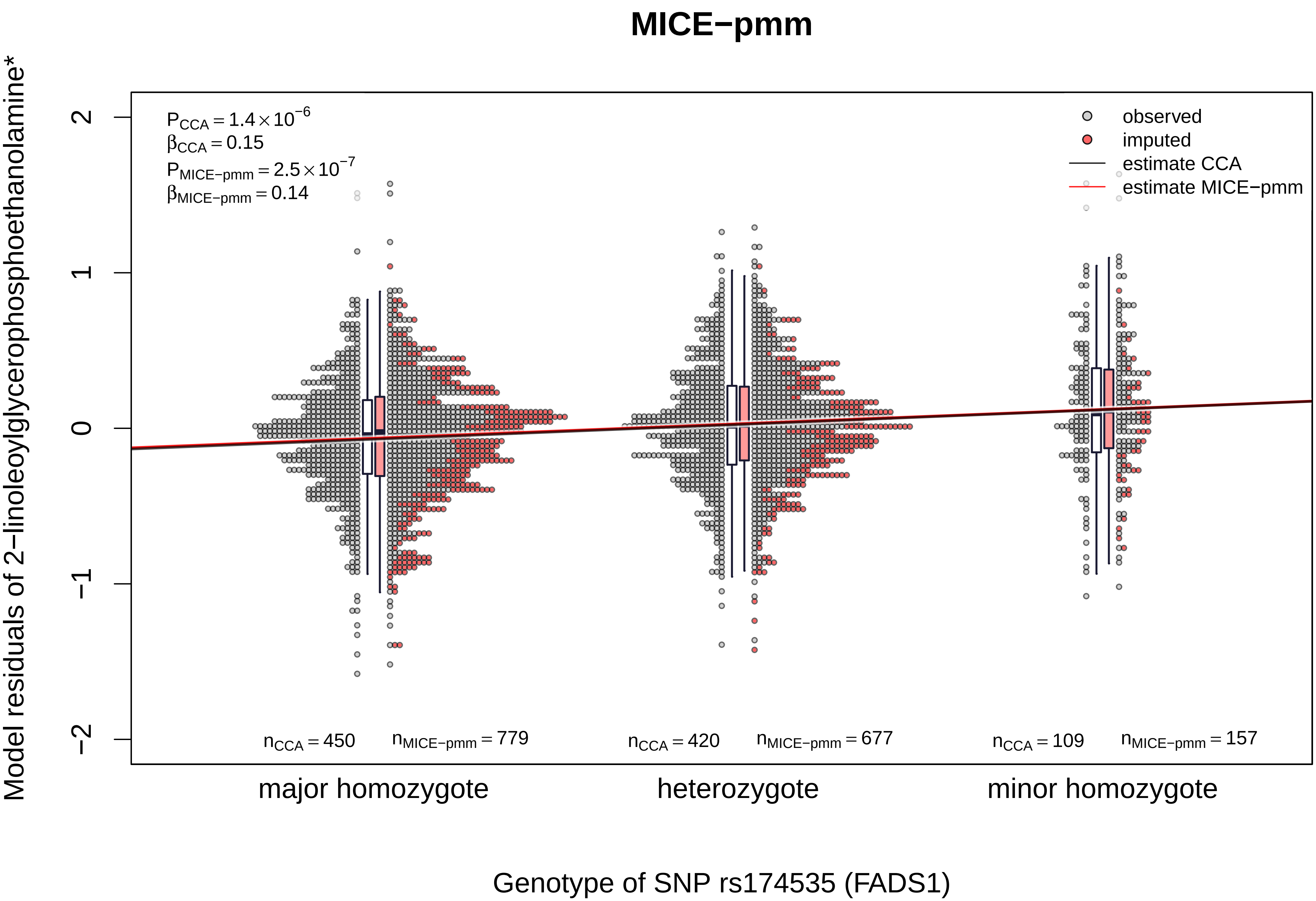
MICE−pmm
$P_{CCA} = 1.4 \times 10^{-6}$
$\beta_{CCA} = 0.15$
$P_{MICE-pmm} = 2.5 \times 10^{-7}$
$\beta_{MICE-pmm} = 0.14$
observed
imputed
estimate CCA
estimate MICE−pmm
$n_{CCA} = 450$
$n_{MICE-pmm} = 779$
$n_{CCA} = 420$
$n_{MICE-pmm} = 677$
$n_{CCA} = 109$
$n_{MICE-pmm} = 157$
Model residuals of 2−linoleoylglycerophosphoethanolamine*
2
1
0
−1
−2
major homozygote
heterozygote
minor homozygote
Genotype of SNP rs174535 (FADS1)

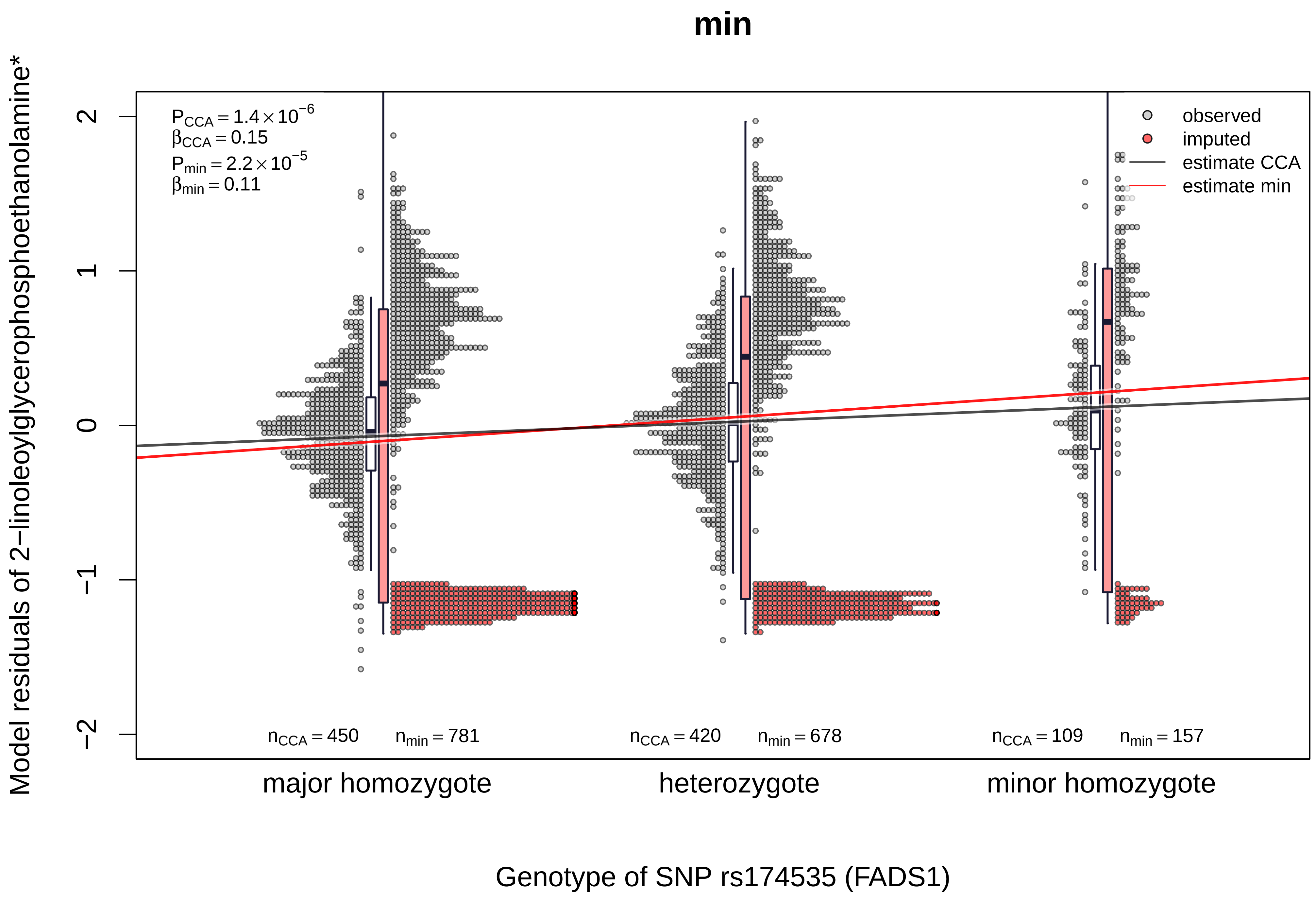

min
$P_{CCA} = 1.4 \times 10^{-6}$
$\beta_{CCA} = 0.15$
$P_{min} = 2.2 \times 10^{-5}$
$\beta_{min} = 0.11$
observed
imputed
estimate CCA
estimate min
Model residuals of 2−linoleoylglycerophosphoethanolamine*
2
1
0
−1
−2
$n_{CCA} = 450$
$n_{min} = 781$
$n_{CCA} = 420$
$n_{min} = 678$
$n_{CCA} = 109$
$n_{min} = 157$
major homozygote
heterozygote
minor homozygote
Genotype of SNP rs174535 (FADS1)

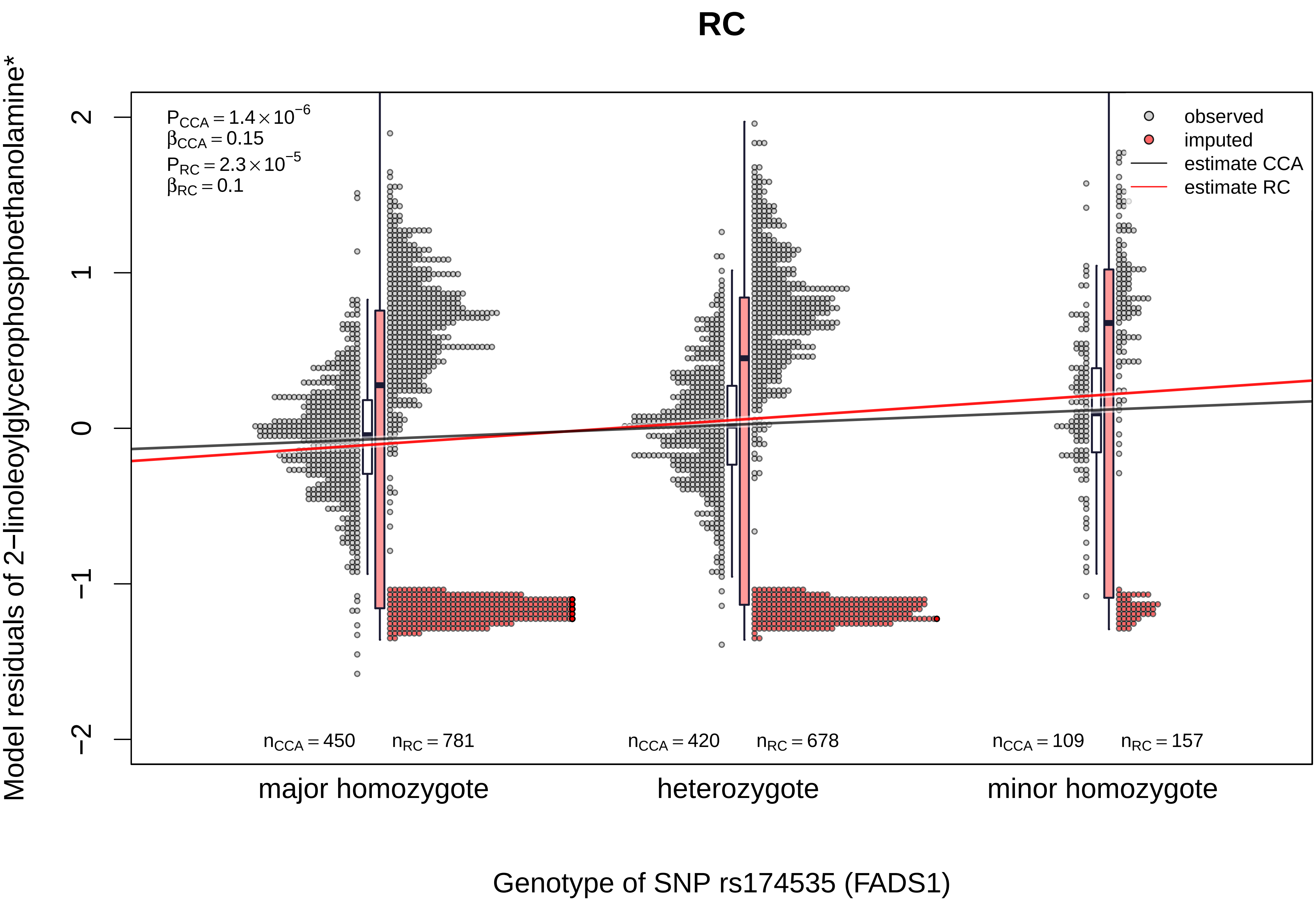

RC
$P_{CCA} = 1.4 \times 10^{-6}$
$\beta_{CCA} = 0.15$
$P_{RC} = 2.3 \times 10^{-5}$
$\beta_{RC} = 0.1$
observed
imputed
estimate CCA
estimate RC
2
1
0
−1
−2
$n_{CCA} = 450$
$n_{RC} = 781$
$n_{CCA} = 420$
$n_{RC} = 678$
$n_{CCA} = 109$
$n_{RC} = 157$
major homozygote
heterozygote
minor homozygote
Genotype of SNP rs174535 (FADS1)
Model residuals of 2−linoleoylglycerophosphoethanolamine*

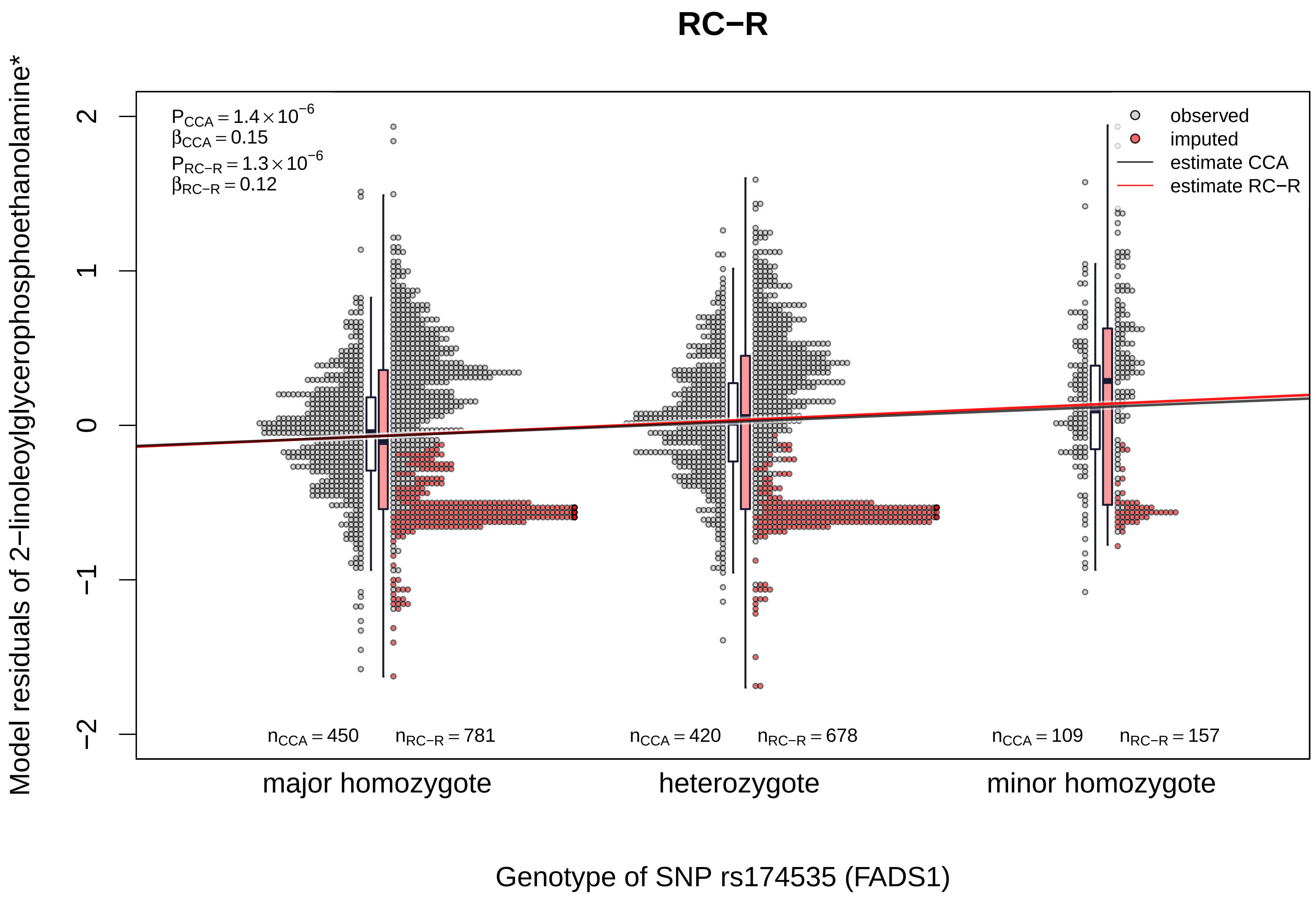
RC−R
$P_{CCA} = 1.4 \times 10^{-6}$
$\beta_{CCA} = 0.15$
$P_{RC-R} = 1.3 \times 10^{-6}$
$\beta_{RC-R} = 0.12$
observed
imputed
estimate CCA
estimate RC−R
$n_{CCA} = 450$
$n_{RC-R} = 781$
$n_{CCA} = 420$
$n_{RC-R} = 678$
$n_{CCA} = 109$
$n_{RC-R} = 157$
2
1
0
−1
−2
Model residuals of 2−linoleoylglycerophosphoethanolamine*
major homozygote
heterozygote
minor homozygote
Genotype of SNP rs174535 (FADS1)

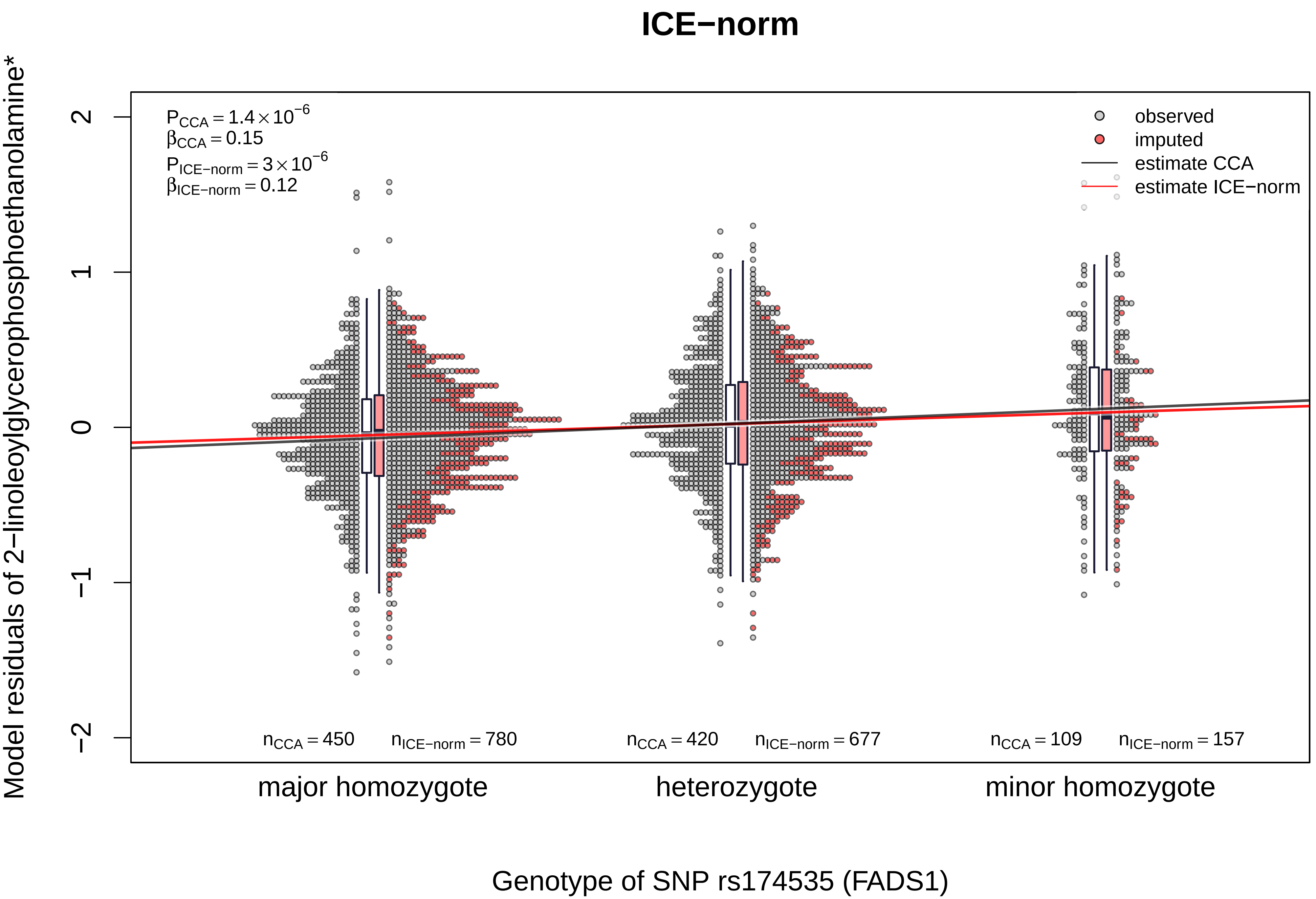

ICE−norm
$P_{CCA} = 1.4 \times 10^{-6}$
$\beta_{CCA} = 0.15$
$P_{ICE-norm} = 3 \times 10^{-6}$
$\beta_{ICE-norm} = 0.12$
observed
imputed
estimate CCA
estimate ICE−norm
Model residuals of 2−linoleoylglycerophosphoethanolamine*
2
1
0
−1
−2
$n_{CCA} = 450$
$n_{ICE-norm} = 780$
$n_{CCA} = 420$
$n_{ICE-norm} = 677$
$n_{CCA} = 109$
$n_{ICE-norm} = 157$
major homozygote
heterozygote
minor homozygote
Genotype of SNP rs174535 (FADS1)

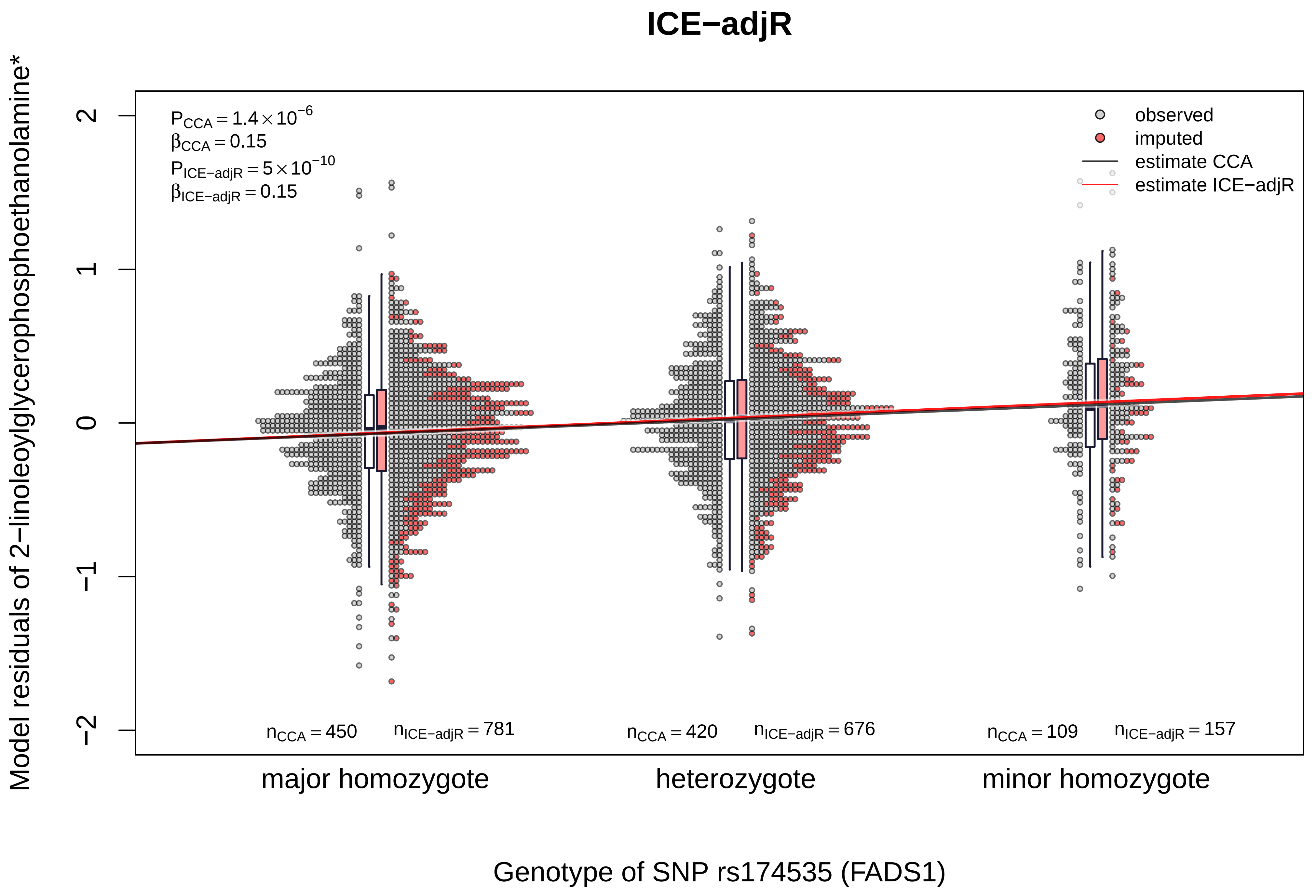

ICE−adjR
P_CCA = 1.4 × 10^−6
β_CCA = 0.15
P_ICE−adjR = 5 × 10^−10
β_ICE−adjR = 0.15
observed
imputed
estimate CCA
estimate ICE−adjR
2
1
0
−1
−2
n_CCA = 450
n_ICE−adjR = 781
n_CCA = 420
n_ICE−adjR = 676
n_CCA = 109
n_ICE−adjR = 157
major homozygote
heterozygote
minor homozygote
Model residuals of 2−linoleoylglycerophosphoethanolamine*
Genotype of SNP rs174535 (FADS1)

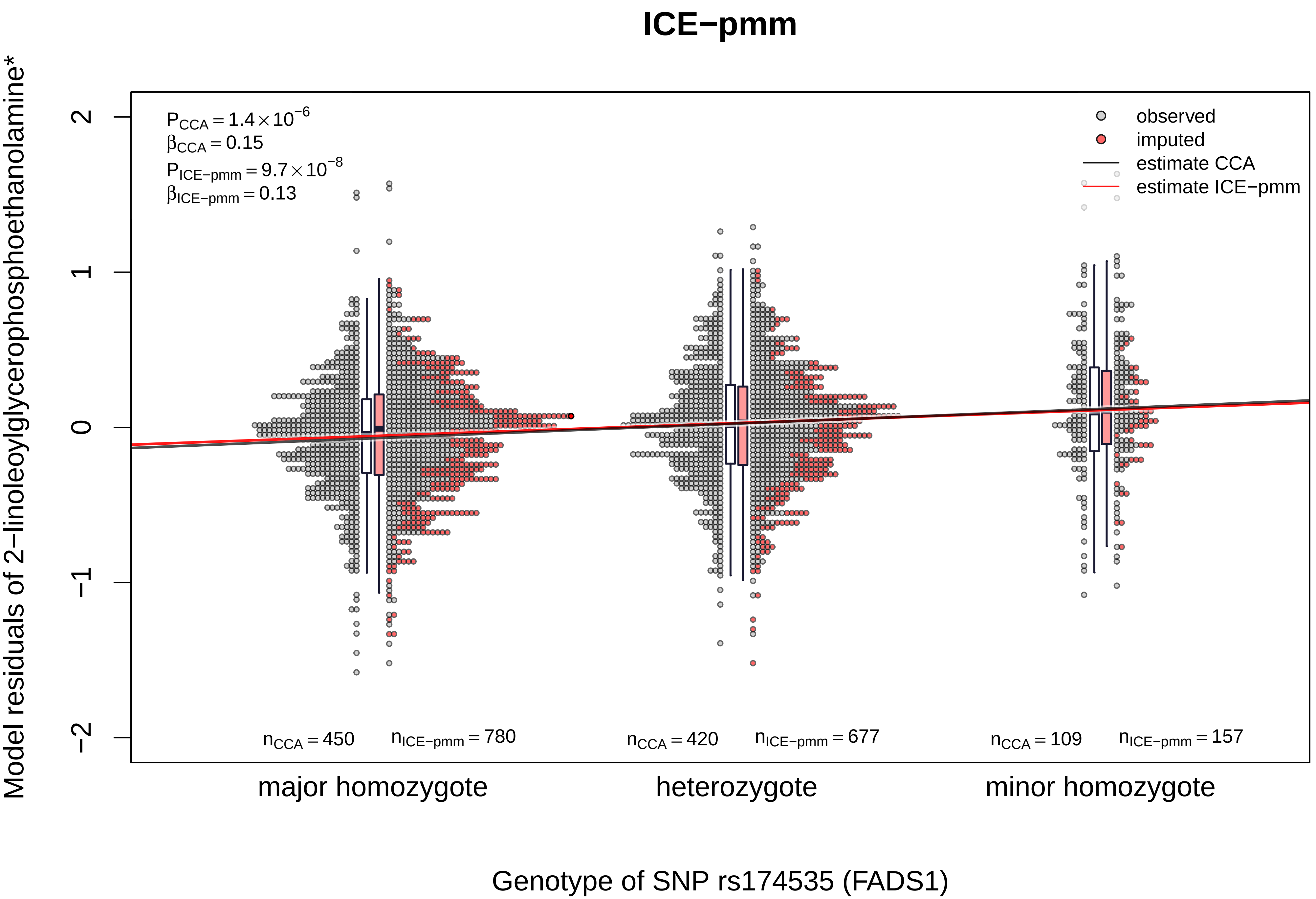

ICE−pmm
$P_{CCA} = 1.4 \times 10^{-6}$
$\beta_{CCA} = 0.15$
$P_{ICE-pmm} = 9.7 \times 10^{-8}$
$\beta_{ICE-pmm} = 0.13$
observed
imputed
estimate CCA
estimate ICE−pmm
2
1
0
−1
−2
Model residuals of 2−linoleoylglycerophosphoethanolamine*
$n_{CCA} = 450$
$n_{ICE-pmm} = 780$
$n_{CCA} = 420$
$n_{ICE-pmm} = 677$
$n_{CCA} = 109$
$n_{ICE-pmm} = 157$
major homozygote
heterozygote
minor homozygote
Genotype of SNP rs174535 (FADS1)

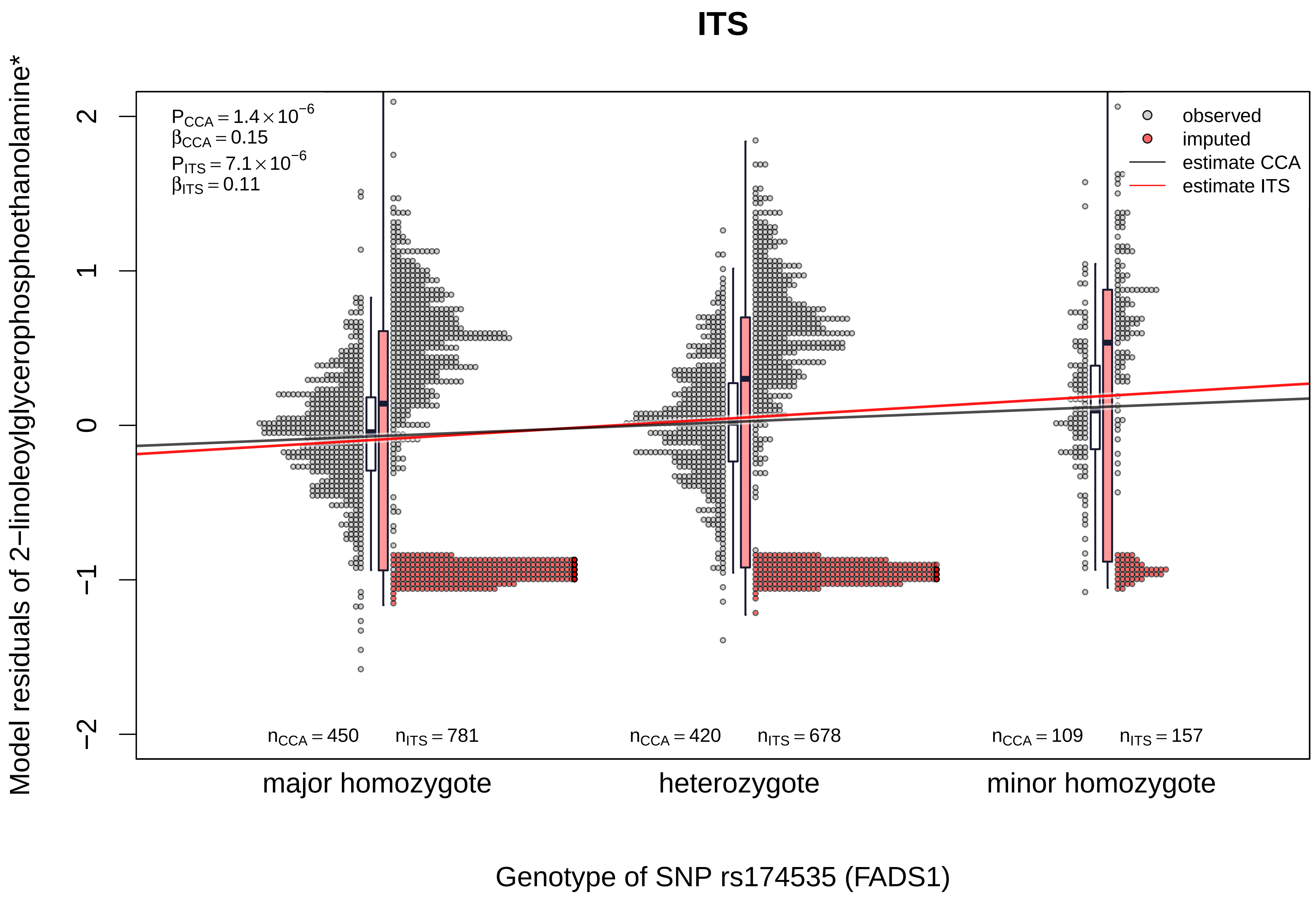

ITS
$P_{CCA} = 1.4 \times 10^{-6}$
$\beta_{CCA} = 0.15$
$P_{ITS} = 7.1 \times 10^{-6}$
$\beta_{ITS} = 0.11$
observed
imputed
estimate CCA
estimate ITS
$n_{CCA} = 450$
$n_{ITS} = 781$
$n_{CCA} = 420$
$n_{ITS} = 678$
$n_{CCA} = 109$
$n_{ITS} = 157$
2
1
0
−1
−2
major homozygote
heterozygote
minor homozygote
Model residuals of 2−linoleoylglycerophosphoethanolamine*
Genotype of SNP rs174535 (FADS1)

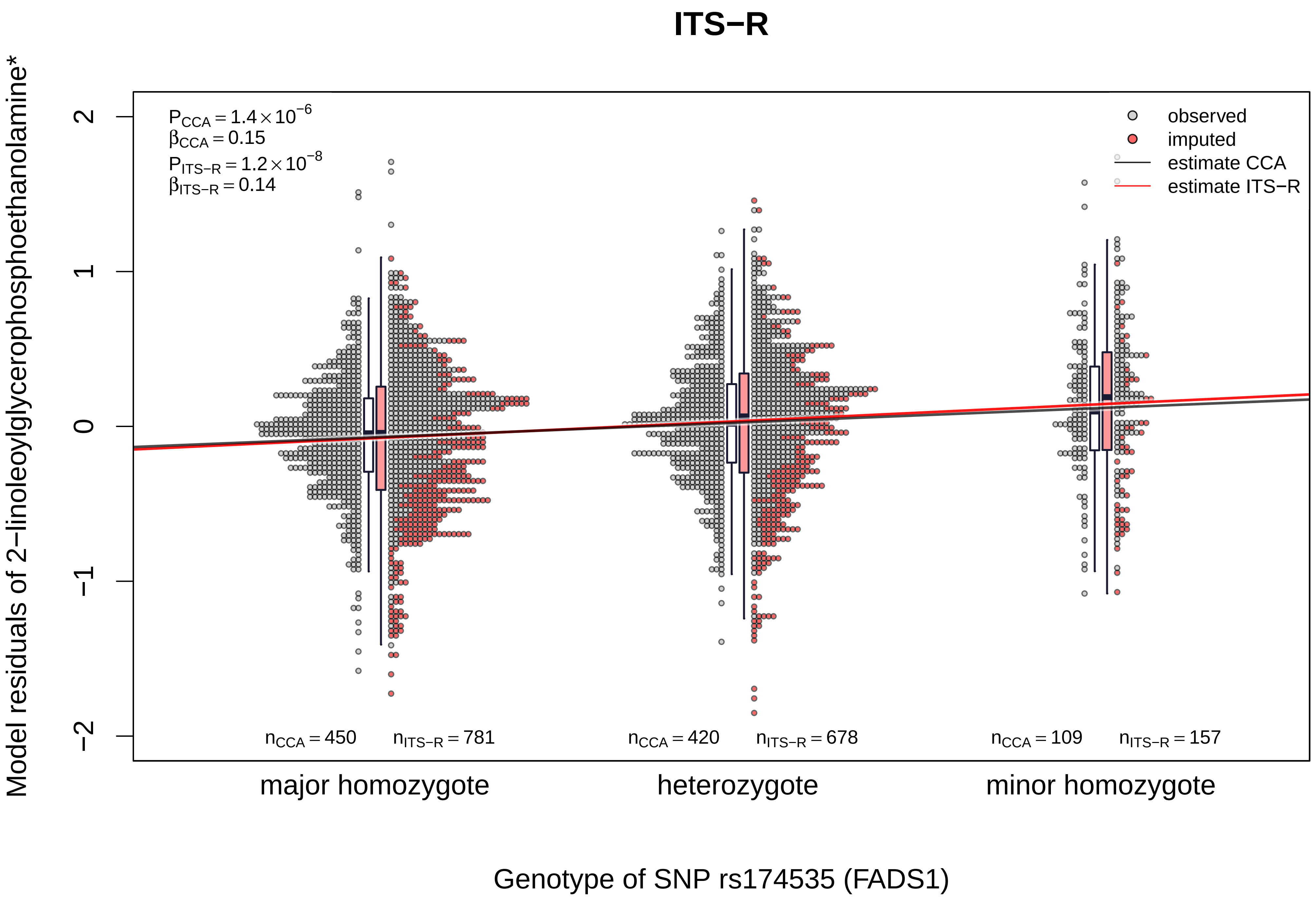
ITS−R
$P_{CCA} = 1.4 \times 10^{-6}$
$\beta_{CCA} = 0.15$
$P_{ITS-R} = 1.2 \times 10^{-8}$
$\beta_{ITS-R} = 0.14$
observed
imputed
estimate CCA
estimate ITS−R
$n_{CCA} = 450$
$n_{ITS-R} = 781$
$n_{CCA} = 420$
$n_{ITS-R} = 678$
$n_{CCA} = 109$
$n_{ITS-R} = 157$
2
1
0
−1
−2
major homozygote
heterozygote
minor homozygote
Model residuals of 2−linoleoylglycerophosphoethanolamine*
Genotype of SNP rs174535 (FADS1)

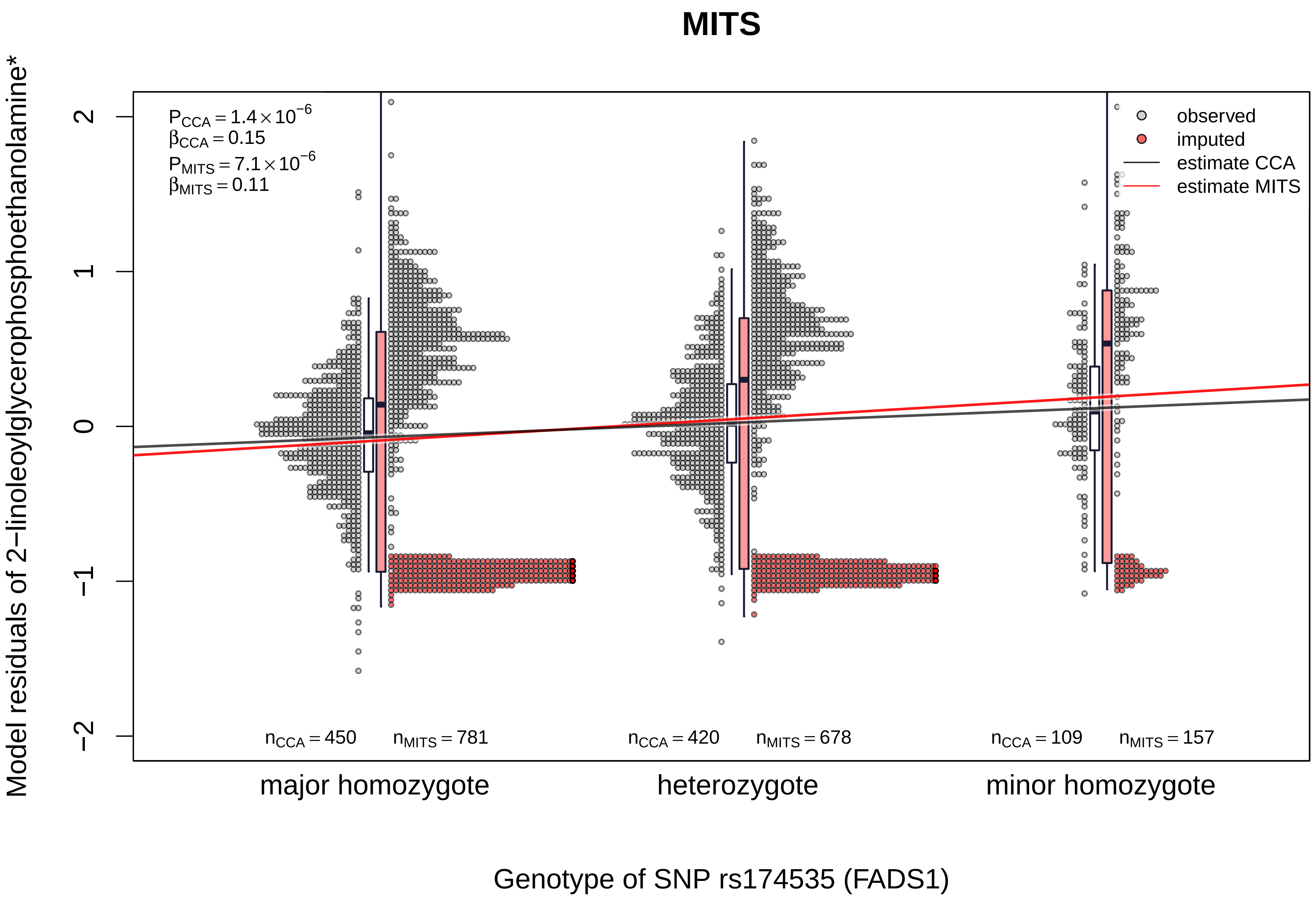

MITS
$P_{CCA} = 1.4 \times 10^{-6}$
$\beta_{CCA} = 0.15$
$P_{MITS} = 7.1 \times 10^{-6}$
$\beta_{MITS} = 0.11$
observed
imputed
estimate CCA
estimate MITS
$n_{CCA} = 450$
$n_{MITS} = 781$
$n_{CCA} = 420$
$n_{MITS} = 678$
$n_{CCA} = 109$
$n_{MITS} = 157$
major homozygote
heterozygote
minor homozygote
Genotype of SNP rs174535 (FADS1)
Model residuals of 2−linoleoylglycerophosphoethanolamine*
2
1
0
−1
−2

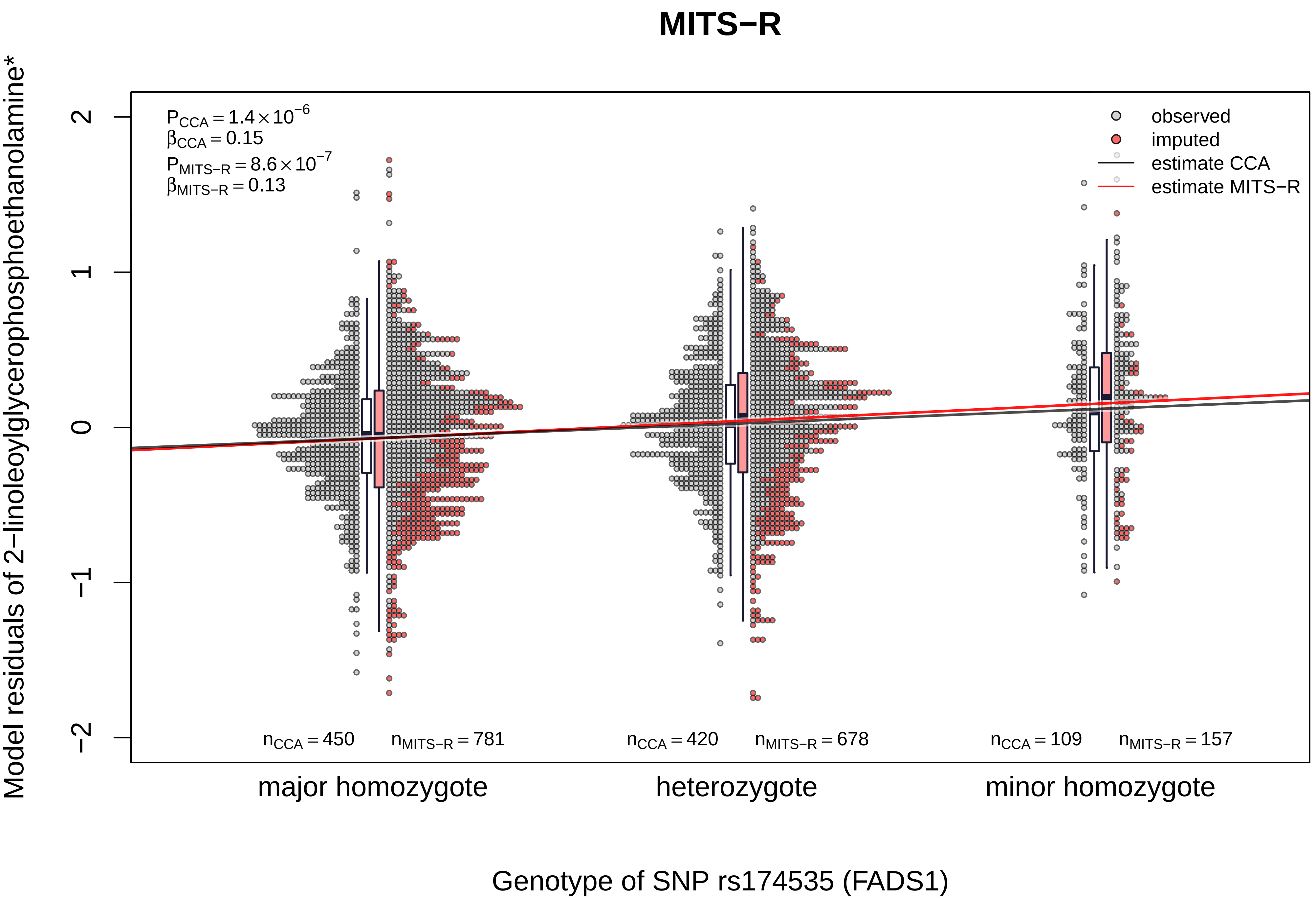

MITS−R
$P_{CCA} = 1.4 \times 10^{-6}$
$\beta_{CCA} = 0.15$
$P_{MITS-R} = 8.6 \times 10^{-7}$
$\beta_{MITS-R} = 0.13$
observed
imputed
estimate CCA
estimate MITS−R
$n_{CCA} = 450$
$n_{MITS-R} = 781$
$n_{CCA} = 420$
$n_{MITS-R} = 678$
$n_{CCA} = 109$
$n_{MITS-R} = 157$
2
1
0
−1
−2
Model residuals of 2−linoleoylglycerophosphoethanolamine*
major homozygote
heterozygote
minor homozygote
Genotype of SNP rs174535 (FADS1)

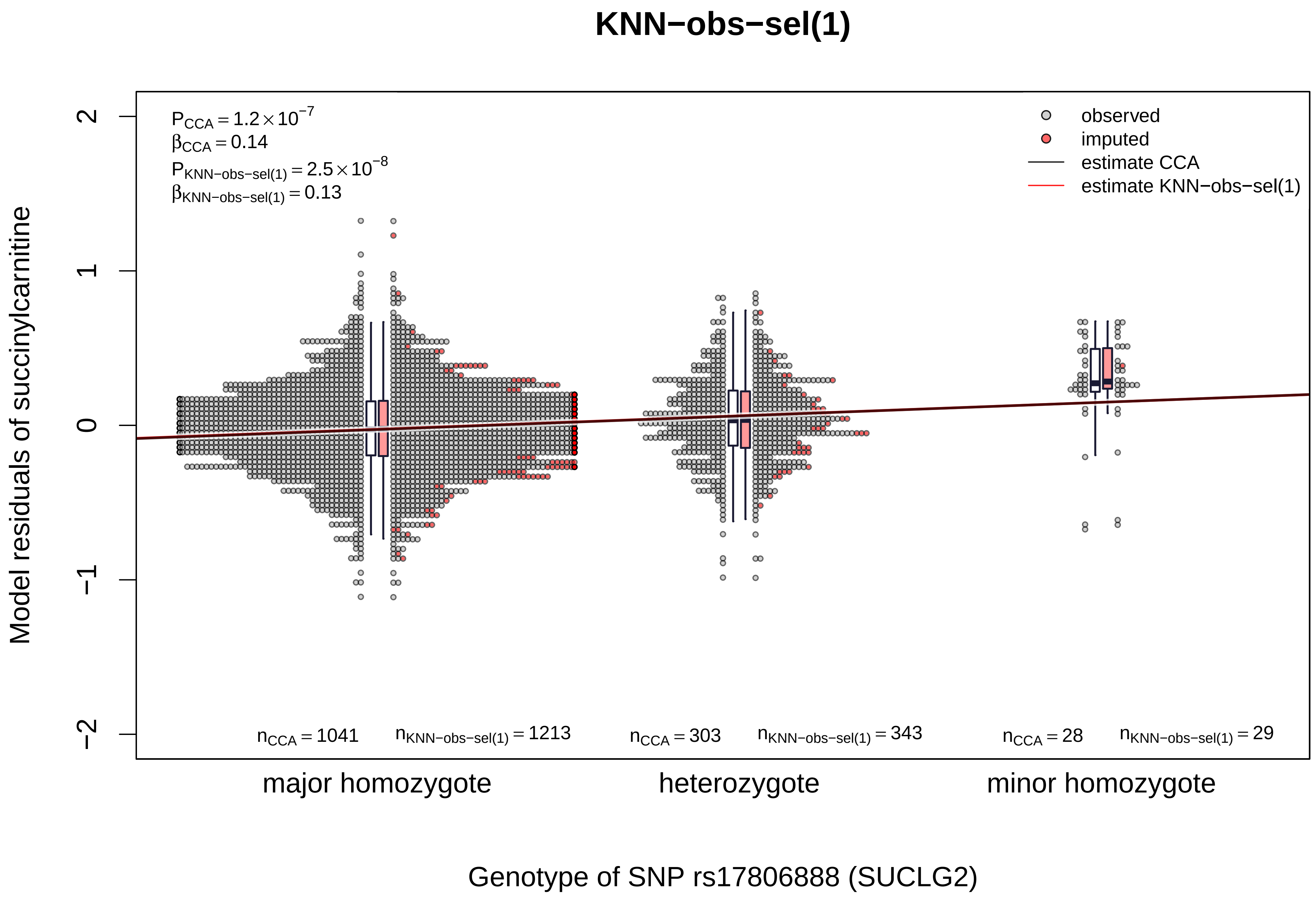
KNN−obs−sel(1)
$P_{CCA} = 1.2 \times 10^{-7}$
$\beta_{CCA} = 0.14$
$P_{KNN-obs-sel(1)} = 2.5 \times 10^{-8}$
$\beta_{KNN-obs-sel(1)} = 0.13$
observed
imputed
estimate CCA
estimate KNN−obs−sel(1)
Model residuals of succinylcarnitine
2
1
0
−1
−2
$n_{CCA} = 1041$
$n_{KNN-obs-sel(1)} = 1213$
$n_{CCA} = 303$
$n_{KNN-obs-sel(1)} = 343$
$n_{CCA} = 28$
$n_{KNN-obs-sel(1)} = 29$
major homozygote
heterozygote
minor homozygote
Genotype of SNP rs17806888 (SUCLG2)

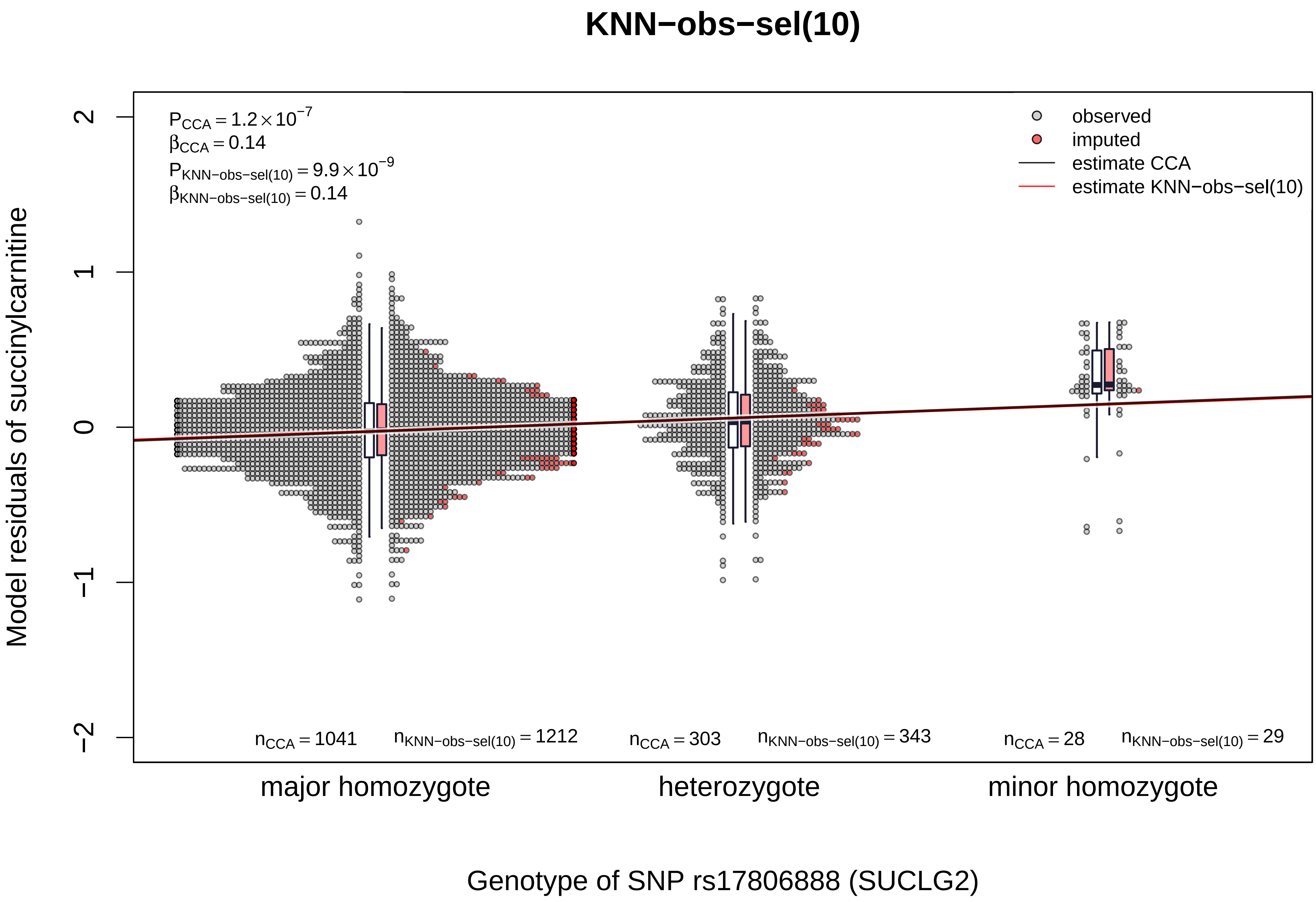

KNN−obs−sel(10)
$P_{CCA} = 1.2 \times 10^{-7}$
$\beta_{CCA} = 0.14$
$P_{KNN-obs-sel(10)} = 9.9 \times 10^{-9}$
$\beta_{KNN-obs-sel(10)} = 0.14$
observed
imputed
estimate CCA
estimate KNN−obs−sel(10)
Model residuals of succinylcarnitine
2
1
0
−1
−2
$n_{CCA} = 1041$
$n_{KNN-obs-sel(10)} = 1212$
$n_{CCA} = 303$
$n_{KNN-obs-sel(10)} = 343$
$n_{CCA} = 28$
$n_{KNN-obs-sel(10)} = 29$
major homozygote
heterozygote
minor homozygote
Genotype of SNP rs17806888 (SUCLG2)

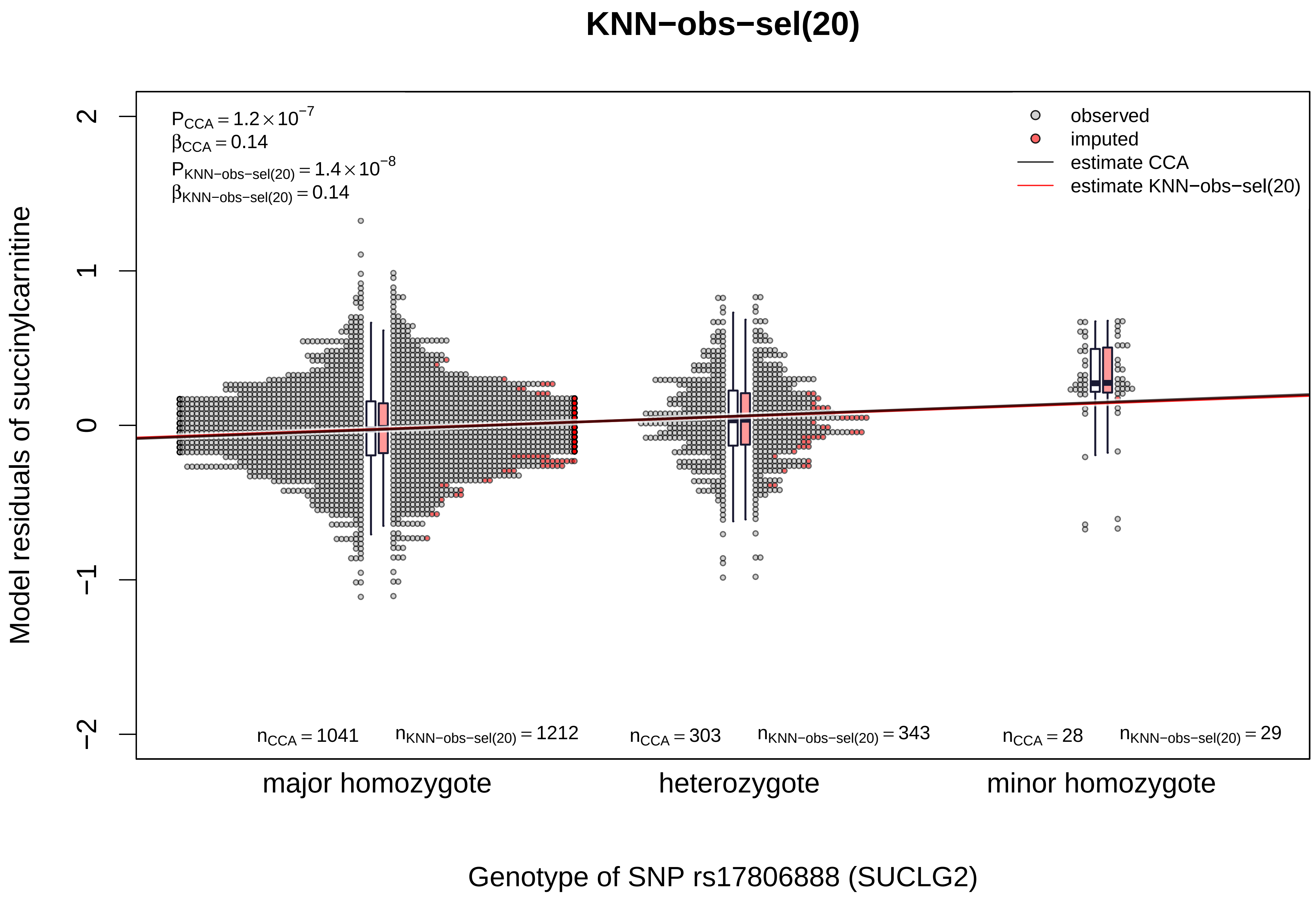
KNN−obs−sel(20)
$P_{CCA} = 1.2 \times 10^{-7}$
$\beta_{CCA} = 0.14$
$P_{KNN-obs-sel(20)} = 1.4 \times 10^{-8}$
$\beta_{KNN-obs-sel(20)} = 0.14$
observed
imputed
estimate CCA
estimate KNN−obs−sel(20)
Model residuals of succinylcarnitine
2
1
0
−1
−2
$n_{CCA} = 1041$
$n_{KNN-obs-sel(20)} = 1212$
$n_{CCA} = 303$
$n_{KNN-obs-sel(20)} = 343$
$n_{CCA} = 28$
$n_{KNN-obs-sel(20)} = 29$
major homozygote
heterozygote
minor homozygote
Genotype of SNP rs17806888 (SUCLG2)

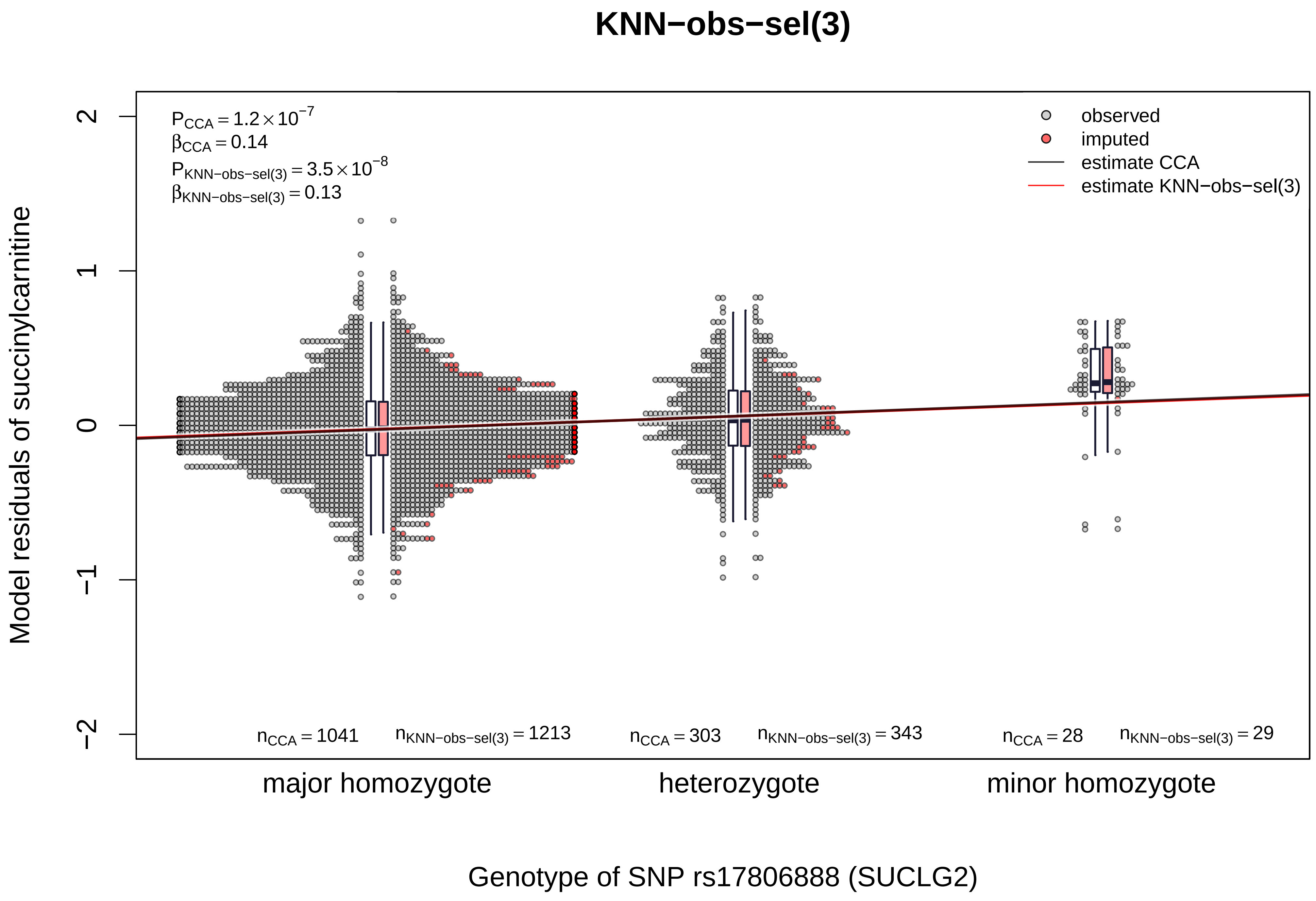

KNN−obs−sel(3)
$P_{CCA} = 1.2 \times 10^{-7}$
$\beta_{CCA} = 0.14$
$P_{KNN-obs-sel(3)} = 3.5 \times 10^{-8}$
$\beta_{KNN-obs-sel(3)} = 0.13$
observed
imputed
estimate CCA
estimate KNN−obs−sel(3)
2
1
0
−1
−2
Model residuals of succinylcarnitine
$n_{CCA} = 1041$
$n_{KNN-obs-sel(3)} = 1213$
$n_{CCA} = 303$
$n_{KNN-obs-sel(3)} = 343$
$n_{CCA} = 28$
$n_{KNN-obs-sel(3)} = 29$
major homozygote
heterozygote
minor homozygote
Genotype of SNP rs17806888 (SUCLG2)

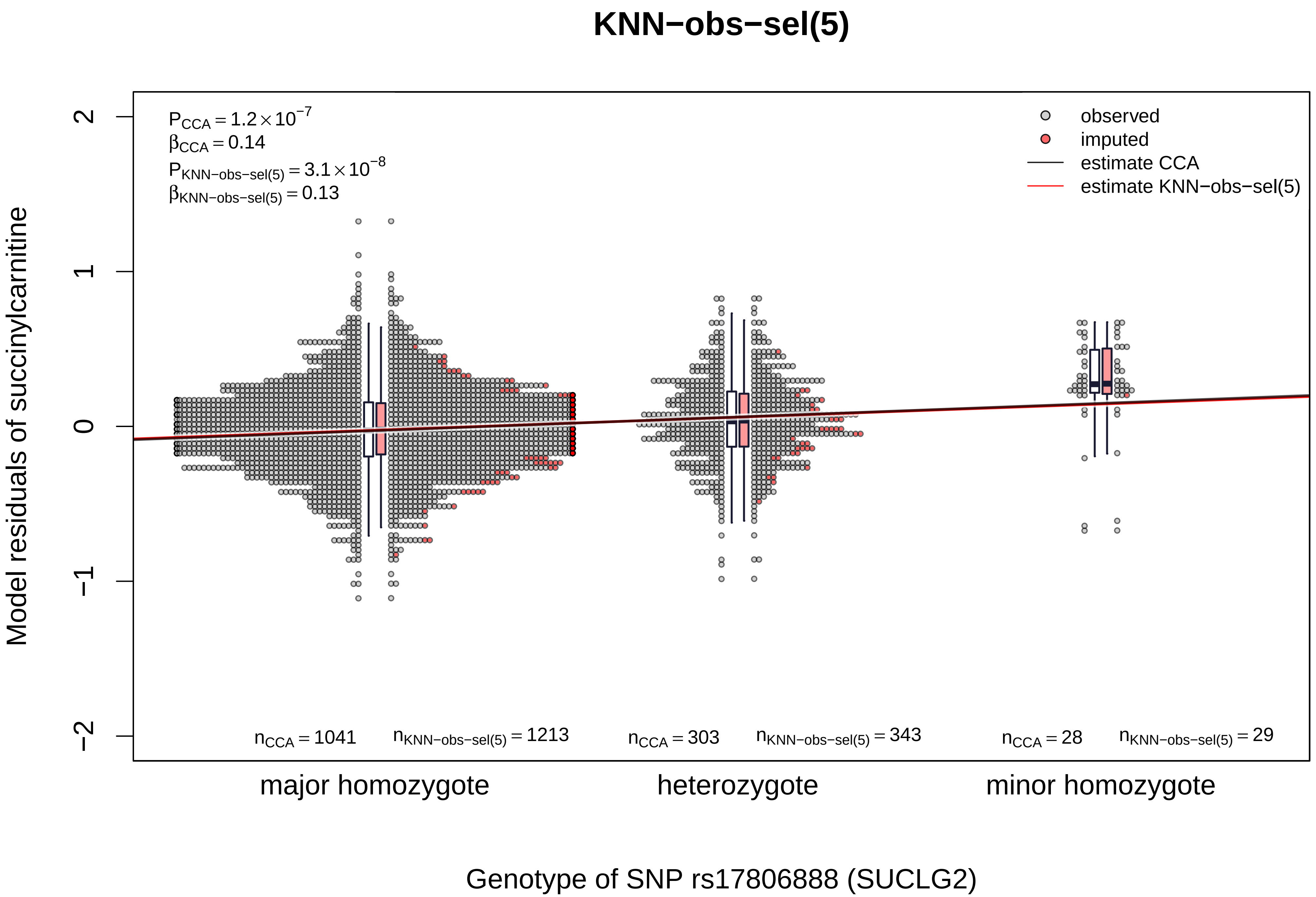

KNN−obs−sel(5)
$P_{CCA} = 1.2 \times 10^{-7}$
$\beta_{CCA} = 0.14$
$P_{KNN-obs-sel(5)} = 3.1 \times 10^{-8}$
$\beta_{KNN-obs-sel(5)} = 0.13$
observed
imputed
estimate CCA
estimate KNN−obs−sel(5)
Model residuals of succinylcarnitine
2
1
0
−1
−2
$n_{CCA} = 1041$
$n_{KNN-obs-sel(5)} = 1213$
$n_{CCA} = 303$
$n_{KNN-obs-sel(5)} = 343$
$n_{CCA} = 28$
$n_{KNN-obs-sel(5)} = 29$
major homozygote
heterozygote
minor homozygote
Genotype of SNP rs17806888 (SUCLG2)

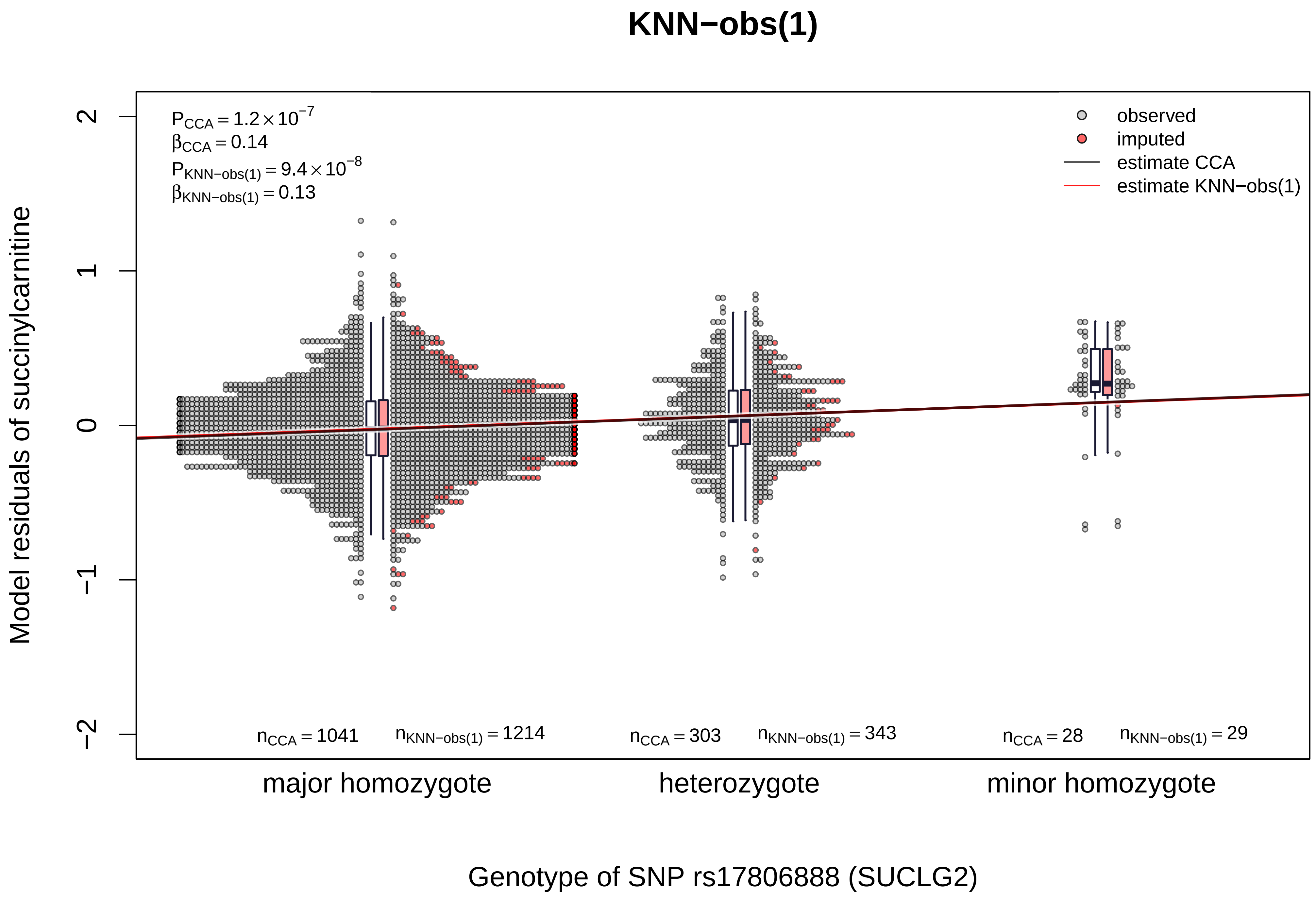

KNN−obs(1)
$P_{CCA} = 1.2 \times 10^{-7}$
$\beta_{CCA} = 0.14$
$P_{KNN-obs(1)} = 9.4 \times 10^{-8}$
$\beta_{KNN-obs(1)} = 0.13$
observed
imputed
estimate CCA
estimate KNN−obs(1)
Model residuals of succinylcarnitine
2
1
0
−1
−2
$n_{CCA} = 1041$
$n_{KNN-obs(1)} = 1214$
$n_{CCA} = 303$
$n_{KNN-obs(1)} = 343$
$n_{CCA} = 28$
$n_{KNN-obs(1)} = 29$
major homozygote
heterozygote
minor homozygote
Genotype of SNP rs17806888 (SUCLG2)

KNN−obs(10)

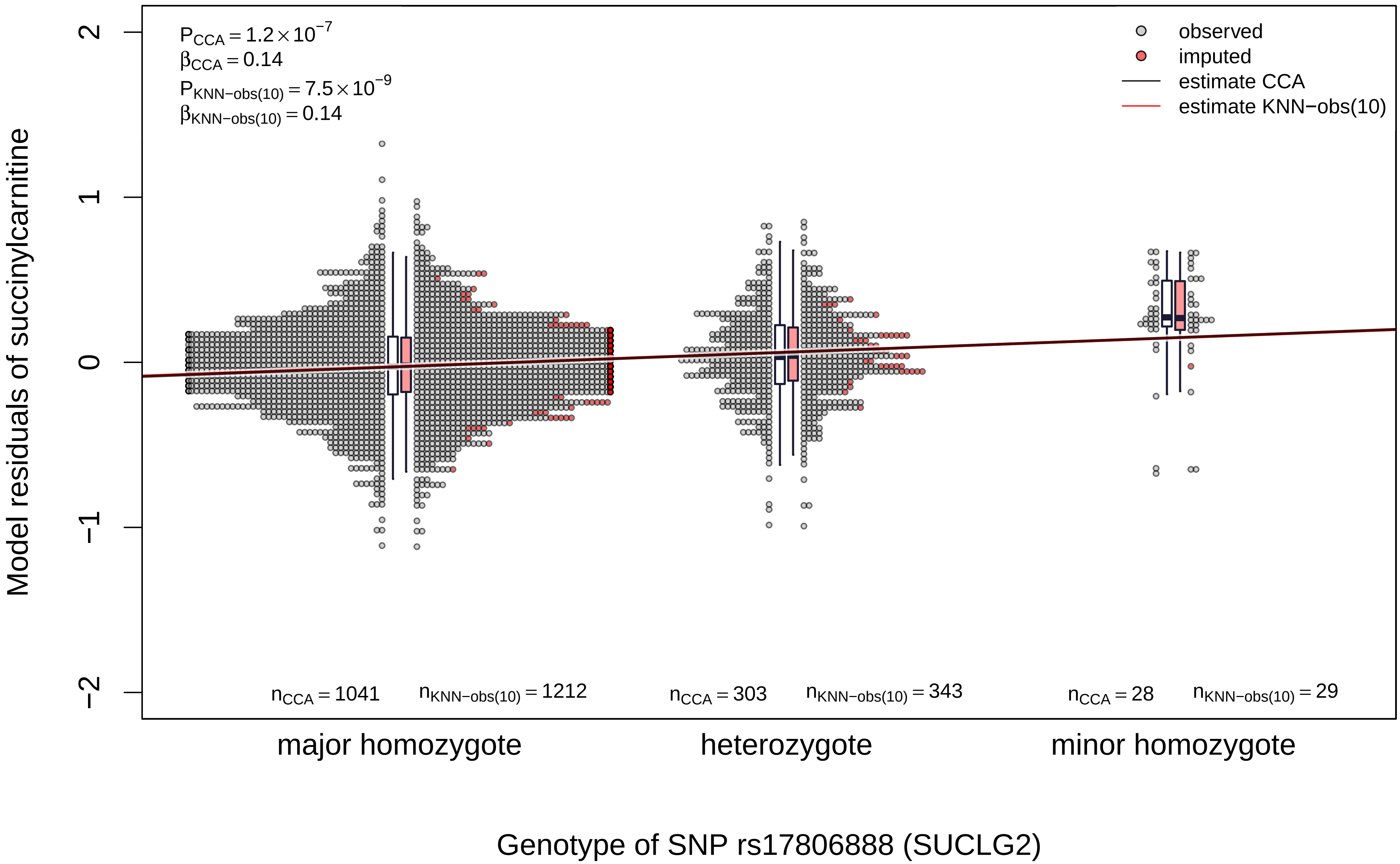

$P_{CCA} = 1.2 \times 10^{-7}$
$\beta_{CCA} = 0.14$
$P_{KNN-obs(10)} = 7.5 \times 10^{-9}$
$\beta_{KNN-obs(10)} = 0.14$
observed
imputed
estimate CCA
estimate KNN−obs(10)
Model residuals of succinylcarnitine
2
1
0
−1
−2
$n_{CCA} = 1041$
$n_{KNN-obs(10)} = 1212$
$n_{CCA} = 303$
$n_{KNN-obs(10)} = 343$
$n_{CCA} = 28$
$n_{KNN-obs(10)} = 29$
major homozygote
heterozygote
minor homozygote
Genotype of SNP rs17806888 (SUCLG2)

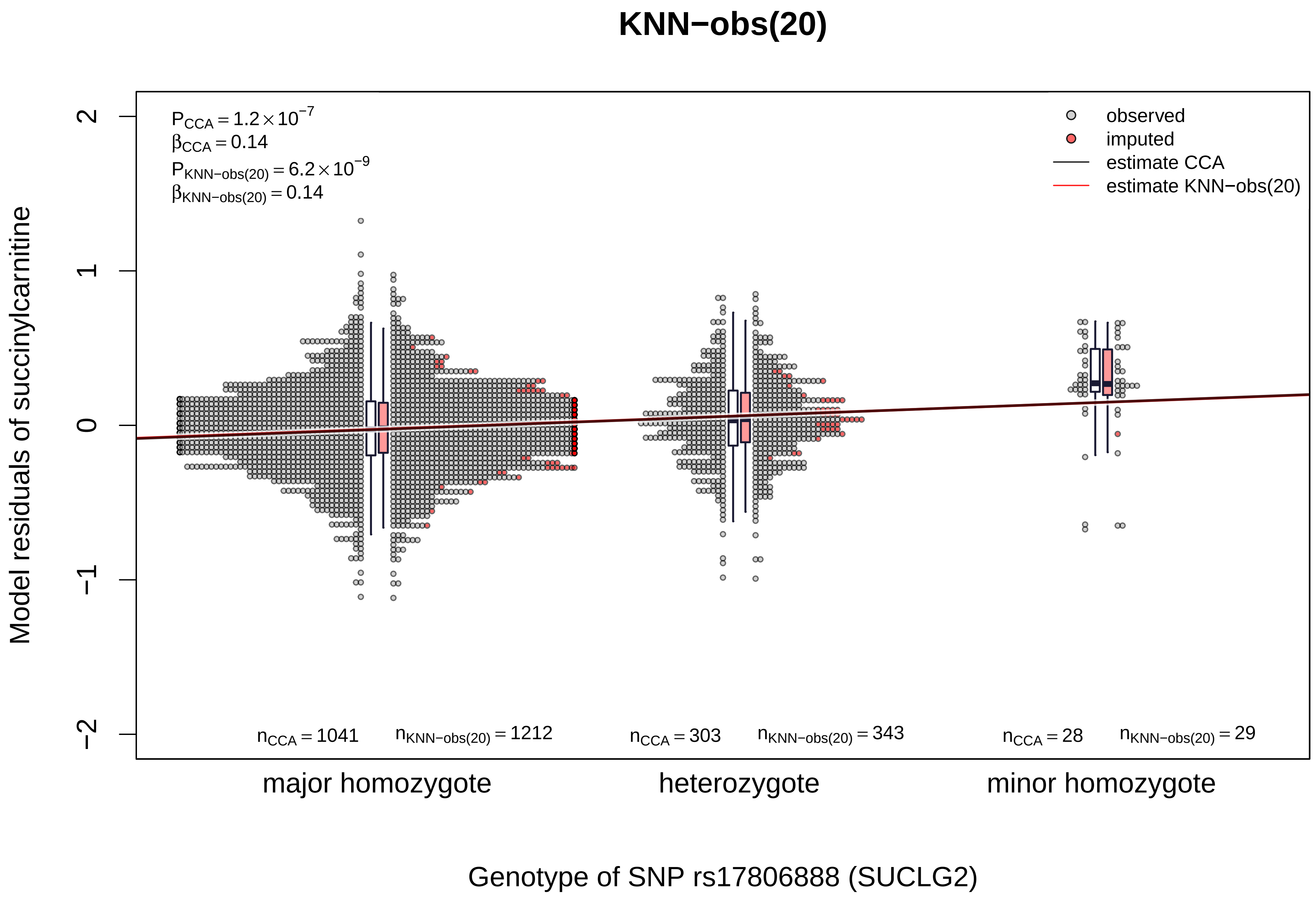

KNN−obs(20)
$P_{CCA} = 1.2 \times 10^{-7}$
$\beta_{CCA} = 0.14$
$P_{KNN-obs(20)} = 6.2 \times 10^{-9}$
$\beta_{KNN-obs(20)} = 0.14$
observed
imputed
estimate CCA
estimate KNN−obs(20)
Model residuals of succinylcarnitine
2
1
0
−1
−2
$n_{CCA} = 1041$
$n_{KNN-obs(20)} = 1212$
$n_{CCA} = 303$
$n_{KNN-obs(20)} = 343$
$n_{CCA} = 28$
$n_{KNN-obs(20)} = 29$
major homozygote
heterozygote
minor homozygote
Genotype of SNP rs17806888 (SUCLG2)

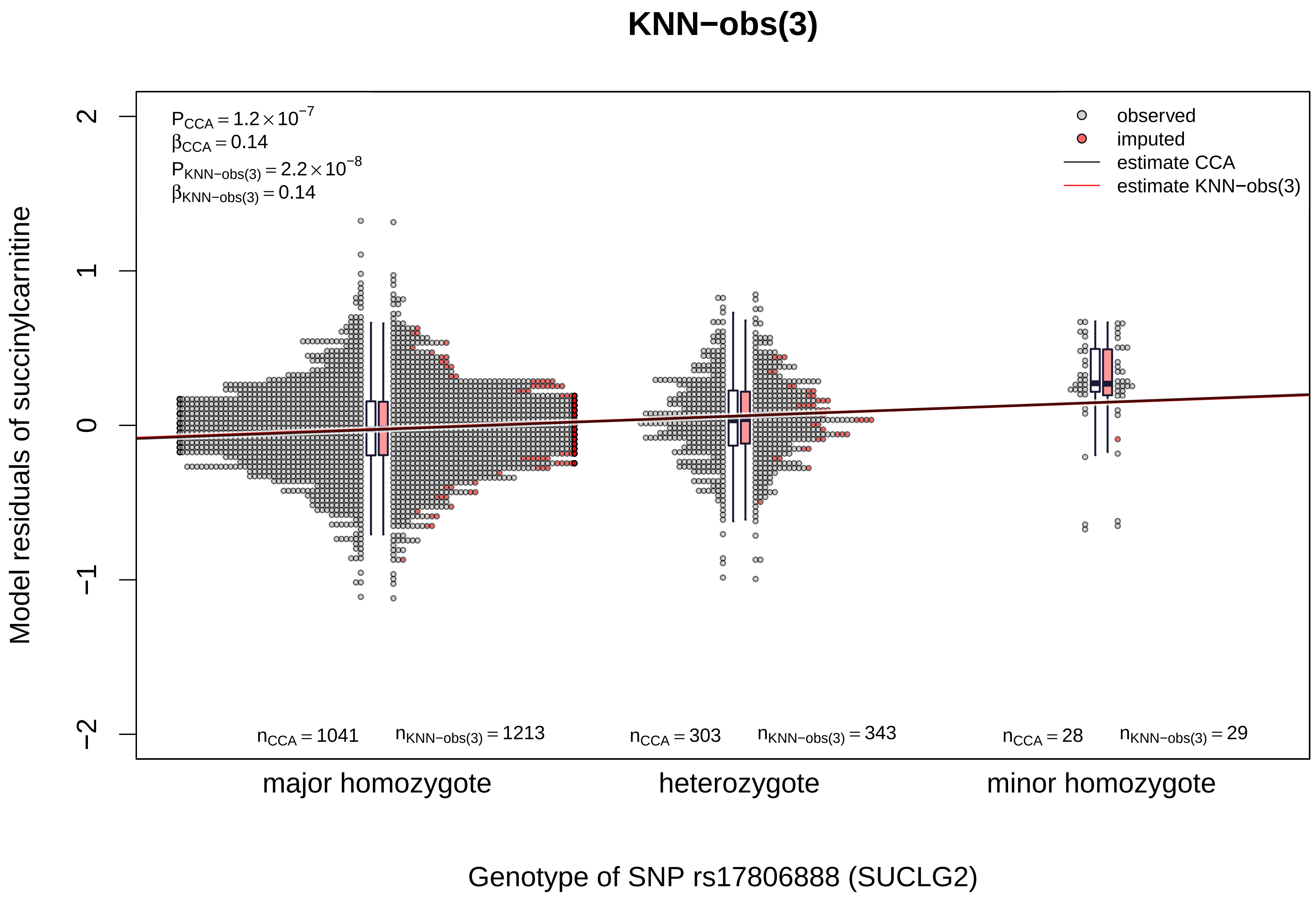

KNN−obs(3)
$P_{CCA} = 1.2 \times 10^{-7}$
$\beta_{CCA} = 0.14$
$P_{KNN-obs(3)} = 2.2 \times 10^{-8}$
$\beta_{KNN-obs(3)} = 0.14$
observed
imputed
estimate CCA
estimate KNN−obs(3)
Model residuals of succinylcarnitine
2
1
0
−1
−2
$n_{CCA} = 1041$
$n_{KNN-obs(3)} = 1213$
$n_{CCA} = 303$
$n_{KNN-obs(3)} = 343$
$n_{CCA} = 28$
$n_{KNN-obs(3)} = 29$
major homozygote
heterozygote
minor homozygote
Genotype of SNP rs17806888 (SUCLG2)

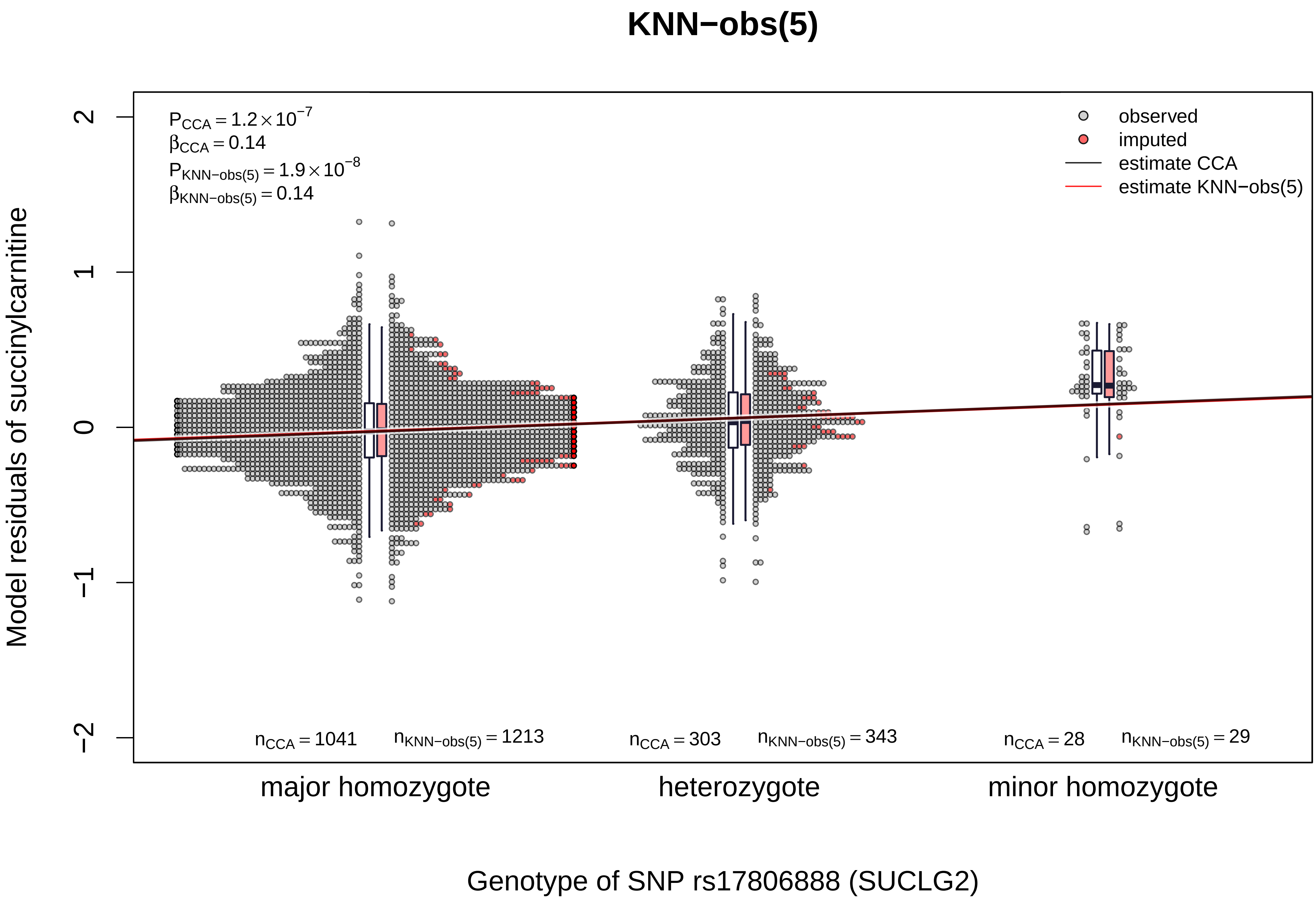
KNN−obs(5)
$P_{CCA} = 1.2 \times 10^{-7}$
$\beta_{CCA} = 0.14$
$P_{KNN-obs(5)} = 1.9 \times 10^{-8}$
$\beta_{KNN-obs(5)} = 0.14$
observed
imputed
estimate CCA
estimate KNN−obs(5)
Model residuals of succinylcarnitine
2
1
0
−1
−2
$n_{CCA} = 1041$
$n_{KNN-obs(5)} = 1213$
$n_{CCA} = 303$
$n_{KNN-obs(5)} = 343$
$n_{CCA} = 28$
$n_{KNN-obs(5)} = 29$
major homozygote
heterozygote
minor homozygote
Genotype of SNP rs17806888 (SUCLG2)

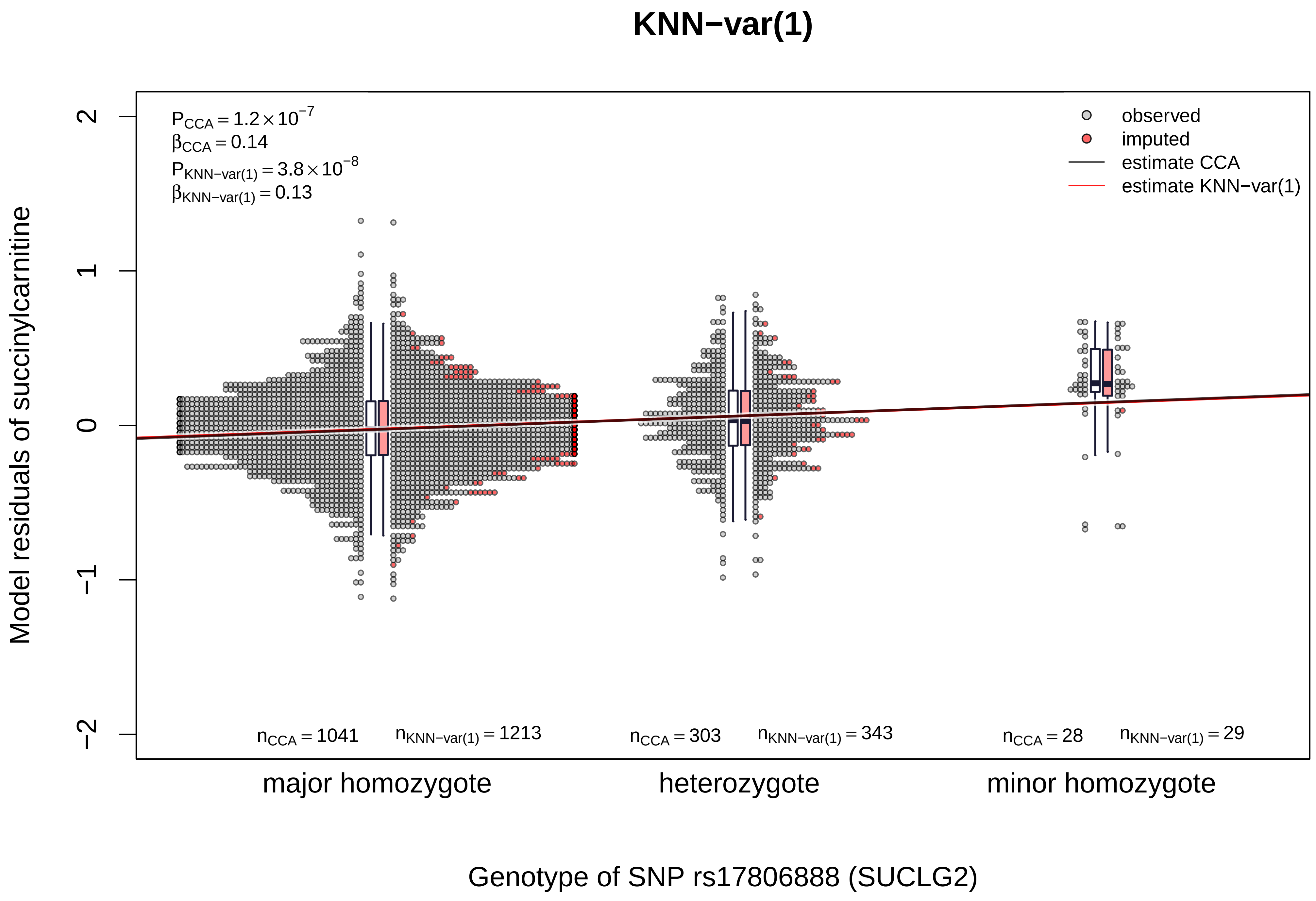

KNN−var(1)
$P_{CCA} = 1.2 \times 10^{-7}$
$\beta_{CCA} = 0.14$
$P_{KNN-var(1)} = 3.8 \times 10^{-8}$
$\beta_{KNN-var(1)} = 0.13$
observed
imputed
estimate CCA
estimate KNN−var(1)
Model residuals of succinylcarnitine
2
1
0
−1
−2
$n_{CCA} = 1041$
$n_{KNN-var(1)} = 1213$
$n_{CCA} = 303$
$n_{KNN-var(1)} = 343$
$n_{CCA} = 28$
$n_{KNN-var(1)} = 29$
major homozygote
heterozygote
minor homozygote
Genotype of SNP rs17806888 (SUCLG2)

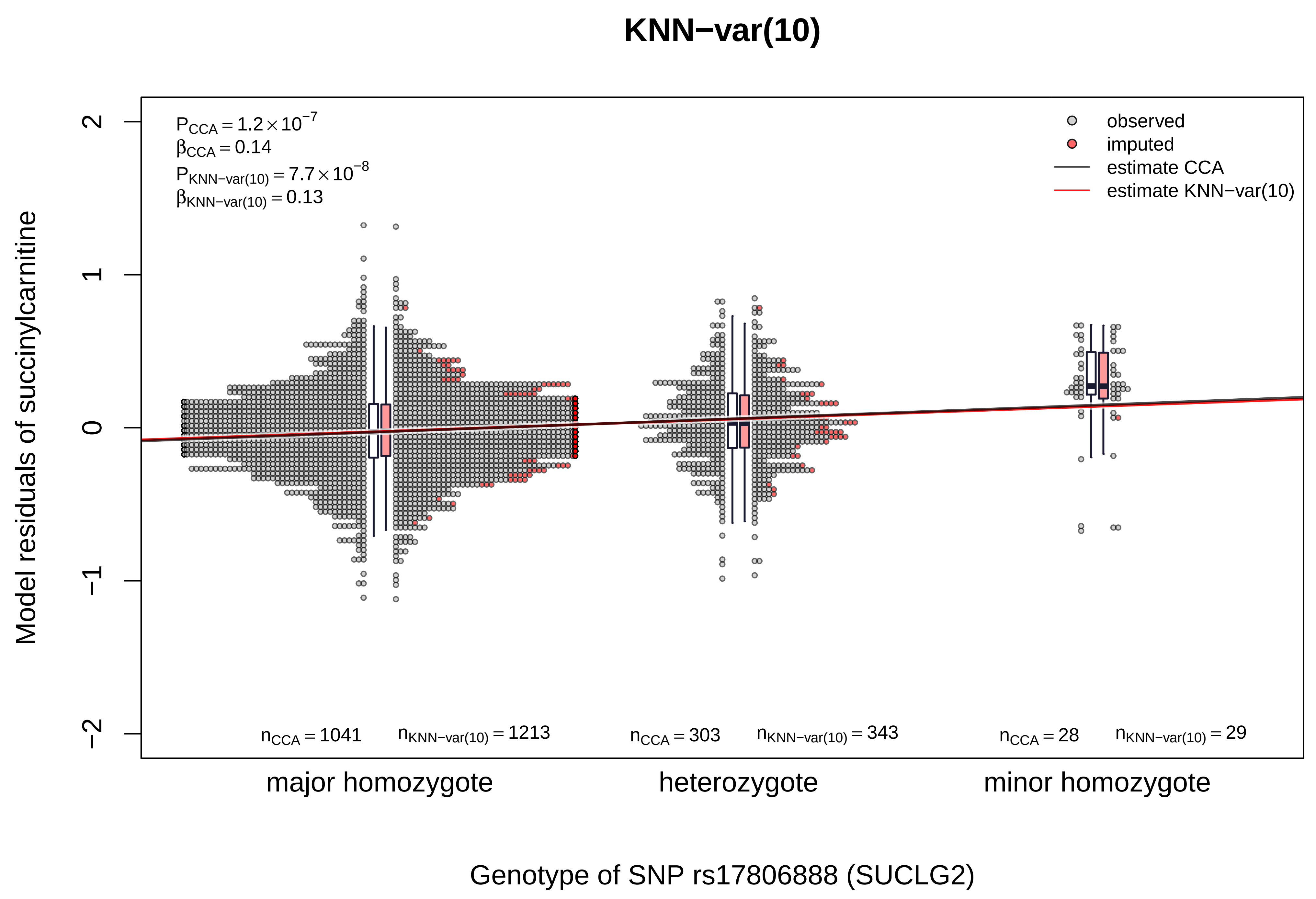

KNN−var(10)
$P_{CCA} = 1.2 \times 10^{-7}$
$\beta_{CCA} = 0.14$
$P_{KNN-var(10)} = 7.7 \times 10^{-8}$
$\beta_{KNN-var(10)} = 0.13$
observed
imputed
estimate CCA
estimate KNN−var(10)
$n_{CCA} = 1041$
$n_{KNN-var(10)} = 1213$
$n_{CCA} = 303$
$n_{KNN-var(10)} = 343$
$n_{CCA} = 28$
$n_{KNN-var(10)} = 29$
2
1
0
−1
−2
Model residuals of succinylcarnitine
major homozygote
heterozygote
minor homozygote
Genotype of SNP rs17806888 (SUCLG2)

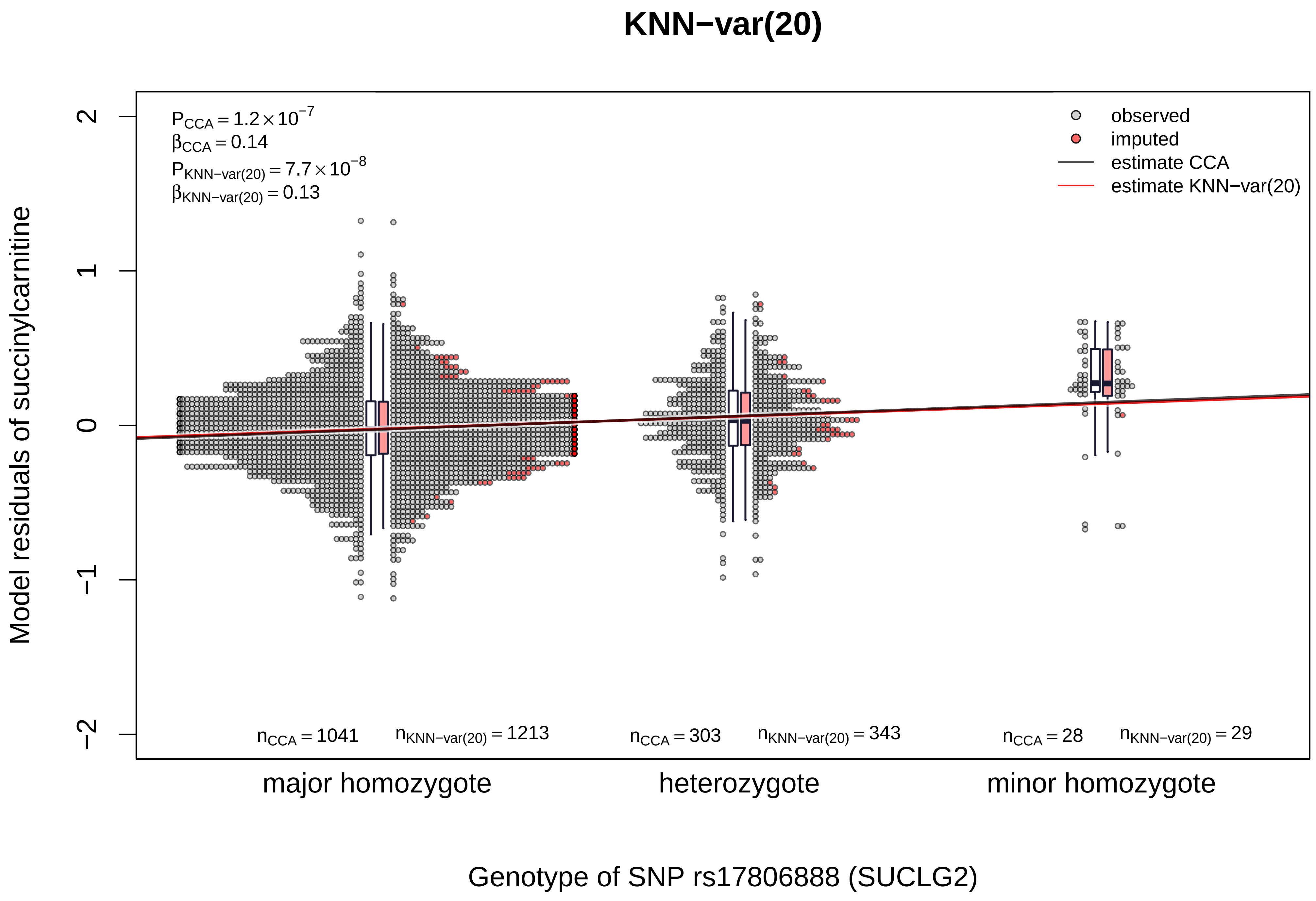

KNN−var(20)
$P_{CCA} = 1.2 \times 10^{-7}$
$\beta_{CCA} = 0.14$
$P_{KNN-var(20)} = 7.7 \times 10^{-8}$
$\beta_{KNN-var(20)} = 0.13$
observed
imputed
estimate CCA
estimate KNN−var(20)
Model residuals of succinylcarnitine
2
1
0
−1
−2
$n_{CCA} = 1041$
$n_{KNN-var(20)} = 1213$
$n_{CCA} = 303$
$n_{KNN-var(20)} = 343$
$n_{CCA} = 28$
$n_{KNN-var(20)} = 29$
major homozygote
heterozygote
minor homozygote
Genotype of SNP rs17806888 (SUCLG2)

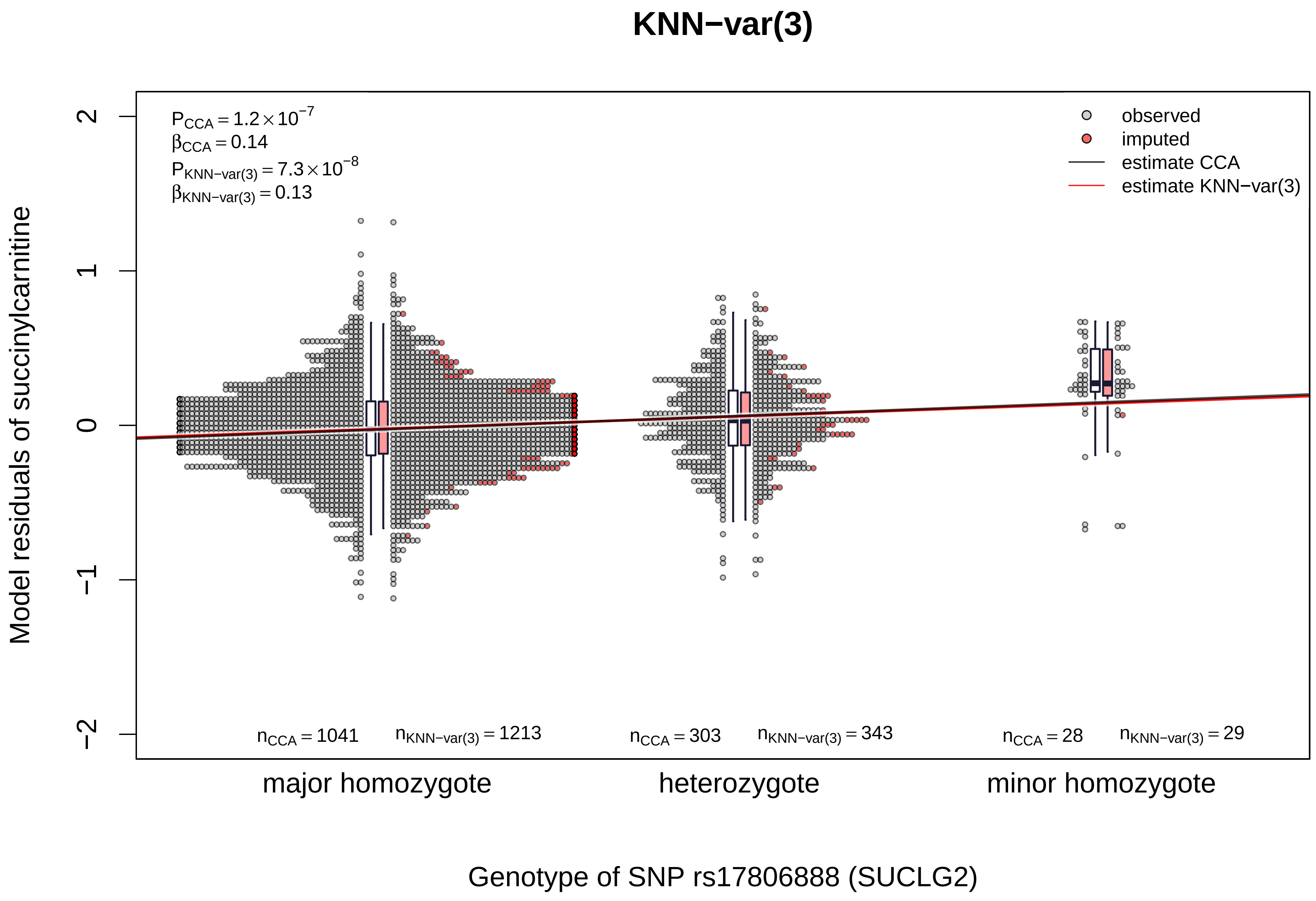

KNN−var(3)
$P_{CCA} = 1.2 \times 10^{-7}$
$\beta_{CCA} = 0.14$
$P_{KNN-var(3)} = 7.3 \times 10^{-8}$
$\beta_{KNN-var(3)} = 0.13$
observed
imputed
estimate CCA
estimate KNN−var(3)
$n_{CCA} = 1041$
$n_{KNN-var(3)} = 1213$
$n_{CCA} = 303$
$n_{KNN-var(3)} = 343$
$n_{CCA} = 28$
$n_{KNN-var(3)} = 29$
2
1
0
−1
−2
Model residuals of succinylcarnitine
major homozygote
heterozygote
minor homozygote
Genotype of SNP rs17806888 (SUCLG2)

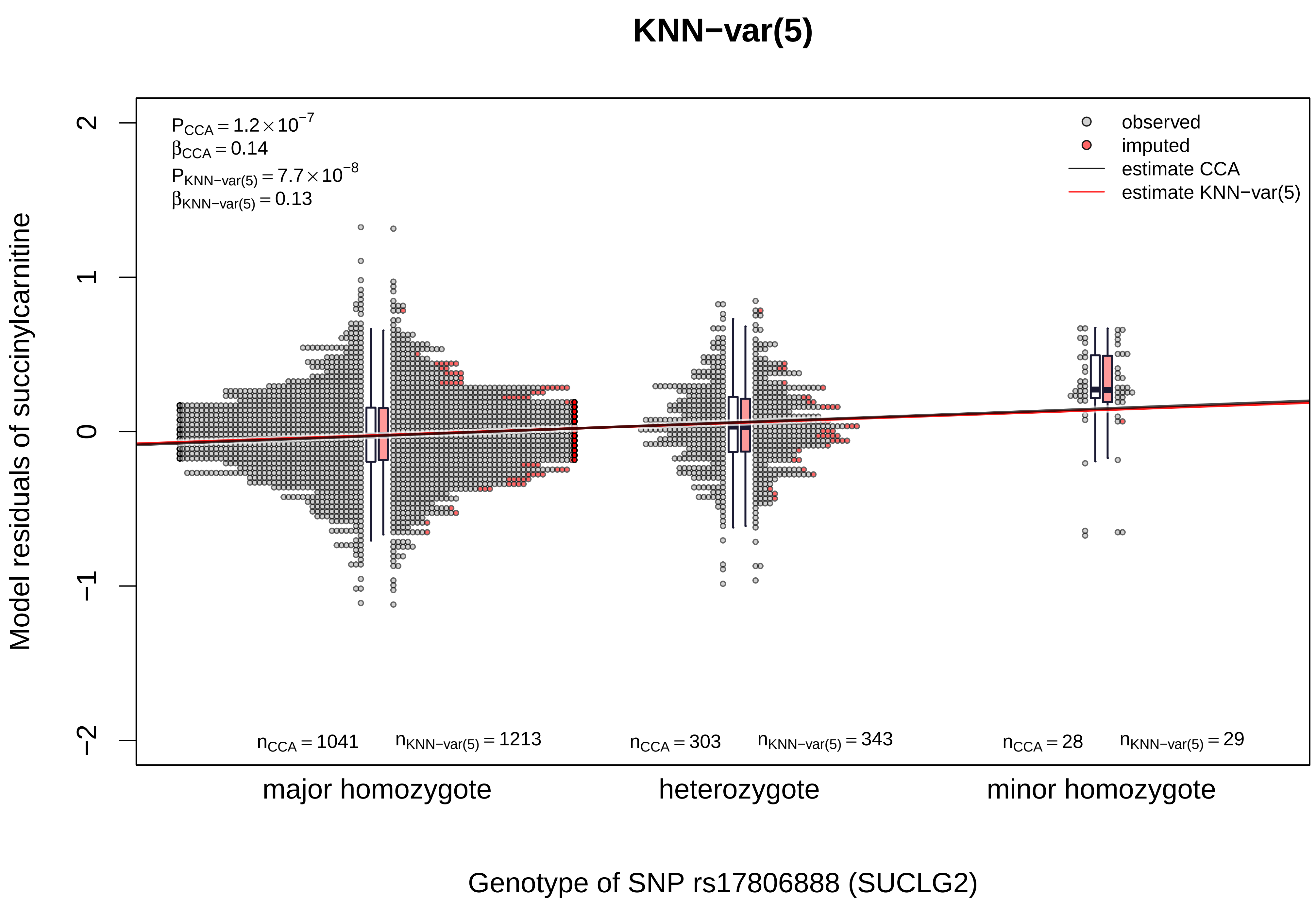

KNN−var(5)
$P_{CCA} = 1.2 \times 10^{-7}$
$\beta_{CCA} = 0.14$
$P_{KNN-var(5)} = 7.7 \times 10^{-8}$
$\beta_{KNN-var(5)} = 0.13$
observed
imputed
estimate CCA
estimate KNN−var(5)
Model residuals of succinylcarnitine
2
1
0
−1
−2
$n_{CCA} = 1041$
$n_{KNN-var(5)} = 1213$
$n_{CCA} = 303$
$n_{KNN-var(5)} = 343$
$n_{CCA} = 28$
$n_{KNN-var(5)} = 29$
major homozygote
heterozygote
minor homozygote
Genotype of SNP rs17806888 (SUCLG2)

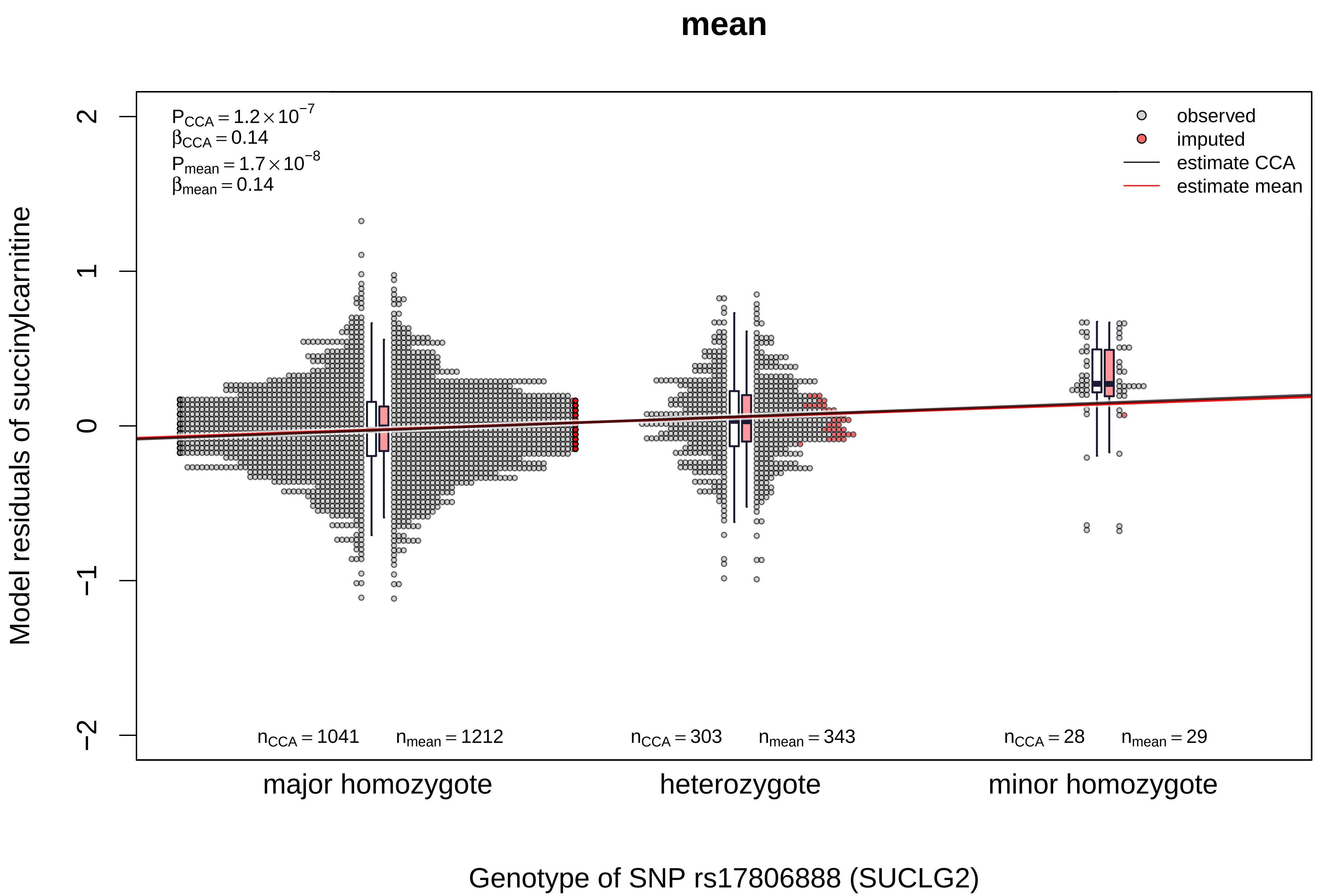

mean
$P_{CCA} = 1.2 \times 10^{-7}$
$\beta_{CCA} = 0.14$
$P_{mean} = 1.7 \times 10^{-8}$
$\beta_{mean} = 0.14$
observed
imputed
estimate CCA
estimate mean
Model residuals of succinylcarnitine
2
1
0
−1
−2
$n_{CCA} = 1041$
$n_{mean} = 1212$
$n_{CCA} = 303$
$n_{mean} = 343$
$n_{CCA} = 28$
$n_{mean} = 29$
major homozygote
heterozygote
minor homozygote
Genotype of SNP rs17806888 (SUCLG2)

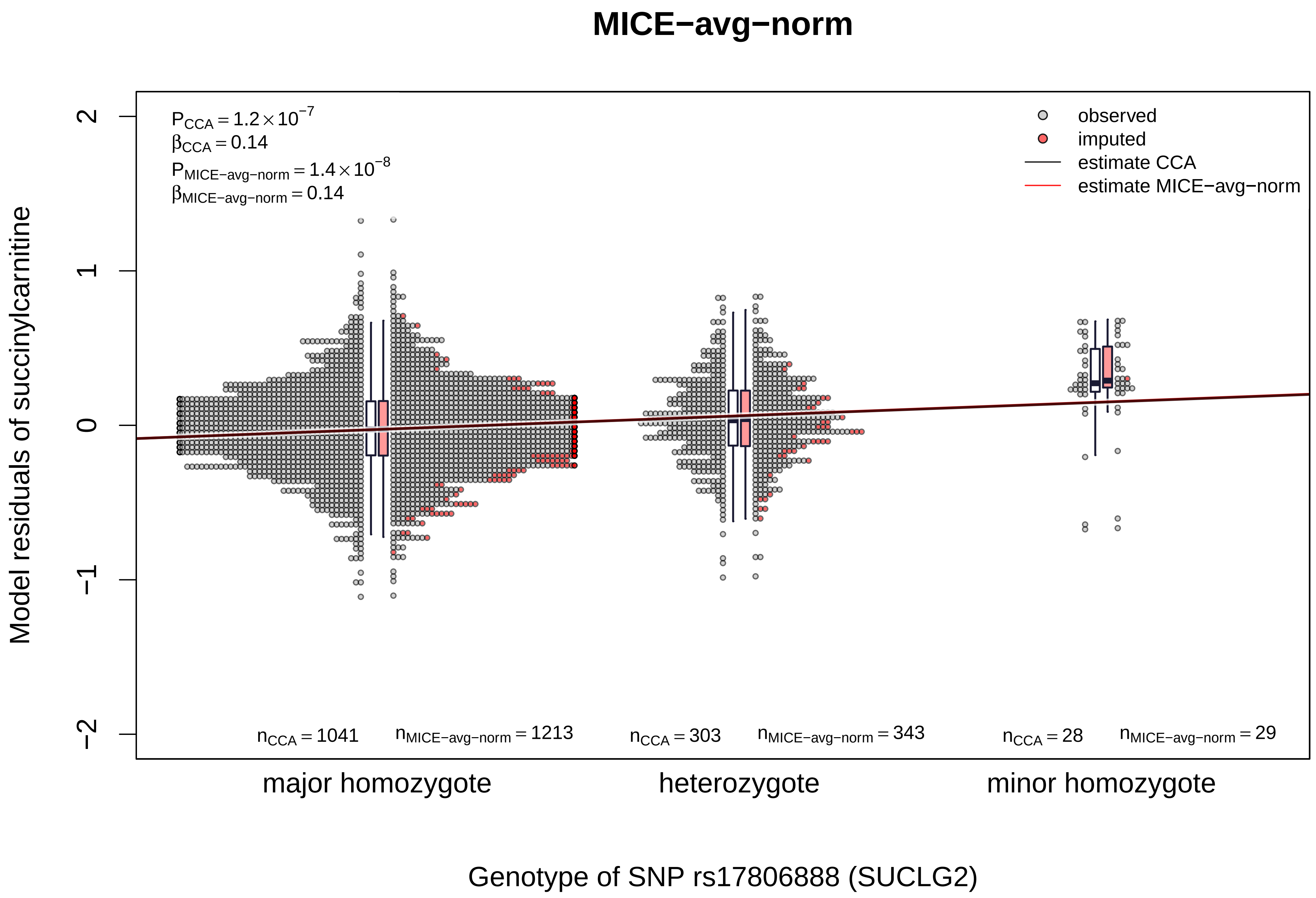

MICE−avg−norm
$P_{CCA} = 1.2 \times 10^{-7}$
$\beta_{CCA} = 0.14$
$P_{MICE-avg-norm} = 1.4 \times 10^{-8}$
$\beta_{MICE-avg-norm} = 0.14$
observed
imputed
estimate CCA
estimate MICE−avg−norm
Model residuals of succinylcarnitine
2
1
0
−1
−2
$n_{CCA} = 1041$
$n_{MICE-avg-norm} = 1213$
$n_{CCA} = 303$
$n_{MICE-avg-norm} = 343$
$n_{CCA} = 28$
$n_{MICE-avg-norm} = 29$
major homozygote
heterozygote
minor homozygote
Genotype of SNP rs17806888 (SUCLG2)

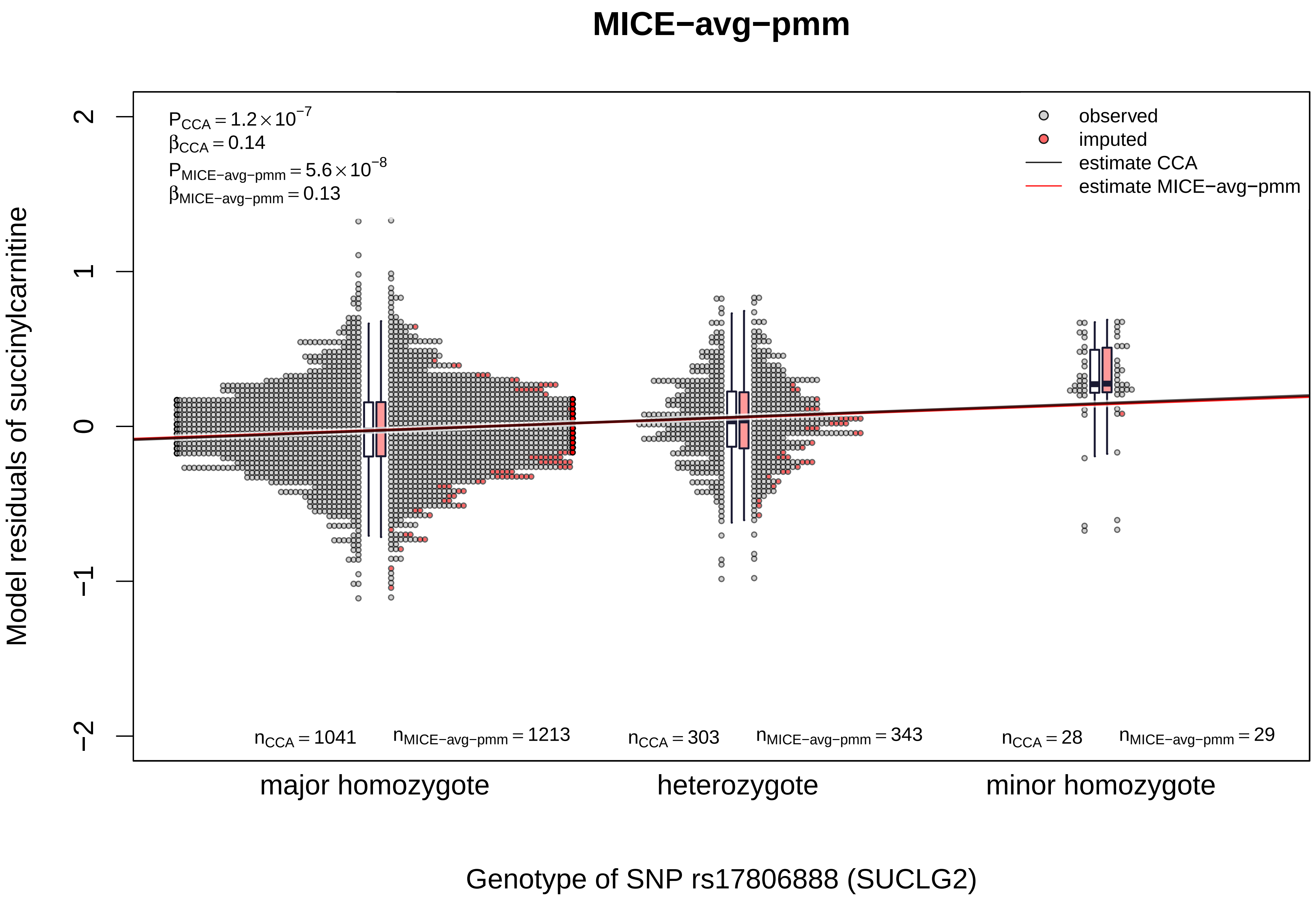

MICE−avg−pmm
$P_{CCA} = 1.2 \times 10^{-7}$
$\beta_{CCA} = 0.14$
$P_{MICE-avg-pmm} = 5.6 \times 10^{-8}$
$\beta_{MICE-avg-pmm} = 0.13$
observed
imputed
estimate CCA
estimate MICE−avg−pmm
Model residuals of succinylcarnitine
2
1
0
−1
−2
$n_{CCA} = 1041$
$n_{MICE-avg-pmm} = 1213$
$n_{CCA} = 303$
$n_{MICE-avg-pmm} = 343$
$n_{CCA} = 28$
$n_{MICE-avg-pmm} = 29$
major homozygote
heterozygote
minor homozygote
Genotype of SNP rs17806888 (SUCLG2)

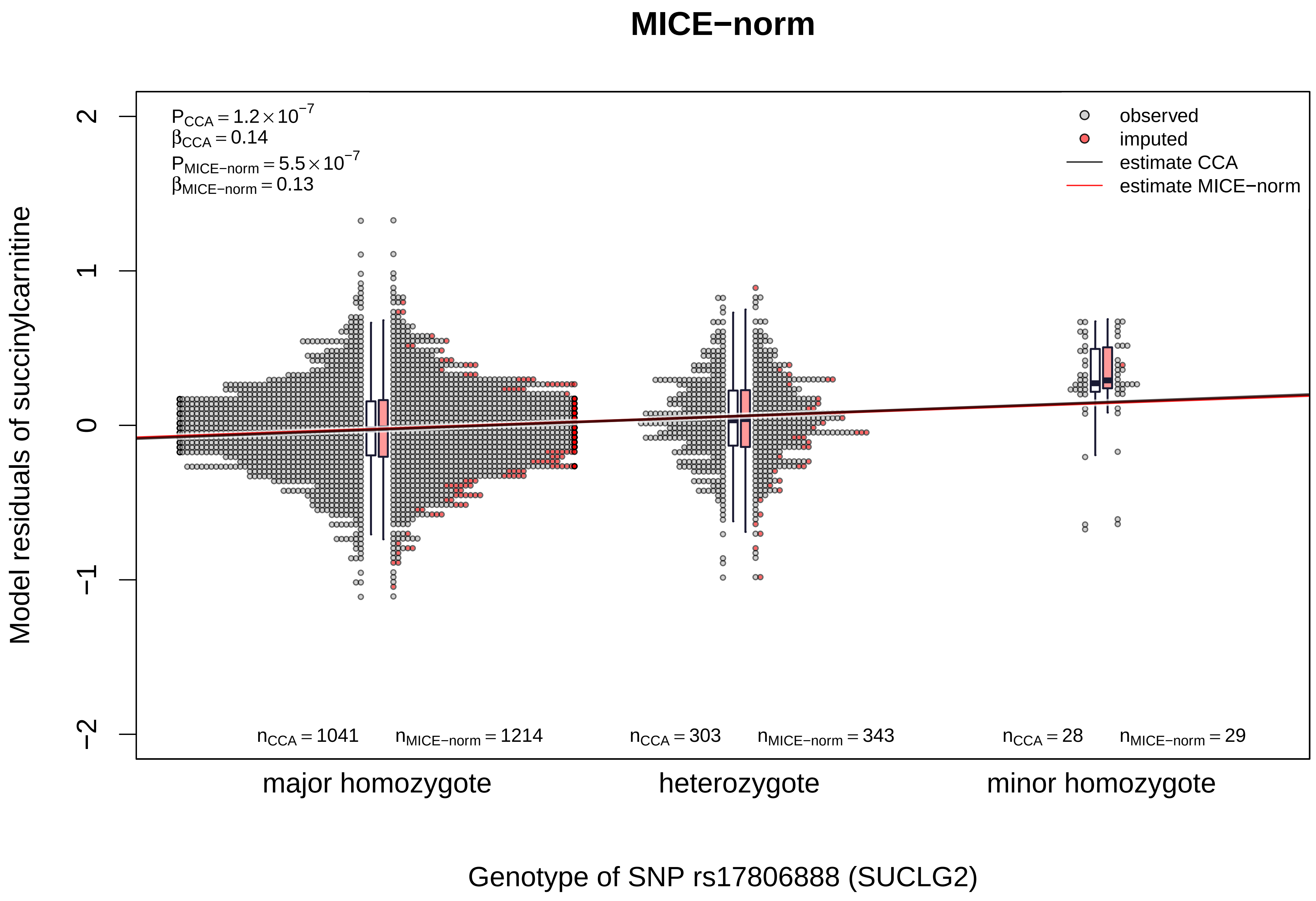
MICE−norm
$P_{CCA} = 1.2 \times 10^{-7}$
$\beta_{CCA} = 0.14$
$P_{MICE-norm} = 5.5 \times 10^{-7}$
$\beta_{MICE-norm} = 0.13$
observed
imputed
estimate CCA
estimate MICE−norm
Model residuals of succinylcarnitine
2
1
0
−1
−2
$n_{CCA} = 1041$
$n_{MICE-norm} = 1214$
$n_{CCA} = 303$
$n_{MICE-norm} = 343$
$n_{CCA} = 28$
$n_{MICE-norm} = 29$
major homozygote
heterozygote
minor homozygote
Genotype of SNP rs17806888 (SUCLG2)

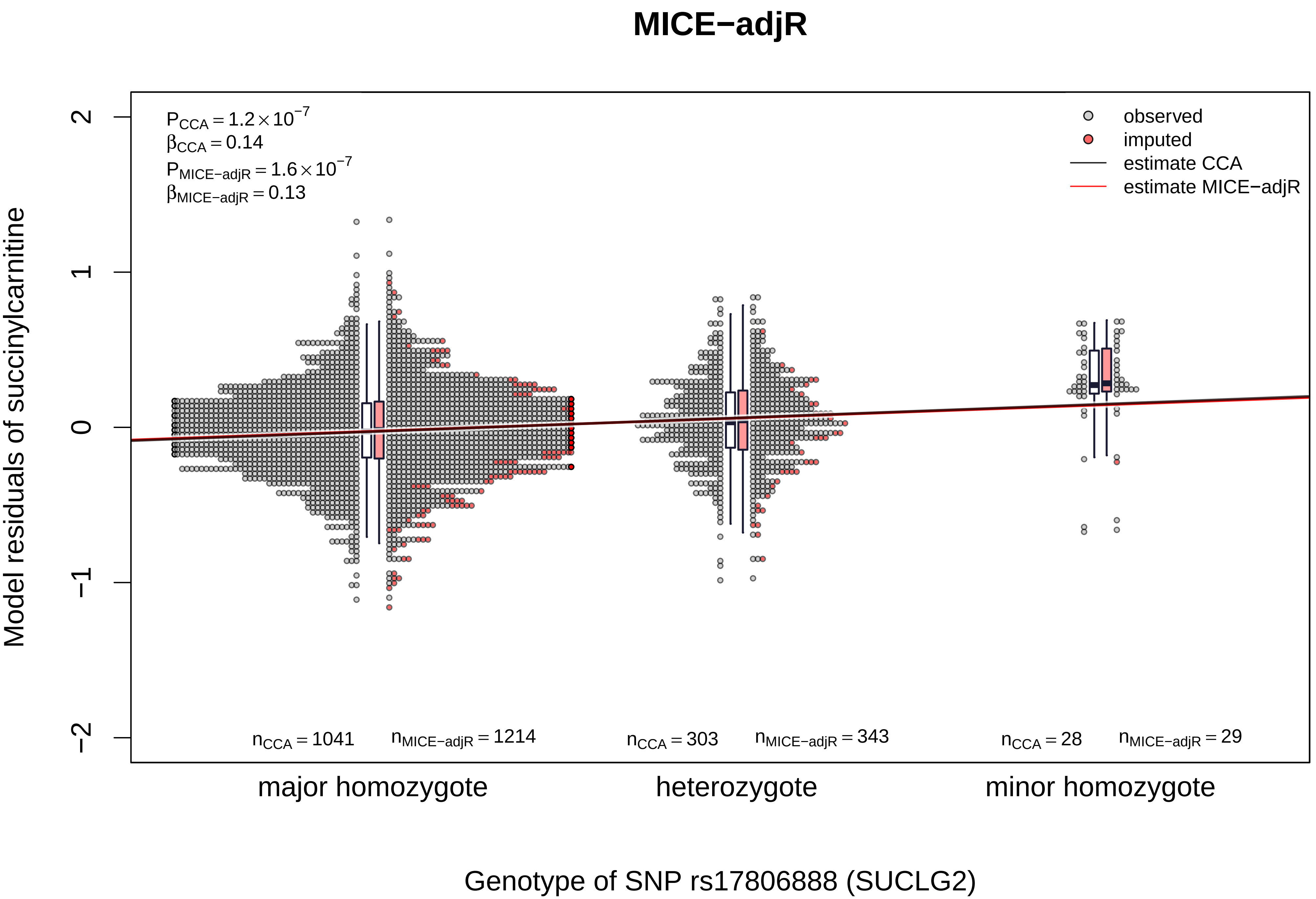

MICE−adjR
$P_{CCA} = 1.2 \times 10^{-7}$
$\beta_{CCA} = 0.14$
$P_{MICE-adjR} = 1.6 \times 10^{-7}$
$\beta_{MICE-adjR} = 0.13$
observed
imputed
estimate CCA
estimate MICE−adjR
Model residuals of succinylcarnitine
2
1
0
−1
−2
$n_{CCA} = 1041$
$n_{MICE-adjR} = 1214$
$n_{CCA} = 303$
$n_{MICE-adjR} = 343$
$n_{CCA} = 28$
$n_{MICE-adjR} = 29$
major homozygote
heterozygote
minor homozygote
Genotype of SNP rs17806888 (SUCLG2)

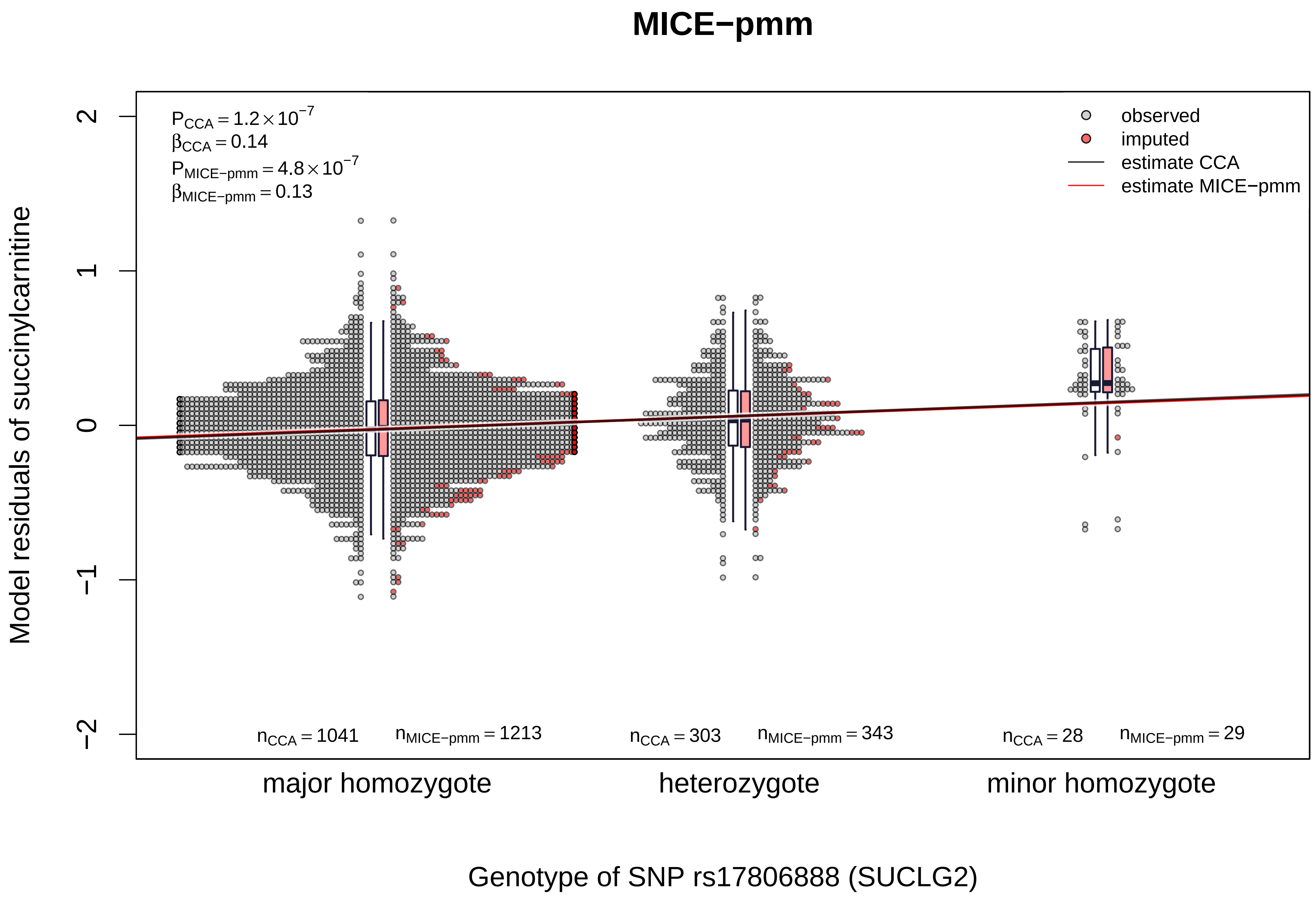

MICE−pmm
$P_{CCA} = 1.2 \times 10^{-7}$
$\beta_{CCA} = 0.14$
$P_{MICE-pmm} = 4.8 \times 10^{-7}$
$\beta_{MICE-pmm} = 0.13$
observed
imputed
estimate CCA
estimate MICE−pmm
$n_{CCA} = 1041$
$n_{MICE-pmm} = 1213$
$n_{CCA} = 303$
$n_{MICE-pmm} = 343$
$n_{CCA} = 28$
$n_{MICE-pmm} = 29$
2
1
0
−1
−2
Model residuals of succinylcarnitine
major homozygote
heterozygote
minor homozygote
Genotype of SNP rs17806888 (SUCLG2)

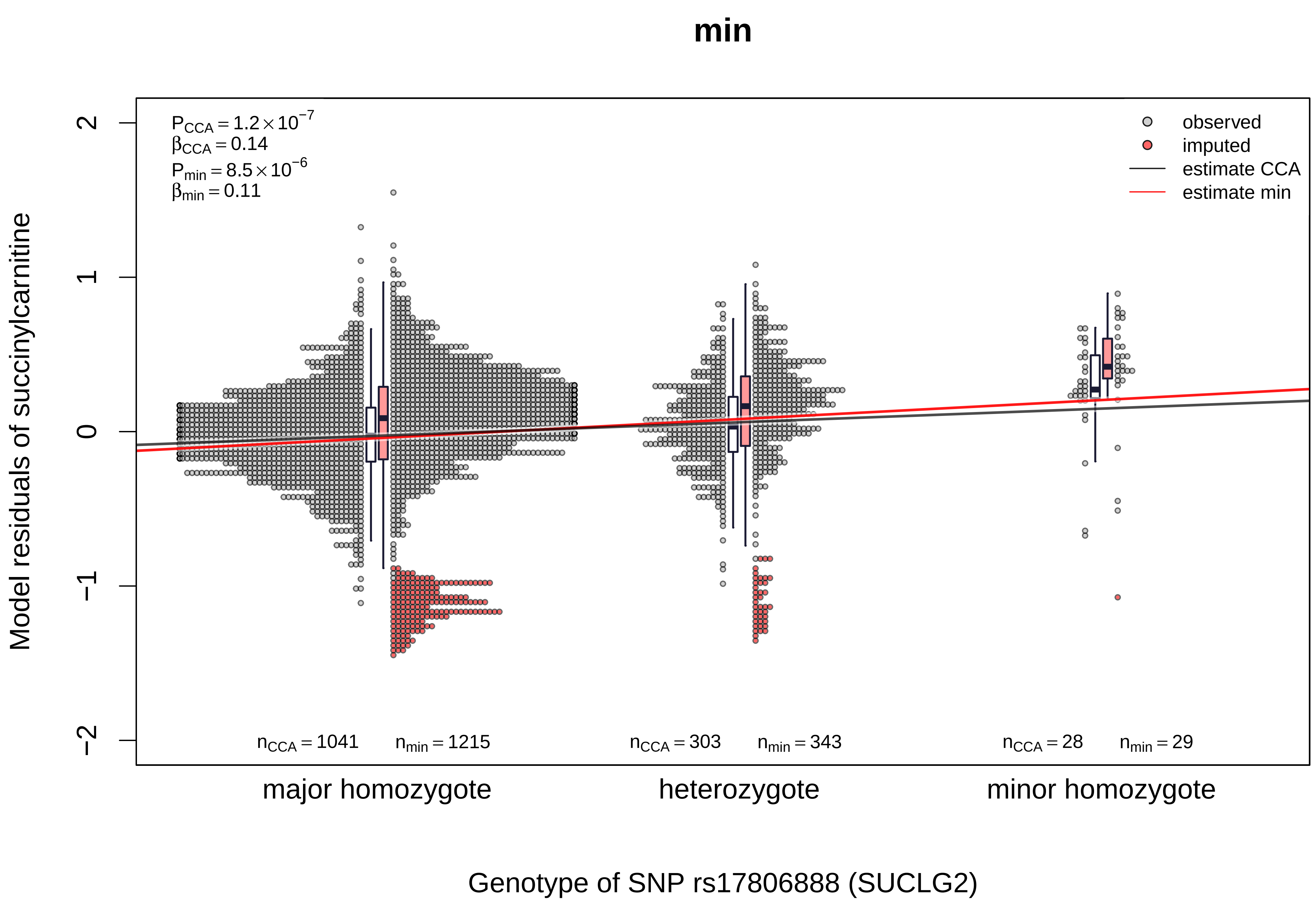
min
$P_{CCA} = 1.2 \times 10^{-7}$
$\beta_{CCA} = 0.14$
$P_{min} = 8.5 \times 10^{-6}$
$\beta_{min} = 0.11$
observed
imputed
estimate CCA
estimate min
2
1
0
−1
−2
Model residuals of succinylcarnitine
$n_{CCA} = 1041$
$n_{min} = 1215$
$n_{CCA} = 303$
$n_{min} = 343$
$n_{CCA} = 28$
$n_{min} = 29$
major homozygote
heterozygote
minor homozygote
Genotype of SNP rs17806888 (SUCLG2)

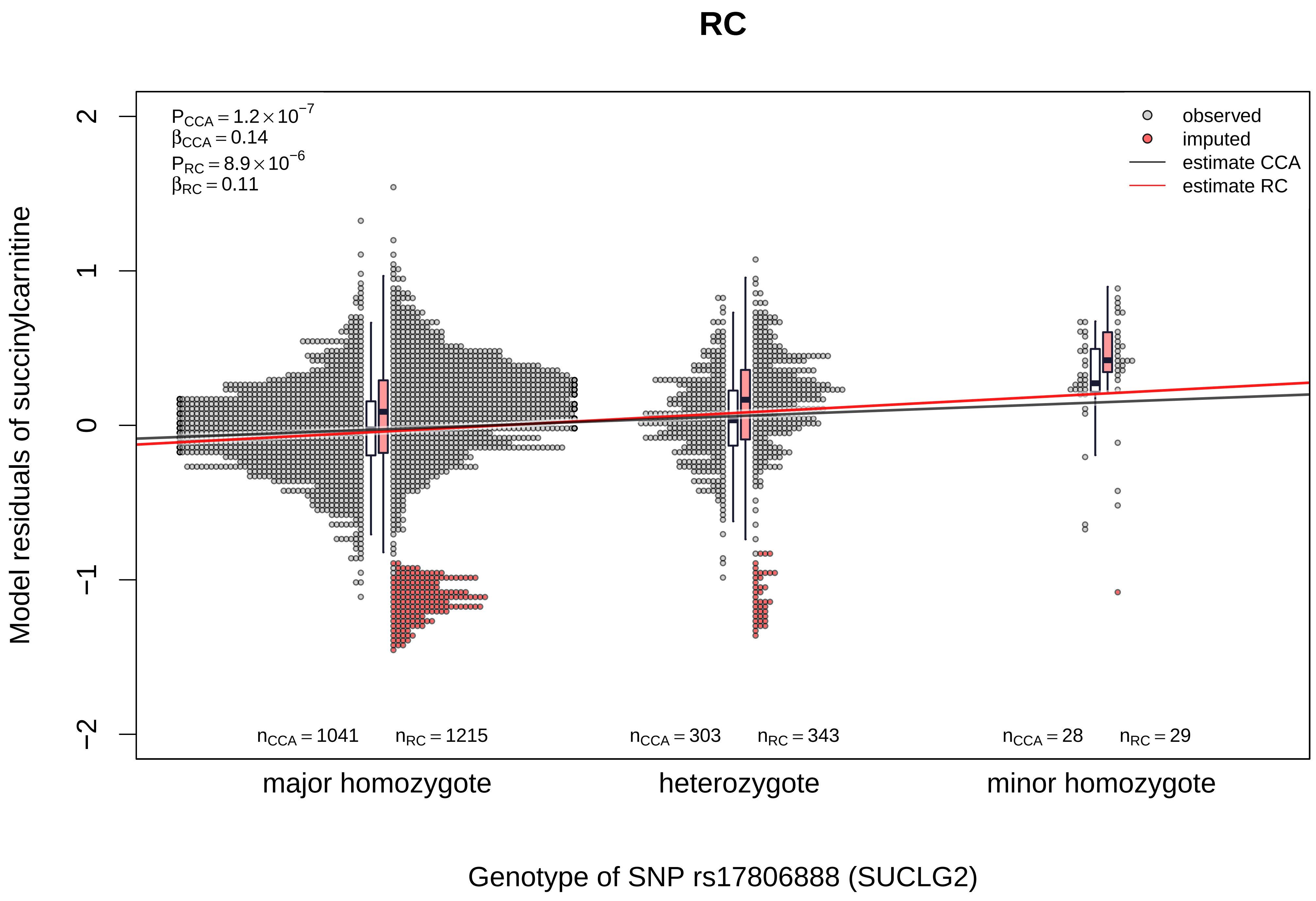

RC
$P_{CCA} = 1.2 \times 10^{-7}$
$\beta_{CCA} = 0.14$
$P_{RC} = 8.9 \times 10^{-6}$
$\beta_{RC} = 0.11$
observed
imputed
estimate CCA
estimate RC
Model residuals of succinylcarnitine
2
1
0
−1
−2
$n_{CCA} = 1041$
$n_{RC} = 1215$
$n_{CCA} = 303$
$n_{RC} = 343$
$n_{CCA} = 28$
$n_{RC} = 29$
major homozygote
heterozygote
minor homozygote
Genotype of SNP rs17806888 (SUCLG2)

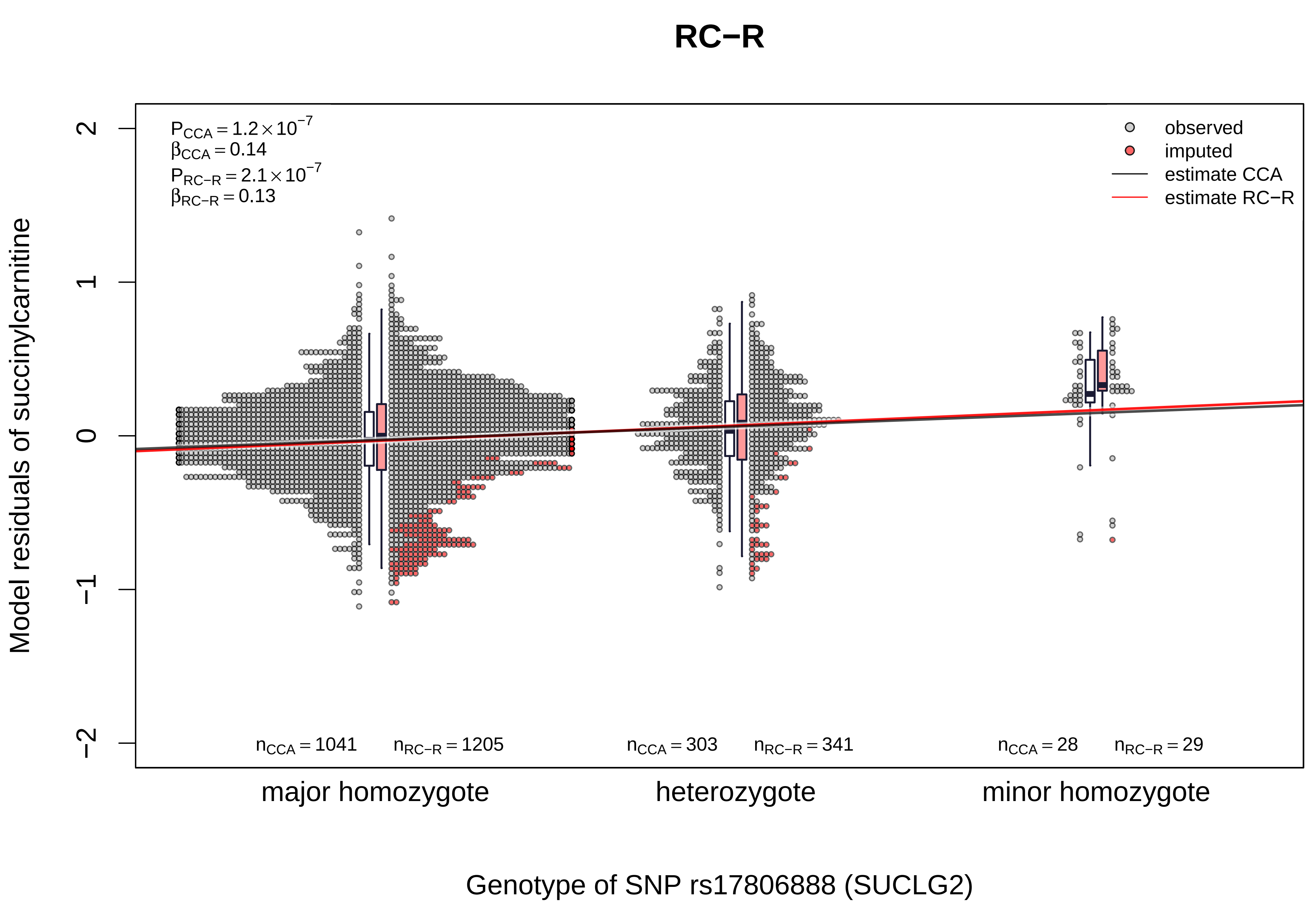

RC−R
$P_{CCA} = 1.2 \times 10^{-7}$
$\beta_{CCA} = 0.14$
$P_{RC-R} = 2.1 \times 10^{-7}$
$\beta_{RC-R} = 0.13$
observed
imputed
estimate CCA
estimate RC−R
Model residuals of succinylcarnitine
2
1
0
−1
−2
$n_{CCA} = 1041$
$n_{RC-R} = 1205$
$n_{CCA} = 303$
$n_{RC-R} = 341$
$n_{CCA} = 28$
$n_{RC-R} = 29$
major homozygote
heterozygote
minor homozygote
Genotype of SNP rs17806888 (SUCLG2)

ICE−norm

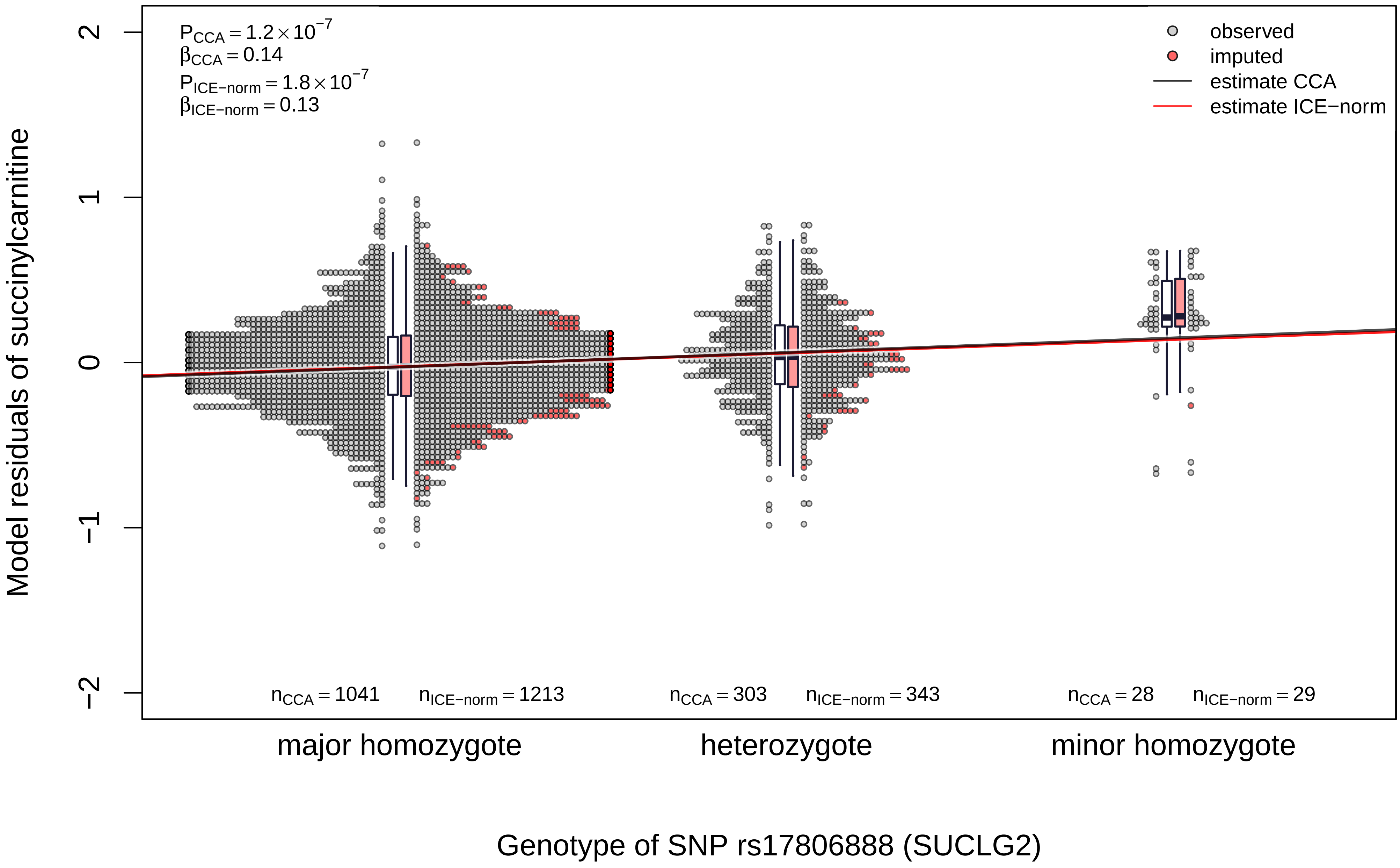

$P_{CCA} = 1.2 \times 10^{-7}$
$\beta_{CCA} = 0.14$
$P_{ICE-norm} = 1.8 \times 10^{-7}$
$\beta_{ICE-norm} = 0.13$
observed
imputed
estimate CCA
estimate ICE−norm
Model residuals of succinylcarnitine
2
1
0
−1
−2
$n_{CCA} = 1041$
$n_{ICE-norm} = 1213$
$n_{CCA} = 303$
$n_{ICE-norm} = 343$
$n_{CCA} = 28$
$n_{ICE-norm} = 29$
major homozygote
heterozygote
minor homozygote
Genotype of SNP rs17806888 (SUCLG2)

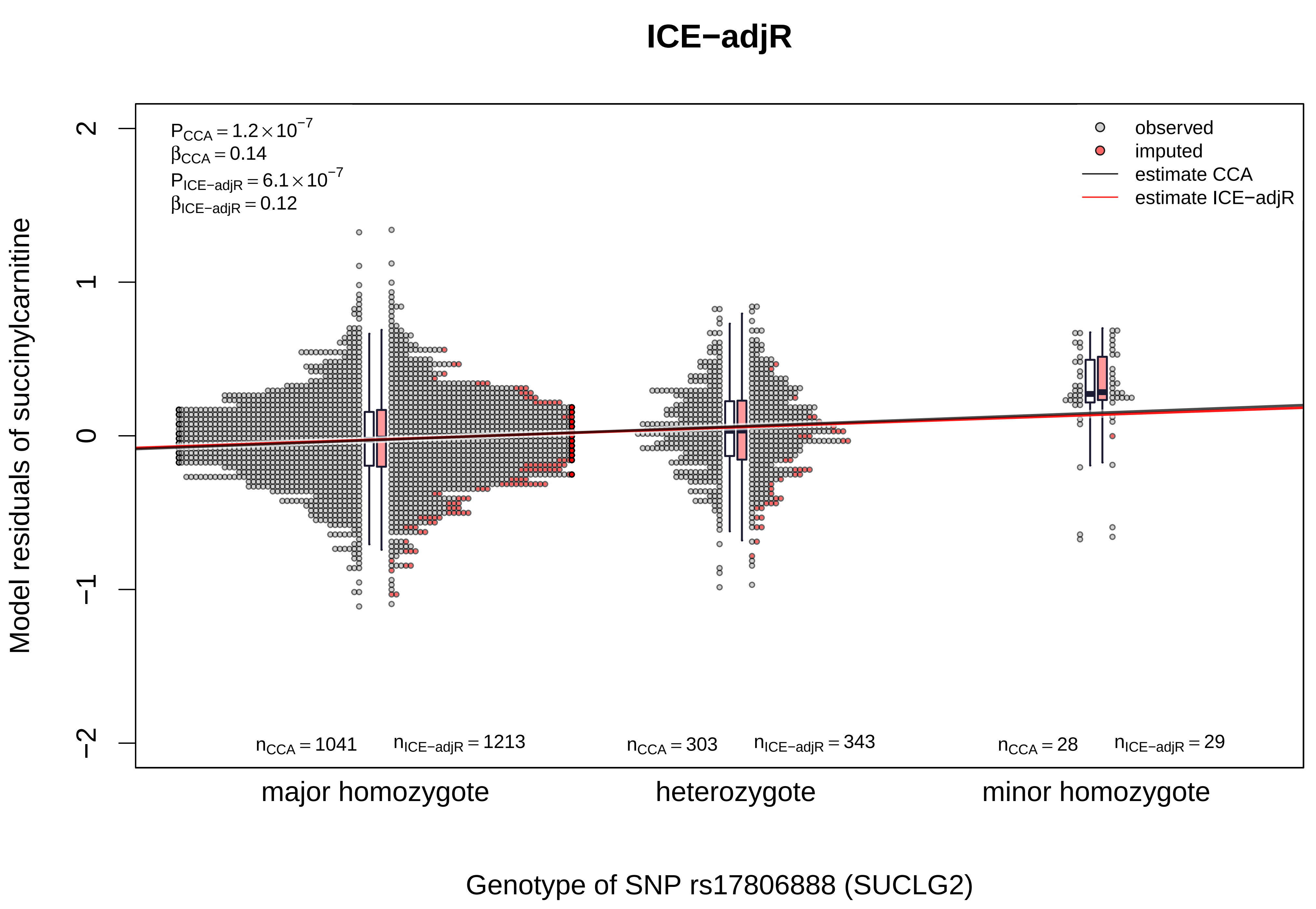

ICE−adjR
$P_{CCA} = 1.2 \times 10^{-7}$
$\beta_{CCA} = 0.14$
$P_{ICE-adjR} = 6.1 \times 10^{-7}$
$\beta_{ICE-adjR} = 0.12$
observed
imputed
estimate CCA
estimate ICE−adjR
$n_{CCA} = 1041$
$n_{ICE-adjR} = 1213$
$n_{CCA} = 303$
$n_{ICE-adjR} = 343$
$n_{CCA} = 28$
$n_{ICE-adjR} = 29$
Model residuals of succinylcarnitine
2
1
0
−1
−2
major homozygote
heterozygote
minor homozygote
Genotype of SNP rs17806888 (SUCLG2)

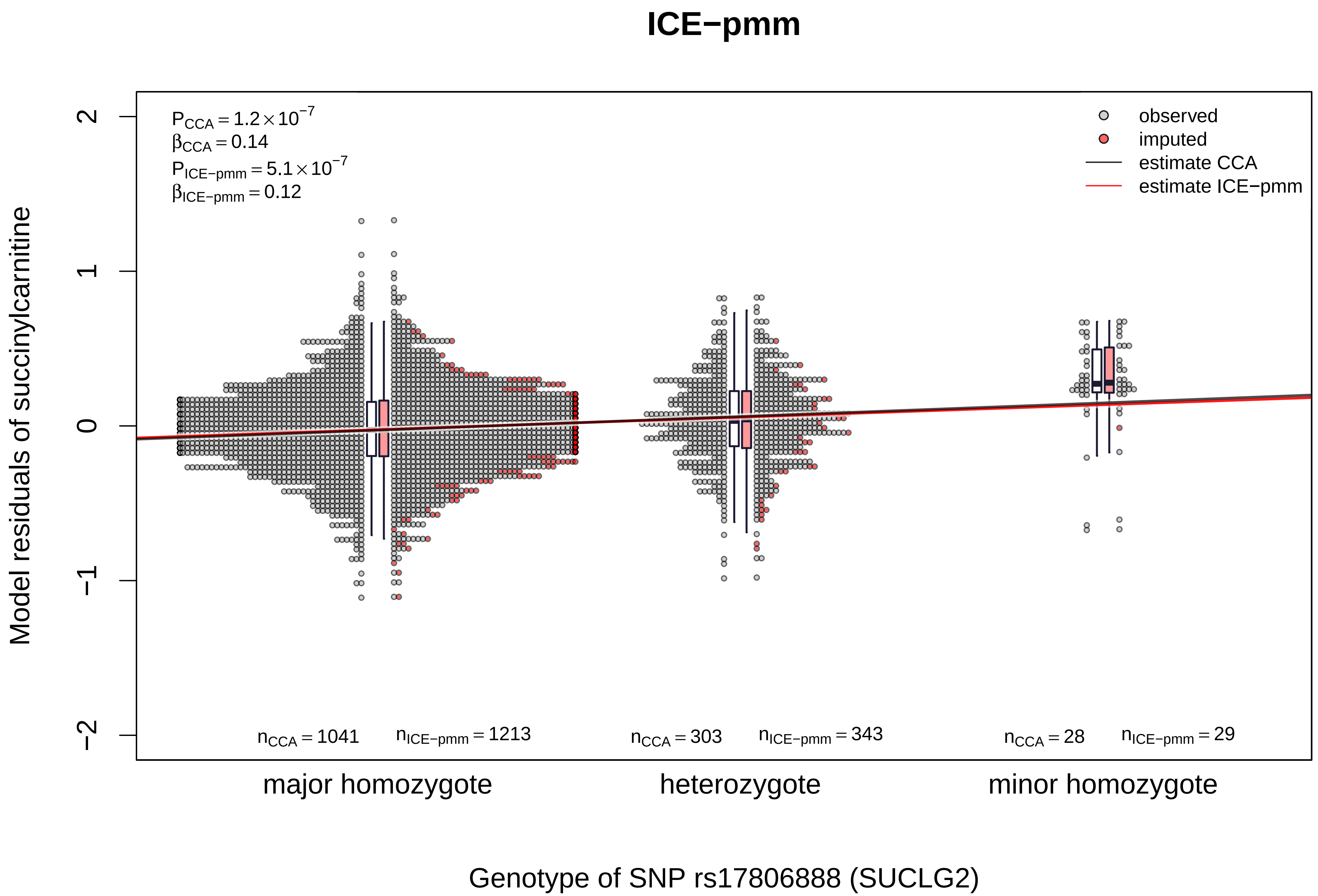

ICE−pmm
$P_{CCA} = 1.2 \times 10^{-7}$
$\beta_{CCA} = 0.14$
$P_{ICE-pmm} = 5.1 \times 10^{-7}$
$\beta_{ICE-pmm} = 0.12$
observed
imputed
estimate CCA
estimate ICE−pmm
Model residuals of succinylcarnitine
2
1
0
−1
−2
$n_{CCA} = 1041$
$n_{ICE-pmm} = 1213$
$n_{CCA} = 303$
$n_{ICE-pmm} = 343$
$n_{CCA} = 28$
$n_{ICE-pmm} = 29$
major homozygote
heterozygote
minor homozygote
Genotype of SNP rs17806888 (SUCLG2)

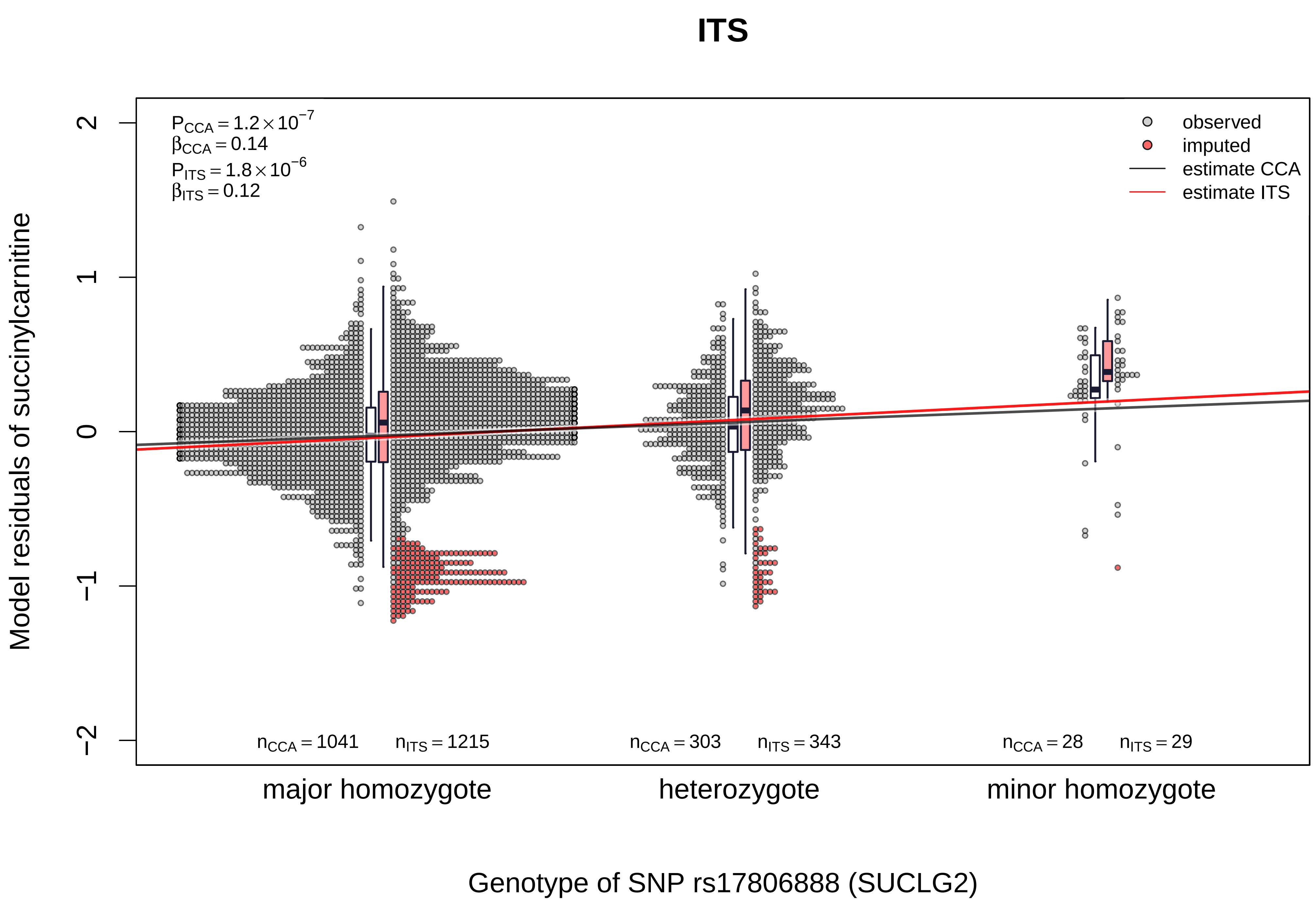

ITS
$P_{CCA} = 1.2 \times 10^{-7}$
$\beta_{CCA} = 0.14$
$P_{ITS} = 1.8 \times 10^{-6}$
$\beta_{ITS} = 0.12$
observed
imputed
estimate CCA
estimate ITS
Model residuals of succinylcarnitine
2
1
0
−1
−2
$n_{CCA} = 1041$
$n_{ITS} = 1215$
$n_{CCA} = 303$
$n_{ITS} = 343$
$n_{CCA} = 28$
$n_{ITS} = 29$
major homozygote
heterozygote
minor homozygote
Genotype of SNP rs17806888 (SUCLG2)

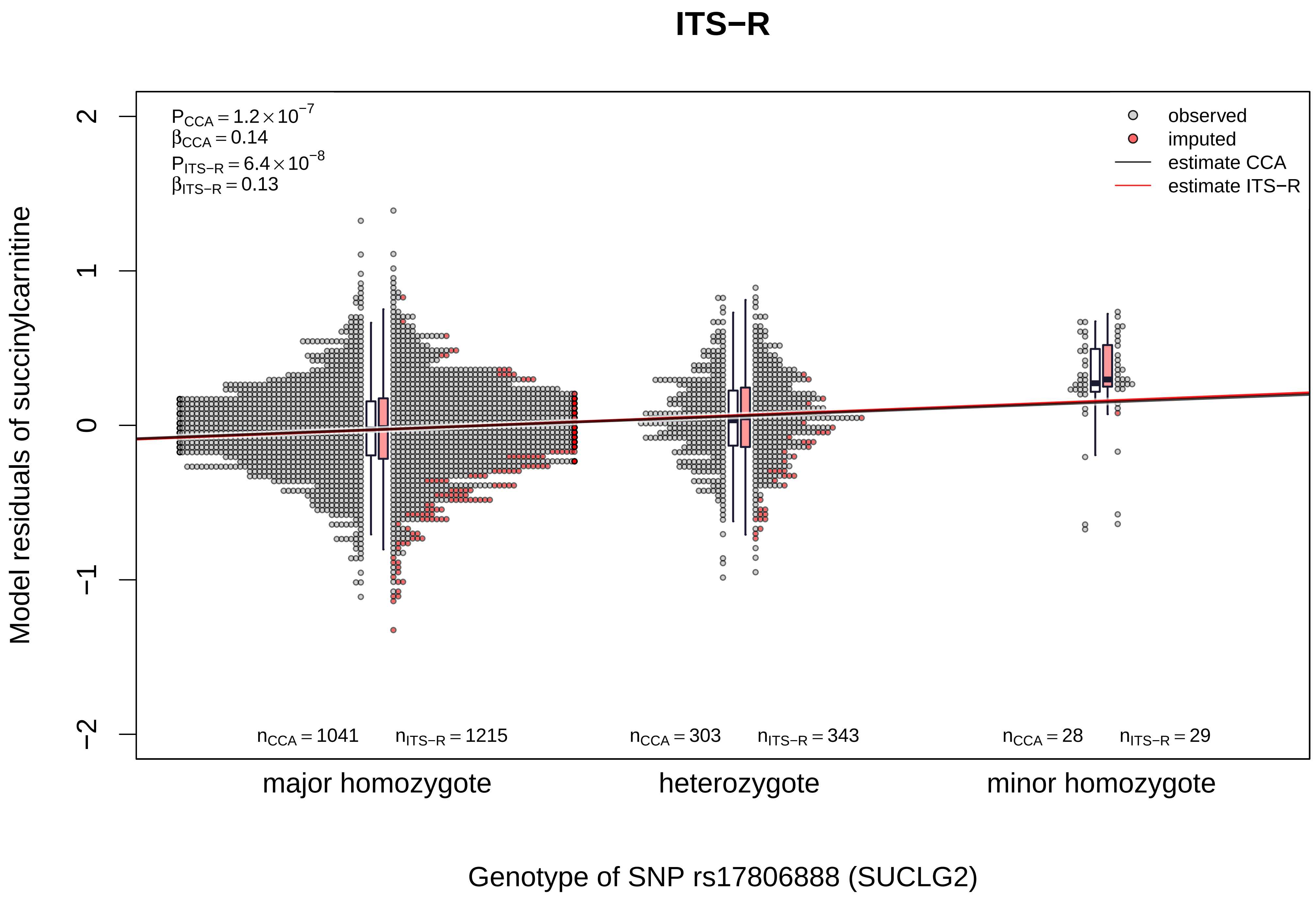

ITS−R
$P_{CCA} = 1.2 \times 10^{-7}$
$\beta_{CCA} = 0.14$
$P_{ITS-R} = 6.4 \times 10^{-8}$
$\beta_{ITS-R} = 0.13$
observed
imputed
estimate CCA
estimate ITS−R
Model residuals of succinylcarnitine
2
1
0
−1
−2
$n_{CCA} = 1041$
$n_{ITS-R} = 1215$
$n_{CCA} = 303$
$n_{ITS-R} = 343$
$n_{CCA} = 28$
$n_{ITS-R} = 29$
major homozygote
heterozygote
minor homozygote
Genotype of SNP rs17806888 (SUCLG2)

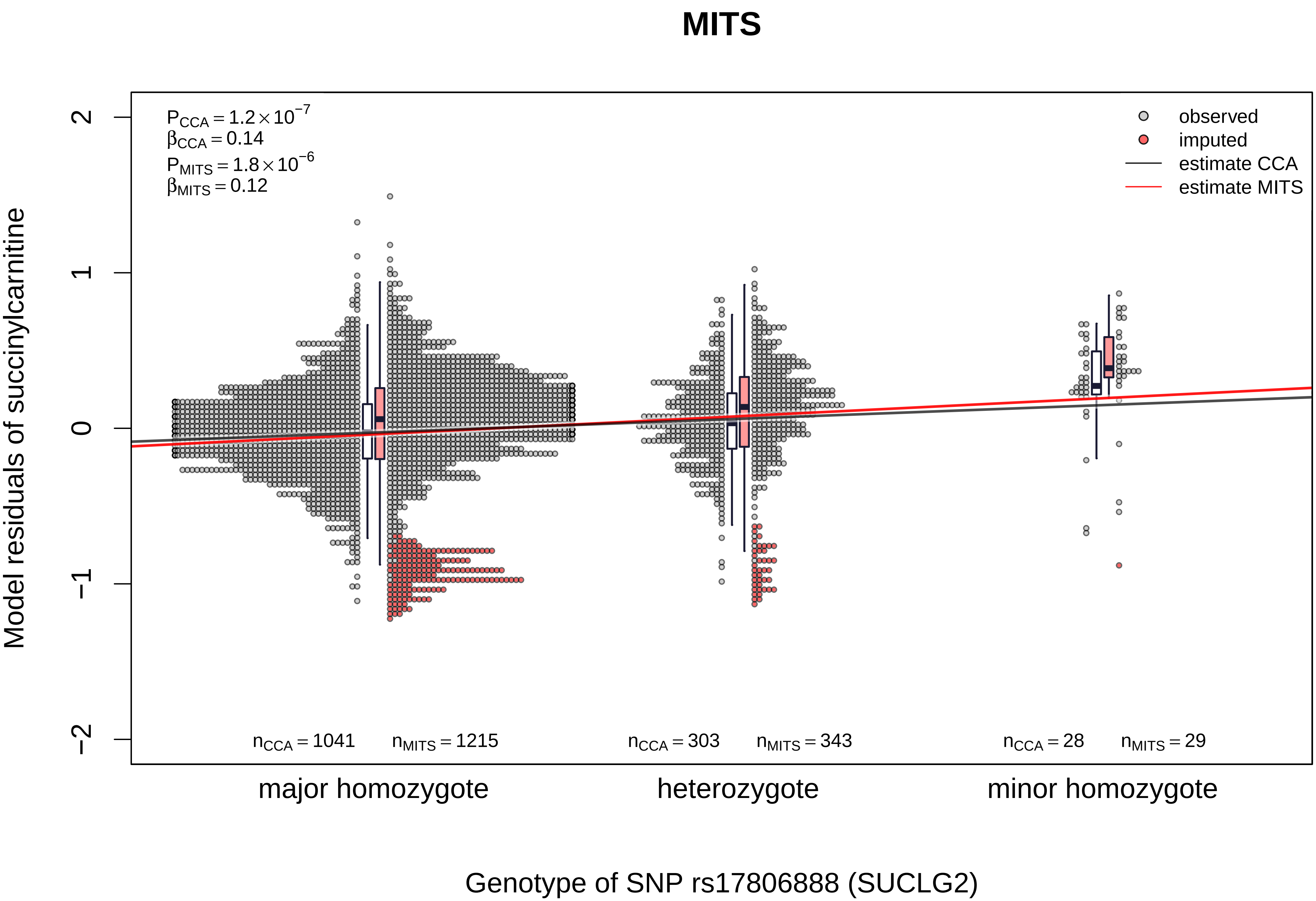

MITS
$P_{CCA} = 1.2 \times 10^{-7}$
$\beta_{CCA} = 0.14$
$P_{MITS} = 1.8 \times 10^{-6}$
$\beta_{MITS} = 0.12$
observed
imputed
estimate CCA
estimate MITS
Model residuals of succinylcarnitine
2
1
0
−1
−2
$n_{CCA} = 1041$
$n_{MITS} = 1215$
$n_{CCA} = 303$
$n_{MITS} = 343$
$n_{CCA} = 28$
$n_{MITS} = 29$
major homozygote
heterozygote
minor homozygote
Genotype of SNP rs17806888 (SUCLG2)

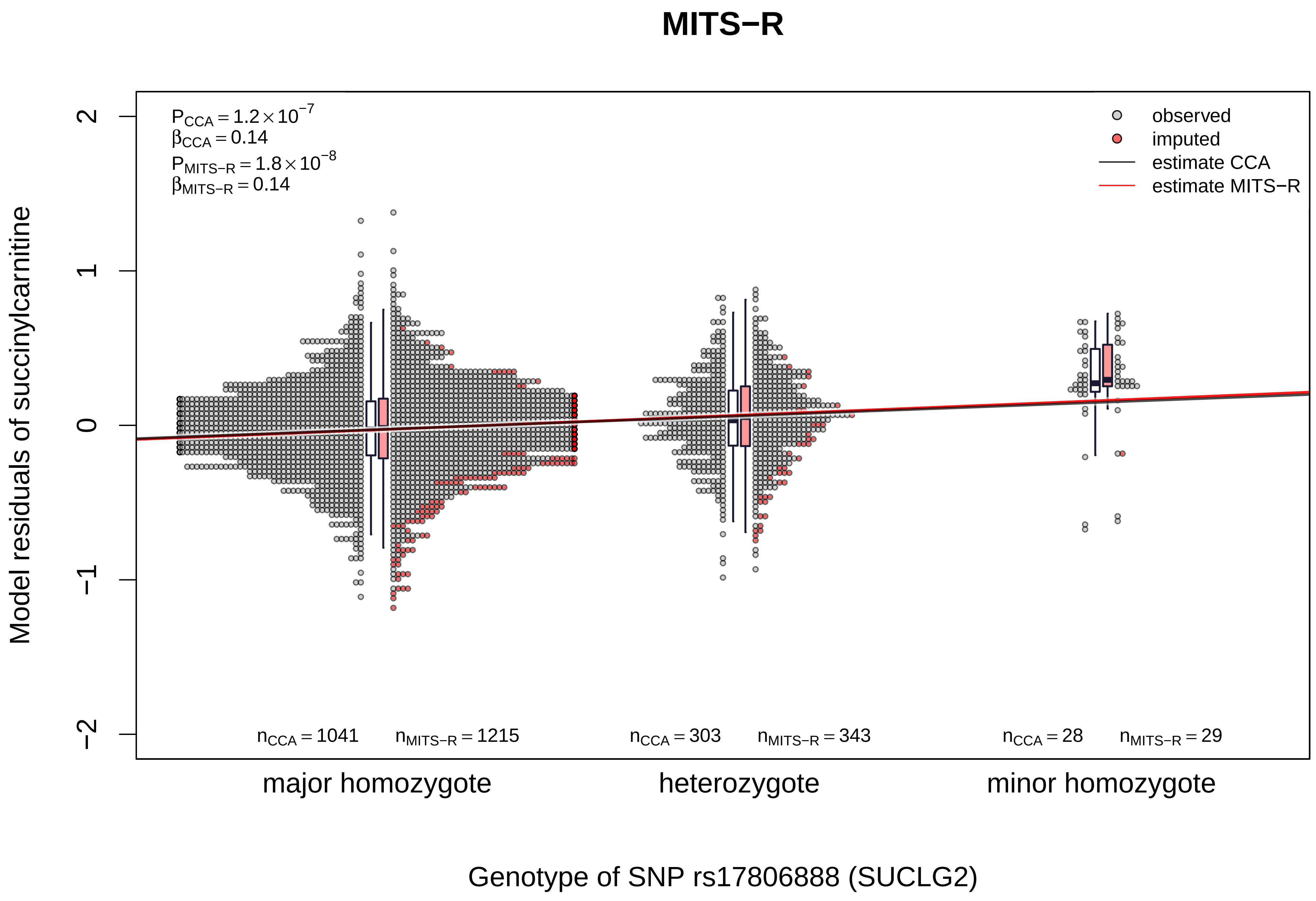

MITS−R
$P_{CCA} = 1.2 \times 10^{-7}$
$\beta_{CCA} = 0.14$
$P_{MITS-R} = 1.8 \times 10^{-8}$
$\beta_{MITS-R} = 0.14$
observed
imputed
estimate CCA
estimate MITS−R
Model residuals of succinylcarnitine
2
1
0
−1
−2
$n_{CCA} = 1041$
$n_{MITS-R} = 1215$
$n_{CCA} = 303$
$n_{MITS-R} = 343$
$n_{CCA} = 28$
$n_{MITS-R} = 29$
major homozygote
heterozygote
minor homozygote
Genotype of SNP rs17806888 (SUCLG2)

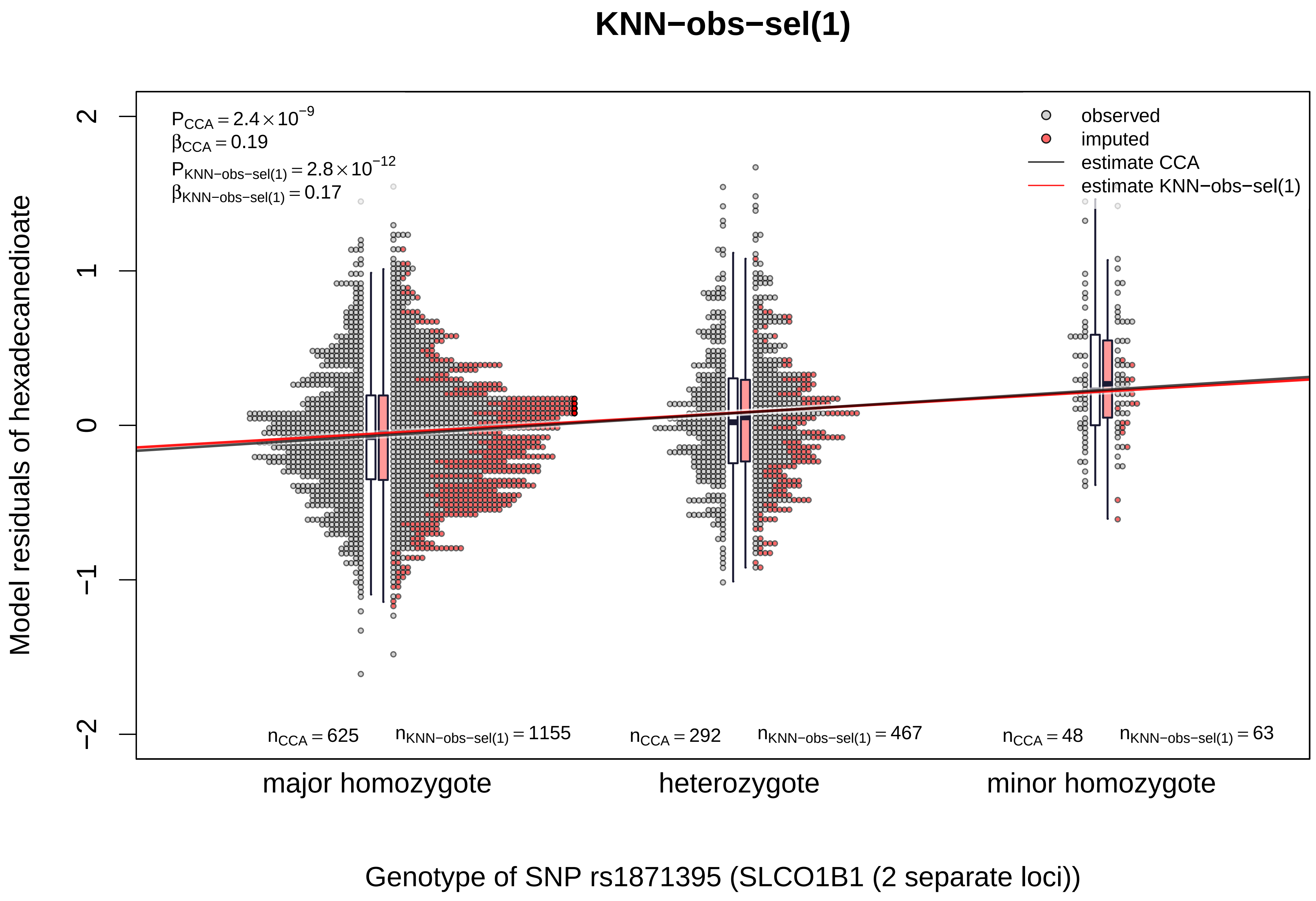
KNN−obs−sel(1)
$P_{CCA} = 2.4 \times 10^{-9}$
$\beta_{CCA} = 0.19$
$P_{KNN-obs-sel(1)} = 2.8 \times 10^{-12}$
$\beta_{KNN-obs-sel(1)} = 0.17$
observed
imputed
estimate CCA
estimate KNN−obs−sel(1)
Model residuals of hexadecanedioate
2
1
0
−1
−2
$n_{CCA} = 625$
$n_{KNN-obs-sel(1)} = 1155$
$n_{CCA} = 292$
$n_{KNN-obs-sel(1)} = 467$
$n_{CCA} = 48$
$n_{KNN-obs-sel(1)} = 63$
major homozygote
heterozygote
minor homozygote
Genotype of SNP rs1871395 (SLCO1B1 (2 separate loci))

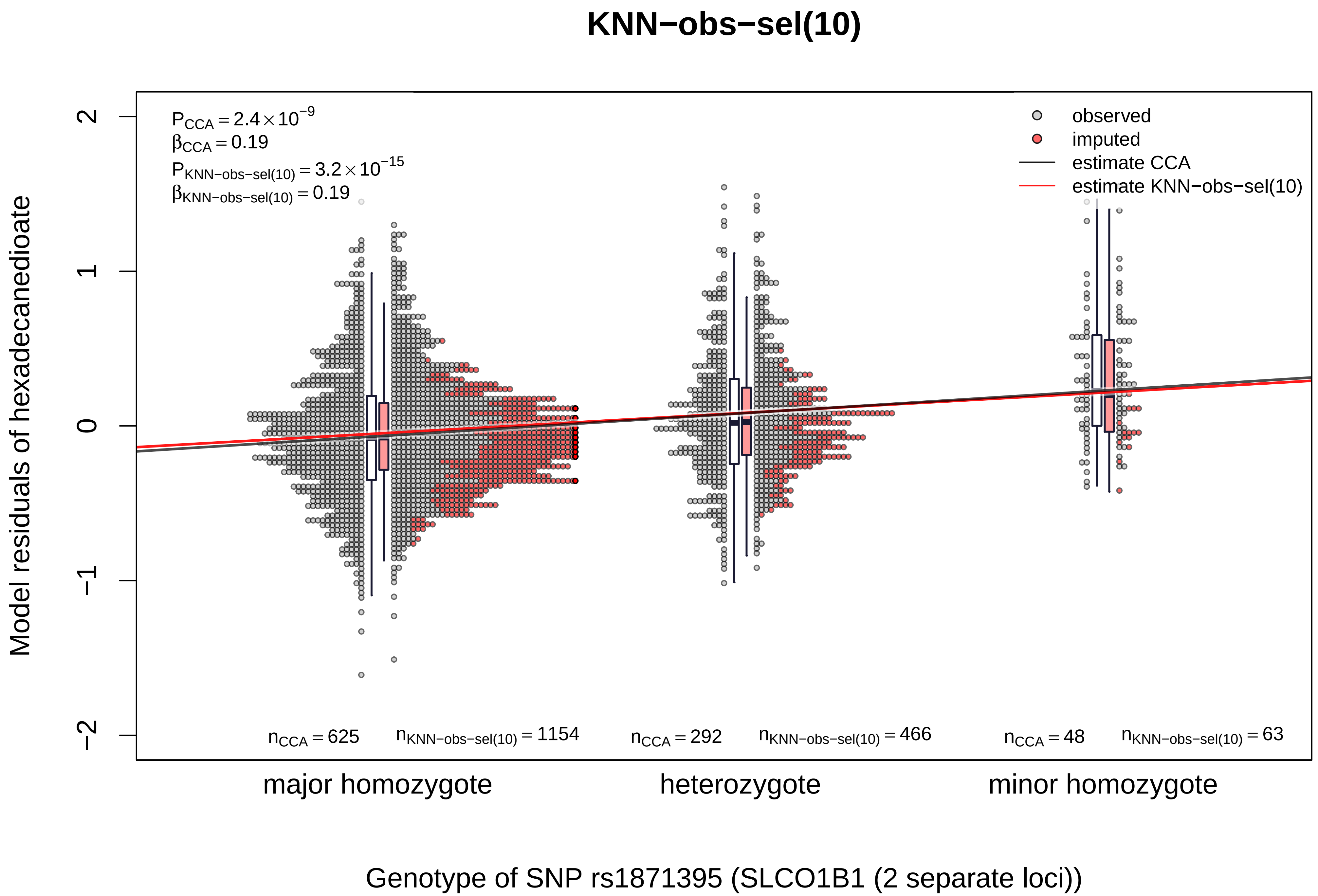

KNN−obs−sel(10)
$P_{CCA} = 2.4 \times 10^{-9}$
$\beta_{CCA} = 0.19$
$P_{KNN-obs-sel(10)} = 3.2 \times 10^{-15}$
$\beta_{KNN-obs-sel(10)} = 0.19$
observed
imputed
estimate CCA
estimate KNN−obs−sel(10)
Model residuals of hexadecanedioate
2
1
0
−1
−2
$n_{CCA} = 625$
$n_{KNN-obs-sel(10)} = 1154$
$n_{CCA} = 292$
$n_{KNN-obs-sel(10)} = 466$
$n_{CCA} = 48$
$n_{KNN-obs-sel(10)} = 63$
major homozygote
heterozygote
minor homozygote
Genotype of SNP rs1871395 (SLCO1B1 (2 separate loci))

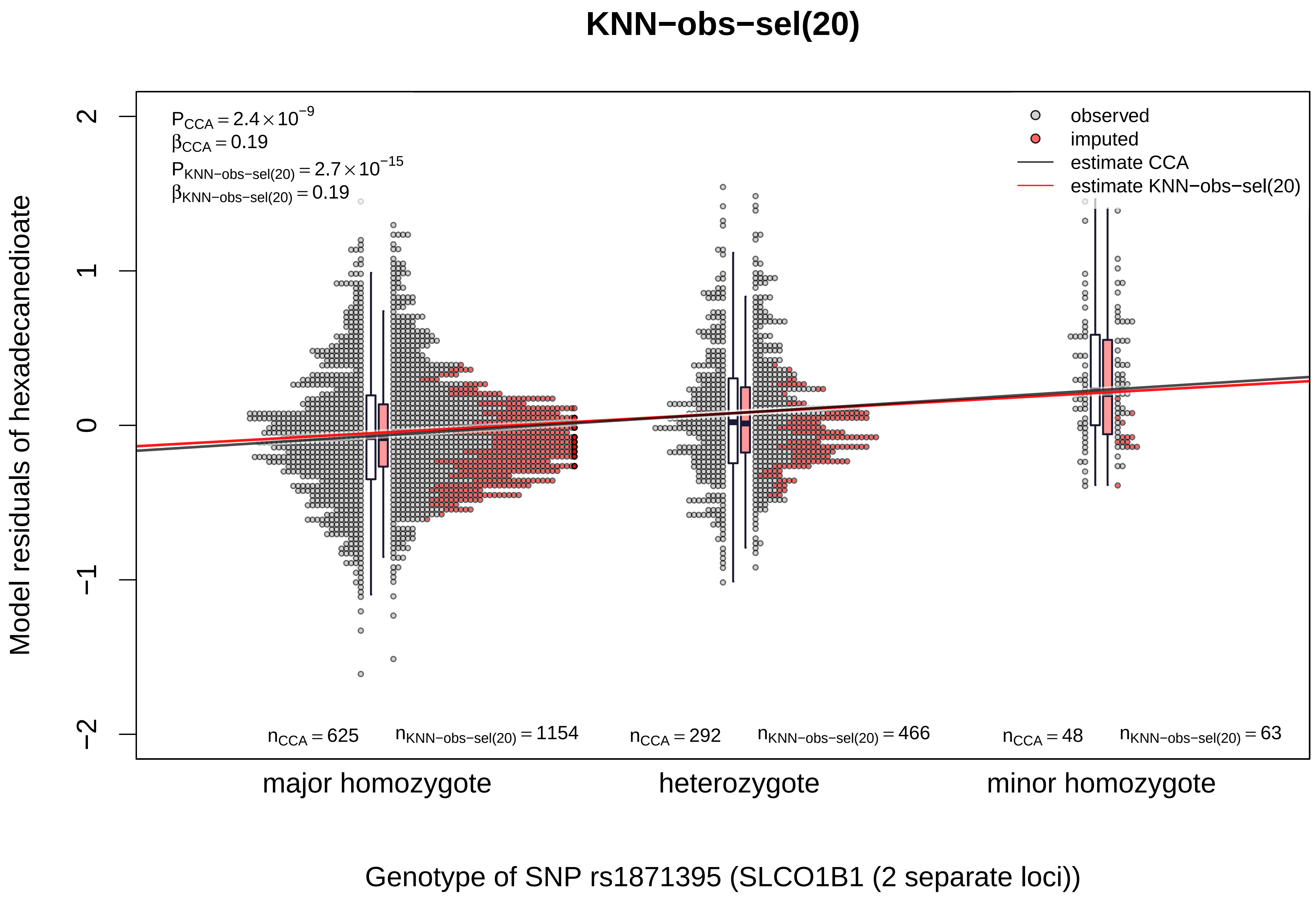

KNN−obs−sel(20)
$P_{CCA} = 2.4 \times 10^{-9}$
$\beta_{CCA} = 0.19$
$P_{KNN-obs-sel(20)} = 2.7 \times 10^{-15}$
$\beta_{KNN-obs-sel(20)} = 0.19$
observed
imputed
estimate CCA
estimate KNN−obs−sel(20)
Model residuals of hexadecanedioate
2
1
0
−1
−2
$n_{CCA} = 625$
$n_{KNN-obs-sel(20)} = 1154$
$n_{CCA} = 292$
$n_{KNN-obs-sel(20)} = 466$
$n_{CCA} = 48$
$n_{KNN-obs-sel(20)} = 63$
major homozygote
heterozygote
minor homozygote
Genotype of SNP rs1871395 (SLCO1B1 (2 separate loci))

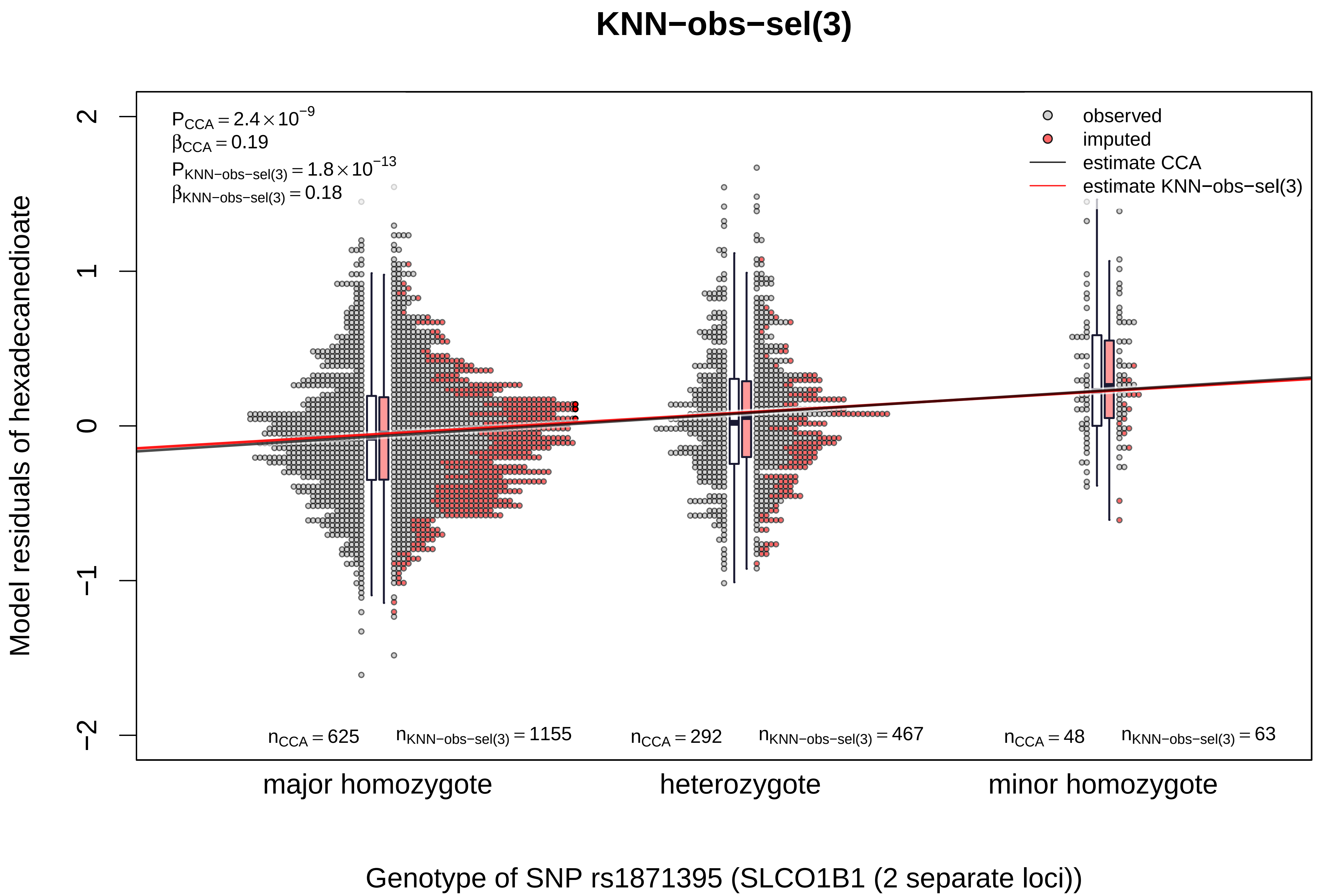

KNN−obs−sel(3)
$P_{CCA} = 2.4 \times 10^{-9}$
$\beta_{CCA} = 0.19$
$P_{KNN-obs-sel(3)} = 1.8 \times 10^{-13}$
$\beta_{KNN-obs-sel(3)} = 0.18$
observed
imputed
estimate CCA
estimate KNN−obs−sel(3)
Model residuals of hexadecanedioate
2
1
0
−1
−2
$n_{CCA} = 625$
$n_{KNN-obs-sel(3)} = 1155$
$n_{CCA} = 292$
$n_{KNN-obs-sel(3)} = 467$
$n_{CCA} = 48$
$n_{KNN-obs-sel(3)} = 63$
major homozygote
heterozygote
minor homozygote
Genotype of SNP rs1871395 (SLCO1B1 (2 separate loci))

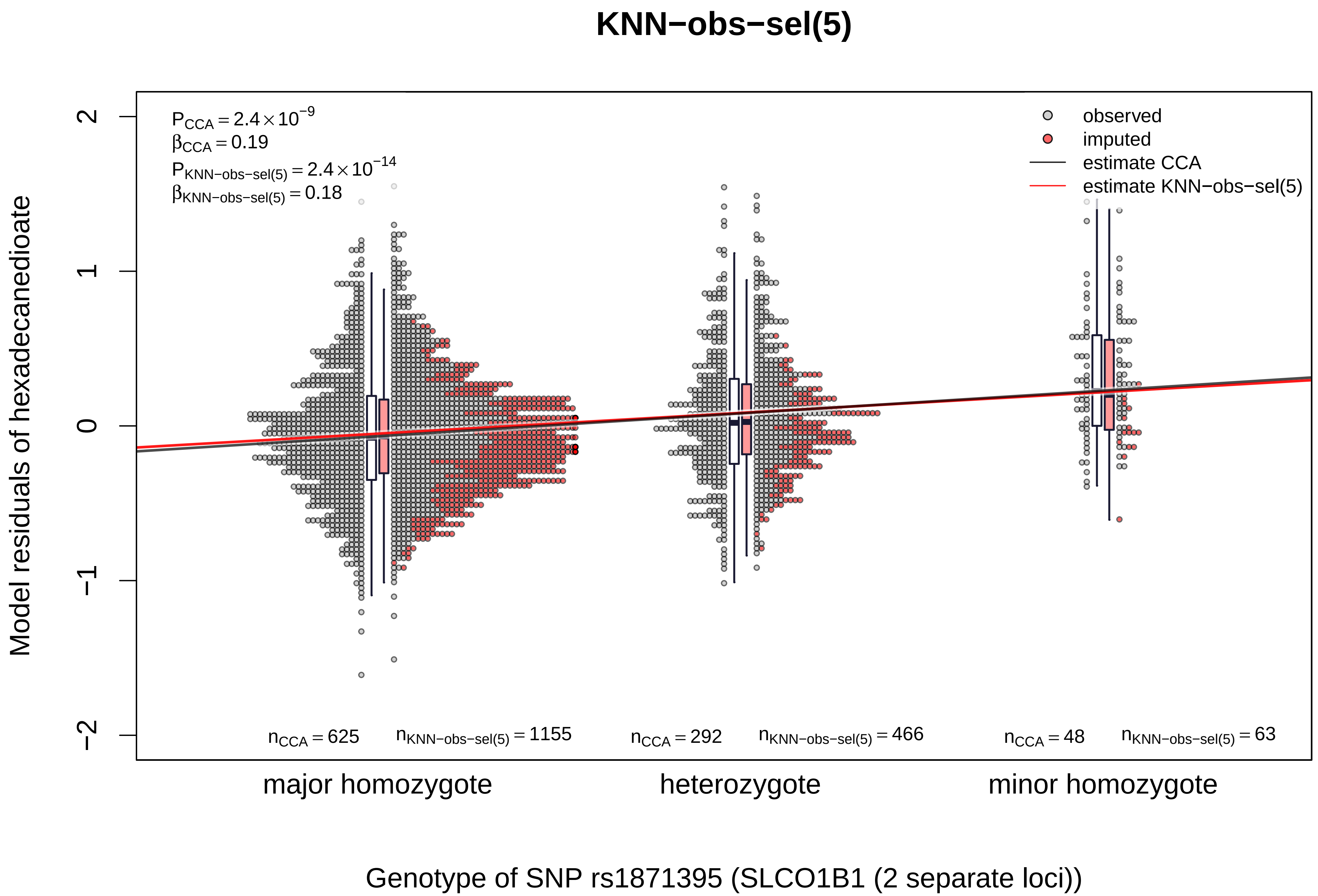

KNN−obs−sel(5)
$P_{CCA} = 2.4 \times 10^{-9}$
$\beta_{CCA} = 0.19$
$P_{KNN-obs-sel(5)} = 2.4 \times 10^{-14}$
$\beta_{KNN-obs-sel(5)} = 0.18$
observed
imputed
estimate CCA
estimate KNN−obs−sel(5)
Model residuals of hexadecanedioate
2
1
0
−1
−2
$n_{CCA} = 625$
$n_{KNN-obs-sel(5)} = 1155$
$n_{CCA} = 292$
$n_{KNN-obs-sel(5)} = 466$
$n_{CCA} = 48$
$n_{KNN-obs-sel(5)} = 63$
major homozygote
heterozygote
minor homozygote
Genotype of SNP rs1871395 (SLCO1B1 (2 separate loci))

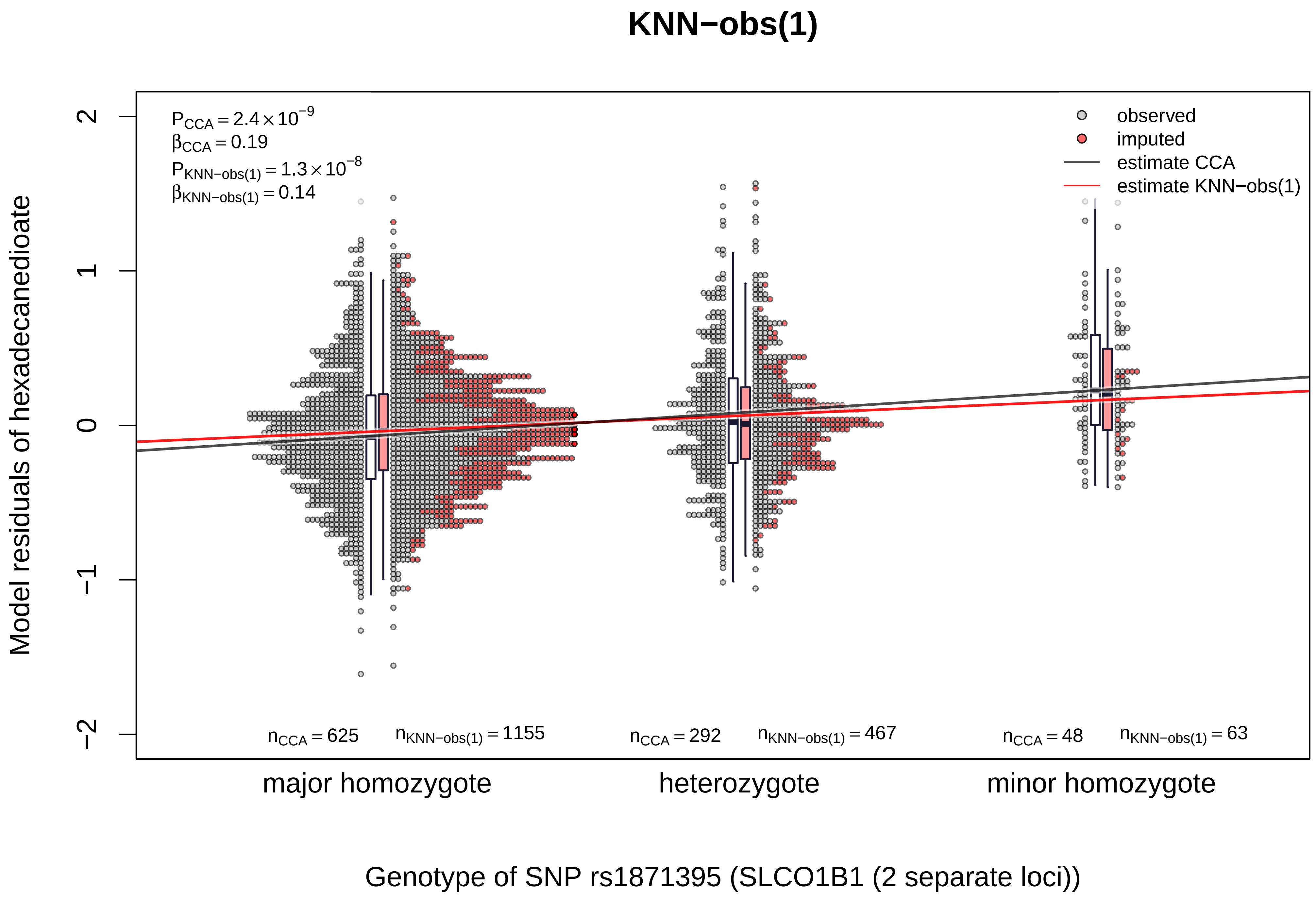

KNN−obs(1)
$P_{CCA} = 2.4 \times 10^{-9}$
$\beta_{CCA} = 0.19$
$P_{KNN-obs(1)} = 1.3 \times 10^{-8}$
$\beta_{KNN-obs(1)} = 0.14$
observed
imputed
estimate CCA
estimate KNN−obs(1)
Model residuals of hexadecanedioate
2
1
0
−1
−2
$n_{CCA} = 625$
$n_{KNN-obs(1)} = 1155$
$n_{CCA} = 292$
$n_{KNN-obs(1)} = 467$
$n_{CCA} = 48$
$n_{KNN-obs(1)} = 63$
major homozygote
heterozygote
minor homozygote
Genotype of SNP rs1871395 (SLCO1B1 (2 separate loci))

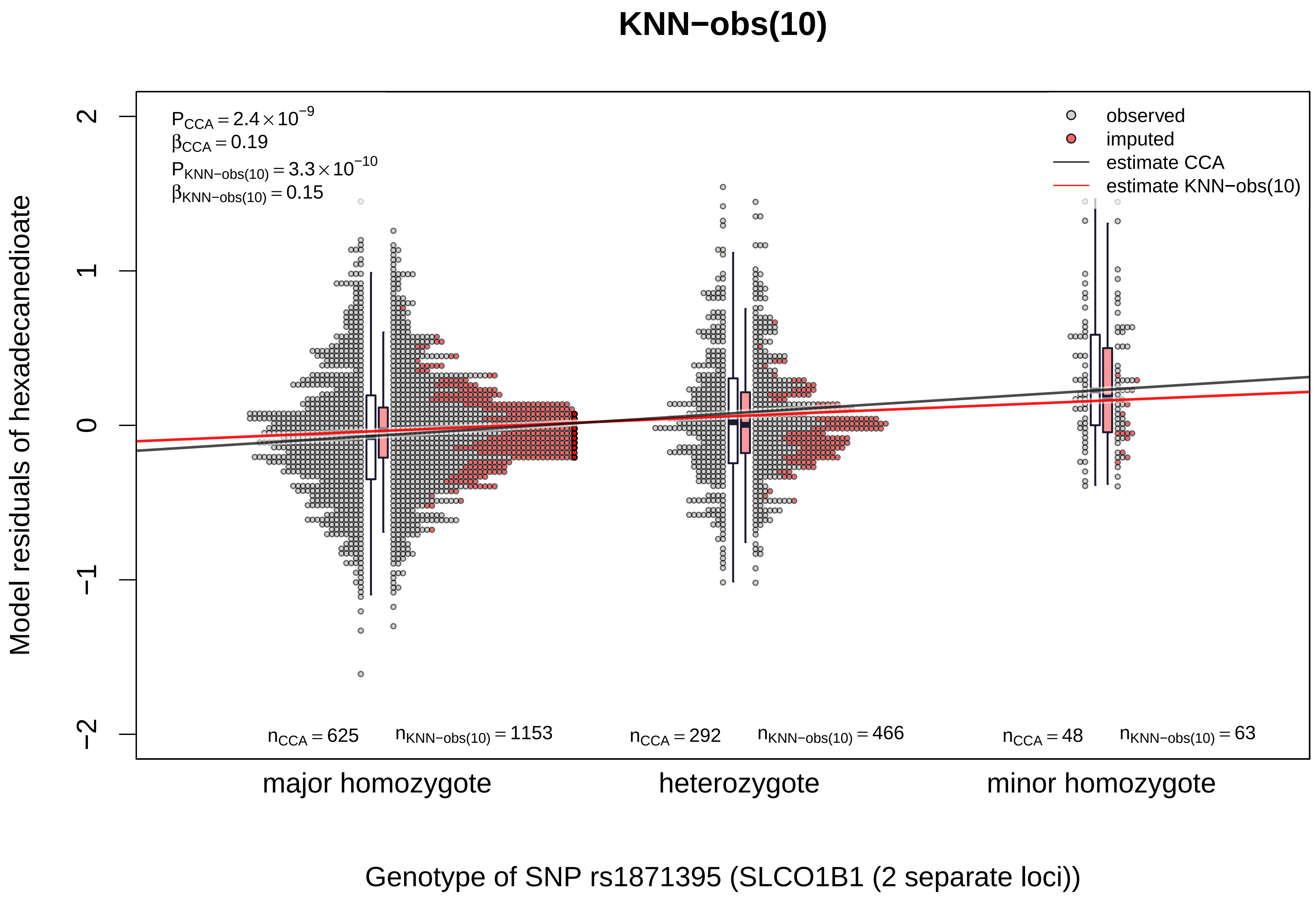
KNN−obs(10)
$P_{CCA} = 2.4 \times 10^{-9}$
$\beta_{CCA} = 0.19$
$P_{KNN-obs(10)} = 3.3 \times 10^{-10}$
$\beta_{KNN-obs(10)} = 0.15$
observed
imputed
estimate CCA
estimate KNN−obs(10)
Model residuals of hexadecanedioate
2
1
0
−1
−2
$n_{CCA} = 625$
$n_{KNN-obs(10)} = 1153$
$n_{CCA} = 292$
$n_{KNN-obs(10)} = 466$
$n_{CCA} = 48$
$n_{KNN-obs(10)} = 63$
major homozygote
heterozygote
minor homozygote
Genotype of SNP rs1871395 (SLCO1B1 (2 separate loci))

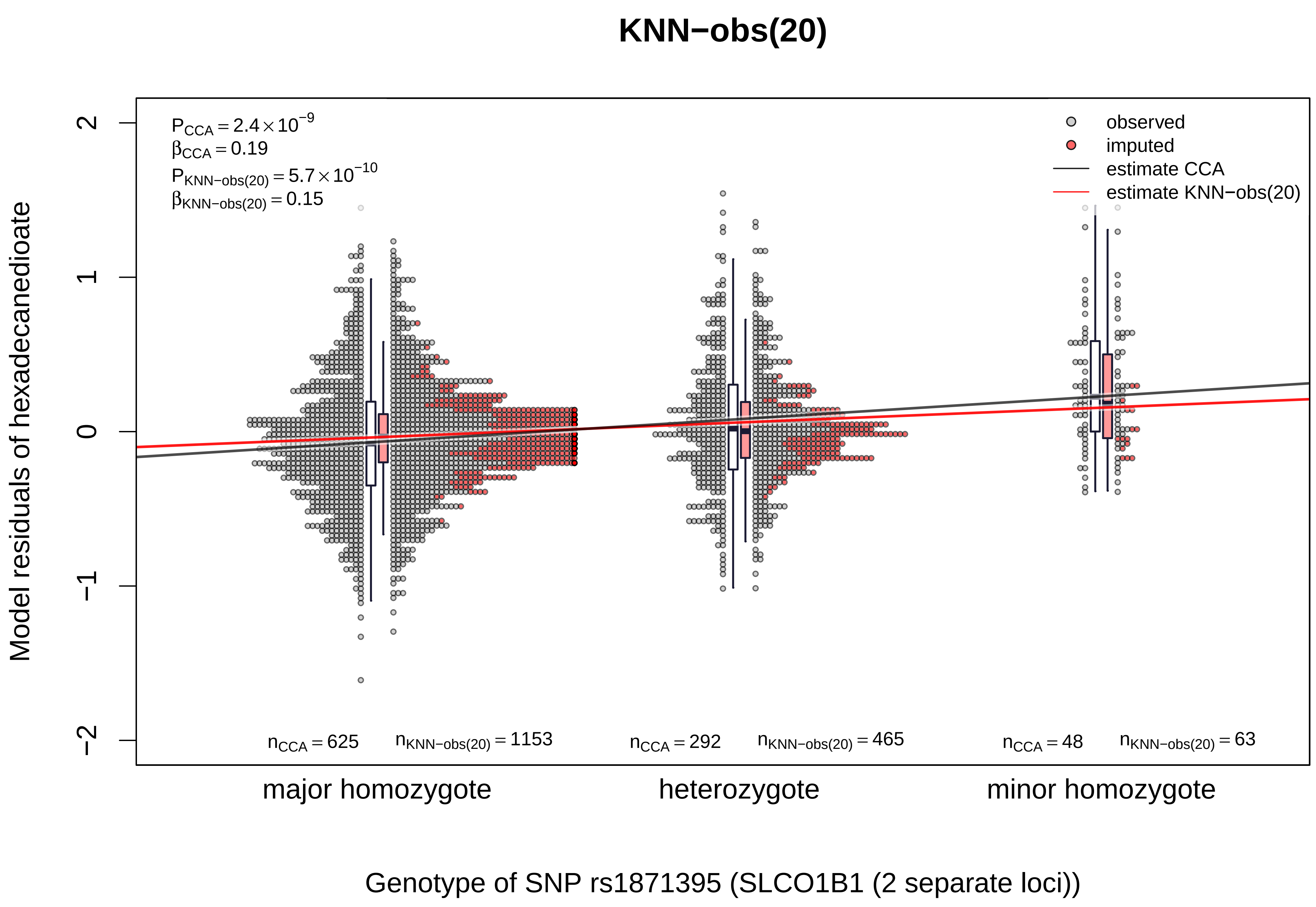

KNN−obs(20)
$P_{CCA} = 2.4 \times 10^{-9}$
$\beta_{CCA} = 0.19$
$P_{KNN-obs(20)} = 5.7 \times 10^{-10}$
$\beta_{KNN-obs(20)} = 0.15$
observed
imputed
estimate CCA
estimate KNN−obs(20)
Model residuals of hexadecanedioate
2
1
0
−1
−2
$n_{CCA} = 625$
$n_{KNN-obs(20)} = 1153$
$n_{CCA} = 292$
$n_{KNN-obs(20)} = 465$
$n_{CCA} = 48$
$n_{KNN-obs(20)} = 63$
major homozygote
heterozygote
minor homozygote
Genotype of SNP rs1871395 (SLCO1B1 (2 separate loci))

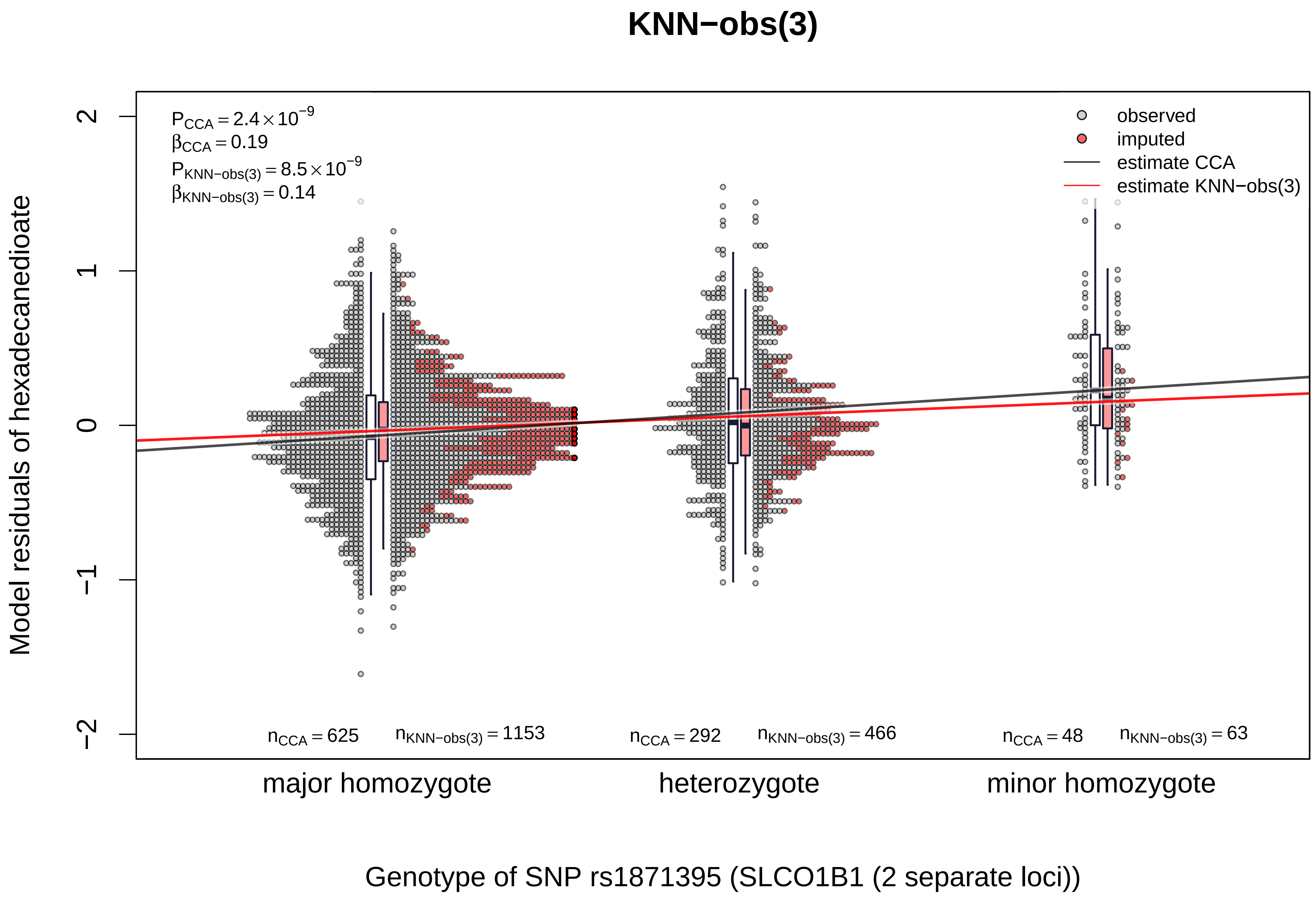

KNN−obs(3)
$P_{CCA} = 2.4 \times 10^{-9}$
$\beta_{CCA} = 0.19$
$P_{KNN-obs(3)} = 8.5 \times 10^{-9}$
$\beta_{KNN-obs(3)} = 0.14$
observed
imputed
estimate CCA
estimate KNN−obs(3)
Model residuals of hexadecanedioate
2
1
0
−1
−2
$n_{CCA} = 625$
$n_{KNN-obs(3)} = 1153$
$n_{CCA} = 292$
$n_{KNN-obs(3)} = 466$
$n_{CCA} = 48$
$n_{KNN-obs(3)} = 63$
major homozygote
heterozygote
minor homozygote
Genotype of SNP rs1871395 (SLCO1B1 (2 separate loci))

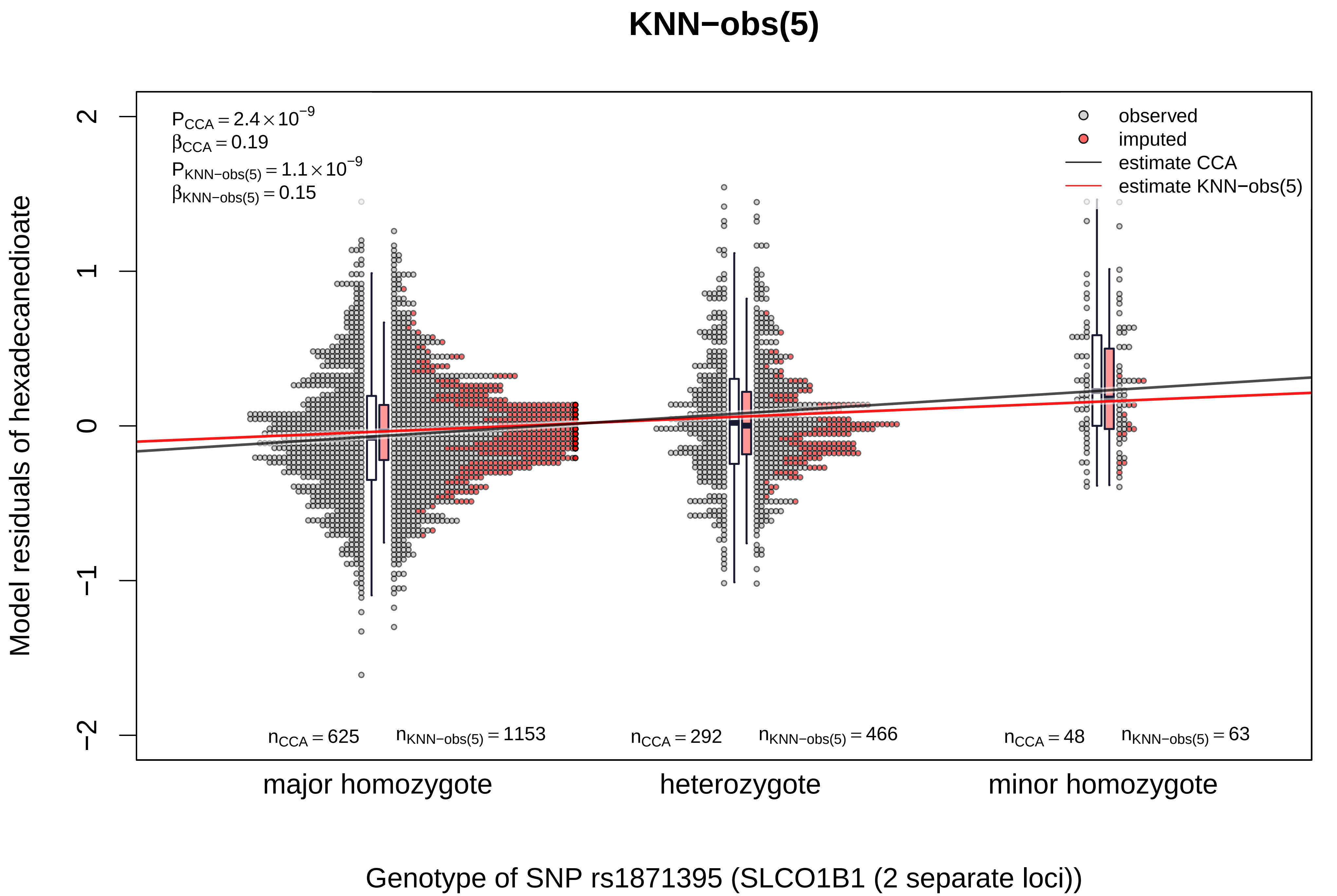

KNN−obs(5)
$P_{CCA} = 2.4 \times 10^{-9}$
$\beta_{CCA} = 0.19$
$P_{KNN-obs(5)} = 1.1 \times 10^{-9}$
$\beta_{KNN-obs(5)} = 0.15$
observed
imputed
estimate CCA
estimate KNN−obs(5)
Model residuals of hexadecanedioate
2
1
0
−1
−2
$n_{CCA} = 625$
$n_{KNN-obs(5)} = 1153$
$n_{CCA} = 292$
$n_{KNN-obs(5)} = 466$
$n_{CCA} = 48$
$n_{KNN-obs(5)} = 63$
major homozygote
heterozygote
minor homozygote
Genotype of SNP rs1871395 (SLCO1B1 (2 separate loci))

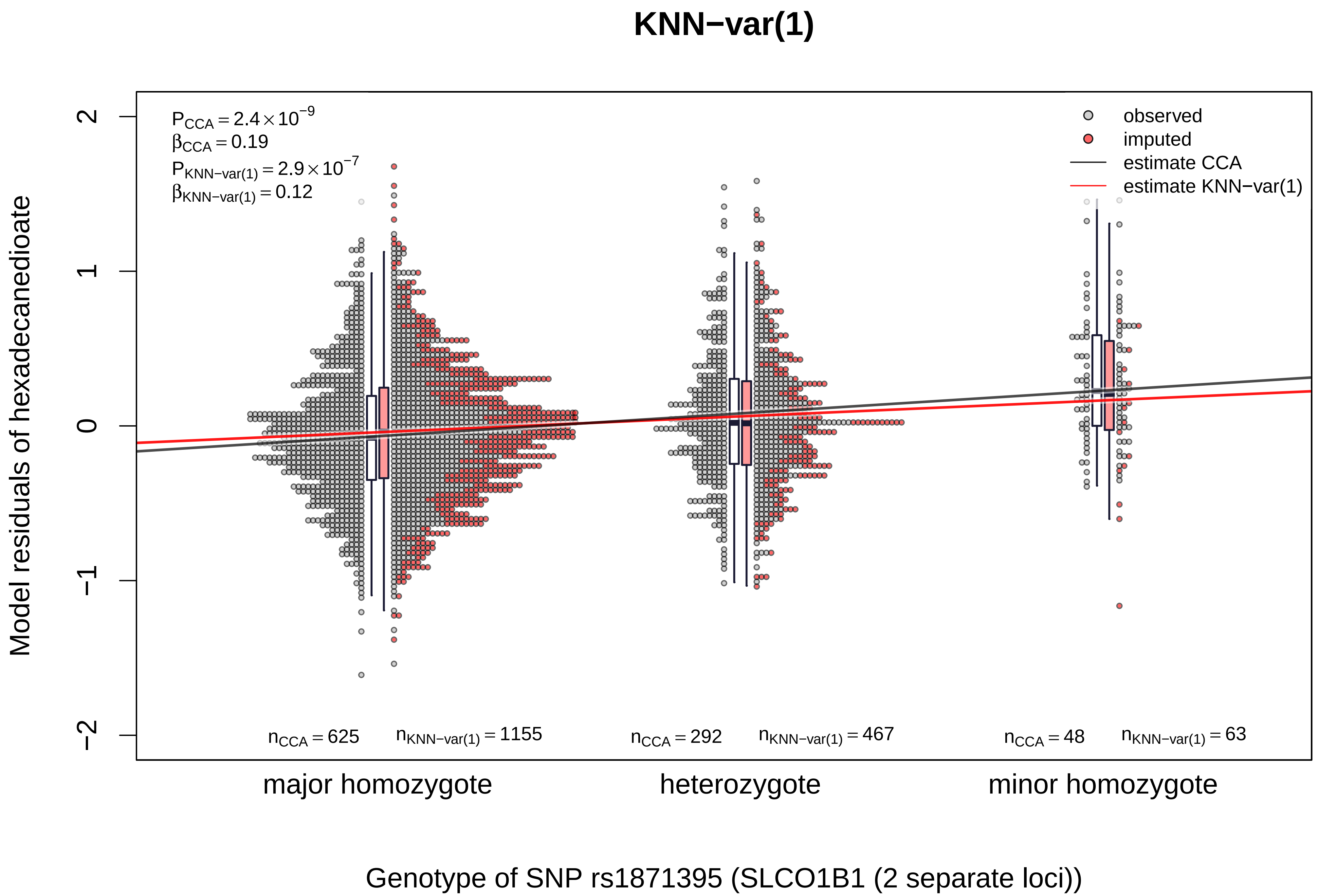

KNN−var(1)
$P_{CCA} = 2.4 \times 10^{-9}$
$\beta_{CCA} = 0.19$
$P_{KNN-var(1)} = 2.9 \times 10^{-7}$
$\beta_{KNN-var(1)} = 0.12$
observed
imputed
estimate CCA
estimate KNN−var(1)
Model residuals of hexadecanedioate
2
1
0
−1
−2
$n_{CCA} = 625$
$n_{KNN-var(1)} = 1155$
$n_{CCA} = 292$
$n_{KNN-var(1)} = 467$
$n_{CCA} = 48$
$n_{KNN-var(1)} = 63$
major homozygote
heterozygote
minor homozygote
Genotype of SNP rs1871395 (SLCO1B1 (2 separate loci))

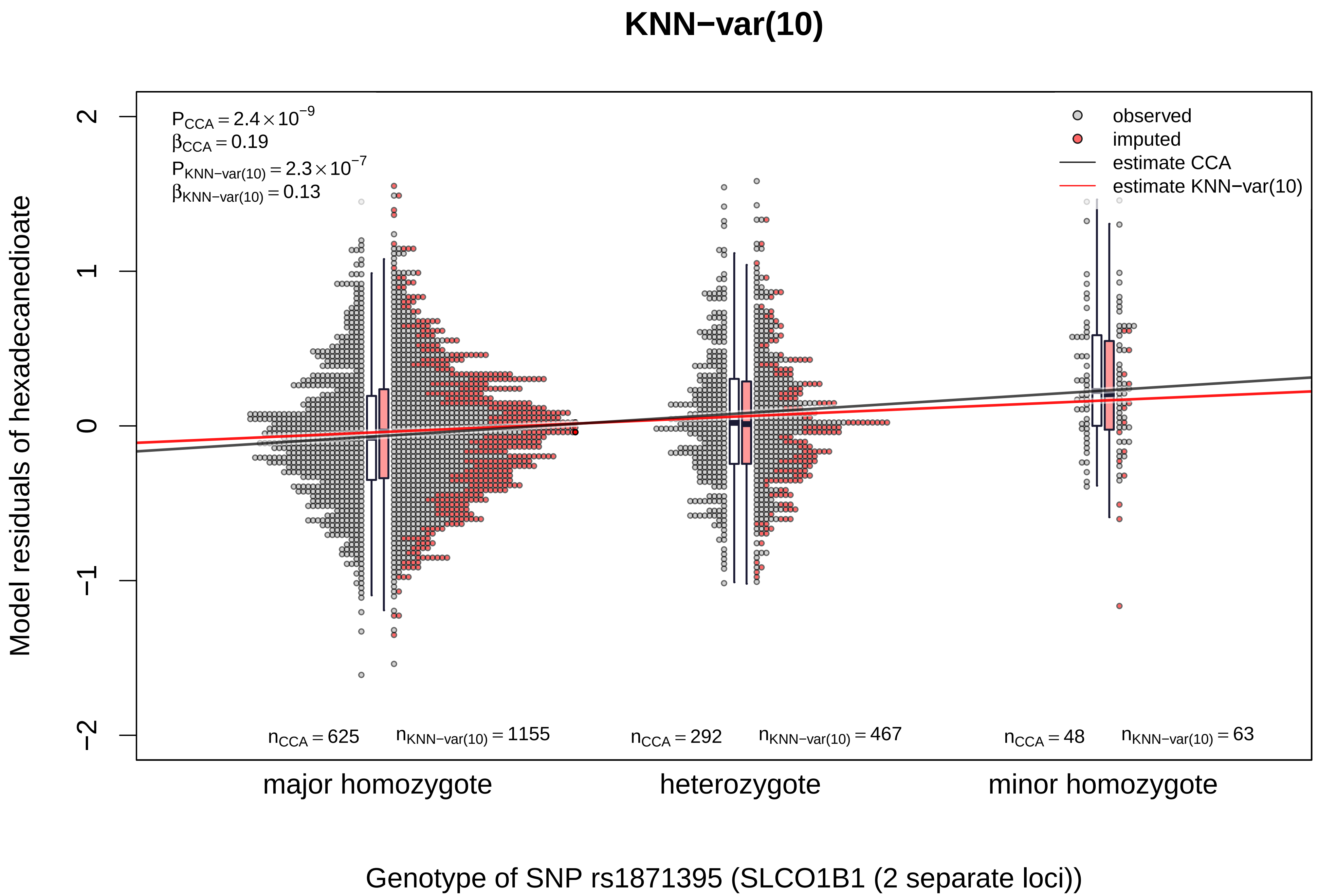

KNN−var(10)
$P_{CCA} = 2.4 \times 10^{-9}$
$\beta_{CCA} = 0.19$
$P_{KNN-var(10)} = 2.3 \times 10^{-7}$
$\beta_{KNN-var(10)} = 0.13$
observed
imputed
estimate CCA
estimate KNN−var(10)
Model residuals of hexadecanedioate
2
1
0
−1
−2
$n_{CCA} = 625$
$n_{KNN-var(10)} = 1155$
$n_{CCA} = 292$
$n_{KNN-var(10)} = 467$
$n_{CCA} = 48$
$n_{KNN-var(10)} = 63$
major homozygote
heterozygote
minor homozygote
Genotype of SNP rs1871395 (SLCO1B1 (2 separate loci))

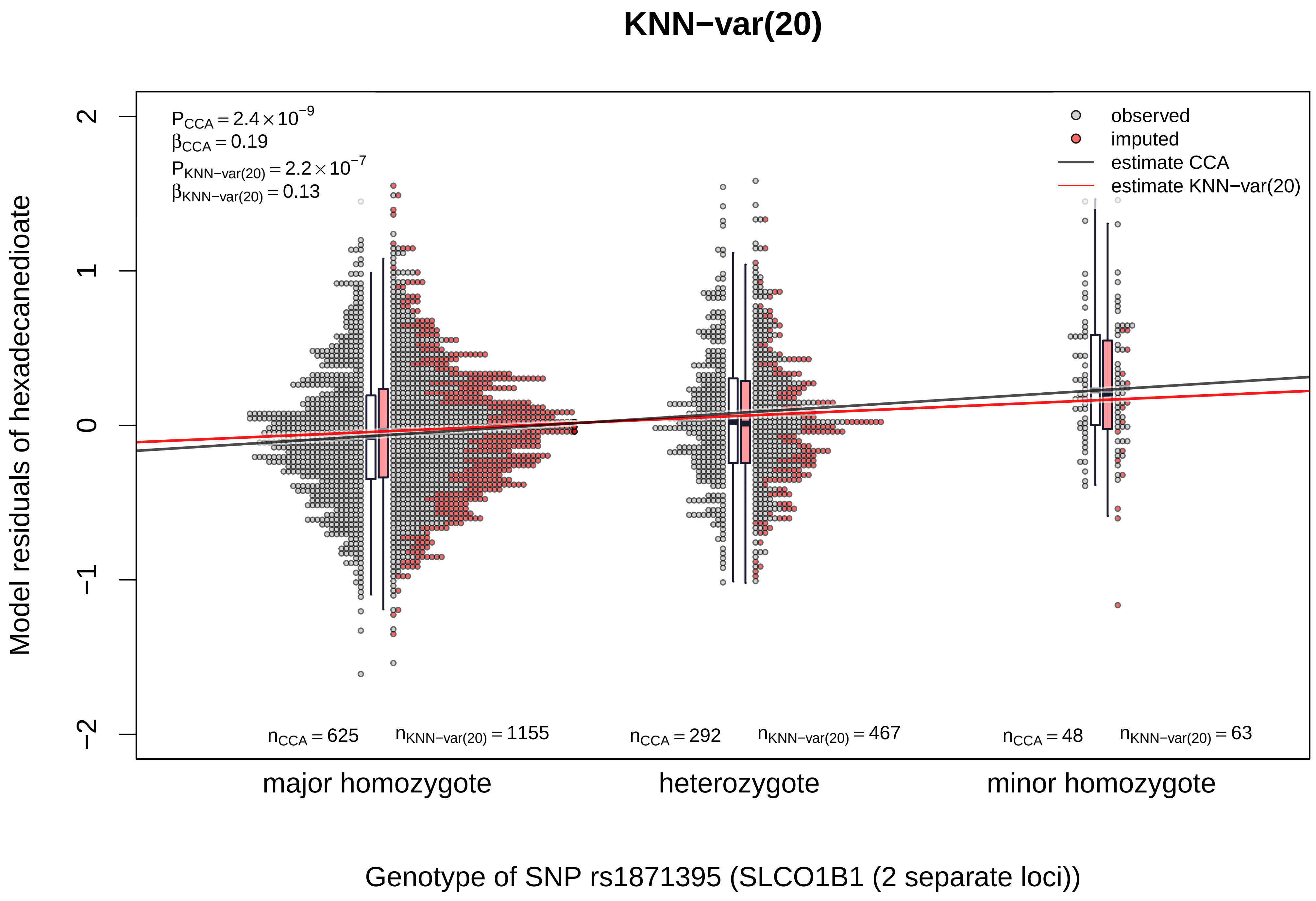

KNN−var(20)
$P_{CCA} = 2.4 \times 10^{-9}$
$\beta_{CCA} = 0.19$
$P_{KNN-var(20)} = 2.2 \times 10^{-7}$
$\beta_{KNN-var(20)} = 0.13$
observed
imputed
estimate CCA
estimate KNN−var(20)
Model residuals of hexadecanedioate
2
1
0
−1
−2
$n_{CCA} = 625$
$n_{KNN-var(20)} = 1155$
$n_{CCA} = 292$
$n_{KNN-var(20)} = 467$
$n_{CCA} = 48$
$n_{KNN-var(20)} = 63$
major homozygote
heterozygote
minor homozygote
Genotype of SNP rs1871395 (SLCO1B1 (2 separate loci))

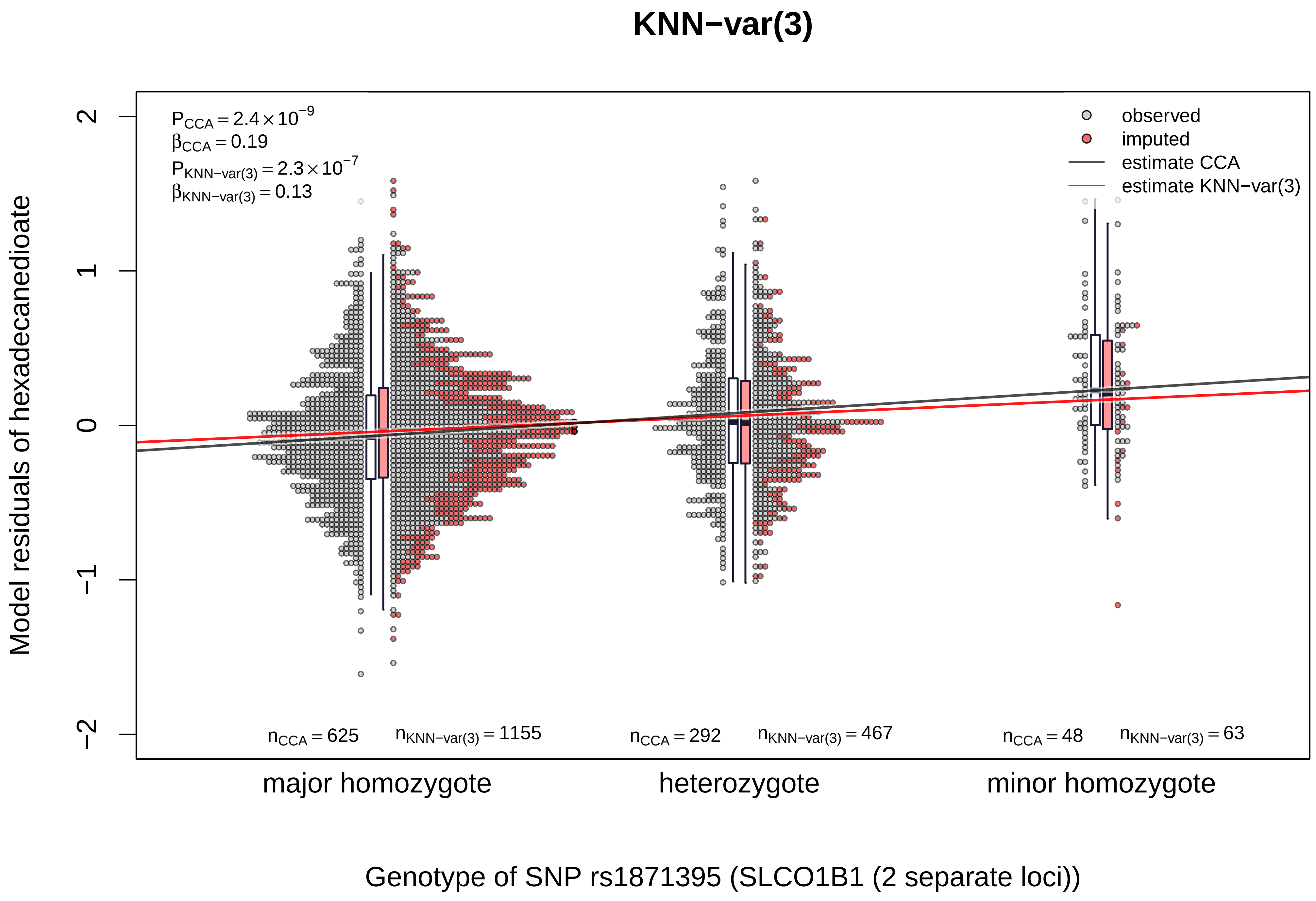

KNN−var(3)
$P_{CCA} = 2.4 \times 10^{-9}$
$\beta_{CCA} = 0.19$
$P_{KNN-var(3)} = 2.3 \times 10^{-7}$
$\beta_{KNN-var(3)} = 0.13$
observed
imputed
estimate CCA
estimate KNN−var(3)
Model residuals of hexadecanedioate
2
1
0
−1
−2
$n_{CCA} = 625$
$n_{KNN-var(3)} = 1155$
$n_{CCA} = 292$
$n_{KNN-var(3)} = 467$
$n_{CCA} = 48$
$n_{KNN-var(3)} = 63$
major homozygote
heterozygote
minor homozygote
Genotype of SNP rs1871395 (SLCO1B1 (2 separate loci))

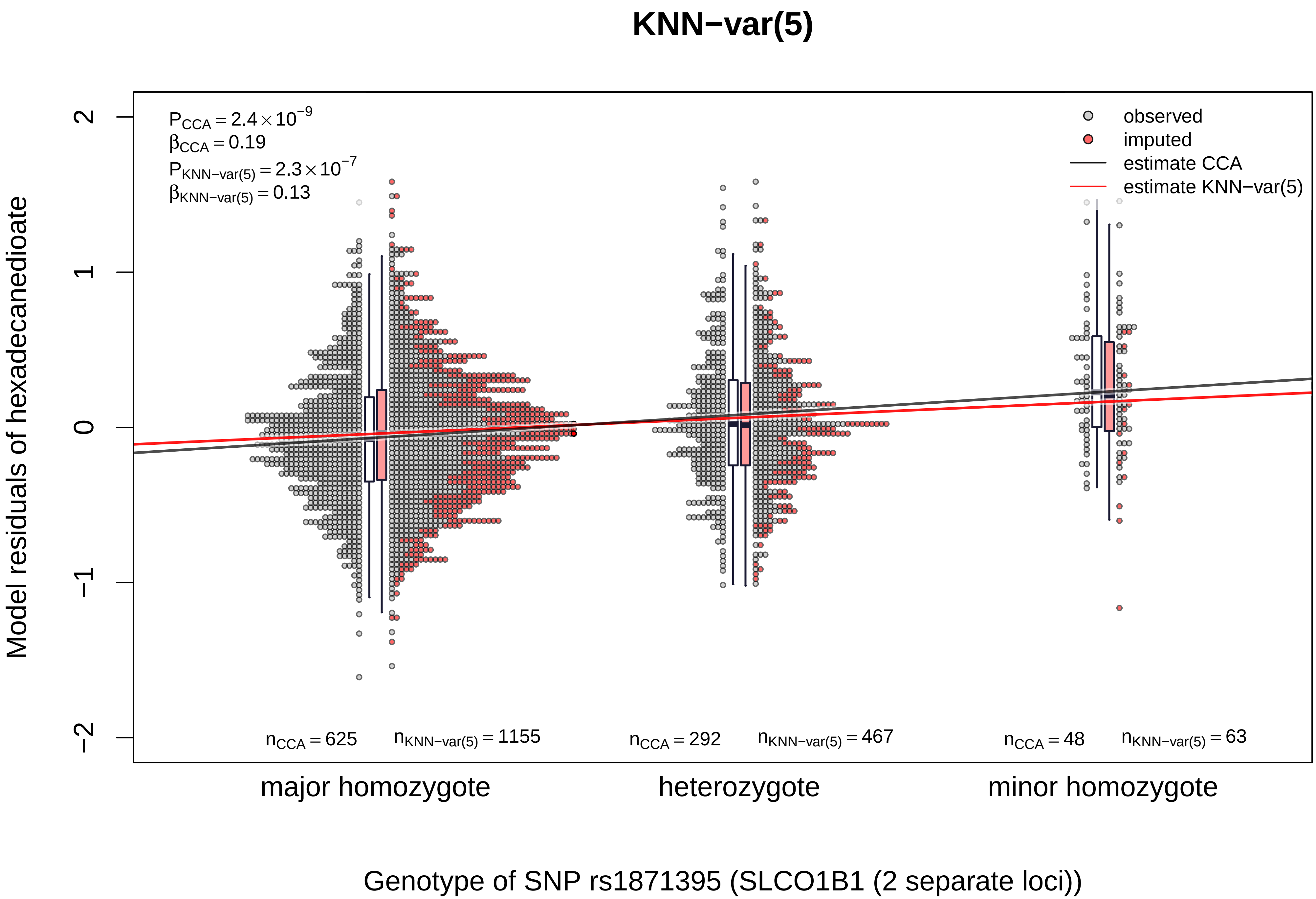

KNN−var(5)
$P_{CCA} = 2.4 \times 10^{-9}$
$\beta_{CCA} = 0.19$
$P_{KNN-var(5)} = 2.3 \times 10^{-7}$
$\beta_{KNN-var(5)} = 0.13$
observed
imputed
estimate CCA
estimate KNN−var(5)
Model residuals of hexadecanedioate
2
1
0
−1
−2
$n_{CCA} = 625$
$n_{KNN-var(5)} = 1155$
$n_{CCA} = 292$
$n_{KNN-var(5)} = 467$
$n_{CCA} = 48$
$n_{KNN-var(5)} = 63$
major homozygote
heterozygote
minor homozygote
Genotype of SNP rs1871395 (SLCO1B1 (2 separate loci))

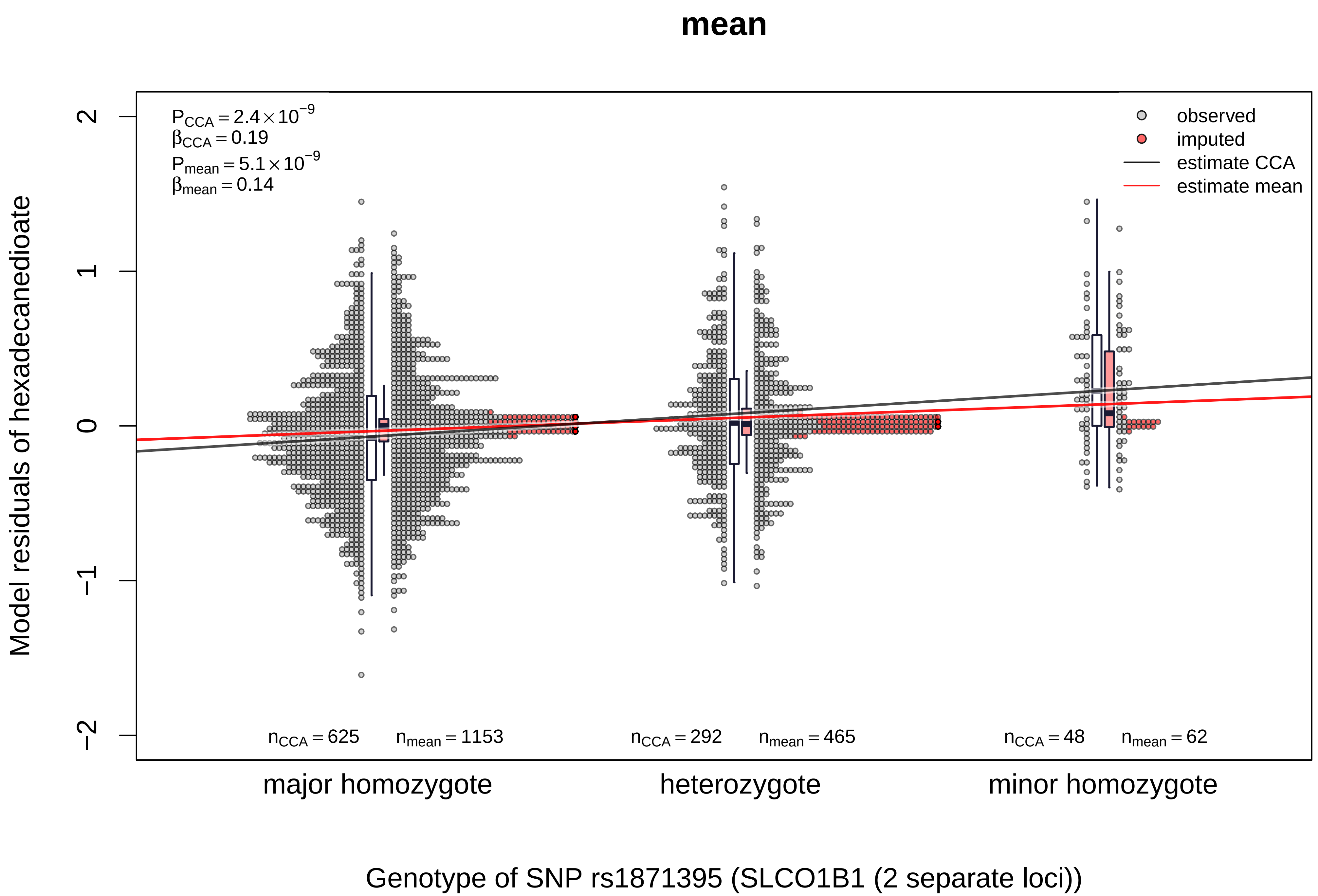

mean
$P_{CCA} = 2.4 \times 10^{-9}$
$\beta_{CCA} = 0.19$
$P_{mean} = 5.1 \times 10^{-9}$
$\beta_{mean} = 0.14$
observed
imputed
estimate CCA
estimate mean
2
1
0
−1
−2
Model residuals of hexadecanedioate
$n_{CCA} = 625$
$n_{mean} = 1153$
$n_{CCA} = 292$
$n_{mean} = 465$
$n_{CCA} = 48$
$n_{mean} = 62$
major homozygote
heterozygote
minor homozygote
Genotype of SNP rs1871395 (SLCO1B1 (2 separate loci))

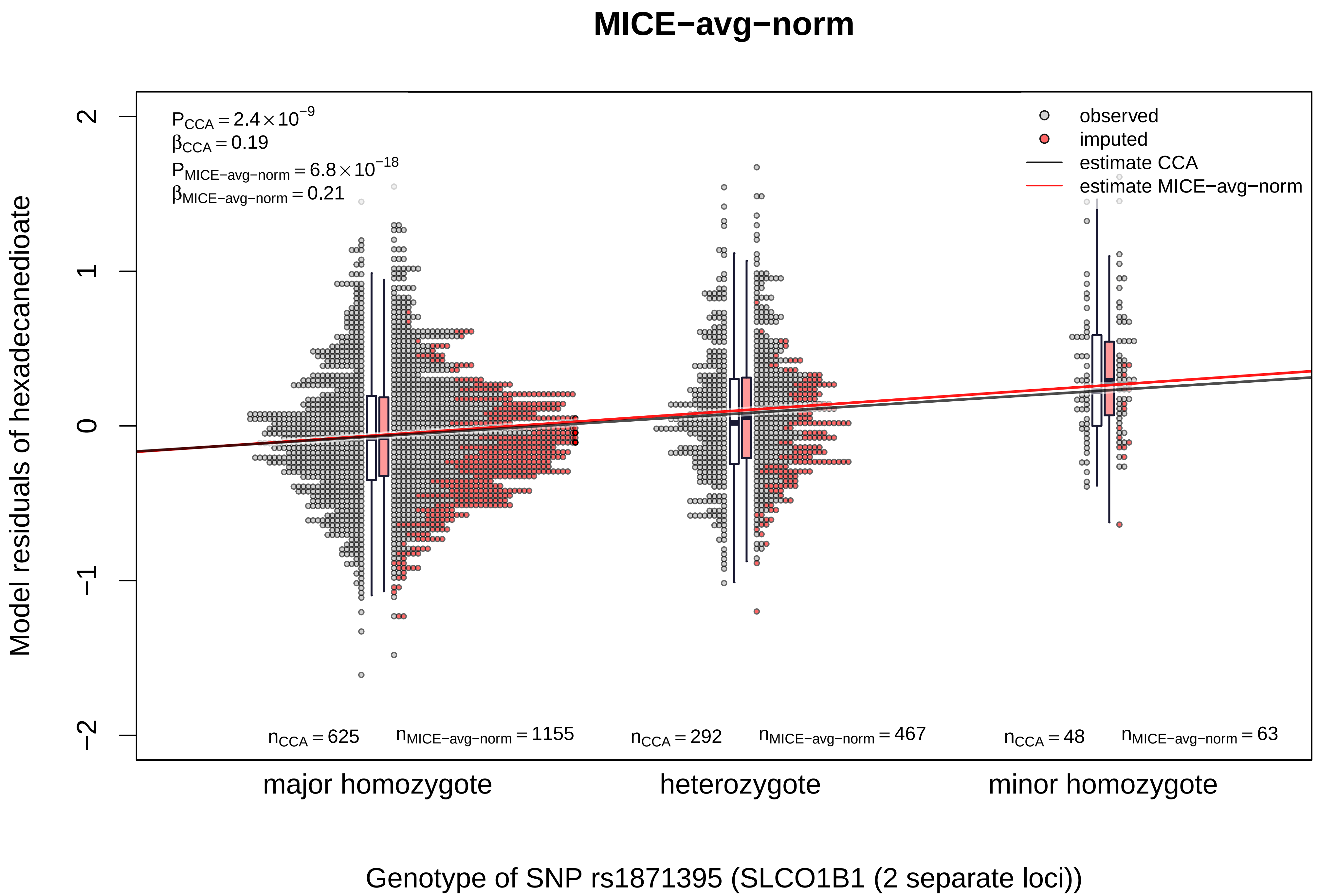
MICE−avg−norm
$P_{CCA} = 2.4 \times 10^{-9}$
$\beta_{CCA} = 0.19$
$P_{MICE-avg-norm} = 6.8 \times 10^{-18}$
$\beta_{MICE-avg-norm} = 0.21$
observed
imputed
estimate CCA
estimate MICE−avg−norm
Model residuals of hexadecanedioate
2
1
0
−1
−2
$n_{CCA} = 625$
$n_{MICE-avg-norm} = 1155$
$n_{CCA} = 292$
$n_{MICE-avg-norm} = 467$
$n_{CCA} = 48$
$n_{MICE-avg-norm} = 63$
major homozygote
heterozygote
minor homozygote
Genotype of SNP rs1871395 (SLCO1B1 (2 separate loci))

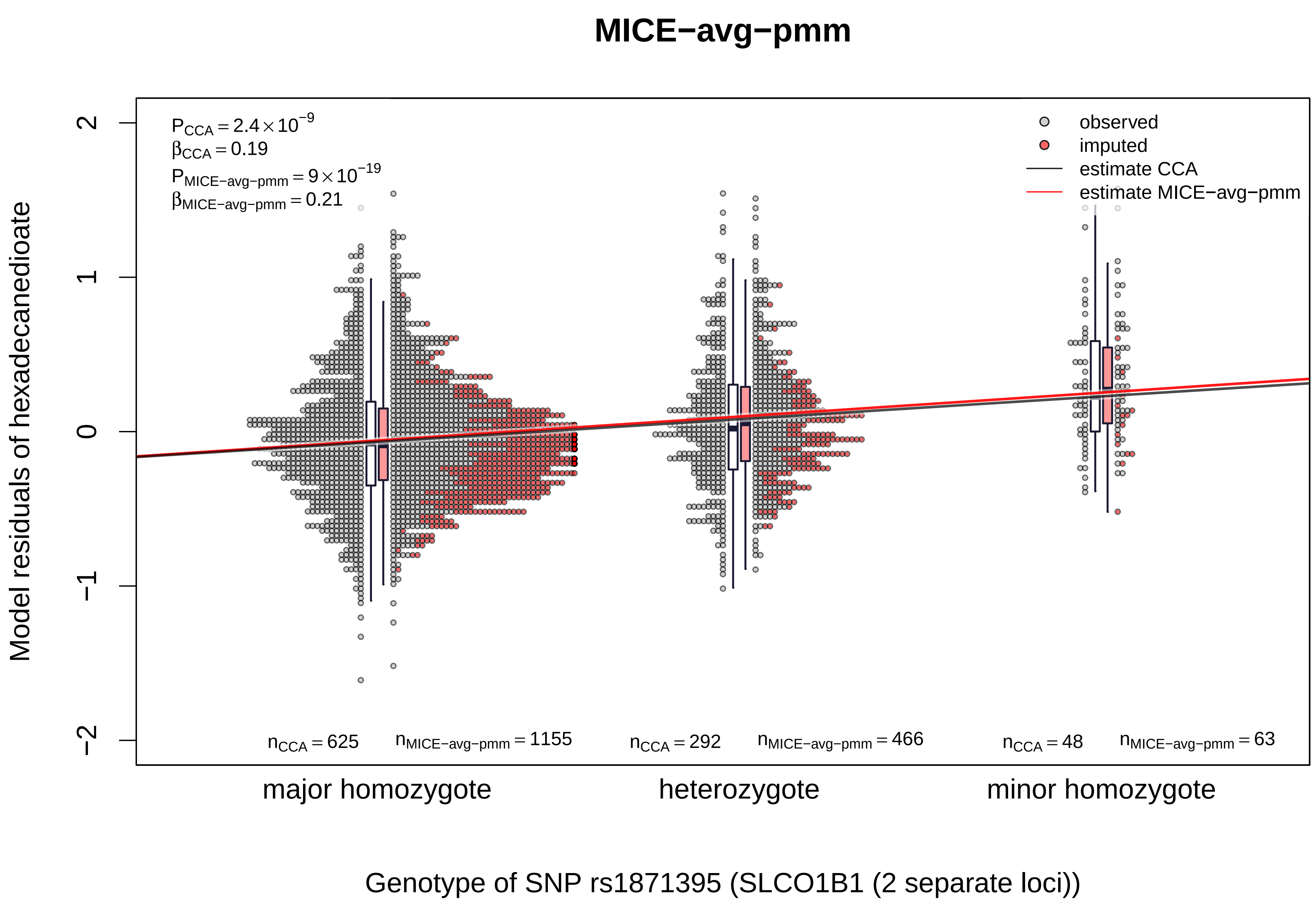

MICE−avg−pmm
$P_{CCA} = 2.4 \times 10^{-9}$
$\beta_{CCA} = 0.19$
$P_{MICE-avg-pmm} = 9 \times 10^{-19}$
$\beta_{MICE-avg-pmm} = 0.21$
observed
imputed
estimate CCA
estimate MICE−avg−pmm
Model residuals of hexadecanedioate
2
1
0
−1
−2
$n_{CCA} = 625$
$n_{MICE-avg-pmm} = 1155$
$n_{CCA} = 292$
$n_{MICE-avg-pmm} = 466$
$n_{CCA} = 48$
$n_{MICE-avg-pmm} = 63$
major homozygote
heterozygote
minor homozygote
Genotype of SNP rs1871395 (SLCO1B1 (2 separate loci))

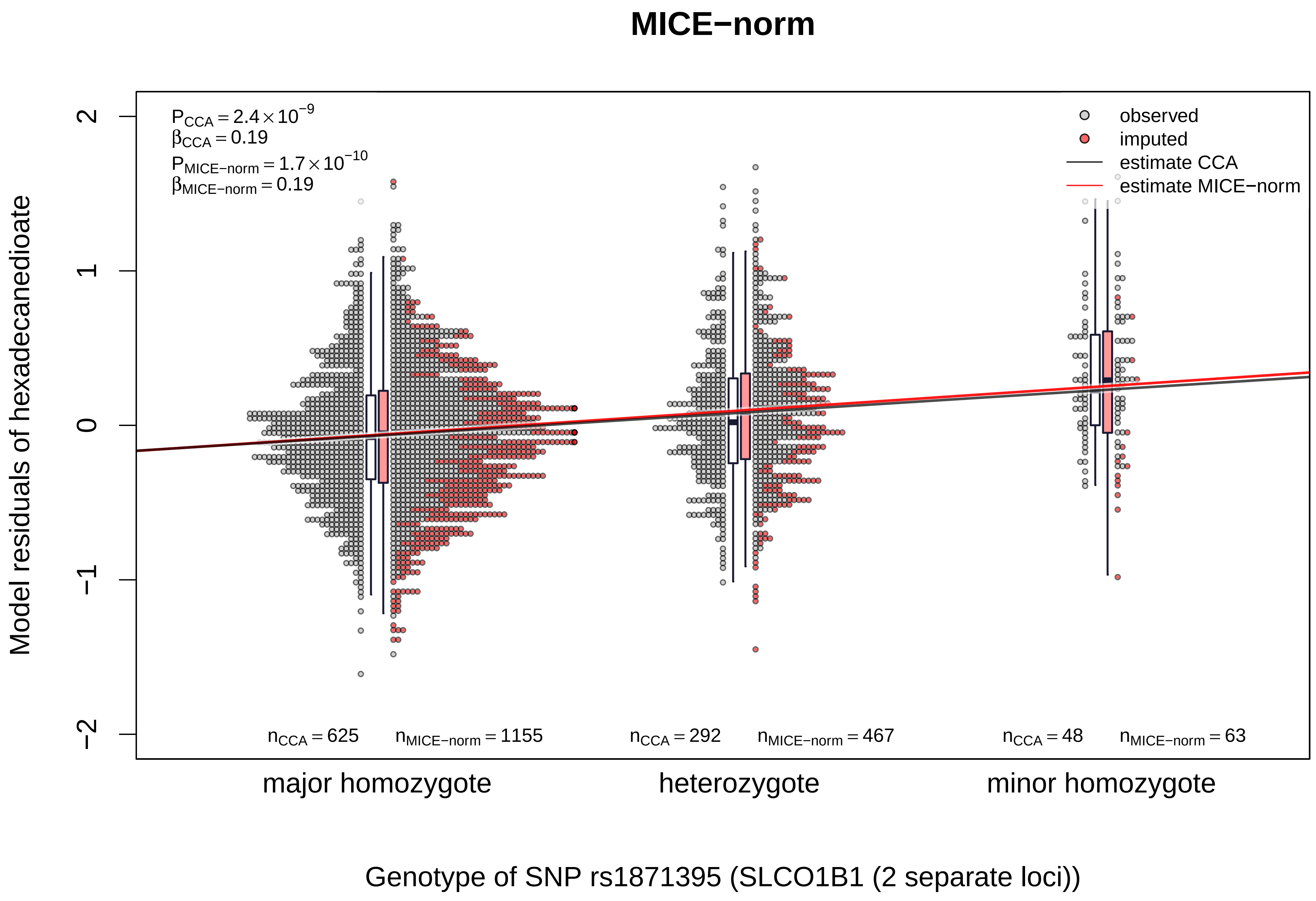

MICE−norm
$P_{CCA} = 2.4 \times 10^{-9}$
$\beta_{CCA} = 0.19$
$P_{MICE-norm} = 1.7 \times 10^{-10}$
$\beta_{MICE-norm} = 0.19$
observed
imputed
estimate CCA
estimate MICE−norm
2
1
0
−1
−2
Model residuals of hexadecanedioate
$n_{CCA} = 625$
$n_{MICE-norm} = 1155$
$n_{CCA} = 292$
$n_{MICE-norm} = 467$
$n_{CCA} = 48$
$n_{MICE-norm} = 63$
major homozygote
heterozygote
minor homozygote
Genotype of SNP rs1871395 (SLCO1B1 (2 separate loci))

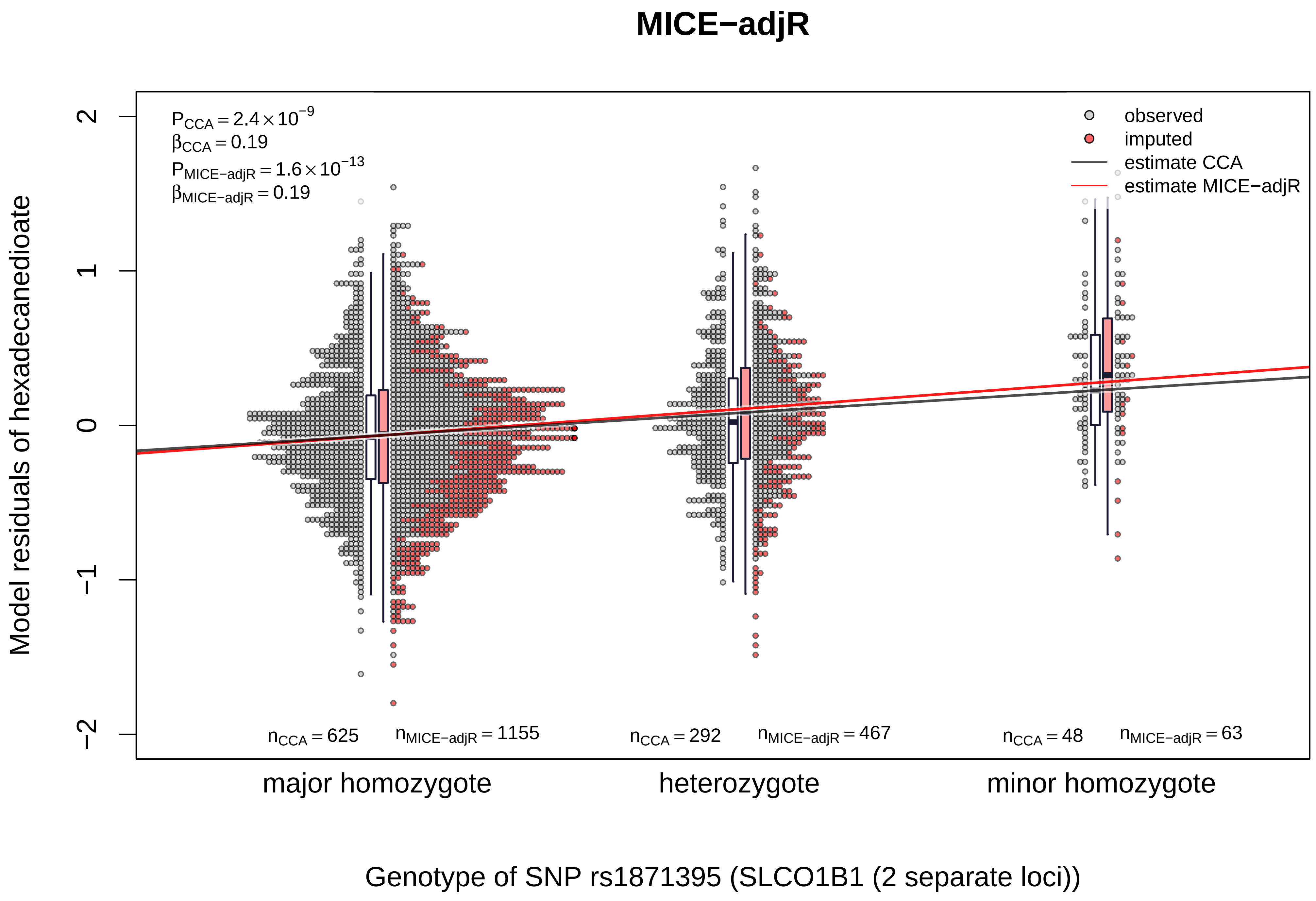

MICE−adjR
$P_{CCA} = 2.4 \times 10^{-9}$
$\beta_{CCA} = 0.19$
$P_{MICE-adjR} = 1.6 \times 10^{-13}$
$\beta_{MICE-adjR} = 0.19$
observed
imputed
estimate CCA
estimate MICE−adjR
$n_{CCA} = 625$
$n_{MICE-adjR} = 1155$
$n_{CCA} = 292$
$n_{MICE-adjR} = 467$
$n_{CCA} = 48$
$n_{MICE-adjR} = 63$
2
1
0
−1
−2
Model residuals of hexadecanedioate
major homozygote
heterozygote
minor homozygote
Genotype of SNP rs1871395 (SLCO1B1 (2 separate loci))

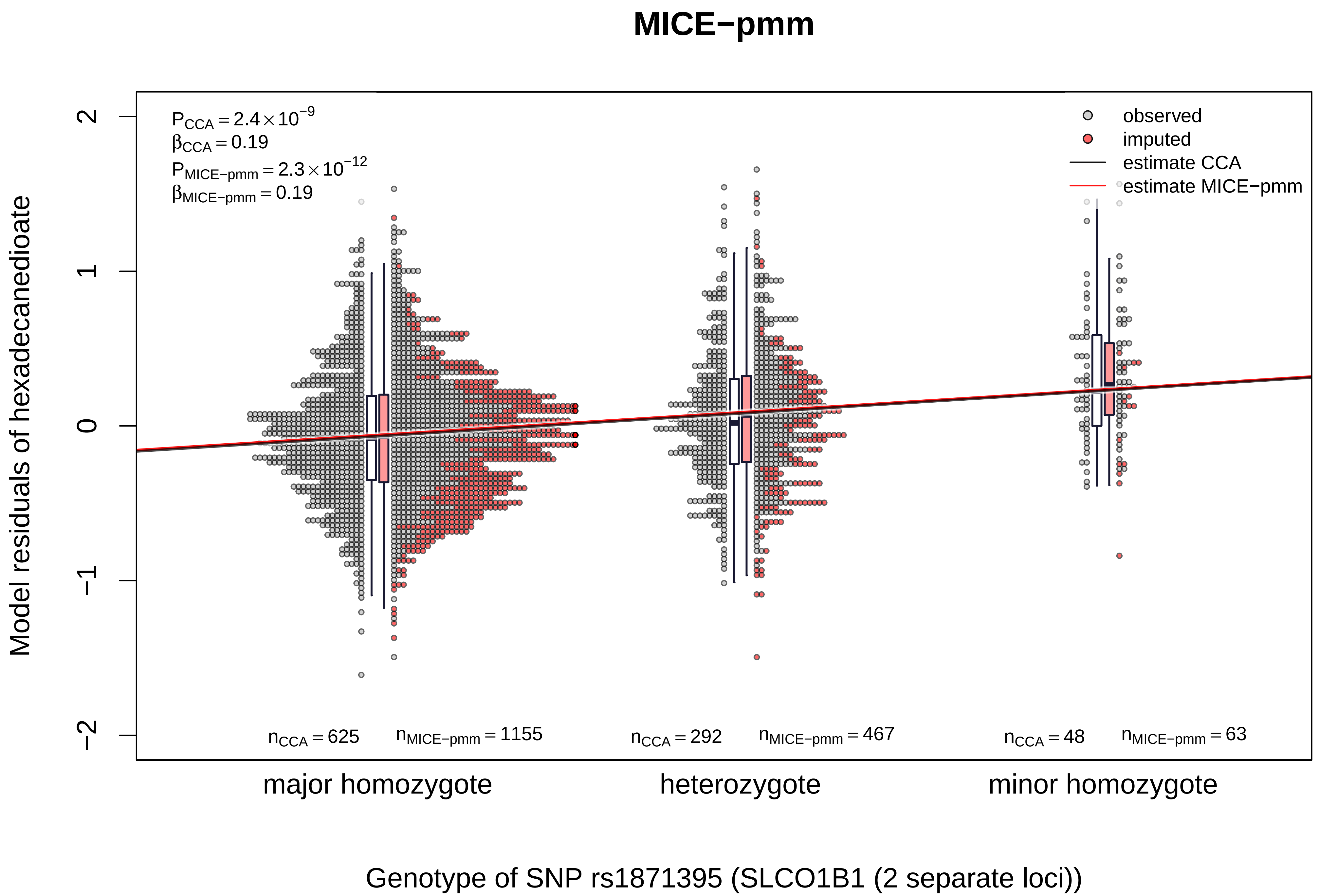

MICE−pmm
$P_{CCA} = 2.4 \times 10^{-9}$
$\beta_{CCA} = 0.19$
$P_{MICE-pmm} = 2.3 \times 10^{-12}$
$\beta_{MICE-pmm} = 0.19$
observed
imputed
estimate CCA
estimate MICE−pmm
Model residuals of hexadecanedioate
2
1
0
−1
−2
$n_{CCA} = 625$
$n_{MICE-pmm} = 1155$
$n_{CCA} = 292$
$n_{MICE-pmm} = 467$
$n_{CCA} = 48$
$n_{MICE-pmm} = 63$
major homozygote
heterozygote
minor homozygote
Genotype of SNP rs1871395 (SLCO1B1 (2 separate loci))

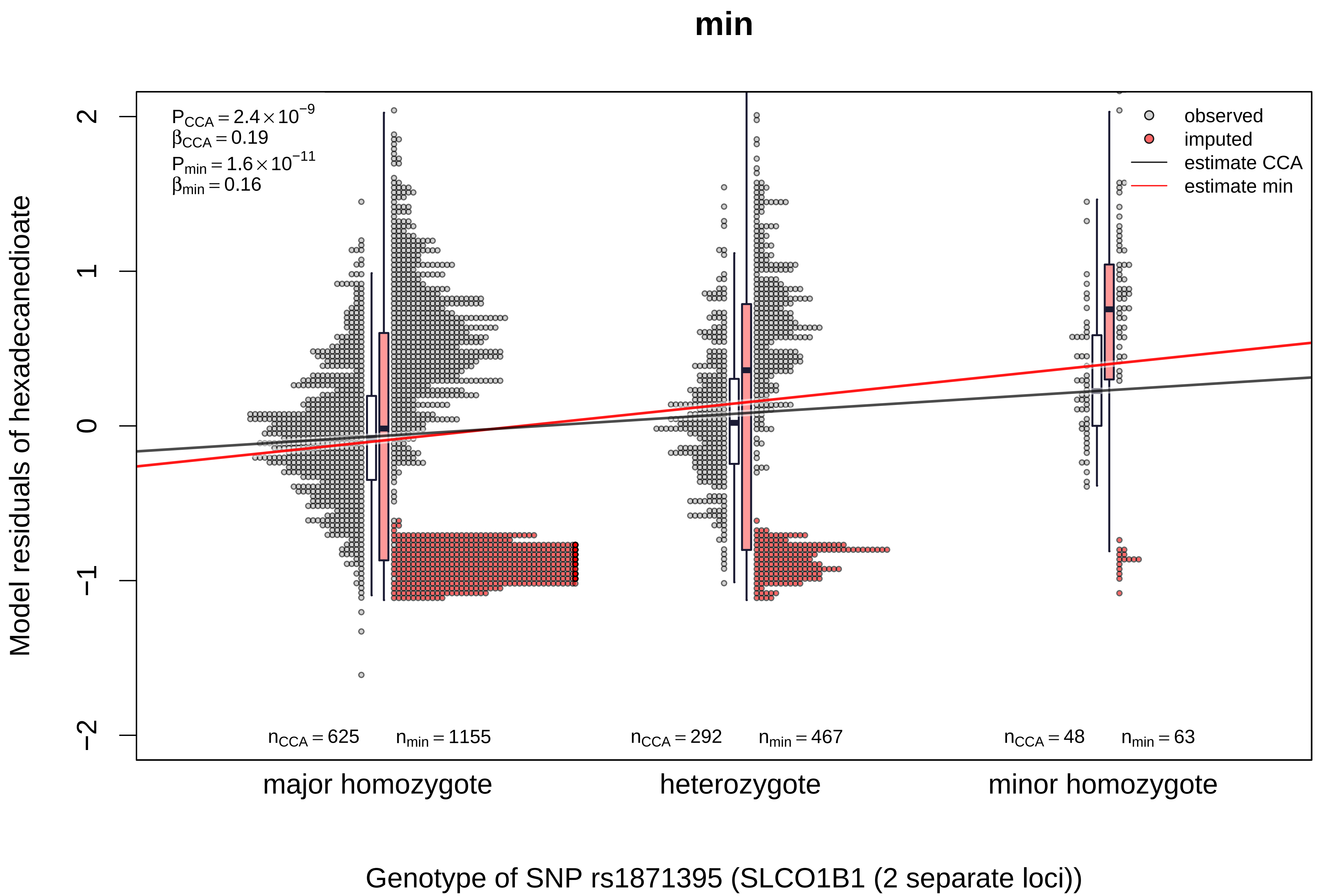

min
$P_{CCA} = 2.4 \times 10^{-9}$
$\beta_{CCA} = 0.19$
$P_{min} = 1.6 \times 10^{-11}$
$\beta_{min} = 0.16$
observed
imputed
estimate CCA
estimate min
2
1
0
−1
−2
Model residuals of hexadecanedioate
$n_{CCA} = 625$
$n_{min} = 1155$
$n_{CCA} = 292$
$n_{min} = 467$
$n_{CCA} = 48$
$n_{min} = 63$
major homozygote
heterozygote
minor homozygote
Genotype of SNP rs1871395 (SLCO1B1 (2 separate loci))

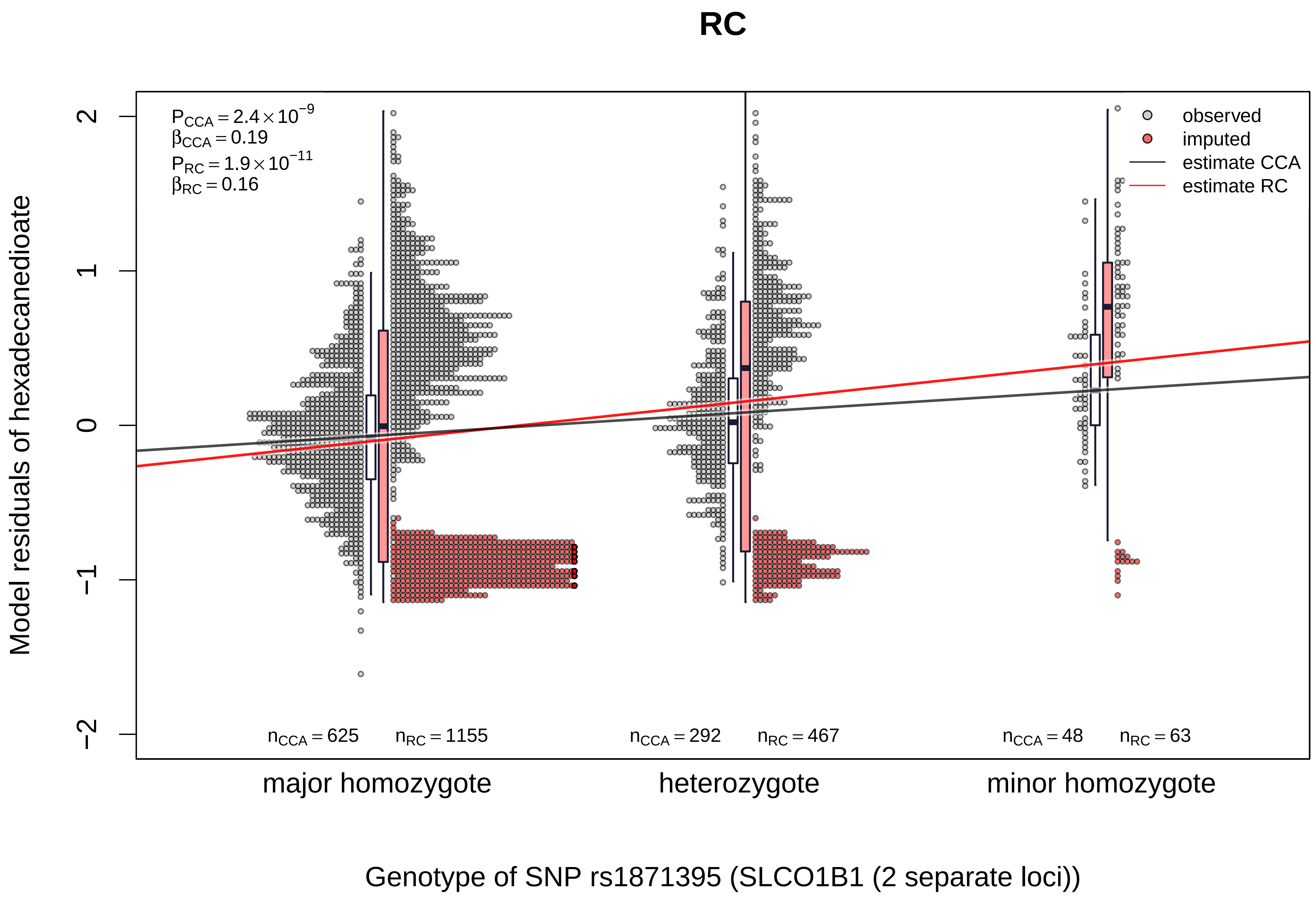

RC
$P_{CCA} = 2.4 \times 10^{-9}$
$\beta_{CCA} = 0.19$
$P_{RC} = 1.9 \times 10^{-11}$
$\beta_{RC} = 0.16$
observed
imputed
estimate CCA
estimate RC
Model residuals of hexadecanedioate
2
1
0
−1
−2
$n_{CCA} = 625$
$n_{RC} = 1155$
$n_{CCA} = 292$
$n_{RC} = 467$
$n_{CCA} = 48$
$n_{RC} = 63$
major homozygote
heterozygote
minor homozygote
Genotype of SNP rs1871395 (SLCO1B1 (2 separate loci))

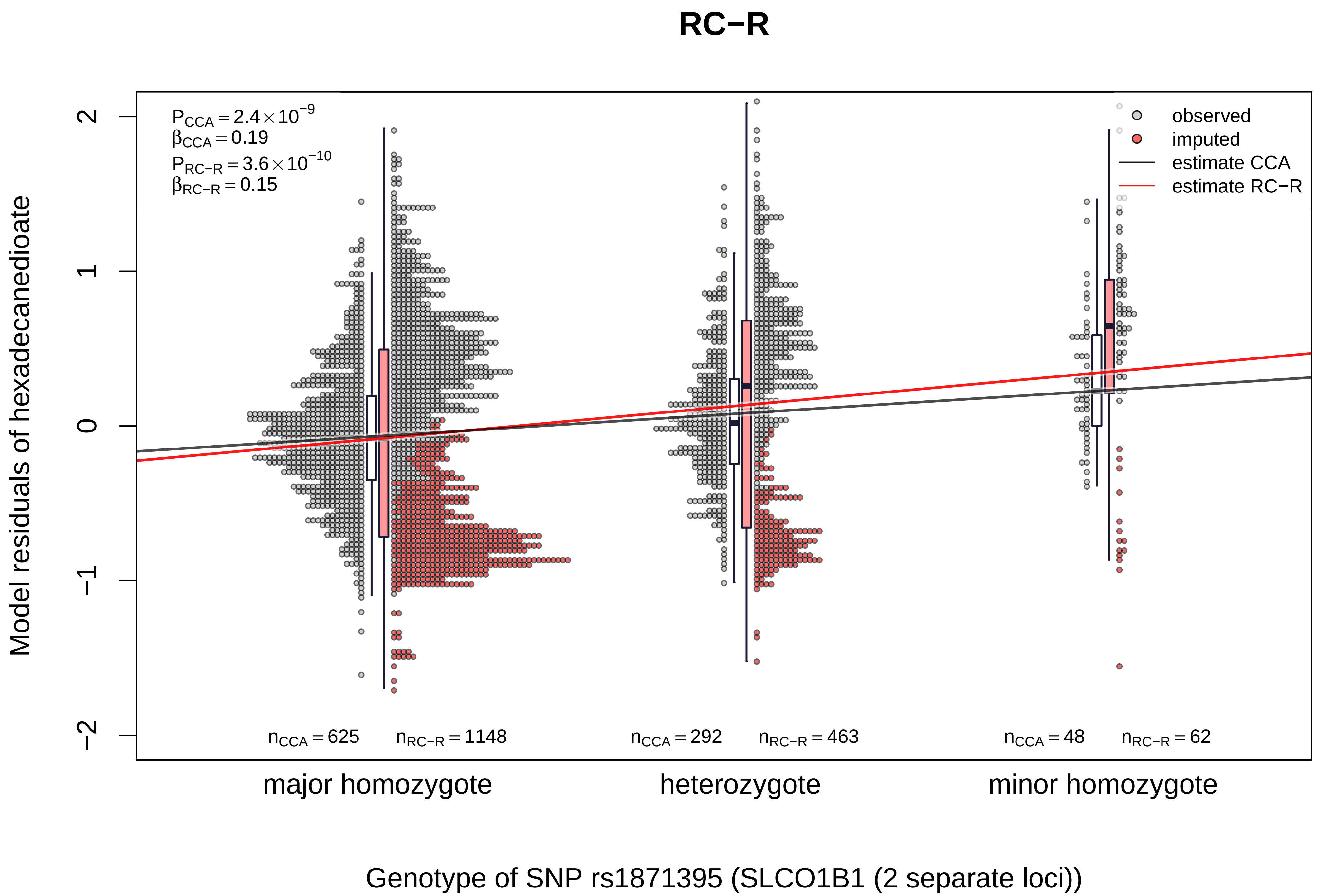

RC−R
$P_{CCA} = 2.4 \times 10^{-9}$
$\beta_{CCA} = 0.19$
$P_{RC-R} = 3.6 \times 10^{-10}$
$\beta_{RC-R} = 0.15$
observed
imputed
estimate CCA
estimate RC−R
Model residuals of hexadecanedioate
2
1
0
−1
−2
$n_{CCA} = 625$
$n_{RC-R} = 1148$
$n_{CCA} = 292$
$n_{RC-R} = 463$
$n_{CCA} = 48$
$n_{RC-R} = 62$
major homozygote
heterozygote
minor homozygote
Genotype of SNP rs1871395 (SLCO1B1 (2 separate loci))

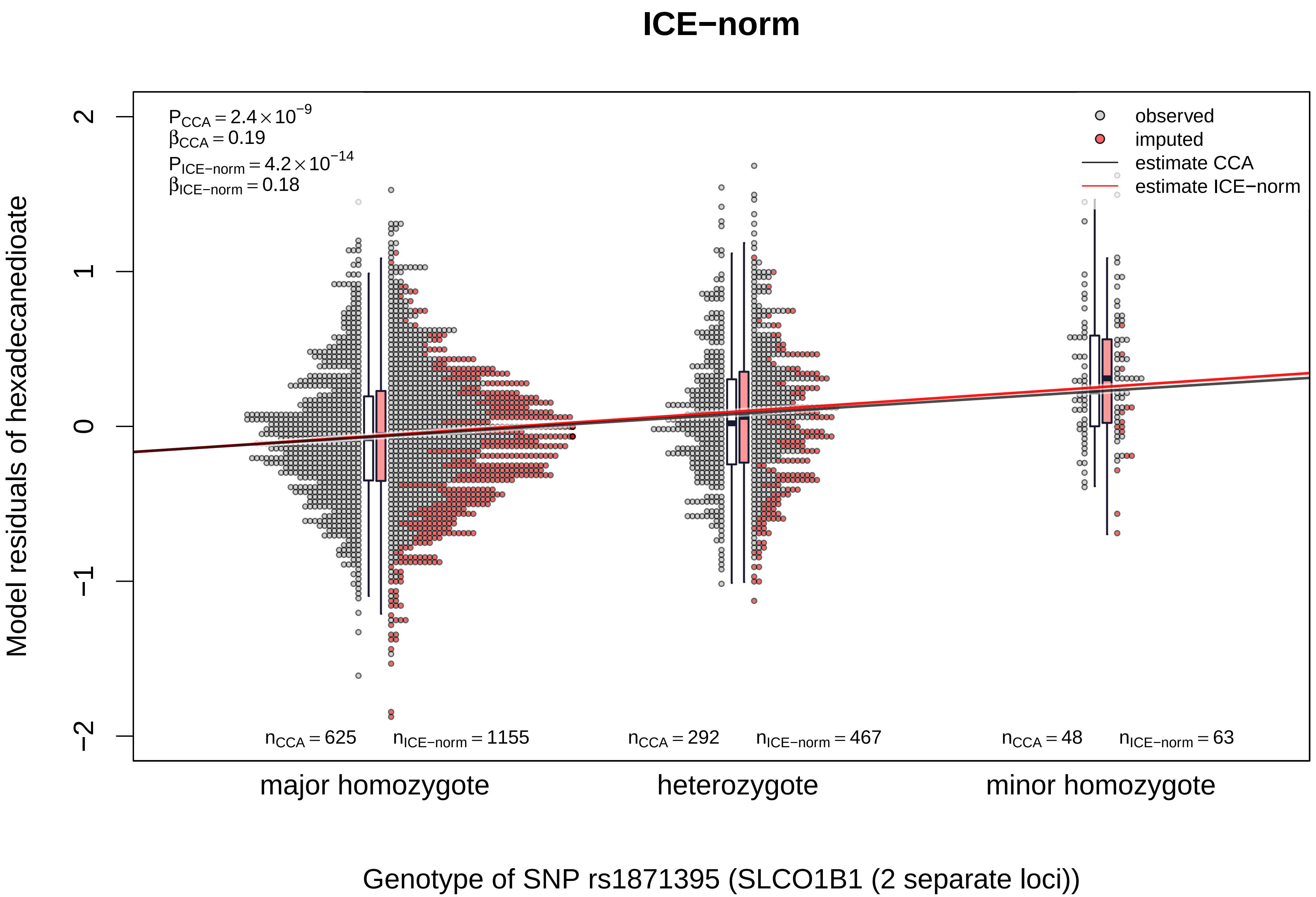
ICE−norm
$P_{CCA} = 2.4 \times 10^{-9}$
$\beta_{CCA} = 0.19$
$P_{ICE-norm} = 4.2 \times 10^{-14}$
$\beta_{ICE-norm} = 0.18$
observed
imputed
estimate CCA
estimate ICE−norm
Model residuals of hexadecanedioate
2
1
0
−1
−2
$n_{CCA} = 625$
$n_{ICE-norm} = 1155$
$n_{CCA} = 292$
$n_{ICE-norm} = 467$
$n_{CCA} = 48$
$n_{ICE-norm} = 63$
major homozygote
heterozygote
minor homozygote
Genotype of SNP rs1871395 (SLCO1B1 (2 separate loci))

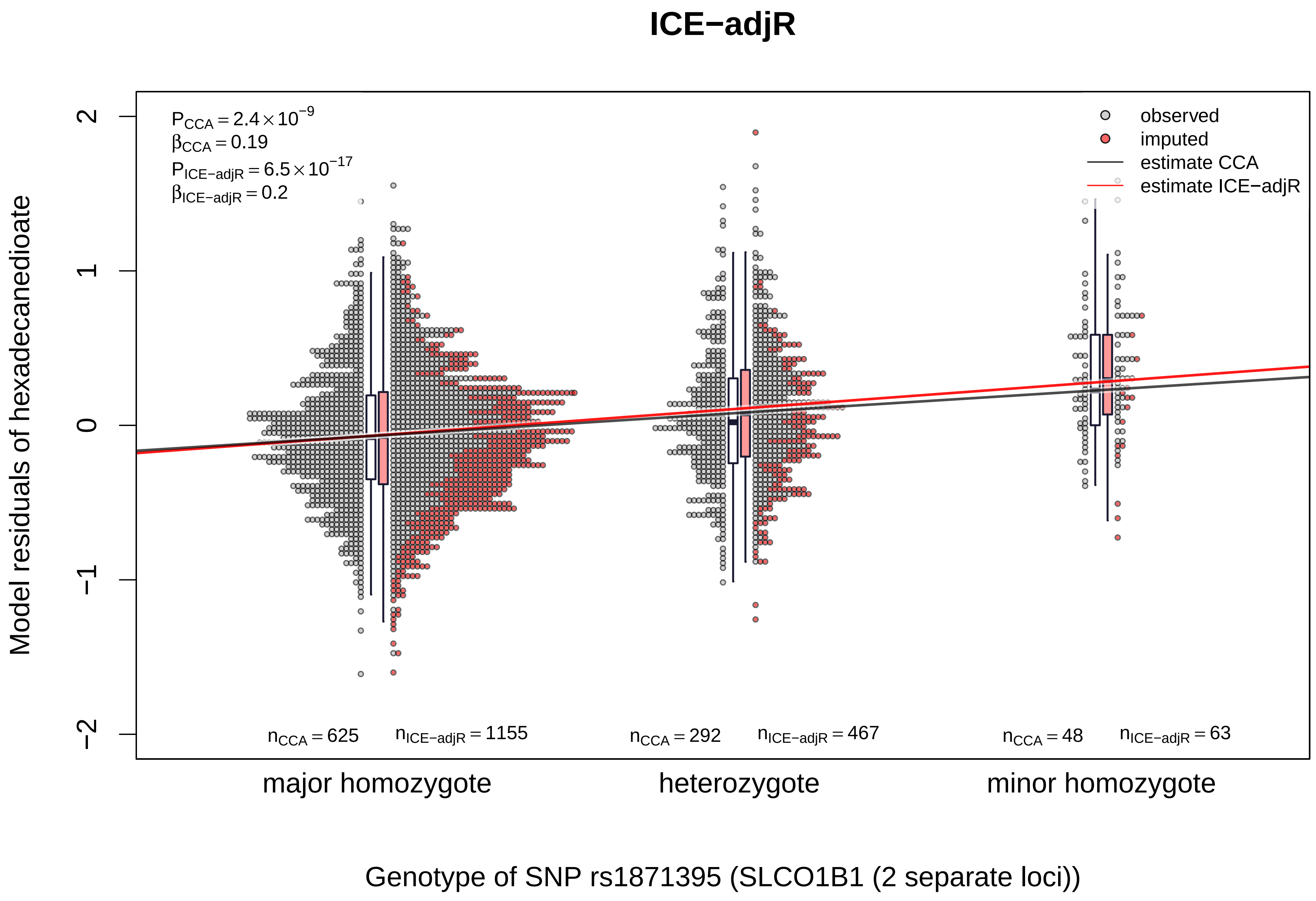
ICE−adjR
$P_{CCA} = 2.4 \times 10^{-9}$
$\beta_{CCA} = 0.19$
$P_{ICE-adjR} = 6.5 \times 10^{-17}$
$\beta_{ICE-adjR} = 0.2$
observed
imputed
estimate CCA
estimate ICE−adjR
Model residuals of hexadecanedioate
2
1
0
−1
−2
$n_{CCA} = 625$
$n_{ICE-adjR} = 1155$
$n_{CCA} = 292$
$n_{ICE-adjR} = 467$
$n_{CCA} = 48$
$n_{ICE-adjR} = 63$
major homozygote
heterozygote
minor homozygote
Genotype of SNP rs1871395 (SLCO1B1 (2 separate loci))

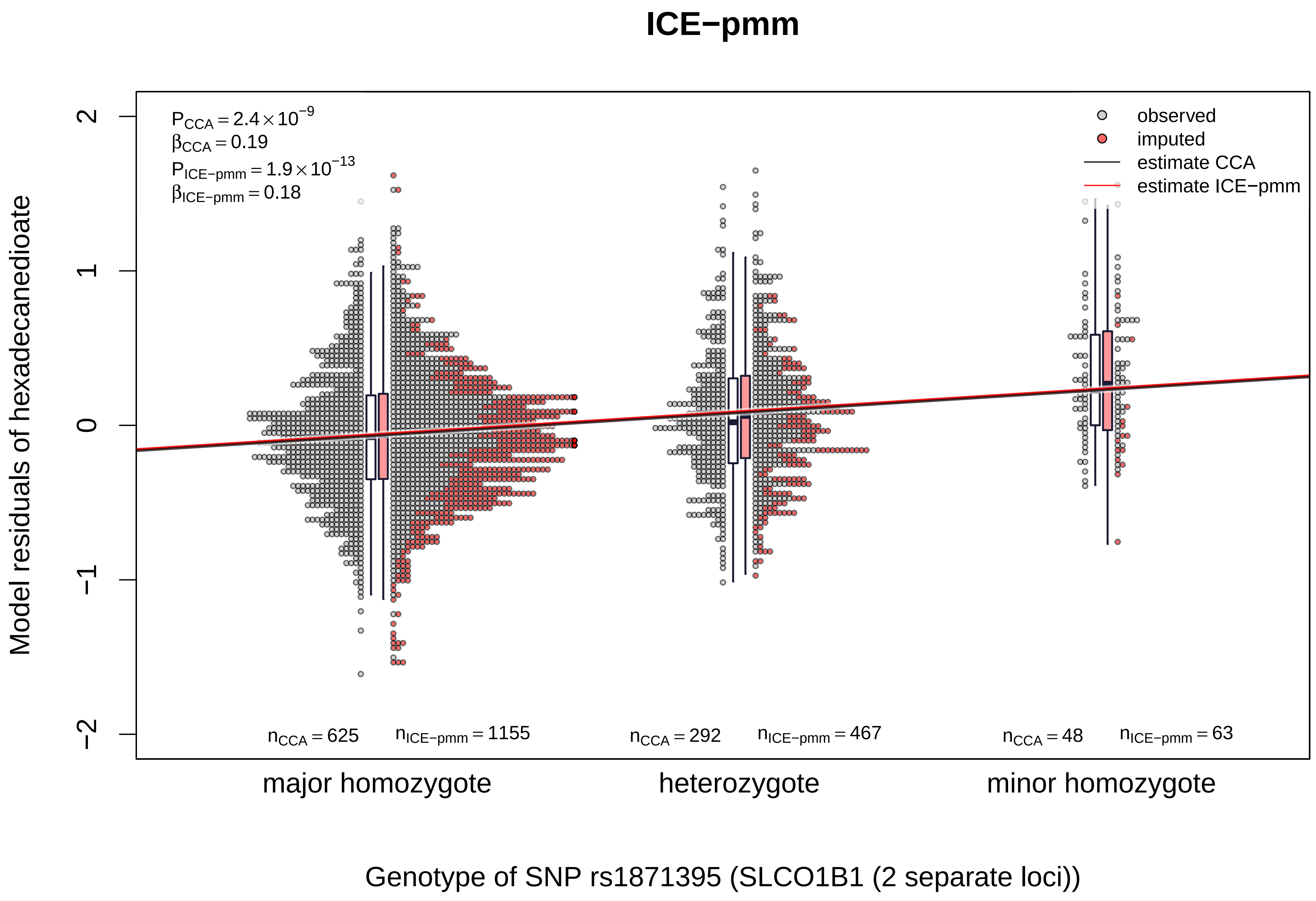
ICE−pmm
$P_{CCA} = 2.4 \times 10^{-9}$
$\beta_{CCA} = 0.19$
$P_{ICE-pmm} = 1.9 \times 10^{-13}$
$\beta_{ICE-pmm} = 0.18$
observed
imputed
estimate CCA
estimate ICE−pmm
Model residuals of hexadecanedioate
2
1
0
−1
−2
$n_{CCA} = 625$
$n_{ICE-pmm} = 1155$
$n_{CCA} = 292$
$n_{ICE-pmm} = 467$
$n_{CCA} = 48$
$n_{ICE-pmm} = 63$
major homozygote
heterozygote
minor homozygote
Genotype of SNP rs1871395 (SLCO1B1 (2 separate loci))

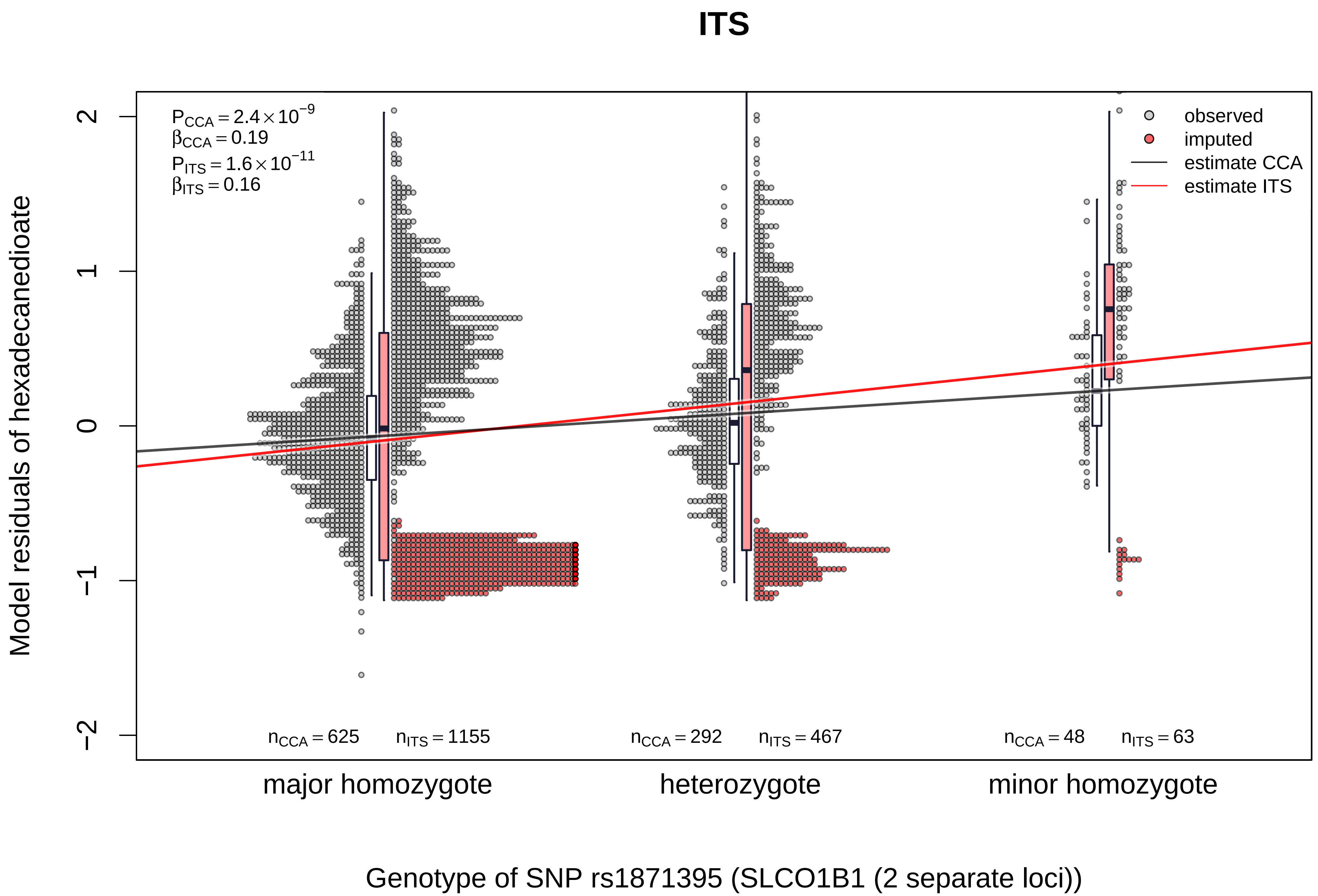
ITS
$P_{CCA} = 2.4 \times 10^{-9}$
$\beta_{CCA} = 0.19$
$P_{ITS} = 1.6 \times 10^{-11}$
$\beta_{ITS} = 0.16$
observed
imputed
estimate CCA
estimate ITS
Model residuals of hexadecanedioate
2
1
0
−1
−2
$n_{CCA} = 625$
$n_{ITS} = 1155$
$n_{CCA} = 292$
$n_{ITS} = 467$
$n_{CCA} = 48$
$n_{ITS} = 63$
major homozygote
heterozygote
minor homozygote
Genotype of SNP rs1871395 (SLCO1B1 (2 separate loci))

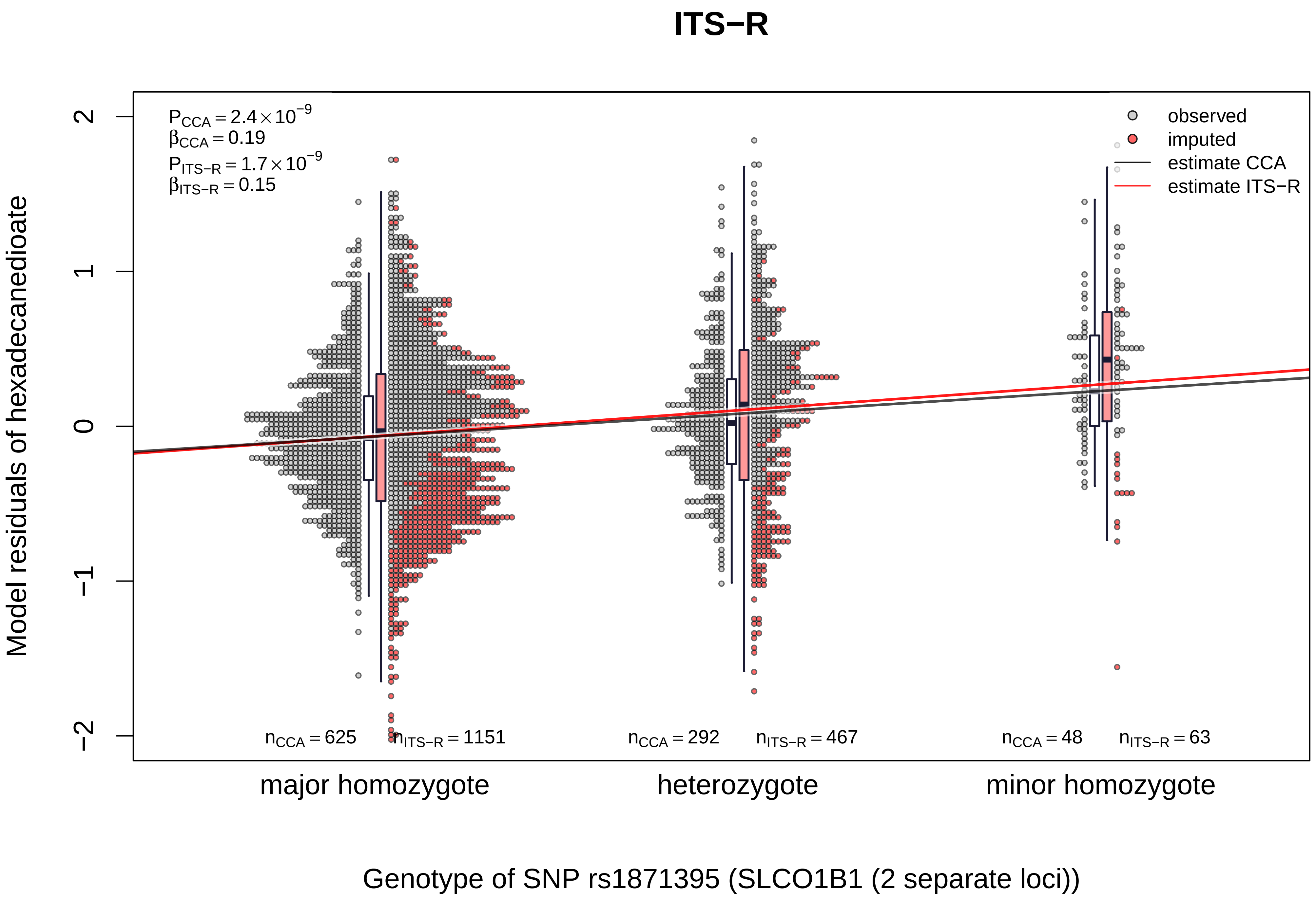

ITS−R
$P_{CCA} = 2.4 \times 10^{-9}$
$\beta_{CCA} = 0.19$
$P_{ITS-R} = 1.7 \times 10^{-9}$
$\beta_{ITS-R} = 0.15$
observed
imputed
estimate CCA
estimate ITS−R
Model residuals of hexadecanediоate
2
1
0
−1
−2
$n_{CCA} = 625$
$n_{ITS-R} = 1151$
$n_{CCA} = 292$
$n_{ITS-R} = 467$
$n_{CCA} = 48$
$n_{ITS-R} = 63$
major homozygote
heterozygote
minor homozygote
Genotype of SNP rs1871395 (SLCO1B1 (2 separate loci))

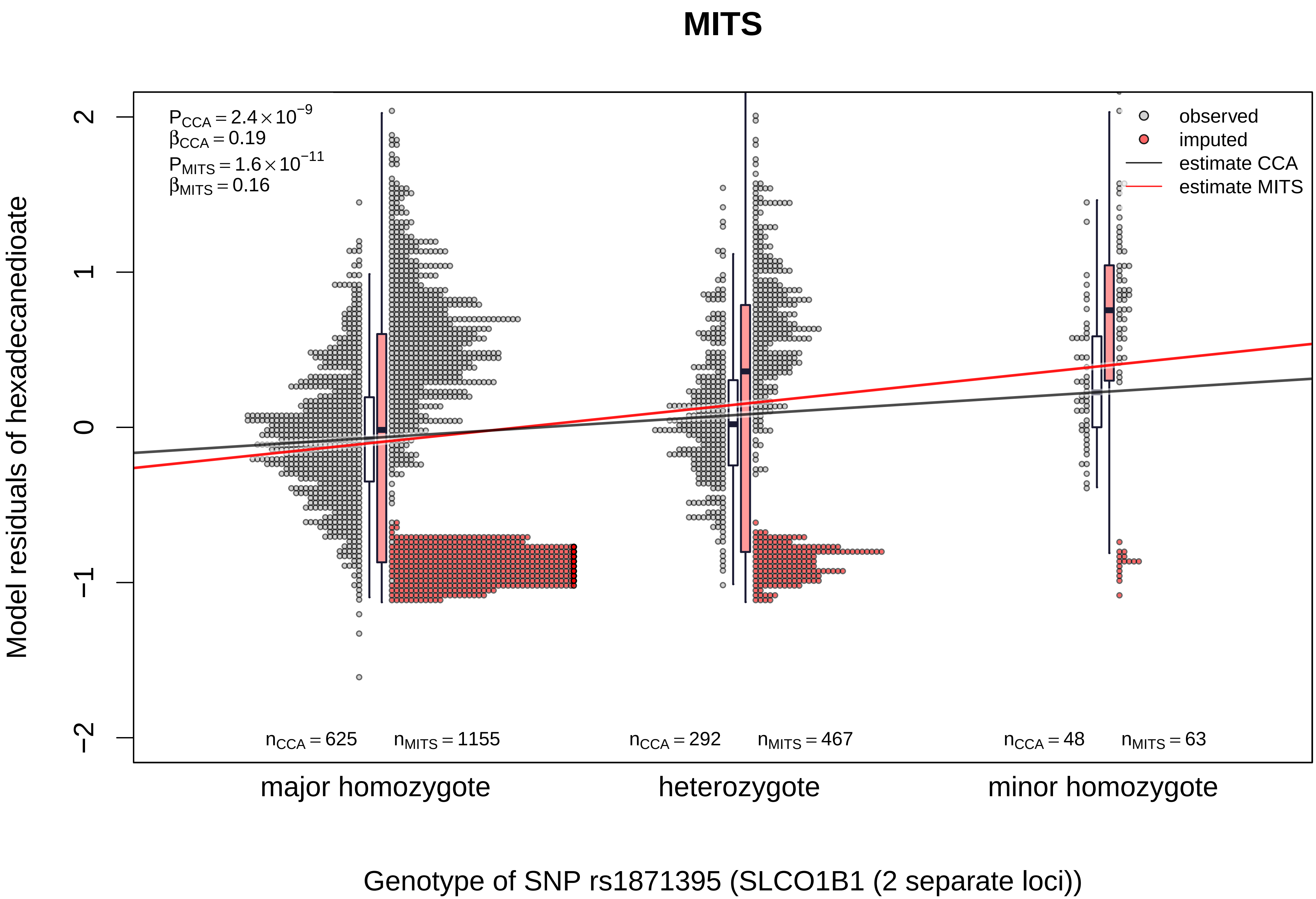

MITS
$P_{CCA} = 2.4 \times 10^{-9}$
$\beta_{CCA} = 0.19$
$P_{MITS} = 1.6 \times 10^{-11}$
$\beta_{MITS} = 0.16$
observed
imputed
estimate CCA
estimate MITS
Model residuals of hexadecanedioate
2
1
0
−1
−2
$n_{CCA} = 625$
$n_{MITS} = 1155$
$n_{CCA} = 292$
$n_{MITS} = 467$
$n_{CCA} = 48$
$n_{MITS} = 63$
major homozygote
heterozygote
minor homozygote
Genotype of SNP rs1871395 (SLCO1B1 (2 separate loci))

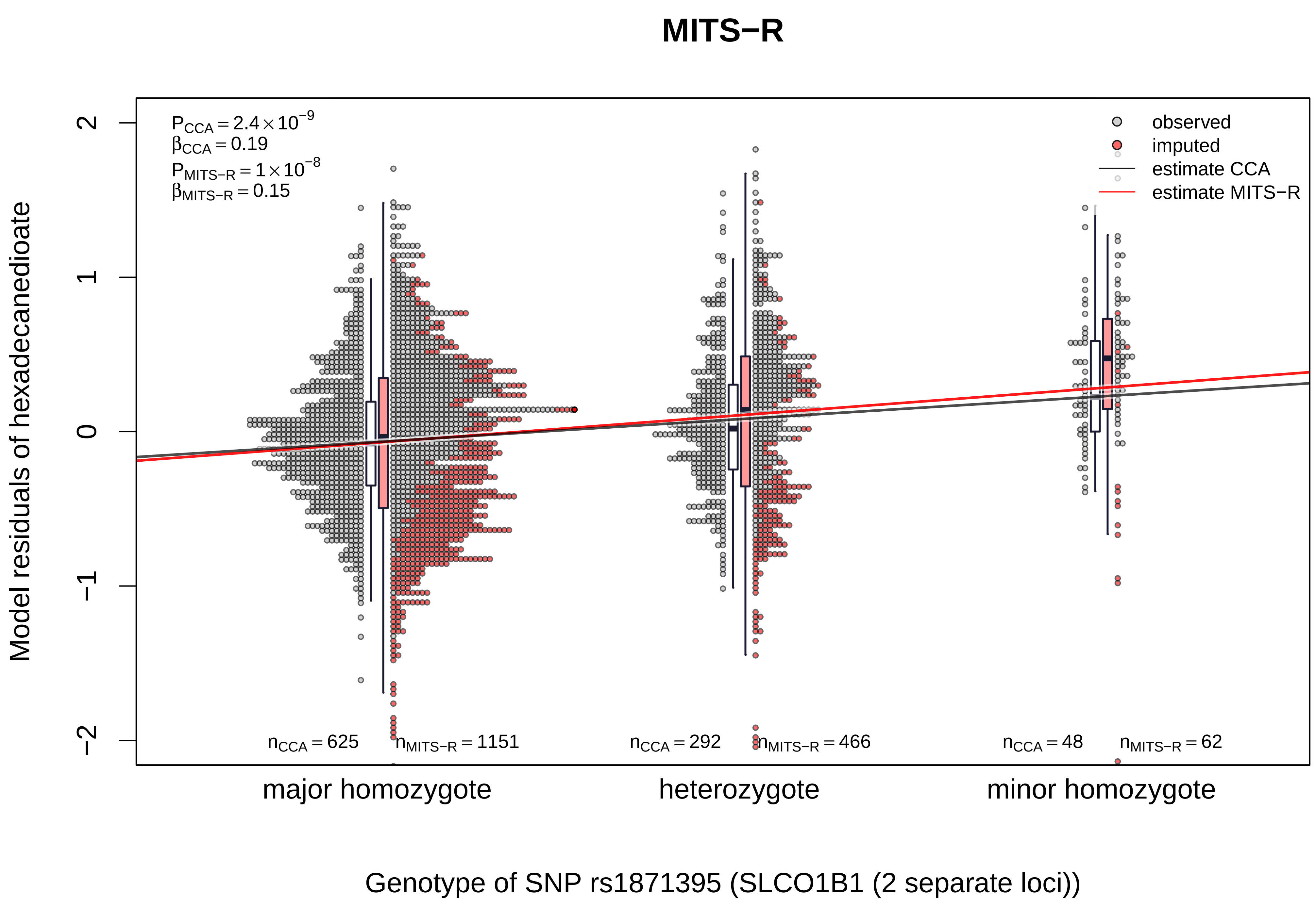

MITS−R
$P_{CCA} = 2.4 \times 10^{-9}$
$\beta_{CCA} = 0.19$
$P_{MITS-R} = 1 \times 10^{-8}$
$\beta_{MITS-R} = 0.15$
observed
imputed
estimate CCA
estimate MITS−R
Model residuals of hexadecanedioate
2
1
0
−1
−2
$n_{CCA} = 625$
$n_{MITS-R} = 1151$
$n_{CCA} = 292$
$n_{MITS-R} = 466$
$n_{CCA} = 48$
$n_{MITS-R} = 62$
major homozygote
heterozygote
minor homozygote
Genotype of SNP rs1871395 (SLCO1B1 (2 separate loci))

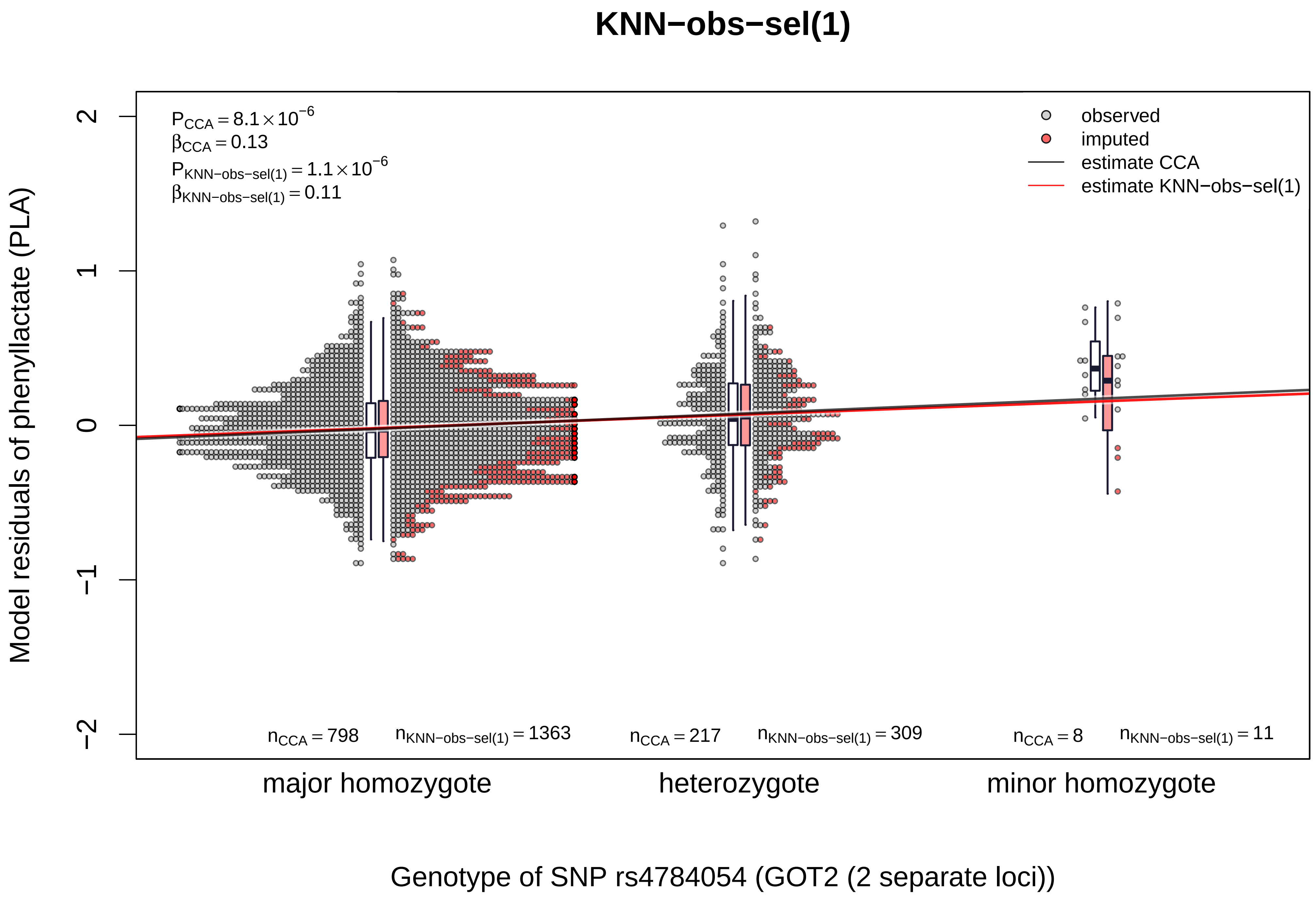
KNN−obs−sel(1)
$P_{CCA} = 8.1 \times 10^{-6}$
$\beta_{CCA} = 0.13$
$P_{KNN-obs-sel(1)} = 1.1 \times 10^{-6}$
$\beta_{KNN-obs-sel(1)} = 0.11$
observed
imputed
estimate CCA
estimate KNN−obs−sel(1)
Model residuals of phenyllactate (PLA)
2
1
0
−1
−2
$n_{CCA} = 798$
$n_{KNN-obs-sel(1)} = 1363$
$n_{CCA} = 217$
$n_{KNN-obs-sel(1)} = 309$
$n_{CCA} = 8$
$n_{KNN-obs-sel(1)} = 11$
major homozygote
heterozygote
minor homozygote
Genotype of SNP rs4784054 (GOT2 (2 separate loci))

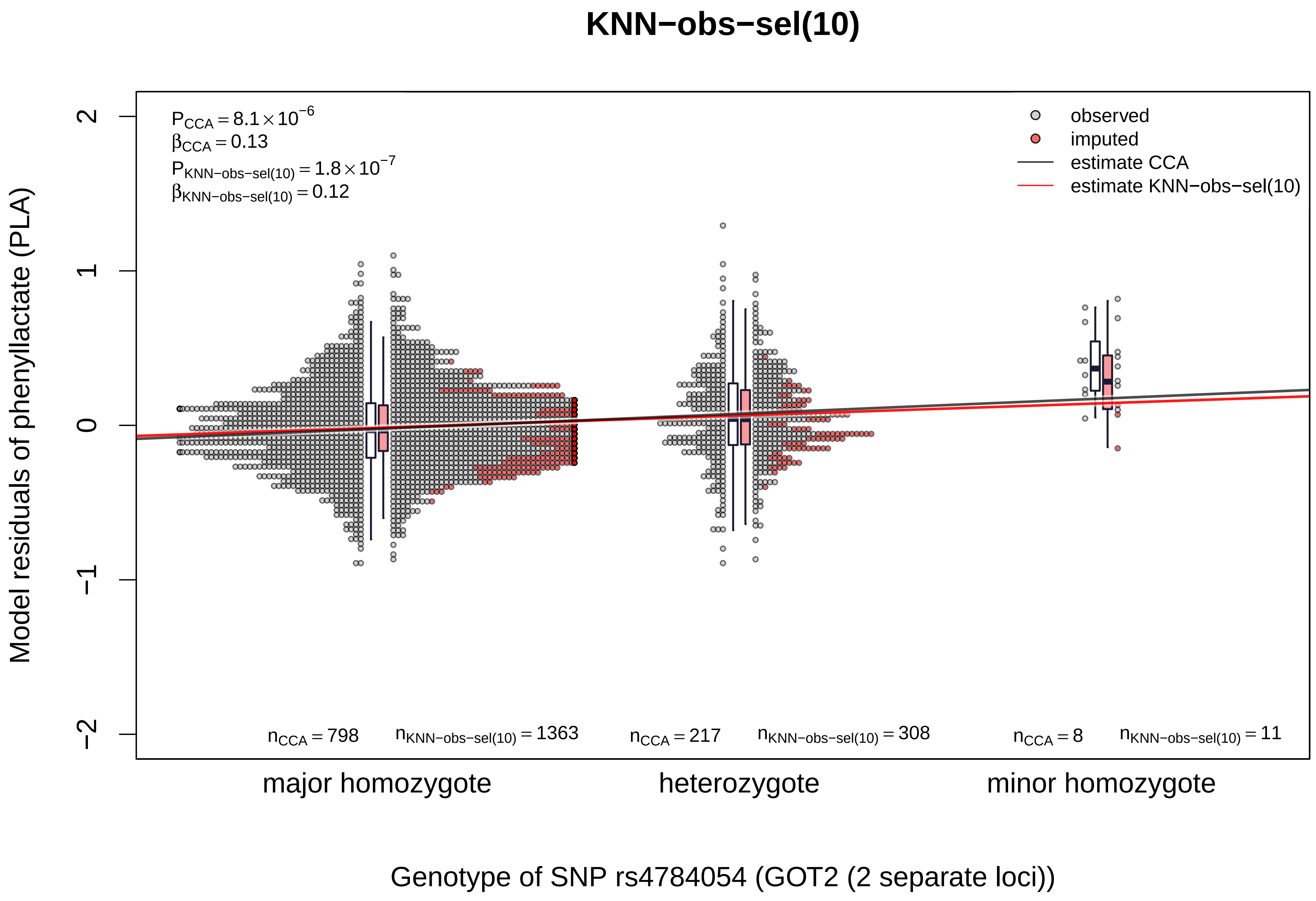

KNN−obs−sel(10)
$P_{CCA} = 8.1 \times 10^{-6}$
$\beta_{CCA} = 0.13$
$P_{KNN-obs-sel(10)} = 1.8 \times 10^{-7}$
$\beta_{KNN-obs-sel(10)} = 0.12$
observed
imputed
estimate CCA
estimate KNN−obs−sel(10)
Model residuals of phenyllactate (PLA)
2
1
0
−1
−2
$n_{CCA} = 798$
$n_{KNN-obs-sel(10)} = 1363$
$n_{CCA} = 217$
$n_{KNN-obs-sel(10)} = 308$
$n_{CCA} = 8$
$n_{KNN-obs-sel(10)} = 11$
major homozygote
heterozygote
minor homozygote
Genotype of SNP rs4784054 (GOT2 (2 separate loci))

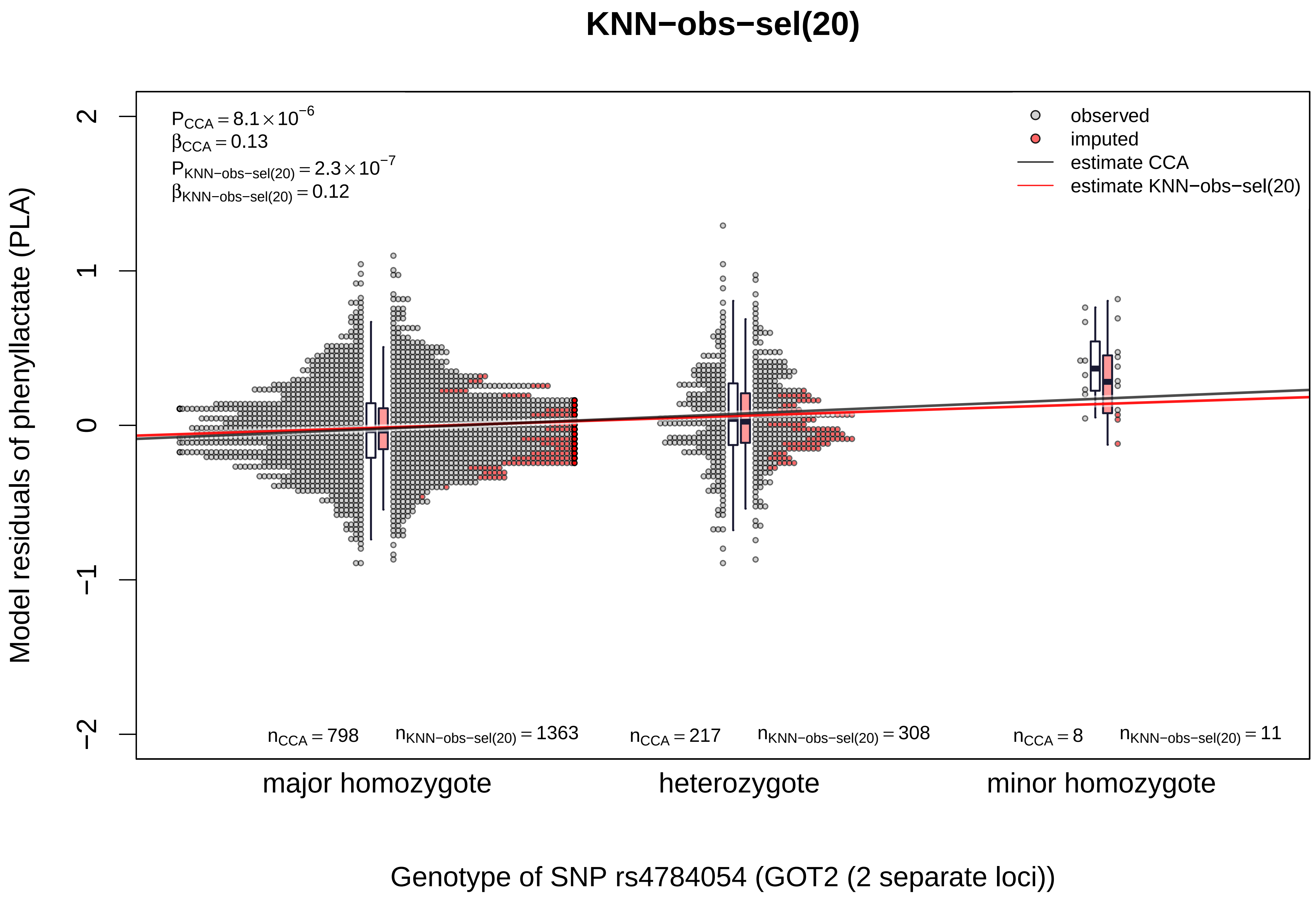

KNN−obs−sel(20)
$P_{CCA} = 8.1 \times 10^{-6}$
$\beta_{CCA} = 0.13$
$P_{KNN-obs-sel(20)} = 2.3 \times 10^{-7}$
$\beta_{KNN-obs-sel(20)} = 0.12$
observed
imputed
estimate CCA
estimate KNN−obs−sel(20)
Model residuals of phenyllactate (PLA)
2
1
0
−1
−2
$n_{CCA} = 798$
$n_{KNN-obs-sel(20)} = 1363$
$n_{CCA} = 217$
$n_{KNN-obs-sel(20)} = 308$
$n_{CCA} = 8$
$n_{KNN-obs-sel(20)} = 11$
major homozygote
heterozygote
minor homozygote
Genotype of SNP rs4784054 (GOT2 (2 separate loci))

KNN−obs−sel(3)

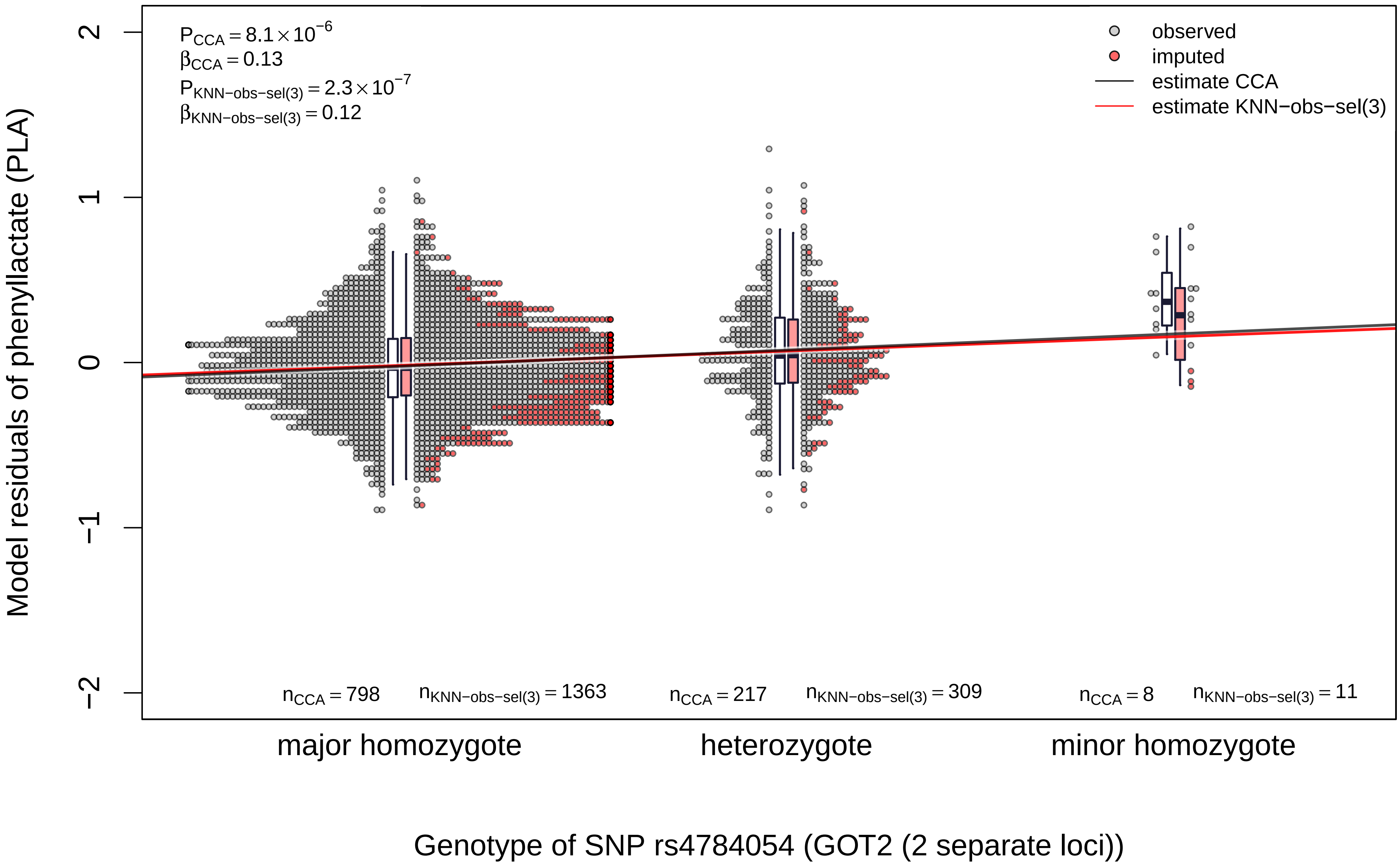

$P_{CCA} = 8.1 \times 10^{-6}$
$\beta_{CCA} = 0.13$
$P_{KNN-obs-sel(3)} = 2.3 \times 10^{-7}$
$\beta_{KNN-obs-sel(3)} = 0.12$
observed
imputed
estimate CCA
estimate KNN−obs−sel(3)
Model residuals of phenyllactate (PLA)
2
1
0
−1
−2
$n_{CCA} = 798$
$n_{KNN-obs-sel(3)} = 1363$
$n_{CCA} = 217$
$n_{KNN-obs-sel(3)} = 309$
$n_{CCA} = 8$
$n_{KNN-obs-sel(3)} = 11$
major homozygote
heterozygote
minor homozygote
Genotype of SNP rs4784054 (GOT2 (2 separate loci))

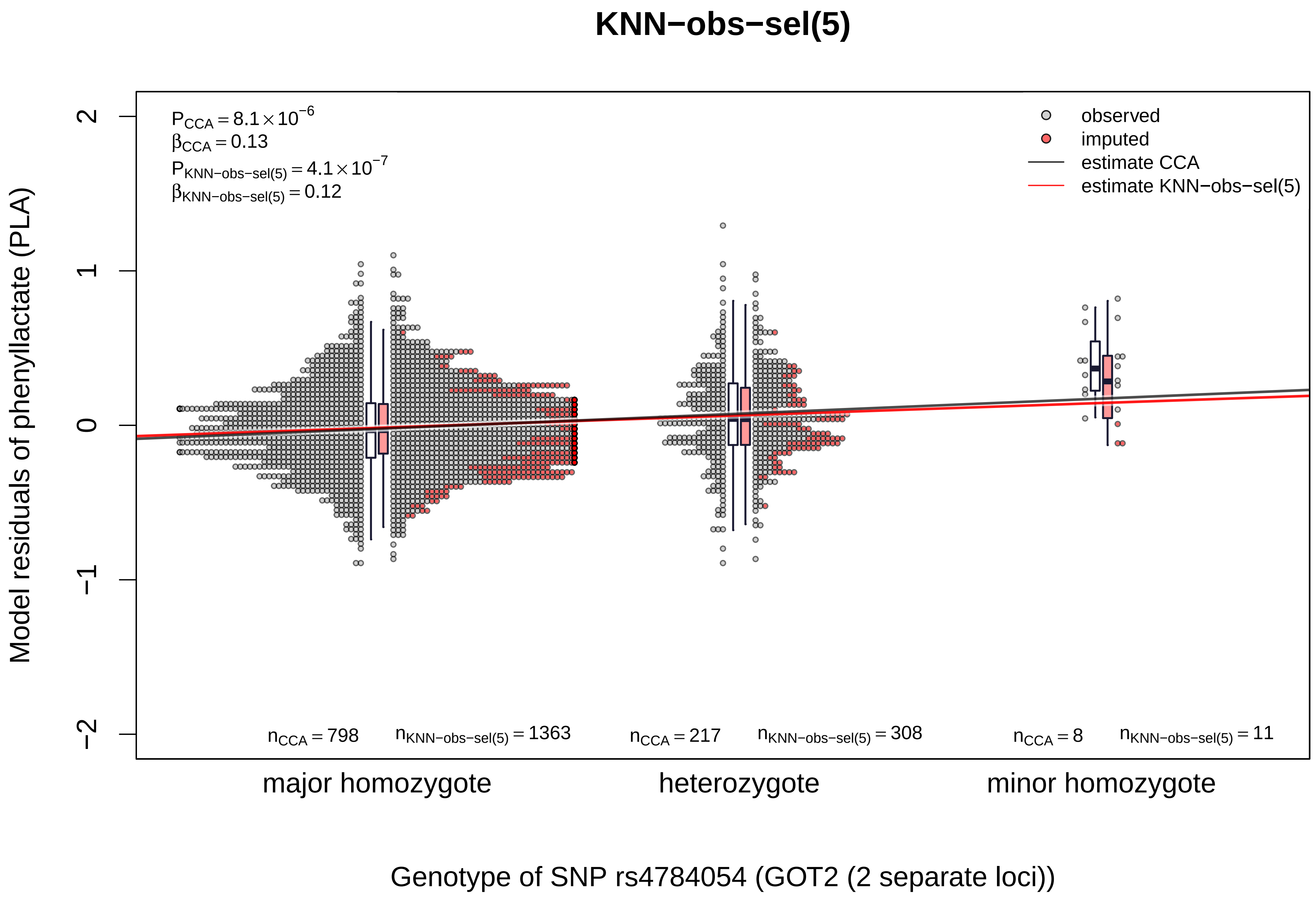

KNN−obs−sel(5)
$P_{CCA} = 8.1 \times 10^{-6}$
$\beta_{CCA} = 0.13$
$P_{KNN-obs-sel(5)} = 4.1 \times 10^{-7}$
$\beta_{KNN-obs-sel(5)} = 0.12$
observed
imputed
estimate CCA
estimate KNN−obs−sel(5)
2
1
0
−1
−2
Model residuals of phenyllactate (PLA)
$n_{CCA} = 798$
$n_{KNN-obs-sel(5)} = 1363$
$n_{CCA} = 217$
$n_{KNN-obs-sel(5)} = 308$
$n_{CCA} = 8$
$n_{KNN-obs-sel(5)} = 11$
major homozygote
heterozygote
minor homozygote
Genotype of SNP rs4784054 (GOT2 (2 separate loci))

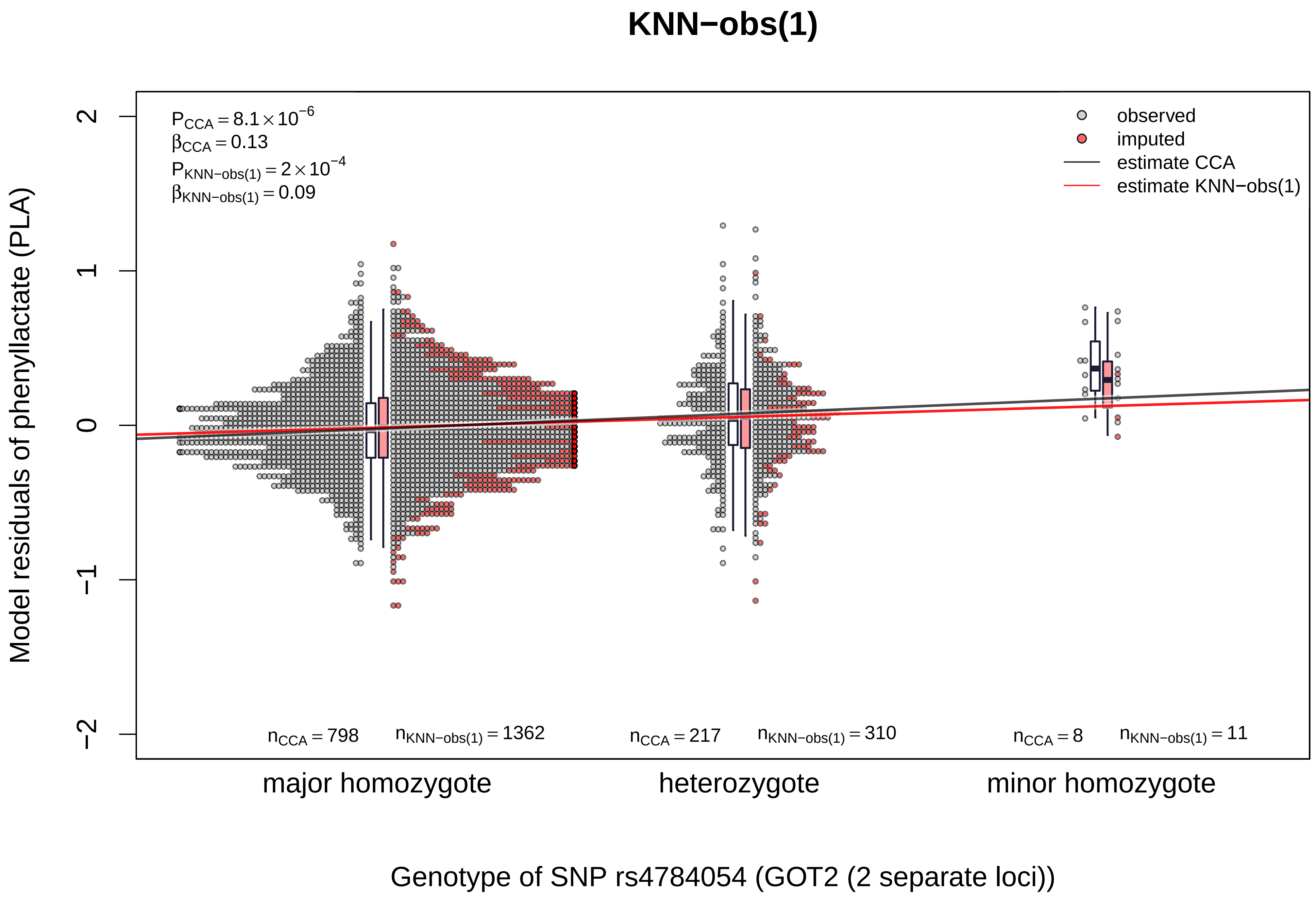
KNN−obs(1)
$P_{CCA} = 8.1 \times 10^{-6}$
$\beta_{CCA} = 0.13$
$P_{KNN-obs(1)} = 2 \times 10^{-4}$
$\beta_{KNN-obs(1)} = 0.09$
observed
imputed
estimate CCA
estimate KNN−obs(1)
Model residuals of phenyllactate (PLA)
2
1
0
−1
−2
$n_{CCA} = 798$
$n_{KNN-obs(1)} = 1362$
$n_{CCA} = 217$
$n_{KNN-obs(1)} = 310$
$n_{CCA} = 8$
$n_{KNN-obs(1)} = 11$
major homozygote
heterozygote
minor homozygote
Genotype of SNP rs4784054 (GOT2 (2 separate loci))

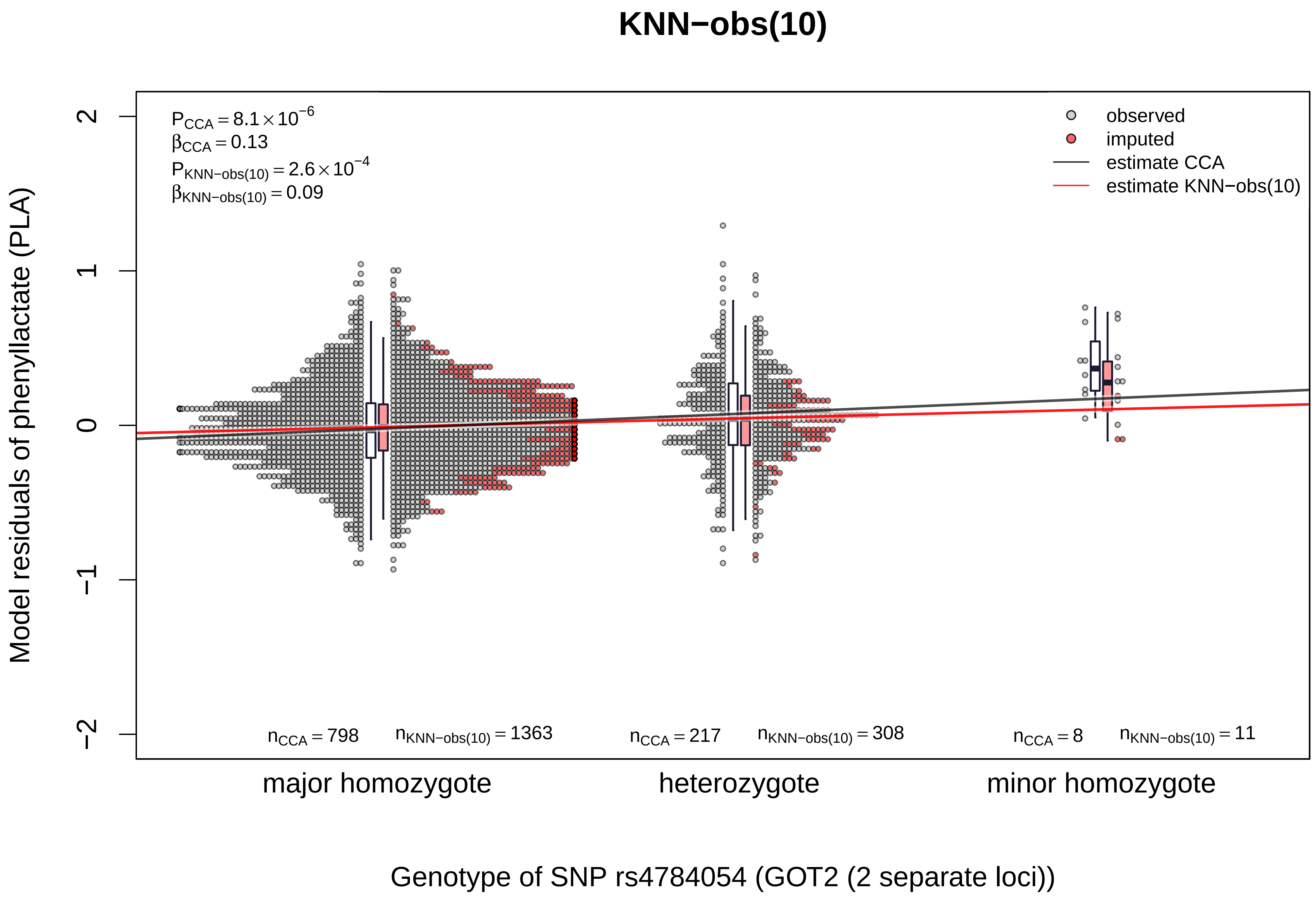

KNN−obs(10)
P_CCA = 8.1 × 10^−6
β_CCA = 0.13
P_KNN−obs(10) = 2.6 × 10^−4
β_KNN−obs(10) = 0.09
observed
imputed
estimate CCA
estimate KNN−obs(10)
2
1
0
−1
−2
Model residuals of phenyllactate (PLA)
n_CCA = 798
n_KNN−obs(10) = 1363
n_CCA = 217
n_KNN−obs(10) = 308
n_CCA = 8
n_KNN−obs(10) = 11
major homozygote
heterozygote
minor homozygote
Genotype of SNP rs4784054 (GOT2 (2 separate loci))

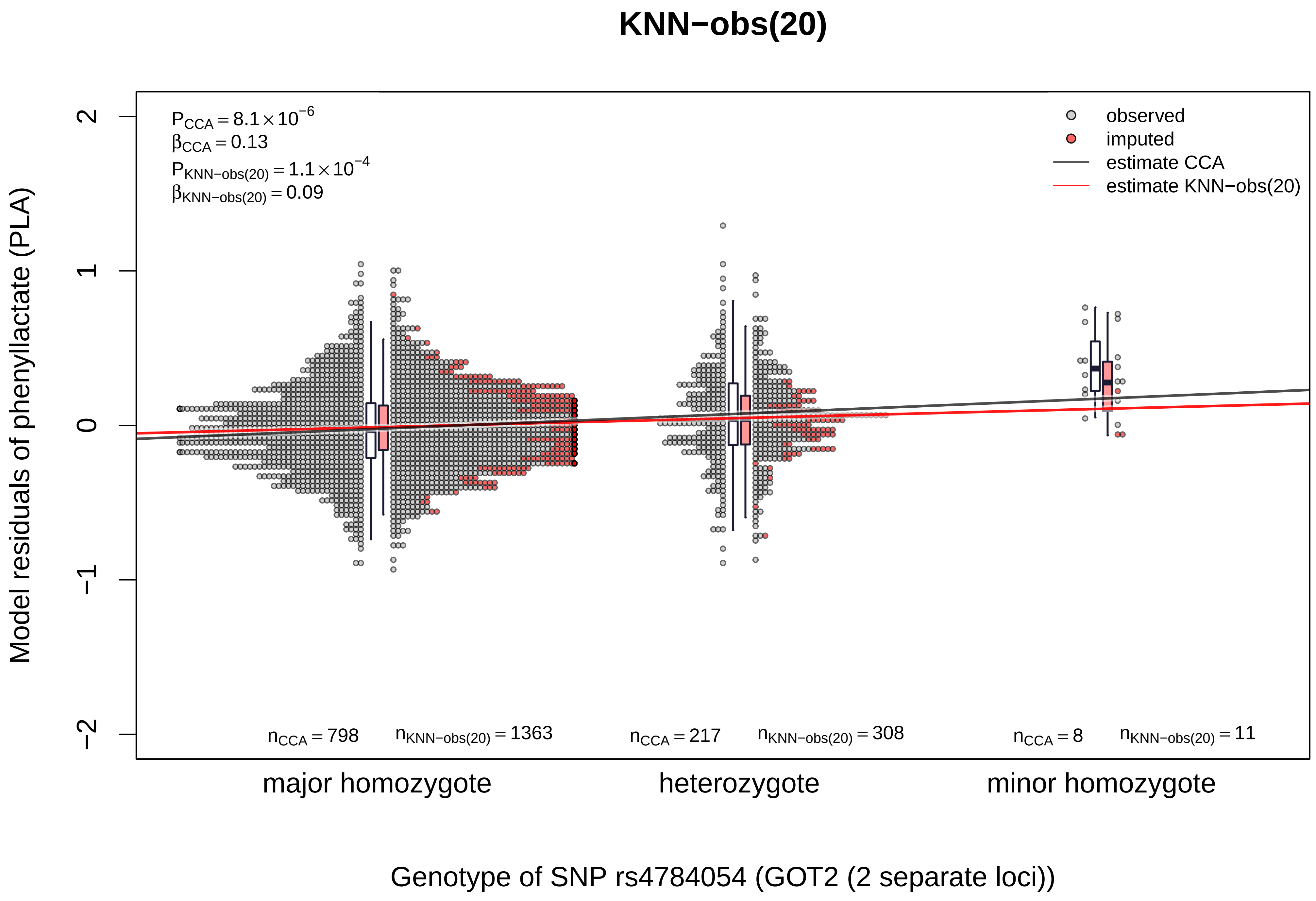

KNN−obs(20)
$P_{CCA} = 8.1 \times 10^{-6}$
$\beta_{CCA} = 0.13$
$P_{KNN-obs(20)} = 1.1 \times 10^{-4}$
$\beta_{KNN-obs(20)} = 0.09$
observed
imputed
estimate CCA
estimate KNN−obs(20)
Model residuals of phenyllactate (PLA)
2
1
0
−1
−2
$n_{CCA} = 798$
$n_{KNN-obs(20)} = 1363$
$n_{CCA} = 217$
$n_{KNN-obs(20)} = 308$
$n_{CCA} = 8$
$n_{KNN-obs(20)} = 11$
major homozygote
heterozygote
minor homozygote
Genotype of SNP rs4784054 (GOT2 (2 separate loci))

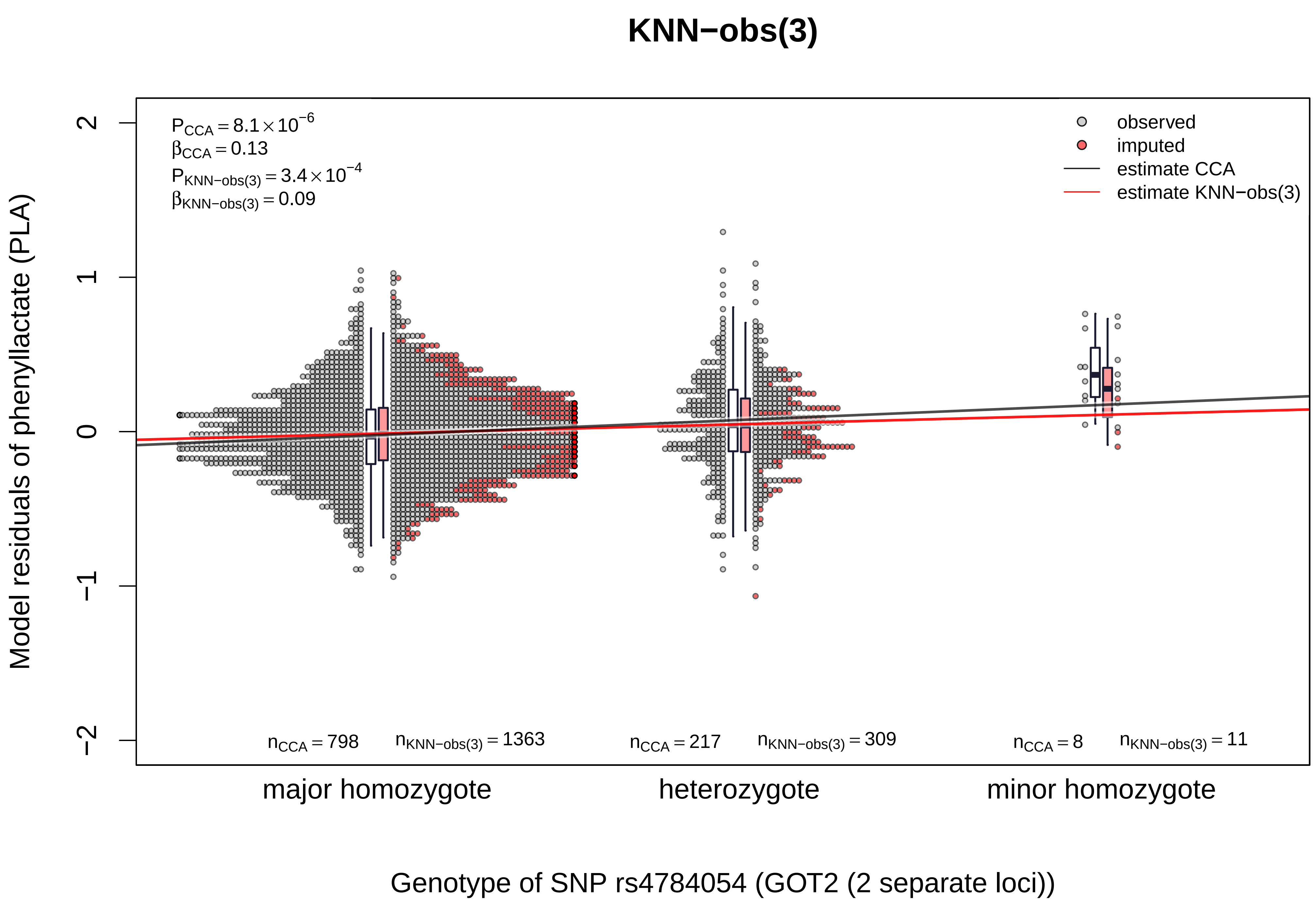

KNN−obs(3)
$P_{CCA} = 8.1 \times 10^{-6}$
$\beta_{CCA} = 0.13$
$P_{KNN-obs(3)} = 3.4 \times 10^{-4}$
$\beta_{KNN-obs(3)} = 0.09$
observed
imputed
estimate CCA
estimate KNN−obs(3)
$n_{CCA} = 798$
$n_{KNN-obs(3)} = 1363$
$n_{CCA} = 217$
$n_{KNN-obs(3)} = 309$
$n_{CCA} = 8$
$n_{KNN-obs(3)} = 11$
2
1
0
−1
−2
Model residuals of phenyllactate (PLA)
major homozygote
heterozygote
minor homozygote
Genotype of SNP rs4784054 (GOT2 (2 separate loci))

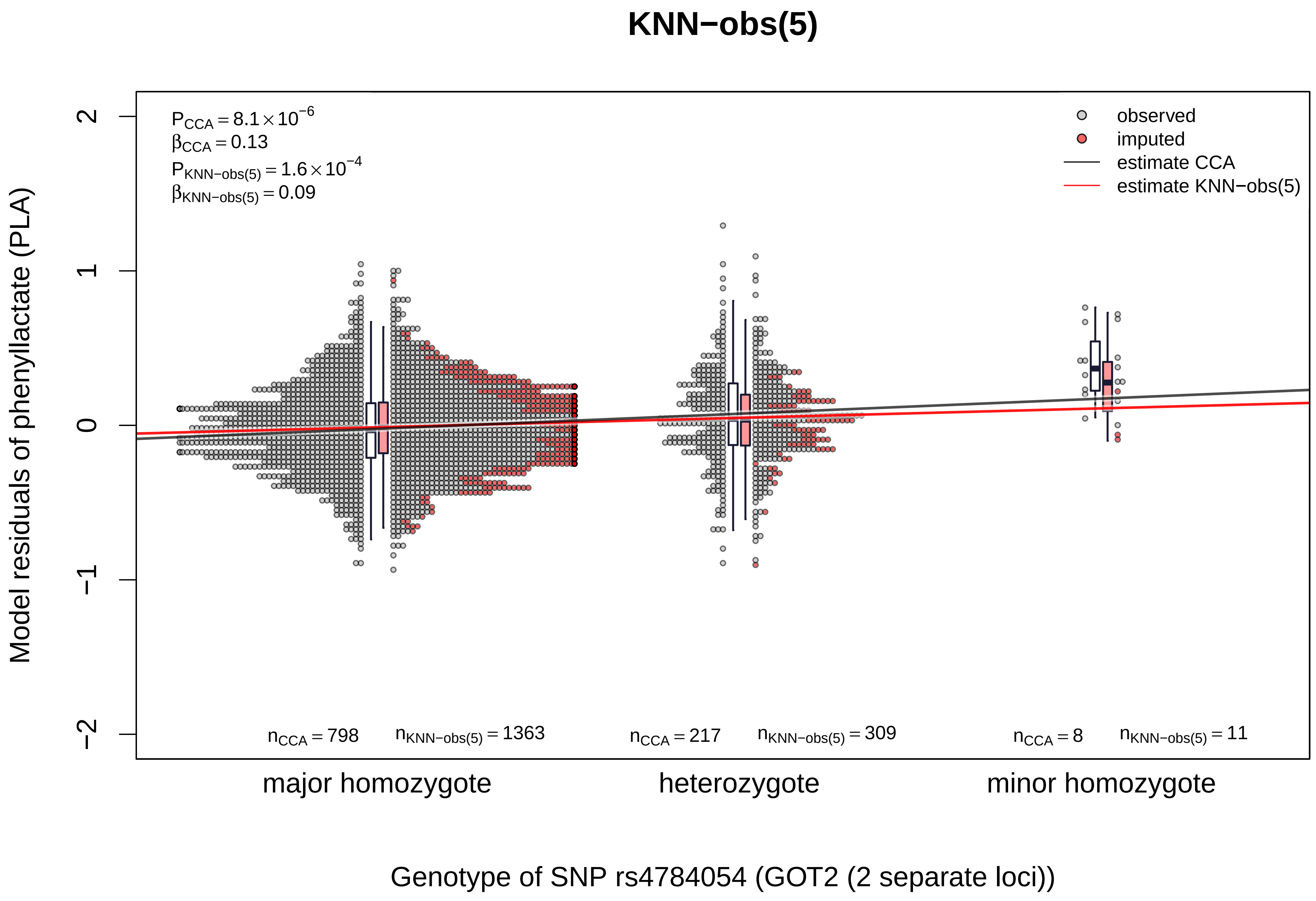

KNN−obs(5)
$P_{CCA} = 8.1 \times 10^{-6}$
$\beta_{CCA} = 0.13$
$P_{KNN-obs(5)} = 1.6 \times 10^{-4}$
$\beta_{KNN-obs(5)} = 0.09$
observed
imputed
estimate CCA
estimate KNN−obs(5)
Model residuals of phenyllactate (PLA)
2
1
0
−1
−2
$n_{CCA} = 798$
$n_{KNN-obs(5)} = 1363$
$n_{CCA} = 217$
$n_{KNN-obs(5)} = 309$
$n_{CCA} = 8$
$n_{KNN-obs(5)} = 11$
major homozygote
heterozygote
minor homozygote
Genotype of SNP rs4784054 (GOT2 (2 separate loci))

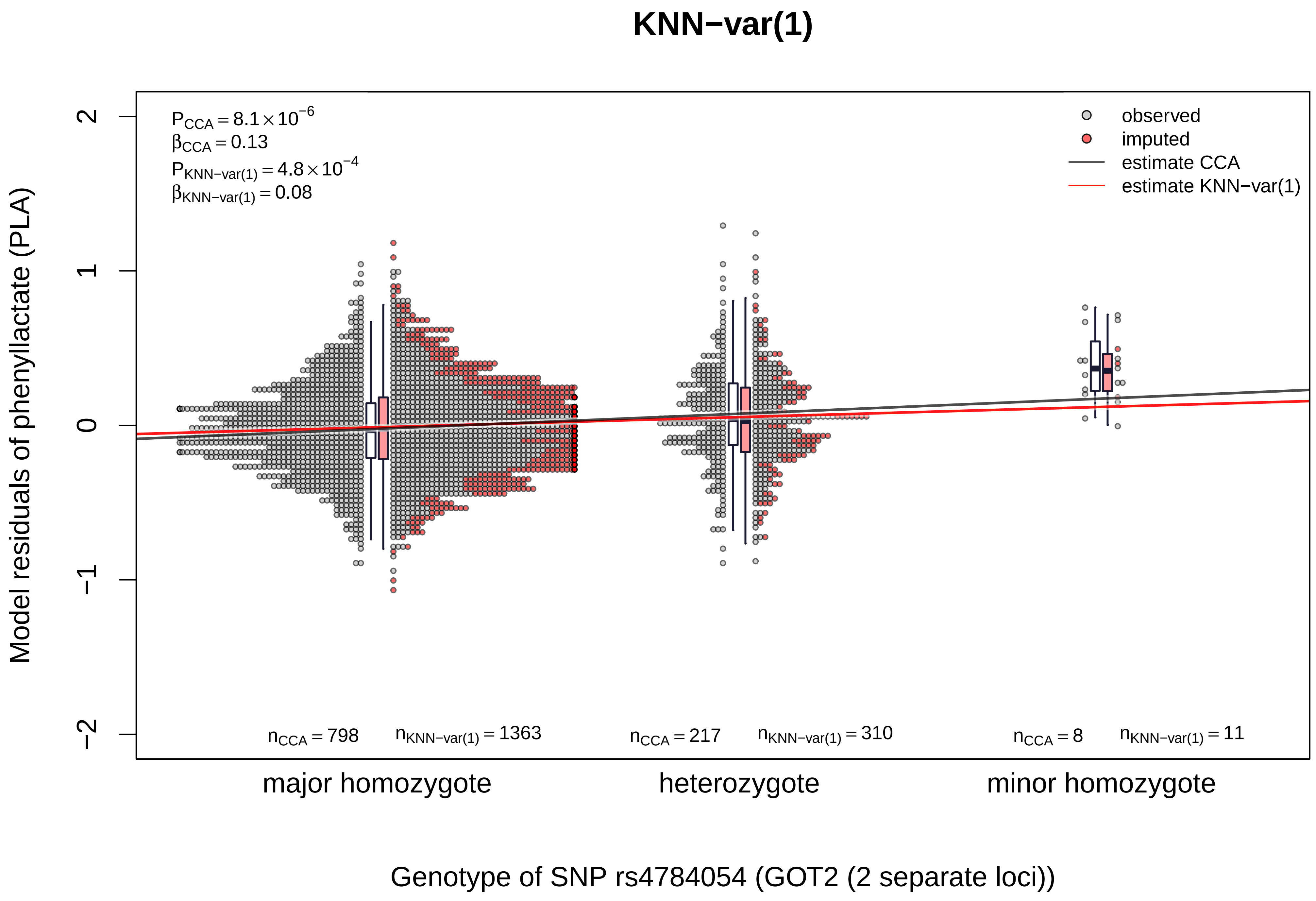
KNN−var(1)
$P_{CCA} = 8.1 \times 10^{-6}$
$\beta_{CCA} = 0.13$
$P_{KNN-var(1)} = 4.8 \times 10^{-4}$
$\beta_{KNN-var(1)} = 0.08$
observed
imputed
estimate CCA
estimate KNN−var(1)
Model residuals of phenyllactate (PLA)
2
1
0
−1
−2
$n_{CCA} = 798$
$n_{KNN-var(1)} = 1363$
$n_{CCA} = 217$
$n_{KNN-var(1)} = 310$
$n_{CCA} = 8$
$n_{KNN-var(1)} = 11$
major homozygote
heterozygote
minor homozygote
Genotype of SNP rs4784054 (GOT2 (2 separate loci))

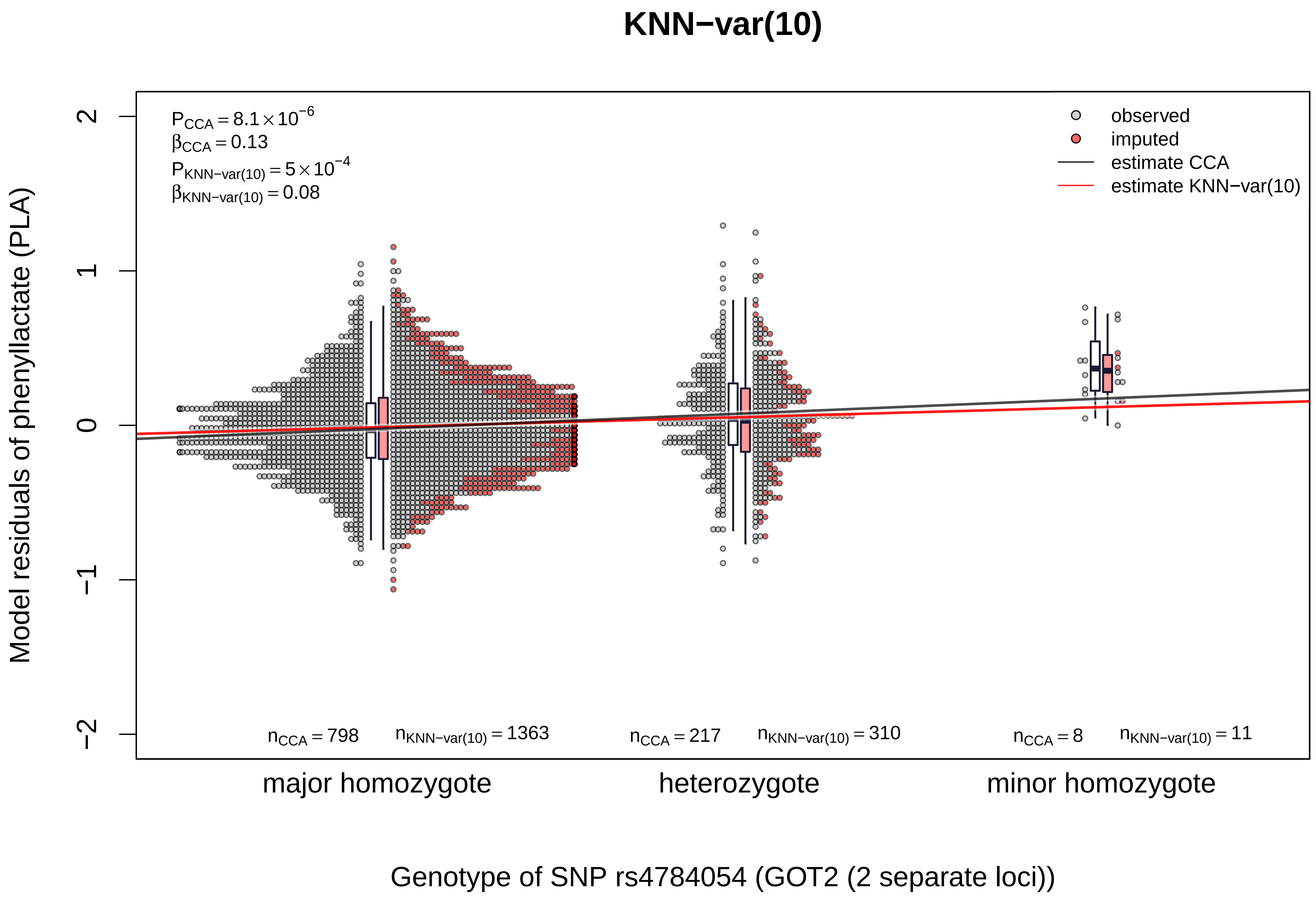

KNN−var(10)
$P_{CCA} = 8.1 \times 10^{-6}$
$\beta_{CCA} = 0.13$
$P_{KNN-var(10)} = 5 \times 10^{-4}$
$\beta_{KNN-var(10)} = 0.08$
observed
imputed
estimate CCA
estimate KNN−var(10)
Model residuals of phenyllactate (PLA)
2
1
0
−1
−2
$n_{CCA} = 798$
$n_{KNN-var(10)} = 1363$
$n_{CCA} = 217$
$n_{KNN-var(10)} = 310$
$n_{CCA} = 8$
$n_{KNN-var(10)} = 11$
major homozygote
heterozygote
minor homozygote
Genotype of SNP rs4784054 (GOT2 (2 separate loci))

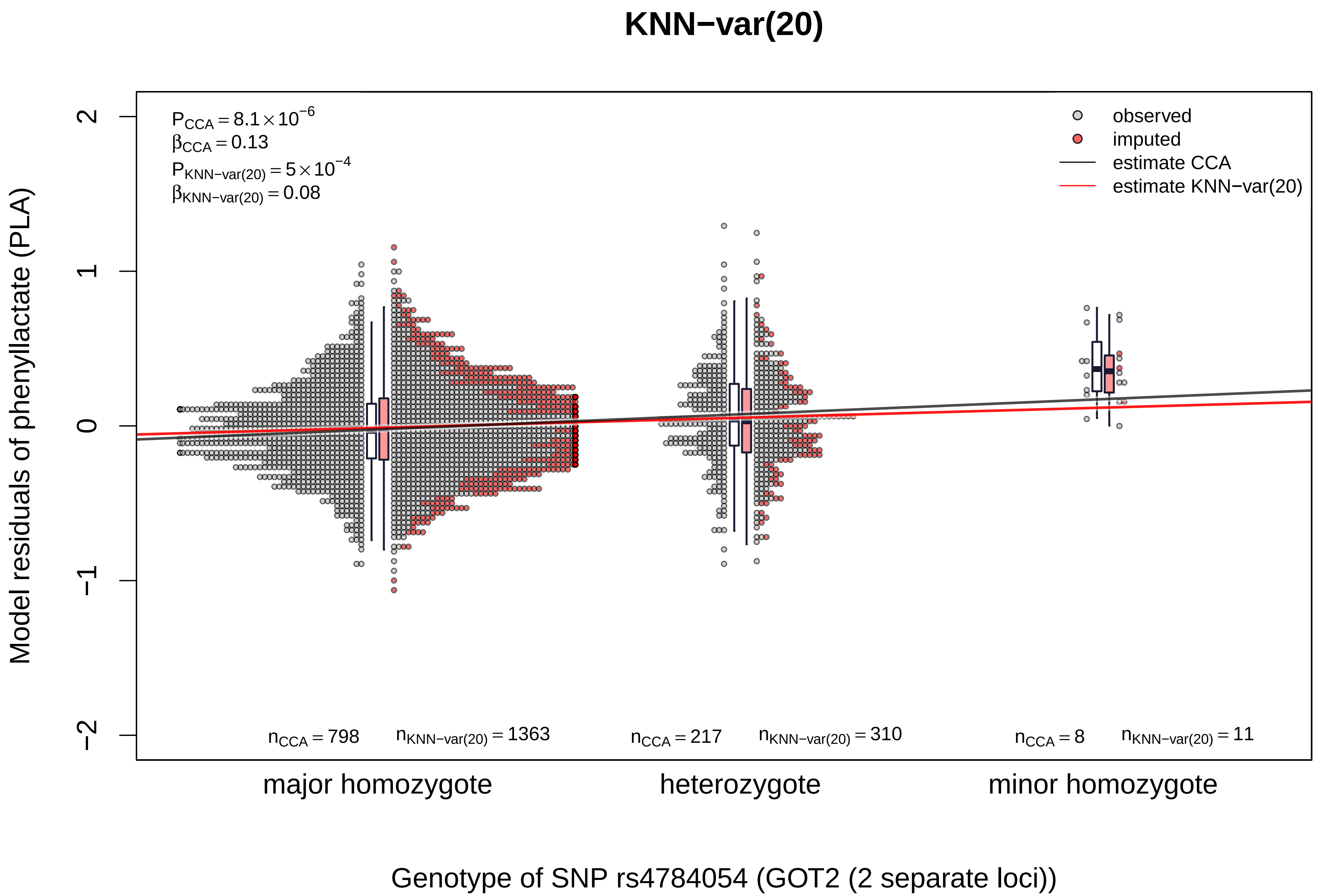
KNN−var(20)
$P_{CCA} = 8.1 \times 10^{-6}$
$\beta_{CCA} = 0.13$
$P_{KNN-var(20)} = 5 \times 10^{-4}$
$\beta_{KNN-var(20)} = 0.08$
observed
imputed
estimate CCA
estimate KNN−var(20)
Model residuals of phenyllactate (PLA)
2
1
0
−1
−2
$n_{CCA} = 798$
$n_{KNN-var(20)} = 1363$
$n_{CCA} = 217$
$n_{KNN-var(20)} = 310$
$n_{CCA} = 8$
$n_{KNN-var(20)} = 11$
major homozygote
heterozygote
minor homozygote
Genotype of SNP rs4784054 (GOT2 (2 separate loci))

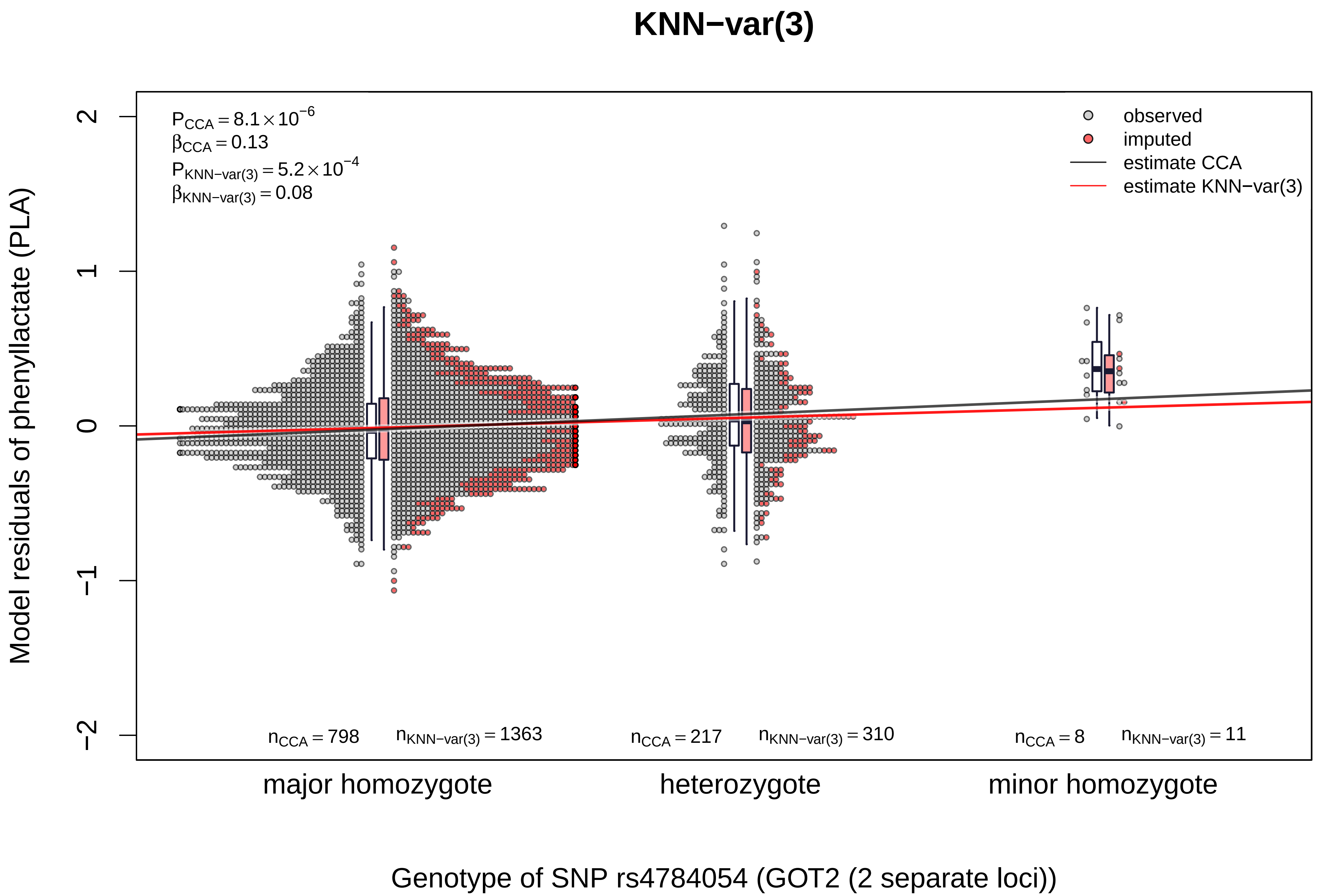

KNN−var(3)
$P_{CCA} = 8.1 \times 10^{-6}$
$\beta_{CCA} = 0.13$
$P_{KNN-var(3)} = 5.2 \times 10^{-4}$
$\beta_{KNN-var(3)} = 0.08$
observed
imputed
estimate CCA
estimate KNN−var(3)
2
1
0
−1
−2
Model residuals of phenyllactate (PLA)
$n_{CCA} = 798$
$n_{KNN-var(3)} = 1363$
$n_{CCA} = 217$
$n_{KNN-var(3)} = 310$
$n_{CCA} = 8$
$n_{KNN-var(3)} = 11$
major homozygote
heterozygote
minor homozygote
Genotype of SNP rs4784054 (GOT2 (2 separate loci))

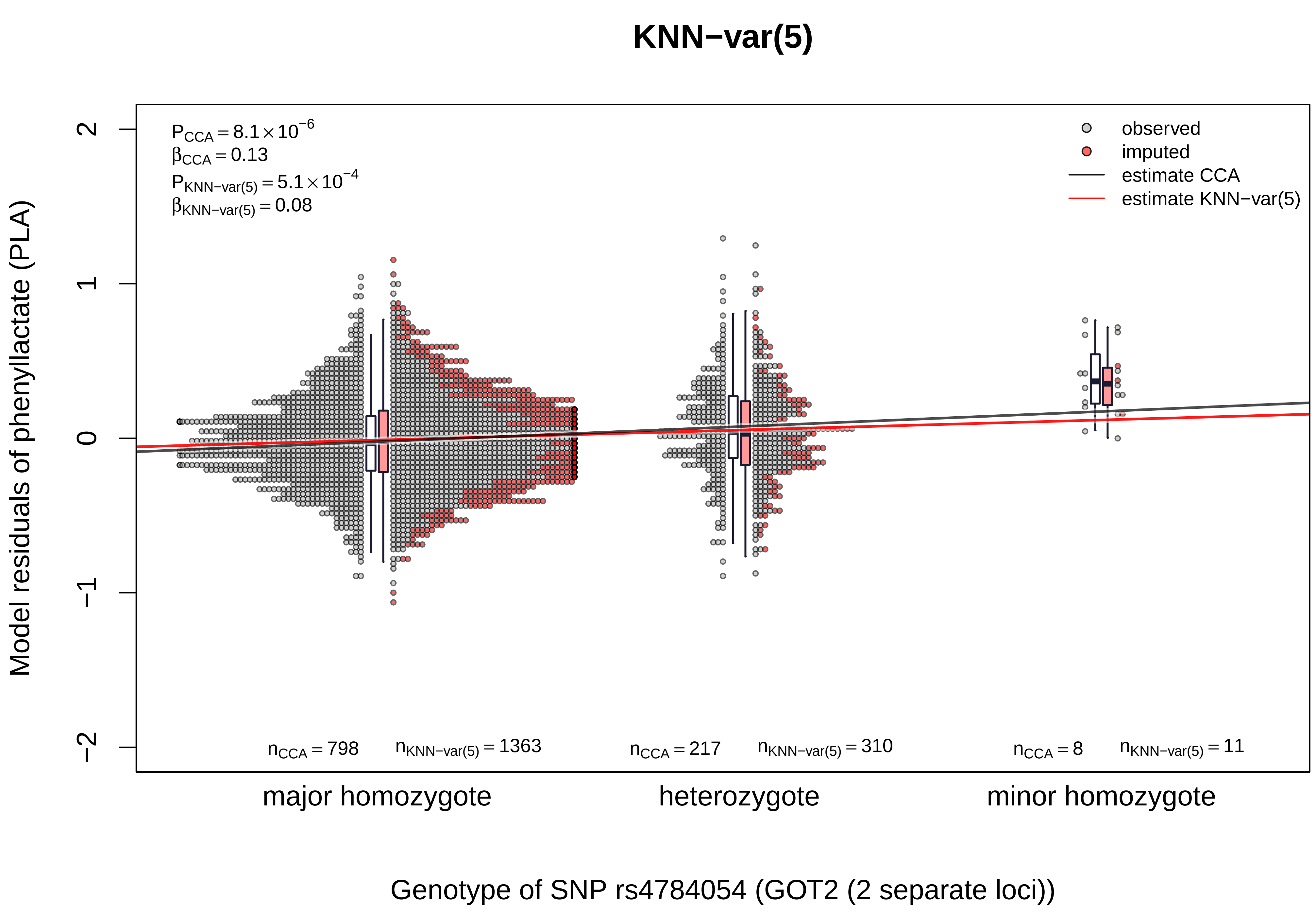
KNN−var(5)
$P_{CCA} = 8.1 \times 10^{-6}$
$\beta_{CCA} = 0.13$
$P_{KNN-var(5)} = 5.1 \times 10^{-4}$
$\beta_{KNN-var(5)} = 0.08$
observed
imputed
estimate CCA
estimate KNN−var(5)
Model residuals of phenyllactate (PLA)
2
1
0
−1
−2
$n_{CCA} = 798$
$n_{KNN-var(5)} = 1363$
$n_{CCA} = 217$
$n_{KNN-var(5)} = 310$
$n_{CCA} = 8$
$n_{KNN-var(5)} = 11$
major homozygote
heterozygote
minor homozygote
Genotype of SNP rs4784054 (GOT2 (2 separate loci))

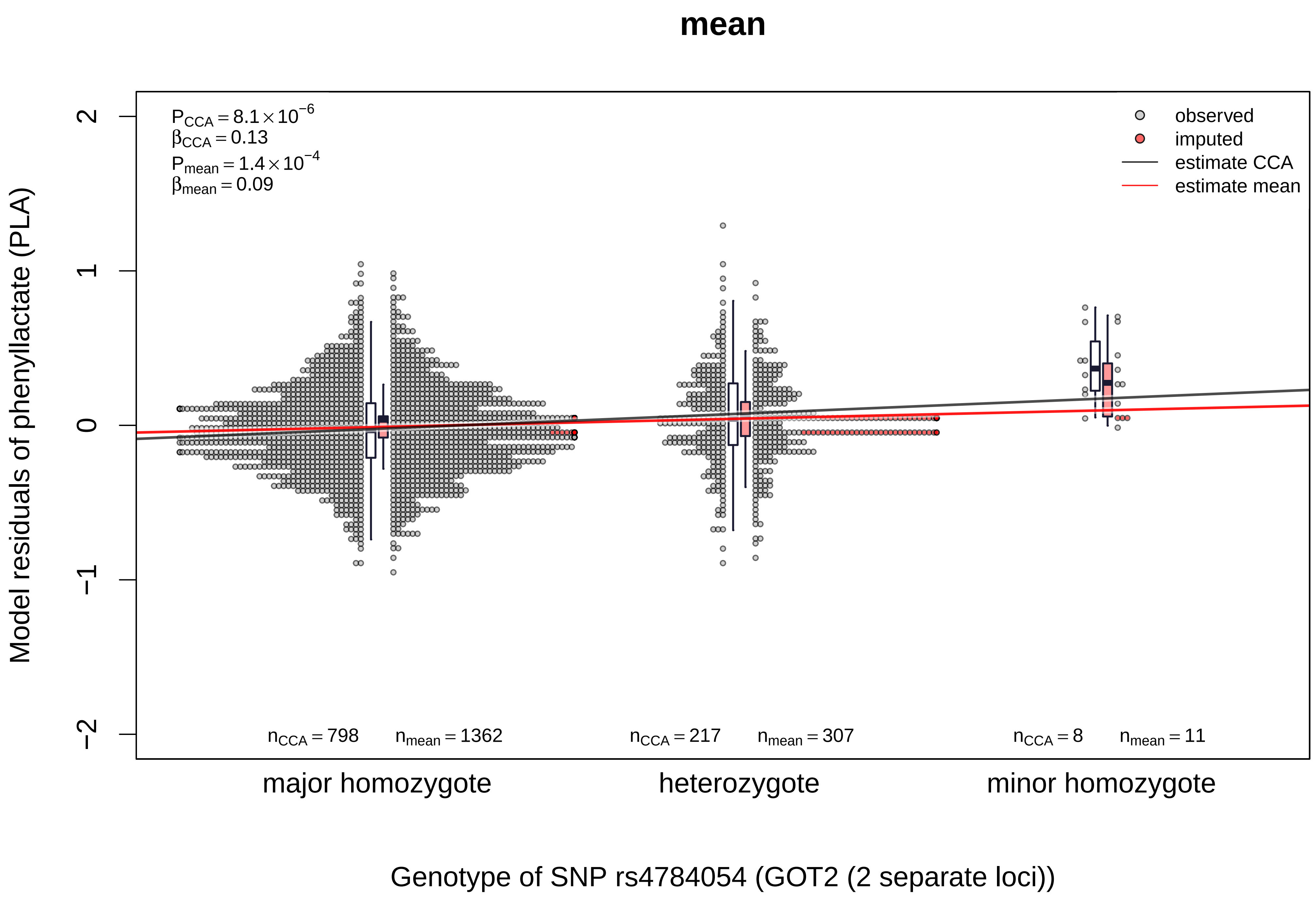

mean
$P_{CCA} = 8.1 \times 10^{-6}$
$\beta_{CCA} = 0.13$
$P_{mean} = 1.4 \times 10^{-4}$
$\beta_{mean} = 0.09$
observed
imputed
estimate CCA
estimate mean
2
1
0
−1
−2
Model residuals of phenyllactate (PLA)
$n_{CCA} = 798$
$n_{mean} = 1362$
$n_{CCA} = 217$
$n_{mean} = 307$
$n_{CCA} = 8$
$n_{mean} = 11$
major homozygote
heterozygote
minor homozygote
Genotype of SNP rs4784054 (GOT2 (2 separate loci))

MICE−avg−norm

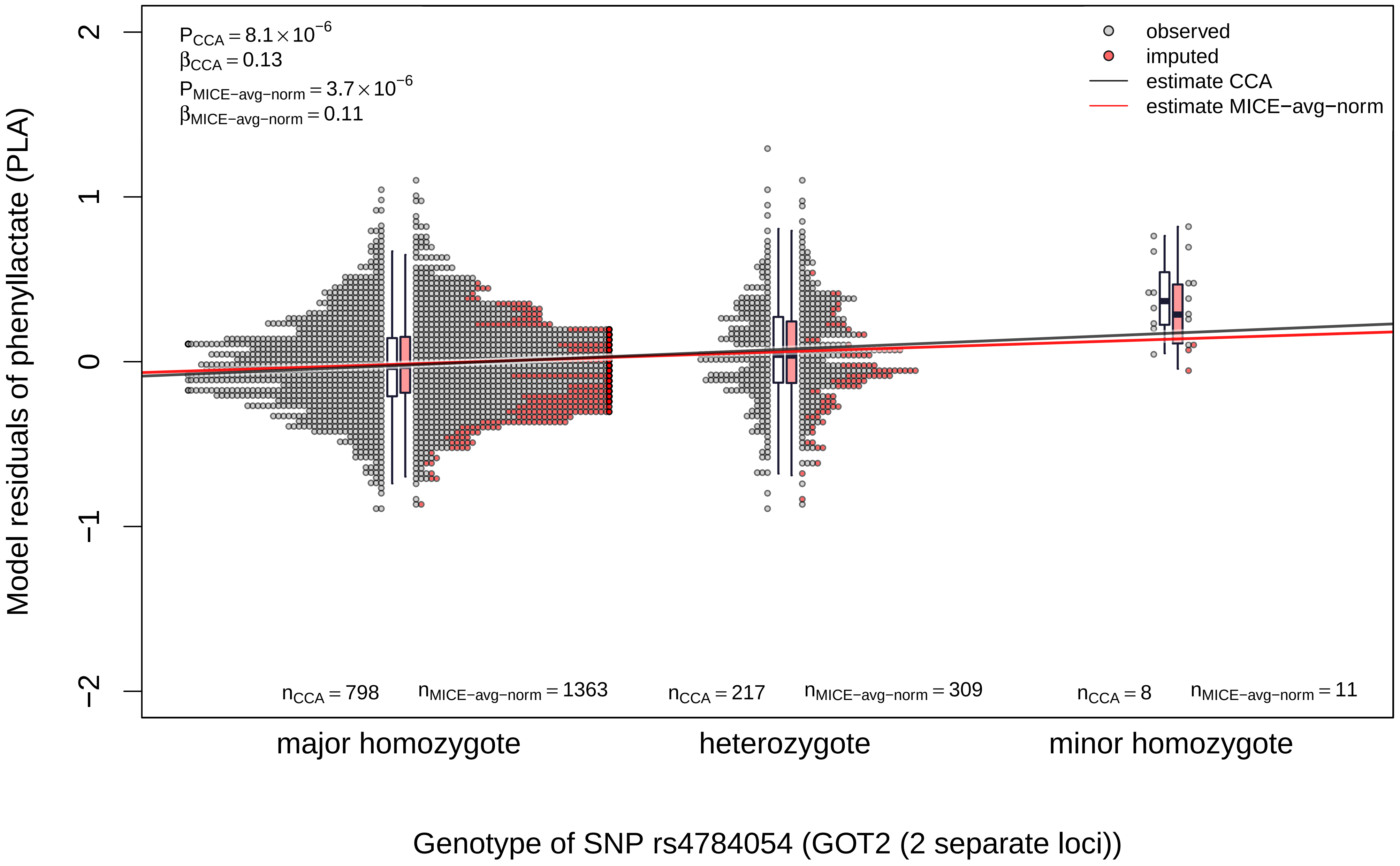

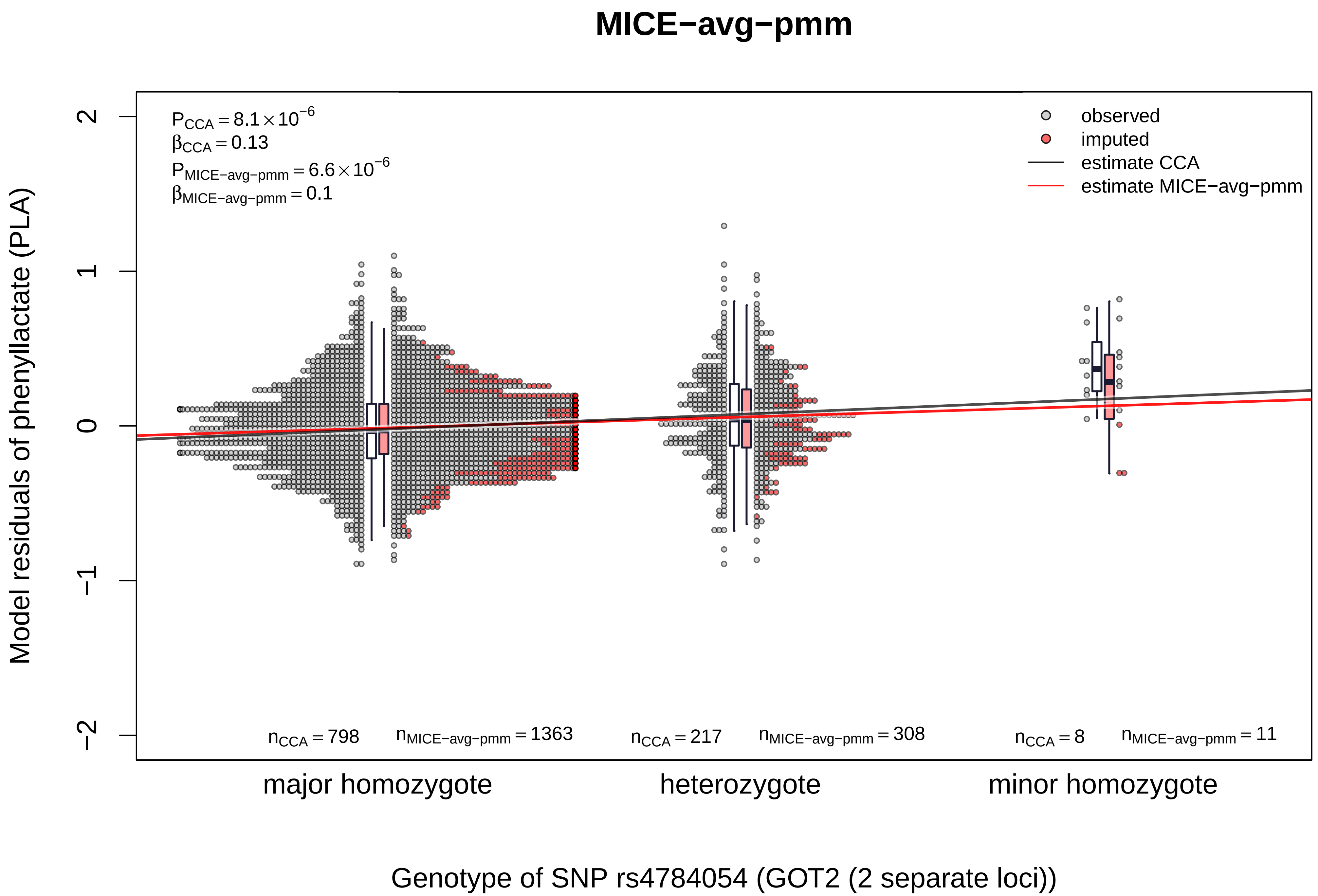
MICE−avg−pmm
$P_{CCA} = 8.1 \times 10^{-6}$
$\beta_{CCA} = 0.13$
$P_{MICE-avg-pmm} = 6.6 \times 10^{-6}$
$\beta_{MICE-avg-pmm} = 0.1$
observed
imputed
estimate CCA
estimate MICE−avg−pmm
2
1
0
−1
−2
Model residuals of phenyllactate (PLA)
$n_{CCA} = 798$
$n_{MICE-avg-pmm} = 1363$
$n_{CCA} = 217$
$n_{MICE-avg-pmm} = 308$
$n_{CCA} = 8$
$n_{MICE-avg-pmm} = 11$
major homozygote
heterozygote
minor homozygote
Genotype of SNP rs4784054 (GOT2 (2 separate loci))

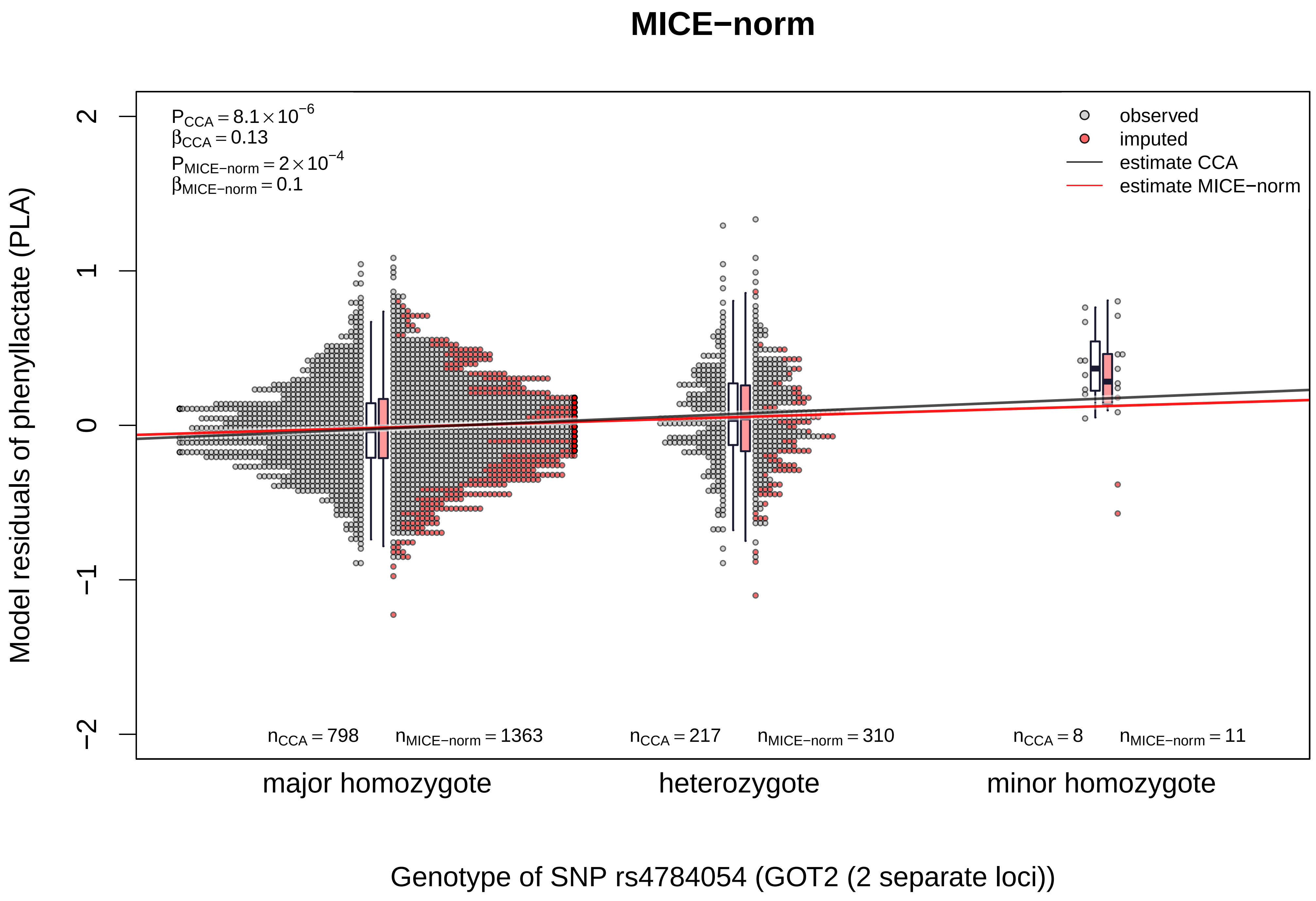
MICE−norm
$P_{CCA} = 8.1 \times 10^{-6}$
$\beta_{CCA} = 0.13$
$P_{MICE-norm} = 2 \times 10^{-4}$
$\beta_{MICE-norm} = 0.1$
observed
imputed
estimate CCA
estimate MICE−norm
2
1
0
−1
−2
Model residuals of phenyllactate (PLA)
$n_{CCA} = 798$
$n_{MICE-norm} = 1363$
$n_{CCA} = 217$
$n_{MICE-norm} = 310$
$n_{CCA} = 8$
$n_{MICE-norm} = 11$
major homozygote
heterozygote
minor homozygote
Genotype of SNP rs4784054 (GOT2 (2 separate loci))

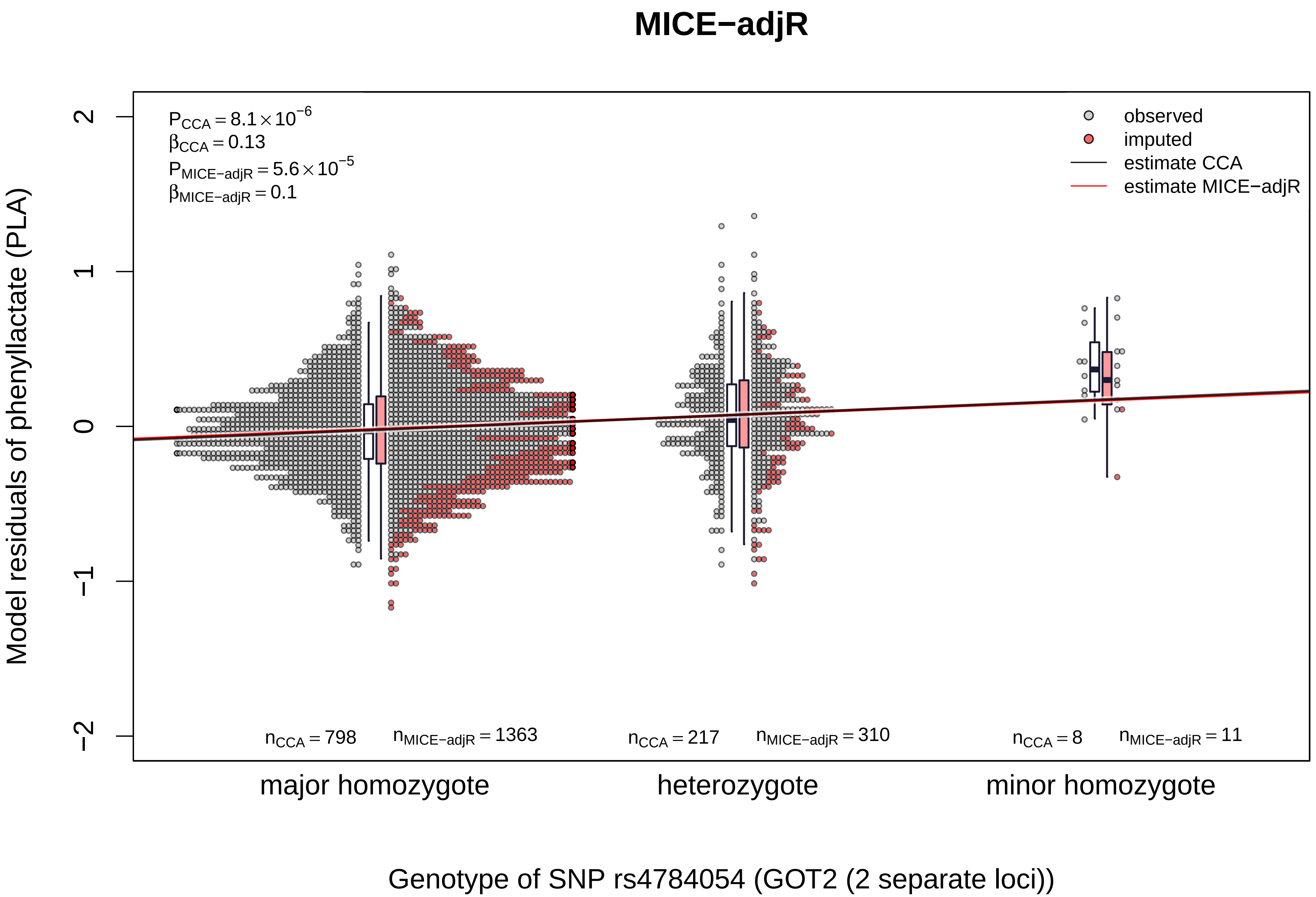

MICE−adjR
$P_{CCA} = 8.1 \times 10^{-6}$
$\beta_{CCA} = 0.13$
$P_{MICE-adjR} = 5.6 \times 10^{-5}$
$\beta_{MICE-adjR} = 0.1$
observed
imputed
estimate CCA
estimate MICE−adjR
2
1
0
−1
−2
Model residuals of phenyllactate (PLA)
$n_{CCA} = 798$
$n_{MICE-adjR} = 1363$
$n_{CCA} = 217$
$n_{MICE-adjR} = 310$
$n_{CCA} = 8$
$n_{MICE-adjR} = 11$
major homozygote
heterozygote
minor homozygote
Genotype of SNP rs4784054 (GOT2 (2 separate loci))

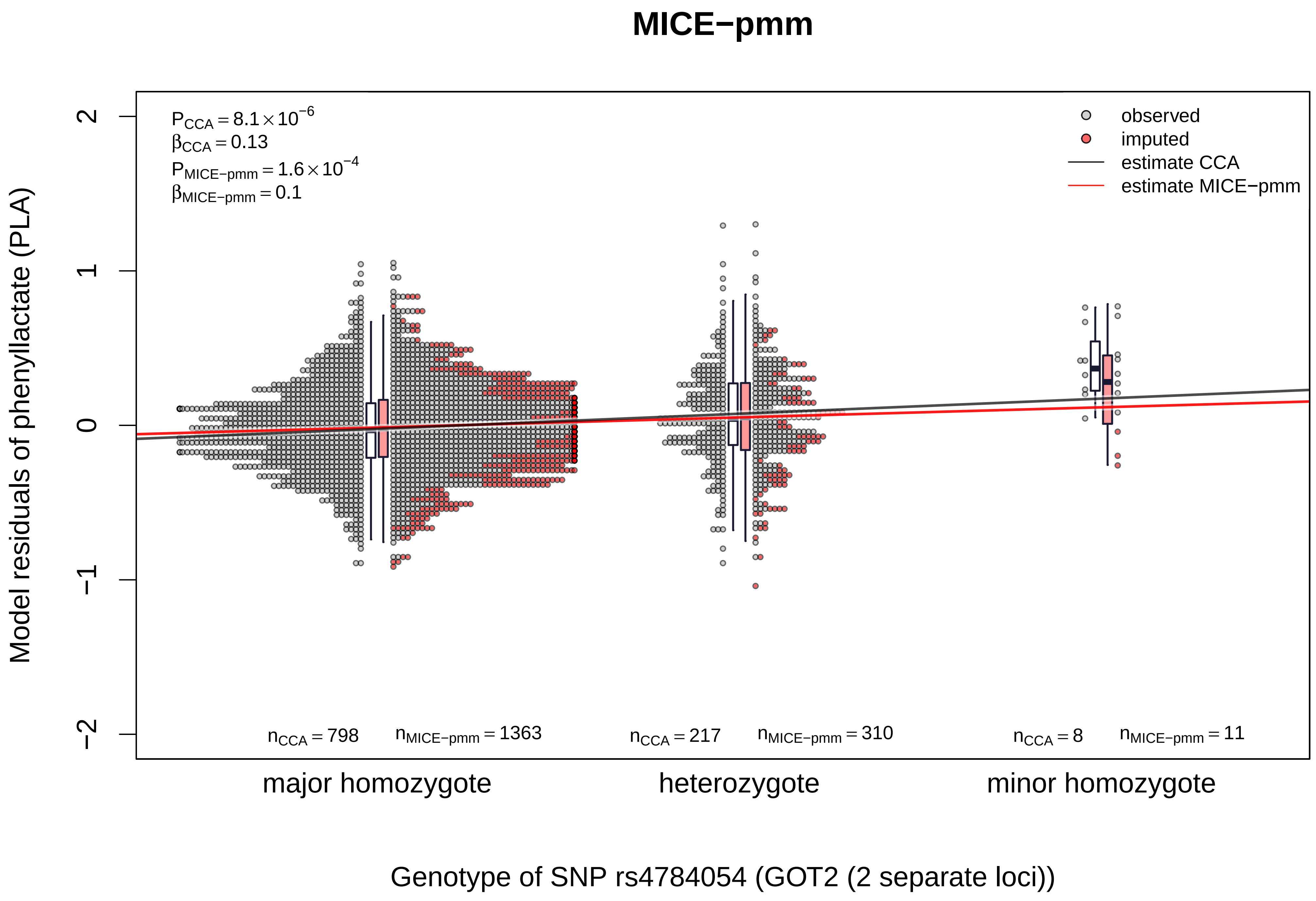

MICE−pmm
$P_{CCA} = 8.1 \times 10^{-6}$
$\beta_{CCA} = 0.13$
$P_{MICE-pmm} = 1.6 \times 10^{-4}$
$\beta_{MICE-pmm} = 0.1$
observed
imputed
estimate CCA
estimate MICE−pmm
Model residuals of phenyllactate (PLA)
2
1
0
−1
−2
$n_{CCA} = 798$
$n_{MICE-pmm} = 1363$
$n_{CCA} = 217$
$n_{MICE-pmm} = 310$
$n_{CCA} = 8$
$n_{MICE-pmm} = 11$
major homozygote
heterozygote
minor homozygote
Genotype of SNP rs4784054 (GOT2 (2 separate loci))

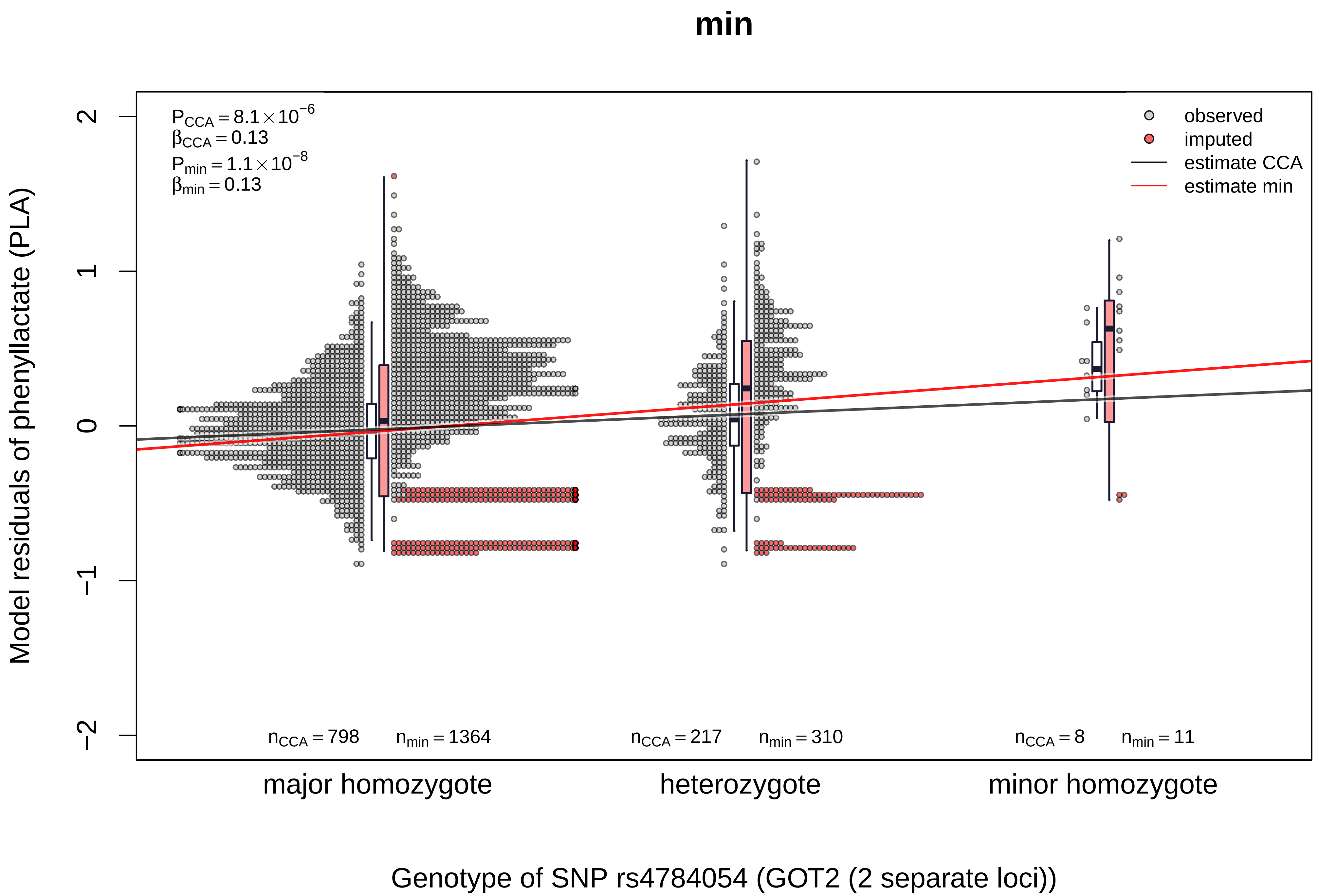

min
$P_{CCA} = 8.1 \times 10^{-6}$
$\beta_{CCA} = 0.13$
$P_{min} = 1.1 \times 10^{-8}$
$\beta_{min} = 0.13$
observed
imputed
estimate CCA
estimate min
Model residuals of phenyllactate (PLA)
2
1
0
−1
−2
$n_{CCA} = 798$
$n_{min} = 1364$
$n_{CCA} = 217$
$n_{min} = 310$
$n_{CCA} = 8$
$n_{min} = 11$
major homozygote
heterozygote
minor homozygote
Genotype of SNP rs4784054 (GOT2 (2 separate loci))

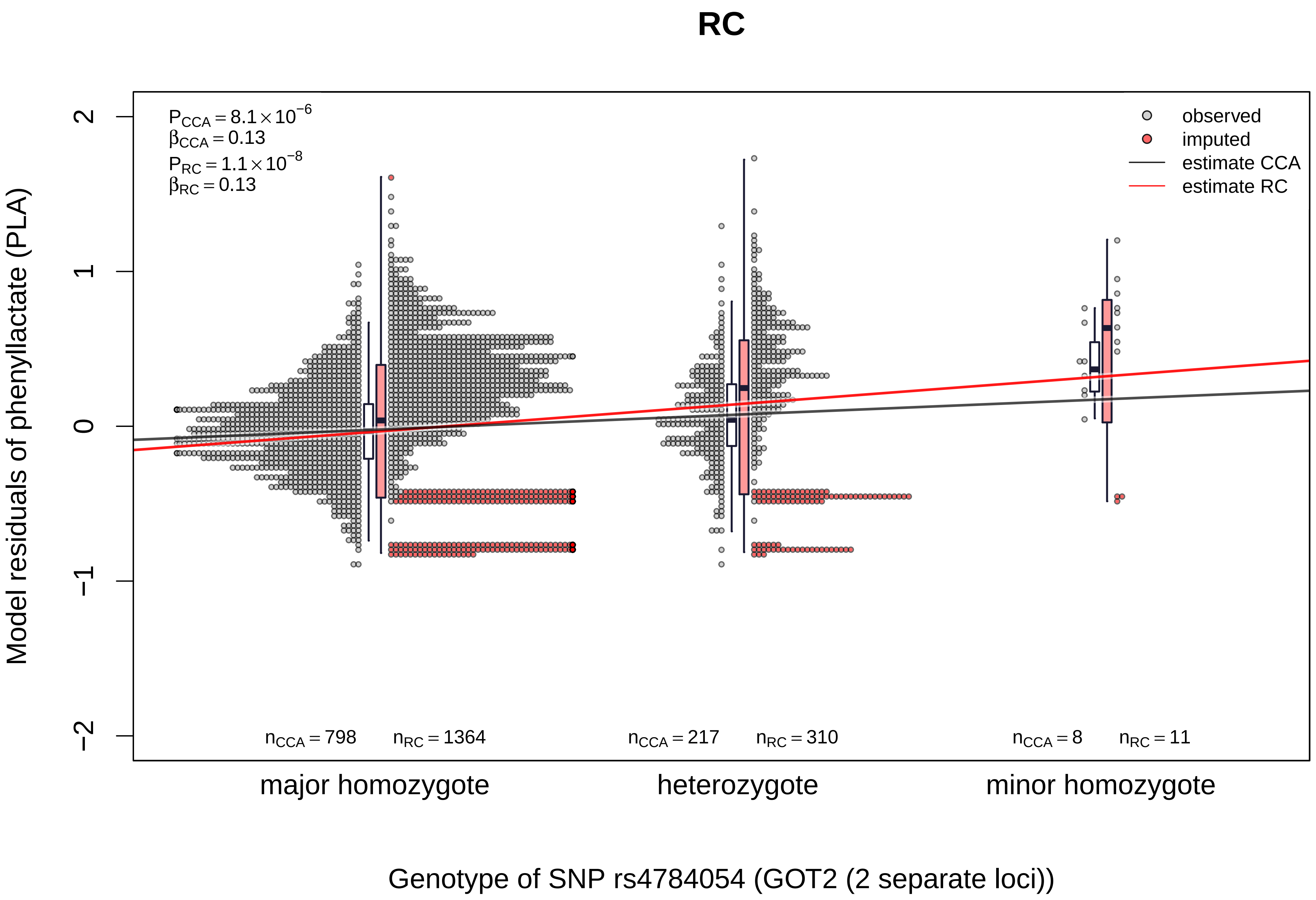

RC
$P_{CCA} = 8.1 \times 10^{-6}$
$\beta_{CCA} = 0.13$
$P_{RC} = 1.1 \times 10^{-8}$
$\beta_{RC} = 0.13$
observed
imputed
estimate CCA
estimate RC
2
1
0
−1
−2
Model residuals of phenyllactate (PLA)
$n_{CCA} = 798$
$n_{RC} = 1364$
$n_{CCA} = 217$
$n_{RC} = 310$
$n_{CCA} = 8$
$n_{RC} = 11$
major homozygote
heterozygote
minor homozygote
Genotype of SNP rs4784054 (GOT2 (2 separate loci))

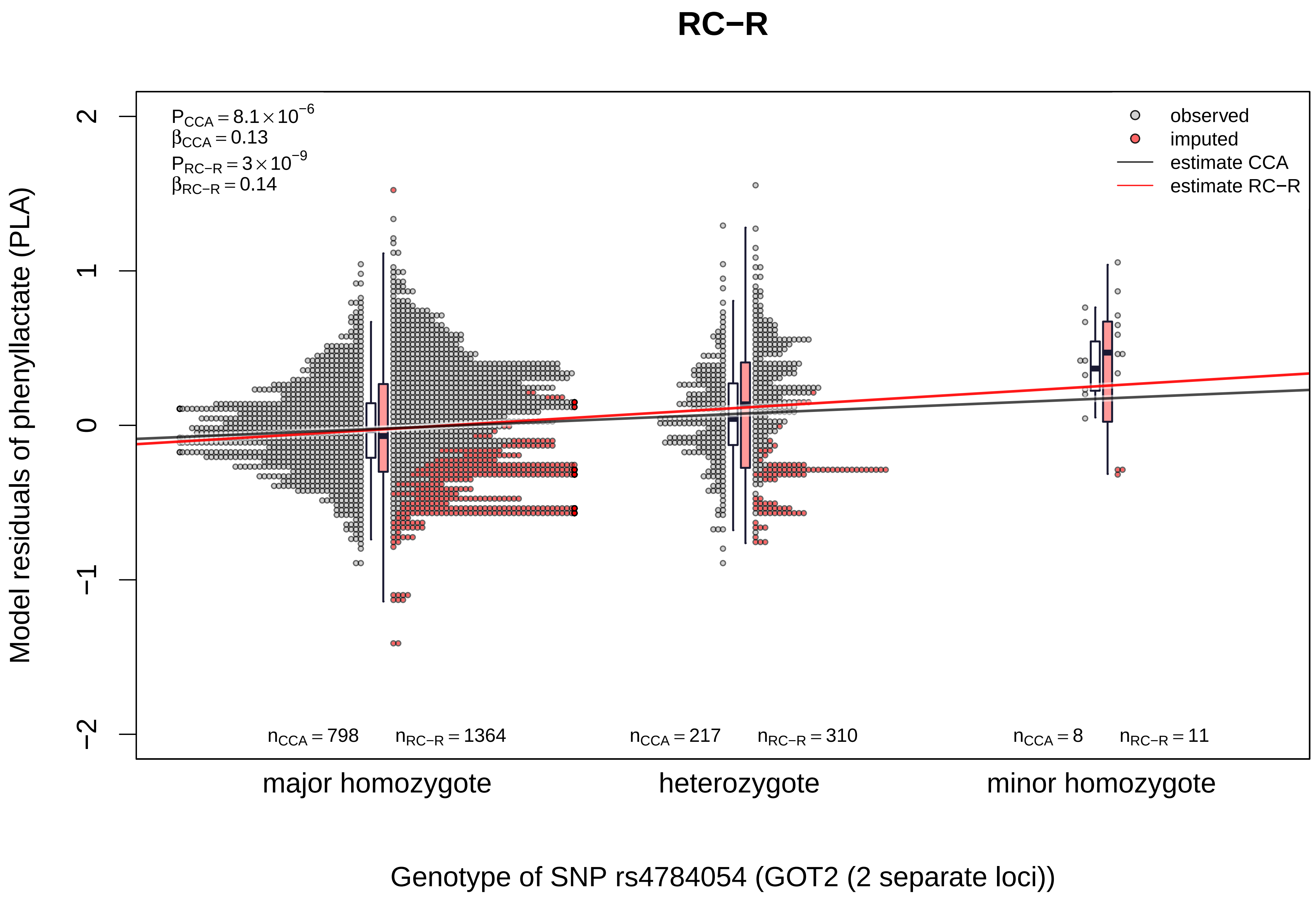

RC−R
$P_{CCA} = 8.1 \times 10^{-6}$
$\beta_{CCA} = 0.13$
$P_{RC-R} = 3 \times 10^{-9}$
$\beta_{RC-R} = 0.14$
observed
imputed
estimate CCA
estimate RC−R
Model residuals of phenyllactate (PLA)
2
1
0
−1
−2
$n_{CCA} = 798$
$n_{RC-R} = 1364$
$n_{CCA} = 217$
$n_{RC-R} = 310$
$n_{CCA} = 8$
$n_{RC-R} = 11$
major homozygote
heterozygote
minor homozygote
Genotype of SNP rs4784054 (GOT2 (2 separate loci))

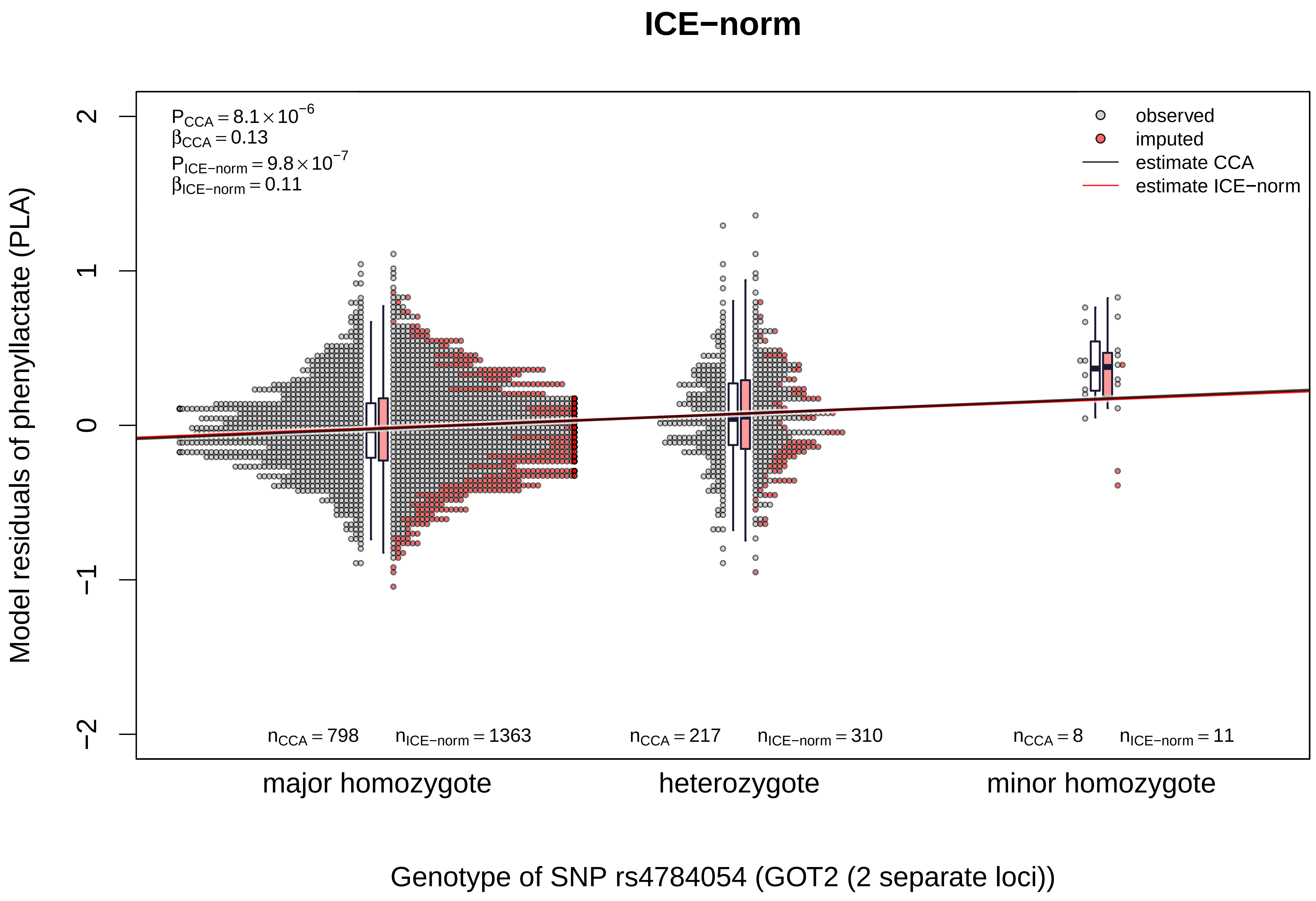
ICE−norm
$P_{CCA} = 8.1 \times 10^{-6}$
$\beta_{CCA} = 0.13$
$P_{ICE-norm} = 9.8 \times 10^{-7}$
$\beta_{ICE-norm} = 0.11$
observed
imputed
estimate CCA
estimate ICE−norm
$n_{CCA} = 798$
$n_{ICE-norm} = 1363$
$n_{CCA} = 217$
$n_{ICE-norm} = 310$
$n_{CCA} = 8$
$n_{ICE-norm} = 11$
2
1
0
−1
−2
Model residuals of phenyllactate (PLA)
major homozygote
heterozygote
minor homozygote
Genotype of SNP rs4784054 (GOT2 (2 separate loci))

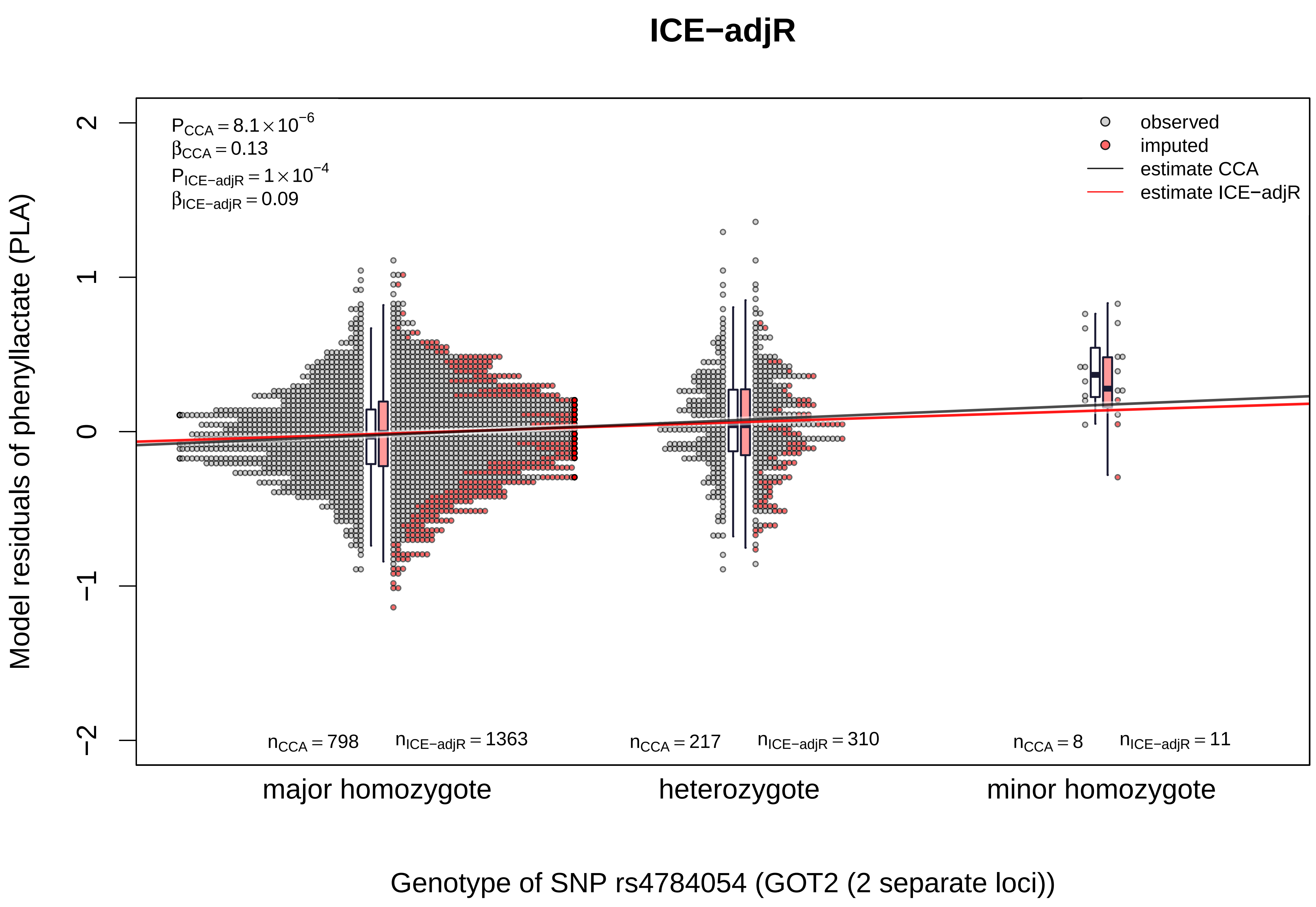

ICE−adjR
$P_{CCA} = 8.1 \times 10^{-6}$
$\beta_{CCA} = 0.13$
$P_{ICE-adjR} = 1 \times 10^{-4}$
$\beta_{ICE-adjR} = 0.09$
observed
imputed
estimate CCA
estimate ICE−adjR
$n_{CCA} = 798$
$n_{ICE-adjR} = 1363$
$n_{CCA} = 217$
$n_{ICE-adjR} = 310$
$n_{CCA} = 8$
$n_{ICE-adjR} = 11$
Model residuals of phenyllactate (PLA)
2
1
0
−1
−2
major homozygote
heterozygote
minor homozygote
Genotype of SNP rs4784054 (GOT2 (2 separate loci))

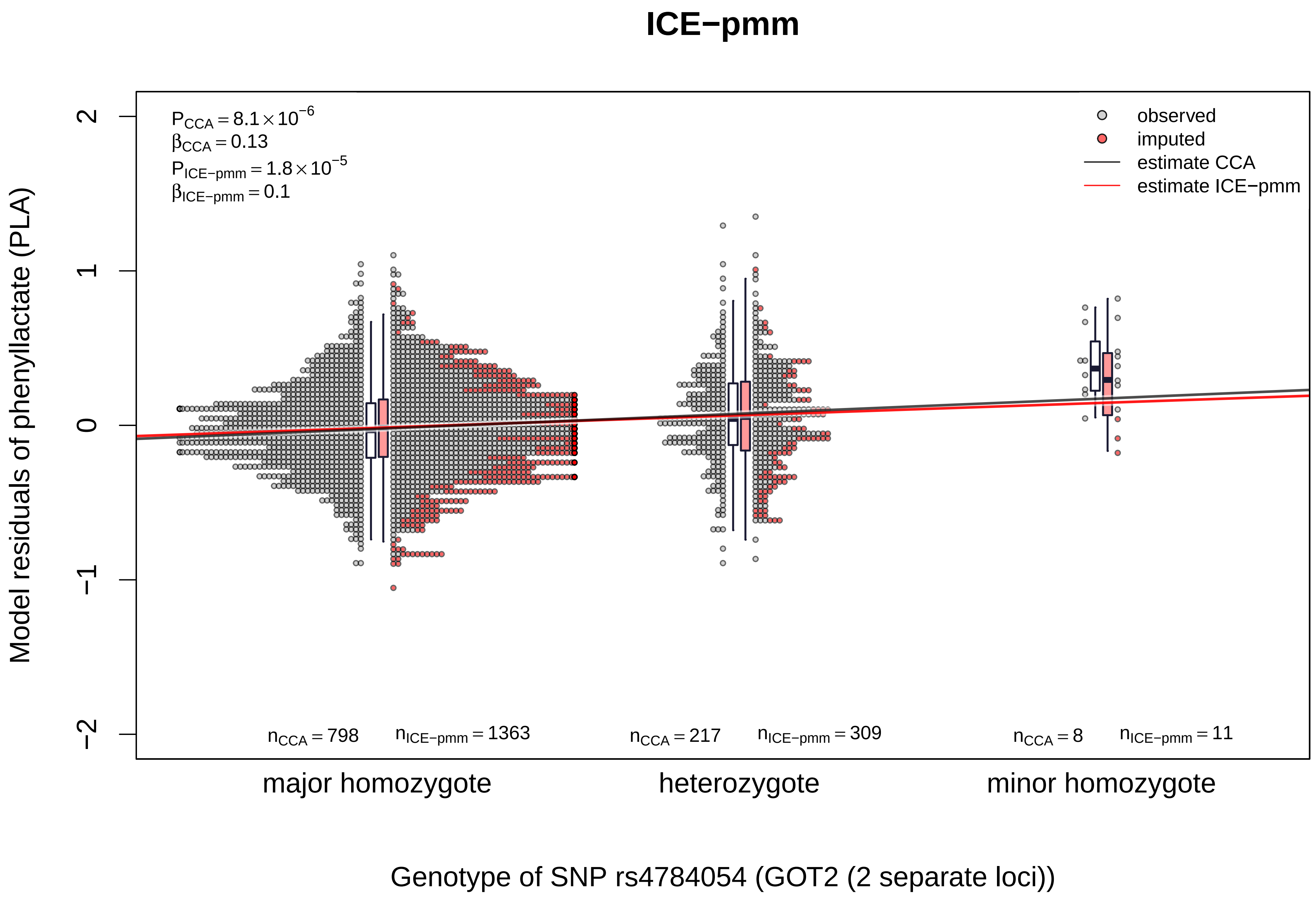
ICE−pmm
$P_{CCA} = 8.1 \times 10^{-6}$
$\beta_{CCA} = 0.13$
$P_{ICE-pmm} = 1.8 \times 10^{-5}$
$\beta_{ICE-pmm} = 0.1$
observed
imputed
estimate CCA
estimate ICE−pmm
Model residuals of phenyllactate (PLA)
2
1
0
−1
−2
$n_{CCA} = 798$
$n_{ICE-pmm} = 1363$
$n_{CCA} = 217$
$n_{ICE-pmm} = 309$
$n_{CCA} = 8$
$n_{ICE-pmm} = 11$
major homozygote
heterozygote
minor homozygote
Genotype of SNP rs4784054 (GOT2 (2 separate loci))

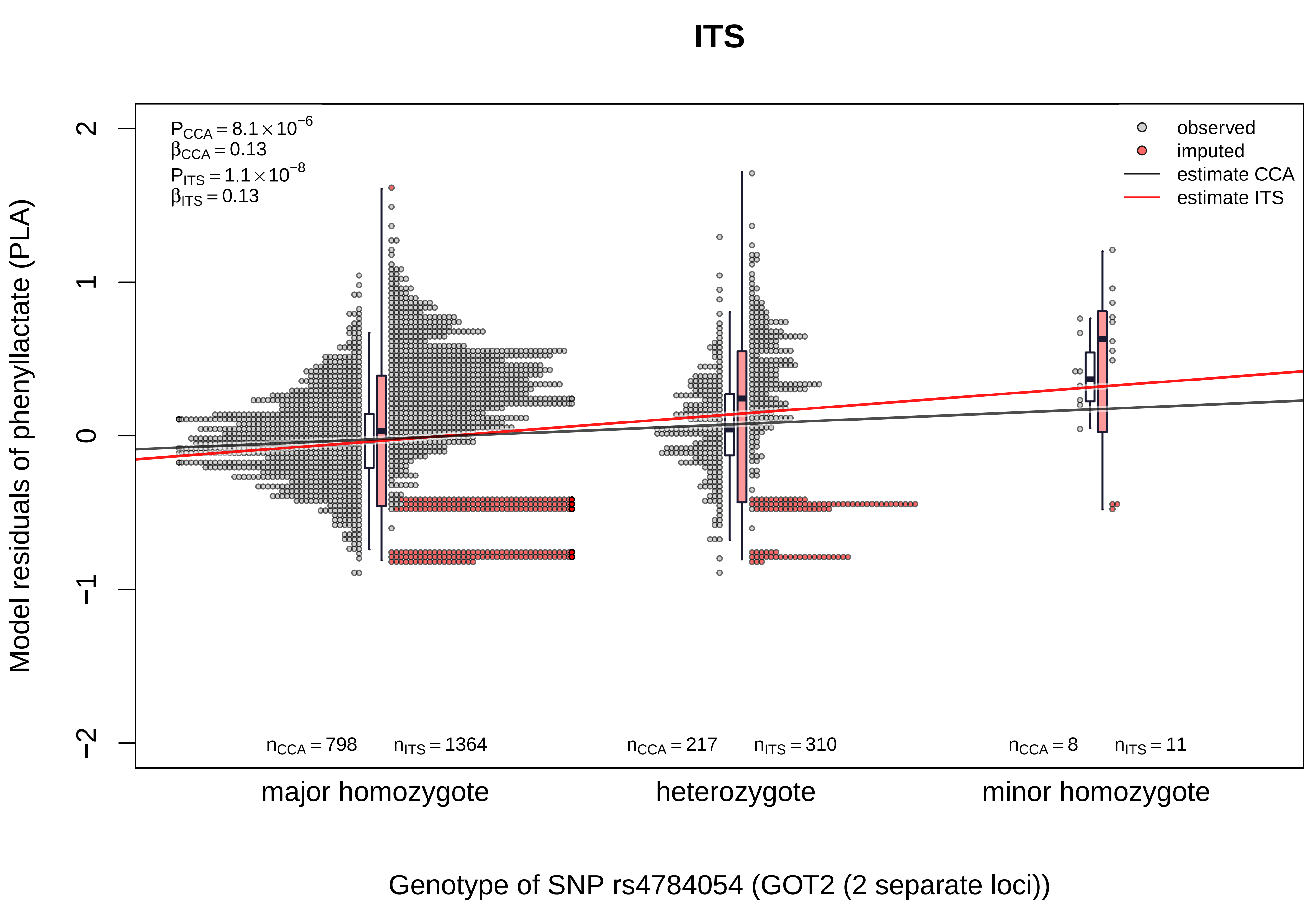

ITS
$P_{CCA} = 8.1 \times 10^{-6}$
$\beta_{CCA} = 0.13$
$P_{ITS} = 1.1 \times 10^{-8}$
$\beta_{ITS} = 0.13$
observed
imputed
estimate CCA
estimate ITS
Model residuals of phenyllactate (PLA)
2
1
0
−1
−2
$n_{CCA} = 798$
$n_{ITS} = 1364$
$n_{CCA} = 217$
$n_{ITS} = 310$
$n_{CCA} = 8$
$n_{ITS} = 11$
major homozygote
heterozygote
minor homozygote
Genotype of SNP rs4784054 (GOT2 (2 separate loci))

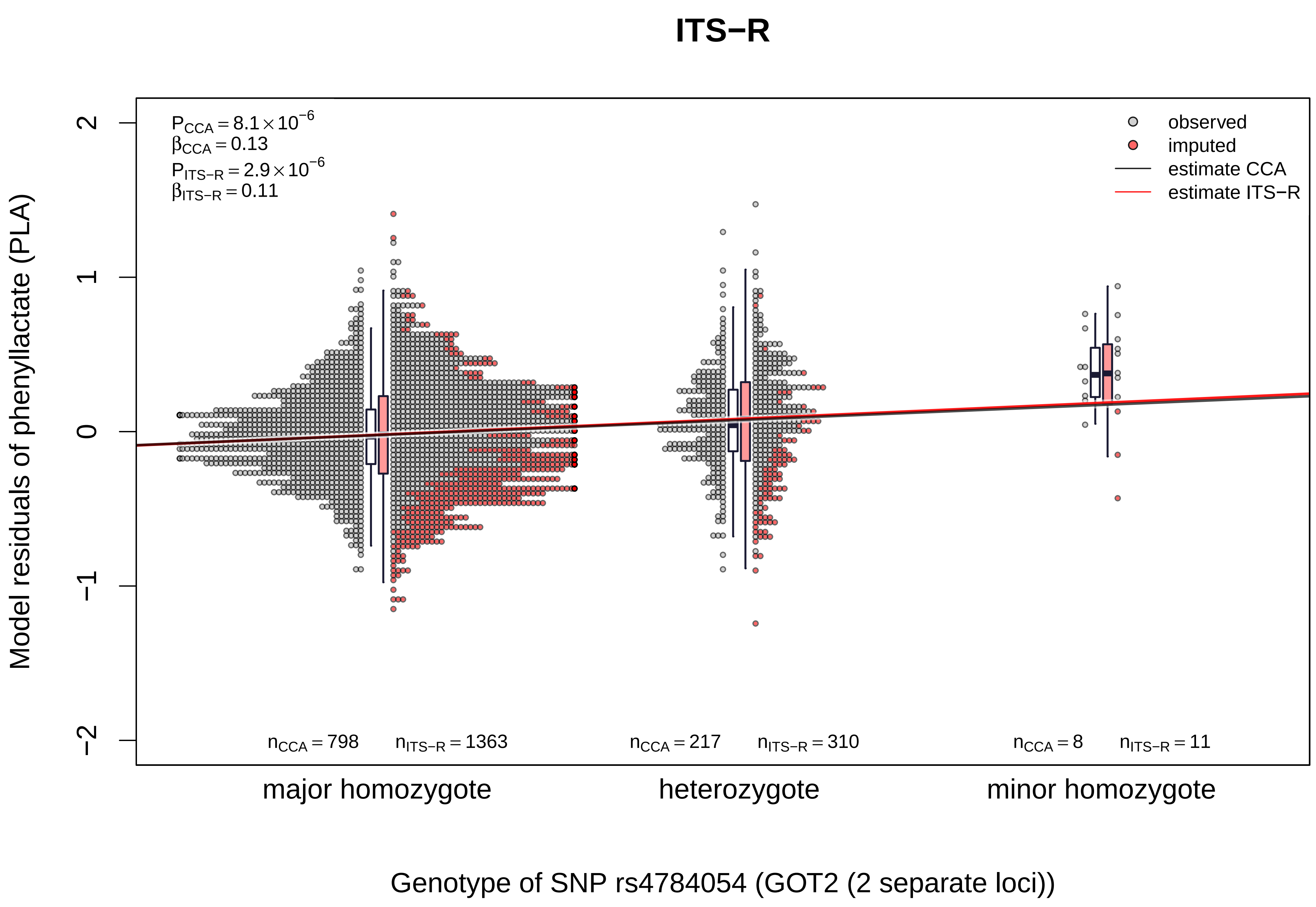
ITS−R
$P_{CCA} = 8.1 \times 10^{-6}$
$\beta_{CCA} = 0.13$
$P_{ITS-R} = 2.9 \times 10^{-6}$
$\beta_{ITS-R} = 0.11$
observed
imputed
estimate CCA
estimate ITS−R
Model residuals of phenyllactate (PLA)
2
1
0
−1
−2
$n_{CCA} = 798$
$n_{ITS-R} = 1363$
$n_{CCA} = 217$
$n_{ITS-R} = 310$
$n_{CCA} = 8$
$n_{ITS-R} = 11$
major homozygote
heterozygote
minor homozygote
Genotype of SNP rs4784054 (GOT2 (2 separate loci))

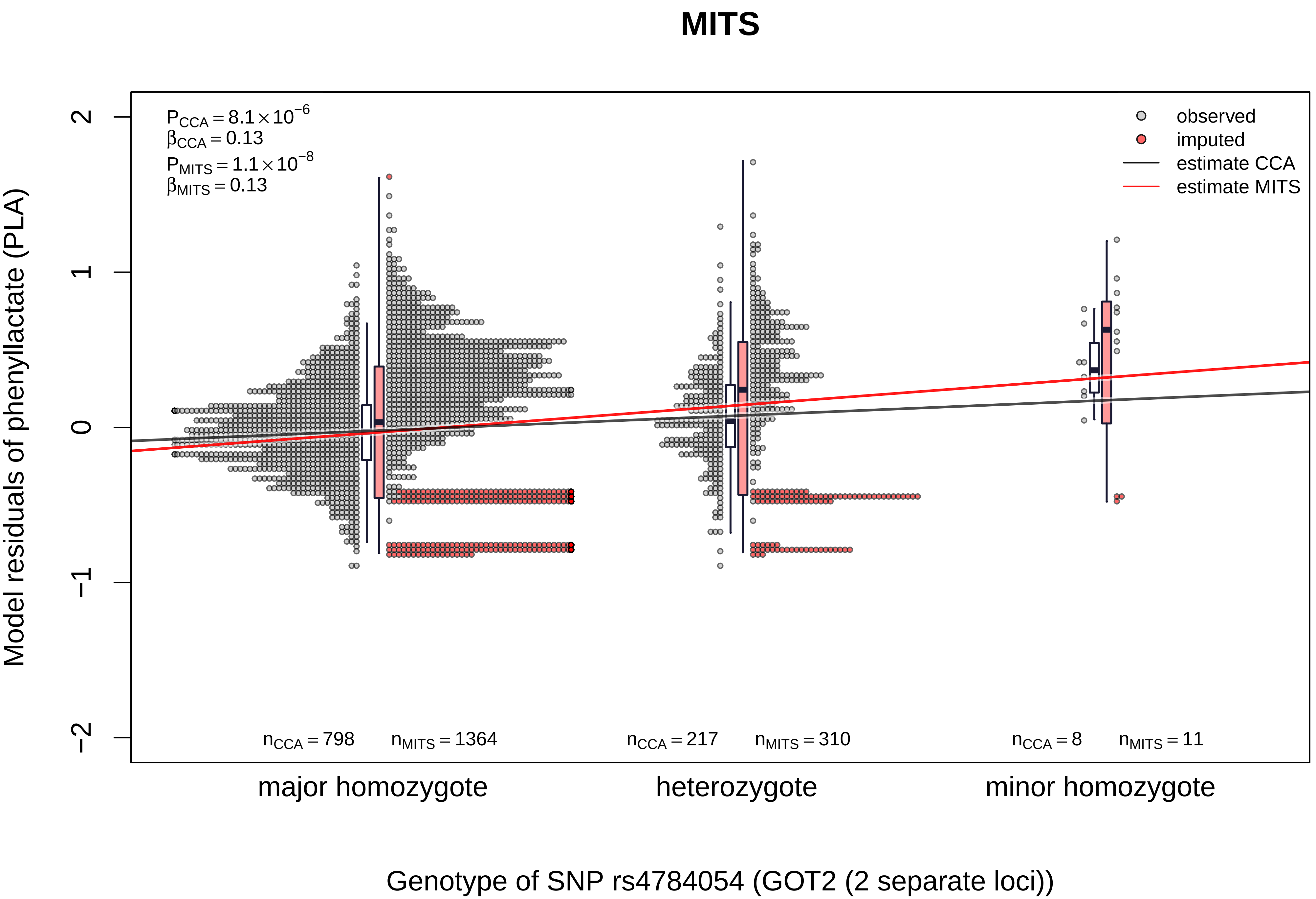
MITS
$P_{CCA} = 8.1 \times 10^{-6}$
$\beta_{CCA} = 0.13$
$P_{MITS} = 1.1 \times 10^{-8}$
$\beta_{MITS} = 0.13$
observed
imputed
estimate CCA
estimate MITS
2
1
0
−1
−2
Model residuals of phenyllactate (PLA)
$n_{CCA} = 798$
$n_{MITS} = 1364$
$n_{CCA} = 217$
$n_{MITS} = 310$
$n_{CCA} = 8$
$n_{MITS} = 11$
major homozygote
heterozygote
minor homozygote
Genotype of SNP rs4784054 (GOT2 (2 separate loci))

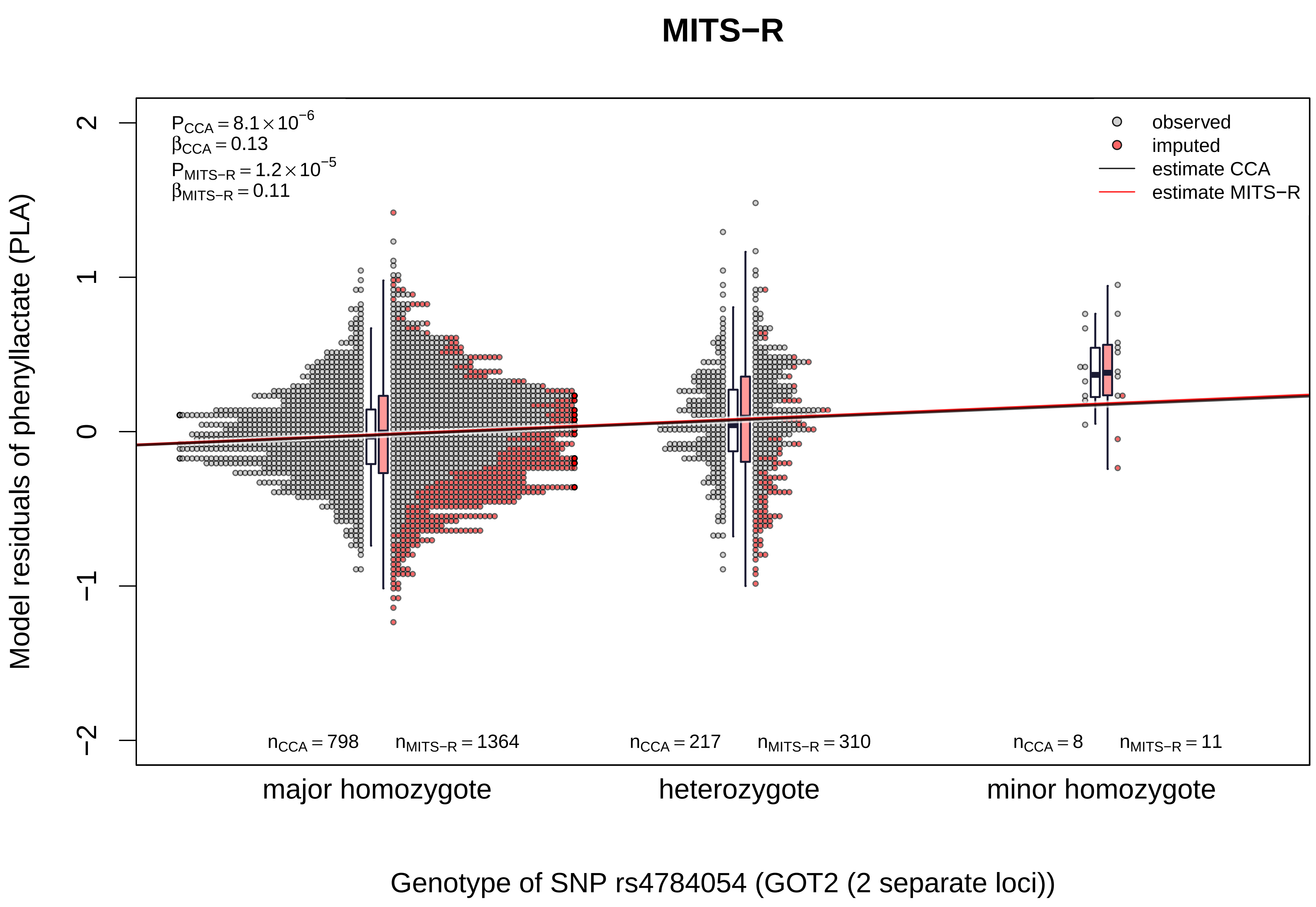
MITS−R
$P_{CCA} = 8.1 \times 10^{-6}$
$\beta_{CCA} = 0.13$
$P_{MITS-R} = 1.2 \times 10^{-5}$
$\beta_{MITS-R} = 0.11$
observed
imputed
estimate CCA
estimate MITS−R
Model residuals of phenyllactate (PLA)
2
1
0
−1
−2
$n_{CCA} = 798$
$n_{MITS-R} = 1364$
$n_{CCA} = 217$
$n_{MITS-R} = 310$
$n_{CCA} = 8$
$n_{MITS-R} = 11$
major homozygote
heterozygote
minor homozygote
Genotype of SNP rs4784054 (GOT2 (2 separate loci))

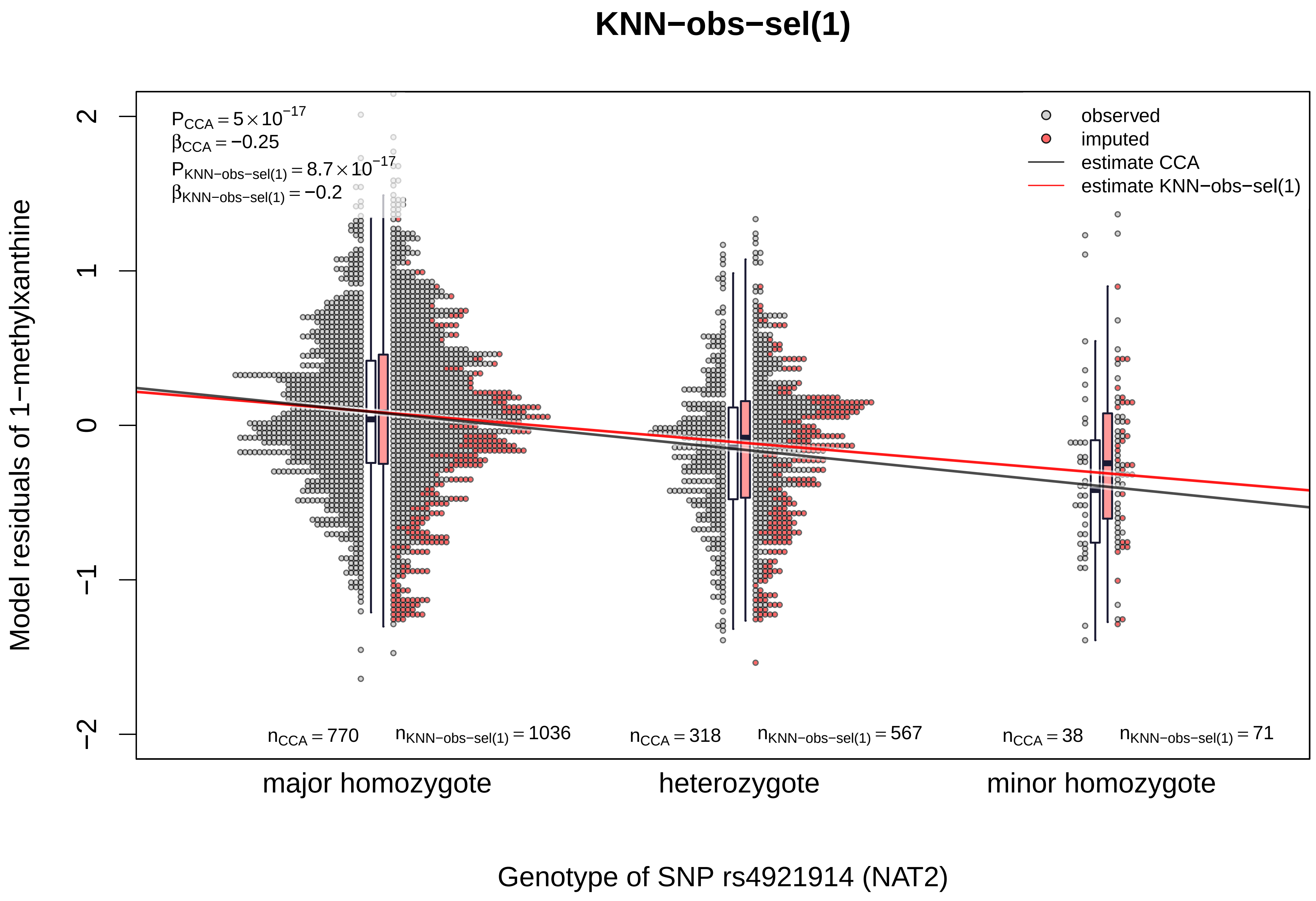

KNN−obs−sel(1)
$P_{CCA} = 5 \times 10^{-17}$
$\beta_{CCA} = -0.25$
$P_{KNN-obs-sel(1)} = 8.7 \times 10^{-17}$
$\beta_{KNN-obs-sel(1)} = -0.2$
observed
imputed
estimate CCA
estimate KNN−obs−sel(1)
Model residuals of 1−methylxanthine
2
1
0
−1
−2
$n_{CCA} = 770$
$n_{KNN-obs-sel(1)} = 1036$
$n_{CCA} = 318$
$n_{KNN-obs-sel(1)} = 567$
$n_{CCA} = 38$
$n_{KNN-obs-sel(1)} = 71$
major homozygote
heterozygote
minor homozygote
Genotype of SNP rs4921914 (NAT2)

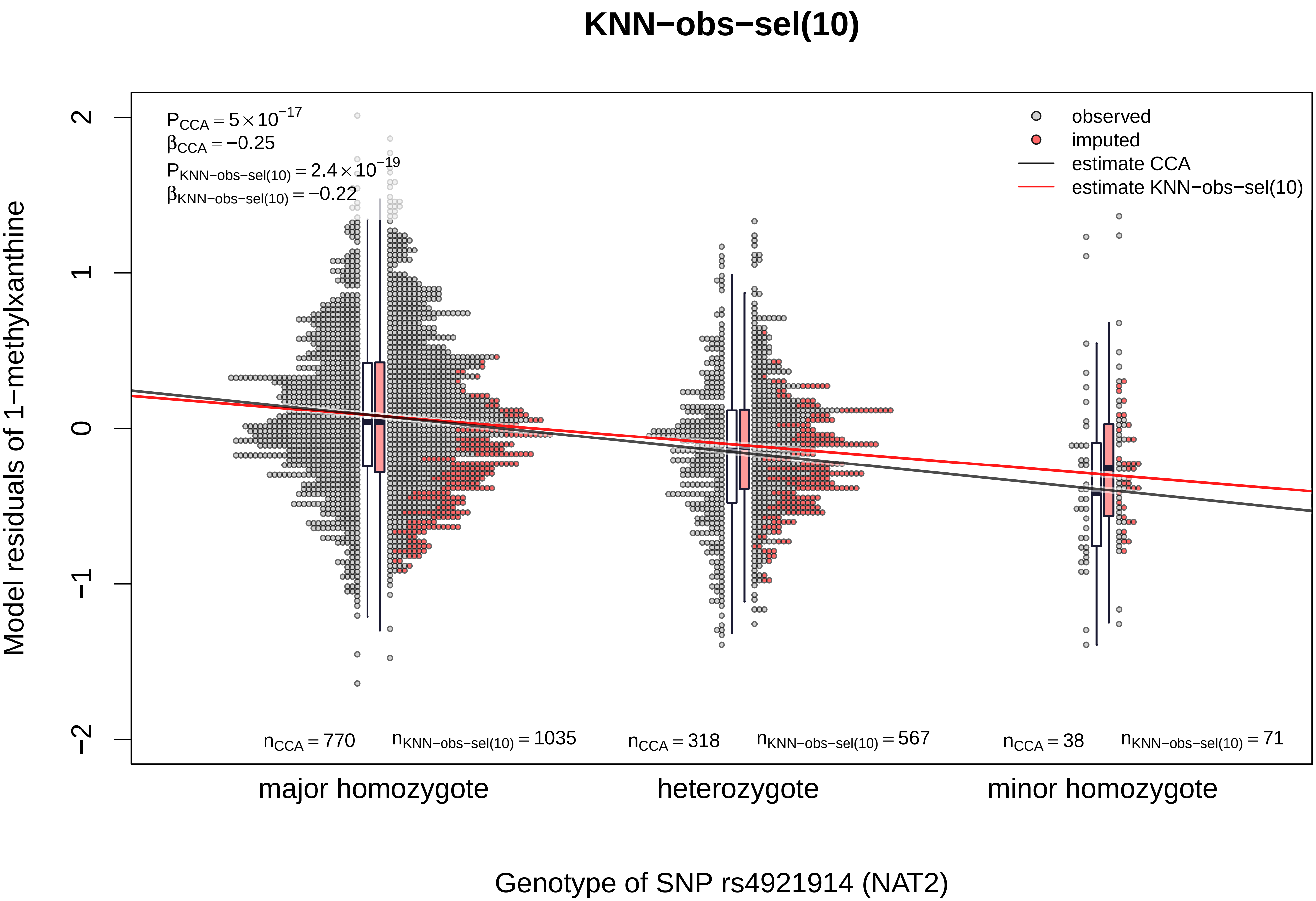

KNN−obs−sel(10)
$P_{CCA} = 5 \times 10^{-17}$
$\beta_{CCA} = -0.25$
$P_{KNN-obs-sel(10)} = 2.4 \times 10^{-19}$
$\beta_{KNN-obs-sel(10)} = -0.22$
observed
imputed
estimate CCA
estimate KNN−obs−sel(10)
Model residuals of 1−methylxanthine
2
1
0
−1
−2
$n_{CCA} = 770$
$n_{KNN-obs-sel(10)} = 1035$
$n_{CCA} = 318$
$n_{KNN-obs-sel(10)} = 567$
$n_{CCA} = 38$
$n_{KNN-obs-sel(10)} = 71$
major homozygote
heterozygote
minor homozygote
Genotype of SNP rs4921914 (NAT2)

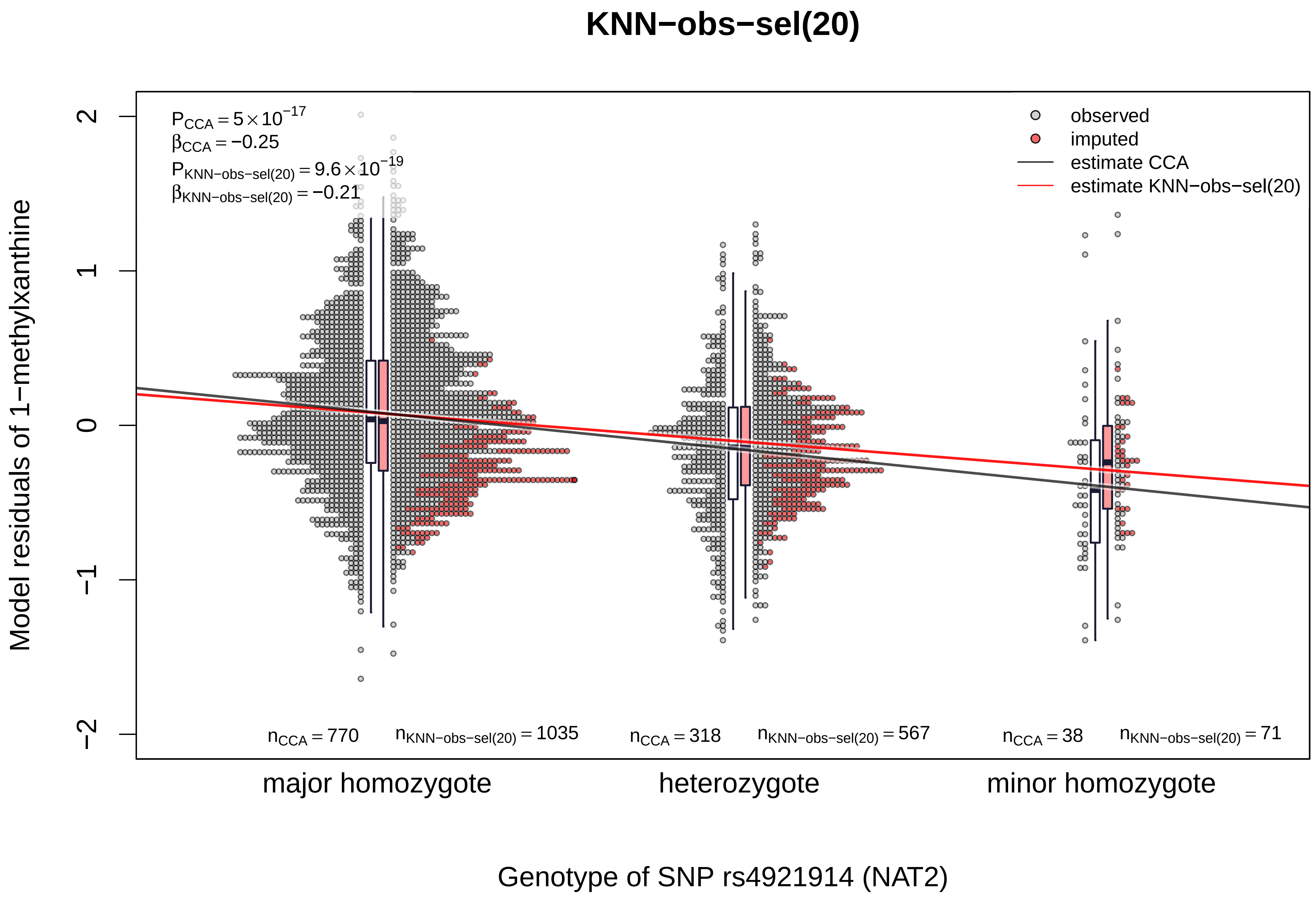

KNN−obs−sel(20)
$P_{CCA} = 5 \times 10^{-17}$
$\beta_{CCA} = -0.25$
$P_{KNN-obs-sel(20)} = 9.6 \times 10^{-19}$
$\beta_{KNN-obs-sel(20)} = -0.21$
observed
imputed
estimate CCA
estimate KNN−obs−sel(20)
Model residuals of 1−methylxanthine
2
1
0
−1
−2
$n_{CCA} = 770$
$n_{KNN-obs-sel(20)} = 1035$
$n_{CCA} = 318$
$n_{KNN-obs-sel(20)} = 567$
$n_{CCA} = 38$
$n_{KNN-obs-sel(20)} = 71$
major homozygote
heterozygote
minor homozygote
Genotype of SNP rs4921914 (NAT2)

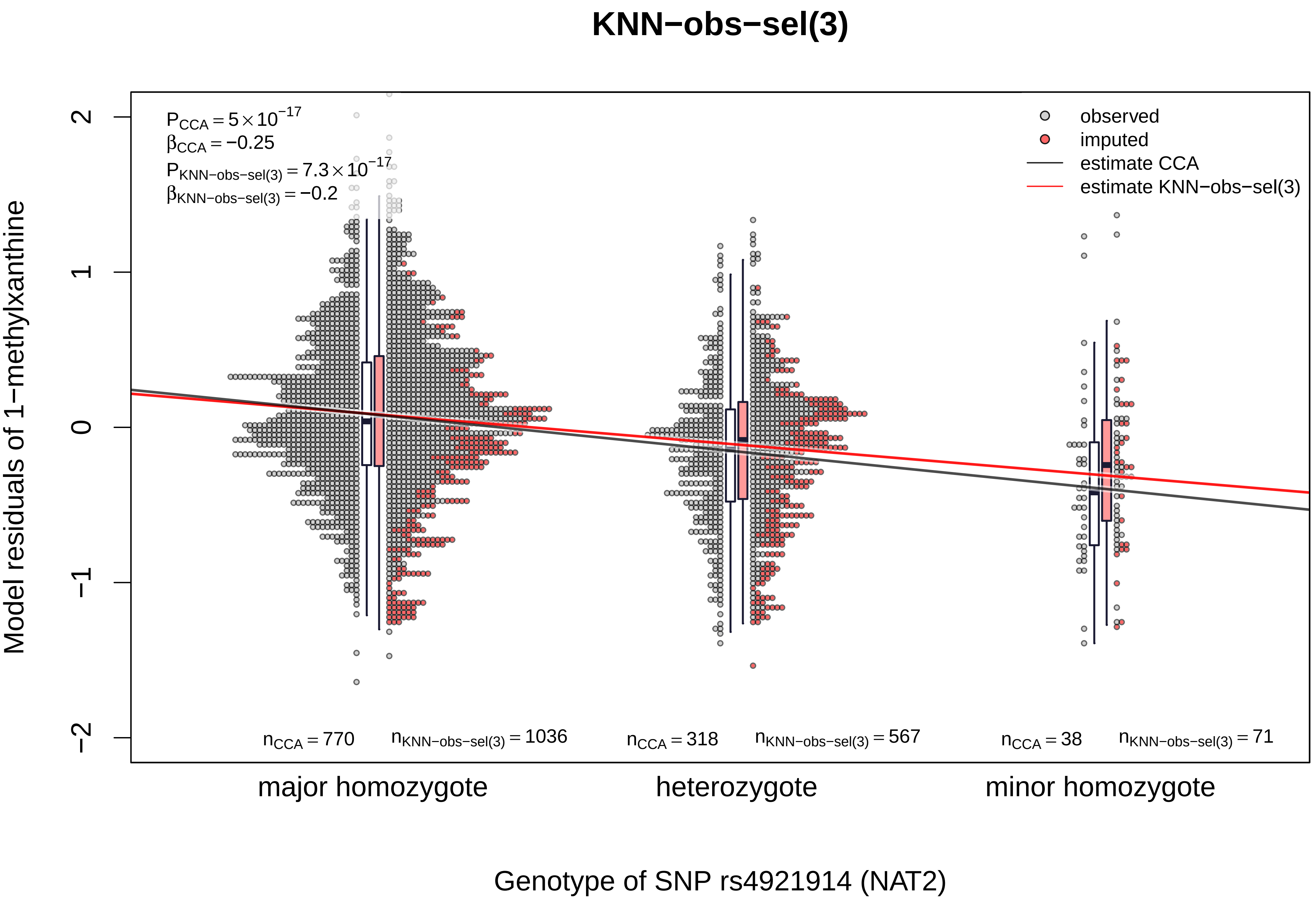

KNN−obs−sel(3)
$P_{CCA} = 5 \times 10^{-17}$
$\beta_{CCA} = -0.25$
$P_{KNN-obs-sel(3)} = 7.3 \times 10^{-17}$
$\beta_{KNN-obs-sel(3)} = -0.2$
observed
imputed
estimate CCA
estimate KNN−obs−sel(3)
Model residuals of 1−methylxanthine
2
1
0
−1
−2
$n_{CCA} = 770$
$n_{KNN-obs-sel(3)} = 1036$
$n_{CCA} = 318$
$n_{KNN-obs-sel(3)} = 567$
$n_{CCA} = 38$
$n_{KNN-obs-sel(3)} = 71$
major homozygote
heterozygote
minor homozygote
Genotype of SNP rs4921914 (NAT2)

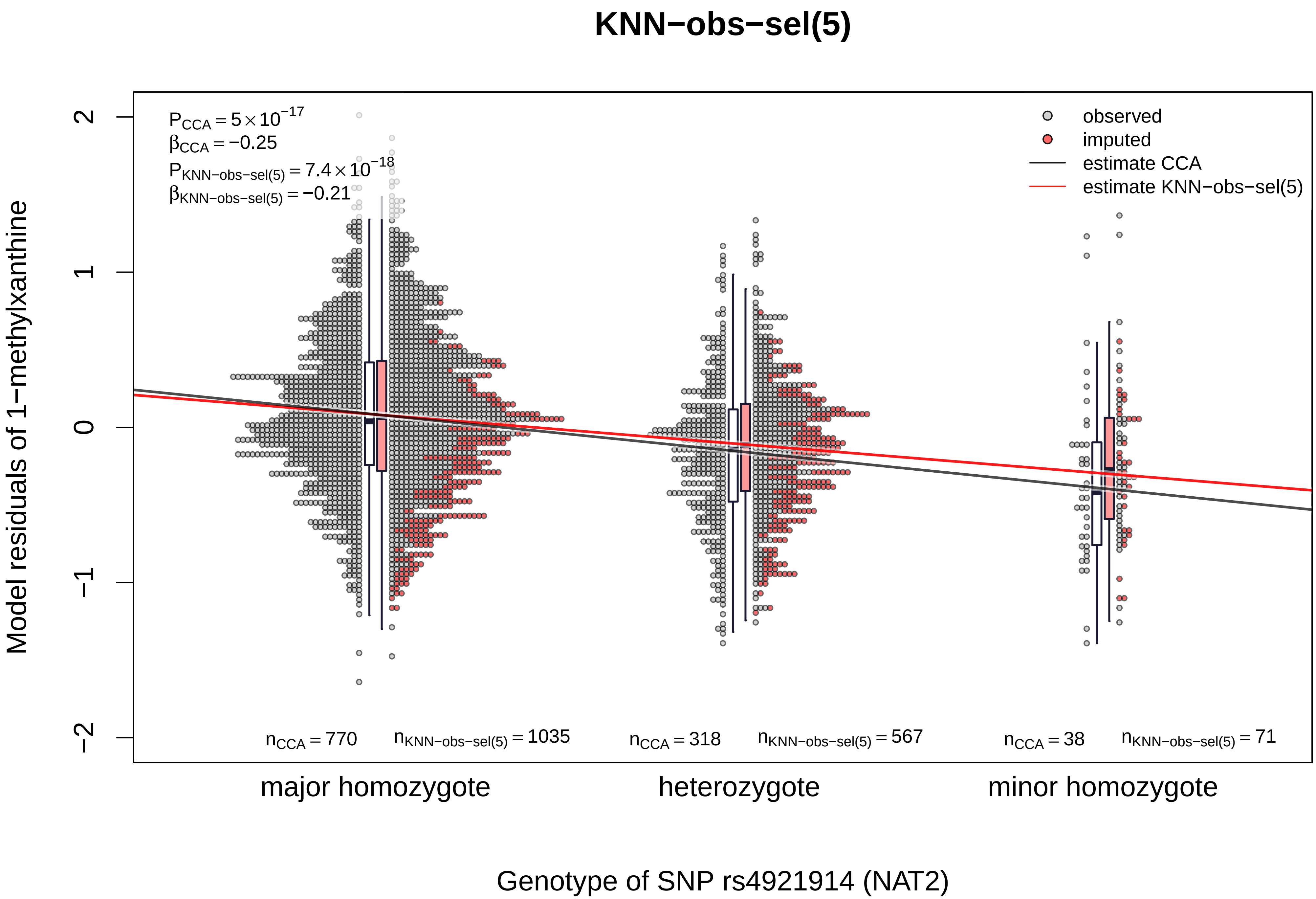
KNN−obs−sel(5)
$P_{CCA} = 5 \times 10^{-17}$
$\beta_{CCA} = -0.25$
$P_{KNN-obs-sel(5)} = 7.4 \times 10^{-18}$
$\beta_{KNN-obs-sel(5)} = -0.21$
observed
imputed
estimate CCA
estimate KNN−obs−sel(5)
Model residuals of 1−methylxanthine
2
1
0
−1
−2
$n_{CCA} = 770$
$n_{KNN-obs-sel(5)} = 1035$
$n_{CCA} = 318$
$n_{KNN-obs-sel(5)} = 567$
$n_{CCA} = 38$
$n_{KNN-obs-sel(5)} = 71$
major homozygote
heterozygote
minor homozygote
Genotype of SNP rs4921914 (NAT2)

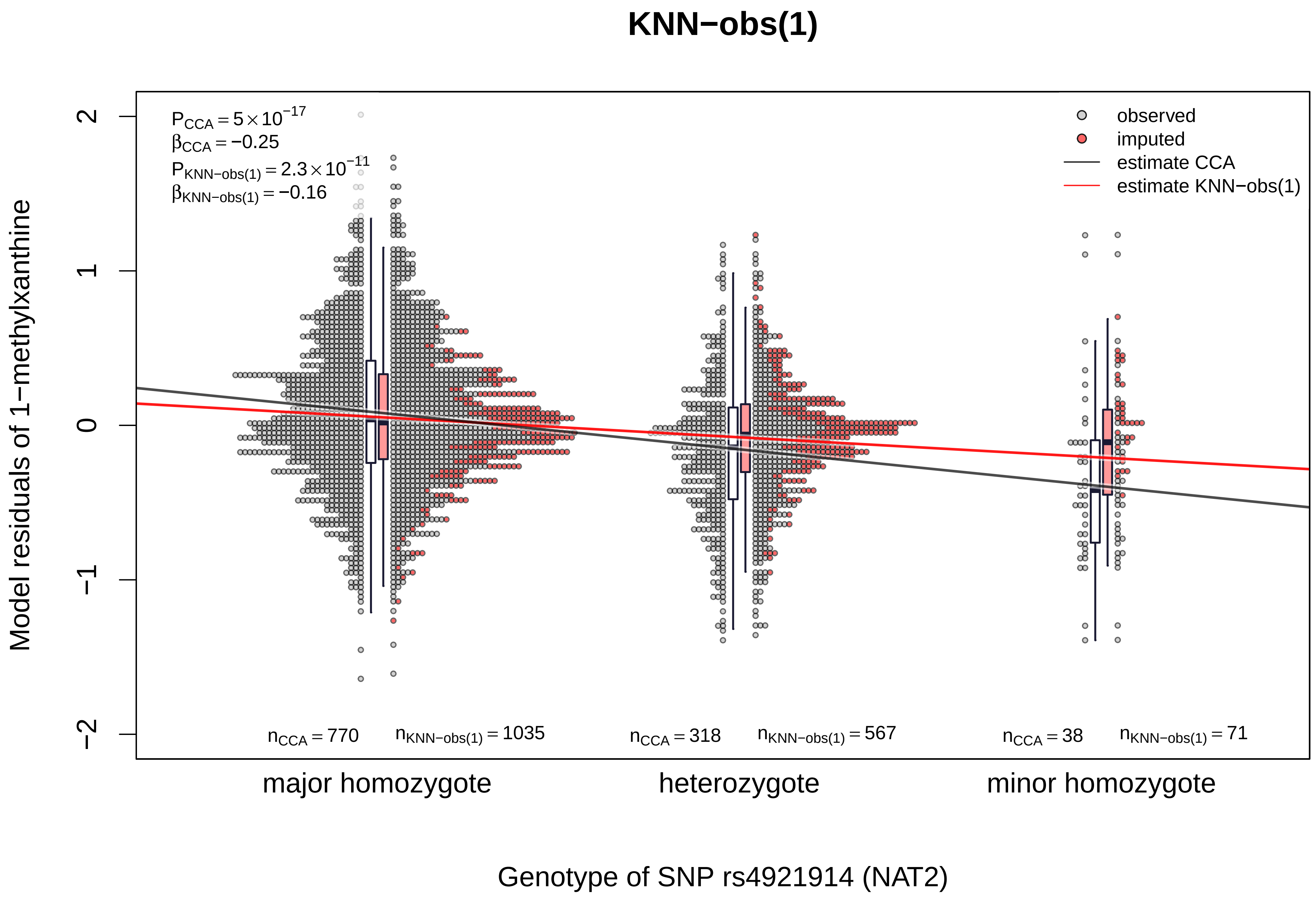

KNN−obs(1)
$P_{CCA} = 5 \times 10^{-17}$
$\beta_{CCA} = -0.25$
$P_{KNN-obs(1)} = 2.3 \times 10^{-11}$
$\beta_{KNN-obs(1)} = -0.16$
observed
imputed
estimate CCA
estimate KNN−obs(1)
Model residuals of 1−methylxanthine
2
1
0
−1
−2
$n_{CCA} = 770$
$n_{KNN-obs(1)} = 1035$
$n_{CCA} = 318$
$n_{KNN-obs(1)} = 567$
$n_{CCA} = 38$
$n_{KNN-obs(1)} = 71$
major homozygote
heterozygote
minor homozygote
Genotype of SNP rs4921914 (NAT2)

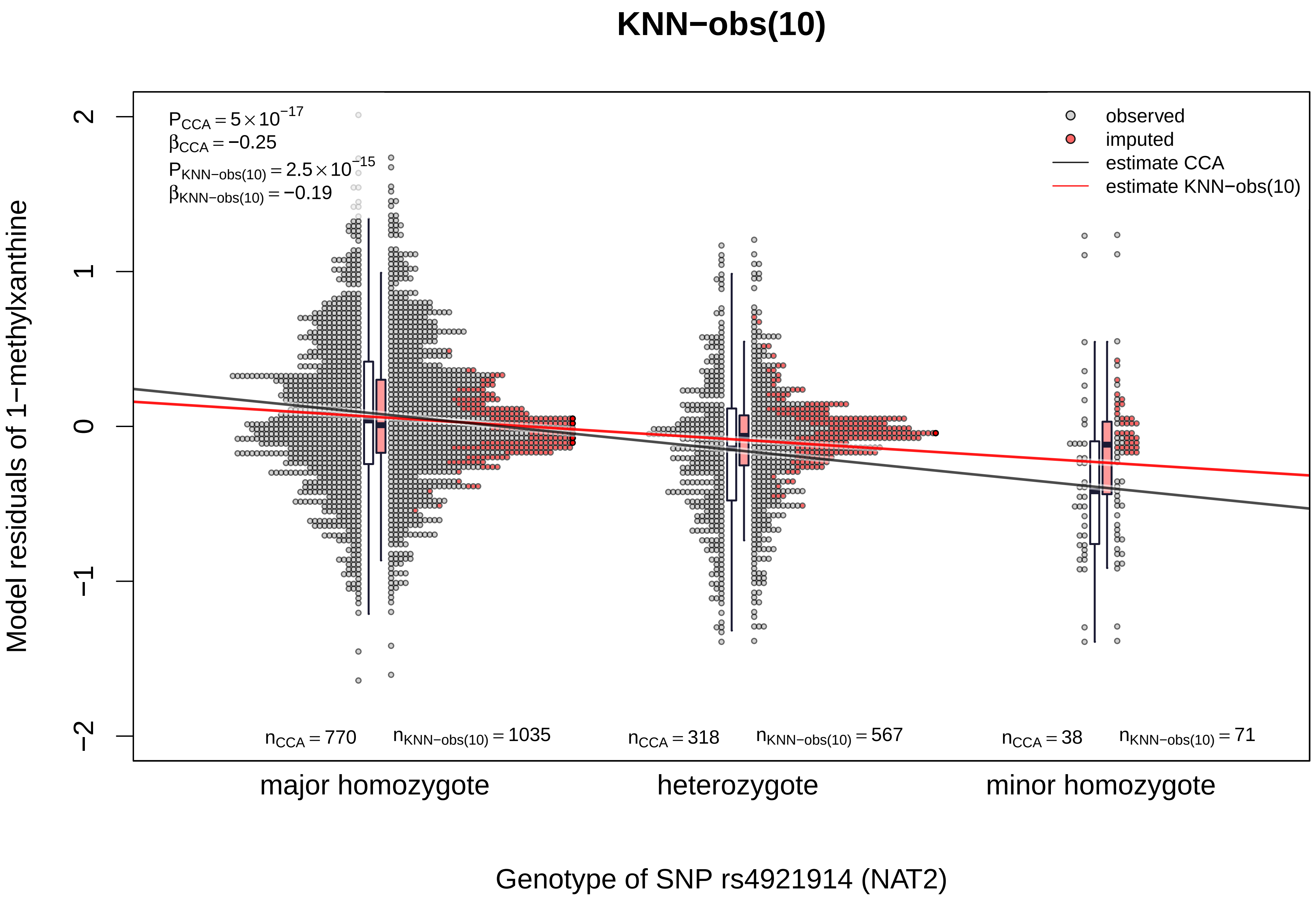
KNN−obs(10)
$P_{CCA} = 5 \times 10^{-17}$
$\beta_{CCA} = -0.25$
$P_{KNN-obs(10)} = 2.5 \times 10^{-15}$
$\beta_{KNN-obs(10)} = -0.19$
observed
imputed
estimate CCA
estimate KNN−obs(10)
Model residuals of 1−methylxanthine
2
1
0
−1
−2
$n_{CCA} = 770$
$n_{KNN-obs(10)} = 1035$
$n_{CCA} = 318$
$n_{KNN-obs(10)} = 567$
$n_{CCA} = 38$
$n_{KNN-obs(10)} = 71$
major homozygote
heterozygote
minor homozygote
Genotype of SNP rs4921914 (NAT2)

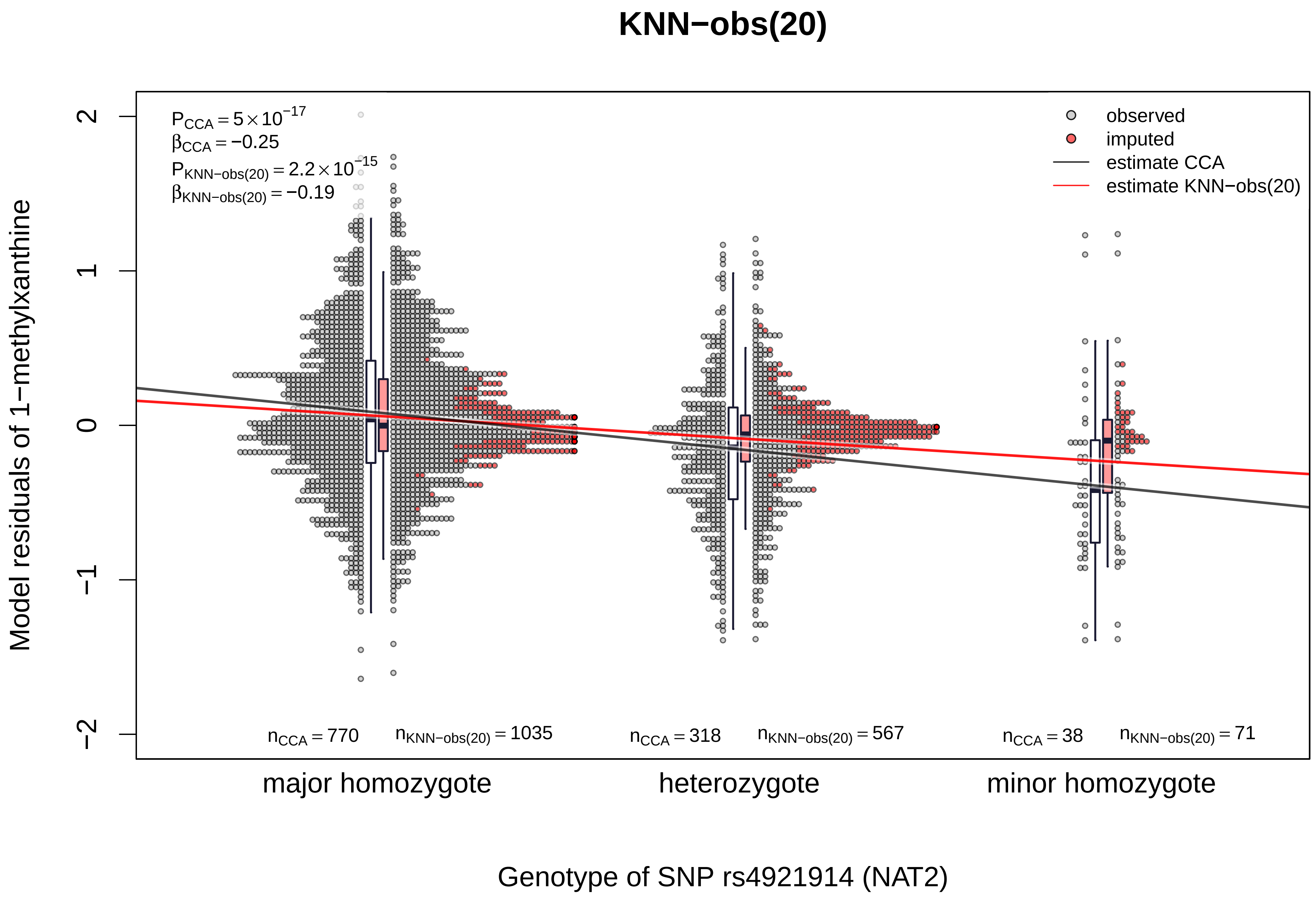
KNN−obs(20)
$P_{CCA} = 5 \times 10^{-17}$
$\beta_{CCA} = -0.25$
$P_{KNN-obs(20)} = 2.2 \times 10^{-15}$
$\beta_{KNN-obs(20)} = -0.19$
observed
imputed
estimate CCA
estimate KNN−obs(20)
Model residuals of 1−methylxanthine
2
1
0
−1
−2
$n_{CCA} = 770$
$n_{KNN-obs(20)} = 1035$
$n_{CCA} = 318$
$n_{KNN-obs(20)} = 567$
$n_{CCA} = 38$
$n_{KNN-obs(20)} = 71$
major homozygote
heterozygote
minor homozygote
Genotype of SNP rs4921914 (NAT2)

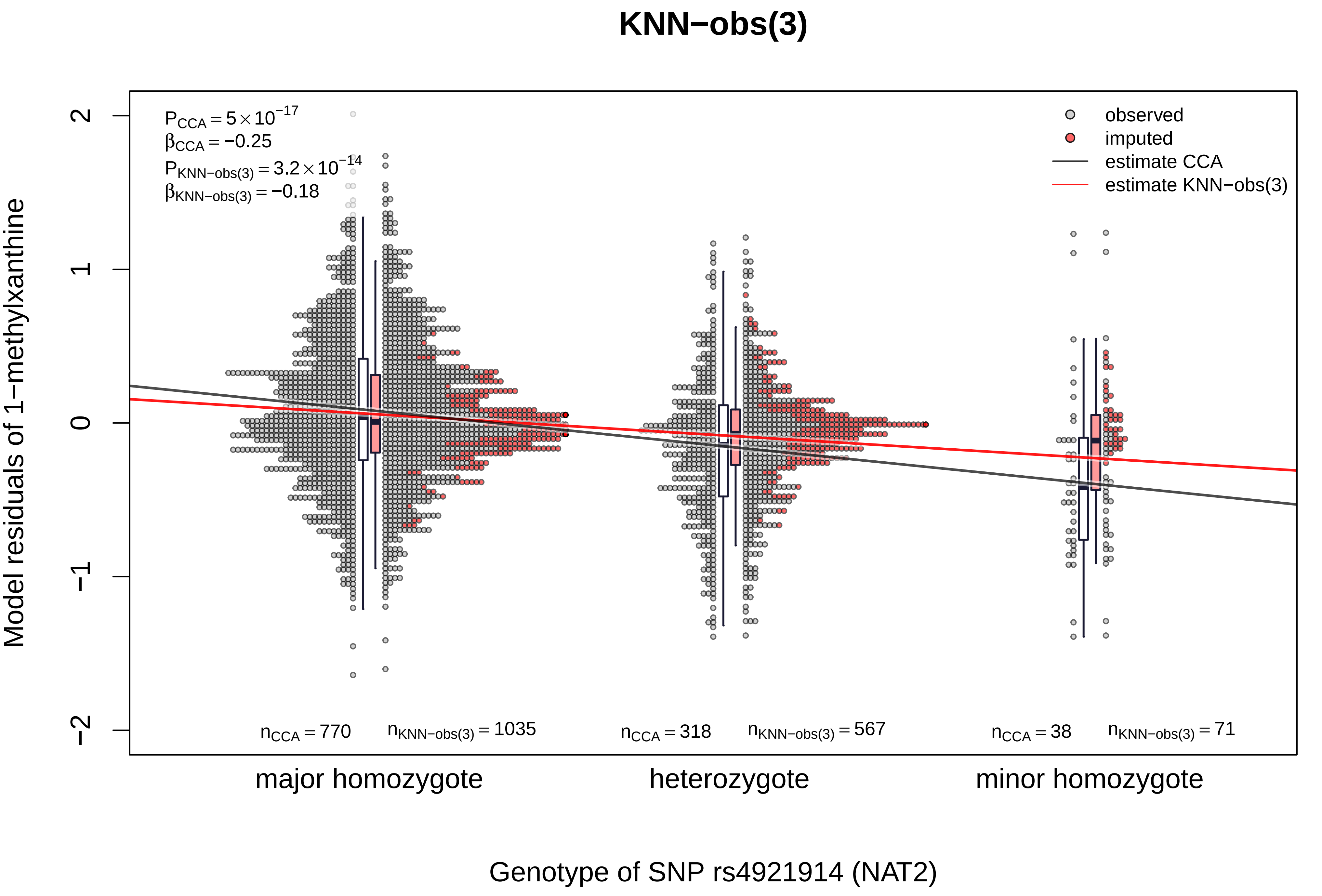

KNN−obs(3)
$P_{CCA} = 5 \times 10^{-17}$
$\beta_{CCA} = -0.25$
$P_{KNN-obs(3)} = 3.2 \times 10^{-14}$
$\beta_{KNN-obs(3)} = -0.18$
observed
imputed
estimate CCA
estimate KNN−obs(3)
2
1
0
−1
−2
Model residuals of 1−methylxanthine
$n_{CCA} = 770$
$n_{KNN-obs(3)} = 1035$
$n_{CCA} = 318$
$n_{KNN-obs(3)} = 567$
$n_{CCA} = 38$
$n_{KNN-obs(3)} = 71$
major homozygote
heterozygote
minor homozygote
Genotype of SNP rs4921914 (NAT2)

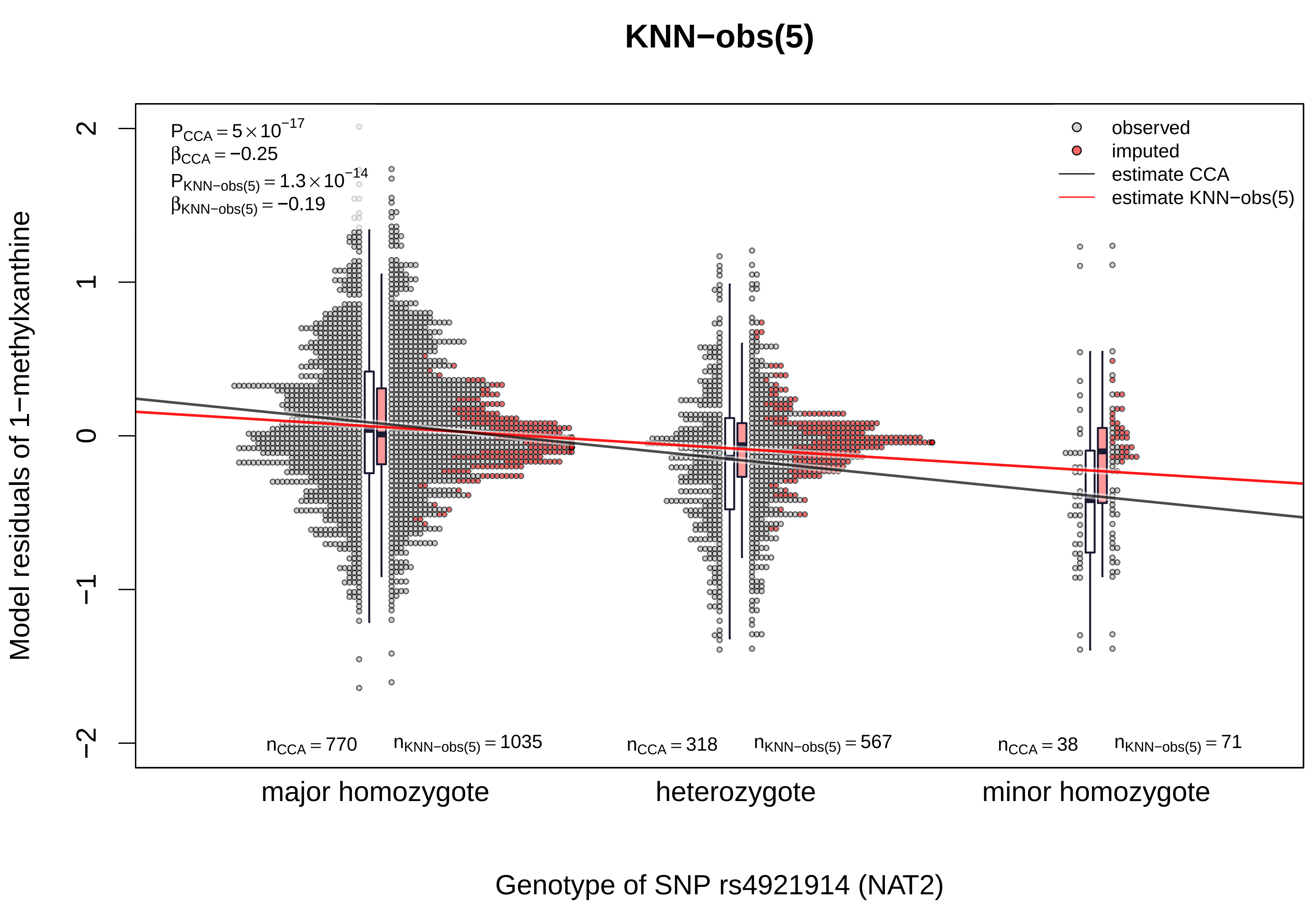

KNN−obs(5)
$P_{CCA} = 5 \times 10^{-17}$
$\beta_{CCA} = -0.25$
$P_{KNN-obs(5)} = 1.3 \times 10^{-14}$
$\beta_{KNN-obs(5)} = -0.19$
observed
imputed
estimate CCA
estimate KNN−obs(5)
2
1
0
−1
−2
Model residuals of 1−methylxanthine
$n_{CCA} = 770$
$n_{KNN-obs(5)} = 1035$
$n_{CCA} = 318$
$n_{KNN-obs(5)} = 567$
$n_{CCA} = 38$
$n_{KNN-obs(5)} = 71$
major homozygote
heterozygote
minor homozygote
Genotype of SNP rs4921914 (NAT2)

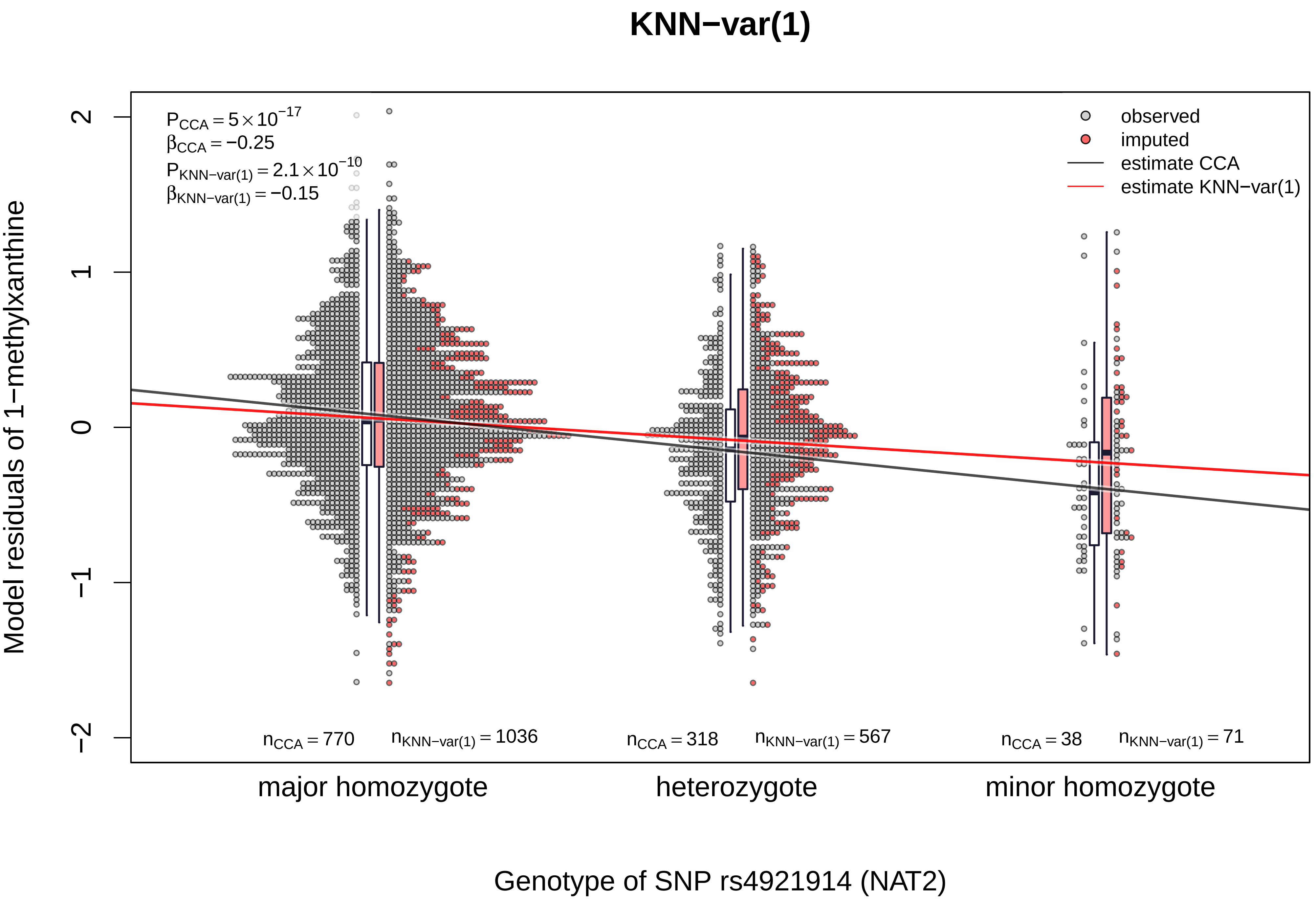

KNN−var(1)
$P_{CCA} = 5 \times 10^{-17}$
$\beta_{CCA} = -0.25$
$P_{KNN-var(1)} = 2.1 \times 10^{-10}$
$\beta_{KNN-var(1)} = -0.15$
observed
imputed
estimate CCA
estimate KNN−var(1)
Model residuals of 1−methylxanthine
2
1
0
−1
−2
$n_{CCA} = 770$
$n_{KNN-var(1)} = 1036$
$n_{CCA} = 318$
$n_{KNN-var(1)} = 567$
$n_{CCA} = 38$
$n_{KNN-var(1)} = 71$
major homozygote
heterozygote
minor homozygote
Genotype of SNP rs4921914 (NAT2)

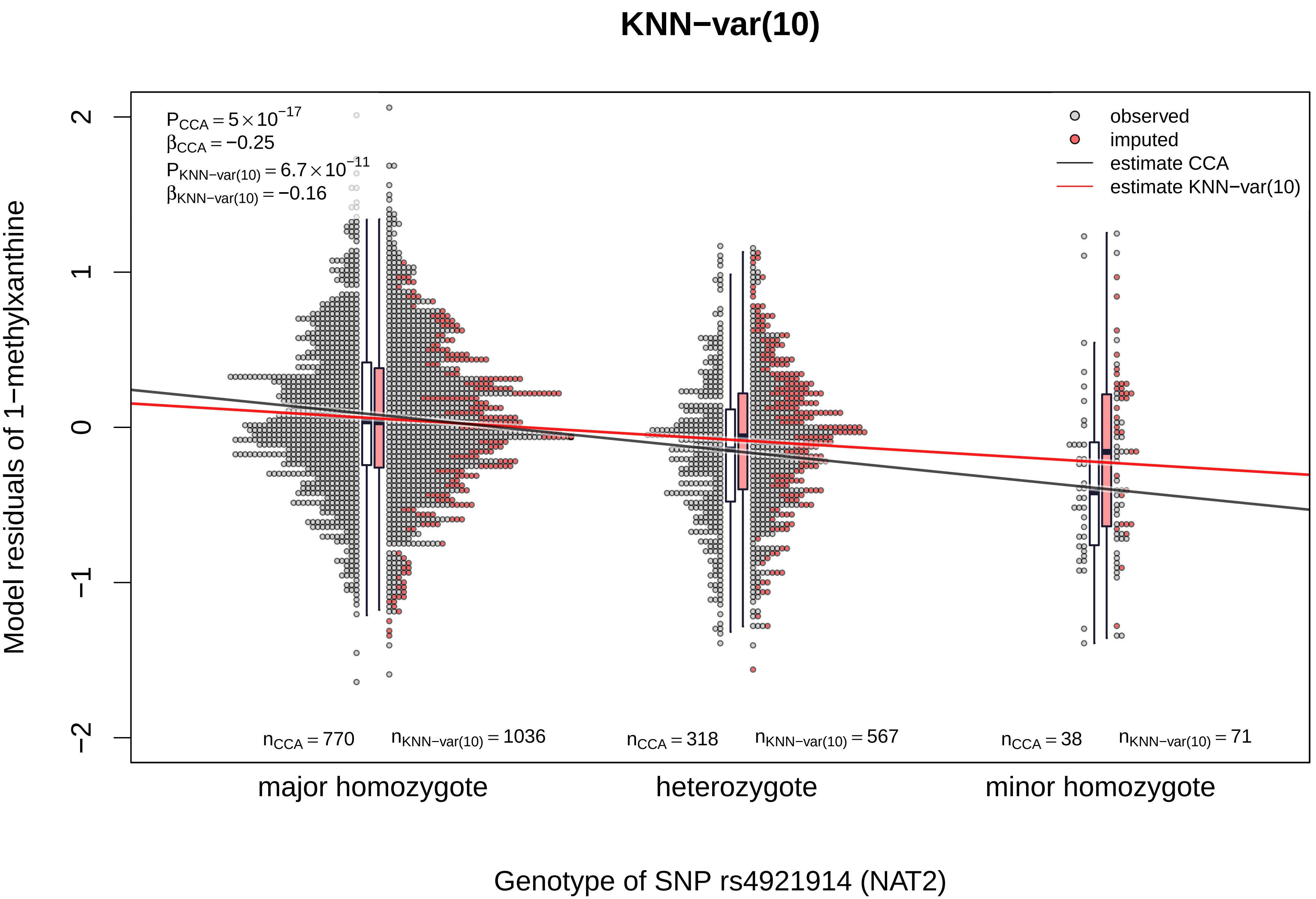

KNN−var(10)
$P_{CCA} = 5 \times 10^{-17}$
$\beta_{CCA} = -0.25$
$P_{KNN-var(10)} = 6.7 \times 10^{-11}$
$\beta_{KNN-var(10)} = -0.16$
observed
imputed
estimate CCA
estimate KNN−var(10)
Model residuals of 1−methylxanthine
2
1
0
−1
−2
$n_{CCA} = 770$
$n_{KNN-var(10)} = 1036$
$n_{CCA} = 318$
$n_{KNN-var(10)} = 567$
$n_{CCA} = 38$
$n_{KNN-var(10)} = 71$
major homozygote
heterozygote
minor homozygote
Genotype of SNP rs4921914 (NAT2)

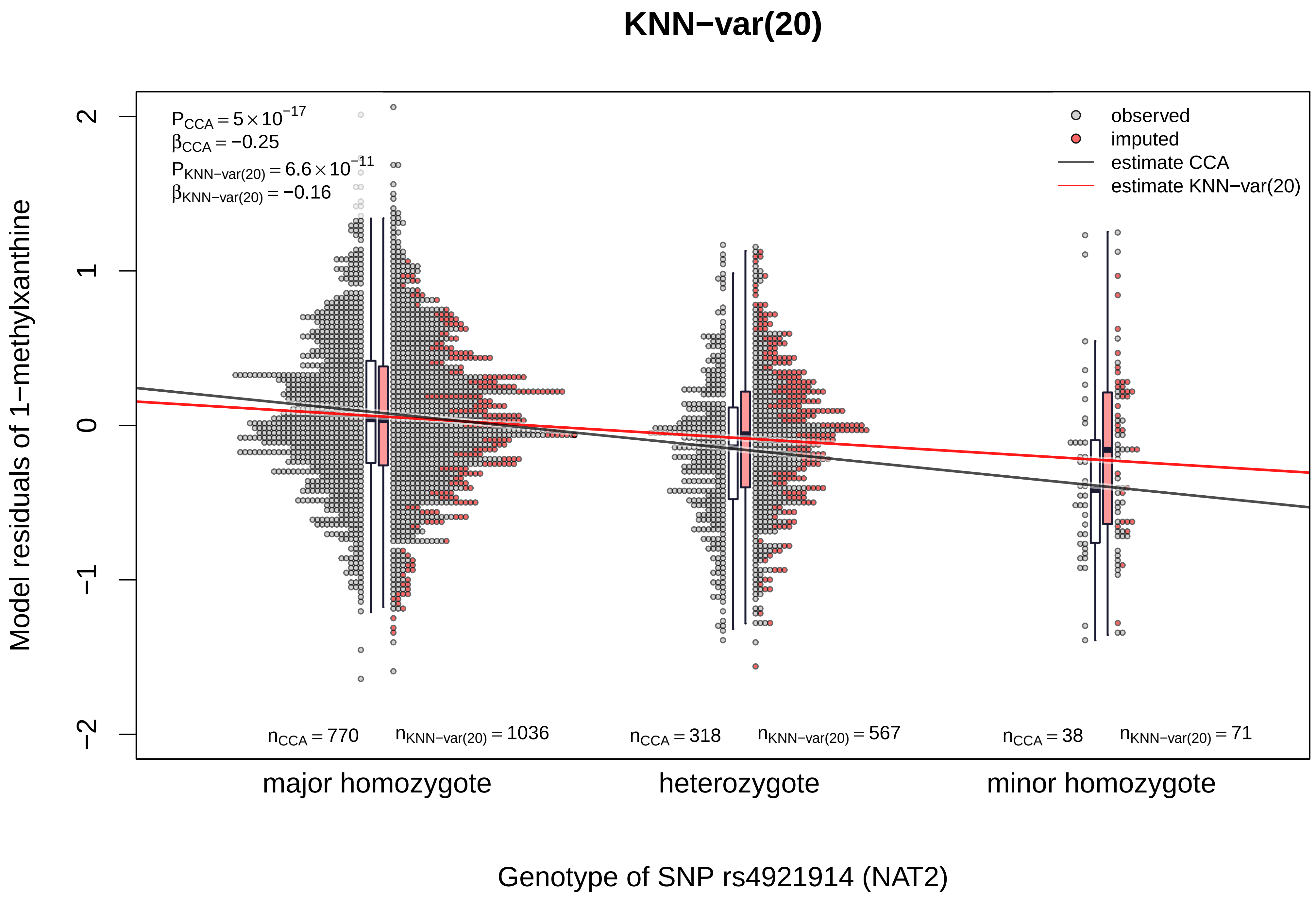

KNN−var(20)
$P_{CCA} = 5 \times 10^{-17}$
$\beta_{CCA} = -0.25$
$P_{KNN-var(20)} = 6.6 \times 10^{-11}$
$\beta_{KNN-var(20)} = -0.16$
observed
imputed
estimate CCA
estimate KNN−var(20)
Model residuals of 1−methylxanthine
2
1
0
−1
−2
$n_{CCA} = 770$
$n_{KNN-var(20)} = 1036$
$n_{CCA} = 318$
$n_{KNN-var(20)} = 567$
$n_{CCA} = 38$
$n_{KNN-var(20)} = 71$
major homozygote
heterozygote
minor homozygote
Genotype of SNP rs4921914 (NAT2)

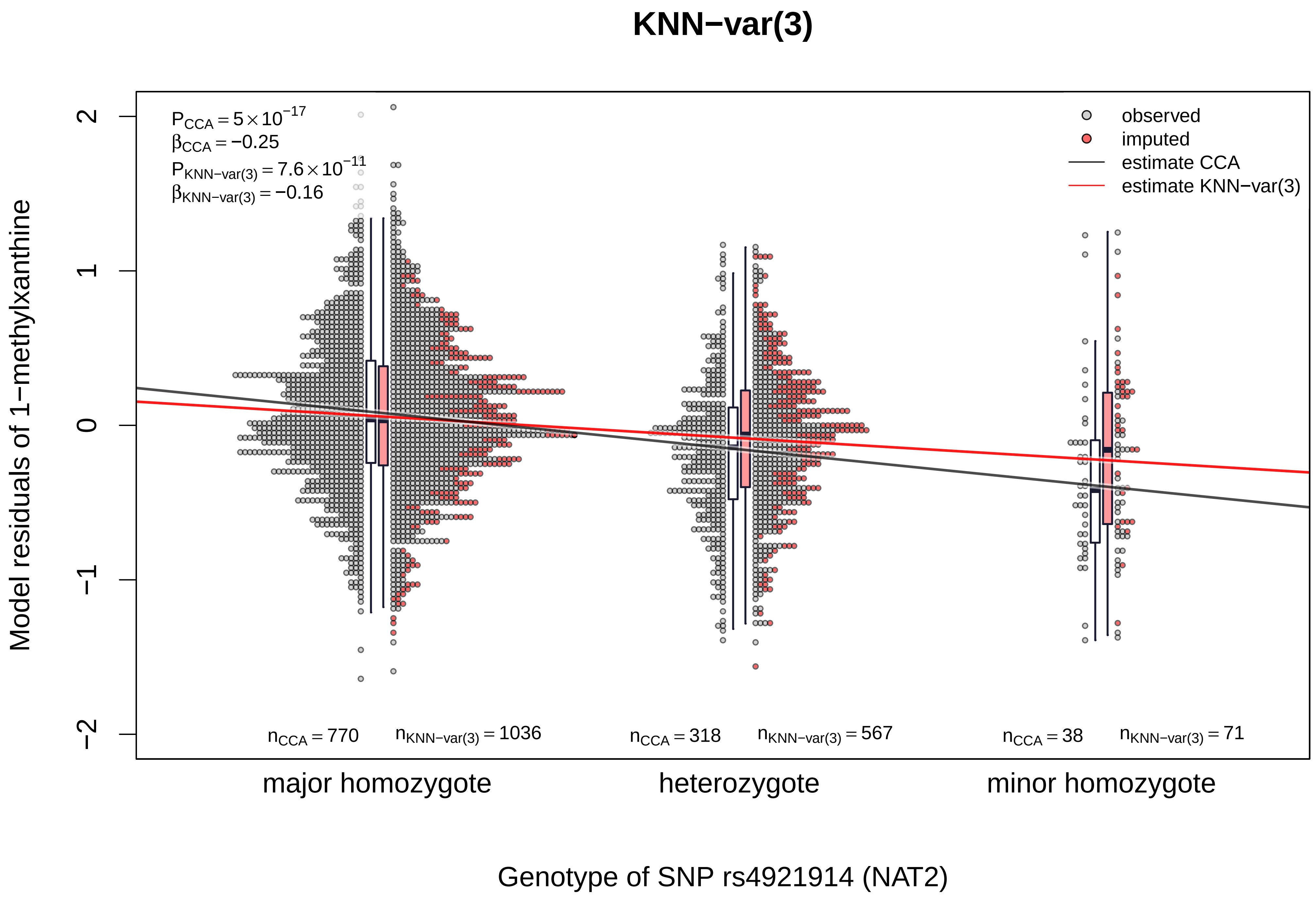

KNN−var(3)
$P_{CCA} = 5 \times 10^{-17}$
$\beta_{CCA} = -0.25$
$P_{KNN-var(3)} = 7.6 \times 10^{-11}$
$\beta_{KNN-var(3)} = -0.16$
observed
imputed
estimate CCA
estimate KNN−var(3)
Model residuals of 1−methylxanthine
2
1
0
−1
−2
$n_{CCA} = 770$
$n_{KNN-var(3)} = 1036$
$n_{CCA} = 318$
$n_{KNN-var(3)} = 567$
$n_{CCA} = 38$
$n_{KNN-var(3)} = 71$
major homozygote
heterozygote
minor homozygote
Genotype of SNP rs4921914 (NAT2)

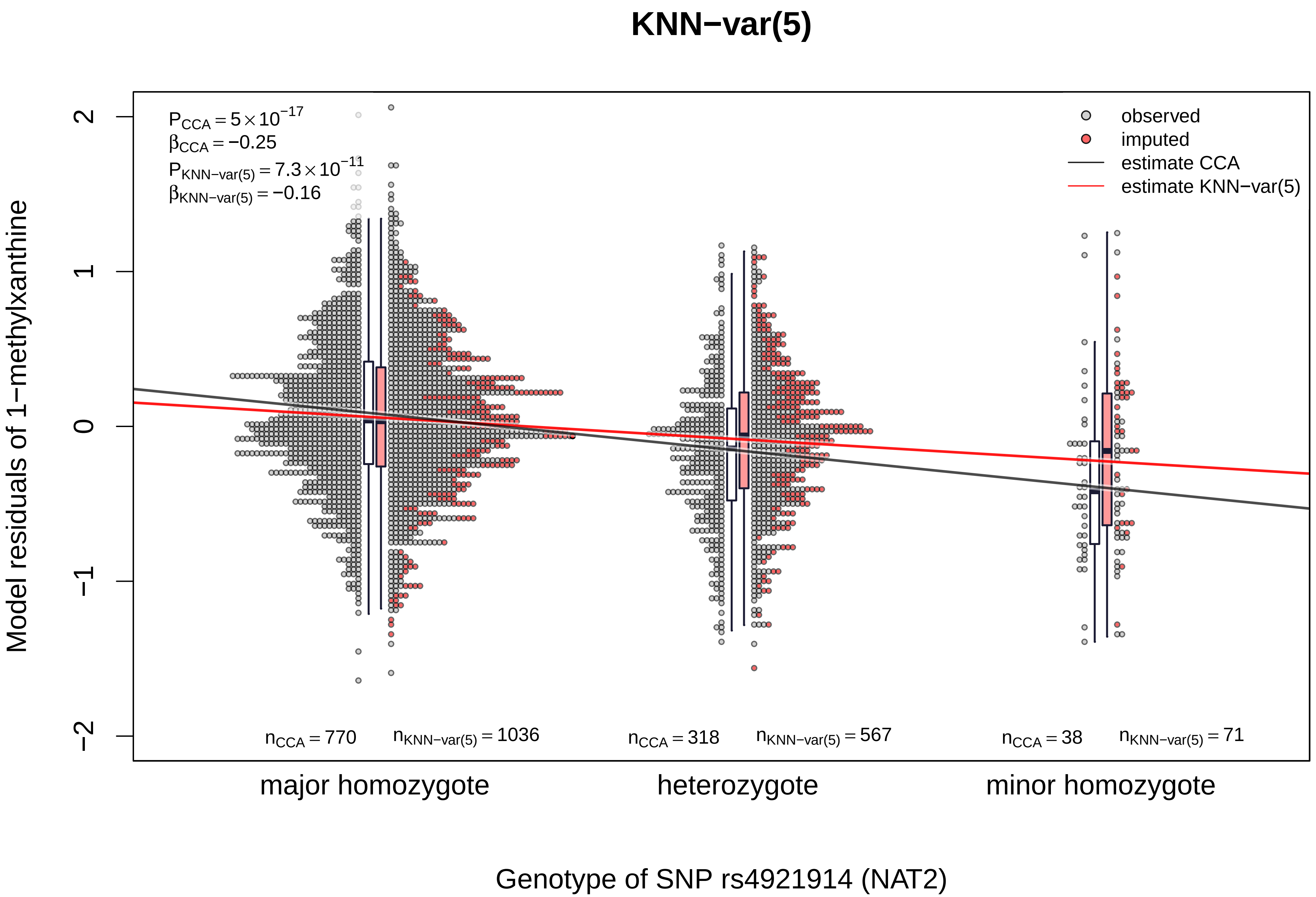
KNN−var(5)
$P_{CCA} = 5 \times 10^{-17}$
$\beta_{CCA} = -0.25$
$P_{KNN-var(5)} = 7.3 \times 10^{-11}$
$\beta_{KNN-var(5)} = -0.16$
observed
imputed
estimate CCA
estimate KNN−var(5)
Model residuals of 1−methylxanthine
2
1
0
−1
−2
$n_{CCA} = 770$
$n_{KNN-var(5)} = 1036$
$n_{CCA} = 318$
$n_{KNN-var(5)} = 567$
$n_{CCA} = 38$
$n_{KNN-var(5)} = 71$
major homozygote
heterozygote
minor homozygote
Genotype of SNP rs4921914 (NAT2)

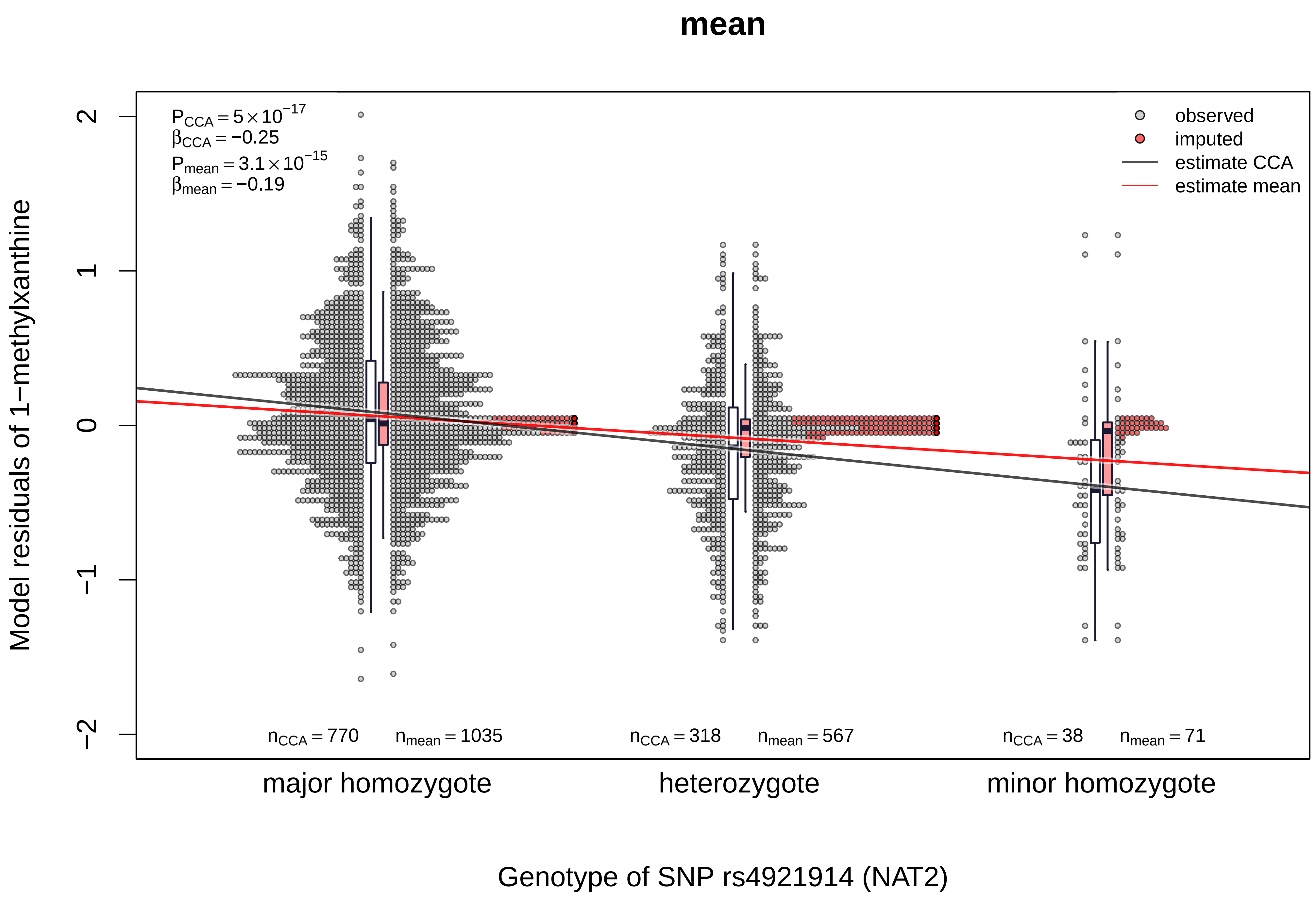

mean
$P_{CCA} = 5 \times 10^{-17}$
$\beta_{CCA} = -0.25$
$P_{mean} = 3.1 \times 10^{-15}$
$\beta_{mean} = -0.19$
observed
imputed
estimate CCA
estimate mean
Model residuals of 1−methylxanthine
2
1
0
−1
−2
$n_{CCA} = 770$
$n_{mean} = 1035$
$n_{CCA} = 318$
$n_{mean} = 567$
$n_{CCA} = 38$
$n_{mean} = 71$
major homozygote
heterozygote
minor homozygote
Genotype of SNP rs4921914 (NAT2)

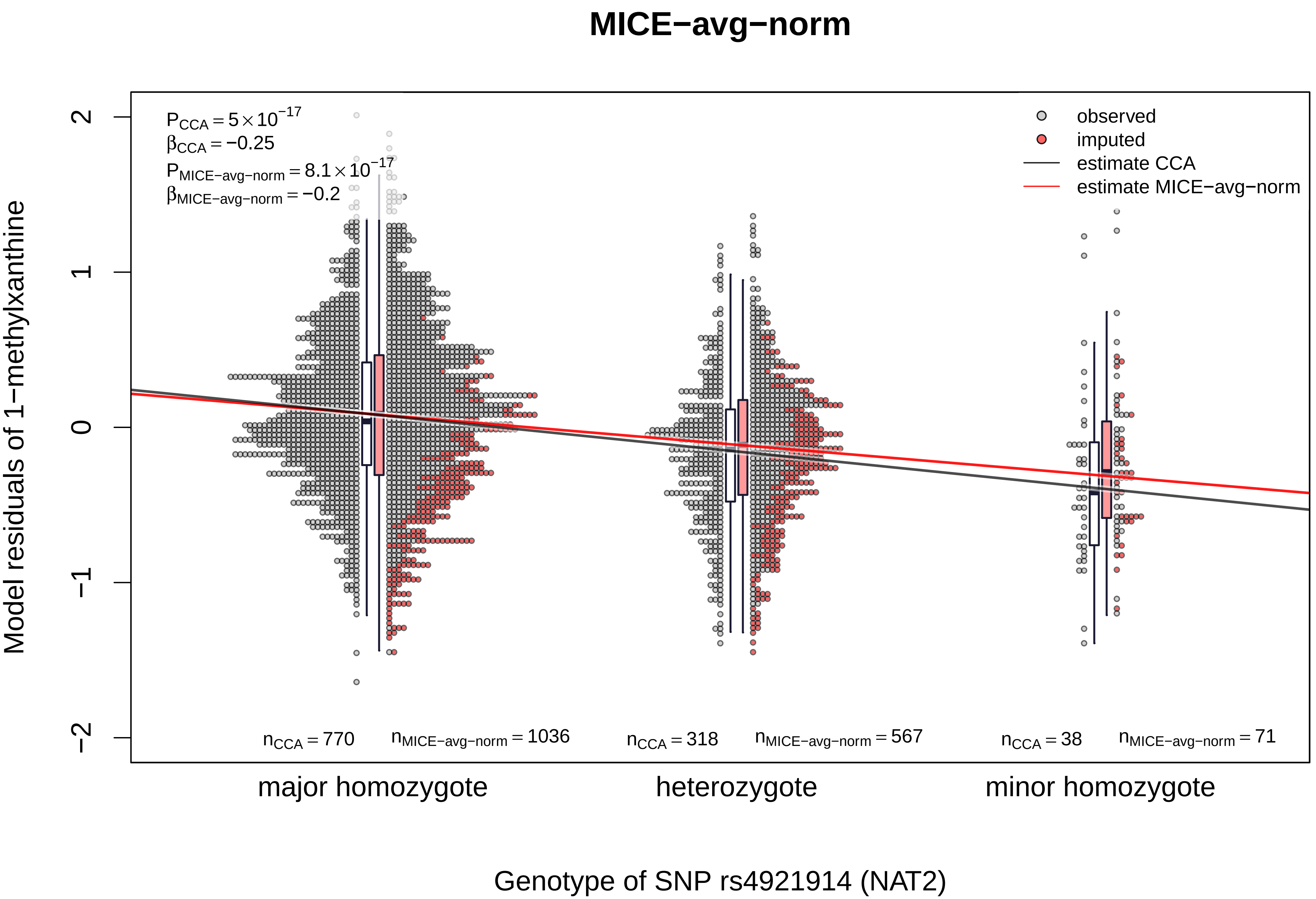

MICE−avg−norm
$P_{CCA} = 5 \times 10^{-17}$
$\beta_{CCA} = -0.25$
$P_{MICE-avg-norm} = 8.1 \times 10^{-17}$
$\beta_{MICE-avg-norm} = -0.2$
observed
imputed
estimate CCA
estimate MICE−avg−norm
Model residuals of 1−methylxanthine
2
1
0
−1
−2
$n_{CCA} = 770$
$n_{MICE-avg-norm} = 1036$
$n_{CCA} = 318$
$n_{MICE-avg-norm} = 567$
$n_{CCA} = 38$
$n_{MICE-avg-norm} = 71$
major homozygote
heterozygote
minor homozygote
Genotype of SNP rs4921914 (NAT2)

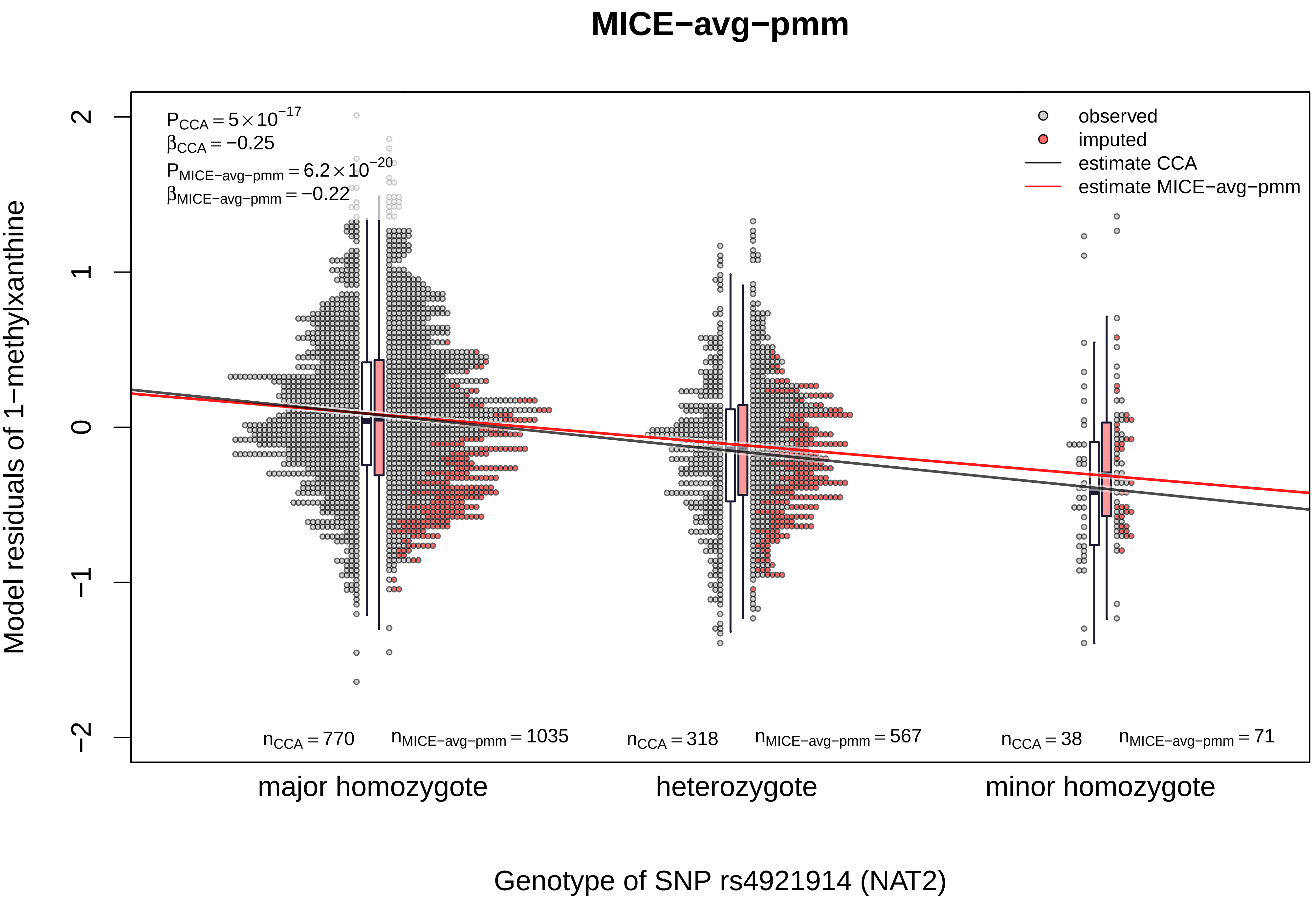

MICE−avg−pmm
$P_{CCA} = 5 \times 10^{-17}$
$\beta_{CCA} = -0.25$
$P_{MICE-avg-pmm} = 6.2 \times 10^{-20}$
$\beta_{MICE-avg-pmm} = -0.22$
observed
imputed
estimate CCA
estimate MICE−avg−pmm
$n_{CCA} = 770$
$n_{MICE-avg-pmm} = 1035$
$n_{CCA} = 318$
$n_{MICE-avg-pmm} = 567$
$n_{CCA} = 38$
$n_{MICE-avg-pmm} = 71$
Model residuals of 1−methylxanthine
2
1
0
−1
−2
major homozygote
heterozygote
minor homozygote
Genotype of SNP rs4921914 (NAT2)

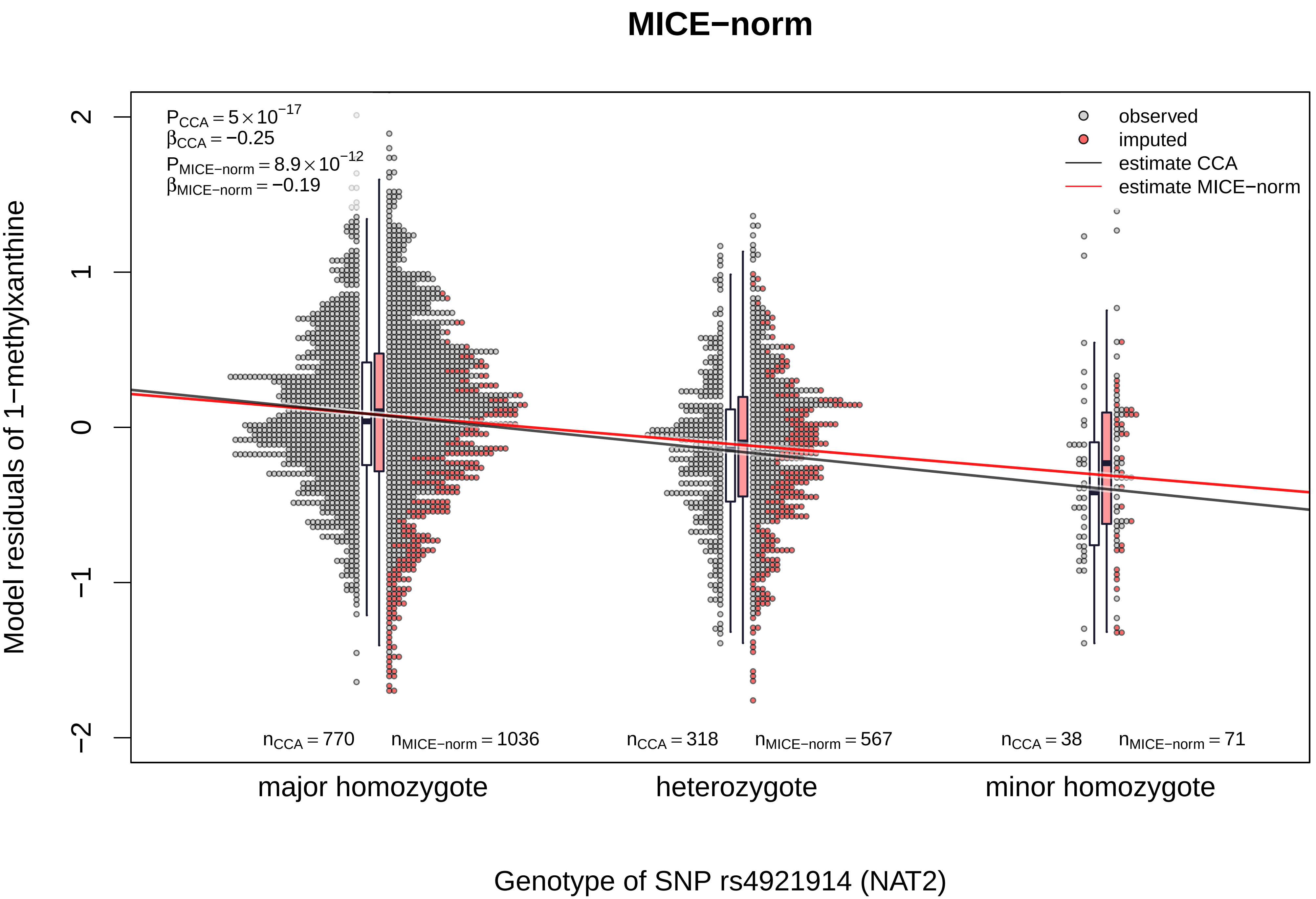

MICE−norm
$P_{CCA} = 5 \times 10^{-17}$
$\beta_{CCA} = -0.25$
$P_{MICE-norm} = 8.9 \times 10^{-12}$
$\beta_{MICE-norm} = -0.19$
observed
imputed
estimate CCA
estimate MICE−norm
Model residuals of 1−methylxanthine
2
1
0
−1
−2
$n_{CCA} = 770$
$n_{MICE-norm} = 1036$
$n_{CCA} = 318$
$n_{MICE-norm} = 567$
$n_{CCA} = 38$
$n_{MICE-norm} = 71$
major homozygote
heterozygote
minor homozygote
Genotype of SNP rs4921914 (NAT2)

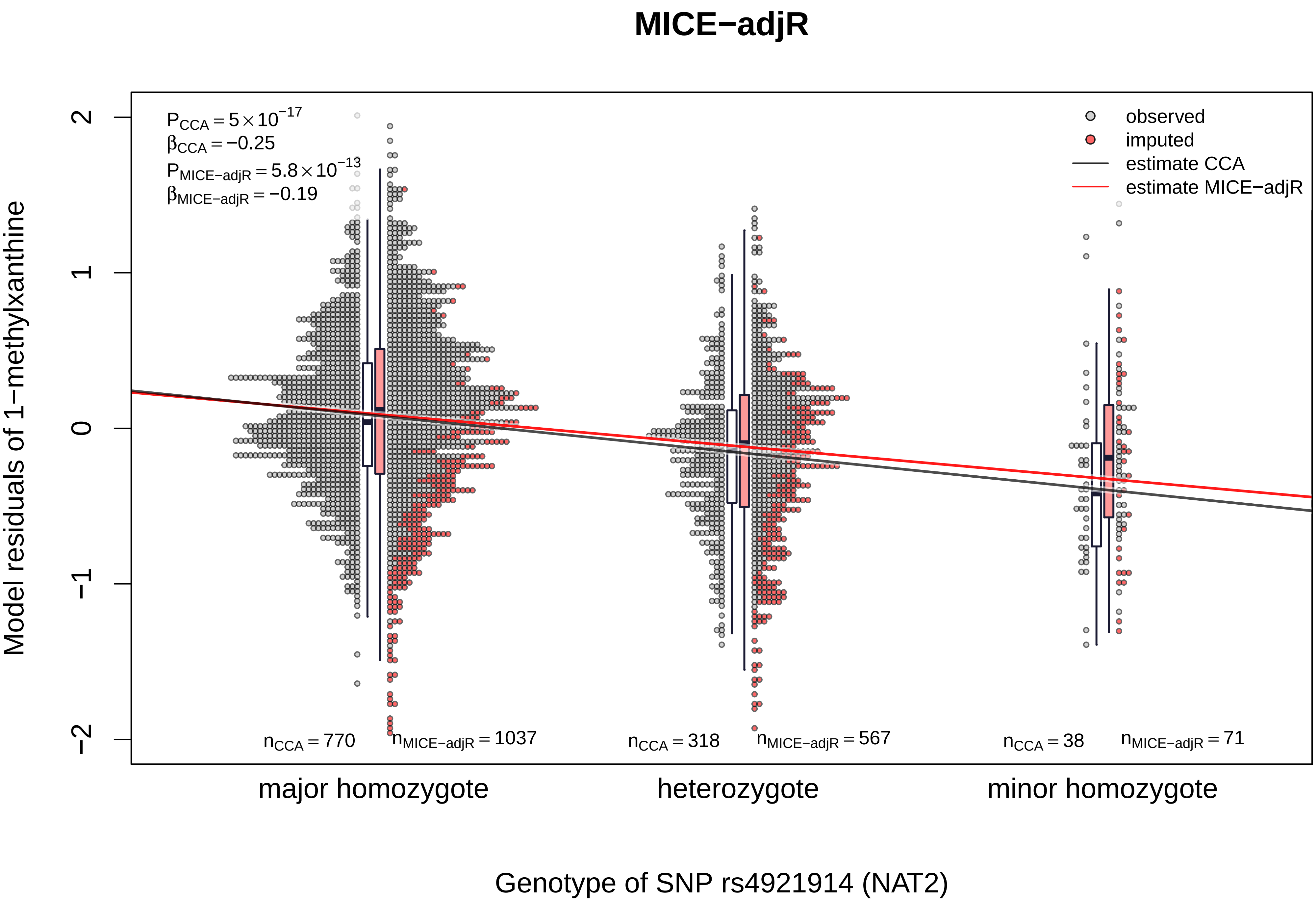

MICE−adjR
$P_{CCA} = 5 \times 10^{-17}$
$\beta_{CCA} = -0.25$
$P_{MICE-adjR} = 5.8 \times 10^{-13}$
$\beta_{MICE-adjR} = -0.19$
observed
imputed
estimate CCA
estimate MICE−adjR
Model residuals of 1−methylxanthine
2
1
0
−1
−2
$n_{CCA} = 770$
$n_{MICE-adjR} = 1037$
$n_{CCA} = 318$
$n_{MICE-adjR} = 567$
$n_{CCA} = 38$
$n_{MICE-adjR} = 71$
major homozygote
heterozygote
minor homozygote
Genotype of SNP rs4921914 (NAT2)

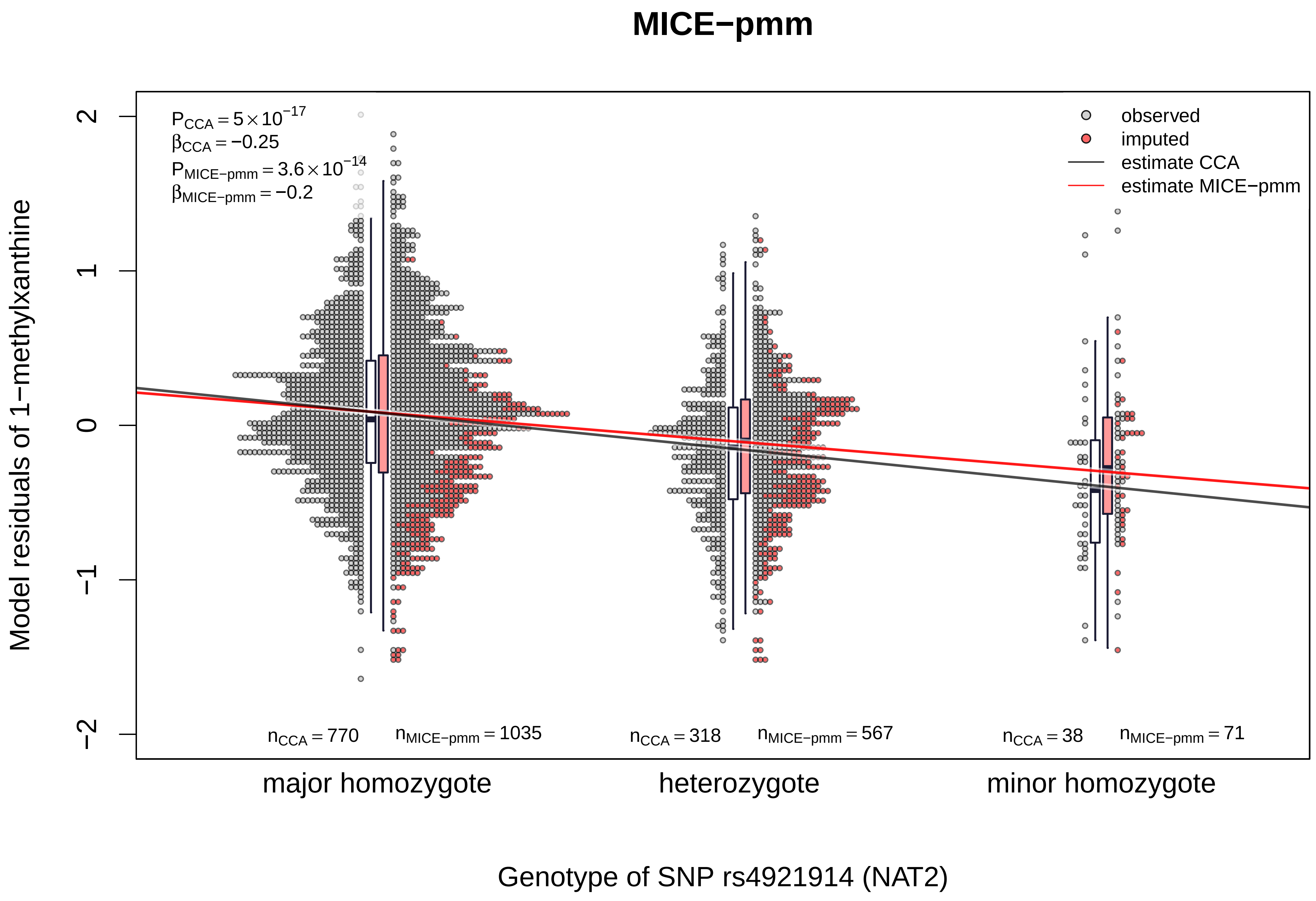

MICE−pmm
$P_{CCA} = 5 \times 10^{-17}$
$\beta_{CCA} = -0.25$
$P_{MICE-pmm} = 3.6 \times 10^{-14}$
$\beta_{MICE-pmm} = -0.2$
observed
imputed
estimate CCA
estimate MICE−pmm
Model residuals of 1−methylxanthine
2
1
0
−1
−2
$n_{CCA} = 770$
$n_{MICE-pmm} = 1035$
$n_{CCA} = 318$
$n_{MICE-pmm} = 567$
$n_{CCA} = 38$
$n_{MICE-pmm} = 71$
major homozygote
heterozygote
minor homozygote
Genotype of SNP rs4921914 (NAT2)

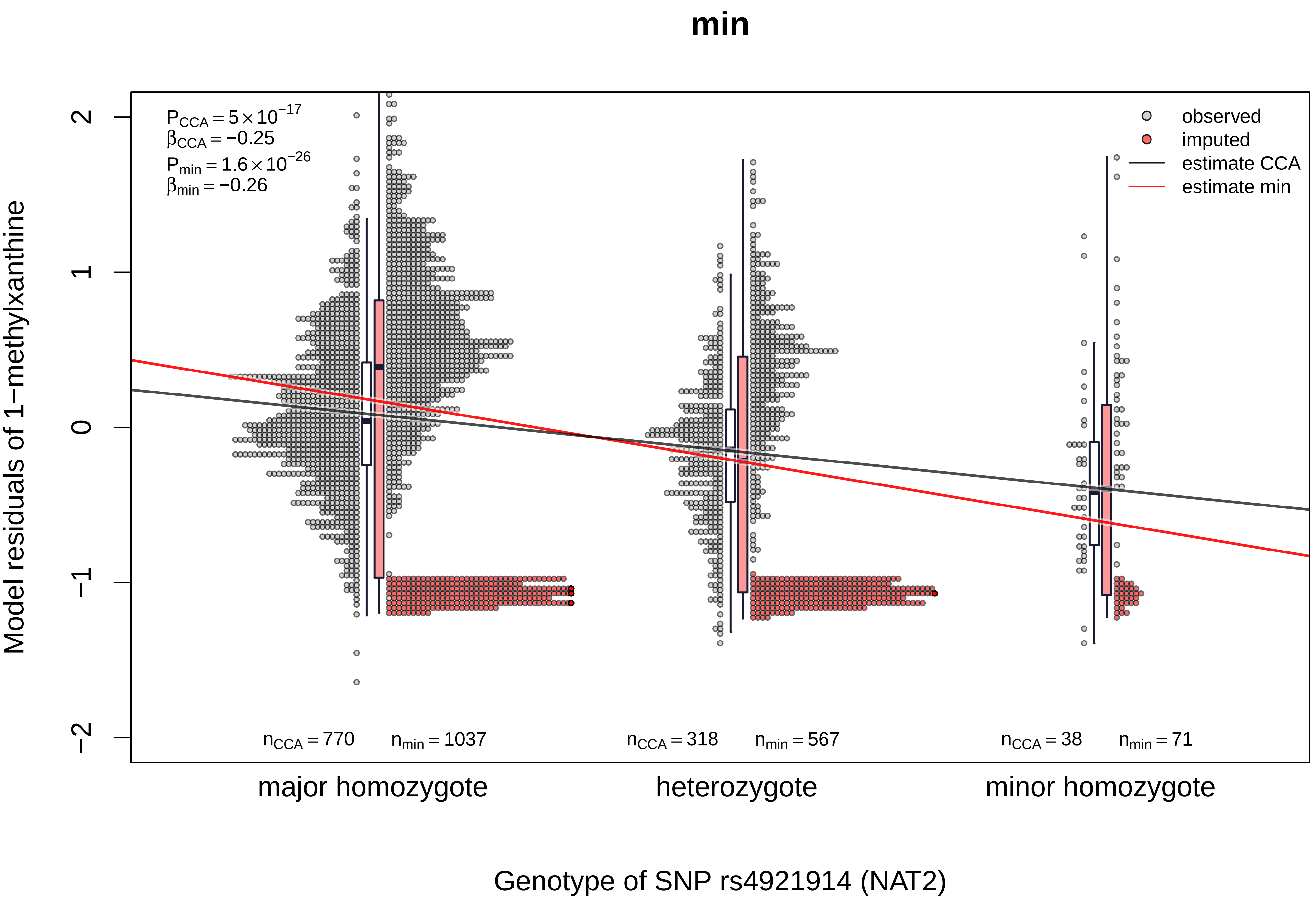

min
$P_{CCA} = 5 \times 10^{-17}$
$\beta_{CCA} = -0.25$
$P_{min} = 1.6 \times 10^{-26}$
$\beta_{min} = -0.26$
observed
imputed
estimate CCA
estimate min
Model residuals of 1−methylxanthine
2
1
0
−1
−2
$n_{CCA} = 770$
$n_{min} = 1037$
$n_{CCA} = 318$
$n_{min} = 567$
$n_{CCA} = 38$
$n_{min} = 71$
major homozygote
heterozygote
minor homozygote
Genotype of SNP rs4921914 (NAT2)

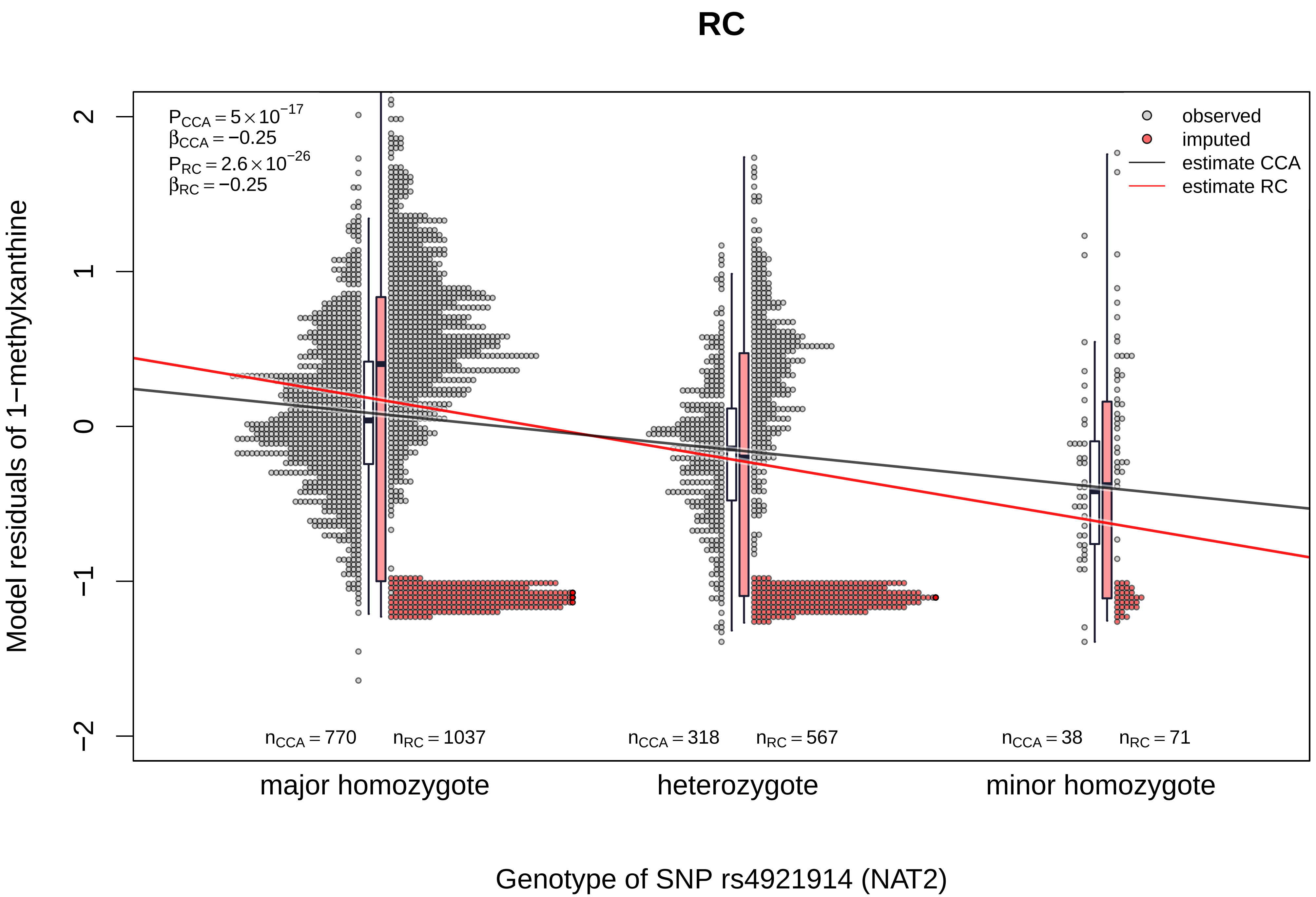

RC
$P_{CCA} = 5 \times 10^{-17}$
$\beta_{CCA} = -0.25$
$P_{RC} = 2.6 \times 10^{-26}$
$\beta_{RC} = -0.25$
observed
imputed
estimate CCA
estimate RC
$n_{CCA} = 770$
$n_{RC} = 1037$
$n_{CCA} = 318$
$n_{RC} = 567$
$n_{CCA} = 38$
$n_{RC} = 71$
2
1
0
−1
−2
Model residuals of 1−methylxanthine
major homozygote
heterozygote
minor homozygote
Genotype of SNP rs4921914 (NAT2)

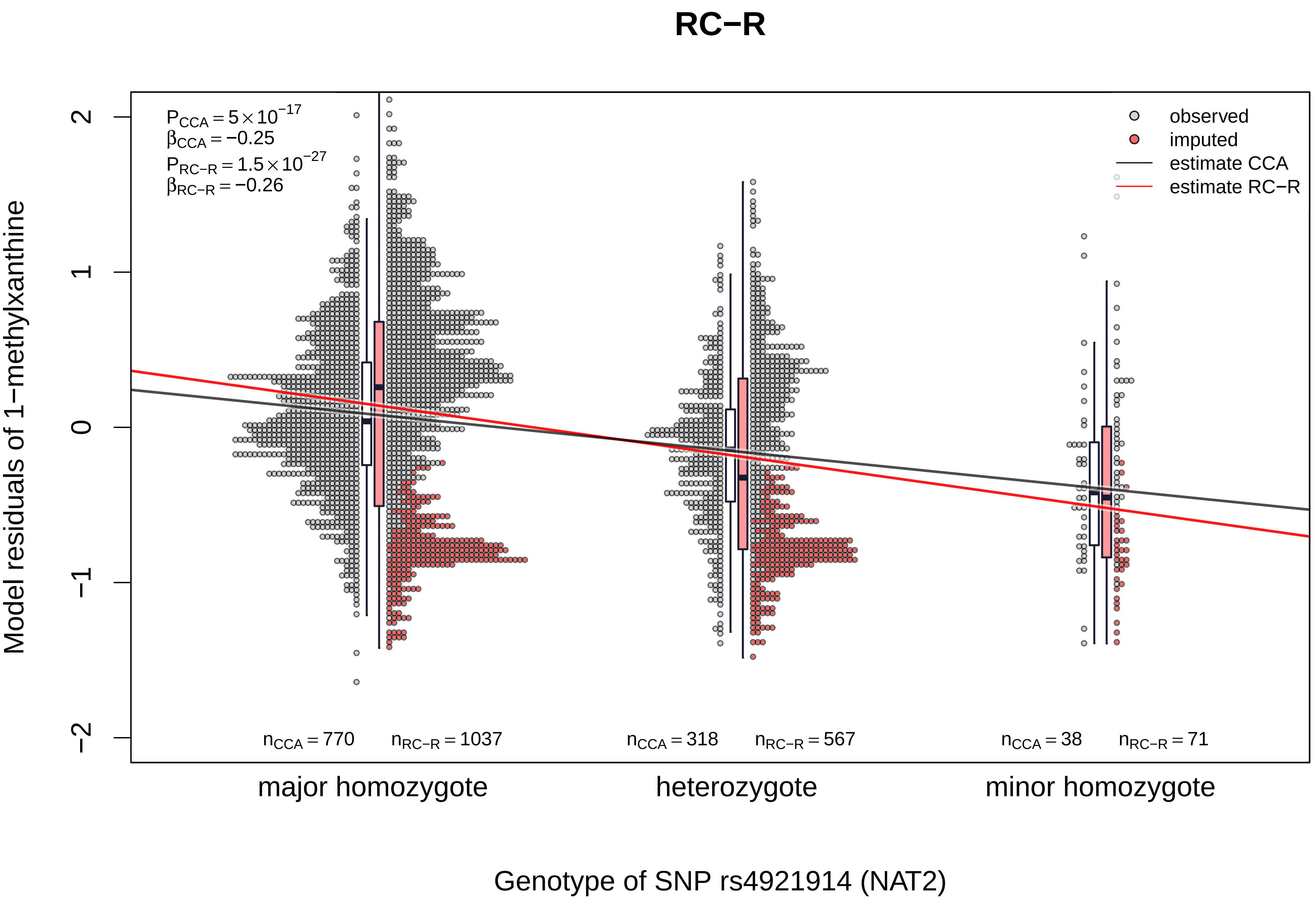

RC−R
$P_{CCA} = 5 \times 10^{-17}$
$\beta_{CCA} = -0.25$
$P_{RC-R} = 1.5 \times 10^{-27}$
$\beta_{RC-R} = -0.26$
observed
imputed
estimate CCA
estimate RC−R
Model residuals of 1−methylxanthine
2
1
0
−1
−2
$n_{CCA} = 770$
$n_{RC-R} = 1037$
$n_{CCA} = 318$
$n_{RC-R} = 567$
$n_{CCA} = 38$
$n_{RC-R} = 71$
major homozygote
heterozygote
minor homozygote
Genotype of SNP rs4921914 (NAT2)

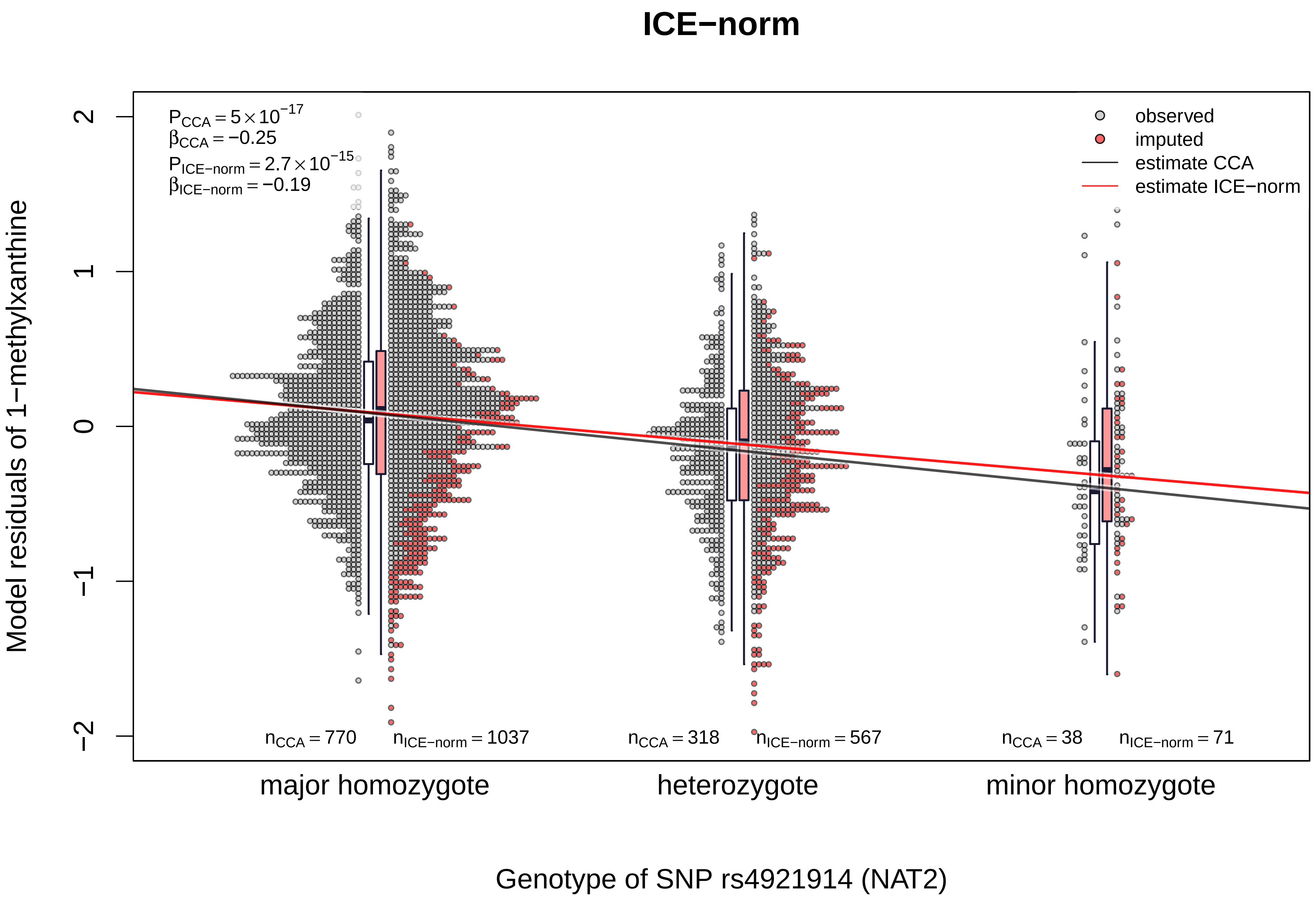

ICE−norm
$P_{CCA} = 5 \times 10^{-17}$
$\beta_{CCA} = -0.25$
$P_{ICE-norm} = 2.7 \times 10^{-15}$
$\beta_{ICE-norm} = -0.19$
observed
imputed
estimate CCA
estimate ICE−norm
Model residuals of 1−methylxanthine
2
1
0
−1
−2
$n_{CCA} = 770$
$n_{ICE-norm} = 1037$
$n_{CCA} = 318$
$n_{ICE-norm} = 567$
$n_{CCA} = 38$
$n_{ICE-norm} = 71$
major homozygote
heterozygote
minor homozygote
Genotype of SNP rs4921914 (NAT2)

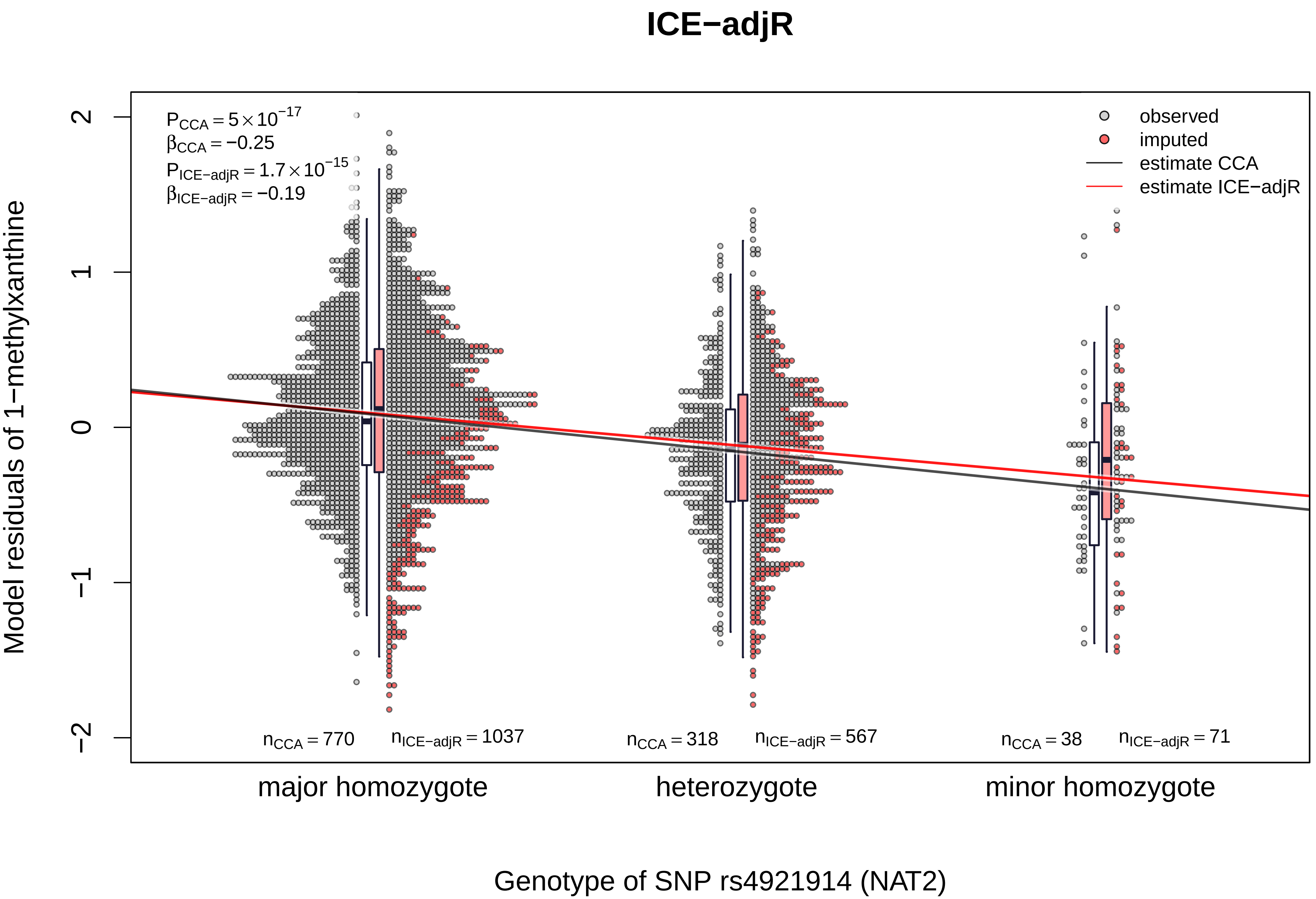

ICE−adjR
$P_{CCA} = 5 \times 10^{-17}$
$\beta_{CCA} = -0.25$
$P_{ICE-adjR} = 1.7 \times 10^{-15}$
$\beta_{ICE-adjR} = -0.19$
observed
imputed
estimate CCA
estimate ICE−adjR
Model residuals of 1−methylxanthine
2
1
0
−1
−2
$n_{CCA} = 770$
$n_{ICE-adjR} = 1037$
$n_{CCA} = 318$
$n_{ICE-adjR} = 567$
$n_{CCA} = 38$
$n_{ICE-adjR} = 71$
major homozygote
heterozygote
minor homozygote
Genotype of SNP rs4921914 (NAT2)

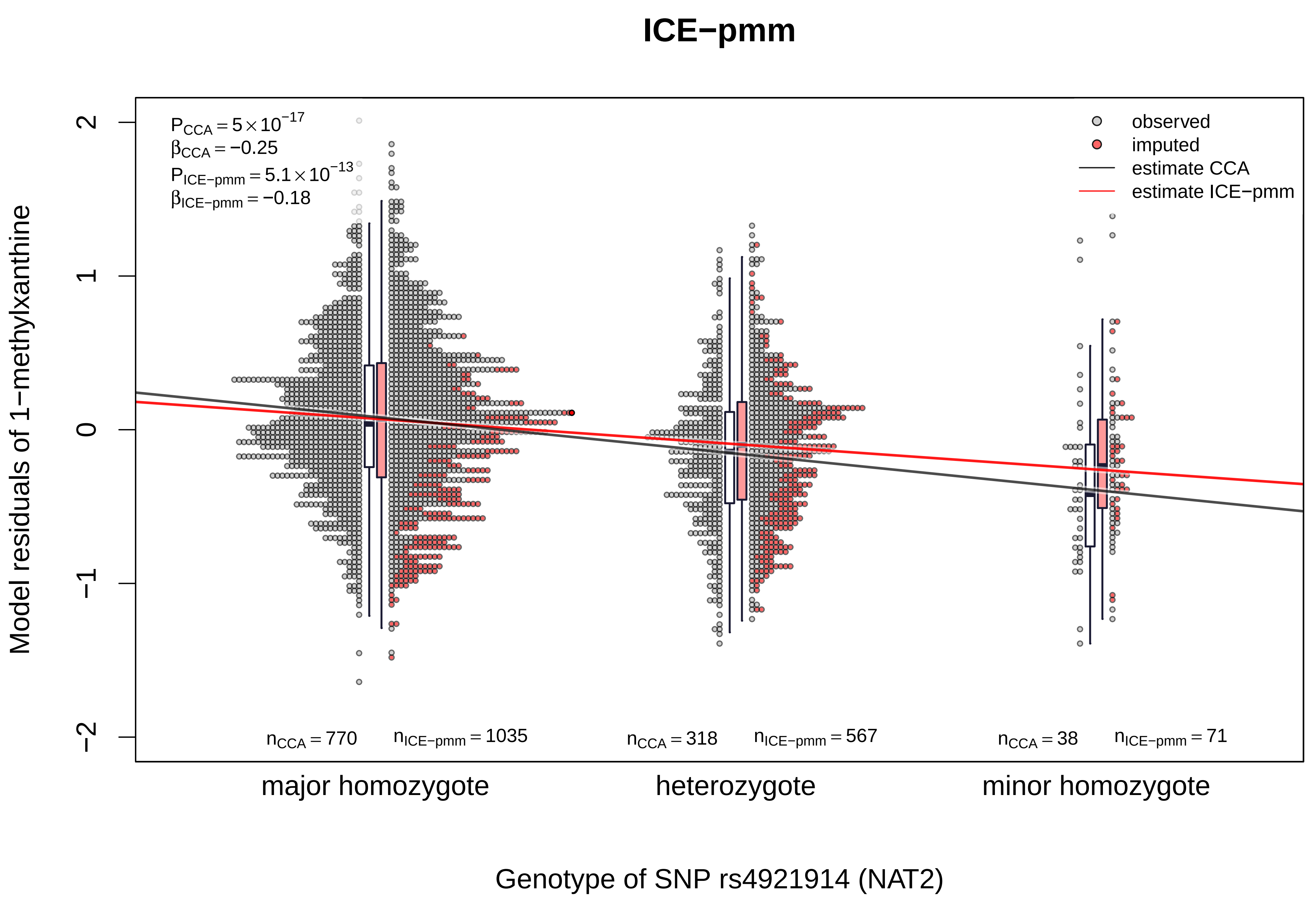

ICE−pmm
$P_{CCA} = 5 \times 10^{-17}$
$\beta_{CCA} = -0.25$
$P_{ICE-pmm} = 5.1 \times 10^{-13}$
$\beta_{ICE-pmm} = -0.18$
observed
imputed
estimate CCA
estimate ICE−pmm
Model residuals of 1−methylxanthine
2
1
0
−1
−2
$n_{CCA} = 770$
$n_{ICE-pmm} = 1035$
$n_{CCA} = 318$
$n_{ICE-pmm} = 567$
$n_{CCA} = 38$
$n_{ICE-pmm} = 71$
major homozygote
heterozygote
minor homozygote
Genotype of SNP rs4921914 (NAT2)

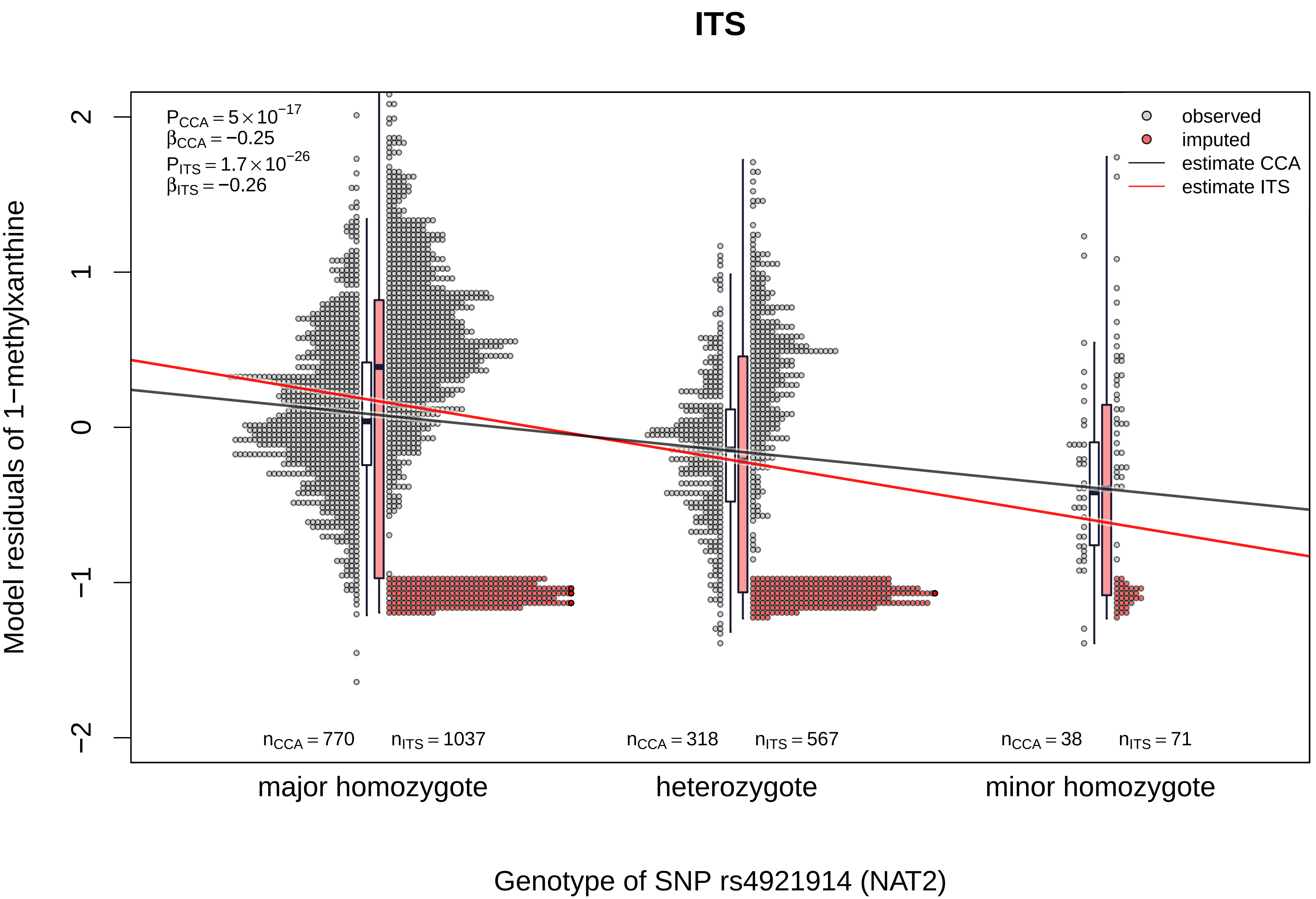

ITS
$P_{CCA} = 5 \times 10^{-17}$
$\beta_{CCA} = -0.25$
$P_{ITS} = 1.7 \times 10^{-26}$
$\beta_{ITS} = -0.26$
observed
imputed
estimate CCA
estimate ITS
2
1
0
−1
−2
Model residuals of 1−methylxanthine
$n_{CCA} = 770$
$n_{ITS} = 1037$
$n_{CCA} = 318$
$n_{ITS} = 567$
$n_{CCA} = 38$
$n_{ITS} = 71$
major homozygote
heterozygote
minor homozygote
Genotype of SNP rs4921914 (NAT2)

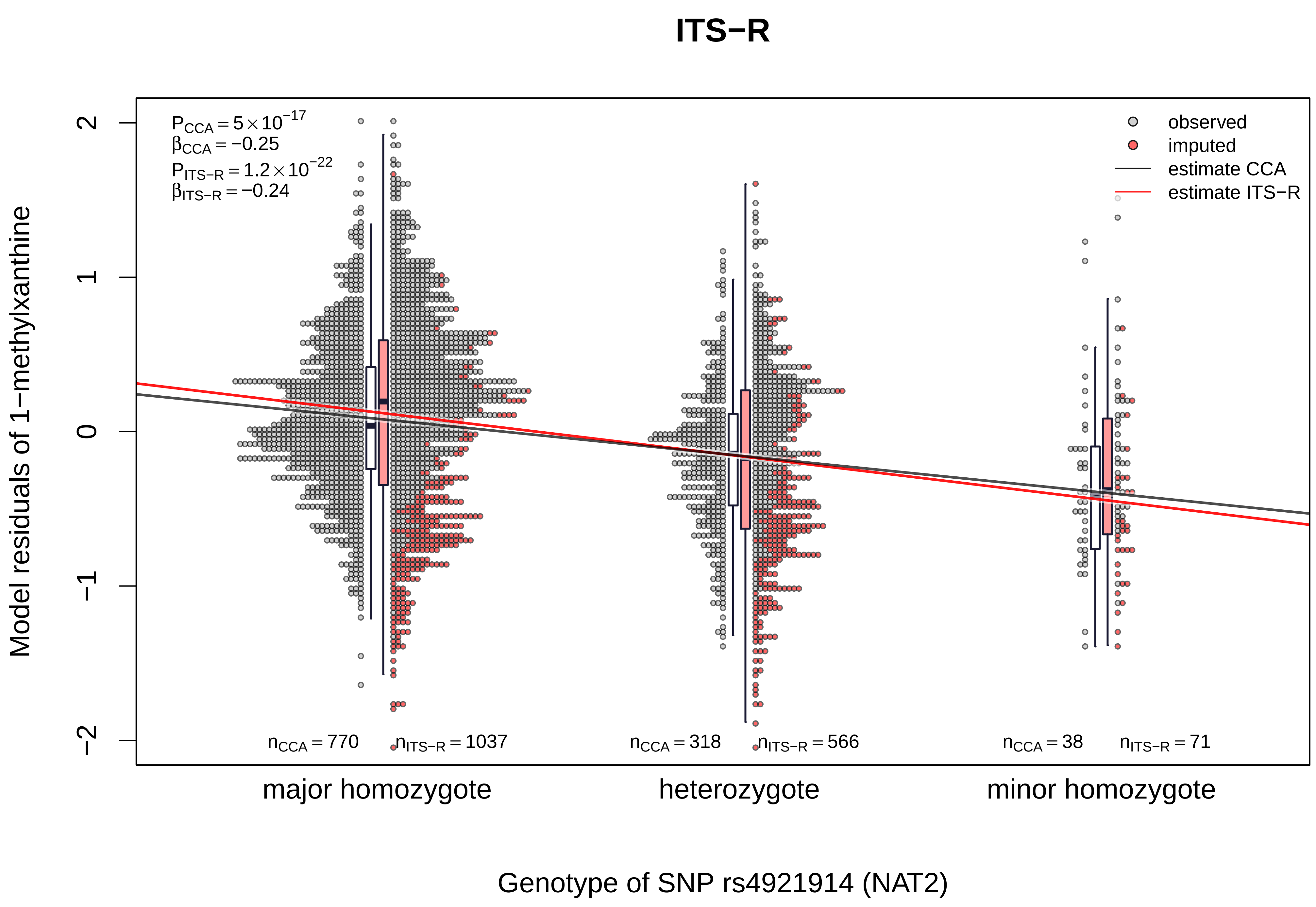

ITS−R
$P_{CCA} = 5 \times 10^{-17}$
$\beta_{CCA} = -0.25$
$P_{ITS-R} = 1.2 \times 10^{-22}$
$\beta_{ITS-R} = -0.24$
observed
imputed
estimate CCA
estimate ITS−R
2
1
0
−1
−2
Model residuals of 1−methylxanthine
$n_{CCA} = 770$
$n_{ITS-R} = 1037$
$n_{CCA} = 318$
$n_{ITS-R} = 566$
$n_{CCA} = 38$
$n_{ITS-R} = 71$
major homozygote
heterozygote
minor homozygote
Genotype of SNP rs4921914 (NAT2)

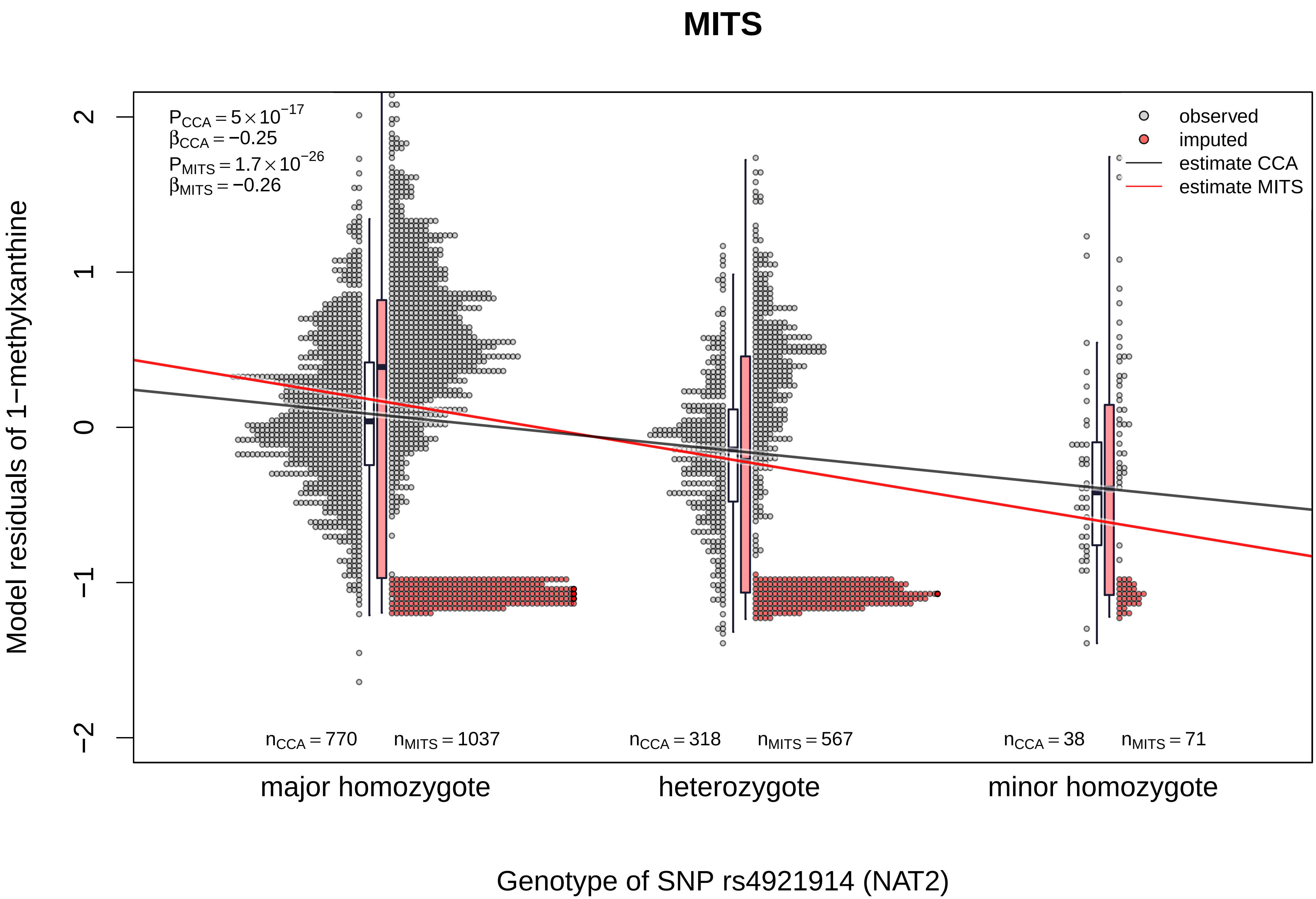

MITS
$P_{CCA} = 5 \times 10^{-17}$
$\beta_{CCA} = -0.25$
$P_{MITS} = 1.7 \times 10^{-26}$
$\beta_{MITS} = -0.26$
observed
imputed
estimate CCA
estimate MITS
Model residuals of 1−methylxanthine
2
1
0
−1
−2
$n_{CCA} = 770$
$n_{MITS} = 1037$
$n_{CCA} = 318$
$n_{MITS} = 567$
$n_{CCA} = 38$
$n_{MITS} = 71$
major homozygote
heterozygote
minor homozygote
Genotype of SNP rs4921914 (NAT2)

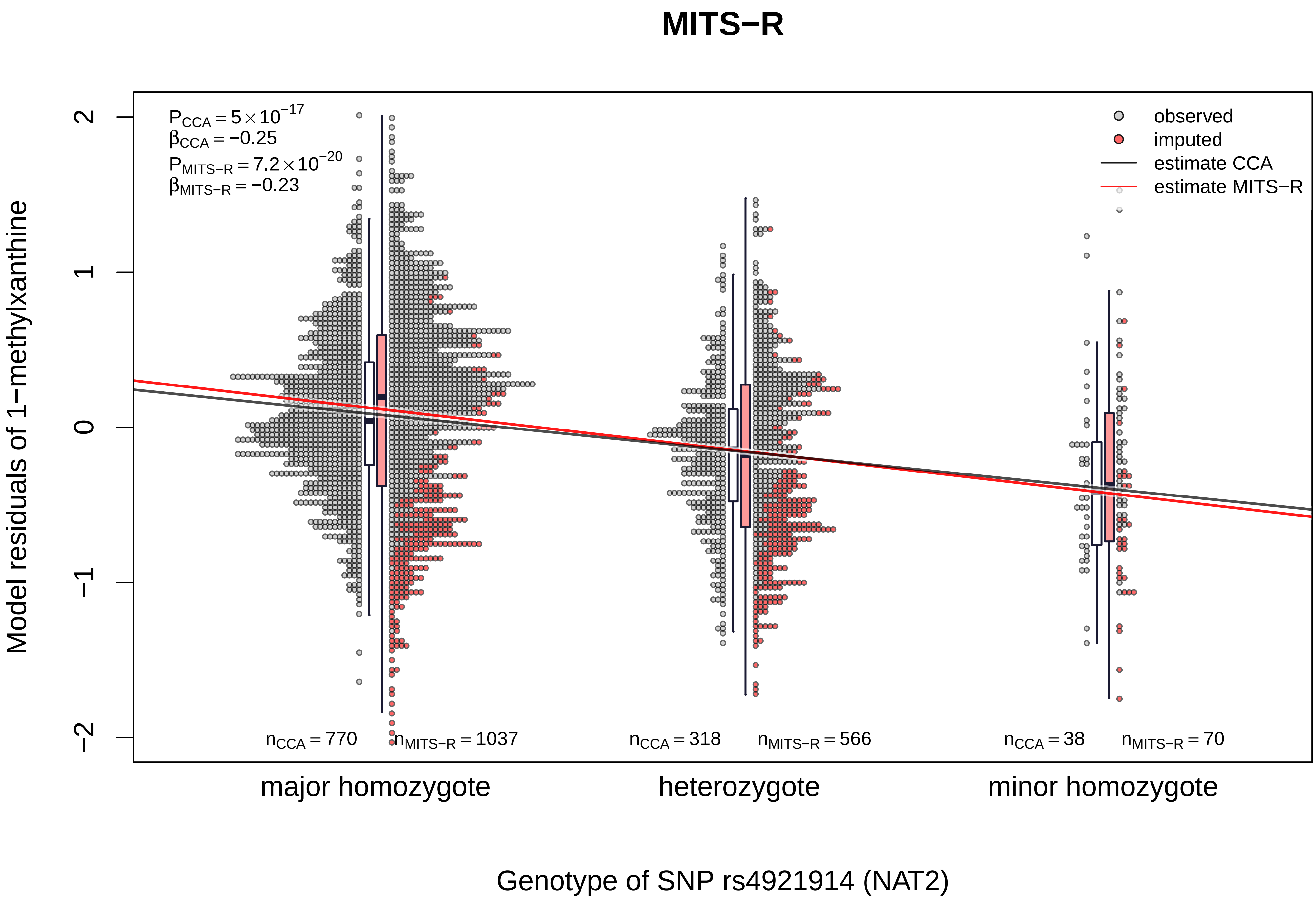
MITS−R
$P_{CCA} = 5 \times 10^{-17}$
$\beta_{CCA} = -0.25$
$P_{MITS-R} = 7.2 \times 10^{-20}$
$\beta_{MITS-R} = -0.23$
observed
imputed
estimate CCA
estimate MITS−R
2
1
0
−1
−2
Model residuals of 1−methylxanthine
$n_{CCA} = 770$
$n_{MITS-R} = 1037$
$n_{CCA} = 318$
$n_{MITS-R} = 566$
$n_{CCA} = 38$
$n_{MITS-R} = 70$
major homozygote
heterozygote
minor homozygote
Genotype of SNP rs4921914 (NAT2)

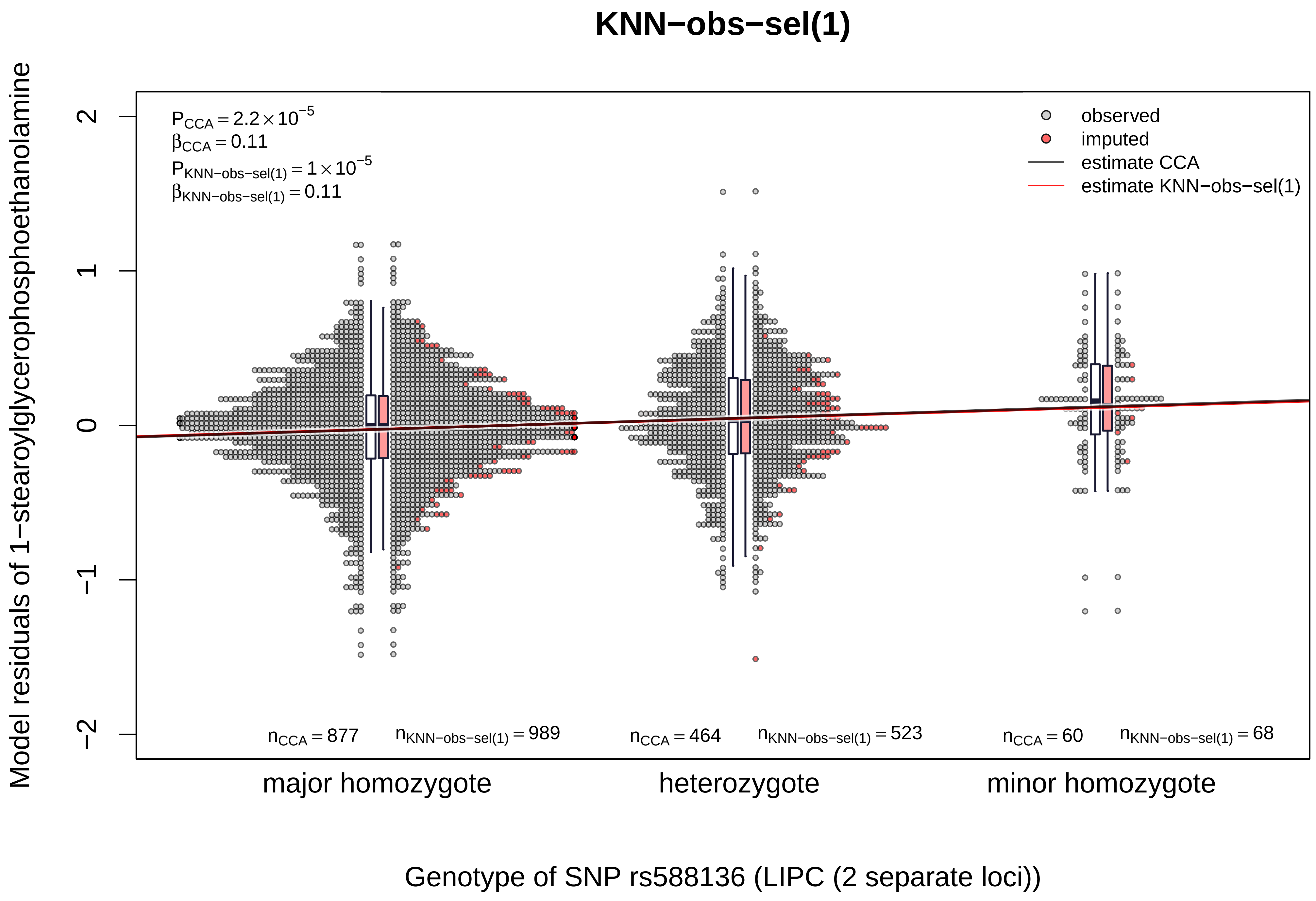

KNN−obs−sel(1)
$P_{CCA} = 2.2 \times 10^{-5}$
$\beta_{CCA} = 0.11$
$P_{KNN-obs-sel(1)} = 1 \times 10^{-5}$
$\beta_{KNN-obs-sel(1)} = 0.11$
observed
imputed
estimate CCA
estimate KNN−obs−sel(1)
Model residuals of 1−stearoylglycerophosphoethanolamine
2
1
0
−1
−2
$n_{CCA} = 877$
$n_{KNN-obs-sel(1)} = 989$
$n_{CCA} = 464$
$n_{KNN-obs-sel(1)} = 523$
$n_{CCA} = 60$
$n_{KNN-obs-sel(1)} = 68$
major homozygote
heterozygote
minor homozygote
Genotype of SNP rs588136 (LIPC (2 separate loci))

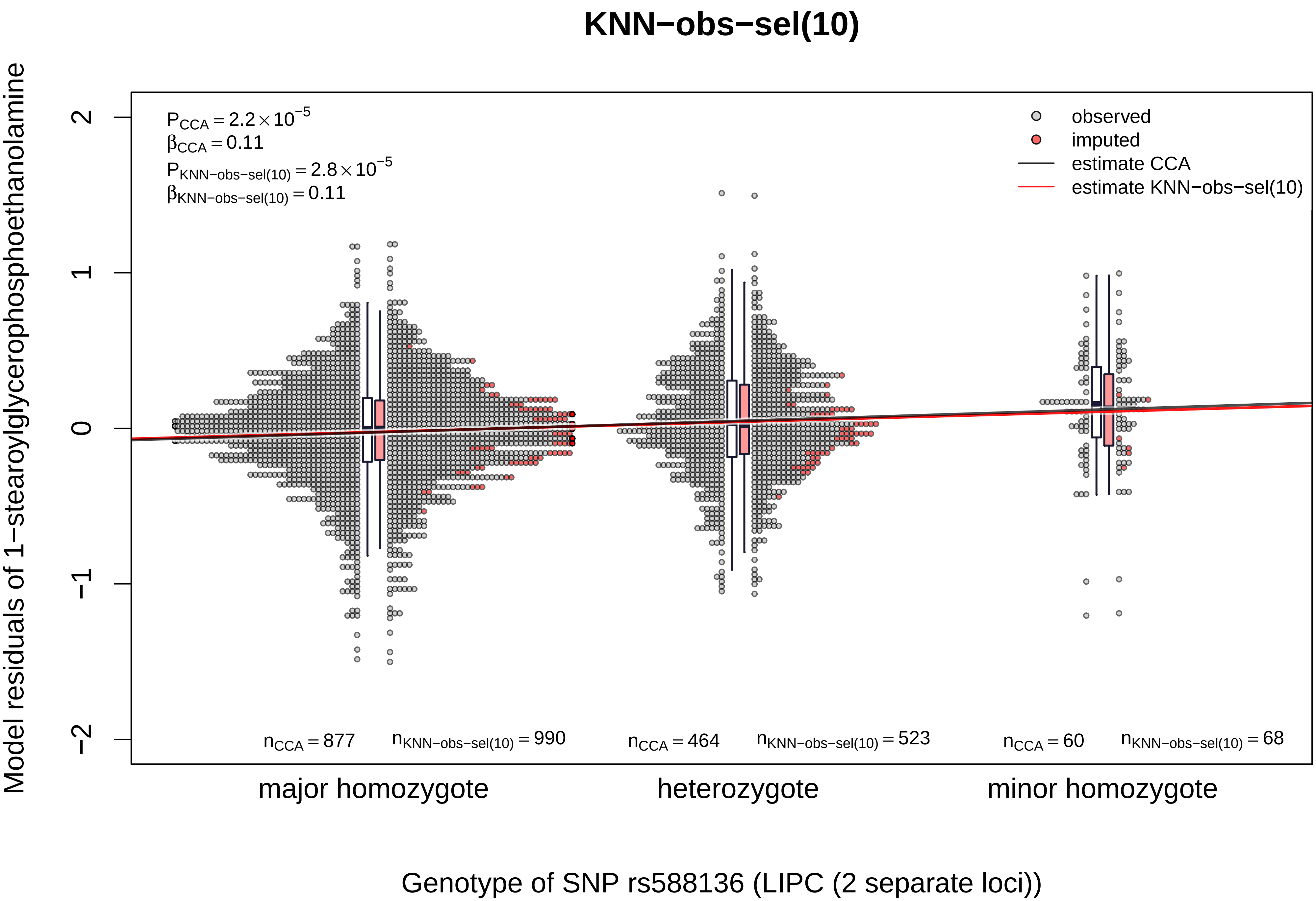

KNN−obs−sel(10)
$P_{CCA} = 2.2 \times 10^{-5}$
$\beta_{CCA} = 0.11$
$P_{KNN-obs-sel(10)} = 2.8 \times 10^{-5}$
$\beta_{KNN-obs-sel(10)} = 0.11$
observed
imputed
estimate CCA
estimate KNN−obs−sel(10)
Model residuals of 1−stearoylglycerophosphoethanolamine
2
1
0
−1
−2
$n_{CCA} = 877$
$n_{KNN-obs-sel(10)} = 990$
$n_{CCA} = 464$
$n_{KNN-obs-sel(10)} = 523$
$n_{CCA} = 60$
$n_{KNN-obs-sel(10)} = 68$
major homozygote
heterozygote
minor homozygote
Genotype of SNP rs588136 (LIPC (2 separate loci))

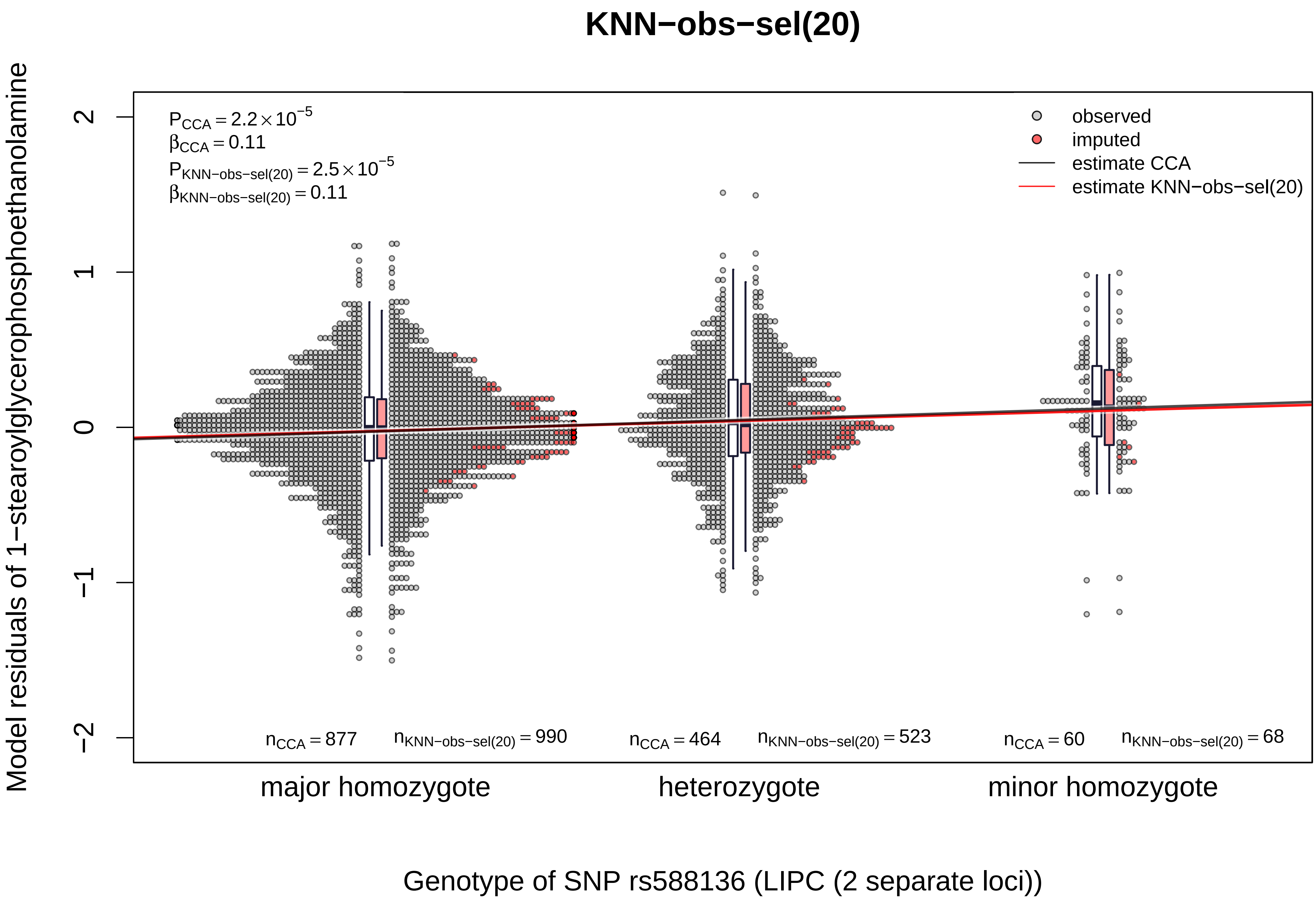

KNN−obs−sel(20)
$P_{CCA} = 2.2 \times 10^{-5}$
$\beta_{CCA} = 0.11$
$P_{KNN-obs-sel(20)} = 2.5 \times 10^{-5}$
$\beta_{KNN-obs-sel(20)} = 0.11$
observed
imputed
estimate CCA
estimate KNN−obs−sel(20)
$n_{CCA} = 877$
$n_{KNN-obs-sel(20)} = 990$
$n_{CCA} = 464$
$n_{KNN-obs-sel(20)} = 523$
$n_{CCA} = 60$
$n_{KNN-obs-sel(20)} = 68$
2
1
0
−1
−2
Model residuals of 1−stearoylglycerophosphoethanolamine
major homozygote
heterozygote
minor homozygote
Genotype of SNP rs588136 (LIPC (2 separate loci))

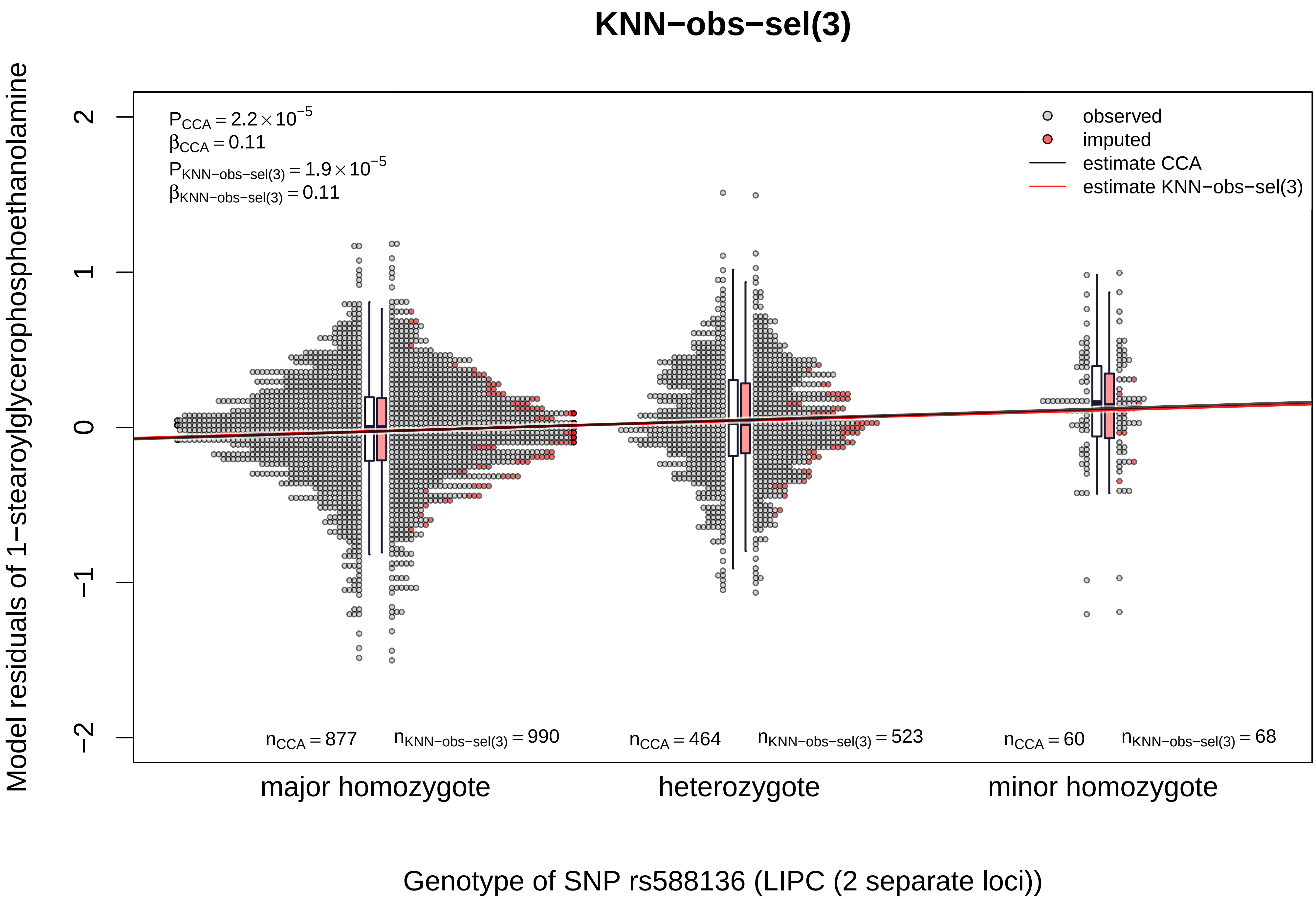

KNN−obs−sel(3)
$P_{CCA} = 2.2 \times 10^{-5}$
$\beta_{CCA} = 0.11$
$P_{KNN-obs-sel(3)} = 1.9 \times 10^{-5}$
$\beta_{KNN-obs-sel(3)} = 0.11$
observed
imputed
estimate CCA
estimate KNN−obs−sel(3)
2
1
0
−1
−2
$n_{CCA} = 877$
$n_{KNN-obs-sel(3)} = 990$
$n_{CCA} = 464$
$n_{KNN-obs-sel(3)} = 523$
$n_{CCA} = 60$
$n_{KNN-obs-sel(3)} = 68$
major homozygote
heterozygote
minor homozygote
Model residuals of 1−stearoylglycerophosphoethanolamine
Genotype of SNP rs588136 (LIPC (2 separate loci))

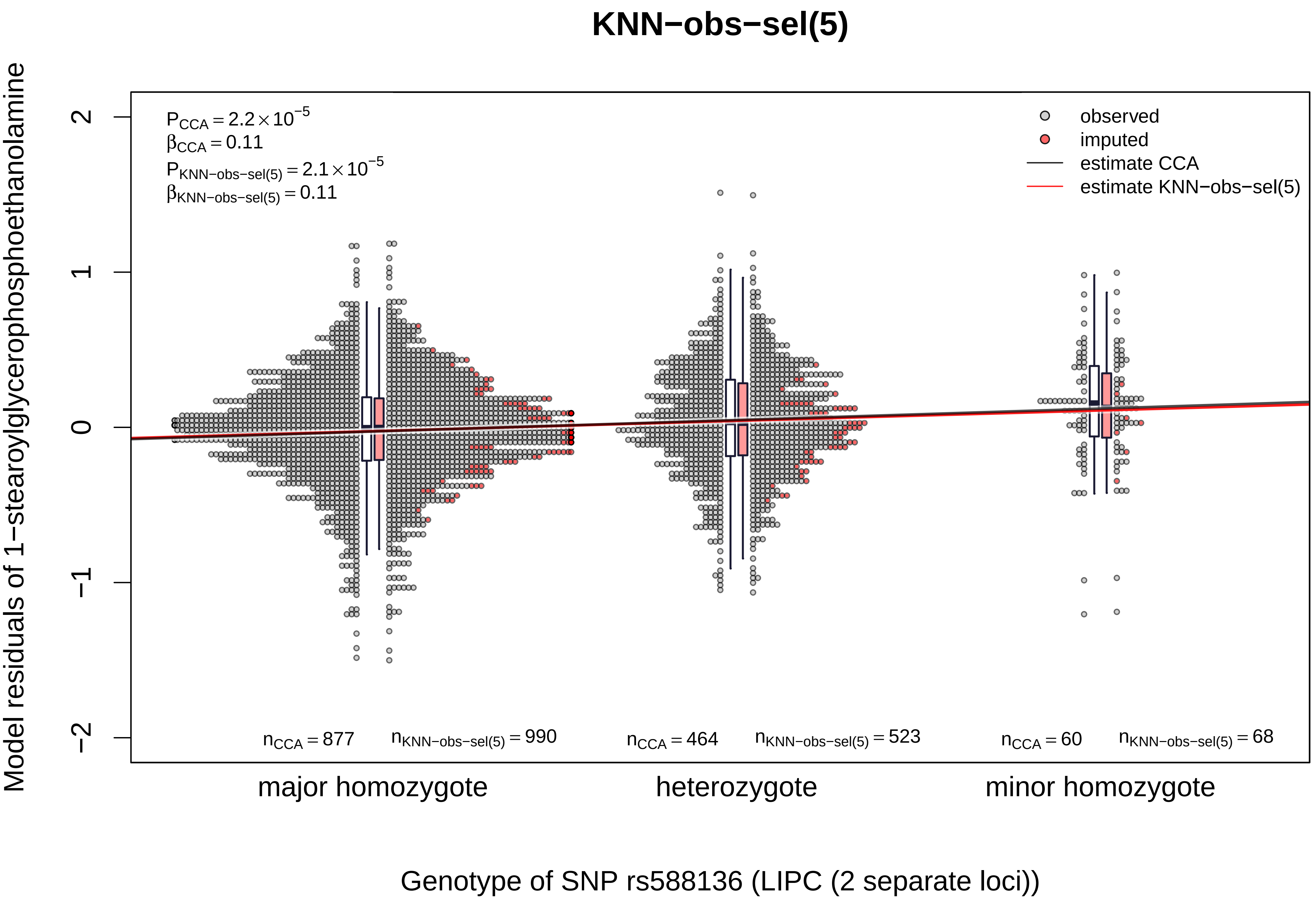

KNN−obs−sel(5)
$P_{CCA} = 2.2 \times 10^{-5}$
$\beta_{CCA} = 0.11$
$P_{KNN-obs-sel(5)} = 2.1 \times 10^{-5}$
$\beta_{KNN-obs-sel(5)} = 0.11$
observed
imputed
estimate CCA
estimate KNN−obs−sel(5)
$n_{CCA} = 877$
$n_{KNN-obs-sel(5)} = 990$
$n_{CCA} = 464$
$n_{KNN-obs-sel(5)} = 523$
$n_{CCA} = 60$
$n_{KNN-obs-sel(5)} = 68$
2
1
0
−1
−2
major homozygote
heterozygote
minor homozygote
Model residuals of 1−stearoylglycerophosphoethanolamine
Genotype of SNP rs588136 (LIPC (2 separate loci))

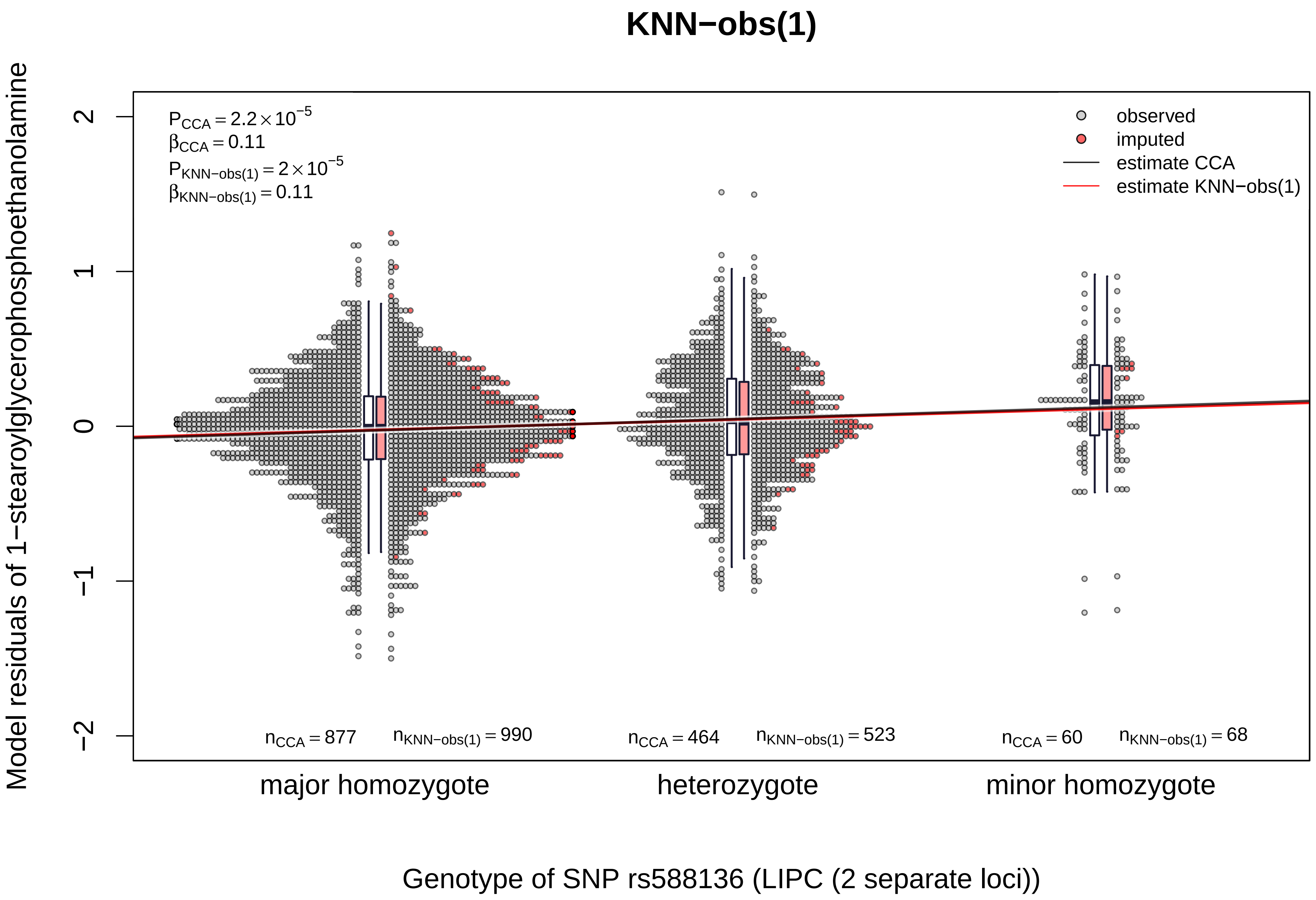
KNN−obs(1)
$P_{CCA} = 2.2 \times 10^{-5}$
$\beta_{CCA} = 0.11$
$P_{KNN-obs(1)} = 2 \times 10^{-5}$
$\beta_{KNN-obs(1)} = 0.11$
observed
imputed
estimate CCA
estimate KNN−obs(1)
2
1
0
−1
−2
Model residuals of 1−stearoylglycerophosphoethanolamine
$n_{CCA} = 877$
$n_{KNN-obs(1)} = 990$
$n_{CCA} = 464$
$n_{KNN-obs(1)} = 523$
$n_{CCA} = 60$
$n_{KNN-obs(1)} = 68$
major homozygote
heterozygote
minor homozygote
Genotype of SNP rs588136 (LIPC (2 separate loci))

KNN−obs(10)

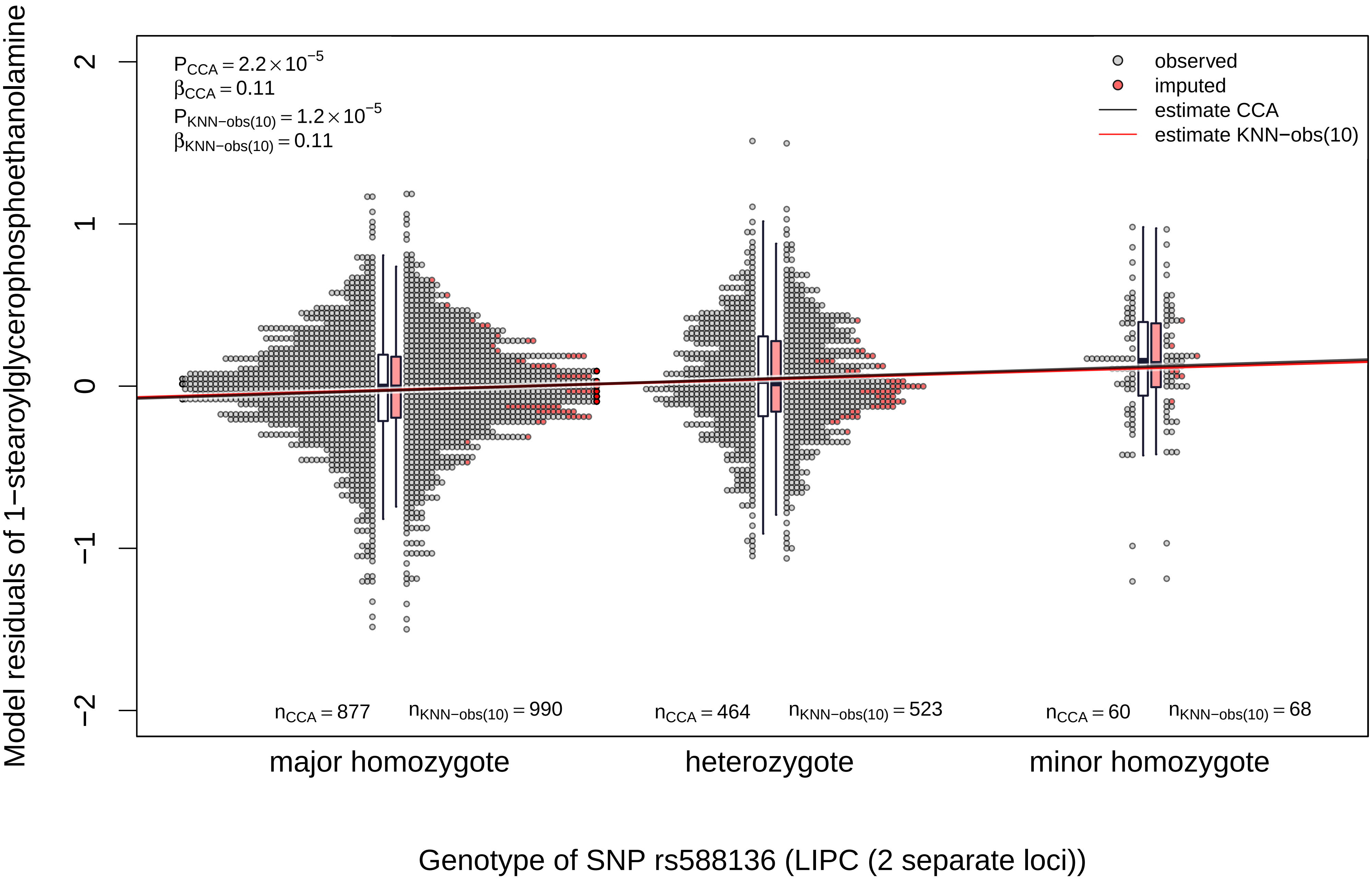

$P_{CCA} = 2.2 \times 10^{-5}$
$\beta_{CCA} = 0.11$
$P_{KNN-obs(10)} = 1.2 \times 10^{-5}$
$\beta_{KNN-obs(10)} = 0.11$
observed
imputed
estimate CCA
estimate KNN−obs(10)
$n_{CCA} = 877$
$n_{KNN-obs(10)} = 990$
$n_{CCA} = 464$
$n_{KNN-obs(10)} = 523$
$n_{CCA} = 60$
$n_{KNN-obs(10)} = 68$
2
1
0
−1
−2
Model residuals of 1−stearoylglycerophosphoethanolamine
major homozygote
heterozygote
minor homozygote
Genotype of SNP rs588136 (LIPC (2 separate loci))

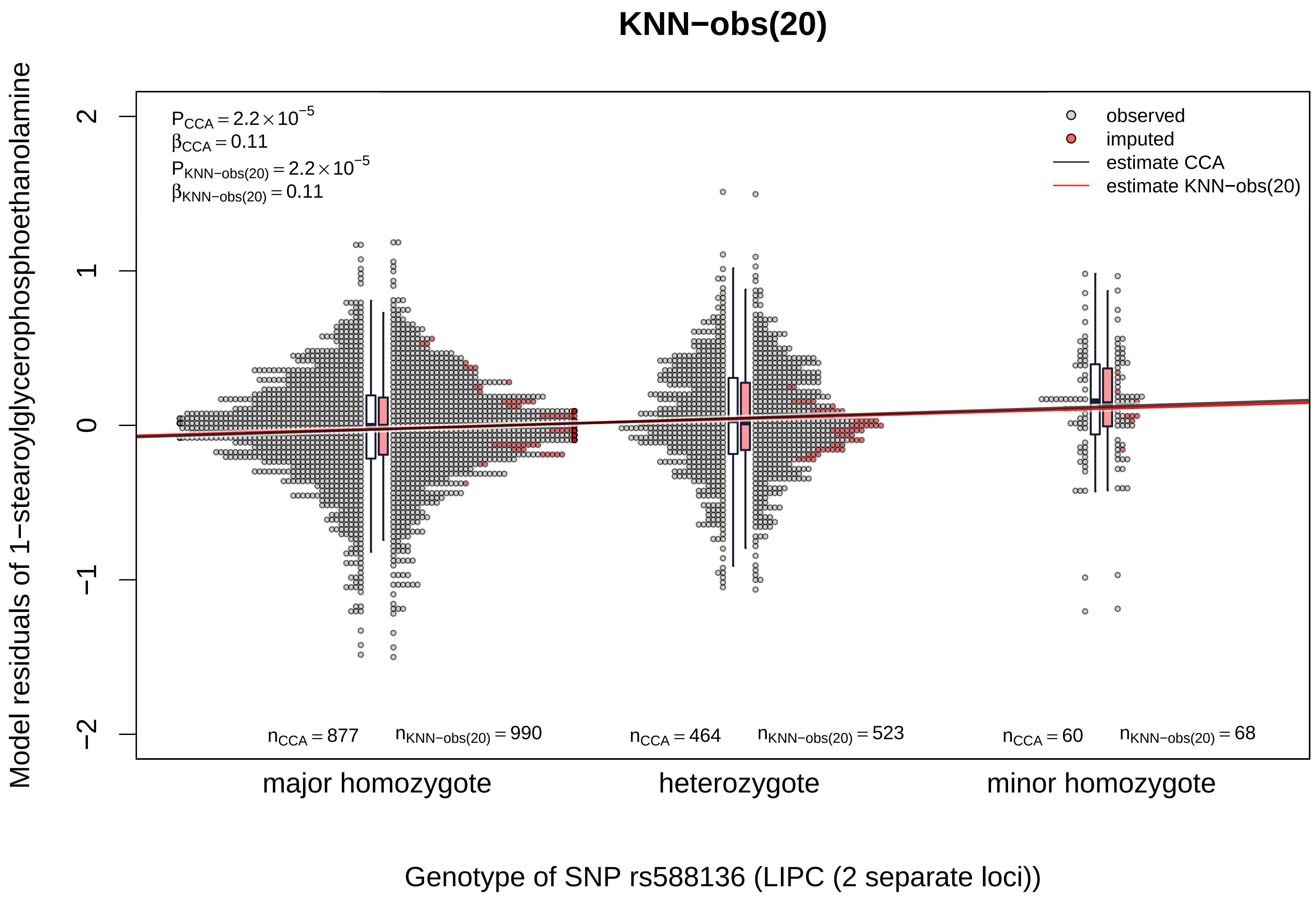

KNN−obs(20)
$P_{CCA} = 2.2 \times 10^{-5}$
$\beta_{CCA} = 0.11$
$P_{KNN-obs(20)} = 2.2 \times 10^{-5}$
$\beta_{KNN-obs(20)} = 0.11$
observed
imputed
estimate CCA
estimate KNN−obs(20)
$n_{CCA} = 877$
$n_{KNN-obs(20)} = 990$
$n_{CCA} = 464$
$n_{KNN-obs(20)} = 523$
$n_{CCA} = 60$
$n_{KNN-obs(20)} = 68$
2
1
0
−1
−2
Model residuals of 1−stearoylglycerophosphoethanolamine
major homozygote
heterozygote
minor homozygote
Genotype of SNP rs588136 (LIPC (2 separate loci))

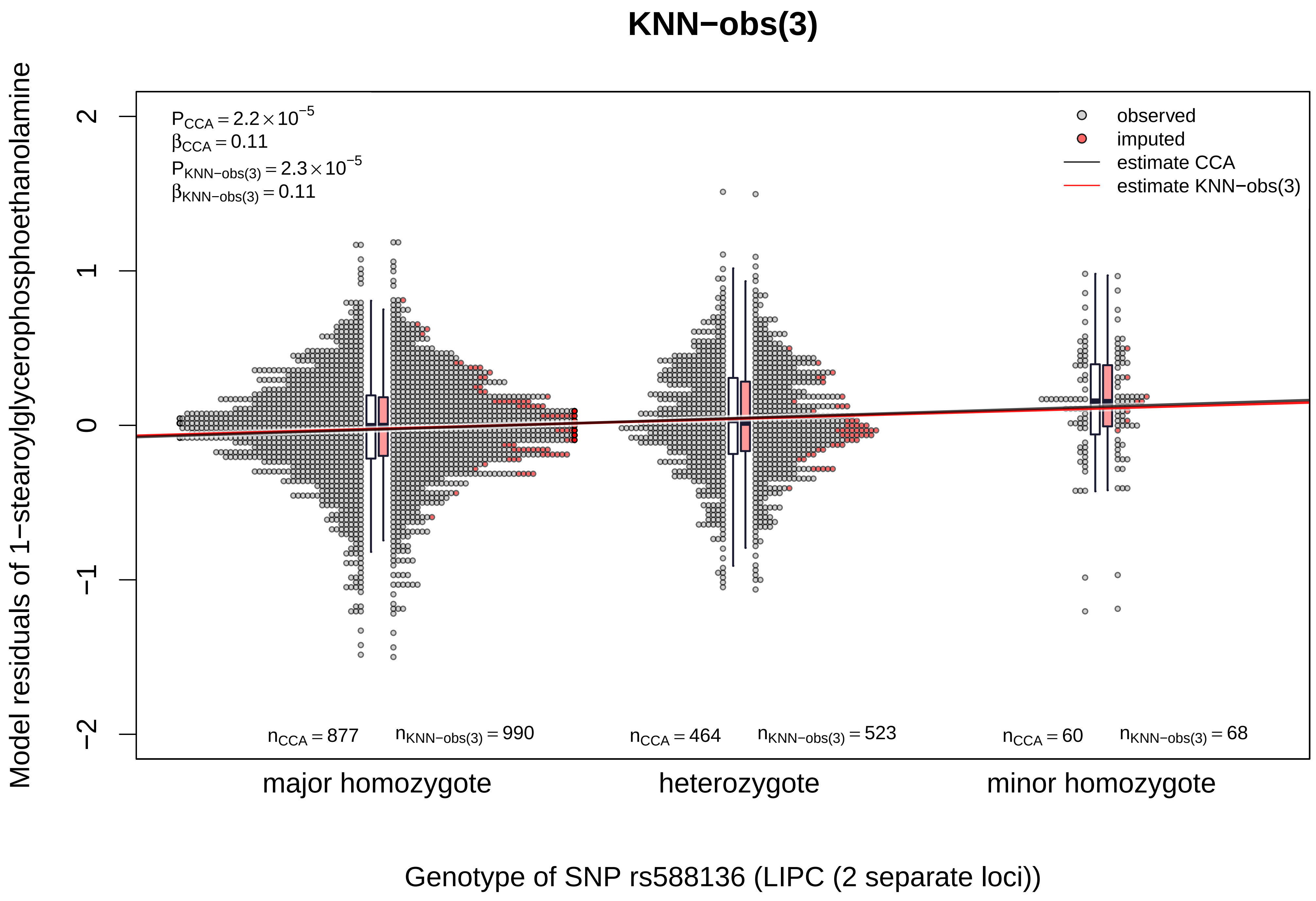

KNN−obs(3)
$P_{CCA} = 2.2 \times 10^{-5}$
$\beta_{CCA} = 0.11$
$P_{KNN-obs(3)} = 2.3 \times 10^{-5}$
$\beta_{KNN-obs(3)} = 0.11$
observed
imputed
estimate CCA
estimate KNN−obs(3)
Model residuals of 1−stearoylglycerophosphoethanolamine
2
1
0
−1
−2
$n_{CCA} = 877$
$n_{KNN-obs(3)} = 990$
$n_{CCA} = 464$
$n_{KNN-obs(3)} = 523$
$n_{CCA} = 60$
$n_{KNN-obs(3)} = 68$
major homozygote
heterozygote
minor homozygote
Genotype of SNP rs588136 (LIPC (2 separate loci))

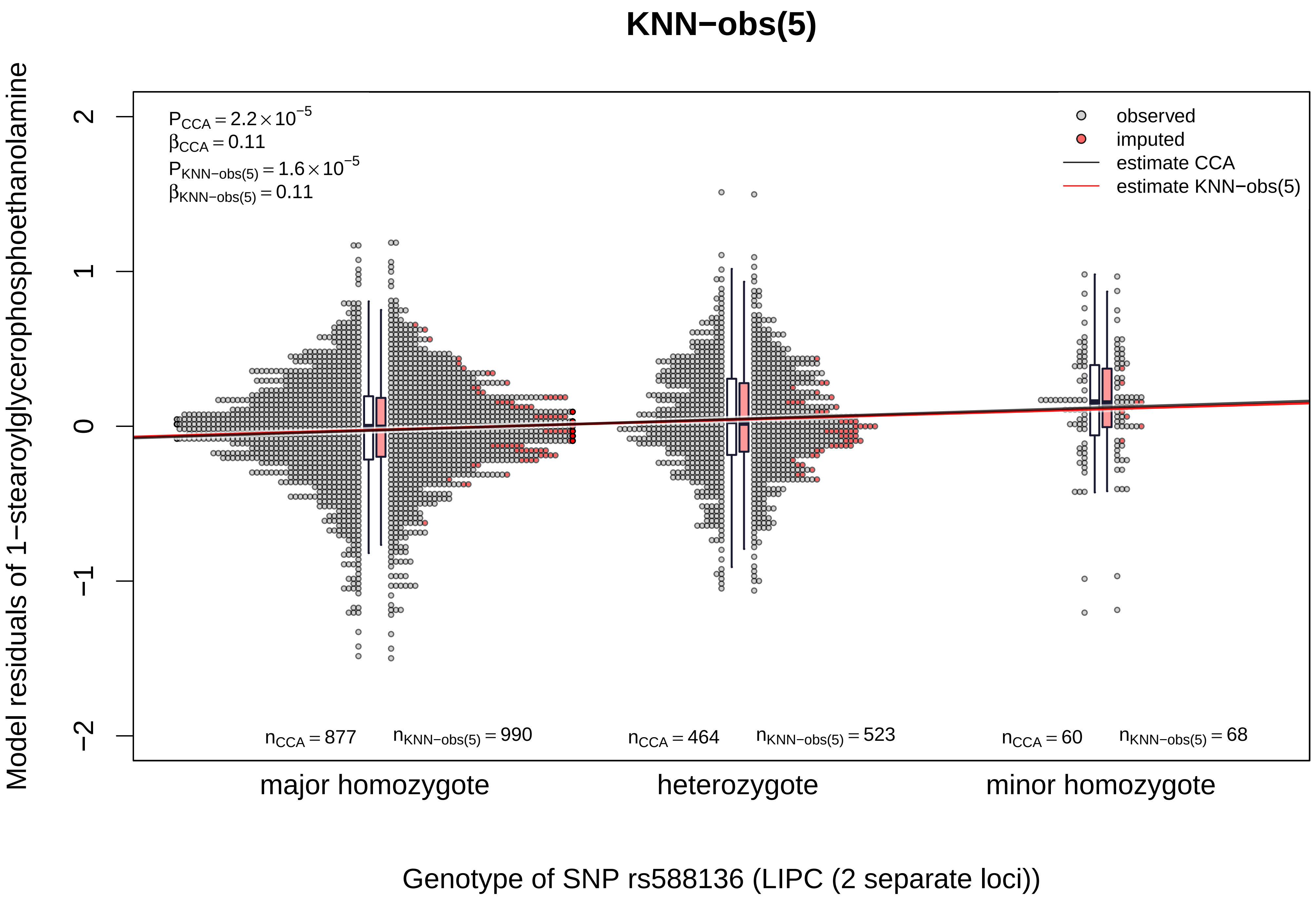

KNN−obs(5)
$P_{CCA} = 2.2 \times 10^{-5}$
$\beta_{CCA} = 0.11$
$P_{KNN-obs(5)} = 1.6 \times 10^{-5}$
$\beta_{KNN-obs(5)} = 0.11$
observed
imputed
estimate CCA
estimate KNN−obs(5)
Model residuals of 1−stearoylglycerophosphoethanolamine
2
1
0
−1
−2
$n_{CCA} = 877$
$n_{KNN-obs(5)} = 990$
$n_{CCA} = 464$
$n_{KNN-obs(5)} = 523$
$n_{CCA} = 60$
$n_{KNN-obs(5)} = 68$
major homozygote
heterozygote
minor homozygote
Genotype of SNP rs588136 (LIPC (2 separate loci))

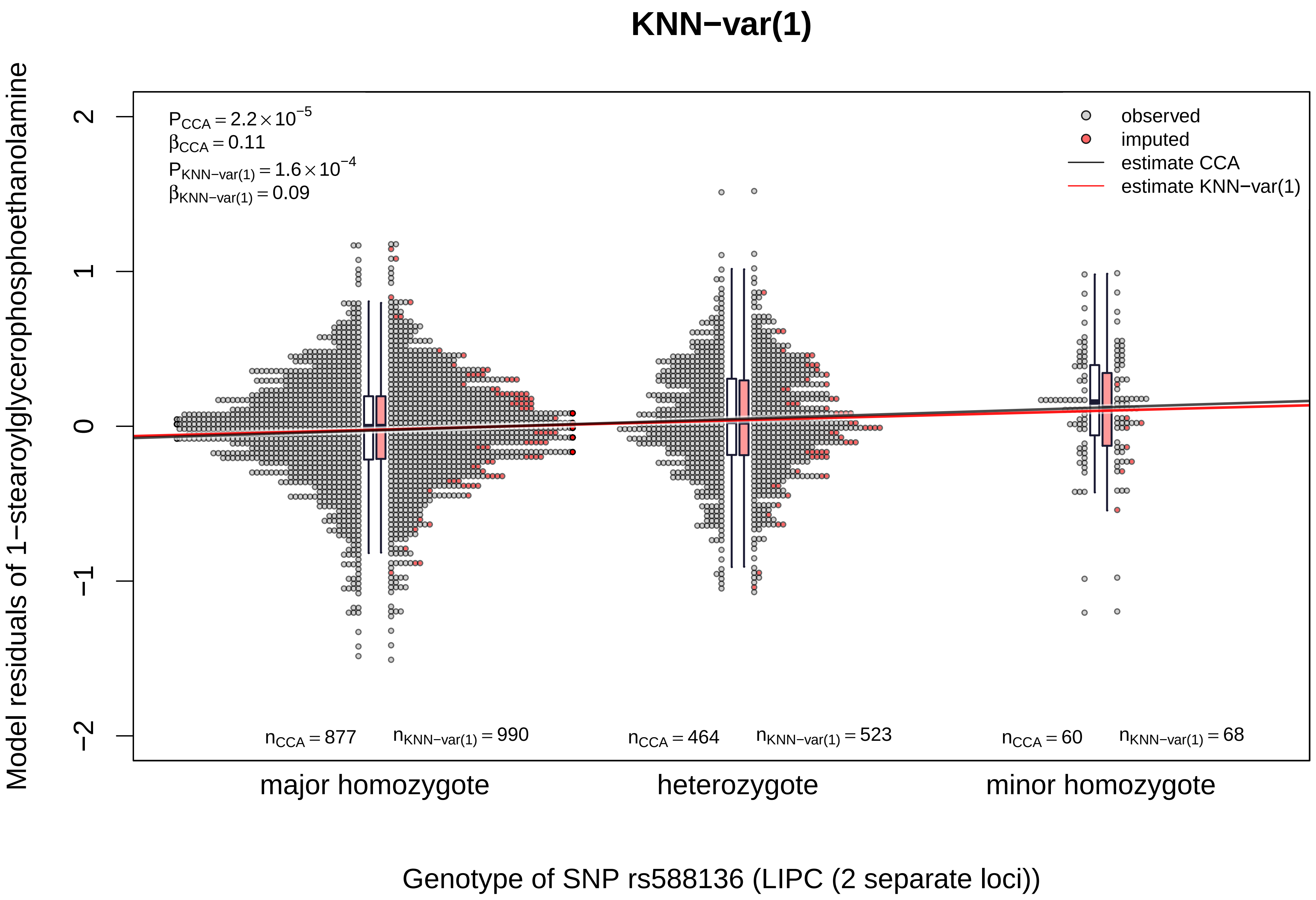
KNN−var(1)
$P_{CCA} = 2.2 \times 10^{-5}$
$\beta_{CCA} = 0.11$
$P_{KNN-var(1)} = 1.6 \times 10^{-4}$
$\beta_{KNN-var(1)} = 0.09$
observed
imputed
estimate CCA
estimate KNN−var(1)
2
1
0
−1
−2
Model residuals of 1−stearoylglycerophosphoethanolamine
$n_{CCA} = 877$
$n_{KNN-var(1)} = 990$
$n_{CCA} = 464$
$n_{KNN-var(1)} = 523$
$n_{CCA} = 60$
$n_{KNN-var(1)} = 68$
major homozygote
heterozygote
minor homozygote
Genotype of SNP rs588136 (LIPC (2 separate loci))

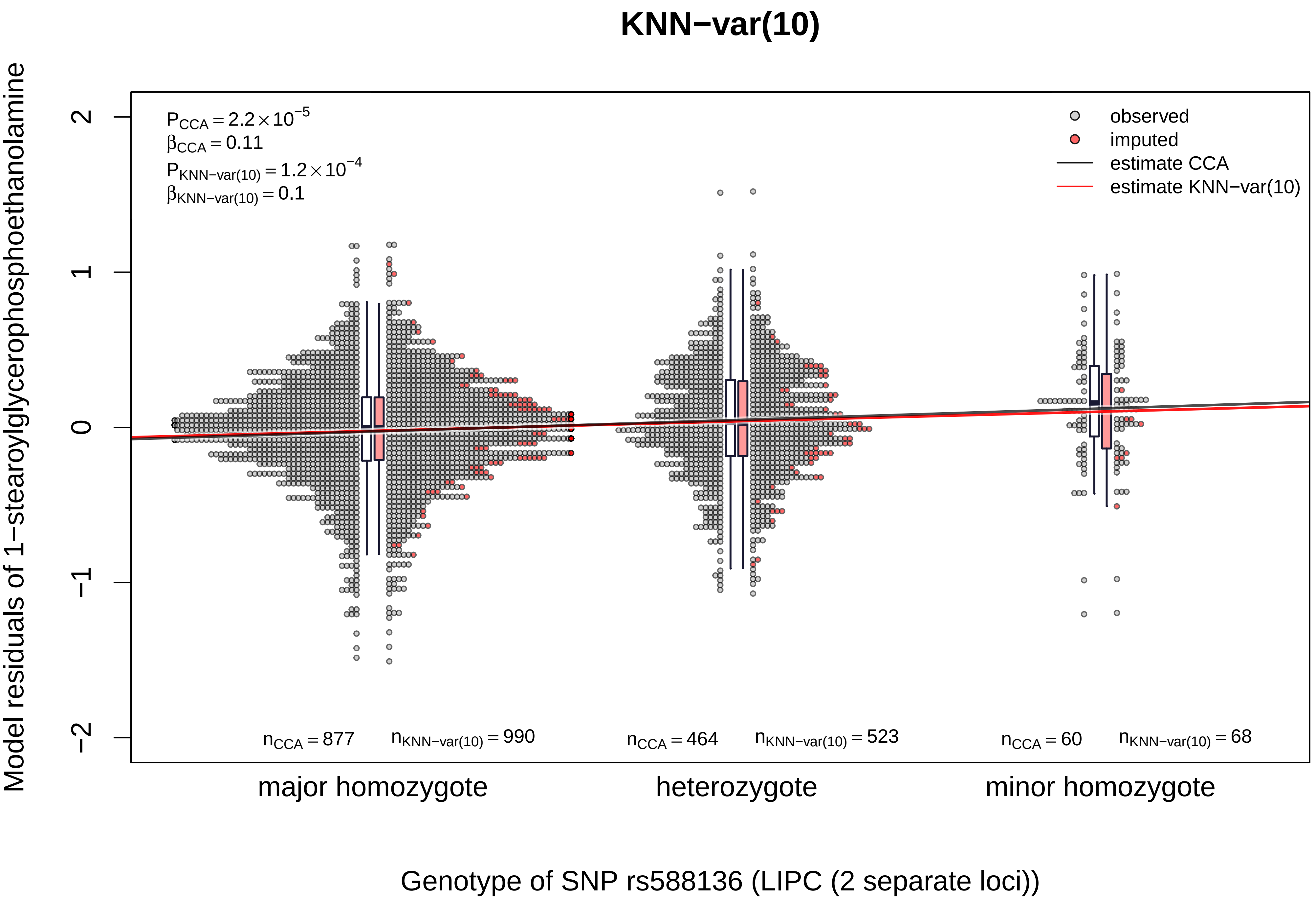

KNN−var(10)
$P_{CCA} = 2.2 \times 10^{-5}$
$\beta_{CCA} = 0.11$
$P_{KNN-var(10)} = 1.2 \times 10^{-4}$
$\beta_{KNN-var(10)} = 0.1$
observed
imputed
estimate CCA
estimate KNN−var(10)
2
1
0
−1
−2
Model residuals of 1−stearoylglycerophosphoethanolamine
$n_{CCA} = 877$
$n_{KNN-var(10)} = 990$
$n_{CCA} = 464$
$n_{KNN-var(10)} = 523$
$n_{CCA} = 60$
$n_{KNN-var(10)} = 68$
major homozygote
heterozygote
minor homozygote
Genotype of SNP rs588136 (LIPC (2 separate loci))

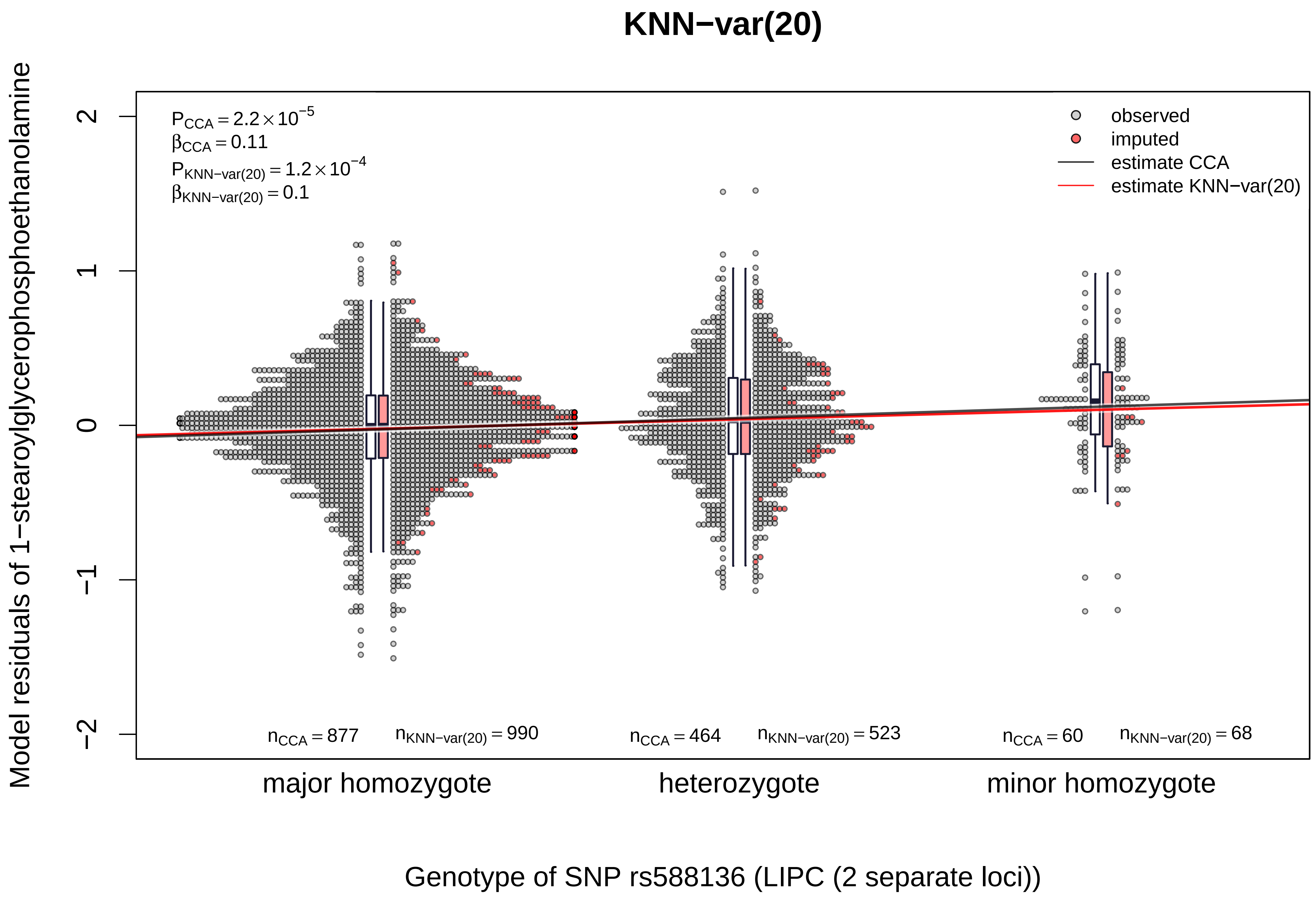
KNN−var(20)
$P_{CCA} = 2.2 \times 10^{-5}$
$\beta_{CCA} = 0.11$
$P_{KNN-var(20)} = 1.2 \times 10^{-4}$
$\beta_{KNN-var(20)} = 0.1$
observed
imputed
estimate CCA
estimate KNN−var(20)
$n_{CCA} = 877$
$n_{KNN-var(20)} = 990$
$n_{CCA} = 464$
$n_{KNN-var(20)} = 523$
$n_{CCA} = 60$
$n_{KNN-var(20)} = 68$
2
1
0
−1
−2
Model residuals of 1−stearoylglycerophosphoethanolamine
major homozygote
heterozygote
minor homozygote
Genotype of SNP rs588136 (LIPC (2 separate loci))

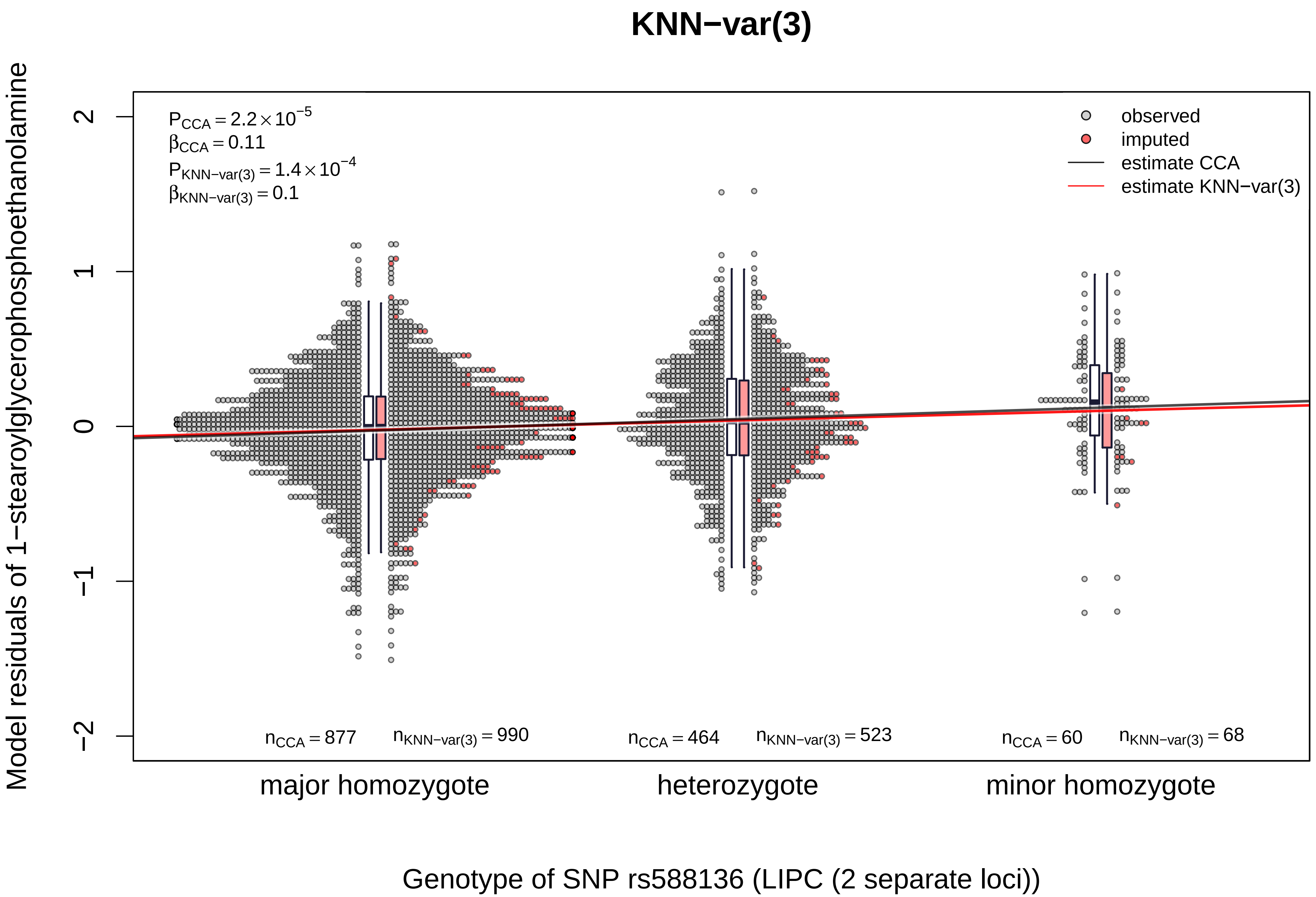

KNN−var(3)
$P_{CCA} = 2.2 \times 10^{-5}$
$\beta_{CCA} = 0.11$
$P_{KNN-var(3)} = 1.4 \times 10^{-4}$
$\beta_{KNN-var(3)} = 0.1$
observed
imputed
estimate CCA
estimate KNN−var(3)
Model residuals of 1−stearoylglycerophosphoethanolamine
2
1
0
−1
−2
$n_{CCA} = 877$
$n_{KNN-var(3)} = 990$
$n_{CCA} = 464$
$n_{KNN-var(3)} = 523$
$n_{CCA} = 60$
$n_{KNN-var(3)} = 68$
major homozygote
heterozygote
minor homozygote
Genotype of SNP rs588136 (LIPC (2 separate loci))

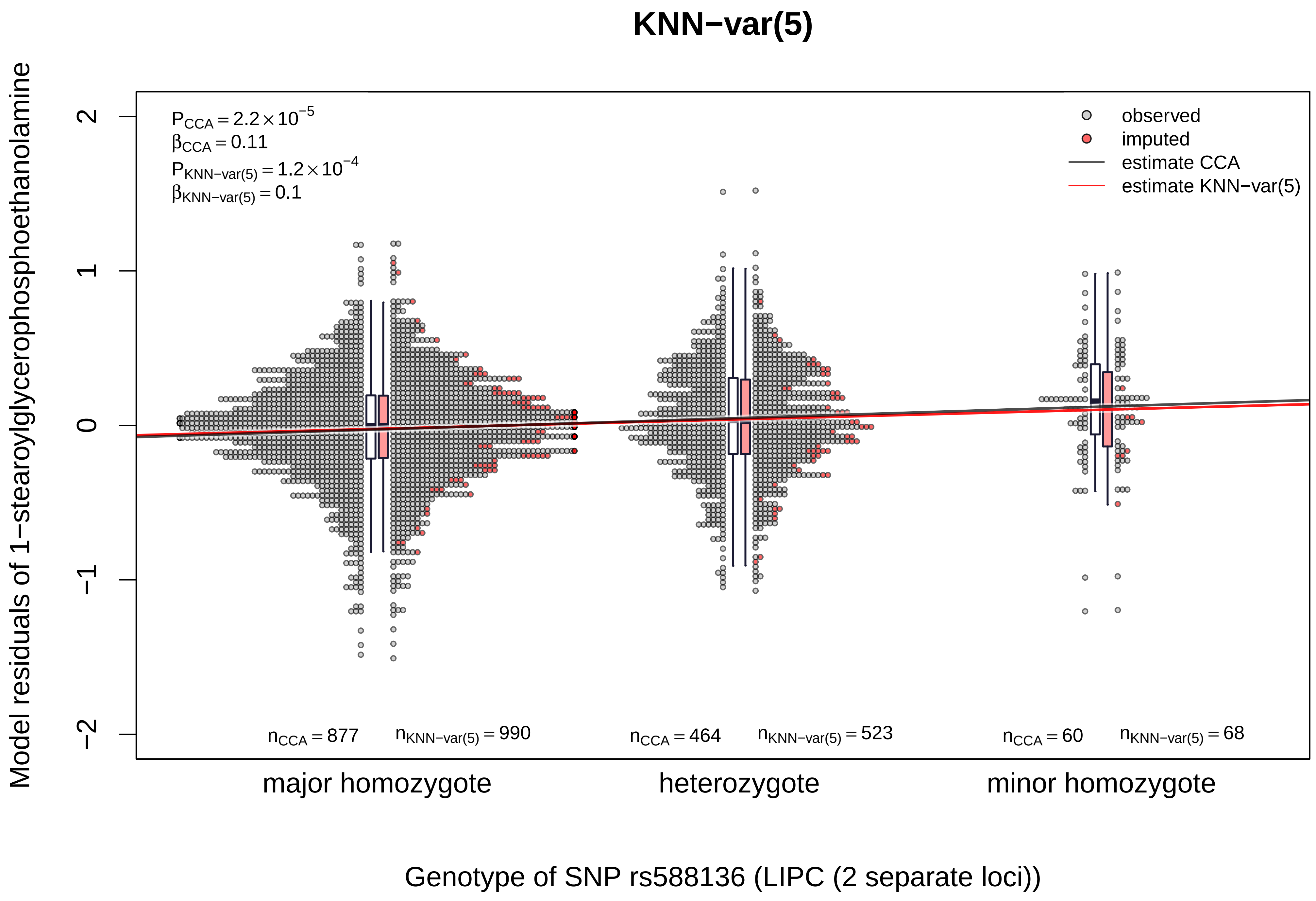

KNN−var(5)
$P_{CCA} = 2.2 \times 10^{-5}$
$\beta_{CCA} = 0.11$
$P_{KNN-var(5)} = 1.2 \times 10^{-4}$
$\beta_{KNN-var(5)} = 0.1$
observed
imputed
estimate CCA
estimate KNN−var(5)
Model residuals of 1−stearoylglycerophosphoethanolamine
2
1
0
−1
−2
$n_{CCA} = 877$
$n_{KNN-var(5)} = 990$
$n_{CCA} = 464$
$n_{KNN-var(5)} = 523$
$n_{CCA} = 60$
$n_{KNN-var(5)} = 68$
major homozygote
heterozygote
minor homozygote
Genotype of SNP rs588136 (LIPC (2 separate loci))

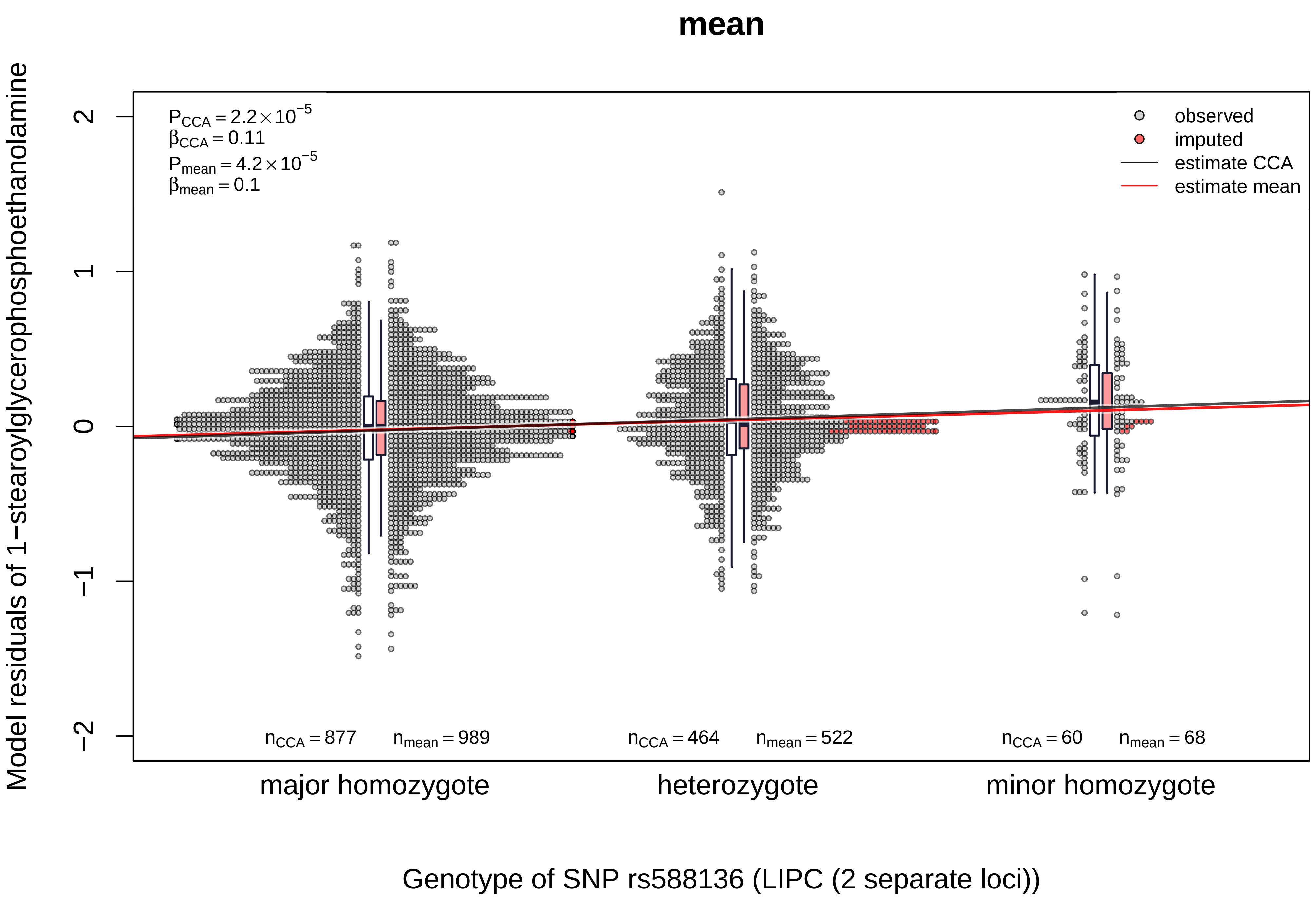

mean
$P_{CCA} = 2.2 \times 10^{-5}$
$\beta_{CCA} = 0.11$
$P_{mean} = 4.2 \times 10^{-5}$
$\beta_{mean} = 0.1$
observed
imputed
estimate CCA
estimate mean
Model residuals of 1−stearoylglycerophosphoethanolamine
2
1
0
−1
−2
$n_{CCA} = 877$
$n_{mean} = 989$
$n_{CCA} = 464$
$n_{mean} = 522$
$n_{CCA} = 60$
$n_{mean} = 68$
major homozygote
heterozygote
minor homozygote
Genotype of SNP rs588136 (LIPC (2 separate loci))

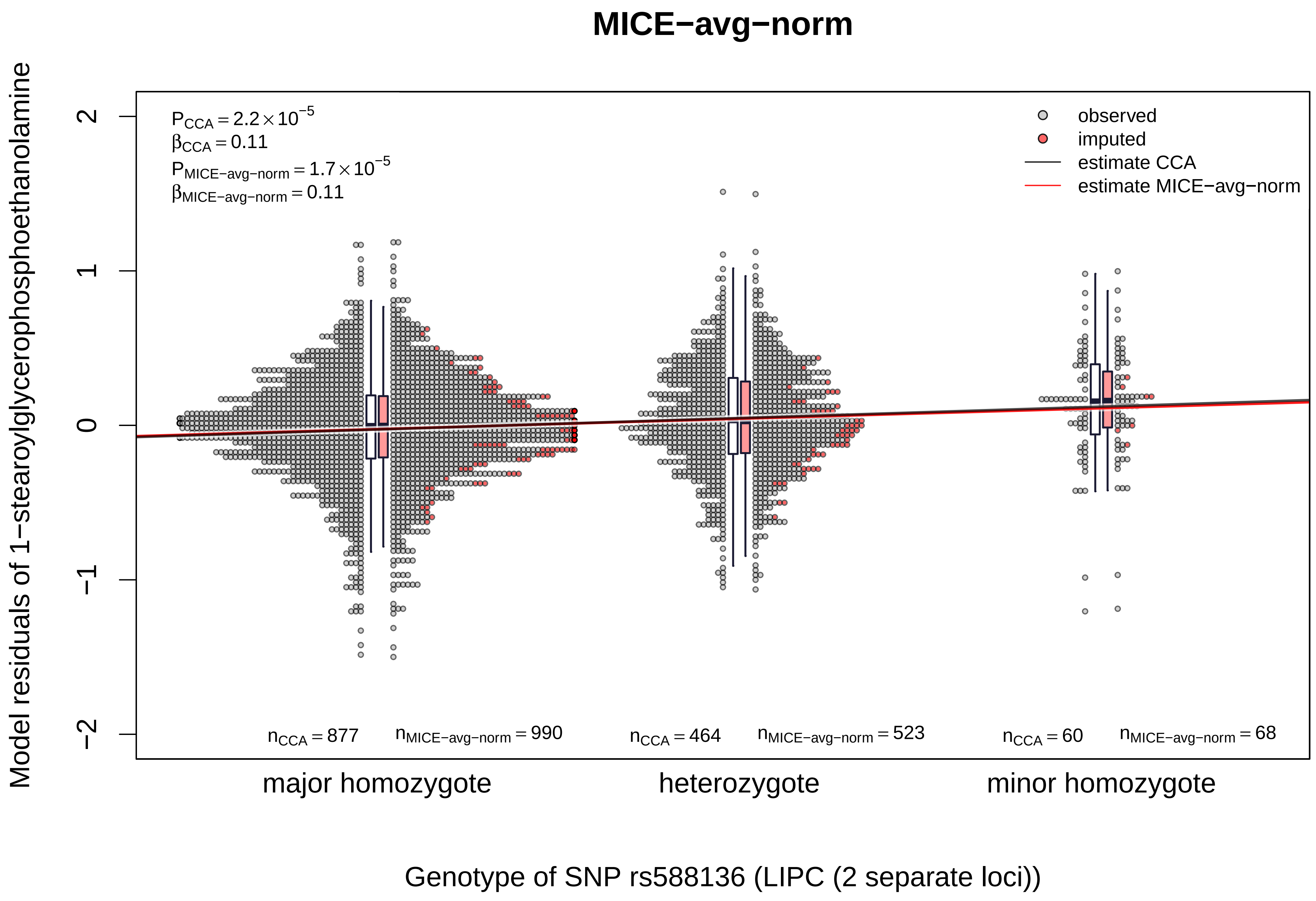
MICE−avg−norm
$P_{CCA} = 2.2 \times 10^{-5}$
$\beta_{CCA} = 0.11$
$P_{MICE-avg-norm} = 1.7 \times 10^{-5}$
$\beta_{MICE-avg-norm} = 0.11$
observed
imputed
estimate CCA
estimate MICE−avg−norm
$n_{CCA} = 877$
$n_{MICE-avg-norm} = 990$
$n_{CCA} = 464$
$n_{MICE-avg-norm} = 523$
$n_{CCA} = 60$
$n_{MICE-avg-norm} = 68$
2
1
0
−1
−2
Model residuals of 1−stearoylglycerophosphoethanolamine
major homozygote
heterozygote
minor homozygote
Genotype of SNP rs588136 (LIPC (2 separate loci))

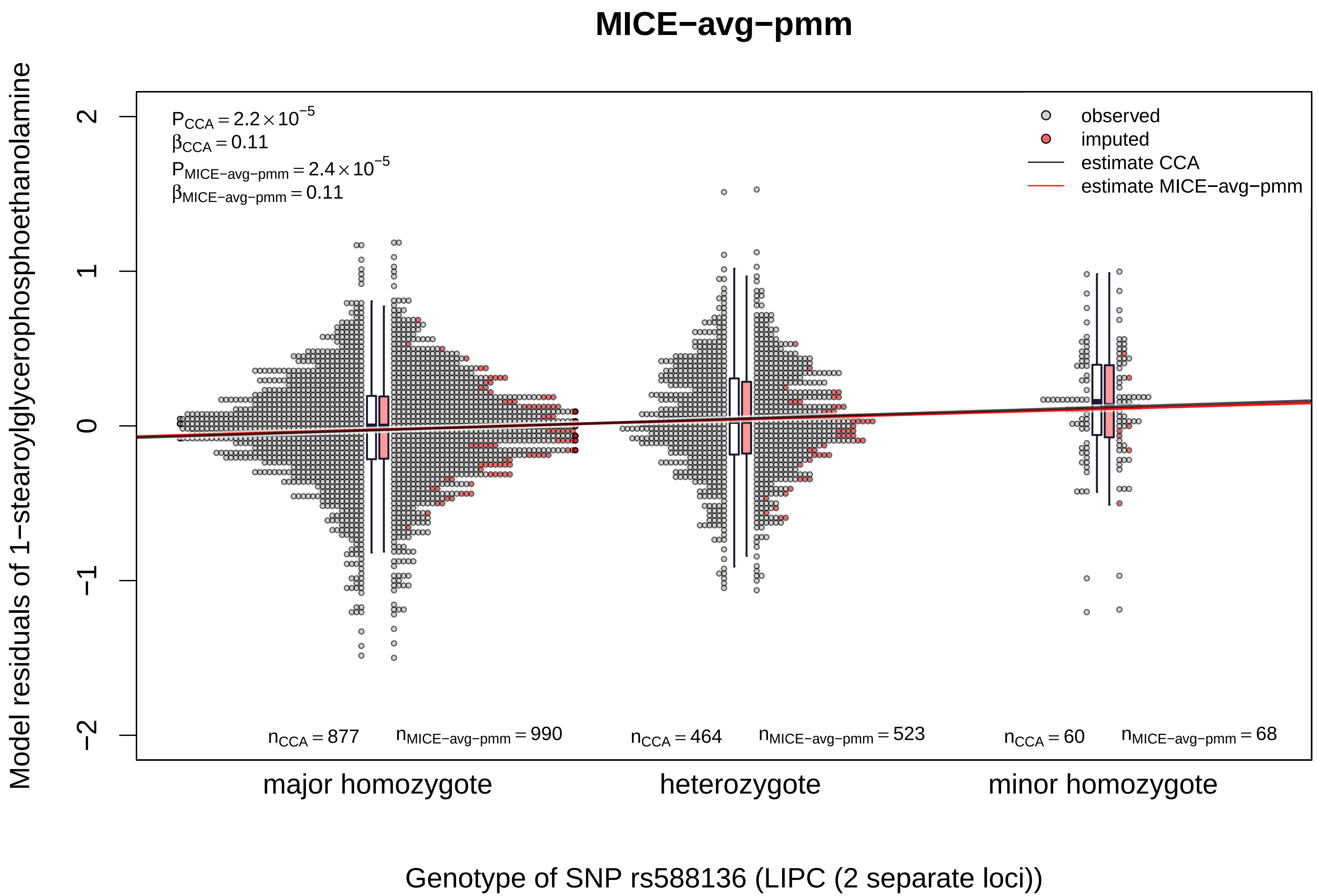
MICE−avg−pmm
$P_{CCA} = 2.2 \times 10^{-5}$
$\beta_{CCA} = 0.11$
$P_{MICE-avg-pmm} = 2.4 \times 10^{-5}$
$\beta_{MICE-avg-pmm} = 0.11$
observed
imputed
estimate CCA
estimate MICE−avg−pmm
$n_{CCA} = 877$
$n_{MICE-avg-pmm} = 990$
$n_{CCA} = 464$
$n_{MICE-avg-pmm} = 523$
$n_{CCA} = 60$
$n_{MICE-avg-pmm} = 68$
2
1
0
−1
−2
Model residuals of 1−stearoylglycerophosphoethanolamine
major homozygote
heterozygote
minor homozygote
Genotype of SNP rs588136 (LIPC (2 separate loci))

MICE−norm

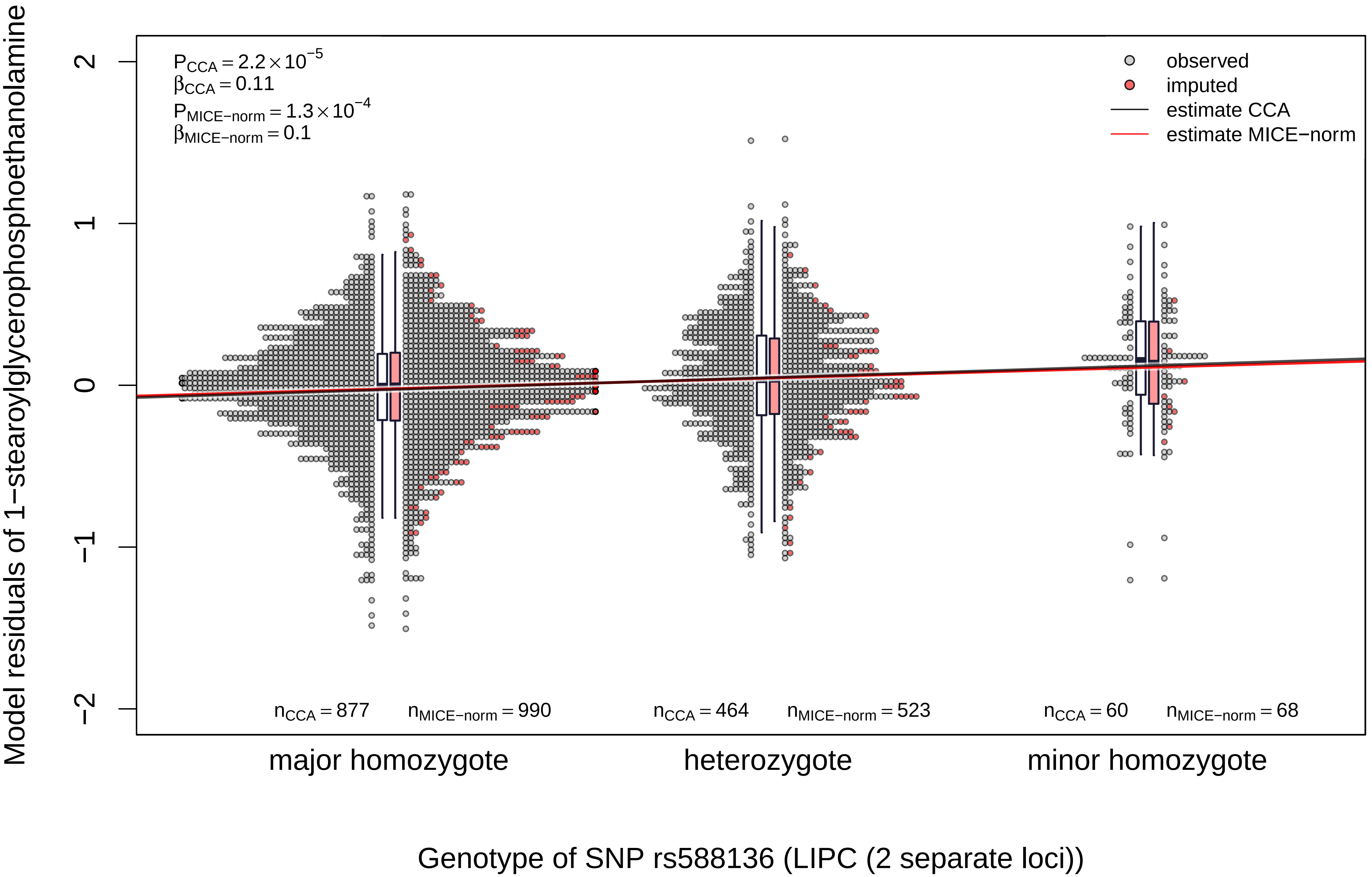

$P_{CCA} = 2.2 \times 10^{-5}$
$\beta_{CCA} = 0.11$
$P_{MICE-norm} = 1.3 \times 10^{-4}$
$\beta_{MICE-norm} = 0.1$
observed
imputed
estimate CCA
estimate MICE−norm
Model residuals of 1−stearoylglycerophosphoethanolamine
2
1
0
−1
−2
$n_{CCA} = 877$
$n_{MICE-norm} = 990$
$n_{CCA} = 464$
$n_{MICE-norm} = 523$
$n_{CCA} = 60$
$n_{MICE-norm} = 68$
major homozygote
heterozygote
minor homozygote
Genotype of SNP rs588136 (LIPC (2 separate loci))

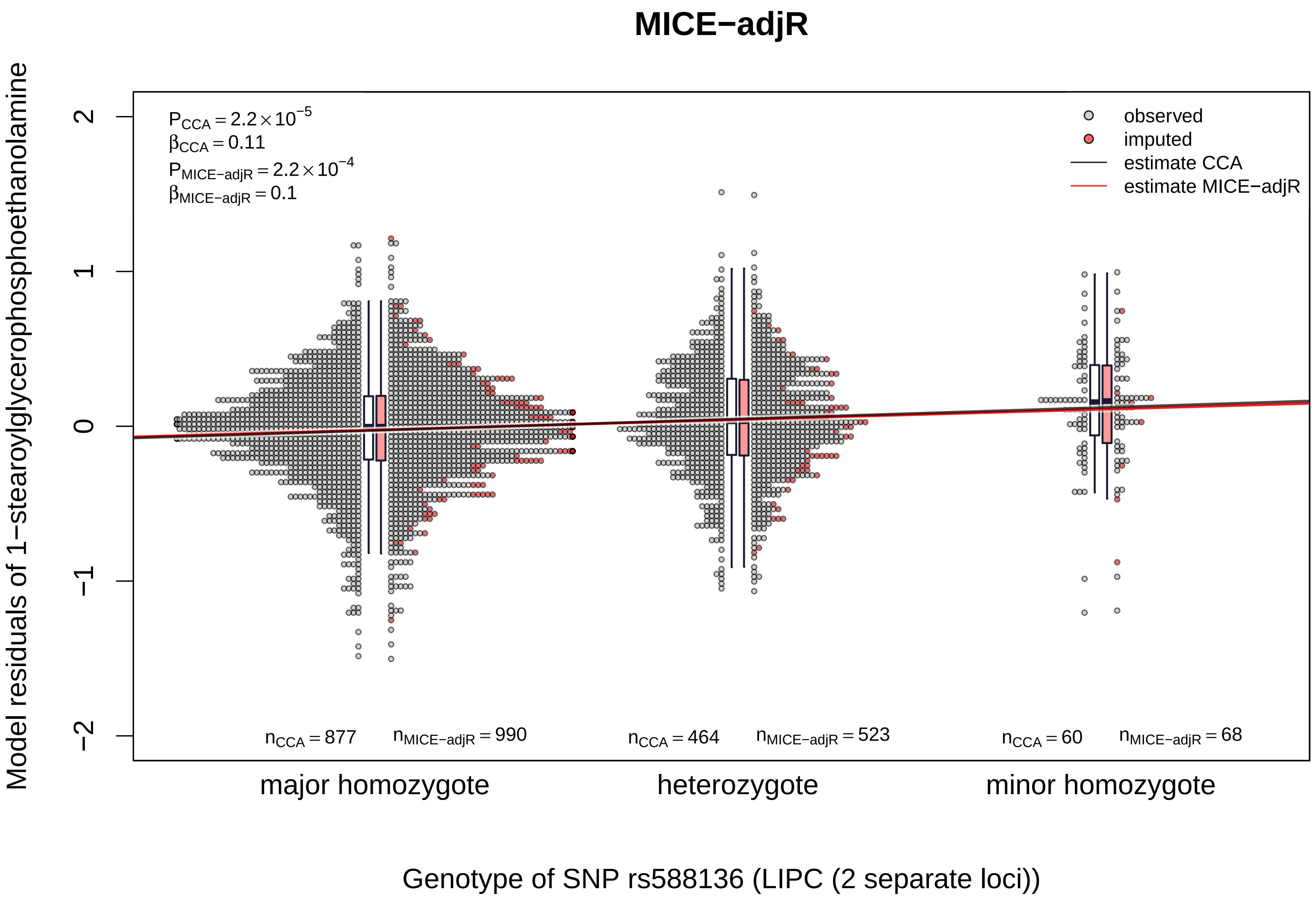
MICE−adjR
$P_{CCA} = 2.2 \times 10^{-5}$
$\beta_{CCA} = 0.11$
$P_{MICE-adjR} = 2.2 \times 10^{-4}$
$\beta_{MICE-adjR} = 0.1$
observed
imputed
estimate CCA
estimate MICE−adjR
2
1
0
−1
−2
$n_{CCA} = 877$
$n_{MICE-adjR} = 990$
$n_{CCA} = 464$
$n_{MICE-adjR} = 523$
$n_{CCA} = 60$
$n_{MICE-adjR} = 68$
major homozygote
heterozygote
minor homozygote
Model residuals of 1−stearoylglycerophosphoethanolamine
Genotype of SNP rs588136 (LIPC (2 separate loci))

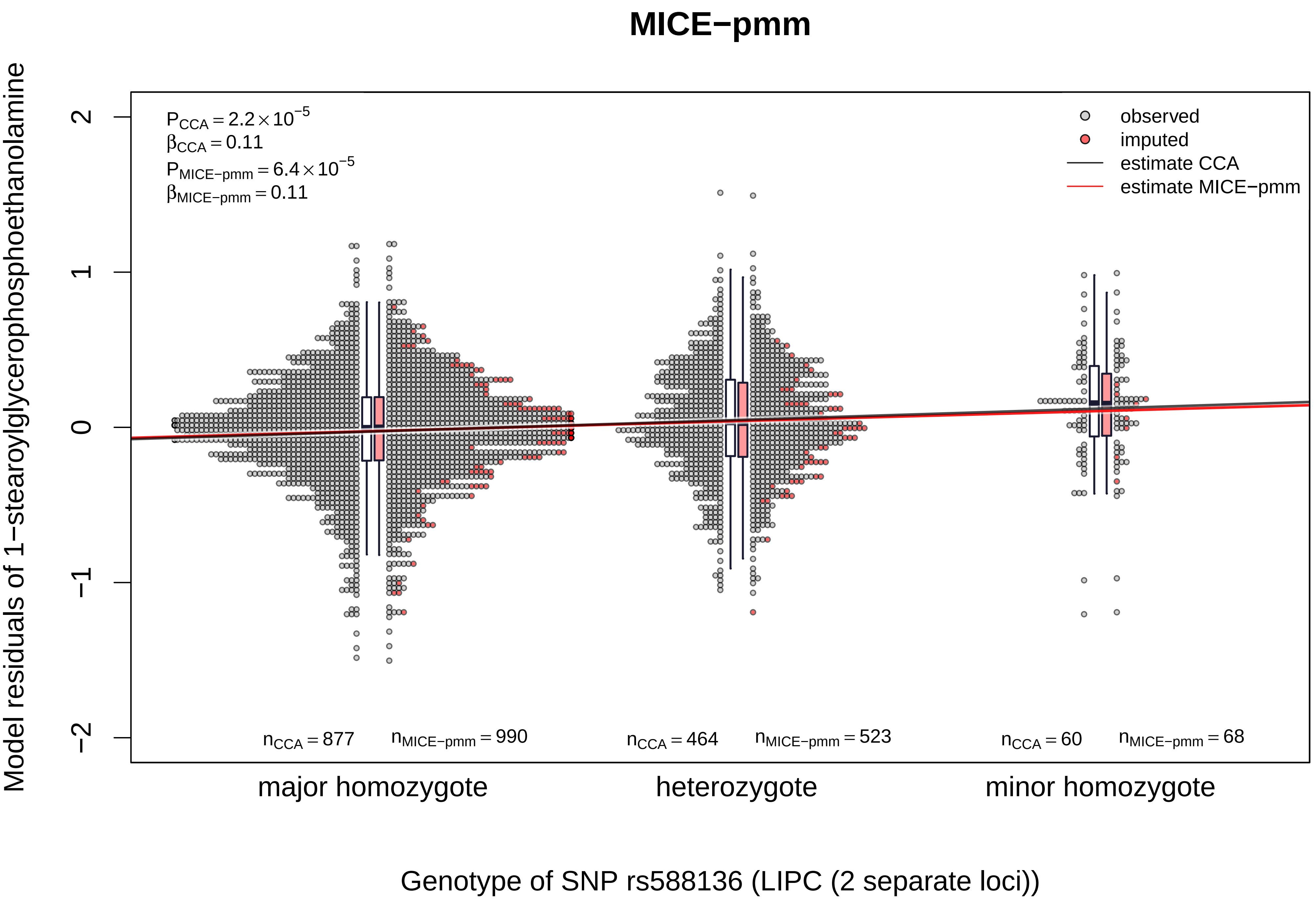

MICE−pmm
$P_{CCA} = 2.2 \times 10^{-5}$
$\beta_{CCA} = 0.11$
$P_{MICE-pmm} = 6.4 \times 10^{-5}$
$\beta_{MICE-pmm} = 0.11$
observed
imputed
estimate CCA
estimate MICE−pmm
$n_{CCA} = 877$
$n_{MICE-pmm} = 990$
$n_{CCA} = 464$
$n_{MICE-pmm} = 523$
$n_{CCA} = 60$
$n_{MICE-pmm} = 68$
2
1
0
−1
−2
Model residuals of 1−stearoylglycerophosphoethanolamine
major homozygote
heterozygote
minor homozygote
Genotype of SNP rs588136 (LIPC (2 separate loci))

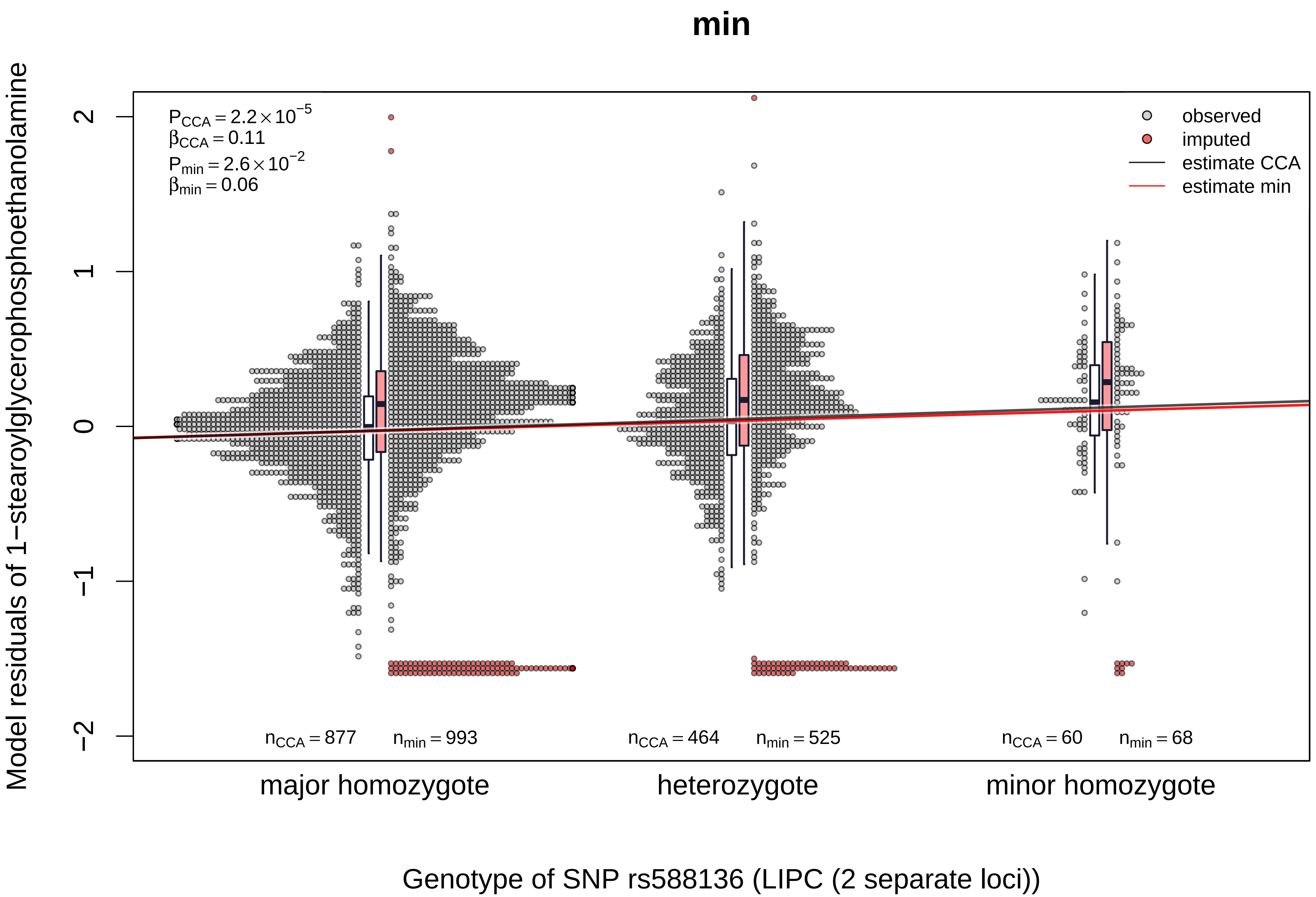

min
$P_{CCA} = 2.2 \times 10^{-5}$
$\beta_{CCA} = 0.11$
$P_{min} = 2.6 \times 10^{-2}$
$\beta_{min} = 0.06$
observed
imputed
estimate CCA
estimate min
2
1
0
−1
−2
$n_{CCA} = 877$
$n_{min} = 993$
$n_{CCA} = 464$
$n_{min} = 525$
$n_{CCA} = 60$
$n_{min} = 68$
major homozygote
heterozygote
minor homozygote
Model residuals of 1−stearoylglycerophosphoethanolamine
Genotype of SNP rs588136 (LIPC (2 separate loci))

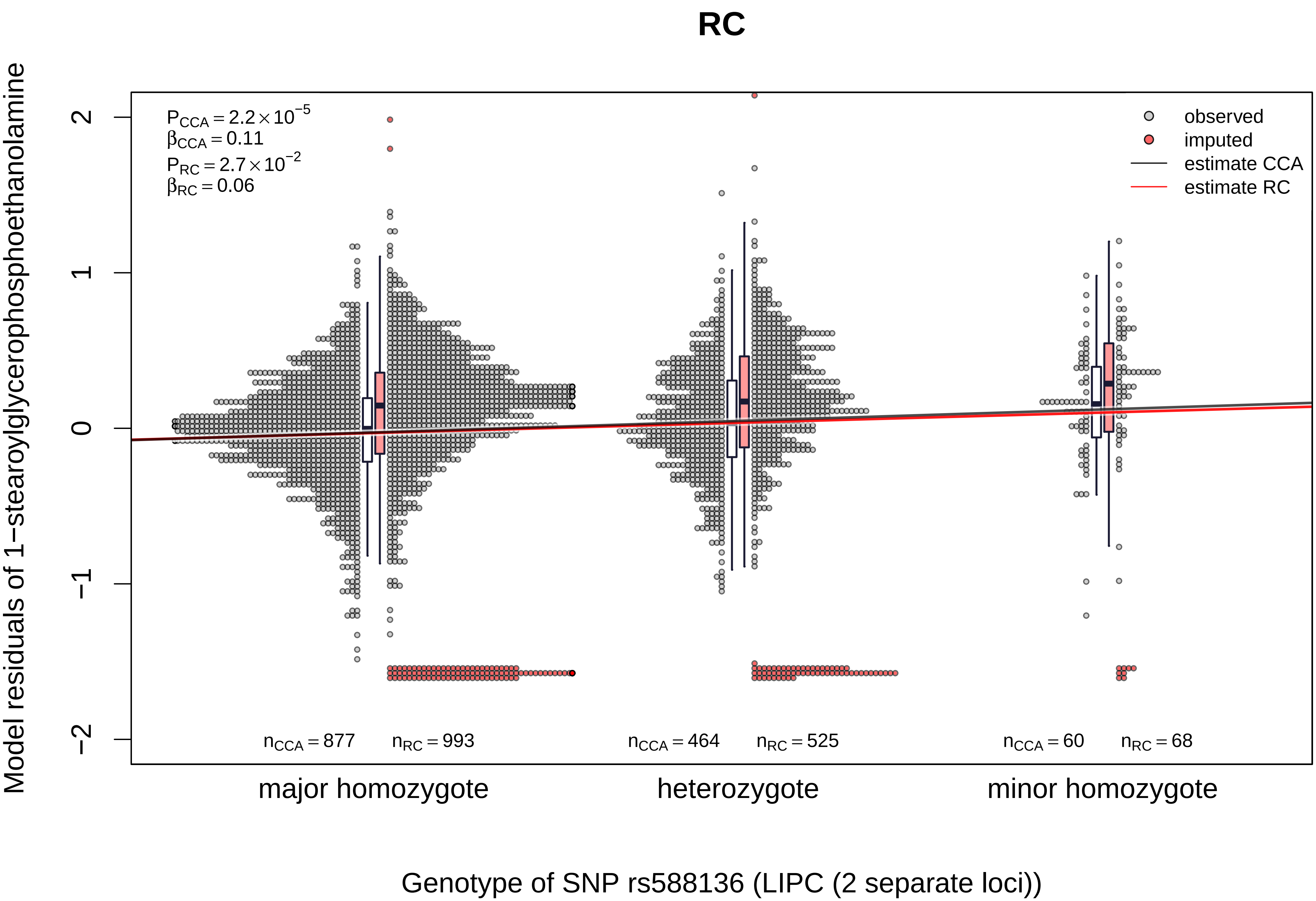

RC
$P_{CCA} = 2.2 \times 10^{-5}$
$\beta_{CCA} = 0.11$
$P_{RC} = 2.7 \times 10^{-2}$
$\beta_{RC} = 0.06$
observed
imputed
estimate CCA
estimate RC
2
1
0
−1
−2
$n_{CCA} = 877$
$n_{RC} = 993$
$n_{CCA} = 464$
$n_{RC} = 525$
$n_{CCA} = 60$
$n_{RC} = 68$
major homozygote
heterozygote
minor homozygote
Model residuals of 1−stearoylglycerophosphoethanolamine
Genotype of SNP rs588136 (LIPC (2 separate loci))

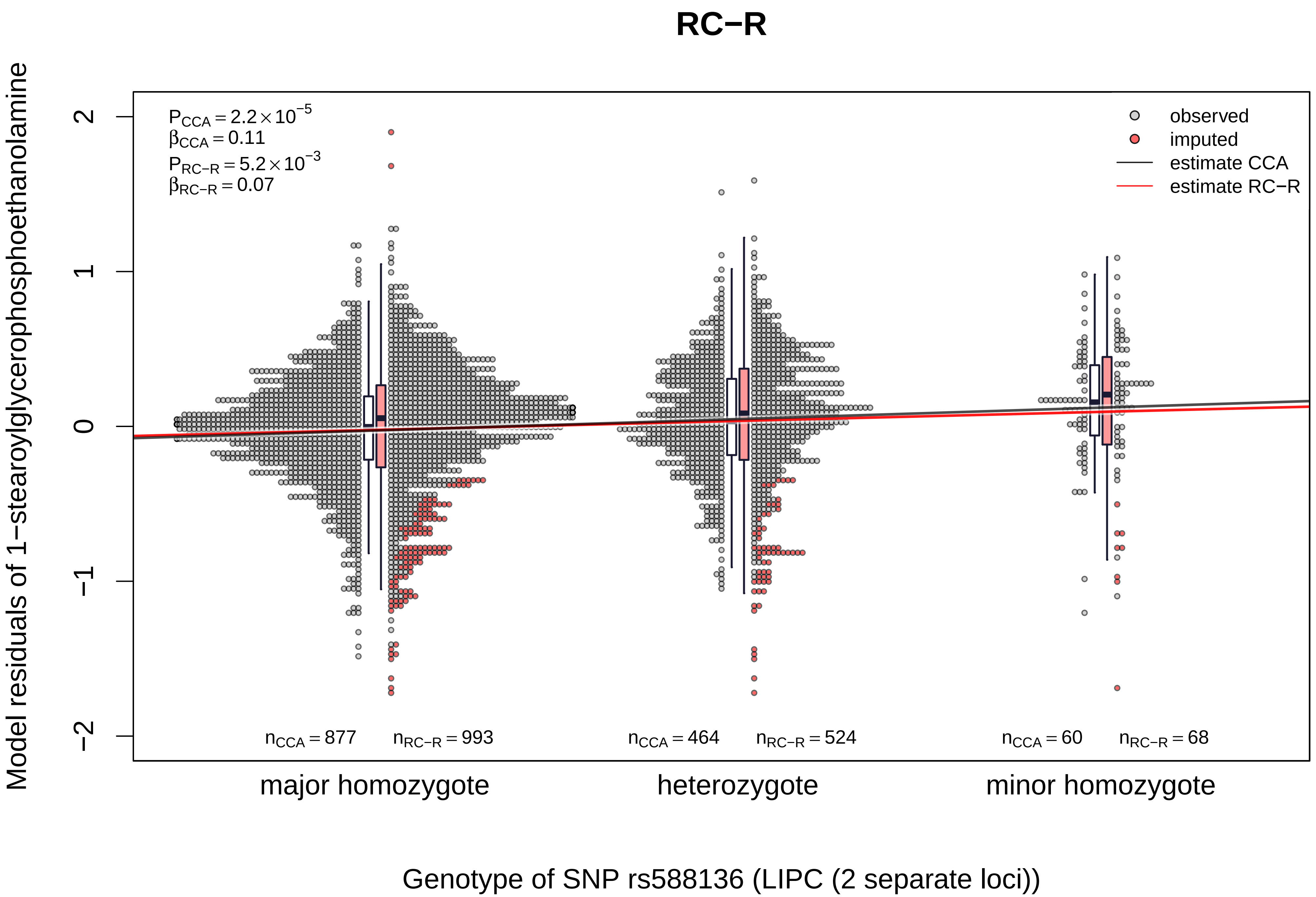

RC−R
$P_{CCA} = 2.2 \times 10^{-5}$
$\beta_{CCA} = 0.11$
$P_{RC-R} = 5.2 \times 10^{-3}$
$\beta_{RC-R} = 0.07$
observed
imputed
estimate CCA
estimate RC−R
$n_{CCA} = 877$
$n_{RC-R} = 993$
$n_{CCA} = 464$
$n_{RC-R} = 524$
$n_{CCA} = 60$
$n_{RC-R} = 68$
2
1
0
−1
−2
Model residuals of 1−stearoylglycerophosphoethanolamine
major homozygote
heterozygote
minor homozygote
Genotype of SNP rs588136 (LIPC (2 separate loci))

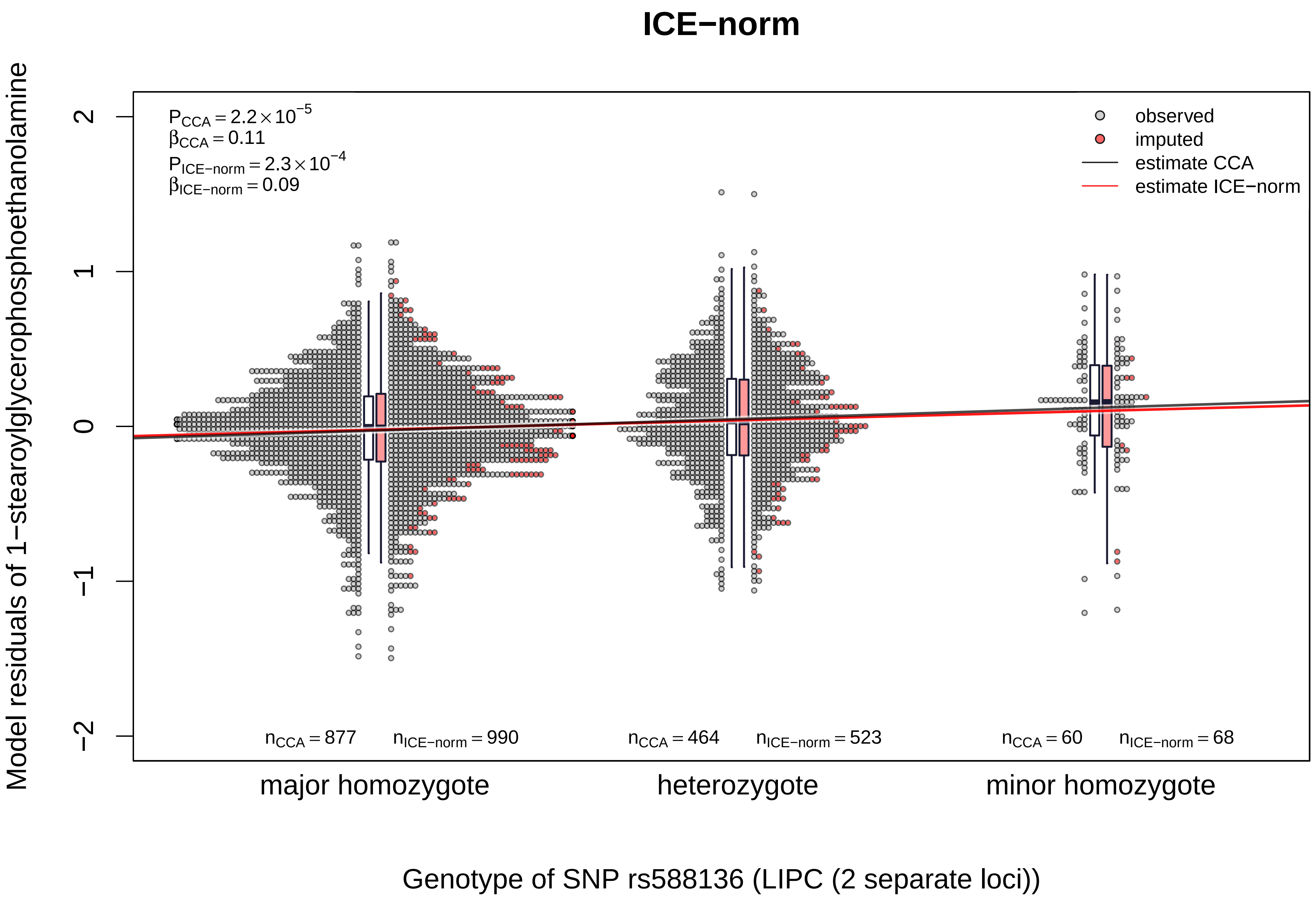

ICE−norm
$P_{CCA} = 2.2 \times 10^{-5}$
$\beta_{CCA} = 0.11$
$P_{ICE-norm} = 2.3 \times 10^{-4}$
$\beta_{ICE-norm} = 0.09$
observed
imputed
estimate CCA
estimate ICE−norm
Model residuals of 1−stearoylglycerophosphoethanolamine
2
1
0
−1
−2
$n_{CCA} = 877$
$n_{ICE-norm} = 990$
$n_{CCA} = 464$
$n_{ICE-norm} = 523$
$n_{CCA} = 60$
$n_{ICE-norm} = 68$
major homozygote
heterozygote
minor homozygote
Genotype of SNP rs588136 (LIPC (2 separate loci))

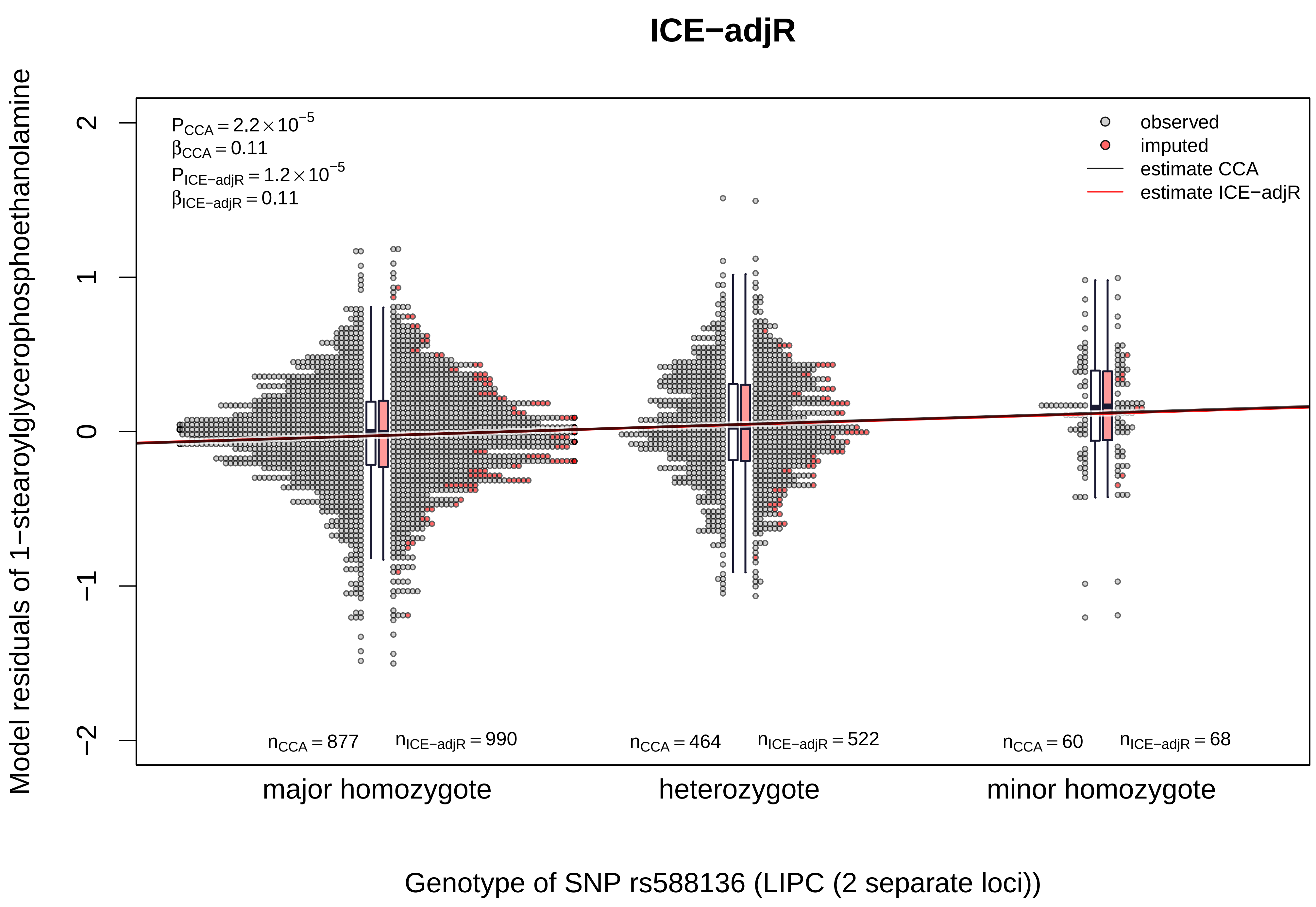
ICE−adjR
$P_{CCA} = 2.2 \times 10^{-5}$
$\beta_{CCA} = 0.11$
$P_{ICE-adjR} = 1.2 \times 10^{-5}$
$\beta_{ICE-adjR} = 0.11$
observed
imputed
estimate CCA
estimate ICE−adjR
$n_{CCA} = 877$
$n_{ICE-adjR} = 990$
$n_{CCA} = 464$
$n_{ICE-adjR} = 522$
$n_{CCA} = 60$
$n_{ICE-adjR} = 68$
2
1
0
−1
−2
Model residuals of 1−stearoylglycerophosphoethanolamine
major homozygote
heterozygote
minor homozygote
Genotype of SNP rs588136 (LIPC (2 separate loci))

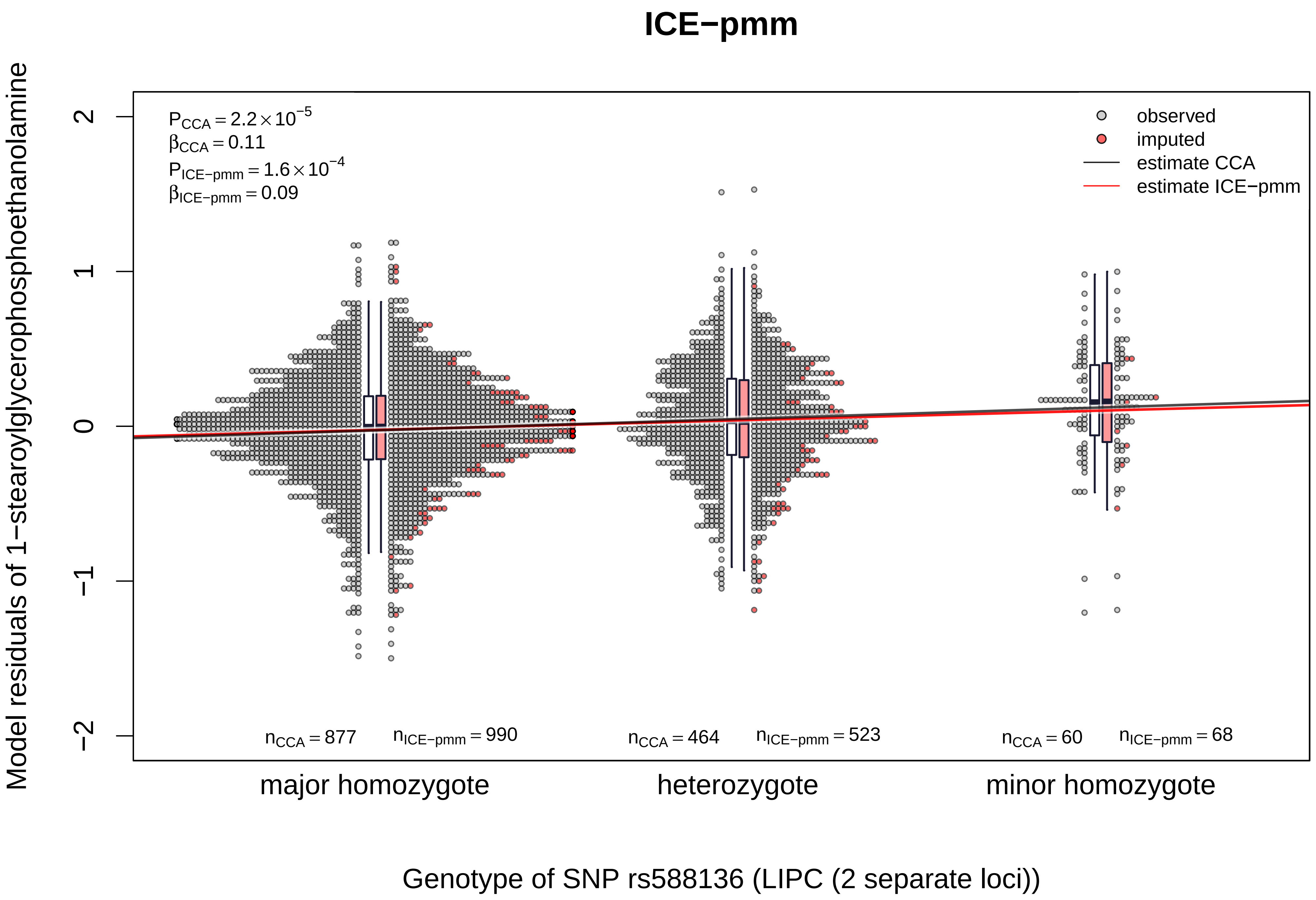

ICE−pmm
$P_{CCA} = 2.2 \times 10^{-5}$
$\beta_{CCA} = 0.11$
$P_{ICE-pmm} = 1.6 \times 10^{-4}$
$\beta_{ICE-pmm} = 0.09$
observed
imputed
estimate CCA
estimate ICE−pmm
Model residuals of 1−stearoylglycerophosphoethanolamine
2
1
0
−1
−2
$n_{CCA} = 877$
$n_{ICE-pmm} = 990$
$n_{CCA} = 464$
$n_{ICE-pmm} = 523$
$n_{CCA} = 60$
$n_{ICE-pmm} = 68$
major homozygote
heterozygote
minor homozygote
Genotype of SNP rs588136 (LIPC (2 separate loci))

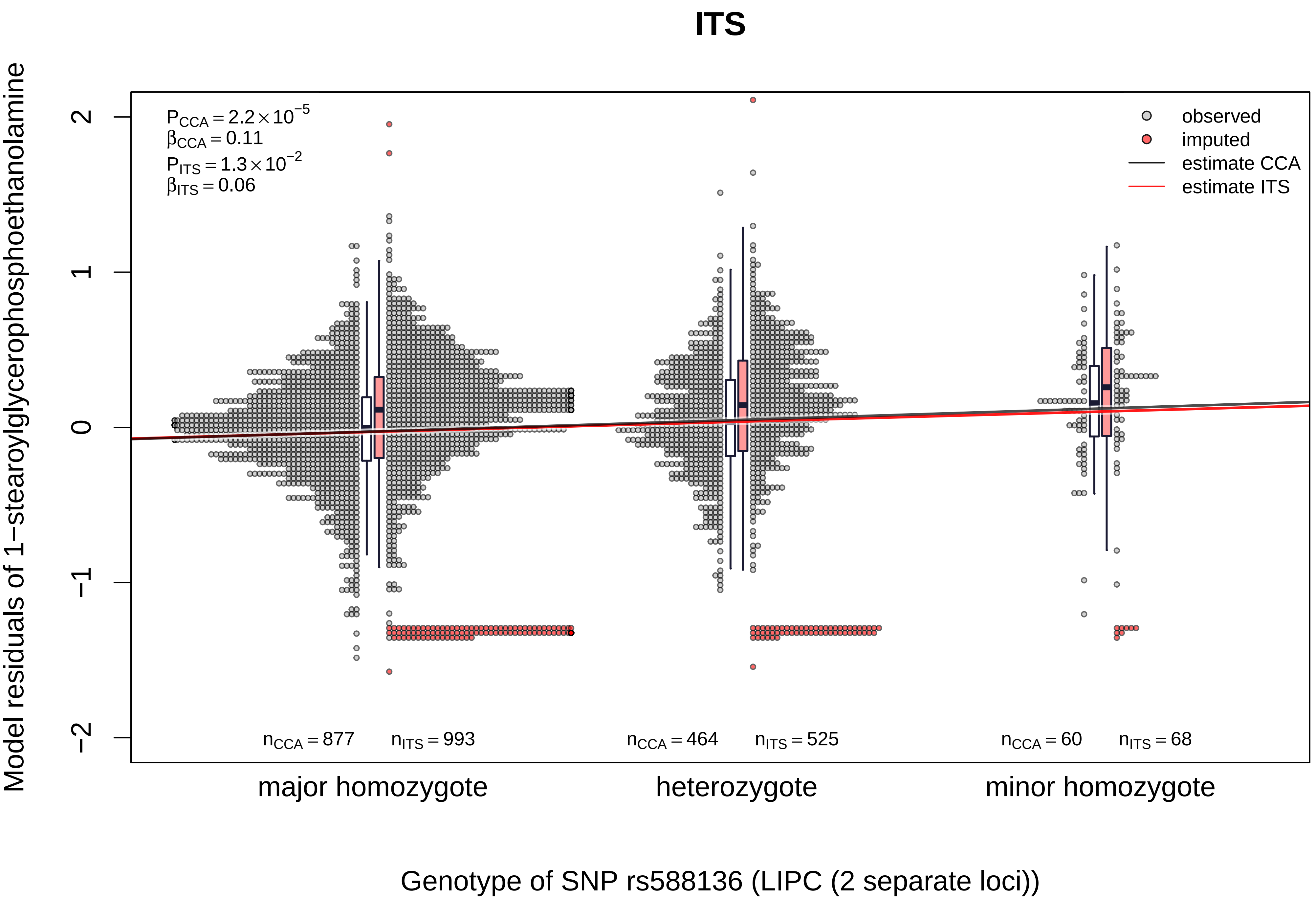
ITS
$P_{CCA} = 2.2 \times 10^{-5}$
$\beta_{CCA} = 0.11$
$P_{ITS} = 1.3 \times 10^{-2}$
$\beta_{ITS} = 0.06$
observed
imputed
estimate CCA
estimate ITS
$n_{CCA} = 877$
$n_{ITS} = 993$
$n_{CCA} = 464$
$n_{ITS} = 525$
$n_{CCA} = 60$
$n_{ITS} = 68$
major homozygote
heterozygote
minor homozygote
Genotype of SNP rs588136 (LIPC (2 separate loci))
Model residuals of 1−stearoylglycerophosphoethanolamine
2
1
0
−1
−2

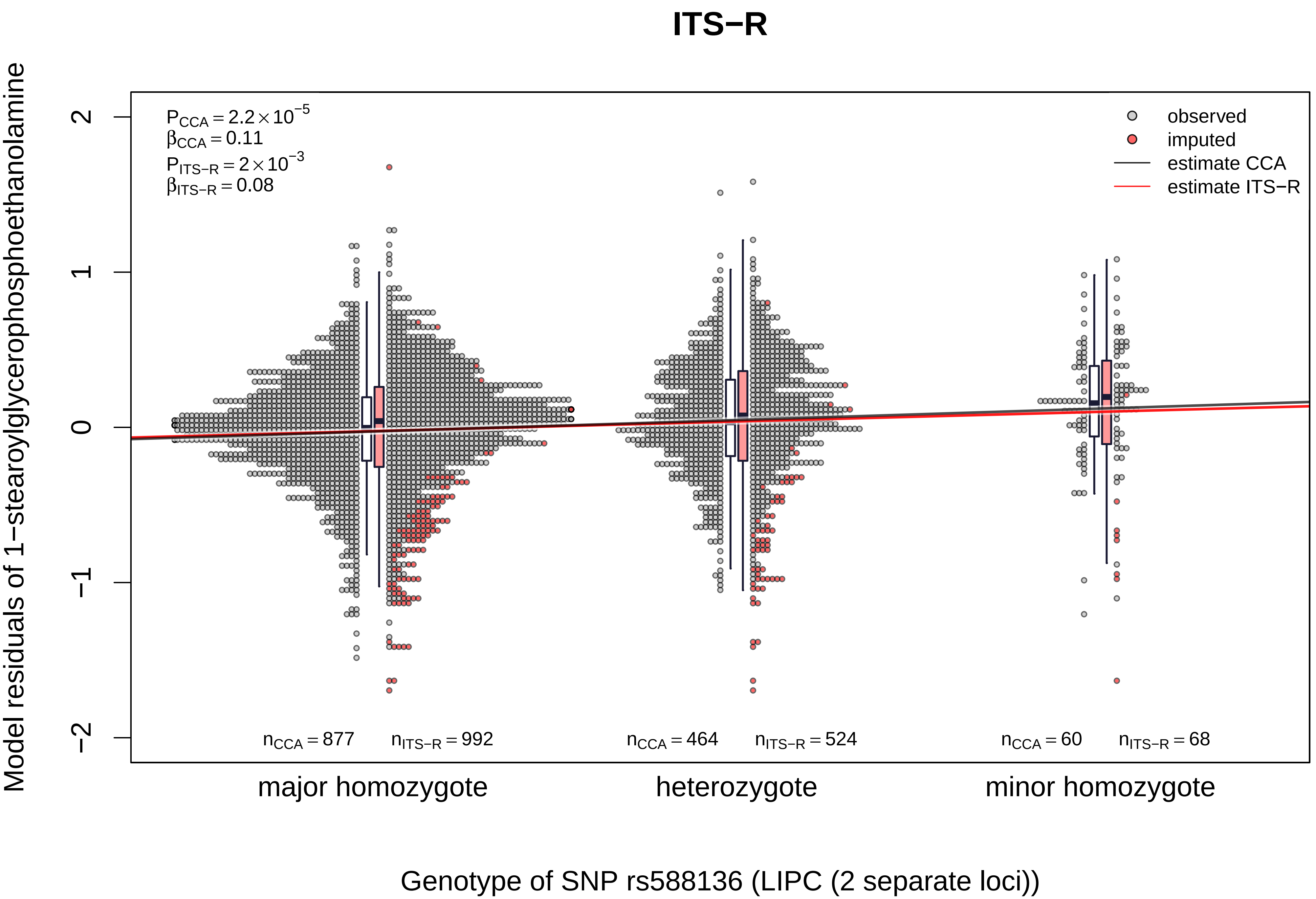

ITS−R
$P_{CCA} = 2.2 \times 10^{-5}$
$\beta_{CCA} = 0.11$
$P_{ITS-R} = 2 \times 10^{-3}$
$\beta_{ITS-R} = 0.08$
observed
imputed
estimate CCA
estimate ITS−R
2
1
0
−1
−2
$n_{CCA} = 877$
$n_{ITS-R} = 992$
$n_{CCA} = 464$
$n_{ITS-R} = 524$
$n_{CCA} = 60$
$n_{ITS-R} = 68$
major homozygote
heterozygote
minor homozygote
Model residuals of 1−stearoylglycerophosphoethanolamine
Genotype of SNP rs588136 (LIPC (2 separate loci))

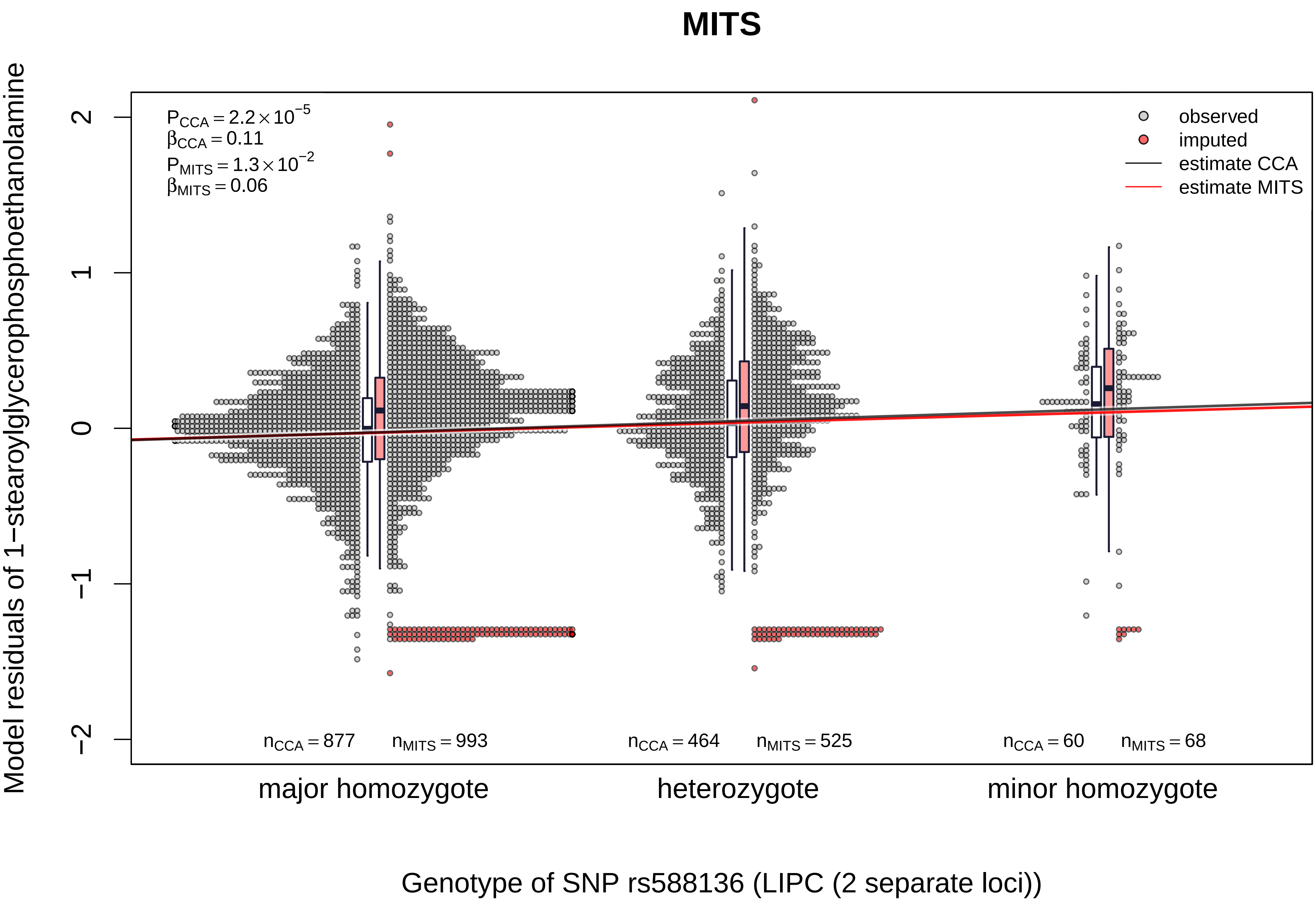

MITS
$P_{CCA} = 2.2 \times 10^{-5}$
$\beta_{CCA} = 0.11$
$P_{MITS} = 1.3 \times 10^{-2}$
$\beta_{MITS} = 0.06$
observed
imputed
estimate CCA
estimate MITS
Model residuals of 1−stearoylglycerophosphoethanolamine
2
1
0
−1
−2
$n_{CCA} = 877$
$n_{MITS} = 993$
$n_{CCA} = 464$
$n_{MITS} = 525$
$n_{CCA} = 60$
$n_{MITS} = 68$
major homozygote
heterozygote
minor homozygote
Genotype of SNP rs588136 (LIPC (2 separate loci))

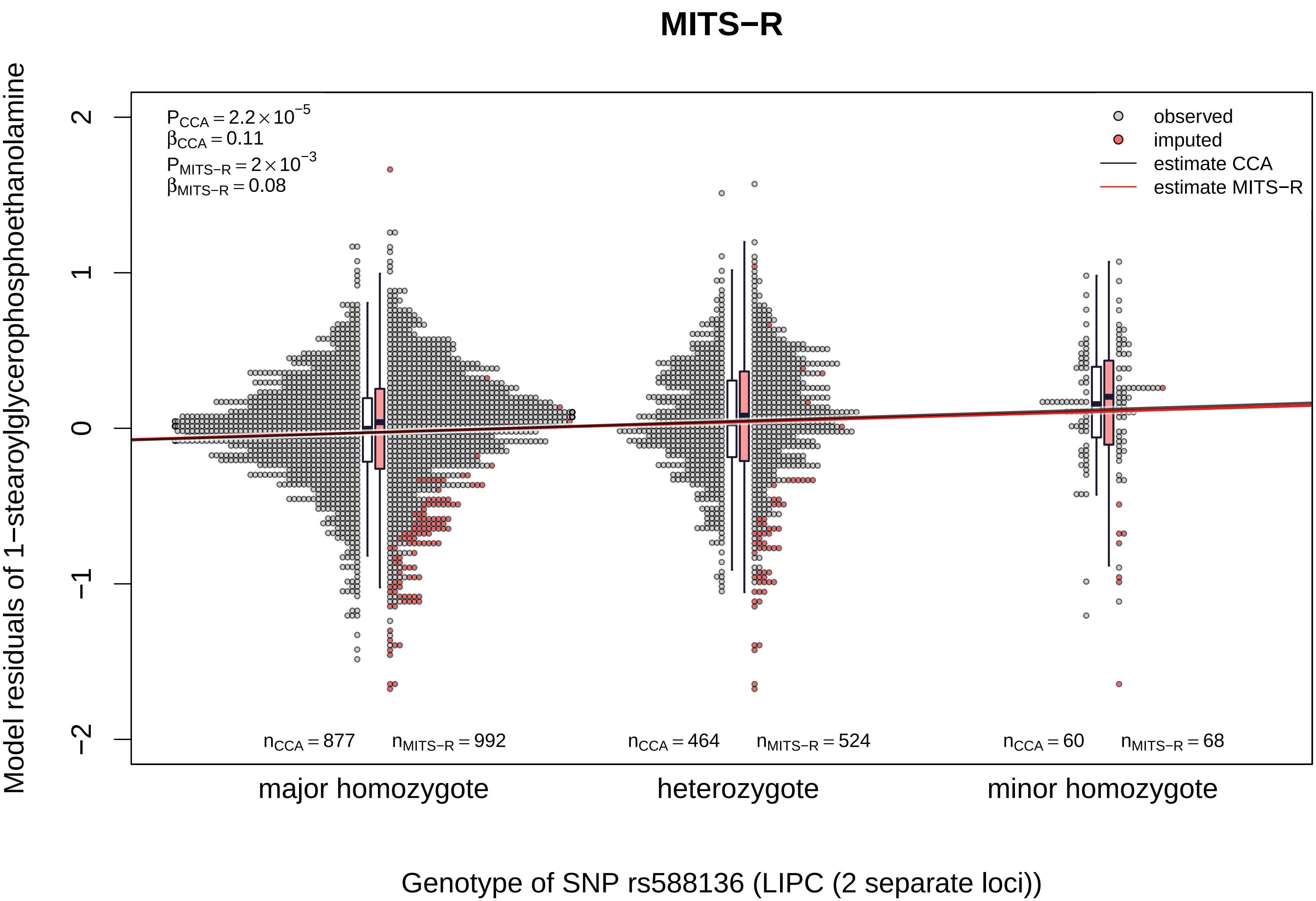

MITS−R
$P_{CCA} = 2.2 \times 10^{-5}$
$\beta_{CCA} = 0.11$
$P_{MITS-R} = 2 \times 10^{-3}$
$\beta_{MITS-R} = 0.08$
observed
imputed
estimate CCA
estimate MITS−R
2
1
0
−1
−2
$n_{CCA} = 877$
$n_{MITS-R} = 992$
$n_{CCA} = 464$
$n_{MITS-R} = 524$
$n_{CCA} = 60$
$n_{MITS-R} = 68$
major homozygote
heterozygote
minor homozygote
Model residuals of 1−stearoylglycerophosphoethanolamine
Genotype of SNP rs588136 (LIPC (2 separate loci))

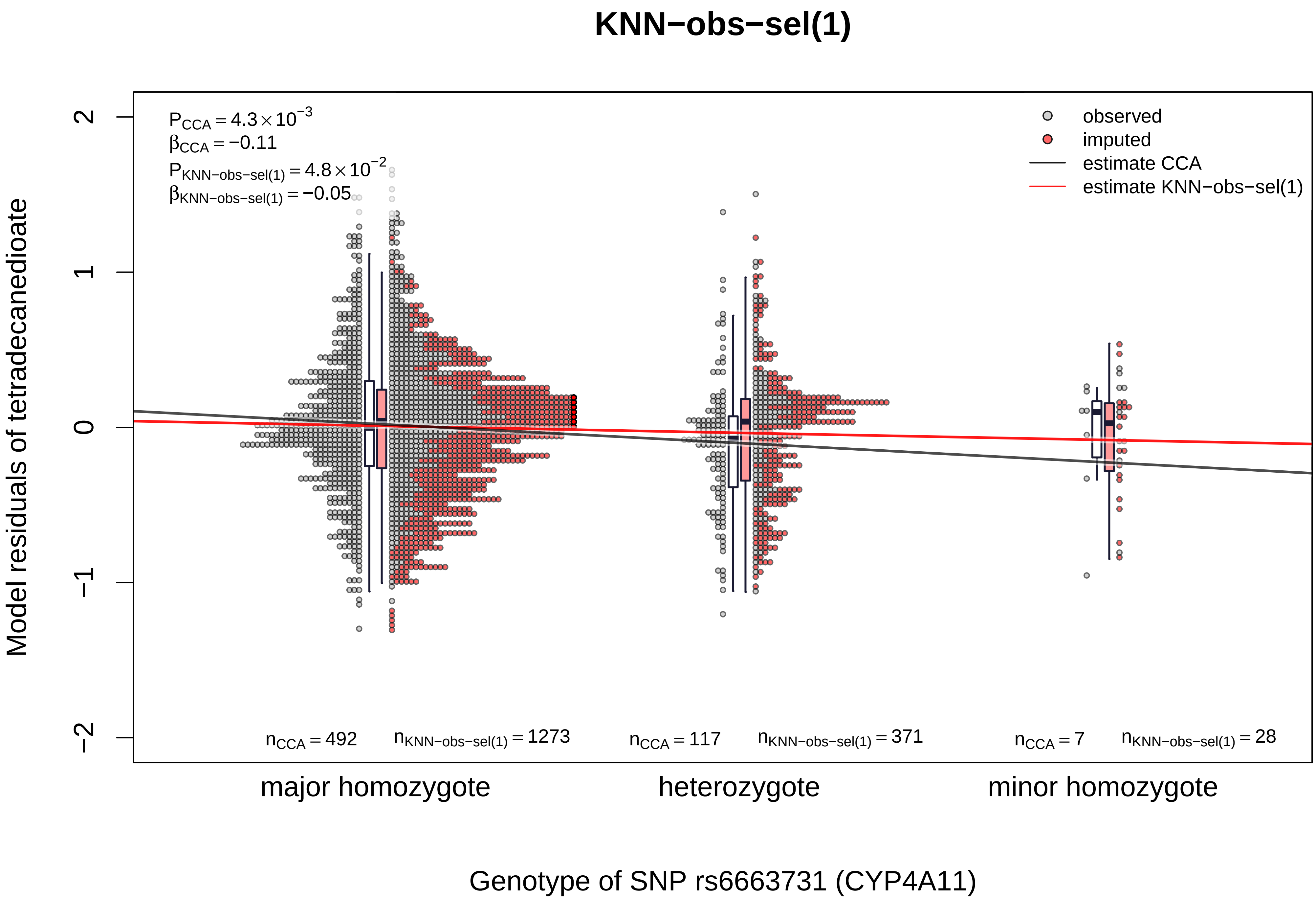
KNN−obs−sel(1)
$P_{CCA} = 4.3 \times 10^{-3}$
$\beta_{CCA} = -0.11$
$P_{KNN-obs-sel(1)} = 4.8 \times 10^{-2}$
$\beta_{KNN-obs-sel(1)} = -0.05$
observed
imputed
estimate CCA
estimate KNN−obs−sel(1)
Model residuals of tetradecanedioate
2
1
0
−1
−2
$n_{CCA} = 492$
$n_{KNN-obs-sel(1)} = 1273$
$n_{CCA} = 117$
$n_{KNN-obs-sel(1)} = 371$
$n_{CCA} = 7$
$n_{KNN-obs-sel(1)} = 28$
major homozygote
heterozygote
minor homozygote
Genotype of SNP rs6663731 (CYP4A11)

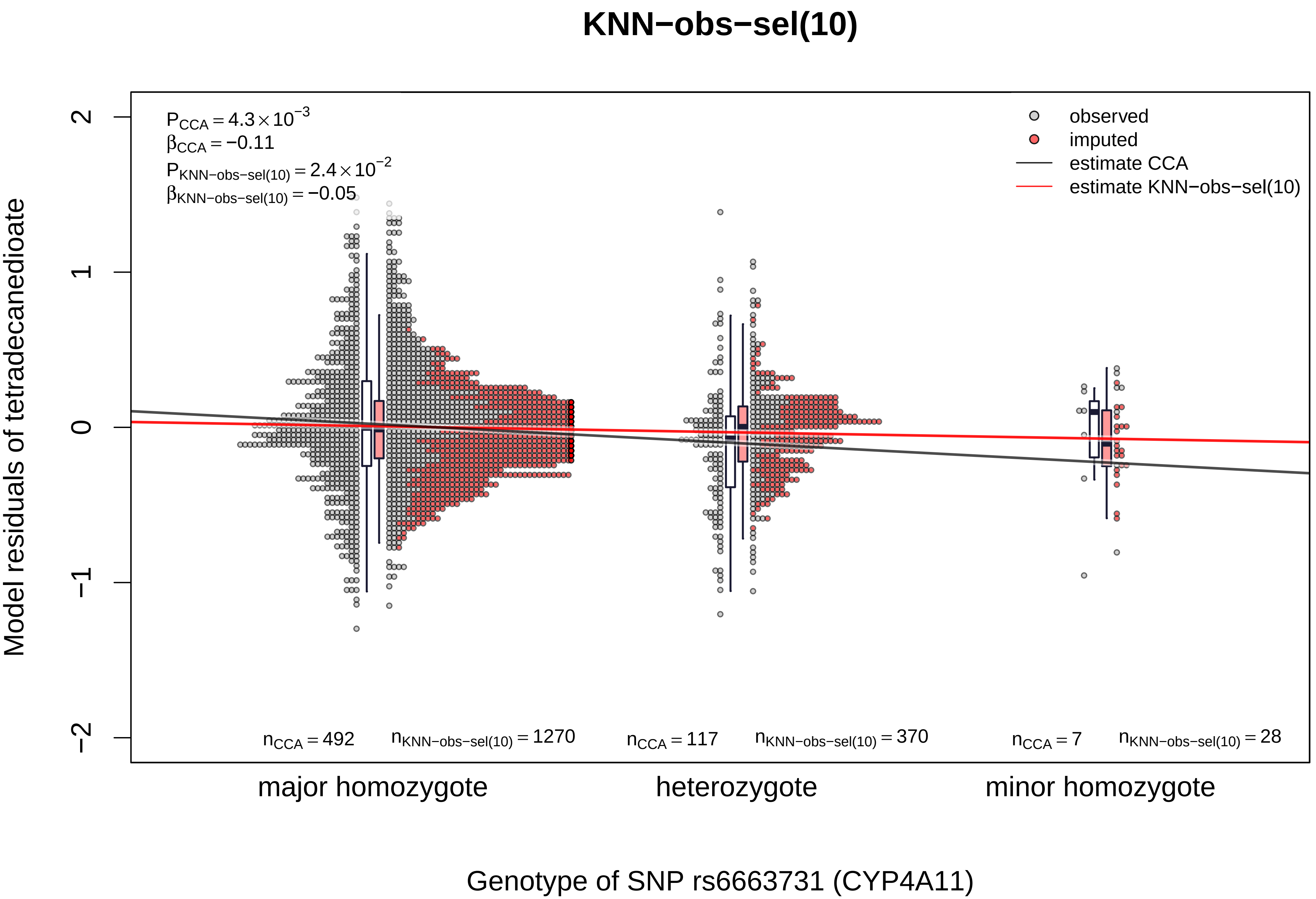

KNN−obs−sel(10)
$P_{CCA} = 4.3 \times 10^{-3}$
$\beta_{CCA} = -0.11$
$P_{KNN-obs-sel(10)} = 2.4 \times 10^{-2}$
$\beta_{KNN-obs-sel(10)} = -0.05$
observed
imputed
estimate CCA
estimate KNN−obs−sel(10)
Model residuals of tetradecanedioate
2
1
0
−1
−2
$n_{CCA} = 492$
$n_{KNN-obs-sel(10)} = 1270$
$n_{CCA} = 117$
$n_{KNN-obs-sel(10)} = 370$
$n_{CCA} = 7$
$n_{KNN-obs-sel(10)} = 28$
major homozygote
heterozygote
minor homozygote
Genotype of SNP rs6663731 (CYP4A11)

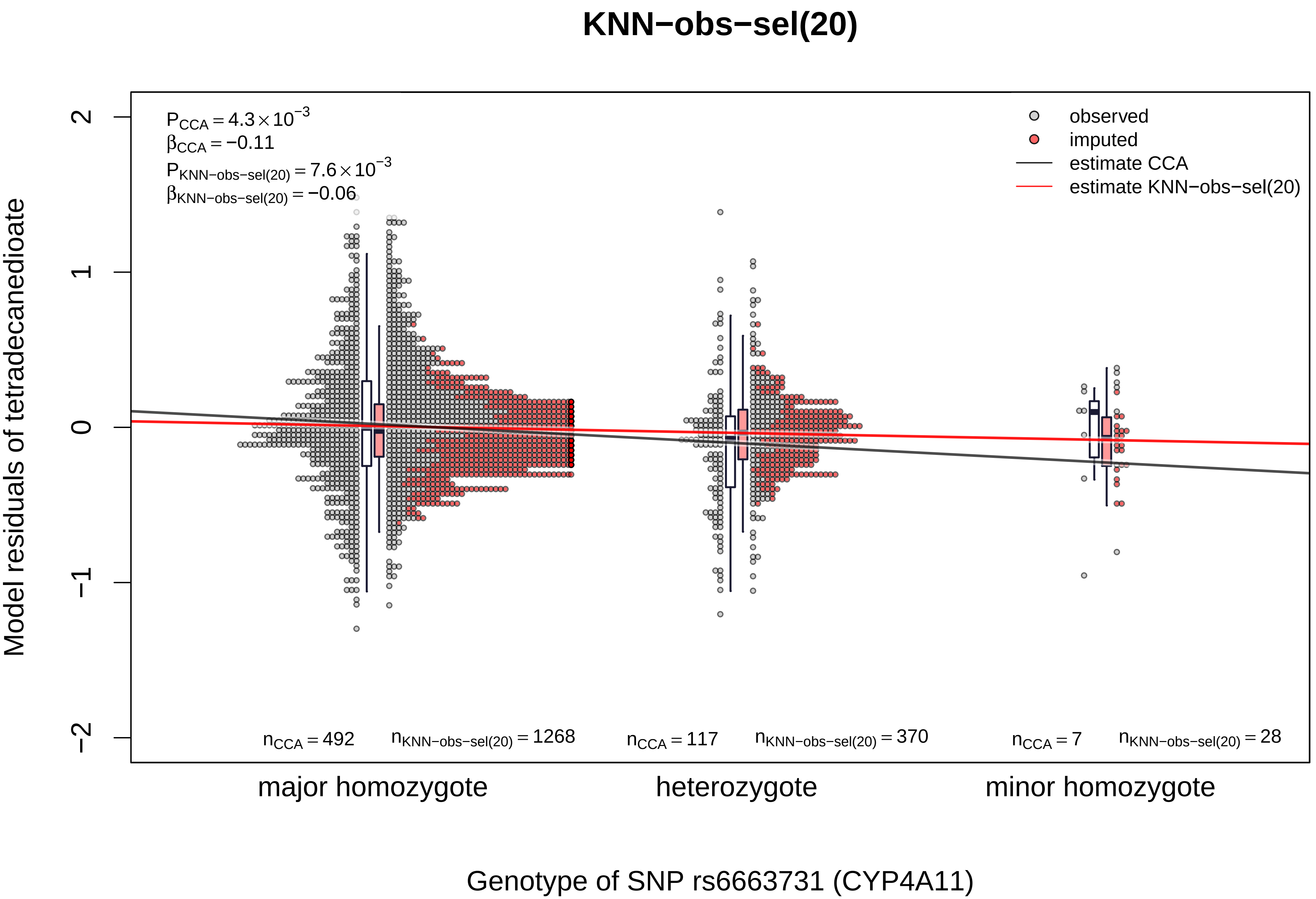

KNN−obs−sel(20)
$P_{CCA} = 4.3 \times 10^{-3}$
$\beta_{CCA} = -0.11$
$P_{KNN-obs-sel(20)} = 7.6 \times 10^{-3}$
$\beta_{KNN-obs-sel(20)} = -0.06$
observed
imputed
estimate CCA
estimate KNN−obs−sel(20)
Model residuals of tetradecanedioate
2
1
0
−1
−2
$n_{CCA} = 492$
$n_{KNN-obs-sel(20)} = 1268$
$n_{CCA} = 117$
$n_{KNN-obs-sel(20)} = 370$
$n_{CCA} = 7$
$n_{KNN-obs-sel(20)} = 28$
major homozygote
heterozygote
minor homozygote
Genotype of SNP rs6663731 (CYP4A11)

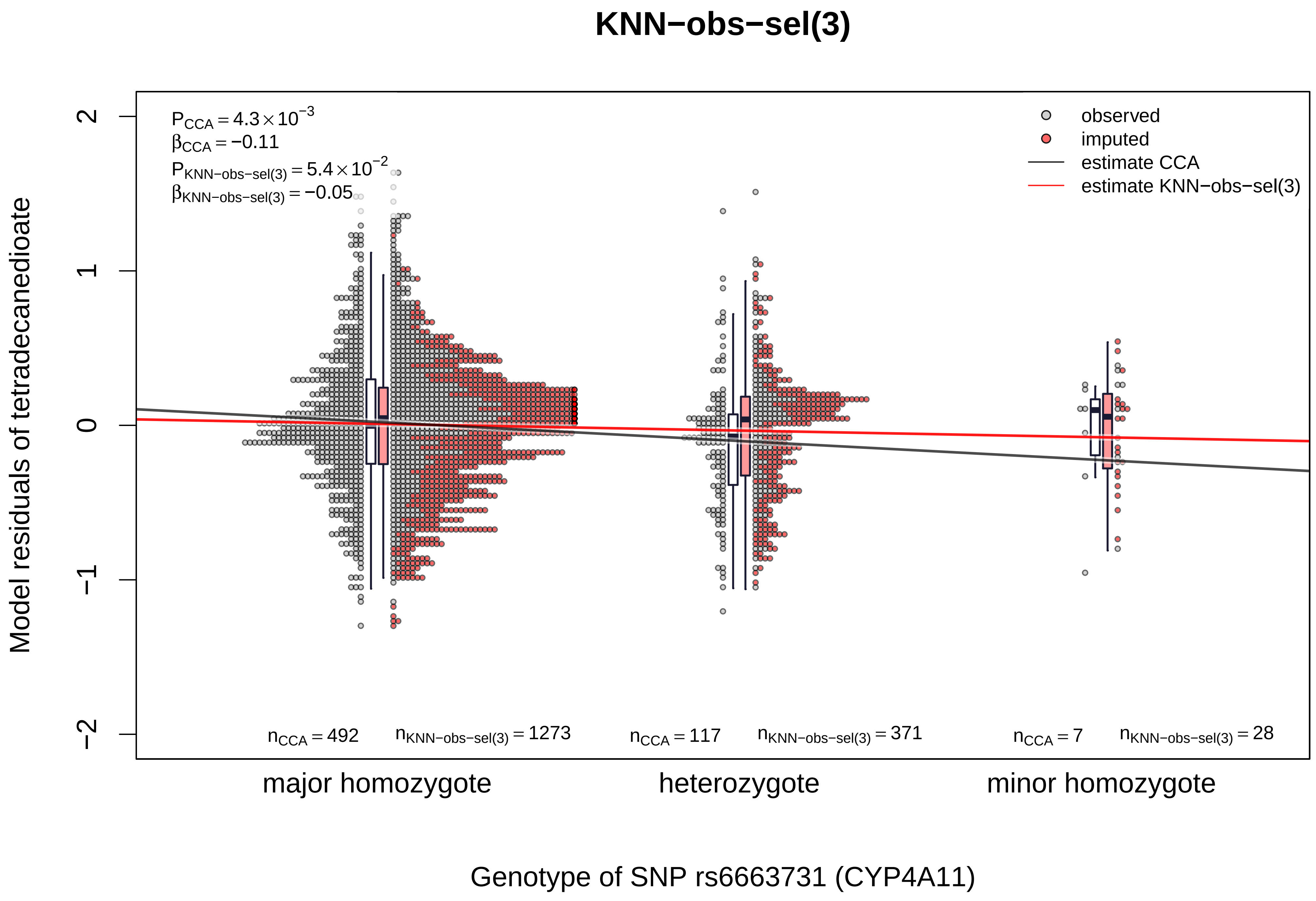

KNN−obs−sel(3)
$P_{CCA} = 4.3 \times 10^{-3}$
$\beta_{CCA} = -0.11$
$P_{KNN-obs-sel(3)} = 5.4 \times 10^{-2}$
$\beta_{KNN-obs-sel(3)} = -0.05$
observed
imputed
estimate CCA
estimate KNN−obs−sel(3)
Model residuals of tetradecanedioate
2
1
0
−1
−2
$n_{CCA} = 492$
$n_{KNN-obs-sel(3)} = 1273$
$n_{CCA} = 117$
$n_{KNN-obs-sel(3)} = 371$
$n_{CCA} = 7$
$n_{KNN-obs-sel(3)} = 28$
major homozygote
heterozygote
minor homozygote
Genotype of SNP rs6663731 (CYP4A11)

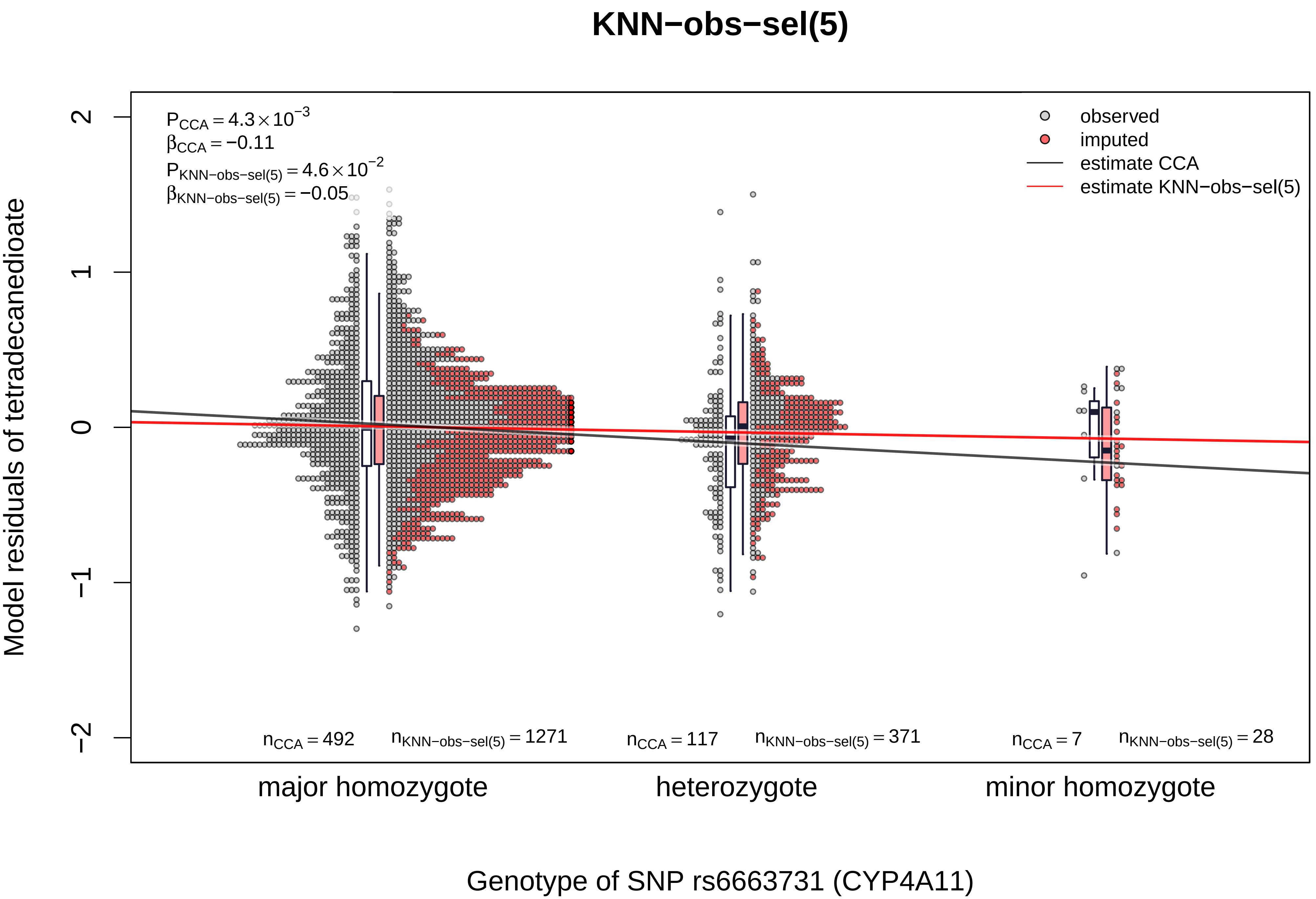

KNN−obs−sel(5)
$P_{CCA} = 4.3 \times 10^{-3}$
$\beta_{CCA} = -0.11$
$P_{KNN-obs-sel(5)} = 4.6 \times 10^{-2}$
$\beta_{KNN-obs-sel(5)} = -0.05$
observed
imputed
estimate CCA
estimate KNN−obs−sel(5)
Model residuals of tetradecanedioate
2
1
0
−1
−2
$n_{CCA} = 492$
$n_{KNN-obs-sel(5)} = 1271$
$n_{CCA} = 117$
$n_{KNN-obs-sel(5)} = 371$
$n_{CCA} = 7$
$n_{KNN-obs-sel(5)} = 28$
major homozygote
heterozygote
minor homozygote
Genotype of SNP rs6663731 (CYP4A11)

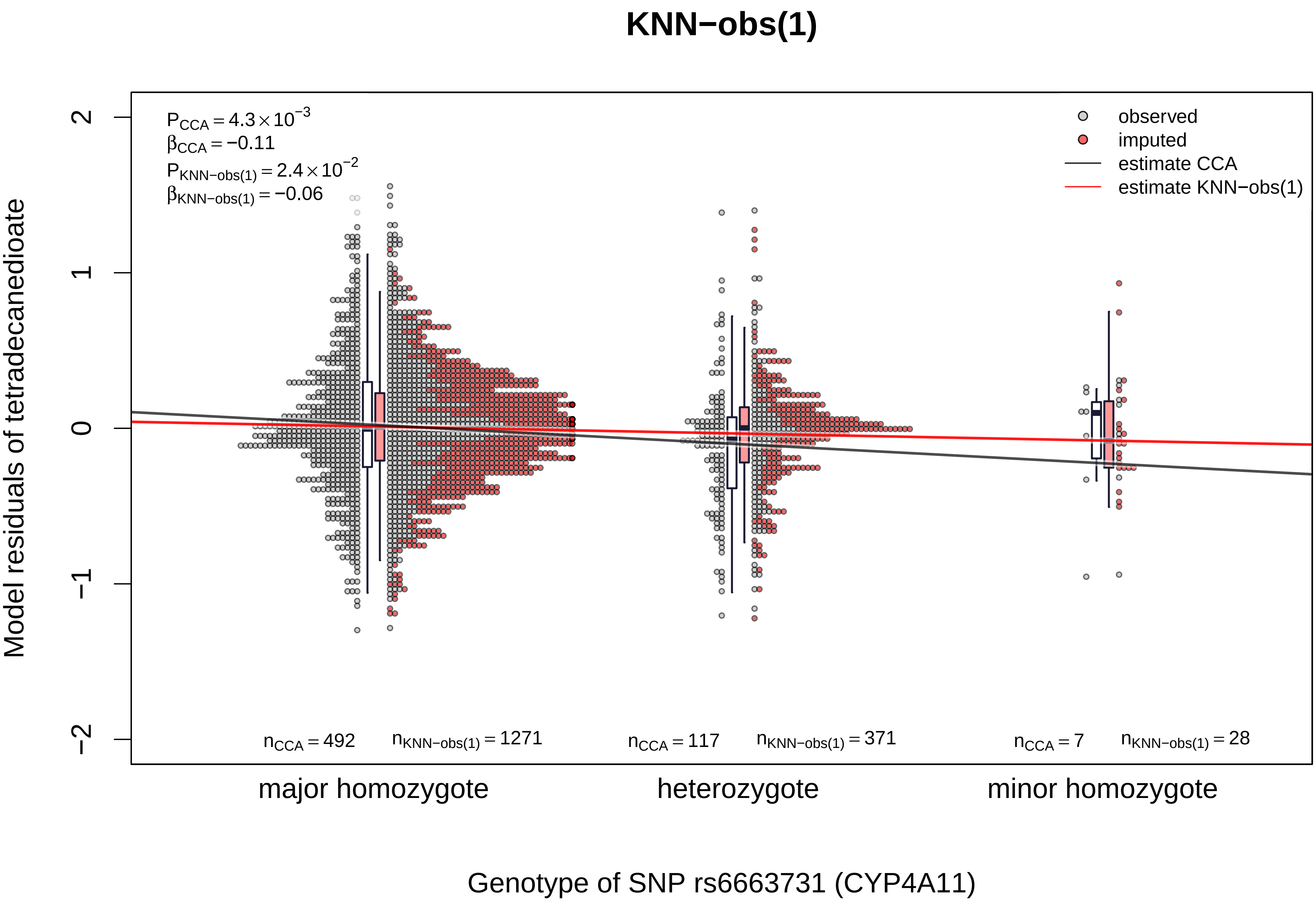

KNN−obs(1)
$P_{CCA} = 4.3 \times 10^{-3}$
$\beta_{CCA} = -0.11$
$P_{KNN-obs(1)} = 2.4 \times 10^{-2}$
$\beta_{KNN-obs(1)} = -0.06$
observed
imputed
estimate CCA
estimate KNN−obs(1)
Model residuals of tetradecanedioate
2
1
0
−1
−2
$n_{CCA} = 492$
$n_{KNN-obs(1)} = 1271$
$n_{CCA} = 117$
$n_{KNN-obs(1)} = 371$
$n_{CCA} = 7$
$n_{KNN-obs(1)} = 28$
major homozygote
heterozygote
minor homozygote
Genotype of SNP rs6663731 (CYP4A11)

KNN−obs(10)

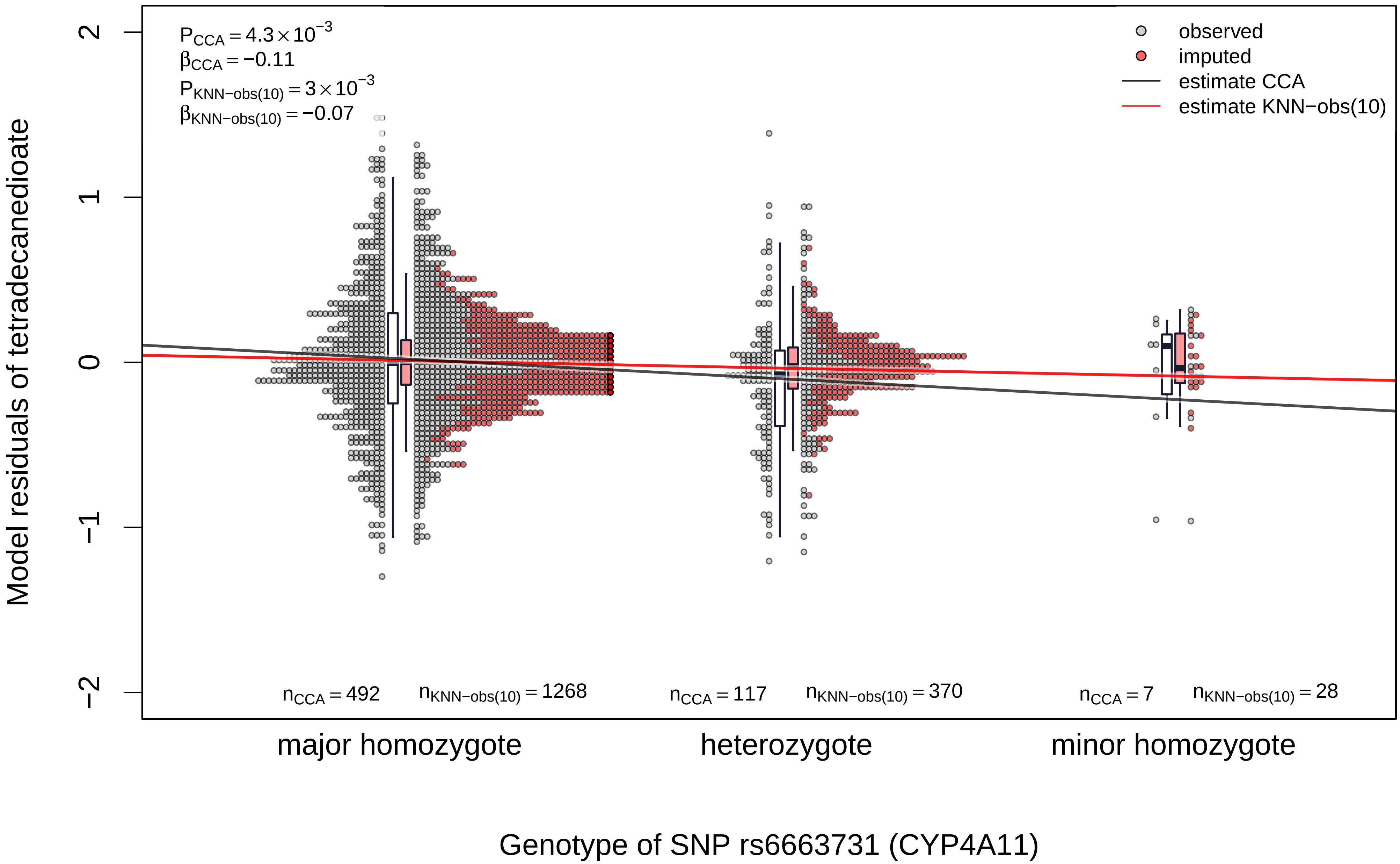

$P_{CCA} = 4.3 \times 10^{-3}$
$\beta_{CCA} = -0.11$
$P_{KNN-obs(10)} = 3 \times 10^{-3}$
$\beta_{KNN-obs(10)} = -0.07$
observed
imputed
estimate CCA
estimate KNN−obs(10)
Model residuals of tetradecanedioate
2
1
0
−1
−2
$n_{CCA} = 492$
$n_{KNN-obs(10)} = 1268$
$n_{CCA} = 117$
$n_{KNN-obs(10)} = 370$
$n_{CCA} = 7$
$n_{KNN-obs(10)} = 28$
major homozygote
heterozygote
minor homozygote
Genotype of SNP rs6663731 (CYP4A11)

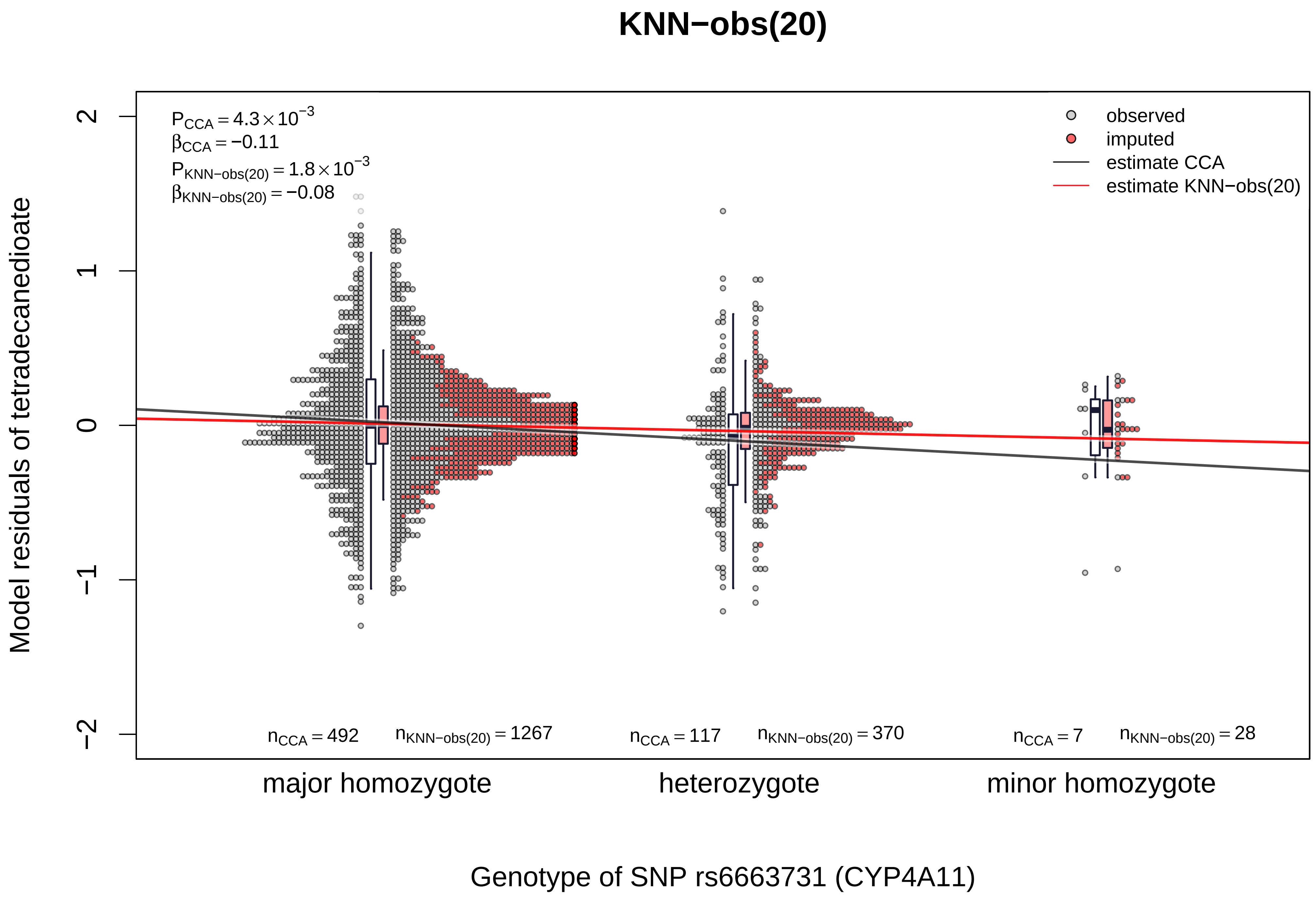

KNN−obs(20)
$P_{CCA} = 4.3 \times 10^{-3}$
$\beta_{CCA} = -0.11$
$P_{KNN-obs(20)} = 1.8 \times 10^{-3}$
$\beta_{KNN-obs(20)} = -0.08$
observed
imputed
estimate CCA
estimate KNN−obs(20)
2
1
0
−1
−2
Model residuals of tetradecanedioate
$n_{CCA} = 492$
$n_{KNN-obs(20)} = 1267$
$n_{CCA} = 117$
$n_{KNN-obs(20)} = 370$
$n_{CCA} = 7$
$n_{KNN-obs(20)} = 28$
major homozygote
heterozygote
minor homozygote
Genotype of SNP rs6663731 (CYP4A11)

KNN−obs(3)

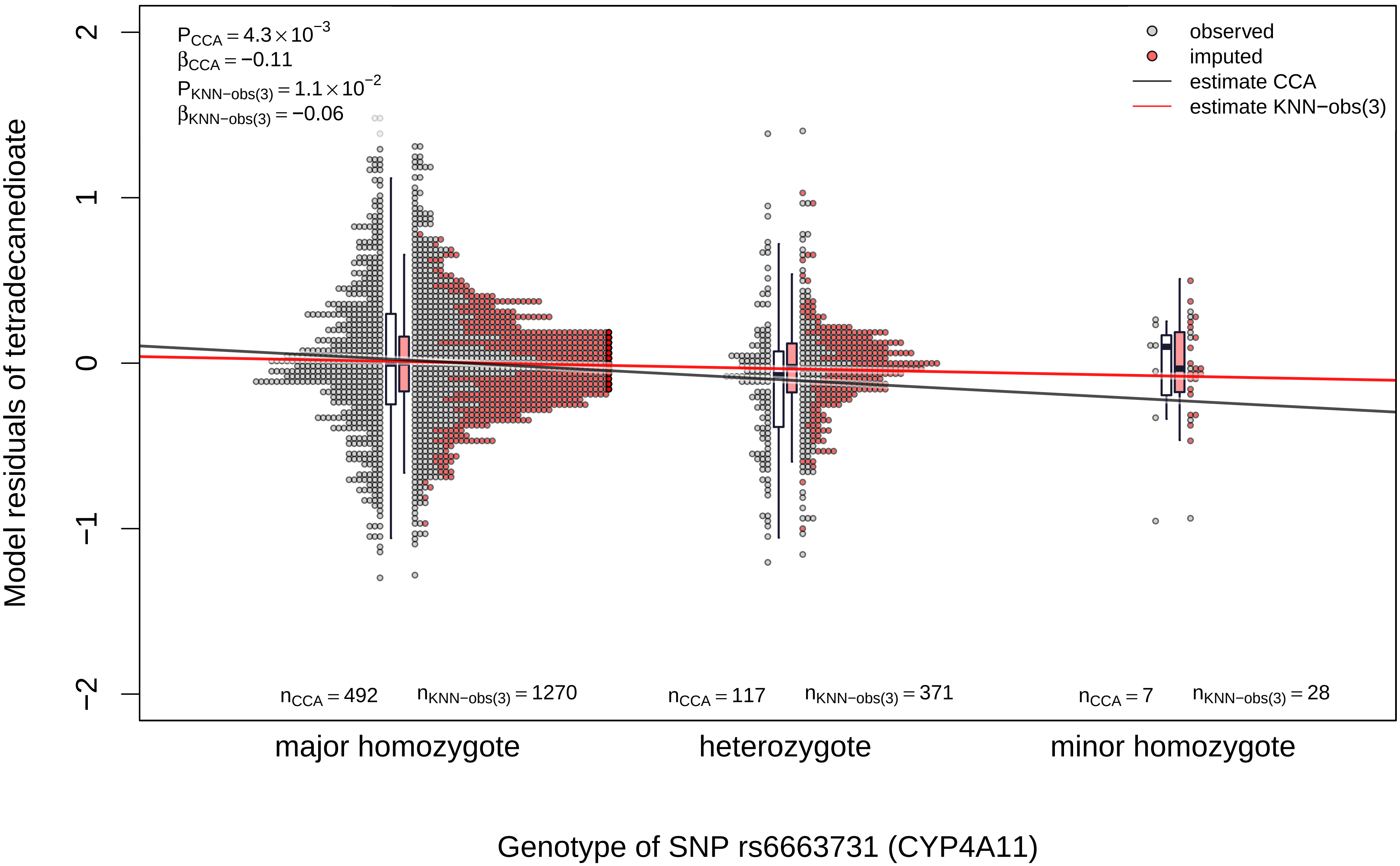

$P_{CCA} = 4.3 \times 10^{-3}$
$\beta_{CCA} = -0.11$
$P_{KNN-obs(3)} = 1.1 \times 10^{-2}$
$\beta_{KNN-obs(3)} = -0.06$
observed
imputed
estimate CCA
estimate KNN−obs(3)
Model residuals of tetradecanedioate
2
1
0
−1
−2
$n_{CCA} = 492$
$n_{KNN-obs(3)} = 1270$
$n_{CCA} = 117$
$n_{KNN-obs(3)} = 371$
$n_{CCA} = 7$
$n_{KNN-obs(3)} = 28$
major homozygote
heterozygote
minor homozygote
Genotype of SNP rs6663731 (CYP4A11)

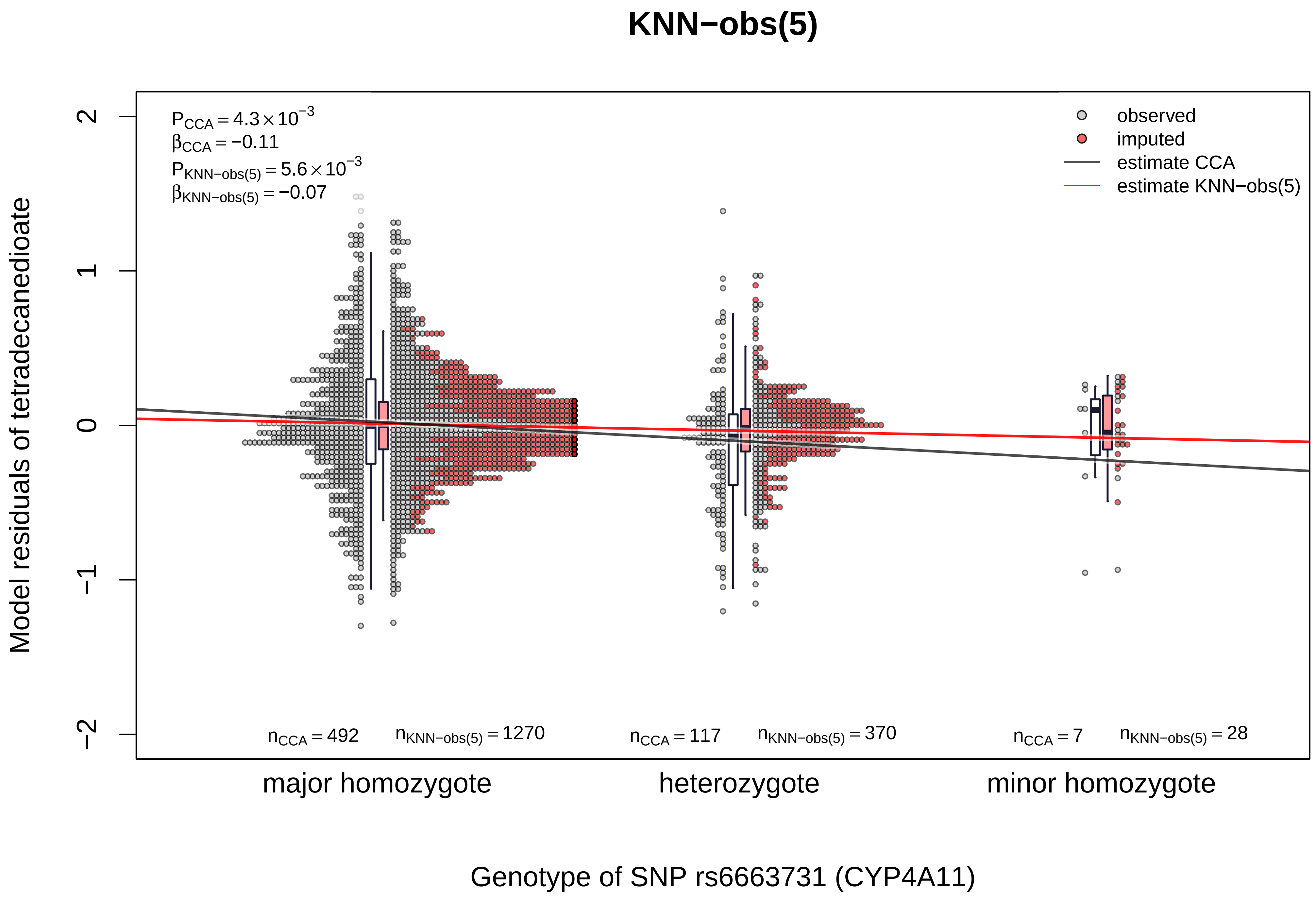

KNN−obs(5)
$P_{CCA} = 4.3 \times 10^{-3}$
$\beta_{CCA} = -0.11$
$P_{KNN-obs(5)} = 5.6 \times 10^{-3}$
$\beta_{KNN-obs(5)} = -0.07$
observed
imputed
estimate CCA
estimate KNN−obs(5)
Model residuals of tetradecanedioate
2
1
0
−1
−2
$n_{CCA} = 492$
$n_{KNN-obs(5)} = 1270$
$n_{CCA} = 117$
$n_{KNN-obs(5)} = 370$
$n_{CCA} = 7$
$n_{KNN-obs(5)} = 28$
major homozygote
heterozygote
minor homozygote
Genotype of SNP rs6663731 (CYP4A11)

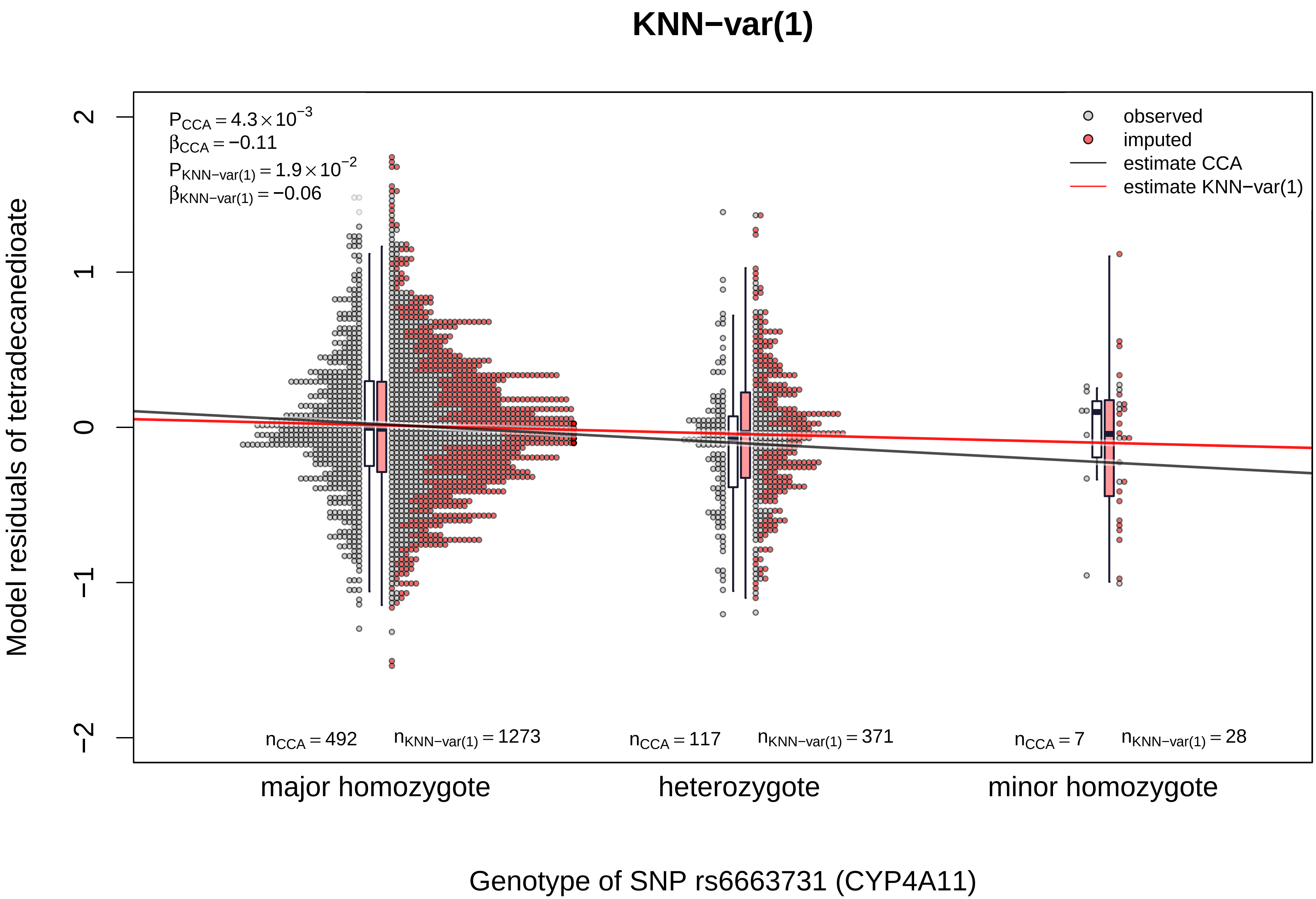

KNN−var(1)
$P_{CCA} = 4.3 \times 10^{-3}$
$\beta_{CCA} = -0.11$
$P_{KNN-var(1)} = 1.9 \times 10^{-2}$
$\beta_{KNN-var(1)} = -0.06$
observed
imputed
estimate CCA
estimate KNN−var(1)
Model residuals of tetradecanedioate
2
1
0
−1
−2
$n_{CCA} = 492$
$n_{KNN-var(1)} = 1273$
$n_{CCA} = 117$
$n_{KNN-var(1)} = 371$
$n_{CCA} = 7$
$n_{KNN-var(1)} = 28$
major homozygote
heterozygote
minor homozygote
Genotype of SNP rs6663731 (CYP4A11)

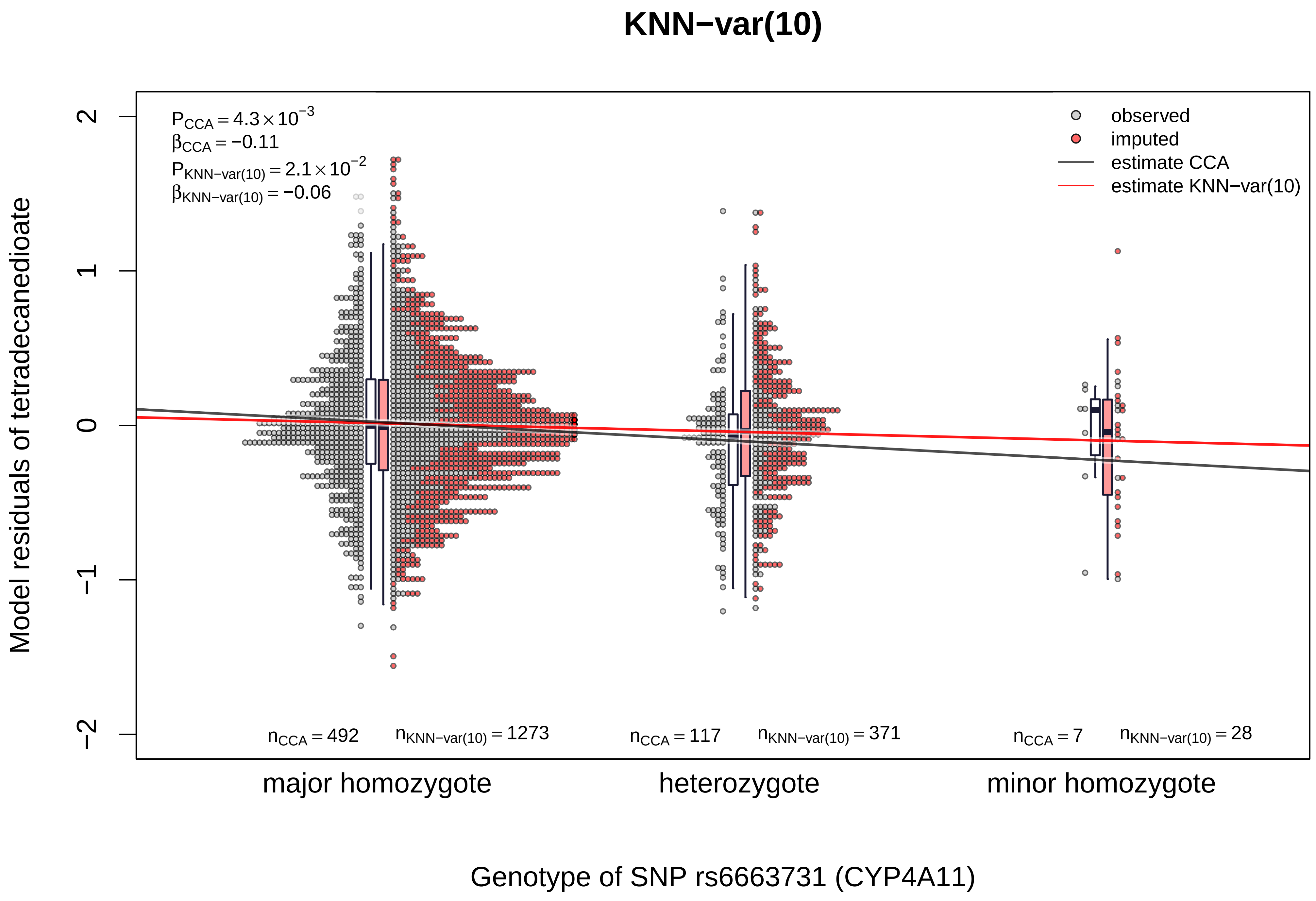
KNN−var(10)
$P_{CCA} = 4.3 \times 10^{-3}$
$\beta_{CCA} = -0.11$
$P_{KNN-var(10)} = 2.1 \times 10^{-2}$
$\beta_{KNN-var(10)} = -0.06$
observed
imputed
estimate CCA
estimate KNN−var(10)
$n_{CCA} = 492$
$n_{KNN-var(10)} = 1273$
$n_{CCA} = 117$
$n_{KNN-var(10)} = 371$
$n_{CCA} = 7$
$n_{KNN-var(10)} = 28$
Model residuals of tetradecanedioate
2
1
0
−1
−2
major homozygote
heterozygote
minor homozygote
Genotype of SNP rs6663731 (CYP4A11)

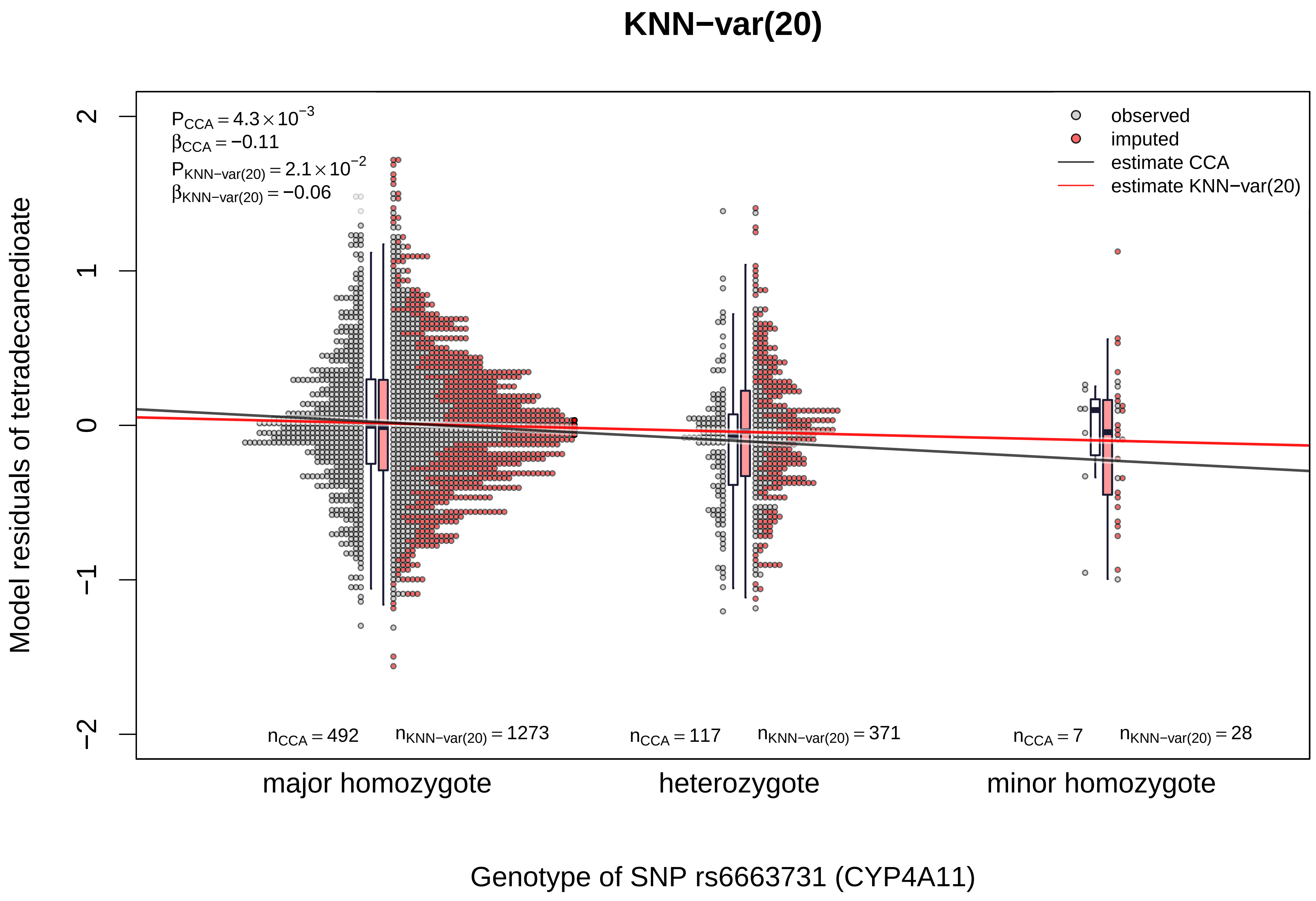
KNN−var(20)
$P_{CCA} = 4.3 \times 10^{-3}$
$\beta_{CCA} = -0.11$
$P_{KNN-var(20)} = 2.1 \times 10^{-2}$
$\beta_{KNN-var(20)} = -0.06$
observed
imputed
estimate CCA
estimate KNN−var(20)
Model residuals of tetradecanedioate
2
1
0
−1
−2
$n_{CCA} = 492$
$n_{KNN-var(20)} = 1273$
$n_{CCA} = 117$
$n_{KNN-var(20)} = 371$
$n_{CCA} = 7$
$n_{KNN-var(20)} = 28$
major homozygote
heterozygote
minor homozygote
Genotype of SNP rs6663731 (CYP4A11)

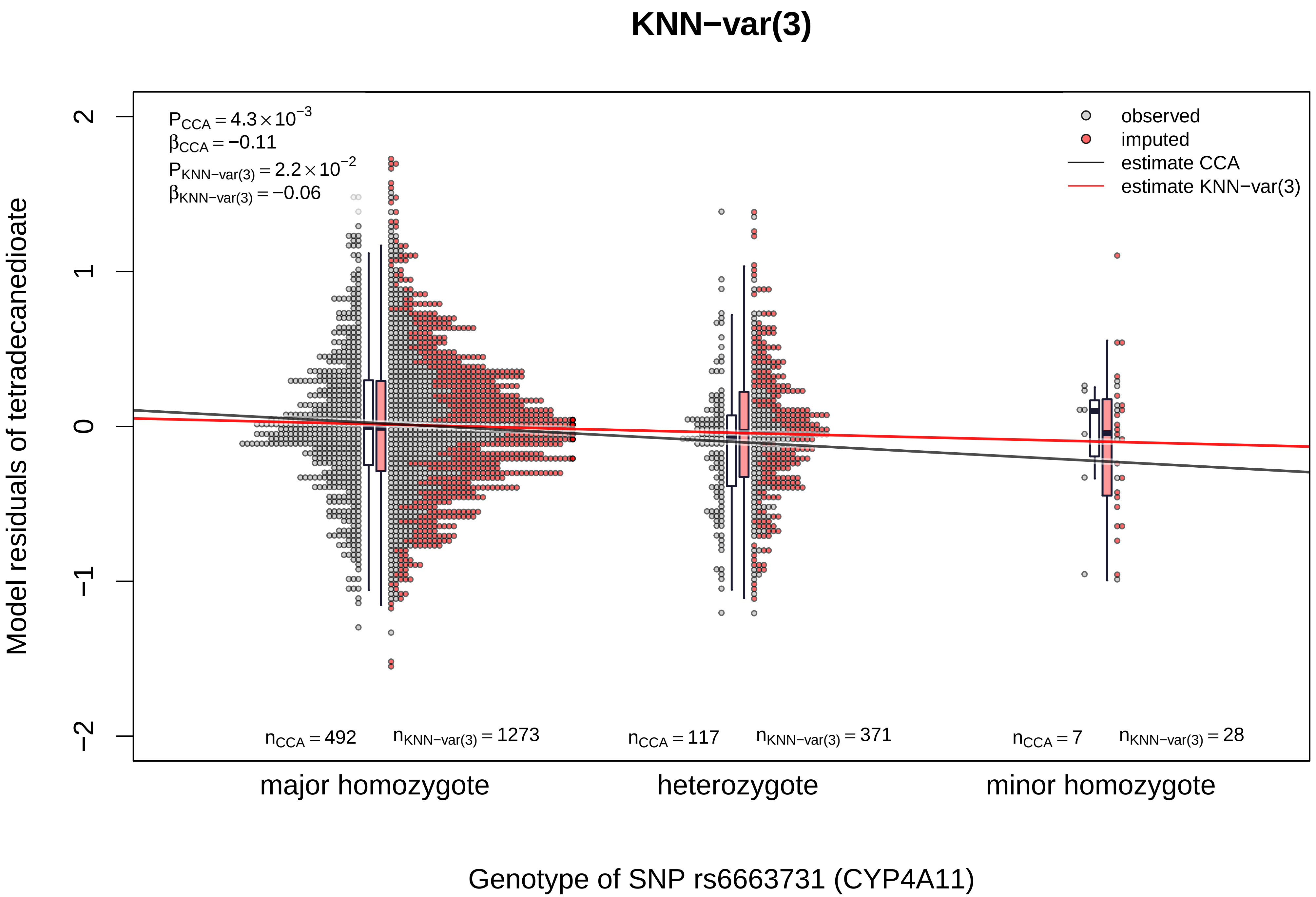
KNN−var(3)
$P_{CCA} = 4.3 \times 10^{-3}$
$\beta_{CCA} = -0.11$
$P_{KNN-var(3)} = 2.2 \times 10^{-2}$
$\beta_{KNN-var(3)} = -0.06$
observed
imputed
estimate CCA
estimate KNN−var(3)
Model residuals of tetradecanedioate
2
1
0
−1
−2
$n_{CCA} = 492$
$n_{KNN-var(3)} = 1273$
$n_{CCA} = 117$
$n_{KNN-var(3)} = 371$
$n_{CCA} = 7$
$n_{KNN-var(3)} = 28$
major homozygote
heterozygote
minor homozygote
Genotype of SNP rs6663731 (CYP4A11)

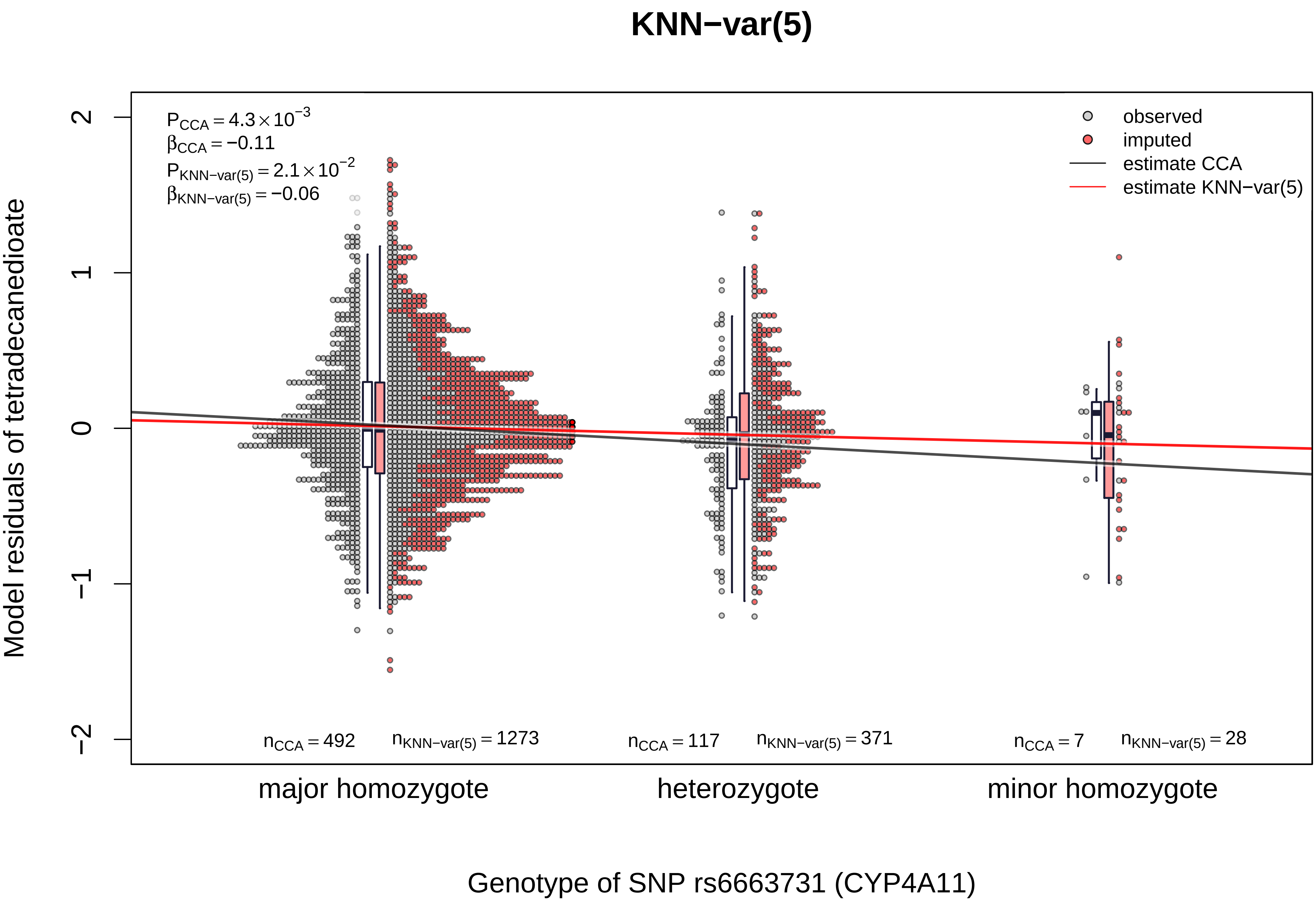
KNN−var(5)
$P_{CCA} = 4.3 \times 10^{-3}$
$\beta_{CCA} = -0.11$
$P_{KNN-var(5)} = 2.1 \times 10^{-2}$
$\beta_{KNN-var(5)} = -0.06$
observed
imputed
estimate CCA
estimate KNN−var(5)
2
1
0
−1
−2
Model residuals of tetradecanedioate
$n_{CCA} = 492$
$n_{KNN-var(5)} = 1273$
$n_{CCA} = 117$
$n_{KNN-var(5)} = 371$
$n_{CCA} = 7$
$n_{KNN-var(5)} = 28$
major homozygote
heterozygote
minor homozygote
Genotype of SNP rs6663731 (CYP4A11)

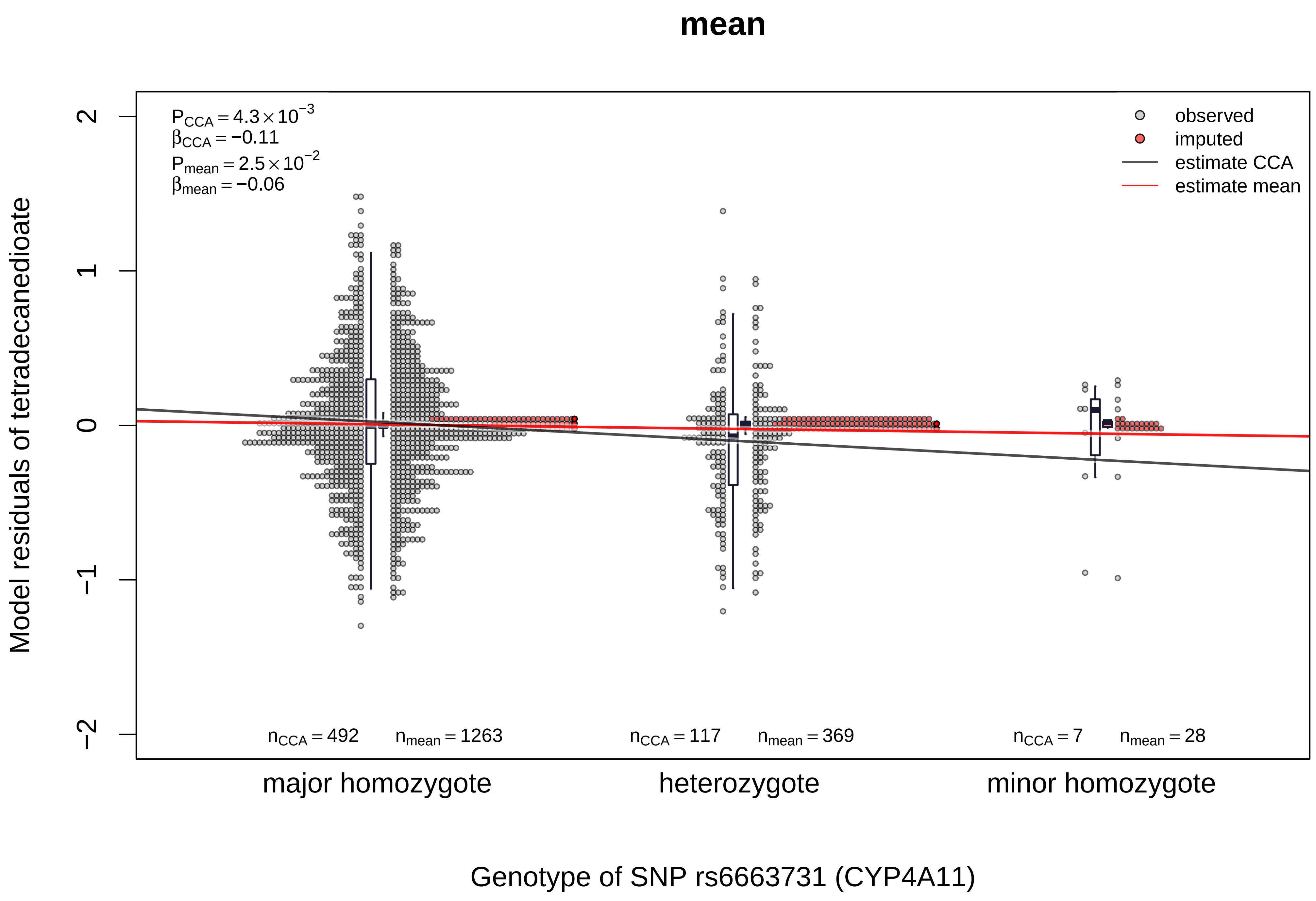

mean
$P_{CCA} = 4.3 \times 10^{-3}$
$\beta_{CCA} = -0.11$
$P_{mean} = 2.5 \times 10^{-2}$
$\beta_{mean} = -0.06$
observed
imputed
estimate CCA
estimate mean
Model residuals of tetradecanedioate
2
1
0
−1
−2
$n_{CCA} = 492$
$n_{mean} = 1263$
$n_{CCA} = 117$
$n_{mean} = 369$
$n_{CCA} = 7$
$n_{mean} = 28$
major homozygote
heterozygote
minor homozygote
Genotype of SNP rs6663731 (CYP4A11)

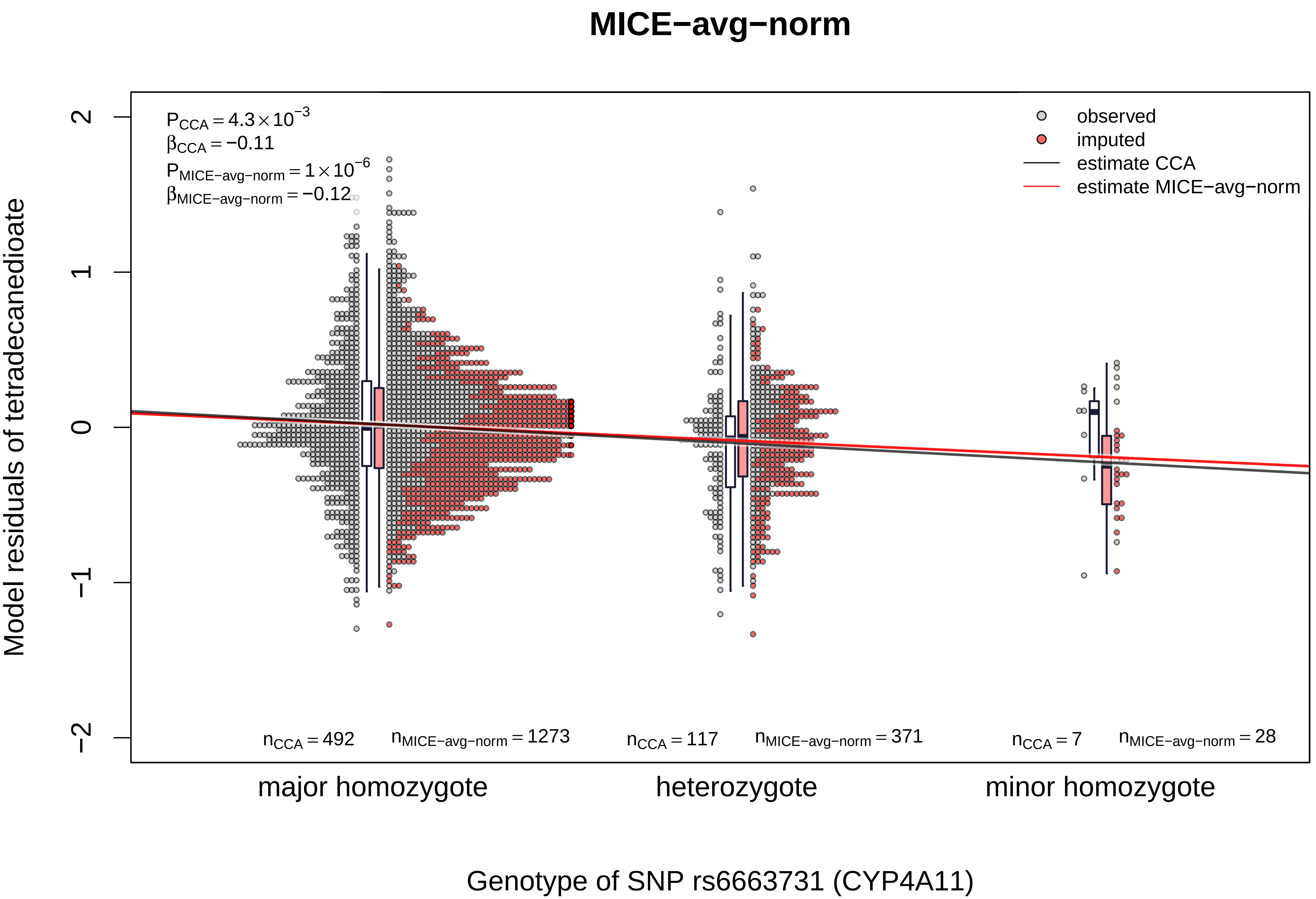
MICE−avg−norm
$P_{CCA} = 4.3 \times 10^{-3}$
$\beta_{CCA} = -0.11$
$P_{MICE-avg-norm} = 1 \times 10^{-6}$
$\beta_{MICE-avg-norm} = -0.12$
observed
imputed
estimate CCA
estimate MICE−avg−norm
$n_{CCA} = 492$
$n_{MICE-avg-norm} = 1273$
$n_{CCA} = 117$
$n_{MICE-avg-norm} = 371$
$n_{CCA} = 7$
$n_{MICE-avg-norm} = 28$
2
1
0
−1
−2
Model residuals of tetradecanedioate
major homozygote
heterozygote
minor homozygote
Genotype of SNP rs6663731 (CYP4A11)

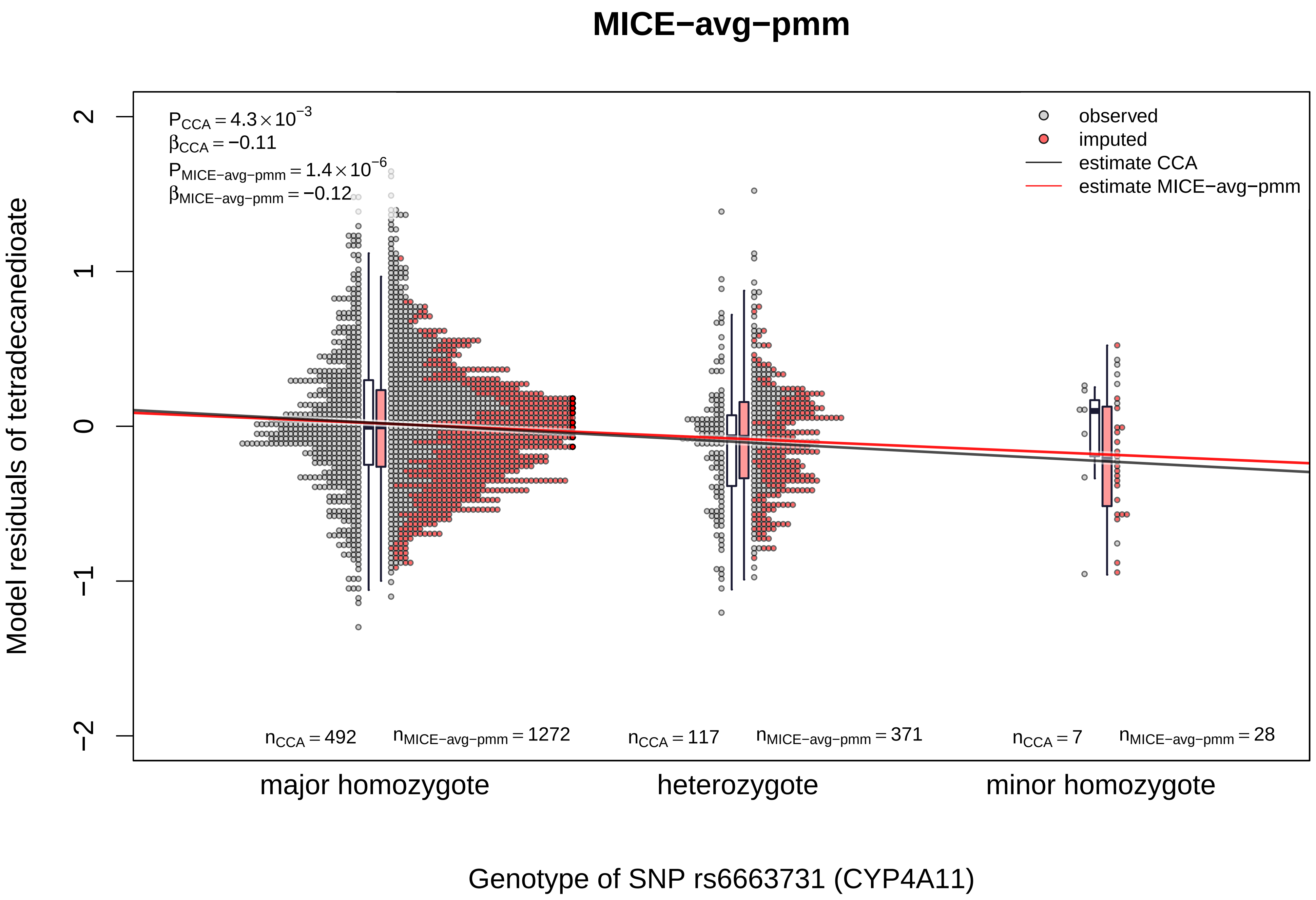

MICE−avg−pmm
$P_{CCA} = 4.3 \times 10^{-3}$
$\beta_{CCA} = -0.11$
$P_{MICE-avg-pmm} = 1.4 \times 10^{-6}$
$\beta_{MICE-avg-pmm} = -0.12$
observed
imputed
estimate CCA
estimate MICE−avg−pmm
$n_{CCA} = 492$
$n_{MICE-avg-pmm} = 1272$
$n_{CCA} = 117$
$n_{MICE-avg-pmm} = 371$
$n_{CCA} = 7$
$n_{MICE-avg-pmm} = 28$
2
1
0
−1
−2
Model residuals of tetradecanedioate
major homozygote
heterozygote
minor homozygote
Genotype of SNP rs6663731 (CYP4A11)

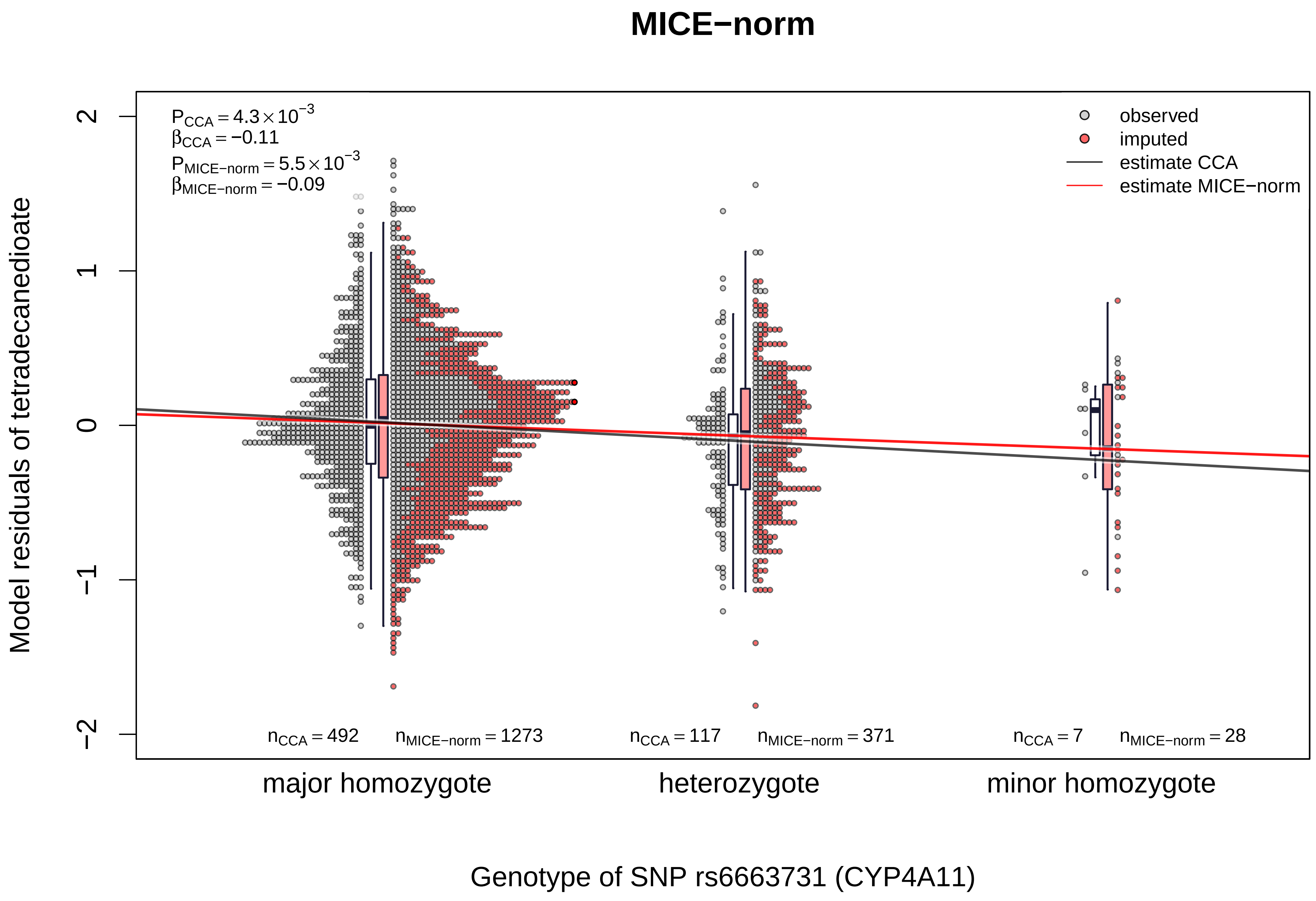

MICE−norm
$P_{CCA} = 4.3 \times 10^{-3}$
$\beta_{CCA} = -0.11$
$P_{MICE-norm} = 5.5 \times 10^{-3}$
$\beta_{MICE-norm} = -0.09$
observed
imputed
estimate CCA
estimate MICE−norm
Model residuals of tetradecanedioate
2
1
0
−1
−2
$n_{CCA} = 492$
$n_{MICE-norm} = 1273$
$n_{CCA} = 117$
$n_{MICE-norm} = 371$
$n_{CCA} = 7$
$n_{MICE-norm} = 28$
major homozygote
heterozygote
minor homozygote
Genotype of SNP rs6663731 (CYP4A11)

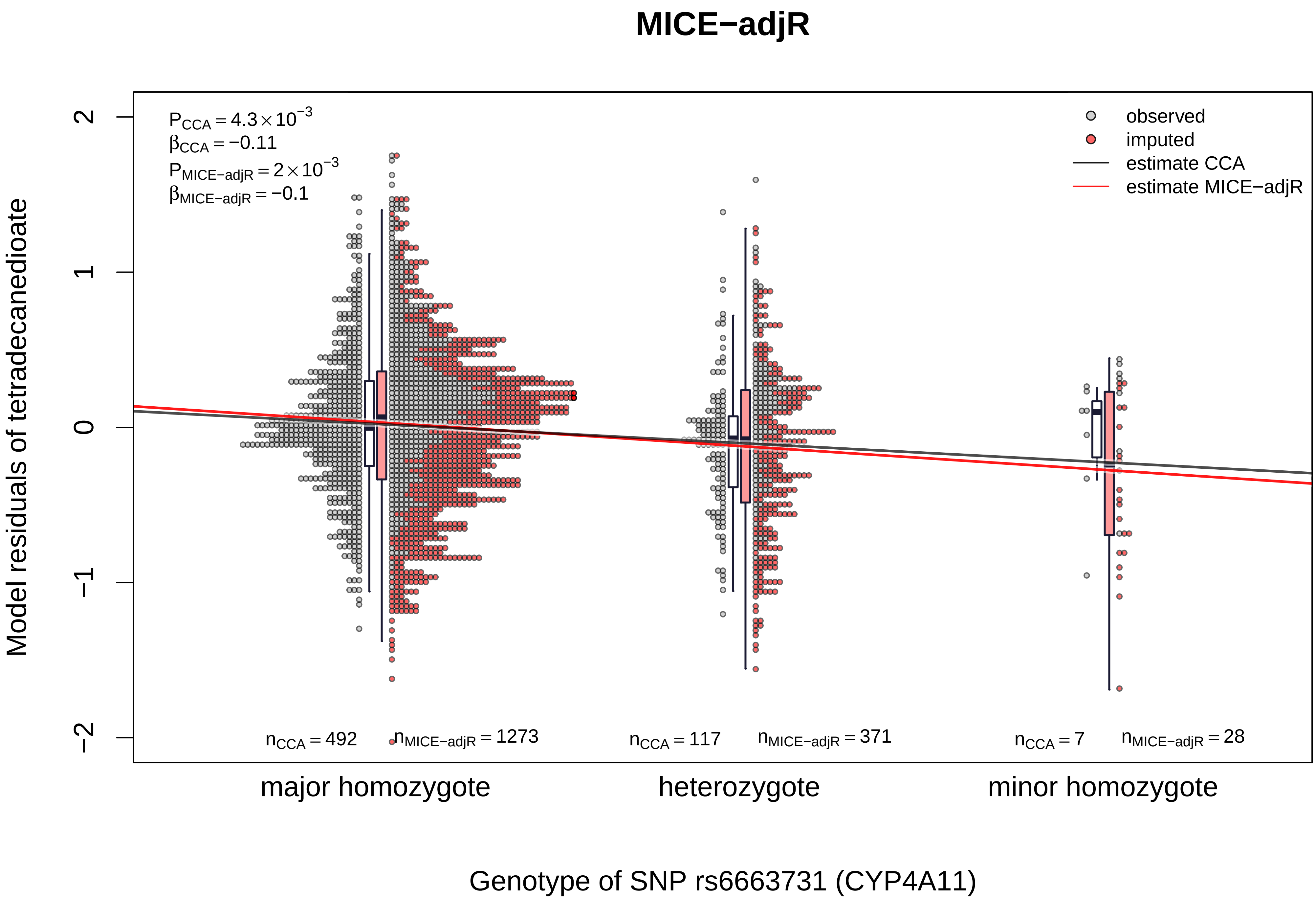
MICE−adjR
$P_{CCA} = 4.3 \times 10^{-3}$
$\beta_{CCA} = -0.11$
$P_{MICE-adjR} = 2 \times 10^{-3}$
$\beta_{MICE-adjR} = -0.1$
observed
imputed
estimate CCA
estimate MICE−adjR
Model residuals of tetradecanedioate
2
1
0
−1
−2
$n_{CCA} = 492$
$n_{MICE-adjR} = 1273$
$n_{CCA} = 117$
$n_{MICE-adjR} = 371$
$n_{CCA} = 7$
$n_{MICE-adjR} = 28$
major homozygote
heterozygote
minor homozygote
Genotype of SNP rs6663731 (CYP4A11)

MICE−pmm

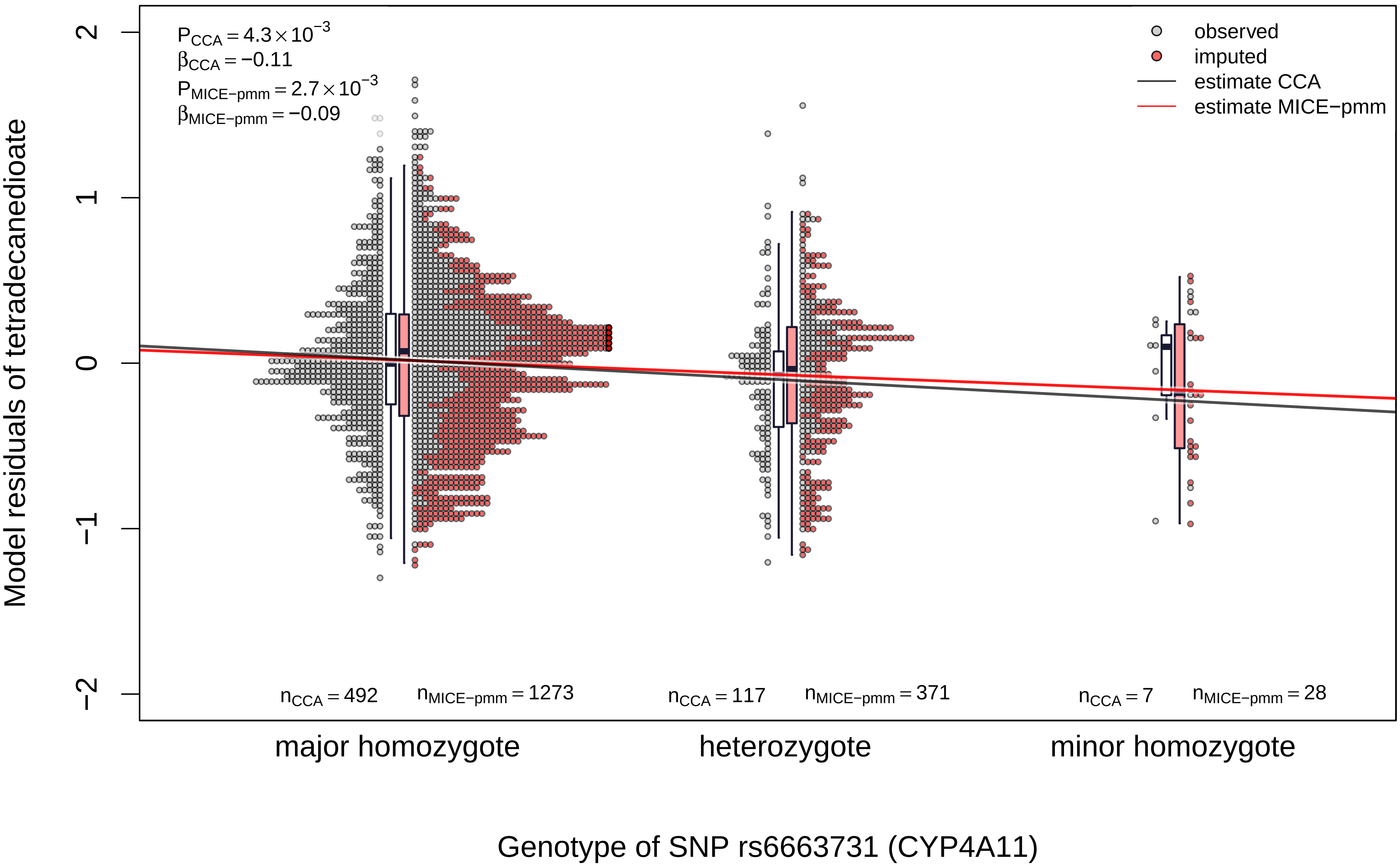

$P_{CCA} = 4.3 \times 10^{-3}$
$\beta_{CCA} = -0.11$
$P_{MICE-pmm} = 2.7 \times 10^{-3}$
$\beta_{MICE-pmm} = -0.09$
observed
imputed
estimate CCA
estimate MICE−pmm
Model residuals of tetradecanedioate
2
1
0
−1
−2
$n_{CCA} = 492$
$n_{MICE-pmm} = 1273$
$n_{CCA} = 117$
$n_{MICE-pmm} = 371$
$n_{CCA} = 7$
$n_{MICE-pmm} = 28$
major homozygote
heterozygote
minor homozygote
Genotype of SNP rs6663731 (CYP4A11)

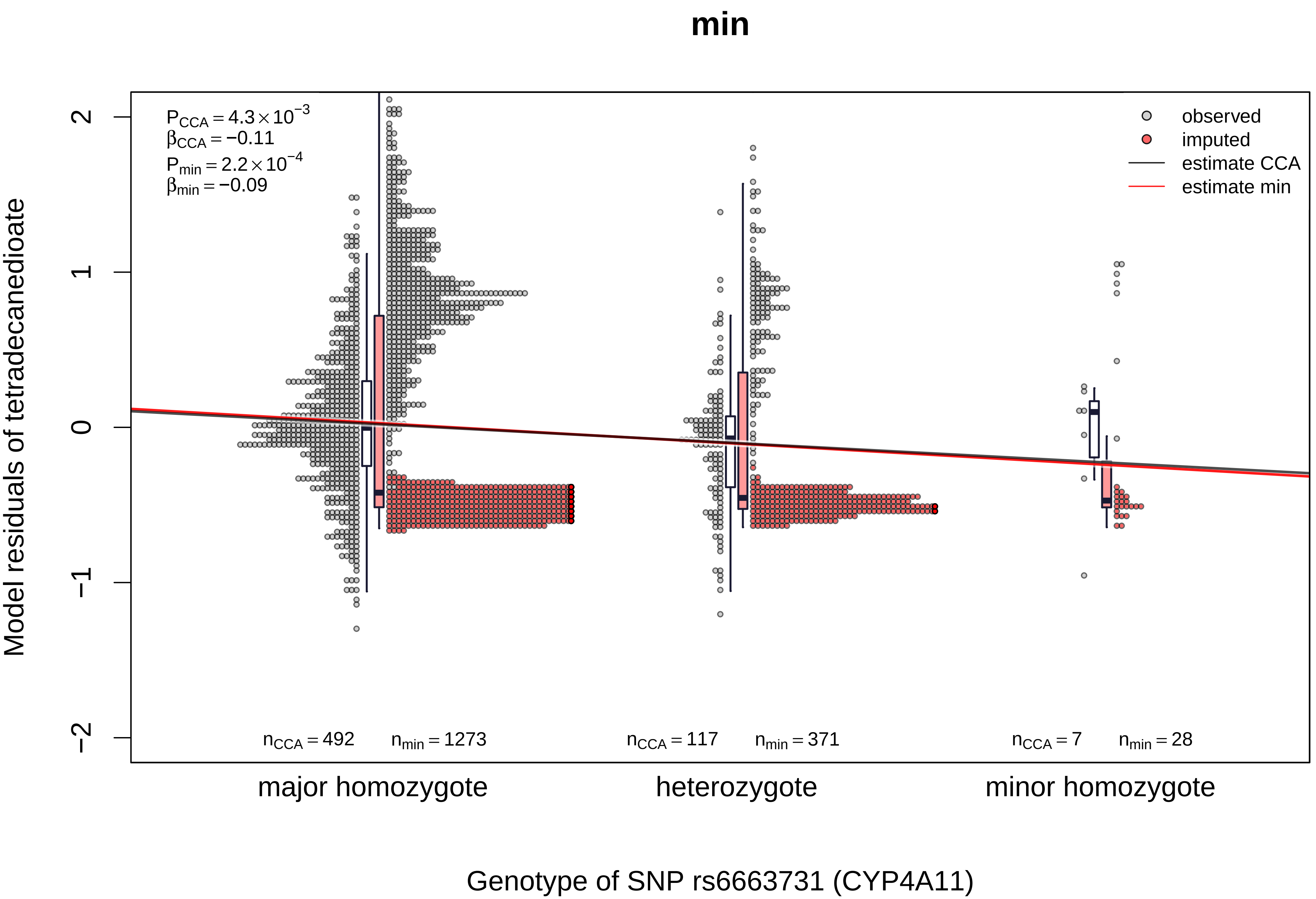

min
$P_{CCA} = 4.3 \times 10^{-3}$
$\beta_{CCA} = -0.11$
$P_{min} = 2.2 \times 10^{-4}$
$\beta_{min} = -0.09$
observed
imputed
estimate CCA
estimate min
Model residuals of tetradecanedioate
2
1
0
−1
−2
$n_{CCA} = 492$
$n_{min} = 1273$
$n_{CCA} = 117$
$n_{min} = 371$
$n_{CCA} = 7$
$n_{min} = 28$
major homozygote
heterozygote
minor homozygote
Genotype of SNP rs6663731 (CYP4A11)

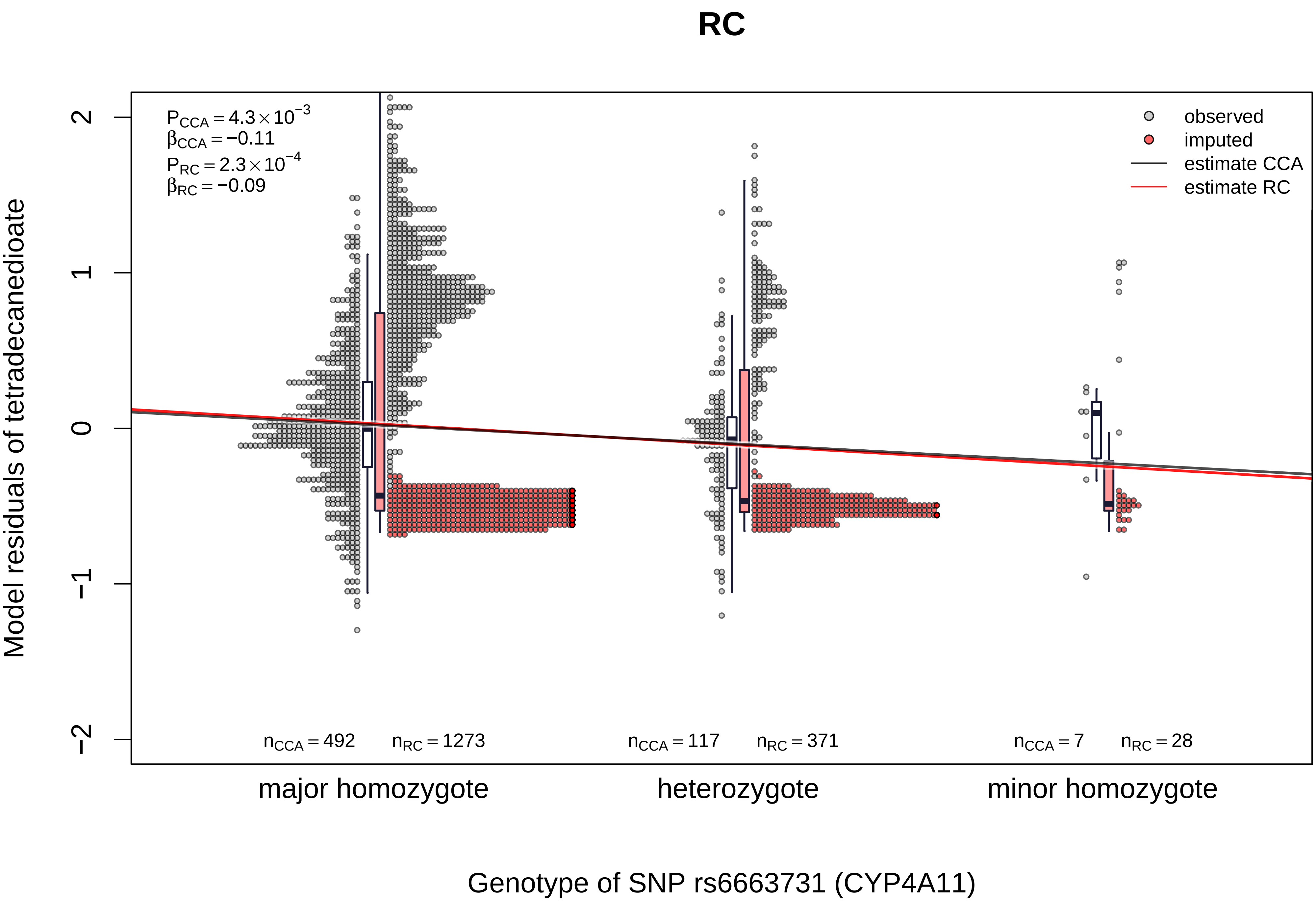

RC
$P_{CCA} = 4.3 \times 10^{-3}$
$\beta_{CCA} = -0.11$
$P_{RC} = 2.3 \times 10^{-4}$
$\beta_{RC} = -0.09$
observed
imputed
estimate CCA
estimate RC
Model residuals of tetradecanedioate
2
1
0
−1
−2
$n_{CCA} = 492$
$n_{RC} = 1273$
$n_{CCA} = 117$
$n_{RC} = 371$
$n_{CCA} = 7$
$n_{RC} = 28$
major homozygote
heterozygote
minor homozygote
Genotype of SNP rs6663731 (CYP4A11)

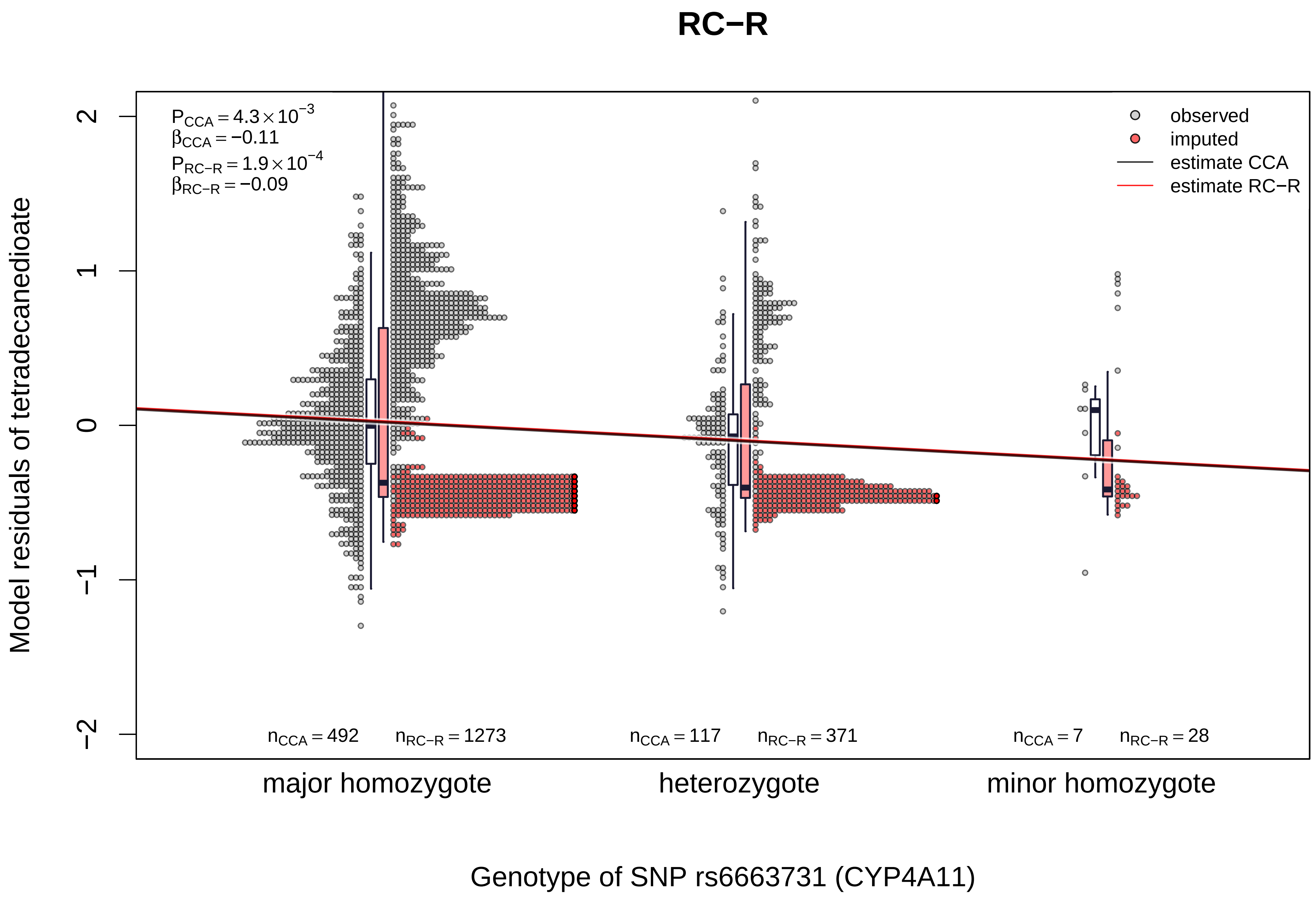

RC−R
$P_{CCA} = 4.3 \times 10^{-3}$
$\beta_{CCA} = -0.11$
$P_{RC-R} = 1.9 \times 10^{-4}$
$\beta_{RC-R} = -0.09$
observed
imputed
estimate CCA
estimate RC−R
Model residuals of tetradecanediodate
2
1
0
−1
−2
$n_{CCA} = 492$
$n_{RC-R} = 1273$
$n_{CCA} = 117$
$n_{RC-R} = 371$
$n_{CCA} = 7$
$n_{RC-R} = 28$
major homozygote
heterozygote
minor homozygote
Genotype of SNP rs6663731 (CYP4A11)

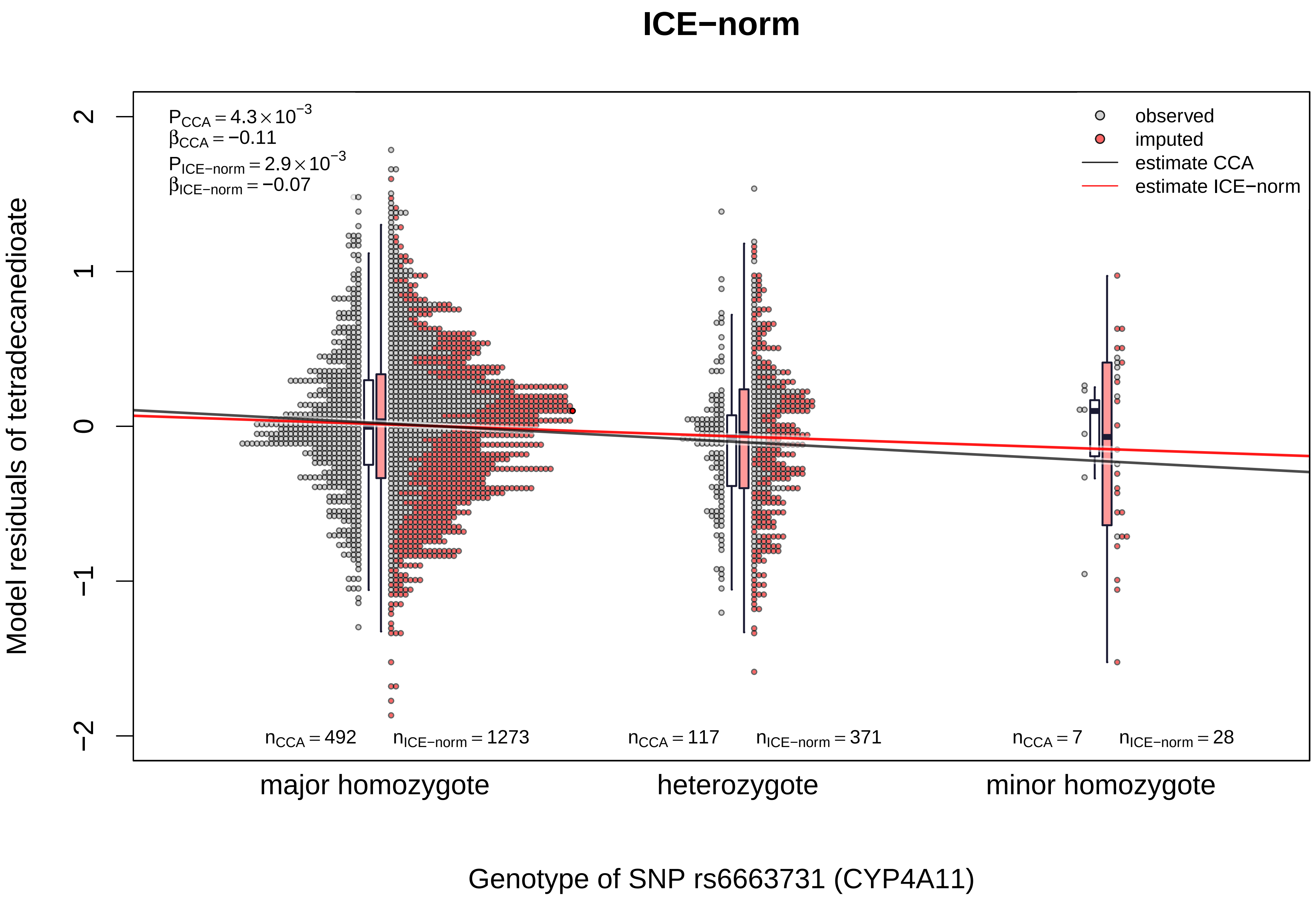

ICE−norm
P_CCA = 4.3 × 10^−3
β_CCA = −0.11
P_ICE−norm = 2.9 × 10^−3
β_ICE−norm = −0.07
observed
imputed
estimate CCA
estimate ICE−norm
Model residuals of tetradecanedioate
2
1
0
−1
−2
n_CCA = 492
n_ICE−norm = 1273
n_CCA = 117
n_ICE−norm = 371
n_CCA = 7
n_ICE−norm = 28
major homozygote
heterozygote
minor homozygote
Genotype of SNP rs6663731 (CYP4A11)

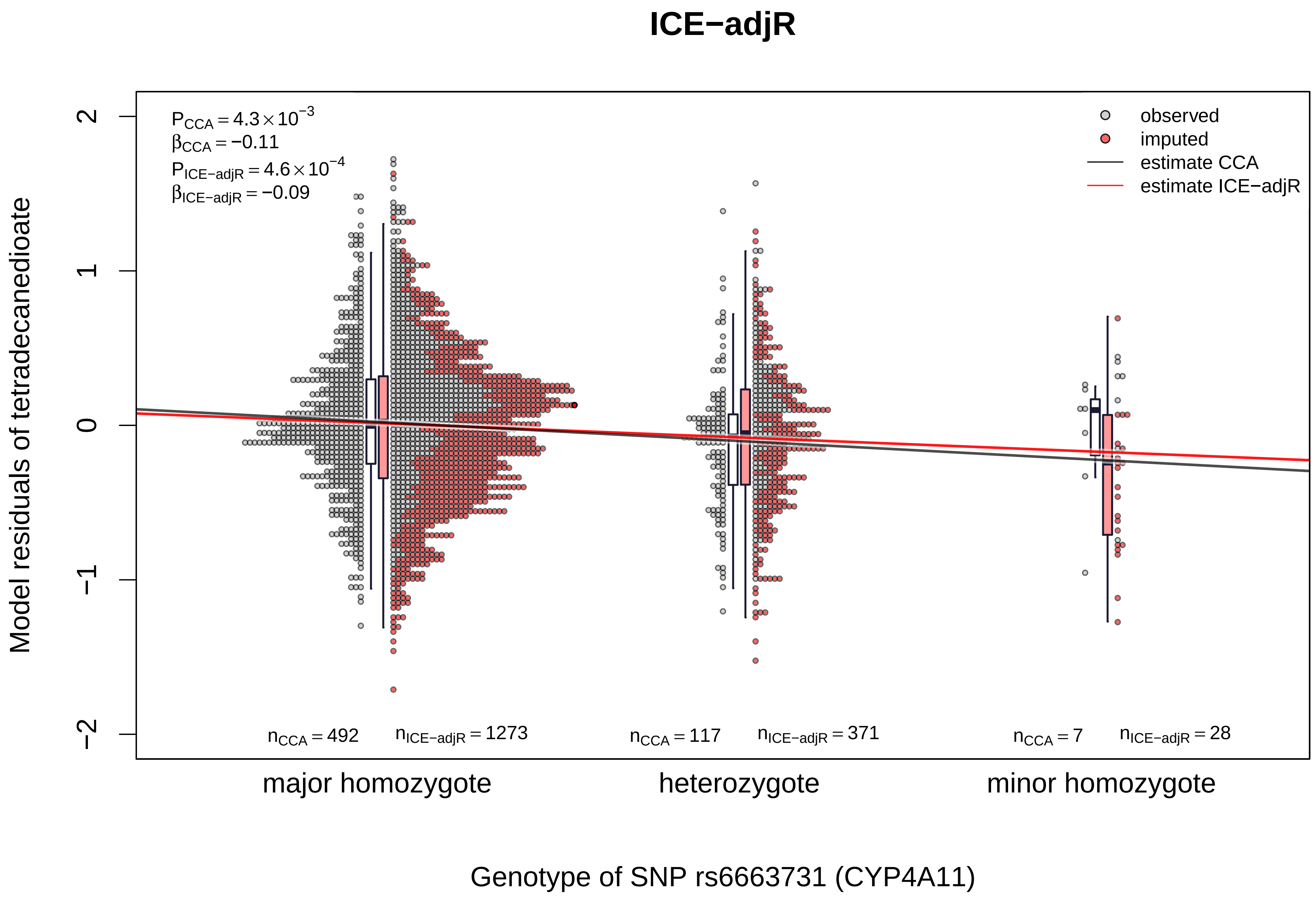

ICE−adjR
$P_{CCA} = 4.3 \times 10^{-3}$
$\beta_{CCA} = -0.11$
$P_{ICE-adjR} = 4.6 \times 10^{-4}$
$\beta_{ICE-adjR} = -0.09$
observed
imputed
estimate CCA
estimate ICE−adjR
2
1
0
−1
−2
Model residuals of tetradecanedioate
$n_{CCA} = 492$
$n_{ICE-adjR} = 1273$
$n_{CCA} = 117$
$n_{ICE-adjR} = 371$
$n_{CCA} = 7$
$n_{ICE-adjR} = 28$
major homozygote
heterozygote
minor homozygote
Genotype of SNP rs6663731 (CYP4A11)

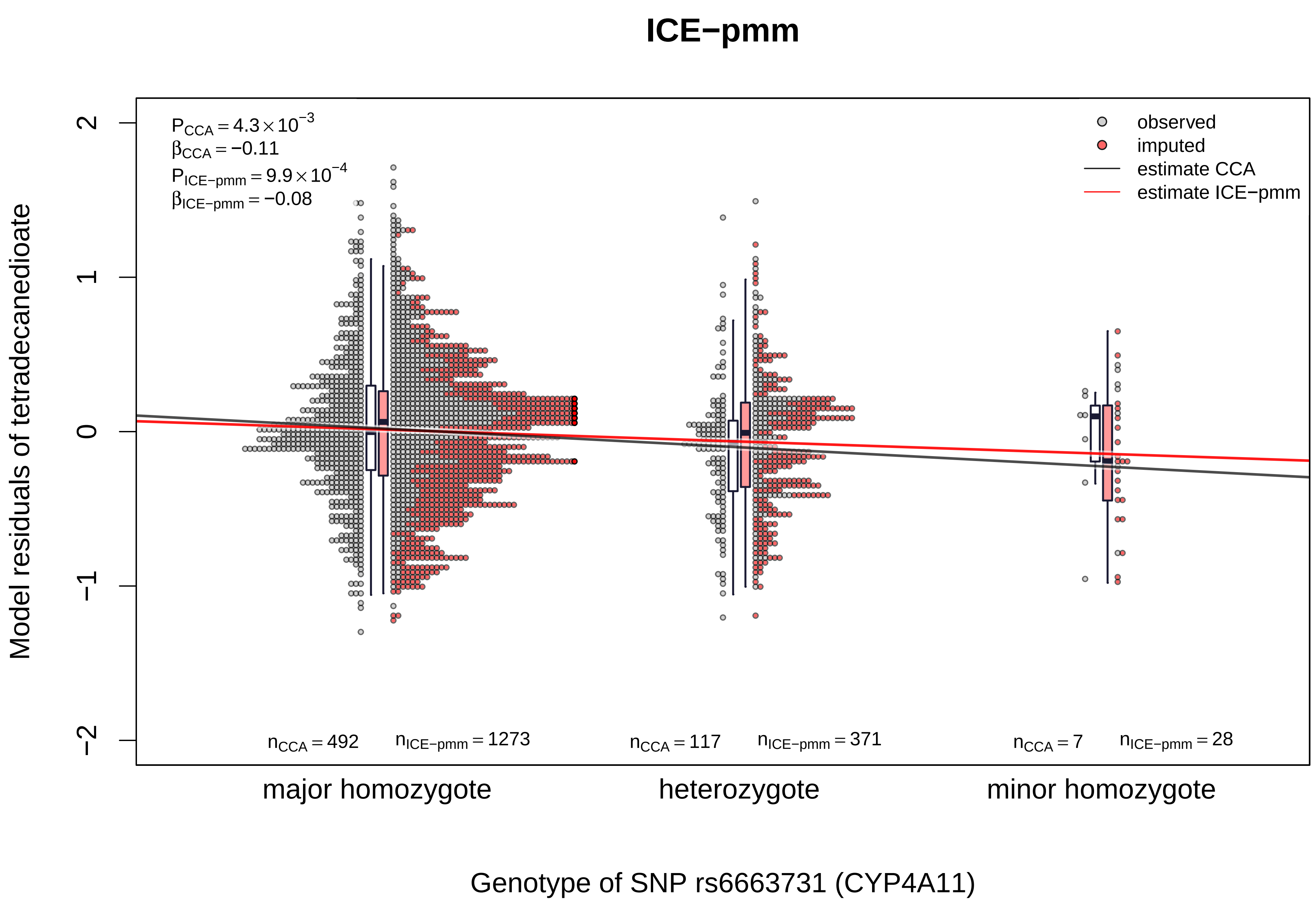

ICE−pmm
$P_{CCA} = 4.3 \times 10^{-3}$
$\beta_{CCA} = -0.11$
$P_{ICE-pmm} = 9.9 \times 10^{-4}$
$\beta_{ICE-pmm} = -0.08$
observed
imputed
estimate CCA
estimate ICE−pmm
Model residuals of tetradecanedioate
2
1
0
−1
−2
$n_{CCA} = 492$
$n_{ICE-pmm} = 1273$
$n_{CCA} = 117$
$n_{ICE-pmm} = 371$
$n_{CCA} = 7$
$n_{ICE-pmm} = 28$
major homozygote
heterozygote
minor homozygote
Genotype of SNP rs6663731 (CYP4A11)

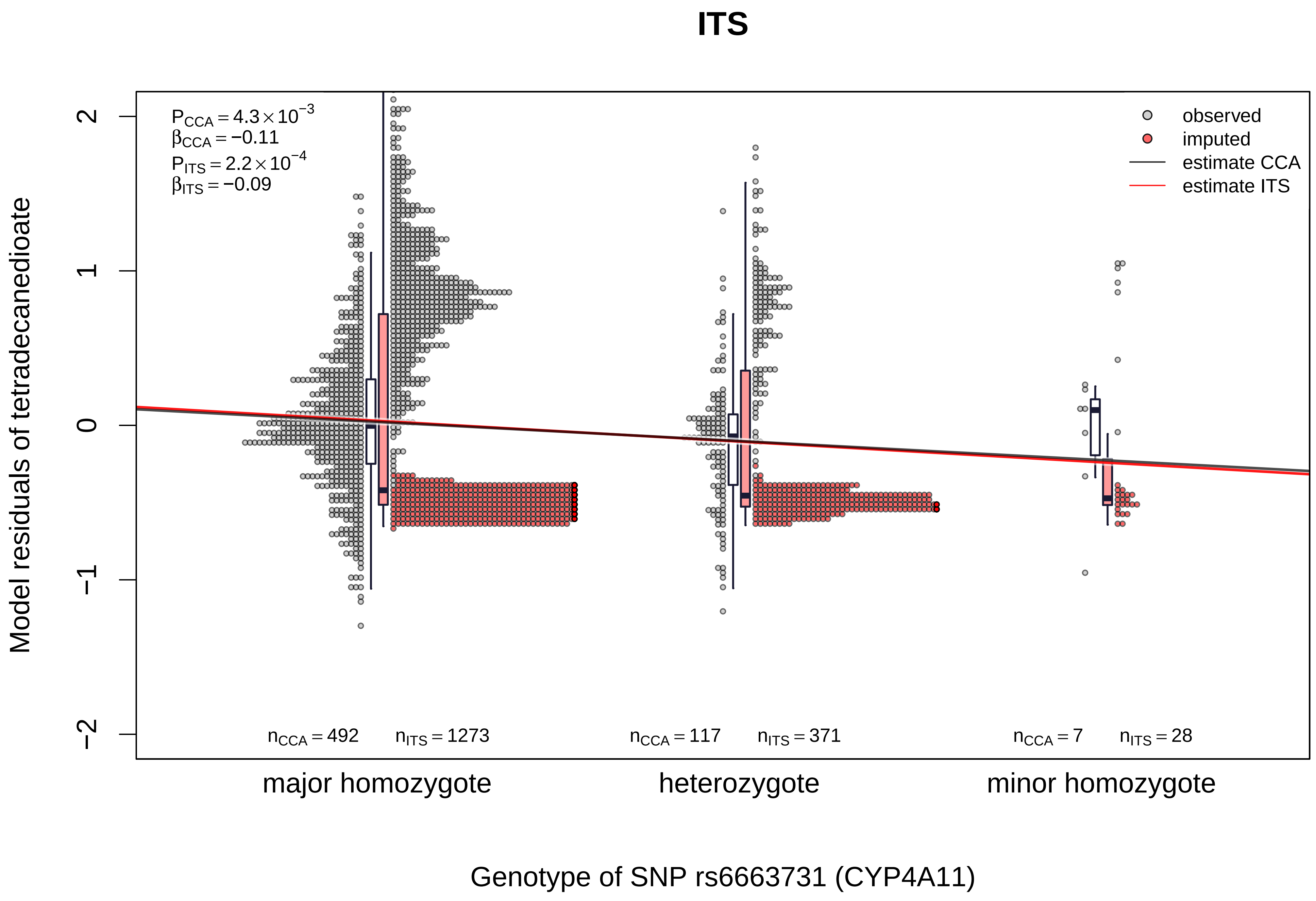

ITS
$P_{CCA} = 4.3 \times 10^{-3}$
$\beta_{CCA} = -0.11$
$P_{ITS} = 2.2 \times 10^{-4}$
$\beta_{ITS} = -0.09$
observed
imputed
estimate CCA
estimate ITS
Model residuals of tetradecanedioate
2
1
0
−1
−2
$n_{CCA} = 492$
$n_{ITS} = 1273$
$n_{CCA} = 117$
$n_{ITS} = 371$
$n_{CCA} = 7$
$n_{ITS} = 28$
major homozygote
heterozygote
minor homozygote
Genotype of SNP rs6663731 (CYP4A11)

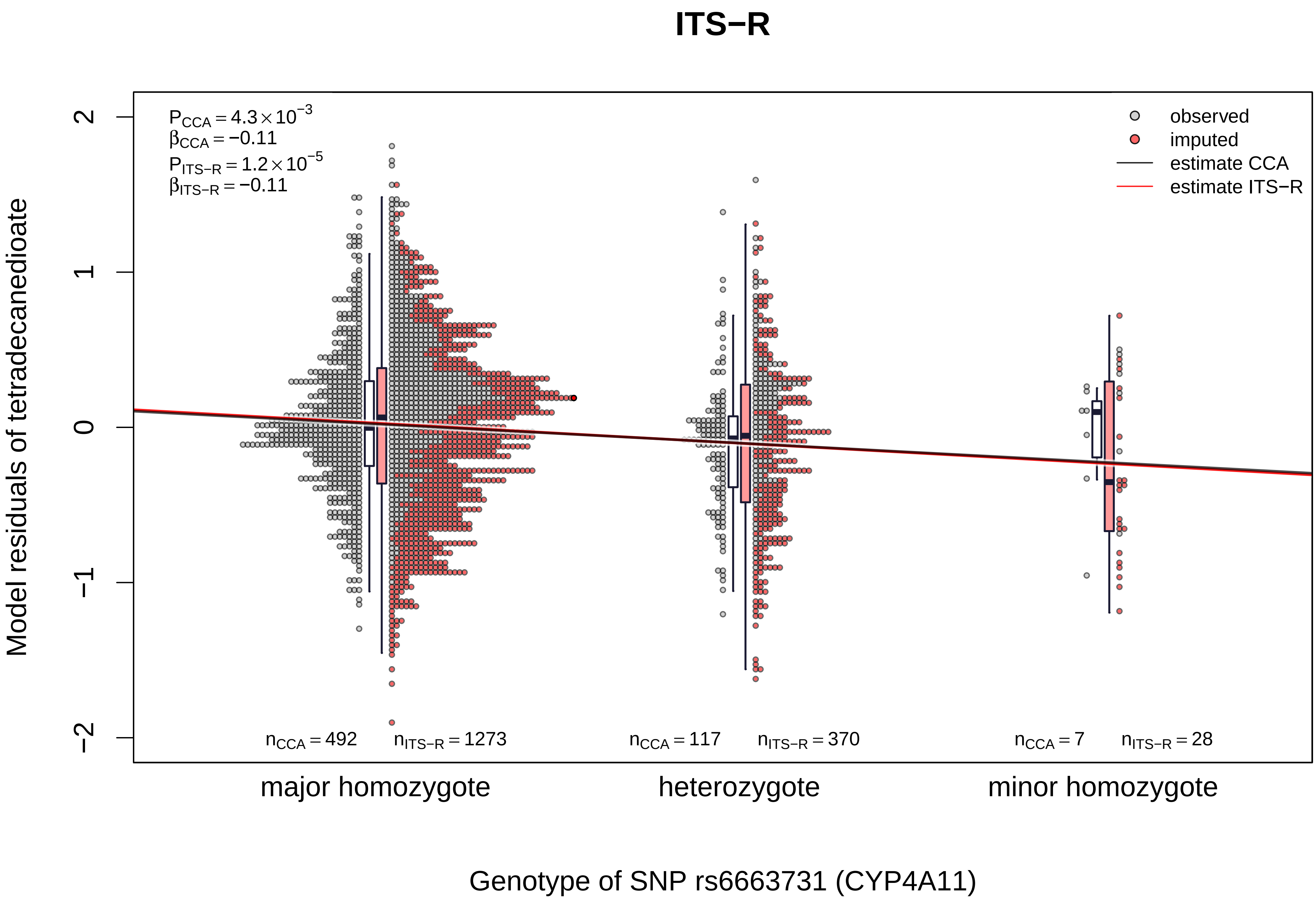

ITS−R
$P_{CCA} = 4.3 \times 10^{-3}$
$\beta_{CCA} = -0.11$
$P_{ITS-R} = 1.2 \times 10^{-5}$
$\beta_{ITS-R} = -0.11$
observed
imputed
estimate CCA
estimate ITS−R
Model residuals of tetradecanedioate
2
1
0
−1
−2
$n_{CCA} = 492$
$n_{ITS-R} = 1273$
$n_{CCA} = 117$
$n_{ITS-R} = 370$
$n_{CCA} = 7$
$n_{ITS-R} = 28$
major homozygote
heterozygote
minor homozygote
Genotype of SNP rs6663731 (CYP4A11)

MITS

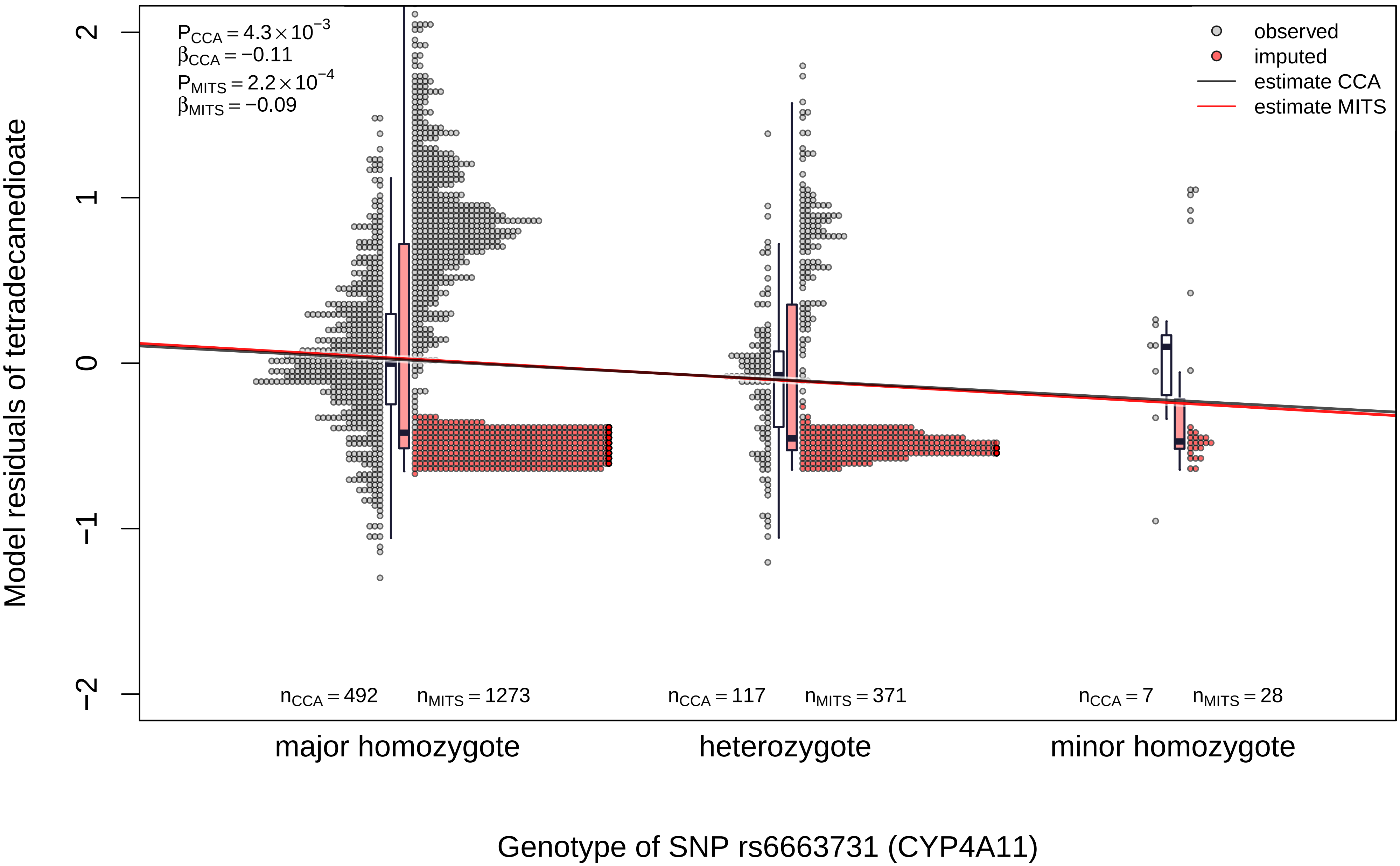

$P_{CCA} = 4.3 \times 10^{-3}$
$\beta_{CCA} = -0.11$
$P_{MITS} = 2.2 \times 10^{-4}$
$\beta_{MITS} = -0.09$
observed
imputed
estimate CCA
estimate MITS
Model residuals of tetradecanedioate
2
1
0
−1
−2
$n_{CCA} = 492$
$n_{MITS} = 1273$
$n_{CCA} = 117$
$n_{MITS} = 371$
$n_{CCA} = 7$
$n_{MITS} = 28$
major homozygote
heterozygote
minor homozygote
Genotype of SNP rs6663731 (CYP4A11)

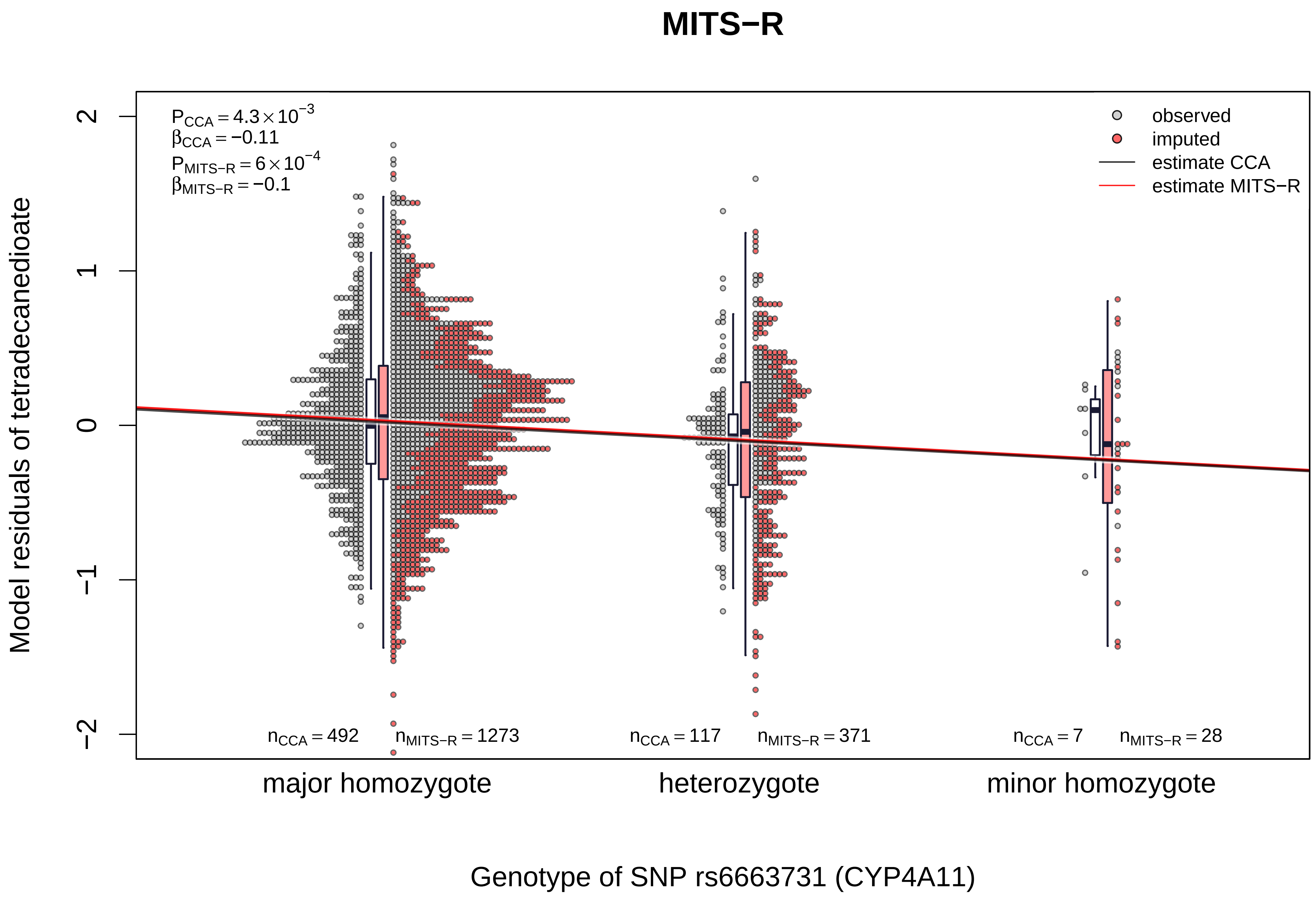

MITS−R
$P_{CCA} = 4.3 \times 10^{-3}$
$\beta_{CCA} = -0.11$
$P_{MITS-R} = 6 \times 10^{-4}$
$\beta_{MITS-R} = -0.1$
observed
imputed
estimate CCA
estimate MITS−R
2
1
0
−1
−2
Model residuals of tetradecanedioate
$n_{CCA} = 492$
$n_{MITS-R} = 1273$
$n_{CCA} = 117$
$n_{MITS-R} = 371$
$n_{CCA} = 7$
$n_{MITS-R} = 28$
major homozygote
heterozygote
minor homozygote
Genotype of SNP rs6663731 (CYP4A11)

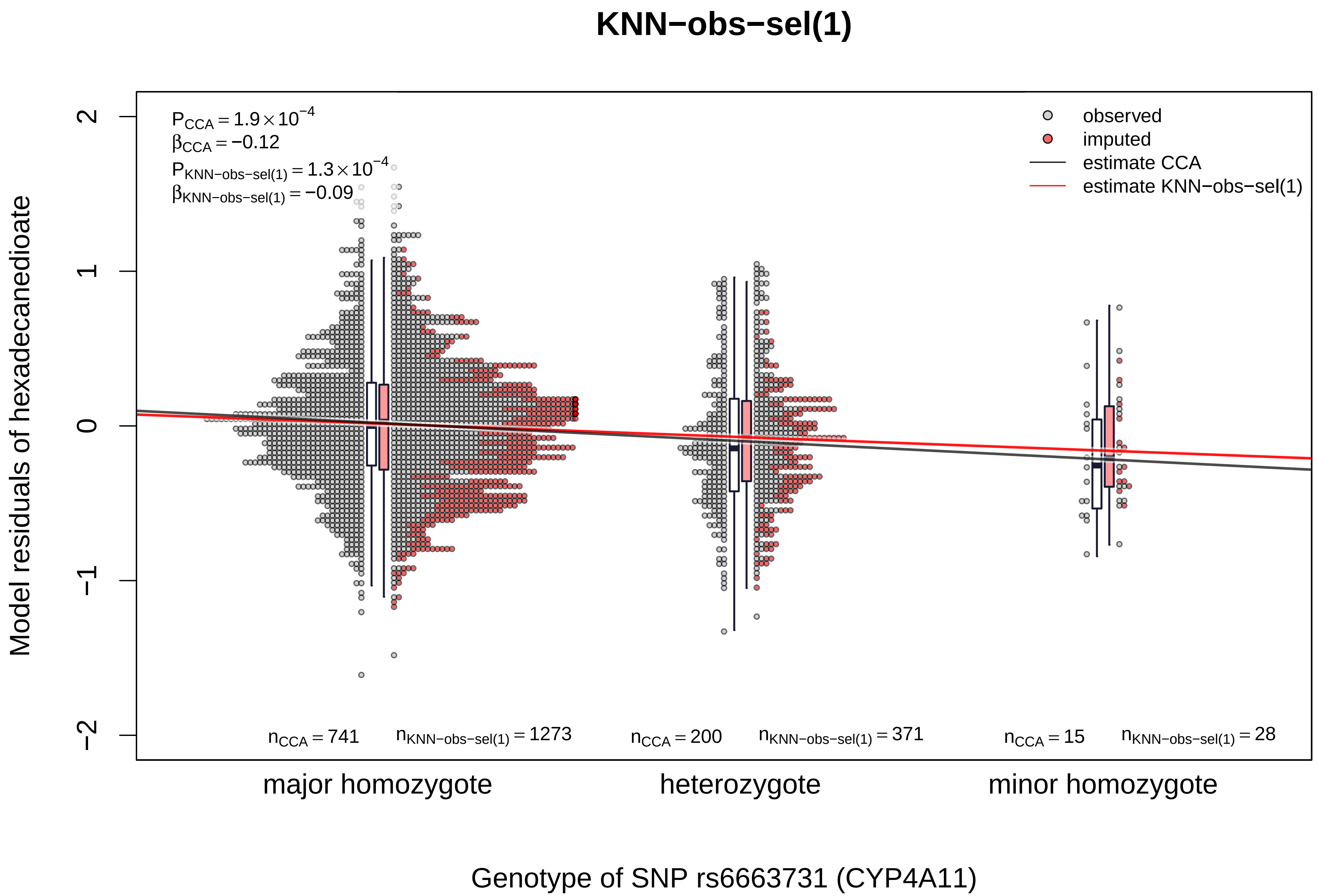

KNN−obs−sel(1)
$P_{CCA} = 1.9 \times 10^{-4}$
$\beta_{CCA} = -0.12$
$P_{KNN-obs-sel(1)} = 1.3 \times 10^{-4}$
$\beta_{KNN-obs-sel(1)} = -0.09$
observed
imputed
estimate CCA
estimate KNN−obs−sel(1)
Model residuals of hexadecanedioate
2
1
0
−1
−2
$n_{CCA} = 741$
$n_{KNN-obs-sel(1)} = 1273$
$n_{CCA} = 200$
$n_{KNN-obs-sel(1)} = 371$
$n_{CCA} = 15$
$n_{KNN-obs-sel(1)} = 28$
major homozygote
heterozygote
minor homozygote
Genotype of SNP rs6663731 (CYP4A11)

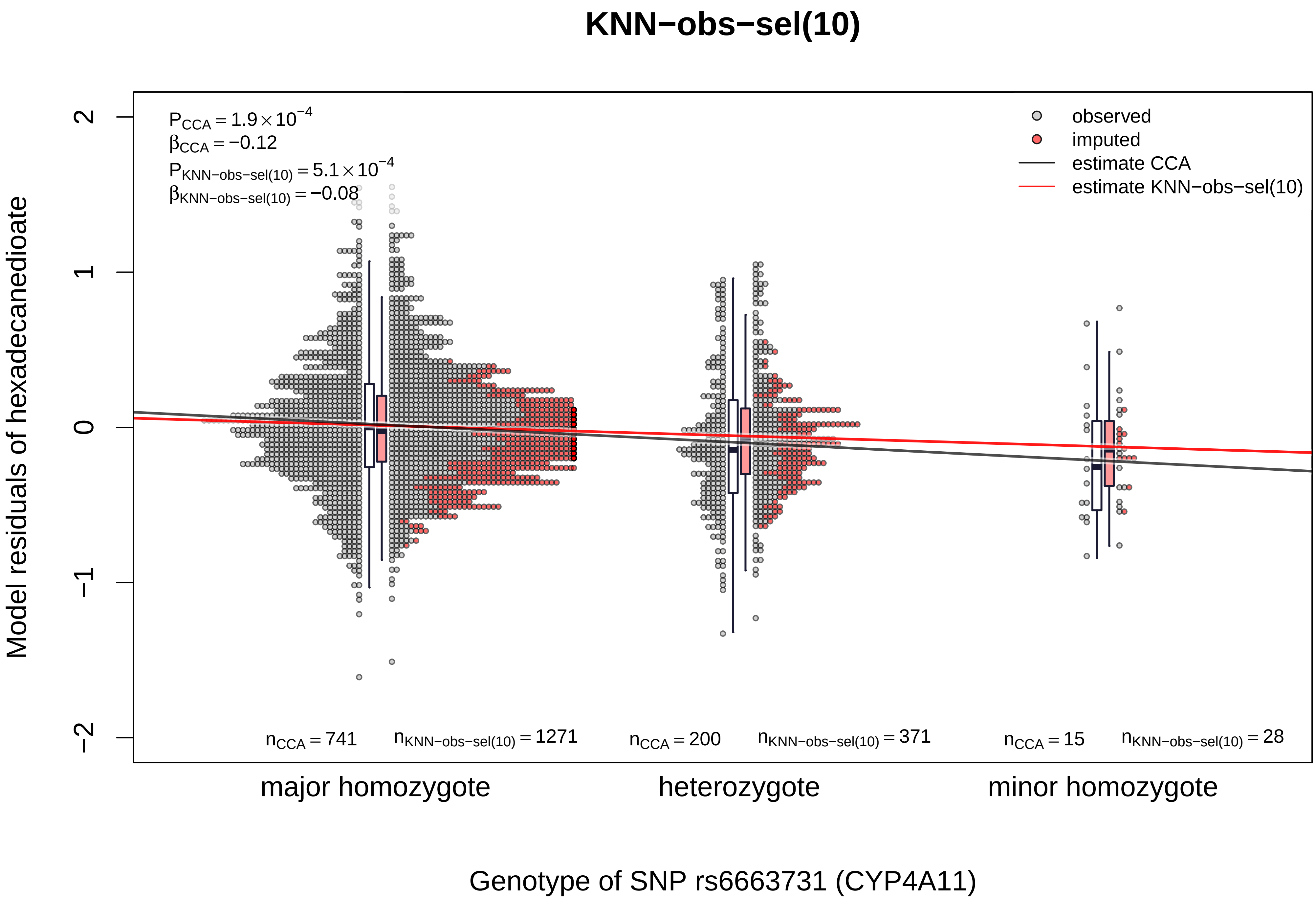

KNN−obs−sel(10)
$P_{CCA} = 1.9 \times 10^{-4}$
$\beta_{CCA} = -0.12$
$P_{KNN-obs-sel(10)} = 5.1 \times 10^{-4}$
$\beta_{KNN-obs-sel(10)} = -0.08$
observed
imputed
estimate CCA
estimate KNN−obs−sel(10)
Model residuals of hexadecanedioate
2
1
0
−1
−2
$n_{CCA} = 741$
$n_{KNN-obs-sel(10)} = 1271$
$n_{CCA} = 200$
$n_{KNN-obs-sel(10)} = 371$
$n_{CCA} = 15$
$n_{KNN-obs-sel(10)} = 28$
major homozygote
heterozygote
minor homozygote
Genotype of SNP rs6663731 (CYP4A11)

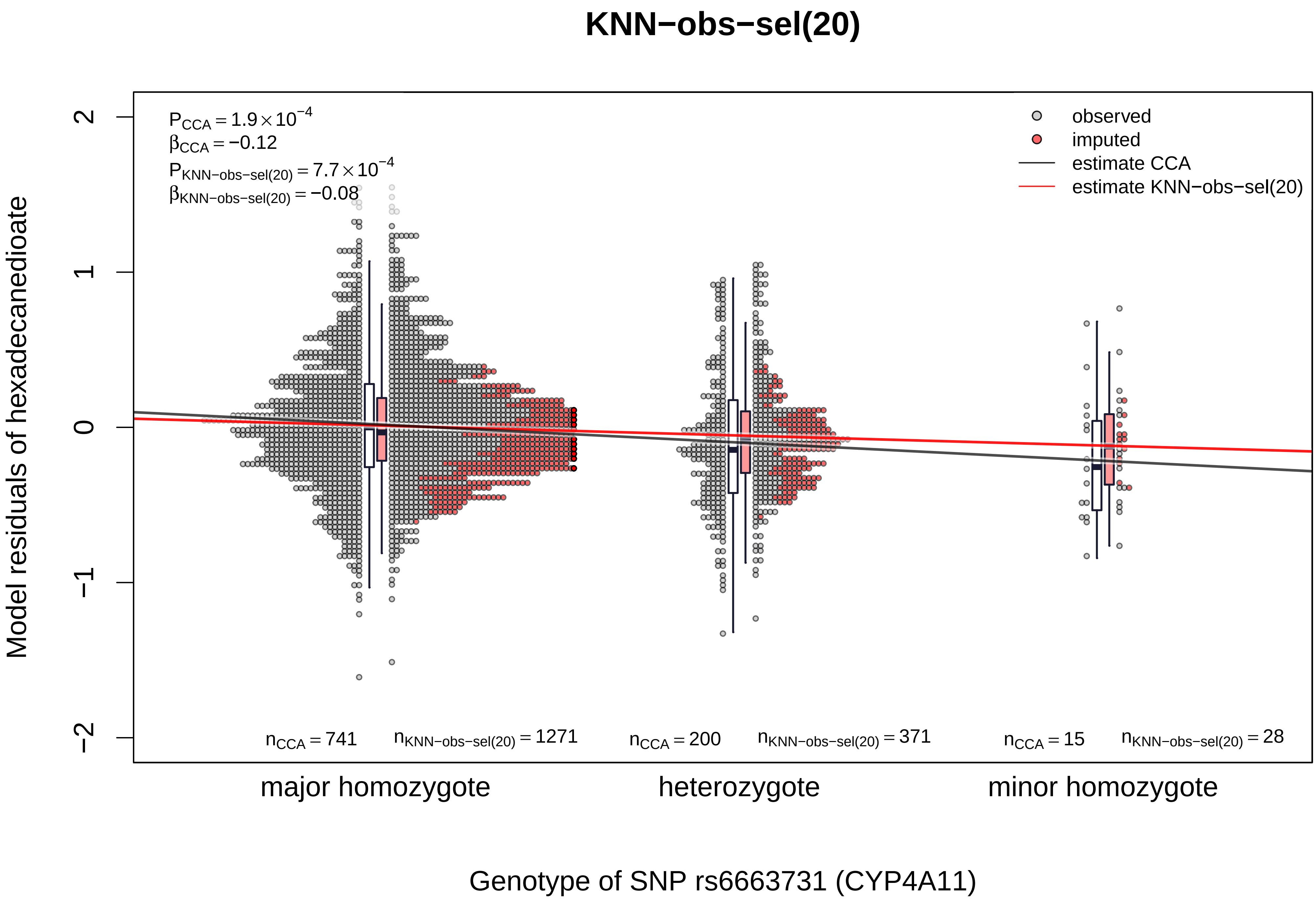

KNN−obs−sel(20)
$P_{CCA} = 1.9 \times 10^{-4}$
$\beta_{CCA} = -0.12$
$P_{KNN-obs-sel(20)} = 7.7 \times 10^{-4}$
$\beta_{KNN-obs-sel(20)} = -0.08$
observed
imputed
estimate CCA
estimate KNN−obs−sel(20)
2
1
0
−1
−2
Model residuals of hexadecanedioate
$n_{CCA} = 741$
$n_{KNN-obs-sel(20)} = 1271$
$n_{CCA} = 200$
$n_{KNN-obs-sel(20)} = 371$
$n_{CCA} = 15$
$n_{KNN-obs-sel(20)} = 28$
major homozygote
heterozygote
minor homozygote
Genotype of SNP rs6663731 (CYP4A11)

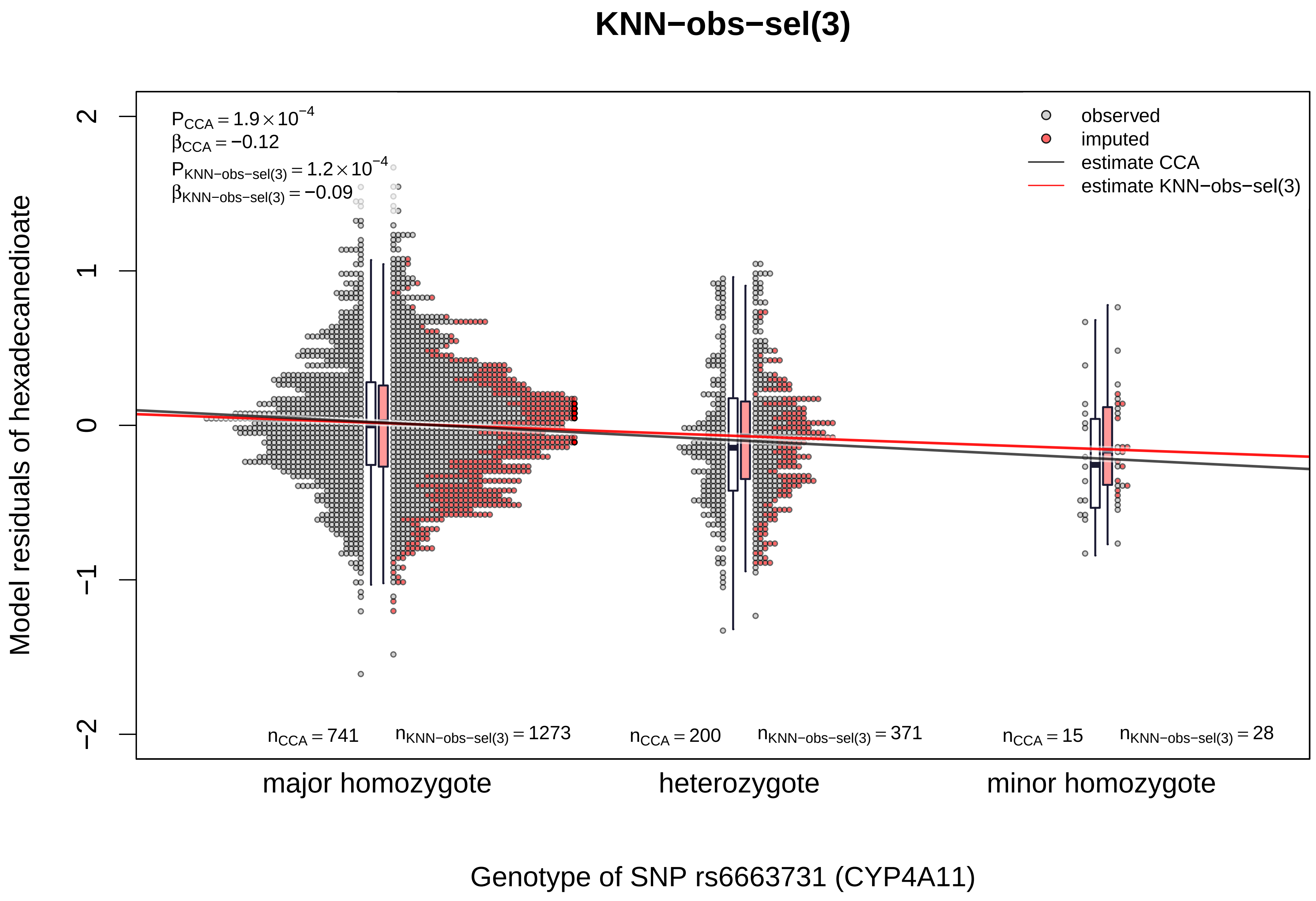
KNN−obs−sel(3)
$P_{CCA} = 1.9 \times 10^{-4}$
$\beta_{CCA} = -0.12$
$P_{KNN-obs-sel(3)} = 1.2 \times 10^{-4}$
$\beta_{KNN-obs-sel(3)} = -0.09$
observed
imputed
estimate CCA
estimate KNN−obs−sel(3)
Model residuals of hexadecanedioate
2
1
0
−1
−2
$n_{CCA} = 741$
$n_{KNN-obs-sel(3)} = 1273$
$n_{CCA} = 200$
$n_{KNN-obs-sel(3)} = 371$
$n_{CCA} = 15$
$n_{KNN-obs-sel(3)} = 28$
major homozygote
heterozygote
minor homozygote
Genotype of SNP rs6663731 (CYP4A11)

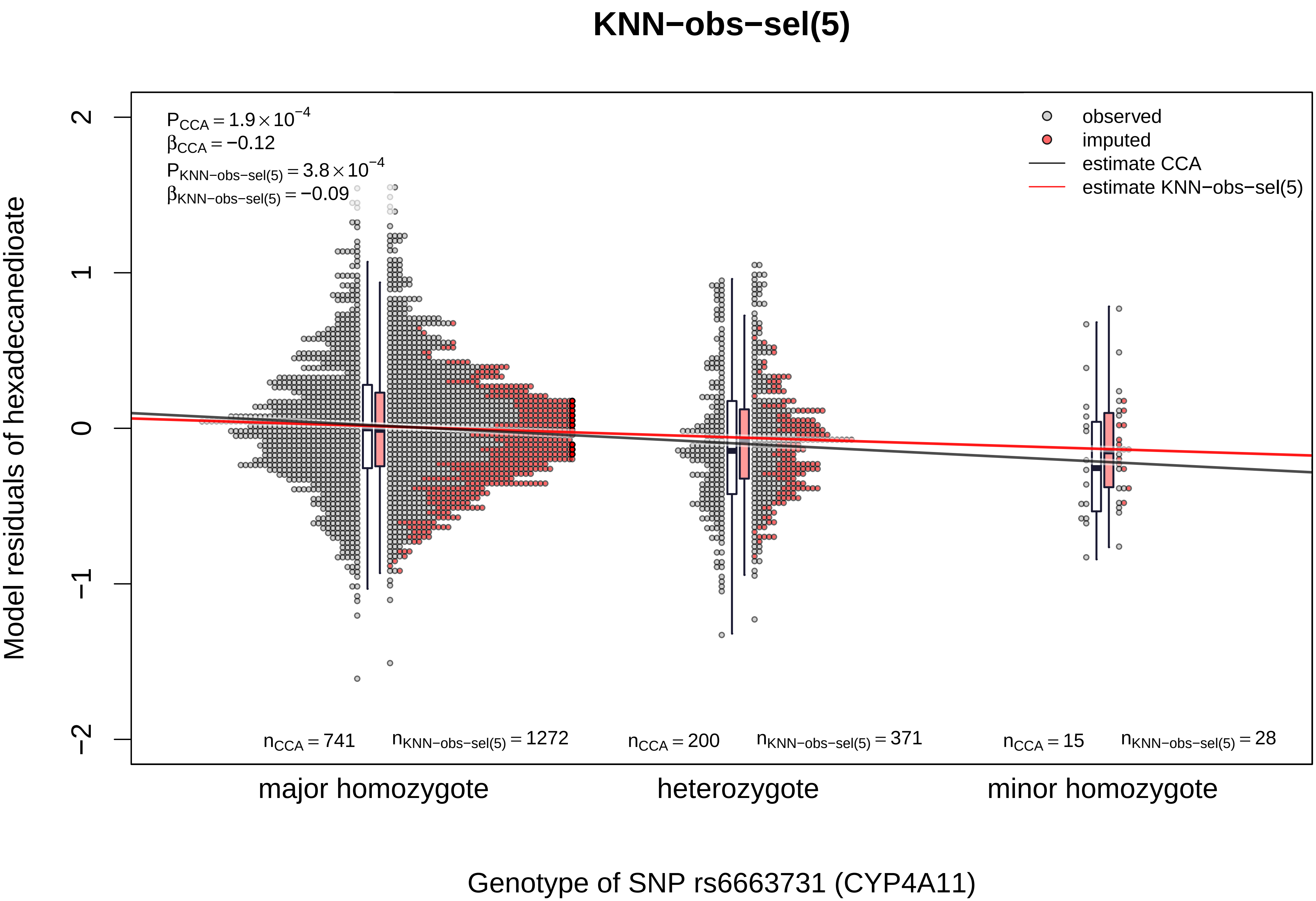

KNN−obs−sel(5)
$P_{CCA} = 1.9 \times 10^{-4}$
$\beta_{CCA} = -0.12$
$P_{KNN-obs-sel(5)} = 3.8 \times 10^{-4}$
$\beta_{KNN-obs-sel(5)} = -0.09$
observed
imputed
estimate CCA
estimate KNN−obs−sel(5)
2
1
0
−1
−2
Model residuals of hexadecanedioate
$n_{CCA} = 741$
$n_{KNN-obs-sel(5)} = 1272$
$n_{CCA} = 200$
$n_{KNN-obs-sel(5)} = 371$
$n_{CCA} = 15$
$n_{KNN-obs-sel(5)} = 28$
major homozygote
heterozygote
minor homozygote
Genotype of SNP rs6663731 (CYP4A11)

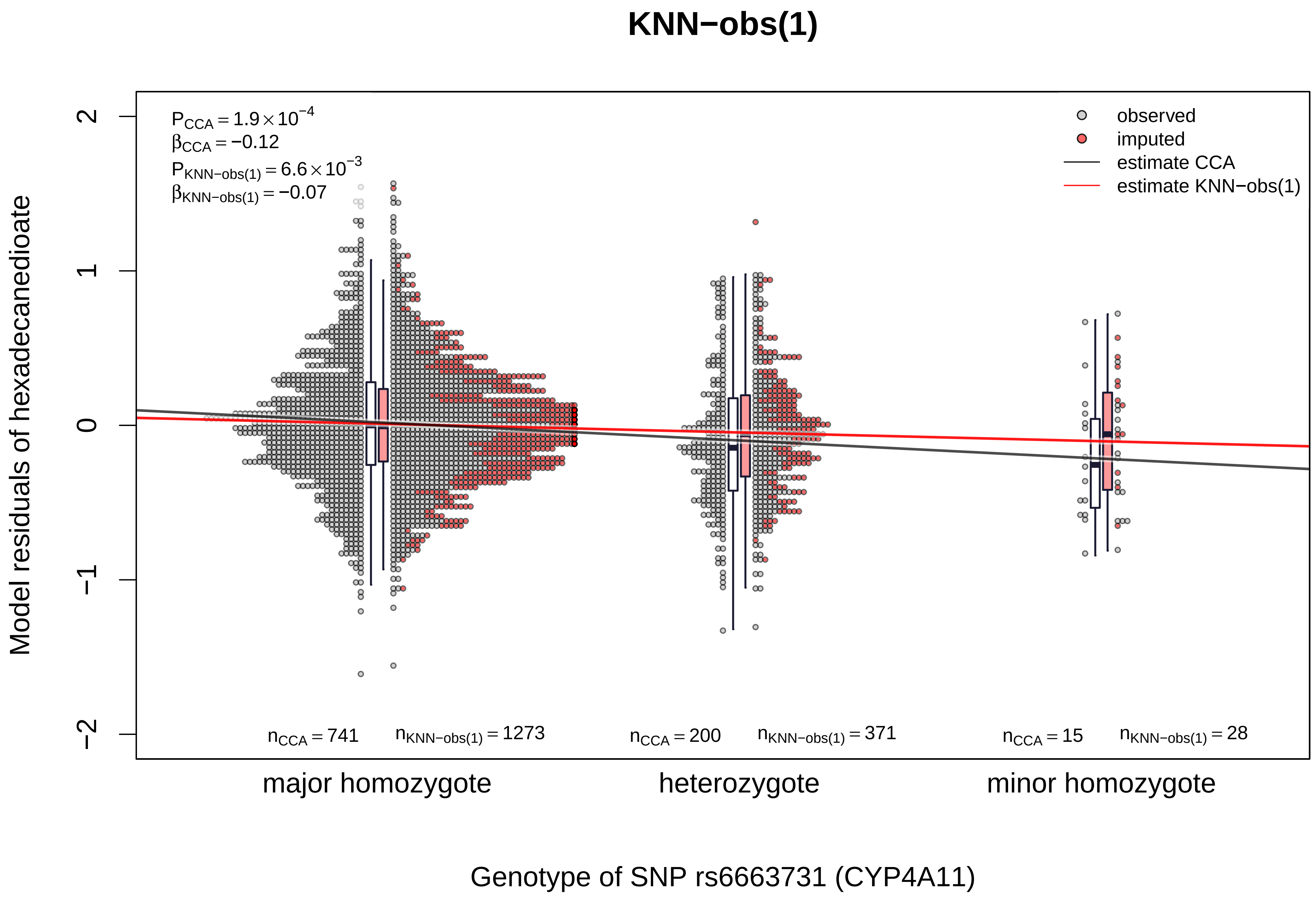
KNN−obs(1)
$P_{CCA} = 1.9 \times 10^{-4}$
$\beta_{CCA} = -0.12$
$P_{KNN-obs(1)} = 6.6 \times 10^{-3}$
$\beta_{KNN-obs(1)} = -0.07$
observed
imputed
estimate CCA
estimate KNN−obs(1)
Model residuals of hexadecanedioate
2
1
0
−1
−2
$n_{CCA} = 741$
$n_{KNN-obs(1)} = 1273$
$n_{CCA} = 200$
$n_{KNN-obs(1)} = 371$
$n_{CCA} = 15$
$n_{KNN-obs(1)} = 28$
major homozygote
heterozygote
minor homozygote
Genotype of SNP rs6663731 (CYP4A11)

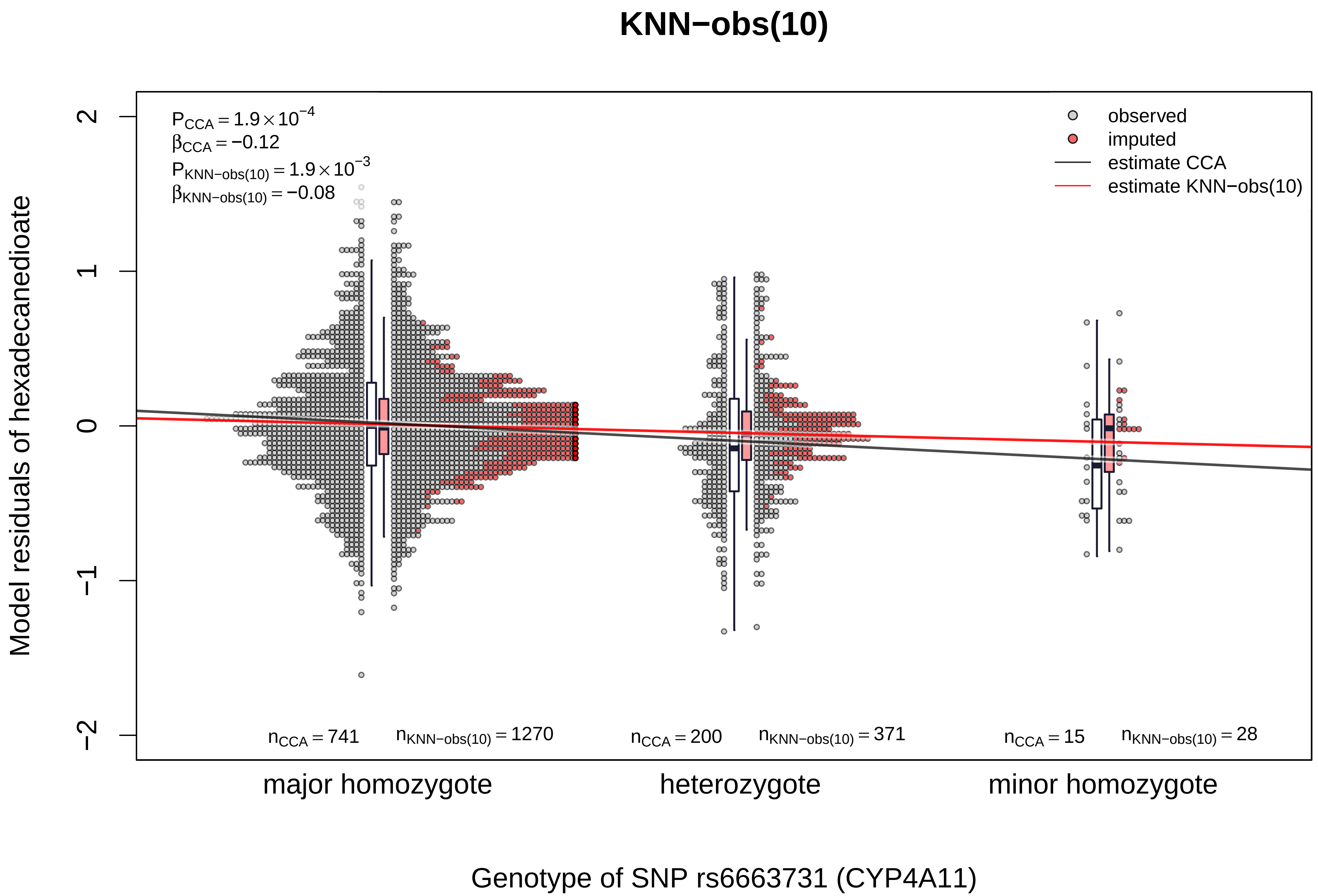

KNN−obs(10)
$P_{CCA} = 1.9 \times 10^{-4}$
$\beta_{CCA} = -0.12$
$P_{KNN-obs(10)} = 1.9 \times 10^{-3}$
$\beta_{KNN-obs(10)} = -0.08$
observed
imputed
estimate CCA
estimate KNN−obs(10)
Model residuals of hexadecanedioate
2
1
0
−1
−2
$n_{CCA} = 741$
$n_{KNN-obs(10)} = 1270$
$n_{CCA} = 200$
$n_{KNN-obs(10)} = 371$
$n_{CCA} = 15$
$n_{KNN-obs(10)} = 28$
major homozygote
heterozygote
minor homozygote
Genotype of SNP rs6663731 (CYP4A11)

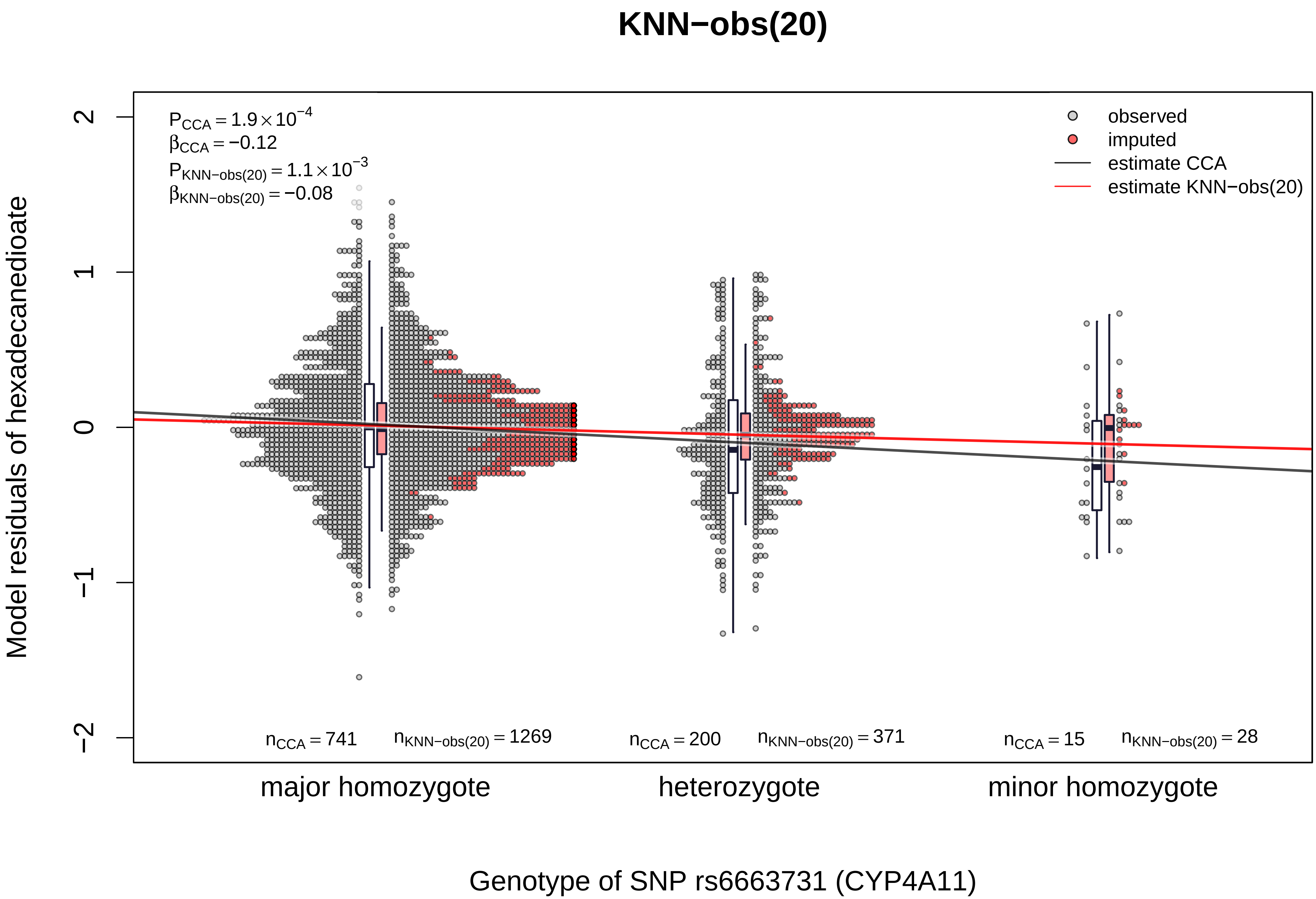
KNN−obs(20)
$P_{CCA} = 1.9 \times 10^{-4}$
$\beta_{CCA} = -0.12$
$P_{KNN-obs(20)} = 1.1 \times 10^{-3}$
$\beta_{KNN-obs(20)} = -0.08$
observed
imputed
estimate CCA
estimate KNN−obs(20)
Model residuals of hexadecanedioate
2
1
0
−1
−2
$n_{CCA} = 741$
$n_{KNN-obs(20)} = 1269$
$n_{CCA} = 200$
$n_{KNN-obs(20)} = 371$
$n_{CCA} = 15$
$n_{KNN-obs(20)} = 28$
major homozygote
heterozygote
minor homozygote
Genotype of SNP rs6663731 (CYP4A11)

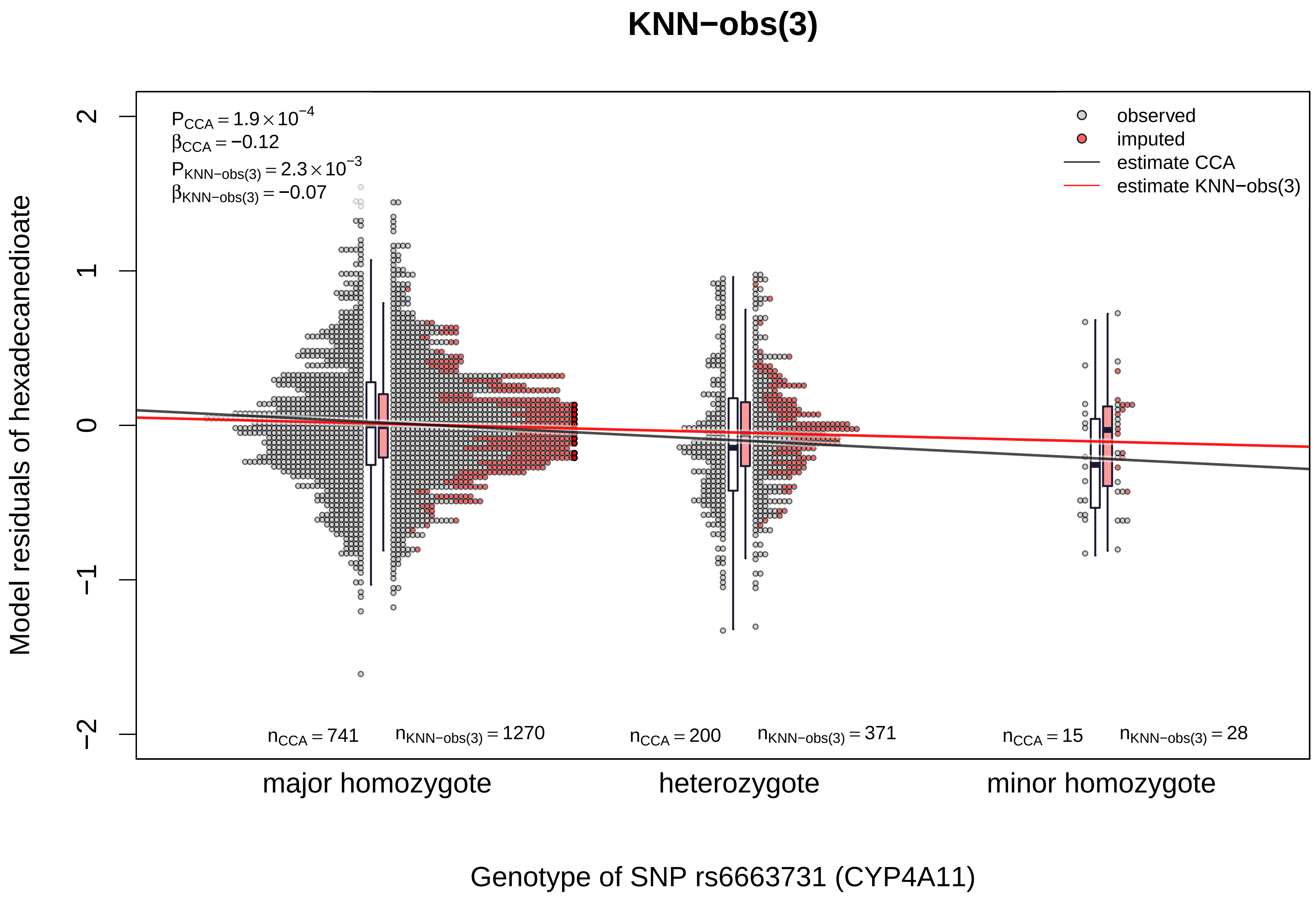

KNN−obs(3)
P_CCA = 1.9 × 10^−4
β_CCA = −0.12
P_KNN−obs(3) = 2.3 × 10^−3
β_KNN−obs(3) = −0.07
observed
imputed
estimate CCA
estimate KNN−obs(3)
Model residuals of hexadecanedioate
2
1
0
−1
−2
n_CCA = 741
n_KNN−obs(3) = 1270
n_CCA = 200
n_KNN−obs(3) = 371
n_CCA = 15
n_KNN−obs(3) = 28
major homozygote
heterozygote
minor homozygote
Genotype of SNP rs6663731 (CYP4A11)

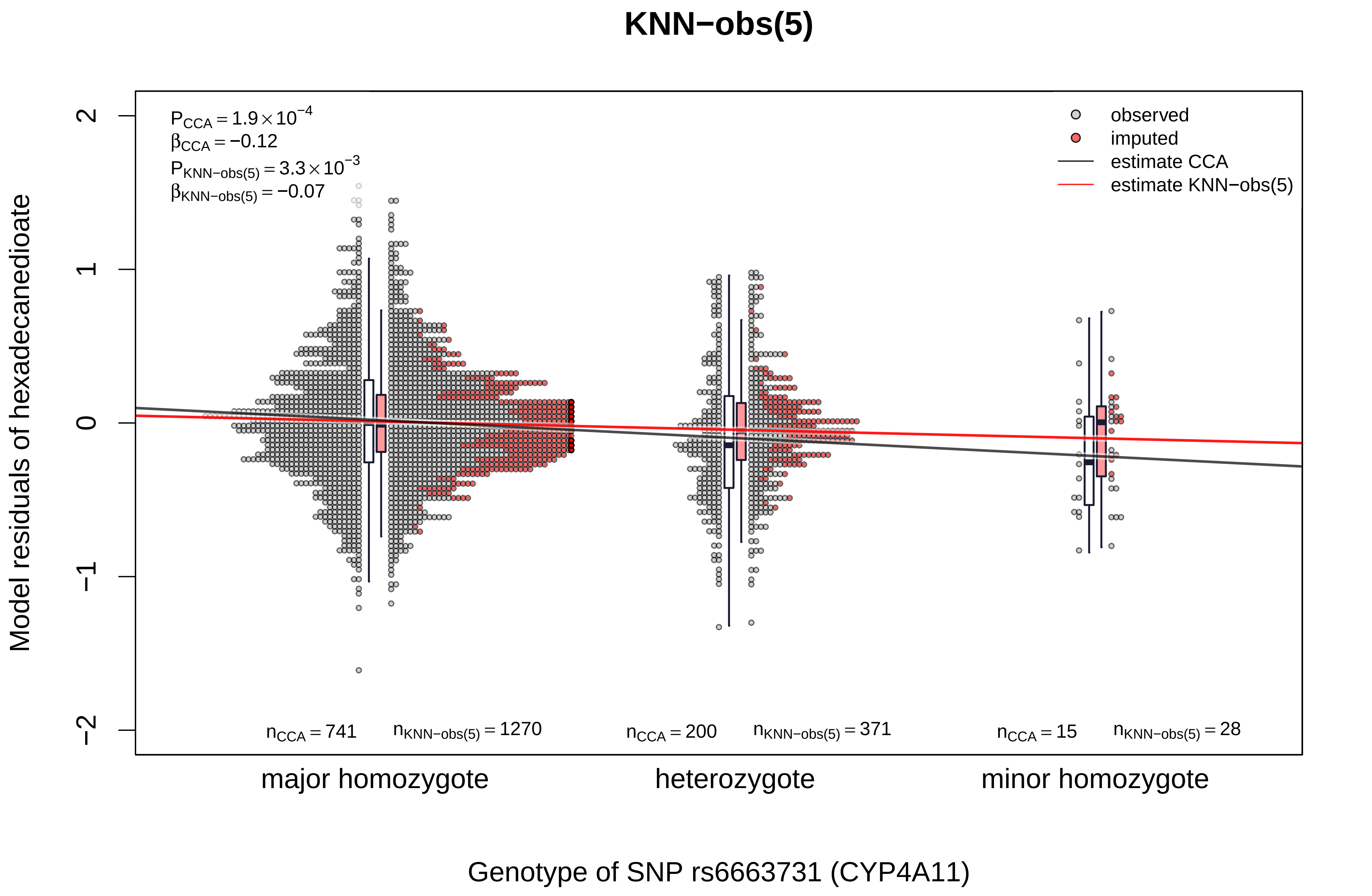

KNN−obs(5)
$P_{CCA} = 1.9 \times 10^{-4}$
$\beta_{CCA} = -0.12$
$P_{KNN-obs(5)} = 3.3 \times 10^{-3}$
$\beta_{KNN-obs(5)} = -0.07$
observed
imputed
estimate CCA
estimate KNN−obs(5)
Model residuals of hexadecanedioate
2
1
0
−1
−2
$n_{CCA} = 741$
$n_{KNN-obs(5)} = 1270$
$n_{CCA} = 200$
$n_{KNN-obs(5)} = 371$
$n_{CCA} = 15$
$n_{KNN-obs(5)} = 28$
major homozygote
heterozygote
minor homozygote
Genotype of SNP rs6663731 (CYP4A11)

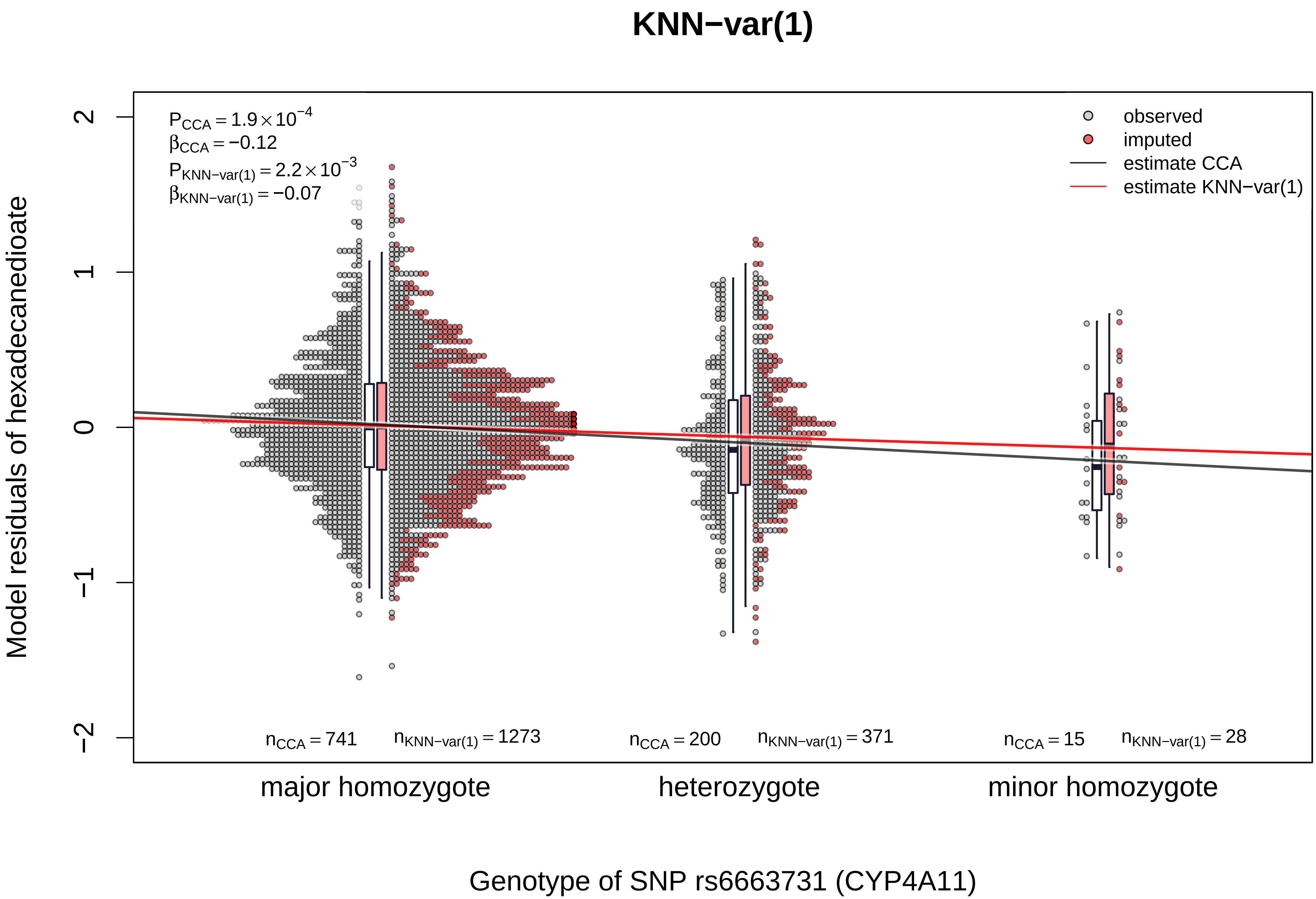

KNN−var(1)
$P_{CCA} = 1.9 \times 10^{-4}$
$\beta_{CCA} = -0.12$
$P_{KNN-var(1)} = 2.2 \times 10^{-3}$
$\beta_{KNN-var(1)} = -0.07$
observed
imputed
estimate CCA
estimate KNN−var(1)
Model residuals of hexadecanedioate
2
1
0
−1
−2
$n_{CCA} = 741$
$n_{KNN-var(1)} = 1273$
$n_{CCA} = 200$
$n_{KNN-var(1)} = 371$
$n_{CCA} = 15$
$n_{KNN-var(1)} = 28$
major homozygote
heterozygote
minor homozygote
Genotype of SNP rs6663731 (CYP4A11)

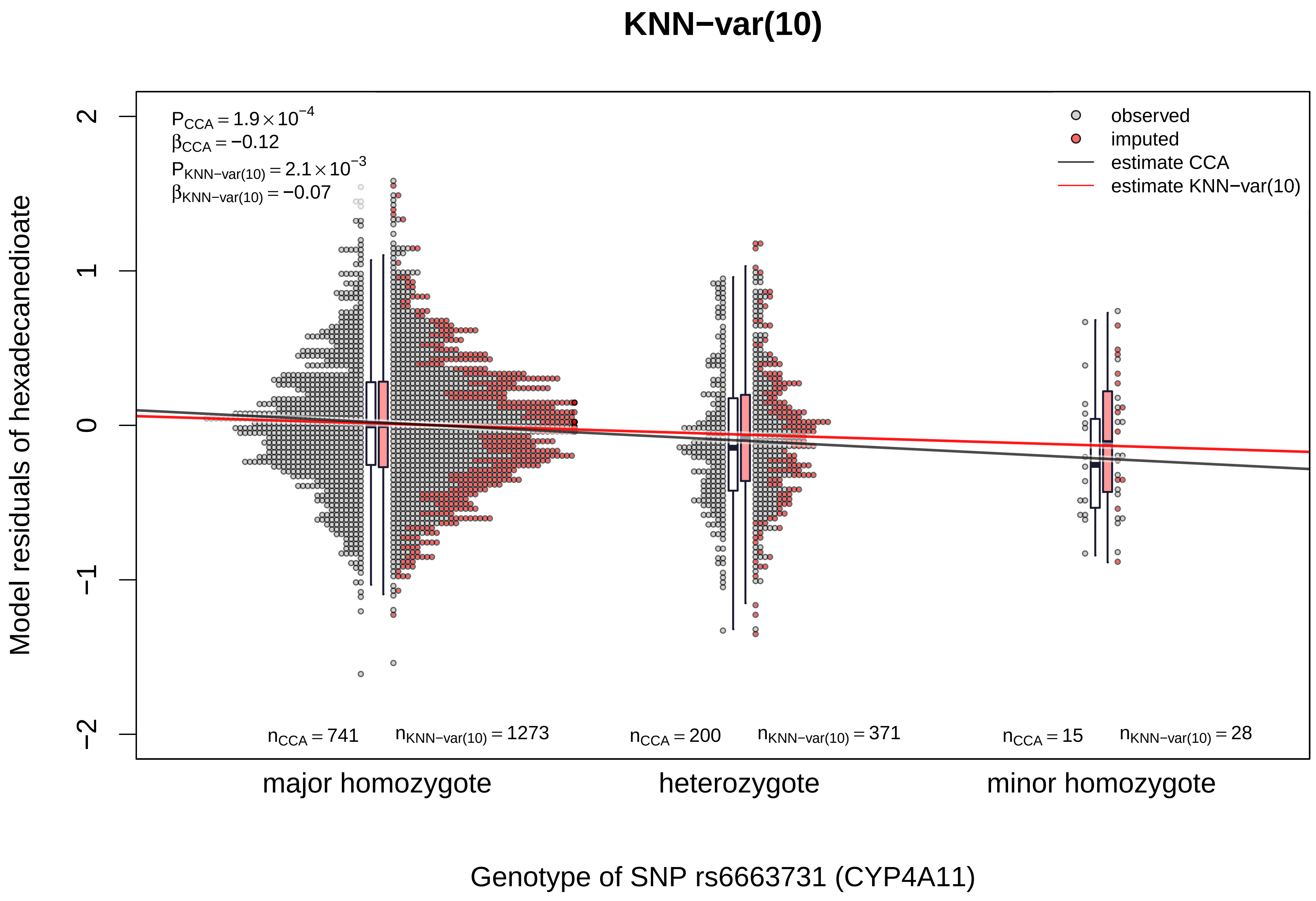

KNN−var(10)
$P_{CCA} = 1.9 \times 10^{-4}$
$\beta_{CCA} = -0.12$
$P_{KNN-var(10)} = 2.1 \times 10^{-3}$
$\beta_{KNN-var(10)} = -0.07$
observed
imputed
estimate CCA
estimate KNN−var(10)
2
1
0
−1
−2
Model residuals of hexadecanedioate
$n_{CCA} = 741$
$n_{KNN-var(10)} = 1273$
$n_{CCA} = 200$
$n_{KNN-var(10)} = 371$
$n_{CCA} = 15$
$n_{KNN-var(10)} = 28$
major homozygote
heterozygote
minor homozygote
Genotype of SNP rs6663731 (CYP4A11)

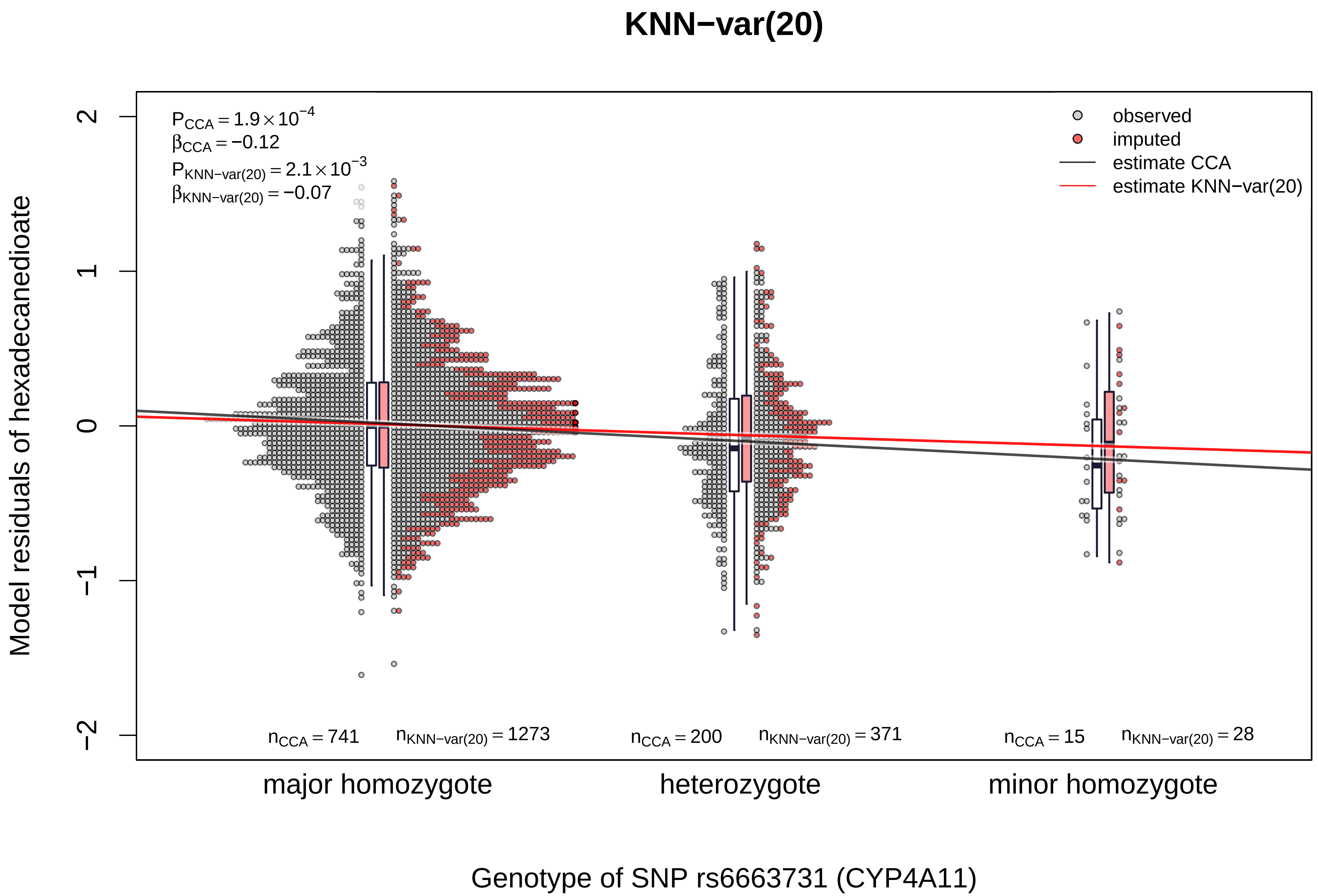
KNN−var(20)
$P_{CCA} = 1.9 \times 10^{-4}$
$\beta_{CCA} = -0.12$
$P_{KNN-var(20)} = 2.1 \times 10^{-3}$
$\beta_{KNN-var(20)} = -0.07$
observed
imputed
estimate CCA
estimate KNN−var(20)
Model residuals of hexadecanedioate
2
1
0
−1
−2
$n_{CCA} = 741$
$n_{KNN-var(20)} = 1273$
$n_{CCA} = 200$
$n_{KNN-var(20)} = 371$
$n_{CCA} = 15$
$n_{KNN-var(20)} = 28$
major homozygote
heterozygote
minor homozygote
Genotype of SNP rs6663731 (CYP4A11)

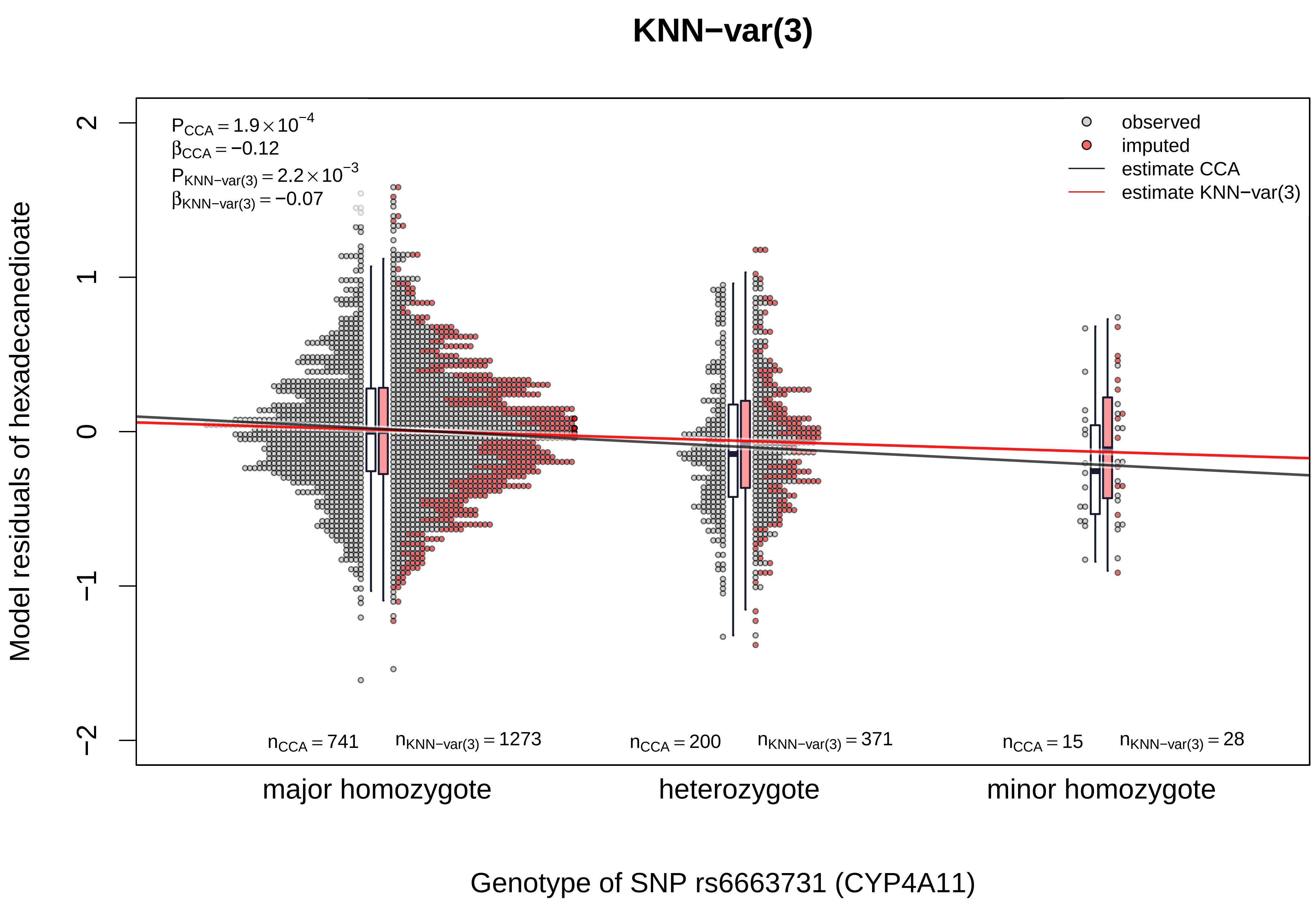

KNN−var(3)
$P_{CCA} = 1.9 \times 10^{-4}$
$\beta_{CCA} = -0.12$
$P_{KNN-var(3)} = 2.2 \times 10^{-3}$
$\beta_{KNN-var(3)} = -0.07$
observed
imputed
estimate CCA
estimate KNN−var(3)
Model residuals of hexadecanedioate
2
1
0
−1
−2
$n_{CCA} = 741$
$n_{KNN-var(3)} = 1273$
$n_{CCA} = 200$
$n_{KNN-var(3)} = 371$
$n_{CCA} = 15$
$n_{KNN-var(3)} = 28$
major homozygote
heterozygote
minor homozygote
Genotype of SNP rs6663731 (CYP4A11)

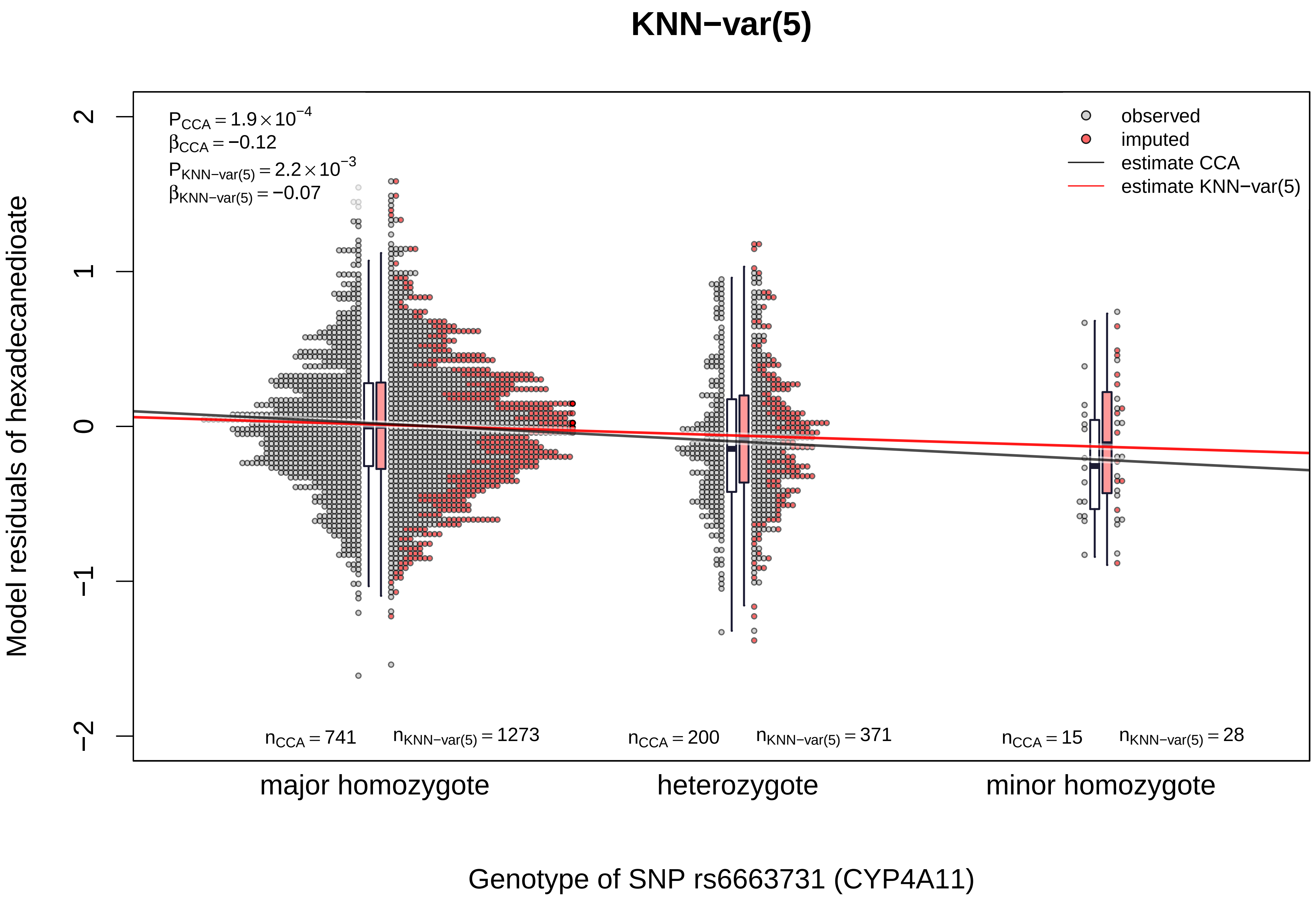

KNN−var(5)
$P_{CCA} = 1.9 \times 10^{-4}$
$\beta_{CCA} = -0.12$
$P_{KNN-var(5)} = 2.2 \times 10^{-3}$
$\beta_{KNN-var(5)} = -0.07$
observed
imputed
estimate CCA
estimate KNN−var(5)
Model residuals of hexadecanedioate
2
1
0
−1
−2
$n_{CCA} = 741$
$n_{KNN-var(5)} = 1273$
$n_{CCA} = 200$
$n_{KNN-var(5)} = 371$
$n_{CCA} = 15$
$n_{KNN-var(5)} = 28$
major homozygote
heterozygote
minor homozygote
Genotype of SNP rs6663731 (CYP4A11)

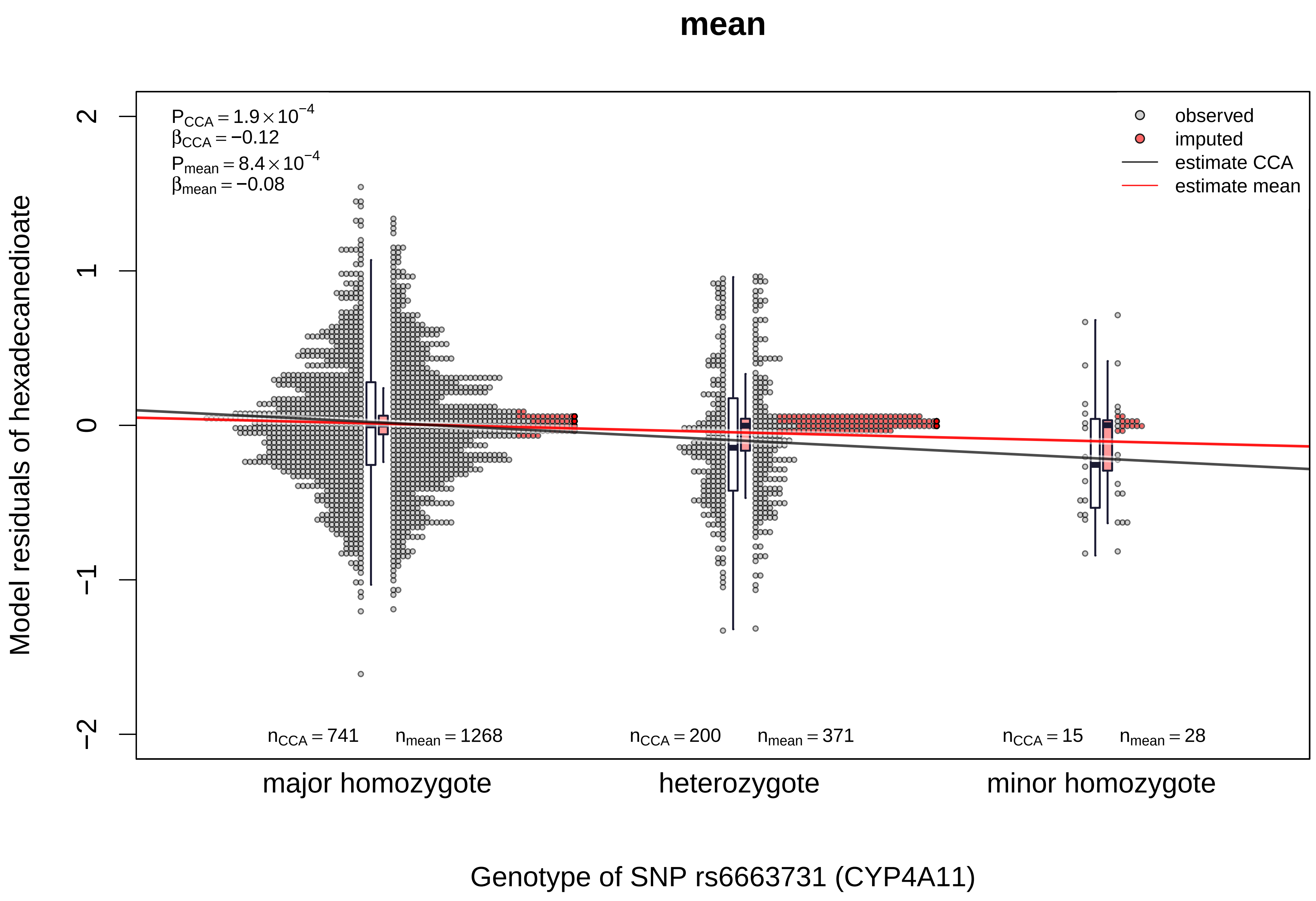
mean
$P_{CCA} = 1.9 \times 10^{-4}$
$\beta_{CCA} = -0.12$
$P_{mean} = 8.4 \times 10^{-4}$
$\beta_{mean} = -0.08$
observed
imputed
estimate CCA
estimate mean
Model residuals of hexadecanedioate
2
1
0
−1
−2
$n_{CCA} = 741$
$n_{mean} = 1268$
$n_{CCA} = 200$
$n_{mean} = 371$
$n_{CCA} = 15$
$n_{mean} = 28$
major homozygote
heterozygote
minor homozygote
Genotype of SNP rs6663731 (CYP4A11)

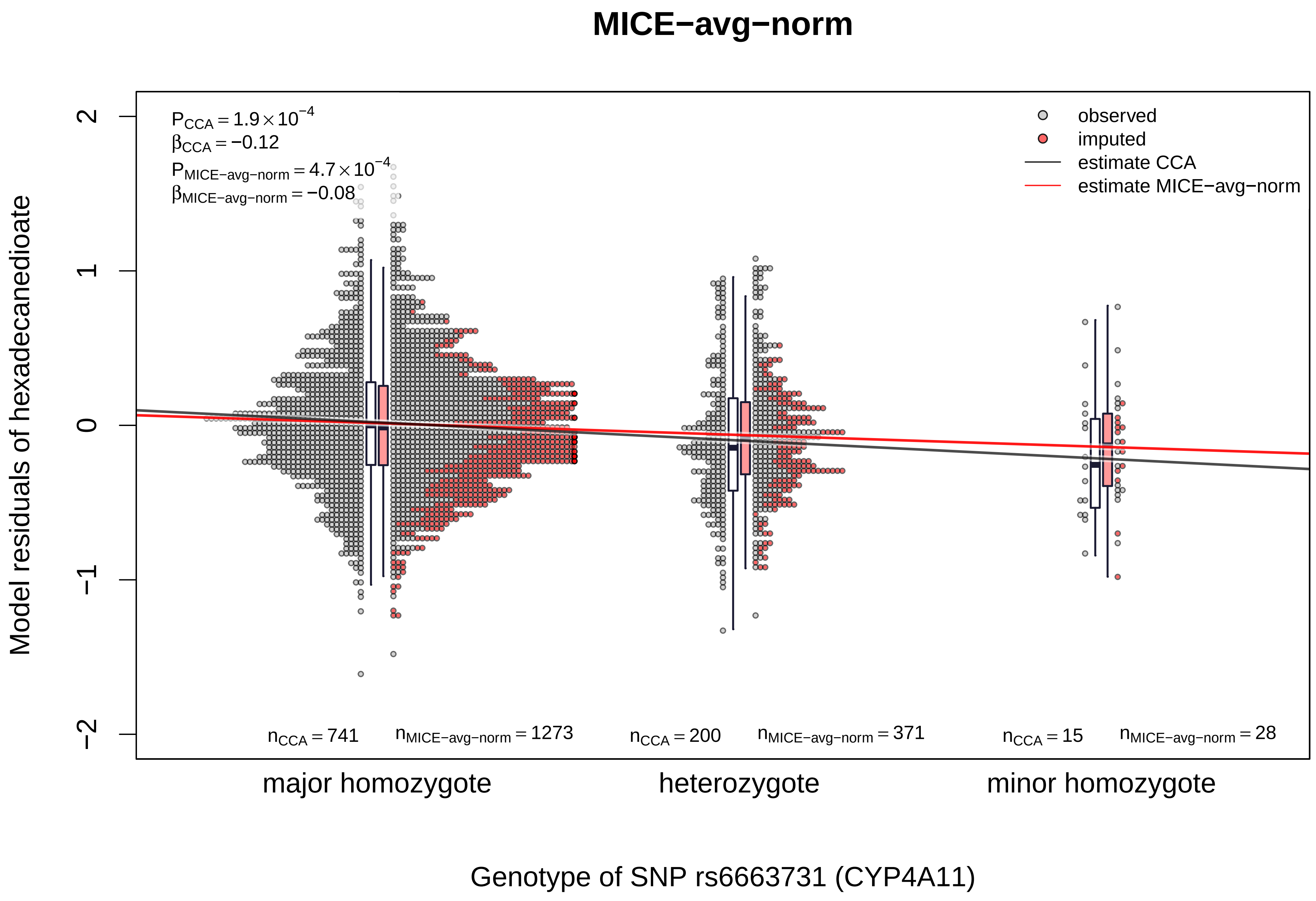
MICE−avg−norm
$P_{CCA} = 1.9 \times 10^{-4}$
$\beta_{CCA} = -0.12$
$P_{MICE-avg-norm} = 4.7 \times 10^{-4}$
$\beta_{MICE-avg-norm} = -0.08$
observed
imputed
estimate CCA
estimate MICE−avg−norm
Model residuals of hexadecanedioate
2
1
0
−1
−2
$n_{CCA} = 741$
$n_{MICE-avg-norm} = 1273$
$n_{CCA} = 200$
$n_{MICE-avg-norm} = 371$
$n_{CCA} = 15$
$n_{MICE-avg-norm} = 28$
major homozygote
heterozygote
minor homozygote
Genotype of SNP rs6663731 (CYP4A11)

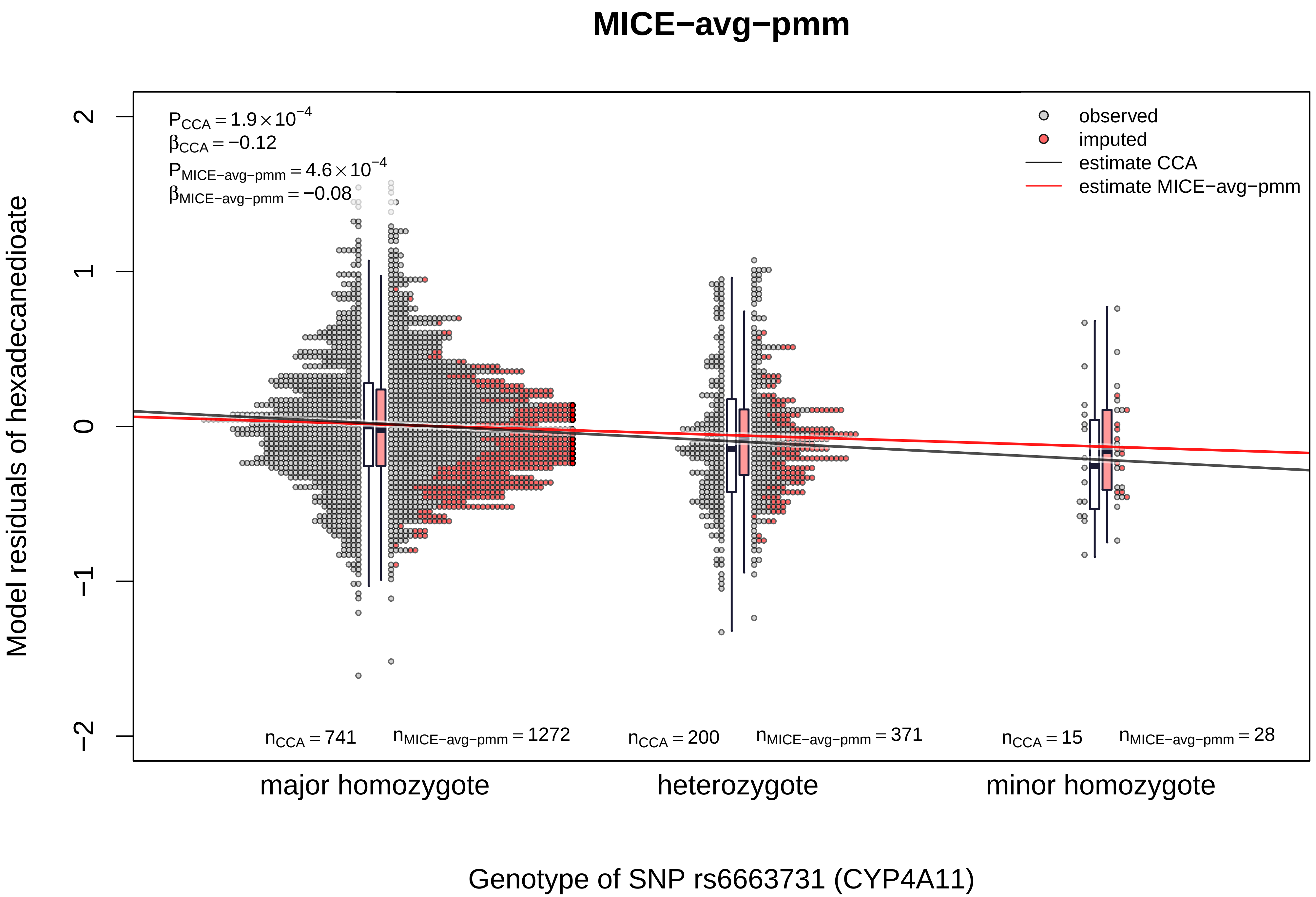

MICE−avg−pmm
$P_{CCA} = 1.9 \times 10^{-4}$
$\beta_{CCA} = -0.12$
$P_{MICE-avg-pmm} = 4.6 \times 10^{-4}$
$\beta_{MICE-avg-pmm} = -0.08$
observed
imputed
estimate CCA
estimate MICE−avg−pmm
Model residuals of hexadecanedioate
2
1
0
−1
−2
$n_{CCA} = 741$
$n_{MICE-avg-pmm} = 1272$
$n_{CCA} = 200$
$n_{MICE-avg-pmm} = 371$
$n_{CCA} = 15$
$n_{MICE-avg-pmm} = 28$
major homozygote
heterozygote
minor homozygote
Genotype of SNP rs6663731 (CYP4A11)

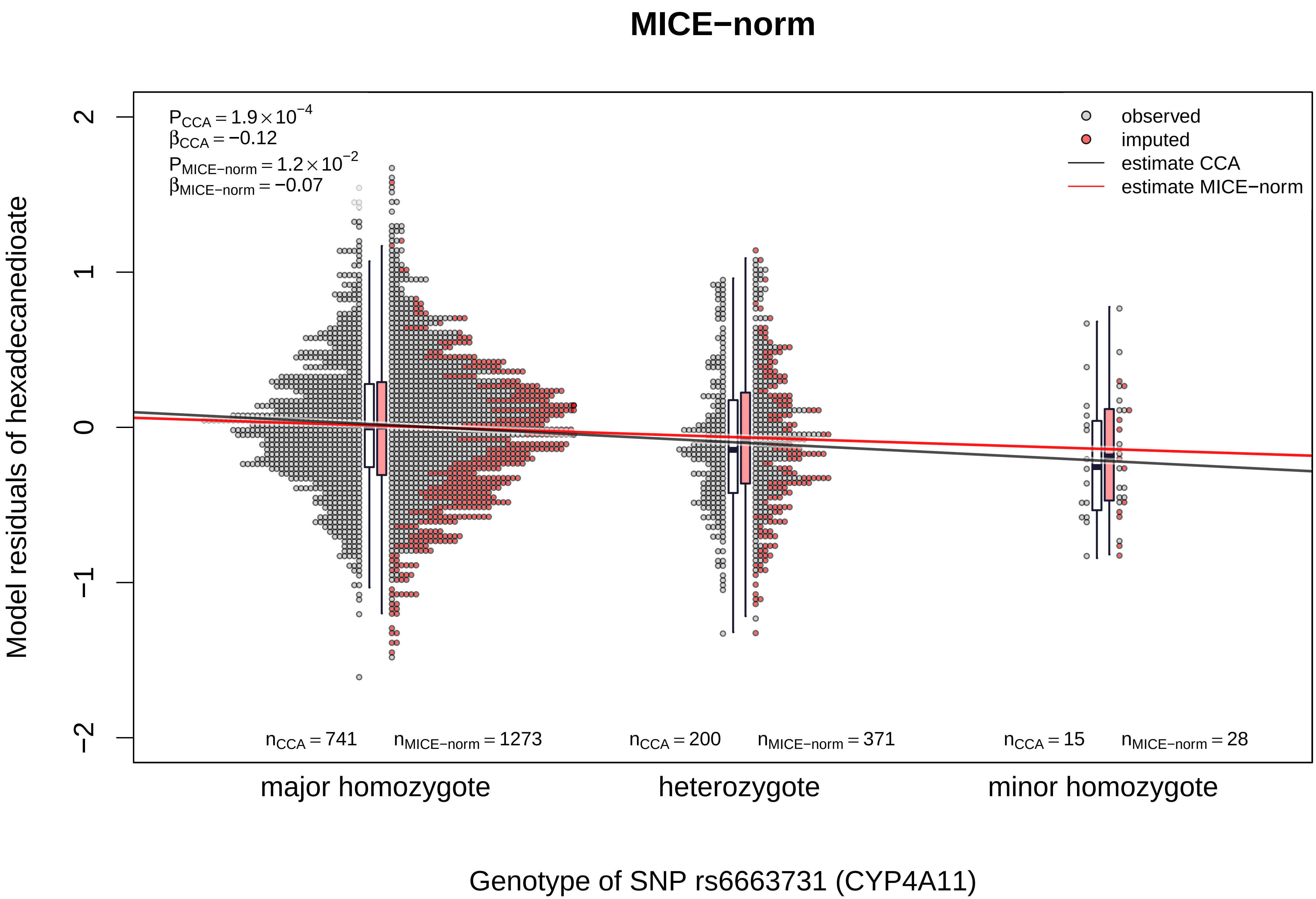
MICE−norm
$P_{CCA} = 1.9 \times 10^{-4}$
$\beta_{CCA} = -0.12$
$P_{MICE-norm} = 1.2 \times 10^{-2}$
$\beta_{MICE-norm} = -0.07$
observed
imputed
estimate CCA
estimate MICE−norm
Model residuals of hexadecanedioate
2
1
0
−1
−2
$n_{CCA} = 741$
$n_{MICE-norm} = 1273$
$n_{CCA} = 200$
$n_{MICE-norm} = 371$
$n_{CCA} = 15$
$n_{MICE-norm} = 28$
major homozygote
heterozygote
minor homozygote
Genotype of SNP rs6663731 (CYP4A11)

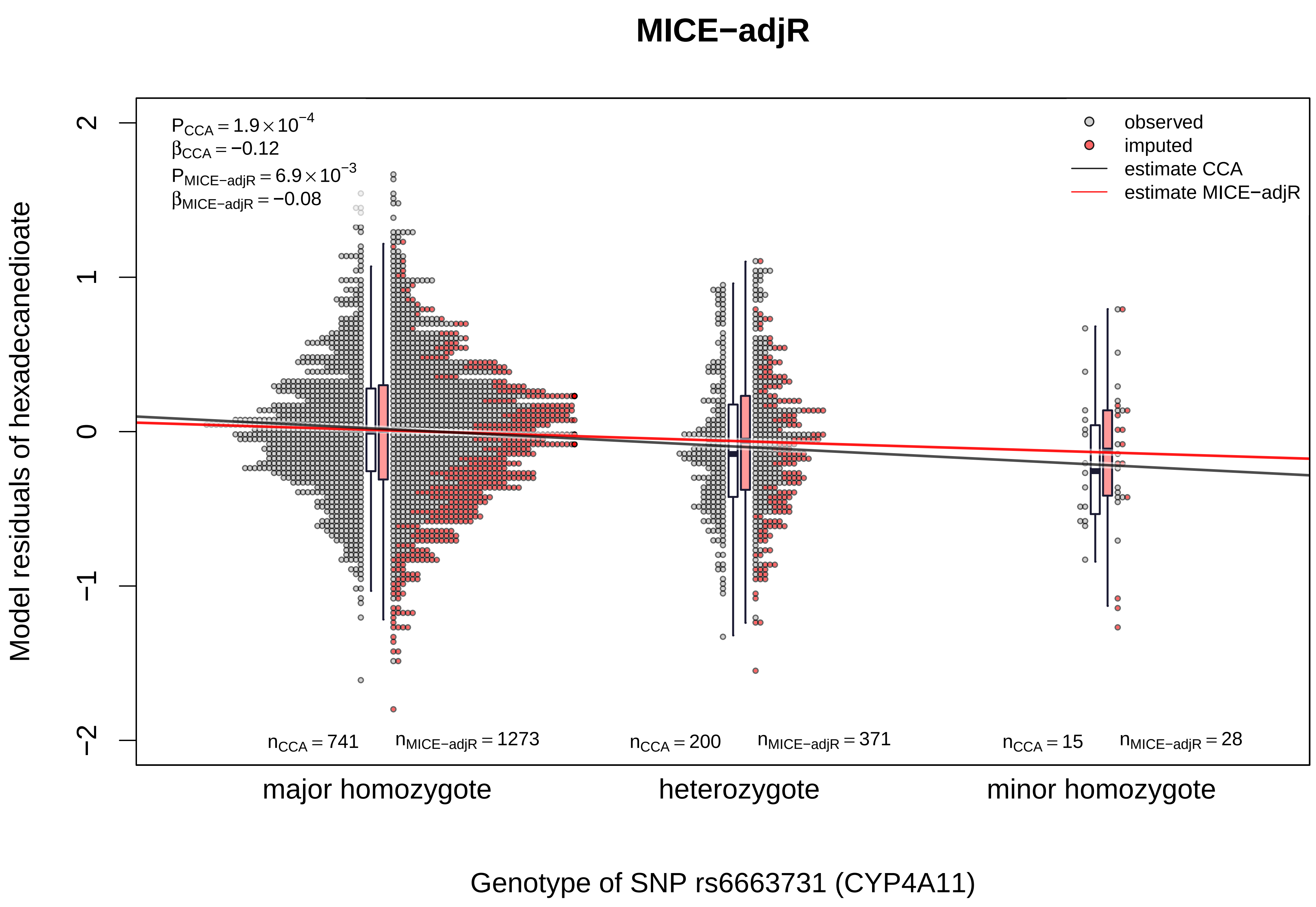
MICE−adjR
$P_{CCA} = 1.9 \times 10^{-4}$
$\beta_{CCA} = -0.12$
$P_{MICE-adjR} = 6.9 \times 10^{-3}$
$\beta_{MICE-adjR} = -0.08$
observed
imputed
estimate CCA
estimate MICE−adjR
Model residuals of hexadecanedioate
2
1
0
−1
−2
$n_{CCA} = 741$
$n_{MICE-adjR} = 1273$
$n_{CCA} = 200$
$n_{MICE-adjR} = 371$
$n_{CCA} = 15$
$n_{MICE-adjR} = 28$
major homozygote
heterozygote
minor homozygote
Genotype of SNP rs6663731 (CYP4A11)

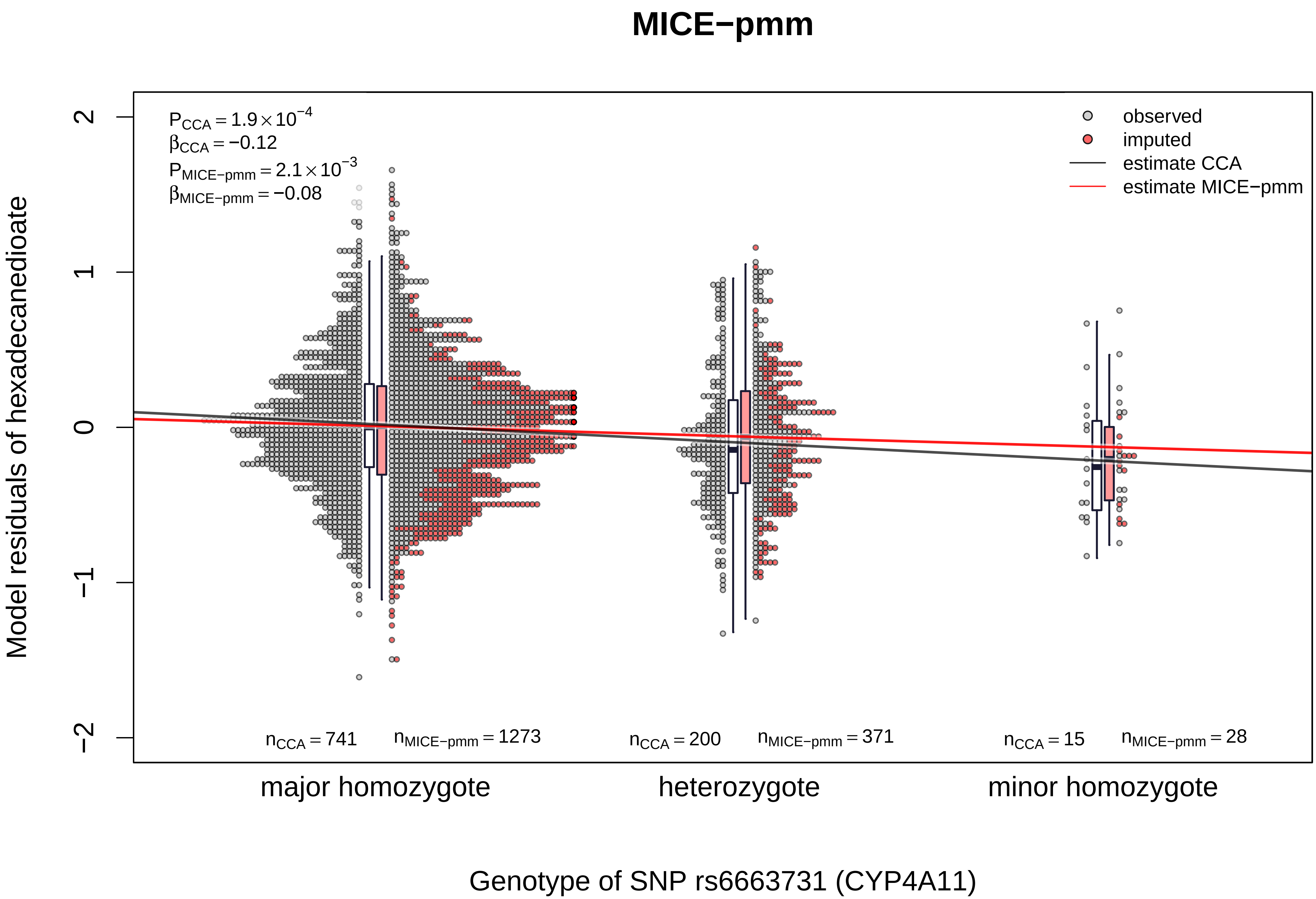

MICE−pmm
$P_{CCA} = 1.9 \times 10^{-4}$
$\beta_{CCA} = -0.12$
$P_{MICE-pmm} = 2.1 \times 10^{-3}$
$\beta_{MICE-pmm} = -0.08$
observed
imputed
estimate CCA
estimate MICE−pmm
$n_{CCA} = 741$
$n_{MICE-pmm} = 1273$
$n_{CCA} = 200$
$n_{MICE-pmm} = 371$
$n_{CCA} = 15$
$n_{MICE-pmm} = 28$
2
1
0
−1
−2
Model residuals of hexadecanedioate
major homozygote
heterozygote
minor homozygote
Genotype of SNP rs6663731 (CYP4A11)

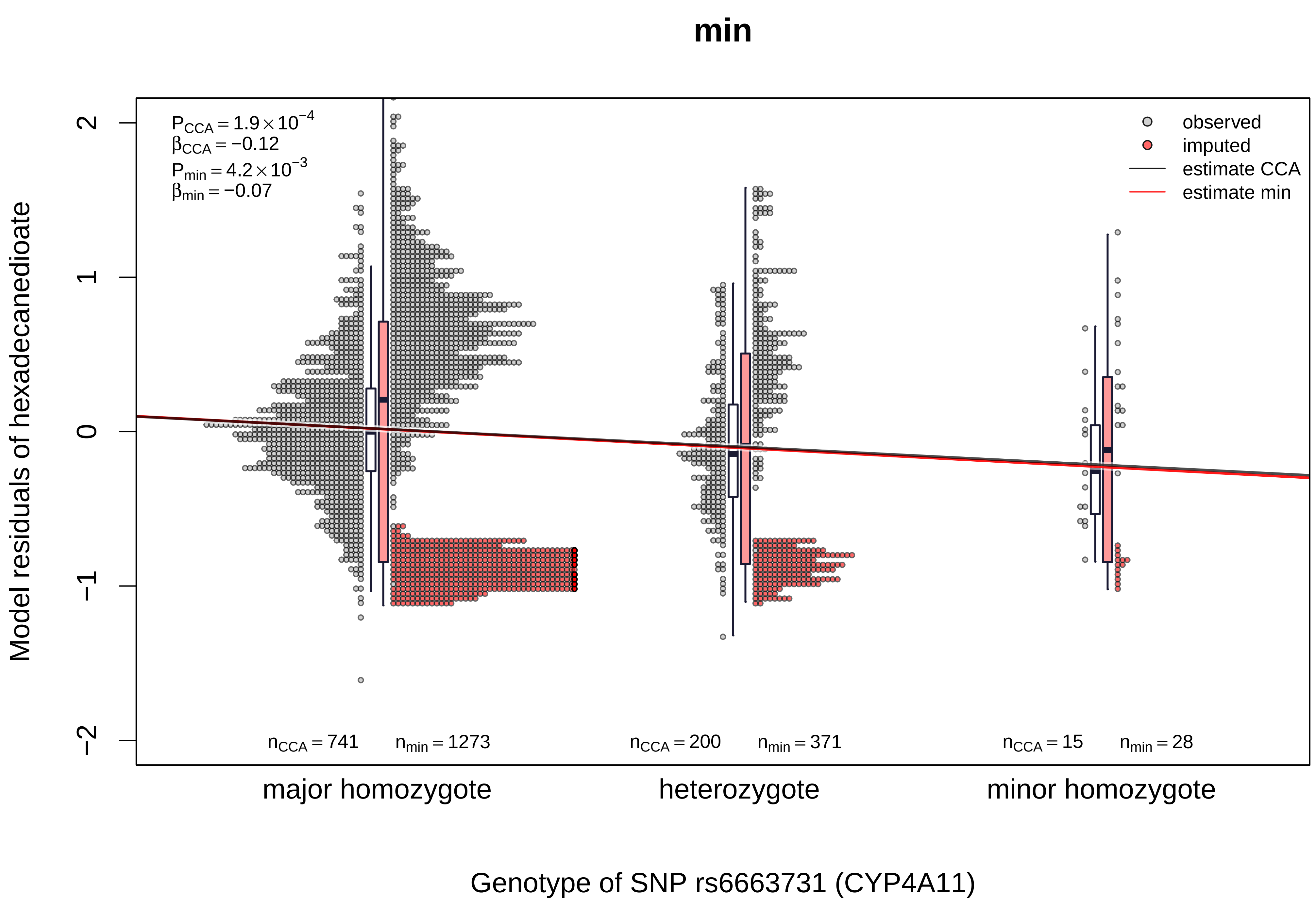

min
$P_{CCA} = 1.9 \times 10^{-4}$
$\beta_{CCA} = -0.12$
$P_{min} = 4.2 \times 10^{-3}$
$\beta_{min} = -0.07$
observed
imputed
estimate CCA
estimate min
2
1
0
−1
−2
Model residuals of hexadecanedioate
$n_{CCA} = 741$
$n_{min} = 1273$
$n_{CCA} = 200$
$n_{min} = 371$
$n_{CCA} = 15$
$n_{min} = 28$
major homozygote
heterozygote
minor homozygote
Genotype of SNP rs6663731 (CYP4A11)

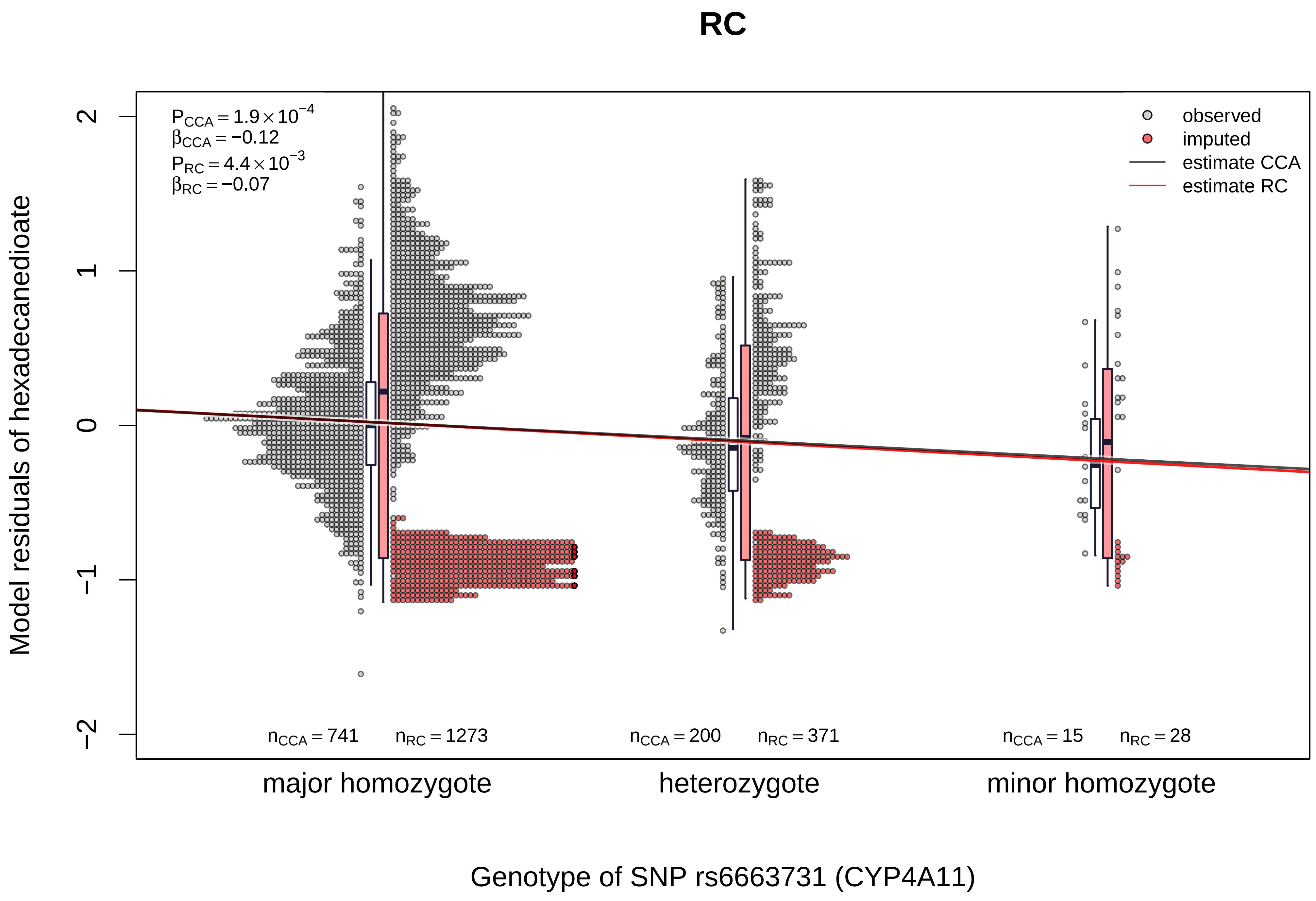

RC
$P_{CCA} = 1.9 \times 10^{-4}$
$\beta_{CCA} = -0.12$
$P_{RC} = 4.4 \times 10^{-3}$
$\beta_{RC} = -0.07$
observed
imputed
estimate CCA
estimate RC
2
1
0
−1
−2
Model residuals of hexadecanedioate
$n_{CCA} = 741$
$n_{RC} = 1273$
$n_{CCA} = 200$
$n_{RC} = 371$
$n_{CCA} = 15$
$n_{RC} = 28$
major homozygote
heterozygote
minor homozygote
Genotype of SNP rs6663731 (CYP4A11)

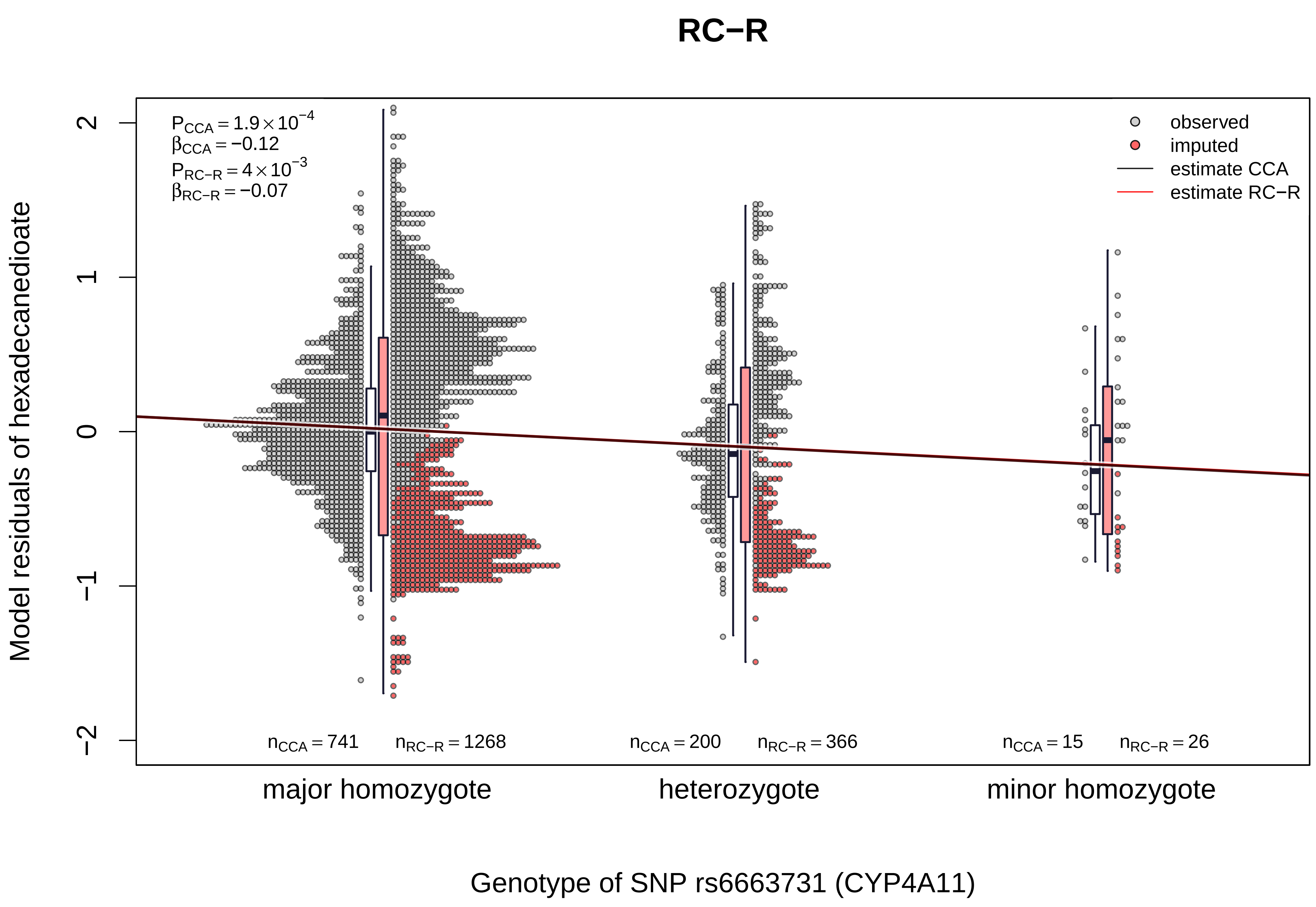

RC−R
$P_{CCA} = 1.9 \times 10^{-4}$
$\beta_{CCA} = -0.12$
$P_{RC-R} = 4 \times 10^{-3}$
$\beta_{RC-R} = -0.07$
observed
imputed
estimate CCA
estimate RC−R
Model residuals of hexadecanedioate
2
1
0
−1
−2
$n_{CCA} = 741$
$n_{RC-R} = 1268$
$n_{CCA} = 200$
$n_{RC-R} = 366$
$n_{CCA} = 15$
$n_{RC-R} = 26$
major homozygote
heterozygote
minor homozygote
Genotype of SNP rs6663731 (CYP4A11)

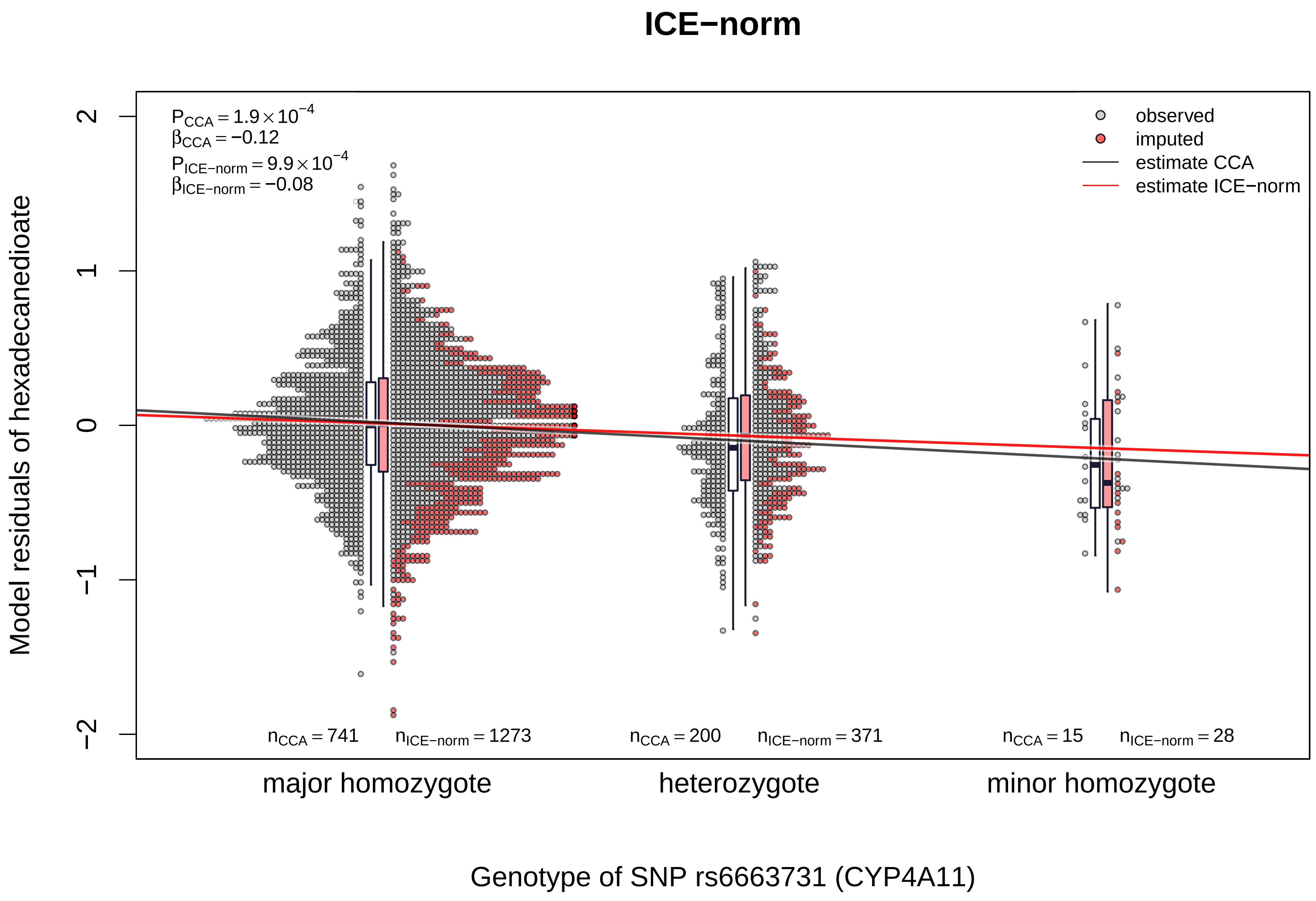

ICE−norm
$P_{CCA} = 1.9 \times 10^{-4}$
$\beta_{CCA} = -0.12$
$P_{ICE-norm} = 9.9 \times 10^{-4}$
$\beta_{ICE-norm} = -0.08$
observed
imputed
estimate CCA
estimate ICE−norm
Model residuals of hexadecanedioate
2
1
0
−1
−2
$n_{CCA} = 741$
$n_{ICE-norm} = 1273$
$n_{CCA} = 200$
$n_{ICE-norm} = 371$
$n_{CCA} = 15$
$n_{ICE-norm} = 28$
major homozygote
heterozygote
minor homozygote
Genotype of SNP rs6663731 (CYP4A11)

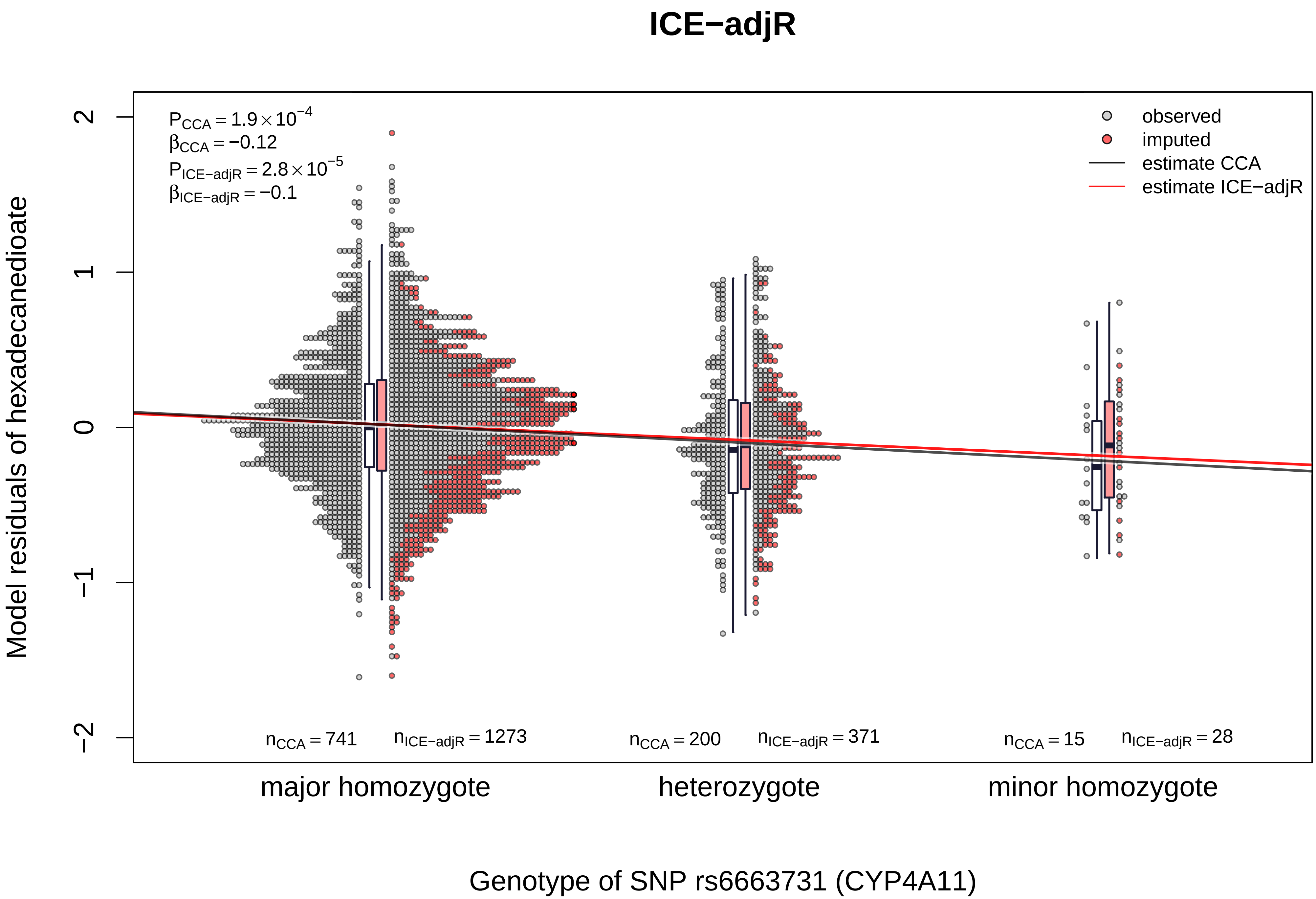

ICE−adjR
$P_{CCA} = 1.9 \times 10^{-4}$
$\beta_{CCA} = -0.12$
$P_{ICE-adjR} = 2.8 \times 10^{-5}$
$\beta_{ICE-adjR} = -0.1$
observed
imputed
estimate CCA
estimate ICE−adjR
Model residuals of hexadecanedioate
2
1
0
−1
−2
$n_{CCA} = 741$
$n_{ICE-adjR} = 1273$
$n_{CCA} = 200$
$n_{ICE-adjR} = 371$
$n_{CCA} = 15$
$n_{ICE-adjR} = 28$
major homozygote
heterozygote
minor homozygote
Genotype of SNP rs6663731 (CYP4A11)

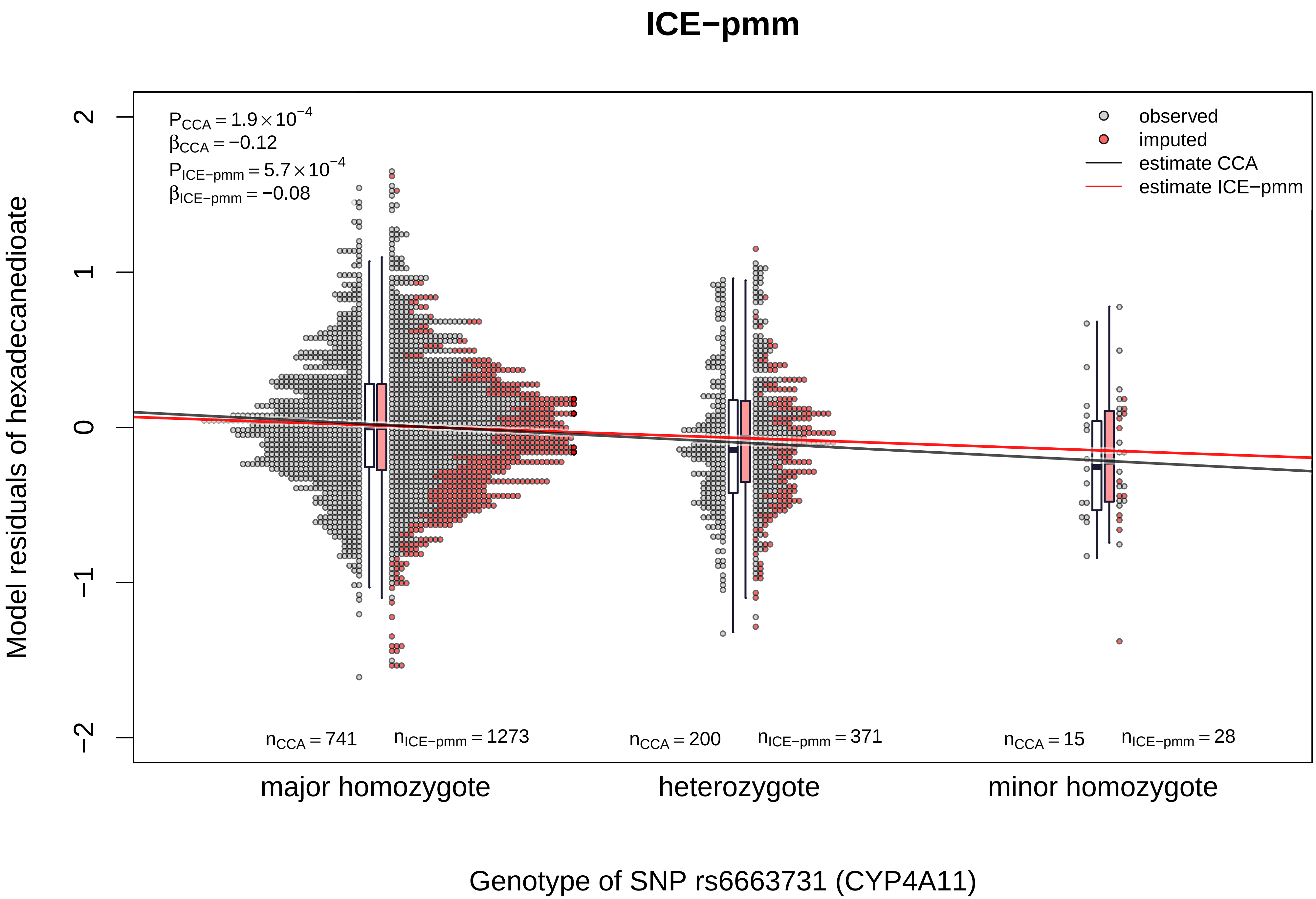

ICE−pmm
$P_{CCA} = 1.9 \times 10^{-4}$
$\beta_{CCA} = -0.12$
$P_{ICE-pmm} = 5.7 \times 10^{-4}$
$\beta_{ICE-pmm} = -0.08$
observed
imputed
estimate CCA
estimate ICE−pmm
Model residuals of hexadecanedioate
2
1
0
−1
−2
$n_{CCA} = 741$
$n_{ICE-pmm} = 1273$
$n_{CCA} = 200$
$n_{ICE-pmm} = 371$
$n_{CCA} = 15$
$n_{ICE-pmm} = 28$
major homozygote
heterozygote
minor homozygote
Genotype of SNP rs6663731 (CYP4A11)

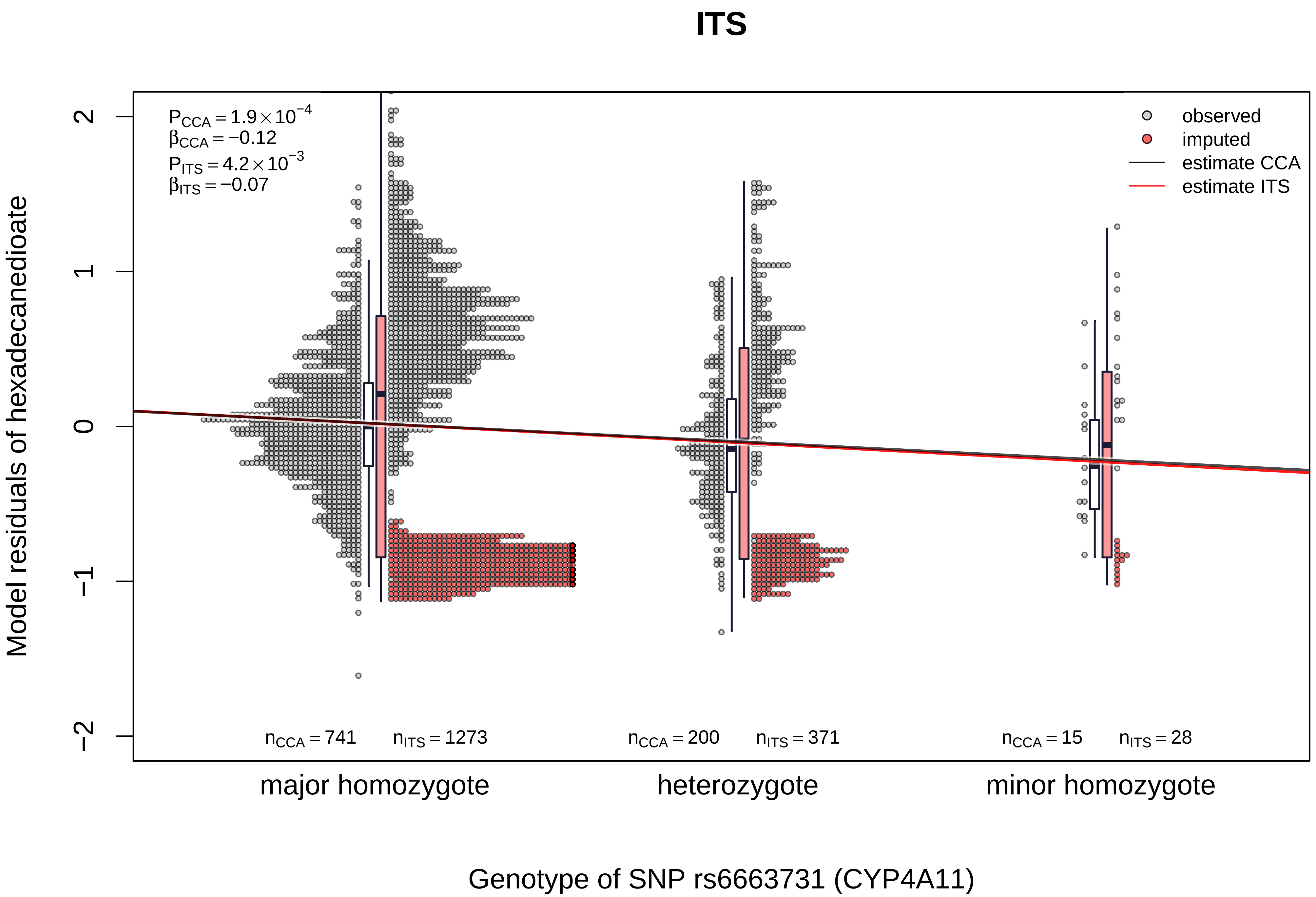

ITS
$P_{CCA} = 1.9 \times 10^{-4}$
$\beta_{CCA} = -0.12$
$P_{ITS} = 4.2 \times 10^{-3}$
$\beta_{ITS} = -0.07$
observed
imputed
estimate CCA
estimate ITS
Model residuals of hexadecanedioate
2
1
0
−1
−2
$n_{CCA} = 741$
$n_{ITS} = 1273$
$n_{CCA} = 200$
$n_{ITS} = 371$
$n_{CCA} = 15$
$n_{ITS} = 28$
major homozygote
heterozygote
minor homozygote
Genotype of SNP rs6663731 (CYP4A11)

ITS−R

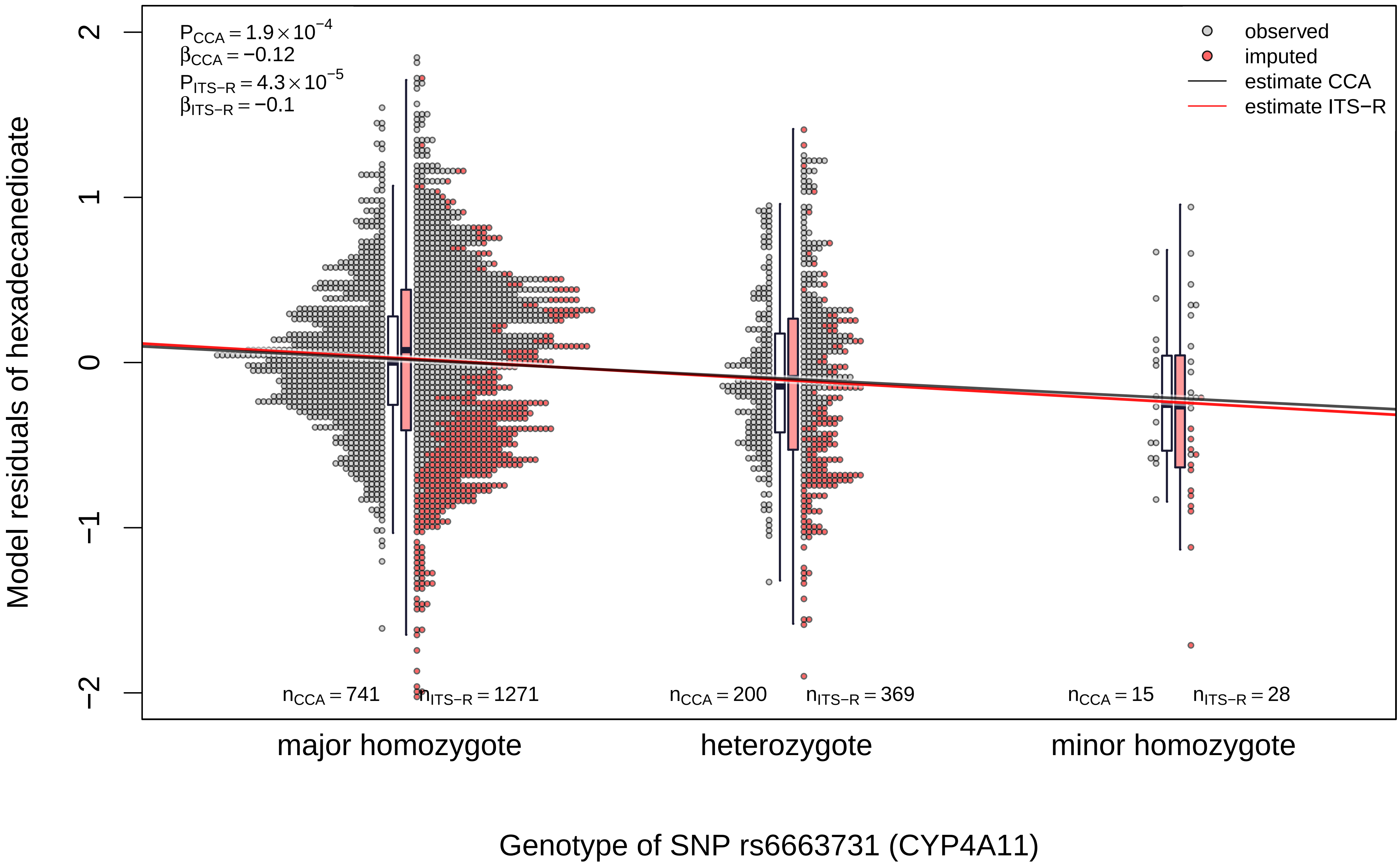

$P_{CCA} = 1.9 \times 10^{-4}$
$\beta_{CCA} = -0.12$
$P_{ITS-R} = 4.3 \times 10^{-5}$
$\beta_{ITS-R} = -0.1$
observed
imputed
estimate CCA
estimate ITS−R
2
1
0
−1
−2
Model residuals of hexadecanedioate
$n_{CCA} = 741$
$n_{ITS-R} = 1271$
$n_{CCA} = 200$
$n_{ITS-R} = 369$
$n_{CCA} = 15$
$n_{ITS-R} = 28$
major homozygote
heterozygote
minor homozygote
Genotype of SNP rs6663731 (CYP4A11)

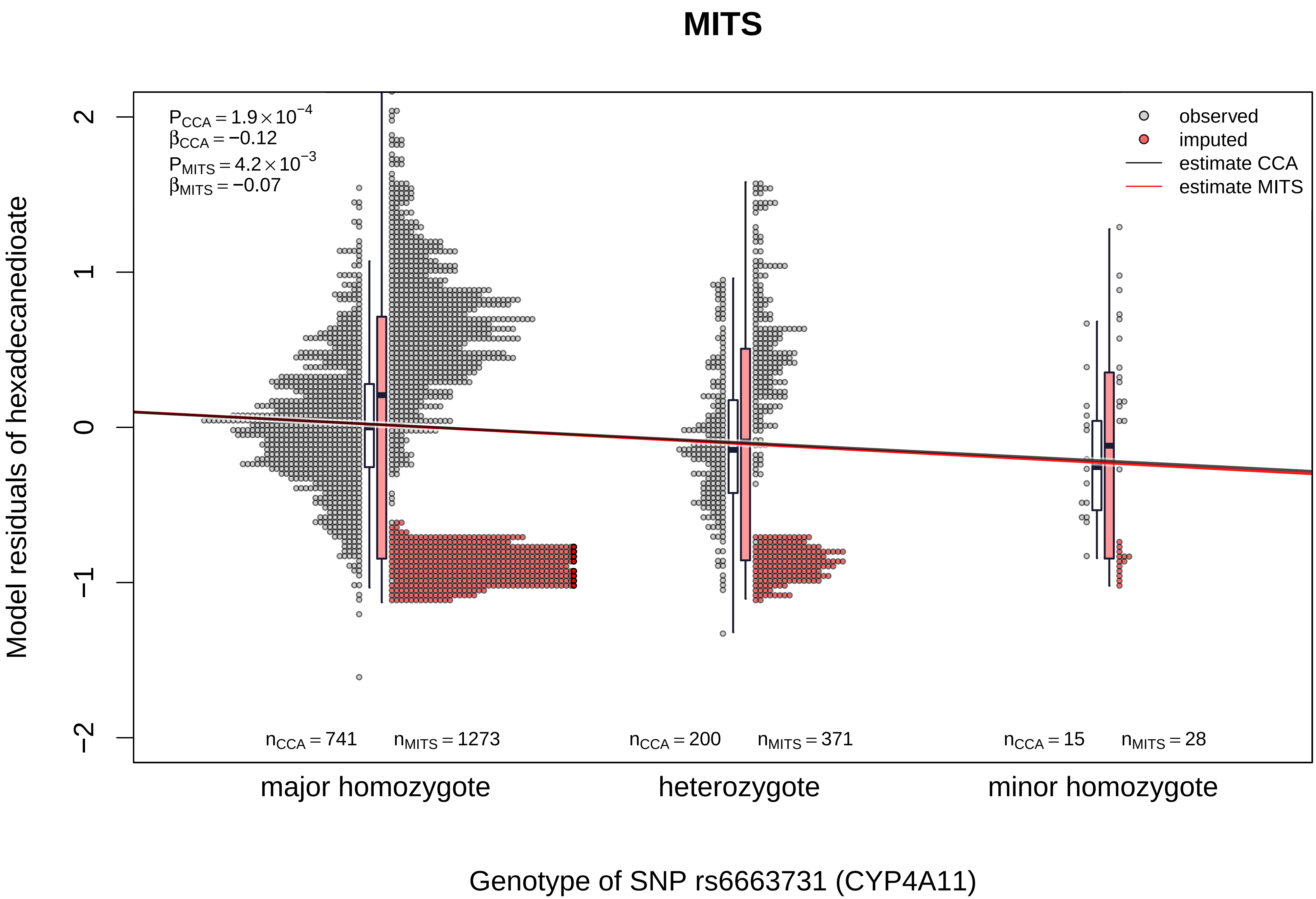
MITS
$P_{CCA} = 1.9 \times 10^{-4}$
$\beta_{CCA} = -0.12$
$P_{MITS} = 4.2 \times 10^{-3}$
$\beta_{MITS} = -0.07$
observed
imputed
estimate CCA
estimate MITS
Model residuals of hexadecanedioate
2
1
0
−1
−2
$n_{CCA} = 741$
$n_{MITS} = 1273$
$n_{CCA} = 200$
$n_{MITS} = 371$
$n_{CCA} = 15$
$n_{MITS} = 28$
major homozygote
heterozygote
minor homozygote
Genotype of SNP rs6663731 (CYP4A11)

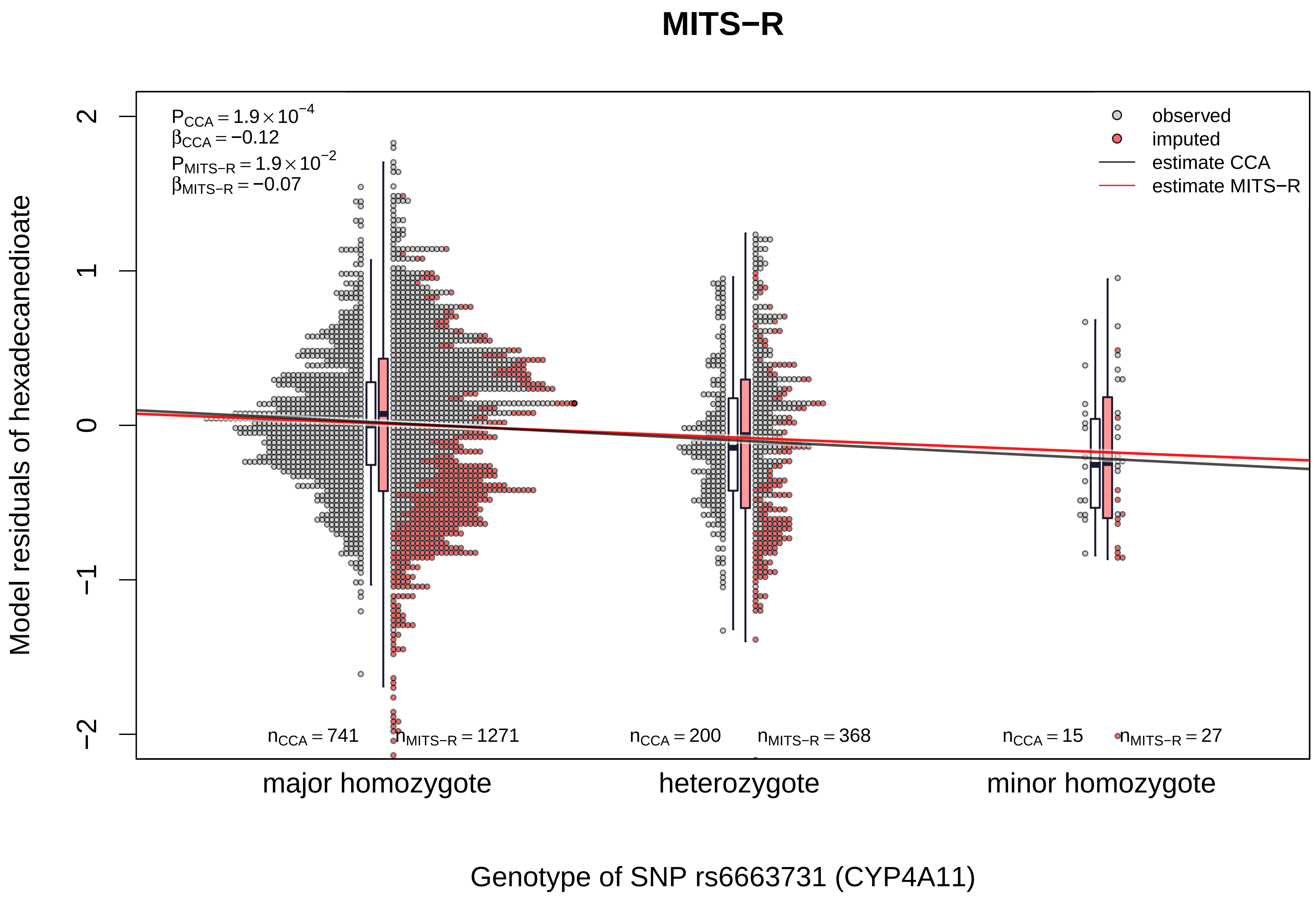

MITS−R
$P_{CCA} = 1.9 \times 10^{-4}$
$\beta_{CCA} = -0.12$
$P_{MITS-R} = 1.9 \times 10^{-2}$
$\beta_{MITS-R} = -0.07$
observed
imputed
estimate CCA
estimate MITS−R
Model residuals of hexadecanedioate
2
1
0
−1
−2
$n_{CCA} = 741$
$n_{MITS-R} = 1271$
$n_{CCA} = 200$
$n_{MITS-R} = 368$
$n_{CCA} = 15$
$n_{MITS-R} = 27$
major homozygote
heterozygote
minor homozygote
Genotype of SNP rs6663731 (CYP4A11)

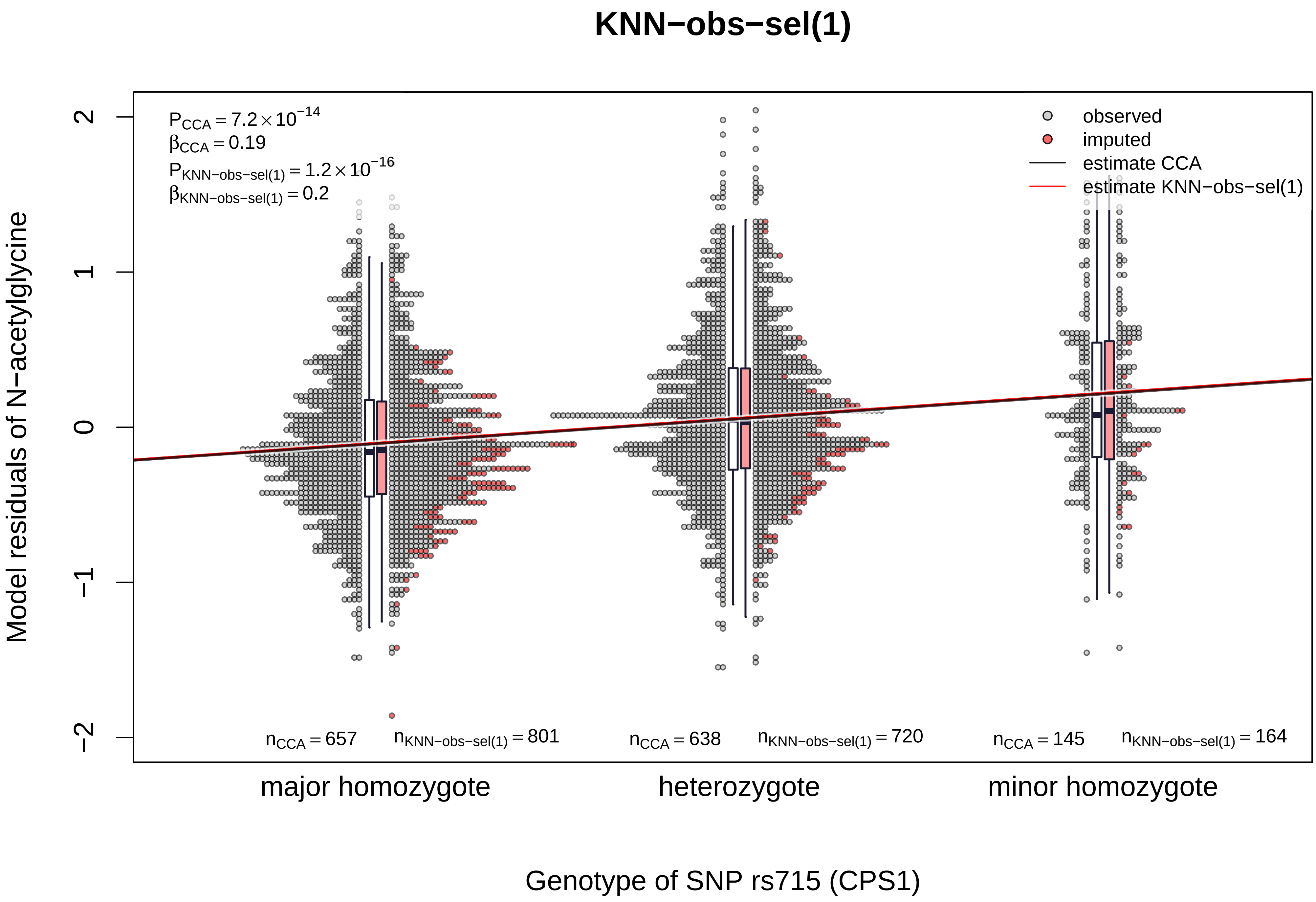

KNN−obs−sel(1)
$P_{CCA} = 7.2 \times 10^{-14}$
$\beta_{CCA} = 0.19$
$P_{KNN-obs-sel(1)} = 1.2 \times 10^{-16}$
$\beta_{KNN-obs-sel(1)} = 0.2$
observed
imputed
estimate CCA
estimate KNN−obs−sel(1)
Model residuals of N−acetylglycine
2
1
0
−1
−2
$n_{CCA} = 657$
$n_{KNN-obs-sel(1)} = 801$
$n_{CCA} = 638$
$n_{KNN-obs-sel(1)} = 720$
$n_{CCA} = 145$
$n_{KNN-obs-sel(1)} = 164$
major homozygote
heterozygote
minor homozygote
Genotype of SNP rs715 (CPS1)

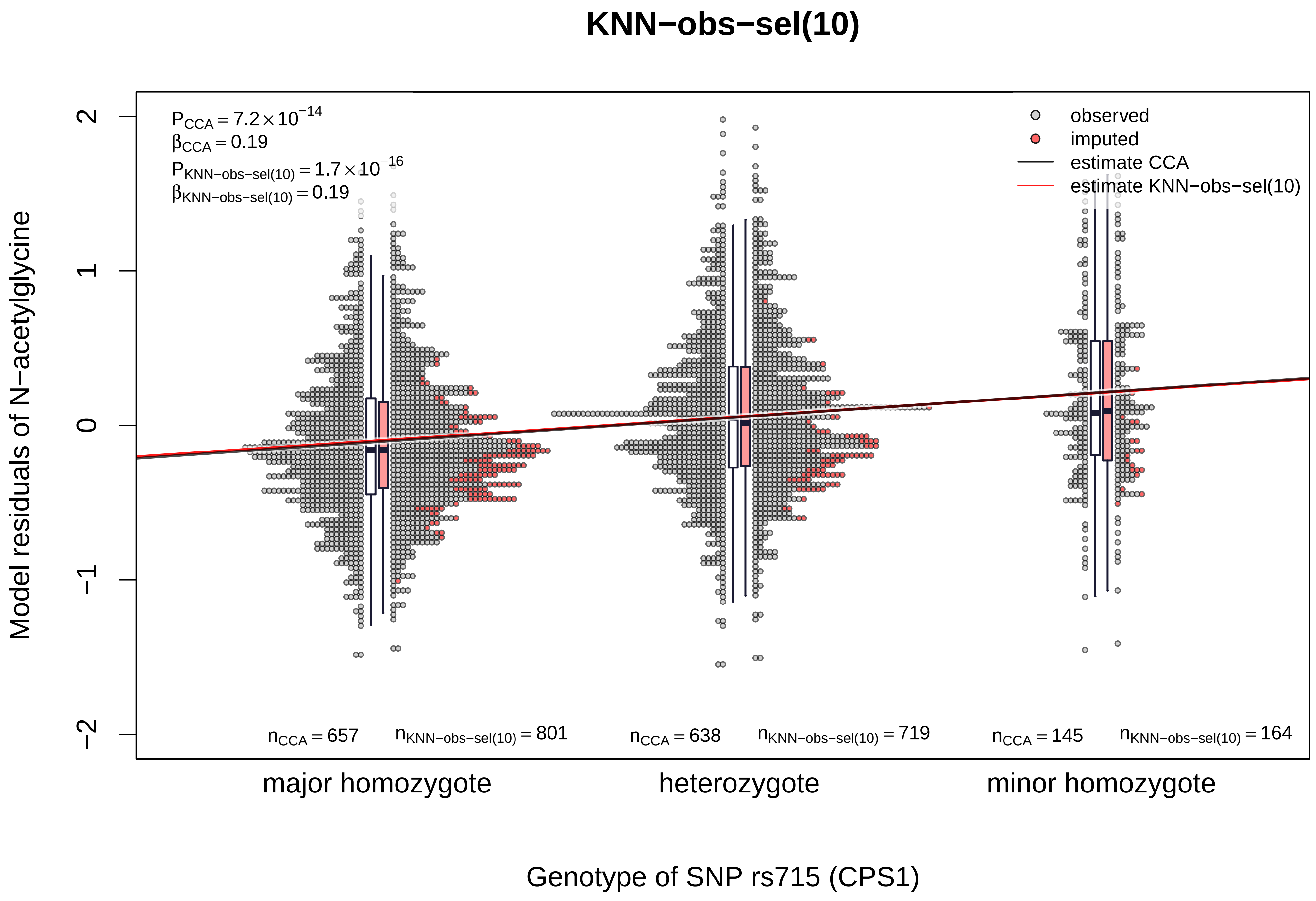

KNN−obs−sel(10)
$P_{CCA} = 7.2 \times 10^{-14}$
$\beta_{CCA} = 0.19$
$P_{KNN-obs-sel(10)} = 1.7 \times 10^{-16}$
$\beta_{KNN-obs-sel(10)} = 0.19$
observed
imputed
estimate CCA
estimate KNN−obs−sel(10)
Model residuals of N−acetylglycine
2
1
0
−1
−2
$n_{CCA} = 657$
$n_{KNN-obs-sel(10)} = 801$
$n_{CCA} = 638$
$n_{KNN-obs-sel(10)} = 719$
$n_{CCA} = 145$
$n_{KNN-obs-sel(10)} = 164$
major homozygote
heterozygote
minor homozygote
Genotype of SNP rs715 (CPS1)

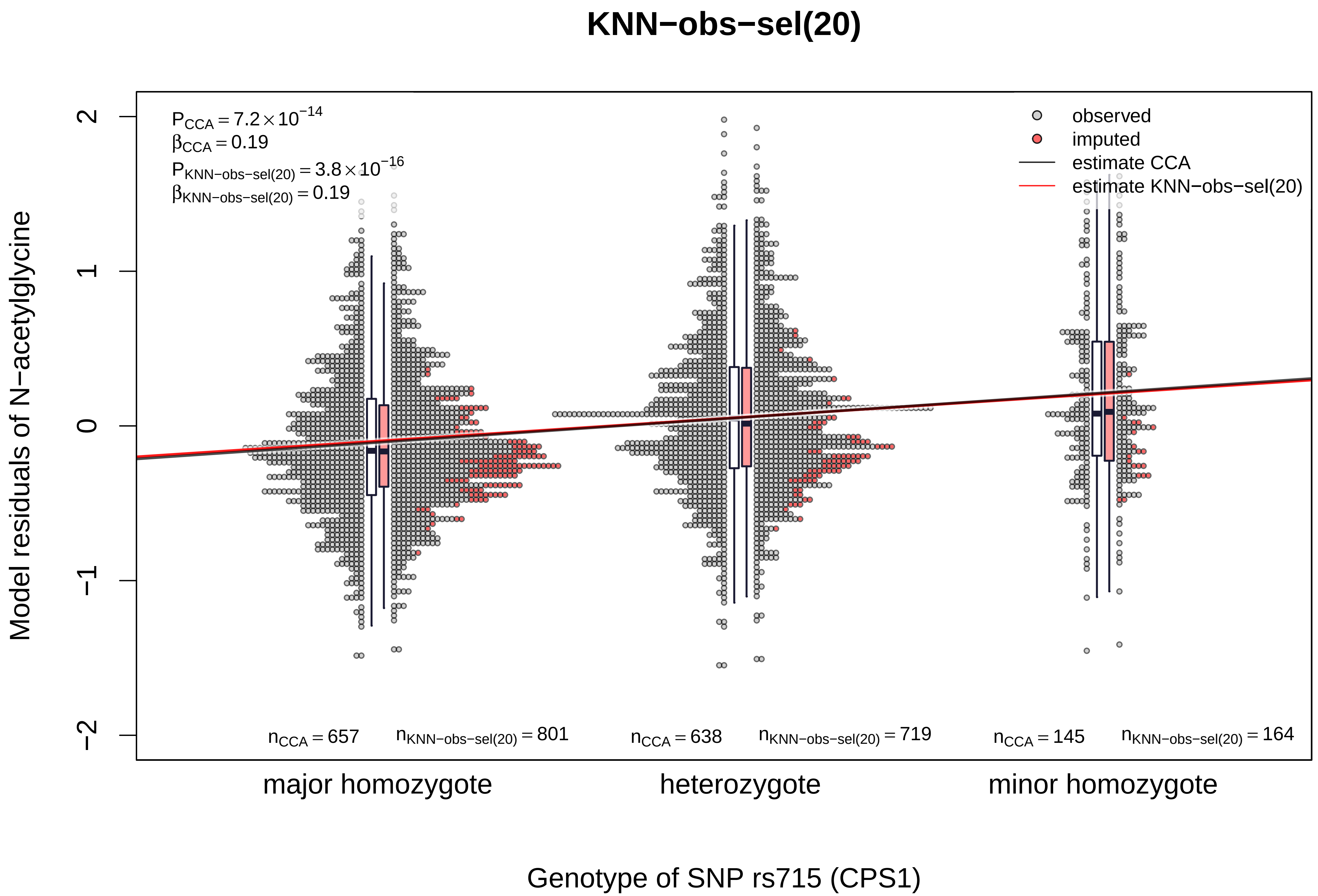

KNN−obs−sel(20)
$P_{CCA} = 7.2 \times 10^{-14}$
$\beta_{CCA} = 0.19$
$P_{KNN-obs-sel(20)} = 3.8 \times 10^{-16}$
$\beta_{KNN-obs-sel(20)} = 0.19$
observed
imputed
estimate CCA
estimate KNN−obs−sel(20)
Model residuals of N−acetylglycine
2
1
0
−1
−2
$n_{CCA} = 657$
$n_{KNN-obs-sel(20)} = 801$
$n_{CCA} = 638$
$n_{KNN-obs-sel(20)} = 719$
$n_{CCA} = 145$
$n_{KNN-obs-sel(20)} = 164$
major homozygote
heterozygote
minor homozygote
Genotype of SNP rs715 (CPS1)

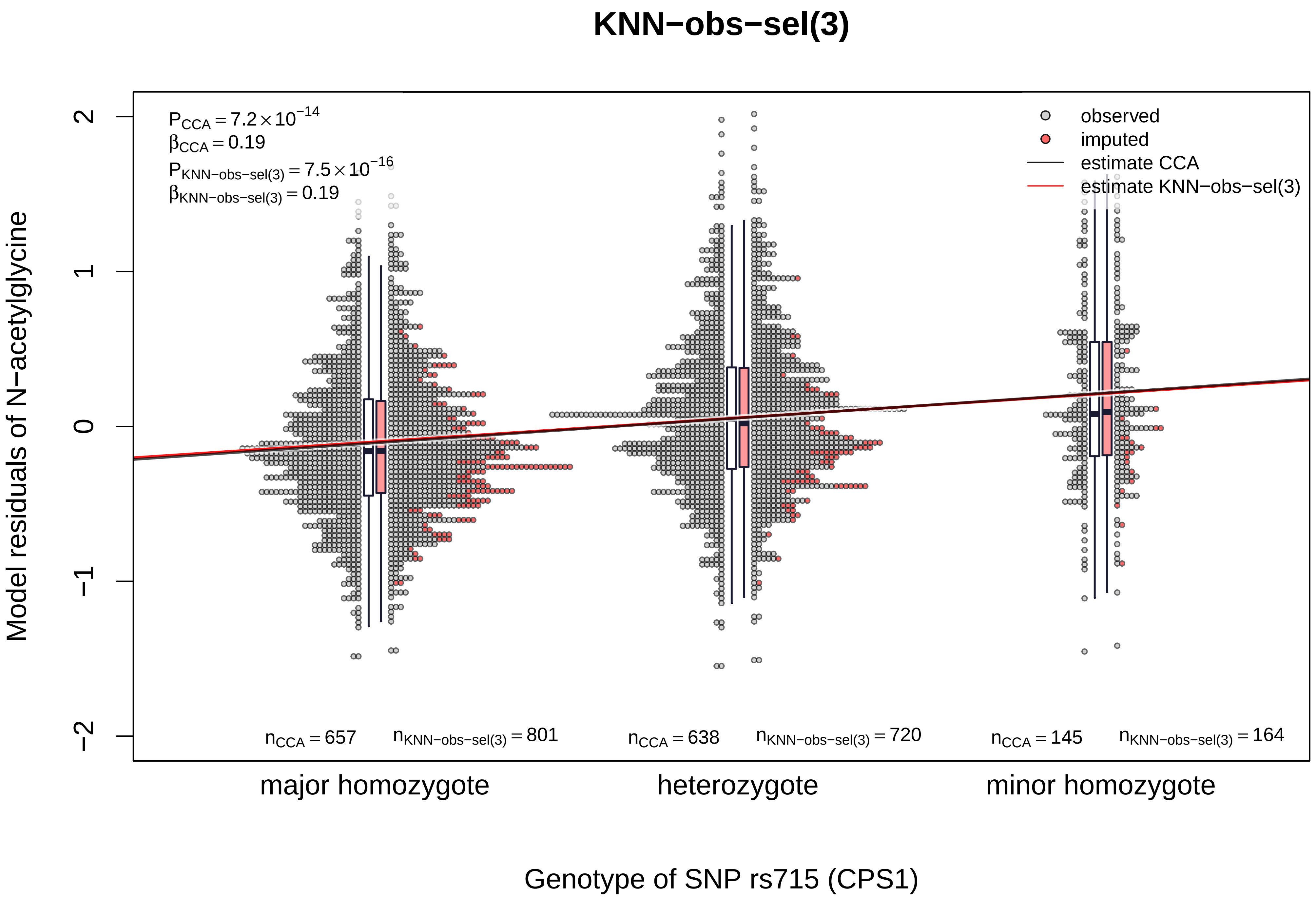
KNN−obs−sel(3)
$P_{CCA} = 7.2 \times 10^{-14}$
$\beta_{CCA} = 0.19$
$P_{KNN-obs-sel(3)} = 7.5 \times 10^{-16}$
$\beta_{KNN-obs-sel(3)} = 0.19$
observed
imputed
estimate CCA
estimate KNN−obs−sel(3)
Model residuals of N−acetylglycine
2
1
0
−1
−2
$n_{CCA} = 657$
$n_{KNN-obs-sel(3)} = 801$
$n_{CCA} = 638$
$n_{KNN-obs-sel(3)} = 720$
$n_{CCA} = 145$
$n_{KNN-obs-sel(3)} = 164$
major homozygote
heterozygote
minor homozygote
Genotype of SNP rs715 (CPS1)

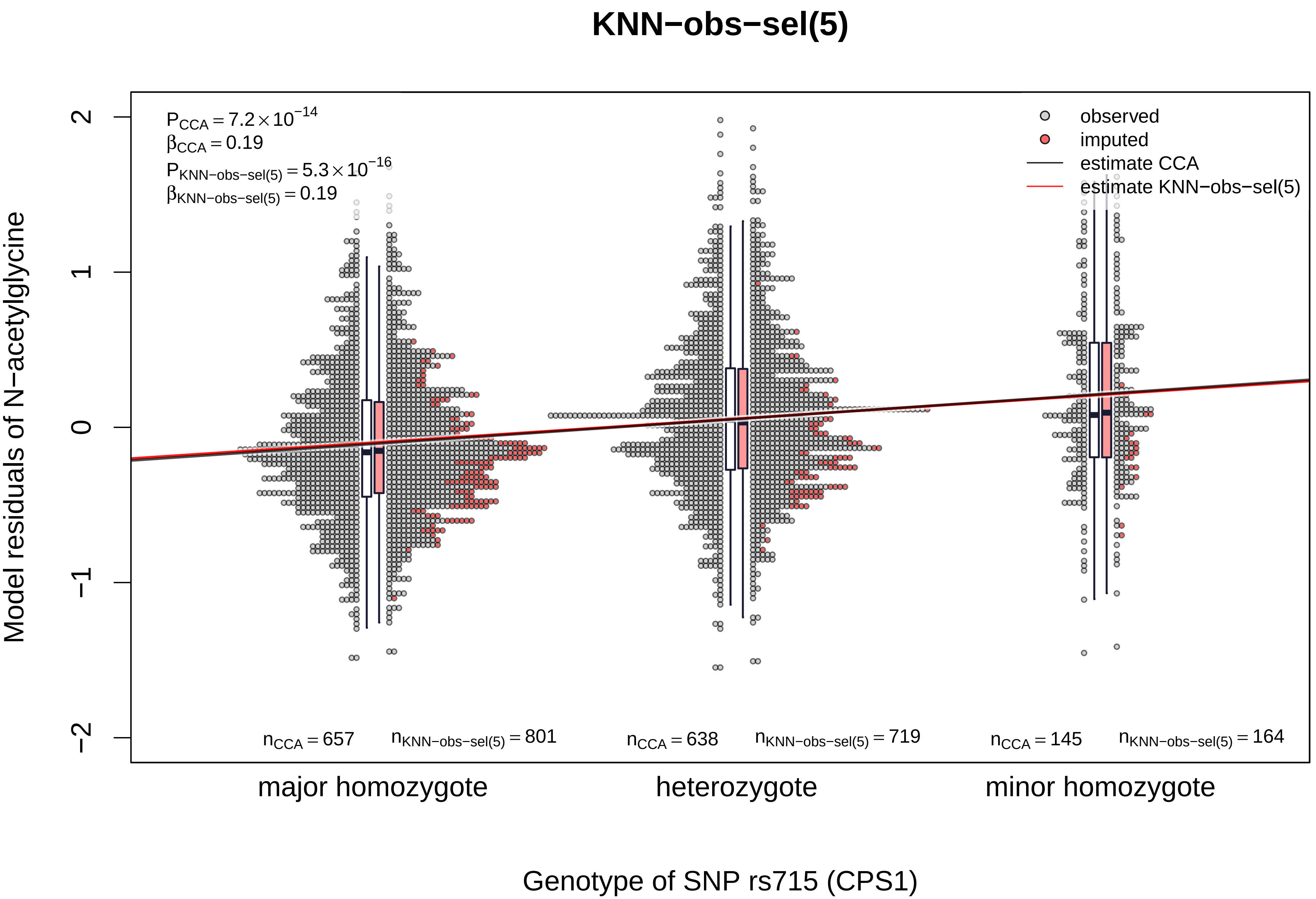

KNN−obs−sel(5)
$P_{CCA} = 7.2 \times 10^{-14}$
$\beta_{CCA} = 0.19$
$P_{KNN-obs-sel(5)} = 5.3 \times 10^{-16}$
$\beta_{KNN-obs-sel(5)} = 0.19$
observed
imputed
estimate CCA
estimate KNN−obs−sel(5)
Model residuals of N−acetylglycine
2
1
0
−1
−2
$n_{CCA} = 657$
$n_{KNN-obs-sel(5)} = 801$
$n_{CCA} = 638$
$n_{KNN-obs-sel(5)} = 719$
$n_{CCA} = 145$
$n_{KNN-obs-sel(5)} = 164$
major homozygote
heterozygote
minor homozygote
Genotype of SNP rs715 (CPS1)

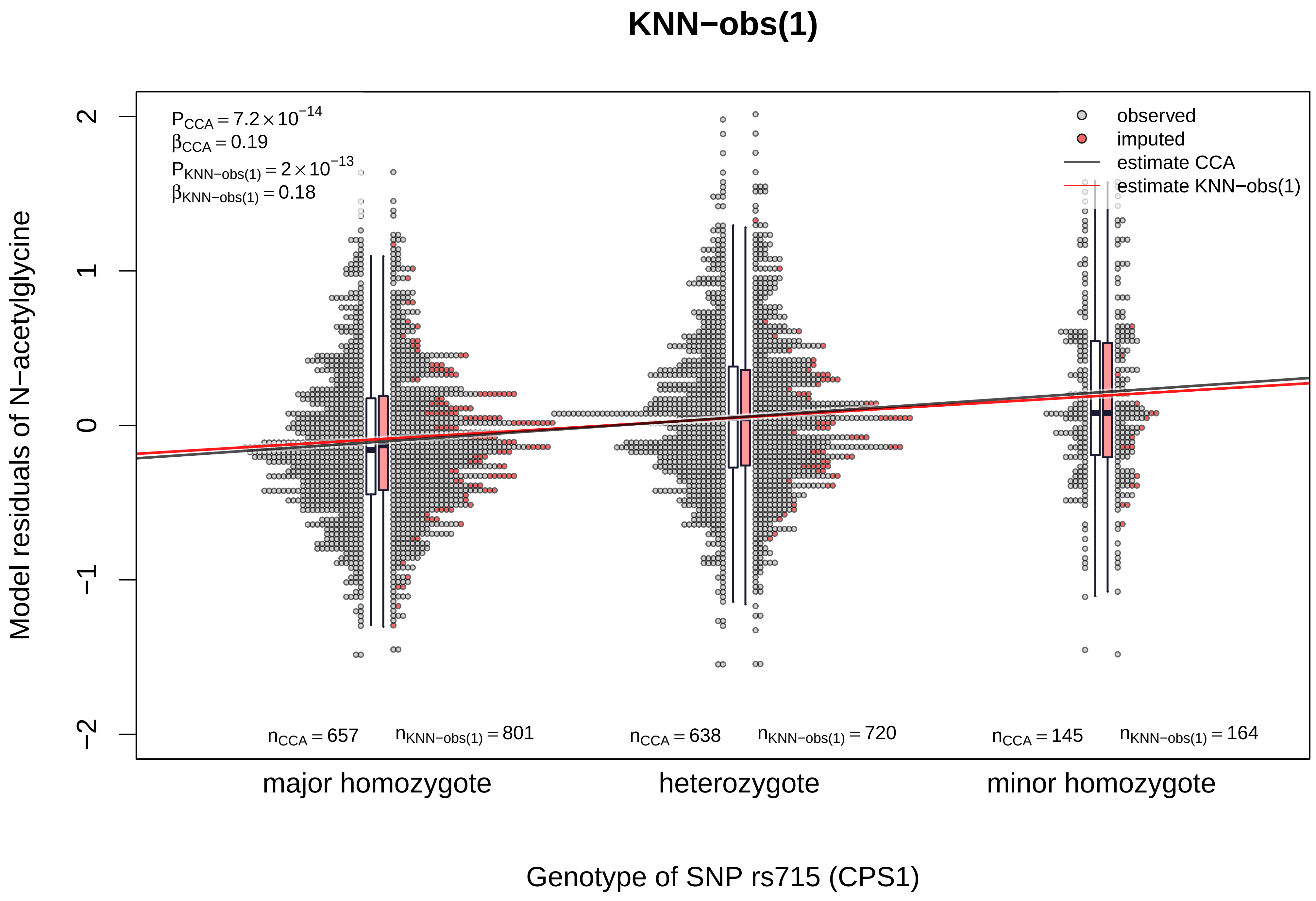
KNN−obs(1)
$P_{CCA} = 7.2 \times 10^{-14}$
$\beta_{CCA} = 0.19$
$P_{KNN-obs(1)} = 2 \times 10^{-13}$
$\beta_{KNN-obs(1)} = 0.18$
observed
imputed
estimate CCA
estimate KNN−obs(1)
2
1
0
−1
−2
Model residuals of N−acetylglycine
$n_{CCA} = 657$
$n_{KNN-obs(1)} = 801$
$n_{CCA} = 638$
$n_{KNN-obs(1)} = 720$
$n_{CCA} = 145$
$n_{KNN-obs(1)} = 164$
major homozygote
heterozygote
minor homozygote
Genotype of SNP rs715 (CPS1)

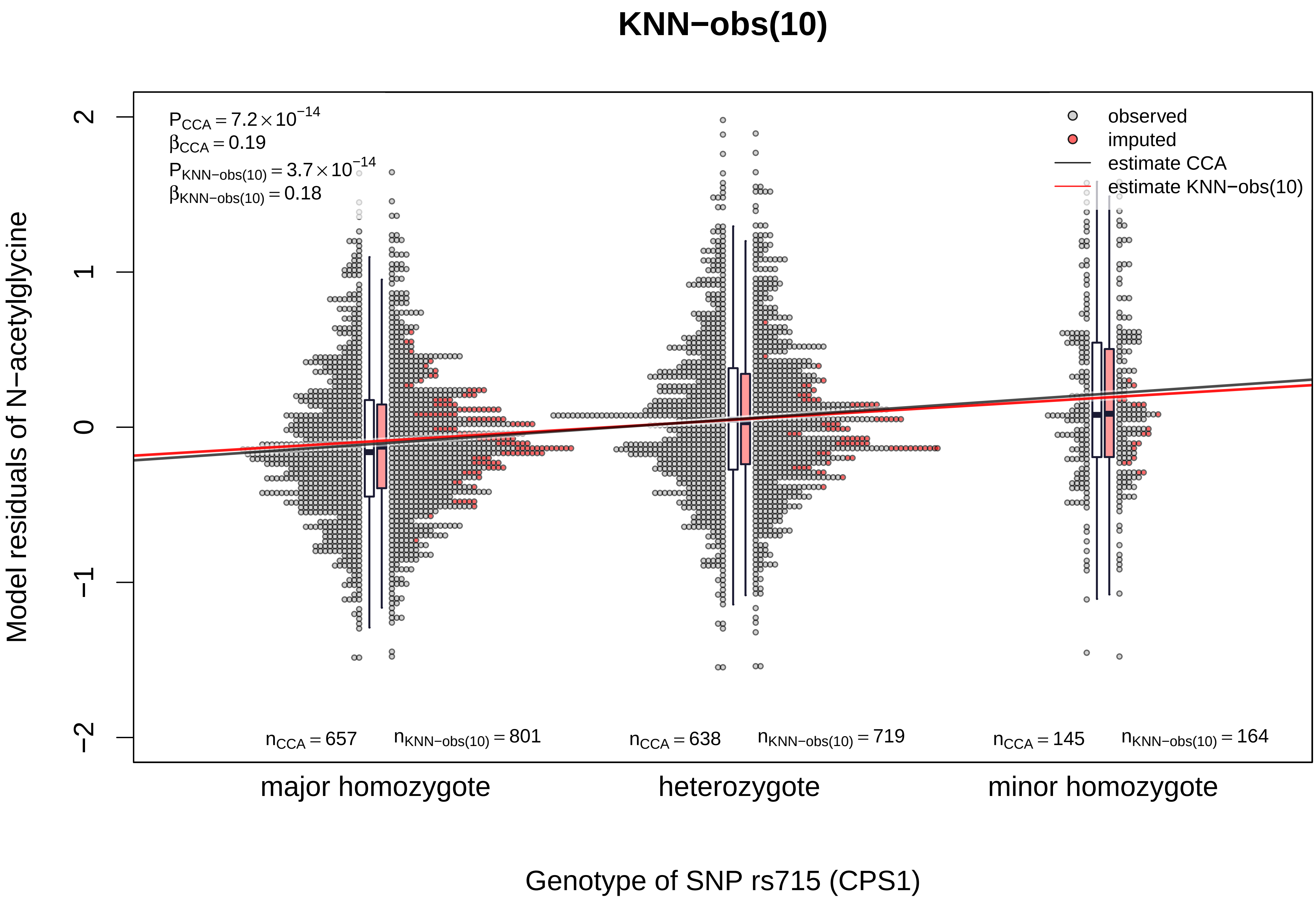
KNN−obs(10)
$P_{CCA} = 7.2 \times 10^{-14}$
$\beta_{CCA} = 0.19$
$P_{KNN-obs(10)} = 3.7 \times 10^{-14}$
$\beta_{KNN-obs(10)} = 0.18$
observed
imputed
estimate CCA
estimate KNN−obs(10)
Model residuals of N−acetylglycine
2
1
0
−1
−2
$n_{CCA} = 657$
$n_{KNN-obs(10)} = 801$
$n_{CCA} = 638$
$n_{KNN-obs(10)} = 719$
$n_{CCA} = 145$
$n_{KNN-obs(10)} = 164$
major homozygote
heterozygote
minor homozygote
Genotype of SNP rs715 (CPS1)

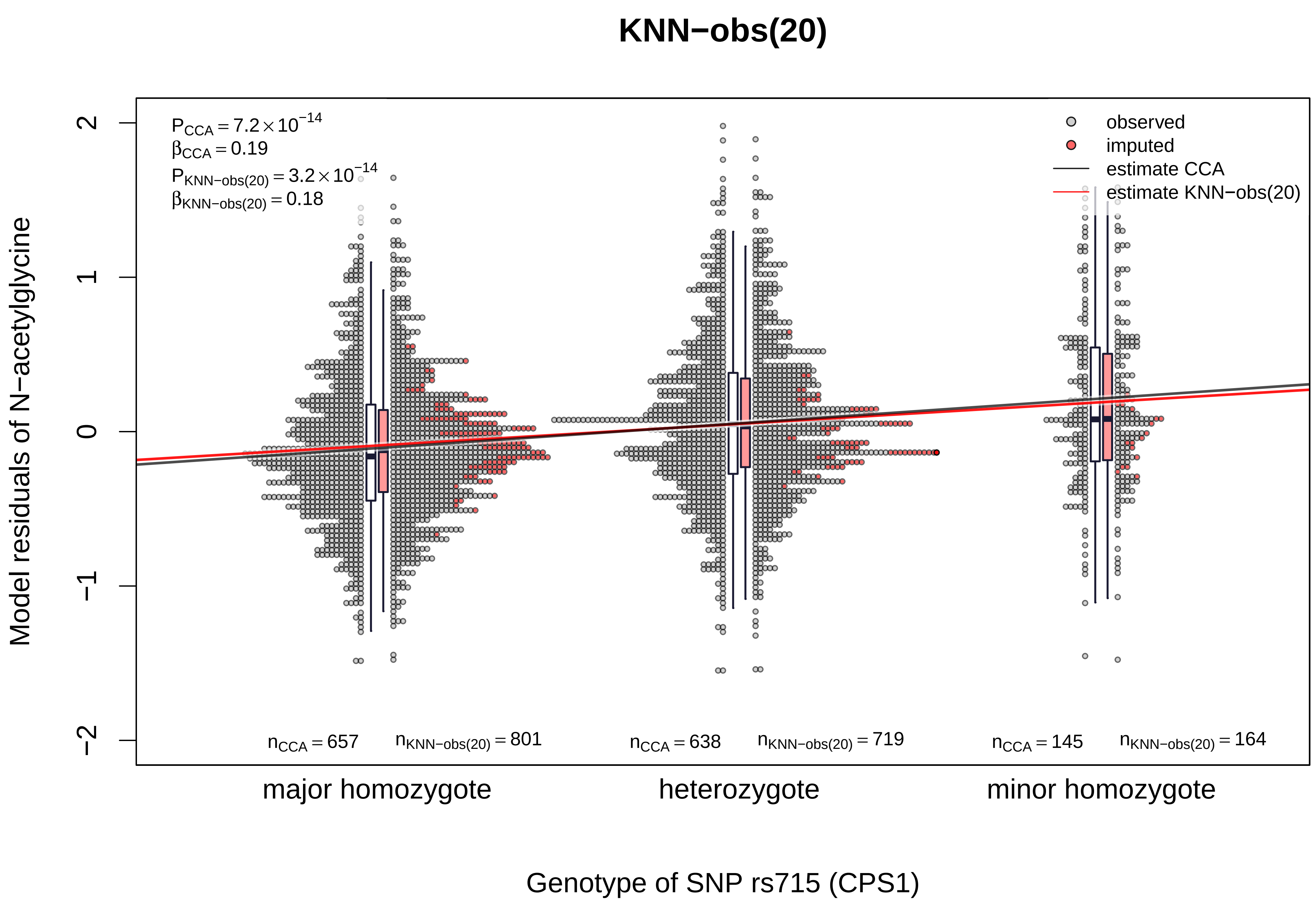

KNN−obs(20)
$P_{CCA} = 7.2 \times 10^{-14}$
$\beta_{CCA} = 0.19$
$P_{KNN-obs(20)} = 3.2 \times 10^{-14}$
$\beta_{KNN-obs(20)} = 0.18$
observed
imputed
estimate CCA
estimate KNN−obs(20)
2
1
0
−1
−2
Model residuals of N−acetylglycine
$n_{CCA} = 657$
$n_{KNN-obs(20)} = 801$
$n_{CCA} = 638$
$n_{KNN-obs(20)} = 719$
$n_{CCA} = 145$
$n_{KNN-obs(20)} = 164$
major homozygote
heterozygote
minor homozygote
Genotype of SNP rs715 (CPS1)

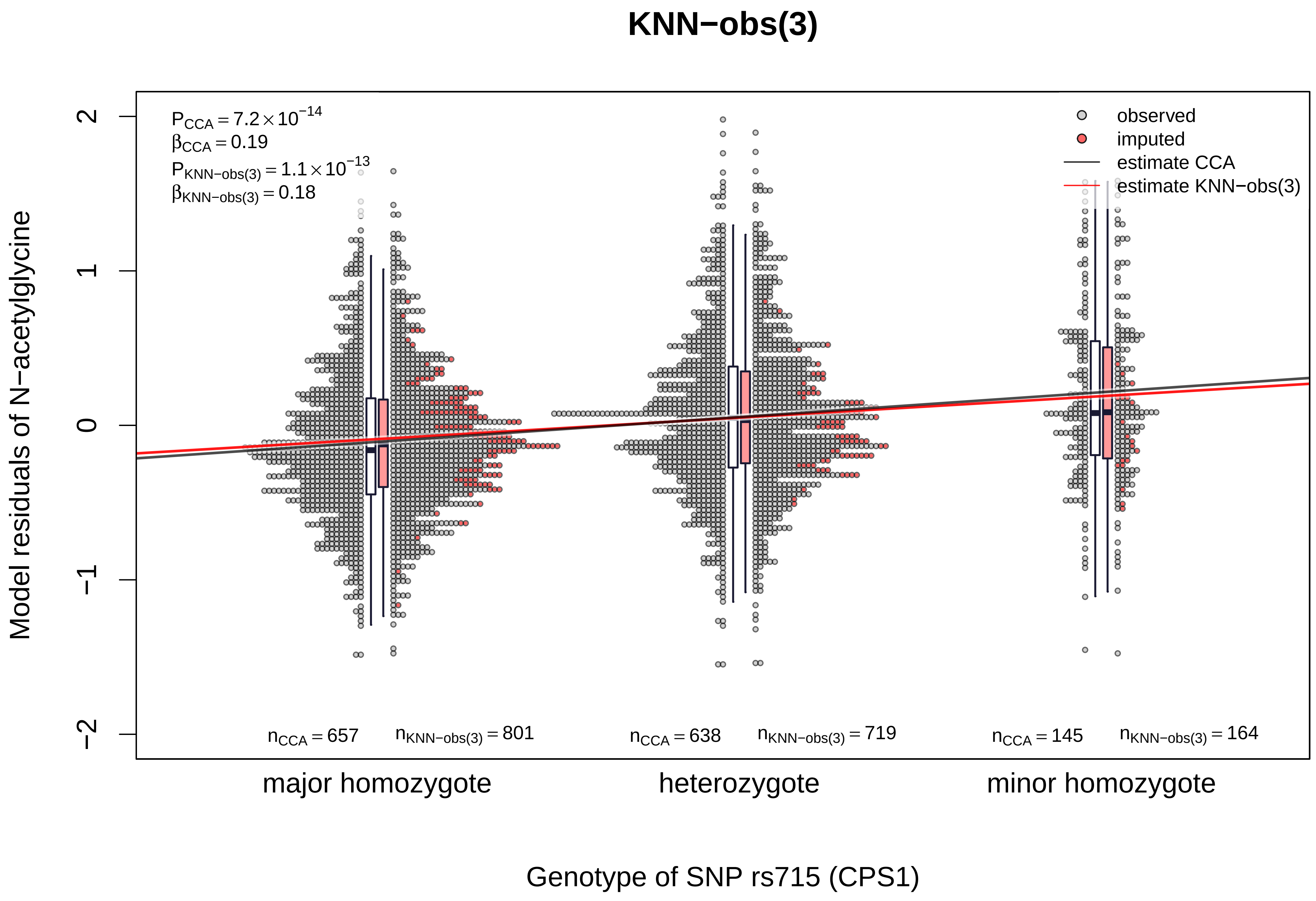

KNN−obs(3)
$P_{CCA} = 7.2 \times 10^{-14}$
$\beta_{CCA} = 0.19$
$P_{KNN-obs(3)} = 1.1 \times 10^{-13}$
$\beta_{KNN-obs(3)} = 0.18$
observed
imputed
estimate CCA
estimate KNN−obs(3)
Model residuals of N−acetylglycine
2
1
0
−1
−2
$n_{CCA} = 657$
$n_{KNN-obs(3)} = 801$
$n_{CCA} = 638$
$n_{KNN-obs(3)} = 719$
$n_{CCA} = 145$
$n_{KNN-obs(3)} = 164$
major homozygote
heterozygote
minor homozygote
Genotype of SNP rs715 (CPS1)

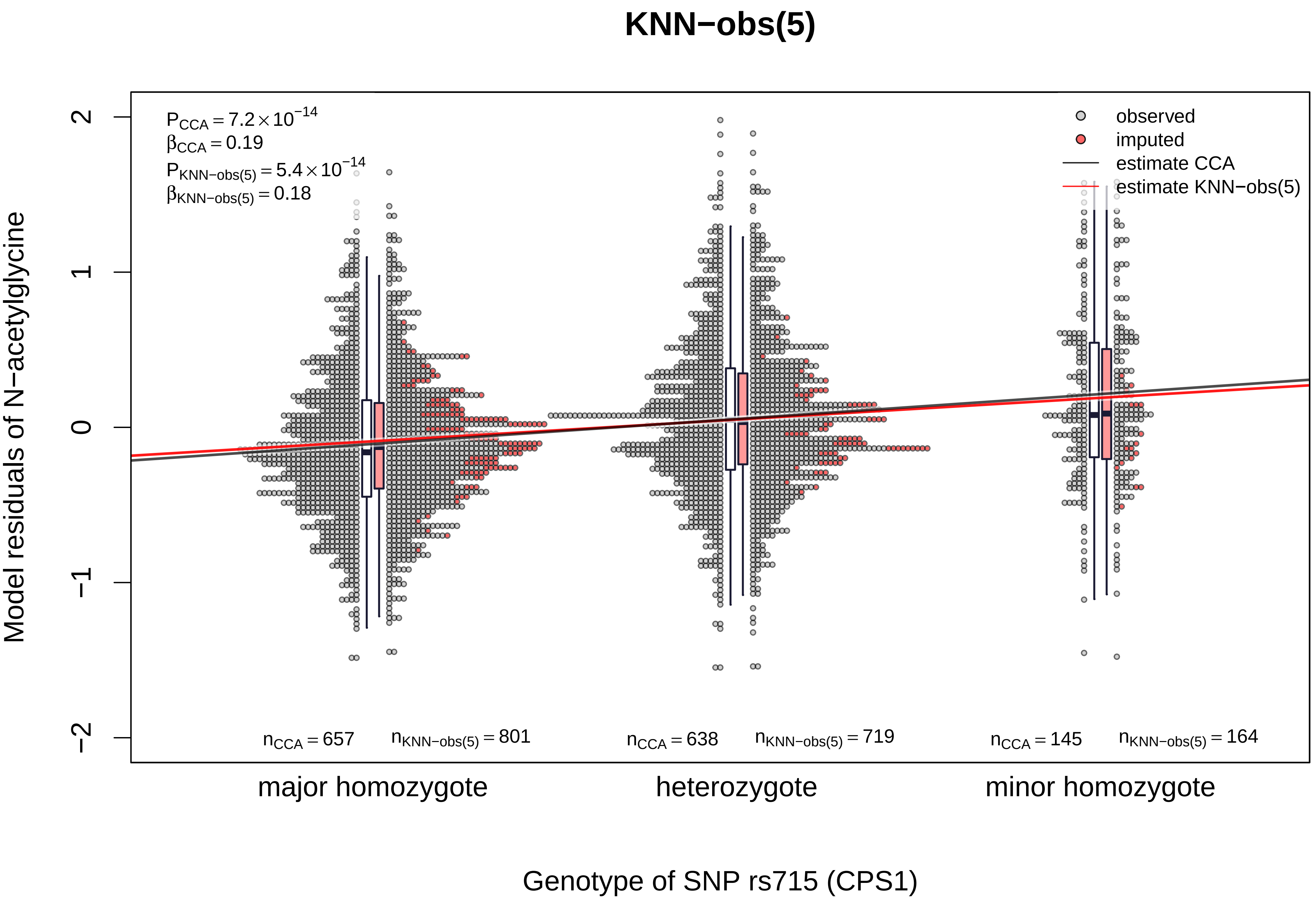

KNN−obs(5)
$P_{CCA} = 7.2 \times 10^{-14}$
$\beta_{CCA} = 0.19$
$P_{KNN-obs(5)} = 5.4 \times 10^{-14}$
$\beta_{KNN-obs(5)} = 0.18$
observed
imputed
estimate CCA
estimate KNN−obs(5)
Model residuals of N−acetylglycine
2
1
0
−1
−2
$n_{CCA} = 657$
$n_{KNN-obs(5)} = 801$
$n_{CCA} = 638$
$n_{KNN-obs(5)} = 719$
$n_{CCA} = 145$
$n_{KNN-obs(5)} = 164$
major homozygote
heterozygote
minor homozygote
Genotype of SNP rs715 (CPS1)

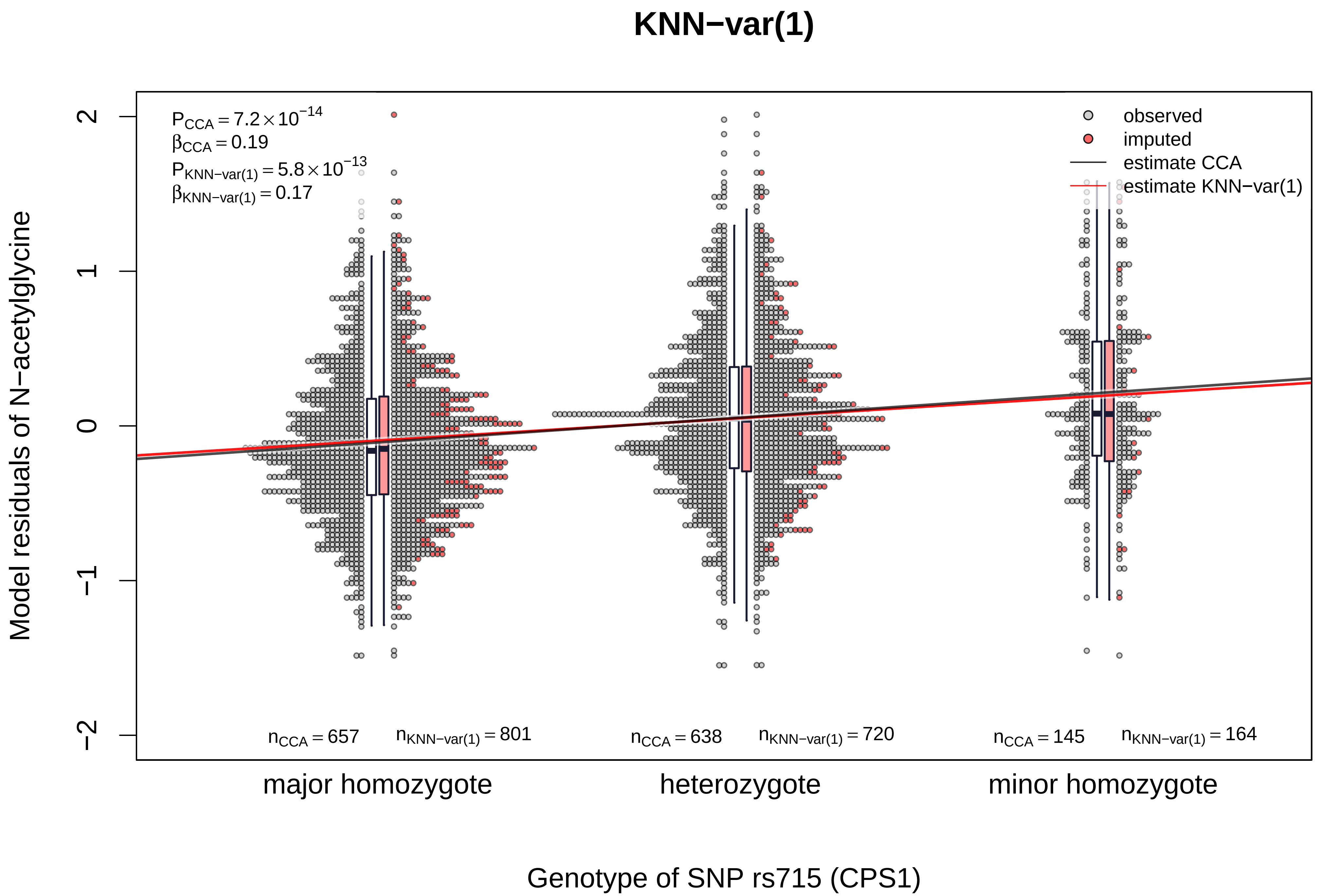

KNN−var(1)
$P_{CCA} = 7.2 \times 10^{-14}$
$\beta_{CCA} = 0.19$
$P_{KNN-var(1)} = 5.8 \times 10^{-13}$
$\beta_{KNN-var(1)} = 0.17$
observed
imputed
estimate CCA
estimate KNN−var(1)
Model residuals of N−acetylglycine
2
1
0
−1
−2
$n_{CCA} = 657$
$n_{KNN-var(1)} = 801$
$n_{CCA} = 638$
$n_{KNN-var(1)} = 720$
$n_{CCA} = 145$
$n_{KNN-var(1)} = 164$
major homozygote
heterozygote
minor homozygote
Genotype of SNP rs715 (CPS1)

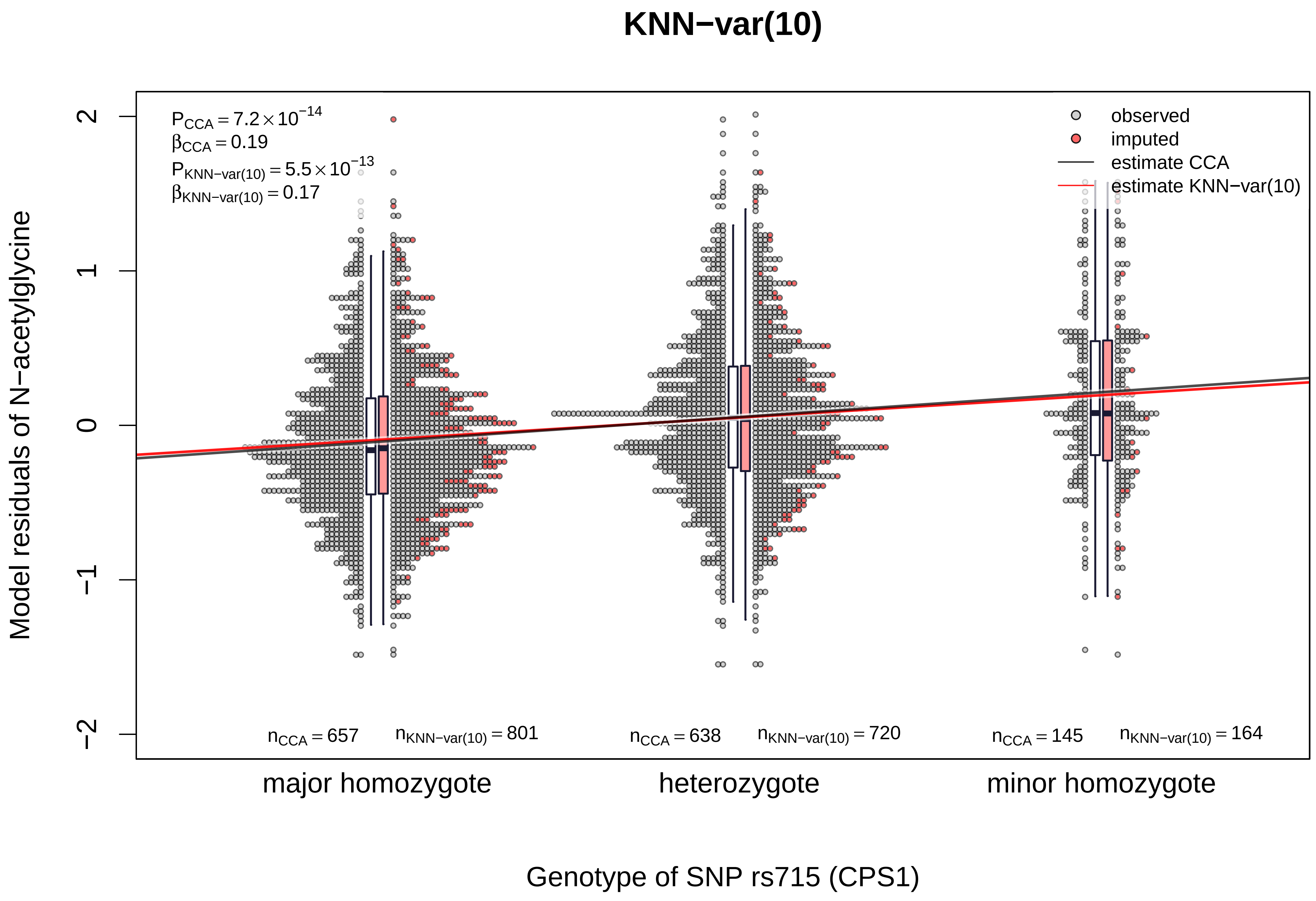

KNN−var(10)
$P_{CCA} = 7.2 \times 10^{-14}$
$\beta_{CCA} = 0.19$
$P_{KNN-var(10)} = 5.5 \times 10^{-13}$
$\beta_{KNN-var(10)} = 0.17$
observed
imputed
estimate CCA
estimate KNN−var(10)
Model residuals of N−acetylglycine
2
1
0
−1
−2
$n_{CCA} = 657$
$n_{KNN-var(10)} = 801$
$n_{CCA} = 638$
$n_{KNN-var(10)} = 720$
$n_{CCA} = 145$
$n_{KNN-var(10)} = 164$
major homozygote
heterozygote
minor homozygote
Genotype of SNP rs715 (CPS1)

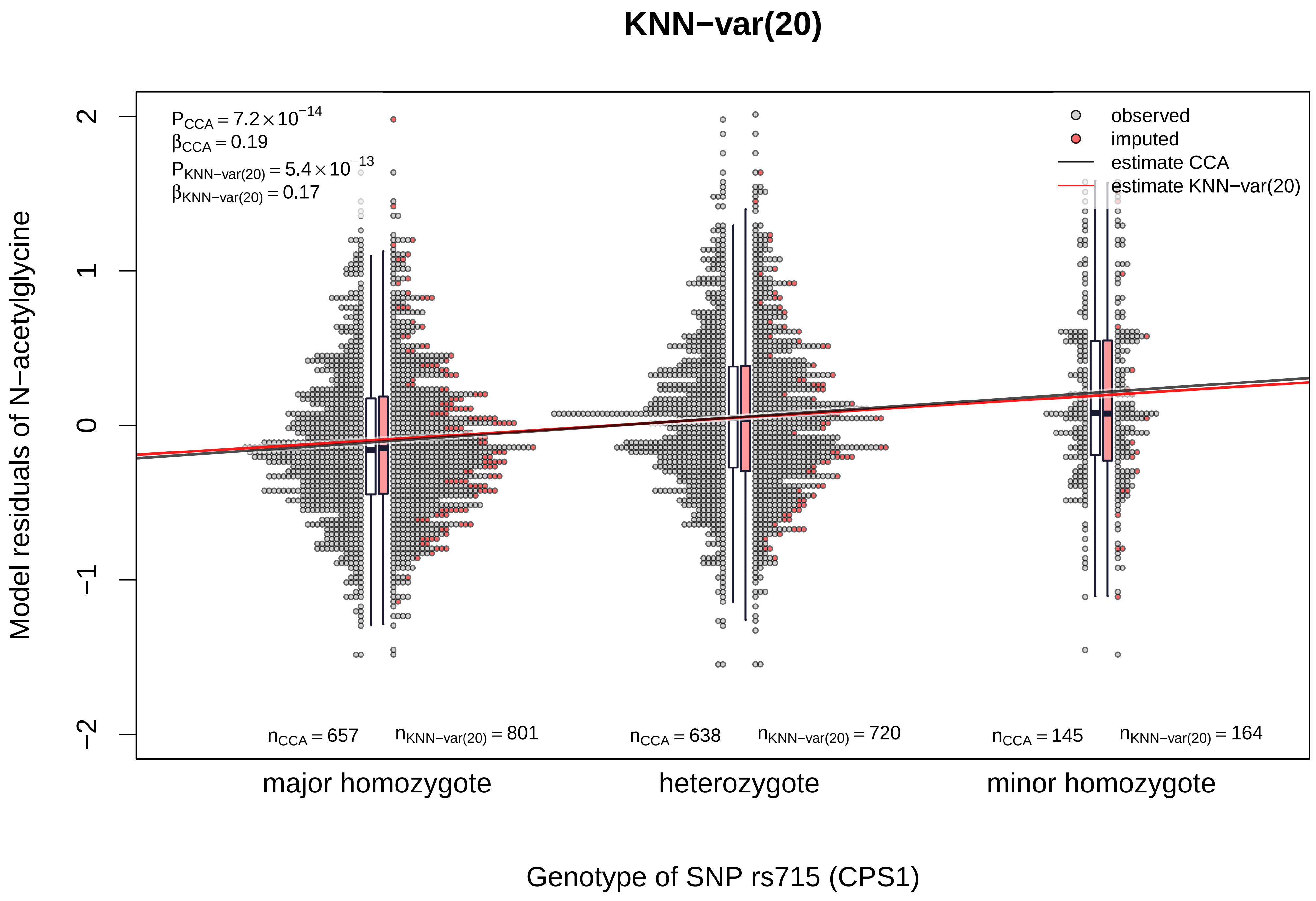

KNN−var(20)
$P_{CCA} = 7.2 \times 10^{-14}$
$\beta_{CCA} = 0.19$
$P_{KNN-var(20)} = 5.4 \times 10^{-13}$
$\beta_{KNN-var(20)} = 0.17$
observed
imputed
estimate CCA
estimate KNN−var(20)
Model residuals of N−acetylglycine
2
1
0
−1
−2
$n_{CCA} = 657$
$n_{KNN-var(20)} = 801$
$n_{CCA} = 638$
$n_{KNN-var(20)} = 720$
$n_{CCA} = 145$
$n_{KNN-var(20)} = 164$
major homozygote
heterozygote
minor homozygote
Genotype of SNP rs715 (CPS1)

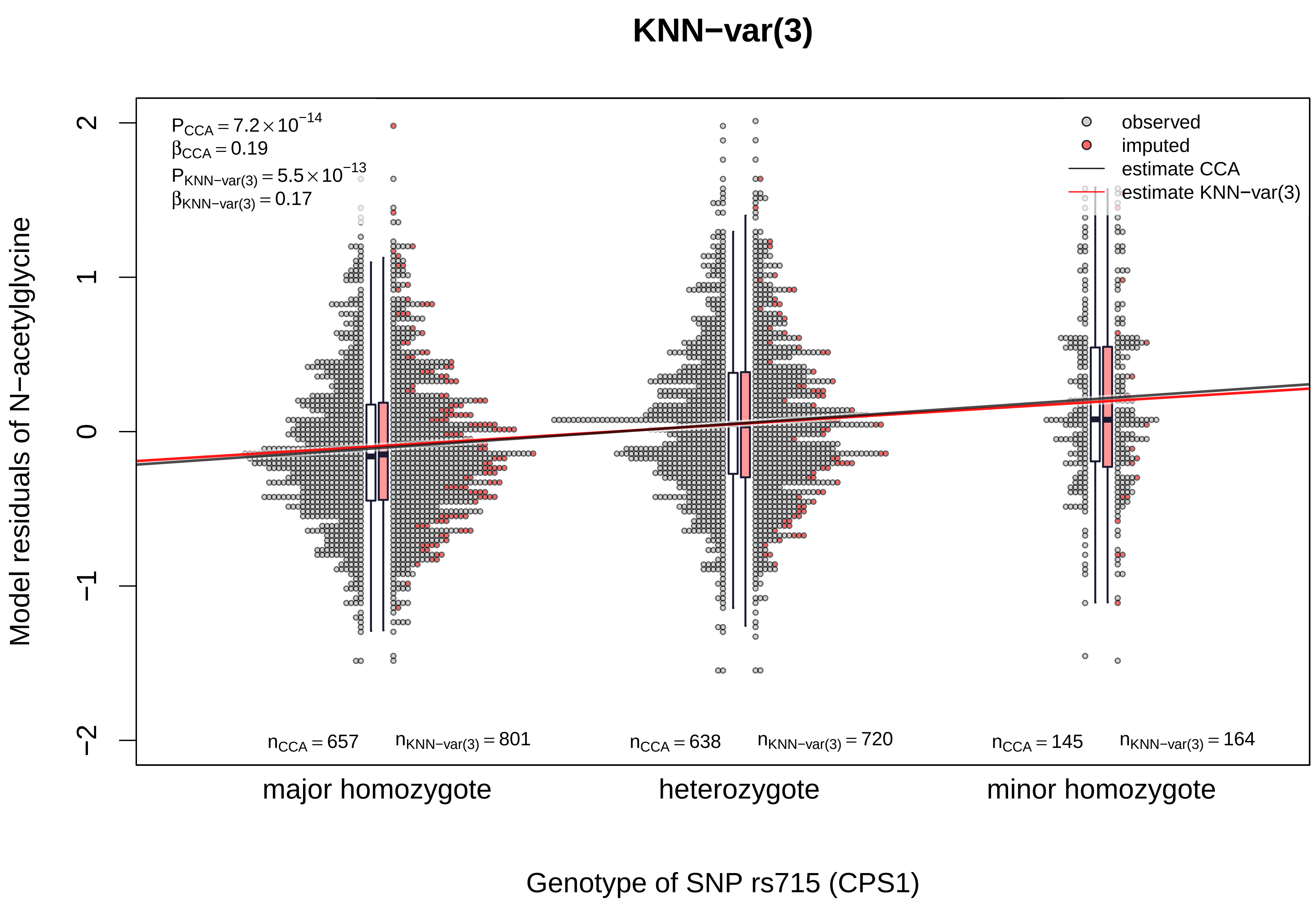

KNN−var(3)
$P_{CCA} = 7.2 \times 10^{-14}$
$\beta_{CCA} = 0.19$
$P_{KNN-var(3)} = 5.5 \times 10^{-13}$
$\beta_{KNN-var(3)} = 0.17$
observed
imputed
estimate CCA
estimate KNN−var(3)
Model residuals of N−acetylglycine
2
1
0
−1
−2
$n_{CCA} = 657$
$n_{KNN-var(3)} = 801$
$n_{CCA} = 638$
$n_{KNN-var(3)} = 720$
$n_{CCA} = 145$
$n_{KNN-var(3)} = 164$
major homozygote
heterozygote
minor homozygote
Genotype of SNP rs715 (CPS1)

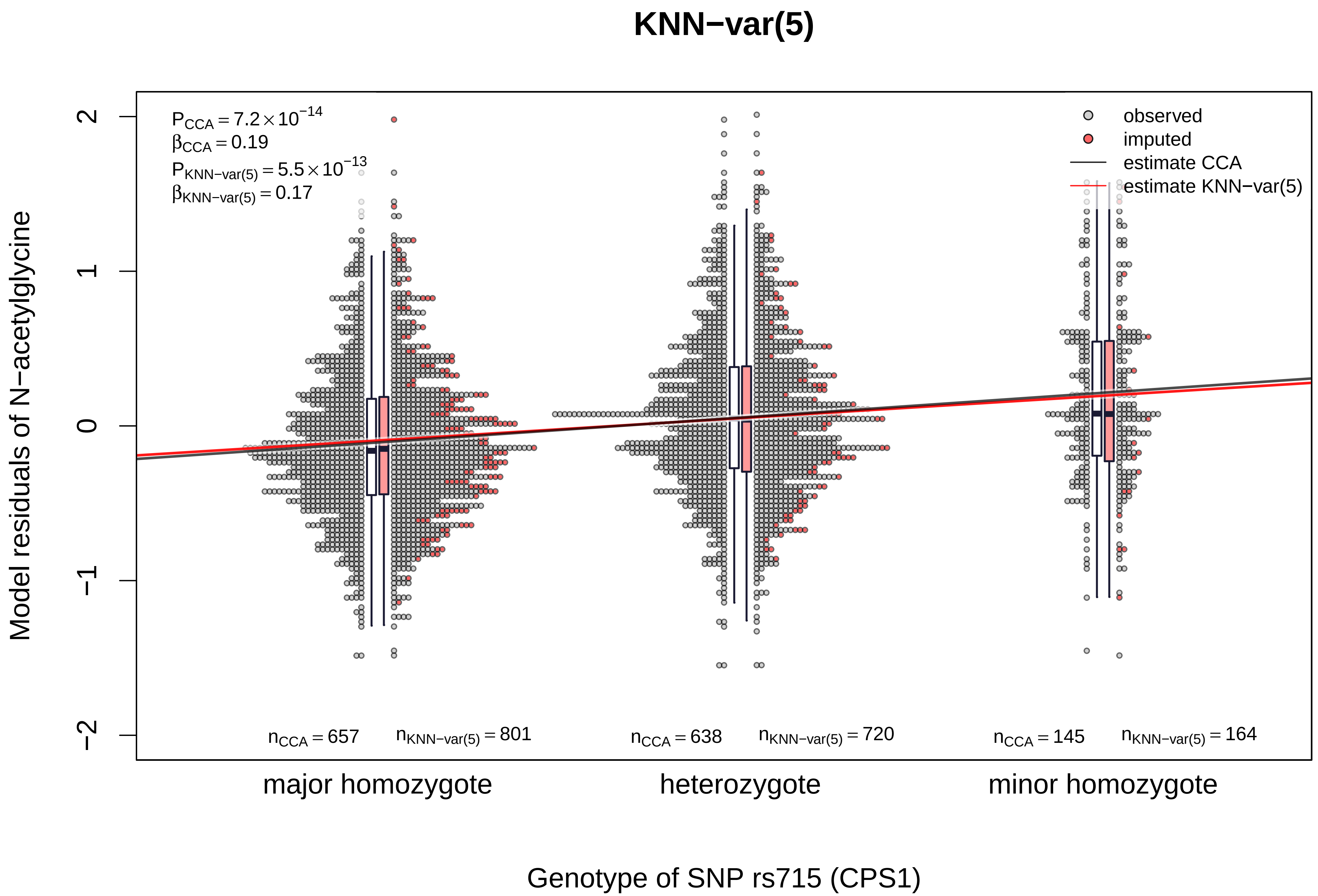

KNN−var(5)
$P_{CCA} = 7.2 \times 10^{-14}$
$\beta_{CCA} = 0.19$
$P_{KNN-var(5)} = 5.5 \times 10^{-13}$
$\beta_{KNN-var(5)} = 0.17$
observed
imputed
estimate CCA
estimate KNN−var(5)
Model residuals of N−acetylglycine
2
1
0
−1
−2
$n_{CCA} = 657$
$n_{KNN-var(5)} = 801$
$n_{CCA} = 638$
$n_{KNN-var(5)} = 720$
$n_{CCA} = 145$
$n_{KNN-var(5)} = 164$
major homozygote
heterozygote
minor homozygote
Genotype of SNP rs715 (CPS1)

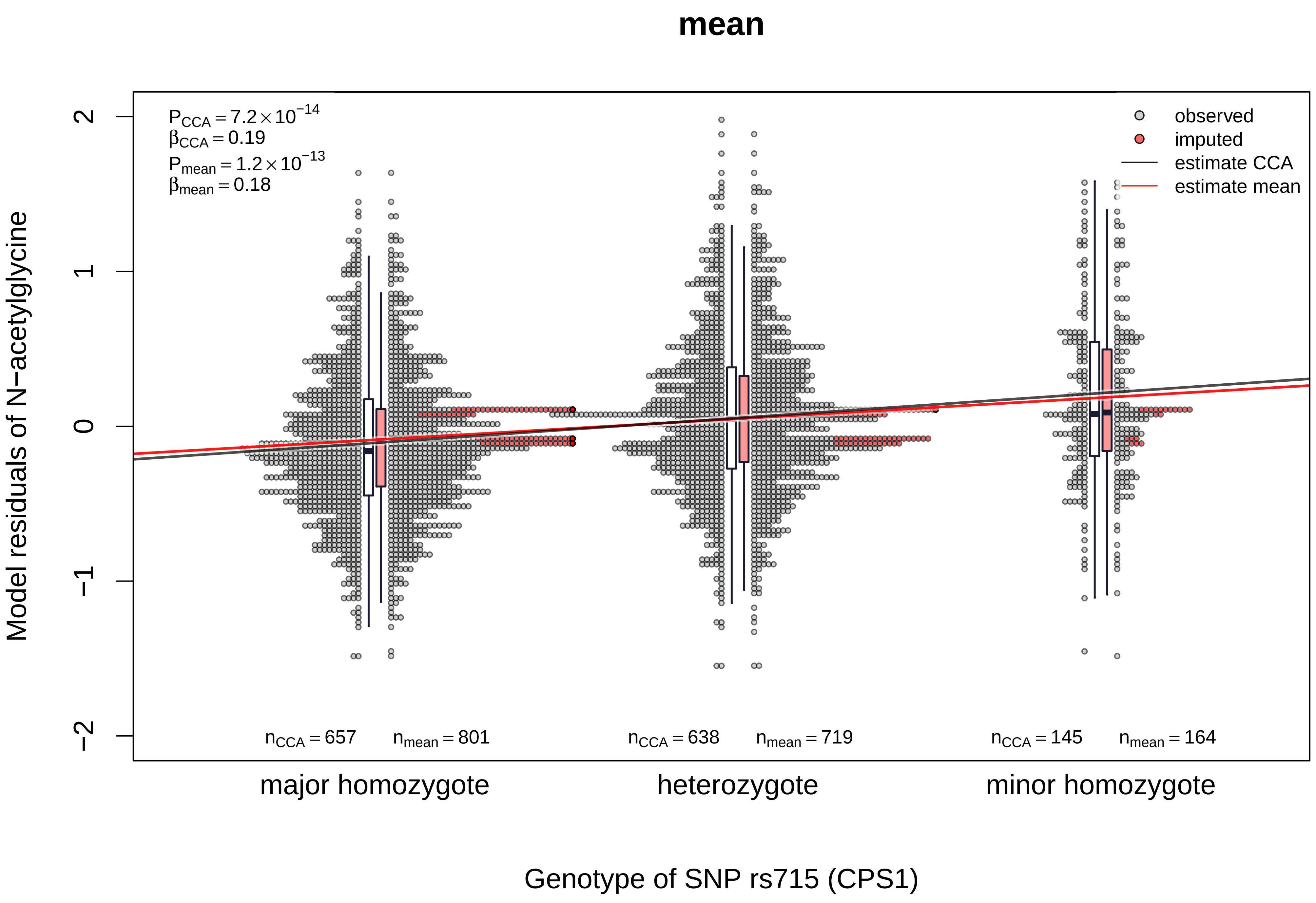

mean
$P_{CCA} = 7.2 \times 10^{-14}$
$\beta_{CCA} = 0.19$
$P_{mean} = 1.2 \times 10^{-13}$
$\beta_{mean} = 0.18$
observed
imputed
estimate CCA
estimate mean
2
1
0
−1
−2
Model residuals of N−acetylglycine
$n_{CCA} = 657$
$n_{mean} = 801$
$n_{CCA} = 638$
$n_{mean} = 719$
$n_{CCA} = 145$
$n_{mean} = 164$
major homozygote
heterozygote
minor homozygote
Genotype of SNP rs715 (CPS1)

MICE−avg−norm

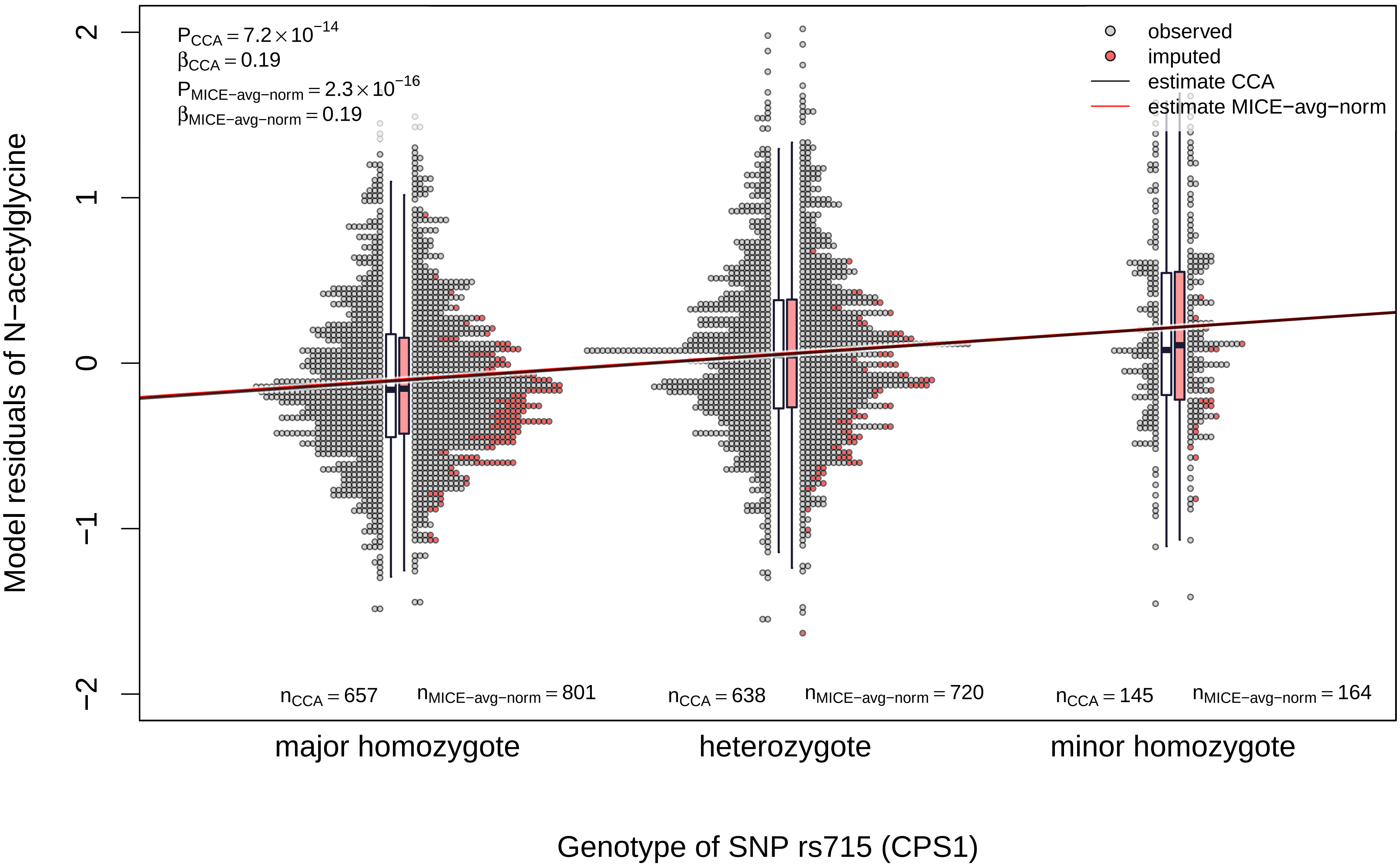

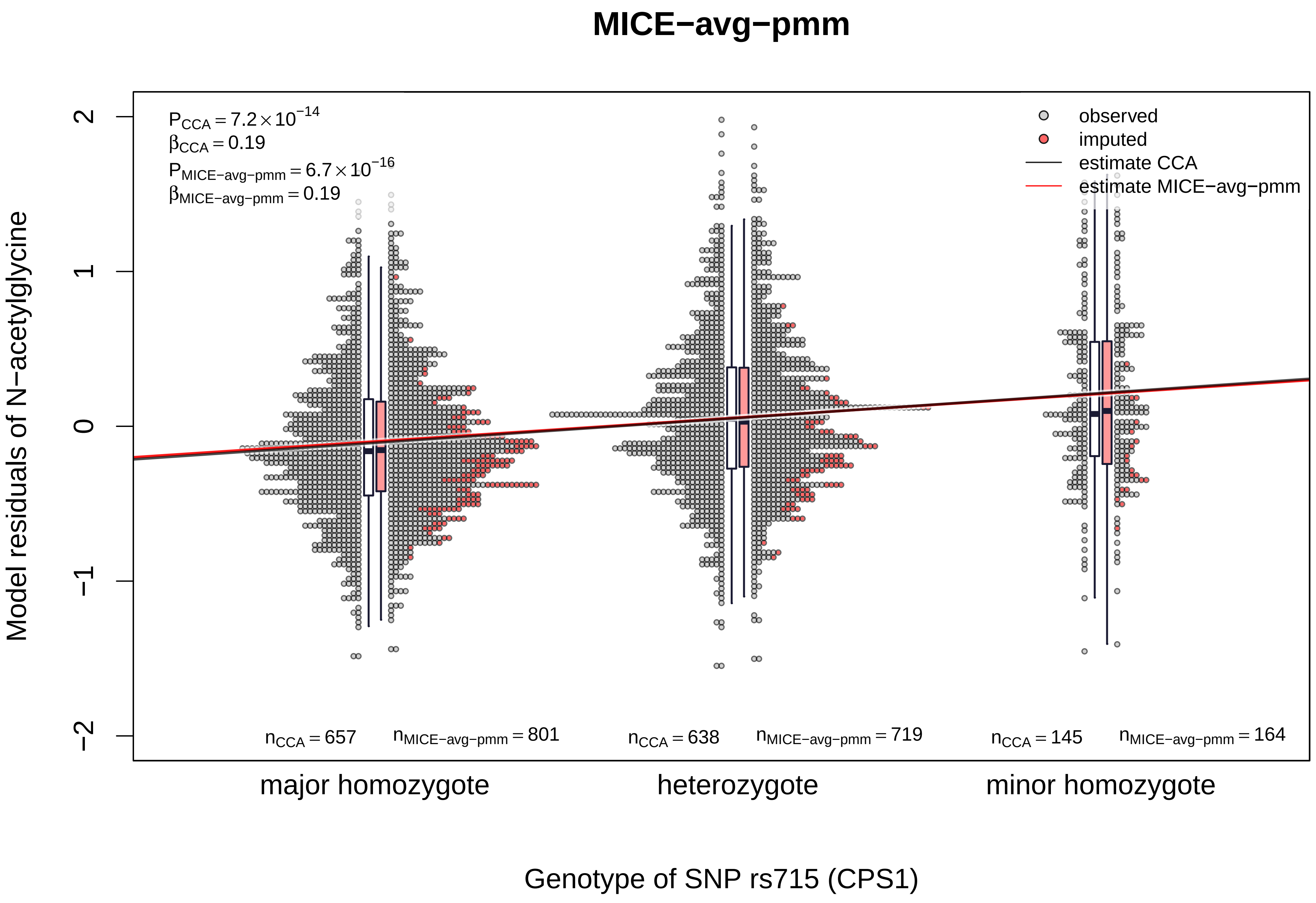

MICE−avg−pmm
$P_{CCA} = 7.2 \times 10^{-14}$
$\beta_{CCA} = 0.19$
$P_{MICE-avg-pmm} = 6.7 \times 10^{-16}$
$\beta_{MICE-avg-pmm} = 0.19$
observed
imputed
estimate CCA
estimate MICE−avg−pmm
2
1
0
−1
−2
Model residuals of N−acetylglycine
$n_{CCA} = 657$
$n_{MICE-avg-pmm} = 801$
$n_{CCA} = 638$
$n_{MICE-avg-pmm} = 719$
$n_{CCA} = 145$
$n_{MICE-avg-pmm} = 164$
major homozygote
heterozygote
minor homozygote
Genotype of SNP rs715 (CPS1)

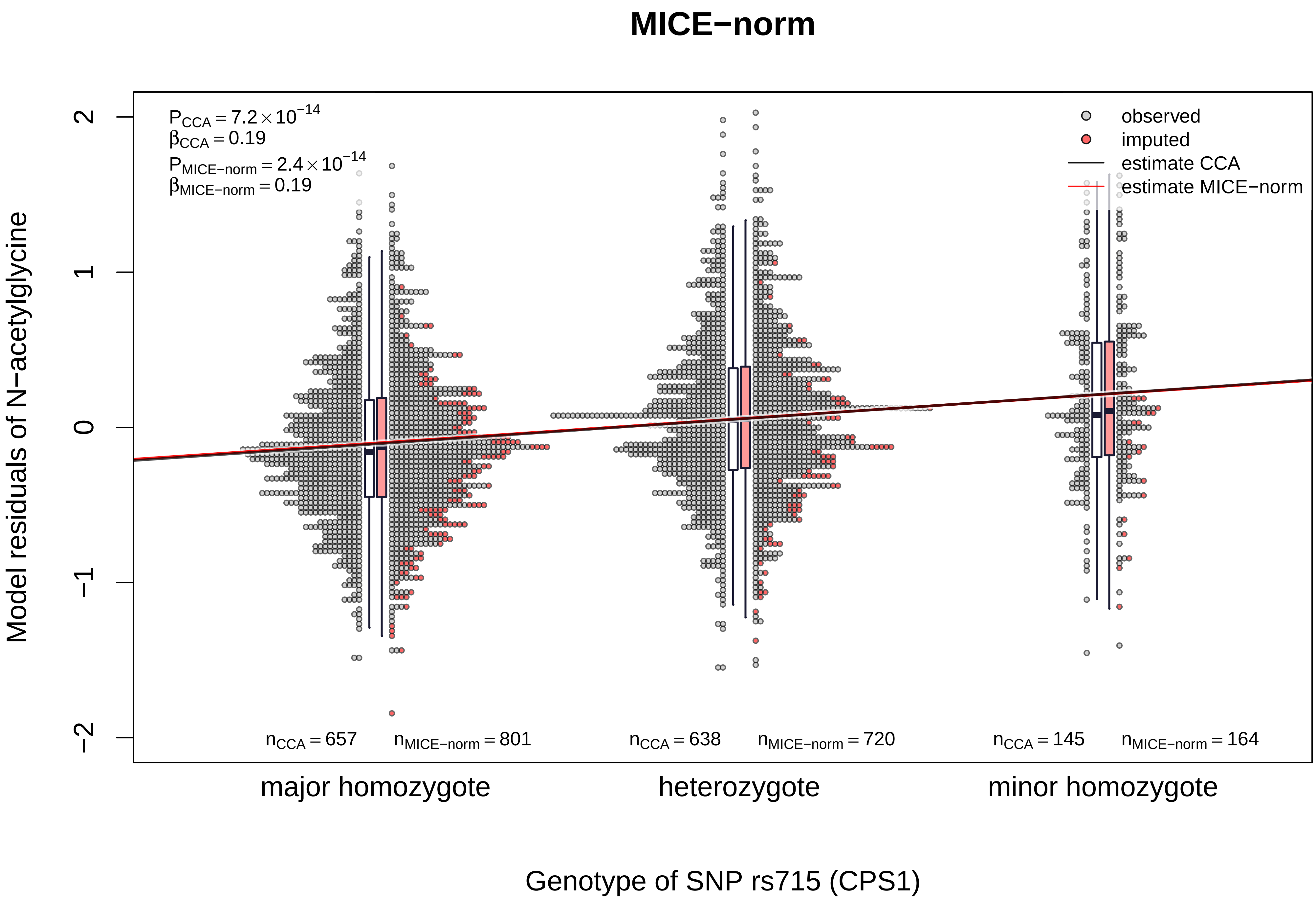
MICE−norm
$P_{CCA} = 7.2 \times 10^{-14}$
$\beta_{CCA} = 0.19$
$P_{MICE-norm} = 2.4 \times 10^{-14}$
$\beta_{MICE-norm} = 0.19$
observed
imputed
estimate CCA
estimate MICE−norm
Model residuals of N−acetylglycine
2
1
0
−1
−2
$n_{CCA} = 657$
$n_{MICE-norm} = 801$
$n_{CCA} = 638$
$n_{MICE-norm} = 720$
$n_{CCA} = 145$
$n_{MICE-norm} = 164$
major homozygote
heterozygote
minor homozygote
Genotype of SNP rs715 (CPS1)

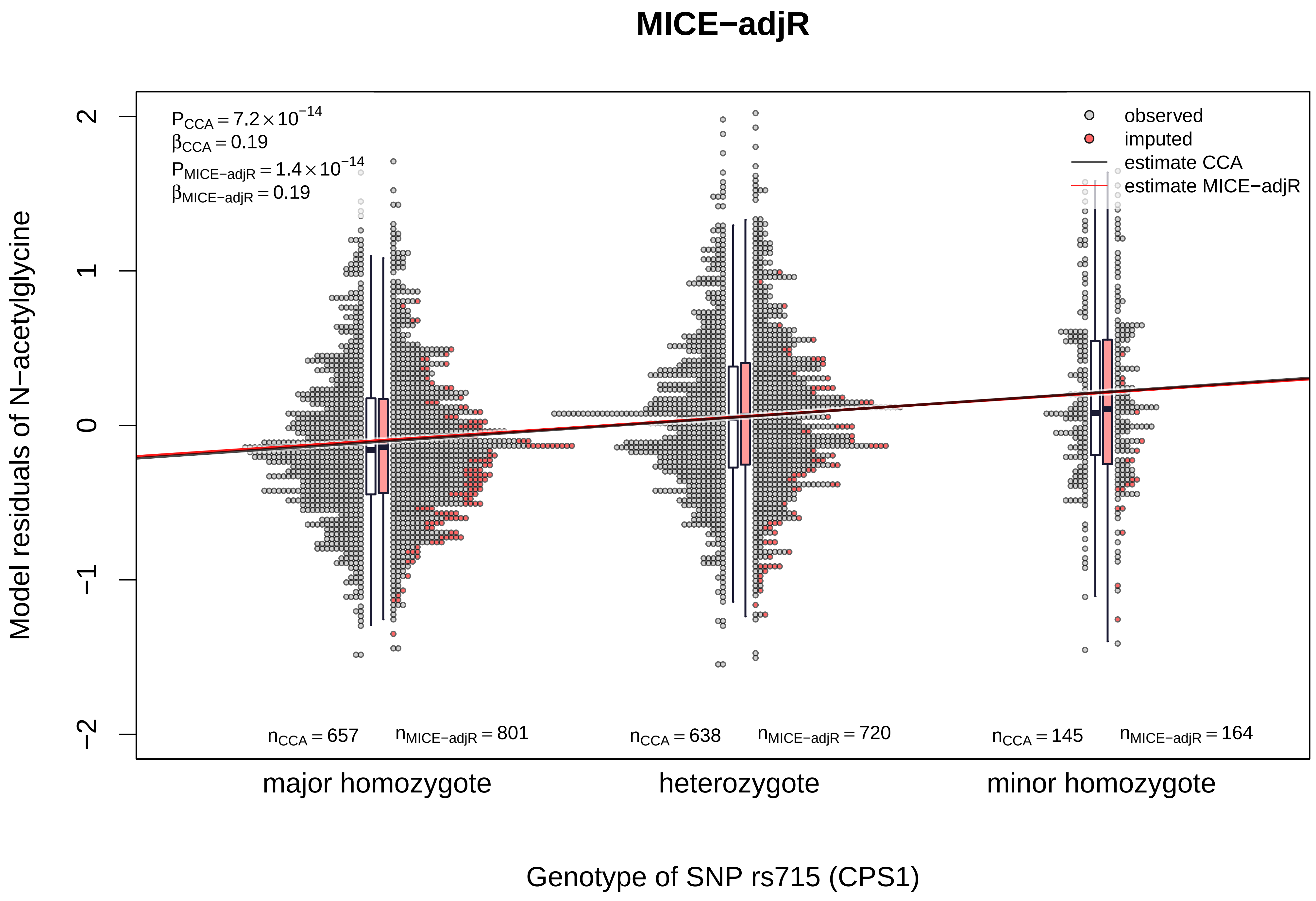

MICE−adjR
$P_{CCA} = 7.2 \times 10^{-14}$
$\beta_{CCA} = 0.19$
$P_{MICE-adjR} = 1.4 \times 10^{-14}$
$\beta_{MICE-adjR} = 0.19$
observed
imputed
estimate CCA
estimate MICE−adjR
Model residuals of N−acetylglycine
2
1
0
−1
−2
$n_{CCA} = 657$
$n_{MICE-adjR} = 801$
$n_{CCA} = 638$
$n_{MICE-adjR} = 720$
$n_{CCA} = 145$
$n_{MICE-adjR} = 164$
major homozygote
heterozygote
minor homozygote
Genotype of SNP rs715 (CPS1)

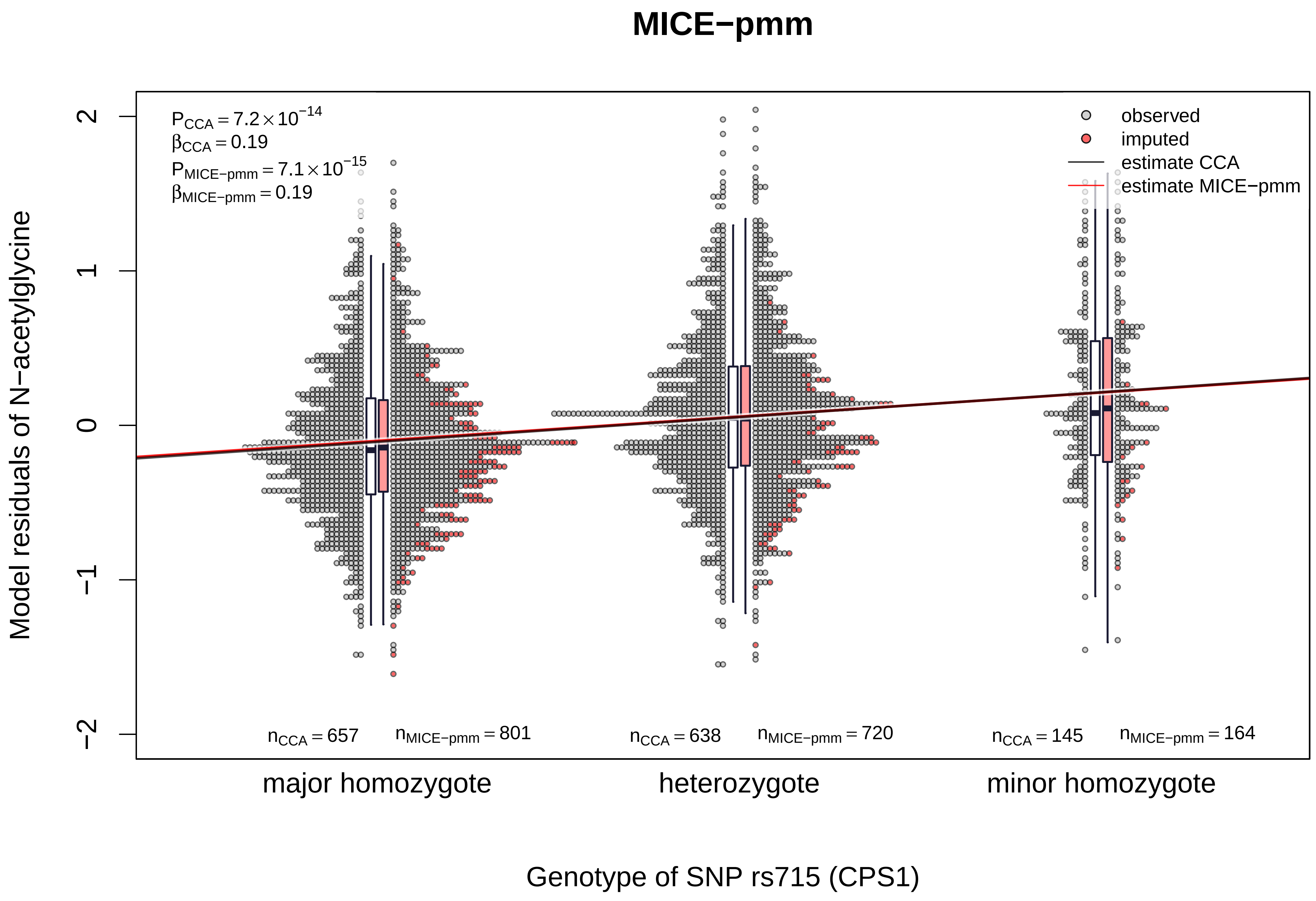

MICE−pmm
$P_{CCA} = 7.2 \times 10^{-14}$
$\beta_{CCA} = 0.19$
$P_{MICE-pmm} = 7.1 \times 10^{-15}$
$\beta_{MICE-pmm} = 0.19$
observed
imputed
estimate CCA
estimate MICE−pmm
Model residuals of N−acetylglycine
2
1
0
−1
−2
$n_{CCA} = 657$
$n_{MICE-pmm} = 801$
$n_{CCA} = 638$
$n_{MICE-pmm} = 720$
$n_{CCA} = 145$
$n_{MICE-pmm} = 164$
major homozygote
heterozygote
minor homozygote
Genotype of SNP rs715 (CPS1)

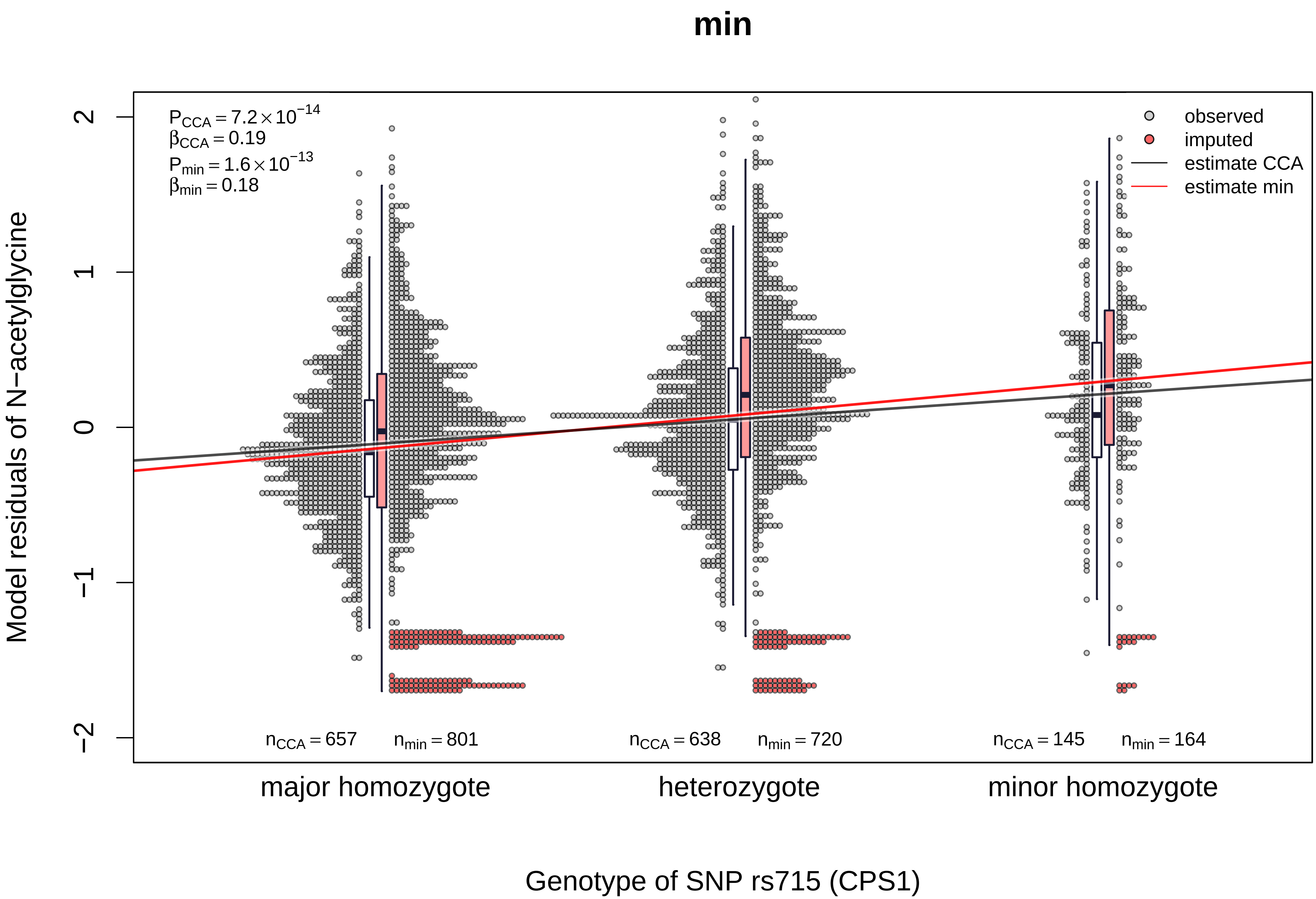

min
$P_{CCA} = 7.2 \times 10^{-14}$
$\beta_{CCA} = 0.19$
$P_{min} = 1.6 \times 10^{-13}$
$\beta_{min} = 0.18$
observed
imputed
estimate CCA
estimate min
Model residuals of N−acetylglycine
2
1
0
−1
−2
$n_{CCA} = 657$
$n_{min} = 801$
$n_{CCA} = 638$
$n_{min} = 720$
$n_{CCA} = 145$
$n_{min} = 164$
major homozygote
heterozygote
minor homozygote
Genotype of SNP rs715 (CPS1)

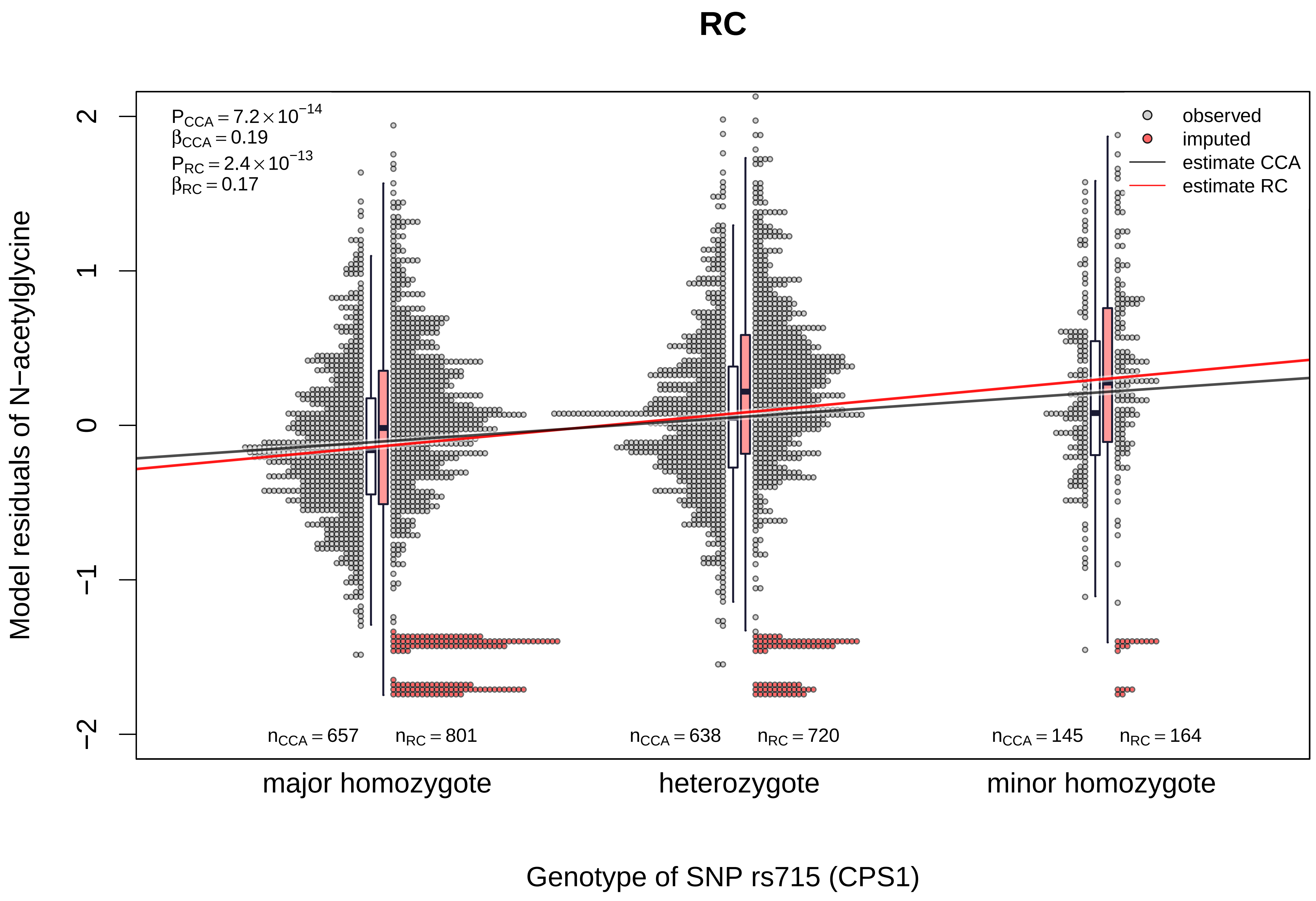
RC
$P_{CCA} = 7.2 \times 10^{-14}$
$\beta_{CCA} = 0.19$
$P_{RC} = 2.4 \times 10^{-13}$
$\beta_{RC} = 0.17$
observed
imputed
estimate CCA
estimate RC
Model residuals of N−acetylglycine
2
1
0
−1
−2
$n_{CCA} = 657$
$n_{RC} = 801$
$n_{CCA} = 638$
$n_{RC} = 720$
$n_{CCA} = 145$
$n_{RC} = 164$
major homozygote
heterozygote
minor homozygote
Genotype of SNP rs715 (CPS1)

RC−R

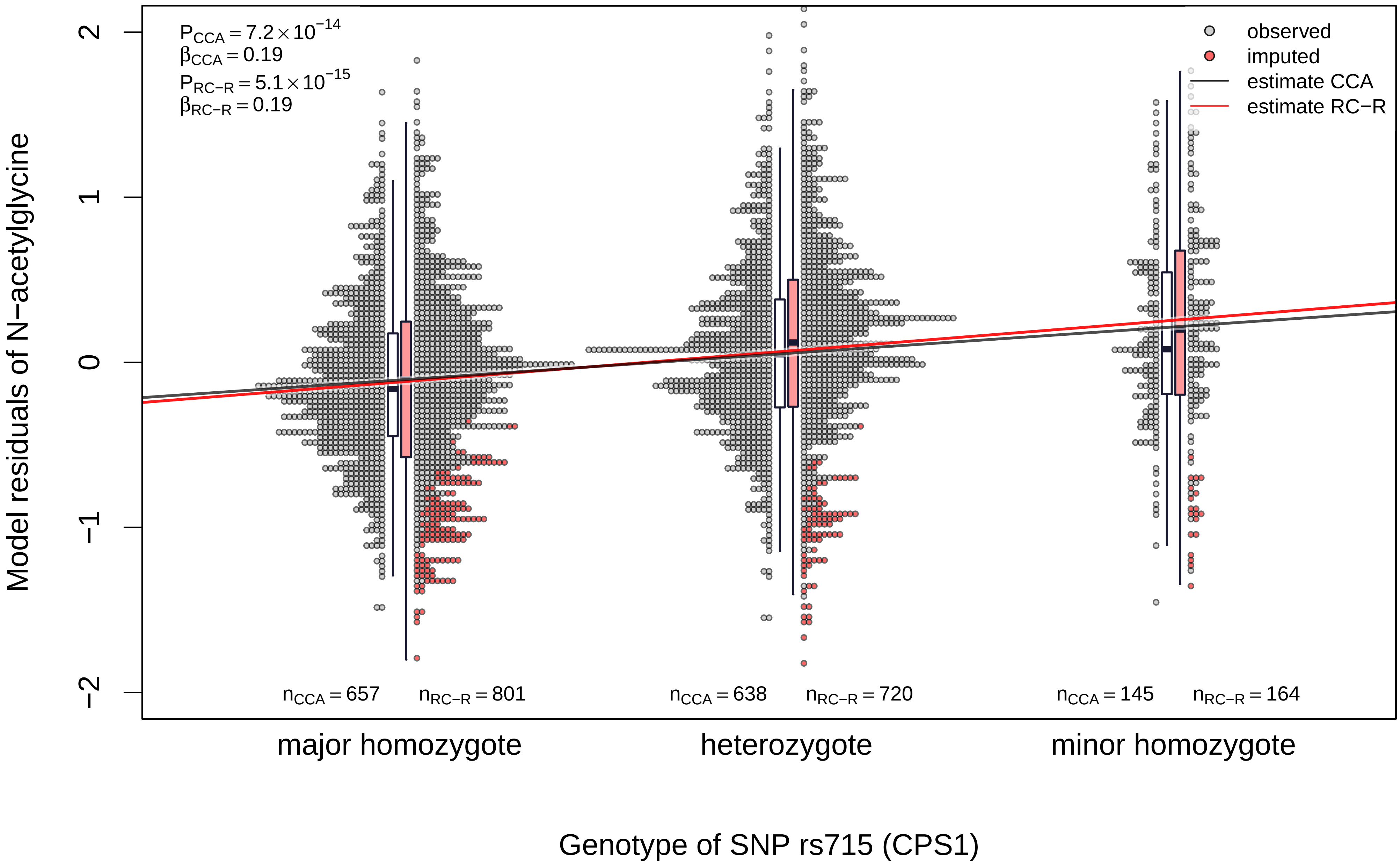

$P_{CCA} = 7.2 \times 10^{-14}$
$\beta_{CCA} = 0.19$
$P_{RC-R} = 5.1 \times 10^{-15}$
$\beta_{RC-R} = 0.19$
observed
imputed
estimate CCA
estimate RC−R
Model residuals of N−acetylglycine
2
1
0
−1
−2
$n_{CCA} = 657$
$n_{RC-R} = 801$
$n_{CCA} = 638$
$n_{RC-R} = 720$
$n_{CCA} = 145$
$n_{RC-R} = 164$
major homozygote
heterozygote
minor homozygote
Genotype of SNP rs715 (CPS1)

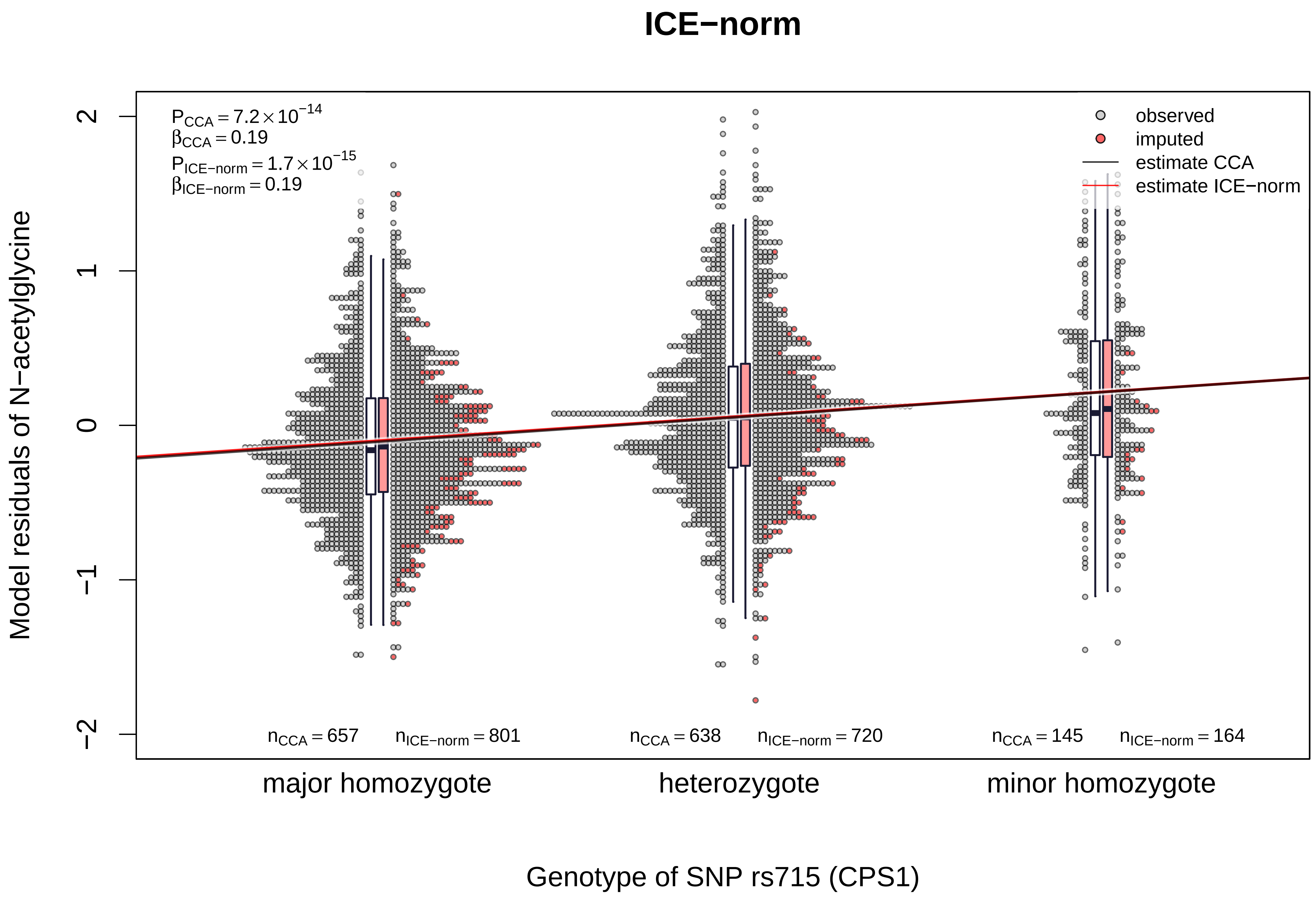

ICE−norm
$P_{CCA} = 7.2 \times 10^{-14}$
$\beta_{CCA} = 0.19$
$P_{ICE-norm} = 1.7 \times 10^{-15}$
$\beta_{ICE-norm} = 0.19$
observed
imputed
estimate CCA
estimate ICE−norm
Model residuals of N−acetylglycine
2
1
0
−1
−2
$n_{CCA} = 657$
$n_{ICE-norm} = 801$
$n_{CCA} = 638$
$n_{ICE-norm} = 720$
$n_{CCA} = 145$
$n_{ICE-norm} = 164$
major homozygote
heterozygote
minor homozygote
Genotype of SNP rs715 (CPS1)

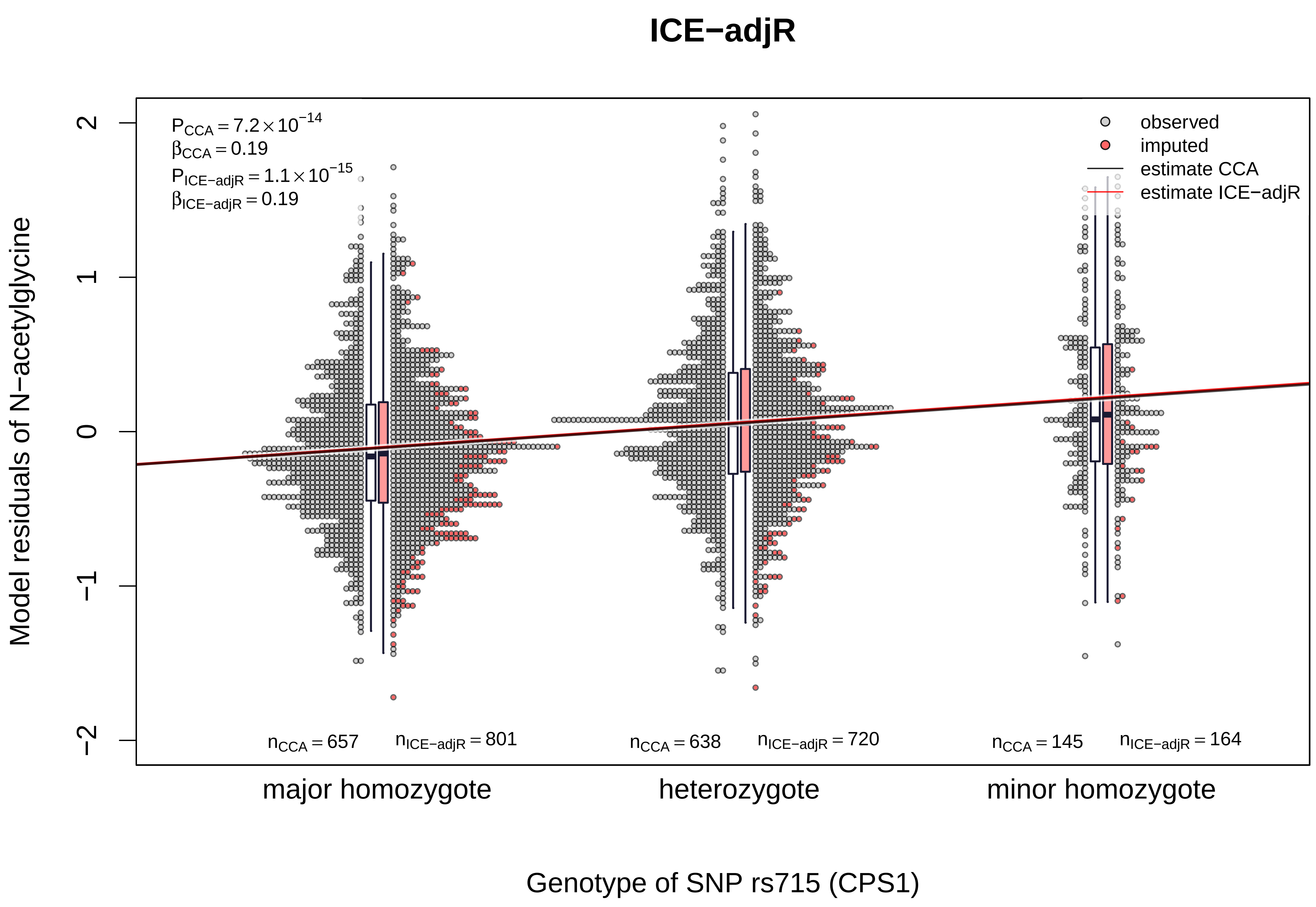

ICE−adjR
P_CCA = 7.2 × 10^−14
β_CCA = 0.19
P_ICE−adjR = 1.1 × 10^−15
β_ICE−adjR = 0.19
observed
imputed
estimate CCA
estimate ICE−adjR
2
1
0
−1
−2
Model residuals of N−acetylglycine
n_CCA = 657
n_ICE−adjR = 801
n_CCA = 638
n_ICE−adjR = 720
n_CCA = 145
n_ICE−adjR = 164
major homozygote
heterozygote
minor homozygote
Genotype of SNP rs715 (CPS1)

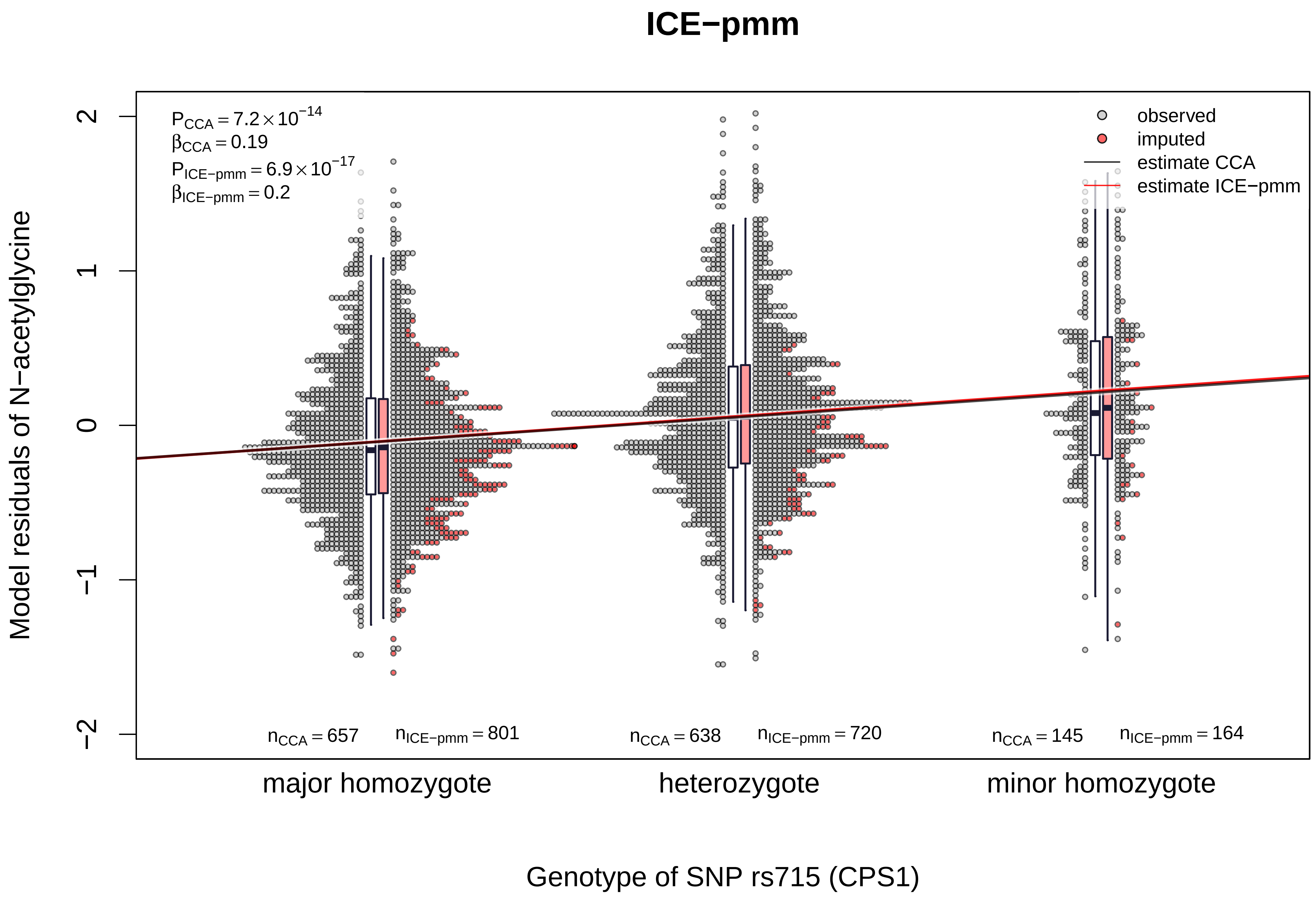
ICE−pmm
$P_{CCA} = 7.2 \times 10^{-14}$
$\beta_{CCA} = 0.19$
$P_{ICE-pmm} = 6.9 \times 10^{-17}$
$\beta_{ICE-pmm} = 0.2$
observed
imputed
estimate CCA
estimate ICE−pmm
Model residuals of N−acetylglycine
2
1
0
−1
−2
$n_{CCA} = 657$
$n_{ICE-pmm} = 801$
$n_{CCA} = 638$
$n_{ICE-pmm} = 720$
$n_{CCA} = 145$
$n_{ICE-pmm} = 164$
major homozygote
heterozygote
minor homozygote
Genotype of SNP rs715 (CPS1)

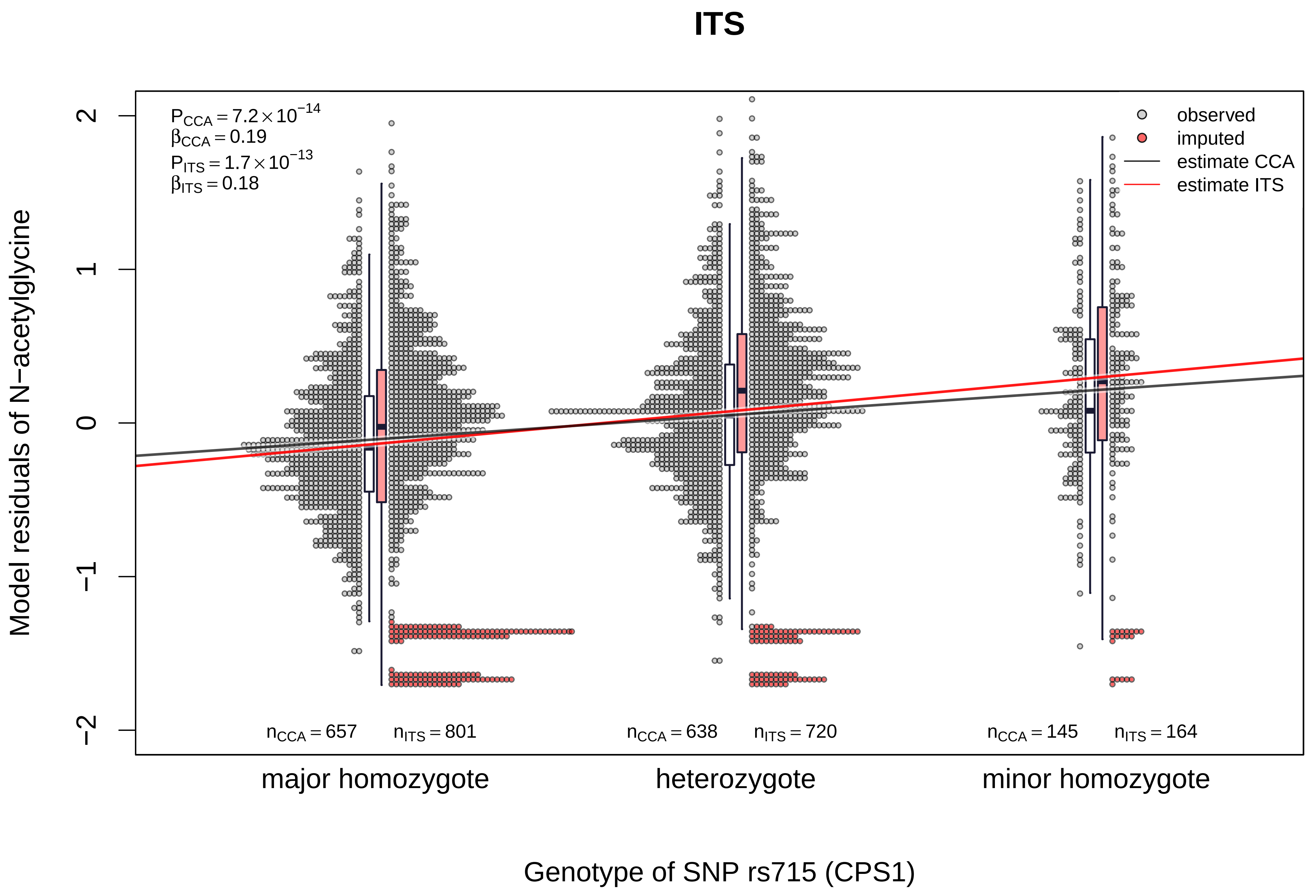

ITS
$P_{CCA} = 7.2 \times 10^{-14}$
$\beta_{CCA} = 0.19$
$P_{ITS} = 1.7 \times 10^{-13}$
$\beta_{ITS} = 0.18$
observed
imputed
estimate CCA
estimate ITS
Model residuals of N−acetylglycine
2
1
0
−1
−2
$n_{CCA} = 657$
$n_{ITS} = 801$
$n_{CCA} = 638$
$n_{ITS} = 720$
$n_{CCA} = 145$
$n_{ITS} = 164$
major homozygote
heterozygote
minor homozygote
Genotype of SNP rs715 (CPS1)

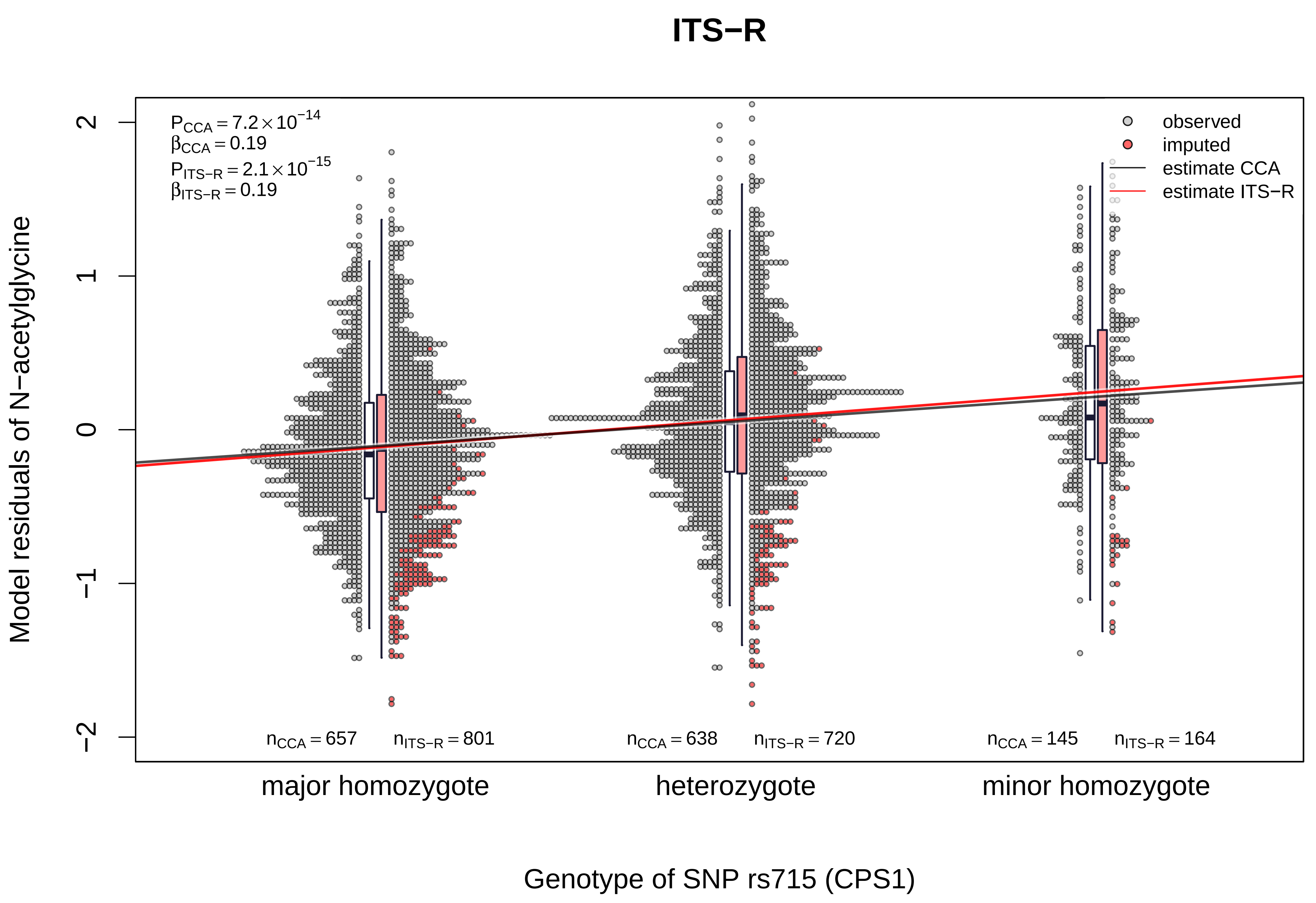

ITS−R
$P_{CCA} = 7.2 \times 10^{-14}$
$\beta_{CCA} = 0.19$
$P_{ITS-R} = 2.1 \times 10^{-15}$
$\beta_{ITS-R} = 0.19$
observed
imputed
estimate CCA
estimate ITS−R
Model residuals of N−acetylglycine
2
1
0
−1
−2
$n_{CCA} = 657$
$n_{ITS-R} = 801$
$n_{CCA} = 638$
$n_{ITS-R} = 720$
$n_{CCA} = 145$
$n_{ITS-R} = 164$
major homozygote
heterozygote
minor homozygote
Genotype of SNP rs715 (CPS1)

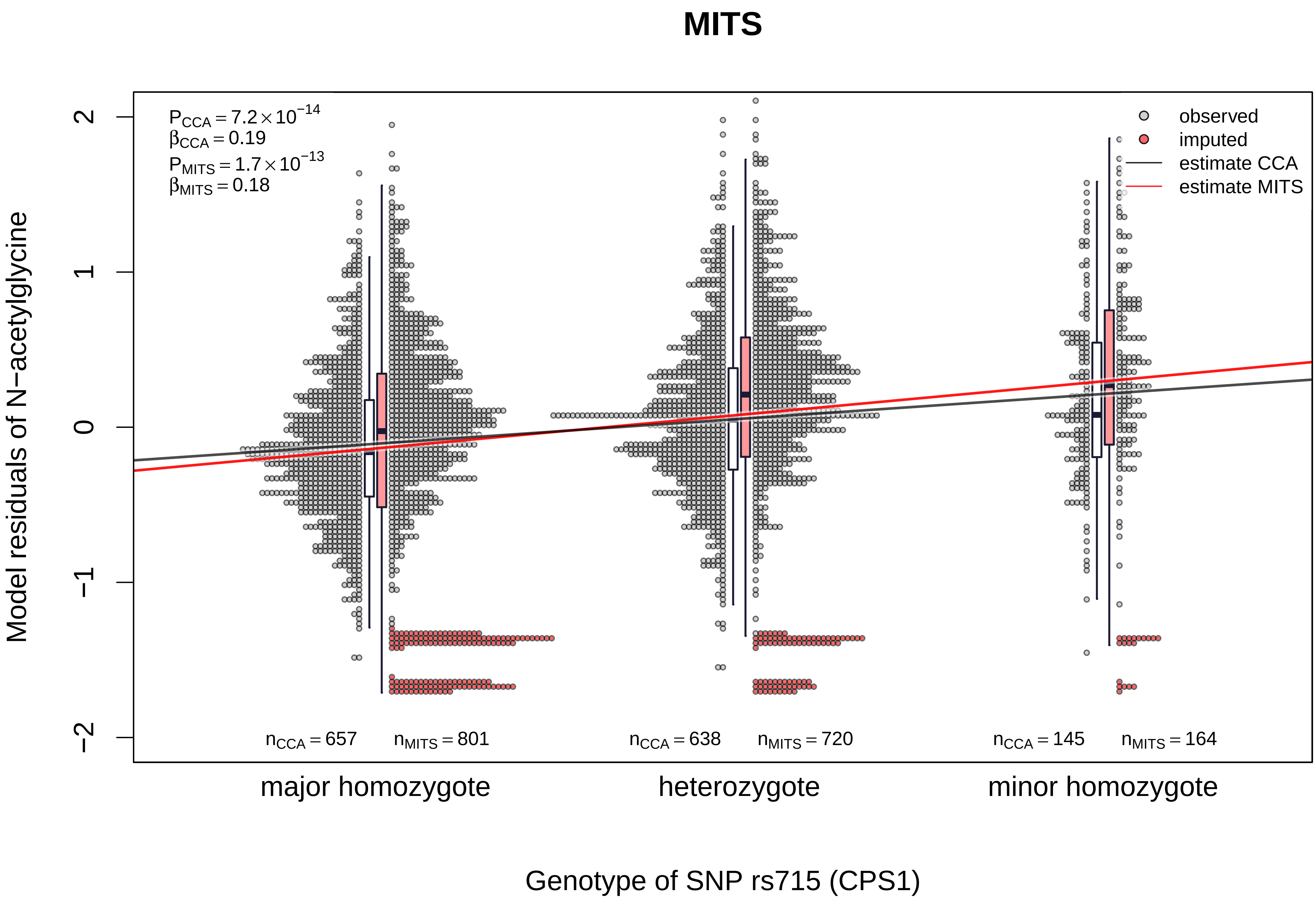

MITS
$P_{CCA} = 7.2 \times 10^{-14}$
$\beta_{CCA} = 0.19$
$P_{MITS} = 1.7 \times 10^{-13}$
$\beta_{MITS} = 0.18$
observed
imputed
estimate CCA
estimate MITS
$n_{CCA} = 657$
$n_{MITS} = 801$
$n_{CCA} = 638$
$n_{MITS} = 720$
$n_{CCA} = 145$
$n_{MITS} = 164$
2
1
0
−1
−2
Model residuals of N−acetylglycine
major homozygote
heterozygote
minor homozygote
Genotype of SNP rs715 (CPS1)

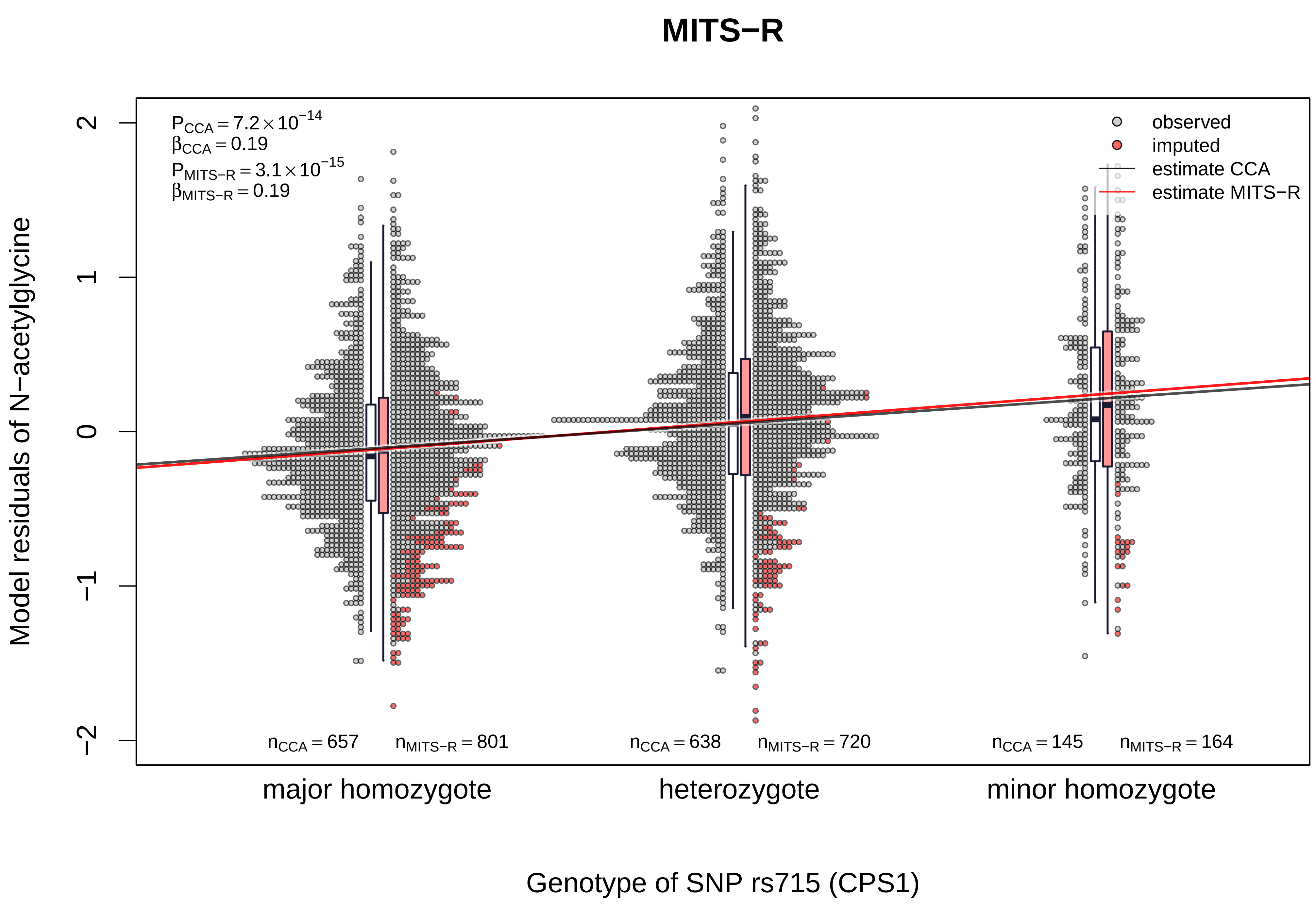

MITS−R
$P_{CCA} = 7.2 \times 10^{-14}$
$\beta_{CCA} = 0.19$
$P_{MITS-R} = 3.1 \times 10^{-15}$
$\beta_{MITS-R} = 0.19$
observed
imputed
estimate CCA
estimate MITS−R
Model residuals of N−acetylglycine
2
1
0
−1
−2
$n_{CCA} = 657$
$n_{MITS-R} = 801$
$n_{CCA} = 638$
$n_{MITS-R} = 720$
$n_{CCA} = 145$
$n_{MITS-R} = 164$
major homozygote
heterozygote
minor homozygote
Genotype of SNP rs715 (CPS1)

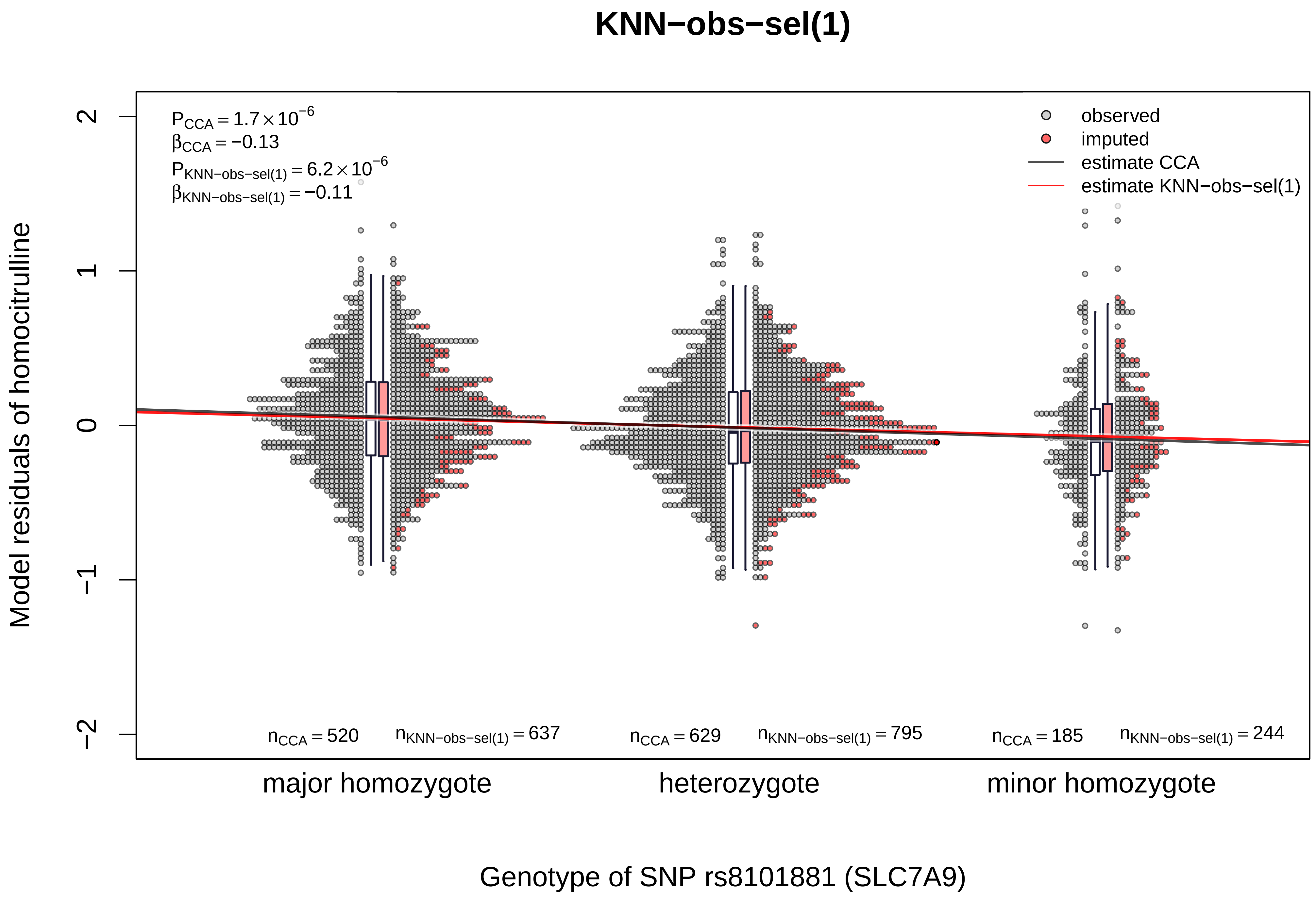
KNN−obs−sel(1)
$P_{CCA} = 1.7 \times 10^{-6}$
$\beta_{CCA} = -0.13$
$P_{KNN-obs-sel(1)} = 6.2 \times 10^{-6}$
$\beta_{KNN-obs-sel(1)} = -0.11$
observed
imputed
estimate CCA
estimate KNN−obs−sel(1)
Model residuals of homocitrulline
2
1
0
−1
−2
$n_{CCA} = 520$
$n_{KNN-obs-sel(1)} = 637$
$n_{CCA} = 629$
$n_{KNN-obs-sel(1)} = 795$
$n_{CCA} = 185$
$n_{KNN-obs-sel(1)} = 244$
major homozygote
heterozygote
minor homozygote
Genotype of SNP rs8101881 (SLC7A9)

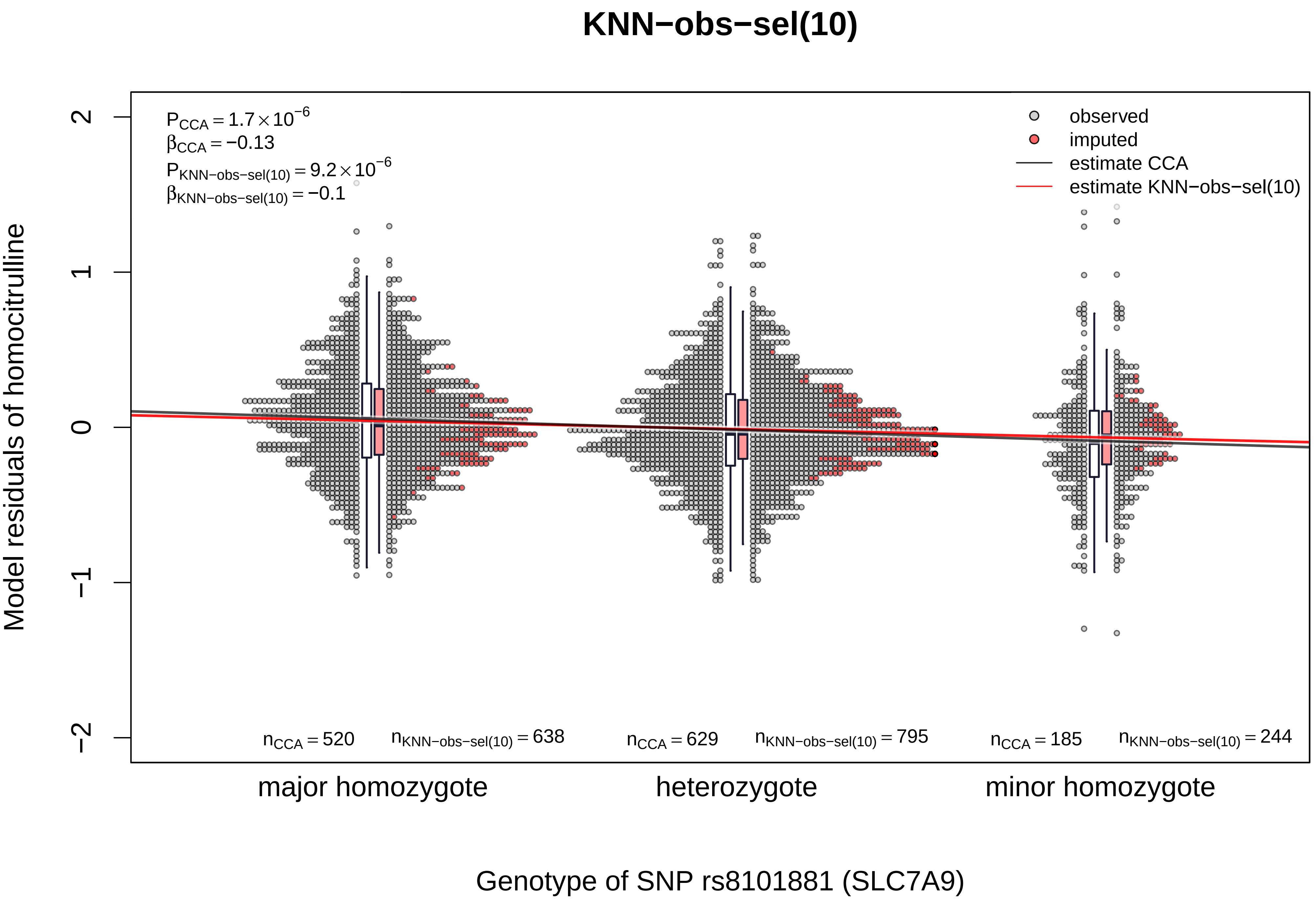

KNN−obs−sel(10)
$P_{CCA} = 1.7 \times 10^{-6}$
$\beta_{CCA} = -0.13$
$P_{KNN-obs-sel(10)} = 9.2 \times 10^{-6}$
$\beta_{KNN-obs-sel(10)} = -0.1$
observed
imputed
estimate CCA
estimate KNN−obs−sel(10)
Model residuals of homocitrulline
2
1
0
−1
−2
$n_{CCA} = 520$
$n_{KNN-obs-sel(10)} = 638$
$n_{CCA} = 629$
$n_{KNN-obs-sel(10)} = 795$
$n_{CCA} = 185$
$n_{KNN-obs-sel(10)} = 244$
major homozygote
heterozygote
minor homozygote
Genotype of SNP rs8101881 (SLC7A9)

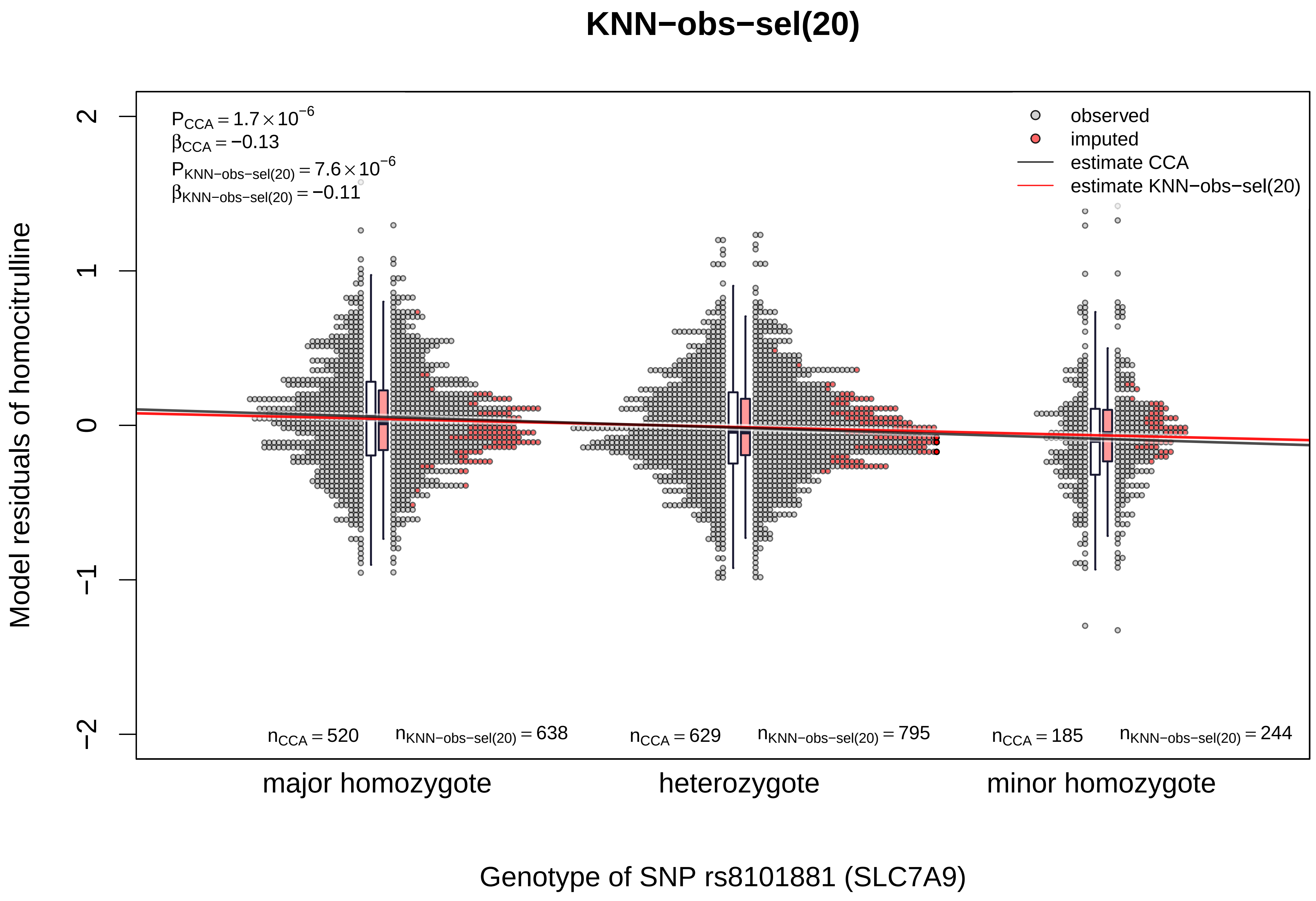
KNN−obs−sel(20)
$P_{CCA} = 1.7 \times 10^{-6}$
$\beta_{CCA} = -0.13$
$P_{KNN-obs-sel(20)} = 7.6 \times 10^{-6}$
$\beta_{KNN-obs-sel(20)} = -0.11$
observed
imputed
estimate CCA
estimate KNN−obs−sel(20)
2
1
0
−1
−2
Model residuals of homocitrulline
$n_{CCA} = 520$
$n_{KNN-obs-sel(20)} = 638$
$n_{CCA} = 629$
$n_{KNN-obs-sel(20)} = 795$
$n_{CCA} = 185$
$n_{KNN-obs-sel(20)} = 244$
major homozygote
heterozygote
minor homozygote
Genotype of SNP rs8101881 (SLC7A9)

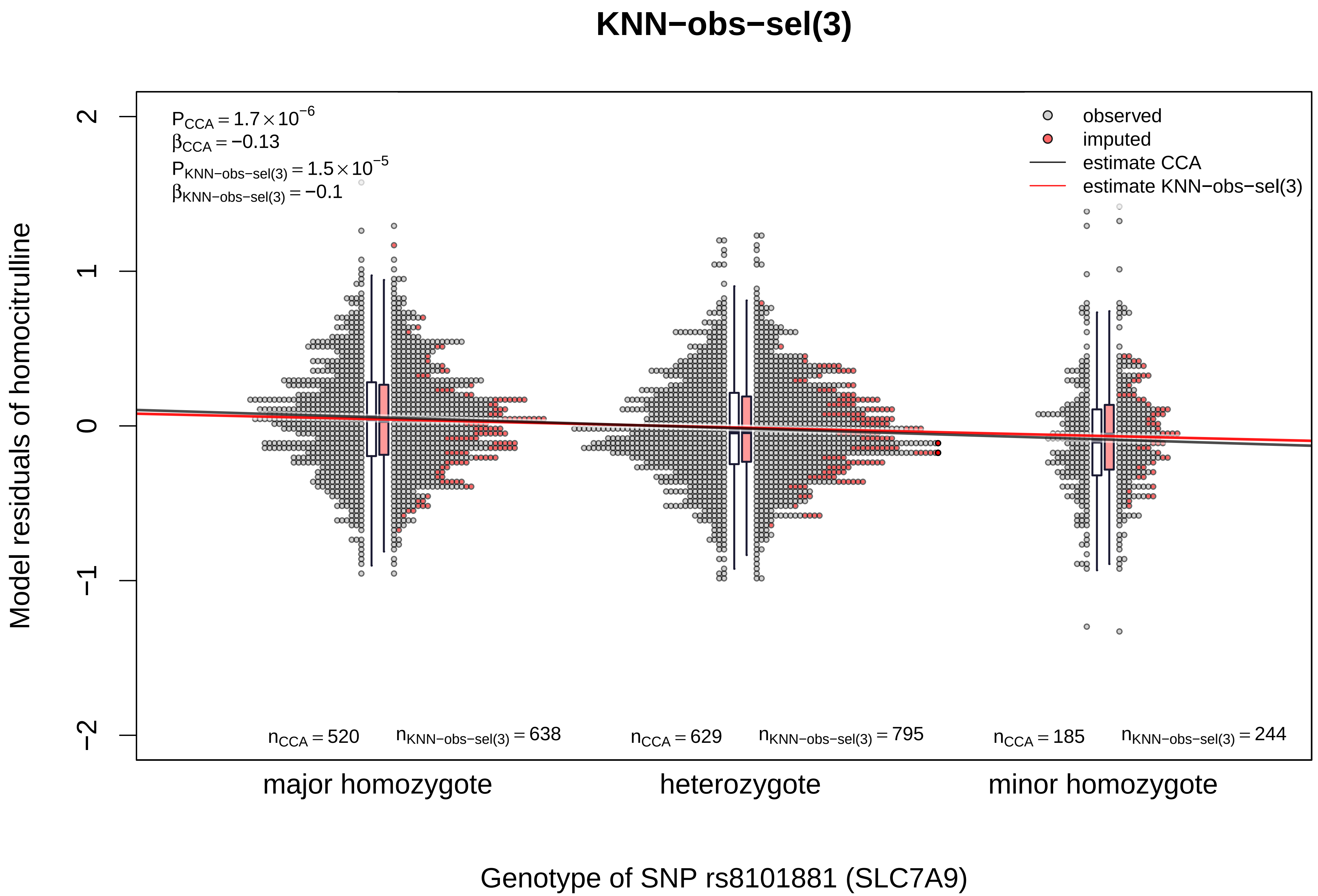

KNN−obs−sel(3)
$P_{CCA} = 1.7 \times 10^{-6}$
$\beta_{CCA} = -0.13$
$P_{KNN-obs-sel(3)} = 1.5 \times 10^{-5}$
$\beta_{KNN-obs-sel(3)} = -0.1$
observed
imputed
estimate CCA
estimate KNN−obs−sel(3)
Model residuals of homocitrulline
2
1
0
−1
−2
$n_{CCA} = 520$
$n_{KNN-obs-sel(3)} = 638$
$n_{CCA} = 629$
$n_{KNN-obs-sel(3)} = 795$
$n_{CCA} = 185$
$n_{KNN-obs-sel(3)} = 244$
major homozygote
heterozygote
minor homozygote
Genotype of SNP rs8101881 (SLC7A9)

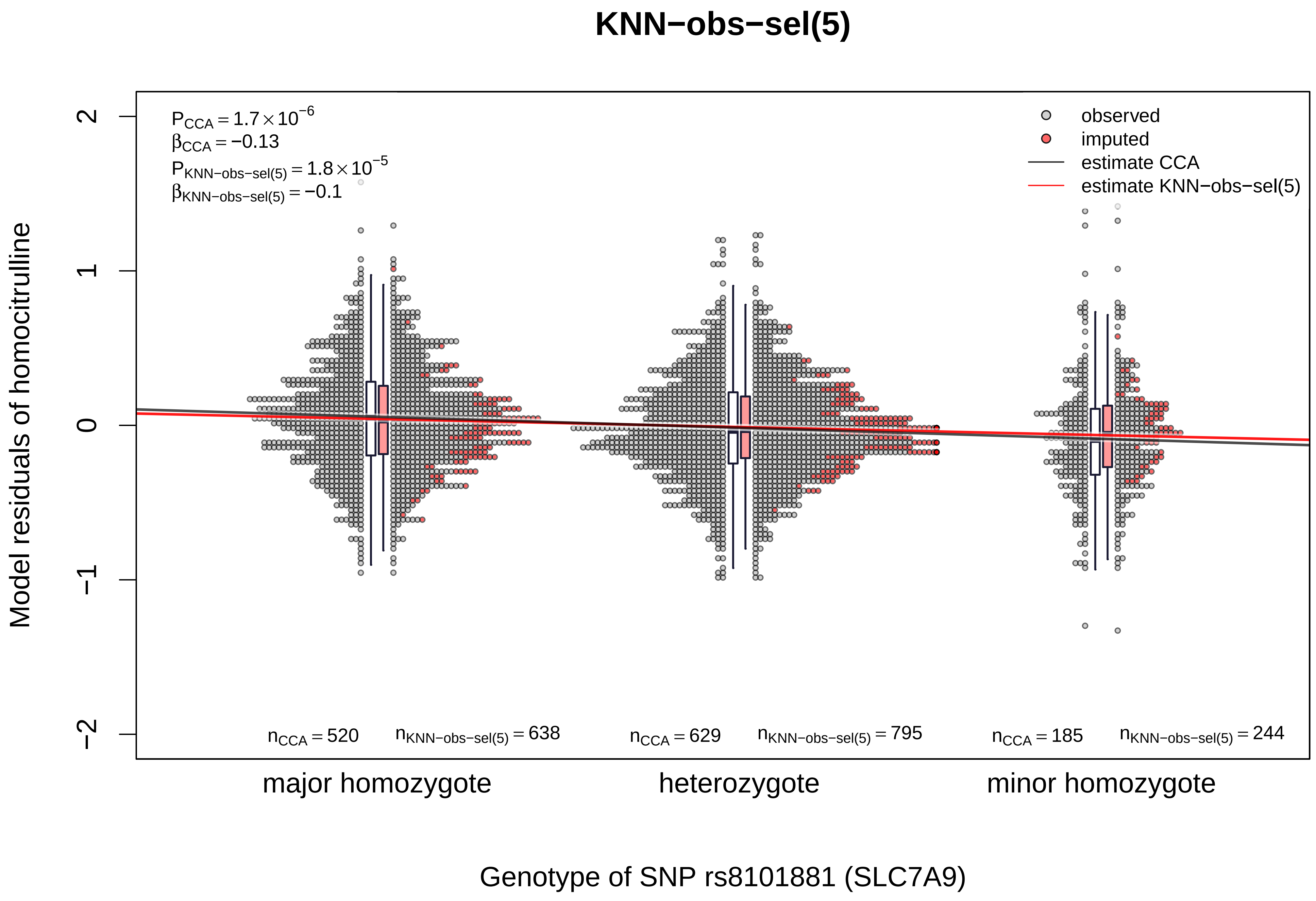

KNN−obs−sel(5)
$P_{CCA} = 1.7 \times 10^{-6}$
$\beta_{CCA} = -0.13$
$P_{KNN-obs-sel(5)} = 1.8 \times 10^{-5}$
$\beta_{KNN-obs-sel(5)} = -0.1$
observed
imputed
estimate CCA
estimate KNN−obs−sel(5)
Model residuals of homocitrulline
2
1
0
−1
−2
$n_{CCA} = 520$
$n_{KNN-obs-sel(5)} = 638$
$n_{CCA} = 629$
$n_{KNN-obs-sel(5)} = 795$
$n_{CCA} = 185$
$n_{KNN-obs-sel(5)} = 244$
major homozygote
heterozygote
minor homozygote
Genotype of SNP rs8101881 (SLC7A9)

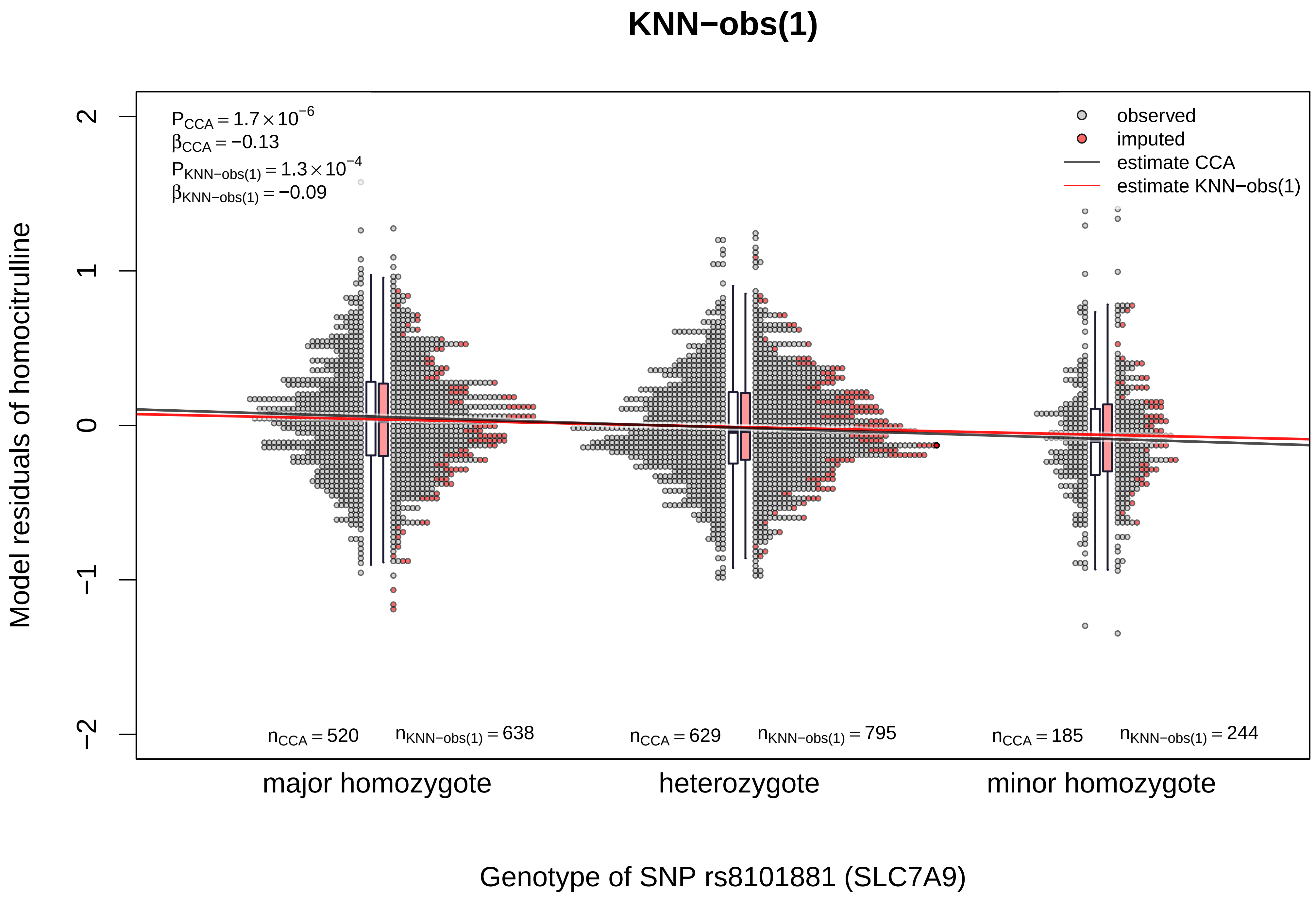

KNN−obs(1)
$P_{CCA} = 1.7 \times 10^{-6}$
$\beta_{CCA} = -0.13$
$P_{KNN-obs(1)} = 1.3 \times 10^{-4}$
$\beta_{KNN-obs(1)} = -0.09$
observed
imputed
estimate CCA
estimate KNN−obs(1)
Model residuals of homocitrulline
2
1
0
−1
−2
$n_{CCA} = 520$
$n_{KNN-obs(1)} = 638$
$n_{CCA} = 629$
$n_{KNN-obs(1)} = 795$
$n_{CCA} = 185$
$n_{KNN-obs(1)} = 244$
major homozygote
heterozygote
minor homozygote
Genotype of SNP rs8101881 (SLC7A9)

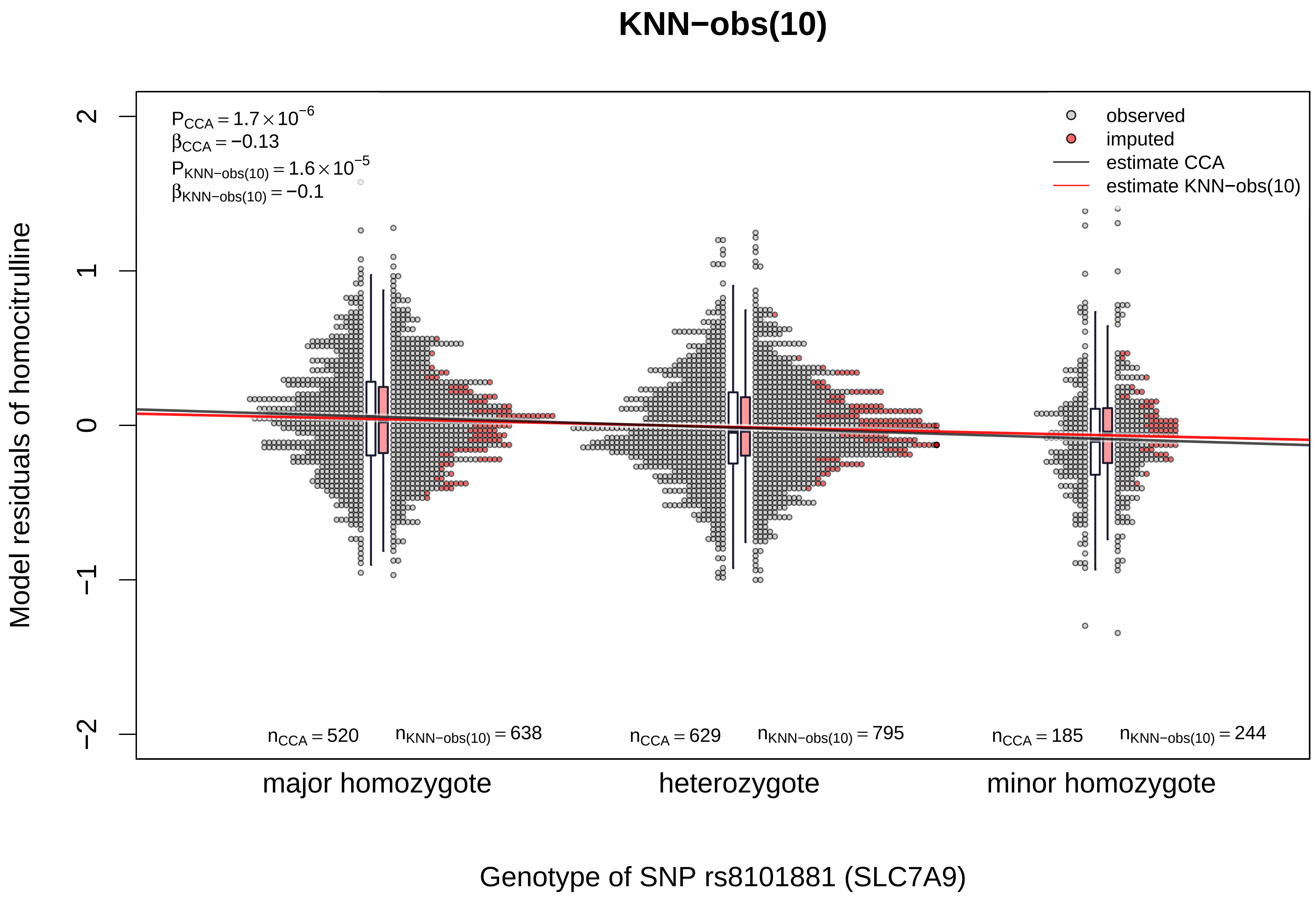

KNN−obs(10)
$P_{CCA} = 1.7 \times 10^{-6}$
$\beta_{CCA} = -0.13$
$P_{KNN-obs(10)} = 1.6 \times 10^{-5}$
$\beta_{KNN-obs(10)} = -0.1$
observed
imputed
estimate CCA
estimate KNN−obs(10)
Model residuals of homocitrulline
2
1
0
−1
−2
$n_{CCA} = 520$
$n_{KNN-obs(10)} = 638$
$n_{CCA} = 629$
$n_{KNN-obs(10)} = 795$
$n_{CCA} = 185$
$n_{KNN-obs(10)} = 244$
major homozygote
heterozygote
minor homozygote
Genotype of SNP rs8101881 (SLC7A9)

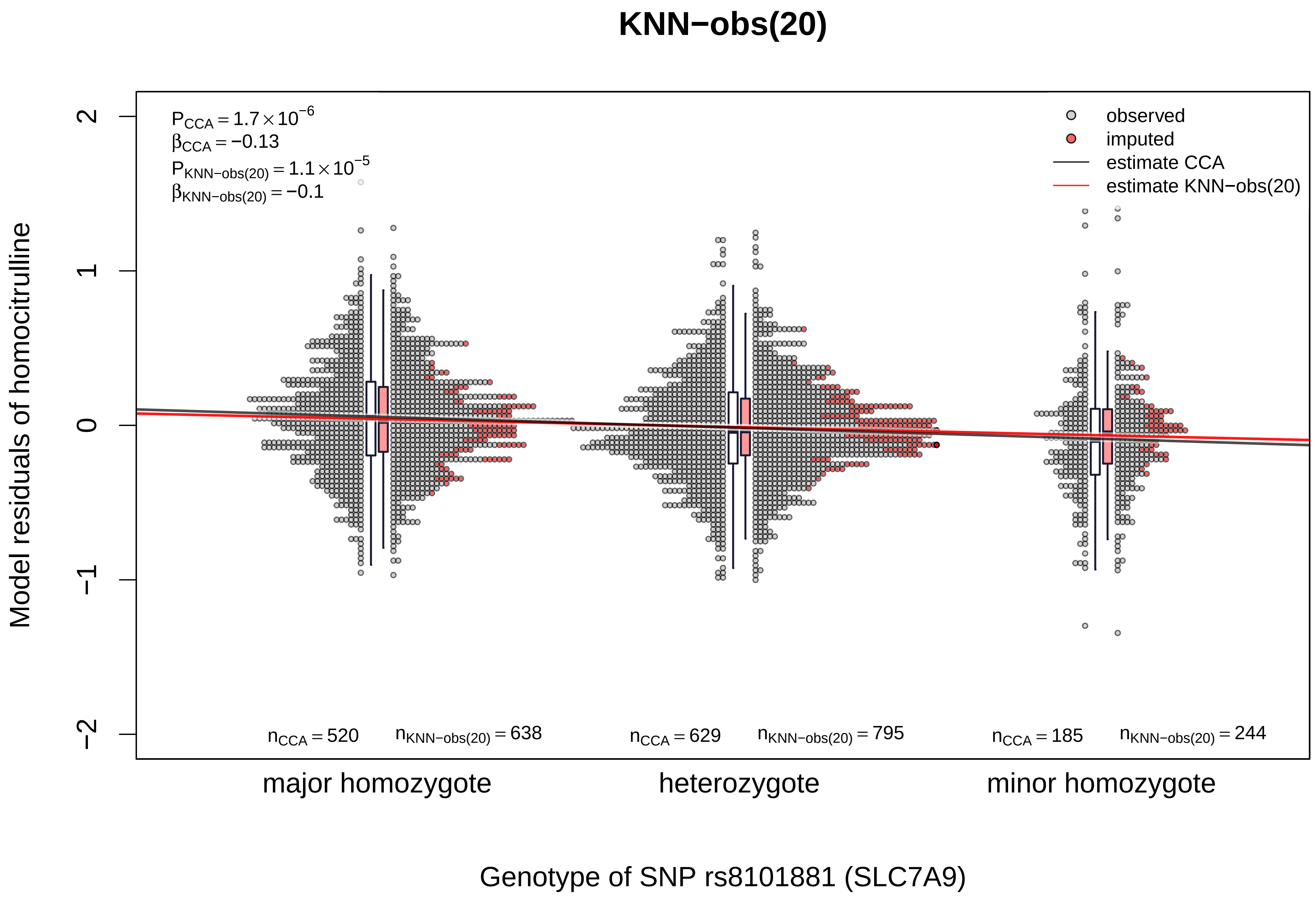

KNN−obs(20)
$P_{CCA} = 1.7 \times 10^{-6}$
$\beta_{CCA} = -0.13$
$P_{KNN-obs(20)} = 1.1 \times 10^{-5}$
$\beta_{KNN-obs(20)} = -0.1$
observed
imputed
estimate CCA
estimate KNN−obs(20)
Model residuals of homocitrulline
2
1
0
−1
−2
$n_{CCA} = 520$
$n_{KNN-obs(20)} = 638$
$n_{CCA} = 629$
$n_{KNN-obs(20)} = 795$
$n_{CCA} = 185$
$n_{KNN-obs(20)} = 244$
major homozygote
heterozygote
minor homozygote
Genotype of SNP rs8101881 (SLC7A9)

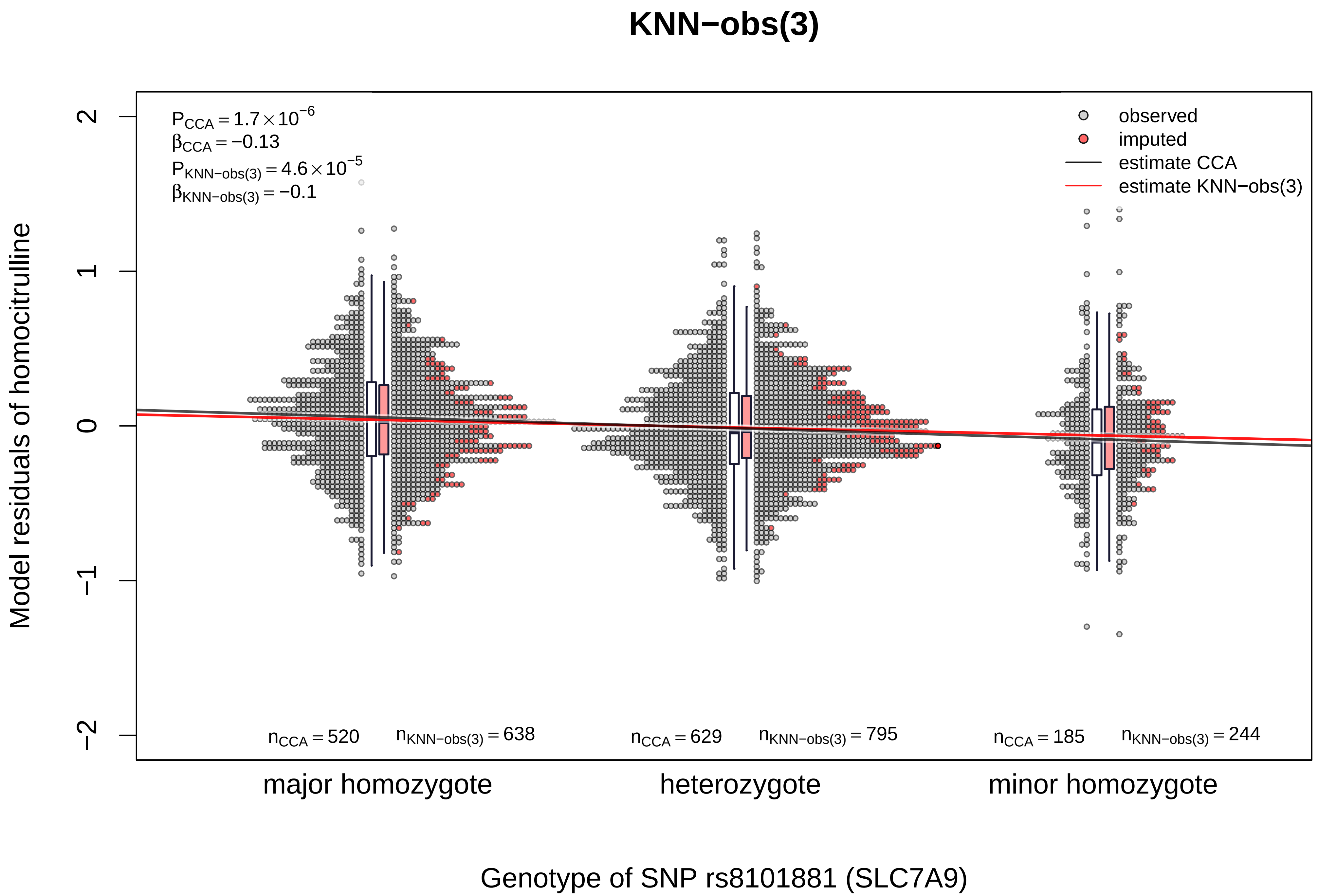
KNN−obs(3)
$P_{CCA} = 1.7 \times 10^{-6}$
$\beta_{CCA} = -0.13$
$P_{KNN-obs(3)} = 4.6 \times 10^{-5}$
$\beta_{KNN-obs(3)} = -0.1$
observed
imputed
estimate CCA
estimate KNN−obs(3)
$n_{CCA} = 520$
$n_{KNN-obs(3)} = 638$
$n_{CCA} = 629$
$n_{KNN-obs(3)} = 795$
$n_{CCA} = 185$
$n_{KNN-obs(3)} = 244$
2
1
0
−1
−2
major homozygote
heterozygote
minor homozygote
Model residuals of homocitrulline
Genotype of SNP rs8101881 (SLC7A9)

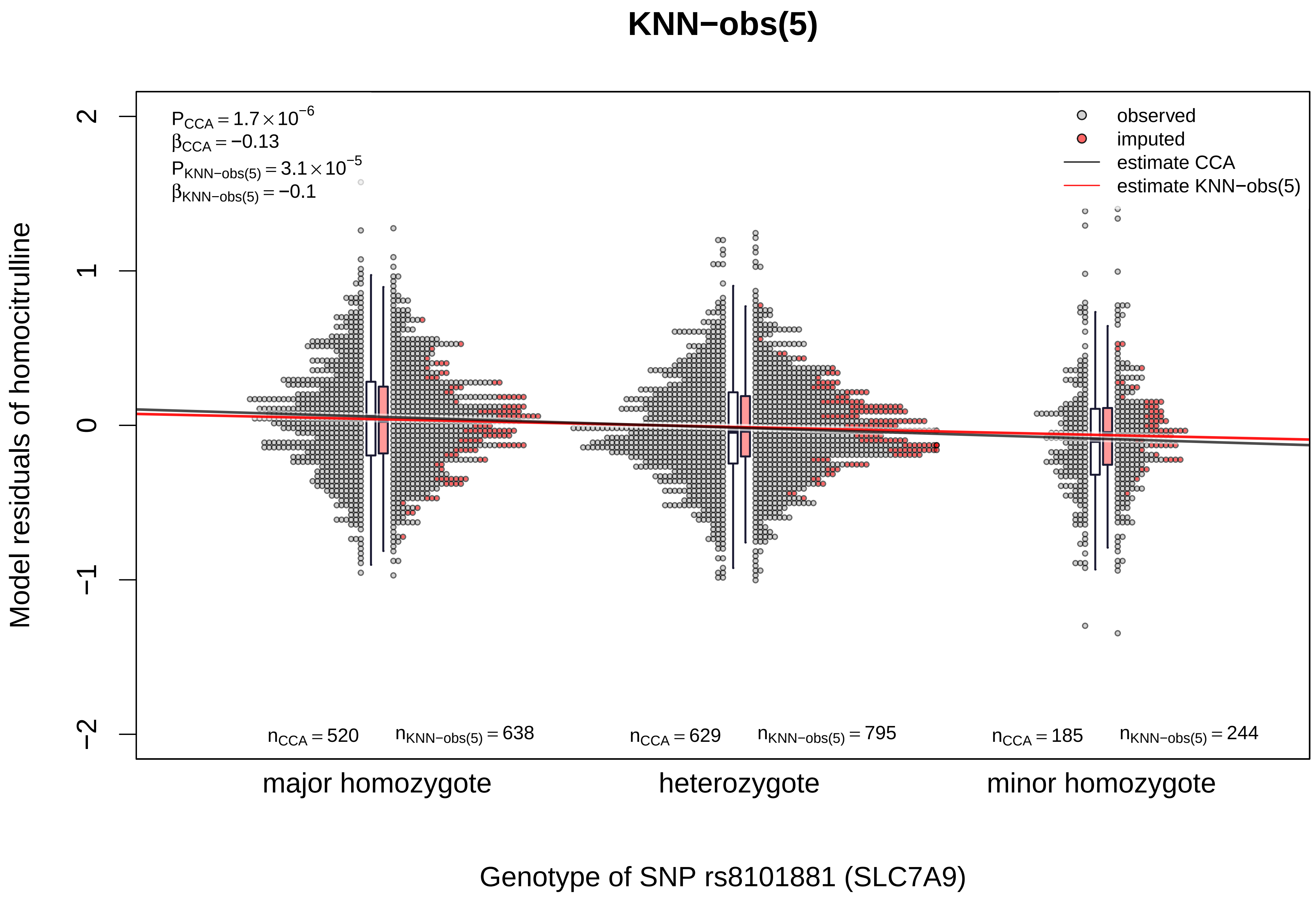

KNN−obs(5)
$P_{CCA} = 1.7 \times 10^{-6}$
$\beta_{CCA} = -0.13$
$P_{KNN-obs(5)} = 3.1 \times 10^{-5}$
$\beta_{KNN-obs(5)} = -0.1$
observed
imputed
estimate CCA
estimate KNN−obs(5)
2
1
0
−1
−2
Model residuals of homocitrulline
$n_{CCA} = 520$
$n_{KNN-obs(5)} = 638$
$n_{CCA} = 629$
$n_{KNN-obs(5)} = 795$
$n_{CCA} = 185$
$n_{KNN-obs(5)} = 244$
major homozygote
heterozygote
minor homozygote
Genotype of SNP rs8101881 (SLC7A9)

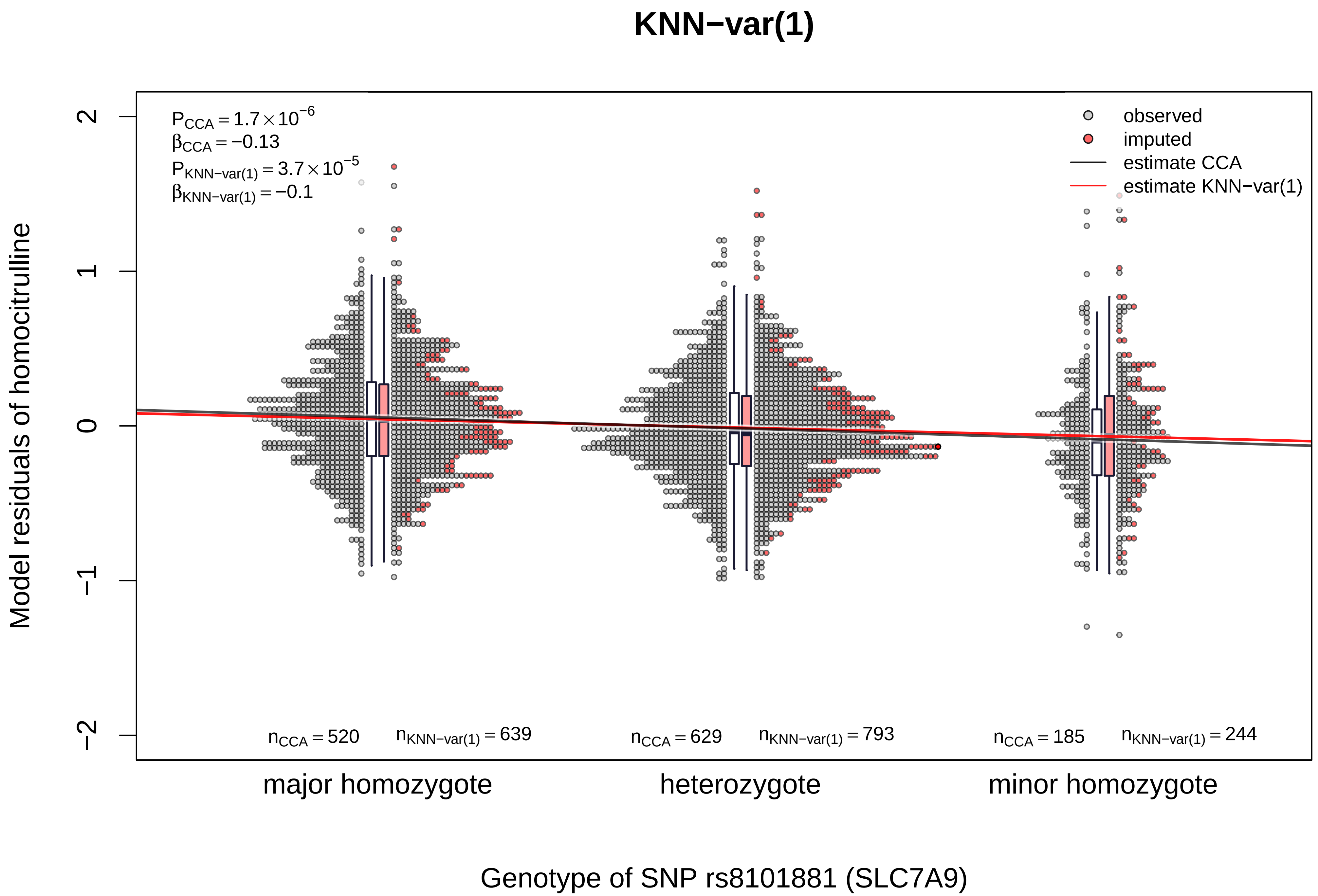

KNN−var(1)
$P_{CCA} = 1.7 \times 10^{-6}$
$\beta_{CCA} = -0.13$
$P_{KNN-var(1)} = 3.7 \times 10^{-5}$
$\beta_{KNN-var(1)} = -0.1$
observed
imputed
estimate CCA
estimate KNN−var(1)
Model residuals of homocitrulline
2
1
0
−1
−2
$n_{CCA} = 520$
$n_{KNN-var(1)} = 639$
$n_{CCA} = 629$
$n_{KNN-var(1)} = 793$
$n_{CCA} = 185$
$n_{KNN-var(1)} = 244$
major homozygote
heterozygote
minor homozygote
Genotype of SNP rs8101881 (SLC7A9)

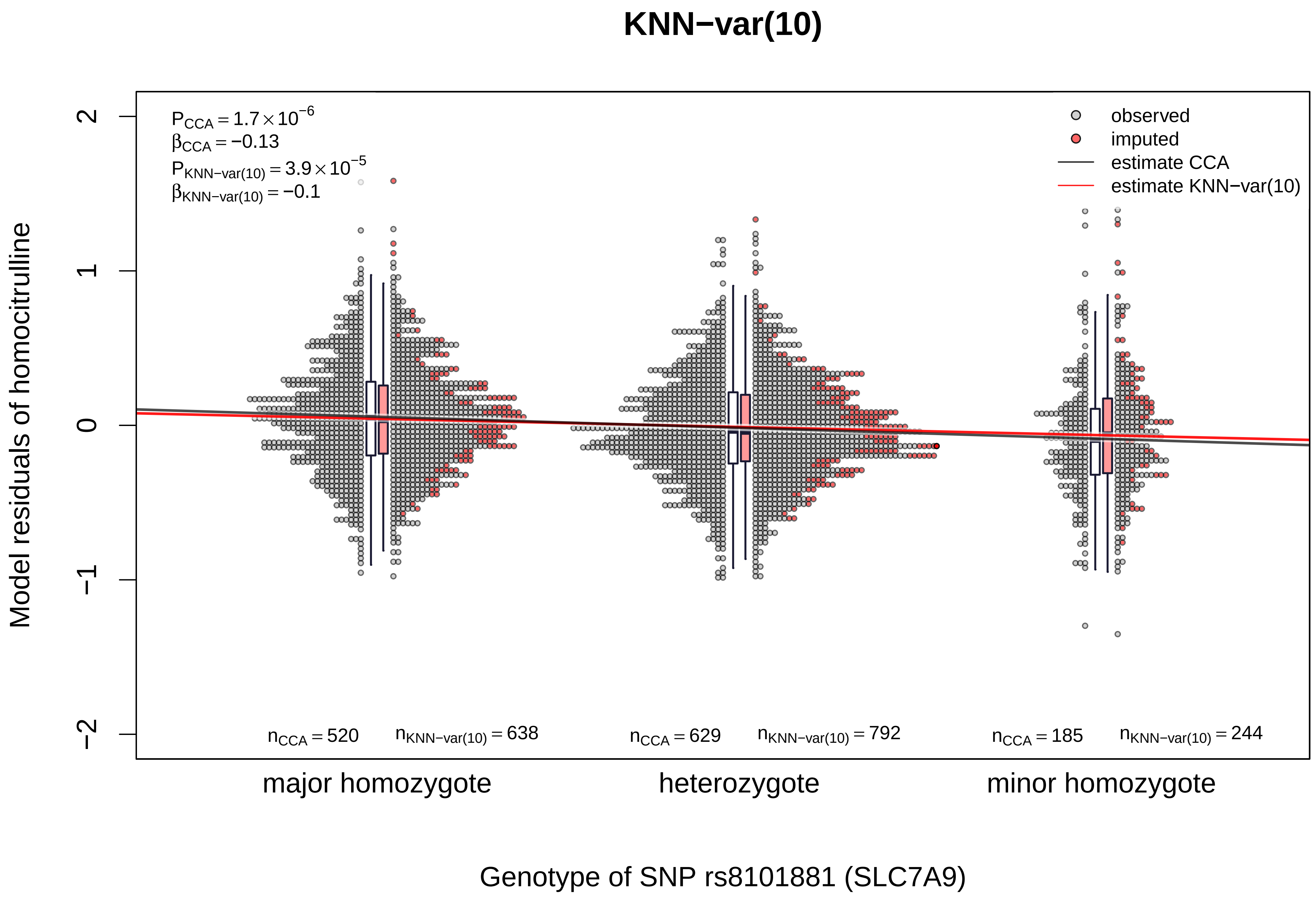

KNN−var(10)
$P_{CCA} = 1.7 \times 10^{-6}$
$\beta_{CCA} = -0.13$
$P_{KNN-var(10)} = 3.9 \times 10^{-5}$
$\beta_{KNN-var(10)} = -0.1$
observed
imputed
estimate CCA
estimate KNN−var(10)
Model residuals of homocitrulline
2
1
0
−1
−2
$n_{CCA} = 520$
$n_{KNN-var(10)} = 638$
$n_{CCA} = 629$
$n_{KNN-var(10)} = 792$
$n_{CCA} = 185$
$n_{KNN-var(10)} = 244$
major homozygote
heterozygote
minor homozygote
Genotype of SNP rs8101881 (SLC7A9)

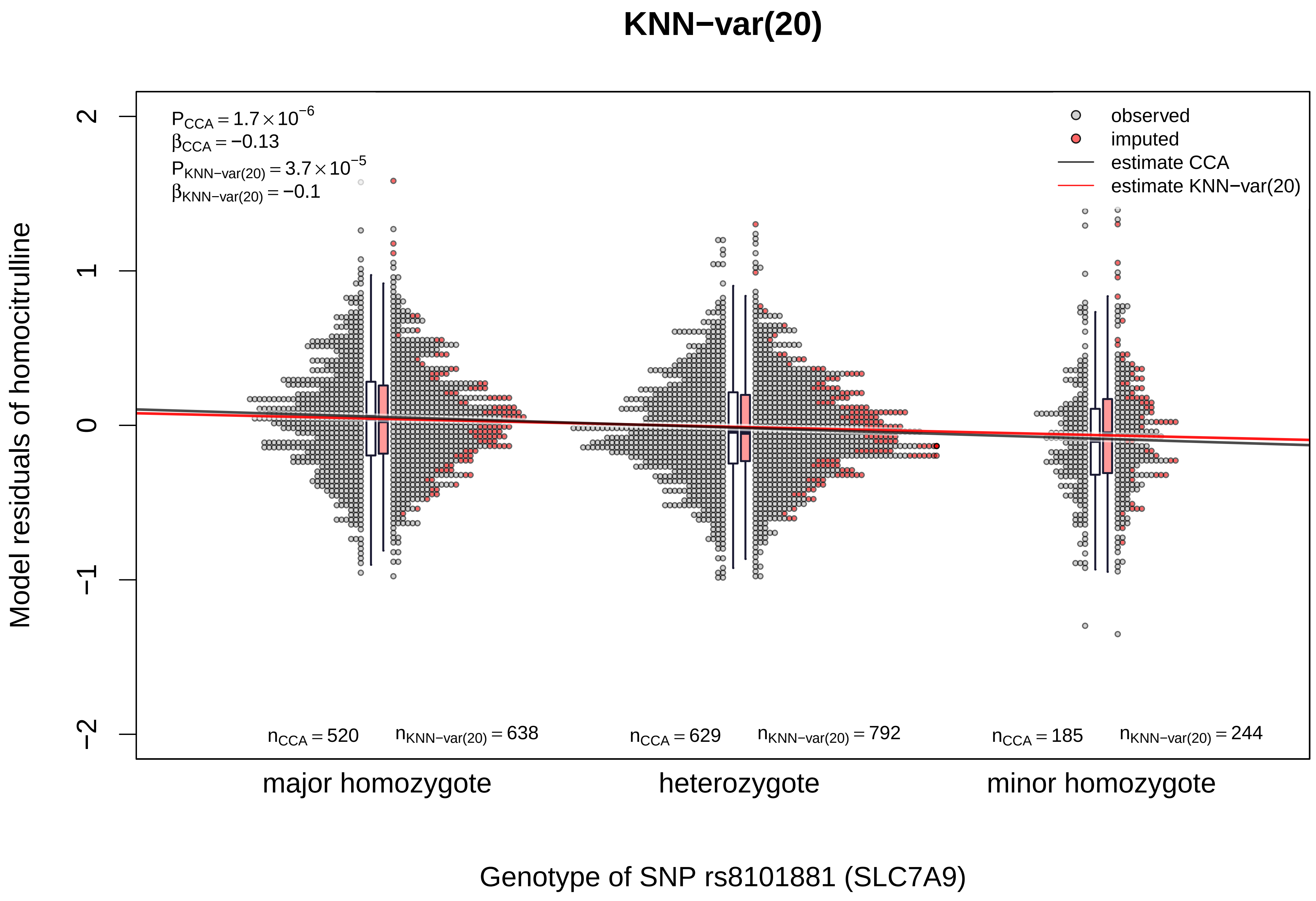

KNN−var(20)
$P_{CCA} = 1.7 \times 10^{-6}$
$\beta_{CCA} = -0.13$
$P_{KNN-var(20)} = 3.7 \times 10^{-5}$
$\beta_{KNN-var(20)} = -0.1$
observed
imputed
estimate CCA
estimate KNN−var(20)
Model residuals of homocitrulline
2
1
0
−1
−2
$n_{CCA} = 520$
$n_{KNN-var(20)} = 638$
$n_{CCA} = 629$
$n_{KNN-var(20)} = 792$
$n_{CCA} = 185$
$n_{KNN-var(20)} = 244$
major homozygote
heterozygote
minor homozygote
Genotype of SNP rs8101881 (SLC7A9)

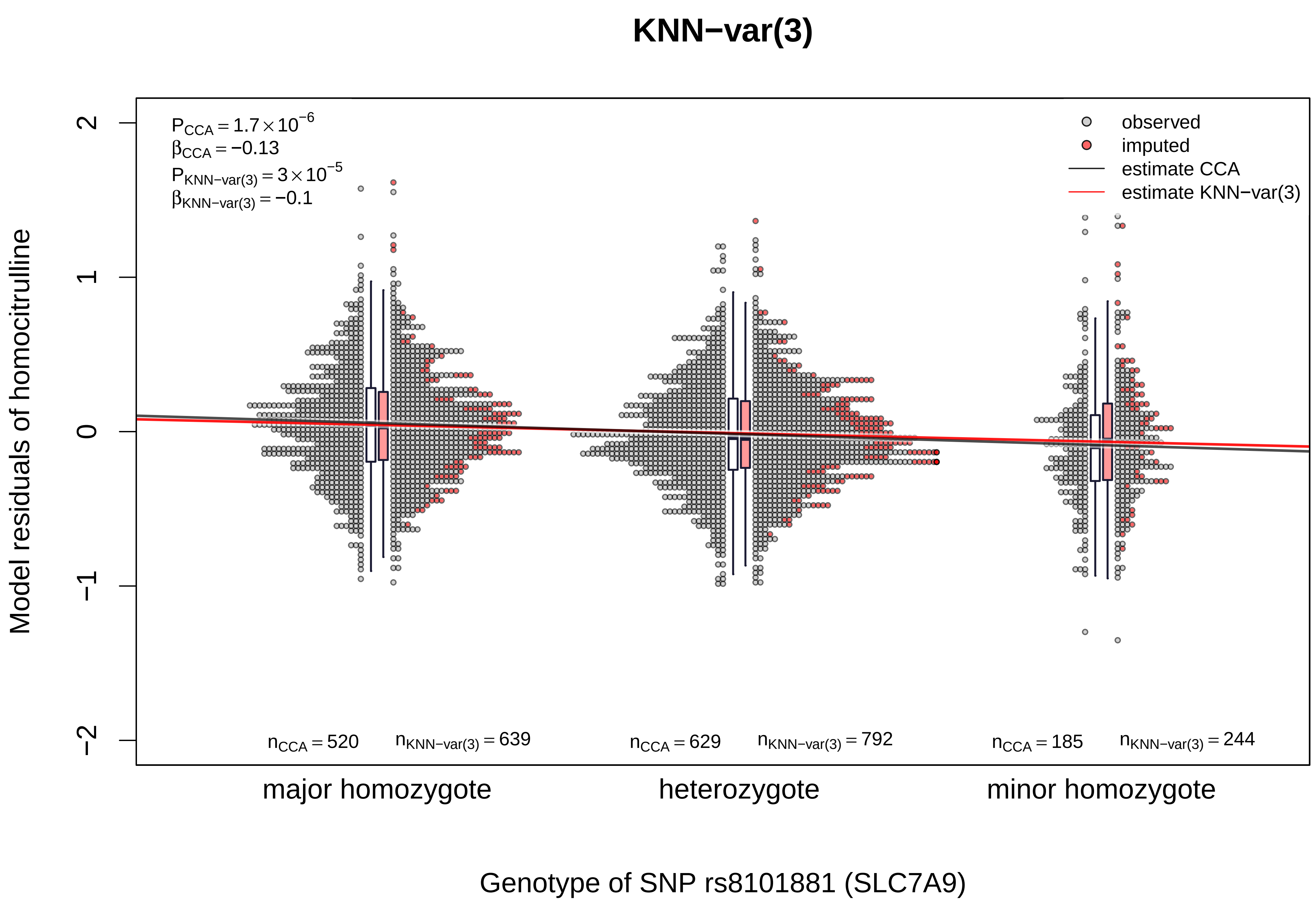
KNN−var(3)
$P_{CCA} = 1.7 \times 10^{-6}$
$\beta_{CCA} = -0.13$
$P_{KNN-var(3)} = 3 \times 10^{-5}$
$\beta_{KNN-var(3)} = -0.1$
observed
imputed
estimate CCA
estimate KNN−var(3)
Model residuals of homocitrulline
2
1
0
−1
−2
$n_{CCA} = 520$
$n_{KNN-var(3)} = 639$
$n_{CCA} = 629$
$n_{KNN-var(3)} = 792$
$n_{CCA} = 185$
$n_{KNN-var(3)} = 244$
major homozygote
heterozygote
minor homozygote
Genotype of SNP rs8101881 (SLC7A9)

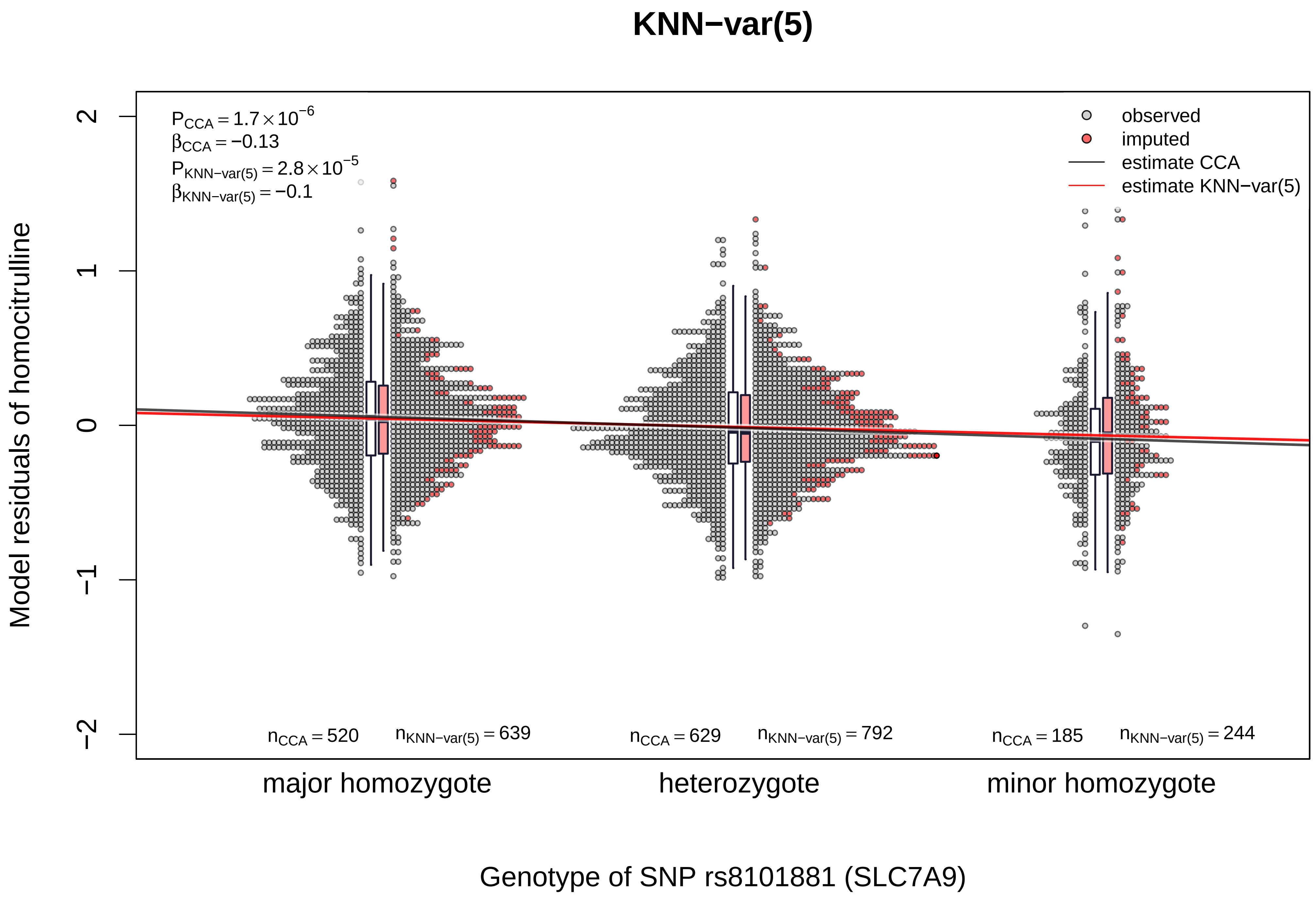

KNN−var(5)
$P_{CCA} = 1.7 \times 10^{-6}$
$\beta_{CCA} = -0.13$
$P_{KNN-var(5)} = 2.8 \times 10^{-5}$
$\beta_{KNN-var(5)} = -0.1$
observed
imputed
estimate CCA
estimate KNN−var(5)
Model residuals of homocitrulline
2
1
0
−1
−2
$n_{CCA} = 520$
$n_{KNN-var(5)} = 639$
$n_{CCA} = 629$
$n_{KNN-var(5)} = 792$
$n_{CCA} = 185$
$n_{KNN-var(5)} = 244$
major homozygote
heterozygote
minor homozygote
Genotype of SNP rs8101881 (SLC7A9)

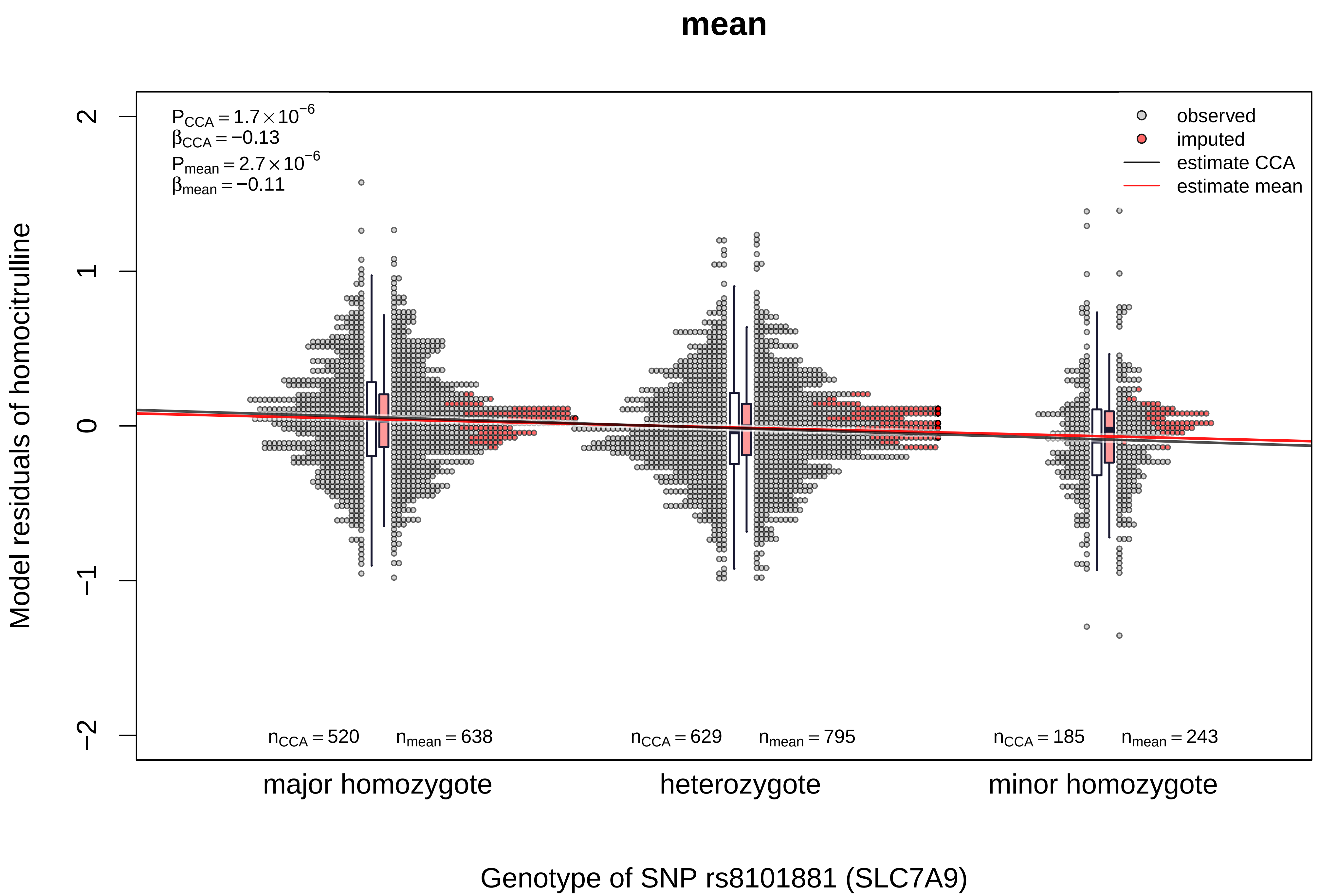

mean
$P_{CCA} = 1.7 \times 10^{-6}$
$\beta_{CCA} = -0.13$
$P_{mean} = 2.7 \times 10^{-6}$
$\beta_{mean} = -0.11$
observed
imputed
estimate CCA
estimate mean
Model residuals of homocitrulline
2
1
0
−1
−2
$n_{CCA} = 520$
$n_{mean} = 638$
$n_{CCA} = 629$
$n_{mean} = 795$
$n_{CCA} = 185$
$n_{mean} = 243$
major homozygote
heterozygote
minor homozygote
Genotype of SNP rs8101881 (SLC7A9)

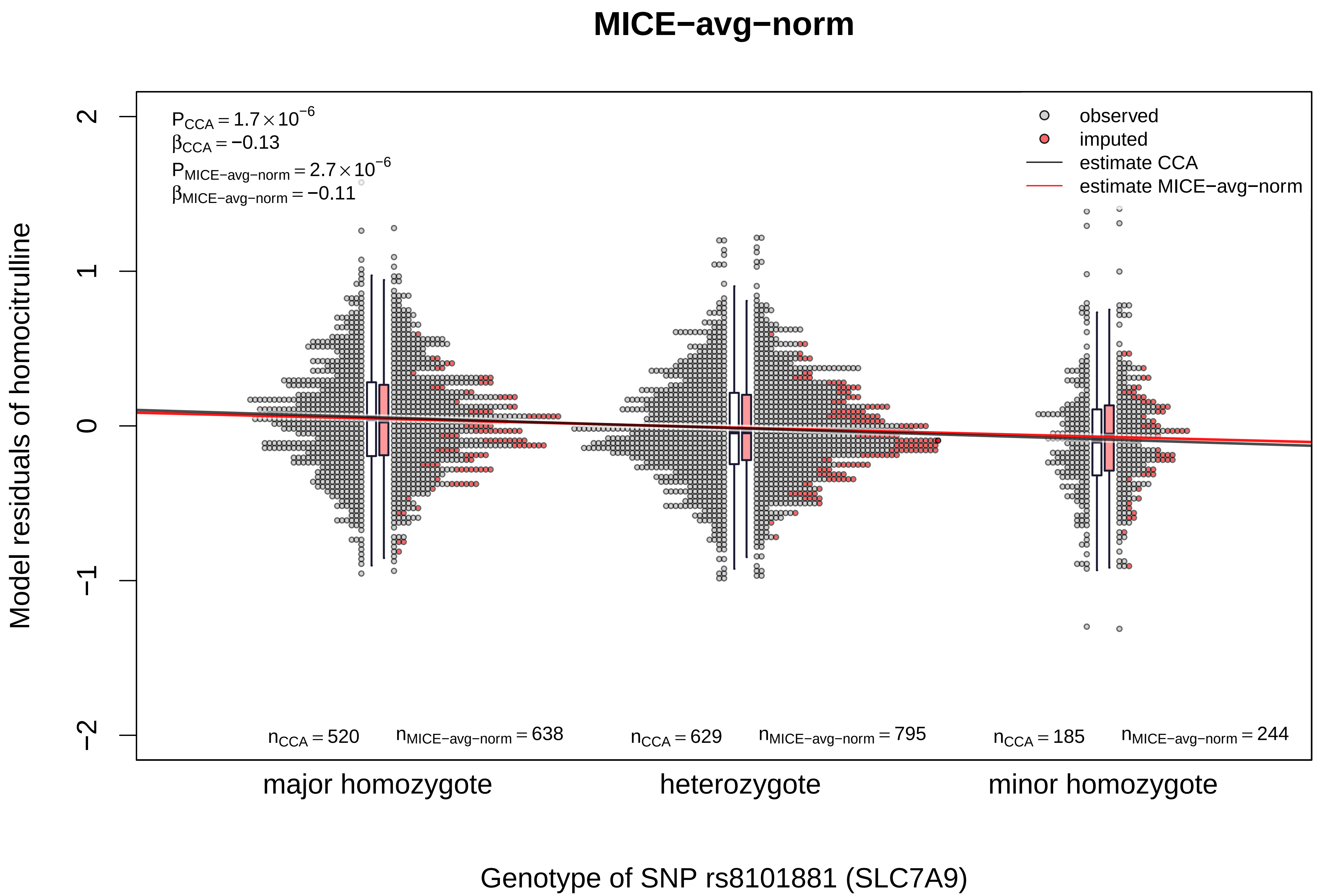

MICE−avg−norm
$P_{CCA} = 1.7 \times 10^{-6}$
$\beta_{CCA} = -0.13$
$P_{MICE-avg-norm} = 2.7 \times 10^{-6}$
$\beta_{MICE-avg-norm} = -0.11$
observed
imputed
estimate CCA
estimate MICE−avg−norm
$n_{CCA} = 520$
$n_{MICE-avg-norm} = 638$
$n_{CCA} = 629$
$n_{MICE-avg-norm} = 795$
$n_{CCA} = 185$
$n_{MICE-avg-norm} = 244$
Model residuals of homocitrulline
2
1
0
−1
−2
major homozygote
heterozygote
minor homozygote
Genotype of SNP rs8101881 (SLC7A9)

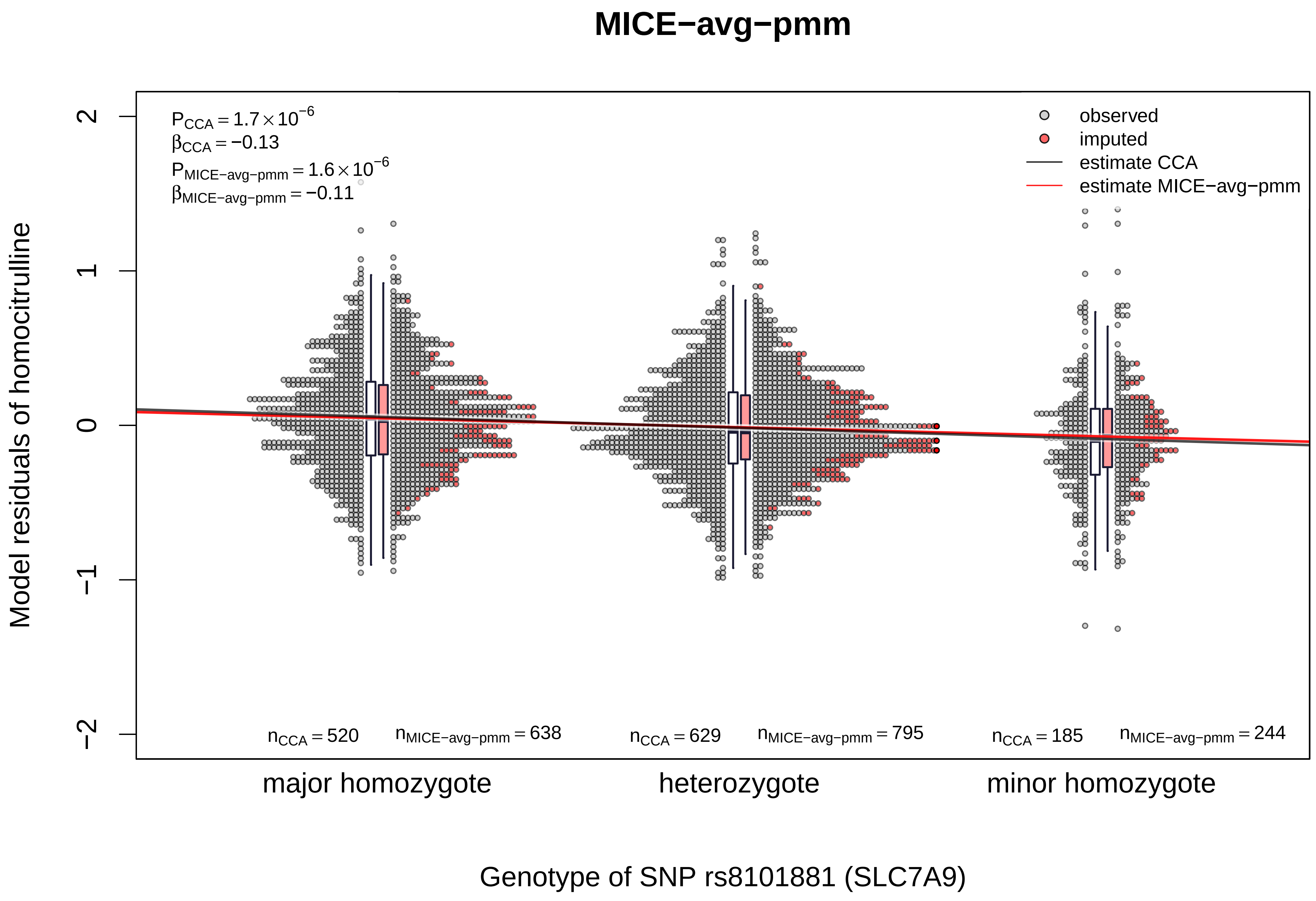

MICE−avg−pmm
$P_{CCA} = 1.7 \times 10^{-6}$
$\beta_{CCA} = -0.13$
$P_{MICE-avg-pmm} = 1.6 \times 10^{-6}$
$\beta_{MICE-avg-pmm} = -0.11$
observed
imputed
estimate CCA
estimate MICE−avg−pmm
Model residuals of homocitrulline
2
1
0
−1
−2
$n_{CCA} = 520$
$n_{MICE-avg-pmm} = 638$
$n_{CCA} = 629$
$n_{MICE-avg-pmm} = 795$
$n_{CCA} = 185$
$n_{MICE-avg-pmm} = 244$
major homozygote
heterozygote
minor homozygote
Genotype of SNP rs8101881 (SLC7A9)

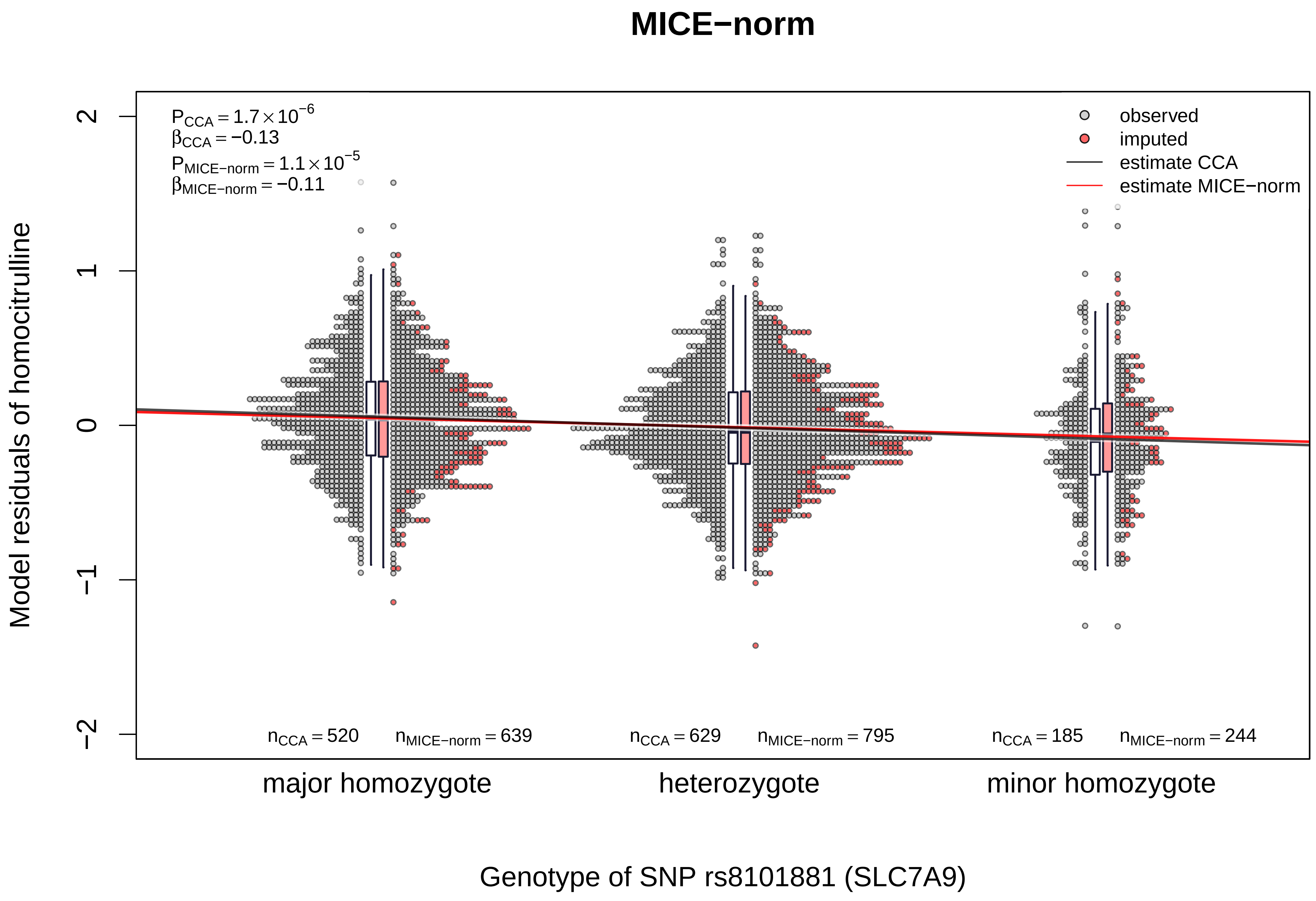
MICE−norm
$P_{CCA} = 1.7 \times 10^{-6}$
$\beta_{CCA} = -0.13$
$P_{MICE-norm} = 1.1 \times 10^{-5}$
$\beta_{MICE-norm} = -0.11$
observed
imputed
estimate CCA
estimate MICE−norm
Model residuals of homocitrulline
2
1
0
−1
−2
$n_{CCA} = 520$
$n_{MICE-norm} = 639$
$n_{CCA} = 629$
$n_{MICE-norm} = 795$
$n_{CCA} = 185$
$n_{MICE-norm} = 244$
major homozygote
heterozygote
minor homozygote
Genotype of SNP rs8101881 (SLC7A9)

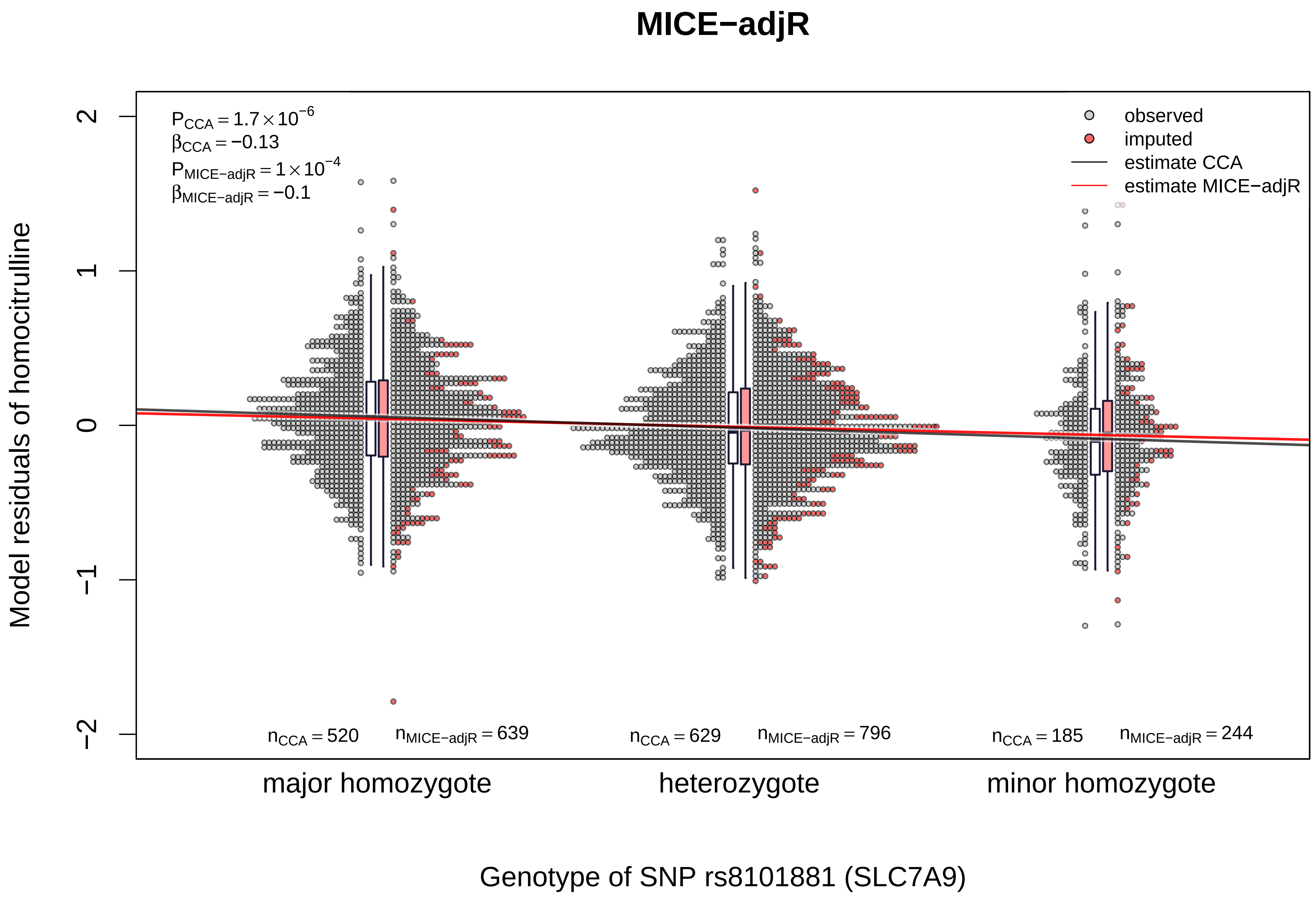

MICE−adjR
$P_{CCA} = 1.7 \times 10^{-6}$
$\beta_{CCA} = -0.13$
$P_{MICE-adjR} = 1 \times 10^{-4}$
$\beta_{MICE-adjR} = -0.1$
observed
imputed
estimate CCA
estimate MICE−adjR
2
1
0
−1
−2
Model residuals of homocitrulline
$n_{CCA} = 520$
$n_{MICE-adjR} = 639$
$n_{CCA} = 629$
$n_{MICE-adjR} = 796$
$n_{CCA} = 185$
$n_{MICE-adjR} = 244$
major homozygote
heterozygote
minor homozygote
Genotype of SNP rs8101881 (SLC7A9)

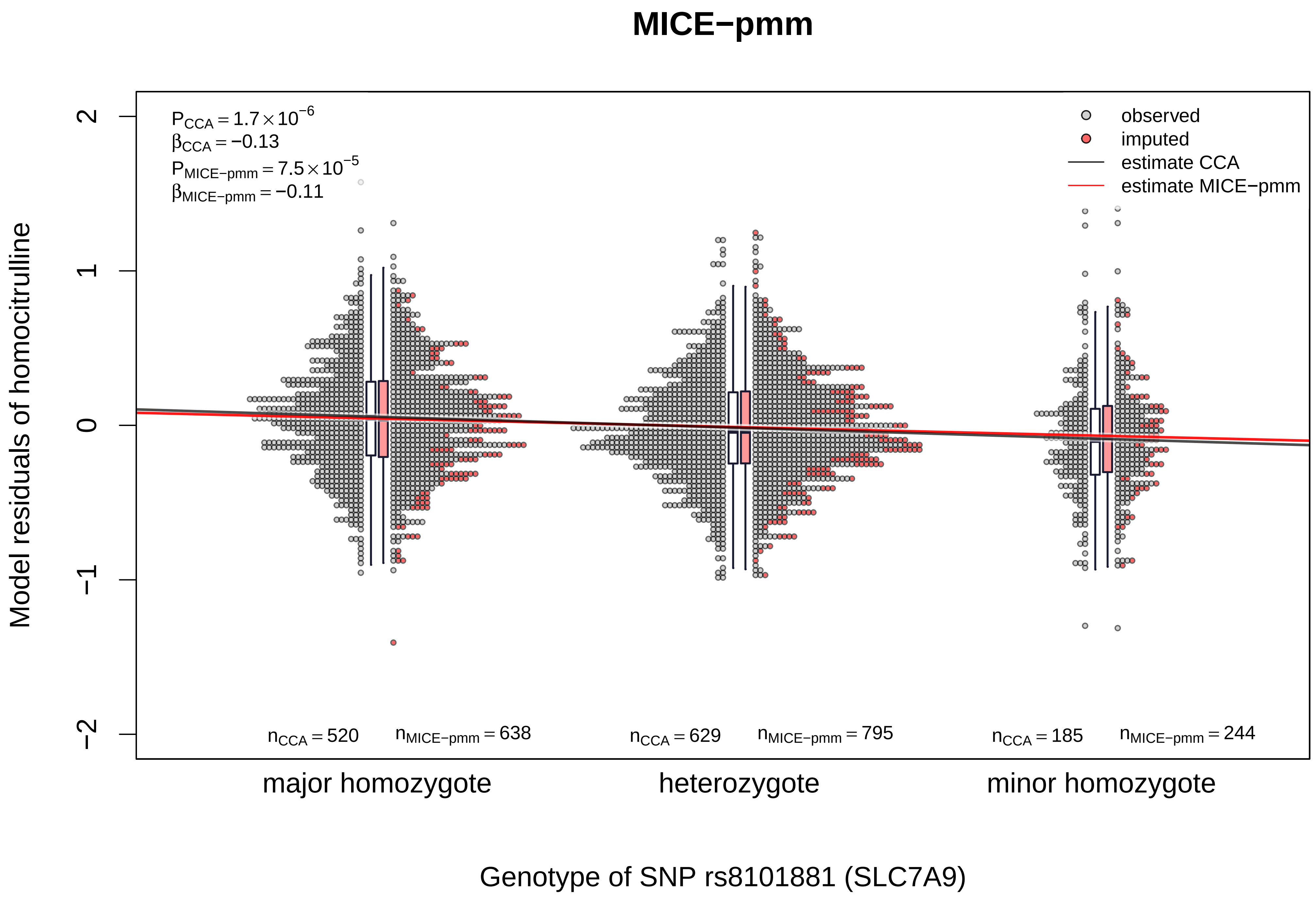

MICE−pmm
$P_{CCA} = 1.7 \times 10^{-6}$
$\beta_{CCA} = -0.13$
$P_{MICE-pmm} = 7.5 \times 10^{-5}$
$\beta_{MICE-pmm} = -0.11$
observed
imputed
estimate CCA
estimate MICE−pmm
2
1
0
−1
−2
Model residuals of homocitrulline
$n_{CCA} = 520$
$n_{MICE-pmm} = 638$
$n_{CCA} = 629$
$n_{MICE-pmm} = 795$
$n_{CCA} = 185$
$n_{MICE-pmm} = 244$
major homozygote
heterozygote
minor homozygote
Genotype of SNP rs8101881 (SLC7A9)

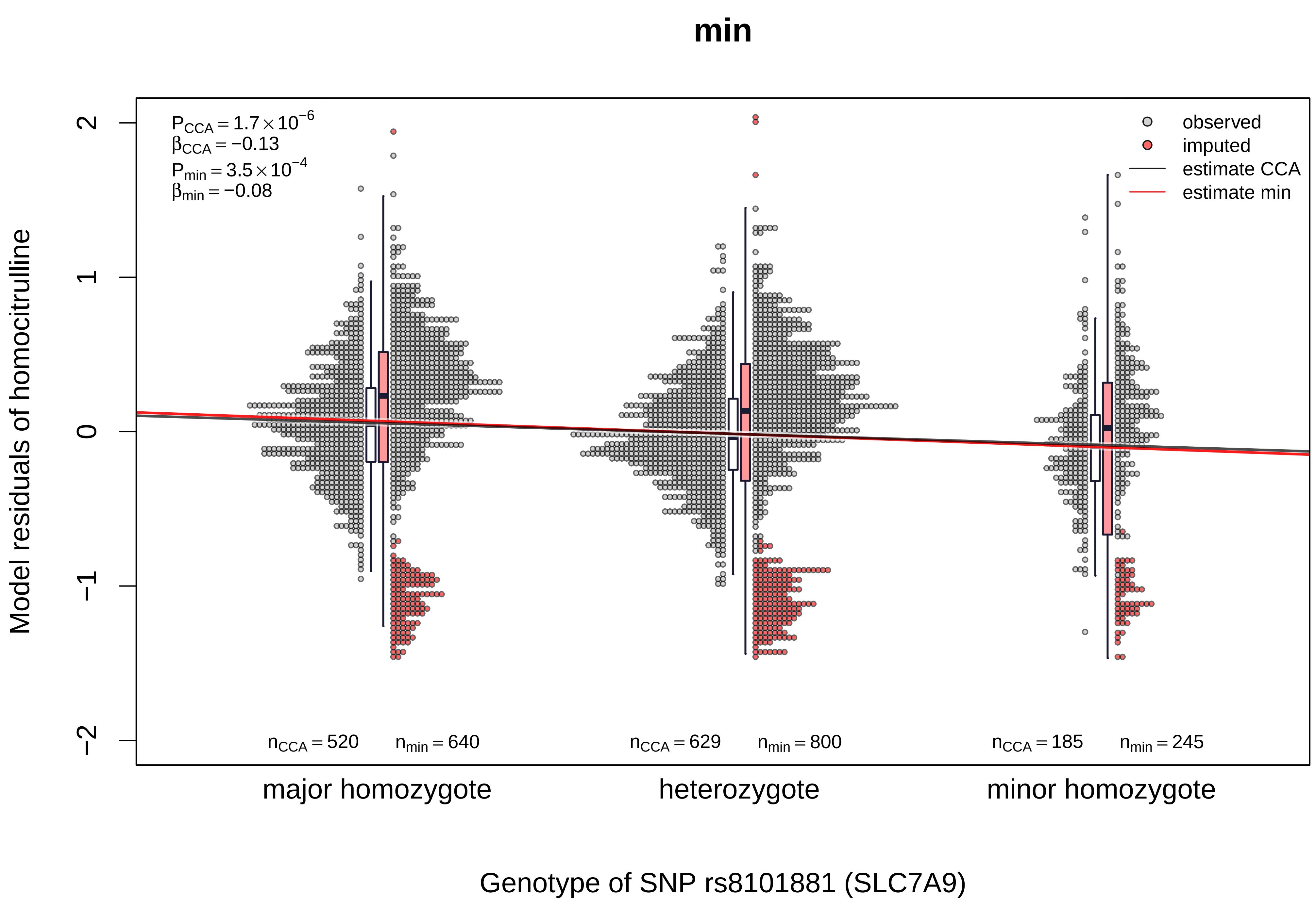

min
$P_{CCA} = 1.7 \times 10^{-6}$
$\beta_{CCA} = -0.13$
$P_{min} = 3.5 \times 10^{-4}$
$\beta_{min} = -0.08$
observed
imputed
estimate CCA
estimate min
Model residuals of homocitrulline
2
1
0
−1
−2
$n_{CCA} = 520$
$n_{min} = 640$
$n_{CCA} = 629$
$n_{min} = 800$
$n_{CCA} = 185$
$n_{min} = 245$
major homozygote
heterozygote
minor homozygote
Genotype of SNP rs8101881 (SLC7A9)

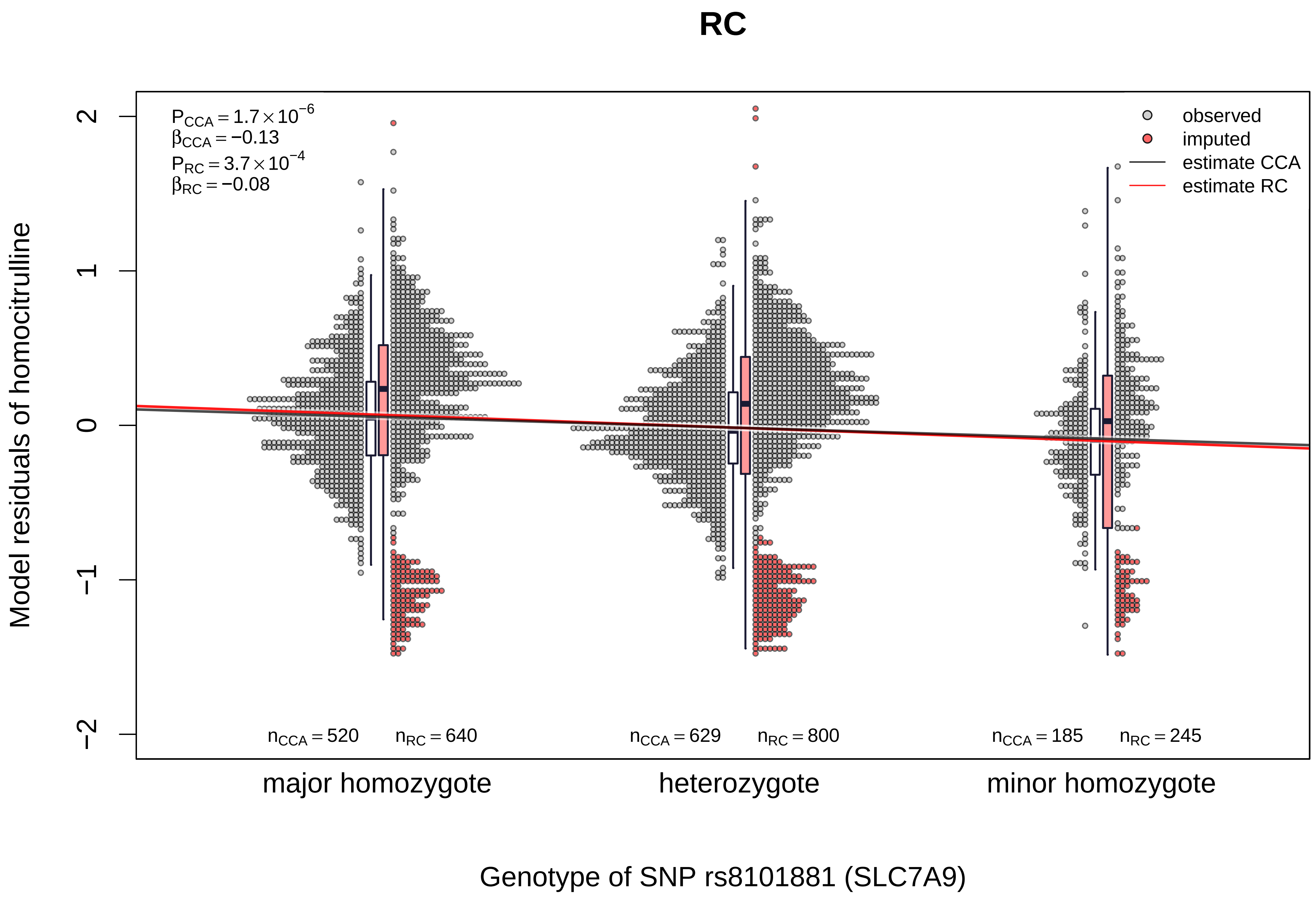
RC
$P_{CCA} = 1.7 \times 10^{-6}$
$\beta_{CCA} = -0.13$
$P_{RC} = 3.7 \times 10^{-4}$
$\beta_{RC} = -0.08$
observed
imputed
estimate CCA
estimate RC
Model residuals of homocitrulline
2
1
0
−1
−2
$n_{CCA} = 520$
$n_{RC} = 640$
$n_{CCA} = 629$
$n_{RC} = 800$
$n_{CCA} = 185$
$n_{RC} = 245$
major homozygote
heterozygote
minor homozygote
Genotype of SNP rs8101881 (SLC7A9)

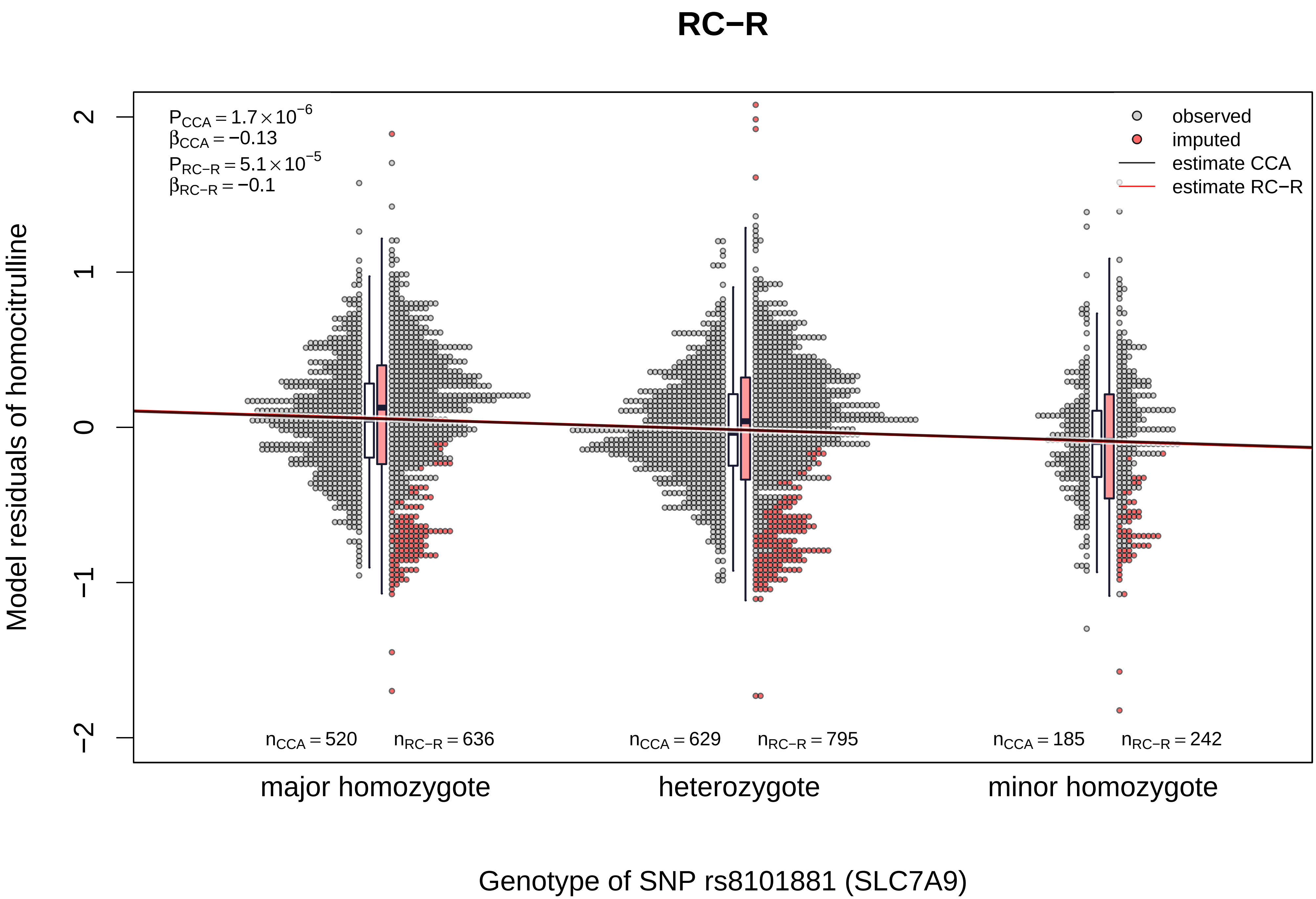
RC−R
$P_{CCA} = 1.7 \times 10^{-6}$
$\beta_{CCA} = -0.13$
$P_{RC-R} = 5.1 \times 10^{-5}$
$\beta_{RC-R} = -0.1$
observed
imputed
estimate CCA
estimate RC−R
Model residuals of homocitrulline
2
1
0
−1
−2
$n_{CCA} = 520$
$n_{RC-R} = 636$
$n_{CCA} = 629$
$n_{RC-R} = 795$
$n_{CCA} = 185$
$n_{RC-R} = 242$
major homozygote
heterozygote
minor homozygote
Genotype of SNP rs8101881 (SLC7A9)

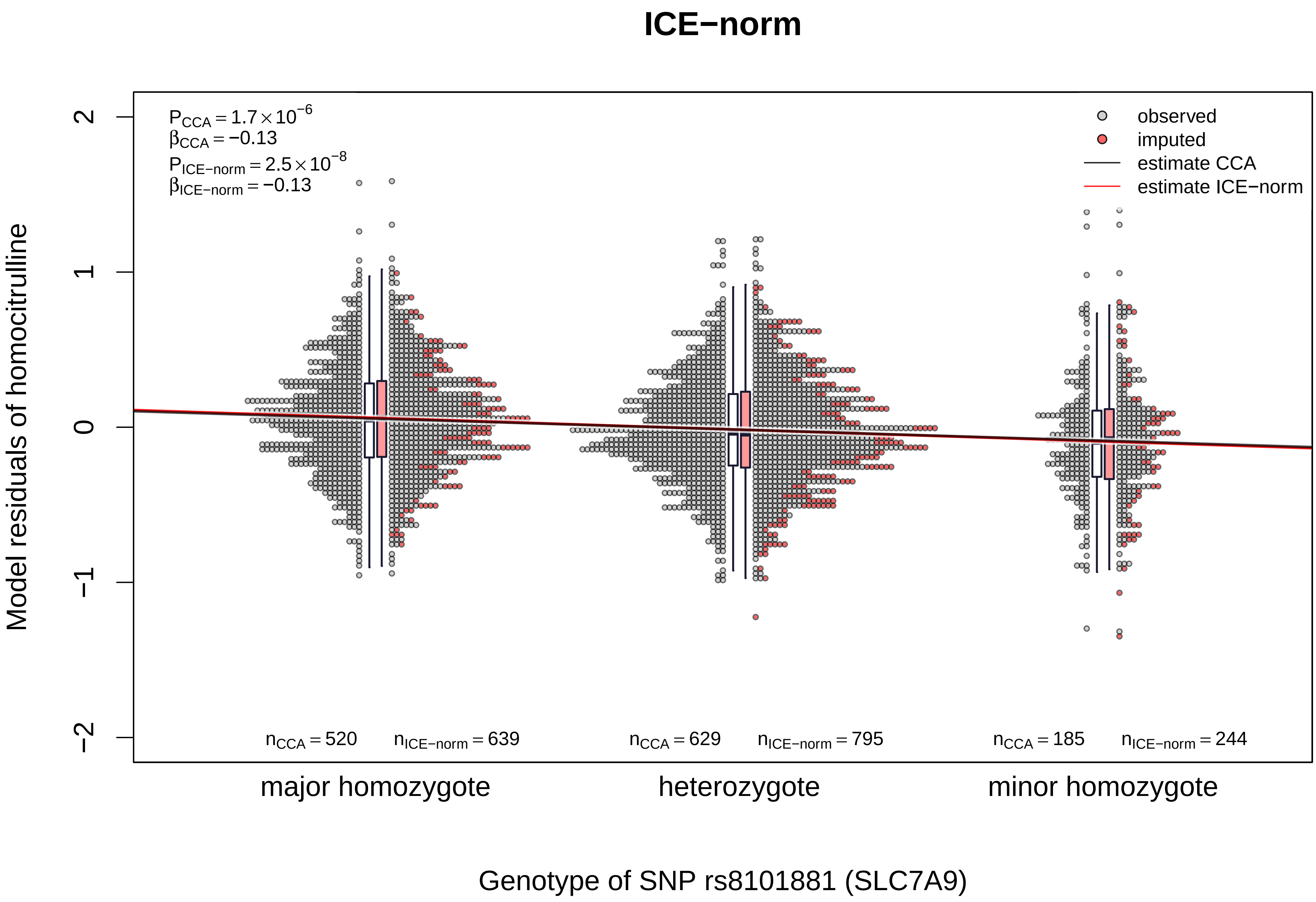
ICE−norm
$P_{CCA} = 1.7 \times 10^{-6}$
$\beta_{CCA} = -0.13$
$P_{ICE-norm} = 2.5 \times 10^{-8}$
$\beta_{ICE-norm} = -0.13$
observed
imputed
estimate CCA
estimate ICE−norm
$n_{CCA} = 520$
$n_{ICE-norm} = 639$
$n_{CCA} = 629$
$n_{ICE-norm} = 795$
$n_{CCA} = 185$
$n_{ICE-norm} = 244$
2
1
0
−1
−2
Model residuals of homocitrulline
major homozygote
heterozygote
minor homozygote
Genotype of SNP rs8101881 (SLC7A9)

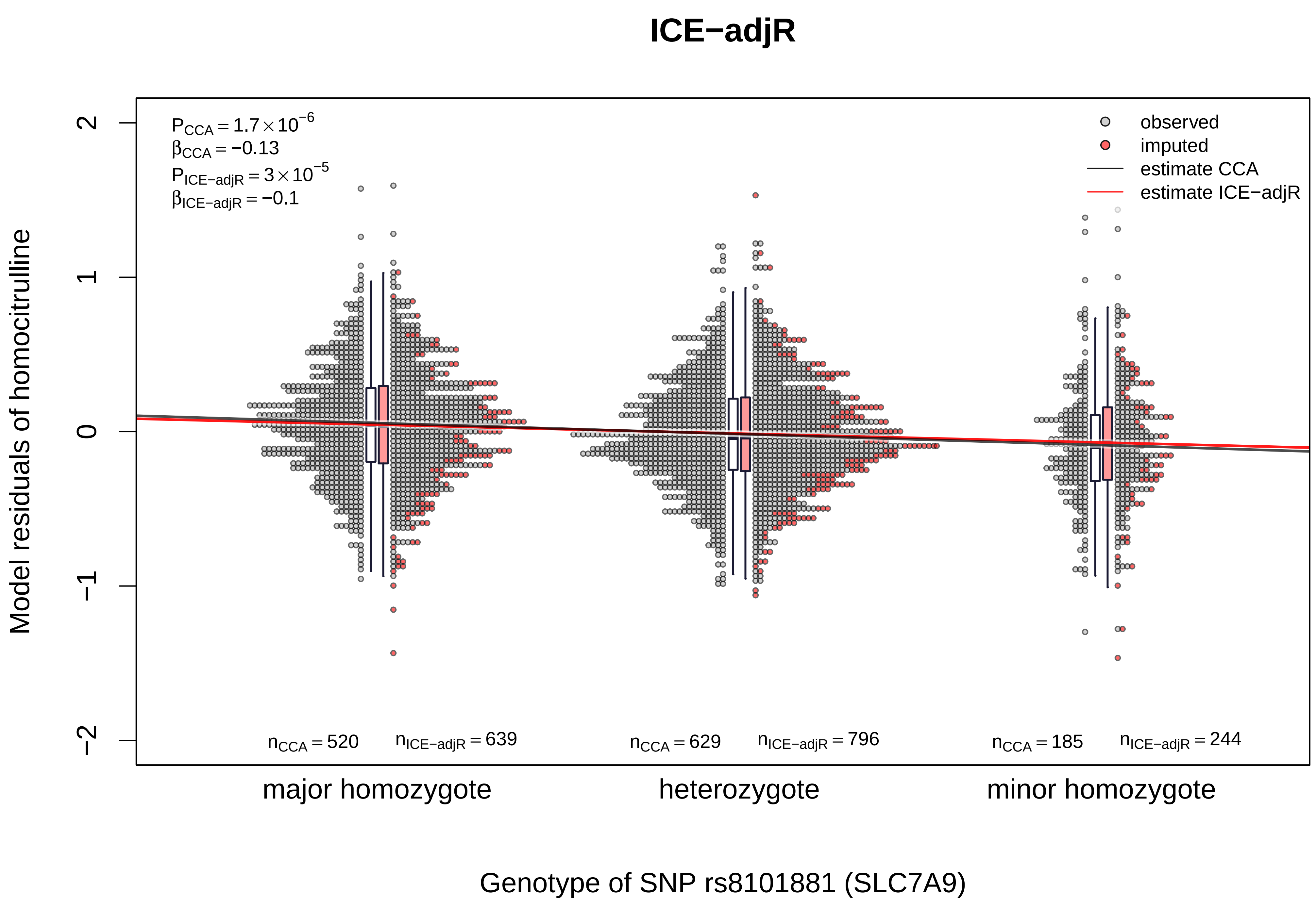
ICE−adjR
$P_{CCA} = 1.7 \times 10^{-6}$
$\beta_{CCA} = -0.13$
$P_{ICE-adjR} = 3 \times 10^{-5}$
$\beta_{ICE-adjR} = -0.1$
observed
imputed
estimate CCA
estimate ICE−adjR
Model residuals of homocitrulline
2
1
0
−1
−2
$n_{CCA} = 520$
$n_{ICE-adjR} = 639$
$n_{CCA} = 629$
$n_{ICE-adjR} = 796$
$n_{CCA} = 185$
$n_{ICE-adjR} = 244$
major homozygote
heterozygote
minor homozygote
Genotype of SNP rs8101881 (SLC7A9)

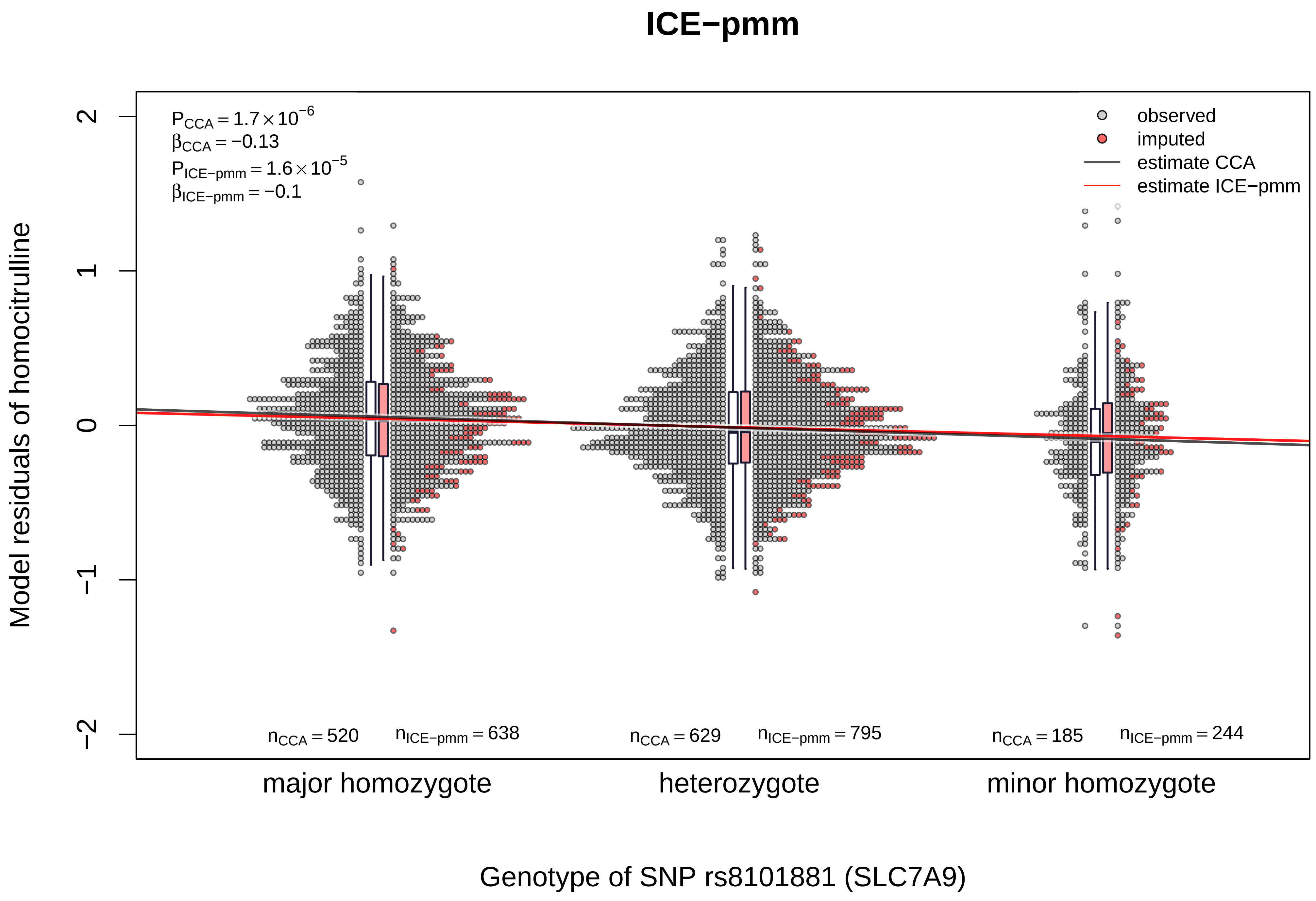

ICE−pmm
$P_{CCA} = 1.7 \times 10^{-6}$
$\beta_{CCA} = -0.13$
$P_{ICE-pmm} = 1.6 \times 10^{-5}$
$\beta_{ICE-pmm} = -0.1$
observed
imputed
estimate CCA
estimate ICE−pmm
Model residuals of homocitrulline
2
1
0
−1
−2
$n_{CCA} = 520$
$n_{ICE-pmm} = 638$
$n_{CCA} = 629$
$n_{ICE-pmm} = 795$
$n_{CCA} = 185$
$n_{ICE-pmm} = 244$
major homozygote
heterozygote
minor homozygote
Genotype of SNP rs8101881 (SLC7A9)

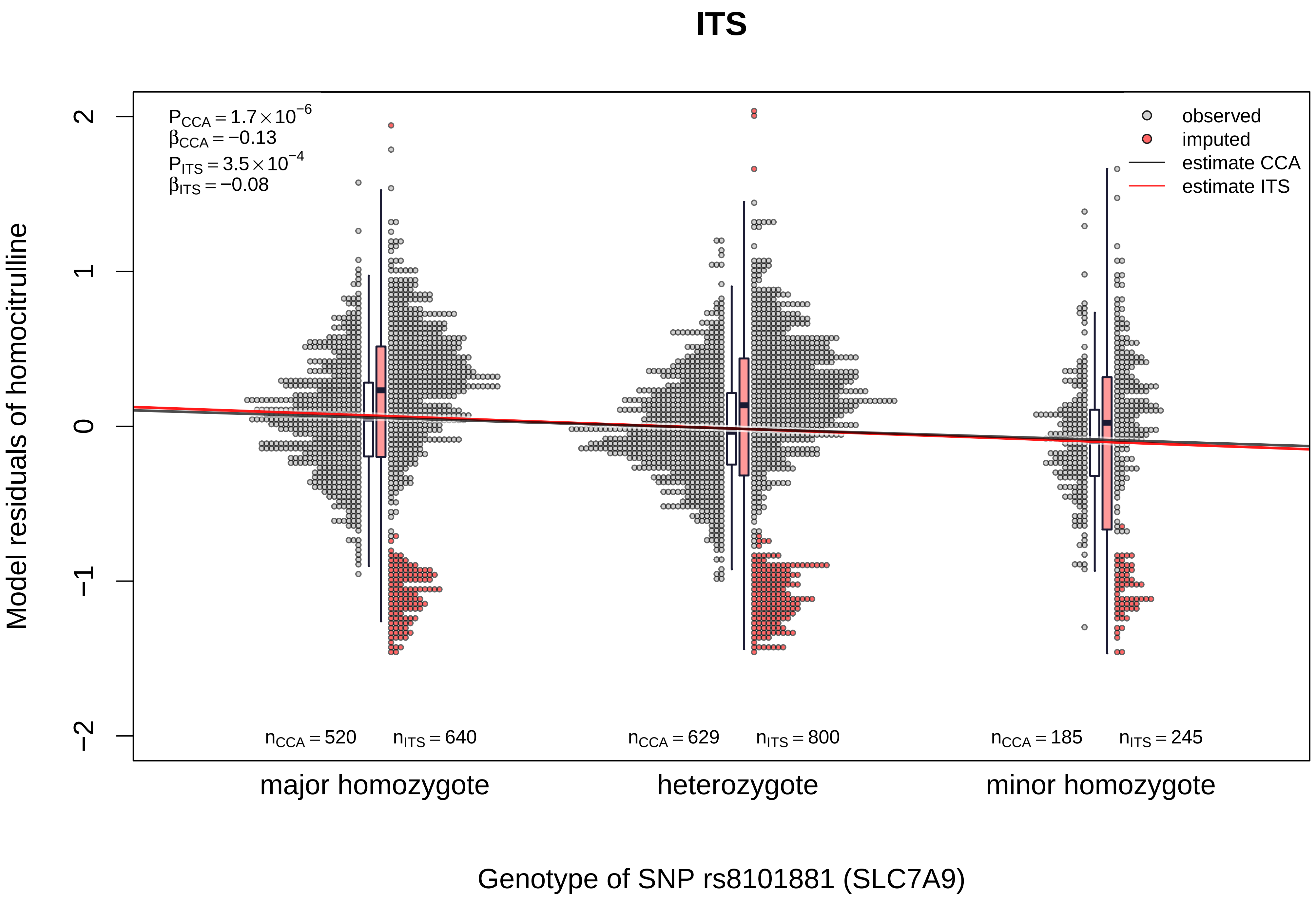

ITS
$P_{CCA} = 1.7 \times 10^{-6}$
$\beta_{CCA} = -0.13$
$P_{ITS} = 3.5 \times 10^{-4}$
$\beta_{ITS} = -0.08$
observed
imputed
estimate CCA
estimate ITS
2
1
0
−1
−2
Model residuals of homocitrulline
$n_{CCA} = 520$
$n_{ITS} = 640$
$n_{CCA} = 629$
$n_{ITS} = 800$
$n_{CCA} = 185$
$n_{ITS} = 245$
major homozygote
heterozygote
minor homozygote
Genotype of SNP rs8101881 (SLC7A9)

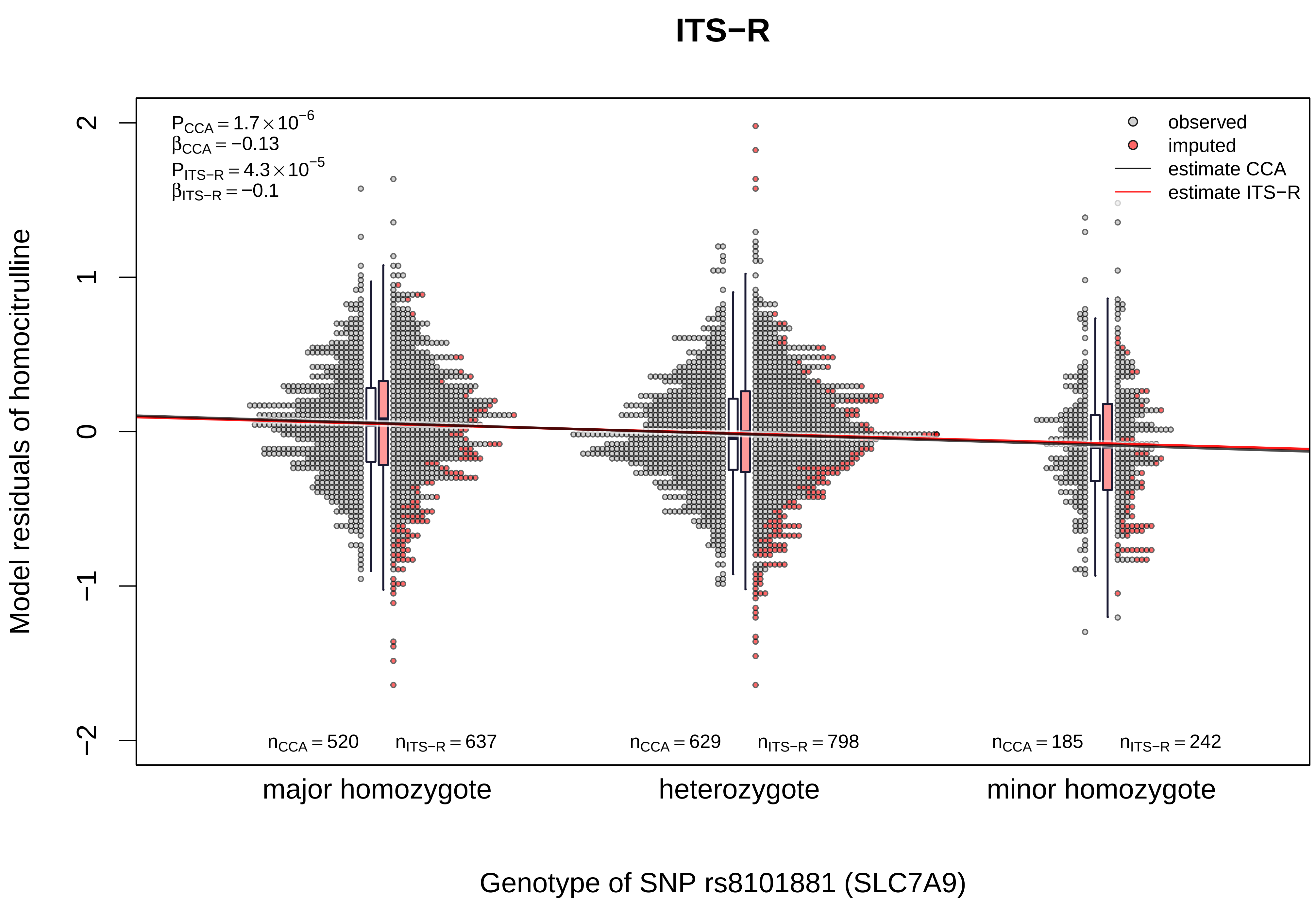
ITS−R
$P_{CCA} = 1.7 \times 10^{-6}$
$\beta_{CCA} = -0.13$
$P_{ITS-R} = 4.3 \times 10^{-5}$
$\beta_{ITS-R} = -0.1$
observed
imputed
estimate CCA
estimate ITS−R
Model residuals of homocitrulline
2
1
0
−1
−2
$n_{CCA} = 520$
$n_{ITS-R} = 637$
$n_{CCA} = 629$
$n_{ITS-R} = 798$
$n_{CCA} = 185$
$n_{ITS-R} = 242$
major homozygote
heterozygote
minor homozygote
Genotype of SNP rs8101881 (SLC7A9)

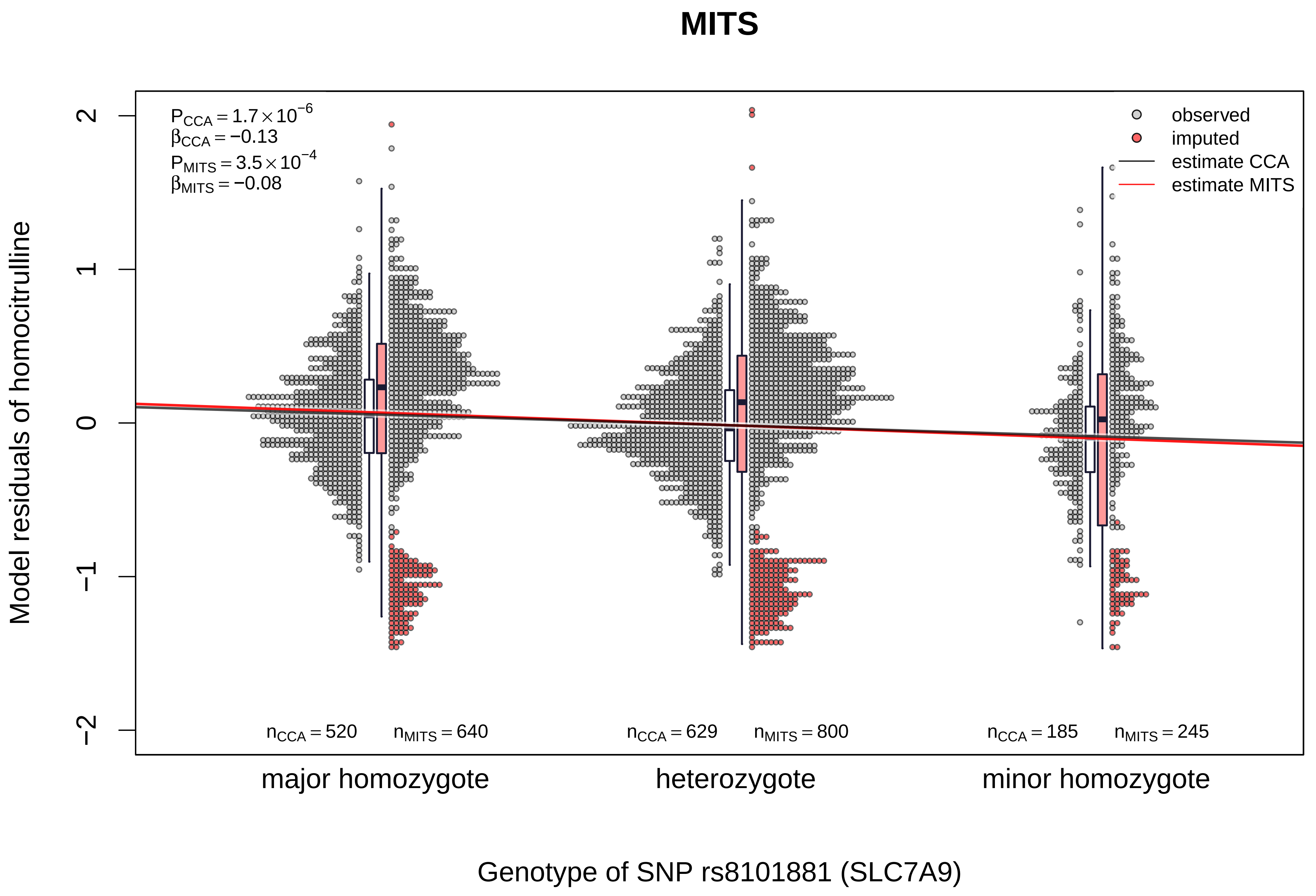

MITS
$P_{CCA} = 1.7 \times 10^{-6}$
$\beta_{CCA} = -0.13$
$P_{MITS} = 3.5 \times 10^{-4}$
$\beta_{MITS} = -0.08$
observed
imputed
estimate CCA
estimate MITS
Model residuals of homocitrulline
2
1
0
−1
−2
$n_{CCA} = 520$
$n_{MITS} = 640$
$n_{CCA} = 629$
$n_{MITS} = 800$
$n_{CCA} = 185$
$n_{MITS} = 245$
major homozygote
heterozygote
minor homozygote
Genotype of SNP rs8101881 (SLC7A9)

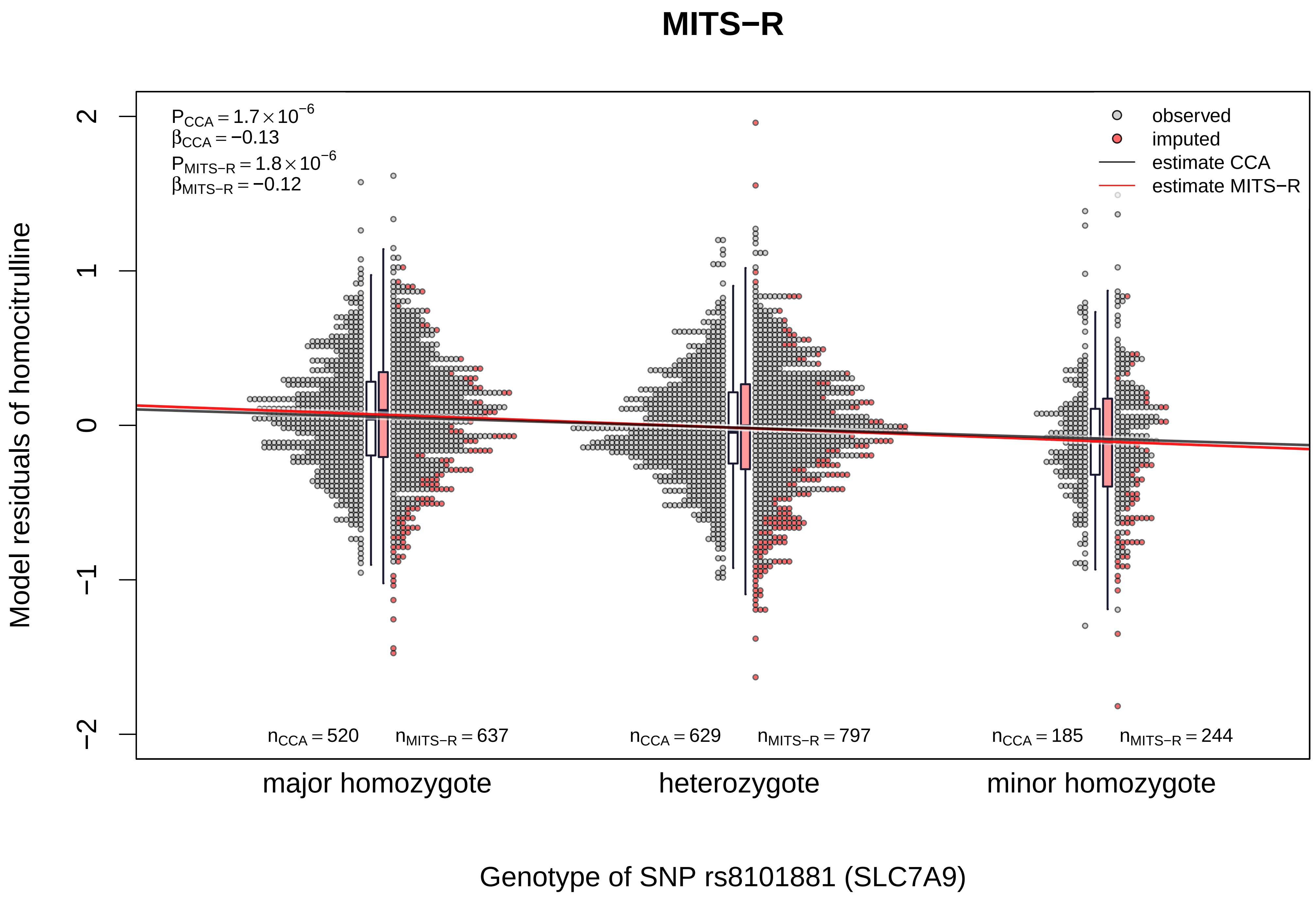

MITS−R
$P_{CCA} = 1.7 \times 10^{-6}$
$\beta_{CCA} = -0.13$
$P_{MITS-R} = 1.8 \times 10^{-6}$
$\beta_{MITS-R} = -0.12$
observed
imputed
estimate CCA
estimate MITS−R
Model residuals of homocitrulline
2
1
0
−1
−2
$n_{CCA} = 520$
$n_{MITS-R} = 637$
$n_{CCA} = 629$
$n_{MITS-R} = 797$
$n_{CCA} = 185$
$n_{MITS-R} = 244$
major homozygote
heterozygote
minor homozygote
Genotype of SNP rs8101881 (SLC7A9)

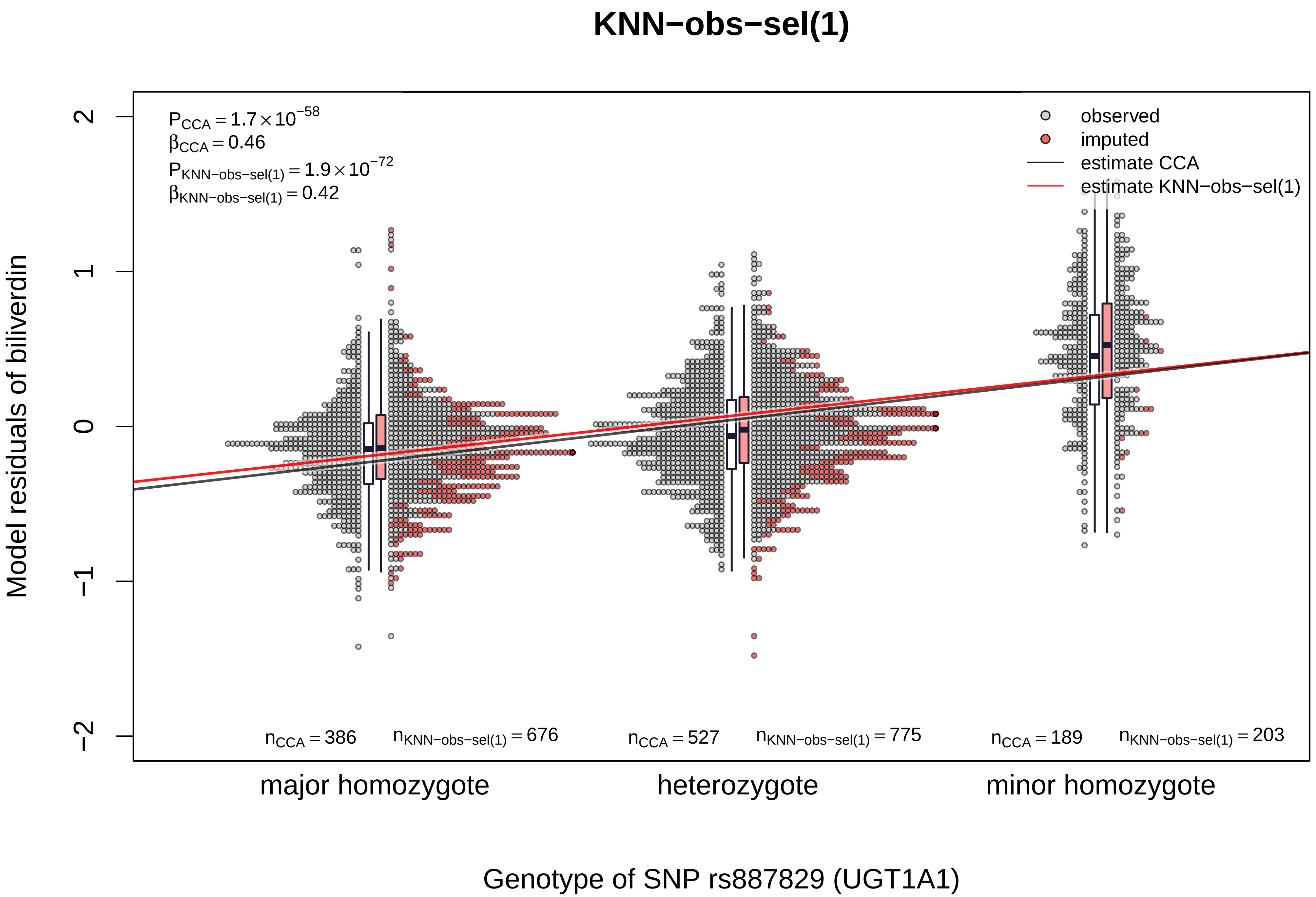
KNN−obs−sel(1)
$P_{CCA} = 1.7 \times 10^{-58}$
$\beta_{CCA} = 0.46$
$P_{KNN-obs-sel(1)} = 1.9 \times 10^{-72}$
$\beta_{KNN-obs-sel(1)} = 0.42$
observed
imputed
estimate CCA
estimate KNN−obs−sel(1)
Model residuals of biliverdin
2
1
0
−1
−2
$n_{CCA} = 386$
$n_{KNN-obs-sel(1)} = 676$
$n_{CCA} = 527$
$n_{KNN-obs-sel(1)} = 775$
$n_{CCA} = 189$
$n_{KNN-obs-sel(1)} = 203$
major homozygote
heterozygote
minor homozygote
Genotype of SNP rs887829 (UGT1A1)

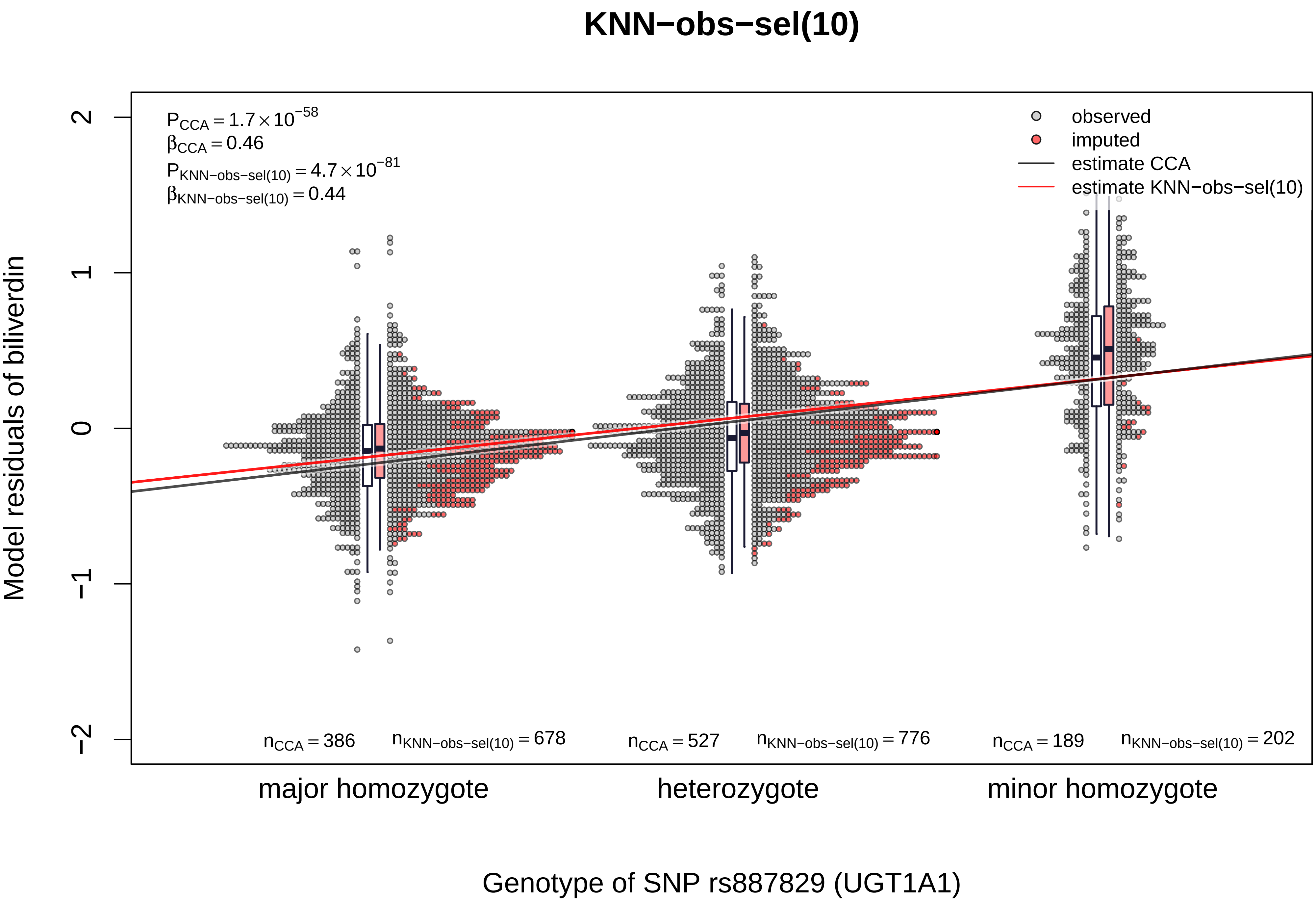

KNN−obs−sel(10)
$P_{CCA} = 1.7 \times 10^{-58}$
$\beta_{CCA} = 0.46$
$P_{KNN-obs-sel(10)} = 4.7 \times 10^{-81}$
$\beta_{KNN-obs-sel(10)} = 0.44$
observed
imputed
estimate CCA
estimate KNN−obs−sel(10)
Model residuals of biliverdin
2
1
0
−1
−2
$n_{CCA} = 386$
$n_{KNN-obs-sel(10)} = 678$
$n_{CCA} = 527$
$n_{KNN-obs-sel(10)} = 776$
$n_{CCA} = 189$
$n_{KNN-obs-sel(10)} = 202$
major homozygote
heterozygote
minor homozygote
Genotype of SNP rs887829 (UGT1A1)

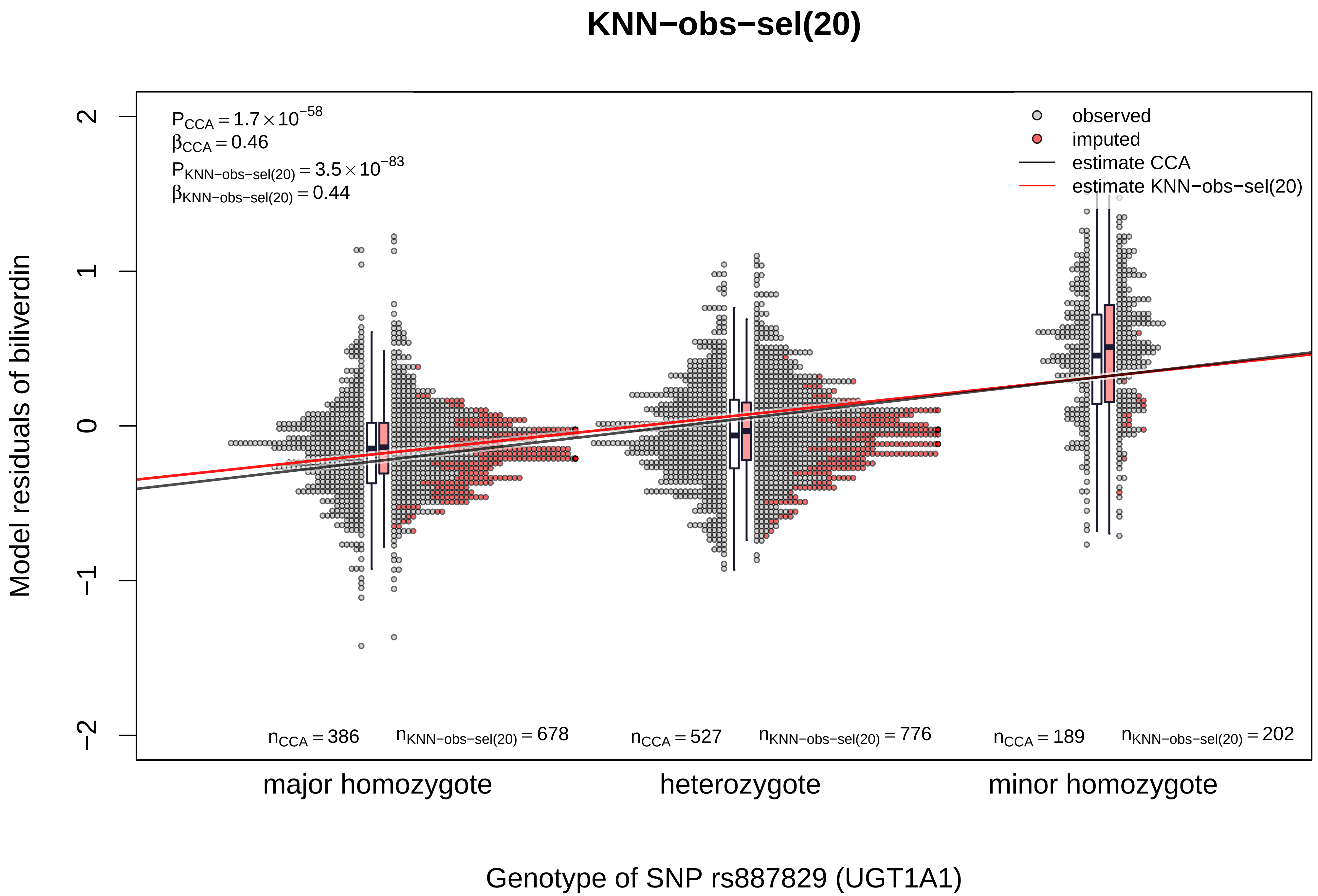

KNN−obs−sel(20)
$P_{CCA} = 1.7 \times 10^{-58}$
$\beta_{CCA} = 0.46$
$P_{KNN-obs-sel(20)} = 3.5 \times 10^{-83}$
$\beta_{KNN-obs-sel(20)} = 0.44$
observed
imputed
estimate CCA
estimate KNN−obs−sel(20)
Model residuals of biliverdin
2
1
0
−1
−2
$n_{CCA} = 386$
$n_{KNN-obs-sel(20)} = 678$
$n_{CCA} = 527$
$n_{KNN-obs-sel(20)} = 776$
$n_{CCA} = 189$
$n_{KNN-obs-sel(20)} = 202$
major homozygote
heterozygote
minor homozygote
Genotype of SNP rs887829 (UGT1A1)

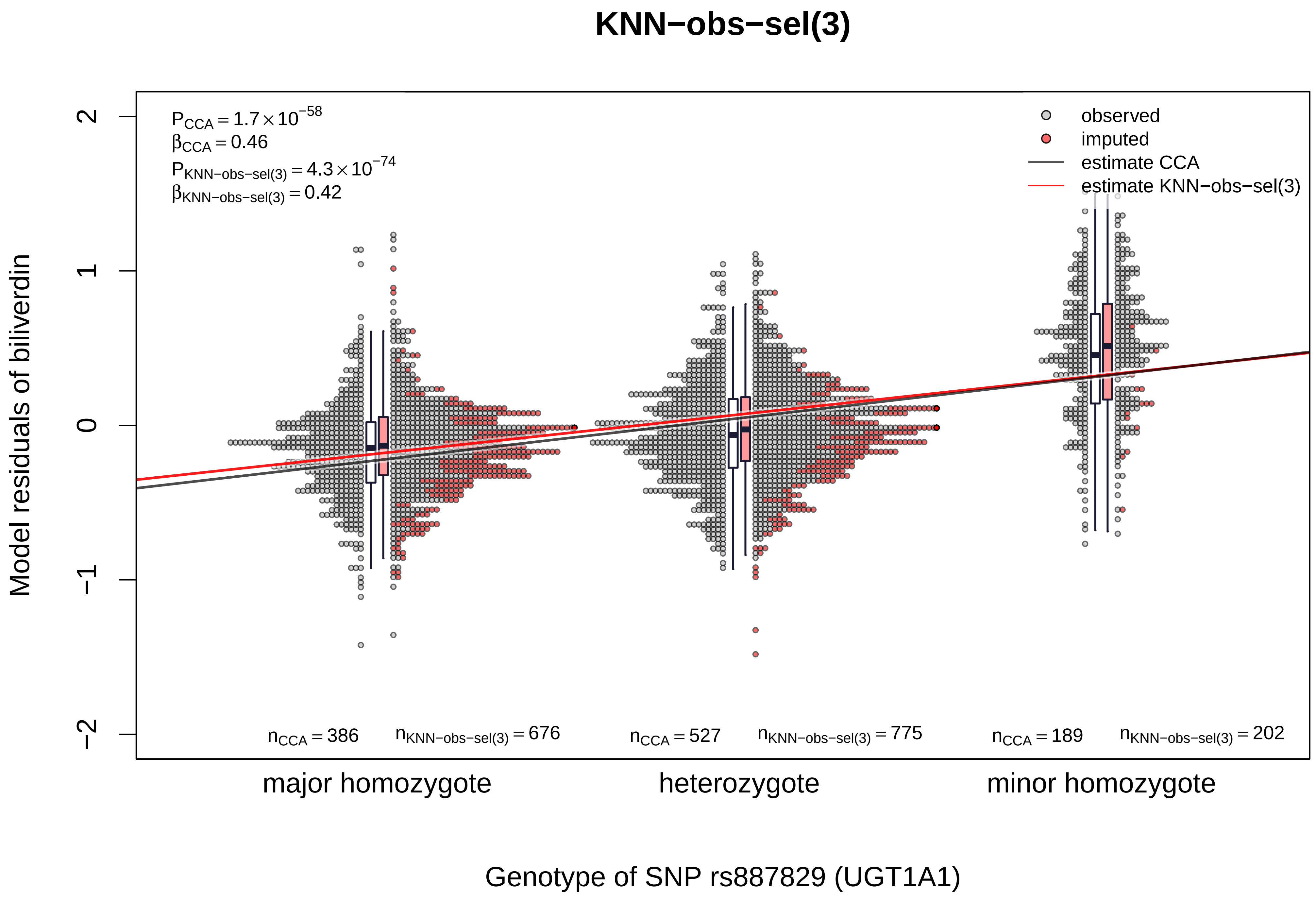

KNN−obs−sel(3)
$P_{CCA} = 1.7 \times 10^{-58}$
$\beta_{CCA} = 0.46$
$P_{KNN-obs-sel(3)} = 4.3 \times 10^{-74}$
$\beta_{KNN-obs-sel(3)} = 0.42$
observed
imputed
estimate CCA
estimate KNN−obs−sel(3)
2
1
0
−1
−2
Model residuals of biliverdin
$n_{CCA} = 386$
$n_{KNN-obs-sel(3)} = 676$
$n_{CCA} = 527$
$n_{KNN-obs-sel(3)} = 775$
$n_{CCA} = 189$
$n_{KNN-obs-sel(3)} = 202$
major homozygote
heterozygote
minor homozygote
Genotype of SNP rs887829 (UGT1A1)

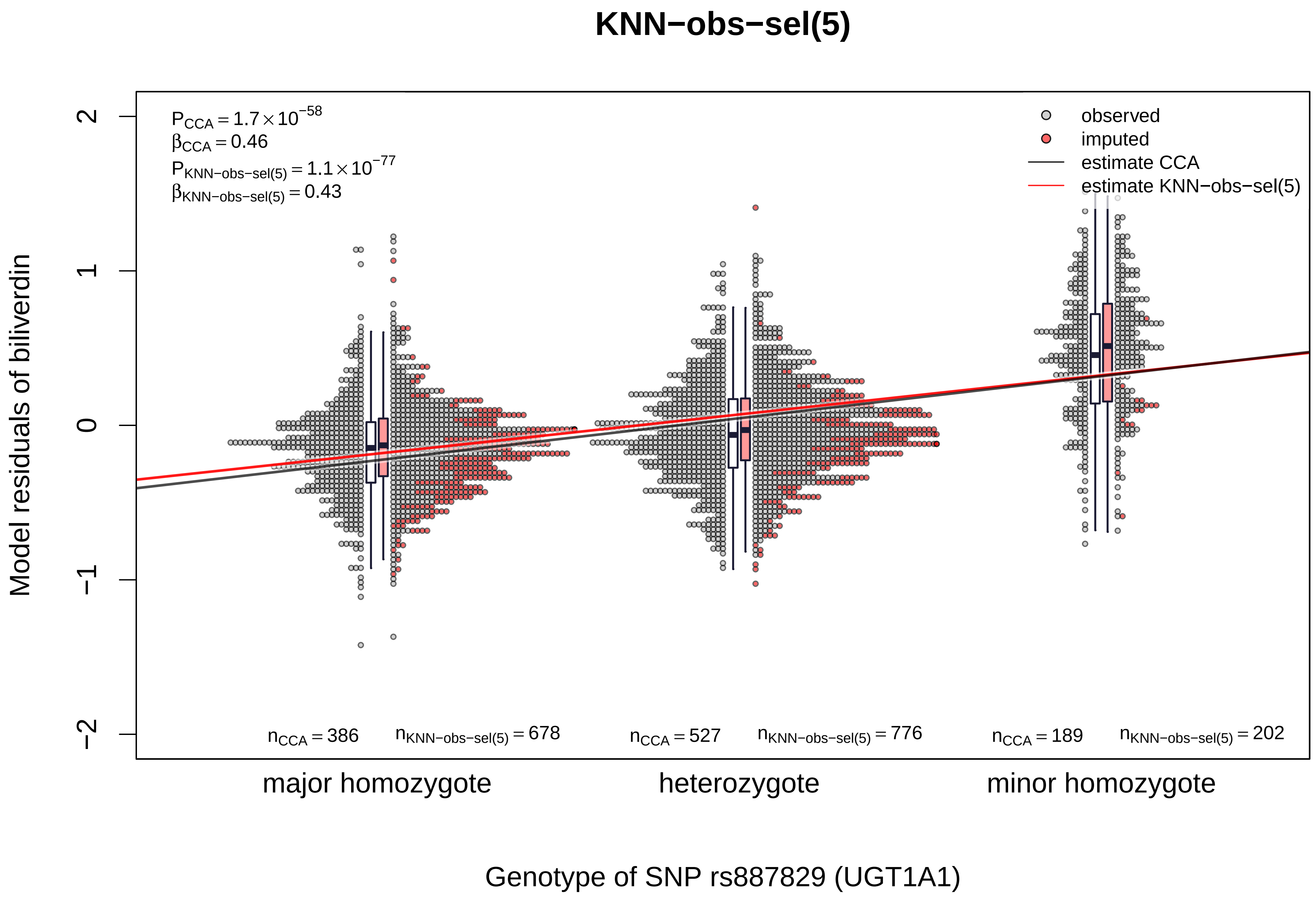

KNN−obs−sel(5)
$P_{CCA} = 1.7 \times 10^{-58}$
$\beta_{CCA} = 0.46$
$P_{KNN-obs-sel(5)} = 1.1 \times 10^{-77}$
$\beta_{KNN-obs-sel(5)} = 0.43$
observed
imputed
estimate CCA
estimate KNN−obs−sel(5)
Model residuals of biliverdin
2
1
0
−1
−2
$n_{CCA} = 386$
$n_{KNN-obs-sel(5)} = 678$
$n_{CCA} = 527$
$n_{KNN-obs-sel(5)} = 776$
$n_{CCA} = 189$
$n_{KNN-obs-sel(5)} = 202$
major homozygote
heterozygote
minor homozygote
Genotype of SNP rs887829 (UGT1A1)

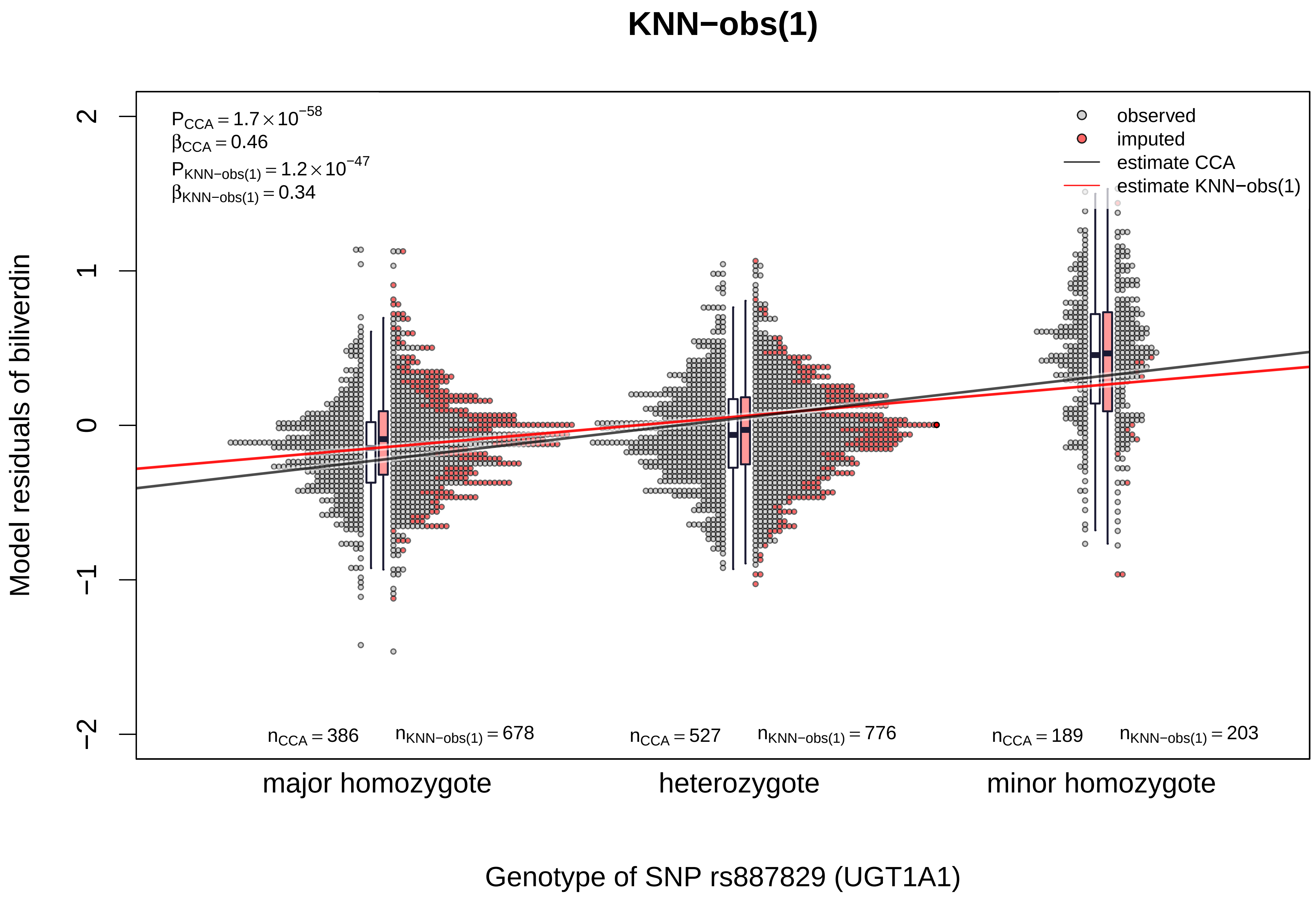
KNN−obs(1)
$P_{CCA} = 1.7 \times 10^{-58}$
$\beta_{CCA} = 0.46$
$P_{KNN-obs(1)} = 1.2 \times 10^{-47}$
$\beta_{KNN-obs(1)} = 0.34$
observed
imputed
estimate CCA
estimate KNN−obs(1)
Model residuals of biliverdin
2
1
0
−1
−2
$n_{CCA} = 386$
$n_{KNN-obs(1)} = 678$
$n_{CCA} = 527$
$n_{KNN-obs(1)} = 776$
$n_{CCA} = 189$
$n_{KNN-obs(1)} = 203$
major homozygote
heterozygote
minor homozygote
Genotype of SNP rs887829 (UGT1A1)

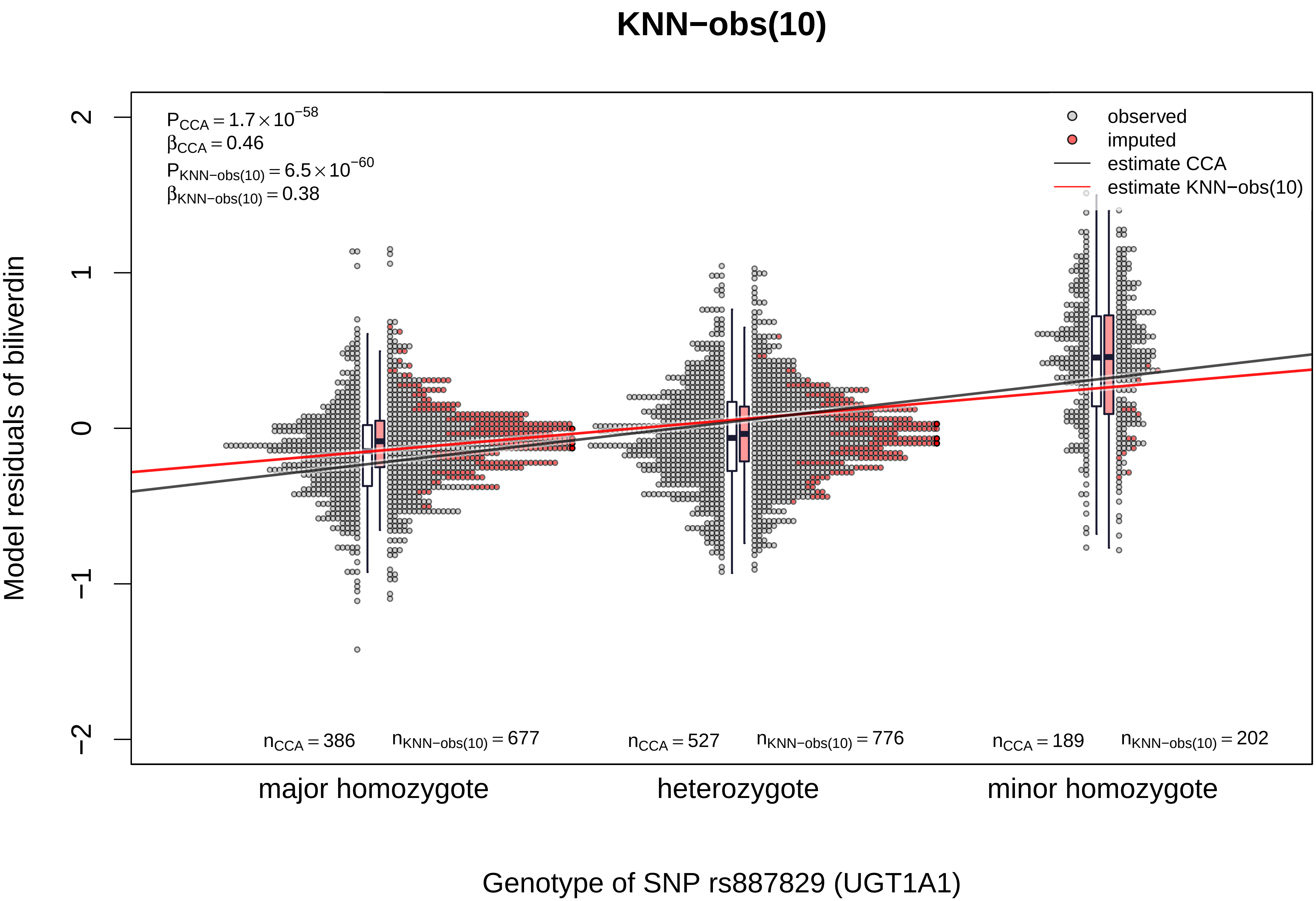
KNN−obs(10)
$P_{CCA} = 1.7 \times 10^{-58}$
$\beta_{CCA} = 0.46$
$P_{KNN-obs(10)} = 6.5 \times 10^{-60}$
$\beta_{KNN-obs(10)} = 0.38$
observed
imputed
estimate CCA
estimate KNN−obs(10)
Model residuals of biliverdin
2
1
0
−1
−2
$n_{CCA} = 386$
$n_{KNN-obs(10)} = 677$
$n_{CCA} = 527$
$n_{KNN-obs(10)} = 776$
$n_{CCA} = 189$
$n_{KNN-obs(10)} = 202$
major homozygote
heterozygote
minor homozygote
Genotype of SNP rs887829 (UGT1A1)

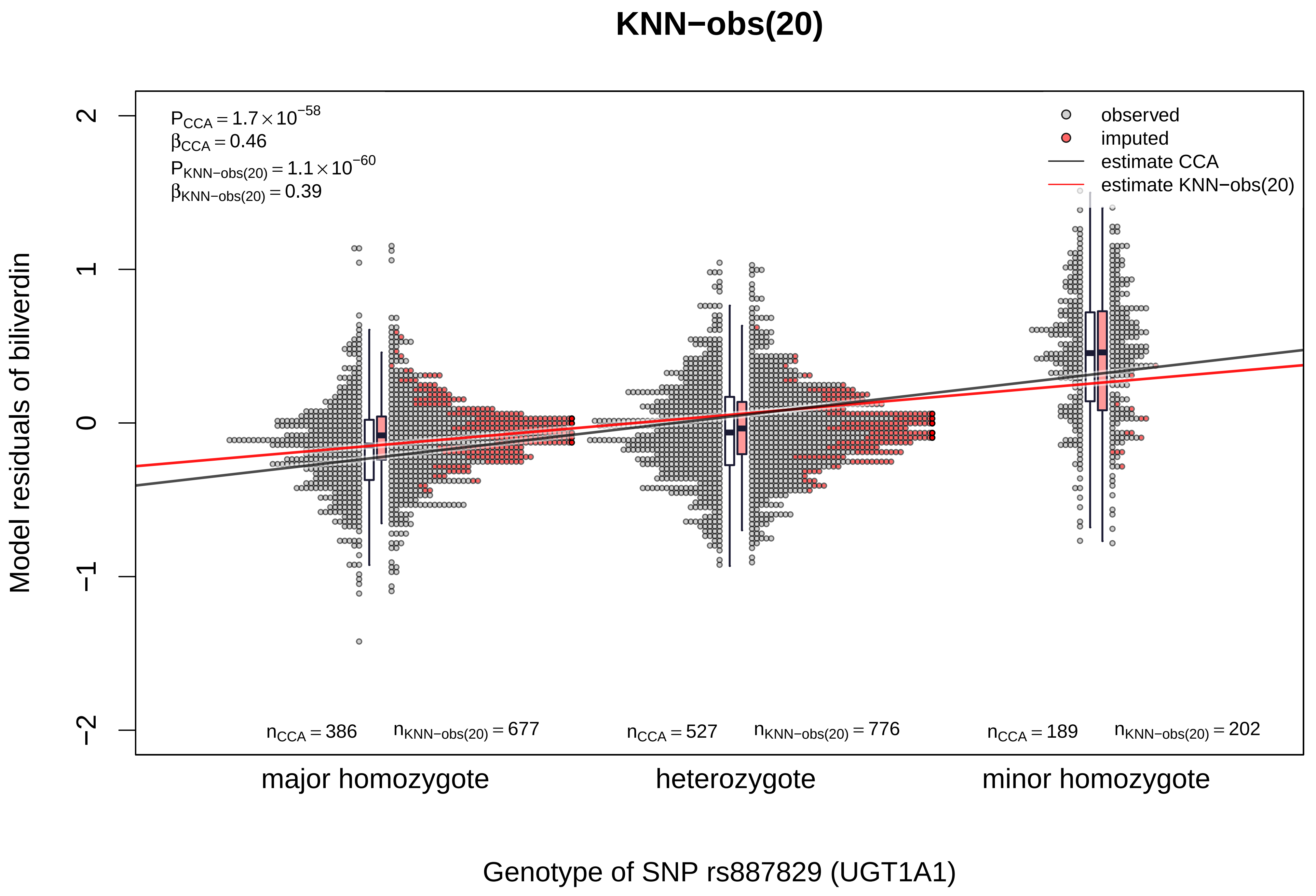

KNN−obs(20)
$P_{CCA} = 1.7 \times 10^{-58}$
$\beta_{CCA} = 0.46$
$P_{KNN-obs(20)} = 1.1 \times 10^{-60}$
$\beta_{KNN-obs(20)} = 0.39$
observed
imputed
estimate CCA
estimate KNN−obs(20)
Model residuals of biliverdin
2
1
0
−1
−2
$n_{CCA} = 386$
$n_{KNN-obs(20)} = 677$
$n_{CCA} = 527$
$n_{KNN-obs(20)} = 776$
$n_{CCA} = 189$
$n_{KNN-obs(20)} = 202$
major homozygote
heterozygote
minor homozygote
Genotype of SNP rs887829 (UGT1A1)

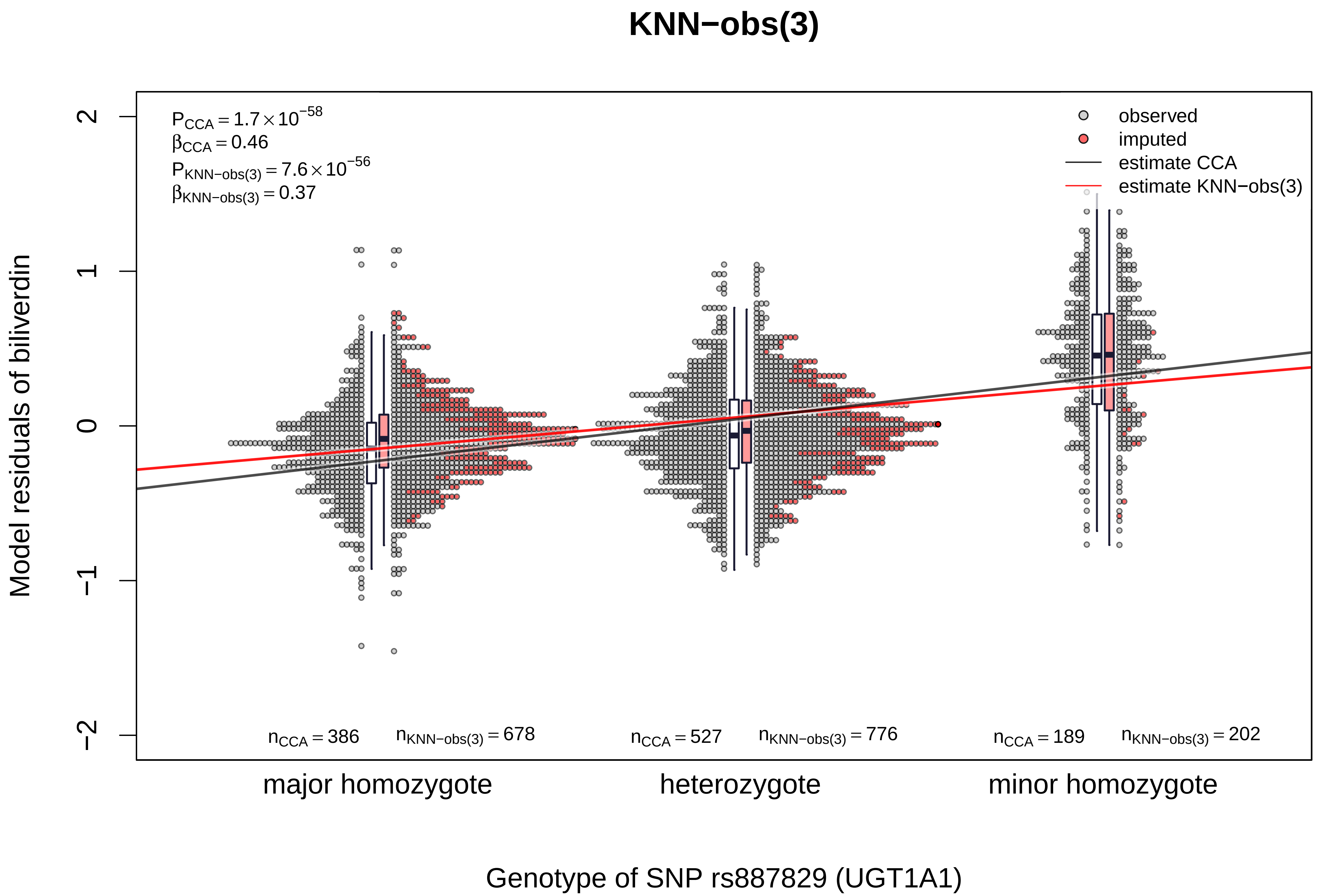

KNN−obs(3)
$P_{CCA} = 1.7 \times 10^{-58}$
$\beta_{CCA} = 0.46$
$P_{KNN-obs(3)} = 7.6 \times 10^{-56}$
$\beta_{KNN-obs(3)} = 0.37$
observed
imputed
estimate CCA
estimate KNN−obs(3)
Model residuals of biliverdin
2
1
0
−1
−2
$n_{CCA} = 386$
$n_{KNN-obs(3)} = 678$
$n_{CCA} = 527$
$n_{KNN-obs(3)} = 776$
$n_{CCA} = 189$
$n_{KNN-obs(3)} = 202$
major homozygote
heterozygote
minor homozygote
Genotype of SNP rs887829 (UGT1A1)

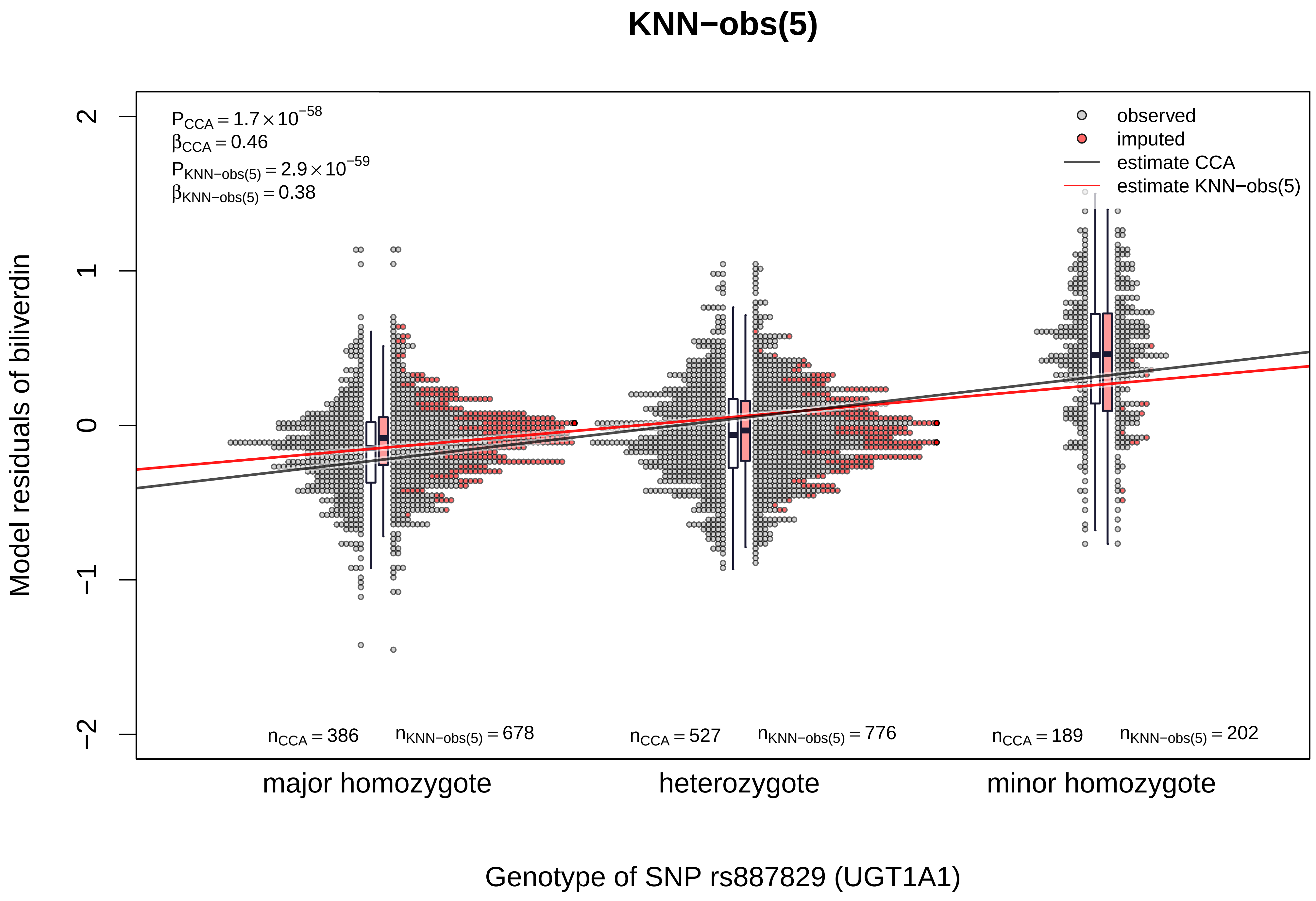

KNN−obs(5)
$P_{CCA} = 1.7 \times 10^{-58}$
$\beta_{CCA} = 0.46$
$P_{KNN-obs(5)} = 2.9 \times 10^{-59}$
$\beta_{KNN-obs(5)} = 0.38$
observed
imputed
estimate CCA
estimate KNN−obs(5)
Model residuals of biliverdin
2
1
0
−1
−2
$n_{CCA} = 386$
$n_{KNN-obs(5)} = 678$
$n_{CCA} = 527$
$n_{KNN-obs(5)} = 776$
$n_{CCA} = 189$
$n_{KNN-obs(5)} = 202$
major homozygote
heterozygote
minor homozygote
Genotype of SNP rs887829 (UGT1A1)

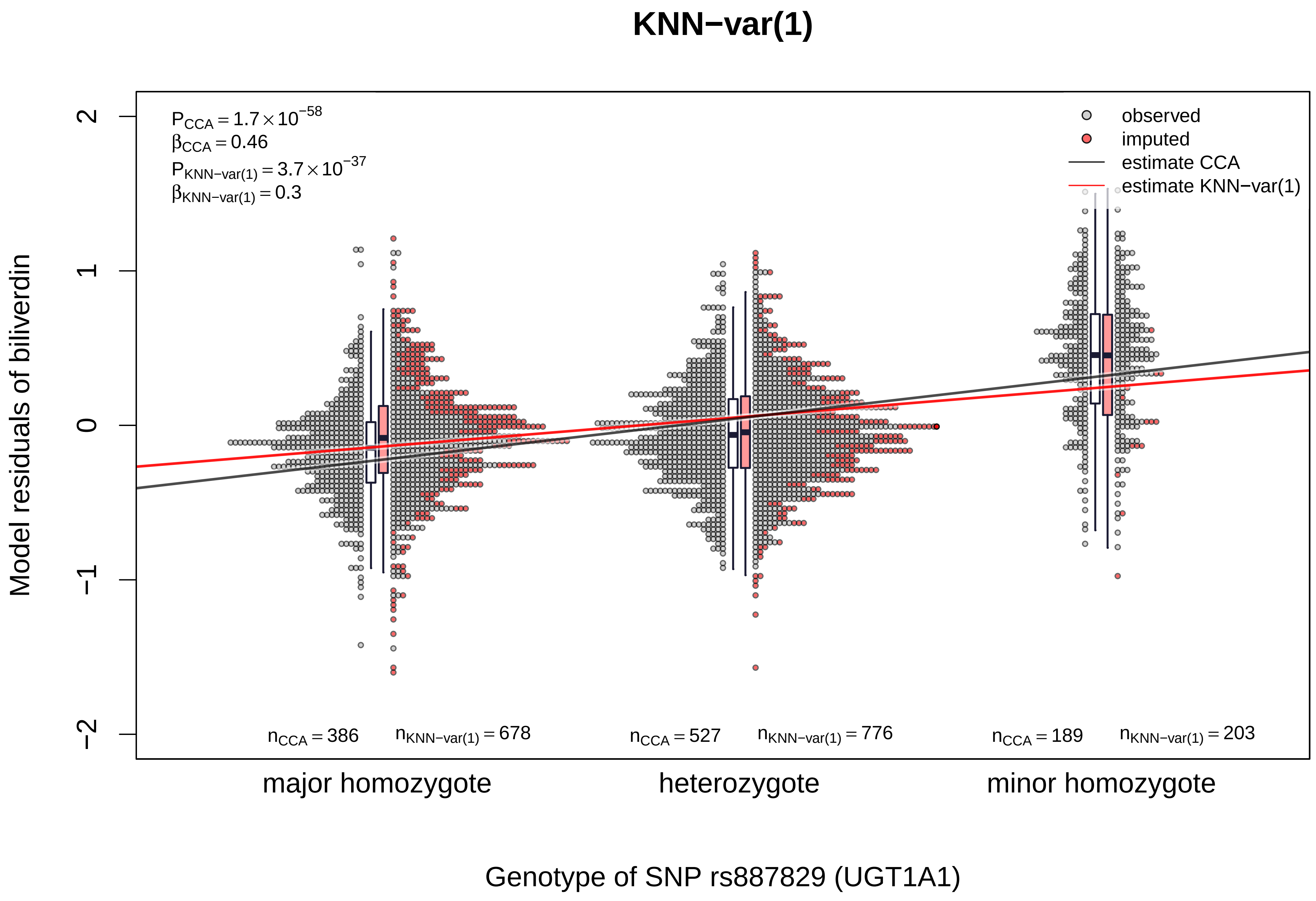

KNN−var(1)
P_CCA = 1.7 × 10^−58
β_CCA = 0.46
P_KNN−var(1) = 3.7 × 10^−37
β_KNN−var(1) = 0.3
observed
imputed
estimate CCA
estimate KNN−var(1)
Model residuals of biliverdin
2
1
0
−1
−2
n_CCA = 386
n_KNN−var(1) = 678
n_CCA = 527
n_KNN−var(1) = 776
n_CCA = 189
n_KNN−var(1) = 203
major homozygote
heterozygote
minor homozygote
Genotype of SNP rs887829 (UGT1A1)

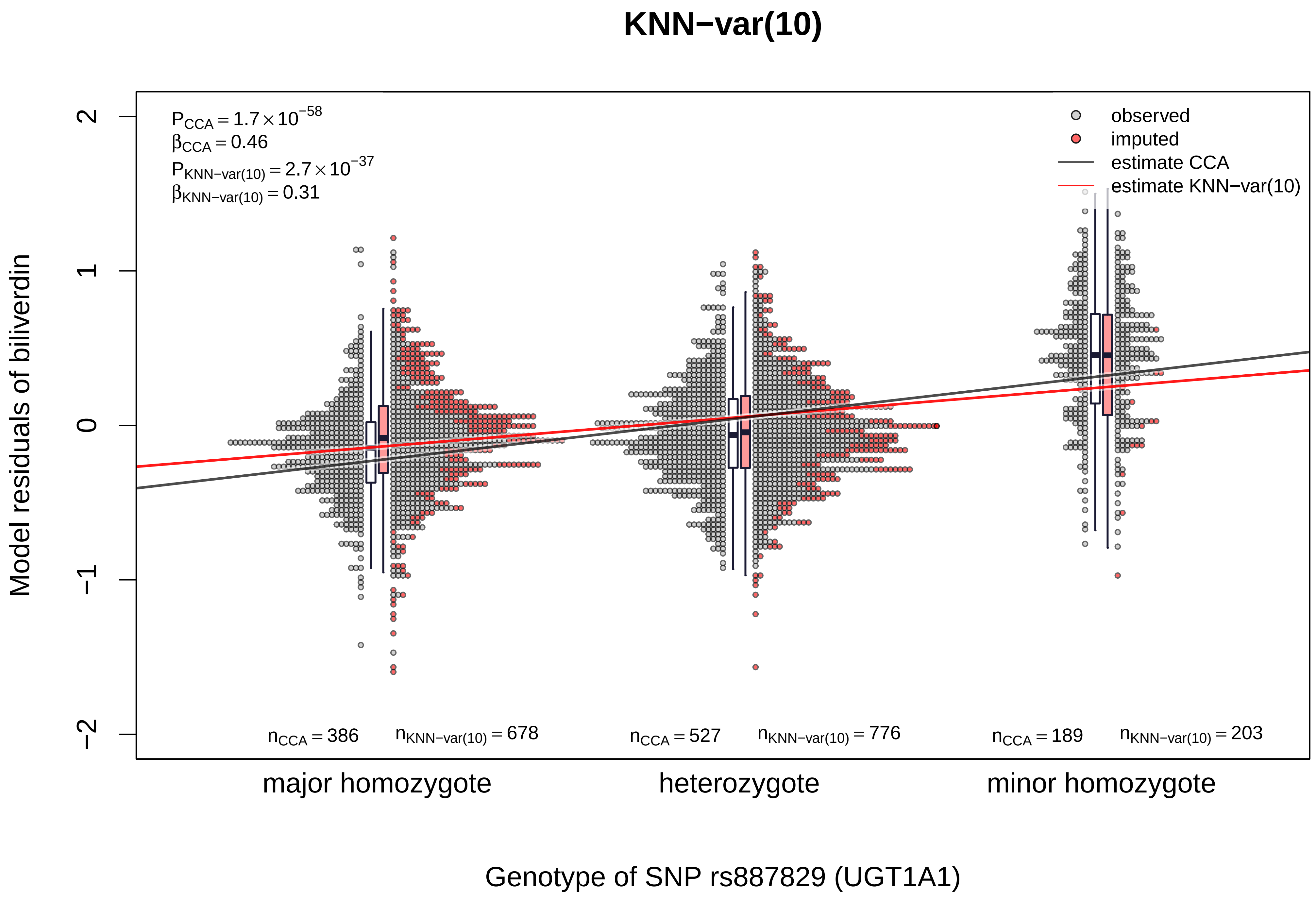

KNN−var(10)
$P_{CCA} = 1.7 \times 10^{-58}$
$\beta_{CCA} = 0.46$
$P_{KNN-var(10)} = 2.7 \times 10^{-37}$
$\beta_{KNN-var(10)} = 0.31$
observed
imputed
estimate CCA
estimate KNN−var(10)
Model residuals of biliverdin
2
1
0
−1
−2
$n_{CCA} = 386$
$n_{KNN-var(10)} = 678$
$n_{CCA} = 527$
$n_{KNN-var(10)} = 776$
$n_{CCA} = 189$
$n_{KNN-var(10)} = 203$
major homozygote
heterozygote
minor homozygote
Genotype of SNP rs887829 (UGT1A1)

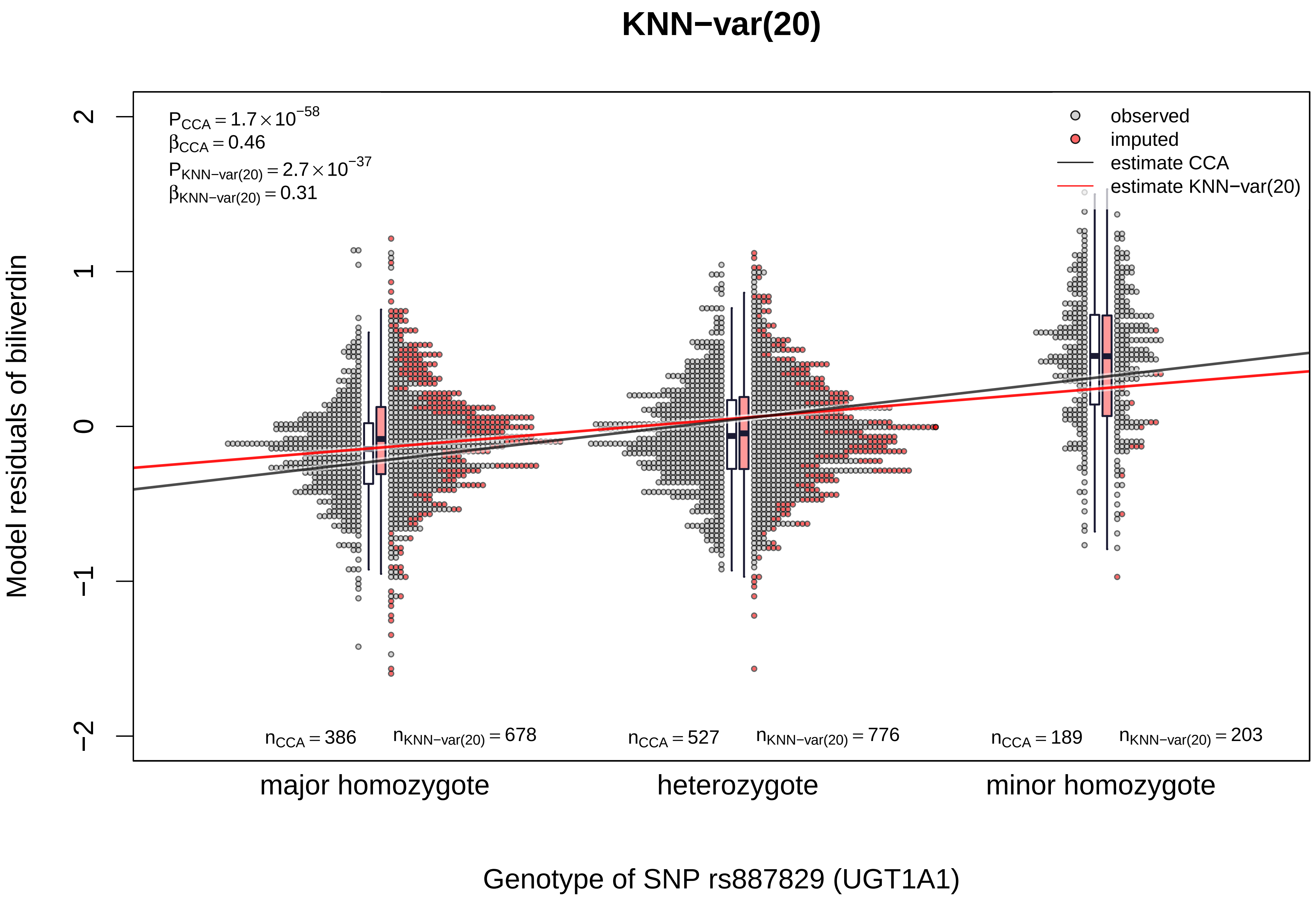

KNN−var(20)
$P_{CCA} = 1.7 \times 10^{-58}$
$\beta_{CCA} = 0.46$
$P_{KNN-var(20)} = 2.7 \times 10^{-37}$
$\beta_{KNN-var(20)} = 0.31$
observed
imputed
estimate CCA
estimate KNN−var(20)
Model residuals of biliverdin
2
1
0
−1
−2
$n_{CCA} = 386$
$n_{KNN-var(20)} = 678$
$n_{CCA} = 527$
$n_{KNN-var(20)} = 776$
$n_{CCA} = 189$
$n_{KNN-var(20)} = 203$
major homozygote
heterozygote
minor homozygote
Genotype of SNP rs887829 (UGT1A1)

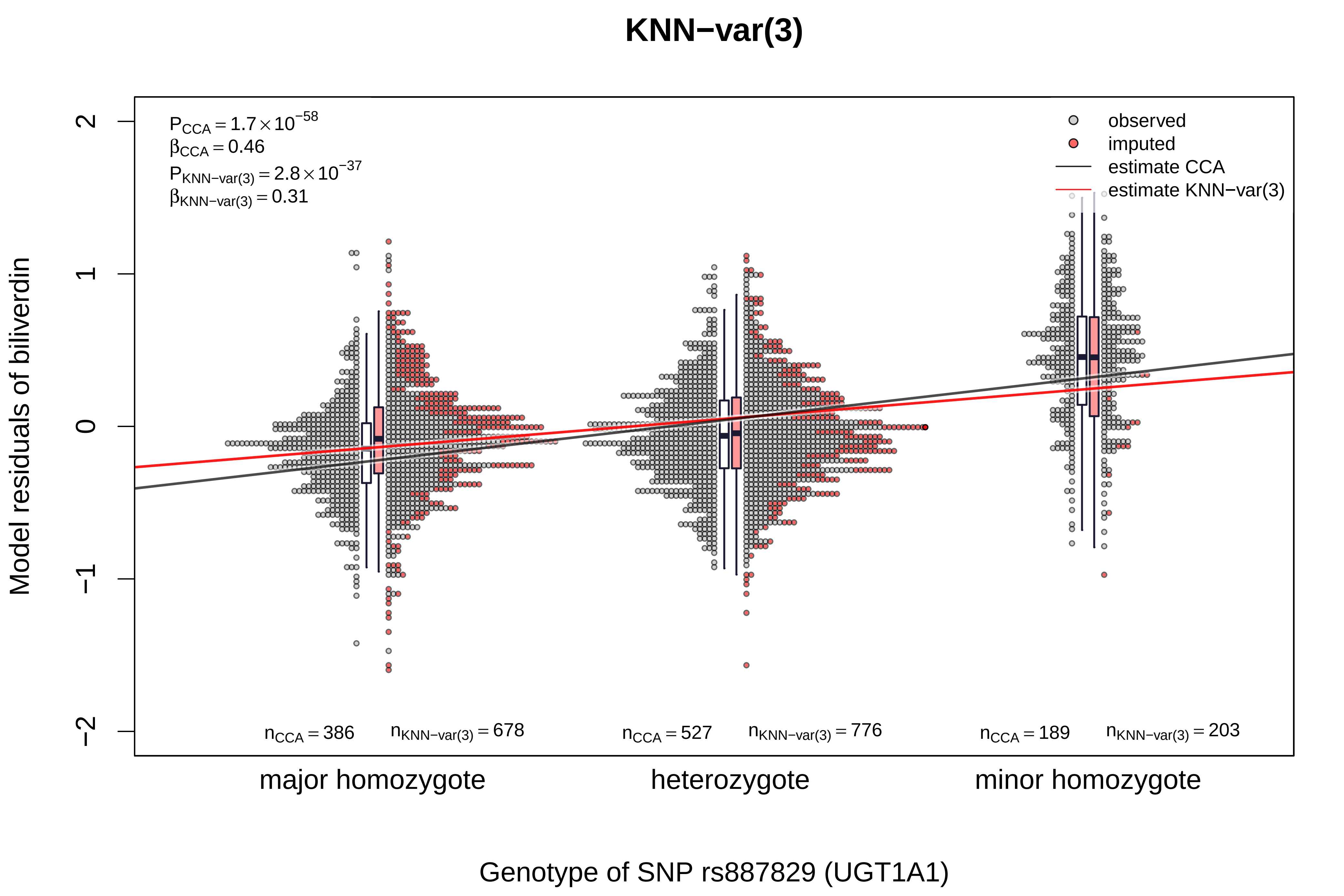
KNN−var(3)
P_CCA = 1.7 × 10^−58
β_CCA = 0.46
P_KNN−var(3) = 2.8 × 10^−37
β_KNN−var(3) = 0.31
observed
imputed
estimate CCA
estimate KNN−var(3)
Model residuals of biliverdin
2
1
0
−1
−2
n_CCA = 386
n_KNN−var(3) = 678
n_CCA = 527
n_KNN−var(3) = 776
n_CCA = 189
n_KNN−var(3) = 203
major homozygote
heterozygote
minor homozygote
Genotype of SNP rs887829 (UGT1A1)

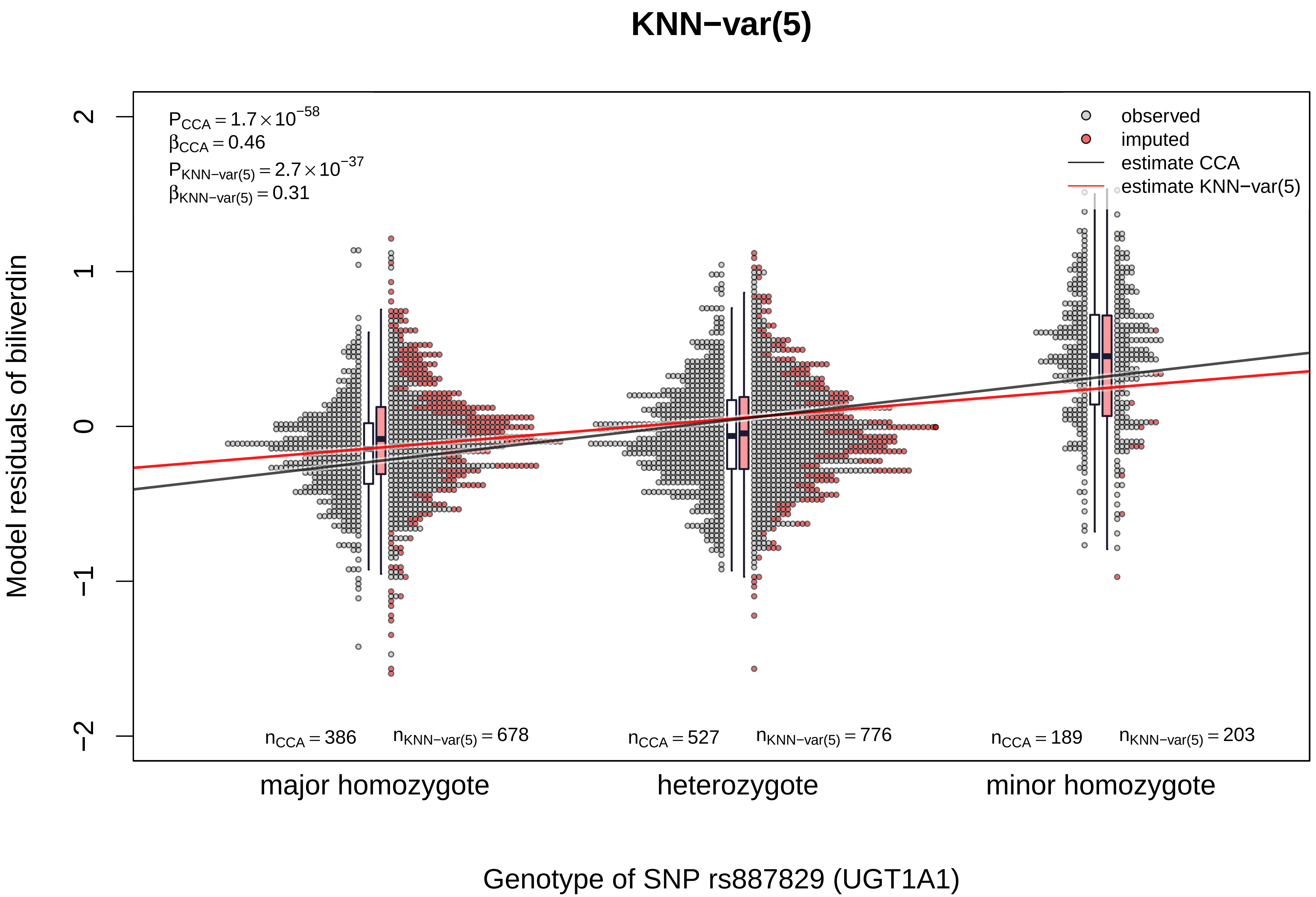

KNN−var(5)
$P_{CCA} = 1.7 \times 10^{-58}$
$\beta_{CCA} = 0.46$
$P_{KNN-var(5)} = 2.7 \times 10^{-37}$
$\beta_{KNN-var(5)} = 0.31$
observed
imputed
estimate CCA
estimate KNN−var(5)
Model residuals of biliverdin
2
1
0
−1
−2
$n_{CCA} = 386$
$n_{KNN-var(5)} = 678$
$n_{CCA} = 527$
$n_{KNN-var(5)} = 776$
$n_{CCA} = 189$
$n_{KNN-var(5)} = 203$
major homozygote
heterozygote
minor homozygote
Genotype of SNP rs887829 (UGT1A1)

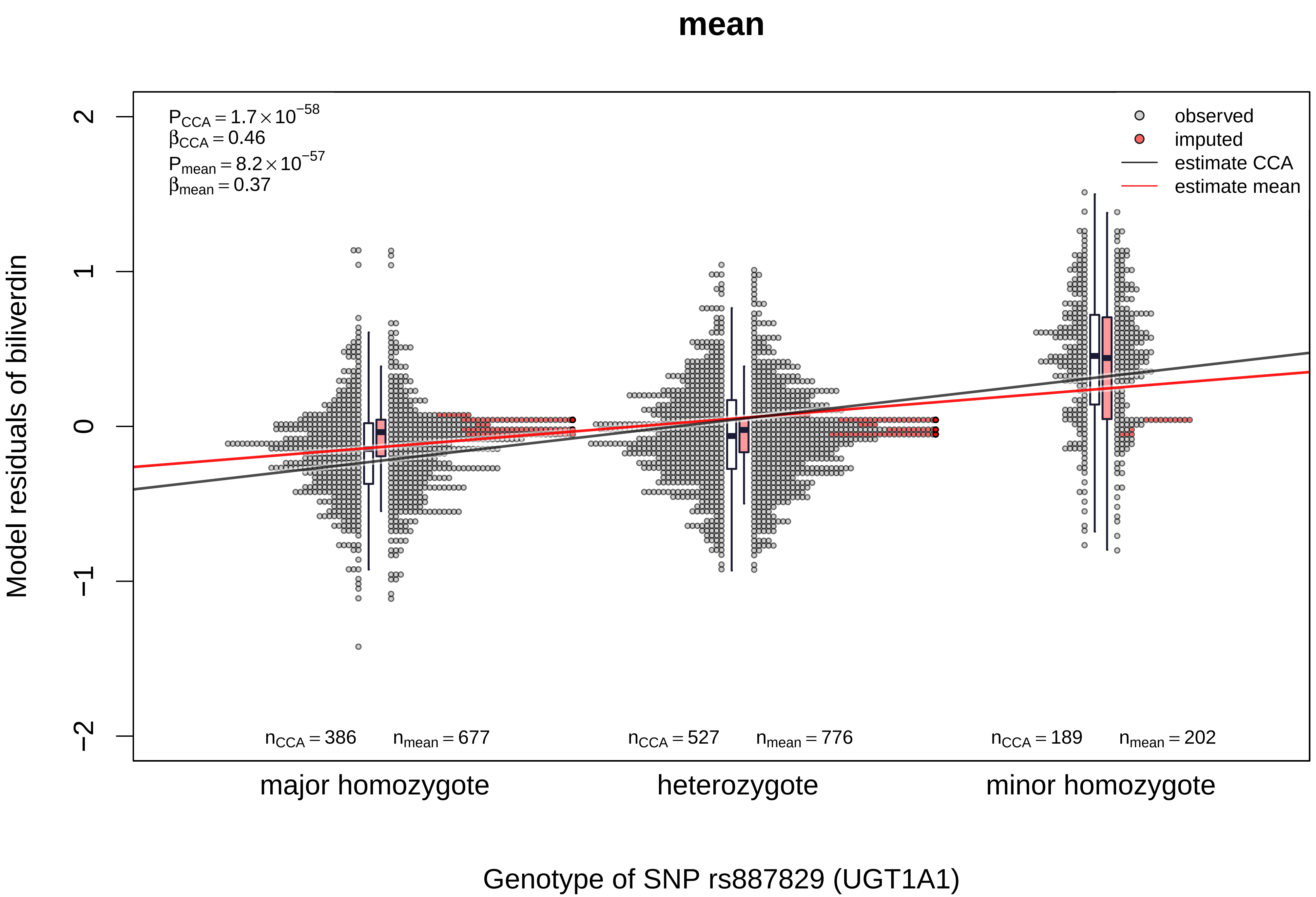
mean
$P_{CCA} = 1.7 \times 10^{-58}$
$\beta_{CCA} = 0.46$
$P_{mean} = 8.2 \times 10^{-57}$
$\beta_{mean} = 0.37$
observed
imputed
estimate CCA
estimate mean
Model residuals of biliverdin
2
1
0
−1
−2
$n_{CCA} = 386$
$n_{mean} = 677$
$n_{CCA} = 527$
$n_{mean} = 776$
$n_{CCA} = 189$
$n_{mean} = 202$
major homozygote
heterozygote
minor homozygote
Genotype of SNP rs887829 (UGT1A1)

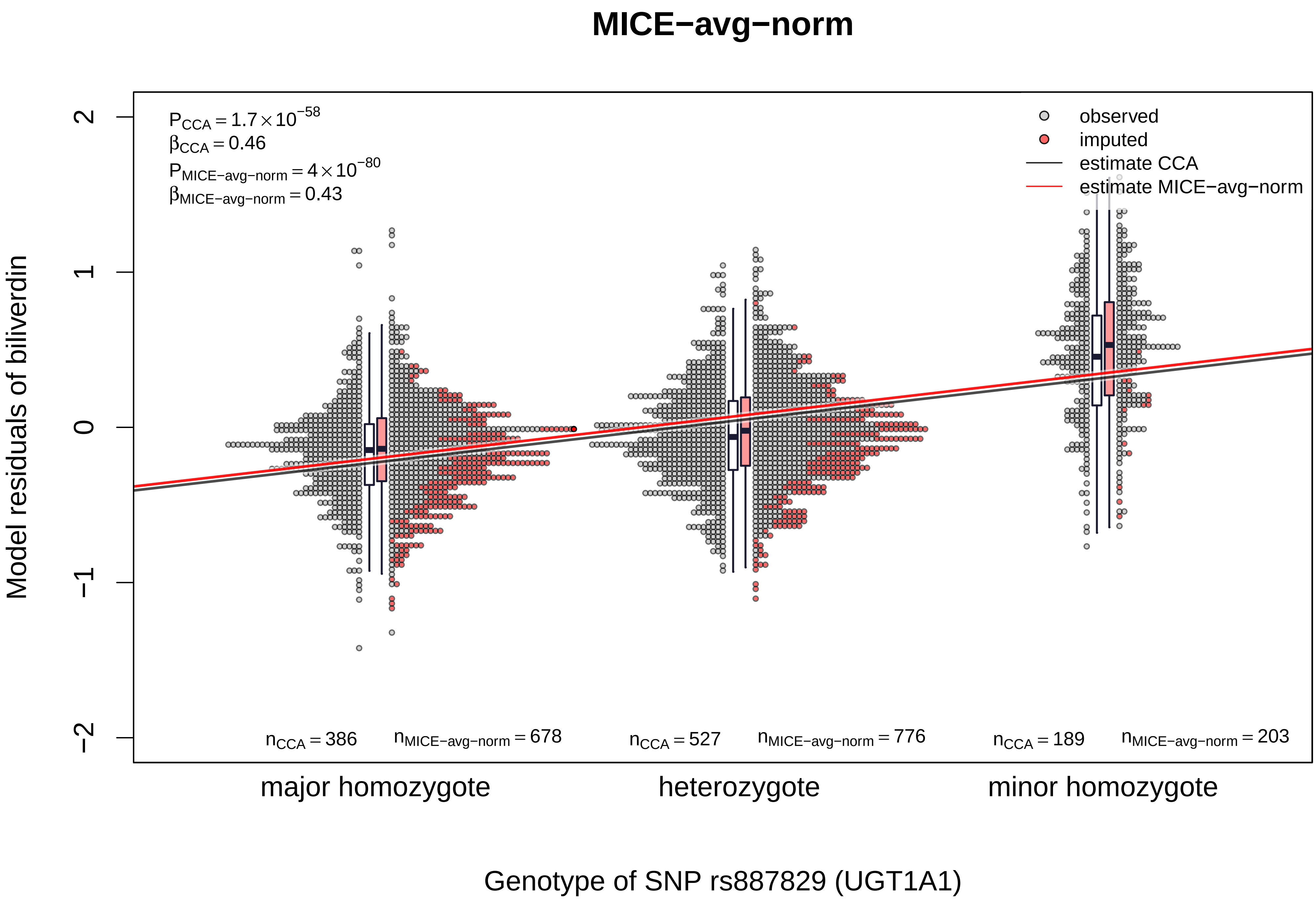
MICE−avg−norm
$P_{CCA} = 1.7 \times 10^{-58}$
$\beta_{CCA} = 0.46$
$P_{MICE-avg-norm} = 4 \times 10^{-80}$
$\beta_{MICE-avg-norm} = 0.43$
observed
imputed
estimate CCA
estimate MICE−avg−norm
Model residuals of biliverdin
2
1
0
−1
−2
$n_{CCA} = 386$
$n_{MICE-avg-norm} = 678$
$n_{CCA} = 527$
$n_{MICE-avg-norm} = 776$
$n_{CCA} = 189$
$n_{MICE-avg-norm} = 203$
major homozygote
heterozygote
minor homozygote
Genotype of SNP rs887829 (UGT1A1)

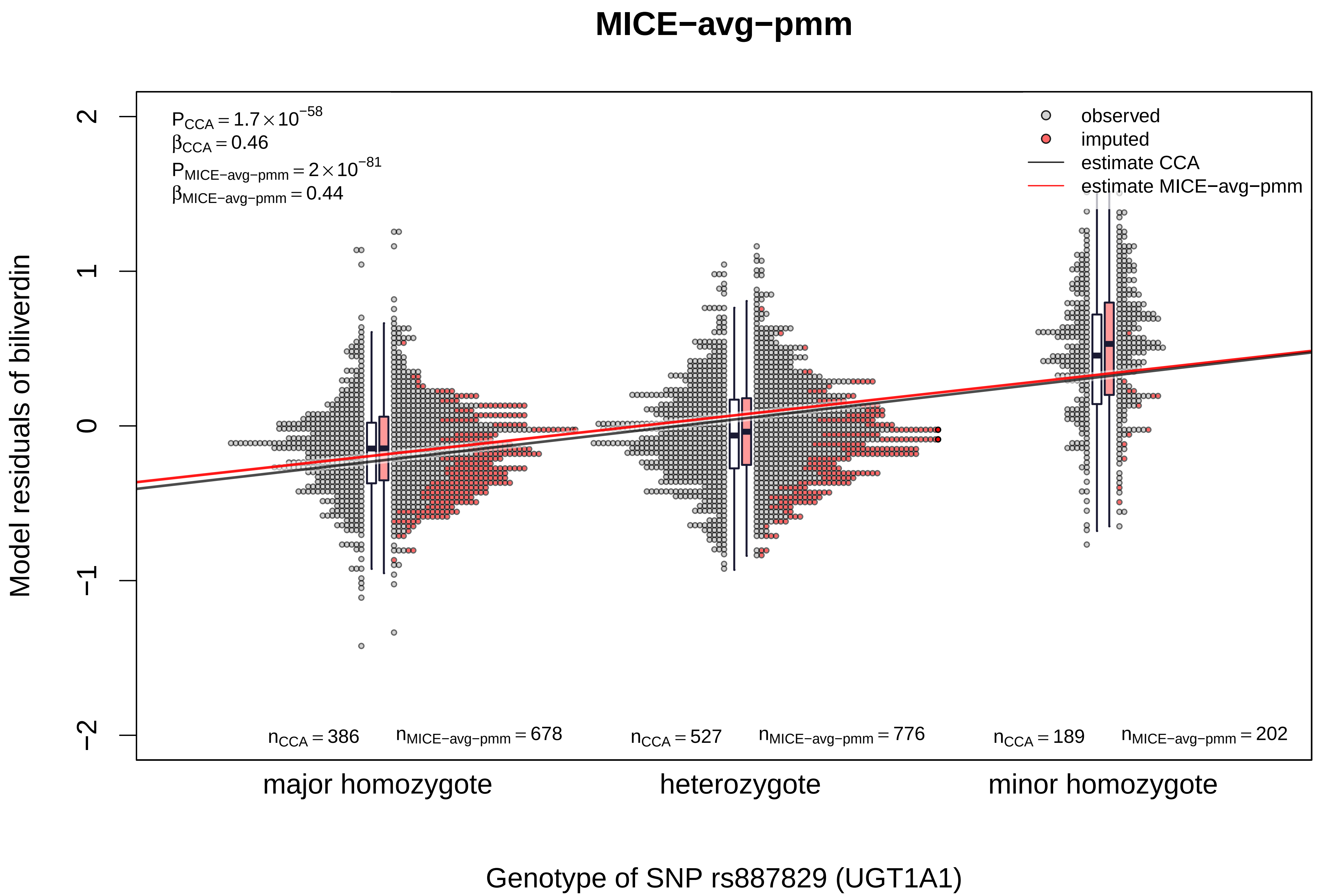

MICE−avg−pmm
$P_{CCA} = 1.7 \times 10^{-58}$
$\beta_{CCA} = 0.46$
$P_{MICE-avg-pmm} = 2 \times 10^{-81}$
$\beta_{MICE-avg-pmm} = 0.44$
observed
imputed
estimate CCA
estimate MICE−avg−pmm
Model residuals of biliverdin
2
1
0
−1
−2
$n_{CCA} = 386$
$n_{MICE-avg-pmm} = 678$
$n_{CCA} = 527$
$n_{MICE-avg-pmm} = 776$
$n_{CCA} = 189$
$n_{MICE-avg-pmm} = 202$
major homozygote
heterozygote
minor homozygote
Genotype of SNP rs887829 (UGT1A1)

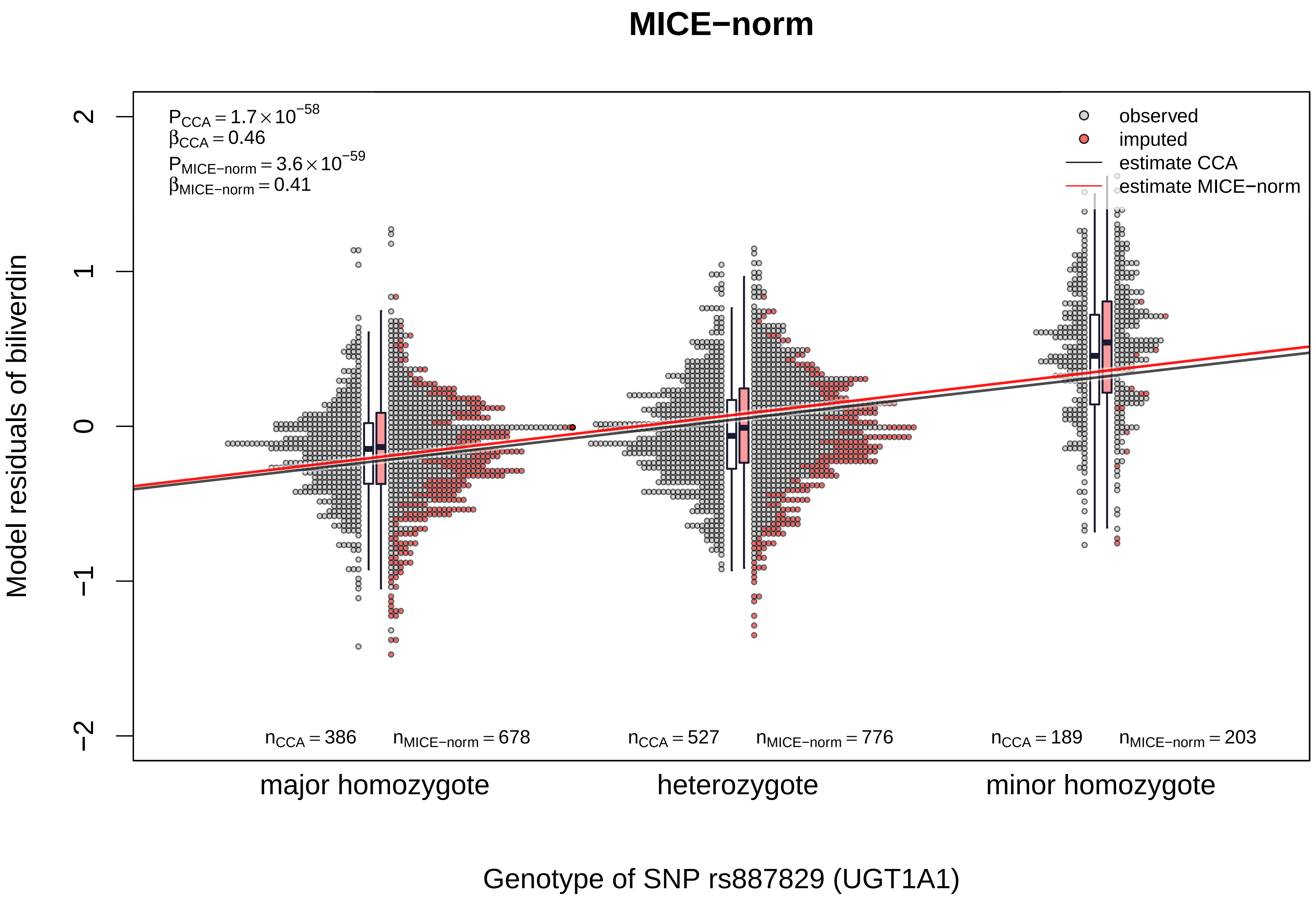

MICE−norm
$P_{CCA} = 1.7 \times 10^{-58}$
$\beta_{CCA} = 0.46$
$P_{MICE-norm} = 3.6 \times 10^{-59}$
$\beta_{MICE-norm} = 0.41$
observed
imputed
estimate CCA
estimate MICE−norm
Model residuals of biliverdin
2
1
0
−1
−2
$n_{CCA} = 386$
$n_{MICE-norm} = 678$
$n_{CCA} = 527$
$n_{MICE-norm} = 776$
$n_{CCA} = 189$
$n_{MICE-norm} = 203$
major homozygote
heterozygote
minor homozygote
Genotype of SNP rs887829 (UGT1A1)

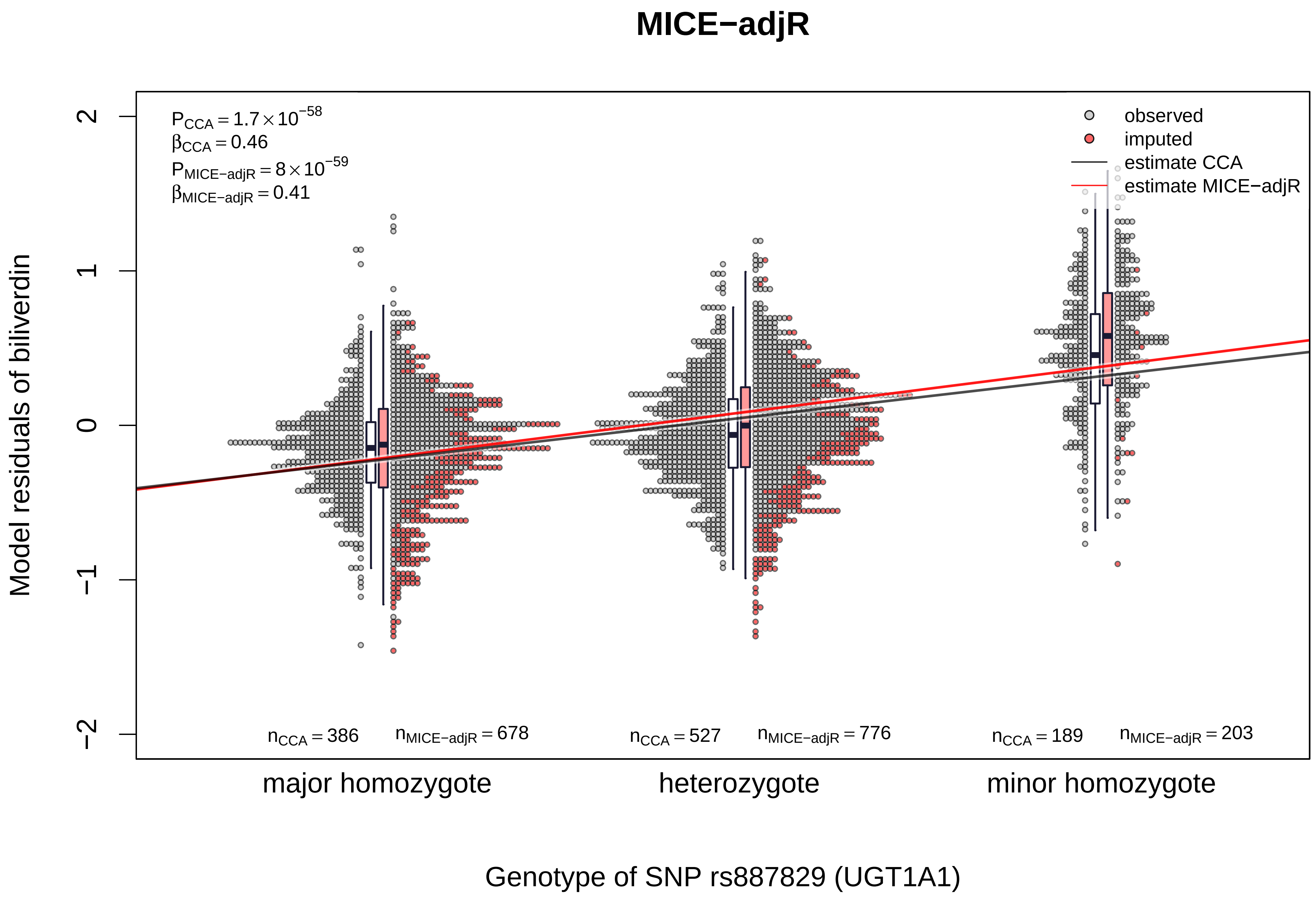

MICE−adjR
$P_{CCA} = 1.7 \times 10^{-58}$
$\beta_{CCA} = 0.46$
$P_{MICE-adjR} = 8 \times 10^{-59}$
$\beta_{MICE-adjR} = 0.41$
observed
imputed
estimate CCA
estimate MICE−adjR
Model residuals of biliverdin
2
1
0
−1
−2
$n_{CCA} = 386$
$n_{MICE-adjR} = 678$
$n_{CCA} = 527$
$n_{MICE-adjR} = 776$
$n_{CCA} = 189$
$n_{MICE-adjR} = 203$
major homozygote
heterozygote
minor homozygote
Genotype of SNP rs887829 (UGT1A1)

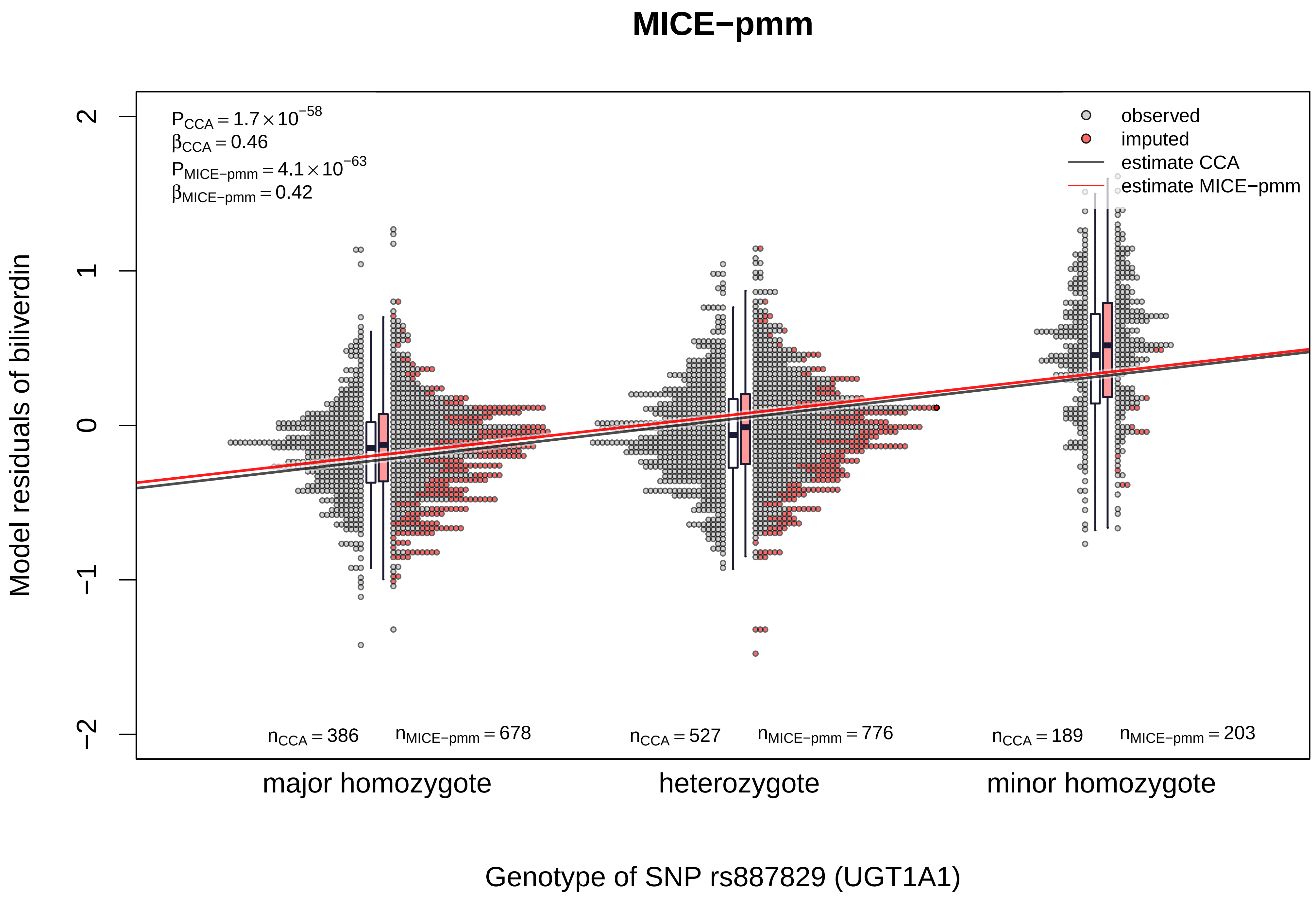

MICE−pmm
$P_{CCA} = 1.7 \times 10^{-58}$
$\beta_{CCA} = 0.46$
$P_{MICE-pmm} = 4.1 \times 10^{-63}$
$\beta_{MICE-pmm} = 0.42$
observed
imputed
estimate CCA
estimate MICE−pmm
Model residuals of biliverdin
2
1
0
−1
−2
$n_{CCA} = 386$
$n_{MICE-pmm} = 678$
$n_{CCA} = 527$
$n_{MICE-pmm} = 776$
$n_{CCA} = 189$
$n_{MICE-pmm} = 203$
major homozygote
heterozygote
minor homozygote
Genotype of SNP rs887829 (UGT1A1)

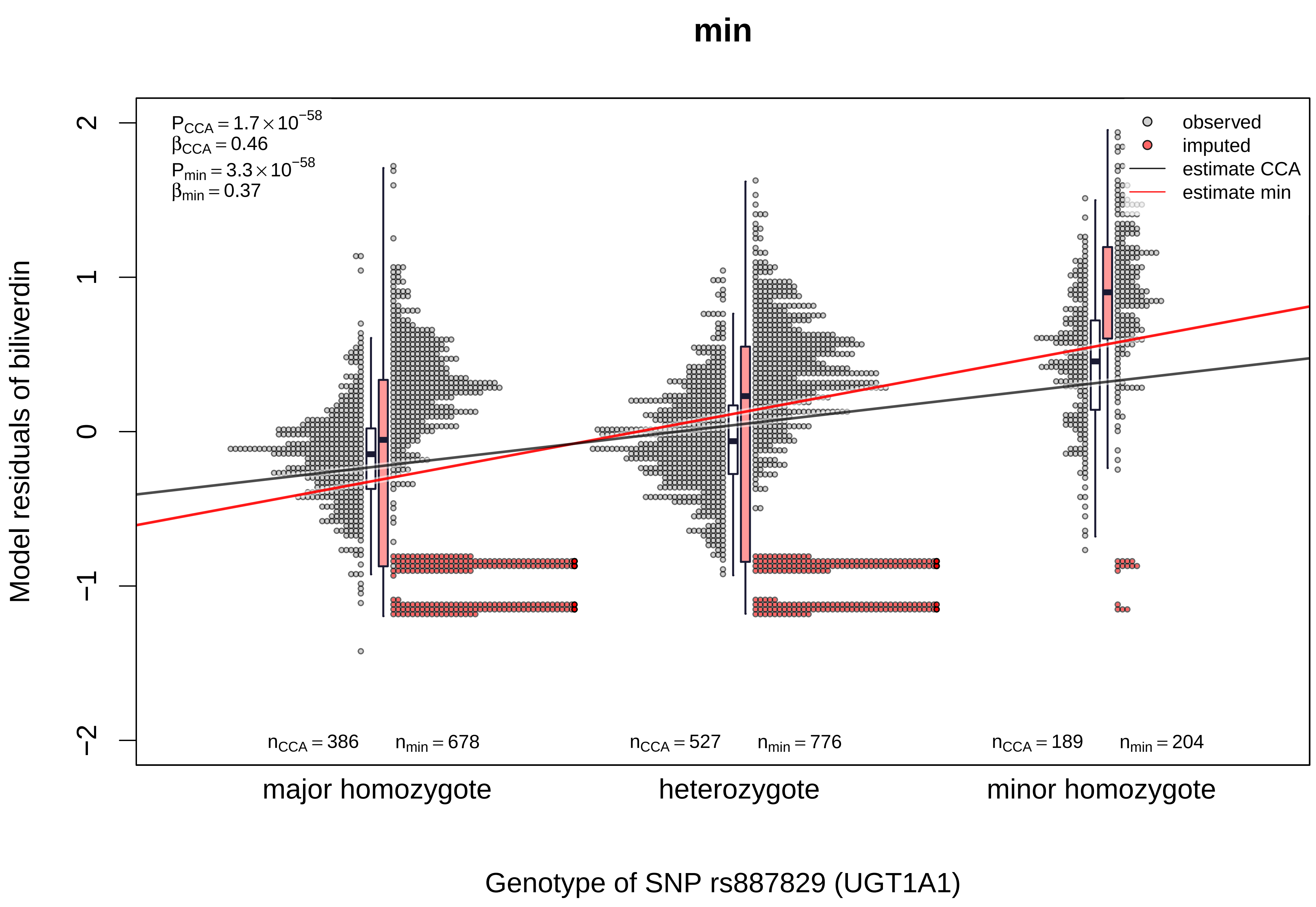

min
P_CCA = 1.7 × 10^−58
β_CCA = 0.46
P_min = 3.3 × 10^−58
β_min = 0.37
observed
imputed
estimate CCA
estimate min
Model residuals of biliverdin
2
1
0
−1
−2
n_CCA = 386
n_min = 678
n_CCA = 527
n_min = 776
n_CCA = 189
n_min = 204
major homozygote
heterozygote
minor homozygote
Genotype of SNP rs887829 (UGT1A1)

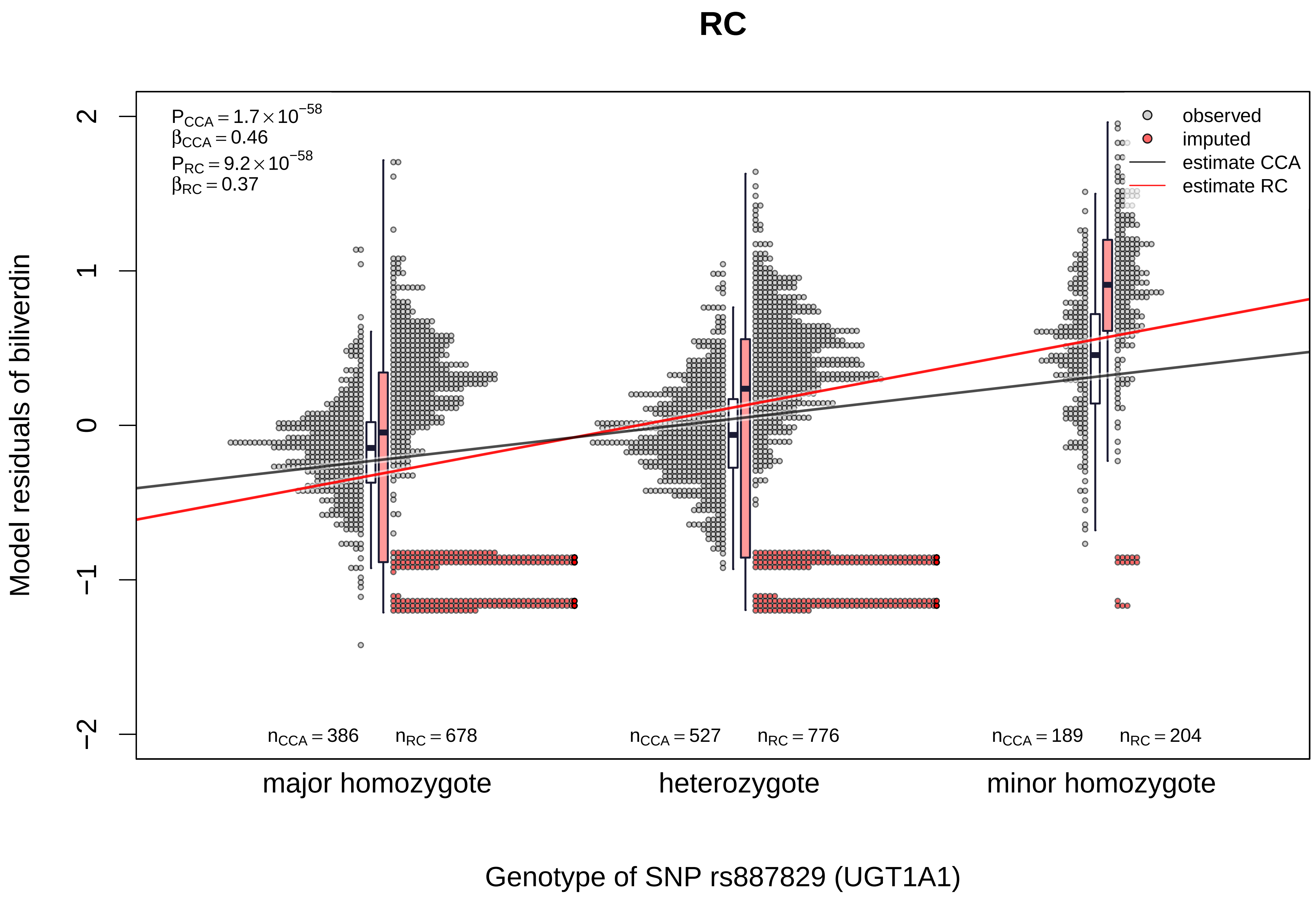

RC
$P_{CCA} = 1.7 \times 10^{-58}$
$\beta_{CCA} = 0.46$
$P_{RC} = 9.2 \times 10^{-58}$
$\beta_{RC} = 0.37$
observed
imputed
estimate CCA
estimate RC
Model residuals of biliverdin
2
1
0
−1
−2
$n_{CCA} = 386$
$n_{RC} = 678$
$n_{CCA} = 527$
$n_{RC} = 776$
$n_{CCA} = 189$
$n_{RC} = 204$
major homozygote
heterozygote
minor homozygote
Genotype of SNP rs887829 (UGT1A1)

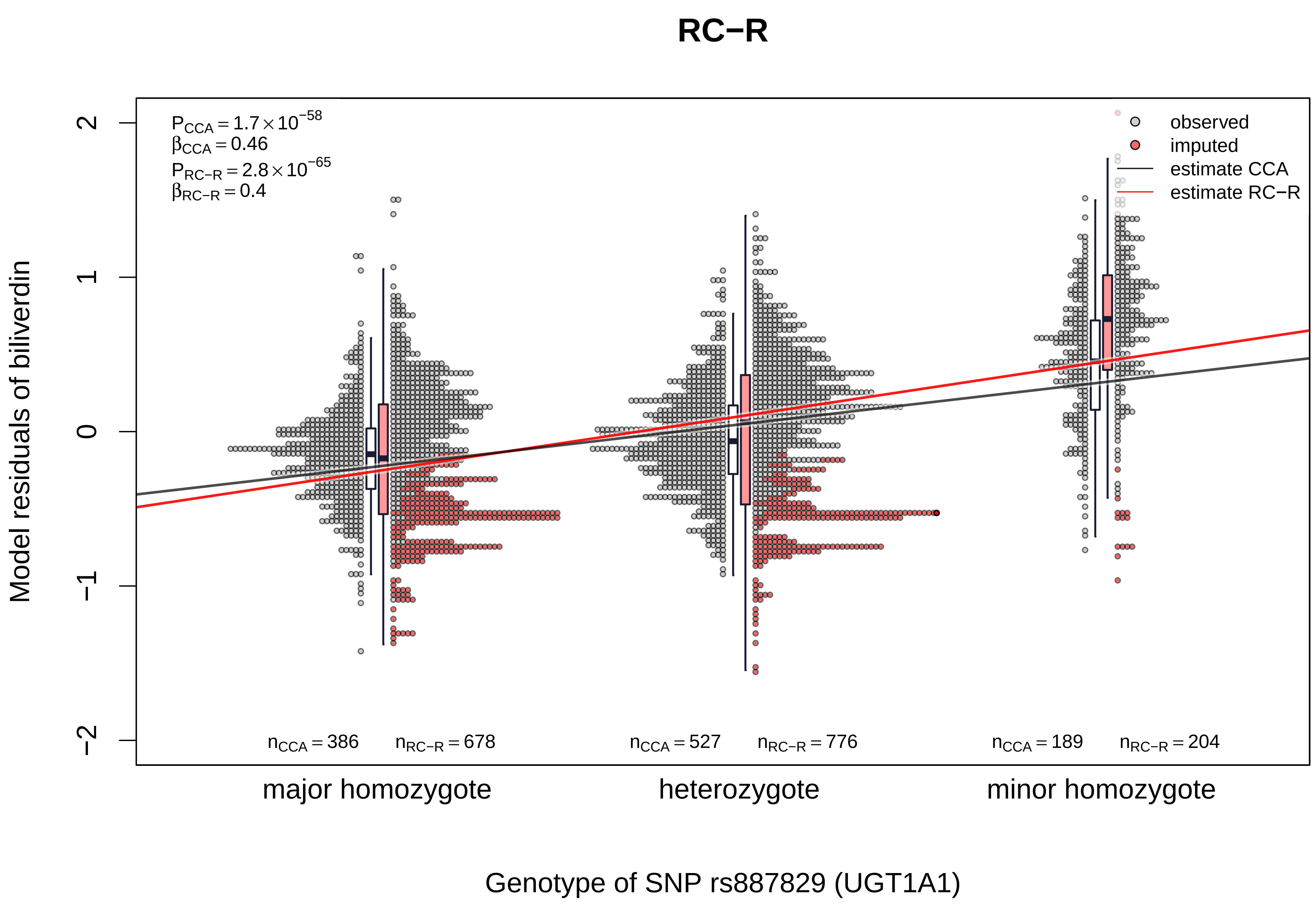

RC−R
$P_{CCA} = 1.7 \times 10^{-58}$
$\beta_{CCA} = 0.46$
$P_{RC-R} = 2.8 \times 10^{-65}$
$\beta_{RC-R} = 0.4$
observed
imputed
estimate CCA
estimate RC−R
Model residuals of biliverdin
2
1
0
−1
−2
$n_{CCA} = 386$
$n_{RC-R} = 678$
$n_{CCA} = 527$
$n_{RC-R} = 776$
$n_{CCA} = 189$
$n_{RC-R} = 204$
major homozygote
heterozygote
minor homozygote
Genotype of SNP rs887829 (UGT1A1)

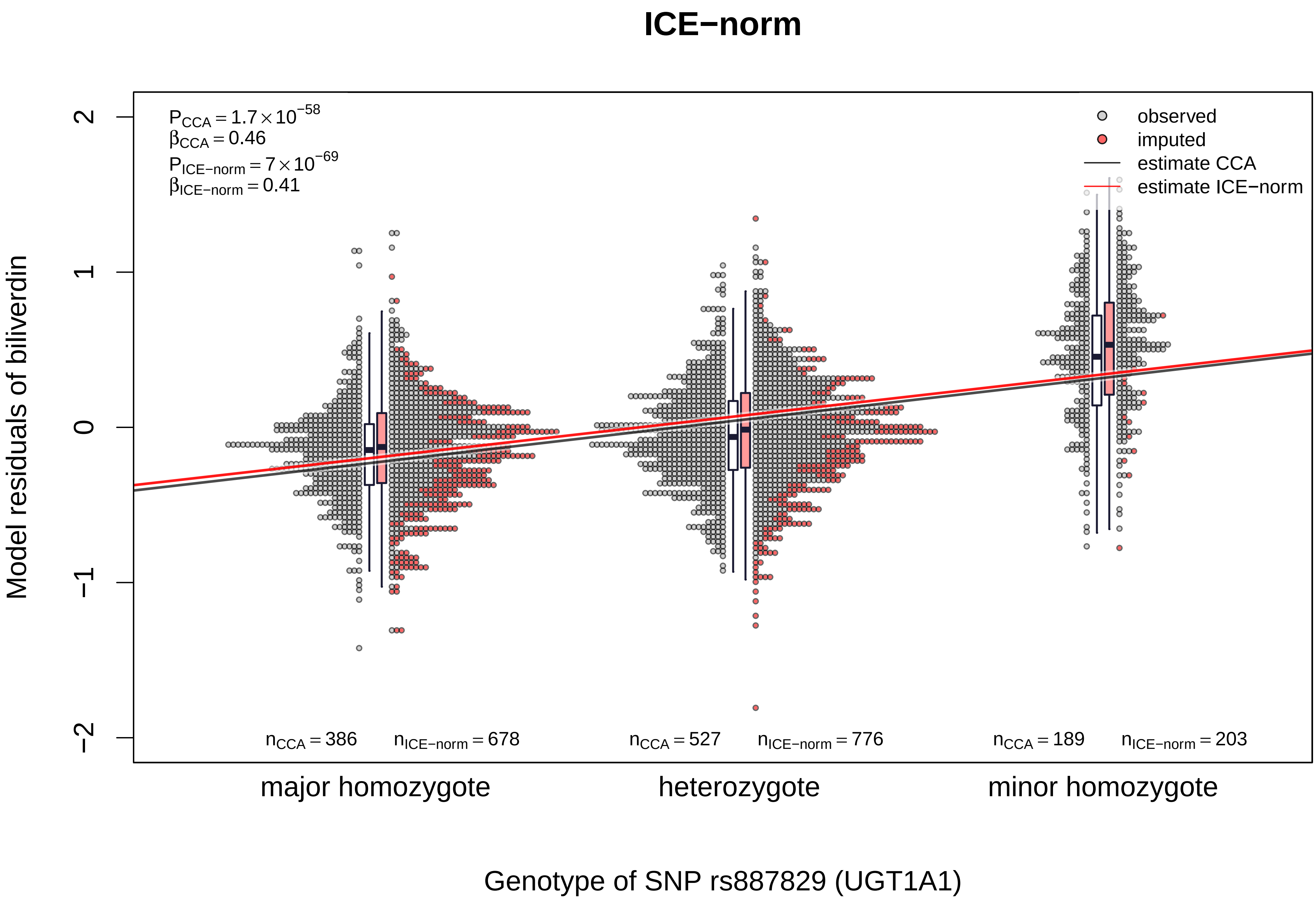

ICE−norm
$P_{CCA} = 1.7 \times 10^{-58}$
$\beta_{CCA} = 0.46$
$P_{ICE-norm} = 7 \times 10^{-69}$
$\beta_{ICE-norm} = 0.41$
observed
imputed
estimate CCA
estimate ICE−norm
Model residuals of biliverdin
2
1
0
−1
−2
$n_{CCA} = 386$
$n_{ICE-norm} = 678$
$n_{CCA} = 527$
$n_{ICE-norm} = 776$
$n_{CCA} = 189$
$n_{ICE-norm} = 203$
major homozygote
heterozygote
minor homozygote
Genotype of SNP rs887829 (UGT1A1)

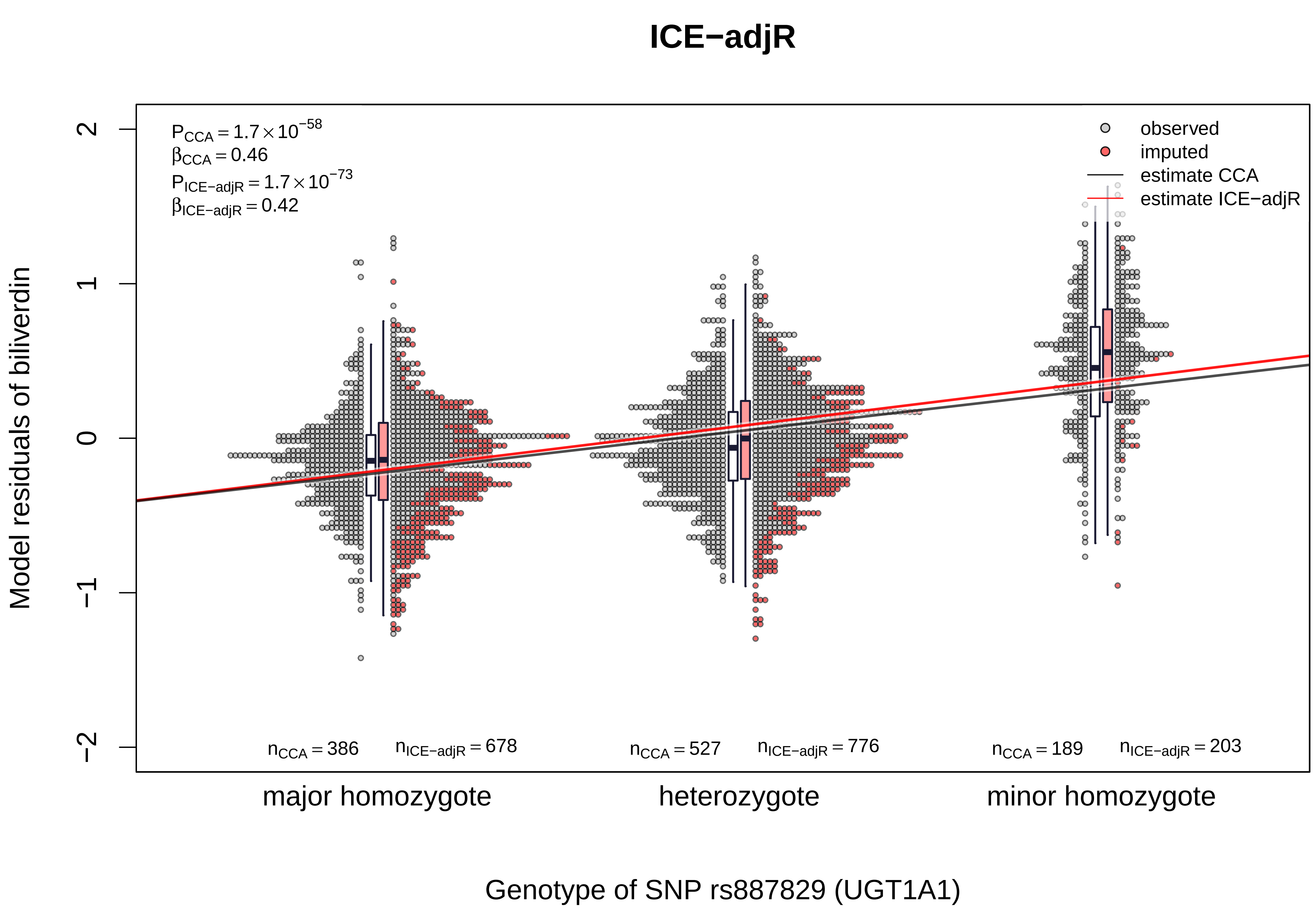

ICE−adjR
$P_{CCA} = 1.7 \times 10^{-58}$
$\beta_{CCA} = 0.46$
$P_{ICE-adjR} = 1.7 \times 10^{-73}$
$\beta_{ICE-adjR} = 0.42$
observed
imputed
estimate CCA
estimate ICE−adjR
Model residuals of biliverdin
2
1
0
−1
−2
$n_{CCA} = 386$
$n_{ICE-adjR} = 678$
$n_{CCA} = 527$
$n_{ICE-adjR} = 776$
$n_{CCA} = 189$
$n_{ICE-adjR} = 203$
major homozygote
heterozygote
minor homozygote
Genotype of SNP rs887829 (UGT1A1)

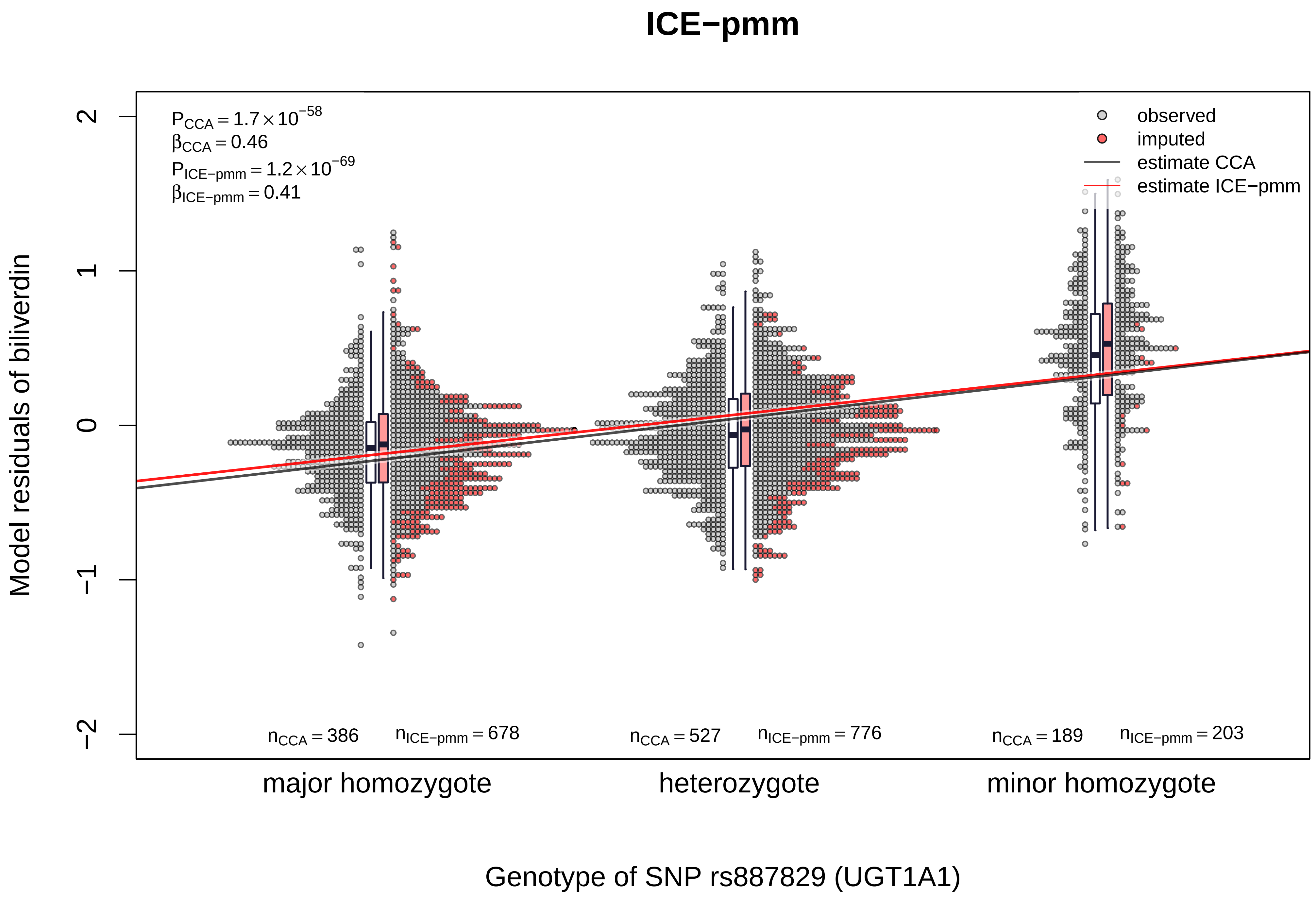

ICE−pmm
$P_{CCA} = 1.7 \times 10^{-58}$
$\beta_{CCA} = 0.46$
$P_{ICE-pmm} = 1.2 \times 10^{-69}$
$\beta_{ICE-pmm} = 0.41$
observed
imputed
estimate CCA
estimate ICE−pmm
Model residuals of biliverdin
2
1
0
−1
−2
$n_{CCA} = 386$
$n_{ICE-pmm} = 678$
$n_{CCA} = 527$
$n_{ICE-pmm} = 776$
$n_{CCA} = 189$
$n_{ICE-pmm} = 203$
major homozygote
heterozygote
minor homozygote
Genotype of SNP rs887829 (UGT1A1)

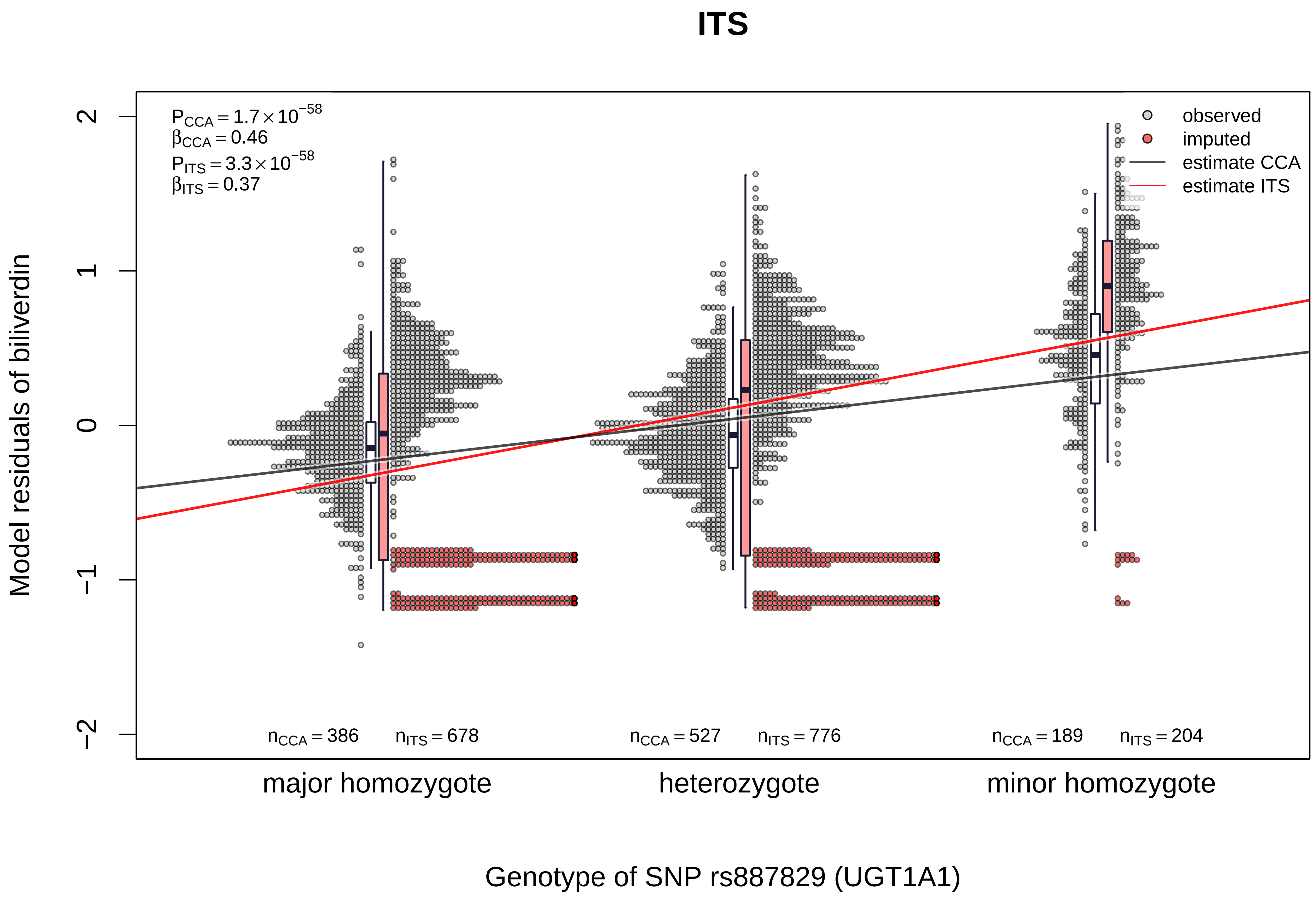

ITS
$P_{CCA} = 1.7 \times 10^{-58}$
$\beta_{CCA} = 0.46$
$P_{ITS} = 3.3 \times 10^{-58}$
$\beta_{ITS} = 0.37$
observed
imputed
estimate CCA
estimate ITS
Model residuals of biliverdin
2
1
0
−1
−2
$n_{CCA} = 386$
$n_{ITS} = 678$
$n_{CCA} = 527$
$n_{ITS} = 776$
$n_{CCA} = 189$
$n_{ITS} = 204$
major homozygote
heterozygote
minor homozygote
Genotype of SNP rs887829 (UGT1A1)

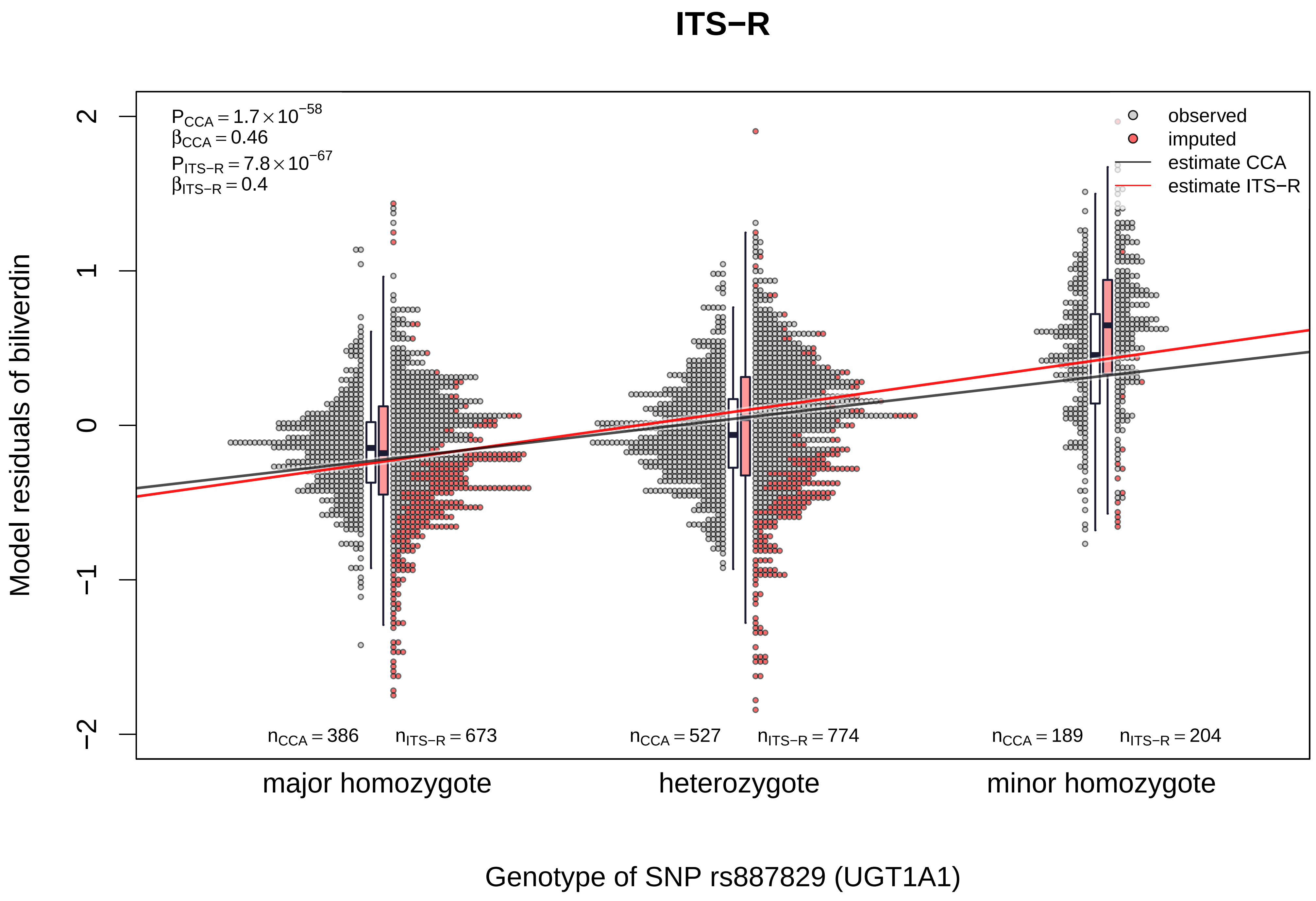
ITS−R
$P_{CCA} = 1.7 \times 10^{-58}$
$\beta_{CCA} = 0.46$
$P_{ITS-R} = 7.8 \times 10^{-67}$
$\beta_{ITS-R} = 0.4$
observed
imputed
estimate CCA
estimate ITS−R
Model residuals of biliverdin
2
1
0
−1
−2
$n_{CCA} = 386$
$n_{ITS-R} = 673$
$n_{CCA} = 527$
$n_{ITS-R} = 774$
$n_{CCA} = 189$
$n_{ITS-R} = 204$
major homozygote
heterozygote
minor homozygote
Genotype of SNP rs887829 (UGT1A1)

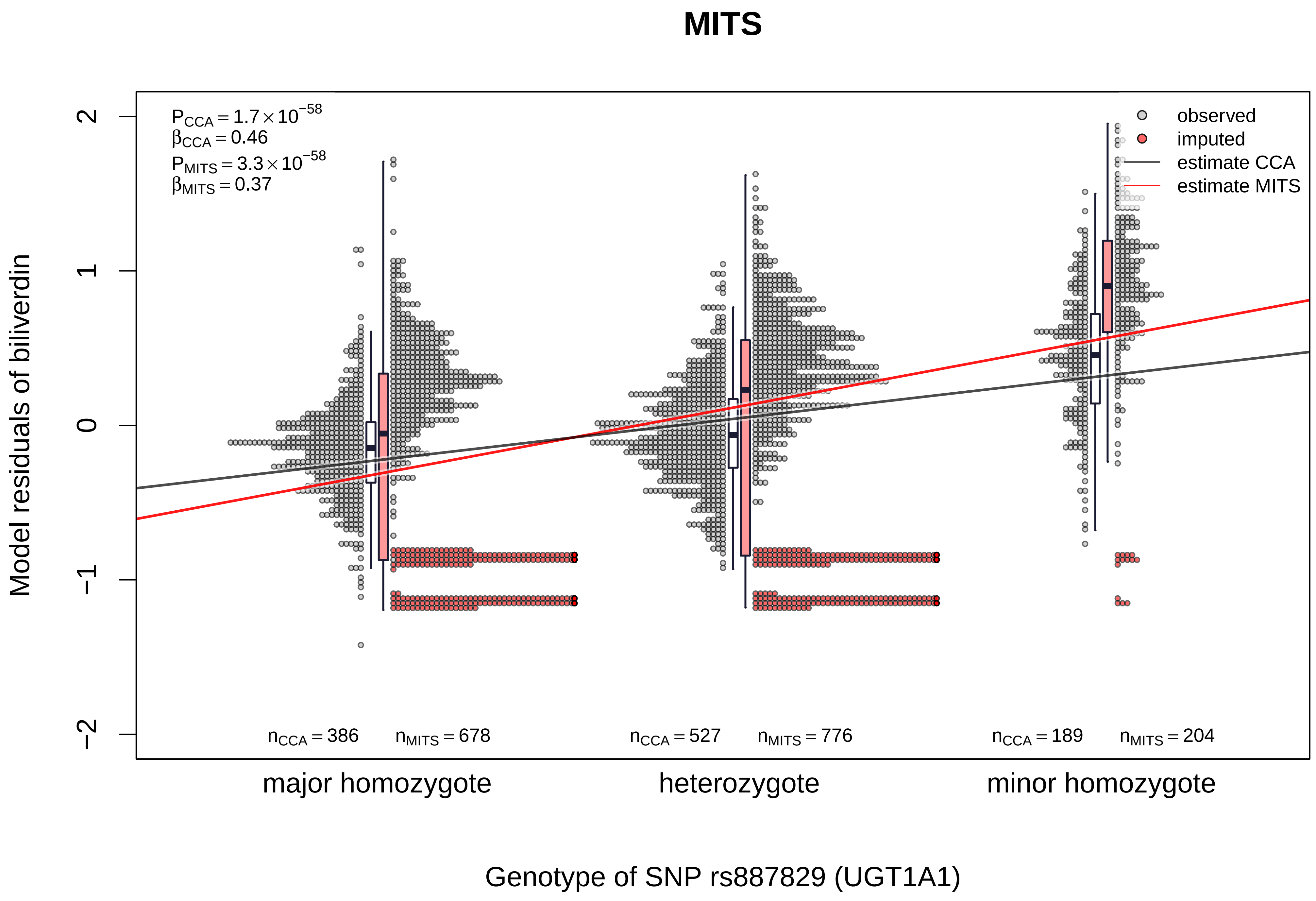

MITS
$P_{CCA} = 1.7 \times 10^{-58}$
$\beta_{CCA} = 0.46$
$P_{MITS} = 3.3 \times 10^{-58}$
$\beta_{MITS} = 0.37$
observed
imputed
estimate CCA
estimate MITS
2
1
0
−1
−2
Model residuals of biliverdin
$n_{CCA} = 386$
$n_{MITS} = 678$
$n_{CCA} = 527$
$n_{MITS} = 776$
$n_{CCA} = 189$
$n_{MITS} = 204$
major homozygote
heterozygote
minor homozygote
Genotype of SNP rs887829 (UGT1A1)

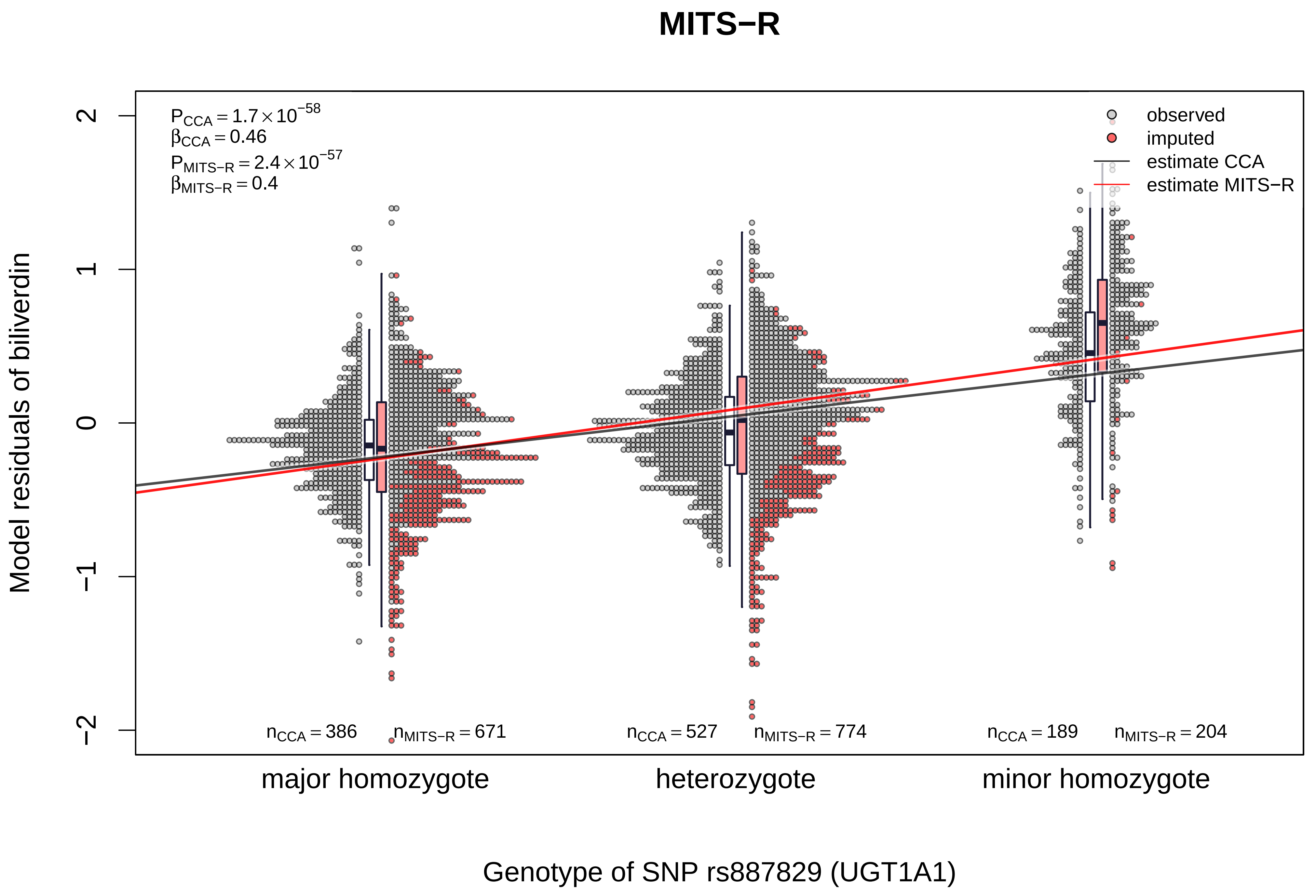

MITS−R
$P_{CCA} = 1.7 \times 10^{-58}$
$\beta_{CCA} = 0.46$
$P_{MITS-R} = 2.4 \times 10^{-57}$
$\beta_{MITS-R} = 0.4$
observed
imputed
estimate CCA
estimate MITS−R
Model residuals of biliverdin
2
1
0
−1
−2
$n_{CCA} = 386$
$n_{MITS-R} = 671$
$n_{CCA} = 527$
$n_{MITS-R} = 774$
$n_{CCA} = 189$
$n_{MITS-R} = 204$
major homozygote
heterozygote
minor homozygote
Genotype of SNP rs887829 (UGT1A1)

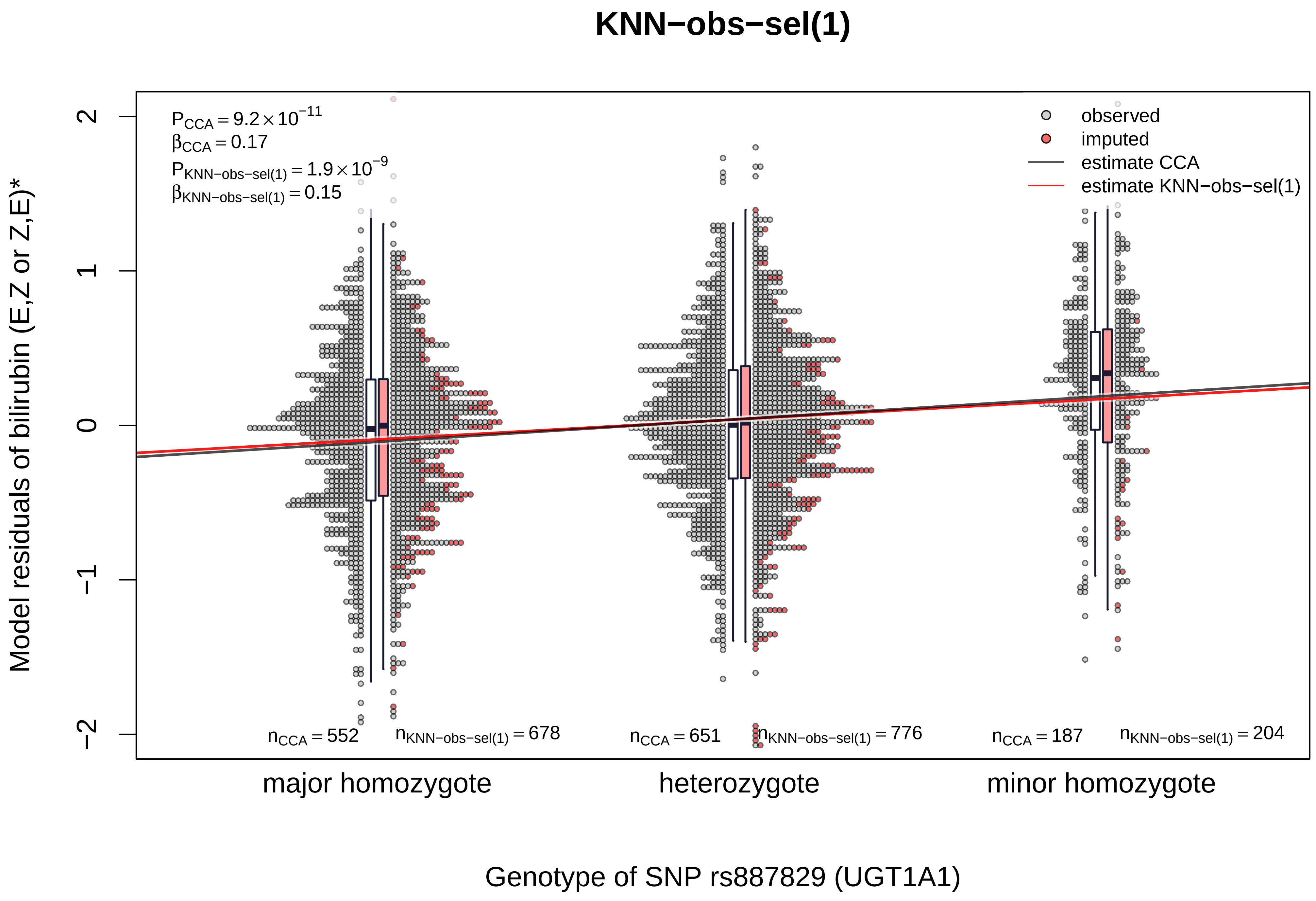

KNN−obs−sel(1)
$P_{CCA} = 9.2 \times 10^{-11}$
$\beta_{CCA} = 0.17$
$P_{KNN-obs-sel(1)} = 1.9 \times 10^{-9}$
$\beta_{KNN-obs-sel(1)} = 0.15$
observed
imputed
estimate CCA
estimate KNN−obs−sel(1)
Model residuals of bilirubin (E,Z or Z,E)*
2
1
0
−1
−2
$n_{CCA} = 552$
$n_{KNN-obs-sel(1)} = 678$
$n_{CCA} = 651$
$n_{KNN-obs-sel(1)} = 776$
$n_{CCA} = 187$
$n_{KNN-obs-sel(1)} = 204$
major homozygote
heterozygote
minor homozygote
Genotype of SNP rs887829 (UGT1A1)

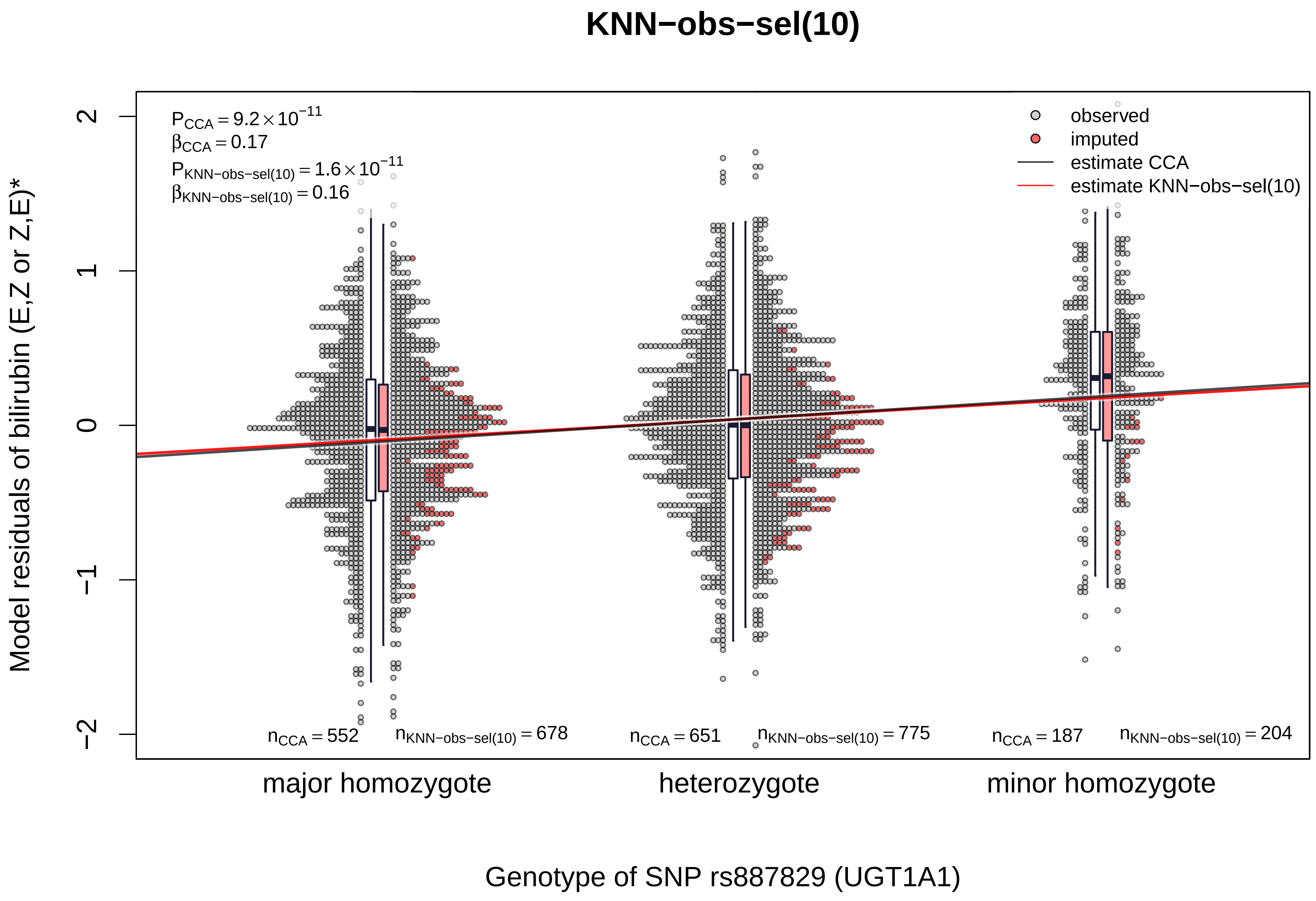

KNN−obs−sel(10)
$P_{CCA} = 9.2 \times 10^{-11}$
$\beta_{CCA} = 0.17$
$P_{KNN-obs-sel(10)} = 1.6 \times 10^{-11}$
$\beta_{KNN-obs-sel(10)} = 0.16$
observed
imputed
estimate CCA
estimate KNN−obs−sel(10)
Model residuals of bilirubin (E,Z or Z,E)*
2
1
0
−1
−2
$n_{CCA} = 552$
$n_{KNN-obs-sel(10)} = 678$
$n_{CCA} = 651$
$n_{KNN-obs-sel(10)} = 775$
$n_{CCA} = 187$
$n_{KNN-obs-sel(10)} = 204$
major homozygote
heterozygote
minor homozygote
Genotype of SNP rs887829 (UGT1A1)

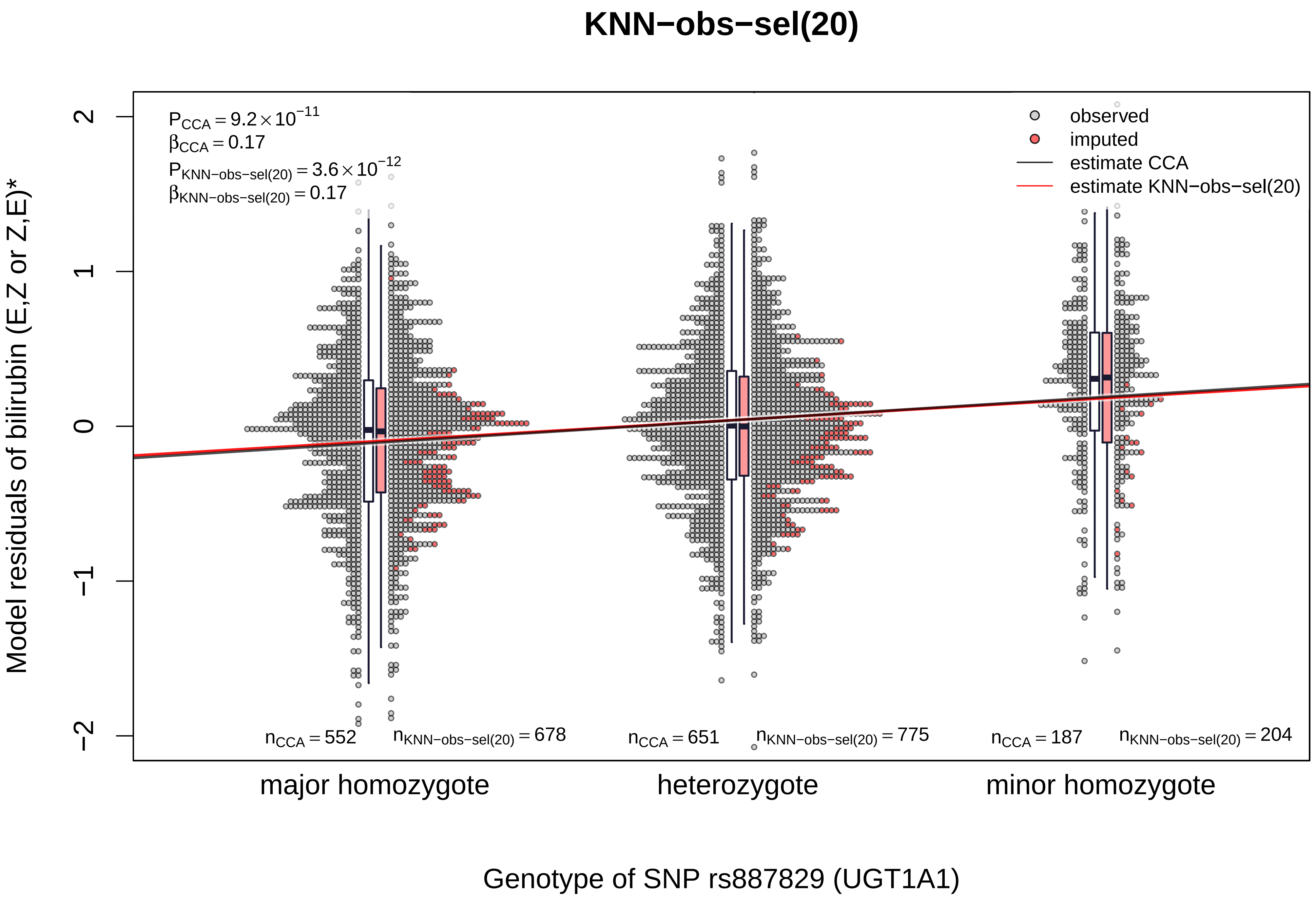

KNN−obs−sel(20)
$P_{CCA} = 9.2 \times 10^{-11}$
$\beta_{CCA} = 0.17$
$P_{KNN-obs-sel(20)} = 3.6 \times 10^{-12}$
$\beta_{KNN-obs-sel(20)} = 0.17$
observed
imputed
estimate CCA
estimate KNN−obs−sel(20)
Model residuals of bilirubin (E,Z or Z,E)*
2
1
0
−1
−2
$n_{CCA} = 552$
$n_{KNN-obs-sel(20)} = 678$
$n_{CCA} = 651$
$n_{KNN-obs-sel(20)} = 775$
$n_{CCA} = 187$
$n_{KNN-obs-sel(20)} = 204$
major homozygote
heterozygote
minor homozygote
Genotype of SNP rs887829 (UGT1A1)

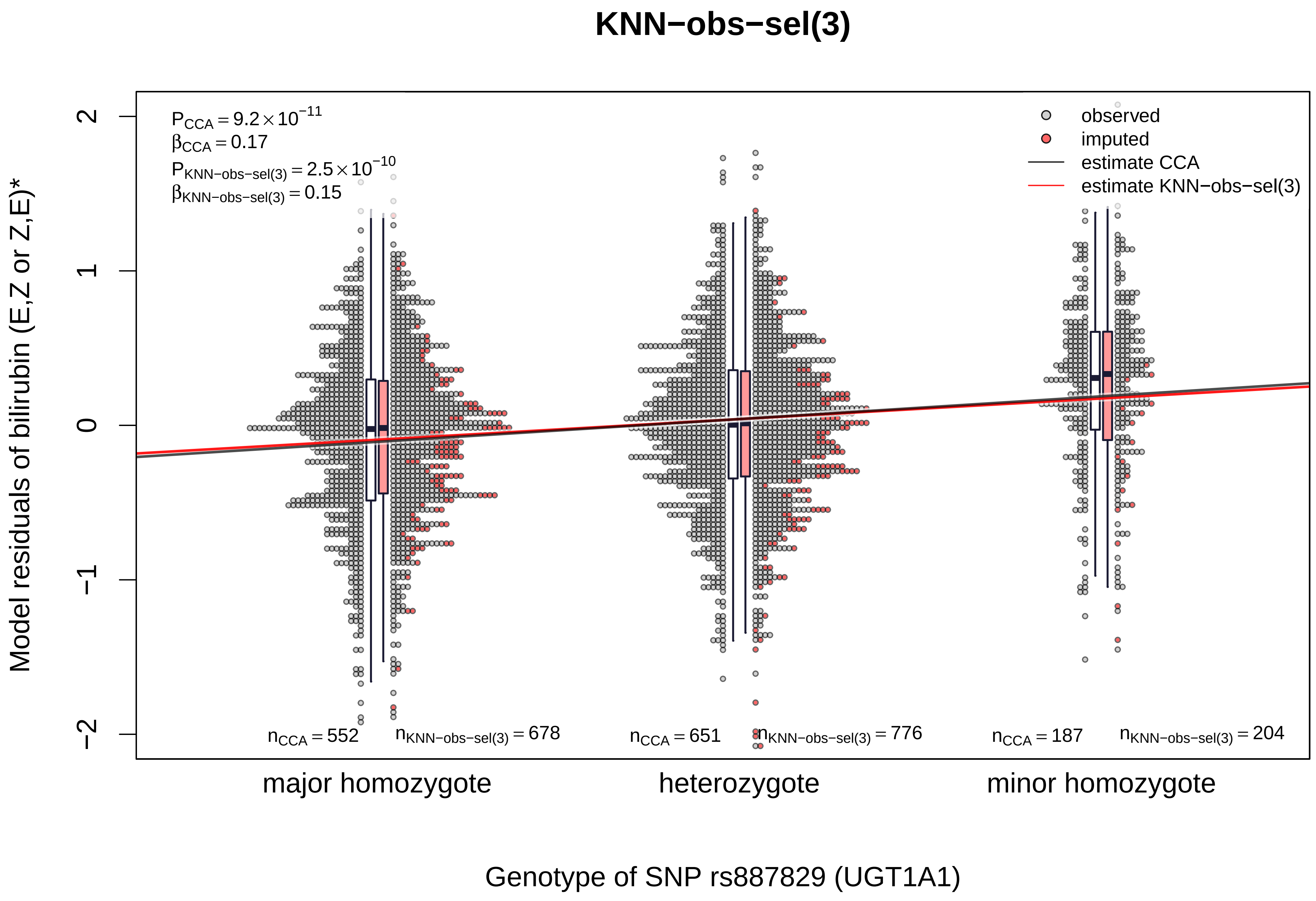
KNN−obs−sel(3)
$P_{CCA} = 9.2 \times 10^{-11}$
$\beta_{CCA} = 0.17$
$P_{KNN-obs-sel(3)} = 2.5 \times 10^{-10}$
$\beta_{KNN-obs-sel(3)} = 0.15$
observed
imputed
estimate CCA
estimate KNN−obs−sel(3)
Model residuals of bilirubin (E,Z or Z,E)*
2
1
0
−1
−2
$n_{CCA} = 552$
$n_{KNN-obs-sel(3)} = 678$
$n_{CCA} = 651$
$n_{KNN-obs-sel(3)} = 776$
$n_{CCA} = 187$
$n_{KNN-obs-sel(3)} = 204$
major homozygote
heterozygote
minor homozygote
Genotype of SNP rs887829 (UGT1A1)

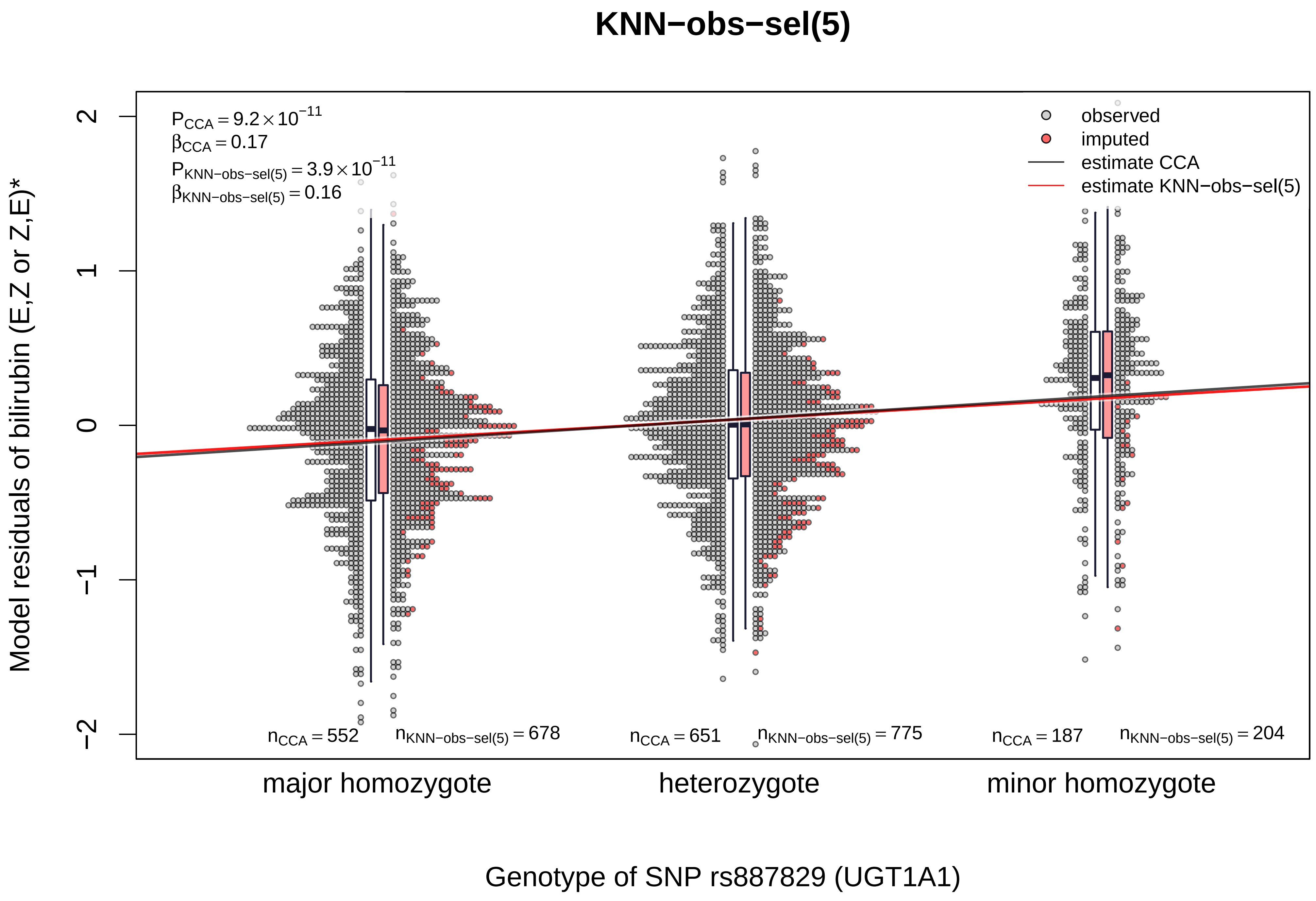

KNN−obs−sel(5)
$P_{CCA} = 9.2 \times 10^{-11}$
$\beta_{CCA} = 0.17$
$P_{KNN-obs-sel(5)} = 3.9 \times 10^{-11}$
$\beta_{KNN-obs-sel(5)} = 0.16$
observed
imputed
estimate CCA
estimate KNN−obs−sel(5)
Model residuals of bilirubin (E,Z or Z,E)*
2
1
0
−1
−2
$n_{CCA} = 552$
$n_{KNN-obs-sel(5)} = 678$
$n_{CCA} = 651$
$n_{KNN-obs-sel(5)} = 775$
$n_{CCA} = 187$
$n_{KNN-obs-sel(5)} = 204$
major homozygote
heterozygote
minor homozygote
Genotype of SNP rs887829 (UGT1A1)

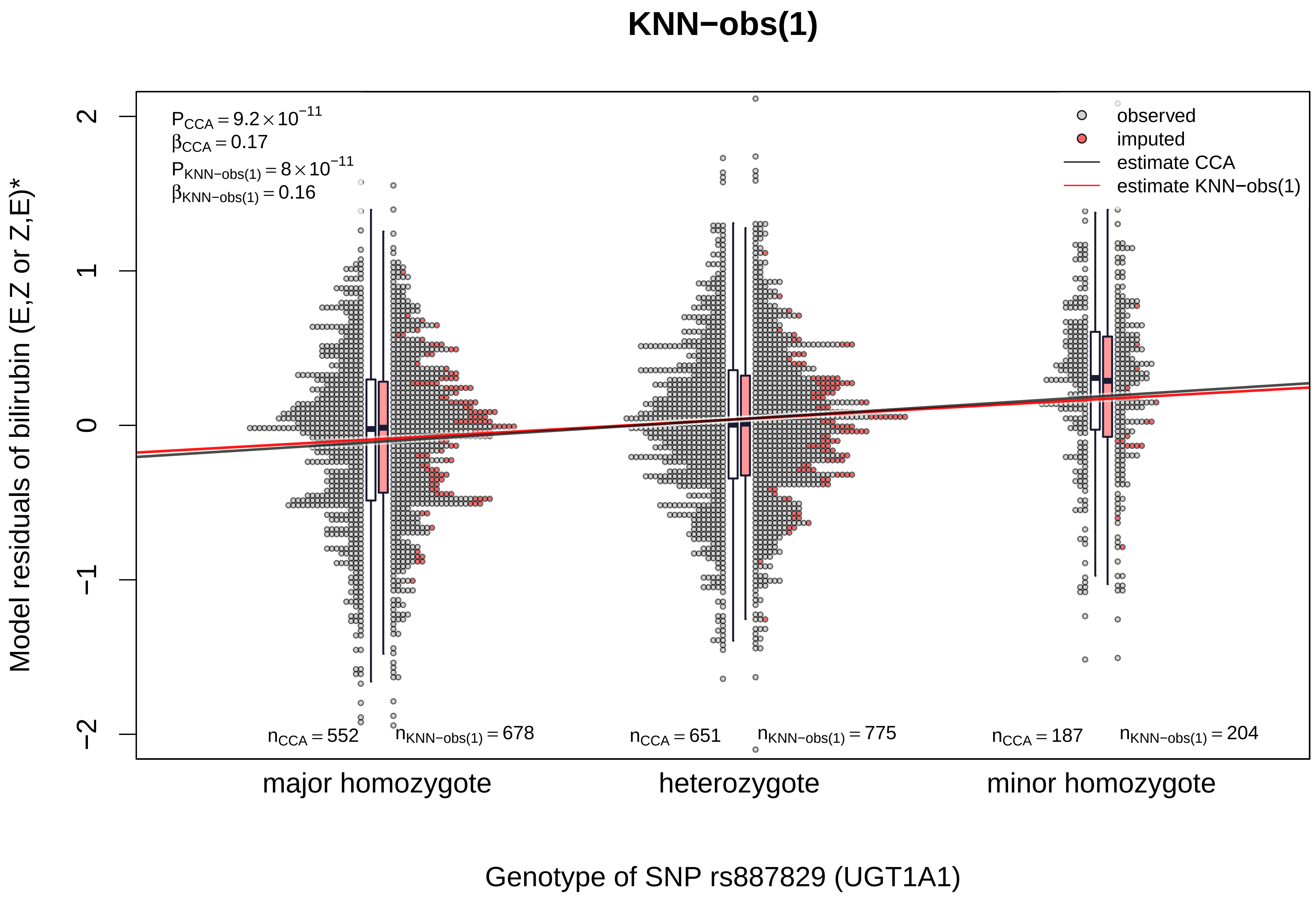

KNN−obs(1)
$P_{CCA} = 9.2 \times 10^{-11}$
$\beta_{CCA} = 0.17$
$P_{KNN-obs(1)} = 8 \times 10^{-11}$
$\beta_{KNN-obs(1)} = 0.16$
observed
imputed
estimate CCA
estimate KNN−obs(1)
Model residuals of bilirubin (E,Z or Z,E)*
2
1
0
−1
−2
$n_{CCA} = 552$
$n_{KNN-obs(1)} = 678$
$n_{CCA} = 651$
$n_{KNN-obs(1)} = 775$
$n_{CCA} = 187$
$n_{KNN-obs(1)} = 204$
major homozygote
heterozygote
minor homozygote
Genotype of SNP rs887829 (UGT1A1)

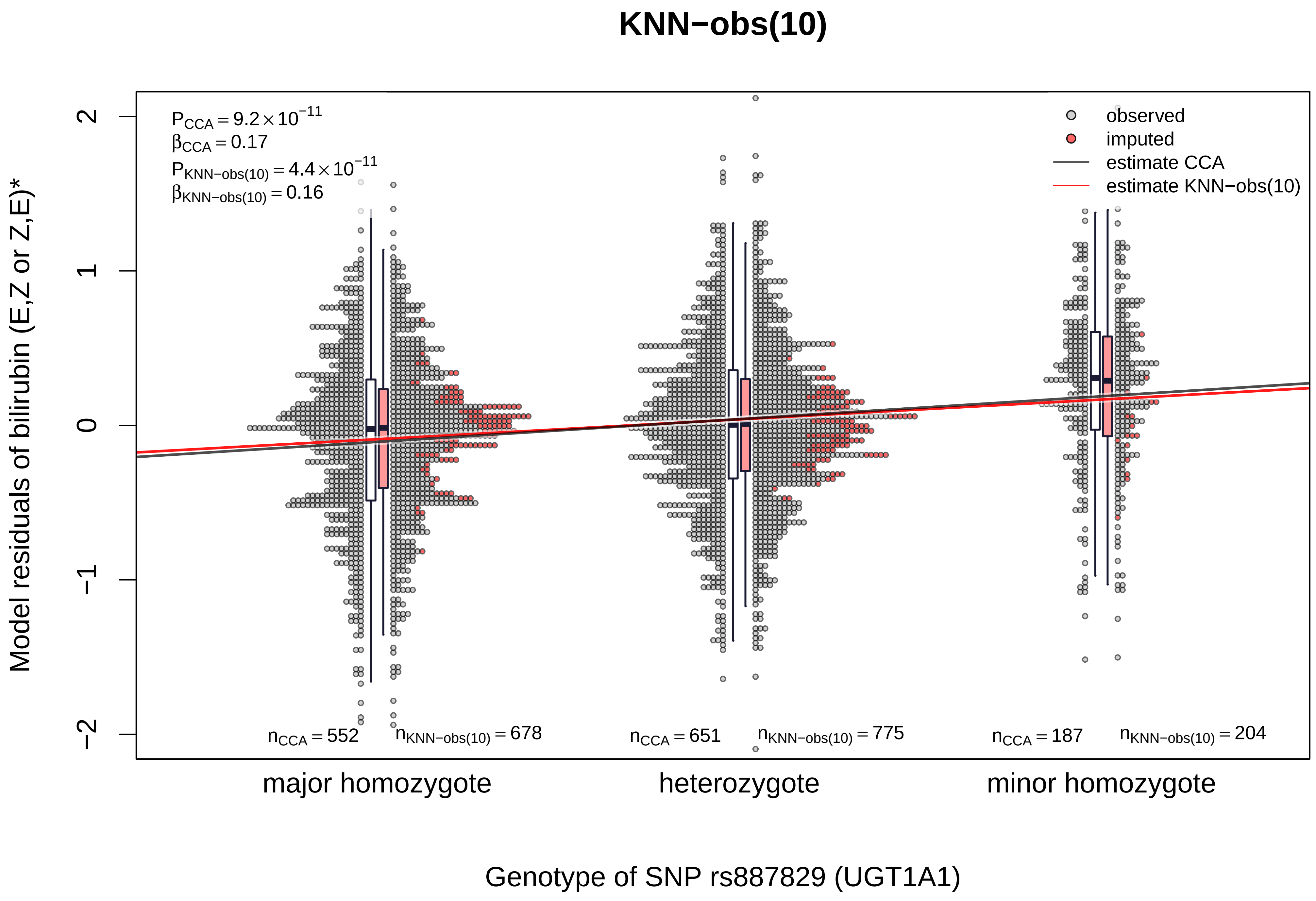
KNN−obs(10)
P_CCA = 9.2 × 10^−11
β_CCA = 0.17
P_KNN−obs(10) = 4.4 × 10^−11
β_KNN−obs(10) = 0.16
observed
imputed
estimate CCA
estimate KNN−obs(10)
n_CCA = 552
n_KNN−obs(10) = 678
n_CCA = 651
n_KNN−obs(10) = 775
n_CCA = 187
n_KNN−obs(10) = 204
major homozygote
heterozygote
minor homozygote
Genotype of SNP rs887829 (UGT1A1)
Model residuals of bilirubin (E,Z or Z,E)*
2
1
0
−1
−2

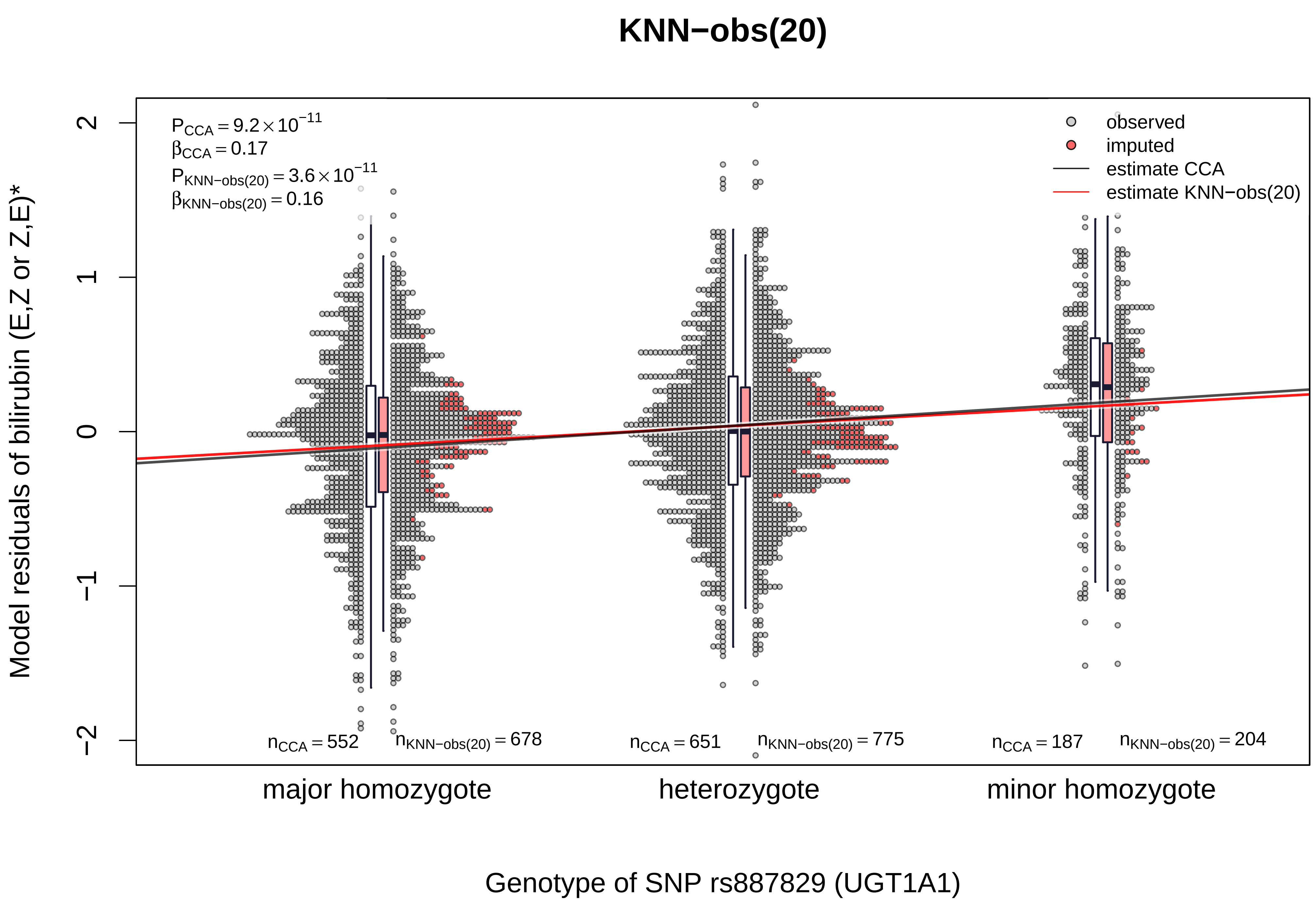
KNN−obs(20)
$P_{CCA} = 9.2 \times 10^{-11}$
$\beta_{CCA} = 0.17$
$P_{KNN-obs(20)} = 3.6 \times 10^{-11}$
$\beta_{KNN-obs(20)} = 0.16$
observed
imputed
estimate CCA
estimate KNN−obs(20)
Model residuals of bilirubin (E,Z or Z,E)*
2
1
0
−1
−2
$n_{CCA} = 552$
$n_{KNN-obs(20)} = 678$
$n_{CCA} = 651$
$n_{KNN-obs(20)} = 775$
$n_{CCA} = 187$
$n_{KNN-obs(20)} = 204$
major homozygote
heterozygote
minor homozygote
Genotype of SNP rs887829 (UGT1A1)

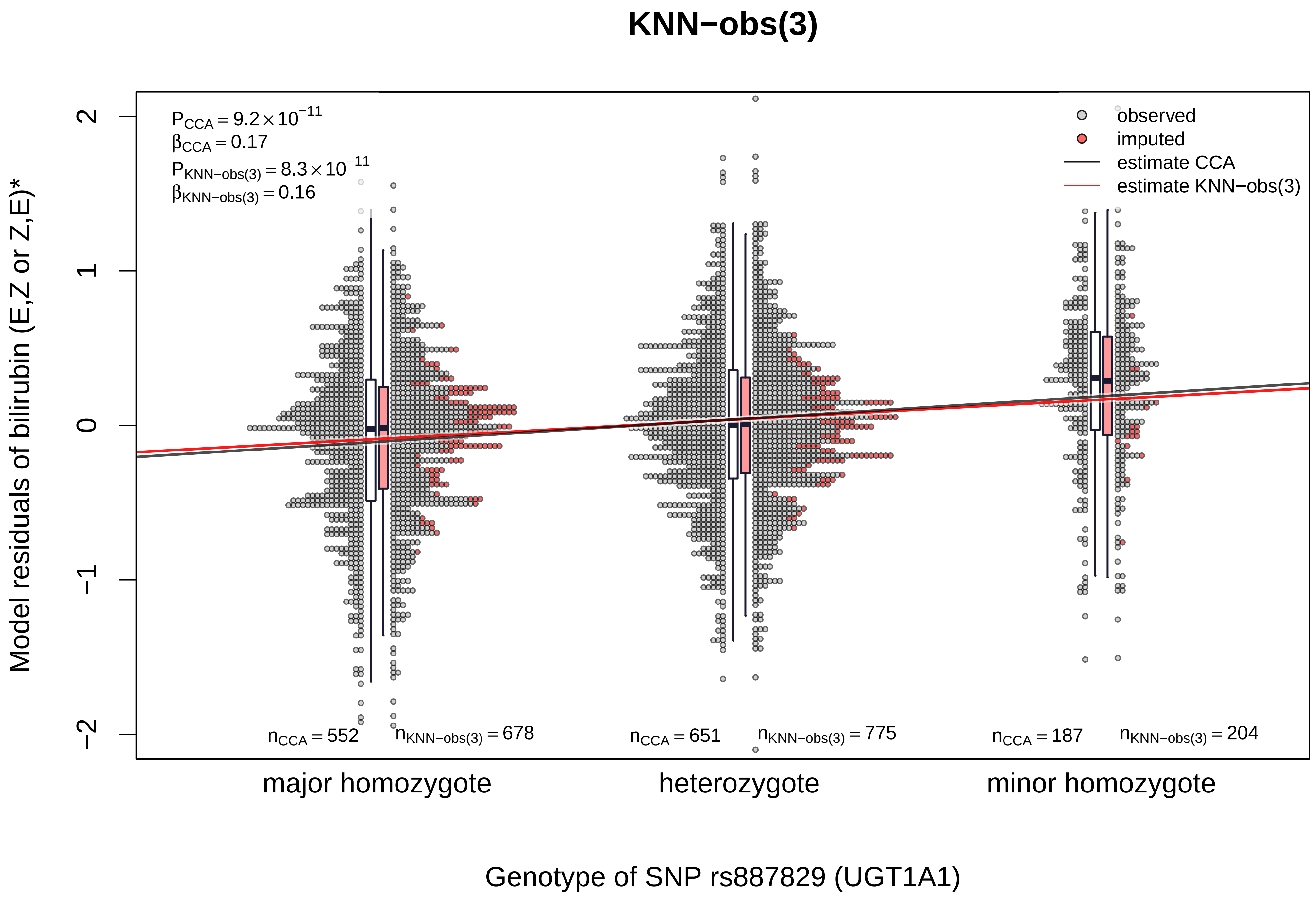

KNN−obs(3)
$P_{CCA} = 9.2 \times 10^{-11}$
$\beta_{CCA} = 0.17$
$P_{KNN-obs(3)} = 8.3 \times 10^{-11}$
$\beta_{KNN-obs(3)} = 0.16$
observed
imputed
estimate CCA
estimate KNN−obs(3)
Model residuals of bilirubin (E,Z or Z,E)*
2
1
0
−1
−2
$n_{CCA} = 552$
$n_{KNN-obs(3)} = 678$
$n_{CCA} = 651$
$n_{KNN-obs(3)} = 775$
$n_{CCA} = 187$
$n_{KNN-obs(3)} = 204$
major homozygote
heterozygote
minor homozygote
Genotype of SNP rs887829 (UGT1A1)

KNN−obs(5)

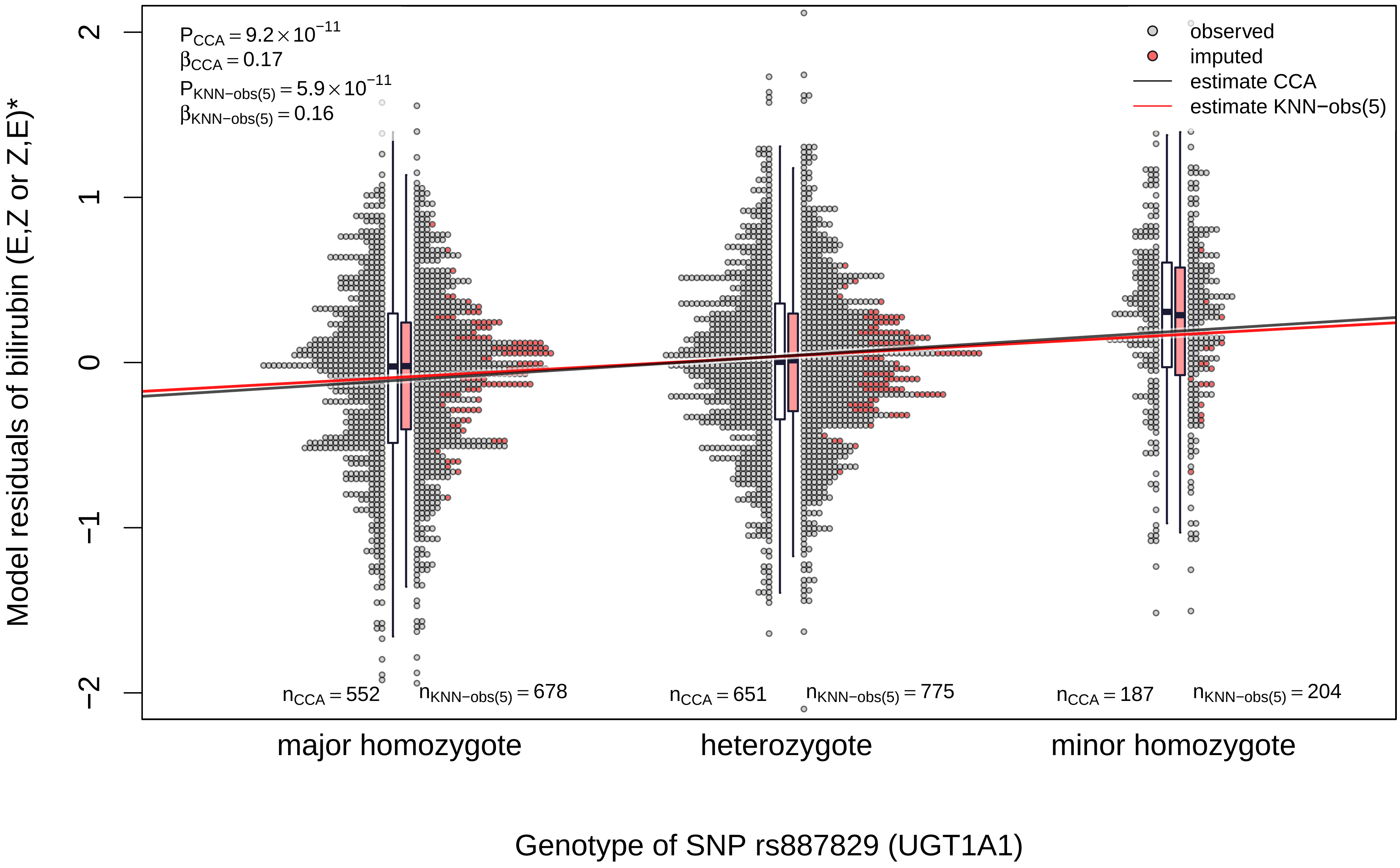

$P_{CCA} = 9.2 \times 10^{-11}$
$\beta_{CCA} = 0.17$
$P_{KNN-obs(5)} = 5.9 \times 10^{-11}$
$\beta_{KNN-obs(5)} = 0.16$
observed
imputed
estimate CCA
estimate KNN−obs(5)
Model residuals of bilirubin (E,Z or Z,E)*
2
1
0
−1
−2
$n_{CCA} = 552$
$n_{KNN-obs(5)} = 678$
$n_{CCA} = 651$
$n_{KNN-obs(5)} = 775$
$n_{CCA} = 187$
$n_{KNN-obs(5)} = 204$
major homozygote
heterozygote
minor homozygote
Genotype of SNP rs887829 (UGT1A1)

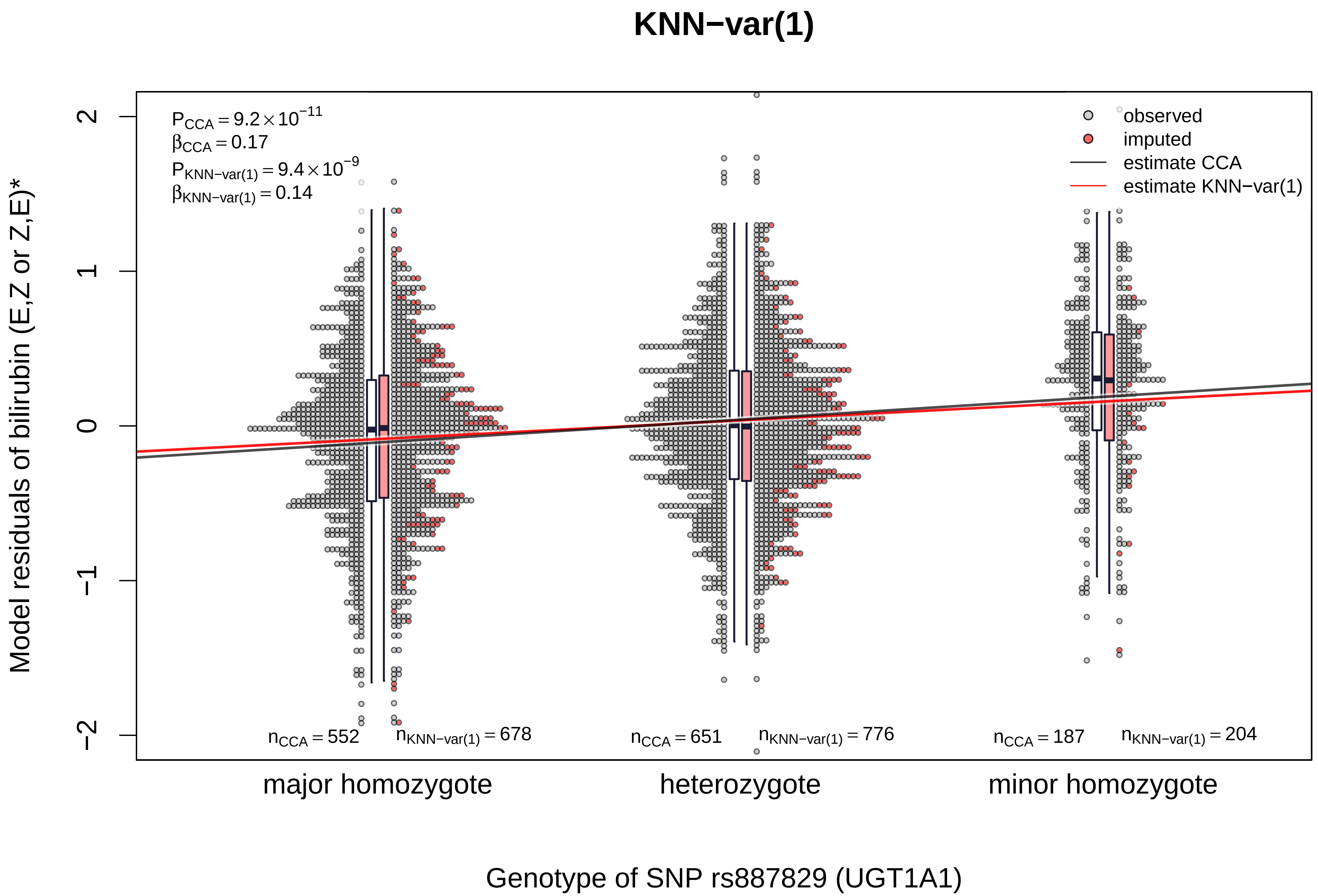

KNN−var(1)
$P_{CCA} = 9.2 \times 10^{-11}$
$\beta_{CCA} = 0.17$
$P_{KNN-var(1)} = 9.4 \times 10^{-9}$
$\beta_{KNN-var(1)} = 0.14$
observed
imputed
estimate CCA
estimate KNN−var(1)
Model residuals of bilirubin (E,Z or Z,E)*
2
1
0
−1
−2
$n_{CCA} = 552$
$n_{KNN-var(1)} = 678$
$n_{CCA} = 651$
$n_{KNN-var(1)} = 776$
$n_{CCA} = 187$
$n_{KNN-var(1)} = 204$
major homozygote
heterozygote
minor homozygote
Genotype of SNP rs887829 (UGT1A1)

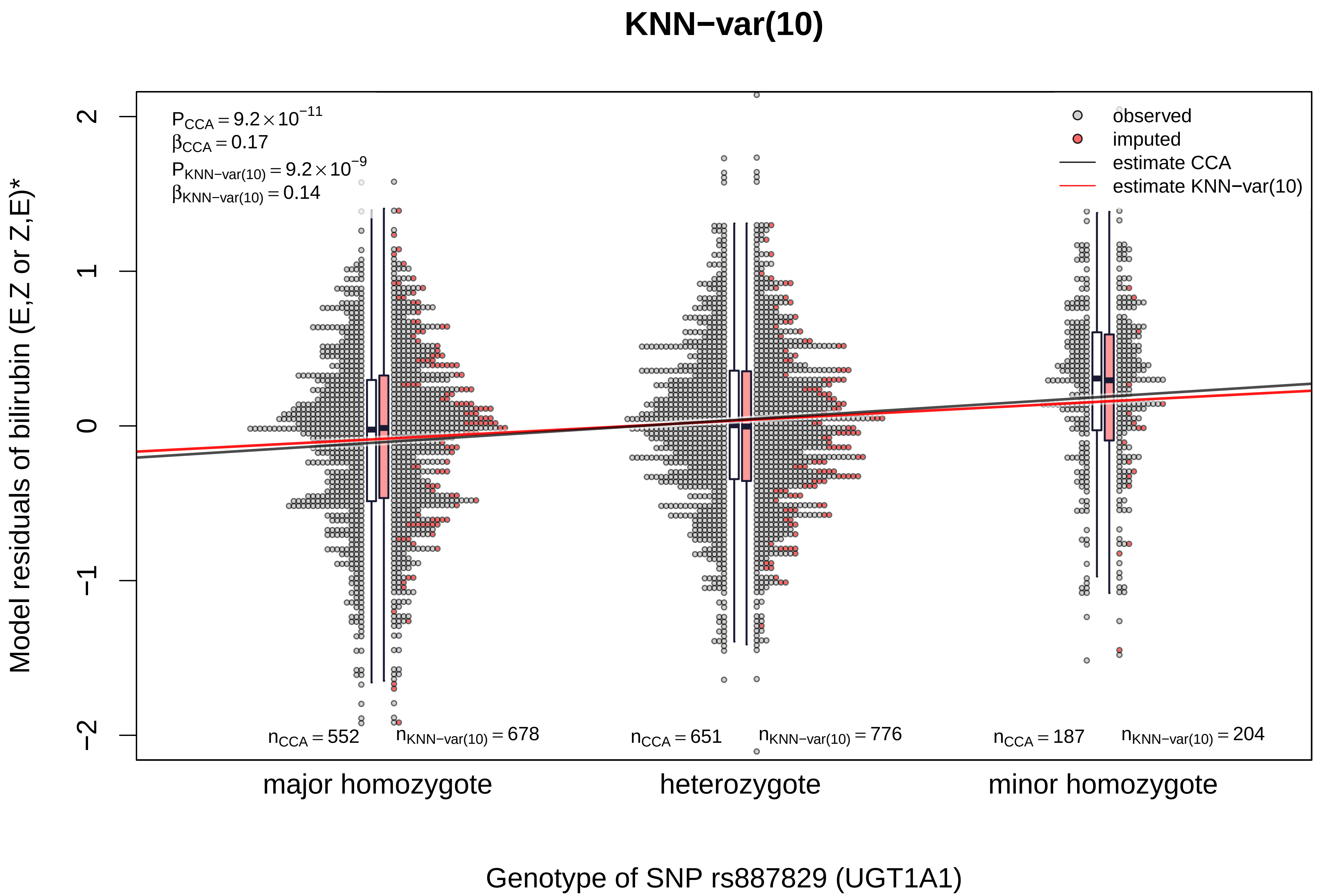

KNN−var(10)
$P_{CCA} = 9.2 \times 10^{-11}$
$\beta_{CCA} = 0.17$
$P_{KNN-var(10)} = 9.2 \times 10^{-9}$
$\beta_{KNN-var(10)} = 0.14$
observed
imputed
estimate CCA
estimate KNN−var(10)
Model residuals of bilirubin (E,Z or Z,E)*
2
1
0
−1
−2
$n_{CCA} = 552$
$n_{KNN-var(10)} = 678$
$n_{CCA} = 651$
$n_{KNN-var(10)} = 776$
$n_{CCA} = 187$
$n_{KNN-var(10)} = 204$
major homozygote
heterozygote
minor homozygote
Genotype of SNP rs887829 (UGT1A1)

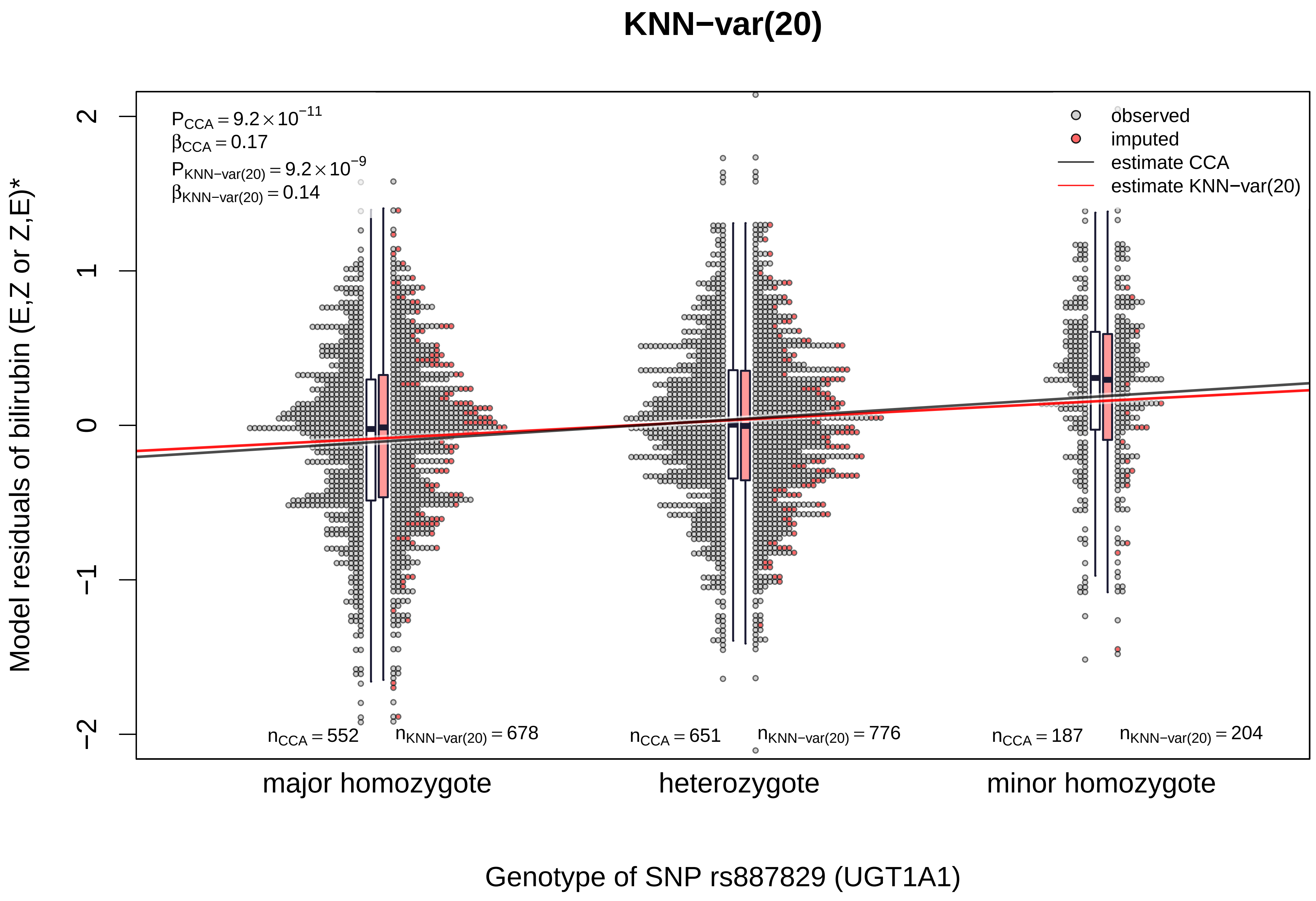

KNN−var(20)
$P_{CCA} = 9.2 \times 10^{-11}$
$\beta_{CCA} = 0.17$
$P_{KNN-var(20)} = 9.2 \times 10^{-9}$
$\beta_{KNN-var(20)} = 0.14$
observed
imputed
estimate CCA
estimate KNN−var(20)
Model residuals of bilirubin (E,Z or Z,E)*
2
1
0
−1
−2
$n_{CCA} = 552$
$n_{KNN-var(20)} = 678$
$n_{CCA} = 651$
$n_{KNN-var(20)} = 776$
$n_{CCA} = 187$
$n_{KNN-var(20)} = 204$
major homozygote
heterozygote
minor homozygote
Genotype of SNP rs887829 (UGT1A1)

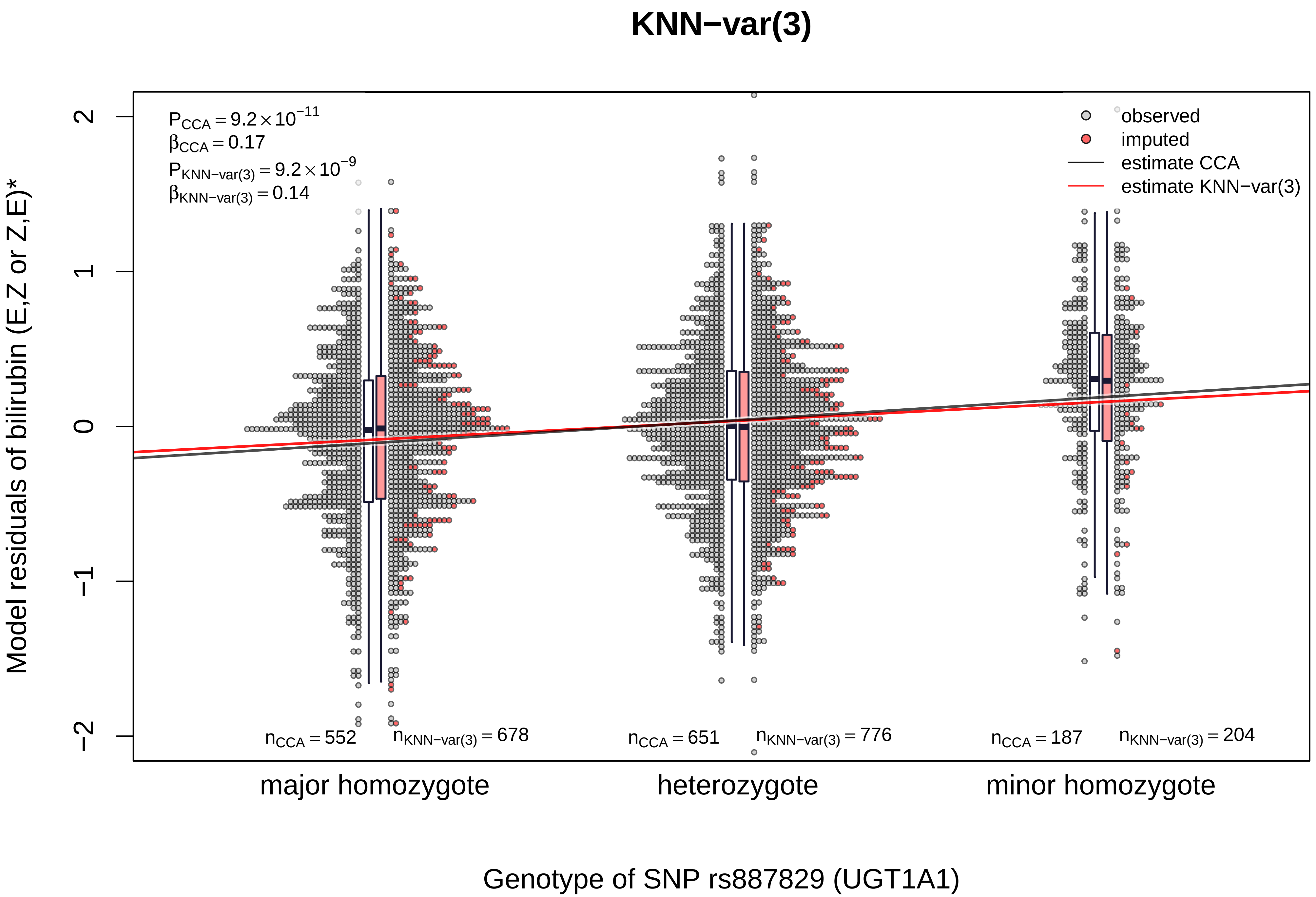

KNN−var(3)
$P_{CCA} = 9.2 \times 10^{-11}$
$\beta_{CCA} = 0.17$
$P_{KNN-var(3)} = 9.2 \times 10^{-9}$
$\beta_{KNN-var(3)} = 0.14$
observed
imputed
estimate CCA
estimate KNN−var(3)
$n_{CCA} = 552$
$n_{KNN-var(3)} = 678$
$n_{CCA} = 651$
$n_{KNN-var(3)} = 776$
$n_{CCA} = 187$
$n_{KNN-var(3)} = 204$
Model residuals of bilirubin (E,Z or Z,E)*
2
1
0
−1
−2
major homozygote
heterozygote
minor homozygote
Genotype of SNP rs887829 (UGT1A1)

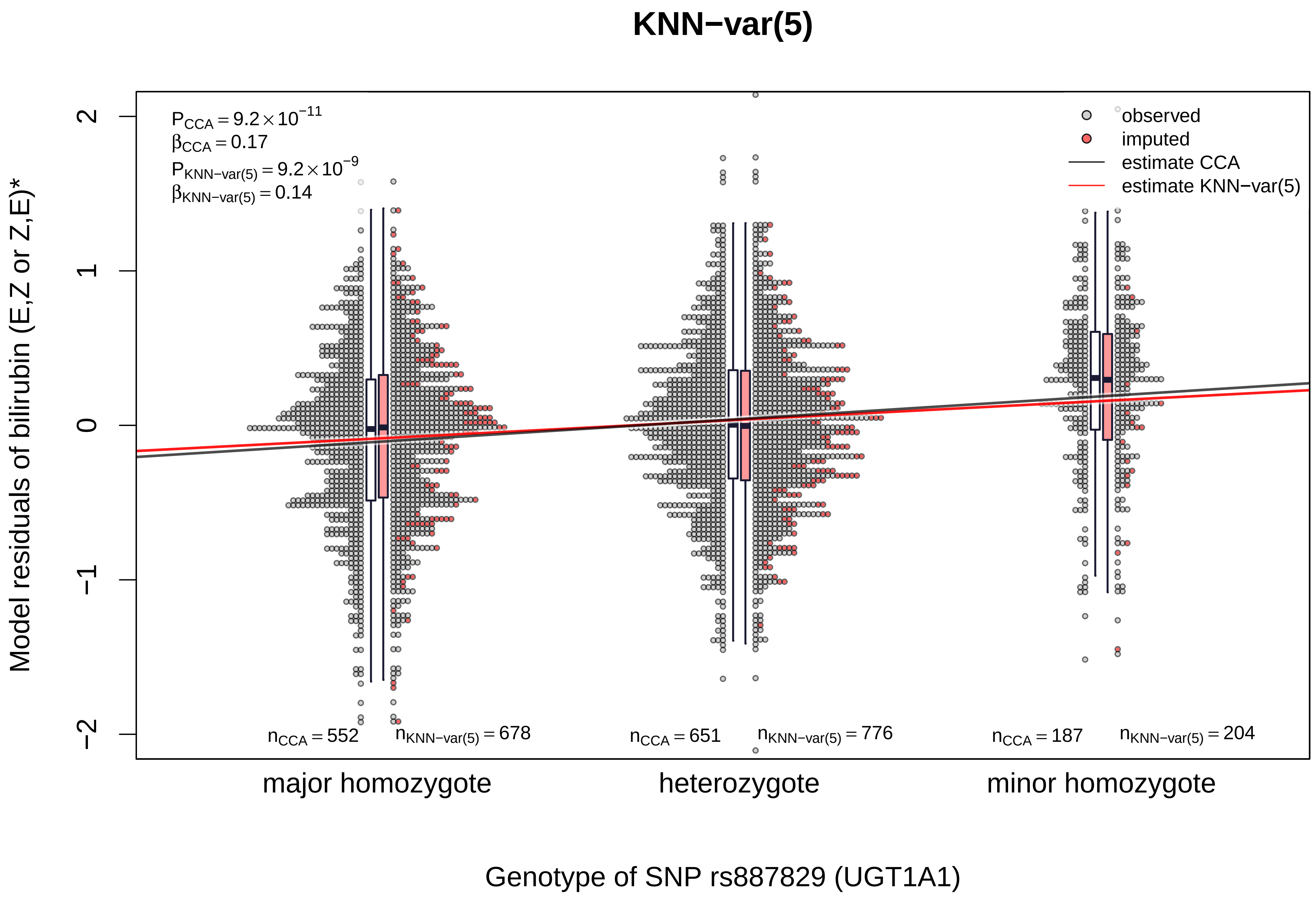

KNN−var(5)
$P_{CCA} = 9.2 \times 10^{-11}$
$\beta_{CCA} = 0.17$
$P_{KNN-var(5)} = 9.2 \times 10^{-9}$
$\beta_{KNN-var(5)} = 0.14$
observed
imputed
estimate CCA
estimate KNN−var(5)
2
1
0
−1
−2
Model residuals of bilirubin (E,Z or Z,E)*
$n_{CCA} = 552$
$n_{KNN-var(5)} = 678$
$n_{CCA} = 651$
$n_{KNN-var(5)} = 776$
$n_{CCA} = 187$
$n_{KNN-var(5)} = 204$
major homozygote
heterozygote
minor homozygote
Genotype of SNP rs887829 (UGT1A1)

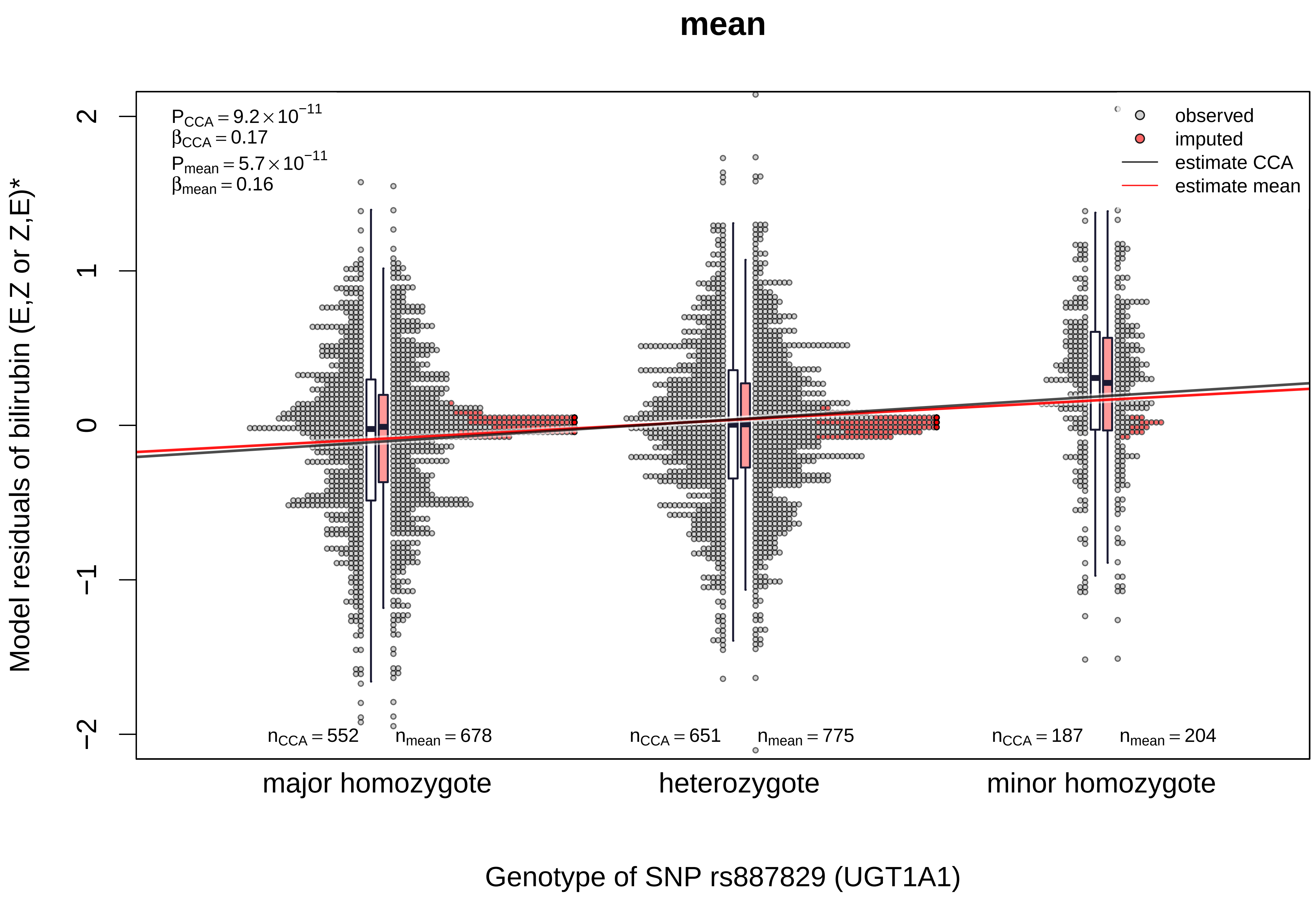

mean
$P_{CCA} = 9.2 \times 10^{-11}$
$\beta_{CCA} = 0.17$
$P_{mean} = 5.7 \times 10^{-11}$
$\beta_{mean} = 0.16$
observed
imputed
estimate CCA
estimate mean
Model residuals of bilirubin (E,Z or Z,E)*
2
1
0
−1
−2
$n_{CCA} = 552$
$n_{mean} = 678$
$n_{CCA} = 651$
$n_{mean} = 775$
$n_{CCA} = 187$
$n_{mean} = 204$
major homozygote
heterozygote
minor homozygote
Genotype of SNP rs887829 (UGT1A1)

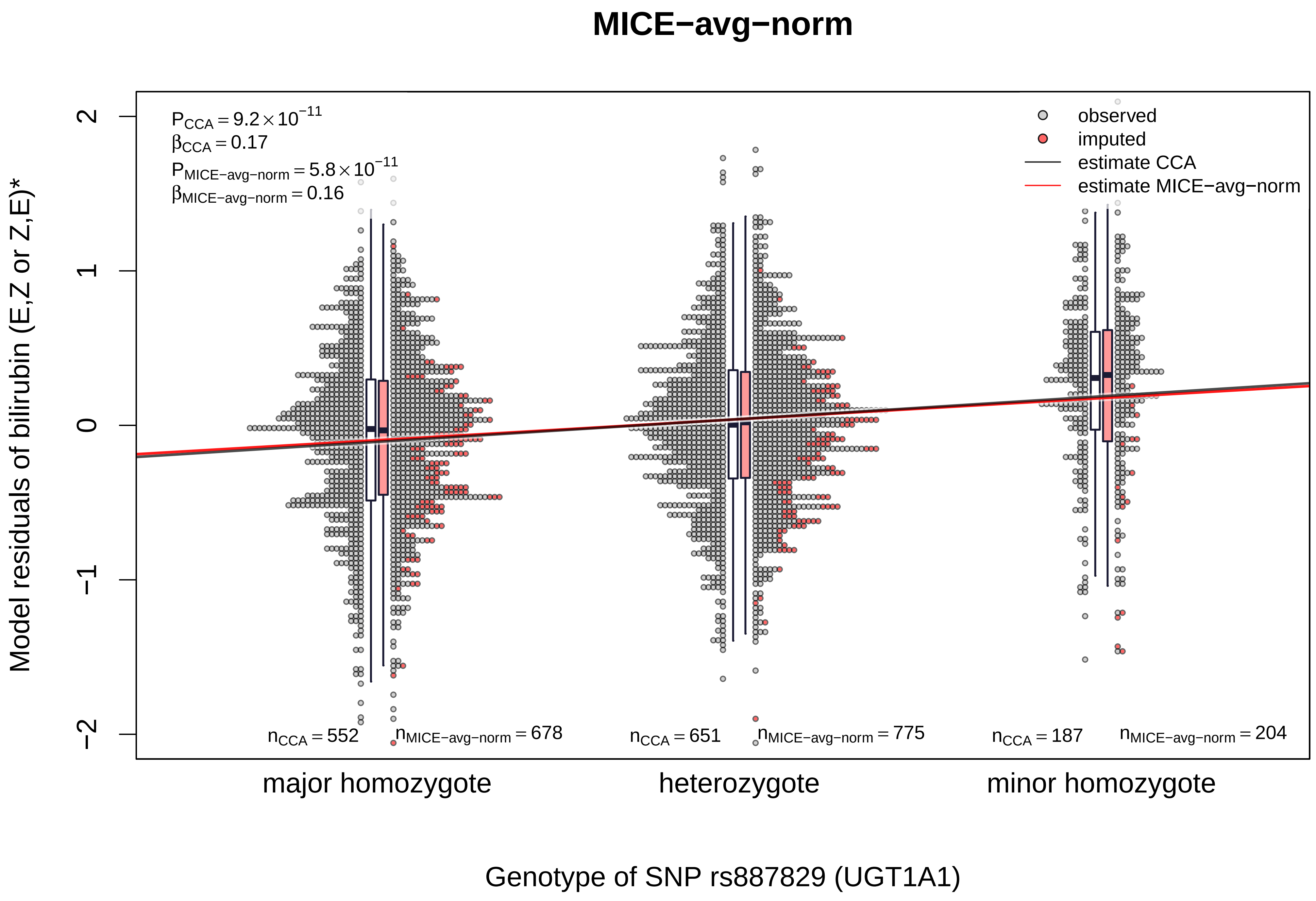
MICE−avg−norm
$P_{CCA} = 9.2 \times 10^{-11}$
$\beta_{CCA} = 0.17$
$P_{MICE-avg-norm} = 5.8 \times 10^{-11}$
$\beta_{MICE-avg-norm} = 0.16$
observed
imputed
estimate CCA
estimate MICE−avg−norm
$n_{CCA} = 552$
$n_{MICE-avg-norm} = 678$
$n_{CCA} = 651$
$n_{MICE-avg-norm} = 775$
$n_{CCA} = 187$
$n_{MICE-avg-norm} = 204$
Model residuals of bilirubin (E,Z or Z,E)*
2
1
0
−1
−2
major homozygote
heterozygote
minor homozygote
Genotype of SNP rs887829 (UGT1A1)

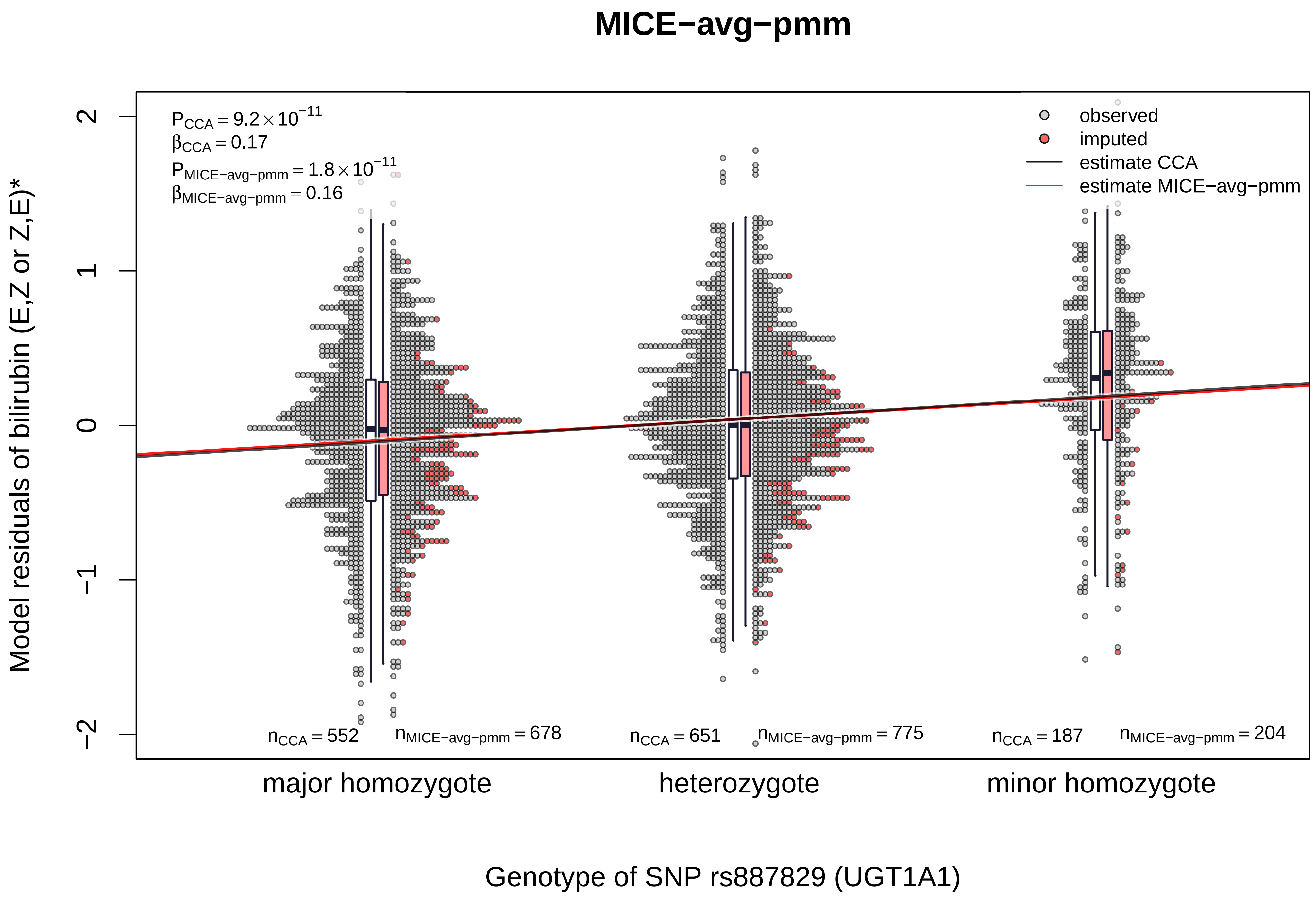
MICE−avg−pmm
$P_{CCA} = 9.2 \times 10^{-11}$
$\beta_{CCA} = 0.17$
$P_{MICE-avg-pmm} = 1.8 \times 10^{-11}$
$\beta_{MICE-avg-pmm} = 0.16$
observed
imputed
estimate CCA
estimate MICE−avg−pmm
2
1
0
−1
−2
Model residuals of bilirubin (E,Z or Z,E)*
$n_{CCA} = 552$
$n_{MICE-avg-pmm} = 678$
$n_{CCA} = 651$
$n_{MICE-avg-pmm} = 775$
$n_{CCA} = 187$
$n_{MICE-avg-pmm} = 204$
major homozygote
heterozygote
minor homozygote
Genotype of SNP rs887829 (UGT1A1)

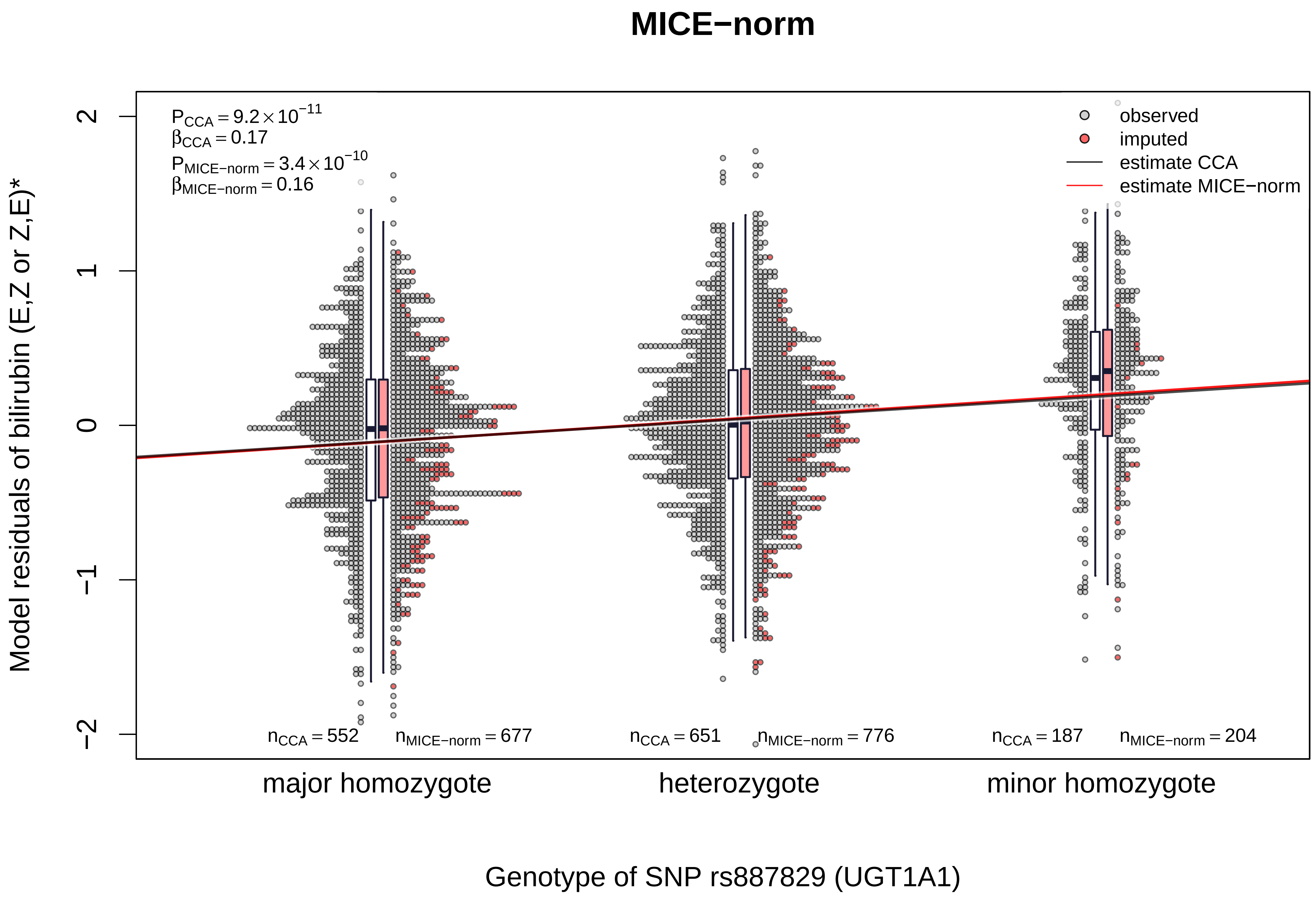
MICE−norm
$P_{CCA} = 9.2 \times 10^{-11}$
$\beta_{CCA} = 0.17$
$P_{MICE-norm} = 3.4 \times 10^{-10}$
$\beta_{MICE-norm} = 0.16$
observed
imputed
estimate CCA
estimate MICE−norm
Model residuals of bilirubin (E,Z or Z,E)*
2
1
0
−1
−2
$n_{CCA} = 552$
$n_{MICE-norm} = 677$
$n_{CCA} = 651$
$n_{MICE-norm} = 776$
$n_{CCA} = 187$
$n_{MICE-norm} = 204$
major homozygote
heterozygote
minor homozygote
Genotype of SNP rs887829 (UGT1A1)

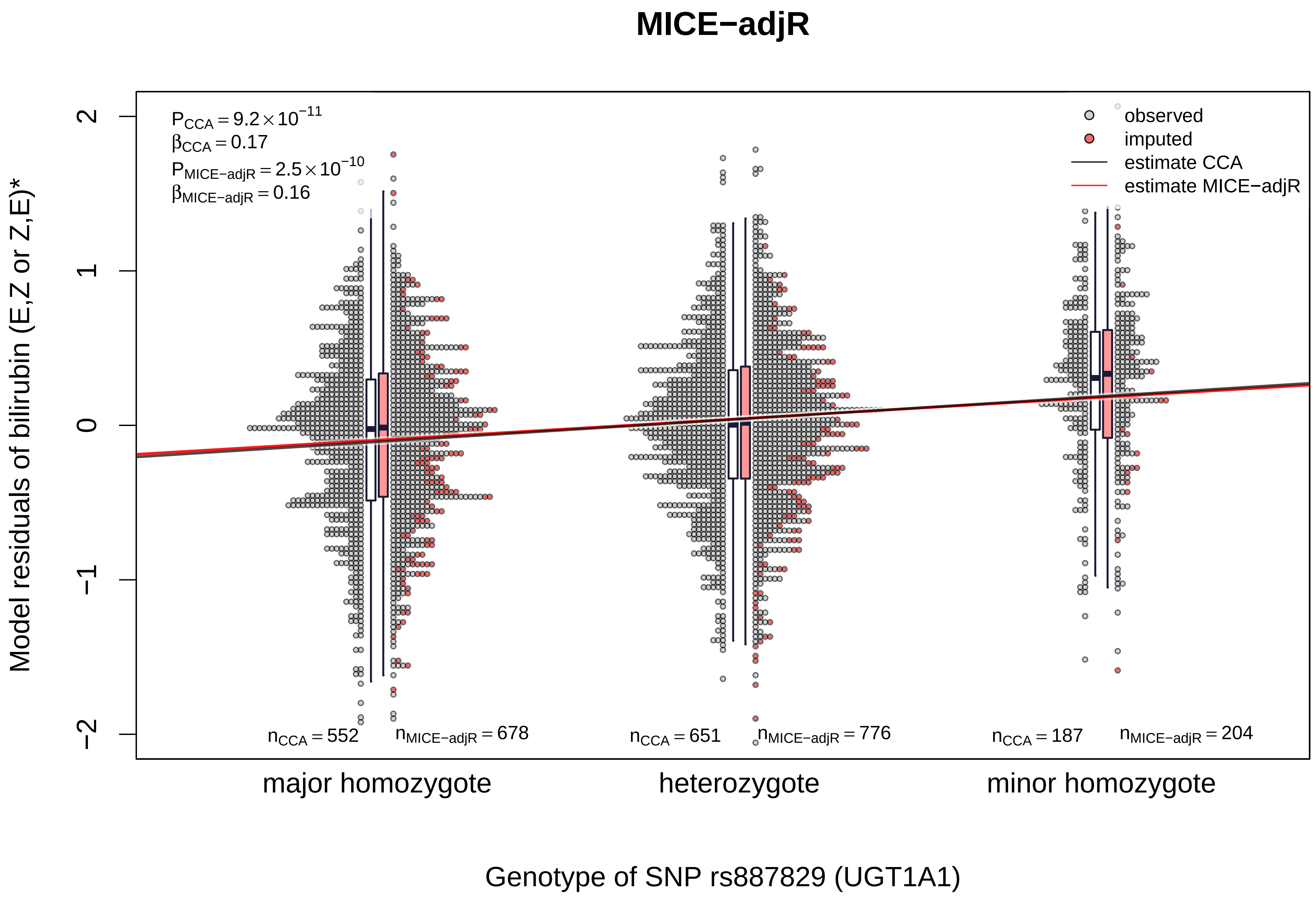

MICE−adjR
$P_{CCA} = 9.2 \times 10^{-11}$
$\beta_{CCA} = 0.17$
$P_{MICE-adjR} = 2.5 \times 10^{-10}$
$\beta_{MICE-adjR} = 0.16$
observed
imputed
estimate CCA
estimate MICE−adjR
Model residuals of bilirubin (E,Z or Z,E)*
2
1
0
−1
−2
$n_{CCA} = 552$
$n_{MICE-adjR} = 678$
$n_{CCA} = 651$
$n_{MICE-adjR} = 776$
$n_{CCA} = 187$
$n_{MICE-adjR} = 204$
major homozygote
heterozygote
minor homozygote
Genotype of SNP rs887829 (UGT1A1)

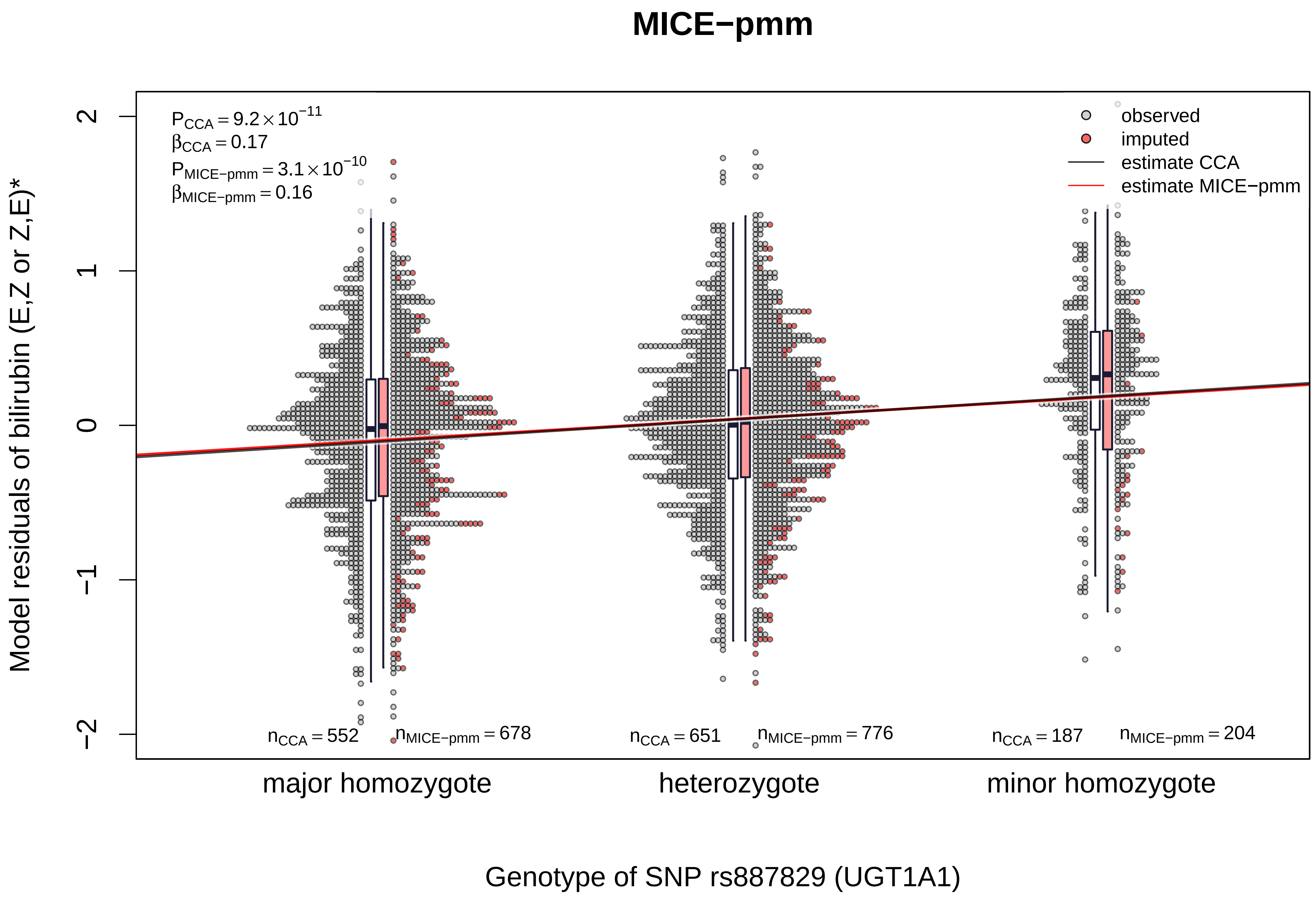

MICE−pmm
$P_{CCA} = 9.2 \times 10^{-11}$
$\beta_{CCA} = 0.17$
$P_{MICE-pmm} = 3.1 \times 10^{-10}$
$\beta_{MICE-pmm} = 0.16$
observed
imputed
estimate CCA
estimate MICE−pmm
Model residuals of bilirubin (E,Z or Z,E)*
2
1
0
−1
−2
$n_{CCA} = 552$
$n_{MICE-pmm} = 678$
$n_{CCA} = 651$
$n_{MICE-pmm} = 776$
$n_{CCA} = 187$
$n_{MICE-pmm} = 204$
major homozygote
heterozygote
minor homozygote
Genotype of SNP rs887829 (UGT1A1)

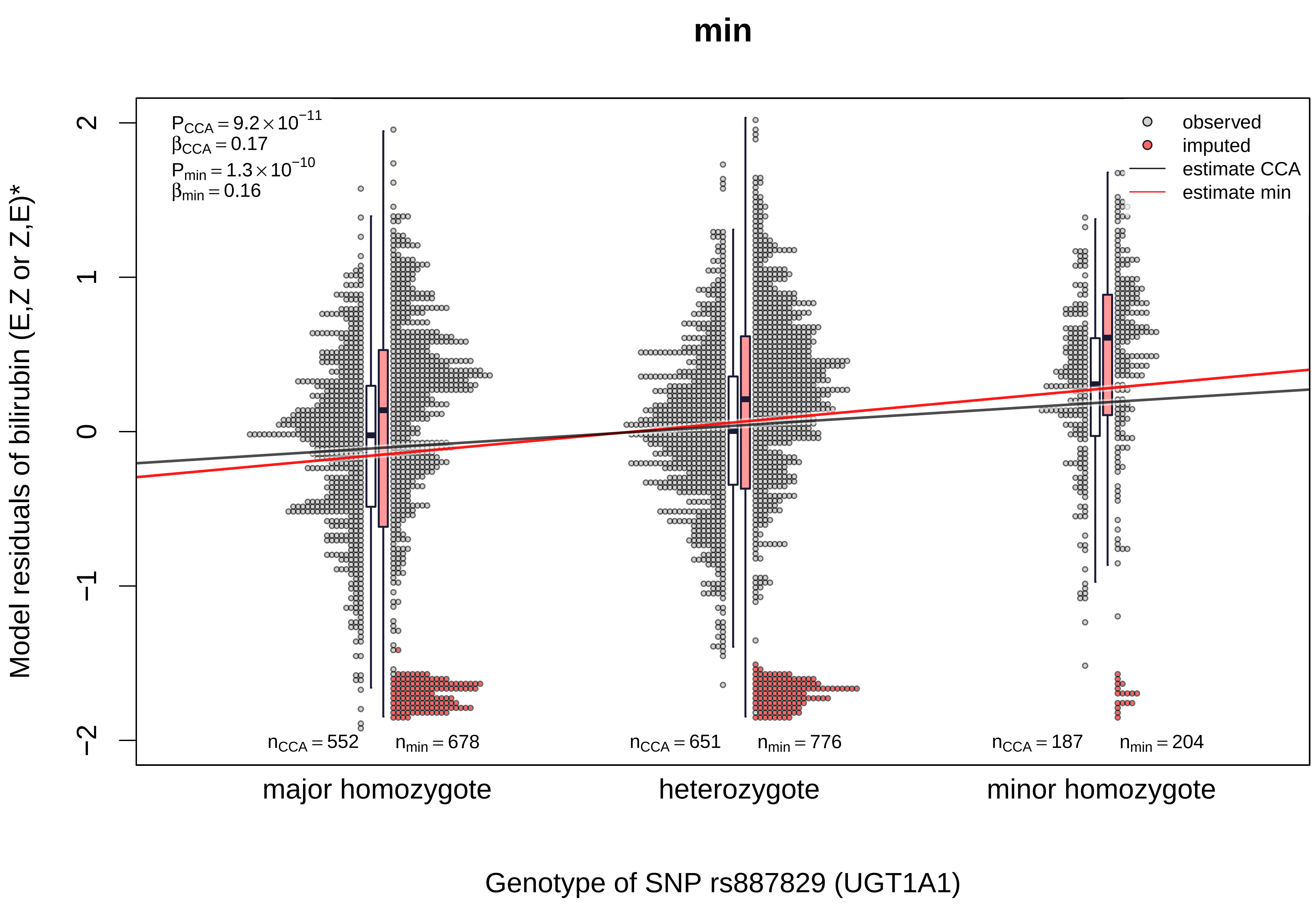
min
$P_{CCA} = 9.2 \times 10^{-11}$
$\beta_{CCA} = 0.17$
$P_{min} = 1.3 \times 10^{-10}$
$\beta_{min} = 0.16$
observed
imputed
estimate CCA
estimate min
Model residuals of bilirubin (E,Z or Z,E)*
2
1
0
−1
−2
$n_{CCA} = 552$
$n_{min} = 678$
$n_{CCA} = 651$
$n_{min} = 776$
$n_{CCA} = 187$
$n_{min} = 204$
major homozygote
heterozygote
minor homozygote
Genotype of SNP rs887829 (UGT1A1)

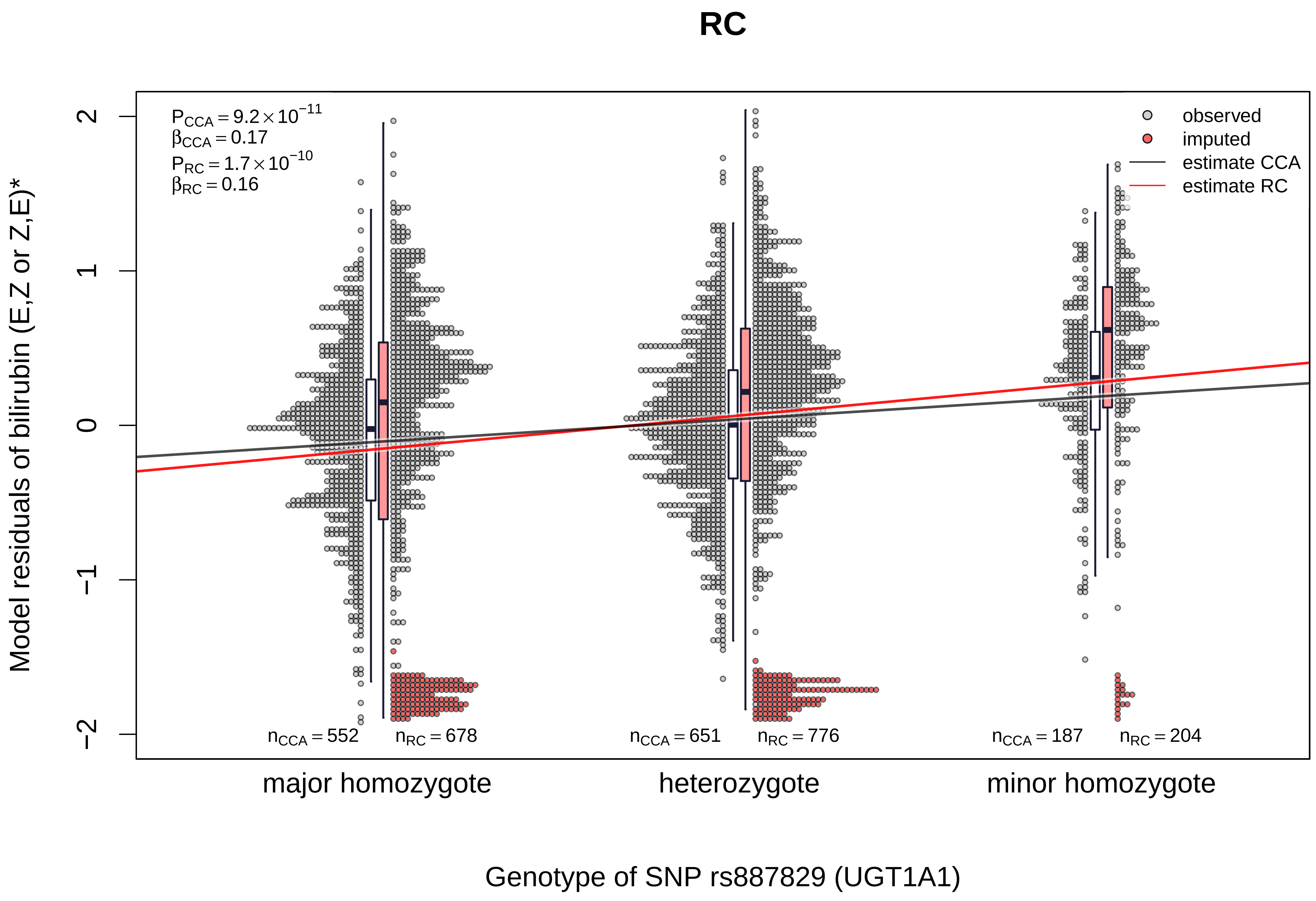

RC
$P_{CCA} = 9.2 \times 10^{-11}$
$\beta_{CCA} = 0.17$
$P_{RC} = 1.7 \times 10^{-10}$
$\beta_{RC} = 0.16$
observed
imputed
estimate CCA
estimate RC
2
1
0
−1
−2
Model residuals of bilirubin (E,Z or Z,E)*
$n_{CCA} = 552$
$n_{RC} = 678$
$n_{CCA} = 651$
$n_{RC} = 776$
$n_{CCA} = 187$
$n_{RC} = 204$
major homozygote
heterozygote
minor homozygote
Genotype of SNP rs887829 (UGT1A1)

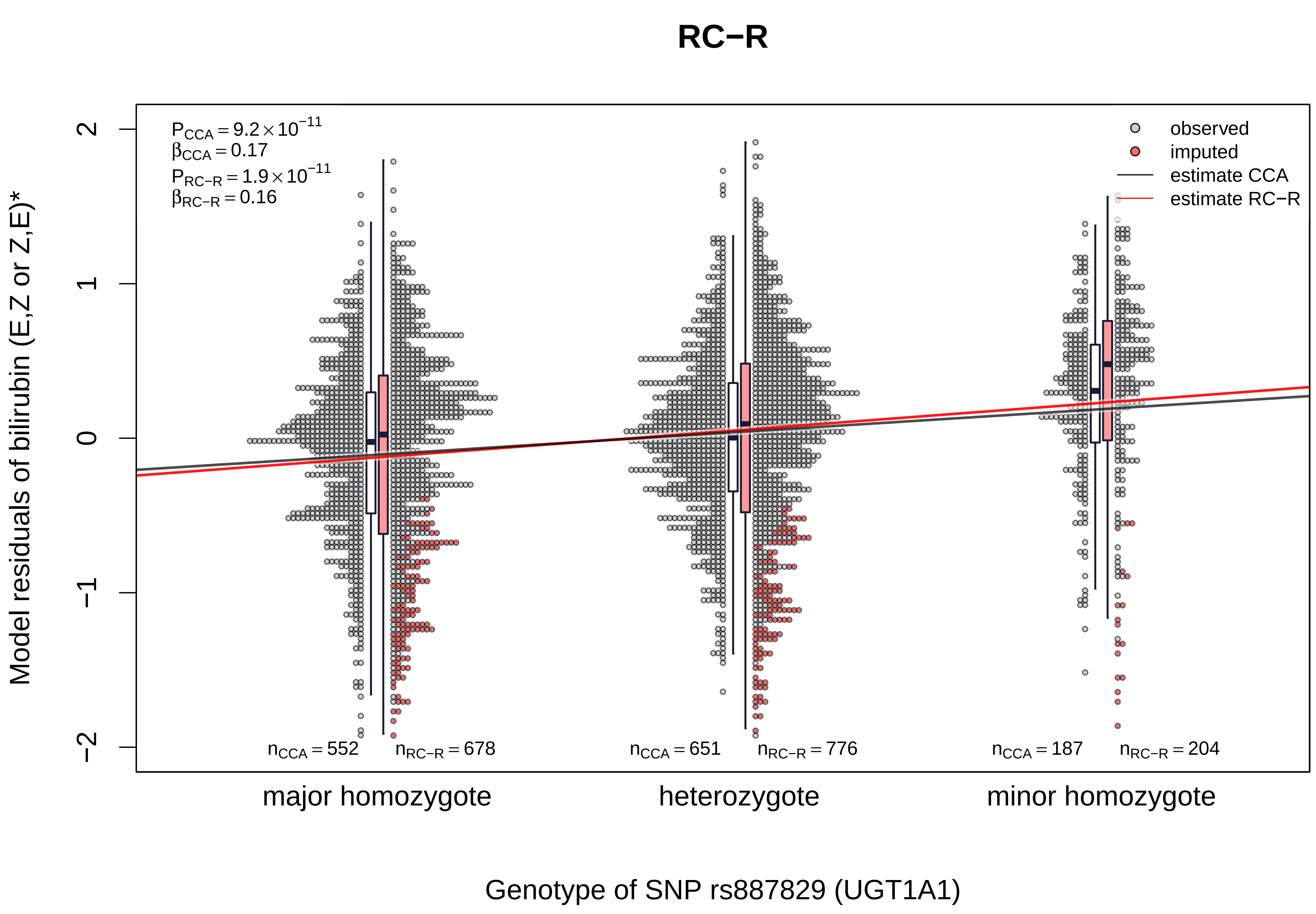

RC−R
$P_{CCA} = 9.2 \times 10^{-11}$
$\beta_{CCA} = 0.17$
$P_{RC-R} = 1.9 \times 10^{-11}$
$\beta_{RC-R} = 0.16$
observed
imputed
estimate CCA
estimate RC−R
$n_{CCA} = 552$
$n_{RC-R} = 678$
$n_{CCA} = 651$
$n_{RC-R} = 776$
$n_{CCA} = 187$
$n_{RC-R} = 204$
Model residuals of bilirubin (E,Z or Z,E)*
2
1
0
−1
−2
major homozygote
heterozygote
minor homozygote
Genotype of SNP rs887829 (UGT1A1)

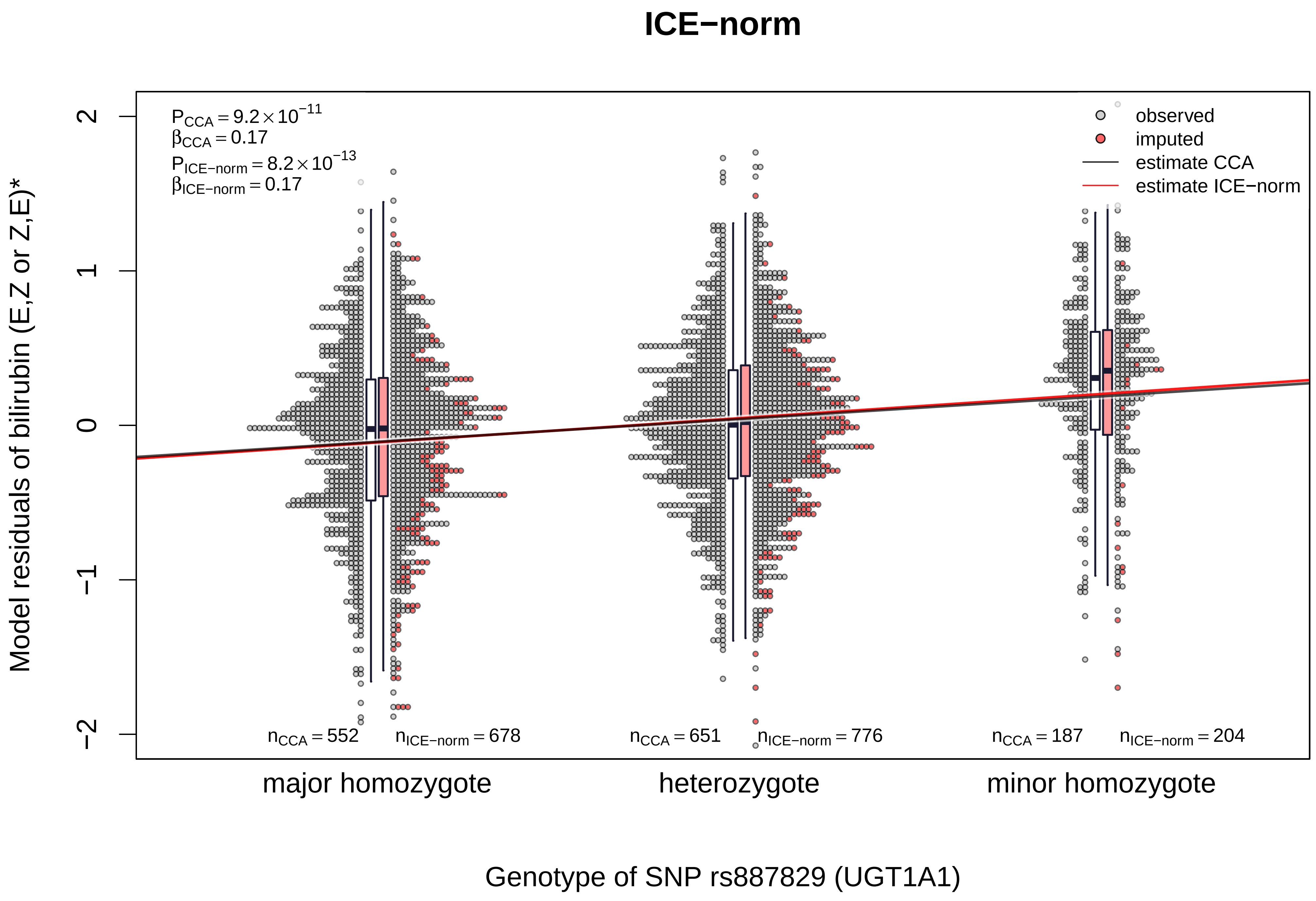
ICE−norm
$P_{CCA} = 9.2 \times 10^{-11}$
$\beta_{CCA} = 0.17$
$P_{ICE-norm} = 8.2 \times 10^{-13}$
$\beta_{ICE-norm} = 0.17$
observed
imputed
estimate CCA
estimate ICE−norm
2
1
0
−1
−2
Model residuals of bilirubin (E,Z or Z,E)*
$n_{CCA} = 552$
$n_{ICE-norm} = 678$
$n_{CCA} = 651$
$n_{ICE-norm} = 776$
$n_{CCA} = 187$
$n_{ICE-norm} = 204$
major homozygote
heterozygote
minor homozygote
Genotype of SNP rs887829 (UGT1A1)

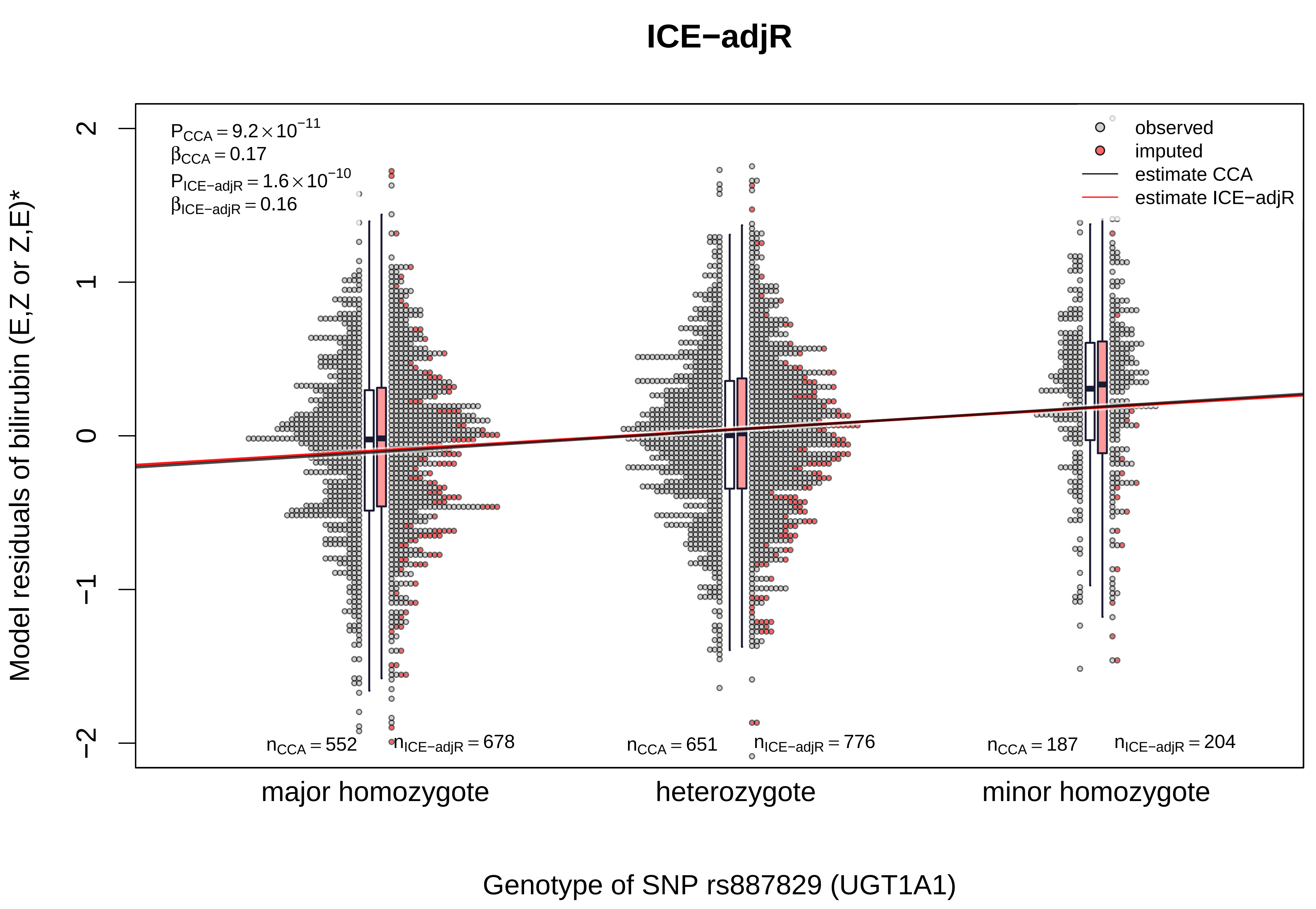

ICE−adjR
$P_{CCA} = 9.2 \times 10^{-11}$
$\beta_{CCA} = 0.17$
$P_{ICE-adjR} = 1.6 \times 10^{-10}$
$\beta_{ICE-adjR} = 0.16$
observed
imputed
estimate CCA
estimate ICE−adjR
Model residuals of bilirubin (E,Z or Z,E)*
2
1
0
−1
−2
$n_{CCA} = 552$
$n_{ICE-adjR} = 678$
$n_{CCA} = 651$
$n_{ICE-adjR} = 776$
$n_{CCA} = 187$
$n_{ICE-adjR} = 204$
major homozygote
heterozygote
minor homozygote
Genotype of SNP rs887829 (UGT1A1)

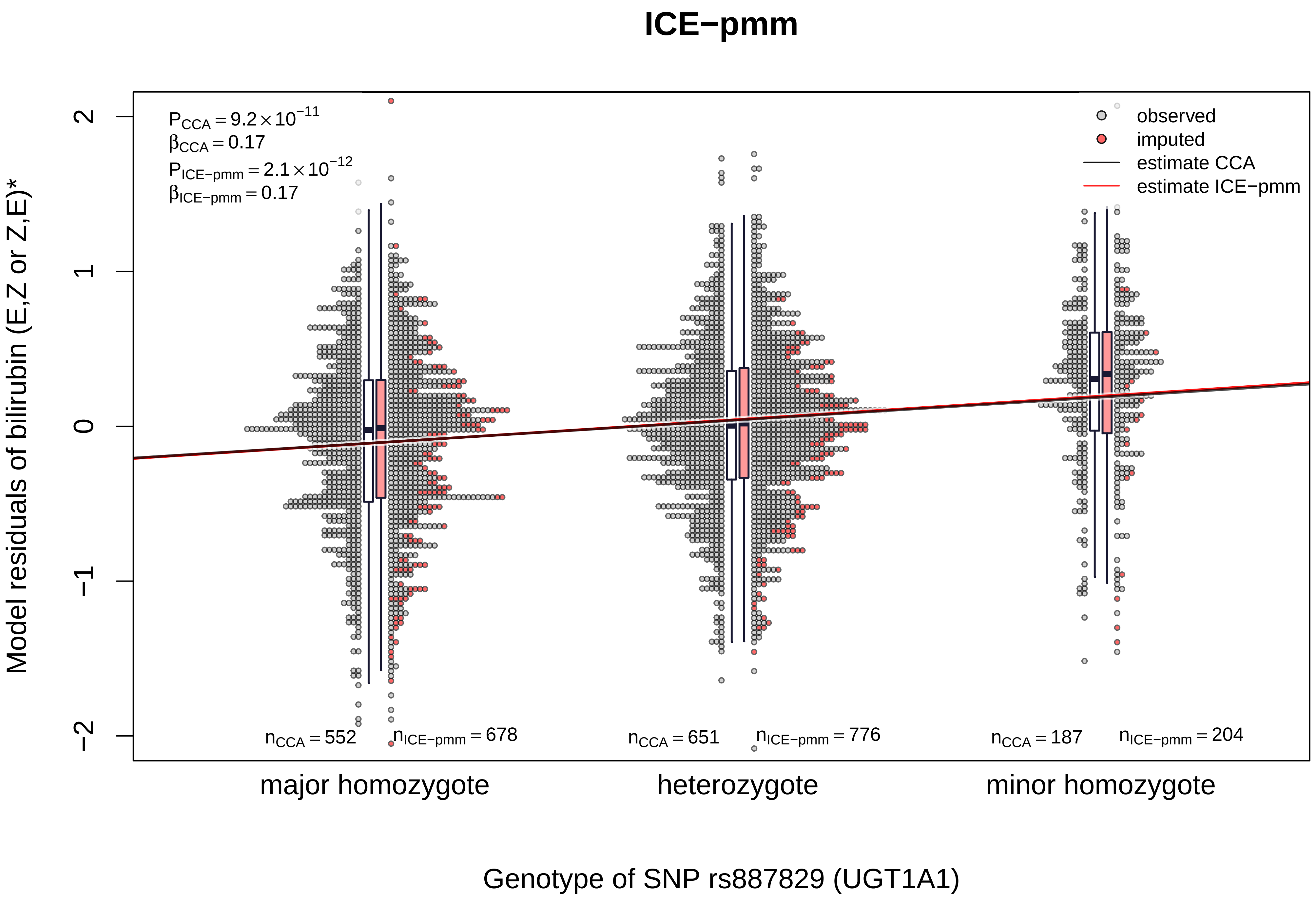
ICE−pmm
$P_{CCA} = 9.2 \times 10^{-11}$
$\beta_{CCA} = 0.17$
$P_{ICE-pmm} = 2.1 \times 10^{-12}$
$\beta_{ICE-pmm} = 0.17$
observed
imputed
estimate CCA
estimate ICE−pmm
Model residuals of bilirubin (E,Z or Z,E)*
2
1
0
−1
−2
$n_{CCA} = 552$
$n_{ICE-pmm} = 678$
$n_{CCA} = 651$
$n_{ICE-pmm} = 776$
$n_{CCA} = 187$
$n_{ICE-pmm} = 204$
major homozygote
heterozygote
minor homozygote
Genotype of SNP rs887829 (UGT1A1)

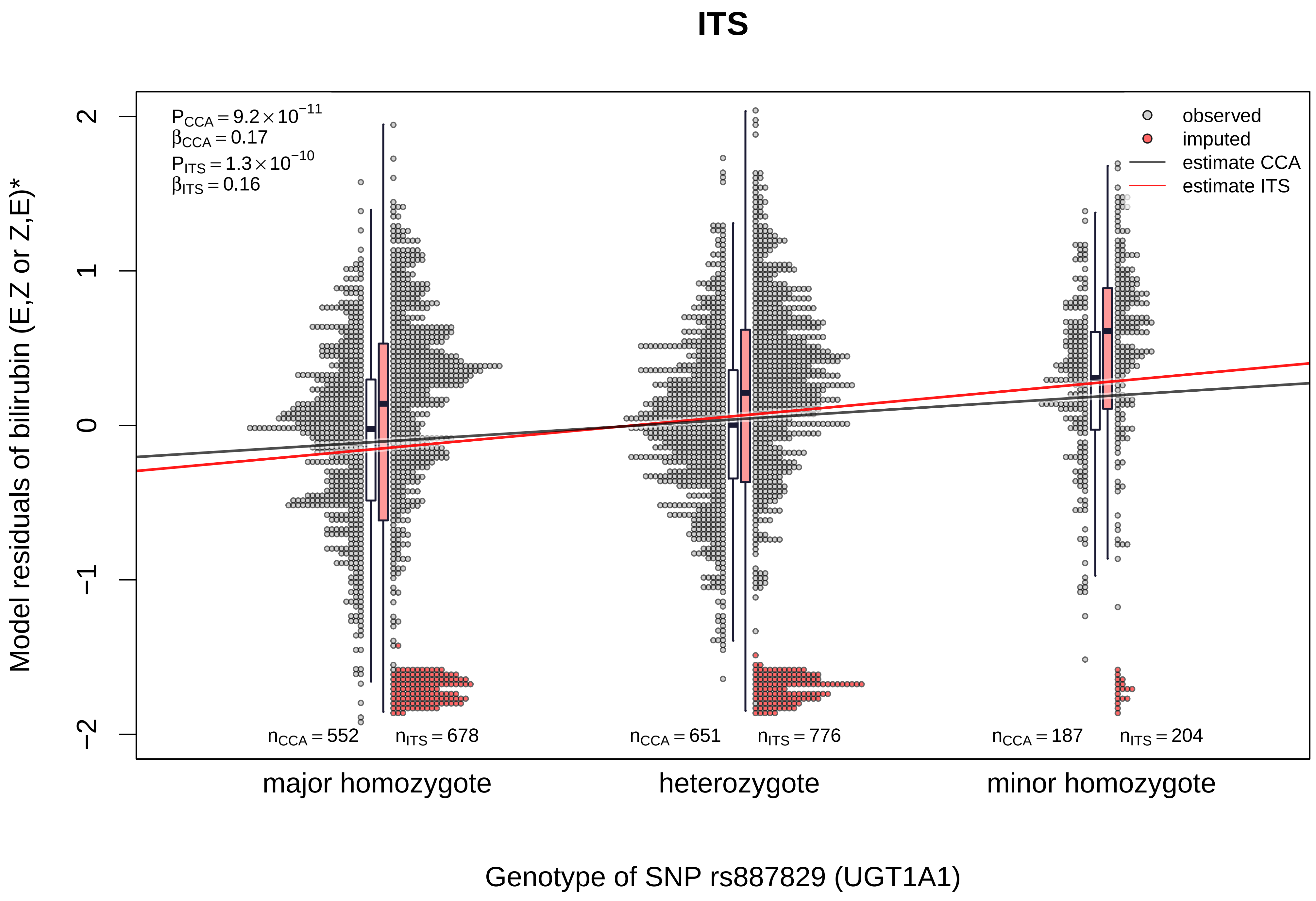
ITS
$P_{CCA} = 9.2 \times 10^{-11}$
$\beta_{CCA} = 0.17$
$P_{ITS} = 1.3 \times 10^{-10}$
$\beta_{ITS} = 0.16$
observed
imputed
estimate CCA
estimate ITS
Model residuals of bilirubin (E,Z or Z,E)*
2
1
0
−1
−2
$n_{CCA} = 552$
$n_{ITS} = 678$
$n_{CCA} = 651$
$n_{ITS} = 776$
$n_{CCA} = 187$
$n_{ITS} = 204$
major homozygote
heterozygote
minor homozygote
Genotype of SNP rs887829 (UGT1A1)

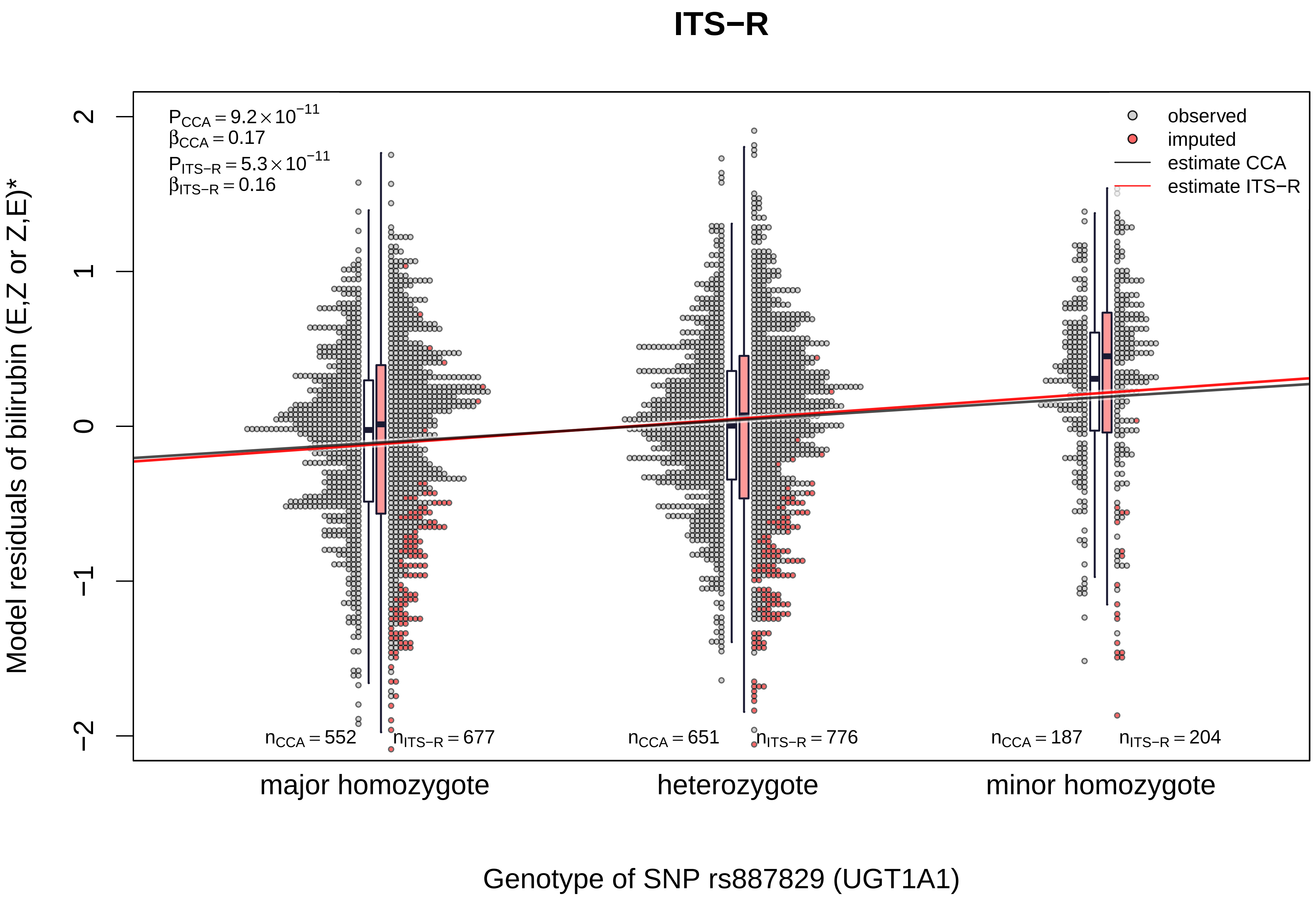

ITS−R
$P_{CCA} = 9.2 \times 10^{-11}$
$\beta_{CCA} = 0.17$
$P_{ITS-R} = 5.3 \times 10^{-11}$
$\beta_{ITS-R} = 0.16$
observed
imputed
estimate CCA
estimate ITS−R
Model residuals of bilirubin (E,Z or Z,E)*
2
1
0
−1
−2
$n_{CCA} = 552$
$n_{ITS-R} = 677$
$n_{CCA} = 651$
$n_{ITS-R} = 776$
$n_{CCA} = 187$
$n_{ITS-R} = 204$
major homozygote
heterozygote
minor homozygote
Genotype of SNP rs887829 (UGT1A1)

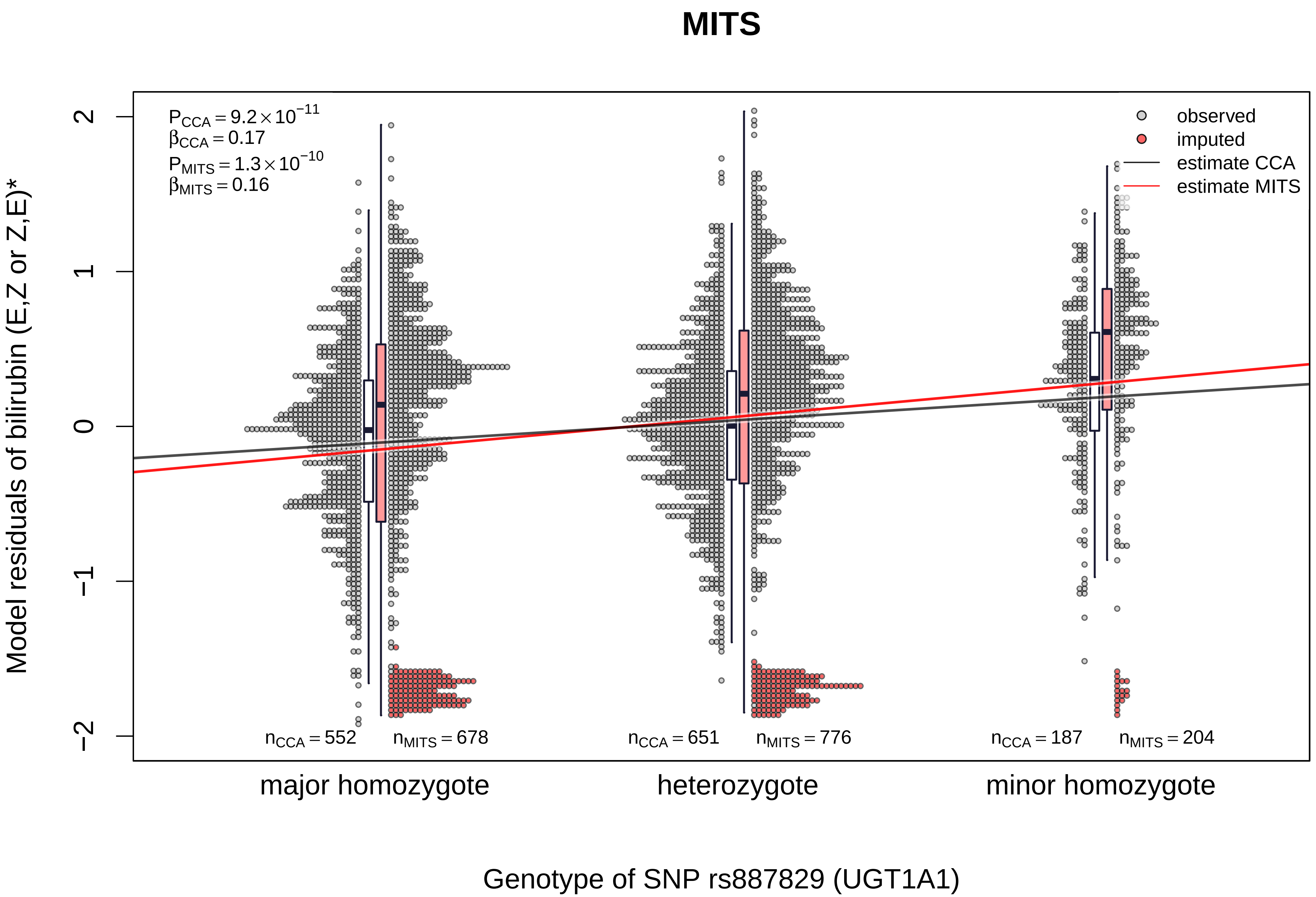

MITS
$P_{CCA} = 9.2 \times 10^{-11}$
$\beta_{CCA} = 0.17$
$P_{MITS} = 1.3 \times 10^{-10}$
$\beta_{MITS} = 0.16$
observed
imputed
estimate CCA
estimate MITS
$n_{CCA} = 552$
$n_{MITS} = 678$
$n_{CCA} = 651$
$n_{MITS} = 776$
$n_{CCA} = 187$
$n_{MITS} = 204$
2
1
0
−1
−2
Model residuals of bilirubin (E,Z or Z,E)*
major homozygote
heterozygote
minor homozygote
Genotype of SNP rs887829 (UGT1A1)

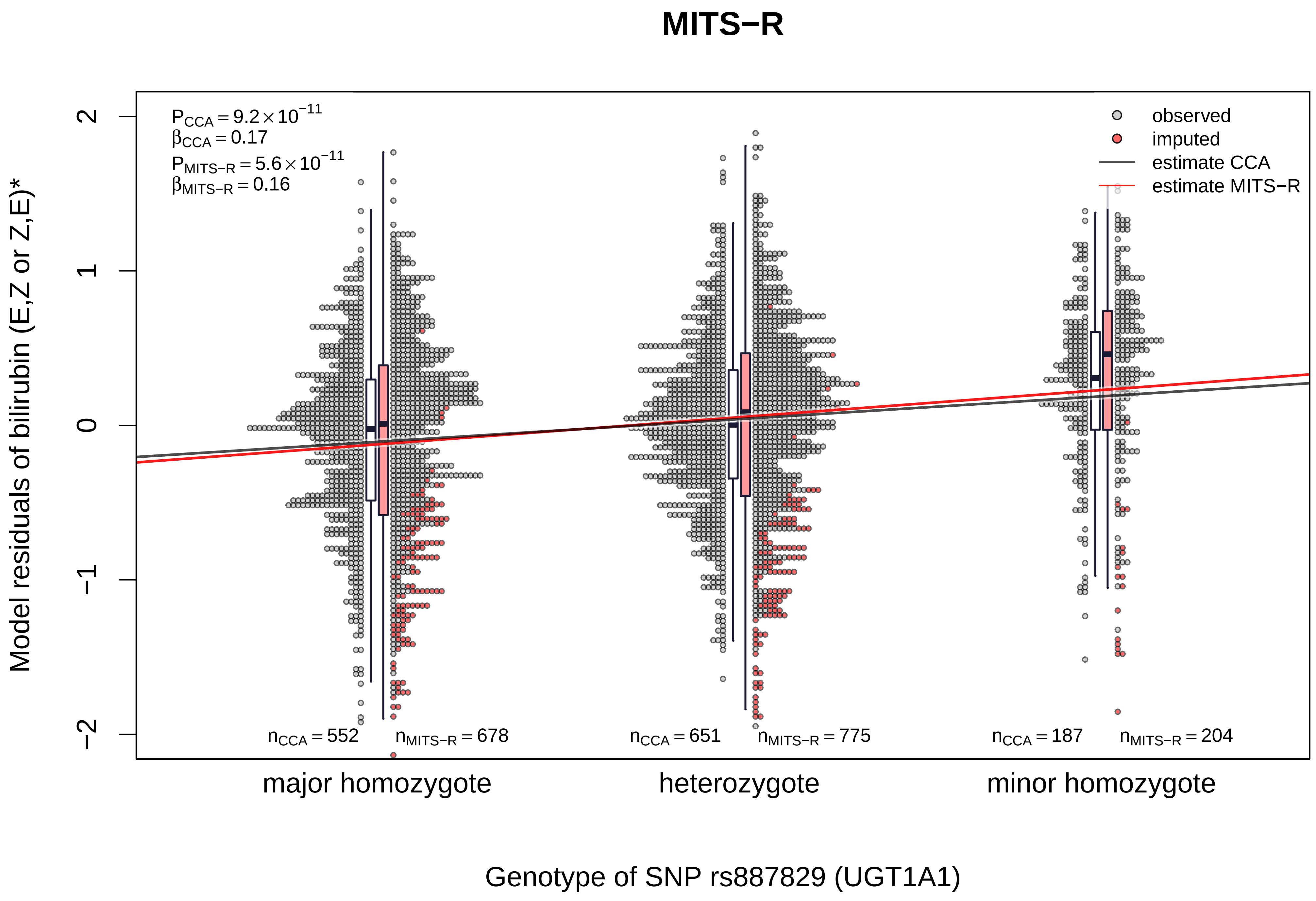
MITS−R
$P_{CCA} = 9.2 \times 10^{-11}$
$\beta_{CCA} = 0.17$
$P_{MITS-R} = 5.6 \times 10^{-11}$
$\beta_{MITS-R} = 0.16$
observed
imputed
estimate CCA
estimate MITS−R
Model residuals of bilirubin (E,Z or Z,E)*
2
1
0
−1
−2
$n_{CCA} = 552$
$n_{MITS-R} = 678$
$n_{CCA} = 651$
$n_{MITS-R} = 775$
$n_{CCA} = 187$
$n_{MITS-R} = 204$
major homozygote
heterozygote
minor homozygote
Genotype of SNP rs887829 (UGT1A1)
